# The Impact II, a Very High-Resolution Quadrupole Time-of-Flight Instrument (QTOF) for Deep Shotgun Proteomics*[Fn FN2]

**DOI:** 10.1074/mcp.M114.047407

**Published:** 2015-05-19

**Authors:** Scarlet Beck, Annette Michalski, Oliver Raether, Markus Lubeck, Stephanie Kaspar, Niels Goedecke, Carsten Baessmann, Daniel Hornburg, Florian Meier, Igor Paron, Nils A. Kulak, Juergen Cox, Matthias Mann

**Affiliations:** From the ‡Proteomics and Signal Transduction, Max-Planck-Institute of Biochemistry, Am Klopferspitz 18, 82152 Martinsried, Germany;; §Bruker Daltonik GmbH, Fahrenheitstr. 4, 28359 Bremen, Germany;; ¶Computational Systems Biochemistry, Max-Planck-Institute of Biochemistry, Am Klopferspitz 18, 82152 Martinsried, Germany

## Abstract

Hybrid quadrupole time-of-flight (QTOF) mass spectrometry is one of the two major principles used in proteomics. Although based on simple fundamentals, it has over the last decades greatly evolved in terms of achievable resolution, mass accuracy, and dynamic range. The Bruker impact platform of QTOF instruments takes advantage of these developments and here we develop and evaluate the impact II for shotgun proteomics applications. Adaption of our heated liquid chromatography system achieved very narrow peptide elution peaks. The impact II is equipped with a new collision cell with both axial and radial ion ejection, more than doubling ion extraction at high tandem MS frequencies. The new reflectron and detector improve resolving power compared with the previous model up to 80%, *i.e.* to 40,000 at *m*/*z* 1222. We analyzed the ion current from the inlet capillary and found very high transmission (>80%) up to the collision cell. Simulation and measurement indicated 60% transfer into the flight tube. We adapted MaxQuant for QTOF data, improving absolute average mass deviations to better than 1.45 ppm. More than 4800 proteins can be identified in a single run of HeLa digest in a 90 min gradient. The workflow achieved high technical reproducibility (R2 > 0.99) and accurate fold change determination in spike-in experiments in complex mixtures. Using label-free quantification we rapidly quantified haploid against diploid yeast and characterized overall proteome differences in mouse cell lines originating from different tissues. Finally, after high pH reversed-phase fractionation we identified 9515 proteins in a triplicate measurement of HeLa peptide mixture and 11,257 proteins in single measurements of cerebellum—the highest proteome coverage reported with a QTOF instrument so far.

Building on the fundamental advance of the soft ionization techniques electrospray ionization and matrix-assisted laser desorption/ionization (1, 2), MS-based proteomics has advanced tremendously over the last two decades (3–6). Bottom-up, shotgun proteomics is usually performed in a liquid chromatography-tandem MS (LC-MS/MS)^1^ format, where nanoscale liquid chromatography is coupled through electrospray ionization to an instrument capable of measuring a mass spectrum and fragmenting the recognized precursor peaks on the chromatographic time scale. Fundamental challenges of shotgun proteomics include the very large numbers of peptides that elute over relatively short periods and peptide abundances that vary by many orders of magnitude. Developments in mass spectrometers toward higher sensitivity, sequencing speed, and resolution were needed and helped to address these critical challenges (7, 8). Especially the introduction of the Orbitrap mass analyzers has advanced the state of the art of the field because of their very high resolution and mass accuracy (9, 10). A popular configuration couples a quadrupole mass filter for precursor selection to the Orbitrap analyzer in a compact benchtop format (11–13).

In addition to the improvements in MS instrumentation, there have been key advances in the entire proteomics workflow, from sample preparation through improved LC systems and in computational proteomics (14–16). Together, such advances are making shotgun proteomics increasingly comprehensive and deep analyses can now be performed in a reasonable time (13, 17–19). Nevertheless, complete analysis of all expressed proteins in a complex system remains extremely challenging and complete measurement of all the peptides produced in shotgun proteomics may not even be possible in principle (20, 21). Therefore, an urgent need for continued improvements in proteomics technology remains.

Besides the Orbitrap analyzer and other ion trap technologies, the main alternative MS technology is time-of-flight, a technology that has been used for many decades in diverse fields. The configuration employed in proteomics laboratories combines a quadrupole mass filter via a collision cell and orthogonal acceleration unit to a reflectron and a multichannel plate (MCP) detector (22). TOF scans are generated in much less than a millisecond (ms), and a number of these “pulses” are added to obtain an MS or MS/MS spectrum with the desired signal to noise ratio. Our own laboratory has used such a quadrupole time-of-flight (QTOF) instrument as the main workhorse in proteomics for many years, but then switched to high-resolution trapping instruments because of their superior resolution and mass accuracy. However, TOF technology has fundamental attractions, such as the extremely high scan speed and the absence of space charge, which limits the number of usable ions in all trapping instruments. In principle, the high spectra rate makes TOF instruments capable of making use of the majority of ions, thus promising optimal sensitivity, dynamic range and hence quantification. It also means that TOF can naturally be interfaced with ion mobility devices, which typically separate ions on the ms time scale. Data independent analysis strategies such as MS^E^, in which all precursors are fragmented simultaneously (23, 24) or SWATH, in which the precursor ion window is rapidly cycled through the entire mass range (25), also make use of the high scanning speed offered by QTOF instruments. It appears that QTOFs are set to make a comeback in proteomics with recent examples showing impressive depth of coverage of complex proteomes. For instance, using a variant of the MS^E^ method, identification of 5468 proteins was reported in HeLa cells in single shots and small sample amounts (26). In another report, employing ion mobility for better transmission of fragment ions to the detector led to the identification of up to 7548 proteins in human ovary tissue (27).

In this paper, we describe the impact II™, a benchtop QTOF instrument from Bruker Daltonics, and its use in shotgun proteomics. This QTOF instrument is a member of an instrument family first introduced in 2008, which consists of the compact, the impact, and the maXis. The original impact was introduced in 2011 and was followed by the impact HD, which was equipped with a better digitizer, expanding the dynamic range of the detector. With the impact II, which became commercially available in 2014, we aimed to achieve a resolution and sequencing speed adequate for demanding shotgun proteomics experiments. To achieve this we developed an improved collision cell, orthogonal accelerator scheme, reflectron, and detector. Here we measure ion transmission characteristics of this instrument and the actually realized resolution and mass accuracy in typical proteomics experiments. Furthermore, we investigated the attainable proteome coverage in single shot analysis and we ask if QTOF performance is now sufficient for very deep characterization of complex cell line and tissue proteomes.

## EXPERIMENTAL PROCEDURES

### 

#### 

##### Preparation of HeLa Lysates

HeLa cells (ATCC, S3 subclone) were cultured in Dulbecco's modified Eagle's medium (DMEM) containing 10% fetal bovine serum, 20 mm glutamine and 1% penicillin-streptomycin (all from PAA Laboratories, Freiburg, Germany). Cells were collected by centrifugation at 200 × *g* for 10 min, washed once with cold phosphate buffered saline (PBS) and centrifuged again. Supernatant was carefully discarded and the cell pellet shock frozen in liquid nitrogen and stored at −80 °C until further use. A pellet containing 5 × 10^7^ cells was resuspended in 1.5 ml of ice cold Milli-Q water, then an equal volume of trifluoroethanol (Sigma-Aldrich, Taufkirchen, Germany) was added. The cell suspension was kept on ice for 10 min, vortexed for 1 min and sonicated for 2 min at 20% duty cycle and output control 3 (Branson Ultrasonics sonifier, Danbury, CT; model 250). After the addition of 200 μl Tris (pH 8.5, final concentration: 100 mm), 400 μl TCEP (final concentration: 10 mm) and 400 μl 2-chloroacetamide (CAA) (final concentration: 40 mm) the lysate was incubated for 10 min at 95 °C. Then the sample was diluted to 15 ml with 50 mm ammonium bicarbonate. The mixture was digested by adding LysC (Wako Chemicals GmbH, Neuss, Germany; ratio 1 μg LysC:100 μg sample protein) for 2 h at 37 °C, followed by adding trypsin (ratio 1 μg trypsin:75 μg sample protein, Promega GmbH, Mannheim, Germany) at 37 °C overnight. After a further digestion with trypsin (ratio 1:125) for 5 h at 37 °C, the digested peptides with an estimated concentration of 1 μg/μl were diluted 1:4 with water and acidified by adding formic acid (FA) (final concentration: 0.2%) and purified on Sep-Pak tC18 cartridges (Waters, Milford, MA) according to manufacturer's instructions. Peptide concentration was determined using a NanoDrop spectrophotometer (Thermo Scientific, Wilmington, DE).

##### Preparation of Yeast Lysates

Saccharomyces cerevisiae strains BY4742 and BY4743 (EUROSCARF) were grown at 30 °C in yeast extract peptone dextrose (YPD) media (10 g/l BactoYeast extract, 20 g/l Bacto^TM^ peptone (BD), 2% w/v glucose). Cells were grown to log phase (OD_600_ of 0.6), harvested by centrifugation at 1600 × *g* for 10 min at 4 °C, washed with cold Milli-Q water and then collected again by centrifugation at 10,000 × *g* for 5 min at 4 °C. Cells were lysed in 1% sodium deoxycholate, 10 mm TCEP, 40 mm CAA in 100 mm Tris pH 8.5, boiled for 10 min at 95 °C and sonicated for 3 min at 30% duty cycle and output control 3 (Branson Ultrasonics sonifier; model 250). Protein concentrations were determined by tryptophan fluorescence emission assay. Cell lysates were diluted 1:2 with Milli-Q water and digested by adding LysC (Wako Chemicals GmbH, ratio 1 μg LysC:50 μg sample protein) for 4 h at 37 °C, followed by adding again LysC (ratio 1:50) overnight at 37 °C. An equal volume of ethyl acetate acidified with 1% TFA was added to the solution, samples were vortexed for 2 min and digested peptides were purified with SDB-RPS StageTips as described in Kulak *et al.* (19). Peptide concentrations were determined using a NanoDrop spectrophotometer.

##### Preparation of MEFs, Hepa, and NSC Cell Line Lysates

Spinal cord neuron-neuroblastoma (NSC-34) (CED-CLU140, Biozol, Eching, Germany), mouse embryonic fibroblasts (MEFs) (American Type Culture Collection, Manassas, VA), and mouse hepatoma (liver cancer, Hepa 1–6) (CRL-1830, American Type Culture Collection) cell lines were cultured and proteins prepared as previously described (28). Briefly, the cells were lysed in lysis buffer (4% SDS, 10 mm Hepes, pH 8.0) during sonication for 15 min (level 5, Bioruptor; Diagenode, Seraing (Ougrée) - Belgium). Cell lysis was followed by reduction of disulfide bonds with 10 mm DTT for 30 min and subsequent alkylation with 55 mm IAA for 45 min. To remove the detergent, cold acetone (−20 °C) was added to 100 μg of proteins to a final concentration of 80% v/v, and proteins were precipitated for at least 2 h at −20 °C. The suspension was centrifuged for 15 min (4 °C, 16,000 × *g*) and the precipitate was washed with 80% acetone (−20 °C) prior to re-suspension in 50 μl of 6 m urea/2 m thiourea, 10 mm Hepes, pH 8.0. An initial digestion step (3 h) was carried out after the addition of 1 μg of LysC, followed by dilution with four volumes of 50 mm ammonium bicarbonate and the final digestion with 1 μg of trypsin overnight at room temperature. The resulting peptide mixtures were desalted on SDB-RPS StageTips (29) and subjected to single shot LC-MS/MS analysis.

##### Preparation of Cerebellum Lysates

Cerebellum from a single mouse (strain: C57Bl6) was homogenized in 4% SDS in 100 mm Tris pH 7.6 using a FastPrep 24 homogenizer (MP Biomedicals, Eschwege, Germany), incubated for 10 min at 95 °C and sonicated for 3 min at 30% duty cycle and output control 3 (Branson Ultrasonics sonifier; model 250). To remove the detergent, acetone (−20 °C) was added to a final concentration of 80% v/v and proteins were precipitated overnight at −20 °C. Supernatants were carefully discarded after centrifugation at 1600 × *g* for 20 min at 4 °C, and the pellets were washed with 80% acetone (−20 °C). The protein pellets were dissolved in 8 m Urea in 10 mm Hepes and protein concentrations were determined by the tryptophan fluorescence emission at 350 nm using an excitation wavelength of 295 nm. Proteins were reduced with 10 mm DTT for 30 min and alkylated with 55 mm iodoacetamide for 20 min. After addition of thiourea to a final concentration of 0.1 m, samples were digested by adding LysC (Wako Chemicals, ratio 1 μg LysC:100 μg sample protein) for 3 h at RT, diluted with four volumes of 50 mm ammonium bicarbonate, and further digested with trypsin (ratio 1 μg trypsin:100 μg sample protein, Promega) at RT overnight. After a further digestion with LysC and trypsin (ratio 1:100) for 8 h at RT, digested peptides were acidified by adding TFA (final concentration: 0.5%) and purified on Sep-Pak tC18 cartridges (Waters) according to manufacturer's instructions. Peptide concentrations were determined using a NanoDrop spectrophotometer.

##### Sample Preparation for Quantification

Universal Proteomics Standard (UPS-1, Sigma-Aldrich) and Proteomics Dynamic Range Standard (UPS-2, Sigma-Aldrich), both containing 48 human proteins, either at equimolar concentrations (UPS-1) or formulated into a dynamic range of concentrations, covering five orders of magnitude (UPS-2), were prepared according to ref (30). Predigested yeast sample (Promega) was re-suspended in 0.1% trifluoroacetic acid to a final concentration of 500 ng/μl. Digested UPS-2 sample was spiked in two different amounts of 250 fmol to 2.5 amol peptide amount for sample 1 and 500 fmol to 5 amol for sample 2 into 500 ng yeast background, thereby creating two samples with a theoretical ratio 1:1 for the yeast proteome and 1:2 for the UPS peptides. In another sample, digested UPS-1 sample (25 fmol for all components) was spiked into 500 ng yeast.

##### High-pH Reverse-Phase Fractionation

We performed high-pH reversed-phase peptide prefractionation with fraction concatenation on 175 μg HeLa or cerebellum peptides on a 2.1 × 300 mm Acquity UPLC Peptide BEH column packed with 130 Å pore, 1.7 μm particle size C_18_ beads (Part No. 186005792, Waters). A gradient of basic reversed-phase buffers (Buffer A: 0.1% formic acid, ammonium hydroxide pH 10; Buffer B: 0.1% formic acid, 80% acetonitrile, ammonium hydroxide pH 10) was run on a Prominence HPLC system (Shimadzu, Duisburg, Germany) at a flow rate of 150 μl/min at 60 °C. The LC run lasted for 240 min with a starting concentration of 5% buffer B increasing to 30% over the initial 120 min and a further increase in concentration to 60% over 70 min. This elution gradient was followed by a 95% wash and re-equilibration. Fraction collection started after 0.2 ml elution and fractions were collected every 140 s resulting in 72 fractions used for concatenation into 24 fractions as described previously (31).

##### Inlet Capillary and CaptiveSpray

In our instrument, in contrast to many other commercial ion source designs, the high voltage for the electrospray (ES) process is applied to the vacuum capillary inlet, whereas the sprayer is kept at ground, which allows for a simpler source design (supplemental Fig. S1*A*). To electrically decouple the ES voltage and the electrical potential of the vacuum section, we use an inlet capillary made from high resistive glass (∼1GOhm). Positioning the ES voltage at the capillary entrance means that the ions are transported opposite to the electrical gradient by the gas flow (32). In this configuration, charged molecules travel somewhat slower than the surrounding gas. According to Bernoulli's law, ions are then focused toward the area of highest gas velocity along the center axis of the capillary. The set-up tends to reduce the contamination of the inner capillary walls (33).

The Bruker CaptiveSpray nanoflow ES source is directly attached to the vacuum inlet capillary via a short capillary extension that can be heated using the instrument's drying gas (Supplemental Fig. S1*A*). The spray tip is automatically mechanically aligned on axis with the capillary inlet without the need for any adjustments. The principle of the CaptiveSpray is a vortex gas (usually air) that sweeps around the emitter spray tip at three different stages. The first one is designed to assist spray formation, the second and third one help to focus the spray plume into the MS inlet capillary. All three flows are created solely by the vacuum of the MS system, which requires that the entire source is vacuum sealed.

The spray emitter consists of a 2 cm long, 20 μm ID fused silica capillary. Its tip is etch-tapered, thus the inner diameter remains constant to the very end of the tip making it very robust against clogging. Furthermore, it also allows using the same emitter at flow rates ranging from 50 nl/min to 5 μl/min, thereby supporting a wide range of column types. Fused silica columns, which are often used for proteomics, are typically connected to the emitter via a low dead volume union (supplemental Fig. S1*A*), which also provides the electrical contact for keeping the electrospray at ground potential.

##### Minimizing Postcolumn Dead Volume

Using the described design, the CaptiveSpray source provides very stable ionization; however, when we initially coupled it to the LC set-up used in the Munich laboratory (17, 34), we observed broader LC peak elution distributions than we normally do (supplemental Fig. S1*B*). Furthermore, we wished to incorporate a column oven and pulled tip columns. We therefore constructed a modified source, which keeps the back end of the CaptiveSpray but replaces the front end by the standard set-up used in our department. The modified set-up incorporating the tip column is displayed in supplemental Fig. S1*C*. The modified design of the column holder allows for exact aligning and fixation of the column inside the CaptiveSpray source. Electrical grounding was applied using a connecting tee at the column head. This setup produced the desired, narrow LC peak distributions (supplemental Fig. S1*D*) and was used for the proteomic analyses described in this article.

##### LC-MS/MS Analysis

We used an Easy nLC-1000 (Thermo Fisher Scientific) on-line coupled to an impact II (Bruker Daltonics) with a CaptiveSpray ion source (Bruker Daltonics). The peptide mixtures (1 μg) were loaded onto an in-house packed column (50 cm, 75 μm inner diameter) filled with C_18_ material (ReproSil-Pur C_18_ AQ 1.9 μm reversed phase resin, Dr. Maisch GmbH, Ammerbuch-Entringen, Germany). Chromatographic separation was carried out using a linear gradient of 5–30% buffer B (80% ACN and 0.1% FA) at a flow rate of 250 nl/min over 90 min. Because of loading and washing steps, the total time for an LC-MS/MS run was about 40 to 50 min longer.

Generally, LC-MS/MS data were acquired using a data-dependent auto-MS/MS method selecting the 17 most abundant precursor ions in cycle for fragmentation and an MS/MS summation time adjusted to the precursor intensity (Compass 1.8 acquisition and processing software, Bruker Daltonics). For the deep proteome measurements of a cell line in combination with peptide fractionation, we used a “dynamic method,” with a fixed cycle time of 3 s. The mass range of the MS scan was set to extend from *m*/*z* 150 to 1750. Dynamic exclusion duration was 0.4 min. Isolation of precursor ions was performed using an *m*/*z* dependent isolation window of 1.5–5 Th. The collision energy was adjusted between 23–65 eV as a function of the *m*/*z* value.

For the quantitative analysis of the UPS standards in yeast we used a trapping column set-up (PepMap pre-column, 2 cm x 100 μm; Thermo Scientific) and a Dionex HPLC pump (Ultimate 3000, Thermo Scientific). For this experiment, peptides were separated on a PepMap UHPLC column (50 cm x 75 μm, 2 μm particles; Thermo Scientific) using a 90 min multistep ACN gradient (buffer A: 0.1% FA; buffer B 100% ACN in 0.1% FA). The unmodified CaptiveSpray ion source (see above) was used to interface the LC system to the impact II. For quantification full scan MS spectra were acquired at a spectra rate of 1Hz followed by acquisition of 1 MS/MS spectrum. Six replicates per sample were acquired. For data acquisition of the UPS-1 in yeast sample, the 17 most intense precursor ions were selected for fragmentation, resulting in a total cycle time of 1.2 s.

##### Intact Protein Analysis

Adalimumab was cleaved at the hinge region with IdeS (FabRICATOR, Genovis) and reduced to obtain the Fc/2, Fd and light chain sub units as recently described in (35). The subunits were separated by chromatography (35) prior to analysis on the impact II. Data was analyzed using the SNAP algorithm to fit the theoretical pattern (36, 37).

##### Development of MaxQuant for QTOF Data

In general all processing steps from the standard MaxQuant computational workflow, which was optimized for the analysis of Orbitrap data, are also applied to QTOF data. The nonlinear mass recalibration algorithm experienced major adaptations. Its original form for the Orbitrap applies a recalibration function with nonlinear dependence on the two variables *m*/*z* and retention time. It has been extended to include the peak intensity as a third dimension that the mass recalibration depends on. This is necessary because of appreciable systematic nonlinear intensity dependent peak mass shifts that are typically found in time of flight data. The intensity dependence is parameterized as a polynomial in the logarithm of peak intensities. The new mass recalibration algorithm allows for high mass accuracy without the use of internal or external calibrants.

We added a new instrument type called “Bruker QTOF” in which several relevant parameters of algorithms for the processing of spectra are set to default values that are suitable for the analysis of data generated by the impact family. These parameters include mass matching windows for the assembly of 3D peaks, mass tolerances for assembling isotope patterns and labeling pairs, initial peptide mass tolerance windows for the Andromeda search and minimum required number of scans per 3D peak. Raw data can be immediately read from the proprietary Bruker binary format and no conversion to intermediate file formats is needed. Peak centroids are utilized as determined by the centroiding algorithms of in the Bruker software. The viewer module of MaxQuant is enabled for QTOF data, among other features allowing to visualize MS data in *m*/*z*-retention time maps and to annotate and export MS/MS spectra to fulfil journal requirements for reporting of spectral evidence.

##### Analysis of Proteomic Data

All data were analyzed with the MaxQuant software (version 1.5.2.8 or version 1.5.0.1) (38, 39) with the Andromeda search engine (38) with the adaptions and developments described above. The false discovery rate (FDR) was set to 1% for both proteins and peptides and we specified a minimum length of seven amino acids. MaxQuant scored peptides for identification based on a search with an initial allowed mass deviation of the precursor ion of up to 0.07 Da after time-dependent recalibration of the precursor masses. The allowed fragment mass deviation was 40 ppm. The Andromeda search engine was used for the MS/MS spectra search against the Uniprot human database (downloaded on June 21, 2014, containing 88,976 entries and 247 contaminants), the Uniprot Saccharomyces cerevisiae database (downloaded on June 21, 2014, containing 6643 entries), the Uniprot mouse database (downloaded on June 21, 2014, containing 51,573 entries) and UPS fasta file provided by Sigma-Aldrich (http://www.sigmaaldrich.com/life-science/proteomics/mass-spectrometry/ups1-and-ups2-proteomic.html) for quantitative study. Enzyme specificity was set as C-terminal to Arg and Lys, also allowing cleavage at proline bonds and a maximum of two missed cleavages. Carbamidomethylation of cysteine was selected as fixed modification and N-terminal protein acetylation and methionine oxidation as variable modifications.

The “match between runs” feature of MaxQuant was used to transfer identifications to other LC-MS/MS runs based on their masses and retention time (maximum deviation 0.7 min) and this was also used in quantification experiments. Quantifications were performed with the label-free algorithms described recently (39). We required a minimum peptide ratio count of two and at least one “razor peptide” for quantification. For cerebellum, the quantification was based on normalized protein intensities. Further analysis of data was performed in the MaxQuant Viewer, in the Perseus post data acquisition package that is part of MaxQuant (all freely available at www.maxquant.org) and in the R statistical computing environment (40).

Potential contaminants as well as proteins identified only by site modification were strictly excluded from further analysis.

For the quantitative analysis of the UPS standards in yeast, entries were only accepted if they had valid values in all 12 replicates. Results were then filtered for Welch-significant regulation of UPS-2 proteins.

Analysis of the yeast samples were based on label-free intensities (LFQ values). After filtering (3 valid values in at least one group), remaining missing values were imputed from a normal distribution (width: 0.3; down shift: 1.8). Two-sample *t* test was performed with a FDR < 0.01.

For global cell line comparison, triplicates were analyzed twice for a total of six single shot measurements per cell line (except the NSC-34 cell line, which was only measured once). For the principal component analysis (PCA) of the different cell lines, we furthermore limited the data set (LFQ intensities) to entries with a minimum of four valid values in at least one group of six replicates. Remaining missing values were imputed from a normal distribution (see above).

Protein intensity (summed peptide intensity) for cerebellum samples were divided by the molecular weight for ranking by proteins abundance. Annotations (GO molecular function, biological process, cellular component; KEGG and Uniprot Keywords) were matched to protein groups with Perseus. We performed a 1D annotation enrichment (41) on the normalized protein intensities. To evaluate the proteome coverage, we counted the occurrence of categories in our sample and compared it to the category count for the complete murine proteome in Perseus.

MS raw data and data for protein and peptide identification and quantification were submitted as supplementary tables to the ProteomeXchange Consortium via the PRIDE partner repository with the data set identifier PXD001592.

## RESULTS AND DISCUSSION

### 

#### 

##### Overview of the Instrument

**Fig. 1.**
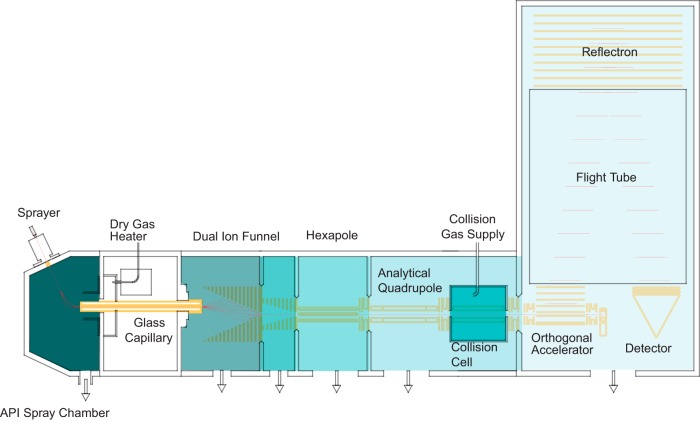
**Schematic of the impact II mass spectrometer (not to scale).**

The Bruker impact II is a QTOF in a benchtop format, featuring several improvements in its design (Fig. 1). Briefly, ions are produced in the CaptiveSpray, which is in an encased nanoelectrospray source that features a well-defined gas stream to guide the ions into the vacuum via a capillary inlet. A double ion funnel, based on principles described by Smith and co-workers (42), is positioned off axis, which prevents neutrals from further transmission along the ion path. The pressure drops by several orders of magnitude from the capillary exit to the postfunnel stage (3 mbar to 3 × 10^−4^ mbar), while the ion current is virtually undiminished (see below). Additionally, the funnel allows for soft transfer based on low electrical field strength independent of the mass (typically 10 V/cm, much lower than in nozzle-skimmer designs). By introducing electrical acceleration in-between the two funnels, in-source fragmentation can still be achieved intentionally. There is a hexapole ion guide between funnel and the analytical quadrupole mass filter, which has a monolithic design based on high precision glass. Precursor ions can be isolated by this quadrupole for subsequent fragmentation in the collision cell. Intact ions or fragments can be stored and extracted from the collision cell and enter the orthogonal deflection region as a very narrowly focused ion beam (< 500 μm). Here they are accelerated into a field-free drift region. A newly designed, two-stage reflectron further compensates the velocity distribution orthogonal to the beam direction. Finally, the ions impinge on an MCP detector coupled to a 10-bit, very high frequency (50 Gbit/s), zero noise digitizer. Data collection is coordinated by the Bruker Compass data system and in the experiments described here, post-acquisition data processing is performed in the MaxQuant environment.

##### Optimization of the Collision Cell

Efficient fragmentation of precursor ions on an LC-MS/MS time scale is a key for the identification of peptides in shotgun proteomics strategies. We optimized several aspects of the collision cell (supplemental Fig. S2*A*): Precise geometrical alignment allows focusing of the ions along the axis of the collision cell, directly translates into well-defined starting conditions for the orthogonal accelerator and is therefore mandatory for high mass resolution. This is implemented via a quadrupolar configuration of the collision cell device providing a narrow pseudo potential well (43). We also introduced a radial ejection step between any two MS or MS/MS experiments, in order to reduce the dead time. The ion path has to be emptied to avoid crosstalk between two consecutive spectra without introducing substantial ion losses. Most importantly, we optimized the time of ion fragmentation and extraction within the fragment spectra to ensure efficient high frequency MS/MS by implementing an electrical axial field gradient. This gradient directs the ions toward the exit of the collision cell, reducing the time it takes for the first ions to reach the extraction lens after quenching the collision cell. From here, they can be rapidly released toward the orthogonal accelerator, forming packages that match the orthogonal pulser frequency. The ion densities after 3 ms (upper red traces in supplemental Fig. S2*B*–S2*C*) reveals two important aspects: Using the axial field gradient results in comparable ion density at the collision cell exit in much shorter time than without, *i.e.* after about 1 ms instead of 3 ms, respectively. This suggests a three times faster ion transfer. Moreover, the overall number of ions within the collision cell is reduced to less than 50% at the moment of quenching, which should reduce ion losses related to quenching accordingly.

The pseudo potential calculations and simulation were confirmed in different experiments comparing the performance of the optimized collision cell on the fragmentation yield of Glu-Fibrino-Peptide B at different MS/MS acquisition rates (supplemental Fig. S2*D*). This revealed that the reduced quench losses in fact improve the number of ions detected at a spectra rate of 16 Hz by a factor of two. We further observed that the axial field gradient improves the stability of the system even in the presence of slight contaminations on the rods.

##### High Transfer Efficiency to the Orthogonal Acceleration Unit

**Fig. 2.**
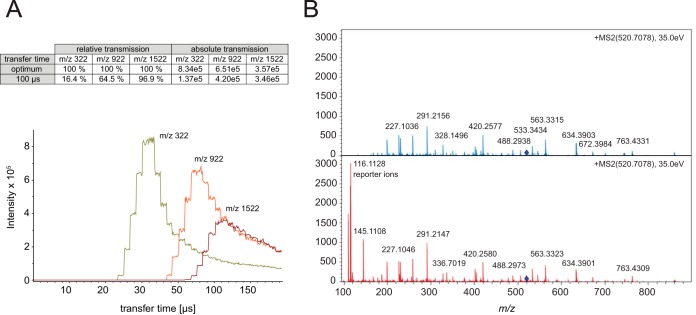
***A*, Absolute ion intensities of *m*/*z* 322, 922 and 1522 as function of the transfer time.** The maxima of the distributions for each *m*/*z* value are the optimal times for efficient transfer into the flight tube. *B*, Fragment spectrum of the iTRAQ labeled peptide LFTGHPETLEK without (blue) and with (red) transfer time stepping that adds the lower *m*/*z* range including the reporter ions without sacrificing intensity in the higher mass range.

The ions travel through the flight tube and require as much time as the largest *m*/*z* species needs to reach the detector, before the HV pulser can send the next ion package toward the detector (typically between 100 and 150 μs). To avoid excessive loss of ions, orthogonal TOF instruments are therefore often operated in a mode in which the ions are stored in the collision cell during the TOF scan and released in time for the next extraction pulse of the orthogonal accelerator. This would allow for 100% duty cycle if all ions were indeed transferred such that they arrive in the orthogonal accelerator at the same time and with the same kinetic energy. In practice, however, the extraction time from the collision cell toward the gate lens is a function of ion mobility. We have analyzed these combined effects by varying the time from opening the gate lens to the extraction pulse of the orthogonal accelerator (transfer time) for different *m*/*z* ratios (Fig. 2*A*). Simulations of ion trajectories and extraction times reveal that about 80% of a single ion species can be accelerated into the drift tube of the TOF analyzer under optimal transfer time conditions. In the impact II the high transfer efficiency is further optimized by reducing the distance between the trapping region and the orthogonal accelerator—it is about four times higher compared with conventional QTOF systems operated in continuous operation mode. Figure 2*A* summarizes the relative transmission efficiency of selected precursors (*m*/*z* = 322 Th, 922 Th and 1522 Th) at a transfer time of 100 μs, which is a standard setting to cover the mass range relevant in shotgun proteomics. The analysis highlights that ions with low *m*/*z* are compromised most, which we have previously counteracted in proteomics experiments by adding special extraction conditions (44). To tackle this problem in a more general way, Bruker introduced the “transfer time stepping” operation mode, where first high mobility species are extracted followed by species with a lower mobility. During the initially short opening times of the gate (typically 50% of the total scan) only the higher mobility, low *m*/*z* ions pass the gate lens, while the lower mobility ions are still accumulated in the collision cell. In the second transfer time steps, extraction times are increased to allow the low mobility, higher *m*/*z* ions to pass the gate lens. Spectra with and without transfer time stepping, reveal the beneficial effect on low mass ions without appreciable loss in the standard mass range (Fig. 2*B*). This is particularly beneficial for labeling experiments that rely on quantification of low mass reporter ions as shown in the figure. These ions are transferred with greater than 60% efficiency during the low *m*/*z* extraction phase and the total amount of ions in the analyzed fragment spectrum is increased by 58%.

Together, our developments led to an overall transmission efficiency of > 60% into the orthogonal acceleration unit. This compares favorably to a recent report, in which ion mobility was performed on fragment ions and their arrival times were synchronized with orthogonal extraction, which lead to an up to 10-fold improvement of detection sensitivities to standard operation (27).

##### Sensitivity and Ion Transfer

**Fig. 3.**
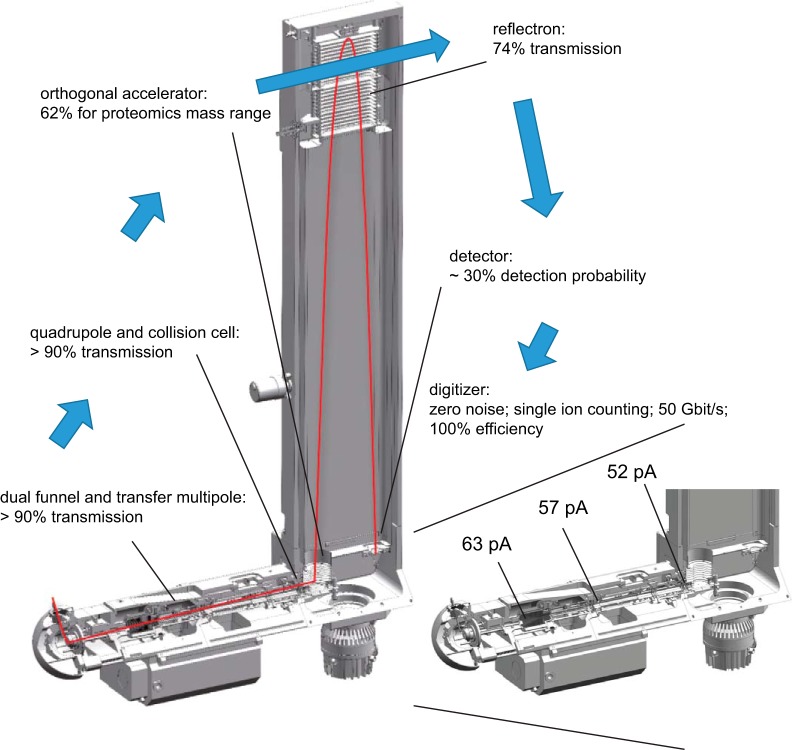
**Ion transfer efficiency of mass range most relevant in shotgun-proteomics experiments (*m*/*z* 100–1500).** Inset shows net analyte ion currents at the indicated measurement points and transfer efficiencies at the orthogonal accelerator at various stages along the flight path of the impact II instrument.

The number of ions that successfully pass through the instrument and are finally recorded determine a mass spectrometer's sensitivity. We were interested in the transfer efficiencies along the ion path, from entering the vacuum system to the detector. To experimentally determine this, we infused a 1 pmol/μl BSA solution or blank solution and measured the difference in the ion current between these conditions. When operating the outlet of the capillary and the funnel region as a Faraday cage, we measured a net ion current of 63 pA, which we defined as the starting value (100%) (Fig. 3). The large ion acceptance aperture of the first funnel efficiently captures in the ion flux leaving the capillary. It transfers the ions to the second funnel, which also passes the ions in an almost lossless manner through the next stage as evidenced by a net current reading of 57 pA (>90%) after the hexapole. Likewise, more than 90% of the ions are transferred through the quadrupole (in nonmass selecting operation) and the collision cell. More than 60% of these ions are transmitted into the flight tube by the orthogonal accelerator (see above). The reflectron contains two grids, which are each passed twice, leading to a geometrically defined overall transmission of 74%. Although all these ions hit the MCP, not all of them enter the channels and not all of them result in secondary electrons (detector quantum yield). However, when secondary ions are generated they are greatly amplified (>10^6^ fold), and can be efficiently discriminated from electronic noise (zero noise detection). Together, this leads to an estimated detection probability of 30% for our MCP detector. Although not all of these measurements and estimates are very precise, they suggest an overall detection probability of ions transmitted into the vacuum system of about 10%. This excellent number is because of the fact that the continuous beam generated by the ES source can be utilized and that all the ion guiding elements have been successfully optimized for high transmission.

##### Resolution and Mass Accuracy

For the impact II several improvements were implemented: symmetrical shielding for better ion focusing; line grids to increase the transmission; low temperature coefficient ceramic spacers to decrease temperature related mass drift and improved axel bearings for precise alignment. Together this led to about 35% increased resolution over the full proteomics mass range. As this improvement is reached solely by better focusing of the ions, mass accuracy and signal to noise are expected to increase accordingly. Improvements to the MCP detector include an increased entrance aperture, higher electron accelerating fields and optimized shielding. Overall, these measures lead to 2-fold faster ion impact transients and 30% higher detection efficiency of the MCP.

**Fig. 4.**
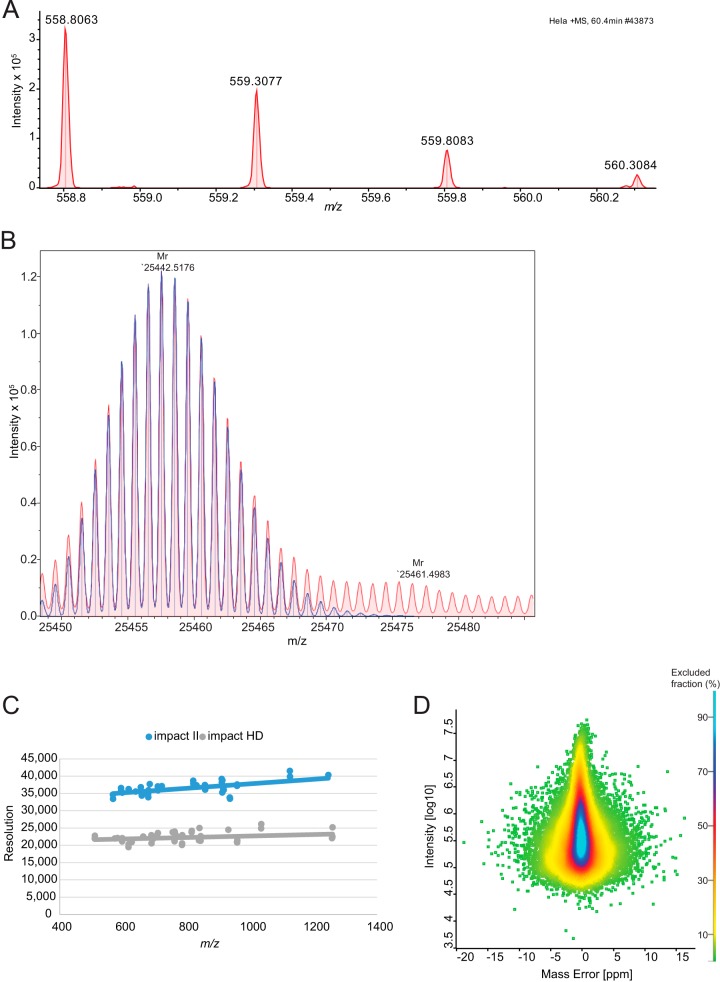
**Resolution and mass accuracy of *A*, a peptide isotope cluster (*m*/*z* 558.8063, *r* = 33k) and *B*, Fd unit of Adalimumab (*m*/*z* 25442.5157, *r* = 63k, 0.26 ppm).** Overall improvement of the resolution with the improved detector, *C*, and the achieved mass accuracy dependent on the summed peptide intensity, *D*, in a shotgun proteomics experiment using the QTOF optimized version of MaxQuant.

In summary, the resolving power of the TOF analyzer is expected to increase by about 70 to 80% by the introduction of the new collision cell, reflectron and detector. To test this experimentally on a standard proteomic sample, we analyzed data from a HeLa digest. Resolution for typical peptides is in excess of 33,000 as illustrated by an example in Fig. 4*A*. Further increase in the resolution can be obtained by the “Focus mode,” which involves real time processing and alignment of successive pulses and increases accuracy of flight time determination, when multiple ions of one species reach the detector at the same time (45). This can be helpful to resolve the isotope distributions of proteins, as shown in Fig. 4*B*, which features a resolution in excess of 60,000 for an antibody subunit (> 25 kDa). In TOF measurements, resolution tends to increase with *m*/*z* (Fig. 4*C*), and reached more than 40,000 for the TuneMix component at *m*/*z* 1222. This constitutes a 70 to 80% improvement over the previous impact model, the impact HD.

This increase in resolving power should also imply better mass accuracy in proteomics samples, which we tested using the software package MaxQuant, which we adapted to QTOF data as described under “Experimental Procedures.” A special feature of MaxQuant is the extraction of individual mass accuracy values (46, 47), which allows to make efficient use of high mass accuracy in the identification of peptides. MaxQuant was originally developed on the basis of data from hybrid Orbitrap instruments. Here we developed MaxQuant further in order to analyze QTOF data and also in this context profit from the high mass accuracy provided by nonlinear mass recalibration algorithms. A special challenge in QTOF data is the drift in the mass scale because of thermal expansion caused by ambient temperature drift. MaxQuant employs a double search strategy, in effect supplying hundreds of reference masses internal to each proteomic sample. This feature efficiently removes any effect of the temperature related mass drift. This “software lock mass” feature makes it unnecessary to use dedicated molecular species for the calibration of spectra (48). With these developments in MaxQuant, we analyzed the peptide mass error distributions over a 90 min gradient run (Fig. 4*D*). This showed an average absolute mass deviation of around 1.45 ppm, which is excellent for a QTOF instrument.

##### Impact II Performance for Single Shot Analysis

**Fig. 5.**
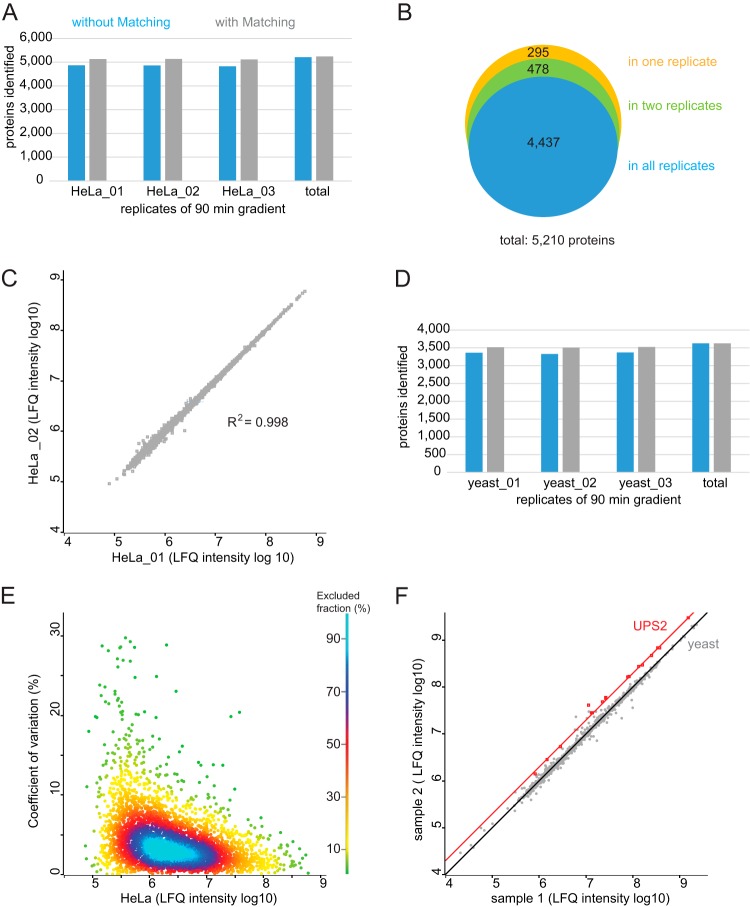
**Triplicate analysis of HeLa and yeast digest using a 90 min gradient for single shot analysis.** *A*, Protein identification numbers of each replicate of 1 μg HeLa digest and *B*, the overlap of protein identities. *C*, Correlation between the rank ordered label-free quantification values of each identified protein in replicate1 (HeLa_01) and replicate2 (HeLa_02) (log10 LFQ intensities). *D*, Protein identification number of 1 μg yeast digest. *E*, Reproducibility across triplicate analysis of HeLa digest. CVs of all three replicates, representing 99% of the data points. *F*, Accuracy in quantification. Results of spike-in experiment showing the UPS-2 standard (orange) and in a yeast proteome background (gray). Yeast lysate was present in equal amounts in sample 1 and sample 2 and the UPS2 protein standard was present in twice the amount in sample 2.

To investigate the performance of the impact II for shotgun proteomics, we first analyzed a complex peptides mixture derived from a mammalian cell line in the single-run format (Experimental Procedures). We separated 1 μg of peptide digest by on-line HPLC with the standard 90 min gradient employed in our laboratory and performed triplicate analysis. The typical data dependent acquisition scheme in bottom-up proteomics consists of an MS scan followed by N MS/MS fragment scans of the most intense precursors (topN method). It is desirable to choose N such that the total cycle is less than a few seconds. For our measurements we aimed at a duty cycle of around 1.3 s and designed a top17 method, consisting of 200 ms for MS acquisition and a MS/MS integration time adapted to the precursor intensity. We found this to be a good balance between acquiring high S/N in the MS and achieving optimal ion intensities in the MS/MS spectra for the HeLa digest. This method reached more than 7000 MS and 79,700 MS/MS scans. In each run MaxQuant identified on average 35,580 unique peptide sequences, which results in total of 48,172 unique peptide sequences in the triplicate analysis (Table I). These peptides mapped to an average of 4854 proteins per run, and a total of 5210 proteins of the HeLa proteome with the three 90 min gradients (Fig. 5*A*), indicating that a deep coverage can be achieved using relatively short, single shot analysis. Transferring identification between the runs based on their mass precision and retention time (“match between runs” feature in MaxQuant) led to around 5100 proteins identifications per single run. Comparing protein identities between the triplicate analyses (without “matching between runs”), we observed that more than 90% of proteins were identified in each of them (Fig. 5*B*). This indicates high reproducibility and a minimal ‘missing value’ problem. This conclusion is further supported by excellent reproducibility (R^2^ = 0.998) in the label-free intensities determined in pair-wise comparison between runs (MaxQuant LFQ values (39, 49)) (Fig. 5*C*). We also determined the number of proteins identified in a single shot run of 1 μg yeast digest. On average, we identified 3352 proteins per single, 90 min gradient, and a total of 3627 proteins when combining the three single shot measurements (Fig. 5*D*).

**Table I:** Identification from HeLa and yeast lysate triplicate analysis using a standard 90 min gradient

	MS scans	Isotope pattern	MS/MS scans	Identification rate [%]	Peptide sequences identified	Proteins identified
HeLa_1	7002	759,774	79,704	47.09	35,547	4870
HeLa_2	7181	769,355	79,876	47.22	35,572	4864
HeLa_3	7272	796,086	81,389	46.57	35,621	4828
Total				46.96	48,172	5210
yeast_1	4873	528,682	63,682	31.08	17,066	3361
yeast_2	4732	541,194	66,441	30.88	16,921	3325
yeast_3	4691	556,675	66,978	31.98	17,494	3369
Total				31.32	24,131	3627

##### Reproducibility and Accuracy of Quantification

To evaluate the reproducibility of the method for label-free quantification, we determined the coefficients of variation (CV) of the label-free intensities, determined in pair-wise comparison between three technical HeLa replicates (see above). For more than 90% of the quantified proteins the CV was smaller than 10% (Fig. 5*E*). For the lowest intensity quantile the median was 0.05 and for the highest one 0.03 (Fig. 5*E*, supplemental Fig. S3). This shows reproducible quantification over four orders of magnitude.

Accurate quantification of differently expressed proteins remains challenging over a wide concentration range and benefits from a very stable analytical platform. QTOF instrumentation has been used widely for label-free quantification, which in principle allows convenient analysis and comparison of an arbitrary number of samples. For evaluation of the label-free quantitative capabilities of the impact II platform in complex mixtures we wished to use a reference sample set with known ratios for a small subset of proteins. We spiked the Universal Protein Standard 2 (UPS-2), consisting of 48 proteins covering a dynamic range of five orders of magnitude, in two different concentrations into the yeast proteome. This generated two samples, in which the yeast peptides should be 1:1 whereas ratios for UPS-2 peptides should be at 1:2. To increase the number of identified peptides we then used the equimolar UPS-1, which we also spiked in a yeast background. This allowed transfer of peptide identifications to unsequenced peptides in the UPS-2 in yeast runs via the ‘match between runs’ algorithm in MaxQuant. We identified all of the 48 human UPS proteins in the sample containing yeast with the equimolar UPS-1 standard. Of these proteins, 23 were identified in each one of the 12 single shot yeast measurements with the dynamic UPS-2 standard either directly via MS/MS or via match between runs and 18 of these proteins showed Welch-significance (Fig. 5*F*). We identified and quantified UPS-2 proteins over more than three orders of magnitude in these relatively fast measurements (90 min gradients). The UPS-2 proteins have an average fold change of 0.49 (± 0.06), which is close to the theoretical ratio of 0.5.

##### Quantification of Changes in the Yeast Proteome

**Fig. 6.**
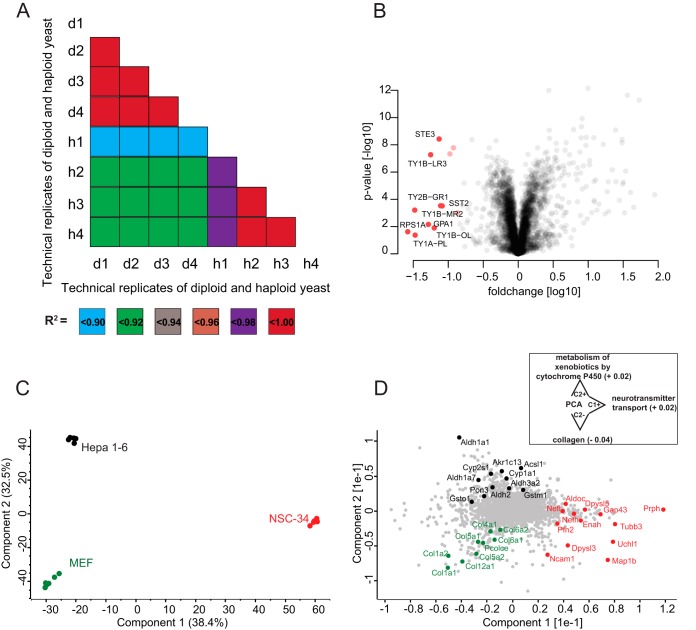
***A*, Correlation of single shot LC-MS/MS measurements of haploid (h) and diploid (d) yeast samples.** All technical replicate correlation values were > 0.98. *B*, Quantitative differences between the haploid and diploid yeast proteome. Proteins marked in red are significantly more abundant in haploid cells. *C*, Principal component analysis (PCA) of LFQ protein expression levels in mouse hepatoma (Hepa 1–6), mouse embryonic fibroblasts (MEF) and motoneuronal cell lines (NSC-34). *D*, Loading of the first two principal components as obtained from the PCA plotted in *C*. The inset indicates significantly enriched annotations along the PC axes (FDR < 0.05). The respective enrichment scores are specified in parentheses.

To test the workflow in a systems biology context, we analyzed proteome changes of diploid and haploid (Matα cell) *S. cerevisiae*. We analyzed 2 μg of yeast digest from haploid and diploid cells in technical quadruplicates with our standard 90 min gradient. This identified 3769 proteins using “match between runs.” For statistical analysis, we only considered LFQ intensities that were detected in at least three replicates of the haploid or diploid groups. After filtering, 3222 proteins remained for further analysis (Experimental Procedures). Remaining missing values were imputed from a normal distribution. The technical replicates correlated much more with each other than they correlated to the other genotype (R^2^ greater 0.98 *versus* R^2^ about 0.92; Fig. 6*A*). As in our previous large-scale analysis on SILAC labeled haploid and diploid yeast (50), we found transposons more abundant in haploid cells than in diploid cells (Fig. 6*B*). Ste3, the pheromone a factor receptor, was specific to haploid yeast, as expected from its mating status. Also absent in diploid but present in haploid cells were Sst2, a GTPase-activating protein for Gpa1 (51), which, consistently, showed higher expression in haploid cells. Conversely, Sps100, which is a sporulation-specific wall maturation protein turned out to be specific for diploid cells. Doing such systems-wide comparisons by traditional methods would have required thousands of individual Western blots. Even compared with our previous large-scale study performed by quantitative MS (50), we here used less than 1% of yeast input material and measurement time. This illustrates the rapidity by which MS-based proteomics is becoming a viable method for answering biological questions.

##### Global Proteomic Comparison of Different Cell Lines

As a second example of typical proteomics experiments, we applied the QTOF-based workflow to the characterization of common cellular disease model systems. For this purpose, we compared the proteomes of spinal cord neuron-neuroblastoma (NSC-34), mouse hepatoma (Hepa 1–6) and mouse embryonic fibroblast (MEF) cell lines in a quantitative manner. All cell lysates were analyzed in single shots using 90 min gradients and subsequently quantified using the MaxQuant label-free quantification algorithm. The observed LFQ intensities were highly reproducible between biological and technical replicates as indicated by Pearson correlations coefficients > 0.97 (supplemental Fig. S4).

After stringent filtering (Experimental Procedures) we performed a principal component analysis (PCA) to evaluate the similarities and dissimilarities of the cell lines on a global scale. Replicates from a single cell line clustered very tightly in the PCA space and the first two principal components accounted for 71% of the cumulative variance within our data set (Fig. 6*C*). Interestingly, the motoneuronal cell line NSC-34 was clearly separated from Hepa 1–6 and MEF cells in the first principle component, whereas the variance between both non-neuronal cell lines was described by the second, orthogonal, principal component. In the latter component, NSC-34 is positioned half-way between Hepa 1–6 and MEF.

To assess individual proteins that are the main drivers for the separation between the three cell lines, we plotted the loadings of the first two principal components (Fig. 6*D*). Hepa 1–6 cells were characterized by gene products involved in regulatory and metabolic processes. As we had found before (52), proteins related to the glycolysis pathway, such as the aldehyde dehydrogenase (Aldh) family, were highly represented in Hepa 1–6. Not surprisingly, proteins driving the separation of MEF were predominantly linked to collagen synthesis, such as the COL gene family and the precollagen C-endopeptidase enhancer 1 (Pcolce). Proteins that differentiate along principal component 1 encompassed various components of the cytoskeleton axons, including Prph and the heavy and light chains of neurofilaments (Nefh and Nefl). In addition, gene products involved in axon guidance (Enah, Dypsl5 and Tubb3) and neuron projection (Uchl1, Gap43) were highly distinctive for NSC-34. Proteins separating NSC-34 from the other cell lines were significantly enriched for neurotransmitter transport while we observed enrichment for collagens and xenobiotic processes for the MEF and Hepa 1–6 cells on component 2, respectively, further showing how proteomics can highlight biological function. This is even more remarkable, given that we previously found that the proteomes of motoneuronal cell lines, including NSC-34, lack distinctive neuronal characteristics, as several key actors in axon growth and guidance were either depleted or low abundant (28). As a result, we had placed motoneuronal cell lines only halfway between *in vivo* motoneurons and non-neuronal controls. Nevertheless, our label-free QTOF-based workflow is very well suited to differentiate subtle alterations in biological systems in a short time of analysis.

##### Impact II Performance for Deep Proteome Analysis of a Cell Line

To evaluate the impact II for deep proteome coverage we performed high pH reverse-phase pre-fractionation with fraction concatenation as described in ref (31). We loaded 175 μg of a HeLa peptide mixture, collected 72 fractions and combined them into 24 fractions (Experimental Procedures). These fractions were analyzed in technical triplicates using a standard 90 min LC-MS/MS gradient (total measurement time of 48 h per replicate). Instead of the simple Top17 method described above, we here used a so-called “dynamic method” with a fixed cycle time and a MS/MS integration time adapted to the precursor intensity. We found that this method helped to generate high quality MS/MS spectra also for low abundant peptides.

**Fig. 7.**
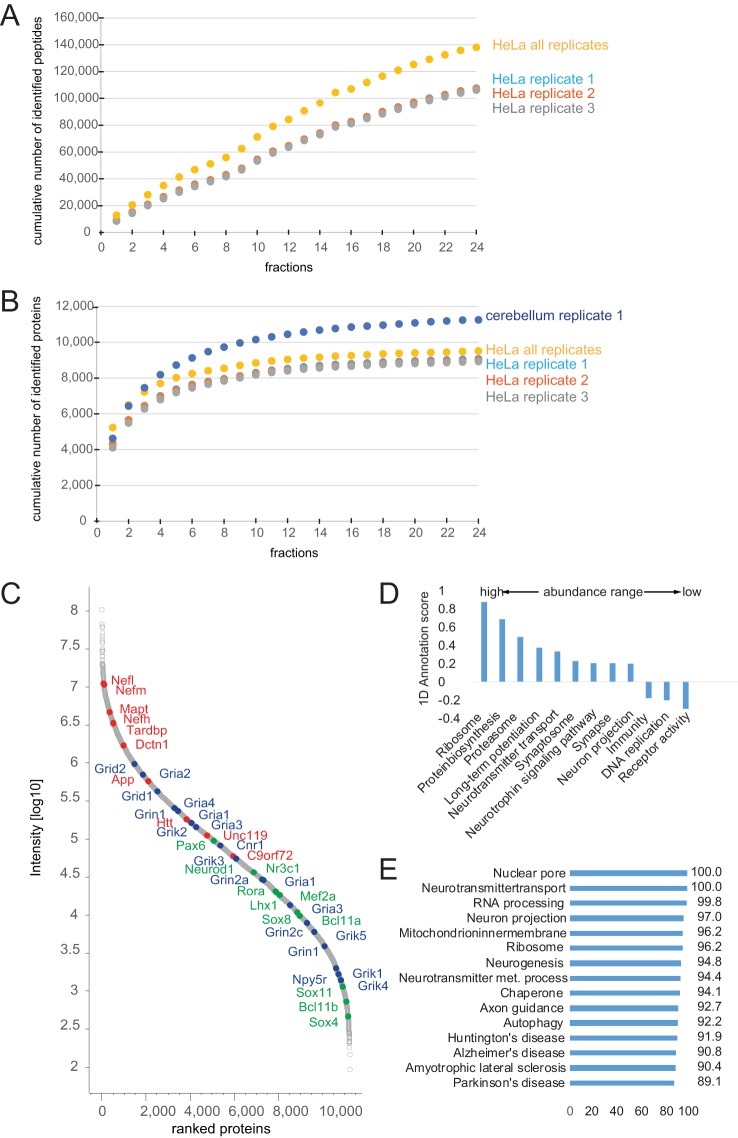
**Triplicate cell line and singlet tissue analysis in 24 high-pH fractions.** *A*, Cumulative number of identified peptides of triplicate analysis of HeLa. *B*, Cumulative number of identified proteins of HeLa replicates and of the singlet analysis of mouse cerebellum (48 h total measurement time). *C*, Rank ordered intensities of each identified protein (log10 intensities) of cerebellum. Proteins involved in neurodegenerative disorders are marked in red; cerebellum specific expressed proteins are marked in blue and a selection of receptors in green. *D*, Protein abundance distribution and *E*, proteome coverage (expressed as a percentage) of different neuronal, metabolic and disease relevant processes in cerebellum.

Figure 7*A* depicts the cumulative number of unique peptides identified as a function of the number of fractions analyzed. The increase is nearly linear, indicating a small overlap of peptide identification between fractions and the good orthogonal separation power as also observed by others using high-pH fractionation (49). The figure also indicates that the reproducibility was very high between the technical replicates. On average we identified 107,038 unique peptides, mapping to around 8995 proteins (protein FDR 1%; Fig. 7*B*). In total we identified 138,086 unique peptides, resulting in 9515 different protein groups. This is to our knowledge the deepest proteome coverage of a human cell line measured with a QTOF instrument.

##### Impact II Performance for Ultra-Deep Proteome Analysis of Tissue

Tissues are more challenging to analyze by proteomics than cell lines, because they are made up of different cell types, the extracellular matrix and other structural and connective elements. Among the different tissues, the brain is thought to be the most complex one. To evaluate the Impact II in this context, we homogenized the cerebellum of a single mouse, digested it using our standard workflow and separated part of the resulting tryptic peptides using high pH fractionation (Experimental Procedures). In total, we identified 11,257 proteins from a single analysis of 24 fractions (2 days of measurement time) (Fig. 7*B*, supplemental Table S1). To our knowledge, this is the deepest proteome measurement of any tissue reported by TOF instrumentation so far. Protein abundance, as indicated by the summed and normalized peptide signal varied by more than five orders of magnitudes (Fig. 7*C*). We identified many high to medium abundant proteins involved in neurodegenerative disorders (Fig. 7*C* marked in red). The transcription factors and DNA binders Pax6, Lhx1/5, Otx1/2, and Neurod1 (Fig. 7*C*, marked in blue) are examples of proteins that have been reported to be specifically expressed in cerebellum (53). Like various neuronal receptors, transcription factors populate the medium to low abundance range (Fig. 7*C*, marked in green). The distribution of molecular functions throughout the abundance range is similar to that observed in previous studies (Fig. 7*D*, supplemental Table S2) (54, 55). The very high depth of this cerebellum proteome is shown by almost complete coverage for neuronal, general metabolic and disease relevant processes (Fig. 7*E*, supplemental Table S2). Rapid estimation of protein abundances in the brain regions of a single mouse could be useful for studying tissue characteristics and disease specific alterations. For instance, knowledge about changes in the complete proteome would provide an additional layer of information on the pathological processes in neurodegeneration.

## CONCLUSION

Here we have described the construction and performance of a state of the art QTOF instrument, the impact II. We documented significant improvements in the ion path, collision cell performance and, in particular, in the performance of the reflectron and detector. The latter allow a mass resolution and mass accuracy that is compatible with the high demands of shotgun proteomics of complex mixtures. For the first time, we measured and modeled the ion transmission from capillary to the detector, which revealed an excellent efficiency of about 10%. The new features of the impact II allow deep characterization in single shots, where we identified more than 5200 proteins in HeLa cells and 3600 proteins in yeast. Using off-line high pH reversed-phase fractionation we identified more than 9500 proteins in HeLa cells and 11,250 proteins in a single cerebellum tissue analysis. These are extremely high numbers for any platform and additional method developments should further improve these results. We also document excellent quantitative reproducibility and accuracy in a label-free format. In concordance with others (26, 27), we conclude that the improvements in QTOF technologies in recent years now clearly enable demanding, in-depth analysis of very complex proteomes.

## Supplementary Material

Supplemental Data

## Footnotes

^1^ The abbreviations used are:

LC-MS/MS: liquid chromatography-tandem MS
CAA: chloroacetamide
ES: electrospray
FDR: false discovery rate
Hepa 1–6: mouse hepatoma
ID: inner diameter
LFQ: label-free quantification
MCP: multichannel plate
MEFs: mouse embryonic fibroblasts
MS/MS: tandem mass spectrometry
NSC-34: spinal cord neuron-neuroblastoma
QTOF: quadrupole time-of-flight
TOF: time-of-flight
UPS: universal protein standard.
