# Supplementary material for: The Impact II, a Very High-Resolution Quadrupole Time-of-Flight Instrument (QTOF) for Deep Shotgun Proteomics
Source: Mol Cell Proteomics. 2015 May 19;14(7):2014–29. doi: 10.1074/mcp.M114.047407 (PMC4587313; doi:10.1074/mcp.M114.047407)
Supplement: Supplemental Data [file supp_14_7_2014__index.html]

The impact II, a very high resolution quadrupole time-of-flight instrument for deep shotgun proteomics — The Impact II, a Very High-Resolution Quadrupole Time-of-Flight Instrument (QTOF) for Deep Shotgun Proteomics — High-Resolution Quadrupole TOF for Deep Shotgun Proteomics — Supplemental Data 

# The Impact II, a Very High-Resolution Quadrupole Time-of-Flight Instrument (QTOF) for Deep Shotgun Proteomics

## Supplemental Data

- Suppl.Figures1-4 - Suppl.Figures1-4
- Supplementary table S1 - Supplementary table S1 proteinGroups Cerebellum highPh experiment
- Supplementary table S2 - Supplementary table S2 Significant categories cerebellum proteome
- descriptions of tables - descriptions of table headers
- singlePeptides\_HeLa\_singleshot - singlePeptides\_HeLa\_singleshot
- singlePeptides\_Yeast\_singleshot - singlePeptides\_Yeast\_singleshot
- singlePeptides\_UPS\_Yeast - singlePeptides\_UPS\_Yeast
- singlePeptides\_Yeast\_haploid\_diploid - singlePeptides\_Yeast\_haploid\_diploid
- singlePeptides\_MotoneuronalCellLines - singlePeptides\_MotoneuronalCellLines
- singlePeptides\_HeLa\_highpH\_replicate1 - singlePeptides\_HeLa\_highpH\_replicate1
- singlePeptides\_HeLa\_highpH\_replicate2 - singlePeptides\_HeLa\_highpH\_replicate2
- singlePeptides\_HeLa\_highpH\_replicate3 - singlePeptides\_HeLa\_highpH\_replicate3
- singlePeptides\_HeLa\_highpH\_allreplicates - singlePeptides\_HeLa\_highpH\_allreplicates
- singlePeptides\_Cerebellum\_highpH\_replicate1 - singlePeptides\_Cerebellum\_highpH\_replicate1
