# Supplementary material for: The Impact II, a Very High-Resolution Quadrupole Time-of-Flight Instrument (QTOF) for Deep Shotgun Proteomics
Source: Mol Cell Proteomics. 2015 May 19;14(7):2014–29. doi: 10.1074/mcp.M114.047407 (PMC4587313; doi:10.1074/mcp.M114.047407)
Supplement: Supplemental Data [file supp_M114.047407_mcp.M114.047407-1.pdf]

Supplementary Figure 1

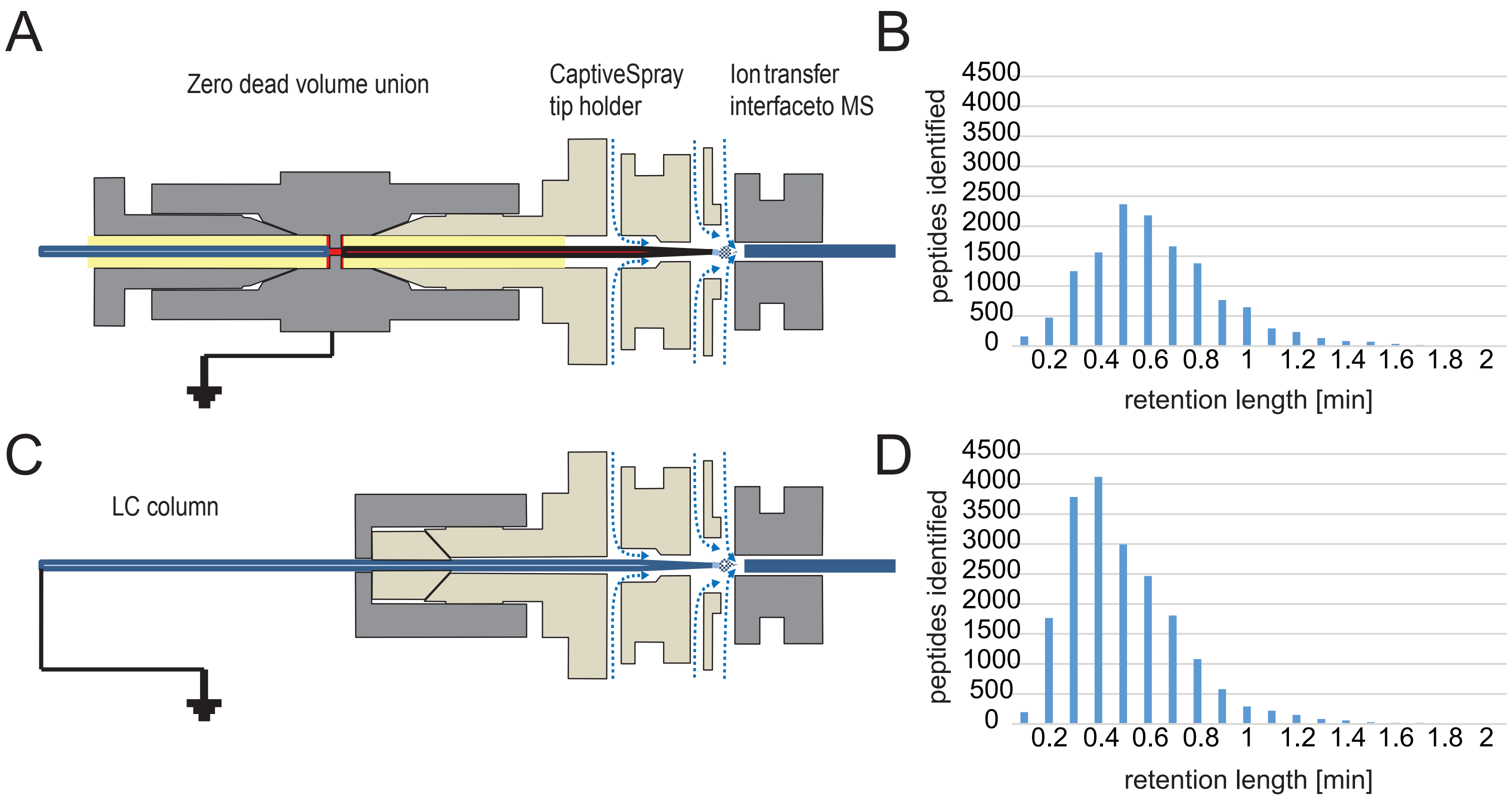

Schematic of the CaptiveSpray ion source in standard set-up (A) and modified setup using a pulled tip column (C). The CaptiveSpray ion source is directly attached to the MS, aligning the spray emitter on axis with the MS inlet capillary via a capillary extension. Filtered ambient air is taken in by the instrument's vacuum system through a set of different channels around the emitter, focusing the spray plume into the inlet orifice. The standard set-up (A) uses an etch-tapered fused silica emitter needle (20 µm ID), that is glued into the tip holder. For connecting a nano LC column a low volume 1/16" metal union is used that also provides the electrical grounding for the electrospray process. Union and emitter result in an unwanted post-column liquid volume. The tip holder in our modified setup (C) was changed in a way that a 1.9 µm ID fused silica column with pulled emitter can be aligned and fixed directly within the tip holder using a ferrule and a clamping nut. Thus any post column volume could be reduced to zero and was used for our proteomics analyses due to reduced retention length (B and D).

Supplementary Figure 2

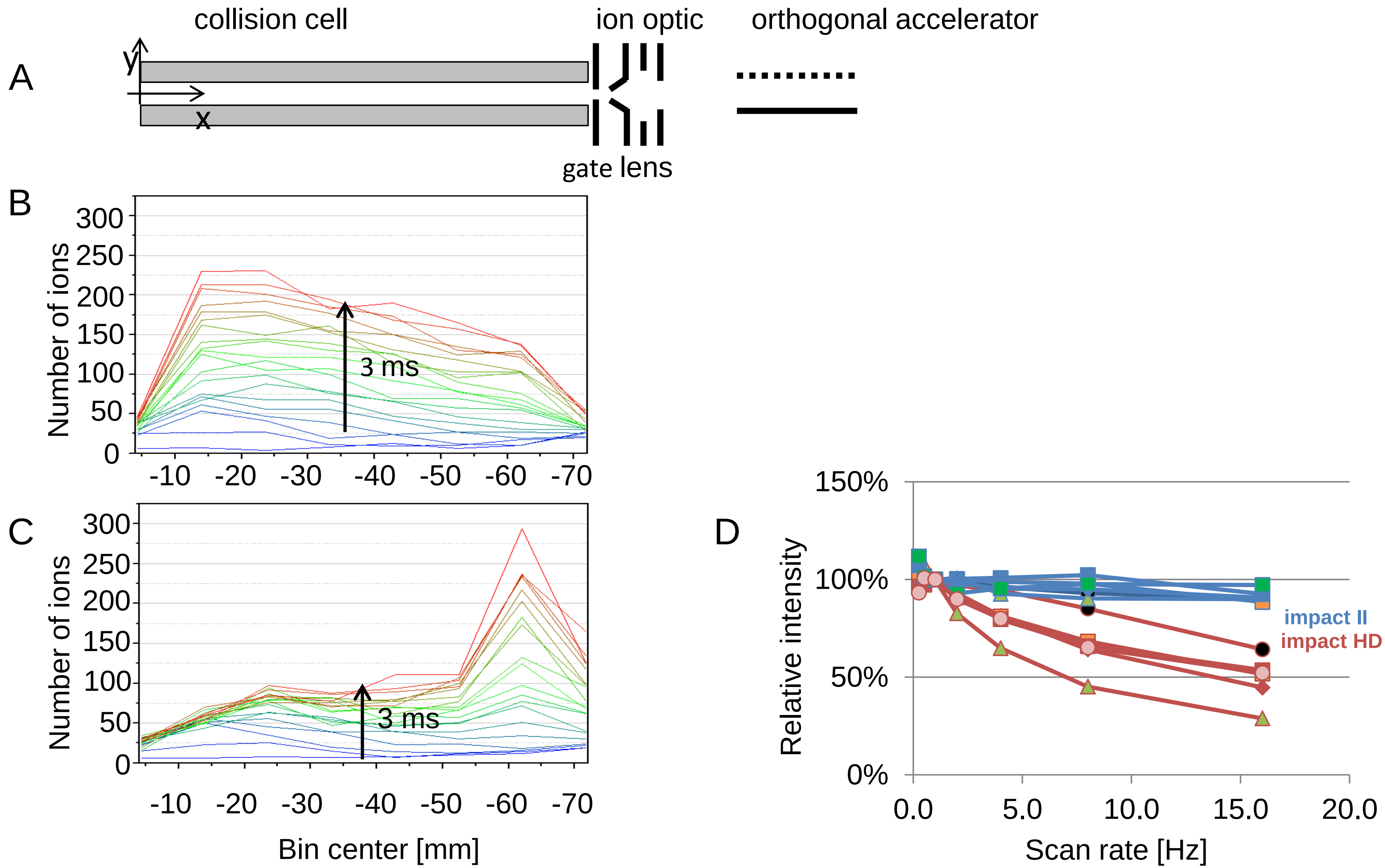

The improved collision cell. (A) Schematic of collision cell coupled to orthogonal accelerator. After an initial RF quench to empty the collision cell, ions are continuously introduced and are periodically blocked and extracted at the exit by alternating the potential of the gate lens. (B+C) Simulations of the ion density distributions across the collision cell within the first 3 ms after the RF quenching. The simulated progress of the ion accumulation is calculated in 20 steps each represented by one line. The initially density profile is colored blue, followed by green, brown and red for the subsequent steps. The distance in time between two consecutive lines is 150 µs. Simulation without axial gradient applied (B) and with axial field gradient (90 mV/cm) applied (C). (D) Series of measurements of five impact HD collision cells (red) and 5 impact II (blue) configurations (improved collision cell with optimized pseudo potential well and field gradient) shows improvement of the detected ions with the new configuration, especially for higher spectra rate. Shown are relative intensities normalized to 1 Hz operation.

Supplementary Figure 3

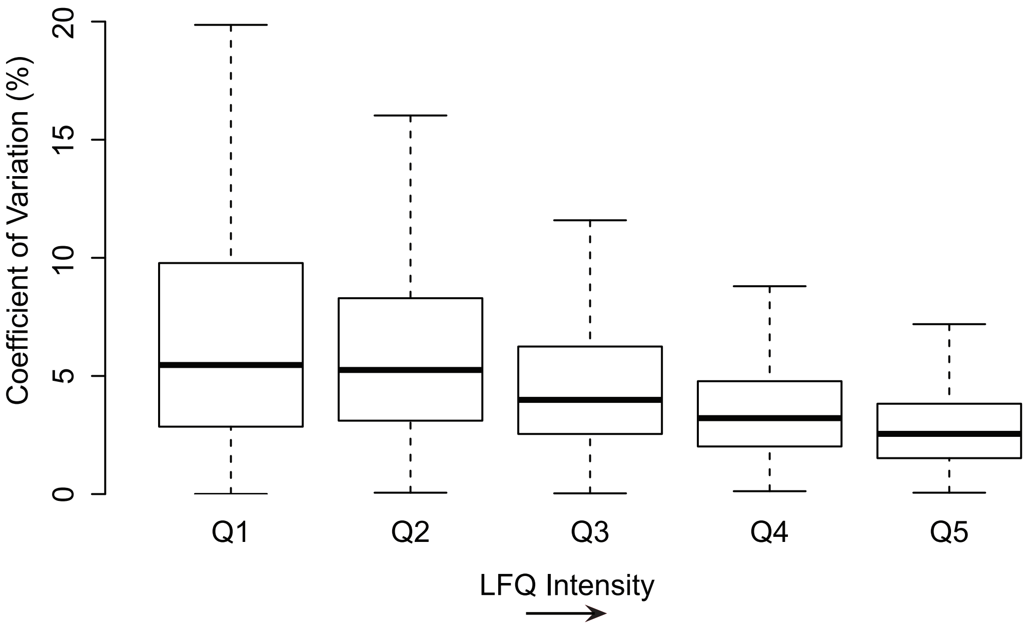

Coefficient of variation of LFQ protein intensities between technical replicates of HeLa single shot analyses: Q1 to Q5 represent LFQ intensity quantiles from the least to the most abundant, respectively. Outliers exceeding 1.5 times the interquartile range are not displayed.

Supplementary Figure 4

A

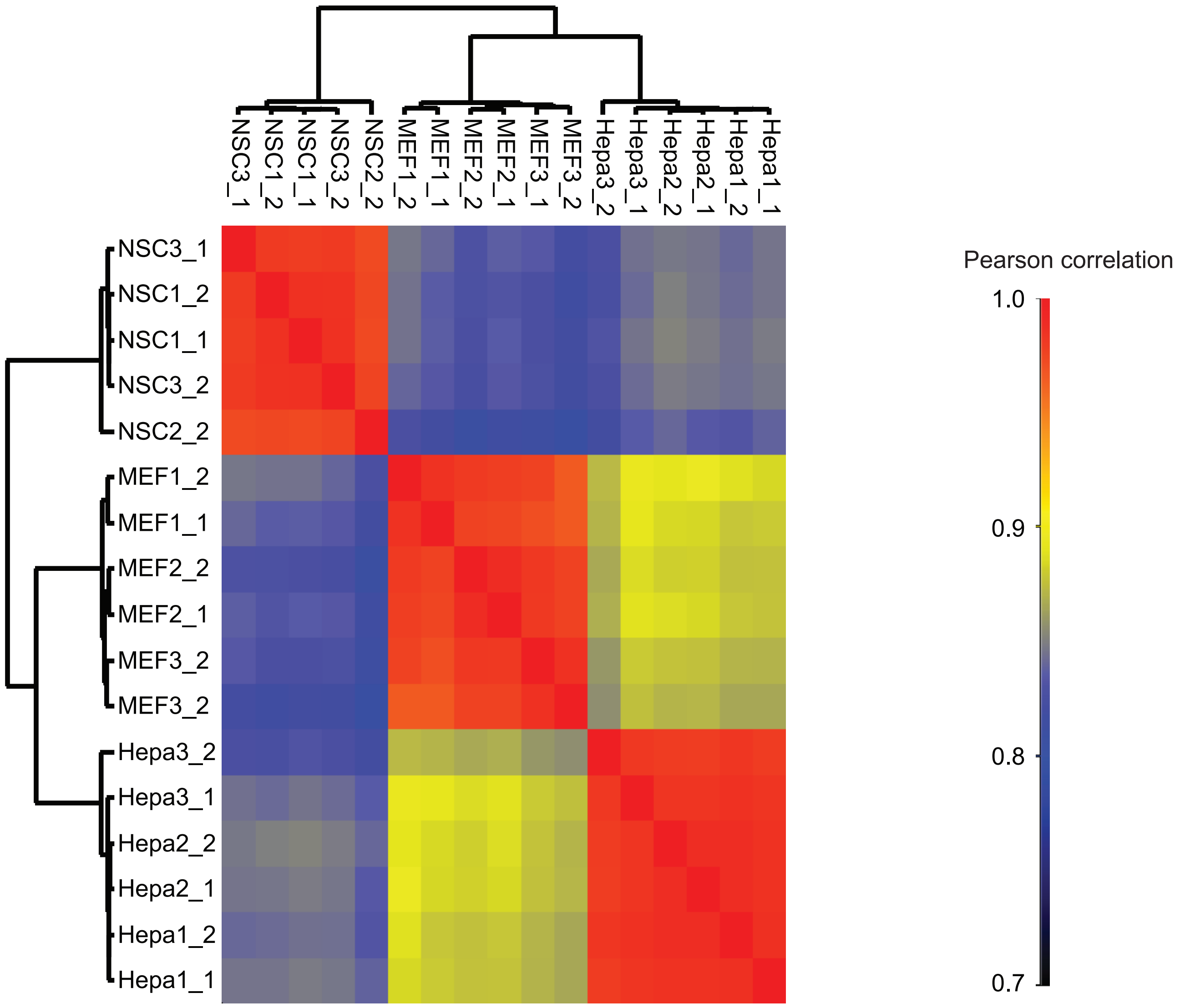

Pearson correlation for LFQ intensities from single shot LC-MS/MS measurements of different cell lines. All replicate correlation values (technical and biological) were greater than 0.97, and the Pearson correlation between different cell lines was at least 0.7.
