# Supplementary material for: The Impact II, a Very High-Resolution Quadrupole Time-of-Flight Instrument (QTOF) for Deep Shotgun Proteomics
Source: Mol Cell Proteomics. 2015 May 19;14(7):2014–29. doi: 10.1074/mcp.M114.047407 (PMC4587313; doi:10.1074/mcp.M114.047407)

Raw file	Scan	Method	Score	m/z	Gene names
20150226_Hela_Top_opt_A3_01_1591	5243	TOF; CID	53.03	670.33	SYF2

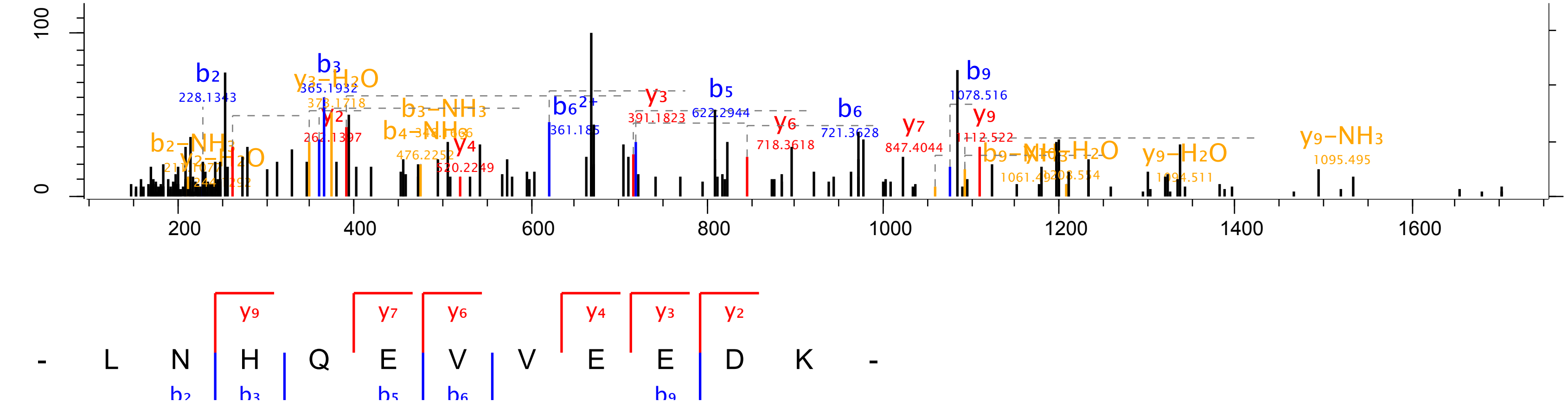

Raw file	Scan	Method	Score	m/z	Gene names
20150226_Hela_Top_opt_A3_01_1591	5388	TOF; CID	58.59	664.3	PPP1R12C

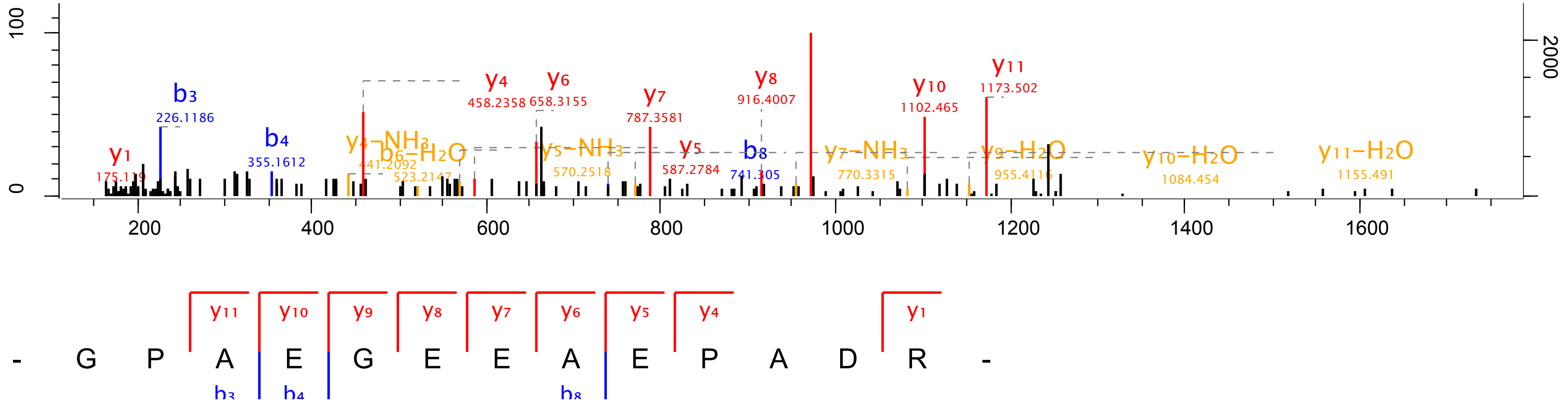

Raw file	Scan	Method	Score	m/z	Gene names
20150226_Hela_Top_opt_A3_01_1591	6262	TOF; CID	45.08	326.17	FAM162B

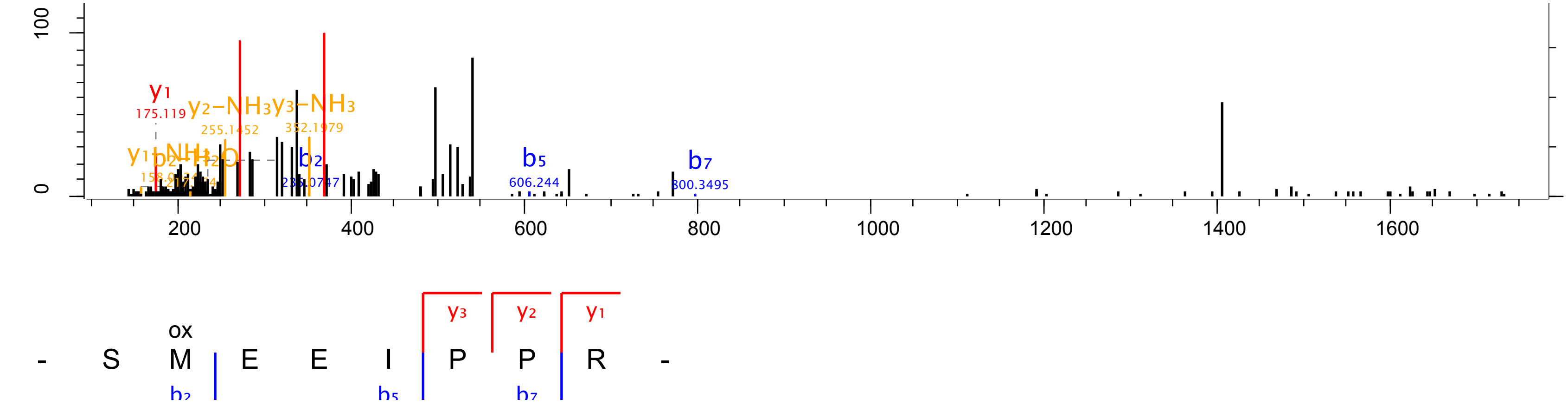

Raw file	Scan	Method	Score	m/z	Gene names
20150226_Hela_Top_opt_A3_01_1591	7469	TOF; CID	51.61	627.31	CUX1

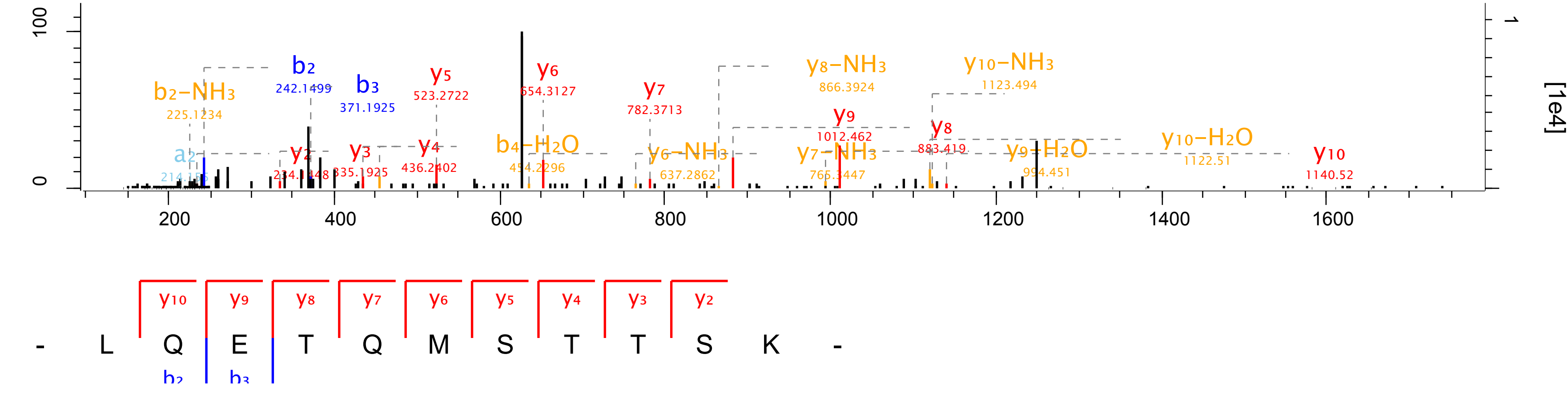

Raw file	Scan	Method	Score	m/z	Gene names
20150226_Hela_Top_opt_A3_01_1591	7709	TOF; CID	70.78	608.96	SP3

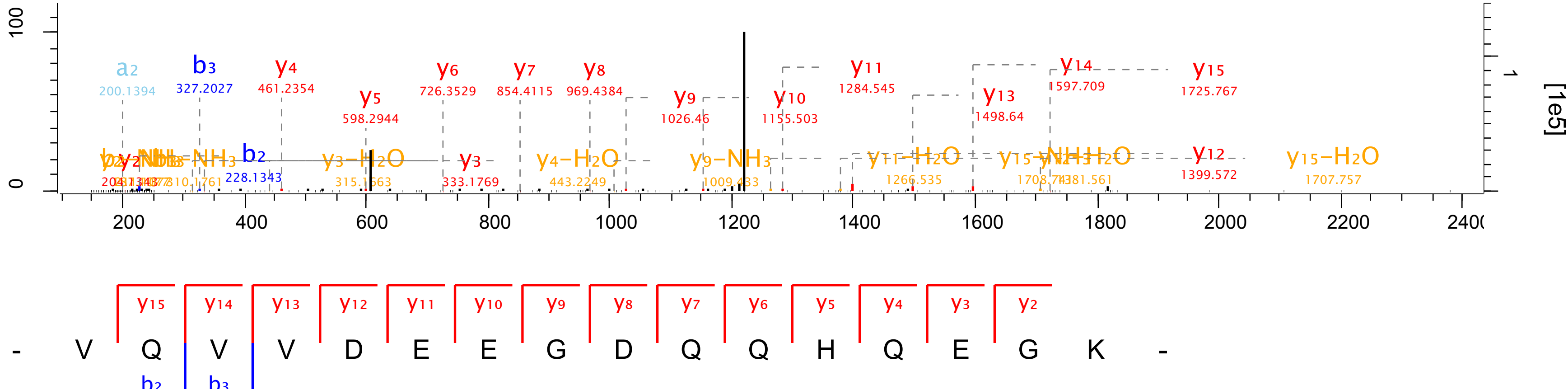

Raw file	Scan	Method	Score	m/z	Gene names
20150226_Hela_Top_opt_A3_01_1591	7884	TOF; CID	38.75	804.35	ATG13

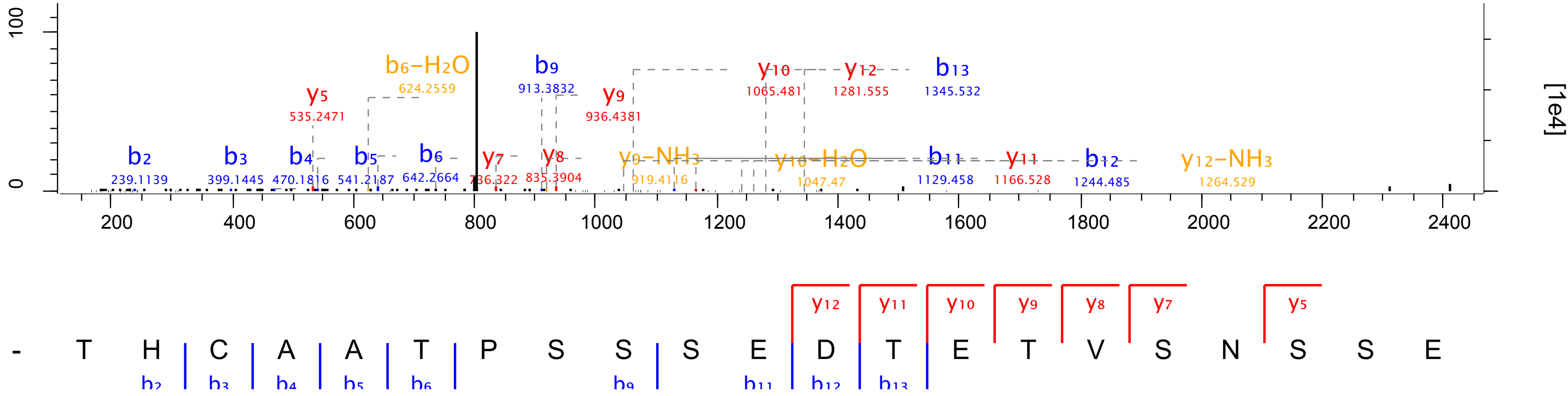

Raw file	Scan	Method	Score	m/z	Gene names
20150226_Hela_Top_opt_A3_01_1591	8733	TOF; CID	55.75	839.88	C5orf22

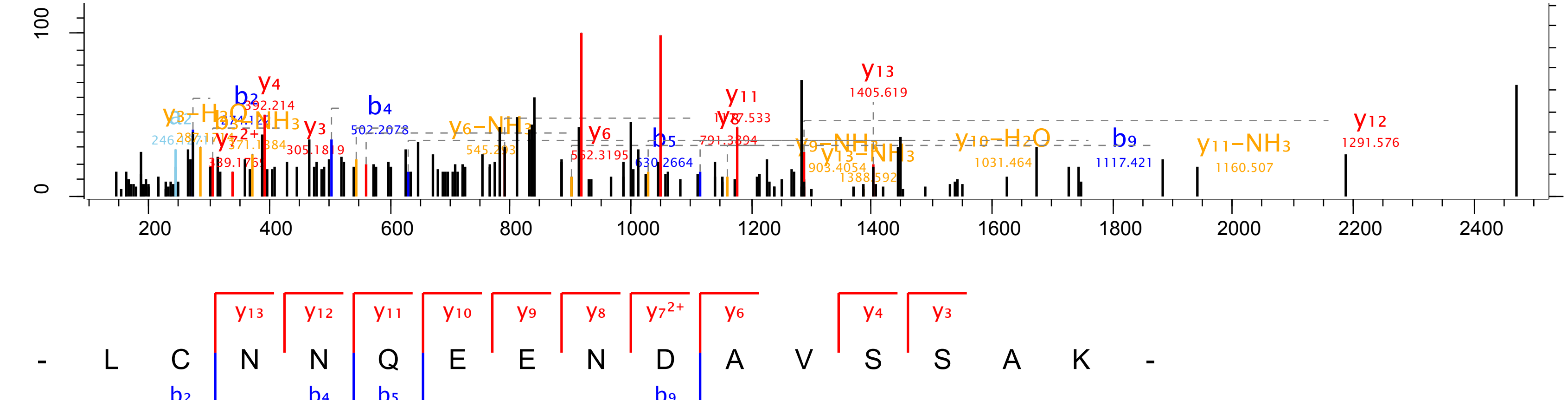

Raw file	Scan	Method	Score	m/z	Gene names
20150226_Hela_Top_opt_A3_01_1591	11054	TOF; CID	94.85	517.76	CTNNAL1

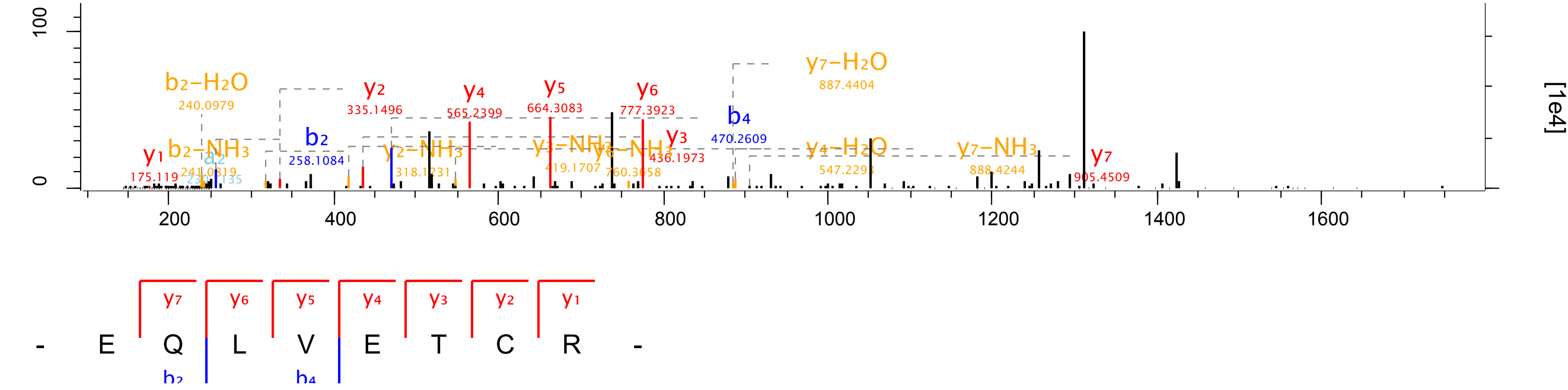

Raw file	Scan	Method	Score	m/z	Gene names
20150226_Hela_Top_opt_A3_01_1591	11129	TOF; CID	63.97	693.86	RRAS2

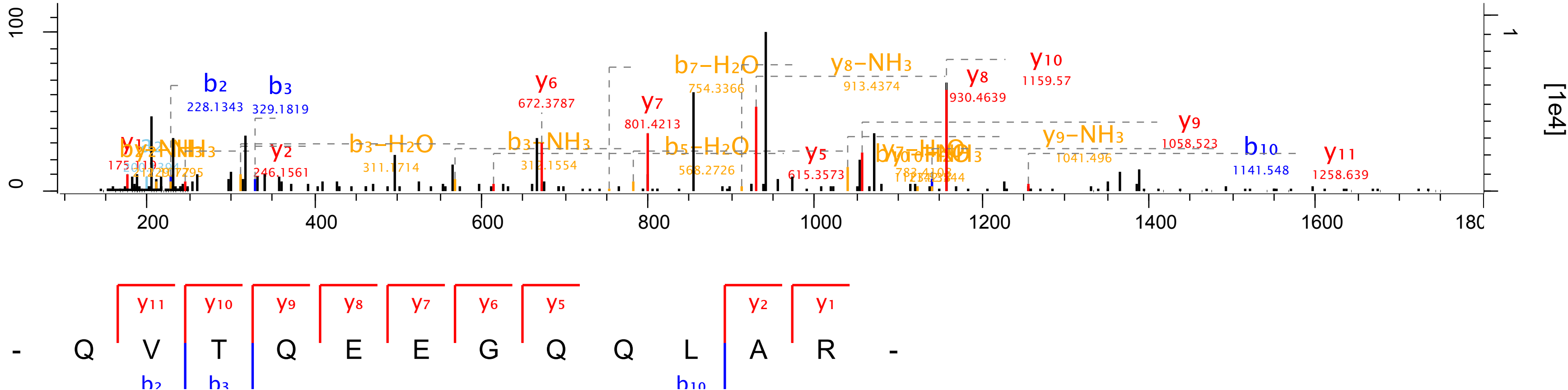

Raw file	Scan	Method	Score	m/z	Gene names
20150226_Hela_Top_opt_A3_01_1591	12892	TOF; CID	78.51	779.39	RNF170

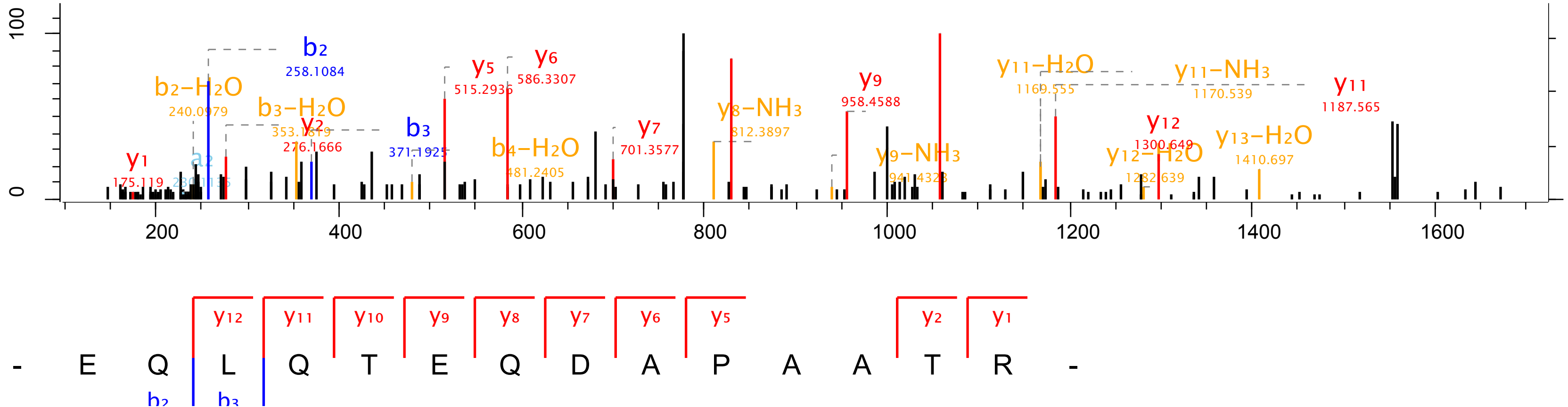

Raw file	Scan	Method	Score	m/z	Gene names
20150226_Hela_Top_opt_A3_01_1591	13227	TOF; CID	125.74	513.79	SMIM12

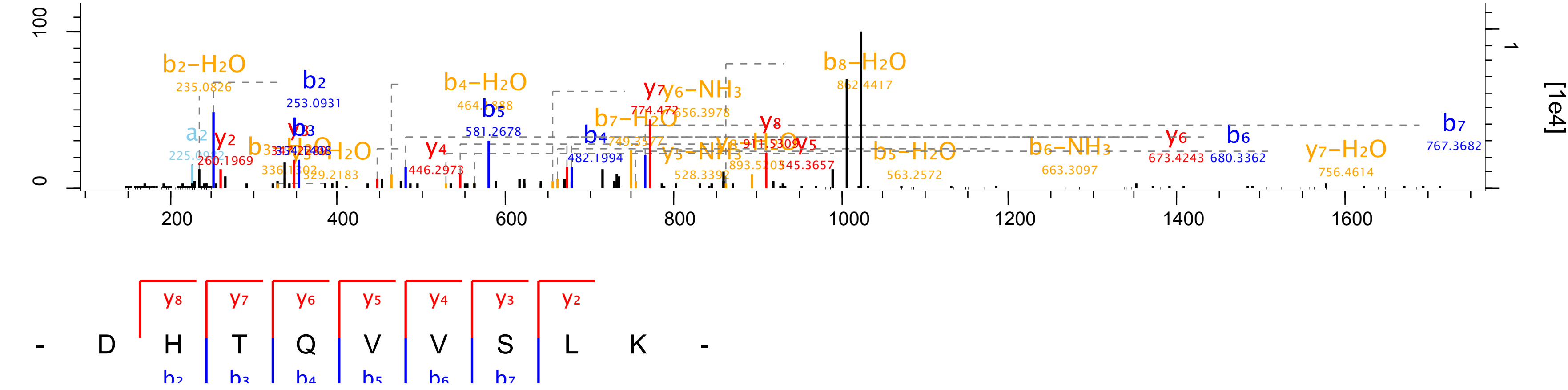

Raw file	Scan	Method	Score	m/z	Gene names
20150226_Hela_Top_opt_A3_01_1591	15191	TOF; CID	51.64	650.83	CENPC;CENPC1

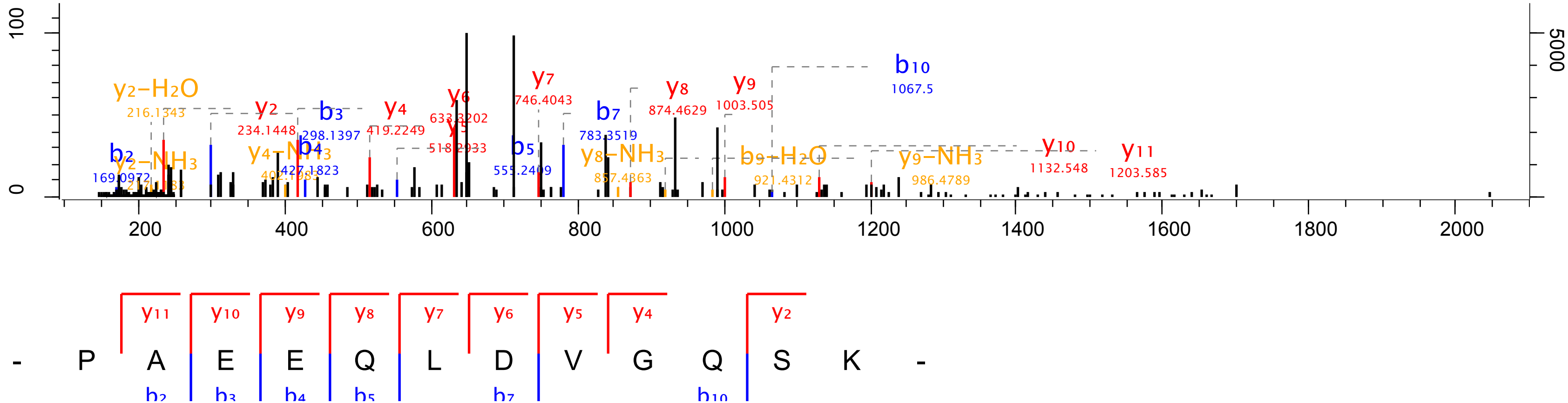

Raw file	Scan	Method	Score	m/z	Gene names
20150226_Hela_Top_opt_A3_01_1591	15820	TOF; CID	116.24	465.25	ALX1

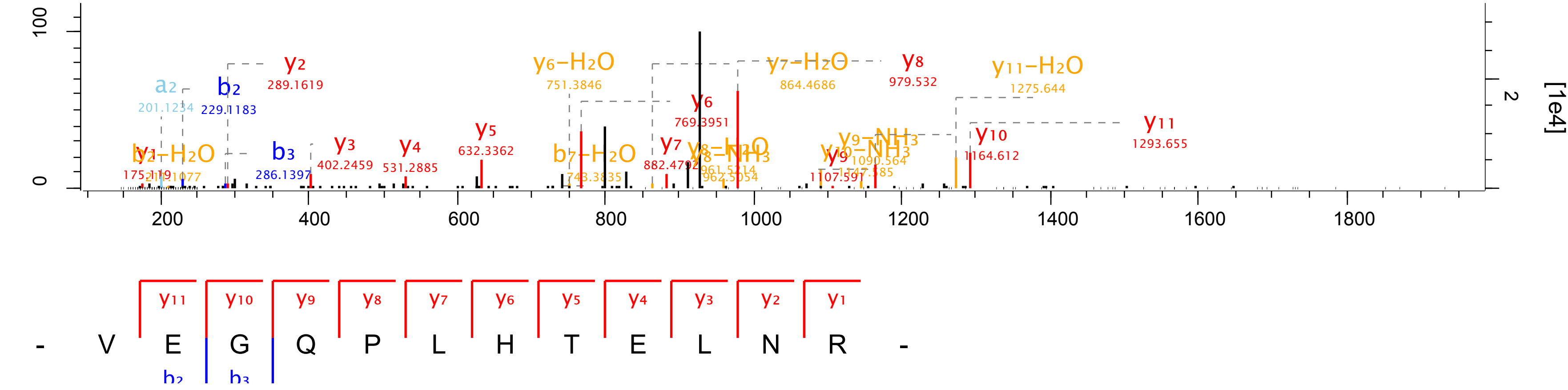

Raw file	Scan	Method	Score	m/z	Gene names
20150226_Hela_Top_opt_A3_01_1591	15982	TOF; CID	102.66	1053.01	GSK3B

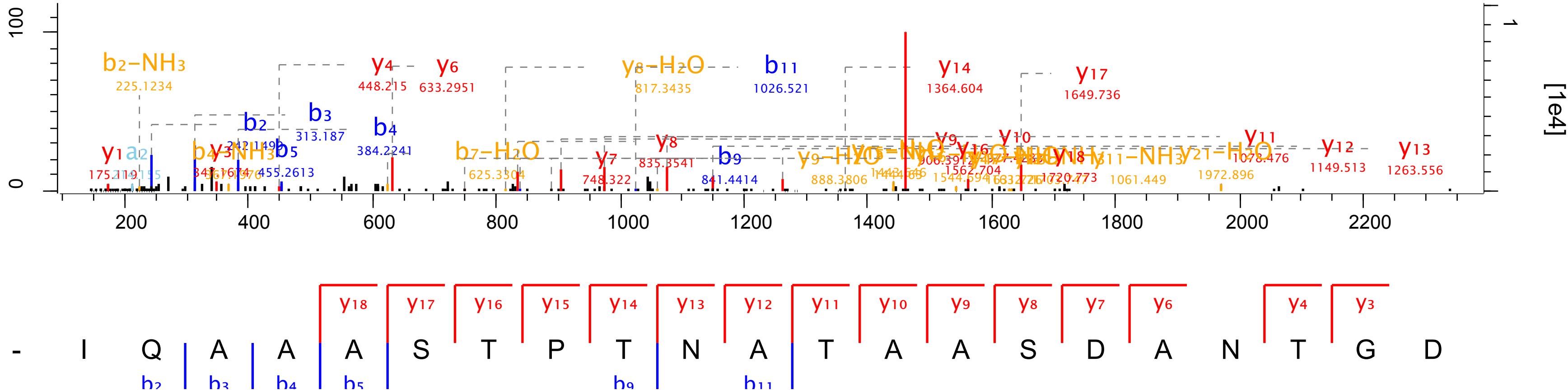

Raw file	Scan	Method	Score	m/z	Gene names
20150226_Hela_Top_opt_A3_01_1591	16147	TOF; CID	113.69	659.35	BANP

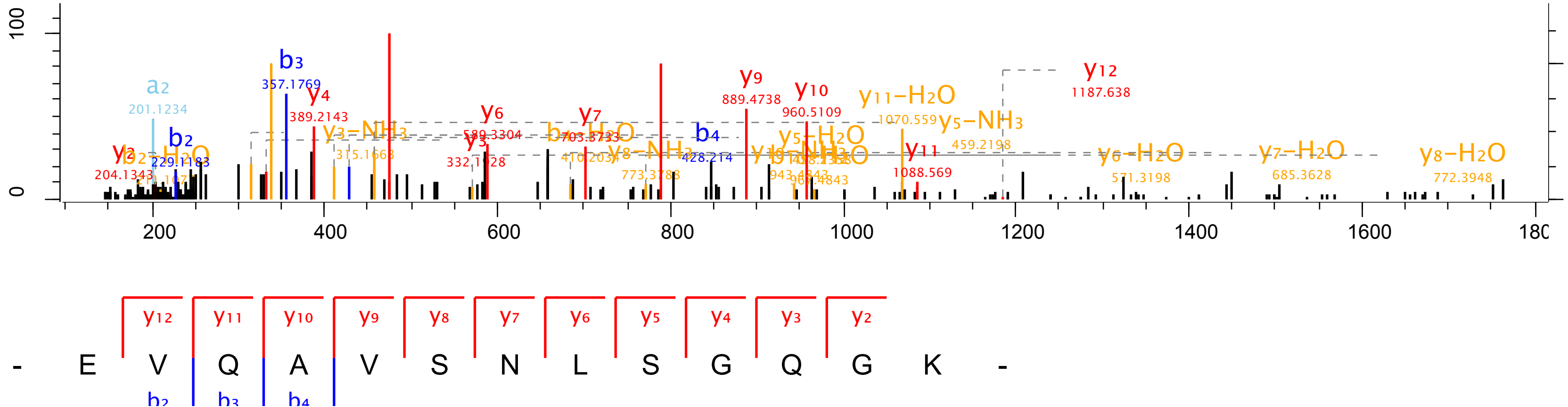

Raw file	Scan	Method	Score	m/z	Gene names
20150226_Hela_Top_opt_A3_01_1591	17924	TOF; CID	40.78	651.33	LAGE3

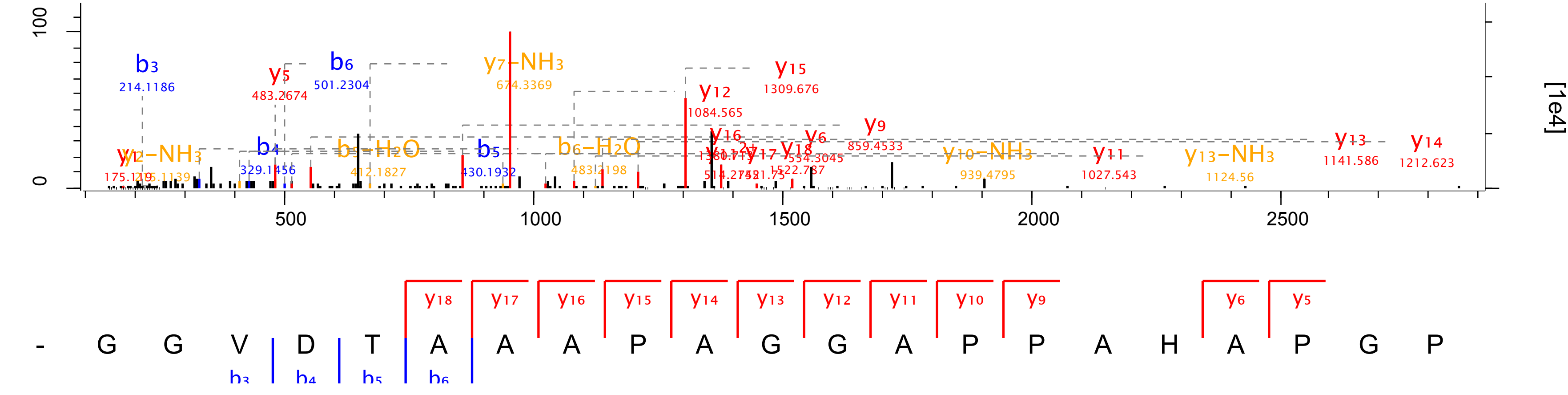

Raw file	Scan	Method	Score	m/z	Gene names
20150226_Hela_Top_opt_A3_01_1591	18258	TOF; CID	51.73	486.26	TRADD

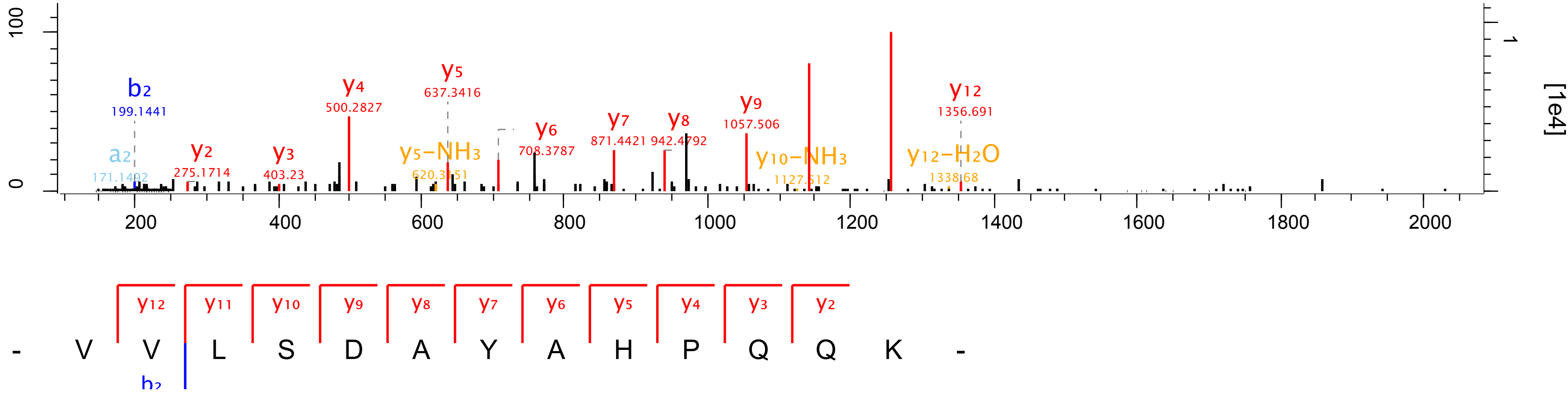

Raw file	Scan	Method	Score	m/z	Gene names
20150226_Hela_Top_opt_A3_01_1591	18920	TOF; CID	104.88	812.89	RNF115

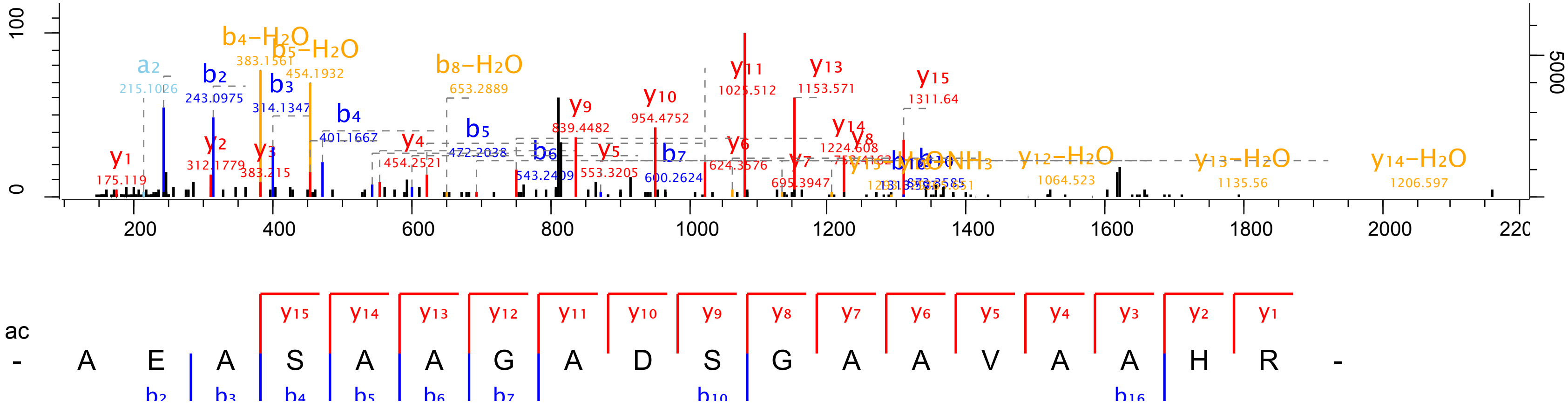

Raw file	Scan	Method	Score	m/z	Gene names
20150226_Hela_Top_opt_A3_01_1591	19502	TOF; CID	78.52	493.76	UTS2

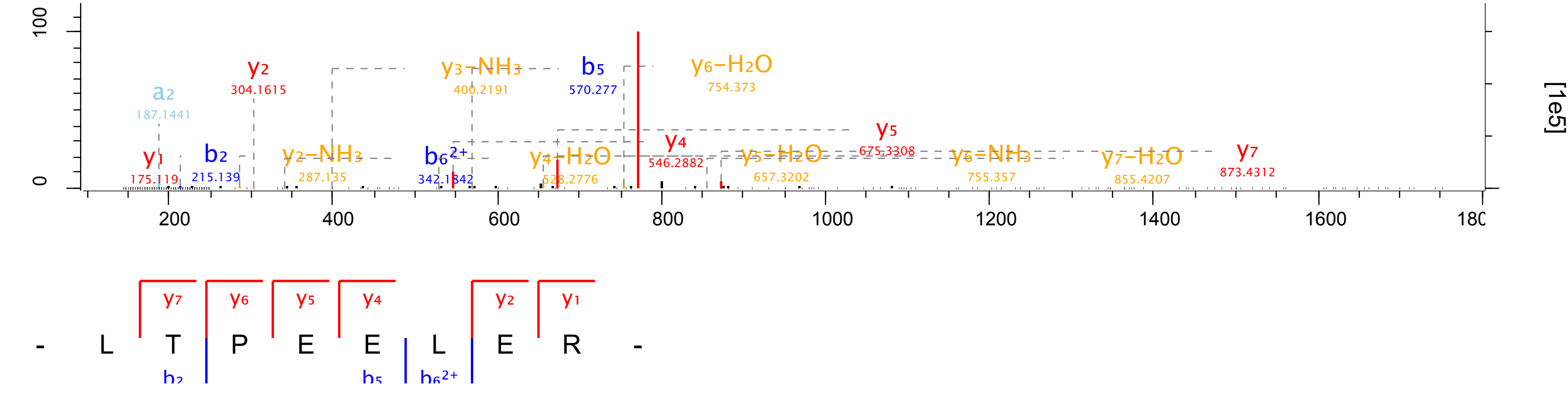

Raw file	Scan	Method	Score	m/z	Gene names
20150226_Hela_Top_opt_A3_01_1591	20086	TOF; CID	62.46	347.23	C10orf115

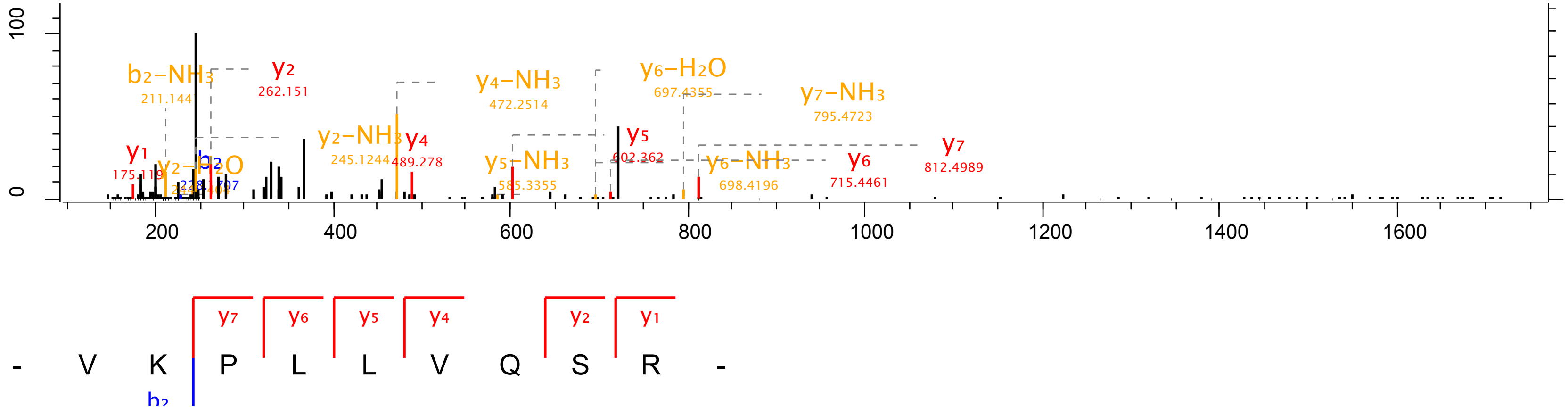

Raw file	Scan	Method	Score	m/z	Gene names
20150226_Hela_Top_opt_A3_01_1591	20666	TOF; CID	71.38	523.78	JADE2

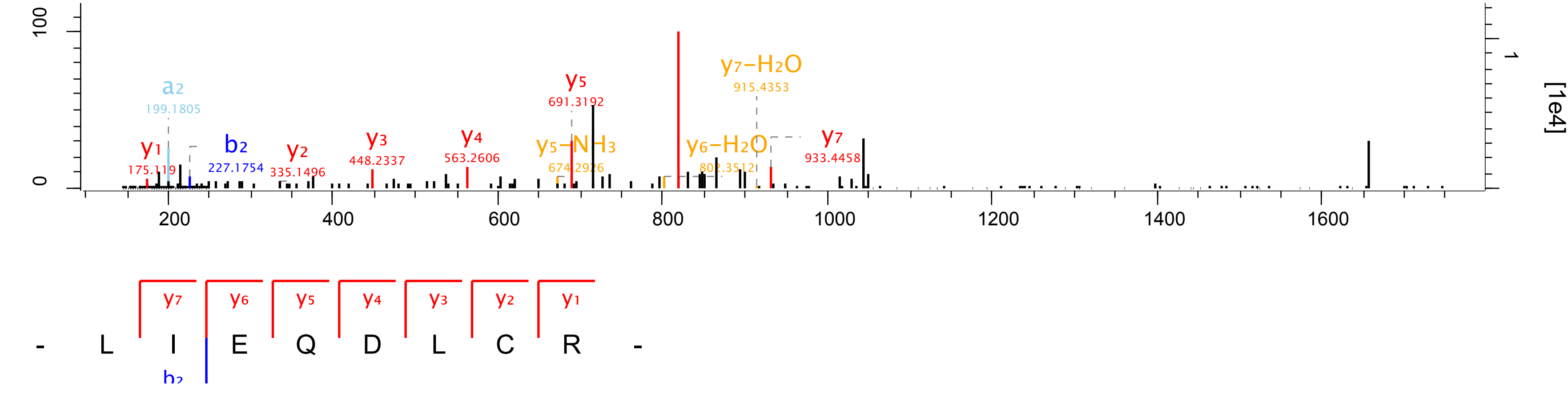

Raw file	Scan	Method	Score	m/z	Gene names
20150226_Hela_Top_opt_A3_01_1591	21244	TOF; CID	62.66	851.38	CLEC2B

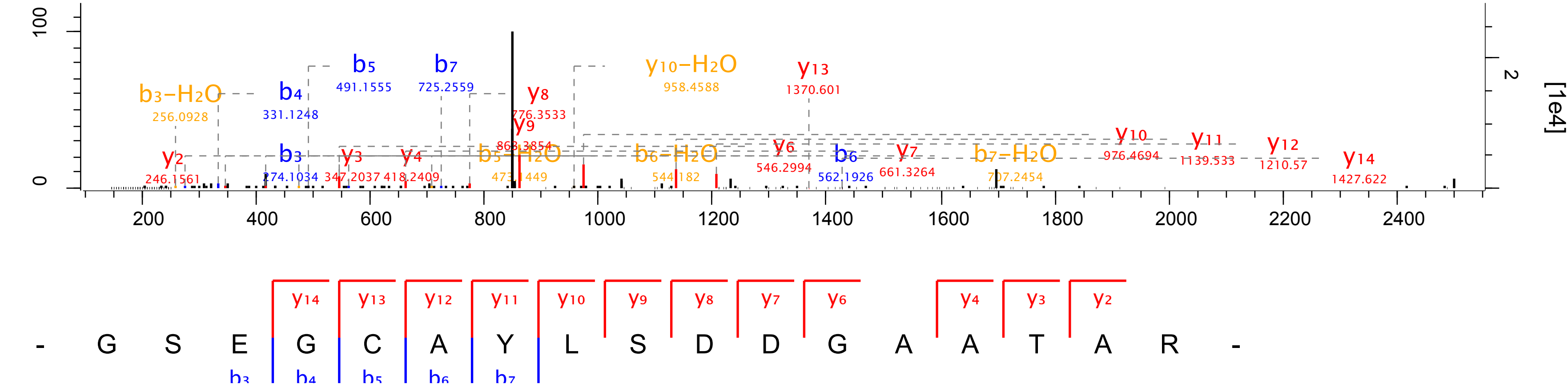

Raw file	Scan	Method	Score	m/z	Gene names
20150226_Hela_Top_opt_A3_01_1591	21618	TOF; CID	67.33	394.55	MICA

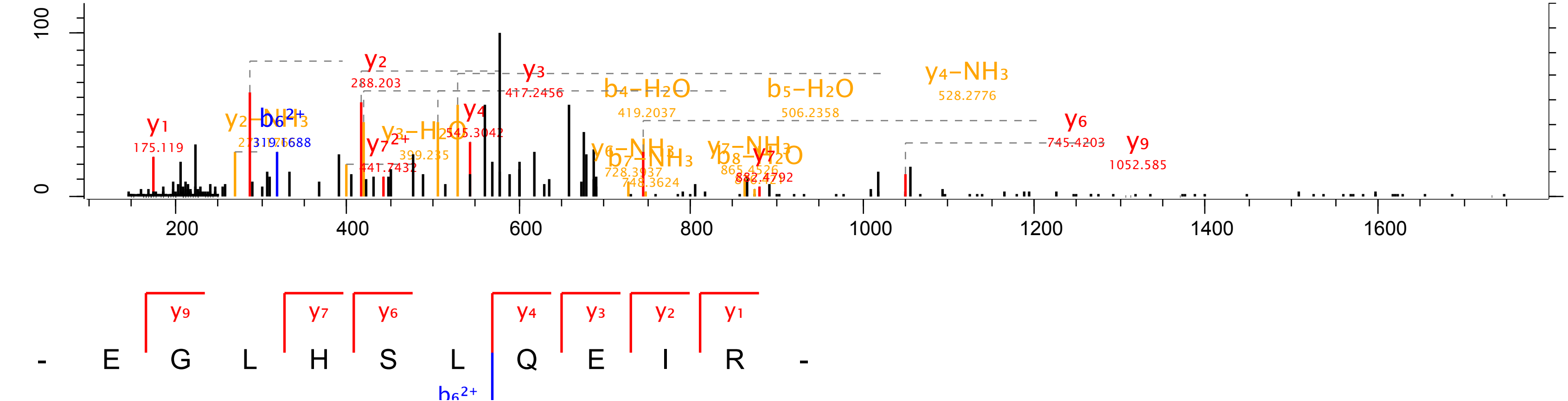

Raw file	Scan	Method	Score	m/z	Gene names
20150226_Hela_Top_opt_A3_01_1591	22114	TOF; CID	110.88	497.26	AKR1D1

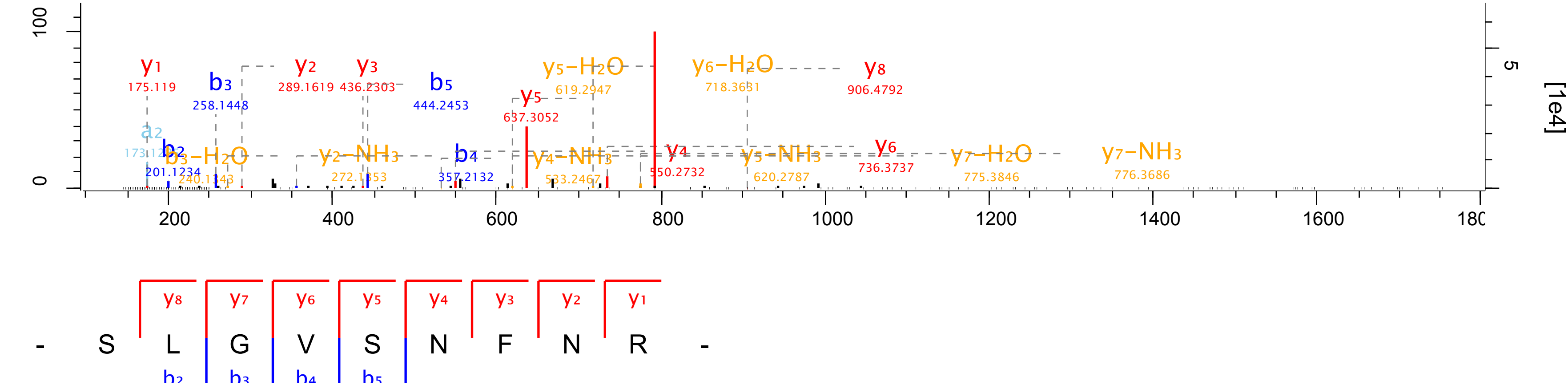

Raw file	Scan	Method	Score	m/z	Gene names
20150226_Hela_Top_opt_A3_01_1591	22649	TOF; CID	77.87	711.31	APOOL

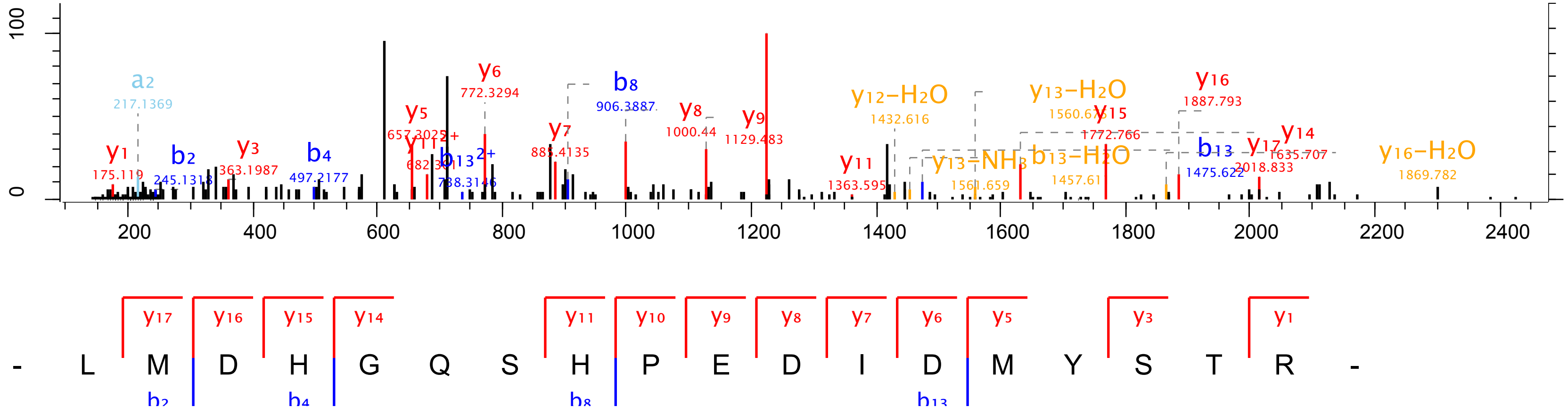

Raw file	Scan	Method	Score	m/z	Gene names
20150226_Hela_Top_opt_A3_01_1591	23162	TOF; CID	78.18	392.76	TSPAN10

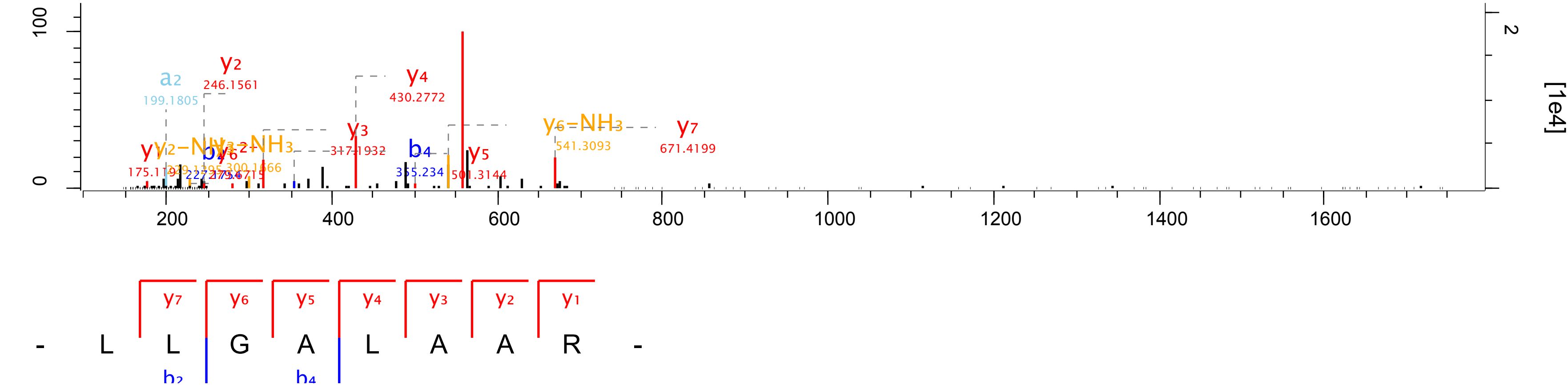

Raw file	Scan	Method	Score	m/z	Gene names
20150226_Hela_Top_opt_A3_01_1591	23312	TOF; CID	47.15	961.94	SLCO4A1

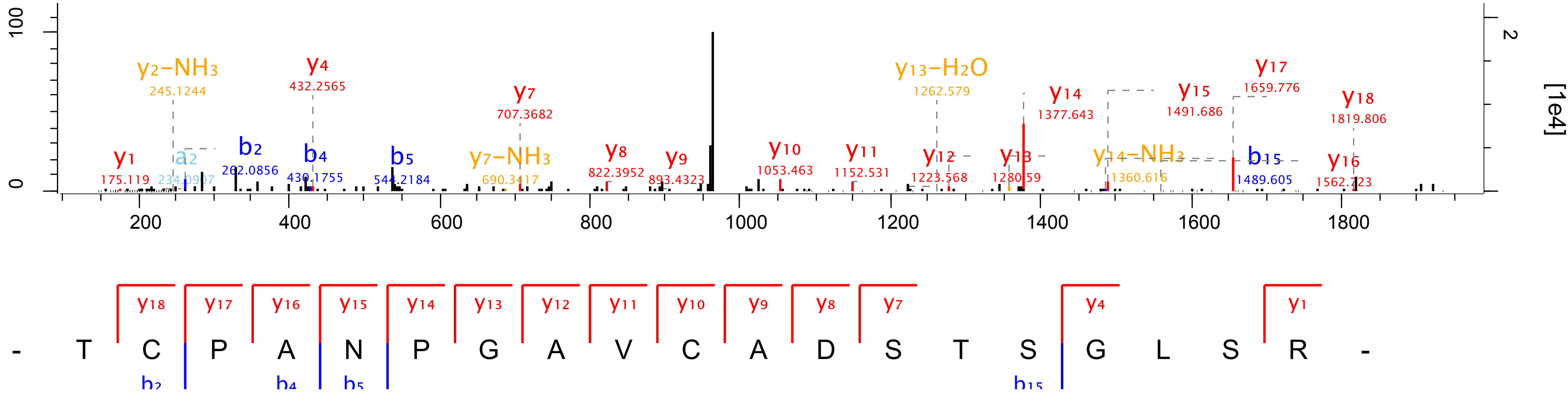

Raw file	Scan	Method	Score	m/z	Gene names
20150226_Hela_Top_opt_A3_01_1591	23528	TOF; CID	64.64	611.31	CCDC127

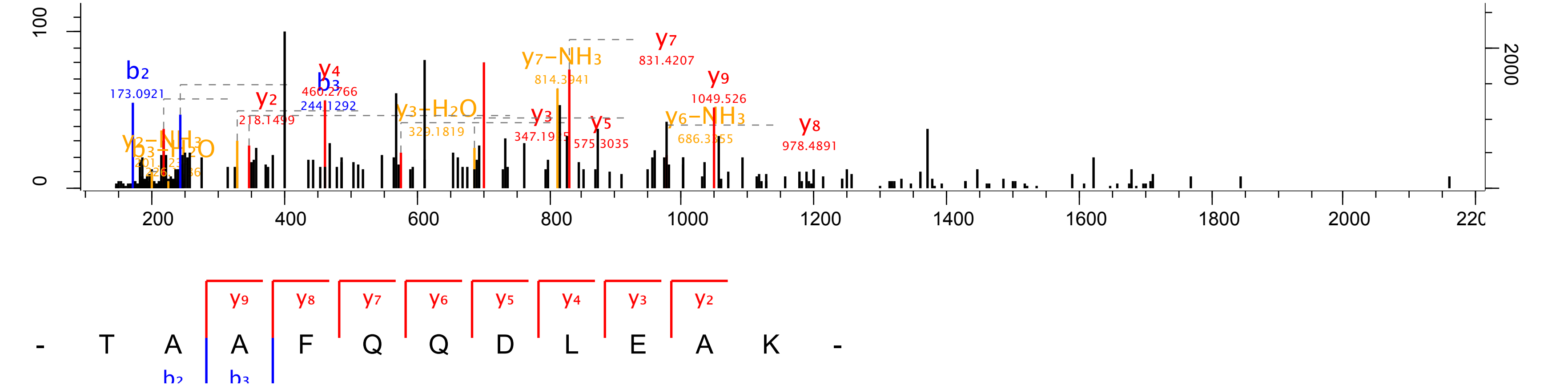

Raw file	Scan	Method	Score	m/z	Gene names
20150226_Hela_Top_opt_A3_01_1591	23634	TOF; CID	71.9	1041	PTDSS2

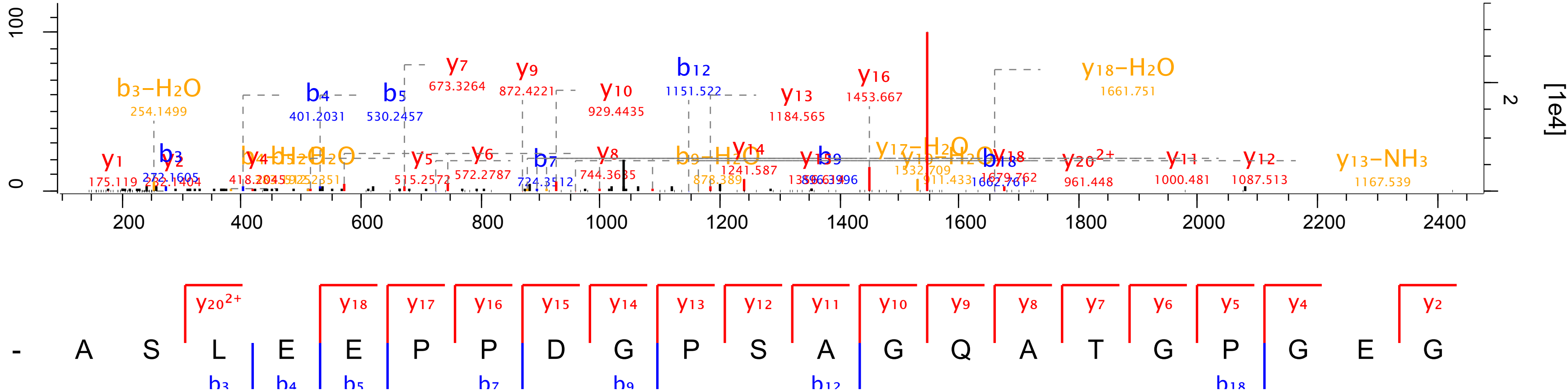

Raw file	Scan	Method	Score	m/z	Gene names
20150226_Hela_Top_opt_A3_01_1591	23669	TOF; CID	41.17	819.4	AMFR

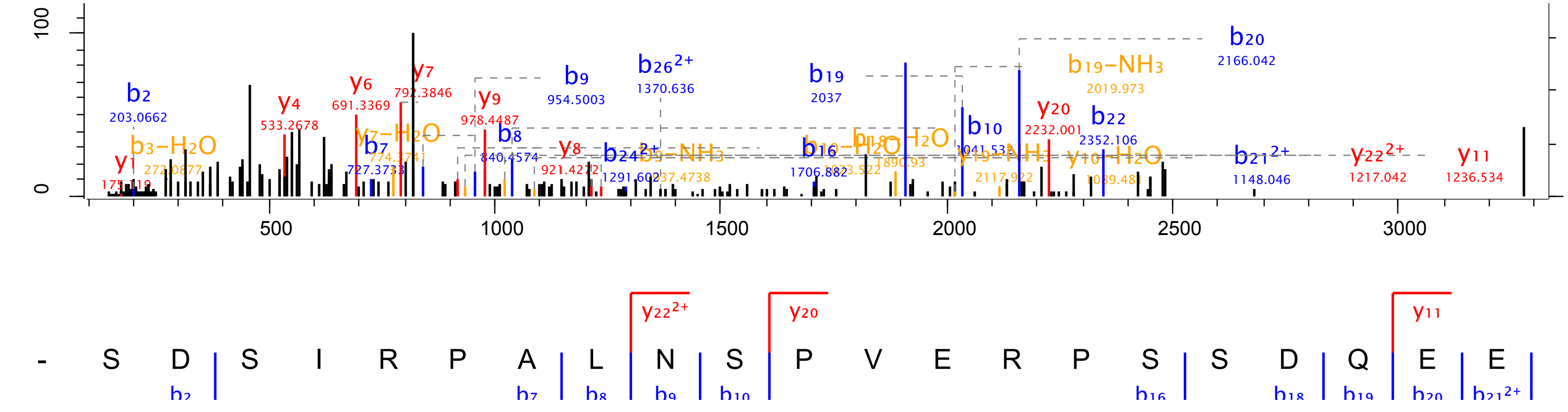

Raw file	Scan	Method	Score	m/z	Gene names
20150226_Hela_Top_opt_A3_01_1591	24341	TOF; CID	54.34	697.33	FAM120B

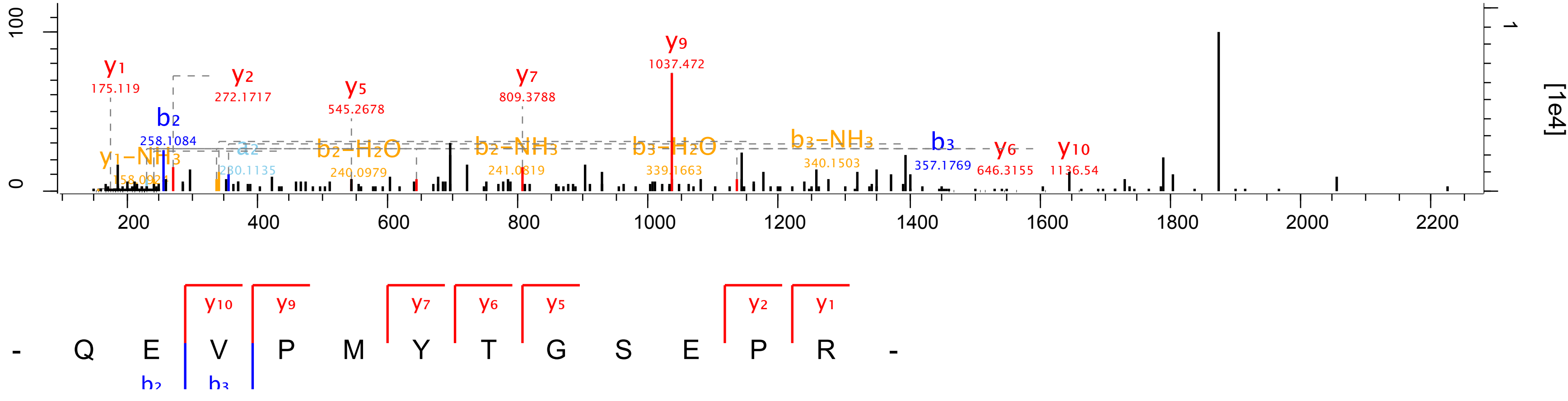

Raw file	Scan	Method	Score	m/z	Gene names
20150226_Hela_Top_opt_A3_01_1591	25203	TOF; CID	61.34	629.27	SLC11A2

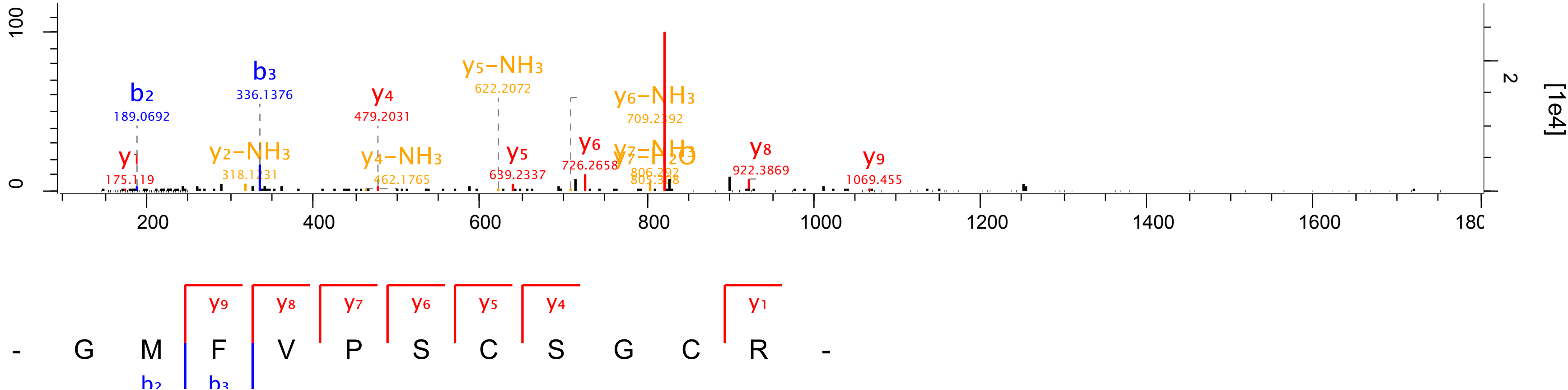

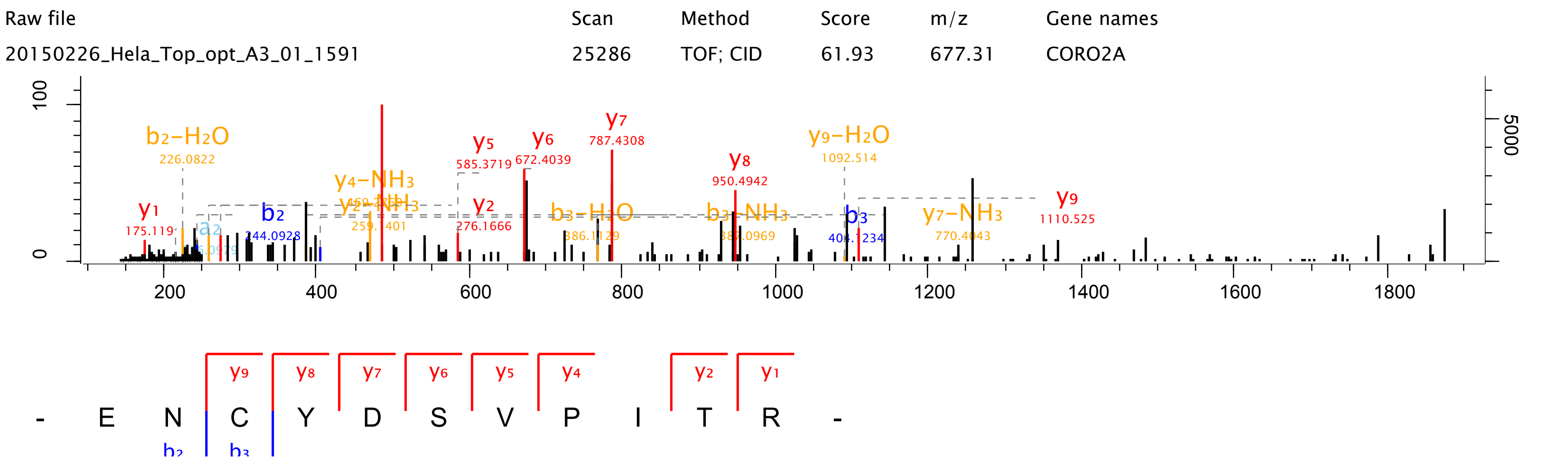

Raw file
Scan
Method
Score
m/z
Gene names
20150226_Hela_Top_opt_A3_01_1591
25286
TOF; CID
61.93
677.31
CORO2A
100
0
5000
b2−H2O
226.0822
y1
175.119
a2
b2
244.0928
y4−NH3
y2−NH3
259.1401
y5
585.3719
y2
276.1666
y6
672.4039
y7
787.4308
b3−H2O
386.1129
y8
950.4942
b3−NH3
y9−H2O
1092.514
b3
y7−NH3
770.4043
y9
1110.525
200
400
600
800
1000
1200
1400
1600
1800
y9
y8
y7
y6
y5
y4
y2
y1
- E N C Y D S V P I T R -
b2
b3

Raw file	Scan	Method	Score	m/z	Gene names
20150226_Hela_Top_opt_A3_01_1591	25344	TOF; CID	45.35	811.43	TPRN

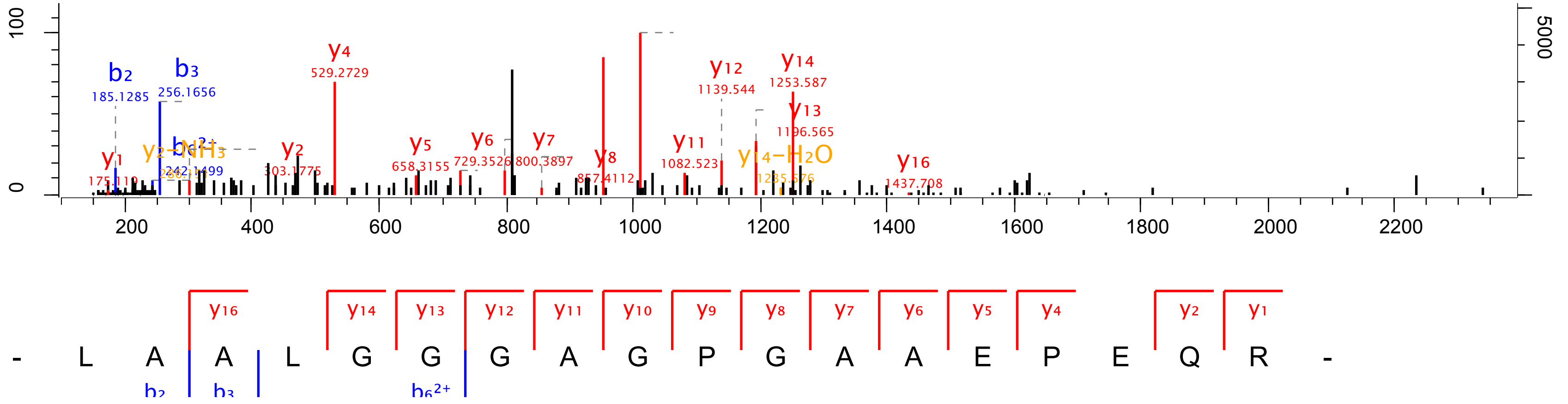

Raw file	Scan	Method	Score	m/z	Gene names
20150226_Hela_Top_opt_A3_01_1591	25602	TOF; CID	72.43	643.34	ERLEC1

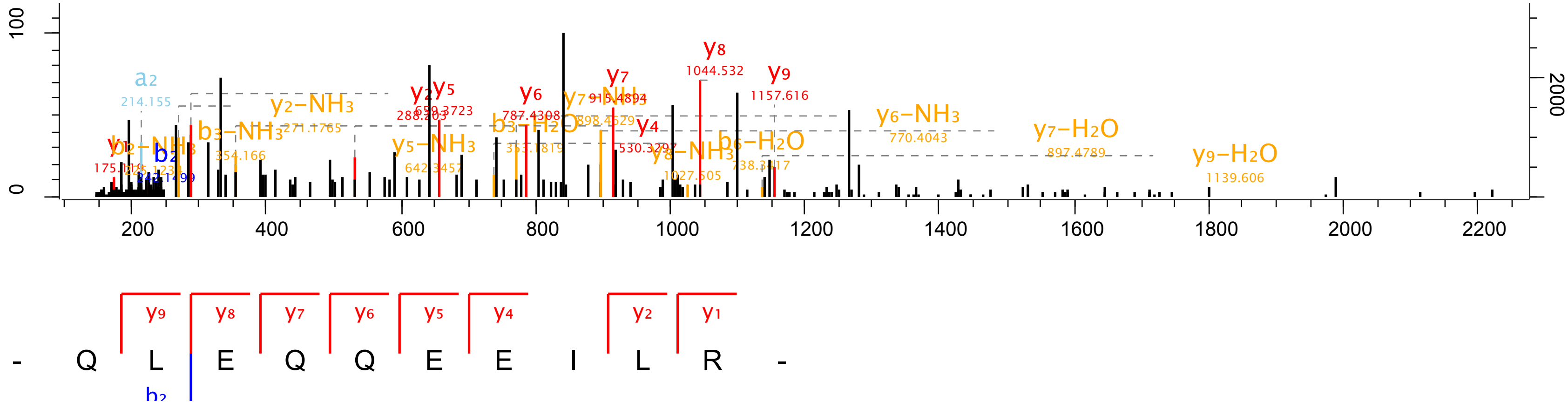

Raw file	Scan	Method	Score	m/z	Gene names
20150226_Hela_Top_opt_A3_01_1591	25966	TOF; CID	52.49	589.82	ARHGAP32

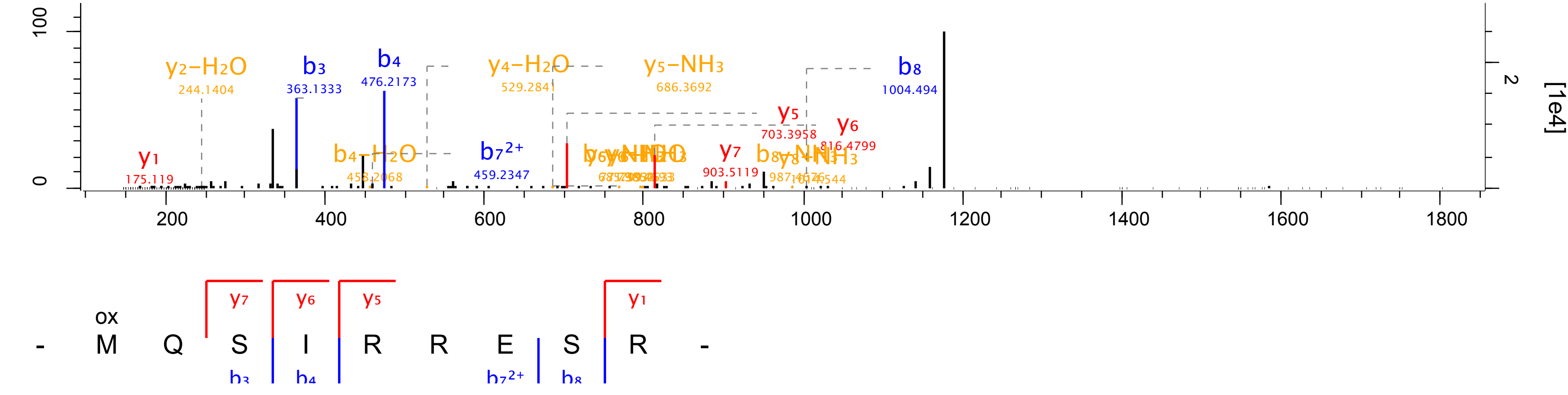

Raw file	Scan	Method	Score	m/z	Gene names
20150226_Hela_Top_opt_A3_01_1591	26681	TOF; CID	120.77	941.4	ZNRD1

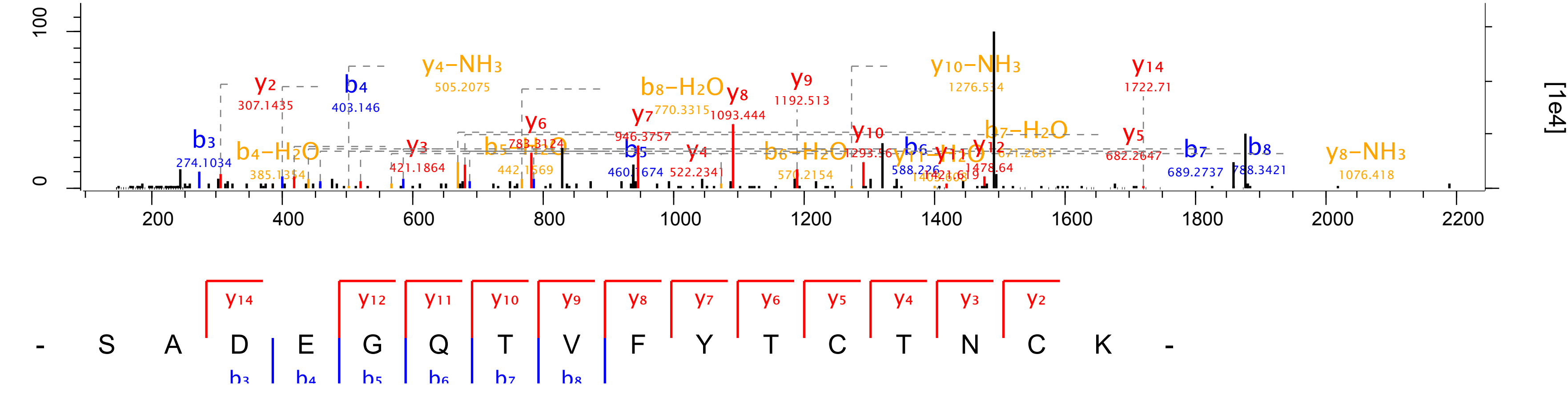

Raw file	Scan	Method	Score	m/z	Gene names
20150226_Hela_Top_opt_A3_01_1591	26696	TOF; CID	137.45	657.31	DYNLT3

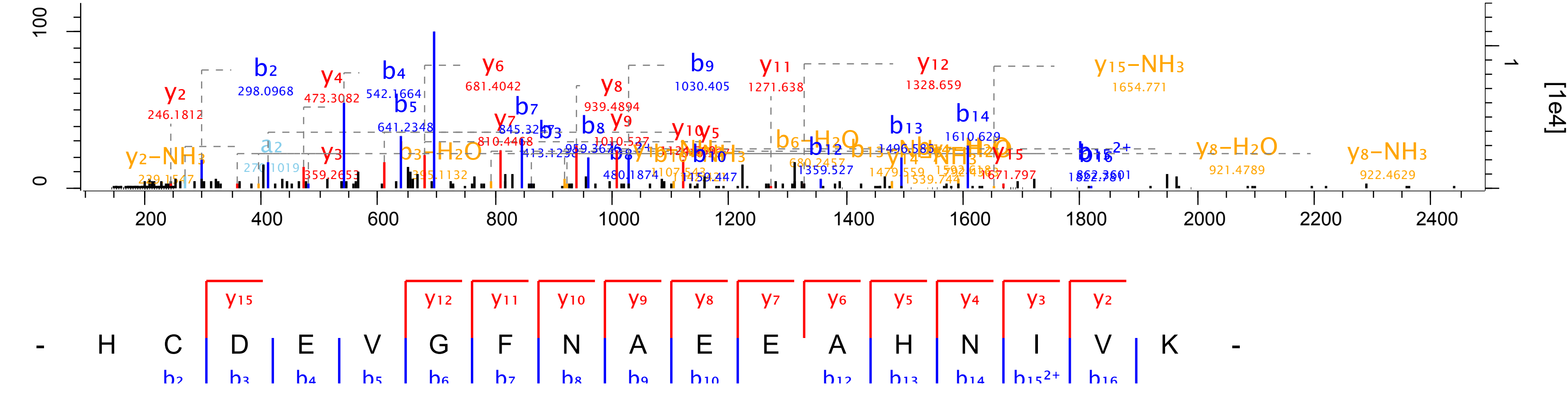

Raw file	Scan	Method	Score	m/z	Gene names
20150226_Hela_Top_opt_A3_01_1591	27112	TOF; CID	53.3	765.39	RILP

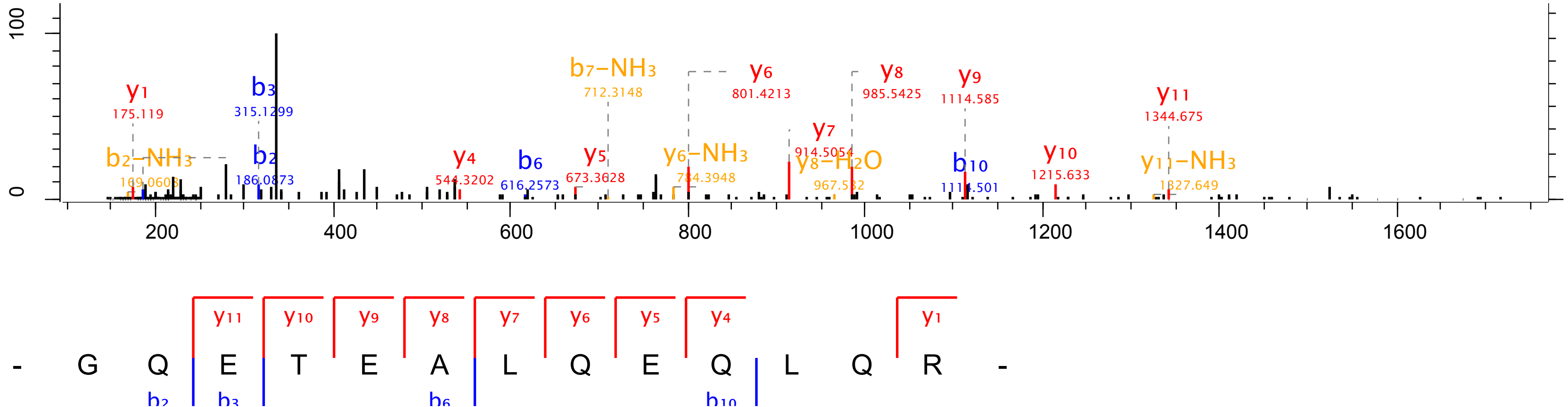

Raw file	Scan	Method	Score	m/z	Gene names
20150226_Hela_Top_opt_A3_01_1591	28137	TOF; CID	48.62	601.31	CST3

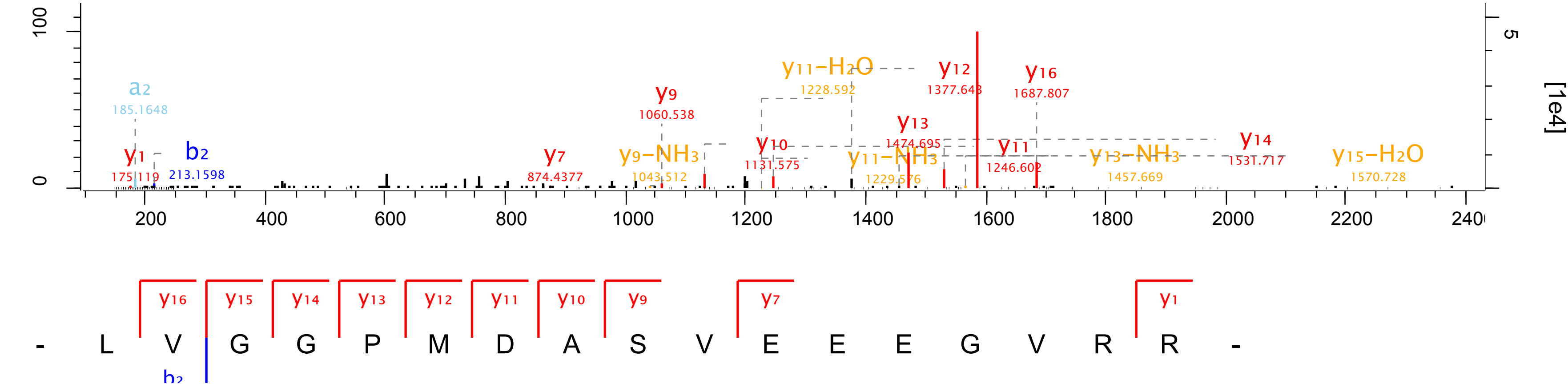

Raw file	Scan	Method	Score	m/z	Gene names
20150226_Hela_Top_opt_A3_01_1591	28460	TOF; CID	103.14	780.36	CSRP2

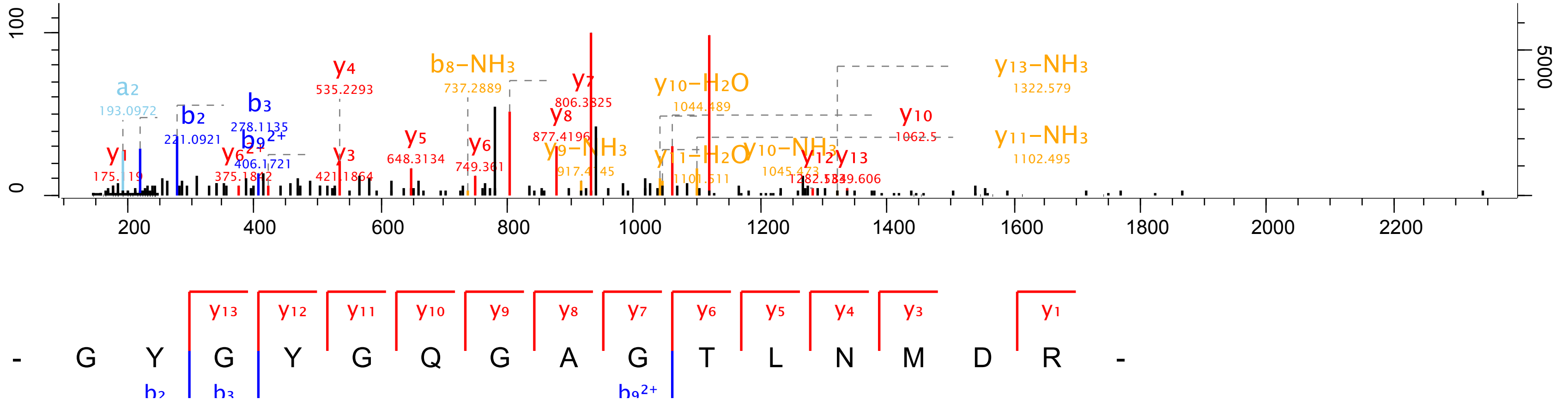

Raw file	Scan	Method	Score	m/z	Gene names
20150226_Hela_Top_opt_A3_01_1591	28896	TOF; CID	58.43	451.26	DOPEY2

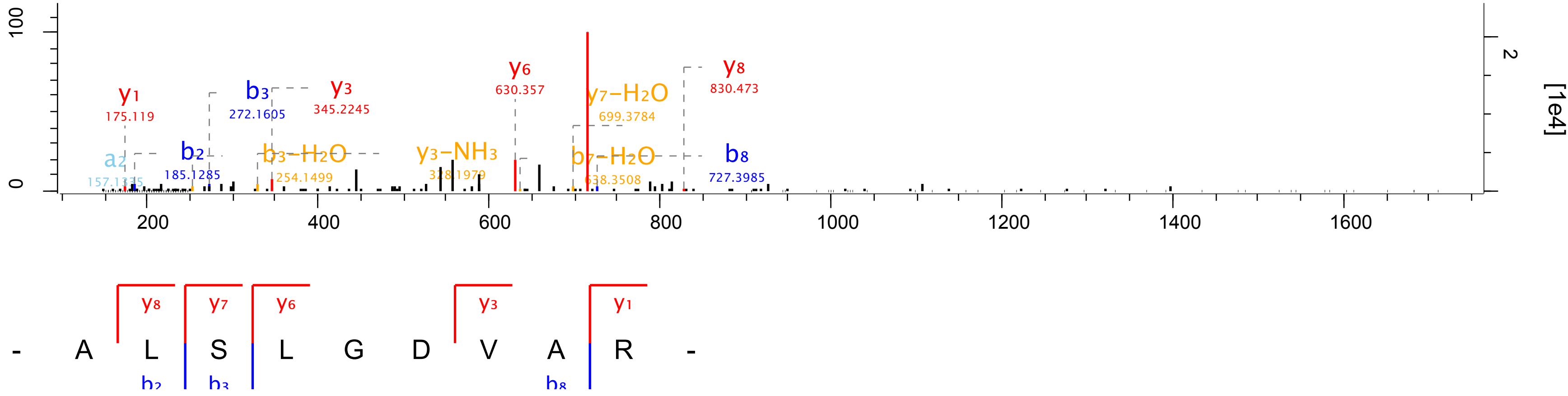

Raw file	Scan	Method	Score	m/z	Gene names
20150226_Hela_Top_opt_A3_01_1591	30278	TOF; CID	114.19	387.25	RPP14

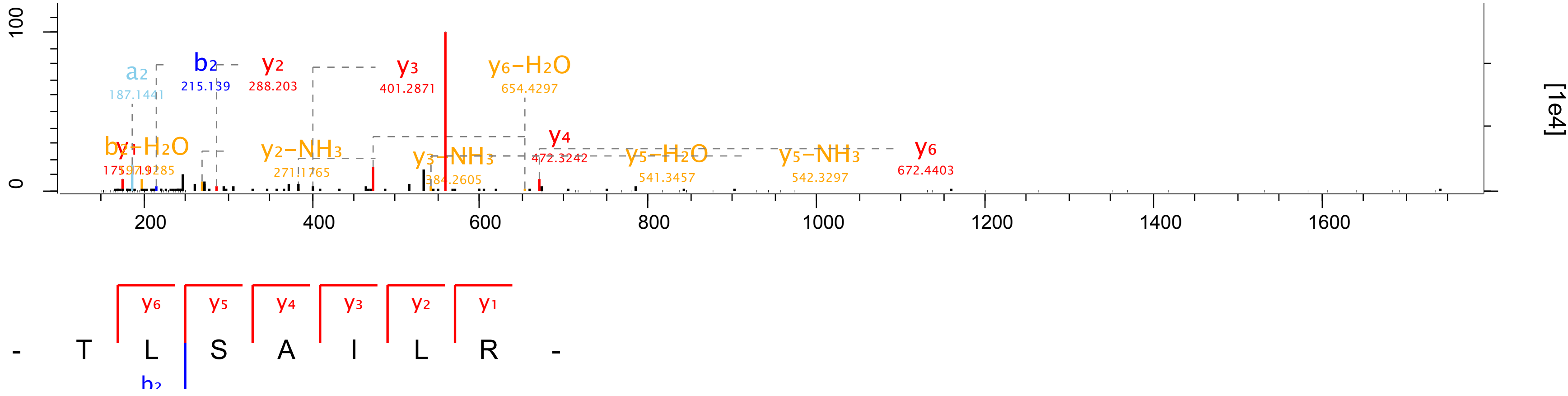

Raw file	Scan	Method	Score	m/z	Gene names
20150226_Hela_Top_opt_A3_01_1591	30403	TOF; CID	85.26	310.87	TLCD1

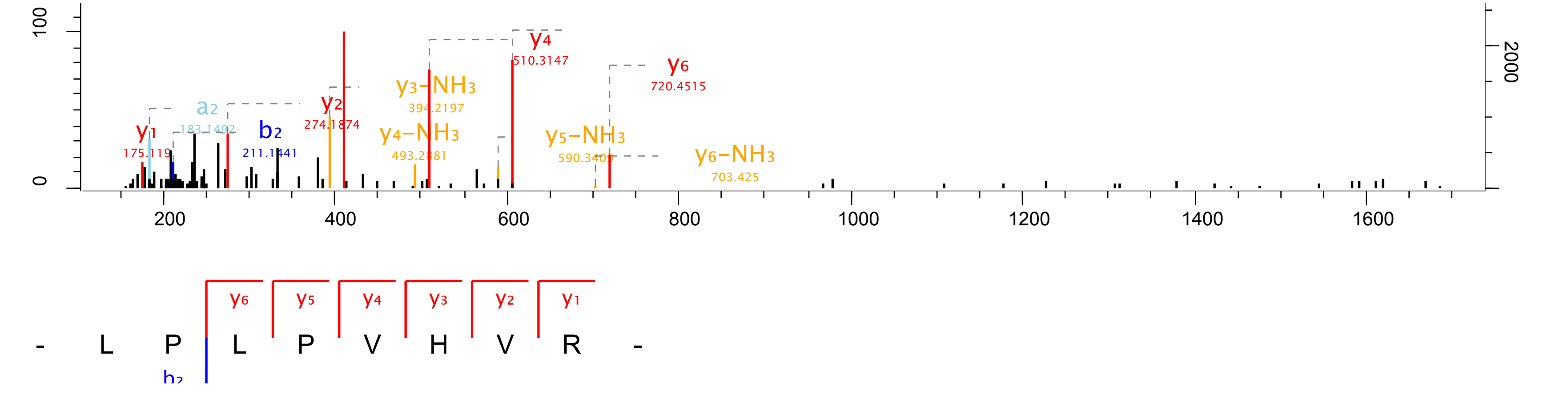

Raw file	Scan	Method	Score	m/z	Gene names
20150226_Hela_Top_opt_A3_01_1591	30966	TOF; CID	58.63	861.41	DPH3

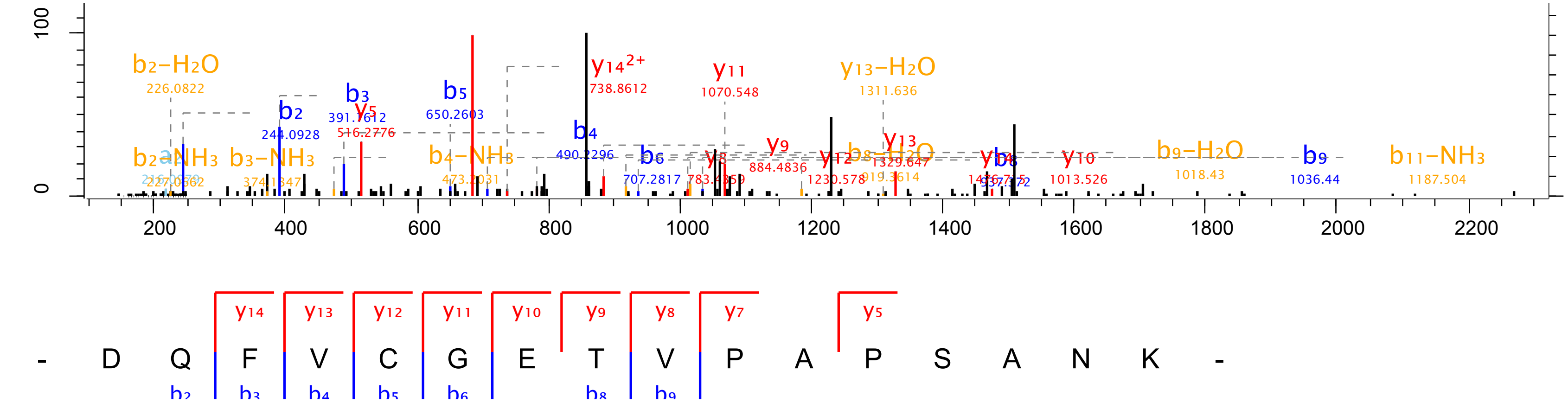

Raw file	Scan	Method	Score	m/z	Gene names
20150226_Hela_Top_opt_A3_01_1591	32368	TOF; CID	92.47	397.75	ZBTB25

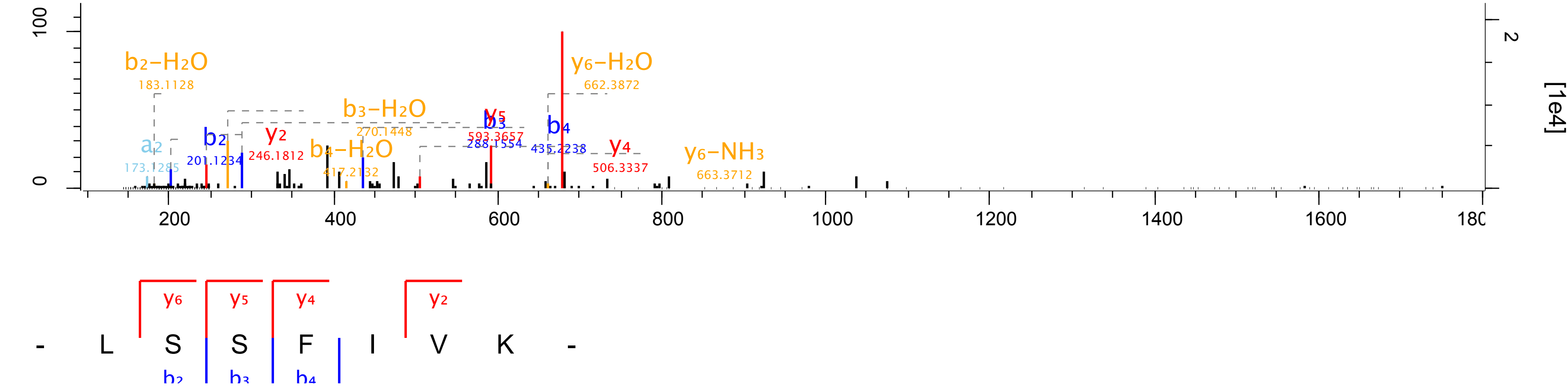

Raw file	Scan	Method	Score	m/z	Gene names
20150226_Hela_Top_opt_A3_01_1591	33011	TOF; CID	95.77	558.8	MTX3

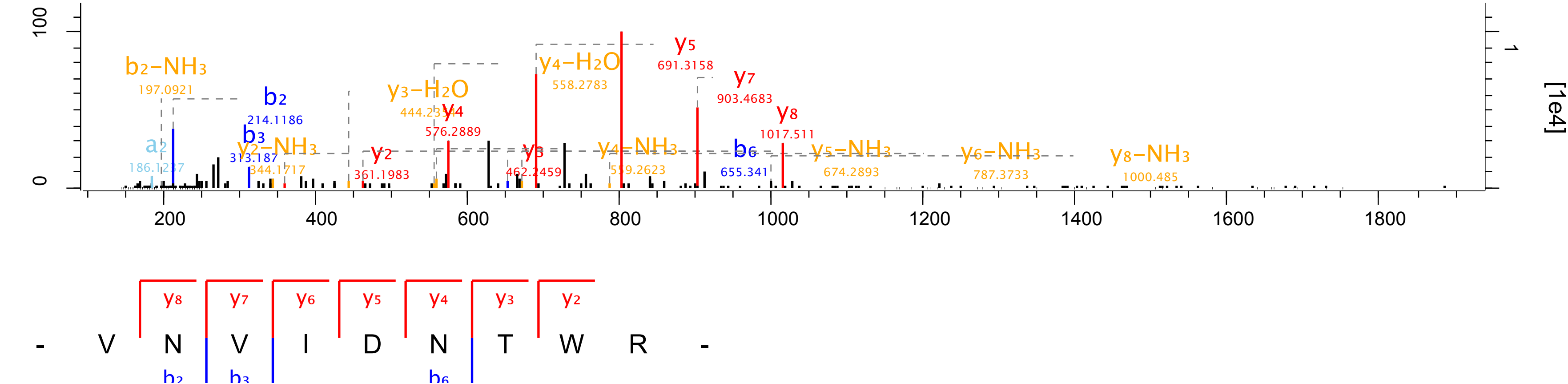

Raw file	Scan	Method	Score	m/z	Gene names
20150226_Hela_Top_opt_A3_01_1591	33222	TOF; CID	100.88	556.82	DCAKD

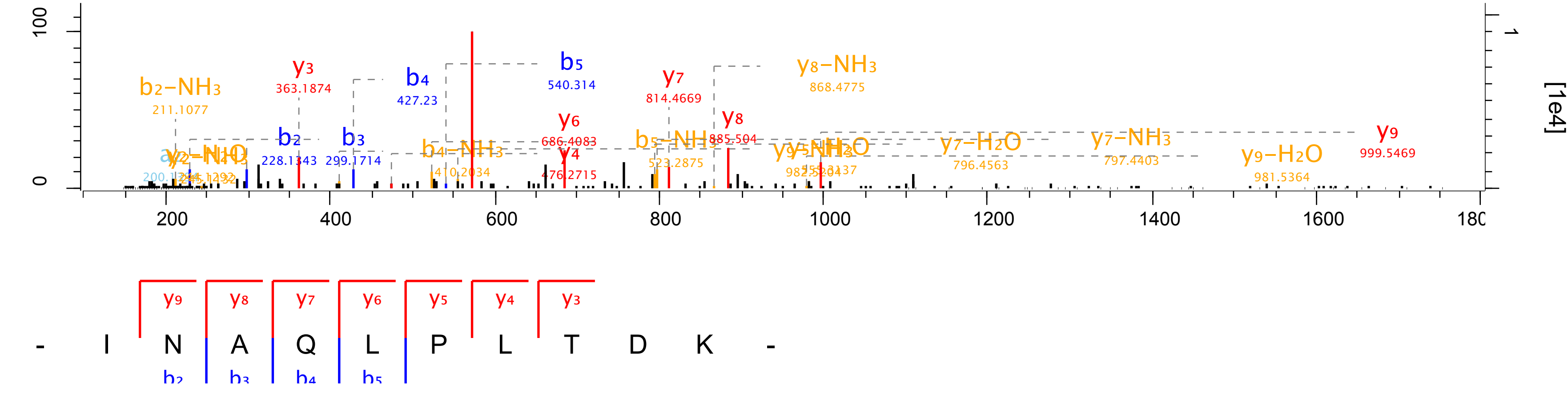

Raw file	Scan	Method	Score	m/z	Gene names
20150226_Hela_Top_opt_A3_01_1591	33381	TOF; CID	67.1	744.39	MGST2

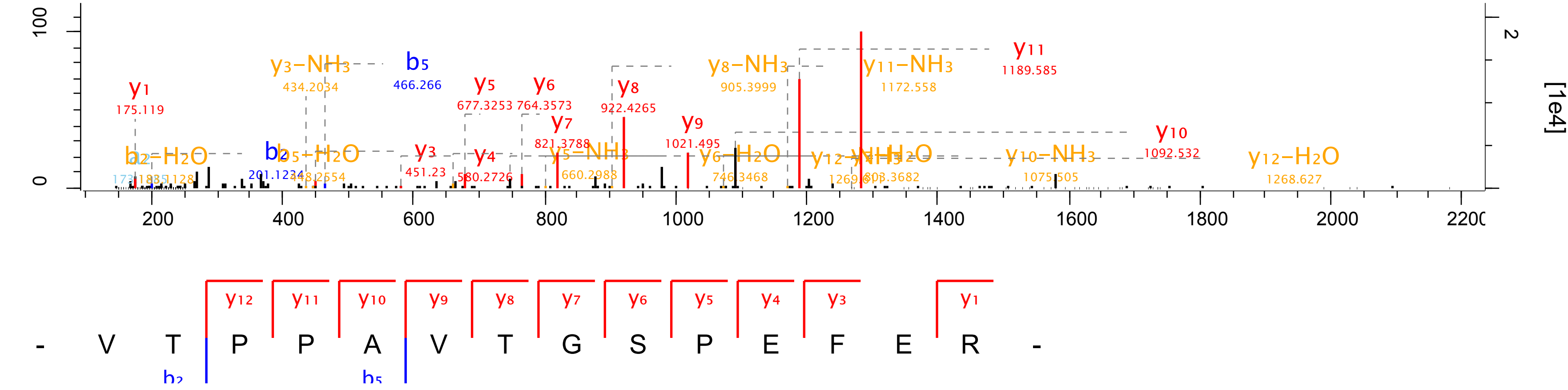

Raw file	Scan	Method	Score	m/z	Gene names
20150226_Hela_Top_opt_A3_01_1591	33468	TOF; CID	107.5	947.43	MID1

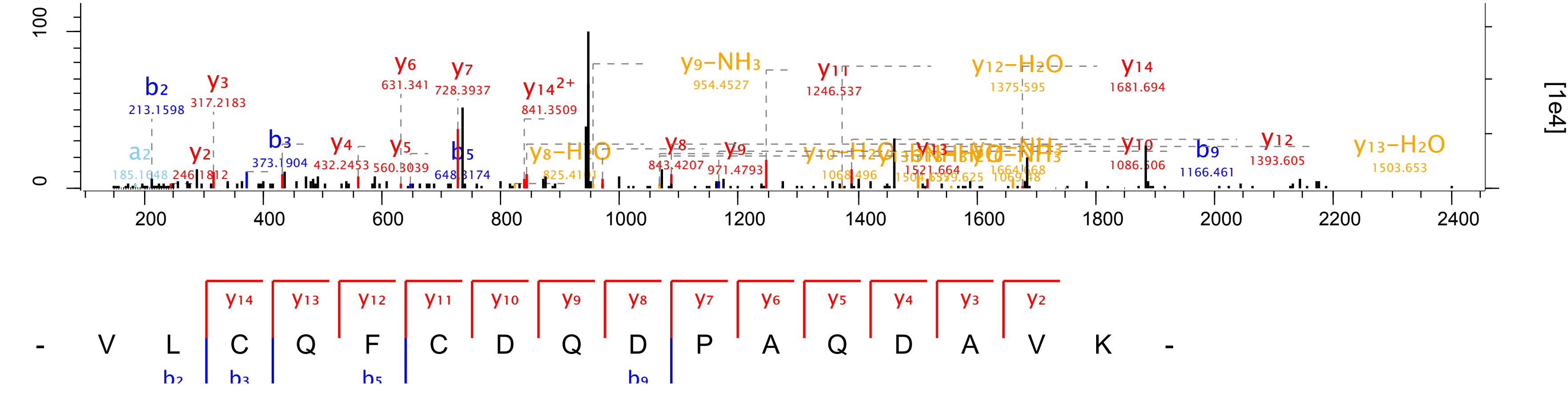

Raw file	Scan	Method	Score	m/z	Gene names
20150226_Hela_Top_opt_A3_01_1591	33666	TOF; CID	60.55	740.37	TRAPPC6B

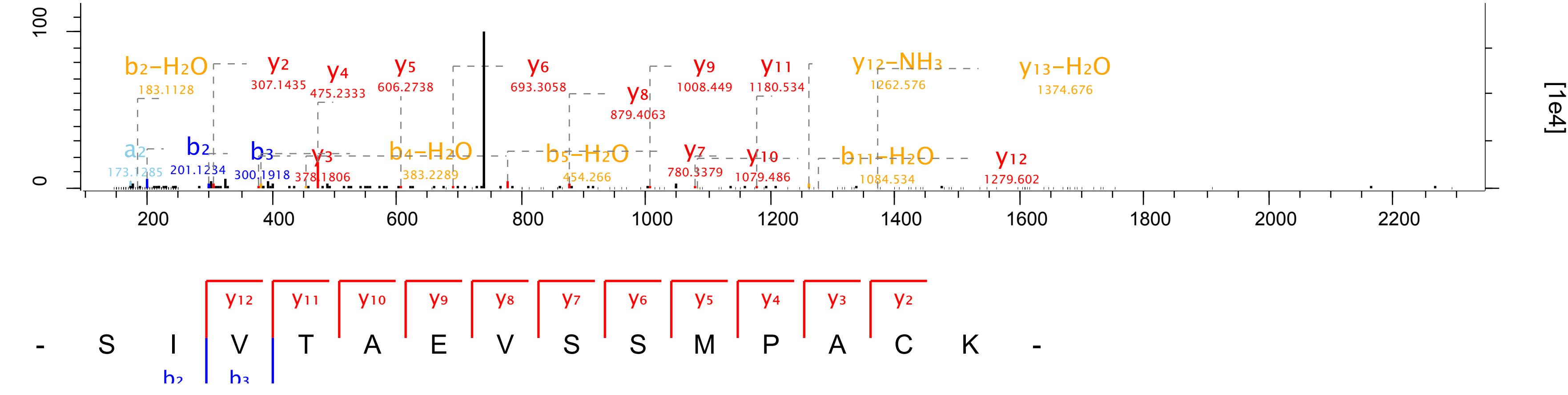

Raw file	Scan	Method	Score	m/z	Gene names
20150226_Hela_Top_opt_A3_01_1591	33881	TOF; CID	80.87	635.83	WWTR1

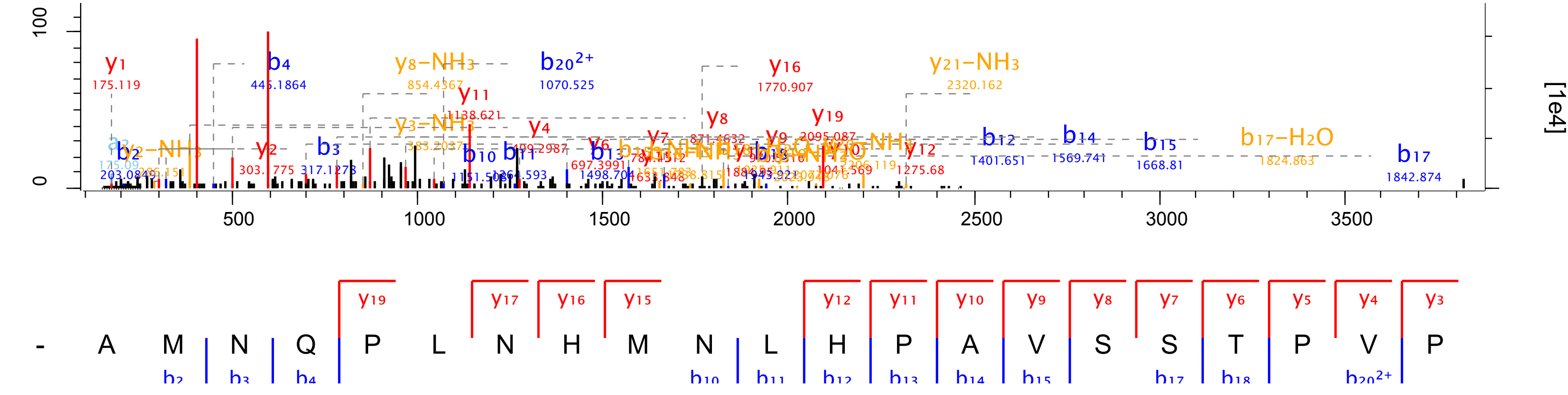

Raw file	Scan	Method	Score	m/z	Gene names
20150226_Hela_Top_opt_A3_01_1591	33934	TOF; CID	64.8	729.87	KCT2

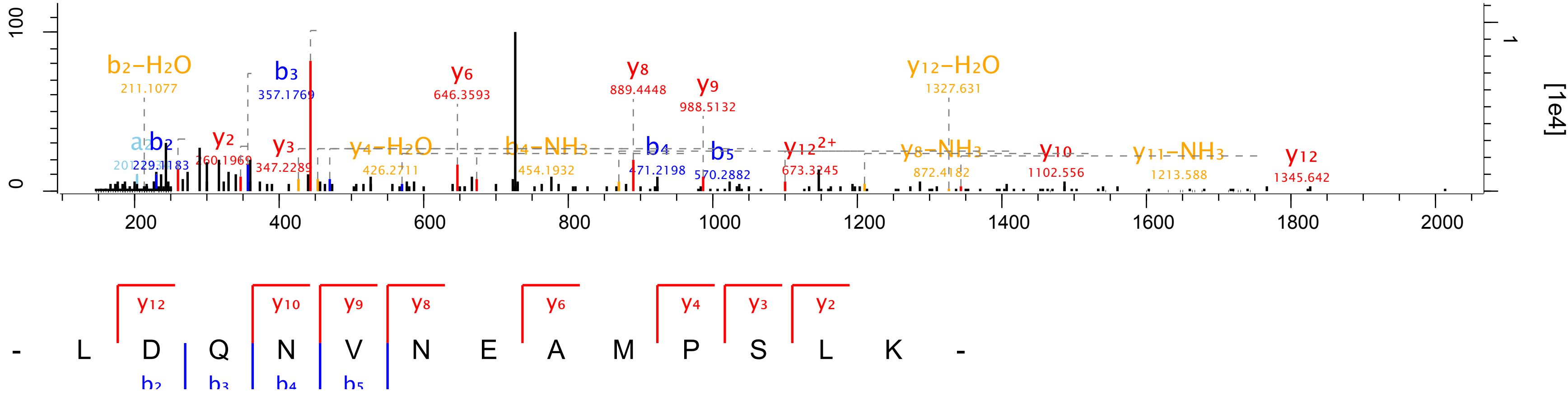

Raw file	Scan	Method	Score	m/z	Gene names
20150226_Hela_Top_opt_A3_01_1591	34175	TOF; CID	69.2	479.23	MAPKAPK5

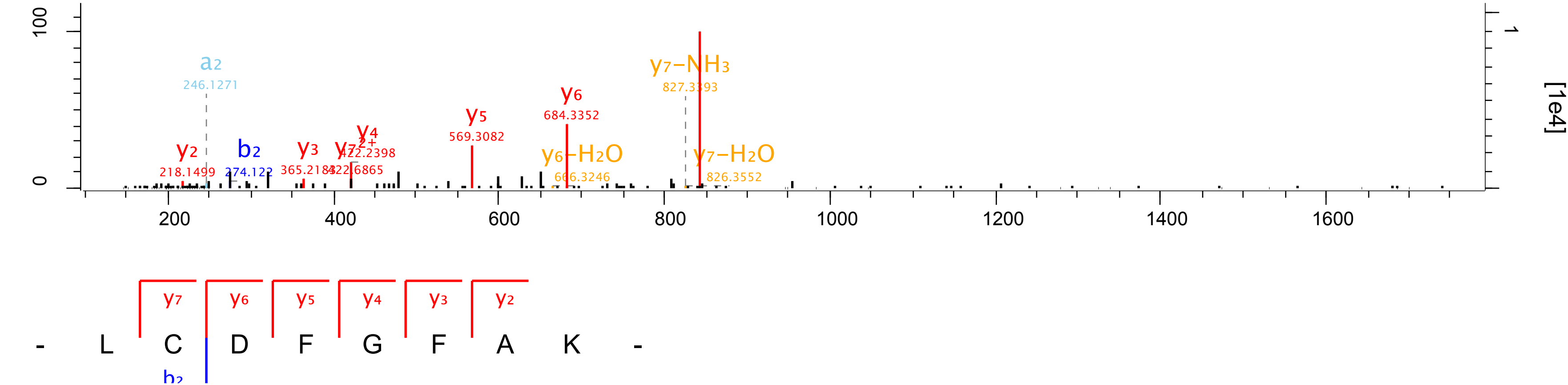

Raw file	Scan	Method	Score	m/z	Gene names
20150226_Hela_Top_opt_A3_01_1591	34255	TOF; CID	52.46	639.84	THTPA

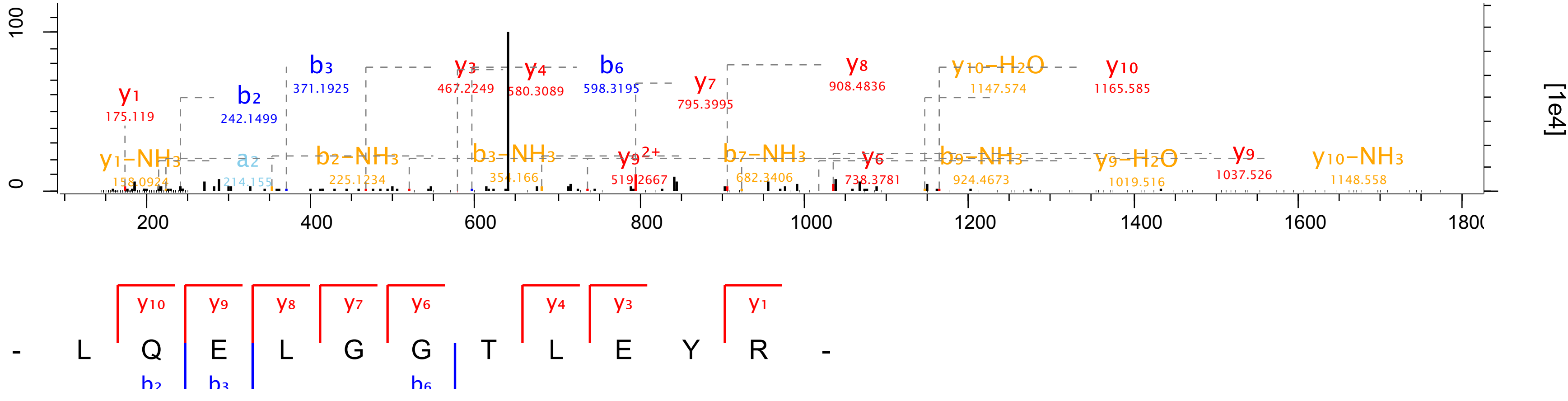

Raw file	Scan	Method	Score	m/z	Gene names
20150226_Hela_Top_opt_A3_01_1591	34923	TOF; CID	47.77	844.91	MYBPC3

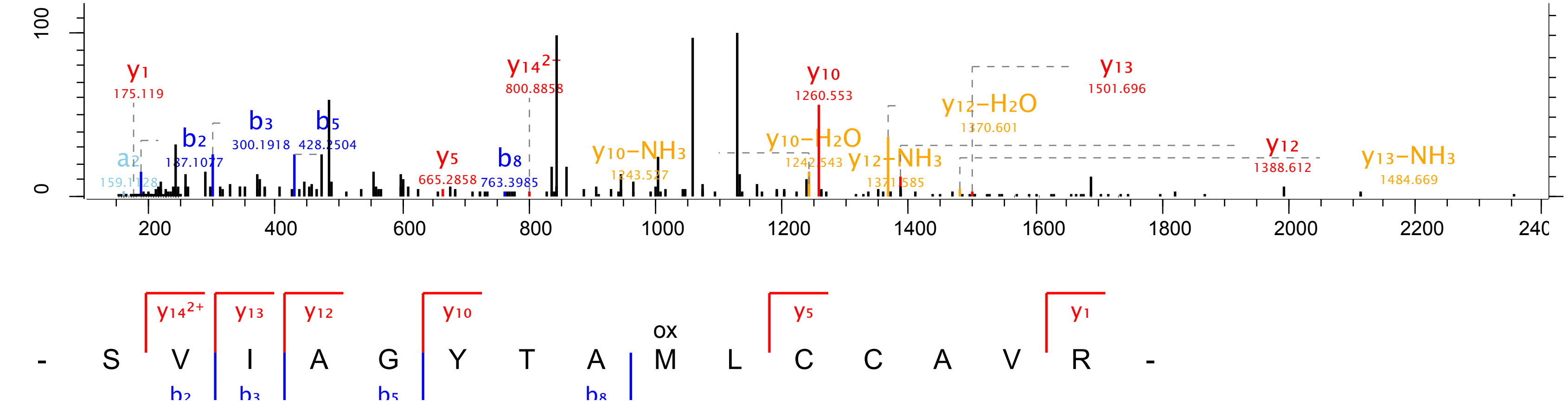

Raw file	Scan	Method	Score	m/z	Gene names
20150226_Hela_Top_opt_A3_01_1591	34924	TOF; CID	110.6	806.39	USMG5

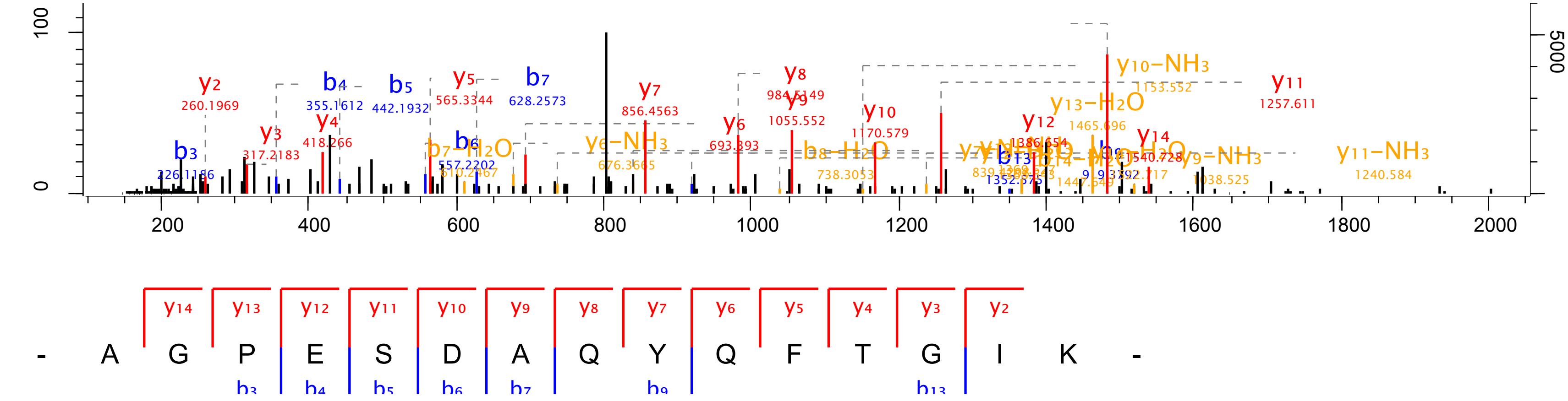

Raw file	Scan	Method	Score	m/z	Gene names
20150226_Hela_Top_opt_A3_01_1591	35132	TOF; CID	55.26	649.35	PVR

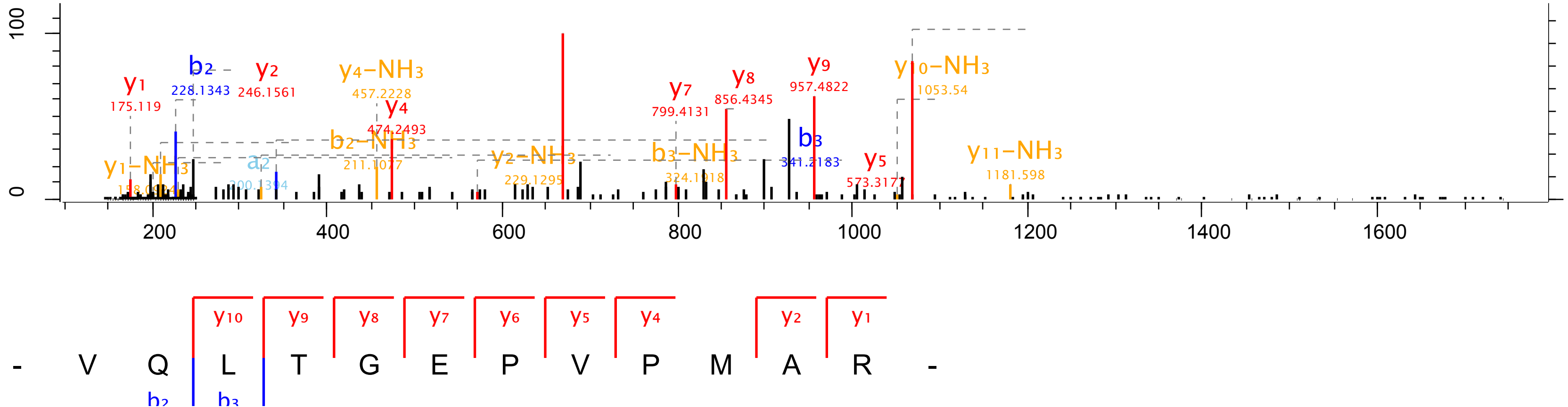

Raw file	Scan	Method	Score	m/z	Gene names
20150226_Hela_Top_opt_A3_01_1591	35586	TOF; CID	68.48	795.41	PIAS1;PIAS2

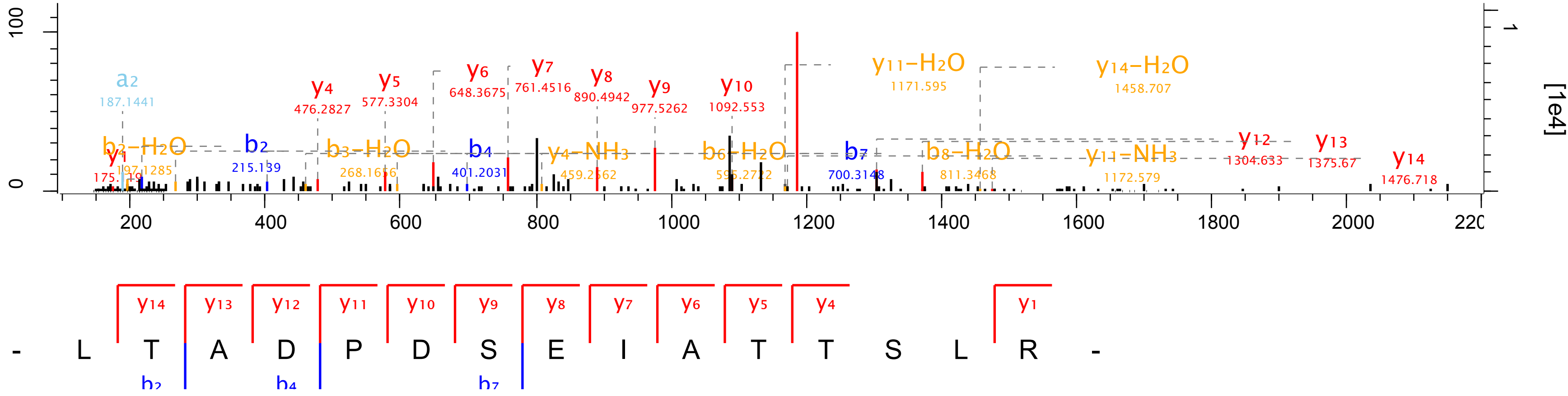

Raw file	Scan	Method	Score	m/z	Gene names
20150226_Hela_Top_opt_A3_01_1591	36084	TOF; CID	84.61	550.81	COX19

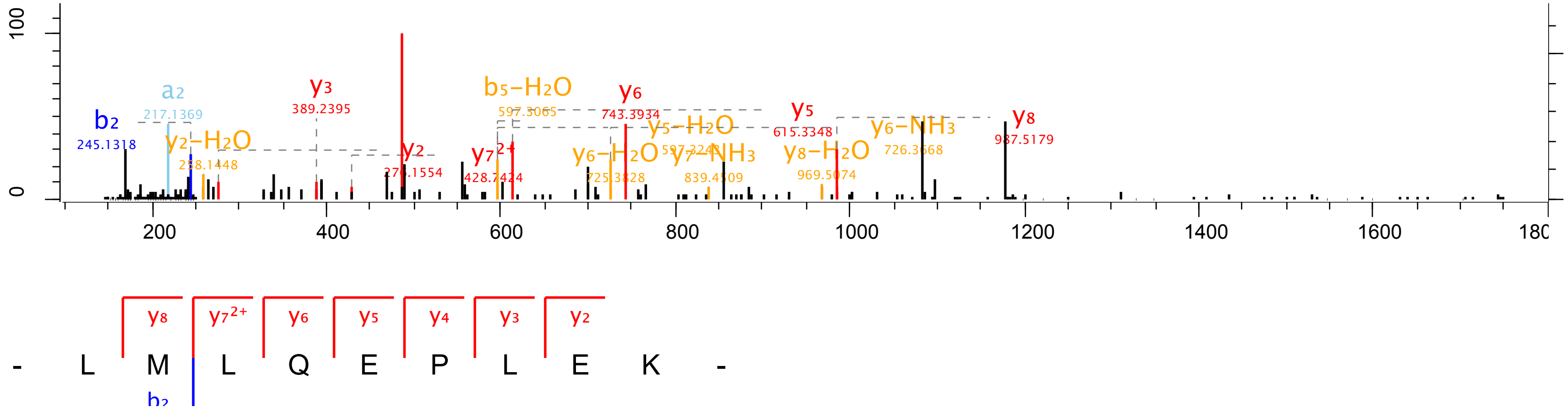

Raw file	Scan	Method	Score	m/z	Gene names
20150226_Hela_Top_opt_A3_01_1591	37959	TOF; CID	110.55	953.11	C6orf120

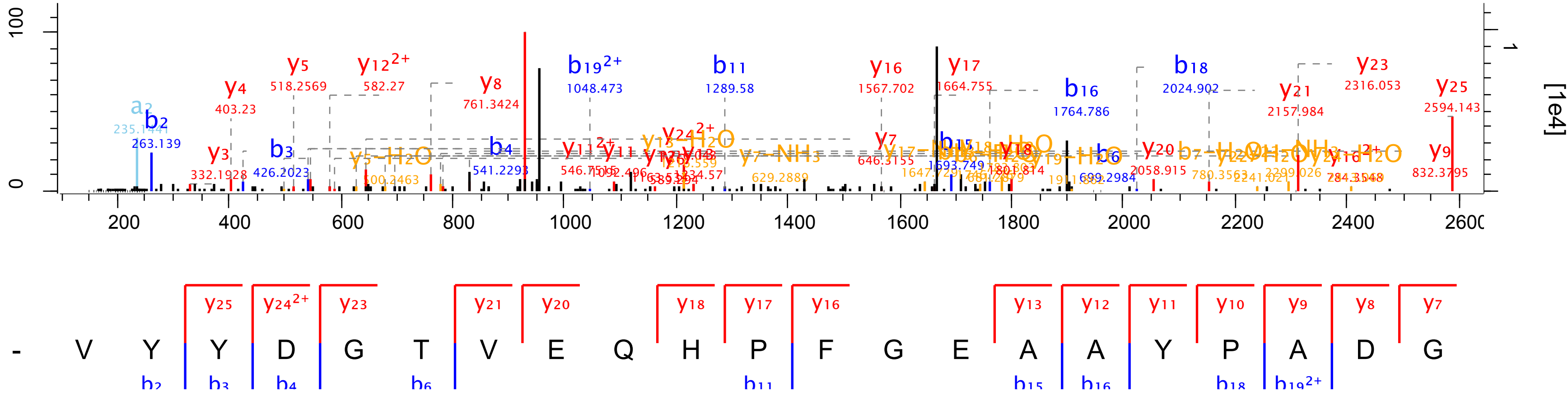

Raw file	Scan	Method	Score	m/z	Gene names
20150226_Hela_Top_opt_A3_01_1591	39376	TOF; CID	93.37	489.77	MRPS24

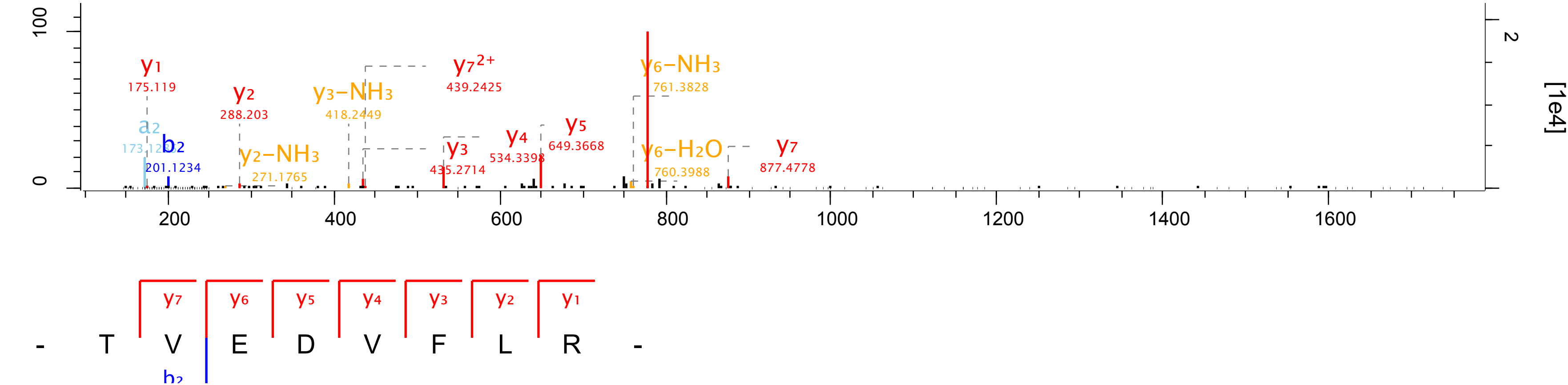

Raw file	Scan	Method	Score	m/z	Gene names
20150226_Hela_Top_opt_A3_01_1591	39888	TOF; CID	53.38	618.86	SLC25A36;SLC25A33

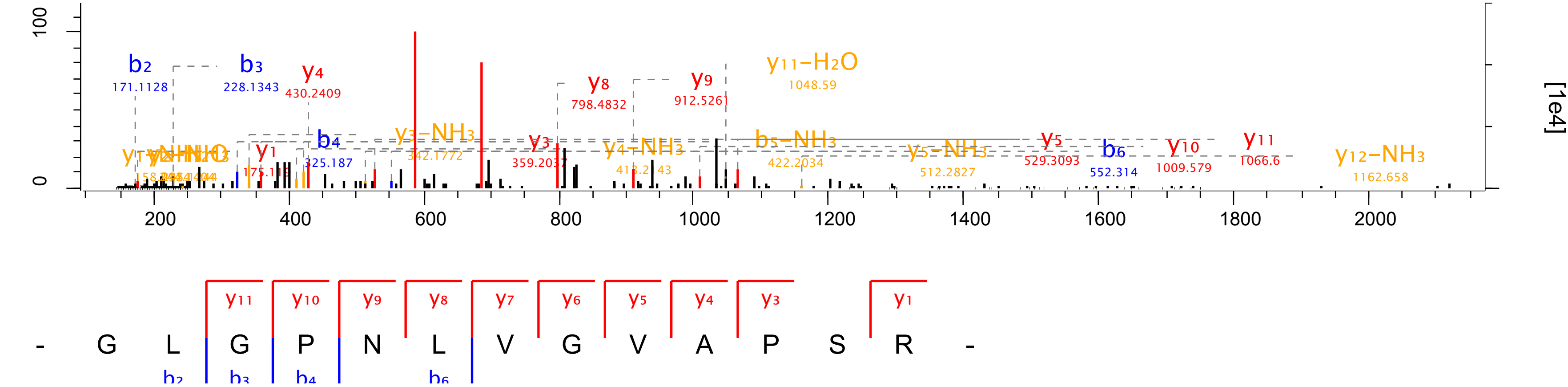

Raw file	Scan	Method	Score	m/z
20150226_Hela_Top_opt_A3_01_1591	39947	TOF; CID	65.24	1004.48

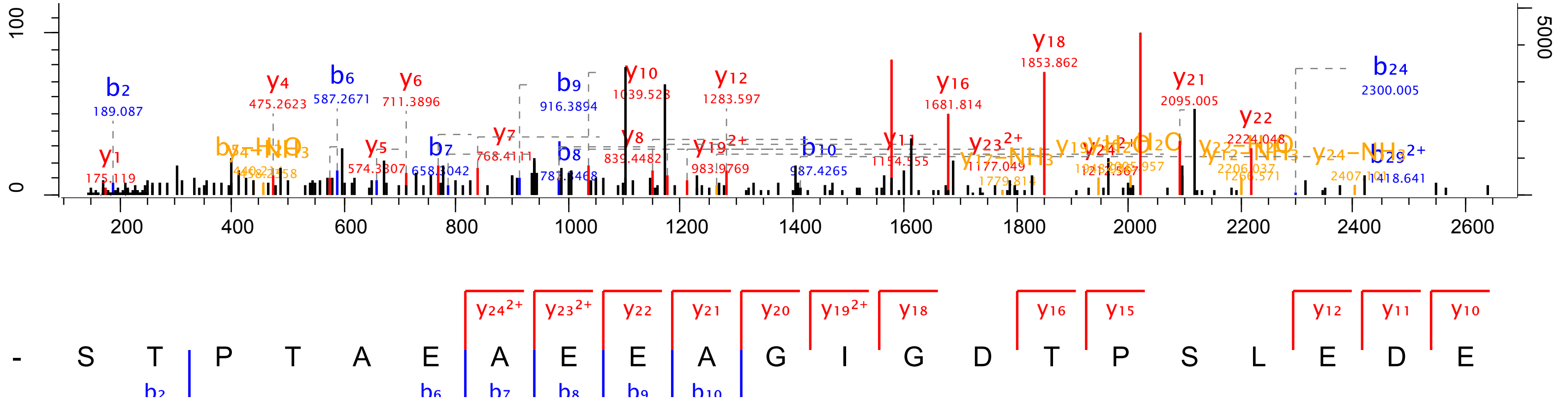

Raw file	Scan	Method	Score	m/z	Gene names
20150226_Hela_Top_opt_A3_01_1591	40053	TOF; CID	50.35	632.83	BECN1

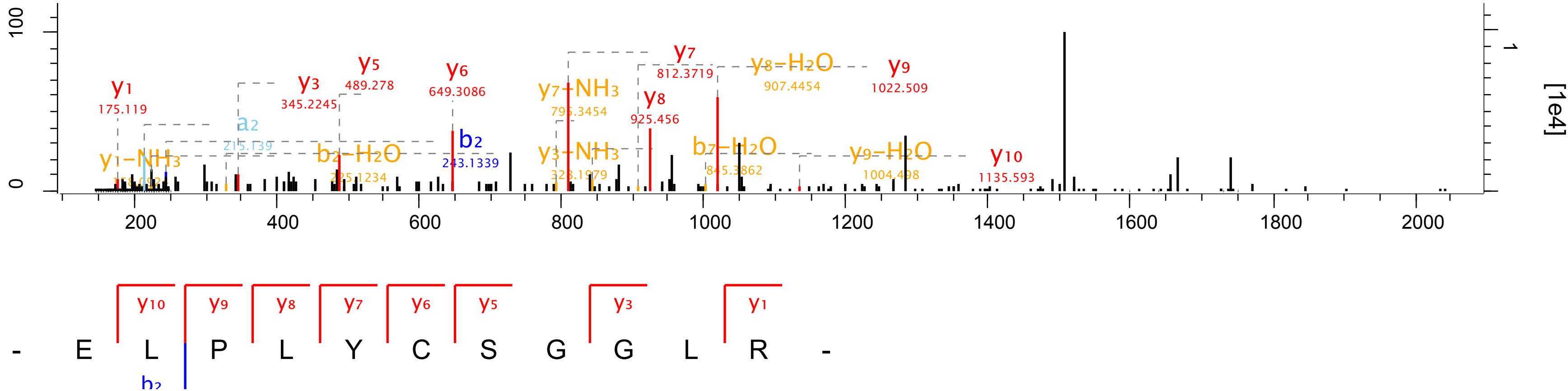

Raw file	Scan	Method	Score	m/z	Gene names
20150226_Hela_Top_opt_A3_01_1591	40613	TOF; CID	68.86	545.95	COX16

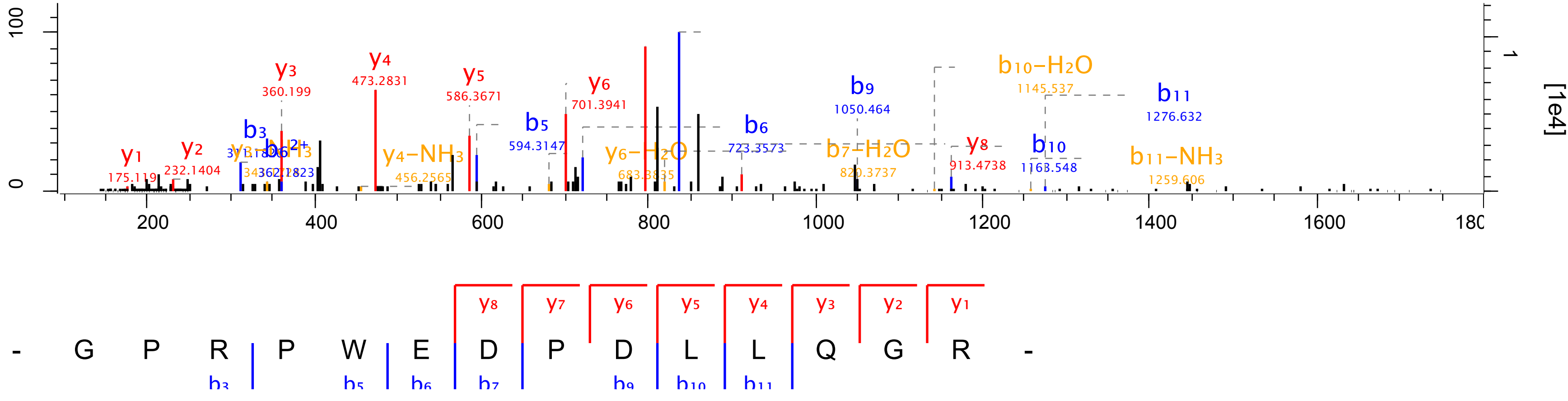

Raw file	Scan	Method	Score	m/z	Gene names
20150226_Hela_Top_opt_A3_01_1591	40746	TOF; CID	140.28	971.94	HIGD1A

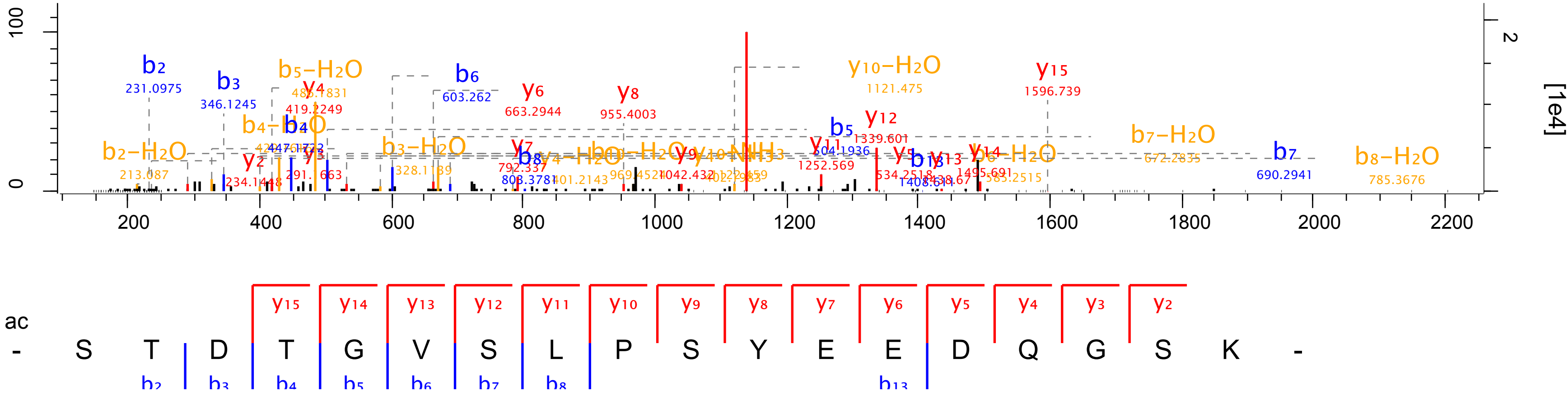

Raw file	Scan	Method	Score	m/z	Gene names
20150226_Hela_Top_opt_A3_01_1591	41012	TOF; CID	82.07	773.88	MEA1

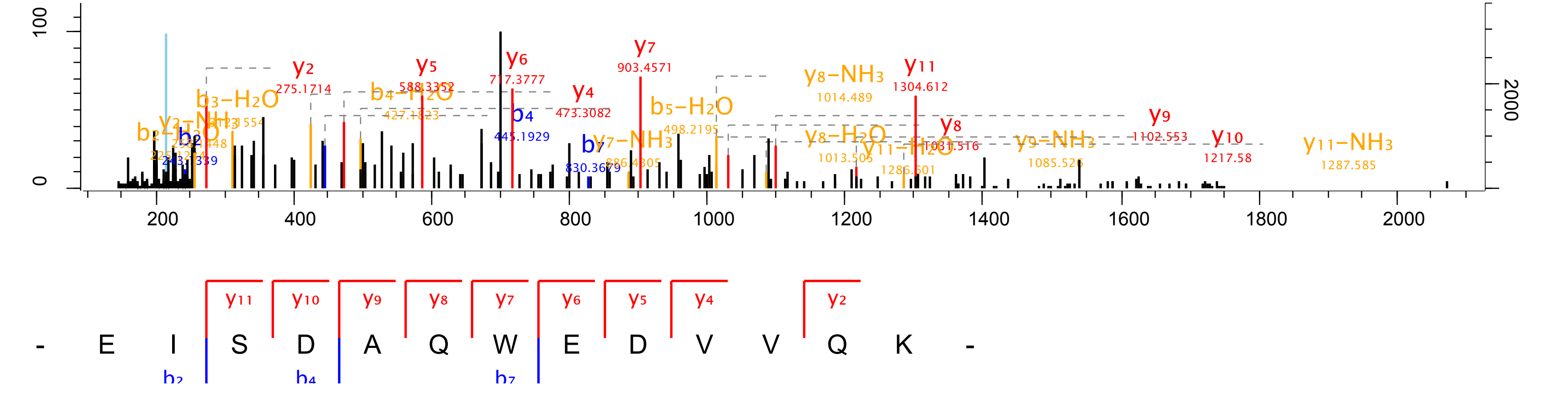

Raw file	Scan	Method	Score	m/z	Gene names
20150226_Hela_Top_opt_A3_01_1591	41308	TOF; CID	66.54	735.91	TRIM11

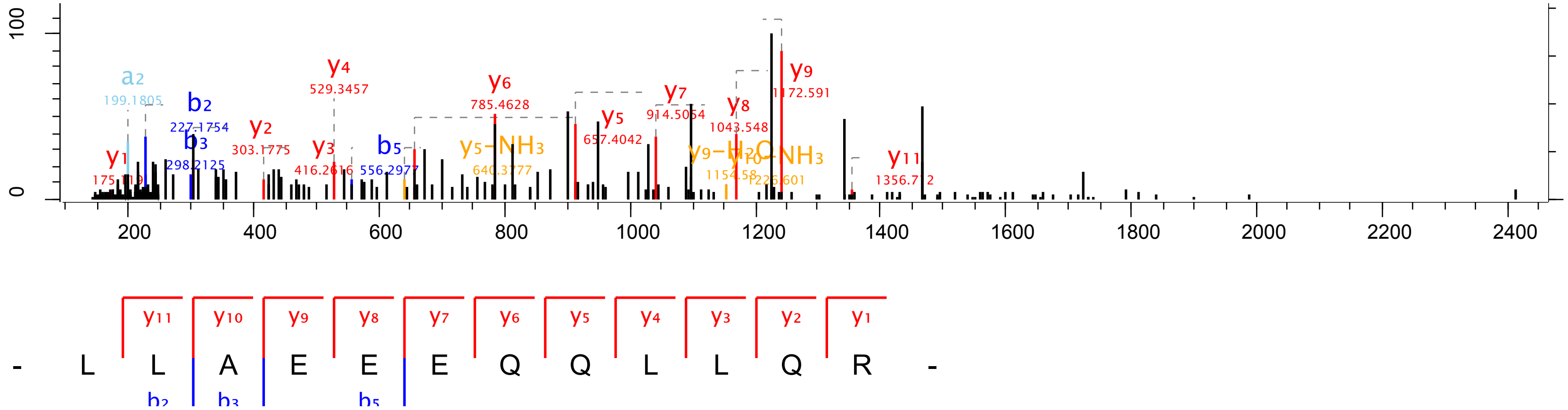

Raw file	Scan	Method	Score	m/z	Gene names
20150226_Hela_Top_opt_A3_01_1591	41658	TOF; CID	66.83	853.4	TMEM179B

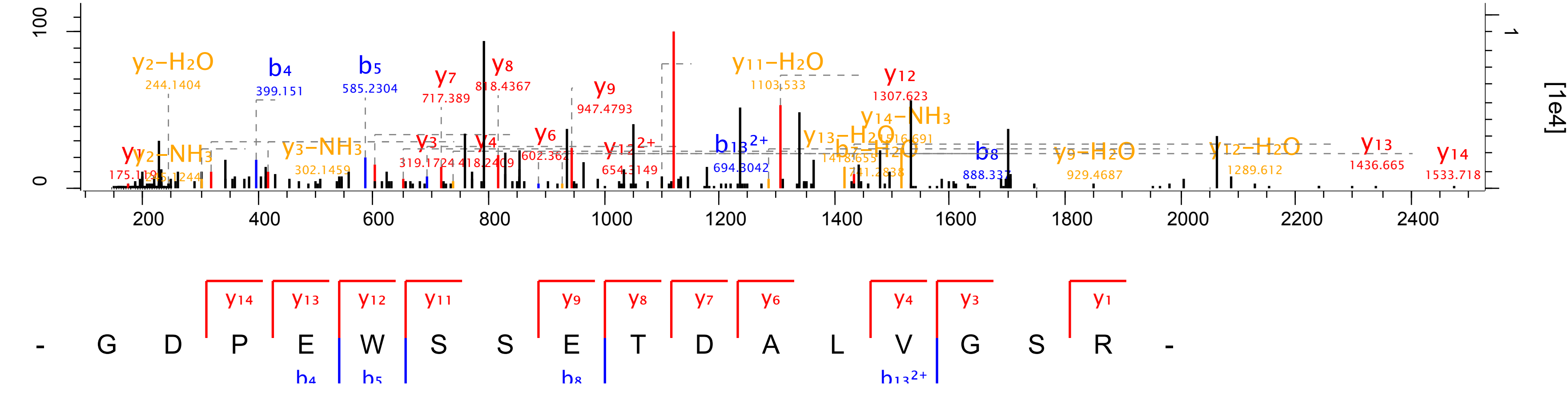

Raw file	Scan	Method	Score	m/z	Gene names
20150226_Hela_Top_opt_A3_01_1591	42436	TOF; CID	75.55	734.66	ELOF1

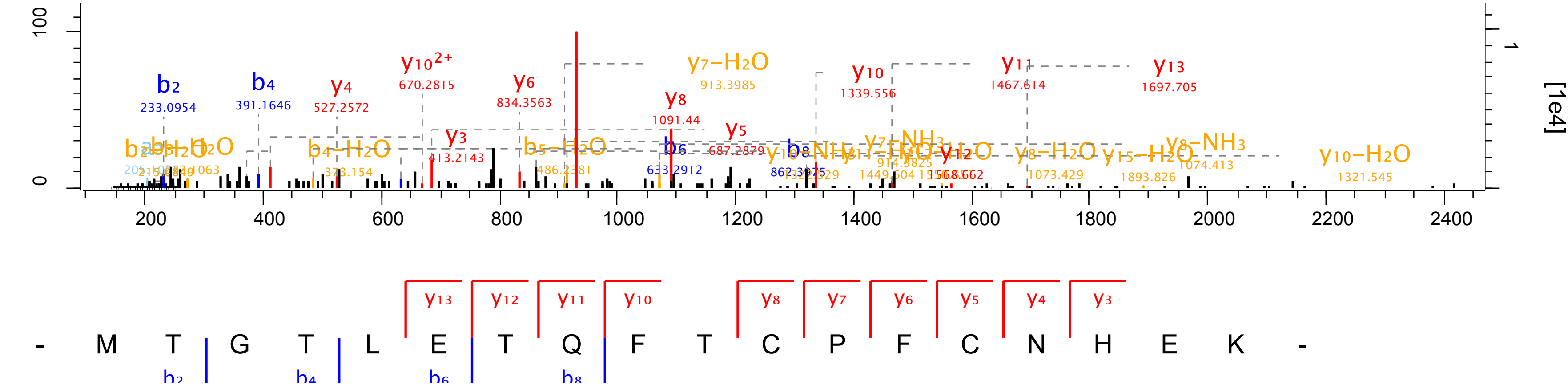

Raw file	Scan	Method	Score	m/z	Gene names
20150226_Hela_Top_opt_A3_01_1591	42476	TOF; CID	151.17	446.28	RRAS

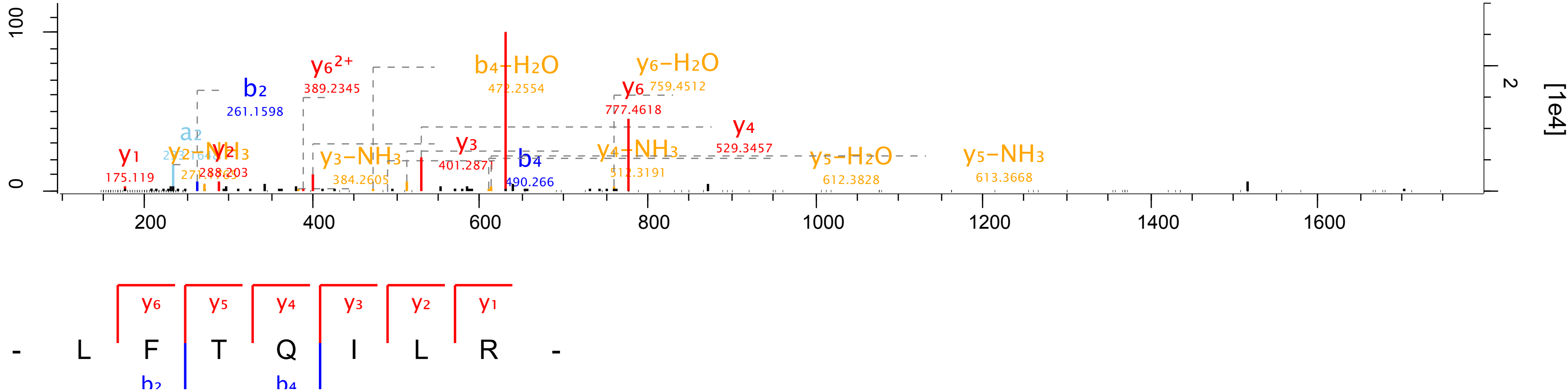

Raw file	Scan	Method	Score	m/z	Gene names
20150226_Hela_Top_opt_A3_01_1591	43914	TOF; CID	57.4	622.8	LIPA

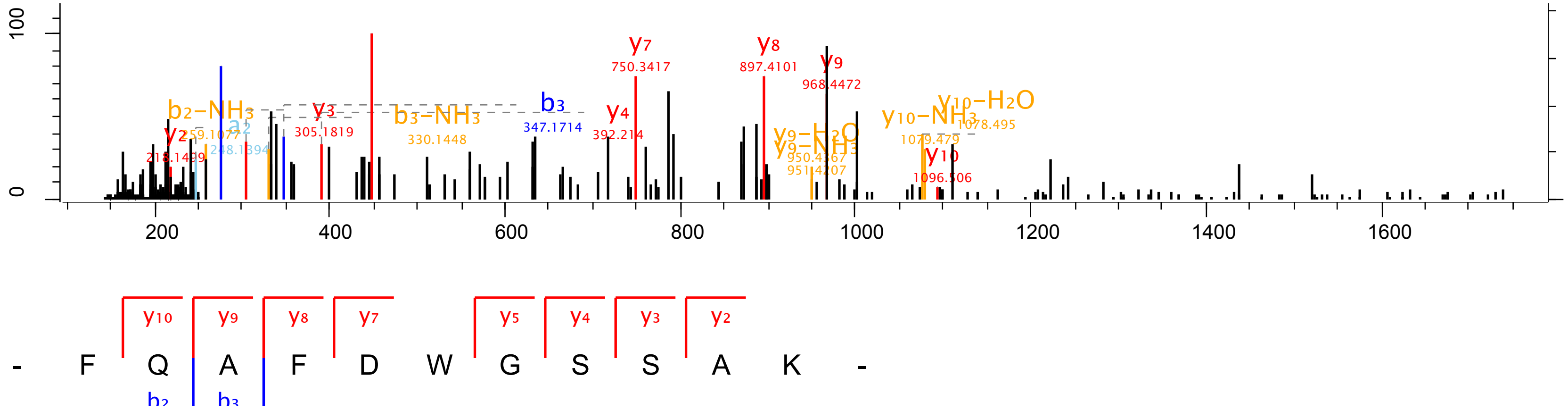

Raw file	Scan	Method	Score	m/z	Gene names
20150226_Hela_Top_opt_A3_01_1591	44157	TOF; CID	46.88	586.84	PHF23

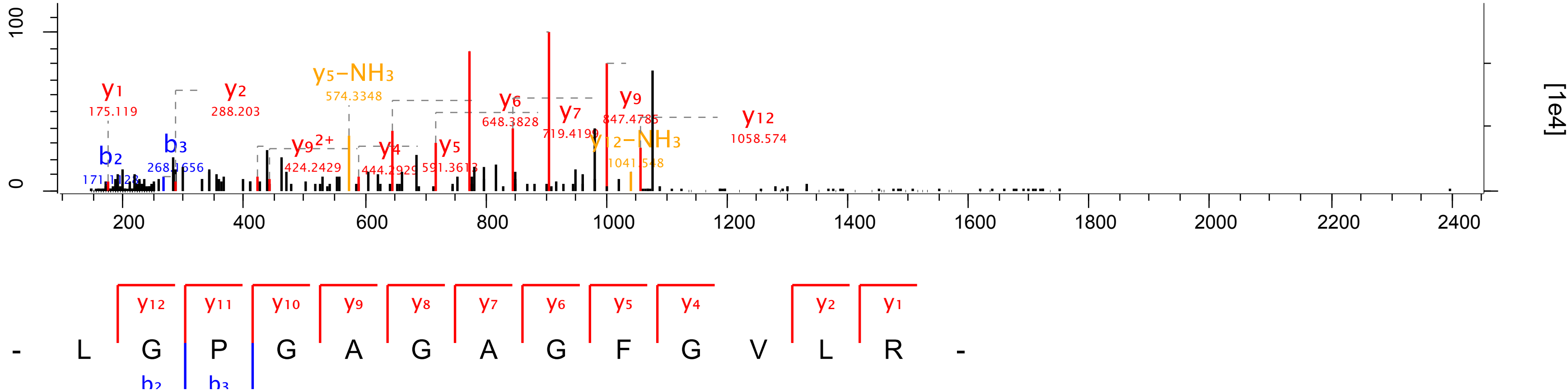

Raw file	Scan	Method	Score	m/z	Gene names
20150226_Hela_Top_opt_A3_01_1591	44654	TOF; CID	87.43	882.45	CTSS

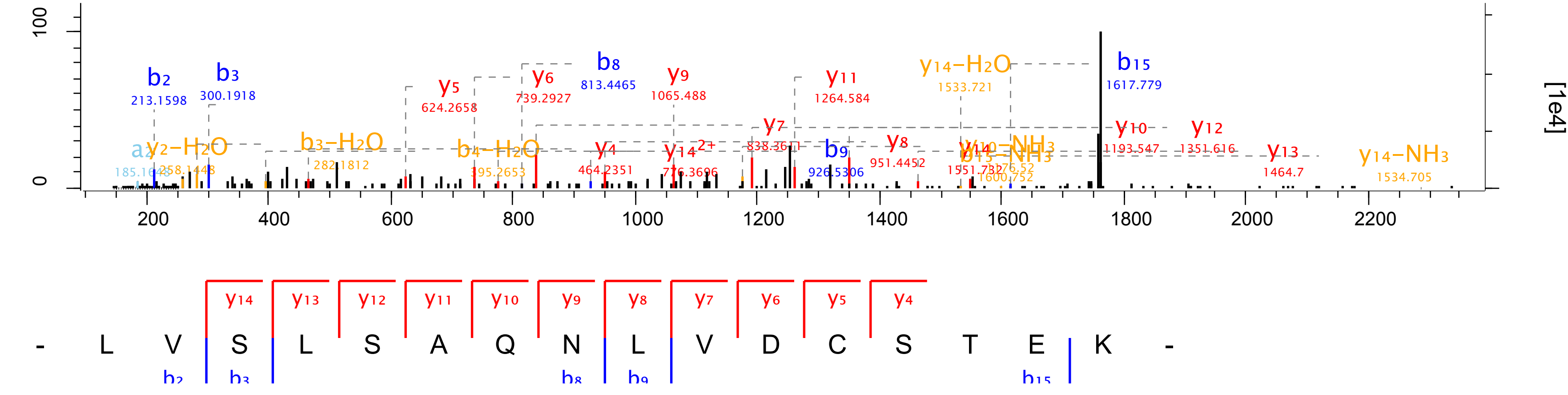

Raw file	Scan	Method	Score	m/z	Gene names
20150226_Hela_Top_opt_A3_01_1591	45851	TOF; CID	116.06	908.46	SLC30A7

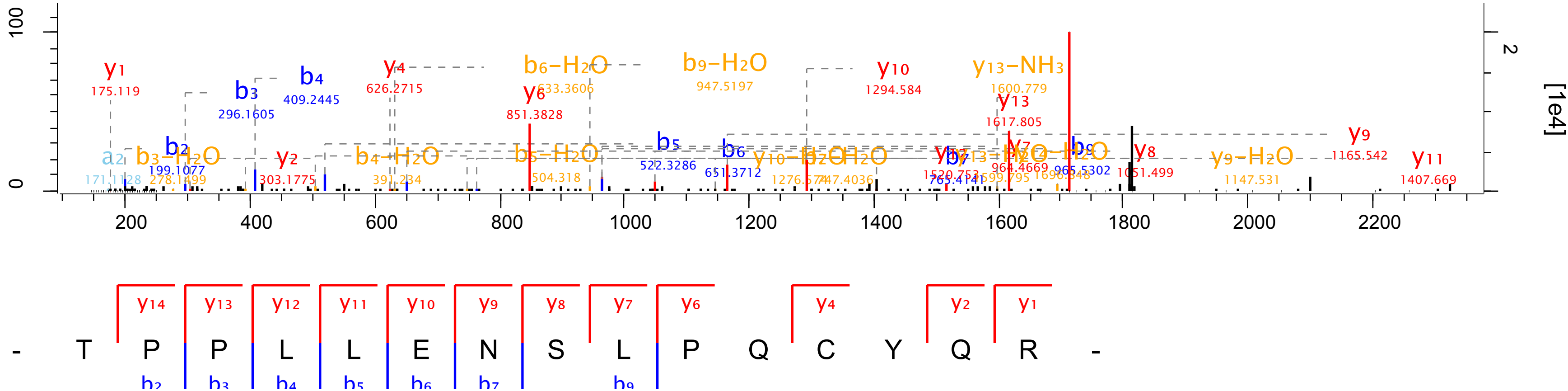

Raw file	Scan	Method	Score	m/z	Gene names
20150226_Hela_Top_opt_A3_01_1591	46308	TOF; CID	56.57	722.41	C15orf40

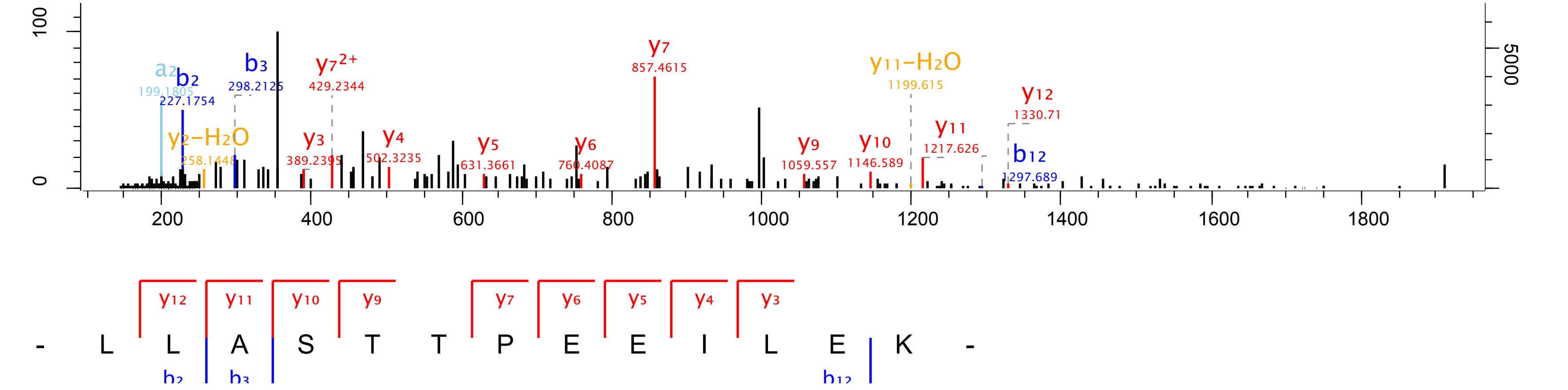

Raw file	Scan	Method	Score	m/z	Gene names
20150226_Hela_Top_opt_A3_01_1591	47650	TOF; CID	133.21	797.39	SLC38A2

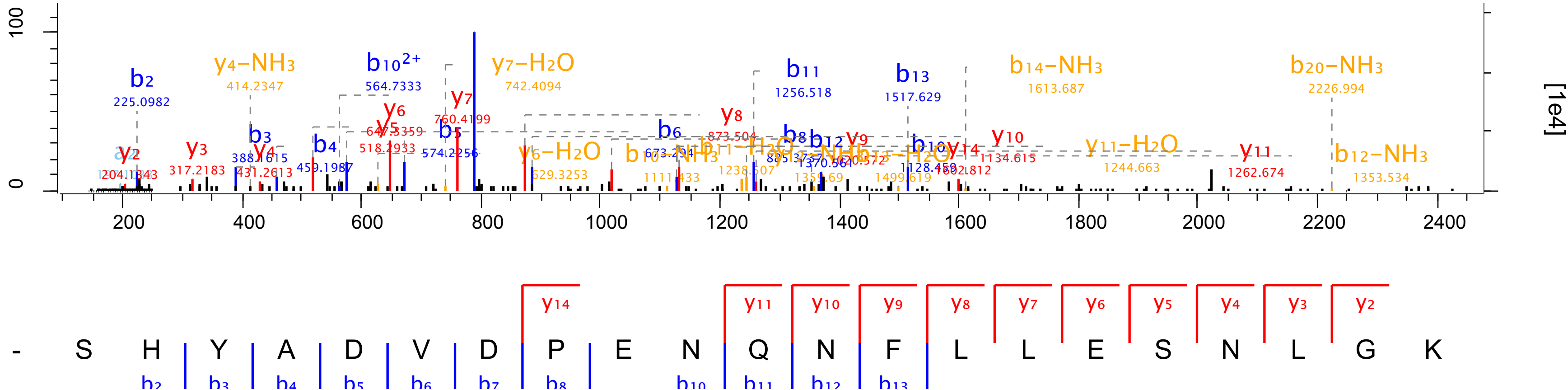

Raw file	Scan	Method	Score	m/z	Gene names
20150226_Hela_Top_opt_A3_01_1591	48943	TOF; CID	62.63	600.82	C16orf70

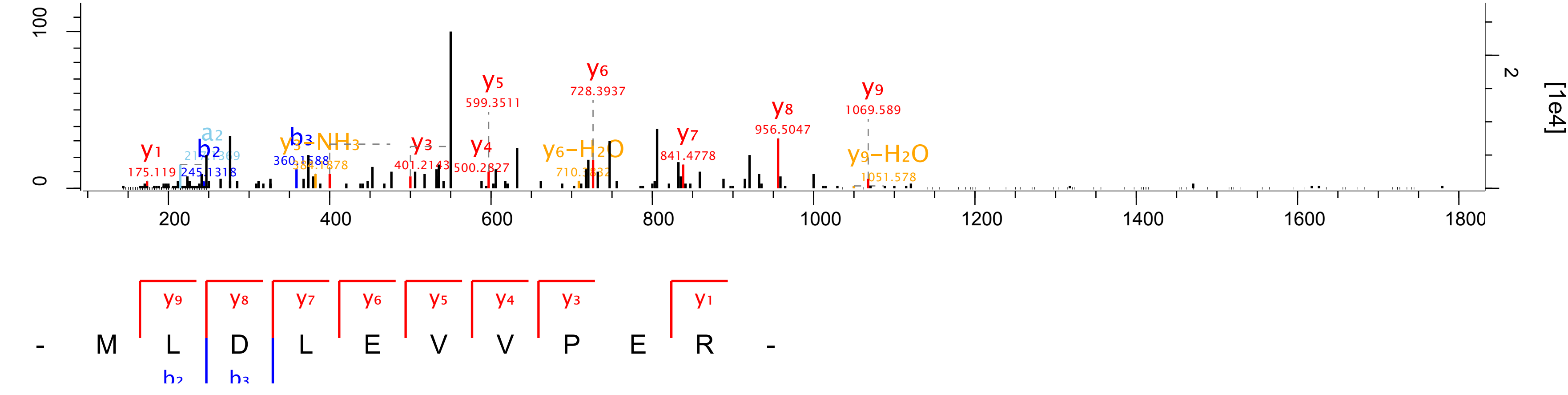

Raw file	Scan	Method	Score	m/z	Gene names
20150226_Hela_Top_opt_A3_01_1591	49082	TOF; CID	105.39	431.27	HS3ST3B1;HS3ST6;ARHGAP40;HS3ST3A1

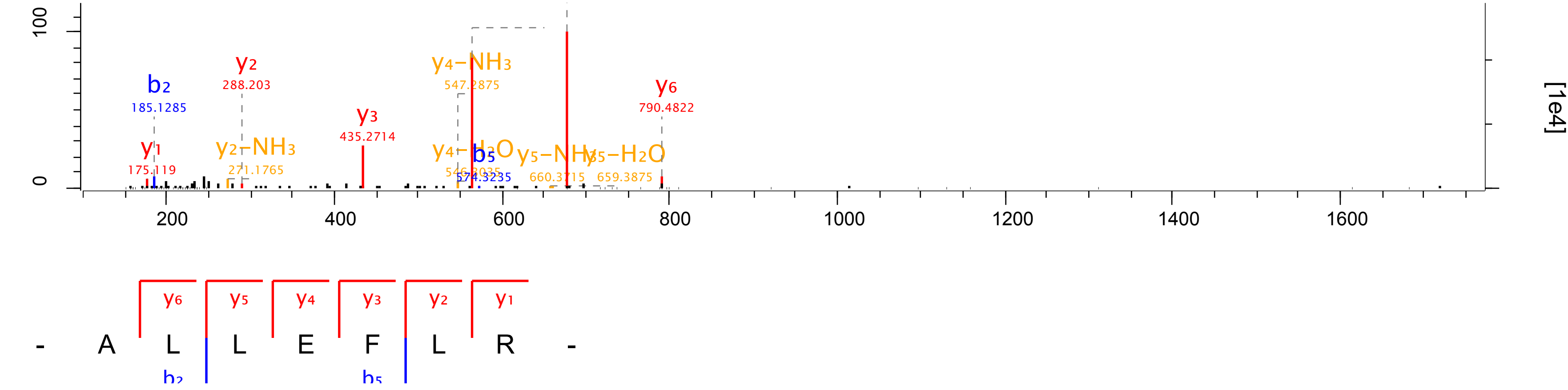

Raw file	Scan	Method	Score	m/z	Gene names
20150226_Hela_Top_opt_A3_01_1591	49341	TOF; CID	55.75	851.96	SEC14L1

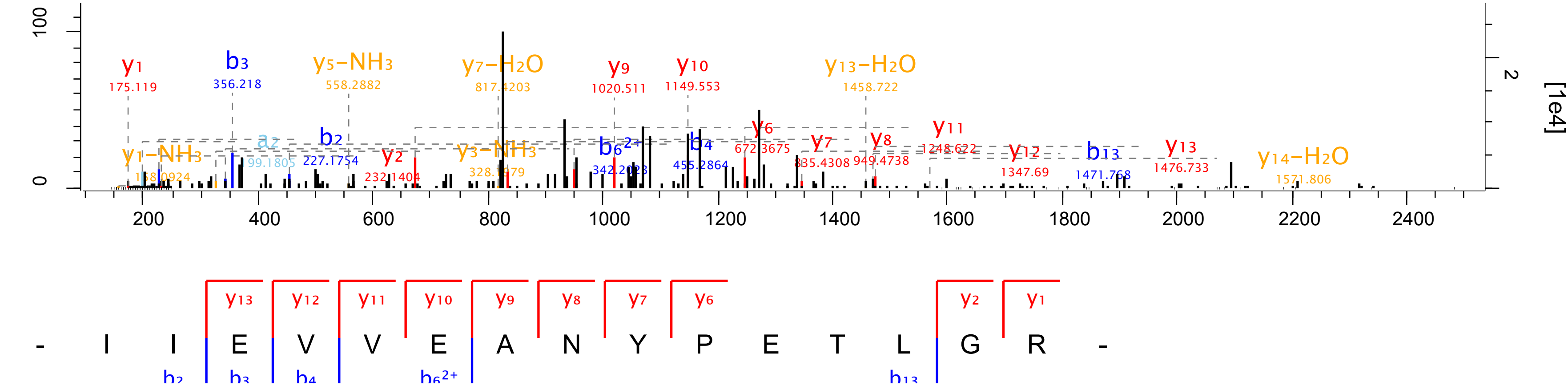

Raw file	Scan	Method	Score	m/z	Gene names
20150226_Hela_Top_opt_A3_01_1591	49343	TOF; CID	39.05	901.45	POGK

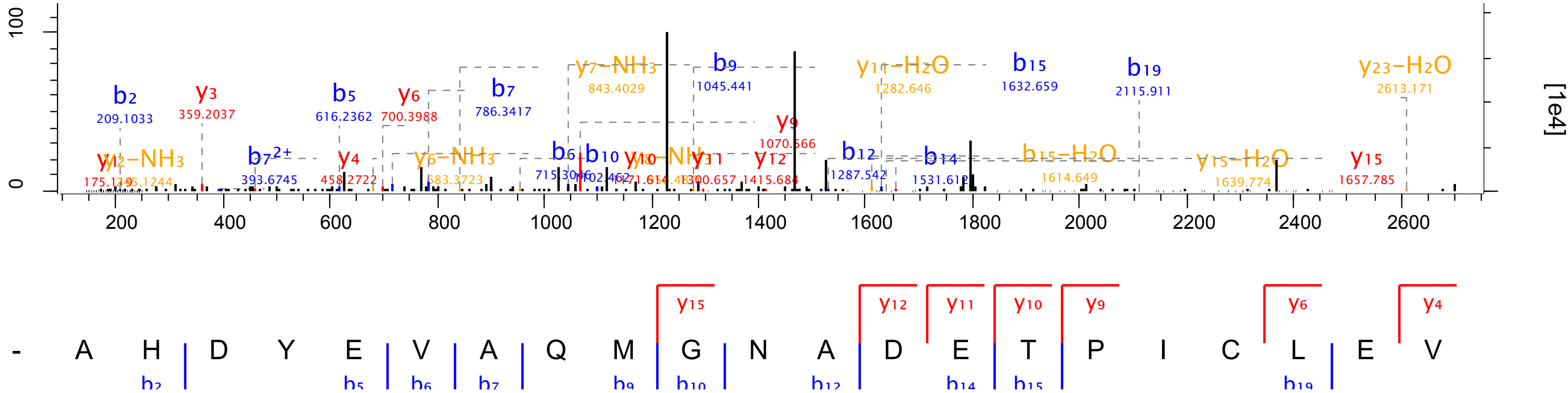

Raw file	Scan	Method	Score	m/z	Gene names
20150226_Hela_Top_opt_A3_01_1591	49516	TOF; CID	88.5	557.83	TRAPPC12

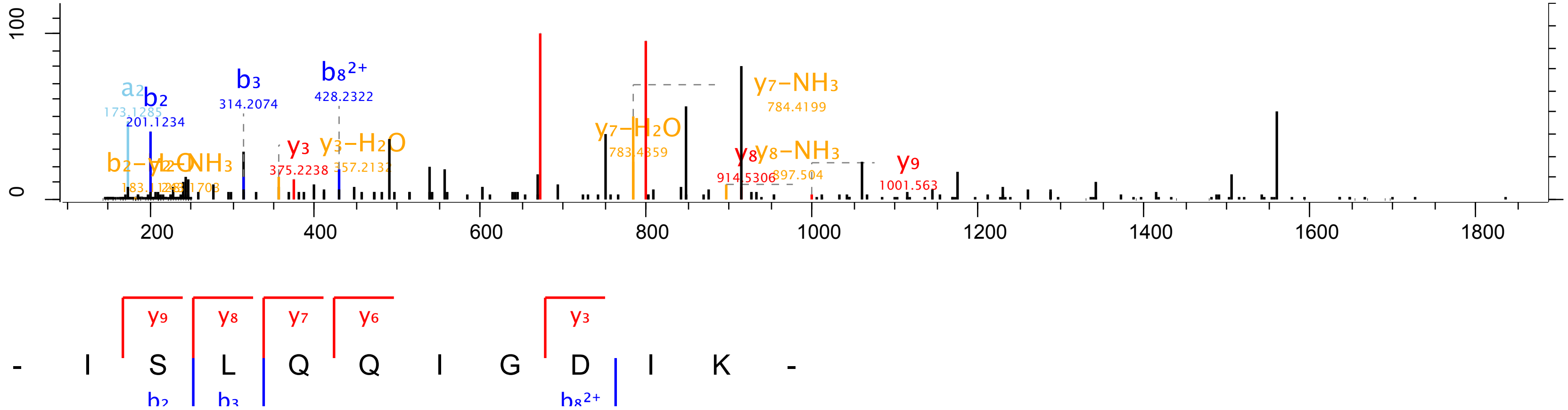

Raw file	Scan	Method	Score	m/z	Gene names
20150226_Hela_Top_opt_A3_01_1591	49820	TOF; CID	56.4	779.45	HUS1

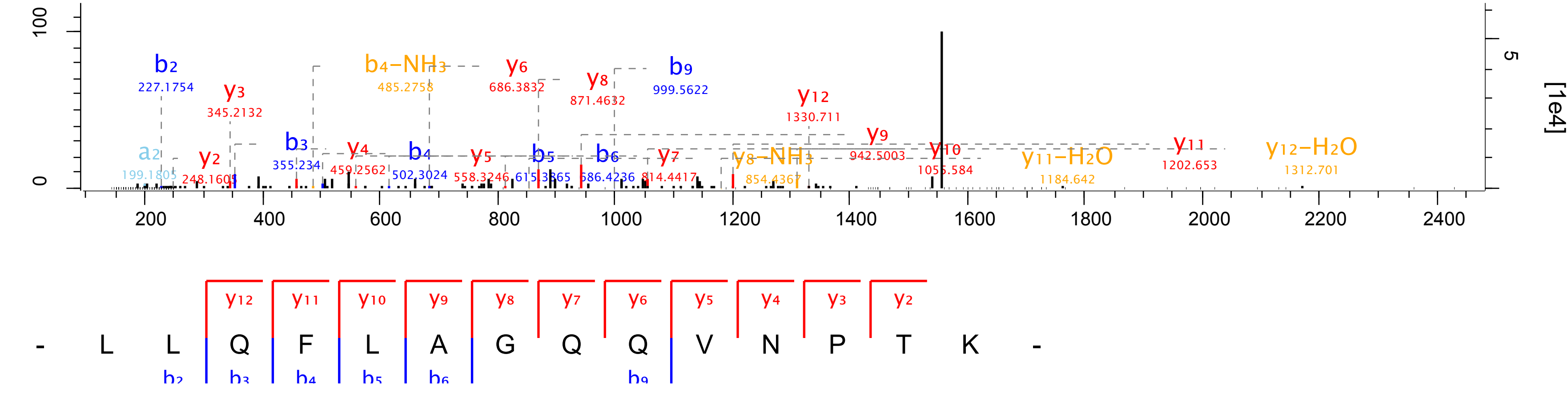

Raw file	Scan	Method	Score	m/z	Gene names
20150226_Hela_Top_opt_A3_01_1591	49860	TOF; CID	84.48	576.85	MAL2

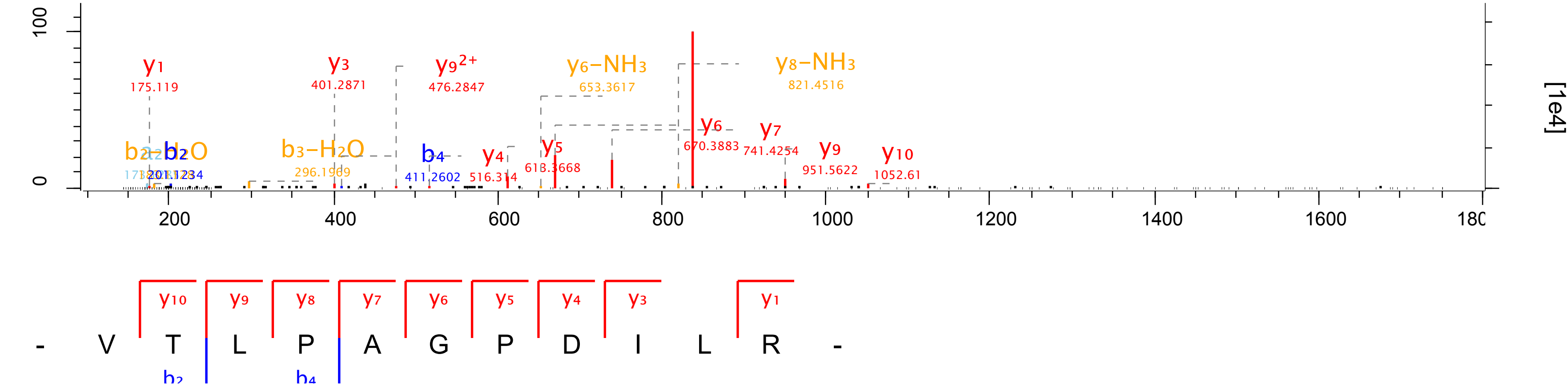

Raw file	Scan	Method	Score	m/z	Gene names
20150226_Hela_Top_opt_A3_01_1591	49880	TOF; CID	105.39	659.36	CASC4

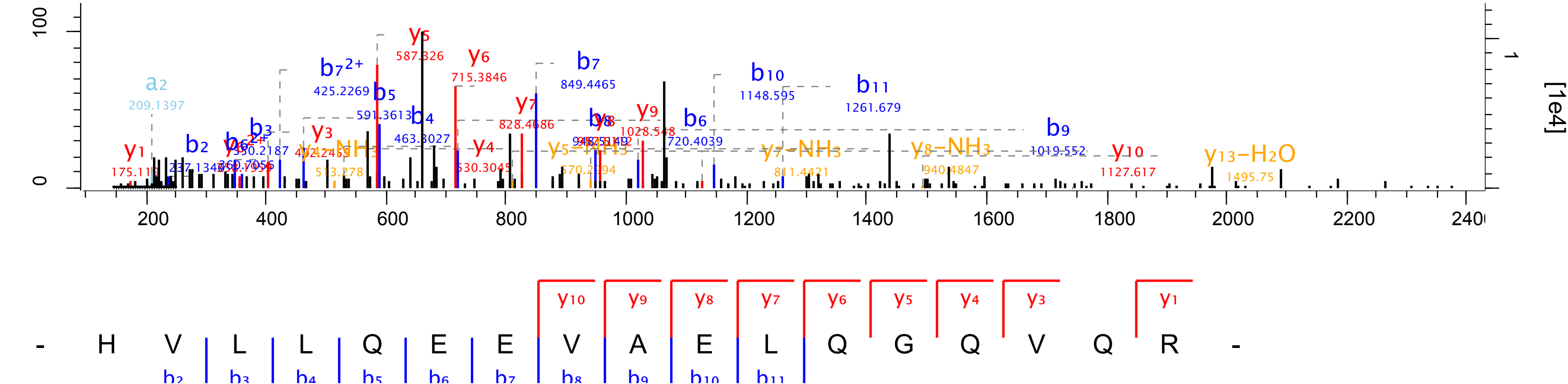

Raw file	Scan	Method	Score	m/z	Gene names
20150226_Hela_Top_opt_A3_01_1591	49943	TOF; CID	76.68	496.76	SEPT3

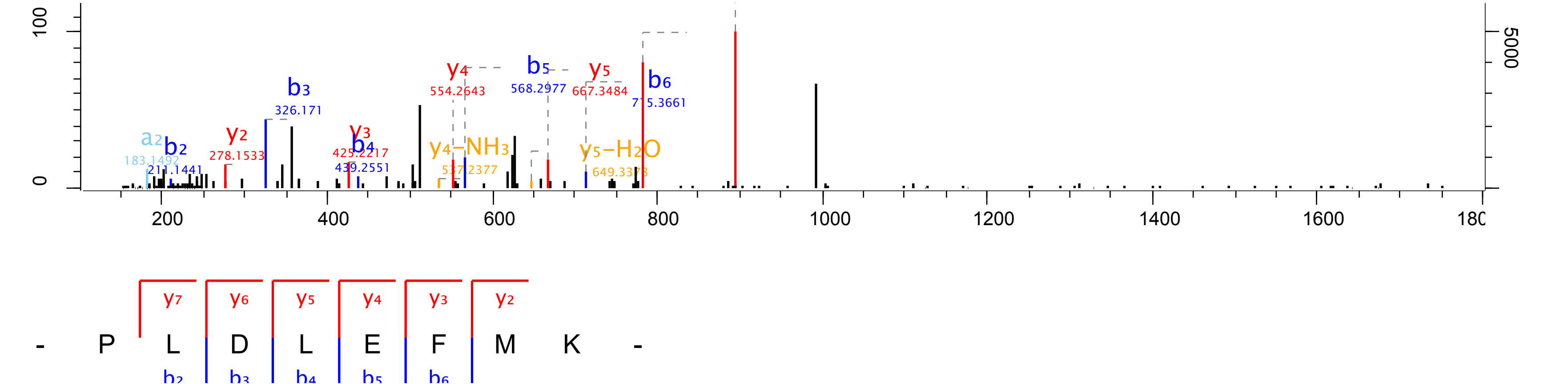

Raw file	Scan	Method	Score	m/z	Gene names
20150226_Hela_Top_opt_A3_01_1591	50128	TOF; CID	61.48	630.36	CT83

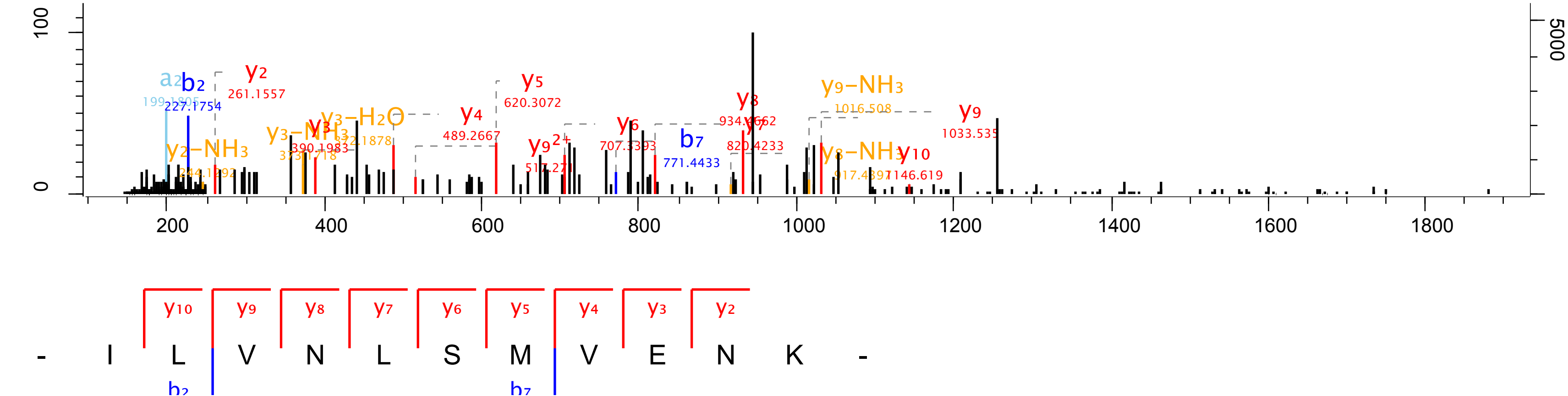

Raw file	Scan	Method	Score	m/z	Gene names
20150226_Hela_Top_opt_A3_01_1591	50897	TOF; CID	55.3	846.48	KLF5

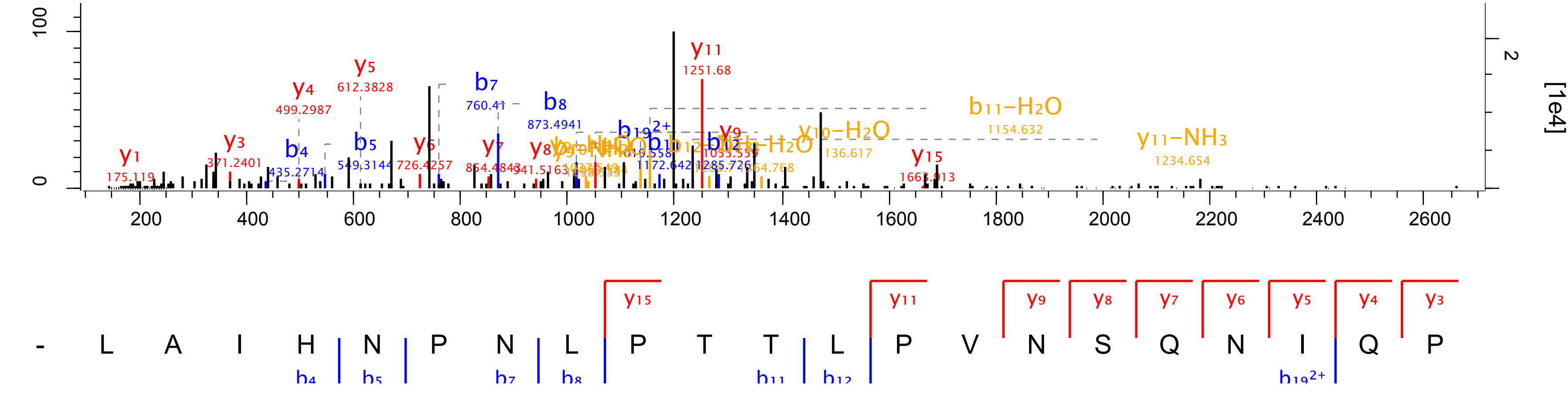

Raw file	Scan	Method	Score	m/z	Gene names
20150226_Hela_Top_opt_A3_01_1591	51226	TOF; CID	65.84	687.86	VTI1A

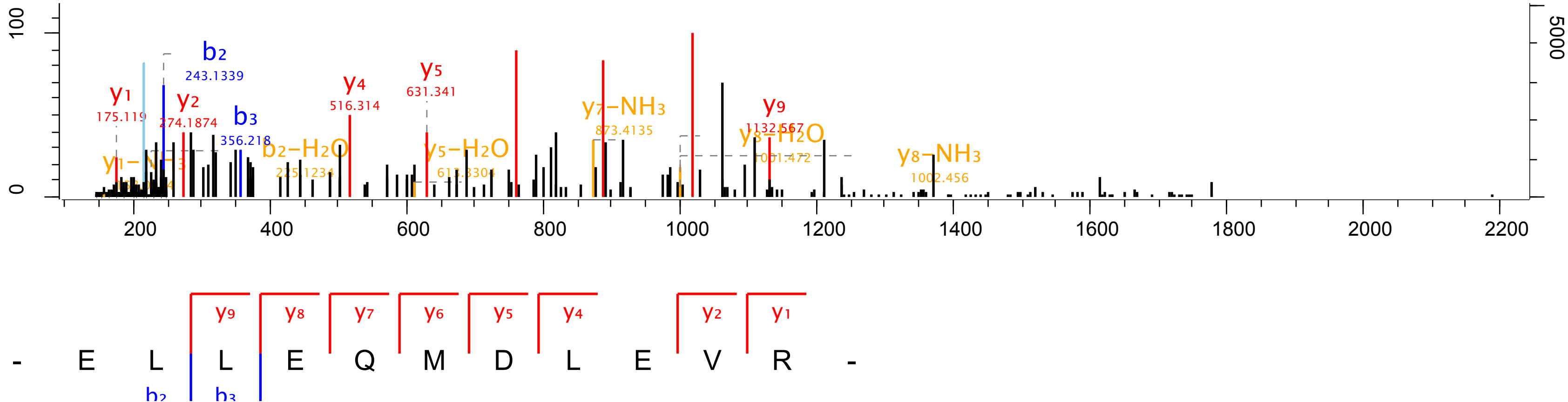

Raw file	Scan	Method	Score	m/z	Gene names
20150226_Hela_Top_opt_A3_01_1591	51422	TOF; CID	43.91	948.45	ABCB1

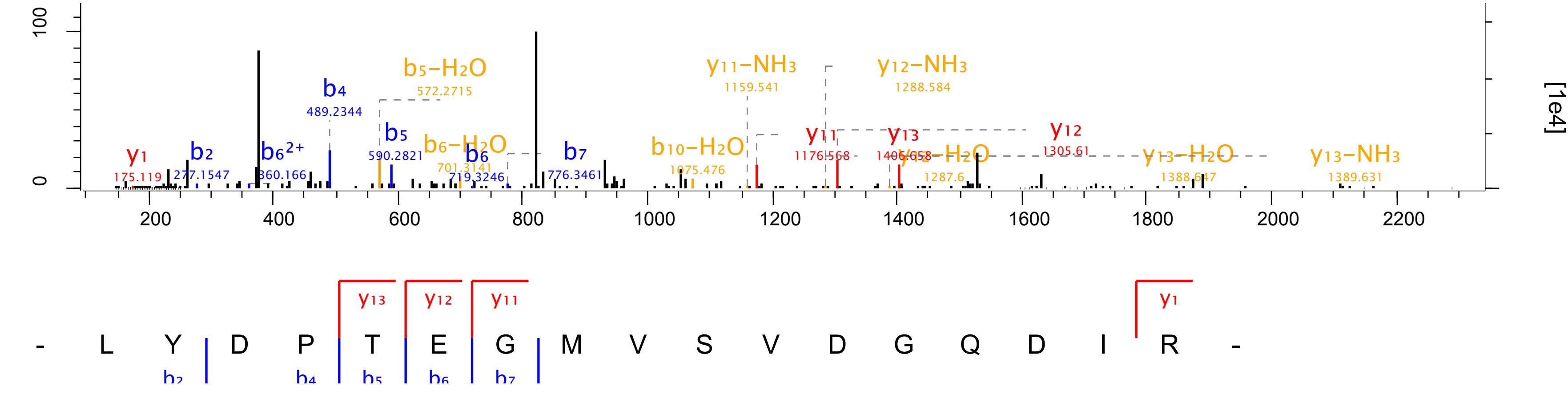

Raw file	Scan	Method	Score	m/z	Gene names
20150226_Hela_Top_opt_A3_01_1591	51557	TOF; CID	110.81	499.28	ITGA6

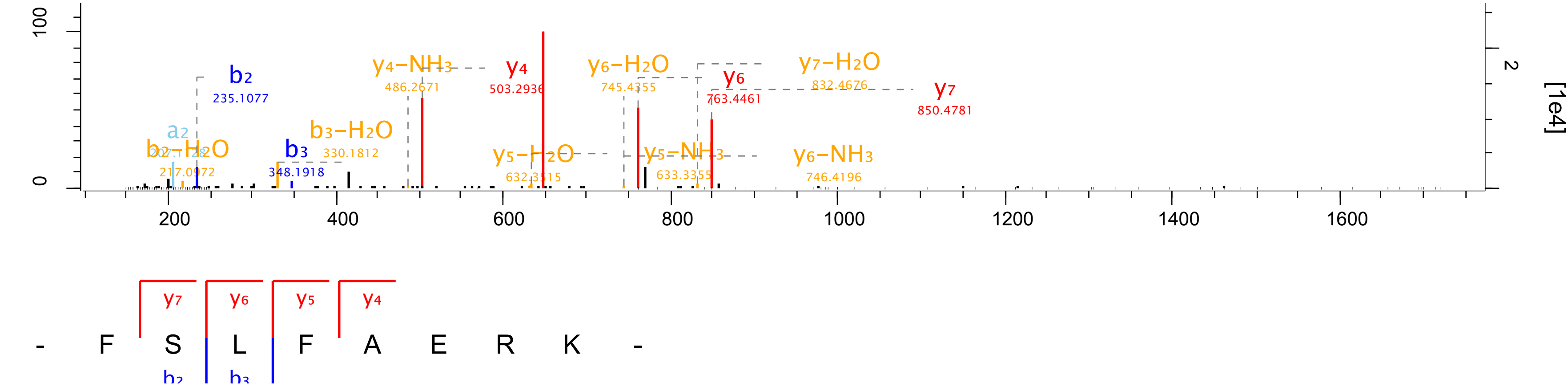

Raw file	Scan	Method	Score	m/z	Gene names
20150226_Hela_Top_opt_A3_01_1591	54321	TOF; CID	78.32	699.36	DPP7

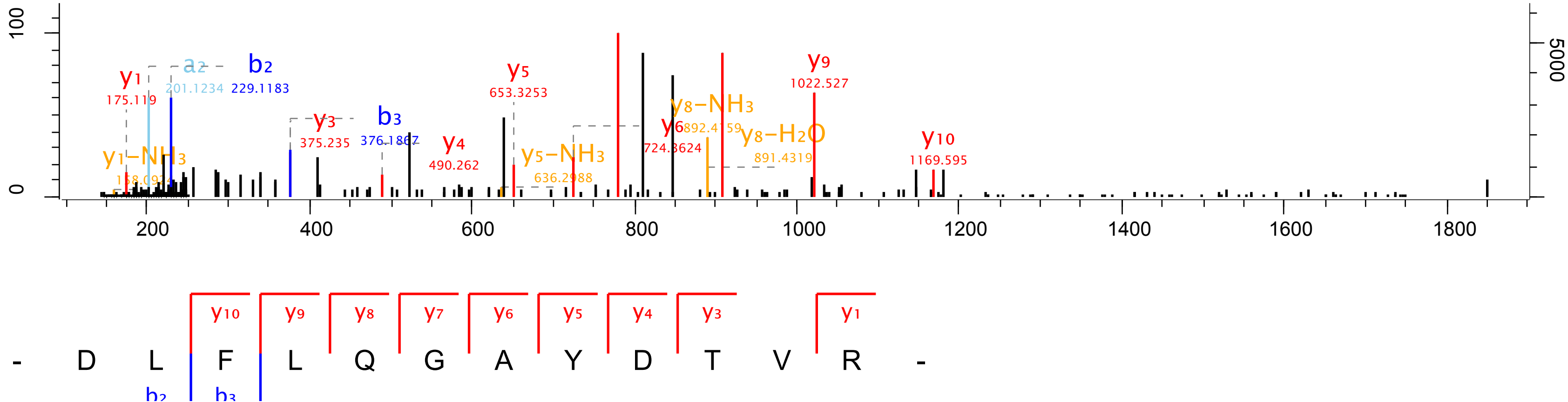

Raw file	Scan	Method	Score	m/z	Gene names
20150226_Hela_Top_opt_A3_01_1591	55072	TOF; CID	74.65	743.94	C20orf27

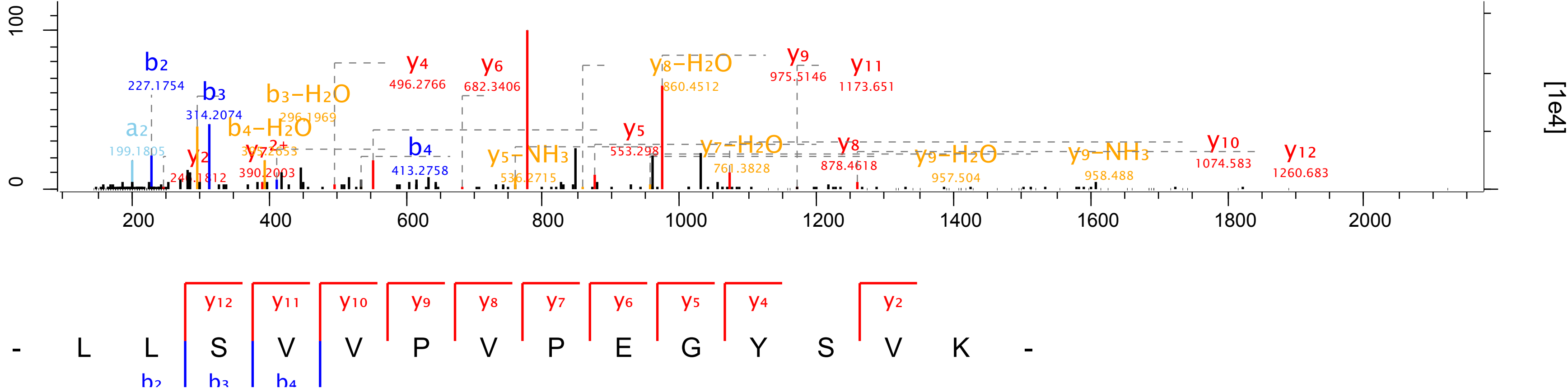

Raw file	Scan	Method	Score	m/z	Gene names
20150226_Hela_Top_opt_A3_01_1591	55094	TOF; CID	135.09	928.95	MAPK1IP1L

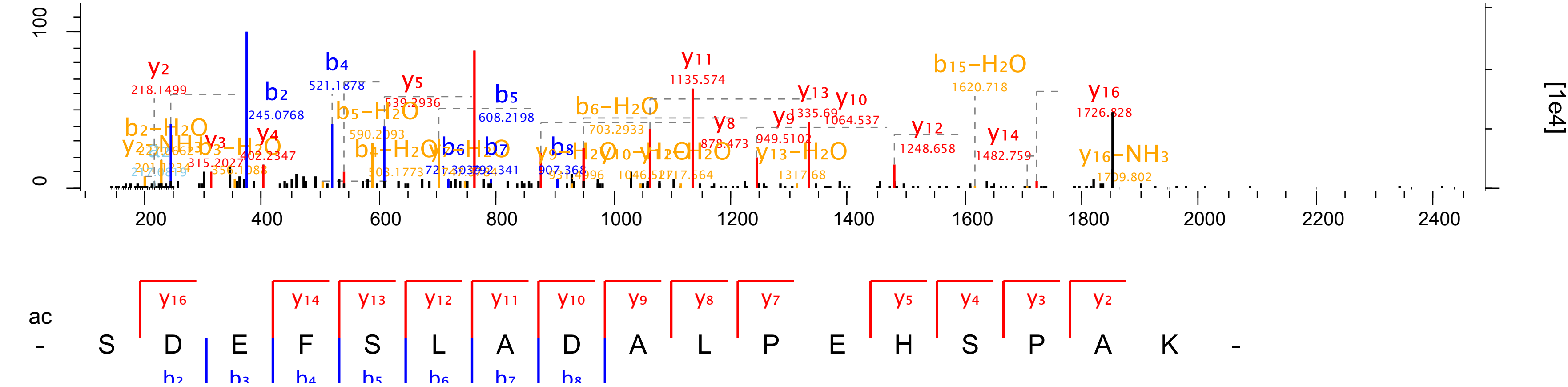

Raw file	Scan	Method	Score	m/z	Gene names
20150226_Hela_Top_opt_A3_01_1591	55101	TOF; CID	63.22	503.8	OARD1

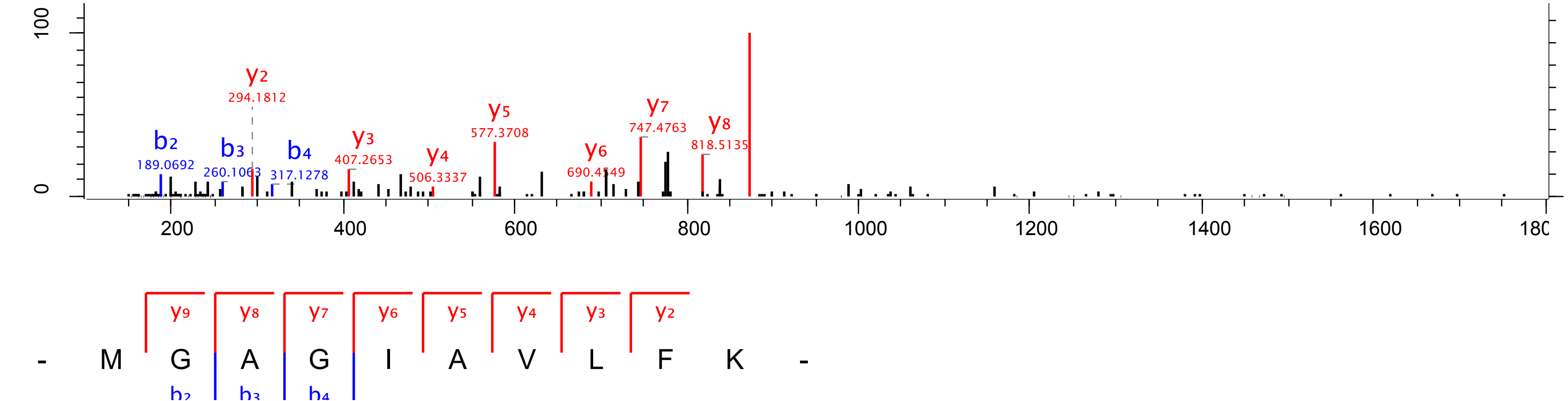

Raw file	Scan	Method	Score	m/z	Gene names
20150226_Hela_Top_opt_A3_01_1591	56668	TOF; CID	70.09	703.89	ISCA2

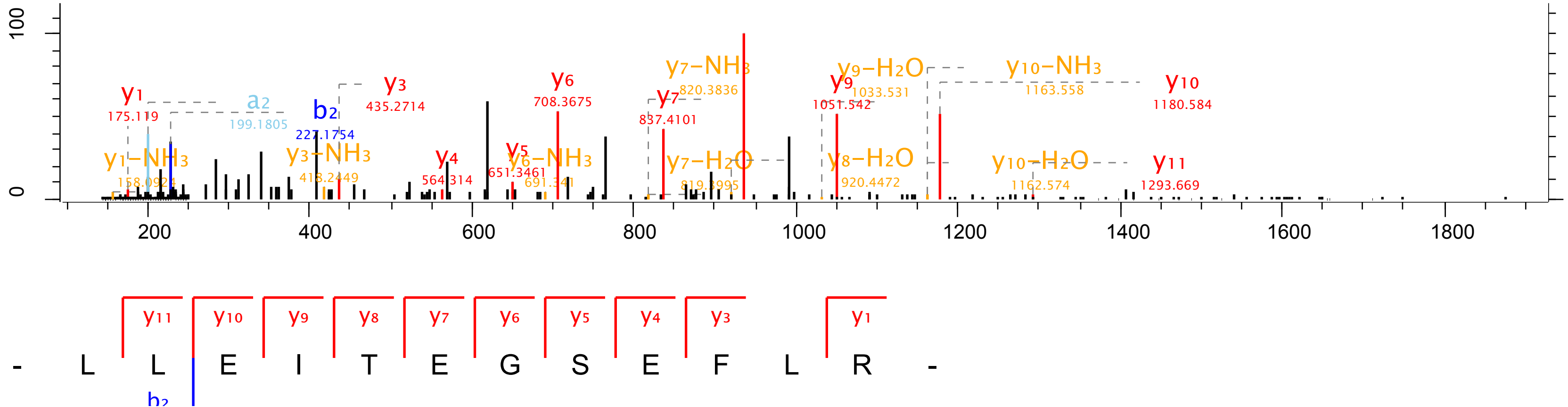

Raw file	Scan	Method	Score	m/z	Gene names
20150226_Hela_Top_opt_A3_01_1591	57582	TOF; CID	104.84	937.51	ZFP36L2

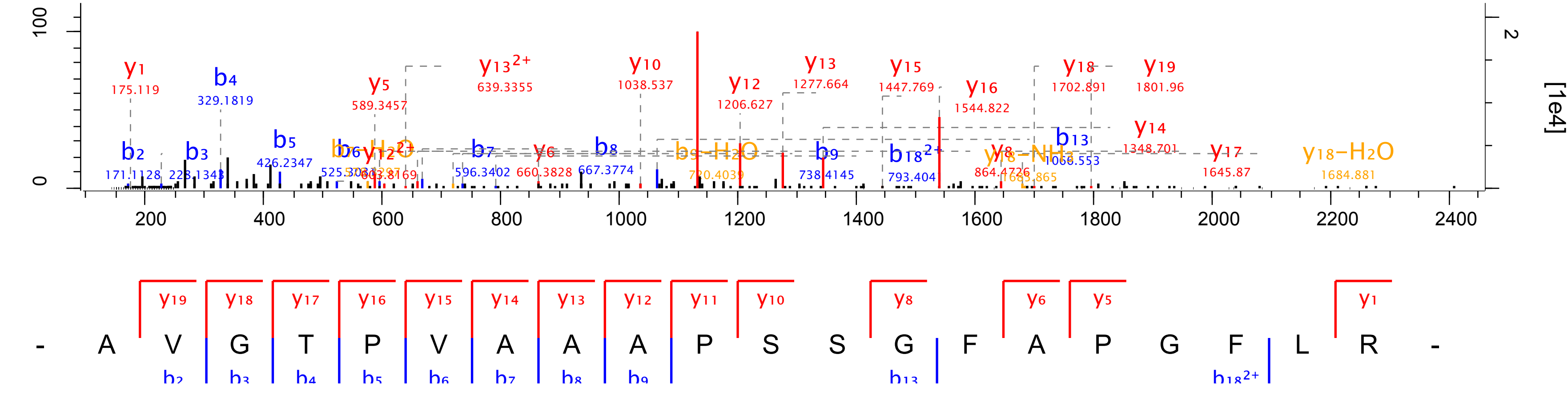

Raw file	Scan	Method	Score	m/z	Gene names
20150226_Hela_Top_opt_A3_01_1591	57585	TOF; CID	58.48	802.07	MTHFSD

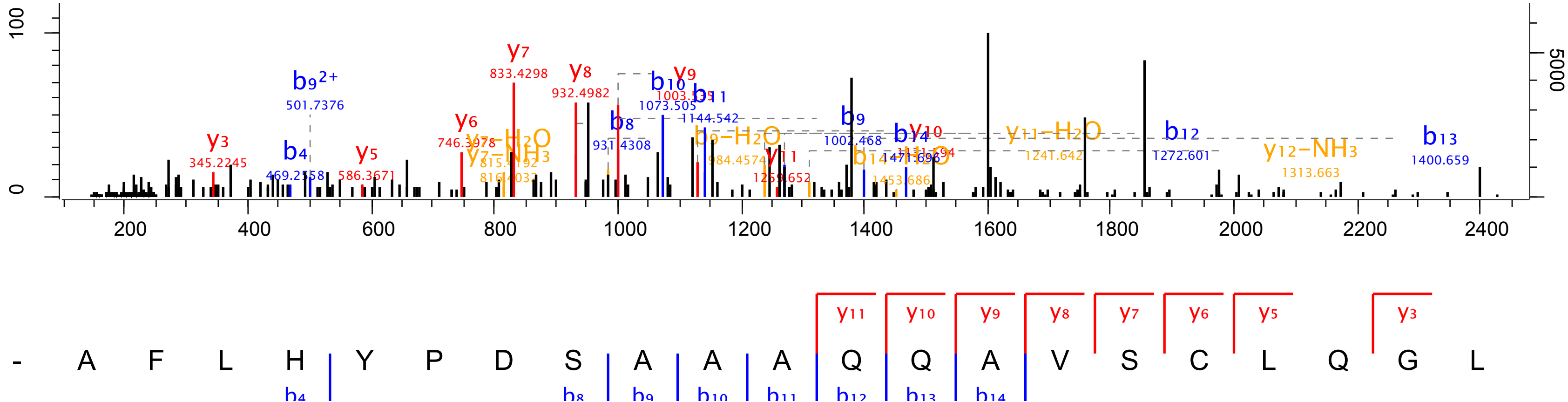

Raw file	Scan	Method	Score	m/z	Gene names
20150226_Hela_Top_opt_A3_01_1591	58106	TOF; CID	118.37	1294.6	MRPL42

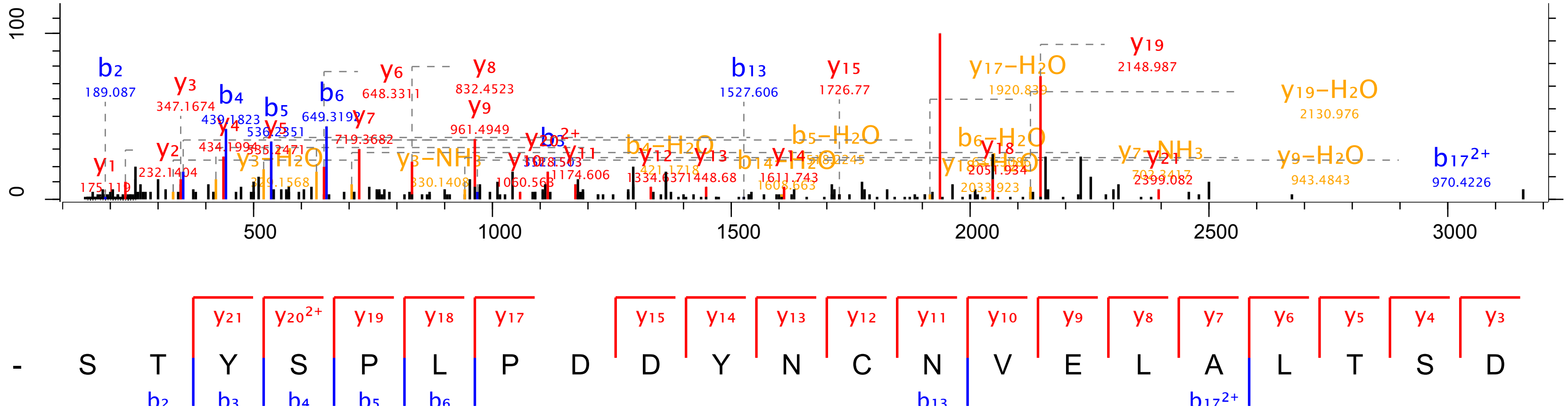

Raw file	Scan	Method	Score	m/z	Gene names
20150226_Hela_Top_opt_A3_01_1591	58828	TOF; CID	78.26	501.31	VRK3

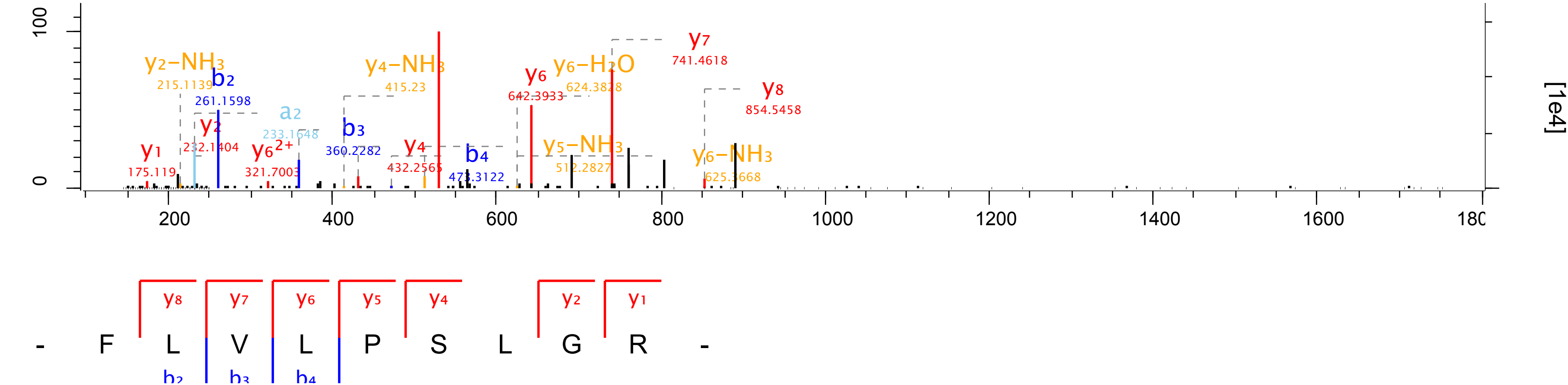

Raw file	Scan	Method	Score	m/z	Gene names
20150226_Hela_Top_opt_A3_01_1591	58835	TOF; CID	119.57	464.81	B4GALT5

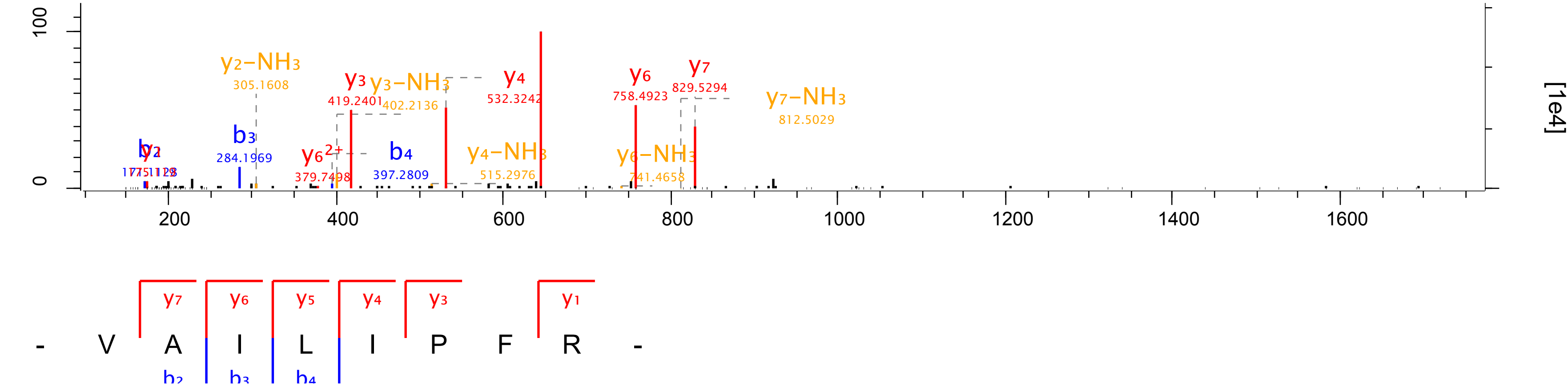

Raw file	Scan	Method	Score	m/z	Gene names
20150226_Hela_Top_opt_A3_01_1591	59090	TOF; CID	87.67	577.35	TMEM65

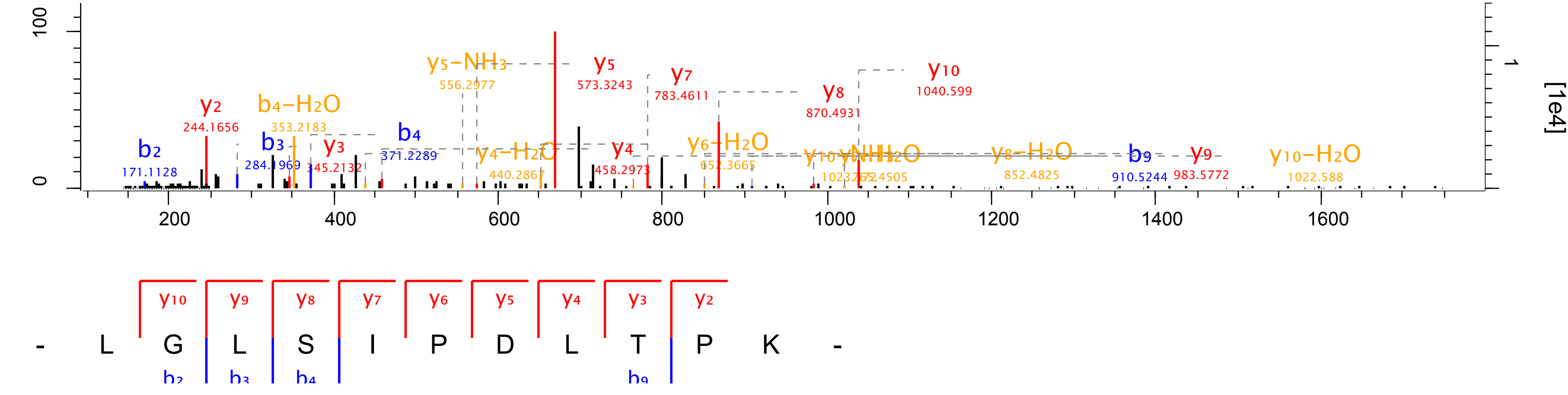

Raw file	Scan	Method	Score	m/z	Gene names
20150226_Hela_Top_opt_A3_01_1591	59172	TOF; CID	51.57	652.86	HSD17B12

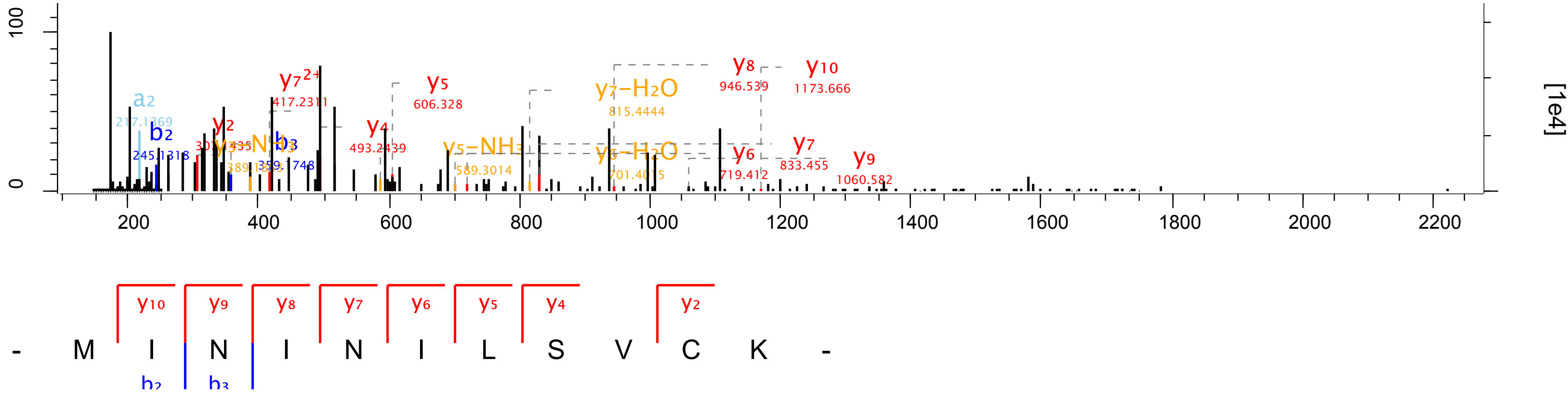

Raw file	Scan	Method	Score	m/z	Gene names
20150226_Hela_Top_opt_A3_01_1591	59348	TOF; CID	91.82	1138.57	FAM195A

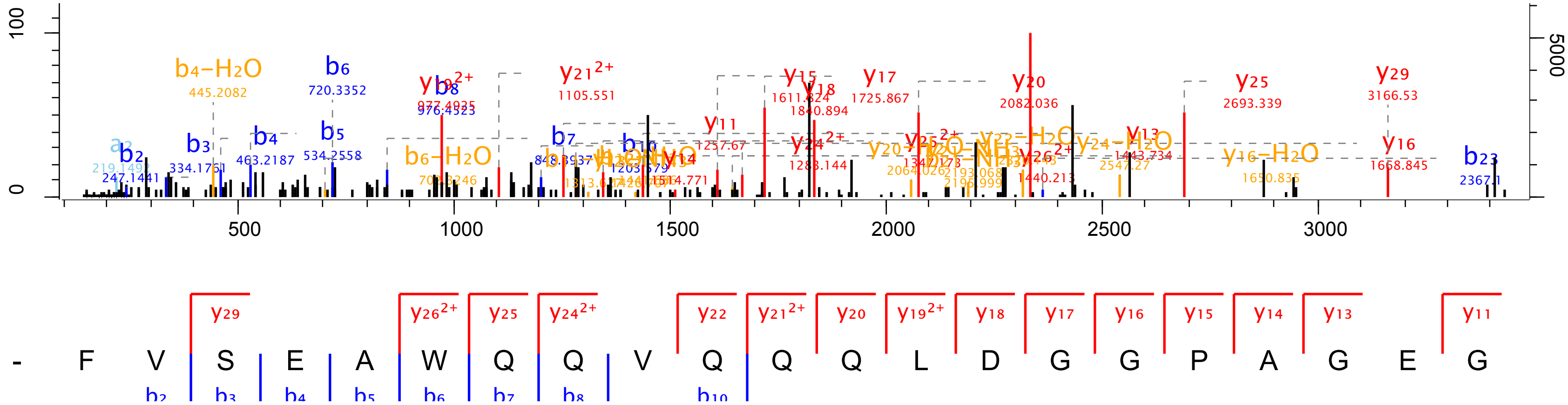

Raw file	Scan	Method	Score	m/z	Gene names
20150226_Hela_Top_opt_A3_01_1591	59665	TOF; CID	38.59	935.17	C1orf85

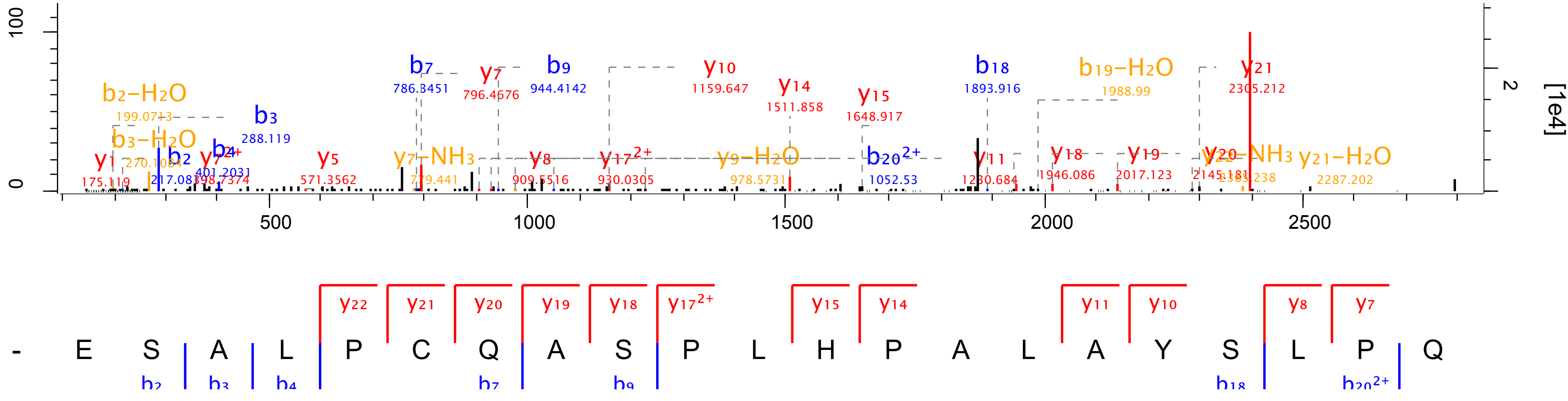

Raw file	Scan	Method	Score	m/z	Gene names
20150226_Hela_Top_opt_A3_01_1591	60044	TOF; CID	77.29	634.88	CEPT1

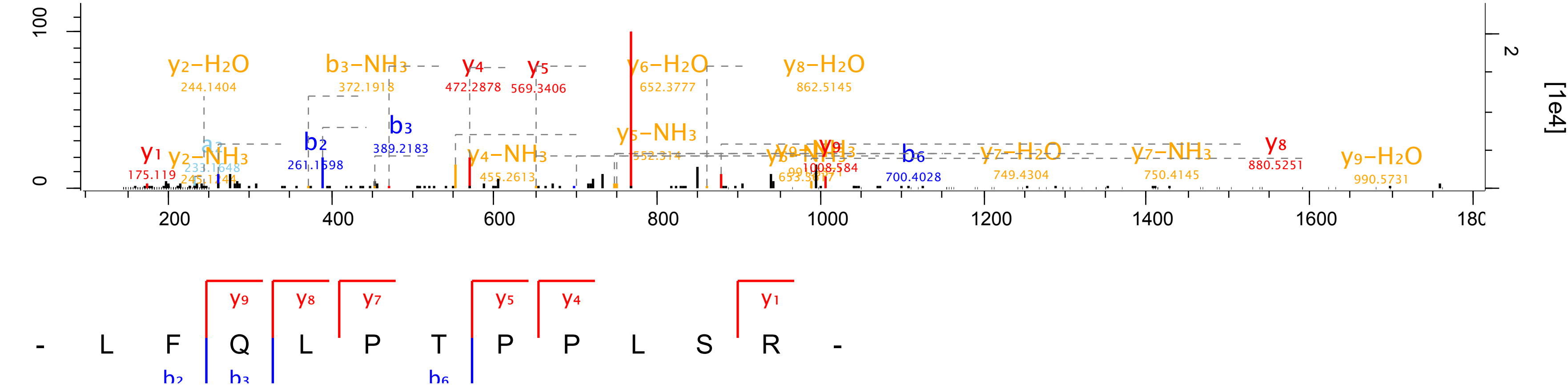

Raw file	Scan	Method	Score	m/z	Gene names
20150226_Hela_Top_opt_A3_01_1591	60104	TOF; CID	98.59	539.29	ZCCHC6

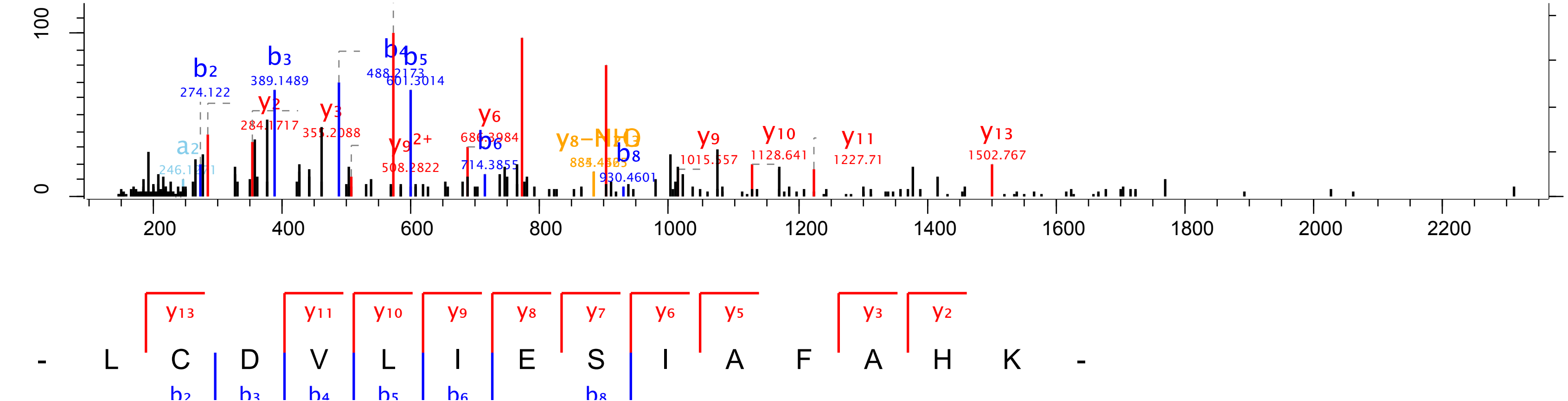

Raw file	Scan	Method	Score	m/z	Gene names
20150226_Hela_Top_opt_A3_01_1591	60243	TOF; CID	76.9	1126.19	DERL2

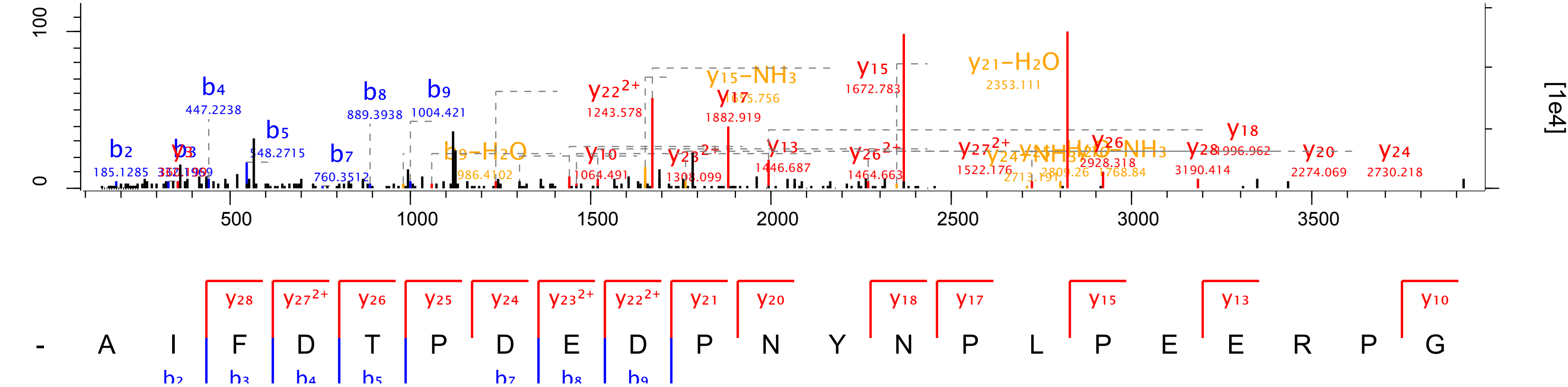

Raw file	Scan	Method	Score	m/z	Gene names
20150226_Hela_Top_opt_A3_01_1591	61175	TOF; CID	87.48	527.83	SLC29A1

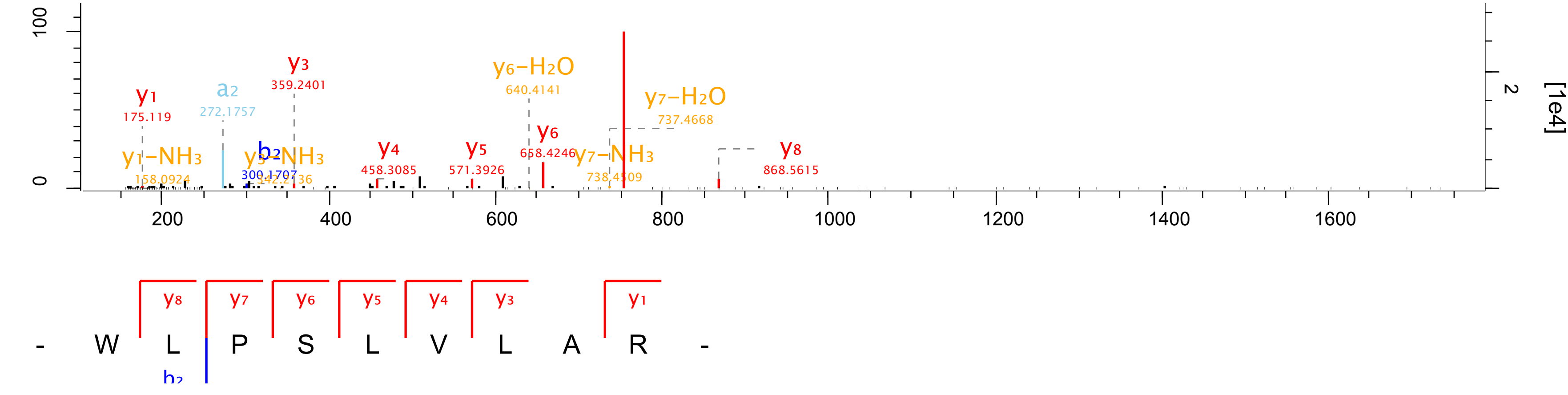

Raw file	Scan	Method	Score	m/z	Gene names
20150226_Hela_Top_opt_A3_01_1591	64049	TOF; CID	57.35	860.42	SWI5

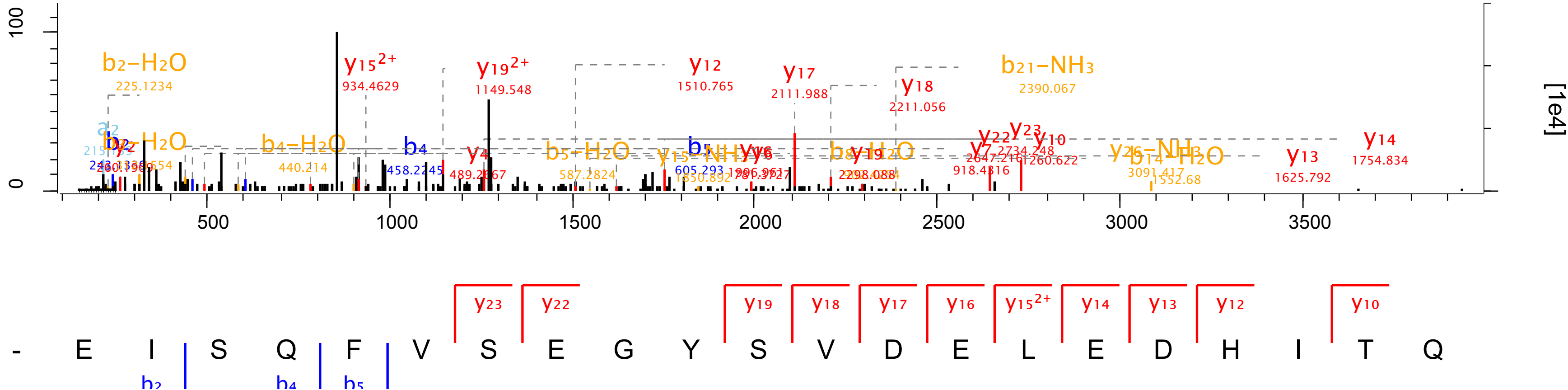

Raw file	Scan	Method	Score	m/z	Gene names
20150226_Hela_Top_opt_A3_01_1591	64207	TOF; CID	87.72	989.53	AES

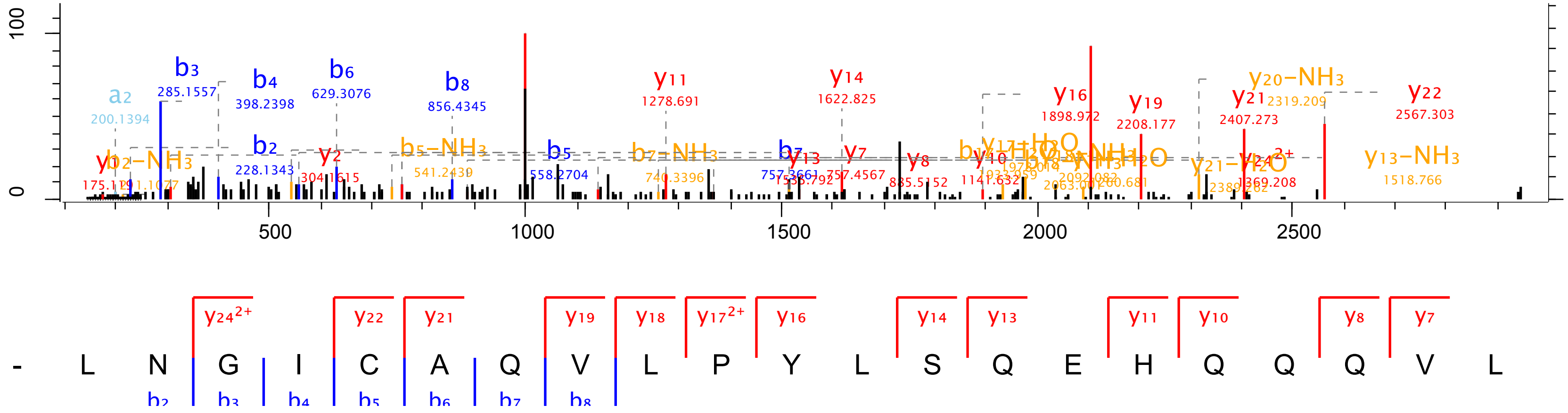

Raw file	Scan	Method	Score	m/z	Gene names
20150226_Hela_Top_opt_A3_01_1591	64248	TOF; CID	65.8	1100.54	MAPK9

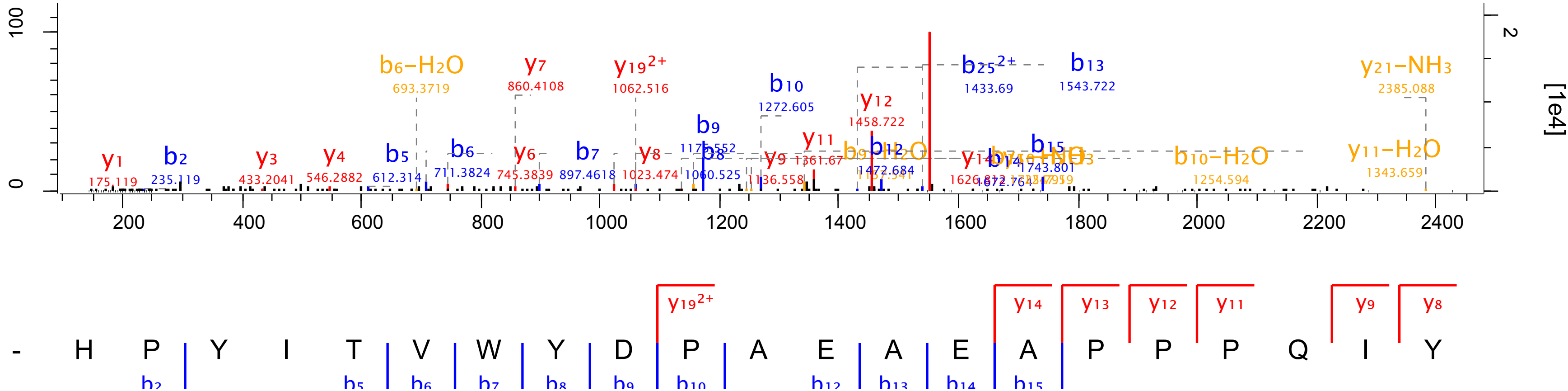

Raw file	Scan	Method	Score	m/z	Gene names
20150226_Hela_Top_opt_A3_01_1591	64961	TOF; CID	61.11	758.42	RILPL1

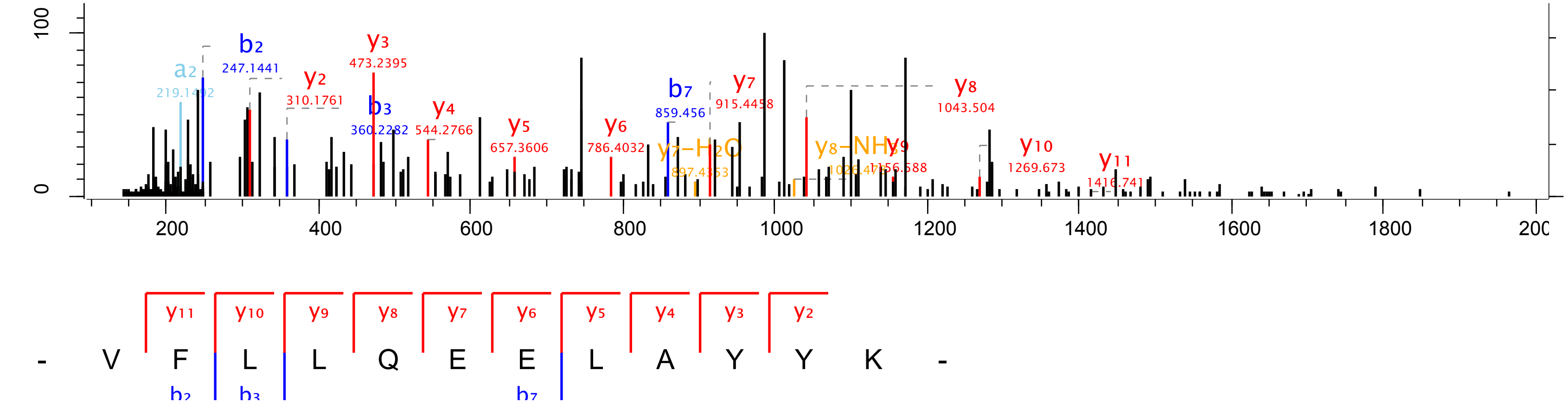

Raw file	Scan	Method	Score	m/z	Gene names
20150226_Hela_Top_opt_A3_01_1591	65069	TOF; CID	50.3	861.46	FAM188A

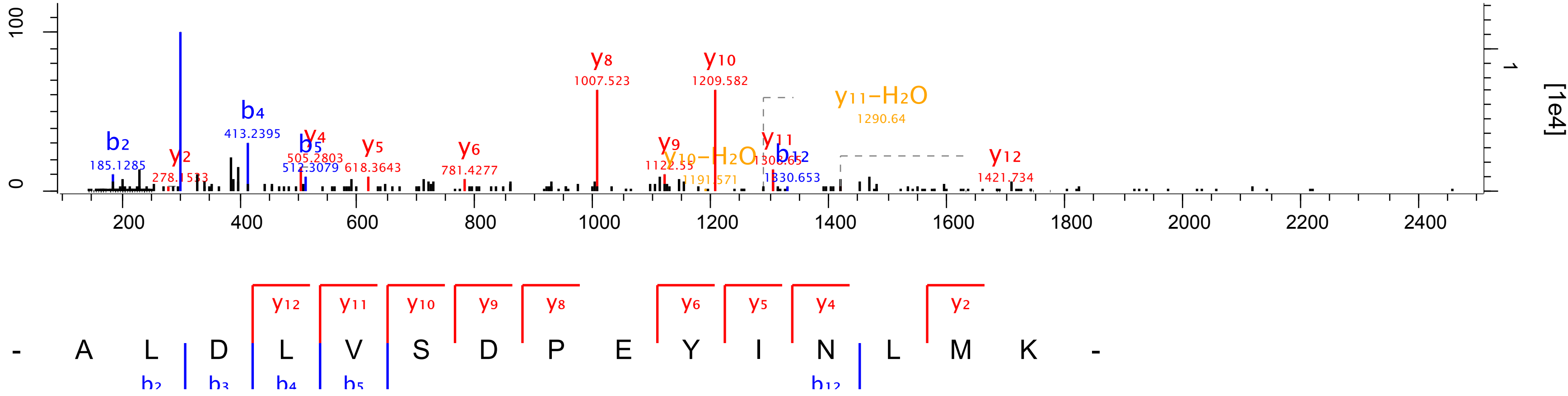

Raw file	Scan	Method	Score	m/z	Gene names
20150226_Hela_Top_opt_A3_01_1591	65129	TOF; CID	70.26	818.43	ZC3H7B

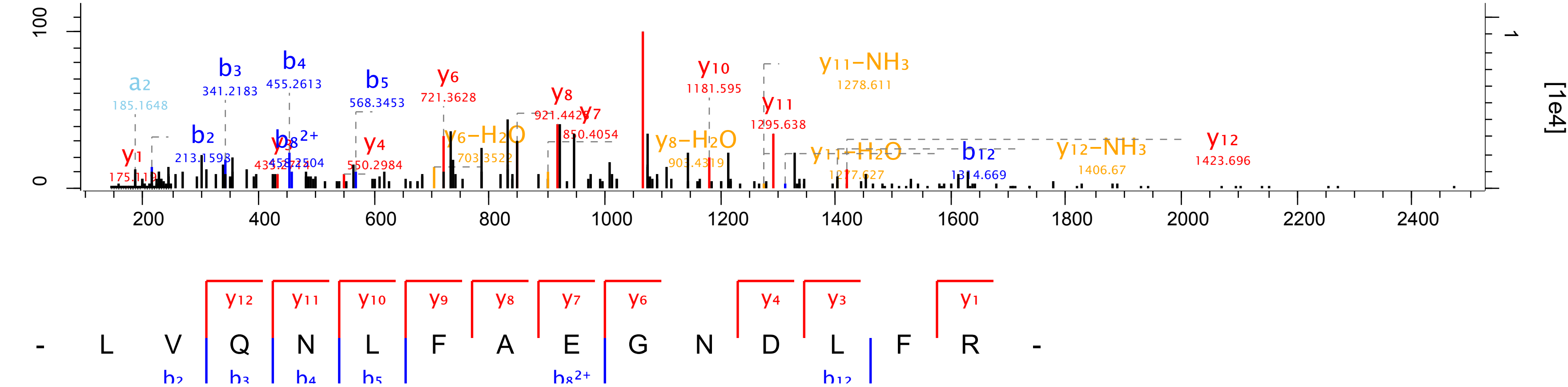

Raw file	Scan	Method	Score	m/z	Gene names
20150226_Hela_Top_opt_A3_01_1591	65247	TOF; CID	66.06	615.83	SNAP29

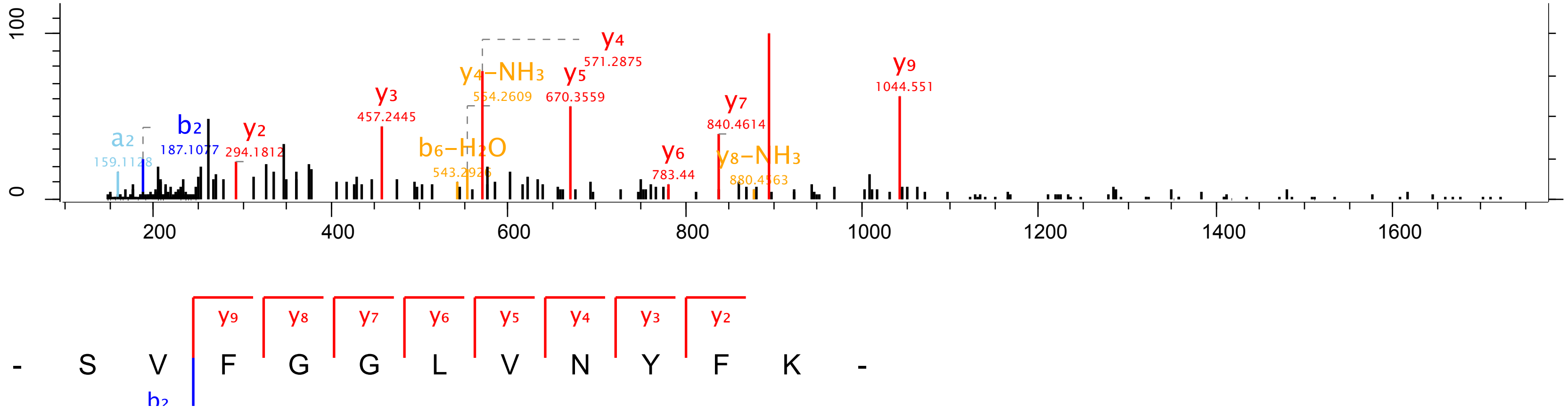

Raw file	Scan	Method	Score	m/z	Gene names
20150226_Hela_Top_opt_A3_01_1591	65587	TOF; CID	65.41	664.34	TRAPPC6A

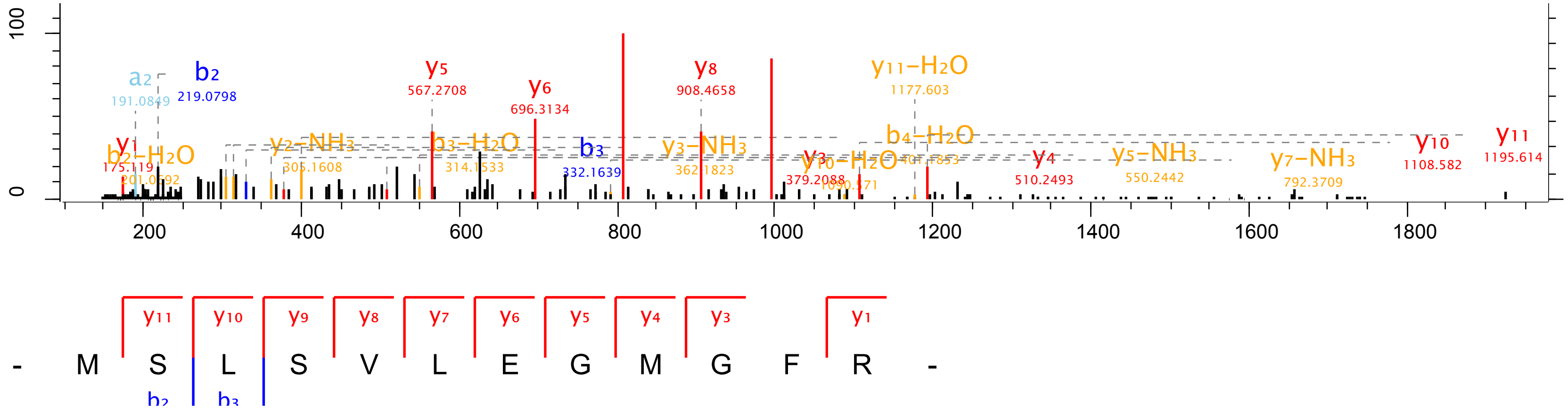

Raw file	Scan	Method	Score	m/z	Gene names
20150226_Hela_Top_opt_A3_01_1591	65669	TOF; CID	72.43	952.47	ZFYVE21

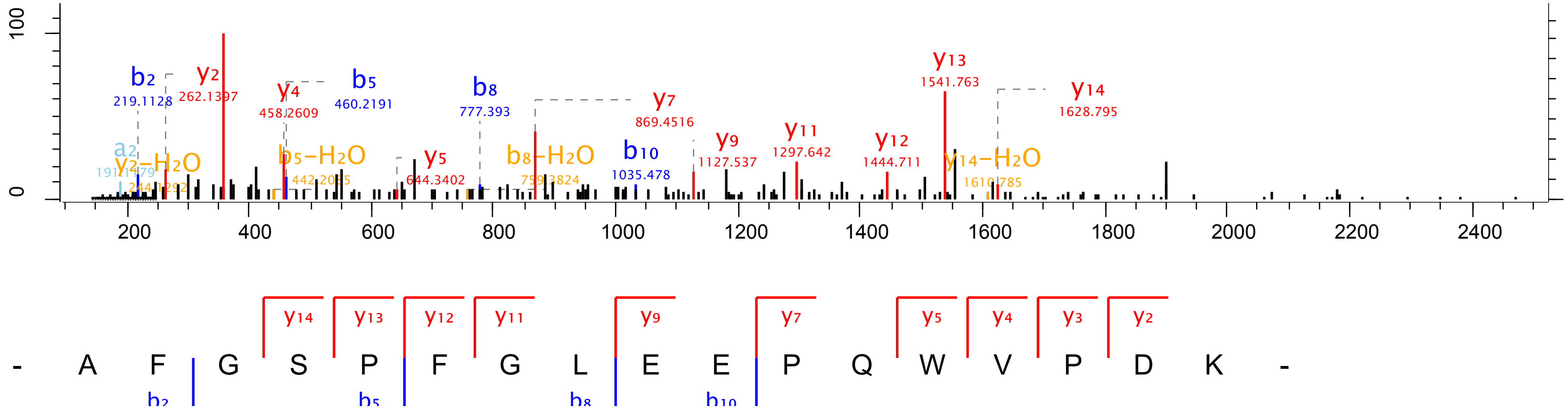

Raw file	Scan	Method	Score	m/z	Gene names
20150226_Hela_Top_opt_A3_01_1591	65859	TOF; CID	84.51	1110.53	TRIO

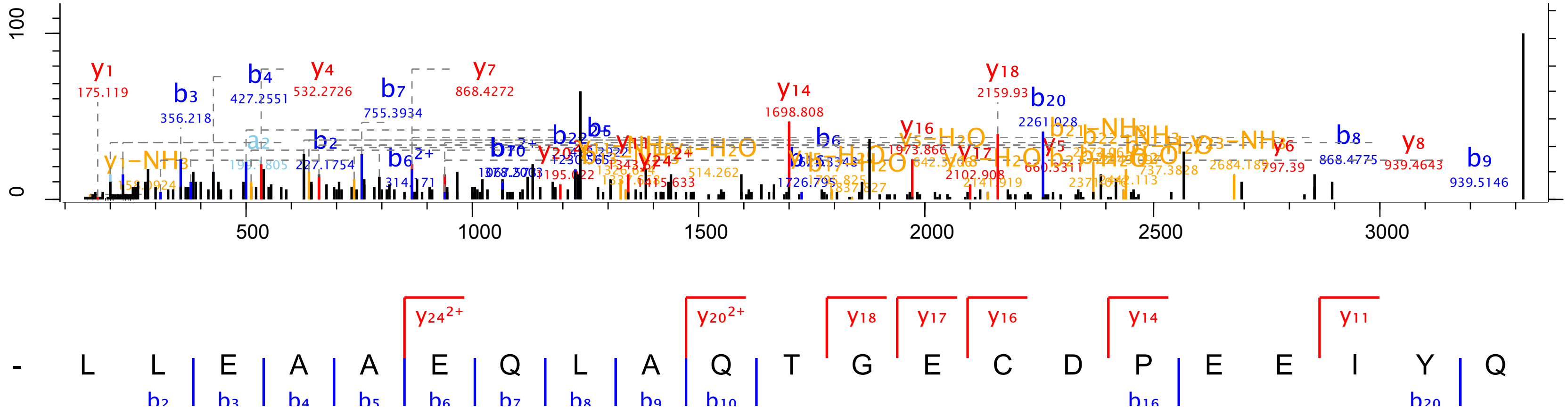

Raw file	Scan	Method	Score	m/z	Gene names
20150226_Hela_Top_opt_A3_01_1591	66206	TOF; CID	48.39	868.45	MTFR1L

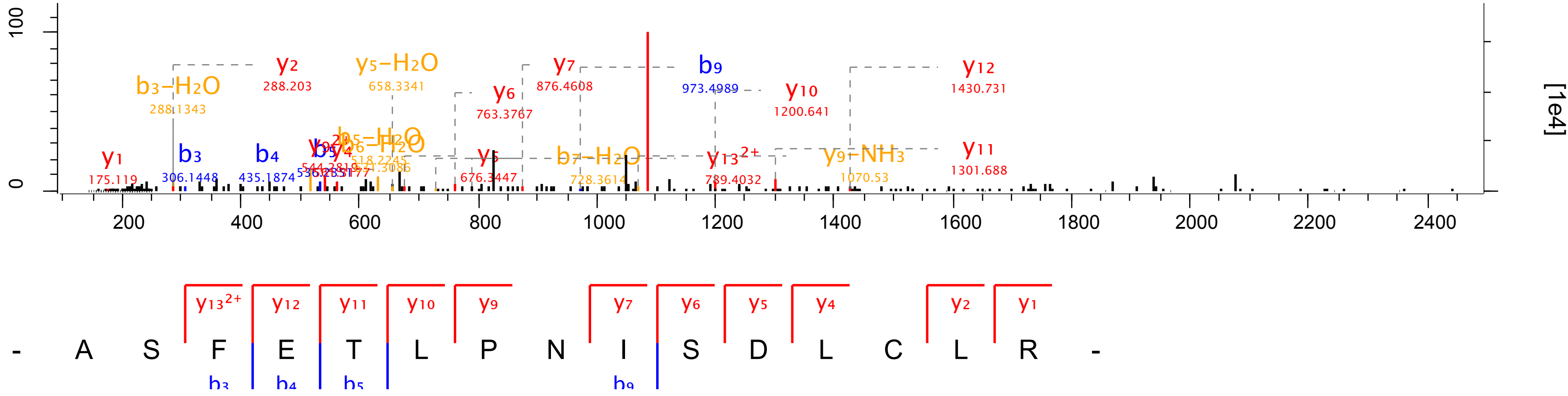

Raw file	Scan	Method	Score	m/z	Gene names
20150226_Hela_Top_opt_A3_01_1591	66453	TOF; CID	81.32	941.49	SLC7A6OS

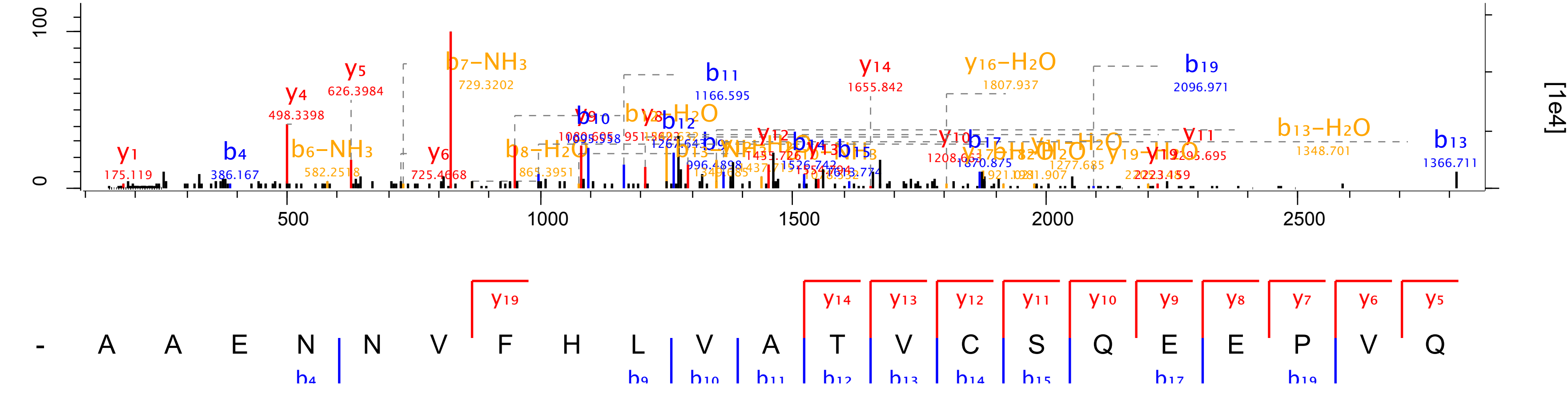

Raw file	Scan	Method	Score	m/z	Gene names
20150226_Hela_Top_opt_A3_01_1591	67279	TOF; CID	83.88	1348.99	C1orf174

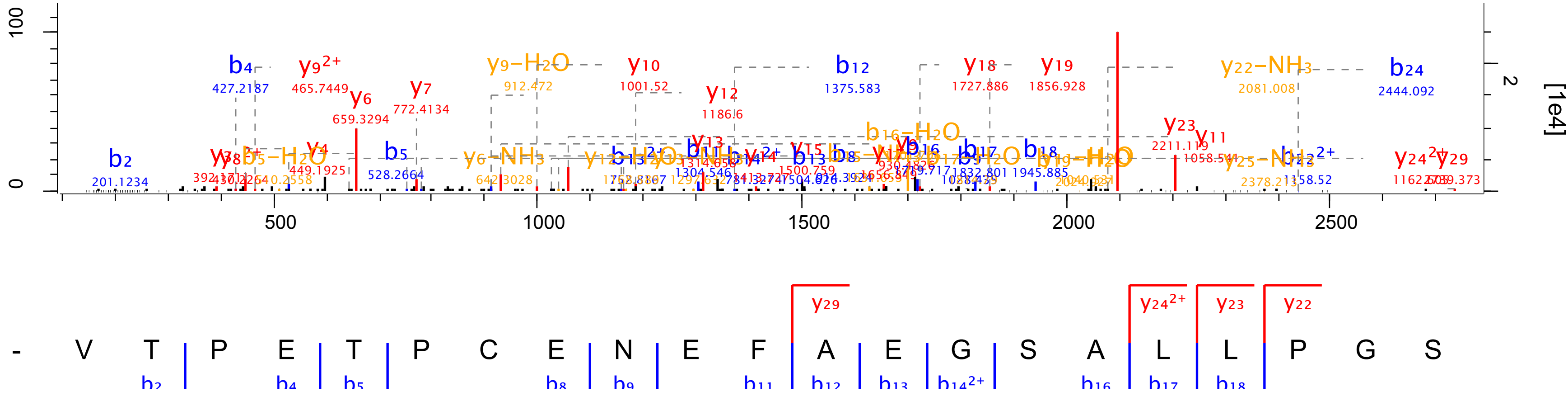

Raw file	Scan	Method	Score	m/z	Gene names
20150226_Hela_Top_opt_A3_01_1591	68249	TOF; CID	63.19	859.45	TRAPPC2L

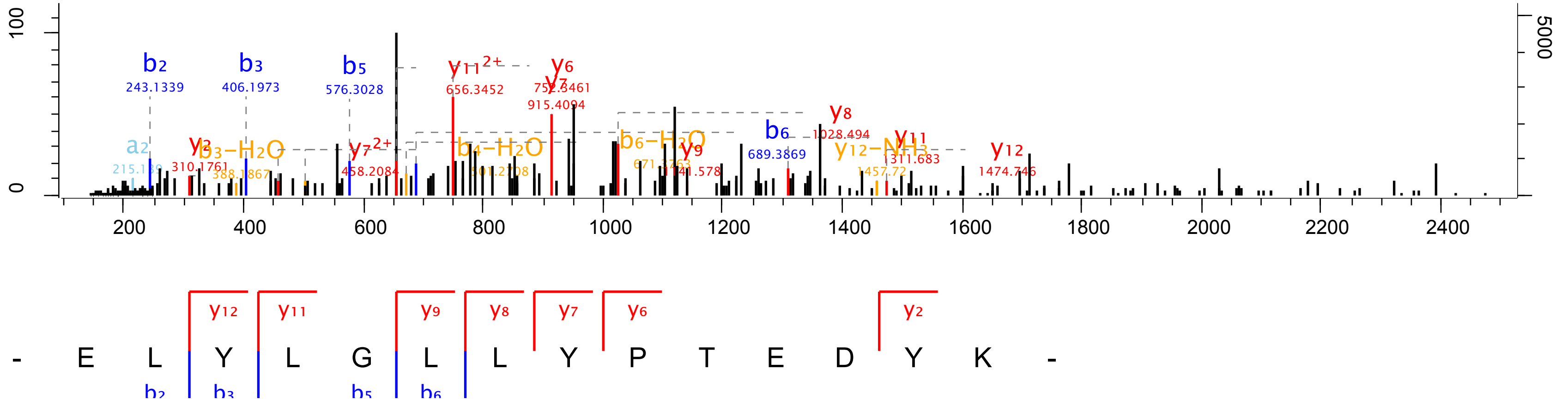

Raw file	Scan	Method	Score	m/z	Gene names
20150226_Hela_Top_opt_A3_01_1591	68849	TOF; CID	86.21	820.96	ATP6V0D2

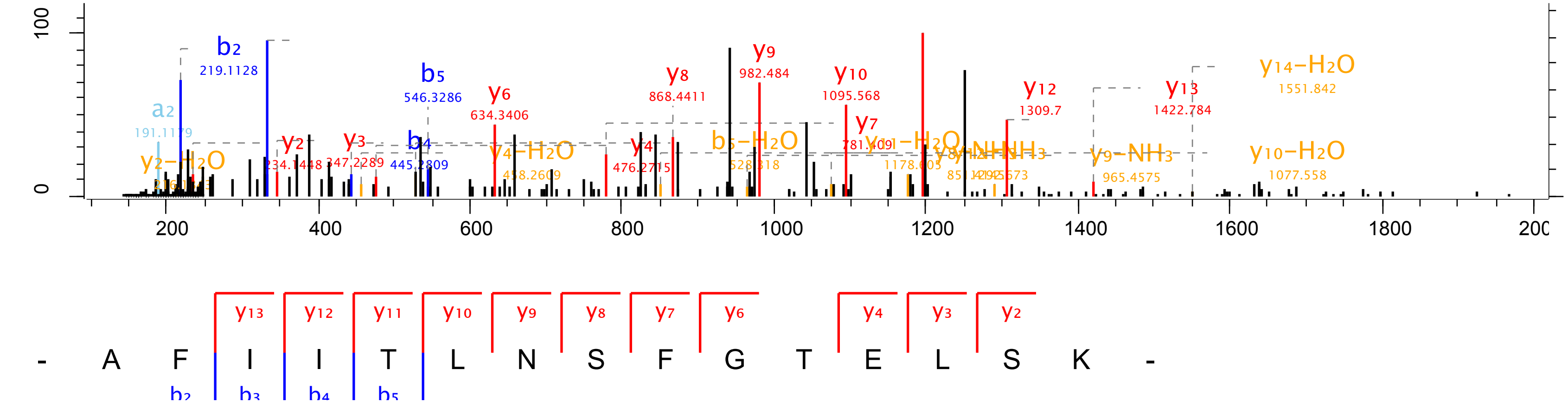

Raw file	Scan	Method	Score	m/z	Gene names
20150226_Hela_Top_opt_A3_01_1591	68941	TOF; CID	44.82	644	ATE1

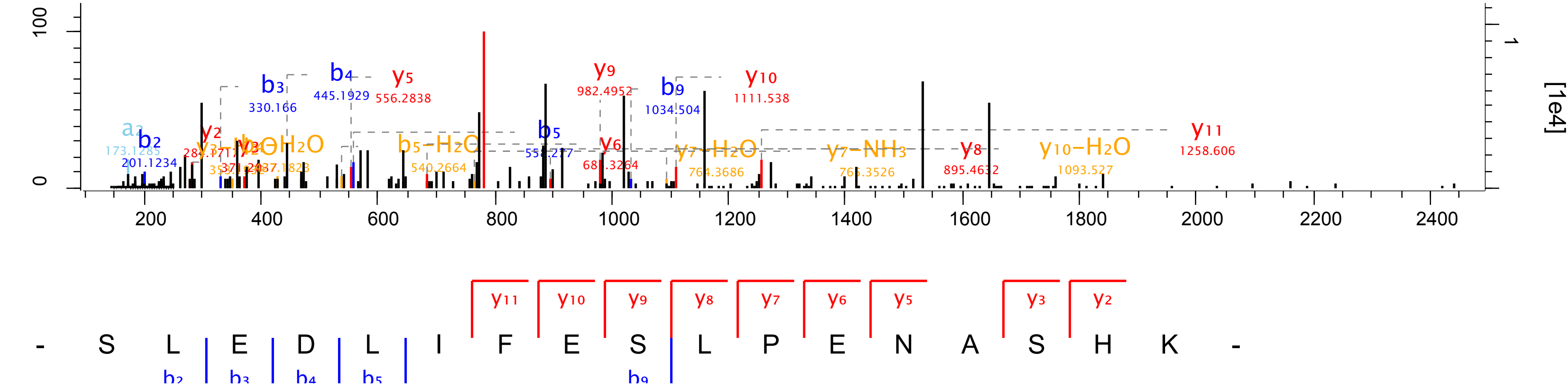

Raw file	Scan	Method	Score	m/z	Gene names
20150226_Hela_Top_opt_A3_01_1591	69258	TOF; CID	82.52	971.84	COX11

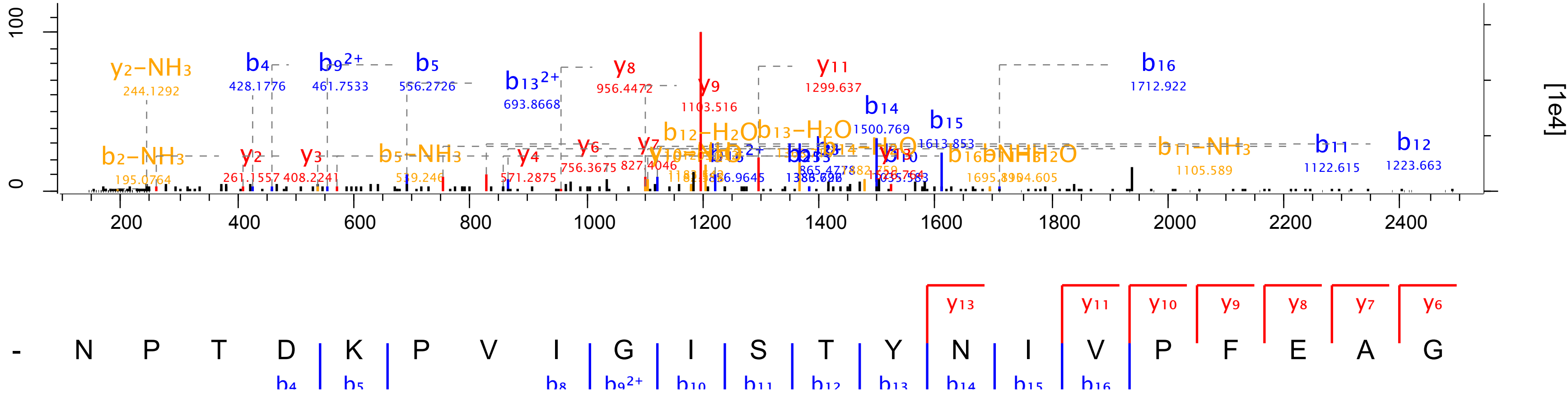

Raw file	Scan	Method	Score	m/z	Gene names
20150226_Hela_Top_opt_A3_01_1591	70583	TOF; CID	135.54	676.36	HAUS8

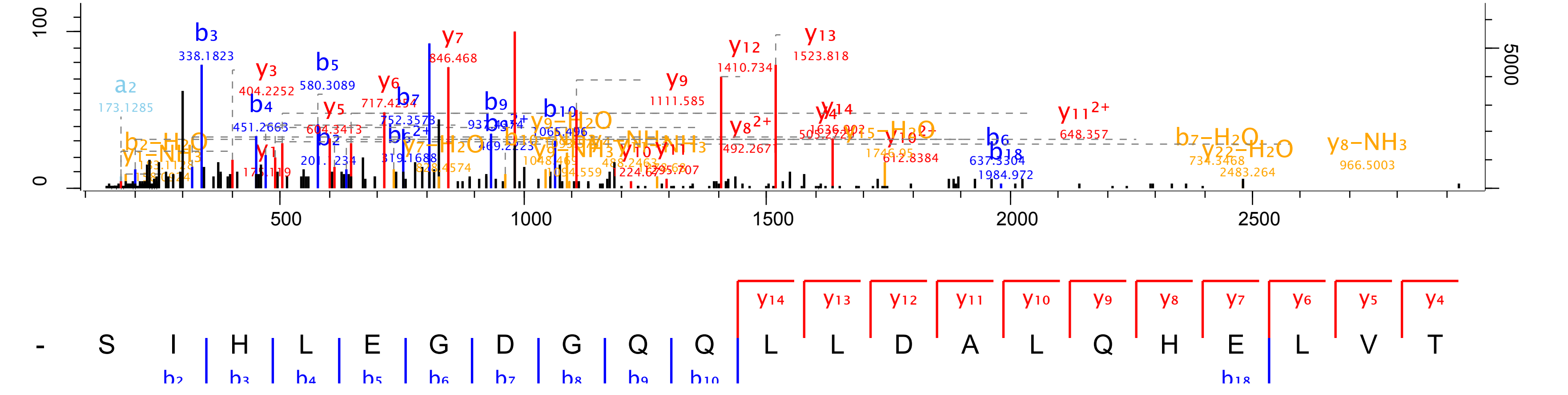

Raw file	Scan	Method	Score	m/z	Gene names
20150226_Hela_Top_opt_A3_01_1591	71104	TOF; CID	45.42	782.09	AP5S1

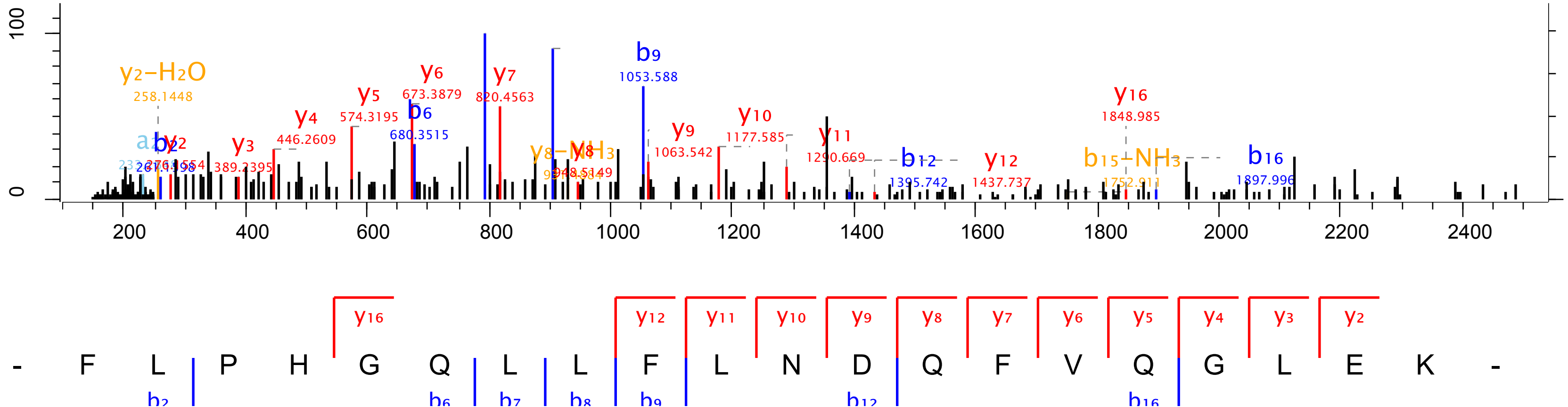

Raw file	Scan	Method	Score	m/z	Gene names
20150226_Hela_Top_opt_A3_01_1591	71216	TOF; CID	74.27	517.81	TMEM167A

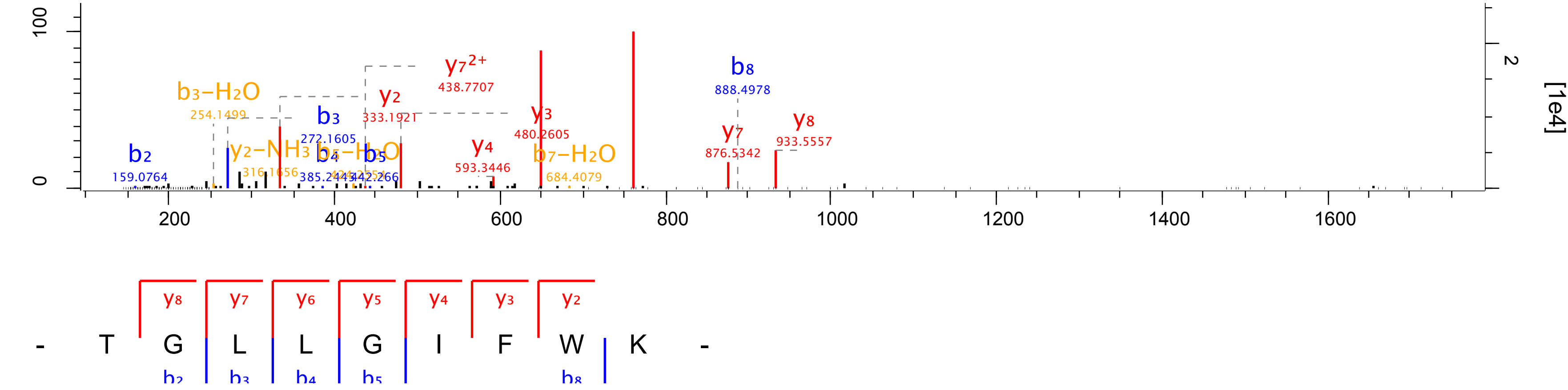

Raw file	Scan	Method	Score	m/z	Gene names
20150226_Hela_Top_opt_A3_01_1591	71484	TOF; CID	58.98	506.34	ZBED1

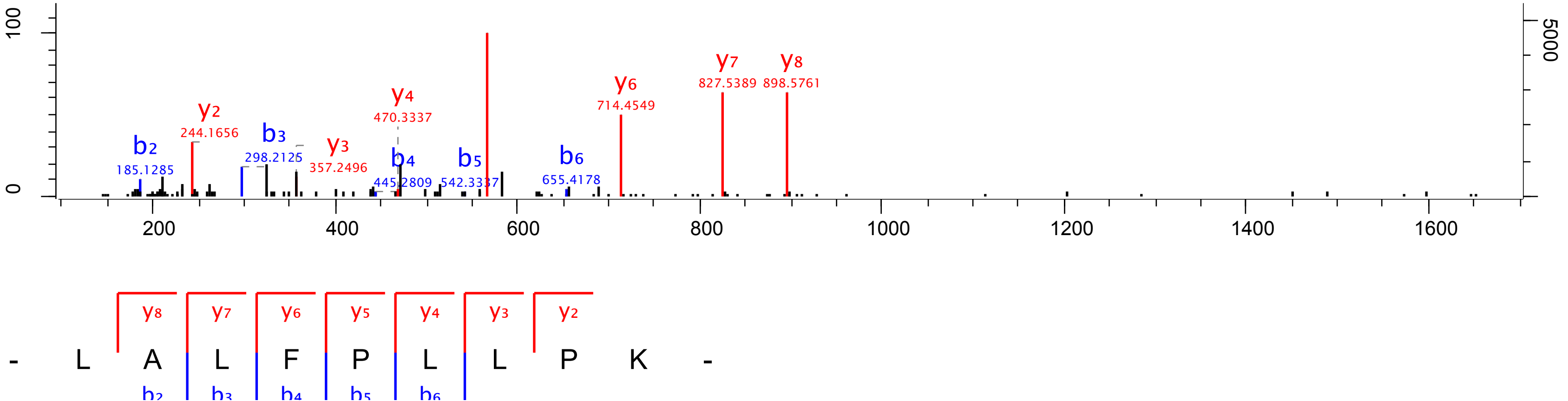

Raw file	Scan	Method	Score	m/z	Gene names
20150226_Hela_Top_opt_A3_01_1591	73279	TOF; CID	78.13	913.85	BCL2L13

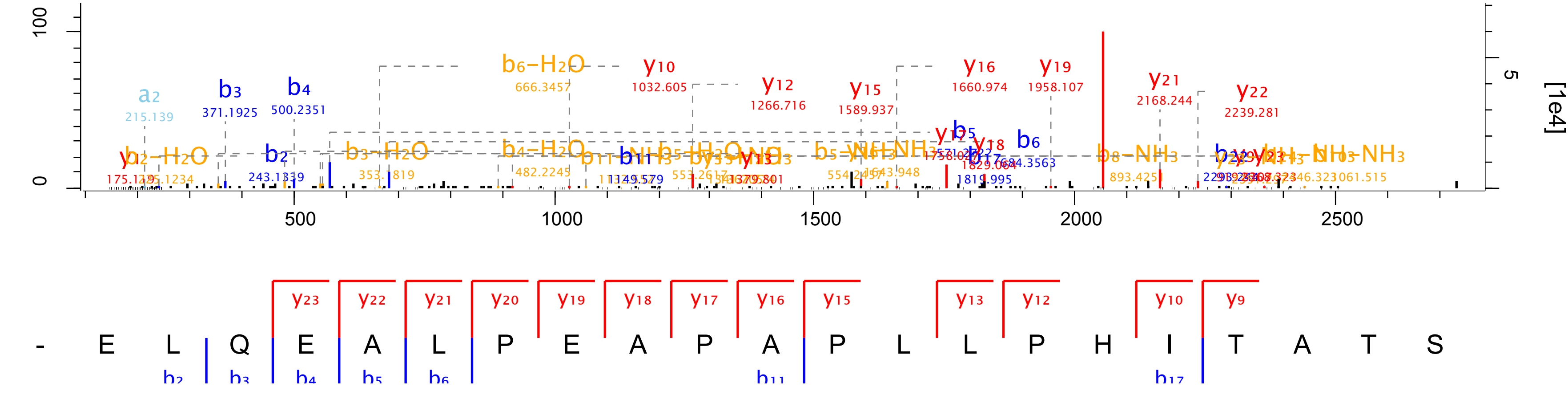

Raw file	Scan	Method	Score	m/z	Gene names
20150226_Hela_Top_opt_A3_01_1591	73482	TOF; CID	78.98	846.43	ADCY3

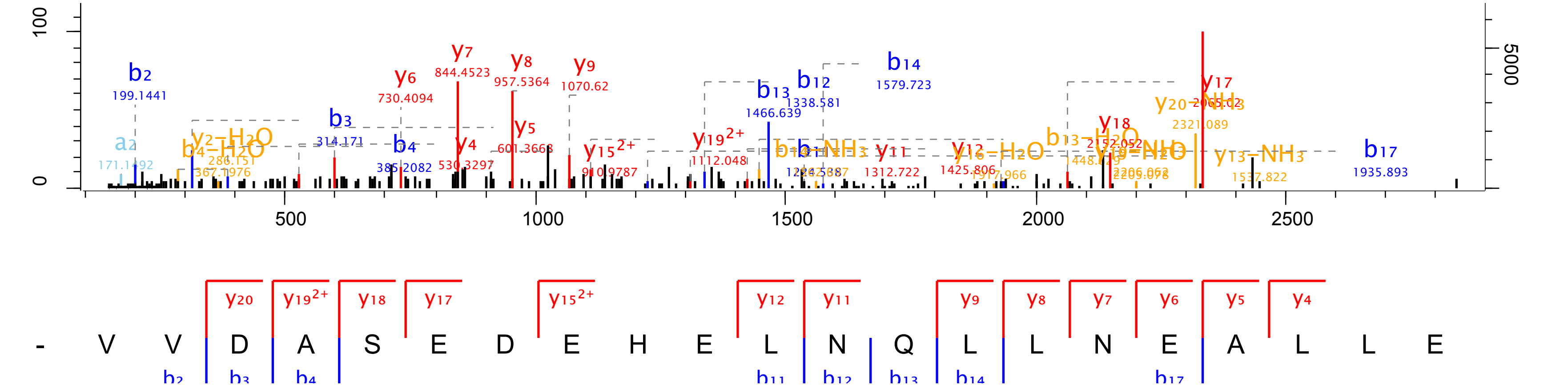

Raw file	Scan	Method	Score	m/z	Gene names
20150226_Hela_Top_opt_A3_01_1591	73709	TOF; CID	79.12	478.83	GXYLT1

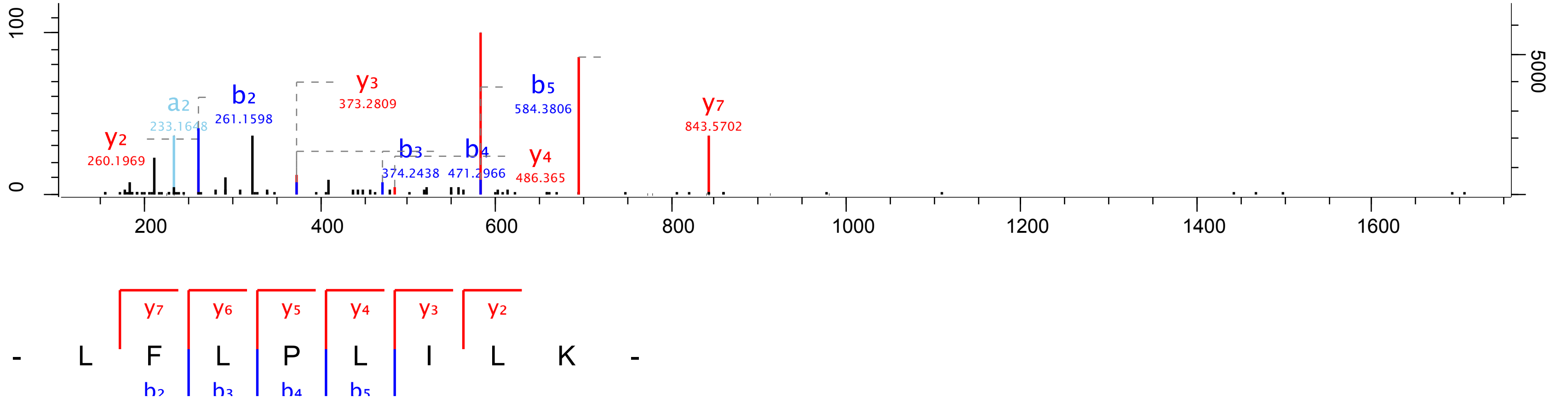

Raw file	Scan	Method	Score	m/z	Gene names
20150226_Hela_Top_opt_A3_01_1591	73968	TOF; CID	67.67	824.44	CCDC117

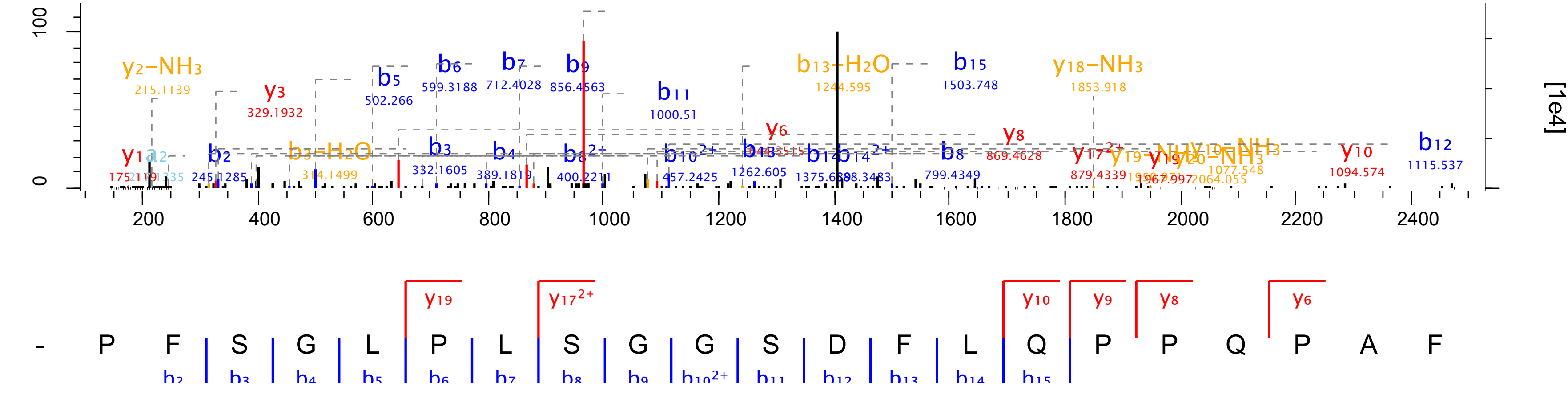

Raw file	Scan	Method	Score	m/z	Gene names
20150226_Hela_Top_opt_A3_01_1591	74366	TOF; CID	99.32	1154.5	VMP1

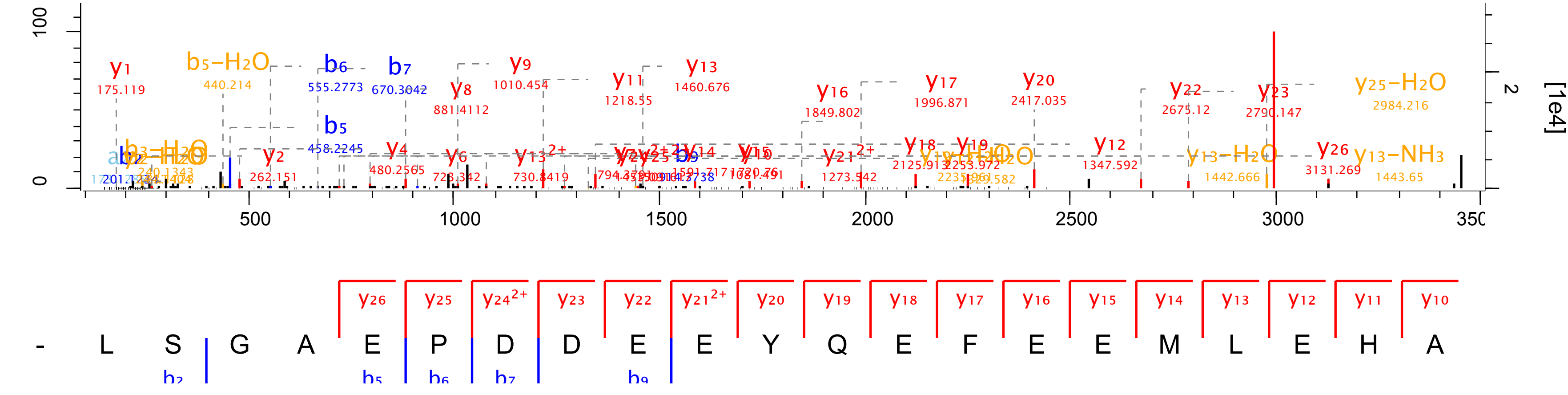

Raw file	Scan	Method	Score	m/z	Gene names
20150226_Hela_Top_opt_A3_01_1591	74777	TOF; CID	92.8	656.02	AMBRA1

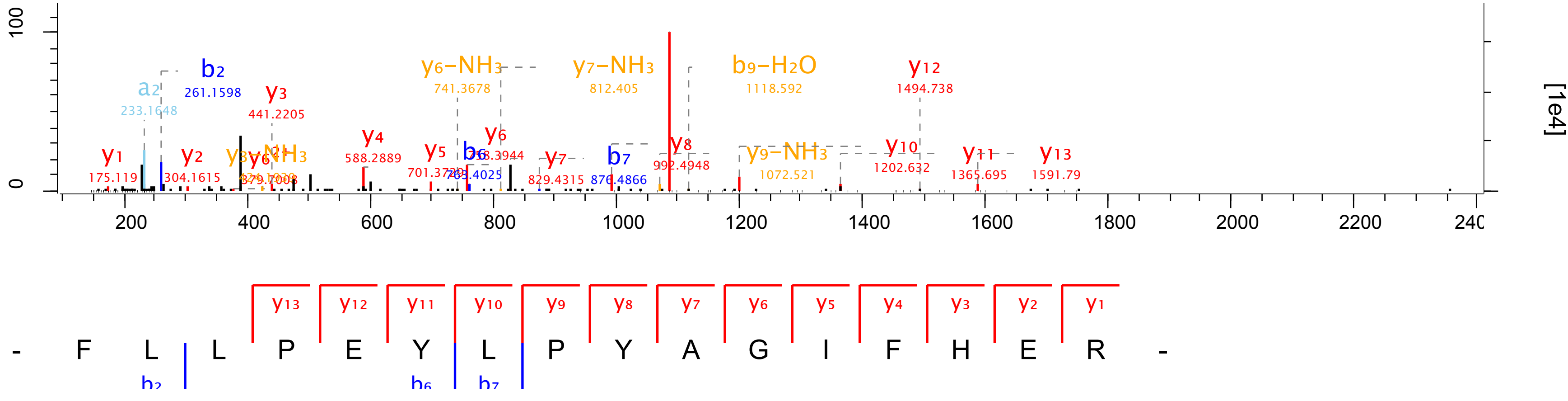

Raw file	Scan	Method	Score	m/z	Gene names
20150226_Hela_Top_opt_A3_01_1591	75199	TOF; CID	58.17	711.88	AAK1;BMP2K

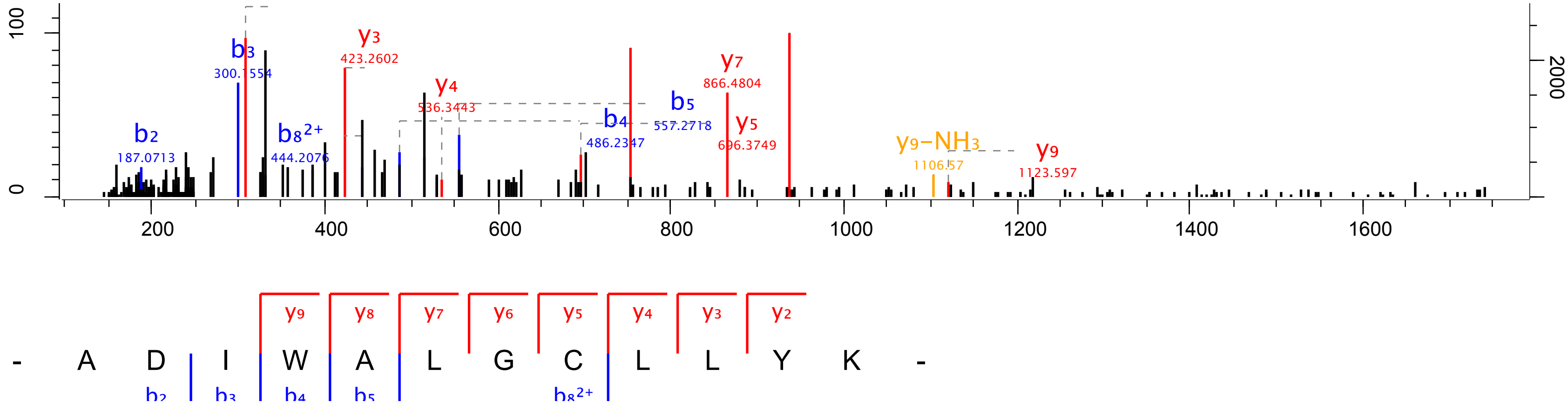

Raw file	Scan	Method	Score	m/z	Gene names
20150226_Hela_Top_opt_A3_01_1591	75695	TOF; CID	47.75	803.07	NDUFAF6

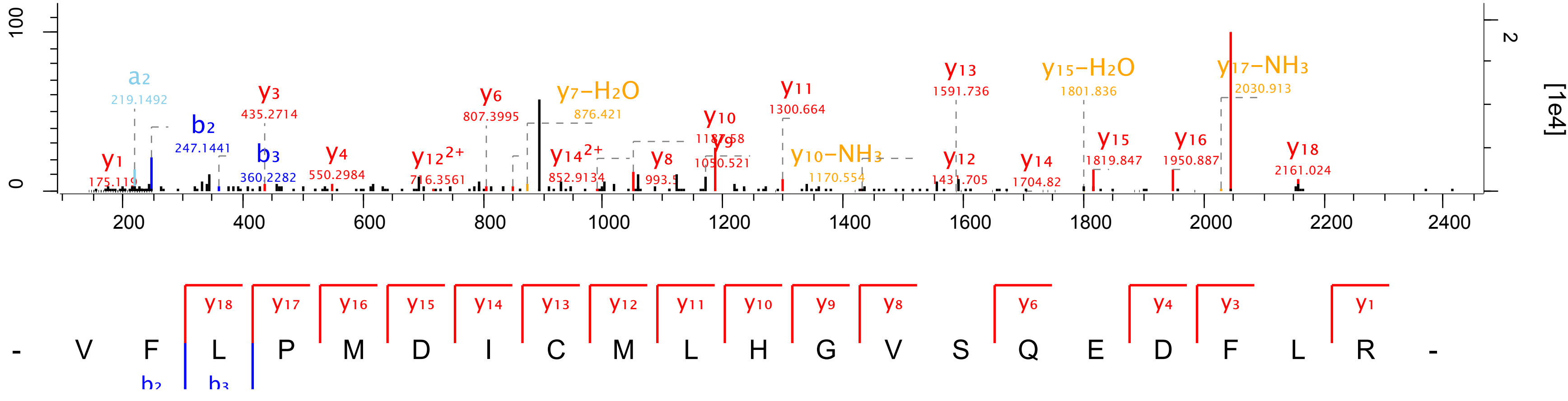

Raw file	Scan	Method	Score	m/z	Gene names
20150226_Hela_Top_opt_A3_01_1591	78485	TOF; CID	102.97	802.43	CEP164

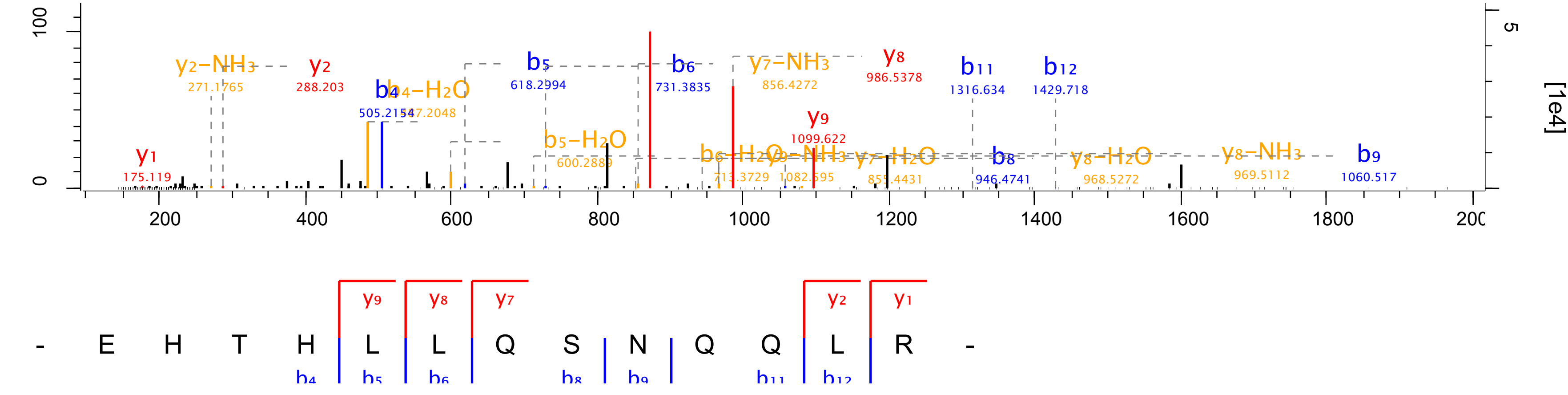

Raw file	Scan	Method	Score	m/z	Gene names
20150226_Hela_Top_opt_A3_01_1591	78836	TOF; CID	93.37	759.37	TRABD

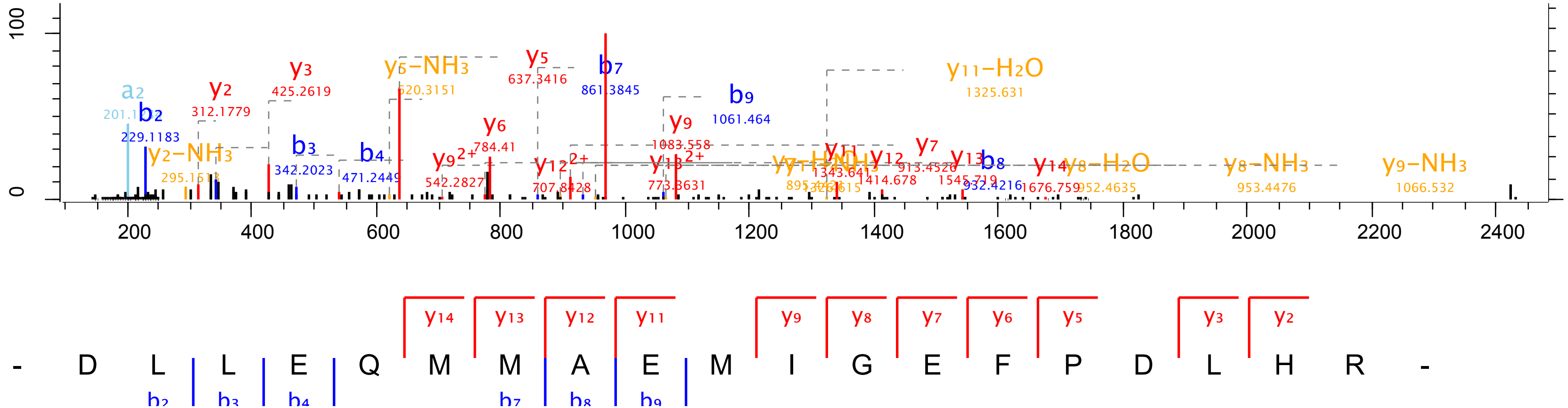

Raw file	Scan	Method	Score	m/z	Gene names
20150226_Hela_Top_opt_A3_01_1593	3374	TOF; CID	82.64	320.86	HMGN2;HMGN3

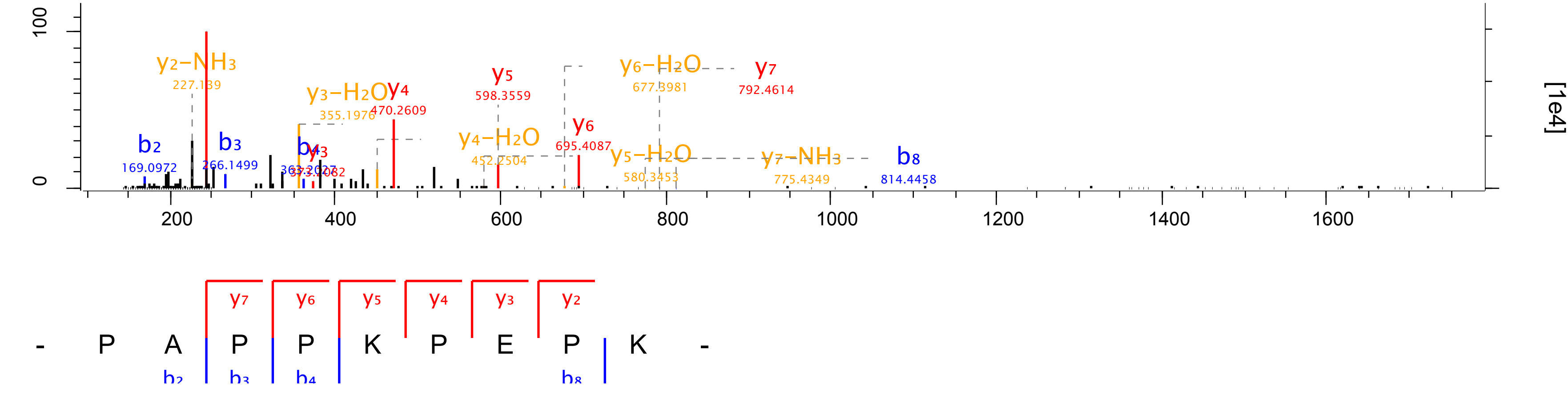

Raw file	Scan	Method	Score	m/z	Gene names
20150226_Hela_Top_opt_A3_01_1593	3407	TOF; CID	71.18	669.81	MANBAL

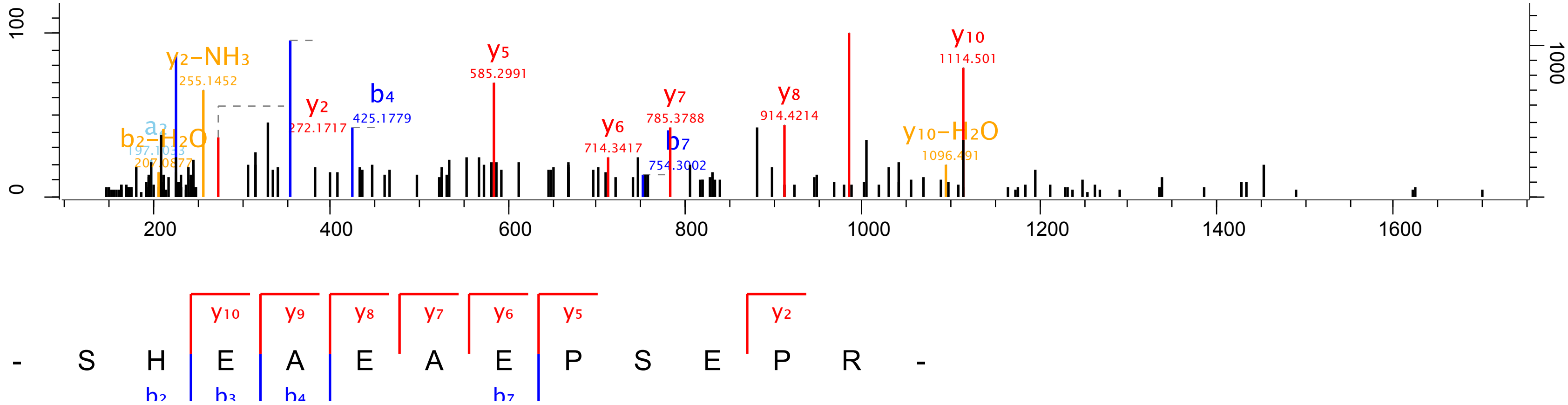

Raw file	Scan	Method	Score	m/z	Gene names
20150226_Hela_Top_opt_A3_01_1593	3704	TOF; CID	94.09	537.27	MT-CO3

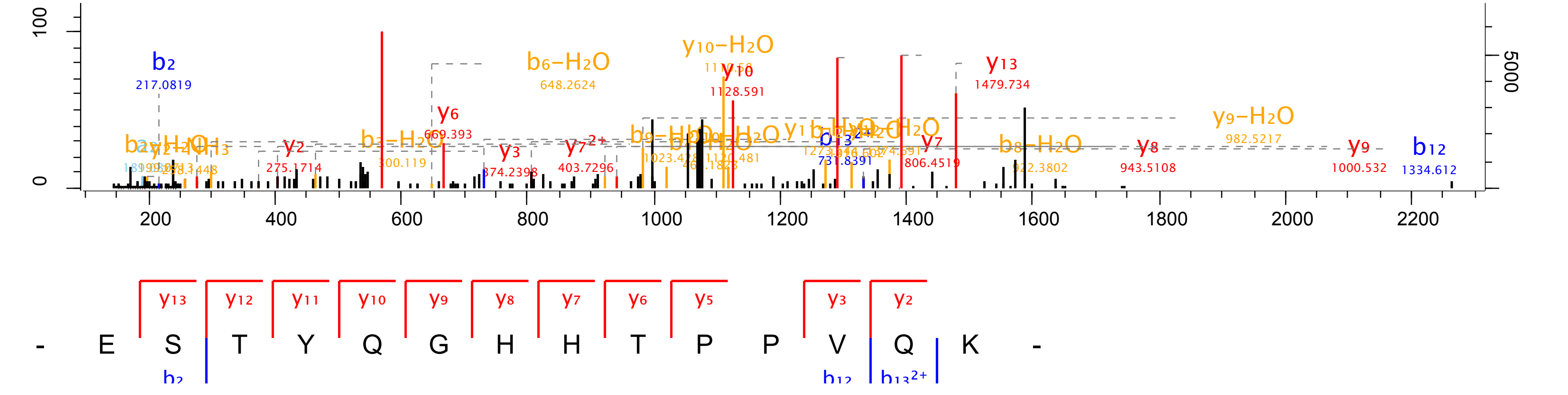

Raw file	Scan	Method	Score	m/z	Gene names
20150226_Hela_Top_opt_A3_01_1593	4089	TOF; CID	78.9	656.82	CCDC82

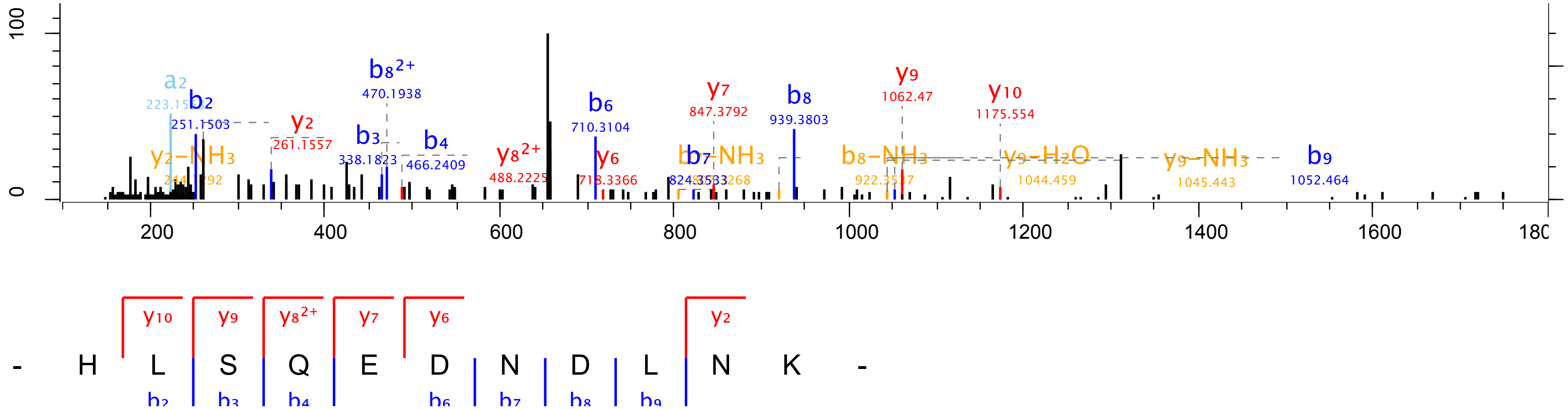

Raw file	Scan	Method	Score	m/z	Gene names
20150226_Hela_Top_opt_A3_01_1593	4313	TOF; CID	58.7	601.82	ZNF557

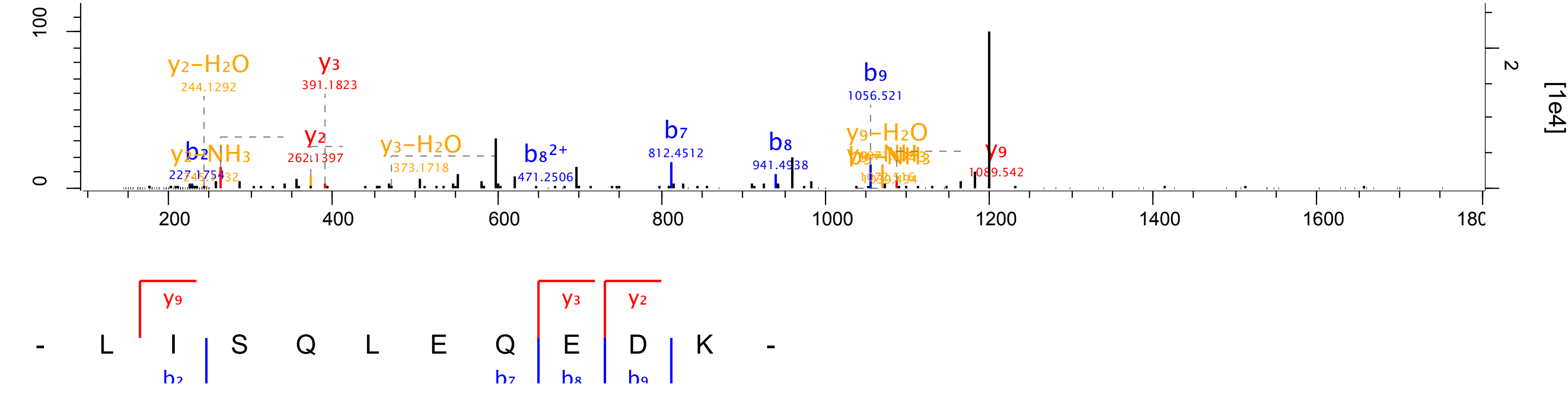

Raw file	Scan	Method	Score	m/z	Gene names
20150226_Hela_Top_opt_A3_01_1593	4843	TOF; CID	128.85	589.8	SLC16A7

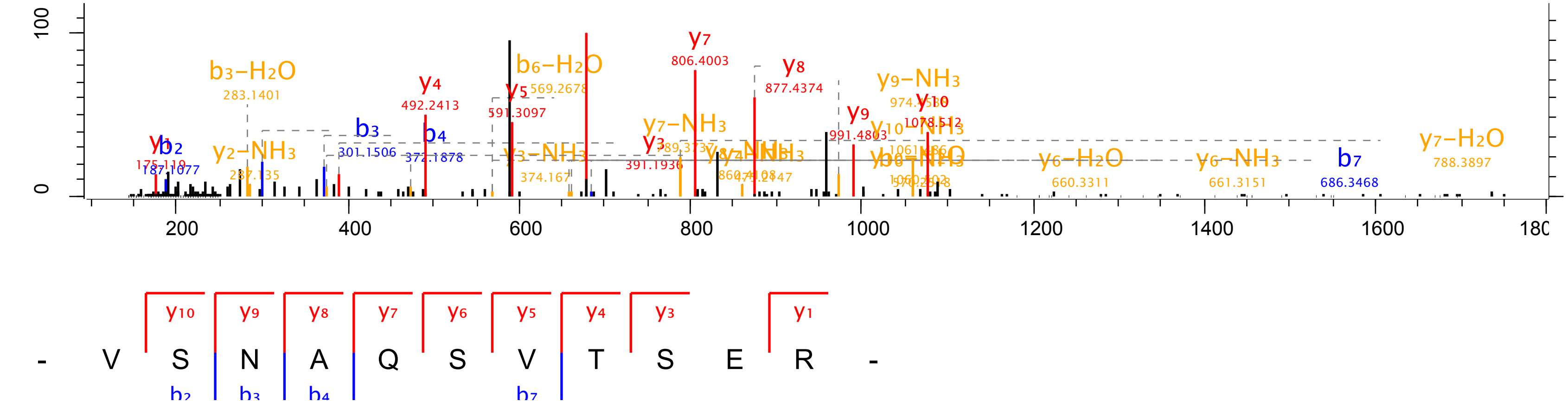

Raw file	Scan	Method	Score	m/z	Gene names
20150226_Hela_Top_opt_A3_01_1593	5402	TOF; CID	63.16	732.36	MAX

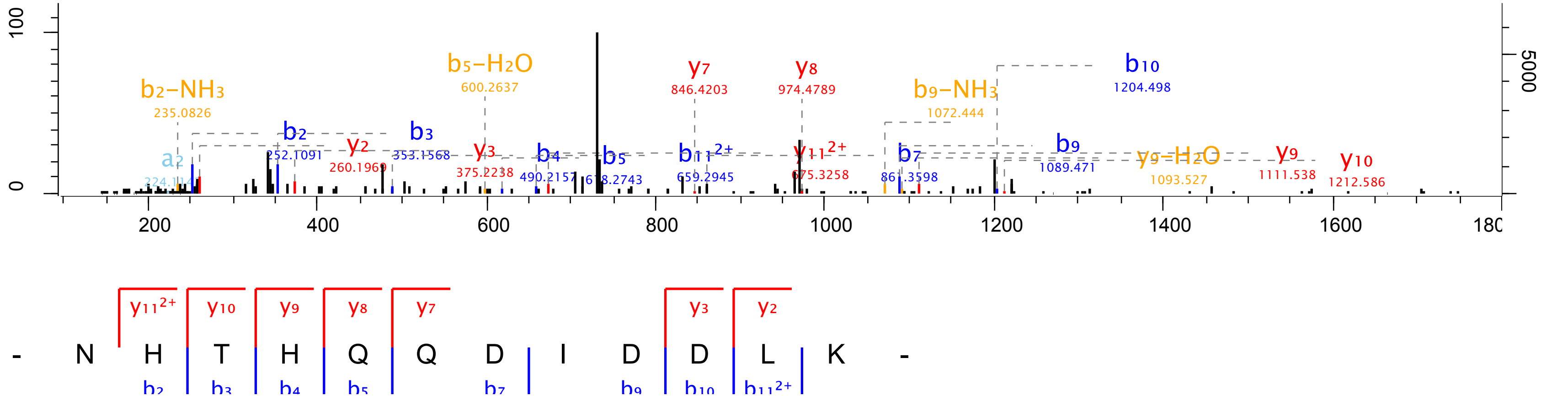

Raw file	Scan	Method	Score	m/z	Gene names
20150226_Hela_Top_opt_A3_01_1593	6215	TOF; CID	57.4	612.78	PDZD8

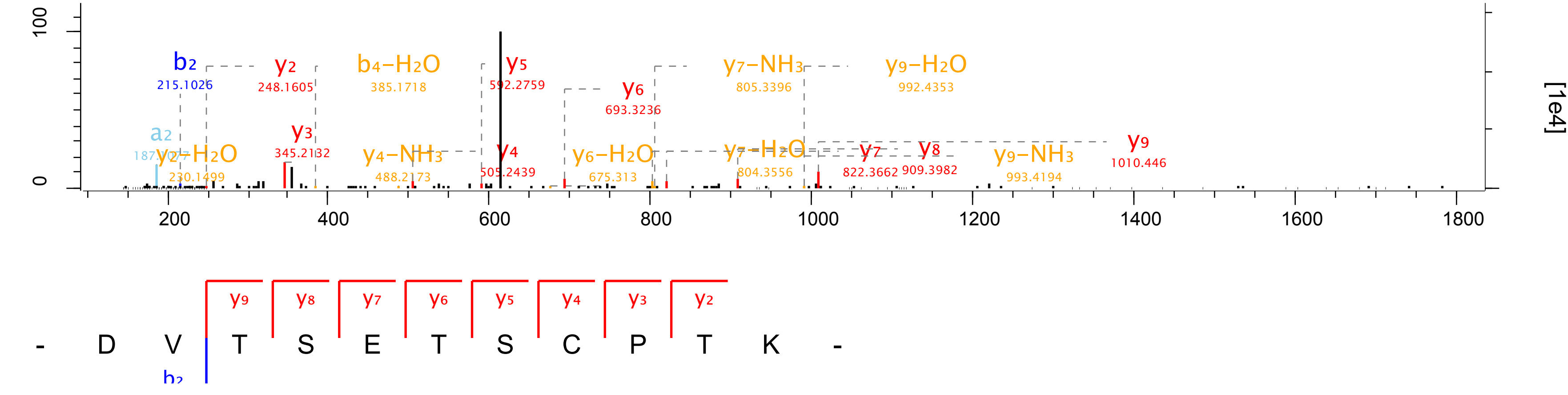

Raw file	Scan	Method	Score	m/z	Gene names
20150226_Hela_Top_opt_A3_01_1593	6290	TOF; CID	162.31	458.76	ZC3H6

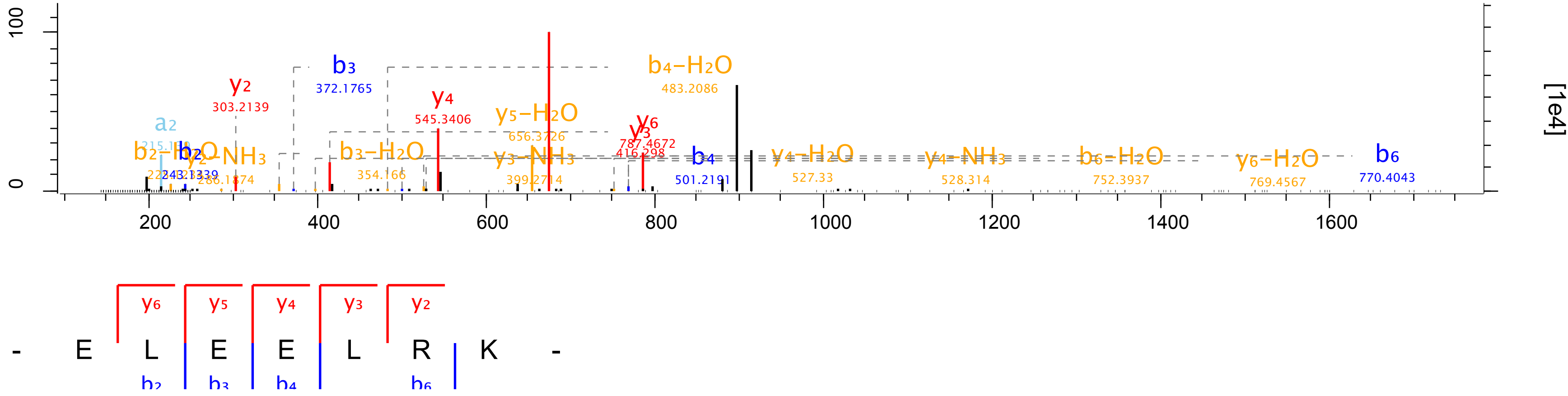

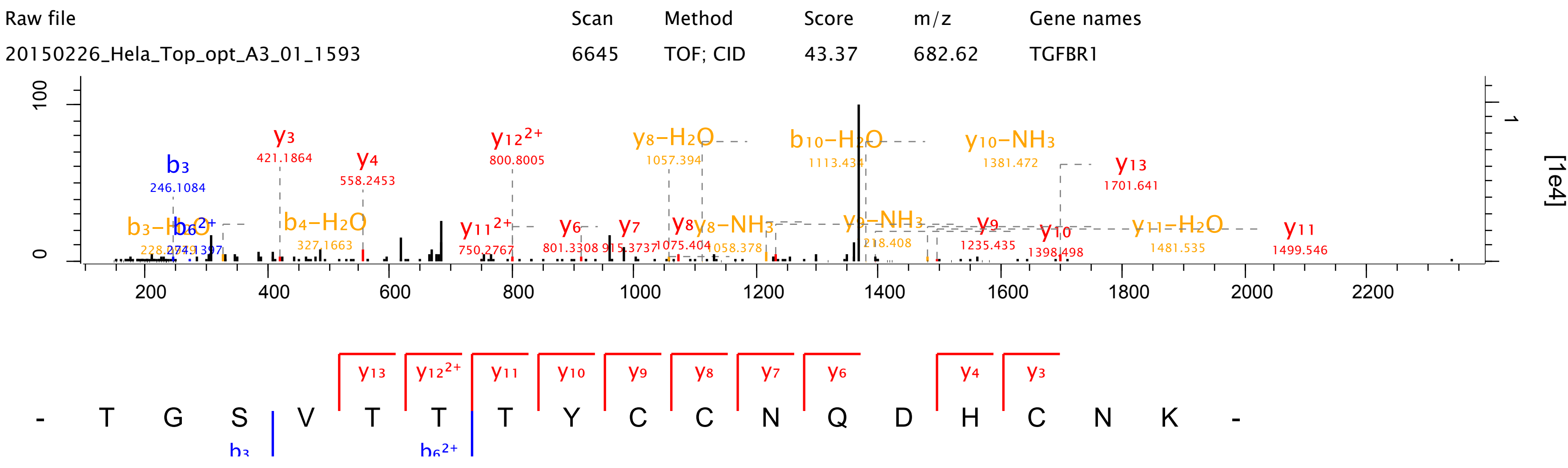
Raw file
20150226_Hela_Top_opt_A3_01_1593
Scan
6645
Method
TOF; CID
Score
43.37
m/z
682.62
Gene names
TGFBR1
100
0
[1e4]
1
200
400
600
800
1000
1200
1400
1600
1800
2000
2200
b3
246.1084
y3
421.1864
y4
558.2453
y12 2+
800.8005
y8−H2O
1057.394
b10−H2O
1113.434
y10−NH3
1381.472
y13
1701.641
b4−H2O
327.1663
y11 2+
750.2767
y6
801.3308
y7
915.3737
y8
1075.404
y8−NH3
1058.378
y9
1235.435
y10
1398.498
y11−H2O
1481.535
y11
1499.546
- T G S V T T T Y C C N Q D H C N K -
y13 y12 2+ y11 y10 y9 y8 y7 y6 y4 y3
b3
b6 2+

Raw file	Scan	Method	Score	m/z	Gene names
20150226_Hela_Top_opt_A3_01_1593	7170	TOF; CID	57.28	867.37	CREBBP

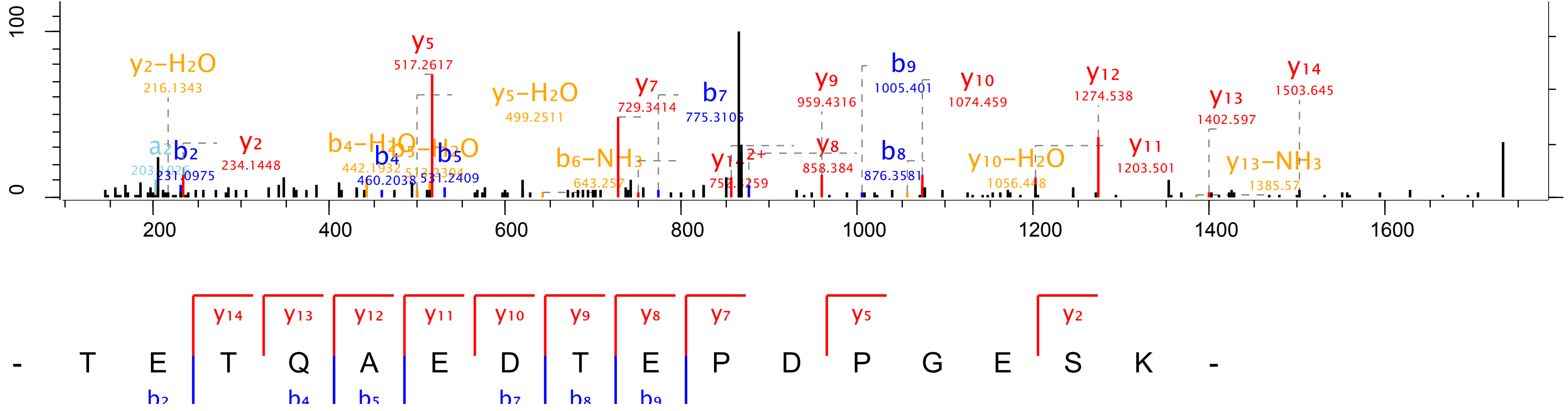

Raw file	Scan	Method	Score	m/z	Gene names
20150226_Hela_Top_opt_A3_01_1593	7995	TOF; CID	60.31	613.27	OSMR

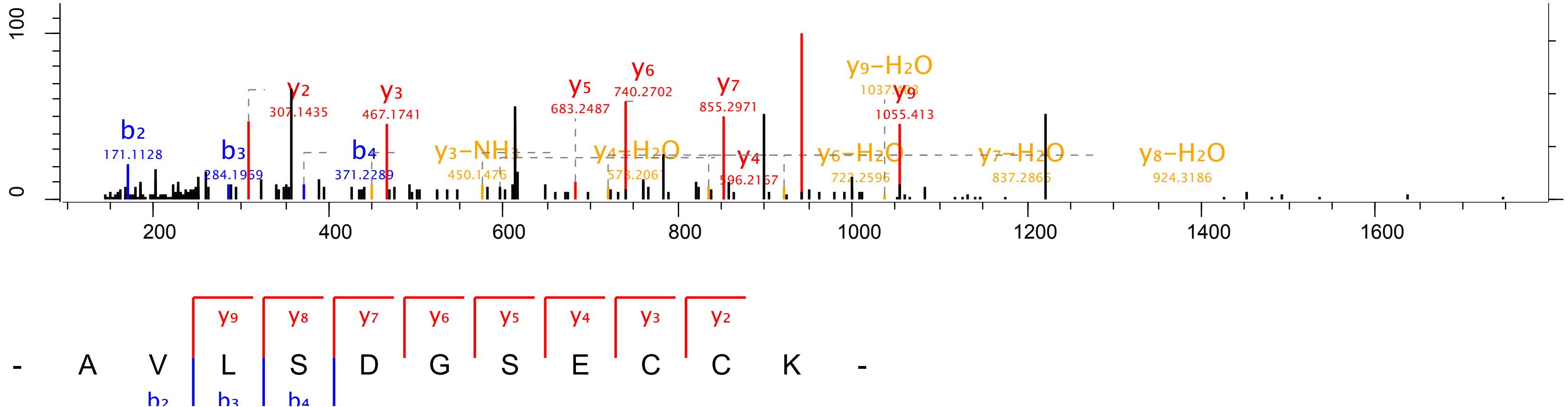

Raw file	Scan	Method	Score	m/z	Gene names
20150226_Hela_Top_opt_A3_01_1593	8655	TOF; CID	88.09	710.36	CKAP2

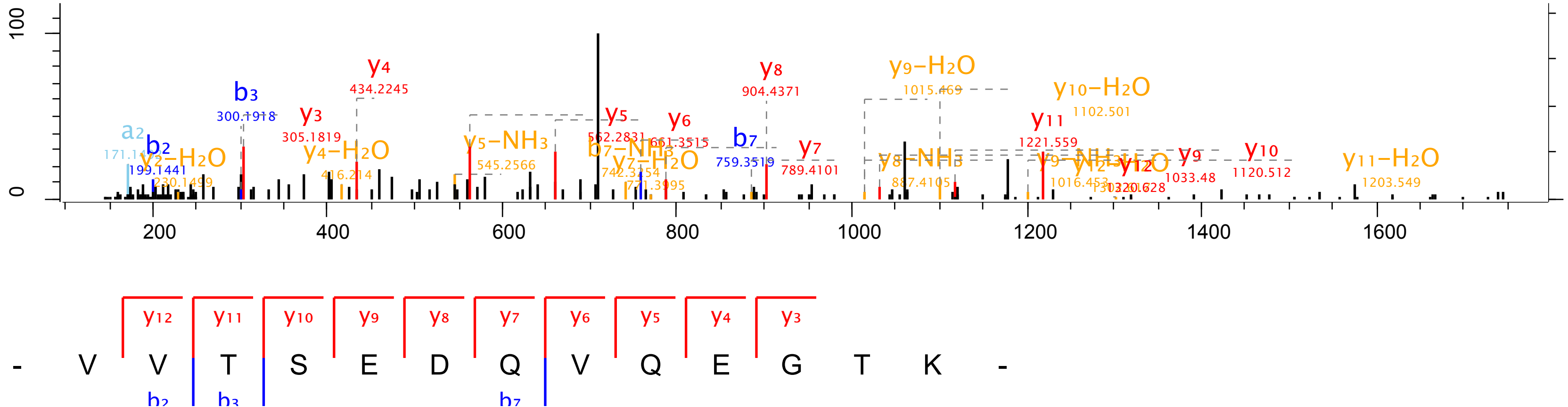

Raw file	Scan	Method	Score	m/z	Gene names
20150226_Hela_Top_opt_A3_01_1593	9504	TOF; CID	47.62	540.91	SLPI

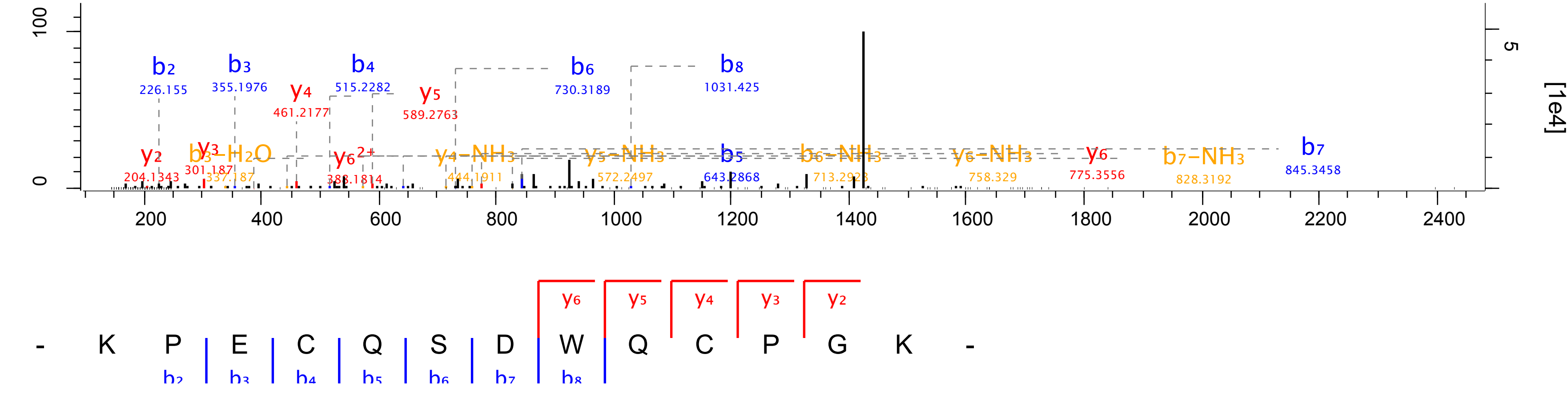

Raw file	Scan	Method	Score	m/z
20150226_Hela_Top_opt_A3_01_1593	9741	TOF; CID	79.87	780.82

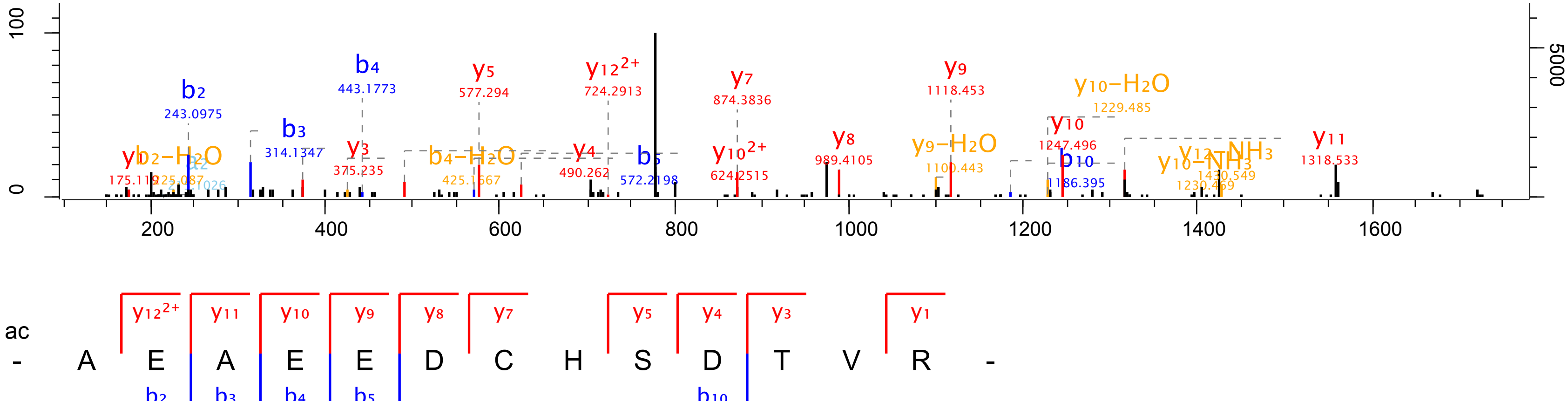

Raw file	Scan	Method	Score	m/z	Gene names
20150226_Hela_Top_opt_A3_01_1593	10263	TOF; CID	55.69	810.4	CIZ1

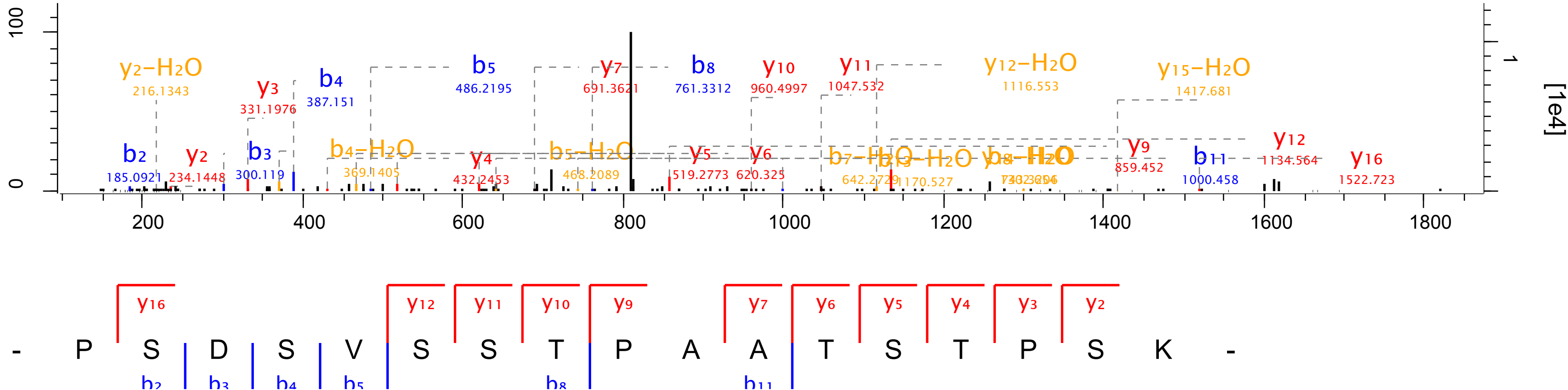

Raw file	Scan	Method	Score	m/z	Gene names
20150226_Hela_Top_opt_A3_01_1593	11652	TOF; CID	60.89	641.3	ARRDC1

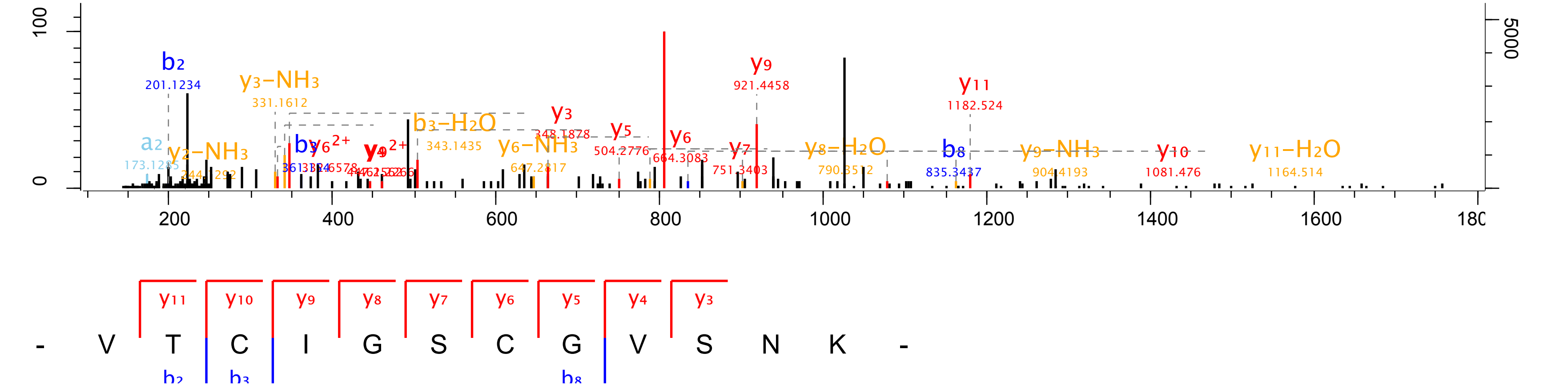

Raw file	Scan	Method	Score	m/z	Gene names
20150226_Hela_Top_opt_A3_01_1593	12894	TOF; CID	67.12	669.82	CEP41

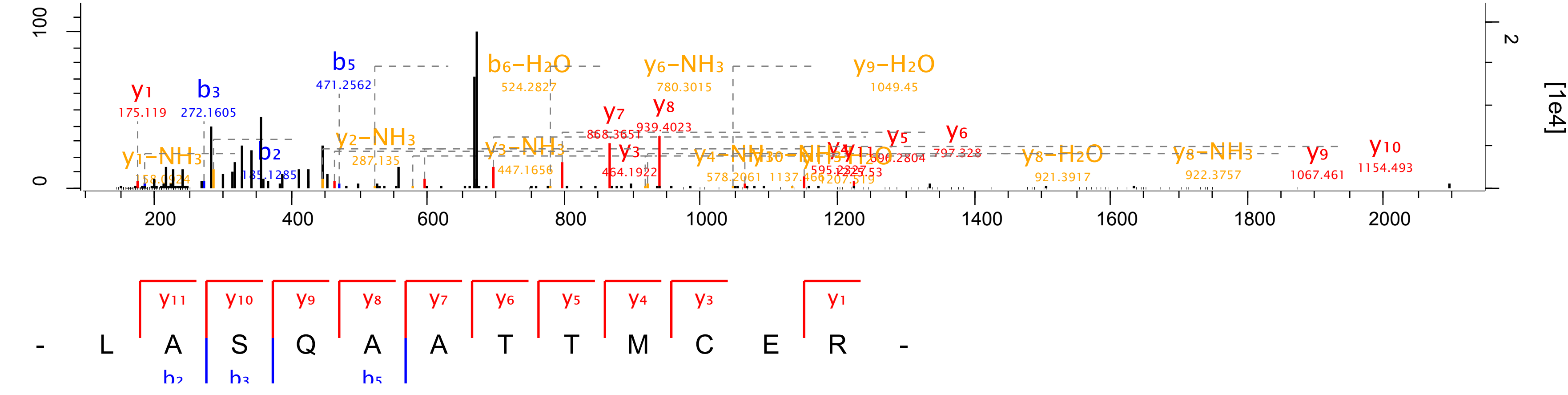

Raw file	Scan	Method	Score	m/z	Gene names
20150226_Hela_Top_opt_A3_01_1593	13742	TOF; CID	95.96	428.77	ARHGEF18

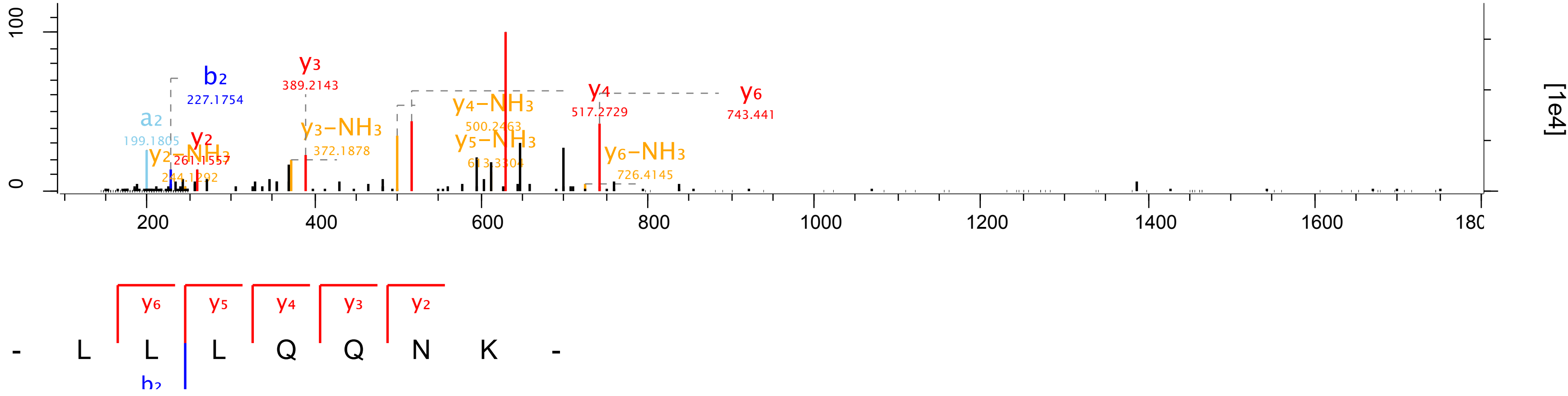

Raw file	Scan	Method	Score	m/z	Gene names
20150226_Hela_Top_opt_A3_01_1593	15030	TOF; CID	72.2	596.76	C5orf24

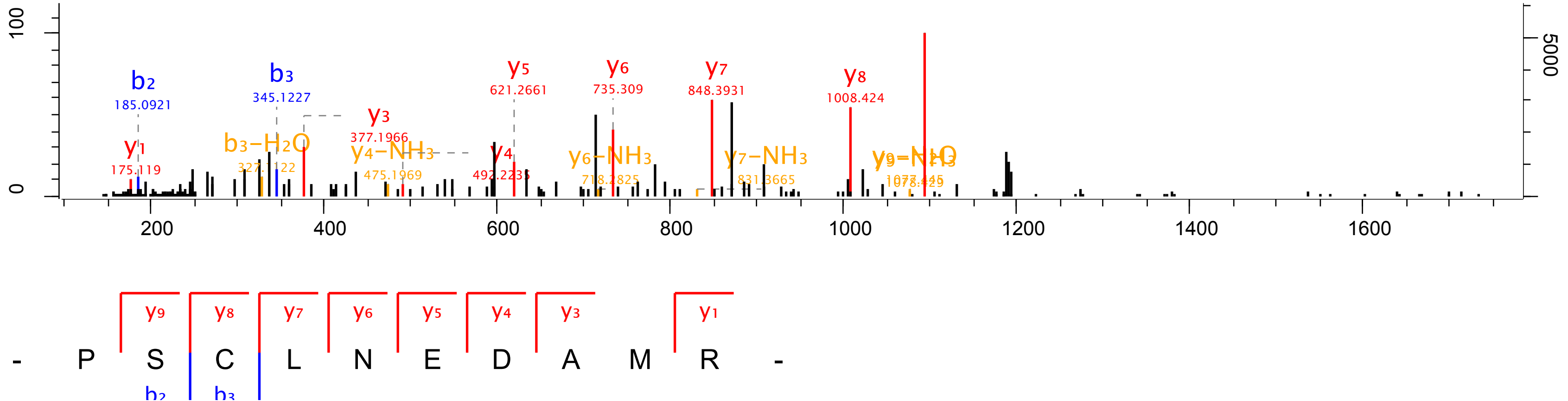

Raw file	Scan	Method	Score	m/z	Gene names
20150226_Hela_Top_opt_A3_01_1593	15252	TOF; CID	46.53	730.36	SLC38A10

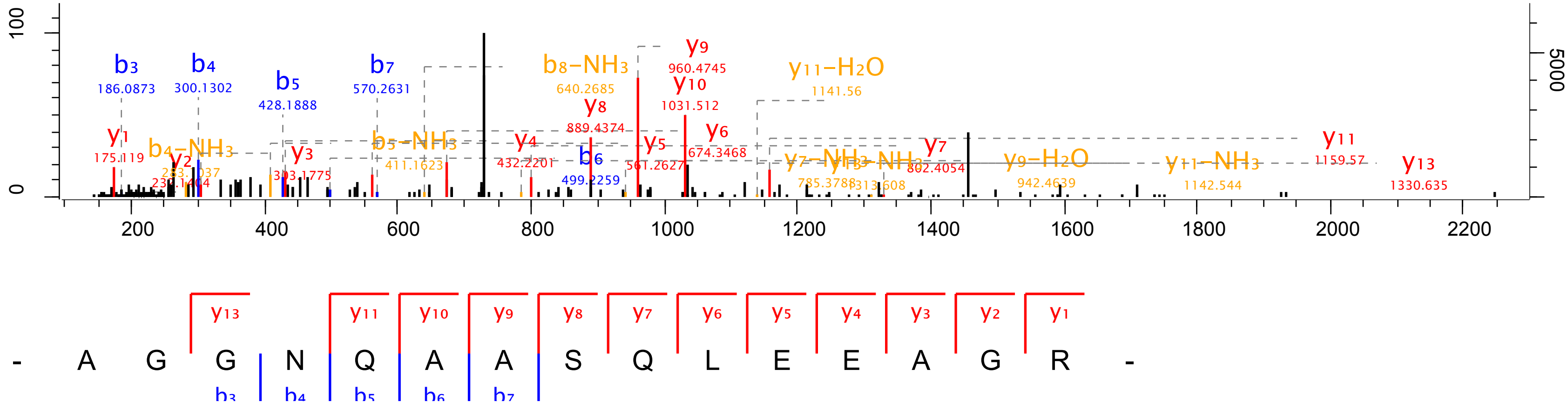

Raw file	Scan	Method	Score	m/z	Gene names
20150226_Hela_Top_opt_A3_01_1593	15356	TOF; CID	71.45	451.26	FAM186B

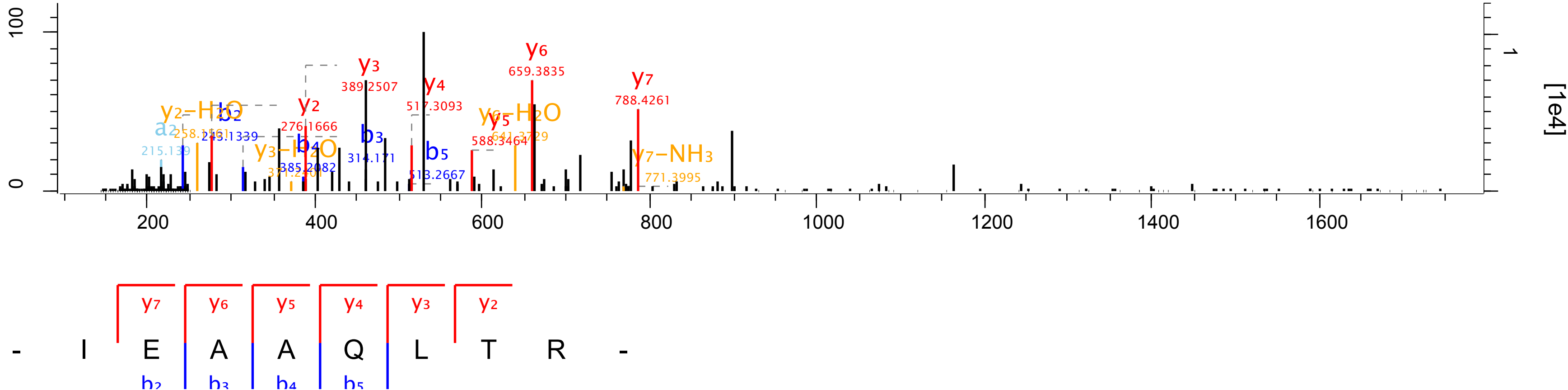

Raw file	Scan	Method	Score	m/z	Gene names
20150226_Hela_Top_opt_A3_01_1593	15895	TOF; CID	60.69	647.77	SUDS3

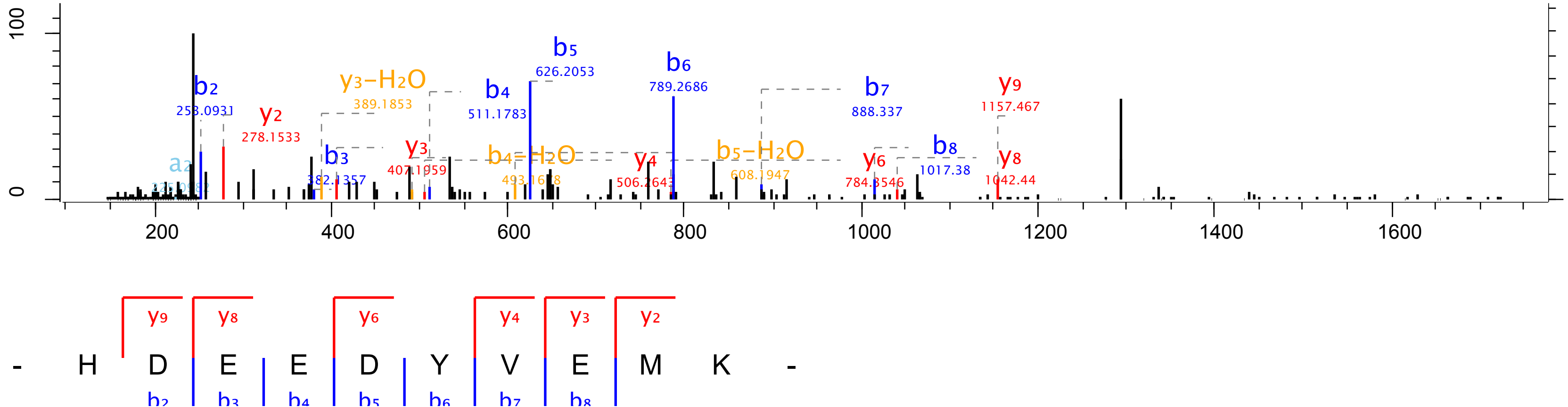

Raw file	Scan	Method	Score	m/z	Gene names
20150226_Hela_Top_opt_A3_01_1593	15911	TOF; CID	93.24	512.29	BLOC1S5;BLOC1S5-TXNDC5

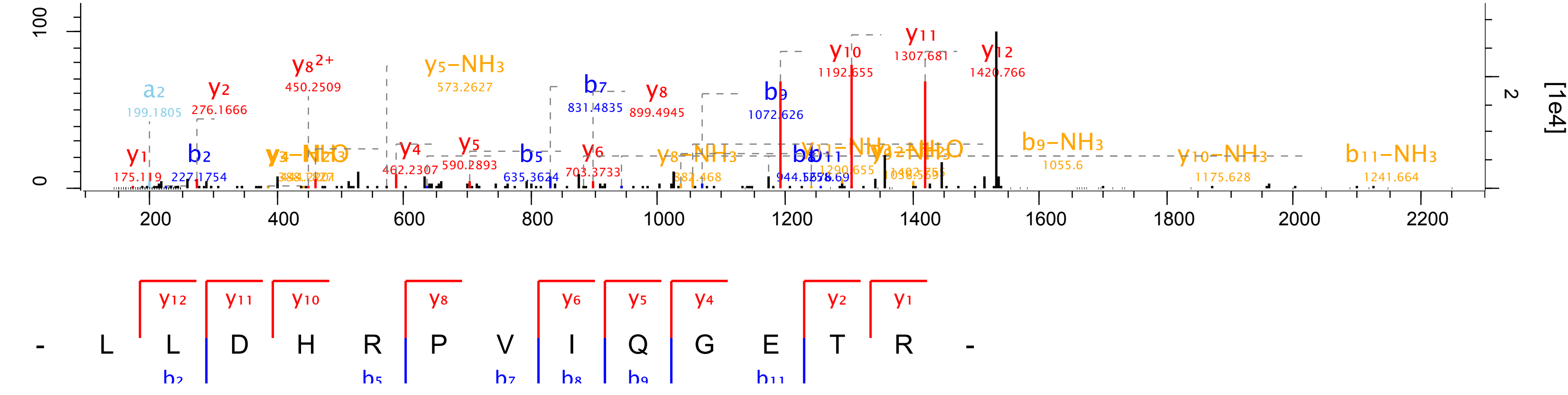

Raw file	Scan	Method	Score	m/z	Gene names
20150226_Hela_Top_opt_A3_01_1593	16007	TOF; CID	81.95	514.3	LENG1

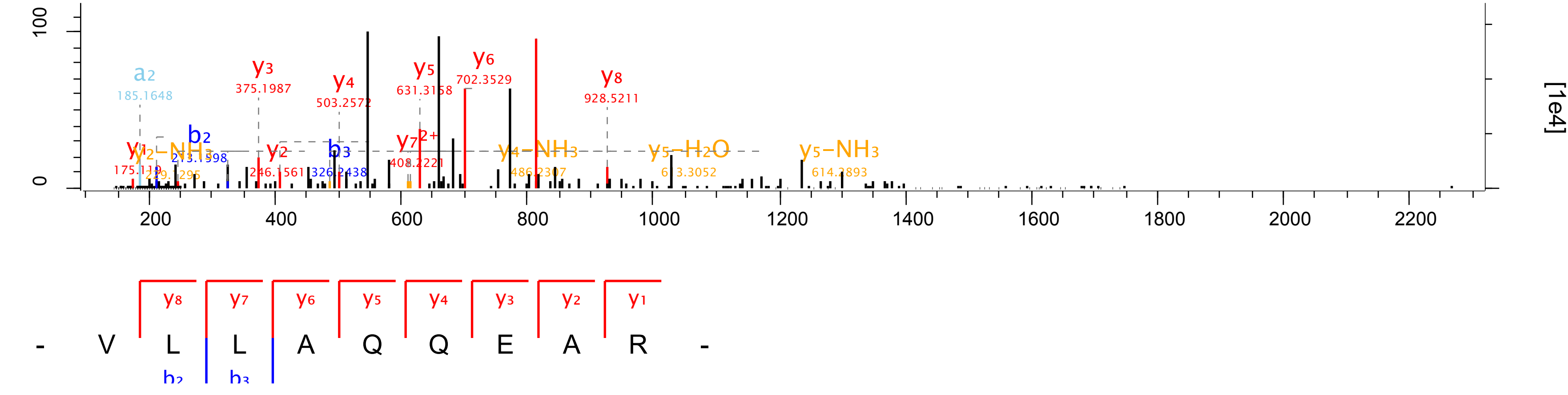

Raw file	Scan	Method	Score	m/z	Gene names
20150226_Hela_Top_opt_A3_01_1593	17205	TOF; CID	56.26	519.27	LRRK2

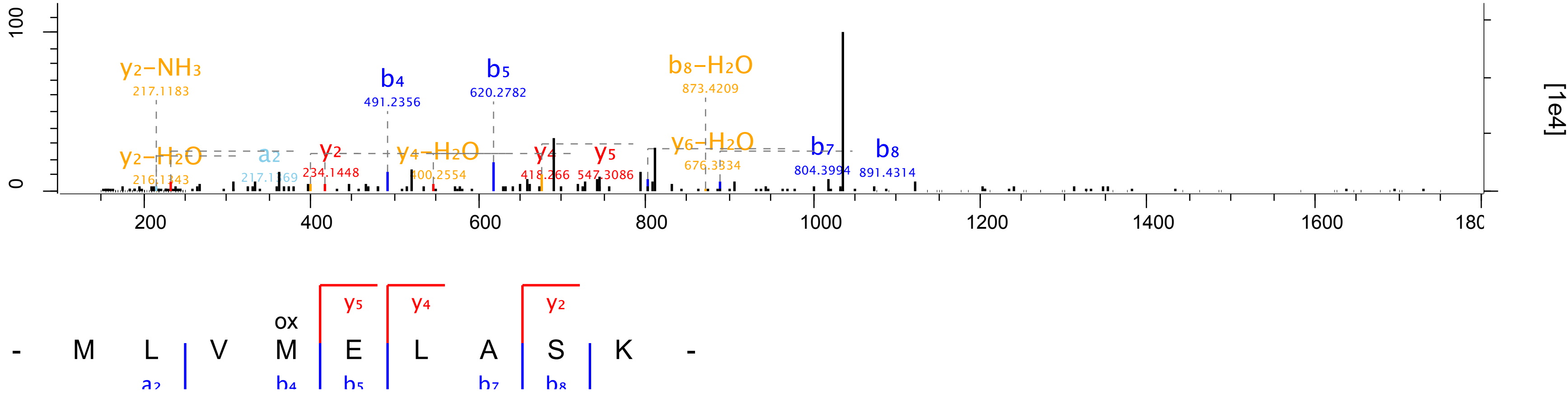

Raw file	Scan	Method	Score	m/z	Gene names
20150226_Hela_Top_opt_A3_01_1593	17585	TOF; CID	51.94	720.32	LEPROTL1

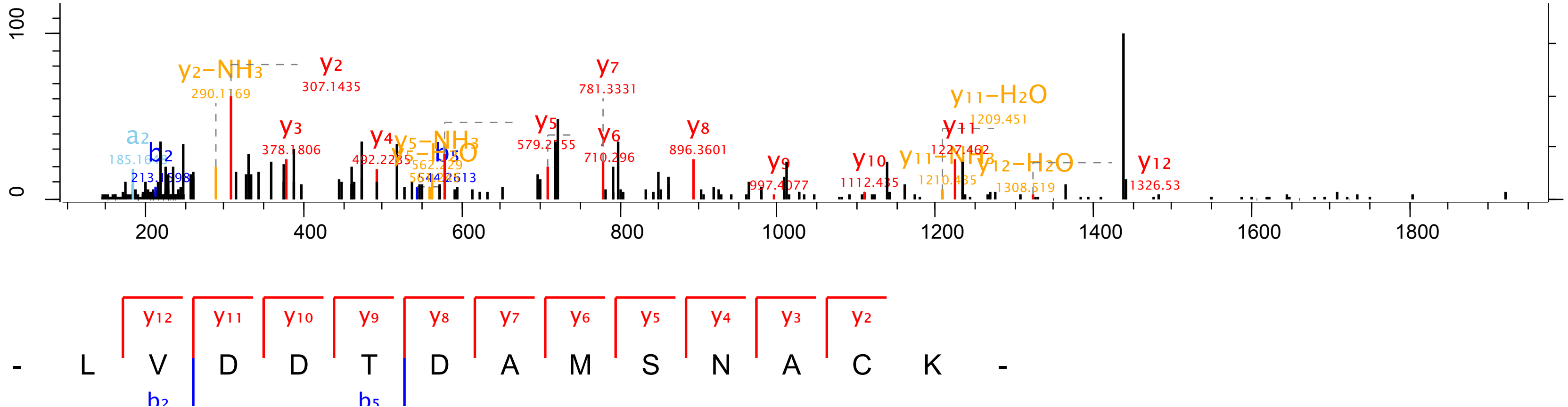

Raw file	Scan	Method	Score	m/z	Gene names
20150226_Hela_Top_opt_A3_01_1593	17646	TOF; CID	91.81	798.36	STX2

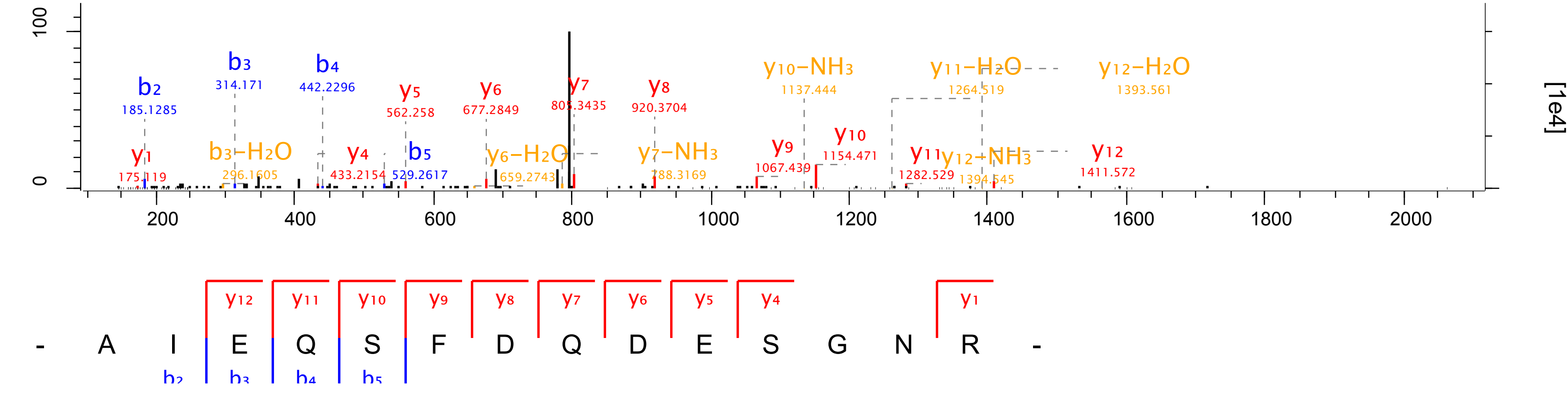

Raw file	Scan	Method	Score	m/z	Gene names
20150226_Hela_Top_opt_A3_01_1593	17866	TOF; CID	65.84	538.29	PTMS

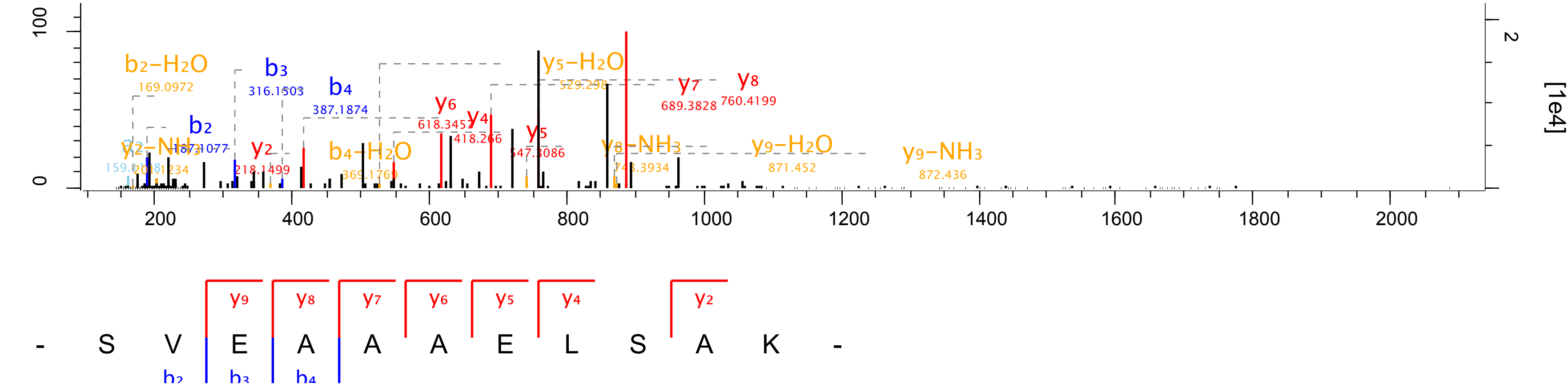

Raw file	Scan	Method	Score	m/z	Gene names
20150226_Hela_Top_opt_A3_01_1593	18111	TOF; CID	55.45	682.97	DNER

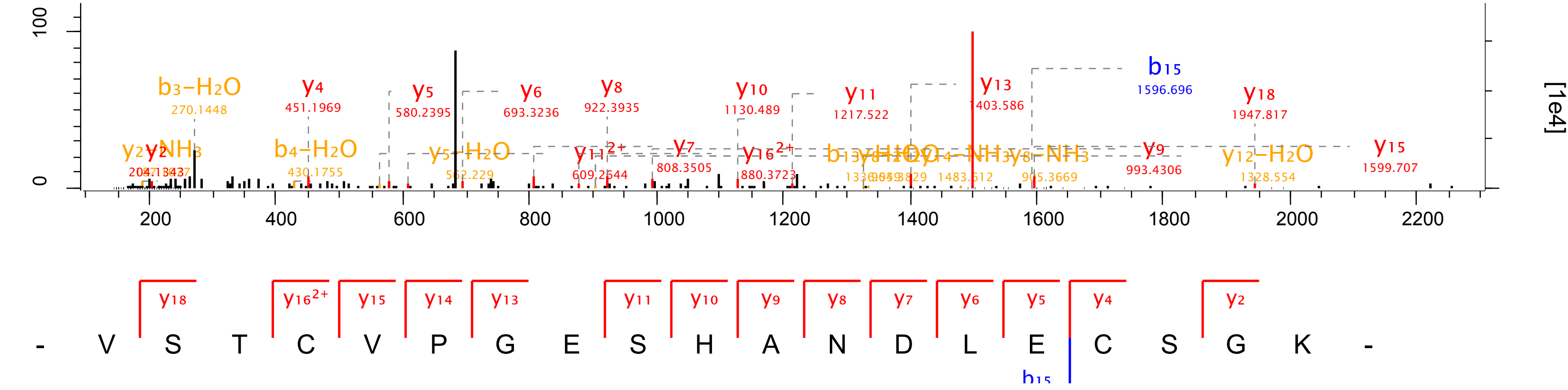

Raw file	Scan	Method	Score	m/z	Gene names
20150226_Hela_Top_opt_A3_01_1593	18921	TOF; CID	103.87	586.28	TMEM126B

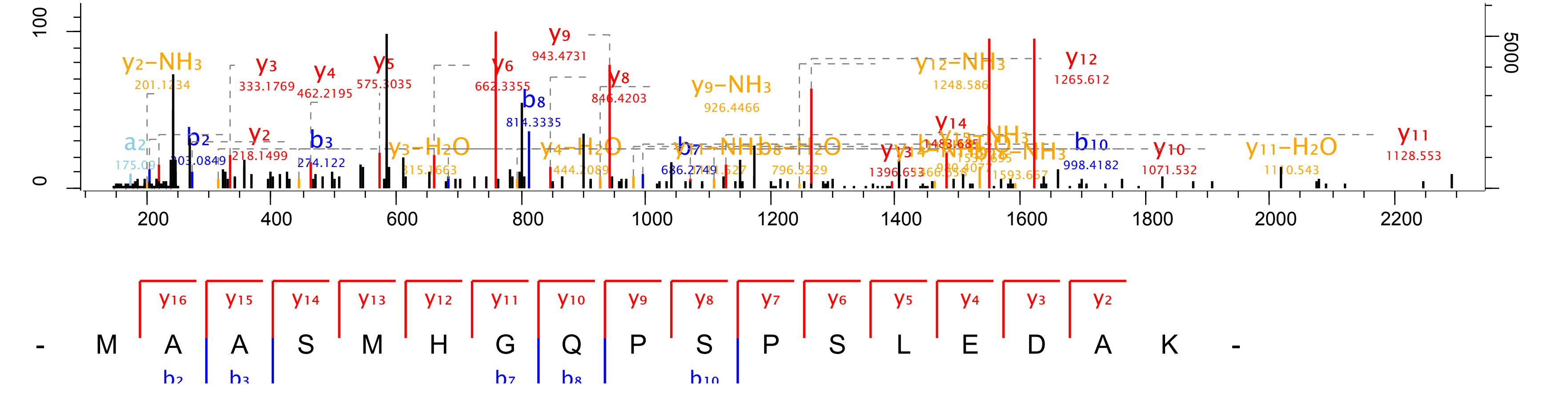

Raw file	Scan	Method	Score	m/z	Gene names
20150226_Hela_Top_opt_A3_01_1593	19140	TOF; CID	55.78	762.38	FAM129A

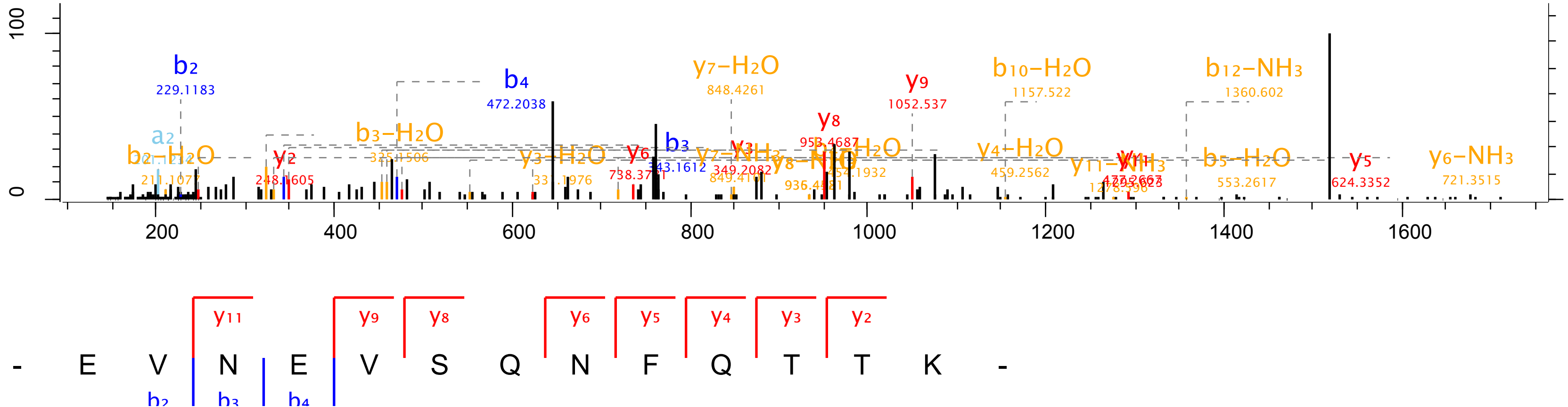

Raw file	Scan	Method	Score	m/z	Gene names
20150226_Hela_Top_opt_A3_01_1593	19607	TOF; CID	81.53	587.6	TIMM10B

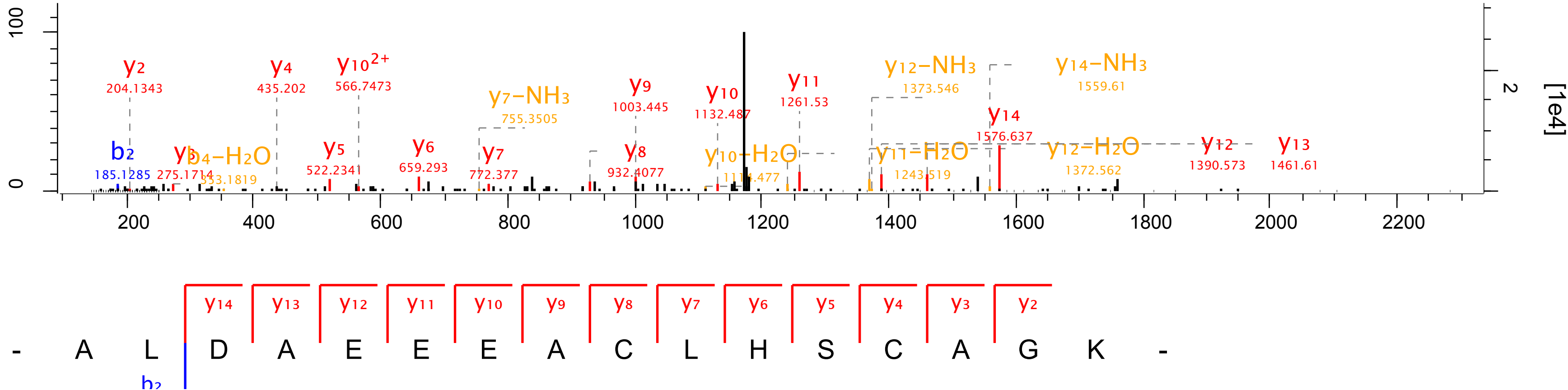

Raw file	Scan	Method	Score	m/z	Gene names
20150226_Hela_Top_opt_A3_01_1593	20634	TOF; CID	45.65	588.79	RBCK1

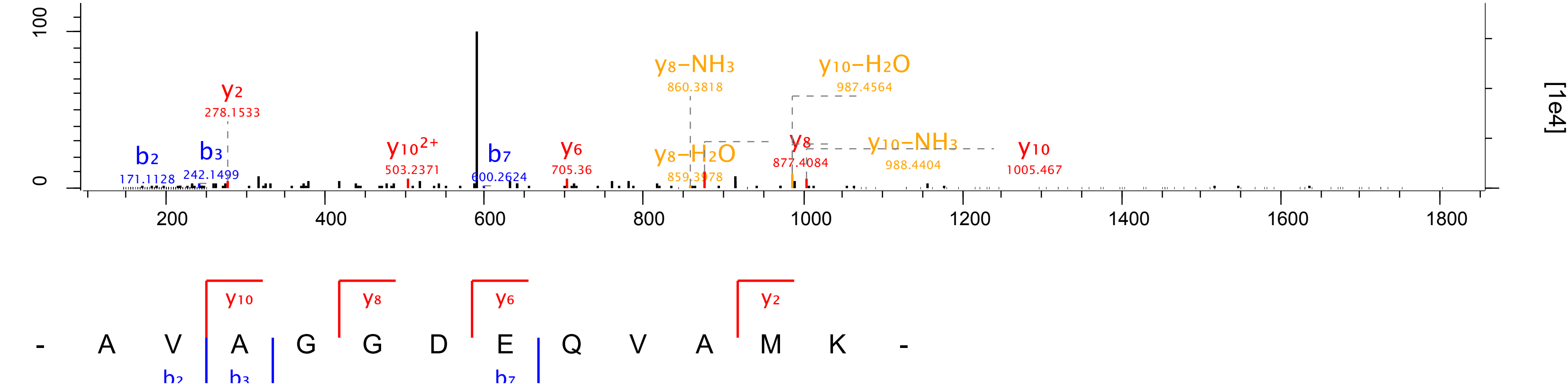

Raw file	Scan	Method	Score	m/z	Gene names
20150226_Hela_Top_opt_A3_01_1593	20994	TOF; CID	57.17	707.78	LGMN

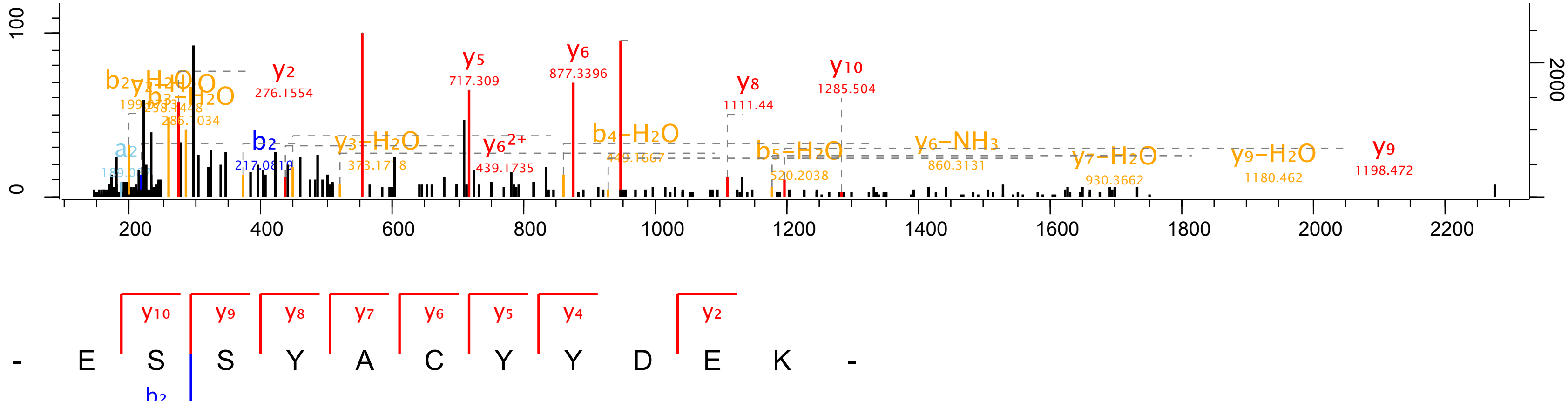

Raw file	Scan	Method	Score	m/z	Gene names
20150226_Hela_Top_opt_A3_01_1593	21090	TOF; CID	54.4	460.57	FAM20B

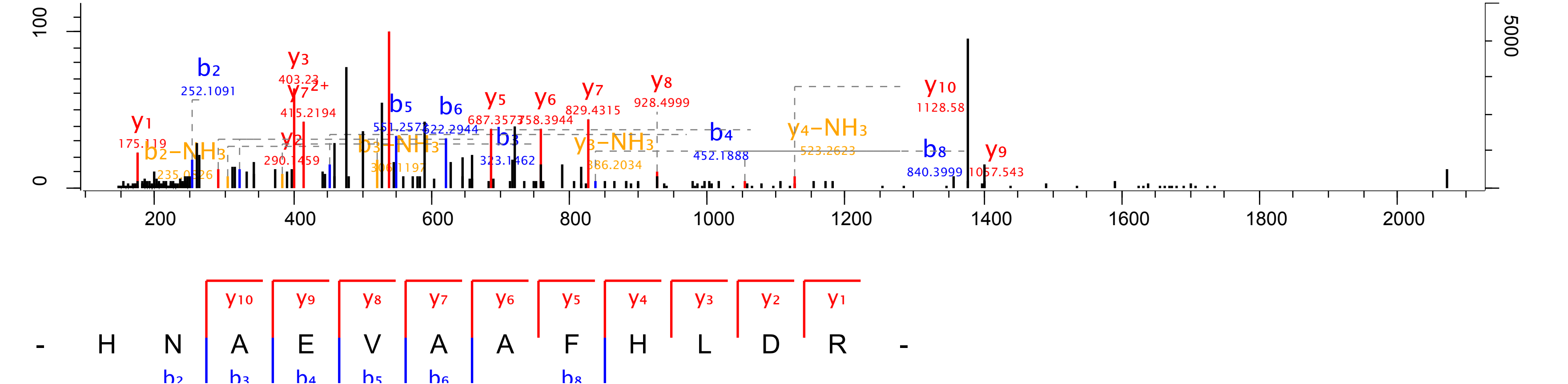

Raw file	Scan	Method	Score	m/z	Gene names
20150226_Hela_Top_opt_A3_01_1593	21295	TOF; CID	103.83	779.4	OIP5

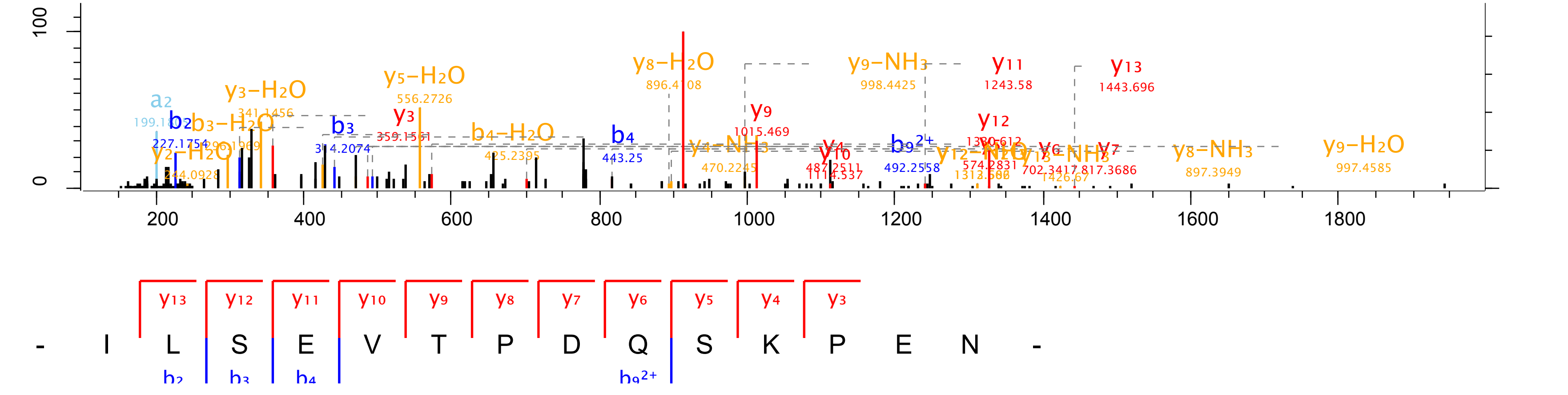

Raw file	Scan	Method	Score	m/z
20150226_Hela_Top_opt_A3_01_1593	21860	TOF; CID	51.25	609.81

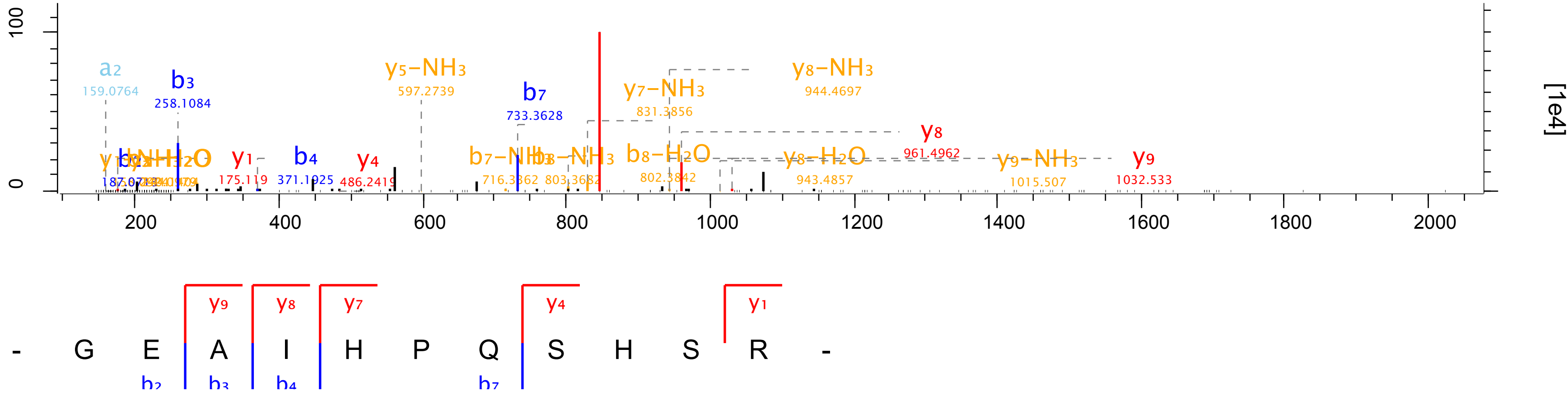

Raw file	Scan	Method	Score	m/z	Gene names
20150226_Hela_Top_opt_A3_01_1593	23662	TOF; CID	104.76	411.26	TRMT2B

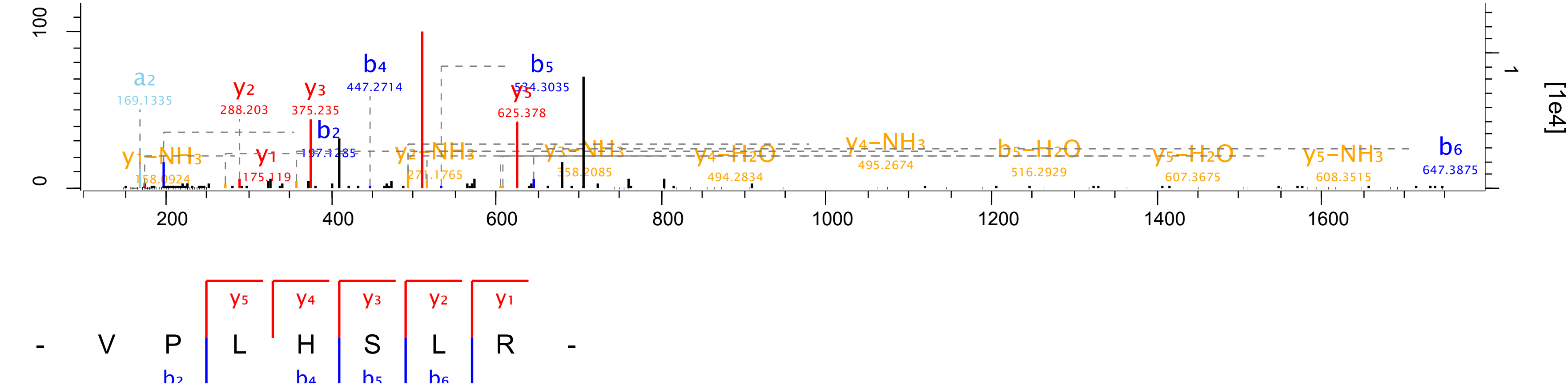

Raw file	Scan	Method	Score	m/z	Gene names
20150226_Hela_Top_opt_A3_01_1593	24096	TOF; CID	83.5	595.26	PPAP2C

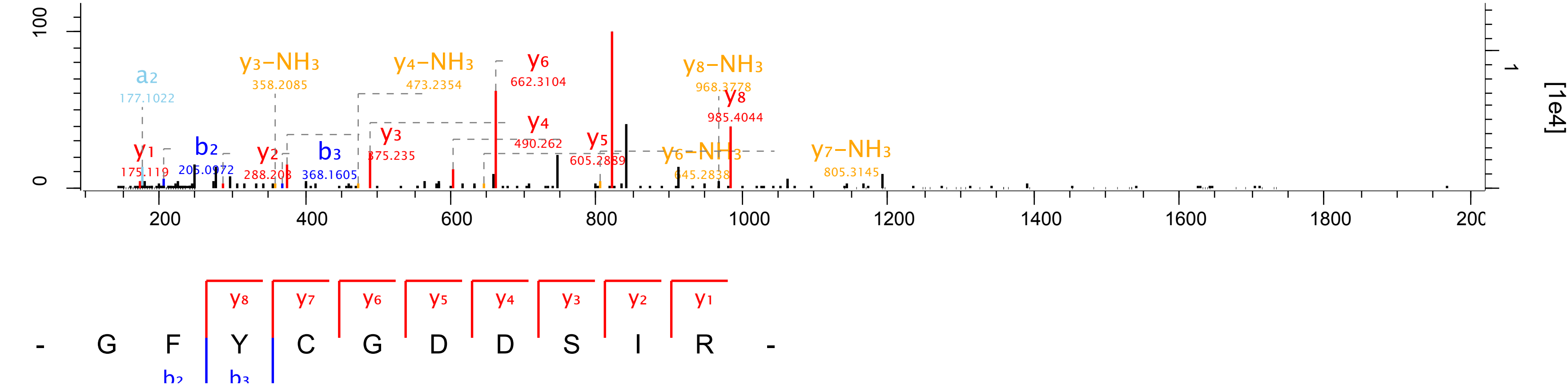

Raw file	Scan	Method	Score	m/z	Gene names
20150226_Hela_Top_opt_A3_01_1593	24251	TOF; CID	73.5	796.4	ST3GAL4

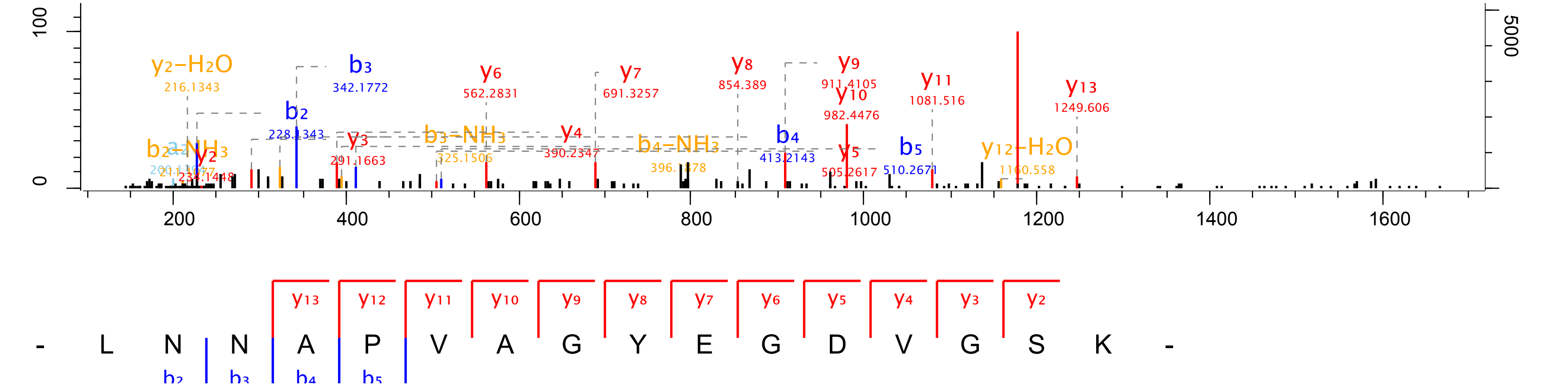

Raw file	Scan	Method	Score	m/z	Gene names
20150226_Hela_Top_opt_A3_01_1593	24772	TOF; CID	111.7	844.41	ELMSAN1

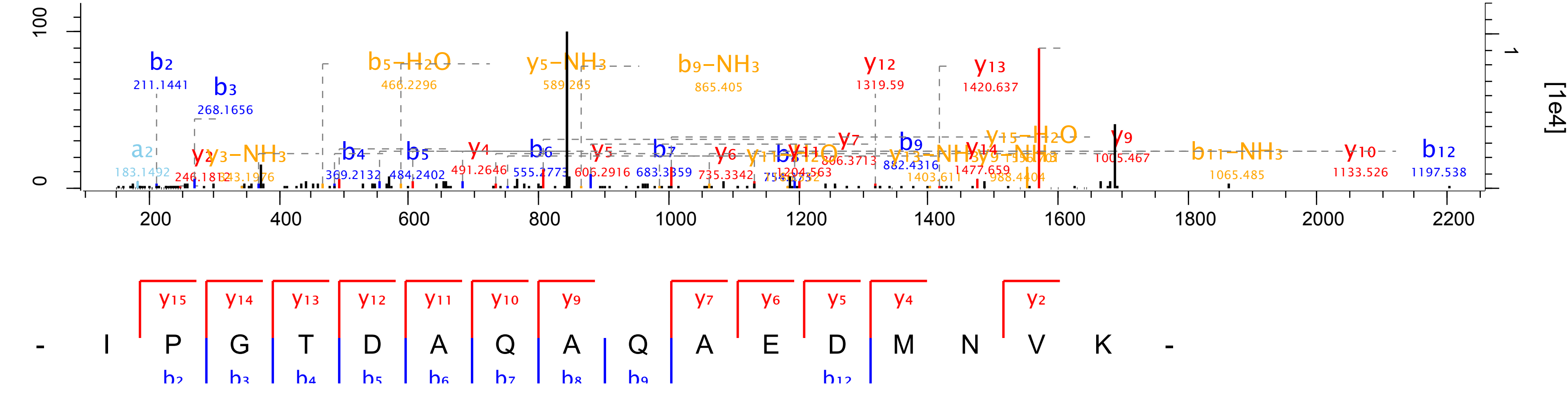

Raw file	Scan	Method	Score	m/z	Gene names
20150226_Hela_Top_opt_A3_01_1593	25198	TOF; CID	91.63	401.24	MEAF6

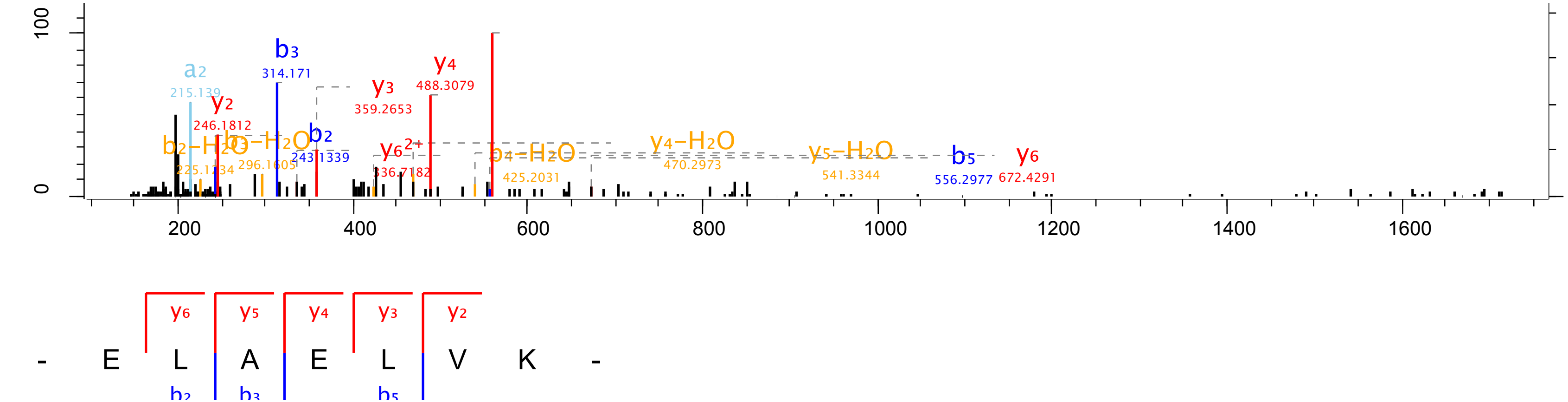

Raw file	Scan	Method	Score	m/z	Gene names
20150226_Hela_Top_opt_A3_01_1593	26653	TOF; CID	74.39	711.32	CETN3

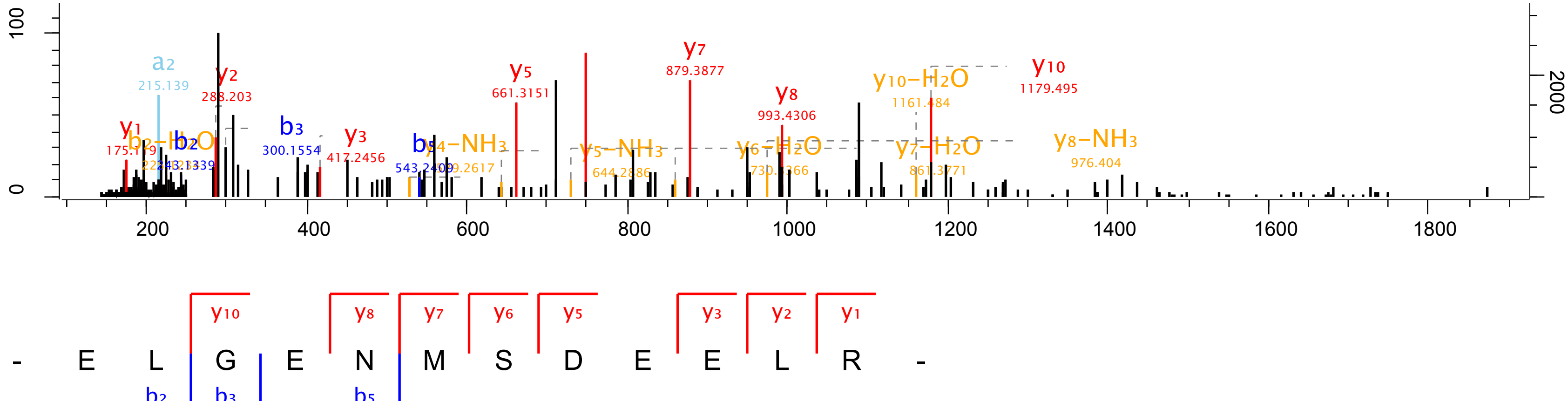

Raw file	Scan	Method	Score	m/z	Gene names
20150226_Hela_Top_opt_A3_01_1593	27017	TOF; CID	106.57	876.45	ZBTB21

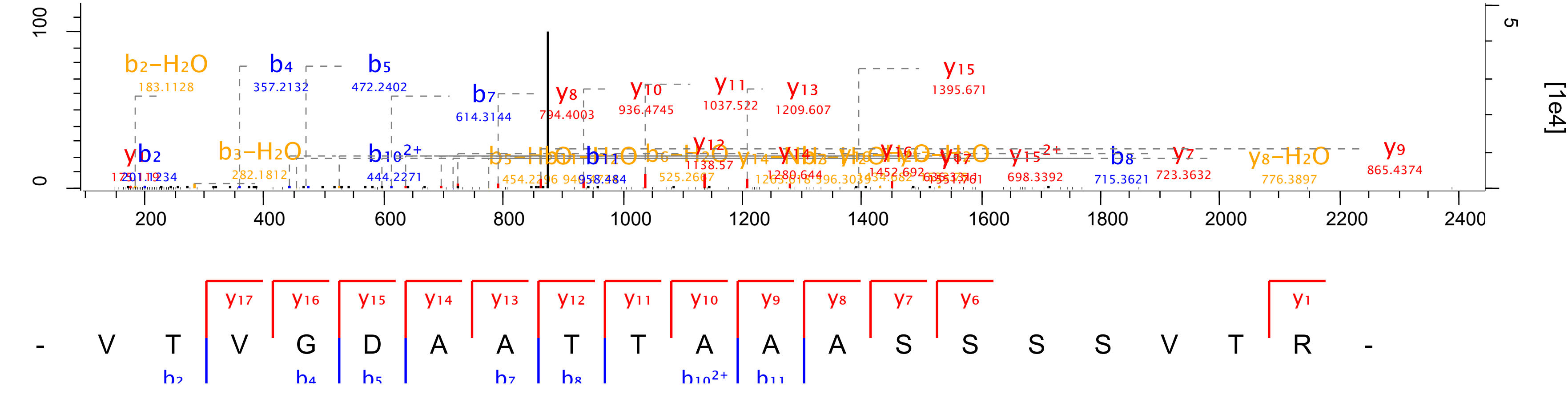

Raw file	Scan	Method	Score	m/z	Gene names
20150226_Hela_Top_opt_A3_01_1593	27510	TOF; CID	57.17	687.84	INSL4

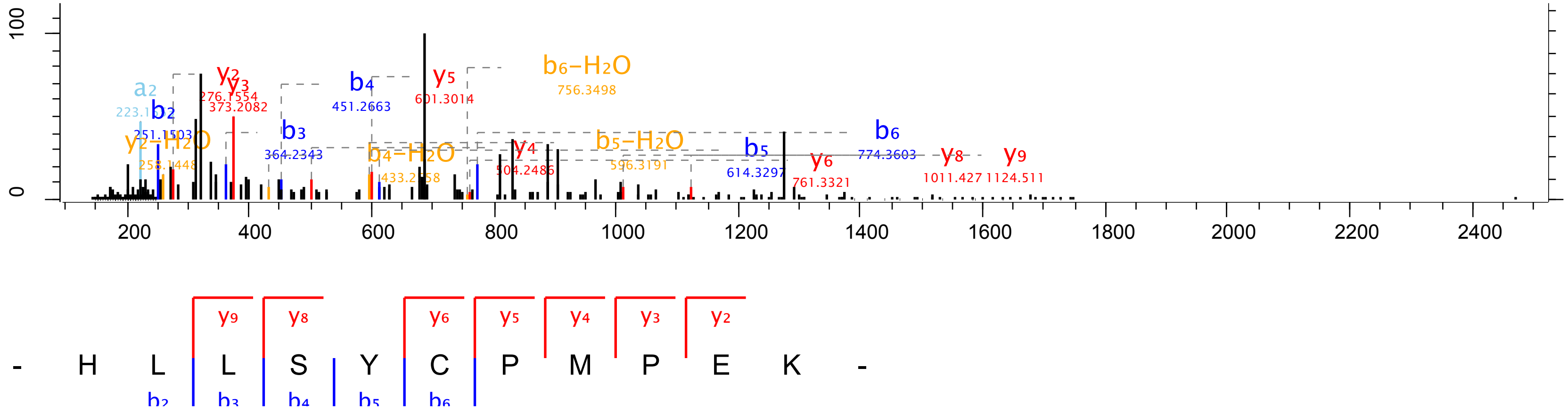

Raw file	Scan	Method	Score	m/z	Gene names
20150226_Hela_Top_opt_A3_01_1593	27814	TOF; CID	41.11	730.37	PLCD3

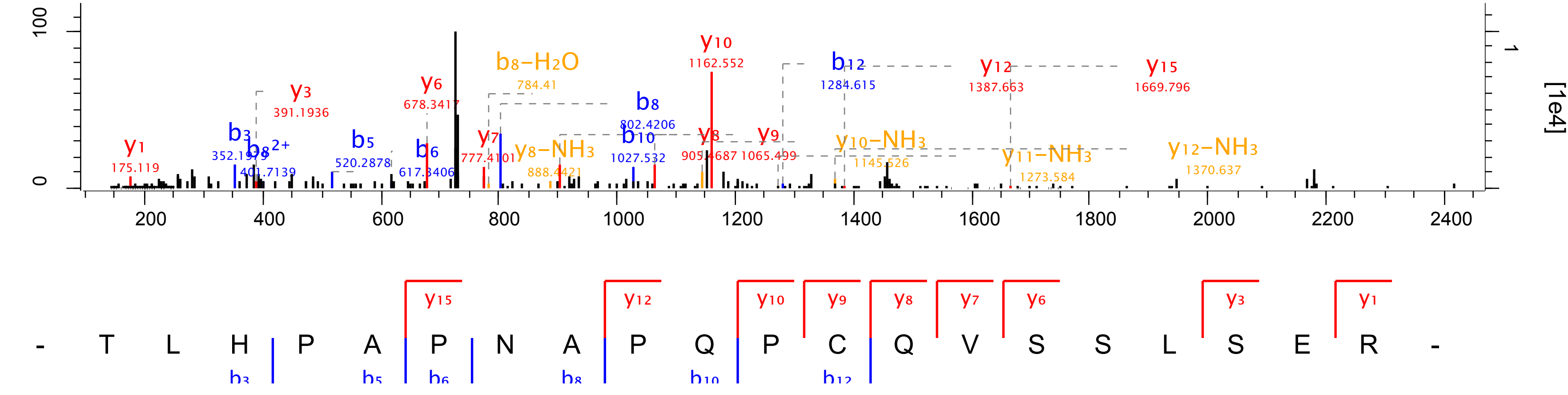

Raw file	Scan	Method	Score	m/z	Gene names
20150226_Hela_Top_opt_A3_01_1593	29177	TOF; CID	86.67	556.8	SFXN2

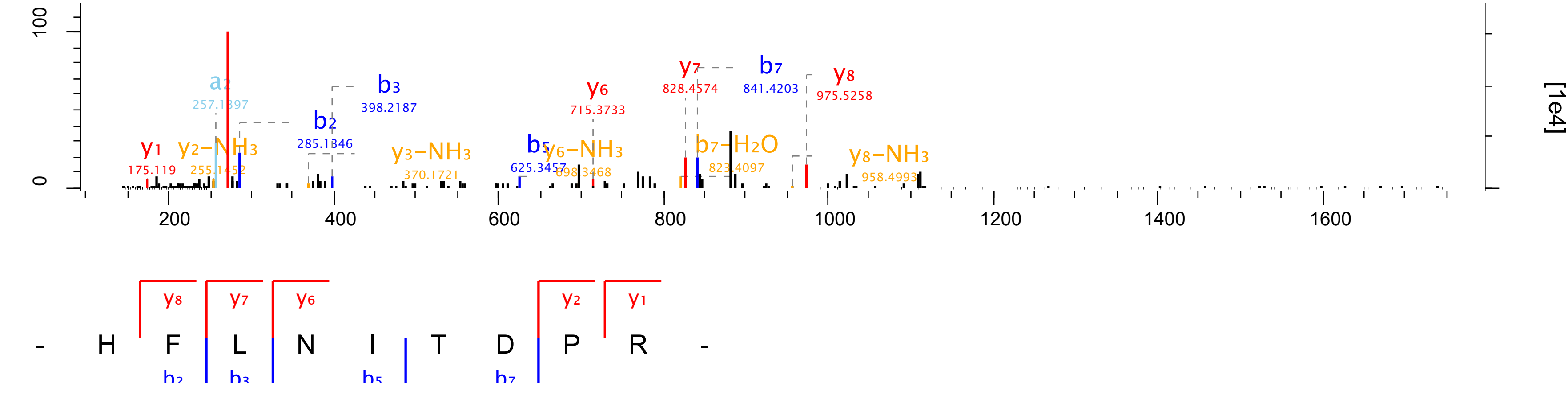

Raw file	Scan	Method	Score	m/z	Gene names
20150226_Hela_Top_opt_A3_01_1593	29183	TOF; CID	84.38	888.46	NDUFV3

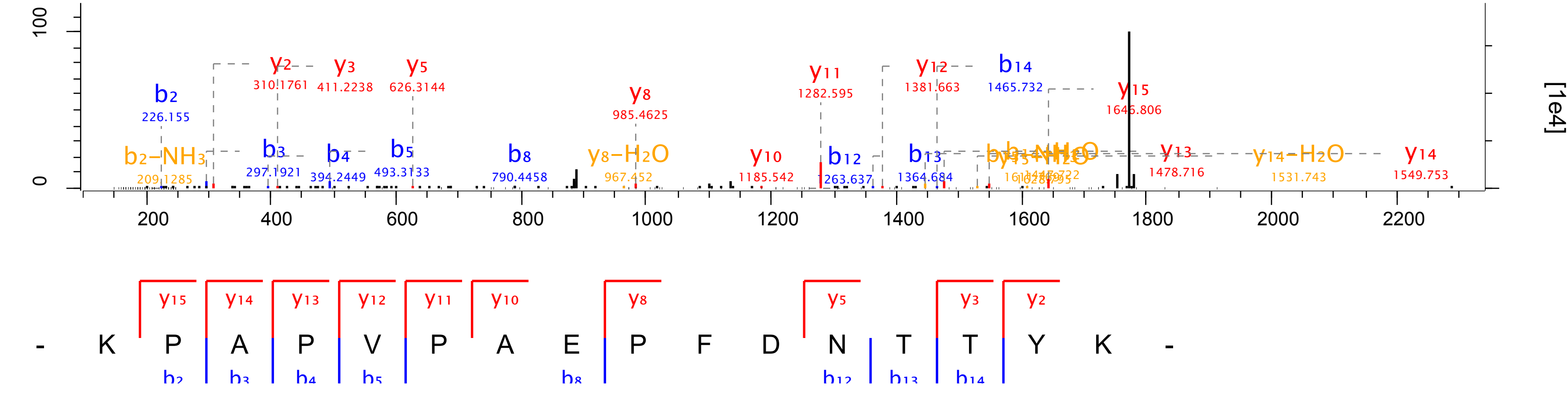

Raw file	Scan	Method	Score	m/z	Gene names
20150226_Hela_Top_opt_A3_01_1593	30871	TOF; CID	60.33	645.33	CRCP

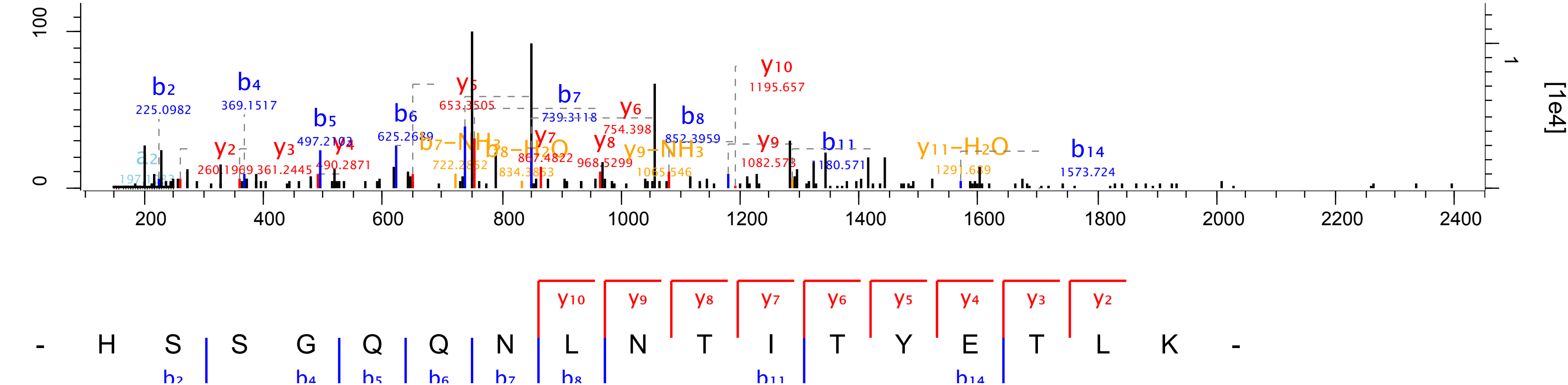

Raw file	Scan	Method	Score	m/z	Gene names
20150226_Hela_Top_opt_A3_01_1593	32146	TOF; CID	58.32	704.38	ARHGAP18

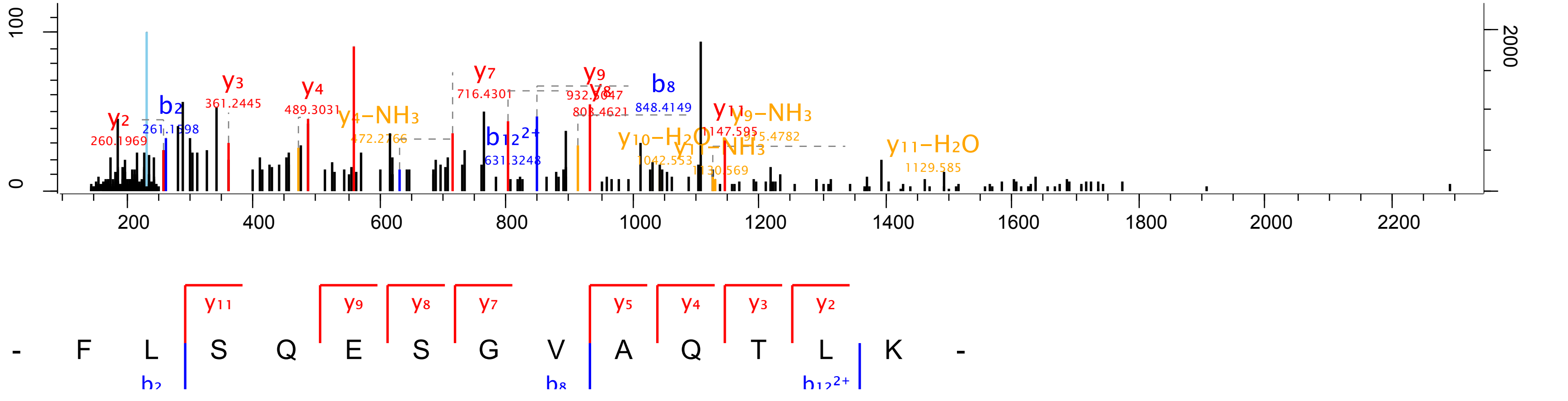

Raw file	Scan	Method	Score	m/z	Gene names
20150226_Hela_Top_opt_A3_01_1593	32331	TOF; CID	54.77	646.36	BRD7

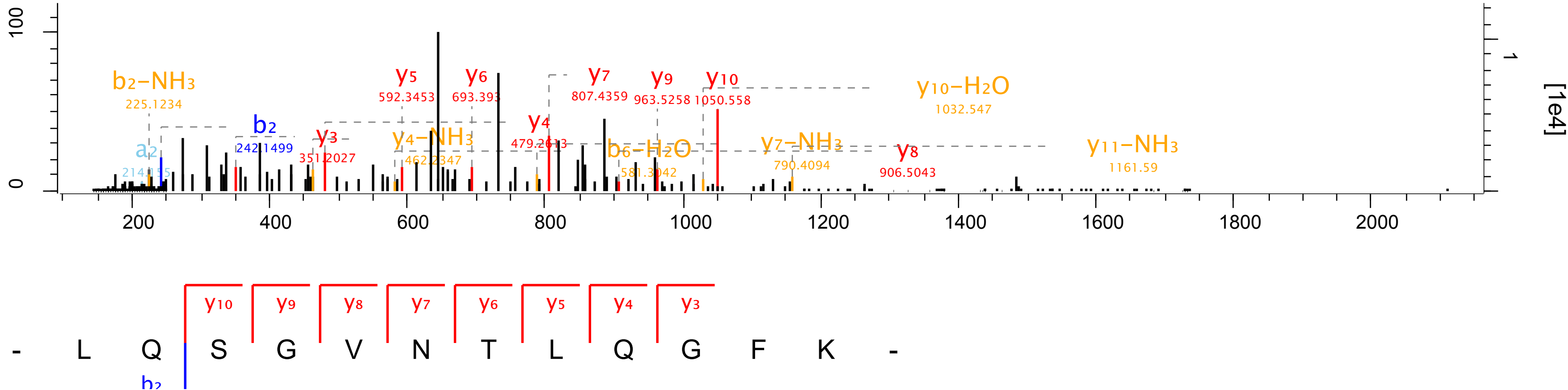

Raw file	Scan	Method	Score	m/z	Gene names
20150226_Hela_Top_opt_A3_01_1593	32440	TOF; CID	51.77	596	DUS2

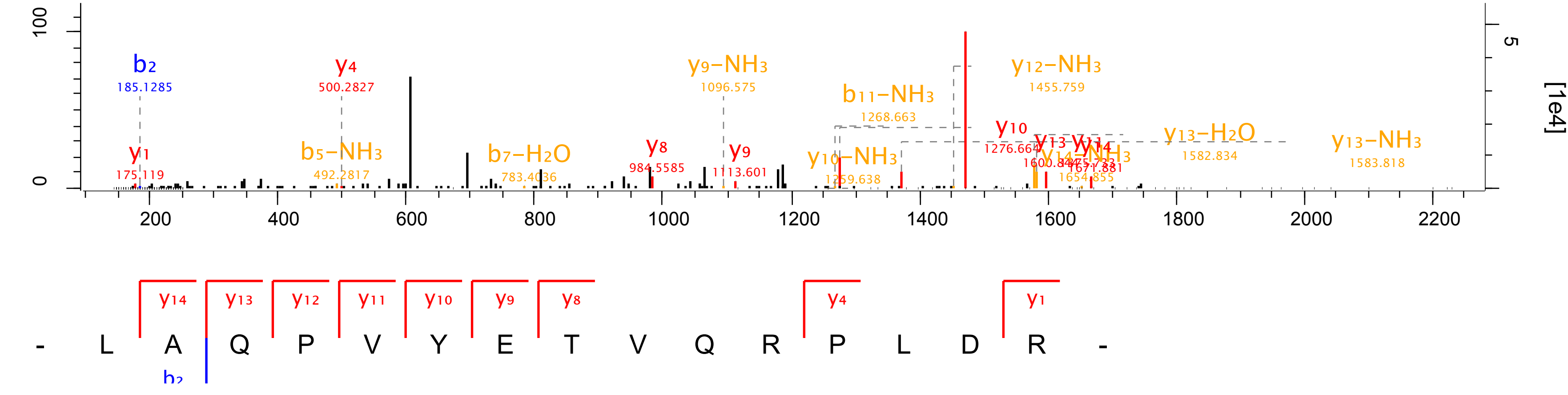

Raw file	Scan	Method	Score	m/z	Gene names
20150226_Hela_Top_opt_A3_01_1593	32741	TOF; CID	53.59	705.36	TMEM69

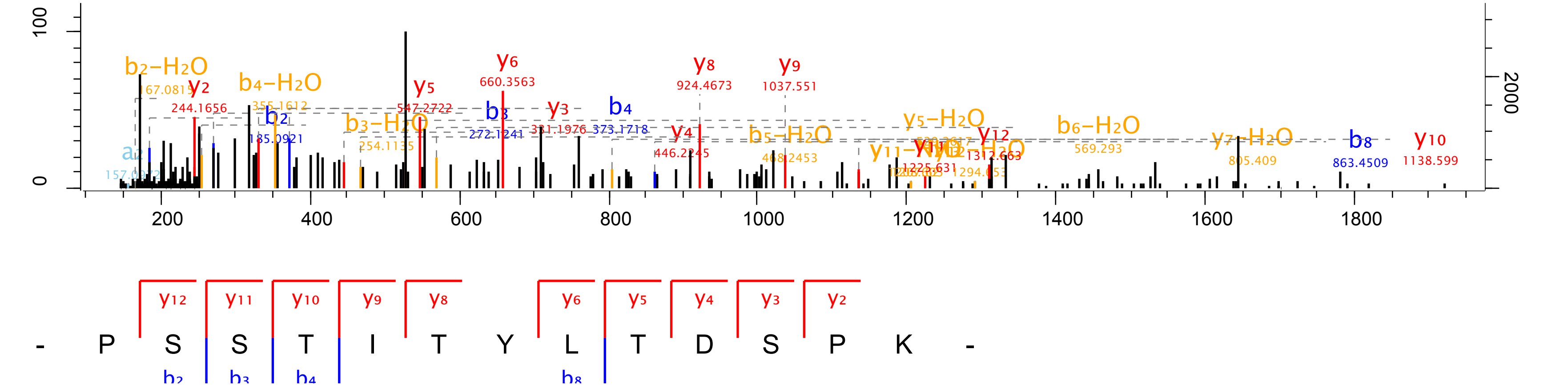

Raw file	Scan	Method	Score	m/z	Gene names
20150226_Hela_Top_opt_A3_01_1593	32853	TOF; CID	90.41	746.36	KIF3A

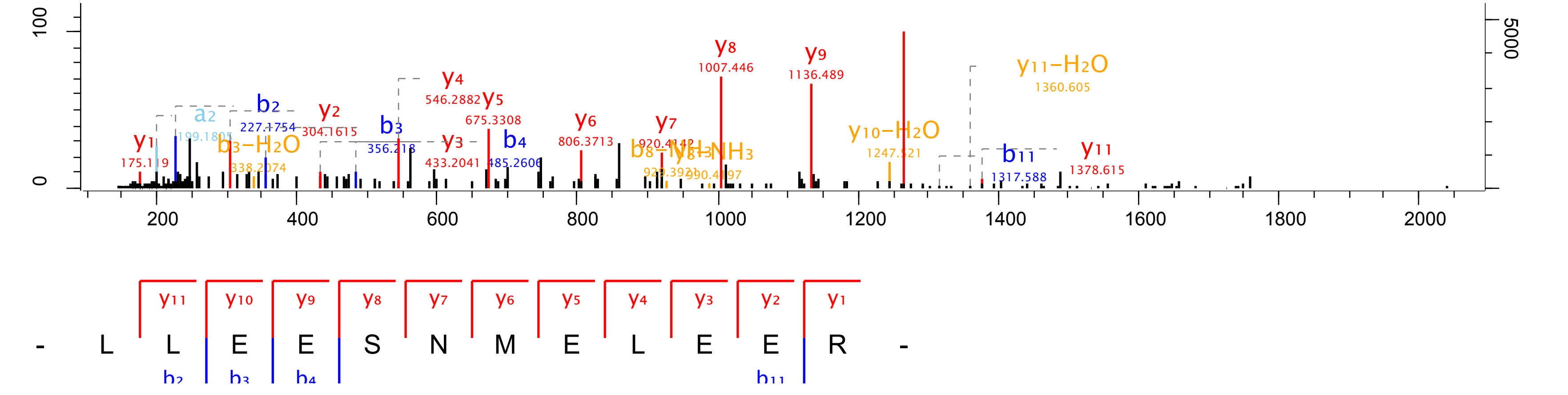

Raw file	Scan	Method	Score	m/z	Gene names
20150226_Hela_Top_opt_A3_01_1593	33119	TOF; CID	103.4	818.4	CEP250

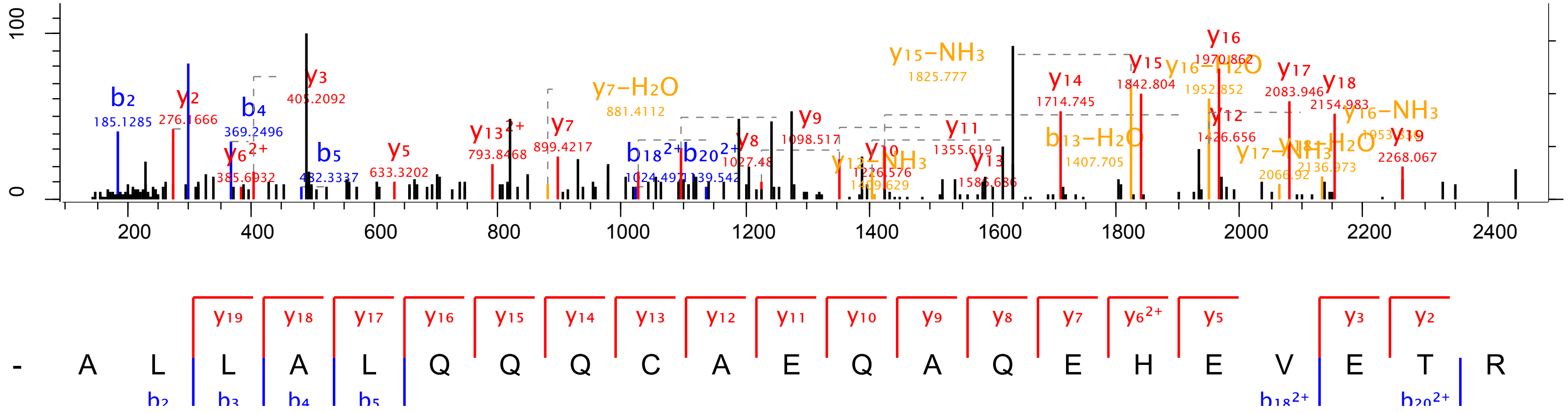

Raw file	Scan	Method	Score	m/z	Gene names
20150226_Hela_Top_opt_A3_01_1593	33295	TOF; CID	143.43	933.45	ROMO1

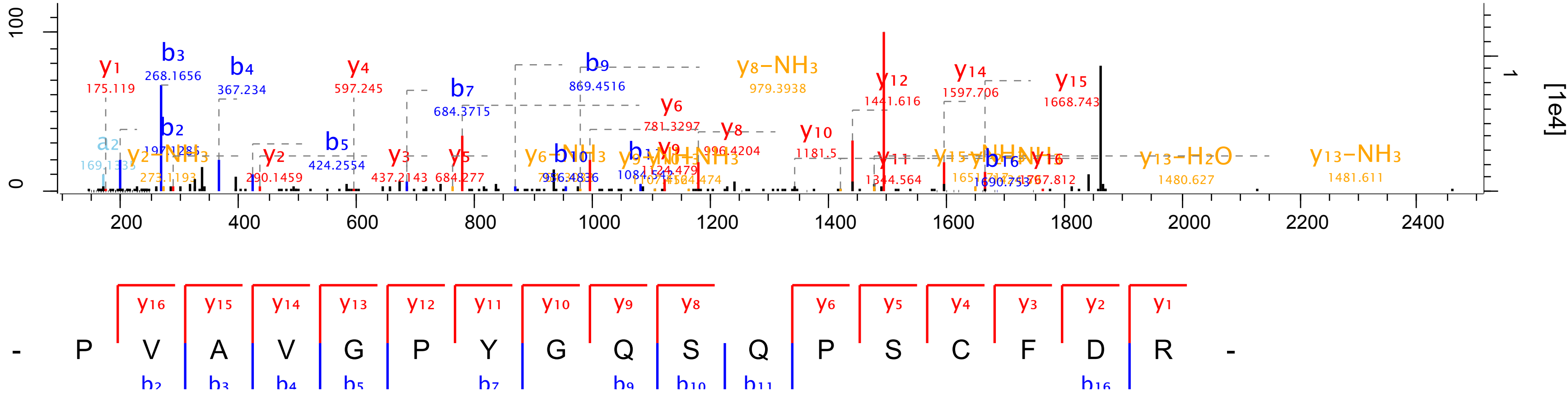

Raw file	Scan	Method	Score	m/z	Gene names
20150226_Hela_Top_opt_A3_01_1593	33520	TOF; CID	116.28	834.42	SLC7A6

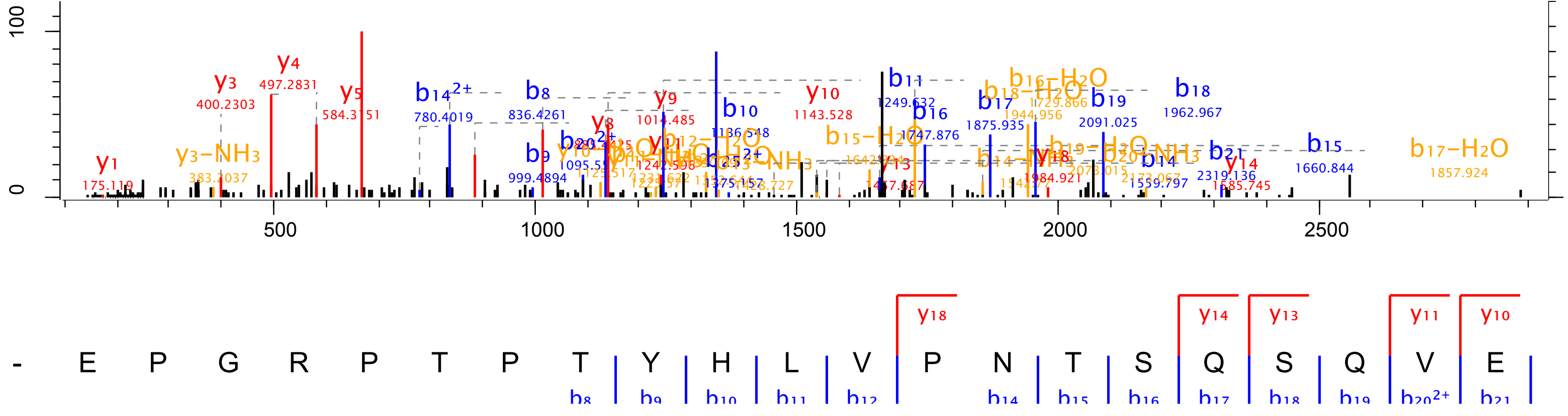

Raw file	Scan	Method	Score	m/z	Gene names
20150226_Hela_Top_opt_A3_01_1593	33731	TOF; CID	110.08	665.36	GDAP1

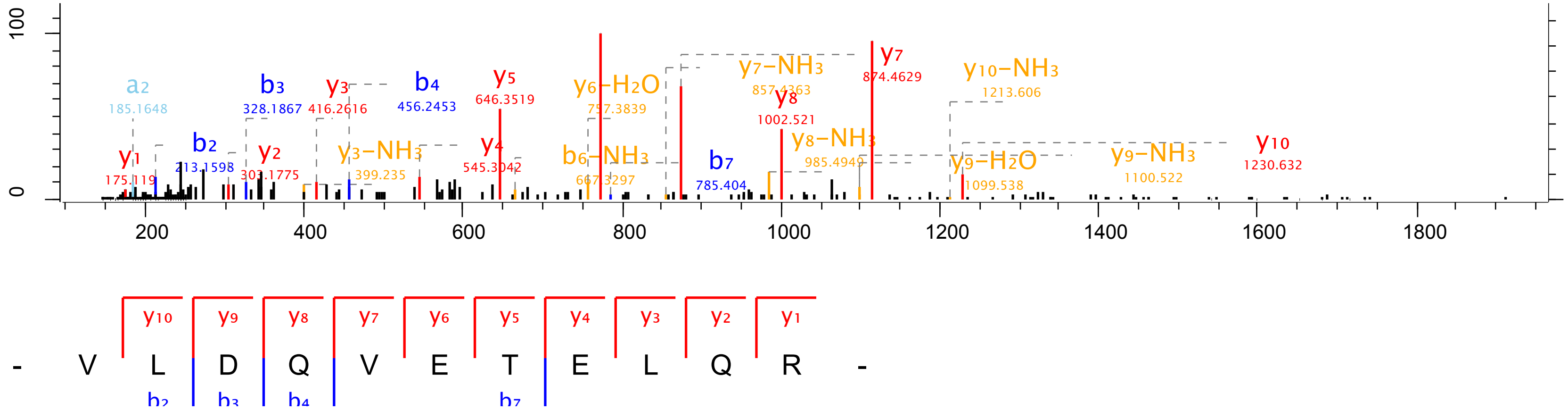

Raw file	Scan	Method	Score	m/z	Gene names
20150226_Hela_Top_opt_A3_01_1593	33950	TOF; CID	54.78	537.32	UBIAD1

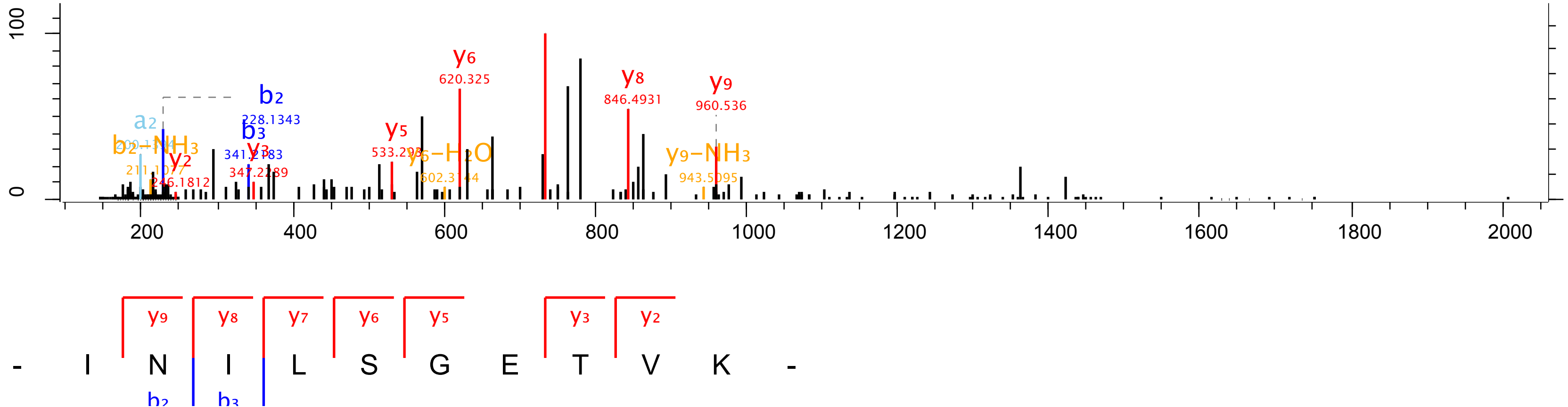

Raw file	Scan	Method	Score	m/z	Gene names
20150226_Hela_Top_opt_A3_01_1593	34401	TOF; CID	65.18	675.34	TBC1D24

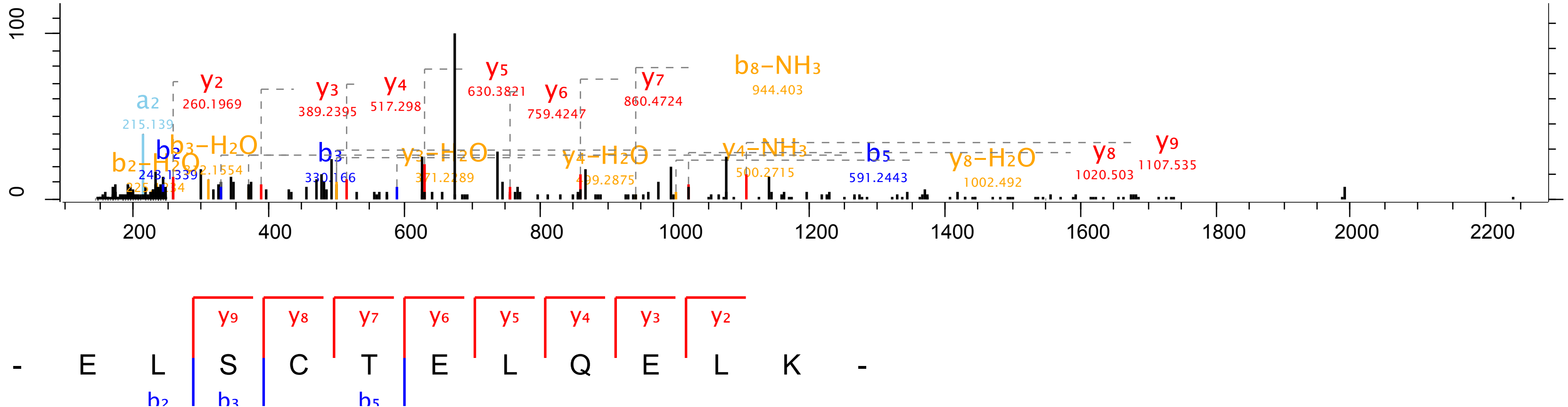

Raw file	Scan	Method	Score	m/z	Gene names
20150226_Hela_Top_opt_A3_01_1593	34650	TOF; CID	77.12	687.85	FOXL2;FOXF1;FOXF2

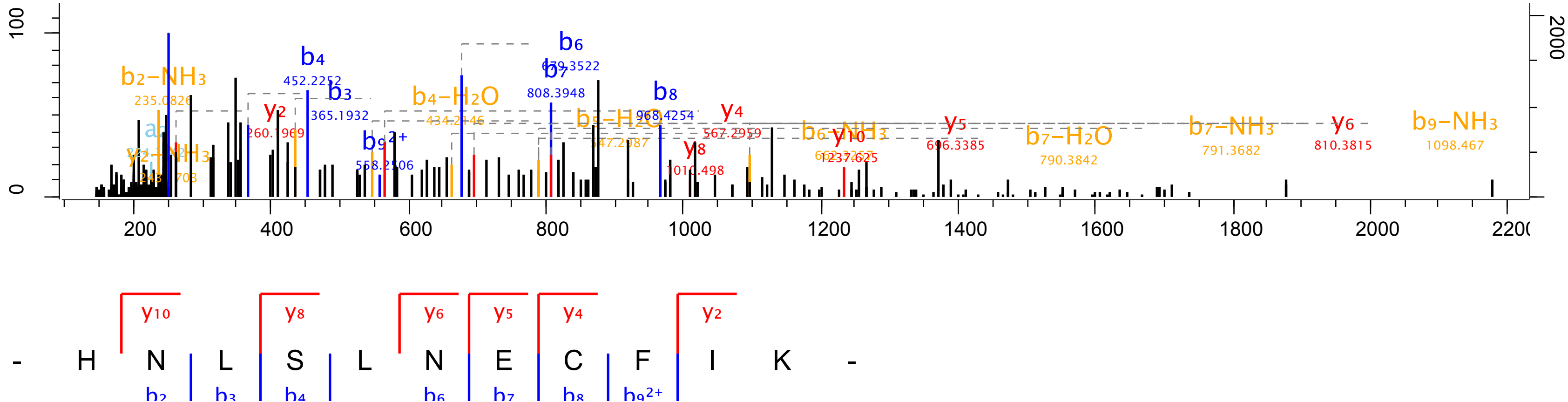

Raw file	Scan	Method	Score	m/z	Gene names
20150226_Hela_Top_opt_A3_01_1593	34773	TOF; CID	115.57	610.31	SLC27A1

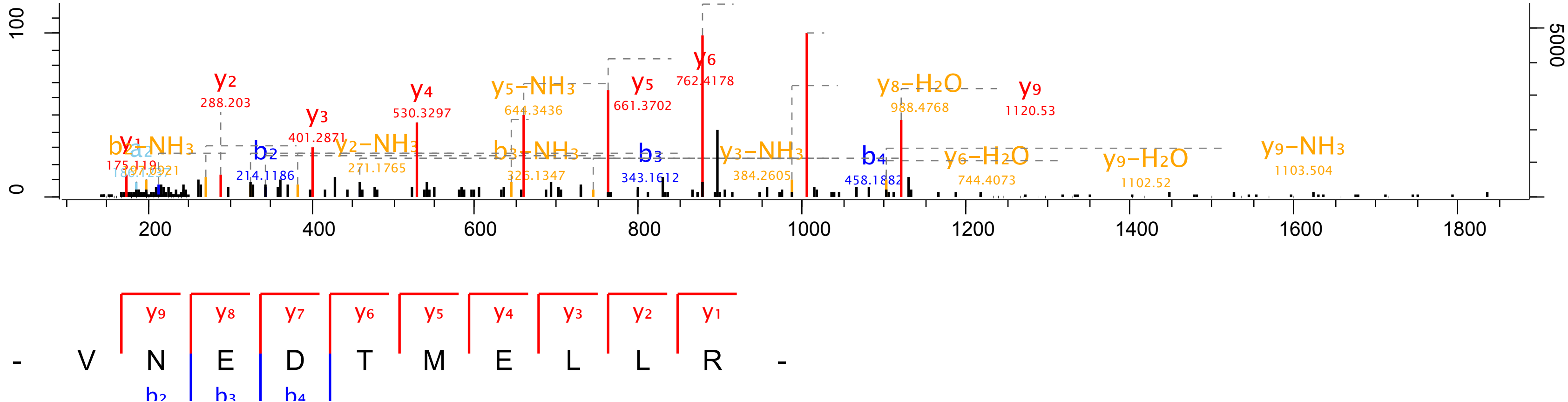

Raw file	Scan	Method	Score	m/z	Gene names
20150226_Hela_Top_opt_A3_01_1593	34884	TOF; CID	83.76	751.35	POLR2K

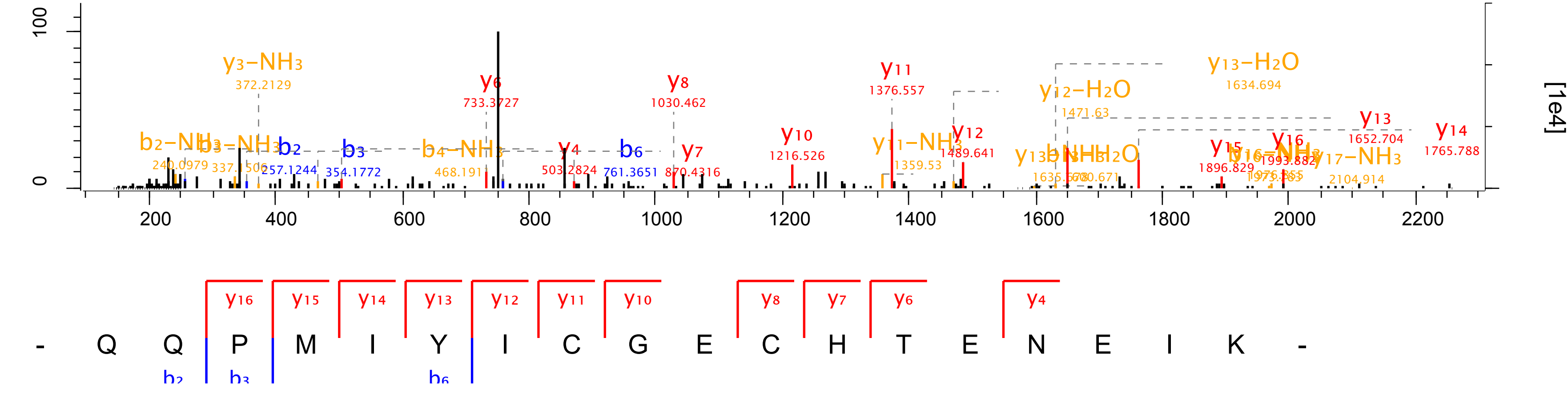

Raw file	Scan	Method	Score	m/z	Gene names
20150226_Hela_Top_opt_A3_01_1593	34938	TOF; CID	53.57	712.32	ZNF330

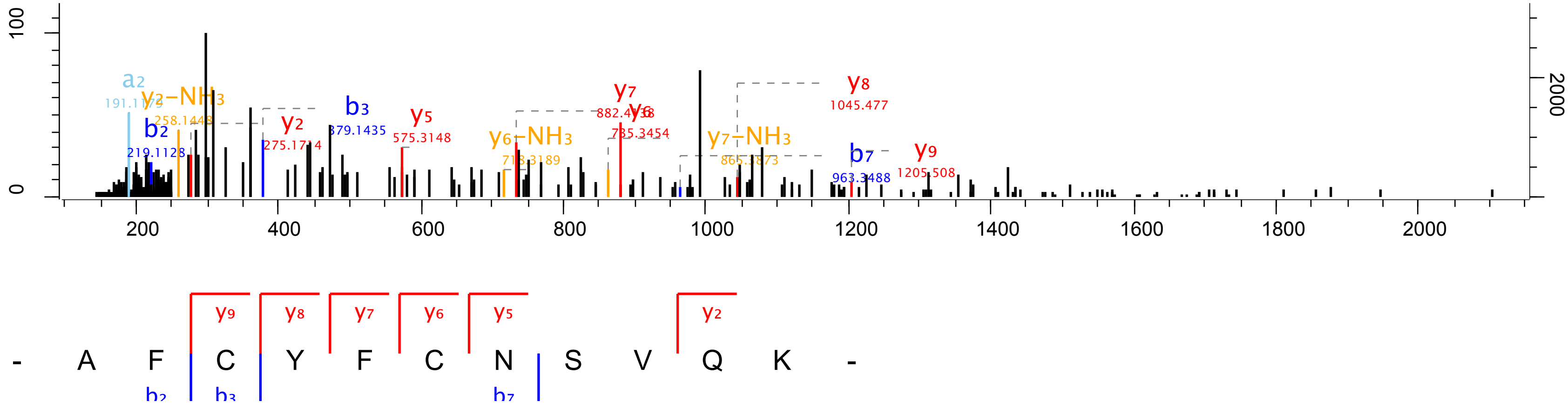

Raw file	Scan	Method	Score	m/z	Gene names
20150226_Hela_Top_opt_A3_01_1593	34959	TOF; CID	59.16	827.4	GOLGA5

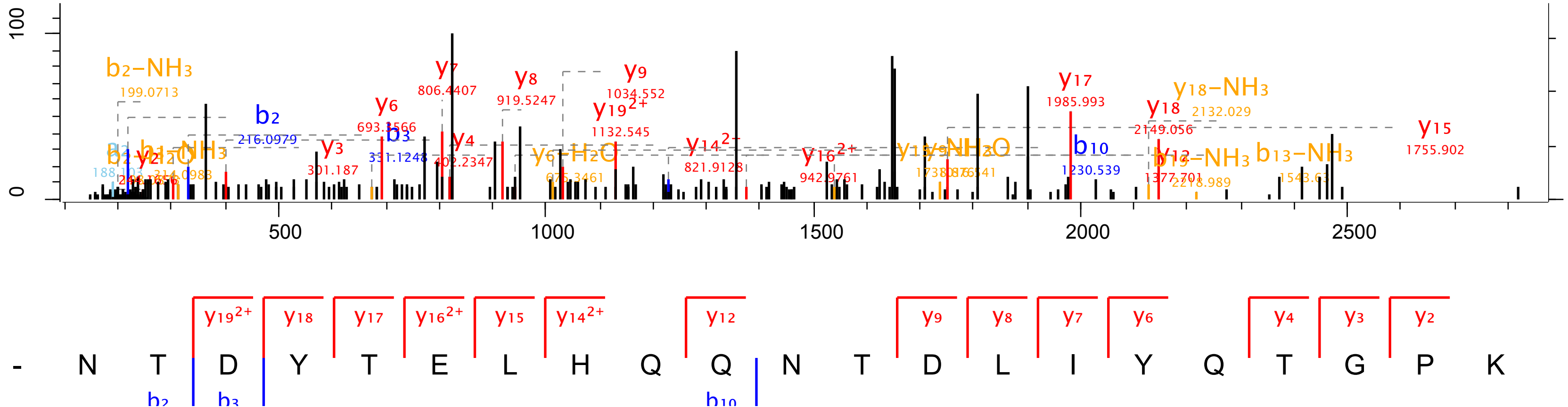

Raw file	Scan	Method	Score	m/z	Gene names
20150226_Hela_Top_opt_A3_01_1593	35352	TOF; CID	62.46	387.89	HDAC9

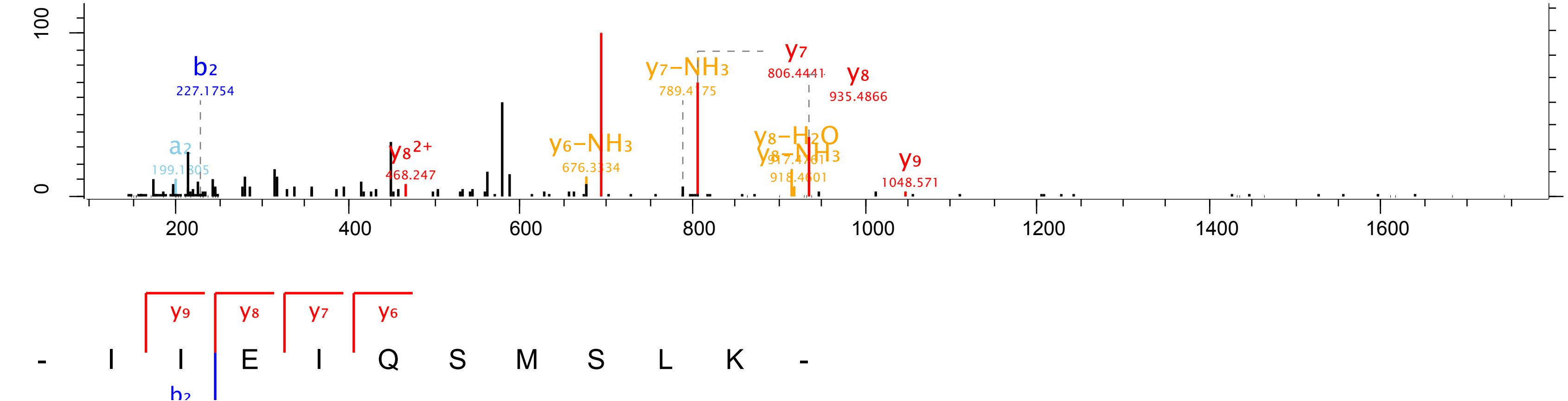

Raw file	Scan	Method	Score	m/z	Gene names
20150226_Hela_Top_opt_A3_01_1593	37014	TOF; CID	75.76	739.37	PROSER2;C10orf47

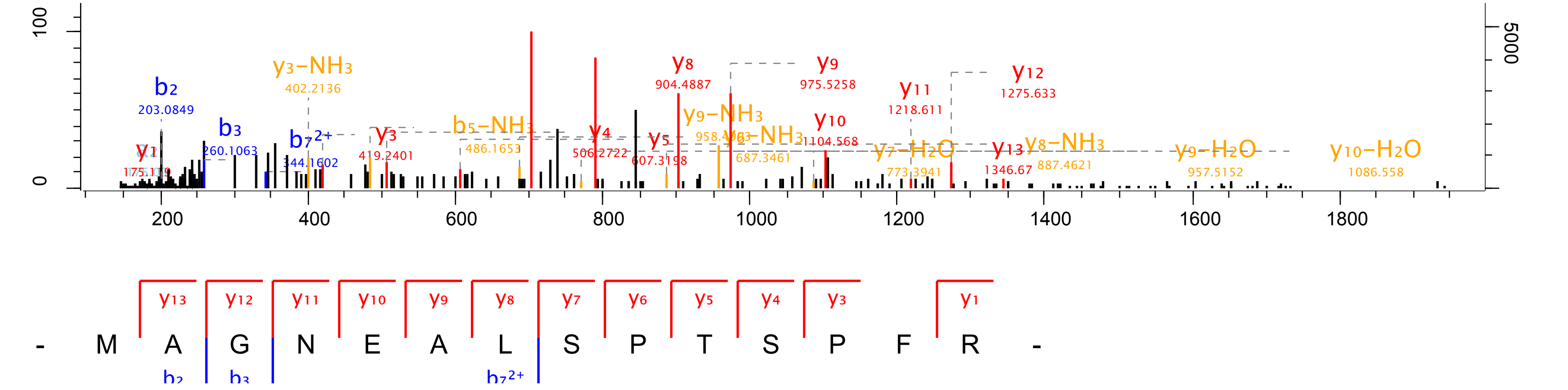

Raw file	Scan	Method	Score	m/z	Gene names
20150226_Hela_Top_opt_A3_01_1593	37352	TOF; CID	75.2	540.79	RAD18

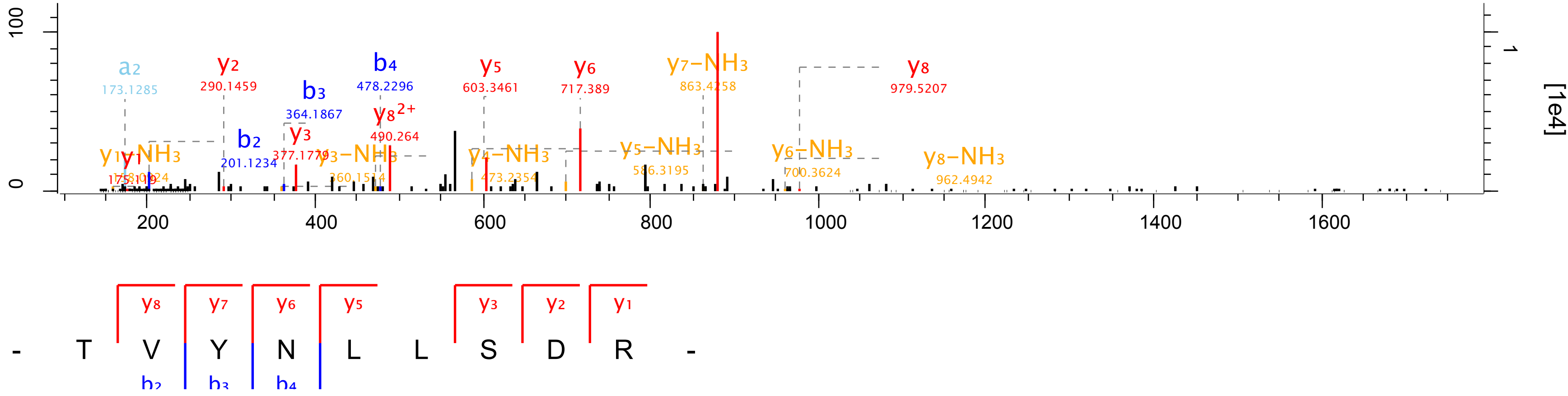

Raw file	Scan	Method	Score	m/z	Gene names
20150226_Hela_Top_opt_A3_01_1593	37434	TOF; CID	70.06	547.3	NARS

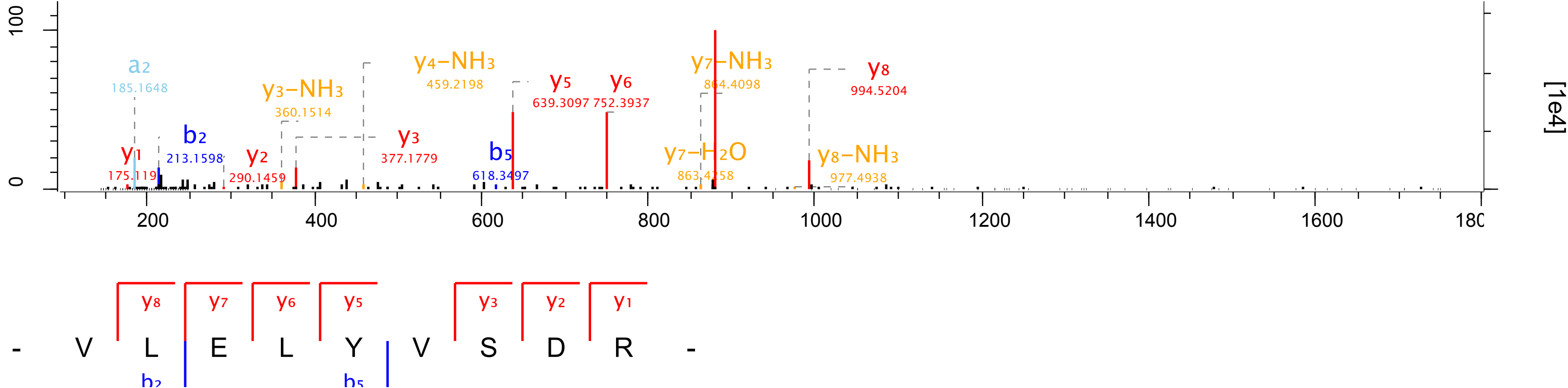

Raw file	Scan	Method	Score	m/z	Gene names
20150226_Hela_Top_opt_A3_01_1593	37749	TOF; CID	122.12	785.39	ZADH2

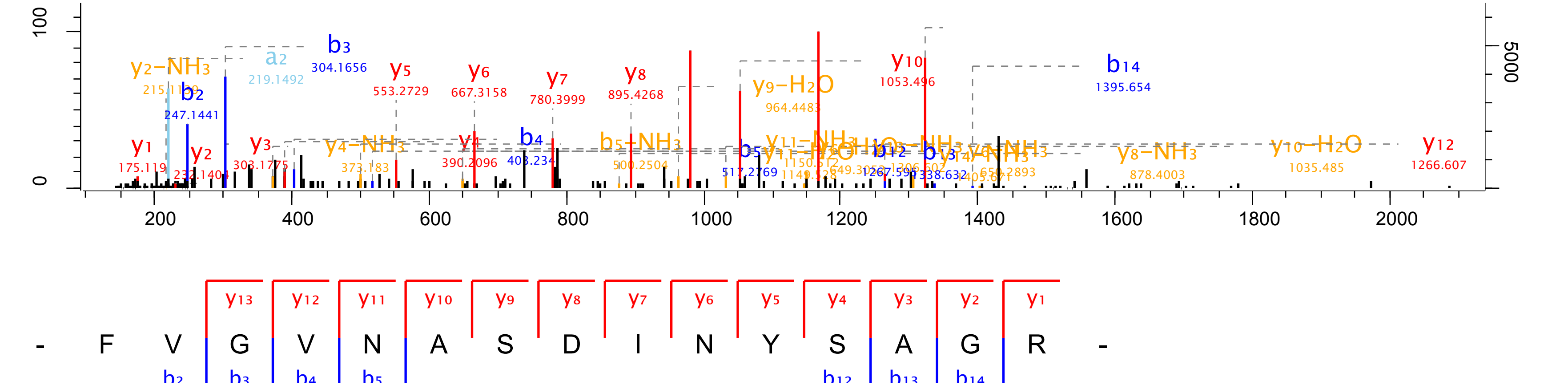

Raw file	Scan	Method	Score	m/z	Gene names
20150226_Hela_Top_opt_A3_01_1593	37968	TOF; CID	50.83	1044.53	POTED

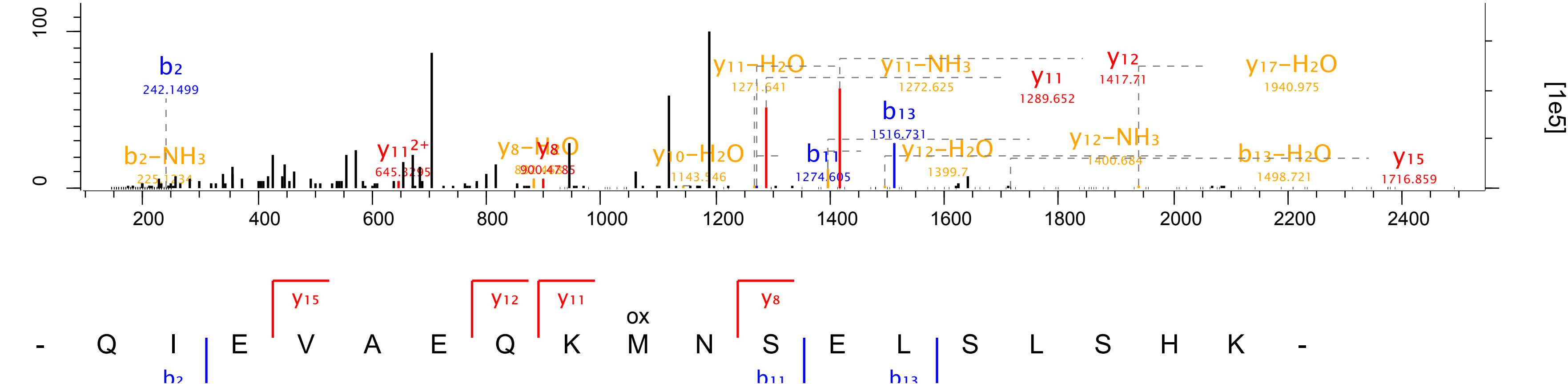

Raw file	Scan	Method	Score	m/z	Gene names
20150226_Hela_Top_opt_A3_01_1593	38175	TOF; CID	74.97	796.42	SYNRG

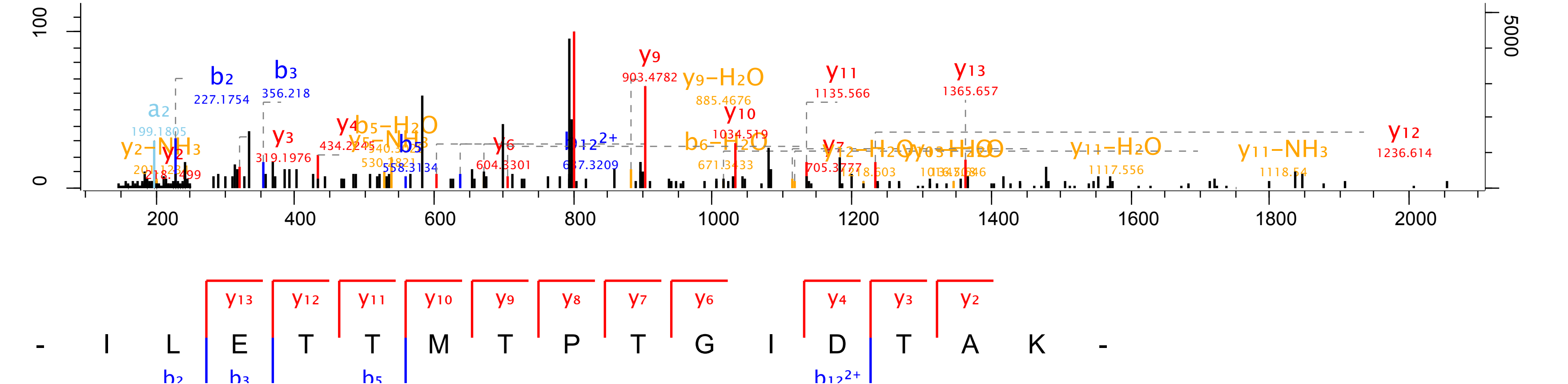

Raw file	Scan	Method	Score	m/z	Gene names
20150226_Hela_Top_opt_A3_01_1593	38544	TOF; CID	79.84	837.41	FAM45A;FAM45B

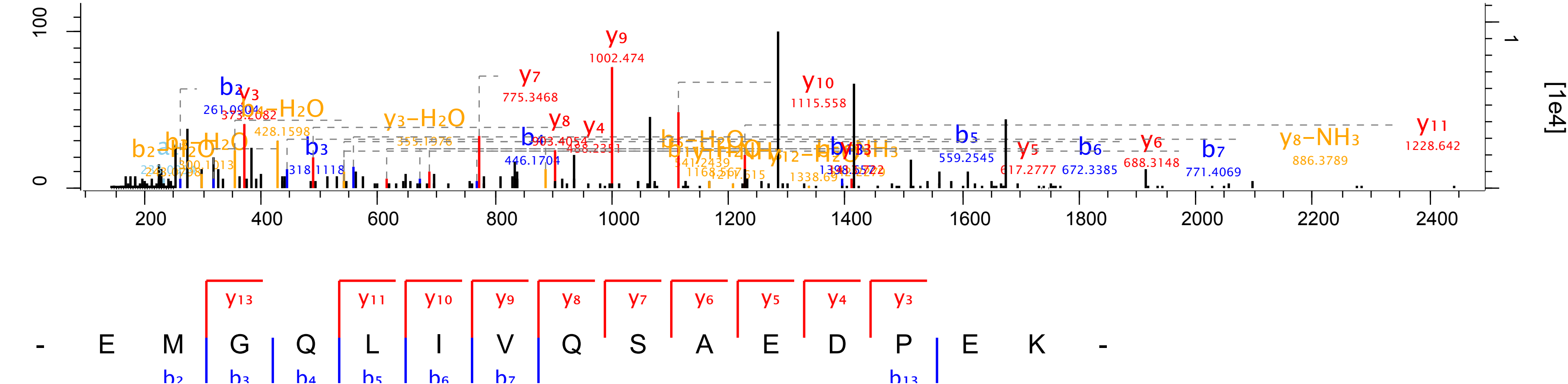

Raw file	Scan	Method	Score	m/z	Gene names
20150226_Hela_Top_opt_A3_01_1593	39636	TOF; CID	106.29	384.26	MYO9A

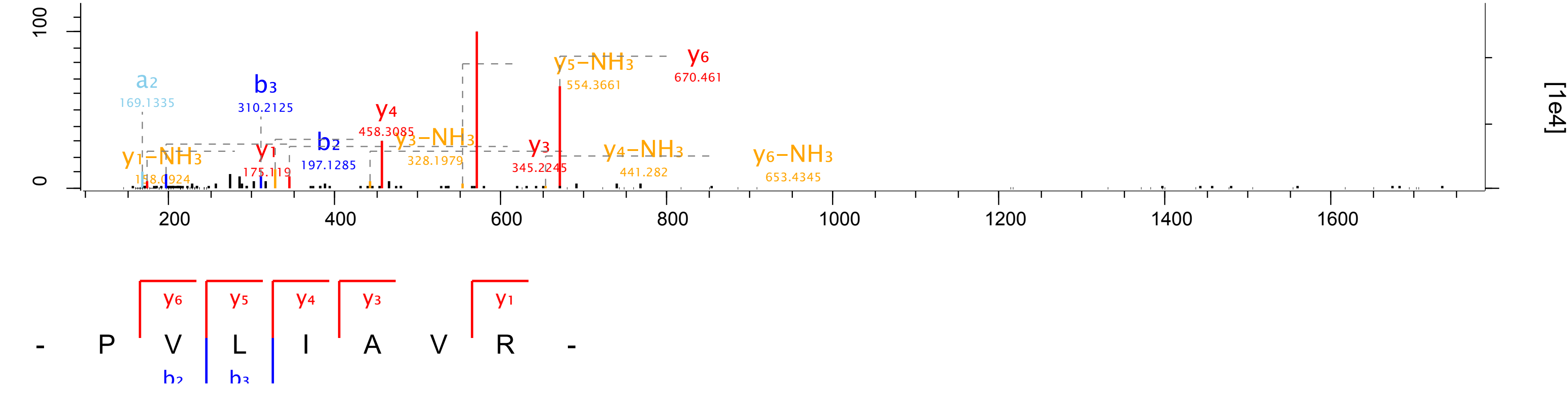

Raw file	Scan	Method	Score	m/z	Gene names
20150226_Hela_Top_opt_A3_01_1593	40243	TOF; CID	62.47	563.81	METTL14

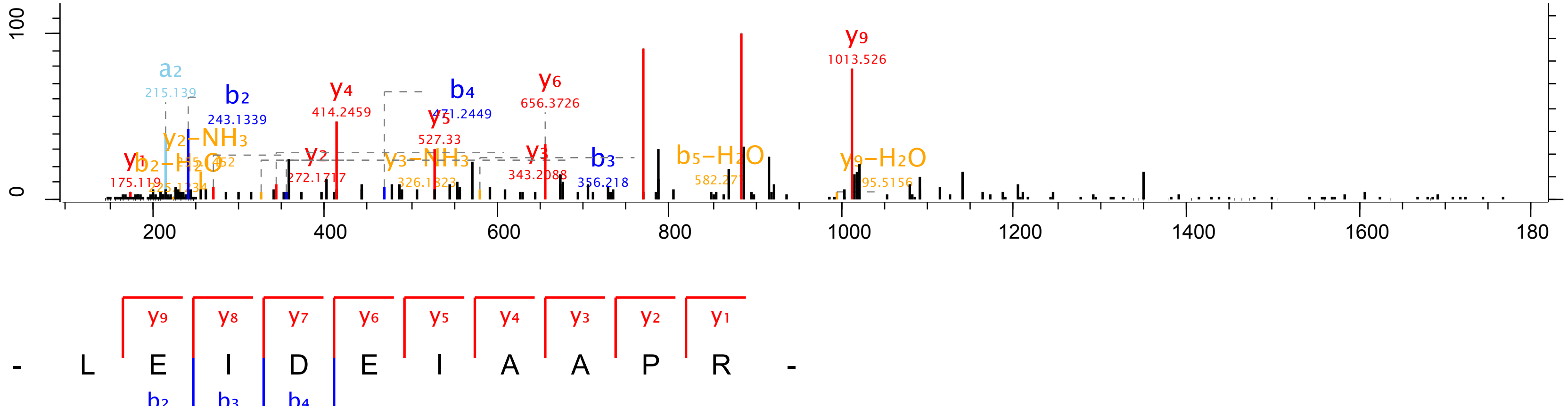

Raw file	Scan	Method	Score	m/z	Gene names
20150226_Hela_Top_opt_A3_01_1593	40282	TOF; CID	62.47	514.83	PLEKHF1

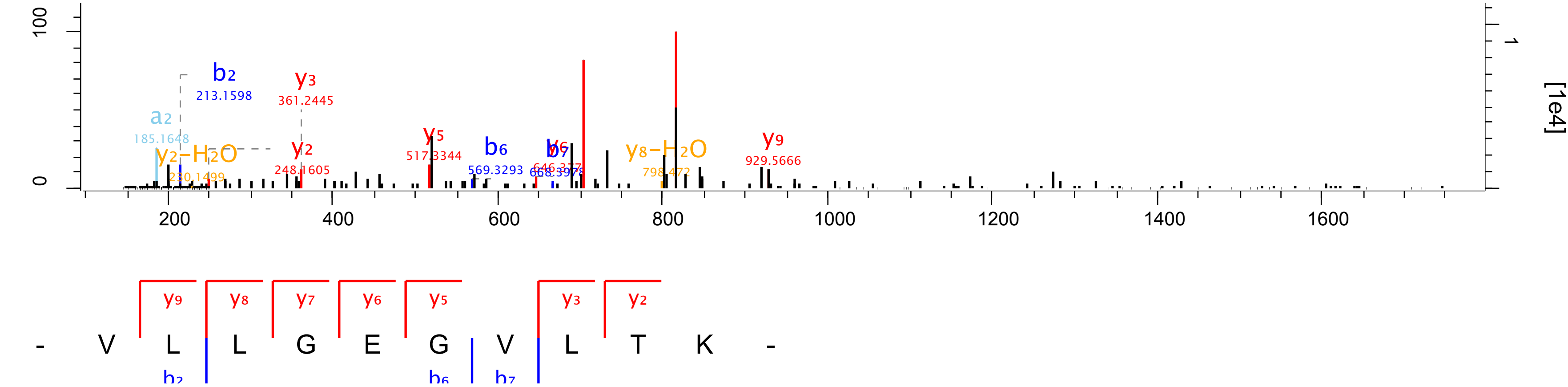

Raw file	Scan	Method	Score	m/z	Gene names
20150226_Hela_Top_opt_A3_01_1593	40552	TOF; CID	81.97	546.81	NAB2;NAB1

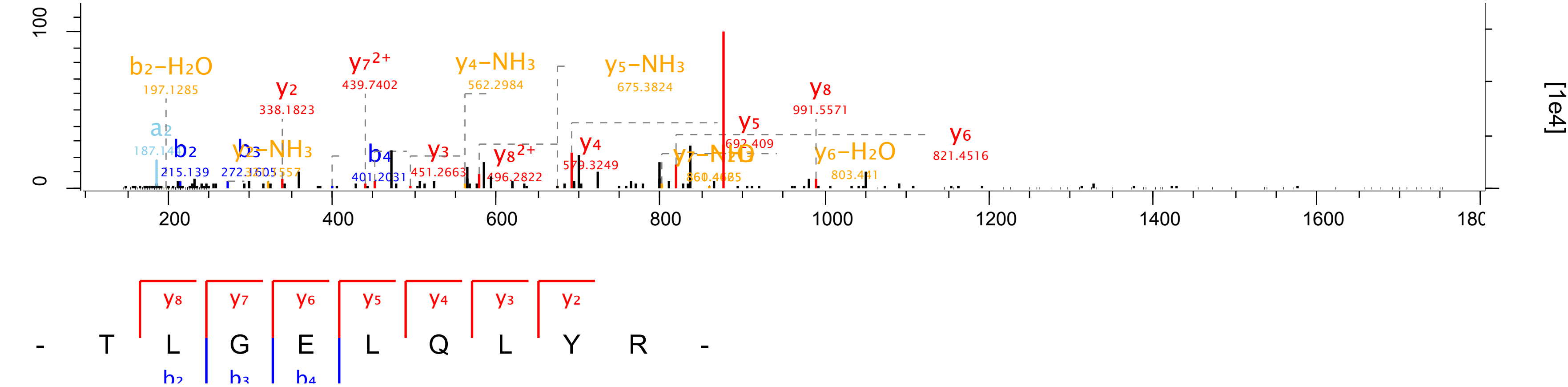

Raw file	Scan	Method	Score	m/z	Gene names
20150226_Hela_Top_opt_A3_01_1593	41521	TOF; CID	71.88	709.39	RPS19BP1

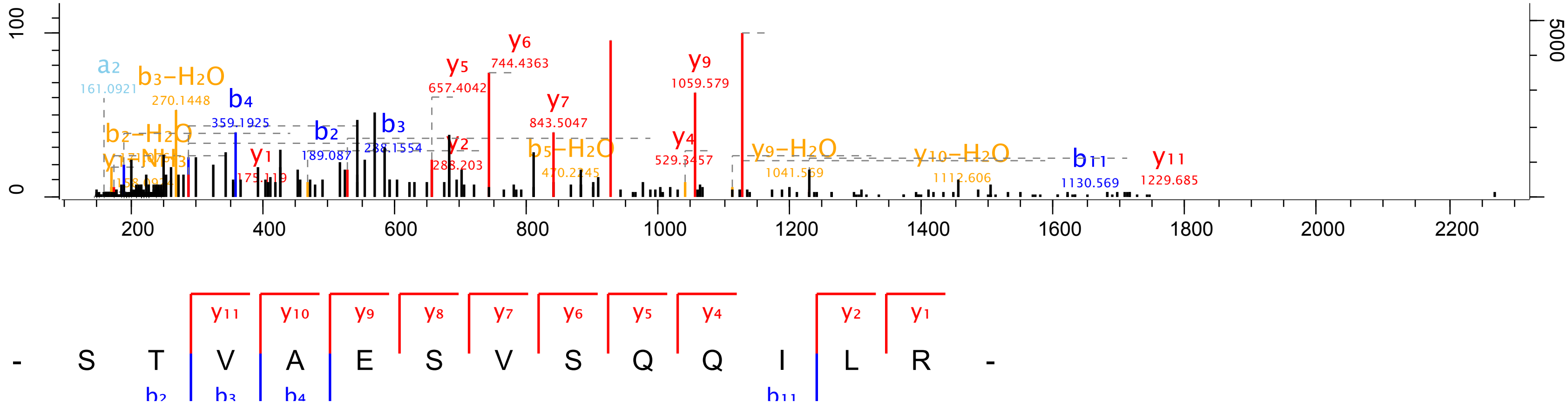

Raw file	Scan	Method	Score	m/z	Gene names
20150226_Hela_Top_opt_A3_01_1593	41800	TOF; CID	57.53	934.96	SDCCAG3

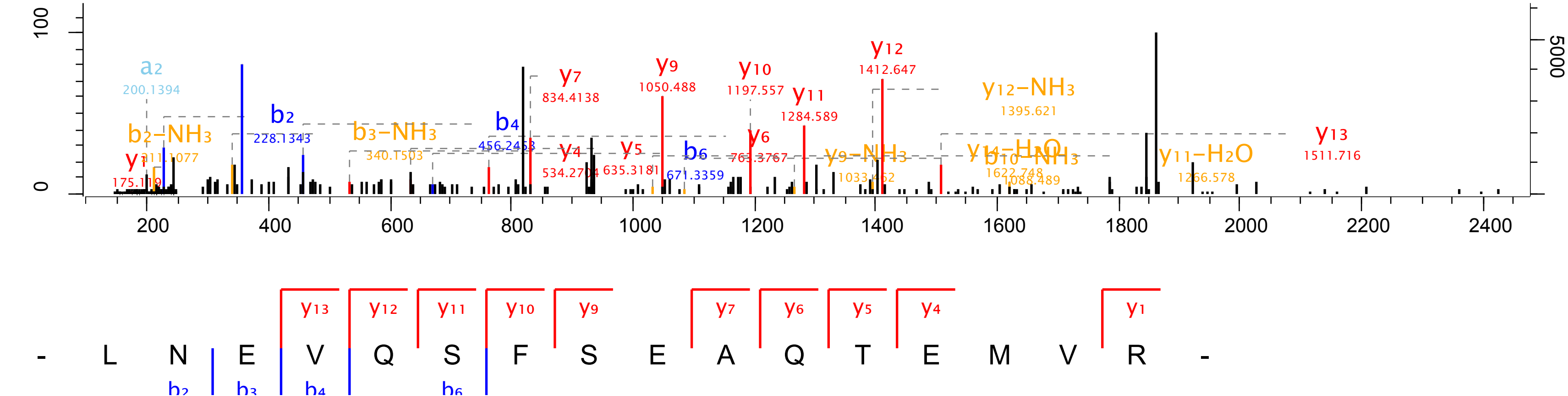

Raw file	Scan	Method	Score	m/z	Gene names
20150226_Hela_Top_opt_A3_01_1593	42012	TOF; CID	93.84	477.26	SAP30

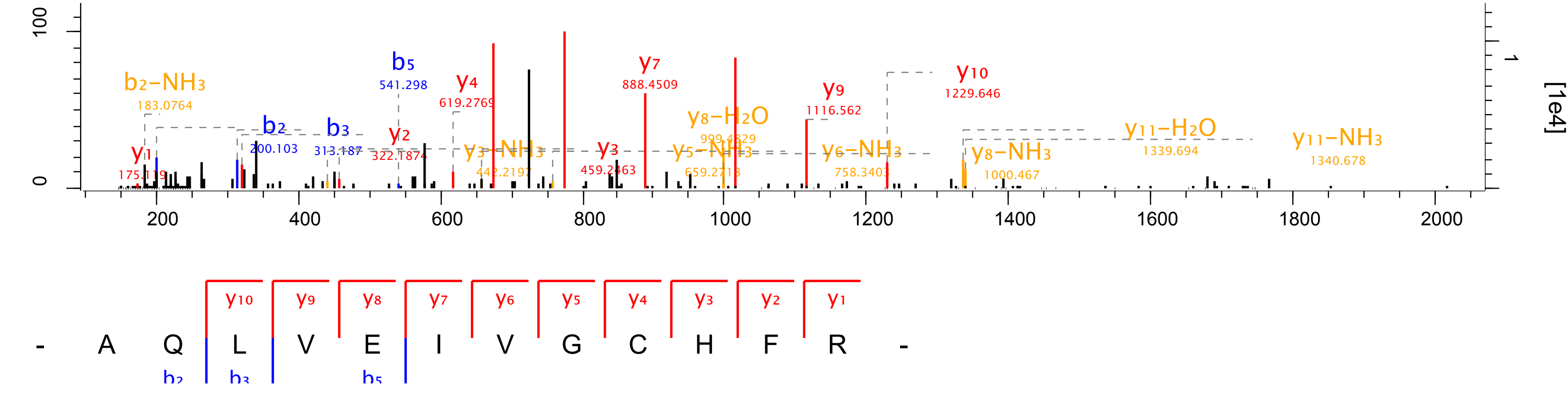

Raw file	Scan	Method	Score	m/z	Gene names
20150226_Hela_Top_opt_A3_01_1593	42630	TOF; CID	58.7	492.77	ITGB6

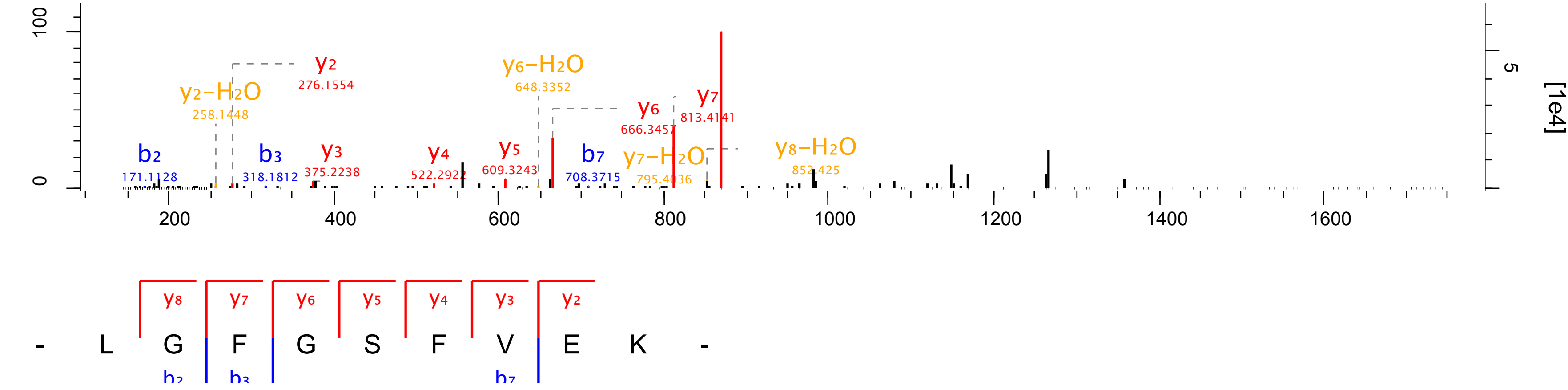

Raw file	Scan	Method	Score	m/z	Gene names
20150226_Hela_Top_opt_A3_01_1593	43187	TOF; CID	66.69	582.81	NEK5

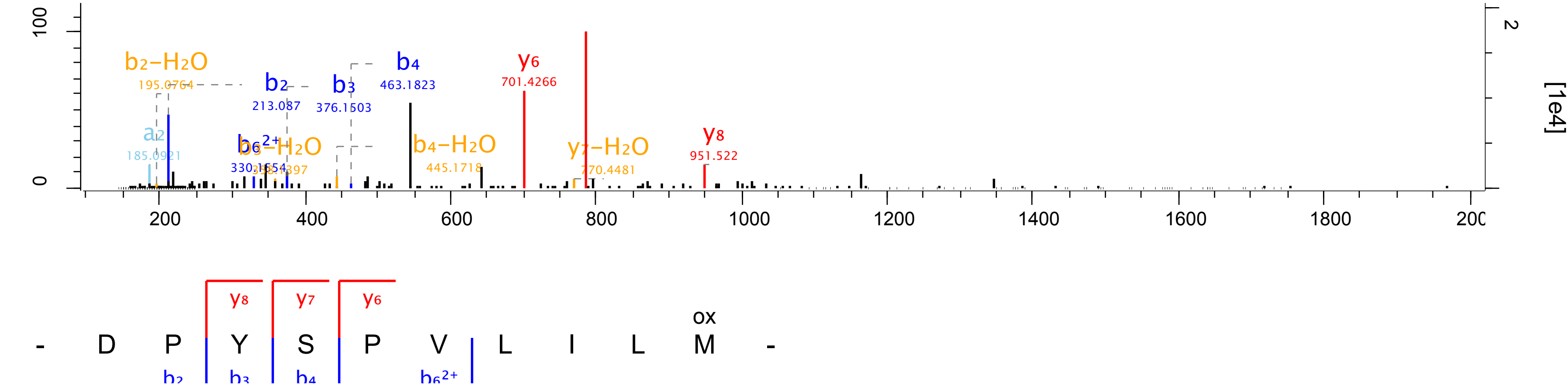

Raw file	Scan	Method	Score	m/z	Gene names
20150226_Hela_Top_opt_A3_01_1593	45000	TOF; CID	43.38	605.33	NAA30

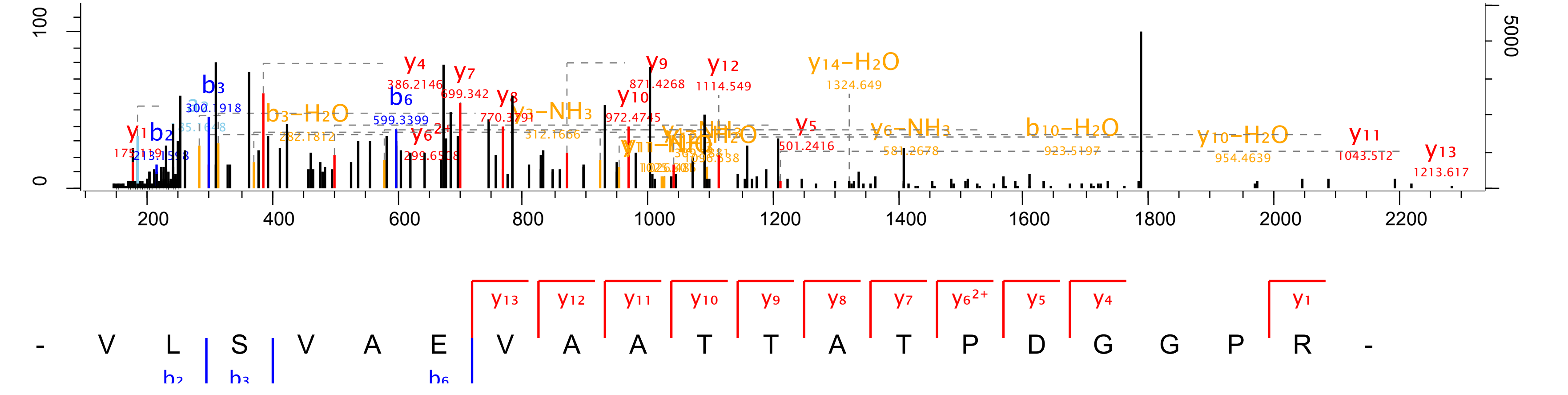

Raw file	Scan	Method	Score	m/z	Gene names
20150226_Hela_Top_opt_A3_01_1593	45216	TOF; CID	83.99	862.42	YIF1B

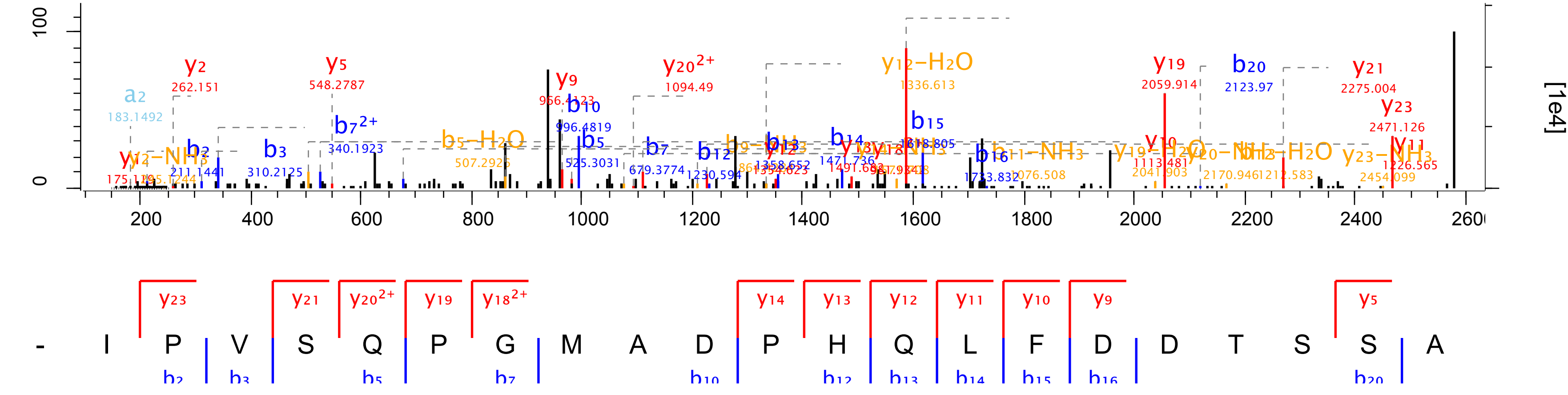

Raw file	Scan	Method	Score	m/z	Gene names
20150226_Hela_Top_opt_A3_01_1593	45304	TOF; CID	80.59	696.35	SLC31A1

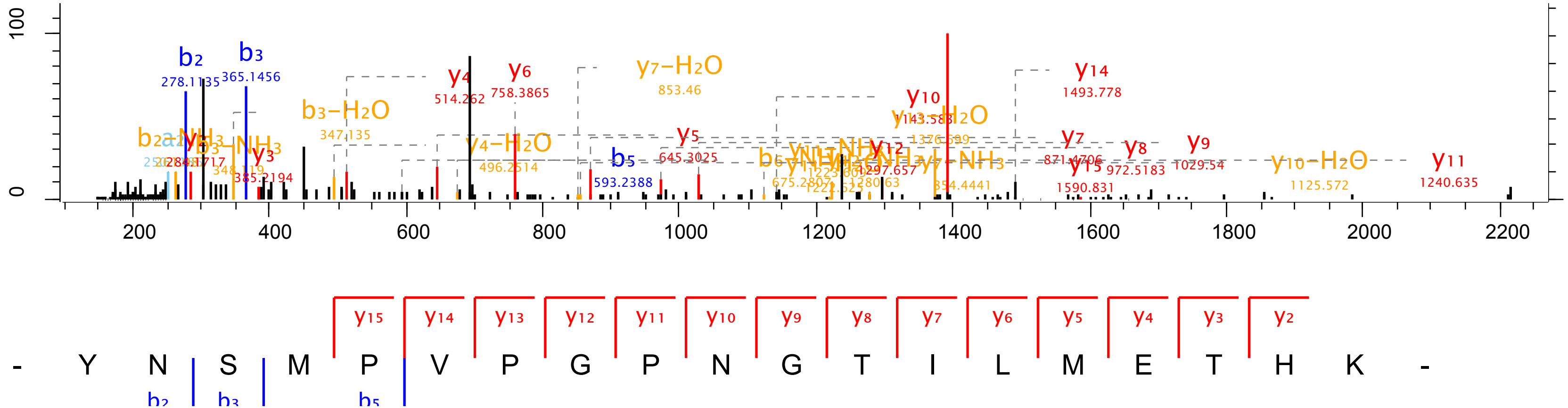

Raw file	Scan	Method	Score	m/z	Gene names
20150226_Hela_Top_opt_A3_01_1593	45993	TOF; CID	101.92	1089.06	ATXN7L3B

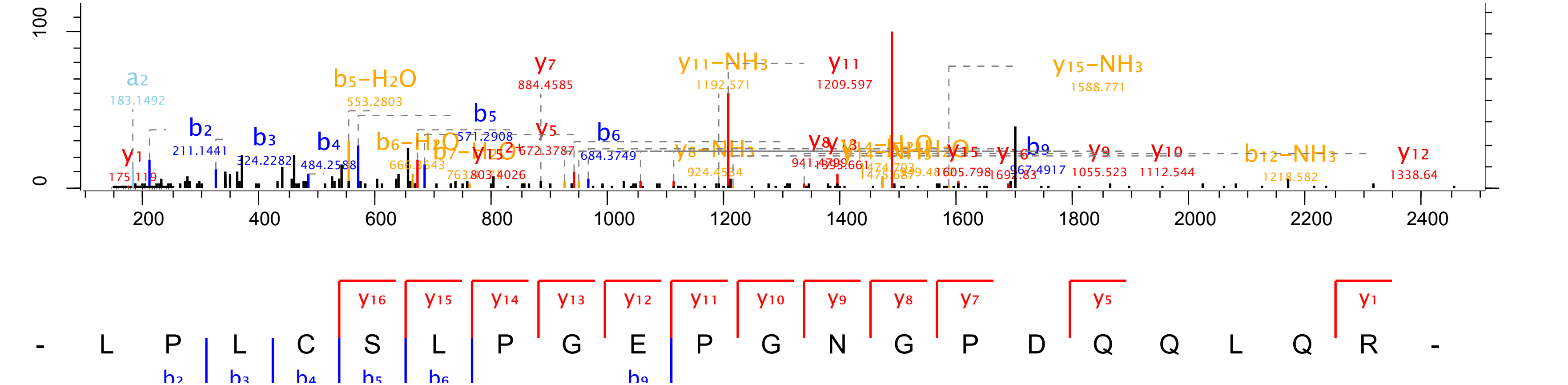

Raw file	Scan	Method	Score	m/z	Gene names
20150226_Hela_Top_opt_A3_01_1593	46492	TOF; CID	69.82	482.79	PLCH1

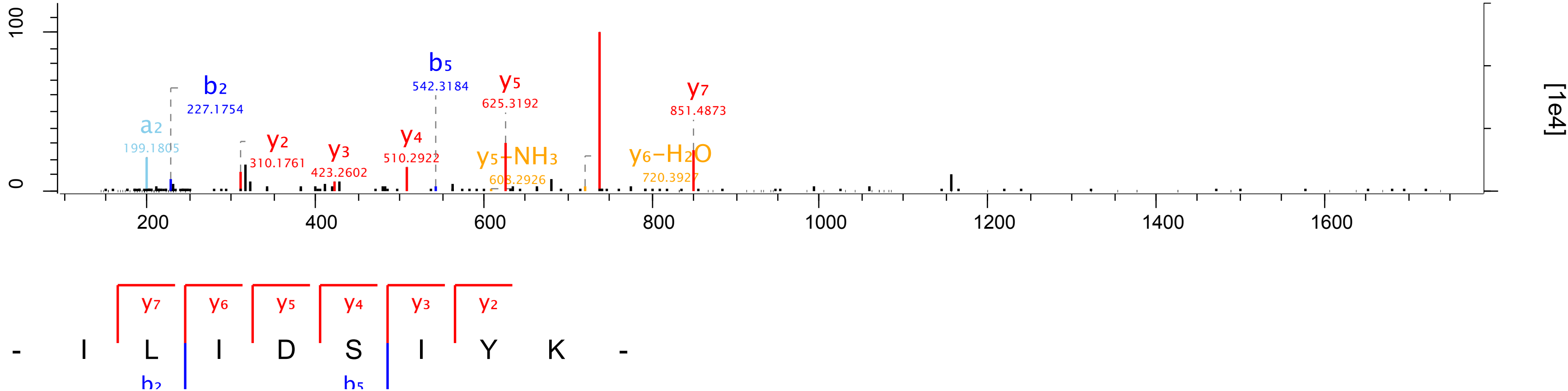

Raw file	Scan	Method	Score	m/z	Gene names
20150226_Hela_Top_opt_A3_01_1593	47961	TOF; CID	76.06	504.73	TMEM258

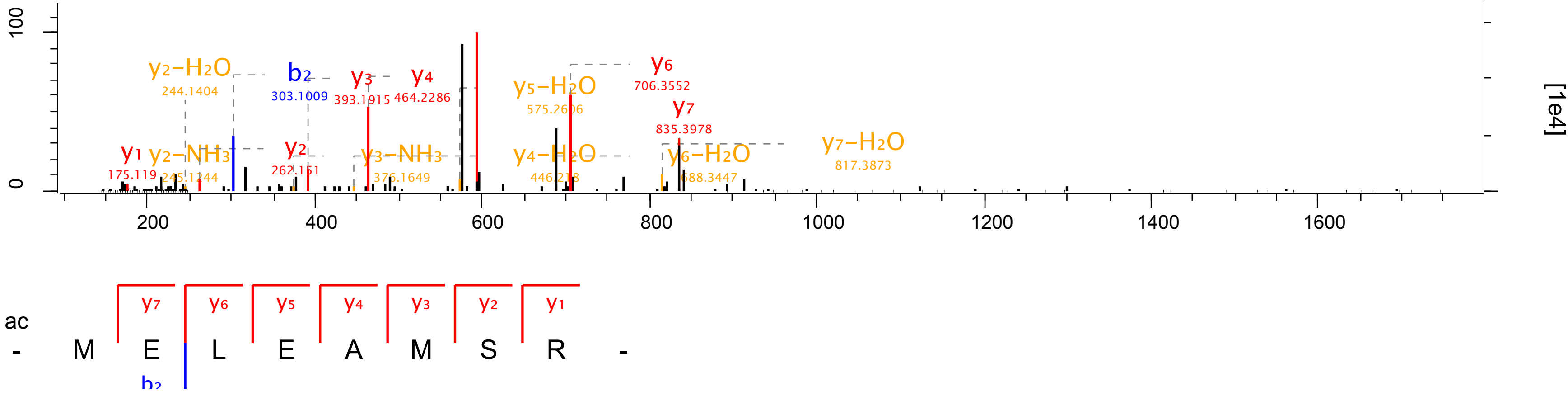

Raw file	Scan	Method	Score	m/z	Gene names
20150226_Hela_Top_opt_A3_01_1593	48045	TOF; CID	56.51	718.42	TIMM22

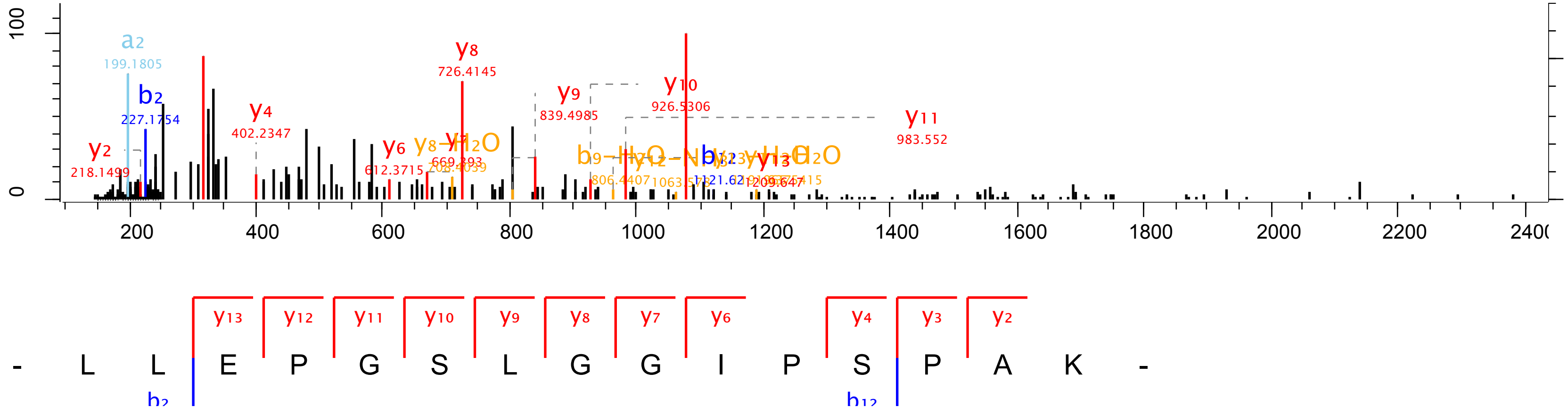

Raw file	Scan	Method	Score	m/z	Gene names
20150226_Hela_Top_opt_A3_01_1593	48259	TOF; CID	82.28	487.3	FAM134A

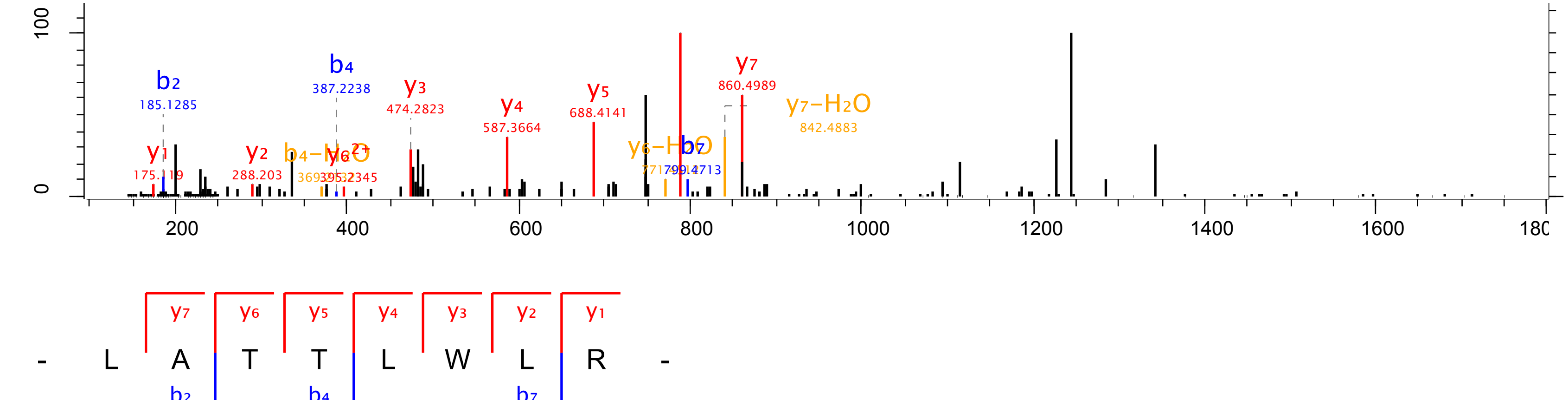

Raw file	Scan	Method	Score	m/z	Gene names
20150226_Hela_Top_opt_A3_01_1593	48784	TOF; CID	41.51	592.02	TFDP1

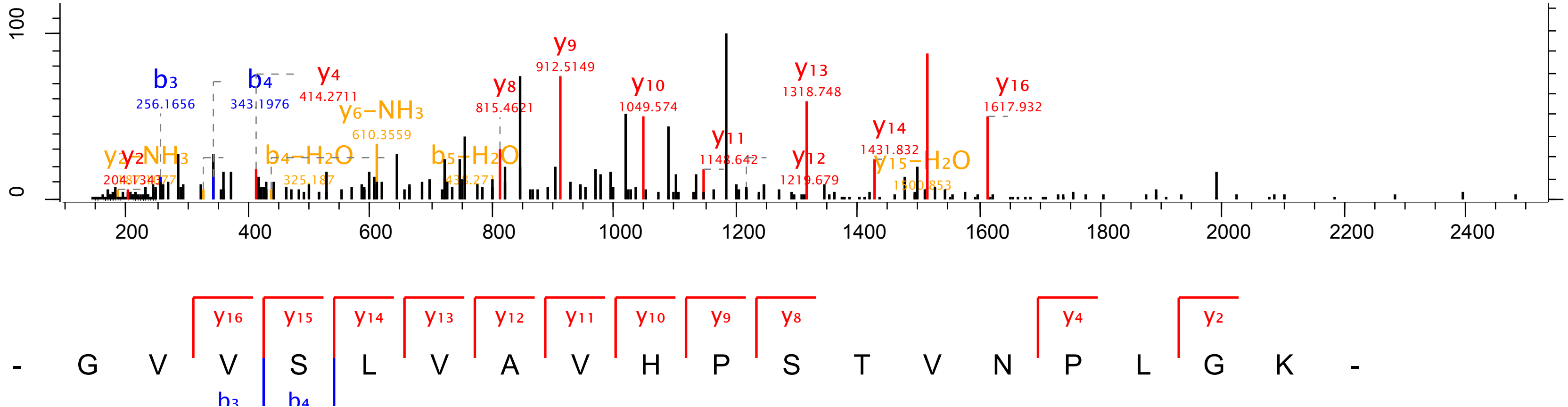

Raw file	Scan	Method	Score	m/z	Gene names
20150226_Hela_Top_opt_A3_01_1593	48858	TOF; CID	66.64	735.04	UBALD1

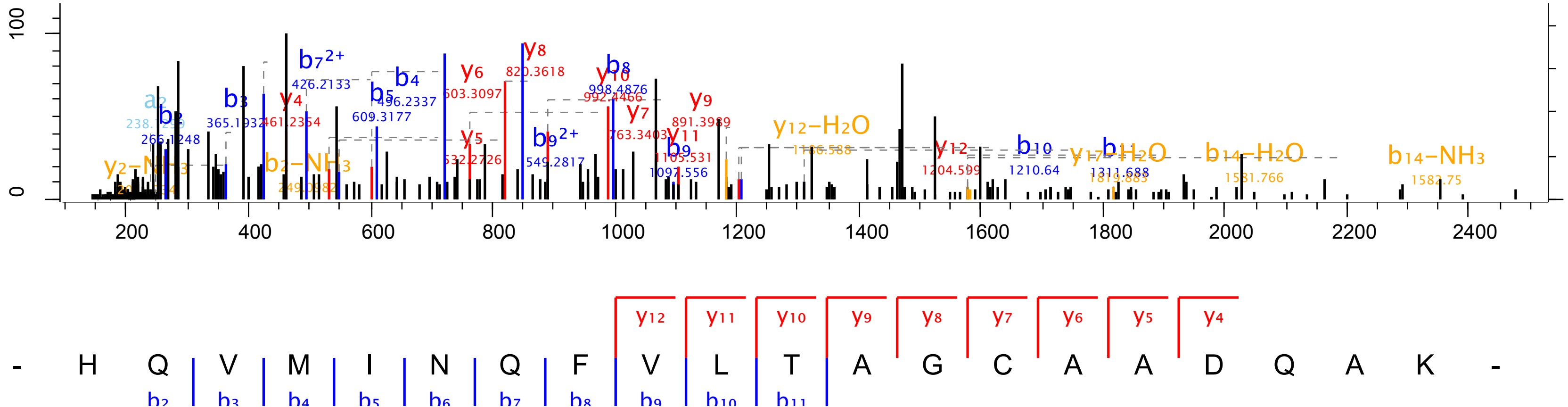

Raw file	Scan	Method	Score	m/z	Gene names
20150226_Hela_Top_opt_A3_01_1593	48930	TOF; CID	80.73	990.45	CDC42EP1

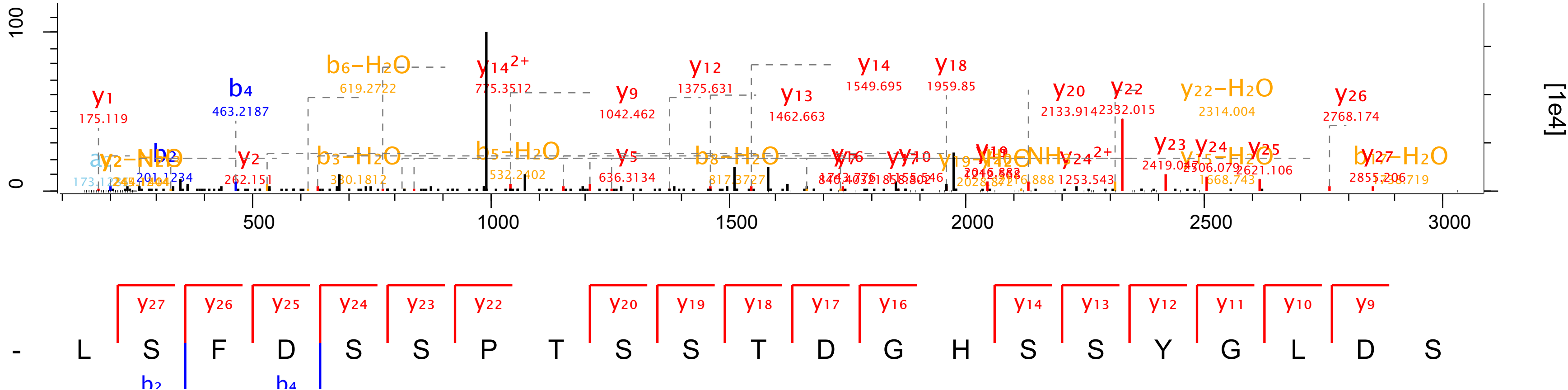

Raw file	Scan	Method	Score	m/z	Gene names
20150226_Hela_Top_opt_A3_01_1593	49425	TOF; CID	53.55	950.46	NKAP

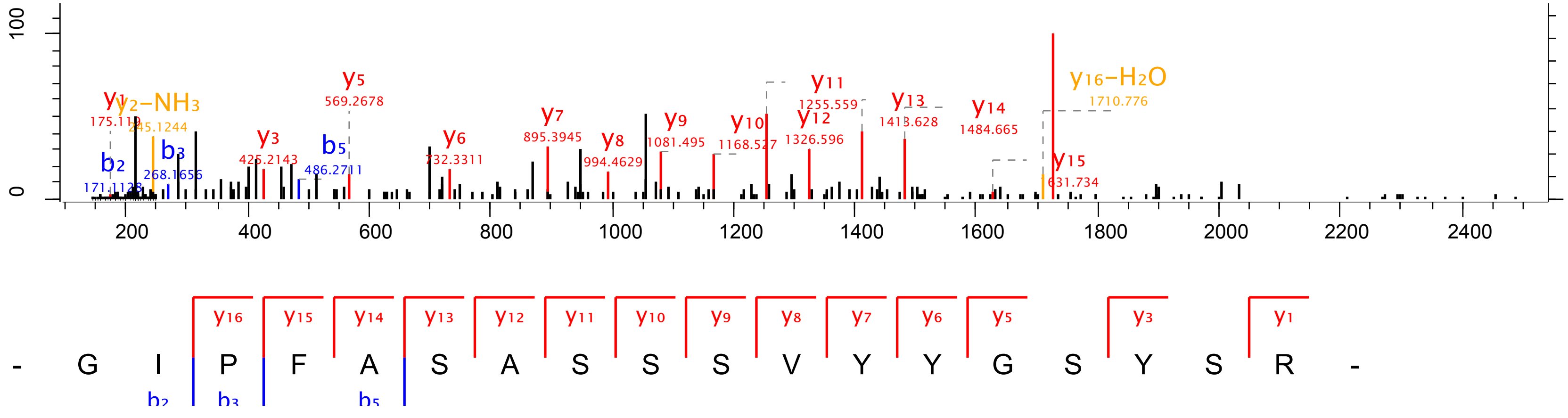

Raw file	Scan	Method	Score	m/z	Gene names
20150226_Hela_Top_opt_A3_01_1593	49448	TOF; CID	55.72	578.35	RFT1

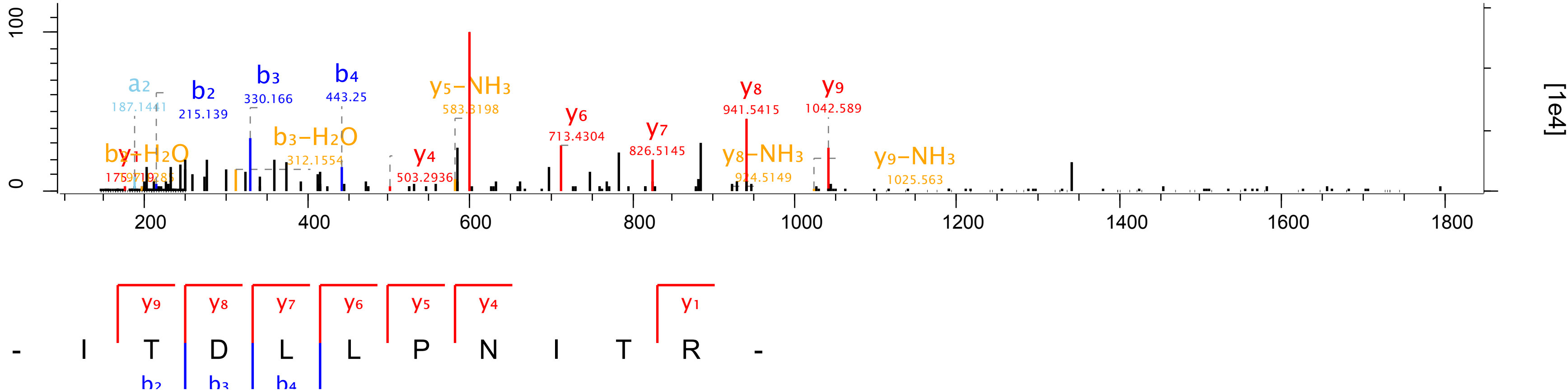

Raw file	Scan	Method	Score	m/z	Gene names
20150226_Hela_Top_opt_A3_01_1593	50268	TOF; CID	96.34	622.38	MT-ATP6

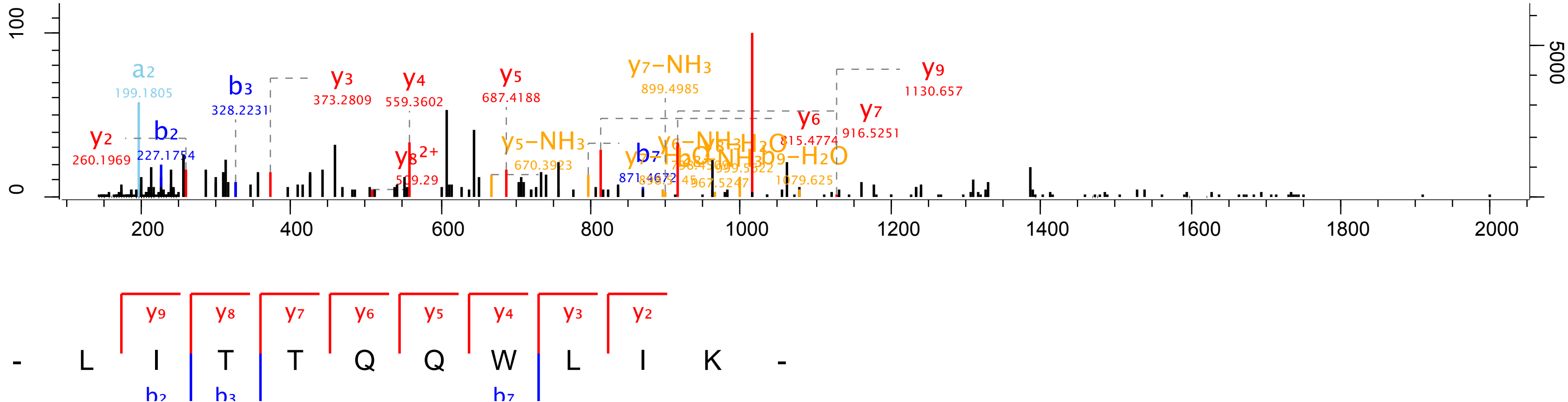

Raw file	Scan	Method	Score	m/z	Gene names
20150226_Hela_Top_opt_A3_01_1593	51798	TOF; CID	88.45	713.04	PDE6D

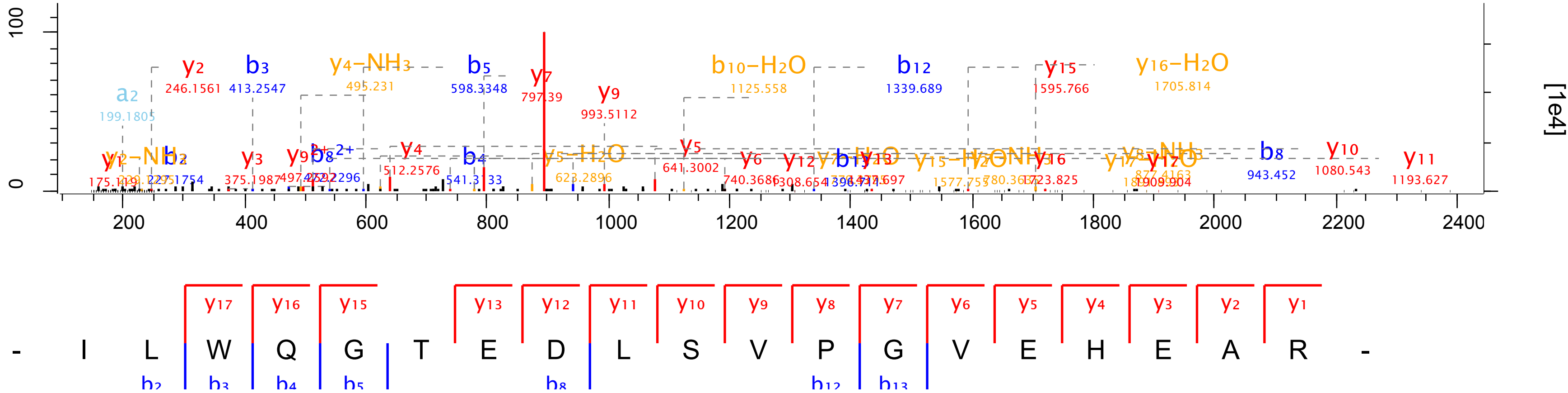

Raw file	Scan	Method	Score	m/z	Gene names
20150226_Hela_Top_opt_A3_01_1593	51816	TOF; CID	61.78	594.31	SLC6A6

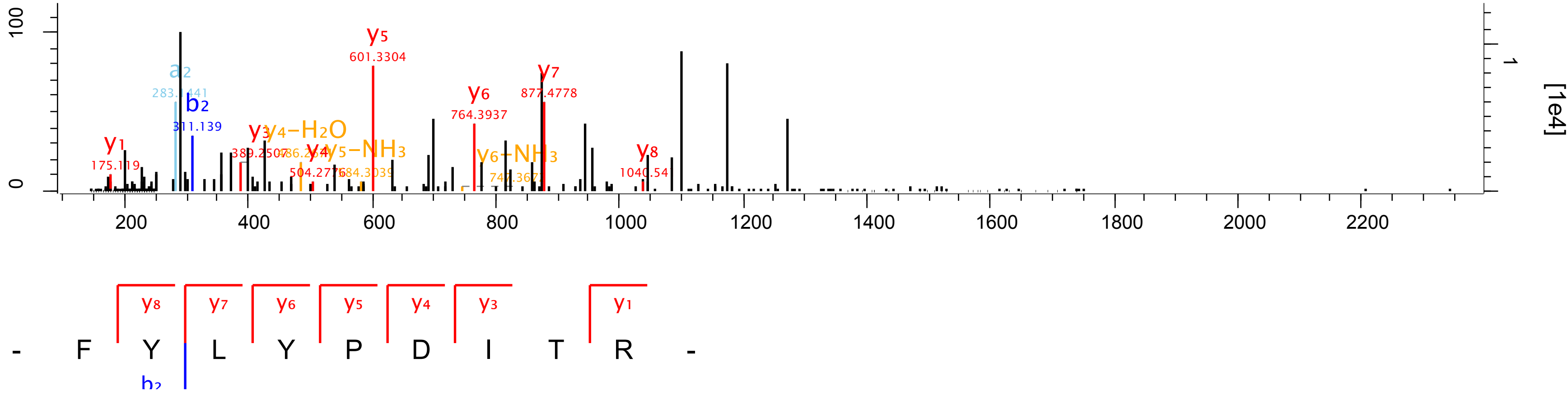

Raw file	Scan	Method	Score	m/z	Gene names
20150226_Hela_Top_opt_A3_01_1593	51842	TOF; CID	75.91	585.34	GATAD1

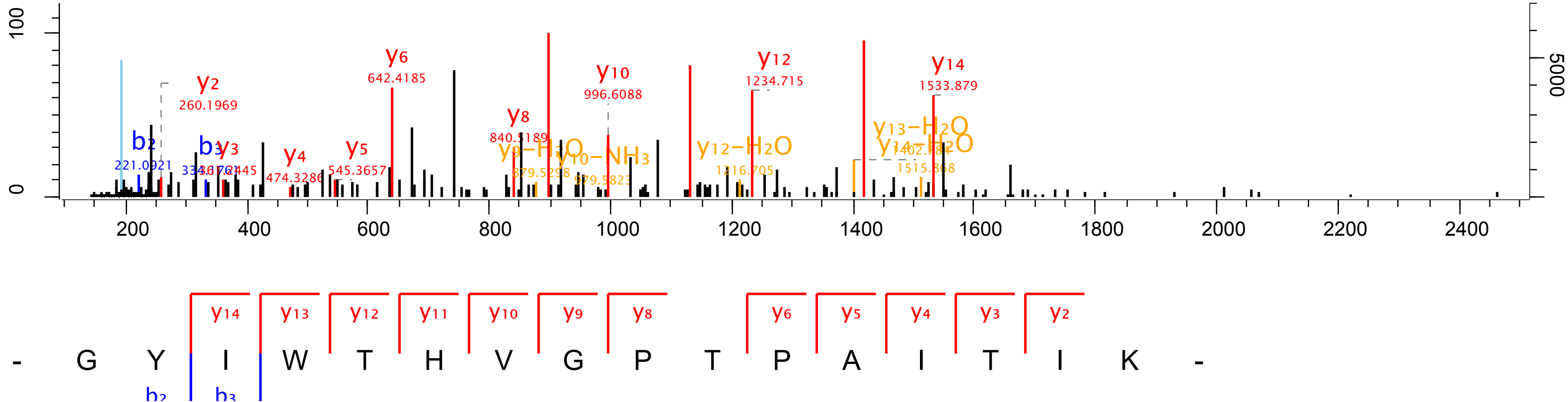

Raw file	Scan	Method	Score	m/z	Gene names
20150226_Hela_Top_opt_A3_01_1593	52203	TOF; CID	109.04	914.43	RAP2C

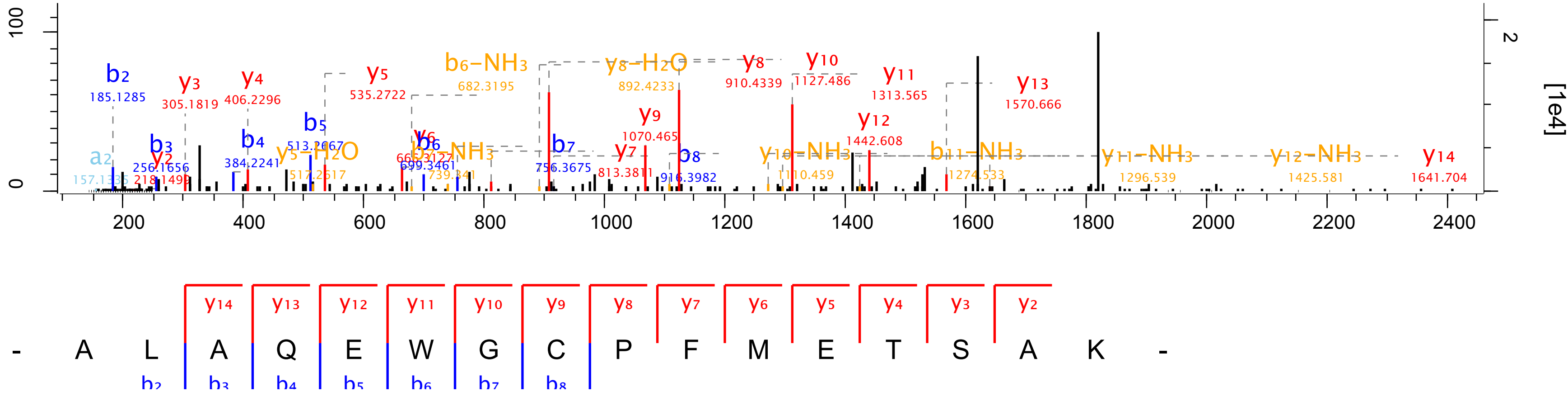

Raw file	Scan	Method	Score	m/z	Gene names
20150226_Hela_Top_opt_A3_01_1593	52472	TOF; CID	62.62	1112.51	EBAG9

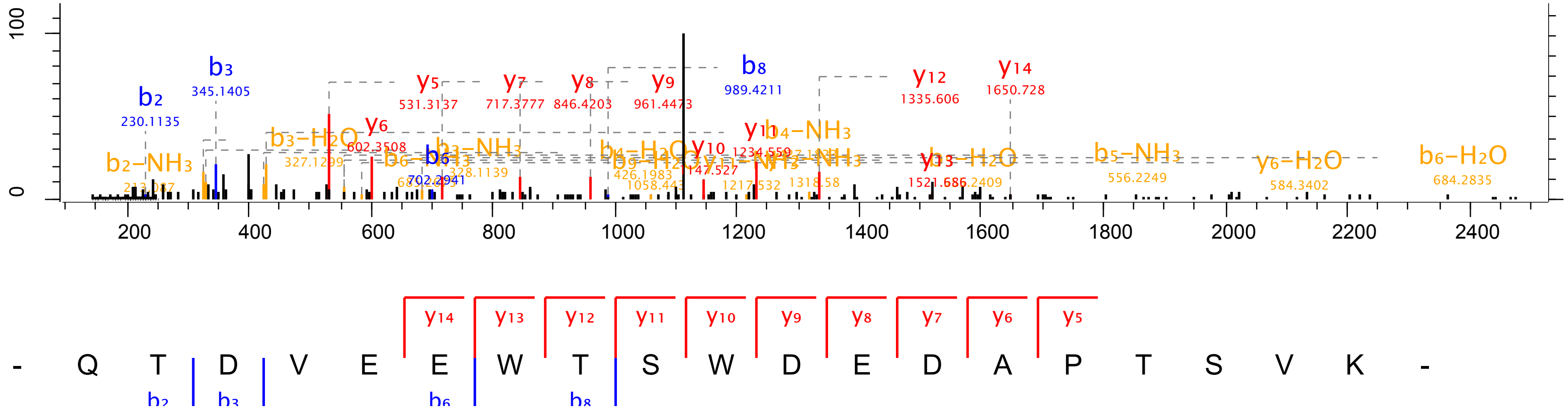

Raw file	Scan	Method	Score	m/z	Gene names
20150226_Hela_Top_opt_A3_01_1593	53372	TOF; CID	49.27	803.43	MEF2D

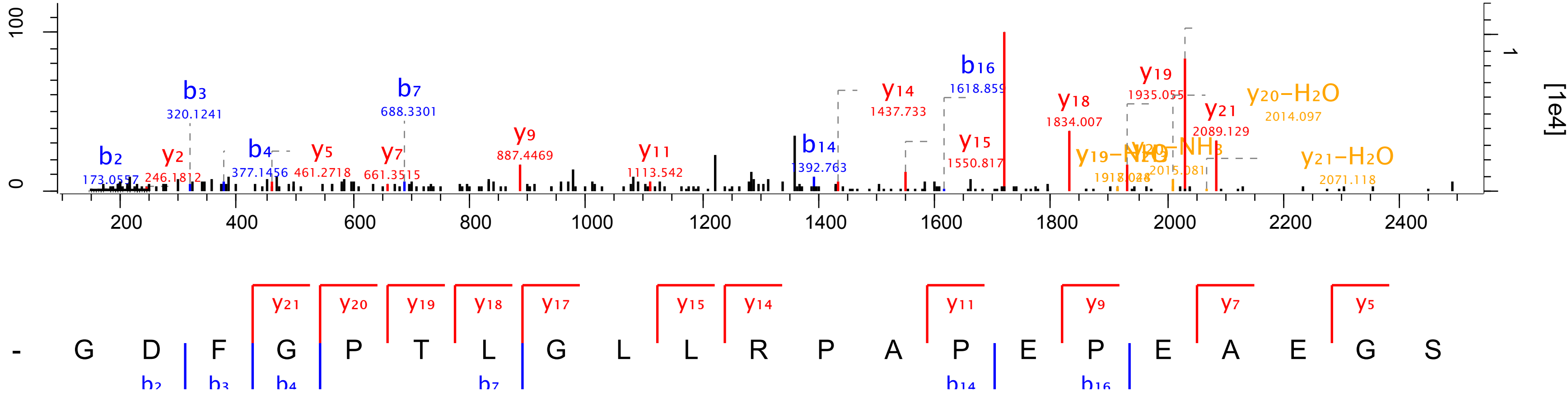

Raw file	Scan	Method	Score	m/z	Gene names
20150226_Hela_Top_opt_A3_01_1593	54545	TOF; CID	69.82	415.77	AKT1

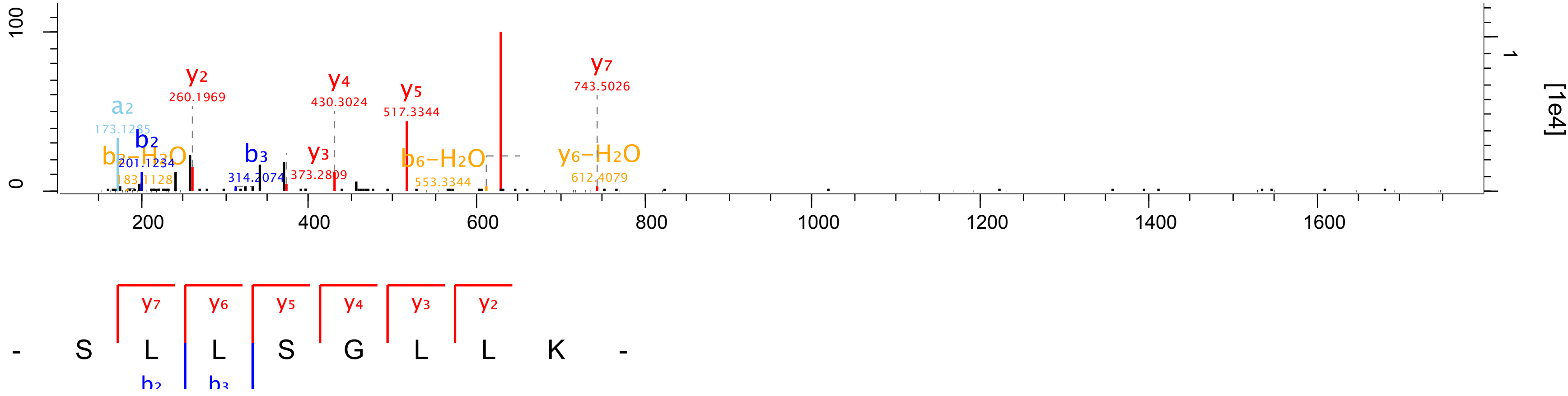

Raw file	Scan	Method	Score	m/z	Gene names
20150226_Hela_Top_opt_A3_01_1593	54672	TOF; CID	57.17	579.34	WRN

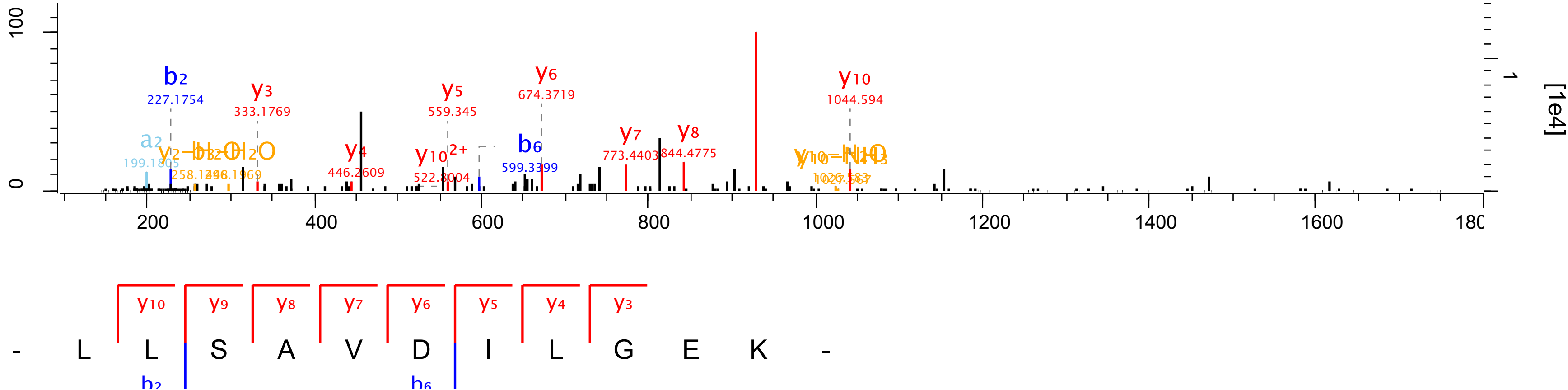

Raw file	Scan	Method	Score	m/z	Gene names
20150226_Hela_Top_opt_A3_01_1593	54707	TOF; CID	87.43	959.91	RYBP

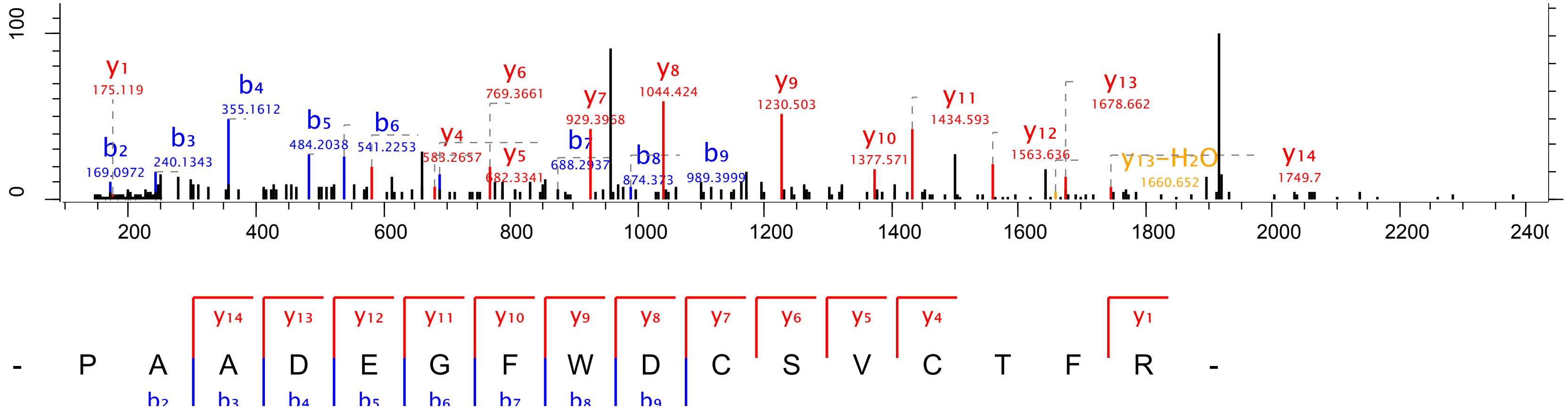

Raw file	Scan	Method	Score	m/z	Gene names
20150226_Hela_Top_opt_A3_01_1593	55766	TOF; CID	50.27	887.75	NDUFA3

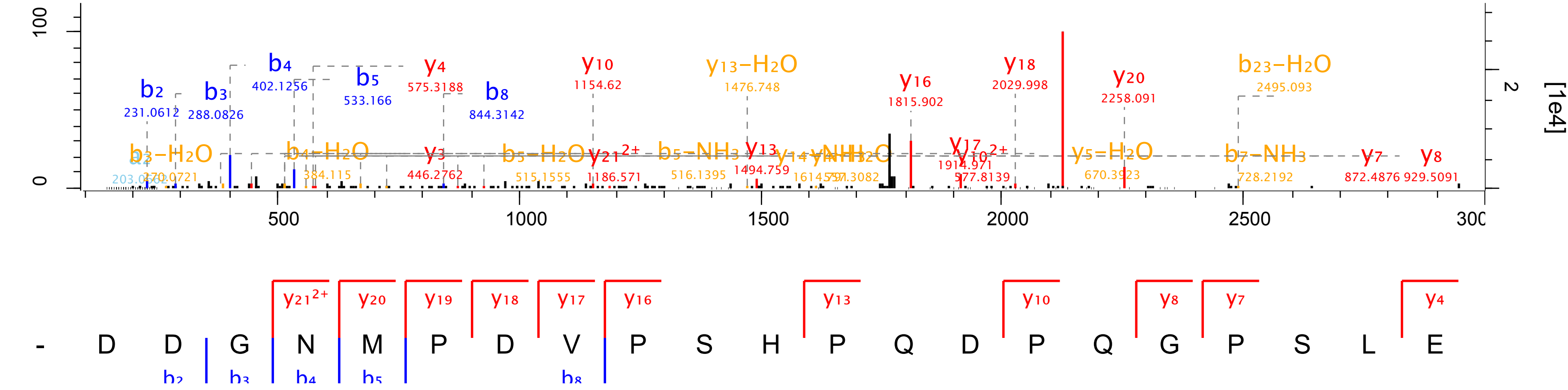

Raw file	Scan	Method	Score	m/z	Gene names
20150226_Hela_Top_opt_A3_01_1593	56075	TOF; CID	80.38	425.74	PRAMEF6;PRAMEF23;PRAMEF5;PRAMEF9;PRAMEF11;PRAMEF4;PRAMEF26

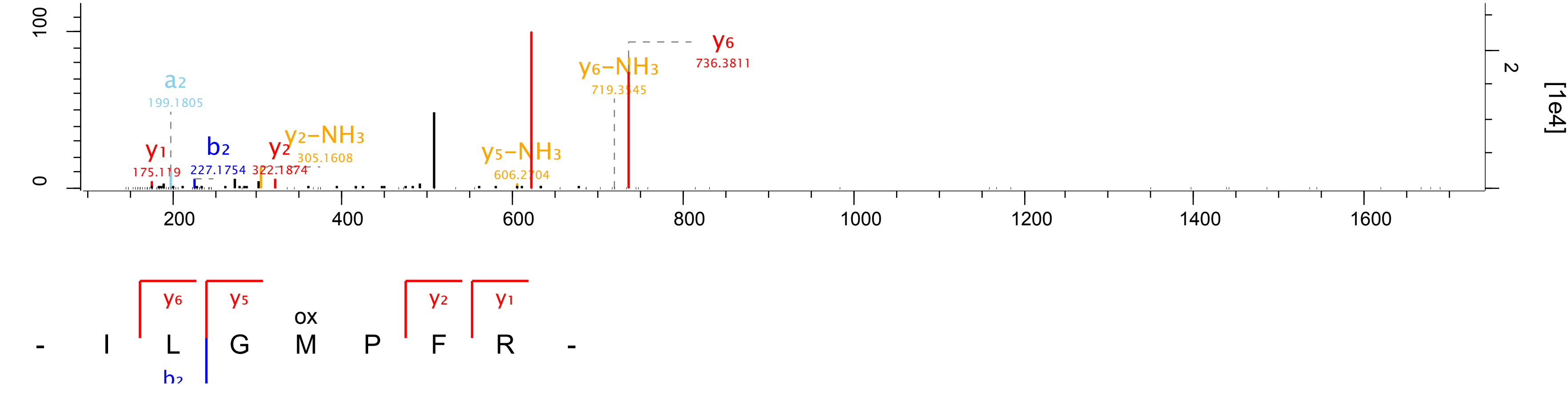

Raw file	Scan	Method	Score	m/z	Gene names
20150226_Hela_Top_opt_A3_01_1593	56734	TOF; CID	81.53	839.44	MUL1

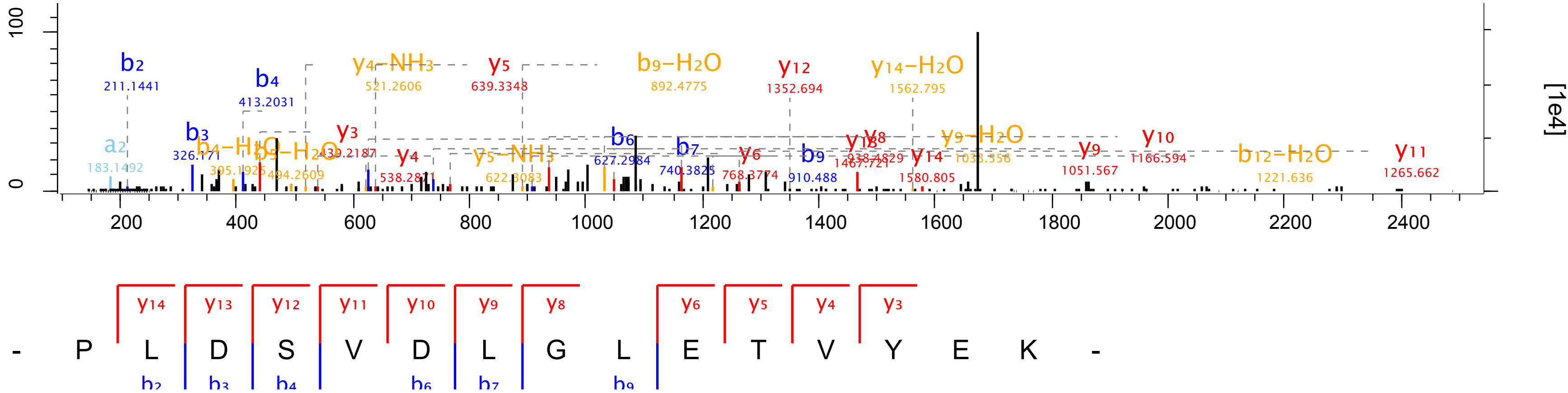

Raw file	Scan	Method	Score	m/z	Gene names
20150226_Hela_Top_opt_A3_01_1593	57383	TOF; CID	83.39	723.06	MITD1

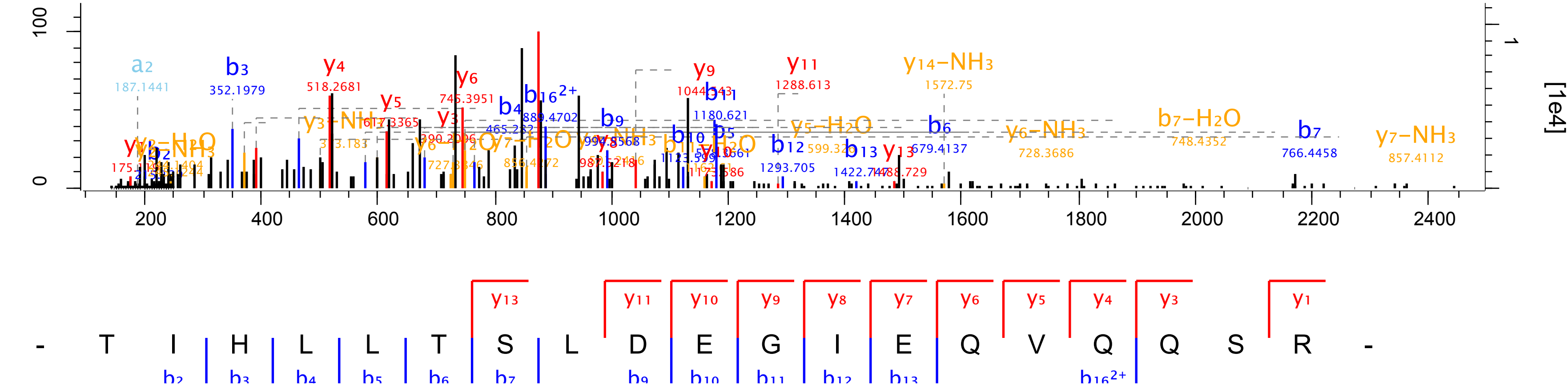

Raw file	Scan	Method	Score	m/z	Gene names
20150226_Hela_Top_opt_A3_01_1593	57812	TOF; CID	81.72	1197.59	BAK1

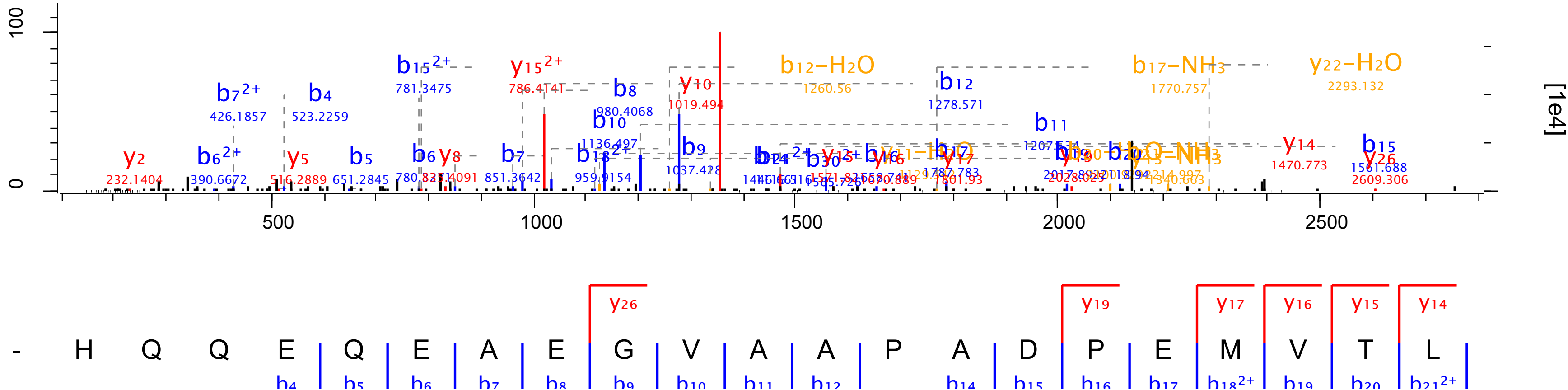

Raw file	Scan	Method	Score	m/z	Gene names
20150226_Hela_Top_opt_A3_01_1593	57822	TOF; CID	61.87	575.97	FBXL15

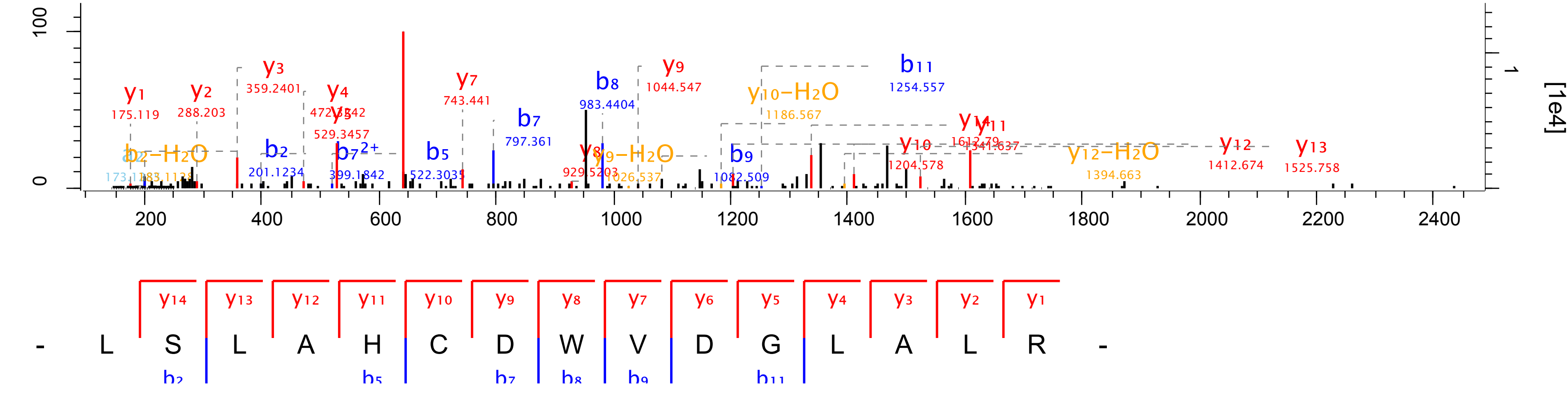

Raw file	Scan	Method	Score	m/z	Gene names
20150226_Hela_Top_opt_A3_01_1593	58360	TOF; CID	85.19	1066.81	TVP23B;TVP23C

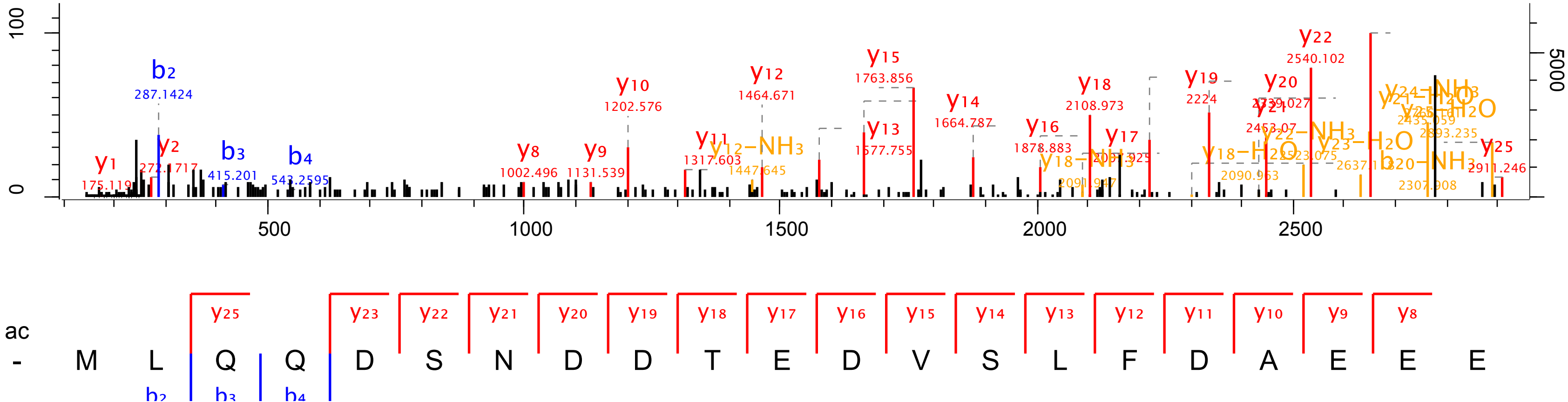

Raw file	Scan	Method	Score	m/z	Gene names
20150226_Hela_Top_opt_A3_01_1593	59325	TOF; CID	93.48	853.93	MTMR12

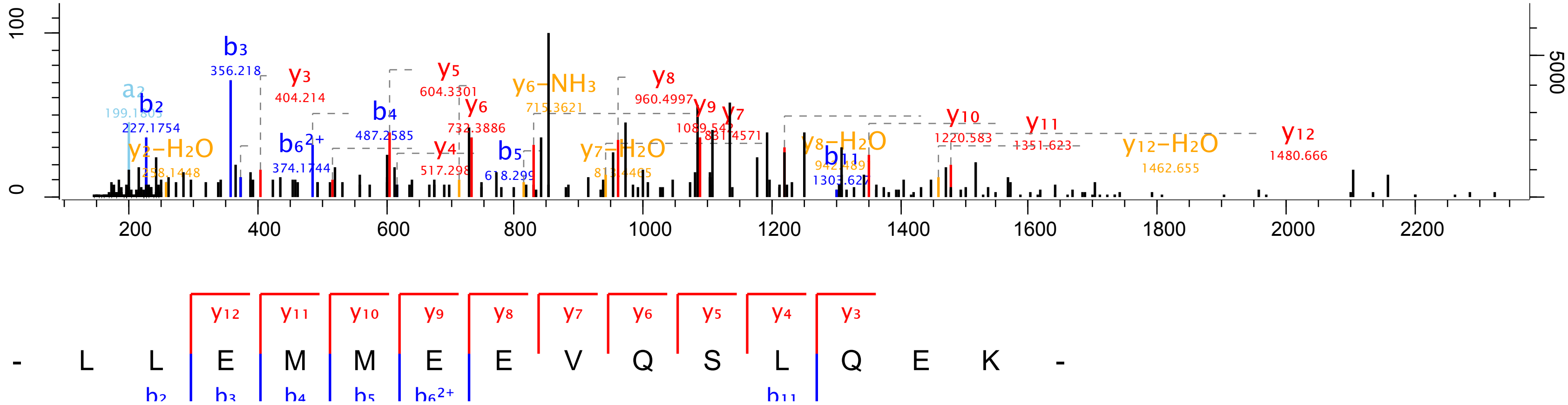

Raw file	Scan	Method	Score	m/z	Gene names
20150226_Hela_Top_opt_A3_01_1593	60357	TOF; CID	85.2	1007.48	CCDC134

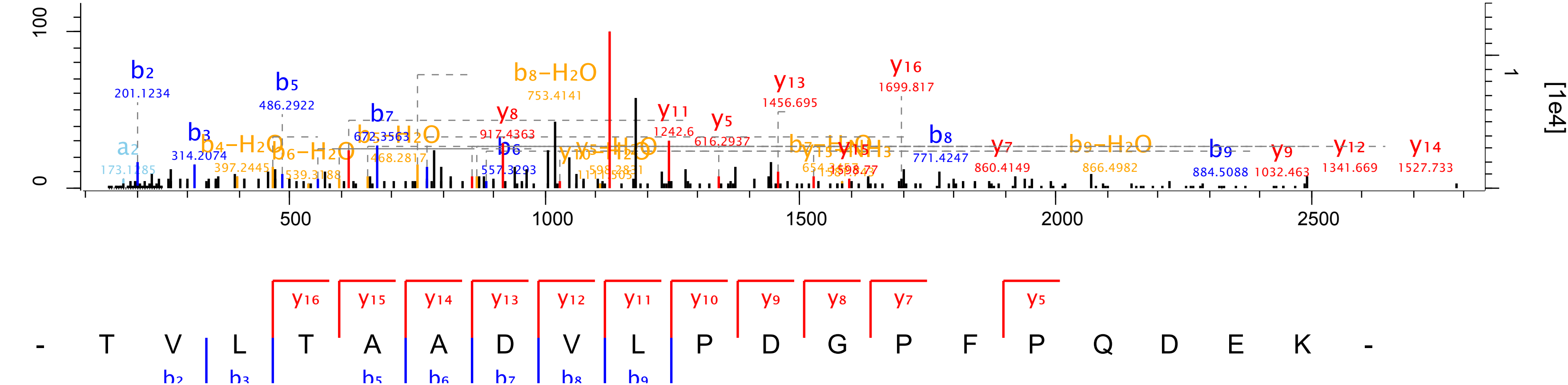

Raw file	Scan	Method	Score	m/z	Gene names
20150226_Hela_Top_opt_A3_01_1593	60880	TOF; CID	70.39	915.82	C19orf52

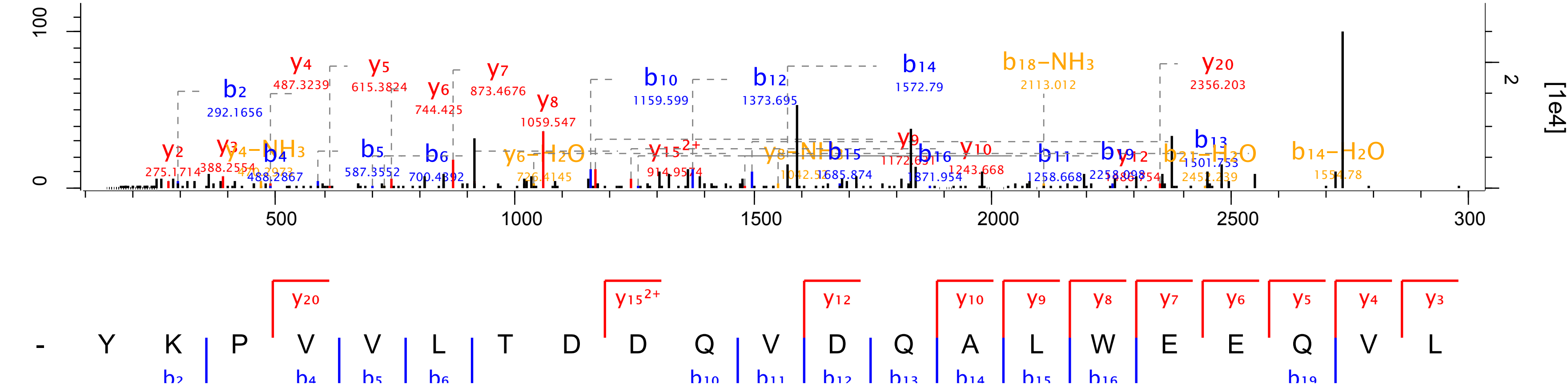

Raw file	Scan	Method	Score	m/z	Gene names
20150226_Hela_Top_opt_A3_01_1593	61565	TOF; CID	91.78	460.75	TMEM50A

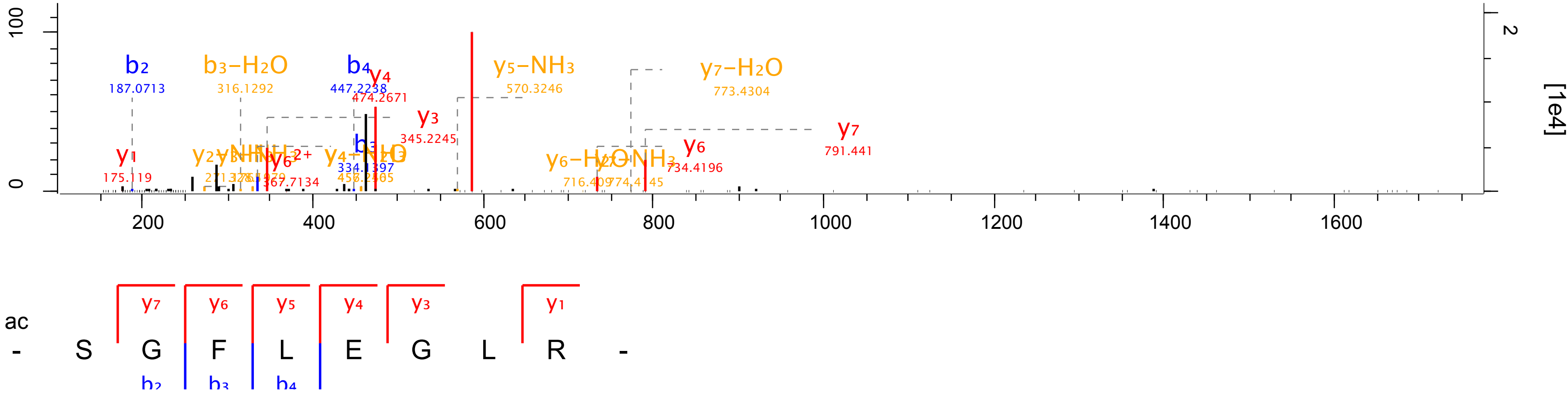

Raw file	Scan	Method	Score	m/z	Gene names
20150226_Hela_Top_opt_A3_01_1593	61666	TOF; CID	57.41	768.37	KIN

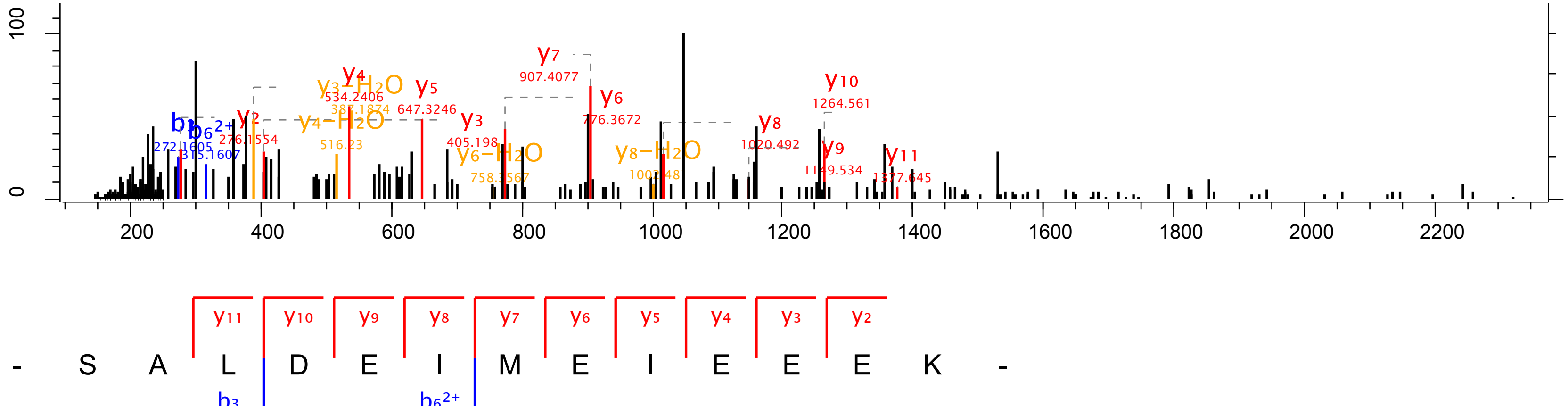

Raw file	Scan	Method	Score	m/z	Gene names
20150226_Hela_Top_opt_A3_01_1593	61743	TOF; CID	100.43	933.43	PITPNC1

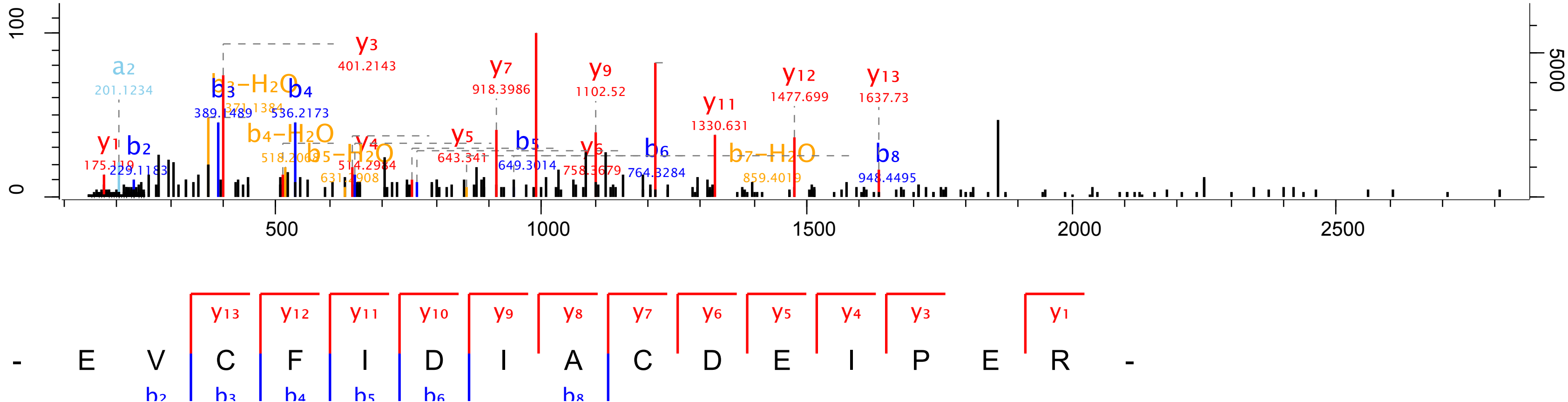

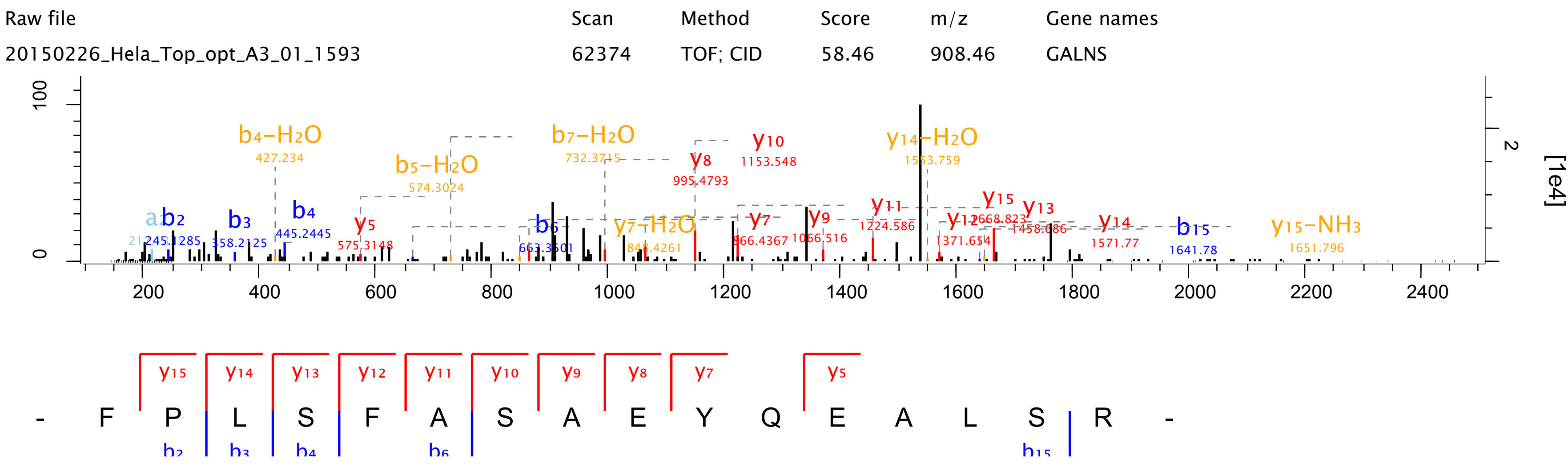

Raw file
20150226_Hela_Top_opt_A3_01_1593
Scan
62374
Method
TOF; CID
Score
58.46
m/z
908.46
Gene names
GALNS
100
0
2
[1e4]
b4−H2O
427.234
b5−H2O
574.3024
b7−H2O
732.3715
y14−H2O
1553.759
y7−H2O
848.4261
y15−NH3
1651.796
y8
995.4793
y10
1153.548
y5
575.3148
y7
866.4367
y9
1066.516
y11
1224.586
y12
1371.654
y15
1668.823
y13
1458.686
y14
1571.77
b2
245.1285
b3
358.2125
b4
445.2445
b6
663.3501
b15
1641.78
200
400
600
800
1000
1200
1400
1600
1800
2000
2200
2400
y15 y14 y13 y12 y11 y10 y9 y8 y7 y5
- F P L S F A S A E Y Q E A L S R -
b2 b3 b4 b6 b15

Raw file	Scan	Method	Score	m/z	Gene names
20150226_Hela_Top_opt_A3_01_1593	62654	TOF; CID	65.18	707.39	TEX11

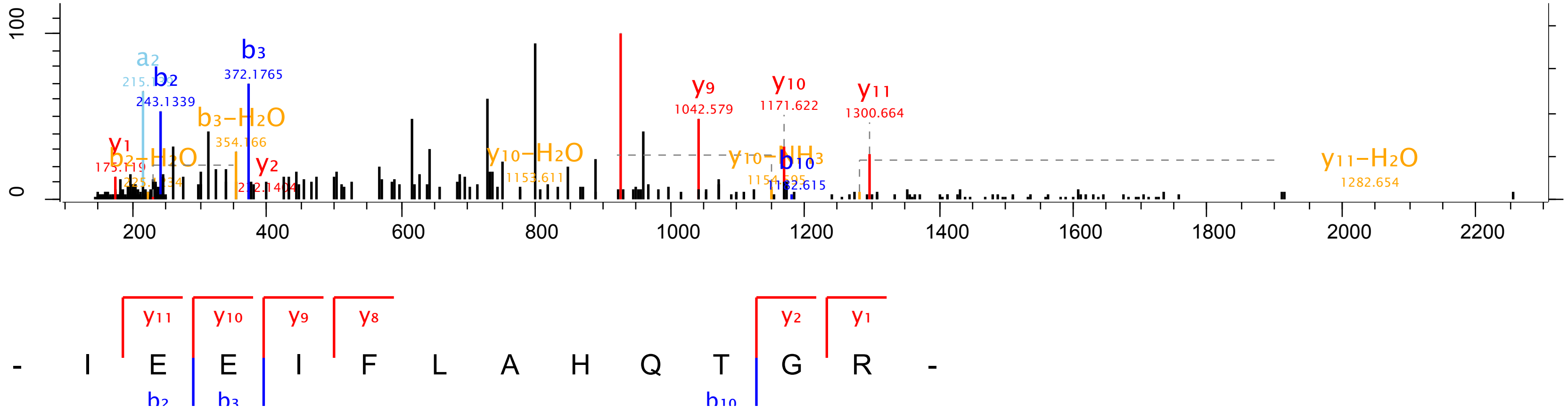

Raw file	Scan	Method	Score	m/z	Gene names
20150226_Hela_Top_opt_A3_01_1593	63291	TOF; CID	43.7	679.38	PIK3R2

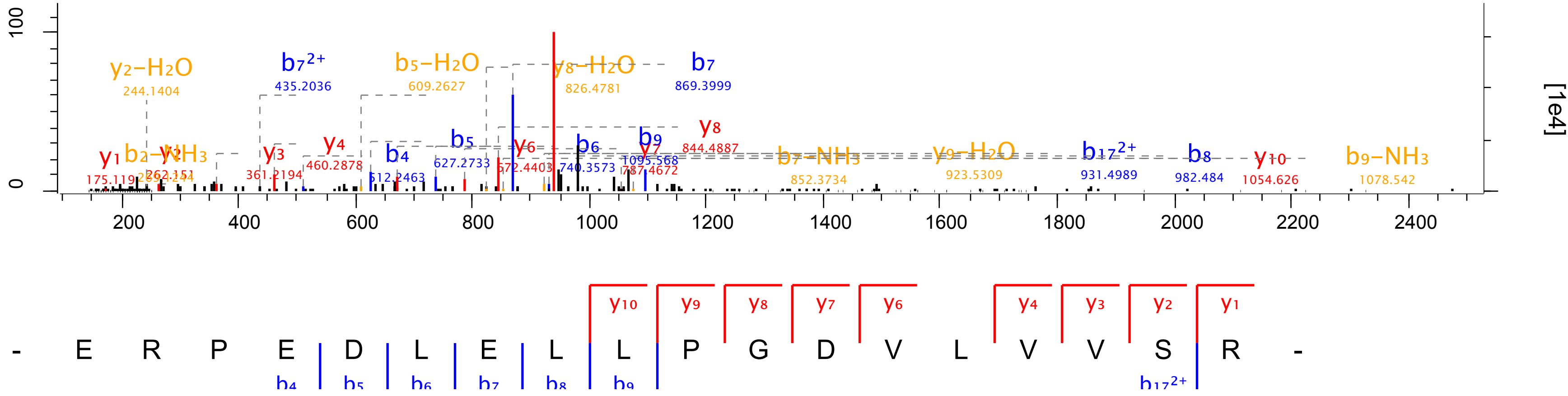

Raw file	Scan	Method	Score	m/z	Gene names
20150226_Hela_Top_opt_A3_01_1593	64406	TOF; CID	88.39	943.48	GLCE

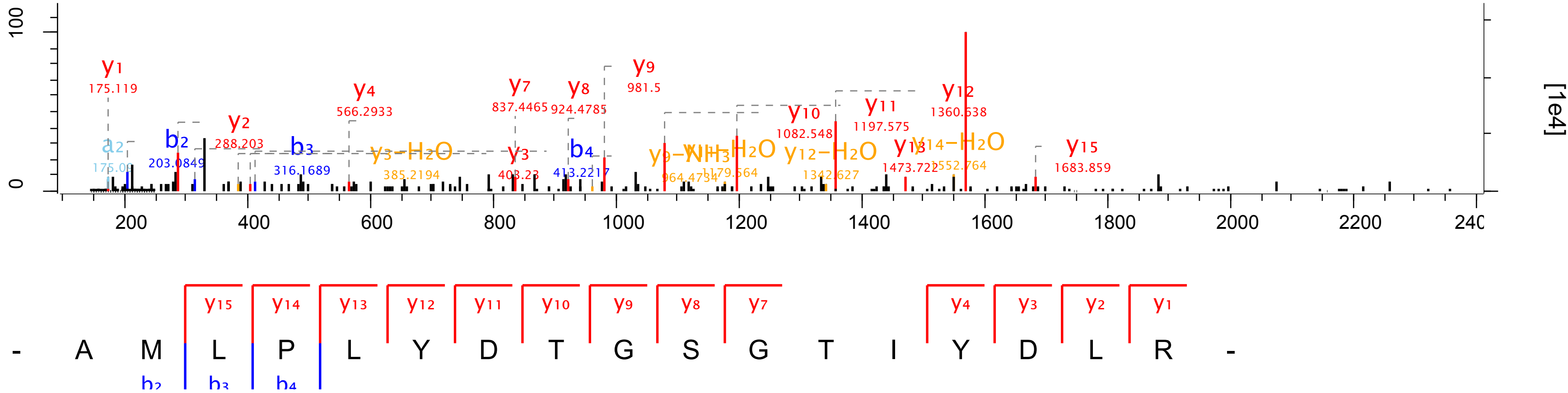

Raw file	Scan	Method	Score	m/z	Gene names
20150226_Hela_Top_opt_A3_01_1593	64481	TOF; CID	39.7	934.47	RNF123

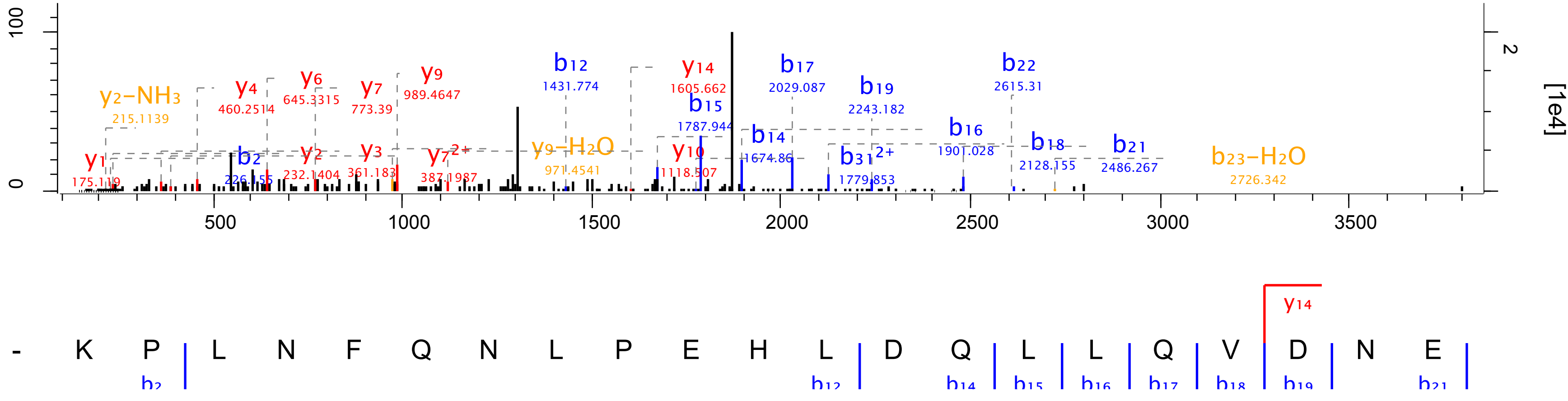

Raw file	Scan	Method	Score	m/z	Gene names
20150226_Hela_Top_opt_A3_01_1593	64711	TOF; CID	56.71	1096.53	DPH2

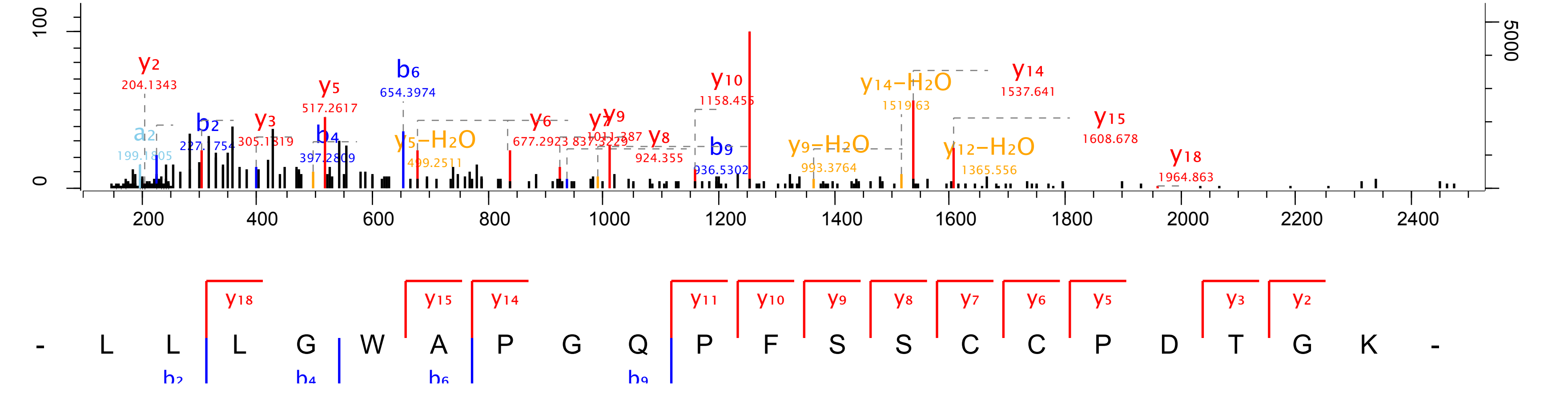

Raw file	Scan	Method	Score	m/z	Gene names
20150226_Hela_Top_opt_A3_01_1593	64974	TOF; CID	59.37	930.51	ABHD17B

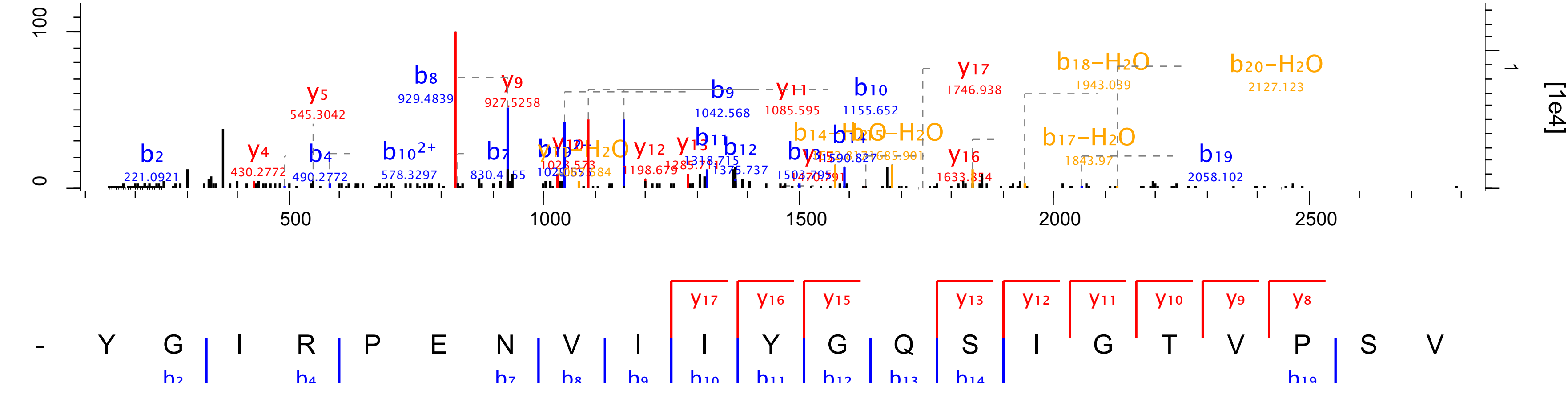

Raw file	Scan	Method	Score	m/z	Gene names
20150226_Hela_Top_opt_A3_01_1593	65073	TOF; CID	82.65	815.92	ANAPC16

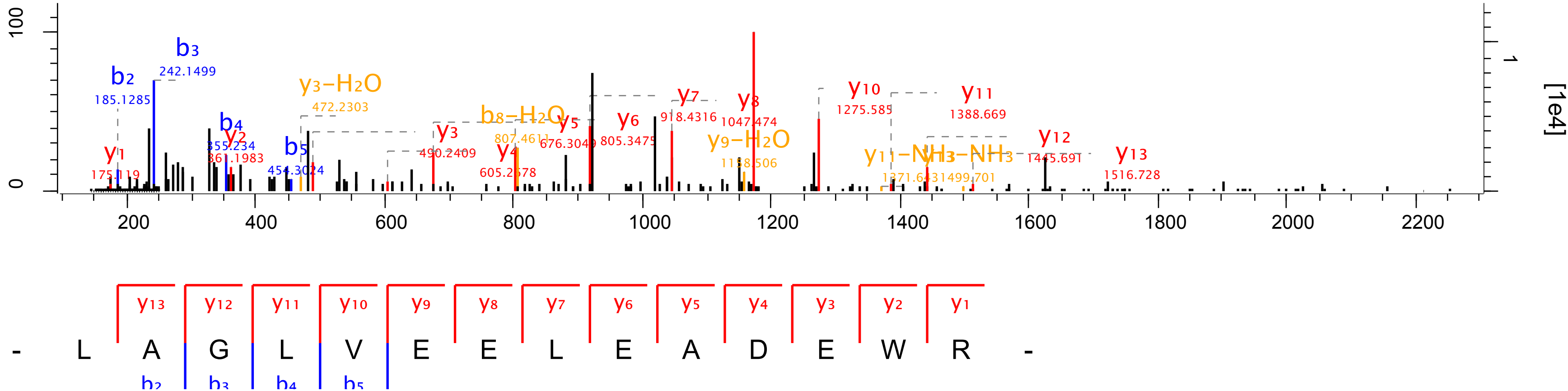

Raw file	Scan	Method	Score	m/z	Gene names
20150226_Hela_Top_opt_A3_01_1593	65180	TOF; CID	79.84	961.47	DEGS1

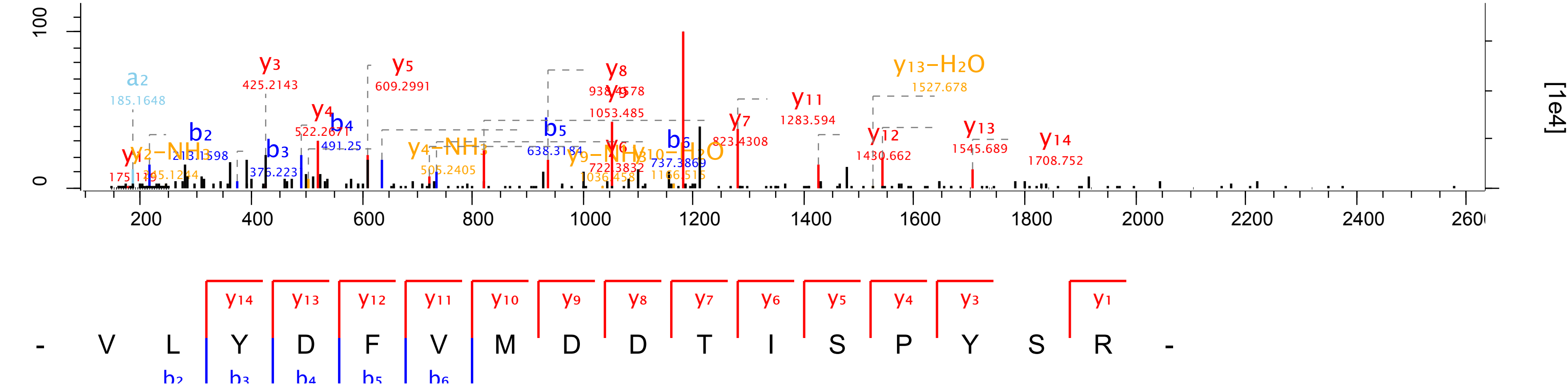

Raw file	Scan	Method	Score	m/z	Gene names
20150226_Hela_Top_opt_A3_01_1593	65414	TOF; CID	36.17	1207.6	USP34

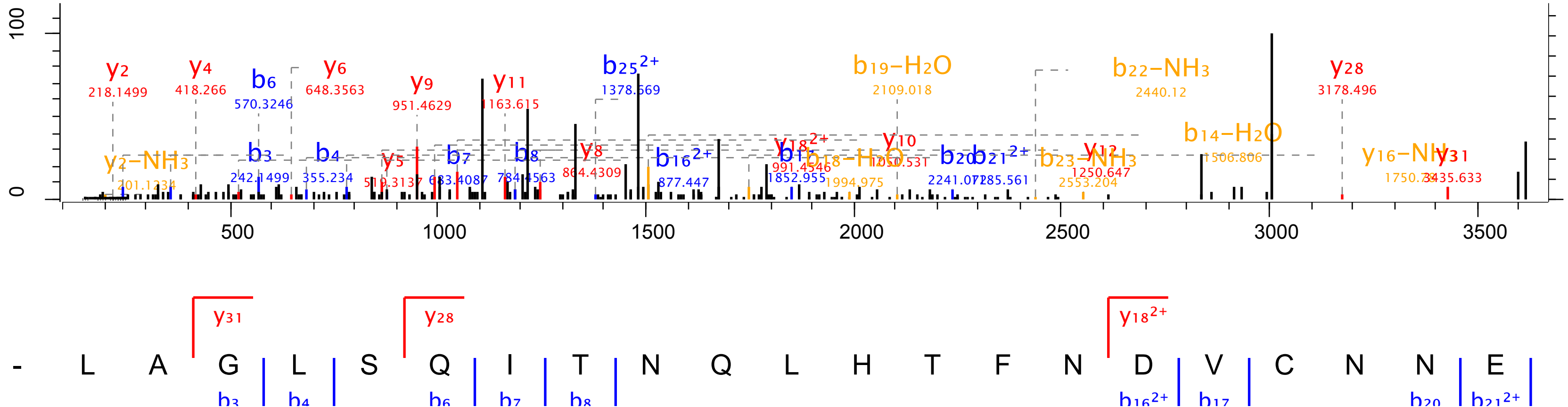

Raw file	Scan	Method	Score	m/z	Gene names
20150226_Hela_Top_opt_A3_01_1593	66533	TOF; CID	125.23	660.37	DFFB

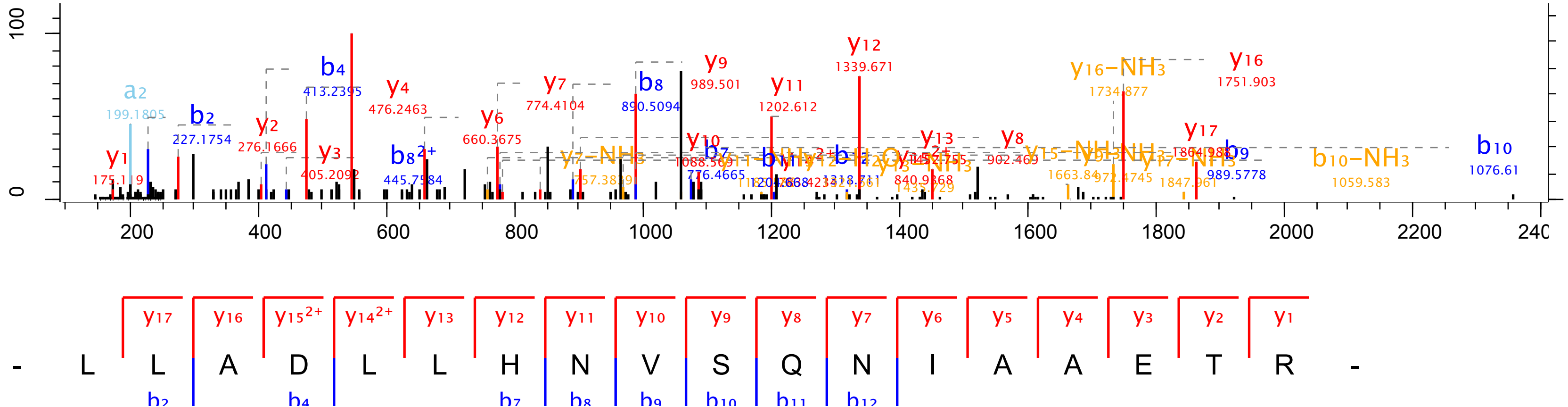

Raw file	Scan	Method	Score	m/z	Gene names
20150226_Hela_Top_opt_A3_01_1593	66693	TOF; CID	87.74	805.9	MID1IP1

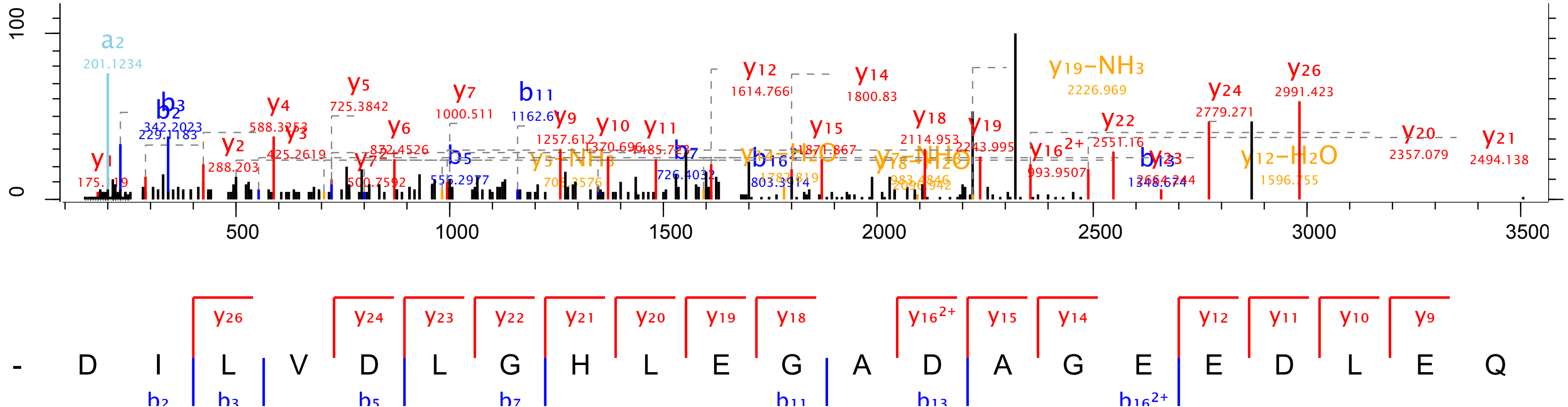

Raw file	Scan	Method	Score	m/z	Gene names
20150226_Hela_Top_opt_A3_01_1593	67336	TOF; CID	47.04	792.42	CCDC91

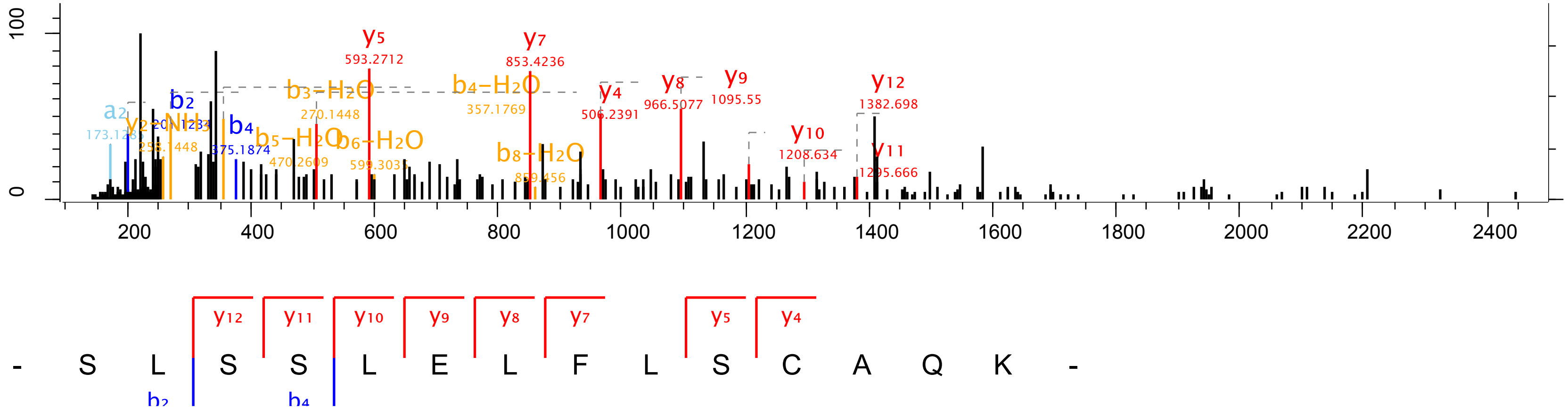

Raw file	Scan	Method	Score	m/z	Gene names
20150226_Hela_Top_opt_A3_01_1593	67444	TOF; CID	96.13	820.42	FAM73A

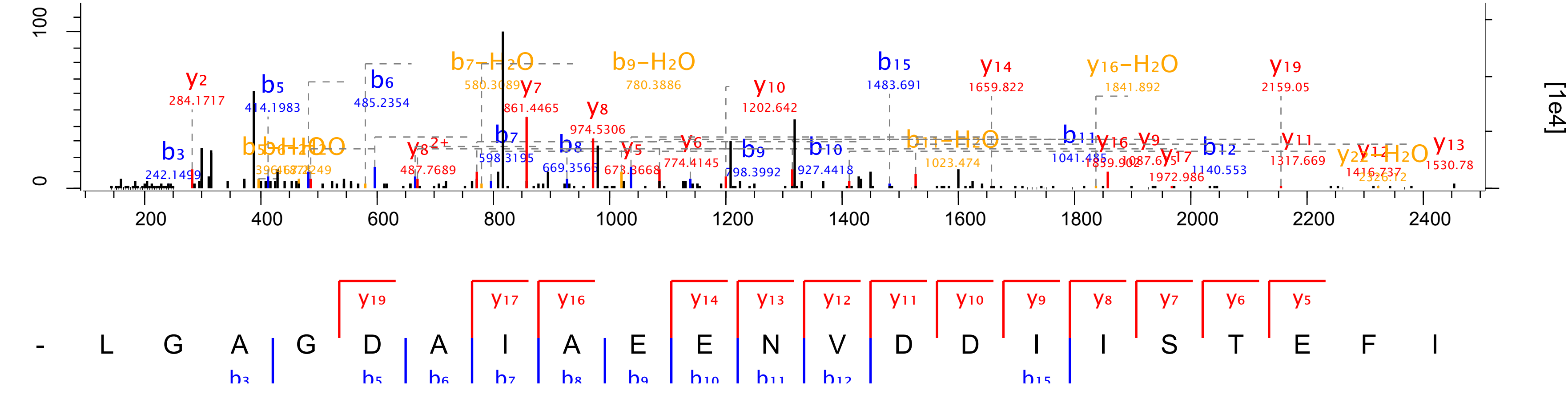

Raw file	Scan	Method	Score	m/z	Gene names
20150226_Hela_Top_opt_A3_01_1593	67646	TOF; CID	79.68	923	RINT1

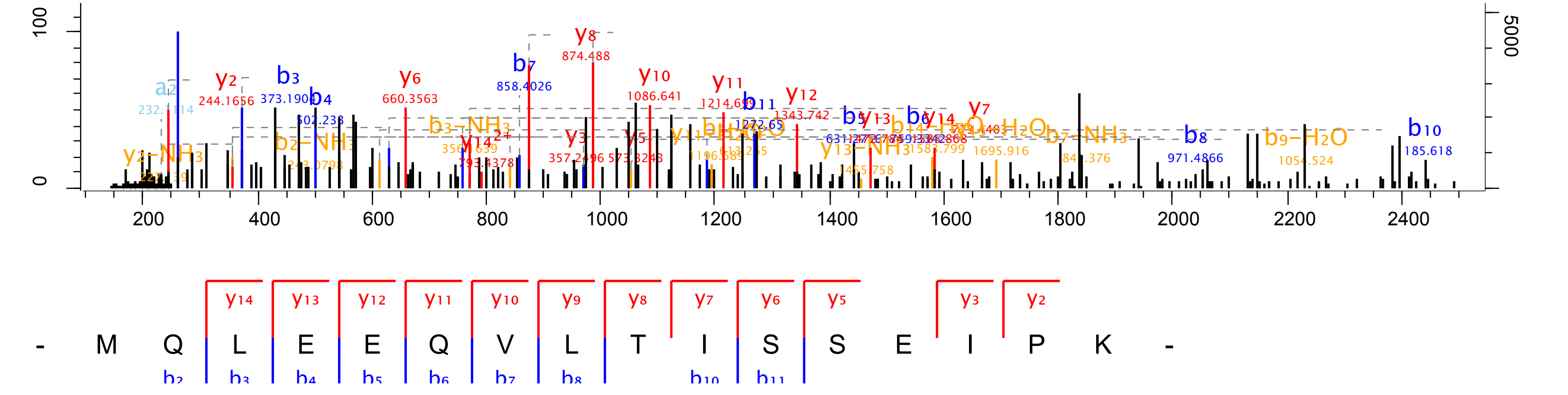

Raw file	Scan	Method	Score	m/z	Gene names
20150226_Hela_Top_opt_A3_01_1593	68386	TOF; CID	91.32	990.01	BTBD1

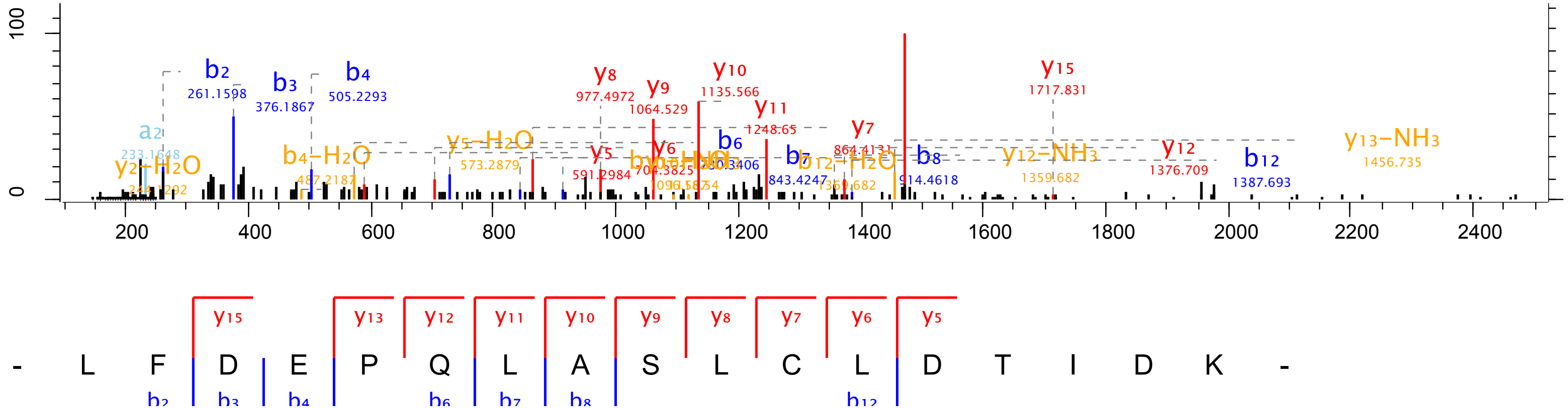

Raw file	Scan	Method	Score	m/z	Gene names
20150226_Hela_Top_opt_A3_01_1593	69026	TOF; CID	119.51	663.67	ABRACL

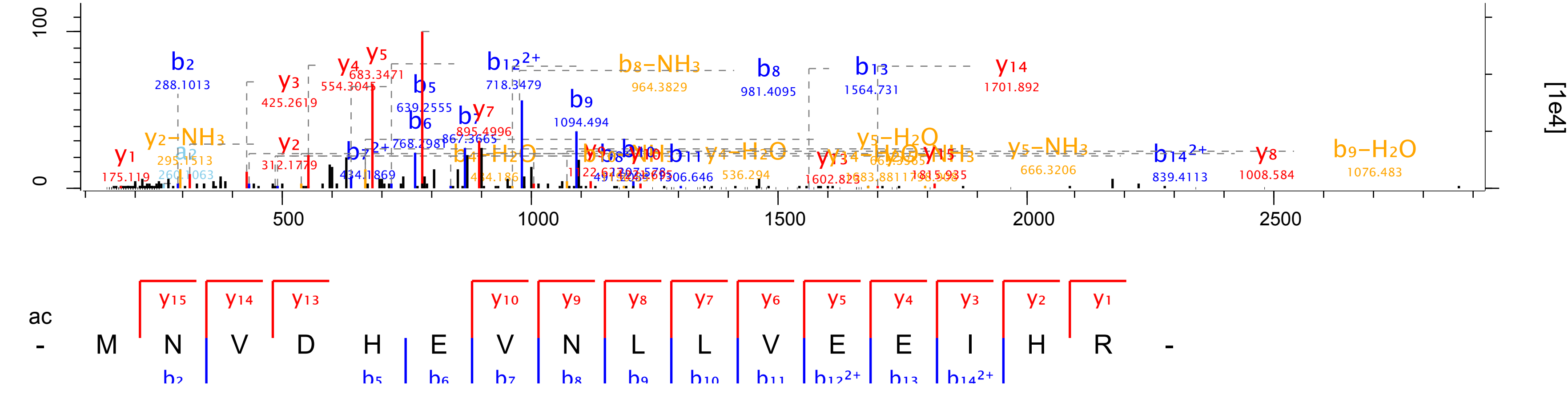

Raw file	Scan	Method	Score	m/z	Gene names
20150226_Hela_Top_opt_A3_01_1593	69087	TOF; CID	74.79	842.45	CIB1

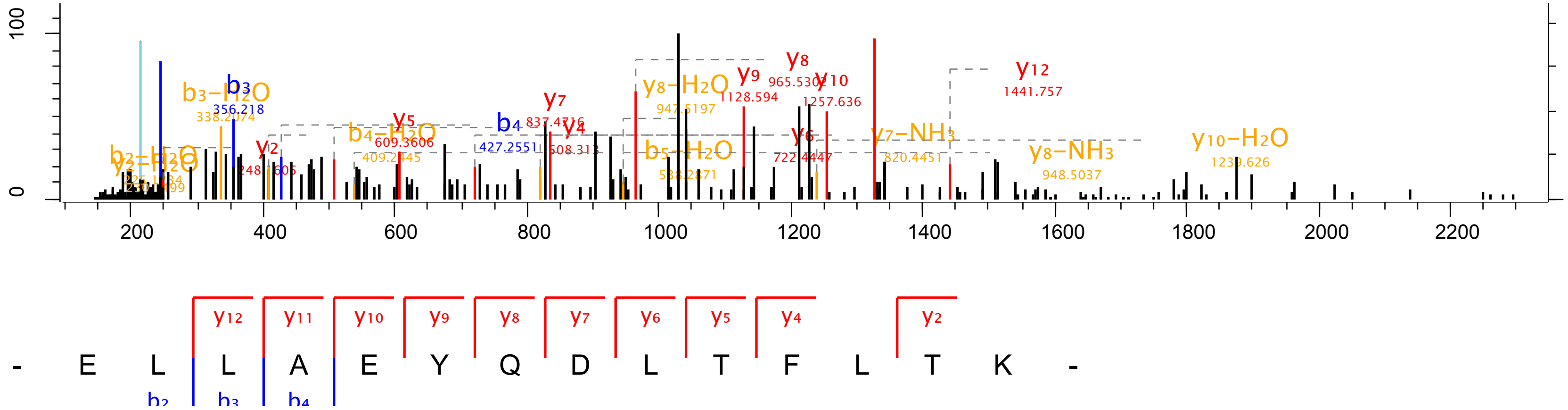

Raw file	Scan	Method	Score	m/z	Gene names
20150226_Hela_Top_opt_A3_01_1593	69126	TOF; CID	38.33	1104.55	ANAPC15

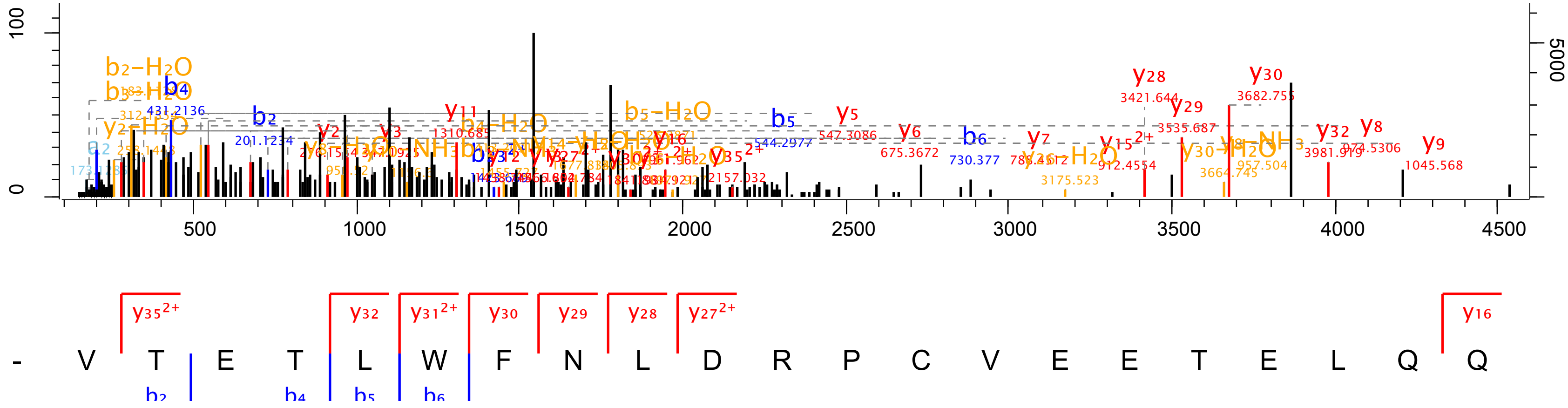

Raw file	Scan	Method	Score	m/z	Gene names
20150226_Hela_Top_opt_A3_01_1593	69609	TOF; CID	96.85	816.12	HERC2

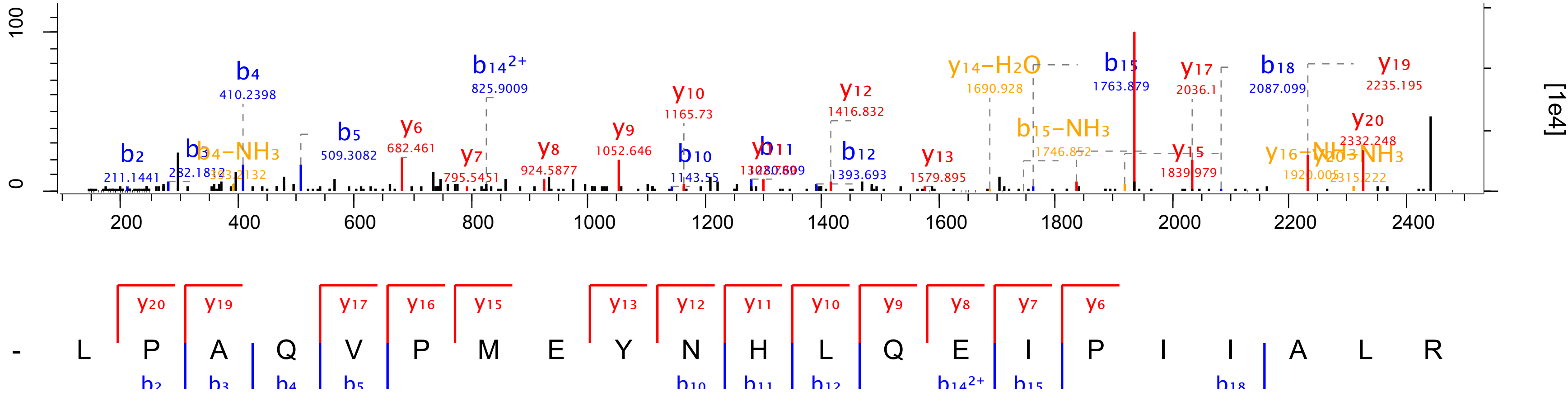

Raw file	Scan	Method	Score	m/z	Gene names
20150226_Hela_Top_opt_A3_01_1593	69982	TOF; CID	100.76	823.43	SAMD9

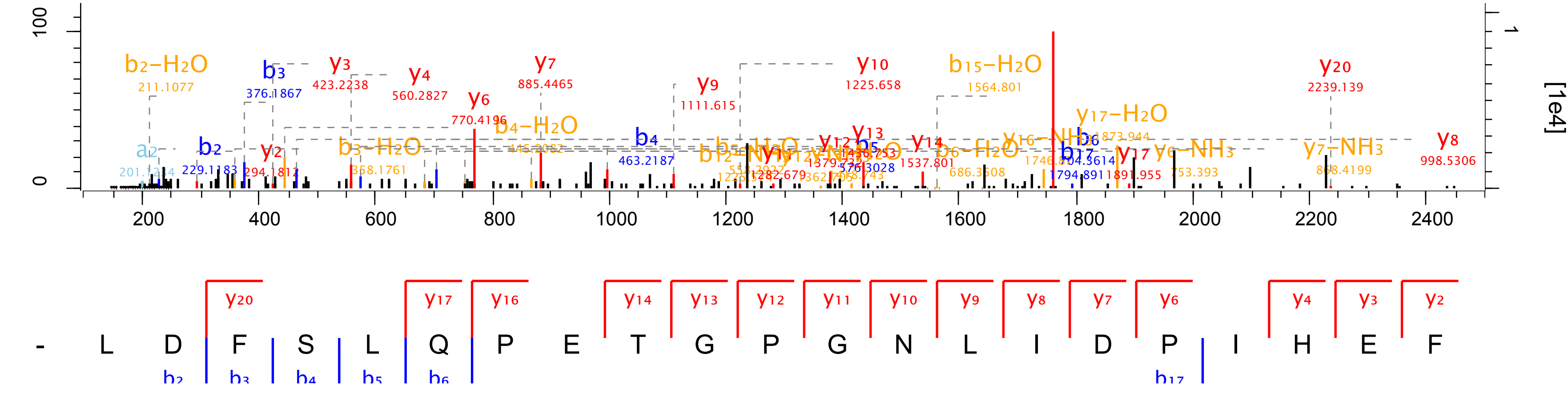

Raw file	Scan	Method	Score	m/z	Gene names
20150226_Hela_Top_opt_A3_01_1593	70366	TOF; CID	38.48	982.51	TSKU

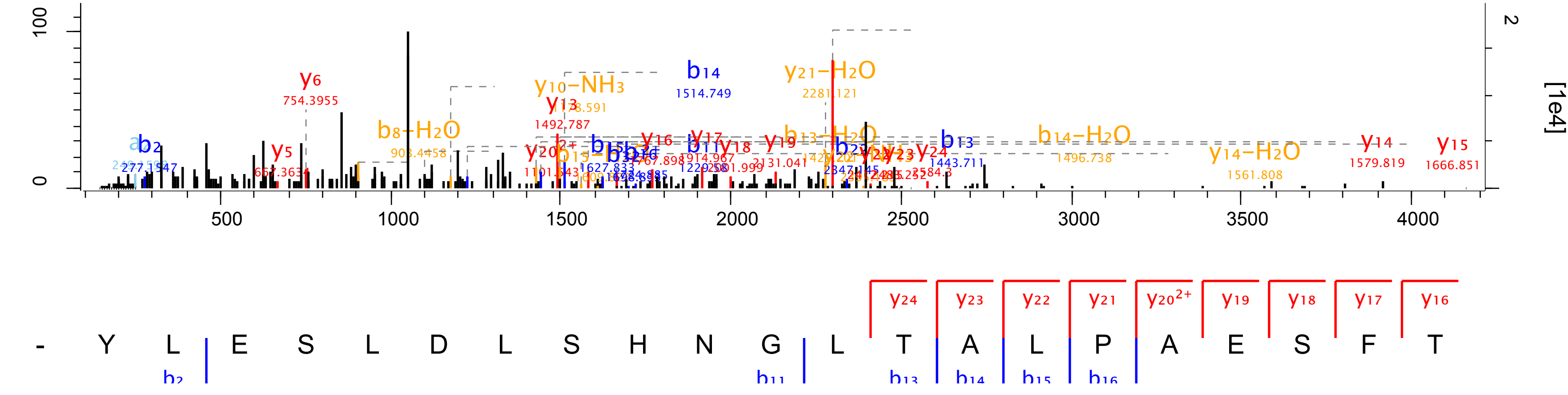

Raw file	Scan	Method	Score	m/z	Gene names
20150226_Hela_Top_opt_A3_01_1593	70537	TOF; CID	43.31	953.83	ALG2

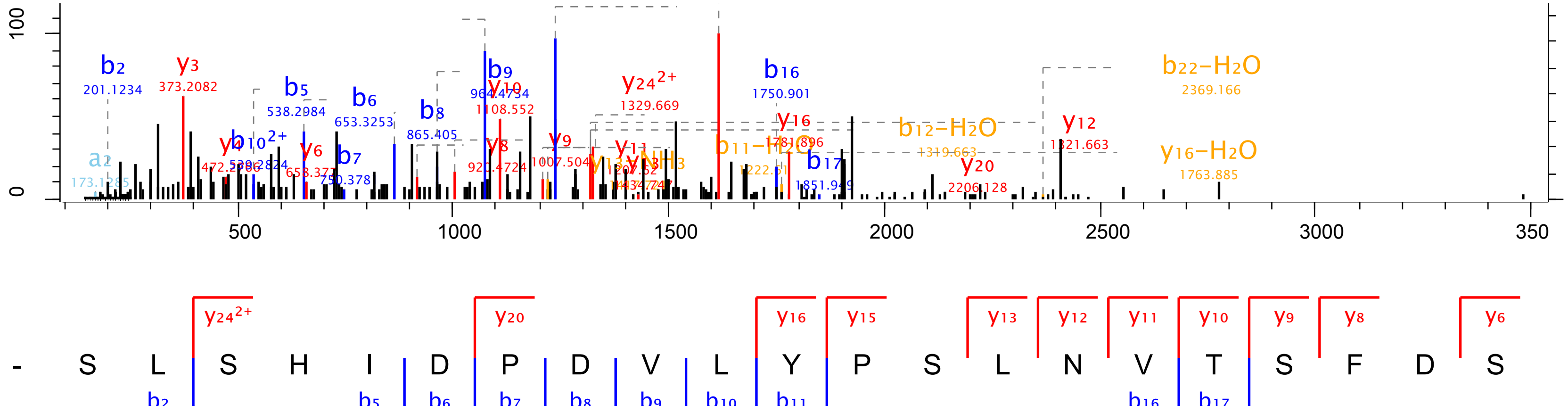

Raw file	Scan	Method	Score	m/z	Gene names
20150226_Hela_Top_opt_A3_01_1593	70633	TOF; CID	49.77	1051.04	COX10

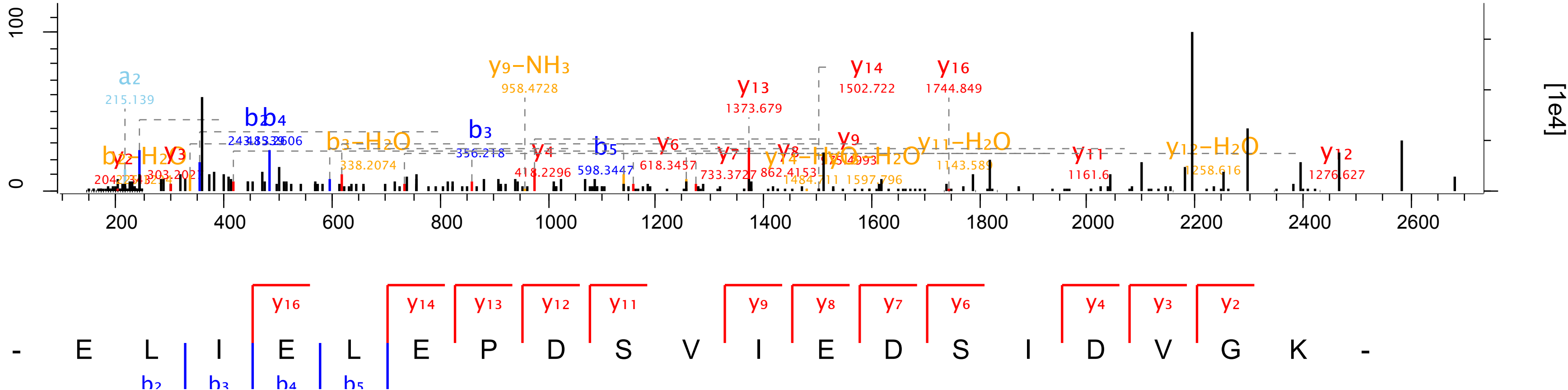

Raw file	Scan	Method	Score	m/z	Gene names
20150226_Hela_Top_opt_A3_01_1593	71645	TOF; CID	42.62	1058.55	UNC50

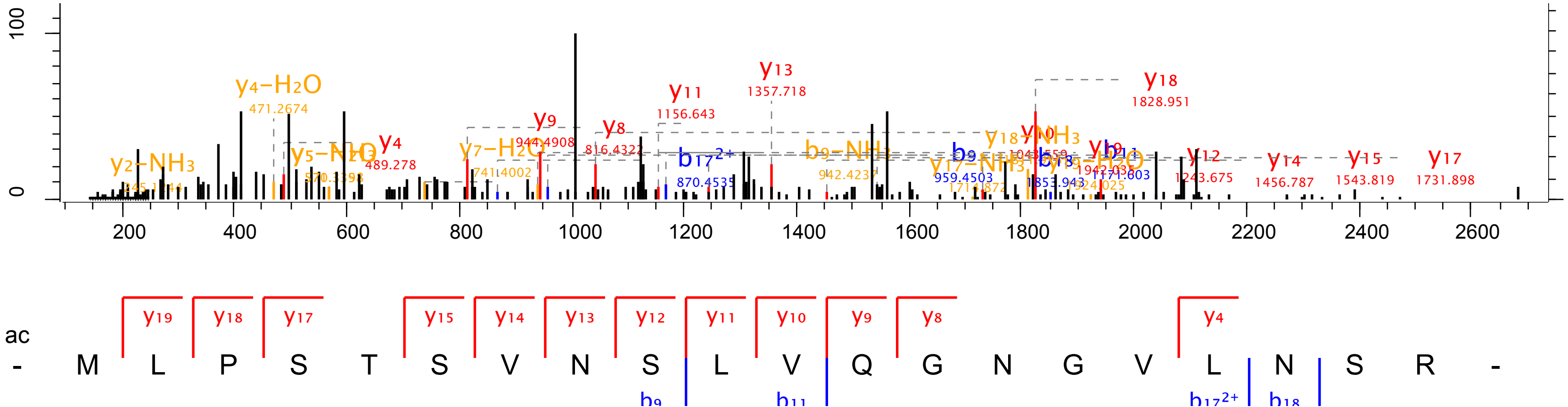

Raw file	Scan	Method	Score	m/z	Gene names
20150226_Hela_Top_opt_A3_01_1593	72326	TOF; CID	85.49	614.33	PDPK1;PDPK2

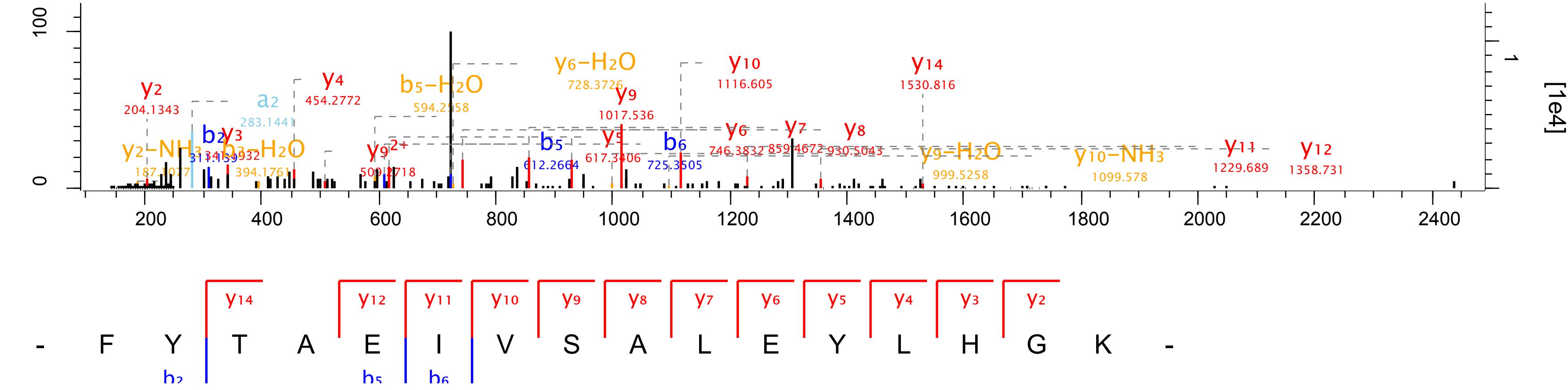

Raw file	Scan	Method	Score	m/z	Gene names
20150226_Hela_Top_opt_A3_01_1593	72329	TOF; CID	43.84	1141.6	BCHE

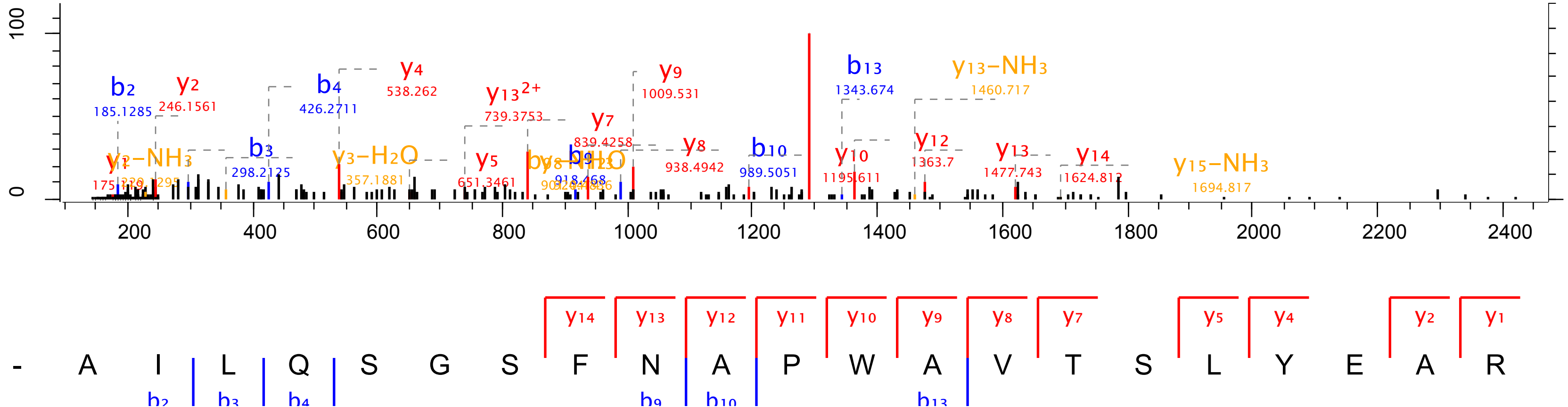

Raw file	Scan	Method	Score	m/z	Gene names
20150226_Hela_Top_opt_A3_01_1593	73405	TOF; CID	95.5	737.05	RALGAPB

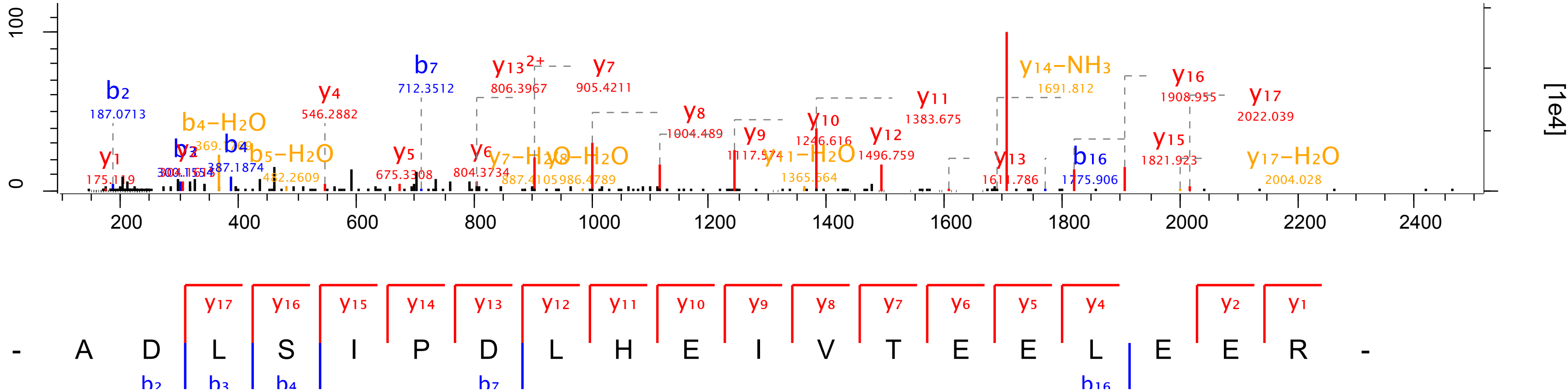

Raw file	Scan	Method	Score	m/z	Gene names
20150226_Hela_Top_opt_A3_01_1593	74142	TOF; CID	74.61	608.38	OCIAD2

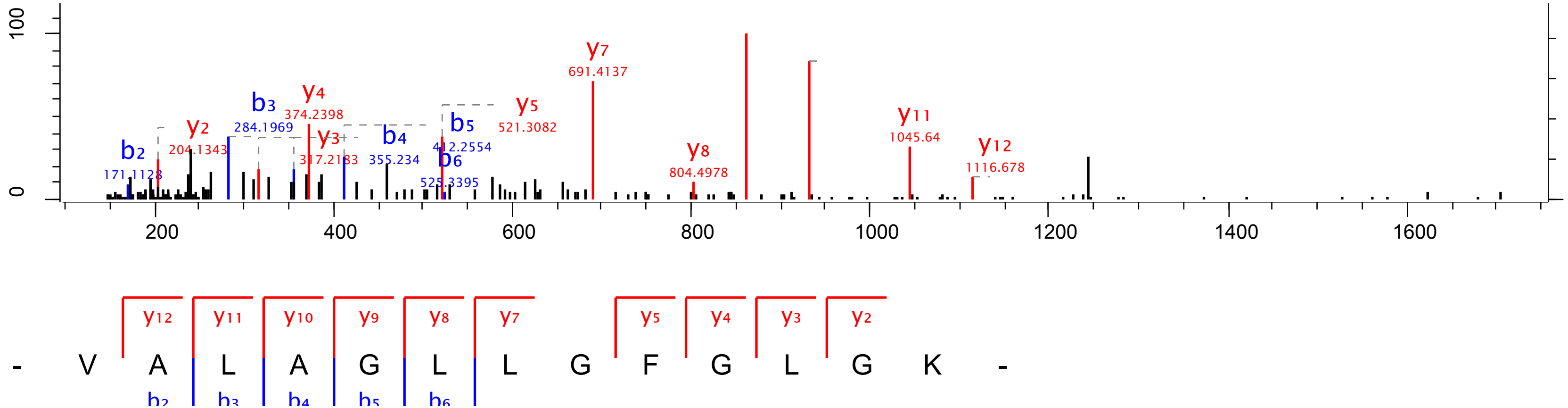

Raw file	Scan	Method	Score	m/z	Gene names
20150226_Hela_Top_opt_A3_01_1593	74449	TOF; CID	51.73	719.88	POLR3H

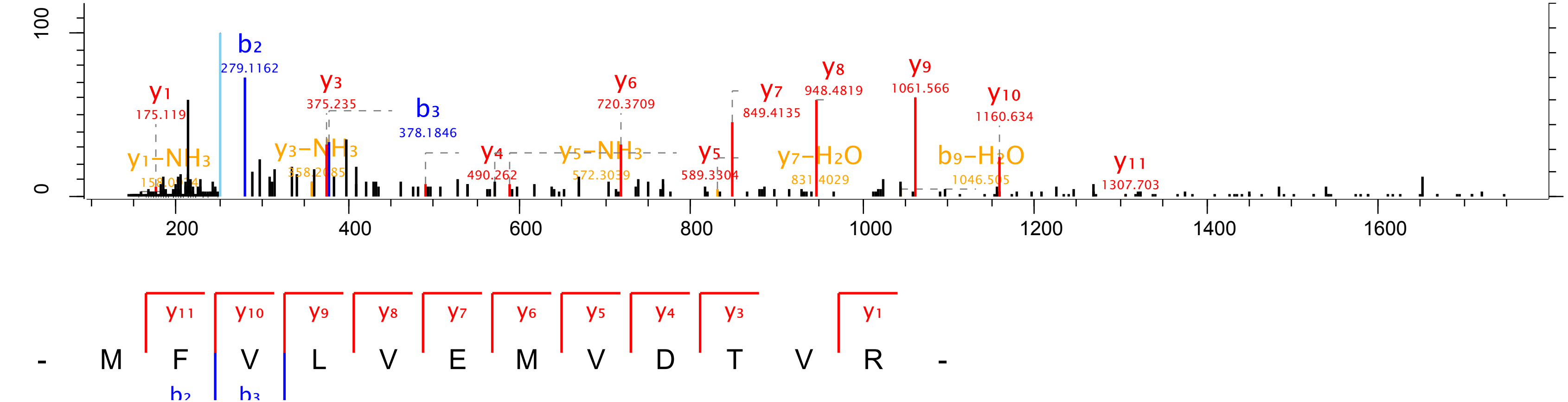

Raw file	Scan	Method	Score	m/z	Gene names
20150226_Hela_Top_opt_A3_01_1593	75332	TOF; CID	70.06	1232.25	TTPAL

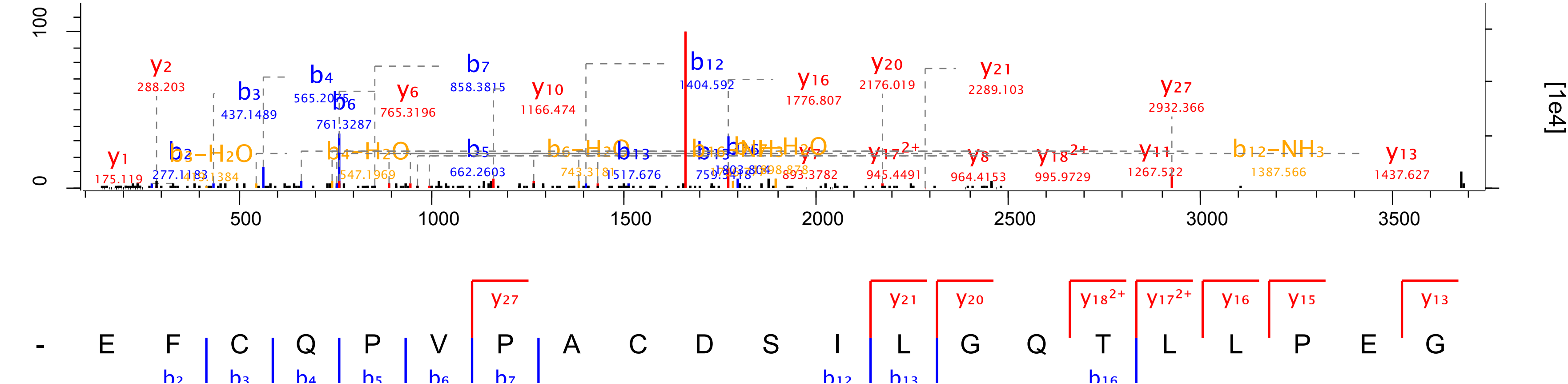

Raw file	Scan	Method	Score	m/z	Gene names
20150226_Hela_Top_opt_A3_01_1593	75371	TOF; CID	107.21	829.45	C11orf83

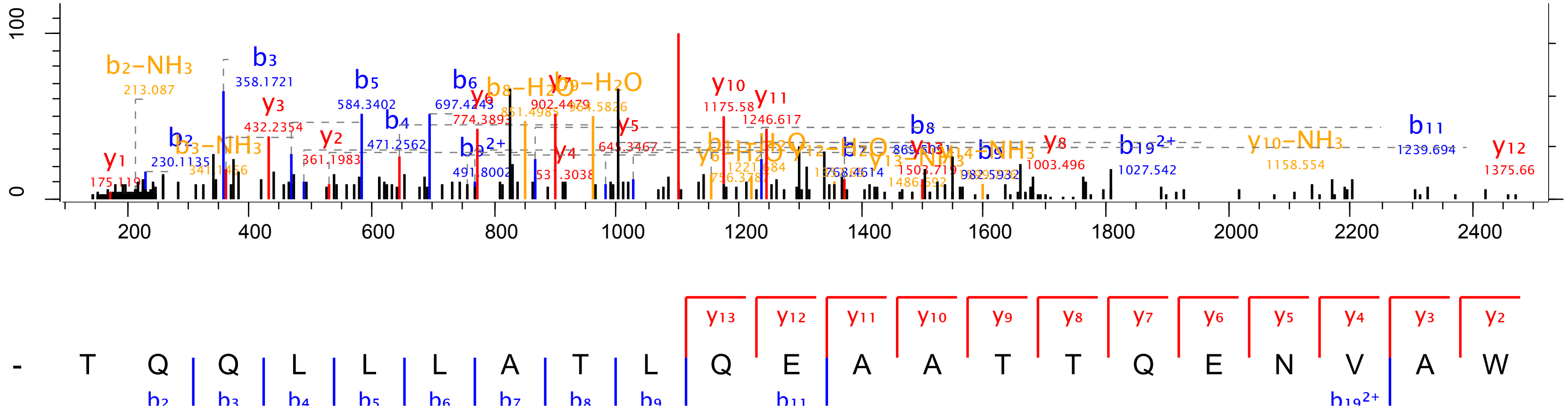

Raw file	Scan	Method	Score	m/z	Gene names
20150226_Hela_Top_opt_A3_01_1593	75406	TOF; CID	87.35	820.03	COL4A5

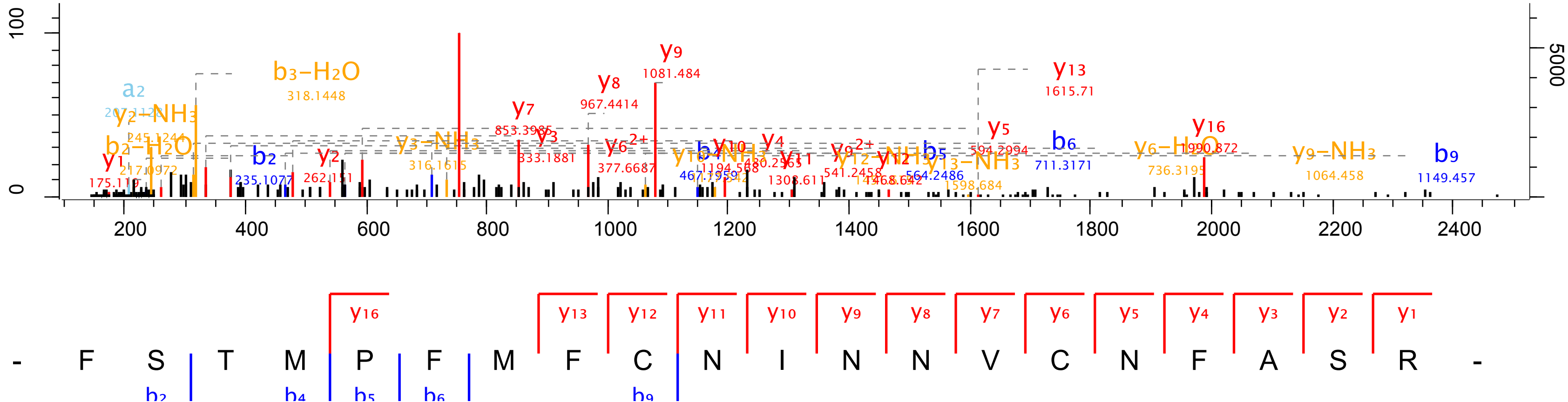

Raw file	Scan	Method	Score	m/z	Gene names
20150226_Hela_Top_opt_A3_01_1593	75407	TOF; CID	47.61	652.87	SLC1A3

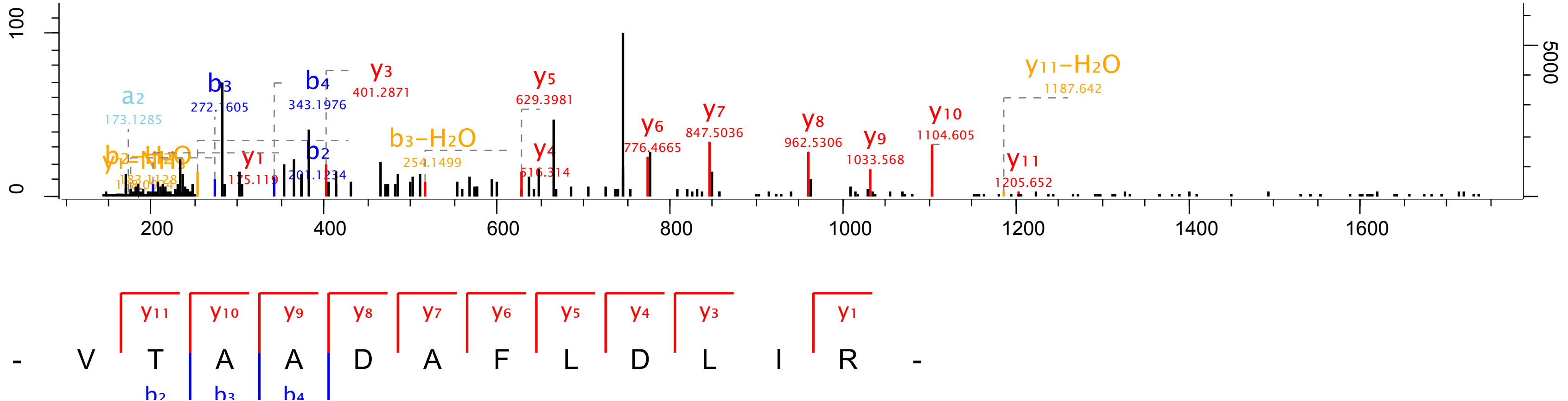

Raw file	Scan	Method	Score	m/z	Gene names
20150226_Hela_Top_opt_A3_01_1593	75444	TOF; CID	66.15	742.7	CLK3

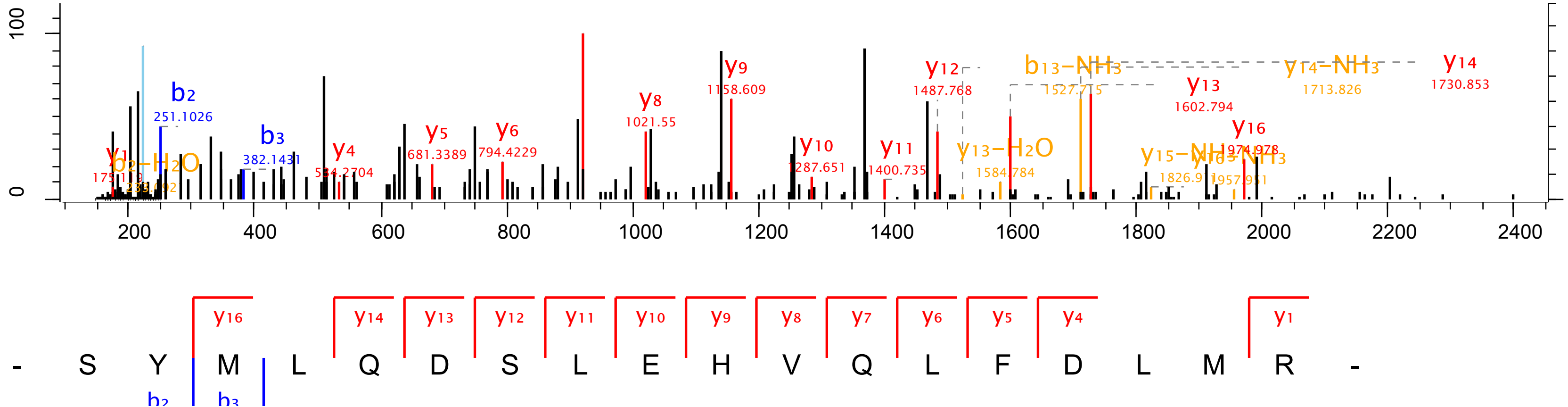

Raw file	Scan	Method	Score	m/z	Gene names
20150226_Hela_Top_opt_A3_01_1593	75619	TOF; CID	92.94	837.95	LPGAT1

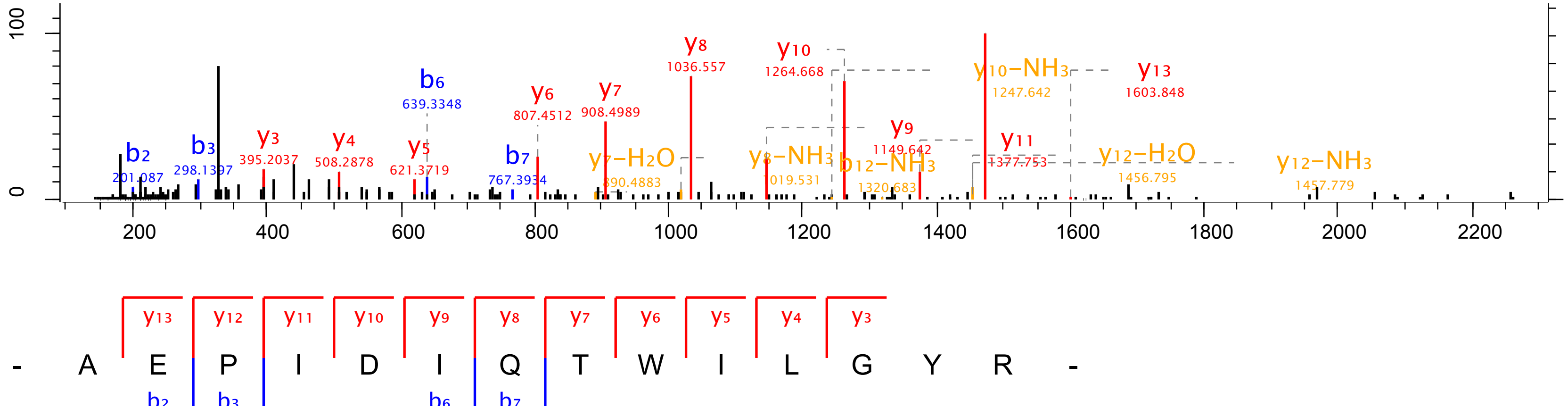

Raw file	Scan	Method	Score	m/z	Gene names
20150226_Hela_Top_opt_A3_01_1593	77937	TOF; CID	53.68	907.81	HIGD2A

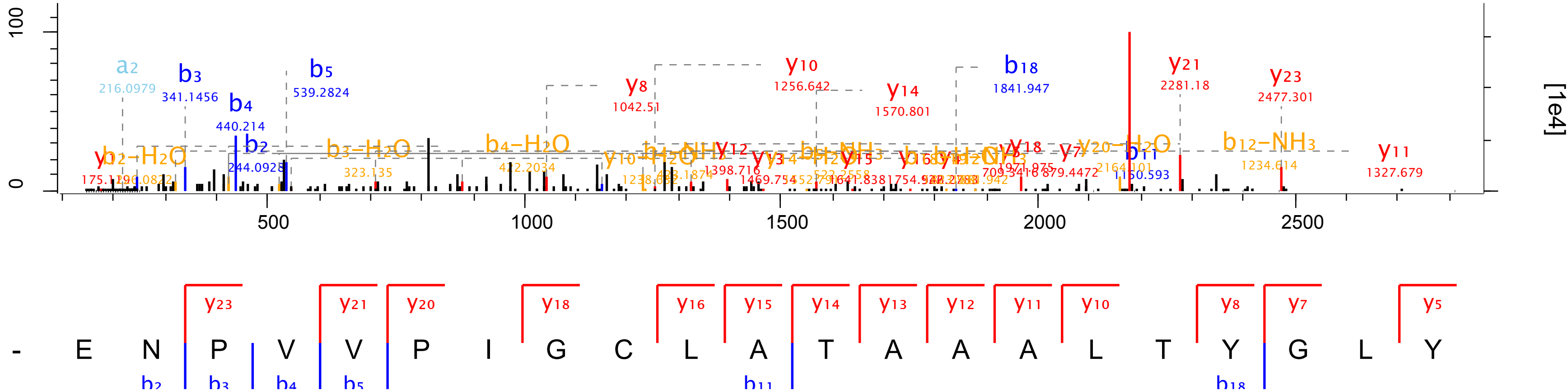

Raw file	Scan	Method	Score	m/z	Gene names
20150226_Hela_Top_opt_A3_01_1593	79484	TOF; CID	95.21	973.52	S100A2

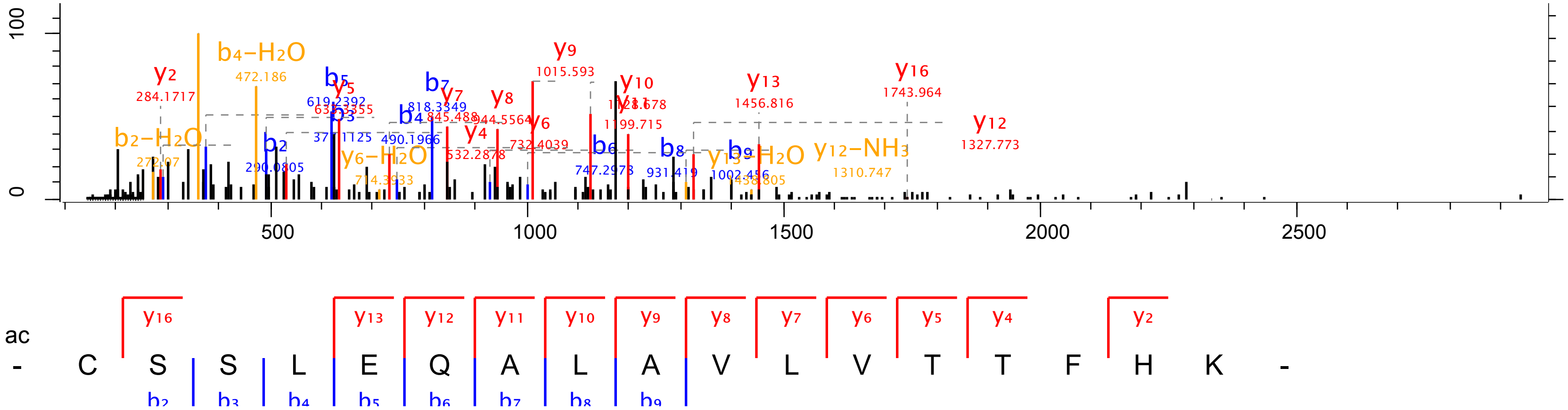

Raw file	Scan	Method	Score	m/z	Gene names
20150226_Hela_Top_opt_A3_01_1593	79852	TOF; CID	80.63	801.42	ATF6B

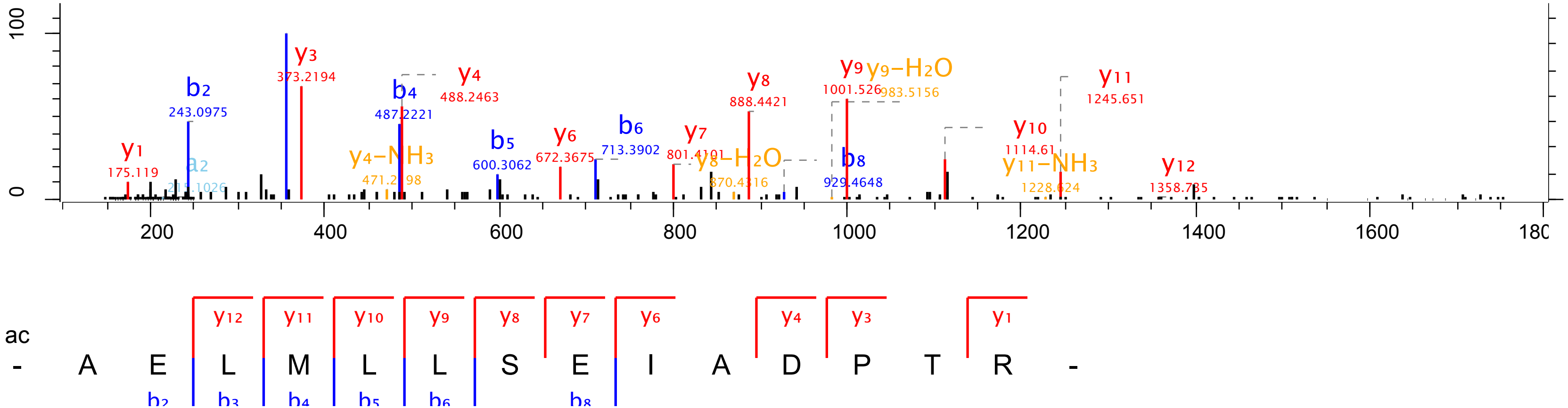

Raw file	Scan	Method	Score	m/z	Gene names
20150226_Hela_Top_opt_A3_01_1593	80882	TOF; CID	179.86	1129.57	LSM5

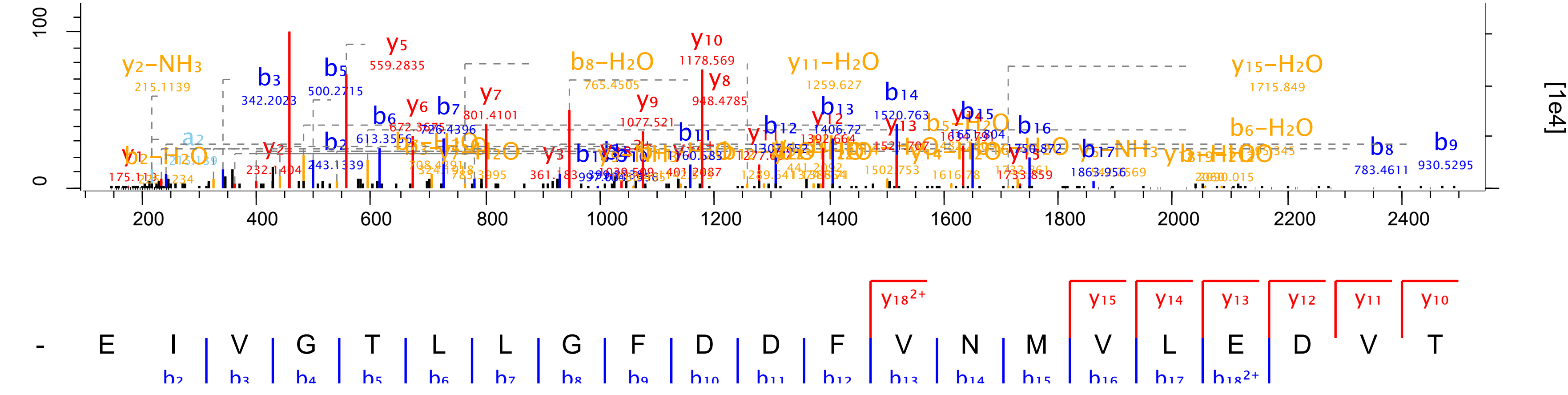

Raw file	Scan	Method	Score	m/z	Gene names
20150226_Hela_Top_opt_A3_01_1595	3116	TOF; CID	65.28	392.54	STX17

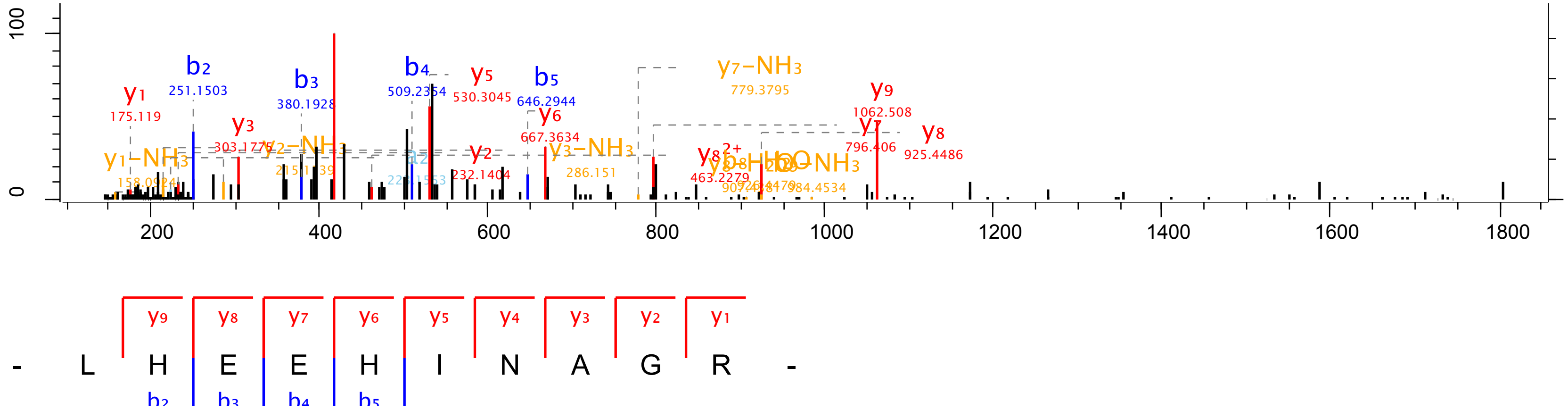

Raw file	Scan	Method	Score	m/z	Gene names
20150226_Hela_Top_opt_A3_01_1595	3184	TOF; CID	123.75	538.76	TM4SF1

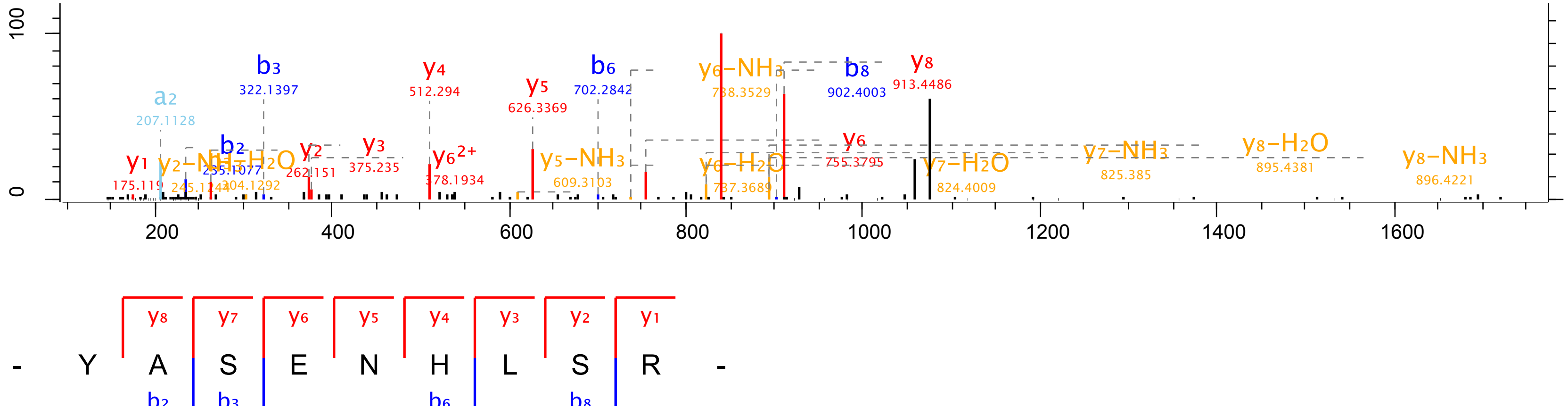

Raw file	Scan	Method	Score	m/z	Gene names
20150226_Hela_Top_opt_A3_01_1595	4657	TOF; CID	49.48	581.29	SCAND1

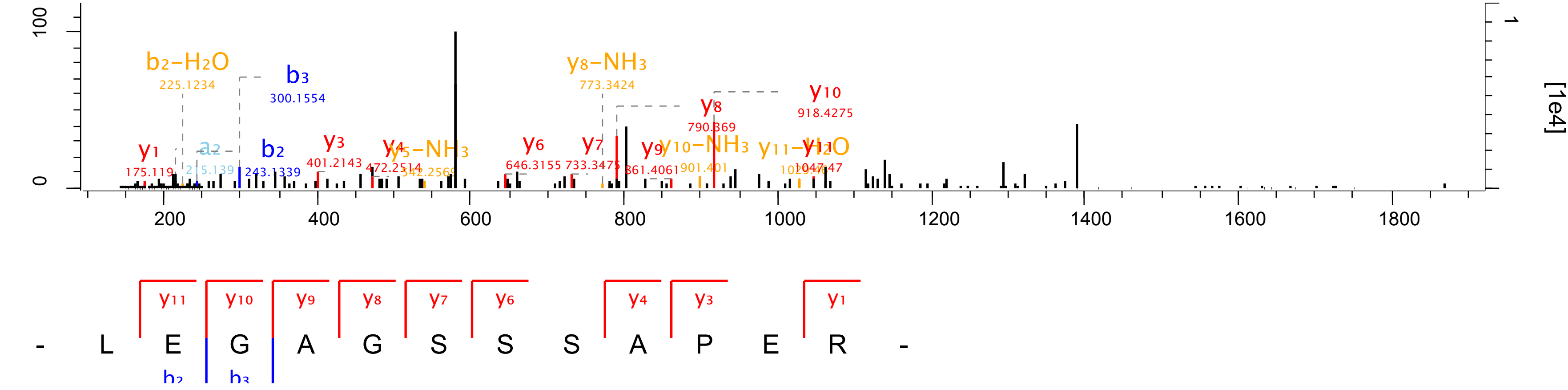

Raw file	Scan	Method	Score	m/z	Gene names
20150226_Hela_Top_opt_A3_01_1595	4696	TOF; CID	66.92	560.27	SFT2D2

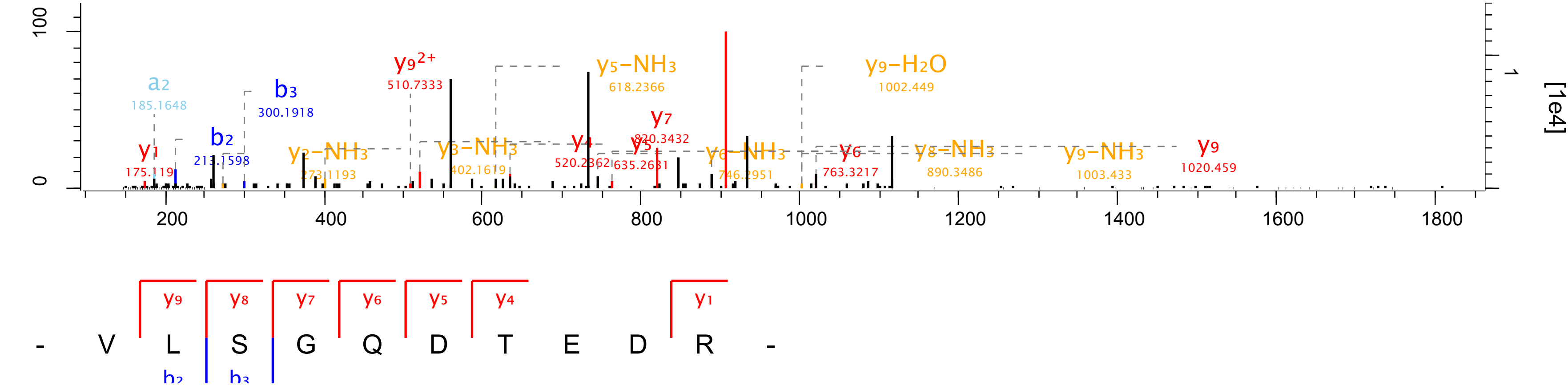

Raw file	Scan	Method	Score	m/z	Gene names
20150226_Hela_Top_opt_A3_01_1595	4939	TOF; CID	115.06	734.86	SLC39A7

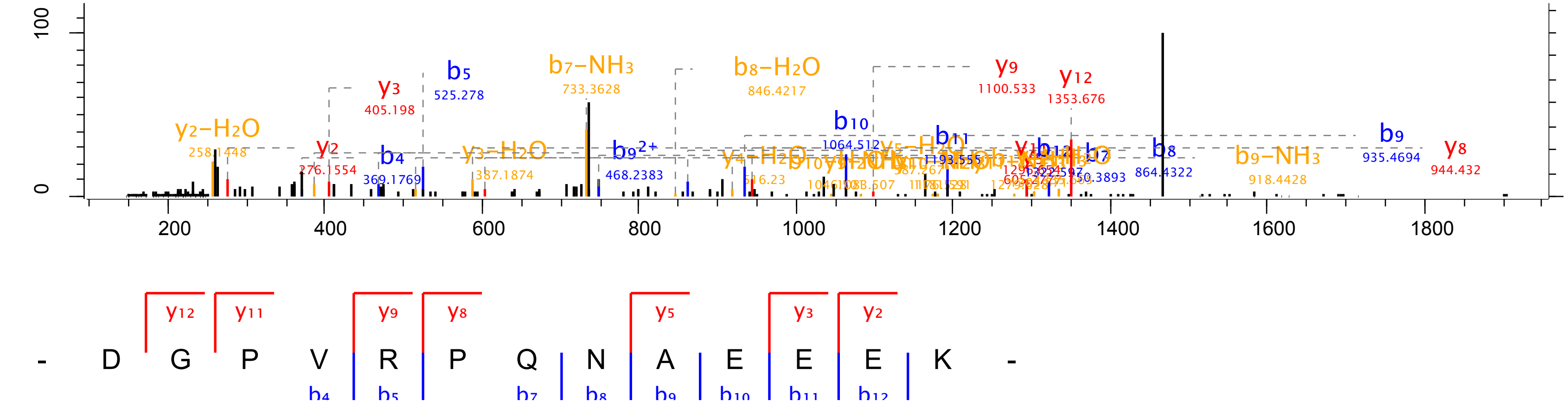

Raw file	Scan	Method	Score	m/z	Gene names
20150226_Hela_Top_opt_A3_01_1595	5087	TOF; CID	89.27	609.33	CDKL2

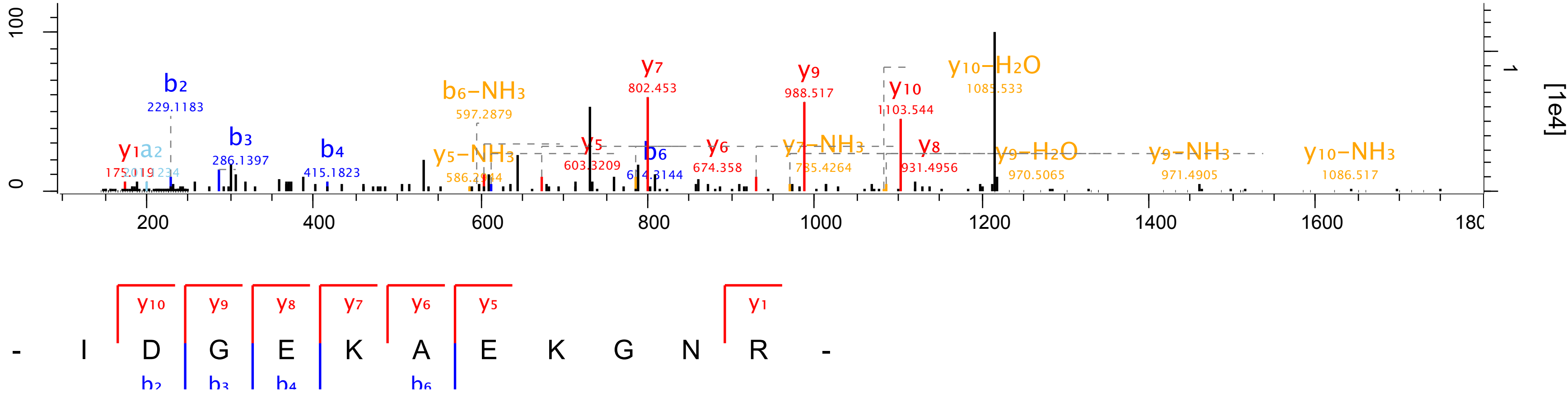

Raw file	Scan	Method	Score	m/z	Gene names
20150226_Hela_Top_opt_A3_01_1595	5322	TOF; CID	74.48	766.37	OFD1

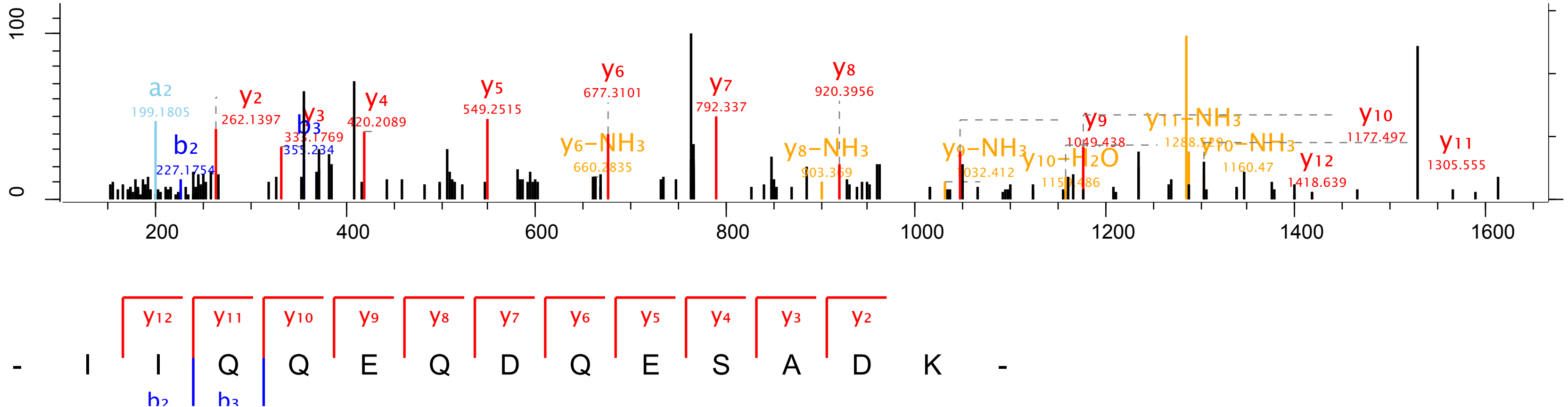

Raw file	Scan	Method	Score	m/z	Gene names
20150226_Hela_Top_opt_A3_01_1595	6915	TOF; CID	84.37	474.74	DPAGT1

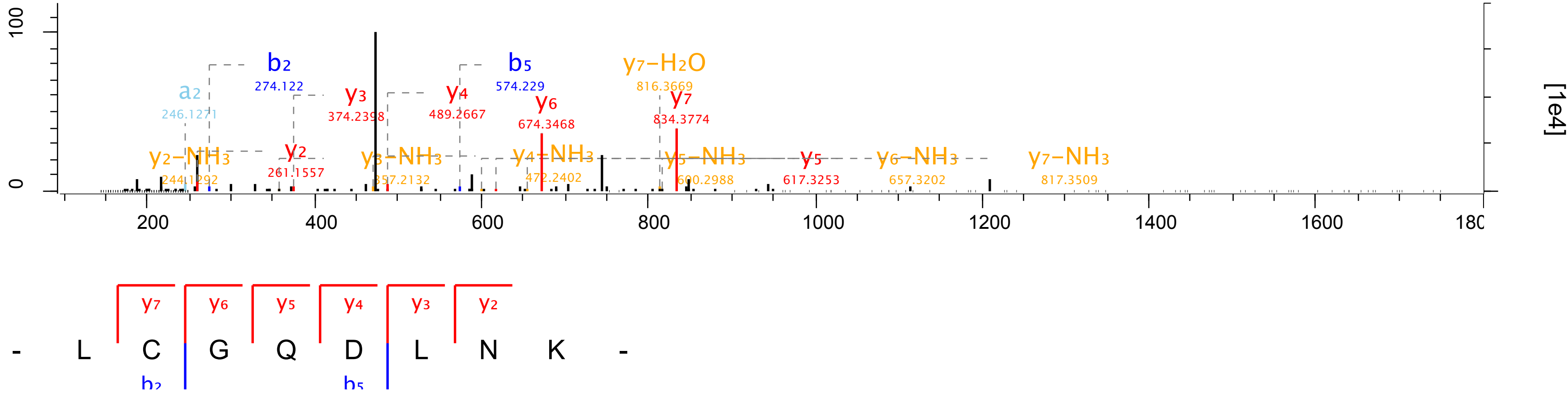

Raw file	Scan	Method	Score	m/z	Gene names
20150226_Hela_Top_opt_A3_01_1595	7876	TOF; CID	65.22	495.23	NKX3-2

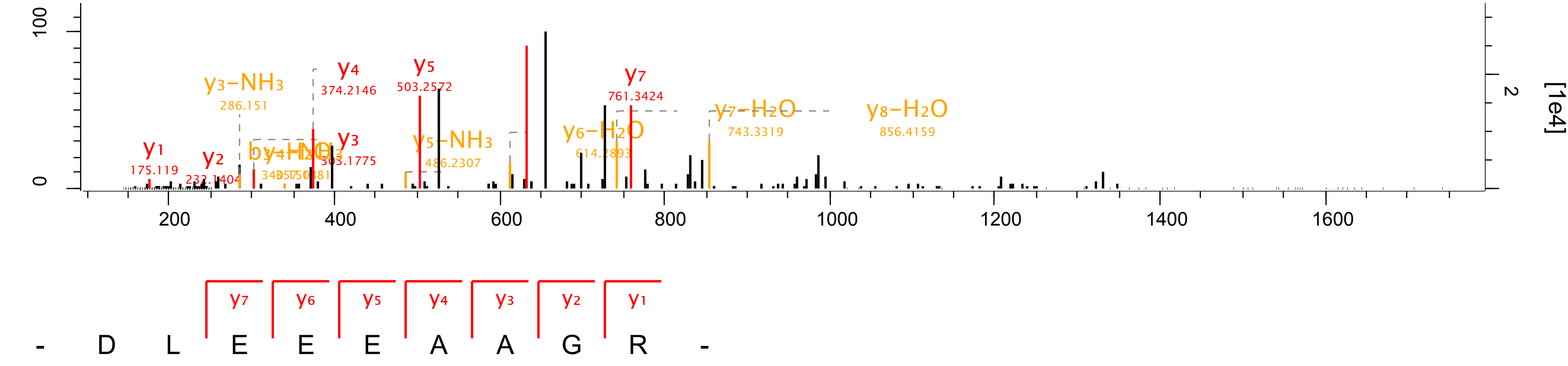

Raw file	Scan	Method	Score	m/z	Gene names
20150226_Hela_Top_opt_A3_01_1595	8306	TOF; CID	72.89	667.83	KIAA1467;DKFZp762C0813

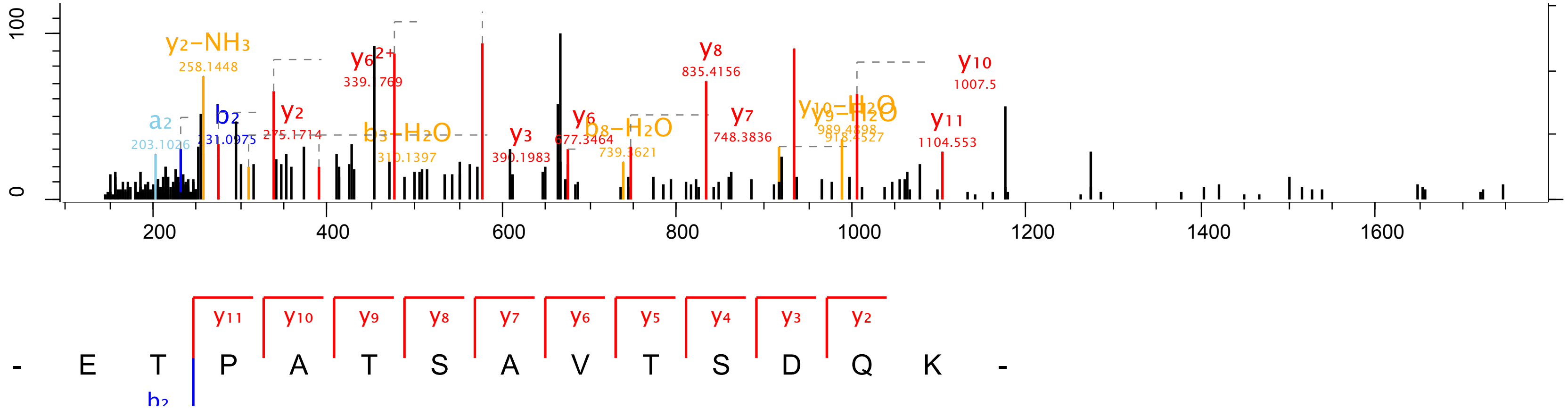

Raw file	Scan	Method	Score	m/z	Gene names
20150226_Hela_Top_opt_A3_01_1595	8484	TOF; CID	64.82	523.29	ORC6

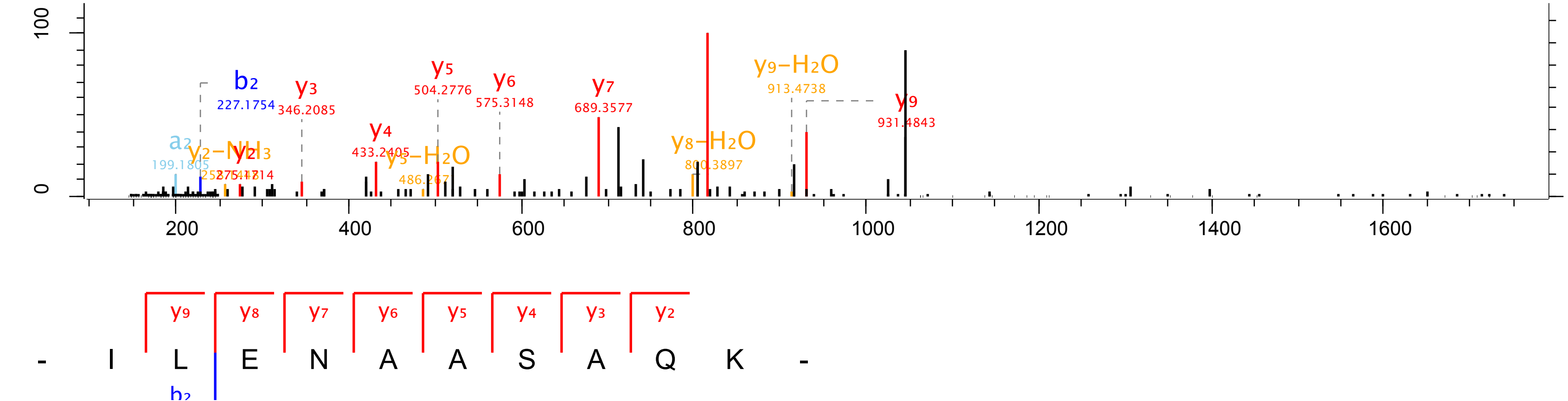

Raw file	Scan	Method	Score	m/z	Gene names
20150226_Hela_Top_opt_A3_01_1595	8995	TOF; CID	59.2	669.8	SPDL1

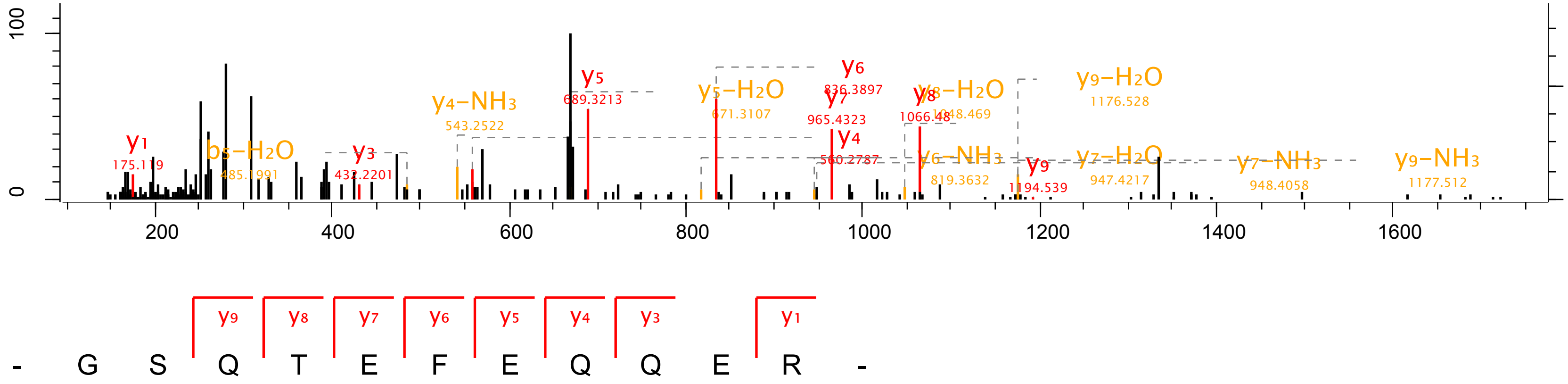

Raw file	Scan	Method	Score	m/z	Gene names
20150226_Hela_Top_opt_A3_01_1595	9965	TOF; CID	117.18	725.35	RFWD2

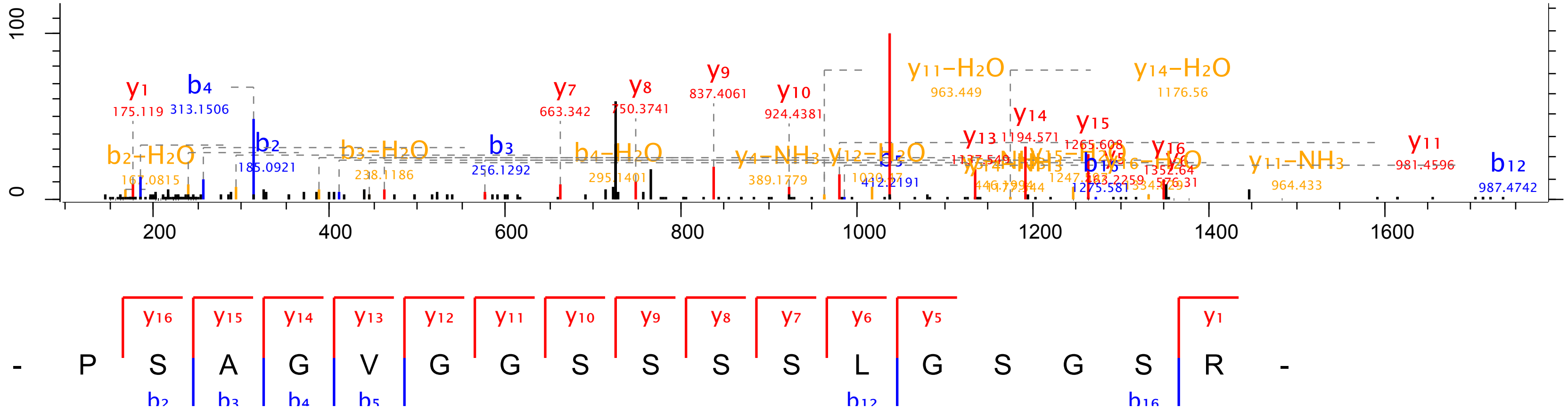

Raw file	Scan	Method	Score	m/z	Gene names
20150226_Hela_Top_opt_A3_01_1595	10034	TOF; CID	69.29	850.4	BRI3BP

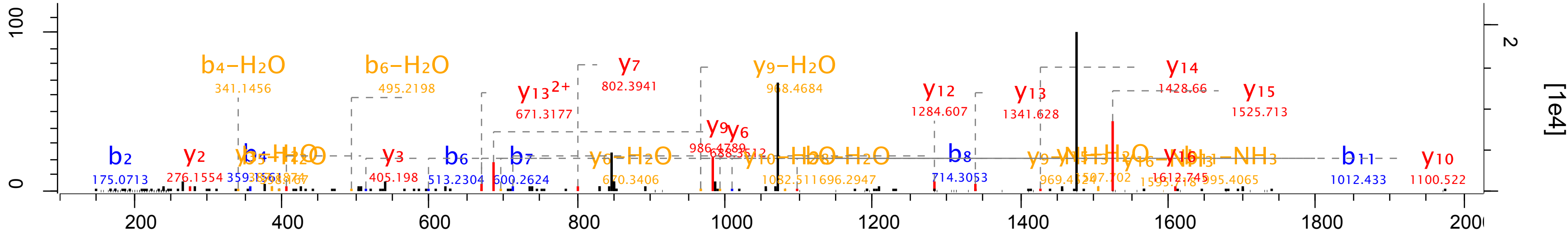

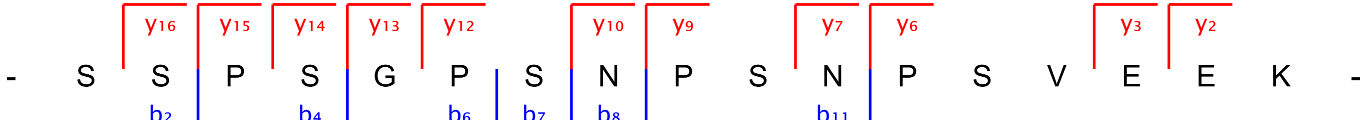

Raw file	Scan	Method	Score	m/z	Gene names
20150226_Hela_Top_opt_A3_01_1595	11619	TOF; CID	85.94	868.89	HOOK1

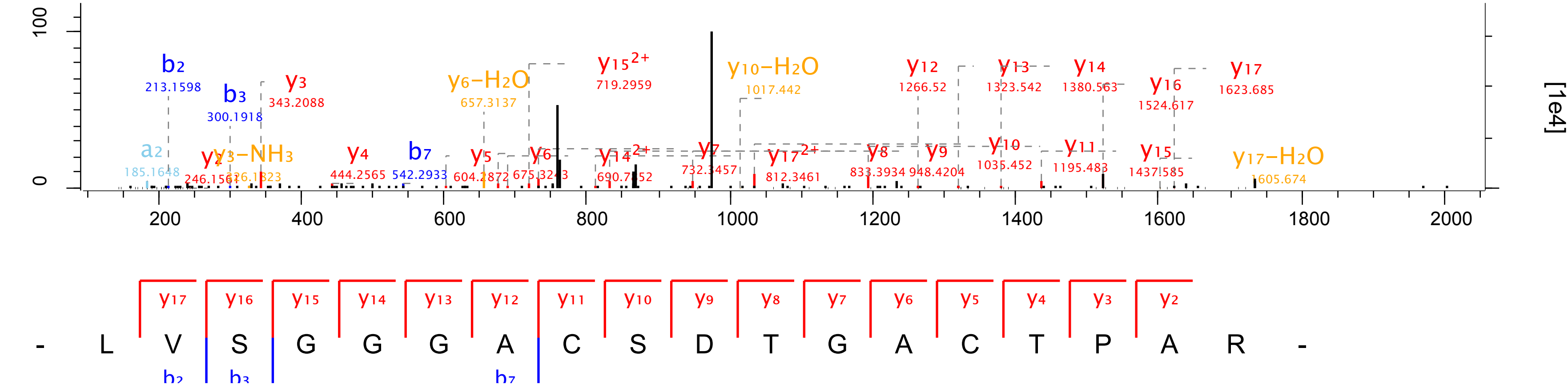

Raw file	Scan	Method	Score	m/z	Gene names
20150226_Hela_Top_opt_A3_01_1595	12049	TOF; CID	105.03	767.36	WDTC1

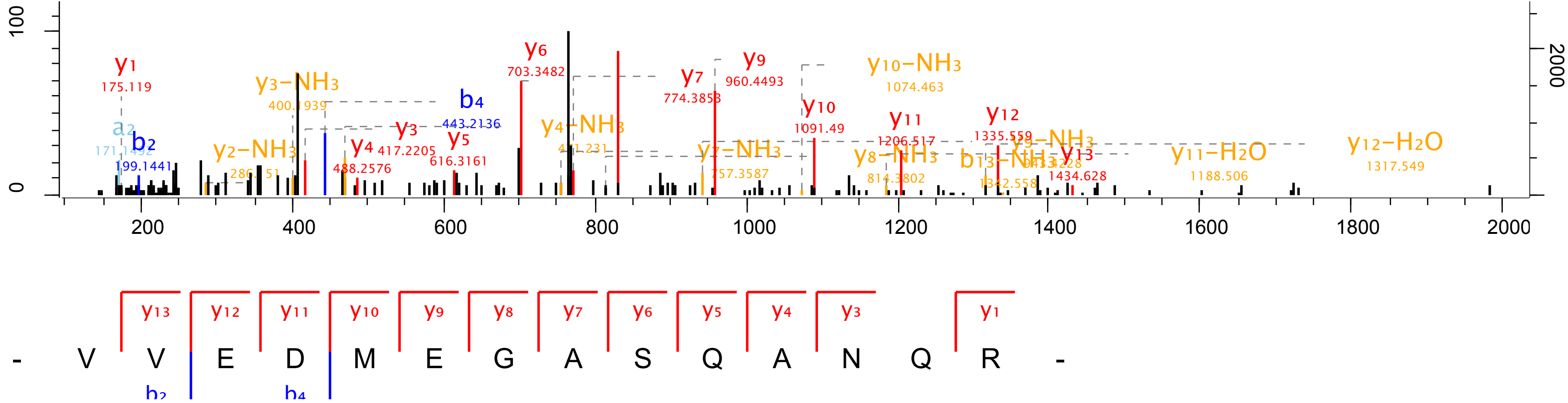

Raw file	Scan	Method	Score	m/z	Gene names
20150226_Hela_Top_opt_A3_01_1595	12397	TOF; CID	76.34	911.89	RFXAP

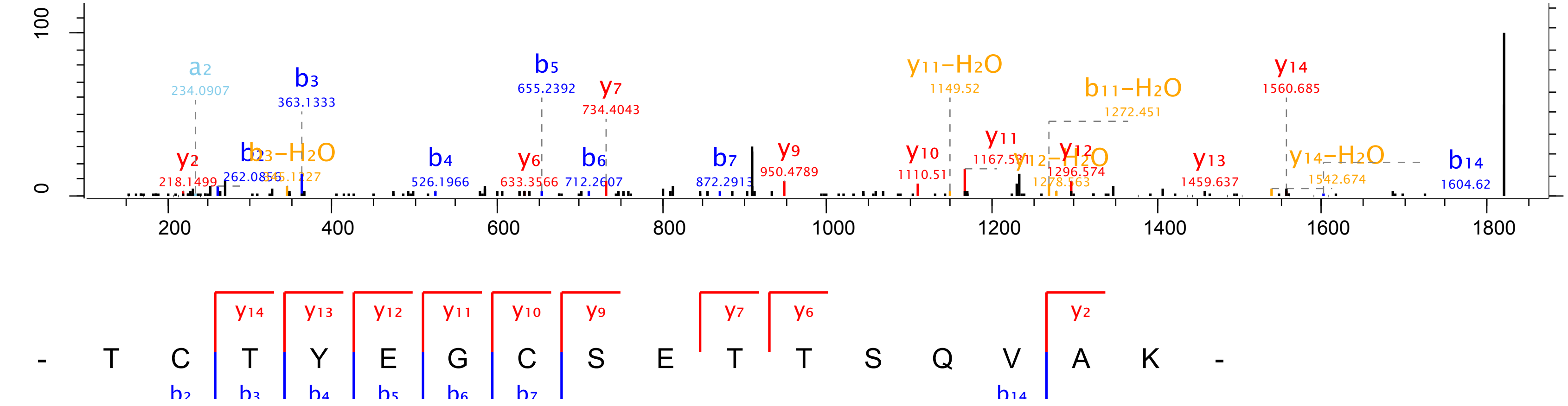

Raw file	Scan	Method	Score	m/z	Gene names
20150226_Hela_Top_opt_A3_01_1595	12421	TOF; CID	114.97	550.79	FLVCR1

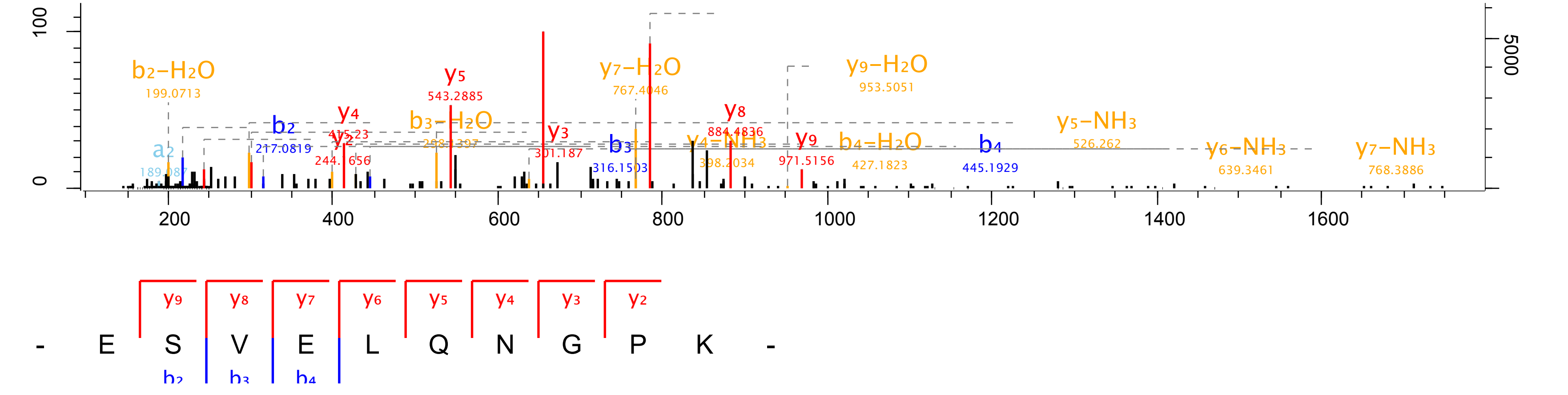

Raw file	Scan	Method	Score	m/z	Gene names
20150226_Hela_Top_opt_A3_01_1595	12607	TOF; CID	141.13	959.41	UNC93B1

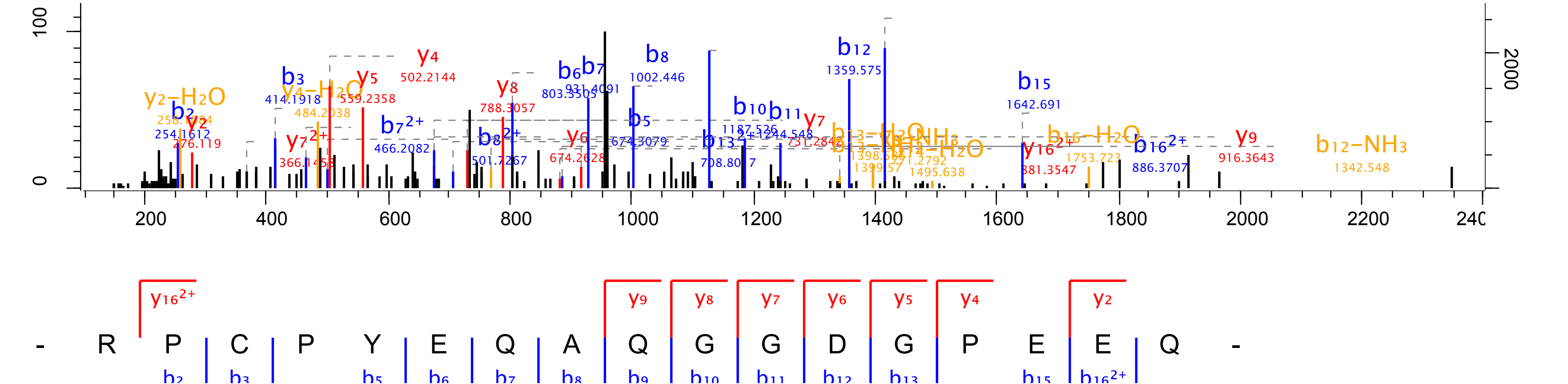

Raw file	Scan	Method	Score	m/z	Gene names
20150226_Hela_Top_opt_A3_01_1595	12757	TOF; CID	83.51	639.3	TAF8

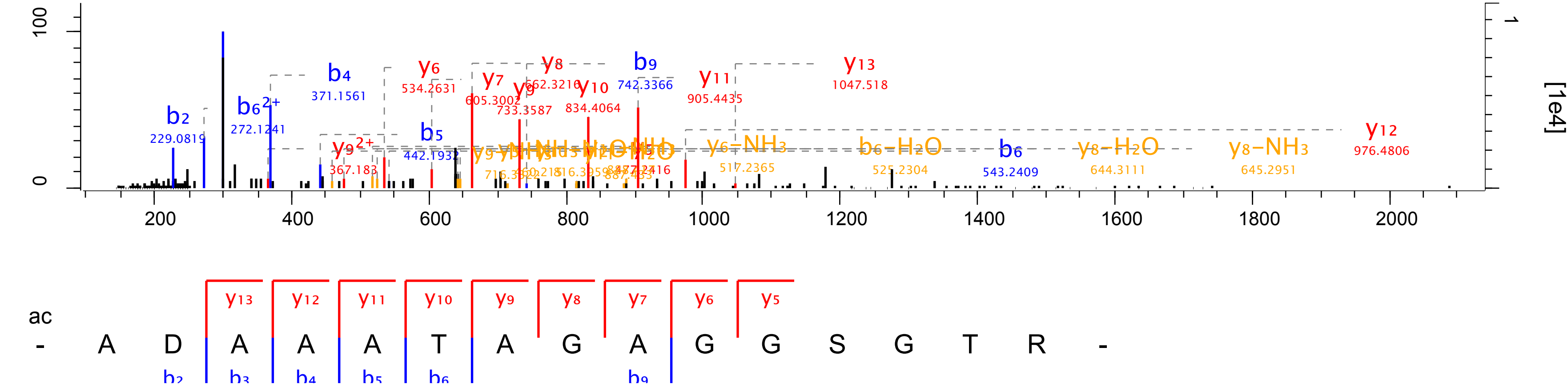

Raw file	Scan	Method	Score	m/z	Gene names
20150226_Hela_Top_opt_A3_01_1595	13187	TOF; CID	65.84	630.32	TIPIN

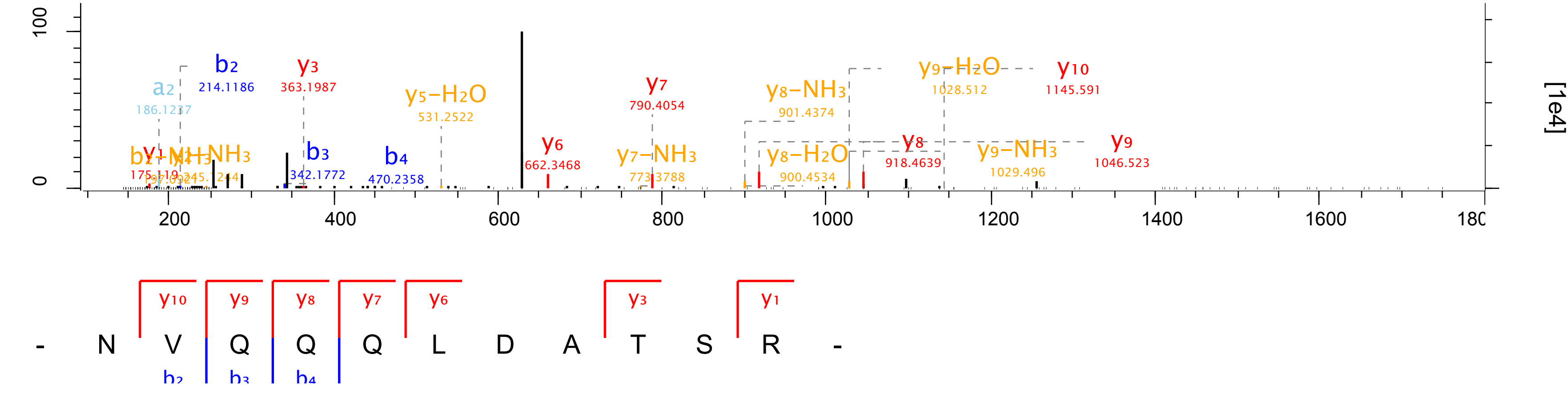

Raw file	Scan	Method	Score	m/z	Gene names
20150226_Hela_Top_opt_A3_01_1595	13544	TOF; CID	96.82	711.29	MRPS33

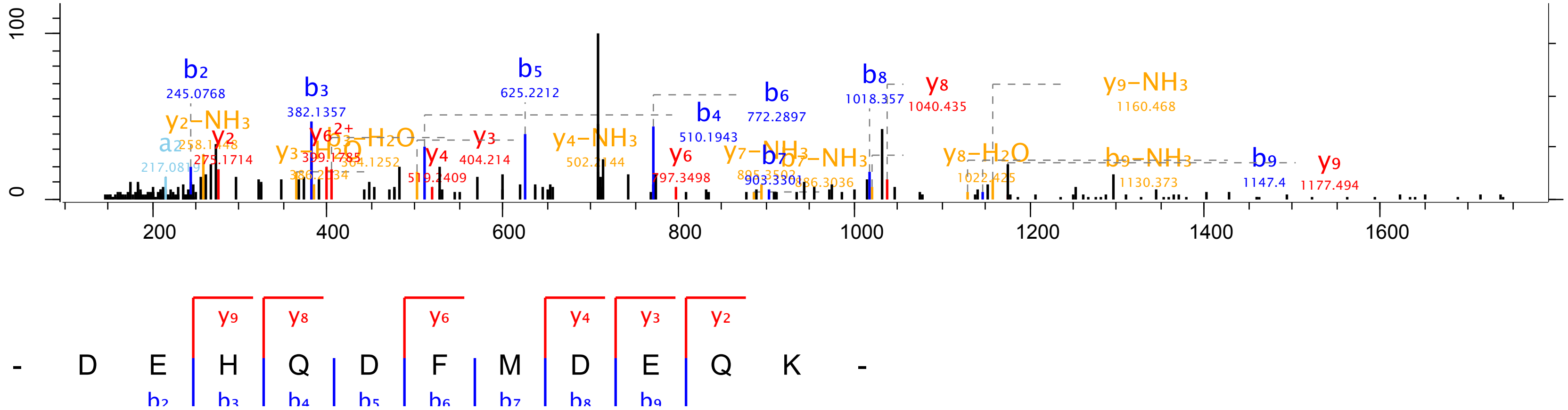

Raw file	Scan	Method	Score	m/z	Gene names
20150226_Hela_Top_opt_A3_01_1595	15336	TOF; CID	72.2	522.28	FUNDC2

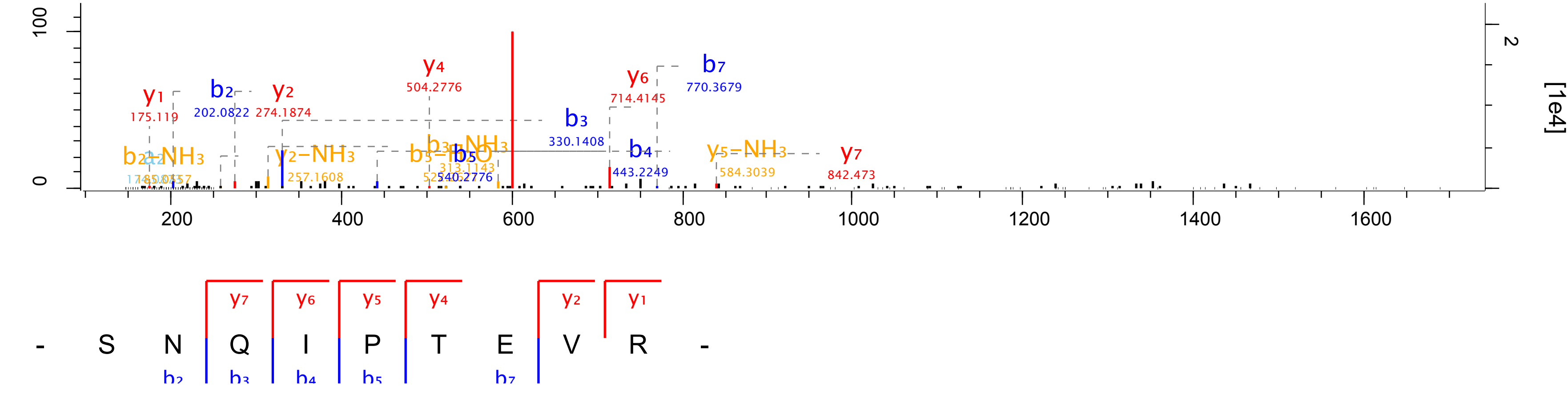

Raw file	Scan	Method	Score	m/z	Gene names
20150226_Hela_Top_opt_A3_01_1595	17145	TOF; CID	51.5	592.67	C20orf24

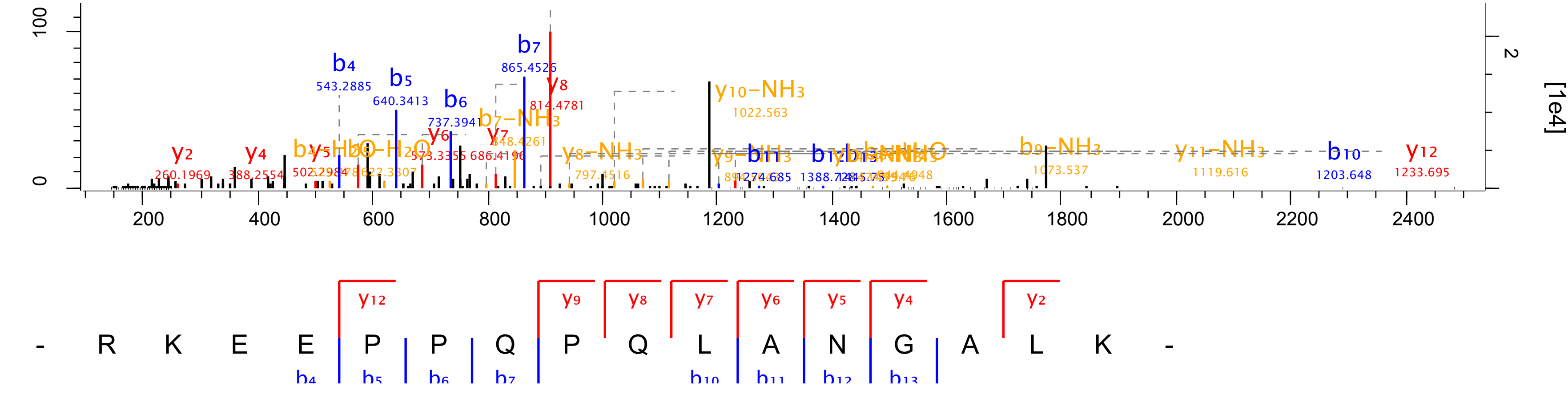

Raw file	Scan	Method	Score	m/z	Gene names
20150226_Hela_Top_opt_A3_01_1595	17253	TOF; CID	65.04	641.35	KLHDC4

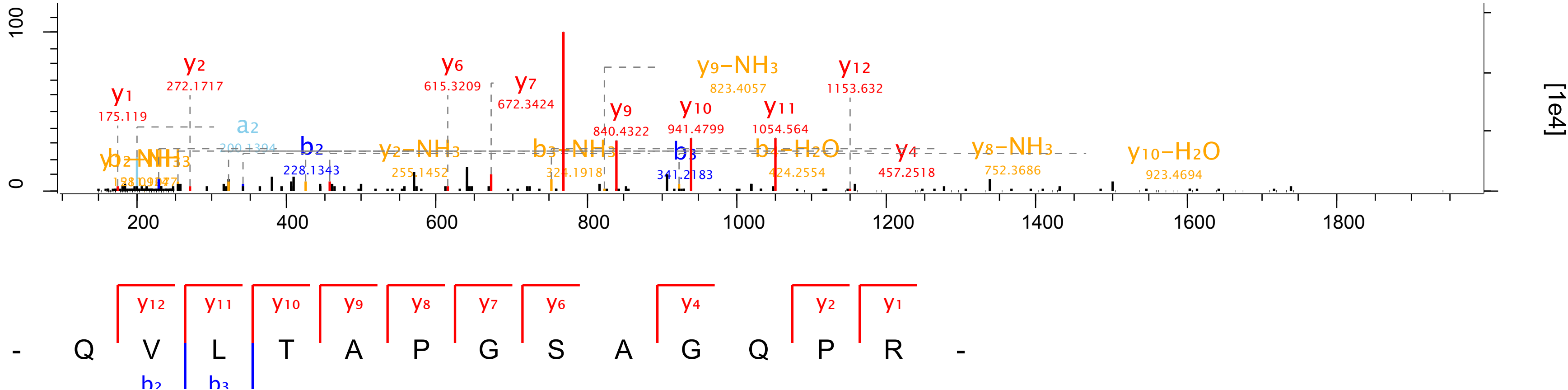

Raw file	Scan	Method	Score	m/z	Gene names
20150226_Hela_Top_opt_A3_01_1595	17939	TOF; CID	96.21	676.31	IFIT1

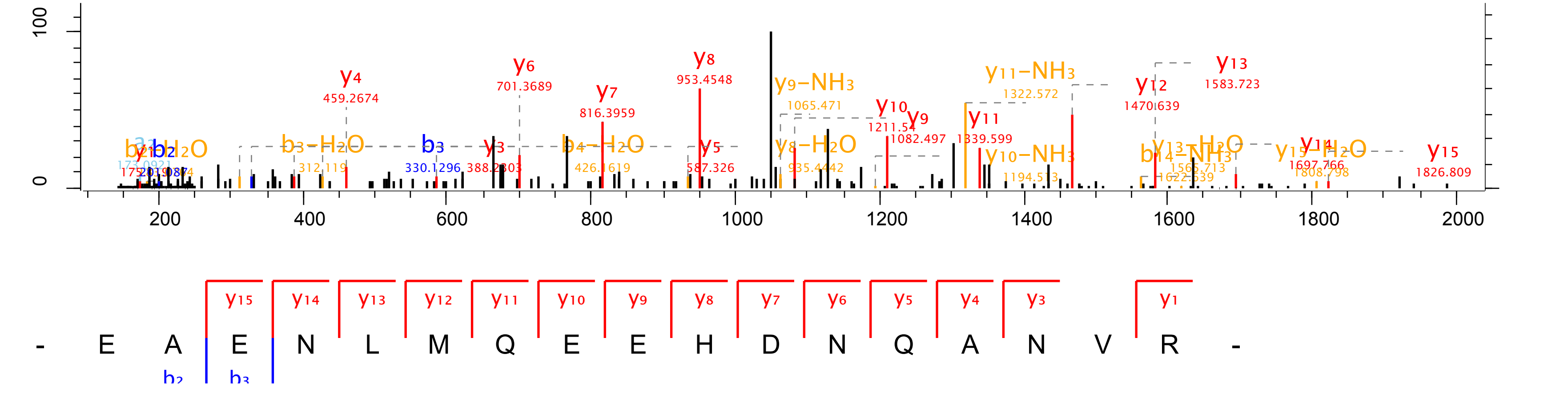

Raw file	Scan	Method	Score	m/z	Gene names
20150226_Hela_Top_opt_A3_01_1595	18154	TOF; CID	152.88	886.09	MPLKIP

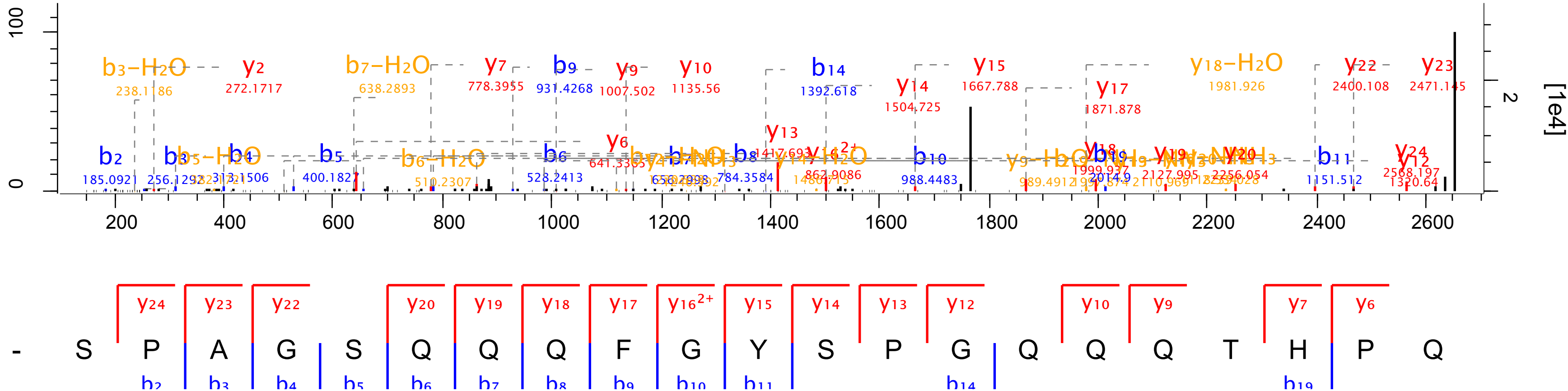

Raw file	Scan	Method	Score	m/z	Gene names
20150226_Hela_Top_opt_A3_01_1595	18347	TOF; CID	52.25	602.81	PBXIP1

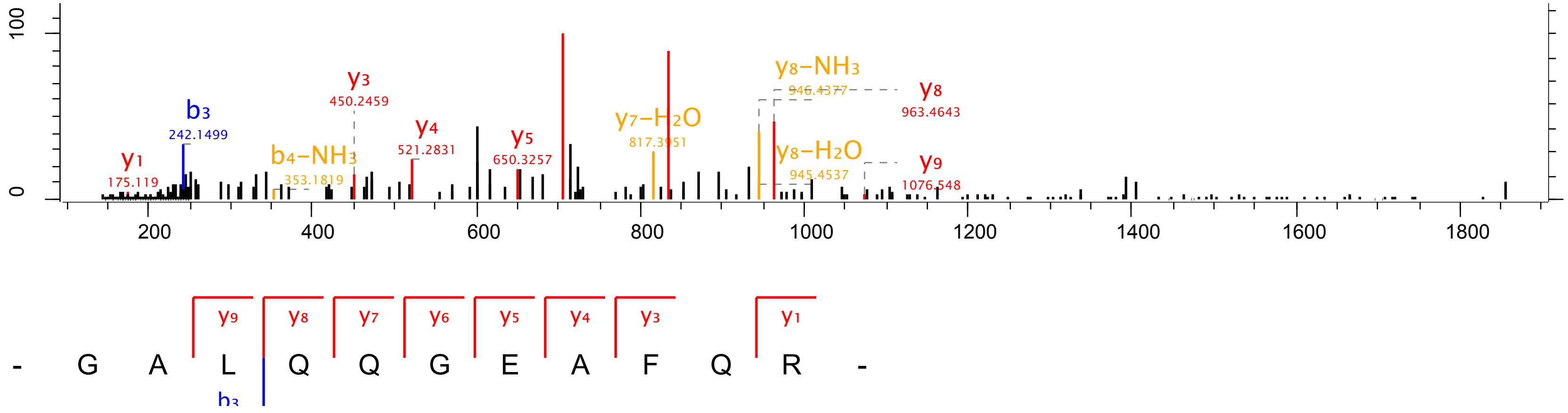

Raw file	Scan	Method	Score	m/z	Gene names
20150226_Hela_Top_opt_A3_01_1595	18674	TOF; CID	115.1	786.42	CACTIN

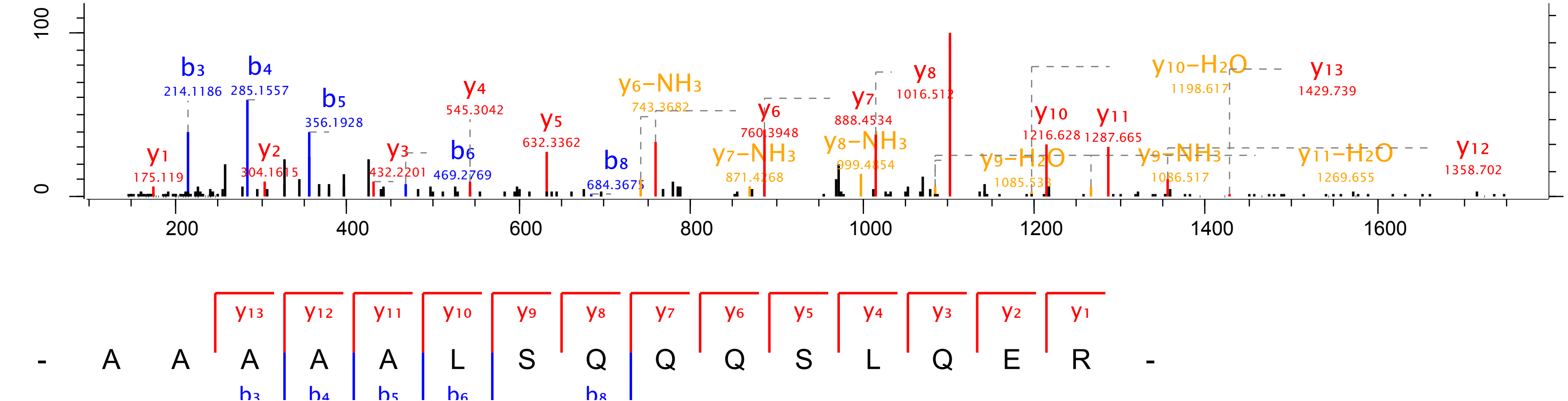

Raw file	Scan	Method	Score	m/z	Gene names
20150226_Hela_Top_opt_A3_01_1595	18796	TOF; CID	111.78	800.39	ENTHD2

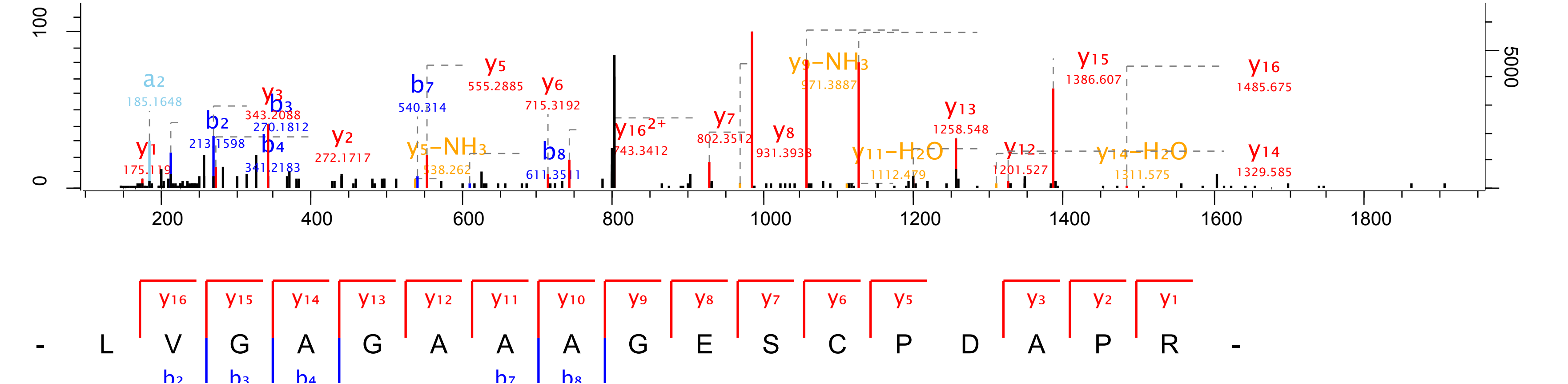

Raw file	Scan	Method	Score	m/z	Gene names
20150226_Hela_Top_opt_A3_01_1595	19289	TOF; CID	47.99	471.28	ZC3H13

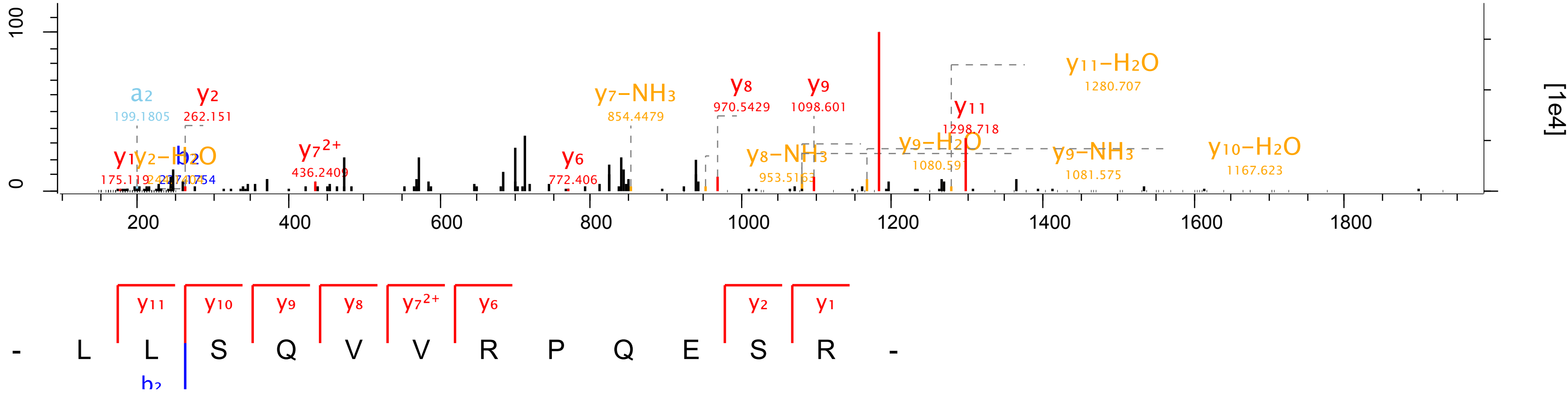

Raw file	Scan	Method	Score	m/z	Gene names
20150226_Hela_Top_opt_A3_01_1595	19795	TOF; CID	59.23	499.28	ZNF24

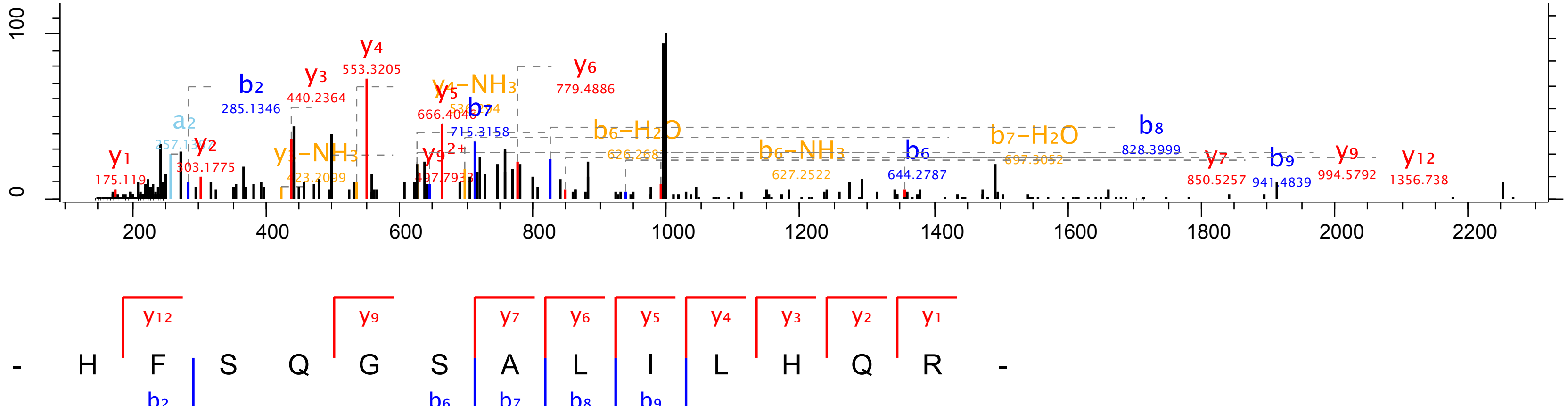

Raw file	Scan	Method	Score	m/z	Gene names
20150226_Hela_Top_opt_A3_01_1595	19970	TOF; CID	115.88	939.47	EYA3

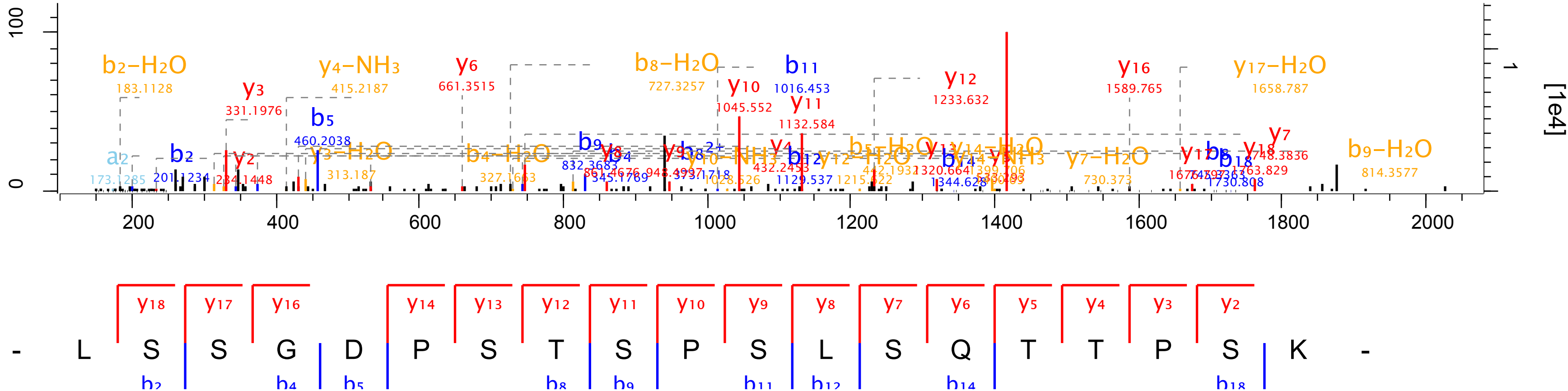

Raw file	Scan	Method	Score	m/z	Gene names
20150226_Hela_Top_opt_A3_01_1595	21455	TOF; CID	61.68	554.3	DNAH7

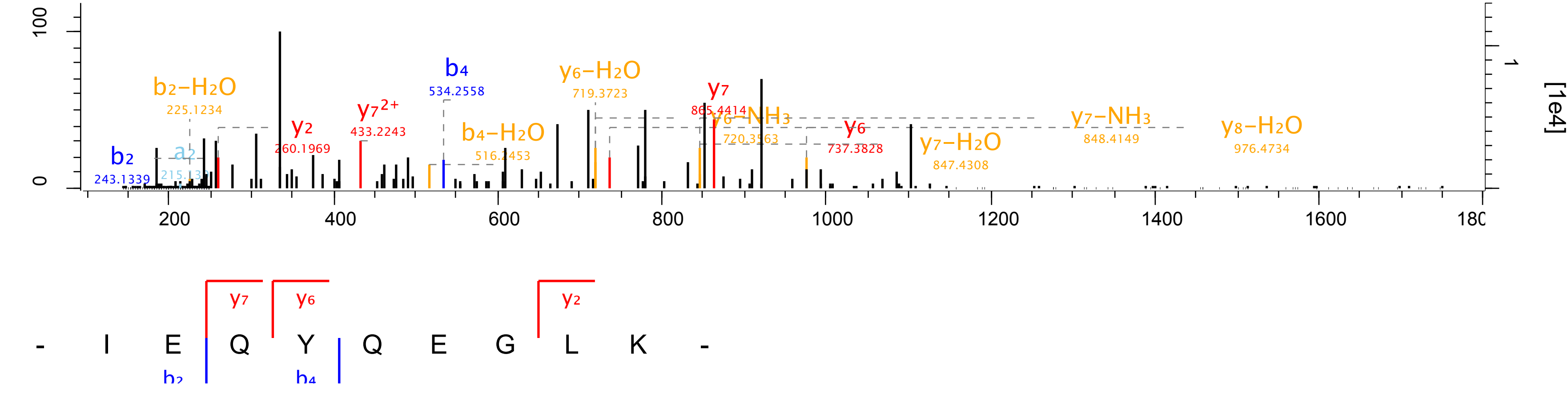

Raw file	Scan	Method	Score	m/z	Gene names
20150226_Hela_Top_opt_A3_01_1595	22112	TOF; CID	48.95	318.2	ZCCHC10

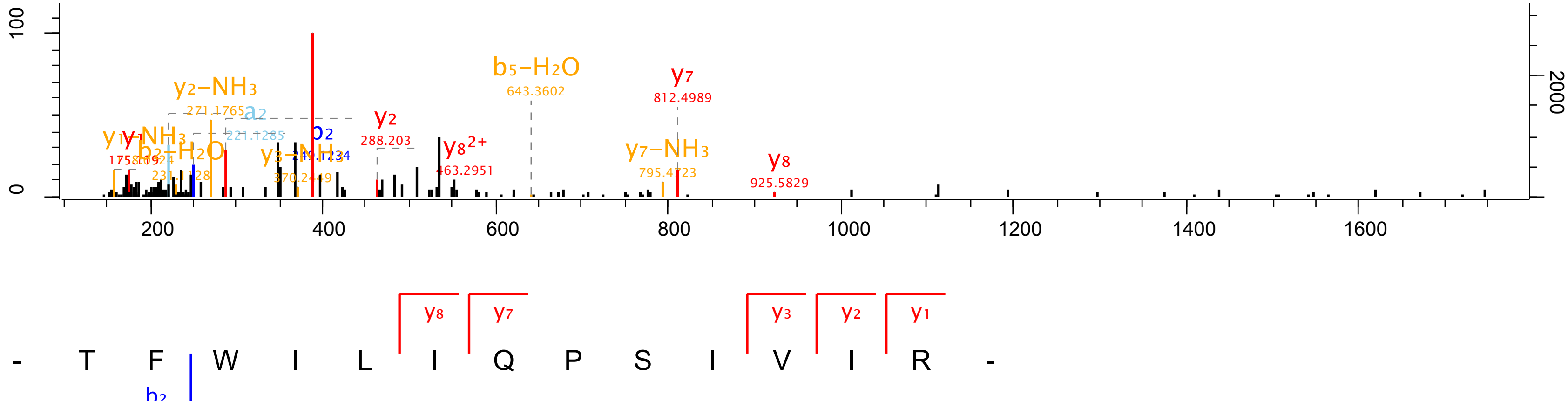

Raw file	Scan	Method	Score	m/z	Gene names
20150226_Hela_Top_opt_A3_01_1595	22155	TOF; CID	76.36	617.3	MYL3;MYL1

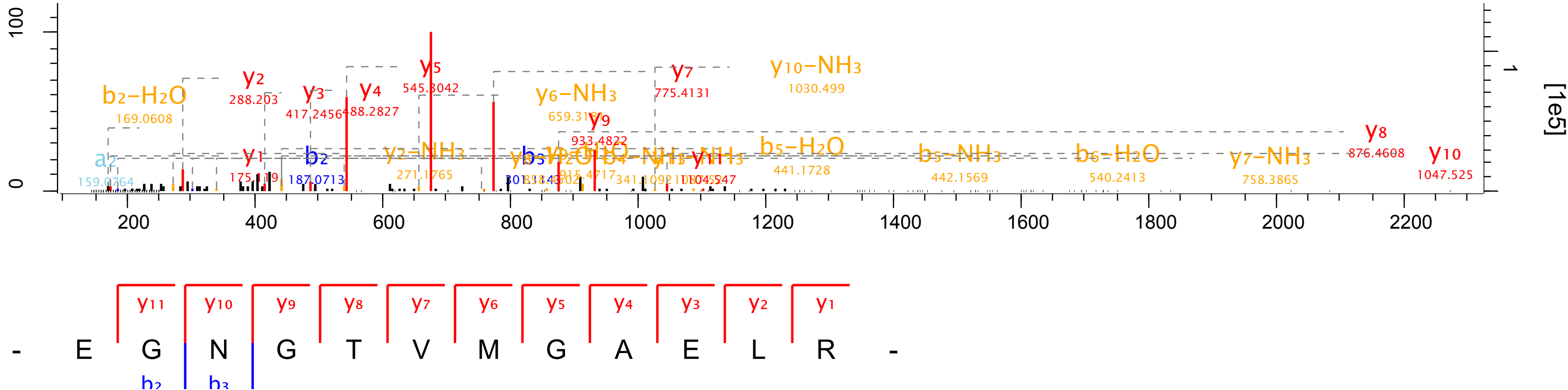

Raw file	Scan	Method	Score	m/z	Gene names
20150226_Hela_Top_opt_A3_01_1595	25570	TOF; CID	84.69	624.86	THG1L

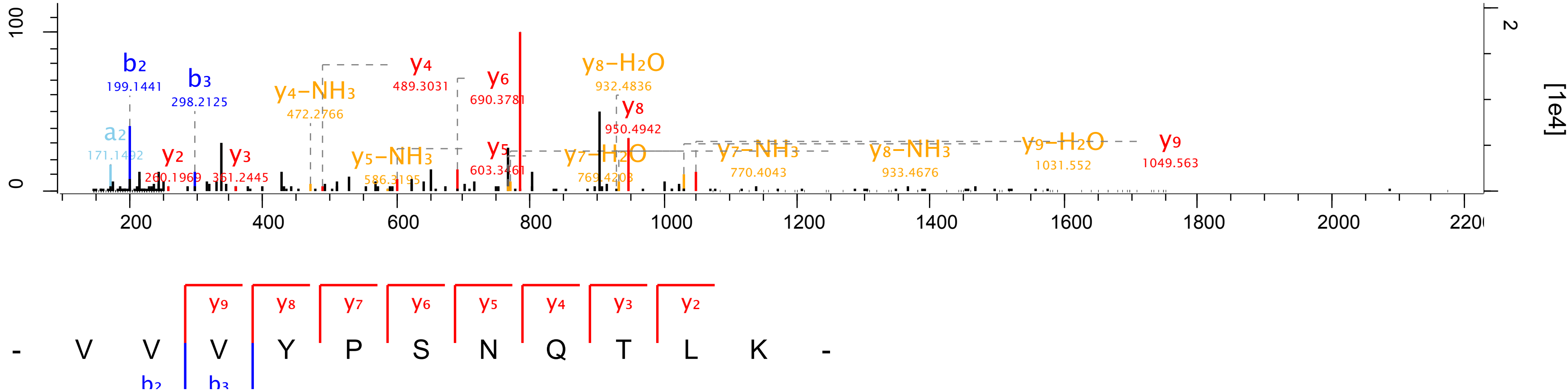

Raw file	Scan	Method	Score	m/z	Gene names
20150226_Hela_Top_opt_A3_01_1595	25688	TOF; CID	91.64	539.8	ITM2B

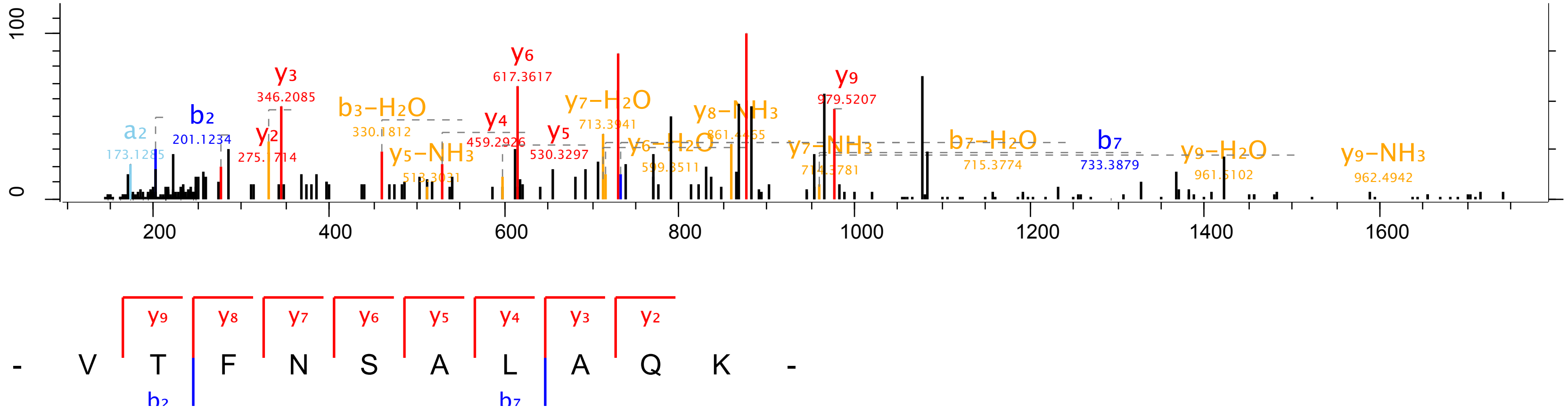

Raw file	Scan	Method	Score	m/z	Gene names
20150226_Hela_Top_opt_A3_01_1595	27112	TOF; CID	73.66	495.24	VLDLR

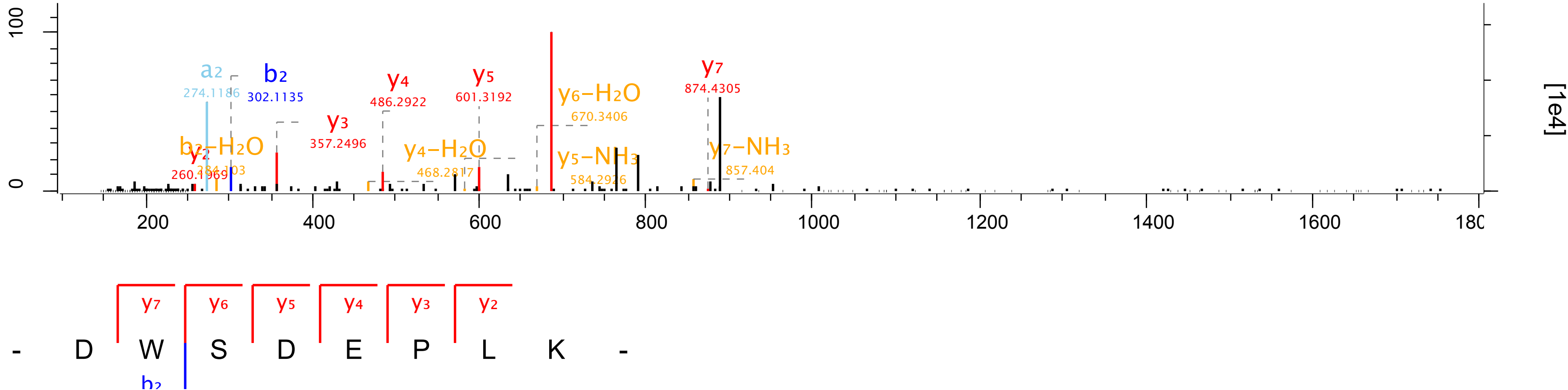

Raw file	Scan	Method	Score	m/z	Gene names
20150226_Hela_Top_opt_A3_01_1595	27782	TOF; CID	76.12	682.66	CHCHD7

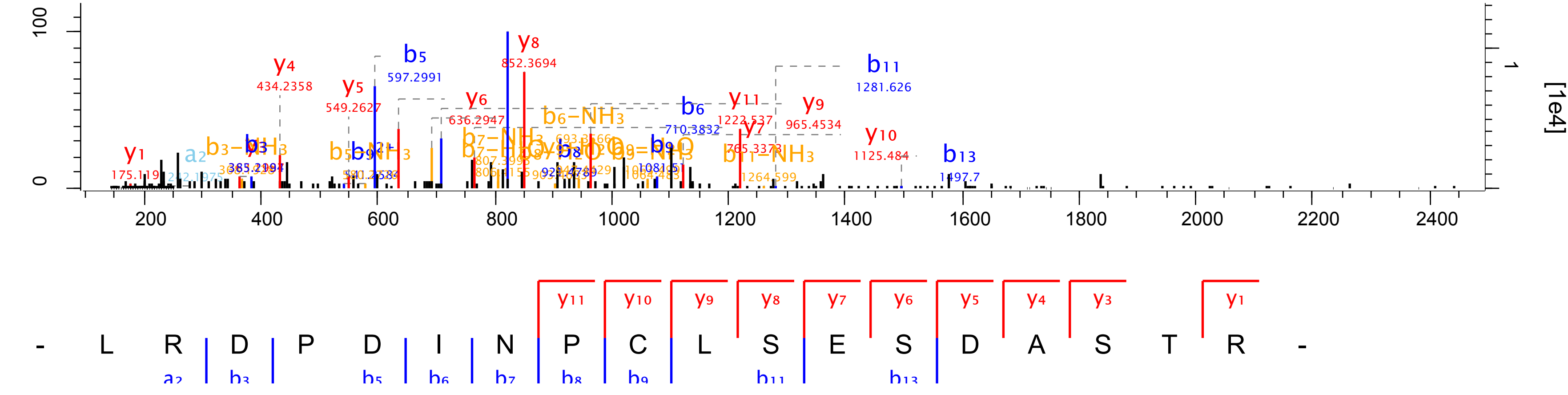

Raw file	Scan	Method	Score	m/z	Gene names
20150226_Hela_Top_opt_A3_01_1595	28219	TOF; CID	60.4	537.81	ATP5SL;DDHD2

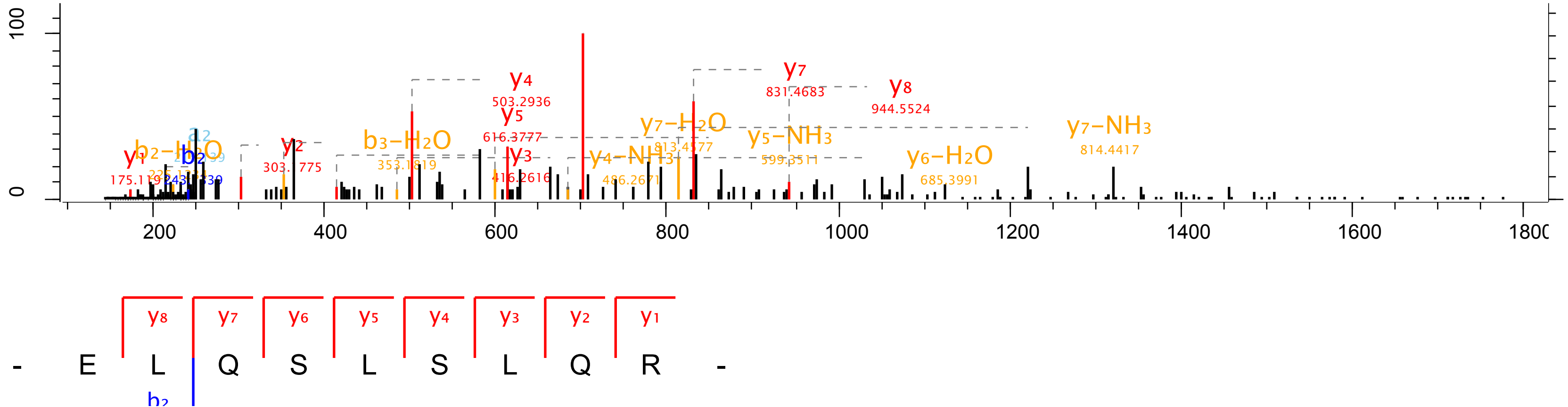

Raw file	Scan	Method	Score	m/z	Gene names
20150226_Hela_Top_opt_A3_01_1595	28356	TOF; CID	76.24	528.27	FAM32A

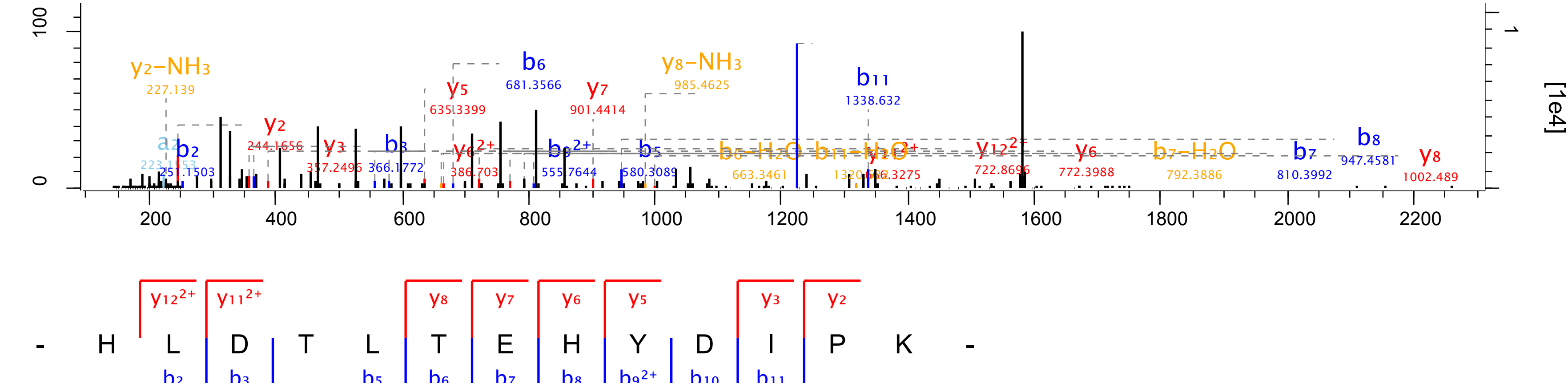

Raw file	Scan	Method	Score	m/z	Gene names
20150226_Hela_Top_opt_A3_01_1595	28720	TOF; CID	69.92	797.4	CHMP6

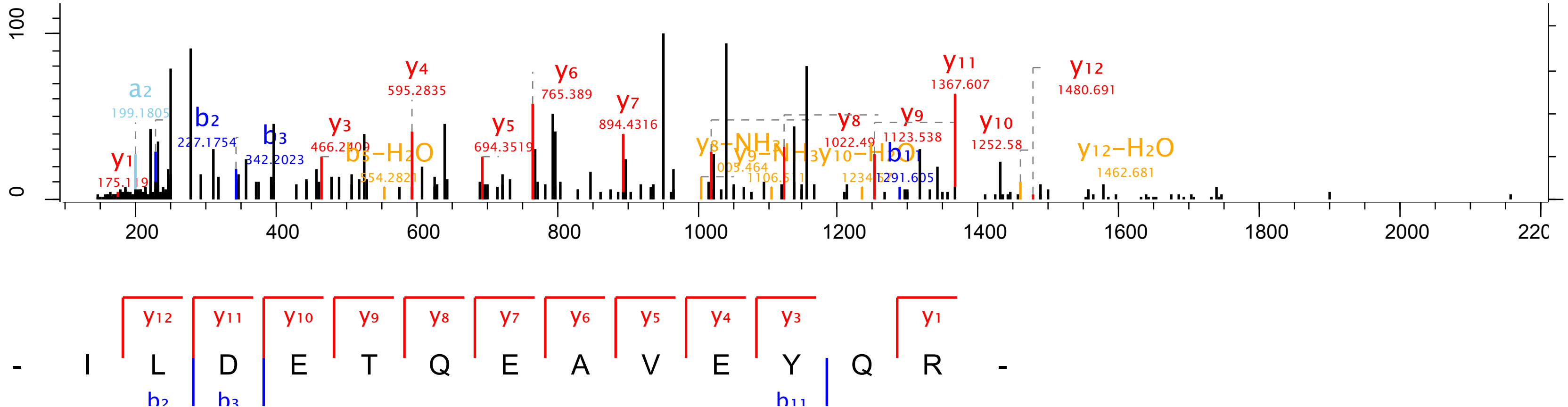

Raw file	Scan	Method	Score	m/z
20150226_Hela_Top_opt_A3_01_1595	30759	TOF; CID	65.22	456.29

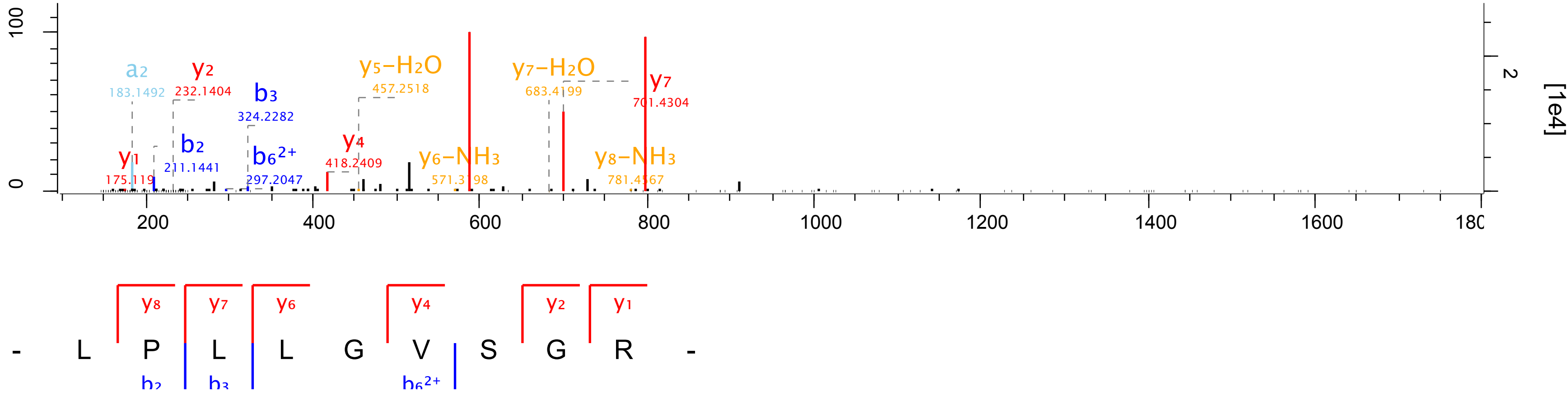

Raw file	Scan	Method	Score	m/z	Gene names
20150226_Hela_Top_opt_A3_01_1595	31801	TOF; CID	71.88	679.36	DAPK3

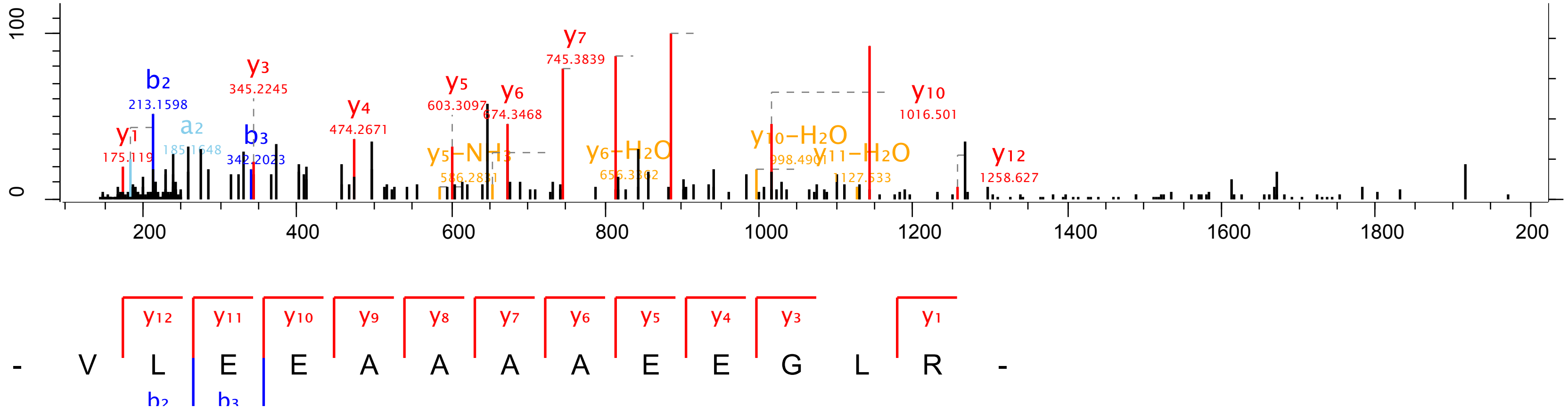

Raw file	Scan	Method	Score	m/z	Gene names
20150226_Hela_Top_opt_A3_01_1595	32946	TOF; CID	63.91	476.27	GOPC

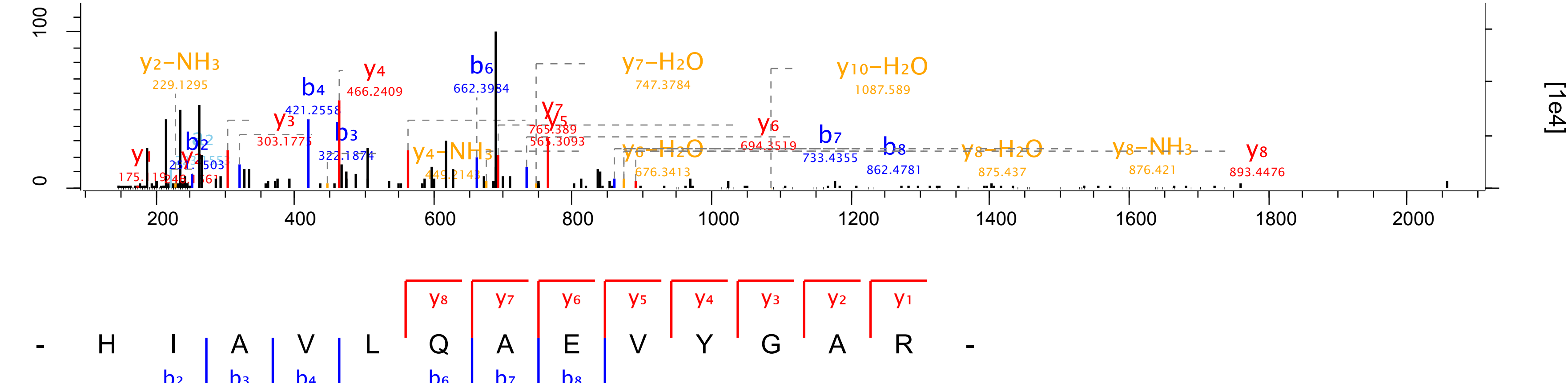

Raw file	Scan	Method	Score	m/z	Gene names
20150226_Hela_Top_opt_A3_01_1595	33483	TOF; CID	69.2	467.27	C1orf43

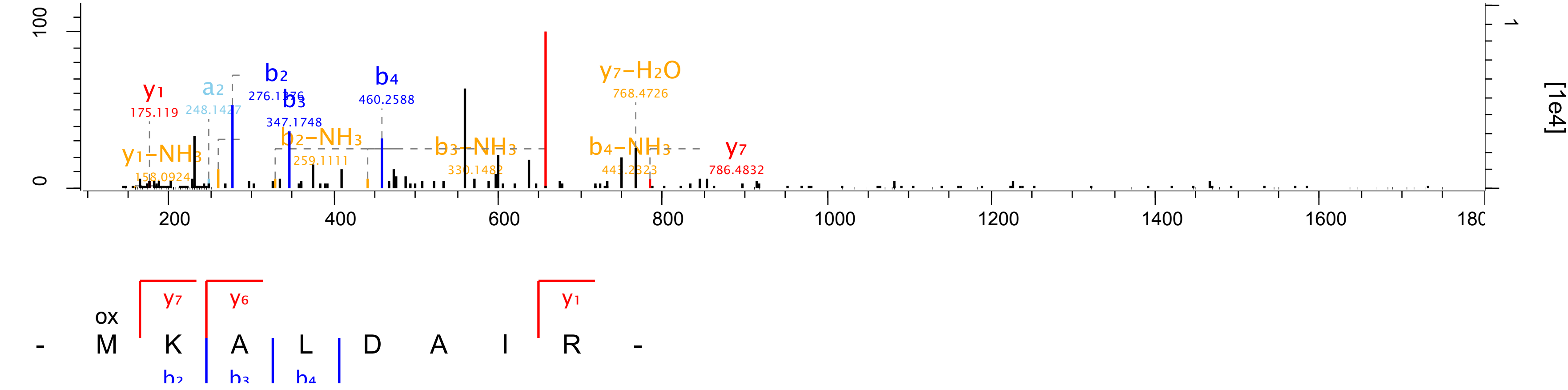

Raw file	Scan	Method	Score	m/z	Gene names
20150226_Hela_Top_opt_A3_01_1595	34288	TOF; CID	93.88	371.26	RTTN

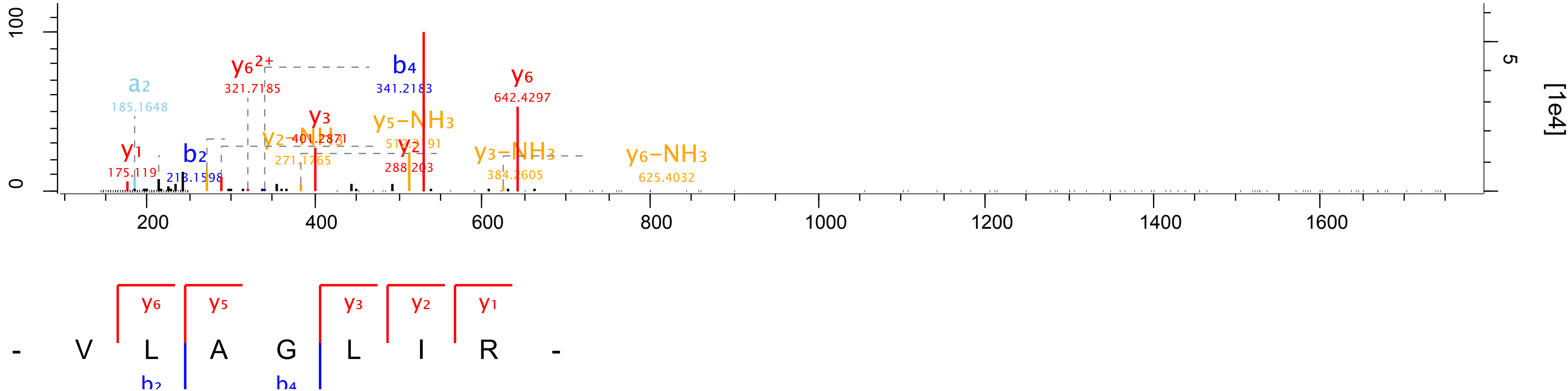

Raw file	Scan	Method	Score	m/z	Gene names
20150226_Hela_Top_opt_A3_01_1595	34685	TOF; CID	66.3	529.29	ALG8

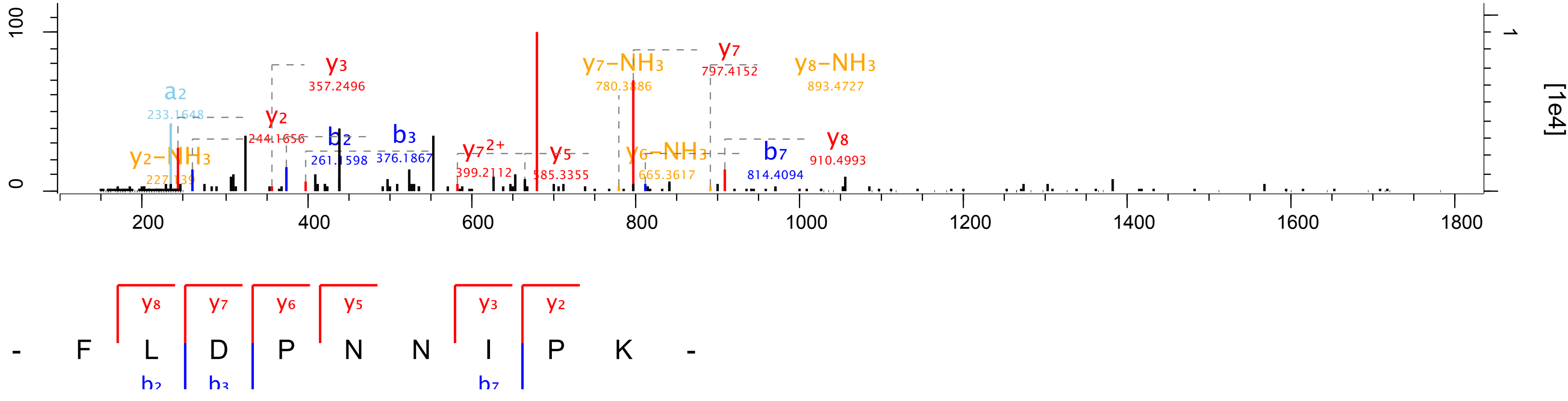

Raw file	Scan	Method	Score	m/z	Gene names
20150226_Hela_Top_opt_A3_01_1595	34786	TOF; CID	84.3	854.36	CXorf56

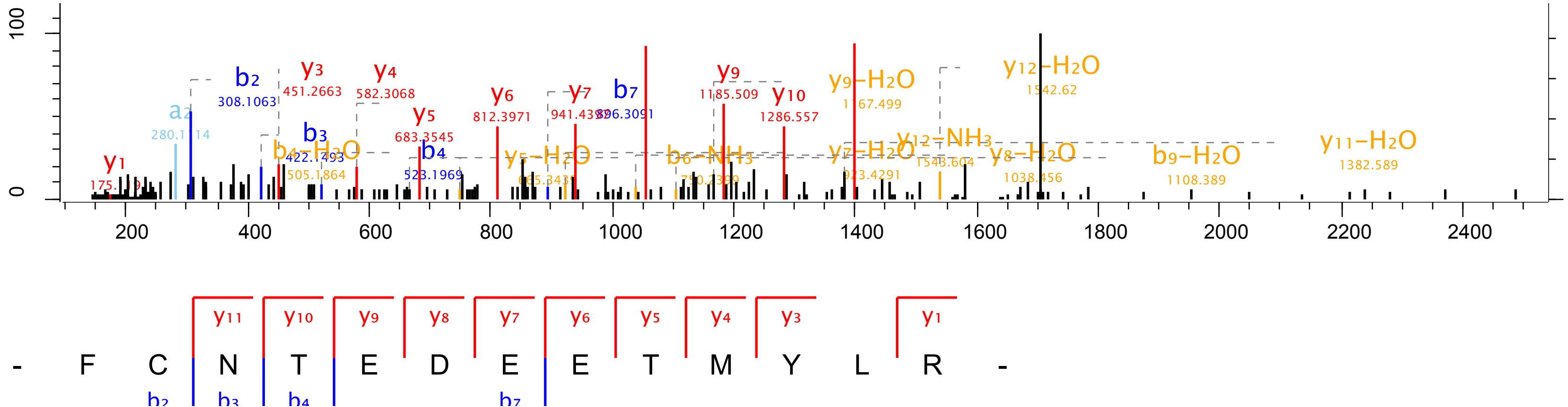

Raw file	Scan	Method	Score	m/z	Gene names
20150226_Hela_Top_opt_A3_01_1595	35524	TOF; CID	76.82	603.63	CDC37L1

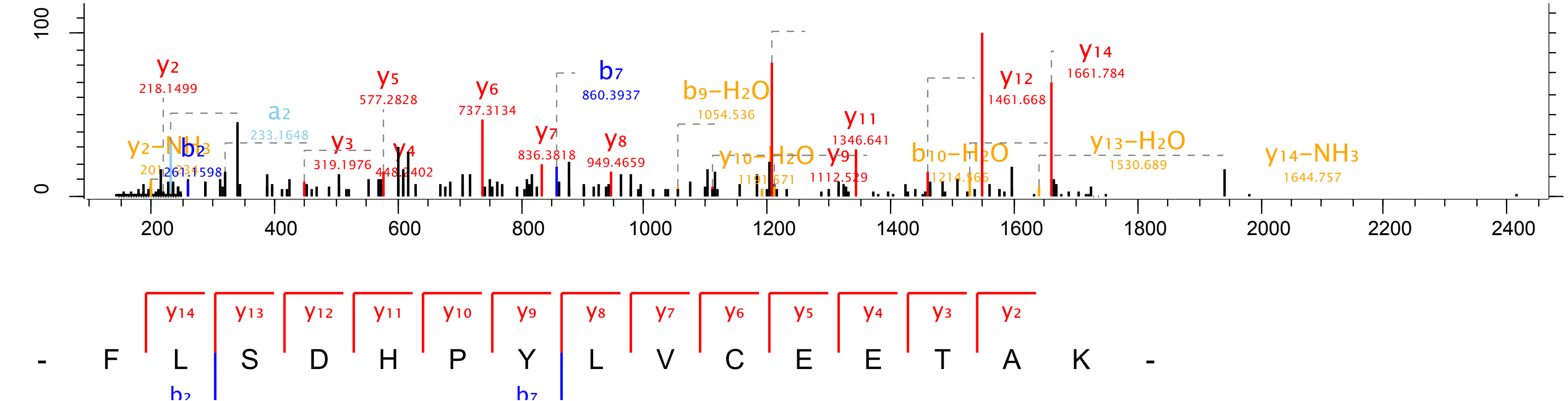

Raw file	Scan	Method	Score	m/z	Gene names
20150226_Hela_Top_opt_A3_01_1595	36367	TOF; CID	127.3	650.35	EIF1AD

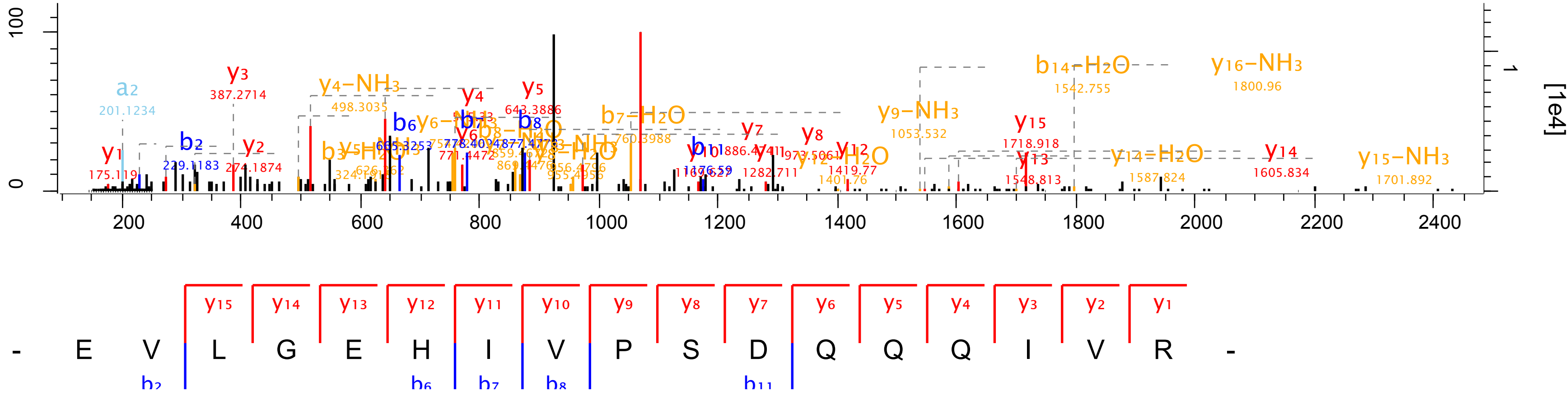

Raw file	Scan	Method	Score	m/z	Gene names
20150226_Hela_Top_opt_A3_01_1595	37297	TOF; CID	63.79	698.85	SLC37A4

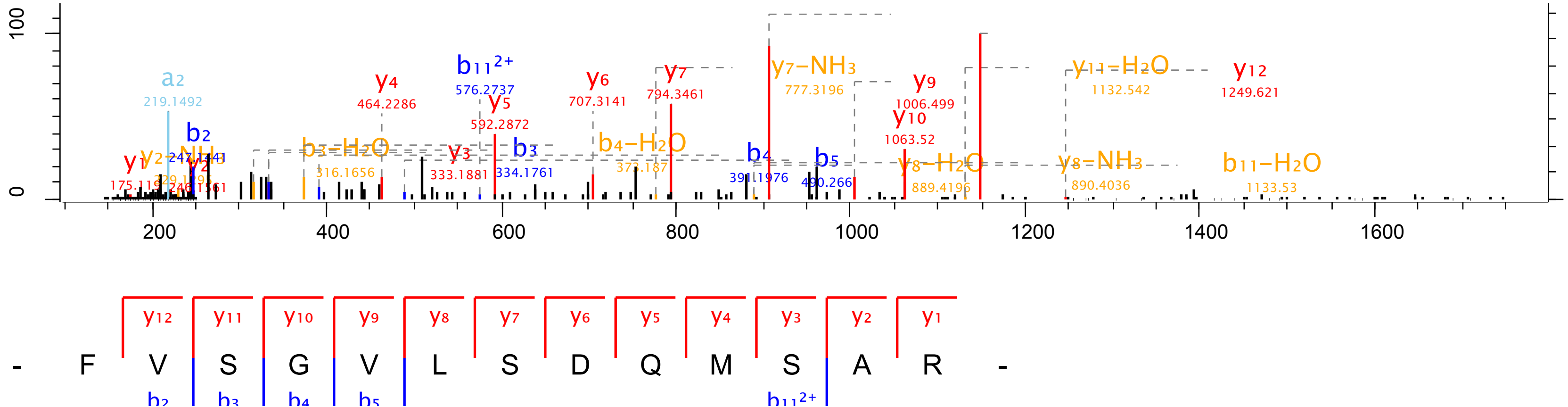

Raw file	Scan	Method	Score	m/z	Gene names
20150226_Hela_Top_opt_A3_01_1595	37970	TOF; CID	48.22	542.31	CDCA8

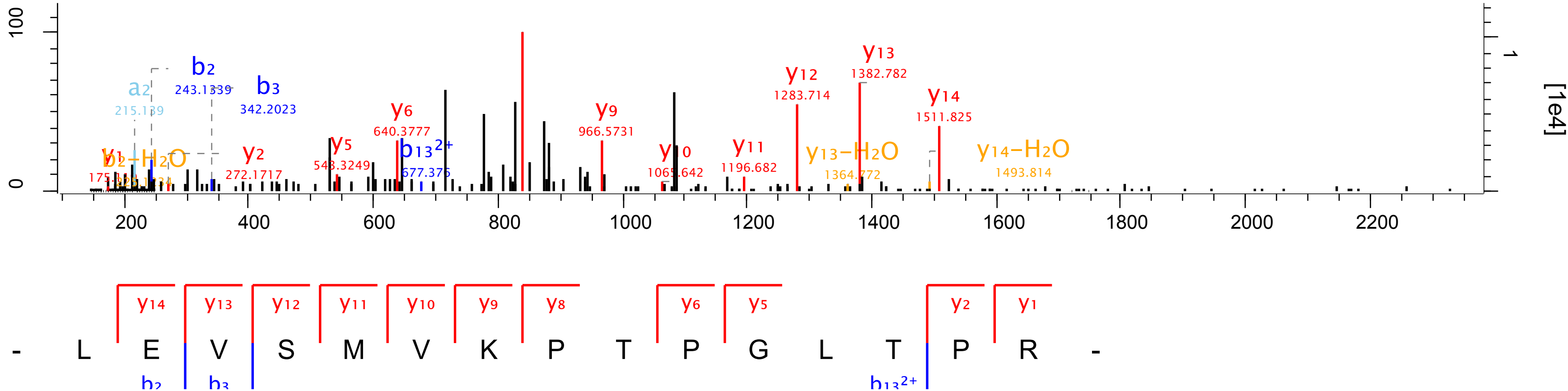

Raw file	Scan	Method	Score	m/z	Gene names
20150226_Hela_Top_opt_A3_01_1595	39090	TOF; CID	46.89	904.43	DCAF16

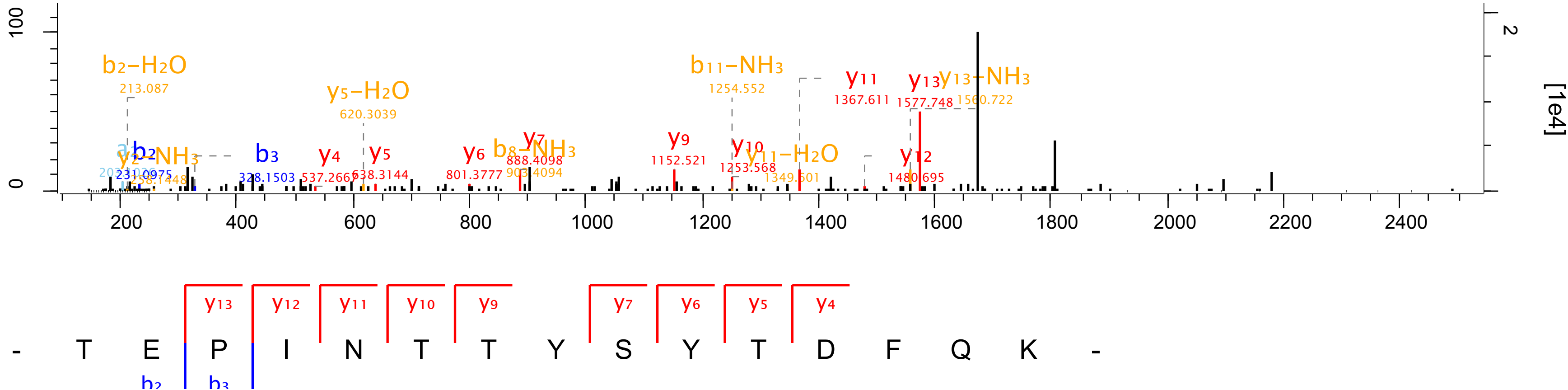

Raw file	Scan	Method	Score	m/z	Gene names
20150226_Hela_Top_opt_A3_01_1595	39300	TOF; CID	79.16	579.3	POLR3K

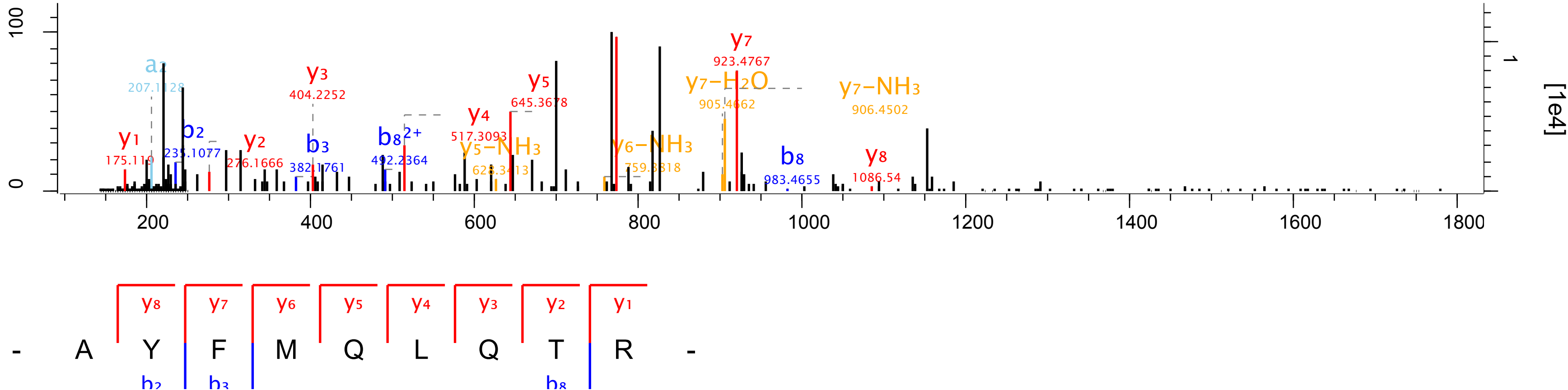

Raw file	Scan	Method	Score	m/z	Gene names
20150226_Hela_Top_opt_A3_01_1595	40423	TOF; CID	89.14	806.43	ERICH1

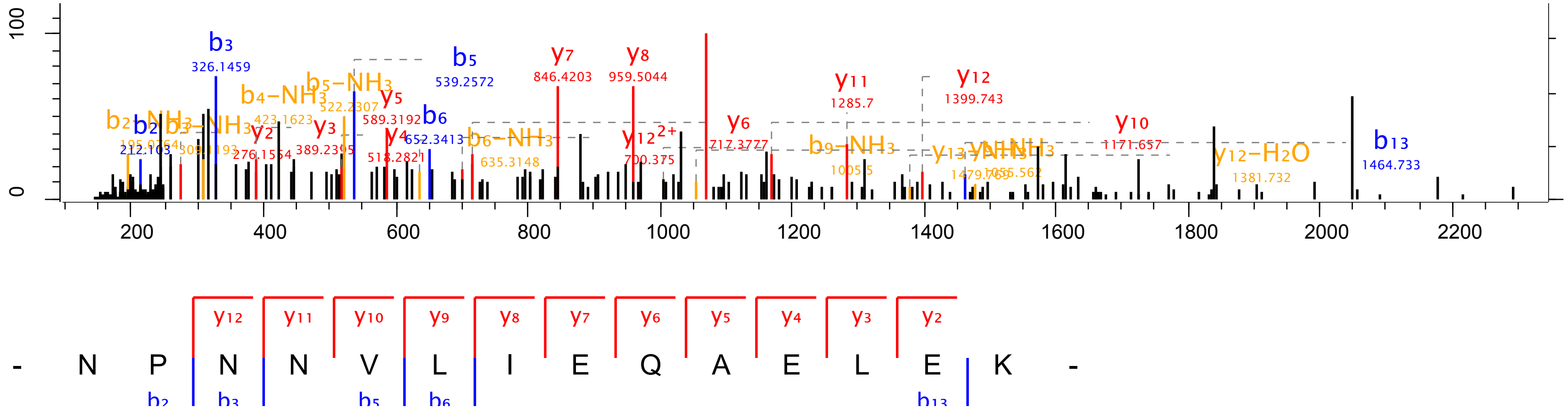

Raw file	Scan	Method	Score	m/z	Gene names
20150226_Hela_Top_opt_A3_01_1595	40639	TOF; CID	78.95	928.47	KLHL7

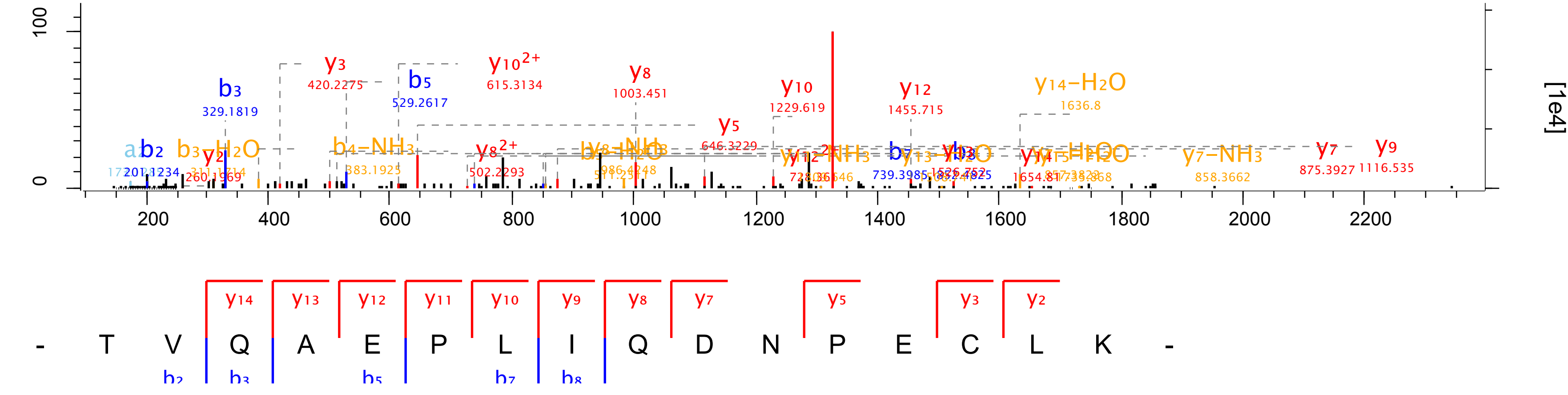

Raw file	Scan	Method	Score	m/z	Gene names
20150226_Hela_Top_opt_A3_01_1595	40657	TOF; CID	38.45	961.7	TCF3

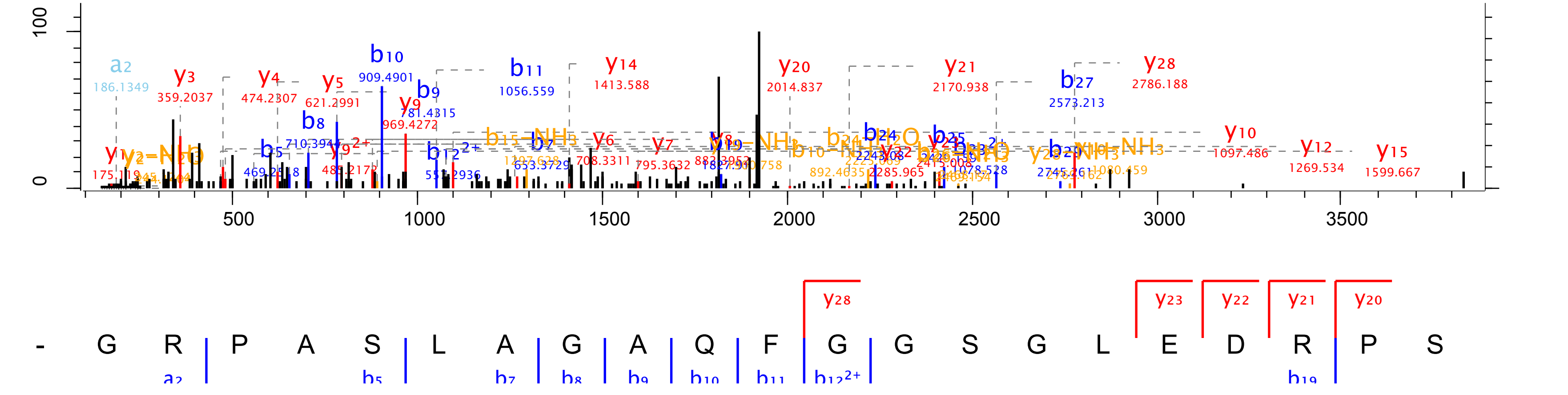

Raw file	Scan	Method	Score	m/z	Gene names
20150226_Hela_Top_opt_A3_01_1595	40943	TOF; CID	57.16	615.34	YTHDC1

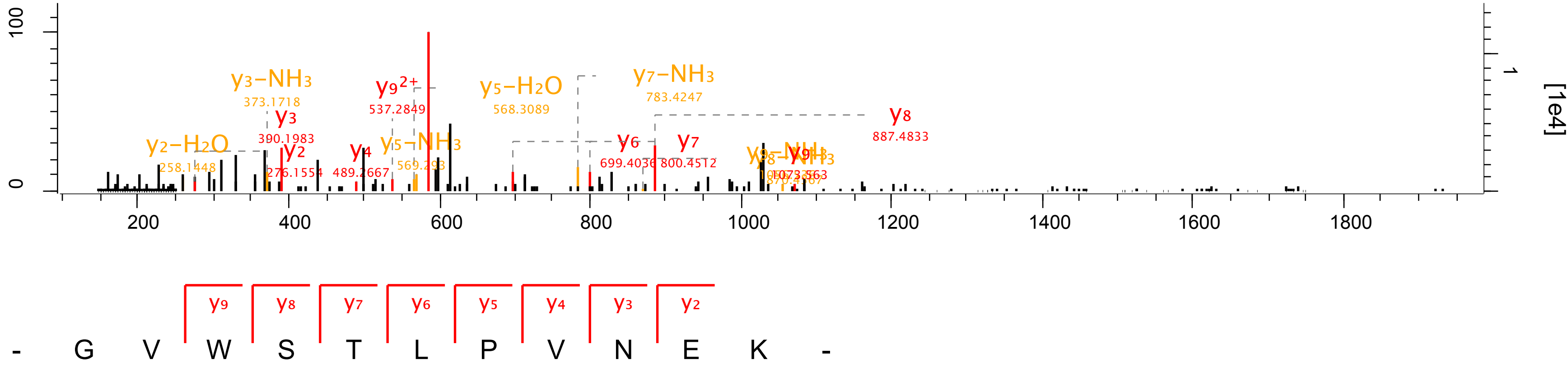

Raw file	Scan	Method	Score	m/z	Gene names
20150226_Hela_Top_opt_A3_01_1595	40992	TOF; CID	69.48	857.87	MT-ND3

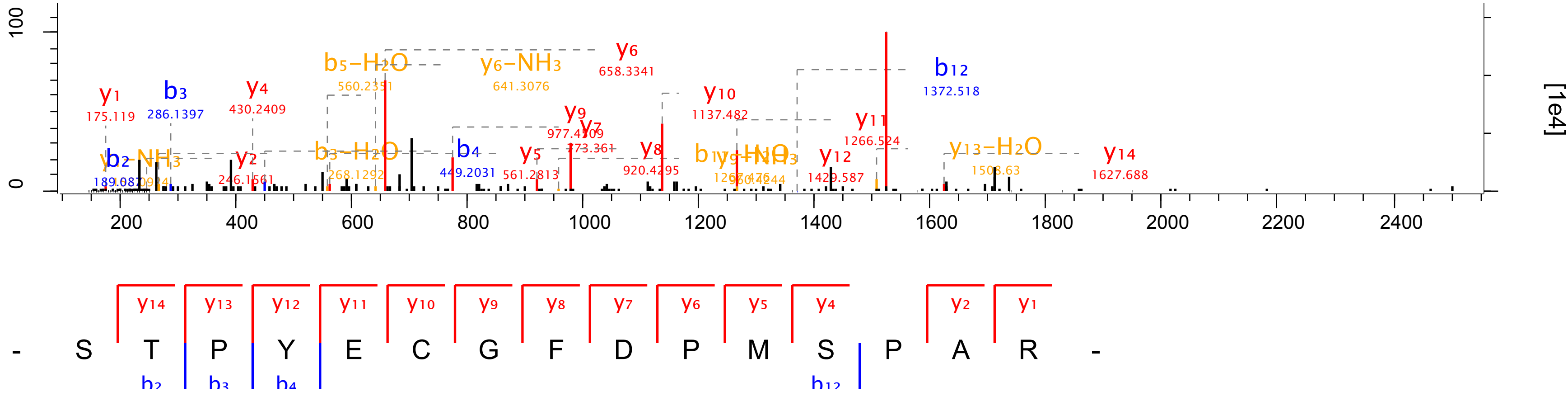

Raw file	Scan	Method	Score	m/z	Gene names
20150226_Hela_Top_opt_A3_01_1595	41403	TOF; CID	100.23	874.44	BRI3

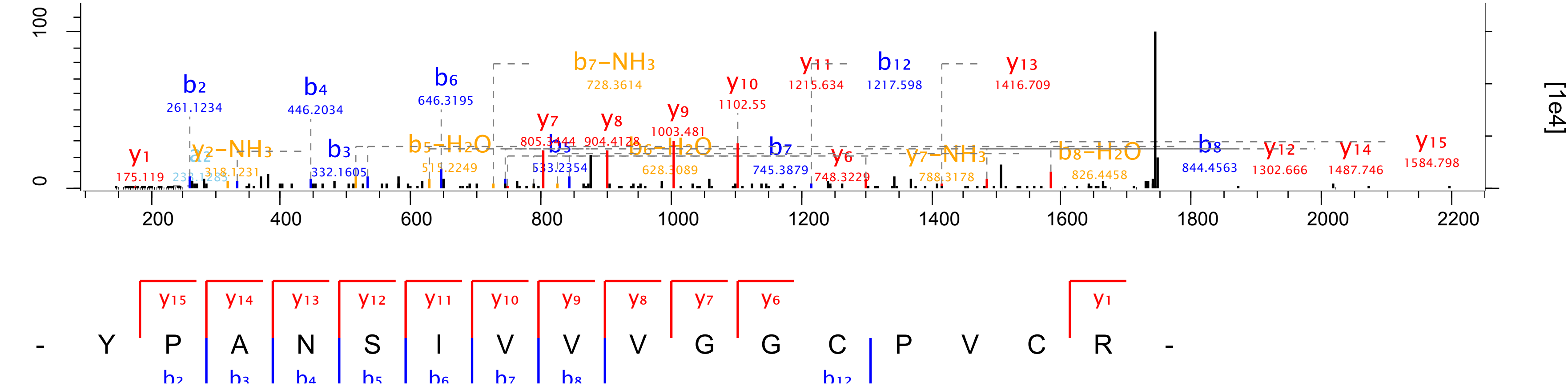

Raw file	Scan	Method	Score	m/z	Gene names
20150226_Hela_Top_opt_A3_01_1595	44724	TOF; CID	91.73	1127.09	MZT2B;MZT2A

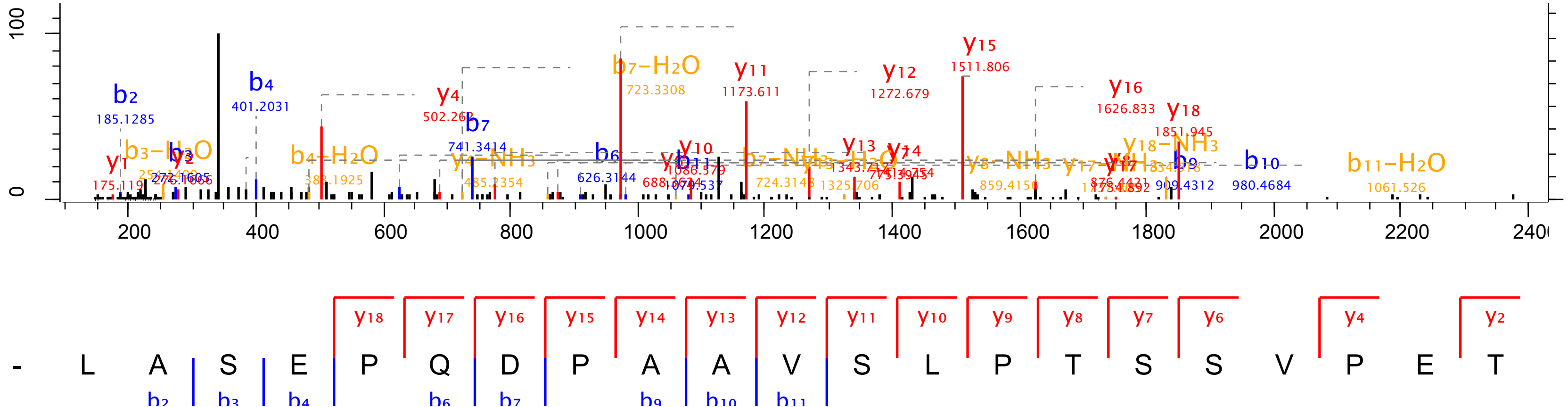

Raw file	Scan	Method	Score	m/z	Gene names
20150226_Hela_Top_opt_A3_01_1595	45267	TOF; CID	92.04	670.9	LOC102288414

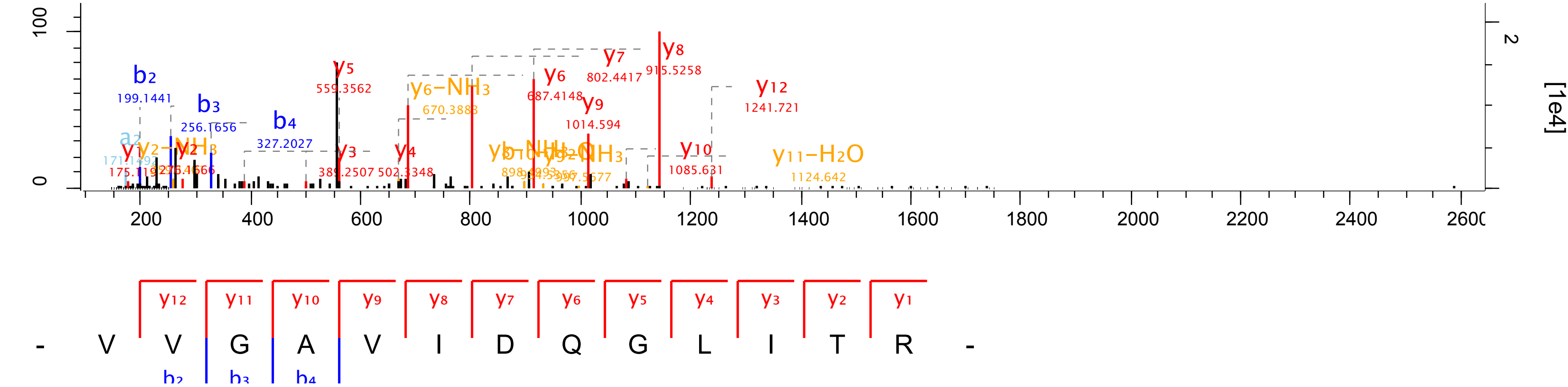

Raw file	Scan	Method	Score	m/z	Gene names
20150226_Hela_Top_opt_A3_01_1595	45342	TOF; CID	72.32	1127.08	MYADM

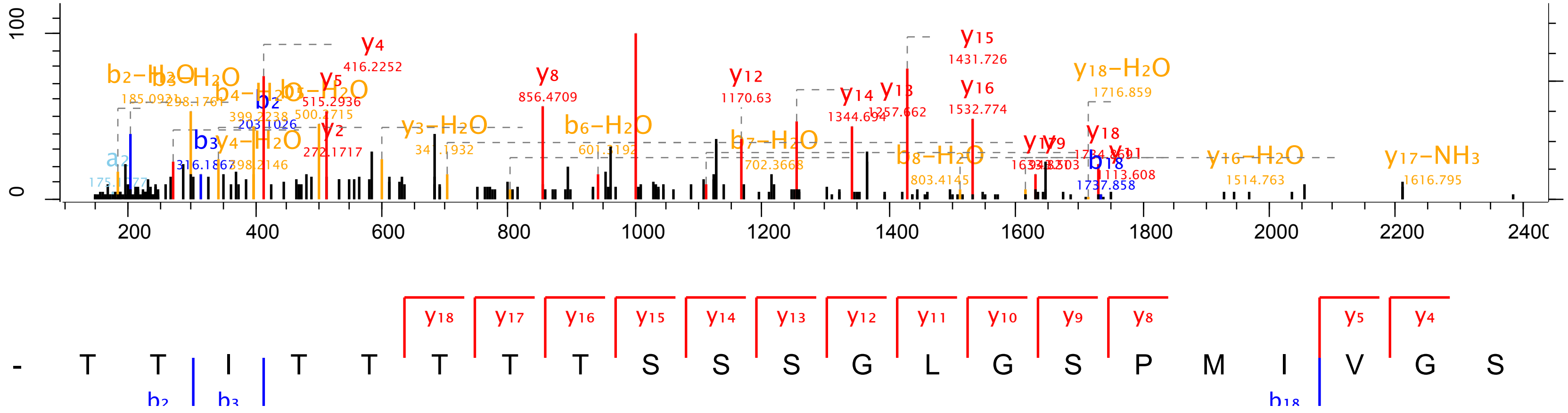

Raw file	Scan	Method	Score	m/z	Gene names
20150226_Hela_Top_opt_A3_01_1595	46462	TOF; CID	58.63	575.79	LYPLAL1

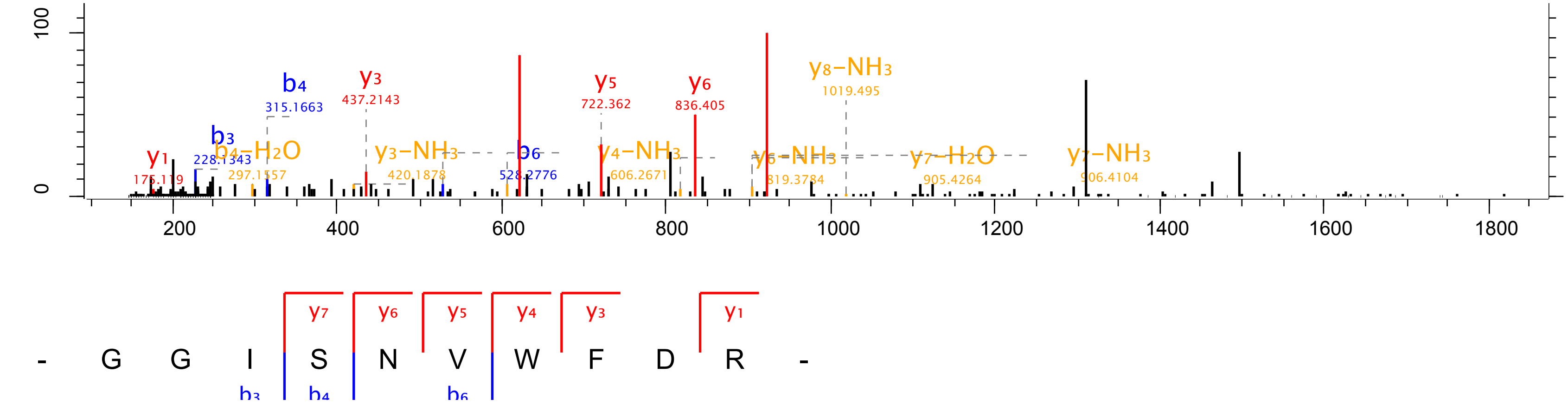

Raw file	Scan	Method	Score	m/z	Gene names
20150226_Hela_Top_opt_A3_01_1595	46499	TOF; CID	65.21	776.4	COG1;ADCK1

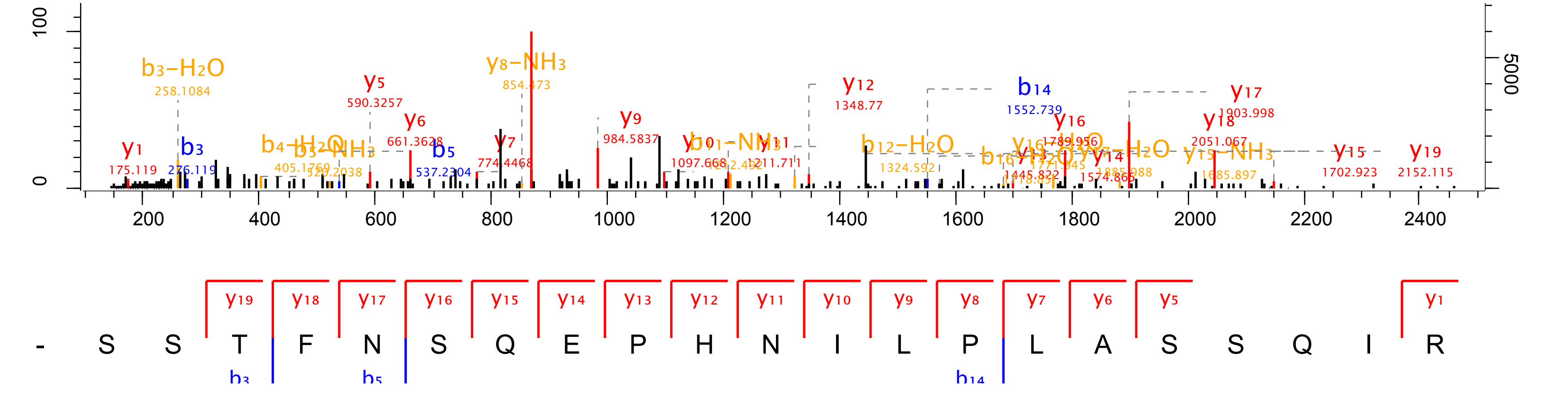

Raw file	Scan	Method	Score	m/z	Gene names
20150226_Hela_Top_opt_A3_01_1595	46625	TOF; CID	59.07	489.28	GATC

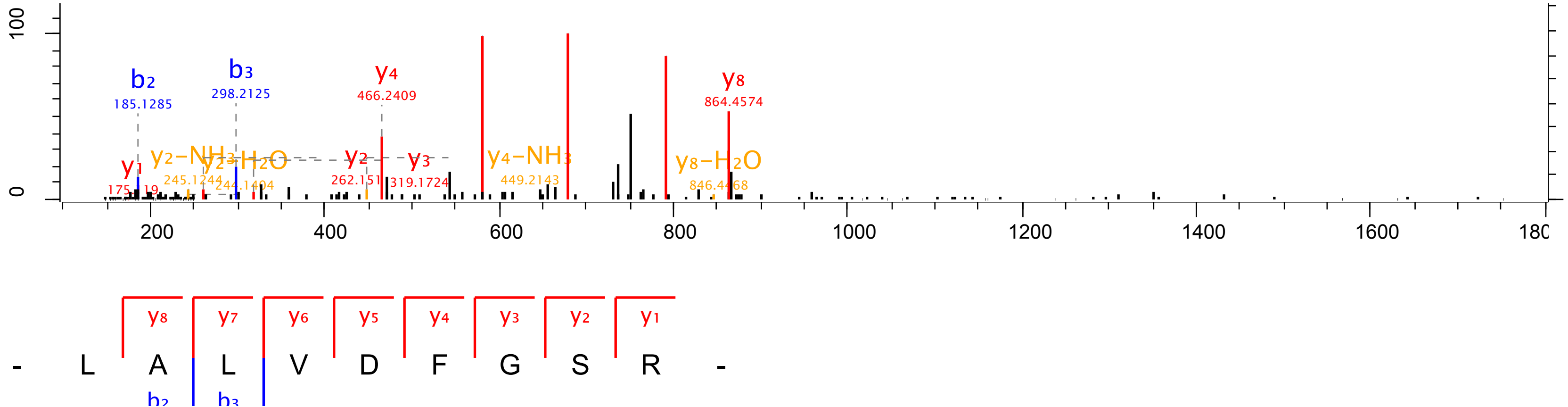

Raw file	Scan	Method	Score	m/z	Gene names
20150226_Hela_Top_opt_A3_01_1595	47331	TOF; CID	43.16	962.47	CDC42SE2

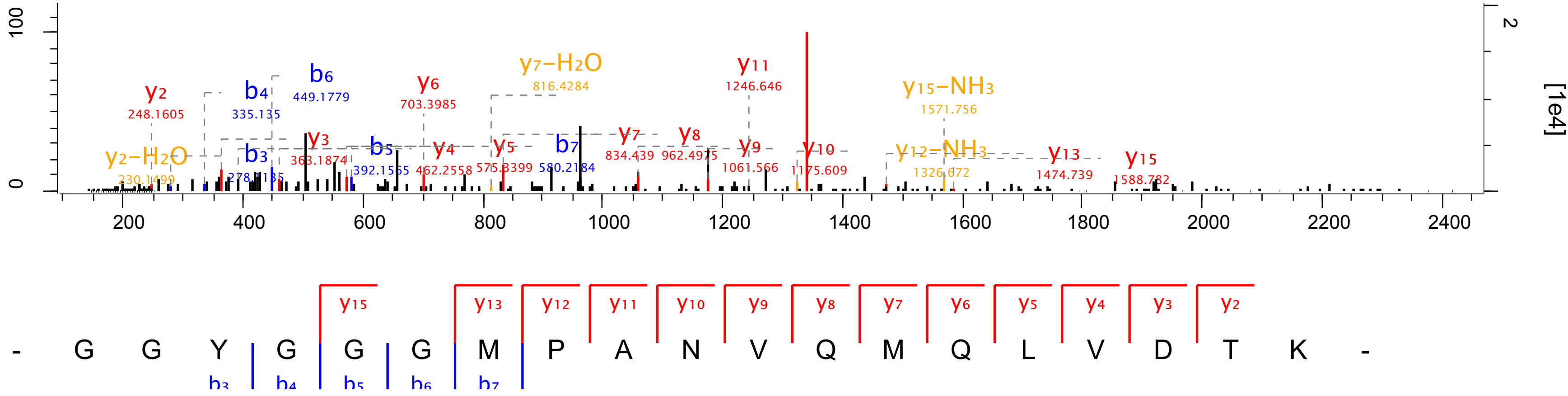

Raw file	Scan	Method	Score	m/z	Gene names
20150226_Hela_Top_opt_A3_01_1595	47513	TOF; CID	96.67	562.96	PHKG2

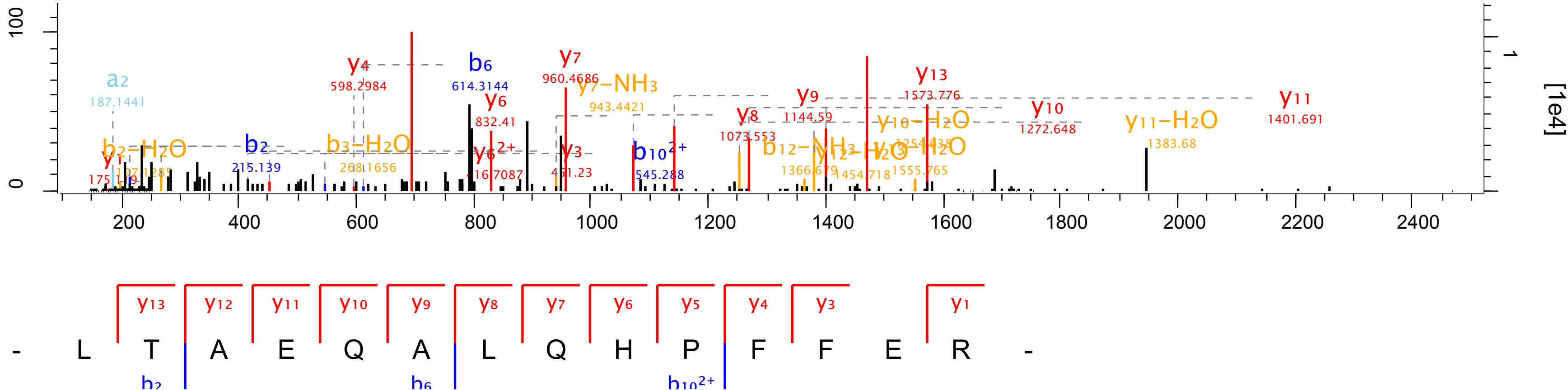

Raw file	Scan	Method	Score	m/z	Gene names
20150226_Hela_Top_opt_A3_01_1595	47727	TOF; CID	56.42	1092.54	FAM107B

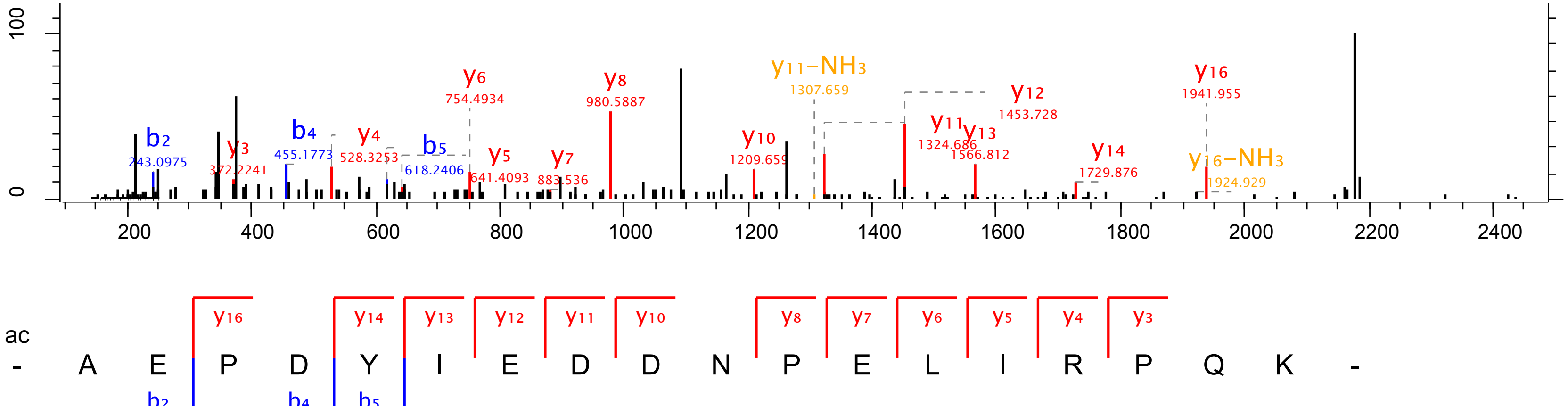

Raw file	Scan	Method	Score	m/z	Gene names
20150226_Hela_Top_opt_A3_01_1595	48541	TOF; CID	108.71	956.99	GTPBP3

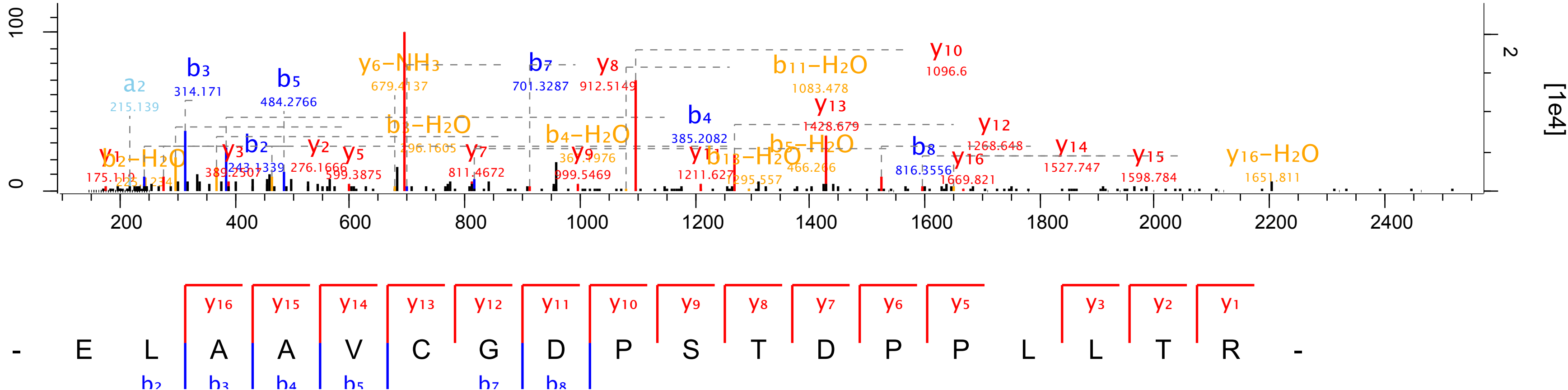

Raw file	Scan	Method	Score	m/z	Gene names
20150226_Hela_Top_opt_A3_01_1595	49913	TOF; CID	94.77	627.39	MRPL34

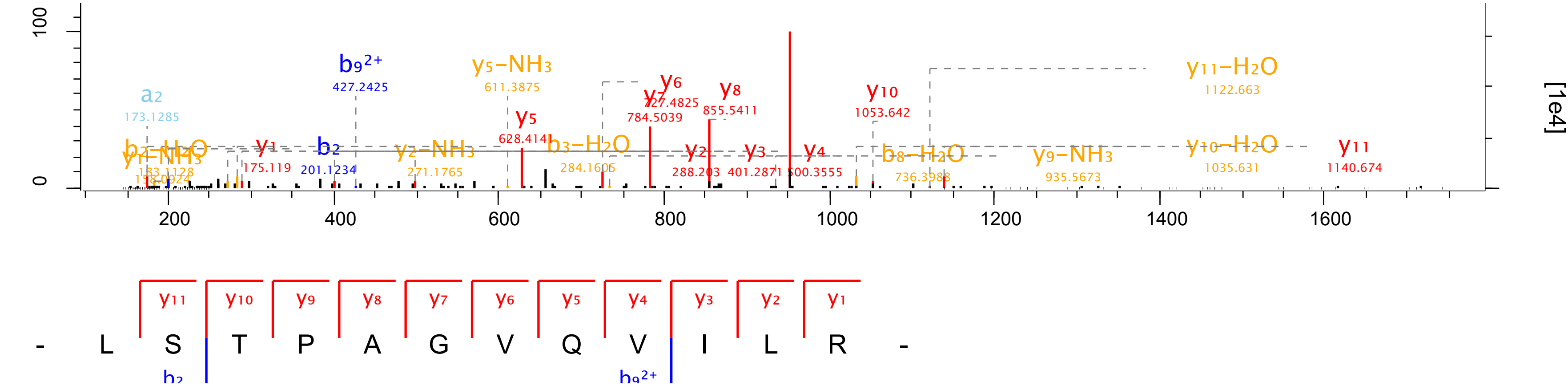

Raw file	Scan	Method	Score	m/z	Gene names
20150226_Hela_Top_opt_A3_01_1595	50753	TOF; CID	71.59	865.99	KRTCAP2

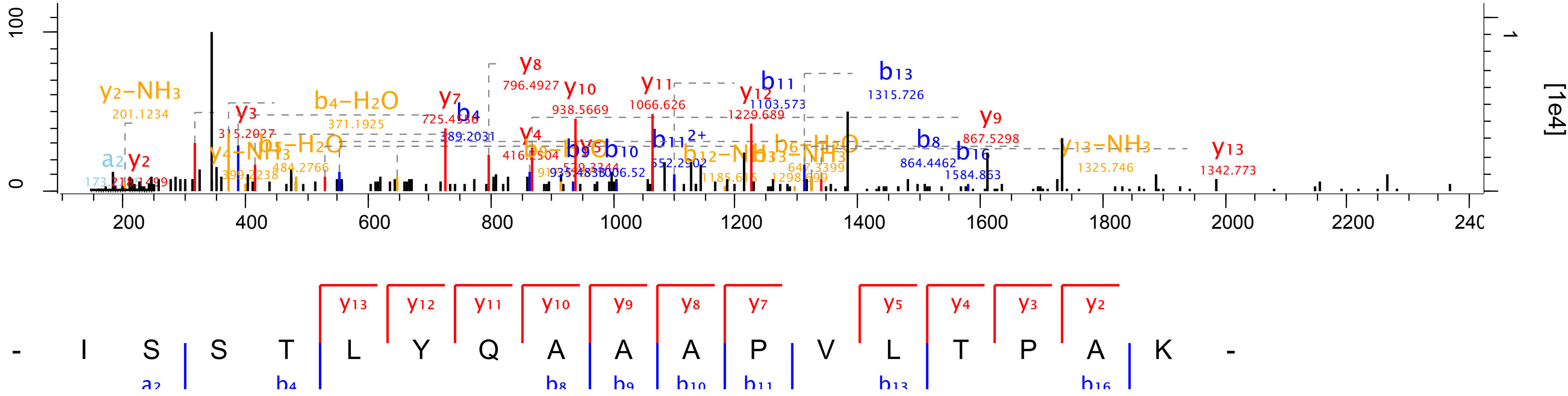

Raw file	Scan	Method	Score	m/z	Gene names
20150226_Hela_Top_opt_A3_01_1595	50898	TOF; CID	60.06	941.45	FAM168B

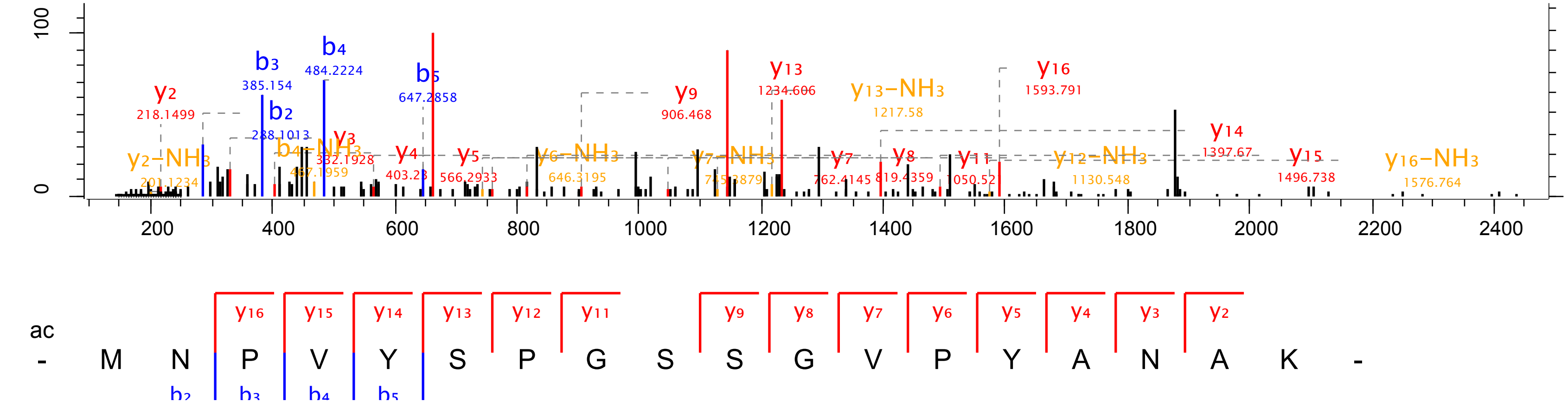

Raw file	Scan	Method	Score	m/z	Gene names
20150226_Hela_Top_opt_A3_01_1595	50934	TOF; CID	140.6	579.02	TMEM11

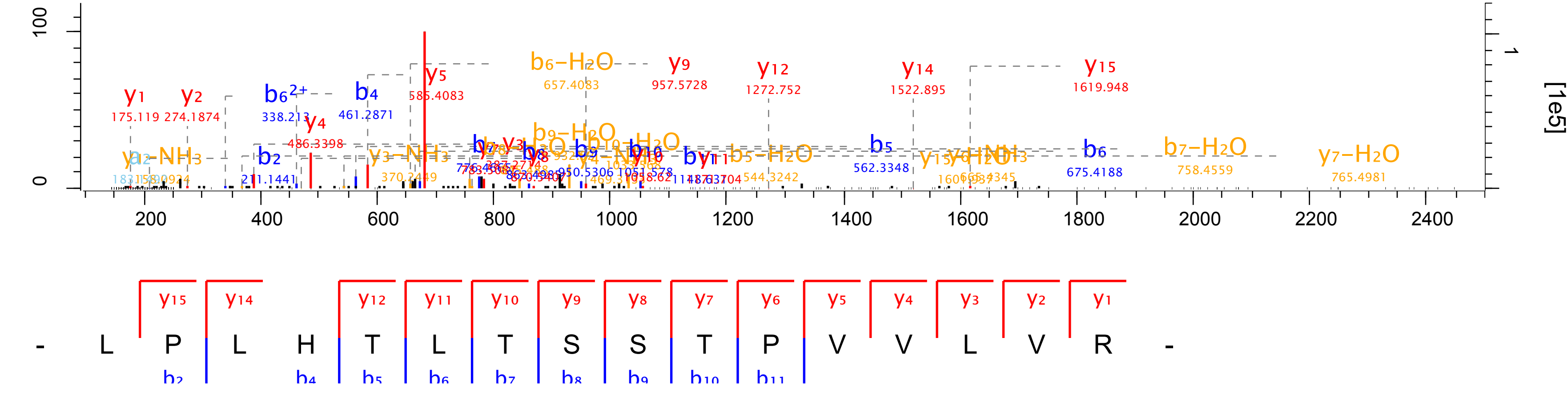

Raw file	Scan	Method	Score	m/z	Gene names
20150226_Hela_Top_opt_A3_01_1595	51058	TOF; CID	86.08	873.45	MCM3AP

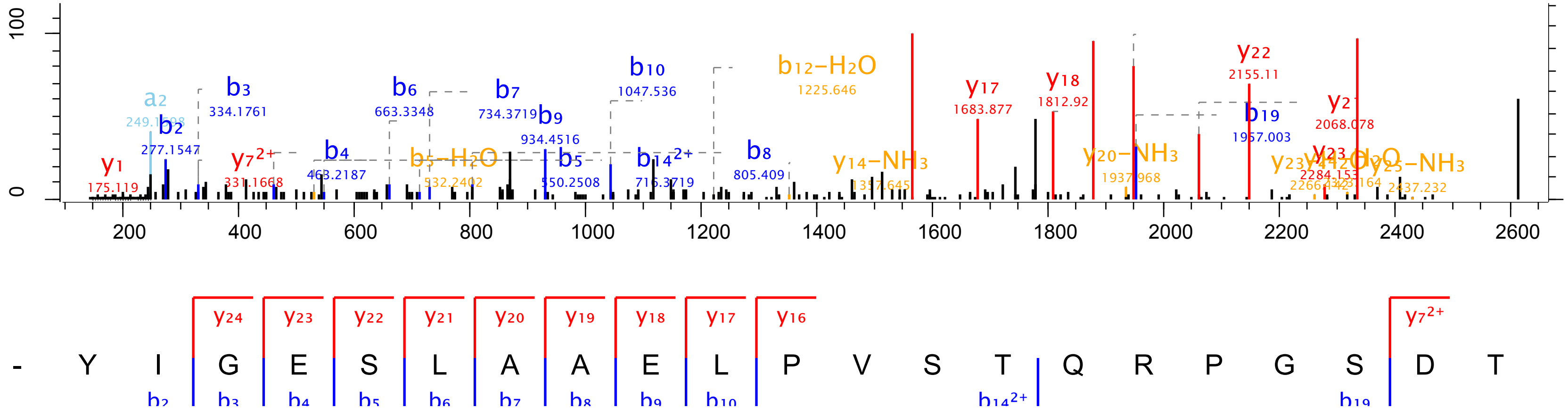

Raw file	Scan	Method	Score	m/z	Gene names
20150226_Hela_Top_opt_A3_01_1595	51538	TOF; CID	73.25	464.78	POC1B;TUWD12

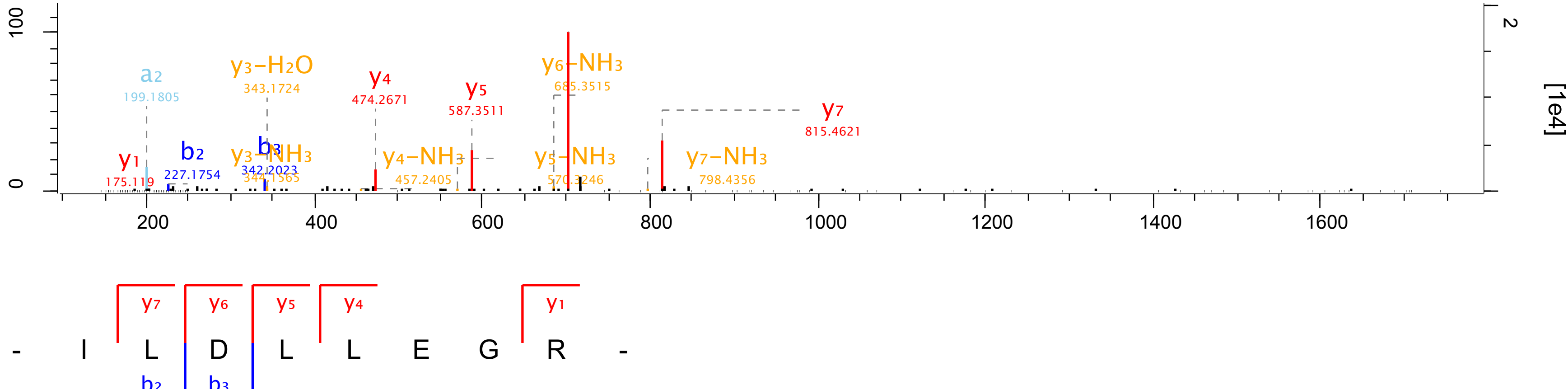

Raw file	Scan	Method	Score	m/z	Gene names
20150226_Hela_Top_opt_A3_01_1595	51725	TOF; CID	52.89	713.4	DTWD2

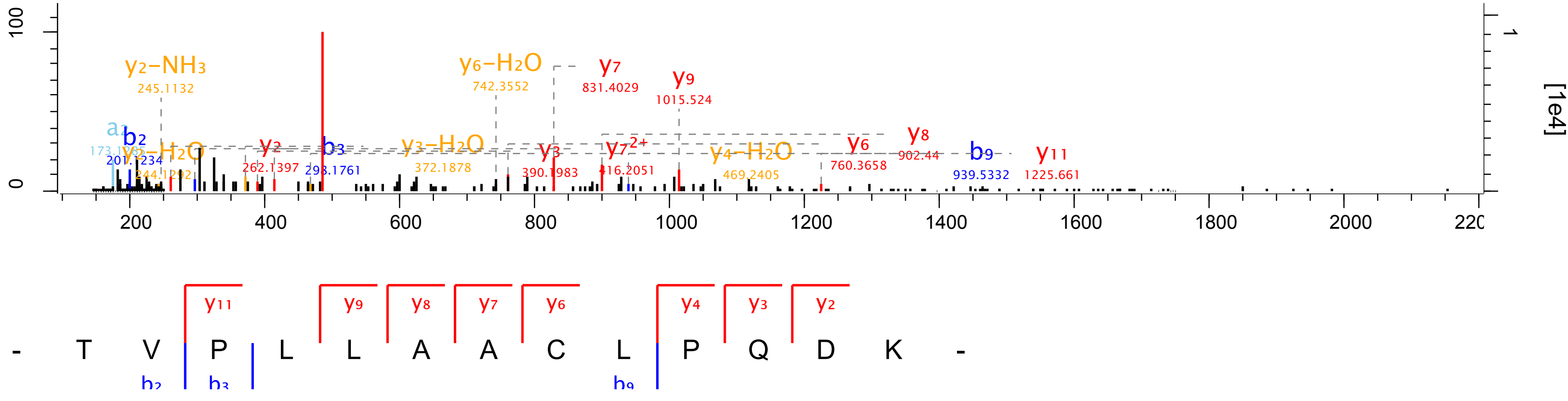

Raw file	Scan	Method	Score	m/z	Gene names
20150226_Hela_Top_opt_A3_01_1595	52240	TOF; CID	48.8	1206.57	DNAJC5

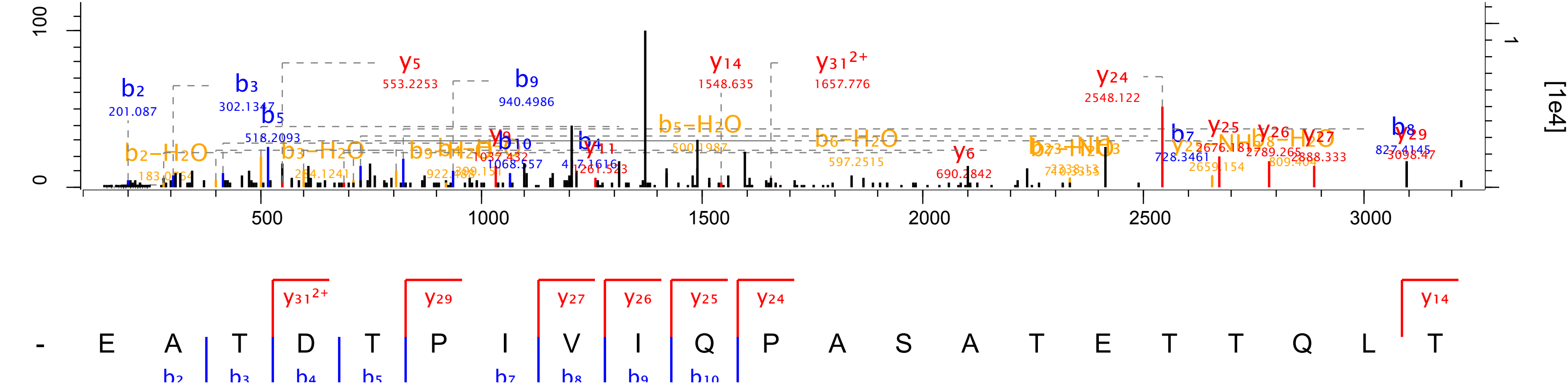

Raw file	Scan	Method	Score	m/z	Gene names
20150226_Hela_Top_opt_A3_01_1595	52823	TOF; CID	40.29	807.42	FYCO1

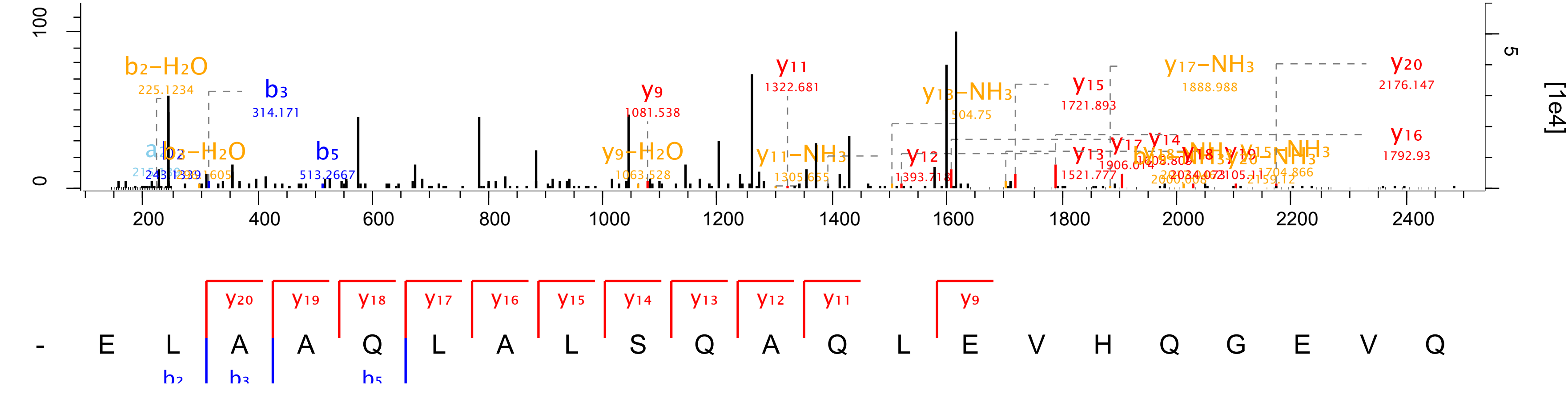

Raw file	Scan	Method	Score	m/z	Gene names
20150226_Hela_Top_opt_A3_01_1595	53411	TOF; CID	52.86	580.64	UFSP2

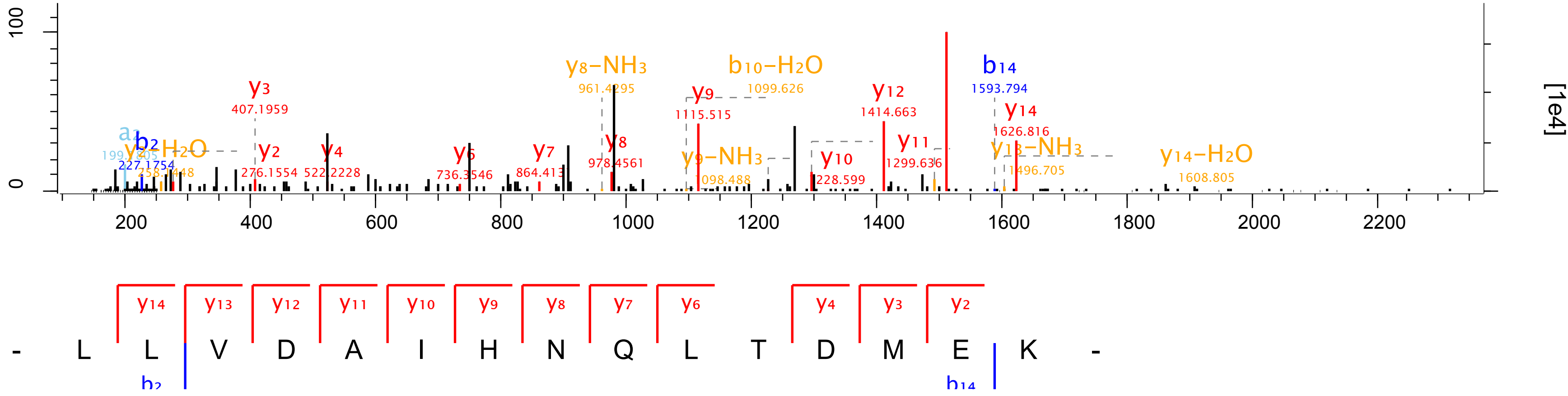

Raw file	Scan	Method	Score	m/z	Gene names
20150226_Hela_Top_opt_A3_01_1595	54460	TOF; CID	63.44	904.46	TAF6L

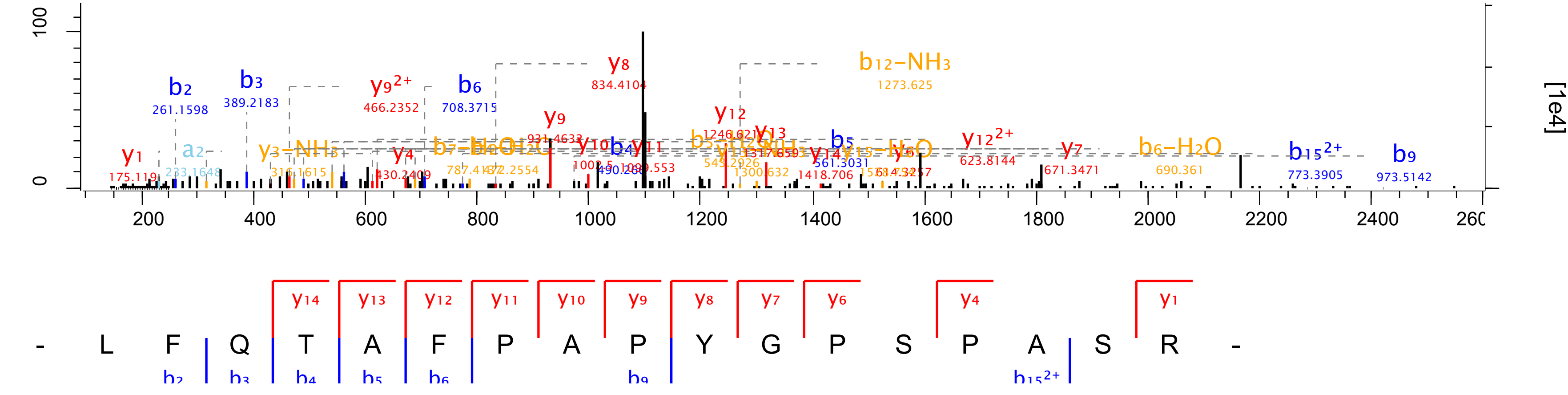

Raw file	Scan	Method	Score	m/z	Gene names
20150226_Hela_Top_opt_A3_01_1595	54625	TOF; CID	105.03	512.96	TOLLIP

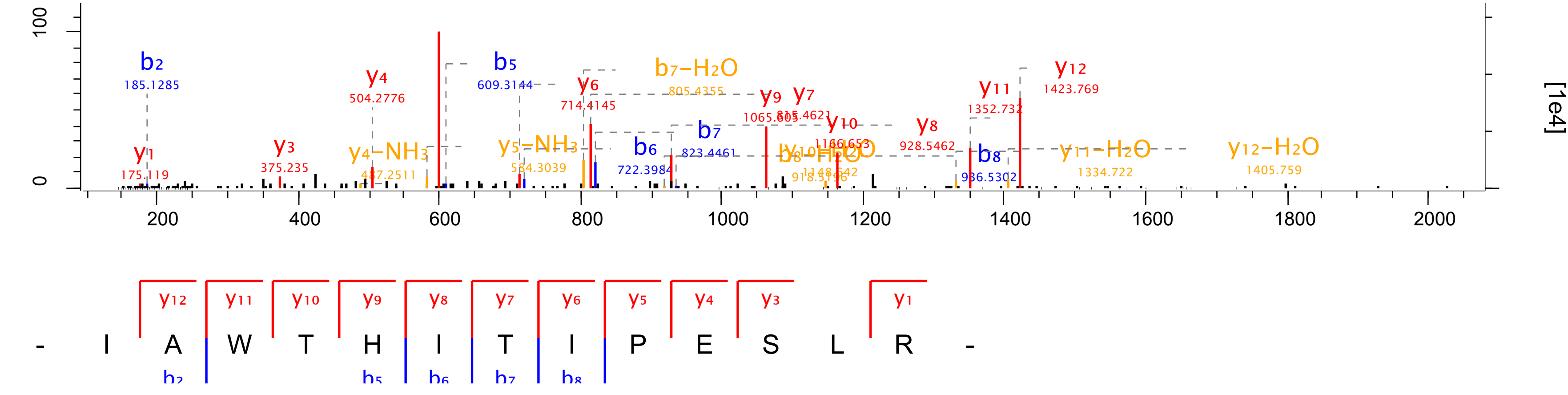

Raw file	Scan	Method	Score	m/z	Gene names
20150226_Hela_Top_opt_A3_01_1595	54874	TOF; CID	69.63	480.26	BNIP2

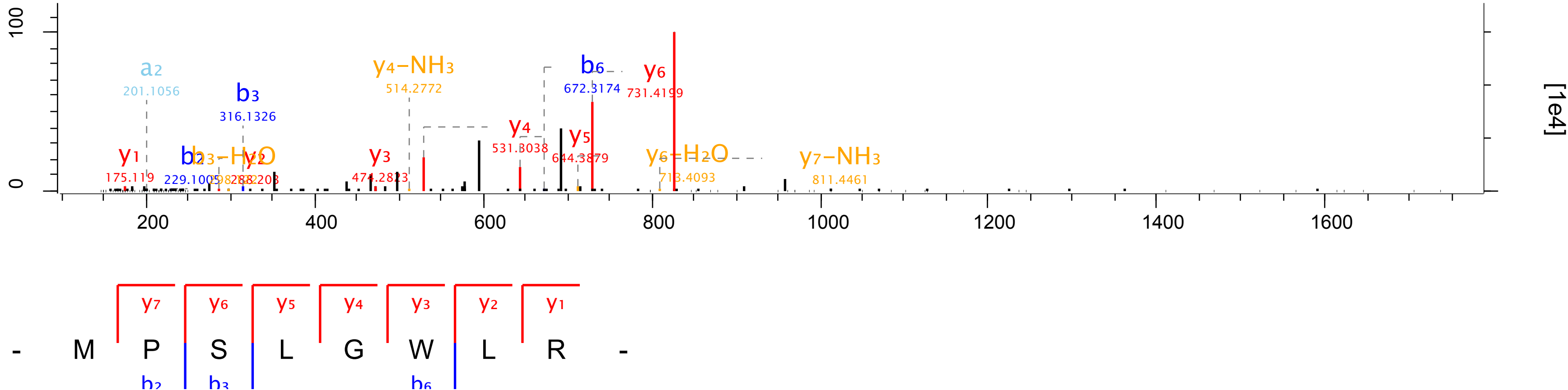

Raw file	Scan	Method	Score	m/z	Gene names
20150226_Hela_Top_opt_A3_01_1595	54985	TOF; CID	50.04	675.35	FZD6

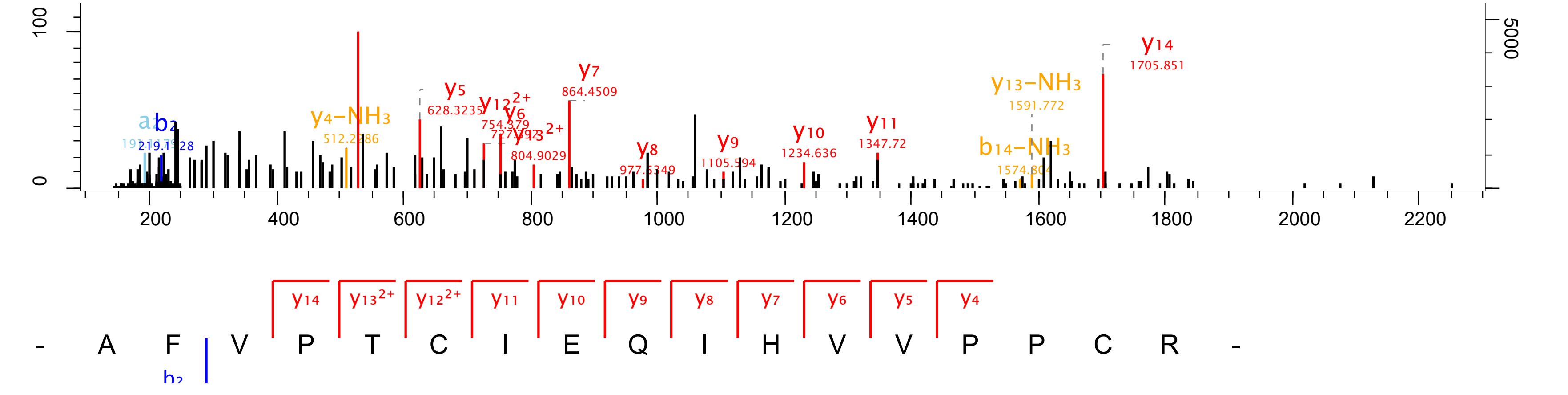

Raw file	Scan	Method	Score	m/z	Gene names
20150226_Hela_Top_opt_A3_01_1595	55397	TOF; CID	114.24	1100.51	CEBPG

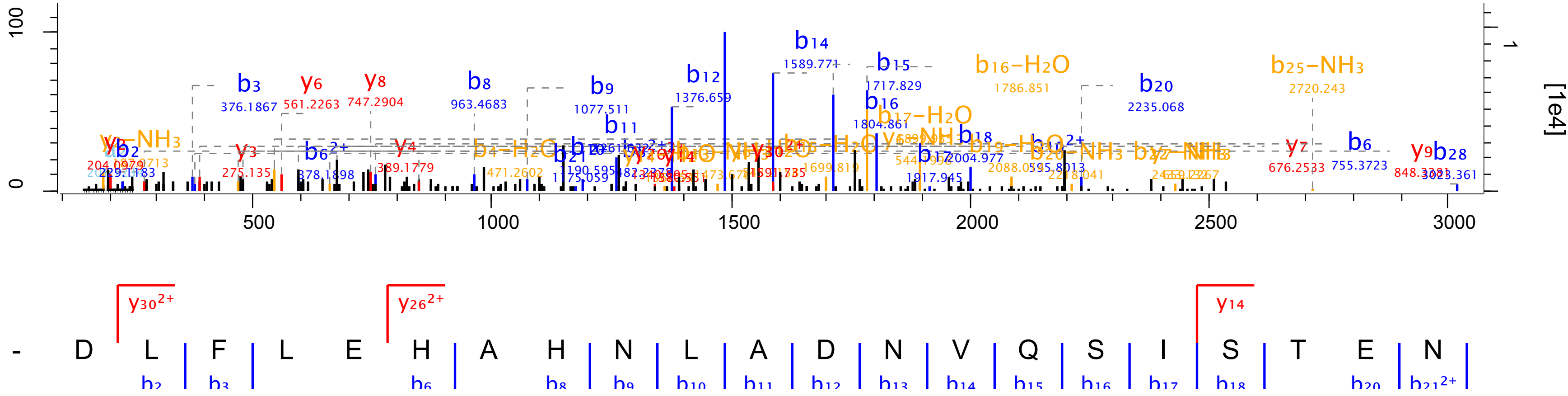

Raw file	Scan	Method	Score	m/z	Gene names
20150226_Hela_Top_opt_A3_01_1595	57504	TOF; CID	49.08	802.41	TOPBP1

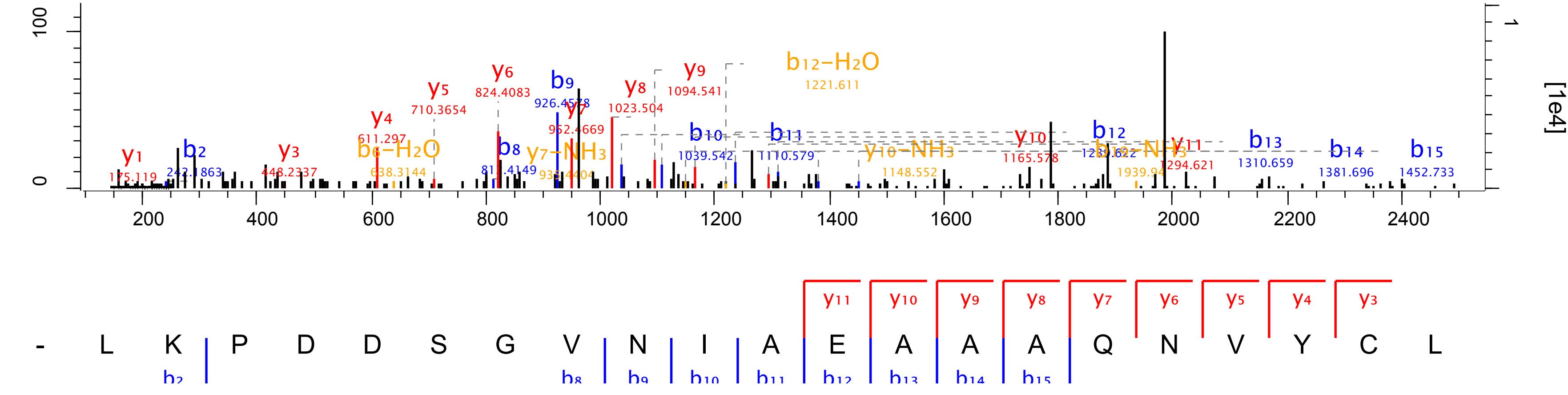

Raw file	Scan	Method	Score	m/z	Gene names
20150226_Hela_Top_opt_A3_01_1595	58096	TOF; CID	58.37	862.46	HELZ2

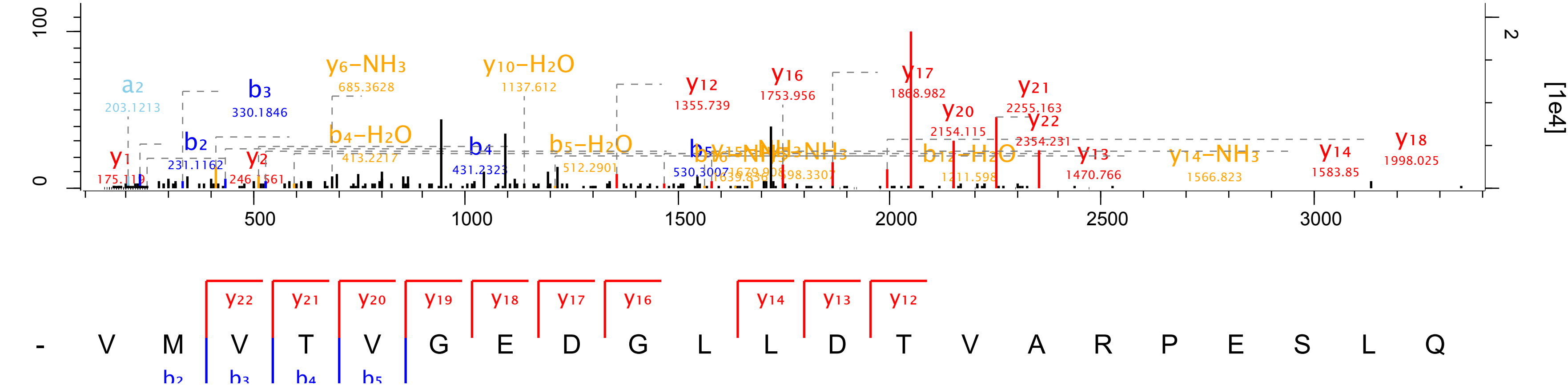

Raw file	Scan	Method	Score	m/z	Gene names
20150226_Hela_Top_opt_A3_01_1595	58194	TOF; CID	71.08	629.84	GTF2A1;HIST1H4F

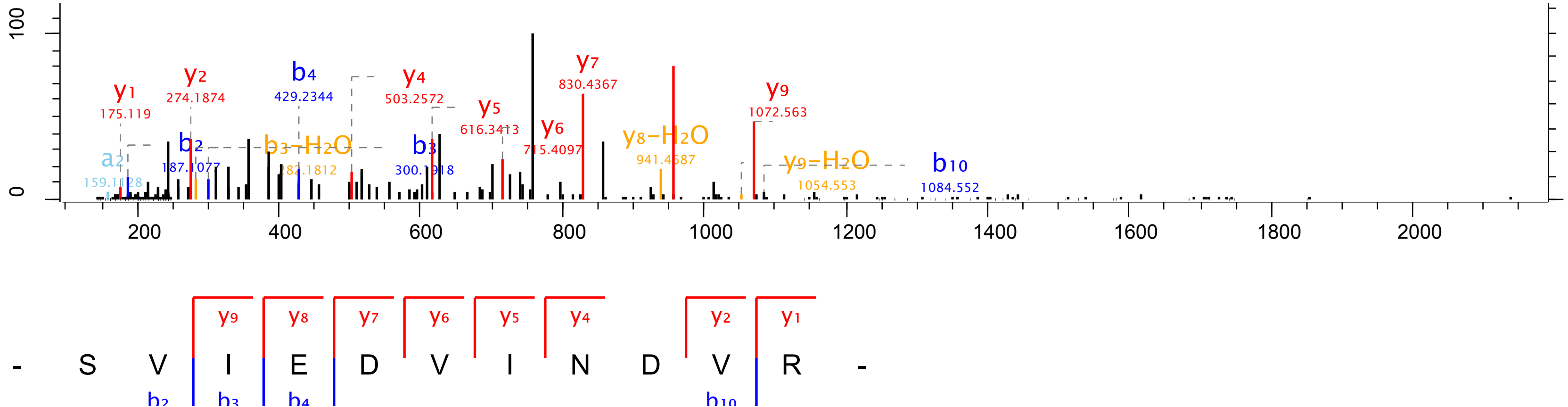

Raw file	Scan	Method	Score	m/z	Gene names
20150226_Hela_Top_opt_A3_01_1595	58213	TOF; CID	92.54	648.88	ASCC1

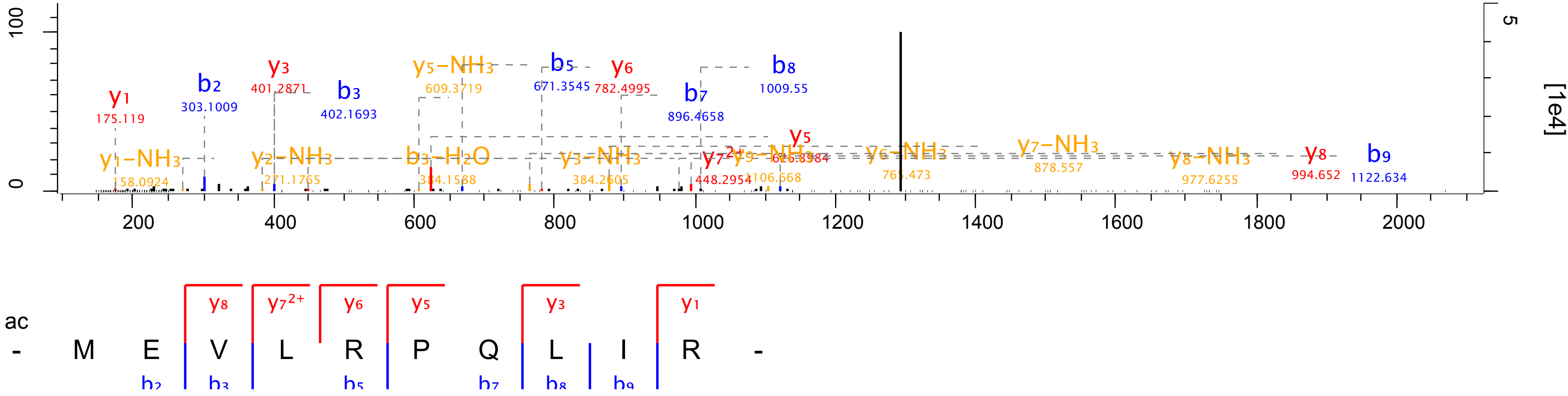

Raw file	Scan	Method	Score	m/z	Gene names
20150226_Hela_Top_opt_A3_01_1595	58417	TOF; CID	53.36	724.4	HPS3

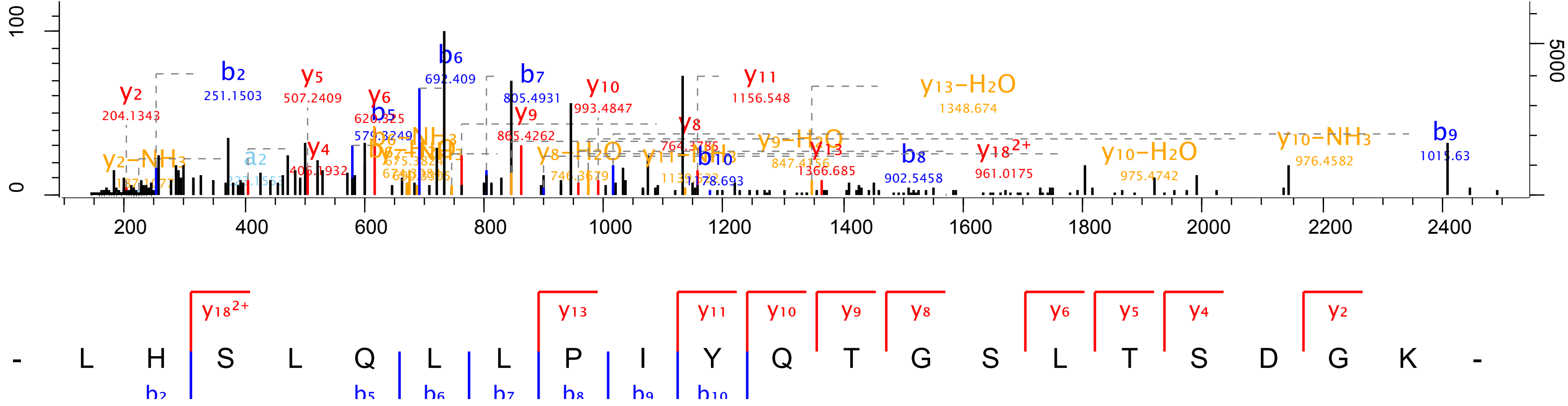

Raw file	Scan	Method	Score	m/z	Gene names
20150226_Hela_Top_opt_A3_01_1595	58755	TOF; CID	76.3	760.04	CNIH4

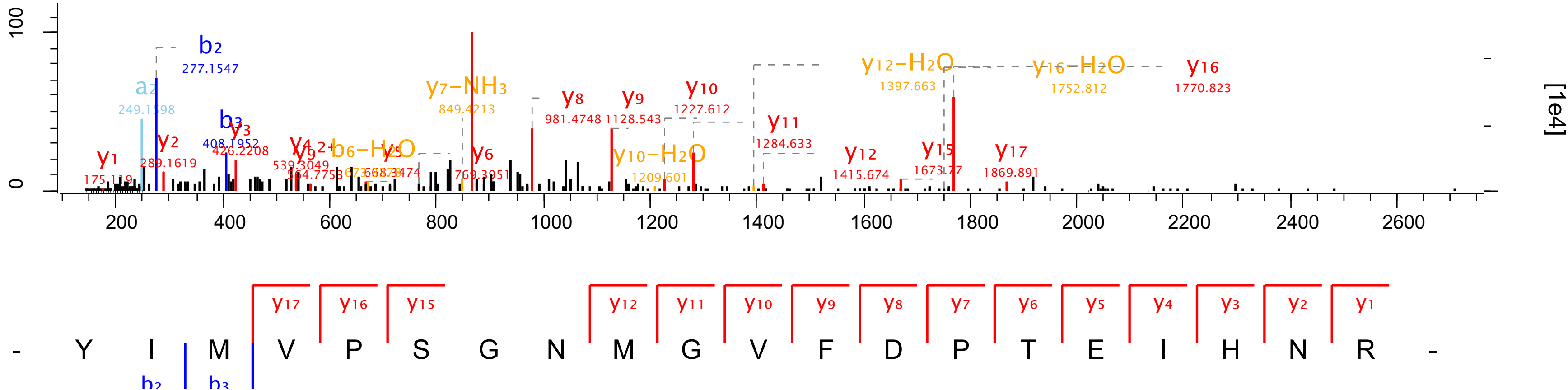

Raw file	Scan	Method	Score	m/z	Gene names
20150226_Hela_Top_opt_A3_01_1595	58872	TOF; CID	139.75	1233.6	PAGR1

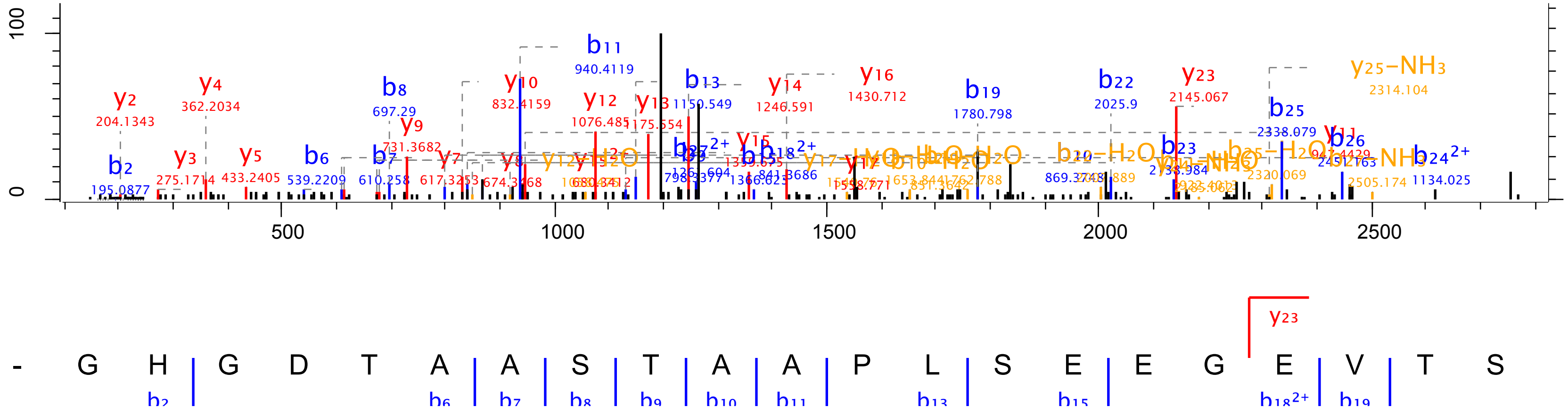

Raw file	Scan	Method	Score	m/z	Gene names
20150226_Hela_Top_opt_A3_01_1595	59374	TOF; CID	80.14	1103.53	SCAMP2

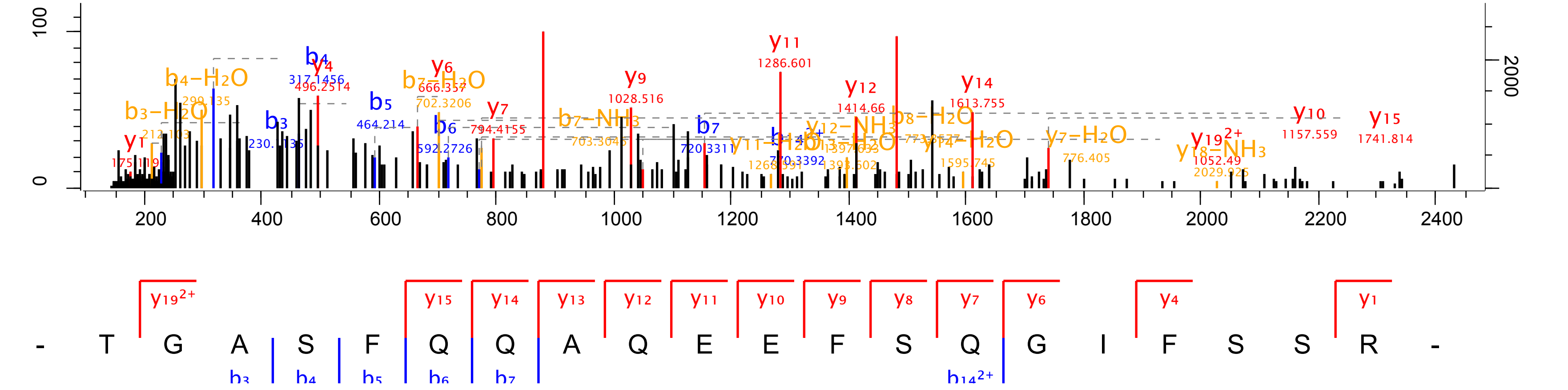

Raw file	Scan	Method	Score	m/z	Gene names
20150226_Hela_Top_opt_A3_01_1595	59722	TOF; CID	117.7	580.86	CNOT6L;CNOT6

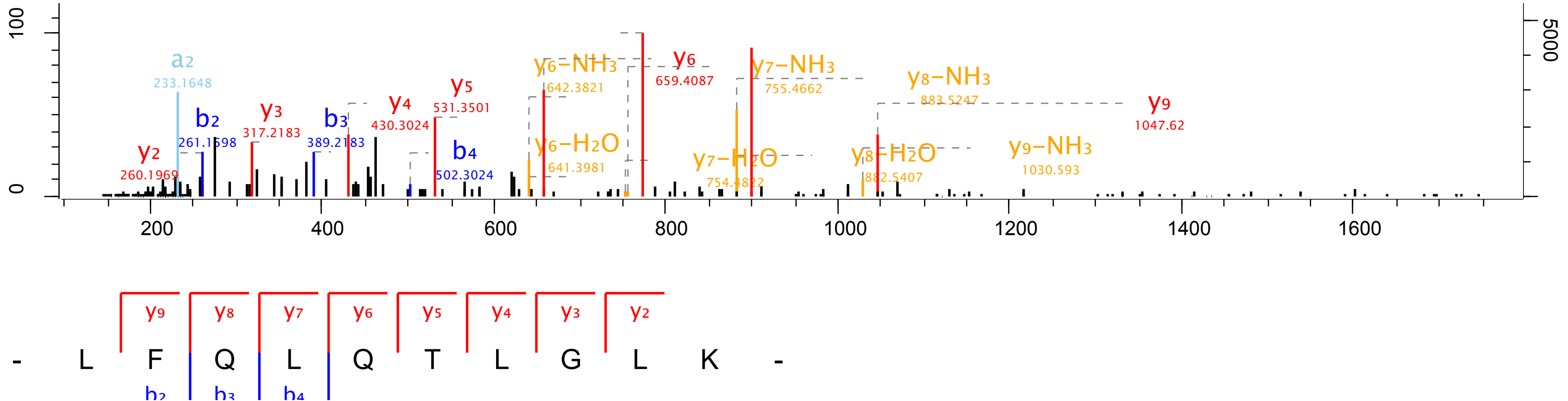

Raw file	Scan	Method	Score	m/z	Gene names
20150226_Hela_Top_opt_A3_01_1595	61393	TOF; CID	59.23	756.4	TTC5

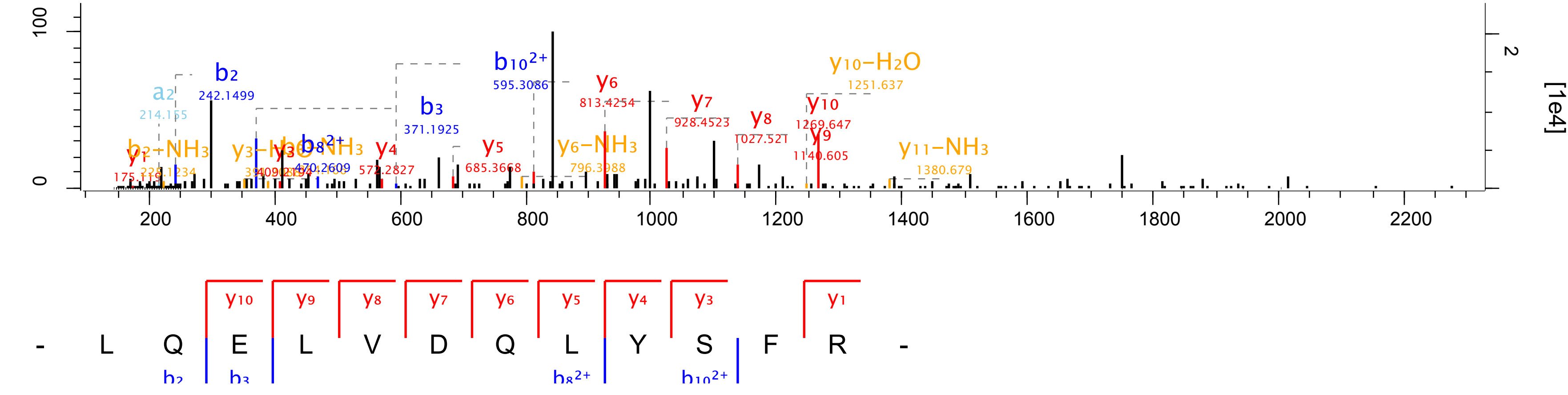

Raw file	Scan	Method	Score	m/z	Gene names
20150226_Hela_Top_opt_A3_01_1595	61550	TOF; CID	111.11	900.43	NUMB

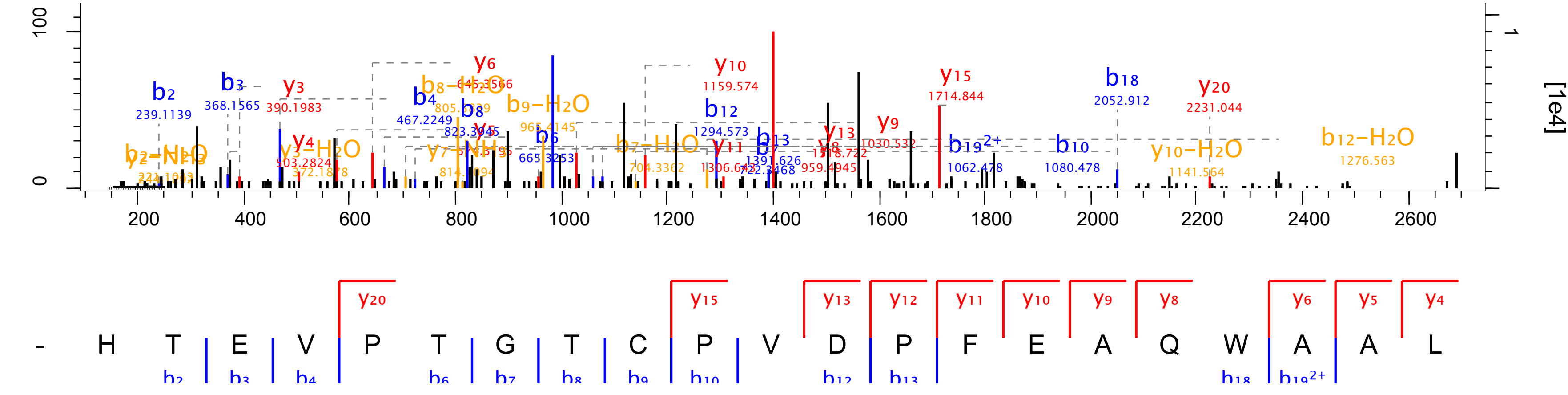

Raw file	Scan	Method	Score	m/z	Gene names
20150226_Hela_Top_opt_A3_01_1595	61822	TOF; CID	75.82	453.26	ERCC1

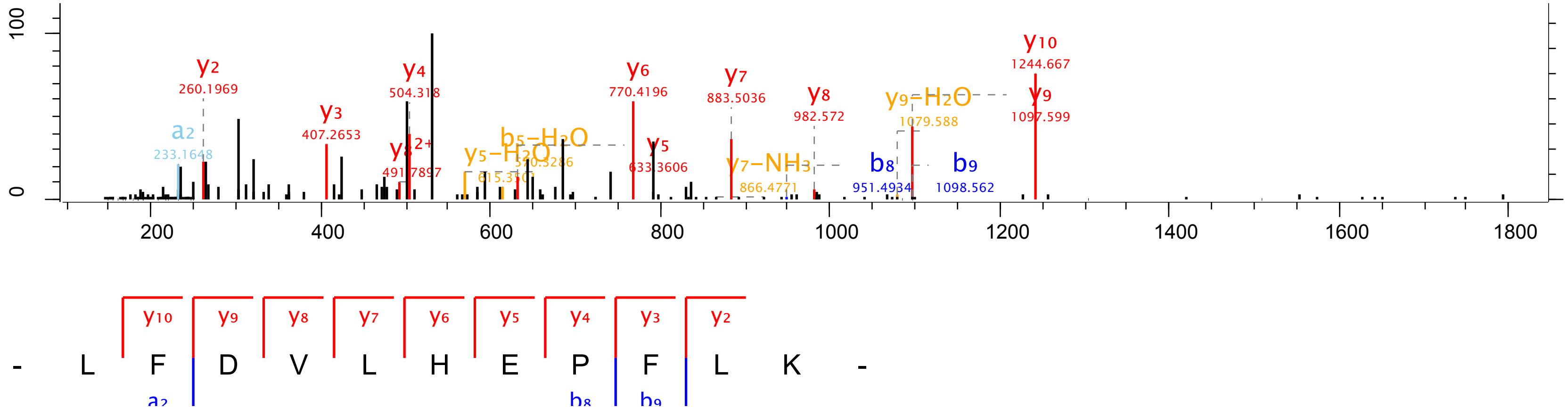

Raw file	Scan	Method	Score	m/z	Gene names
20150226_Hela_Top_opt_A3_01_1595	62434	TOF; CID	83.18	714.42	LOH12CR1

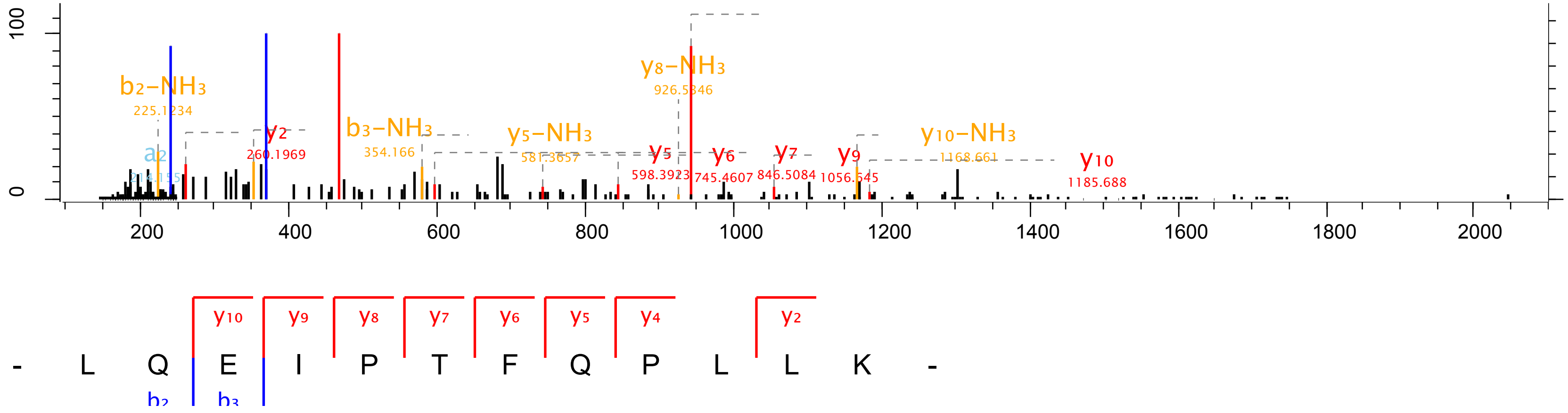

Raw file	Scan	Method	Score	m/z	Gene names
20150226_Hela_Top_opt_A3_01_1595	62507	TOF; CID	53.45	996.51	PTTG1IP

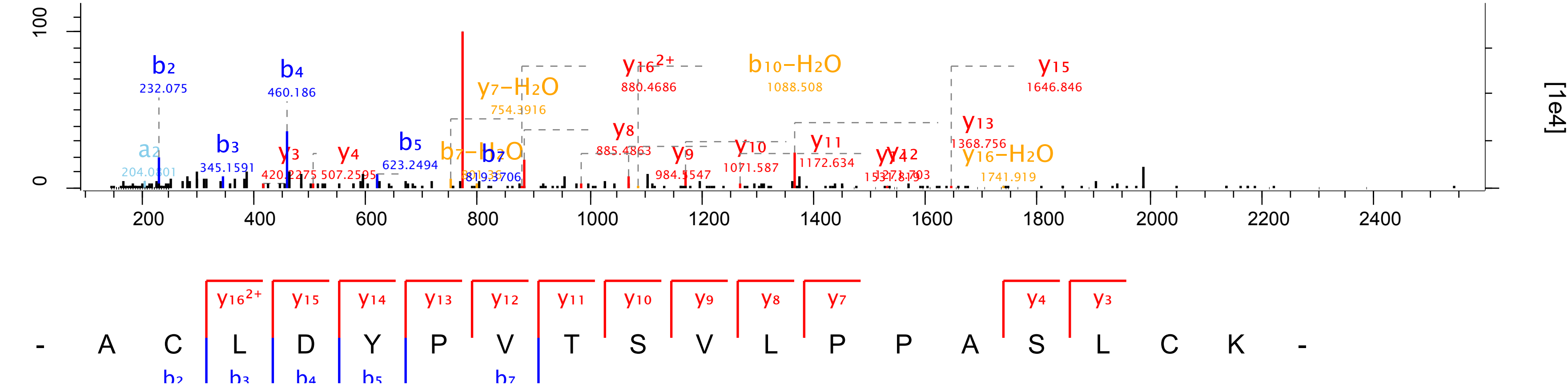

Raw file	Scan	Method	Score	m/z	Gene names
20150226_Hela_Top_opt_A3_01_1595	62703	TOF; CID	67.38	612.87	CYTH2;CYTH1;CYTH3

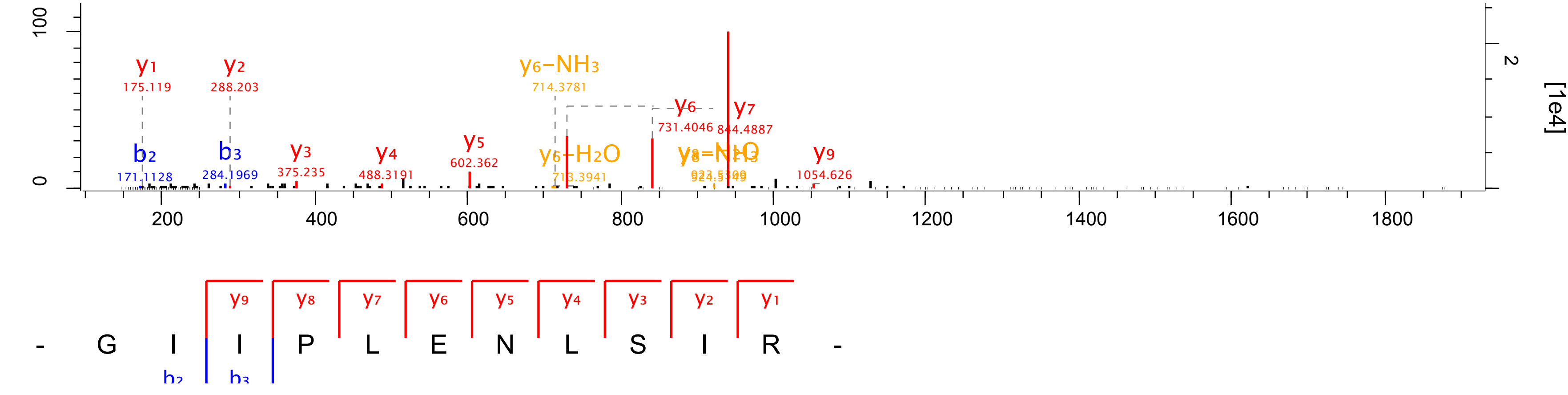

Raw file	Scan	Method	Score	m/z	Gene names
20150226_Hela_Top_opt_A3_01_1595	62730	TOF; CID	85.86	802.7	RABIF

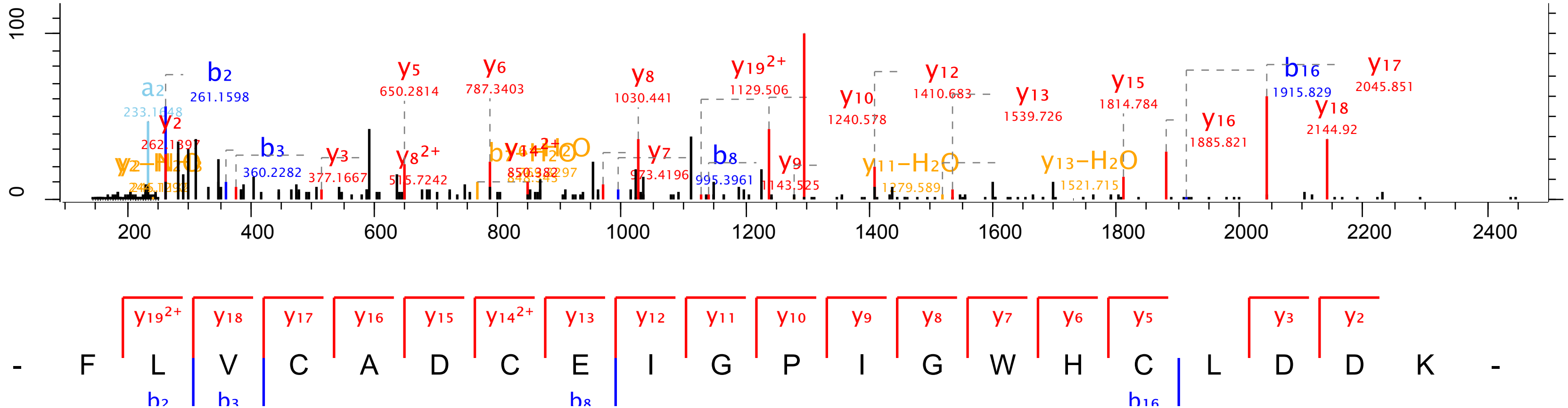

Raw file	Scan	Method	Score	m/z	Gene names
20150226_Hela_Top_opt_A3_01_1595	62945	TOF; CID	74.9	956.79	SCRN3

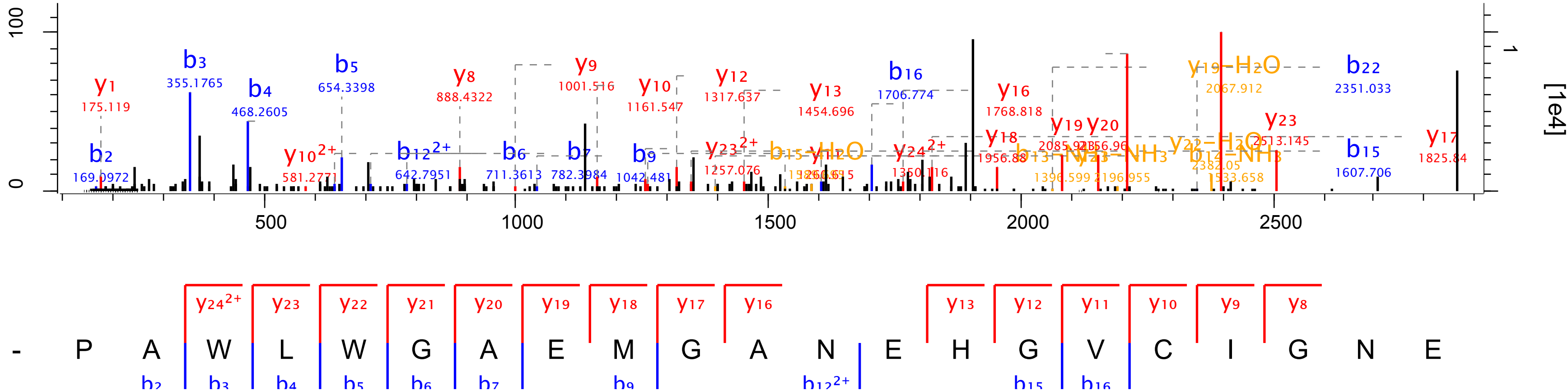

Raw file	Scan	Method	Score	m/z	Gene names
20150226_Hela_Top_opt_A3_01_1595	63771	TOF; CID	107.65	981.17	CPPED1

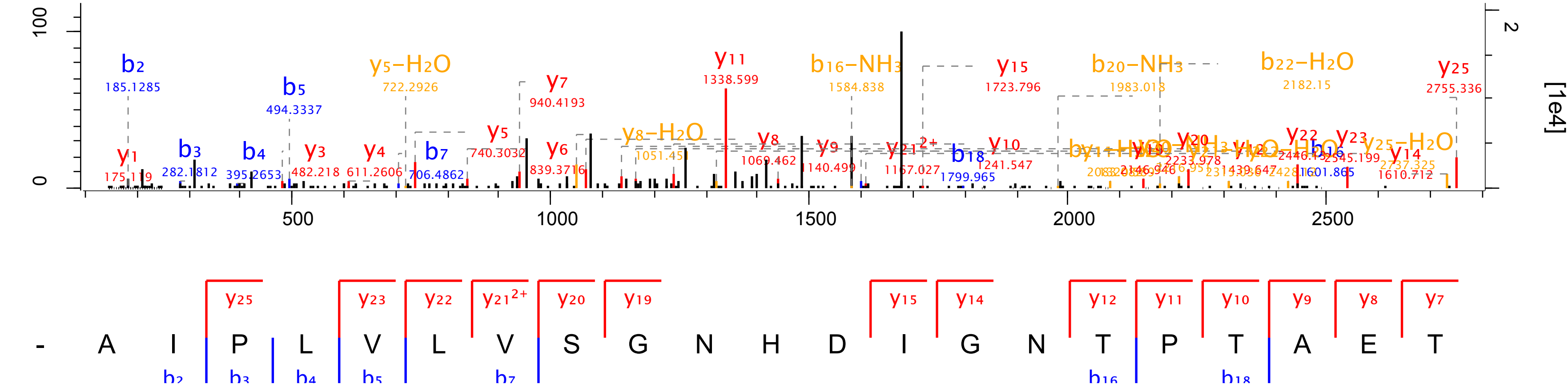

Raw file	Scan	Method	Score	m/z	Gene names
20150226_Hela_Top_opt_A3_01_1595	63942	TOF; CID	66.14	664.84	SLC39A8

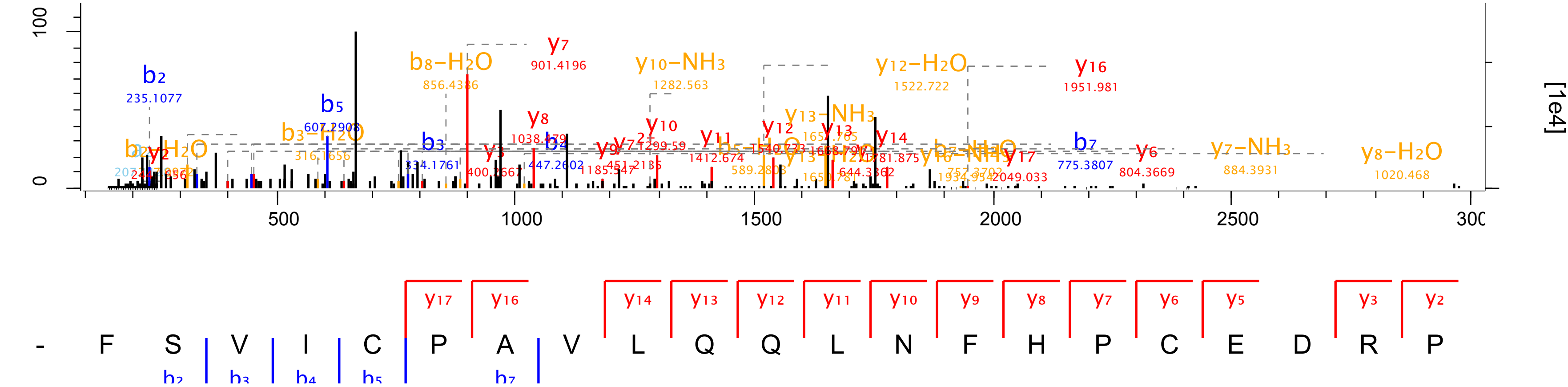

Raw file	Scan	Method	Score	m/z	Gene names
20150226_Hela_Top_opt_A3_01_1595	64050	TOF; CID	51.13	705.87	SMG7

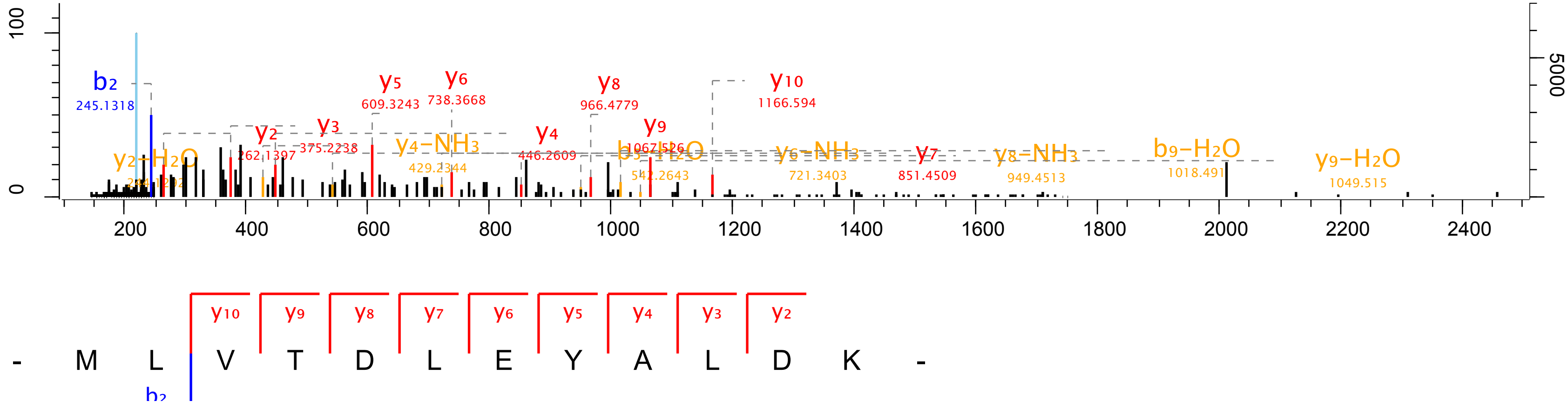

Raw file	Scan	Method	Score	m/z	Gene names
20150226_Hela_Top_opt_A3_01_1595	64345	TOF; CID	96.49	478.28	RAB34

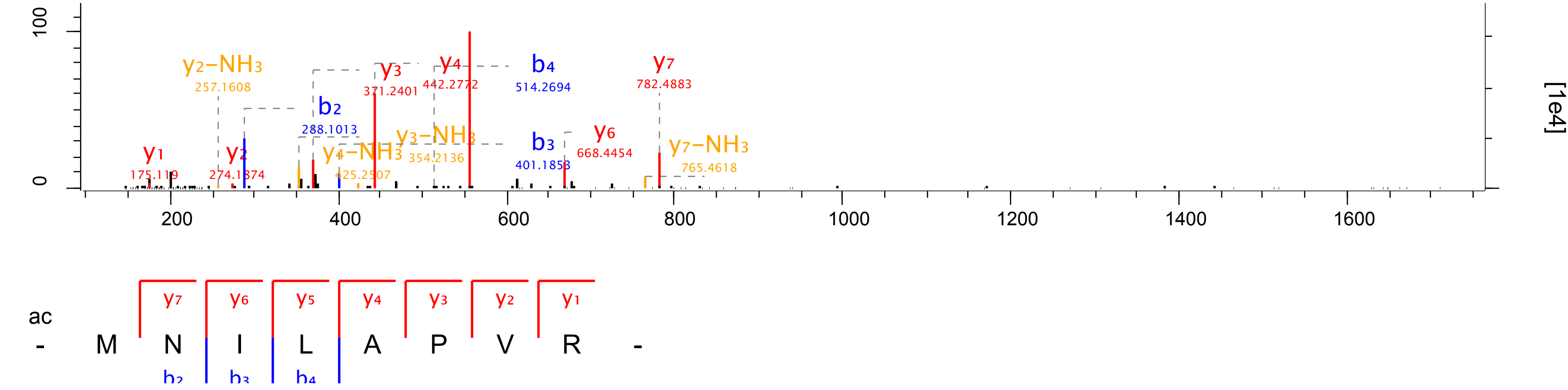

Raw file	Scan	Method	Score	m/z	Gene names
20150226_Hela_Top_opt_A3_01_1595	65261	TOF; CID	45.65	750.36	APOBEC3C

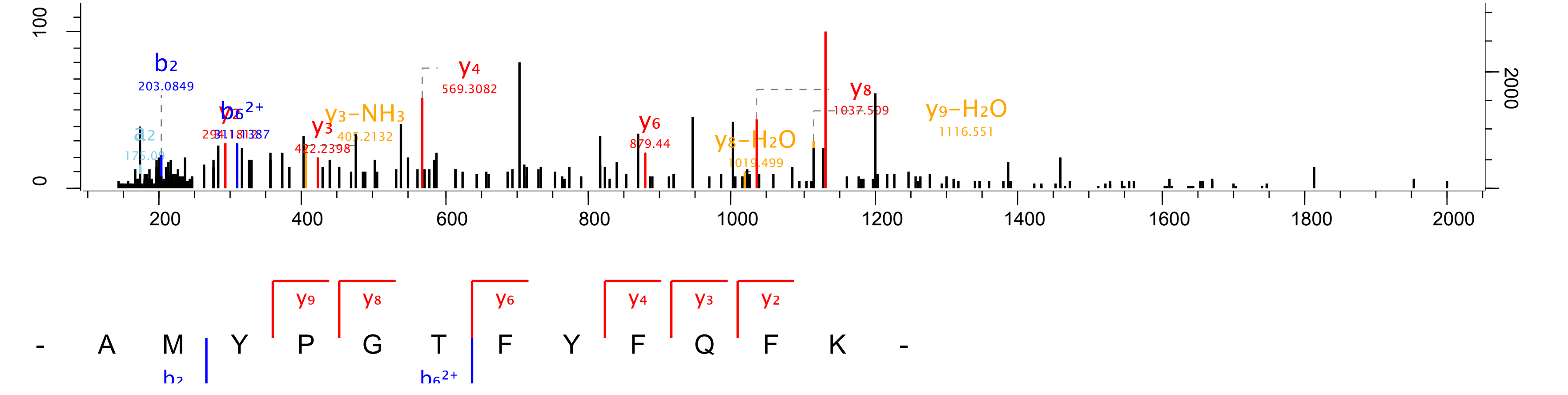

Raw file	Scan	Method	Score	m/z	Gene names
20150226_Hela_Top_opt_A3_01_1595	66609	TOF; CID	83.44	1028.51	FOXRED1

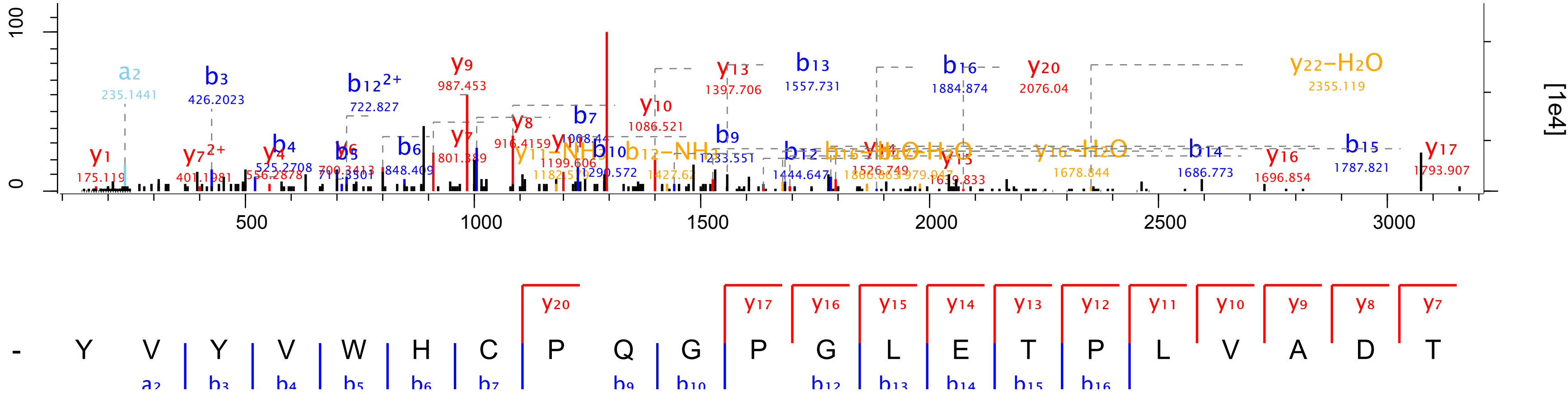

Raw file	Scan	Method	Score	m/z	Gene names
20150226_Hela_Top_opt_A3_01_1595	66738	TOF; CID	92.19	705.91	POMGNT2

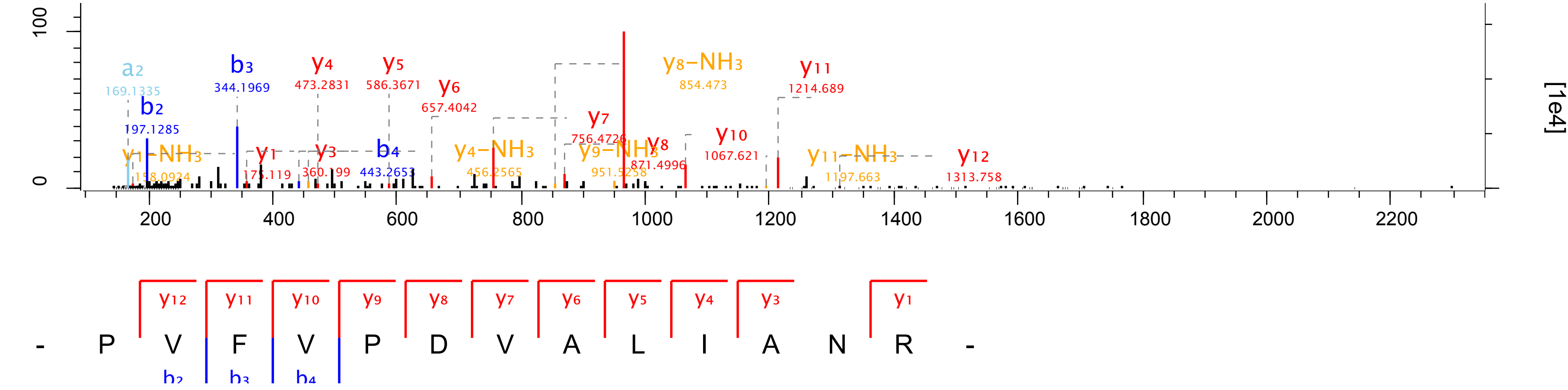

Raw file	Scan	Method	Score	m/z	Gene names
20150226_Hela_Top_opt_A3_01_1595	67039	TOF; CID	73.14	784.41	YIF1A

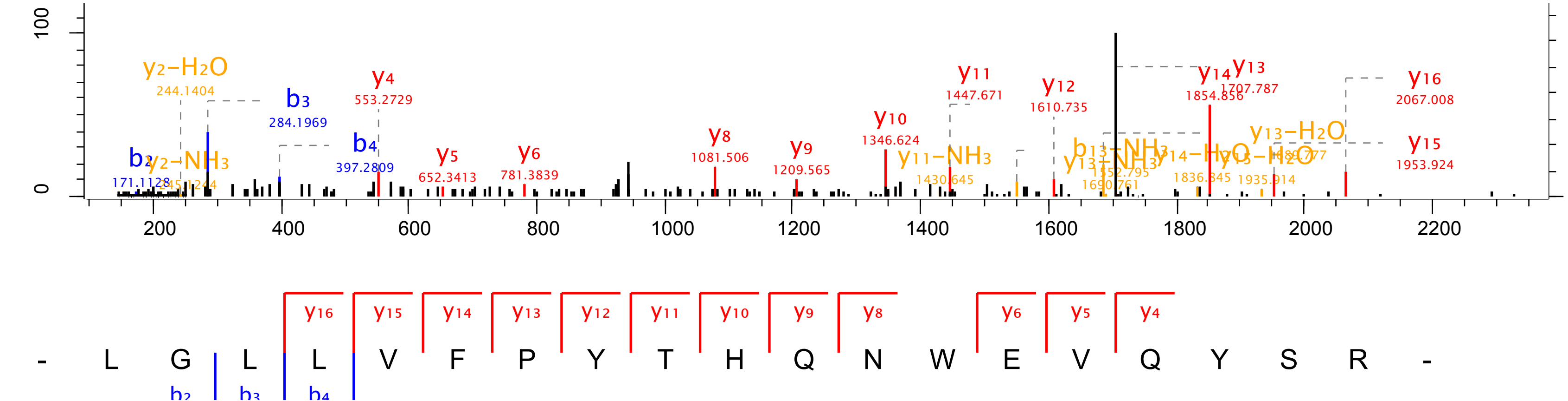

Raw file	Scan	Method	Score	m/z	Gene names
20150226_Hela_Top_opt_A3_01_1595	67379	TOF; CID	45.68	908.43	EPT1

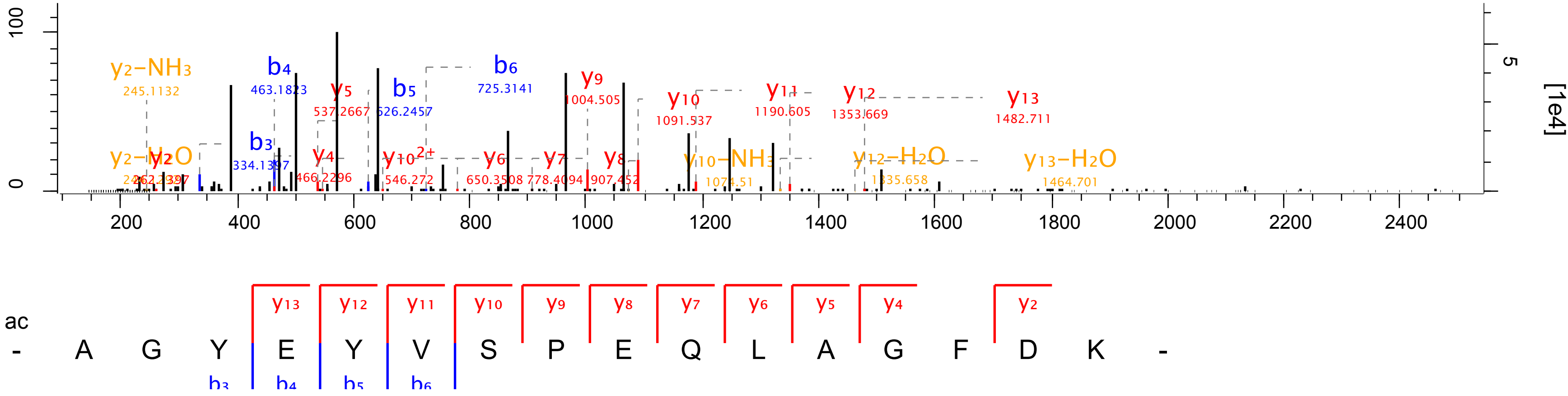

Raw file	Scan	Method	Score	m/z	Gene names
20150226_Hela_Top_opt_A3_01_1595	67839	TOF; CID	77.18	681.38	MRPS21

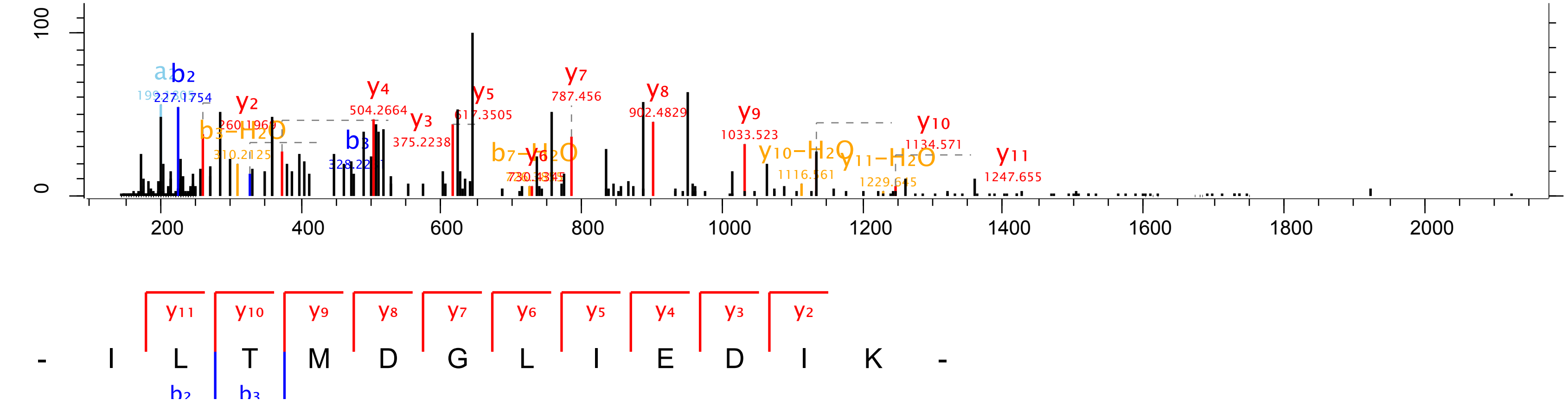

Raw file	Scan	Method	Score	m/z	Gene names
20150226_Hela_Top_opt_A3_01_1595	68700	TOF; CID	55.57	1093.57	GORASP1

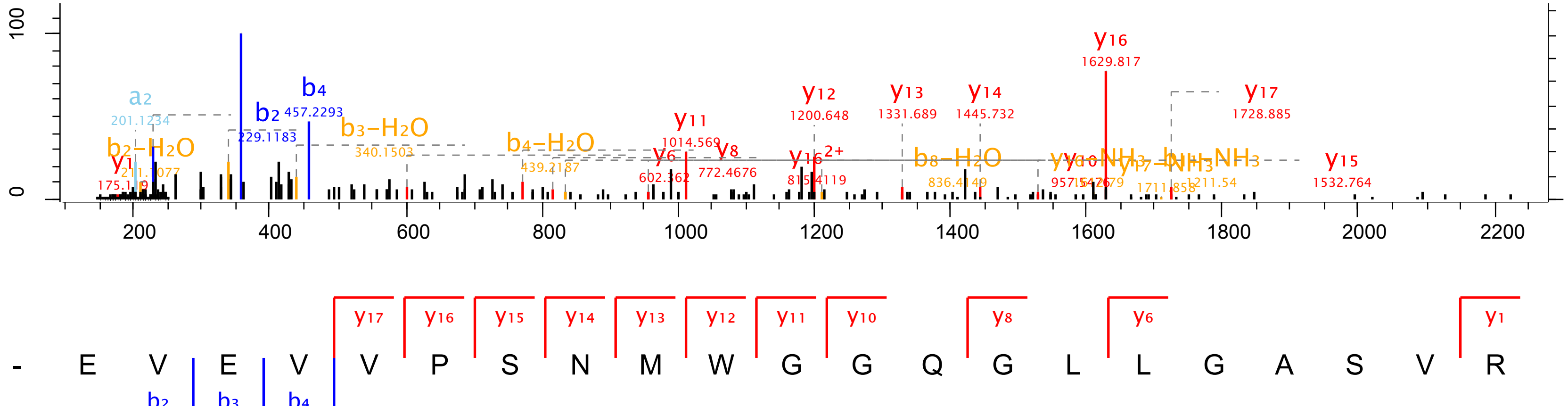

Raw file	Scan	Method	Score	m/z	Gene names
20150226_Hela_Top_opt_A3_01_1595	69445	TOF; CID	70.41	641.69	ASPHD1

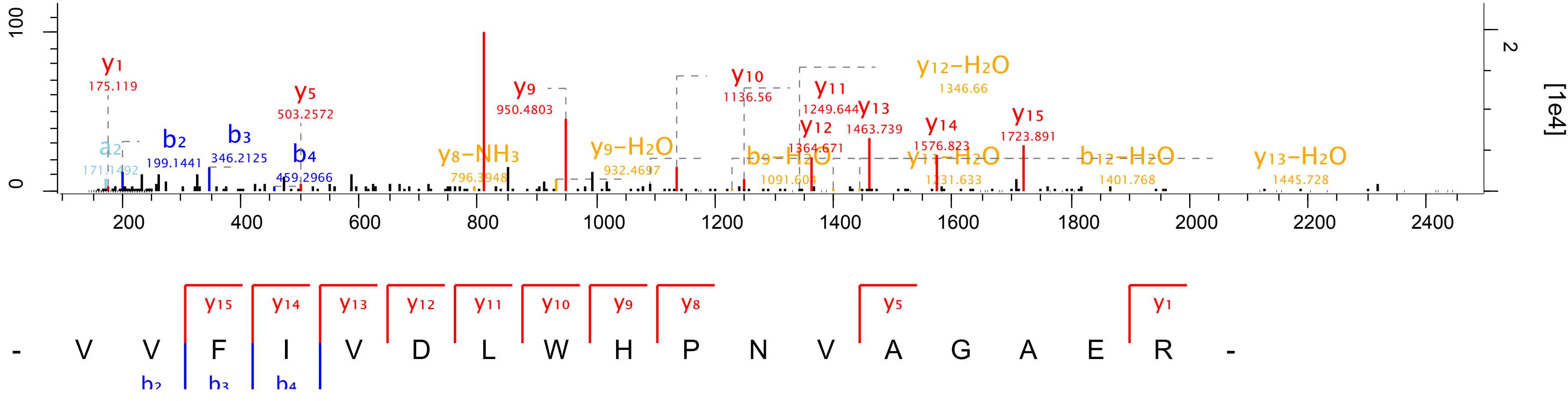

Raw file	Scan	Method	Score	m/z	Gene names
20150226_Hela_Top_opt_A3_01_1595	69598	TOF; CID	72.57	1033.56	C1orf131

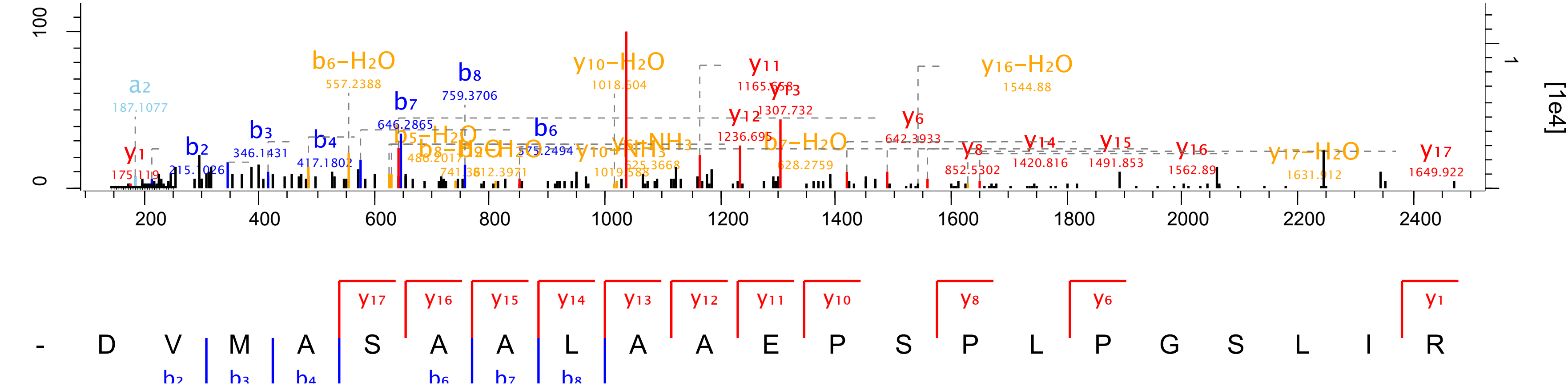

Raw file	Scan	Method	Score	m/z	Gene names
20150226_Hela_Top_opt_A3_01_1595	70383	TOF; CID	95.48	578.86	FAM213A

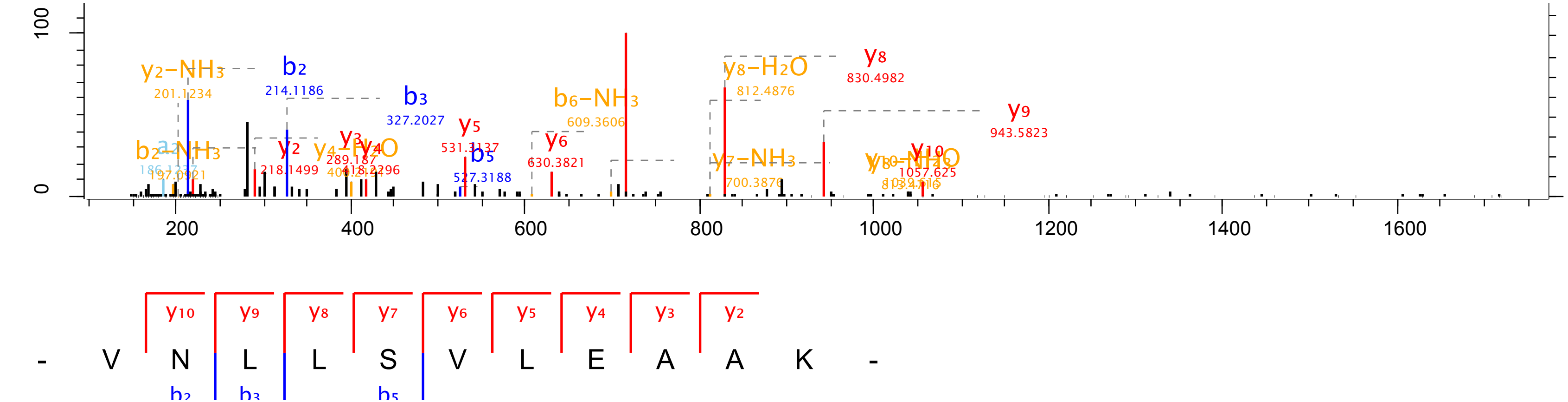

Raw file	Scan	Method	Score	m/z	Gene names
20150226_Hela_Top_opt_A3_01_1595	70654	TOF; CID	57.35	931.51	MED28

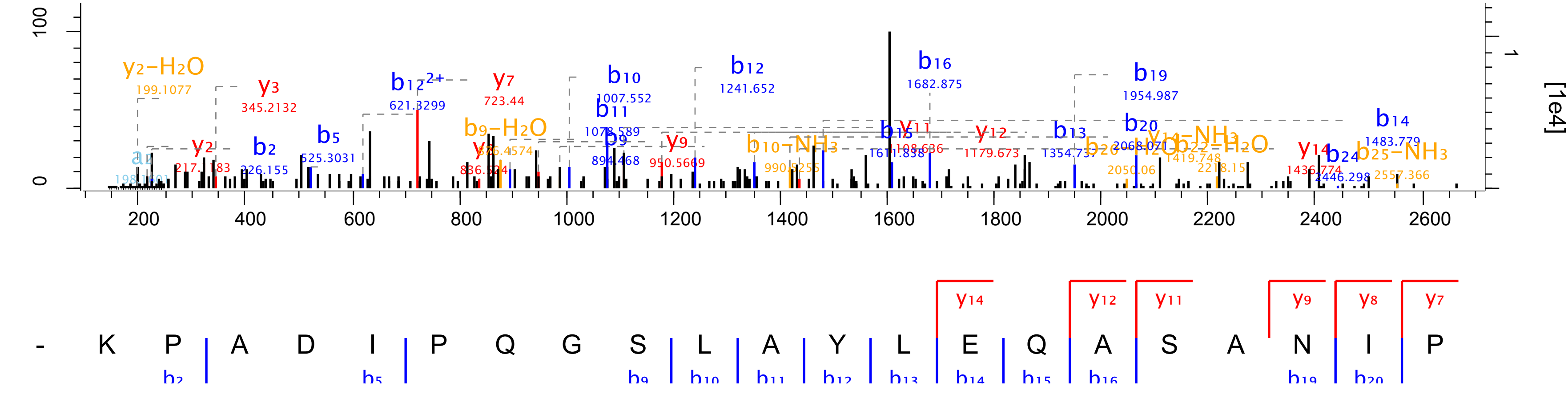

Raw file	Scan	Method	Score	m/z	Gene names
20150226_Hela_Top_opt_A3_01_1595	70890	TOF; CID	79.15	575.82	MED30

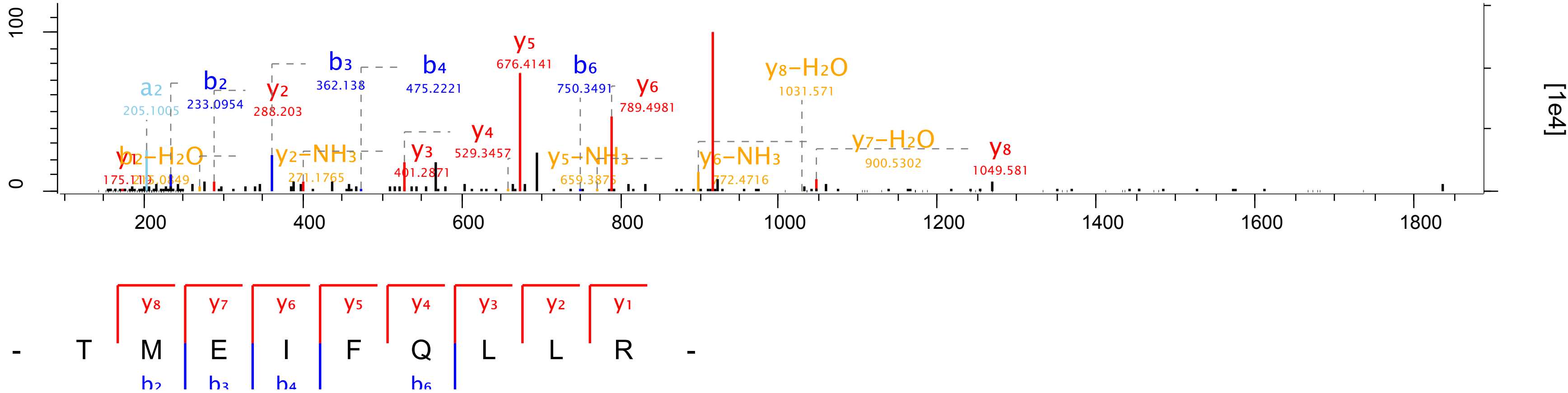

Raw file	Scan	Method	Score	m/z	Gene names
20150226_Hela_Top_opt_A3_01_1595	71037	TOF; CID	58.08	813.43	TXNIP

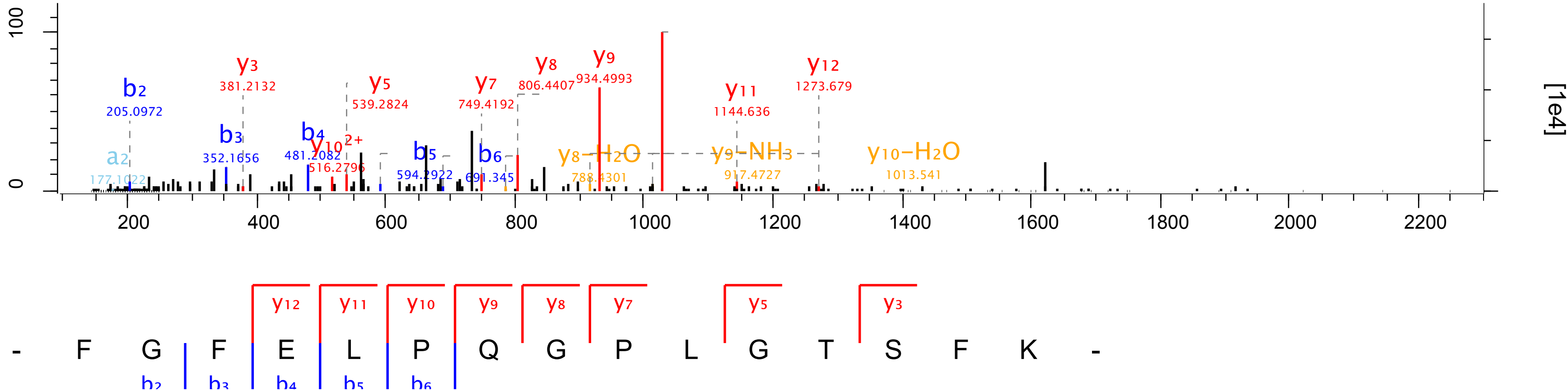

Raw file	Scan	Method	Score	m/z	Gene names
20150226_Hela_Top_opt_A3_01_1595	73127	TOF; CID	58.08	888.43	CRBN

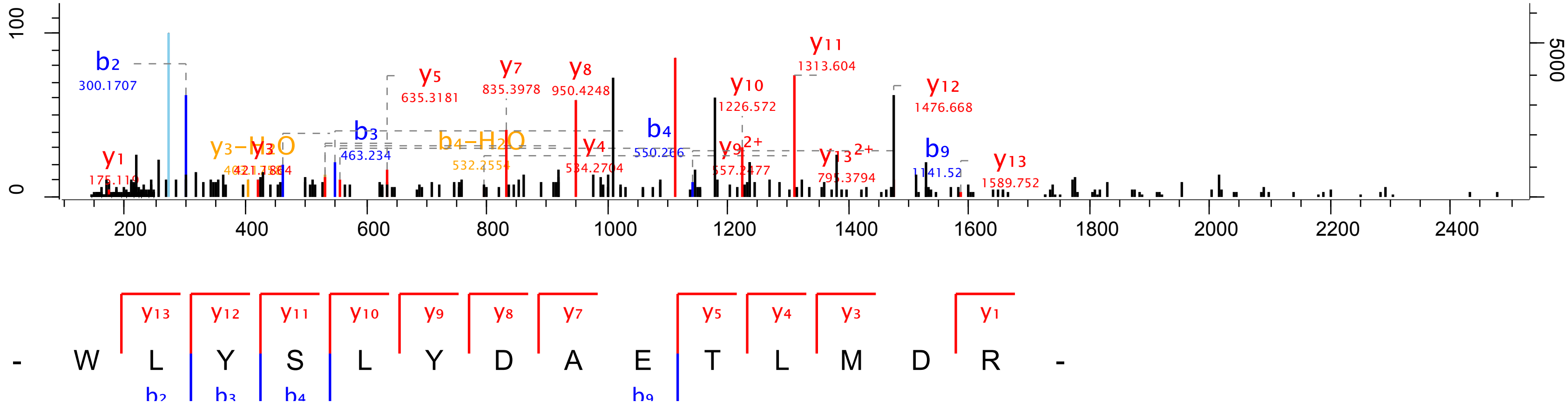

Raw file	Scan	Method	Score	m/z	Gene names
20150226_Hela_Top_opt_A3_01_1595	73230	TOF; CID	34.37	1051.25	TMEM184B

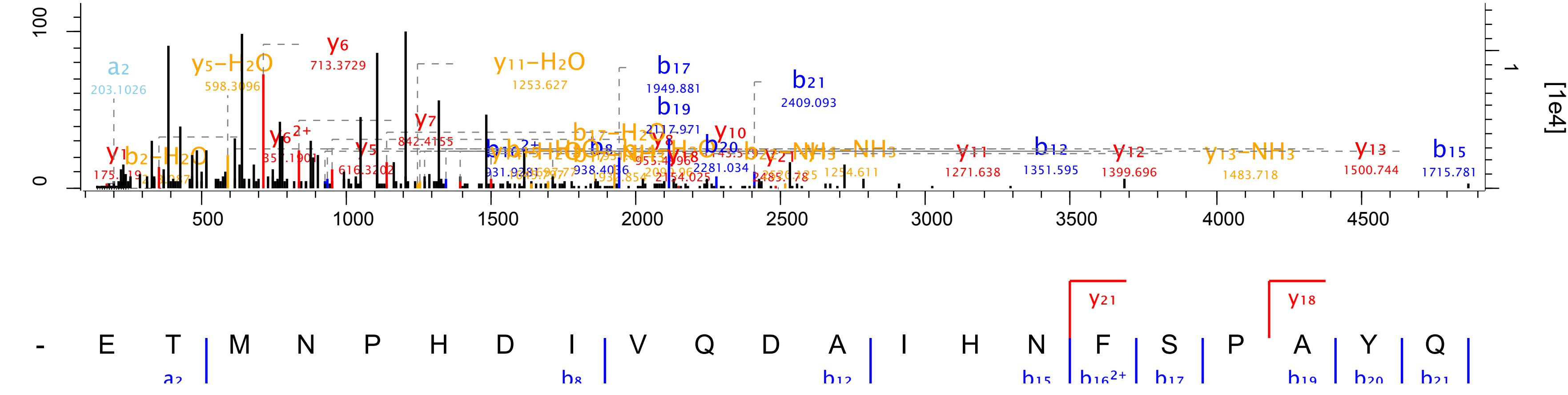

Raw file	Scan	Method	Score	m/z	Gene names
20150226_Hela_Top_opt_A3_01_1595	74196	TOF; CID	116.06	698.39	SCOC

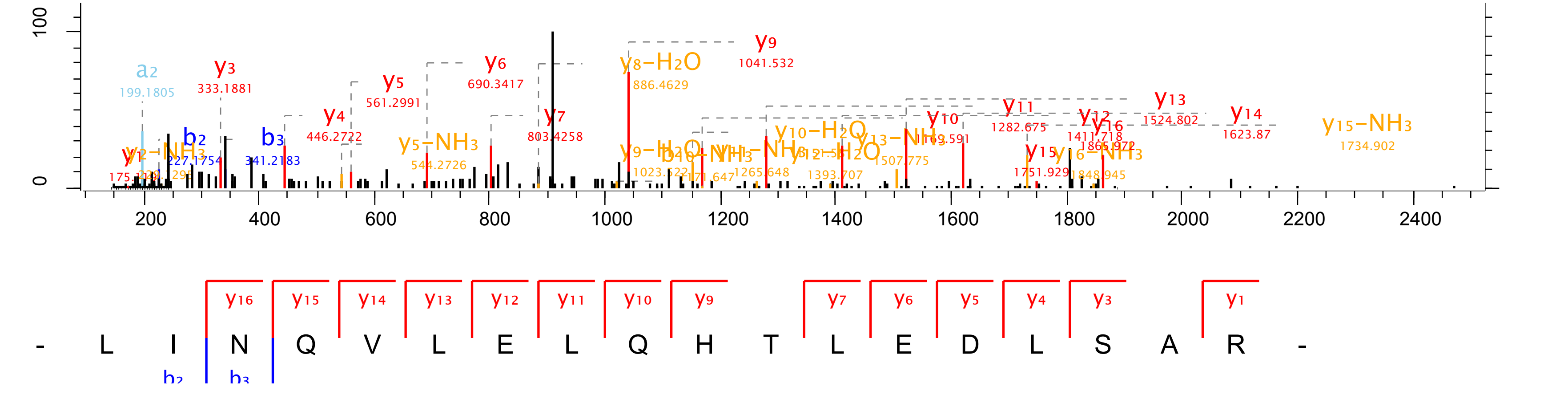

Raw file	Scan	Method	Score	m/z	Gene names
20150226_Hela_Top_opt_A3_01_1595	74593	TOF; CID	67.42	941.81	IL32

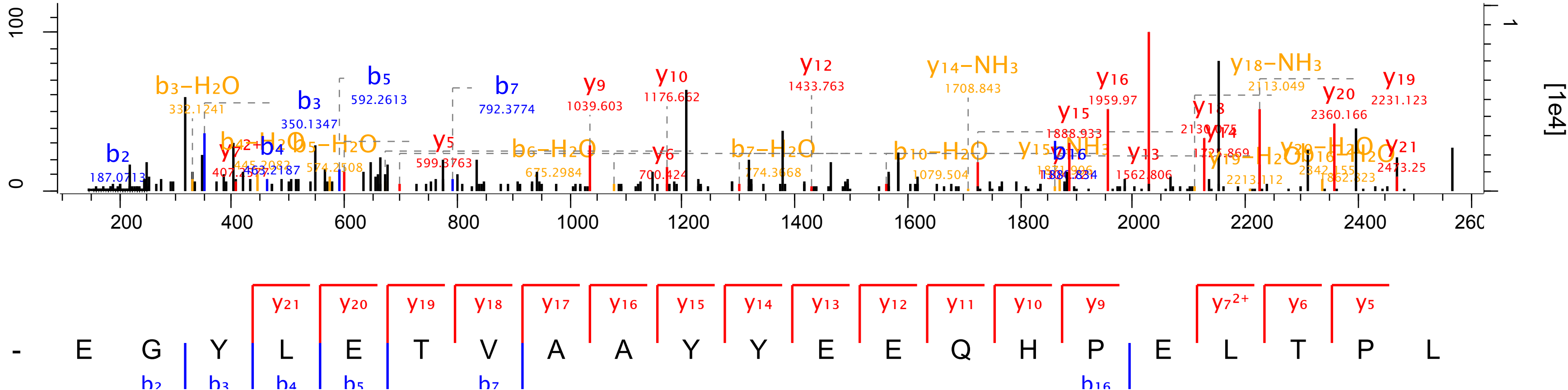

Raw file	Scan	Method	Score	m/z	Gene names
20150226_Hela_Top_opt_A3_01_1595	74639	TOF; CID	32.5	965.96	PYROXD1

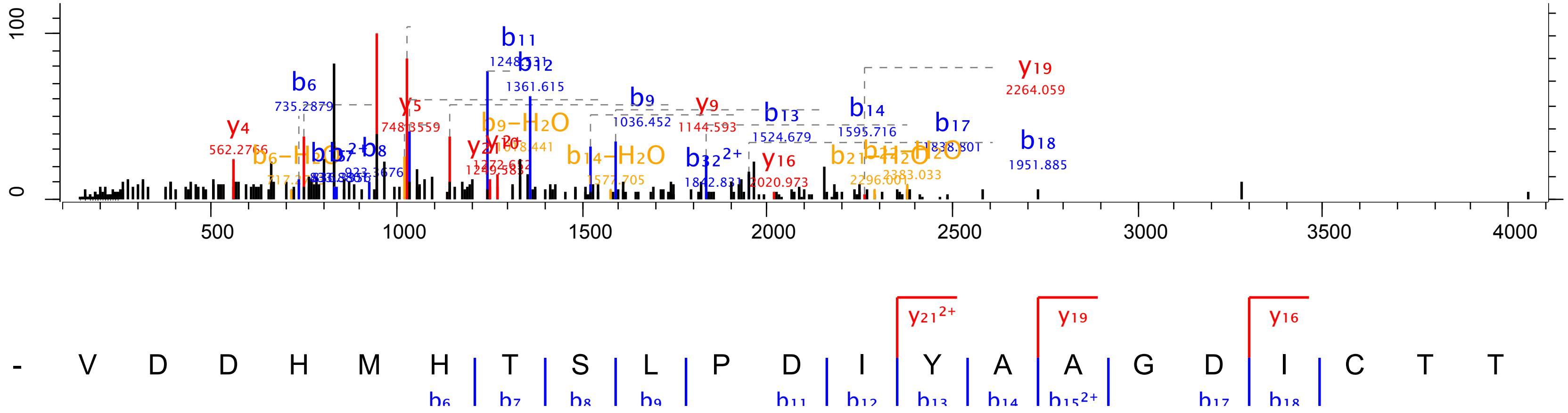

Raw file	Scan	Method	Score	m/z	Gene names
20150226_Hela_Top_opt_A3_01_1595	74974	TOF; CID	47.64	838.46	NSMAF

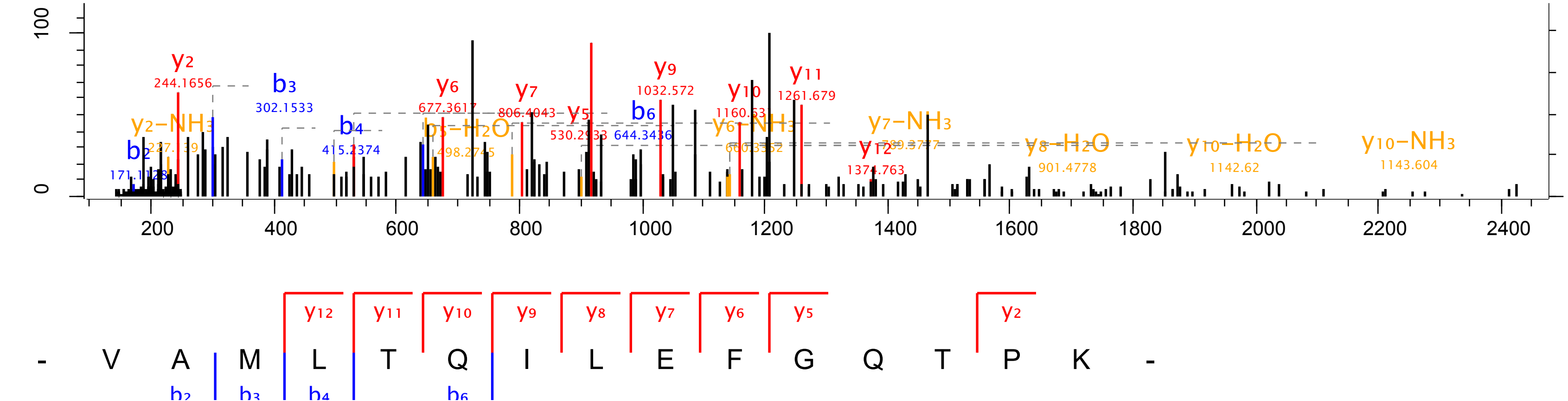

Raw file	Scan	Method	Score	m/z	Gene names
20150226_Hela_Top_opt_A3_01_1595	75070	TOF; CID	117.03	927.45	GMPR2

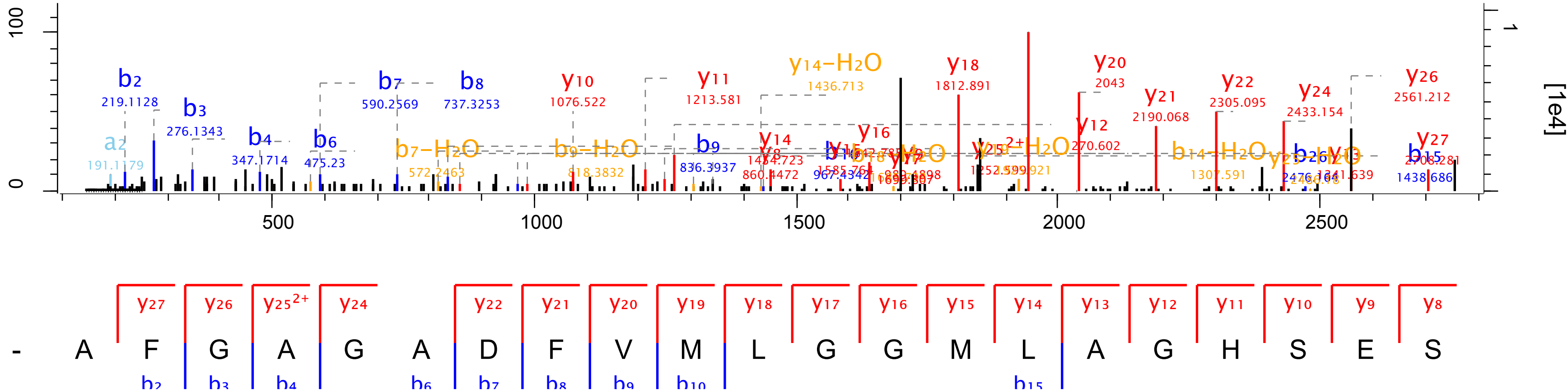

Raw file	Scan	Method	Score	m/z	Gene names
20150226_Hela_Top_opt_A3_01_1595	75226	TOF; CID	86.5	870.54	RHBDD2;WUGSC:H_RG122E10.2a

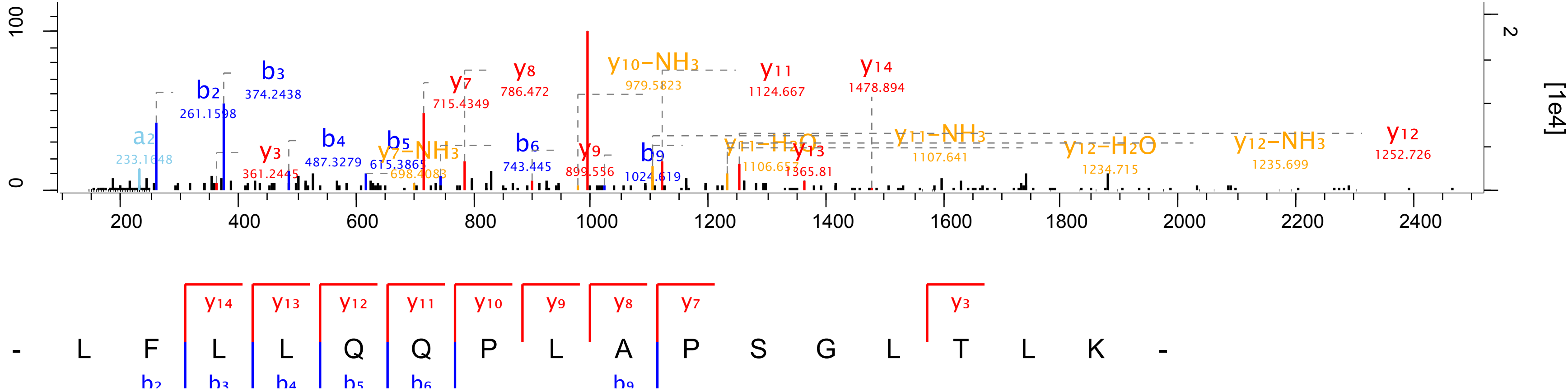

Raw file	Scan	Method	Score	m/z	Gene names
20150226_Hela_Top_opt_A3_01_1595	75228	TOF; CID	81.84	1142.21	LMBRD2

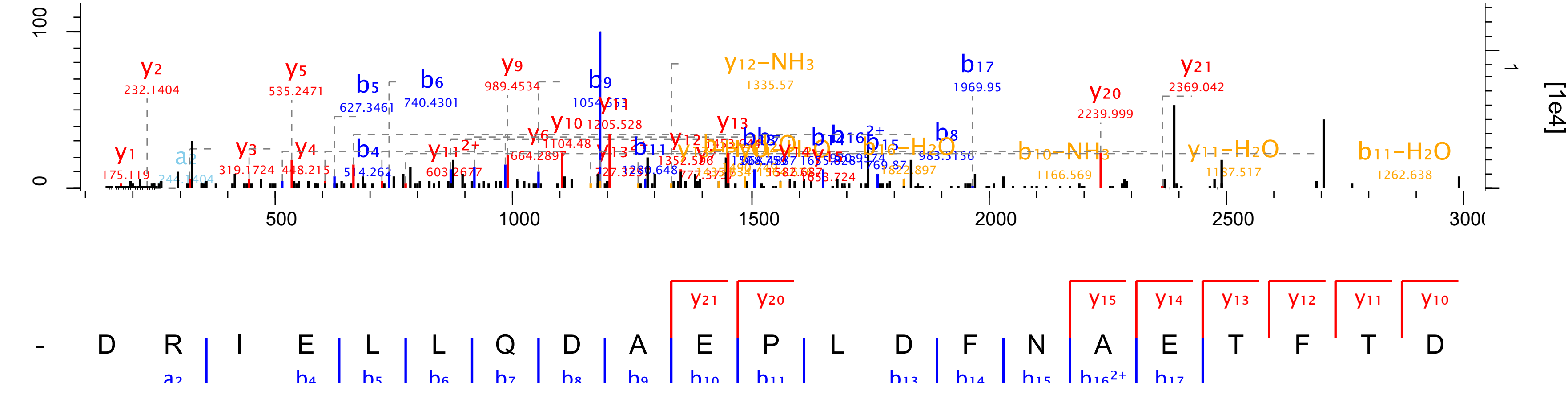

Raw file	Scan	Method	Score	m/z	Gene names
20150226_Hela_Top_opt_A3_01_1595	75607	TOF; CID	53.26	1012.99	NAA38

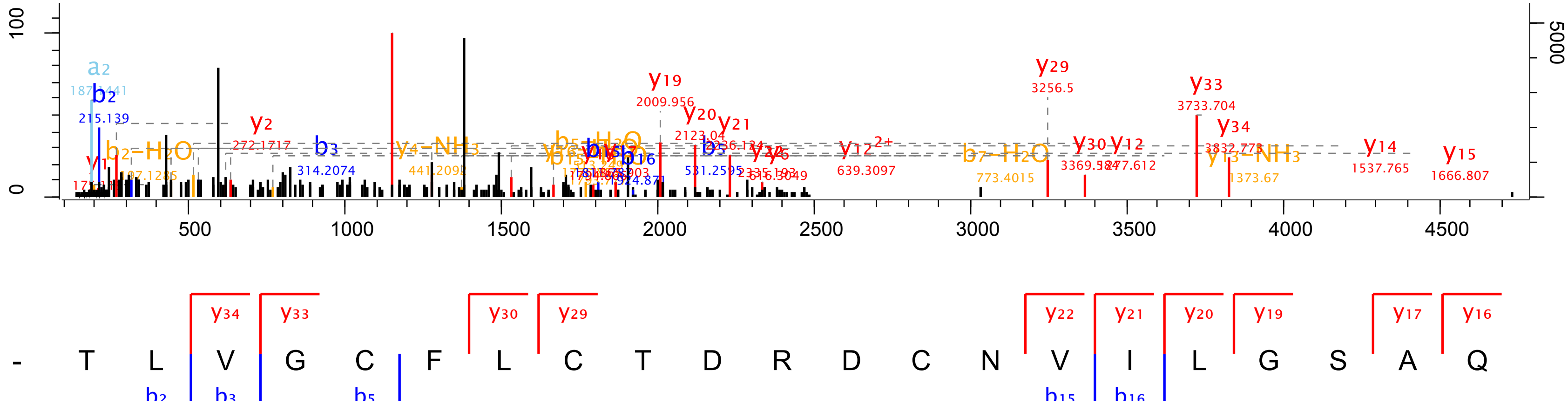

Raw file	Scan	Method	Score	m/z	Gene names
20150226_Hela_Top_opt_A3_01_1595	75625	TOF; CID	52.52	974.5	ZC3H8

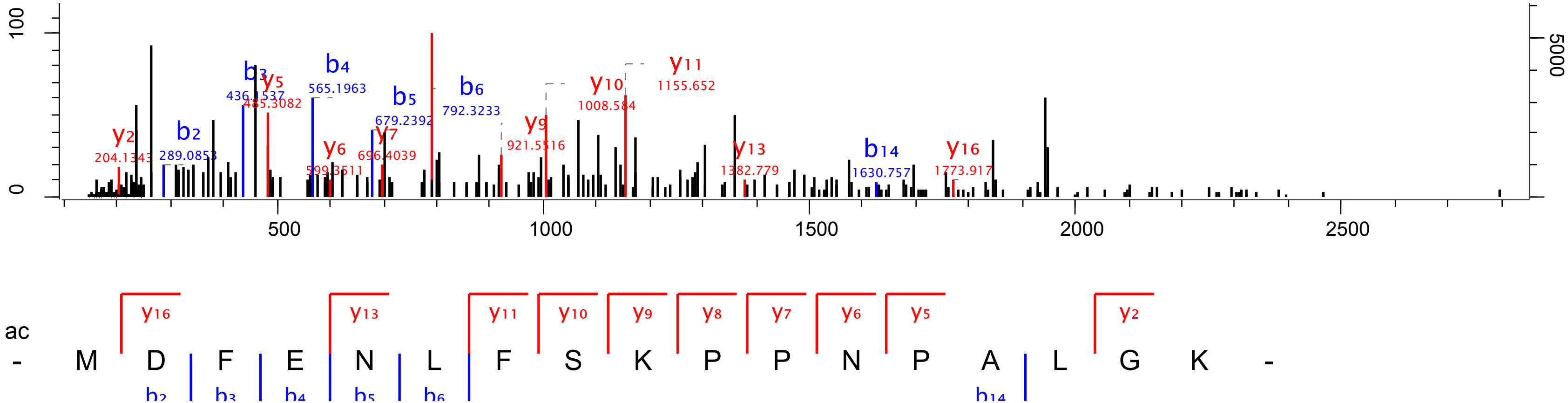

Raw file	Scan	Method	Score	m/z	Gene names
20150226_Hela_Top_opt_A3_01_1595	75810	TOF; CID	53.45	747.41	FMNL2;FMNL3

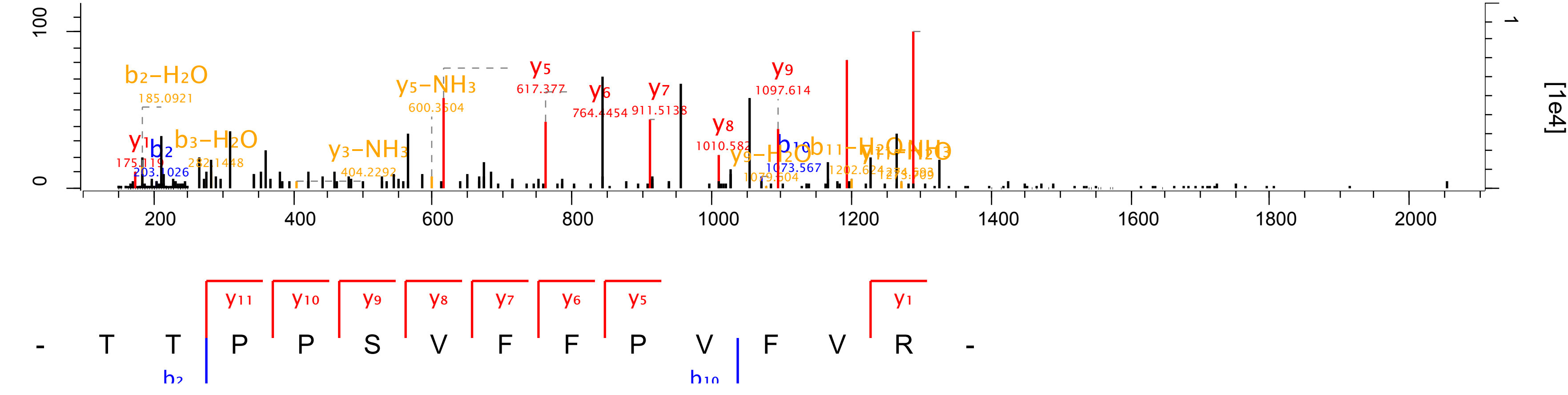

Raw file	Scan	Method	Score	m/z	Gene names
20150226_Hela_Top_opt_A3_01_1595	76337	TOF; CID	94.57	944.98	MRPL51

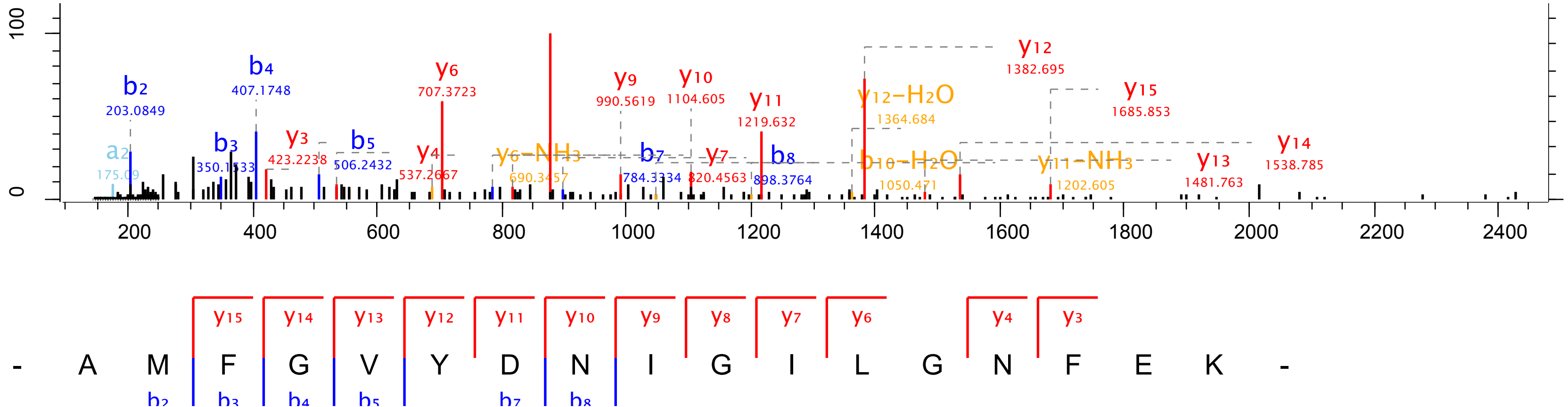

Raw file	Scan	Method	Score	m/z	Gene names
20150226_Hela_Top_opt_A3_01_1595	76448	TOF; CID	59.71	844.4	WDR45B

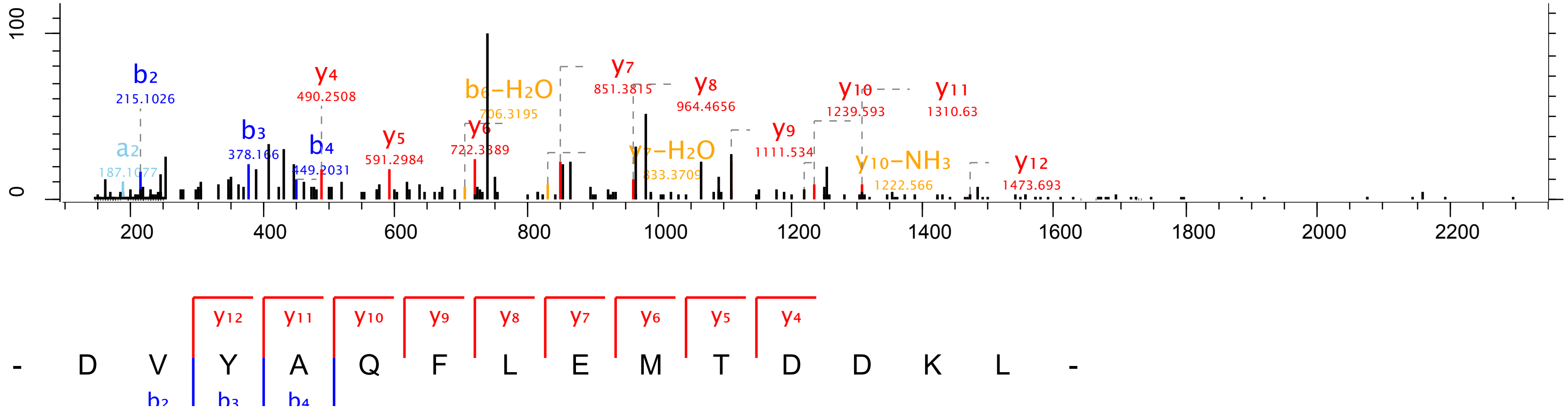

Raw file	Scan	Method	Score	m/z	Gene names
20150226_Hela_Top_opt_A3_01_1595	76681	TOF; CID	60.13	814.42	CENPK

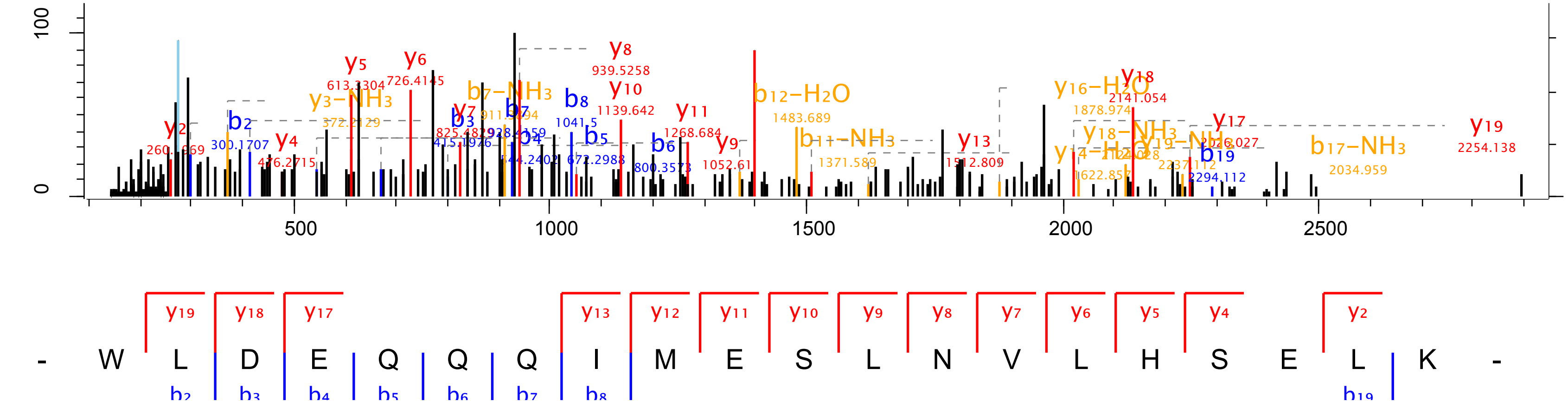

Raw file	Scan	Method	Score	m/z	Gene names
20150226_Hela_Top_opt_A3_01_1595	76735	TOF; CID	96.25	839.91	IMUP

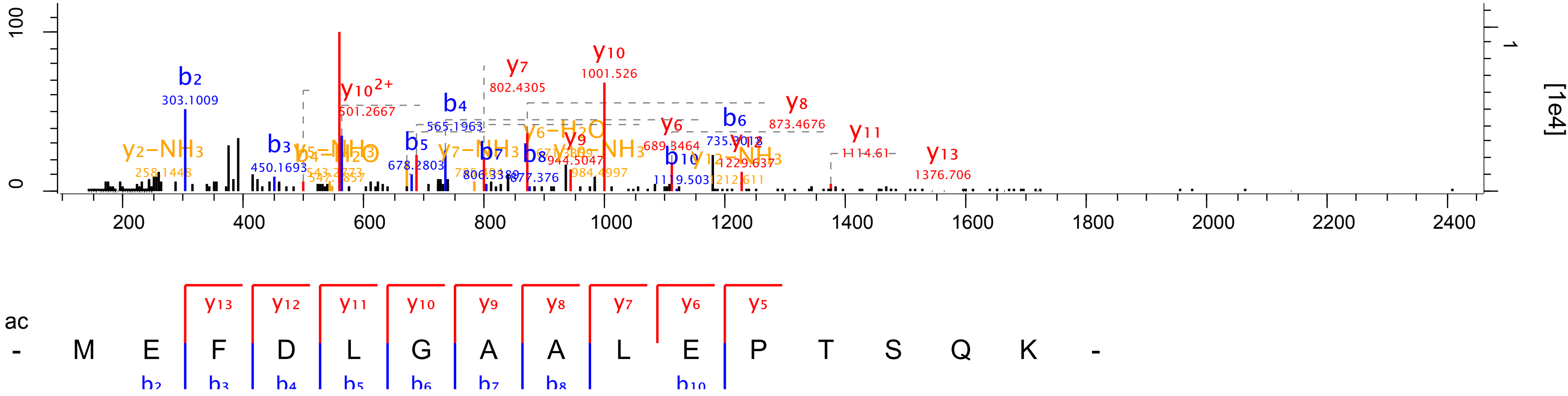

Raw file	Scan	Method	Score	m/z	Gene names
20150226_Hela_Top_opt_A3_01_1595	76834	TOF; CID	108.35	636.38	CRADD

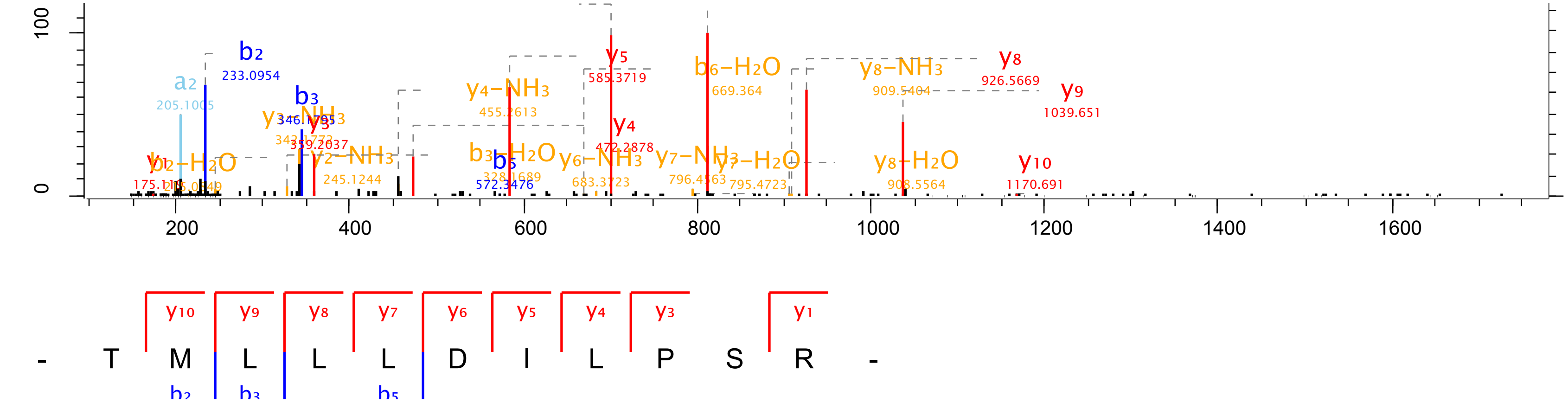

Raw file	Scan	Method	Score	m/z	Gene names
20150226_Hela_Top_opt_A3_01_1595	77016	TOF; CID	118.31	620.02	GIPC2

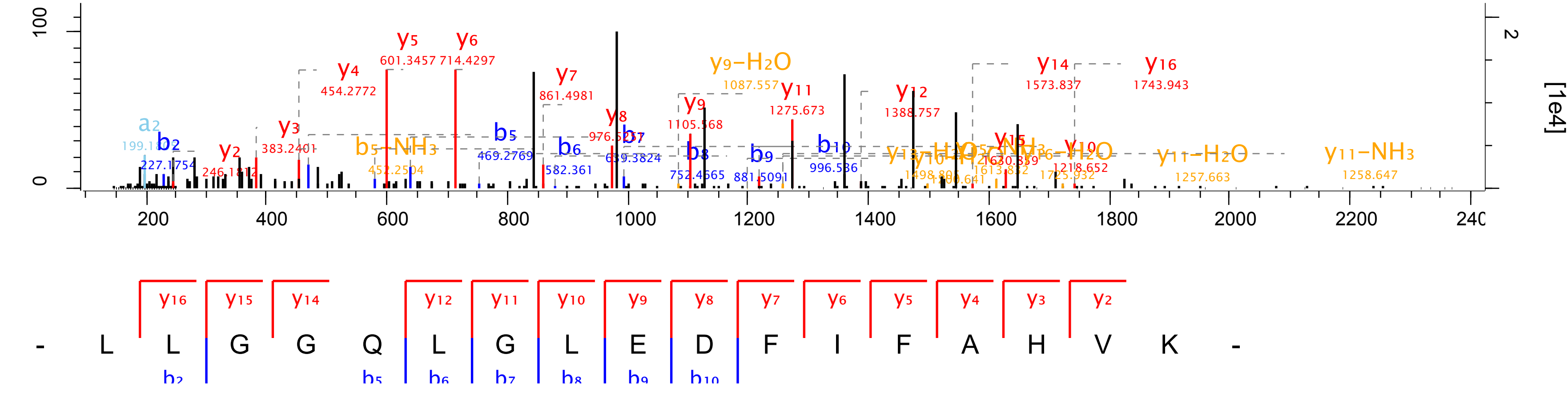

Raw file	Scan	Method	Score	m/z	Gene names
20150226_Hela_Top_opt_A3_01_1595	77475	TOF; CID	59.91	979.85	TBP;TBPL2

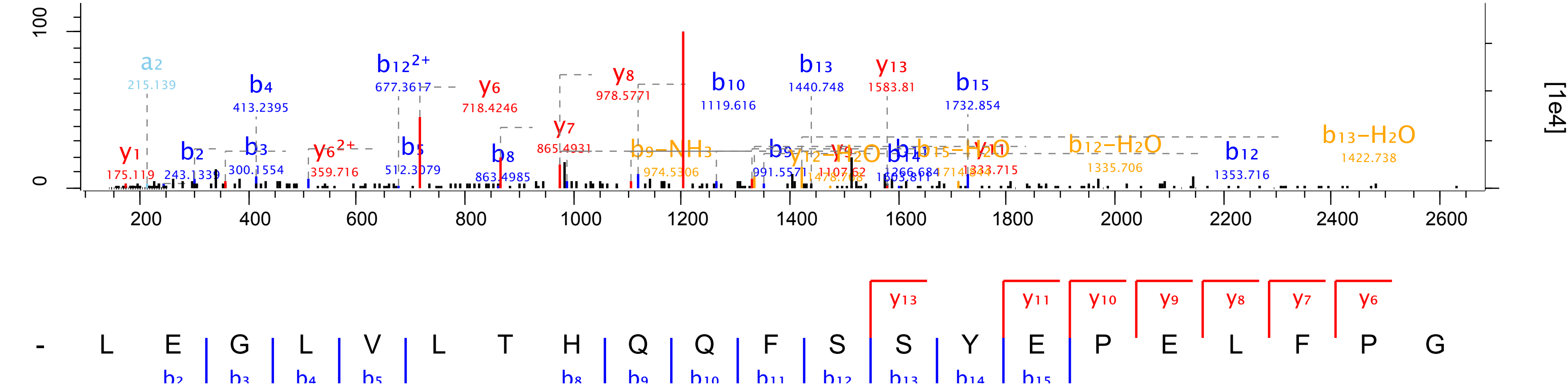

Raw file	Scan	Method	Score	m/z	Gene names
20150226_Hela_Top_opt_A3_01_1595	78115	TOF; CID	69.67	613.83	DOLK

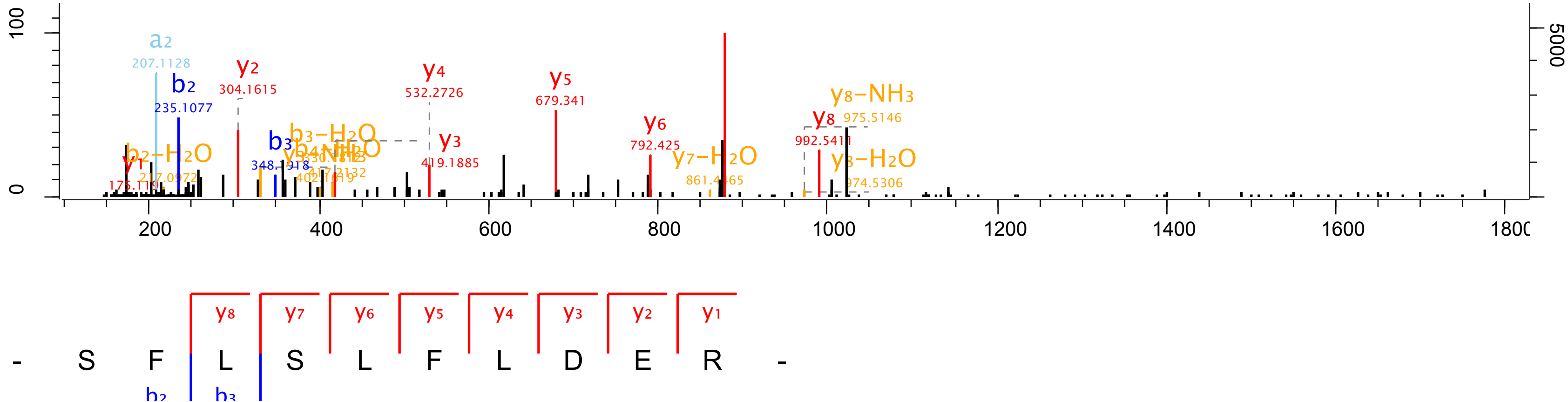

Raw file	Scan	Method	Score	m/z	Gene names
20150226_Hela_Top_opt_A3_01_1595	78257	TOF; CID	89.19	678.86	C8orf82

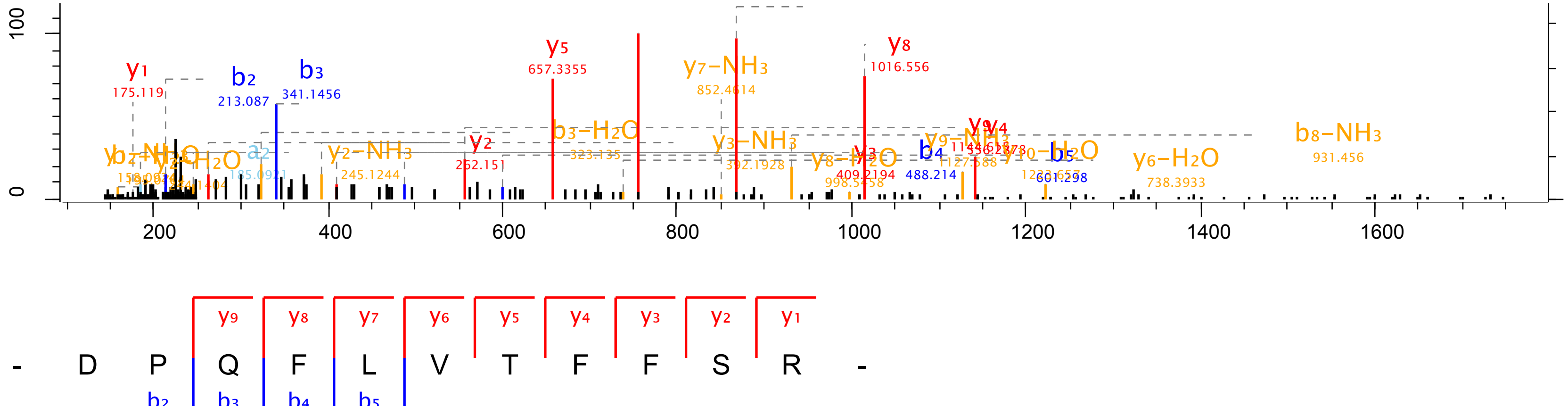

Raw file	Scan	Method	Score	m/z	Gene names
20150226_Hela_Top_opt_A3_01_1595	78341	TOF; CID	36.89	1131.58	OGFOD2

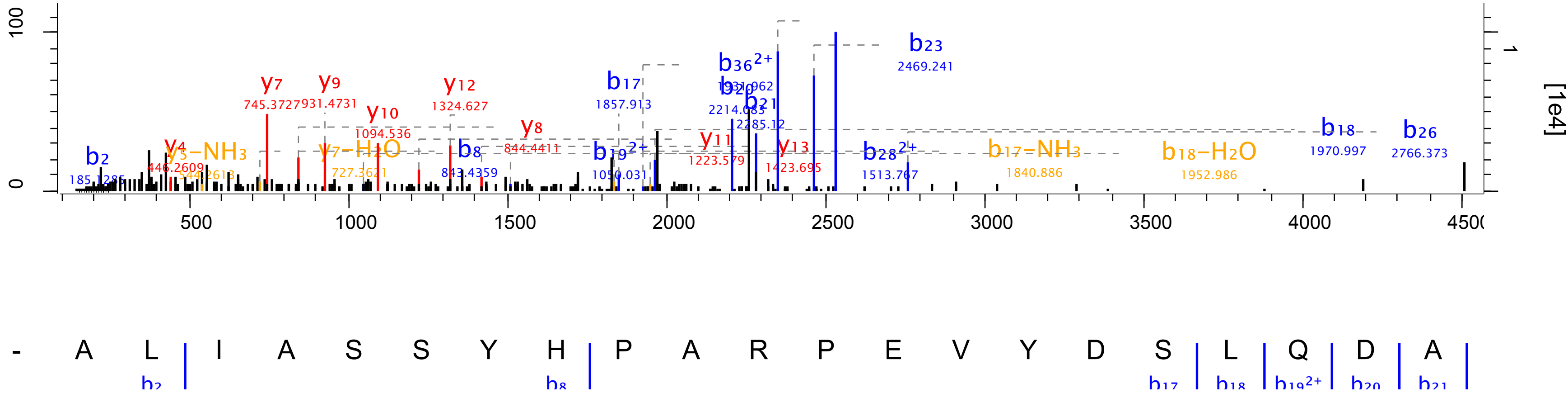

Raw file	Scan	Method	Score	m/z	Gene names
20150226_Hela_Top_opt_A3_01_1595	81542	TOF; CID	85.18	1132.61	SSR2

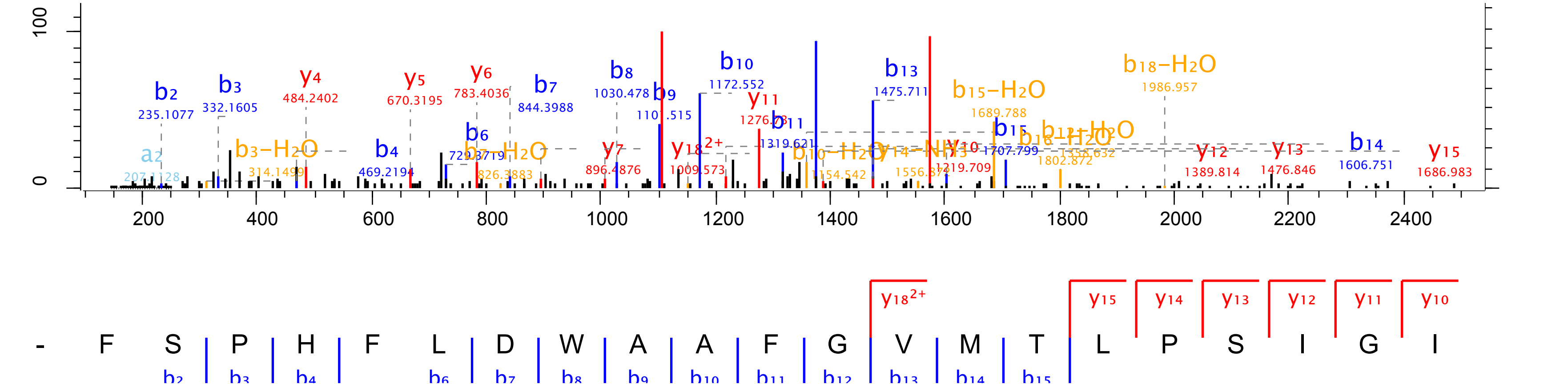

Raw file	Scan	Method	Score	m/z	Gene names
20150226_Hela_Top_opt_A3_01_1595	81677	TOF; CID	67.5	979.51	FANCG

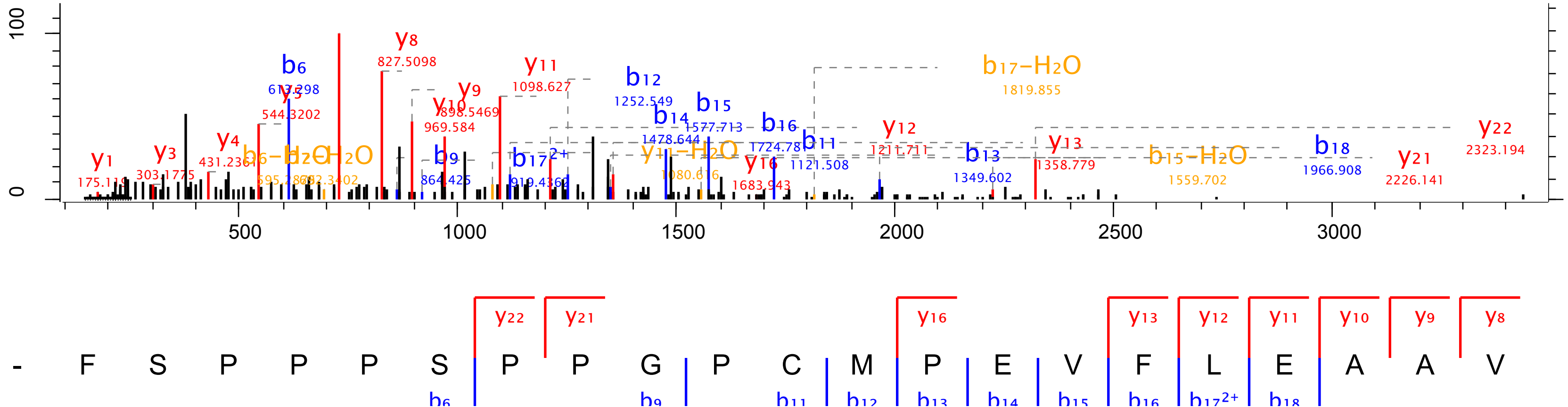

Raw file	Scan	Method	Score	m/z	Gene names
20150226_Hela_Top_opt_A3_01_1595	81909	TOF; CID	79.65	841.43	SENP8

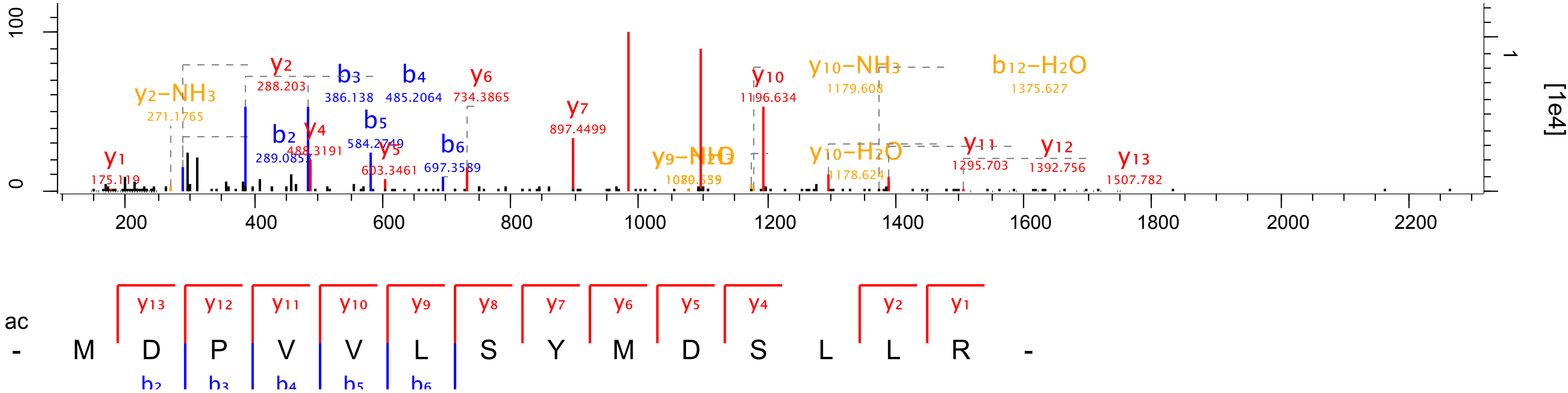

Supplement: Supplemental Data [file supp_M114.047407_mcp.M114.047407-5.pdf]
