# Supplementary material for: The Impact II, a Very High-Resolution Quadrupole Time-of-Flight Instrument (QTOF) for Deep Shotgun Proteomics
Source: Mol Cell Proteomics. 2015 May 19;14(7):2014–29. doi: 10.1074/mcp.M114.047407 (PMC4587313; doi:10.1074/mcp.M114.047407)

Raw file	Scan	Method	Score	m/z	Gene names
20150227_yeast_Top_opt_B1_01_1599	4175	TOF; CID	62.55	379.71	RPS26A

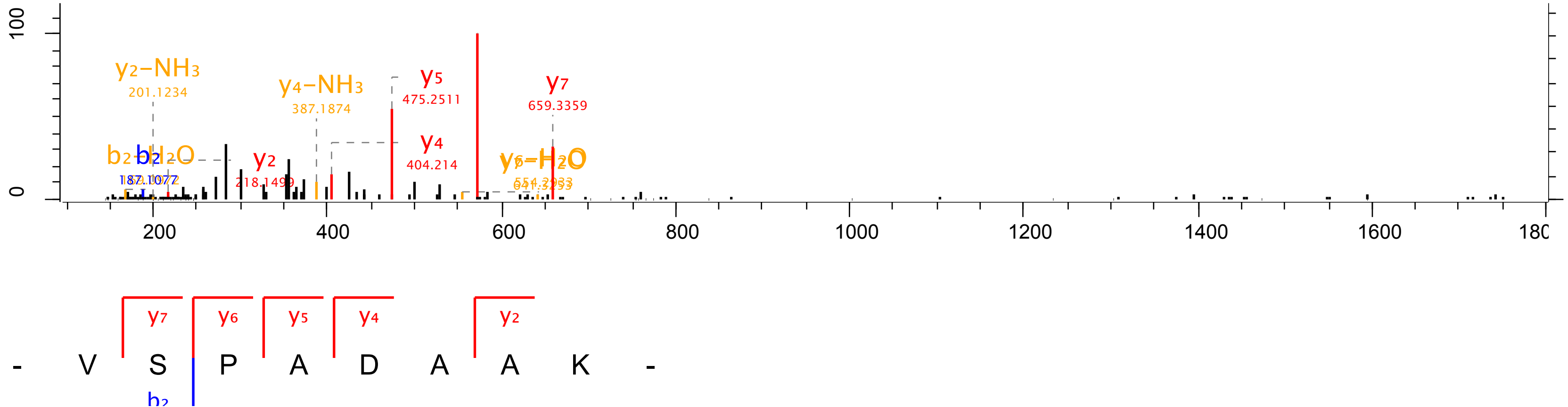

Raw file	Scan	Method	Score	m/z	Gene names
20150227_yeast_Top_opt_B1_01_1599	4839	TOF; CID	47.71	526.25	BTT1

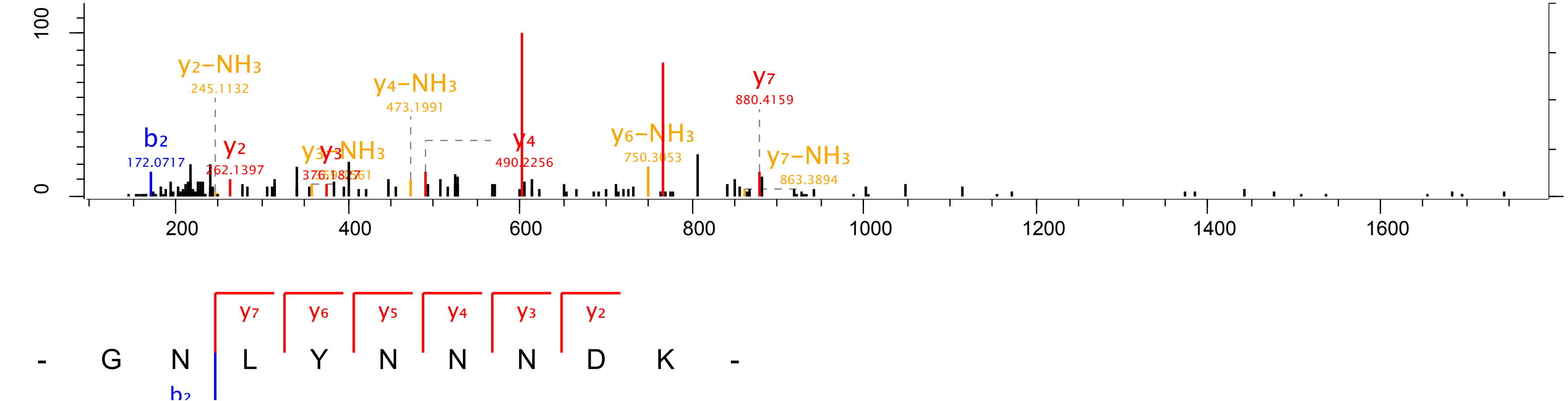

Raw file	Scan	Method	Score	m/z	Gene names
20150227_yeast_Top_opt_B1_01_1599	5971	TOF; CID	86.9	633.29	PEX3

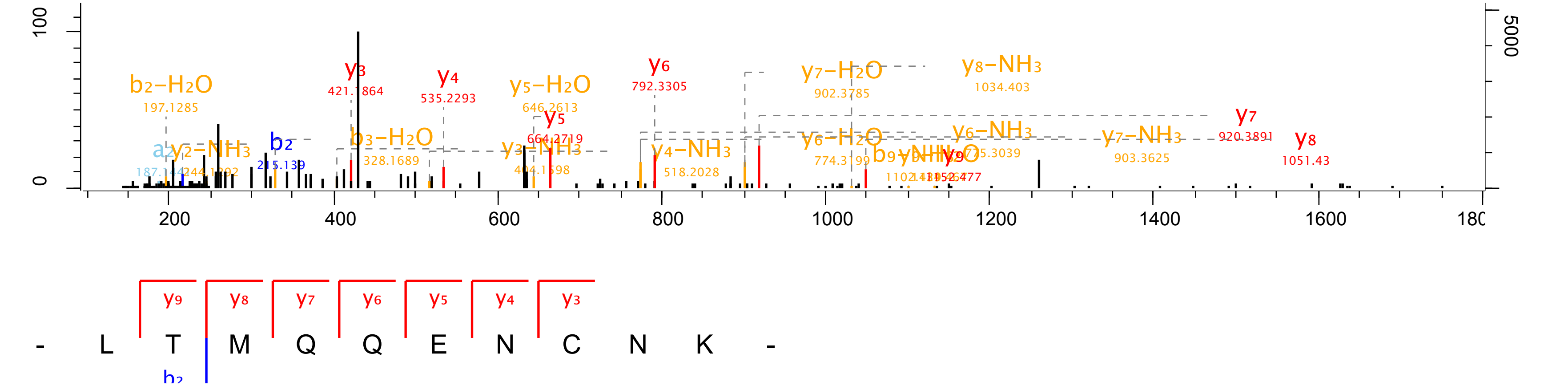

Raw file	Scan	Method	Score	m/z
20150227_yeast_Top_opt_B1_01_1599	6212	TOF; CID	81.02	598.27

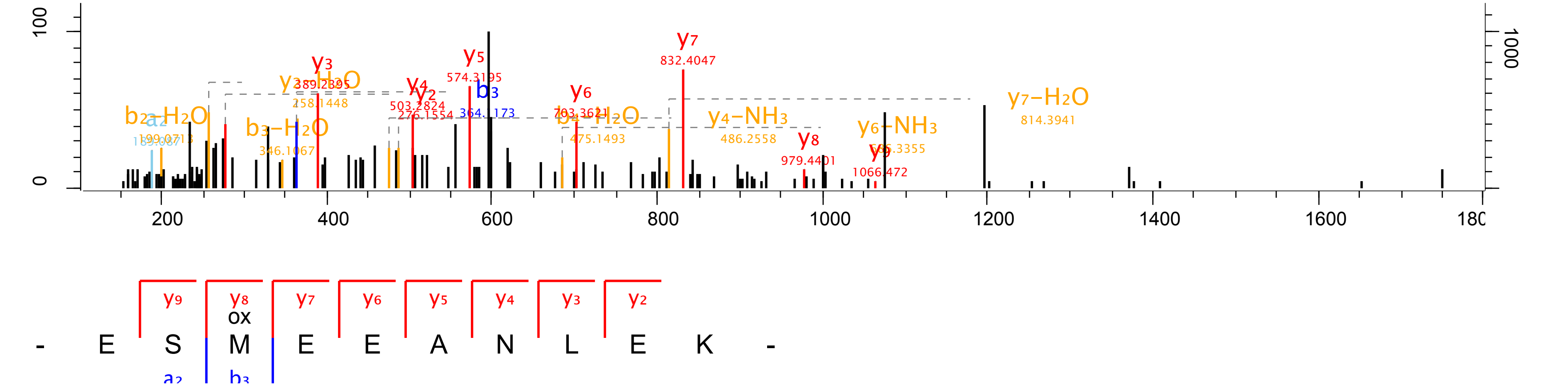

Raw file	Scan	Method	Score	m/z	Gene names
20150227_yeast_Top_opt_B1_01_1599	7040	TOF; CID	54.16	493.77	PDH1

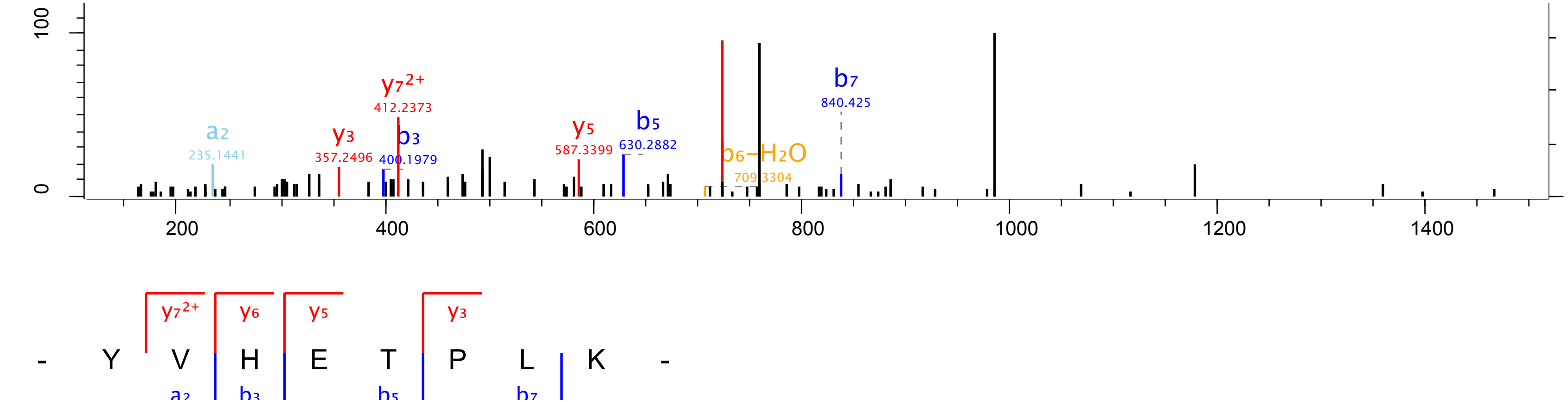

Raw file	Scan	Method	Score	m/z	Gene names
20150227_yeast_Top_opt_B1_01_1599	7436	TOF; CID	51.45	415.23	AMN1

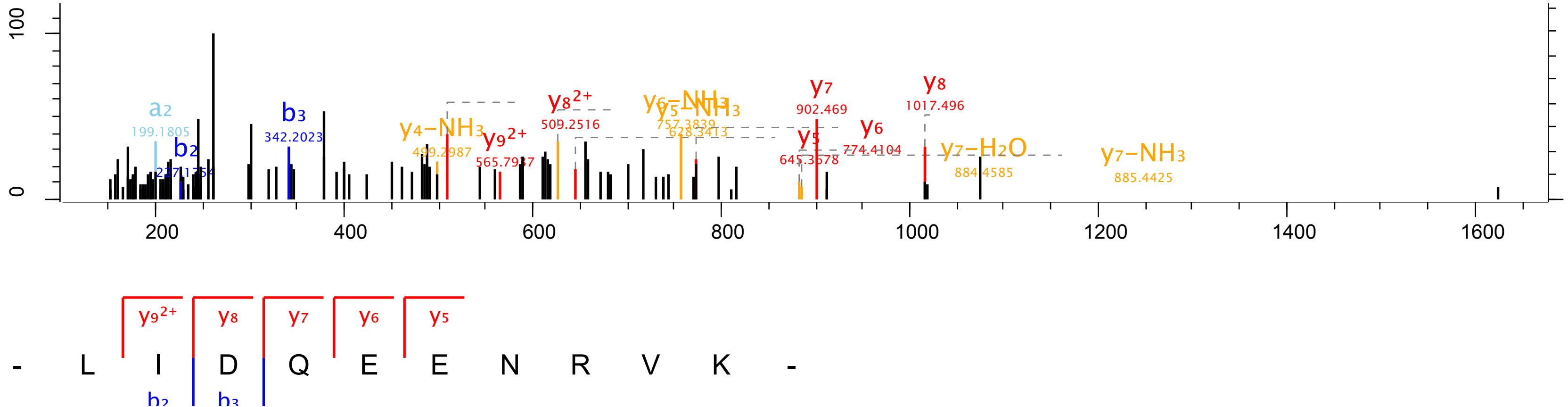

Raw file	Scan	Method	Score	m/z	Gene names
20150227_yeast_Top_opt_B1_01_1599	7956	TOF; CID	61.34	506.26	GAT1

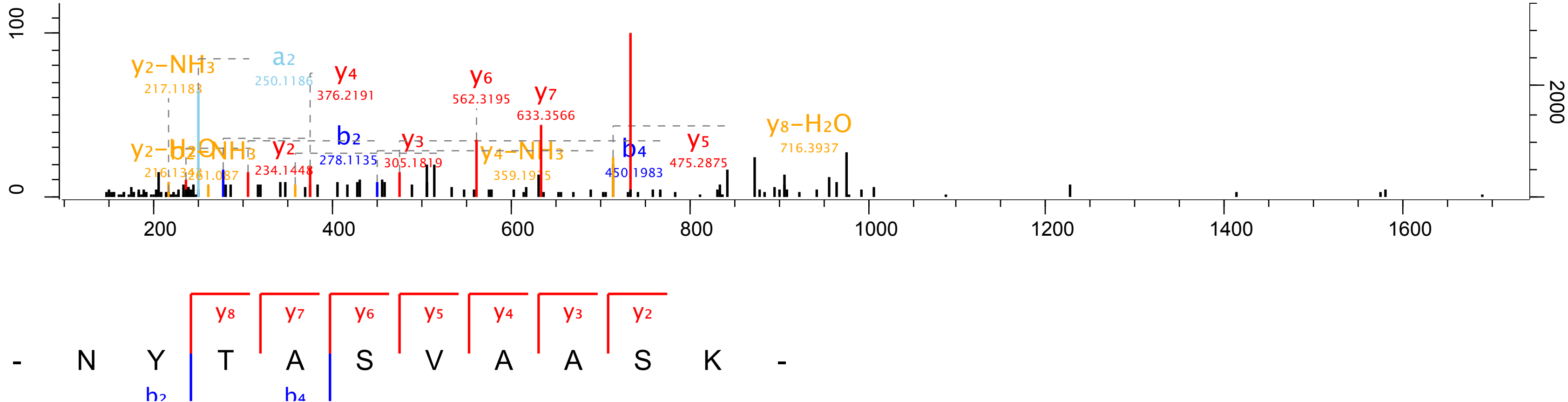

Raw file	Scan	Method	Score	m/z	Gene names
20150227_yeast_Top_opt_B1_01_1599	8184	TOF; CID	64.78	409.25	TEP1

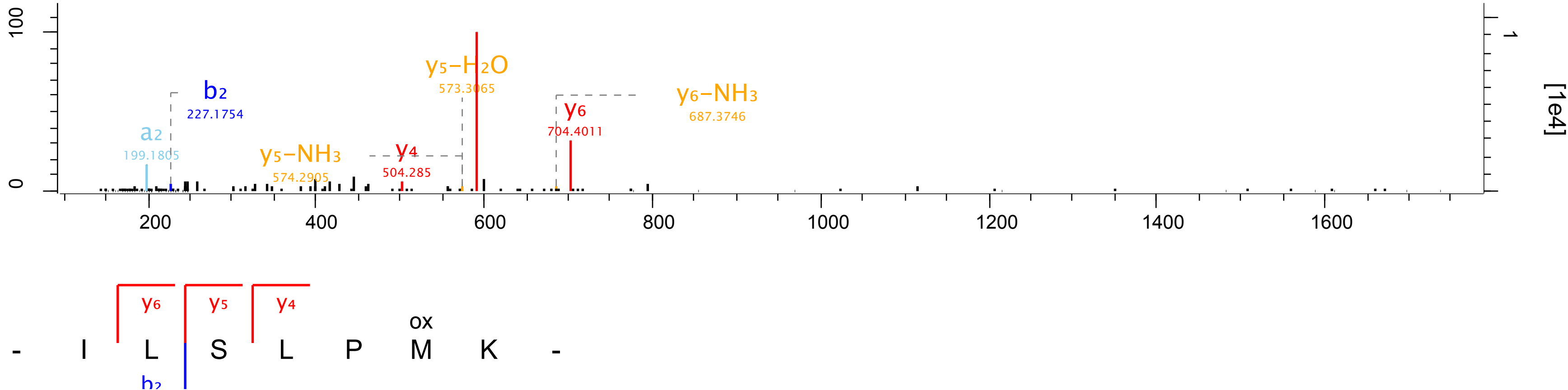

Raw file	Scan	Method	Score	m/z	Gene names
20150227_yeast_Top_opt_B1_01_1599	8698	TOF; CID	137.8	787.37	MHR1

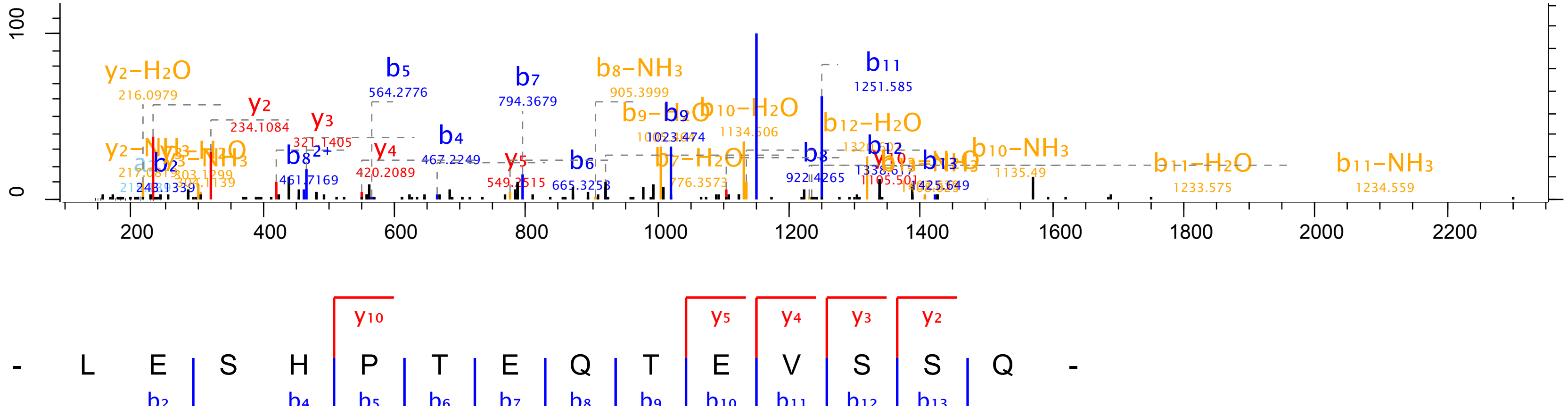

Raw file	Scan	Method	Score	m/z	Gene names
20150227_yeast_Top_opt_B1_01_1599	9114	TOF; CID	74.84	552.79	SBH1

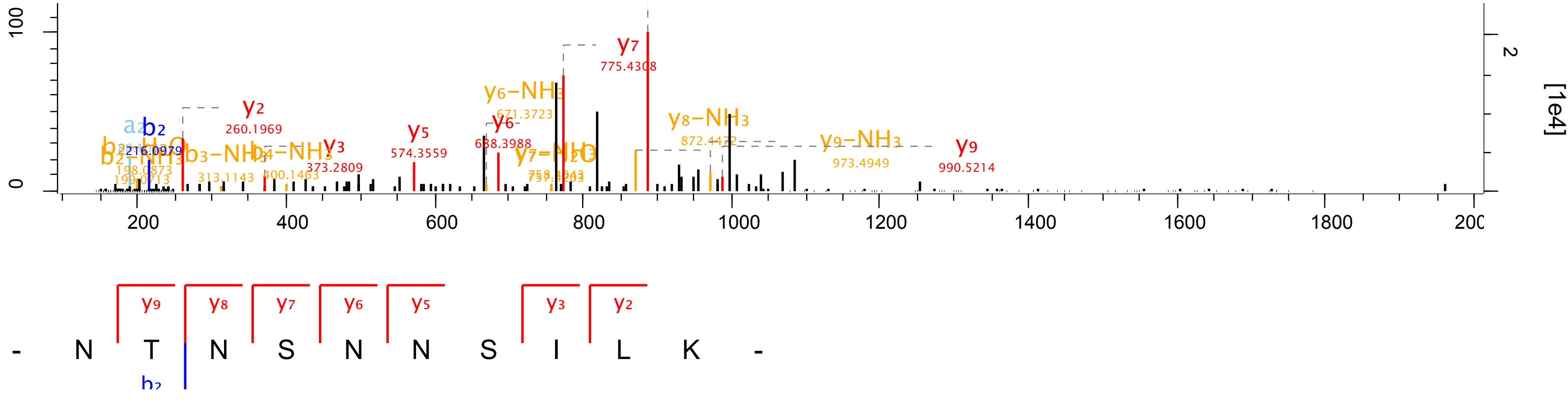

Raw file	Scan	Method	Score	m/z	Gene names
20150227_yeast_Top_opt_B1_01_1599	9775	TOF; CID	50.04	522.32	PES4

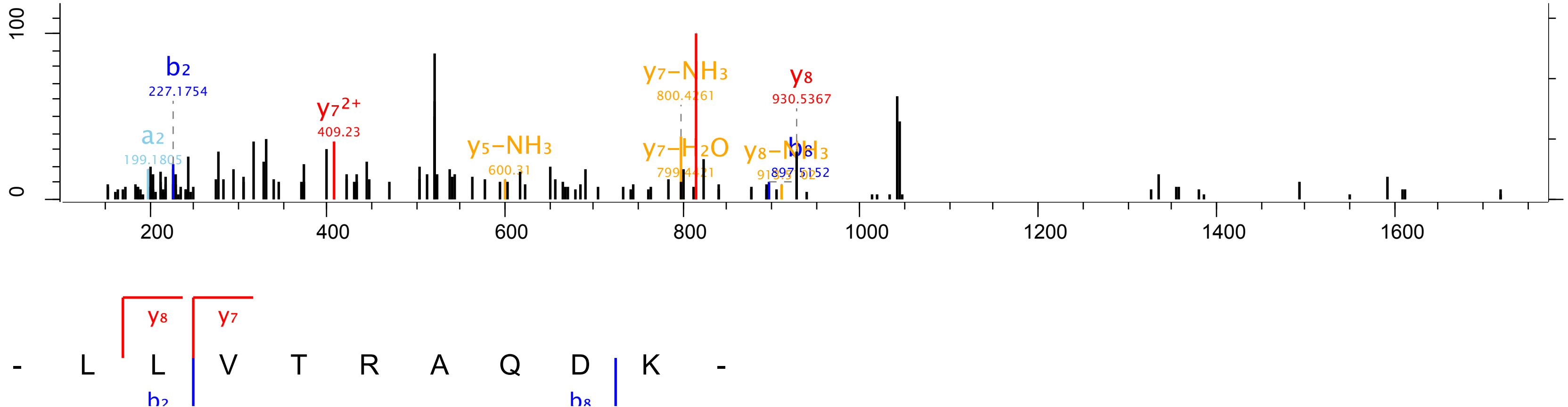

Raw file	Scan	Method	Score	m/z	Gene names
20150227_yeast_Top_opt_B1_01_1599	10171	TOF; CID	82.59	616.32	PSH1

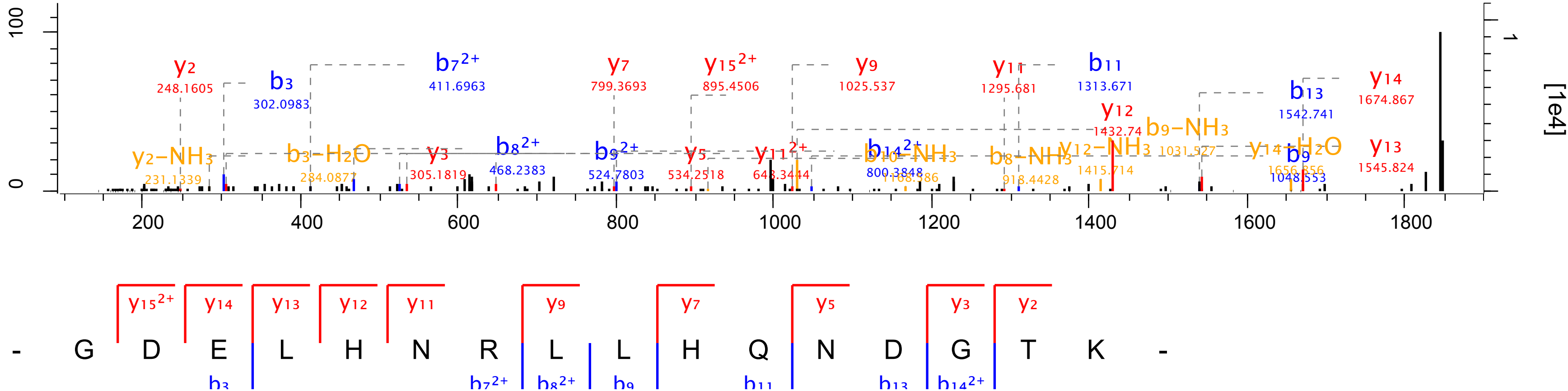

Raw file	Scan	Method	Score	m/z	Gene names
20150227_yeast_Top_opt_B1_01_1599	10980	TOF; CID	46.8	720.83	DSF2

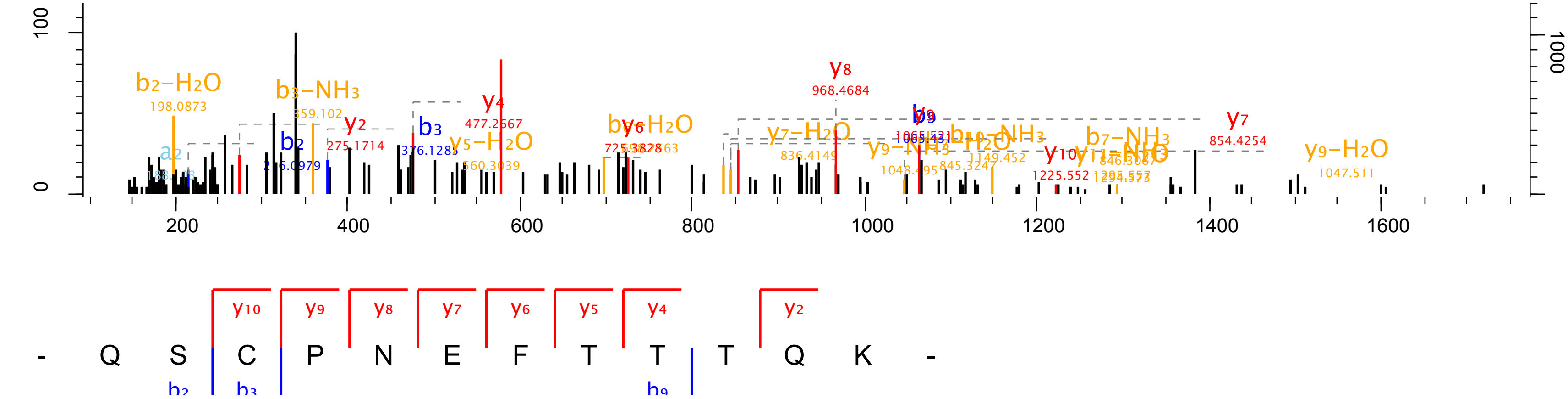

Raw file	Scan	Method	Score	m/z	Gene names
20150227_yeast_Top_opt_B1_01_1599	12023	TOF; CID	78.55	580.79	DLS1

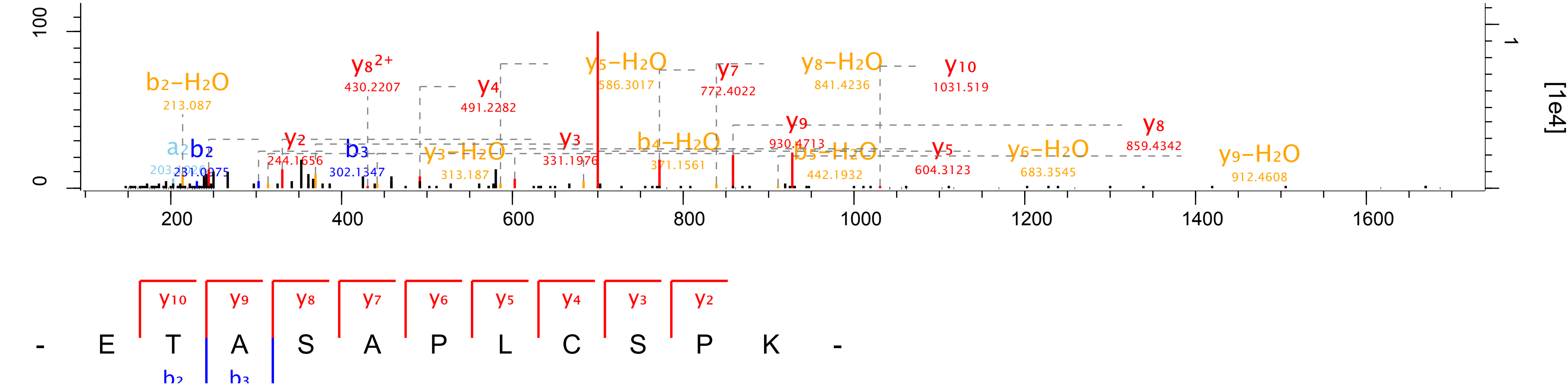

Raw file	Scan	Method	Score	m/z	Gene names
20150227_yeast_Top_opt_B1_01_1599	12126	TOF; CID	56.2	557.26	BAP2

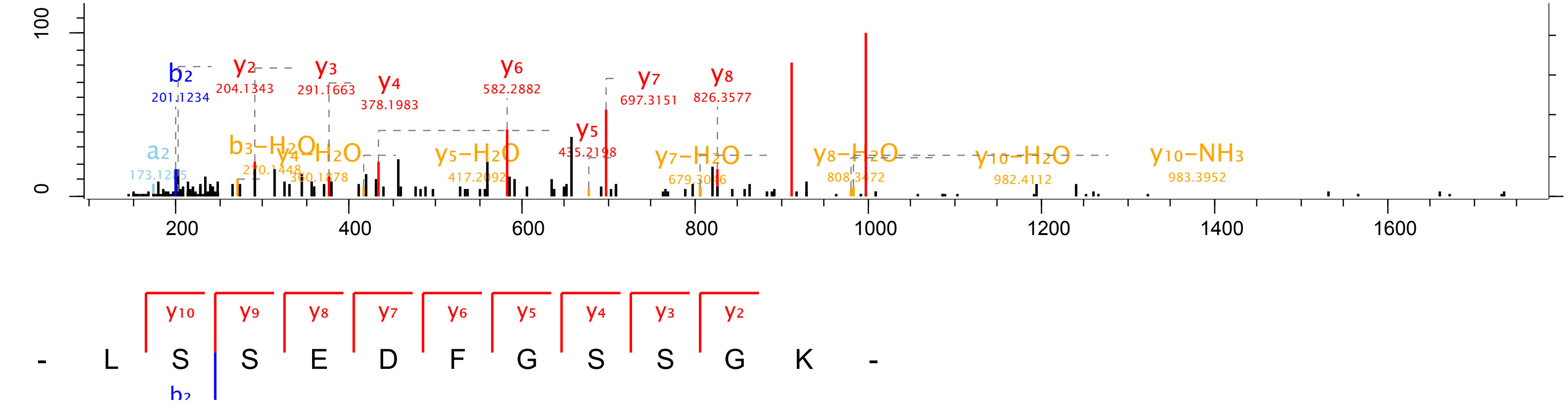

Raw file	Scan	Method	Score	m/z	Gene names
20150227_yeast_Top_opt_B1_01_1599	12380	TOF; CID	42.8	460.76	AVT3

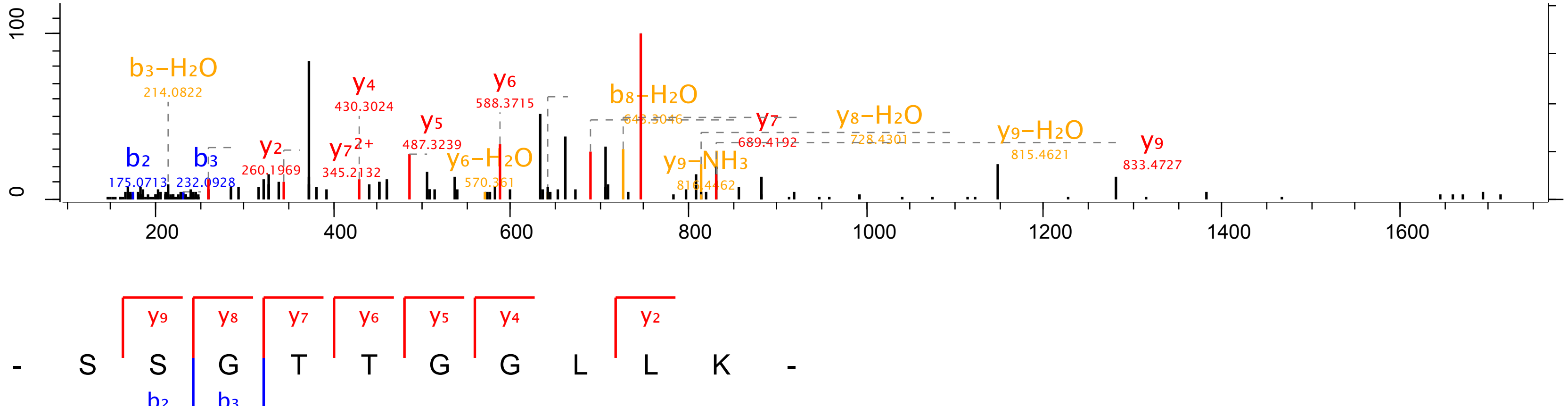

Raw file	Scan	Method	Score	m/z	Gene names
20150227_yeast_Top_opt_B1_01_1599	13003	TOF; CID	145.29	646.32	HST2

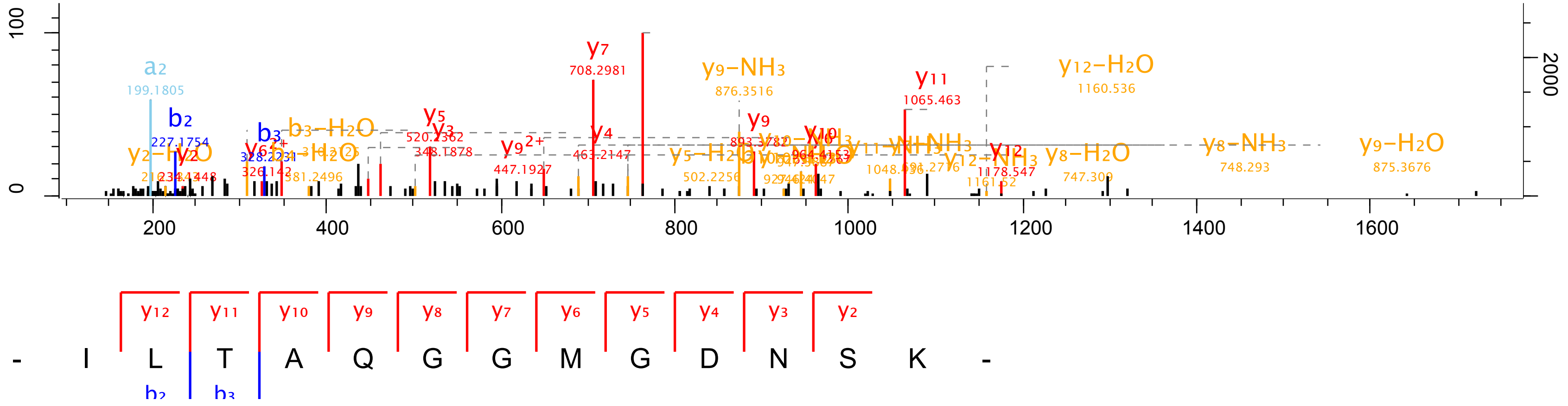

Raw file	Scan	Method	Score	m/z	Gene names
20150227_yeast_Top_opt_B1_01_1599	13251	TOF; CID	74.84	558.31	MDM32

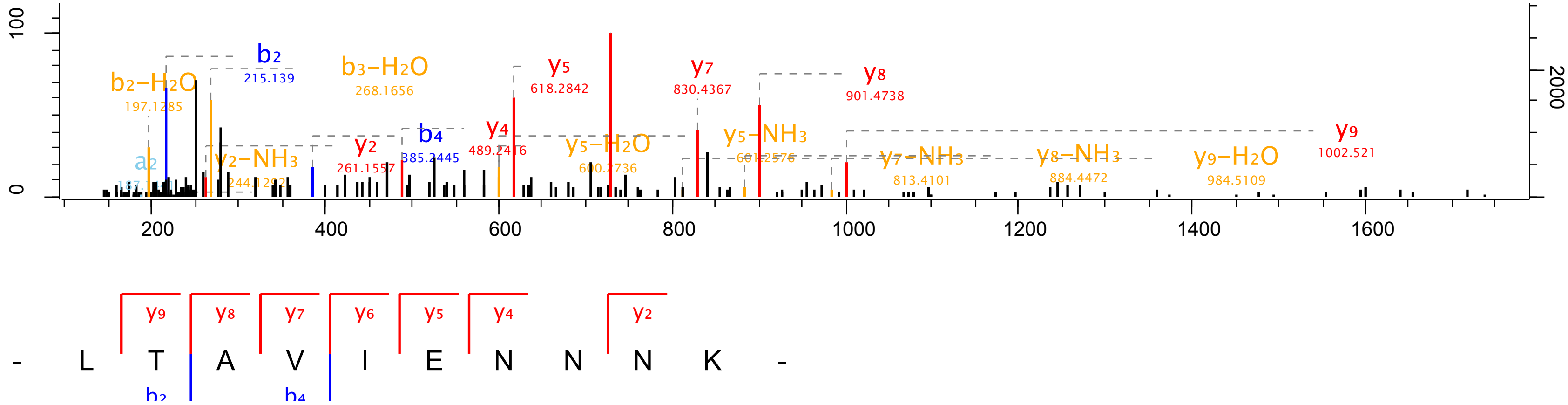

Raw file	Scan	Method	Score	m/z	Gene names
20150227_yeast_Top_opt_B1_01_1599	13944	TOF; CID	70.91	530.8	NAM2

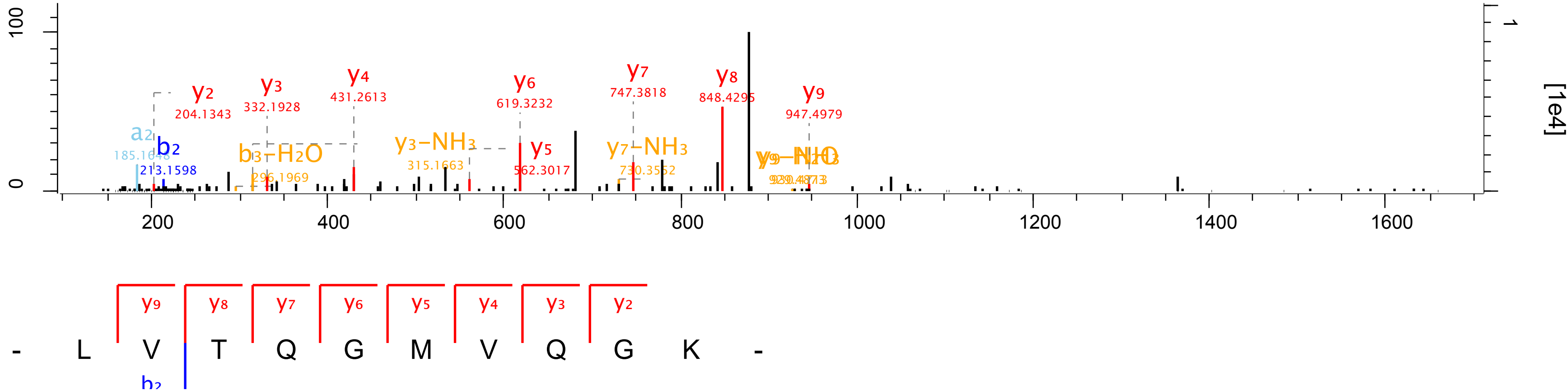

Raw file	Scan	Method	Score	m/z	Gene names
20150227_yeast_Top_opt_B1_01_1599	15802	TOF; CID	68.31	405.21	YDL016C

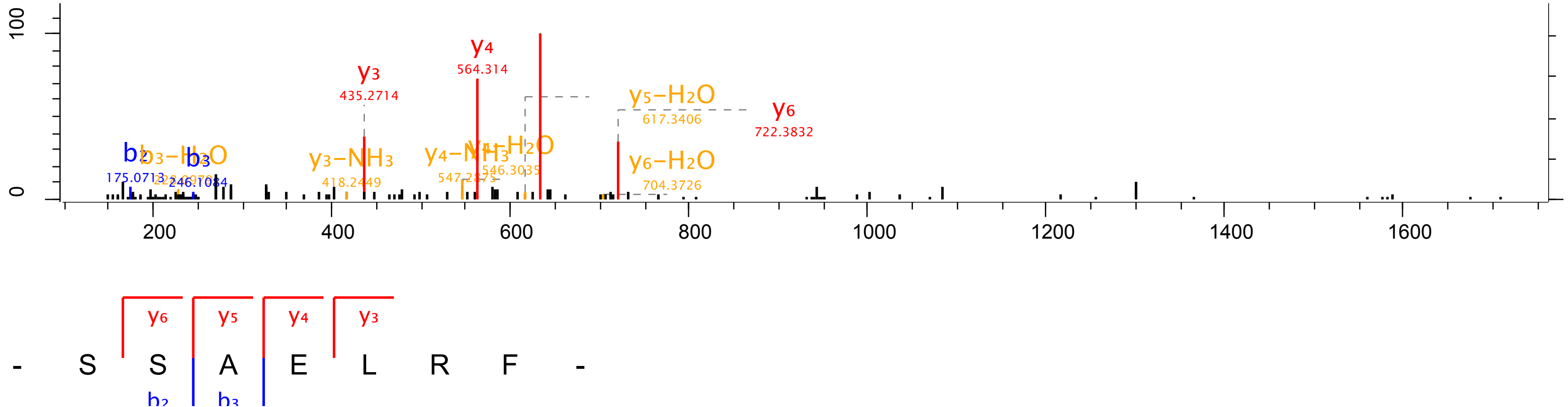

Raw file	Scan	Method	Score	m/z	Gene names
20150227_yeast_Top_opt_B1_01_1599	16018	TOF; CID	68.54	405.91	YIH1

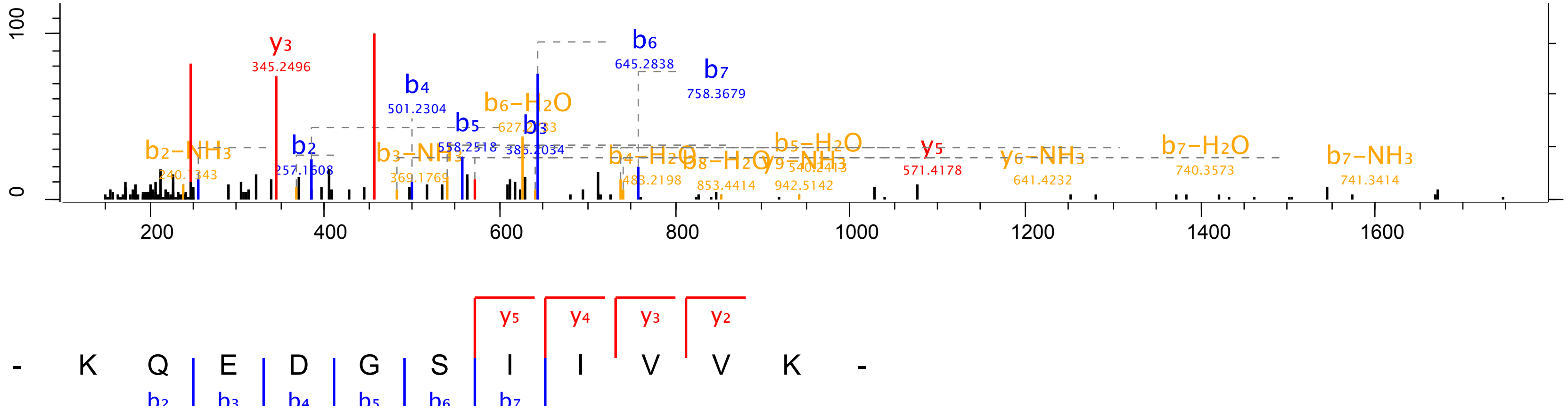

Raw file	Scan	Method	Score	m/z	Gene names
20150227_yeast_Top_opt_B1_01_1599	16166	TOF; CID	63.82	532.77	PSY4

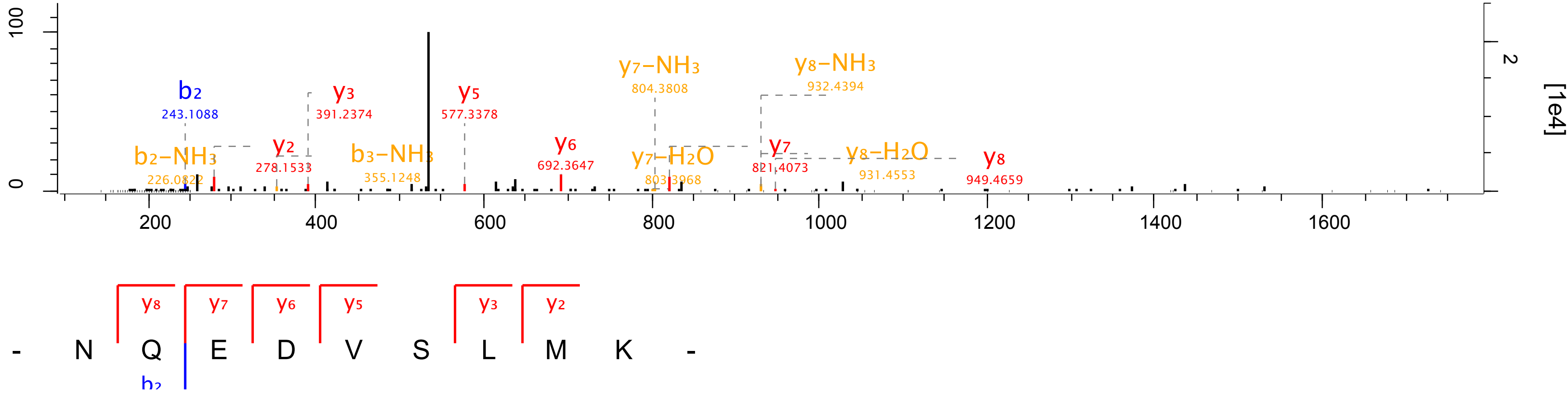

Raw file	Scan	Method	Score	m/z	Gene names
20150227_yeast_Top_opt_B1_01_1599	17164	TOF; CID	68.22	508.74	PHA2

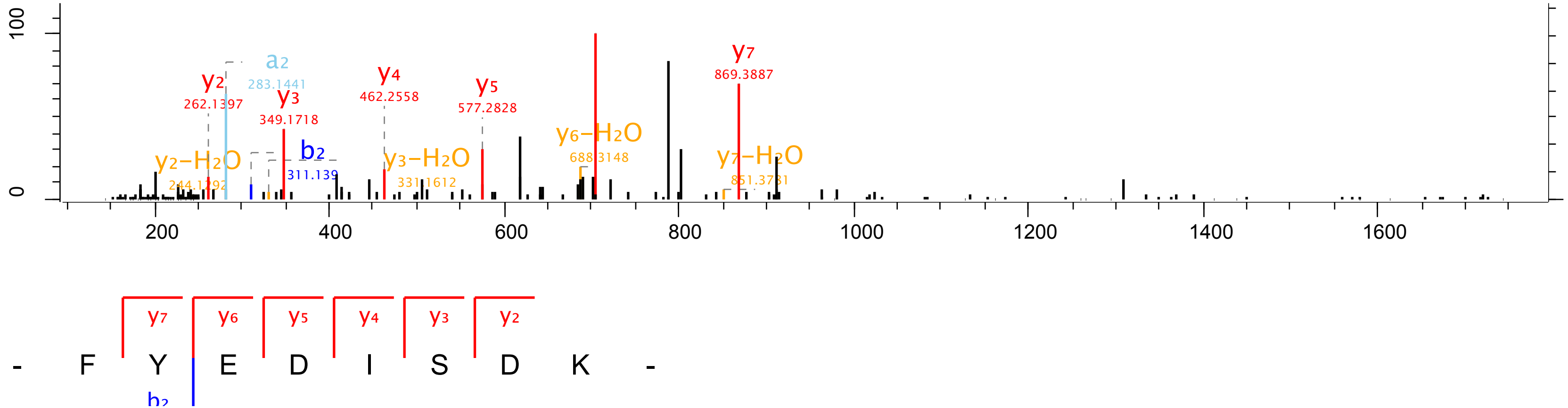

Raw file	Scan	Method	Score	m/z	Gene names
20150227_yeast_Top_opt_B1_01_1599	17208	TOF; CID	39.01	659.84	TCM62

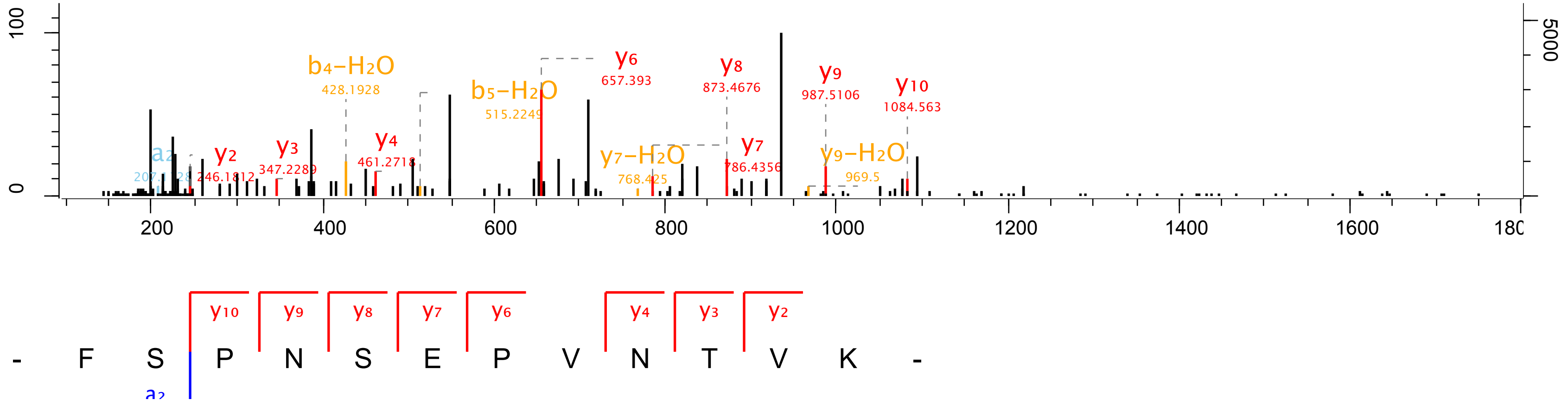

Raw file	Scan	Method	Score	m/z	Gene names
20150227_yeast_Top_opt_B1_01_1599	17292	TOF; CID	93.35	586.28	SSU1

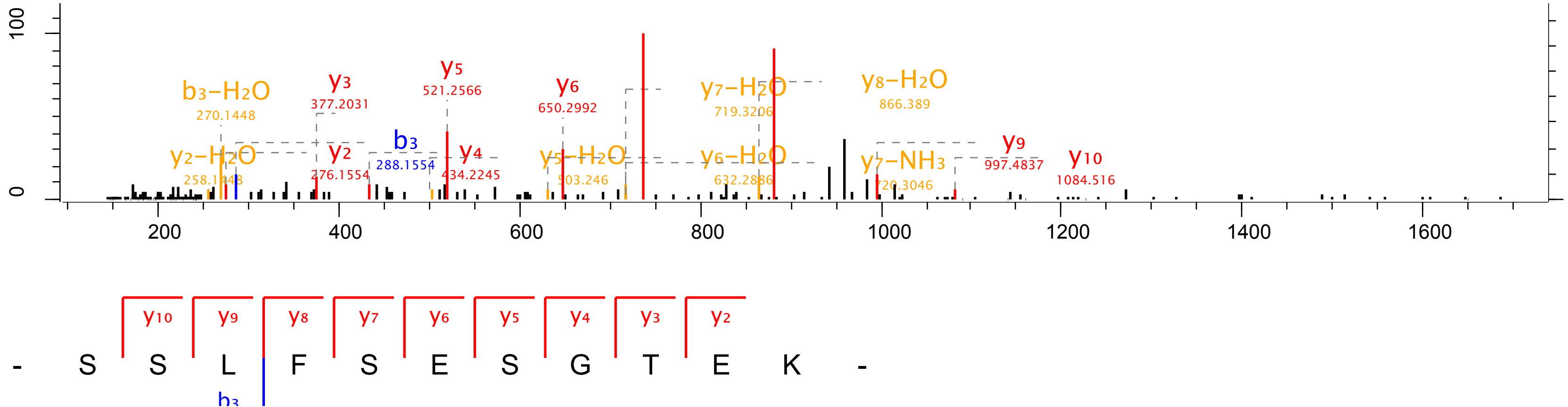

Raw file	Scan	Method	Score	m/z	Gene names
20150227_yeast_Top_opt_B1_01_1599	17646	TOF; CID	67.03	374.24	RAD54

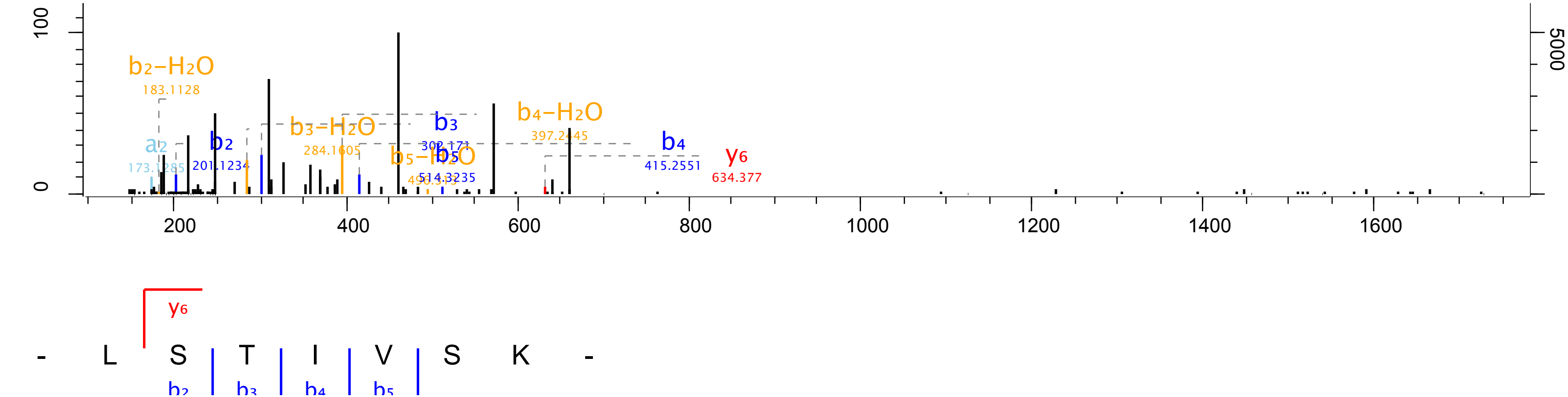

Raw file	Scan	Method	Score	m/z	Gene names
20150227_yeast_Top_opt_B1_01_1599	18435	TOF; CID	105.4	666.29	MRPS17

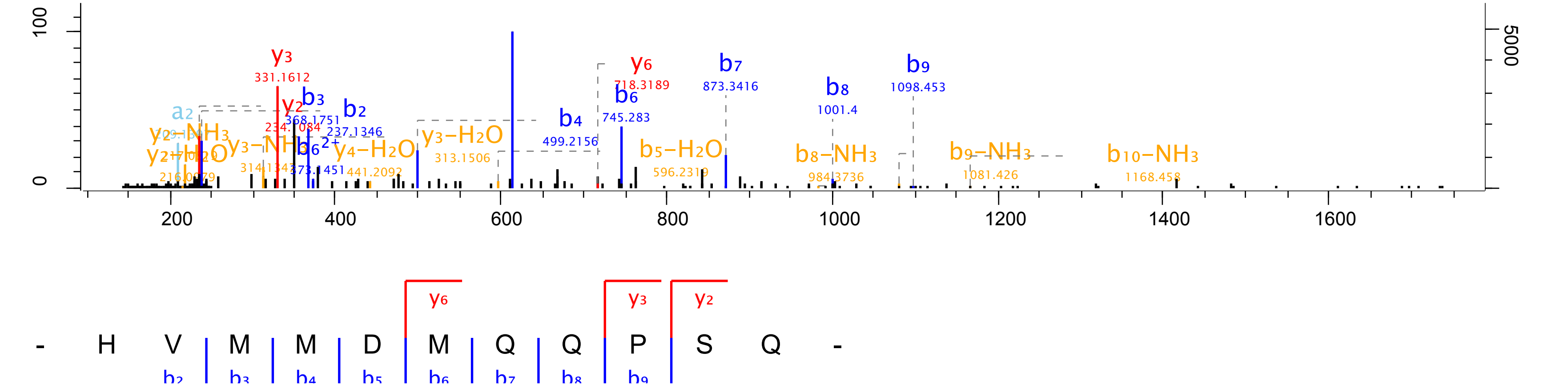

Raw file	Scan	Method	Score	m/z	Gene names
20150227_yeast_Top_opt_B1_01_1599	20694	TOF; CID	92.87	618.82	NGR1

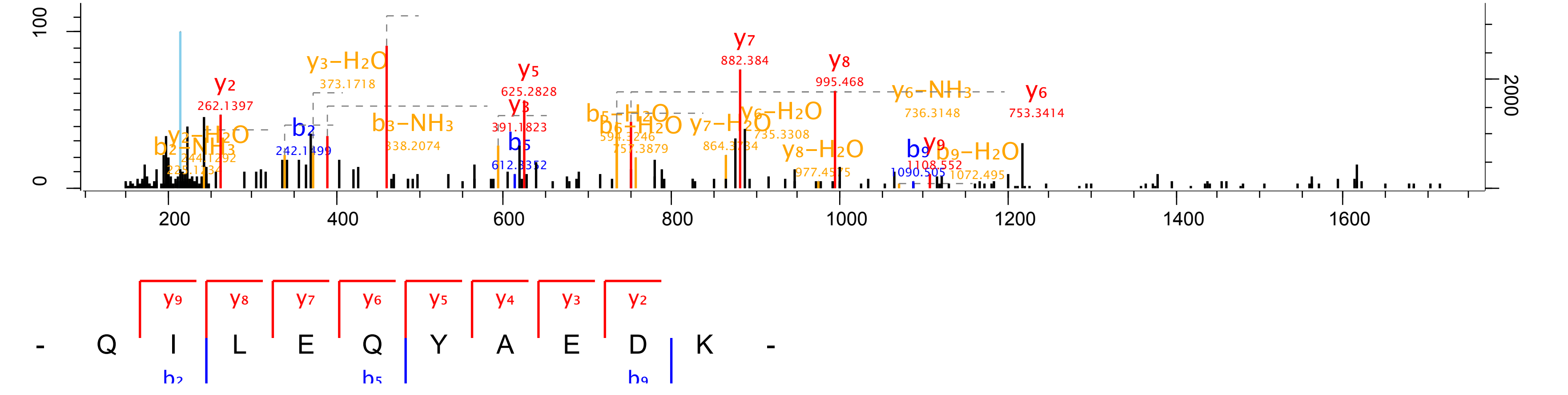

Raw file	Scan	Method	Score	m/z	Gene names
20150227_yeast_Top_opt_B1_01_1599	21328	TOF; CID	60.26	634.33	DOA4

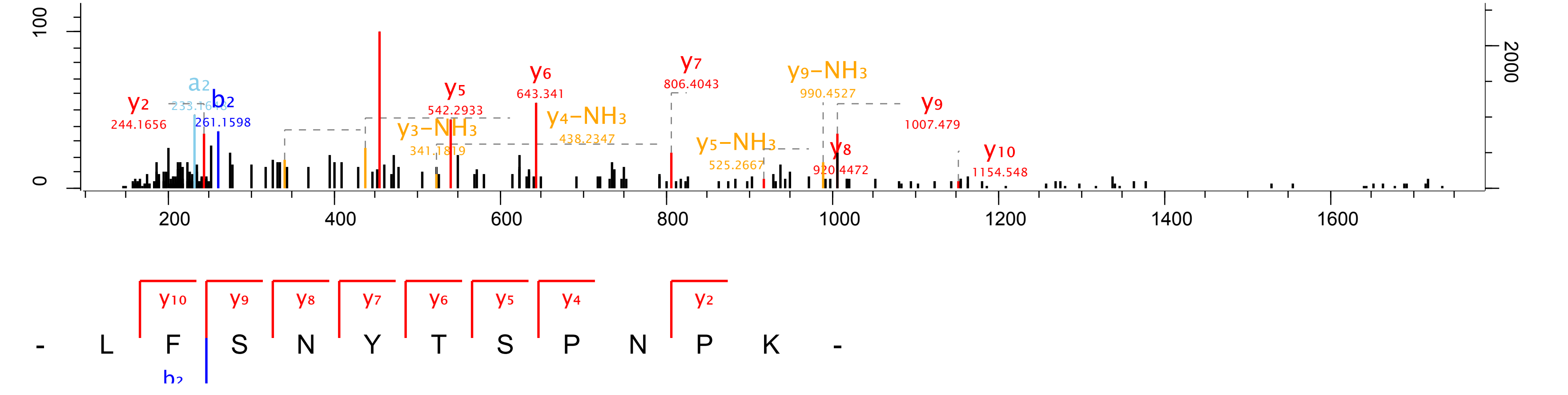

Raw file	Scan	Method	Score	m/z	Gene names
20150227_yeast_Top_opt_B1_01_1599	21451	TOF; CID	96.67	600.31	GLG2

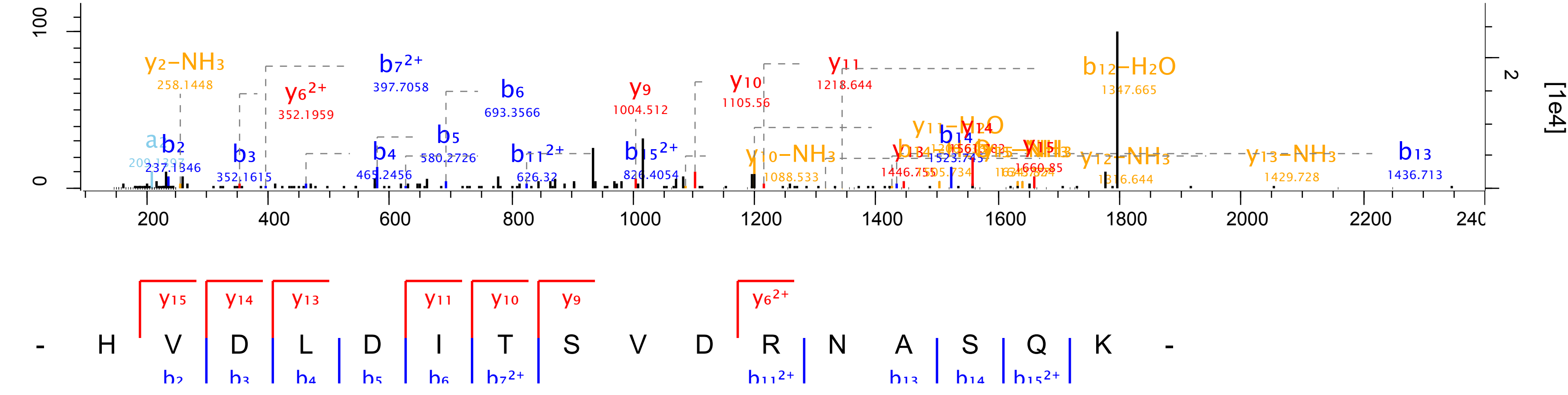

Raw file	Scan	Method	Score	m/z	Gene names
20150227_yeast_Top_opt_B1_01_1599	22227	TOF; CID	78.19	498.75	YKL023W

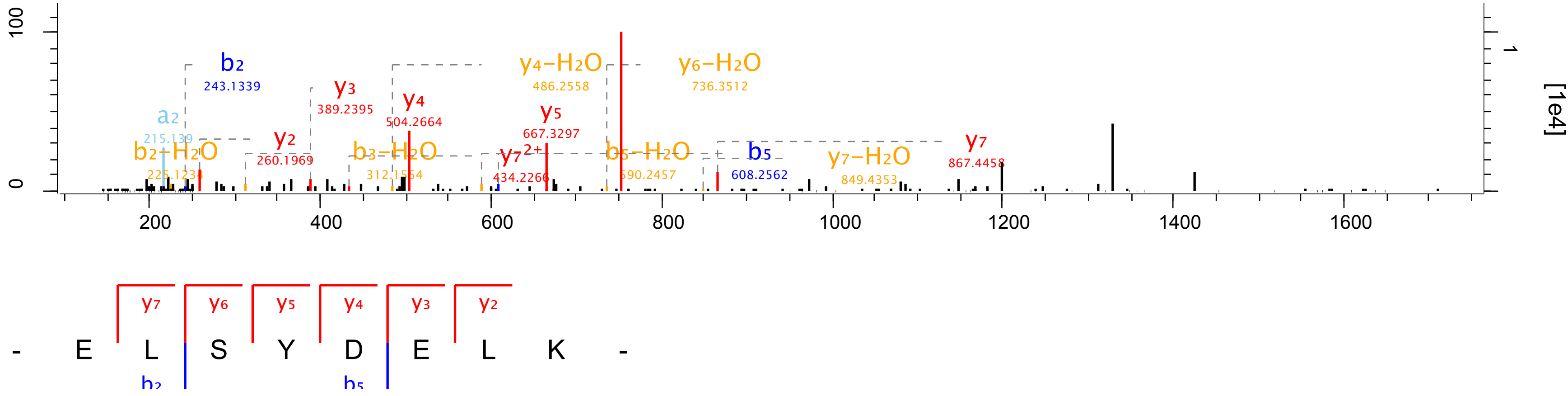

Raw file	Scan	Method	Score	m/z	Gene names
20150227_yeast_Top_opt_B1_01_1599	23216	TOF; CID	60.64	592.3	EGT2

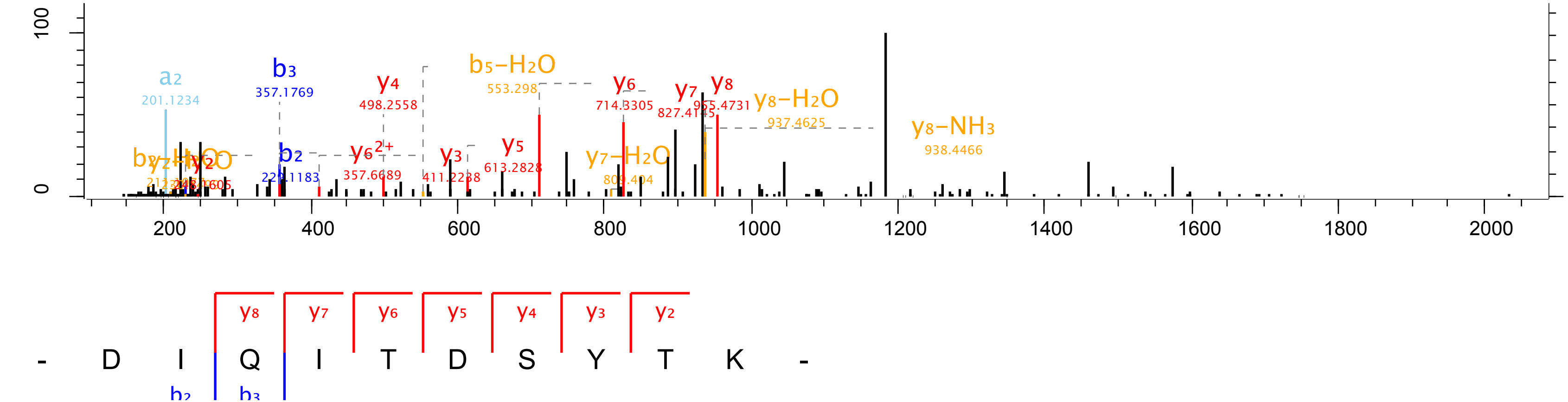

Raw file	Scan	Method	Score	m/z	Gene names
20150227_yeast_Top_opt_B1_01_1599	23346	TOF; CID	74.84	400.54	MSN1

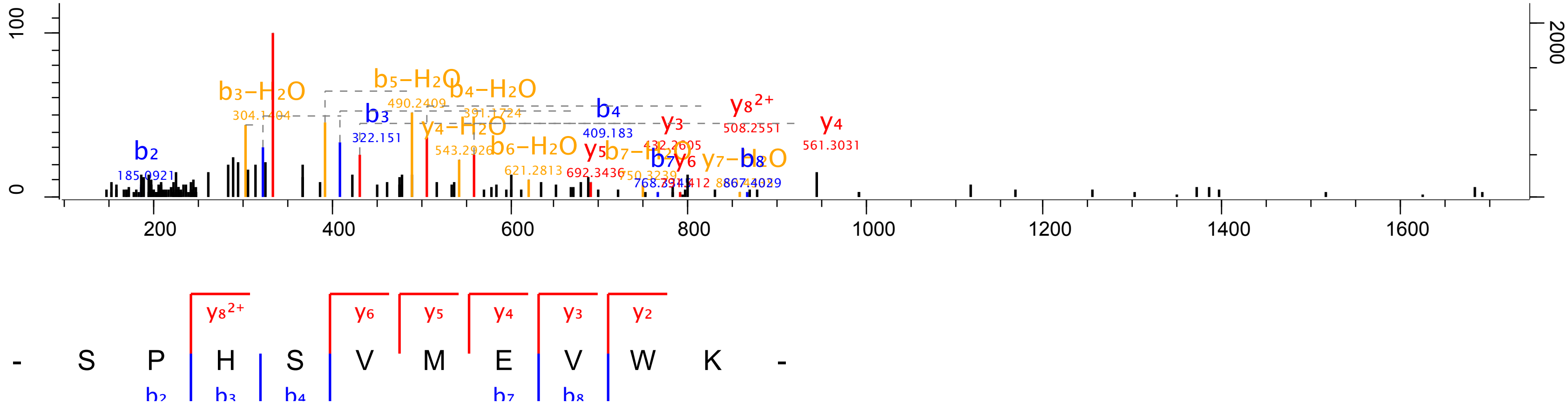

Raw file	Scan	Method	Score	m/z	Gene names
20150227_yeast_Top_opt_B1_01_1599	23347	TOF; CID	128.08	654.85	MSL1

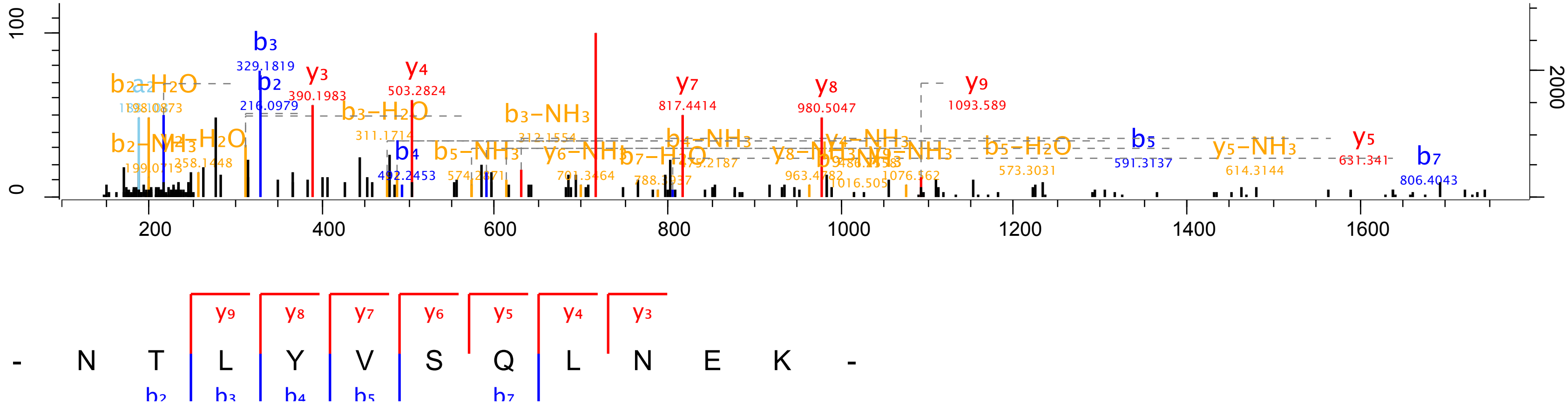

Raw file	Scan	Method	Score	m/z	Gene names
20150227_yeast_Top_opt_B1_01_1599	23573	TOF; CID	59.16	665.35	RDS3

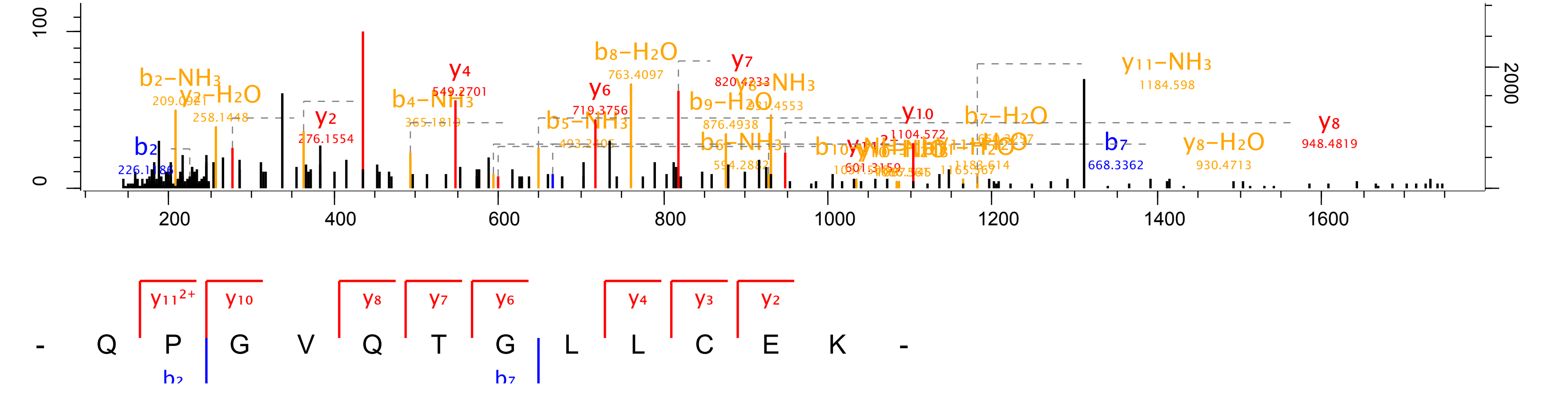

Raw file	Scan	Method	Score	m/z	Gene names
20150227_yeast_Top_opt_B1_01_1599	23908	TOF; CID	73.83	493.63	SRB2

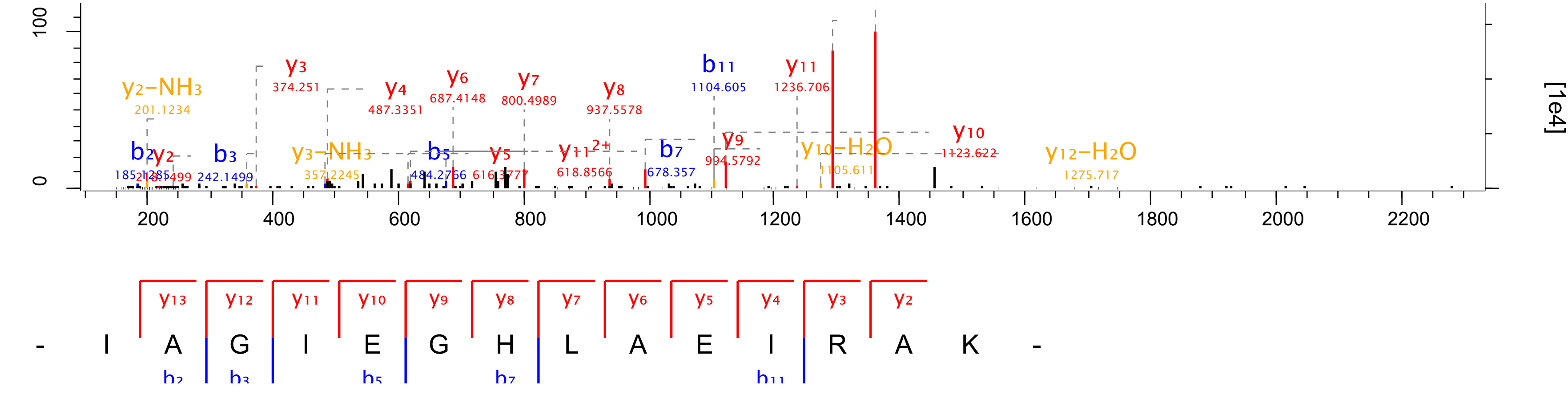

Raw file	Scan	Method	Score	m/z	Gene names
20150227_yeast_Top_opt_B1_01_1599	23930	TOF; CID	73.67	600.34	SDH3

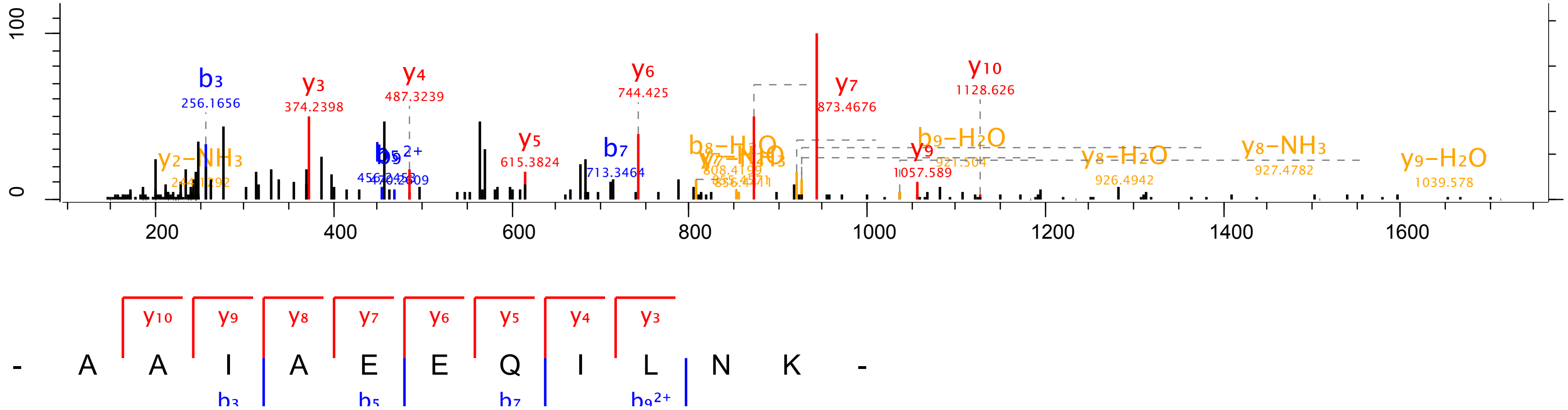

Raw file	Scan	Method	Score	m/z	Gene names
20150227_yeast_Top_opt_B1_01_1599	24496	TOF; CID	83.5	516.78	FMS1

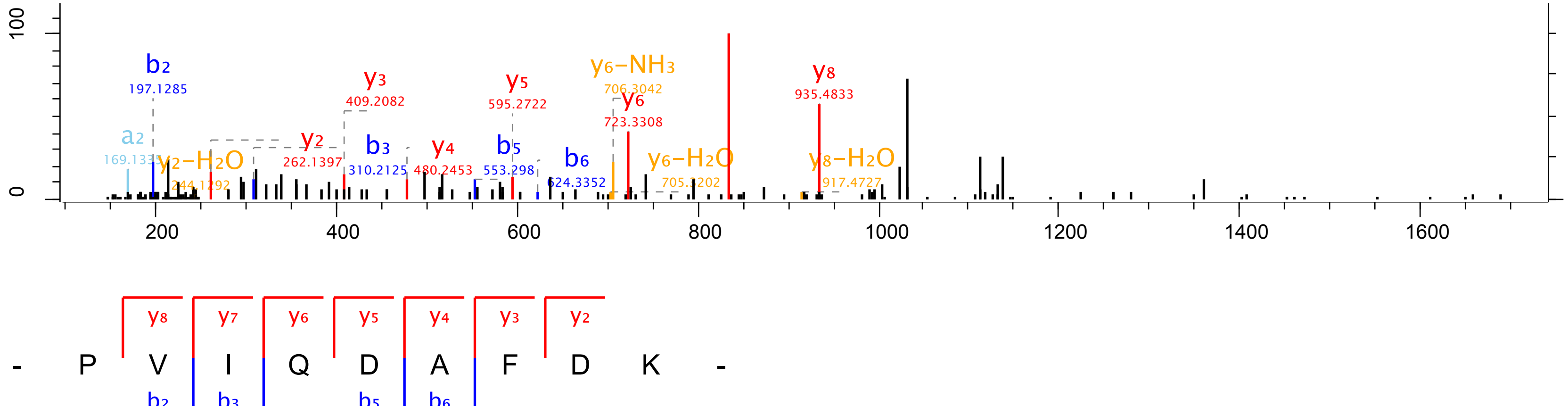

Raw file	Scan	Method	Score	m/z	Gene names
20150227_yeast_Top_opt_B1_01_1599	24805	TOF; CID	46.41	638.33	RNH203

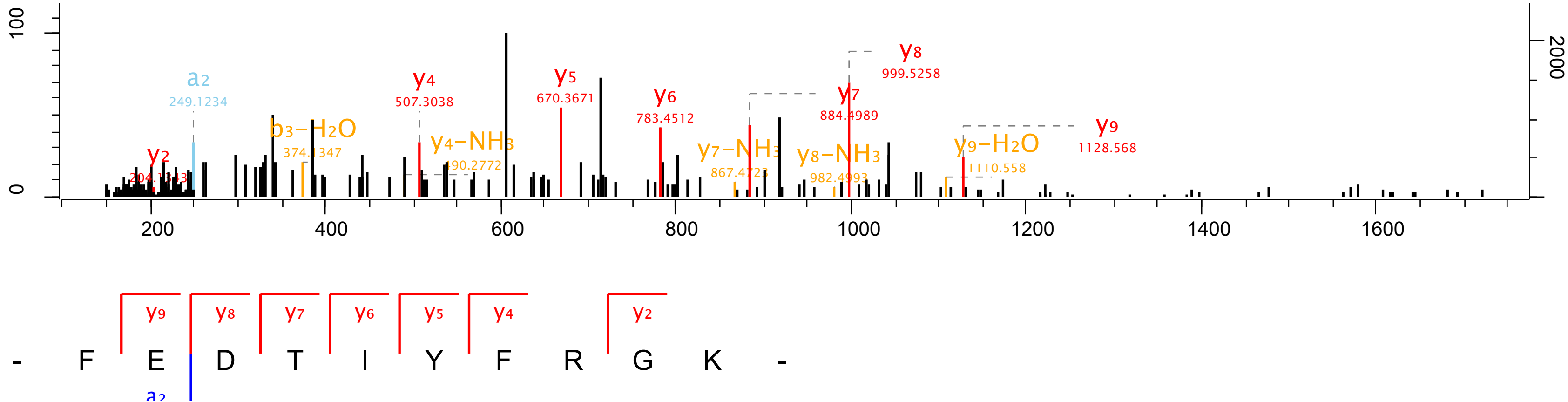

Raw file	Scan	Method	Score	m/z
20150227_yeast_Top_opt_B1_01_1599	25070	TOF; CID	77.22	887.44

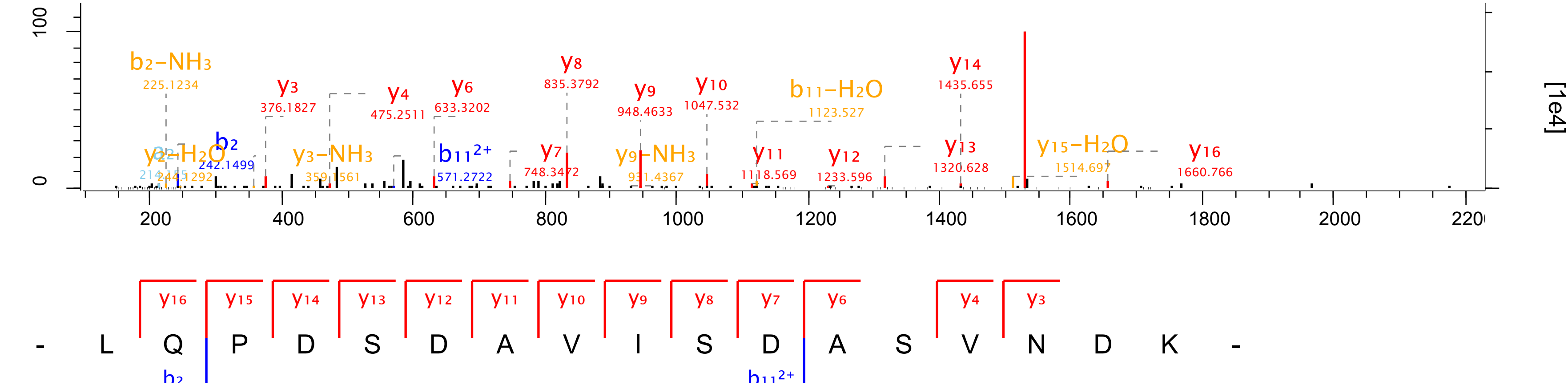

Raw file	Scan	Method	Score	m/z	Gene names
20150227_yeast_Top_opt_B1_01_1599	25380	TOF; CID	91.63	438.75	CGR1

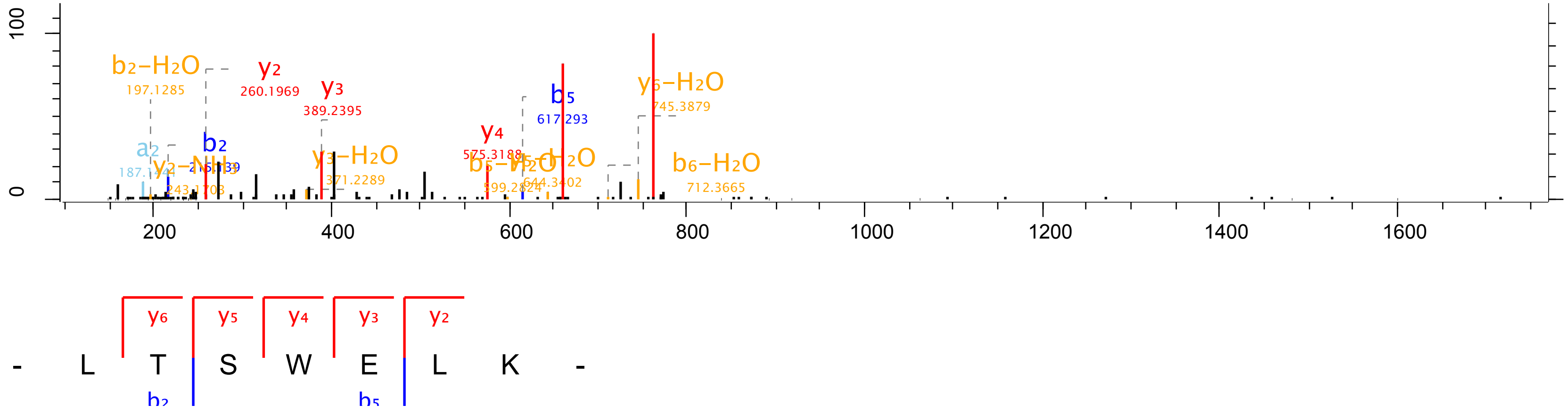

Raw file	Scan	Method	Score	m/z	Gene names
20150227_yeast_Top_opt_B1_01_1599	25473	TOF; CID	75.32	547.6	MON1

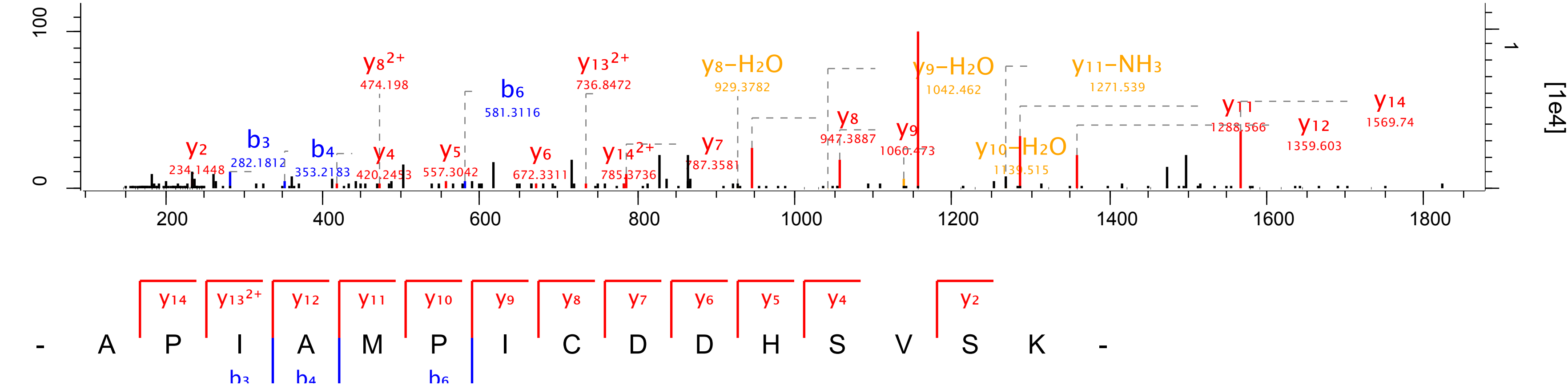

Raw file	Scan	Method	Score	m/z	Gene names
20150227_yeast_Top_opt_B1_01_1599	26100	TOF; CID	59.23	764.36	IDS2

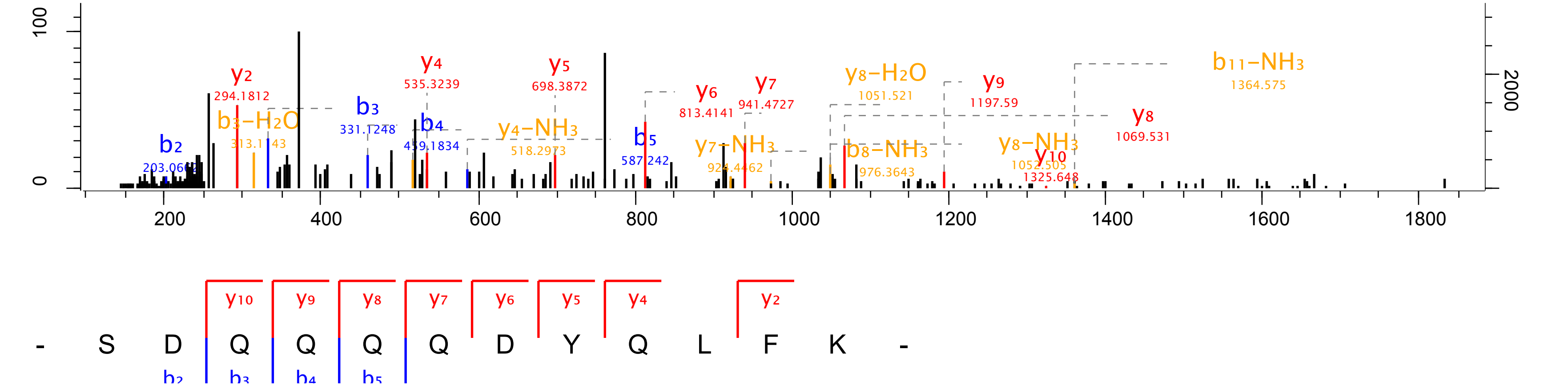

Raw file	Scan	Method	Score	m/z
20150227_yeast_Top_opt_B1_01_1599	26408	TOF; CID	98.94	803.4

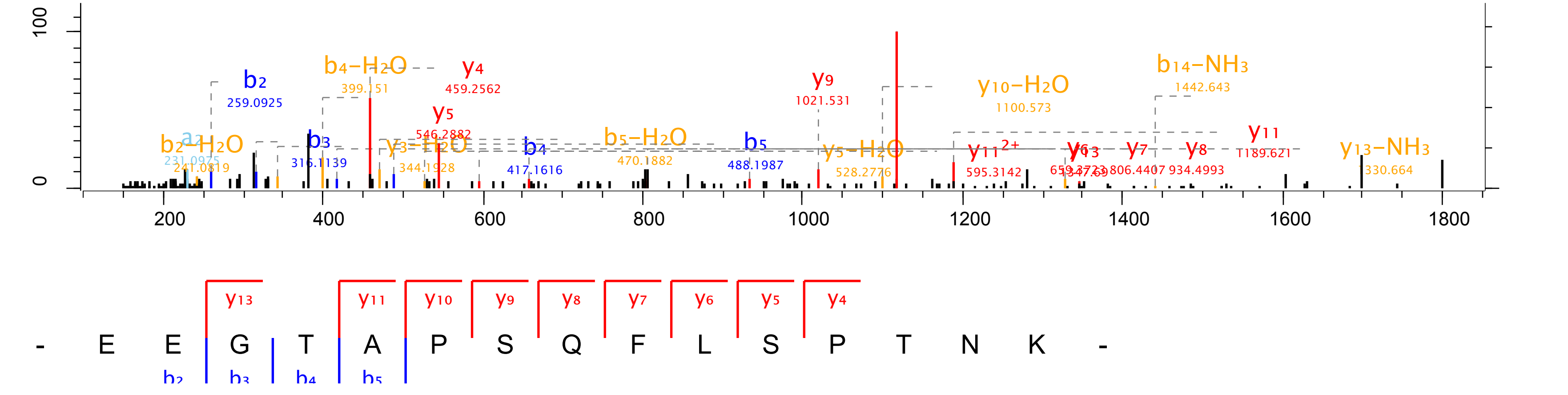

Raw file	Scan	Method	Score	m/z	Gene names
20150227_yeast_Top_opt_B1_01_1599	27274	TOF; CID	56.55	567.32	AVT1

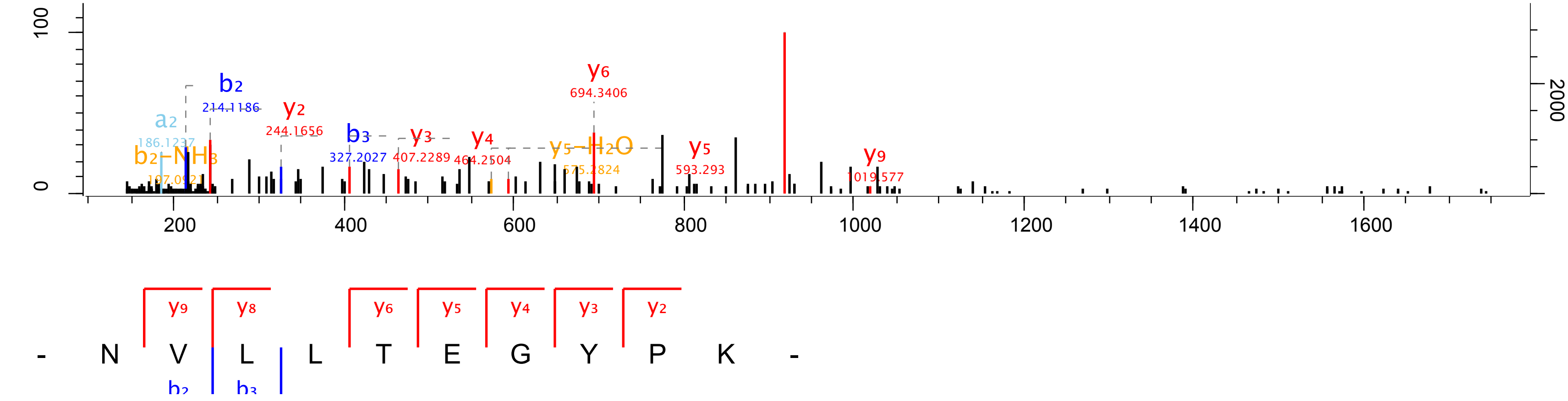

Raw file	Scan	Method	Score	m/z	Gene names
20150227_yeast_Top_opt_B1_01_1599	28065	TOF; CID	91.32	883.43	EAR1

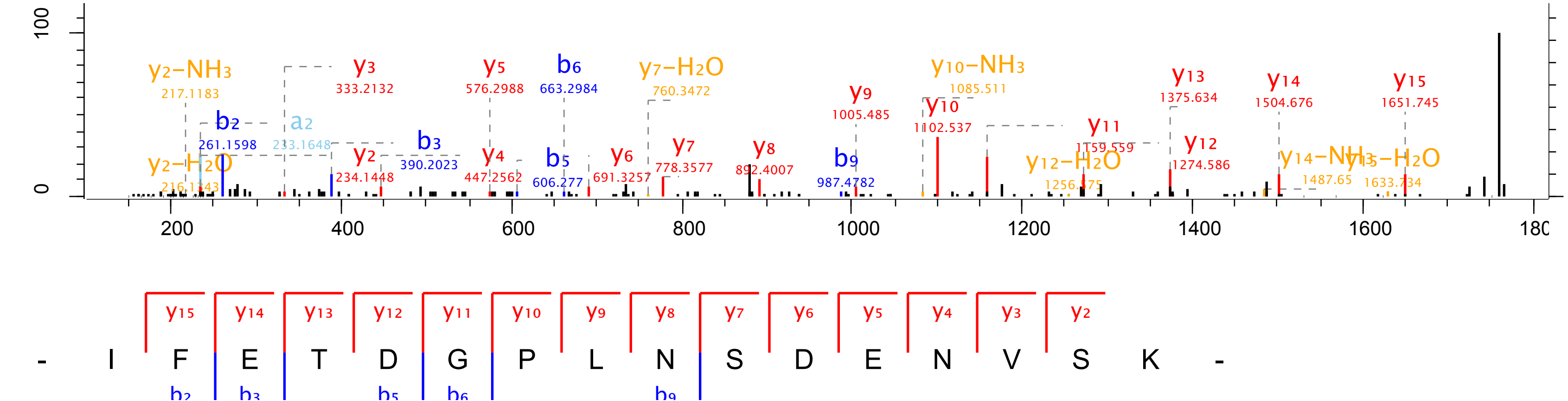

Raw file	Scan	Method	Score	m/z	Gene names
20150227_yeast_Top_opt_B1_01_1599	28093	TOF; CID	62.52	600.3	THP3

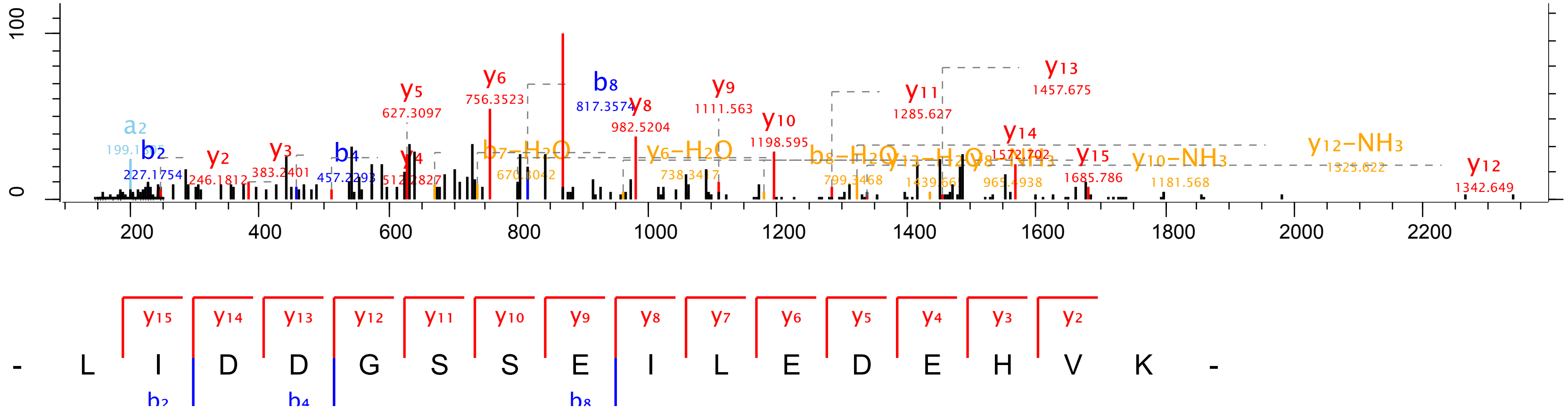

Raw file	Scan	Method	Score	m/z	Gene names
20150227_yeast_Top_opt_B1_01_1599	28262	TOF; CID	46.35	616.33	VBA1

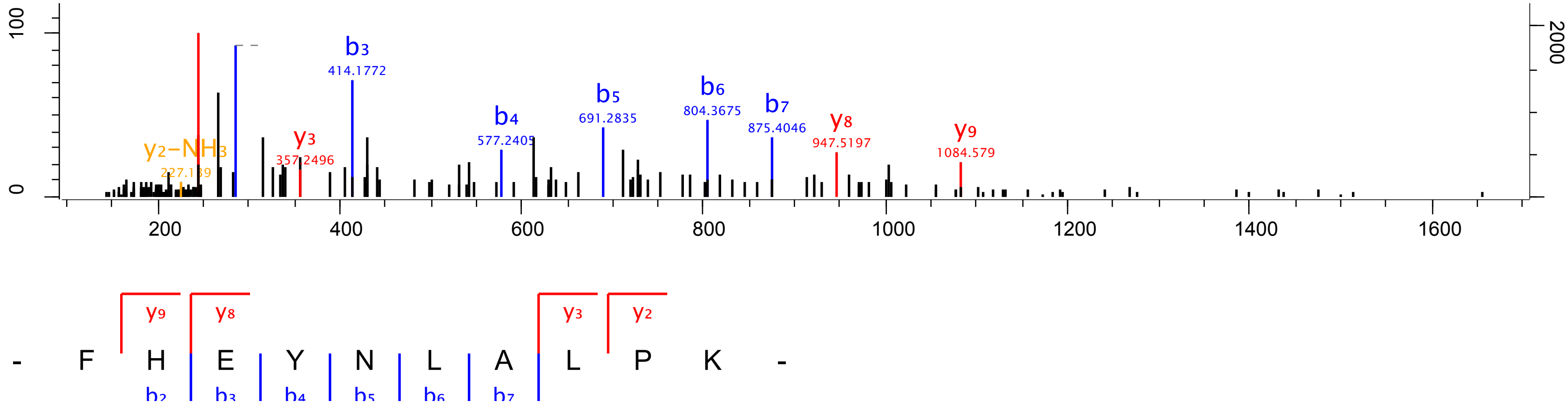

Raw file	Scan	Method	Score	m/z	Gene names
20150227_yeast_Top_opt_B1_01_1599	28600	TOF; CID	50.78	767.37	AKR2

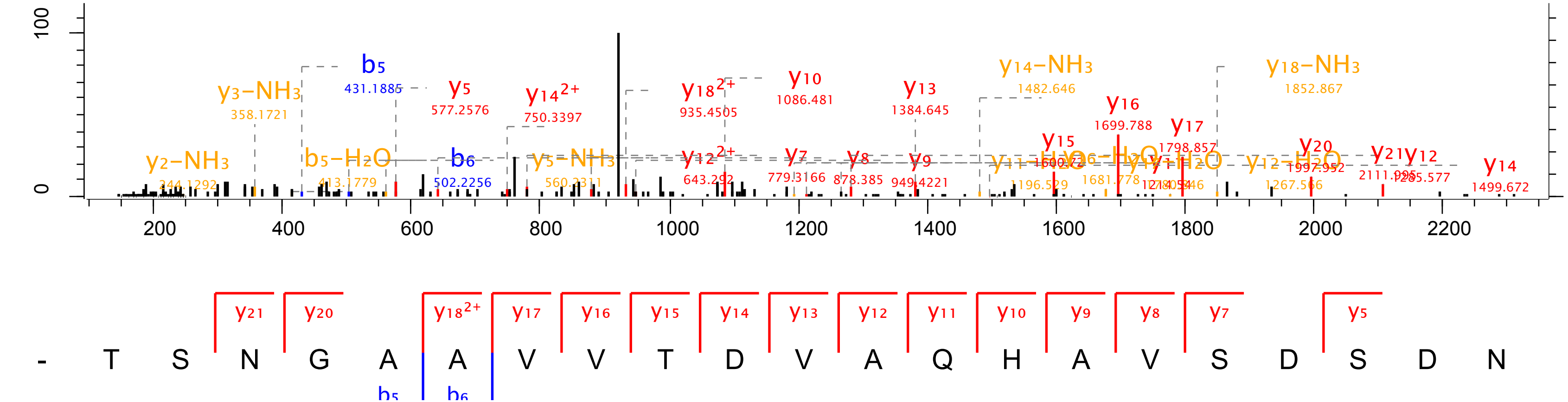

Raw file	Scan	Method	Score	m/z	Gene names
20150227_yeast_Top_opt_B1_01_1599	28662	TOF; CID	81.57	617.83	SNC2

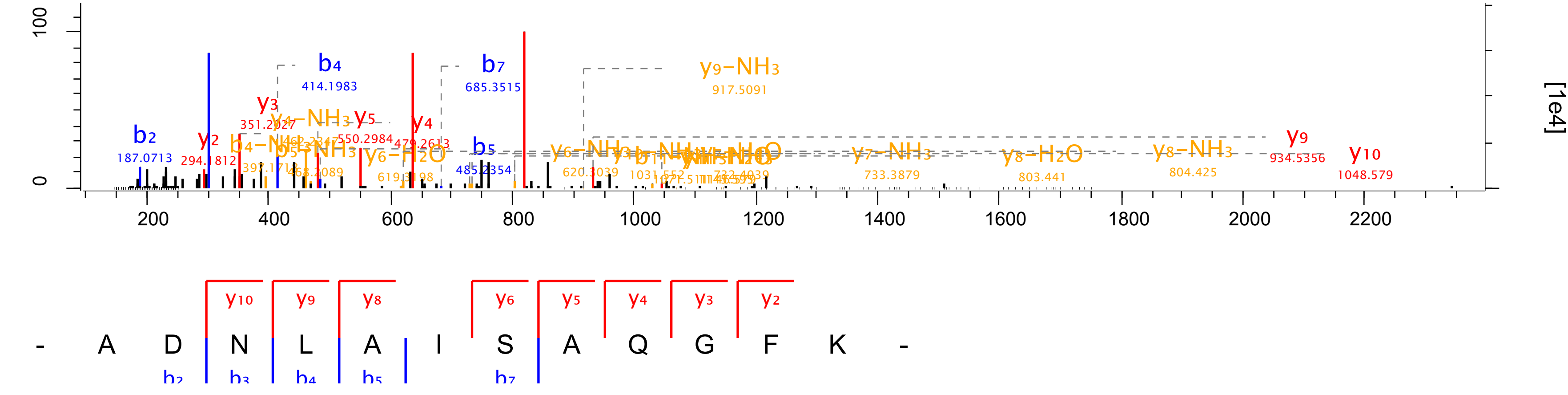

Raw file	Scan	Method	Score	m/z	Gene names
20150227_yeast_Top_opt_B1_01_1599	28764	TOF; CID	39.88	659.33	YPK9

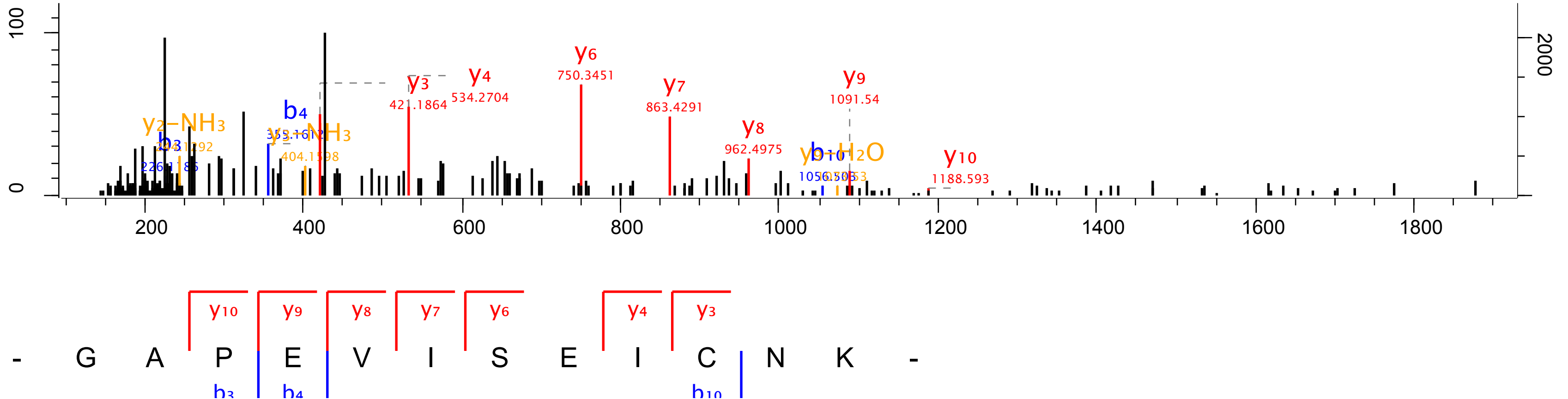

Raw file	Scan	Method	Score	m/z	Gene names
20150227_yeast_Top_opt_B1_01_1599	29140	TOF; CID	83.54	856.46	NBL1

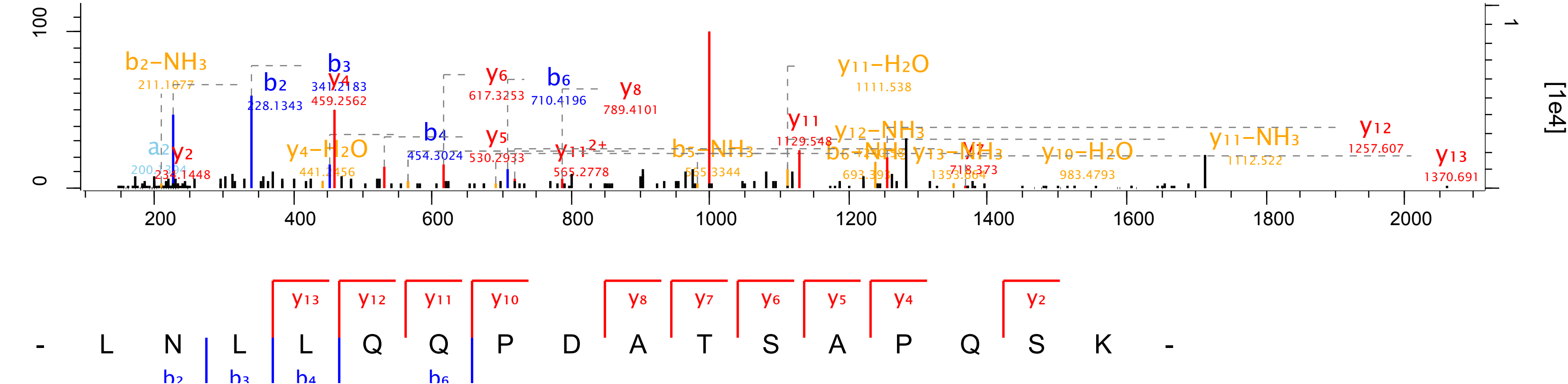

Raw file	Scan	Method	Score	m/z	Gene names
20150227_yeast_Top_opt_B1_01_1599	29574	TOF; CID	54.17	1010.97	ECM22

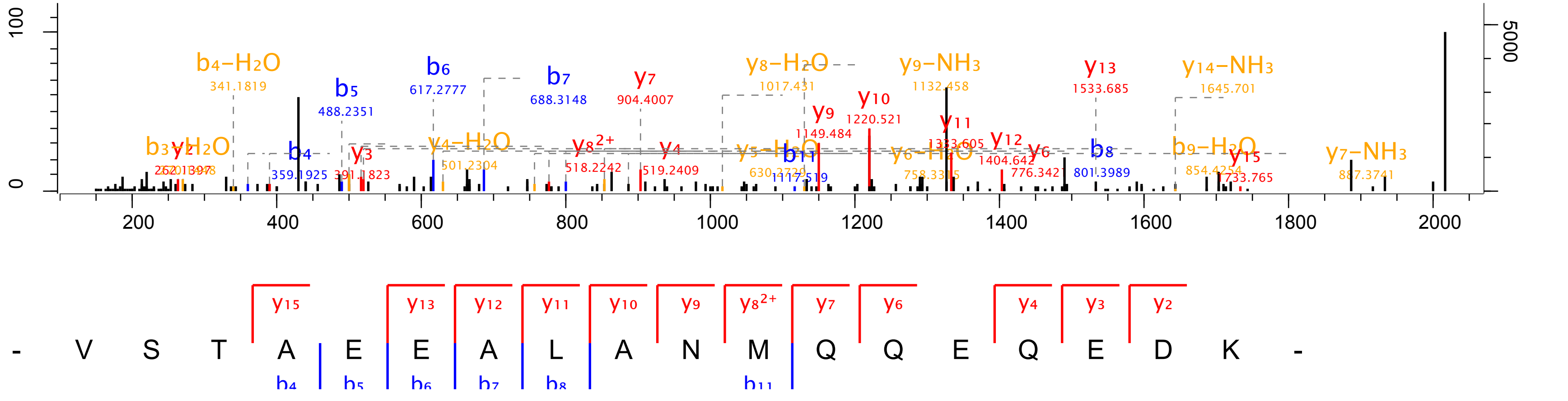

Raw file	Scan	Method	Score	m/z	Gene names
20150227_yeast_Top_opt_B1_01_1599	29808	TOF; CID	47.71	640.32	OAF1

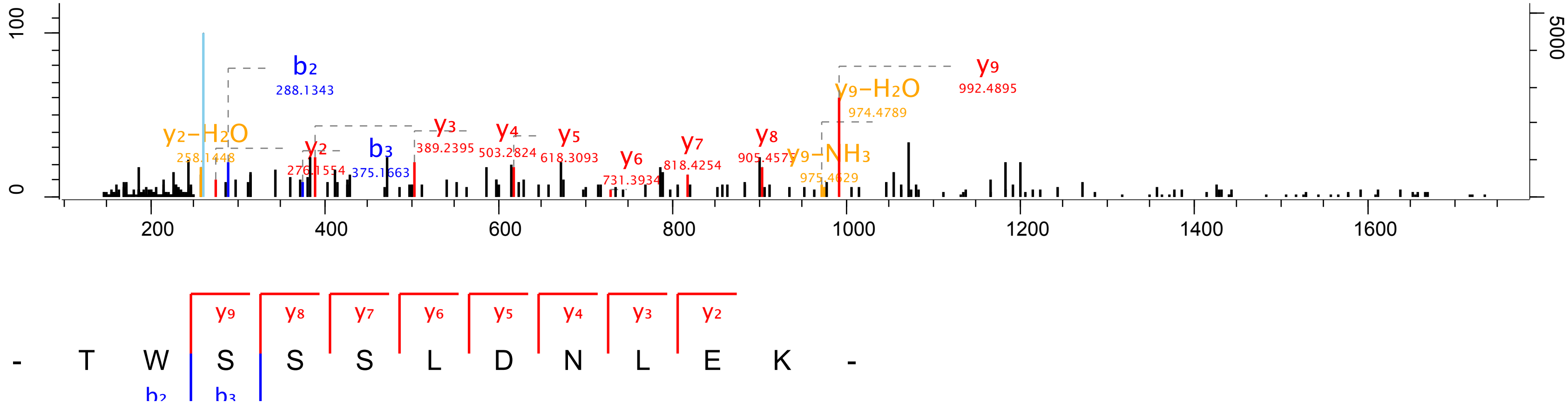

Raw file	Scan	Method	Score	m/z	Gene names
20150227_yeast_Top_opt_B1_01_1599	29857	TOF; CID	54.98	449.3	MRPL10

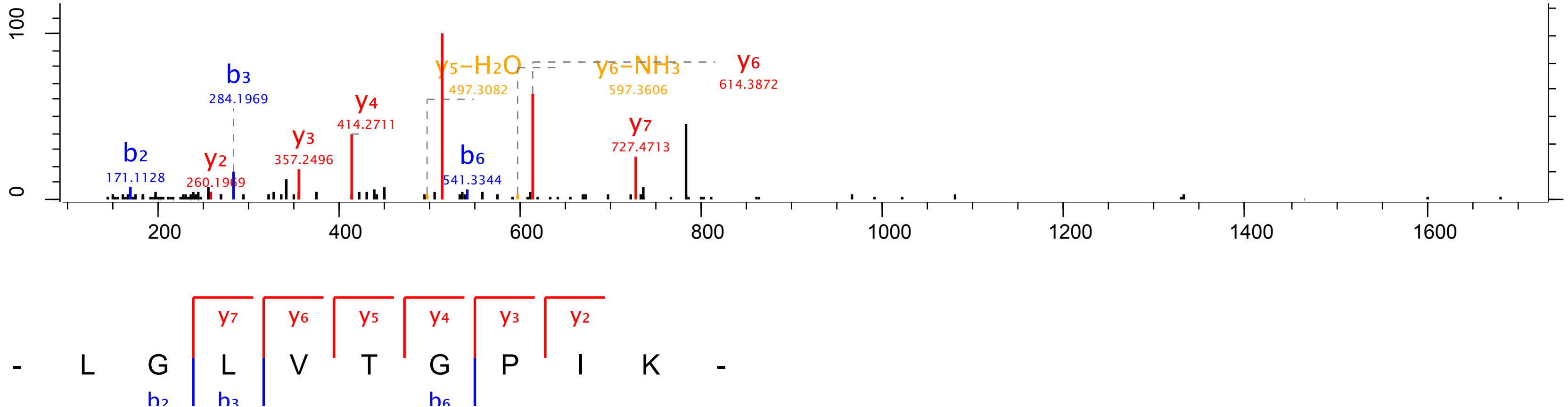

Raw file	Scan	Method	Score	m/z	Gene names
20150227_yeast_Top_opt_B1_01_1599	29954	TOF; CID	52.53	731.89	IRE1

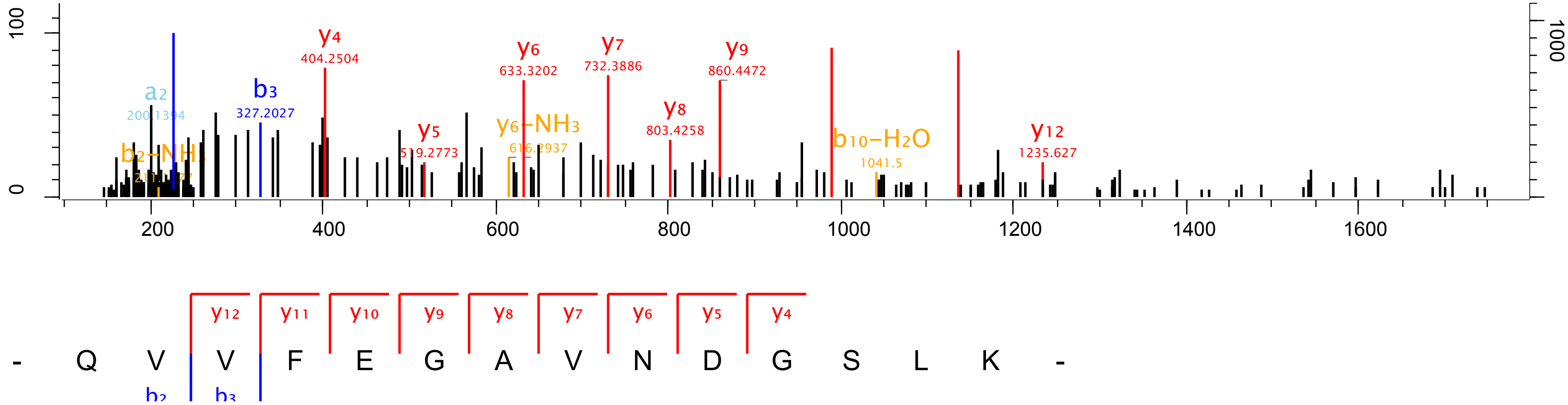

Raw file	Scan	Method	Score	m/z	Gene names
20150227_yeast_Top_opt_B1_01_1599	30085	TOF; CID	148.18	623.33	MOT3

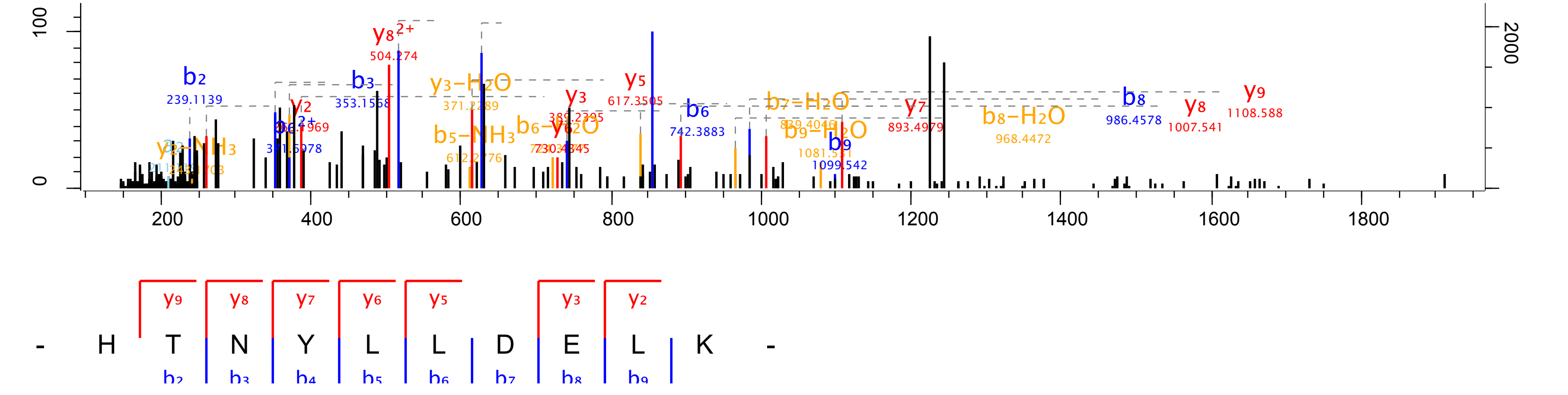

Raw file	Scan	Method	Score	m/z	Gene names
20150227_yeast_Top_opt_B1_01_1599	30927	TOF; CID	47.06	620.78	RNY1

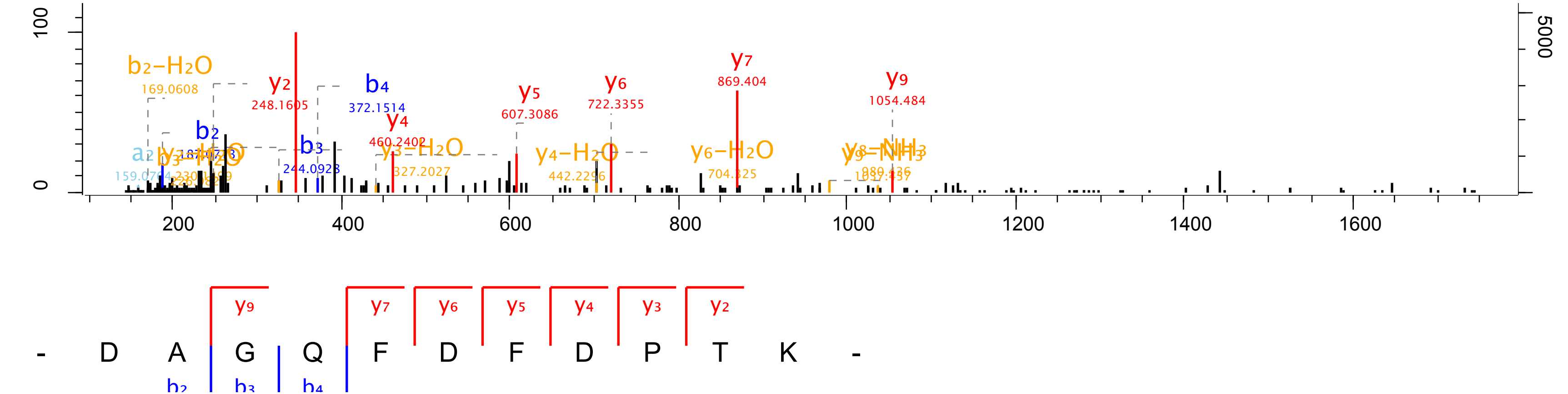

Raw file	Scan	Method	Score	m/z	Gene names
20150227_yeast_Top_opt_B1_01_1599	31835	TOF; CID	49.93	664.83	AST2

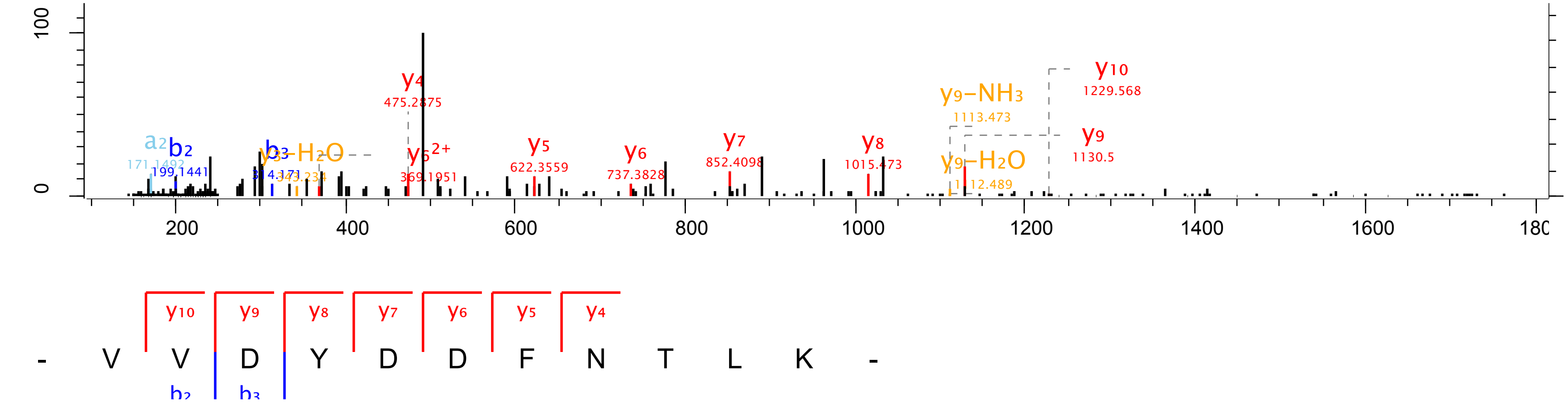

Raw file	Scan	Method	Score	m/z	Gene names
20150227_yeast_Top_opt_B1_01_1599	32241	TOF; CID	55.26	752.36	BRR1

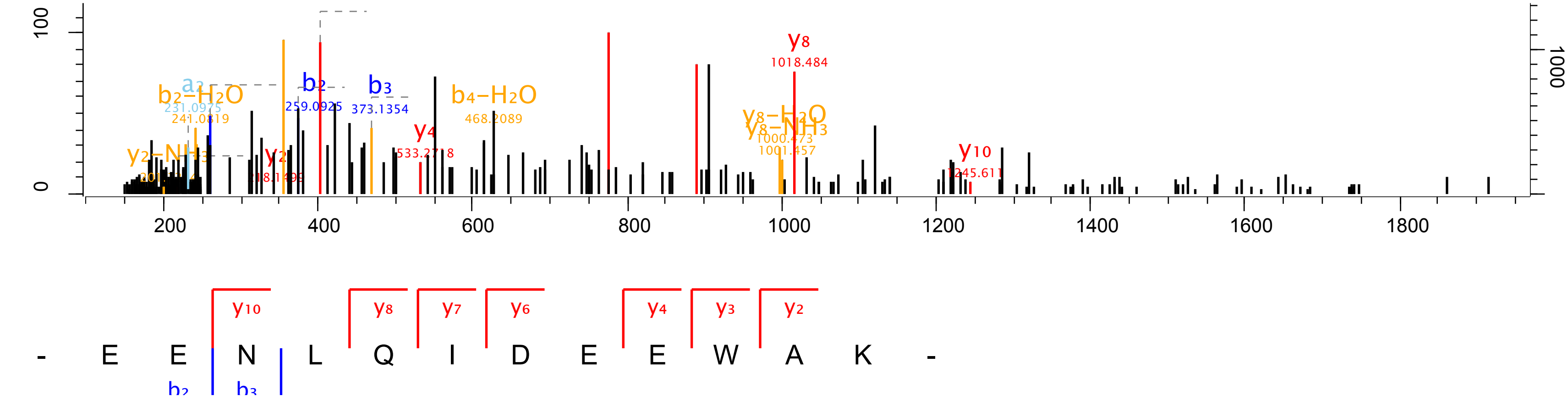

Raw file	Scan	Method	Score	m/z	Gene names
20150227_yeast_Top_opt_B1_01_1599	32289	TOF; CID	47.19	579.33	DAL3

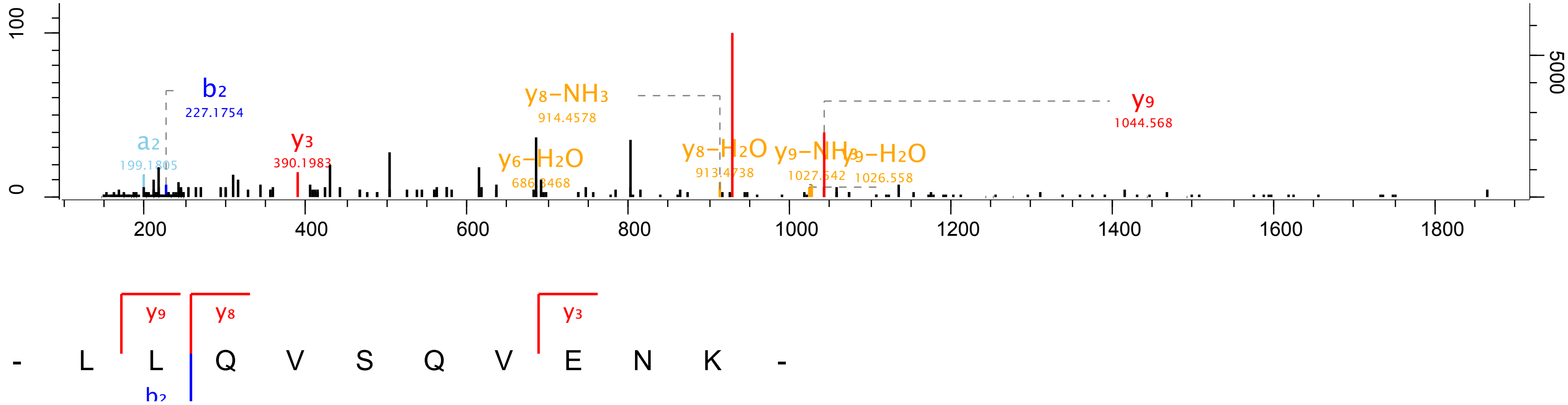

Raw file	Scan	Method	Score	m/z	Gene names
20150227_yeast_Top_opt_B1_01_1599	32433	TOF; CID	104.42	400.88	KSH1

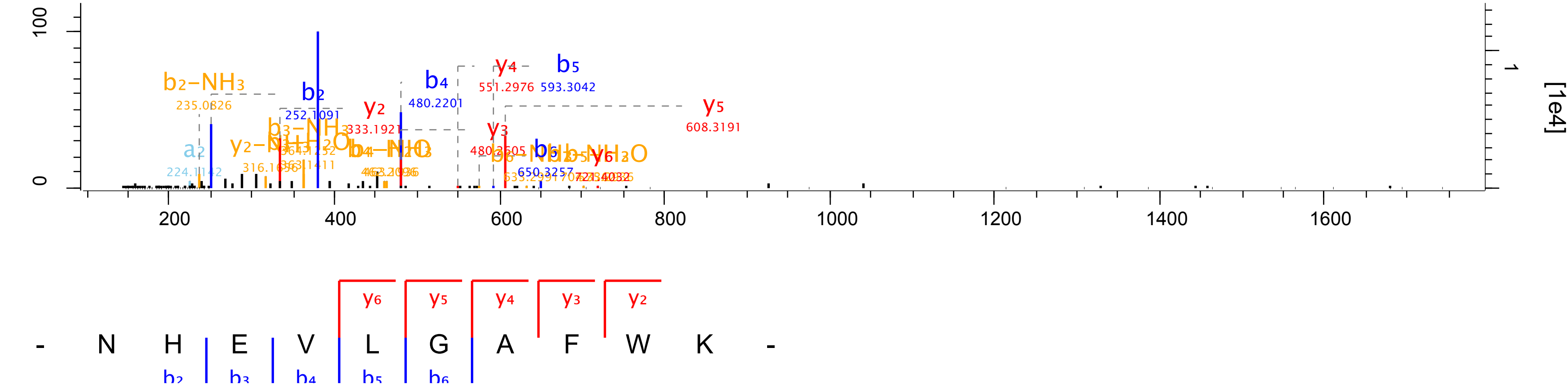

Raw file	Scan	Method	Score	m/z	Gene names
20150227_yeast_Top_opt_B1_01_1599	32754	TOF; CID	82.43	471.74	ELO1

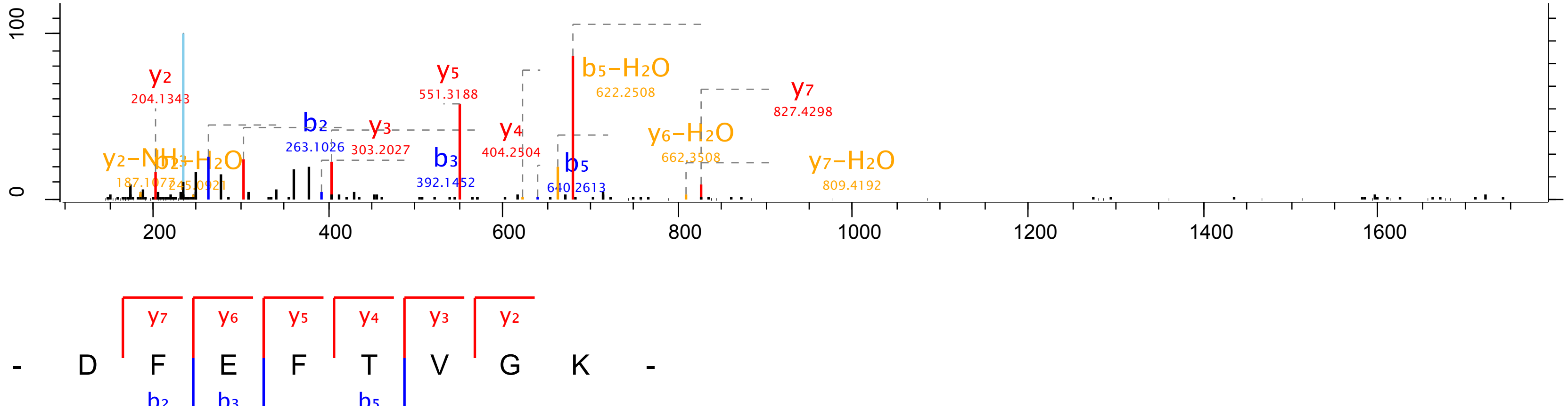

Raw file	Scan	Method	Score	m/z	Gene names
20150227_yeast_Top_opt_B1_01_1599	33399	TOF; CID	70.06	602.34	NAS2

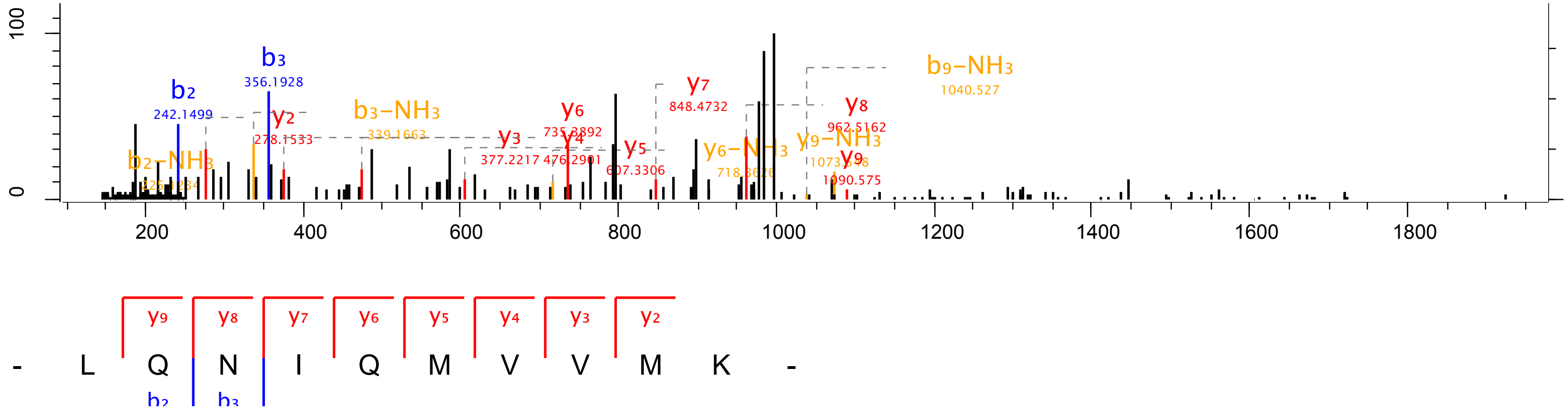

Raw file	Scan	Method	Score	m/z	Gene names
20150227_yeast_Top_opt_B1_01_1599	33400	TOF; CID	105.65	477.27	ERV15

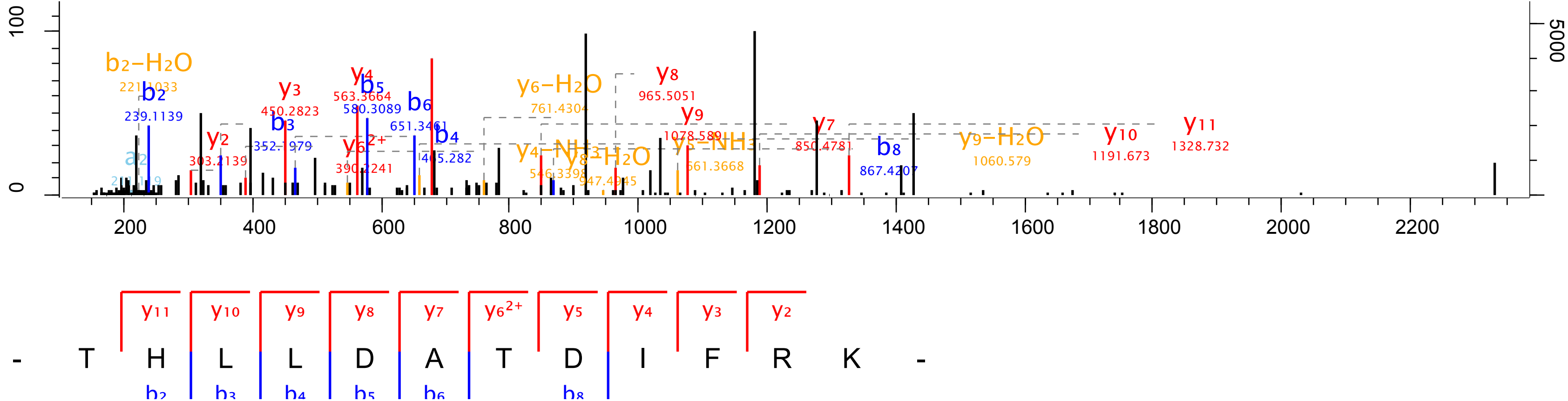

Raw file	Scan	Method	Score	m/z	Gene names
20150227_yeast_Top_opt_B1_01_1599	33820	TOF; CID	62.63	400.56	YHR162W;FMP43

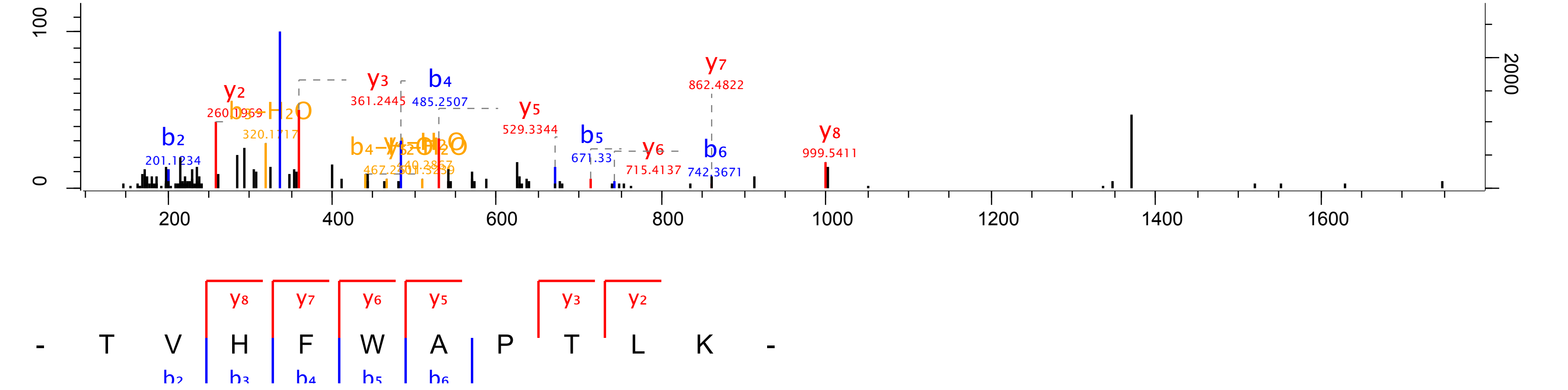

Raw file	Scan	Method	Score	m/z	Gene names
20150227_yeast_Top_opt_B1_01_1599	34343	TOF; CID	69.11	623.31	YPL162C

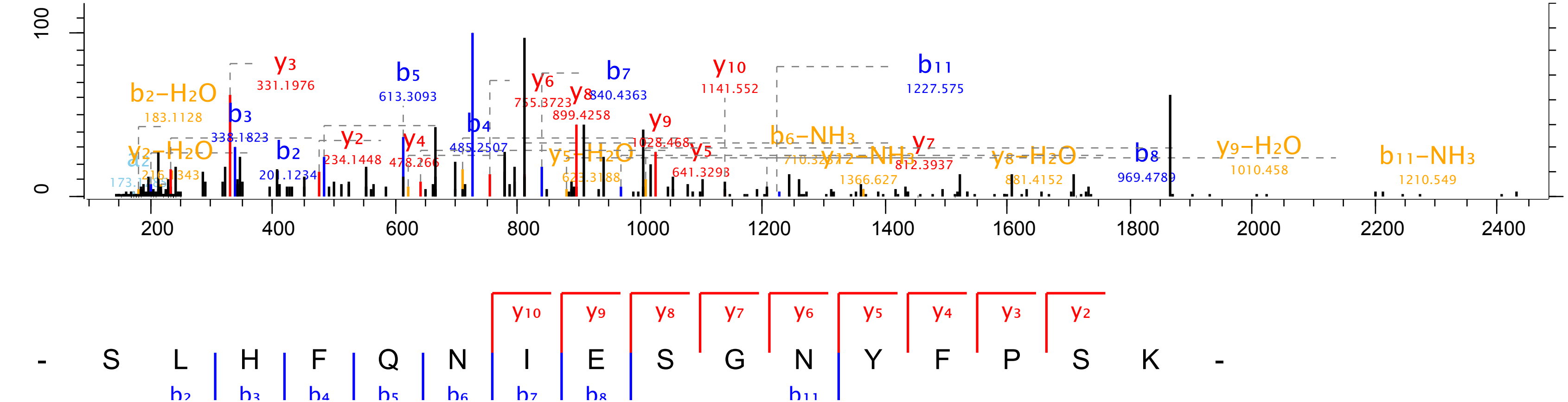

Raw file	Scan	Method	Score	m/z	Gene names
20150227_yeast_Top_opt_B1_01_1599	34440	TOF; CID	40.73	628.87	RDS1

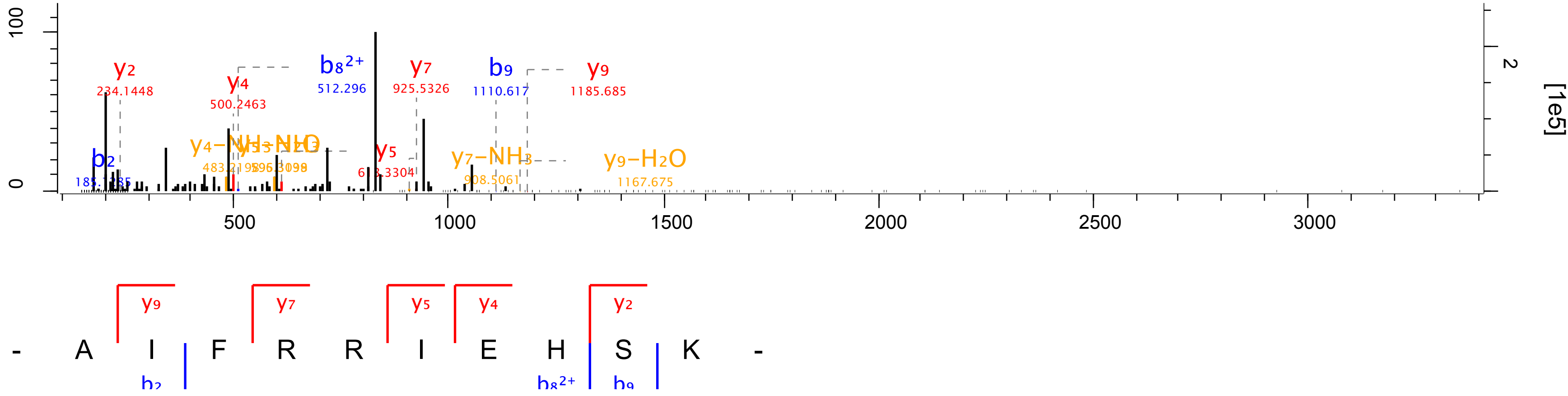

Raw file	Scan	Method	Score	m/z	Gene names
20150227_yeast_Top_opt_B1_01_1599	34471	TOF; CID	121.44	973.03	RFA3

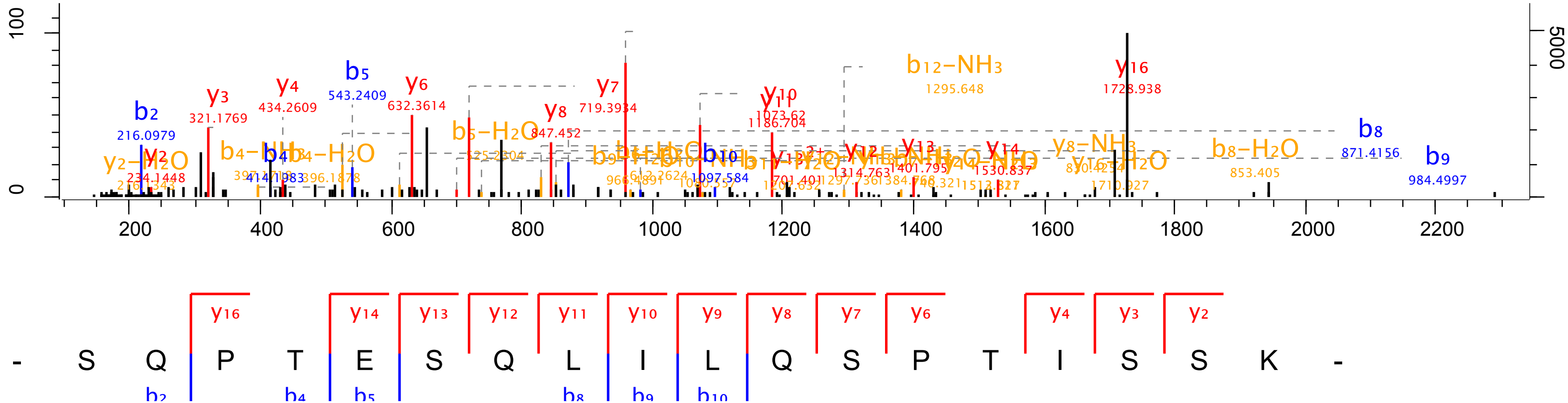

Raw file	Scan	Method	Score	m/z	Gene names
20150227_yeast_Top_opt_B1_01_1599	34490	TOF; CID	37.85	554.52	RGI1

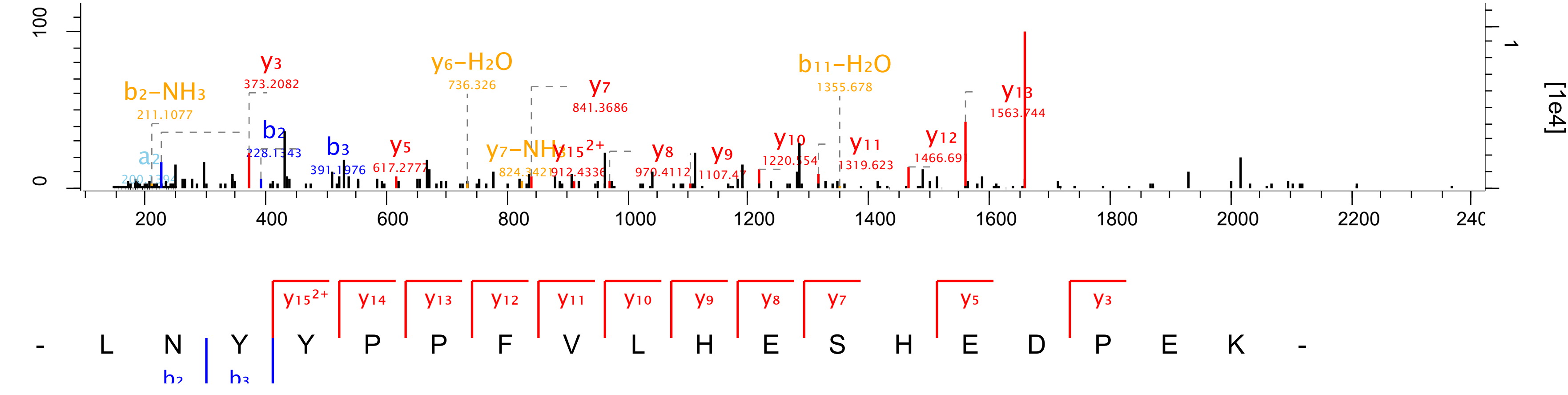

Raw file	Scan	Method	Score	m/z	Gene names
20150227_yeast_Top_opt_B1_01_1599	34585	TOF; CID	73.26	512.78	URE2

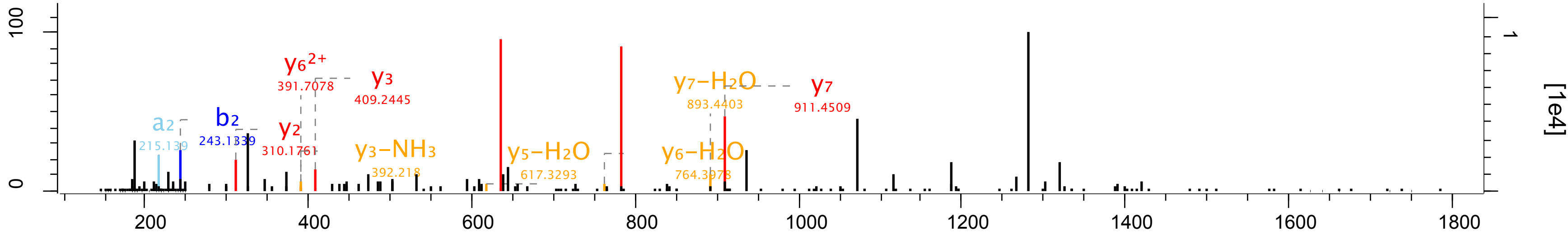

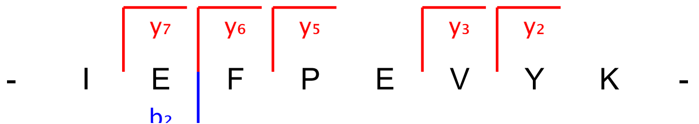

Raw file	Scan	Method	Score	m/z	Gene names
20150227_yeast_Top_opt_B1_01_1599	34670	TOF; CID	39.96	760.37	MCM10

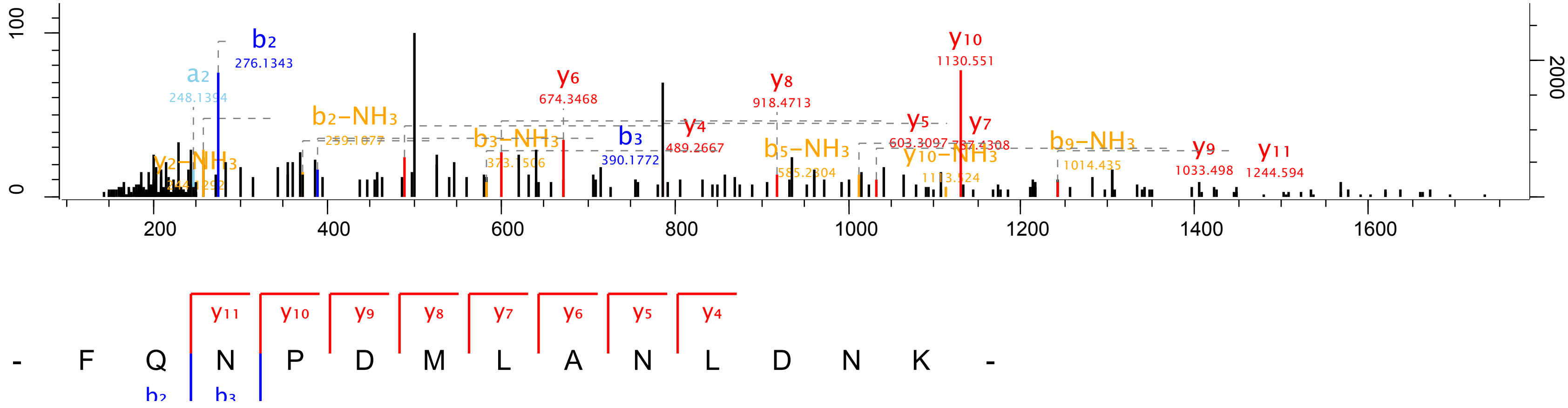

Raw file	Scan	Method	Score	m/z	Gene names
20150227_yeast_Top_opt_B1_01_1599	35124	TOF; CID	48.98	591.3	COX8

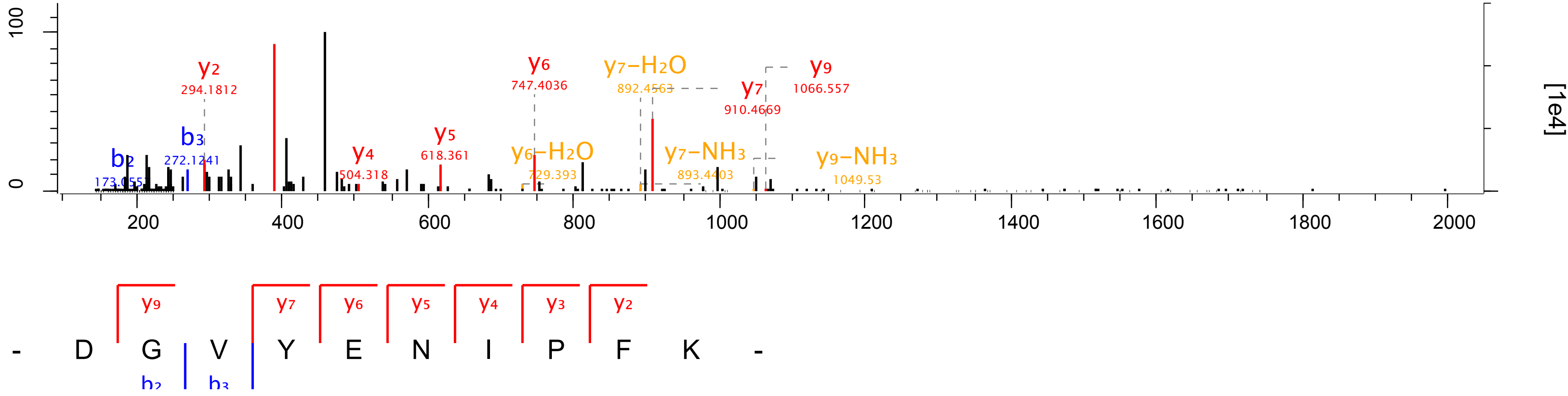

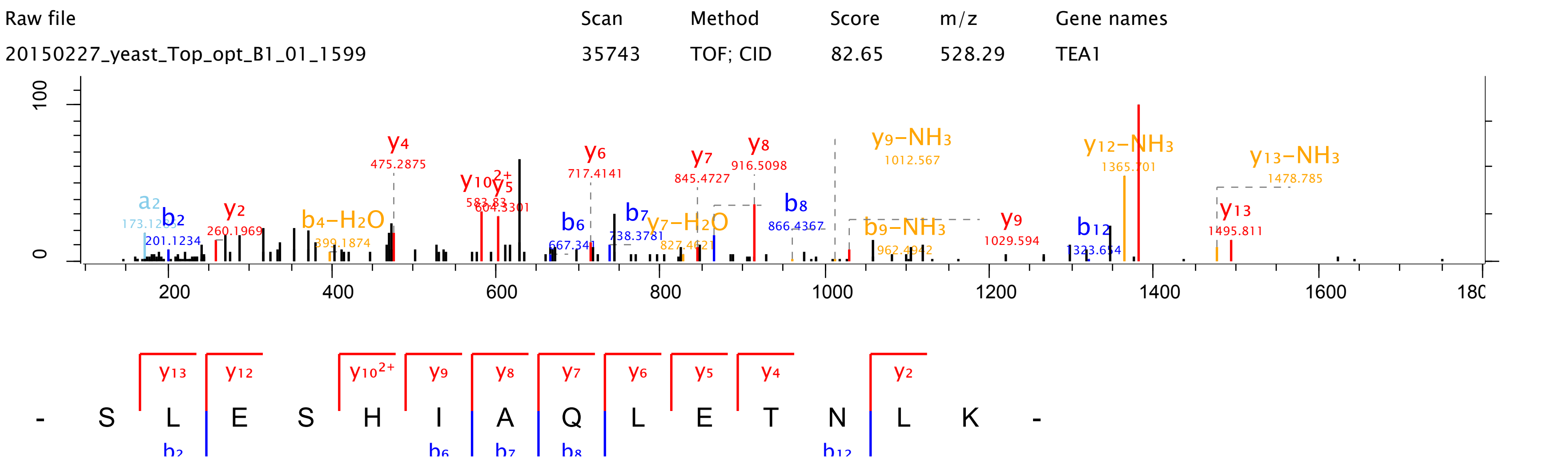

Raw file
20150227_yeast_Top_opt_B1_01_1599
Scan
35743
Method
TOF; CID
Score
82.65
m/z
528.29
Gene names
TEA1
100
0
a2
173.1285
b2
201.1234
y2
260.1969
b4−H2O
399.1874
y4
475.2875
y10 2+
583.83
y5
604.3301
b6
667.341
y6
717.4141
b7
738.3781
y7−H2O
827.4621
y7
845.4727
b8
866.4367
y8
916.5098
b9−NH3
962.4942
y9−NH3
1012.567
y9
1029.594
b12
1323.654
y12−NH3
1365.701
y13
1495.811
y13−NH3
1478.785
200
400
600
800
1000
1200
1400
1600
180
- S L E S H I A Q L E T N L K -
y13 y12 y10 2+ y9 y8 y7 y6 y5 y4 y2
b2 b6 b7 b8 b12

Raw file	Scan	Method	Score	m/z	Gene names
20150227_yeast_Top_opt_B1_01_1599	36137	TOF; CID	163.34	934.96	BIM1

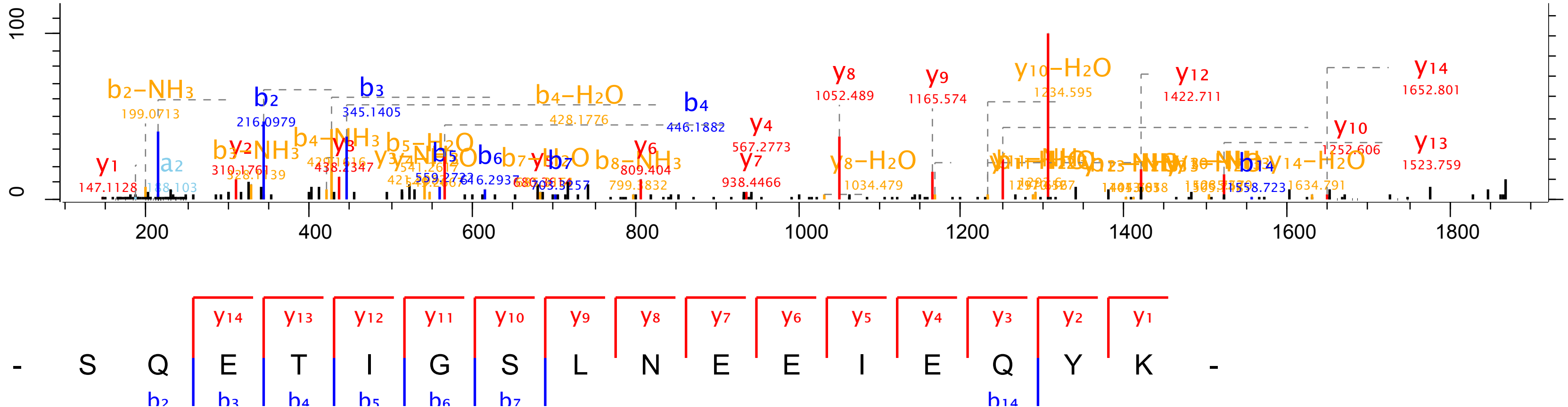

Raw file	Scan	Method	Score	m/z	Gene names
20150227_yeast_Top_opt_B1_01_1599	36344	TOF; CID	63.87	1110.51	KEL2

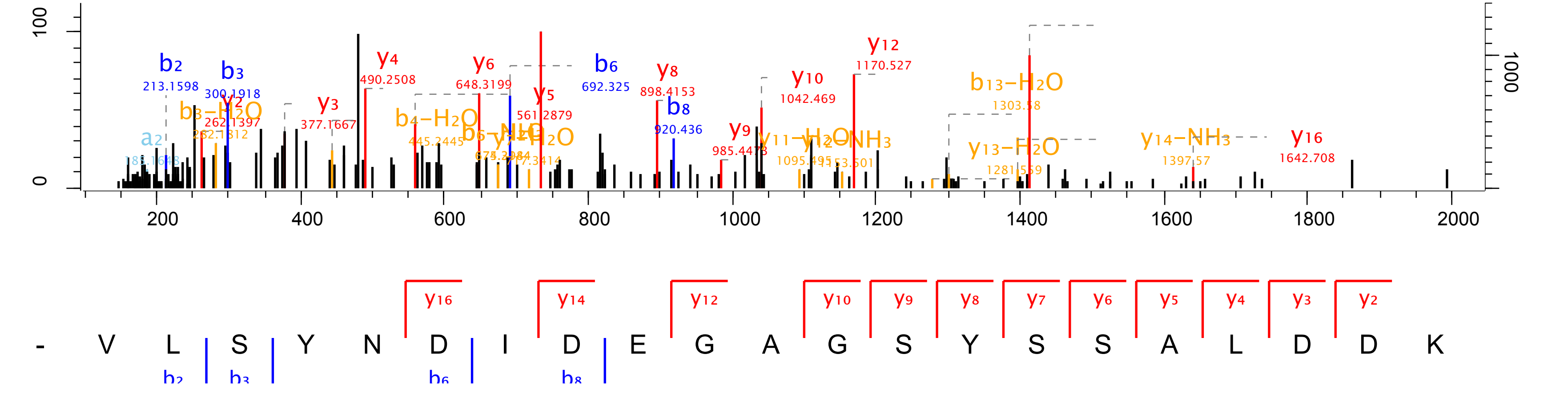

Raw file	Scan	Method	Score	m/z	Gene names
20150227_yeast_Top_opt_B1_01_1599	36544	TOF; CID	44.51	719.41	GUD1

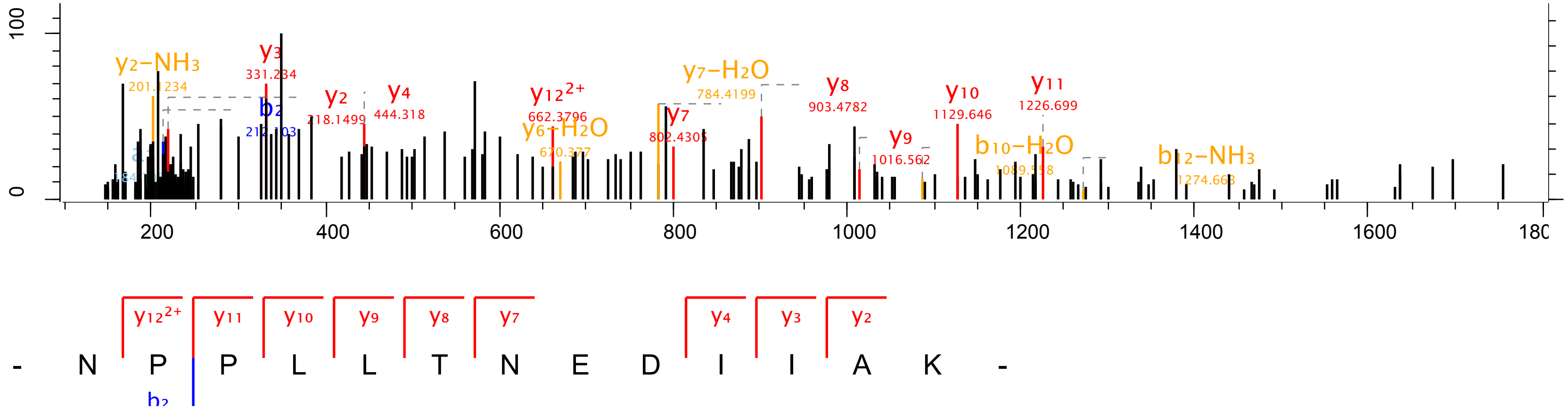

Raw file	Scan	Method	Score	m/z	Gene names
20150227_yeast_Top_opt_B1_01_1599	36572	TOF; CID	66.83	736.91	LST4

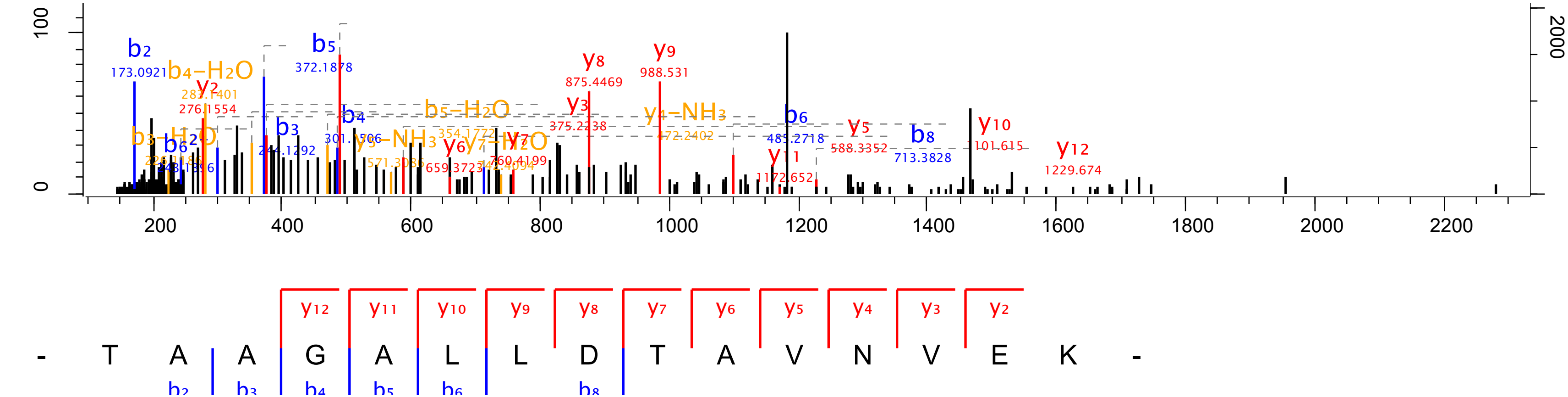

Raw file	Scan	Method	Score	m/z	Gene names
20150227_yeast_Top_opt_B1_01_1599	36811	TOF; CID	54.28	605.31	ECM30

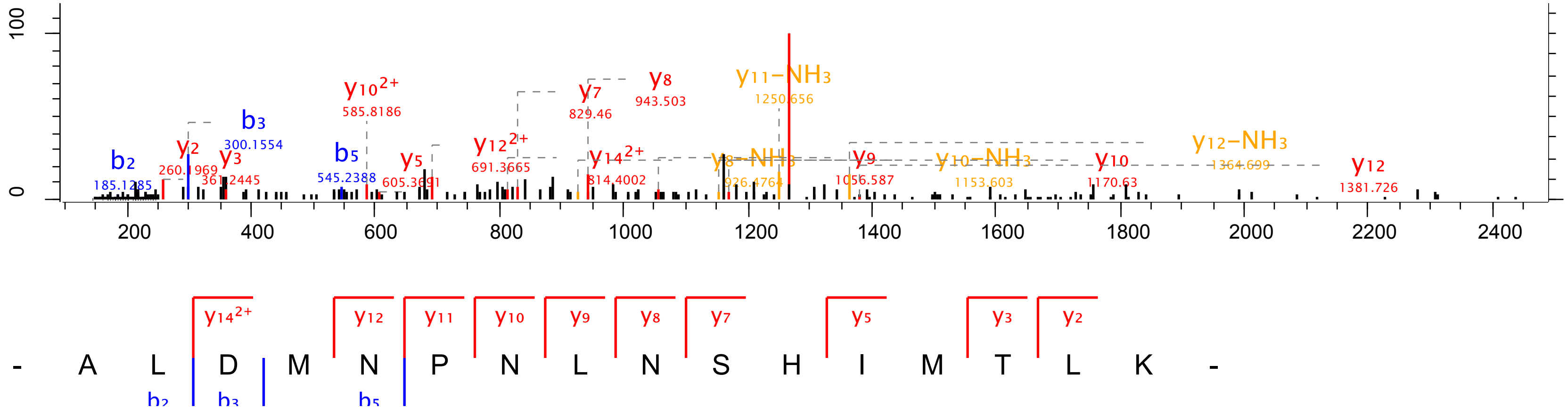

Raw file	Scan	Method	Score	m/z	Gene names
20150227_yeast_Top_opt_B1_01_1599	37530	TOF; CID	55.31	635.81	PSF1

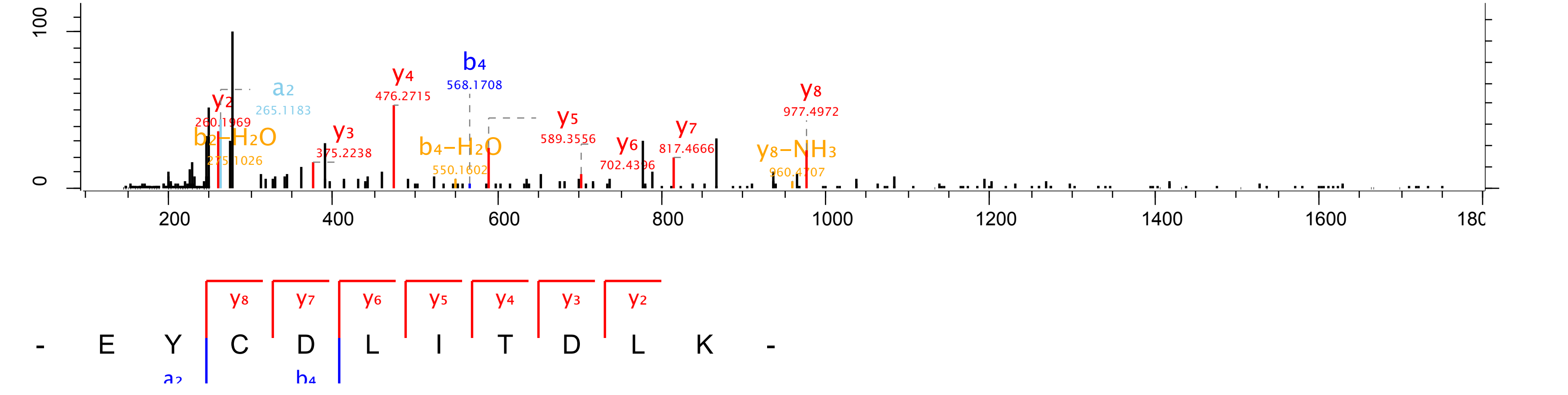

Raw file	Scan	Method	Score	m/z	Gene names
20150227_yeast_Top_opt_B1_01_1599	37826	TOF; CID	142.35	924.44	RPC11

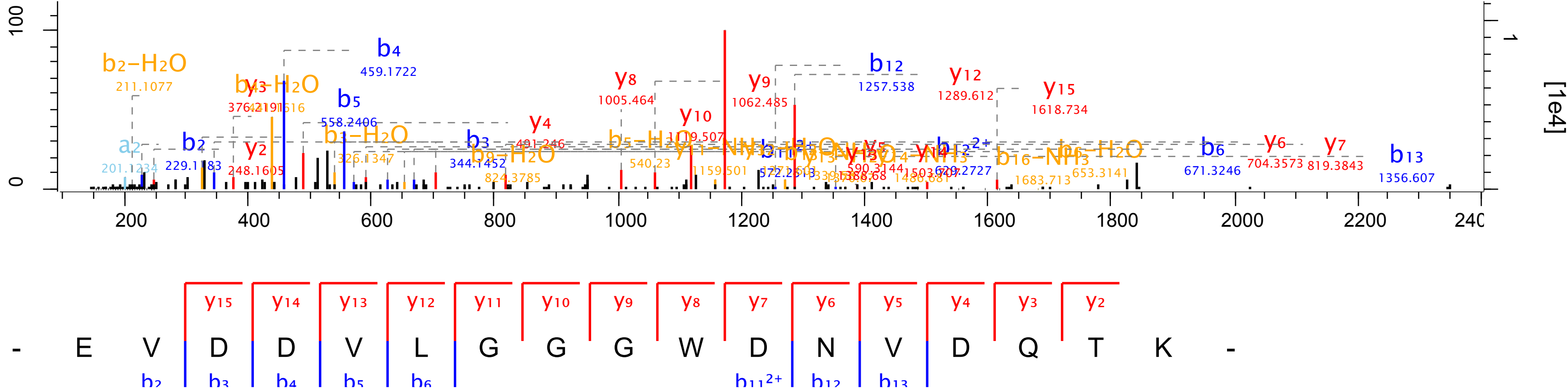

Raw file	Scan	Method	Score	m/z	Gene names
20150227_yeast_Top_opt_B1_01_1599	38269	TOF; CID	84.95	1111.56	TFC6

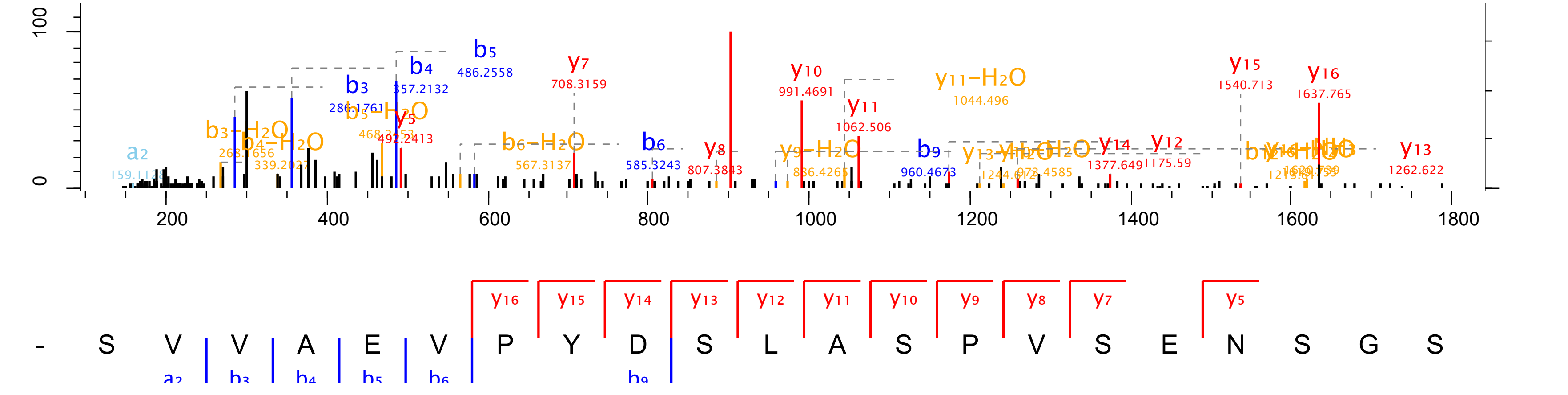

Raw file	Scan	Method	Score	m/z	Gene names
20150227_yeast_Top_opt_B1_01_1599	38307	TOF; CID	39.02	725.36	RSF2

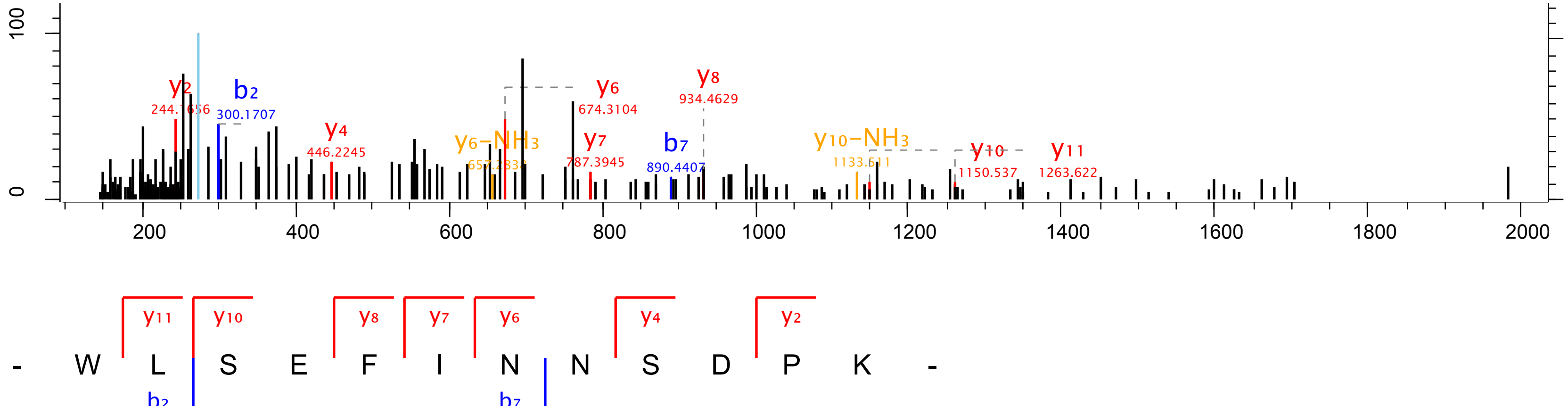

Raw file	Scan	Method	Score	m/z	Gene names
20150227_yeast_Top_opt_B1_01_1599	38640	TOF; CID	67.21	521.83	RLM1

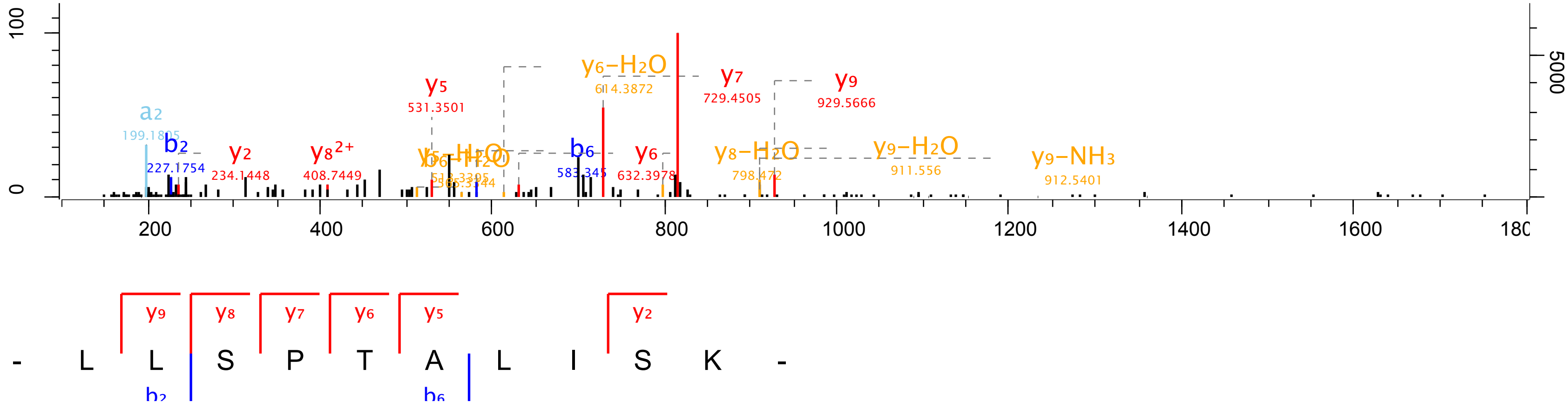

Raw file	Scan	Method	Score	m/z
20150227_yeast_Top_opt_B1_01_1599	39477	TOF; CID	48.91	660.37

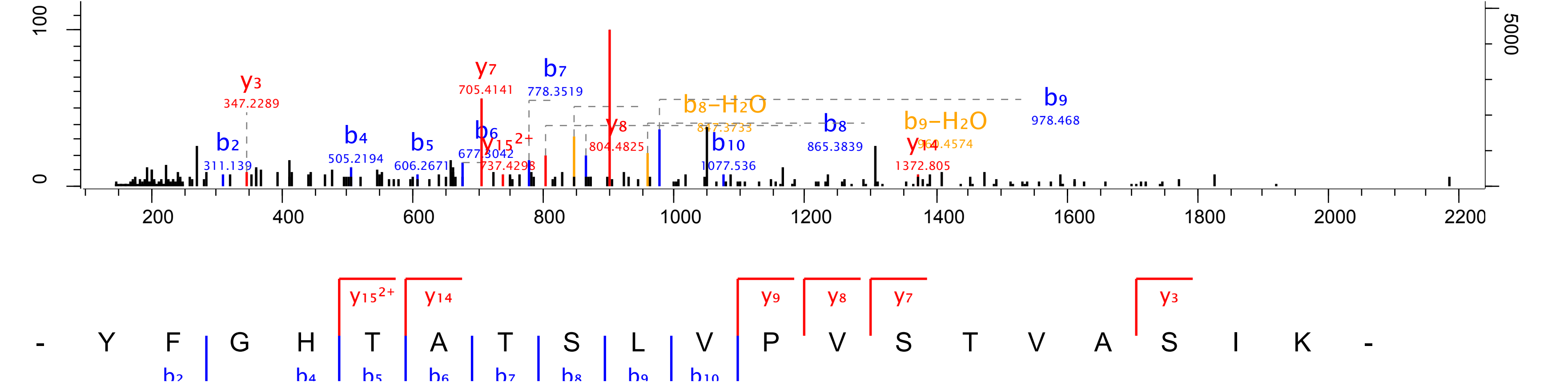

Raw file	Scan	Method	Score	m/z	Gene names
20150227_yeast_Top_opt_B1_01_1599	40108	TOF; CID	64.78	430.79	MET2

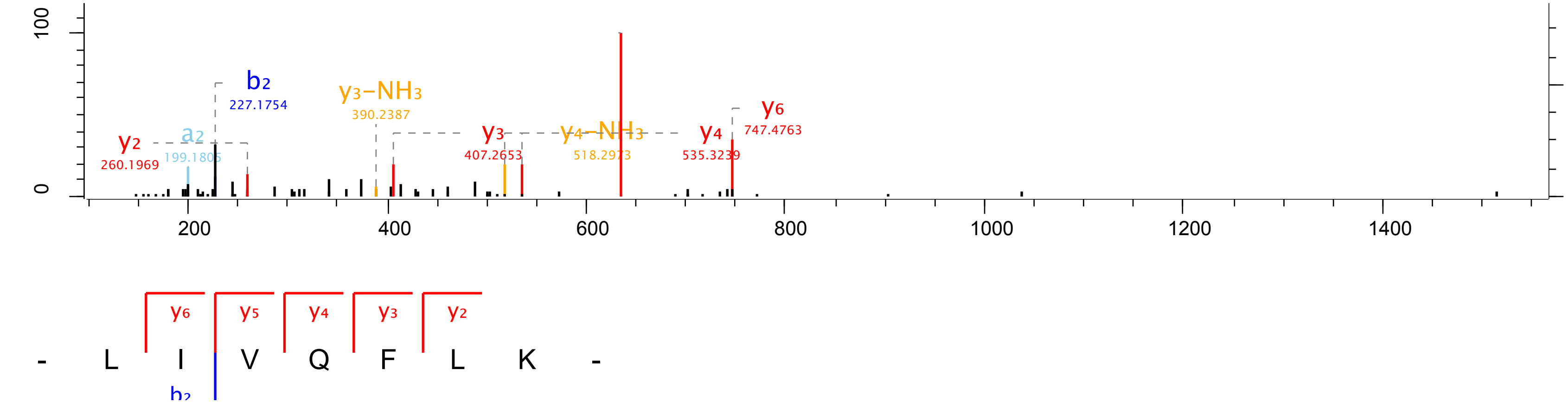

Raw file	Scan	Method	Score	m/z	Gene names
20150227_yeast_Top_opt_B1_01_1599	40462	TOF; CID	39.01	693.36	SYF2

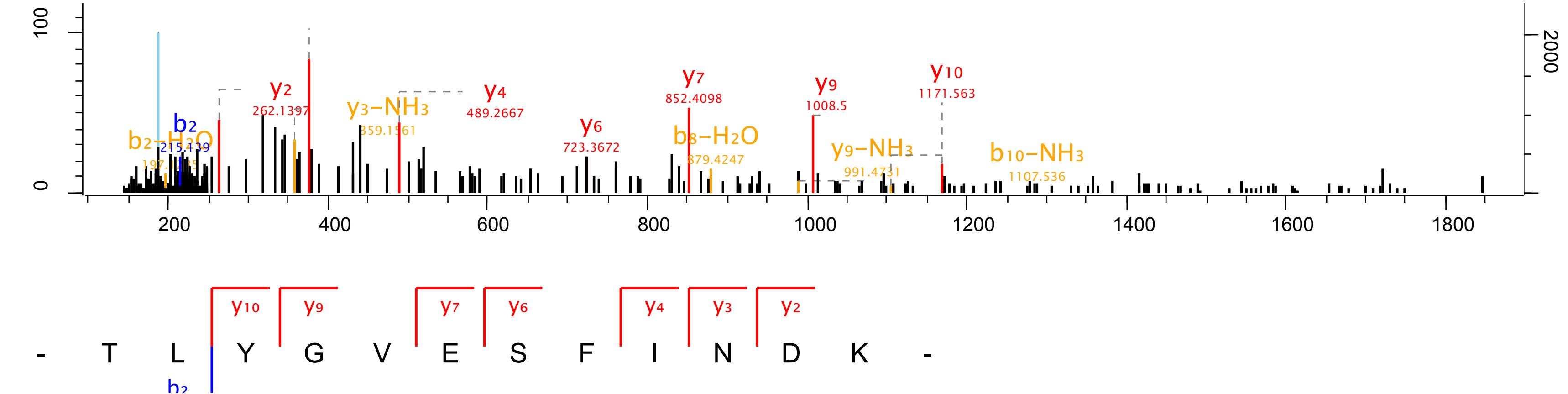

Raw file	Scan	Method	Score	m/z	Gene names
20150227_yeast_Top_opt_B1_01_1599	40760	TOF; CID	82.29	554.81	YPS1

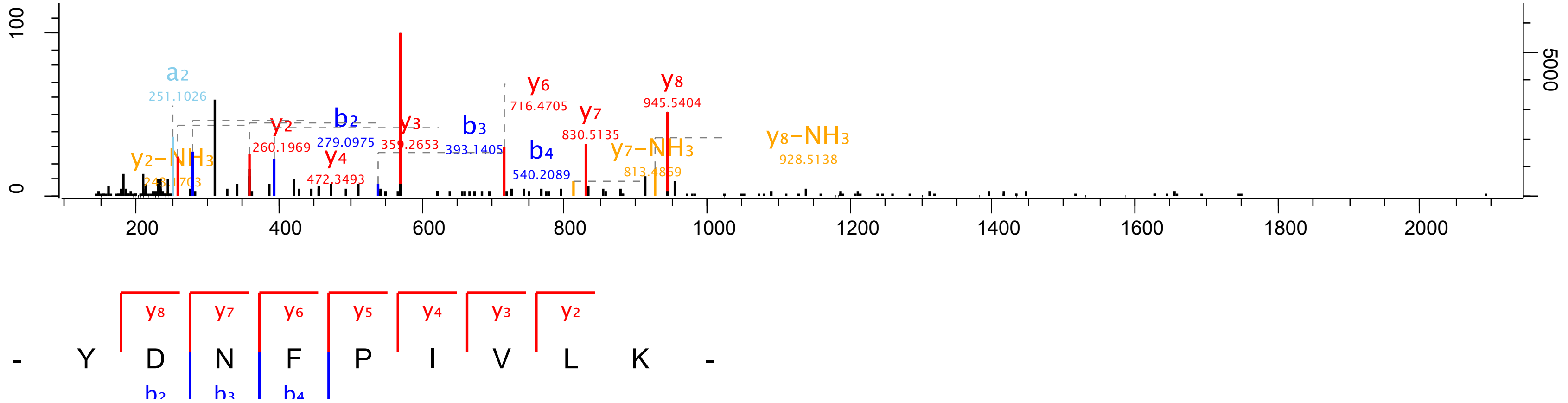

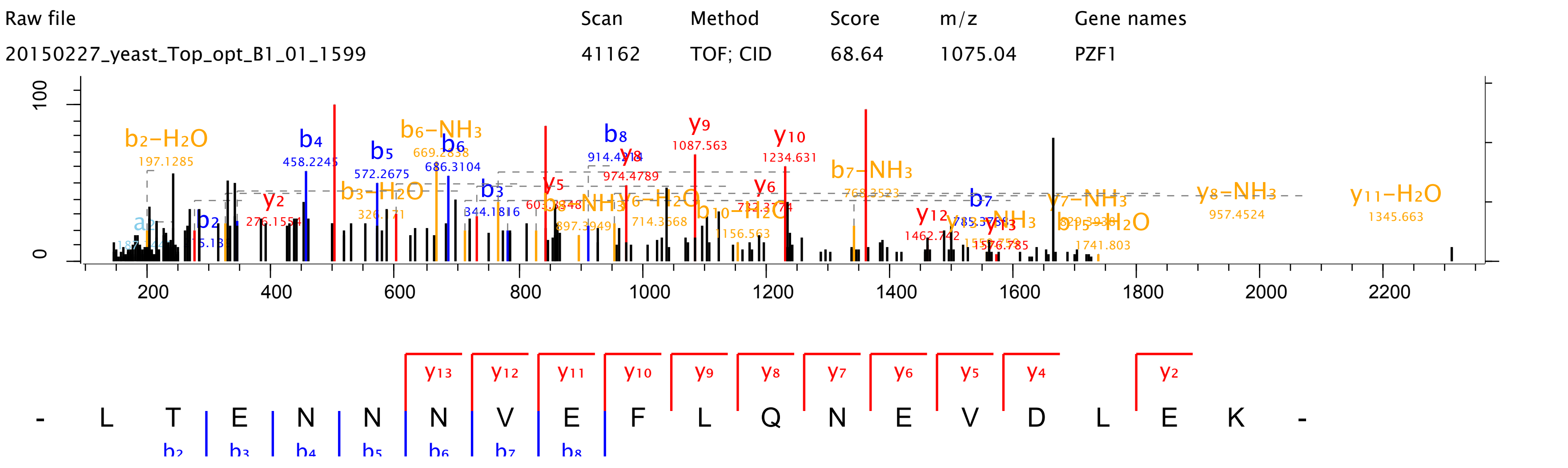

Raw file
20150227_yeast_Top_opt_B1_01_1599
Scan
41162
Method
TOF; CID
Score
68.64
m/z
1075.04
Gene names
PZF1
100
0
200
400
600
800
1000
1200
1400
1600
1800
2000
2200
b2−H2O
197.1285
b4
458.2245
b5
572.2675
b6−NH3
669.2838
b6
686.3104
b8
914.4218
y8
974.4789
y9
1087.563
y10
1234.631
b7−NH3
768.3523
y2
276.1554
b2
b3−H2O
b3
344.1816
y5
y6
732.3774
b10−H2O
1156.563
y6−H2O
714.3568
y12
1462.742
b7
y7−NH3
y8−NH3
957.4524
y11−H2O
1345.663
1741.803
y13 y12 y11 y10 y9 y8 y7 y6 y5 y4 y2
- L T E N N N V E F L Q N E V D L E K -
b2 b3 b4 b5 b6 b7 b8

Raw file	Scan	Method	Score	m/z	Gene names
20150227_yeast_Top_opt_B1_01_1599	41306	TOF; CID	99.82	496.8	TRS130

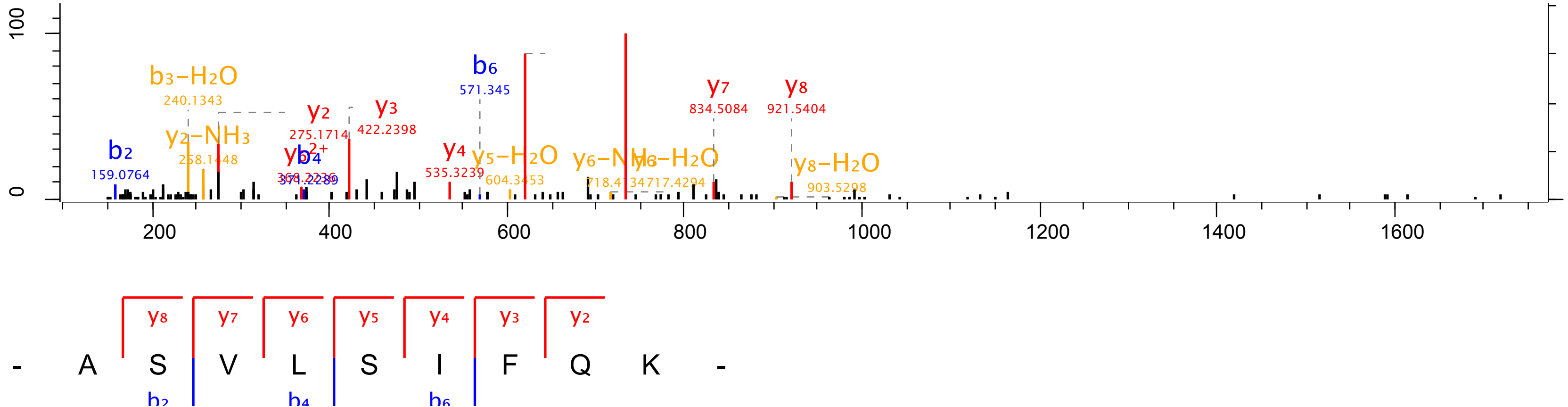

Raw file	Scan	Method	Score	m/z	Gene names
20150227_yeast_Top_opt_B1_01_1599	41614	TOF; CID	35.84	845.43	LDB7

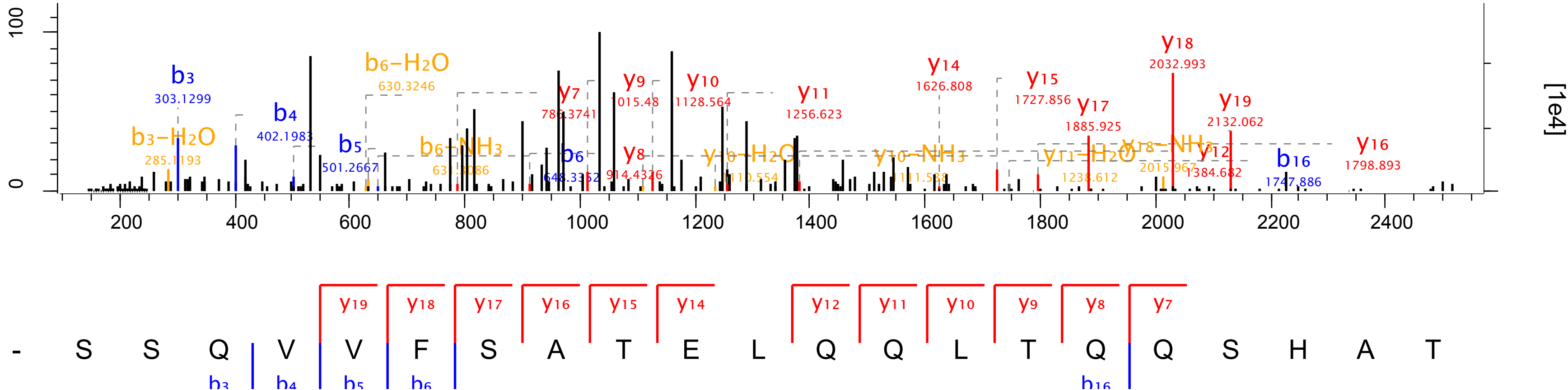

Raw file	Scan	Method	Score	m/z	Gene names
20150227_yeast_Top_opt_B1_01_1599	41755	TOF; CID	76.21	994.51	MIX23

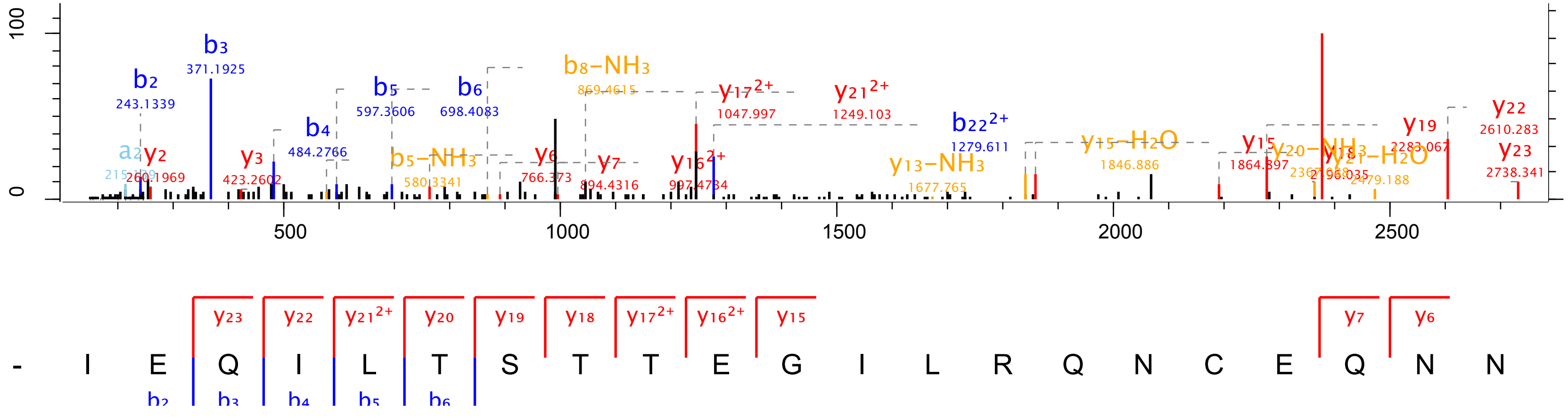

Raw file	Scan	Method	Score	m/z	Gene names
20150227_yeast_Top_opt_B1_01_1599	43257	TOF; CID	54.26	562.81	SMF2

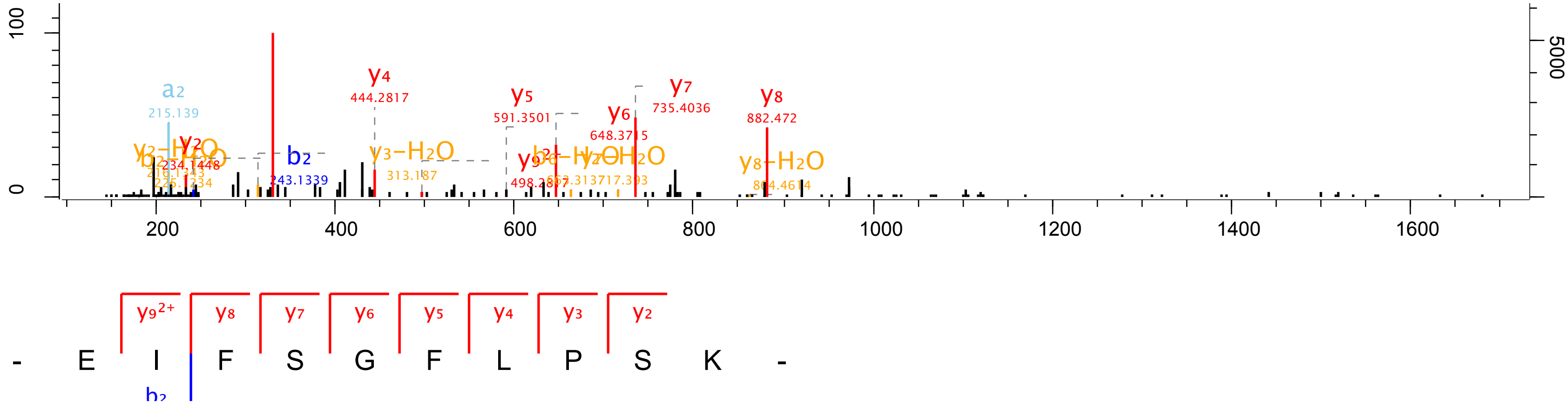

Raw file	Scan	Method	Score	m/z	Gene names
20150227_yeast_Top_opt_B1_01_1599	43868	TOF; CID	62.09	533.96	RHO4

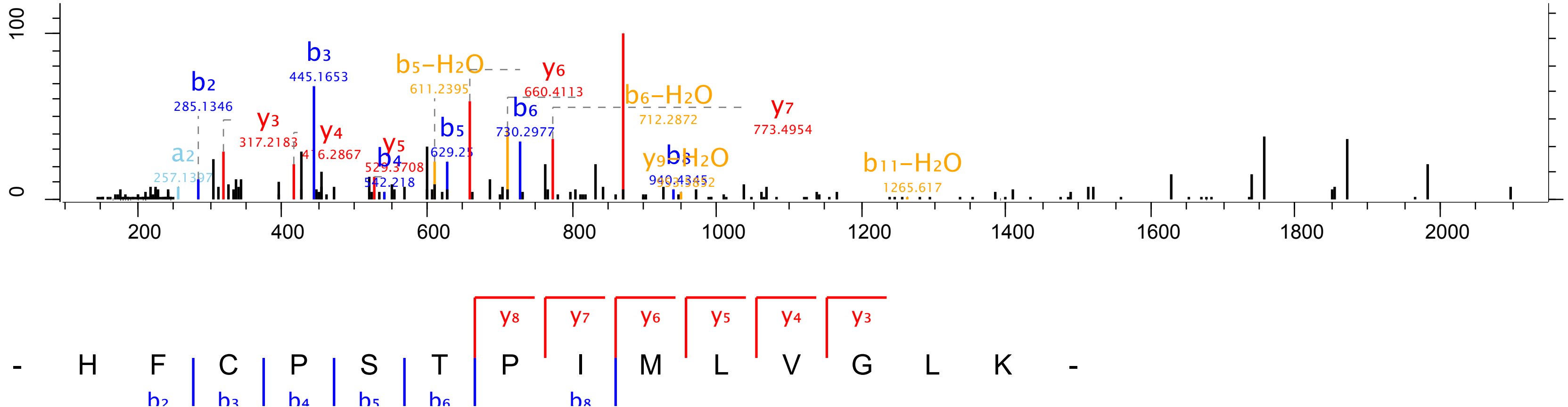

Raw file	Scan	Method	Score	m/z	Gene names
20150227_yeast_Top_opt_B1_01_1599	44079	TOF; CID	97.8	866.45	TIM17

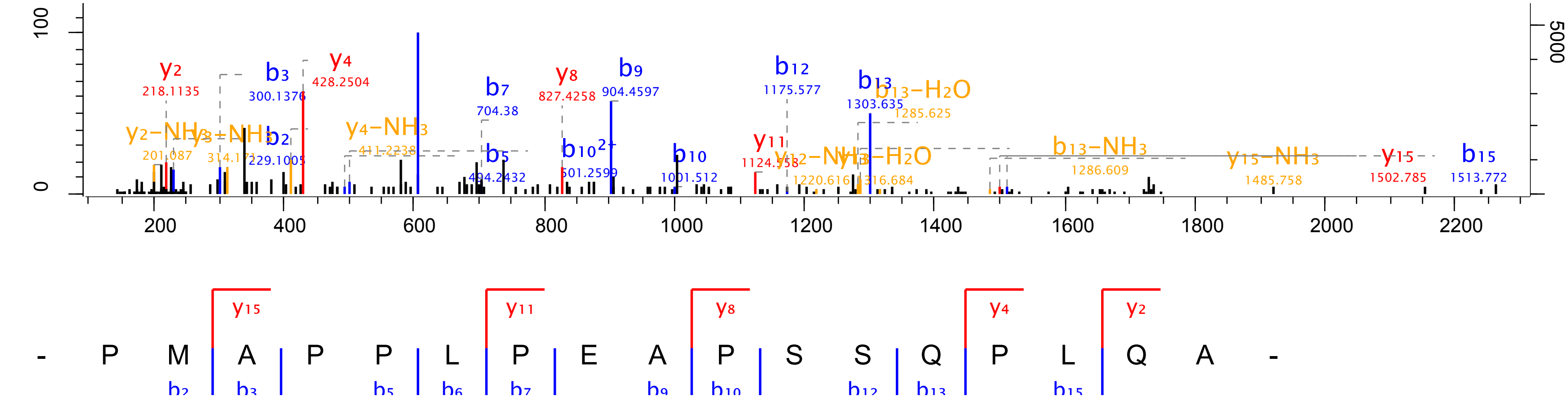

Raw file	Scan	Method	Score	m/z	Gene names
20150227_yeast_Top_opt_B1_01_1599	44165	TOF; CID	116.55	638.35	RSM10

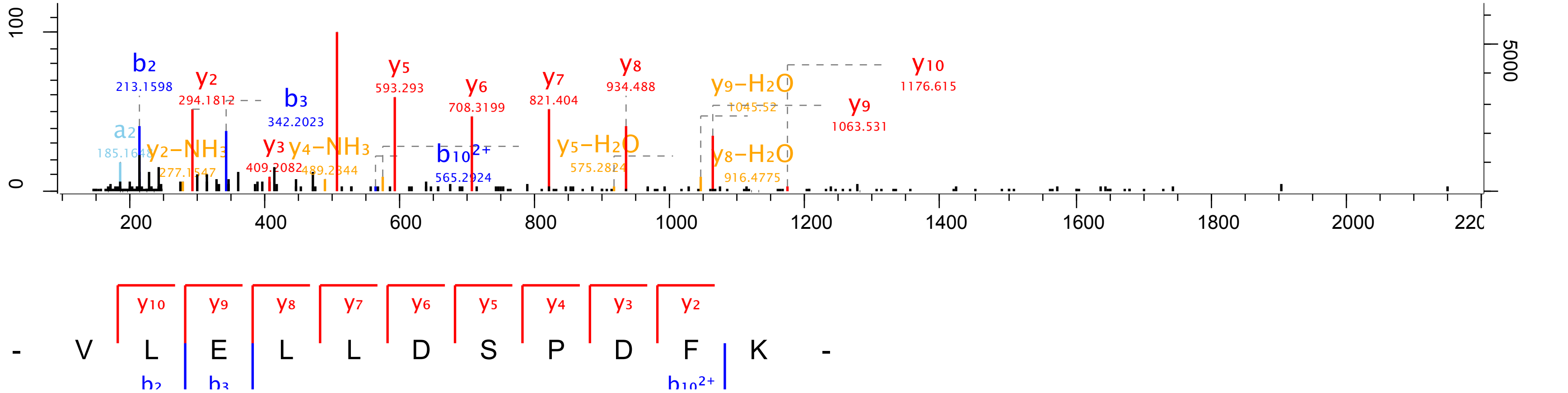

Raw file	Scan	Method	Score	m/z	Gene names
20150227_yeast_Top_opt_B1_01_1599	44241	TOF; CID	74.78	615.82	CUE4

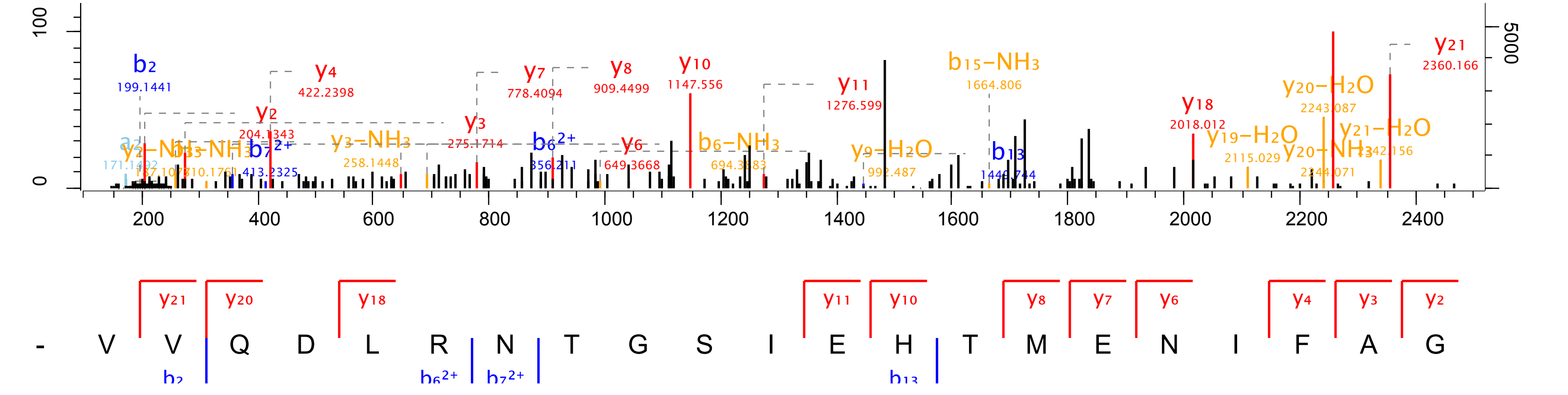

Raw file	Scan	Method	Score	m/z
20150227_yeast_Top_opt_B1_01_1599	45396	TOF; CID	43.84	867.46

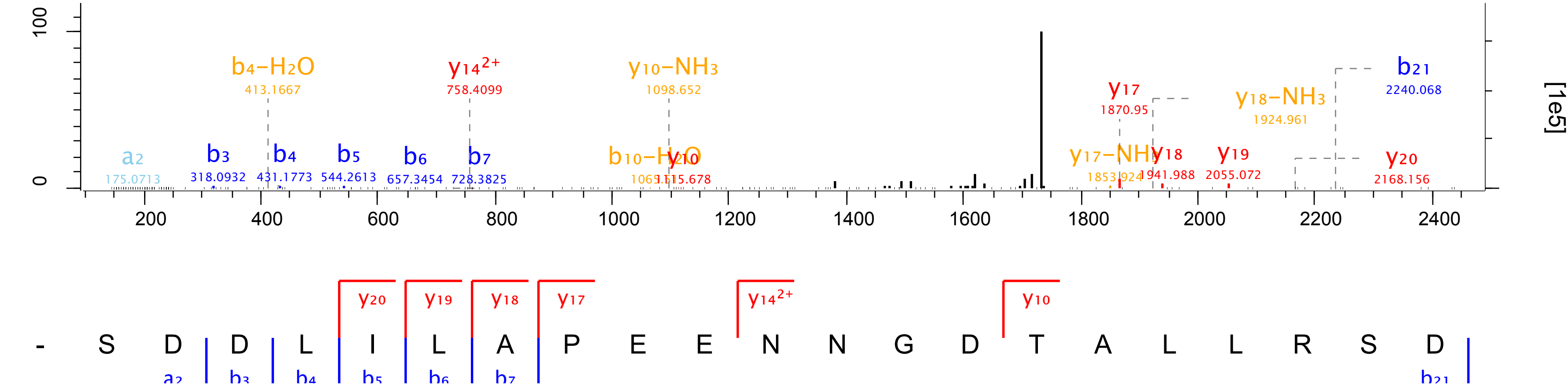

Raw file	Scan	Method	Score	m/z	Gene names
20150227_yeast_Top_opt_B1_01_1599	46076	TOF; CID	55.06	491.32	GIS4

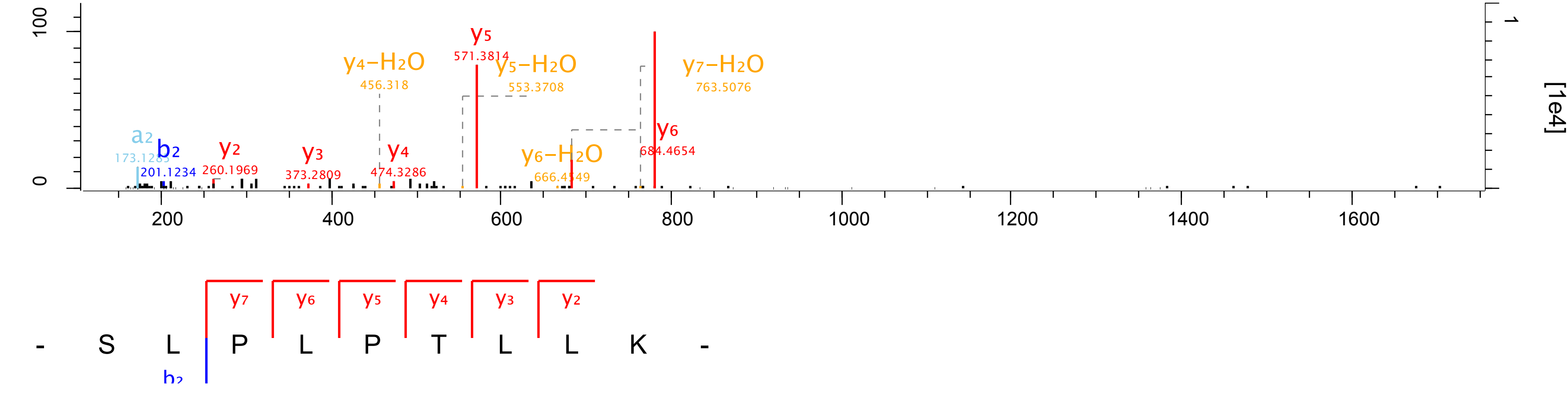

Raw file	Scan	Method	Score	m/z	Gene names
20150227_yeast_Top_opt_B1_01_1599	46148	TOF; CID	61.82	599.33	MRPL49

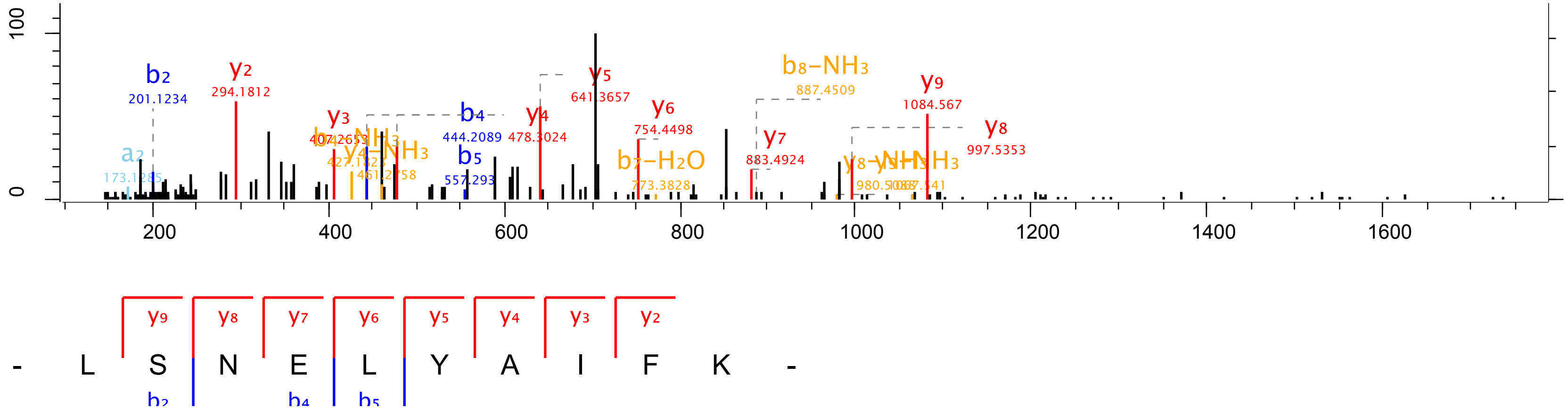

Raw file	Scan	Method	Score	m/z	Gene names
20150227_yeast_Top_opt_B1_01_1599	46750	TOF; CID	47.92	1009.54	SAE2

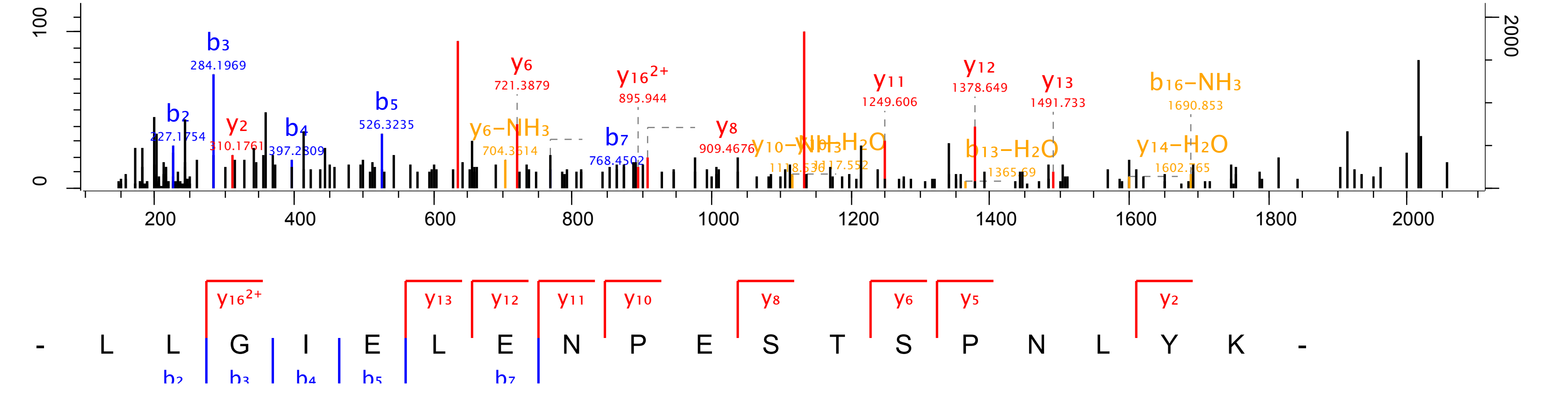

Raw file	Scan	Method	Score	m/z	Gene names
20150227_yeast_Top_opt_B1_01_1599	46788	TOF; CID	61.48	931.47	OST2

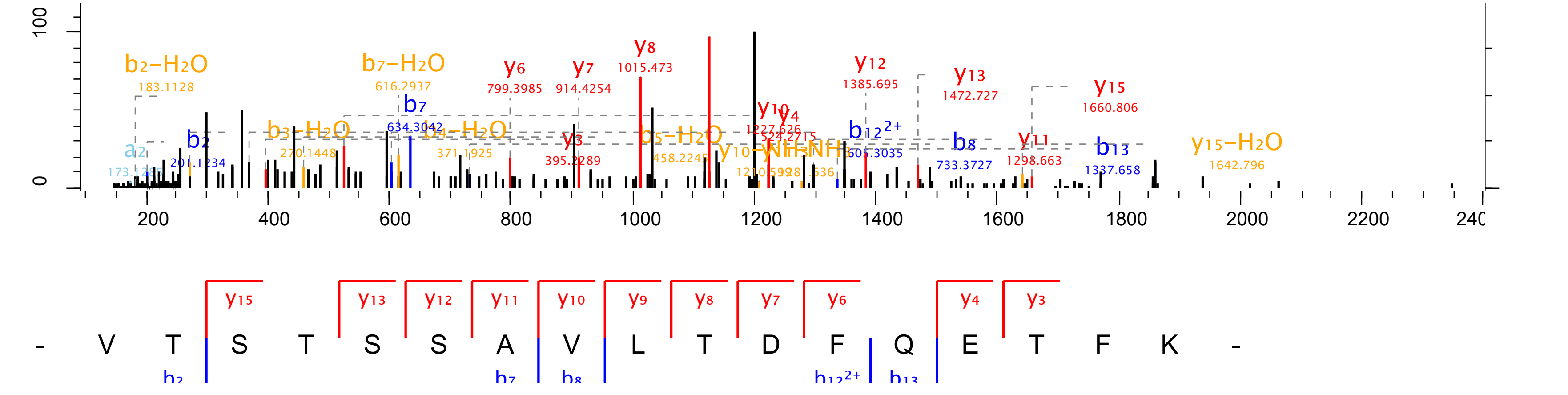

Raw file	Scan	Method	Score	m/z	Gene names
20150227_yeast_Top_opt_B1_01_1599	47288	TOF; CID	44.97	701.9	ATG38

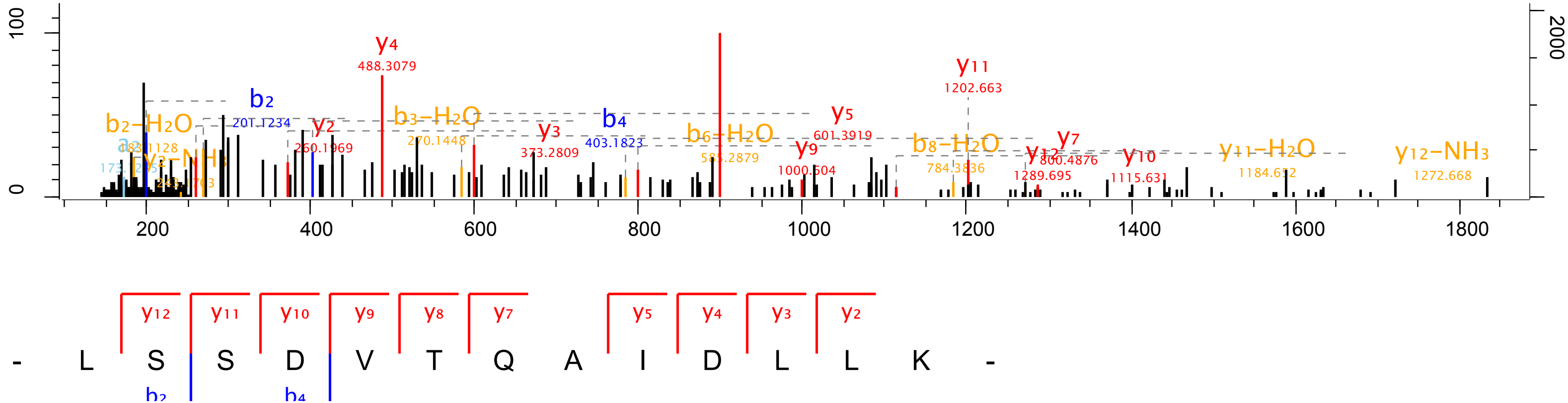

Raw file	Scan	Method	Score	m/z	Gene names
20150227_yeast_Top_opt_B1_01_1599	47625	TOF; CID	51.31	774.71	PEX7

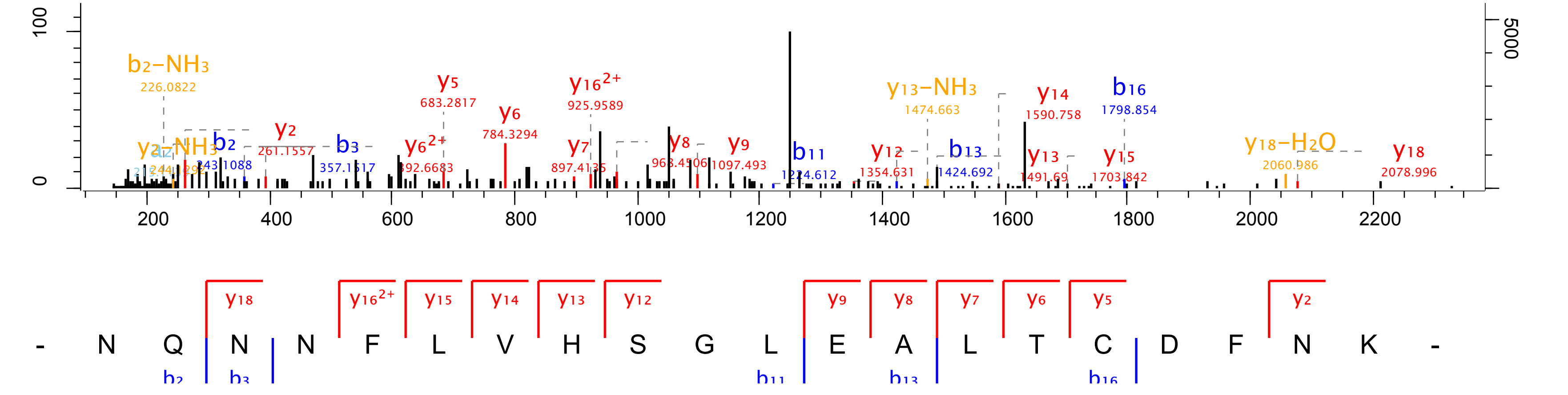

Raw file	Scan	Method	Score	m/z	Gene names
20150227_yeast_Top_opt_B1_01_1599	47750	TOF; CID	39.21	940.49	PFS2

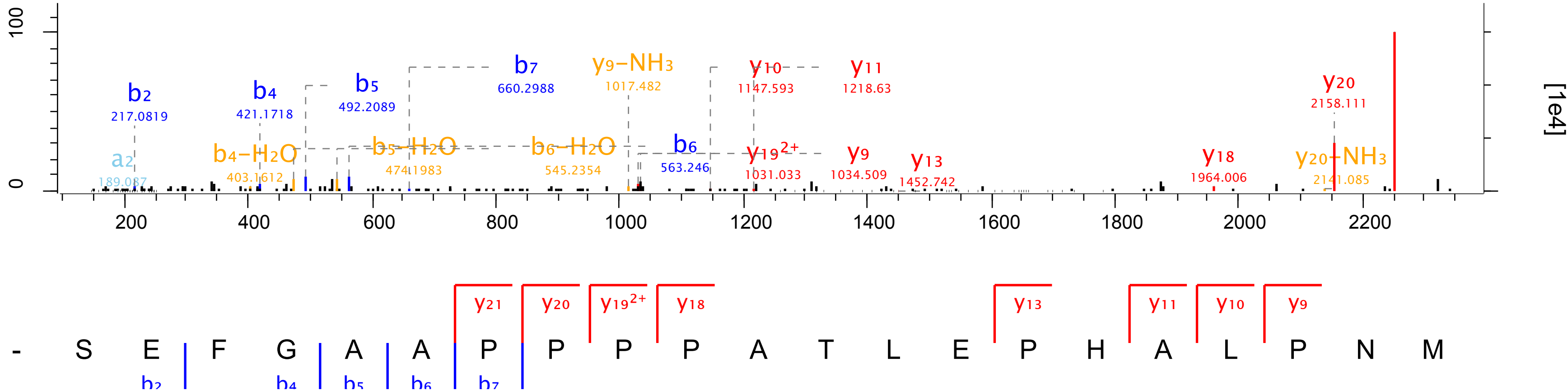

Raw file	Scan	Method	Score	m/z	Gene names
20150227_yeast_Top_opt_B1_01_1599	47865	TOF; CID	109.83	823.92	ART5

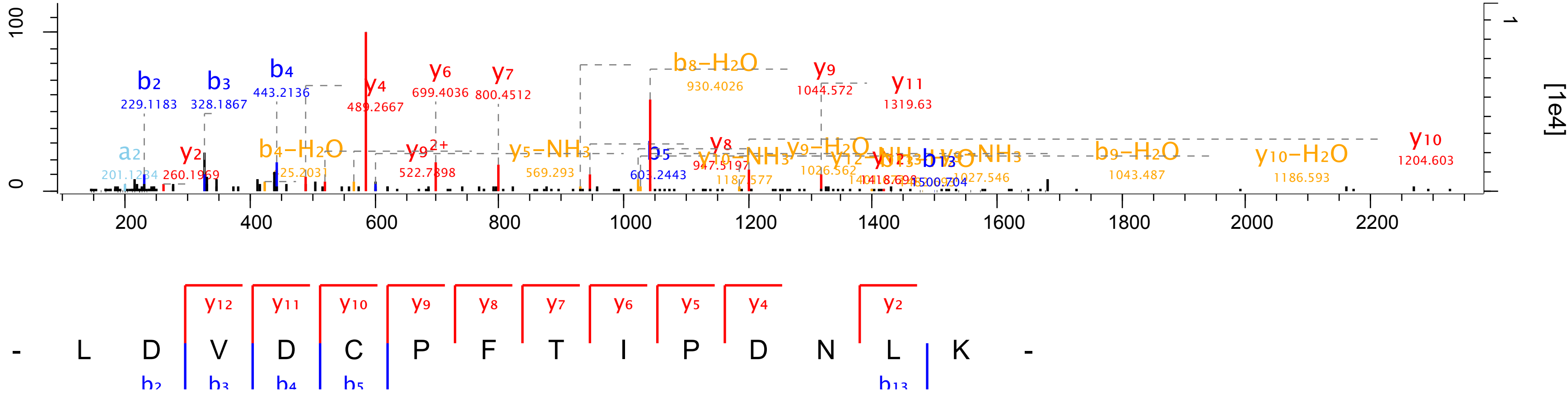

Raw file	Scan	Method	Score	m/z	Gene names
20150227_yeast_Top_opt_B1_01_1599	48640	TOF; CID	56.57	694.42	ARG2

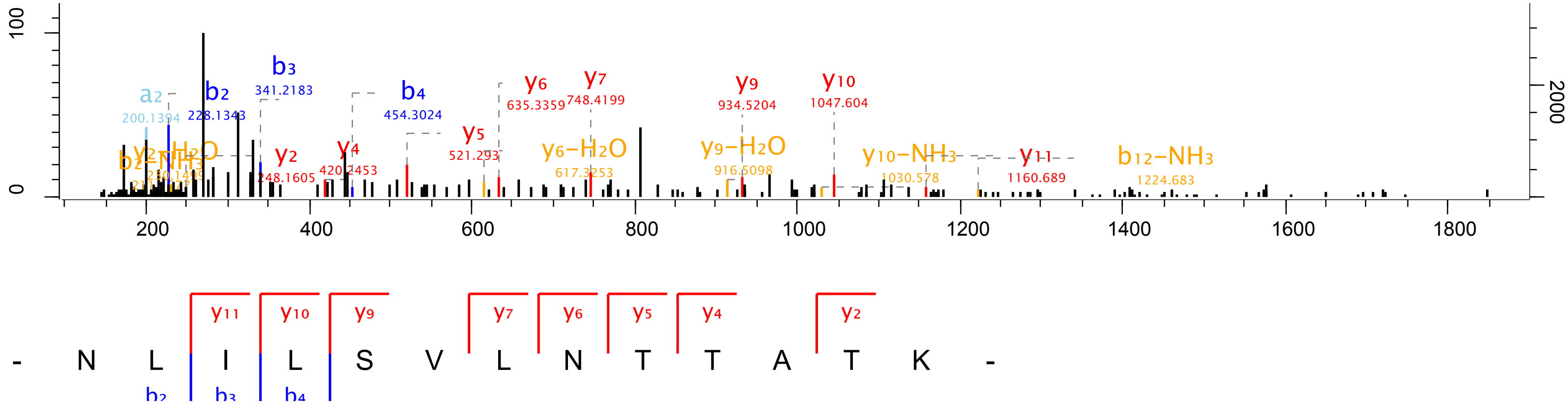

Raw file	Scan	Method	Score	m/z	Gene names
20150227_yeast_Top_opt_B1_01_1599	48925	TOF; CID	109.1	723.39	HIF1

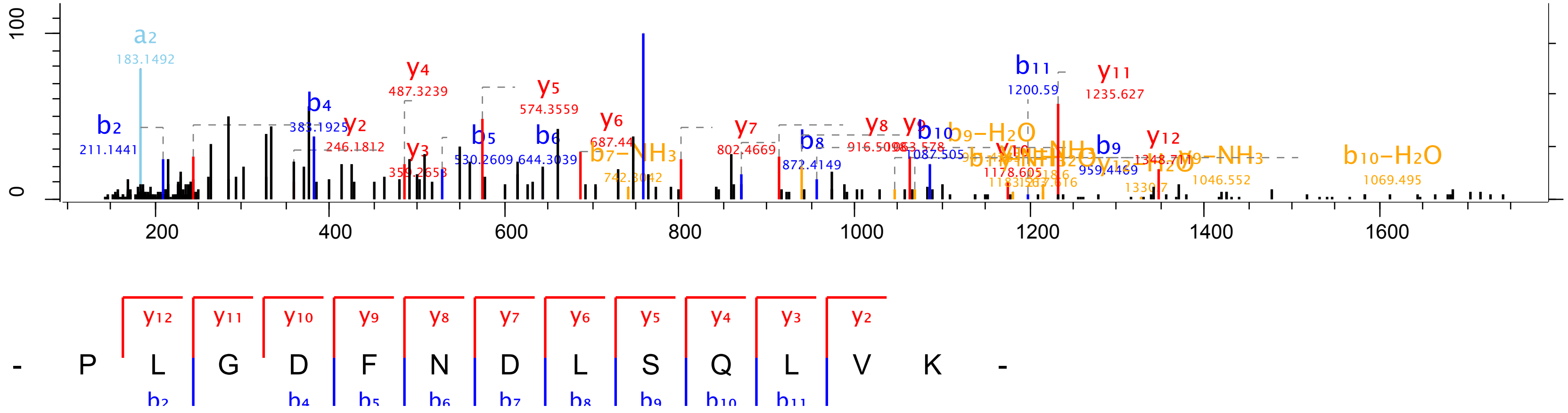

Raw file	Scan	Method	Score	m/z	Gene names
20150227_yeast_Top_opt_B1_01_1599	56502	TOF; CID	42.35	753.41	POR2

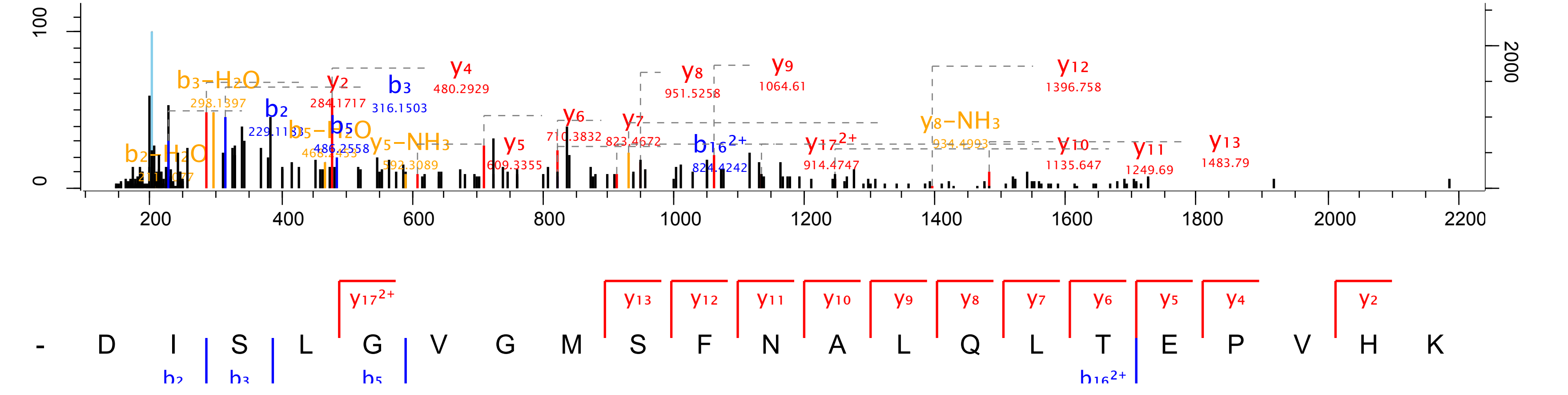

Raw file	Scan	Method	Score	m/z	Gene names
20150227_yeast_Top_opt_B1_01_1599	56955	TOF; CID	111.64	1053.54	PEX4

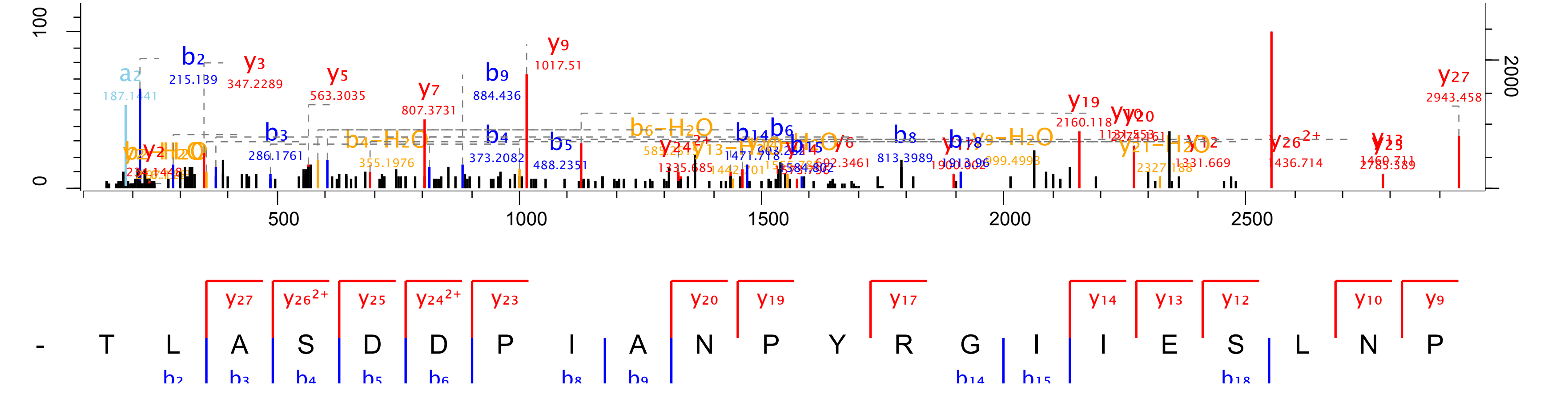

Raw file	Scan	Method	Score	m/z	Gene names
20150227_yeast_Top_opt_B1_01_1599	58045	TOF; CID	92.15	1405.73	YDC1

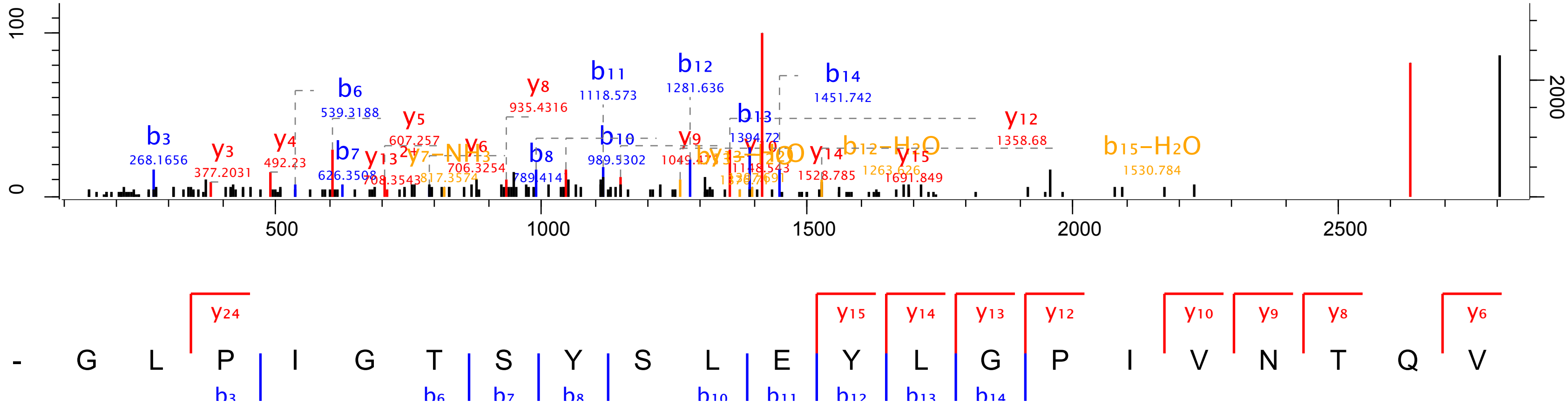

Raw file	Scan	Method	Score	m/z	Gene names
20150227_yeast_Top_opt_B1_01_1599	58416	TOF; CID	54.76	1004.55	PET100

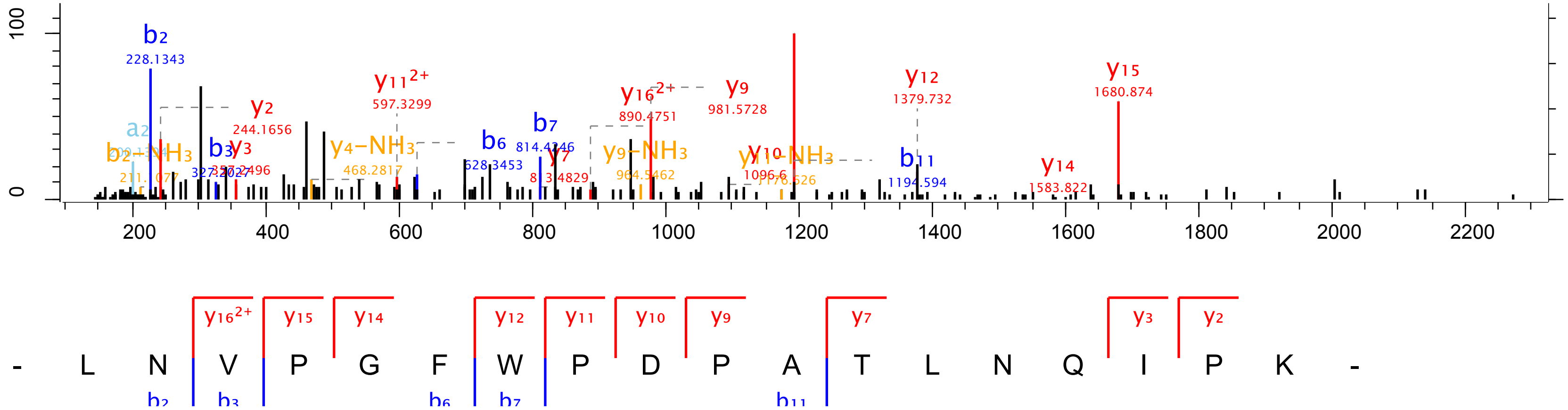

Raw file	Scan	Method	Score	m/z	Gene names
20150227_yeast_Top_opt_B1_01_1599	59282	TOF; CID	58.96	869.78	STE18

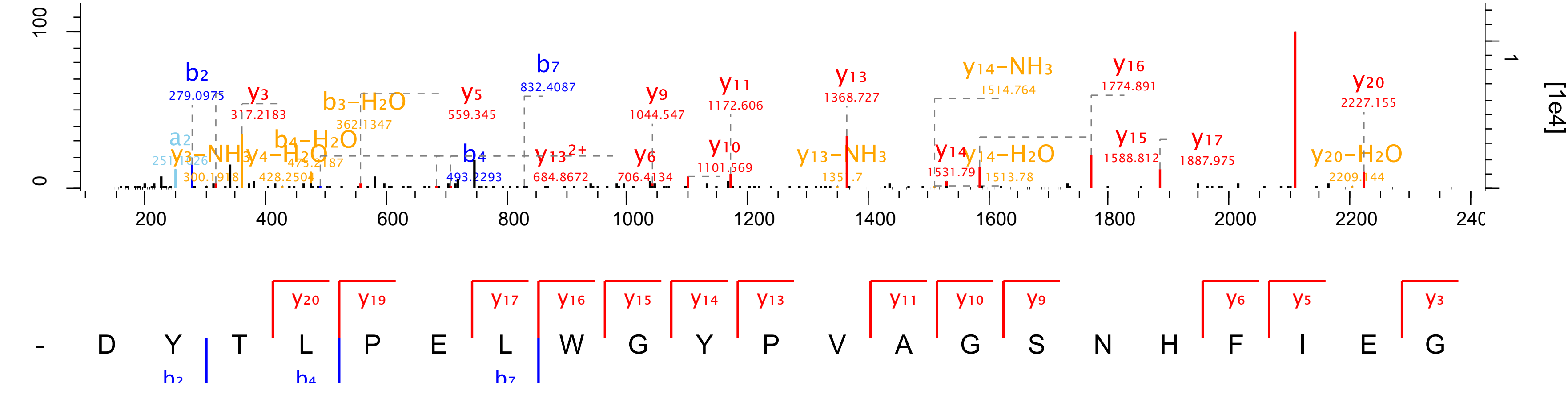

Raw file	Scan	Method	Score	m/z	Gene names
20150227_yeast_Top_opt_B1_01_1599	61423	TOF; CID	54.09	754.44	LSM5

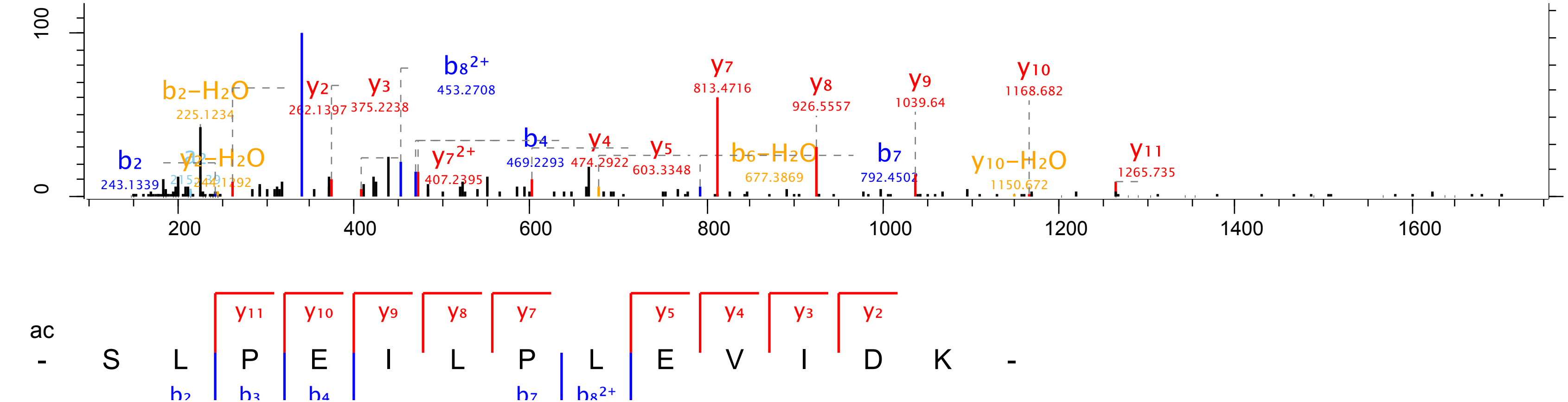

Raw file	Scan	Method	Score	m/z	Gene names
20150228_yeast1_Top_opt_B1_01_1611	1269	TOF; CID	93.16	414.21	ECM15

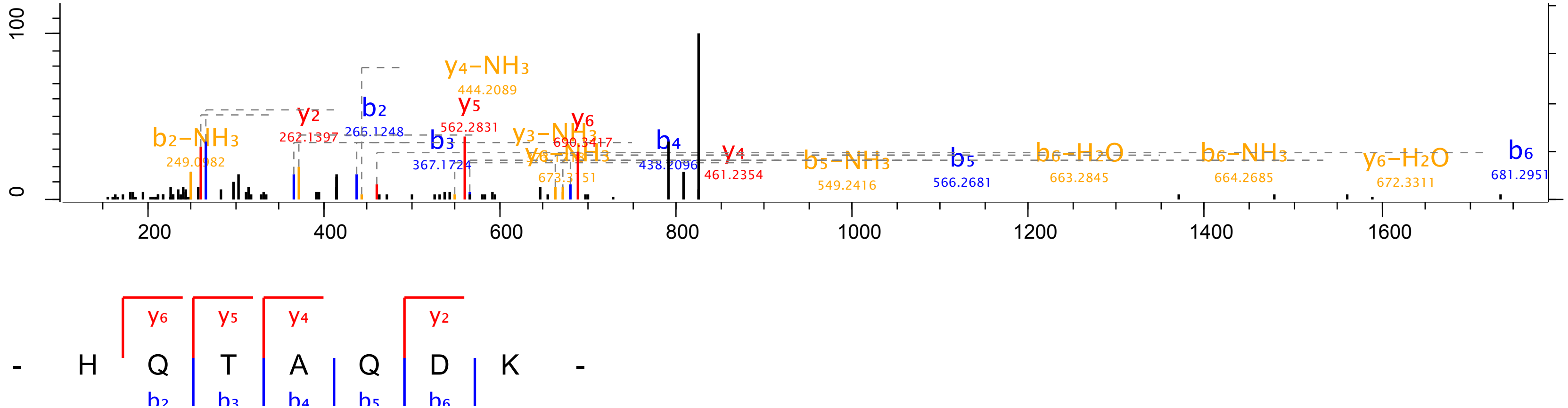

Raw file	Scan	Method	Score	m/z	Gene names
20150228_yeast1_Top_opt_B1_01_1611	2318	TOF; CID	60.79	398.24	RPL29

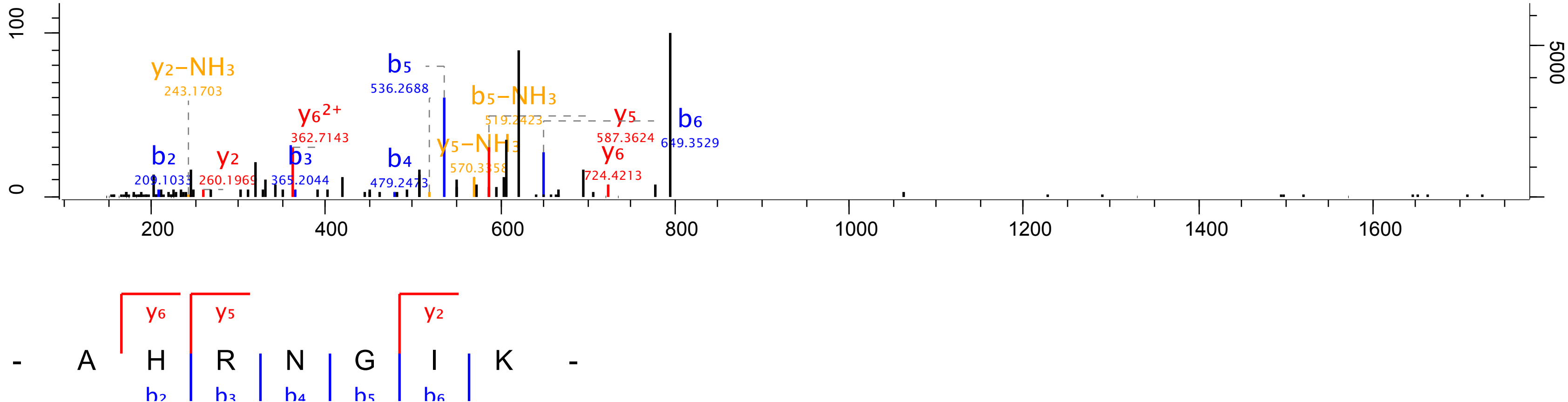

Raw file	Scan	Method	Score	m/z	Gene names
20150228_yeast1_Top_opt_B1_01_1611	4124	TOF; CID	104.81	744.27	CUP1-2;CUP1-1

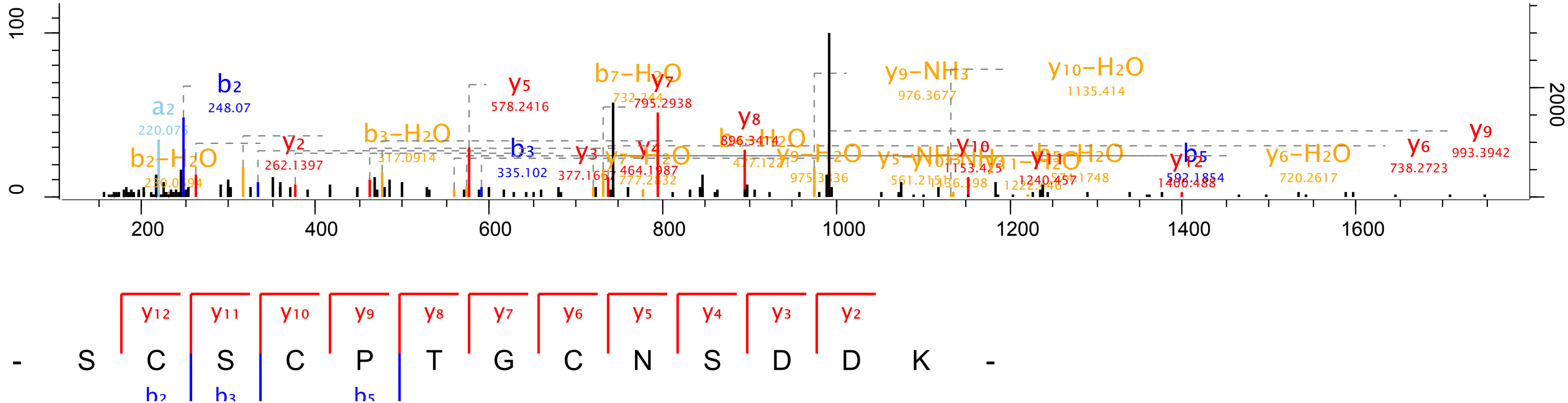

Raw file	Scan	Method	Score	m/z	Gene names
20150228_yeast1_Top_opt_B1_01_1611	4734	TOF; CID	84.17	482.23	AGP1

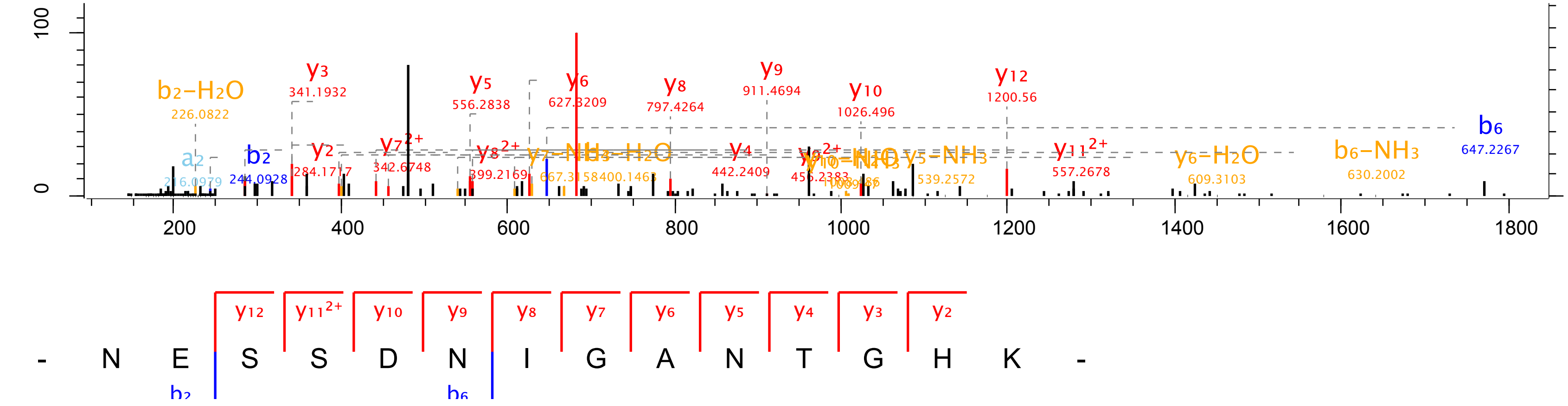

Raw file	Scan	Method	Score	m/z	Gene names
20150228_yeast1_Top_opt_B1_01_1611	5783	TOF; CID	97.07	469.23	TIM9

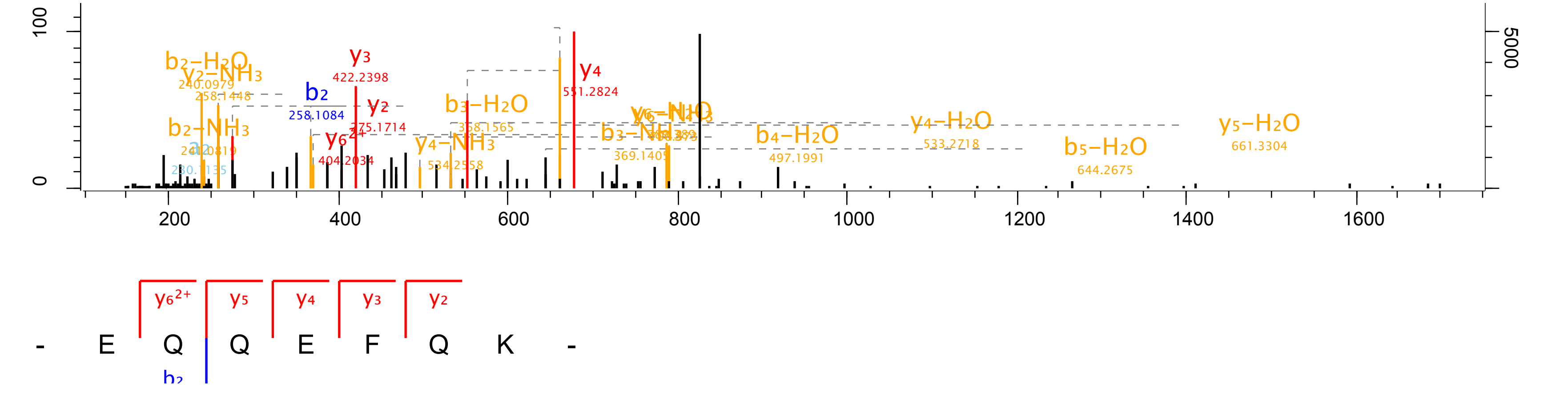

Raw file	Scan	Method	Score	m/z	Gene names
20150228_yeast1_Top_opt_B1_01_1611	5860	TOF; CID	75.74	614.31	PRM5

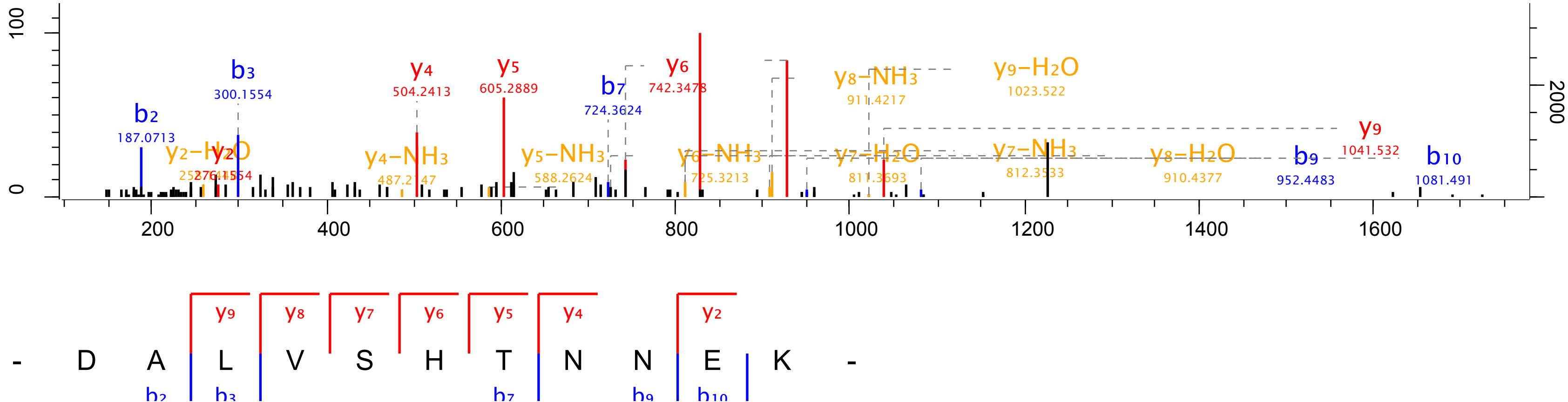

Raw file	Scan	Method	Score	m/z	Gene names
20150228_yeast1_Top_opt_B1_01_1611	6092	TOF; CID	49.45	634.84	RAD53

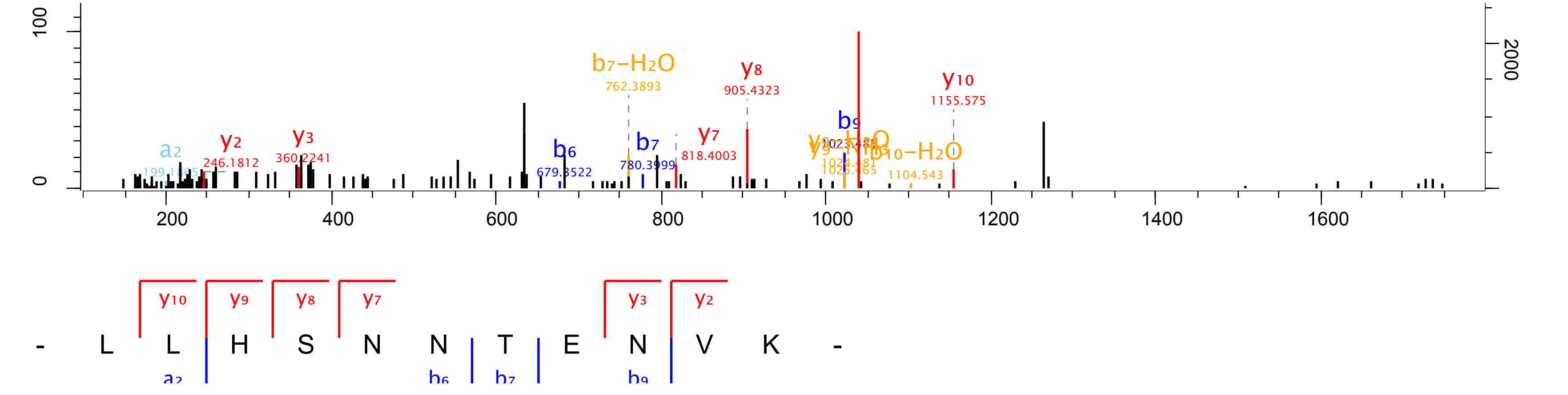

Raw file	Scan	Method	Score	m/z	Gene names
20150228_yeast1_Top_opt_B1_01_1611	6434	TOF; CID	113.62	548.75	MIX17

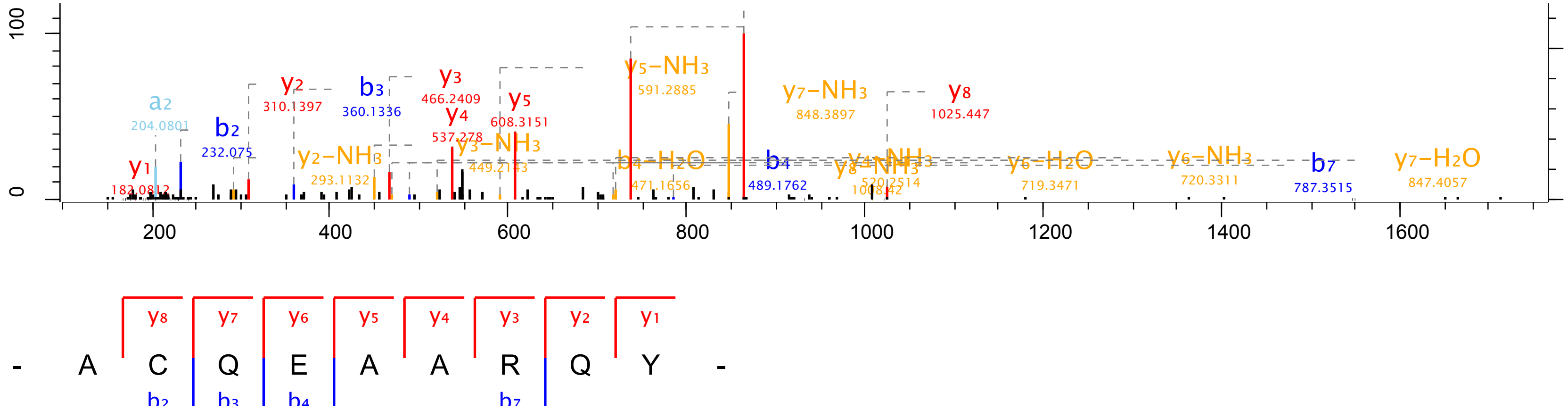

Raw file	Scan	Method	Score	m/z	Gene names
20150228_yeast1_Top_opt_B1_01_1611	6563	TOF; CID	52.6	606.24	ZRT3

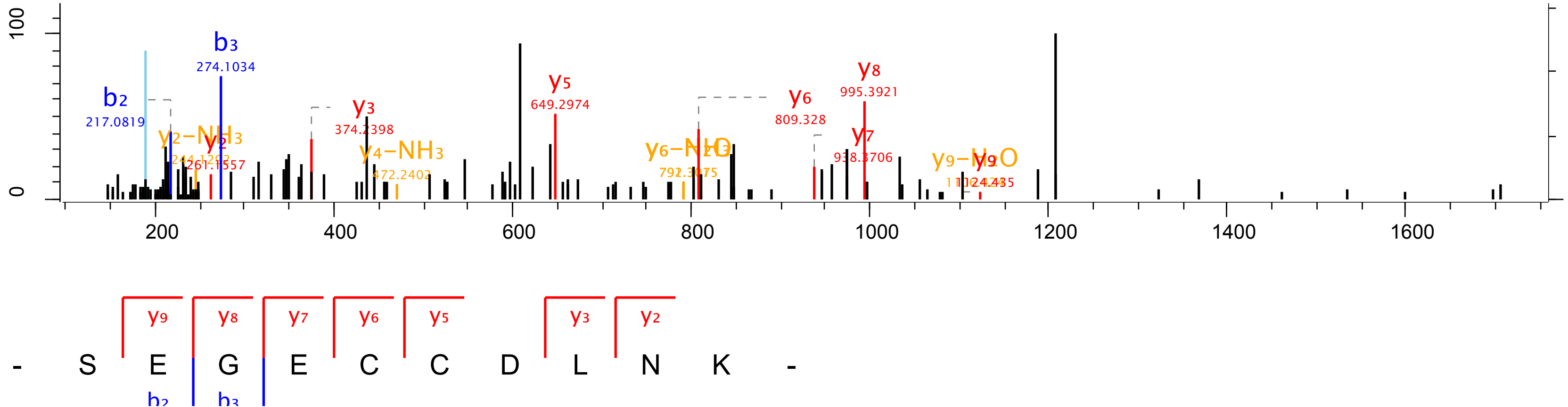

Raw file	Scan	Method	Score	m/z	Gene names
20150228_yeast1_Top_opt_B1_01_1611	6781	TOF; CID	102.52	648.76	POP8

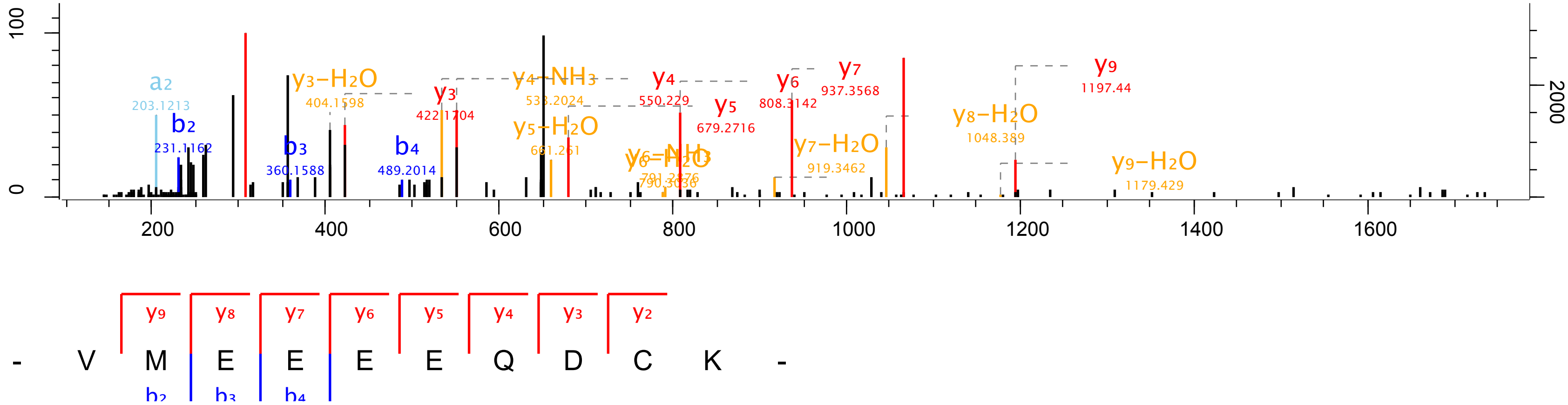

Raw file	Scan	Method	Score	m/z	Gene names
20150228_yeast1_Top_opt_B1_01_1611	7924	TOF; CID	91.91	394.22	NHP6A

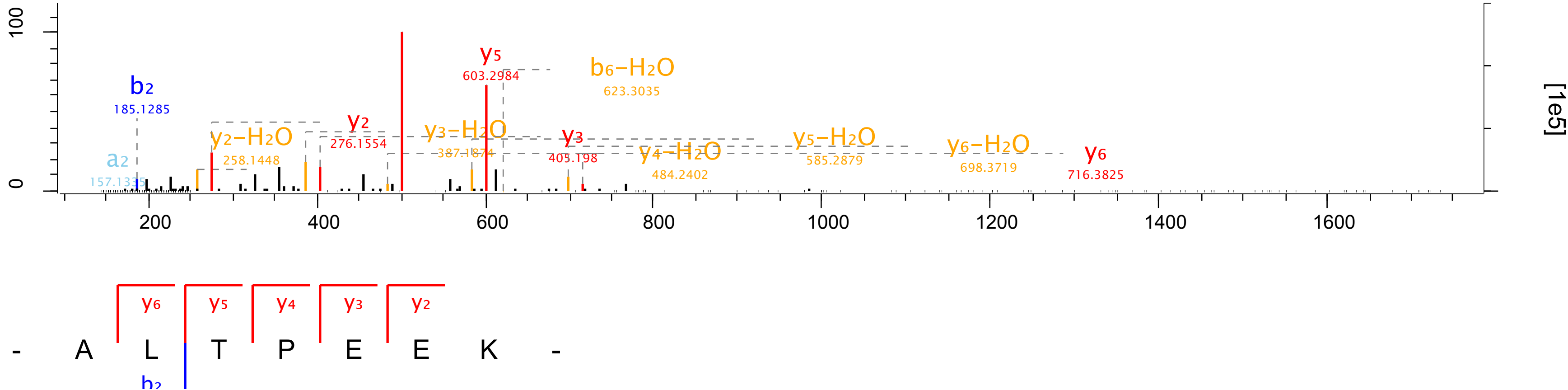

Raw file	Scan	Method	Score	m/z
20150228_yeast1_Top_opt_B1_01_1611	7969	TOF; CID	83.4	918.89

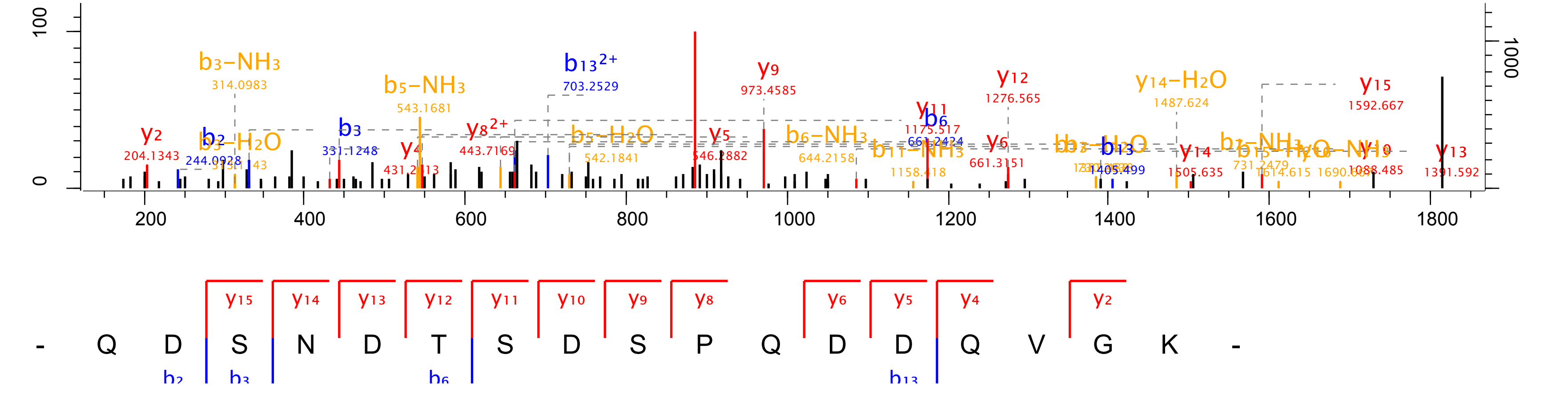

Raw file	Scan	Method	Score	m/z	Gene names
20150228_yeast1_Top_opt_B1_01_1611	8484	TOF; CID	74.17	559.24	RPC10

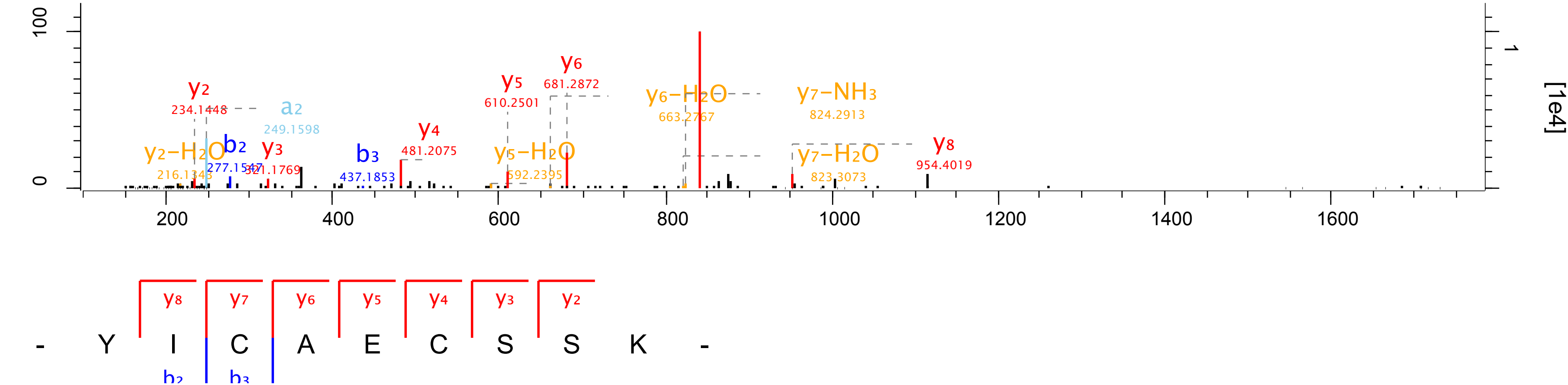

Raw file	Scan	Method	Score	m/z	Gene names
20150228_yeast1_Top_opt_B1_01_1611	9566	TOF; CID	69.63	493.74	RNH1

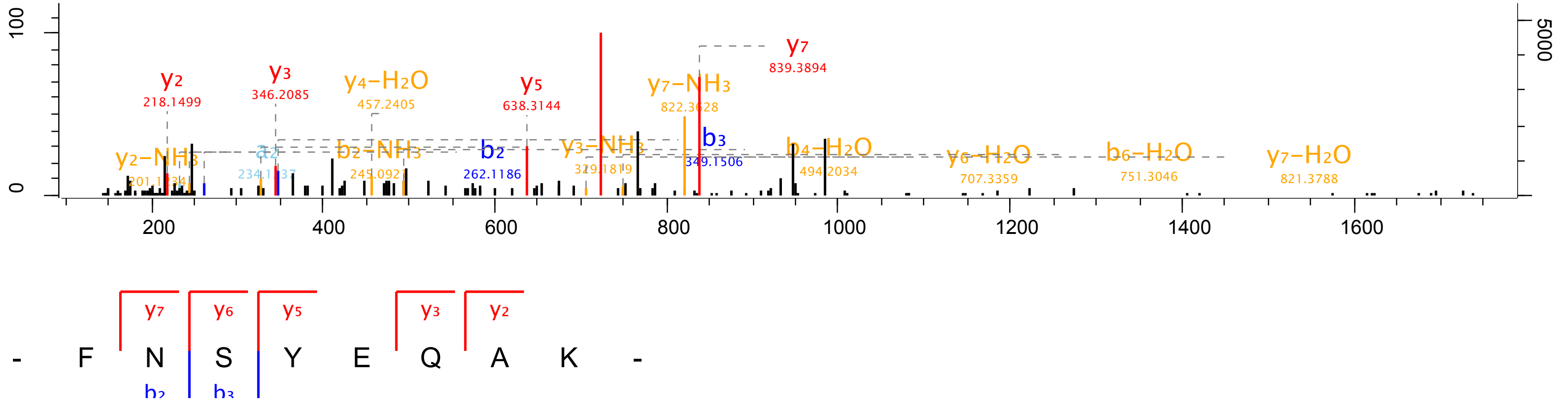

Raw file	Scan	Method	Score	m/z	Gene names
20150228_yeast1_Top_opt_B1_01_1611	10377	TOF; CID	66.3	541.78	TTI1

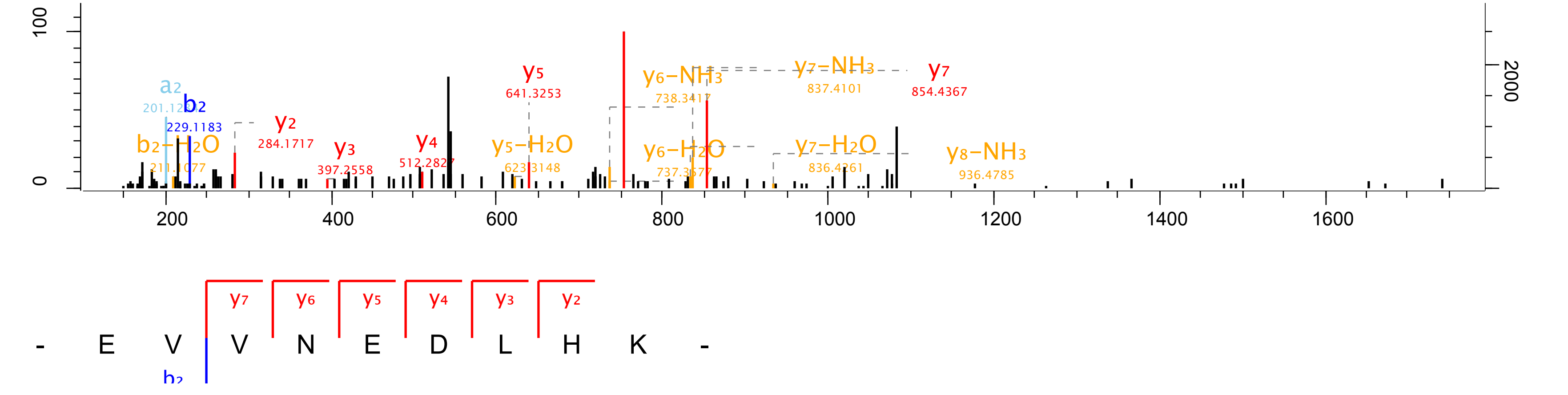

Raw file	Scan	Method	Score	m/z	Gene names
20150228_yeast1_Top_opt_B1_01_1611	10556	TOF; CID	42.34	591.8	SPG5

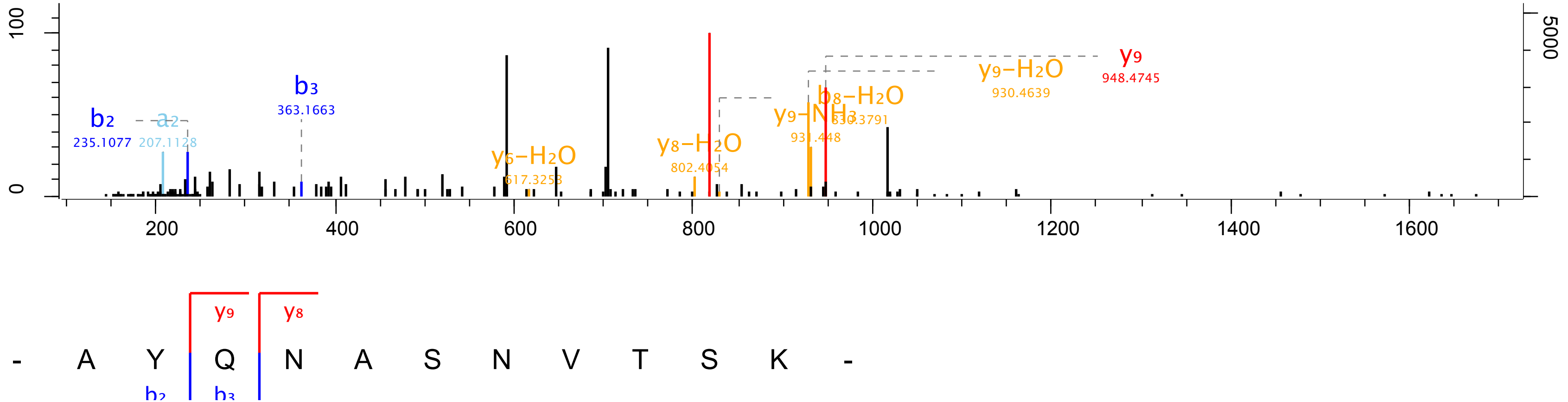

Raw file	Scan	Method	Score	m/z	Gene names
20150228_yeast1_Top_opt_B1_01_1611	10560	TOF; CID	59.91	392.2	CWC22

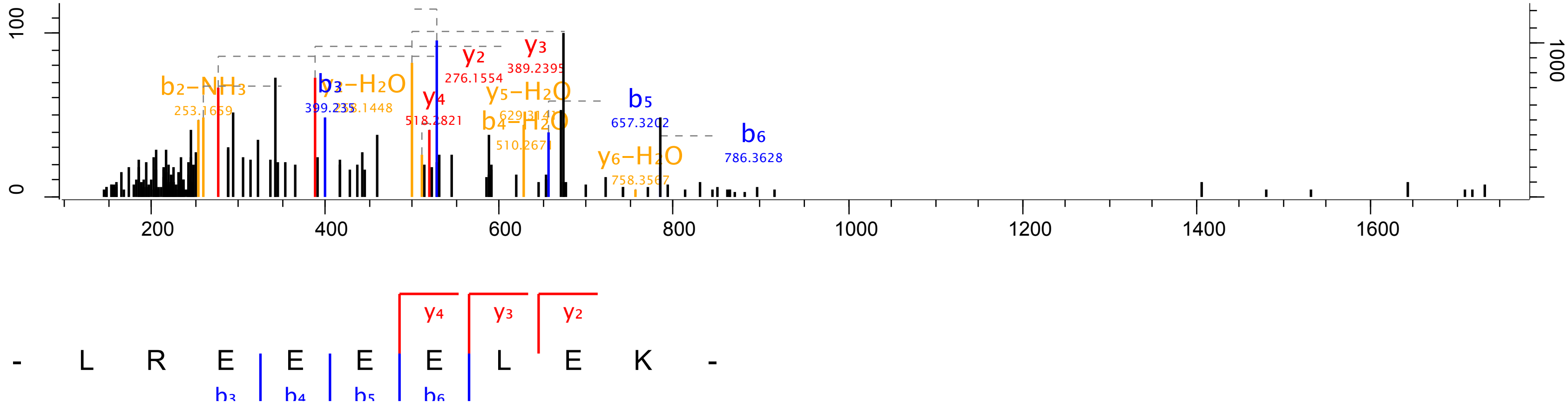

Raw file	Scan	Method	Score	m/z	Gene names
20150228_yeast1_Top_opt_B1_01_1611	10607	TOF; CID	57.35	509.77	EAF6

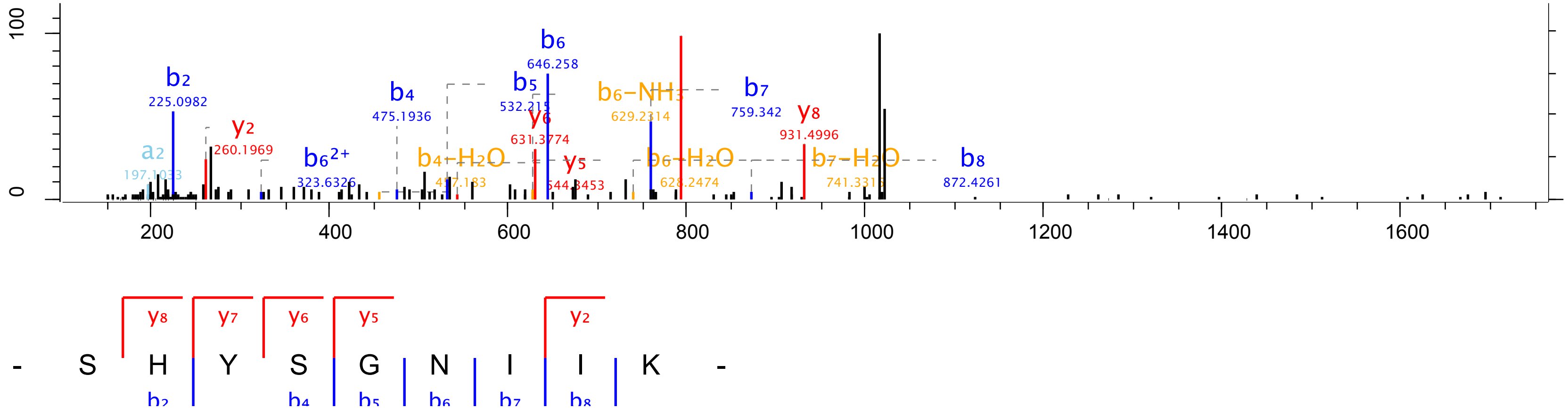

Raw file	Scan	Method	Score	m/z	Gene names
20150228_yeast1_Top_opt_B1_01_1611	10651	TOF; CID	70.09	625.31	ATO3

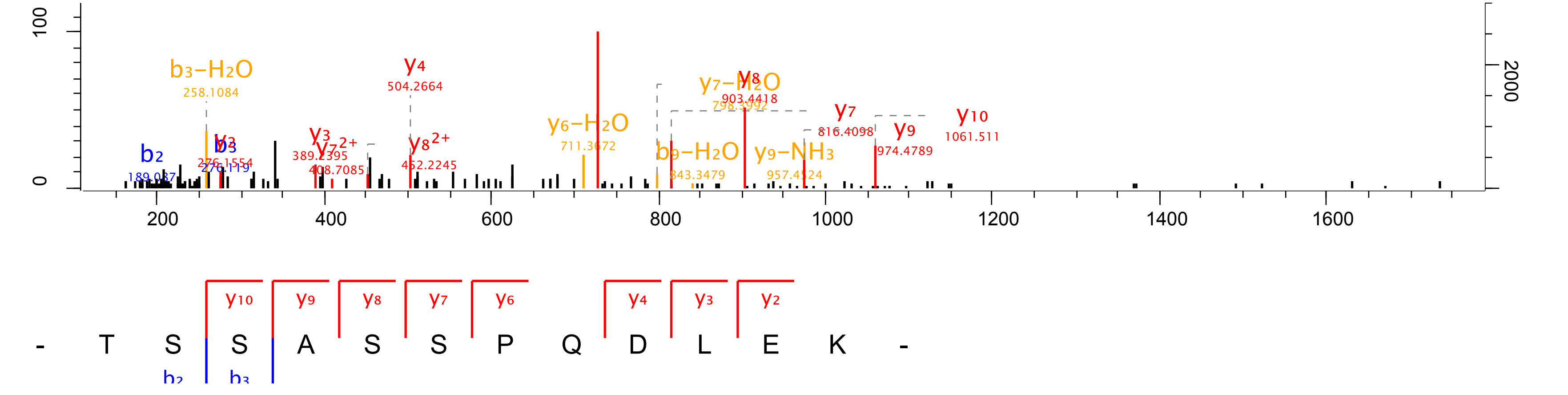

Raw file	Scan	Method	Score	m/z	Gene names
20150228_yeast1_Top_opt_B1_01_1611	10673	TOF; CID	73.26	451.23	RPS29B

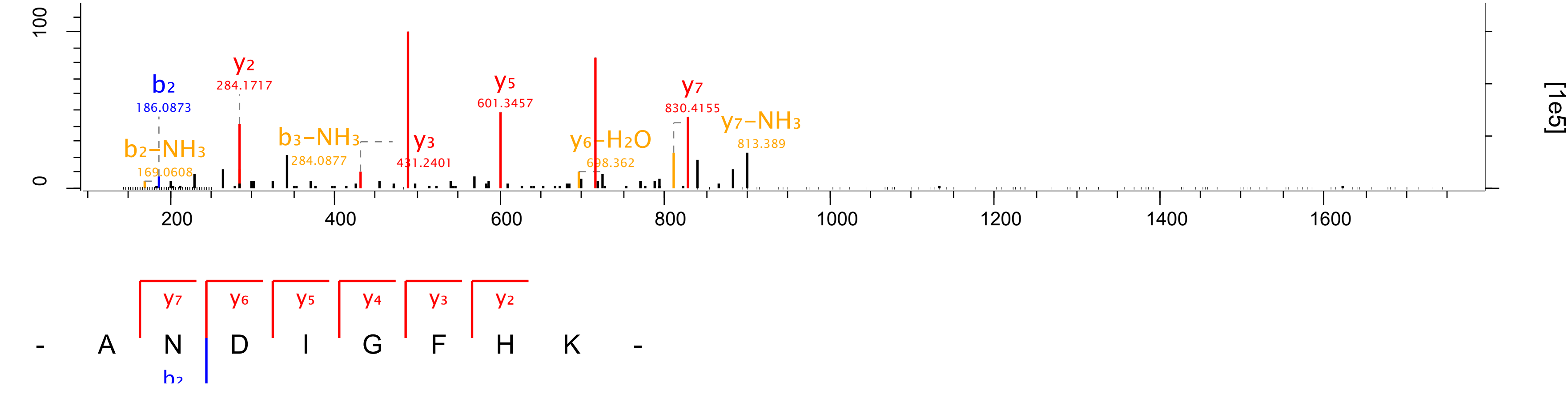

Raw file	Scan	Method	Score	m/z	Gene names
20150228_yeast1_Top_opt_B1_01_1611	10970	TOF; CID	68.66	497.75	FOB1

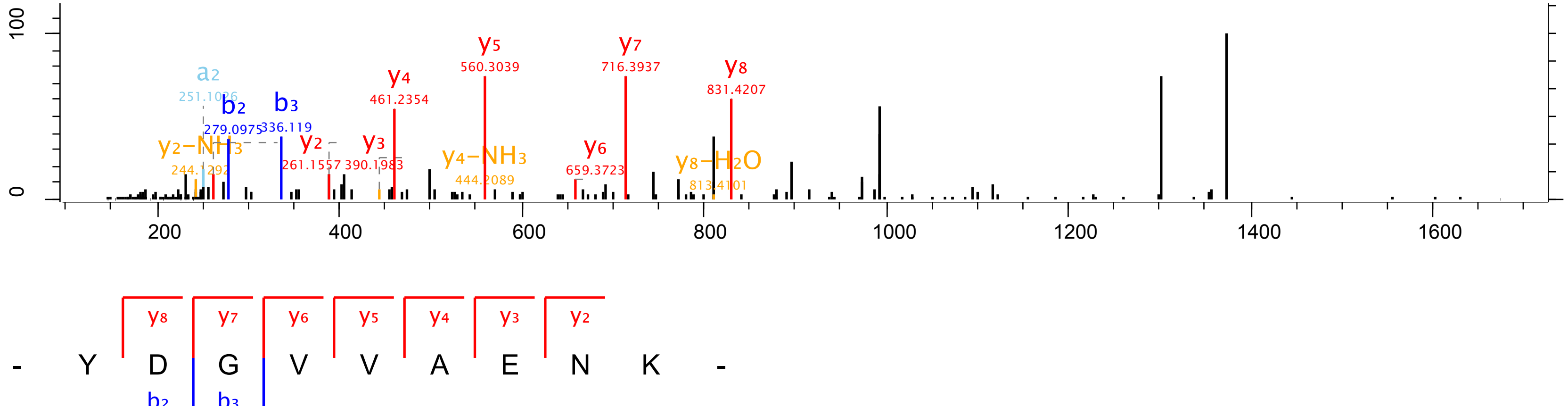

Raw file	Scan	Method	Score	m/z	Gene names
20150228_yeast1_Top_opt_B1_01_1611	11214	TOF; CID	74.42	372.72	SFT1

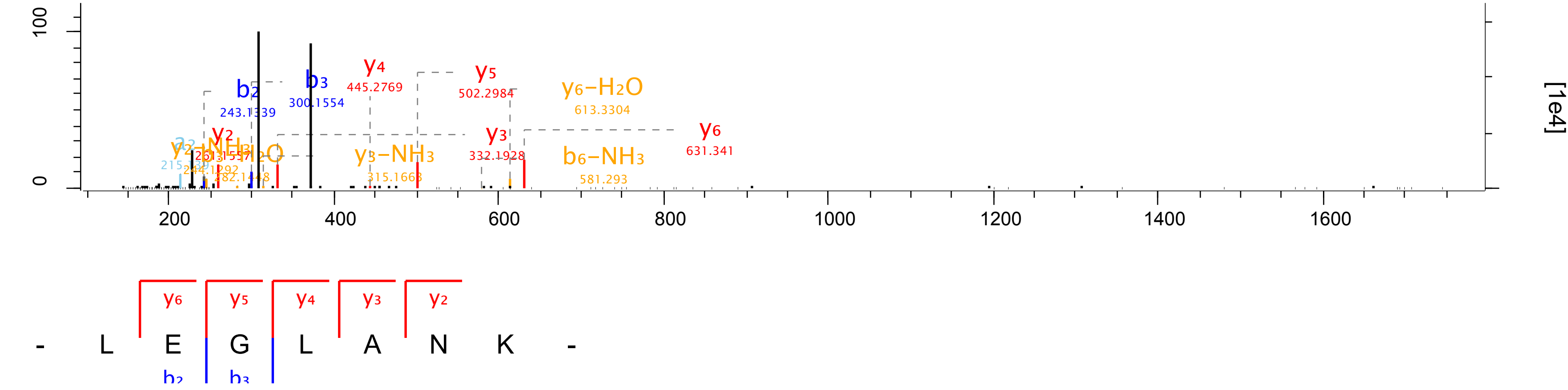

Raw file	Scan	Method	Score	m/z	Gene names
20150228_yeast1_Top_opt_B1_01_1611	11564	TOF; CID	87.18	593.81	UME6

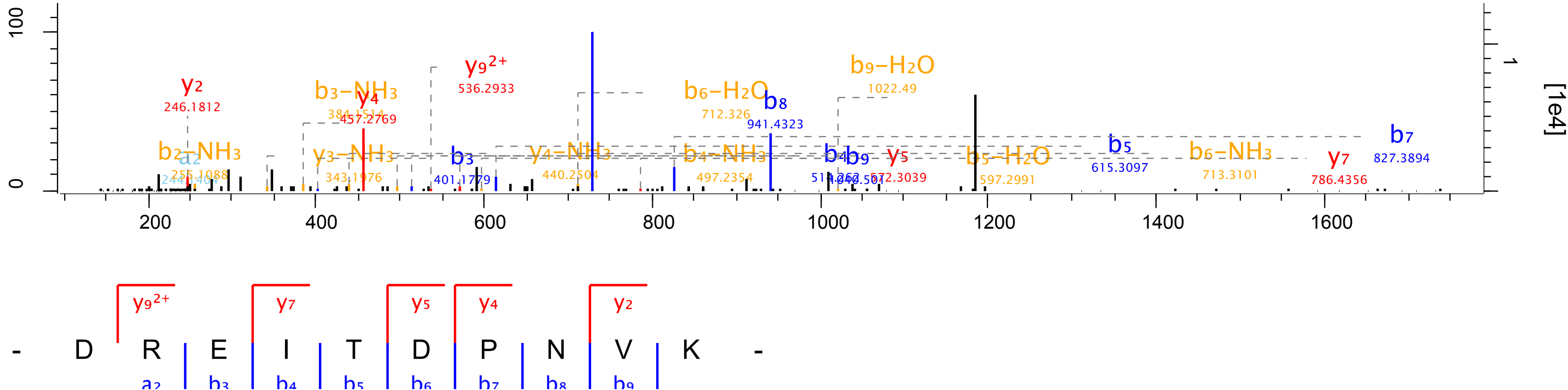

Raw file	Scan	Method	Score	m/z	Gene names
20150228_yeast1_Top_opt_B1_01_1611	11735	TOF; CID	68.66	499.25	YPR098C

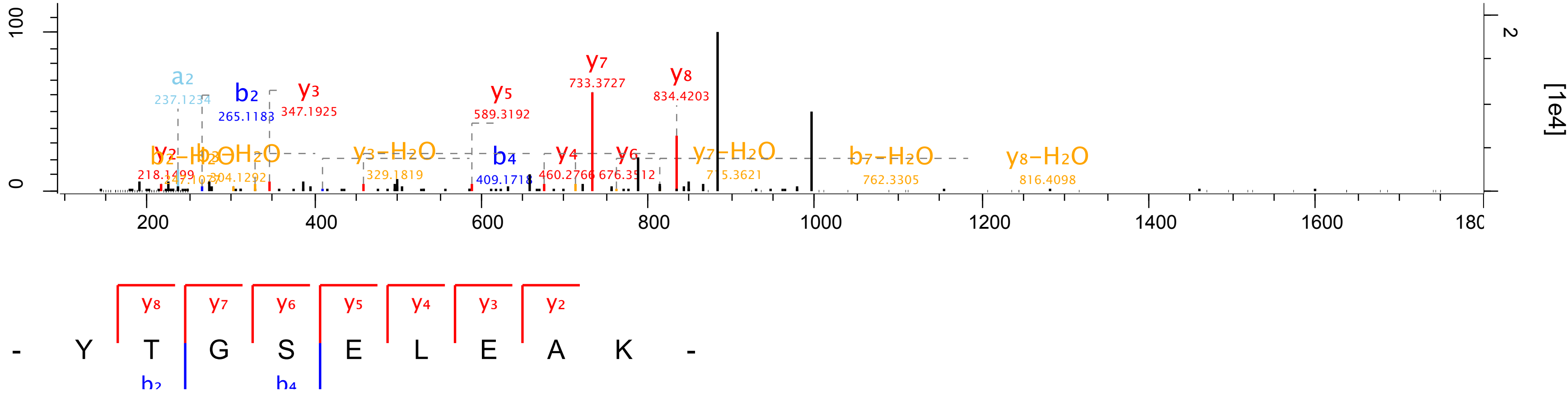

Raw file	Scan	Method	Score	m/z	Gene names
20150228_yeast1_Top_opt_B1_01_1611	12326	TOF; CID	137.81	954.42	DAD2

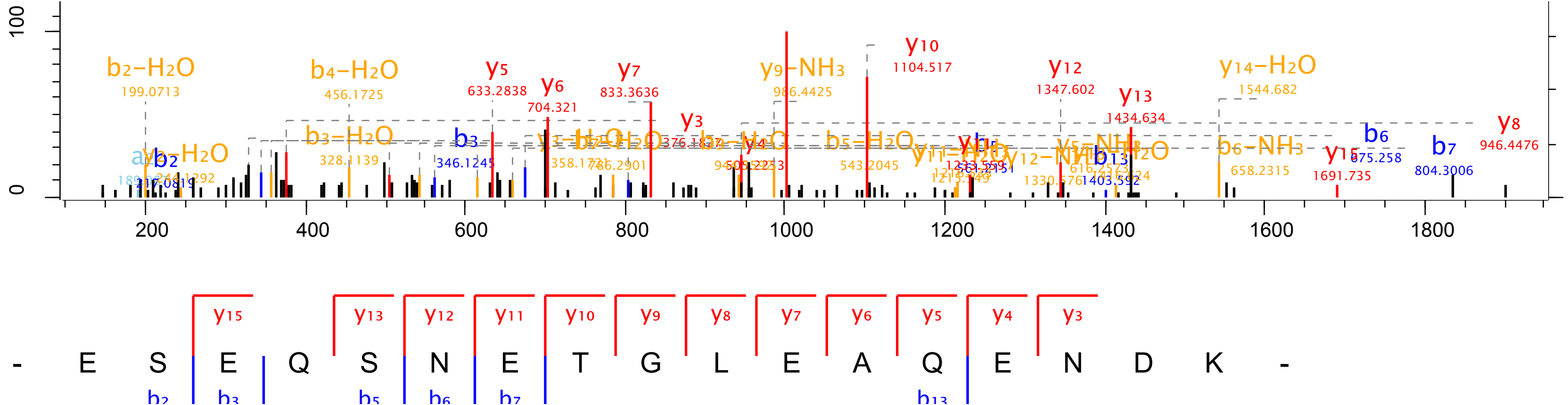

Raw file	Scan	Method	Score	m/z	Gene names
20150228_yeast1_Top_opt_B1_01_1611	12738	TOF; CID	58.31	664.81	ESA1

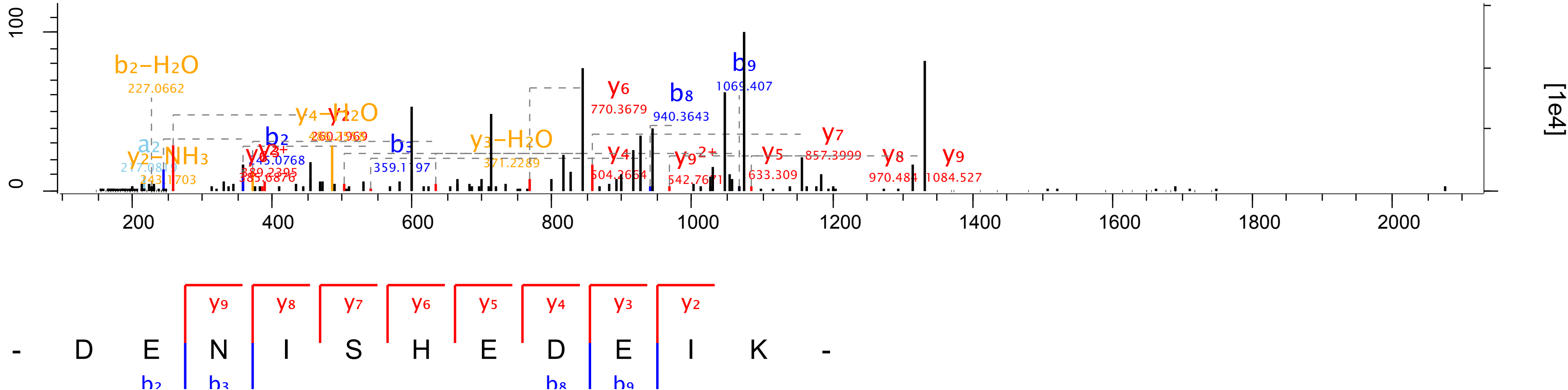

Raw file	Scan	Method	Score	m/z	Gene names
20150228_yeast1_Top_opt_B1_01_1611	13337	TOF; CID	85.36	365.23	RPS28A;RPS28B

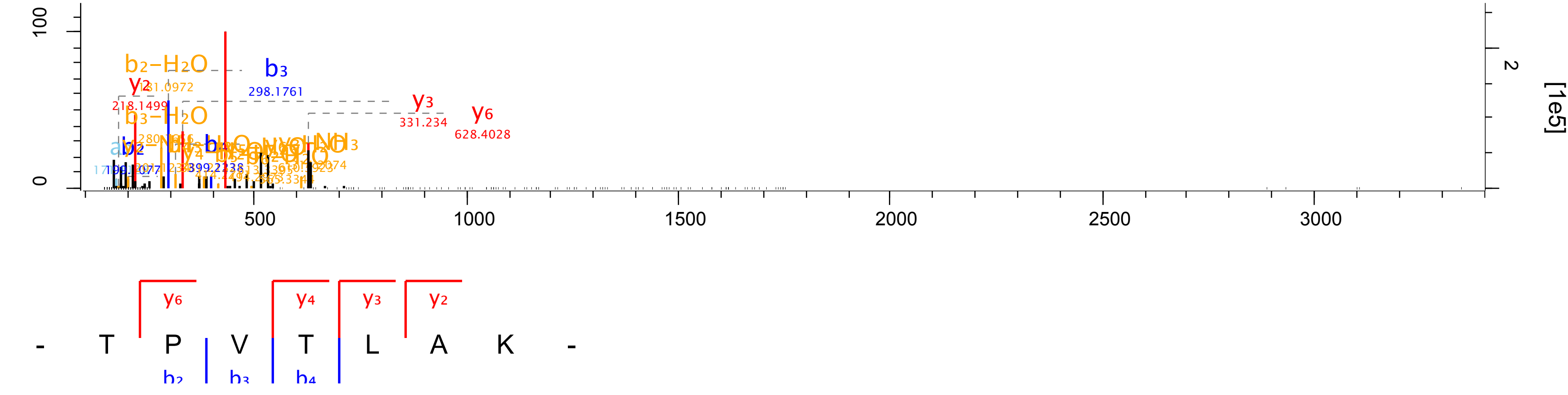

Raw file	Scan	Method	Score	m/z	Gene names
20150228_yeast1_Top_opt_B1_01_1611	13404	TOF; CID	41.88	372.2	SED4

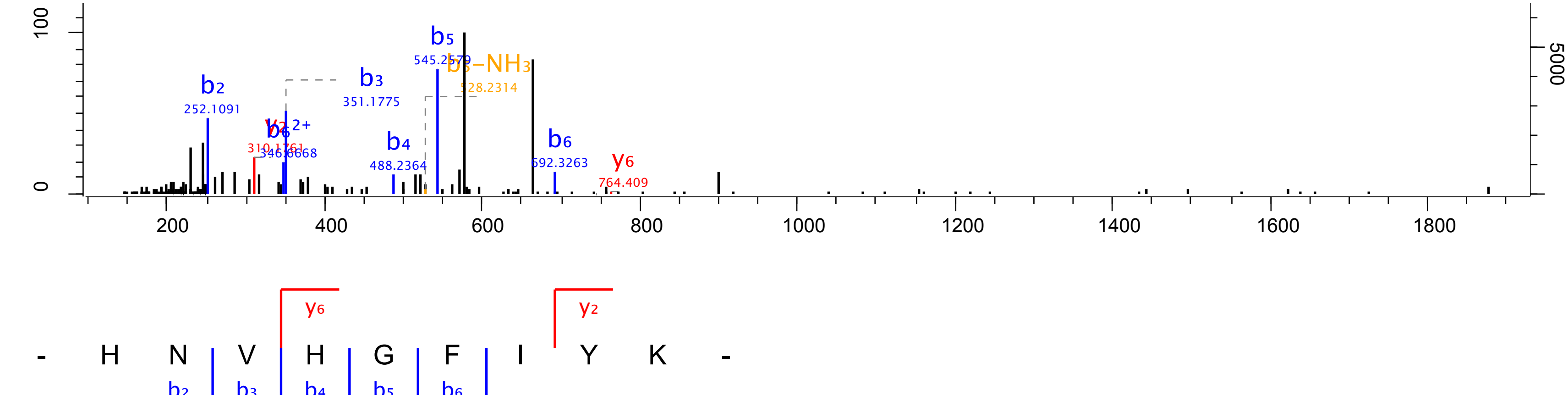

Raw file	Scan	Method	Score	m/z
20150228_yeast1_Top_opt_B1_01_1611	13524	TOF; CID	64.71	522.26

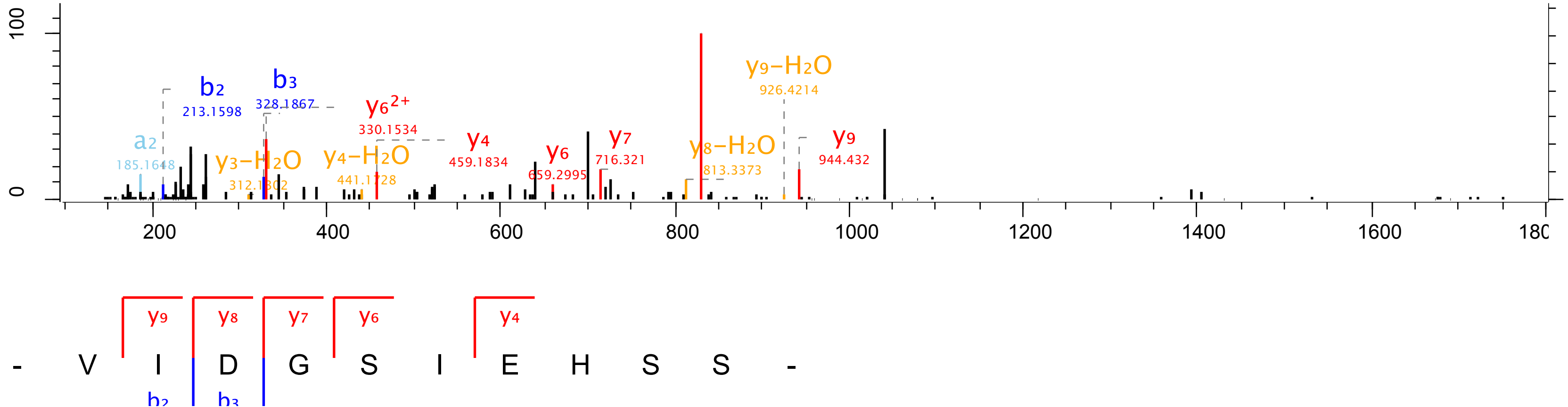

Raw file	Scan	Method	Score	m/z	Gene names
20150228_yeast1_Top_opt_B1_01_1611	13593	TOF; CID	67.7	543.26	PRY3

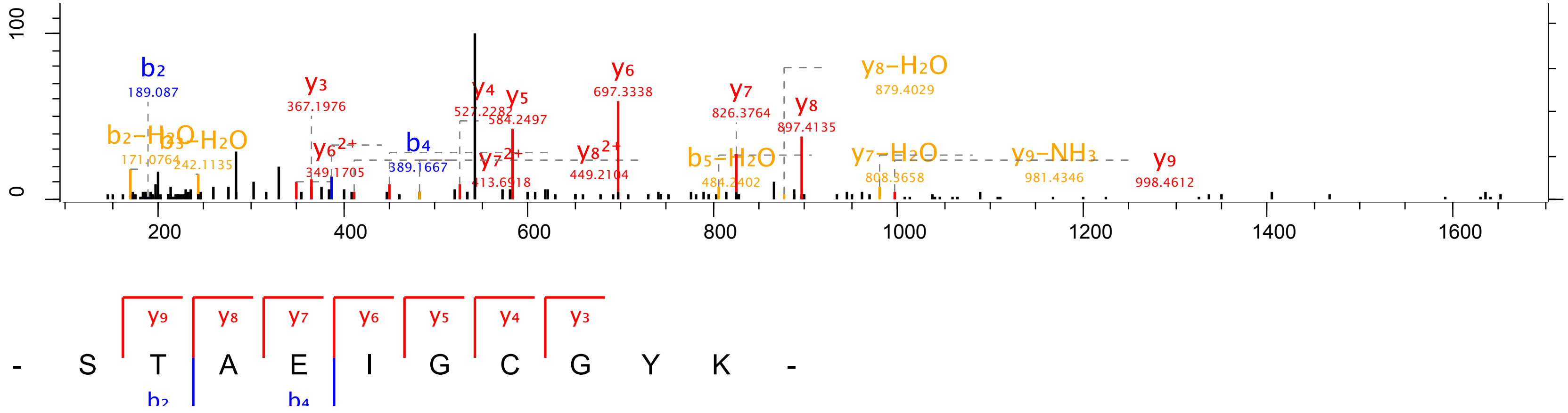

Raw file	Scan	Method	Score	m/z	Gene names
20150228_yeast1_Top_opt_B1_01_1611	14358	TOF; CID	56.88	737.67	TRS85

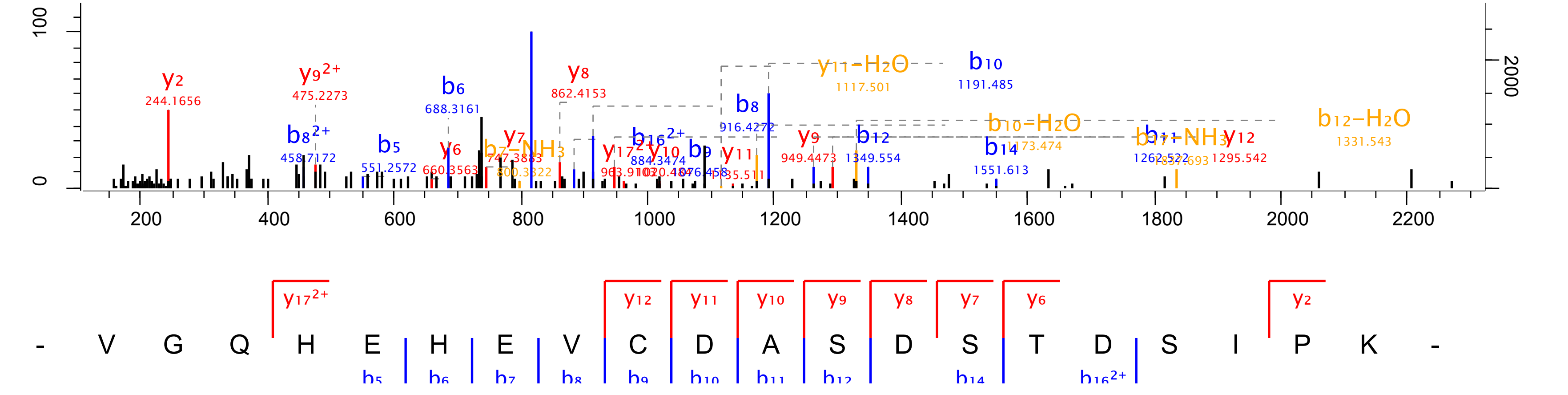

Raw file	Scan	Method	Score	m/z	Gene names
20150228_yeast1_Top_opt_B1_01_1611	14406	TOF; CID	76.06	462.26	ISA1

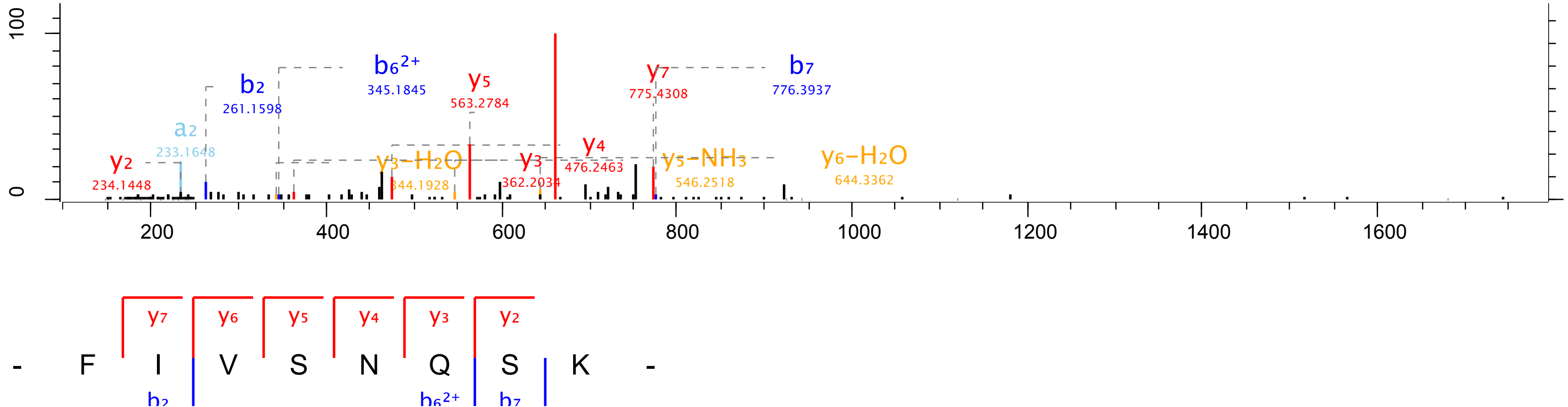

Raw file	Scan	Method	Score	m/z	Gene names
20150228_yeast1_Top_opt_B1_01_1611	14502	TOF; CID	65.84	630.36	TVP38

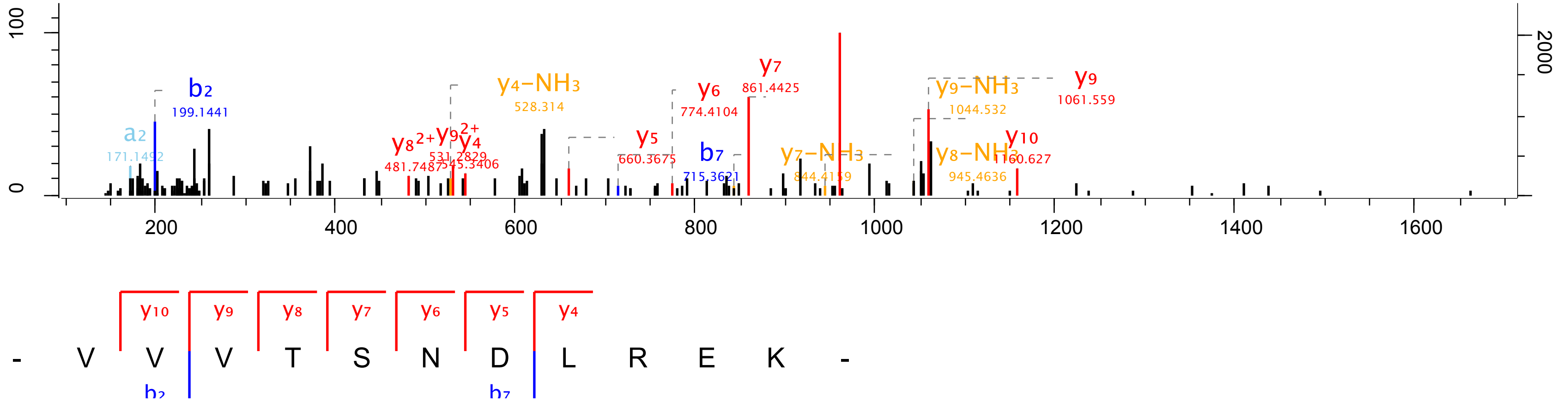

Raw file	Scan	Method	Score	m/z	Gene names
20150228_yeast1_Top_opt_B1_01_1611	14534	TOF; CID	60.6	587.32	PIB1

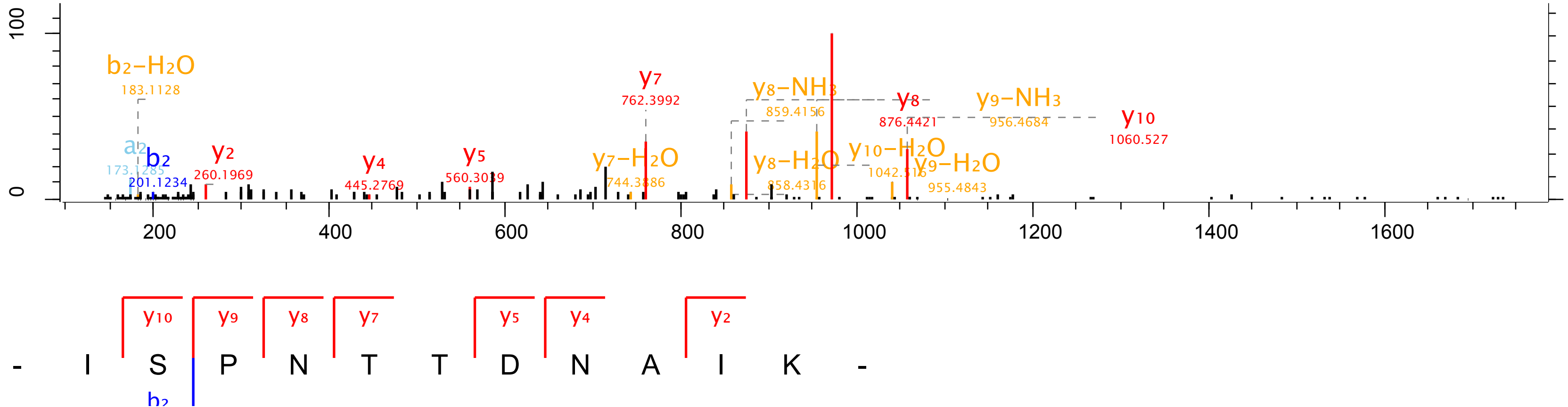

Raw file	Scan	Method	Score	m/z	Gene names
20150228_yeast1_Top_opt_B1_01_1611	14693	TOF; CID	65.84	595.3	YML037C

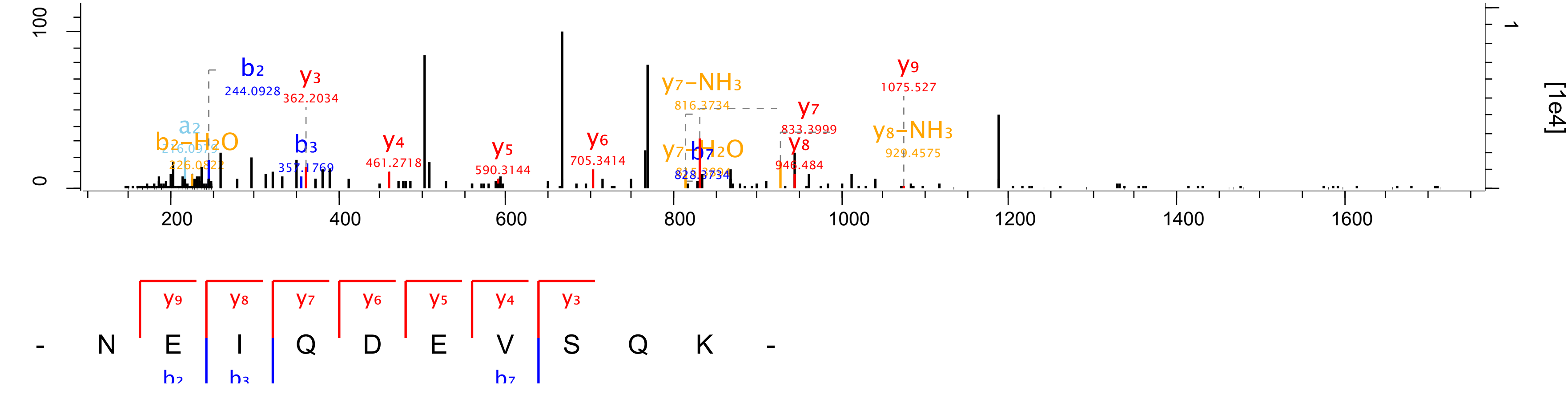

Raw file	Scan	Method	Score	m/z	Gene names
20150228_yeast1_Top_opt_B1_01_1611	14993	TOF; CID	93.99	439.73	RPS29A

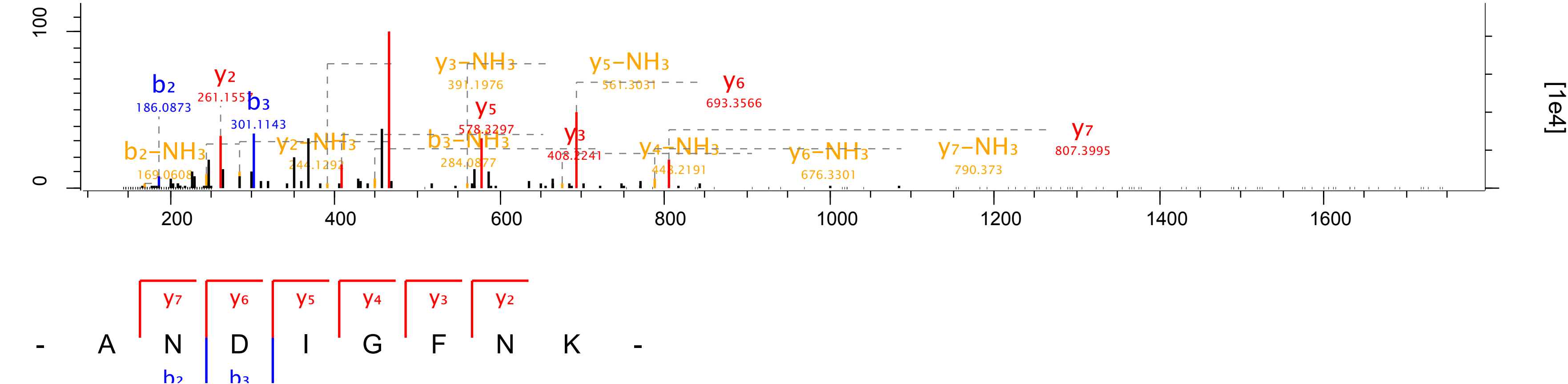

Raw file	Scan	Method	Score	m/z	Gene names
20150228_yeast1_Top_opt_B1_01_1611	15612	TOF; CID	60.02	405.72	CDC43

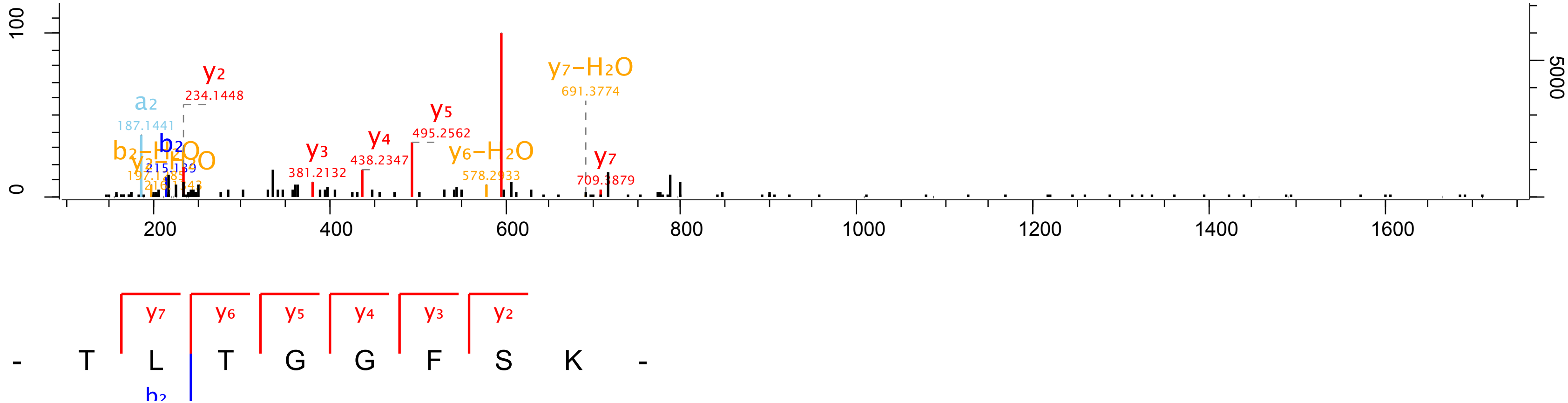

Raw file	Scan	Method	Score	m/z	Gene names
20150228_yeast1_Top_opt_B1_01_1611	16009	TOF; CID	41.74	888.94	AFI1

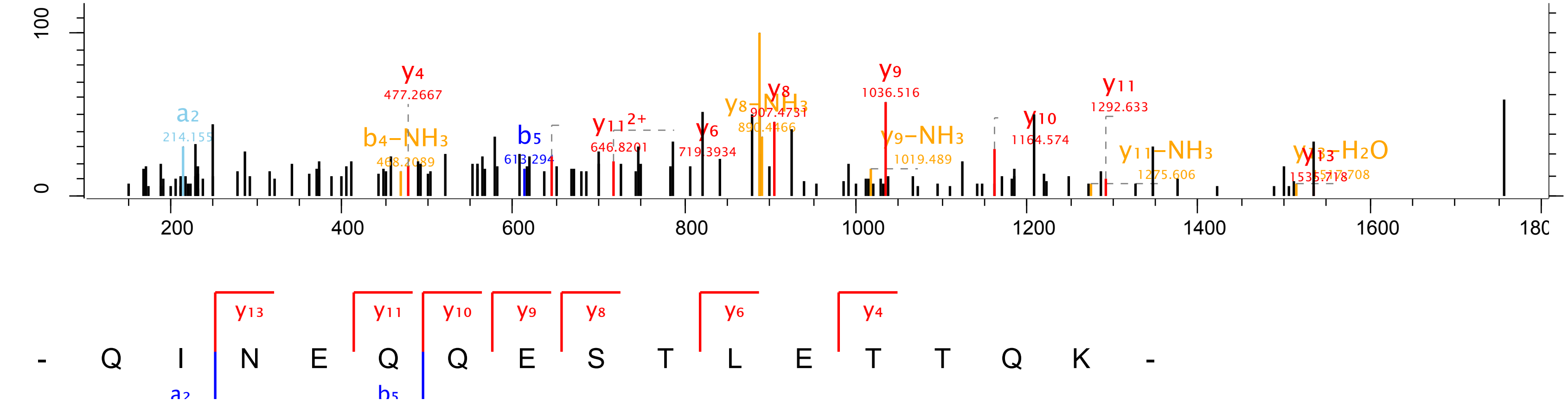

Raw file	Scan	Method	Score	m/z	Gene names
20150228_yeast1_Top_opt_B1_01_1611	16153	TOF; CID	71.45	459.77	RIT1

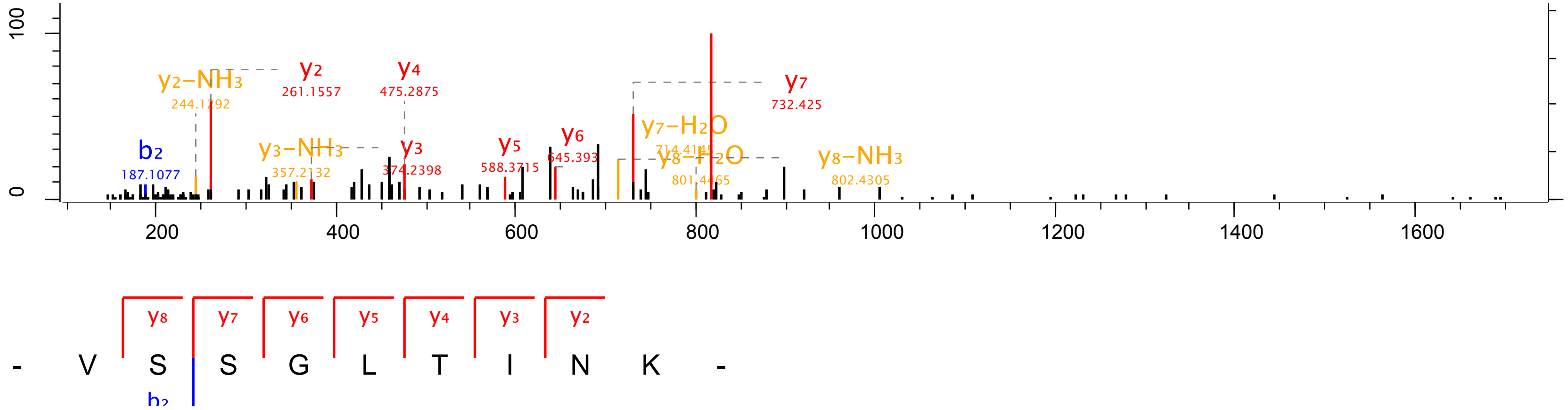

Raw file	Scan	Method	Score	m/z	Gene names
20150228_yeast1_Top_opt_B1_01_1611	16338	TOF; CID	50.47	501.29	BOR1

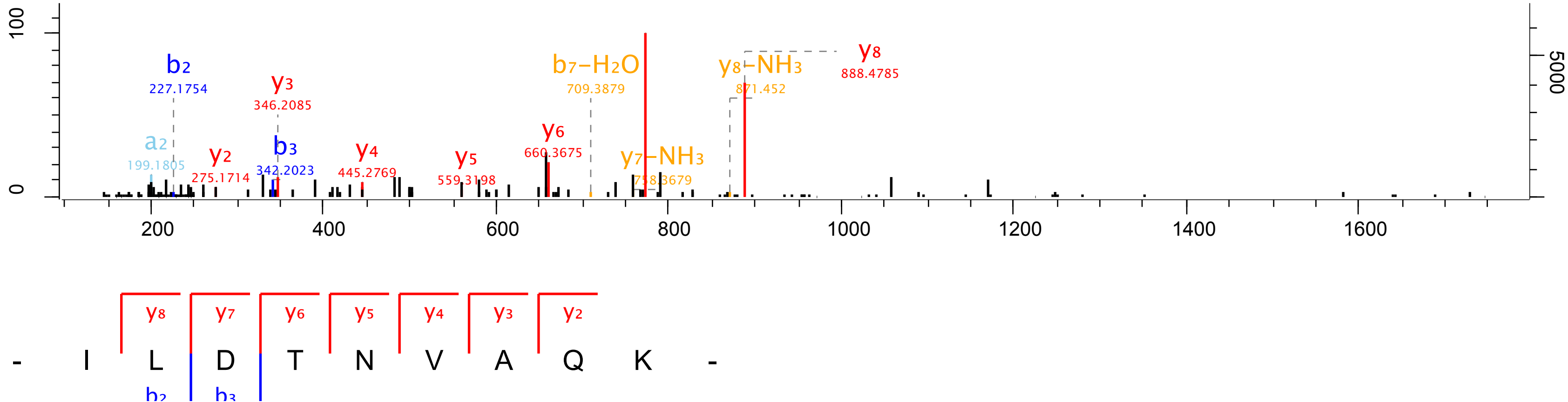

Raw file	Scan	Method	Score	m/z	Gene names
20150228_yeast1_Top_opt_B1_01_1611	16590	TOF; CID	78.81	701.36	ARG80

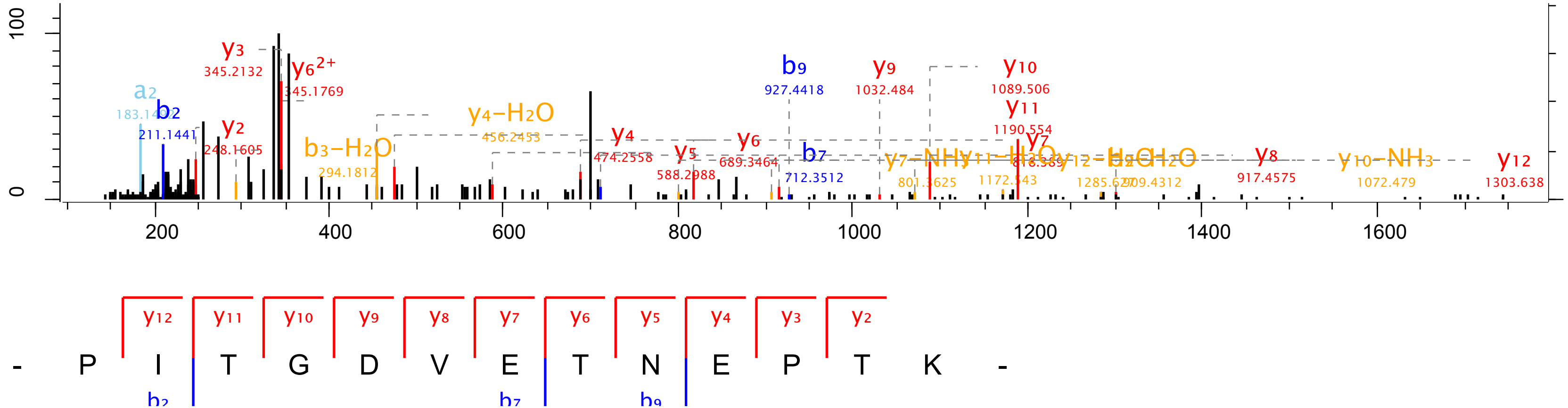

Raw file	Scan	Method	Score	m/z	Gene names
20150228_yeast1_Top_opt_B1_01_1611	16897	TOF; CID	85.53	567.77	APM4

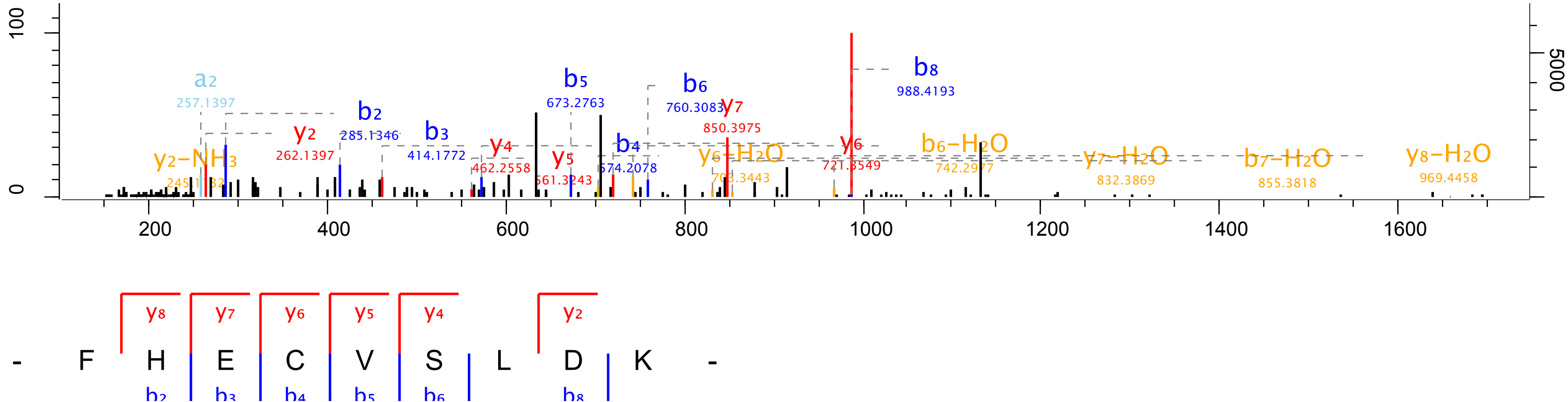

Raw file	Scan	Method	Score	m/z	Gene names
20150228_yeast1_Top_opt_B1_01_1611	17110	TOF; CID	52.26	481.26	ICT1

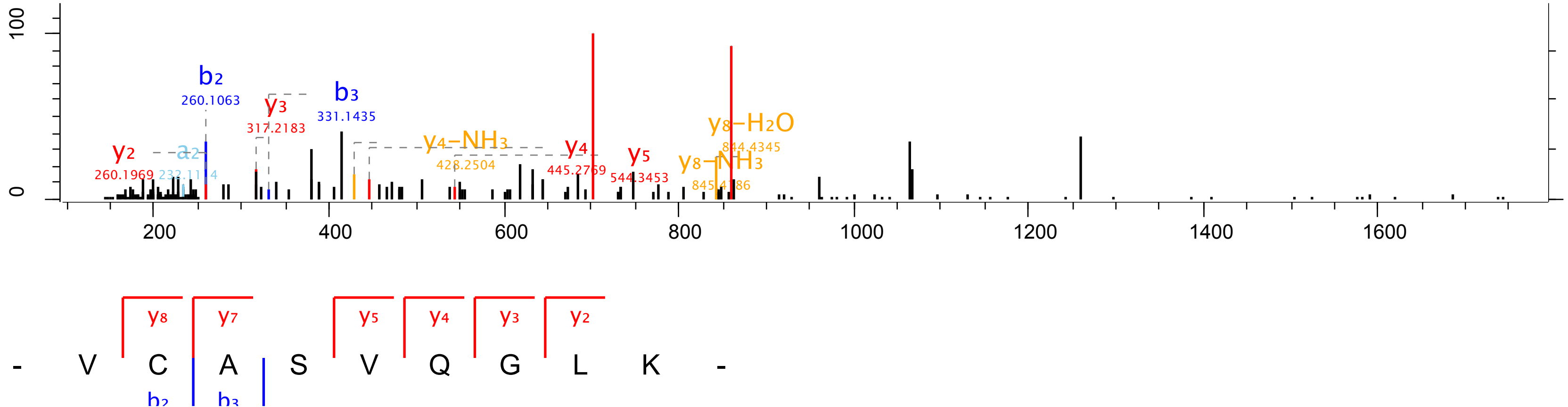

Raw file	Scan	Method	Score	m/z	Gene names
20150228_yeast1_Top_opt_B1_01_1611	17923	TOF; CID	47.82	677.32	MET1

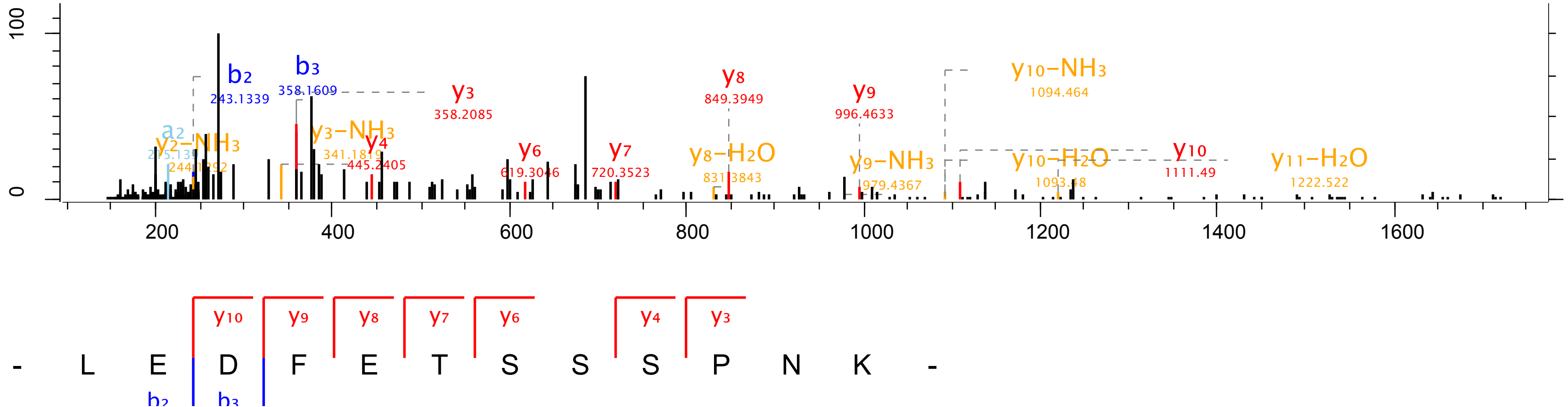

Raw file	Scan	Method	Score	m/z	Gene names
20150228_yeast1_Top_opt_B1_01_1611	18075	TOF; CID	82.42	582.79	ENV7

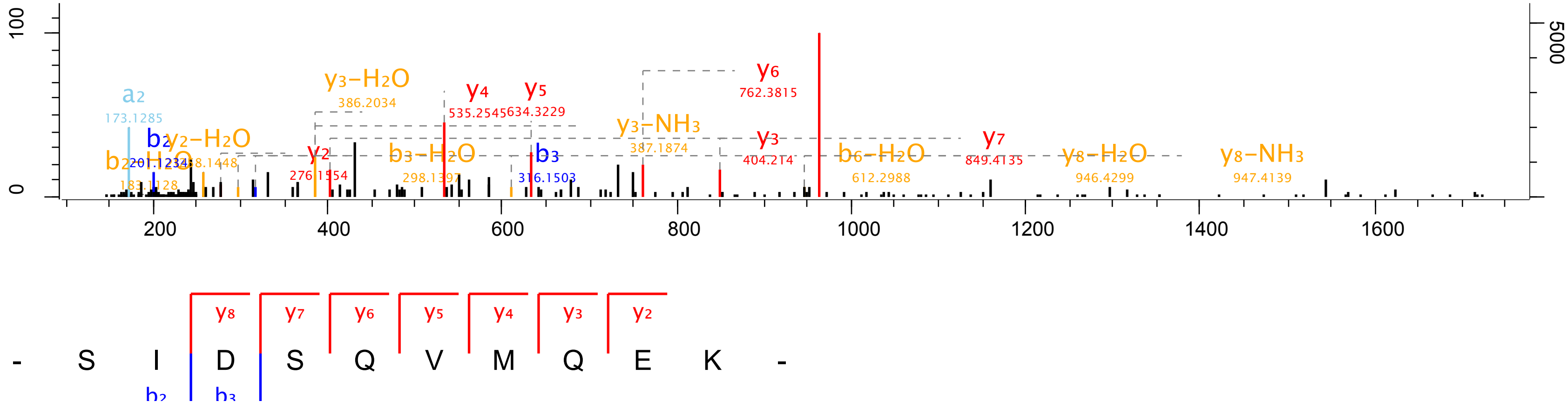

Raw file	Scan	Method	Score	m/z
20150228_yeast1_Top_opt_B1_01_1611	19248	TOF; CID	55.72	687.8

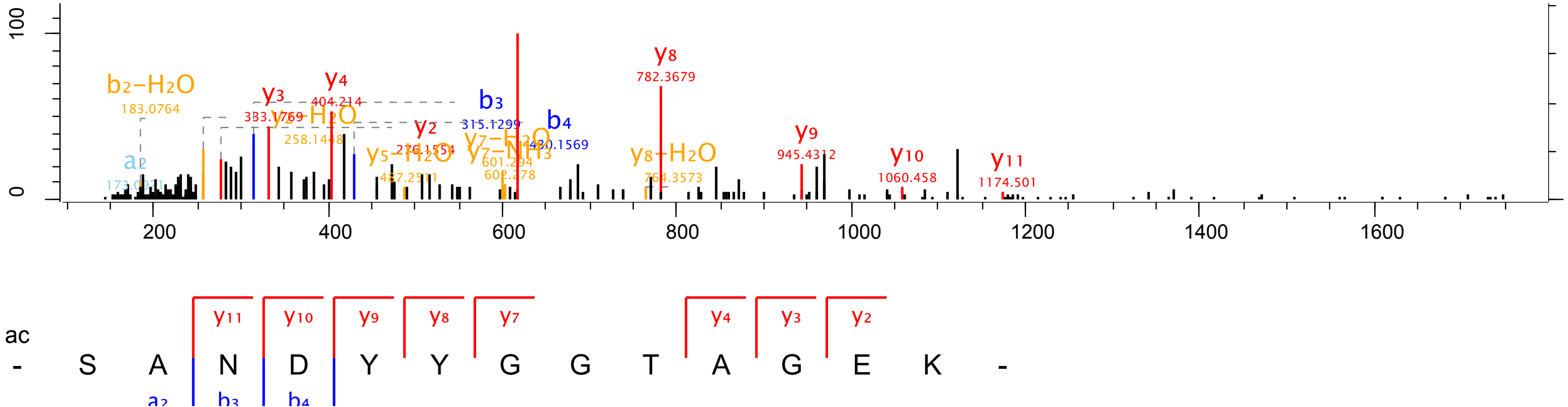

Raw file	Scan	Method	Score	m/z	Gene names
20150228_yeast1_Top_opt_B1_01_1611	19983	TOF; CID	139.86	612.3	HYM1

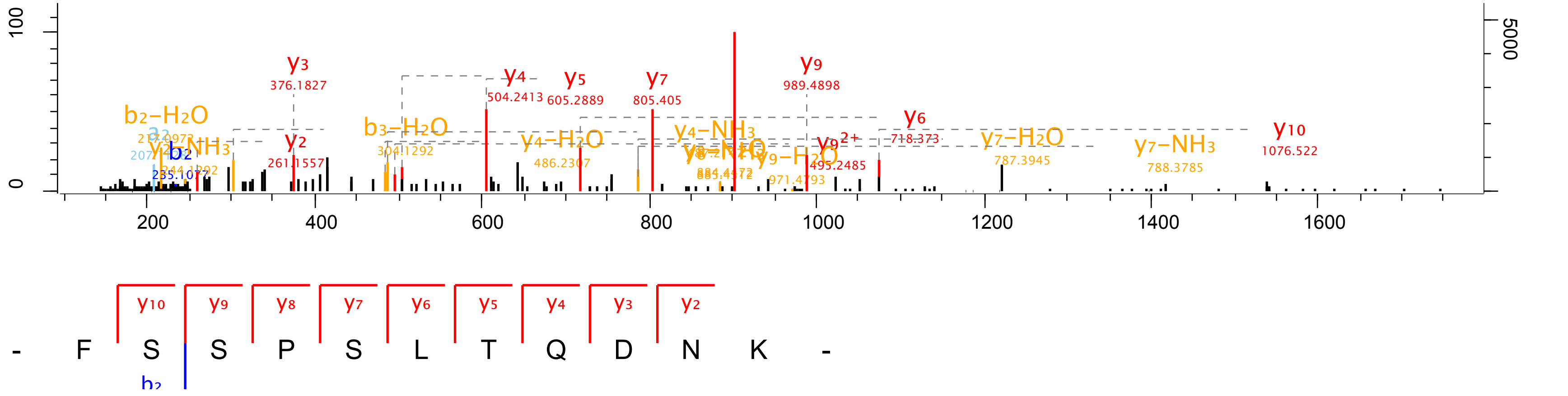

Raw file	Scan	Method	Score	m/z	Gene names
20150228_yeast1_Top_opt_B1_01_1611	20020	TOF; CID	85.81	608.29	TRS31

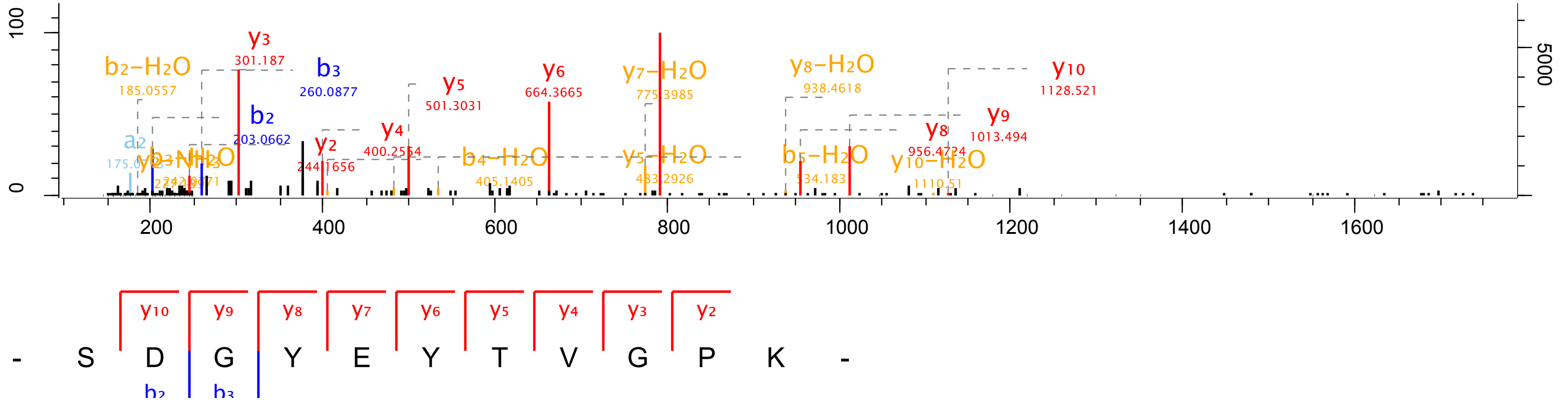

Raw file	Scan	Method	Score	m/z
20150228_yeast1_Top_opt_B1_01_1611	20061	TOF; CID	78.69	791.33

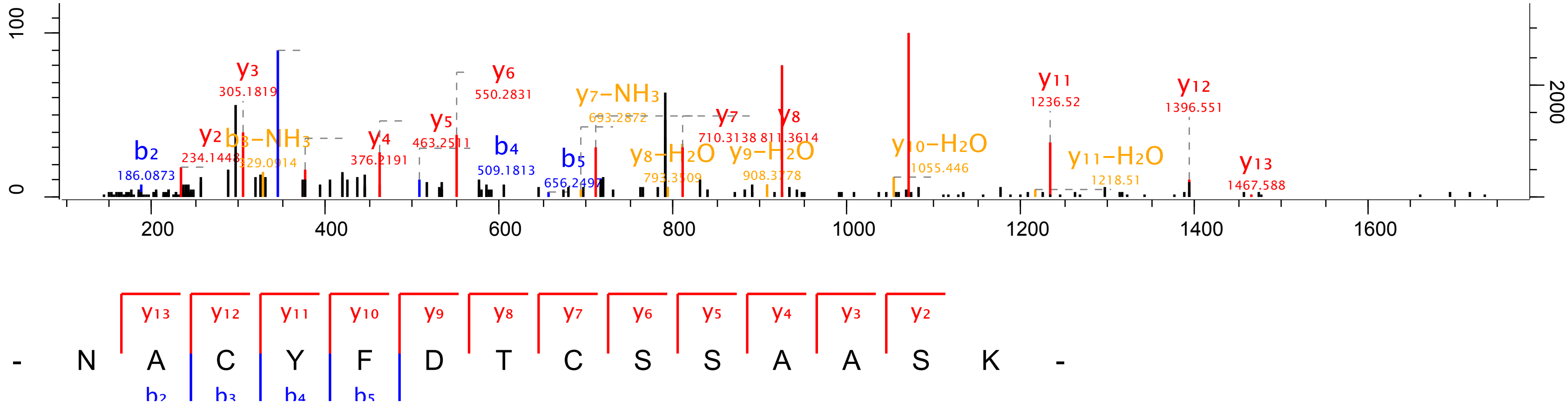

Raw file	Scan	Method	Score	m/z	Gene names
20150228_yeast1_Top_opt_B1_01_1611	20883	TOF; CID	99.34	601.33	QDR3

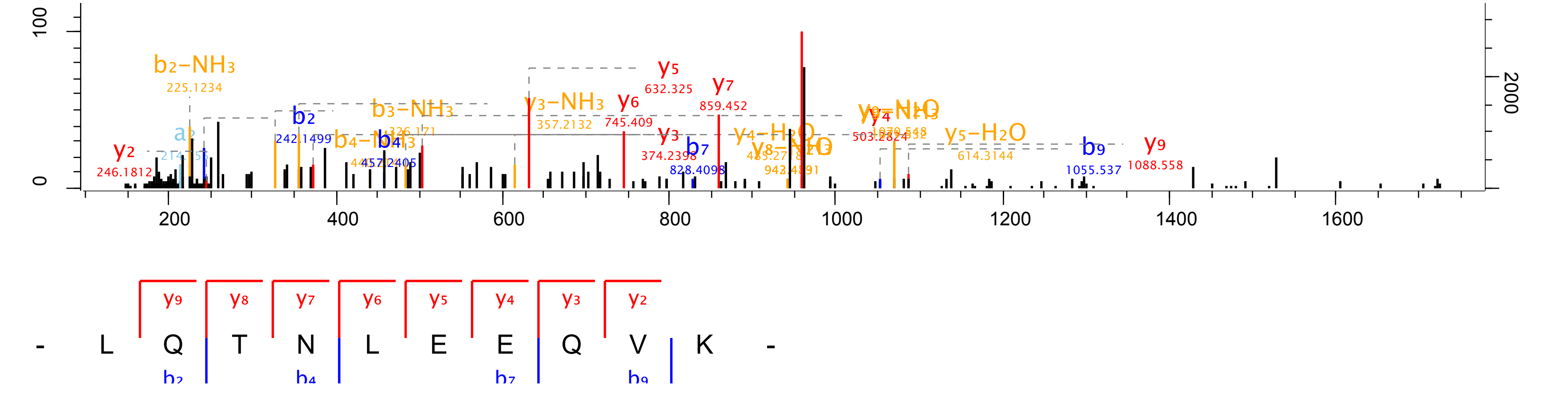

Raw file	Scan	Method	Score	m/z	Gene names
20150228_yeast1_Top_opt_B1_01_1611	21266	TOF; CID	52.58	628.85	PEX17

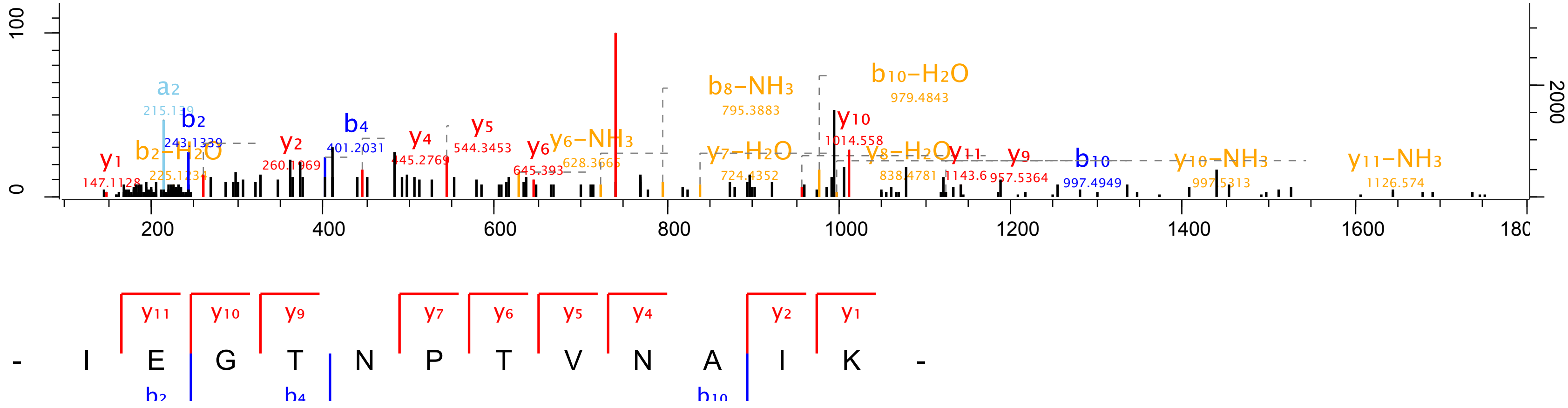

Raw file	Scan	Method	Score	m/z	Gene names
20150228_yeast1_Top_opt_B1_01_1611	21465	TOF; CID	97.78	976.97	SDC1

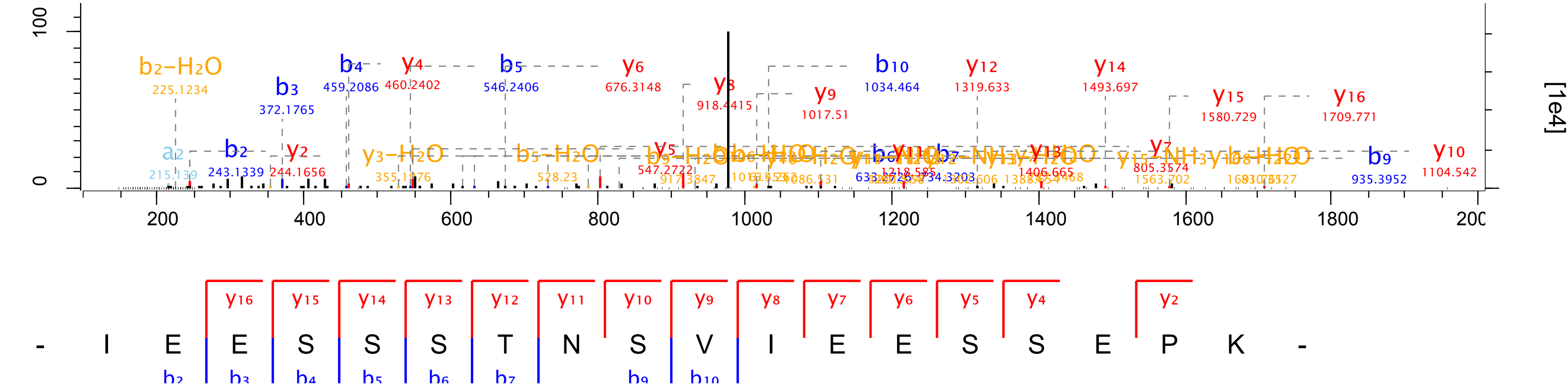

Raw file	Scan	Method	Score	m/z	Gene names
20150228_yeast1_Top_opt_B1_01_1611	21666	TOF; CID	78.52	500.76	YKL077W

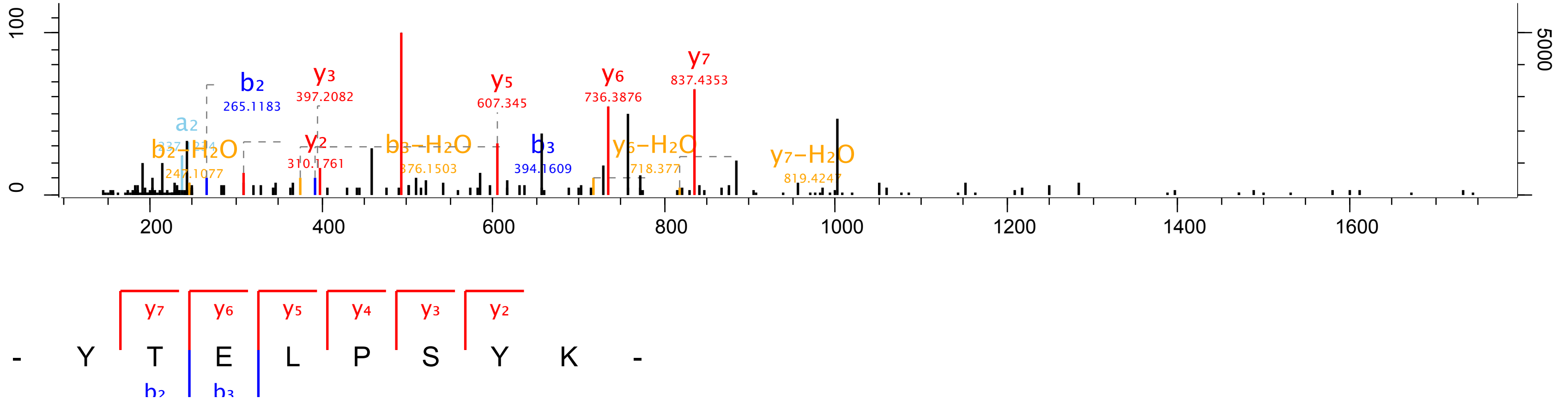

Raw file	Scan	Method	Score	m/z	Gene names
20150228_yeast1_Top_opt_B1_01_1611	21985	TOF; CID	90.05	432.9	LSM8

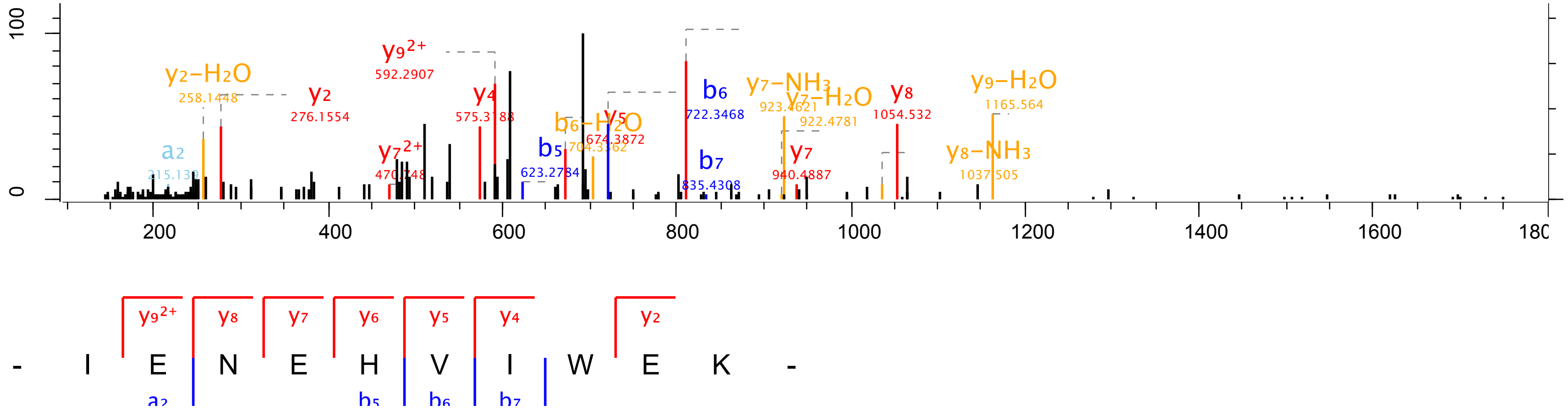

Raw file	Scan	Method	Score	m/z	Gene names
20150228_yeast1_Top_opt_B1_01_1611	22036	TOF; CID	63.31	487.74	COX19

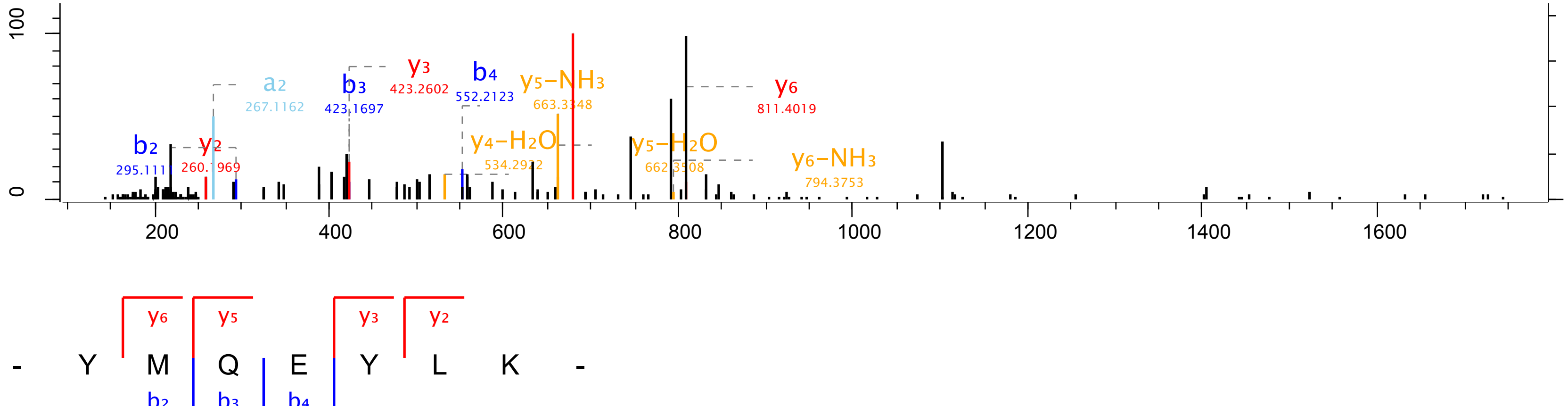

Raw file	Scan	Method	Score	m/z	Gene names
20150228_yeast1_Top_opt_B1_01_1611	22694	TOF; CID	49.1	627.32	SCD5

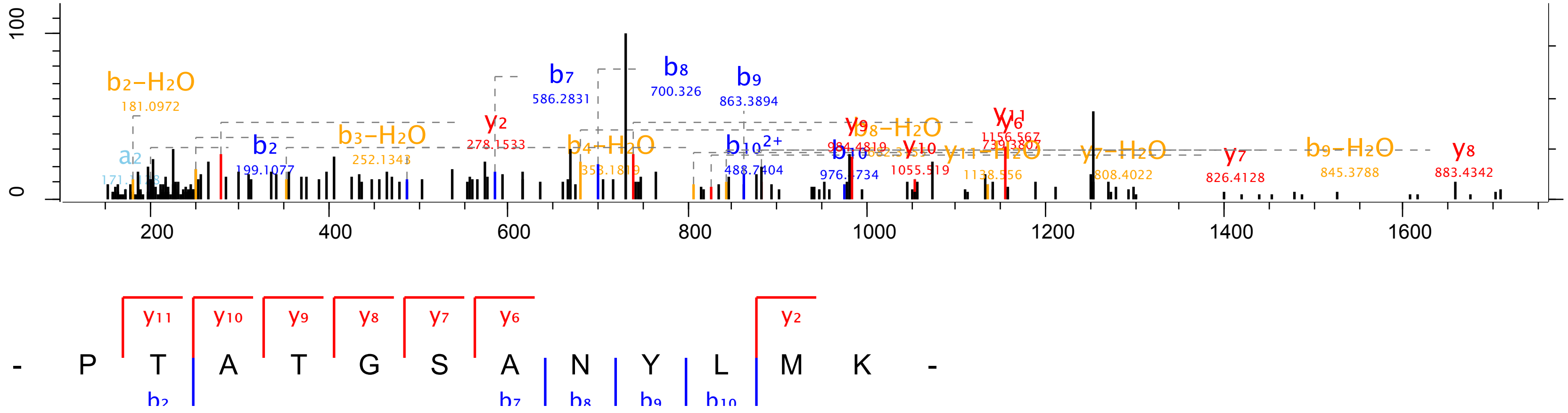

Raw file	Scan	Method	Score	m/z	Gene names
20150228_yeast1_Top_opt_B1_01_1611	22890	TOF; CID	89.67	410.26	PIC2

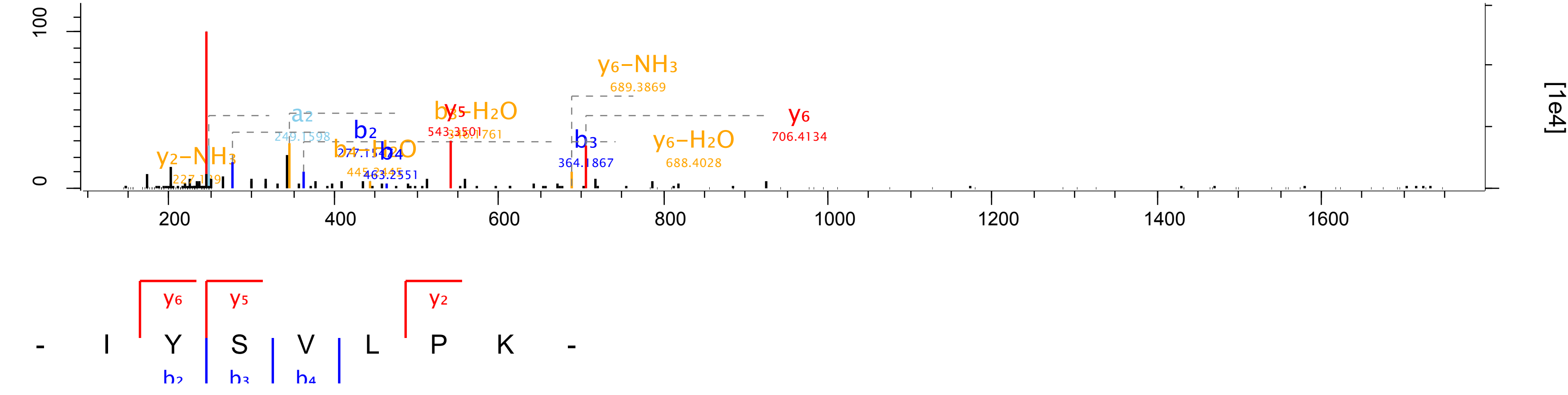

Raw file	Scan	Method	Score	m/z	Gene names
20150228_yeast1_Top_opt_B1_01_1611	23649	TOF; CID	91.55	648.79	FMP37

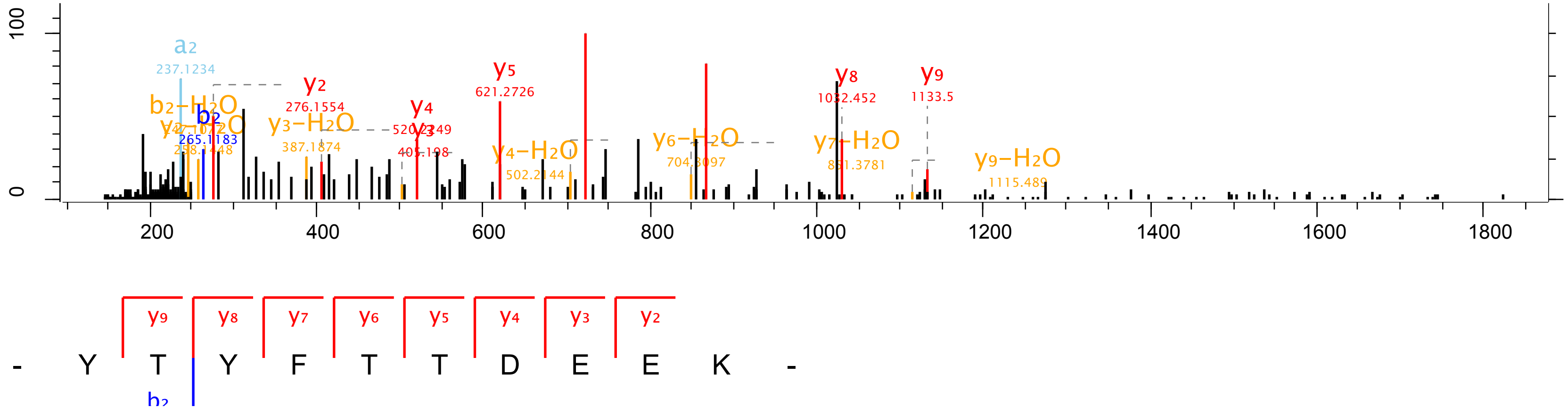

Raw file	Scan	Method	Score	m/z	Gene names
20150228_yeast1_Top_opt_B1_01_1611	25287	TOF; CID	100.97	994.48	CHS6

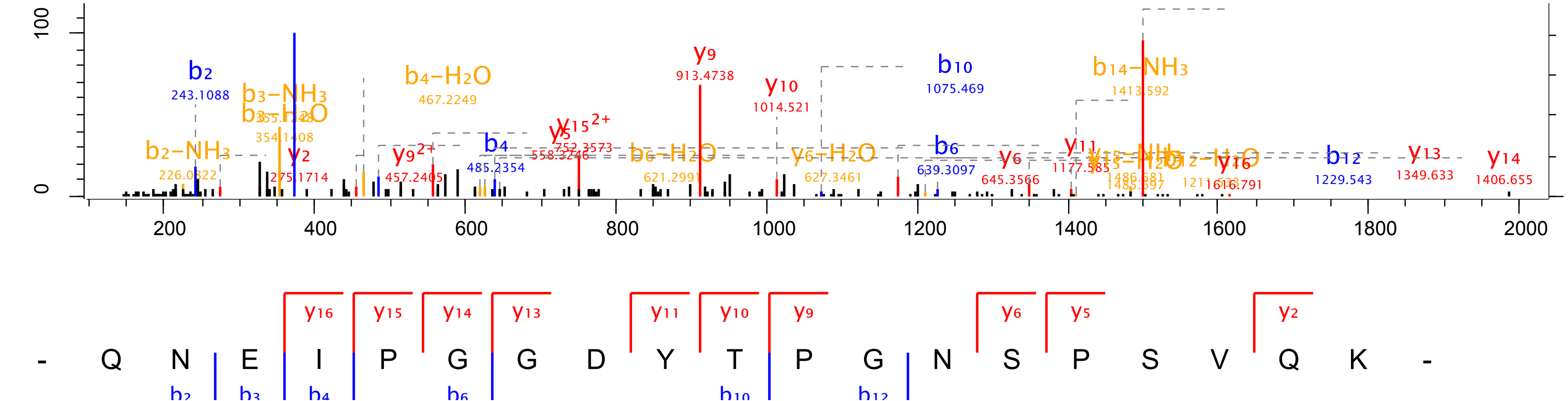

Raw file	Scan	Method	Score	m/z	Gene names
20150228_yeast1_Top_opt_B1_01_1611	25404	TOF; CID	150.91	610.63	MDM35

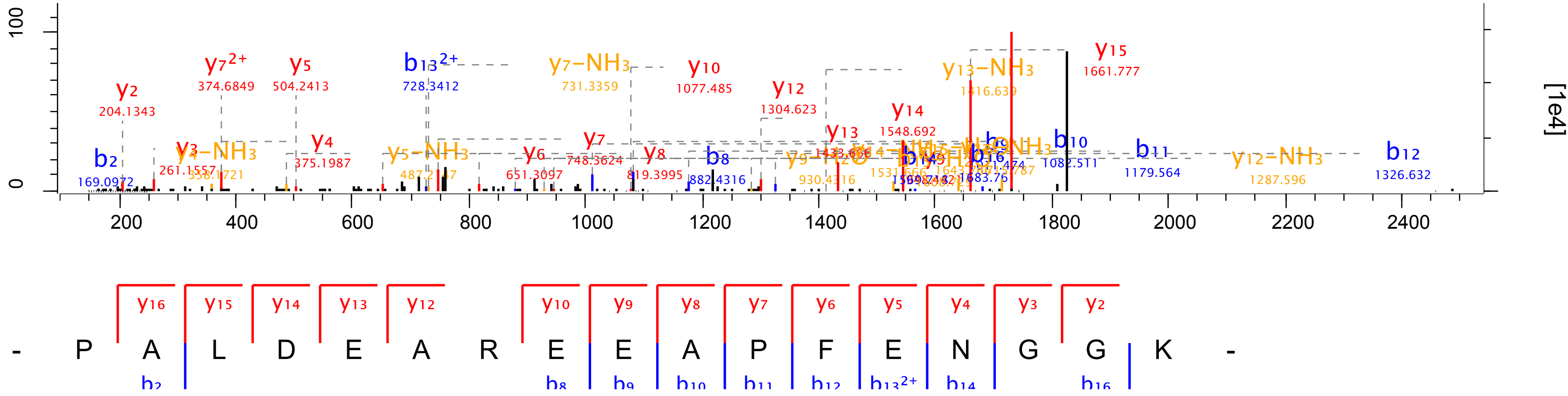

Raw file	Scan	Method	Score	m/z	Gene names
20150228_yeast1_Top_opt_B1_01_1611	25822	TOF; CID	82.26	393.25	TAF8

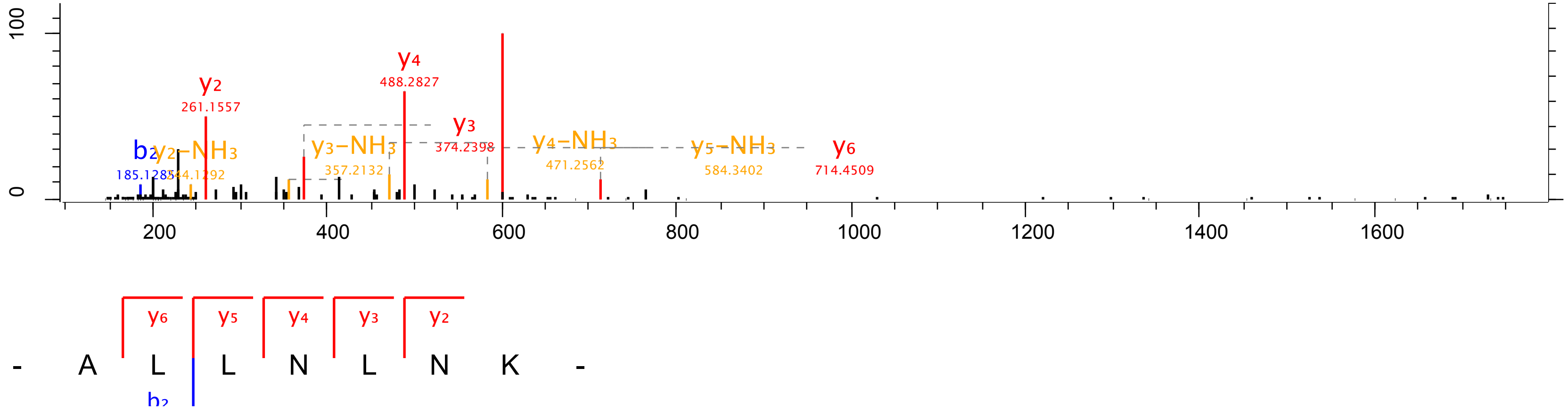

Raw file	Scan	Method	Score	m/z	Gene names
20150228_yeast1_Top_opt_B1_01_1611	26831	TOF; CID	82.01	649.33	MZM1

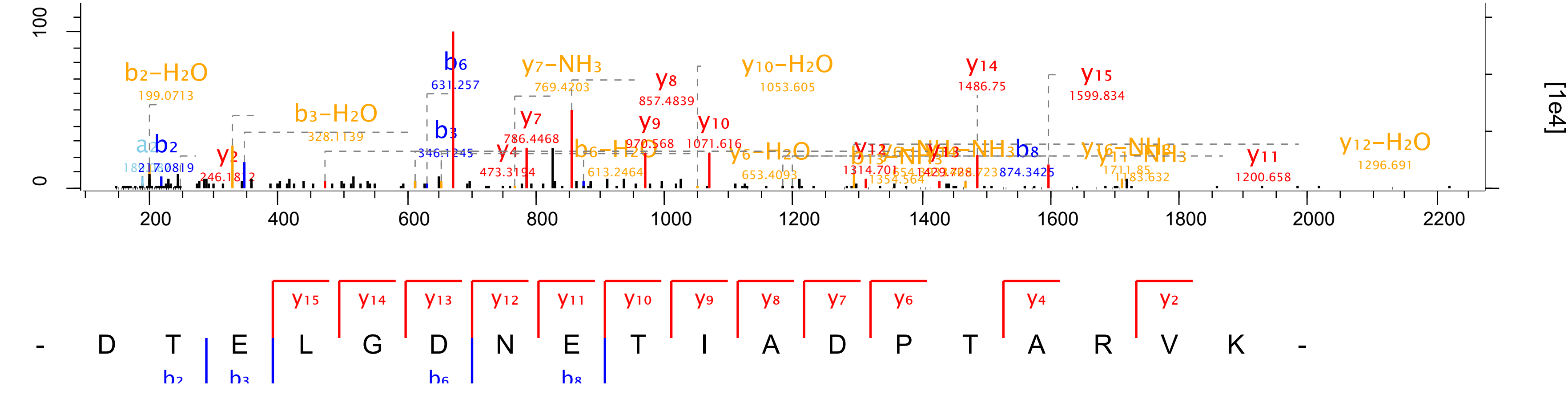

Raw file	Scan	Method	Score	m/z	Gene names
20150228_yeast1_Top_opt_B1_01_1611	26913	TOF; CID	99.94	758.87	PLB1

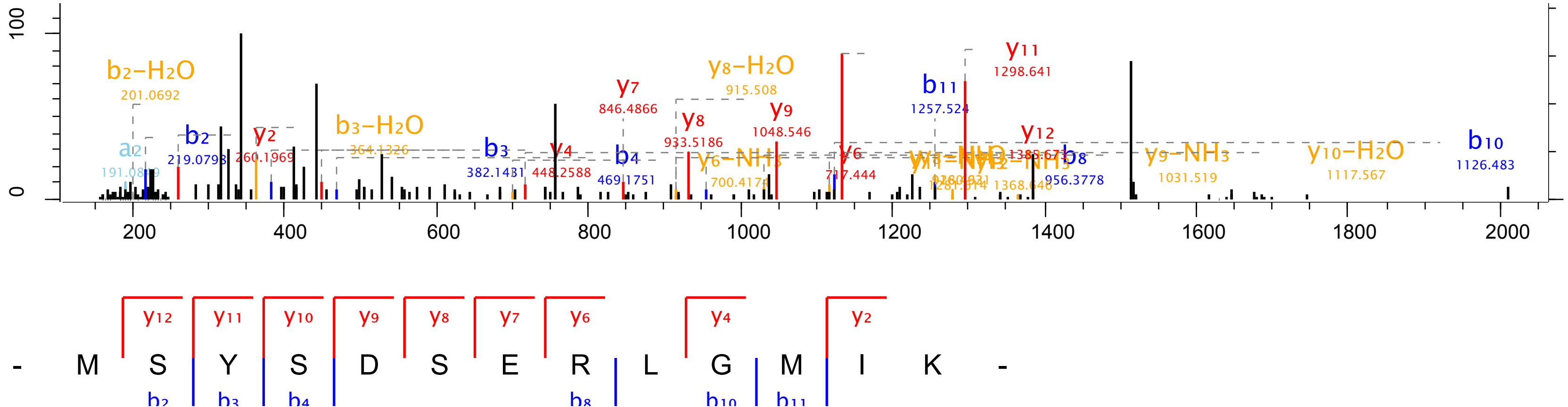

Raw file	Scan	Method	Score	m/z	Gene names
20150228_yeast1_Top_opt_B1_01_1611	27407	TOF; CID	79.16	548.27	CBC2

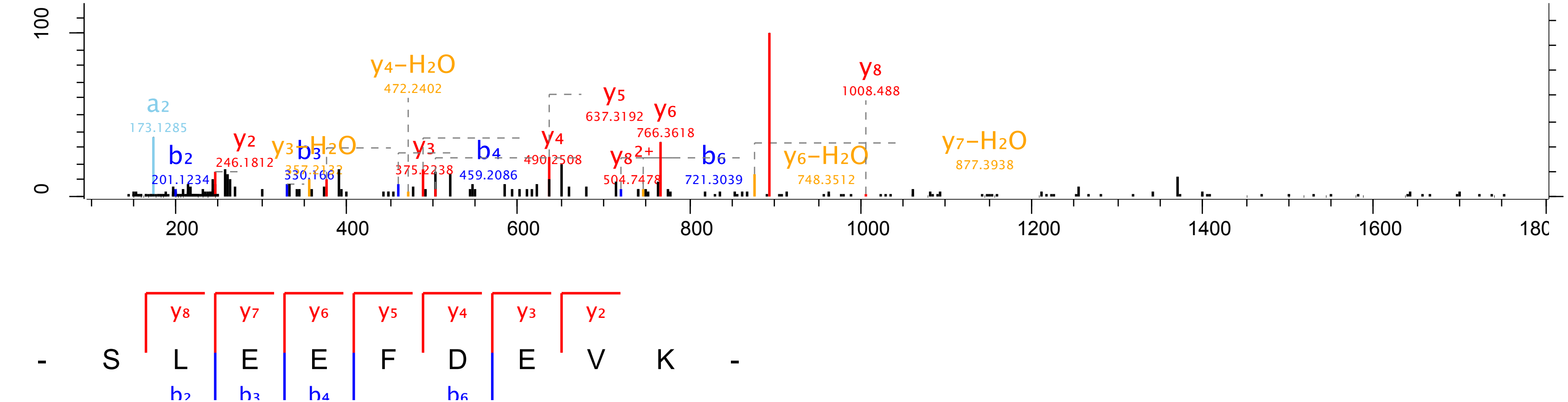

Raw file	Scan	Method	Score	m/z
20150228_yeast1_Top_opt_B1_01_1611	27569	TOF; CID	94.11	458.77

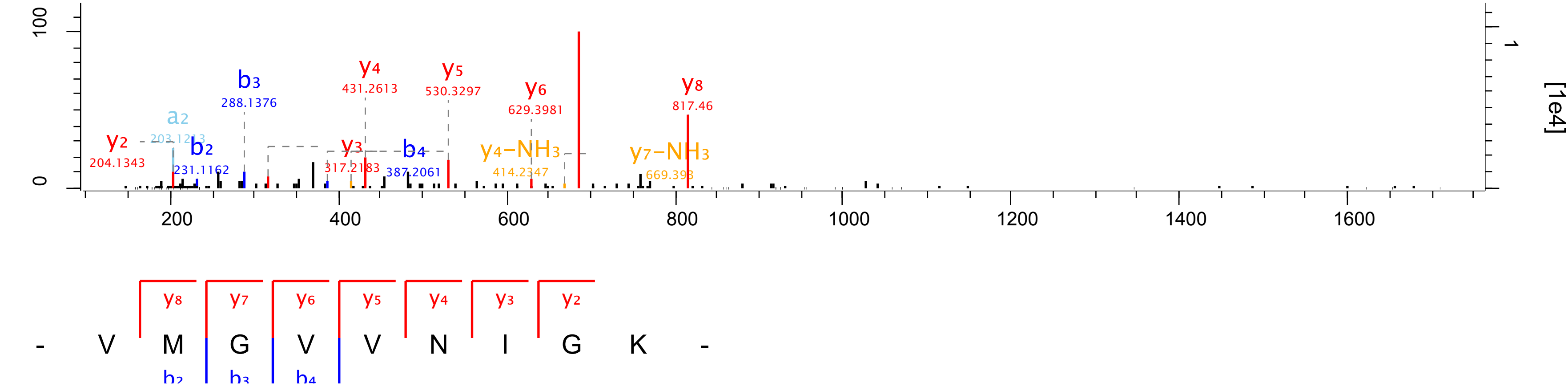

Raw file	Scan	Method	Score	m/z	Gene names
20150228_yeast1_Top_opt_B1_01_1611	27740	TOF; CID	100.02	513.26	OM14

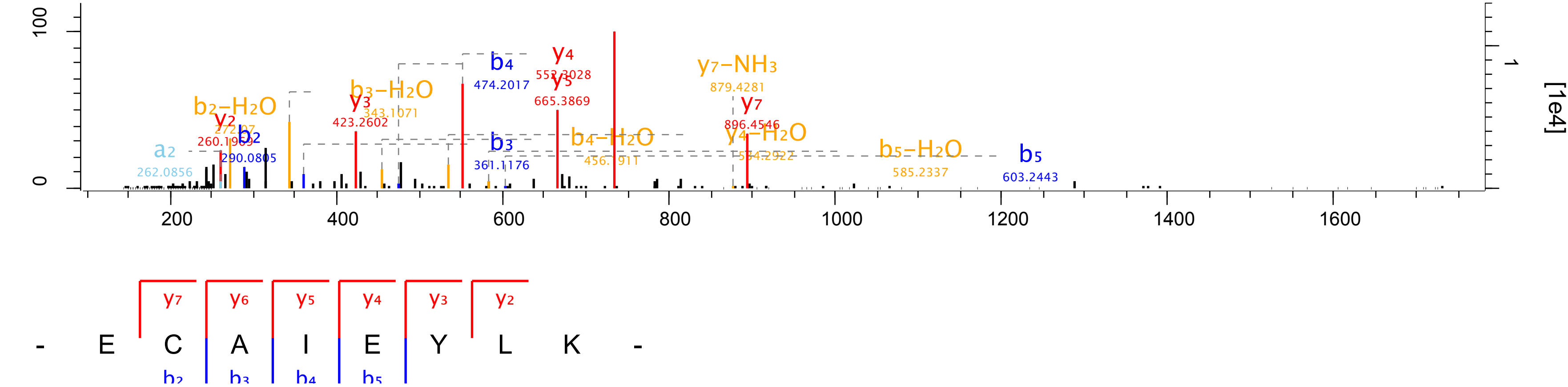

Raw file	Scan	Method	Score	m/z	Gene names
20150228_yeast1_Top_opt_B1_01_1611	27759	TOF; CID	94.58	556.82	MAK31

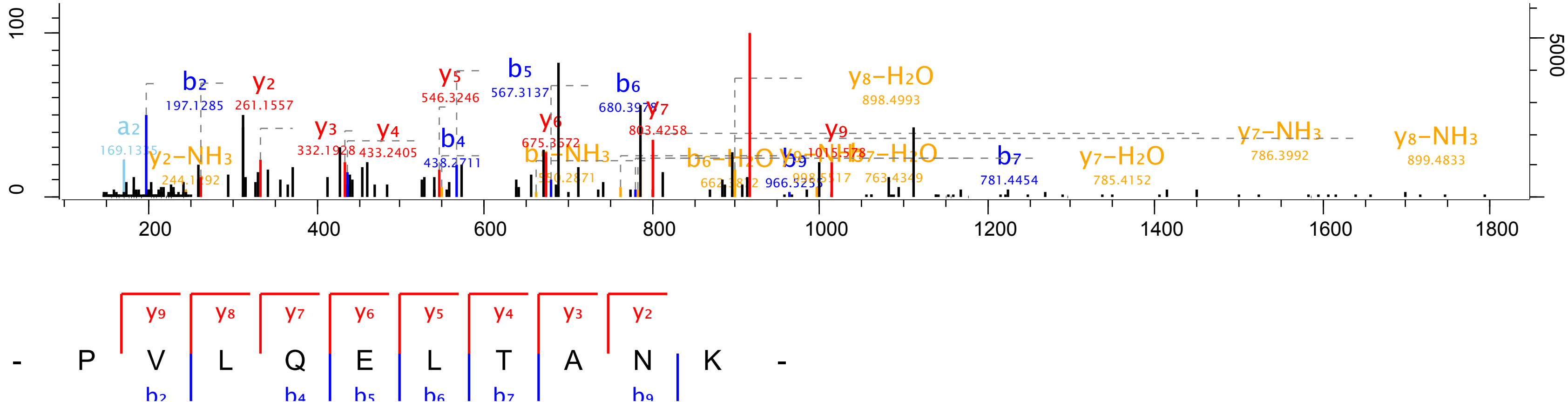

Raw file	Scan	Method	Score	m/z	Gene names
20150228_yeast1_Top_opt_B1_01_1611	28022	TOF; CID	66.06	374.89	MET8

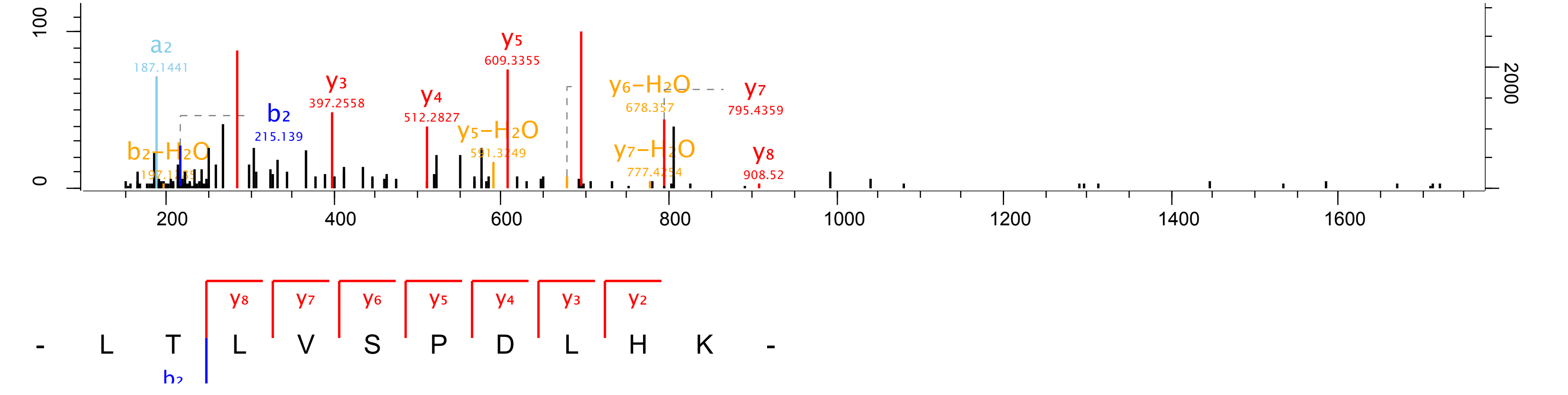

Raw file	Scan	Method	Score	m/z	Gene names
20150228_yeast1_Top_opt_B1_01_1611	28113	TOF; CID	117.08	527.96	MST27;MST28

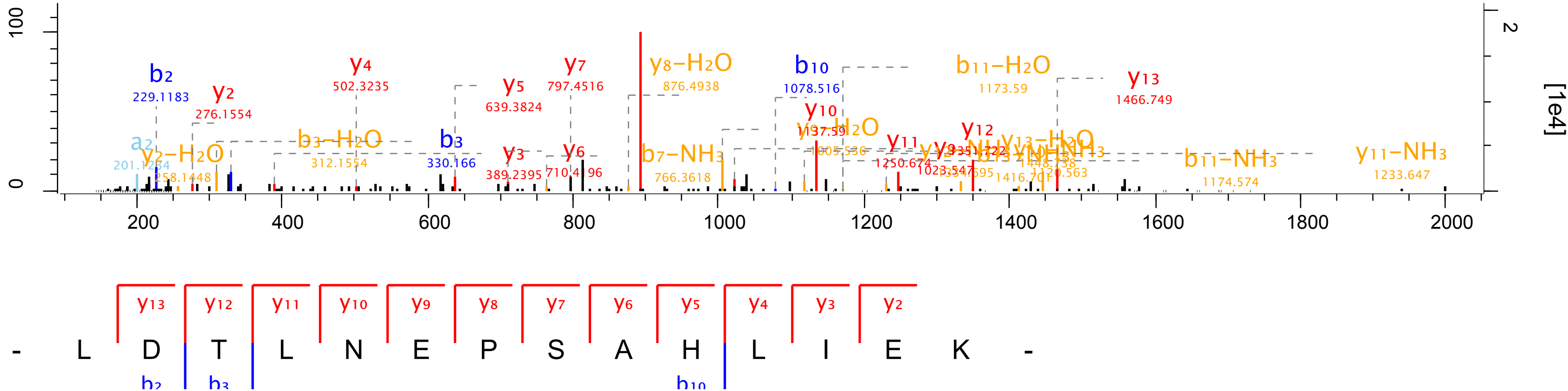

Raw file	Scan	Method	Score	m/z	Gene names
20150228_yeast1_Top_opt_B1_01_1611	28220	TOF; CID	65.18	617.32	MID1

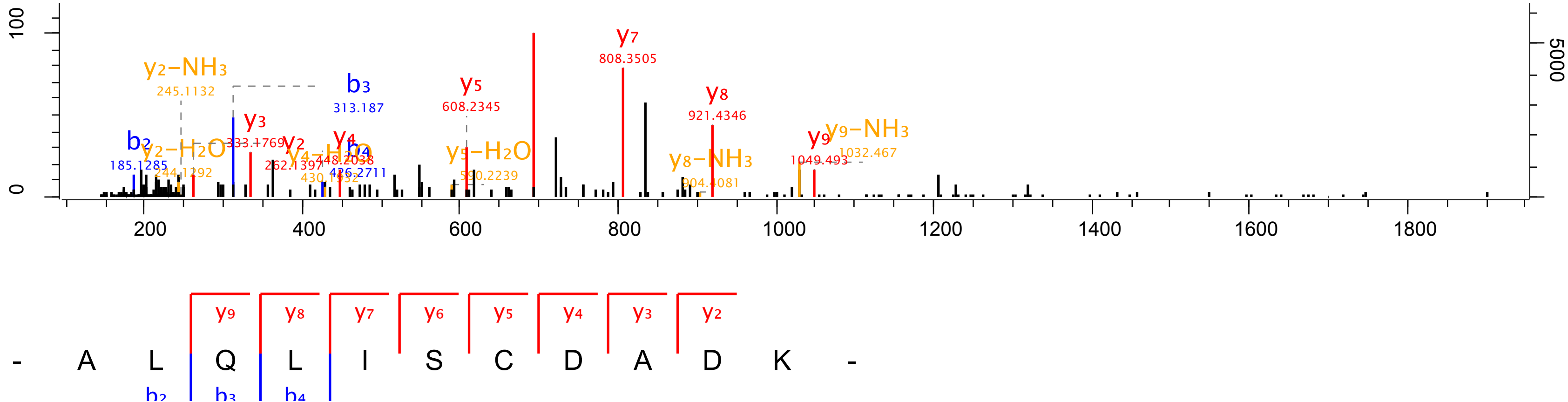

Raw file	Scan	Method	Score	m/z	Gene names
20150228_yeast1_Top_opt_B1_01_1611	28323	TOF; CID	106.86	793.06	STP22

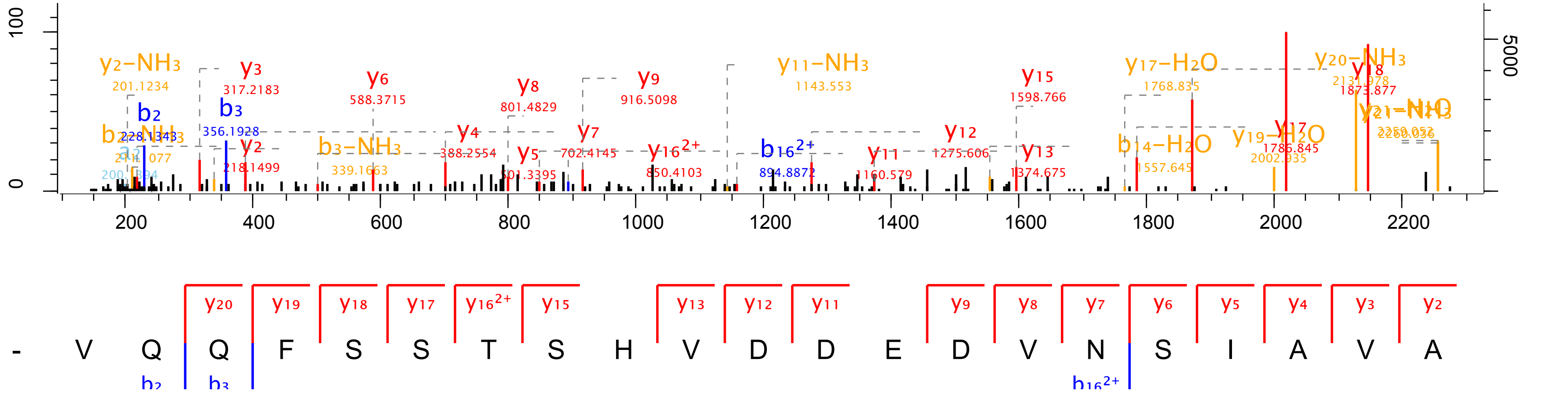

Raw file	Scan	Method	Score	m/z	Gene names
20150228_yeast1_Top_opt_B1_01_1611	28406	TOF; CID	124.12	673.38	MNR2

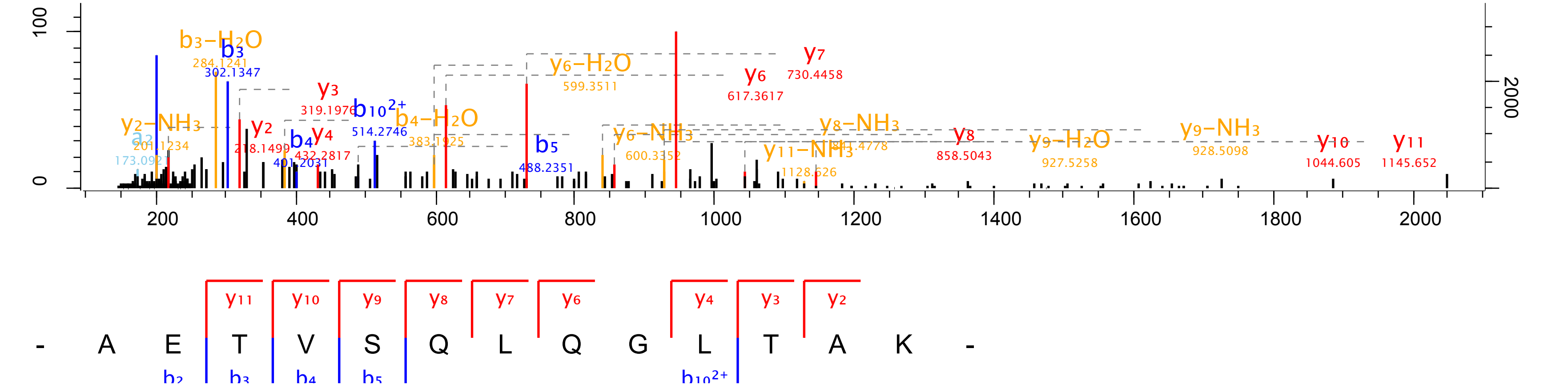

Raw file	Scan	Method	Score	m/z	Gene names
20150228_yeast1_Top_opt_B1_01_1611	28501	TOF; CID	114.28	393.88	YLR361C-A

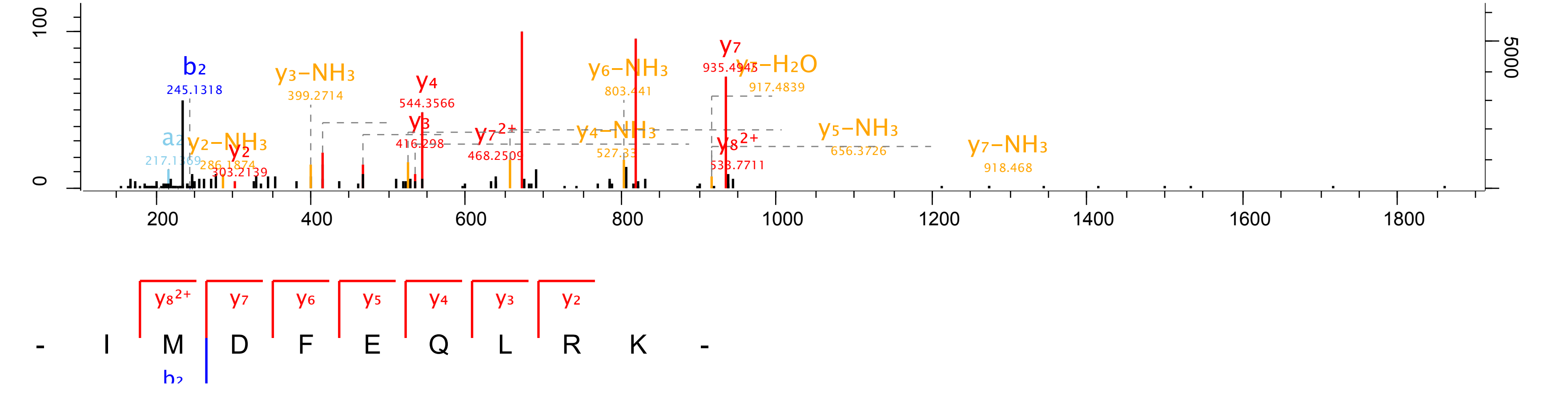

Raw file	Scan	Method	Score	m/z	Gene names
20150228_yeast1_Top_opt_B1_01_1611	29183	TOF; CID	68.22	475.26	RMD1

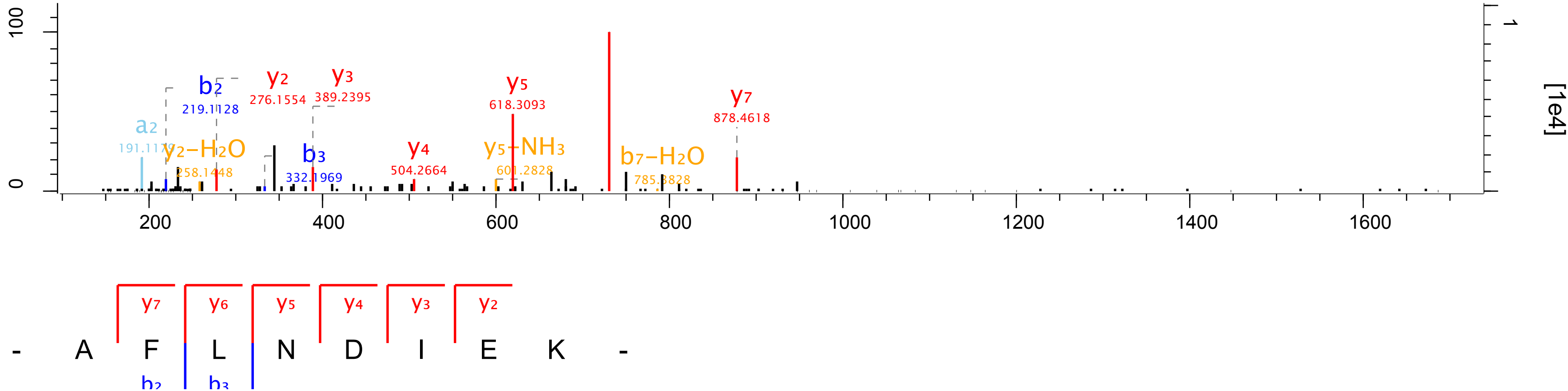

Raw file	Scan	Method	Score	m/z	Gene names
20150228_yeast1_Top_opt_B1_01_1611	30084	TOF; CID	107.82	568.3	YGR169C-A

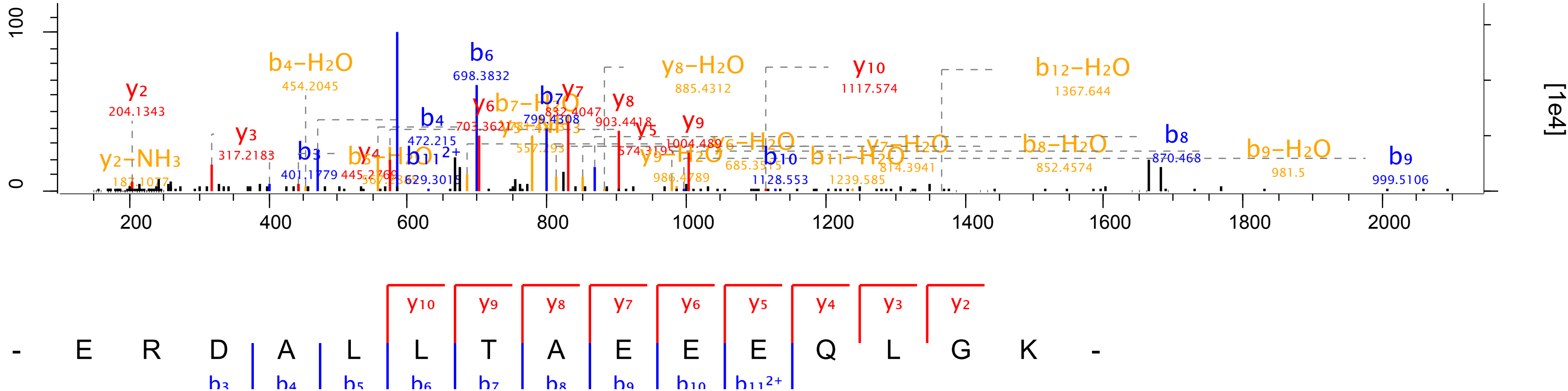

Raw file	Scan	Method	Score	m/z
20150228_yeast1_Top_opt_B1_01_1611	30181	TOF; CID	60.91	505.28

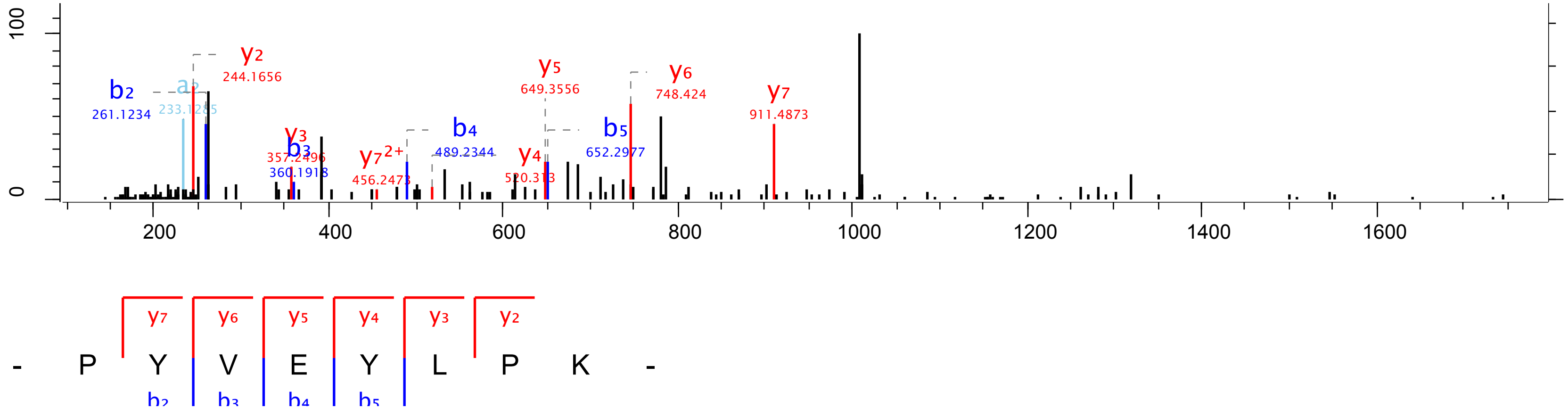

Raw file	Scan	Method	Score	m/z	Gene names
20150228_yeast1_Top_opt_B1_01_1611	30952	TOF; CID	166.77	1006.5	MGR2

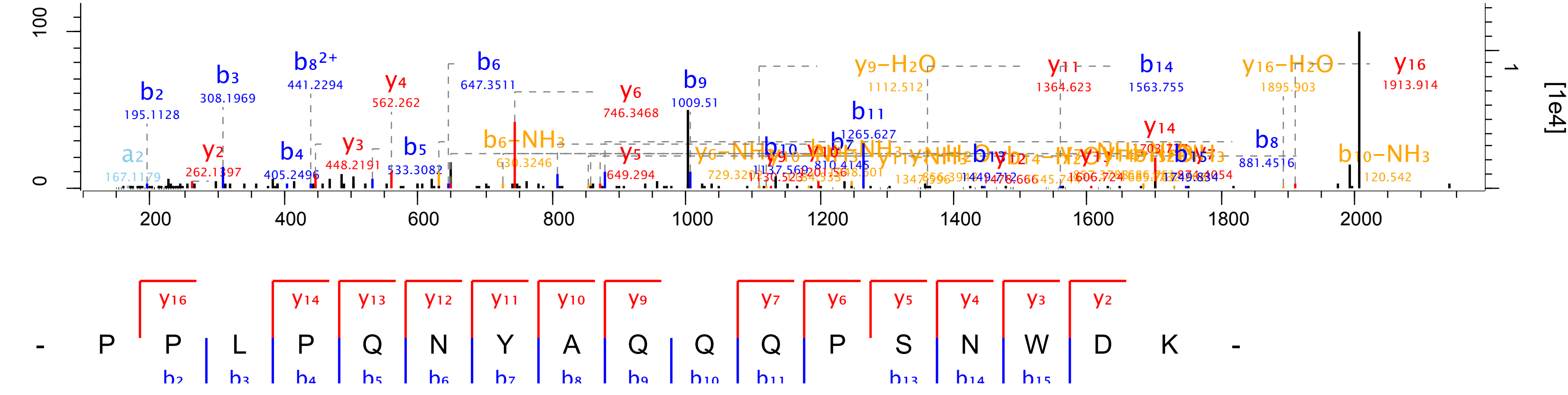

Raw file	Scan	Method	Score	m/z	Gene names
20150228_yeast1_Top_opt_B1_01_1611	31895	TOF; CID	115.12	678.87	RPN13

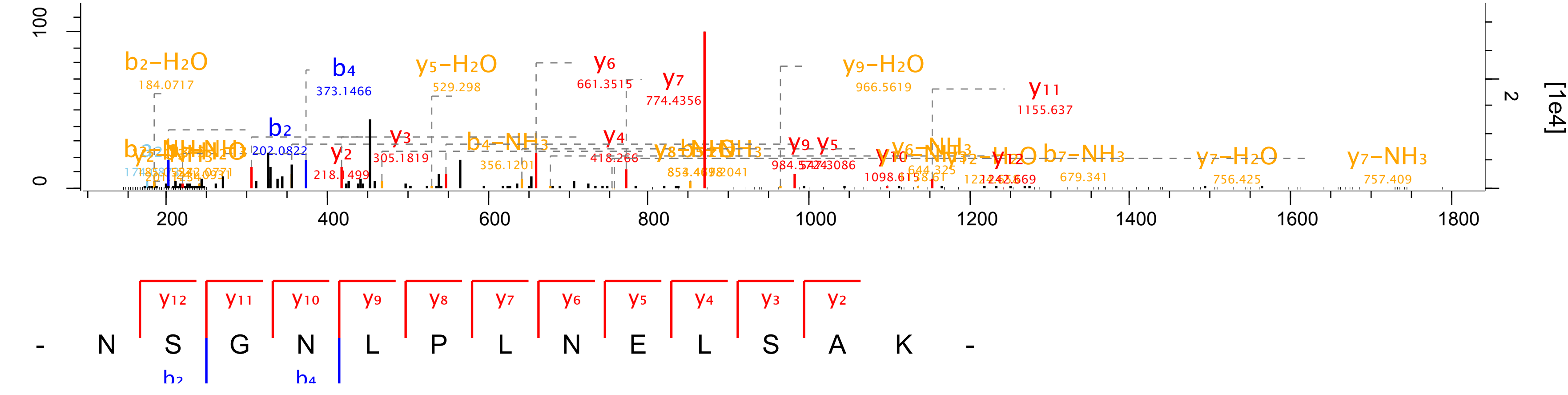

Raw file	Scan	Method	Score	m/z	Gene names
20150228_yeast1_Top_opt_B1_01_1611	31918	TOF; CID	71.38	468.78	BET4

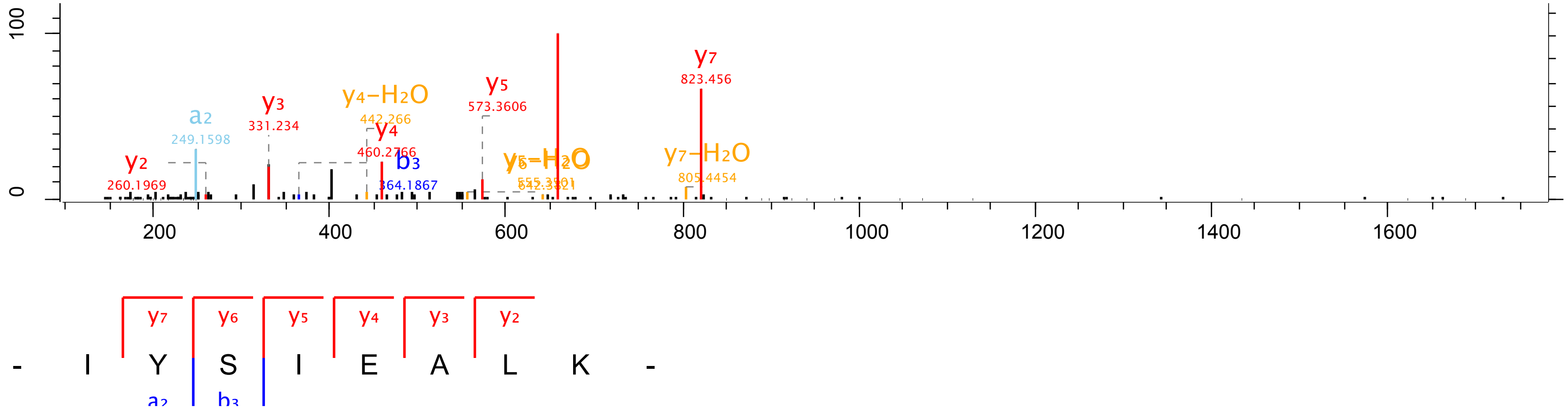

Raw file	Scan	Method	Score	m/z	Gene names
20150228_yeast1_Top_opt_B1_01_1611	32443	TOF; CID	83.05	456.56	ASK1

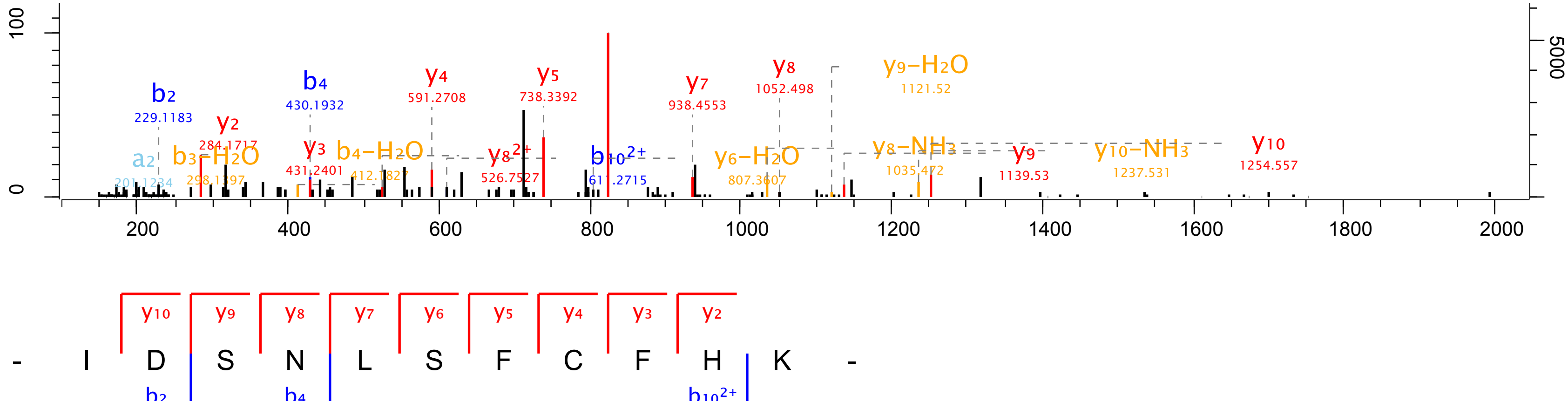

Raw file	Scan	Method	Score	m/z	Gene names
20150228_yeast1_Top_opt_B1_01_1611	33660	TOF; CID	42.86	546.32	TYE7

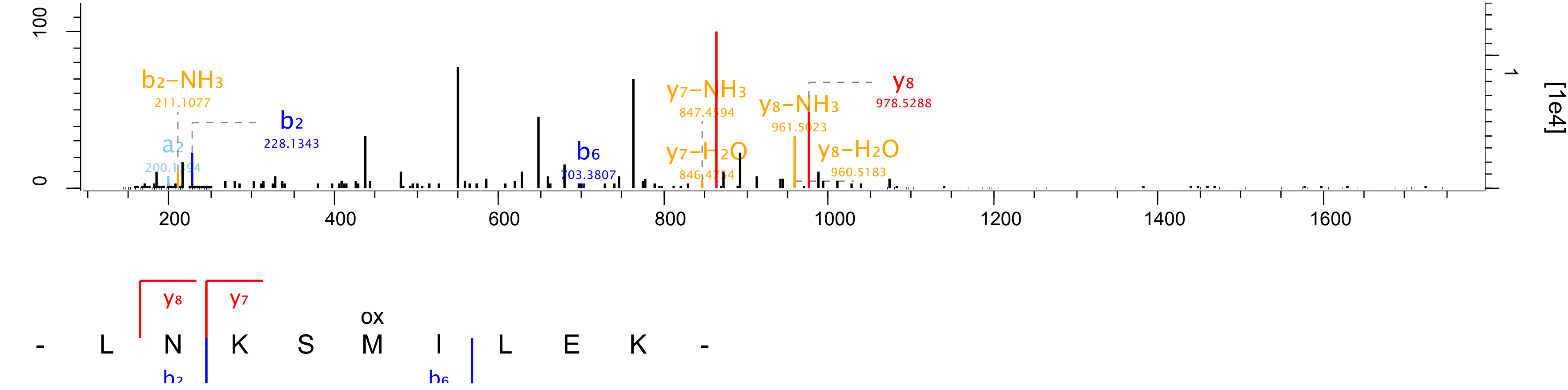

Raw file	Scan	Method	Score	m/z	Gene names
20150228_yeast1_Top_opt_B1_01_1611	33825	TOF; CID	51.73	685.34	LCB3

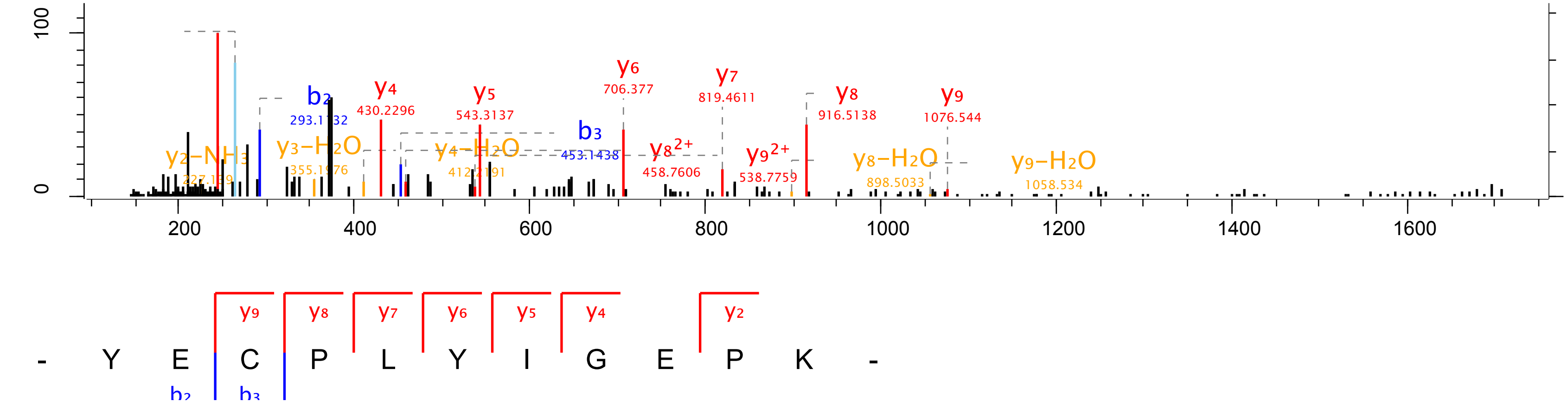

Raw file	Scan	Method	Score	m/z	Gene names
20150228_yeast1_Top_opt_B1_01_1611	33896	TOF; CID	85.68	486.3	PEP12

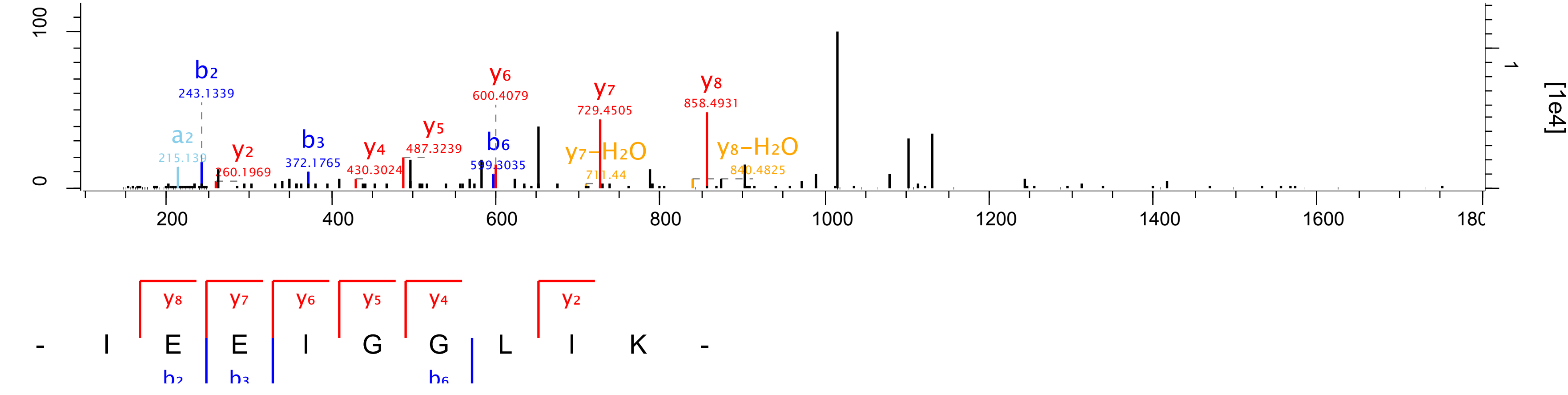

Raw file	Scan	Method	Score	m/z	Gene names
20150228_yeast1_Top_opt_B1_01_1611	34606	TOF; CID	55.98	768.39	JNM1

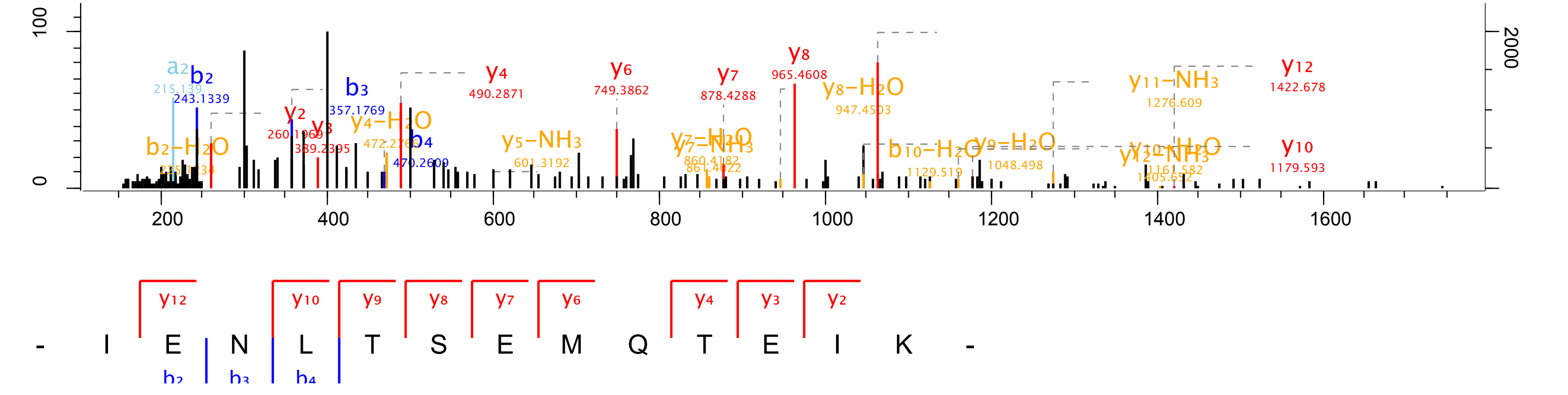

Raw file	Scan	Method	Score	m/z	Gene names
20150228_yeast1_Top_opt_B1_01_1611	34796	TOF; CID	64.82	618.3	AGA2

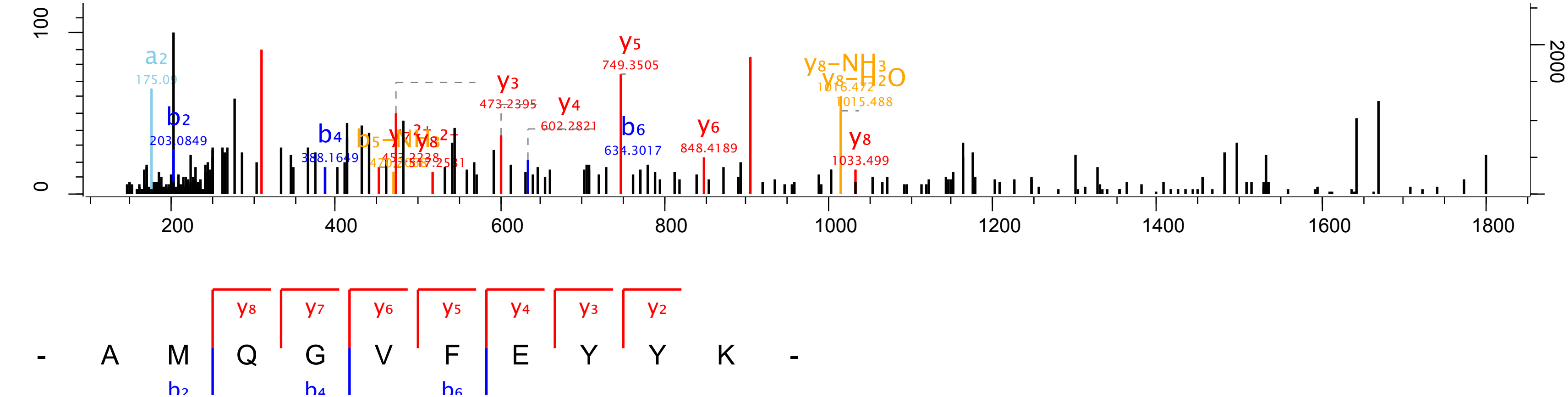

Raw file	Scan	Method	Score	m/z	Gene names
20150228_yeast1_Top_opt_B1_01_1611	34874	TOF; CID	136.26	1158.01	COX23

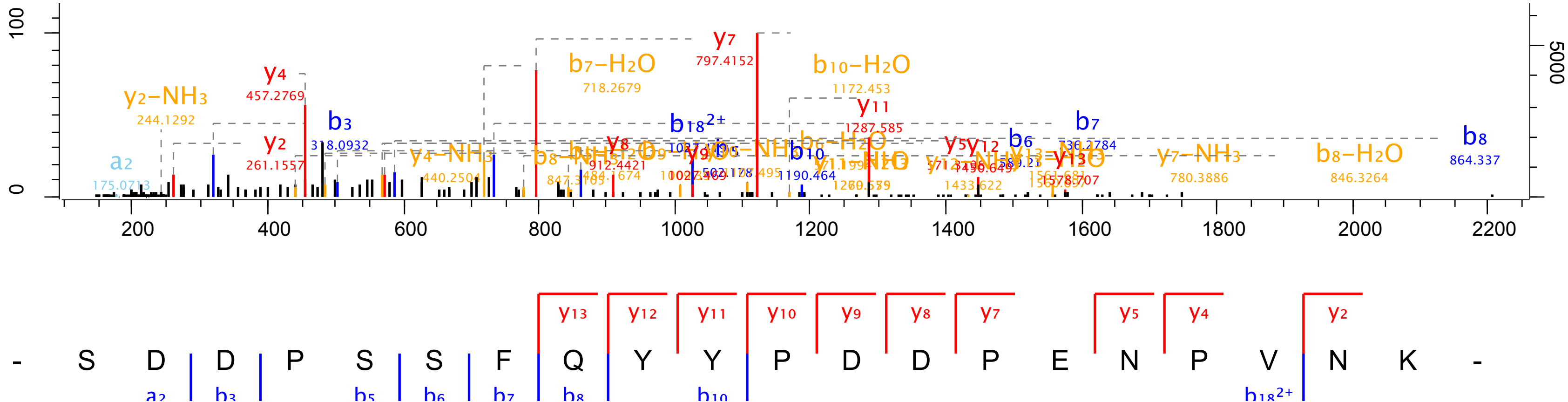

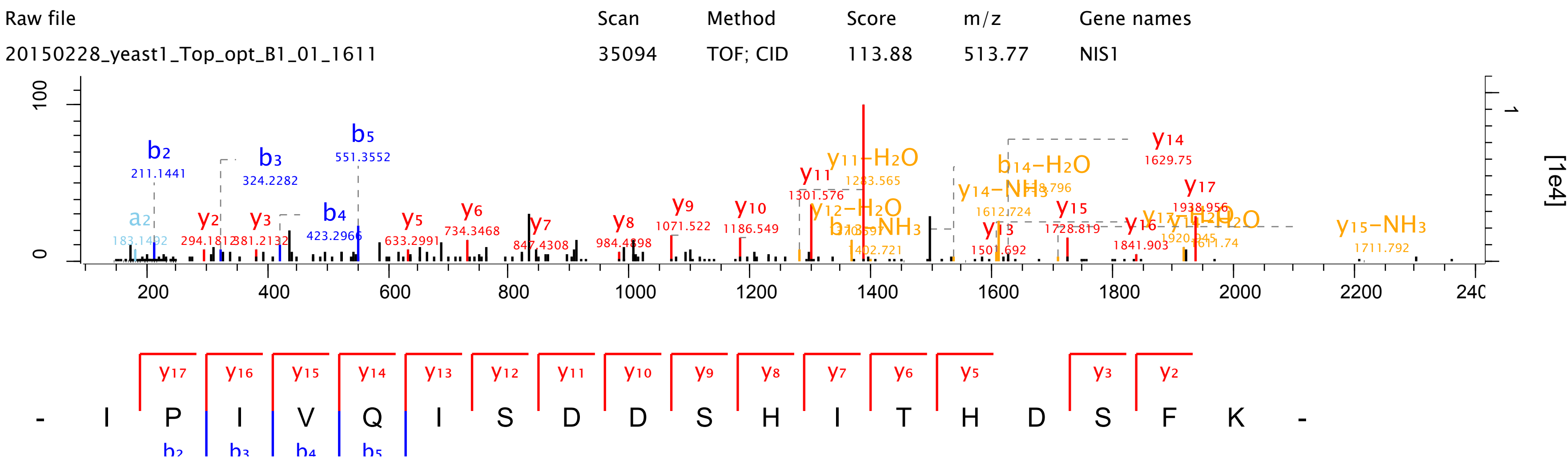

Raw file
20150228_yeast1_Top_opt_B1_01_1611
Scan
35094
Method
TOF; CID
Score
113.88
m/z
513.77
Gene names
NIS1
100
0
[1e4]
1
b2
211.1441
b3
324.2282
b5
551.3552
a2
183.1492
y2
294.1812
y3
381.2132
b4
423.2966
y5
633.2991
y6
734.3468
y7
847.4308
y8
984.4898
y9
1071.522
y10
1186.549
y11
1301.576
y11−H2O
1283.565
y12−H2O
b14−H2O
y14−NH3
1612.724
y14
1629.75
y17
1938.956
y15
1728.819
y13
1501.692
y16
1841.903
y15−NH3
1711.792
1402.721
1920.945
200
400
600
800
1000
1200
1400
1600
1800
2000
2200
y17
y16
y15
y14
y13
y12
y11
y10
y9
y8
y7
y6
y5
y3
y2
- I P I V Q I S D D S H I T H D S F K -
b2
b3
b4
b5

Raw file	Scan	Method	Score	m/z	Gene names
20150228_yeast1_Top_opt_B1_01_1611	35213	TOF; CID	57.43	728.41	INO4

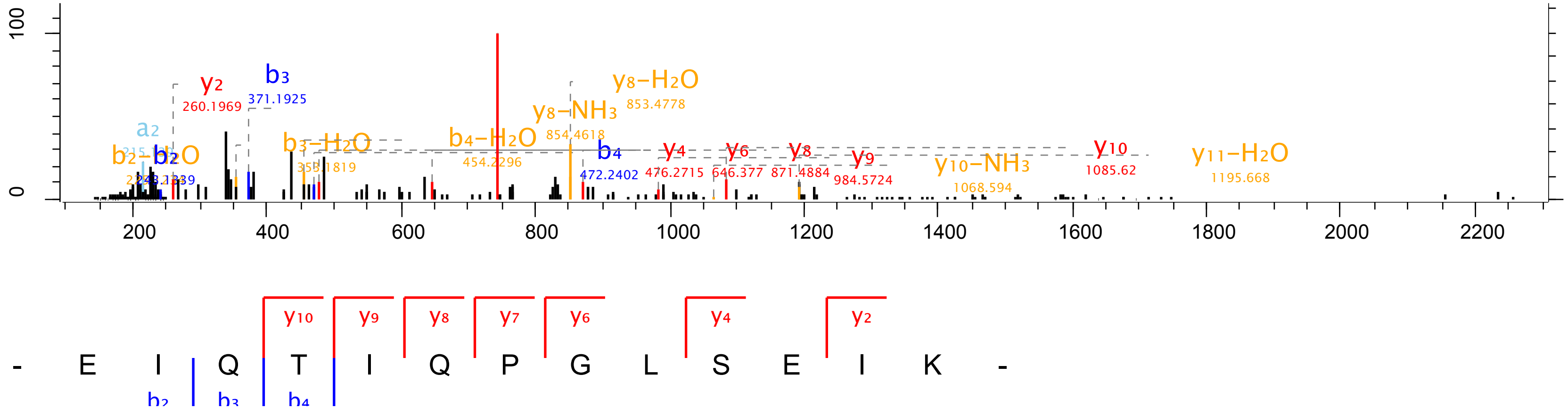

Raw file	Scan	Method	Score	m/z	Gene names
20150228_yeast1_Top_opt_B1_01_1611	36076	TOF; CID	125.97	474.93	ARC19

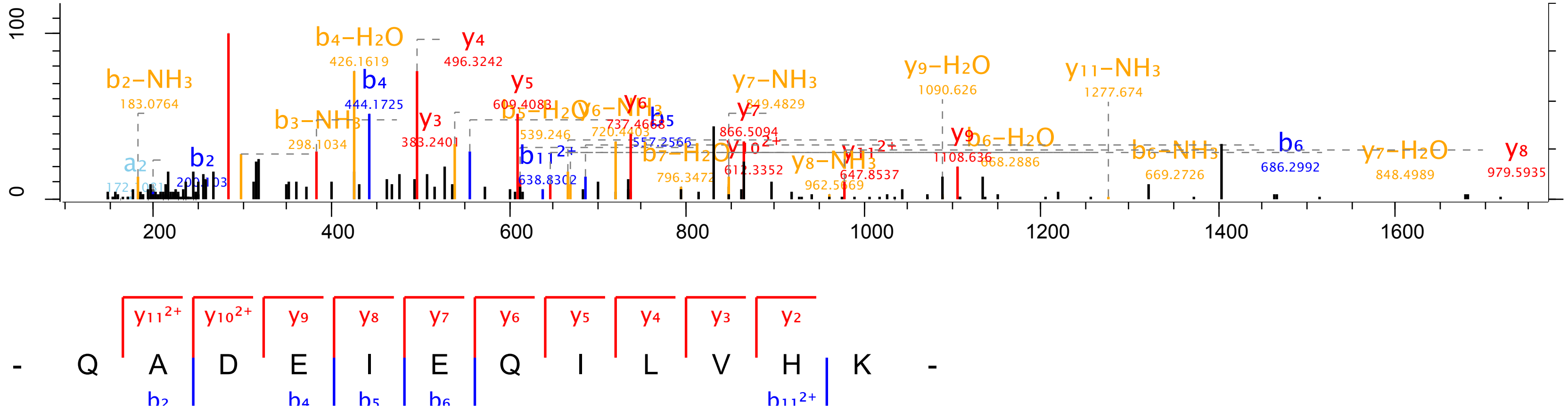

Raw file	Scan	Method	Score	m/z	Gene names
20150228_yeast1_Top_opt_B1_01_1611	37080	TOF; CID	183.23	597.3	MRH1

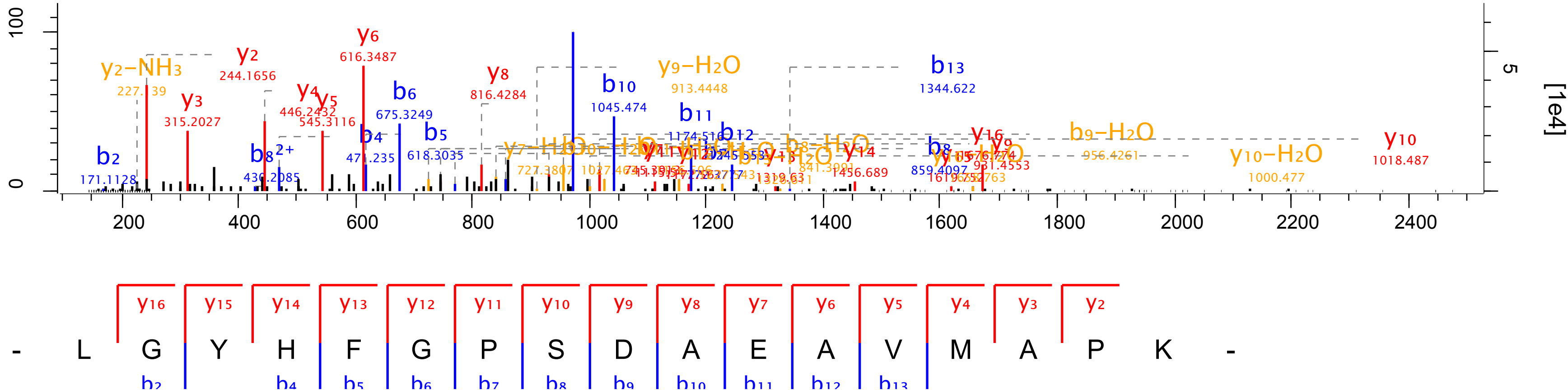

Raw file	Scan	Method	Score	m/z	Gene names
20150228_yeast1_Top_opt_B1_01_1611	37608	TOF; CID	88.71	506.63	SKO1

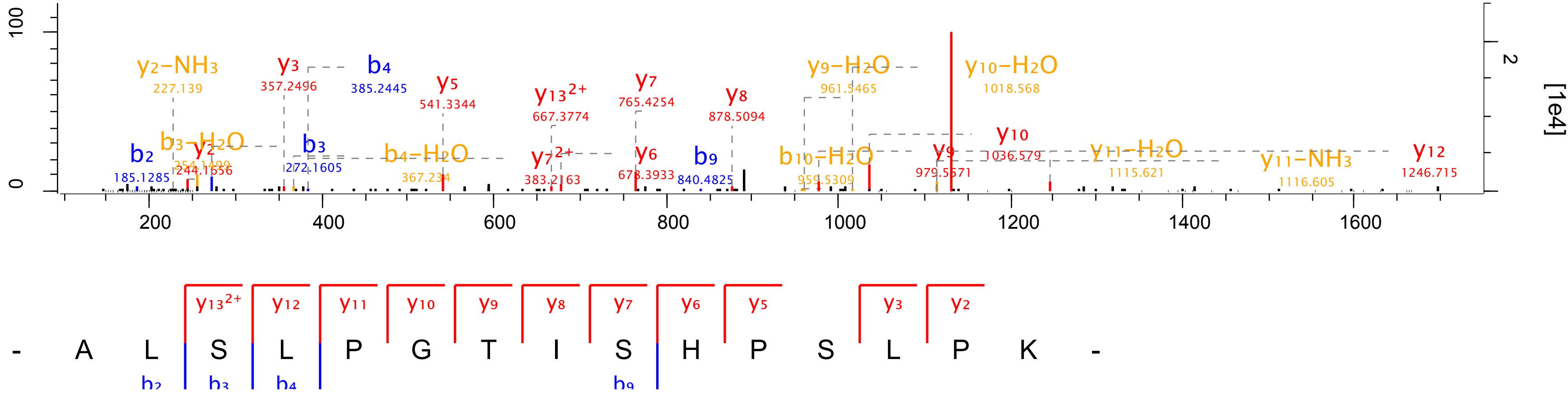

Raw file	Scan	Method	Score	m/z	Gene names
20150228_yeast1_Top_opt_B1_01_1611	38600	TOF; CID	58.12	743.87	VPS20

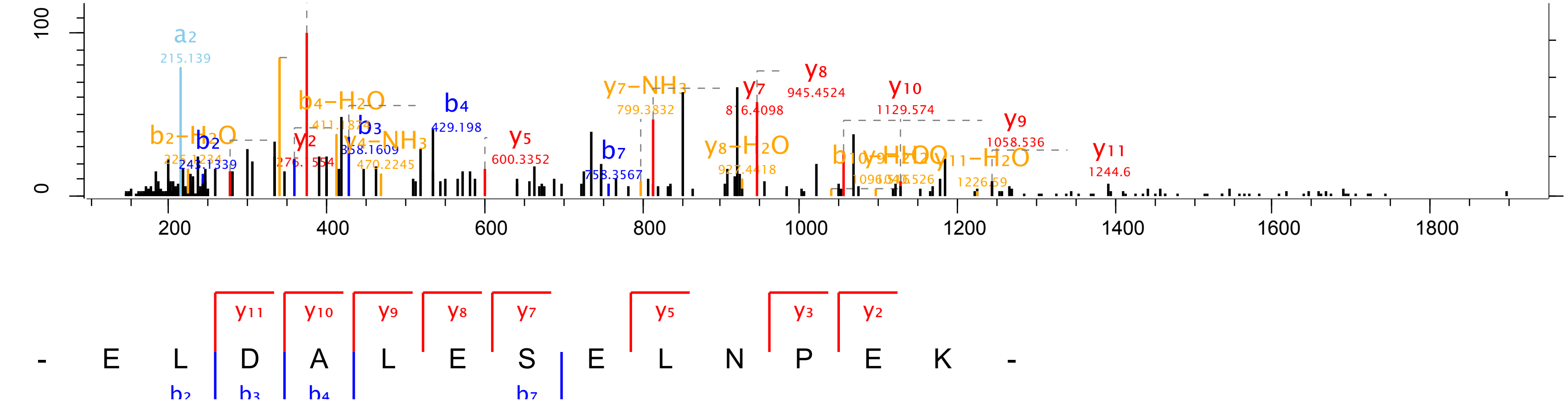

Raw file	Scan	Method	Score	m/z	Gene names
20150228_yeast1_Top_opt_B1_01_1611	39286	TOF; CID	80.51	760.39	MET31

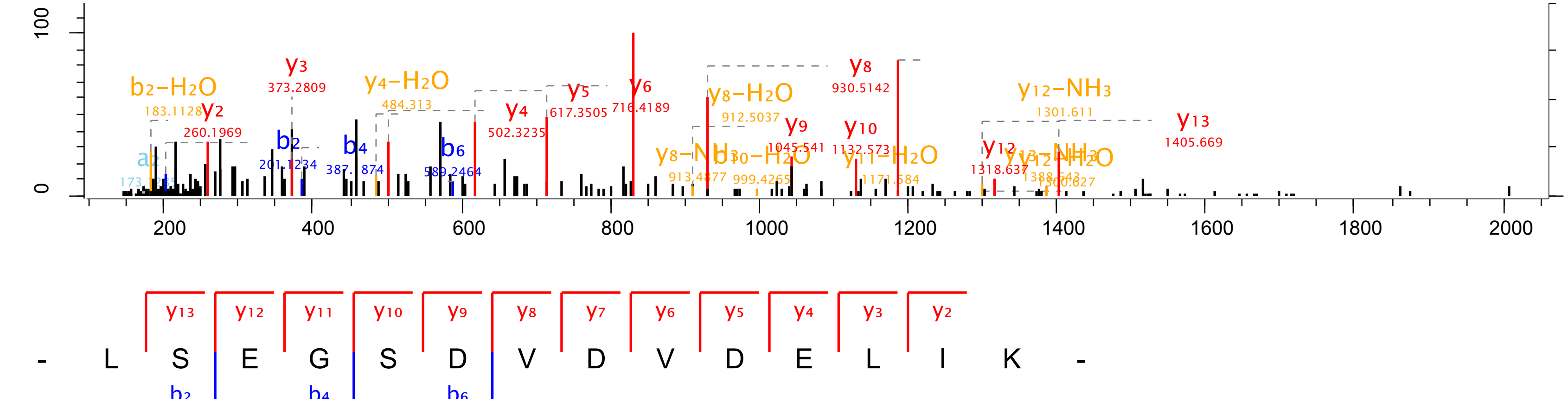

Raw file	Scan	Method	Score	m/z	Gene names
20150228_yeast1_Top_opt_B1_01_1611	40922	TOF; CID	80.44	507.74	SPT2

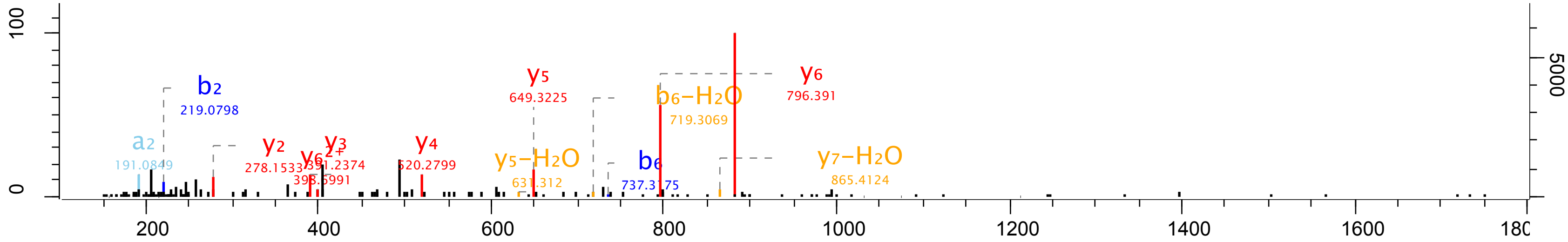

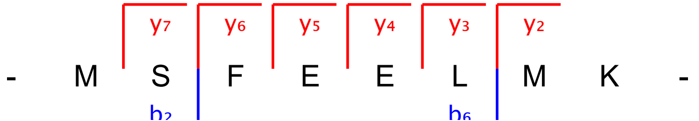

Raw file	Scan	Method	Score	m/z	Gene names
20150228_yeast1_Top_opt_B1_01_1611	41017	TOF; CID	44.61	609.33	RML2

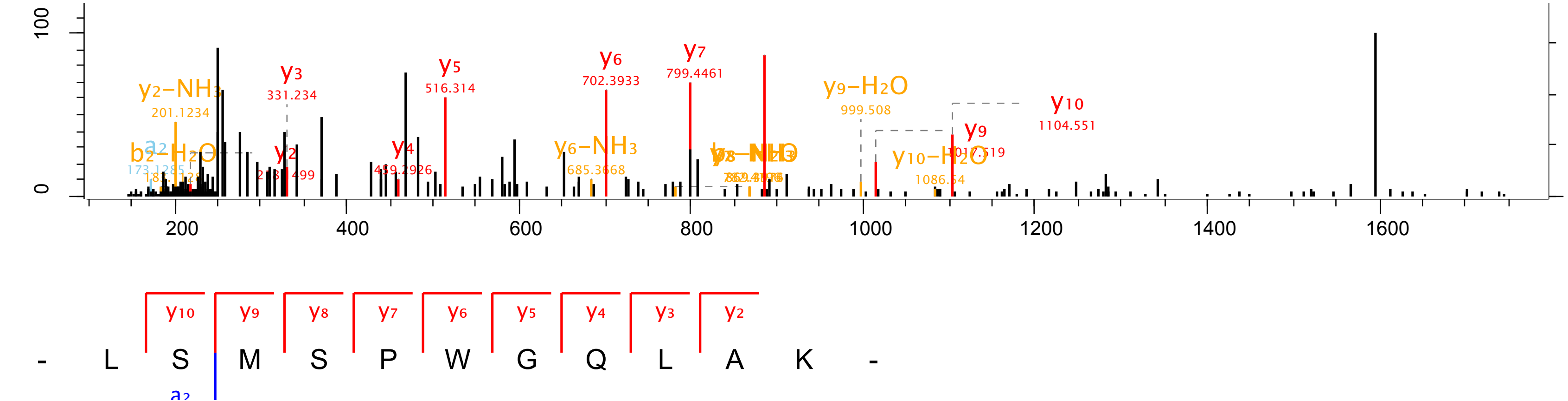

Raw file	Scan	Method	Score	m/z	Gene names
20150228_yeast1_Top_opt_B1_01_1611	41028	TOF; CID	76.55	995.46	CSI2

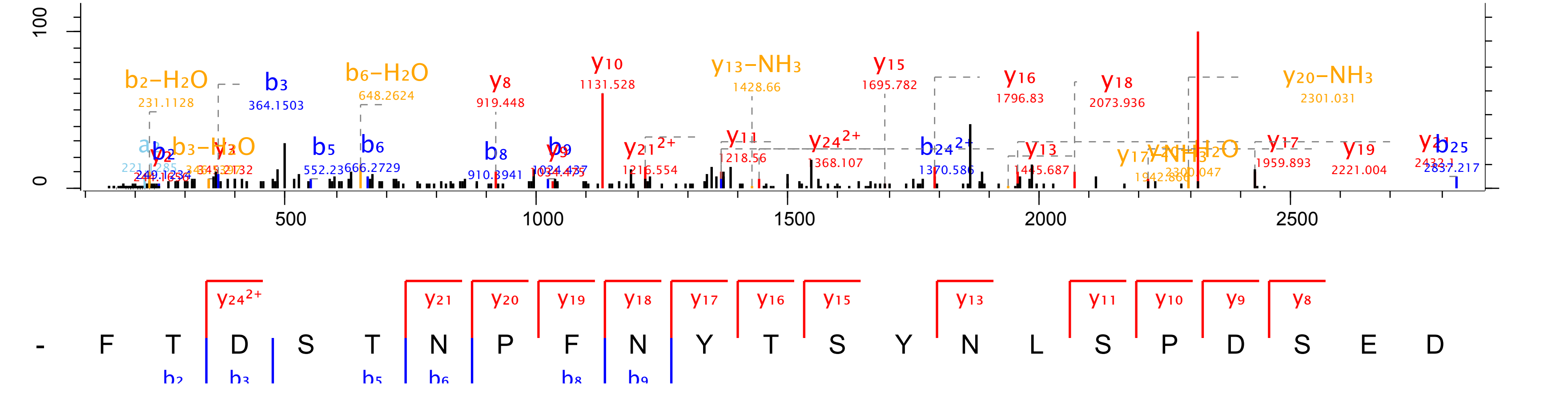

Raw file	Scan	Method	Score	m/z	Gene names
20150228_yeast1_Top_opt_B1_01_1611	41179	TOF; CID	73.07	891.43	YBR255C-A

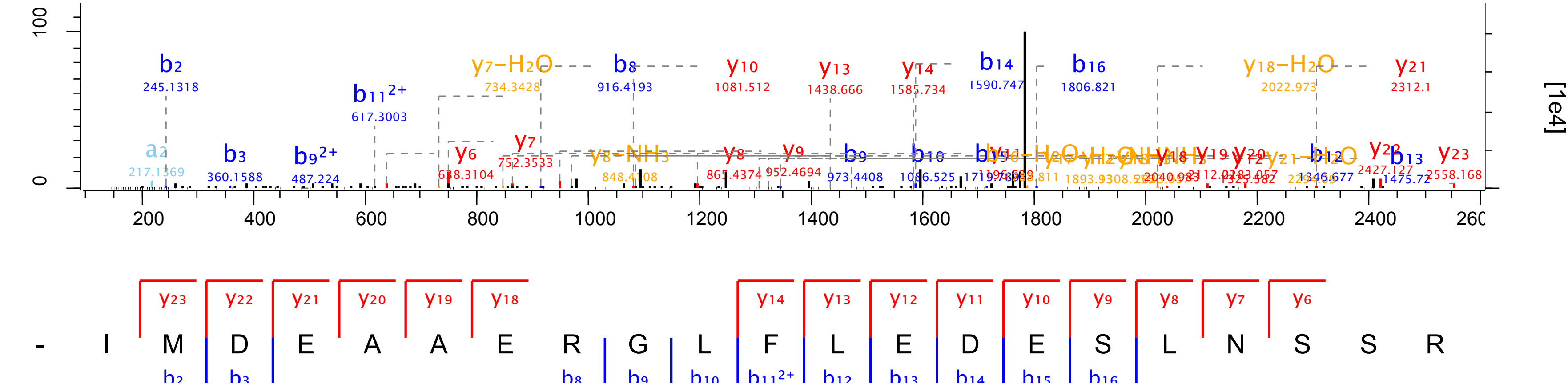

Raw file	Scan	Method	Score	m/z	Gene names
20150228_yeast1_Top_opt_B1_01_1611	41502	TOF; CID	84.36	752.41	SNX41

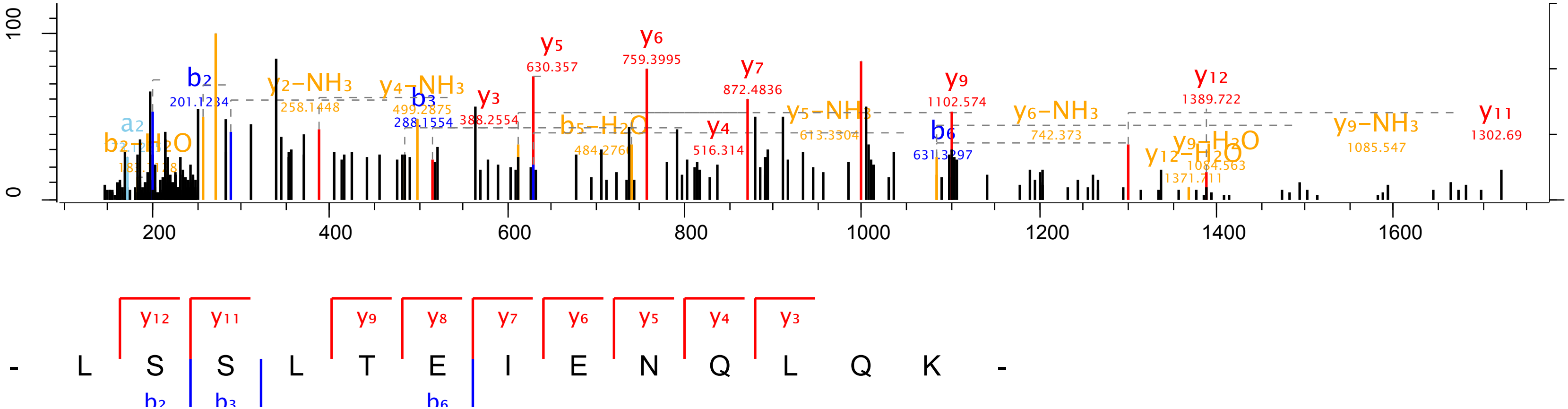

Raw file	Scan	Method	Score	m/z	Gene names
20150228_yeast1_Top_opt_B1_01_1611	41882	TOF; CID	115.63	724.38	COQ8

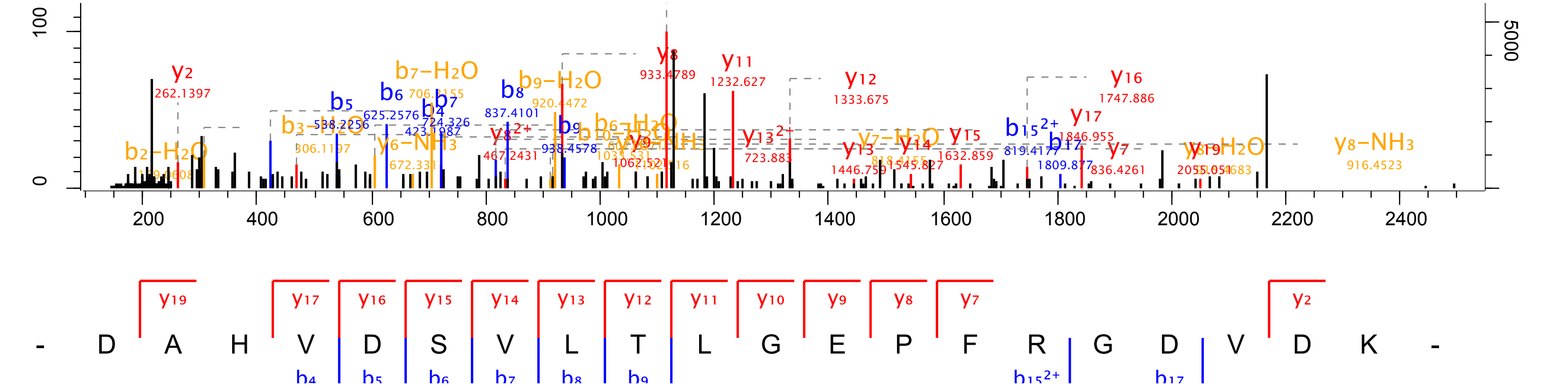

Raw file	Scan	Method	Score	m/z	Gene names
20150228_yeast1_Top_opt_B1_01_1611	42753	TOF; CID	66.02	643.82	UNG1

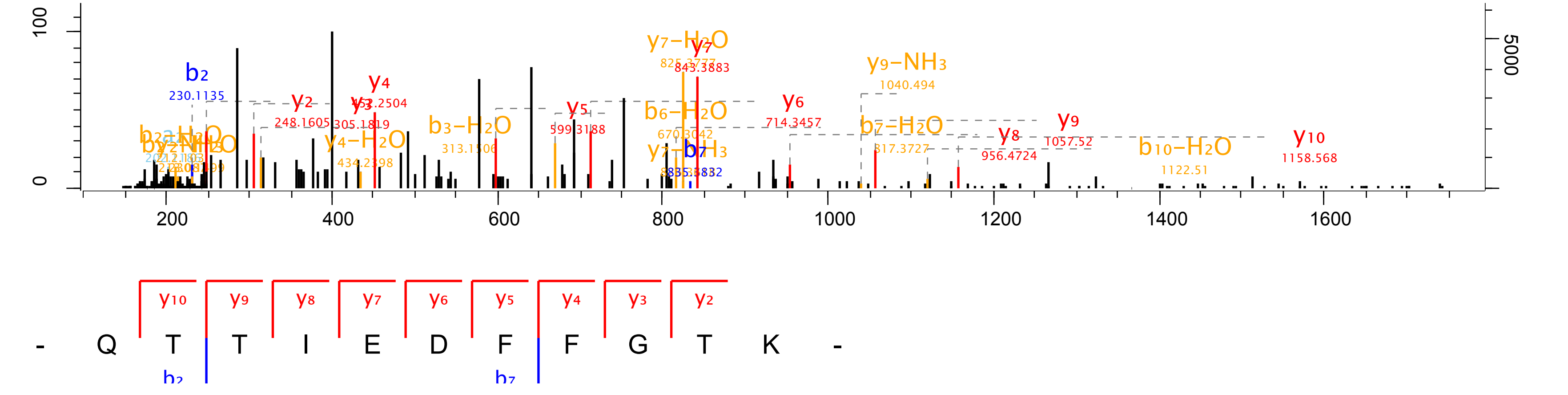

Raw file	Scan	Method	Score	m/z	Gene names
20150228_yeast1_Top_opt_B1_01_1611	43277	TOF; CID	93.1	564.34	PGD1

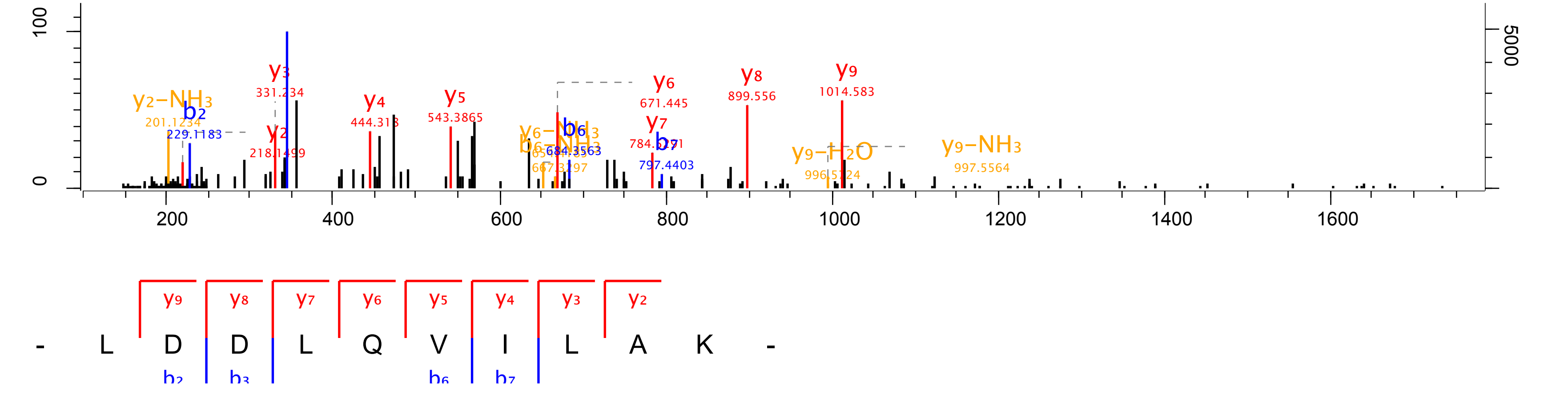

Raw file	Scan	Method	Score	m/z	Gene names
20150228_yeast1_Top_opt_B1_01_1611	43494	TOF; CID	93.37	936.04	PBP2

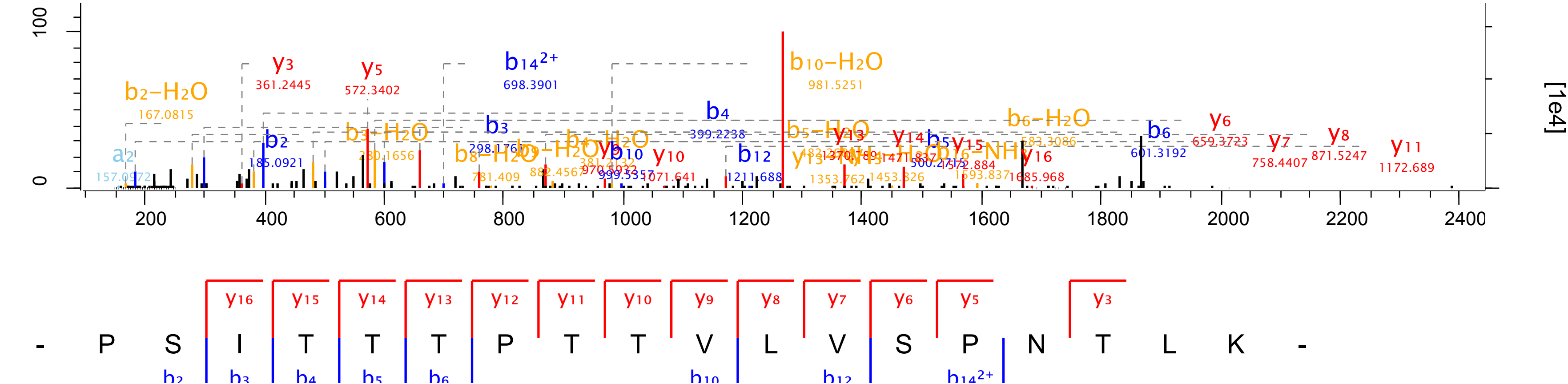

Raw file	Scan	Method	Score	m/z	Gene names
20150228_yeast1_Top_opt_B1_01_1611	43521	TOF; CID	78.9	605.38	SAN1

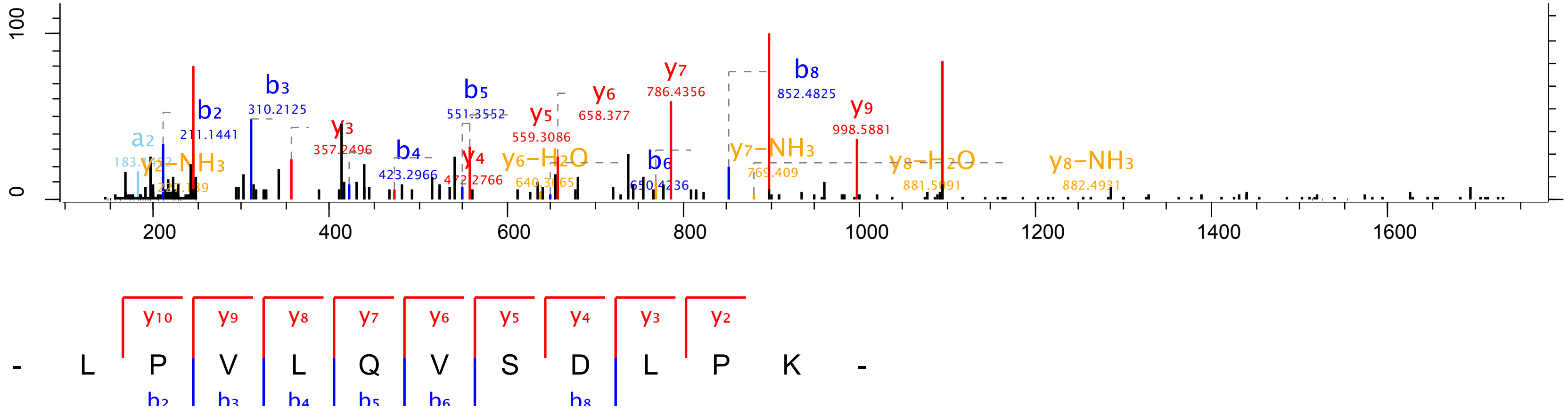

Raw file	Scan	Method	Score	m/z	Gene names
20150228_yeast1_Top_opt_B1_01_1611	43567	TOF; CID	127.3	968.5	ARP1

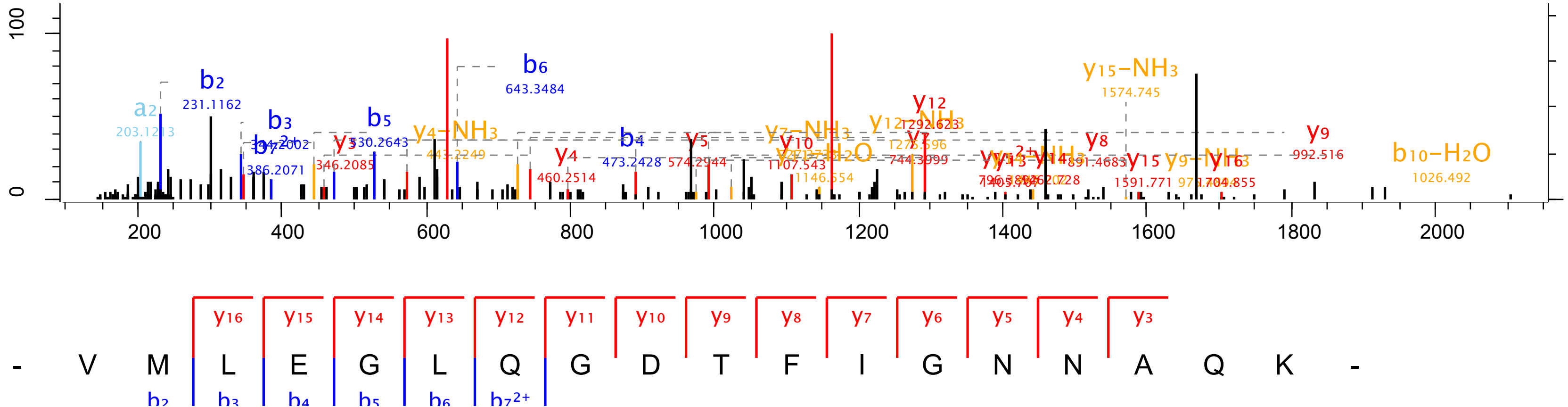

Raw file	Scan	Method	Score	m/z	Gene names
20150228_yeast1_Top_opt_B1_01_1611	43844	TOF; CID	121.21	1176.09	EMC6

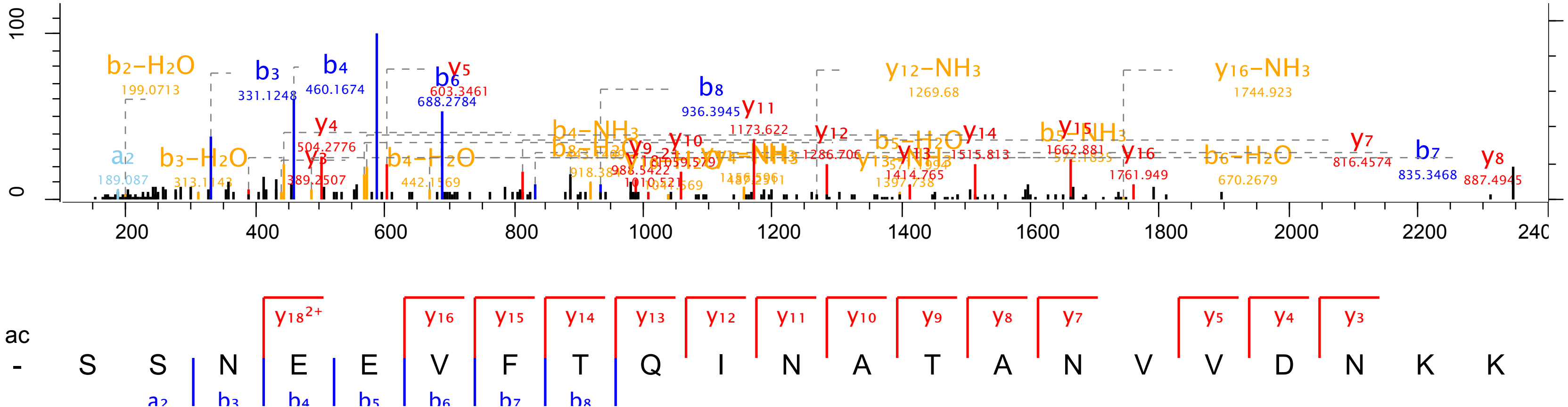

Raw file	Scan	Method	Score	m/z	Gene names
20150228_yeast1_Top_opt_B1_01_1611	44142	TOF; CID	80.23	568.34	NCE101

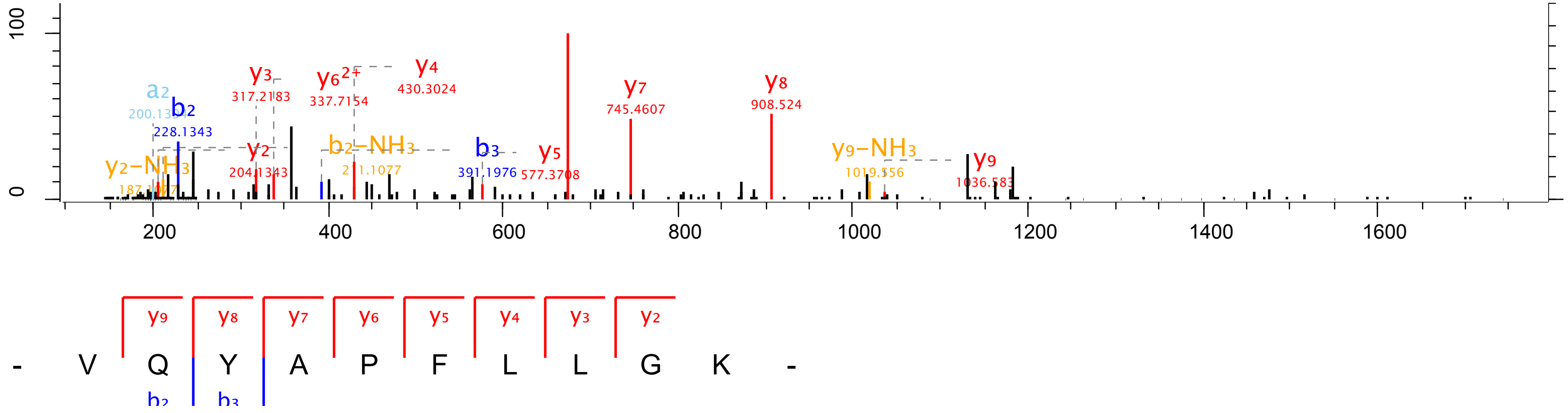

Raw file	Scan	Method	Score	m/z	Gene names
20150228_yeast1_Top_opt_B1_01_1611	44452	TOF; CID	102.21	1147.62	TPO2

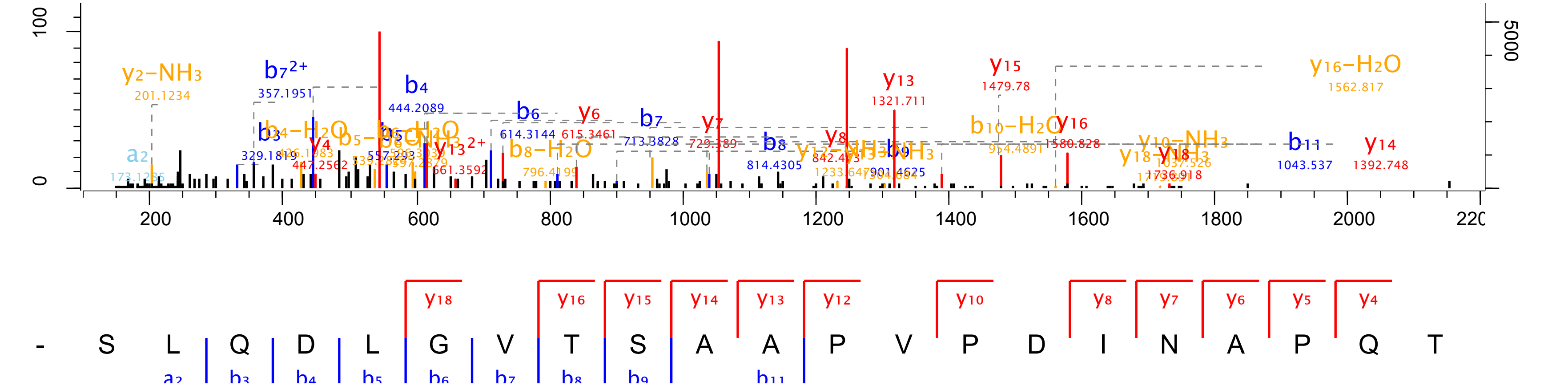

Raw file	Scan	Method	Score	m/z	Gene names
20150228_yeast1_Top_opt_B1_01_1611	45490	TOF; CID	51.47	1106.5	MGA2

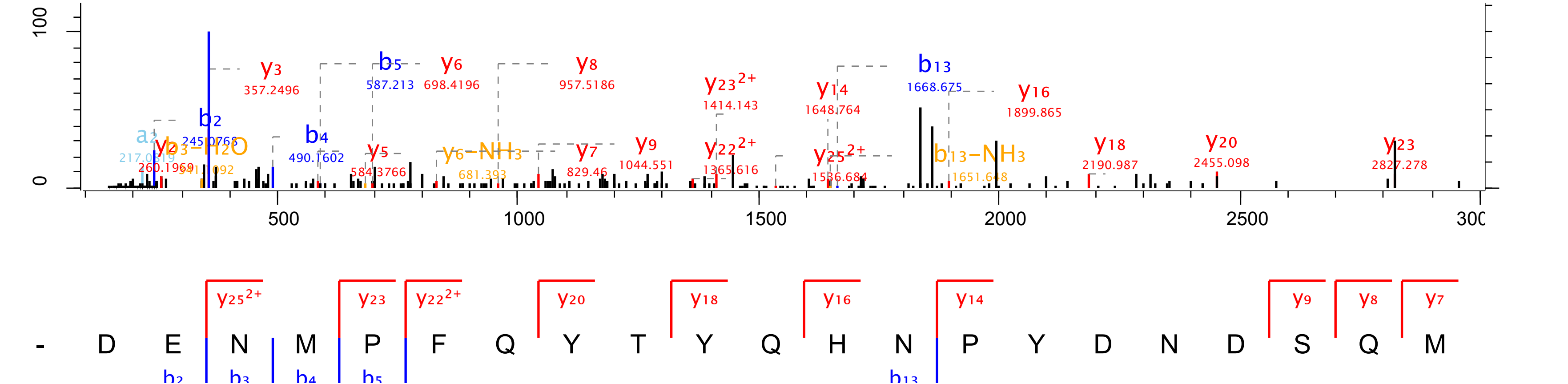

Raw file	Scan	Method	Score	m/z	Gene names
20150228_yeast1_Top_opt_B1_01_1611	45607	TOF; CID	59.25	767.41	SPP2

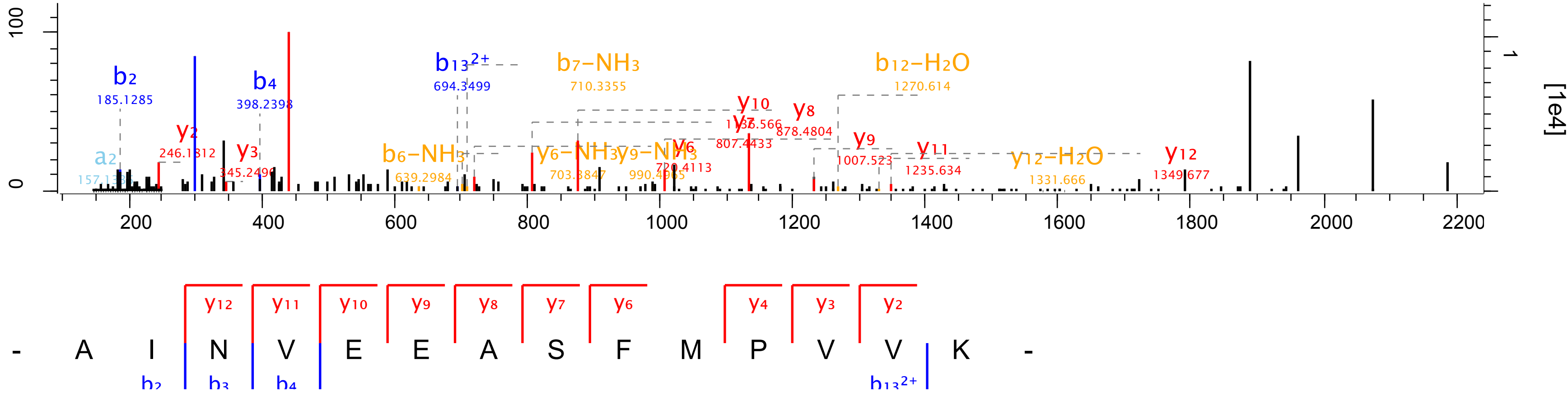

Raw file	Scan	Method	Score	m/z	Gene names
20150228_yeast1_Top_opt_B1_01_1611	46040	TOF; CID	99.41	1046.98	TOM6

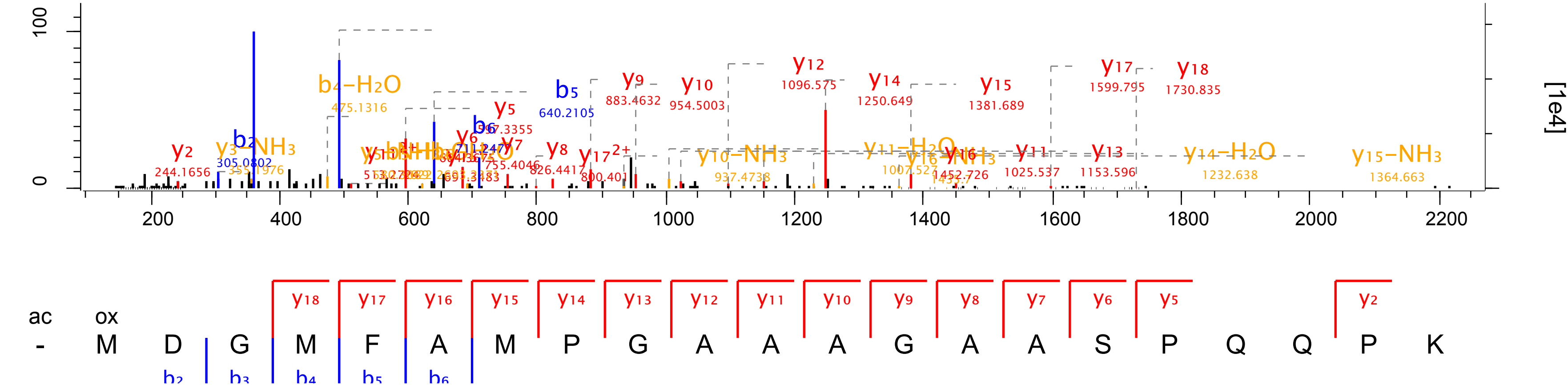

Raw file	Scan	Method	Score	m/z	Gene names
20150228_yeast1_Top_opt_B1_01_1611	47434	TOF; CID	44.83	704.39	CPT1

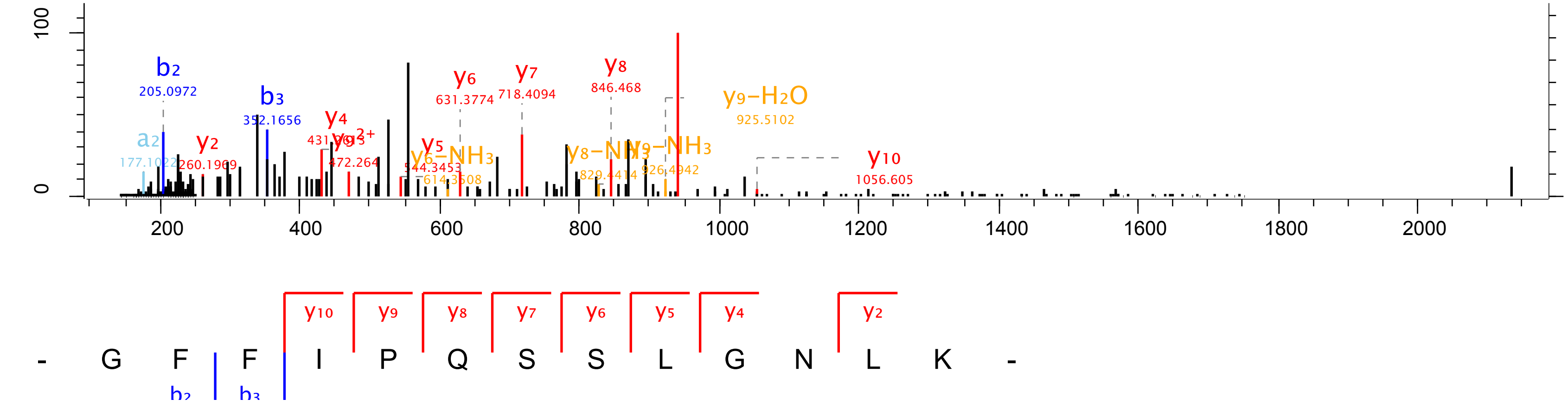

Raw file	Scan	Method	Score	m/z	Gene names
20150228_yeast1_Top_opt_B1_01_1611	47520	TOF; CID	153.82	963.79	NHX1

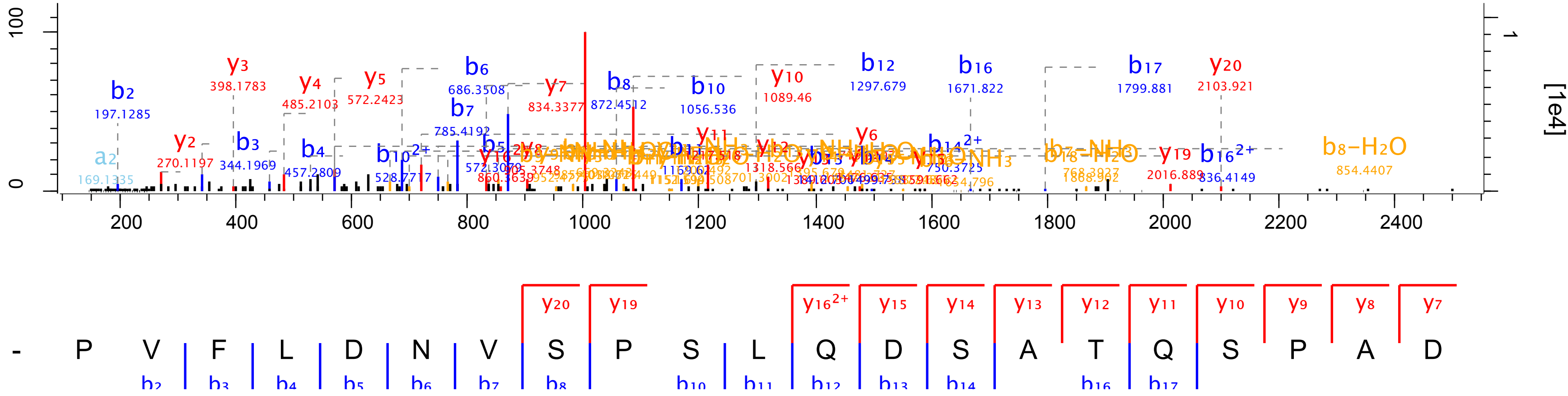

Raw file	Scan	Method	Score	m/z	Gene names
20150228_yeast1_Top_opt_B1_01_1611	47983	TOF; CID	97.9	600.69	TUS1

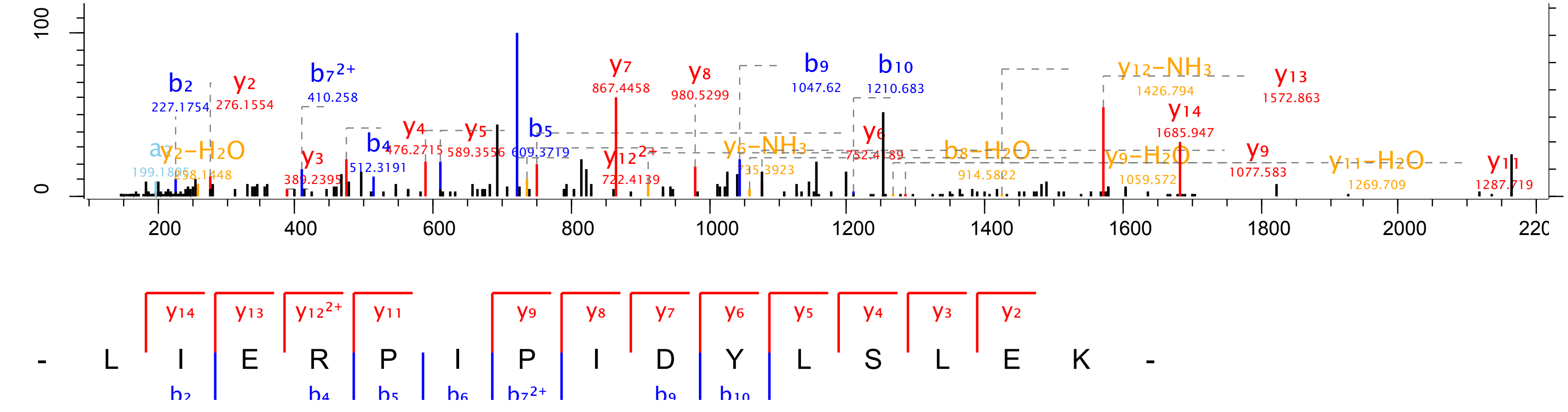

Raw file	Scan	Method	Score	m/z	Gene names
20150228_yeast1_Top_opt_B1_01_1611	49998	TOF; CID	55.46	531.33	POF1

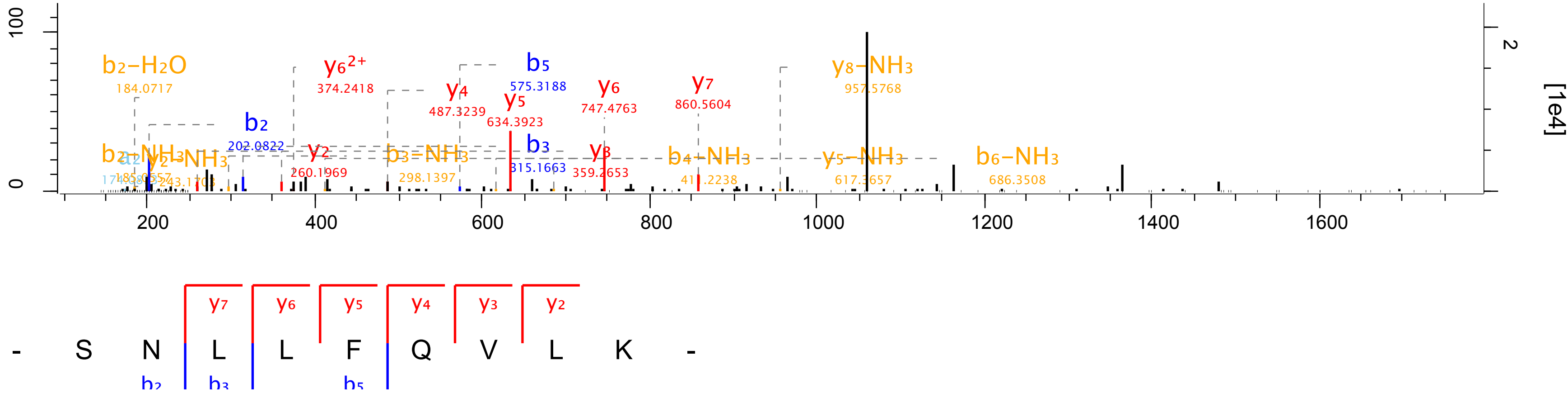

Raw file	Scan	Method	Score	m/z	Gene names
20150228_yeast1_Top_opt_B1_01_1611	50700	TOF; CID	66.02	721.92	YLR352W

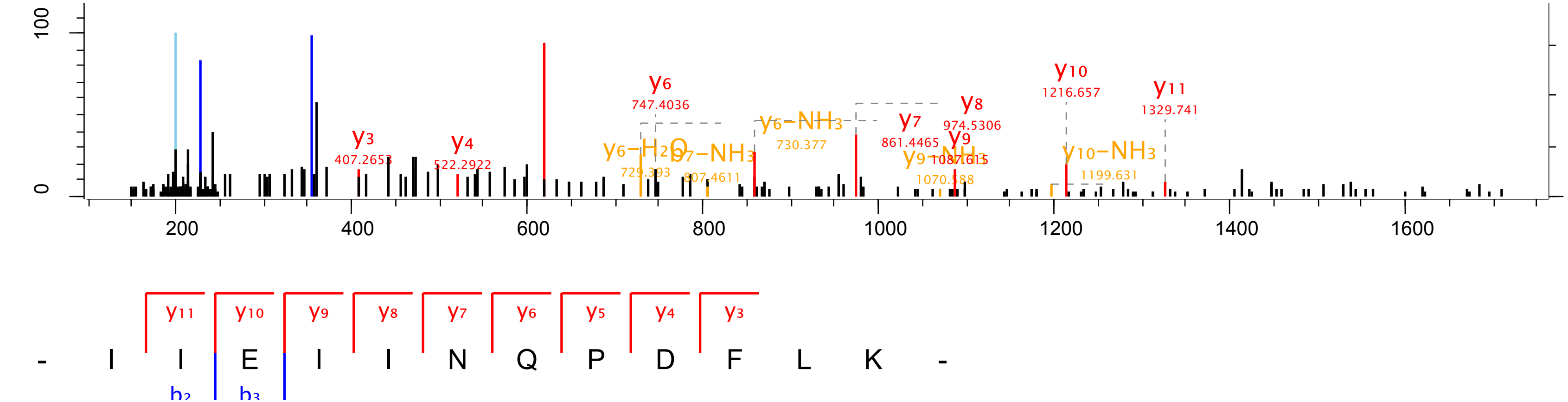

Raw file	Scan	Method	Score	m/z	Gene names
20150228_yeast1_Top_opt_B1_01_1611	52159	TOF; CID	79.59	1340.29	TLG1

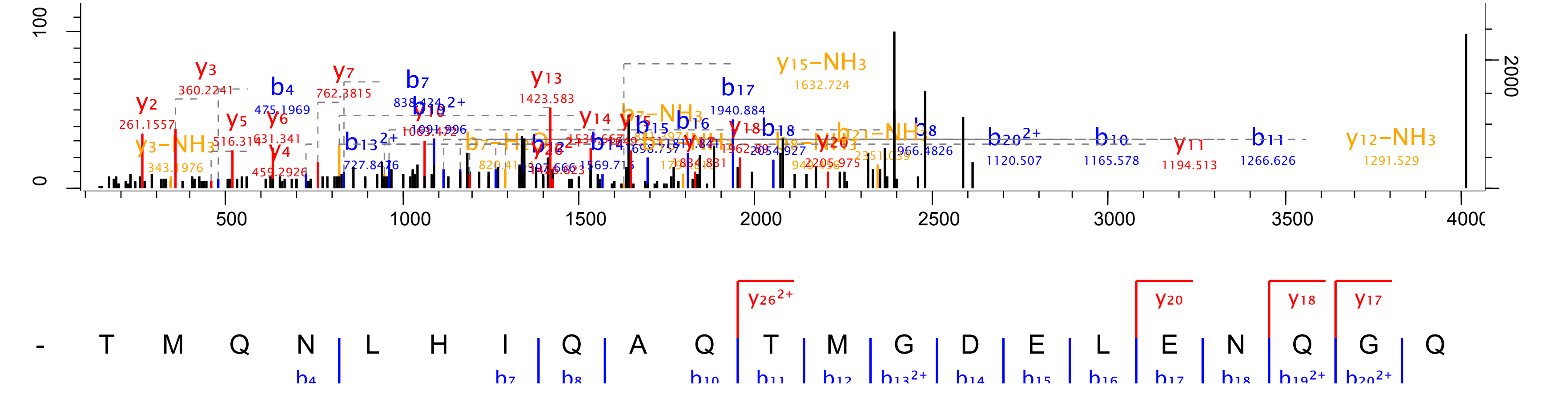

Raw file	Scan	Method	Score	m/z	Gene names
20150228_yeast1_Top_opt_B1_01_1611	53217	TOF; CID	60.27	814.13	SNF11

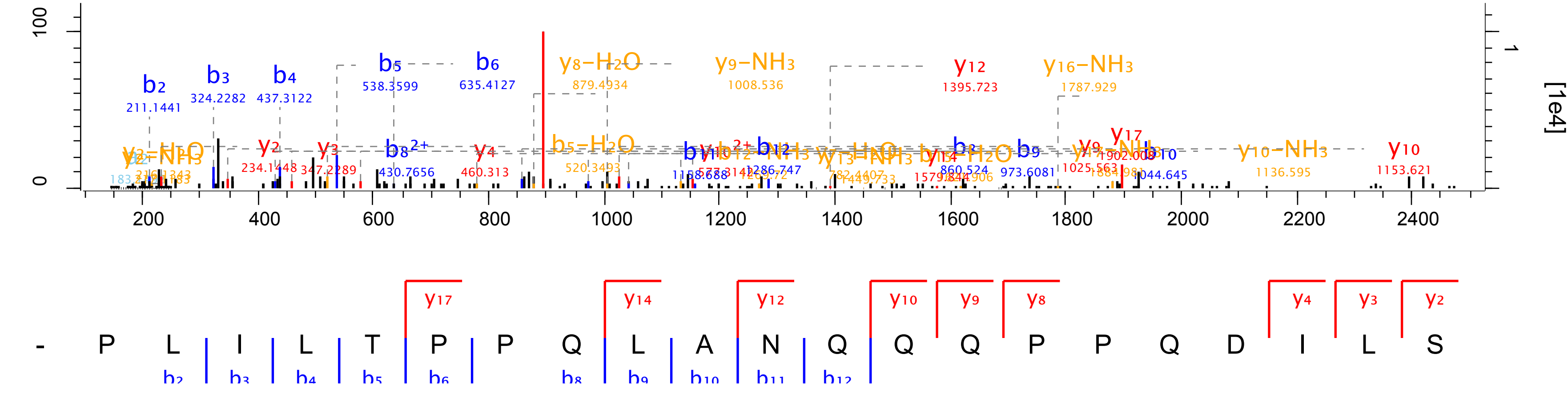

Raw file	Scan	Method	Score	m/z	Gene names
20150228_yeast1_Top_opt_B1_01_1611	53522	TOF; CID	66.94	658.34	YPL108W

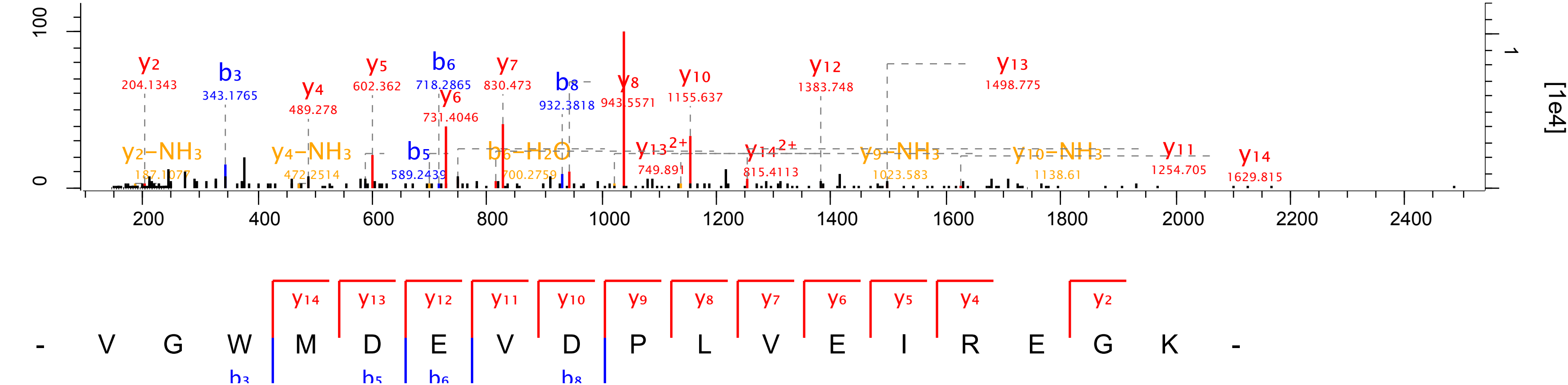

Raw file	Scan	Method	Score	m/z	Gene names
20150228_yeast1_Top_opt_B1_01_1611	55386	TOF; CID	88.57	962.49	IMP2'

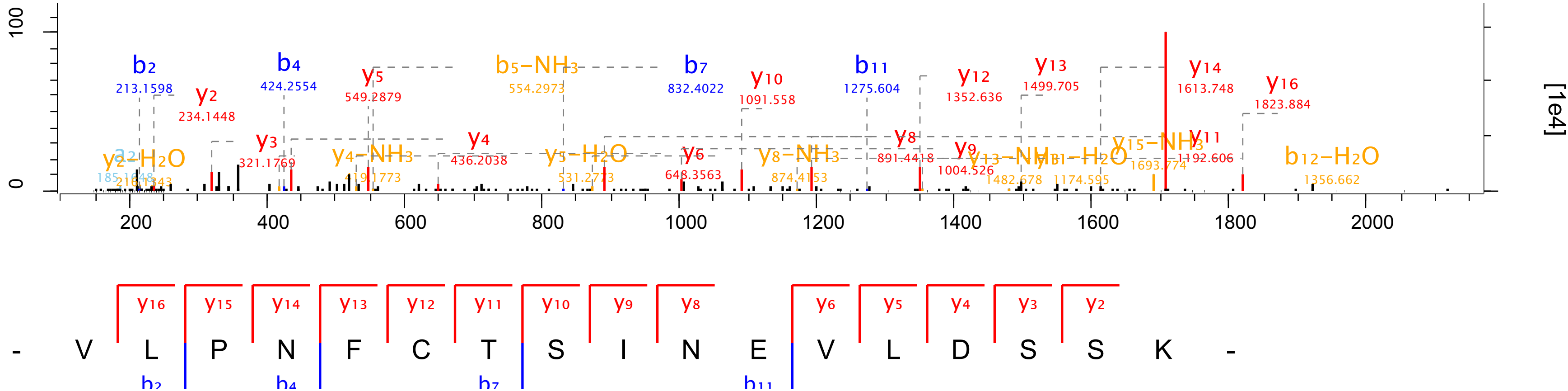

Raw file	Scan	Method	Score	m/z	Gene names
20150228_yeast1_Top_opt_B1_01_1611	56027	TOF; CID	36.33	963.5	ISM1

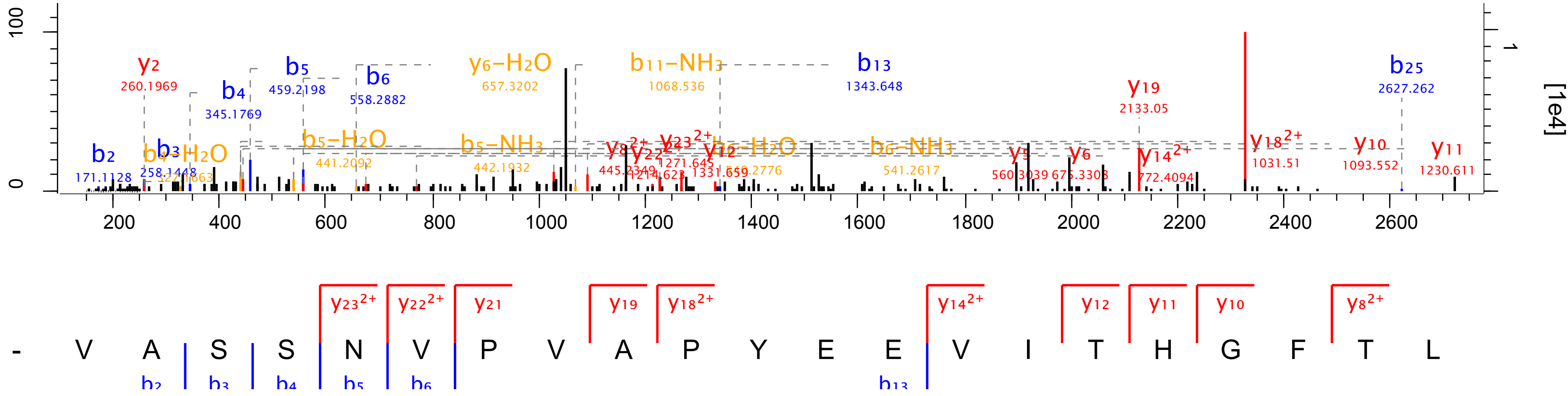

Raw file	Scan	Method	Score	m/z	Gene names
20150228_yeast1_Top_opt_B1_01_1611	56042	TOF; CID	68.04	835.43	NPR2

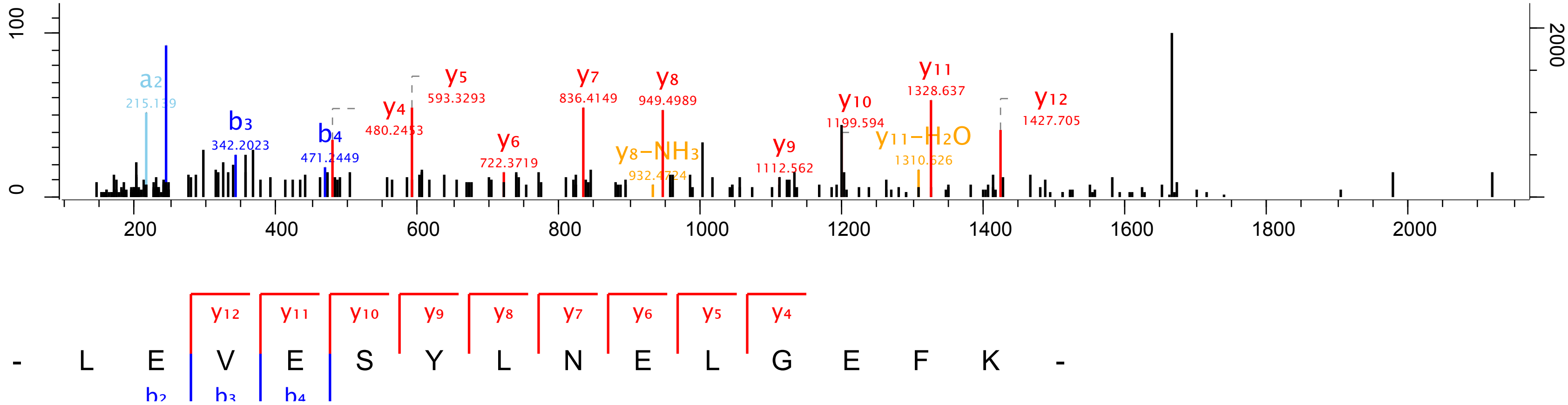

Raw file	Scan	Method	Score	m/z	Gene names
20150228_yeast1_Top_opt_B1_01_1611	57178	TOF; CID	81.63	813.46	MRS1

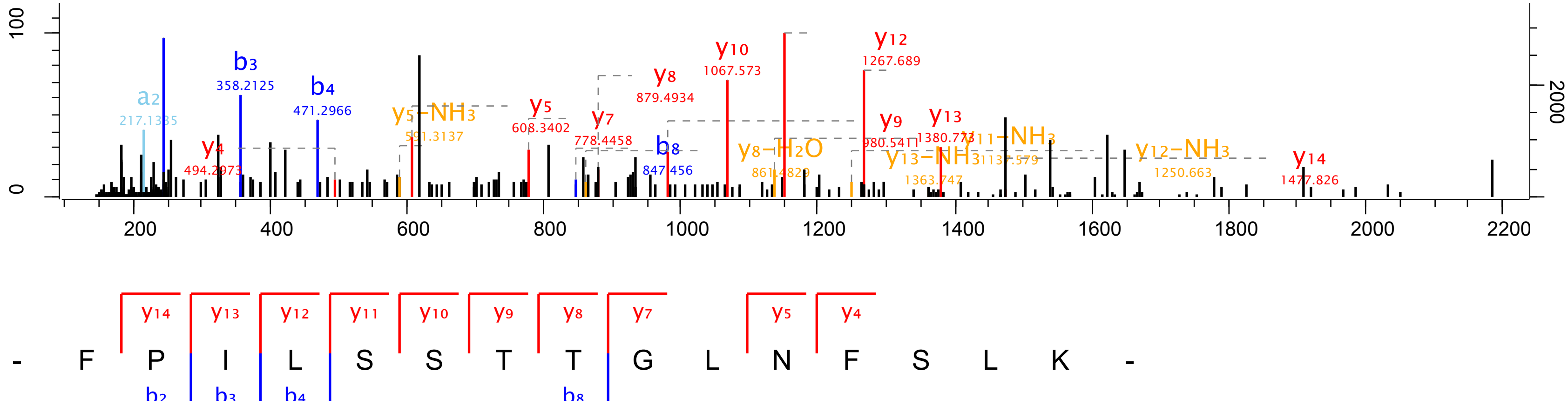

Raw file	Scan	Method	Score	m/z	Gene names
20150228_yeast1_Top_opt_B1_01_1611	57355	TOF; CID	34.32	1018.53	OSW5

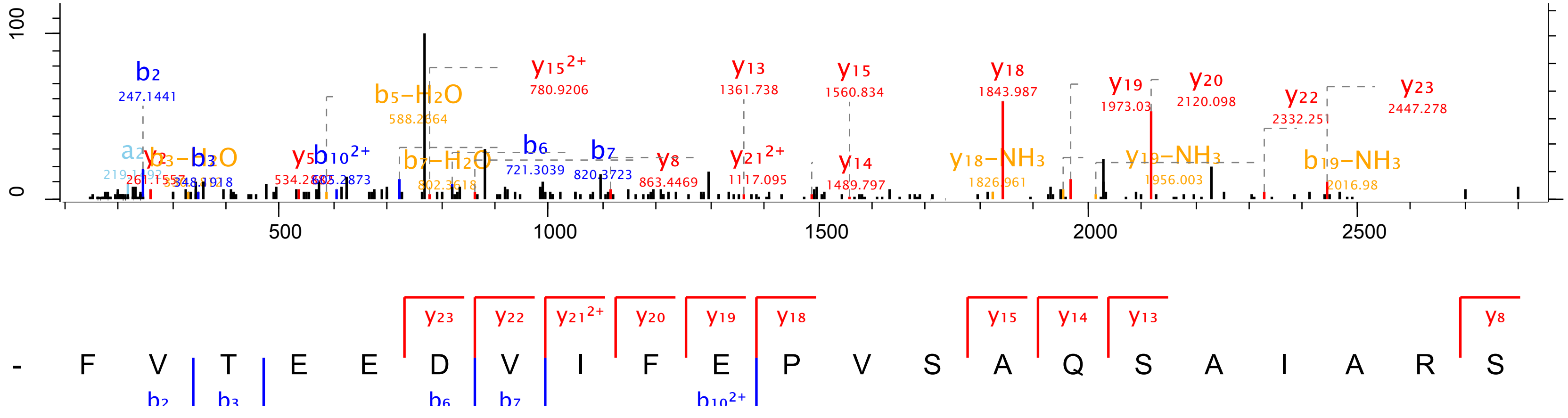

Raw file	Scan	Method	Score	m/z	Gene names
20150228_yeast1_Top_opt_B1_01_1611	57697	TOF; CID	108.7	607.88	YIF1

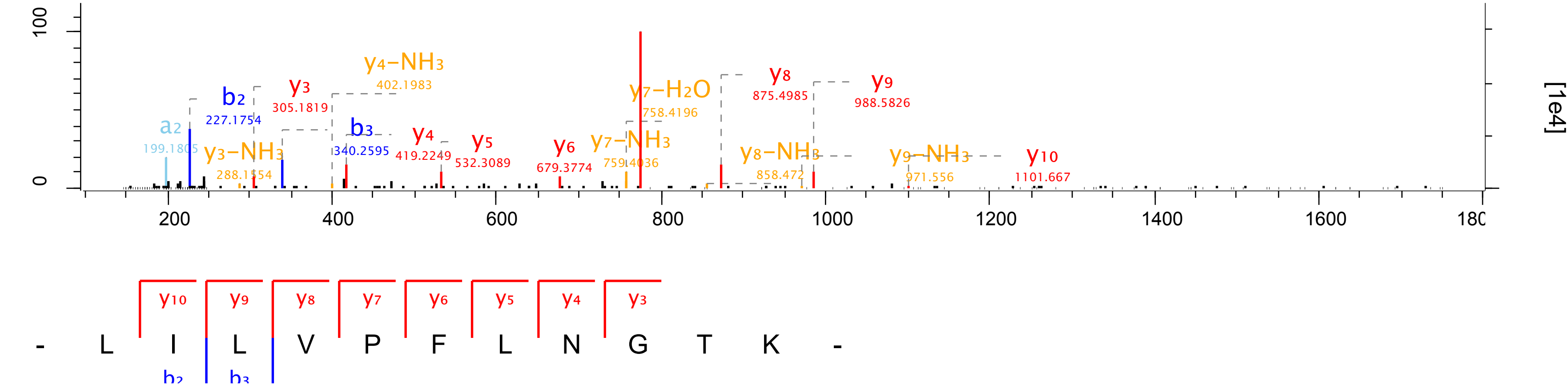

Raw file	Scan	Method	Score	m/z	Gene names
20150228_yeast1_Top_opt_B1_01_1611	58152	TOF; CID	73.44	696.38	MSG5

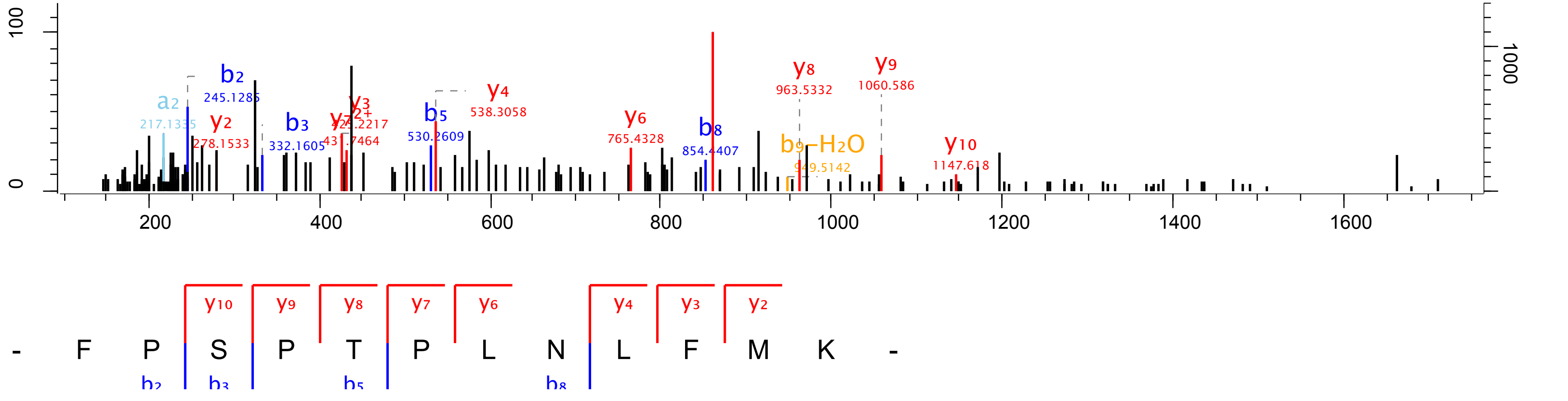

Raw file	Scan	Method	Score	m/z	Gene names
20150228_yeast1_Top_opt_B1_01_1611	59568	TOF; CID	93.58	792.08	SAP30

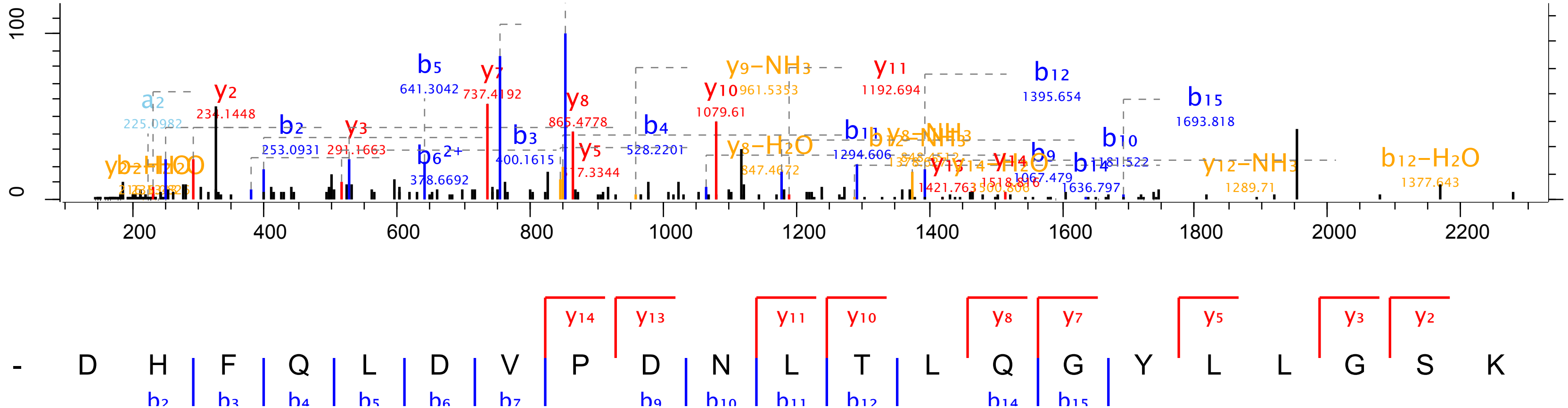

Raw file	Scan	Method	Score	m/z	Gene names
20150228_yeast1_Top_opt_B1_01_1611	60755	TOF; CID	49.09	1144.55	TAD2

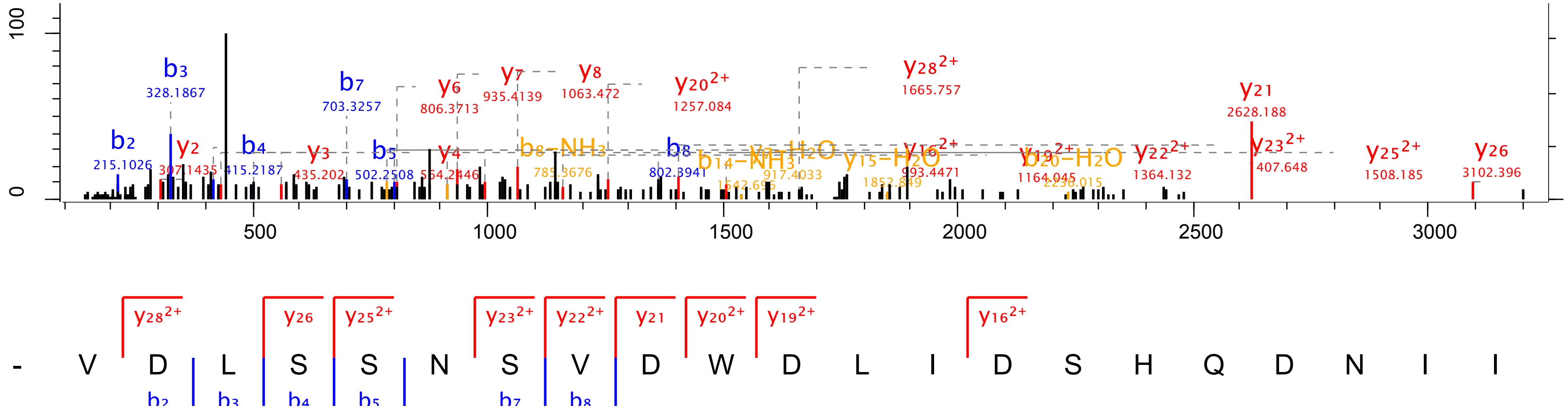

Raw file	Scan	Method	Score	m/z	Gene names
20150228_yeast1_Top_opt_B1_01_1611	61265	TOF; CID	54.93	1084.92	GIS3

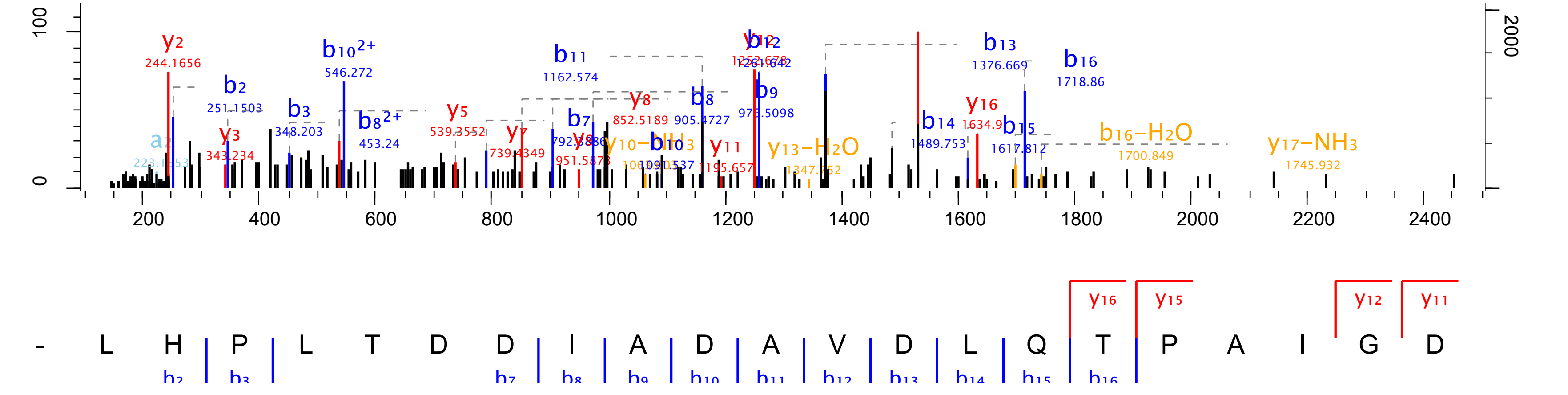

Raw file	Scan	Method	Score	m/z	Gene names
20150228_yeast1_Top_opt_B1_01_1611	61837	TOF; CID	79.64	643.4	TAH11

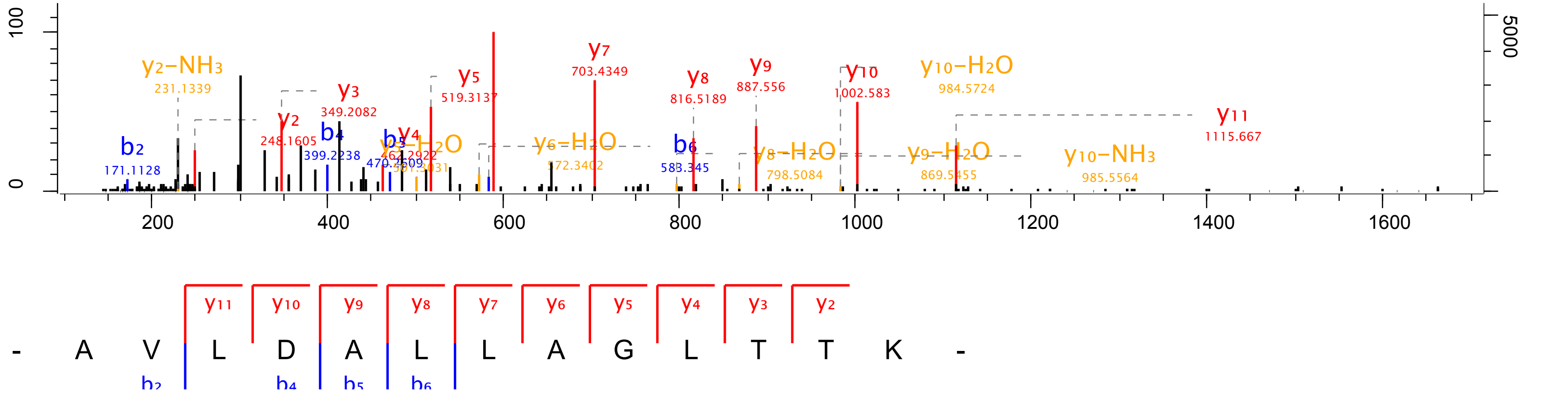

Raw file	Scan	Method	Score	m/z	Gene names
20150228_yeast1_Top_opt_B1_01_1611	61946	TOF; CID	105.9	1095.02	MRPL36

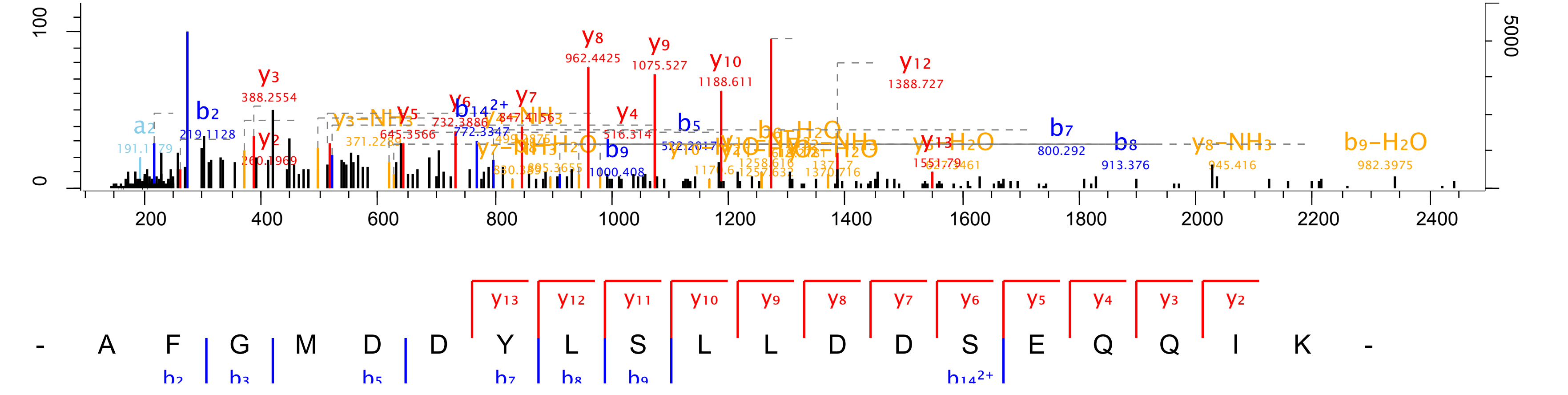

Raw file	Scan	Method	Score	m/z	Gene names
20150228_yeast1_Top_opt_B1_01_1611	62161	TOF; CID	130.21	883.46	PUP3

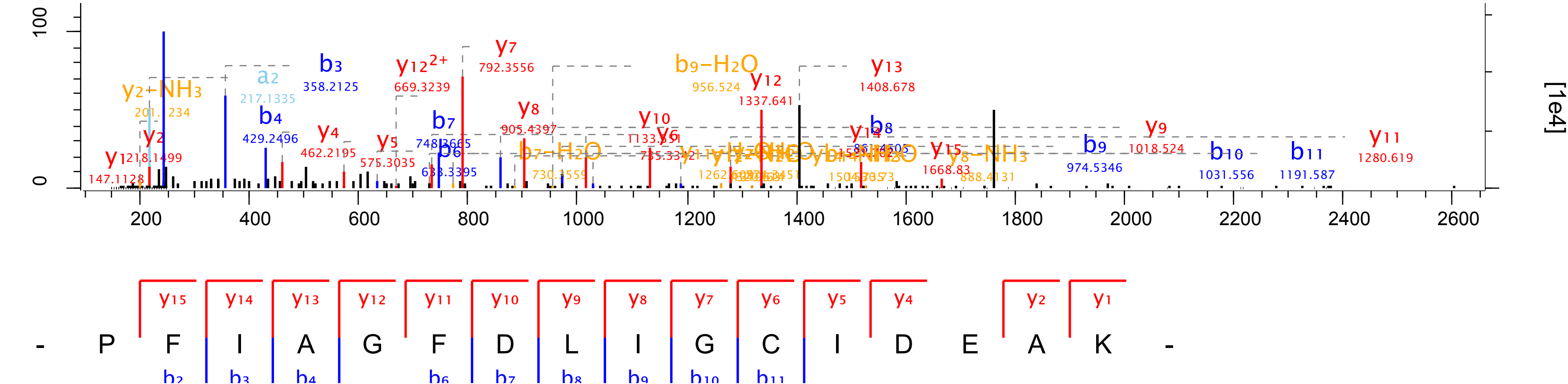

Raw file	Scan	Method	Score	m/z	Gene names
20150228_yeast1_Top_opt_B1_01_1611	63266	TOF; CID	51.32	808.94	MOB2

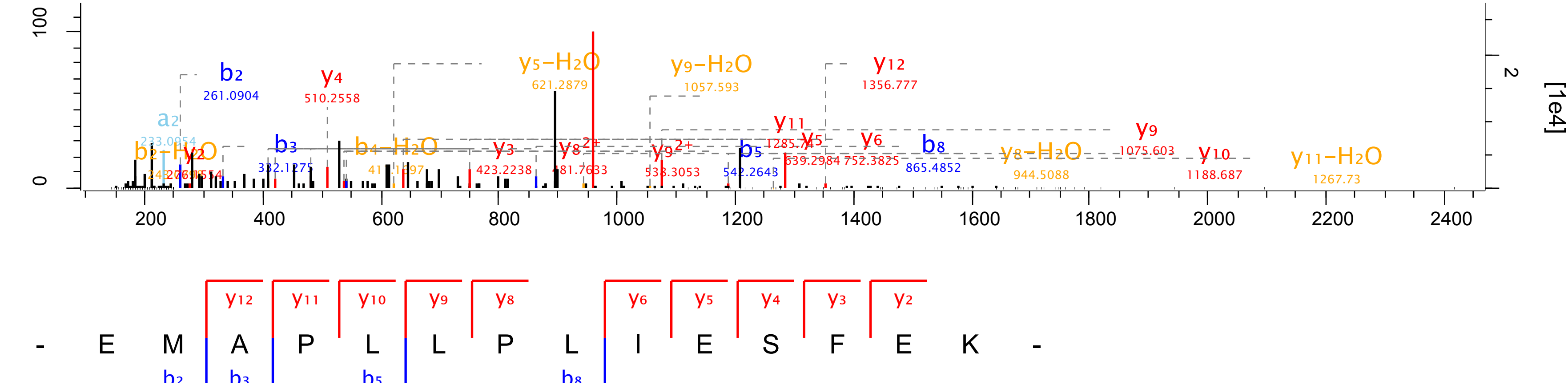

Raw file	Scan	Method	Score	m/z	Gene names
20150228_yeast1_Top_opt_B1_01_1611	63758	TOF; CID	195.02	1015.08	SRP21

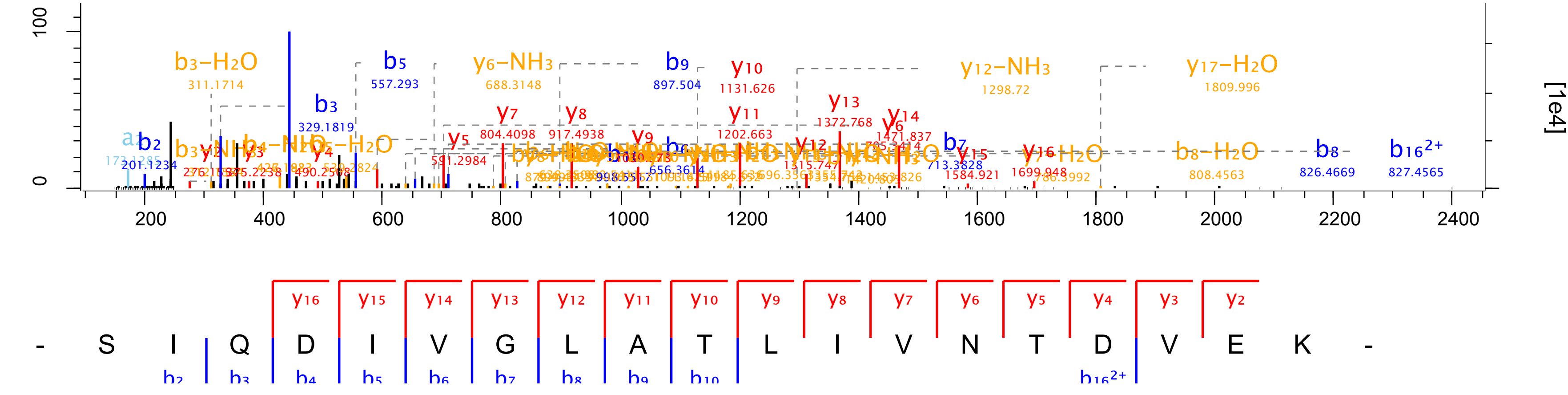

Raw file	Scan	Method	Score	m/z	Gene names
20150228_yeast1_Top_opt_B1_01_1614	2921	TOF; CID	60.79	364.68	MRPL7

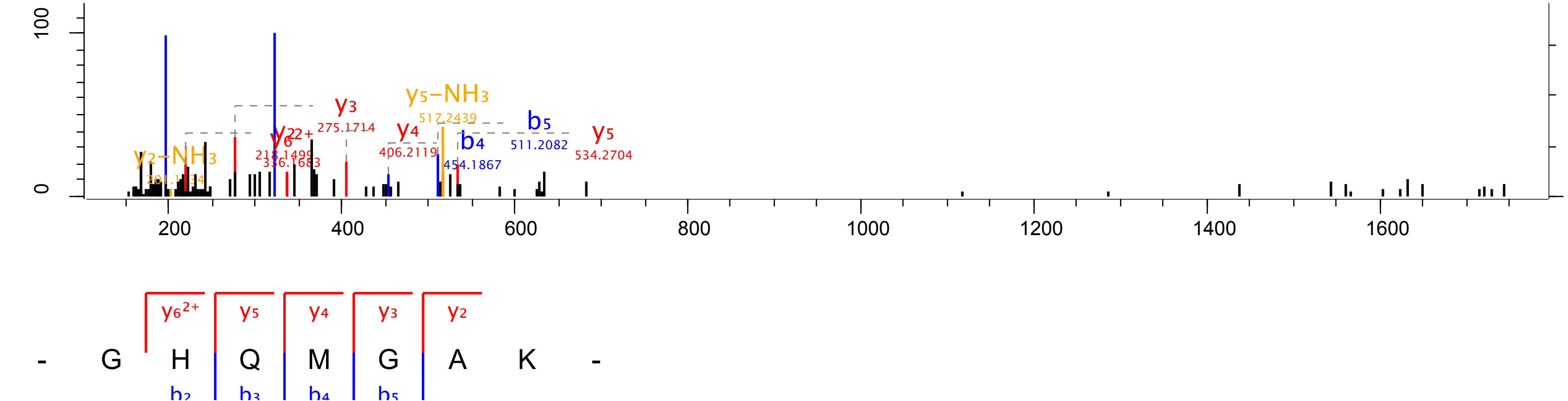

Raw file	Scan	Method	Score	m/z	Gene names
20150228_yeast1_Top_opt_B1_01_1614	4200	TOF; CID	82.61	654.31	YMR295C

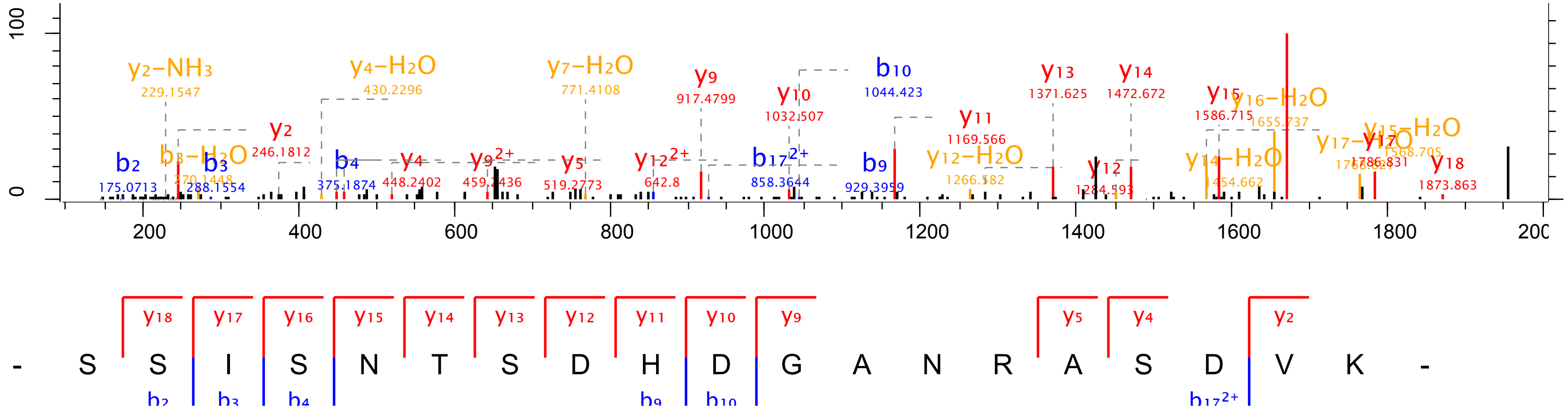

Raw file	Scan	Method	Score	m/z	Gene names
20150228_yeast1_Top_opt_B1_01_1614	4257	TOF; CID	62.56	471.74	PFA4

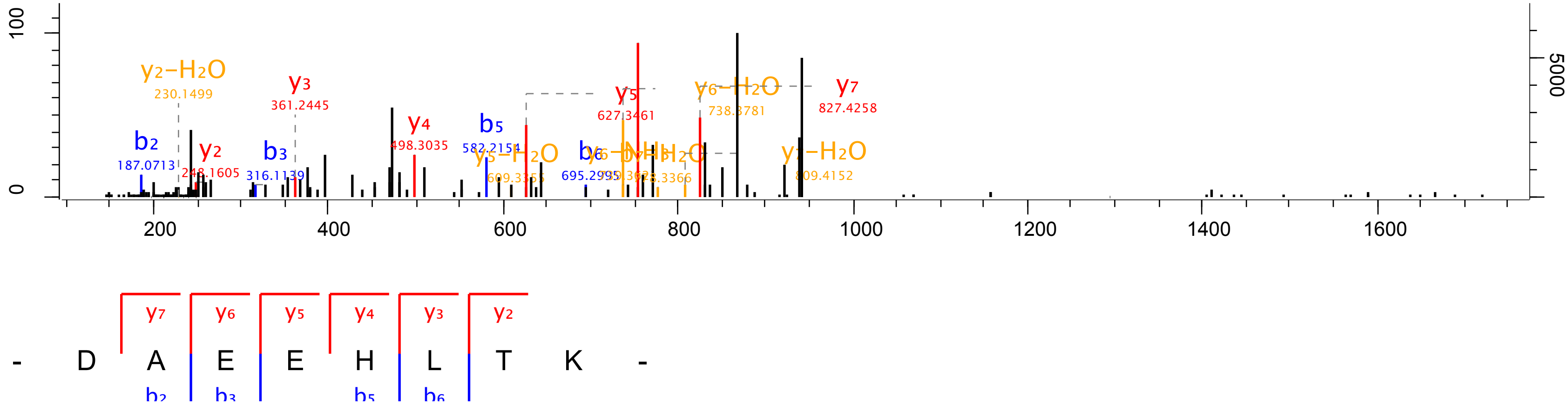

Raw file	Scan	Method	Score	m/z	Gene names
20150228_yeast1_Top_opt_B1_01_1614	4507	TOF; CID	60.65	423.71	LEE1

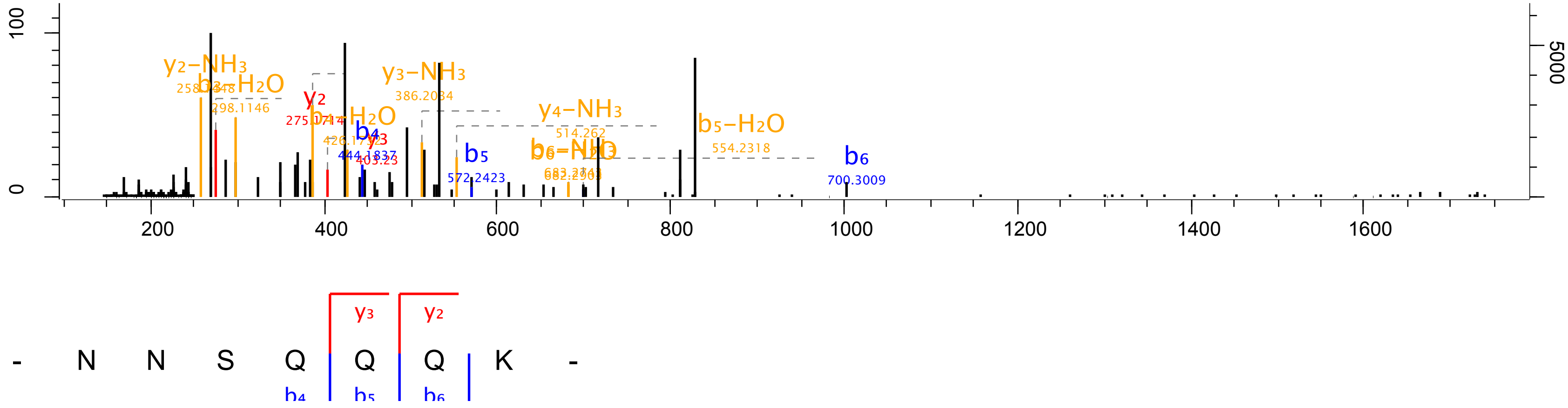

Raw file	Scan	Method	Score	m/z	Gene names
20150228_yeast1_Top_opt_B1_01_1614	4879	TOF; CID	64.73	504.76	WWM1

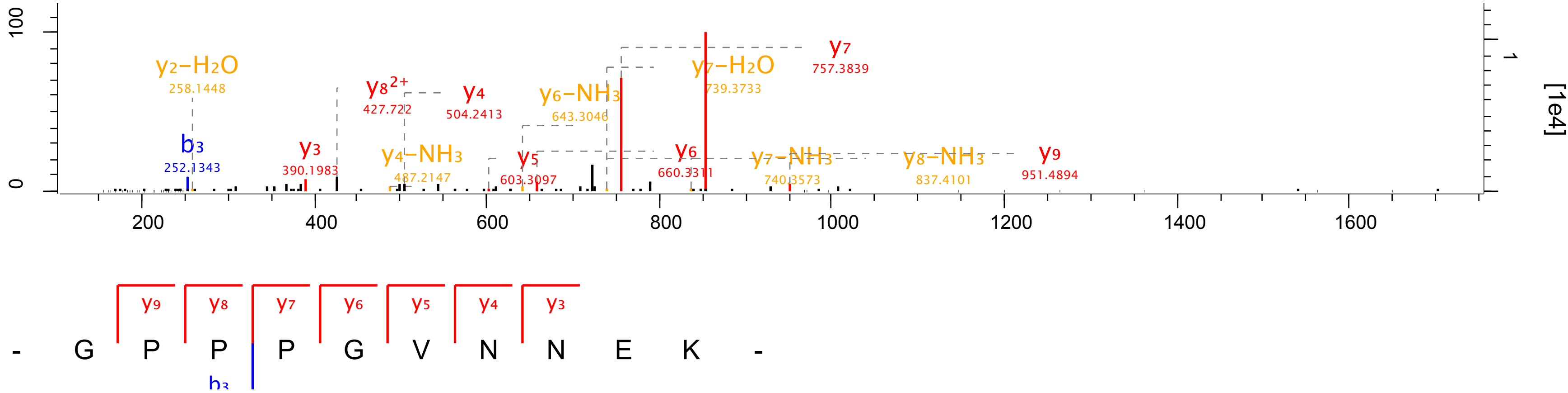

Raw file	Scan	Method	Score	m/z	Gene names
20150228_yeast1_Top_opt_B1_01_1614	4907	TOF; CID	50.3	544.79	YFH1

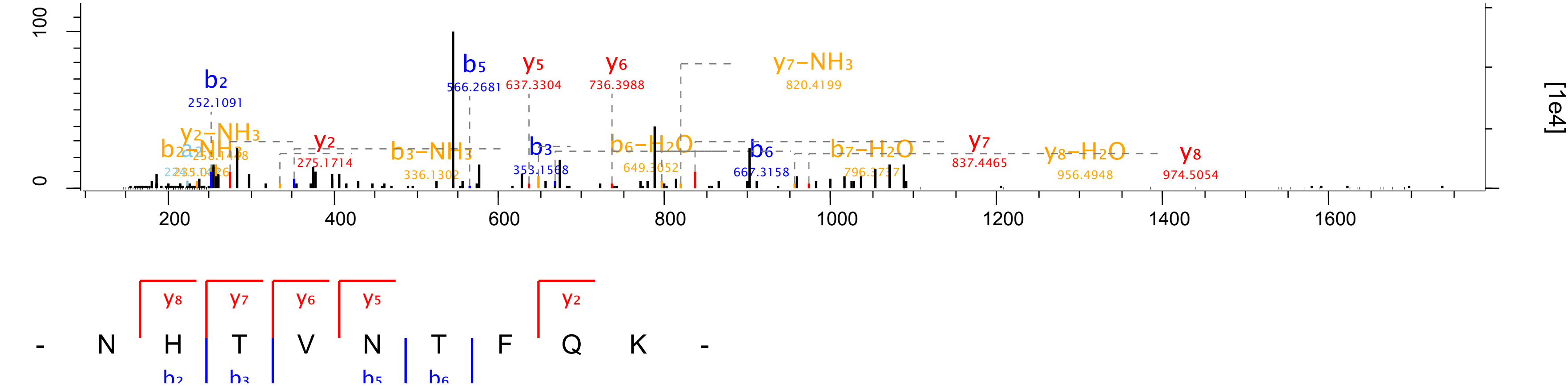

Raw file	Scan	Method	Score	m/z	Gene names
20150228_yeast1_Top_opt_B1_01_1614	4954	TOF; CID	87.32	425.19	PKR1

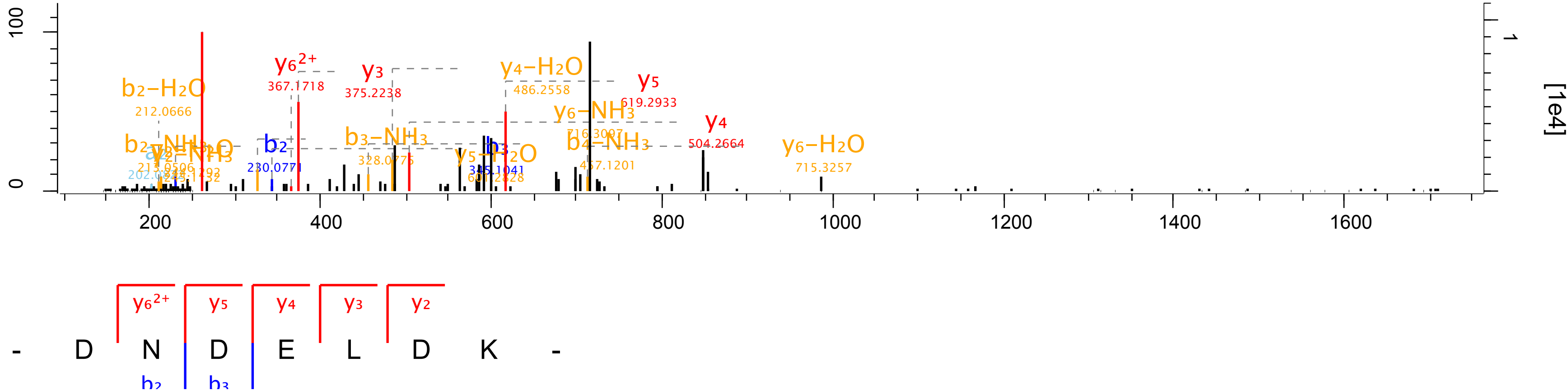

Raw file	Scan	Method	Score	m/z	Gene names
20150228_yeast1_Top_opt_B1_01_1614	5287	TOF; CID	72.61	495.77	GPB1

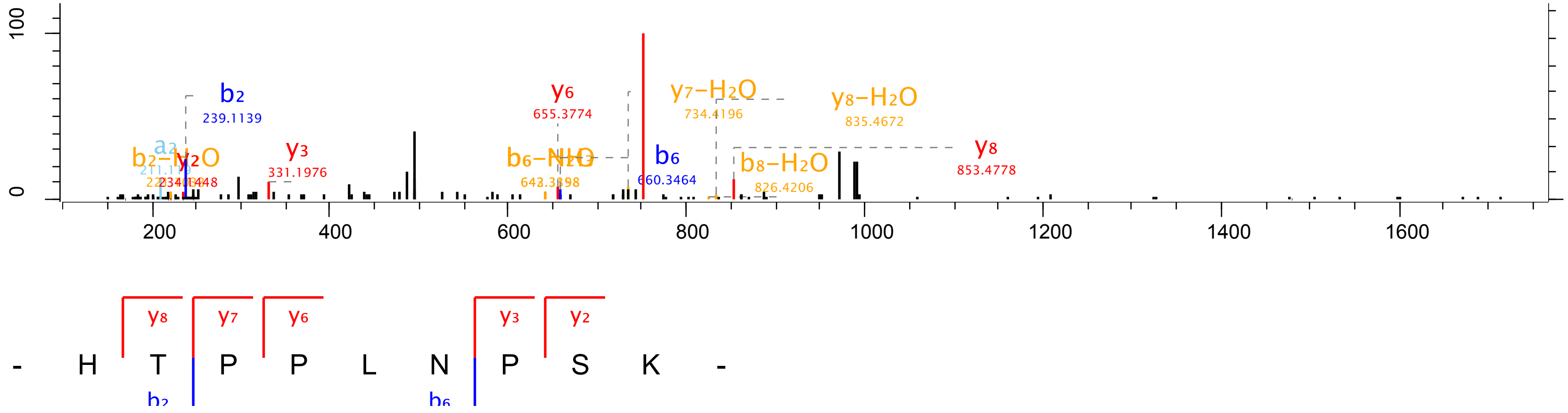

Raw file	Scan	Method	Score	m/z	Gene names
20150228_yeast1_Top_opt_B1_01_1614	5350	TOF; CID	133.13	494.28	STF1

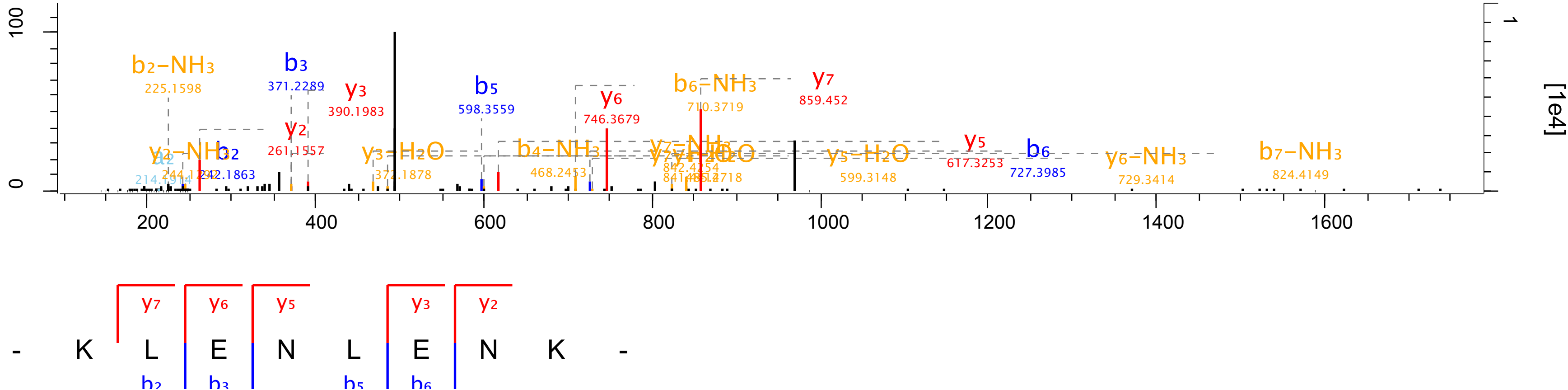

Raw file	Scan	Method	Score	m/z	Gene names
20150228_yeast1_Top_opt_B1_01_1614	6200	TOF; CID	85.55	660.28	QCR9

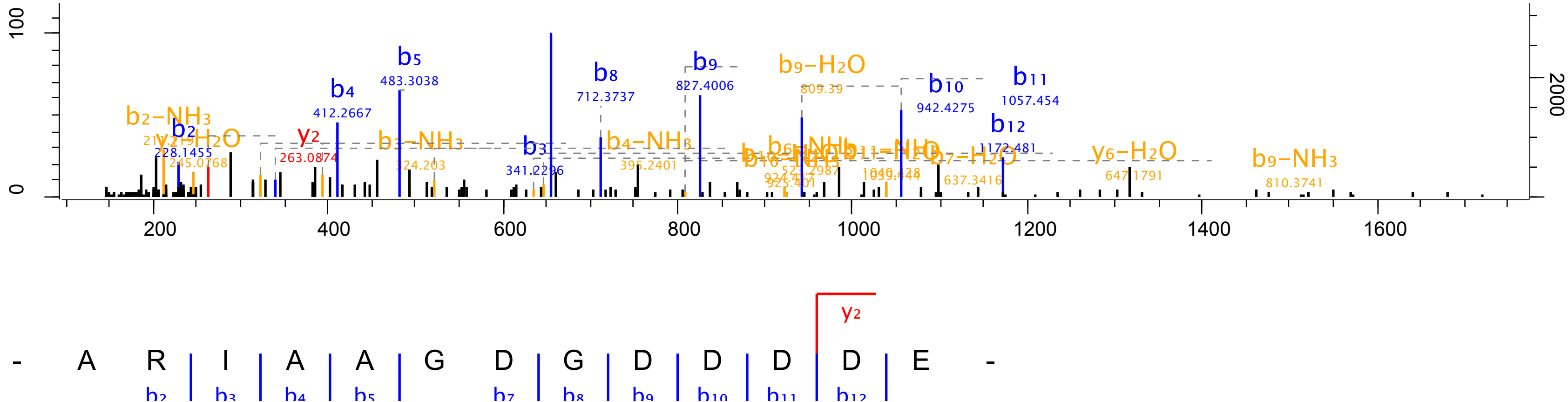

Raw file	Scan	Method	Score	m/z	Gene names
20150228_yeast1_Top_opt_B1_01_1614	6239	TOF; CID	62.72	411.22	MTC5

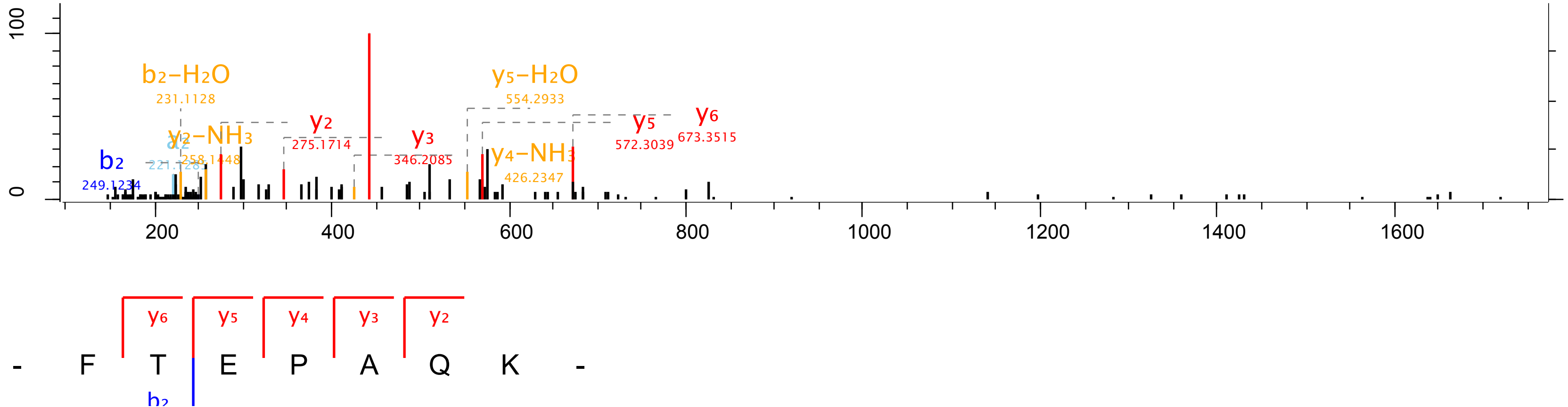

Raw file	Scan	Method	Score	m/z	Gene names
20150228_yeast1_Top_opt_B1_01_1614	6651	TOF; CID	136.7	623.31	GPR1

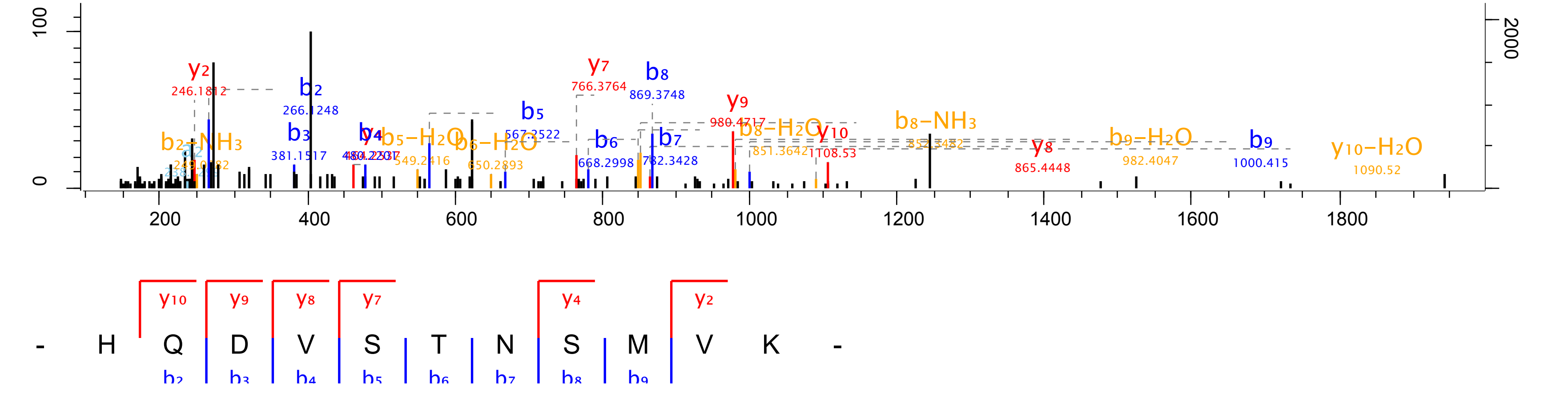

Raw file	Scan	Method	Score	m/z	Gene names
20150228_yeast1_Top_opt_B1_01_1614	7678	TOF; CID	43.42	602.79	SCC4

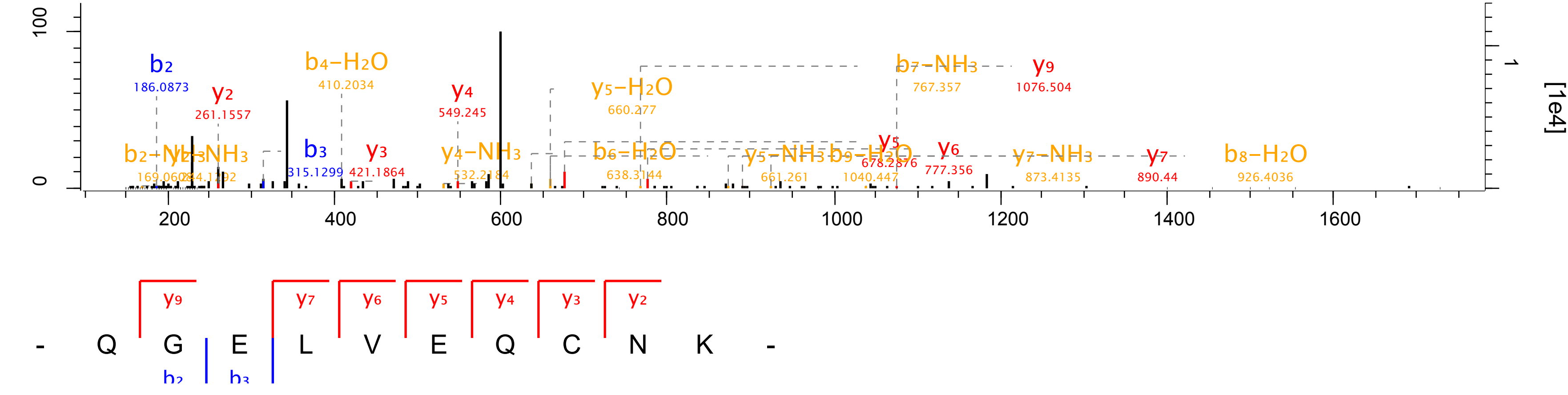

Raw file	Scan	Method	Score	m/z
20150228_yeast1_Top_opt_B1_01_1614	10125	TOF; CID	46.49	923.9

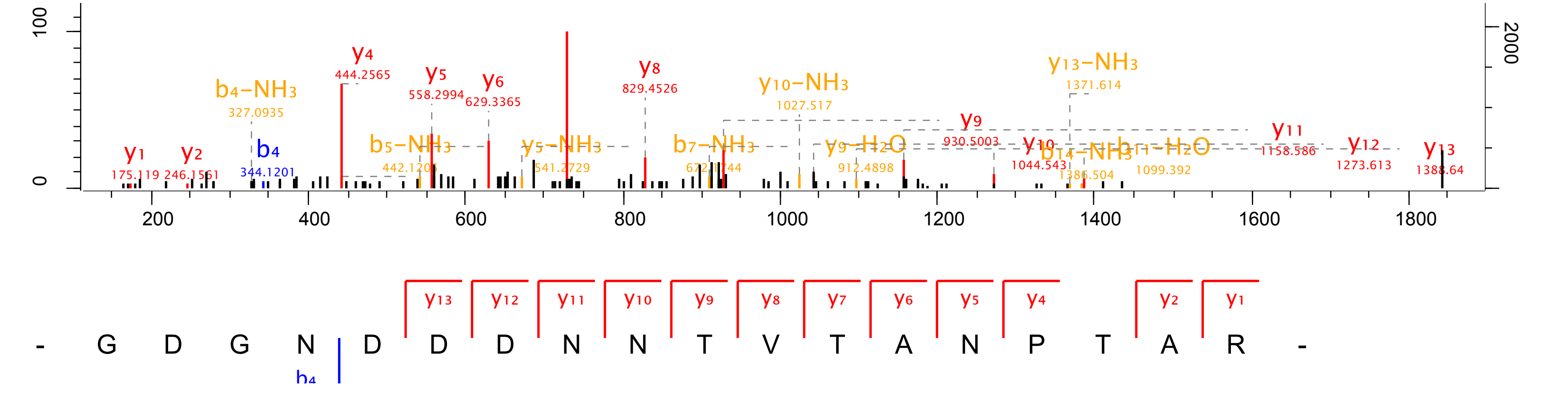

Raw file	Scan	Method	Score	m/z	Gene names
20150228_yeast1_Top_opt_B1_01_1614	10426	TOF; CID	91.96	651.3	FUN26

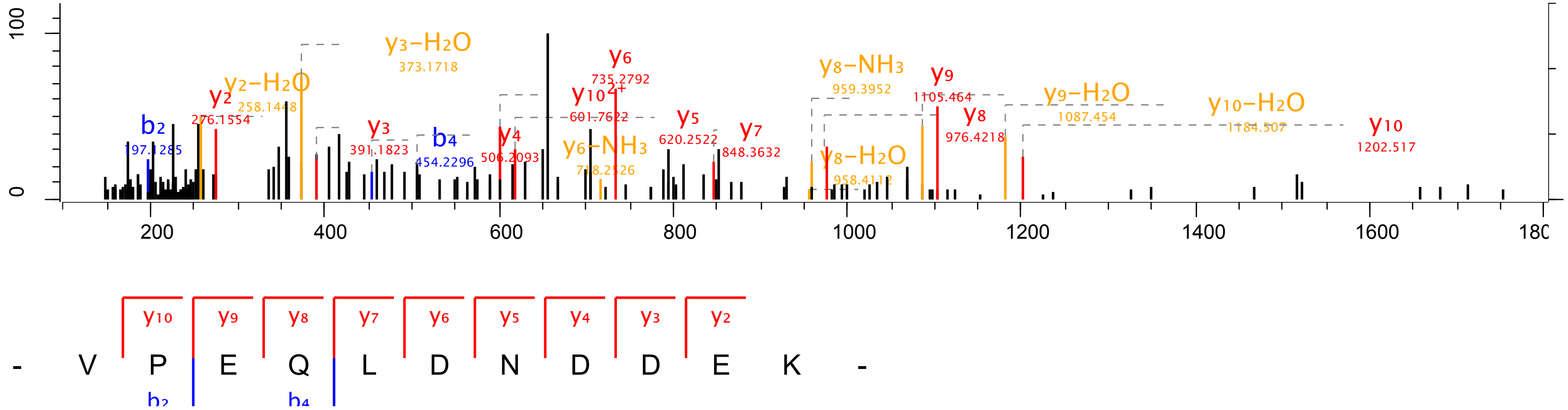

Raw file	Scan	Method	Score	m/z	Gene names
20150228_yeast1_Top_opt_B1_01_1614	10466	TOF; CID	64.8	501.21	INM2

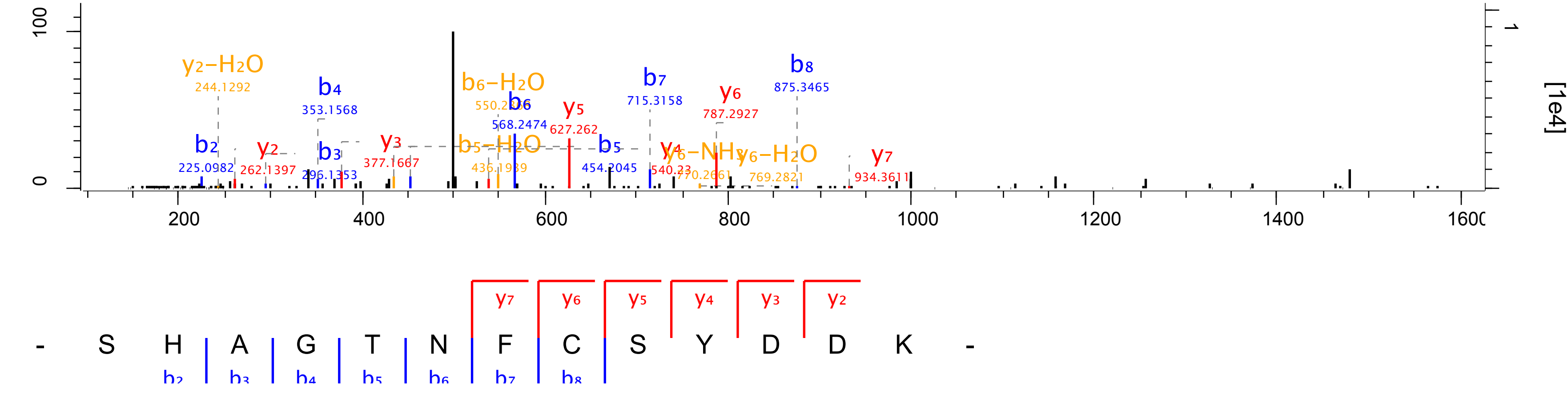

Raw file	Scan	Method	Score	m/z	Gene names
20150228_yeast1_Top_opt_B1_01_1614	11727	TOF; CID	48.11	641.32	UBP9

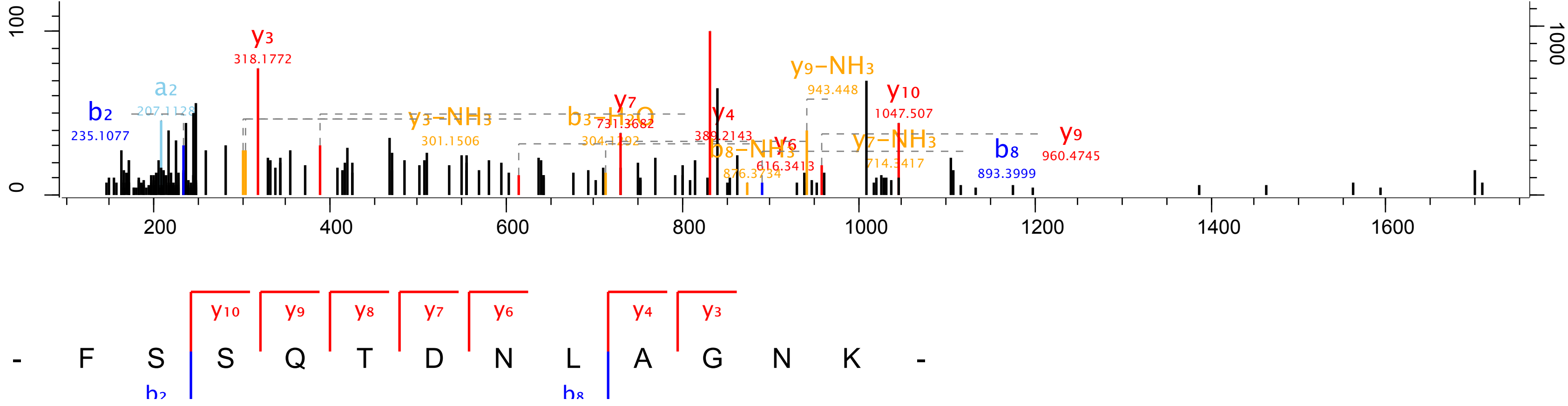

Raw file	Scan	Method	Score	m/z	Gene names
20150228_yeast1_Top_opt_B1_01_1614	11765	TOF; CID	62.46	500.75	RIM2

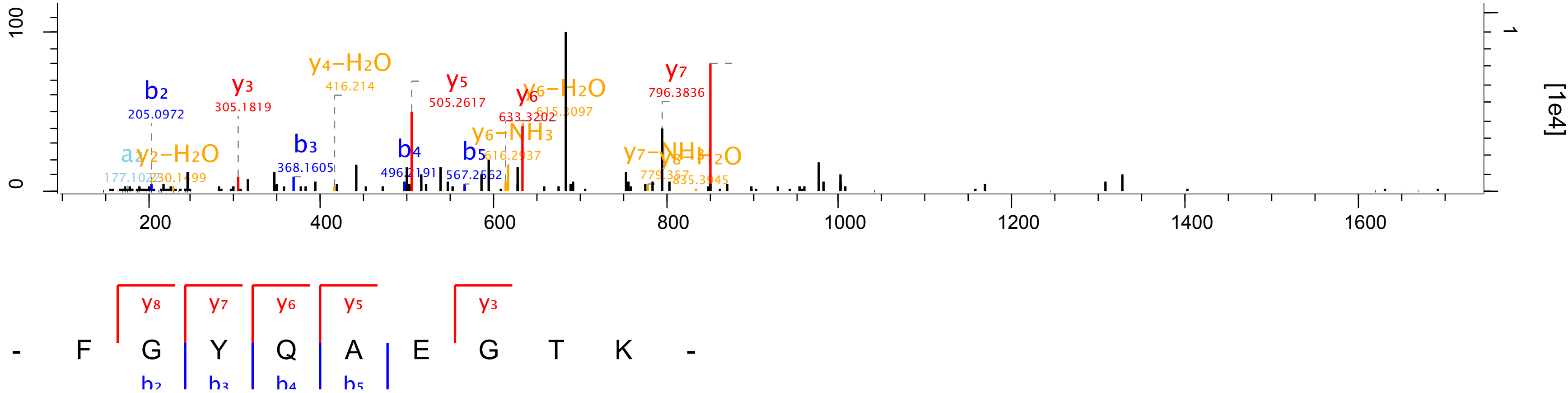

Raw file	Scan	Method	Score	m/z	Gene names
20150228_yeast1_Top_opt_B1_01_1614	11861	TOF; CID	84.61	511.26	RTC3

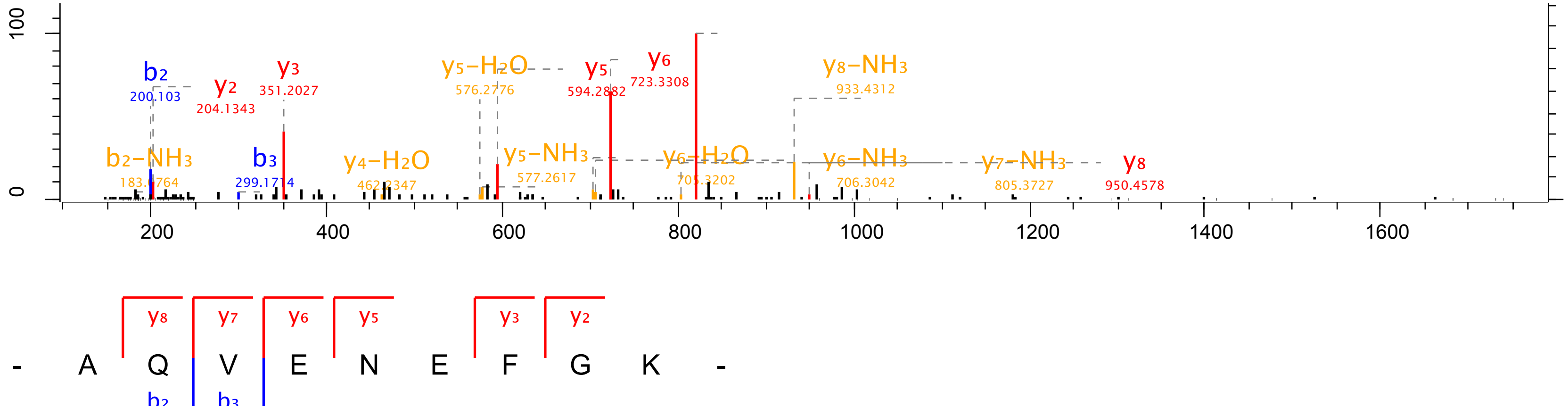

Raw file	Scan	Method	Score	m/z	Gene names
20150228_yeast1_Top_opt_B1_01_1614	12088	TOF; CID	50.18	591.63	CLB2

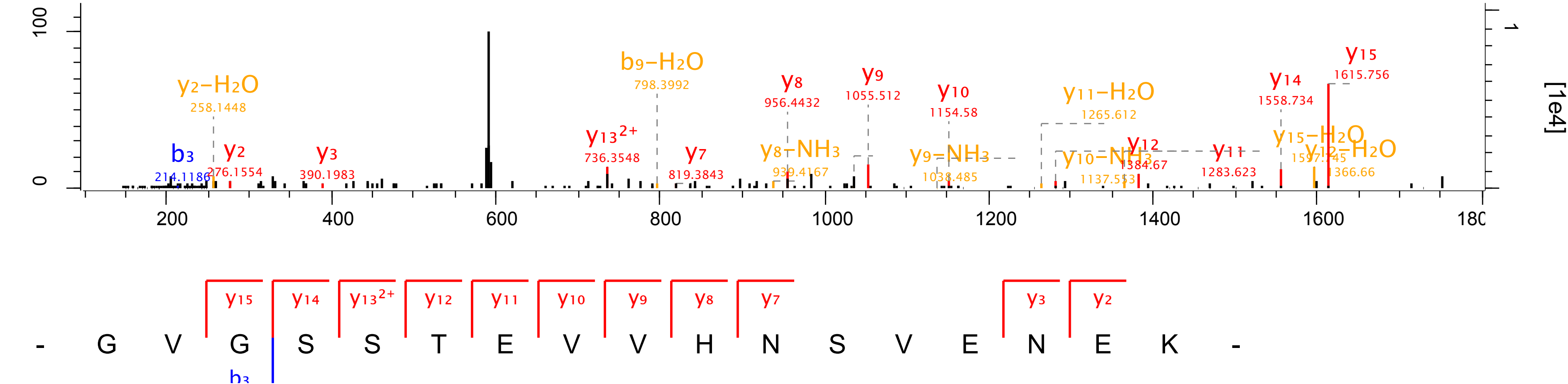

Raw file	Scan	Method	Score	m/z	Gene names
20150228_yeast1_Top_opt_B1_01_1614	12839	TOF; CID	69.03	635.32	MIH1

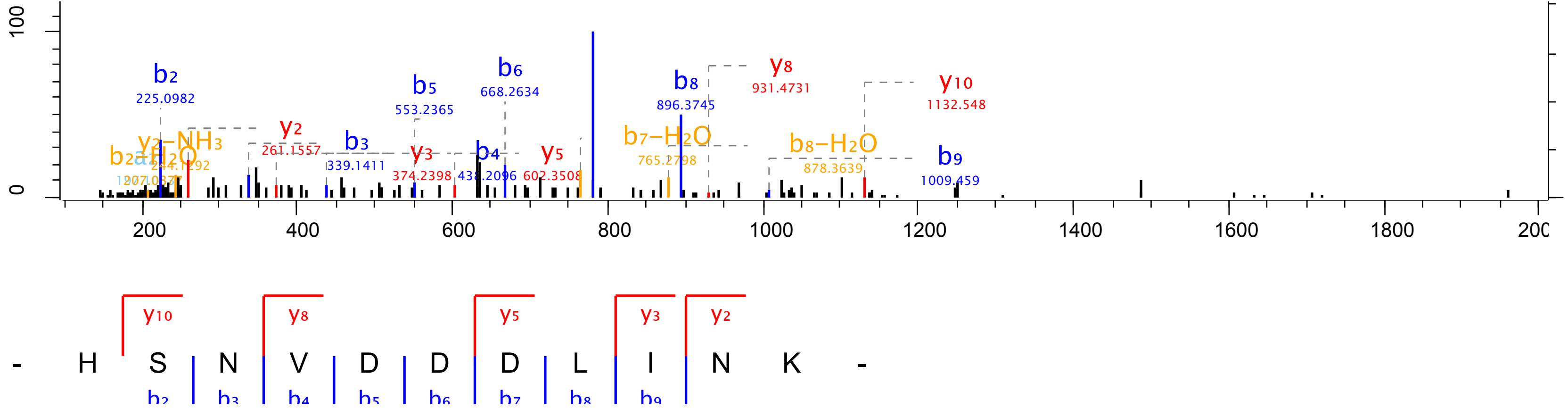

Raw file	Scan	Method	Score	m/z	Gene names
20150228_yeast1_Top_opt_B1_01_1614	13071	TOF; CID	109.44	514.6	PAM17

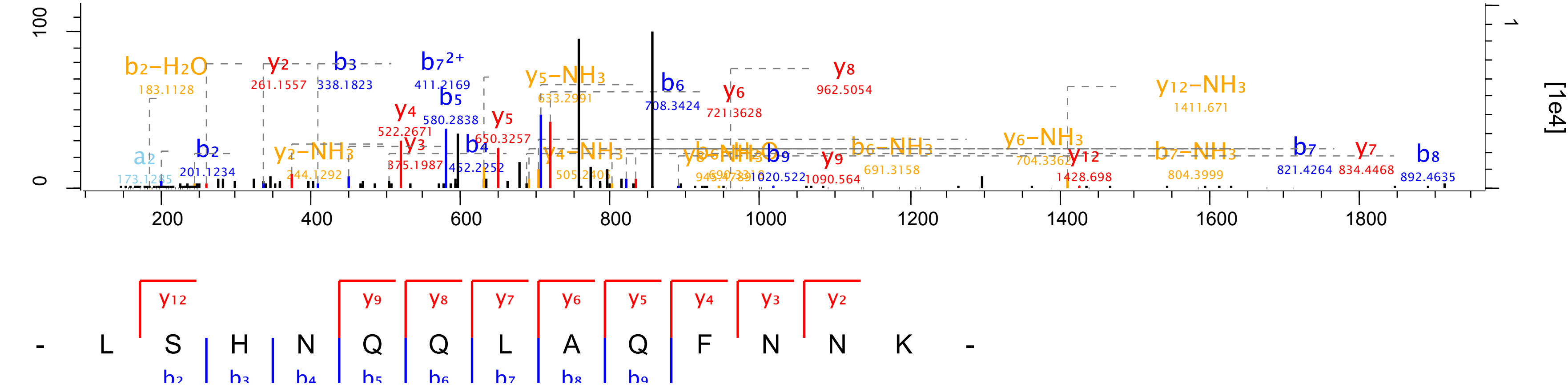

Raw file	Scan	Method	Score	m/z	Gene names
20150228_yeast1_Top_opt_B1_01_1614	13624	TOF; CID	103.43	581.83	RME1

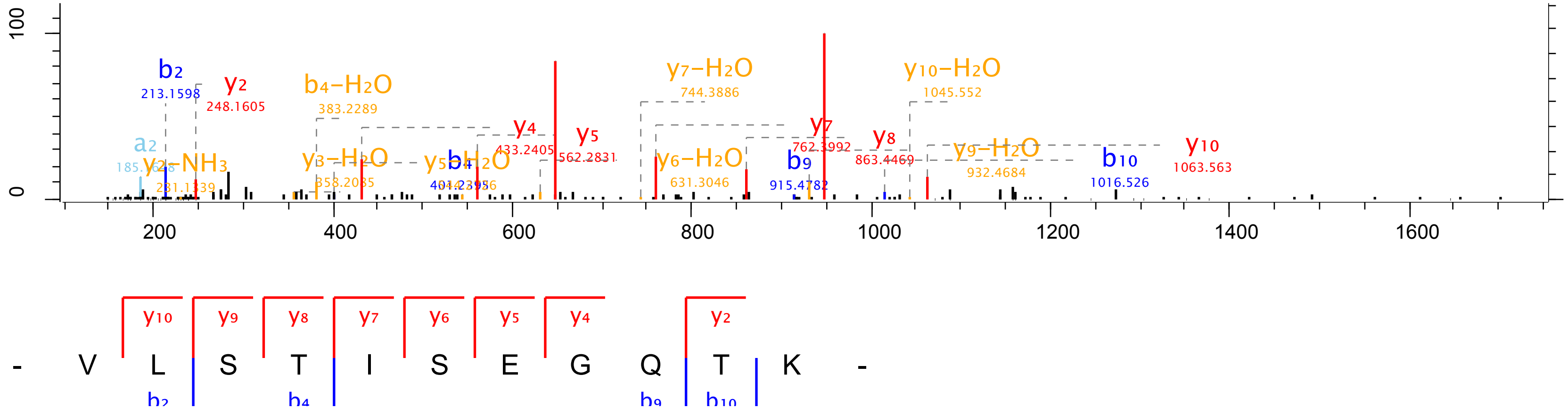

Raw file	Scan	Method	Score	m/z	Gene names
20150228_yeast1_Top_opt_B1_01_1614	14117	TOF; CID	67.51	470.76	NSE3

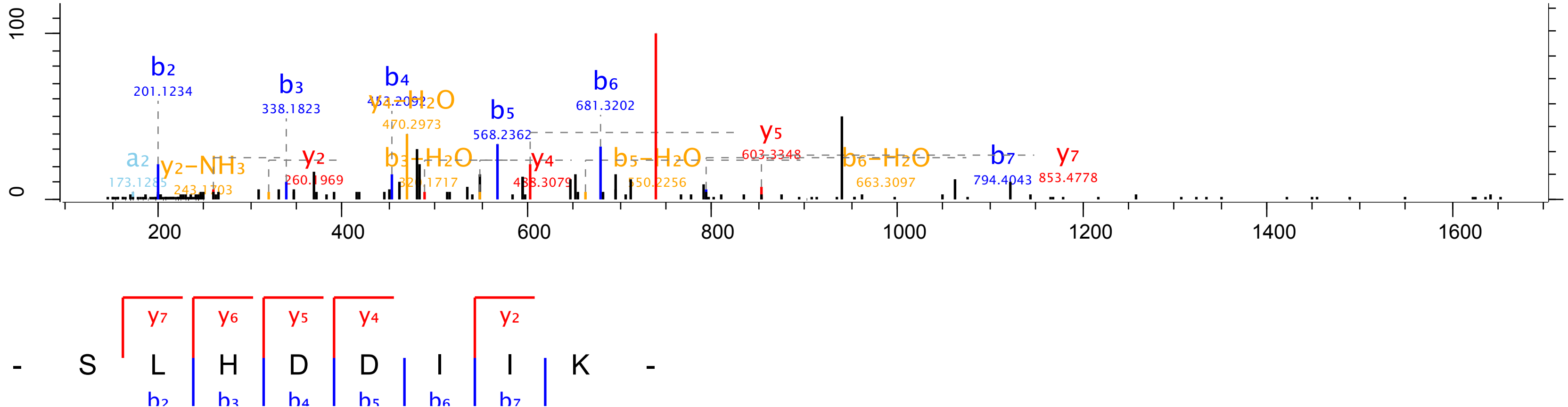

Raw file	Scan	Method	Score	m/z	Gene names
20150228_yeast1_Top_opt_B1_01_1614	14764	TOF; CID	60.44	530.26	PTC4

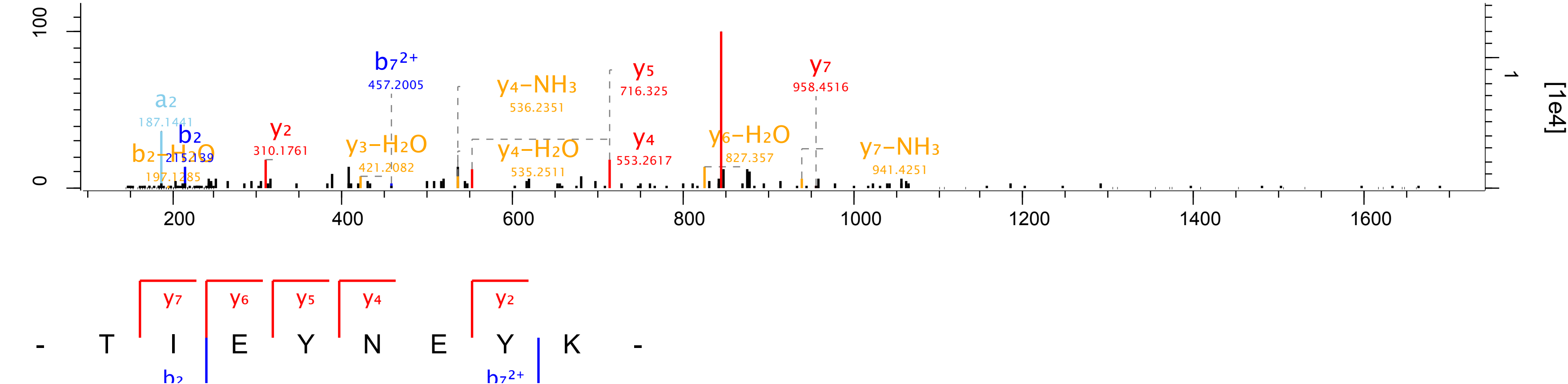

Raw file	Scan	Method	Score	m/z
20150228_yeast1_Top_opt_B1_01_1614	14921	TOF; CID	60.16	703.33

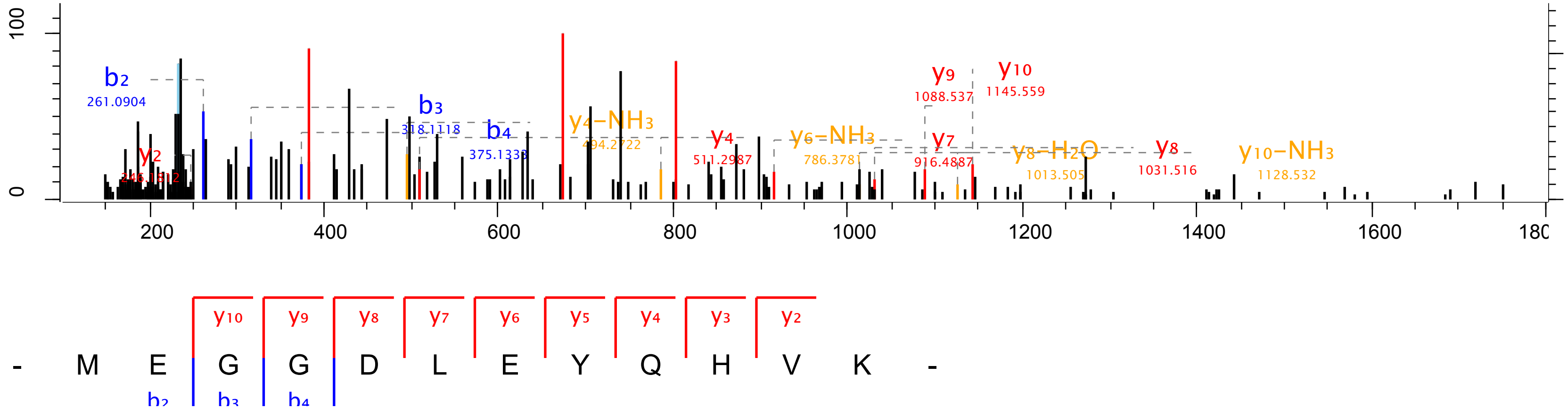

Raw file	Scan	Method	Score	m/z
20150228_yeast1_Top_opt_B1_01_1614	14976	TOF; CID	46.95	624.26

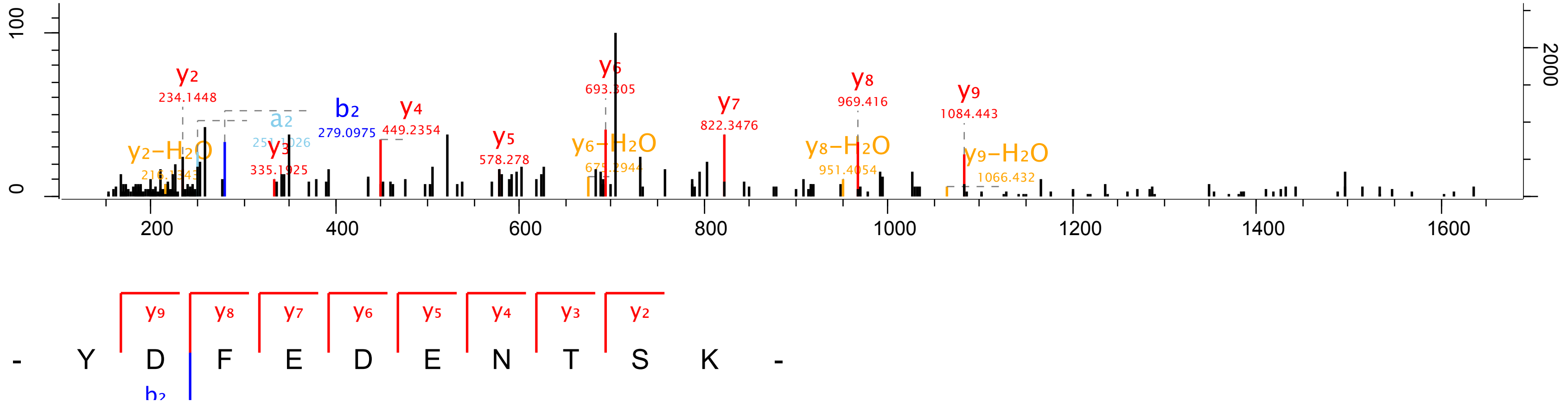

Raw file	Scan	Method	Score	m/z	Gene names
20150228_yeast1_Top_opt_B1_01_1614	15019	TOF; CID	40.5	526.28	MEC1

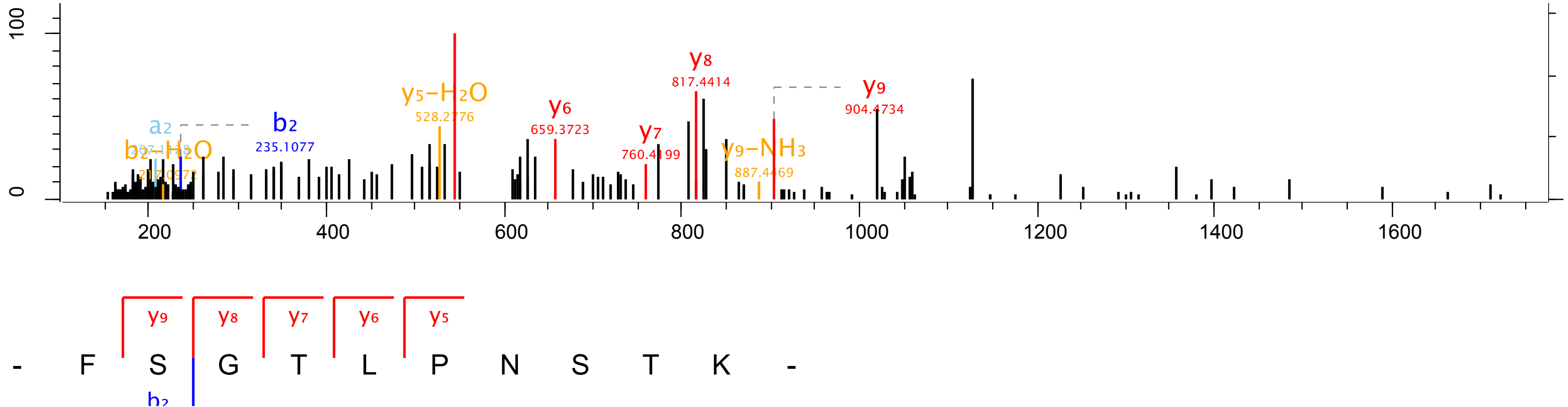

Raw file	Scan	Method	Score	m/z	Gene names
20150228_yeast1_Top_opt_B1_01_1614	15434	TOF; CID	57.14	769.36	ATG23

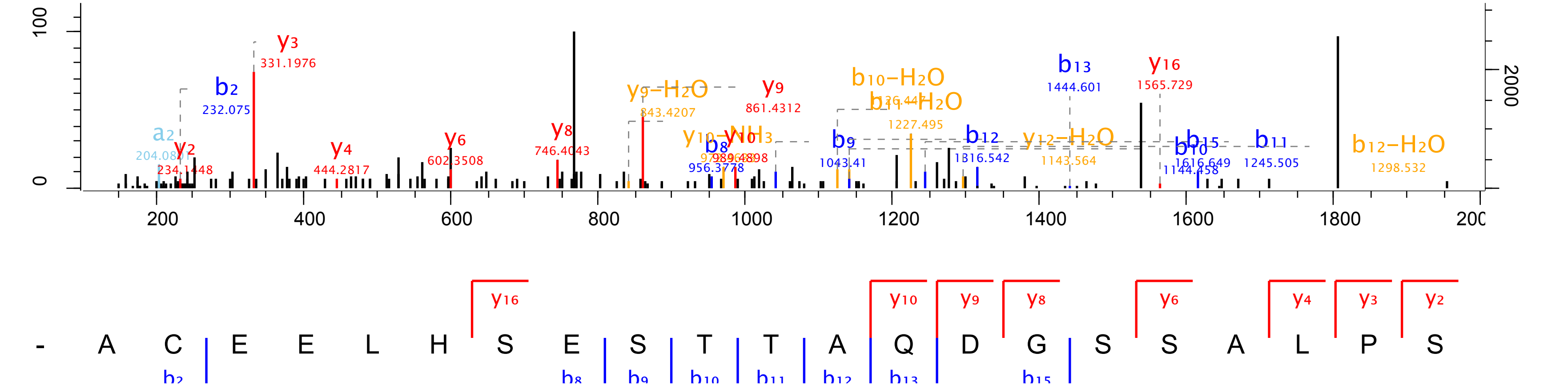

Raw file	Scan	Method	Score	m/z	Gene names
20150228_yeast1_Top_opt_B1_01_1614	16073	TOF; CID	59.35	461.74	TAH1

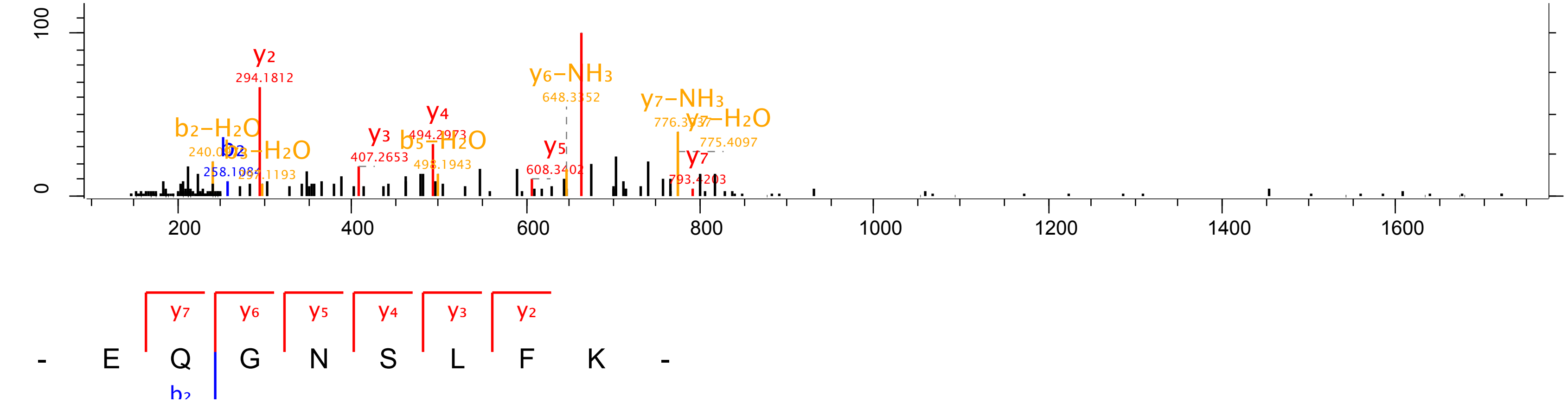

Raw file	Scan	Method	Score	m/z	Gene names
20150228_yeast1_Top_opt_B1_01_1614	16300	TOF; CID	85.47	808.39	AIM43

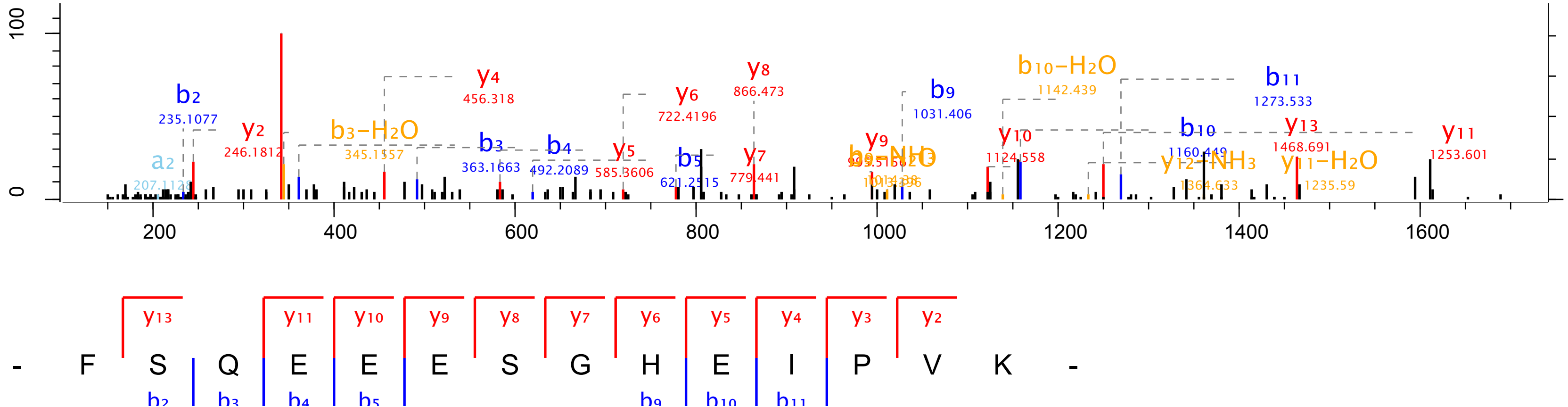

Raw file	Scan	Method	Score	m/z	Gene names
20150228_yeast1_Top_opt_B1_01_1614	16613	TOF; CID	111.52	652.35	SEN34

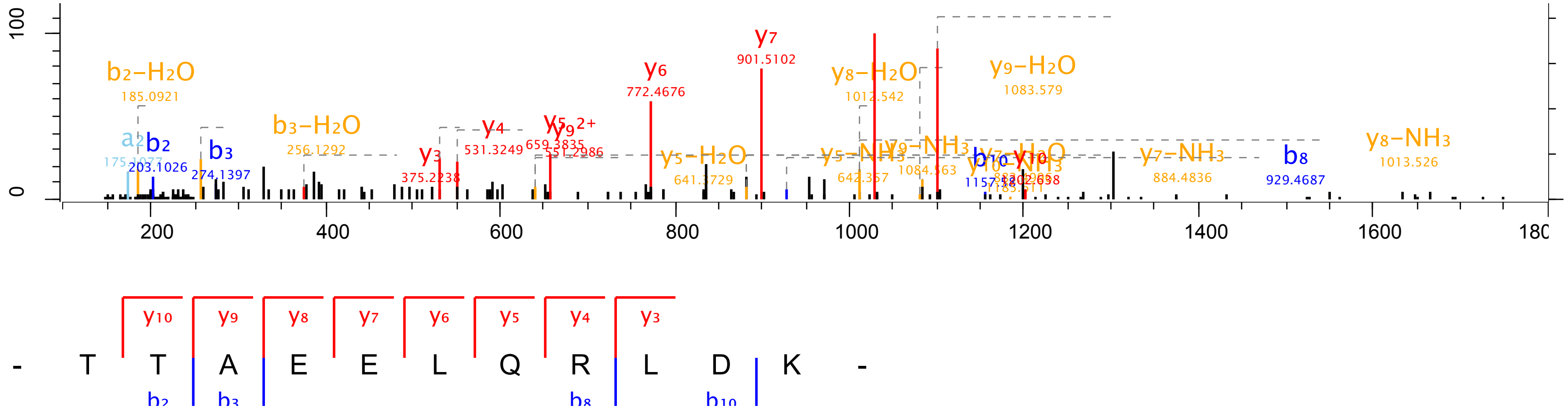

Raw file	Scan	Method	Score	m/z	Gene names
20150228_yeast1_Top_opt_B1_01_1614	16652	TOF; CID	155.56	857.41	SHR3

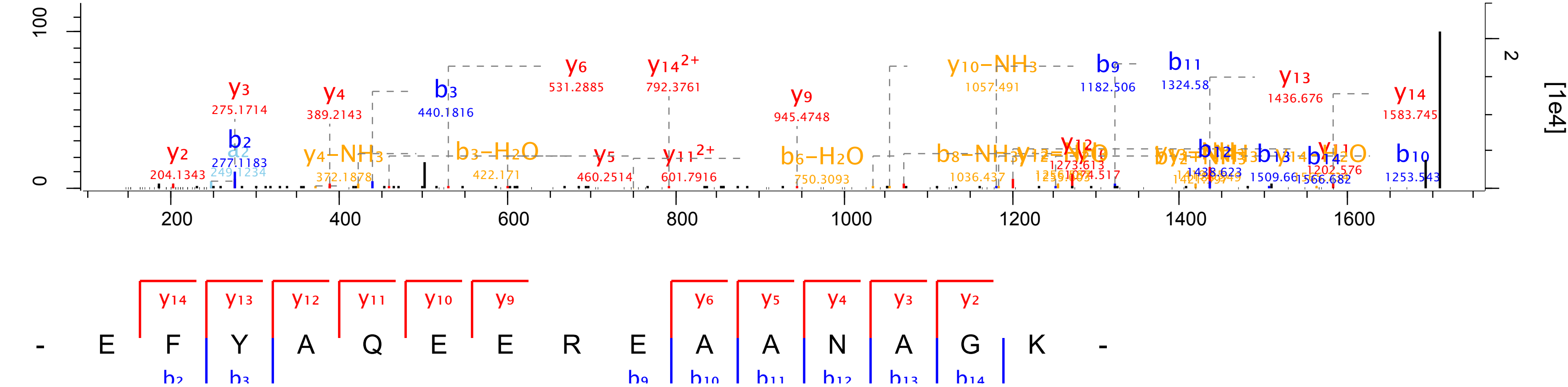

Raw file	Scan	Method	Score	m/z	Gene names
20150228_yeast1_Top_opt_B1_01_1614	16743	TOF; CID	79.09	669.35	MTR2

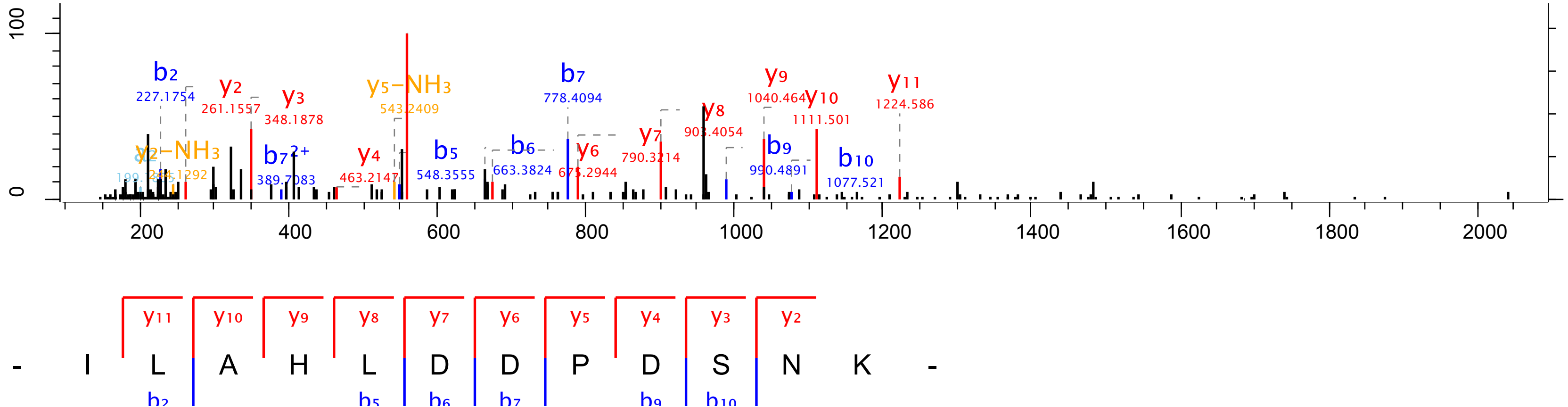

Raw file	Scan	Method	Score	m/z	Gene names
20150228_yeast1_Top_opt_B1_01_1614	17087	TOF; CID	57.79	667.34	ALK2

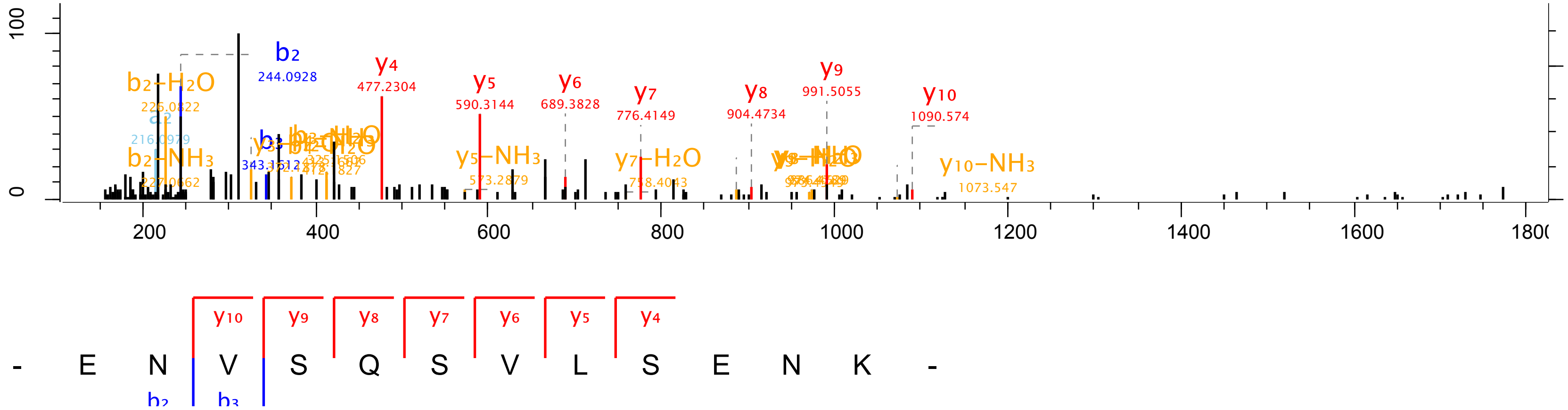

Raw file	Scan	Method	Score	m/z	Gene names
20150228_yeast1_Top_opt_B1_01_1614	17749	TOF; CID	77.74	531.28	ATE1

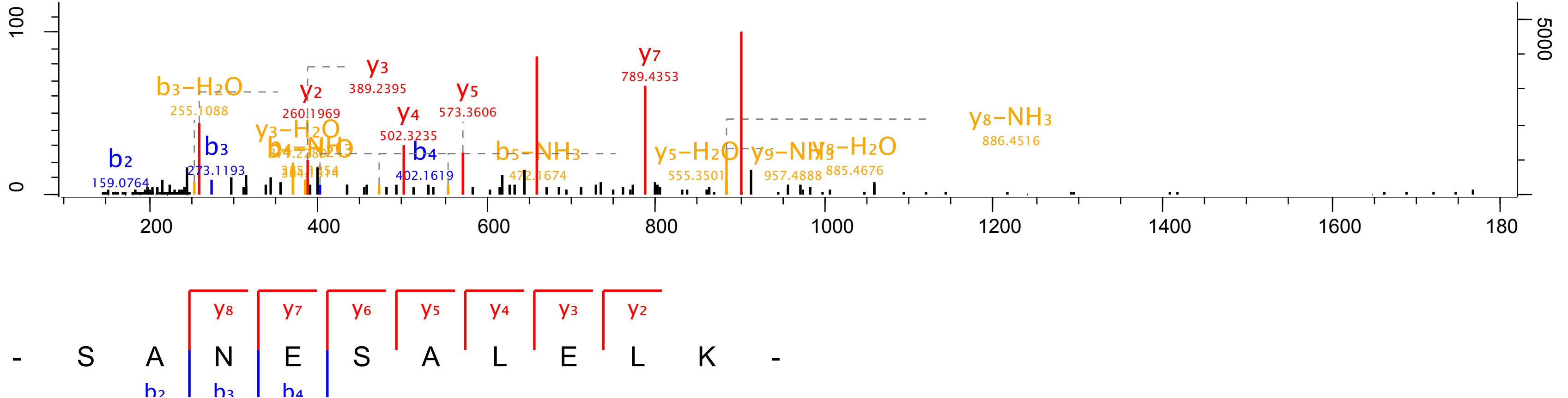

Raw file	Scan	Method	Score	m/z	Gene names
20150228_yeast1_Top_opt_B1_01_1614	17895	TOF; CID	71.98	659.83	SIZ1

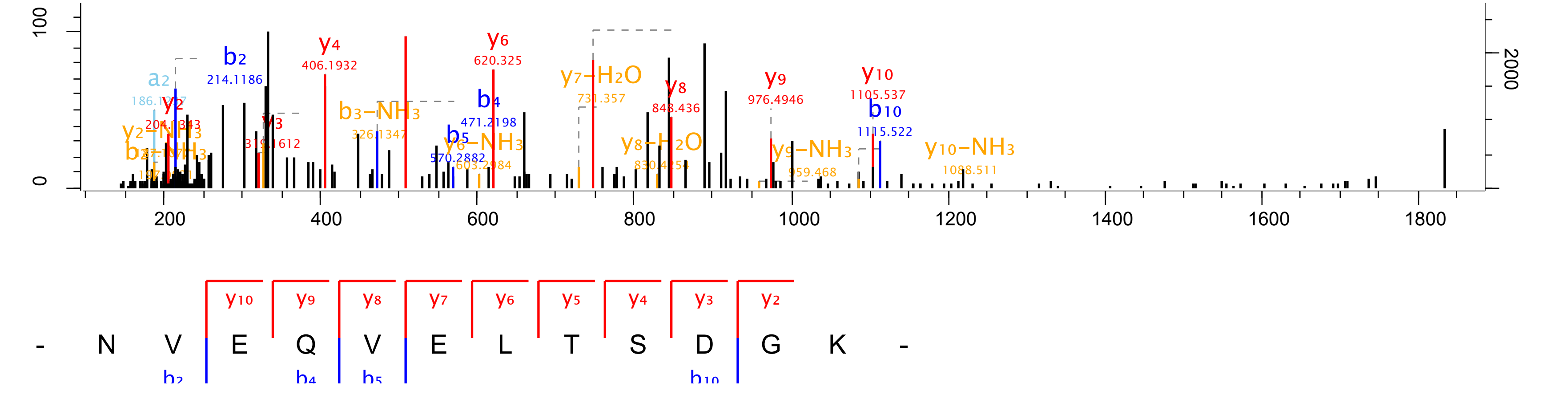

Raw file	Scan	Method	Score	m/z	Gene names
20150228_yeast1_Top_opt_B1_01_1614	18674	TOF; CID	50.29	1095.5	SPT3

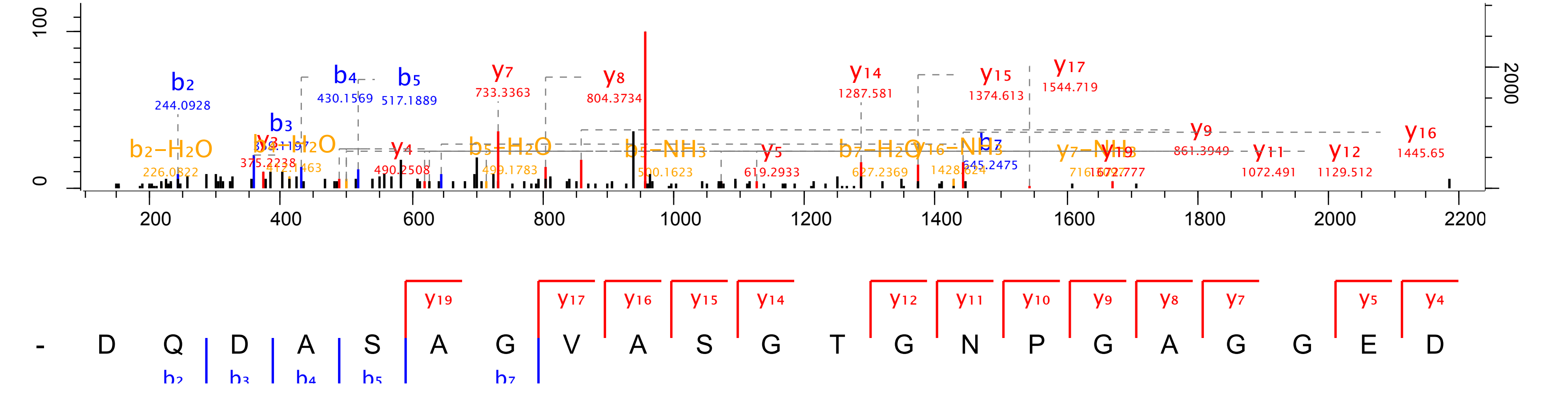

Raw file	Scan	Method	Score	m/z	Gene names
20150228_yeast1_Top_opt_B1_01_1614	18744	TOF; CID	111.34	808.42	FLC3

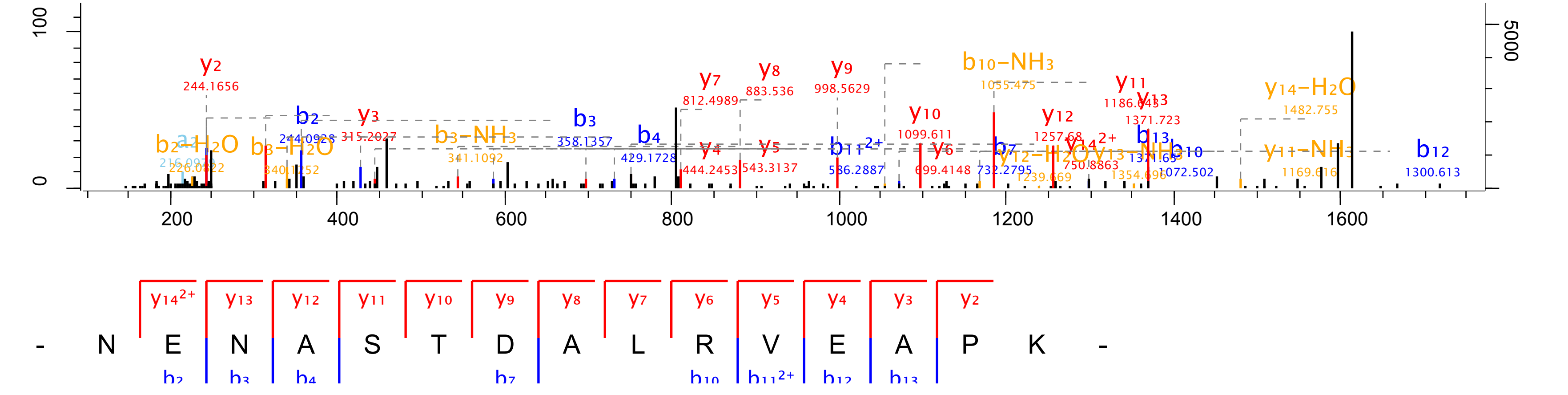

Raw file	Scan	Method	Score	m/z	Gene names
20150228_yeast1_Top_opt_B1_01_1614	18782	TOF; CID	52.58	755.34	SEC59

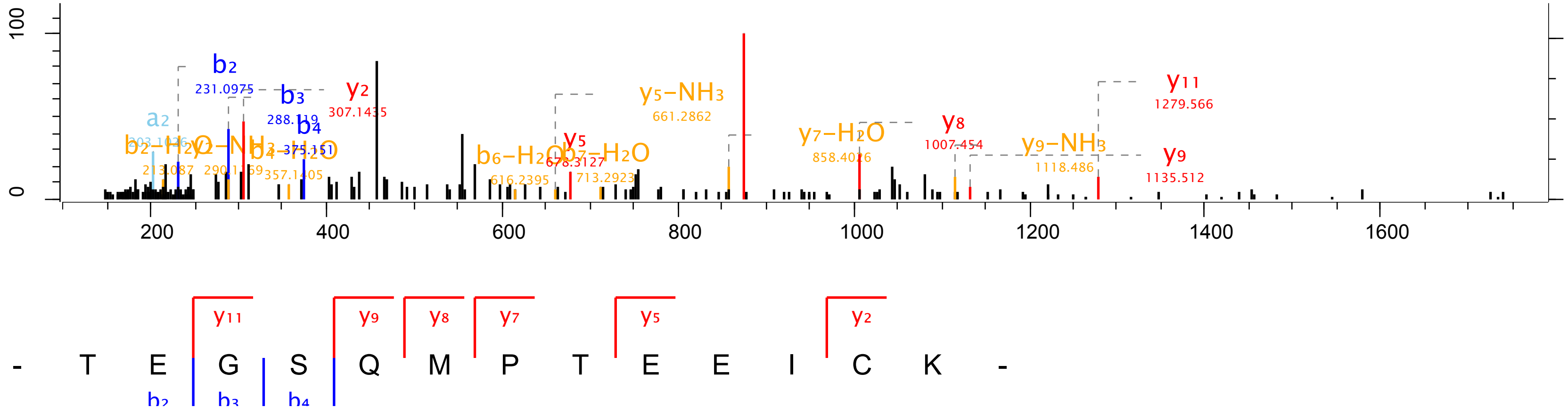

Raw file	Scan	Method	Score	m/z	Gene names
20150228_yeast1_Top_opt_B1_01_1614	19171	TOF; CID	73.04	402.74	YOL107W

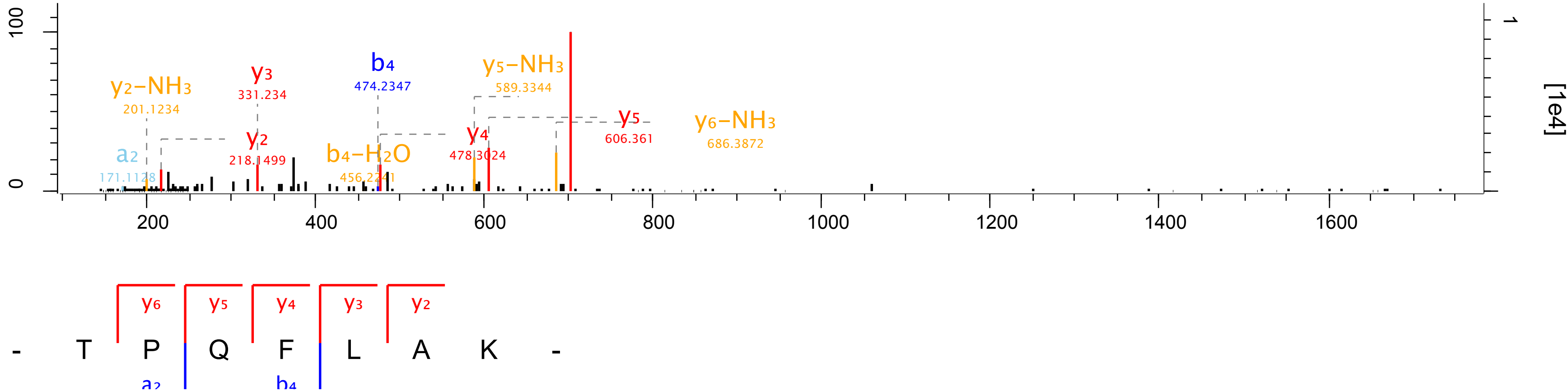

Raw file	Scan	Method	Score	m/z	Gene names
20150228_yeast1_Top_opt_B1_01_1614	19233	TOF; CID	96.14	833.9	RAD10

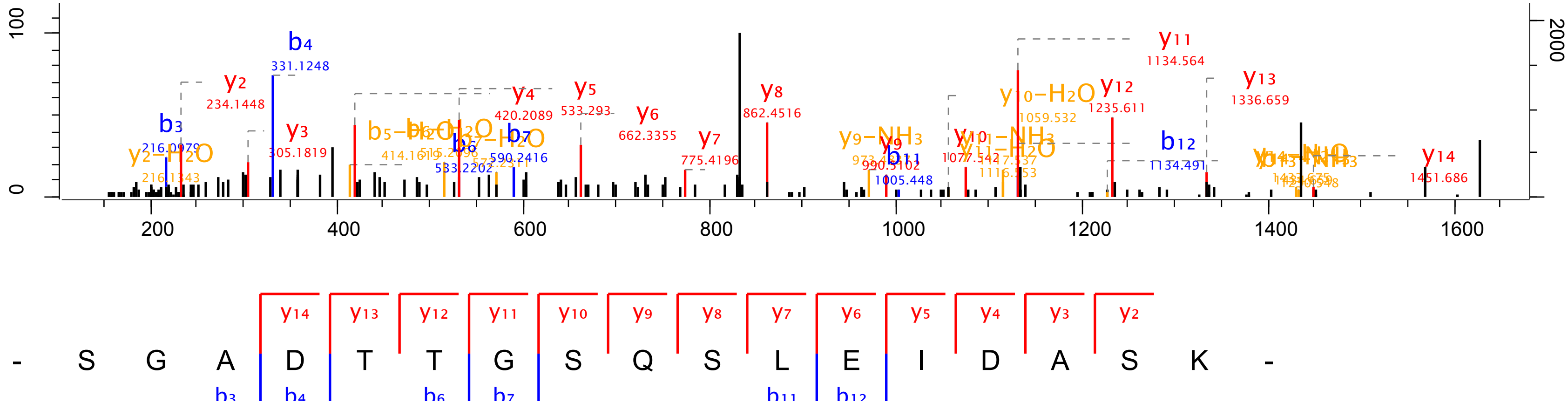

Raw file	Scan	Method	Score	m/z	Gene names
20150228_yeast1_Top_opt_B1_01_1614	19474	TOF; CID	43.42	523.8	FAU1

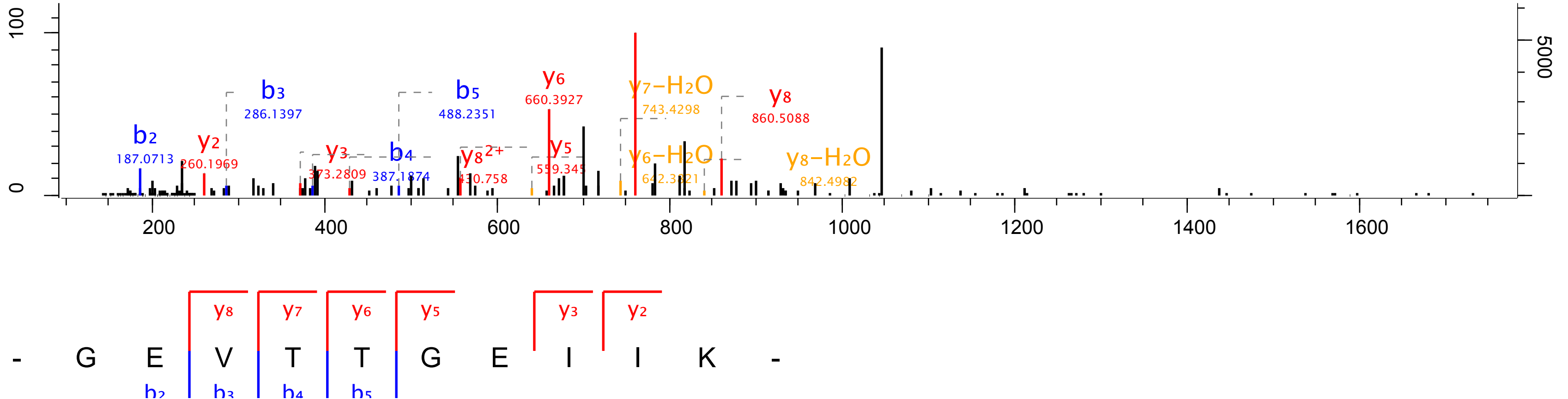

Raw file	Scan	Method	Score	m/z	Gene names
20150228_yeast1_Top_opt_B1_01_1614	19480	TOF; CID	50.35	448.25	OCA1

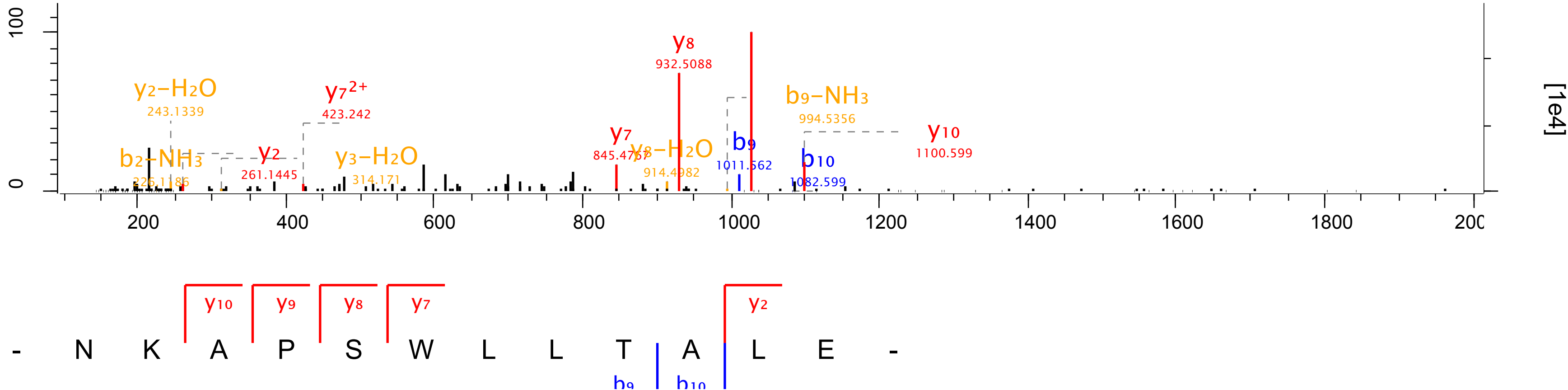

Raw file	Scan	Method	Score	m/z	Gene names
20150228_yeast1_Top_opt_B1_01_1614	19694	TOF; CID	57.79	481.27	YSC83

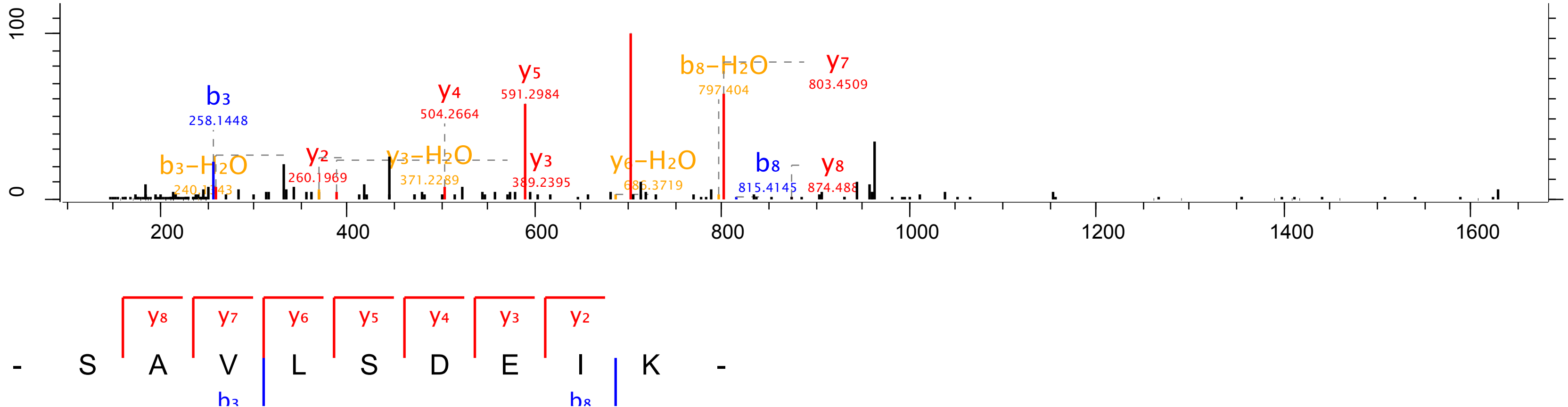

Raw file	Scan	Method	Score	m/z	Gene names
20150228_yeast1_Top_opt_B1_01_1614	20585	TOF; CID	46.25	812.39	MMT2

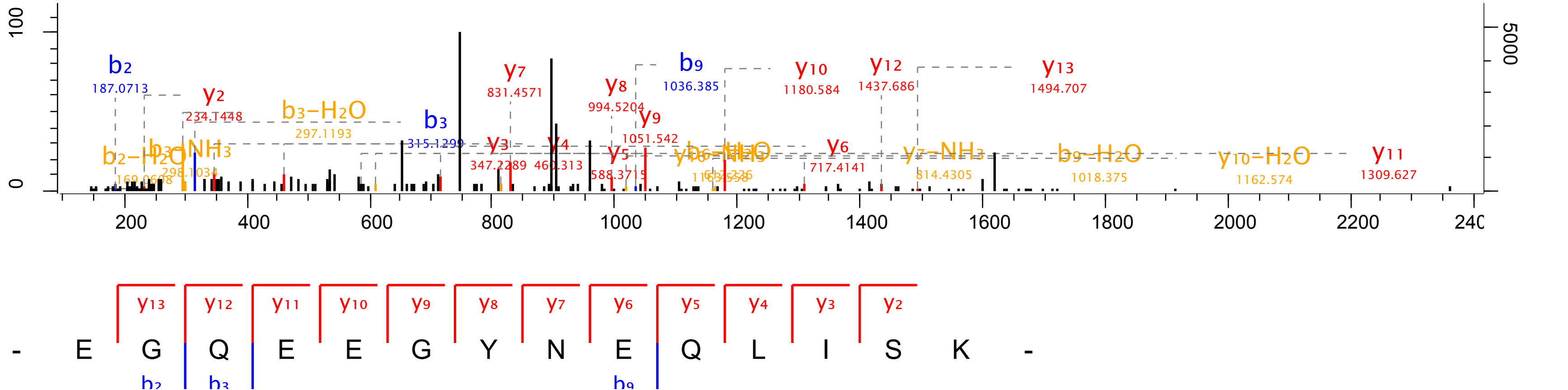

Raw file	Scan	Method	Score	m/z	Gene names
20150228_yeast1_Top_opt_B1_01_1614	21142	TOF; CID	66.27	628.8	MRPL31

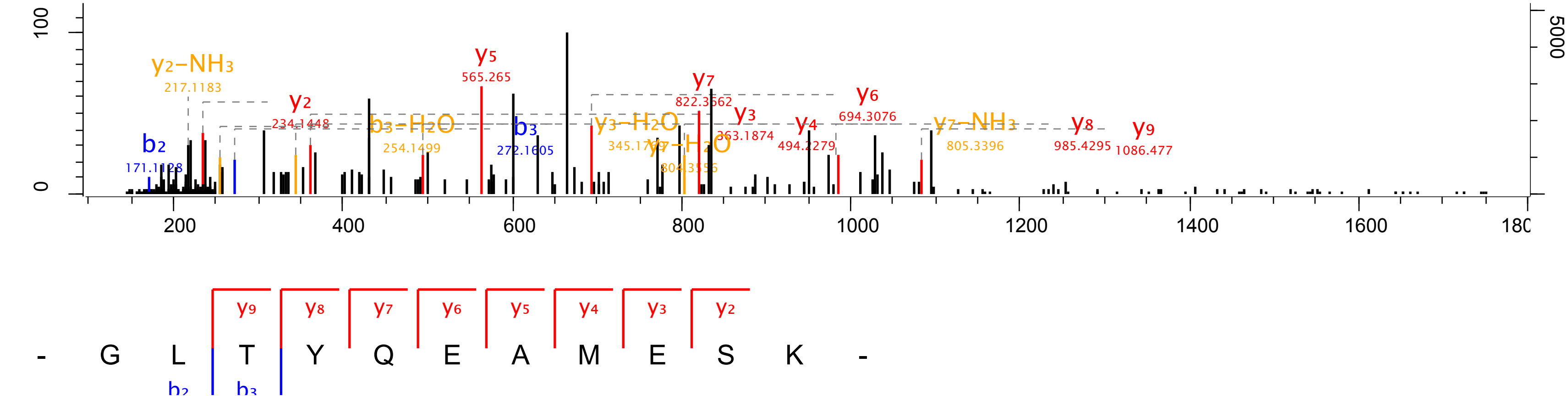

Raw file	Scan	Method	Score	m/z	Gene names
20150228_yeast1_Top_opt_B1_01_1614	21517	TOF; CID	83.69	704.86	RAD2

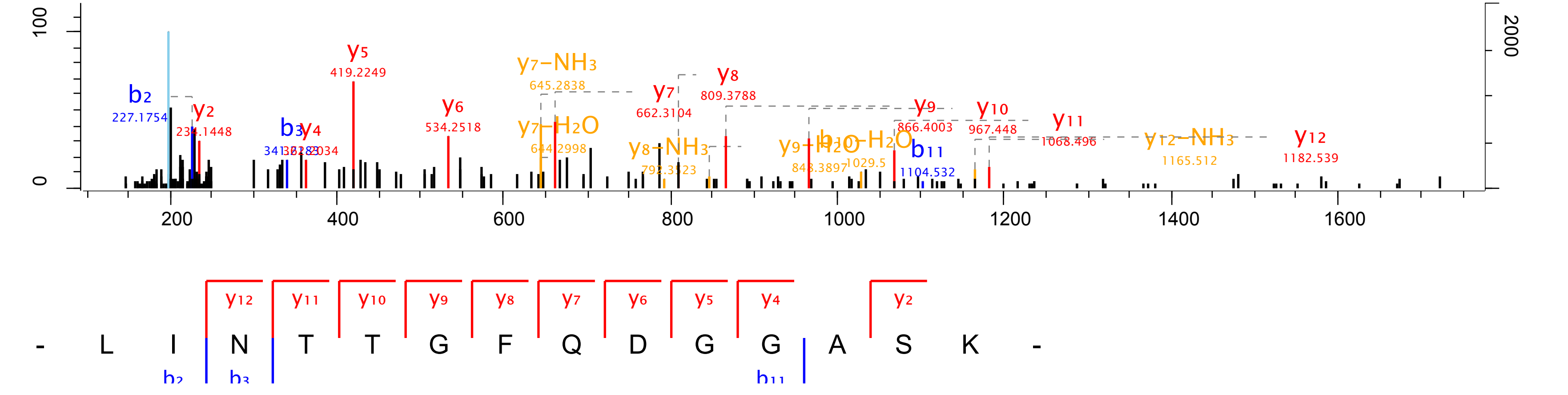

Raw file	Scan	Method	Score	m/z	Gene names
20150228_yeast1_Top_opt_B1_01_1614	22036	TOF; CID	67.76	754.72	HRD1

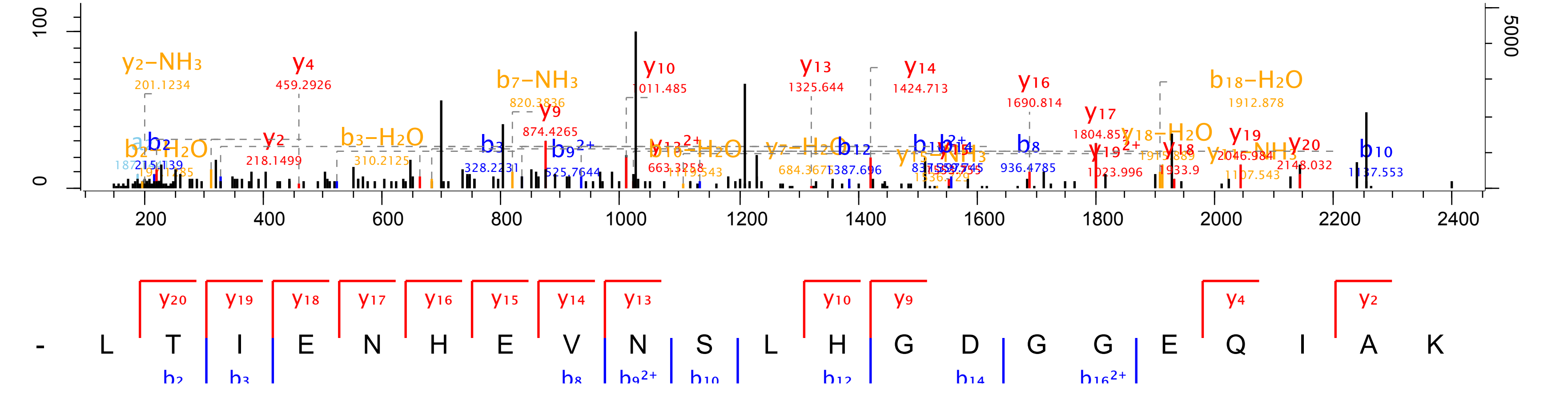

Raw file	Scan	Method	Score	m/z	Gene names
20150228_yeast1_Top_opt_B1_01_1614	22487	TOF; CID	108.98	434.91	EDC1

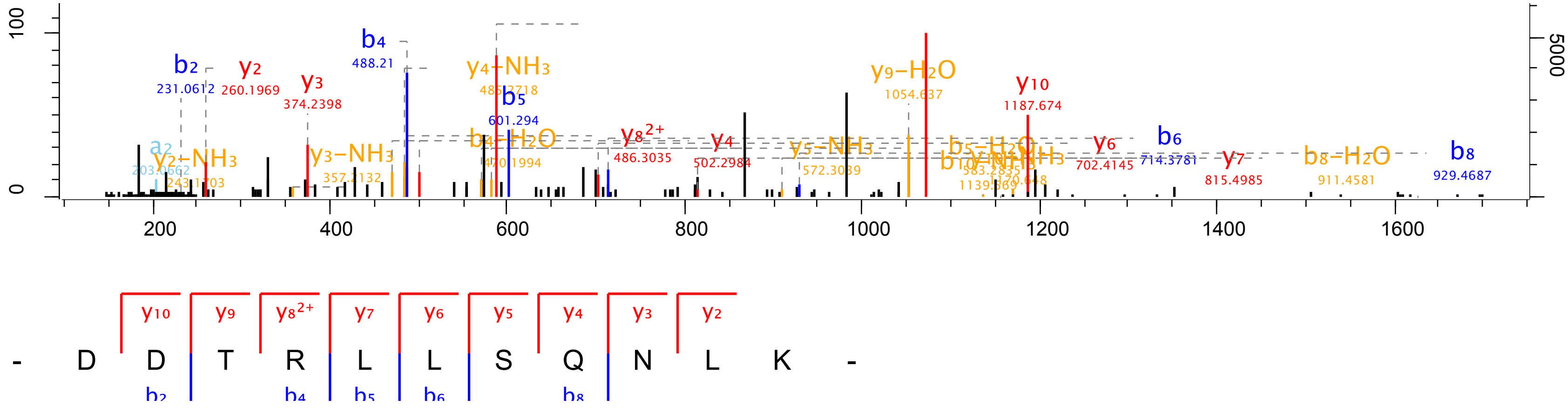

Raw file	Scan	Method	Score	m/z	Gene names
20150228_yeast1_Top_opt_B1_01_1614	23048	TOF; CID	96.71	794.4	VPS36

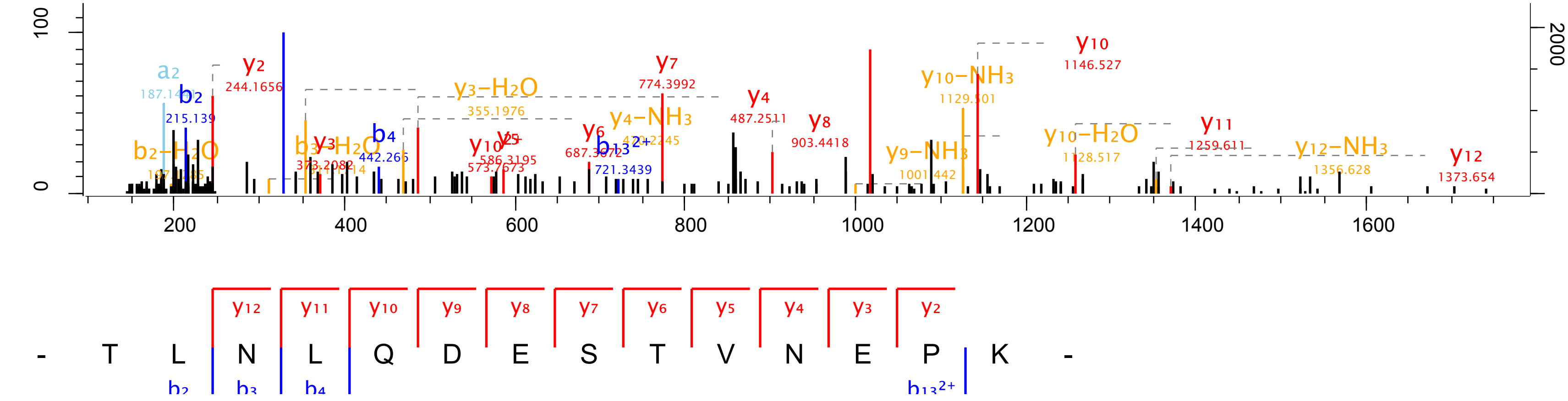

Raw file	Scan	Method	Score	m/z	Gene names
20150228_yeast1_Top_opt_B1_01_1614	23121	TOF; CID	58.08	824.4	YLR036C

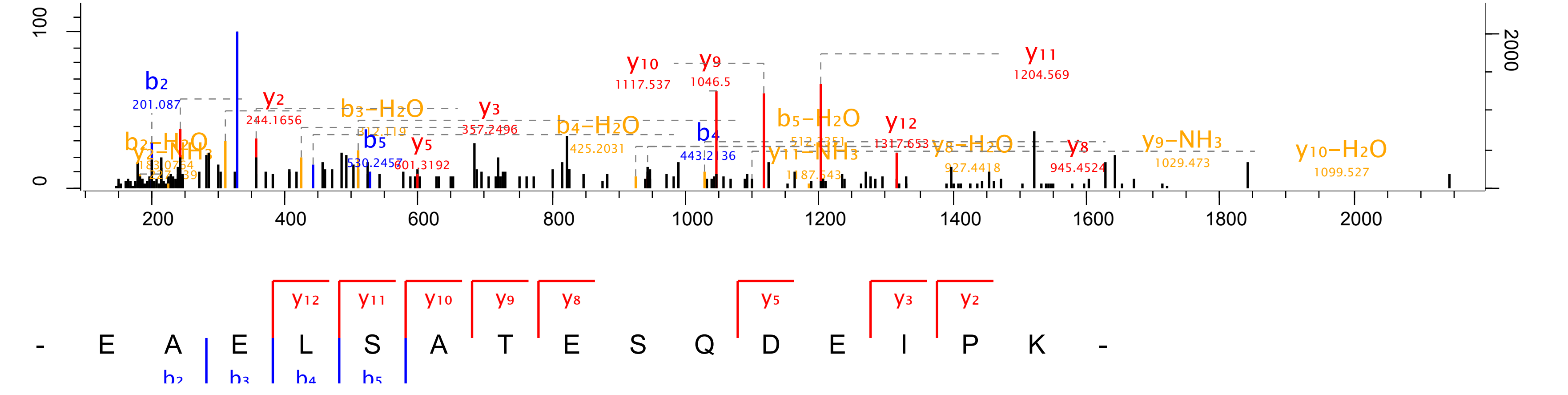

Raw file	Scan	Method	Score	m/z	Gene names
20150228_yeast1_Top_opt_B1_01_1614	23693	TOF; CID	57.17	680.35	HSK3

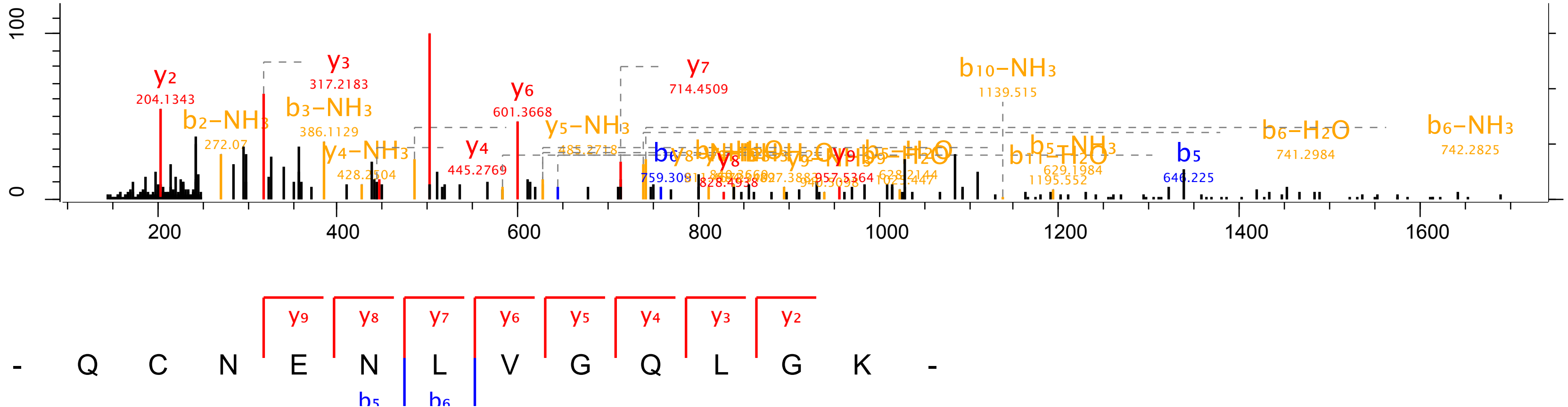

Raw file	Scan	Method	Score	m/z	Gene names
20150228_yeast1_Top_opt_B1_01_1614	23726	TOF; CID	95.07	812.88	HLR1

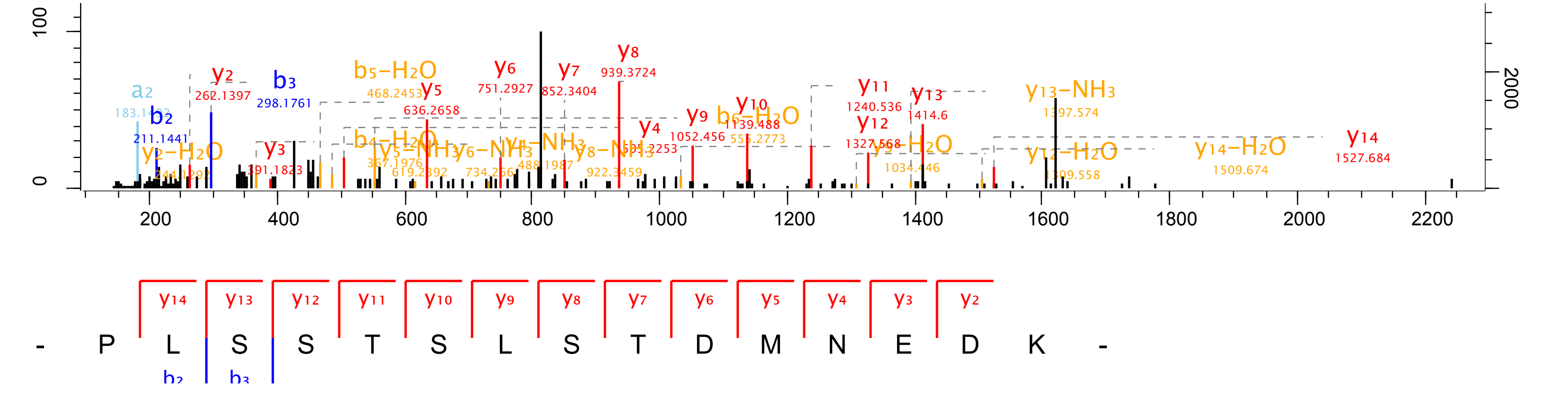

Raw file	Scan	Method	Score	m/z	Gene names
20150228_yeast1_Top_opt_B1_01_1614	23913	TOF; CID	48.29	568.62	NQM1

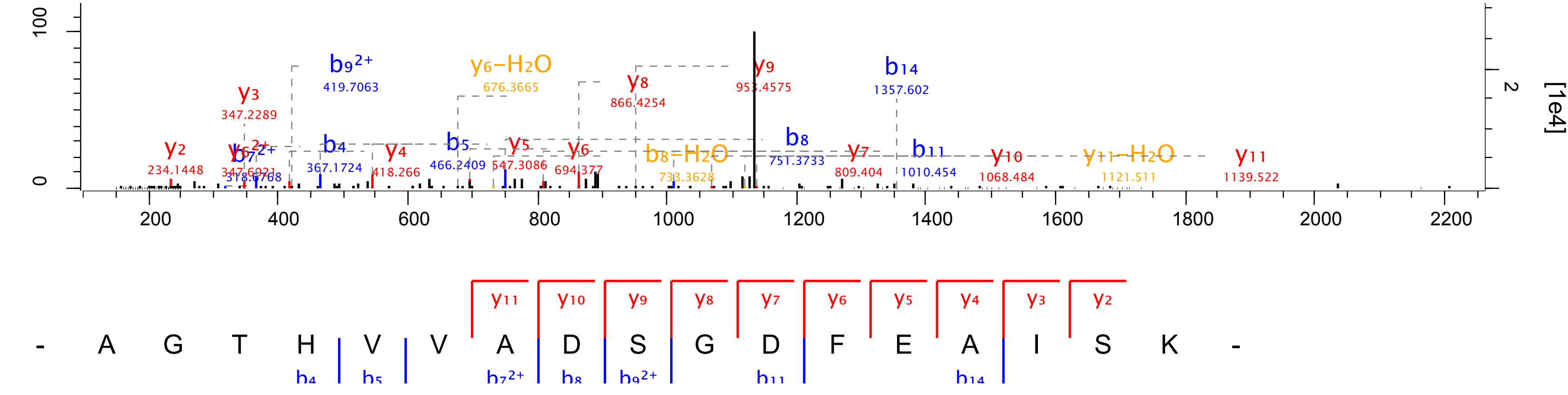

Raw file	Scan	Method	Score	m/z	Gene names
20150228_yeast1_Top_opt_B1_01_1614	24091	TOF; CID	88.34	651.37	MUD1

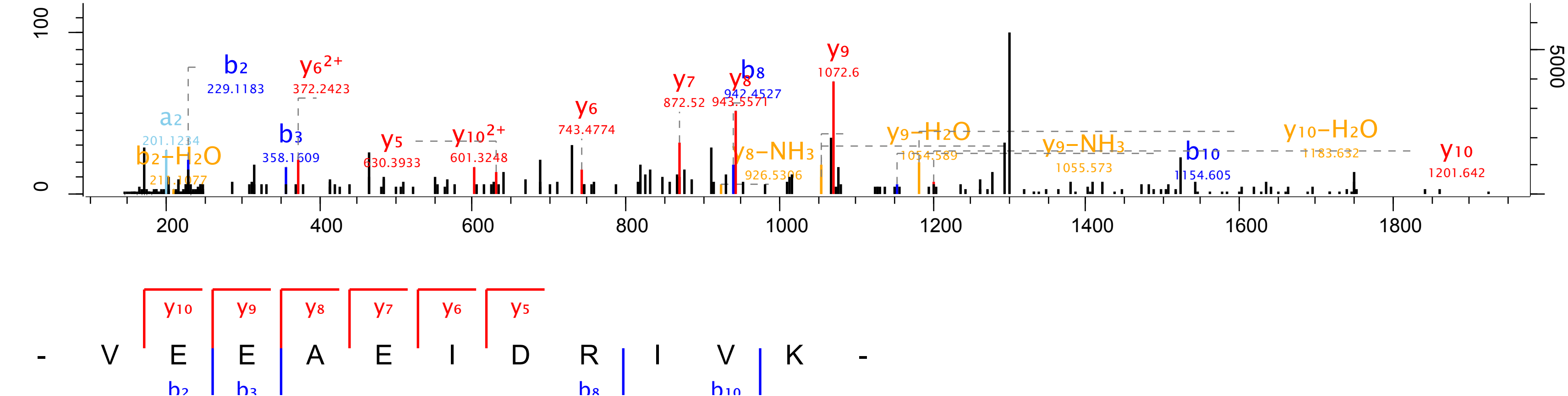

Raw file	Scan	Method	Score	m/z	Gene names
20150228_yeast1_Top_opt_B1_01_1614	24748	TOF; CID	50.09	536.28	KEI1

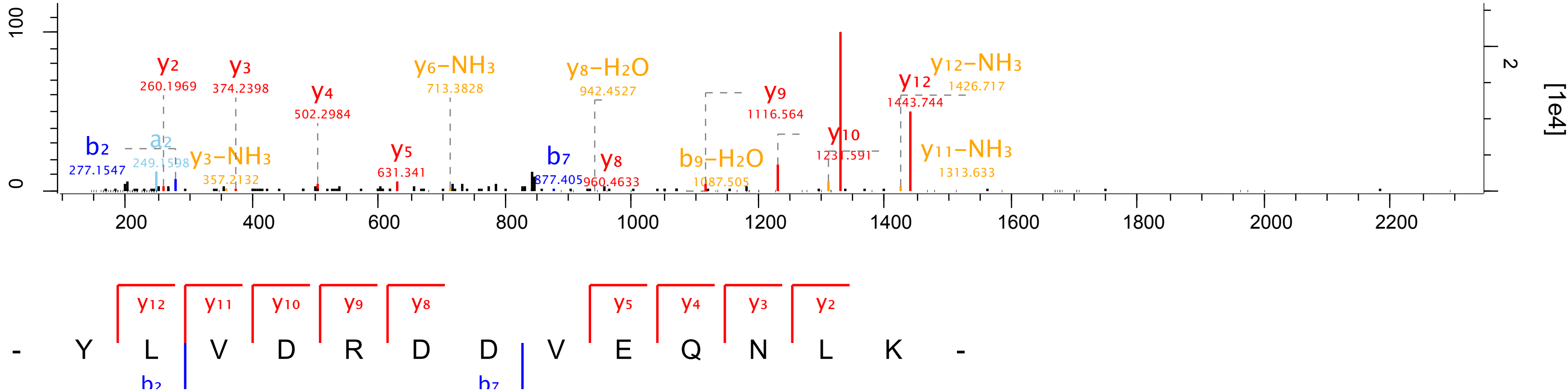

Raw file	Scan	Method	Score	m/z	Gene names
20150228_yeast1_Top_opt_B1_01_1614	25650	TOF; CID	77.06	640.83	CTP1

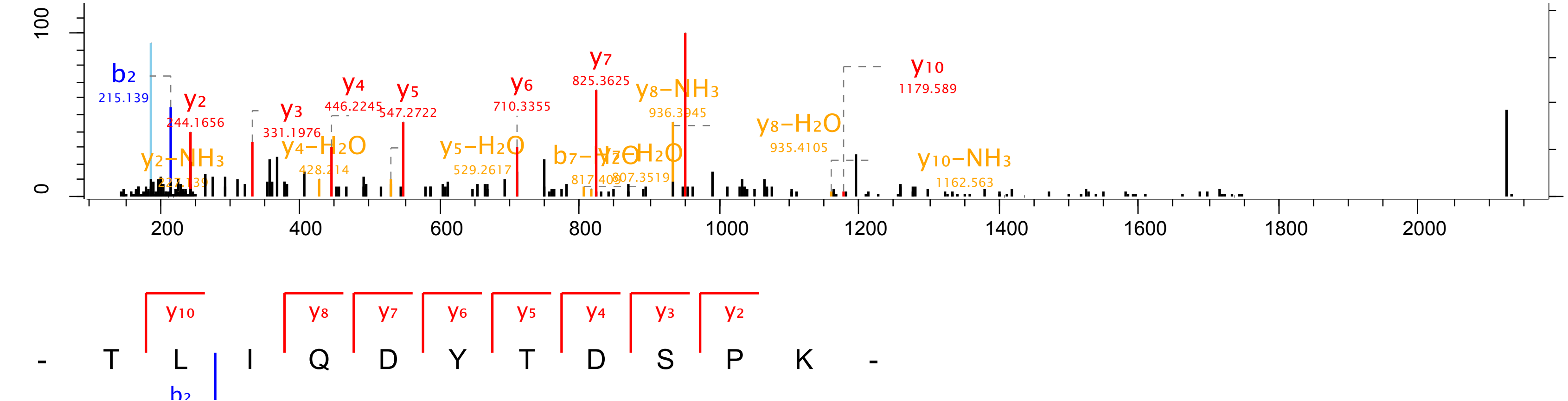

Raw file	Scan	Method	Score	m/z	Gene names
20150228_yeast1_Top_opt_B1_01_1614	25832	TOF; CID	86.94	571.31	YAP1802

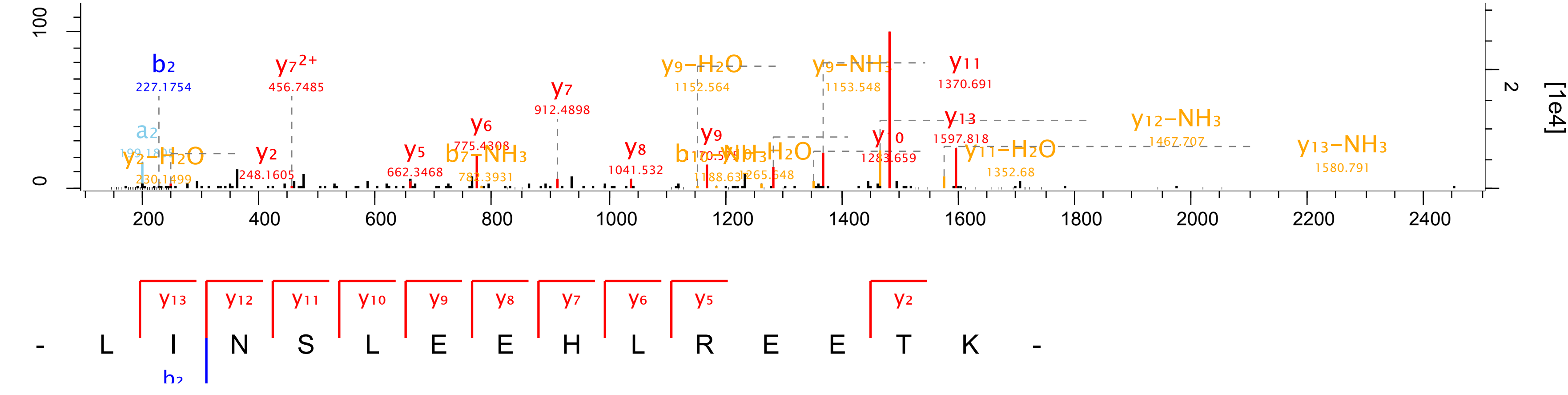

Raw file	Scan	Method	Score	m/z	Gene names
20150228_yeast1_Top_opt_B1_01_1614	26201	TOF; CID	77.06	591.8	GLN3

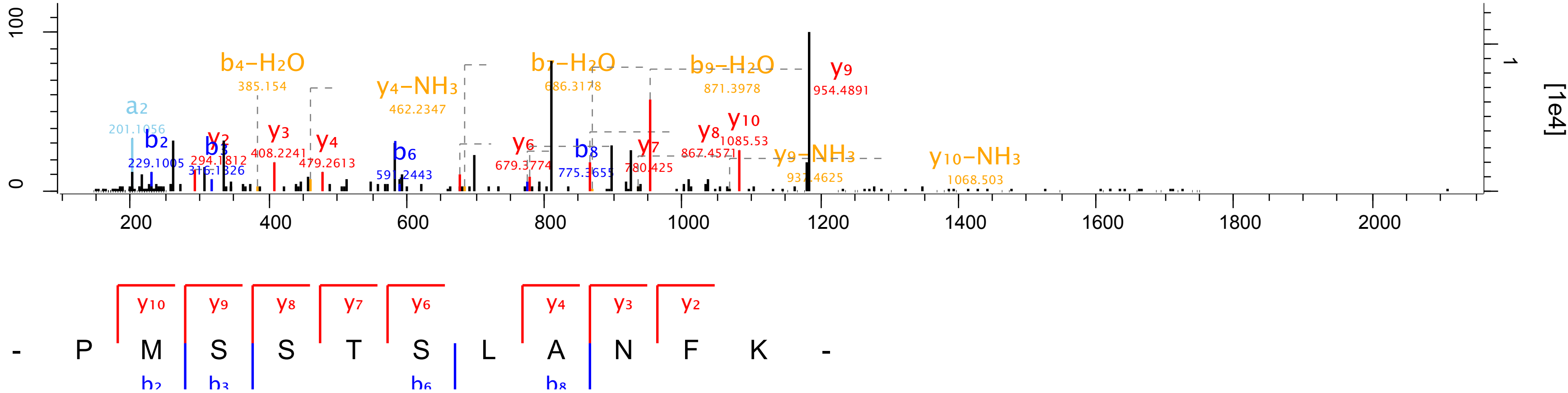

Raw file	Scan	Method	Score	m/z	Gene names
20150228_yeast1_Top_opt_B1_01_1614	26336	TOF; CID	58.27	453.25	DMA2

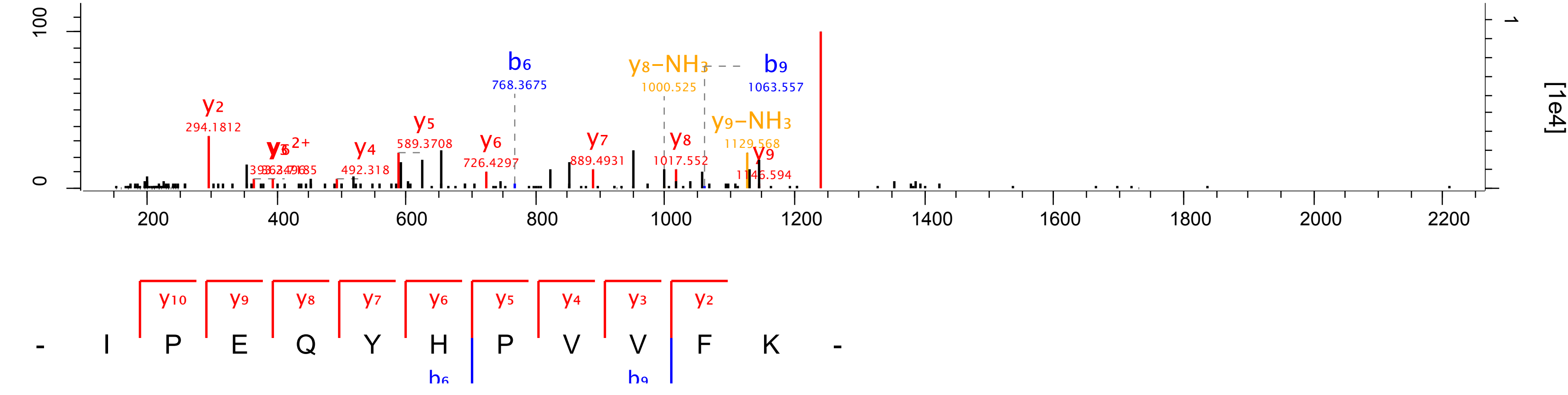

Raw file	Scan	Method	Score	m/z	Gene names
20150228_yeast1_Top_opt_B1_01_1614	27223	TOF; CID	94.45	487.25	NSG2

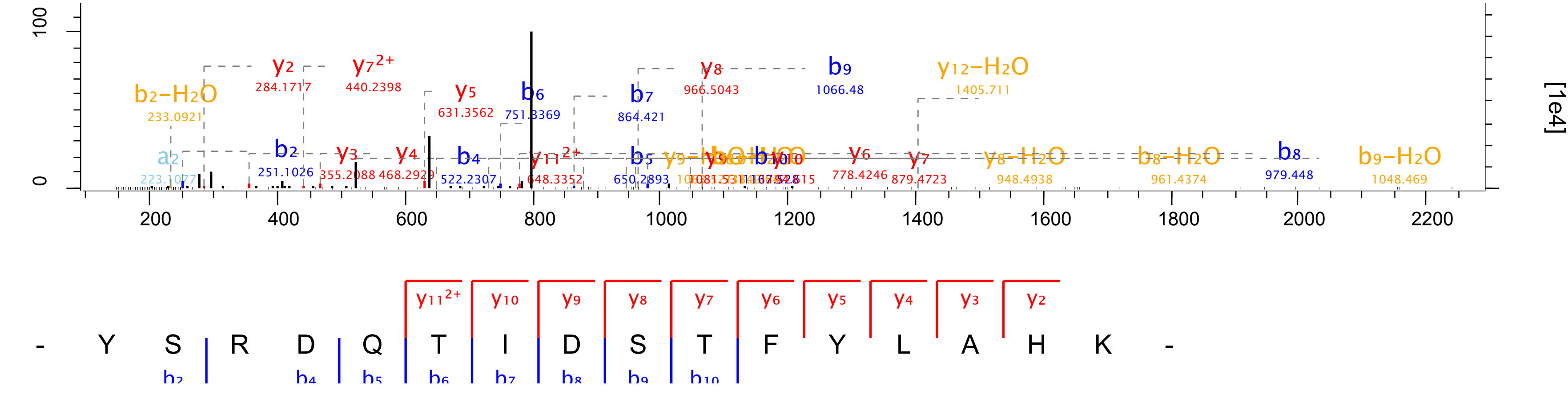

Raw file	Scan	Method	Score	m/z	Gene names
20150228_yeast1_Top_opt_B1_01_1614	27321	TOF; CID	141.18	955.01	RPL20B;RPL20A

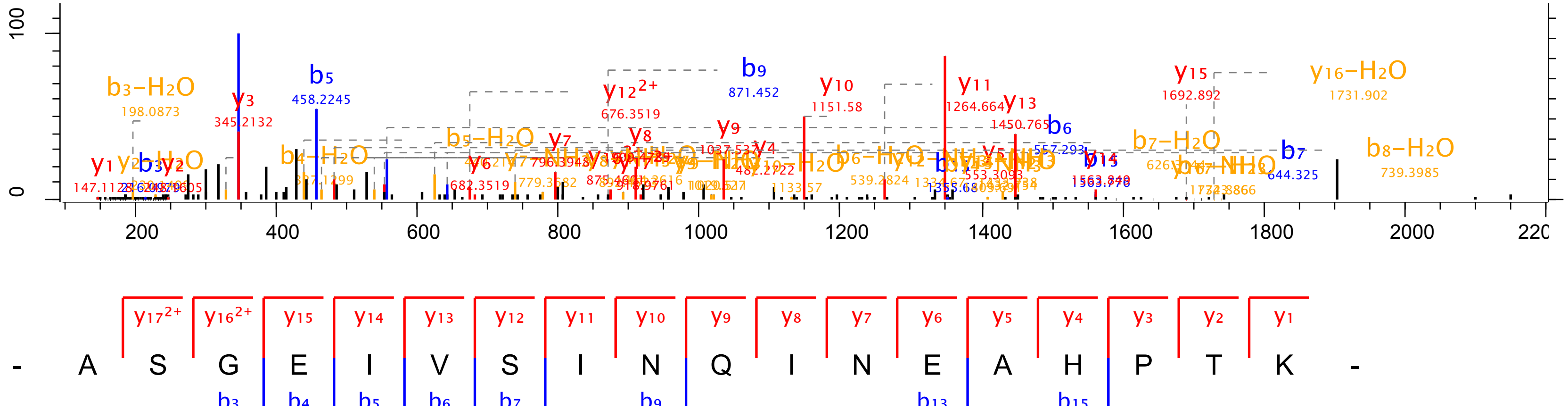

Raw file	Scan	Method	Score	m/z	Gene names
20150228_yeast1_Top_opt_B1_01_1614	27449	TOF; CID	92.27	565.65	MRN1

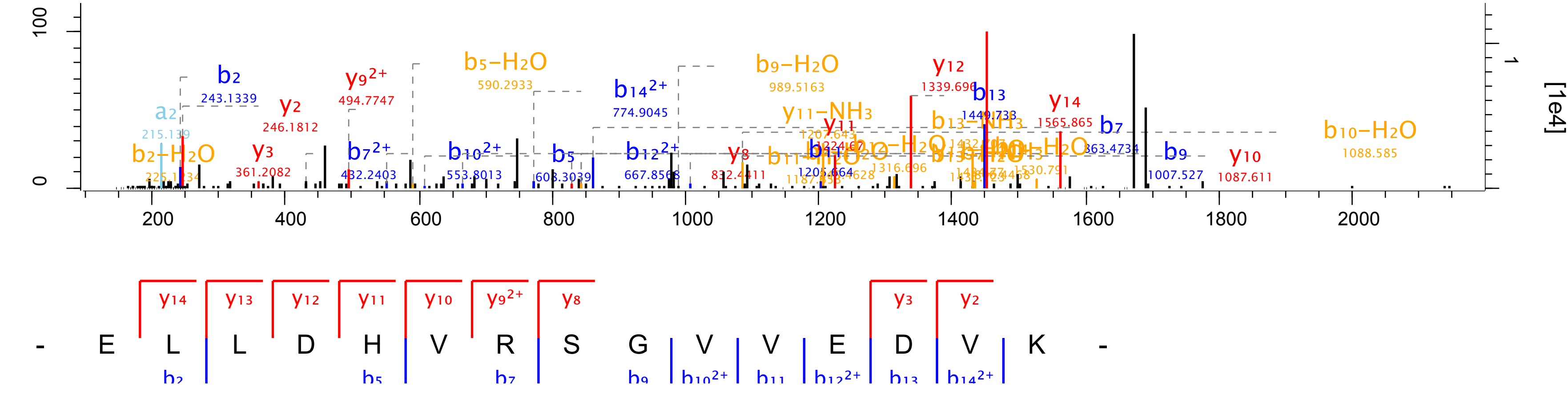

Raw file	Scan	Method	Score	m/z	Gene names
20150228_yeast1_Top_opt_B1_01_1614	27654	TOF; CID	55.68	428.27	PEX22

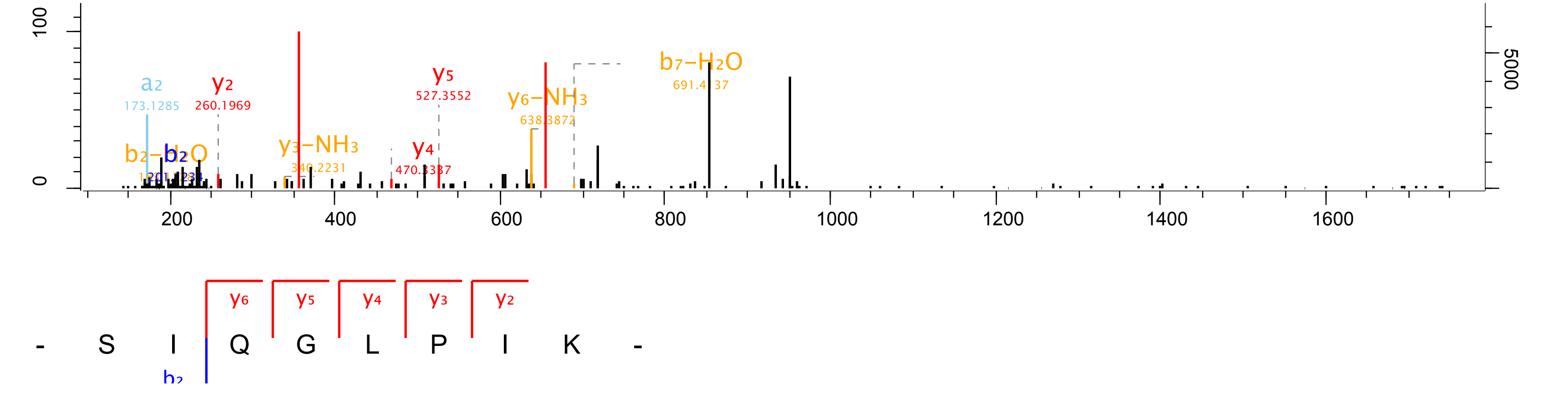

Raw file	Scan	Method	Score	m/z	Gene names
20150228_yeast1_Top_opt_B1_01_1614	27751	TOF; CID	86.26	689.36	ERG2

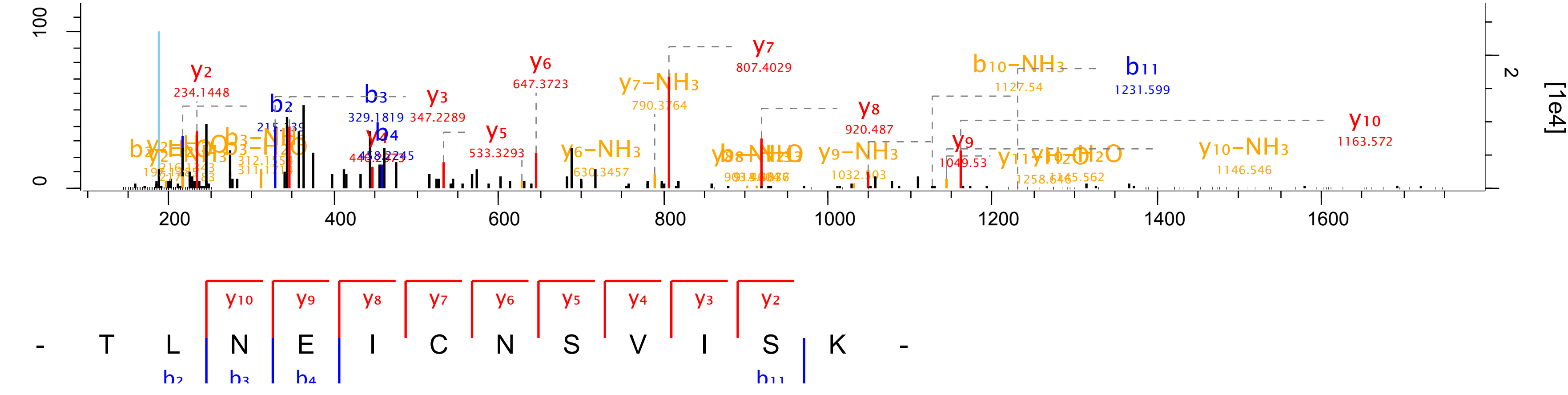

Raw file	Scan	Method	Score	m/z	Gene names
20150228_yeast1_Top_opt_B1_01_1614	27837	TOF; CID	69.35	726.39	BCS1

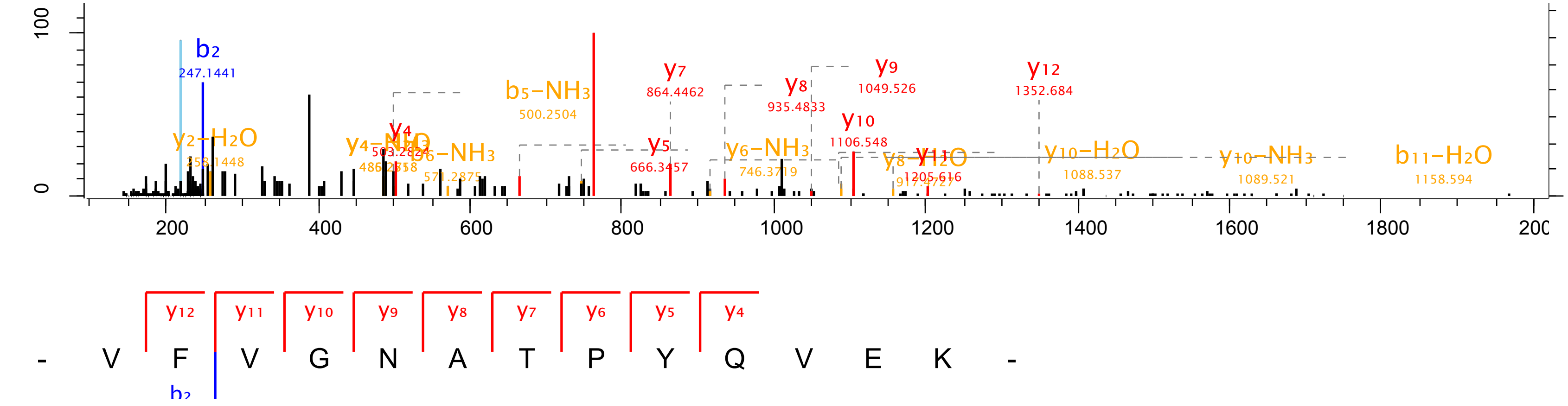

Raw file	Scan	Method	Score	m/z	Gene names
20150228_yeast1_Top_opt_B1_01_1614	27895	TOF; CID	72.14	419.24	YKL071W

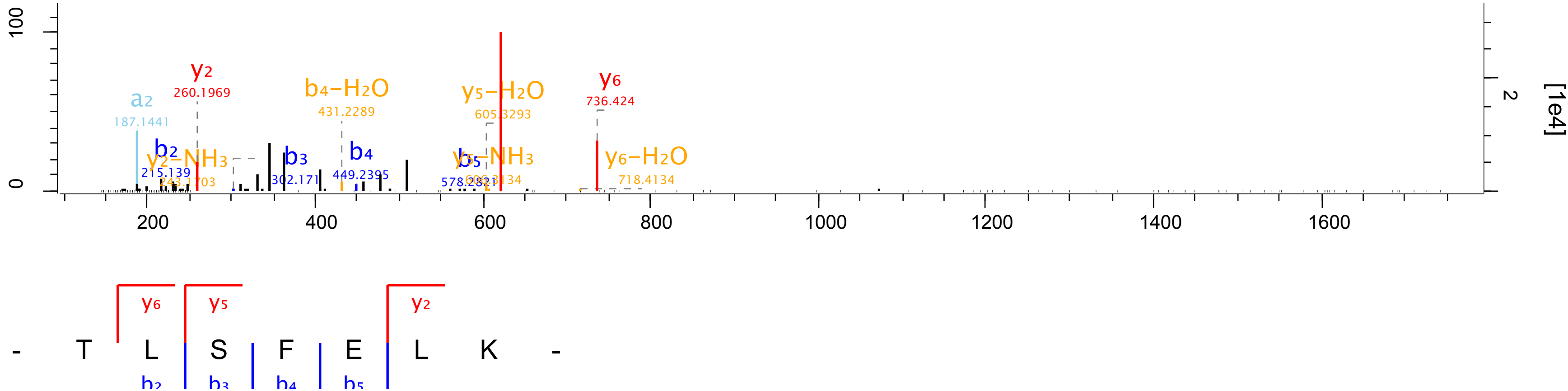

Raw file	Scan	Method	Score	m/z	Gene names
20150228_yeast1_Top_opt_B1_01_1614	28414	TOF; CID	111.12	866.4	MID2

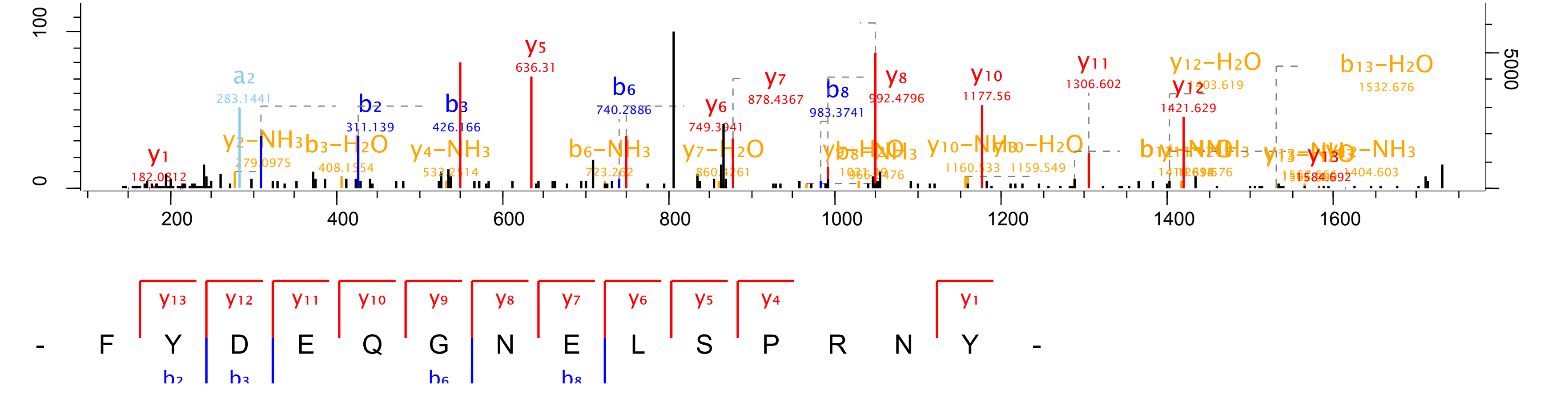

Raw file	Scan	Method	Score	m/z	Gene names
20150228_yeast1_Top_opt_B1_01_1614	28985	TOF; CID	44.02	583.32	YML020W

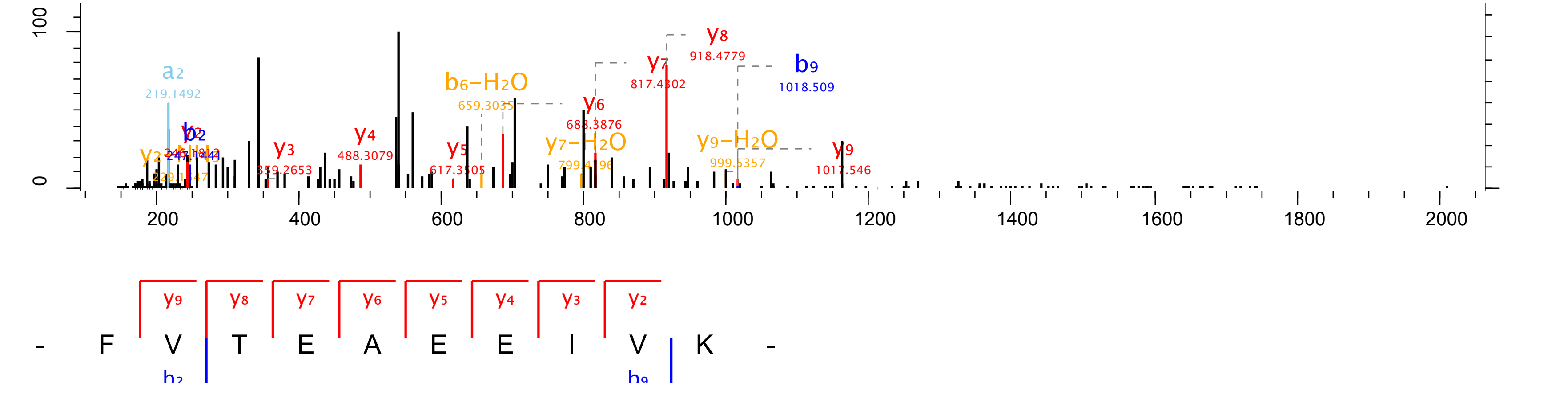

Raw file	Scan	Method	Score	m/z	Gene names
20150228_yeast1_Top_opt_B1_01_1614	29167	TOF; CID	80.44	514.26	RPC25

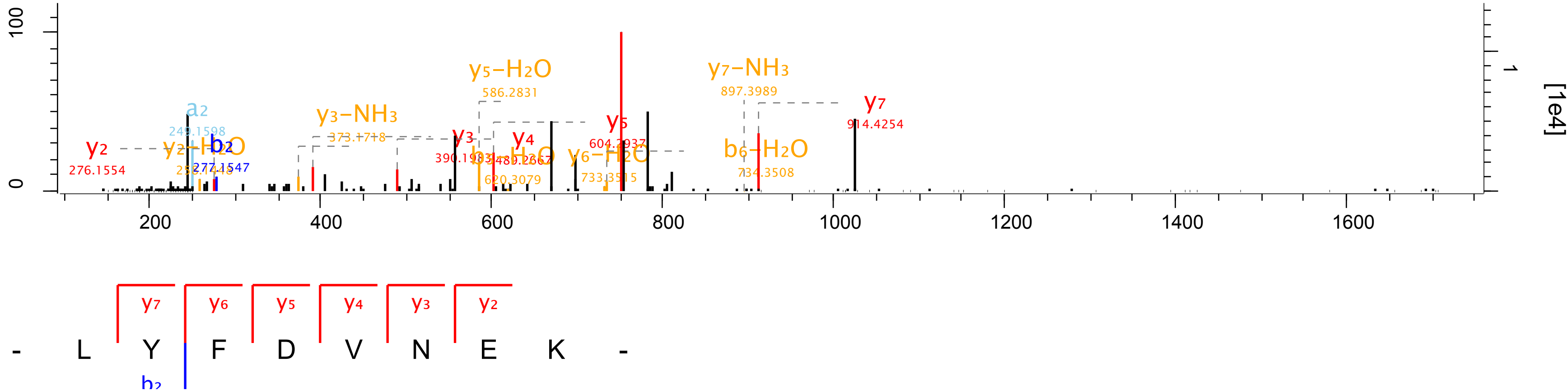

Raw file	Scan	Method	Score	m/z	Gene names
20150228_yeast1_Top_opt_B1_01_1614	29763	TOF; CID	69.26	781.41	PRP38

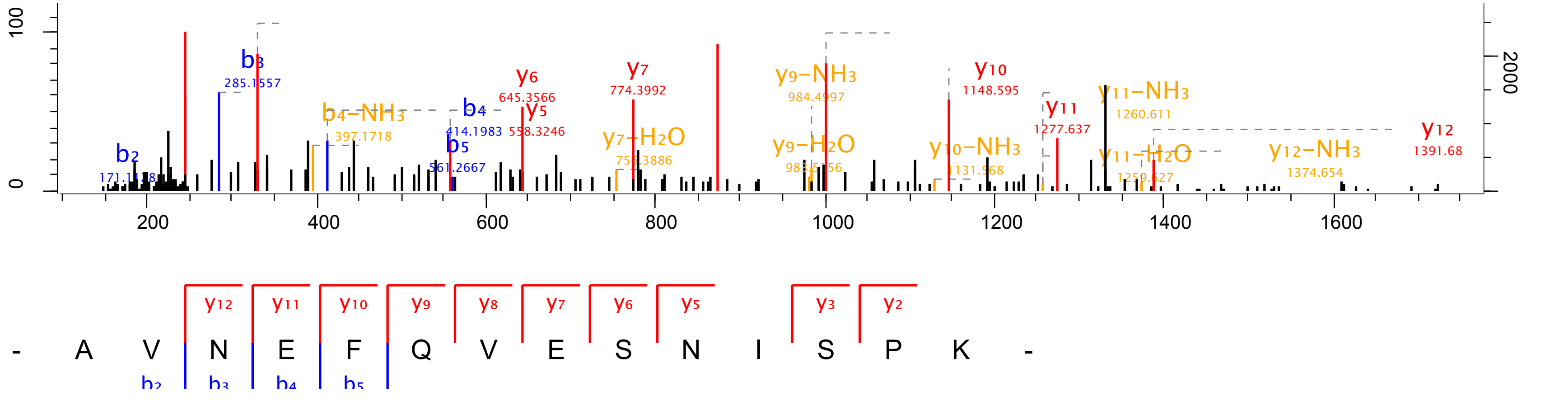

Raw file	Scan	Method	Score	m/z	Gene names
20150228_yeast1_Top_opt_B1_01_1614	29812	TOF; CID	60.73	629.29	ATG18

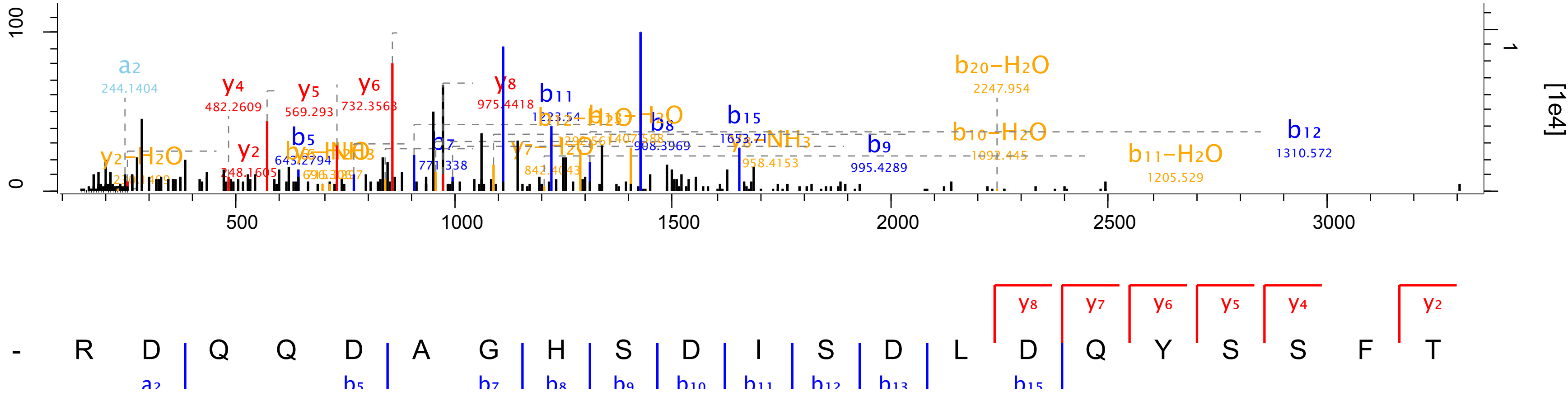

Raw file	Scan	Method	Score	m/z	Gene names
20150228_yeast1_Top_opt_B1_01_1614	29815	TOF; CID	77.32	602.99	SBE2

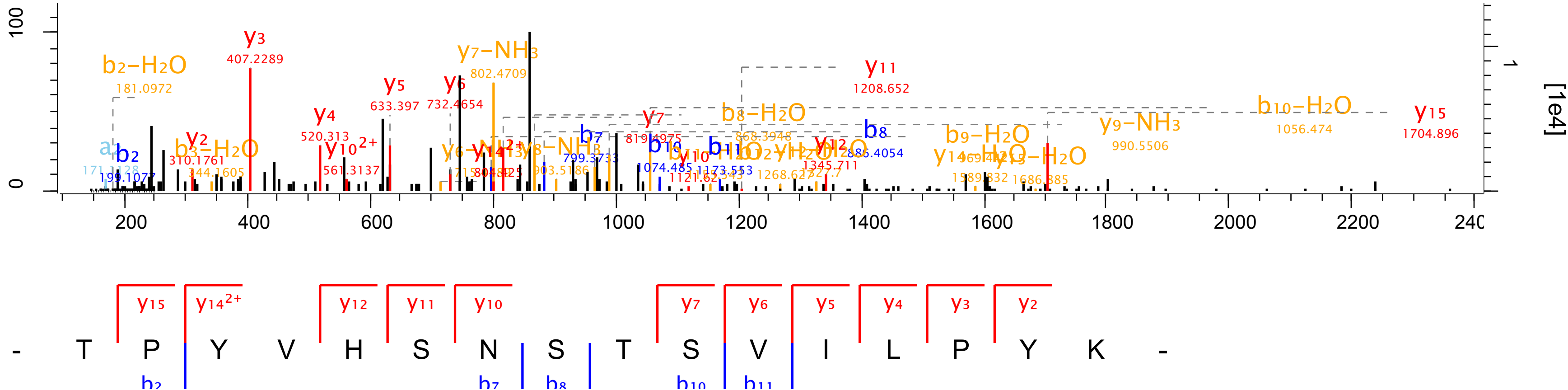

Raw file	Scan	Method	Score	m/z	Gene names
20150228_yeast1_Top_opt_B1_01_1614	29816	TOF; CID	78.15	512.26	DBR1

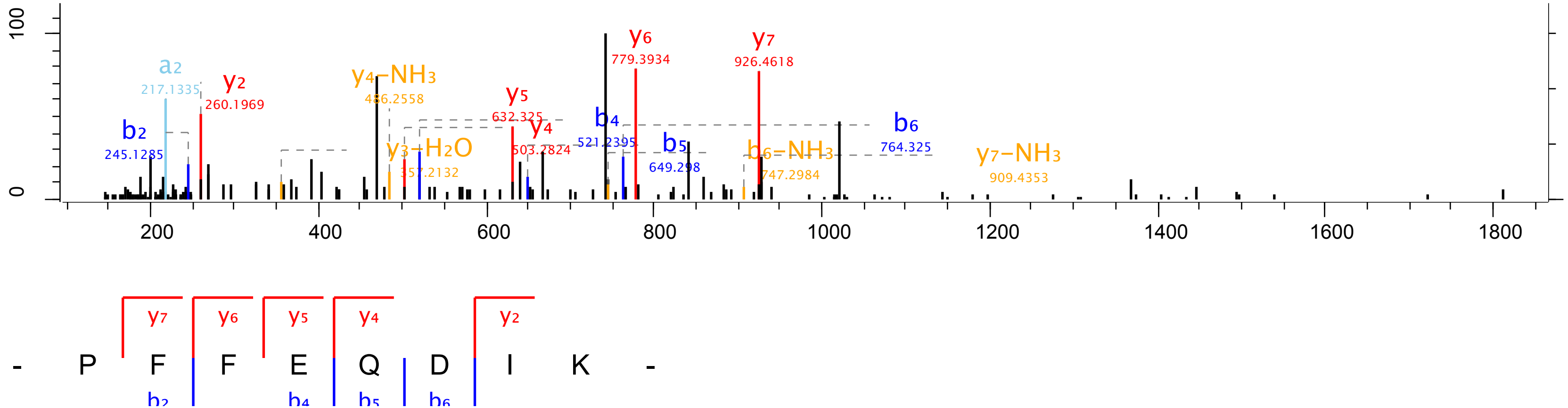

Raw file	Scan	Method	Score	m/z	Gene names
20150228_yeast1_Top_opt_B1_01_1614	30697	TOF; CID	41.29	722.4	SLM5

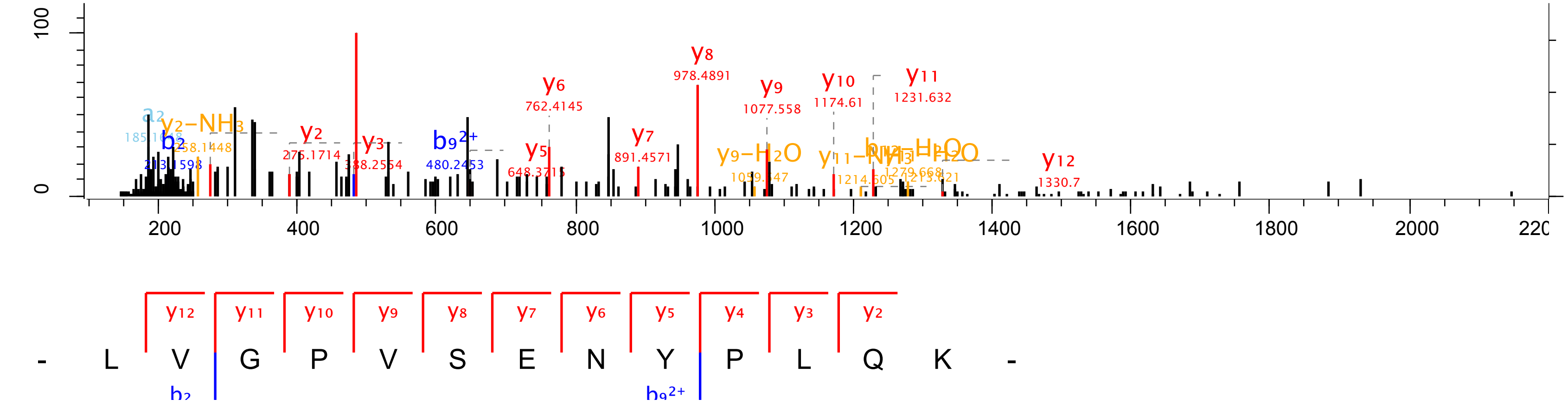

Raw file	Scan	Method	Score	m/z	Gene names
20150228_yeast1_Top_opt_B1_01_1614	30945	TOF; CID	73.5	548.79	MPS2

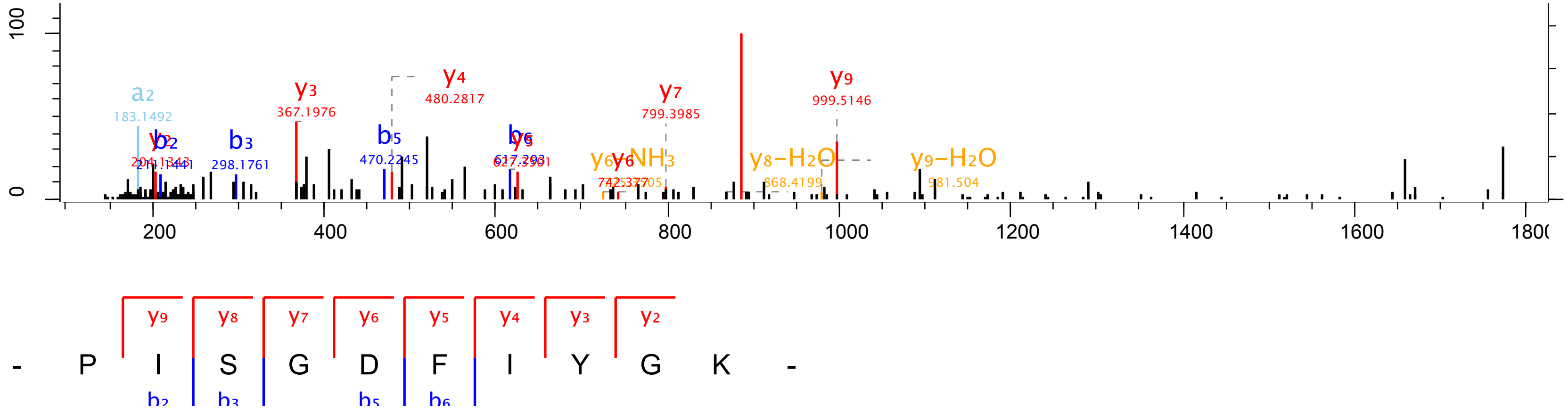

Raw file	Scan	Method	Score	m/z	Gene names
20150228_yeast1_Top_opt_B1_01_1614	31423	TOF; CID	47.99	660.84	UBA4

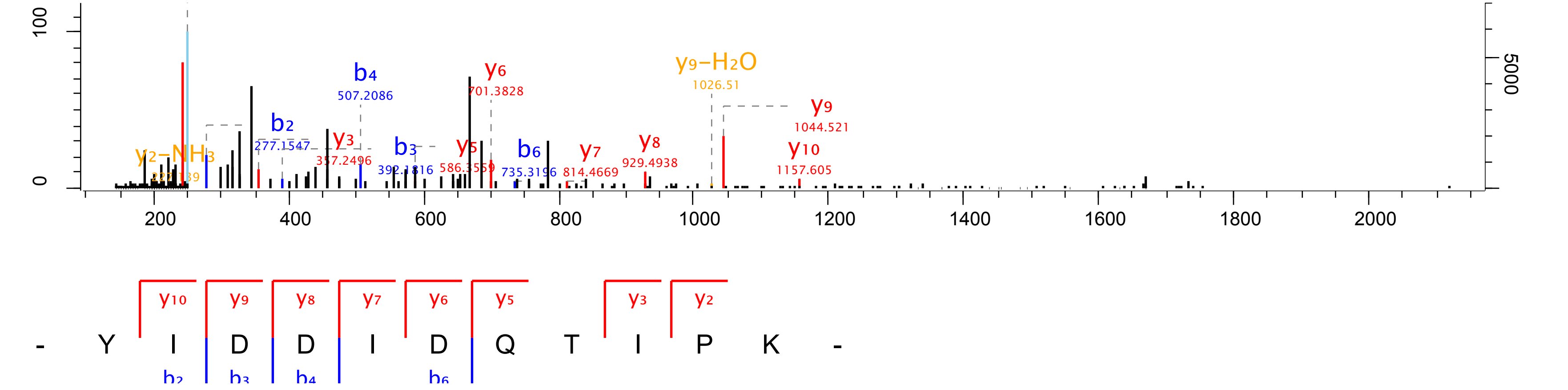

Raw file	Scan	Method	Score	m/z	Gene names
20150228_yeast1_Top_opt_B1_01_1614	31454	TOF; CID	65.22	482.27	DIB1

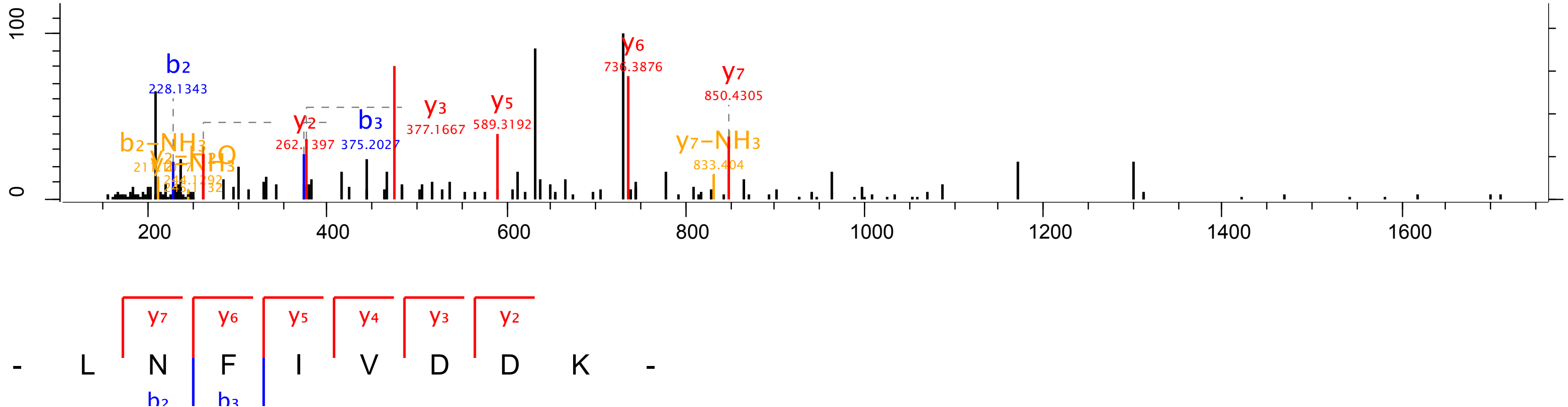

Raw file	Scan	Method	Score	m/z	Gene names
20150228_yeast1_Top_opt_B1_01_1614	31745	TOF; CID	48.57	438.57	YPR084W

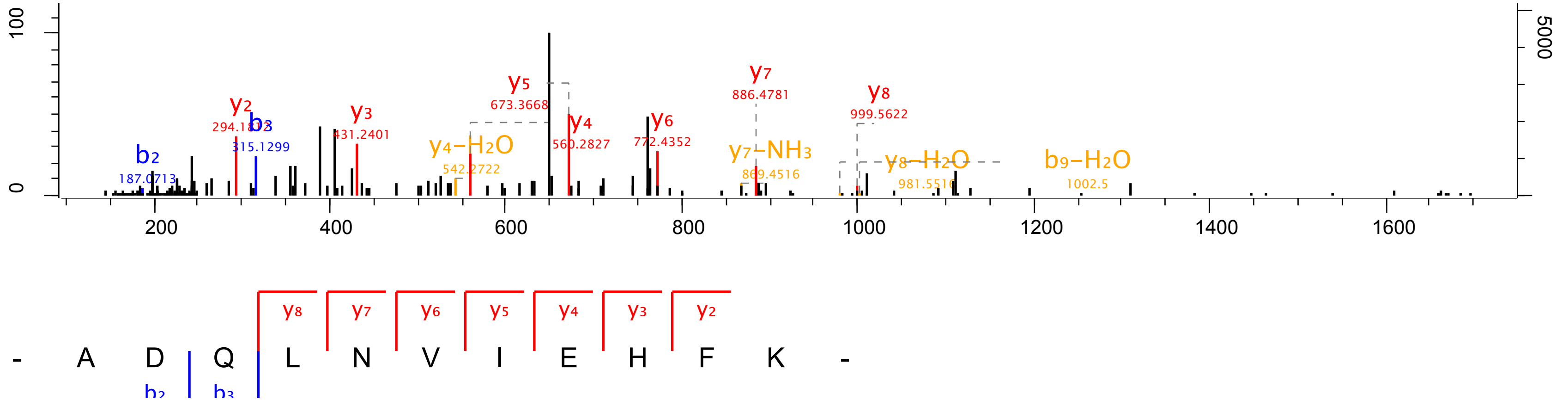

Raw file	Scan	Method	Score	m/z	Gene names
20150228_yeast1_Top_opt_B1_01_1614	32208	TOF; CID	49.3	545.77	TAT2

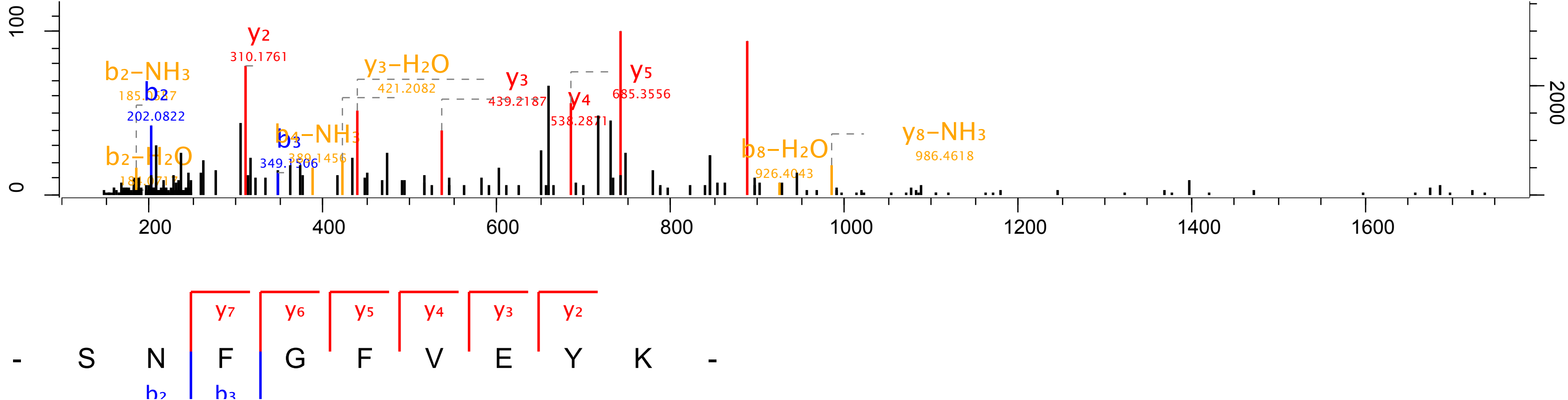

Raw file	Scan	Method	Score	m/z	Gene names
20150228_yeast1_Top_opt_B1_01_1614	32788	TOF; CID	82.28	451.29	STB4

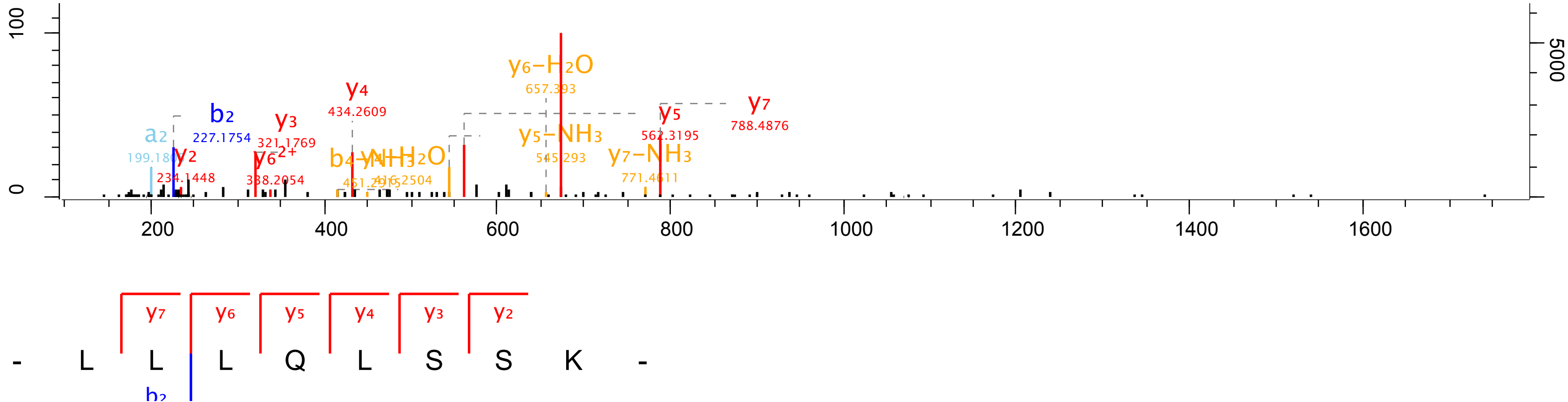

Raw file	Scan	Method	Score	m/z	Gene names
20150228_yeast1_Top_opt_B1_01_1614	33214	TOF; CID	43.4	694.87	YEL020C

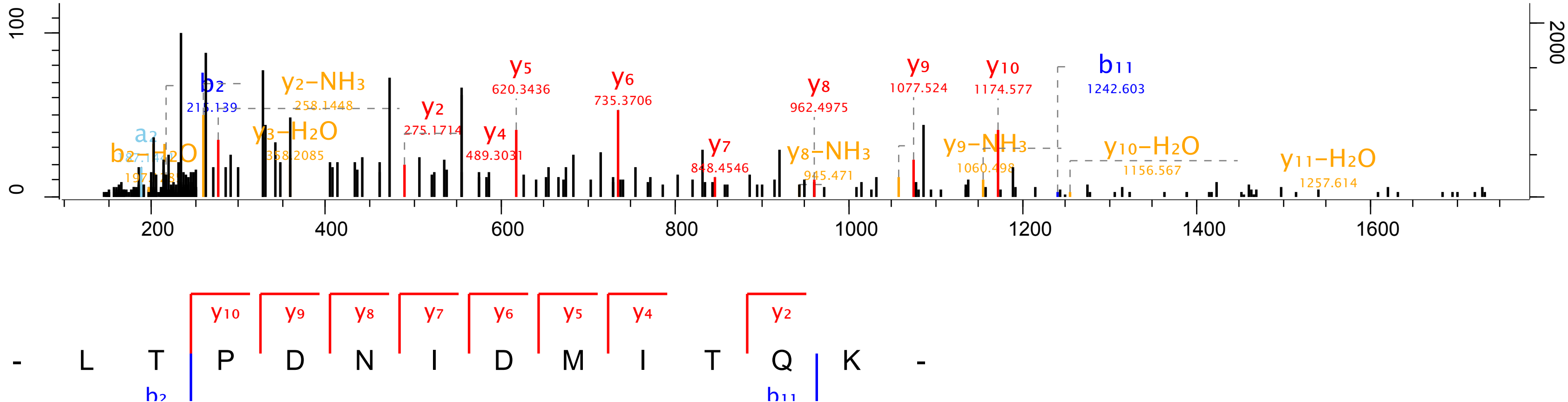

Raw file	Scan	Method	Score	m/z	Gene names
20150228_yeast1_Top_opt_B1_01_1614	33501	TOF; CID	50.09	709.37	HSH49

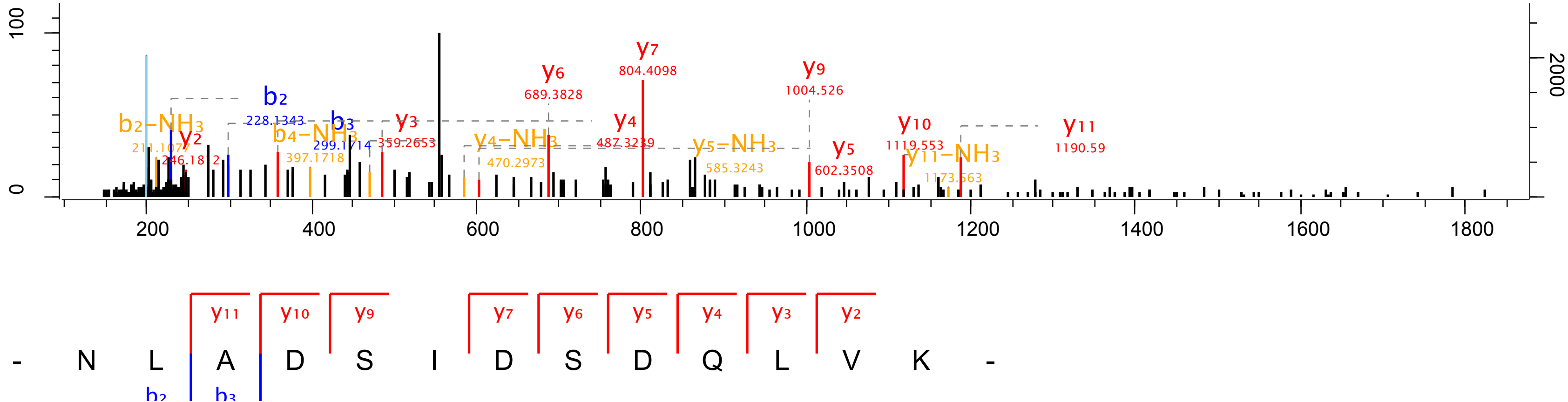

Raw file	Scan	Method	Score	m/z	Gene names
20150228_yeast1_Top_opt_B1_01_1614	34116	TOF; CID	64.12	618.87	PTP1

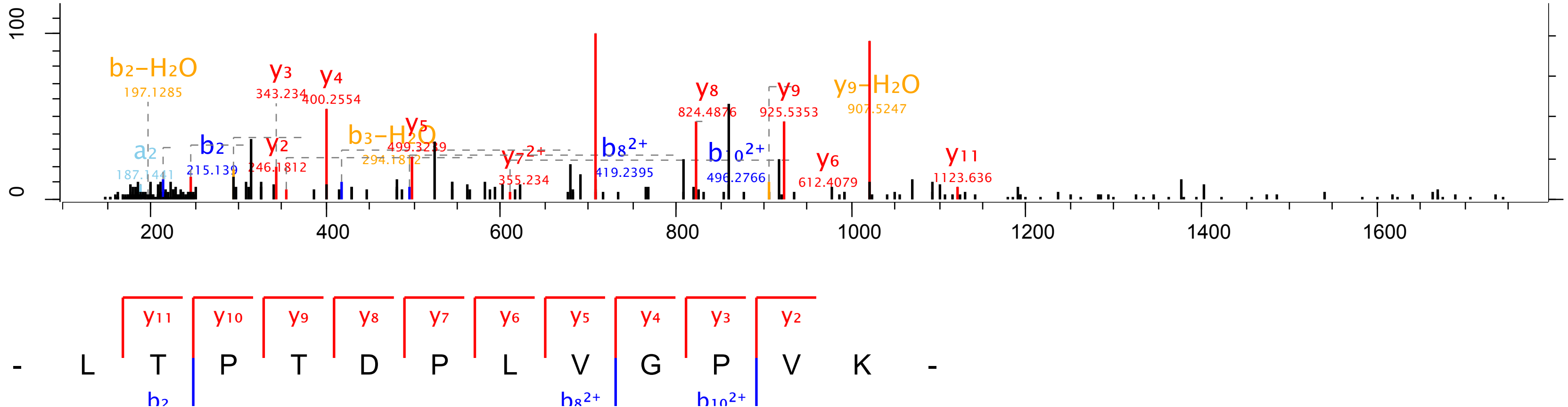

Raw file	Scan	Method	Score	m/z
20150228_yeast1_Top_opt_B1_01_1614	34496	TOF; CID	100.43	497.63

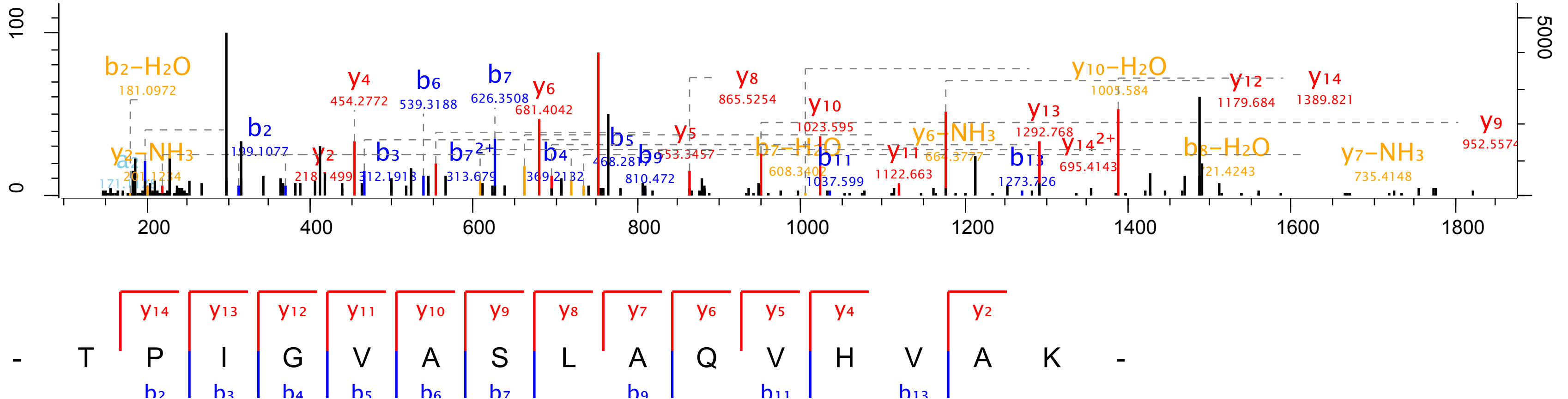

Raw file	Scan	Method	Score	m/z	Gene names
20150228_yeast1_Top_opt_B1_01_1614	34702	TOF; CID	94.12	725.88	OPT1

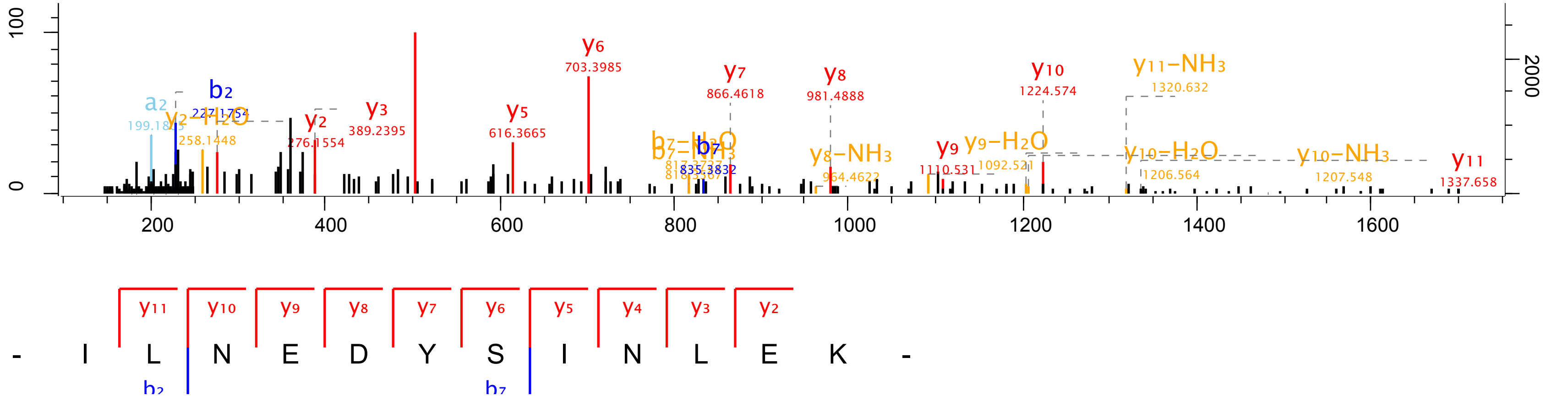

Raw file	Scan	Method	Score	m/z	Gene names
20150228_yeast1_Top_opt_B1_01_1614	34931	TOF; CID	136.24	495.26	IRA1

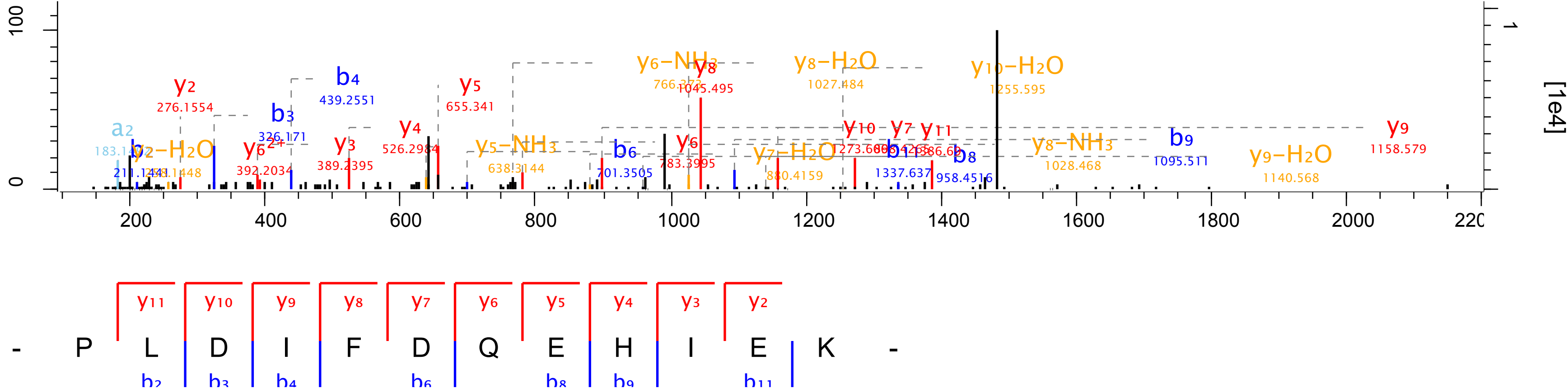

Raw file	Scan	Method	Score	m/z	Gene names
20150228_yeast1_Top_opt_B1_01_1614	35116	TOF; CID	86.47	432.27	BET2

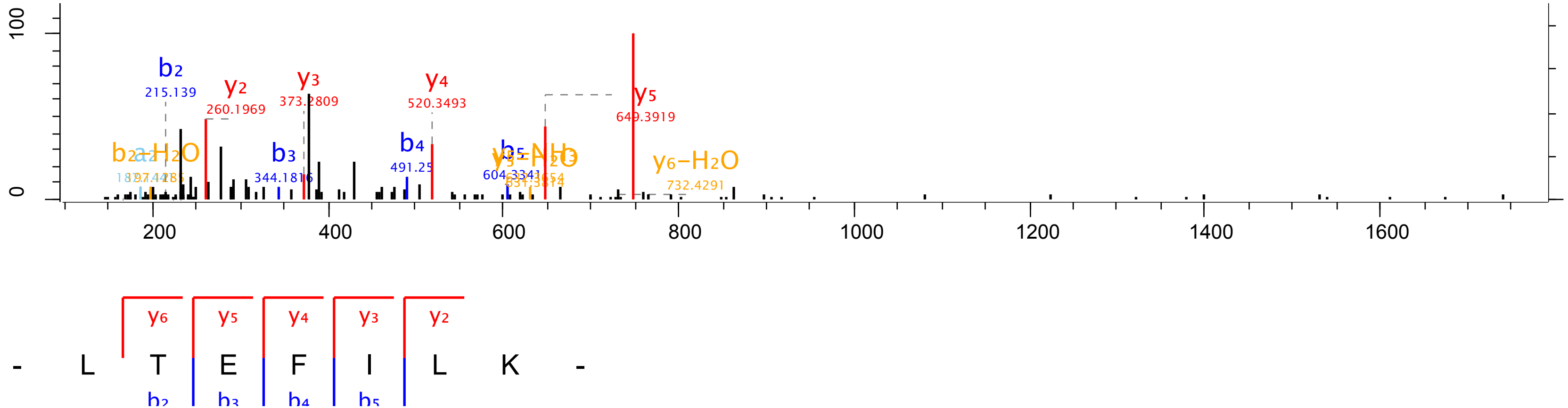

Raw file	Scan	Method	Score	m/z	Gene names
20150228_yeast1_Top_opt_B1_01_1614	35339	TOF; CID	127.83	910.49	BMT2

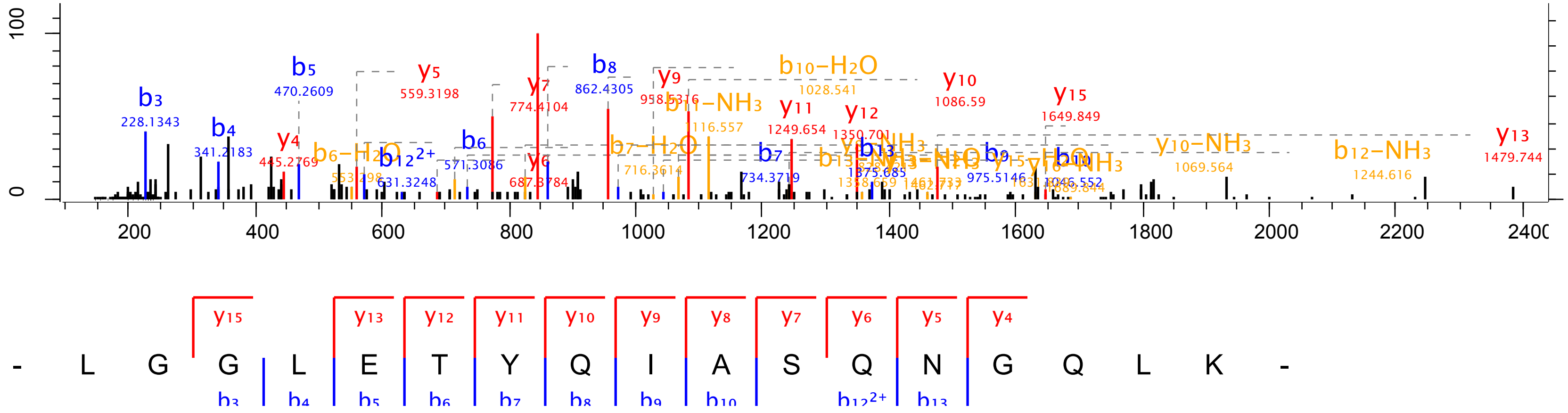

Raw file	Scan	Method	Score	m/z	Gene names
20150228_yeast1_Top_opt_B1_01_1614	35416	TOF; CID	56.94	445.6	DOC1

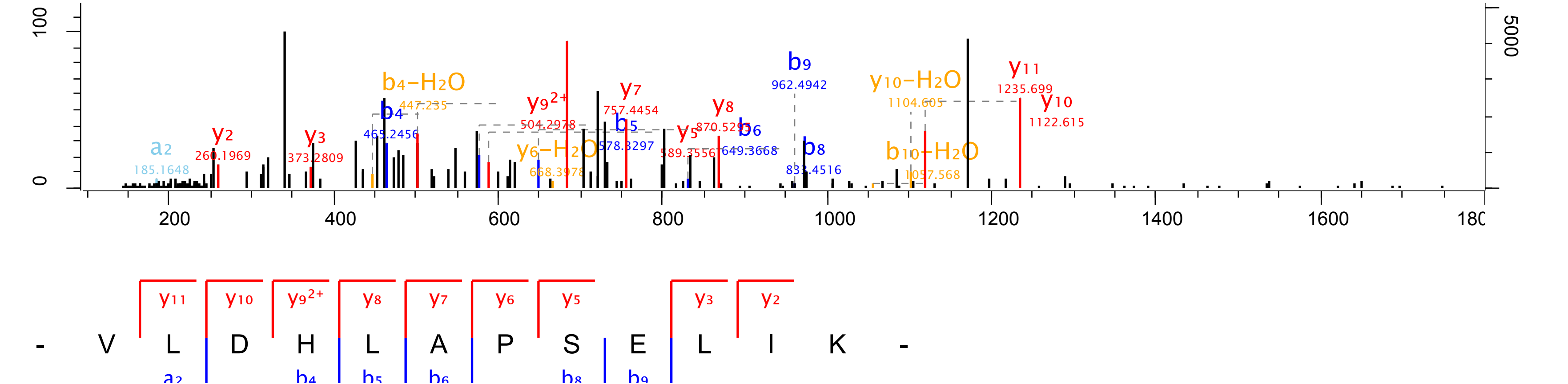

Raw file	Scan	Method	Score	m/z	Gene names
20150228_yeast1_Top_opt_B1_01_1614	35626	TOF; CID	55.37	460.78	SIC1

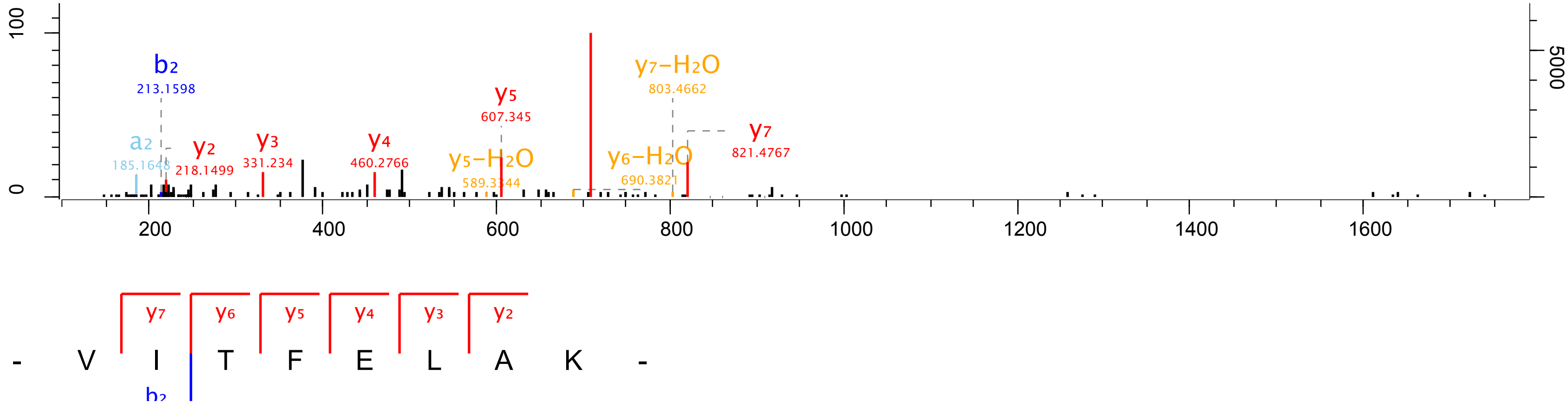

Raw file	Scan	Method	Score	m/z	Gene names
20150228_yeast1_Top_opt_B1_01_1614	36334	TOF; CID	50.09	506.25	SAF1

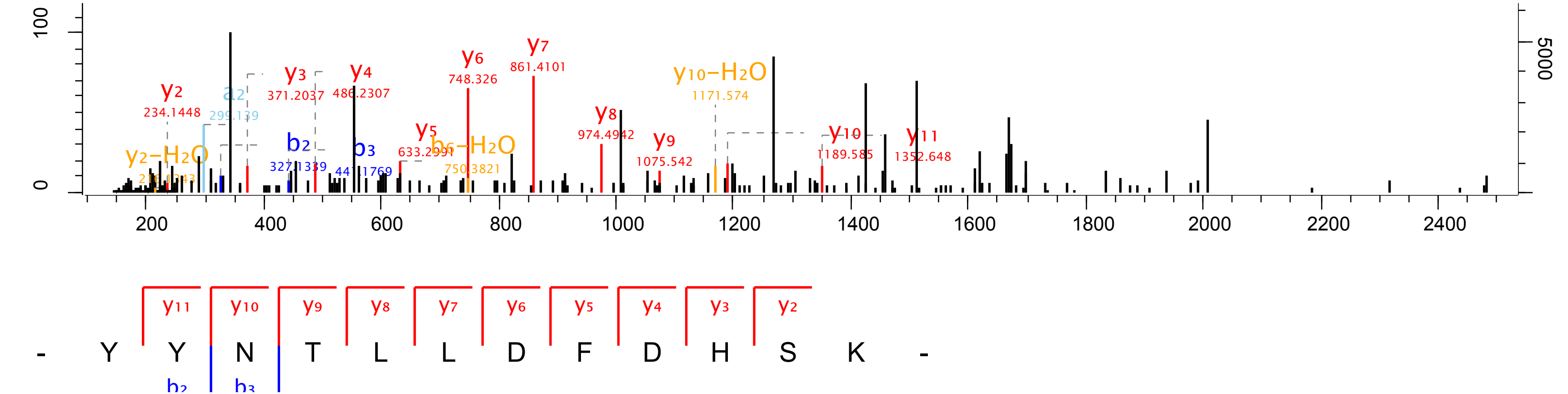

Raw file	Scan	Method	Score	m/z	Gene names
20150228_yeast1_Top_opt_B1_01_1614	36420	TOF; CID	120.11	985.49	TIF6

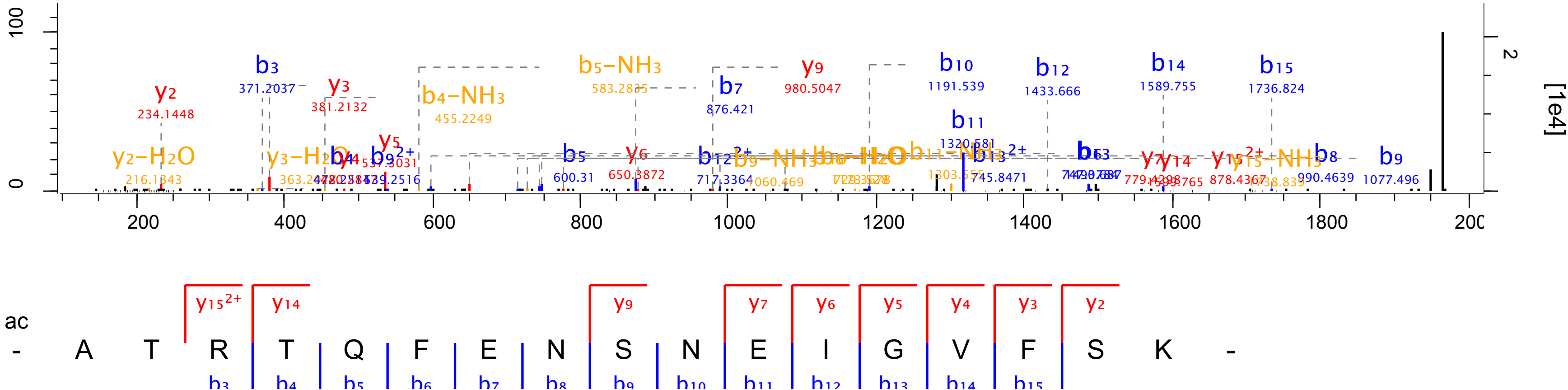

Raw file	Scan	Method	Score	m/z	Gene names
20150228_yeast1_Top_opt_B1_01_1614	37058	TOF; CID	124.83	466.29	NTF2

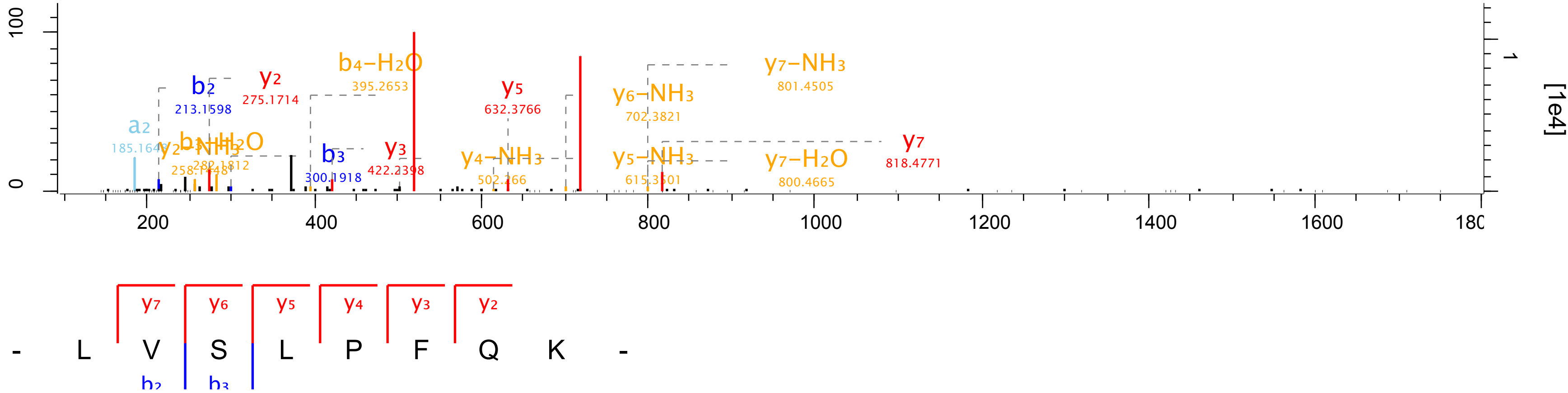

Raw file	Scan	Method	Score	m/z	Gene names
20150228_yeast1_Top_opt_B1_01_1614	37073	TOF; CID	61.96	443.3	YGR127W

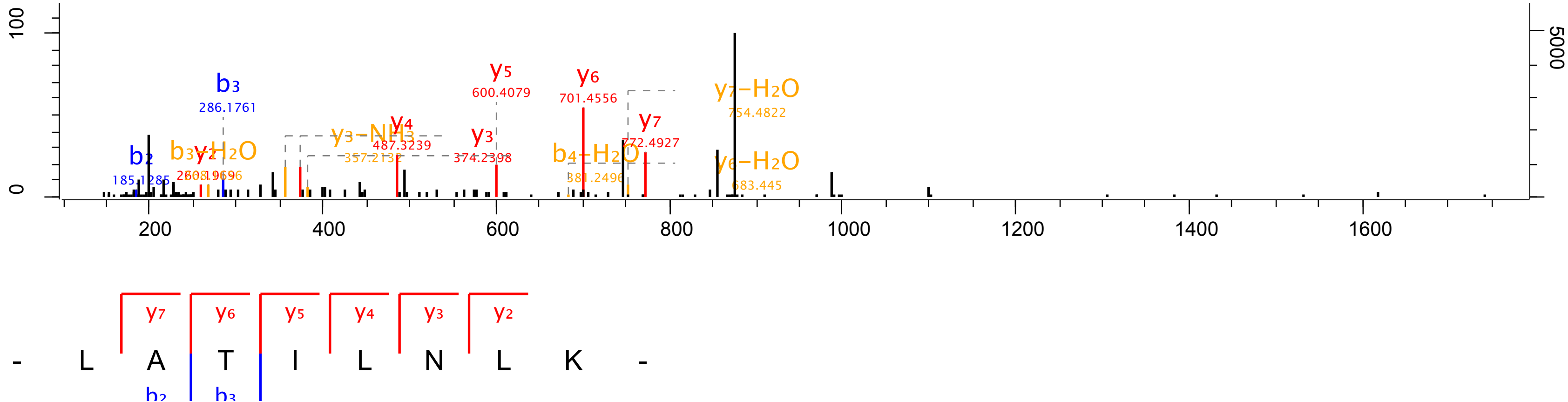

Raw file	Scan	Method	Score	m/z
20150228_yeast1_Top_opt_B1_01_1614	37777	TOF; CID	91.96	666.33

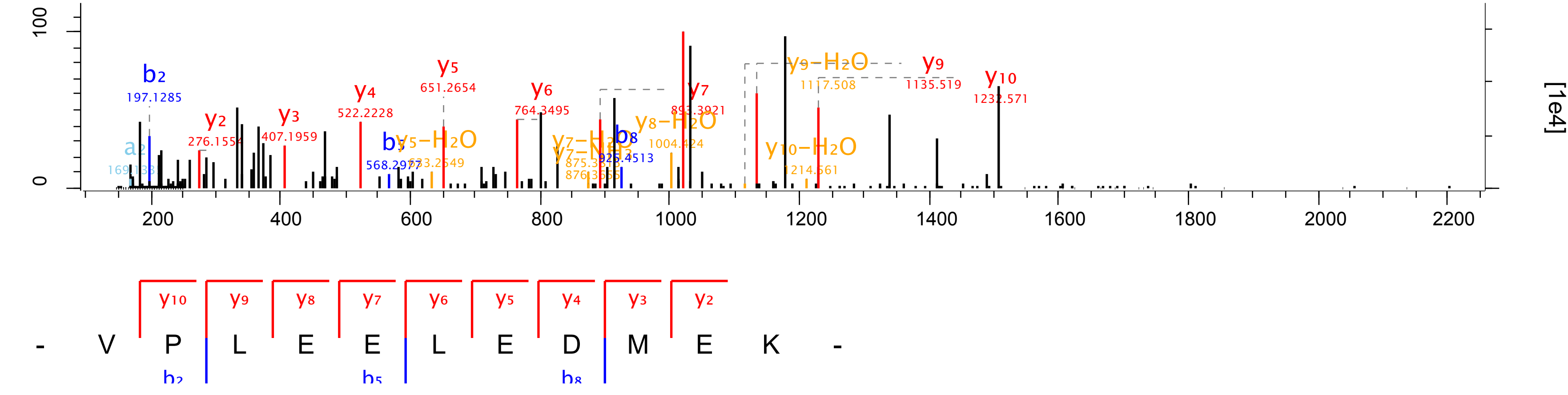

Raw file	Scan	Method	Score	m/z	Gene names
20150228_yeast1_Top_opt_B1_01_1614	37879	TOF; CID	109.15	514.94	MRPL16

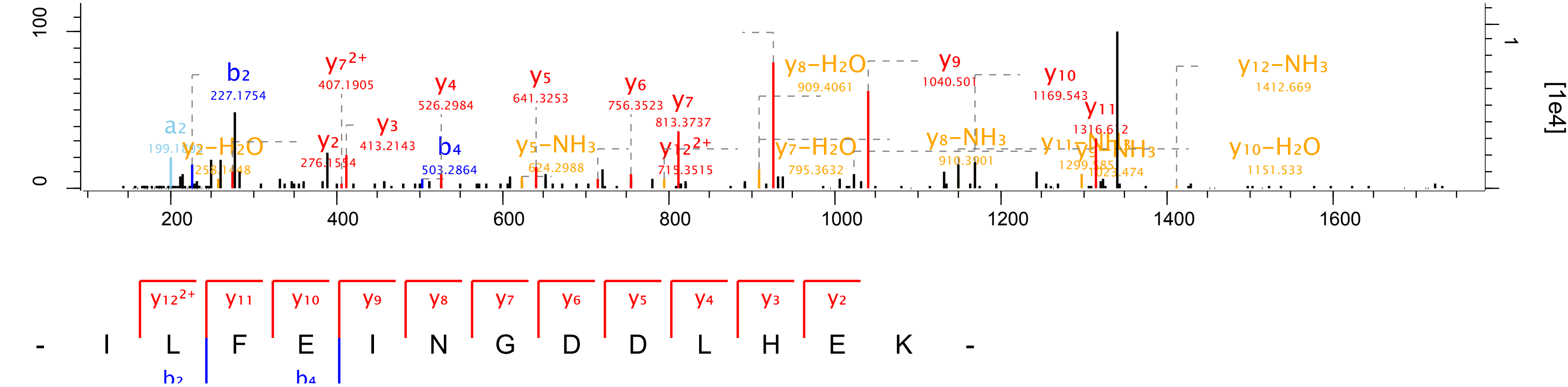

Raw file	Scan	Method	Score	m/z	Gene names
20150228_yeast1_Top_opt_B1_01_1614	37979	TOF; CID	66	648.33	TFC4

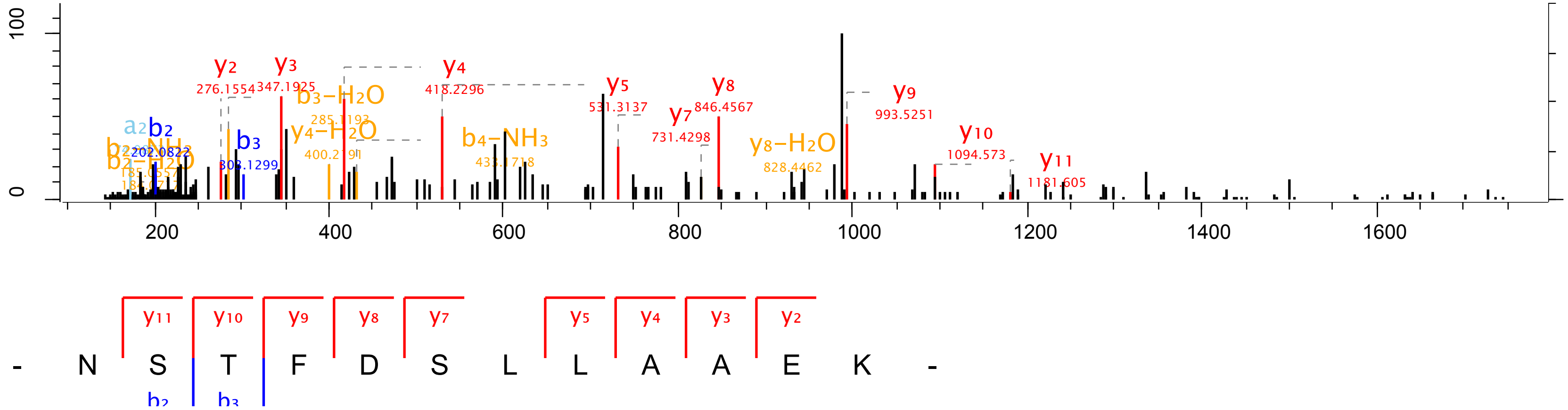

Raw file	Scan	Method	Score	m/z	Gene names
20150228_yeast1_Top_opt_B1_01_1614	38199	TOF; CID	68	675.83	IBD2

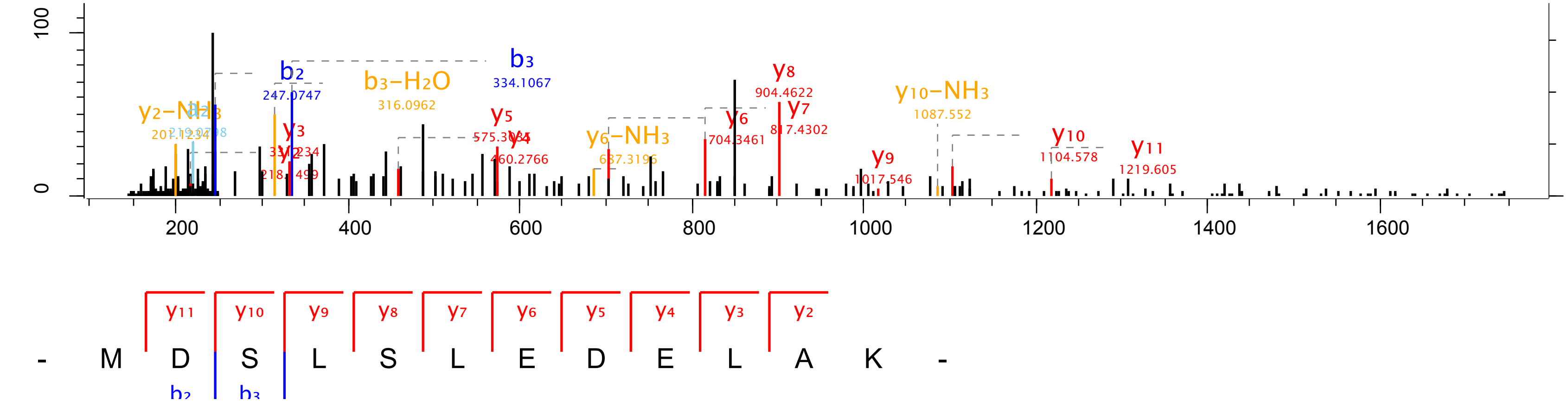

Raw file	Scan	Method	Score	m/z	Gene names
20150228_yeast1_Top_opt_B1_01_1614	38578	TOF; CID	69.82	609.83	YHP1

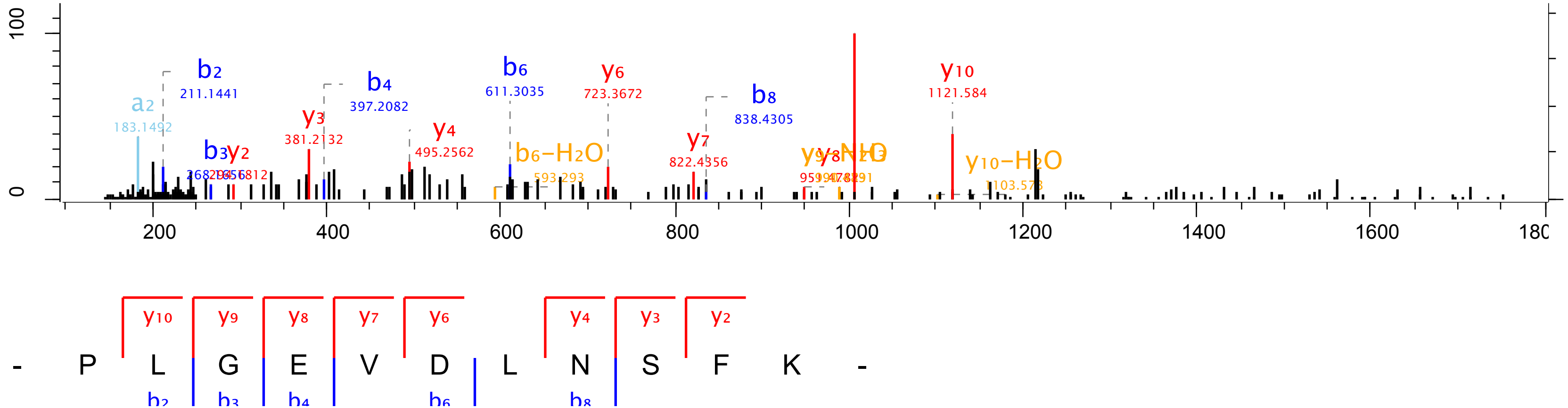

Raw file	Scan	Method	Score	m/z	Gene names
20150228_yeast1_Top_opt_B1_01_1614	39516	TOF; CID	38.3	522.29	SRB6

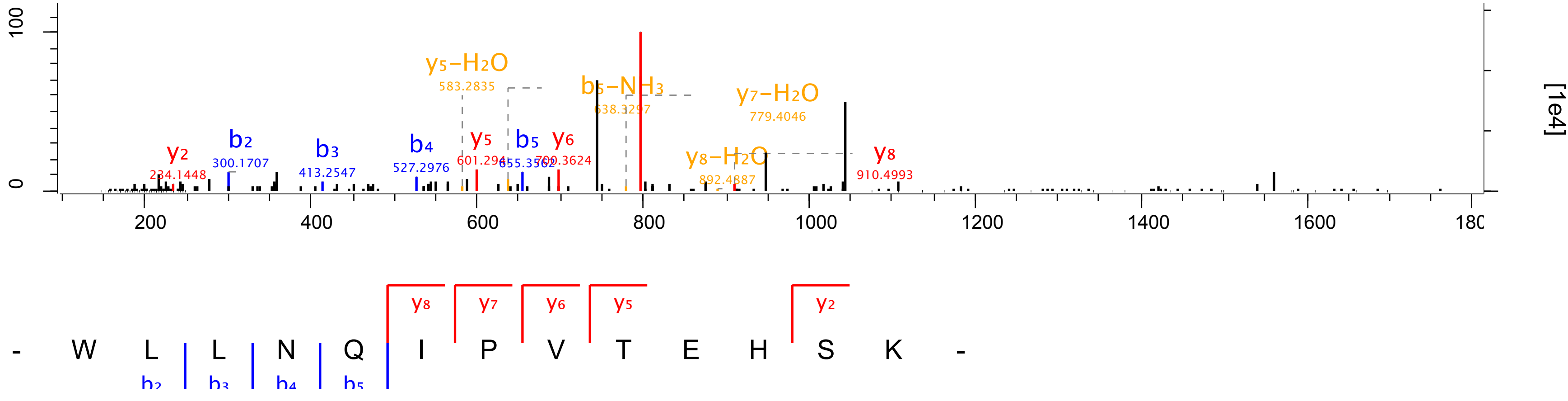

Raw file	Scan	Method	Score	m/z	Gene names
20150228_yeast1_Top_opt_B1_01_1614	39609	TOF; CID	134.91	933.14	ESC8

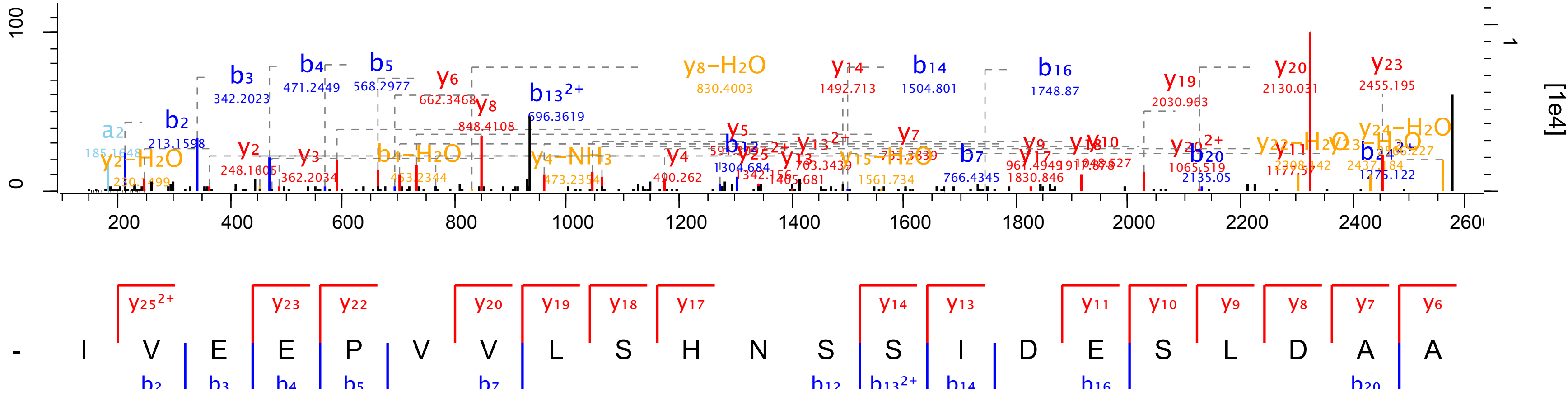

Raw file	Scan	Method	Score	m/z	Gene names
20150228_yeast1_Top_opt_B1_01_1614	40364	TOF; CID	65.37	796.94	SEF1

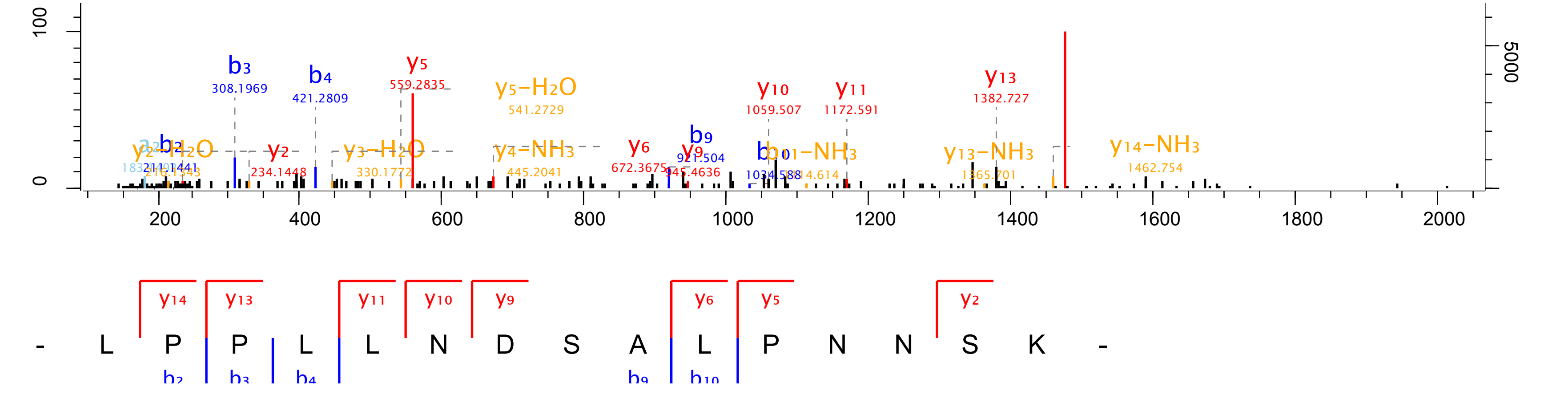

Raw file	Scan	Method	Score	m/z	Gene names
20150228_yeast1_Top_opt_B1_01_1614	40428	TOF; CID	105.65	767.91	LOT5

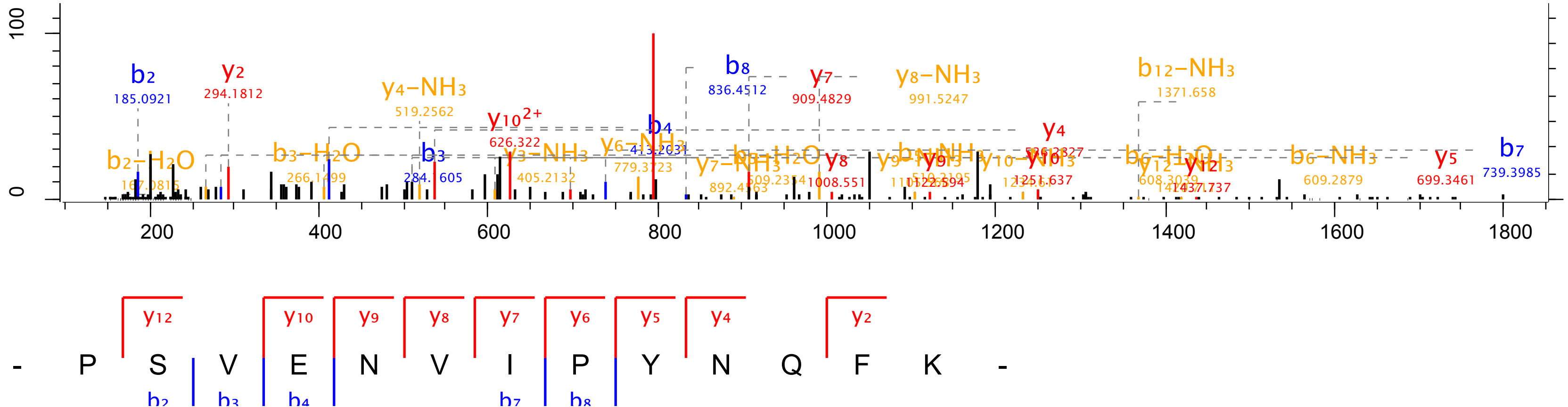

Raw file	Scan	Method	Score	m/z	Gene names
20150228_yeast1_Top_opt_B1_01_1614	40657	TOF; CID	90.48	851.44	RFX1

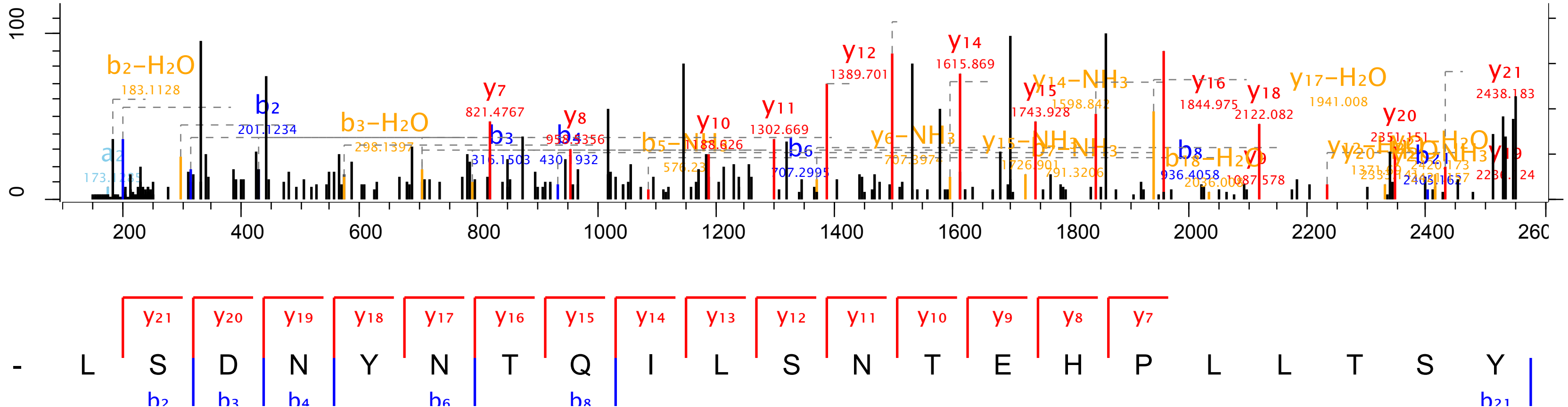

Raw file	Scan	Method	Score	m/z	Gene names
20150228_yeast1_Top_opt_B1_01_1614	41749	TOF; CID	97.8	582.62	RSM19

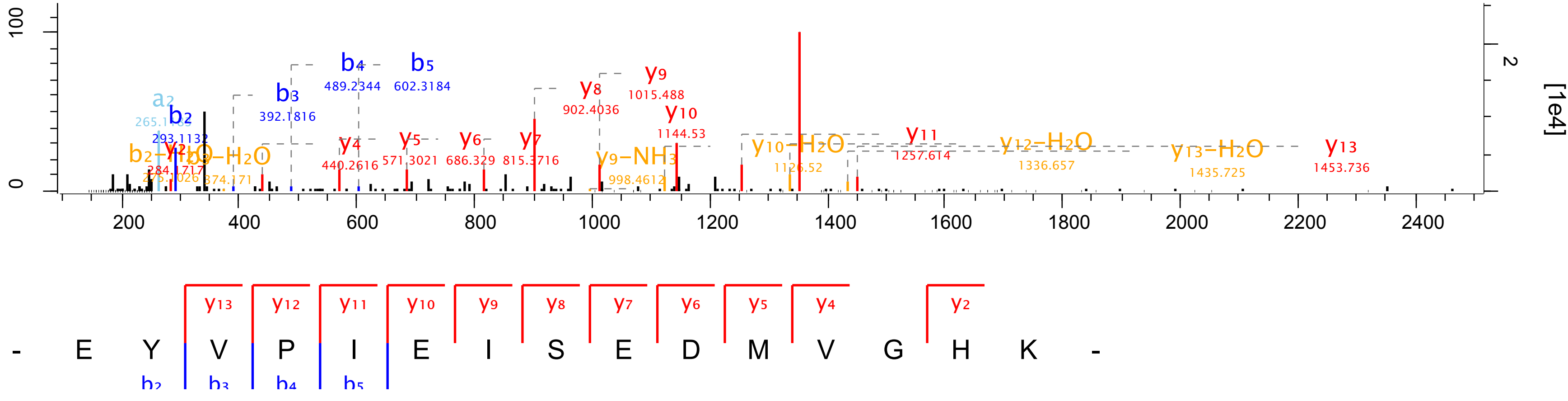

Raw file	Scan	Method	Score	m/z	Gene names
20150228_yeast1_Top_opt_B1_01_1614	41825	TOF; CID	70.03	553.81	ALK1

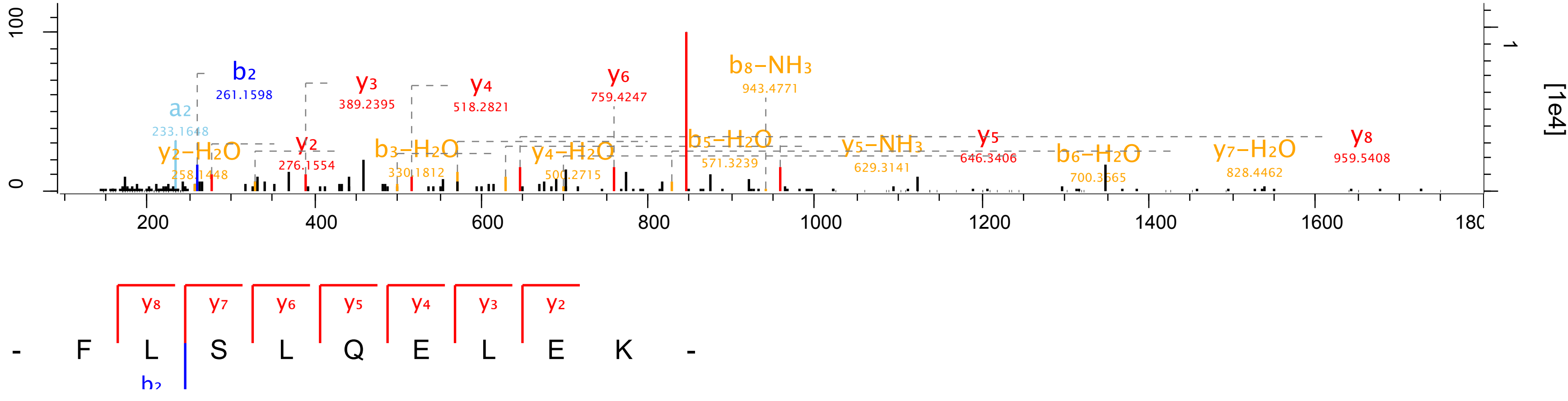

Raw file	Scan	Method	Score	m/z	Gene names
20150228_yeast1_Top_opt_B1_01_1614	41855	TOF; CID	41.54	559.96	BNA7

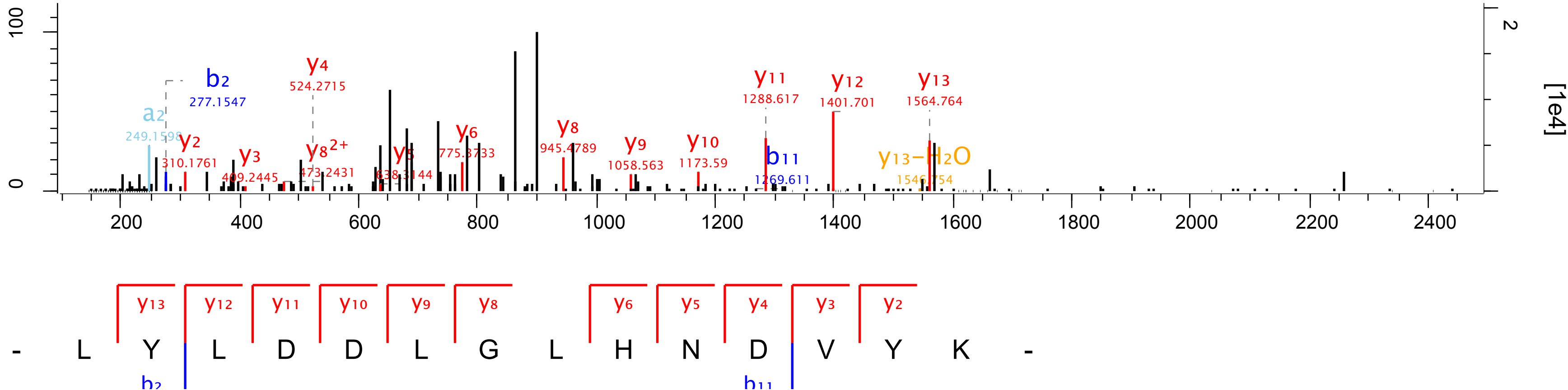

Raw file	Scan	Method	Score	m/z	Gene names
20150228_yeast1_Top_opt_B1_01_1614	42111	TOF; CID	74.99	694.38	YHR122W

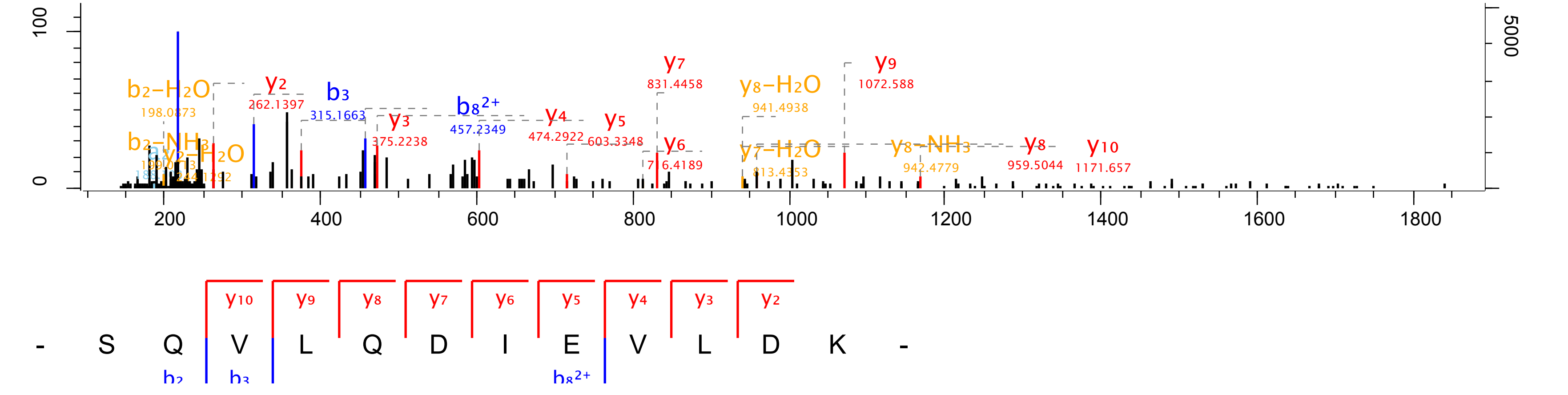

Raw file	Scan	Method	Score	m/z	Gene names
20150228_yeast1_Top_opt_B1_01_1614	43246	TOF; CID	54.76	903.44	UFO1

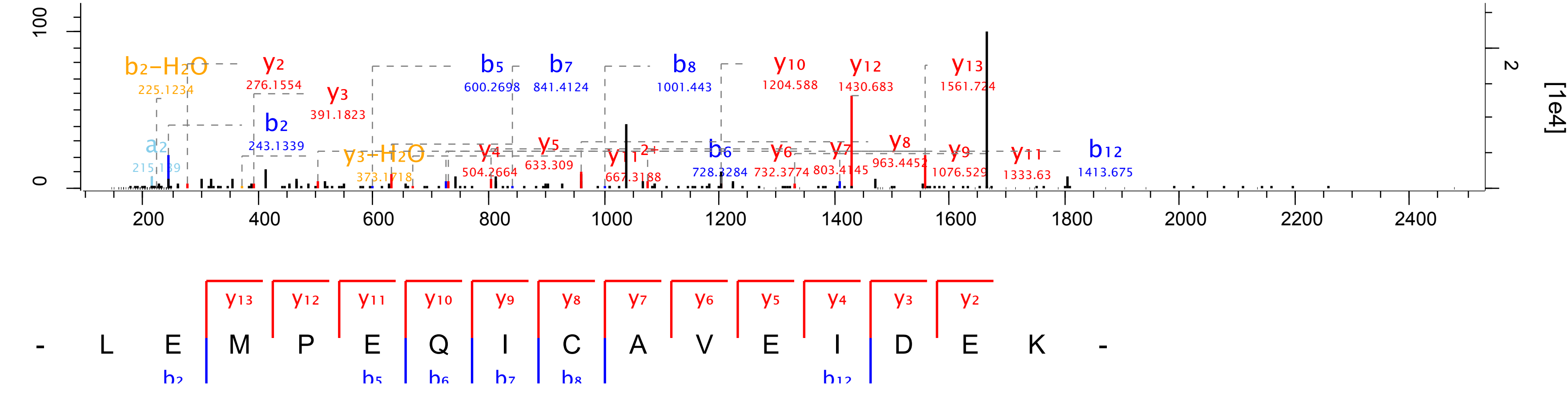

Raw file	Scan	Method	Score	m/z	Gene names
20150228_yeast1_Top_opt_B1_01_1614	43277	TOF; CID	86.9	515.32	SKI6

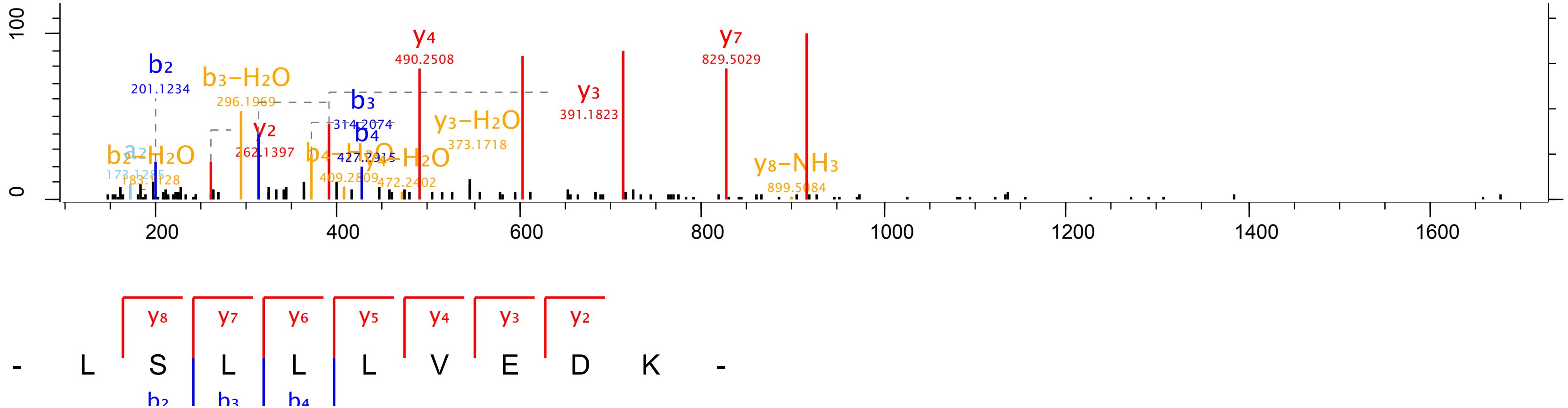

Raw file	Scan	Method	Score	m/z	Gene names
20150228_yeast1_Top_opt_B1_01_1614	43511	TOF; CID	74.38	550.3	MPA43

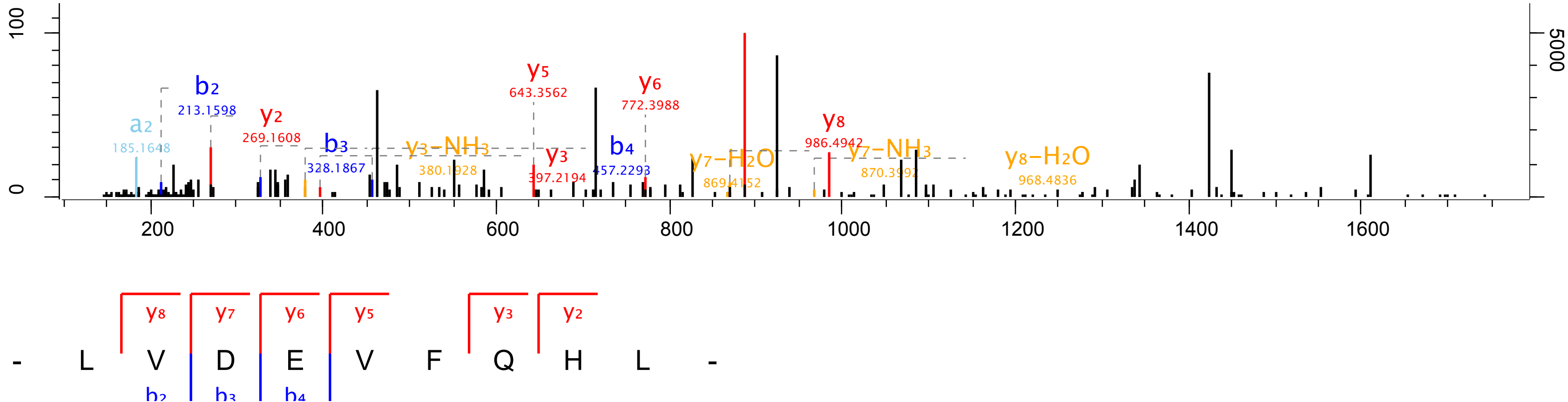

Raw file	Scan	Method	Score	m/z	Gene names
20150228_yeast1_Top_opt_B1_01_1614	43808	TOF; CID	59.17	772.89	FYV4

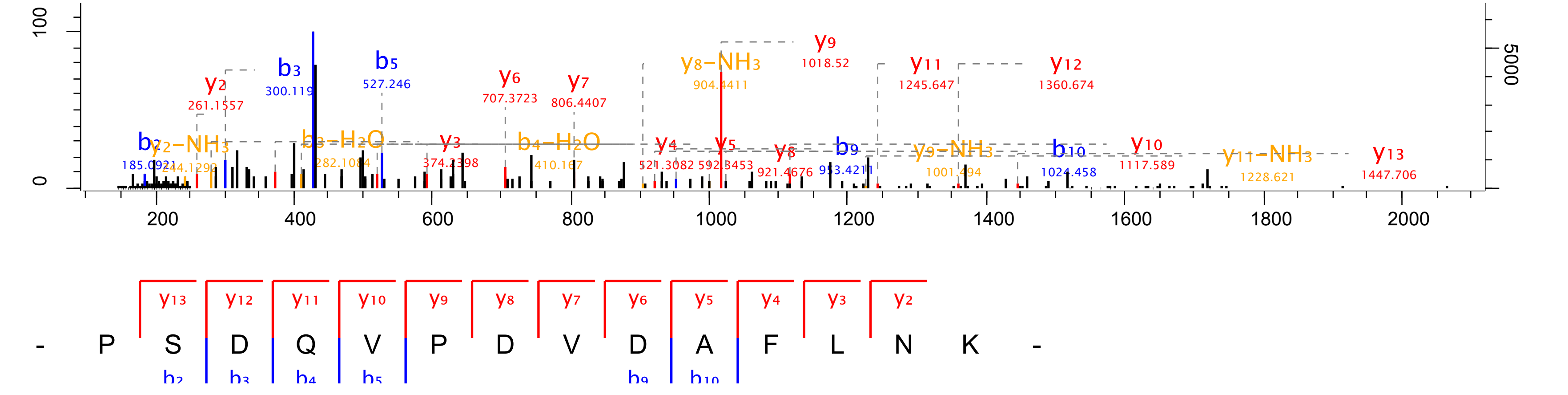

Raw file	Scan	Method	Score	m/z	Gene names
20150228_yeast1_Top_opt_B1_01_1614	44195	TOF; CID	109.44	568.29	LSM1

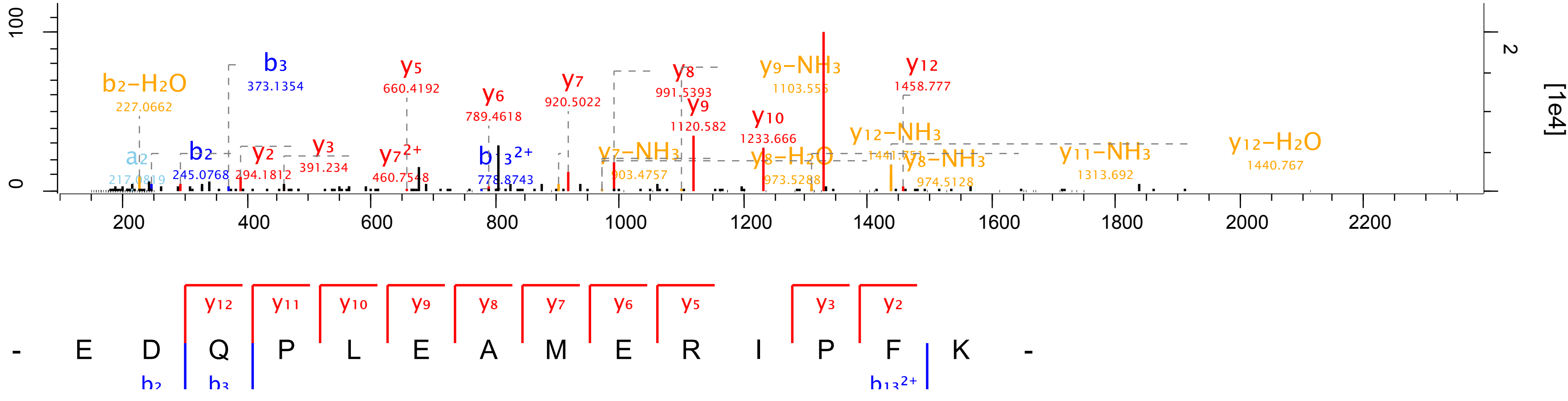

Raw file	Scan	Method	Score	m/z	Gene names
20150228_yeast1_Top_opt_B1_01_1614	44588	TOF; CID	84.48	490.61	MRP20

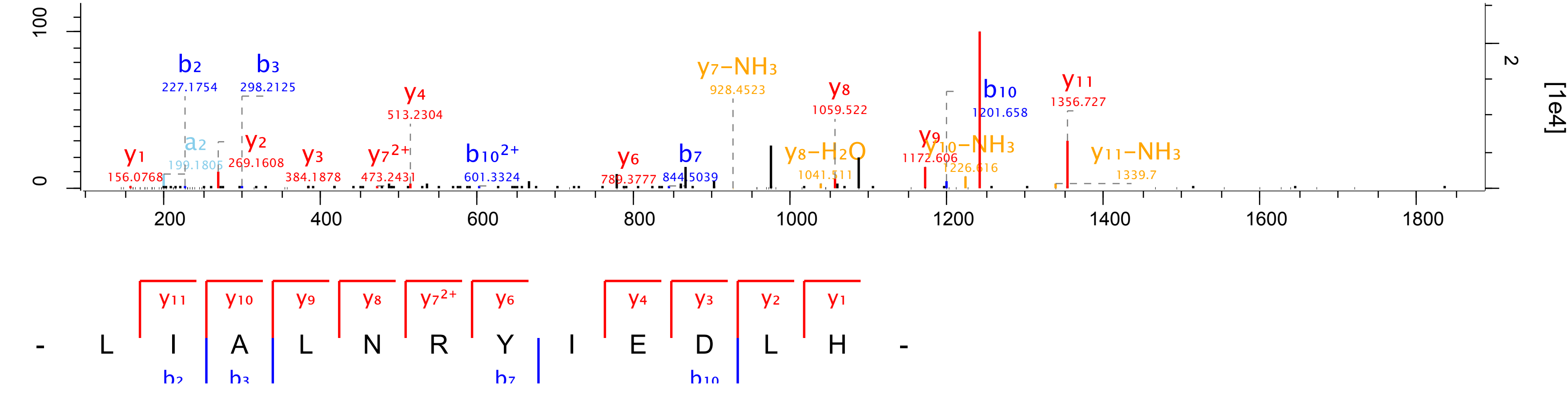

Raw file	Scan	Method	Score	m/z	Gene names
20150228_yeast1_Top_opt_B1_01_1614	44953	TOF; CID	51.64	782.92	APP1

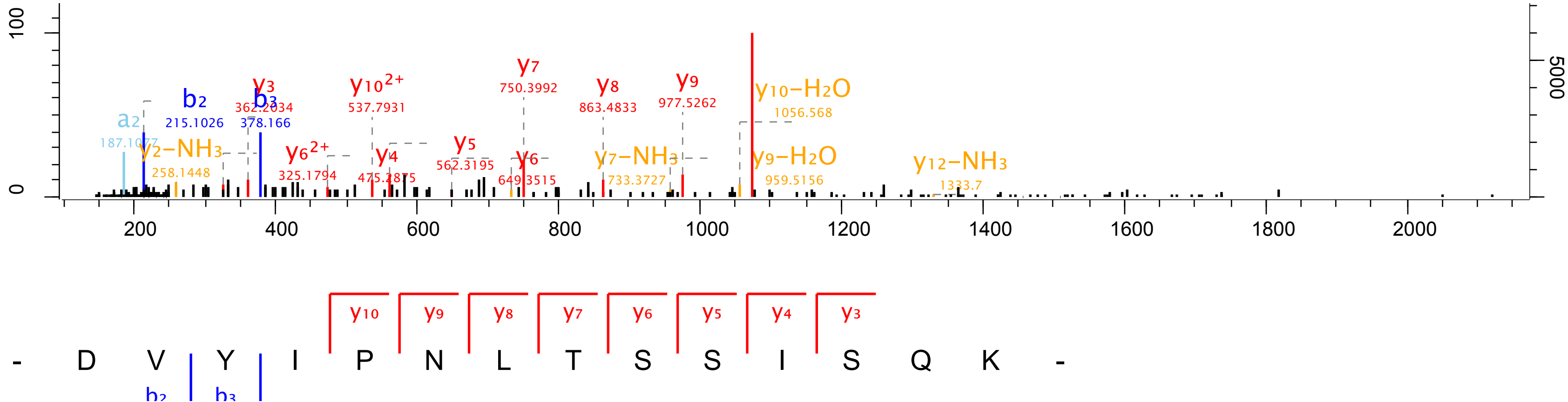

Raw file	Scan	Method	Score	m/z	Gene names
20150228_yeast1_Top_opt_B1_01_1614	45396	TOF; CID	80.68	468.79	HST1

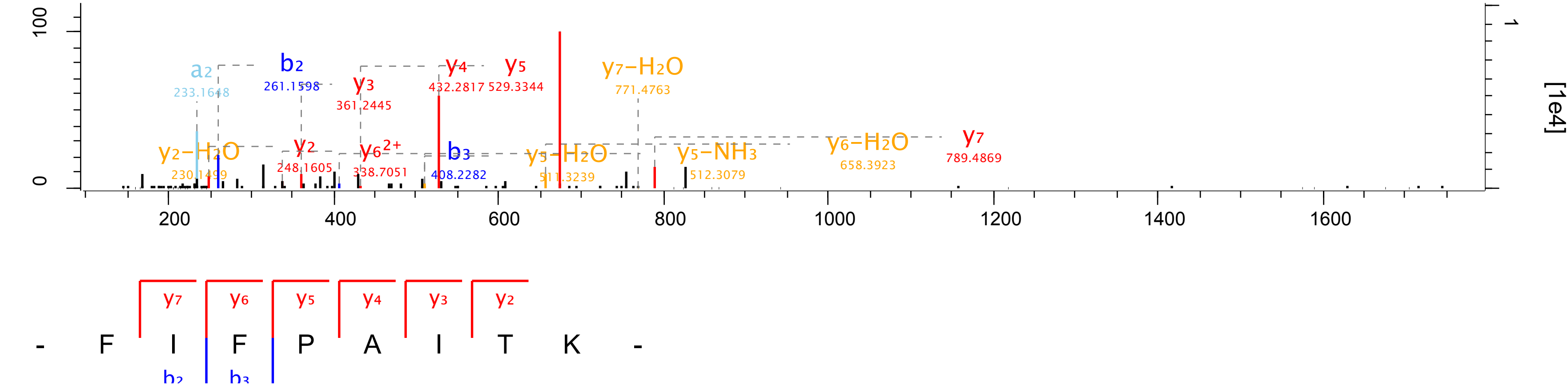

Raw file	Scan	Method	Score	m/z	Gene names
20150228_yeast1_Top_opt_B1_01_1614	46479	TOF; CID	96.02	1287.01	ERV2

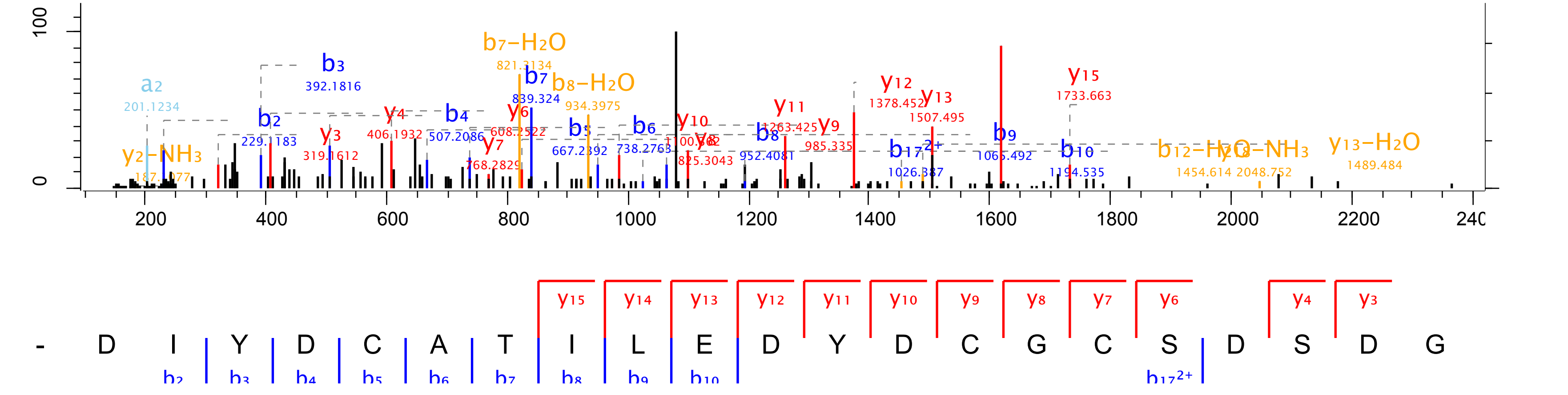

Raw file	Scan	Method	Score	m/z	Gene names
20150228_yeast1_Top_opt_B1_01_1614	46513	TOF; CID	96.21	828.96	IMG1

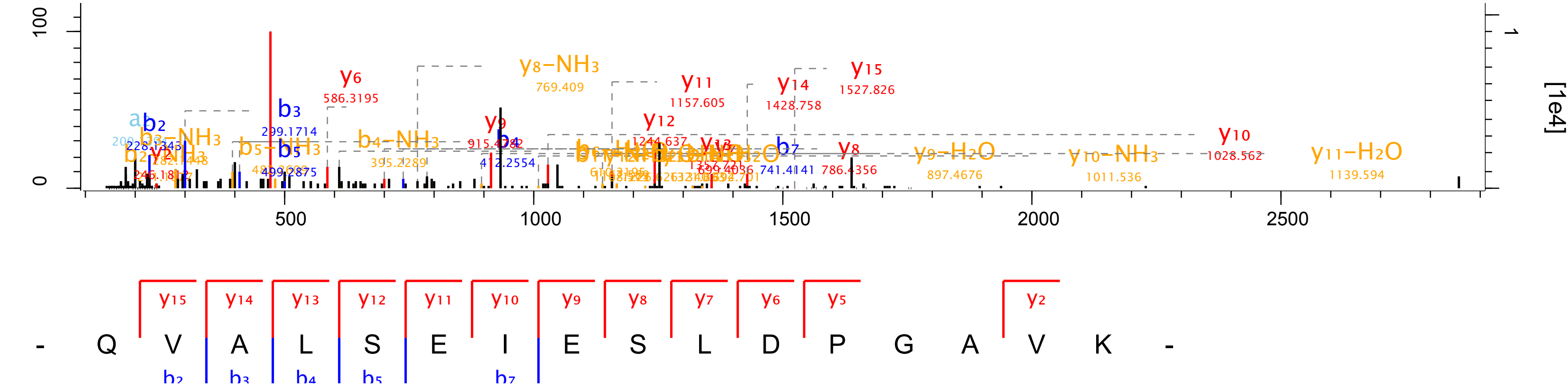

Raw file	Scan	Method	Score	m/z	Gene names
20150228_yeast1_Top_opt_B1_01_1614	46636	TOF; CID	48.28	650.31	TNA1

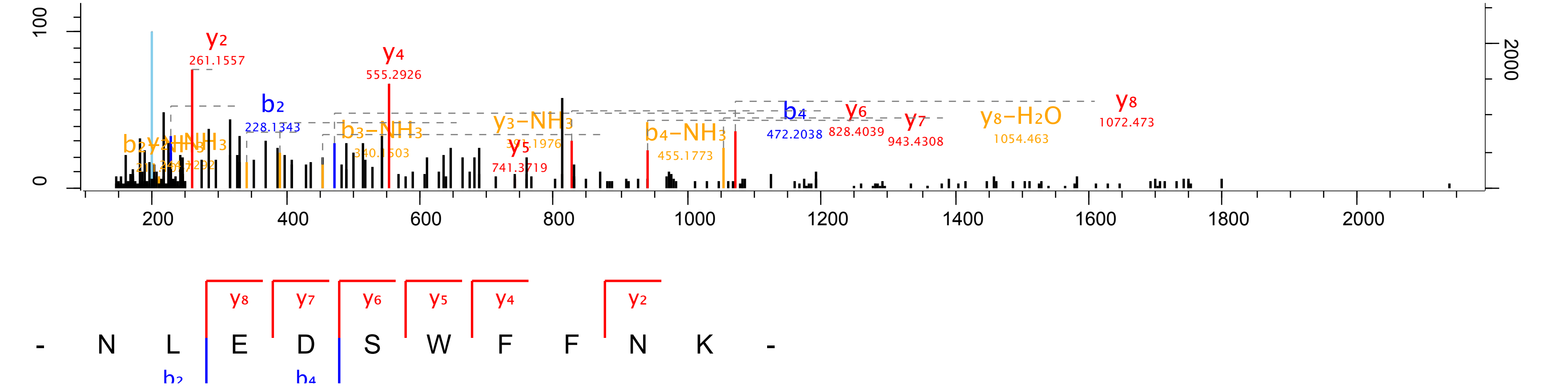

Raw file	Scan	Method	Score	m/z	Gene names
20150228_yeast1_Top_opt_B1_01_1614	47871	TOF; CID	94.69	540.81	RHO1

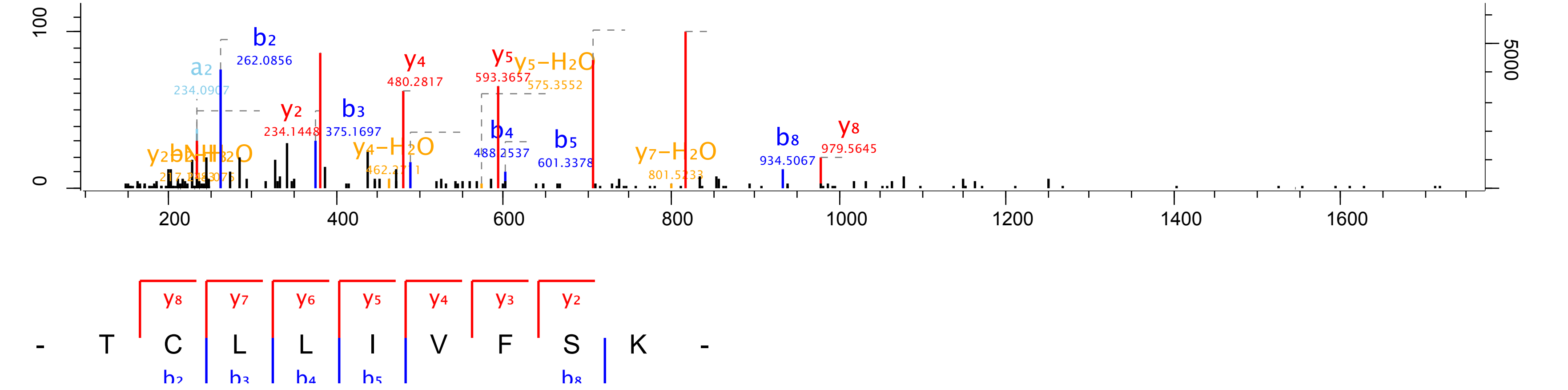

Raw file	Scan	Method	Score	m/z	Gene names
20150228_yeast1_Top_opt_B1_01_1614	49122	TOF; CID	56.14	697.86	SPC97

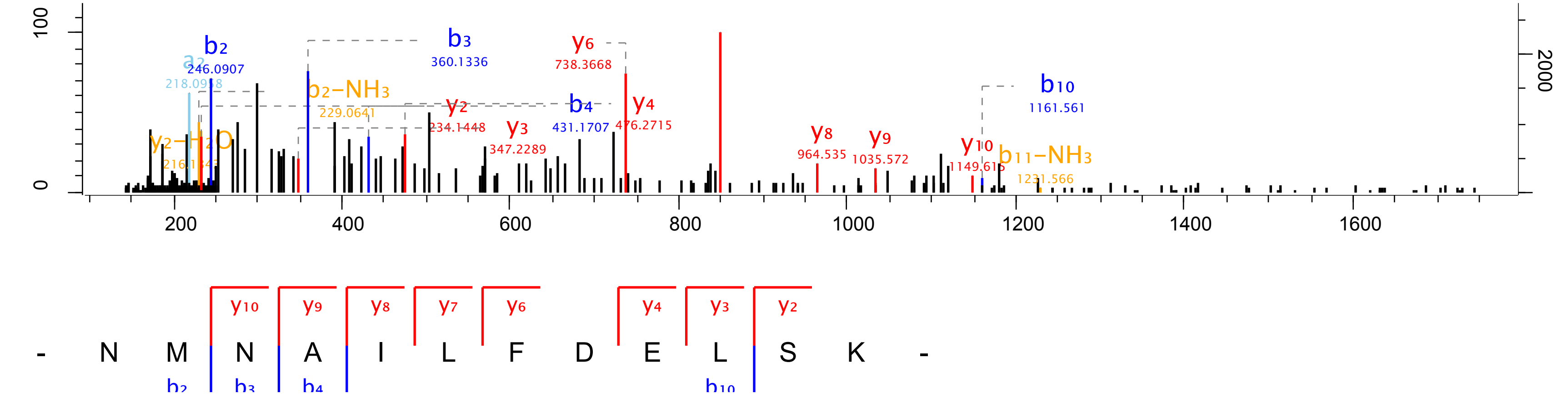

Raw file	Scan	Method	Score	m/z	Gene names
20150228_yeast1_Top_opt_B1_01_1614	49389	TOF; CID	59.2	632.83	YOR020W-A

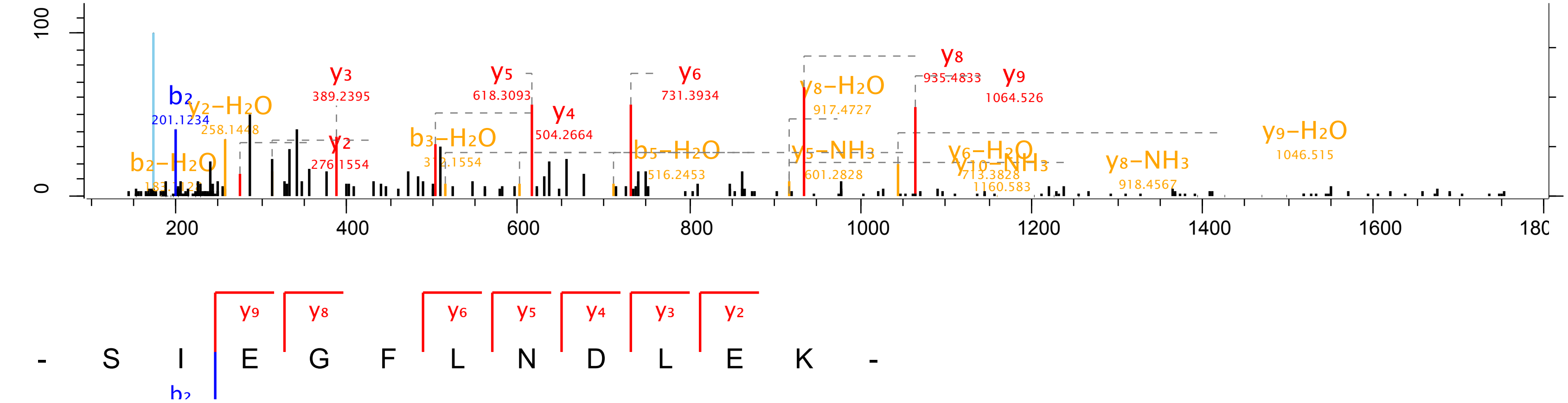

Raw file	Scan	Method	Score	m/z	Gene names
20150228_yeast1_Top_opt_B1_01_1614	49468	TOF; CID	87.09	1033.14	UMP1

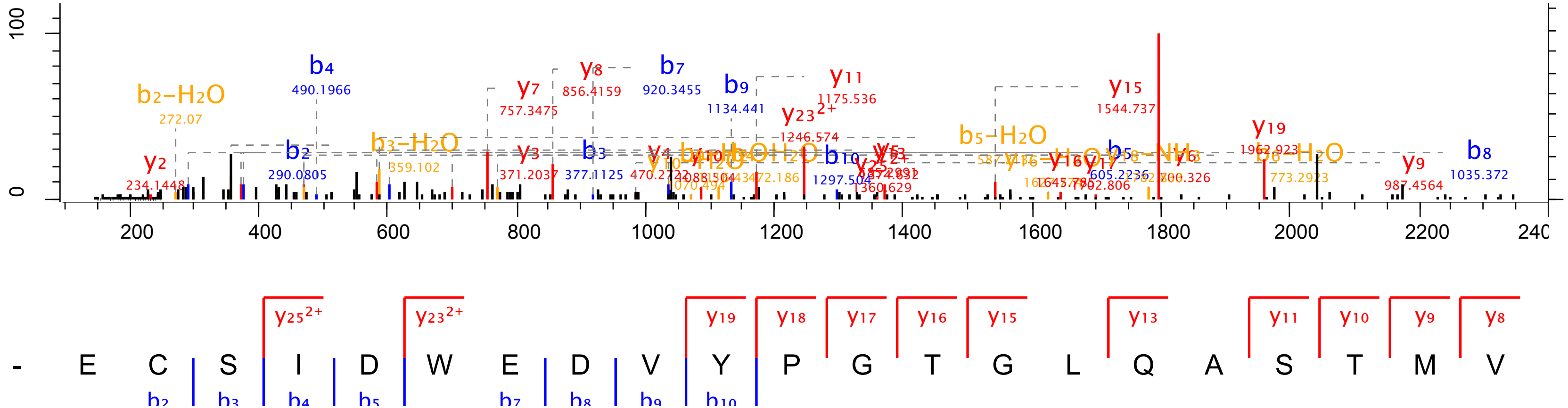

Raw file	Scan	Method	Score	m/z	Gene names
20150228_yeast1_Top_opt_B1_01_1614	50357	TOF; CID	197.4	1369.17	DAD1

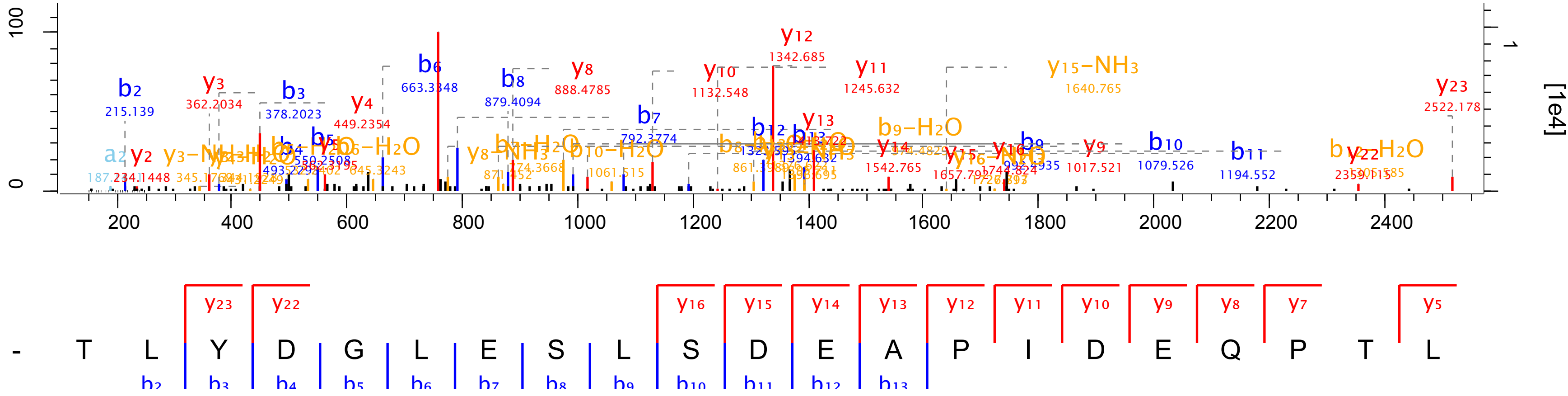

Raw file	Scan	Method	Score	m/z	Gene names
20150228_yeast1_Top_opt_B1_01_1614	50451	TOF; CID	177.12	1212.05	ILM1

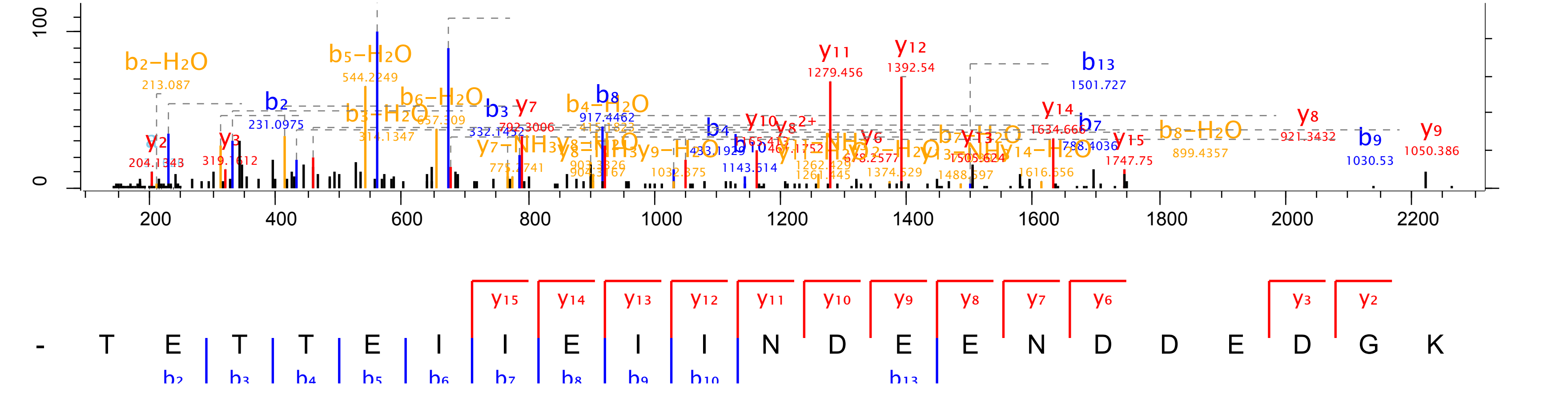

Raw file	Scan	Method	Score	m/z	Gene names
20150228_yeast1_Top_opt_B1_01_1614	50479	TOF; CID	101.78	925.46	SLY41

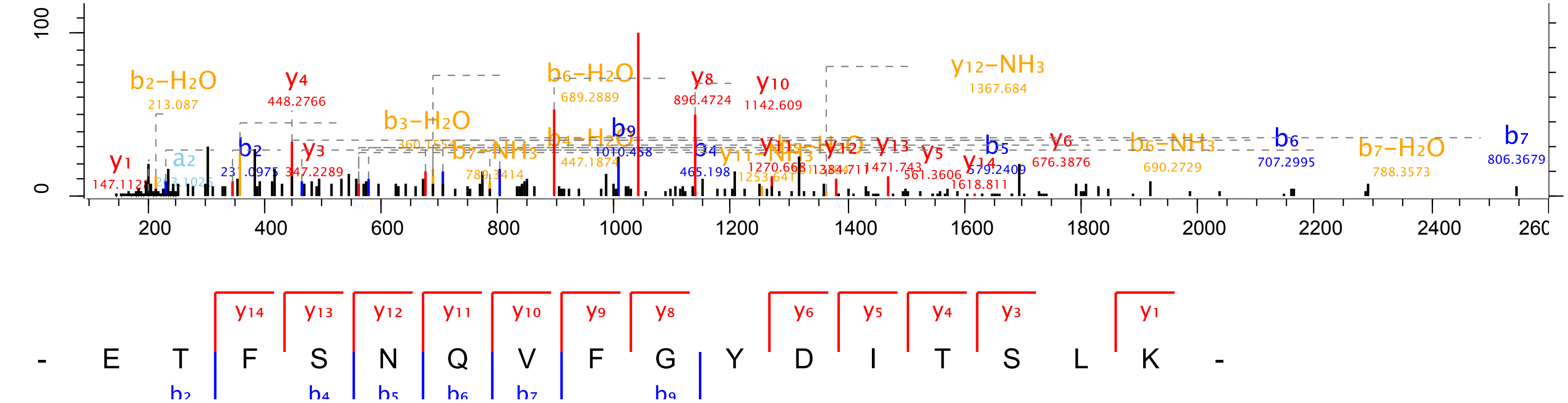

Raw file	Scan	Method	Score	m/z	Gene names
20150228_yeast1_Top_opt_B1_01_1614	50804	TOF; CID	49.27	713.64	MDM10

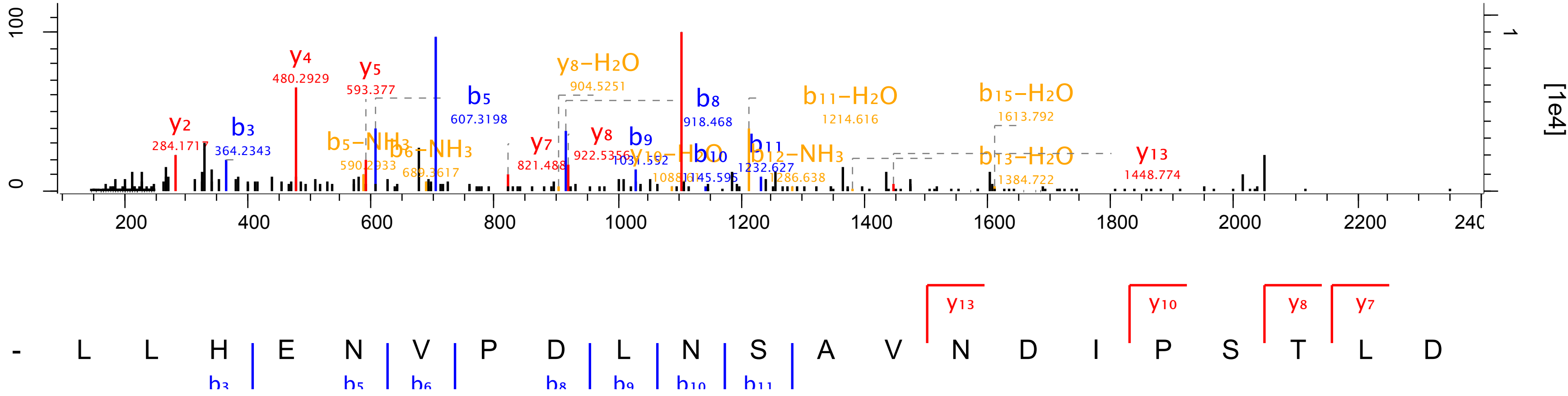

Raw file	Scan	Method	Score	m/z	Gene names
20150228_yeast1_Top_opt_B1_01_1614	51034	TOF; CID	67.58	808.41	PPM1

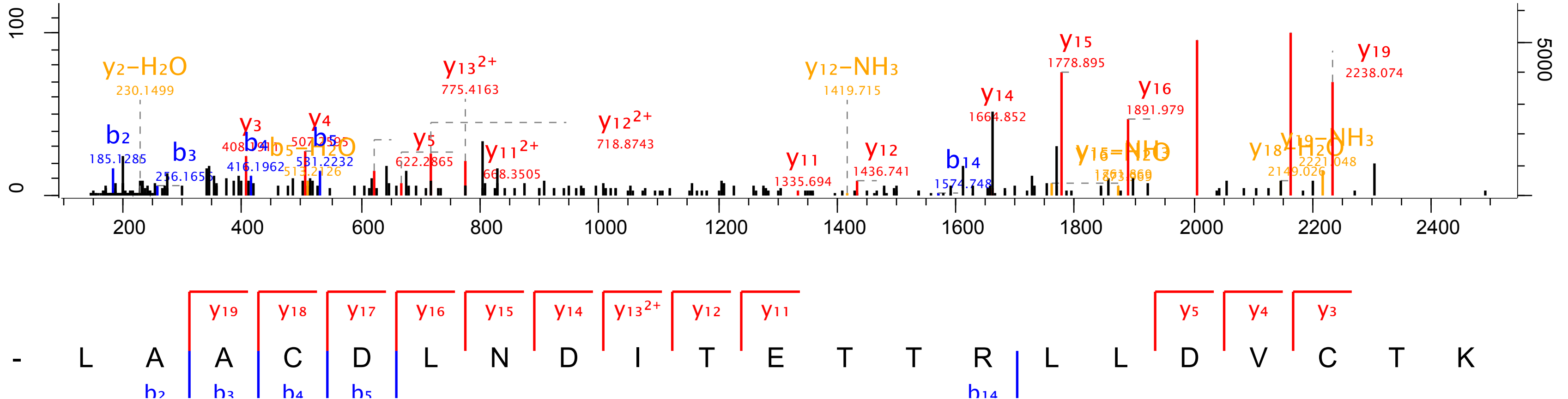

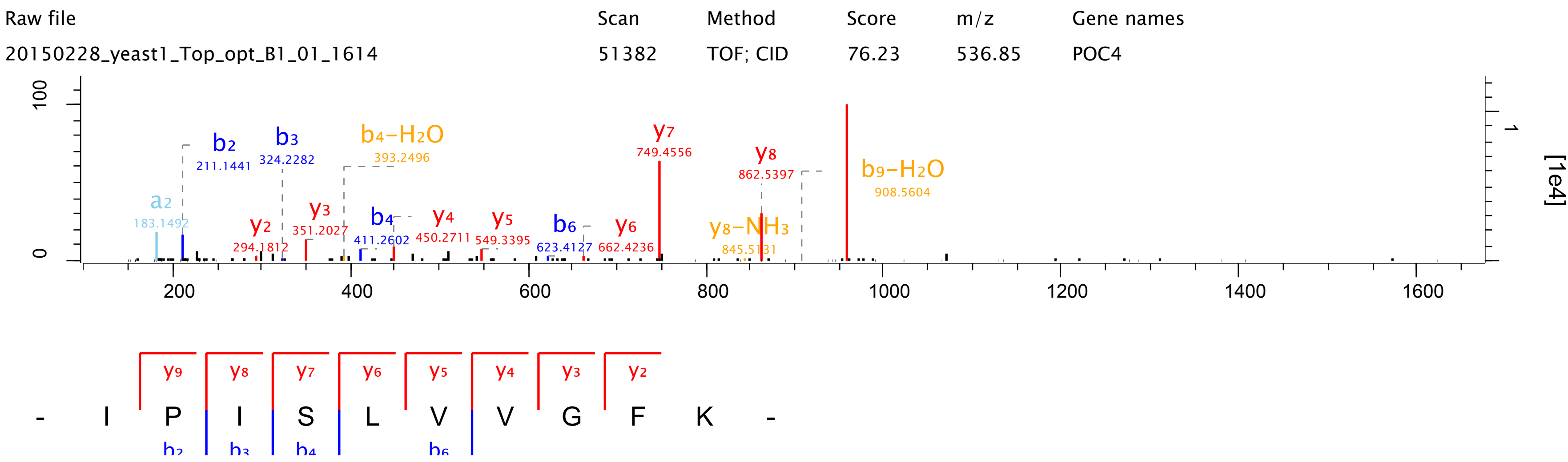

Raw file
20150228_yeast1_Top_opt_B1_01_1614
Scan
51382
Method
TOF; CID
Score
76.23
m/z
536.85
Gene names
POC4
100
0
1
[1e4]
a2 183.1492
b2 211.1441
b3 324.2282
y2 294.1812
y3 351.2027
b4−H2O 393.2496
b4 411.2602
y4 450.2711
y5 549.3395
b6 623.4127
y6 662.4236
y7 749.4556
y8−NH3 845.5131
y8 862.5397
b9−H2O 908.5604
200
400
600
800
1000
1200
1400
1600
y9 y8 y7 y6 y5 y4 y3 y2
- I P I S L V V G F K -
b2 b3 b4 b6

Raw file	Scan	Method	Score	m/z	Gene names
20150228_yeast1_Top_opt_B1_01_1614	52265	TOF; CID	61.96	539.77	COX2

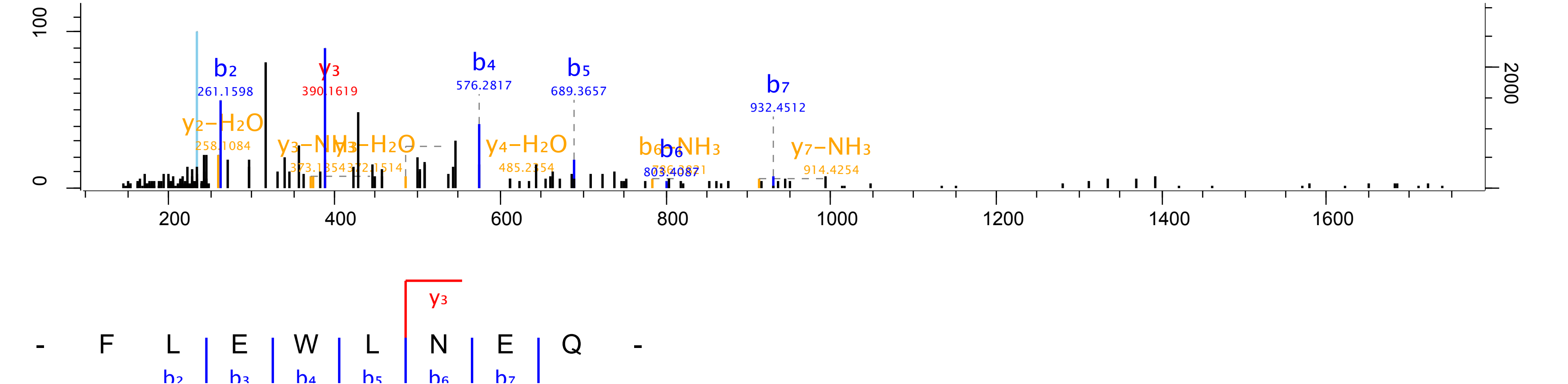

Raw file	Scan	Method	Score	m/z	Gene names
20150228_yeast1_Top_opt_B1_01_1614	53398	TOF; CID	64.27	945.49	UBP16

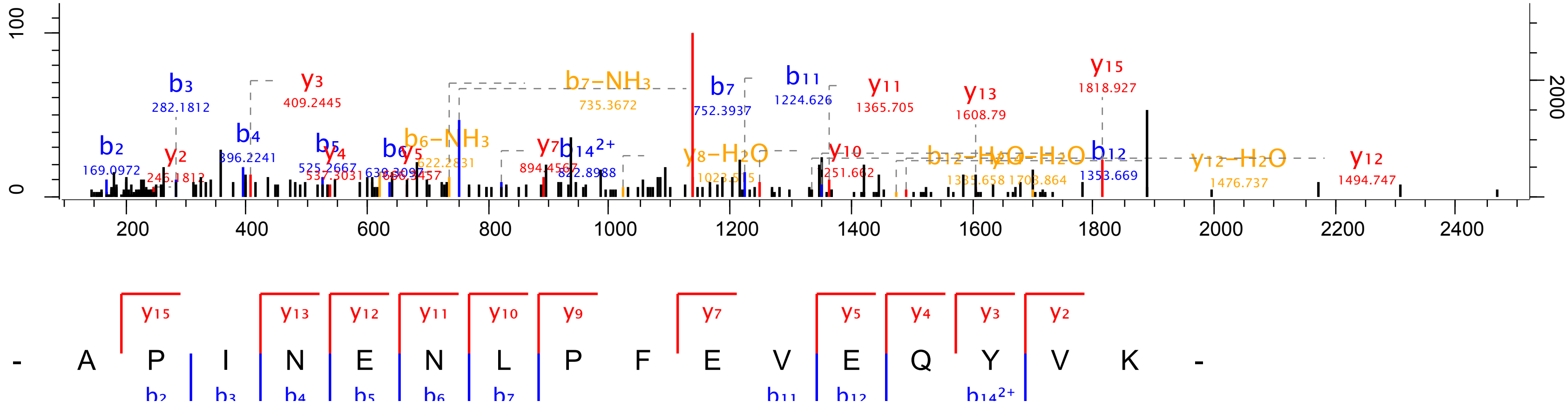

Raw file	Scan	Method	Score	m/z	Gene names
20150228_yeast1_Top_opt_B1_01_1614	53552	TOF; CID	81.16	611.69	VMA11

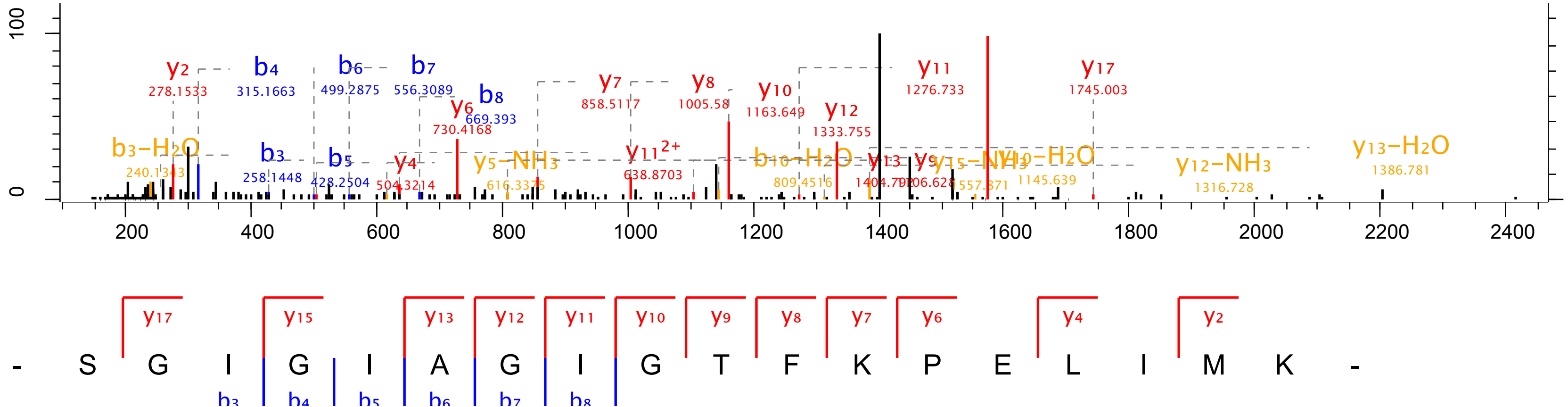

Raw file	Scan	Method	Score	m/z	Gene names
20150228_yeast1_Top_opt_B1_01_1614	54587	TOF; CID	72.93	522.33	SMD3

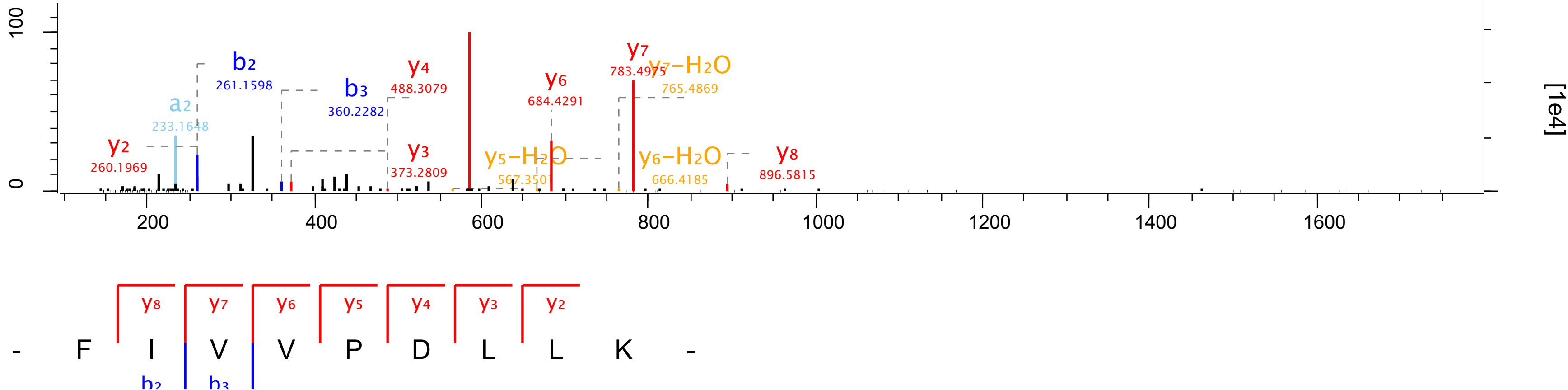

Raw file	Scan	Method	Score	m/z	Gene names
20150228_yeast1_Top_opt_B1_01_1614	54693	TOF; CID	40.43	669.36	VMA3

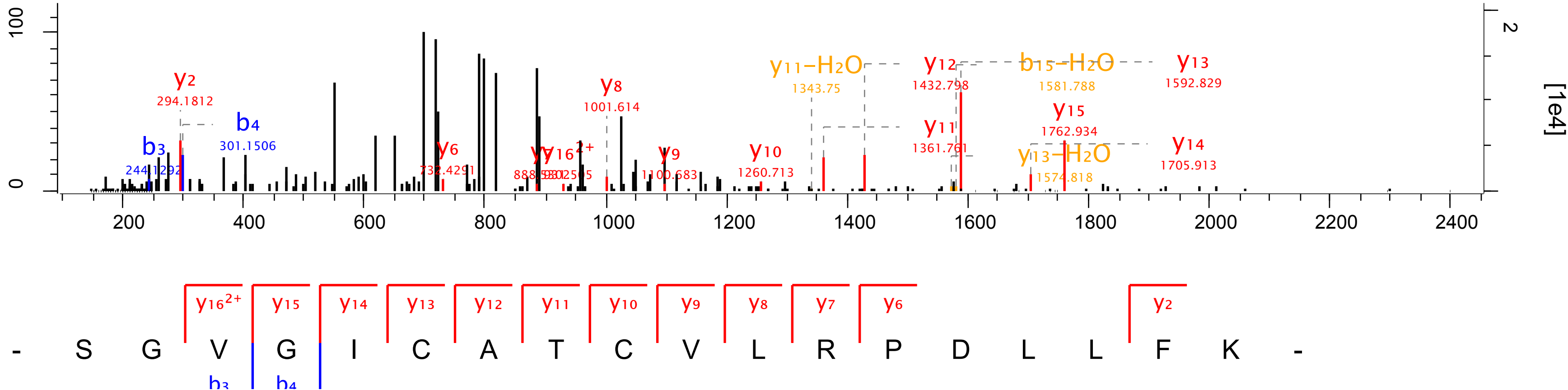

Raw file	Scan	Method	Score	m/z	Gene names
20150228_yeast1_Top_opt_B1_01_1614	55817	TOF; CID	91.66	627.98	VPS68

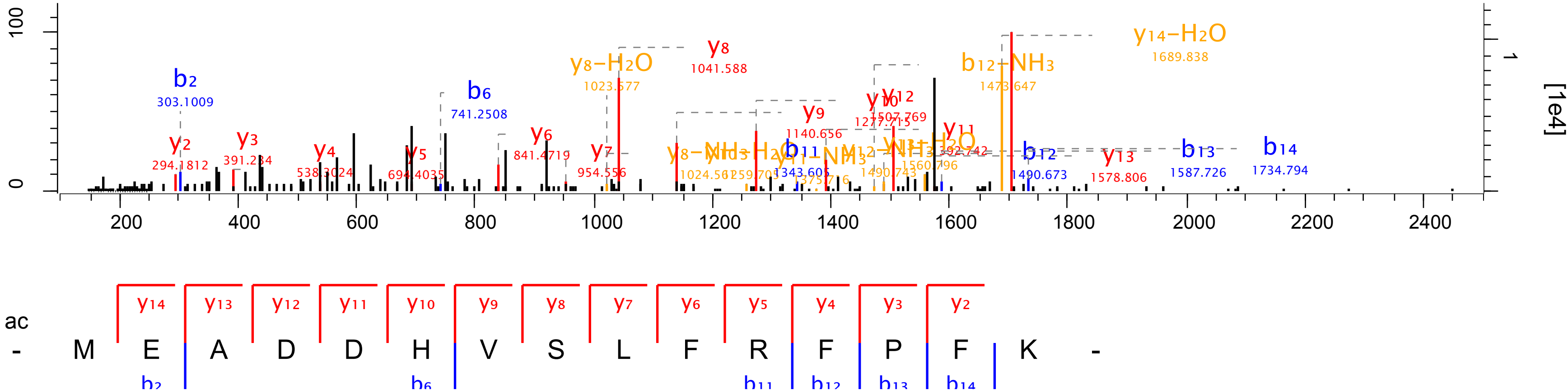

Raw file	Scan	Method	Score	m/z	Gene names
20150228_yeast1_Top_opt_B1_01_1614	56138	TOF; CID	124.07	735.39	TOM7

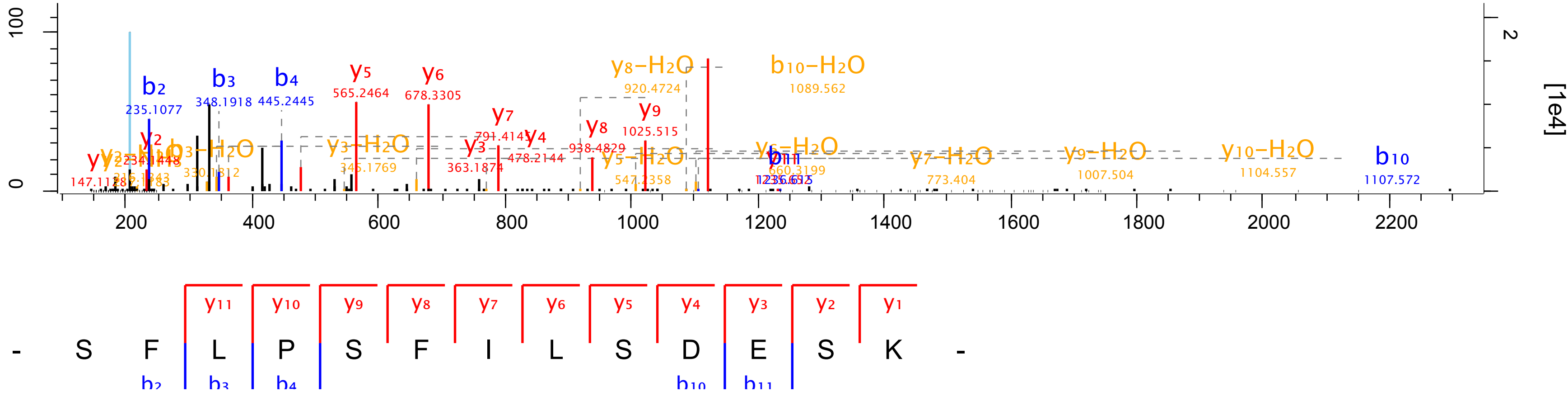

Raw file	Scan	Method	Score	m/z	Gene names
20150228_yeast1_Top_opt_B1_01_1614	56439	TOF; CID	87.82	840.94	CBP2

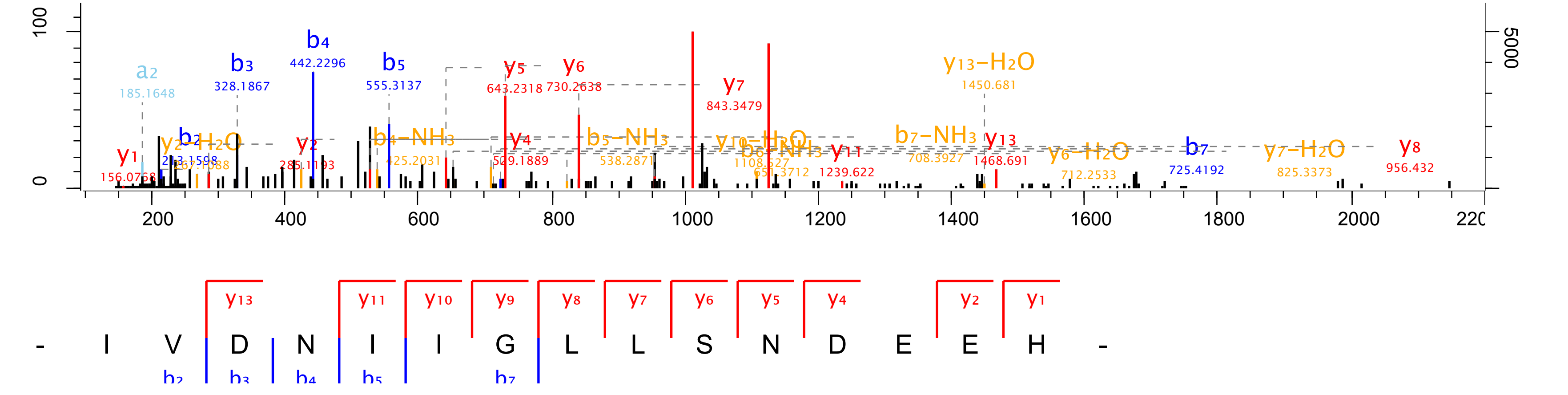

Raw file	Scan	Method	Score	m/z	Gene names
20150228_yeast1_Top_opt_B1_01_1614	57267	TOF; CID	49.04	1032.52	ADK2

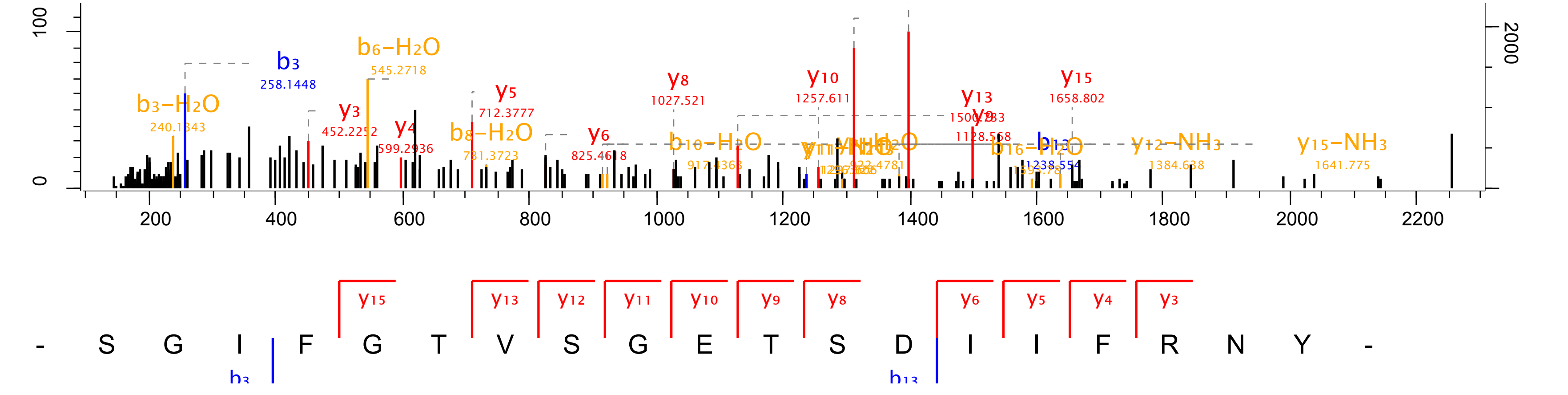

Raw file	Scan	Method	Score	m/z	Gene names
20150228_yeast1_Top_opt_B1_01_1614	57456	TOF; CID	50.35	659.37	MIC10

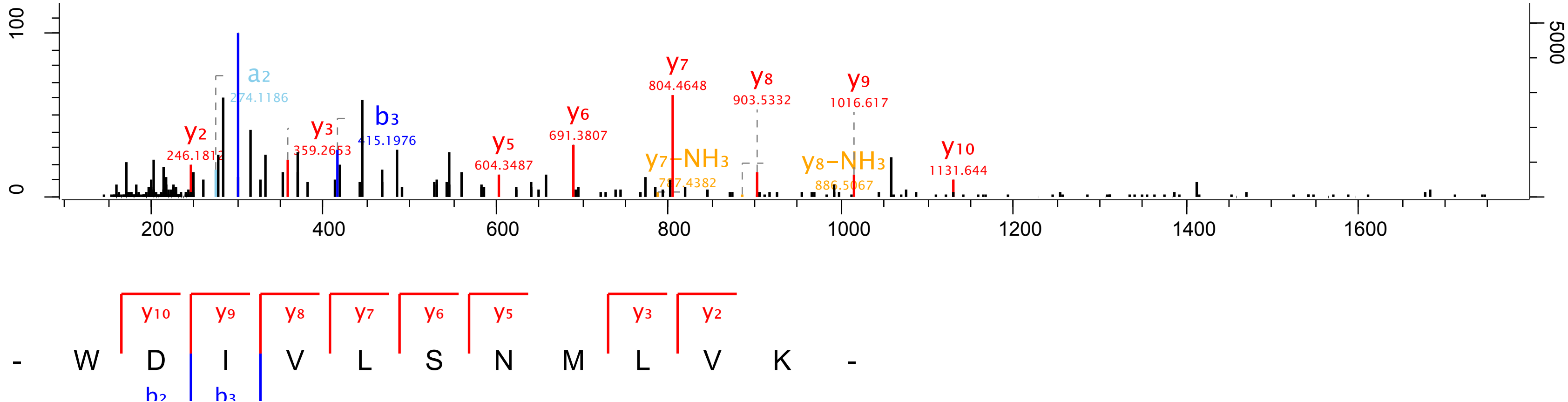

Raw file	Scan	Method	Score	m/z	Gene names
20150228_yeast1_Top_opt_B1_01_1614	57695	TOF; CID	128.35	592.84	APT2

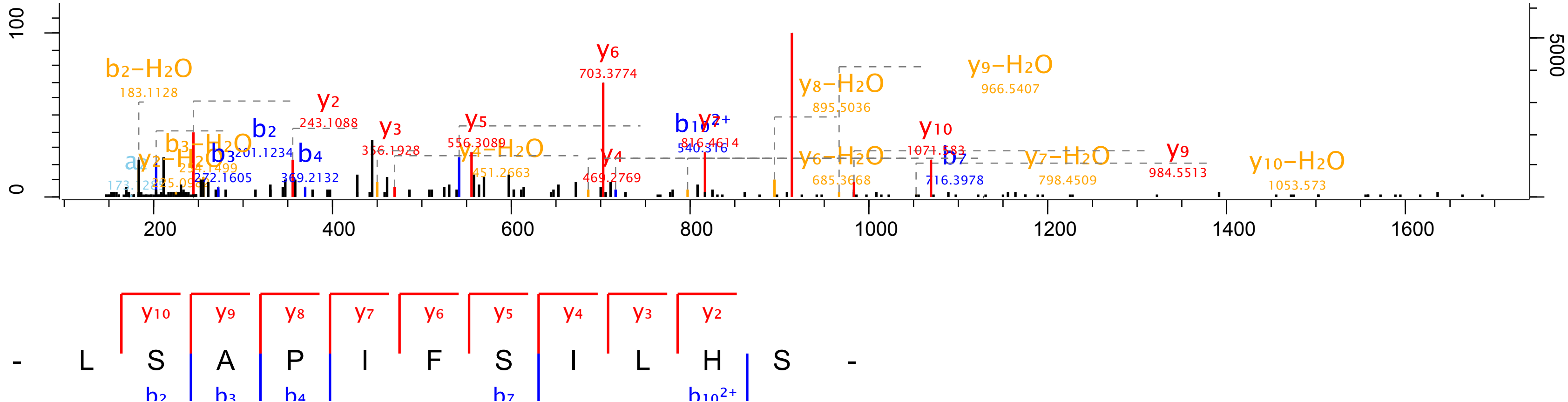

Raw file	Scan	Method	Score	m/z	Gene names
20150228_yeast1_Top_opt_B1_01_1614	58056	TOF; CID	52.87	1078.07	CPD1

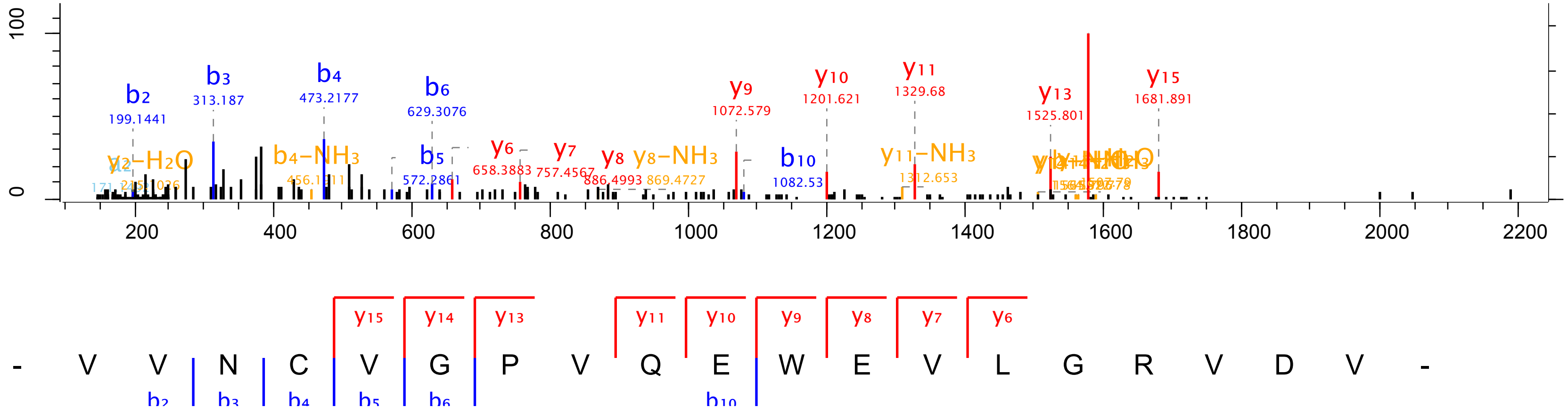

Raw file	Scan	Method	Score	m/z	Gene names
20150228_yeast1_Top_opt_B1_01_1614	58877	TOF; CID	180.48	568.67	ERV14

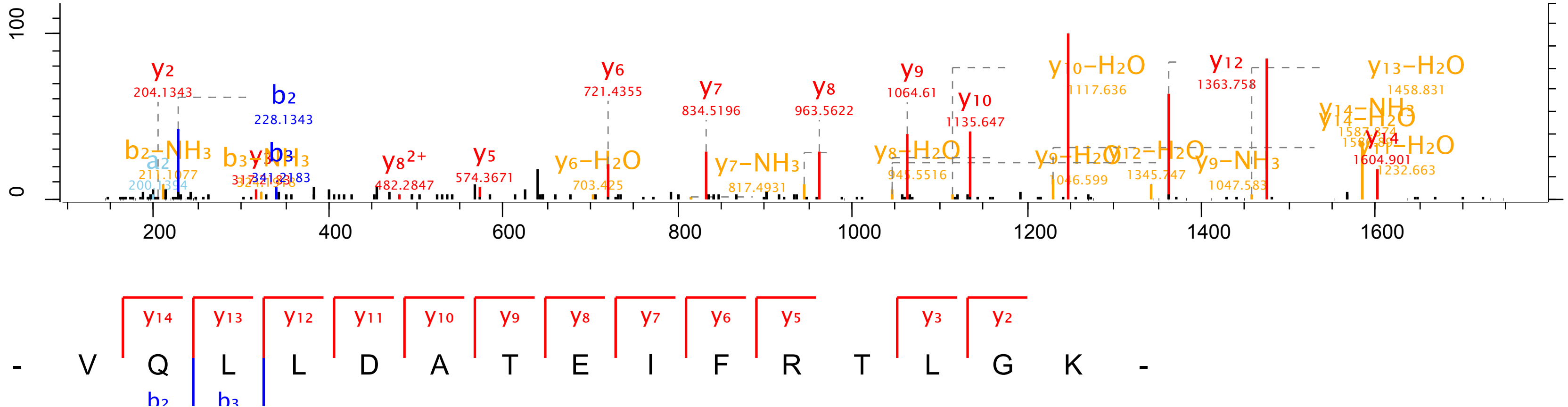

Raw file	Scan	Method	Score	m/z	Gene names
20150228_yeast1_Top_opt_B1_01_1614	59939	TOF; CID	71.98	823.43	PEX15

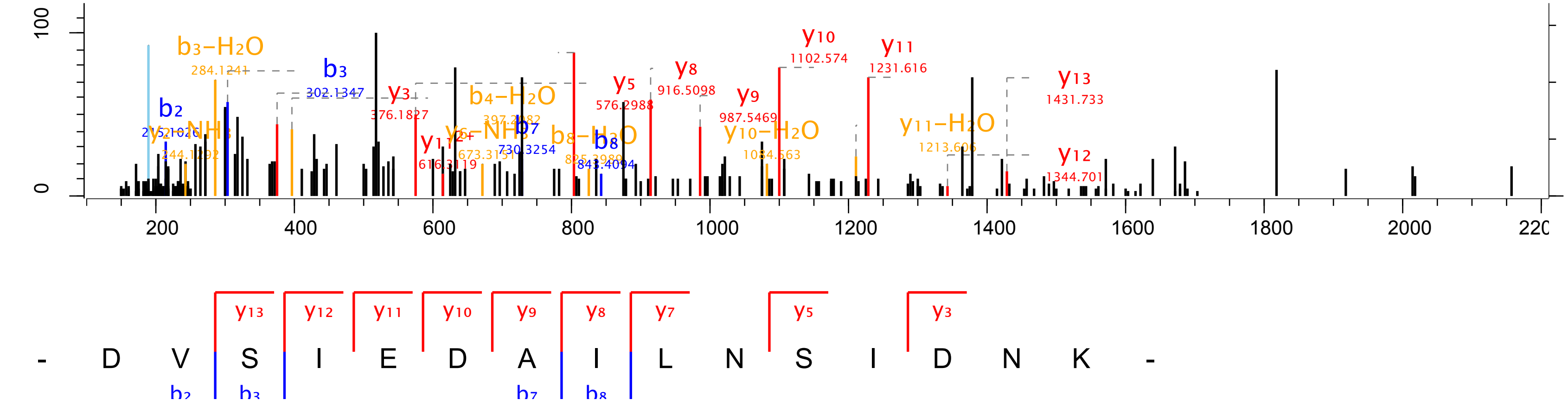

Raw file	Scan	Method	Score	m/z	Gene names
20150228_yeast1_Top_opt_B1_01_1614	60040	TOF; CID	49.09	1188.56	TRM12

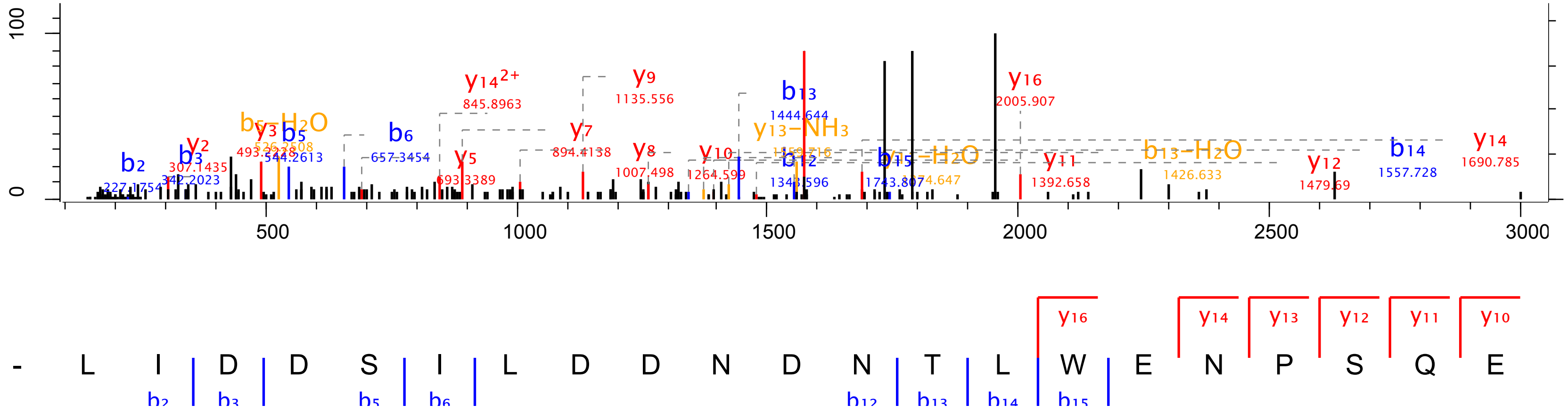

Raw file	Scan	Method	Score	m/z	Gene names
20150228_yeast1_Top_opt_B1_01_1614	60254	TOF; CID	65.1	1077.53	APQ12

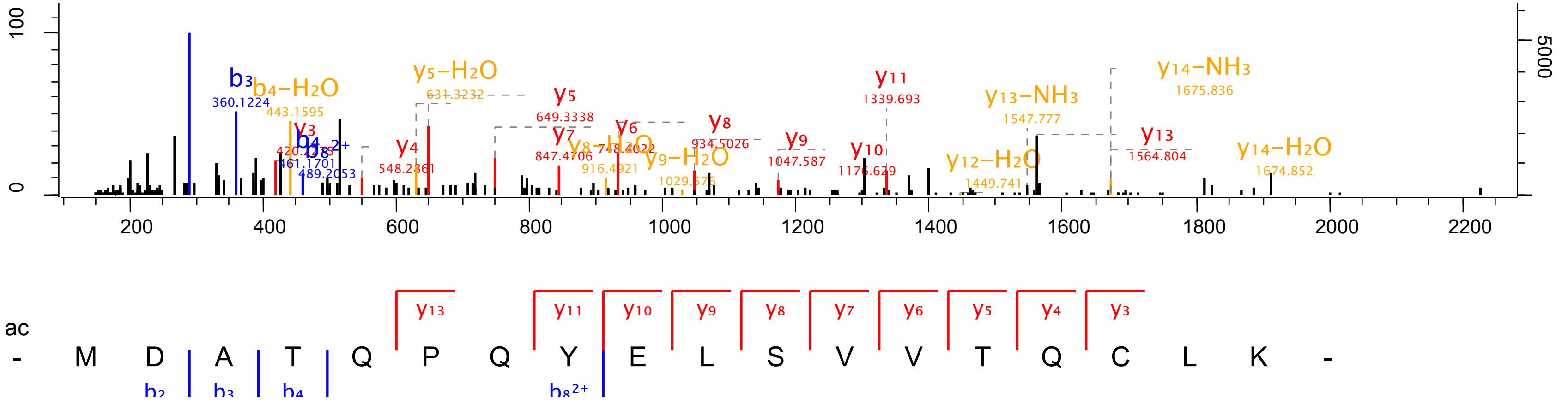

Raw file	Scan	Method	Score	m/z	Gene names
20150228_yeast1_Top_opt_B1_01_1614	64548	TOF; CID	73.88	729.9	CWP2

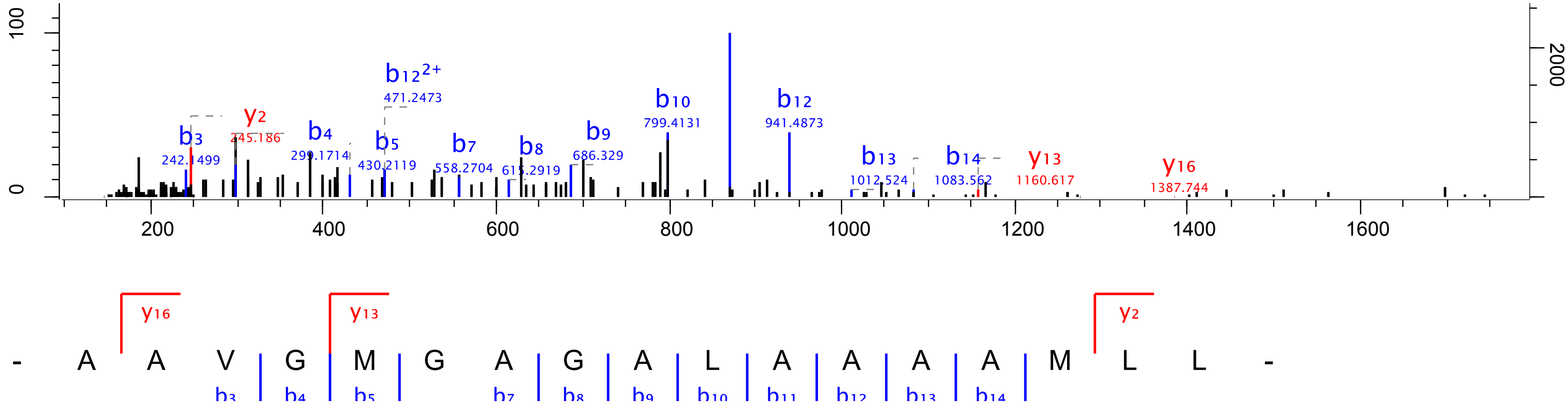

Raw file	Scan	Method	Score	m/z	Gene names
20150228_yeast1_Top_opt_B1_01_1614	65011	TOF; CID	103.55	577.8	DID4

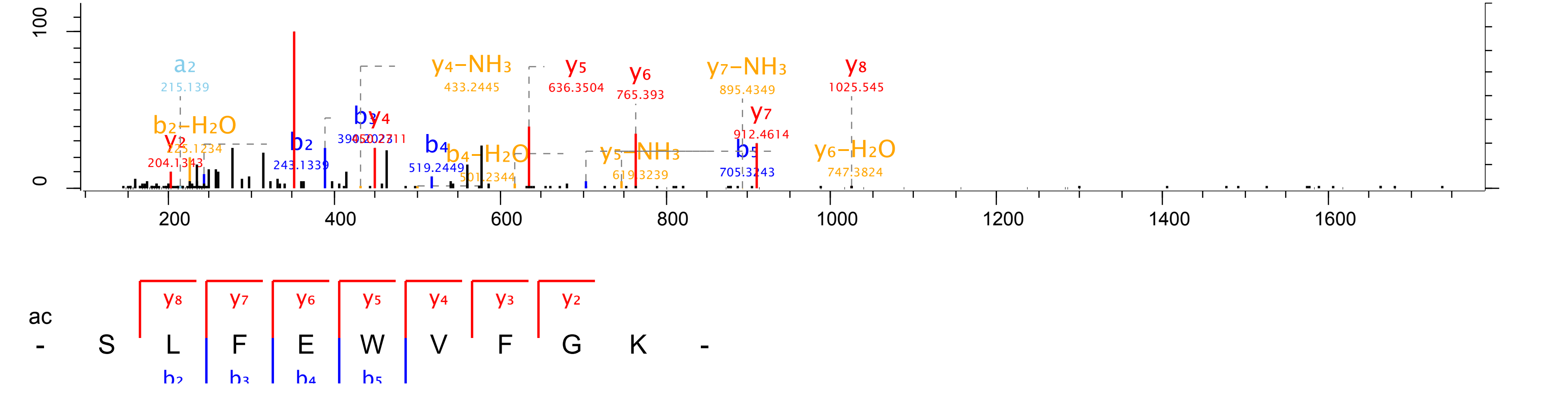

Supplement: Supplemental Data [file supp_M114.047407_mcp.M114.047407-6.pdf]
