# Supplementary material for: The Impact II, a Very High-Resolution Quadrupole Time-of-Flight Instrument (QTOF) for Deep Shotgun Proteomics
Source: Mol Cell Proteomics. 2015 May 19;14(7):2014–29. doi: 10.1074/mcp.M114.047407 (PMC4587313; doi:10.1074/mcp.M114.047407)

Raw file	Scan	Method	Score	Mass
025pmolUPS2_500ngY_90min1Hz1pr_BC1_01_350	2192	TOF; CID	58.82	986.52

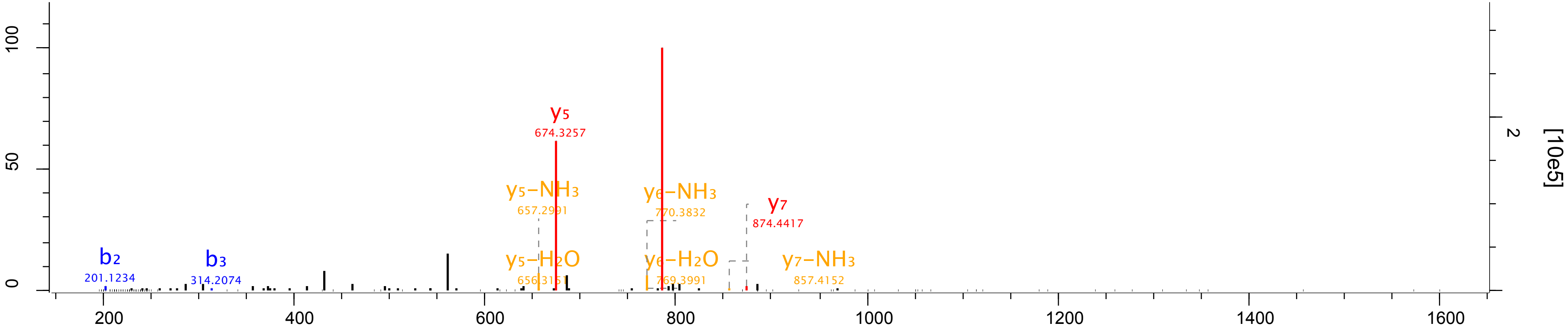

Raw file	Scan	Method	Score	Mass	Gene names
05pmolUPS2_500ngY_90min1Hz1pr_BC2_01_351	2836	TOF; CID	64.76	793.4	RAD30

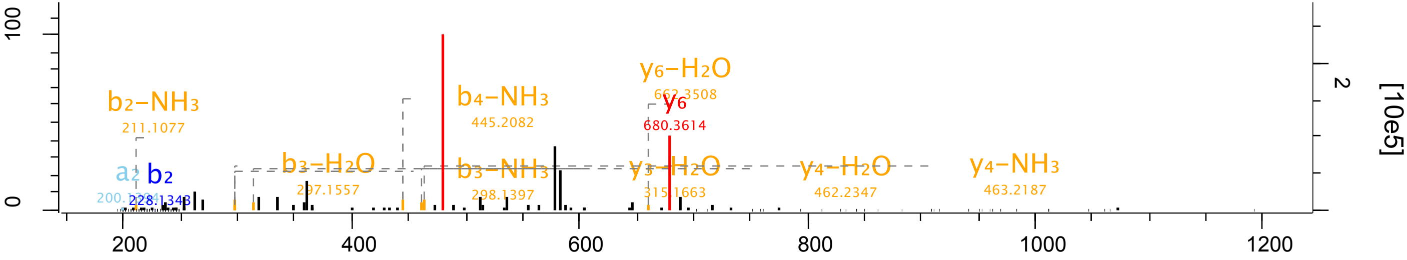

Raw file	Scan	Method	Score	Mass	Gene names
025pmolUPS2_500ngY_90min1Hz1pr_BC1_01_353	3914	TOF; CID	177.83	1366.62	YOP1

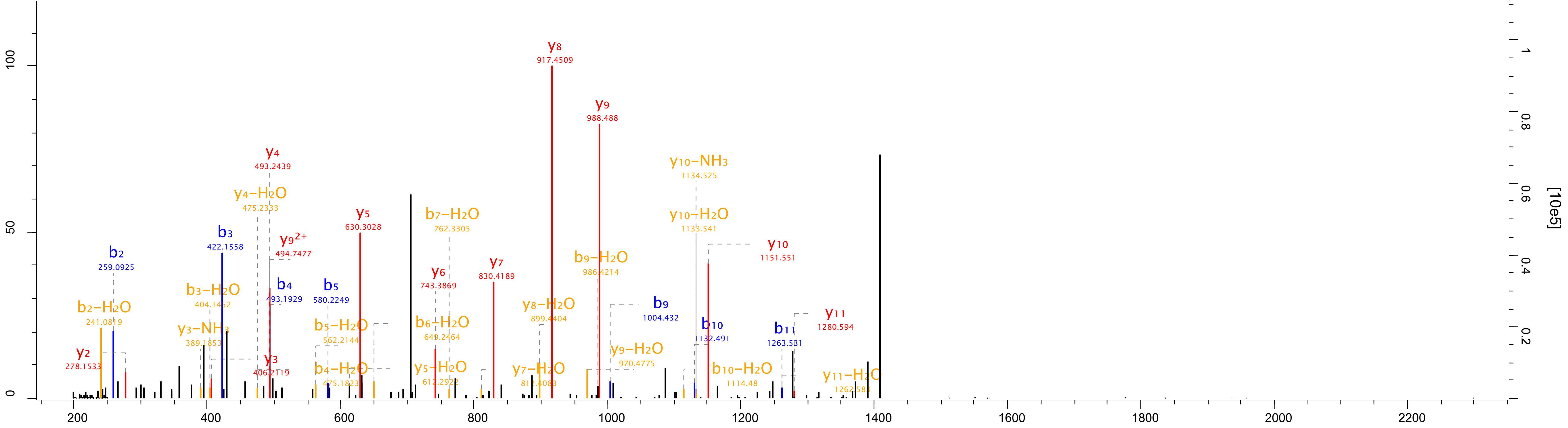

Raw file	Scan	Method	Score	Mass	Gene names
025pmolUPS2_500ngY_90min1Hz1pr_BC1_01_341	4042	TOF; CID	179.51	1306.73	RPL39

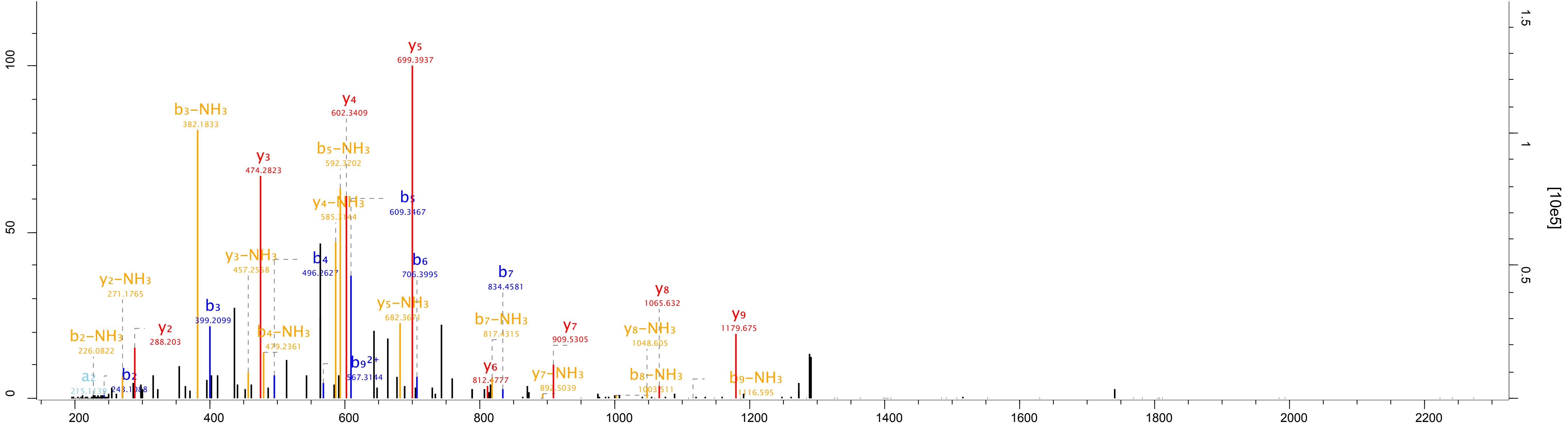

Raw file	Scan	Method	Score	Mass	Gene names
025pmolUPS2_500ngY_90min1Hz1pr_BC1_01_338	4528	TOF; CID	112.24	1313.68	VMA10

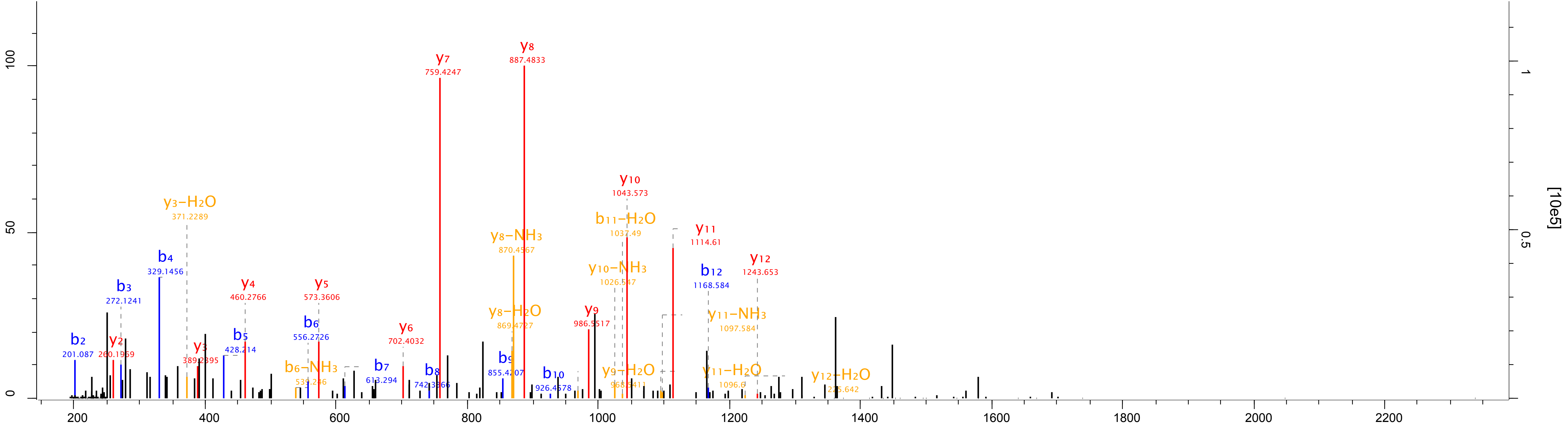

Raw file	Scan	Method	Score	Mass	Gene names
05pmolUPS2_500ngY_90min1Hz1pr_BC2_01_345	4668	TOF; CID	124.98	1055.53	SSA1;SSA2

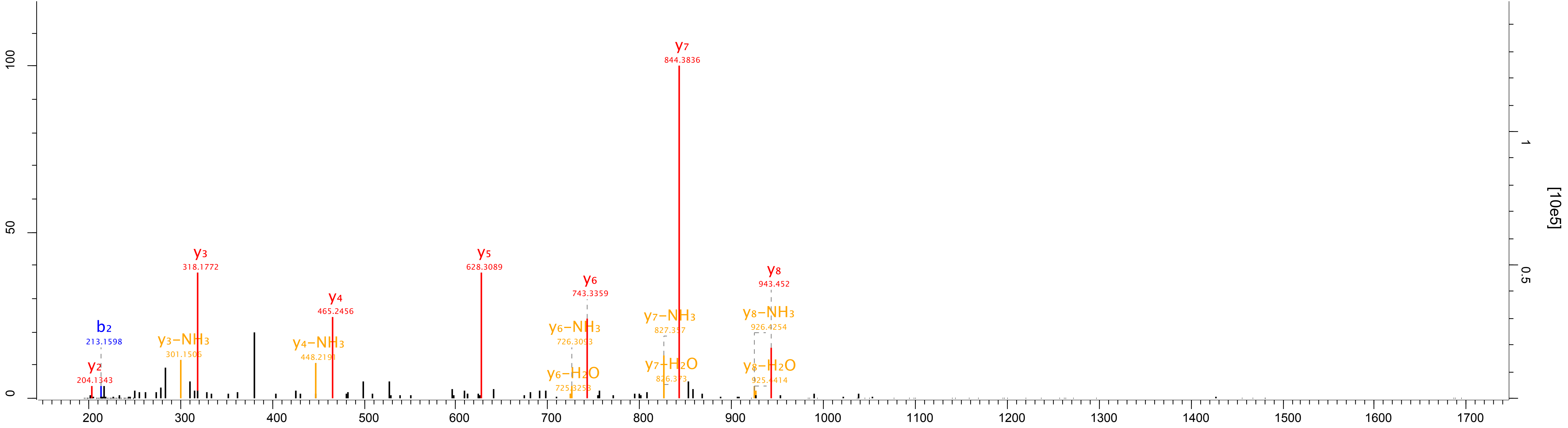

Raw file	Scan	Method	Score	Mass	Gene names
05pmolUPS2_500ngY_90min1Hz1pr_BC2_01_345	5262	TOF; CID	85.36	869.53	VPS30

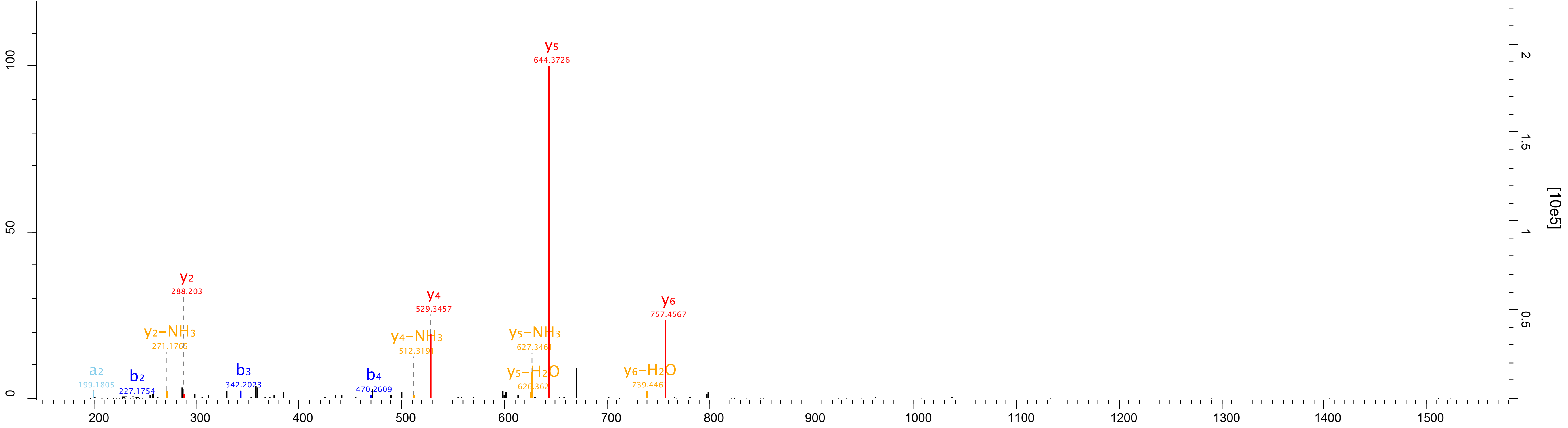

Raw file	Scan	Method	Score	Mass
05pmolUPS2_500ngY_90min1Hz1pr_BC2_01_354	5620	TOF; CID	103.53	2078.9

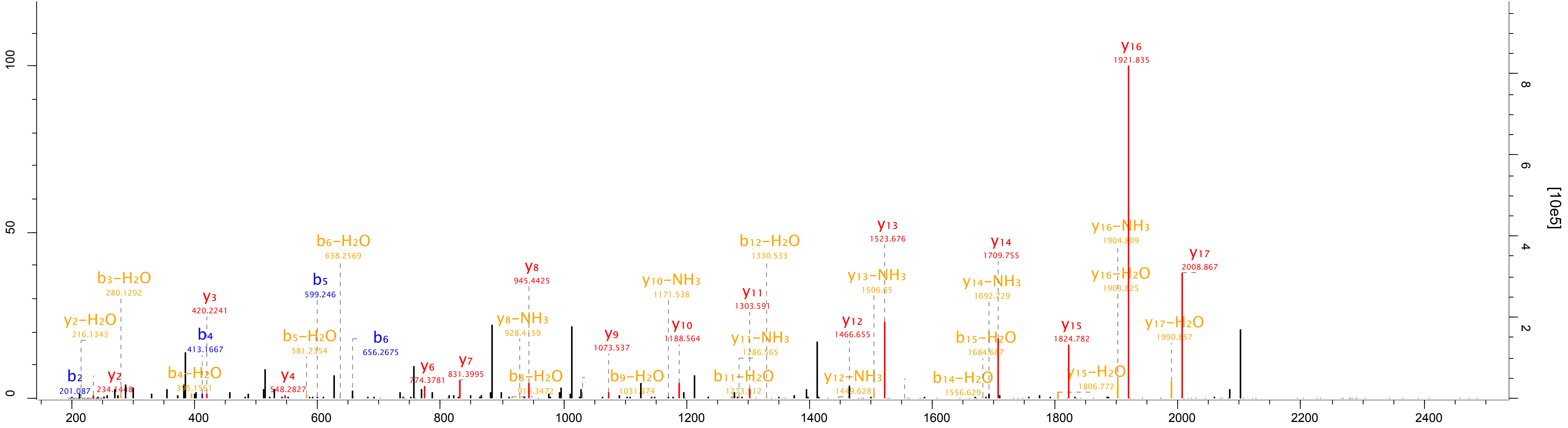

Raw file	Scan	Method	Score	Mass	Gene names
05pmolUPS2_500ngY_90min1Hz1pr_BC2_01_354	5894	TOF; CID	95.09	1244.64	UBC5;UBC4

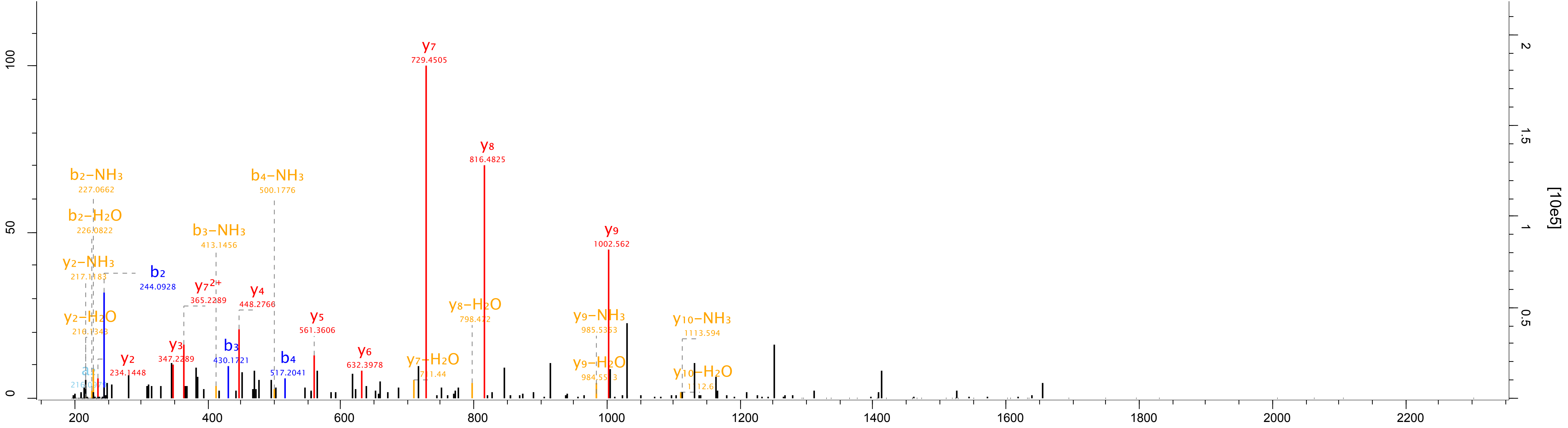

Raw file	Scan	Method	Score	Mass	Gene names
05pmolUPS2_500ngY_90min1Hz1pr_BC2_01_351	6114	TOF; CID	145.08	1184.64	NCE102

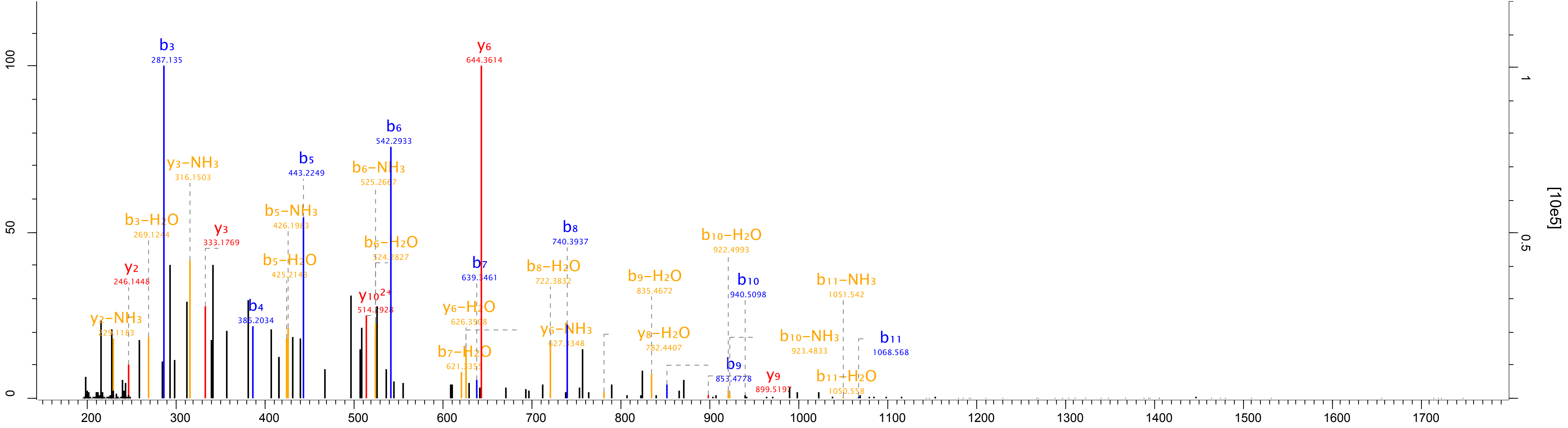

Raw file	Scan	Method	Score	Mass	Gene names
05pmolUPS2_500ngY_90min1Hz1pr_BC2_01_351	7210	TOF; CID	60.76	798.47	YHM2

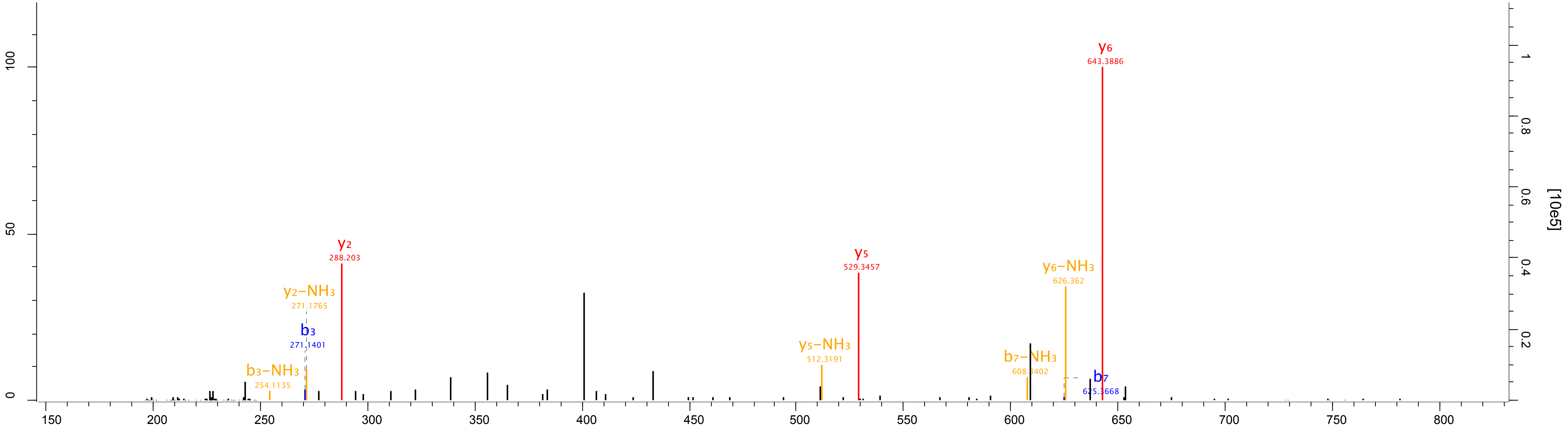

Raw file	Scan	Method	Score	Mass	Gene names
025pmolUPS2_500ngY_90min1Hz1pr_BC1_01_347	7606	TOF; CID	124.48	1468.74	TOM7

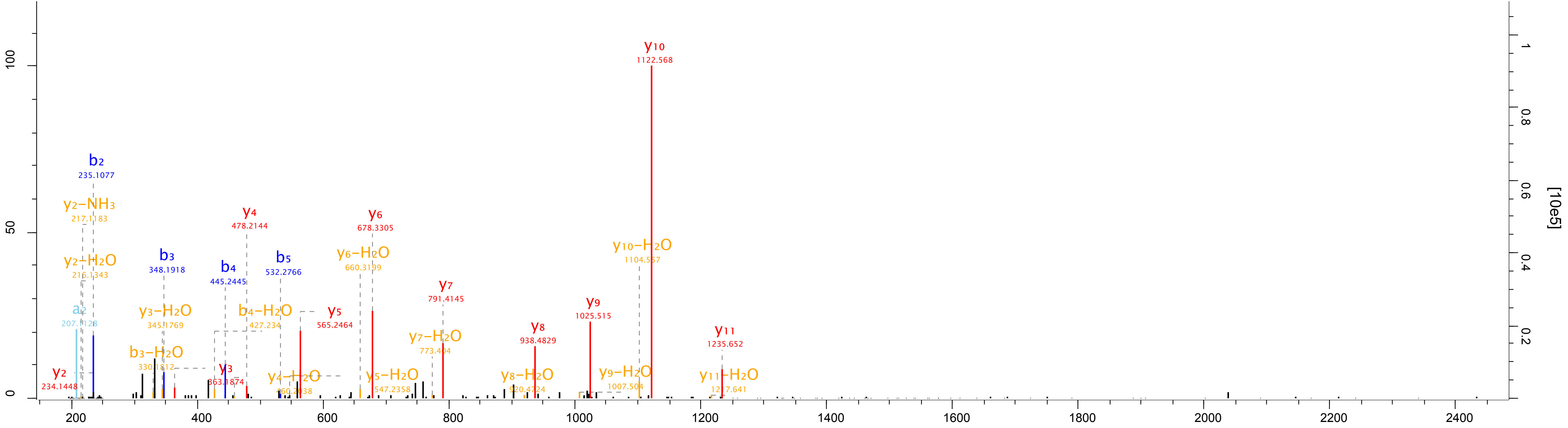

Raw file	Scan	Method	Score	Mass	Gene names
05pmolUPS2_500ngY_90min1Hz1pr_BC2_01_339	8826	TOF; CID	75.68	2056.08	ATX1

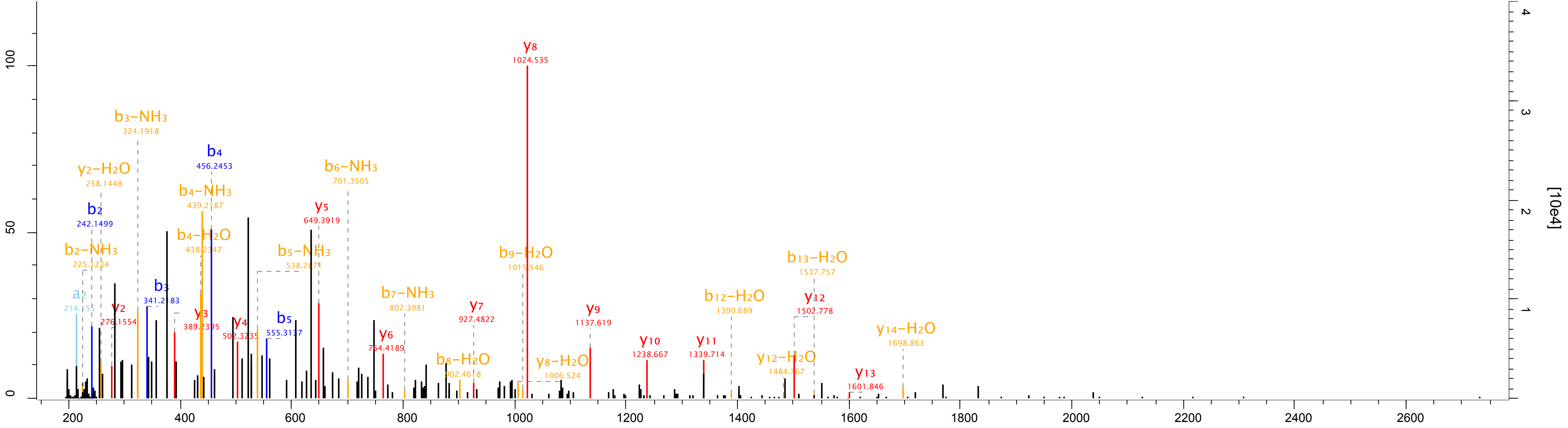

Raw file	Scan	Method	Score	Mass	Gene names
05pmolUPS2_500ngY_90min1Hz1pr_BC2_01_354	9388	TOF; CID	58.63	2084.93	RPB11

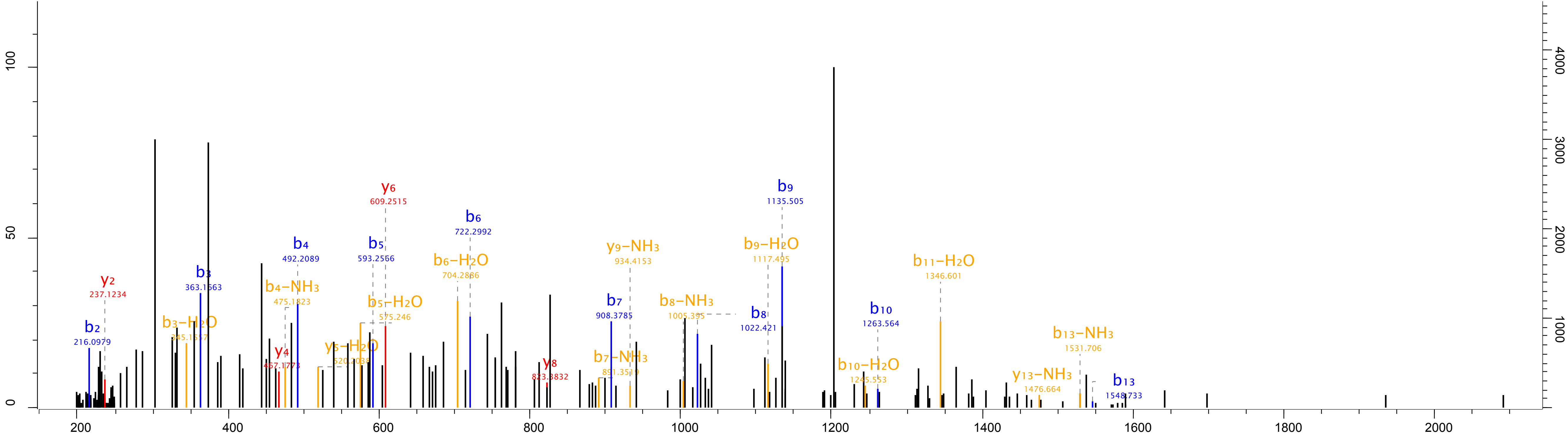

Raw file	Scan	Method	Score	Mass	Gene names
05pmolUPS2_500ngY_90min1Hz1pr_BC2_01_351	10028	TOF; CID	112.06	3179.5	FET3

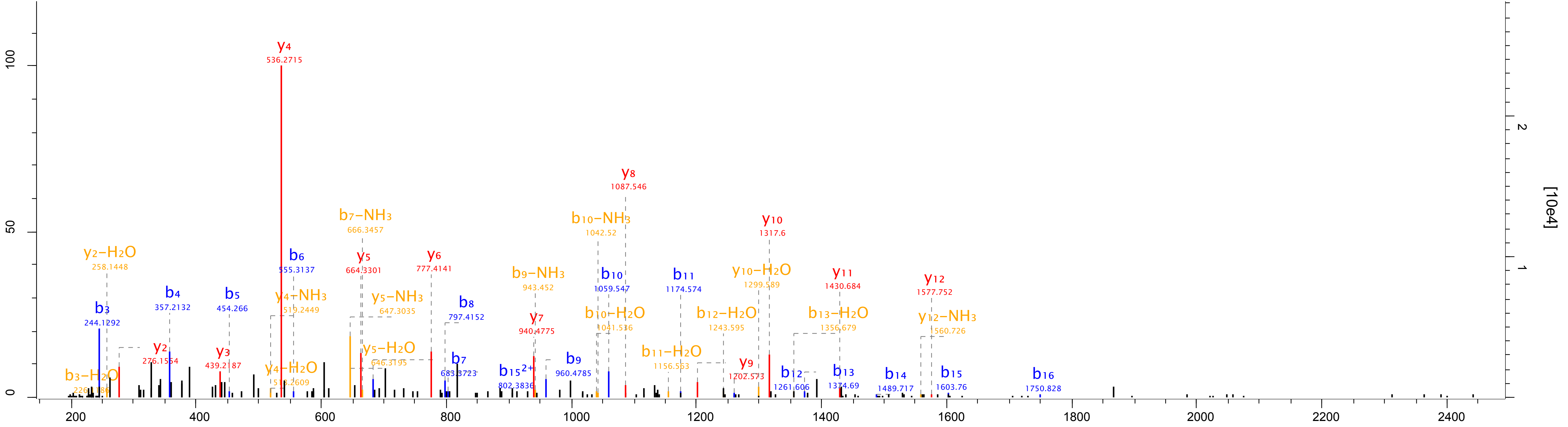

Raw file	Scan	Method	Score	Mass	Gene names
025pmolUPS2_500ngY_90min1Hz1pr_BC1_01_350	10326	TOF; CID	190.35	1464.84	LSM5

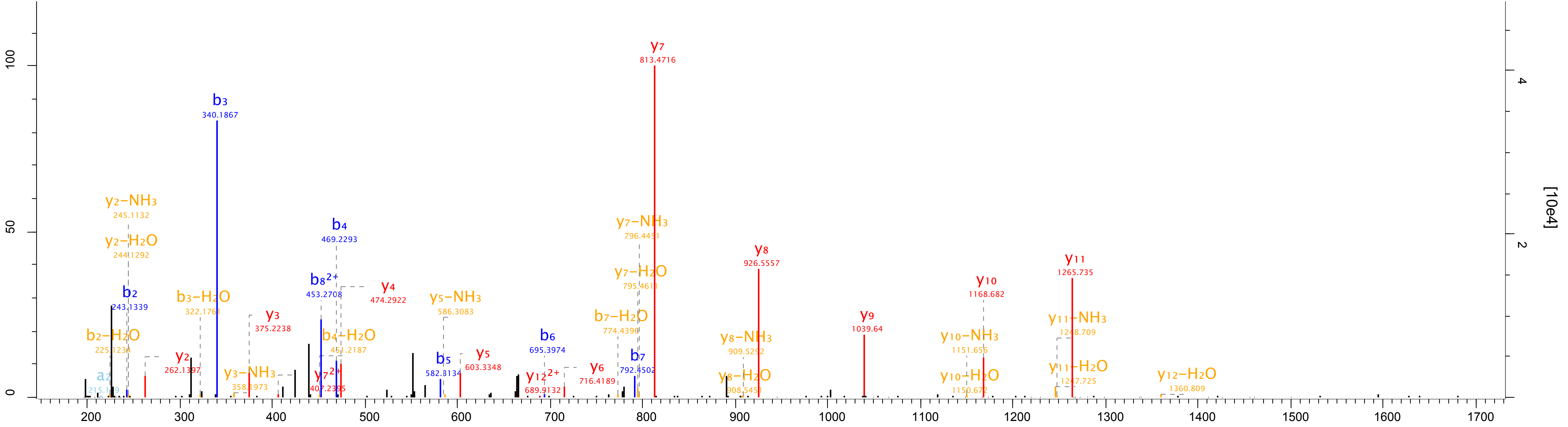

Raw file	Scan	Method	Score	Mass	Gene names
025pmolUPS2_500ngY_90min1Hz1pr_BC1_01_338	10442	TOF; CID	195.28	1900.05	GUP1

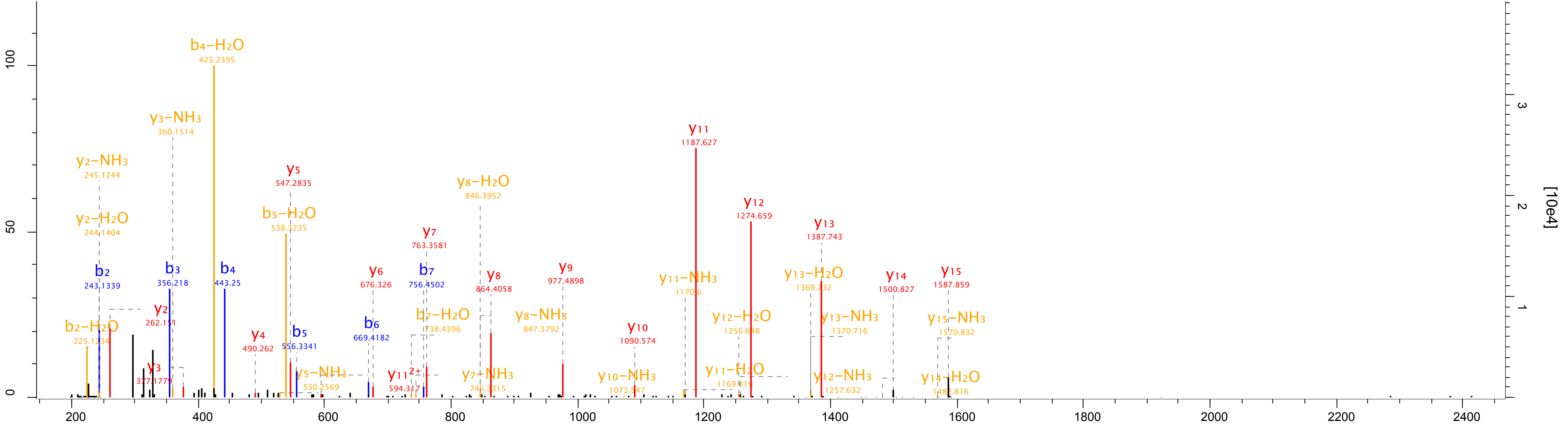

Raw file	Scan	Method	Score	Mass	Gene names
UPS1+500ngY_90minTop17_BC4_01_358	14907	TOF; CID	73.76	1861.79	NOP4

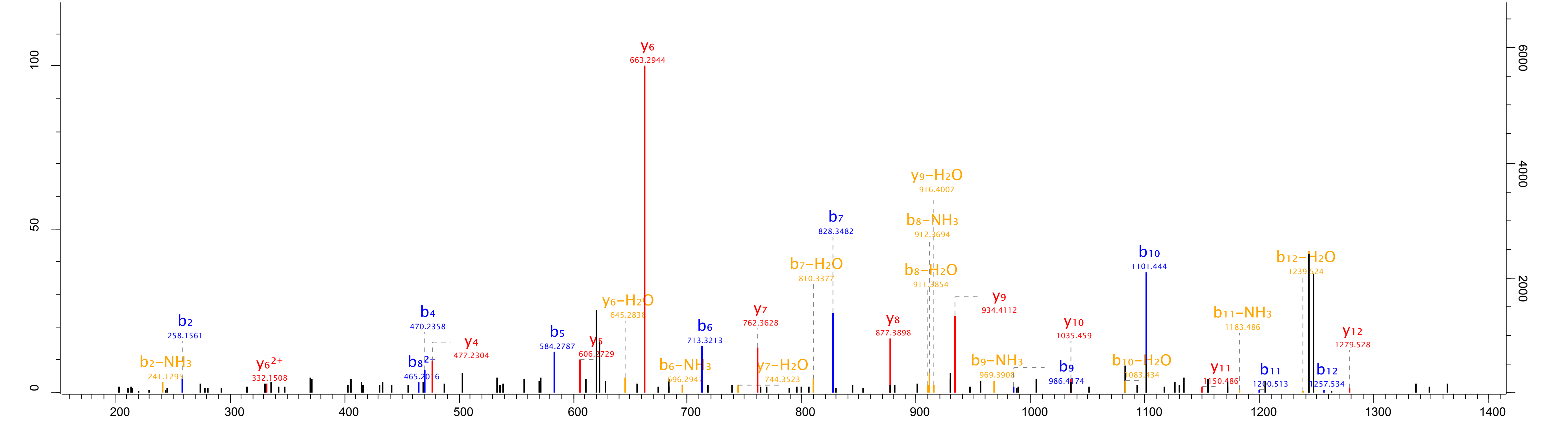

Raw file	Scan	Method	Score	Mass	Gene names
UPS1+500ngY_90minTop17_BC4_01_358	14990	TOF; CID	67.23	1343.63	IRC24

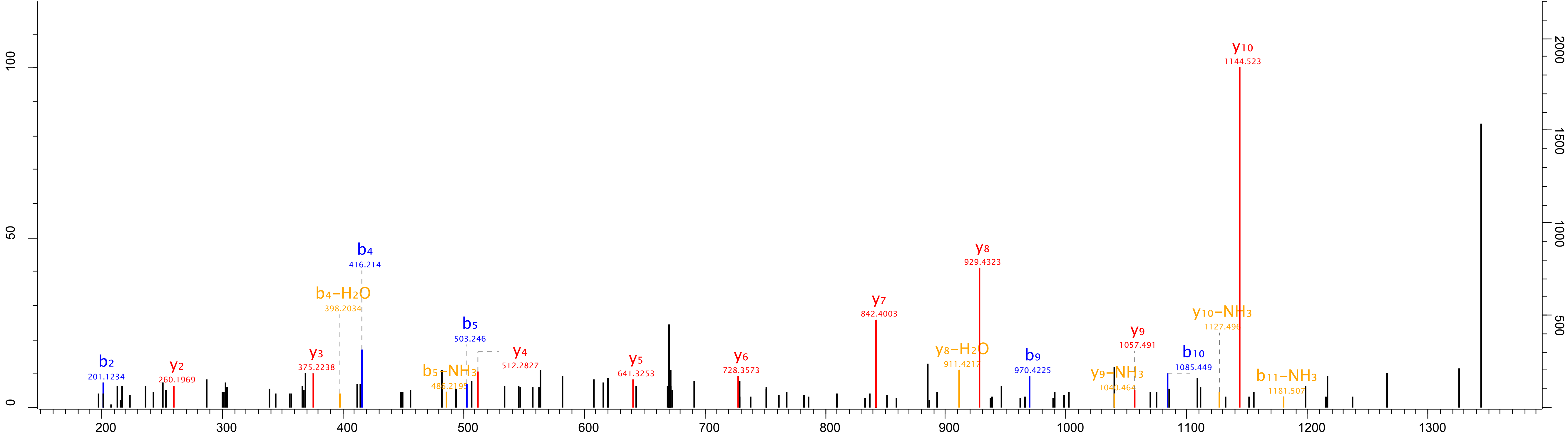

Raw file	Scan	Method	Score	Mass	Gene names
UPS1+500ngY_90minTop17_BC4_01_358	16231	TOF; CID	87.08	1550.63	UTP9

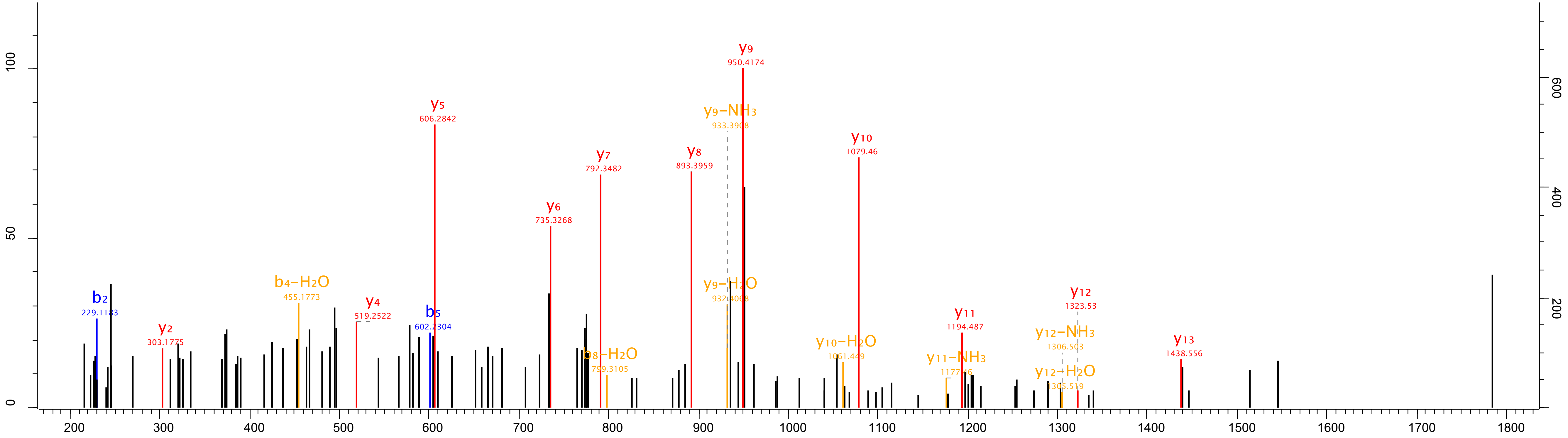

Raw file	Scan	Method	Score	Mass	Gene names
UPS1+500ngY_90minTop17_BC4_01_358	16231	TOF; CID	87.08	1550.63	UTP9

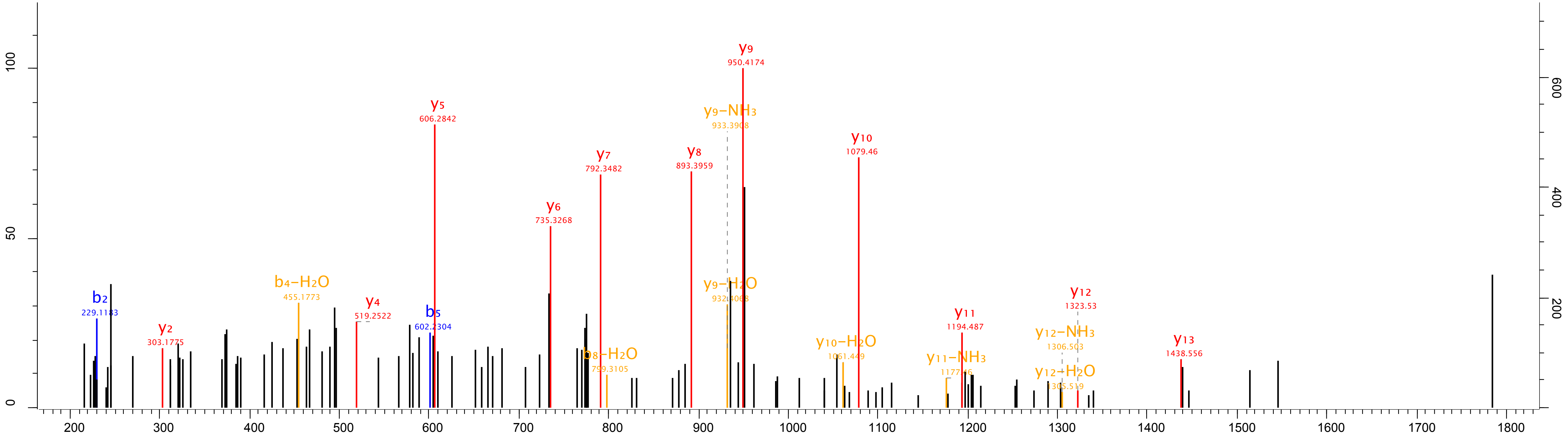

Raw file	Scan	Method	Score	Mass	Gene names
UPS1+500ngY_90minTop17_BC4_01_358	16421	TOF; CID	62.94	1810.73	YME2

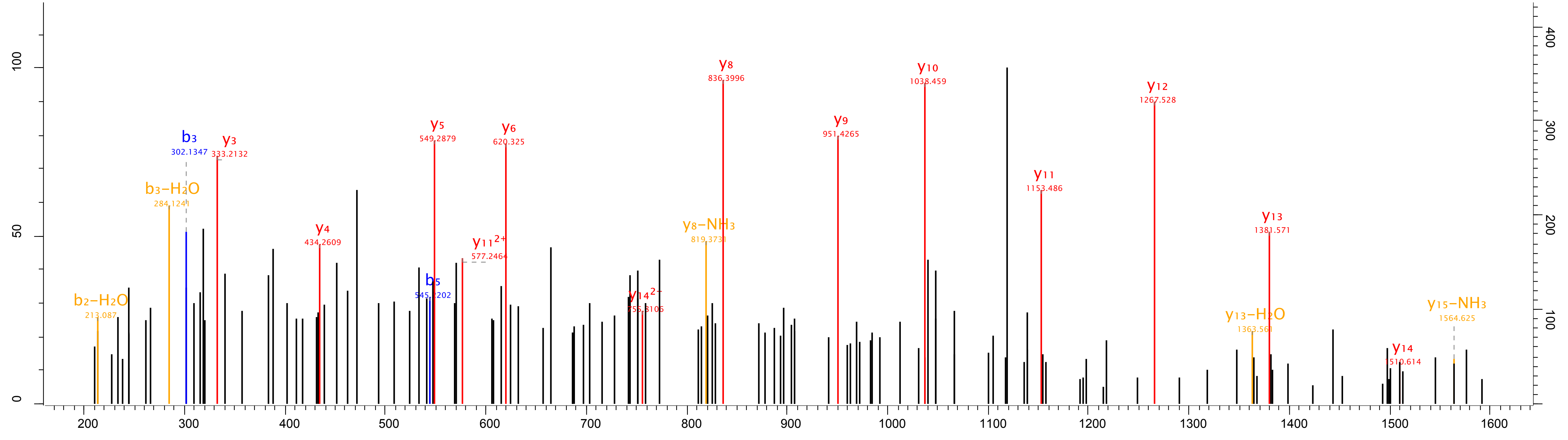

Raw file	Scan	Method	Score	Mass	Gene names
UPS1+500ngY_90minTop17_BC4_01_358	16600	TOF; CID	72.21	2063.96	RIB1

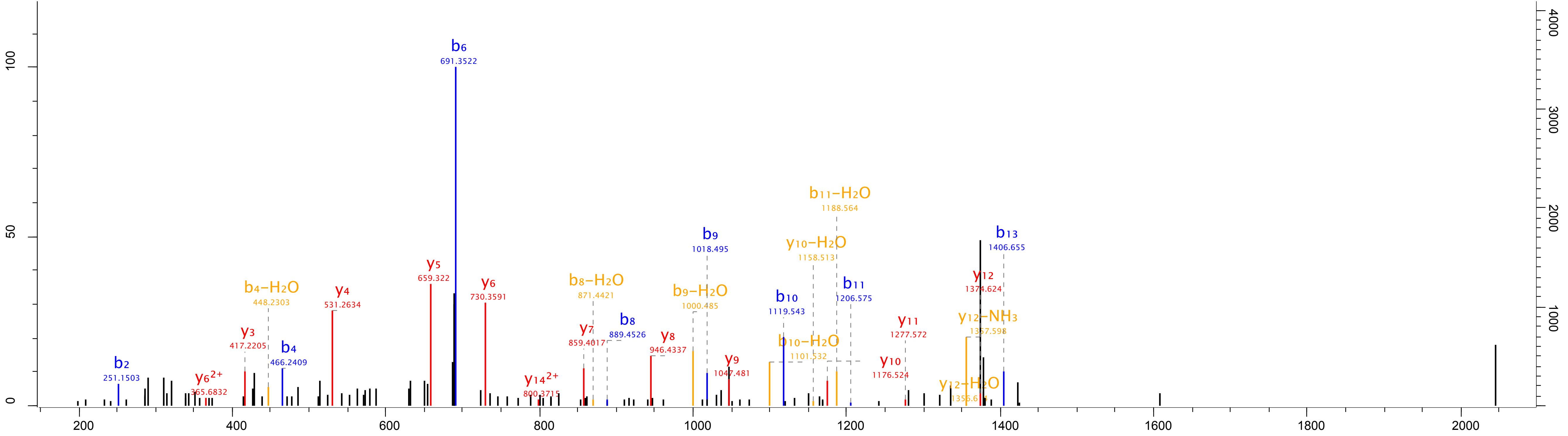

Raw file	Scan	Method	Score	Mass	Gene names
UPS1+500ngY_90minTop17_BC4_01_358	16649	TOF; CID	101.32	1519.72	SEC9

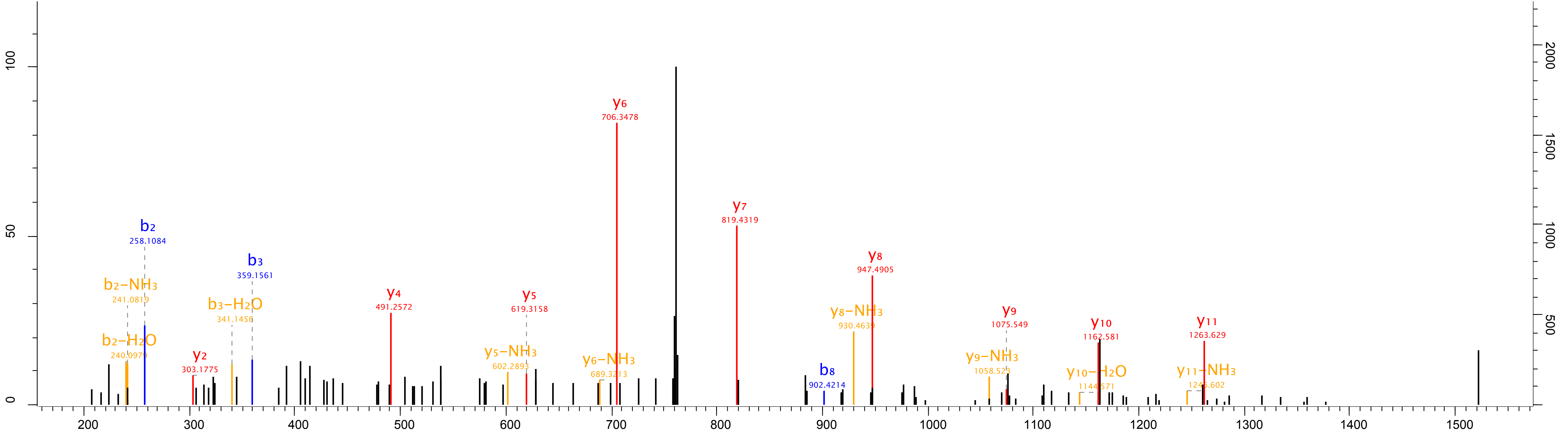

Raw file	Scan	Method	Score	Mass	Gene names
UPS1+500ngY_90minTop17_BC4_01_358	17206	TOF; CID	58.78	960.46	ESF1

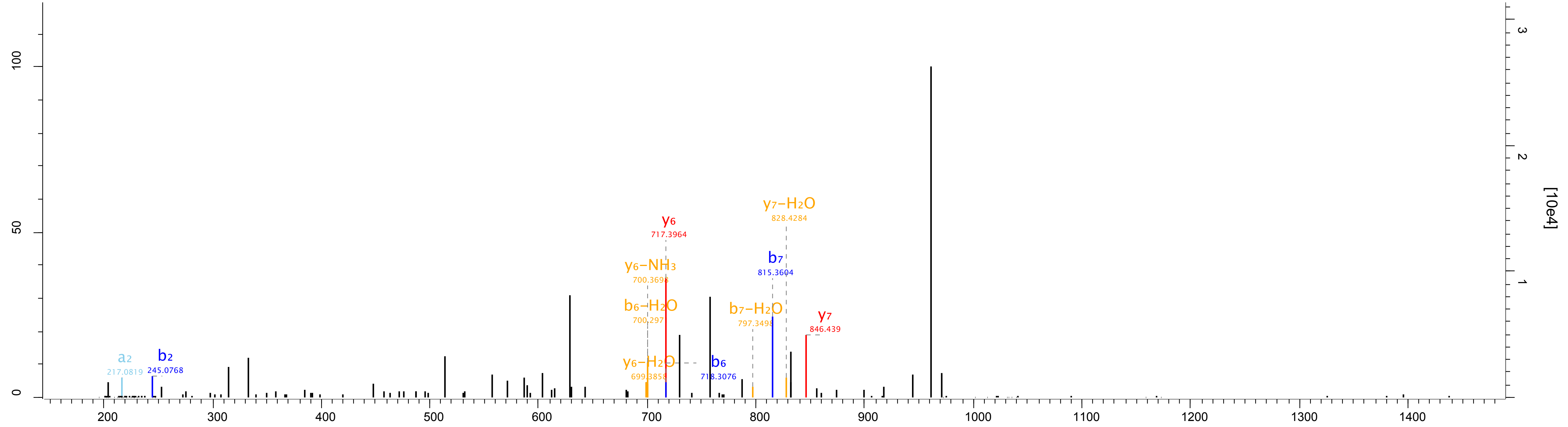

Raw file	Scan	Method	Score	Mass	Gene names
UPS1+500ngY_90minTop17_BC4_01_358	17451	TOF; CID	49.3	1135.53	CEG1

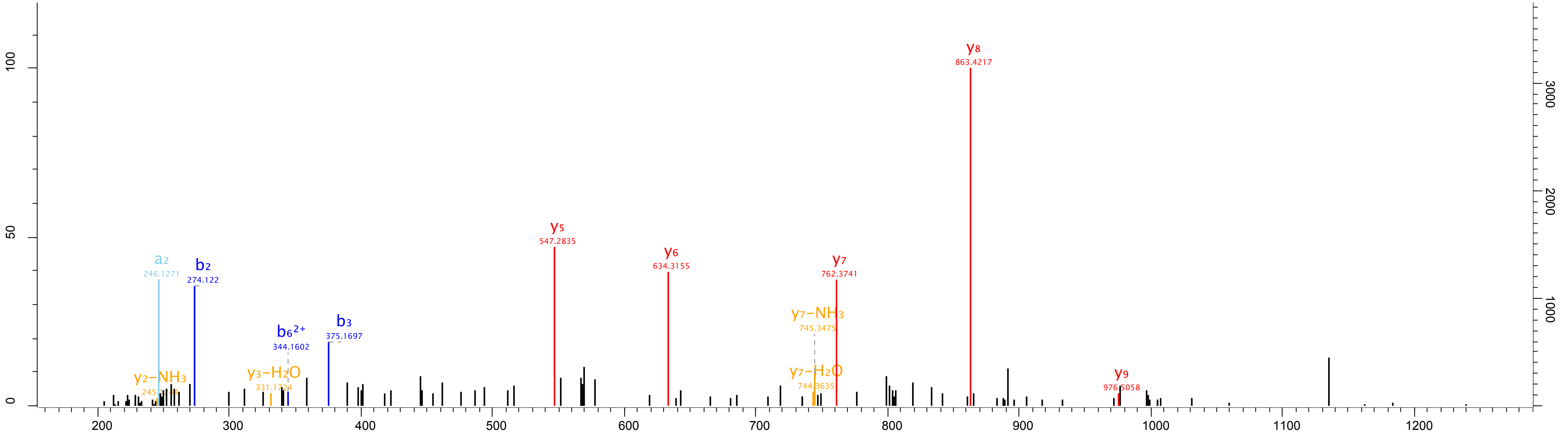

Raw file	Scan	Method	Score	Mass	Gene names
UPS1+500ngY_90minTop17_BC4_01_358	17674	TOF; CID	56.12	1655.76	PEP8

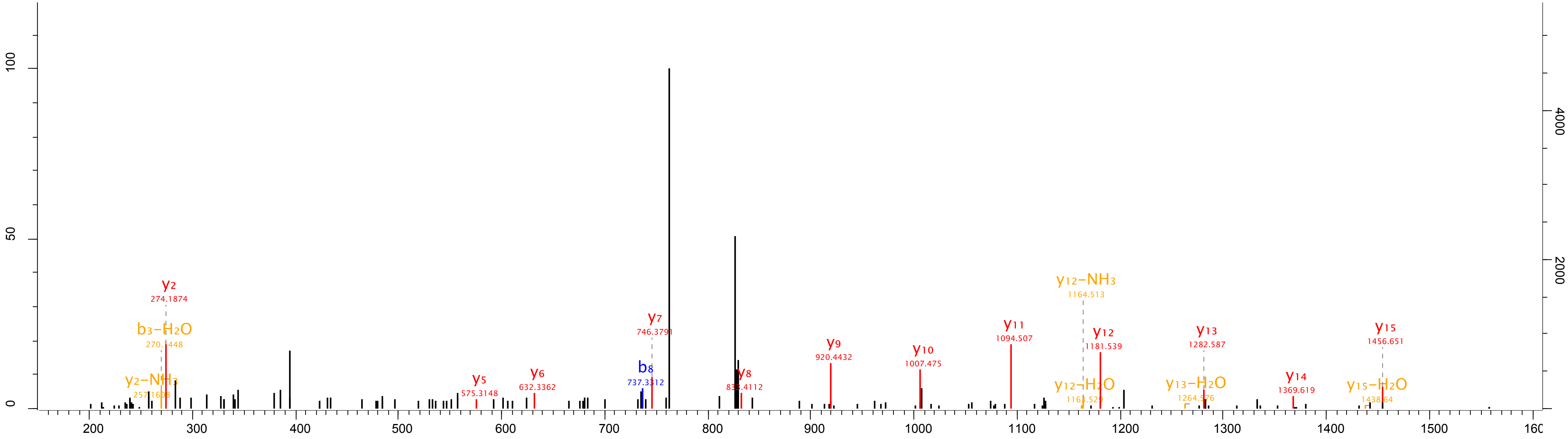

Raw file	Scan	Method	Score	Mass	Gene names
UPS1+500ngY_90minTop17_BC4_01_358	17890	TOF; CID	71.15	2262.86	HAL5

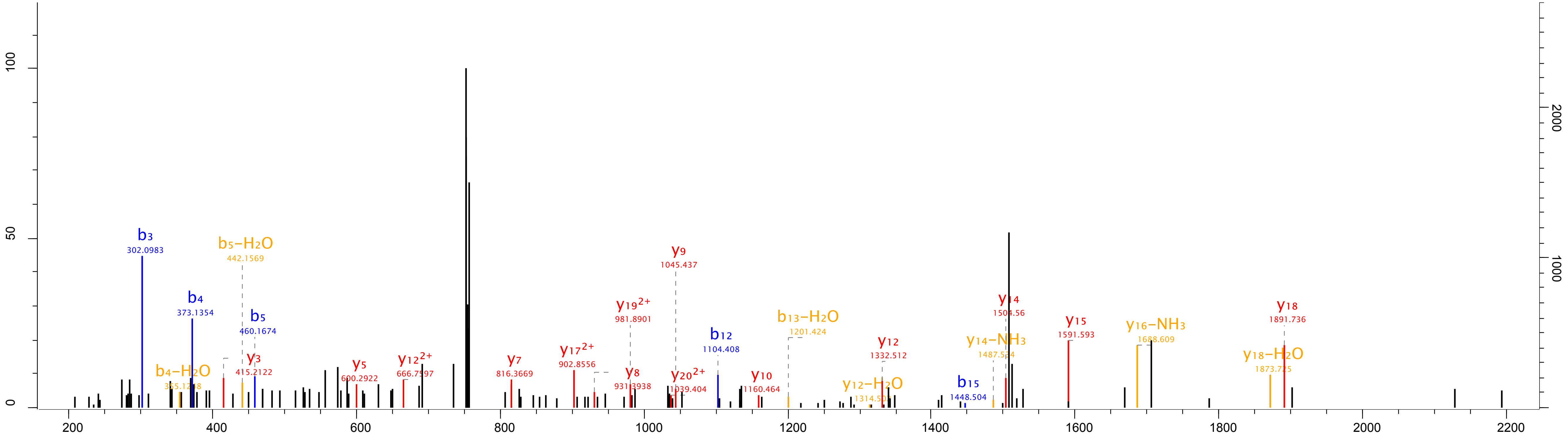

Raw file	Scan	Method	Score	Mass	Gene names
UPS1+500ngY_90minTop17_BC4_01_358	17936	TOF; CID	58.17	1134.59	CEX1

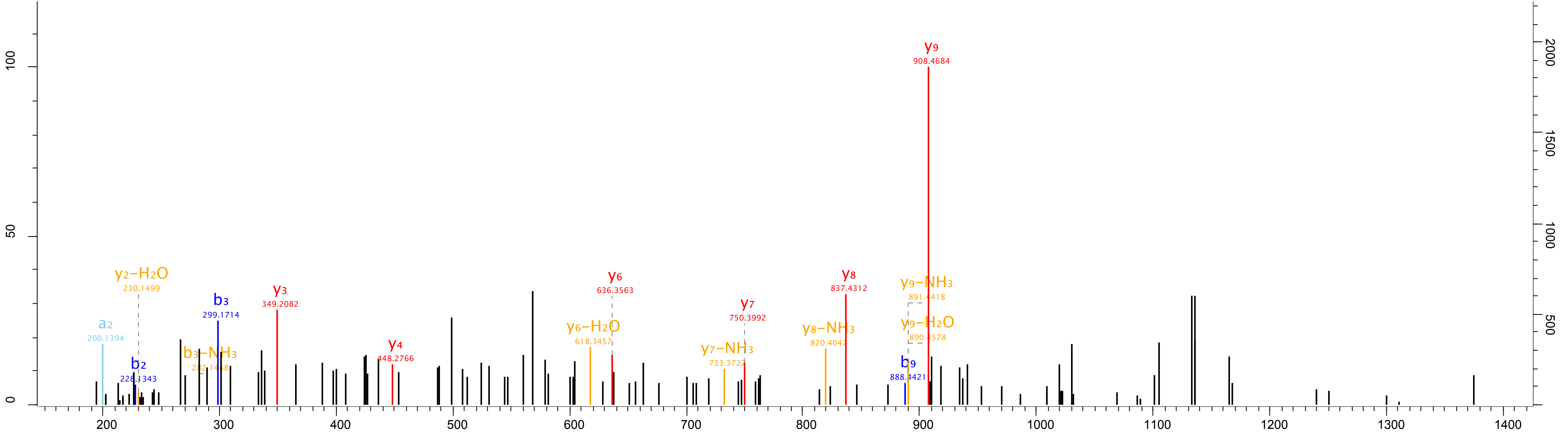

Raw file	Scan	Method	Score	Mass	Gene names
UPS1+500ngY_90minTop17_BC4_01_358	18291	TOF; CID	92.61	1315.63	SML1

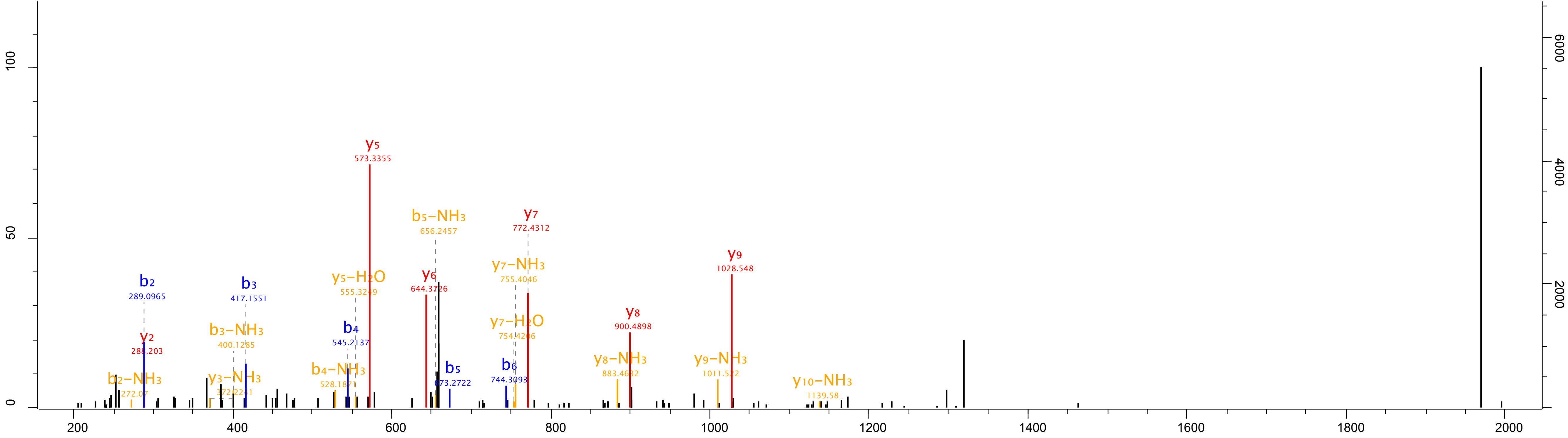

Raw file	Scan	Method	Score	Mass	Gene names
UPS1+500ngY_90minTop17_BC4_01_358	18390	TOF; CID	83.18	1228.51	LSB5

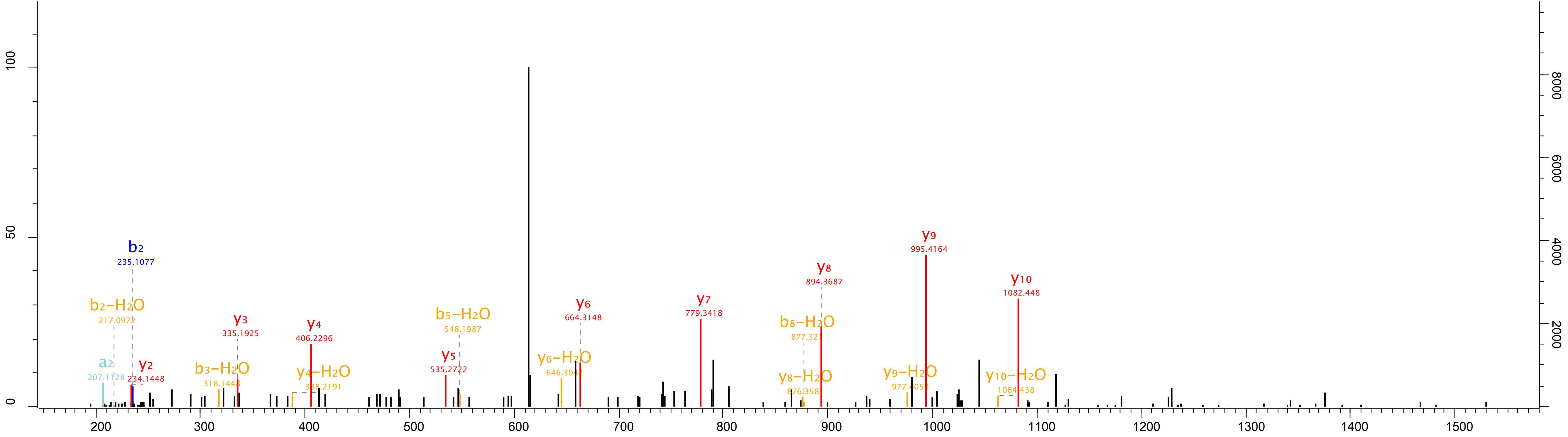

Raw file	Scan	Method	Score	Mass	Gene names
UPS1+500ngY_90minTop17_BC4_01_358	18413	TOF; CID	53.26	1536.74	CMP2

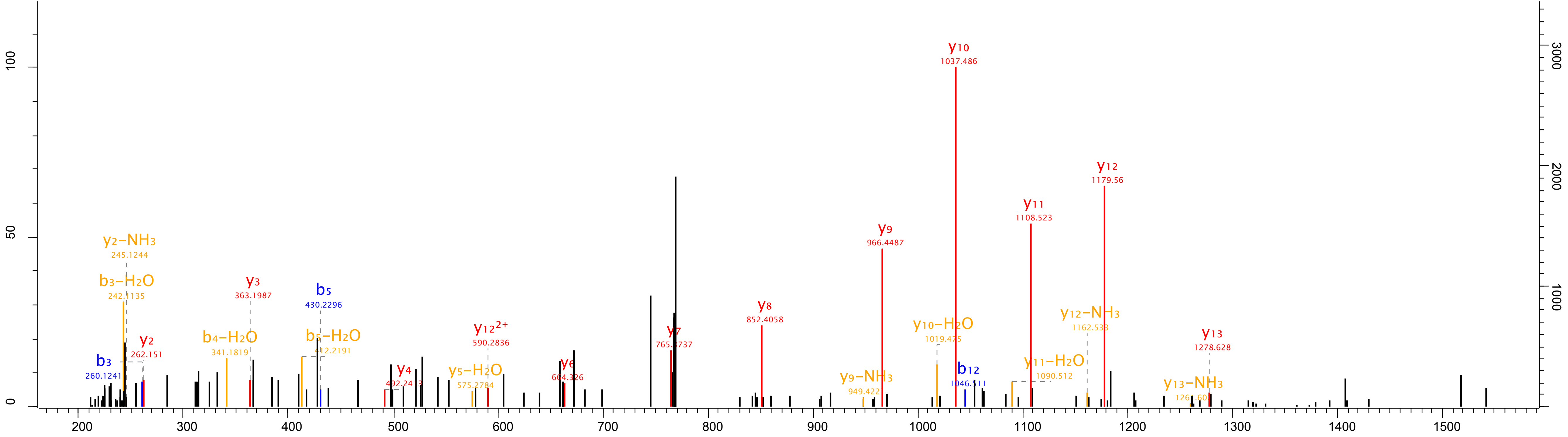

Raw file	Scan	Method	Score	Mass	Gene names
UPS1+500ngY_90minTop17_BC4_01_358	18492	TOF; CID	106.32	1388.62	PAR32

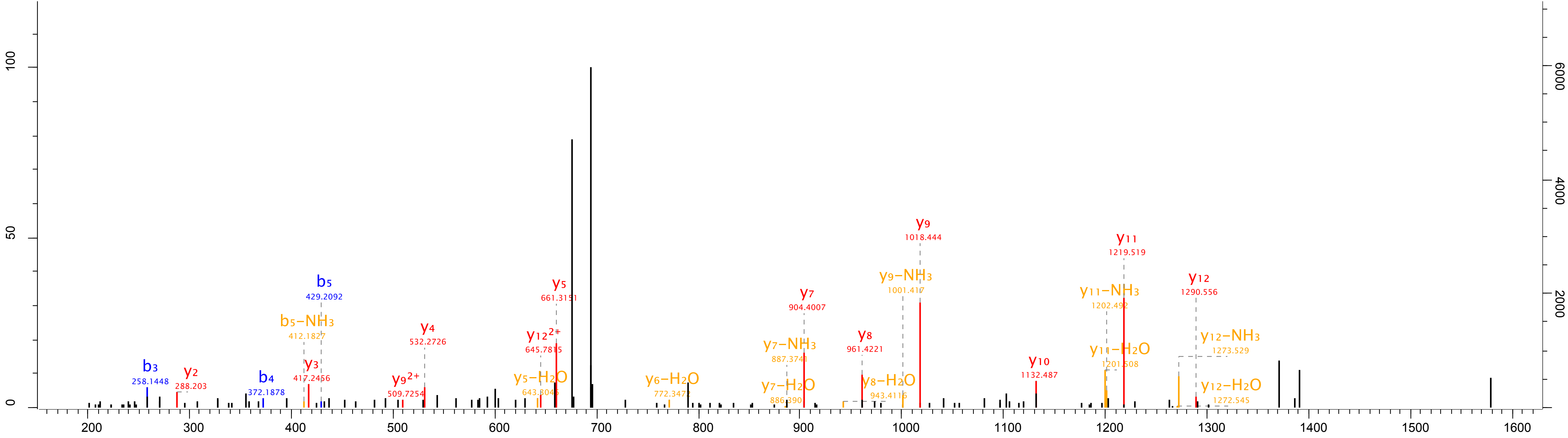

Raw file	Scan	Method	Score	Mass	Gene names
UPS1+500ngY_90minTop17_BC4_01_358	18662	TOF; CID	98.1	1356.67	NUP57

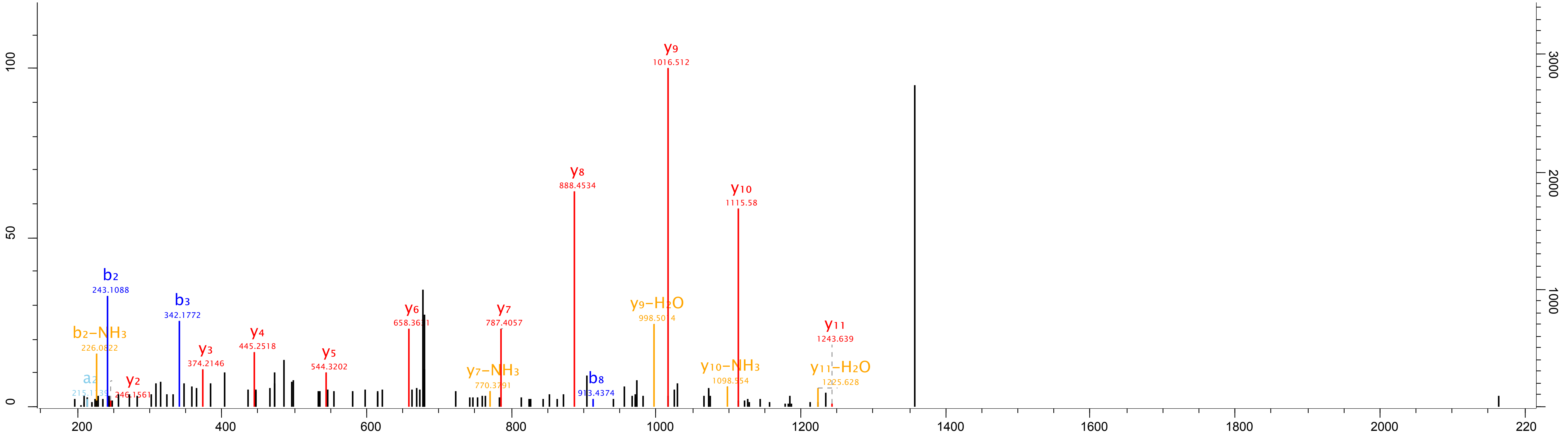

Raw file	Scan	Method	Score	Mass	Gene names
UPS1+500ngY_90minTop17_BC4_01_358	19006	TOF; CID	50.35	1168.49	CYR1

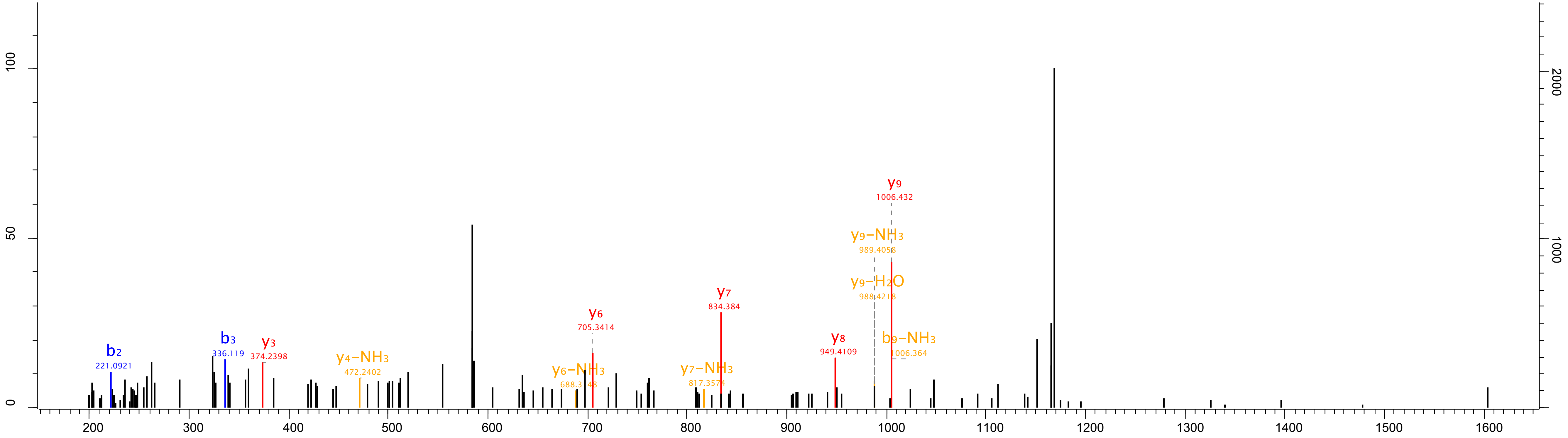

Raw file	Scan	Method	Score	Mass	Gene names
UPS1+500ngY_90minTop17_BC4_01_358	19192	TOF; CID	68.97	1417.68	PNO1

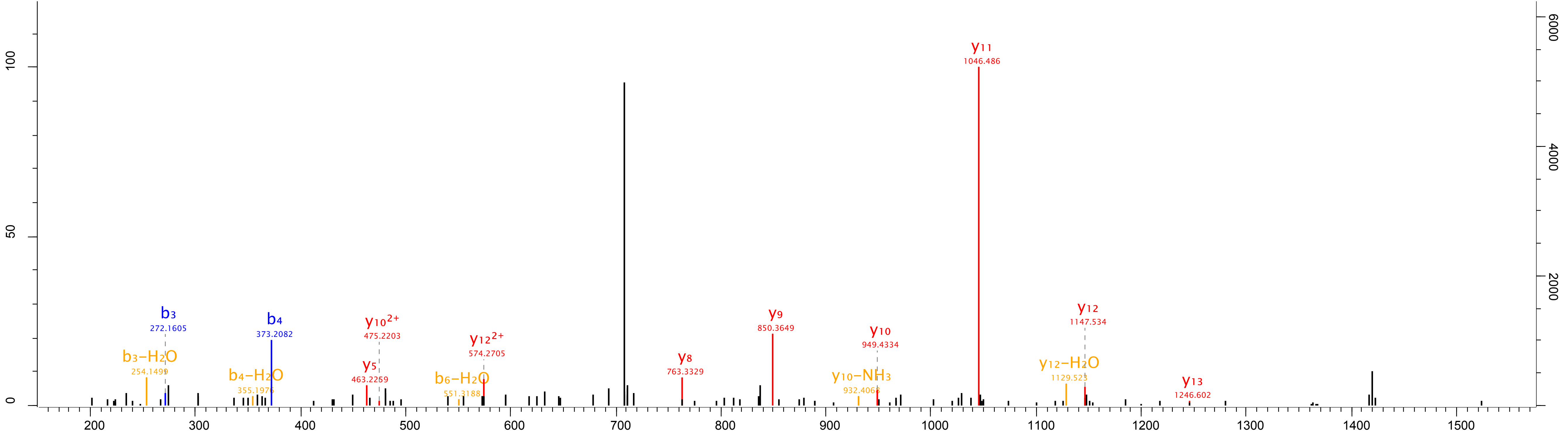

Raw file	Scan	Method	Score	Mass	Gene names
UPS1+500ngY_90minTop17_BC4_01_358	19832	TOF; CID	59.84	1014.53	PUP1

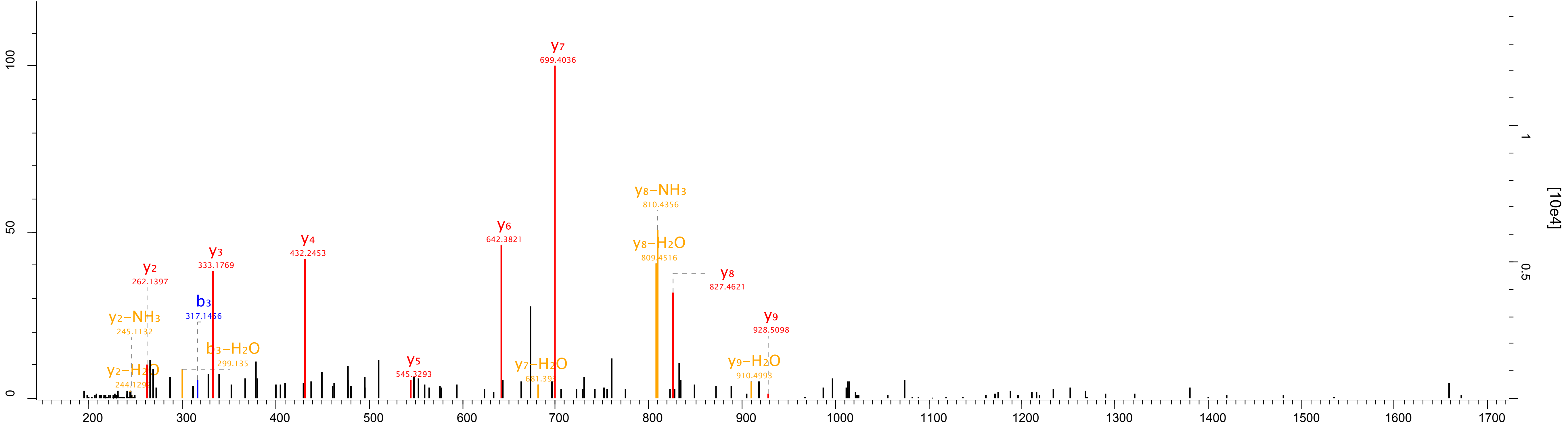

Raw file	Scan	Method	Score	Mass	Gene names
UPS1+500ngY_90minTop17_BC4_01_358	20027	TOF; CID	80.24	2216.85	SLG1

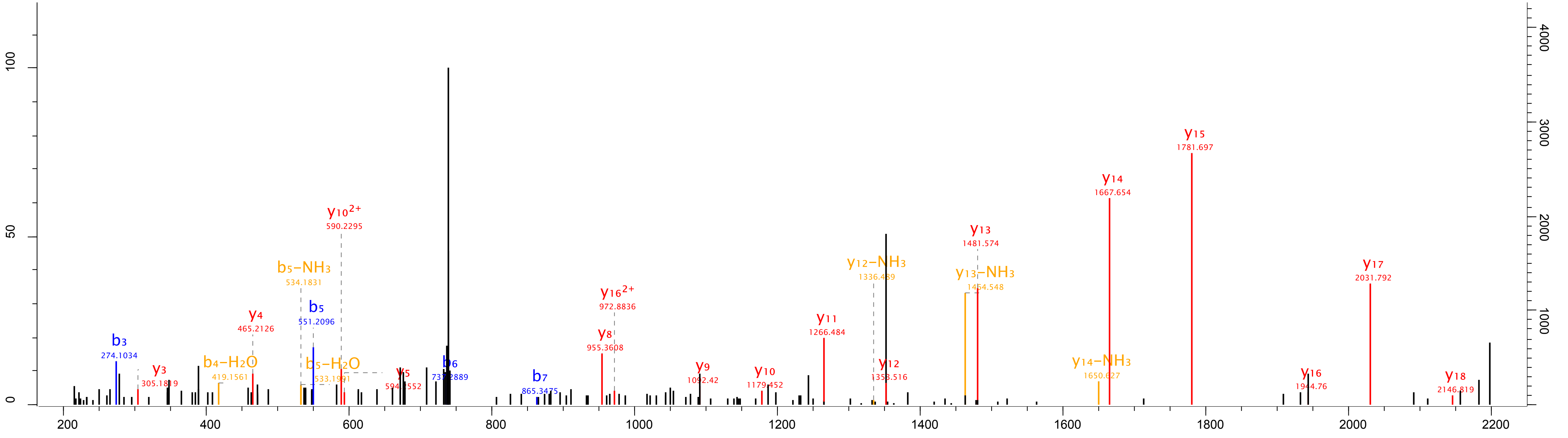

Raw file	Scan	Method	Score	Mass	Gene names
UPS1+500ngY_90minTop17_BC4_01_358	20078	TOF; CID	54.02	1274.62	CIR2

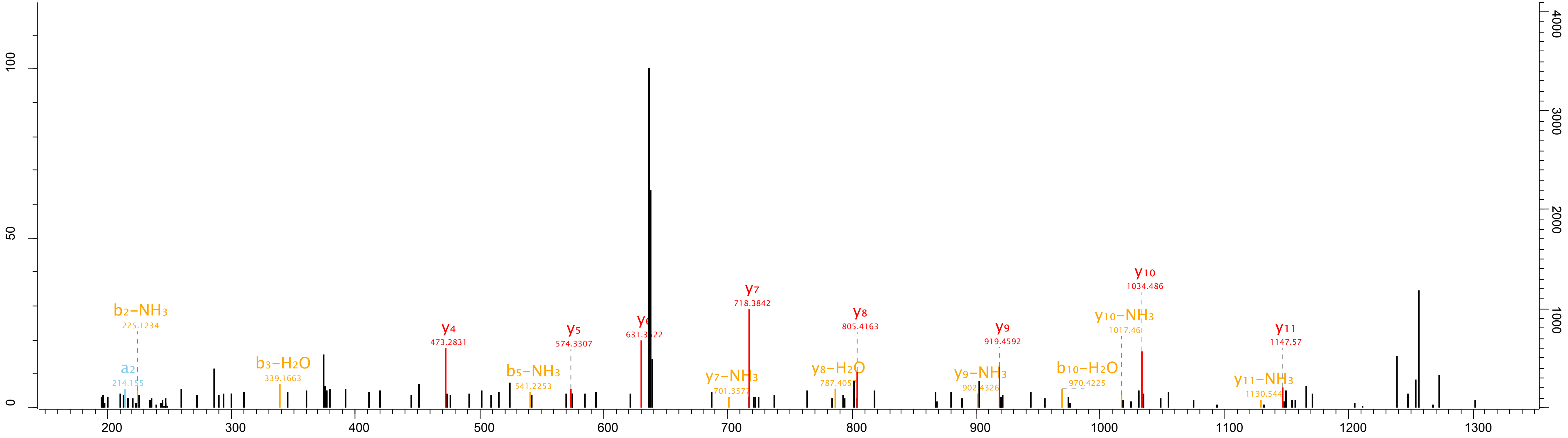

Raw file	Scan	Method	Score	Mass	Gene names
UPS1+500ngY_90minTop17_BC4_01_358	20200	TOF; CID	66.96	1166.56	FMP10

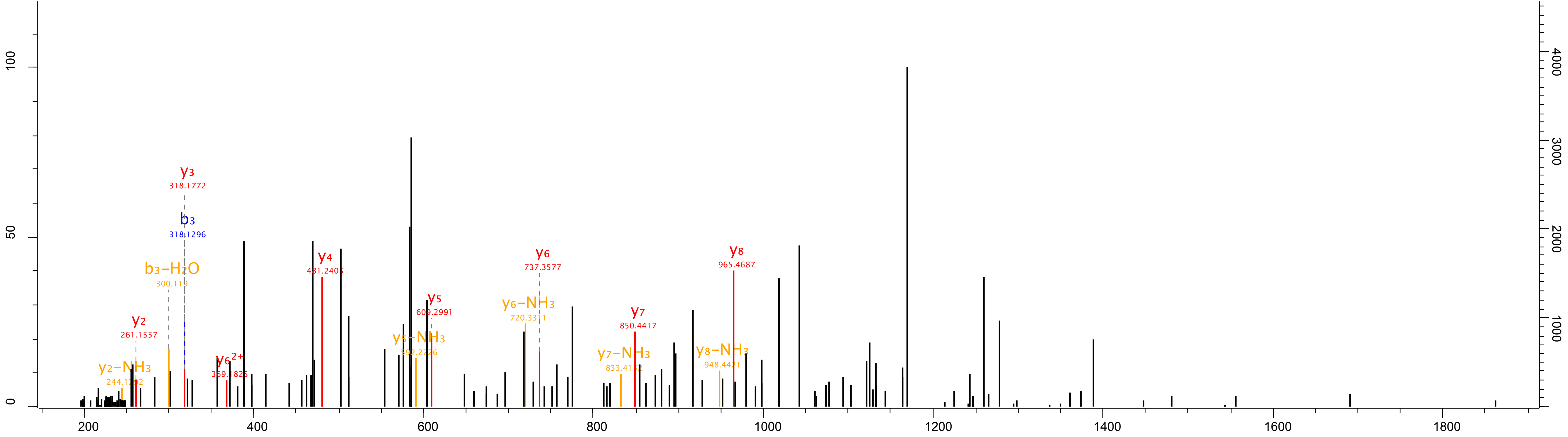

Raw file	Scan	Method	Score	Mass	Gene names
UPS1+500ngY_90minTop17_BC4_01_358	22234	TOF; CID	71.38	1070.47	SHR3

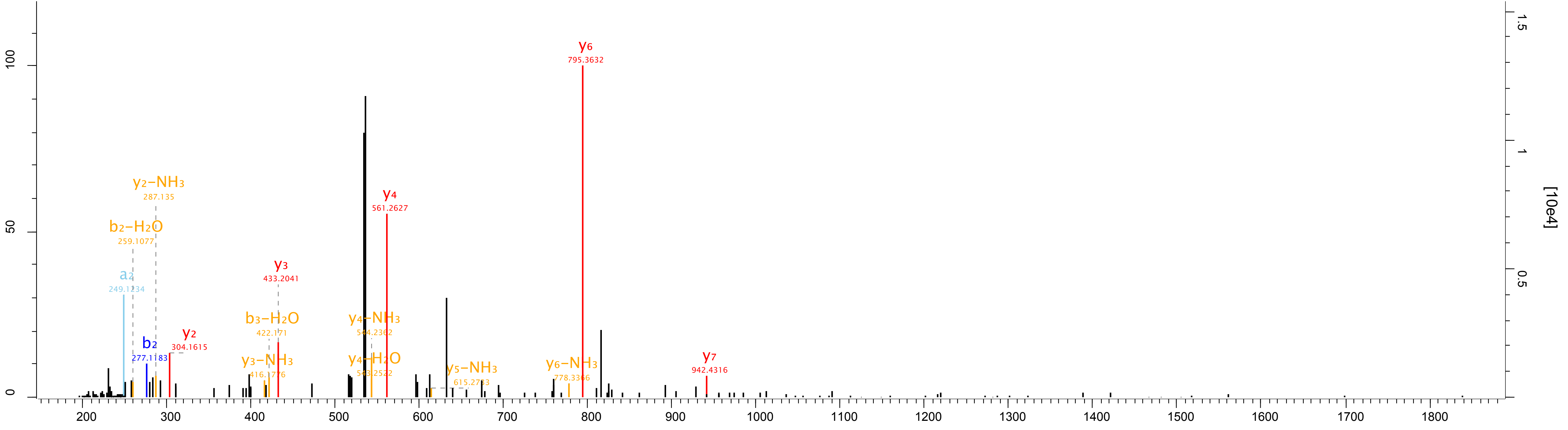

Raw file	Scan	Method	Score	Mass	Gene names
UPS1+500ngY_90minTop17_BC4_01_358	22844	TOF; CID	122.97	1422.61	RRP1

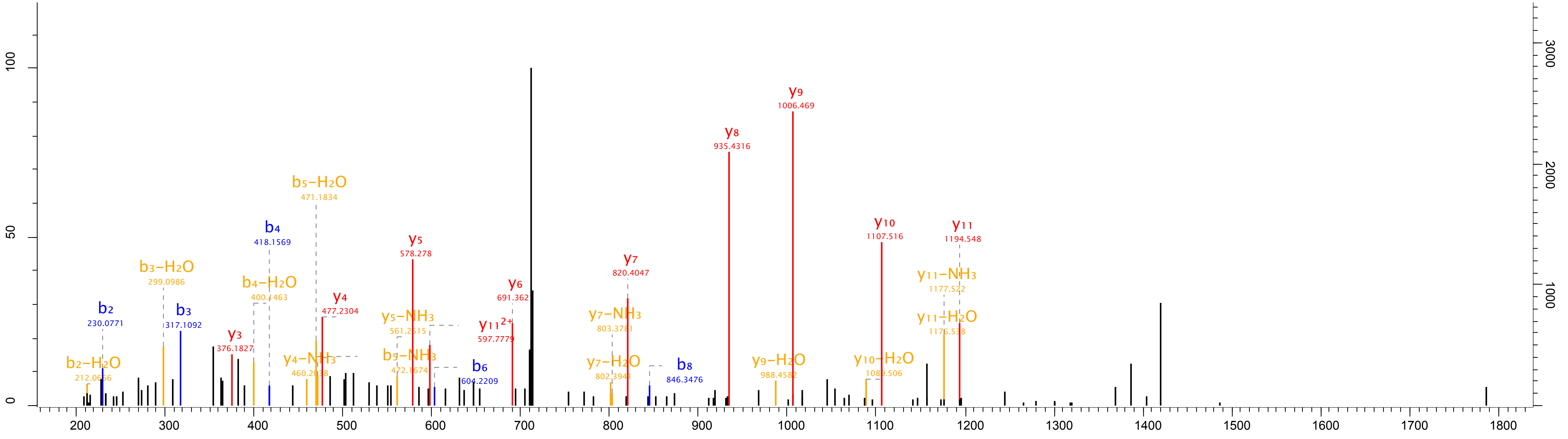

Raw file	Scan	Method	Score	Mass	Gene names
UPS1+500ngY_90minTop17_BC4_01_358	23805	TOF; CID	88.34	1304.6	FMP16

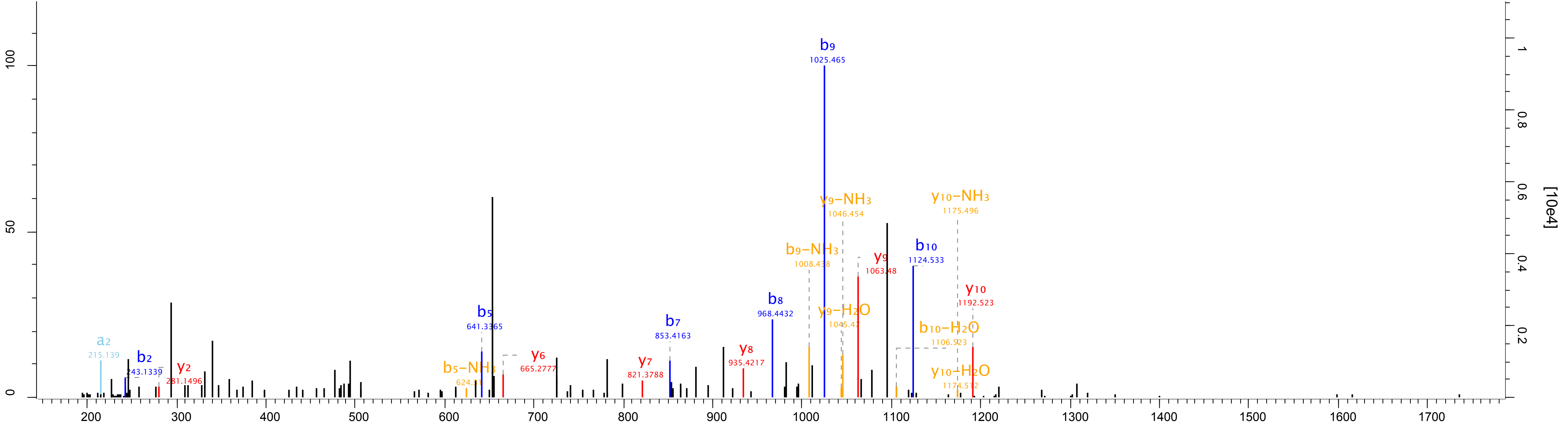

Raw file	Scan	Method	Score	Mass	Gene names
UPS1+500ngY_90minTop17_BC4_01_358	24771	TOF; CID	118.31	1344.69	POM33

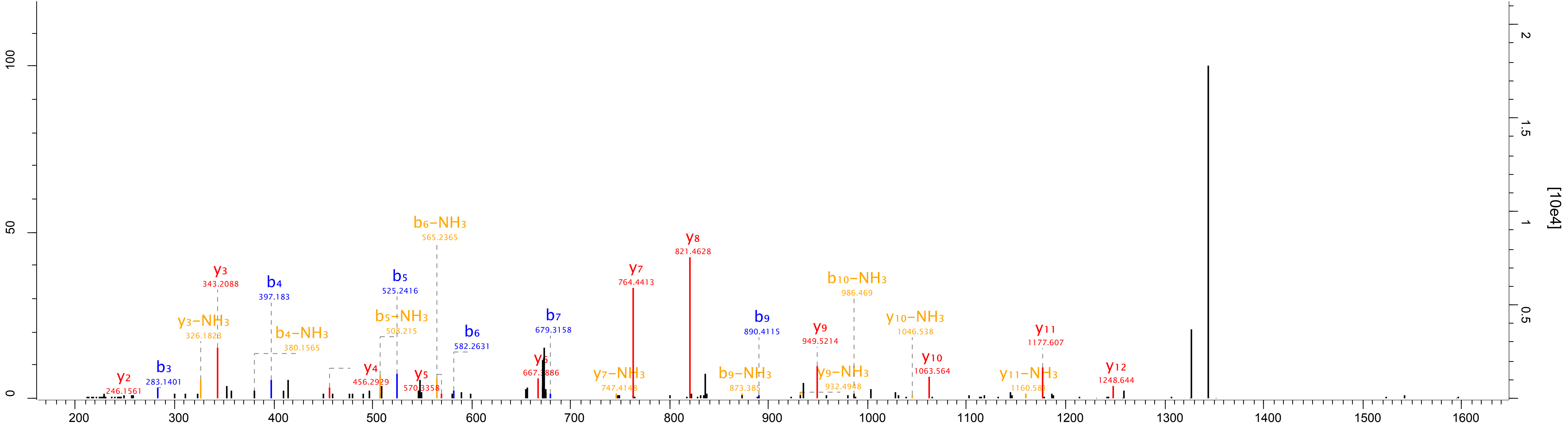

Raw file	Scan	Method	Score	Mass	Gene names
UPS1+500ngY_90minTop17_BC4_01_358	24796	TOF; CID	54.26	1186.56	AVT1

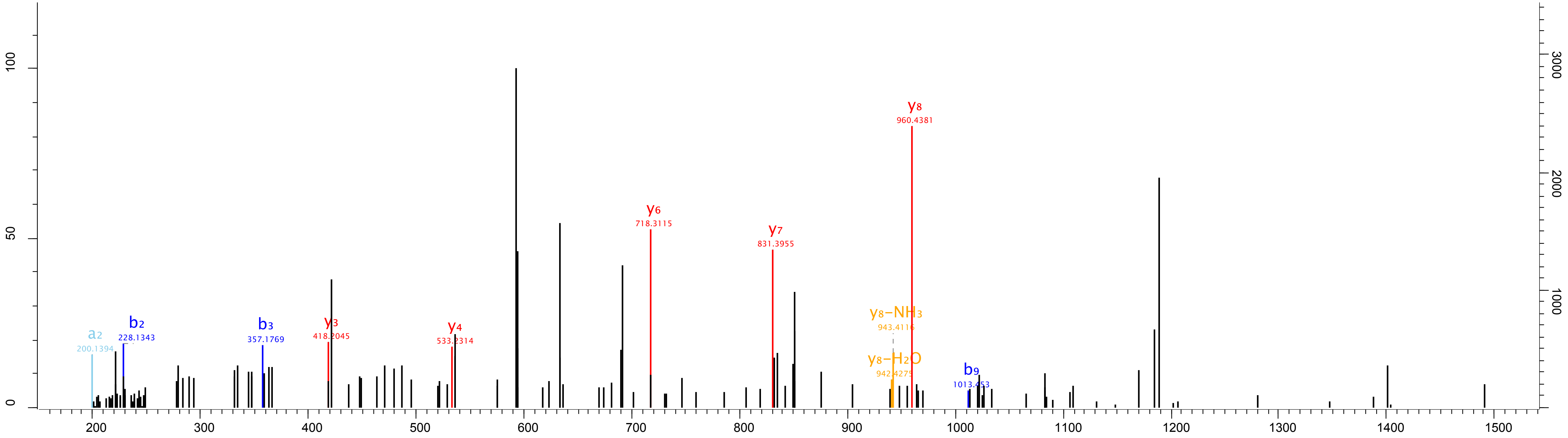

Raw file	Scan	Method	Score	Mass	Gene names
UPS1+500ngY_90minTop17_BC4_01_358	24908	TOF; CID	42.91	1920.87	GPA2

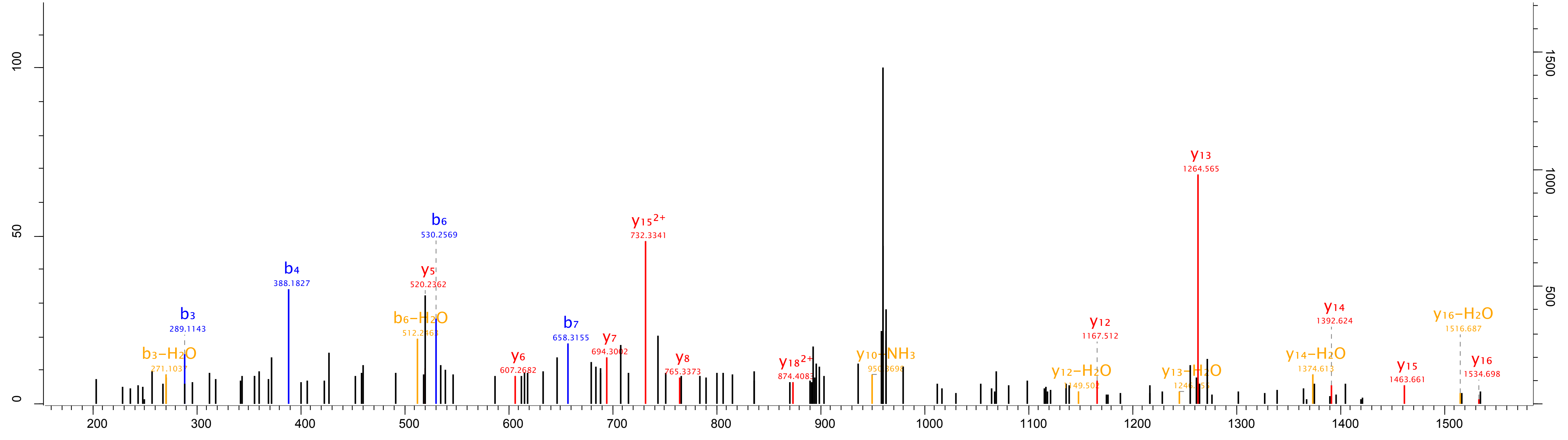

Raw file	Scan	Method	Score	Mass	Gene names
UPS1+500ngY_90minTop17_BC4_01_358	25699	TOF; CID	107.69	1415.69	ARL1

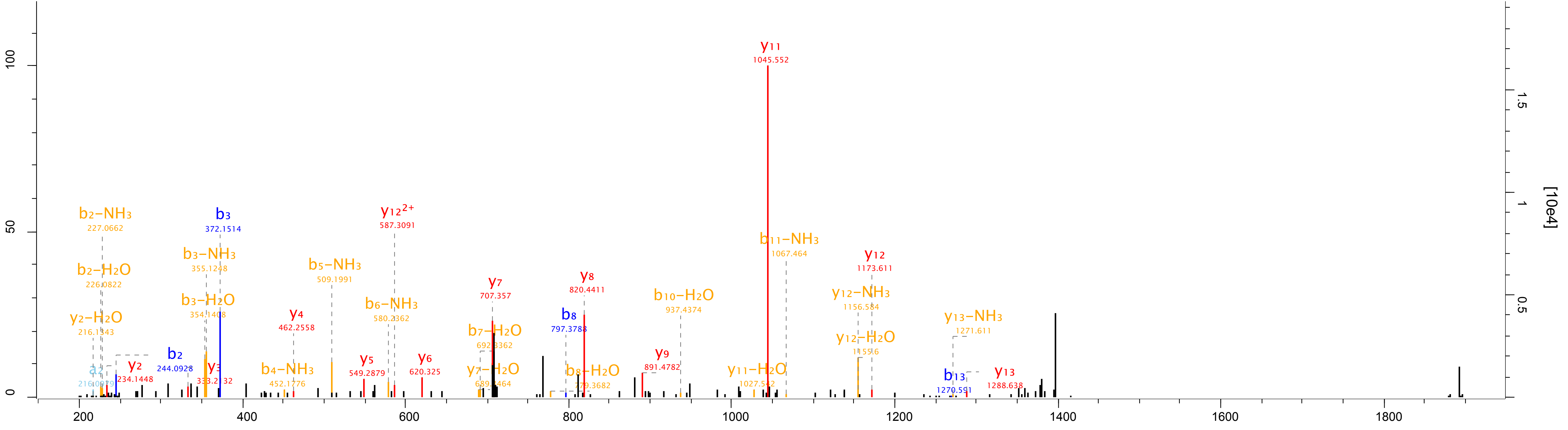

Raw file	Scan	Method	Score	Mass	Gene names
UPS1+500ngY_90minTop17_BC4_01_358	25755	TOF; CID	87.08	1328.7	EMC5

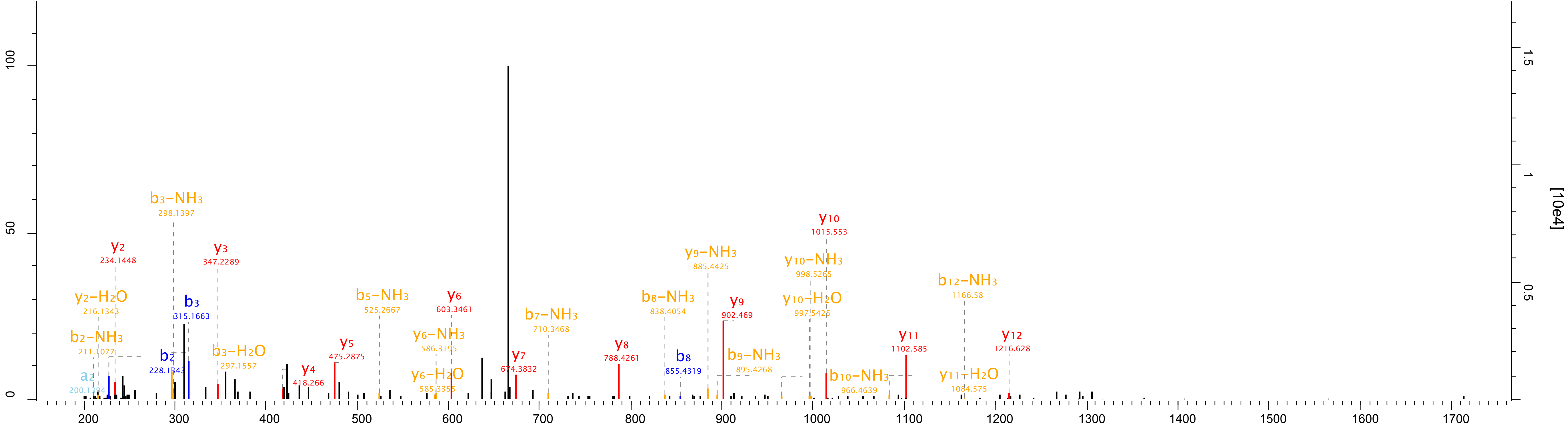

Raw file	Scan	Method	Score	Mass	Gene names
UPS1+500ngY_90minTop17_BC4_01_358	26063	TOF; CID	68	1478.63	RPC17

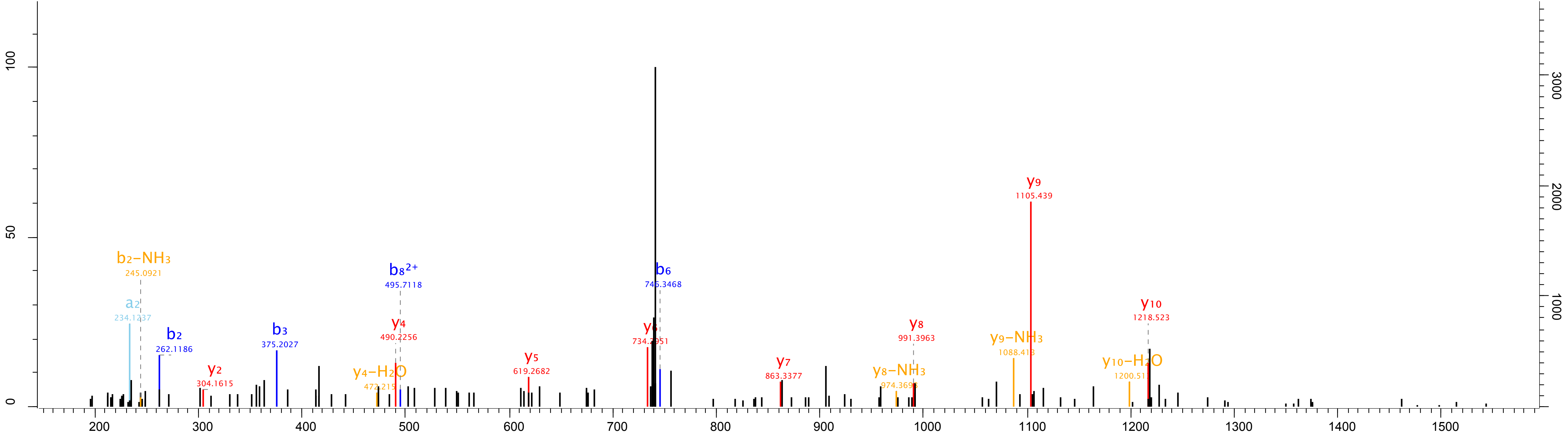

Raw file	Scan	Method	Score	Mass	Gene names
UPS1+500ngY_90minTop17_BC4_01_358	26251	TOF; CID	54.28	1330.67	TRM8

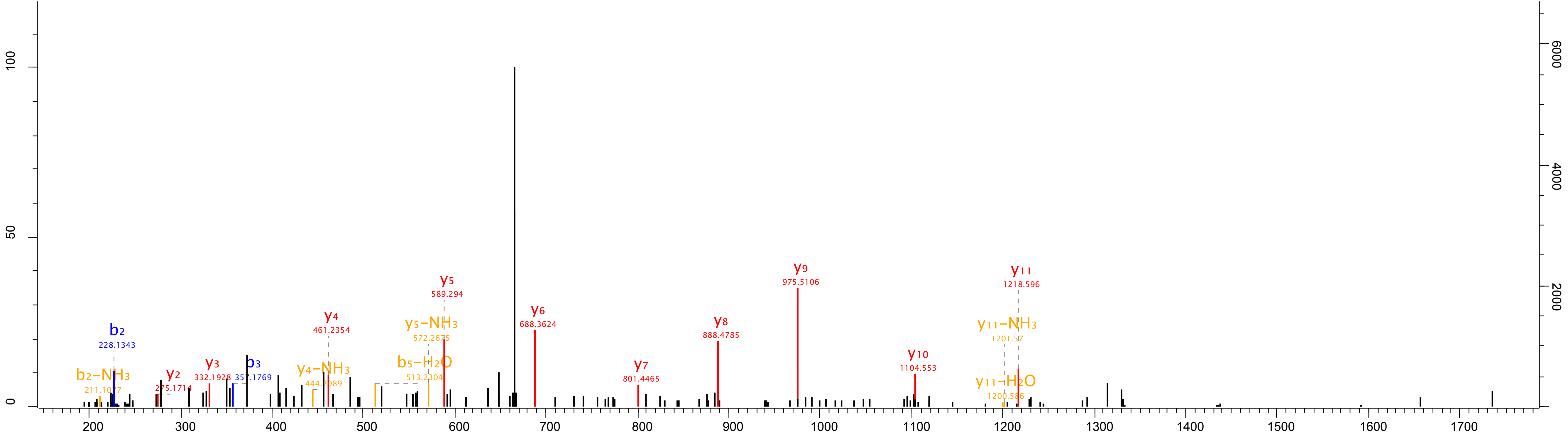

Raw file	Scan	Method	Score	Mass	Gene names
UPS1+500ngY_90minTop17_BC4_01_358	26594	TOF; CID	84.3	1253.63	SBH2

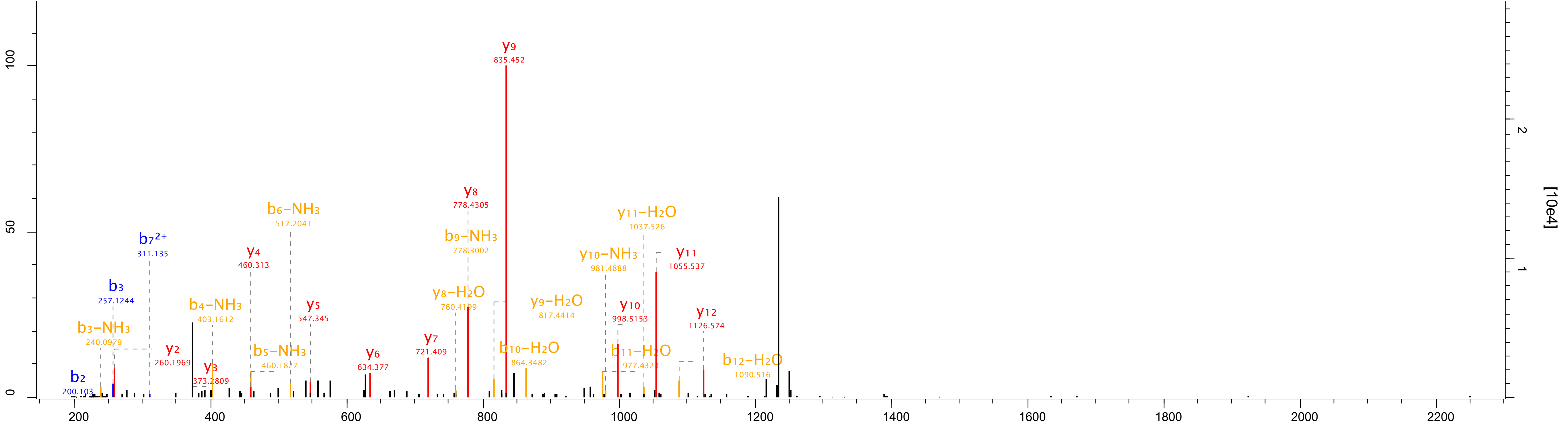

Raw file	Scan	Method	Score	Mass	Gene names
UPS1+500ngY_90minTop17_BC4_01_358	26725	TOF; CID	75.32	1462.66	YGR169C-A

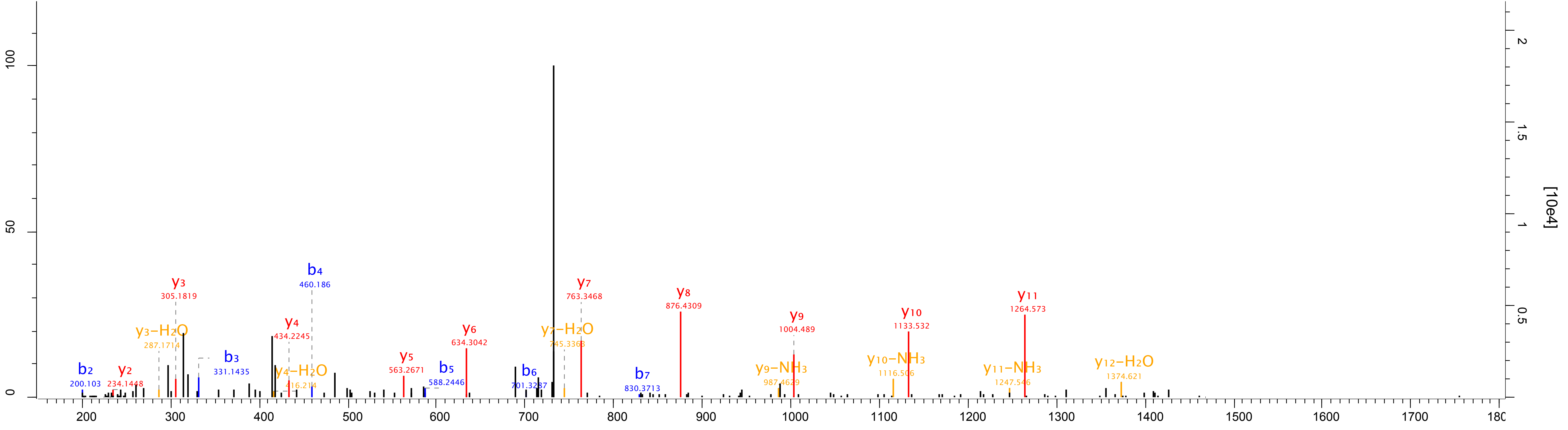

Raw file	Scan	Method	Score	Mass	Gene names
UPS1+500ngY_90minTop17_BC4_01_358	28395	TOF; CID	110.56	1779.79	YOS1

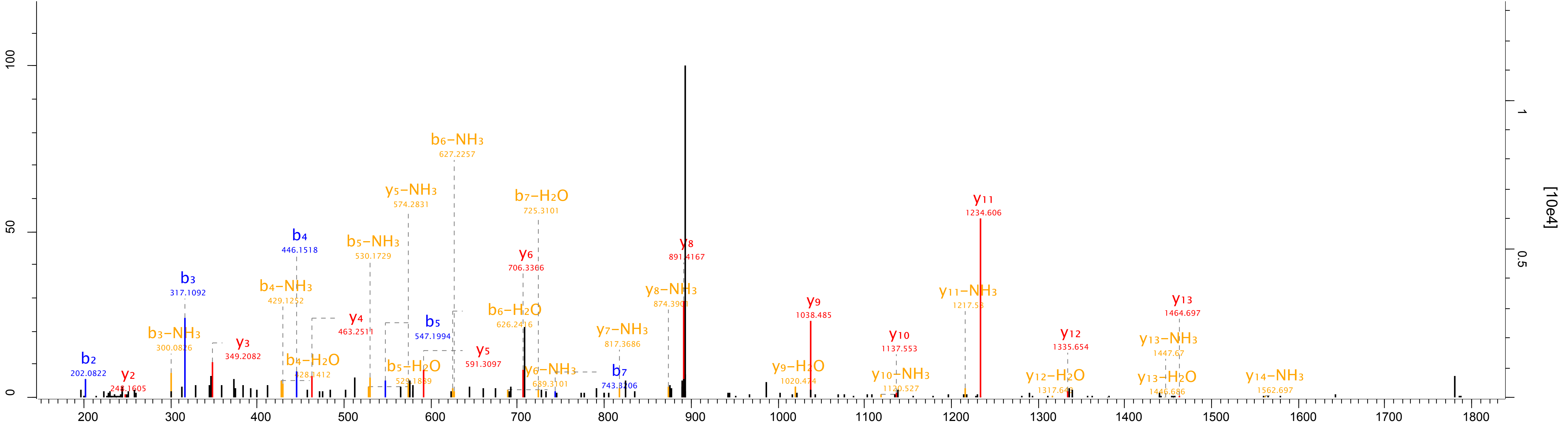

Raw file	Scan	Method	Score	Mass	Gene names
UPS1+500ngY_90minTop17_BC4_01_358	28410	TOF; CID	74.79	1718.84	DFM1

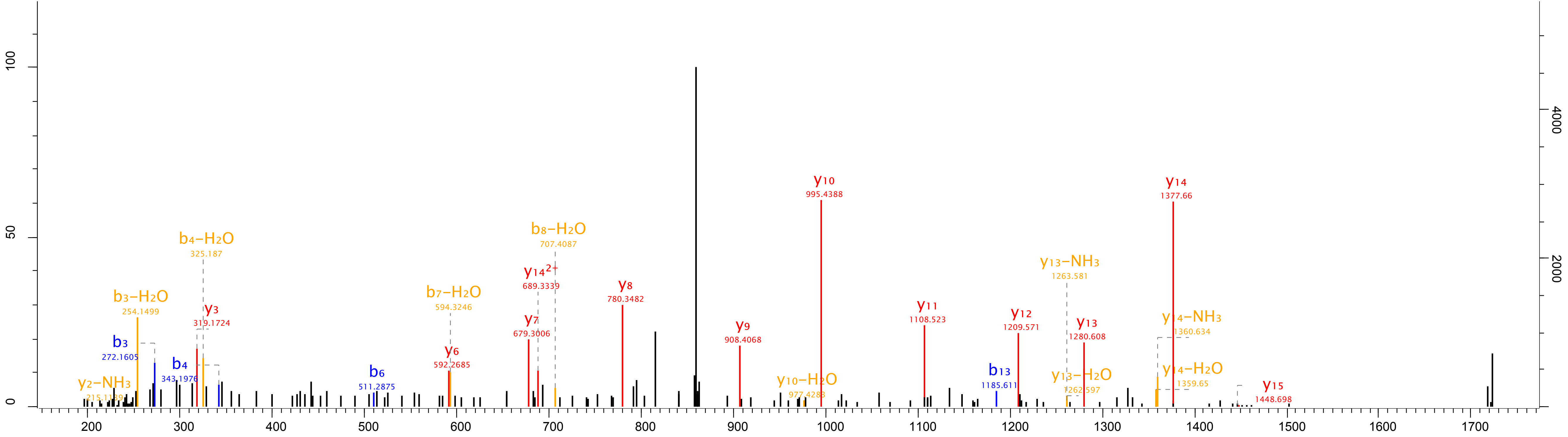

Raw file	Scan	Method	Score	Mass	Gene names
UPS1+500ngY_90minTop17_BC4_01_358	28470	TOF; CID	75.89	1300.59	NUP133

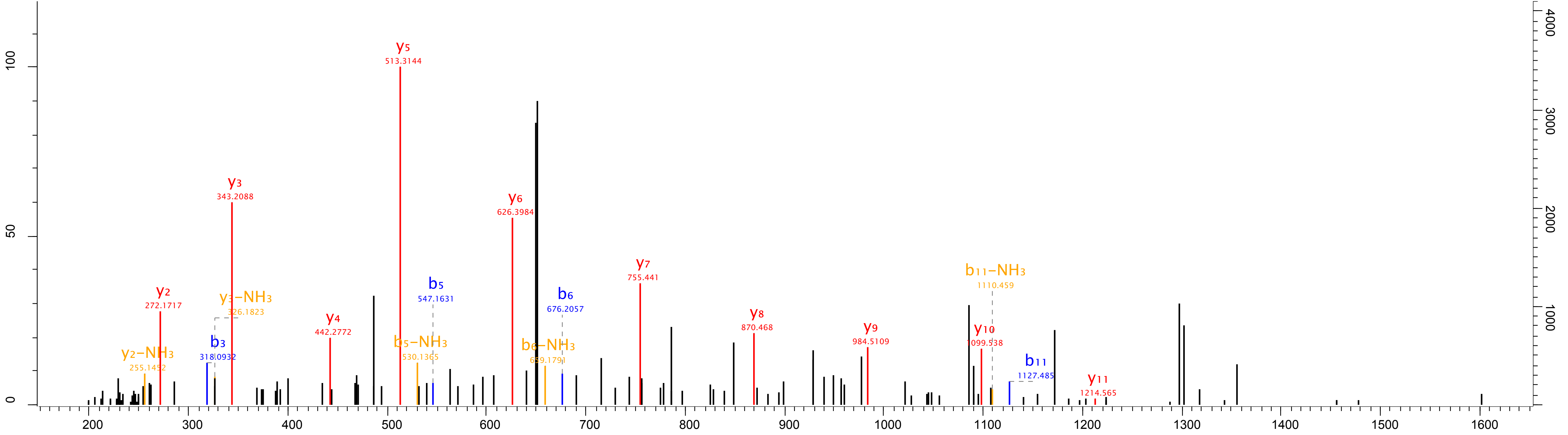

Raw file	Scan	Method	Score	Mass
UPS1+500ngY_90minTop17_BC4_01_358	29814	TOF; CID	68.17	1582.66

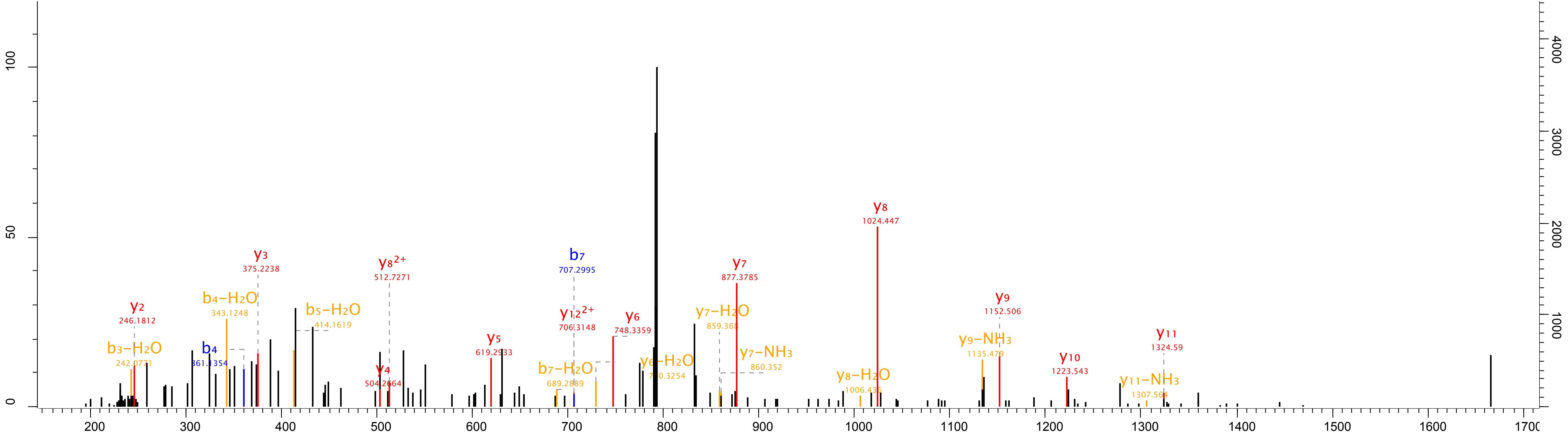

Raw file	Scan	Method	Score	Mass	Gene names
UPS1+500ngY_90minTop17_BC4_01_358	30820	TOF; CID	64.22	1227.58	CWP1

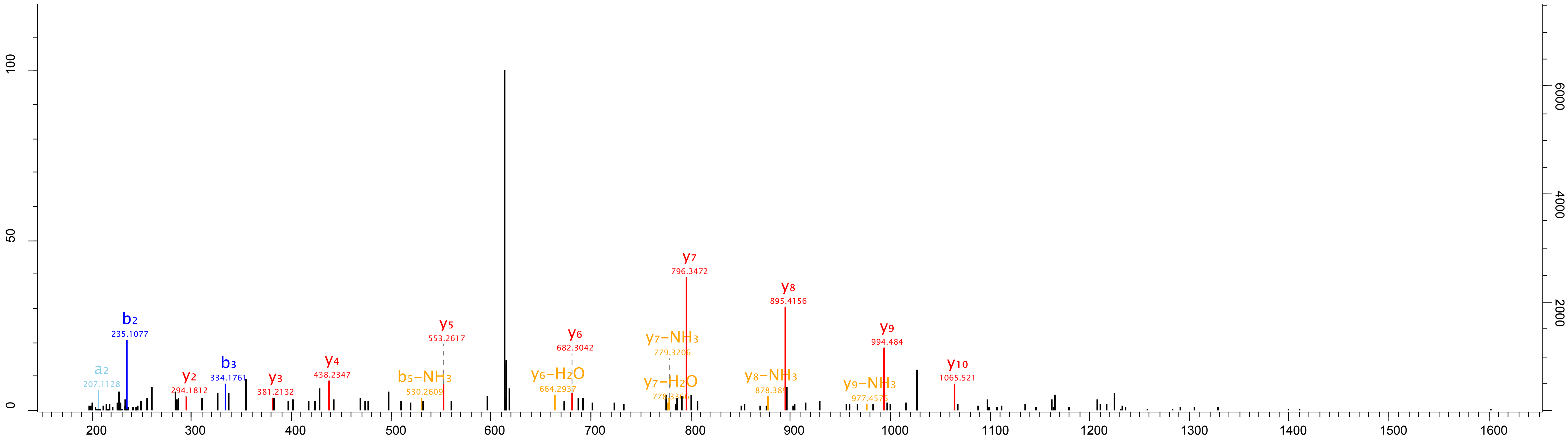

Raw file	Scan	Method	Score	Mass	Gene names
UPS1+500ngY_90minTop17_BC4_01_358	31028	TOF; CID	49.59	1564.71	SEC11

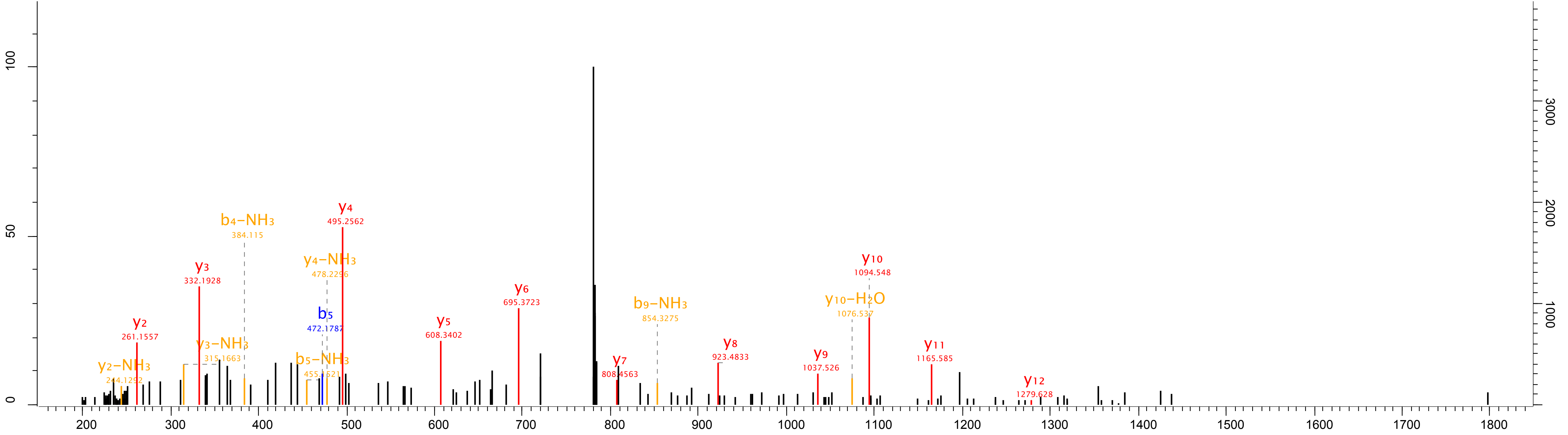

Raw file	Scan	Method	Score	Mass	Gene names
UPS1+500ngY_90minTop17_BC4_01_358	31118	TOF; CID	62.09	1453.65	MID2

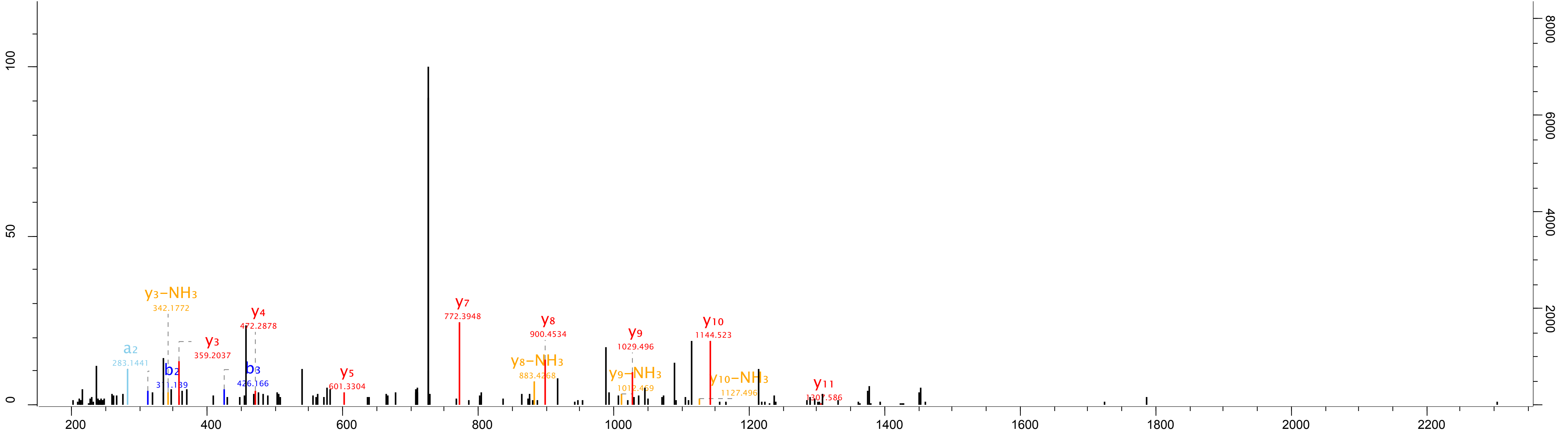

Raw file	Scan	Method	Score	Mass	Gene names
UPS1+500ngY_90minTop17_BC4_01_358	31363	TOF; CID	74.39	1350.61	ATP15

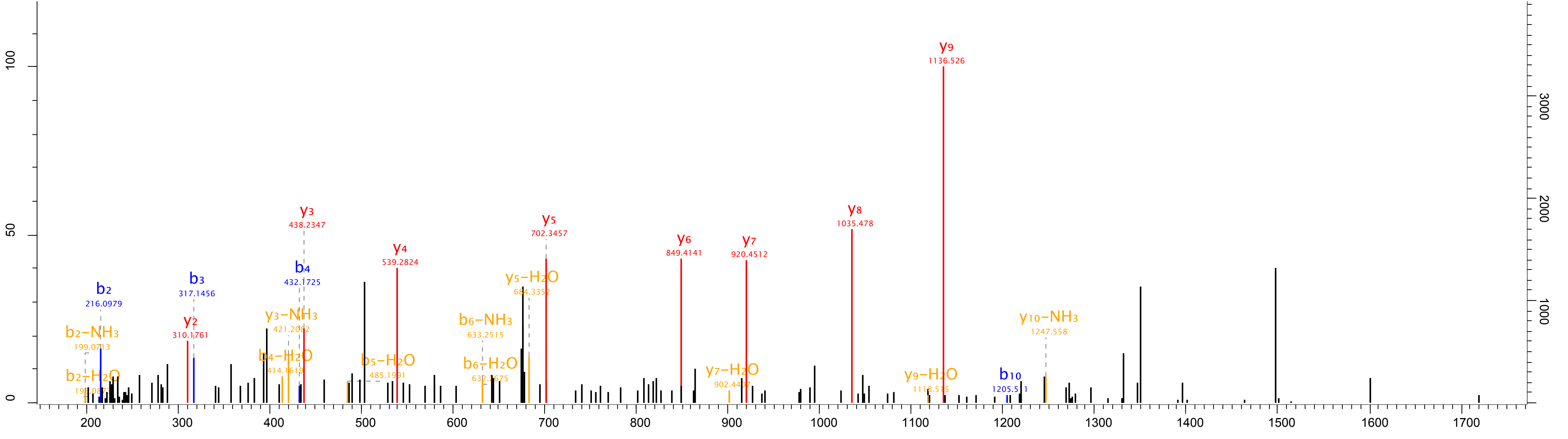

Raw file	Scan	Method	Score	Mass	Gene names
UPS1+500ngY_90minTop17_BC4_01_358	31766	TOF; CID	84.3	1614.68	SNA2

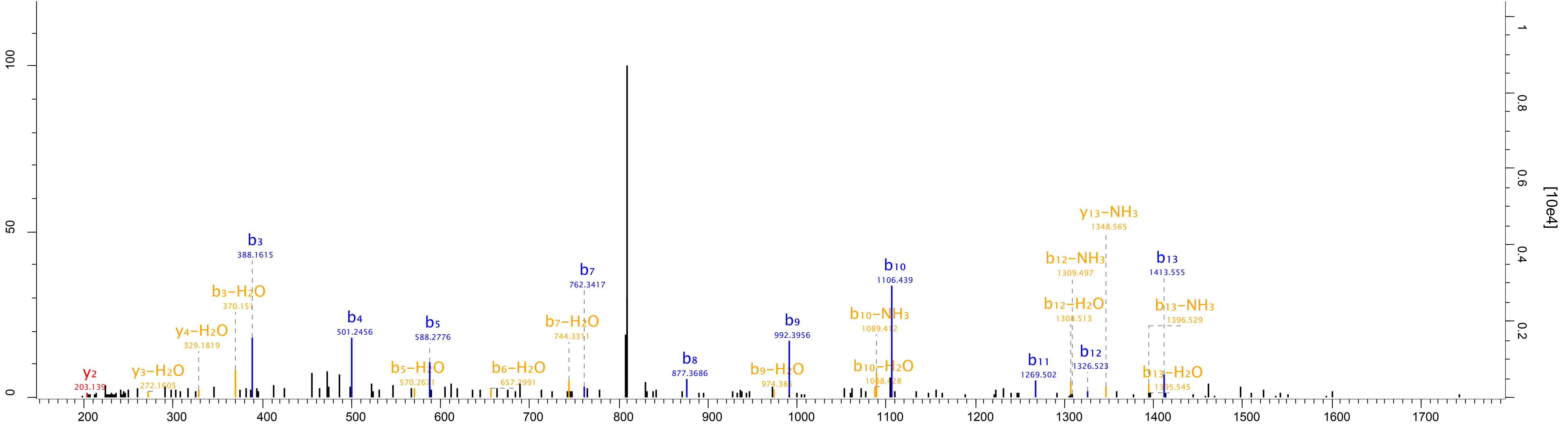

Raw file	Scan	Method	Score	Mass	Gene names
UPS1+500ngY_90minTop17_BC4_01_358	32280	TOF; CID	78.81	1568.7	XKS1

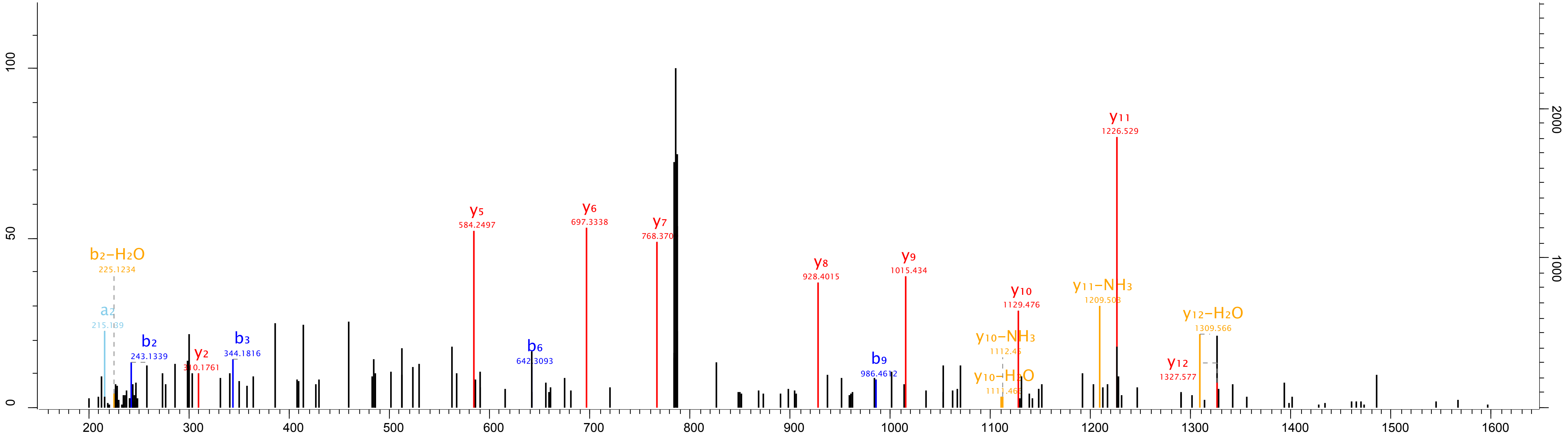

Raw file	Scan	Method	Score	Mass	Gene names
UPS1+500ngY_90minTop17_BC4_01_358	32331	TOF; CID	94.78	1556.64	CDC73

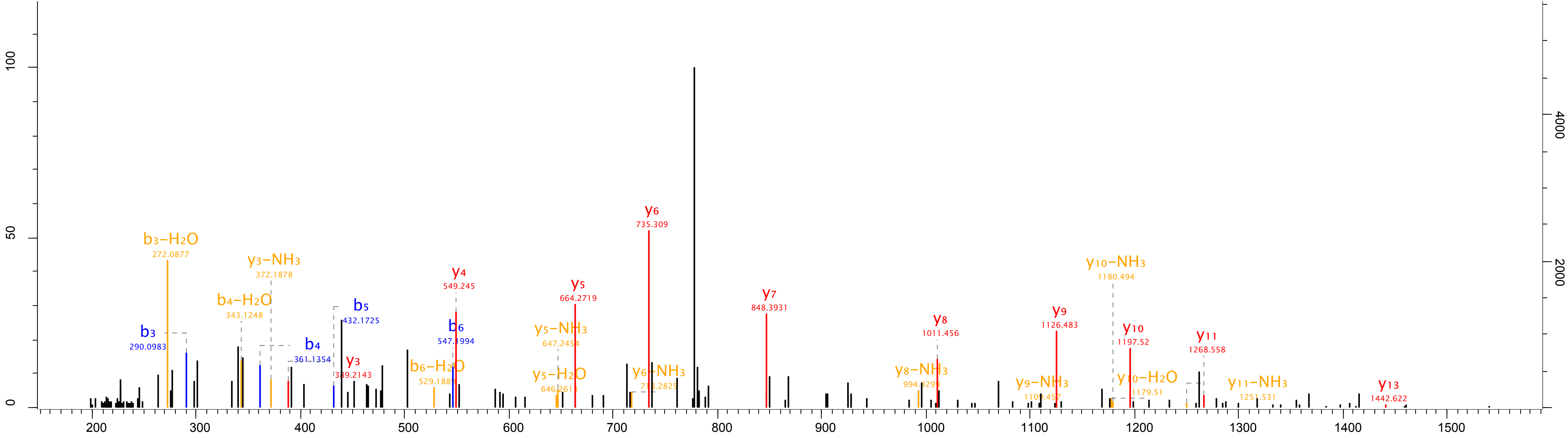

Raw file	Scan	Method	Score	Mass	Gene names
UPS1+500ngY_90minTop17_BC4_01_358	32485	TOF; CID	54.34	1164.57	HLJ1

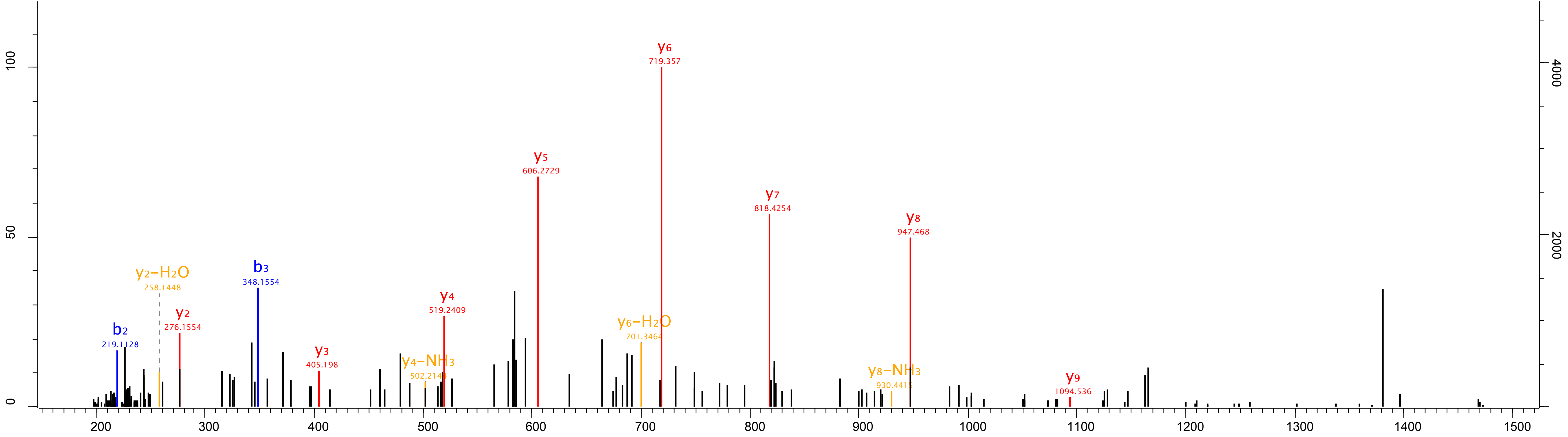

Raw file	Scan	Method	Score	Mass	Gene names
UPS1+500ngY_90minTop17_BC4_01_358	32523	TOF; CID	62.16	1437.67	SED4

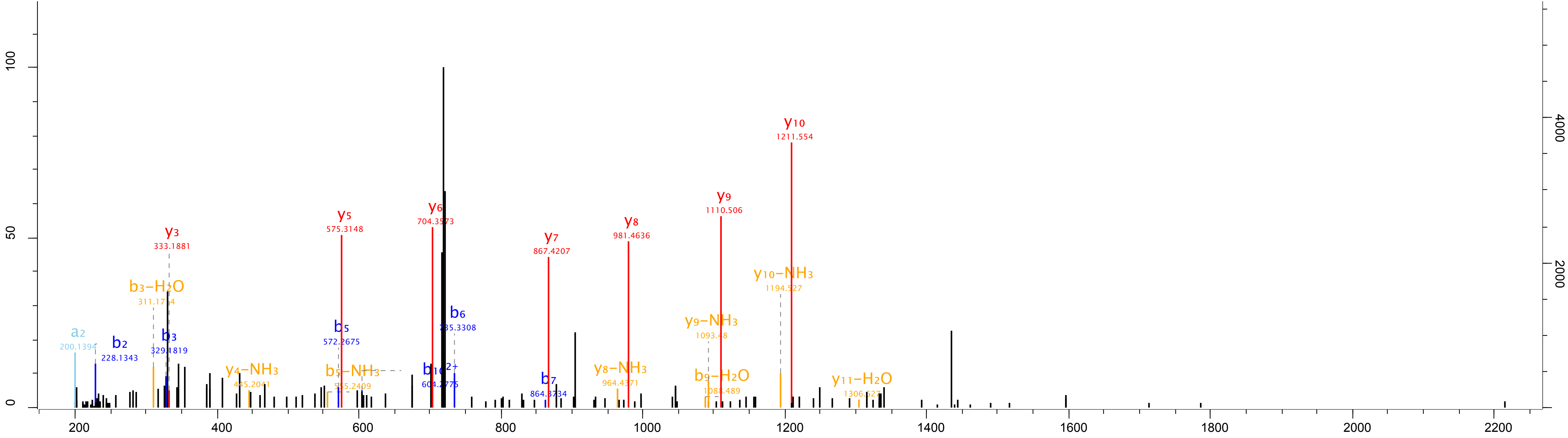

Raw file	Scan	Method	Score	Mass	Gene names
UPS1+500ngY_90minTop17_BC4_01_358	32729	TOF; CID	55.26	1260.66	CWH43

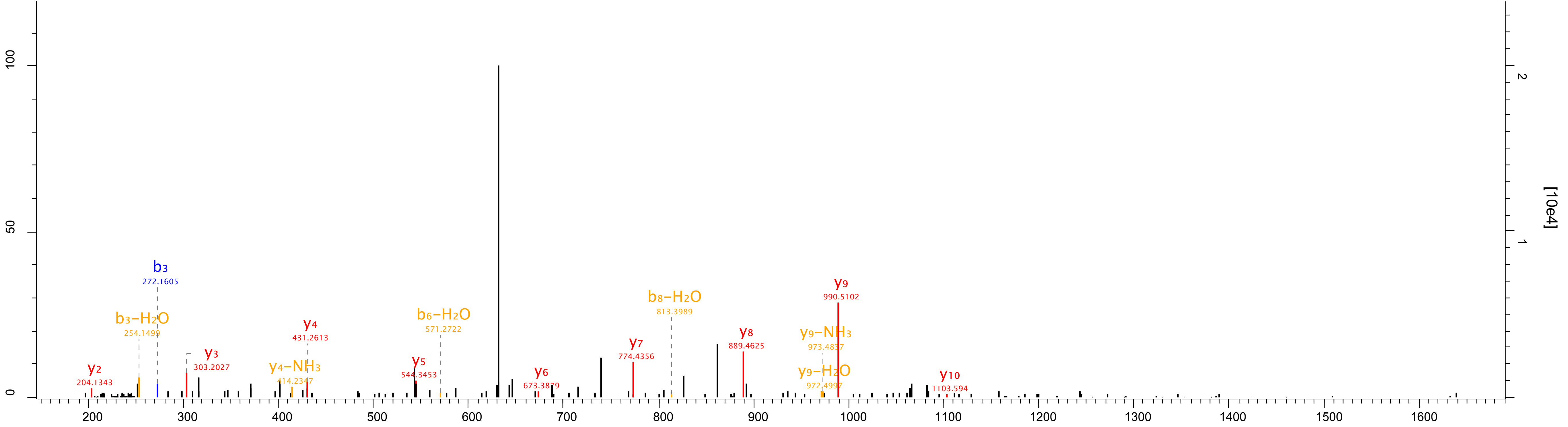

Raw file	Scan	Method	Score	Mass	Gene names
UPS1+500ngY_90minTop17_BC4_01_358	32899	TOF; CID	83.09	1487.75	TIM9

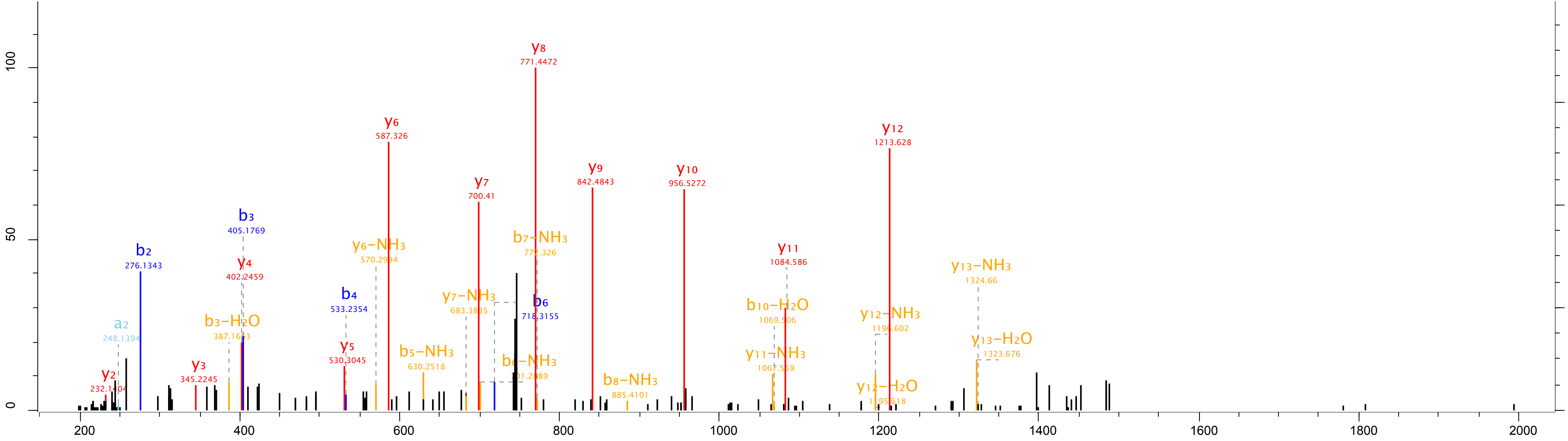

Raw file	Scan	Method	Score	Mass	Gene names
UPS1+500ngY_90minTop17_BC4_01_358	33007	TOF; CID	47.71	1334.56	PLB1

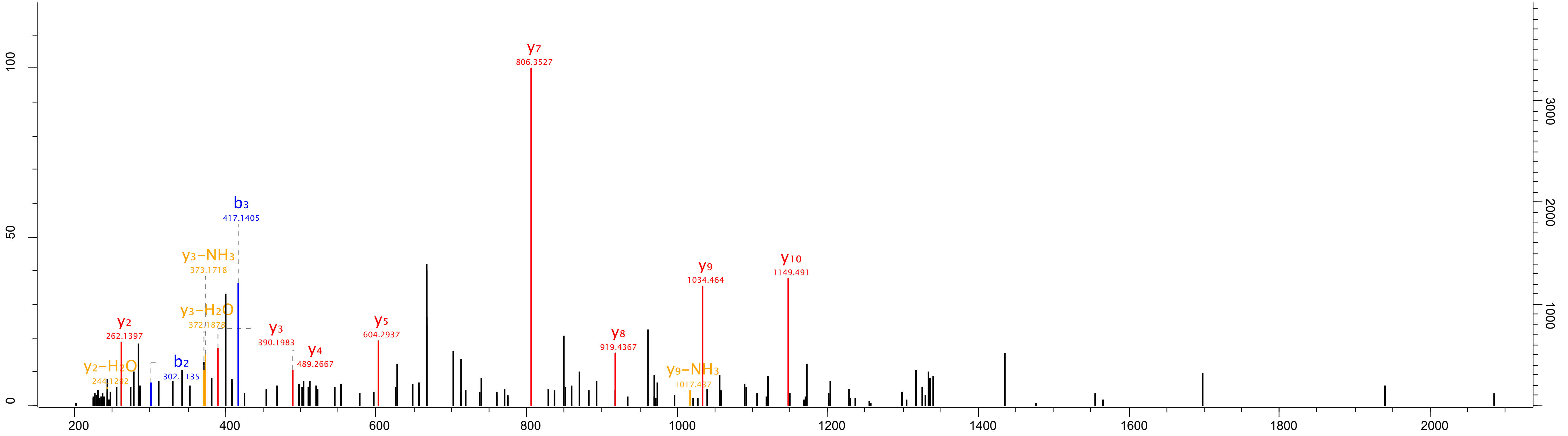

Raw file	Scan	Method	Score	Mass	Gene names
UPS1+500ngY_90minTop17_BC4_01_358	33237	TOF; CID	69.28	1076.53	GIM3

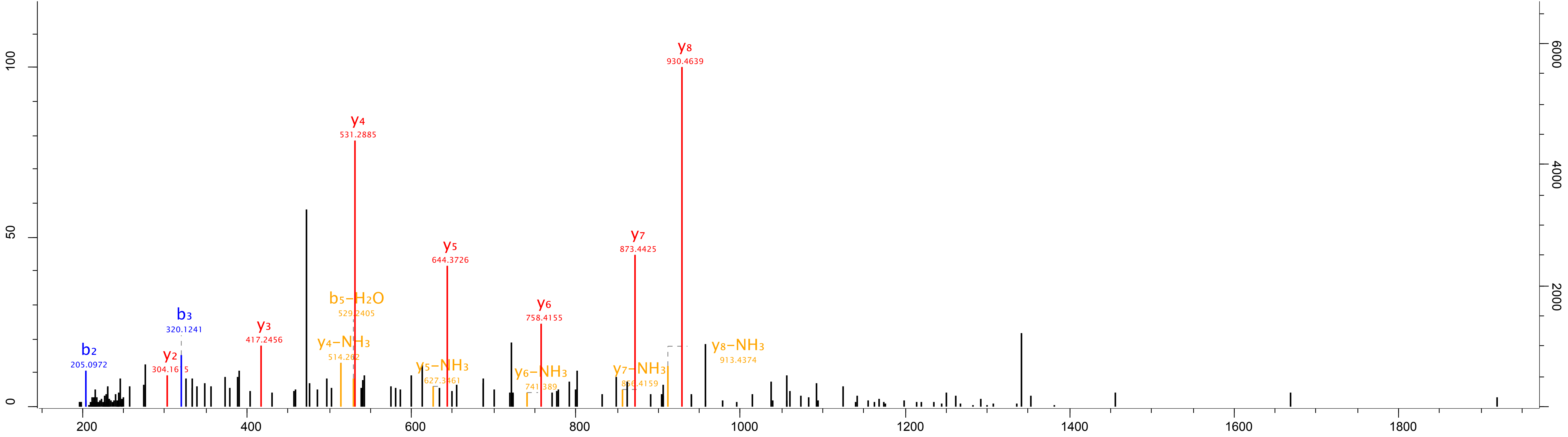

Raw file	Scan	Method	Score	Mass	Gene names
UPS1+500ngY_90minTop17_BC4_01_358	33320	TOF; CID	70.06	1073.59	TRX3

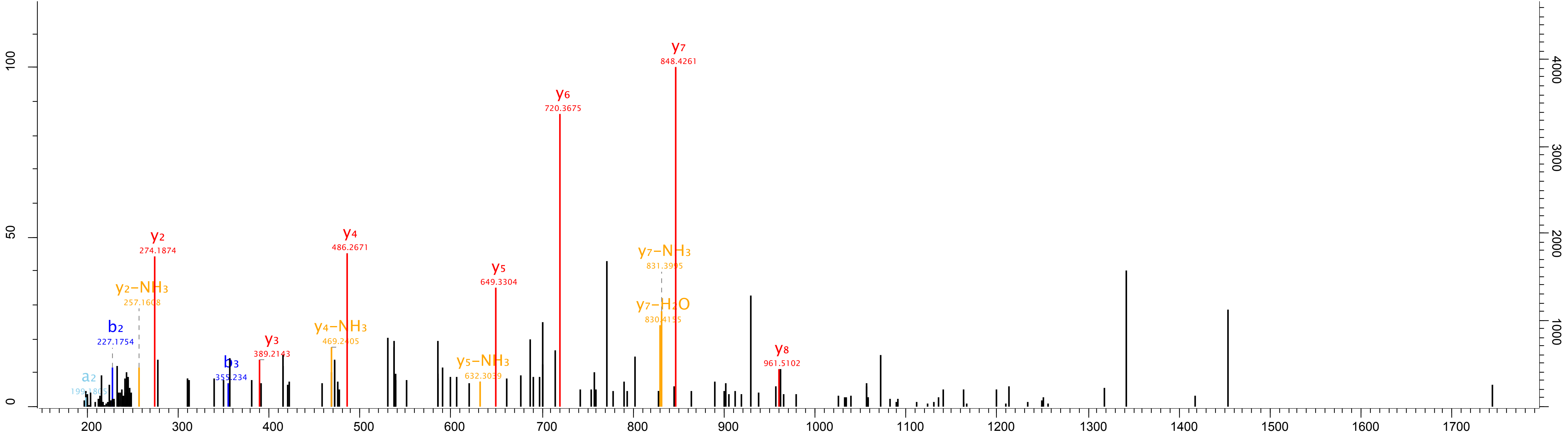

Raw file	Scan	Method	Score	Mass	Gene names
UPS1+500ngY_90minTop17_BC4_01_358	33628	TOF; CID	51.11	1028.55	HMS2

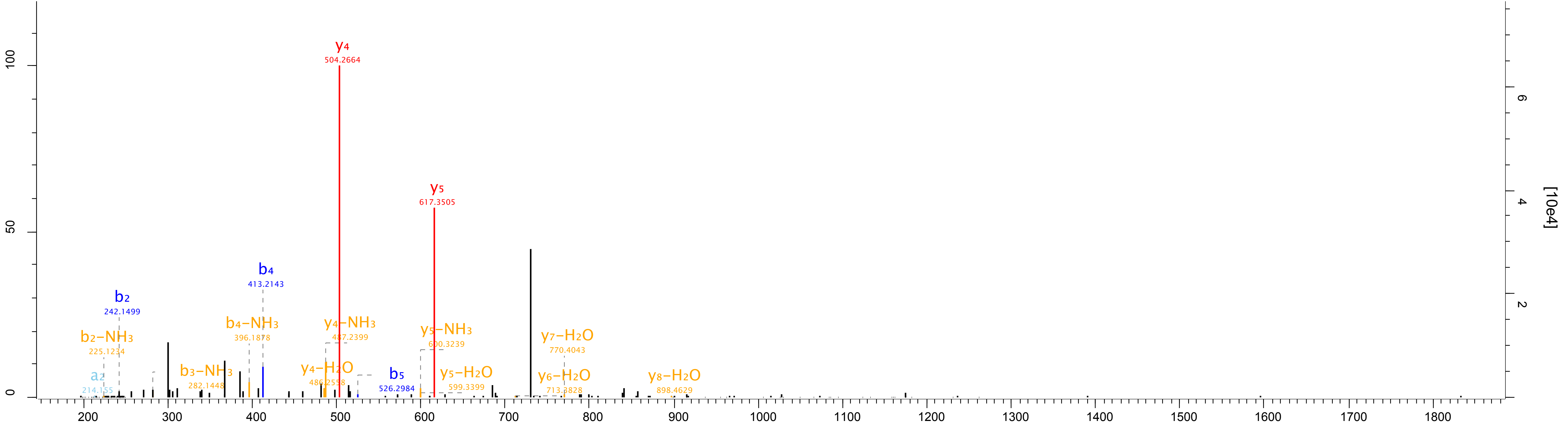

Raw file	Scan	Method	Score	Mass	Gene names
UPS1+500ngY_90minTop17_BC4_01_358	33683	TOF; CID	64.83	1407.62	NOP16

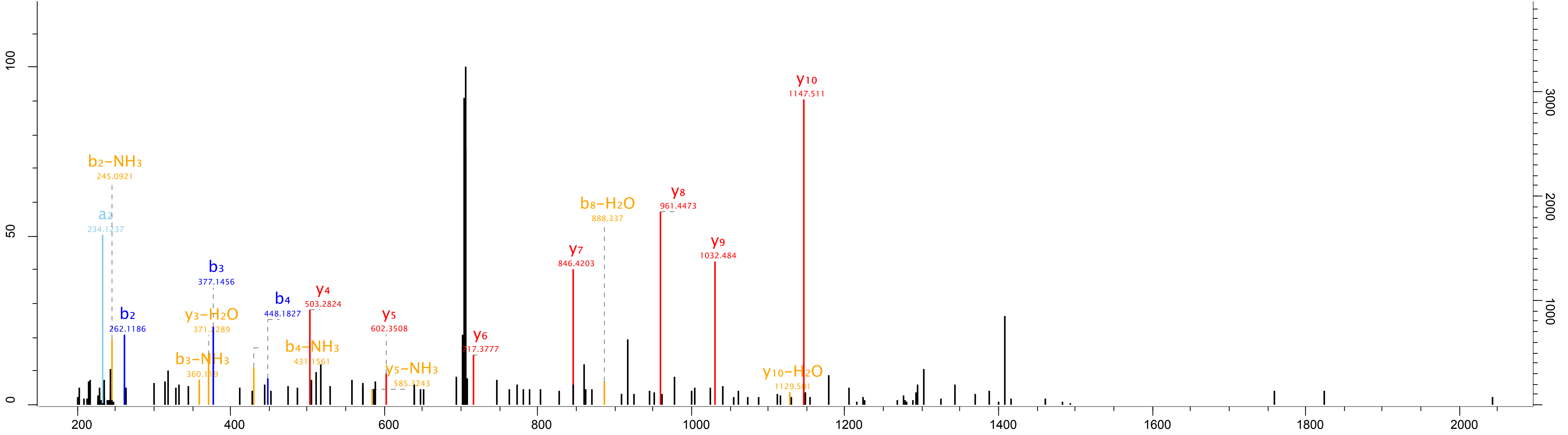

Raw file	Scan	Method	Score	Mass	Gene names
UPS1+500ngY_90minTop17_BC4_01_358	33697	TOF; CID	71.18	1343.72	TIP41

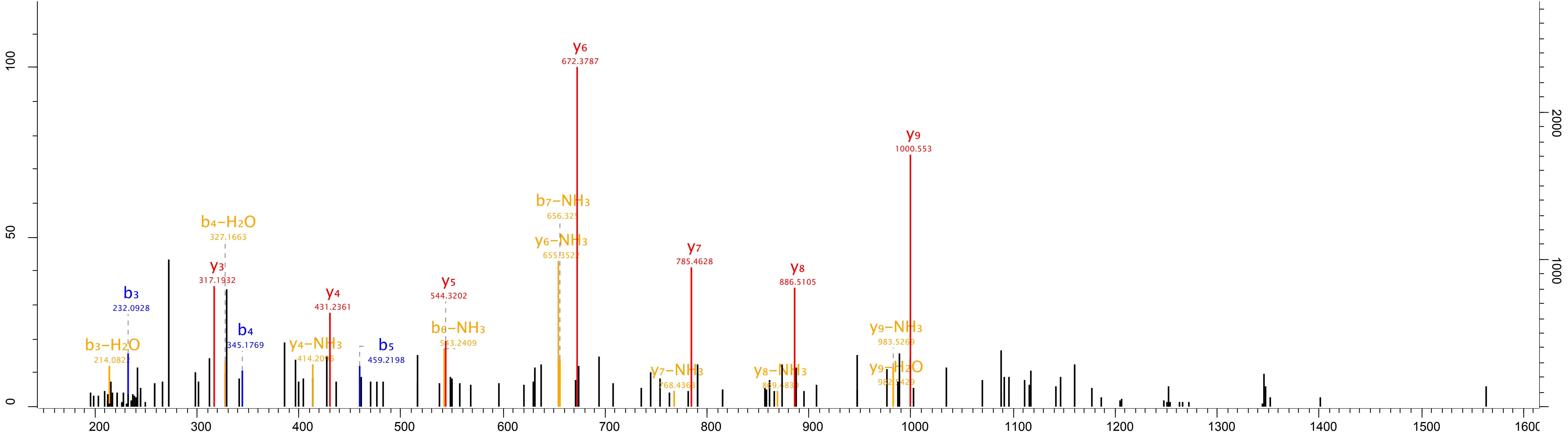

Raw file	Scan	Method	Score	Mass	Gene names
UPS1+500ngY_90minTop17_BC4_01_358	33741	TOF; CID	57.53	1691.7	RPD3

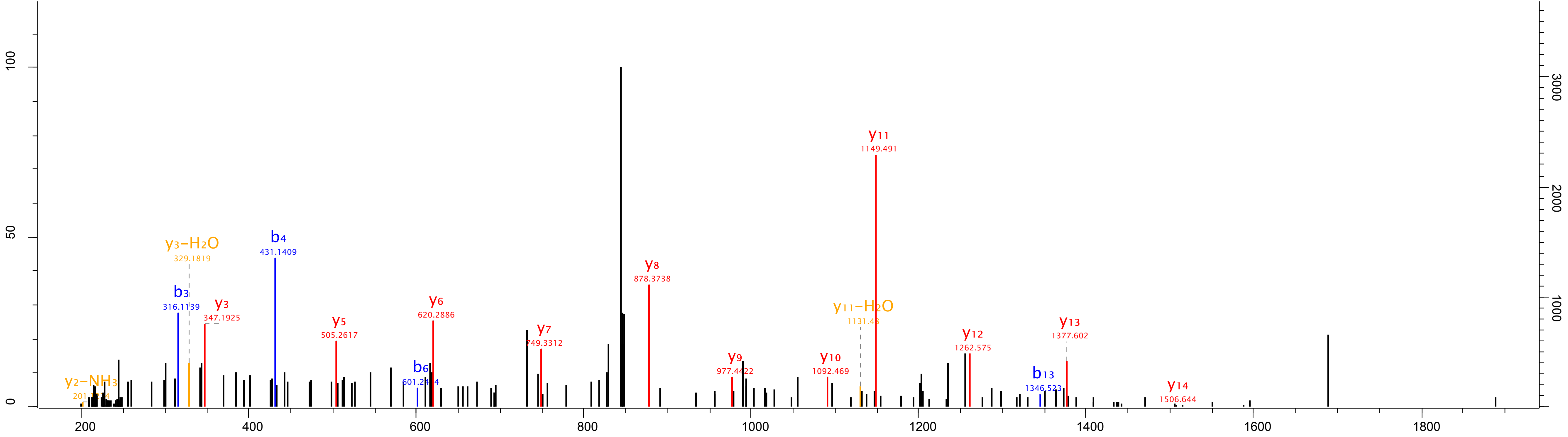

Raw file	Scan	Method	Score	Mass	Gene names
UPS1+500ngY_90minTop17_BC4_01_358	33767	TOF; CID	58.36	2642.21	VPS60

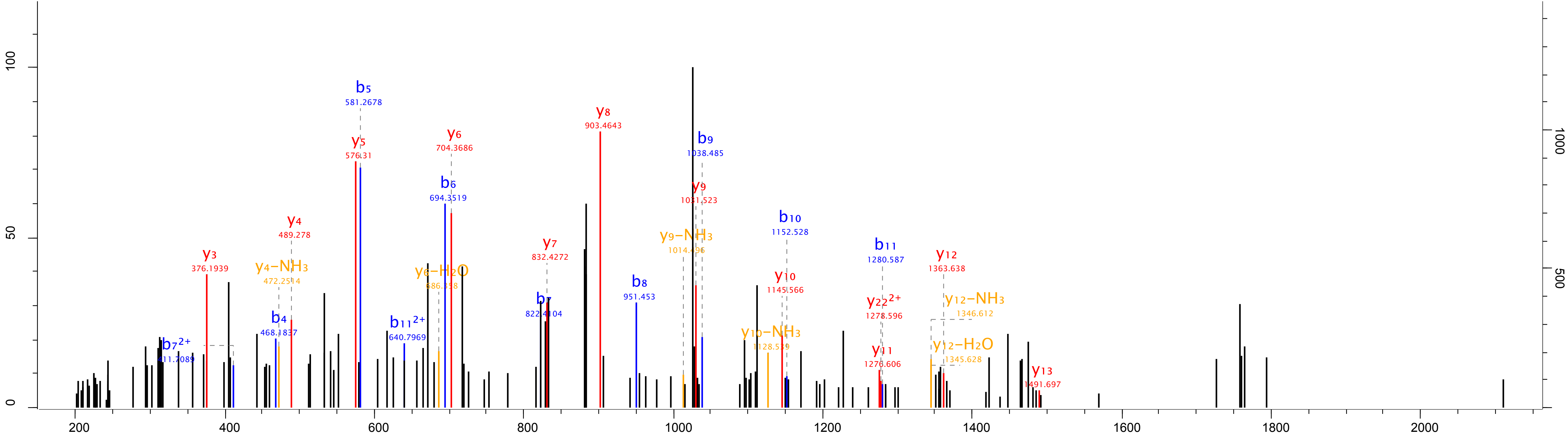

Raw file	Scan	Method	Score	Mass	Gene names
UPS1+500ngY_90minTop17_BC4_01_358	34096	TOF; CID	93.14	1024.49	OM14

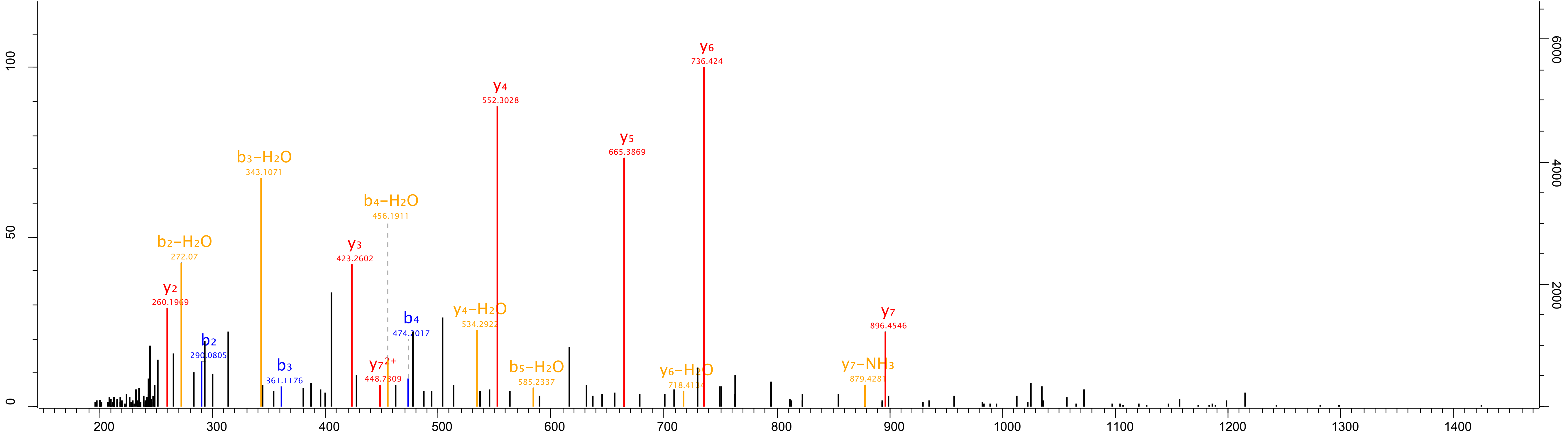

Raw file	Scan	Method	Score	Mass	Gene names
UPS1+500ngY_90minTop17_BC4_01_358	34203	TOF; CID	47.68	1723.86	REB1

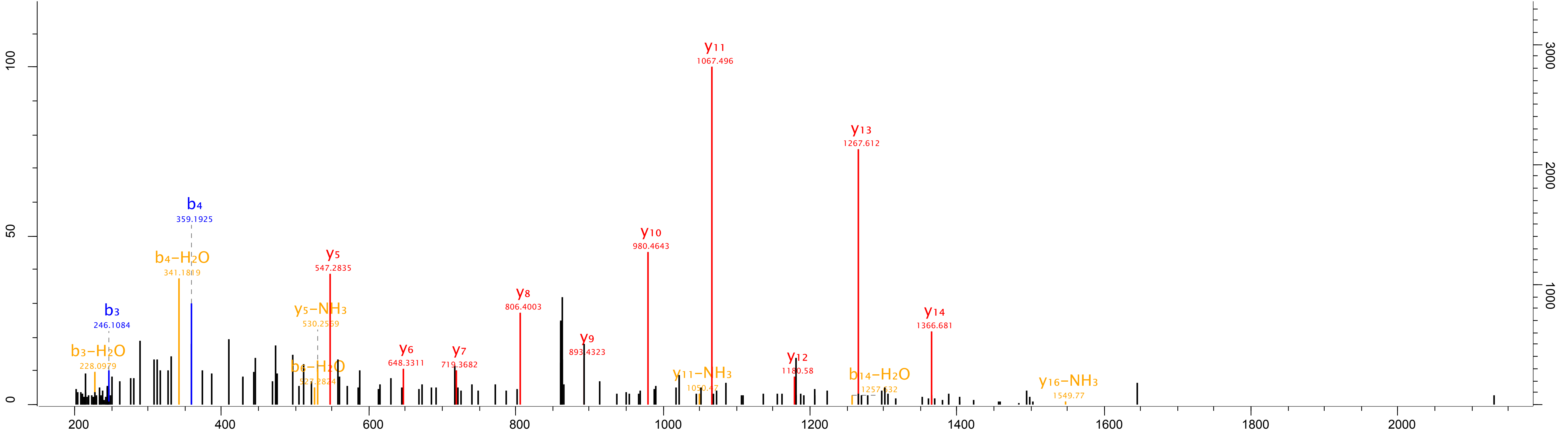

Raw file	Scan	Method	Score	Mass	Gene names
UPS1+500ngY_90minTop17_BC4_01_358	35883	TOF; CID	106.3	1711.79	CUE1

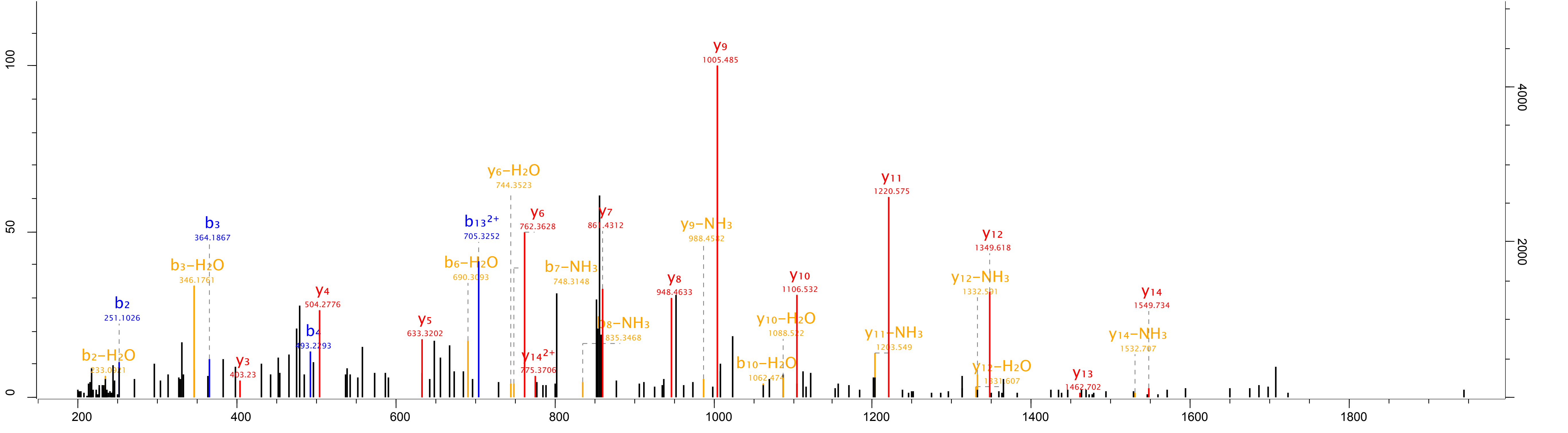

Raw file	Scan	Method	Score	Mass	Gene names
UPS1+500ngY_90minTop17_BC4_01_358	35930	TOF; CID	56.72	1020.5	SVP26

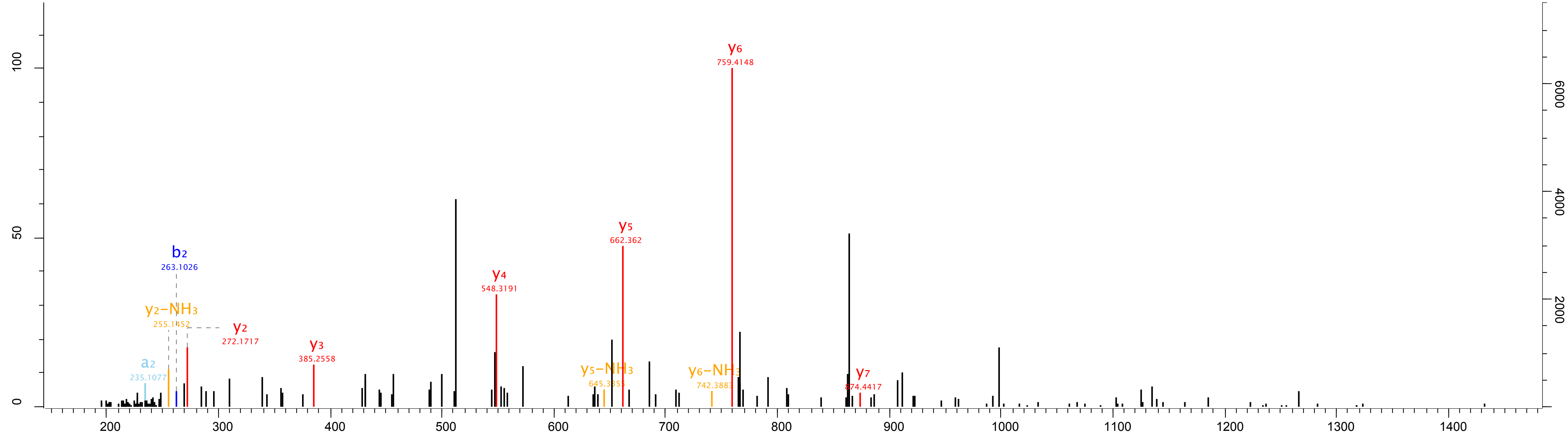

Raw file	Scan	Method	Score	Mass
UPS1+500ngY_90minTop17_BC4_01_358	36028	TOF; CID	61.58	938.55

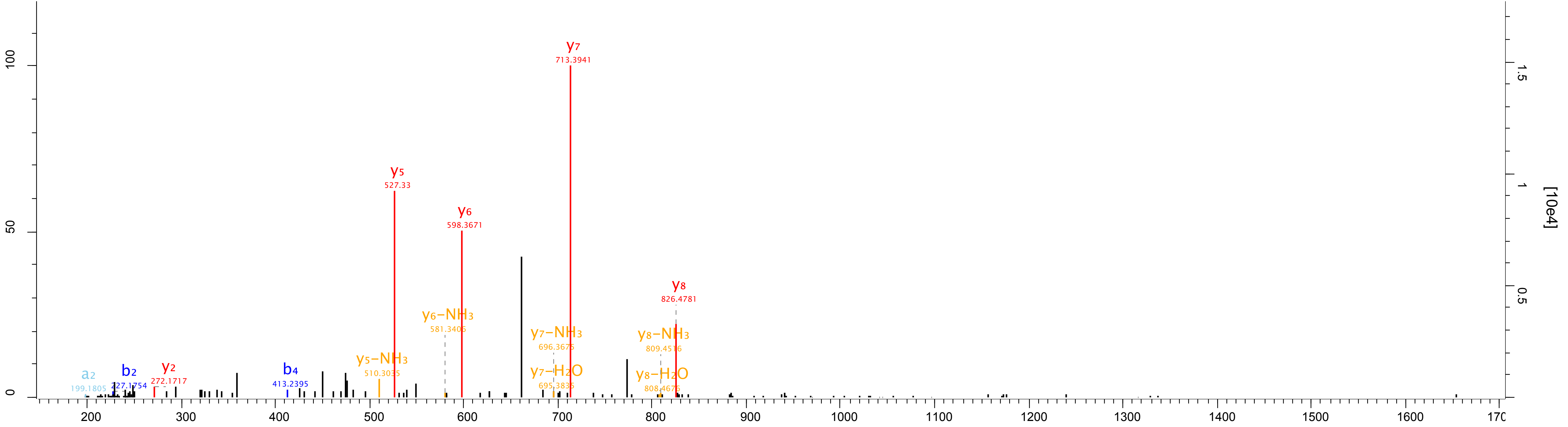

Raw file	Scan	Method	Score	Mass	Gene names
UPS1+500ngY_90minTop17_BC4_01_358	36912	TOF; CID	42.95	1744.77	NAB2

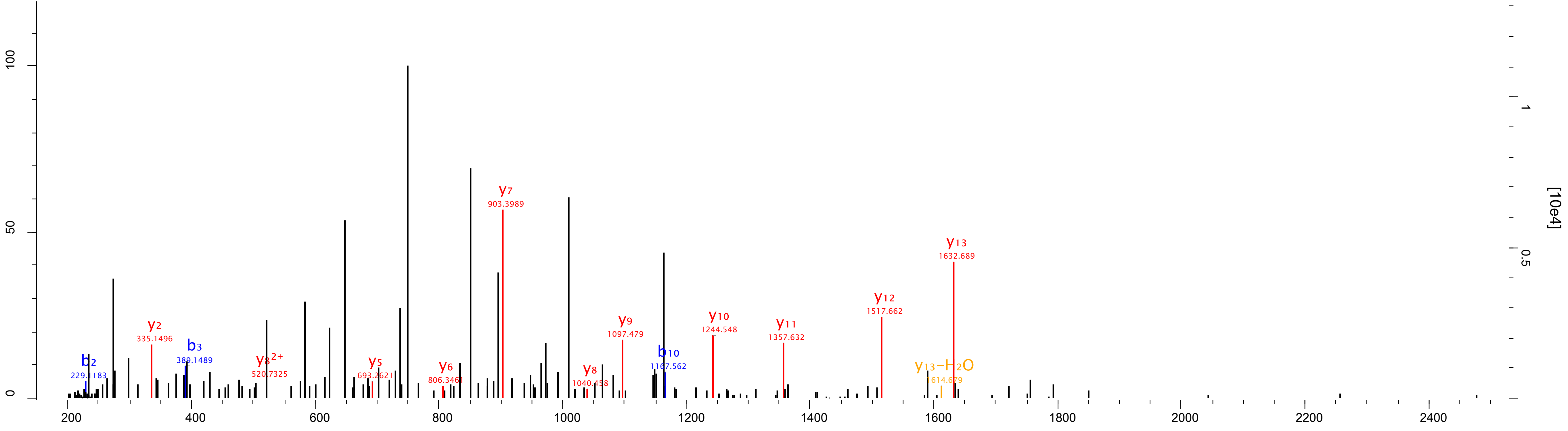

Raw file	Scan	Method	Score	Mass	Gene names
UPS1+500ngY_90minTop17_BC4_01_358	37063	TOF; CID	49.47	1131.58	NCB2

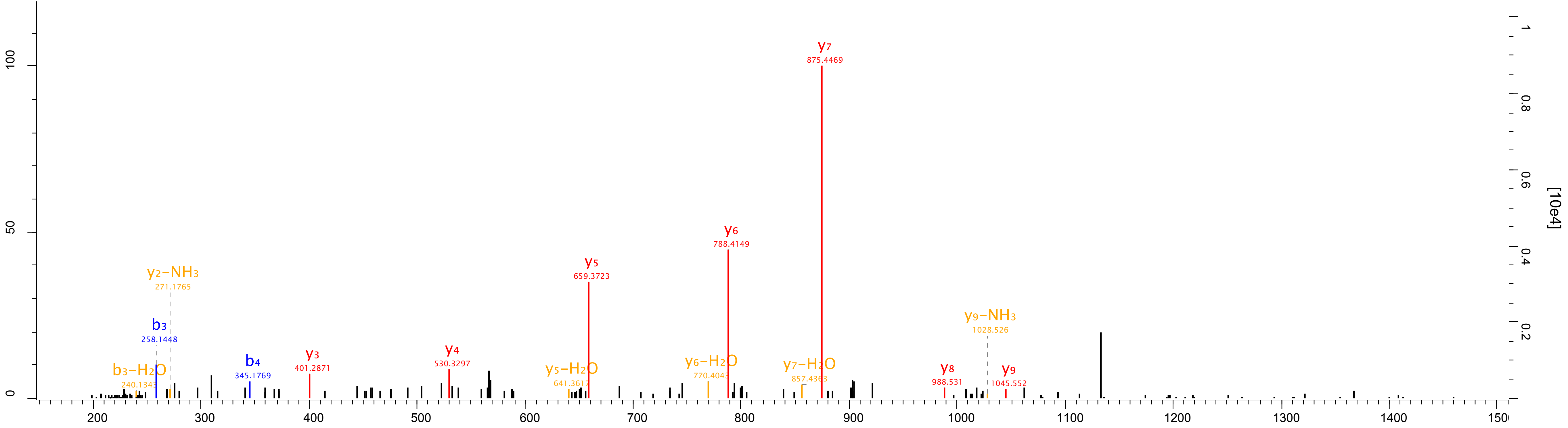

Raw file	Scan	Method	Score	Mass	Gene names
UPS1+500ngY_90minTop17_BC4_01_358	37420	TOF; CID	54.81	1636.83	RPC19

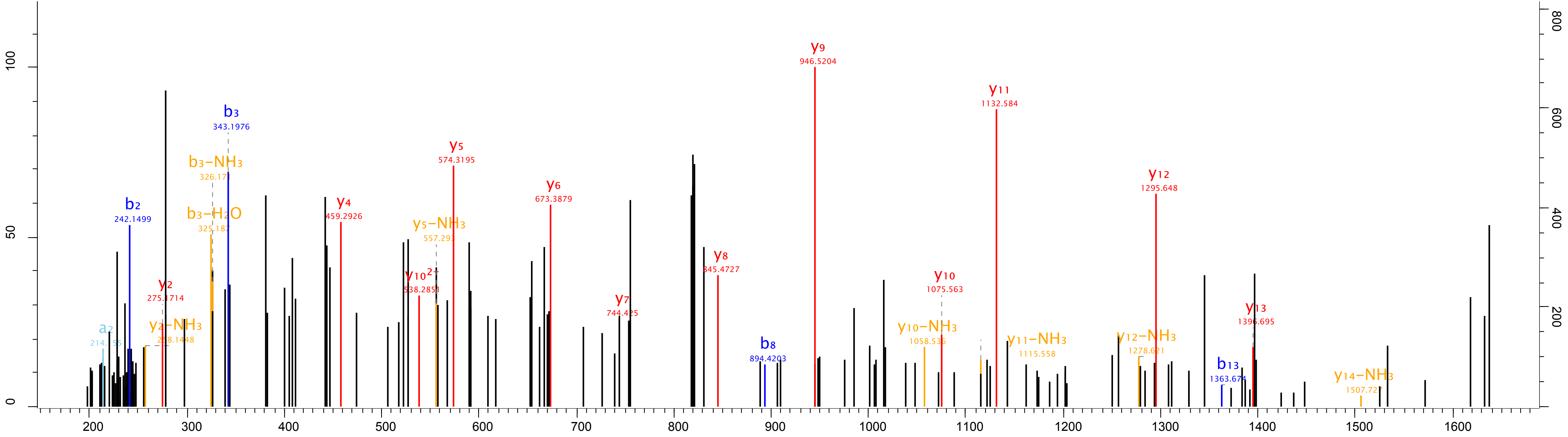

Raw file	Scan	Method	Score	Mass	Gene names
UPS1+500ngY_90minTop17_BC4_01_358	37507	TOF; CID	66.74	2009.96	MGR2

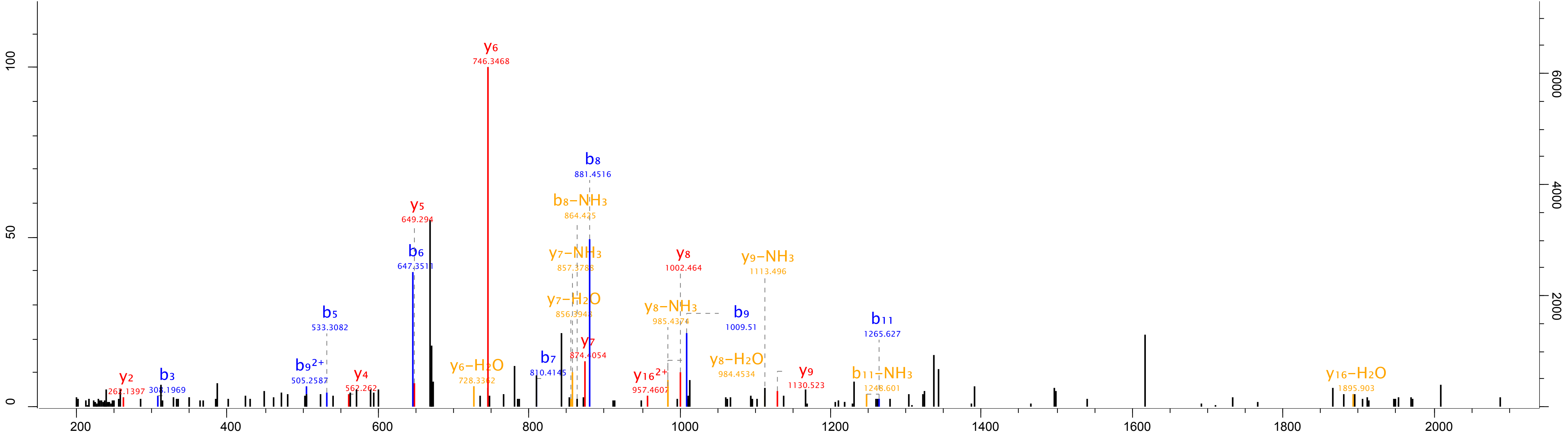

Raw file	Scan	Method	Score	Mass	Gene names
UPS1+500ngY_90minTop17_BC4_01_358	38082	TOF; CID	50.48	1133.61	AIM2

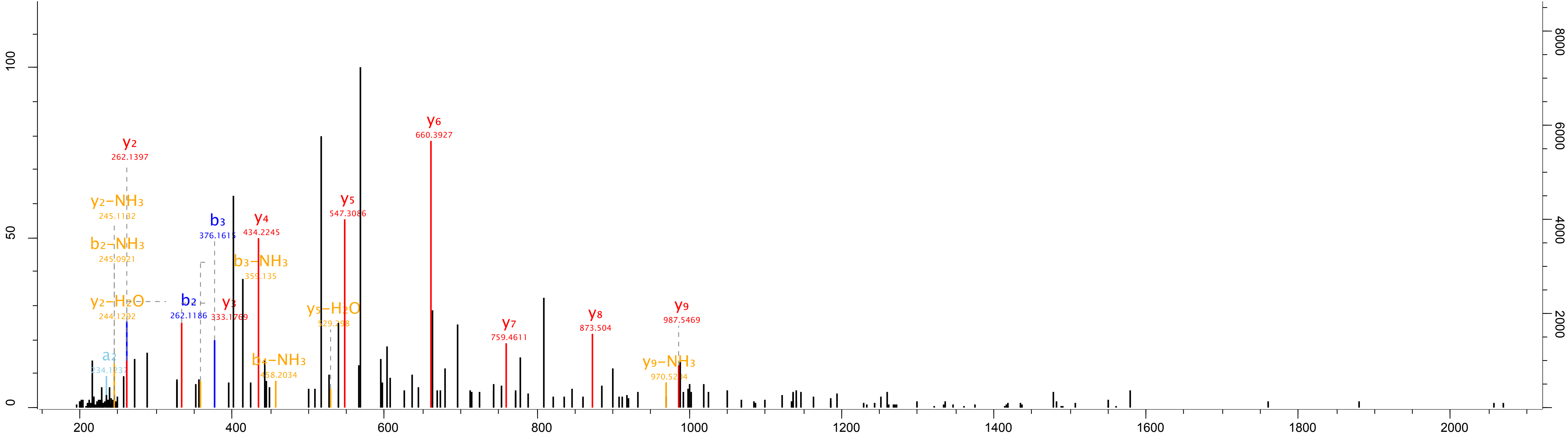

Raw file	Scan	Method	Score	Mass	Gene names
UPS1+500ngY_90minTop17_BC4_01_358	38992	TOF; CID	55.01	1631.84	ATP4

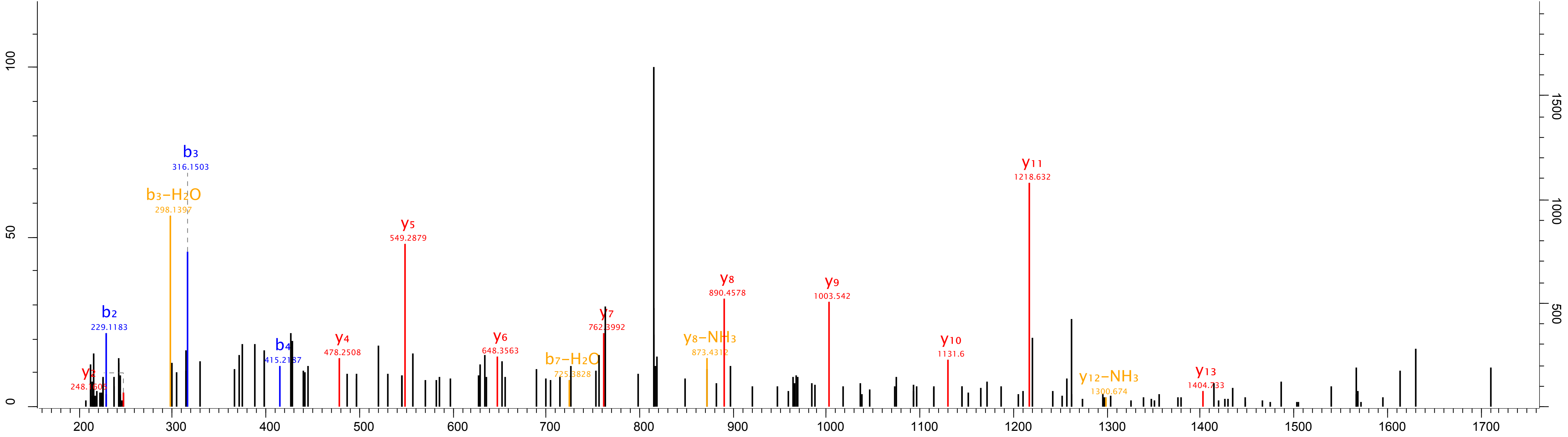

Raw file	Scan	Method	Score	Mass	Gene names
UPS1+500ngY_90minTop17_BC4_01_358	39118	TOF; CID	42.72	1153.51	YER134C

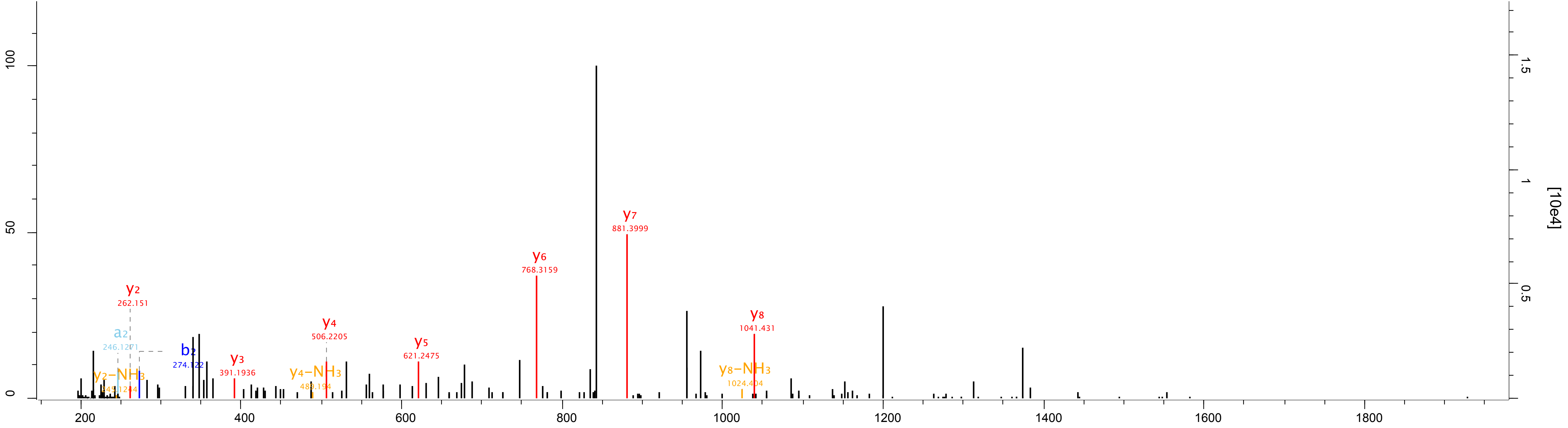

Raw file	Scan	Method	Score	Mass	Gene names
UPS1+500ngY_90minTop17_BC4_01_358	39830	TOF; CID	57.17	1185.64	SWP1

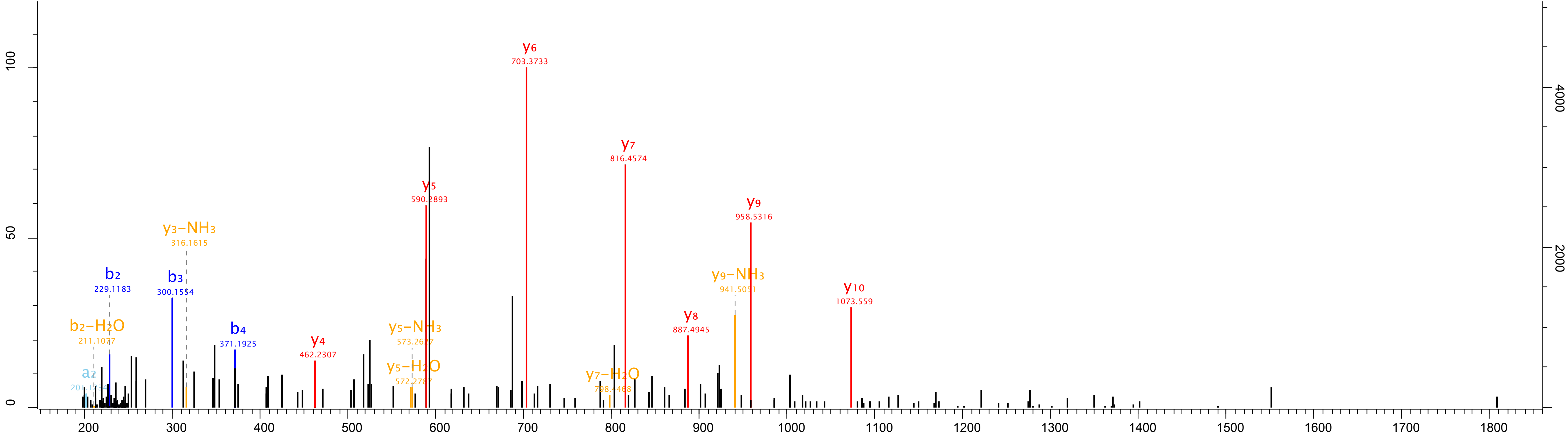

Raw file	Scan	Method	Score	Mass	Gene names
UPS1+500ngY_90minTop17_BC4_01_358	40348	TOF; CID	54.2	1426.72	FCY1

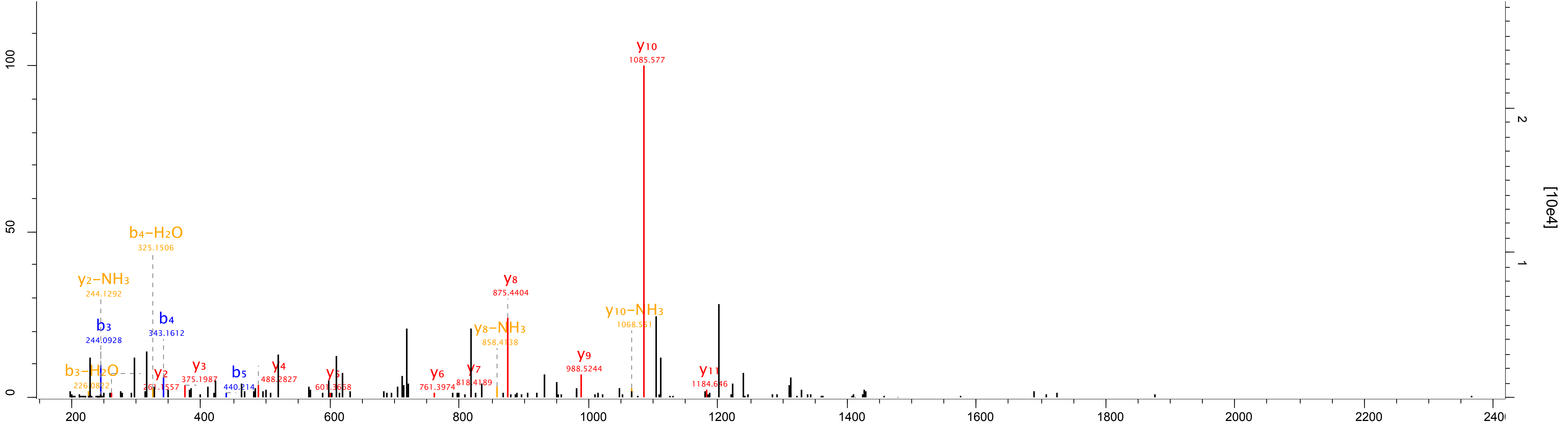

Raw file	Scan	Method	Score	Mass
UPS1+500ngY_90minTop17_BC4_01_358	40543	TOF; CID	76.33	1473.64

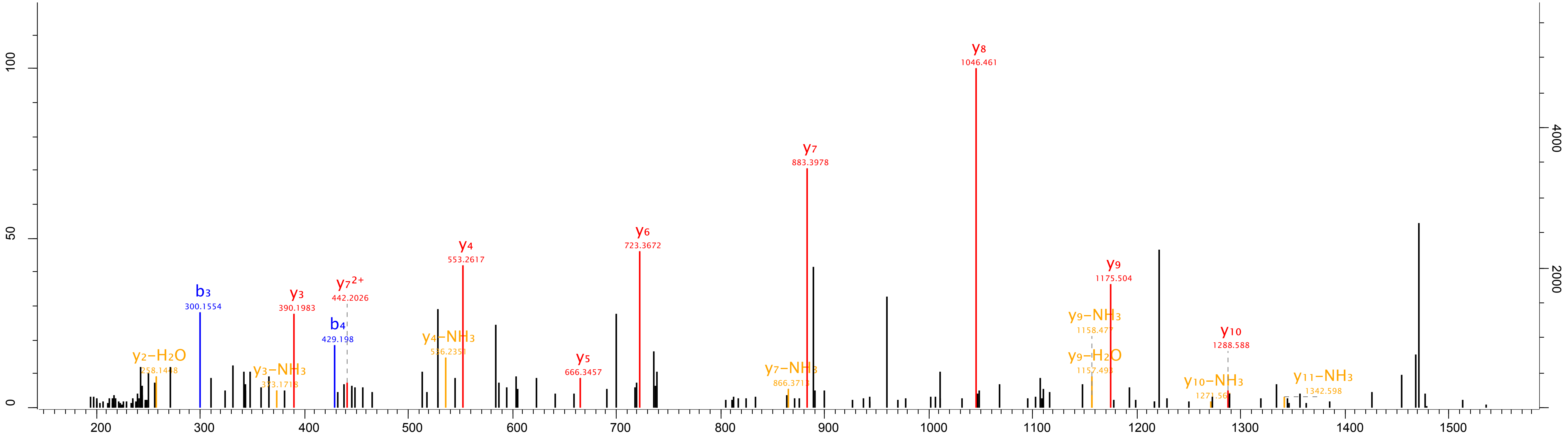

Raw file	Scan	Method	Score	Mass	Gene names
UPS1+500ngY_90minTop17_BC4_01_358	40612	TOF; CID	44.67	1931.9	NPL6

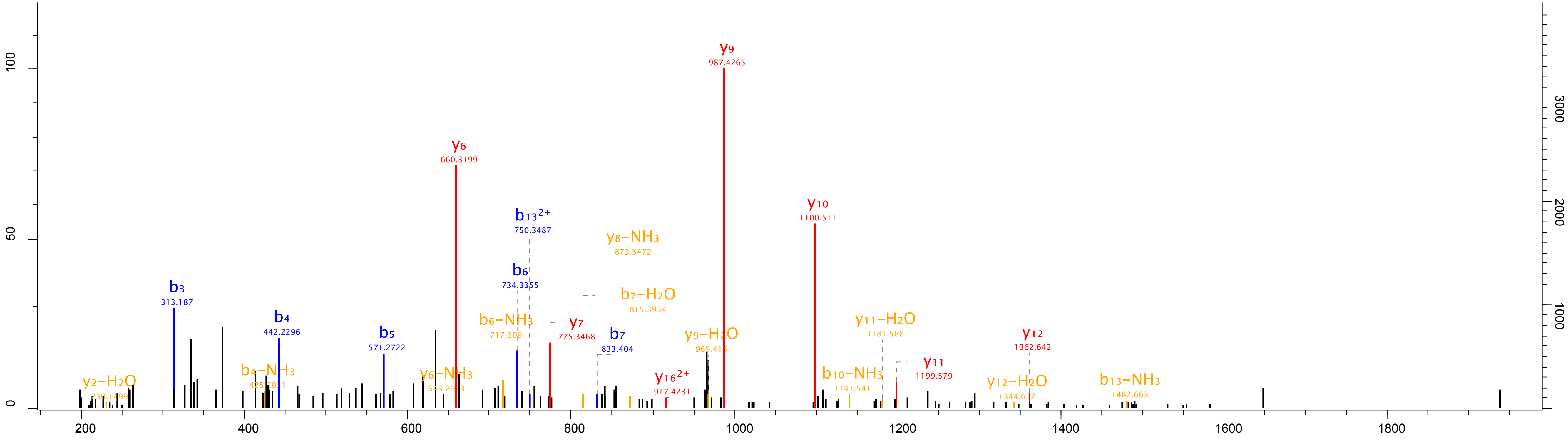

Raw file	Scan	Method	Score	Mass	Gene names
UPS1+500ngY_90minTop17_BC4_01_358	40902	TOF; CID	47.19	1060.56	CKA1

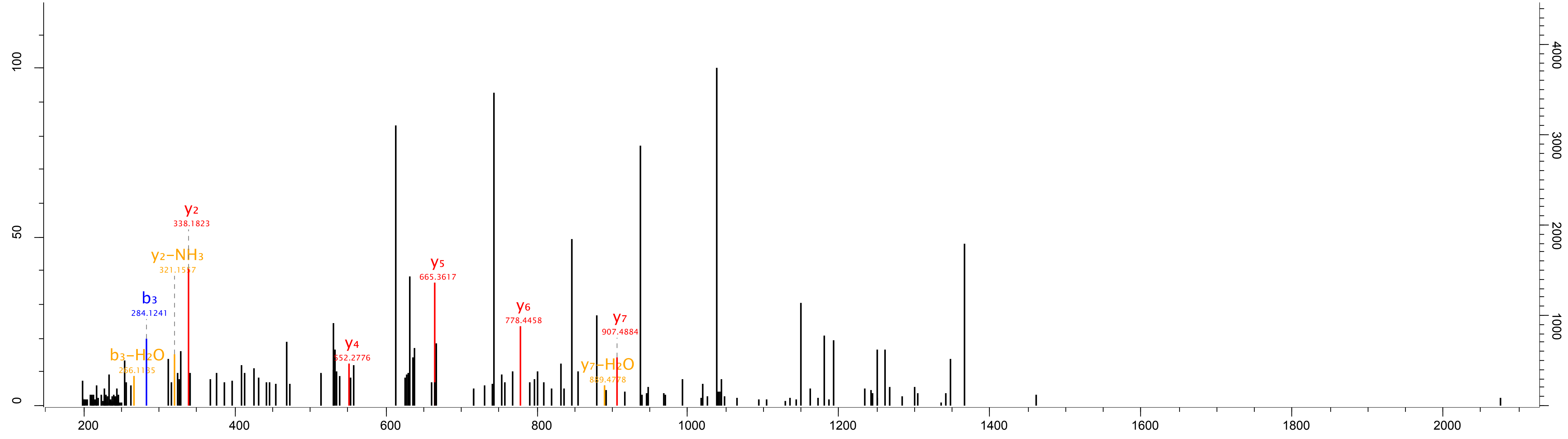

Raw file	Scan	Method	Score	Mass	Gene names
UPS1+500ngY_90minTop17_BC4_01_358	40931	TOF; CID	47.6	970.54	UTP4

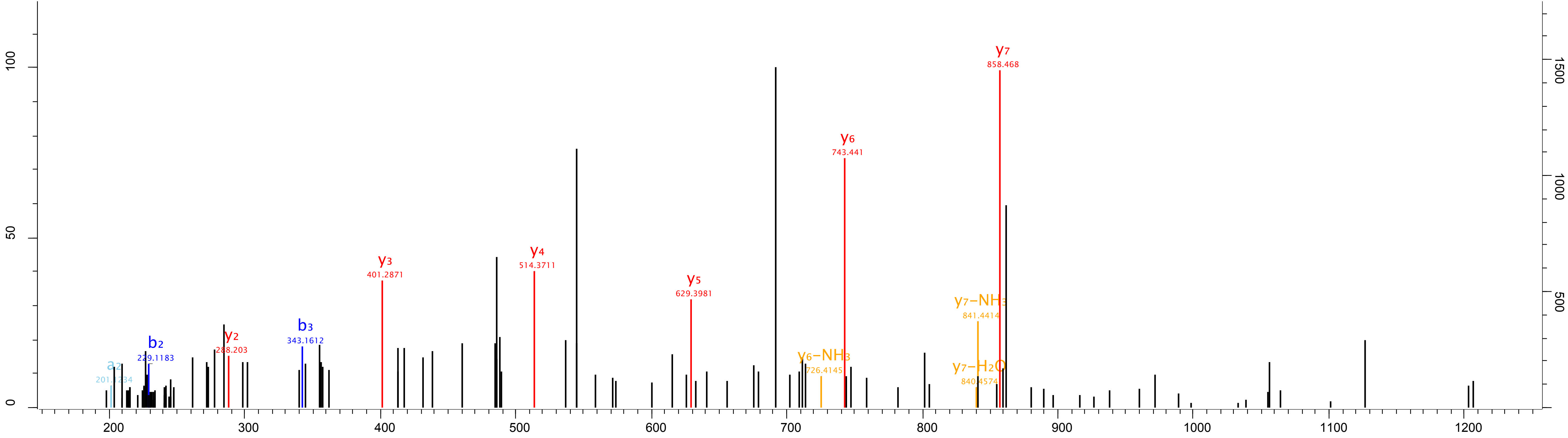

Raw file	Scan	Method	Score	Mass	Gene names
UPS1+500ngY_90minTop17_BC4_01_358	41013	TOF; CID	41.99	1974.9	TLG2

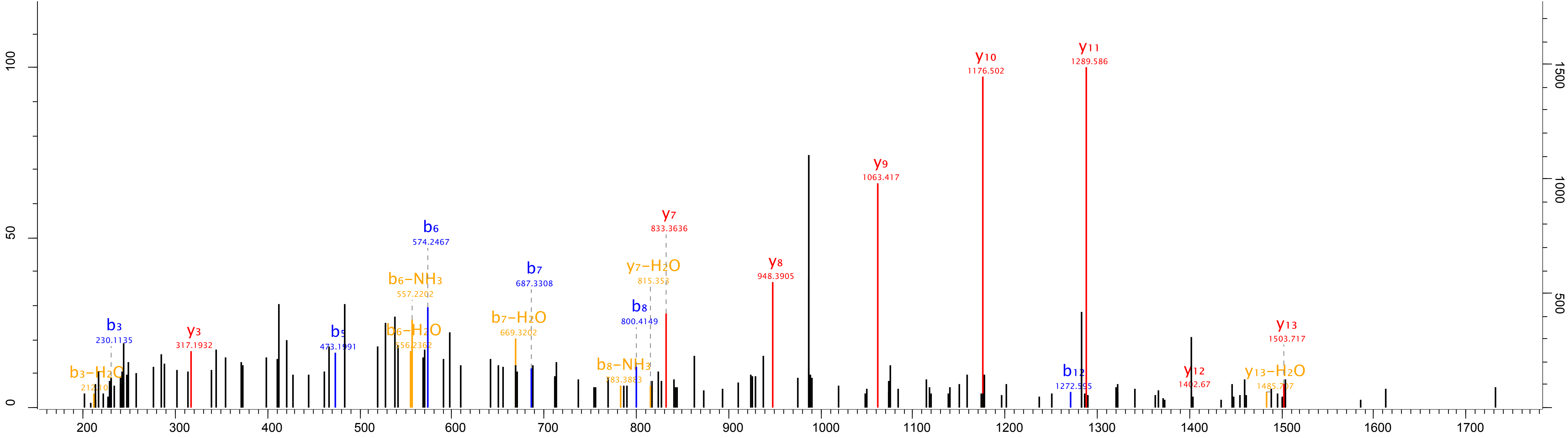

Raw file	Scan	Method	Score	Mass	Gene names
UPS1+500ngY_90minTop17_BC4_01_358	41308	TOF; CID	72.32	1692.83	ALG6

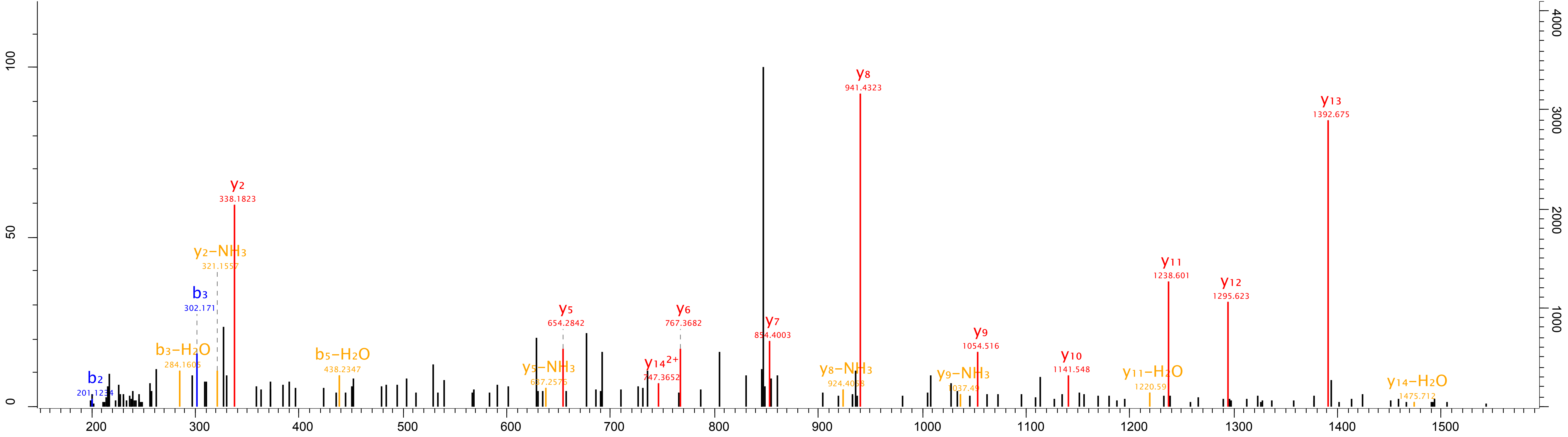

Raw file	Scan	Method	Score	Mass	Gene names
UPS1+500ngY_90minTop17_BC4_01_358	41318	TOF; CID	101.75	1729.8	RPB9

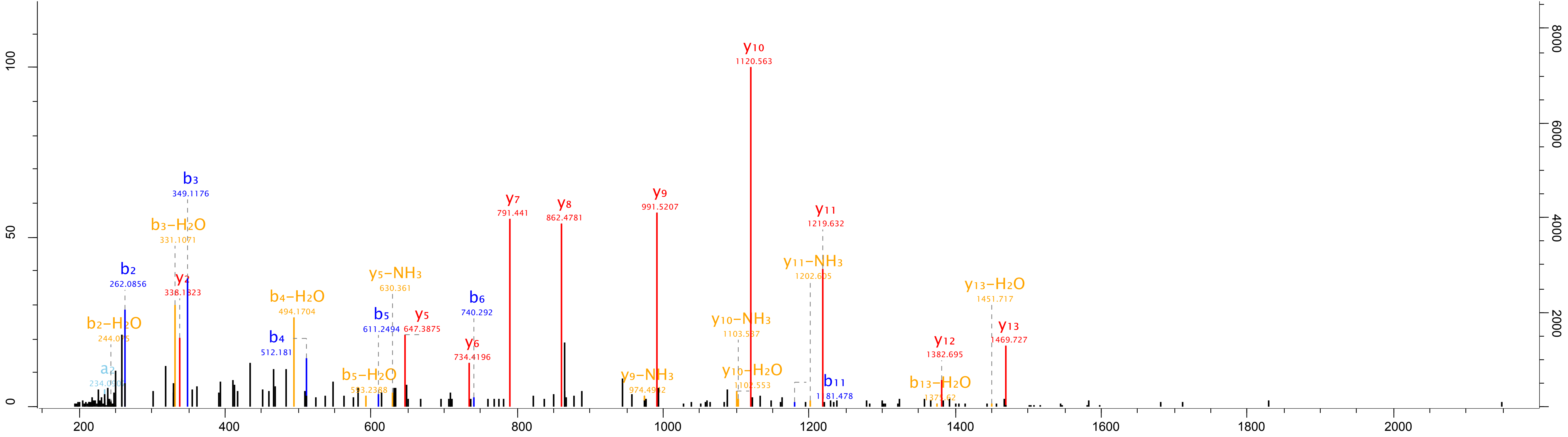

Raw file	Scan	Method	Score	Mass	Gene names
UPS1+500ngY_90minTop17_BC4_01_358	41320	TOF; CID	47.57	913.55	NKP1

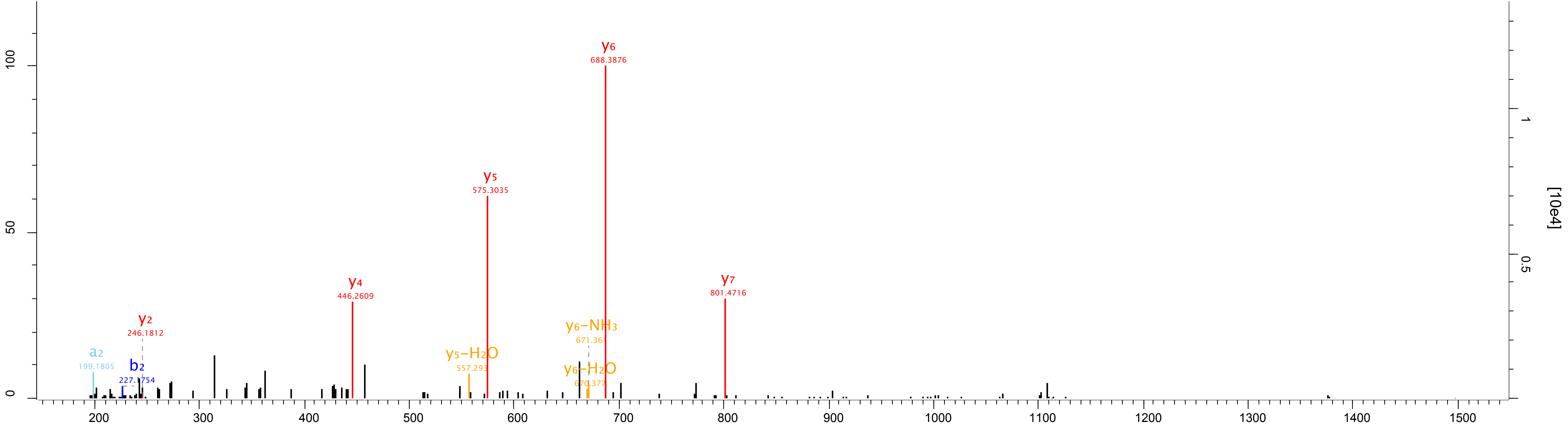

Raw file	Scan	Method	Score	Mass	Gene names
UPS1+500ngY_90minTop17_BC4_01_358	41323	TOF; CID	64.58	1825.91	SMF3

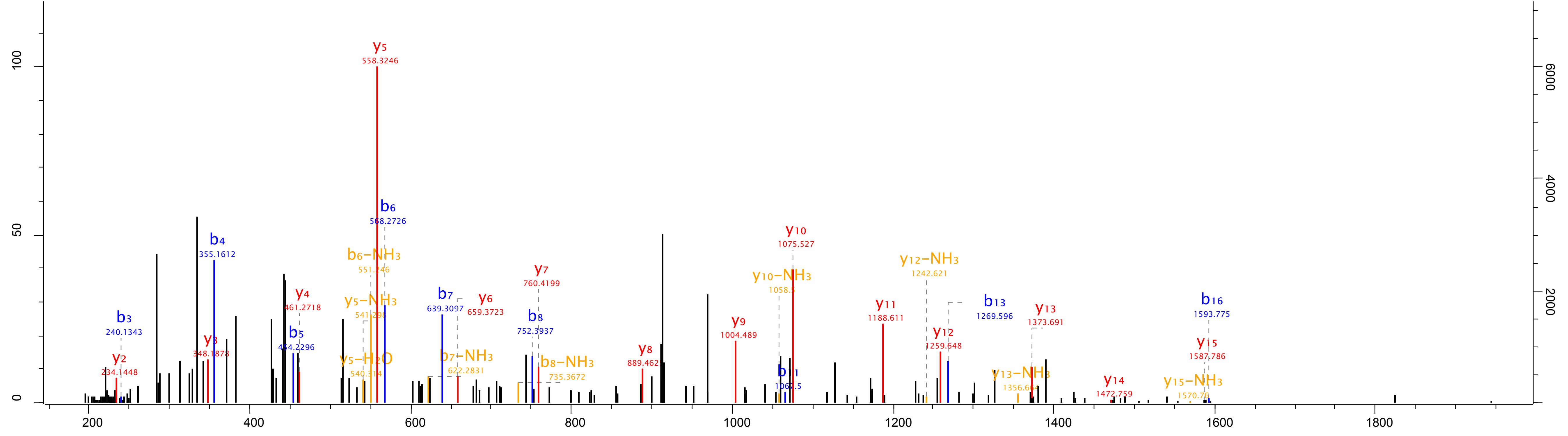

Raw file	Scan	Method	Score	Mass	Gene names
UPS1+500ngY_90minTop17_BC4_01_358	41364	TOF; CID	64.83	1481.81	FSH3

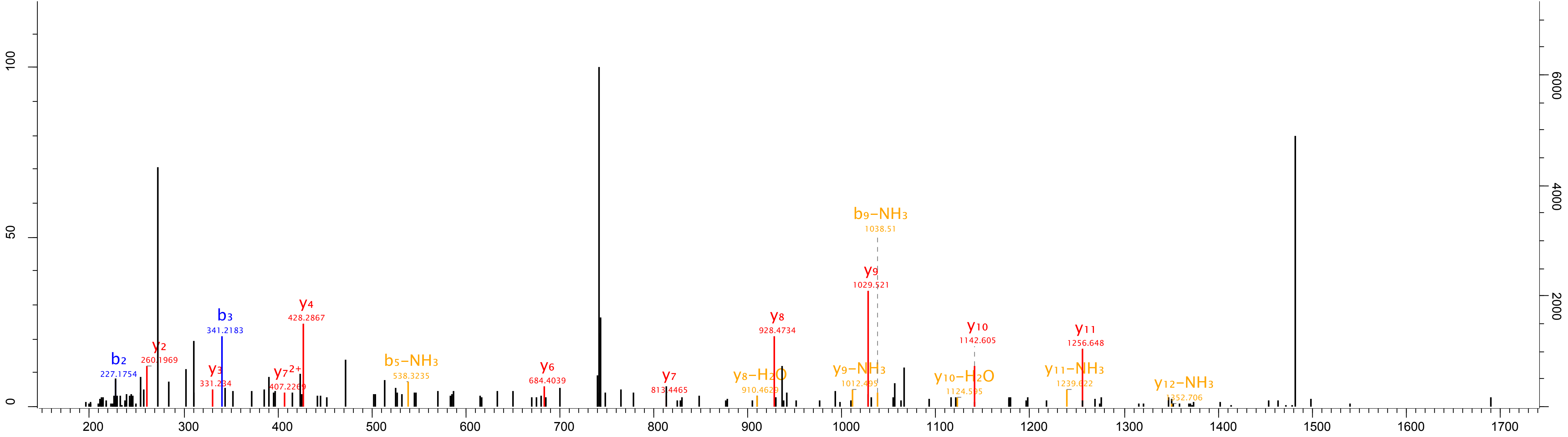

Raw file	Scan	Method	Score	Mass	Gene names
UPS1+500ngY_90minTop17_BC4_01_358	41995	TOF; CID	98.9	1626.63	MDM35

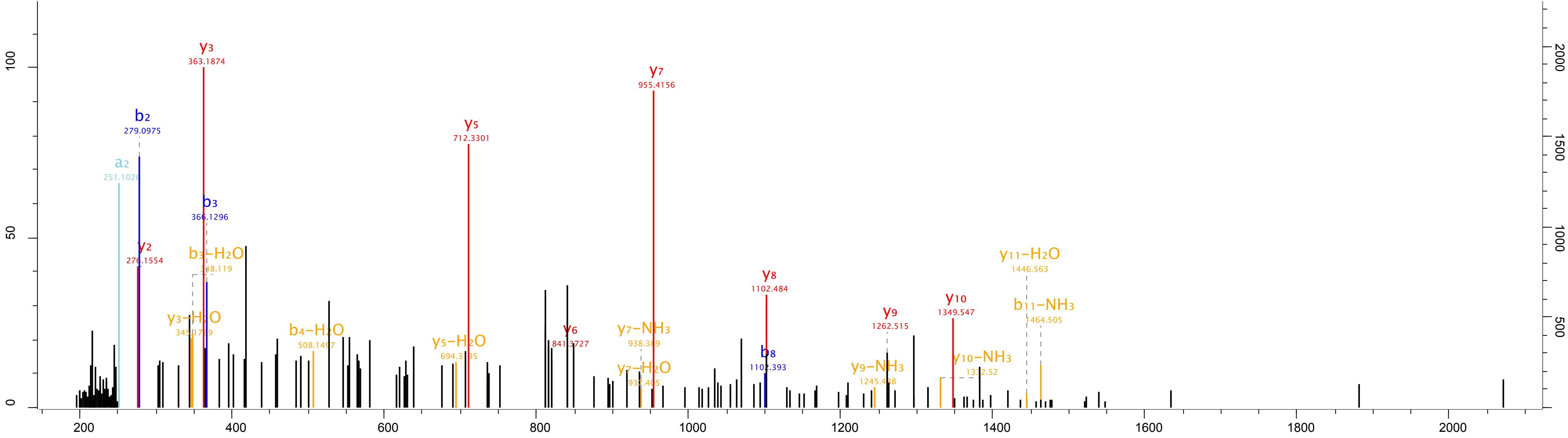

Raw file	Scan	Method	Score	Mass	Gene names
UPS1+500ngY_90minTop17_BC4_01_358	42186	TOF; CID	64.12	1260.62	ENT1

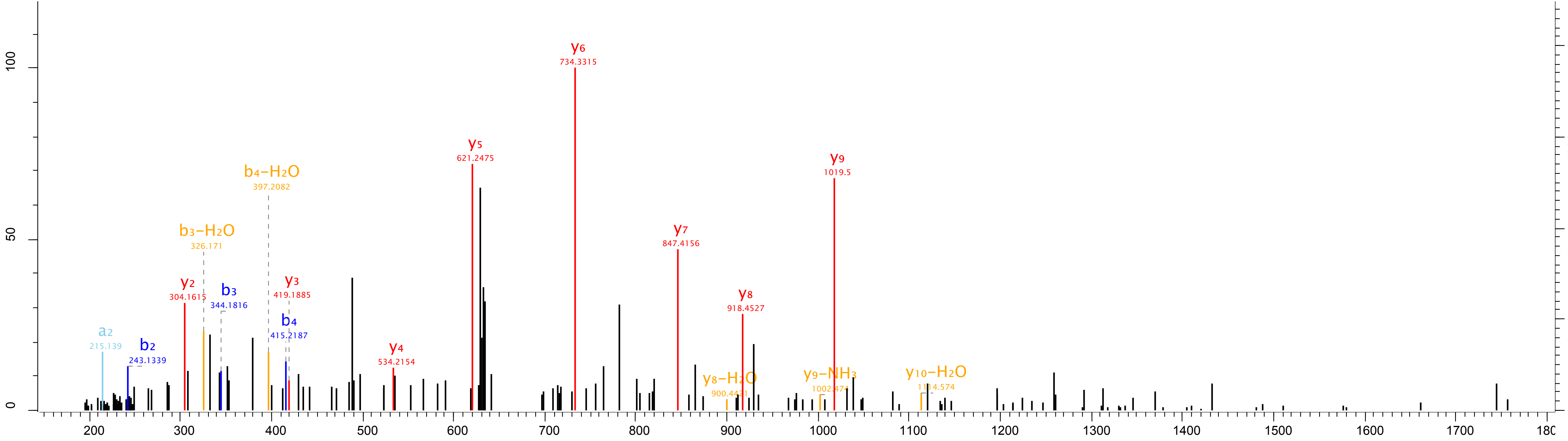

Raw file	Scan	Method	Score	Mass	Gene names
UPS1+500ngY_90minTop17_BC4_01_358	42325	TOF; CID	113.25	1649.76	TOM5

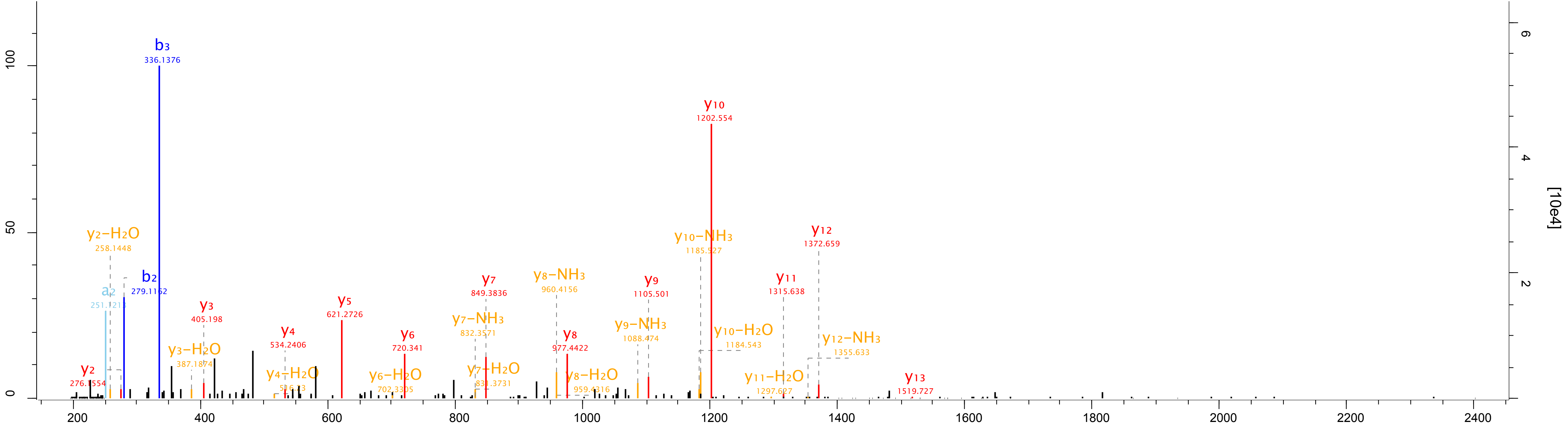

Raw file	Scan	Method	Score	Mass	Gene names
UPS1+500ngY_90minTop17_BC4_01_358	42371	TOF; CID	56.72	2088.93	PAM17

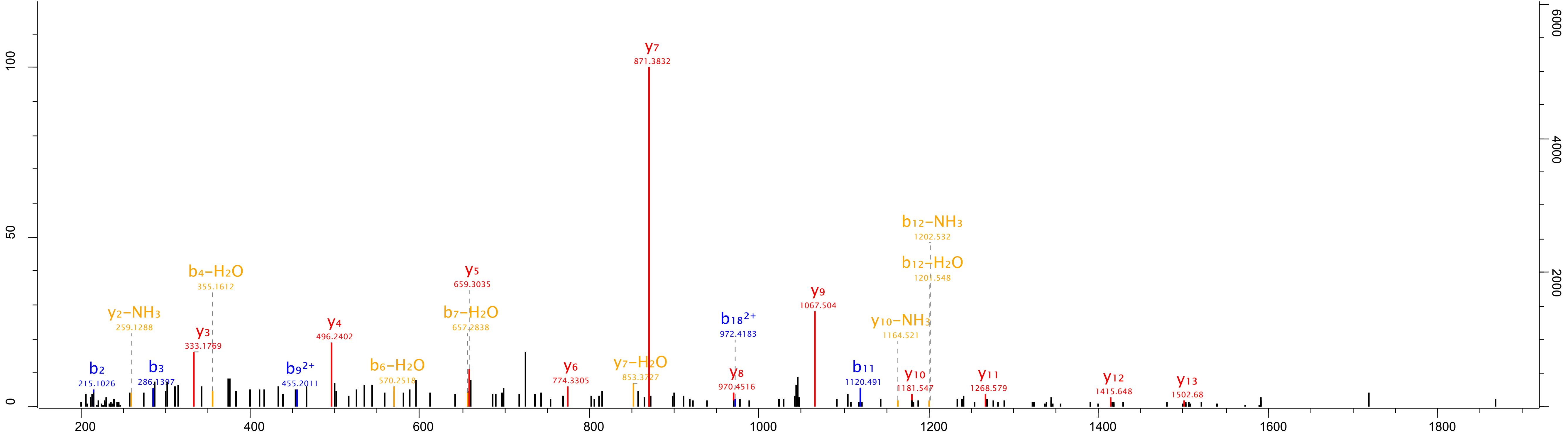

Raw file	Scan	Method	Score	Mass	Gene names
UPS1+500ngY_90minTop17_BC4_01_358	42856	TOF; CID	57.43	1488.75	MEX67

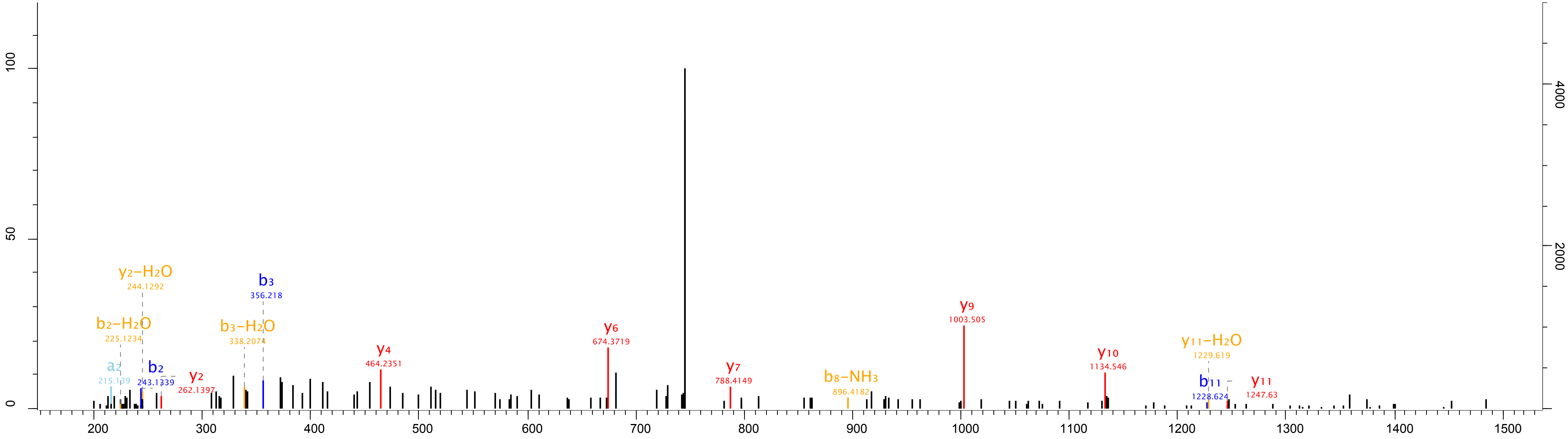

Raw file	Scan	Method	Score	Mass	Gene names
UPS1+500ngY_90minTop17_BC4_01_358	42896	TOF; CID	66.83	1762.82	OPI3

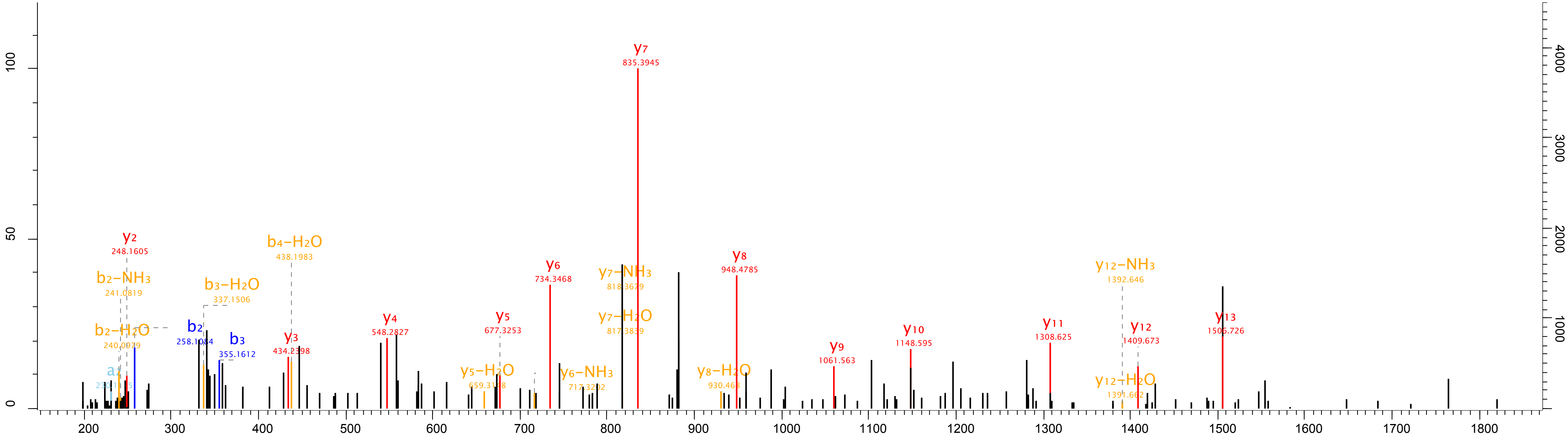

Raw file	Scan	Method	Score	Mass	Gene names
UPS1+500ngY_90minTop17_BC4_01_358	43049	TOF; CID	43.85	1858.88	CNS1

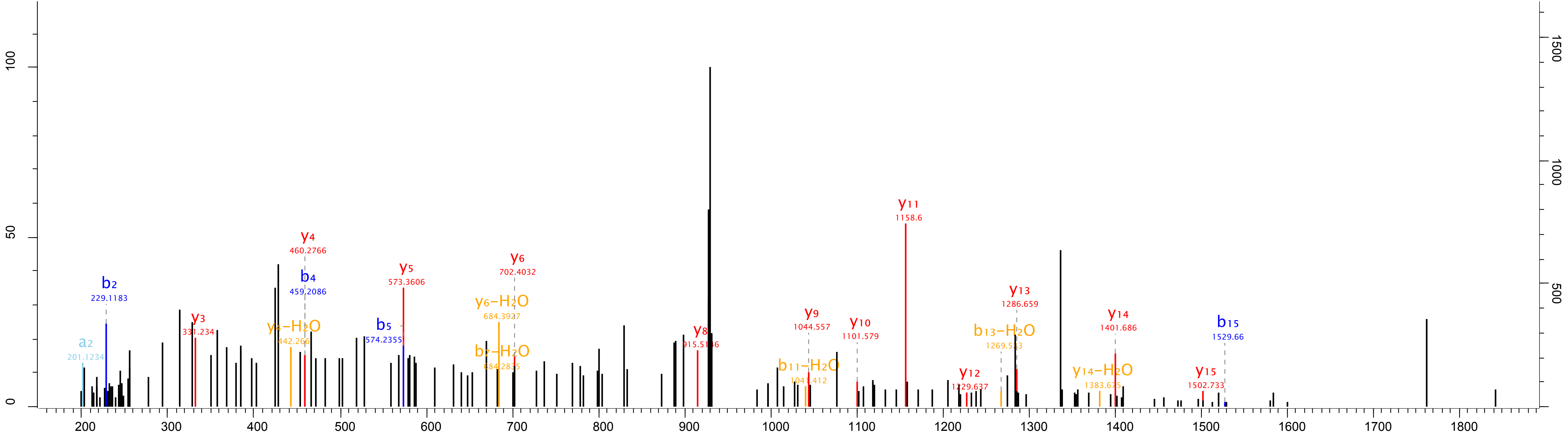

Raw file	Scan	Method	Score	Mass	Gene names
UPS1+500ngY_90minTop17_BC4_01_358	43452	TOF; CID	56.08	2085.89	MGM101

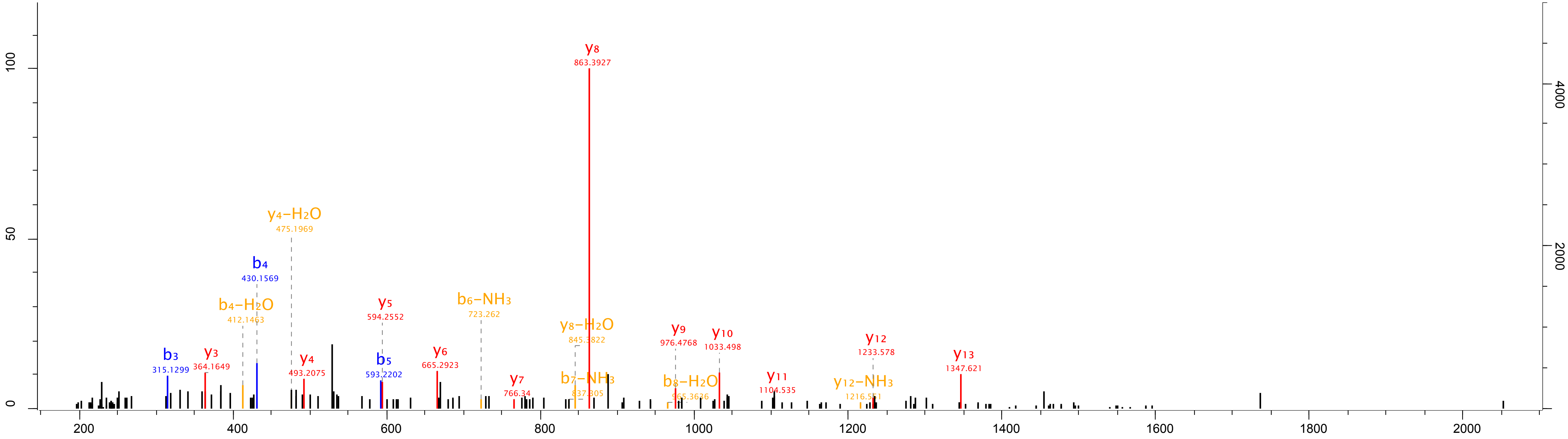

Raw file	Scan	Method	Score	Mass	Gene names
UPS1+500ngY_90minTop17_BC4_01_358	43576	TOF; CID	45.92	2224.94	RER1

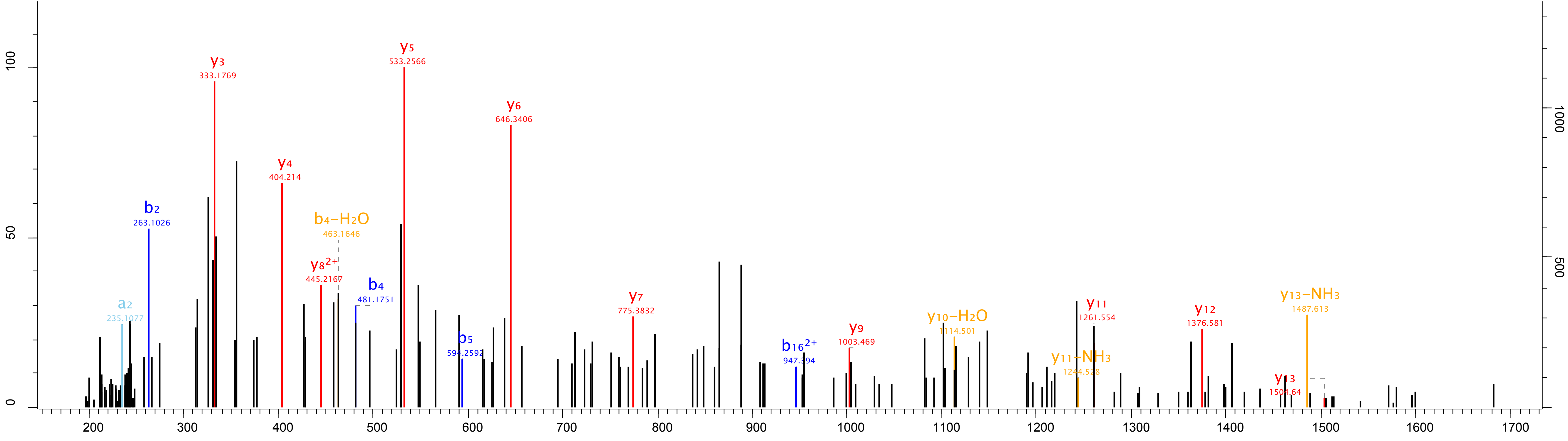

Raw file	Scan	Method	Score	Mass	Gene names
UPS1+500ngY_90minTop17_BC4_01_358	44204	TOF; CID	57.15	1691.78	VTI1

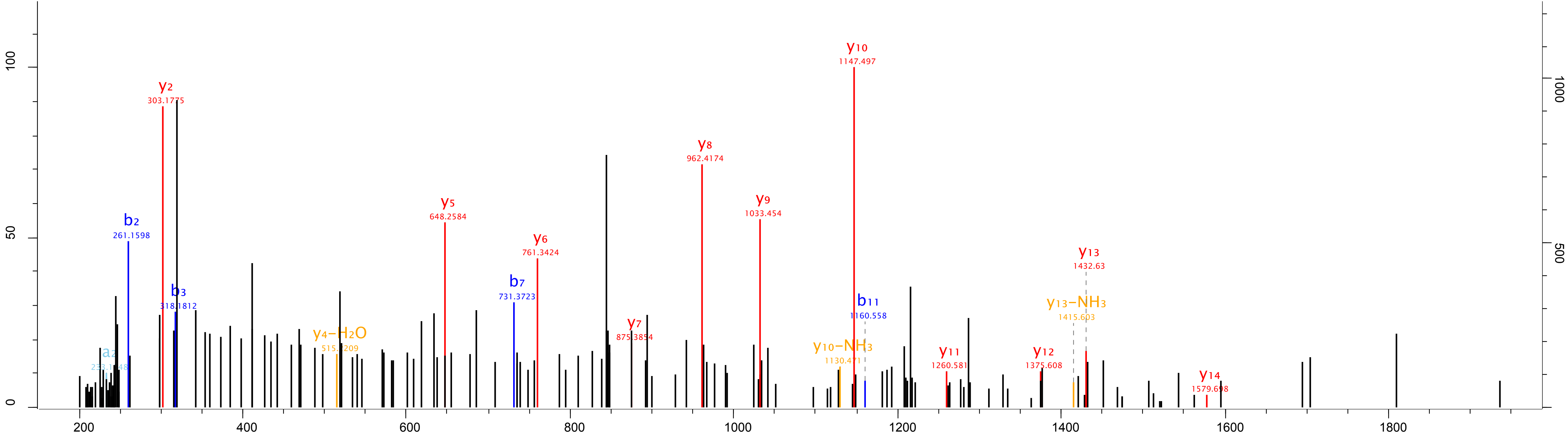

Raw file	Scan	Method	Score	Mass	Gene names
UPS1+500ngY_90minTop17_BC4_01_358	44570	TOF; CID	118.73	1545.73	TMA7

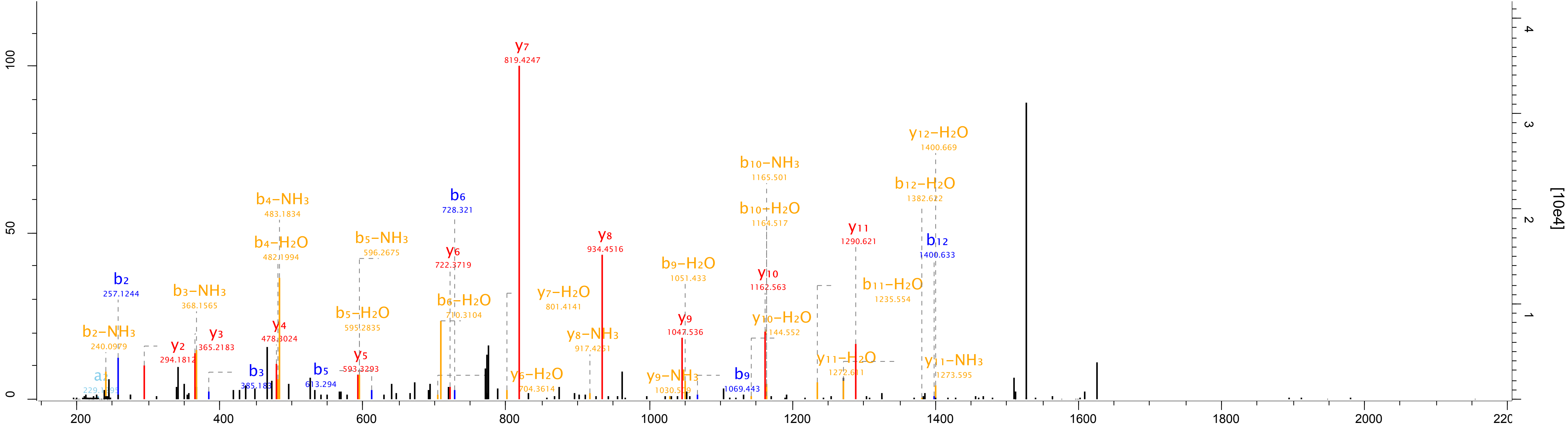

Raw file	Scan	Method	Score	Mass	Gene names
UPS1+500ngY_90minTop17_BC4_01_358	44603	TOF; CID	78.34	1298.6	PPT1

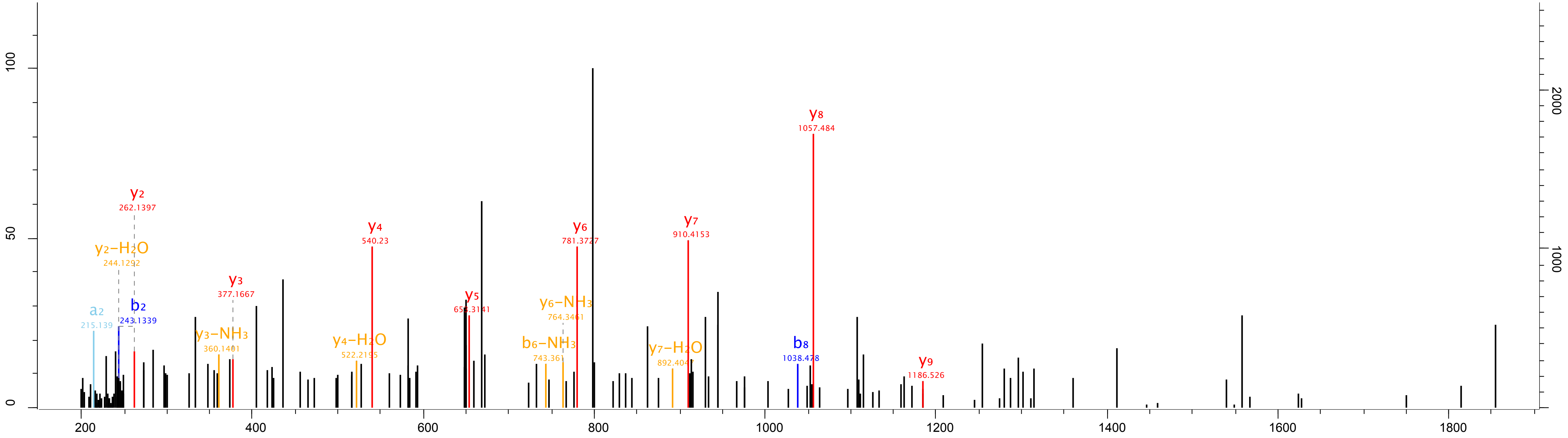

Raw file	Scan	Method	Score	Mass	Gene names
UPS1+500ngY_90minTop17_BC4_01_358	44808	TOF; CID	63.09	1365.71	AGE2

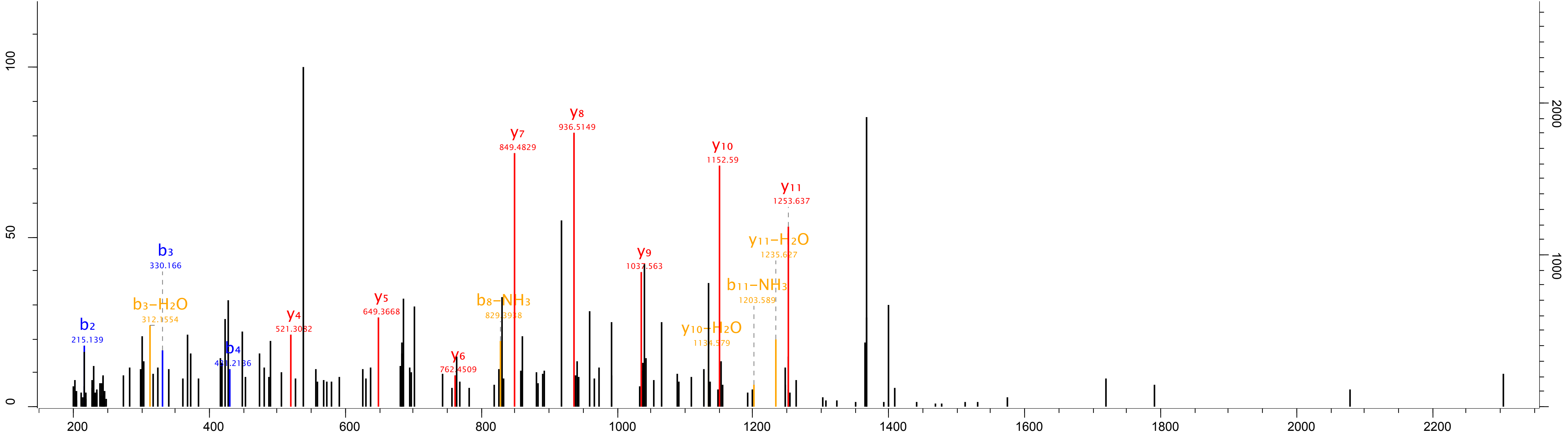

Raw file	Scan	Method	Score	Mass	Gene names
UPS1+500ngY_90minTop17_BC4_01_358	45011	TOF; CID	87.67	1365.65	IRC22

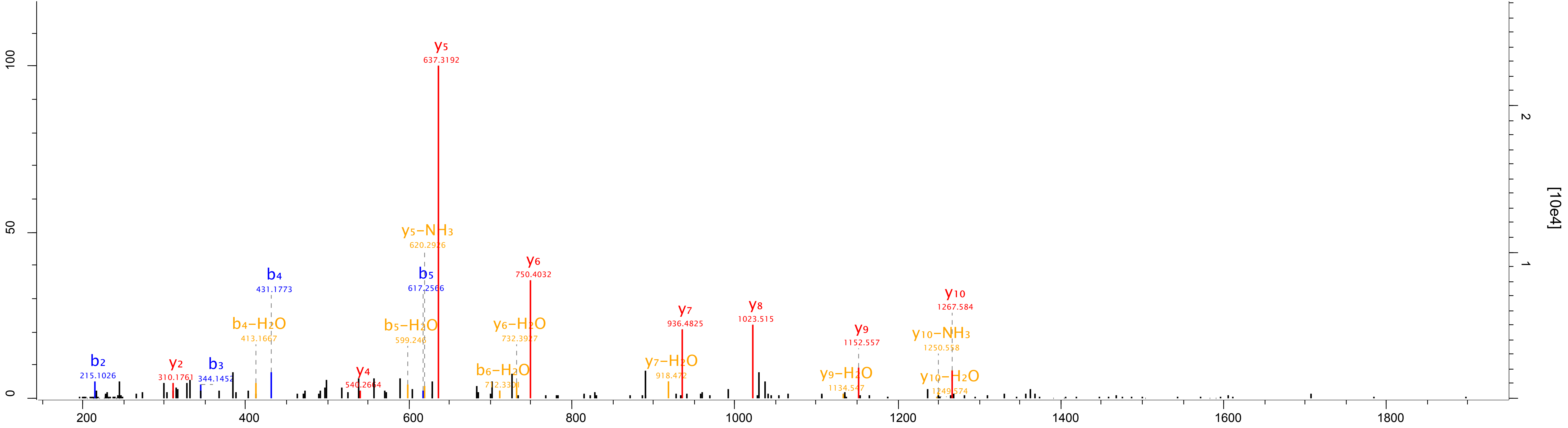

Raw file	Scan	Method	Score	Mass	Gene names
UPS1+500ngY_90minTop17_BC4_01_358	45184	TOF; CID	61.53	1656.75	CKB2

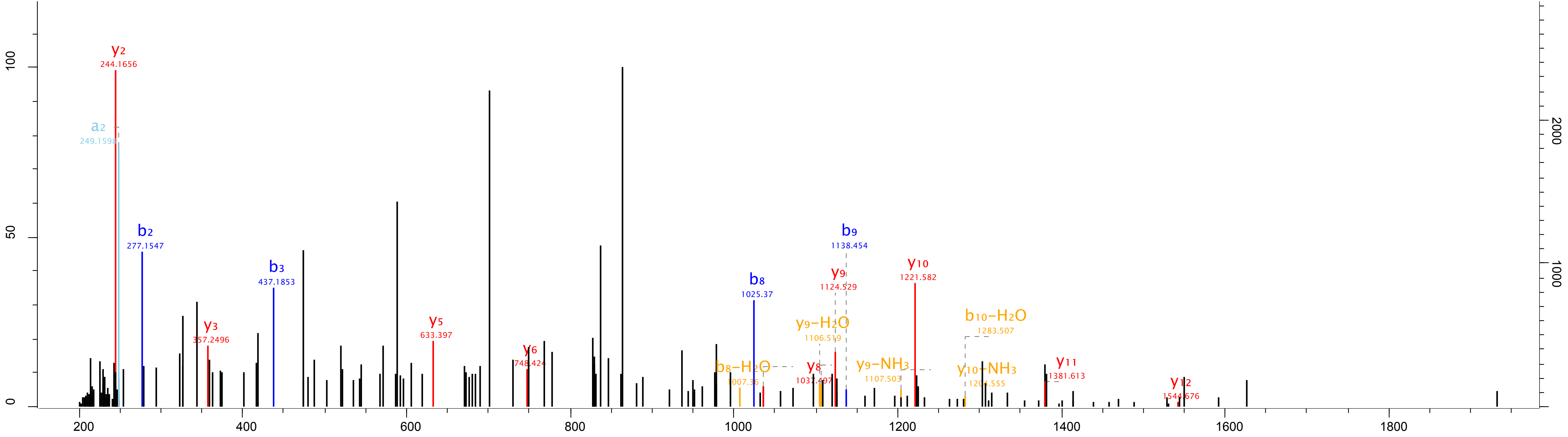

Raw file	Scan	Method	Score	Mass	Gene names
UPS1+500ngY_90minTop17_BC4_01_358	45881	TOF; CID	54.76	1937.94	YJU3

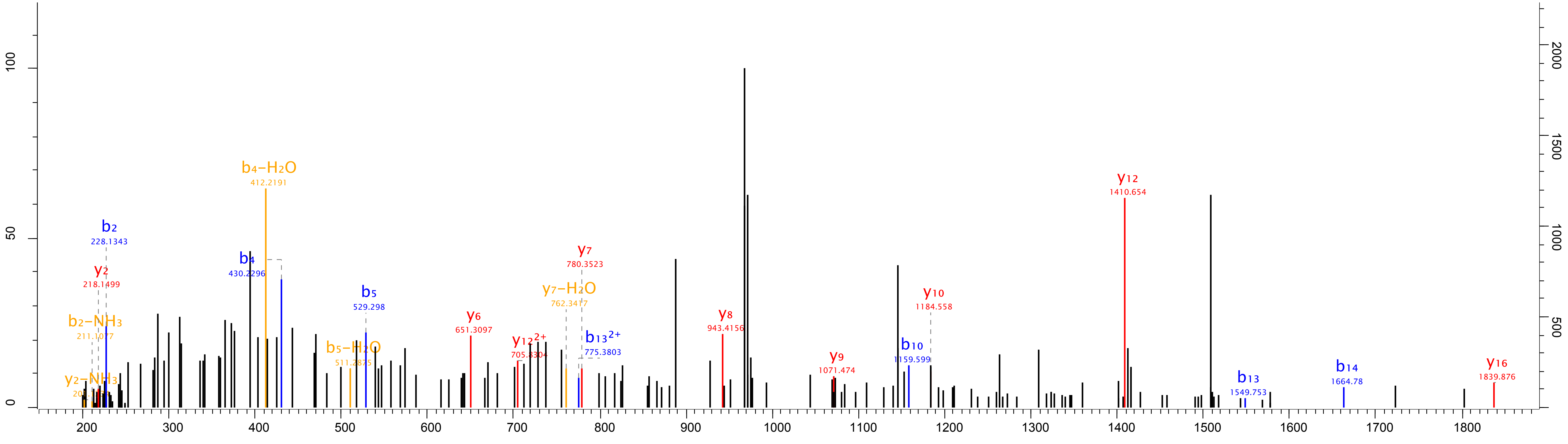

Raw file	Scan	Method	Score	Mass	Gene names
UPS1+500ngY_90minTop17_BC4_01_358	45979	TOF; CID	116.38	2924.33	ERP2

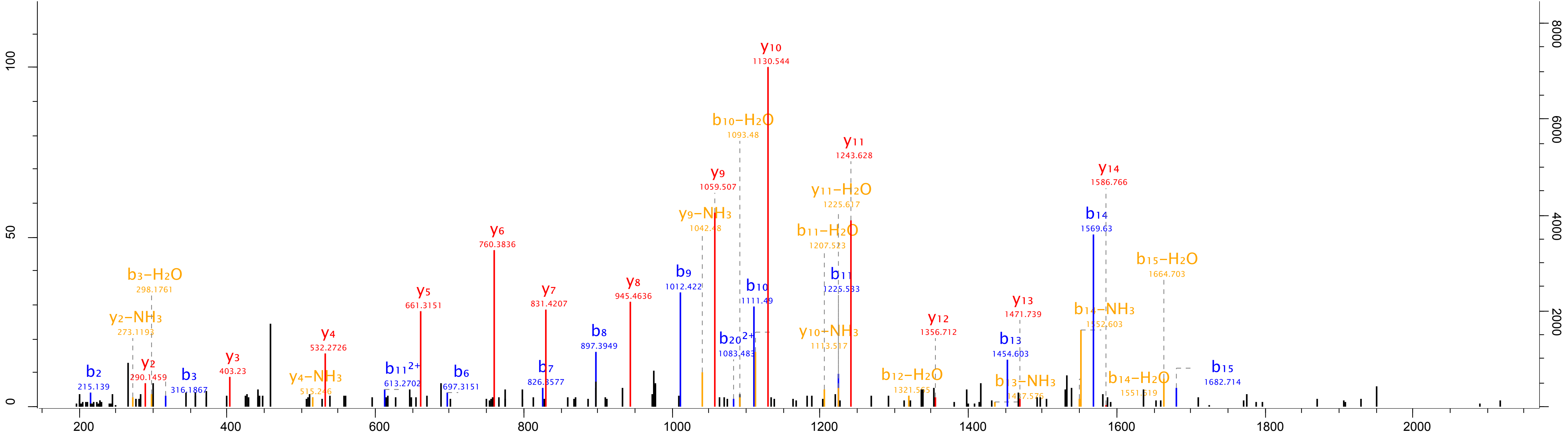

Raw file	Scan	Method	Score	Mass	Gene names
UPS1+500ngY_90minTop17_BC4_01_358	46575	TOF; CID	65.25	1034.54	SEC22

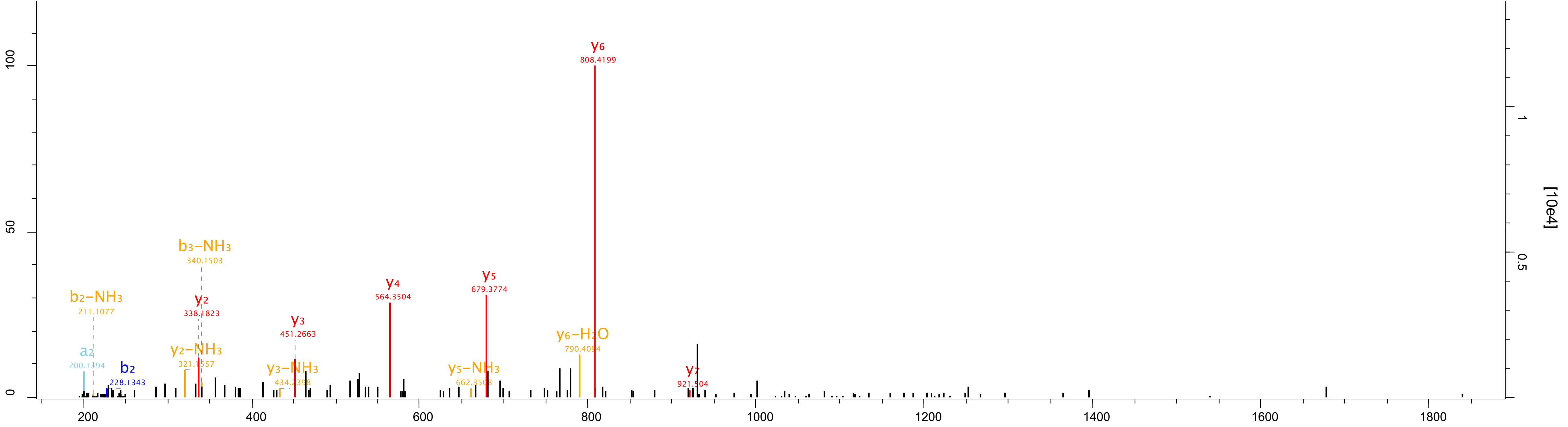

Raw file	Scan	Method	Score	Mass	Gene names
UPS1+500ngY_90minTop17_BC4_01_358	46619	TOF; CID	47.73	2130.9	CCW14

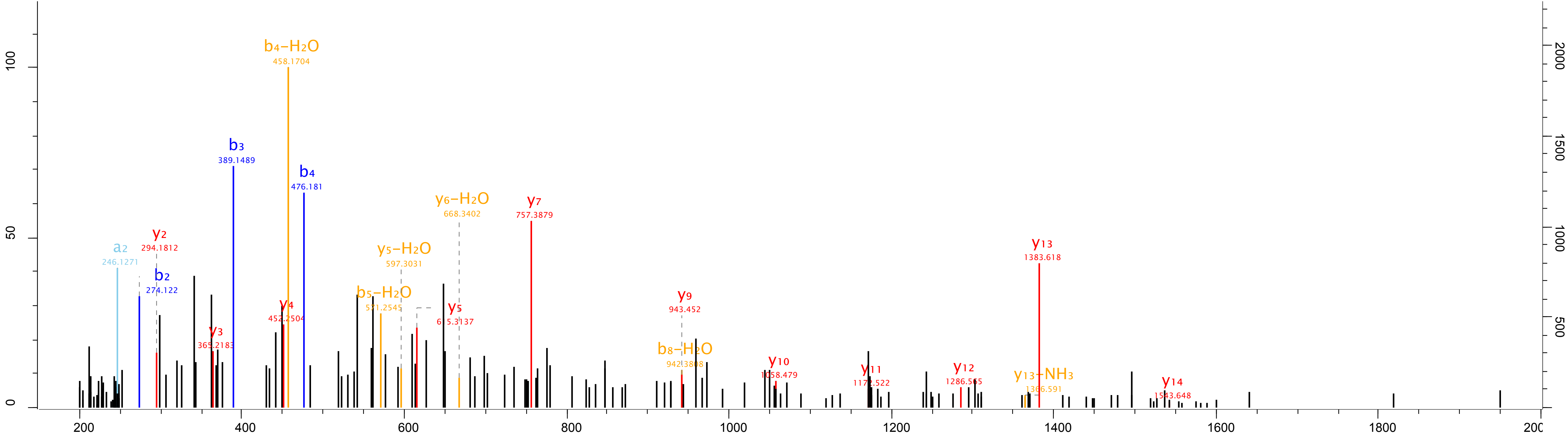

Raw file	Scan	Method	Score	Mass	Gene names
UPS1+500ngY_90minTop17_BC4_01_358	46769	TOF; CID	45.84	2299.13	NUP116

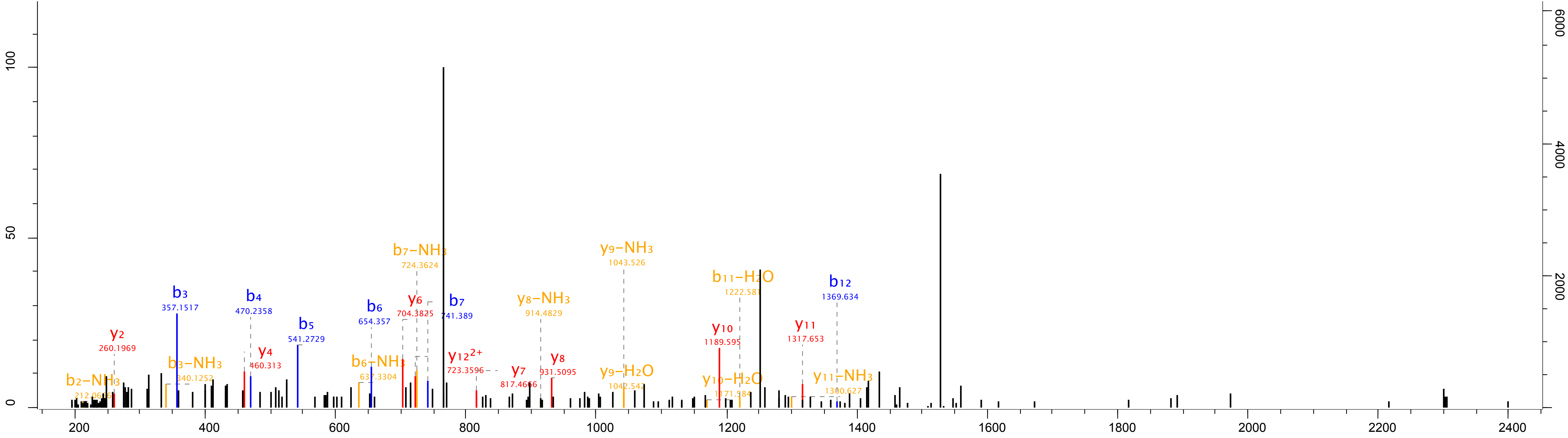

Raw file	Scan	Method	Score	Mass	Gene names
UPS1+500ngY_90minTop17_BC4_01_358	46838	TOF; CID	74.3	1069.53	DTD1

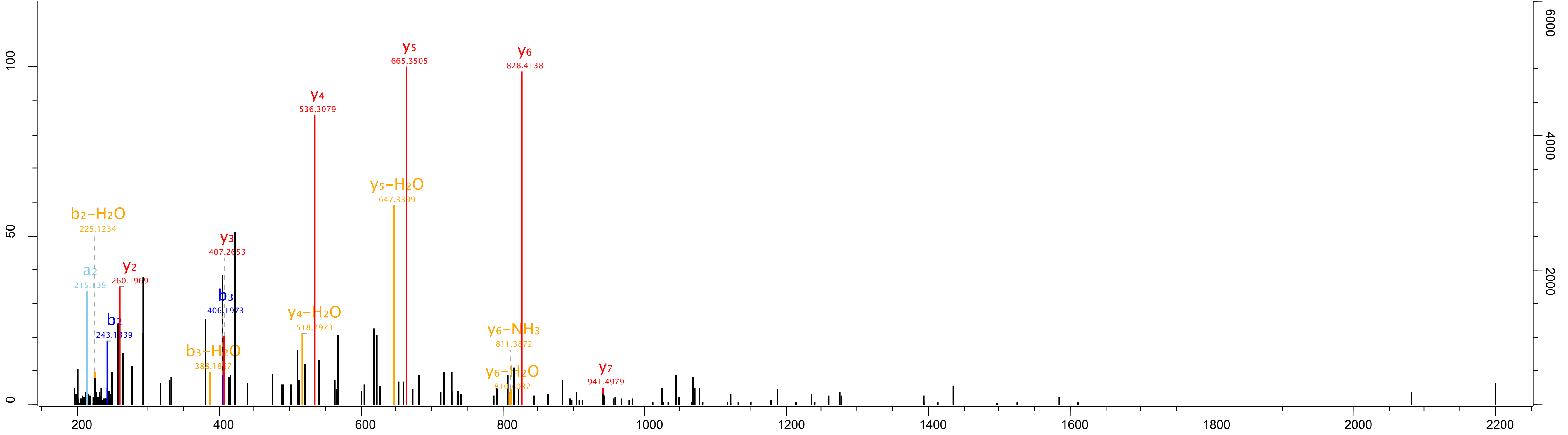

Raw file	Scan	Method	Score	Mass	Gene names
UPS1+500ngY_90minTop17_BC4_01_358	46891	TOF; CID	50.27	2677.18	FBP26

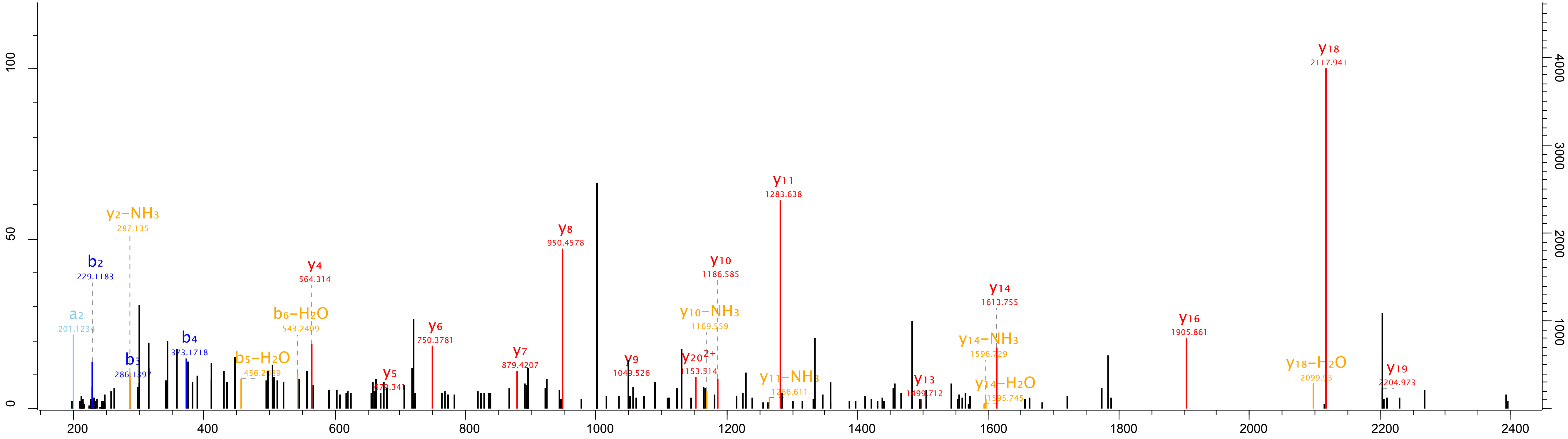

Raw file	Scan	Method	Score	Mass
UPS1+500ngY_90minTop17_BC4_01_358	47095	TOF; CID	67.93	2100.98

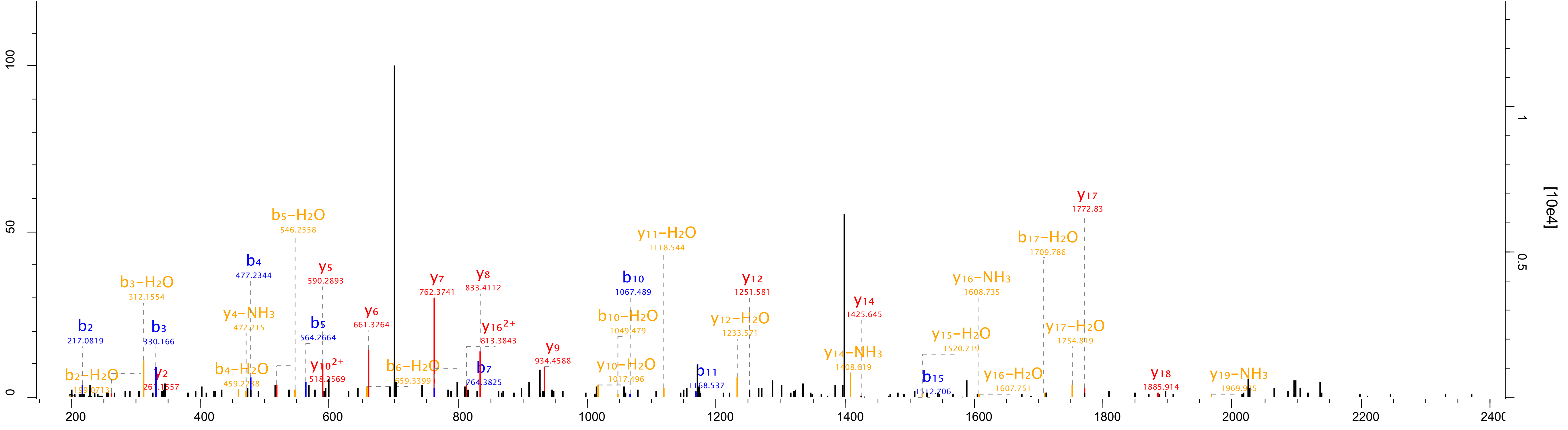

Raw file	Scan	Method	Score	Mass	Gene names
UPS1+500ngY_90minTop17_BC4_01_358	47117	TOF; CID	46.42	1945.01	SVL3

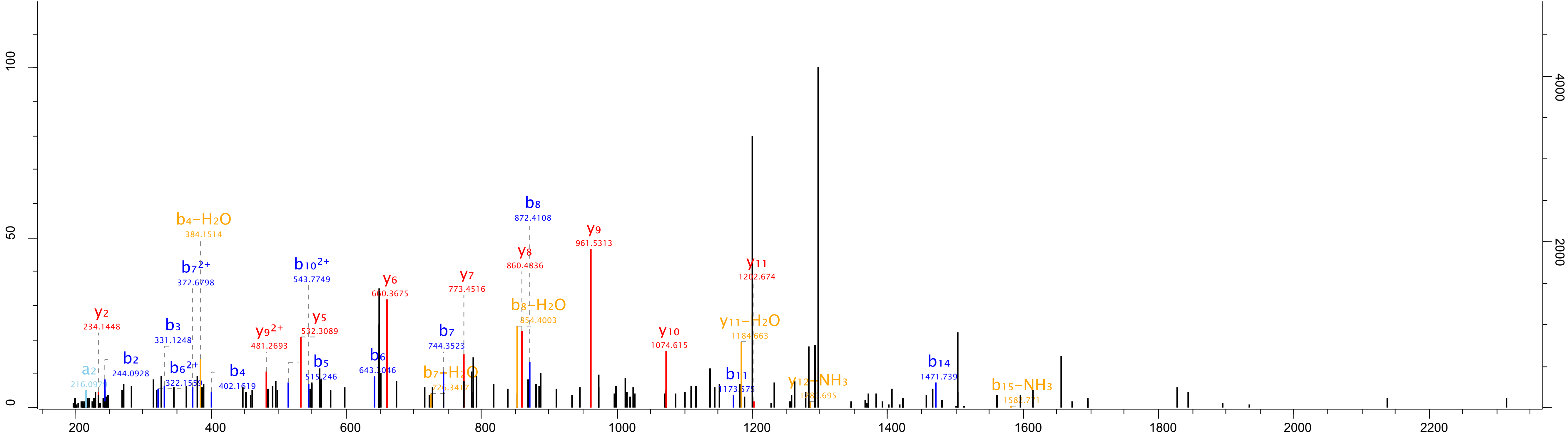

Raw file	Scan	Method	Score	Mass	Gene names
UPS1+500ngY_90minTop17_BC4_01_358	47511	TOF; CID	108.43	1350.58	YKL077W

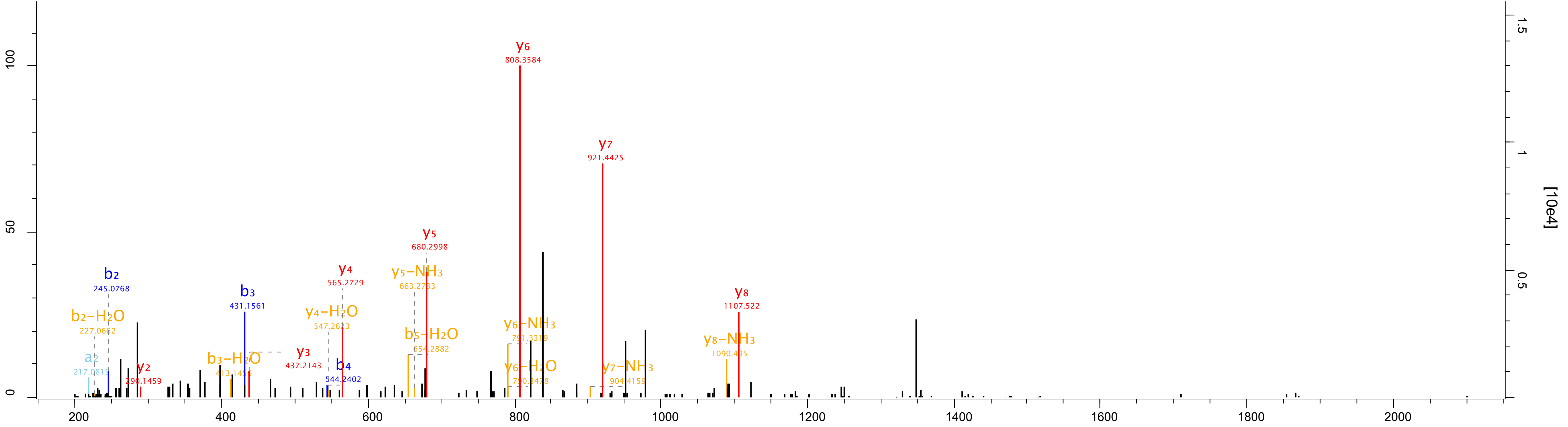

Raw file	Scan	Method	Score	Mass	Gene names
UPS1+500ngY_90minTop17_BC4_01_358	47814	TOF; CID	88.02	1318.6	VPS68

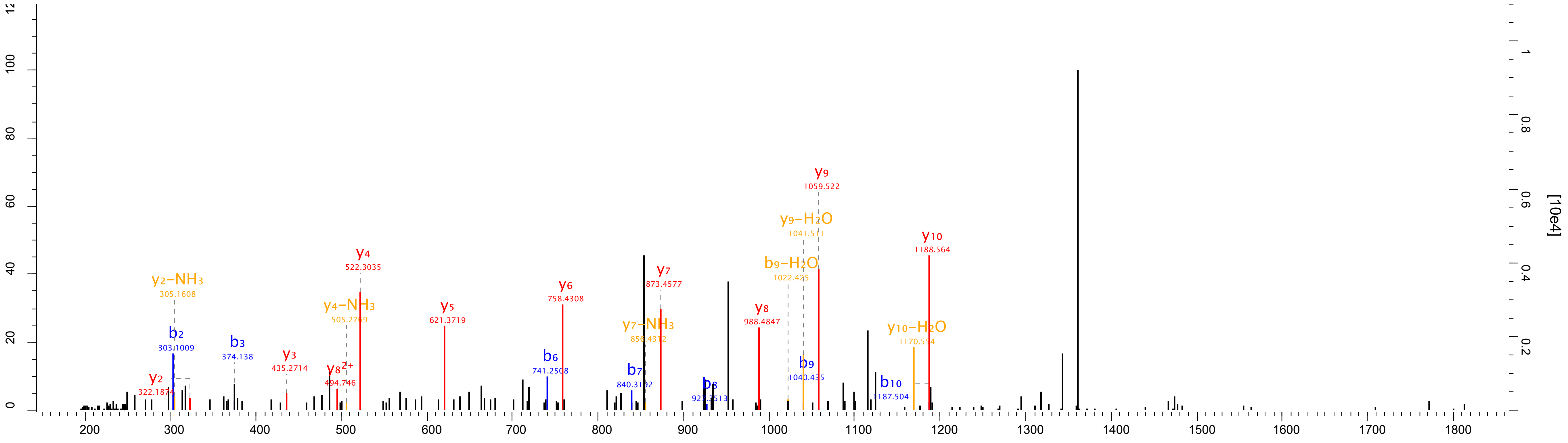

Raw file	Scan	Method	Score	Mass	Gene names
UPS1+500ngY_90minTop17_BC4_01_358	47890	TOF; CID	59.34	1953.99	PGC1

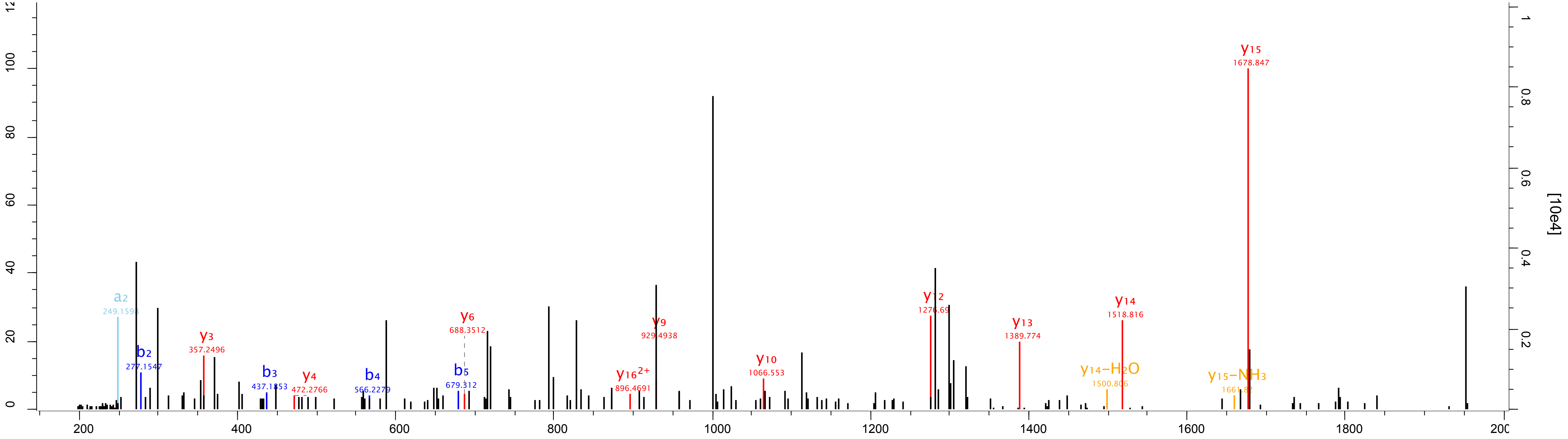

Raw file	Scan	Method	Score	Mass	Gene names
UPS1+500ngY_90minTop17_BC4_01_358	48534	TOF; CID	45	2020.94	ECM4

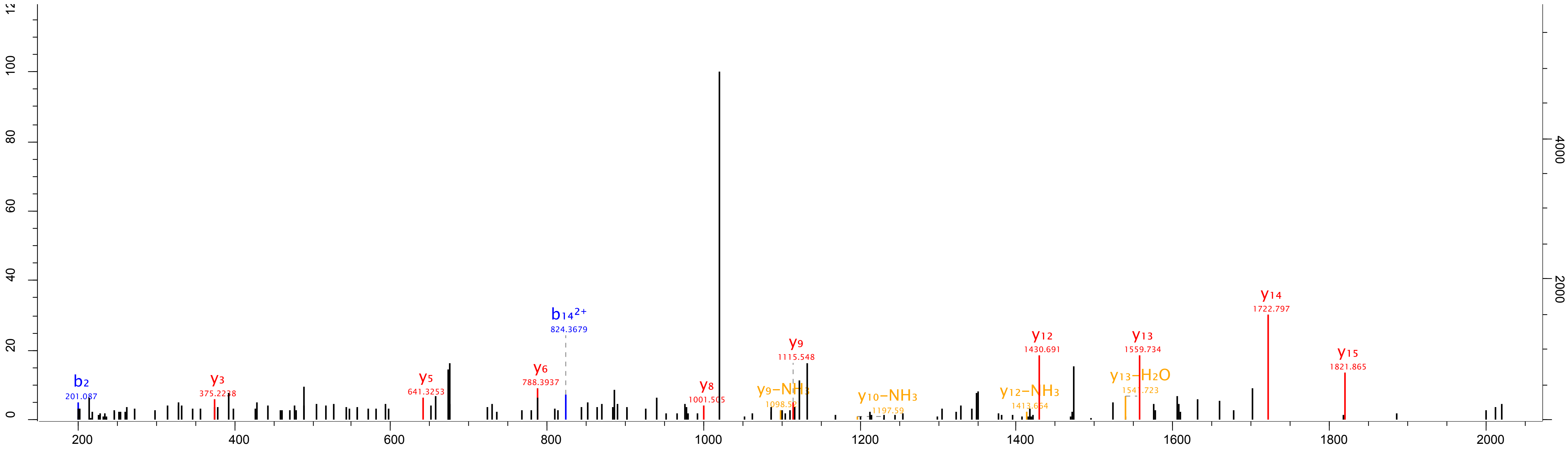

Raw file	Scan	Method	Score	Mass	Gene names
UPS1+500ngY_90minTop17_BC4_01_358	49427	TOF; CID	71.5	1109.48	SNX3

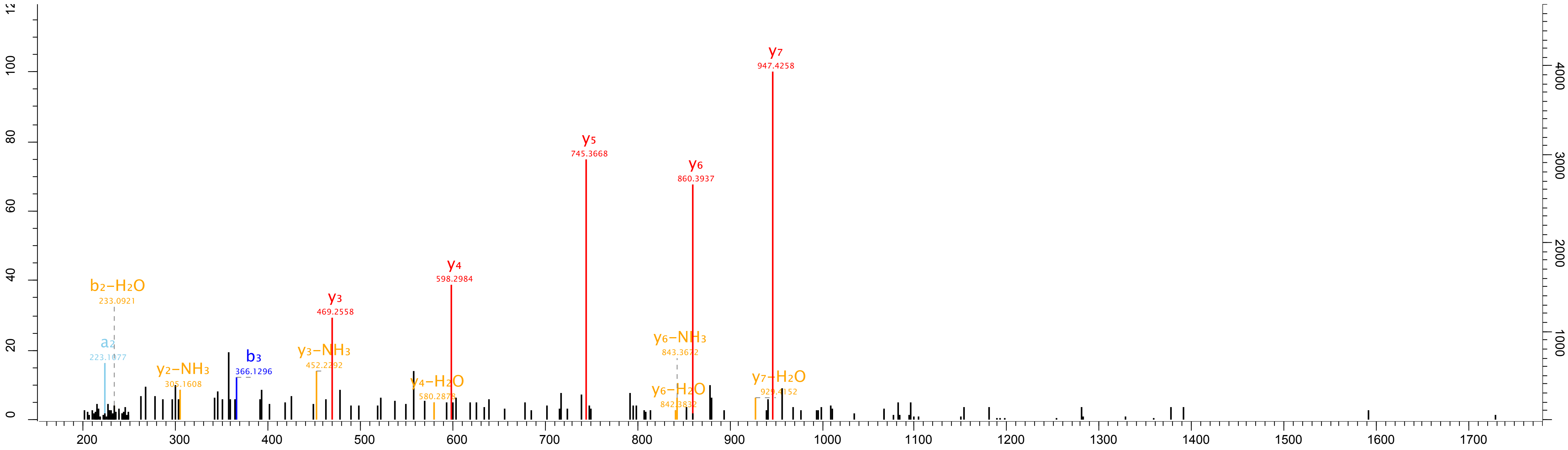

Raw file	Scan	Method	Score	Mass	Gene names
UPS1+500ngY_90minTop17_BC4_01_358	49510	TOF; CID	57.17	1116.64	PCC1

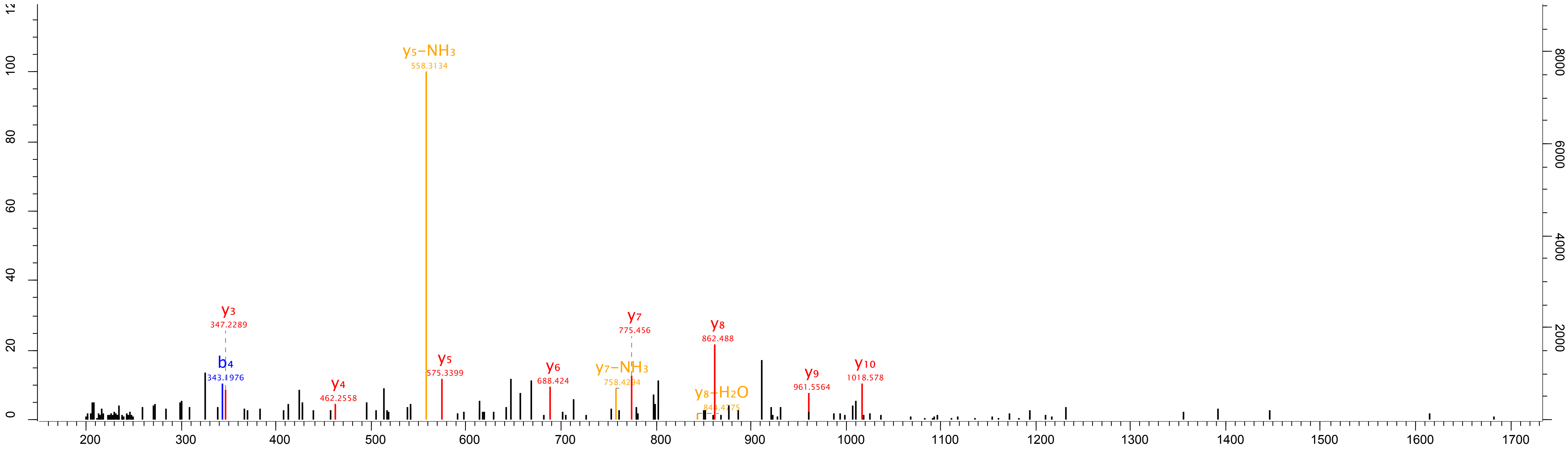

Raw file	Scan	Method	Score	Mass	Gene names
UPS1+500ngY_90minTop17_BC4_01_358	49731	TOF; CID	61.26	1829.87	MET14

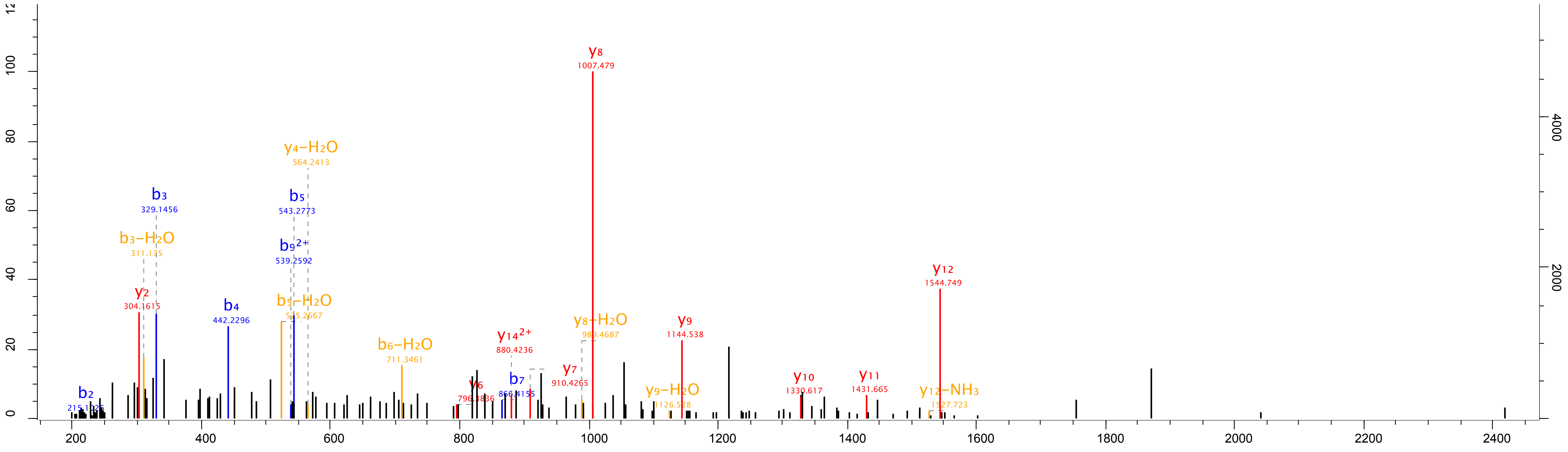

Raw file	Scan	Method	Score	Mass	Gene names
UPS1+500ngY_90minTop17_BC4_01_358	49917	TOF; CID	46.34	1963.9	TOA1

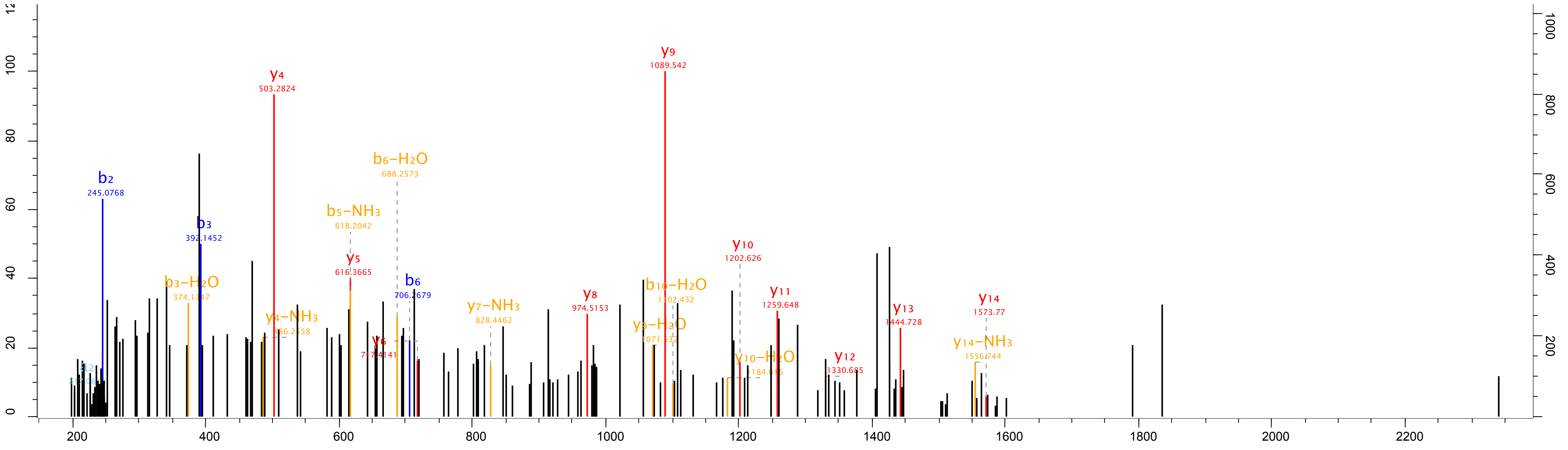

Raw file	Scan	Method	Score	Mass	Gene names
UPS1+500ngY_90minTop17_BC4_01_358	50129	TOF; CID	64.27	2779.27	MDE1

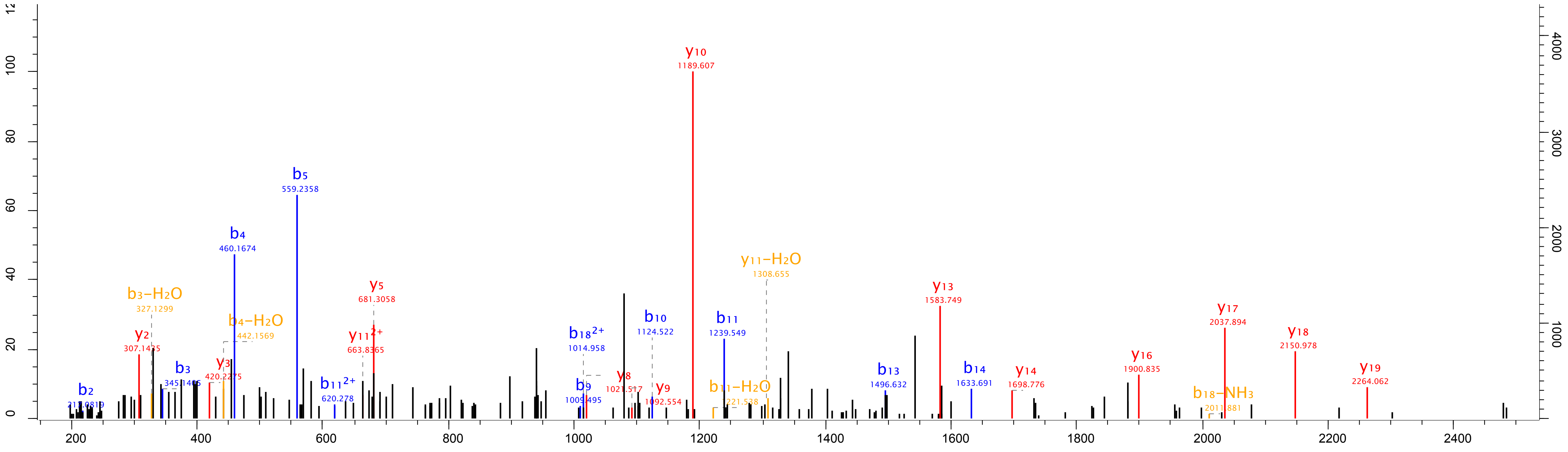

Raw file	Scan	Method	Score	Mass	Gene names
UPS1+500ngY_90minTop17_BC4_01_358	50644	TOF; CID	78.96	1366.75	CPD1

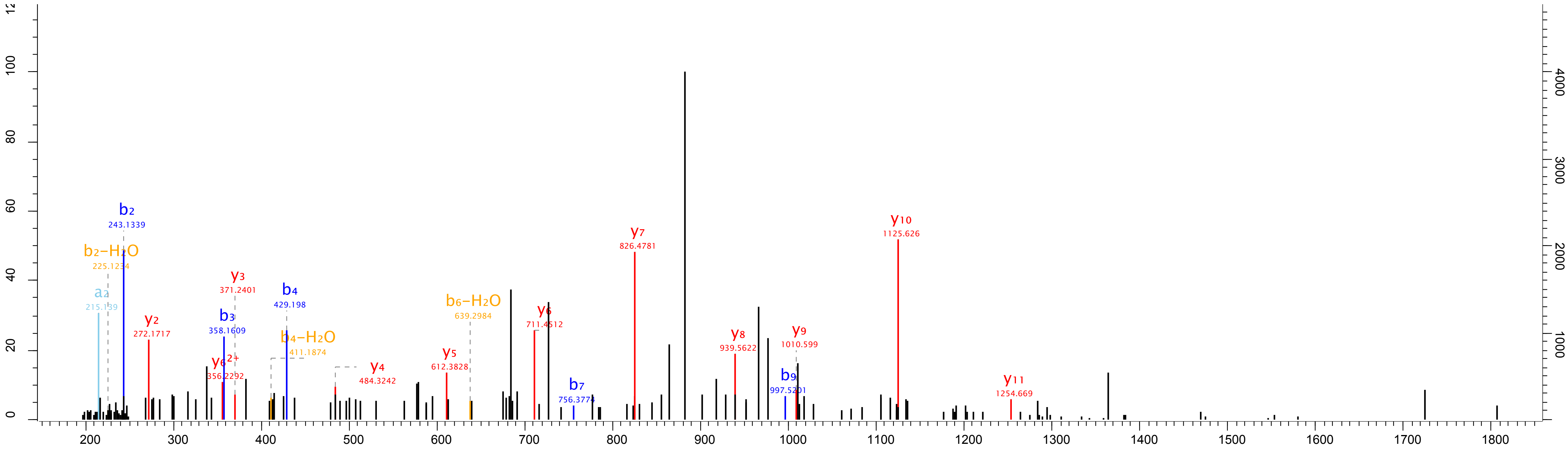

Raw file	Scan	Method	Score	Mass	Gene names
UPS1+500ngY_90minTop17_BC4_01_358	50892	TOF; CID	103.31	2016.99	RPC10

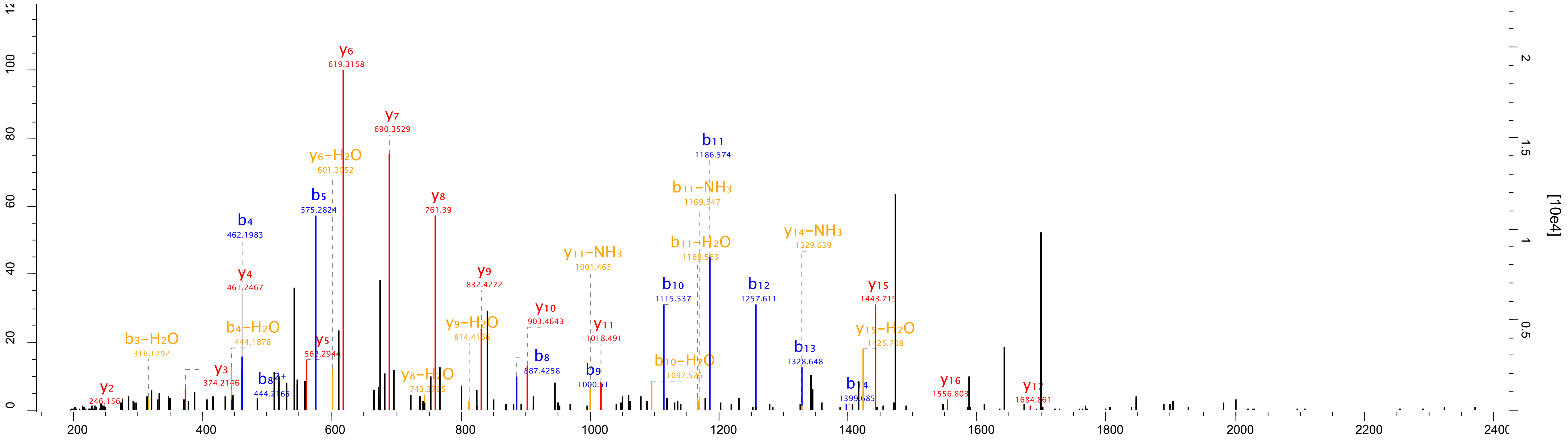

Raw file	Scan	Method	Score	Mass	Gene names
UPS1+500ngY_90minTop17_BC4_01_358	51955	TOF; CID	64.1	1282.71	NUP85

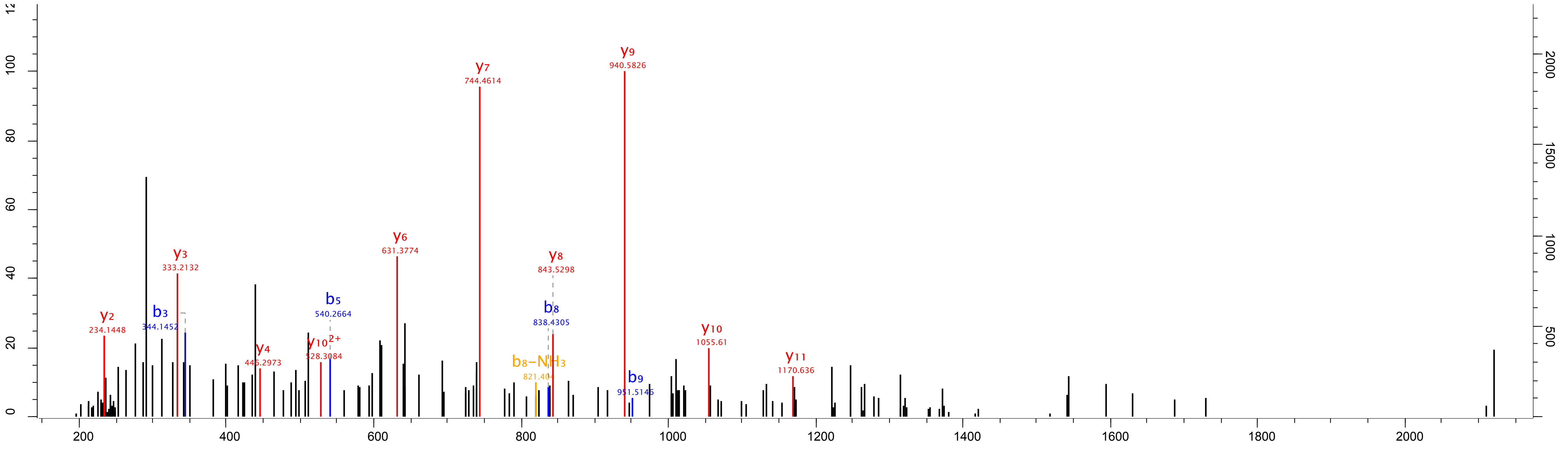

Raw file	Scan	Method	Score	Mass	Gene names
UPS1+500ngY_90minTop17_BC4_01_358	52006	TOF; CID	48.28	1076.55	AIM29

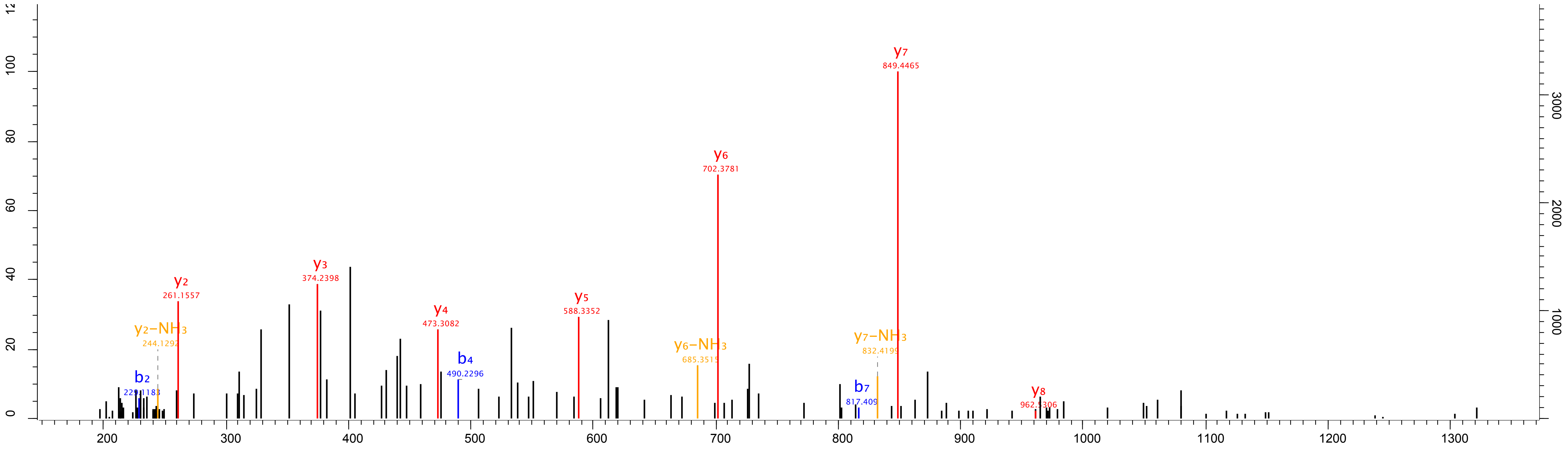

Raw file	Scan	Method	Score	Mass	Gene names
UPS1+500ngY_90minTop17_BC4_01_358	52600	TOF; CID	57.59	1125.55	GGC1

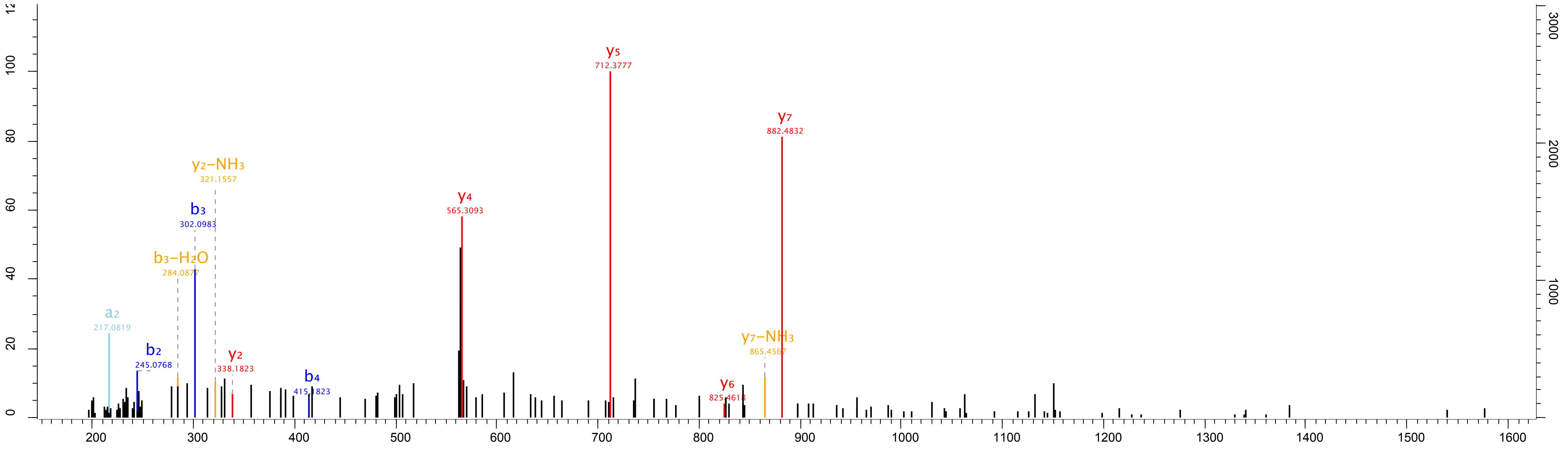

Raw file	Scan	Method	Score	Mass	Gene names
UPS1+500ngY_90minTop17_BC4_01_358	52724	TOF; CID	66.89	1930.91	ERP3

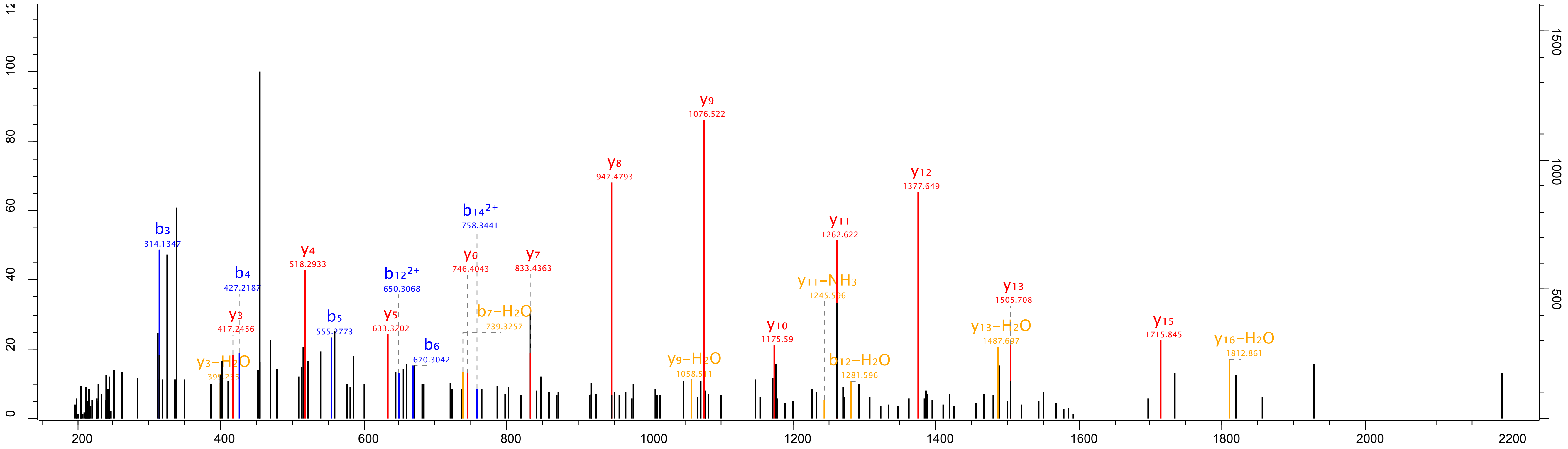

Raw file	Scan	Method	Score	Mass	Gene names
UPS1+500ngY_90minTop17_BC4_01_358	52783	TOF; CID	39.9	2103.93	IES2

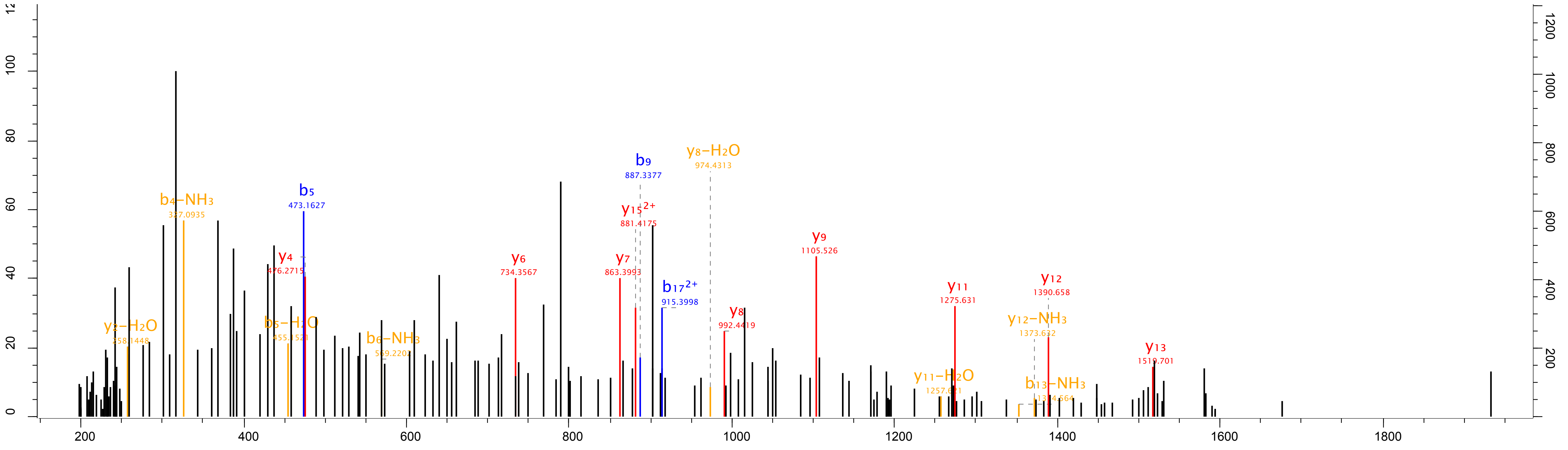

Raw file	Scan	Method	Score	Mass	Gene names
UPS1+500ngY_90minTop17_BC4_01_358	52814	TOF; CID	87.68	1992.95	PRS3

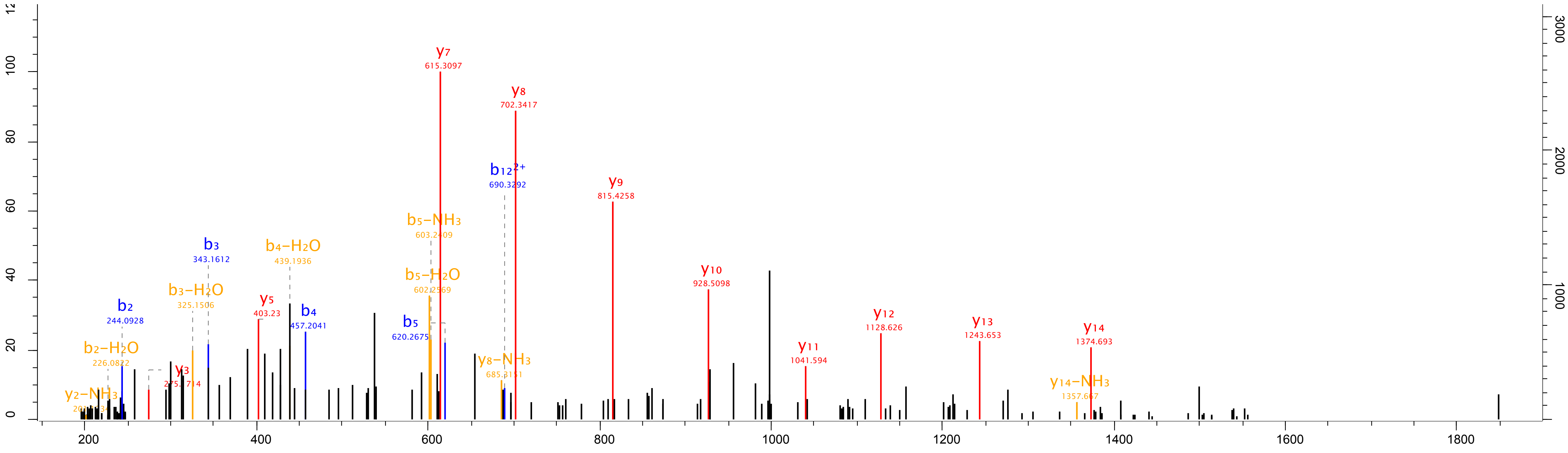

Raw file	Scan	Method	Score	Mass	Gene names
UPS1+500ngY_90minTop17_BC4_01_358	53158	TOF; CID	84.03	2504.21	RNQ1

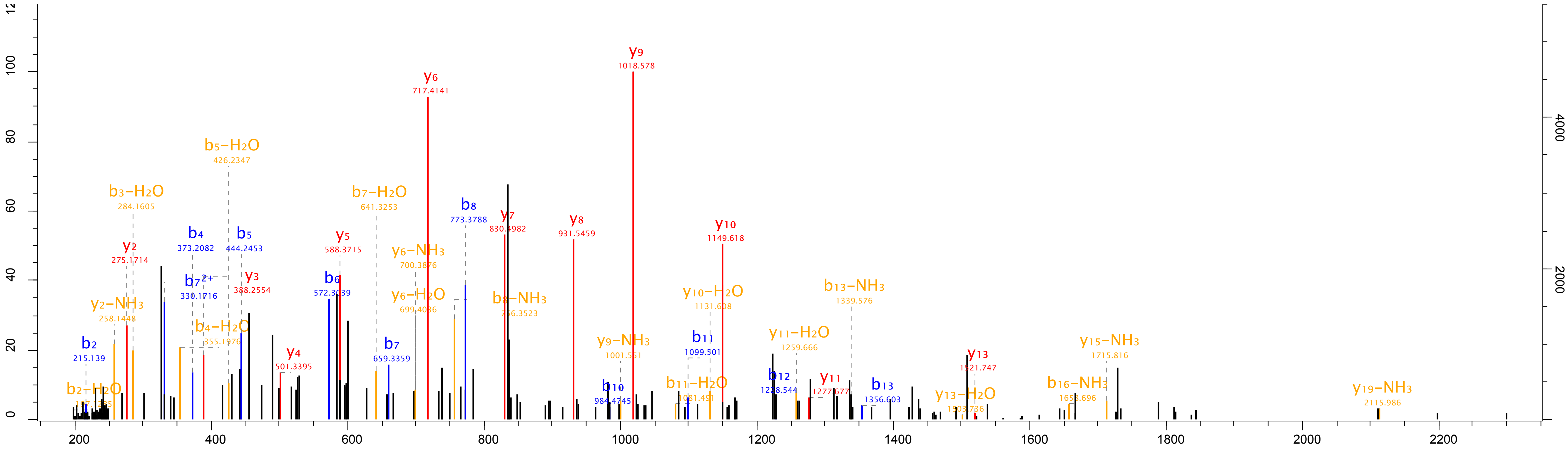

Raw file	Scan	Method	Score	Mass	Gene names
UPS1+500ngY_90minTop17_BC4_01_358	53331	TOF; CID	77.51	1793.91	VCX1

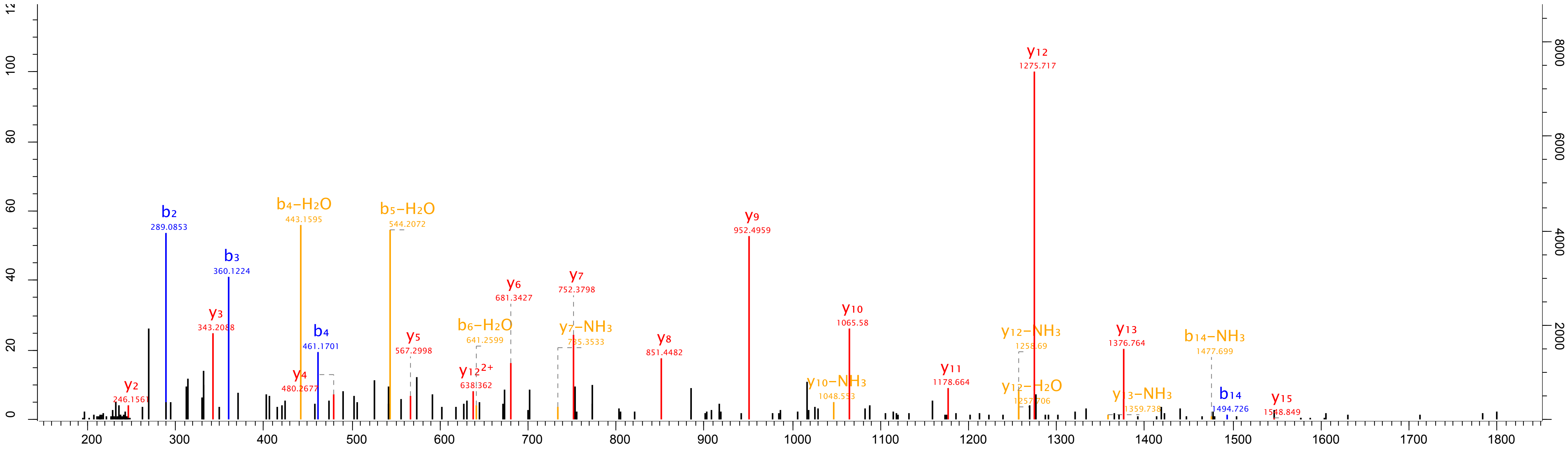

Raw file	Scan	Method	Score	Mass	Gene names
UPS1+500ngY_90minTop17_BC4_01_358	53421	TOF; CID	51.29	1249.67	KTI12

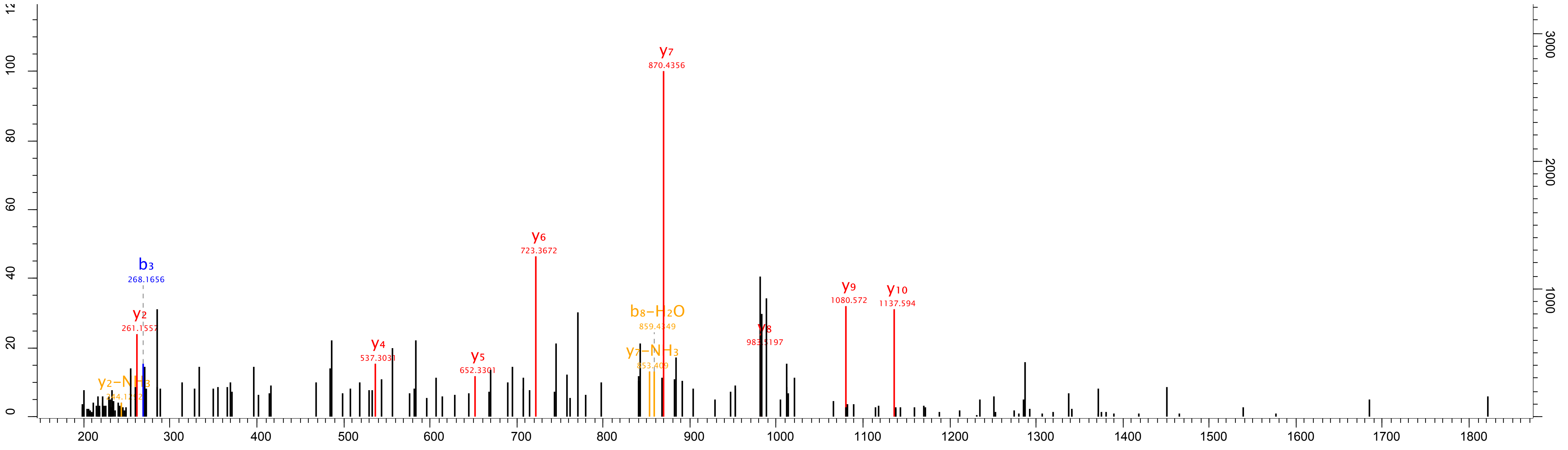

Raw file	Scan	Method	Score	Mass
UPS1+500ngY_90minTop17_BC4_01_358	53457	TOF; CID	46.42	2317.99

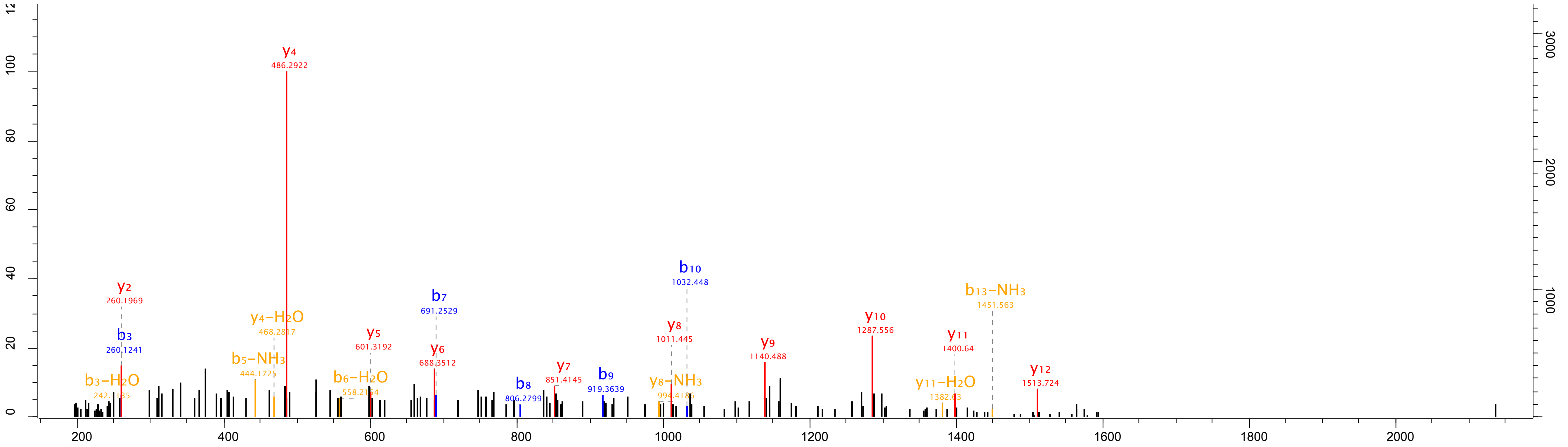

Raw file	Scan	Method	Score	Mass	Gene names
UPS1+500ngY_90minTop17_BC4_01_358	53525	TOF; CID	70	882.49	SRP54

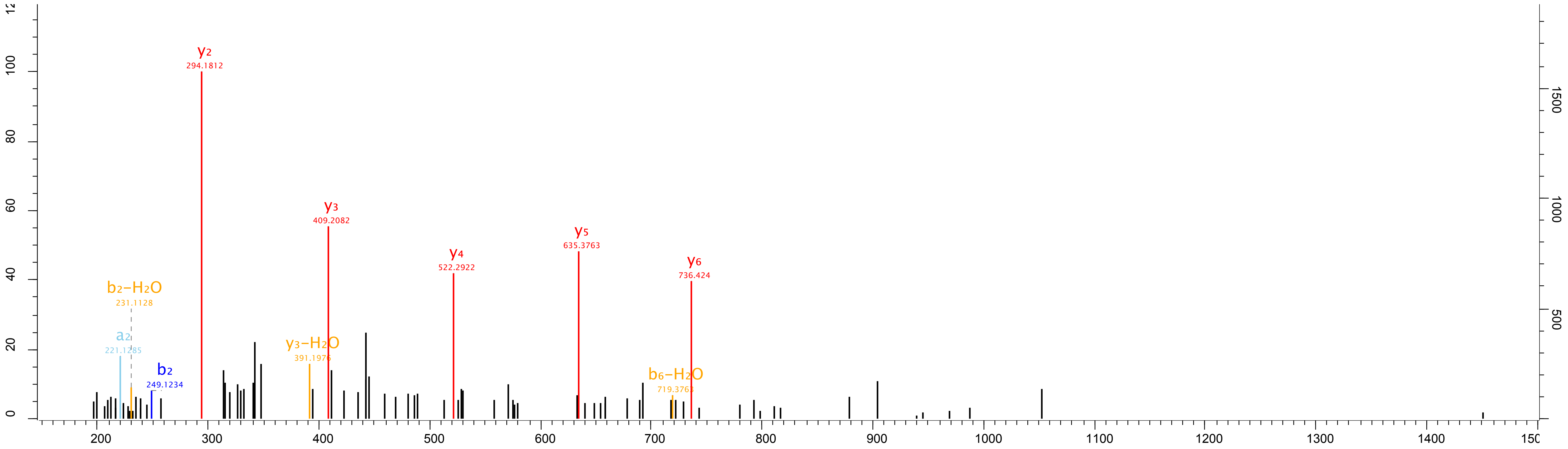

Raw file	Scan	Method	Score	Mass
UPS1+500ngY_90minTop17_BC4_01_358	53968	TOF; CID	52.86	1941.92

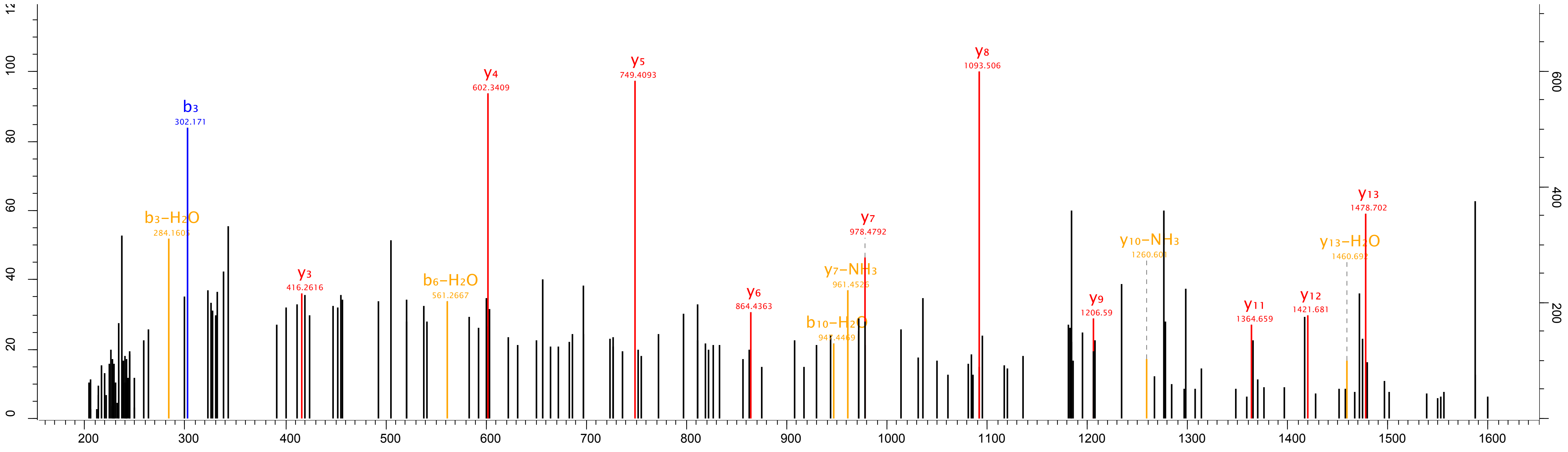

Raw file	Scan	Method	Score	Mass	Gene names
UPS1+500ngY_90minTop17_BC4_01_358	54089	TOF; CID	53.3	1547.75	CBC2

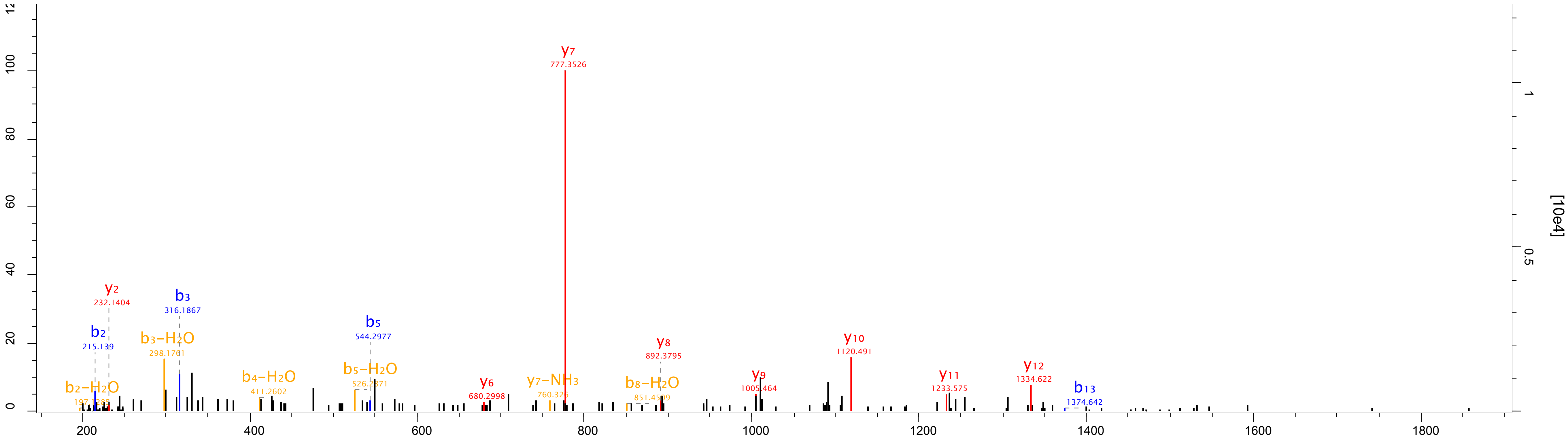

Raw file	Scan	Method	Score	Mass	Gene names
UPS1+500ngY_90minTop17_BC4_01_358	54294	TOF; CID	124.2	1945.96	ADE8

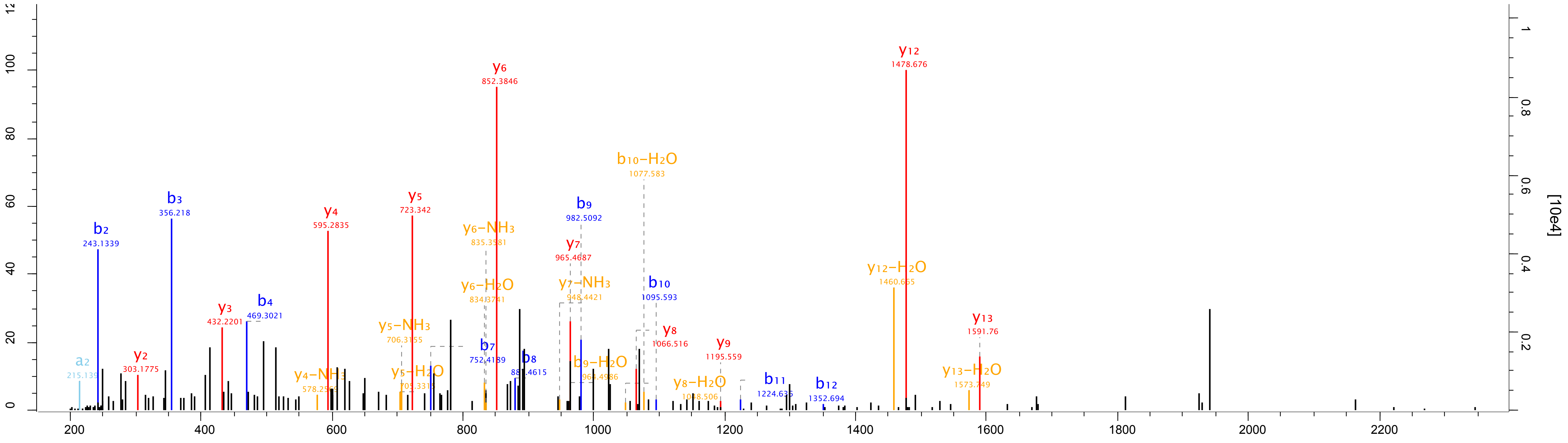

Raw file	Scan	Method	Score	Mass	Gene names
UPS1+500ngY_90minTop17_BC4_01_358	54635	TOF; CID	83.14	1427.71	GLO1

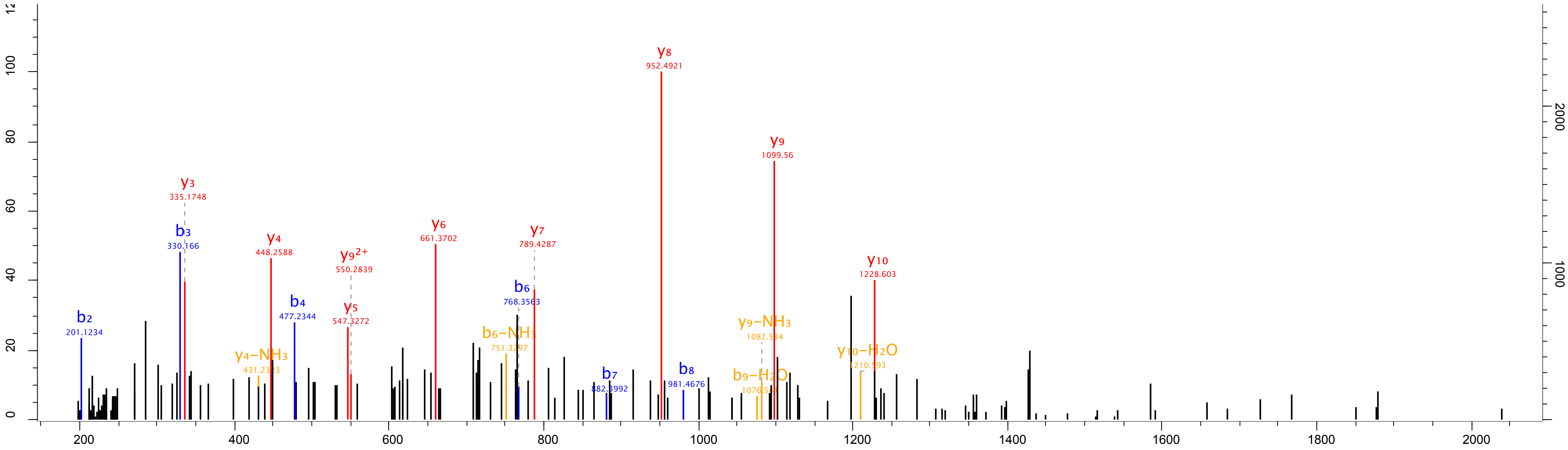

Raw file	Scan	Method	Score	Mass	Gene names
UPS1+500ngY_90minTop17_BC4_01_358	55289	TOF; CID	44.86	1121.54	TMT1

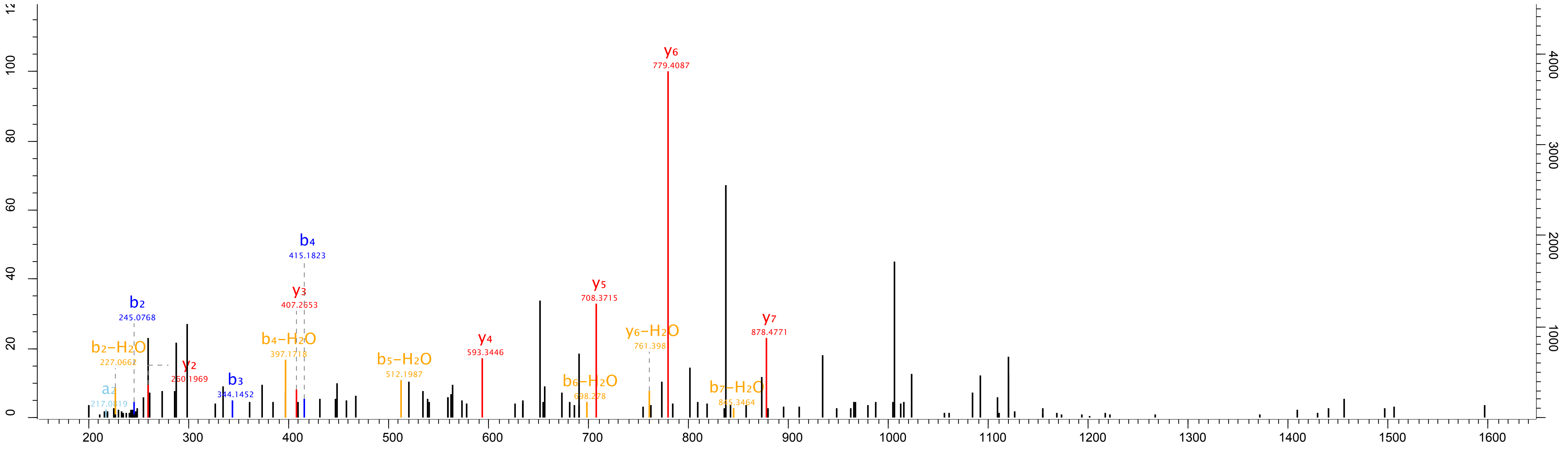

Raw file	Scan	Method	Score	Mass	Gene names
UPS1+500ngY_90minTop17_BC4_01_358	55296	TOF; CID	77.51	2089.07	CBP4

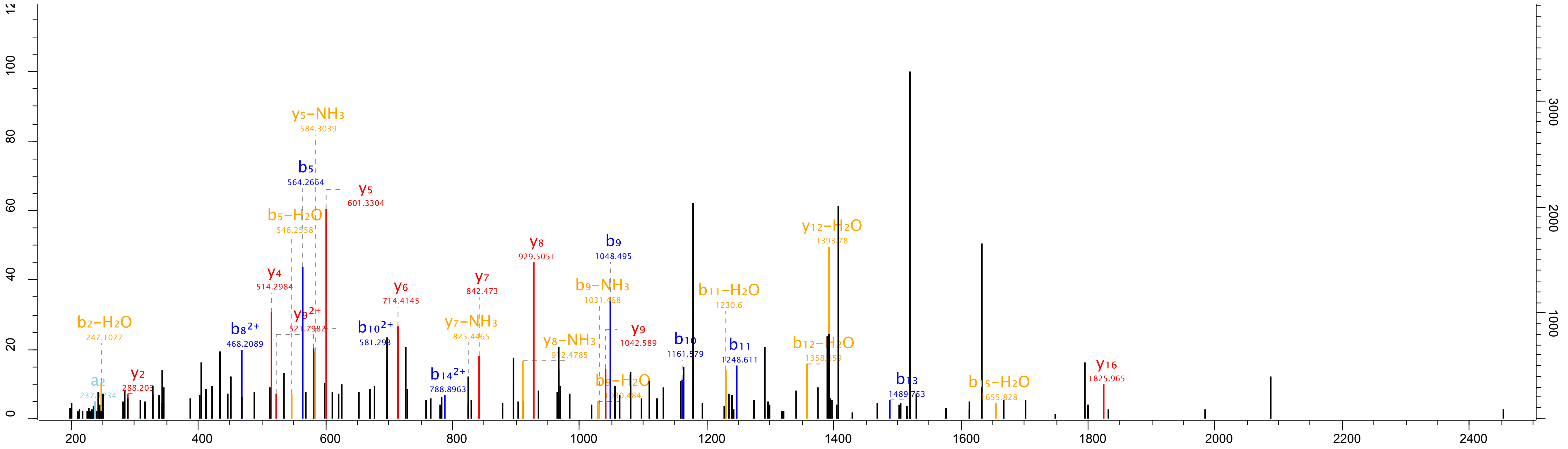

Raw file	Scan	Method	Score	Mass	Gene names
UPS1+500ngY_90minTop17_BC4_01_358	55488	TOF; CID	200.29	2408.39	HTA2;HTA1

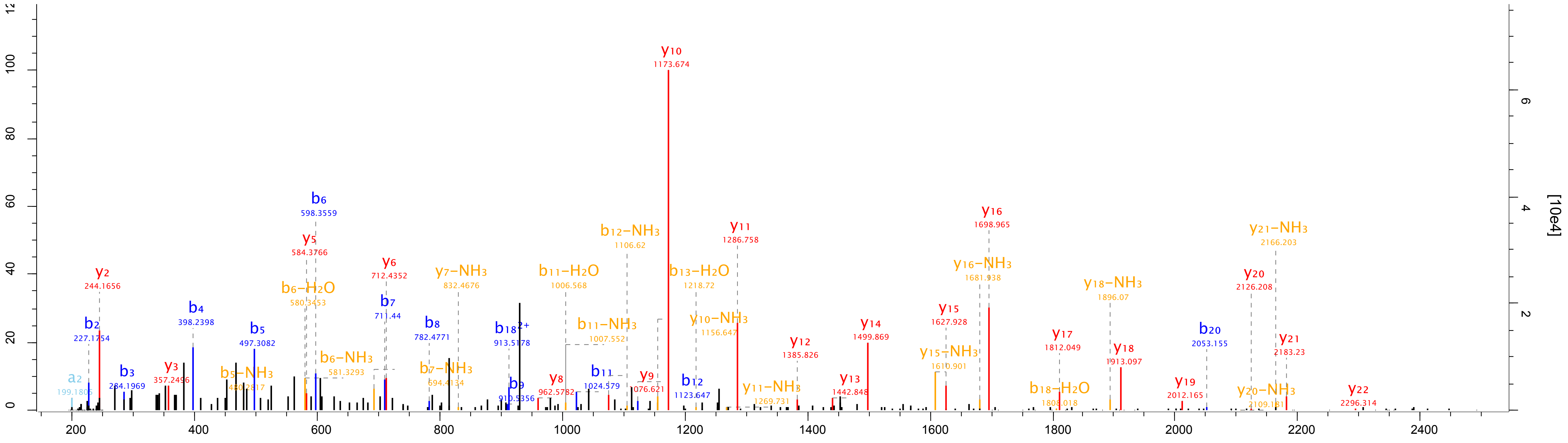

Raw file	Scan	Method	Score	Mass	Gene names
UPS1+500ngY_90minTop17_BC4_01_358	55551	TOF; CID	47.77	1552.78	PPA2

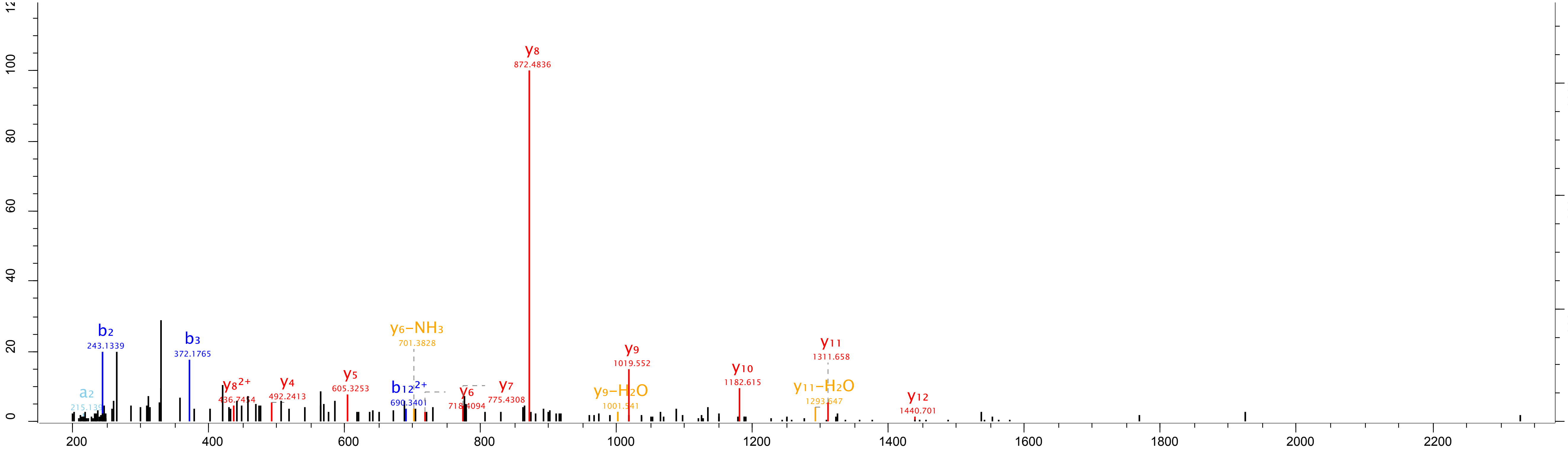

	Raw file	Scan	Method	Score	Mass
peptide 1	UPS1+500ngY_90minTop17_BC4_01_358	55577	TOF; CID	52.07	1450.68
peptide 2	UPS1+500ngY_90minTop17_BC4_01_358	55577	TOF; CID	41.58	1448.72

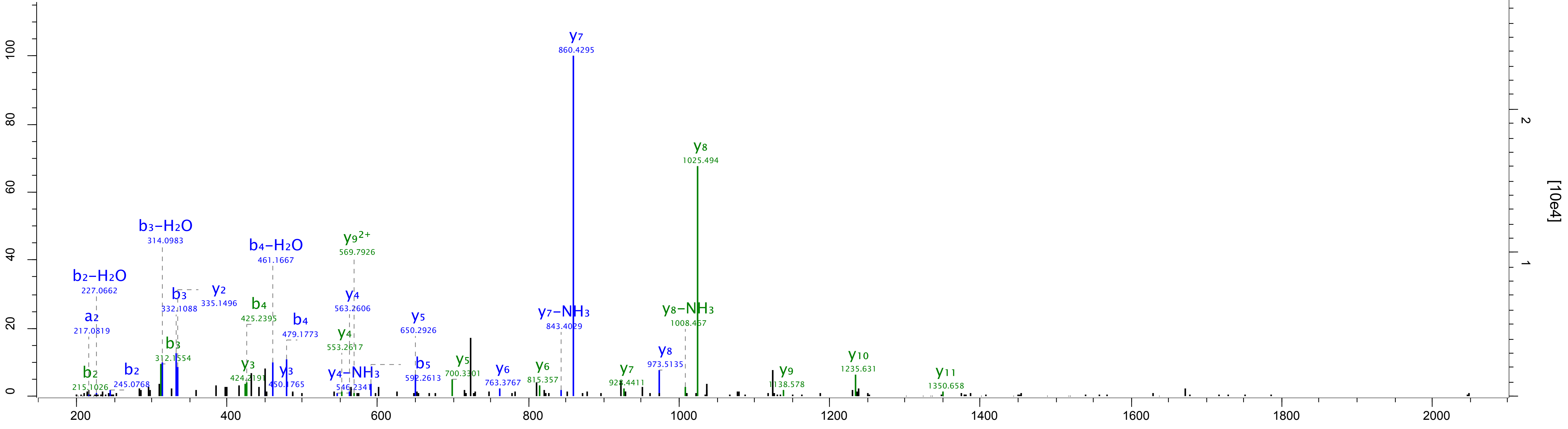

Raw file	Scan	Method	Score	Mass	Gene names
UPS1+500ngY_90minTop17_BC4_01_358	56003	TOF; CID	51.45	996.62	KAP104

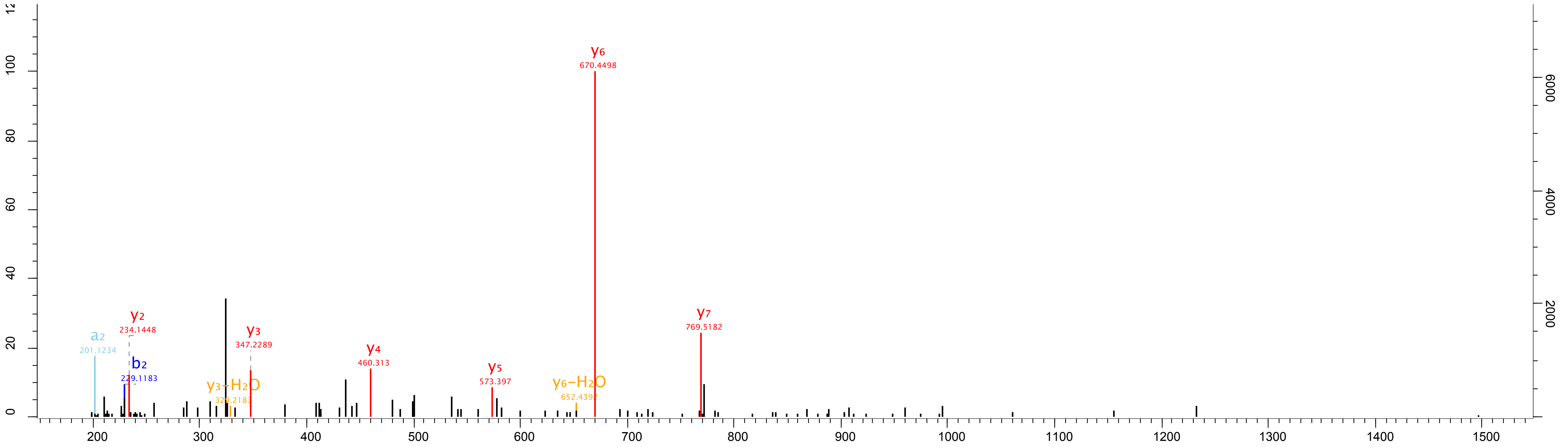

Raw file	Scan	Method	Score	Mass	Gene names
UPS1+500ngY_90minTop17_BC4_01_358	56164	TOF; CID	68.26	1579.79	FET5

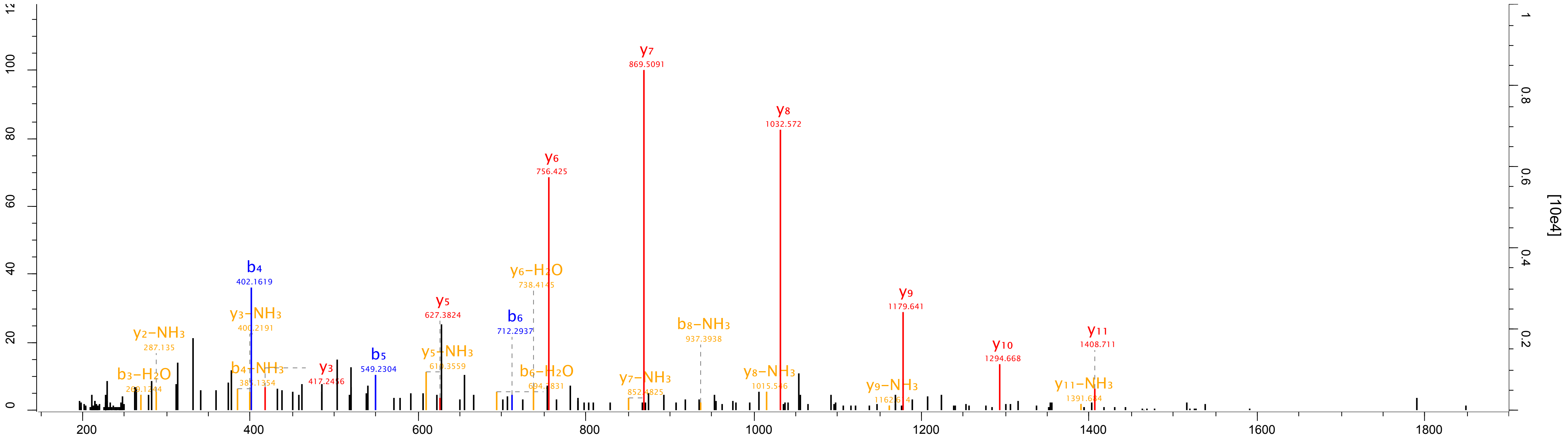

Raw file	Scan	Method	Score	Mass	Gene names
UPS1+500ngY_90minTop17_BC4_01_358	56312	TOF; CID	51.5	1844.89	UBP3

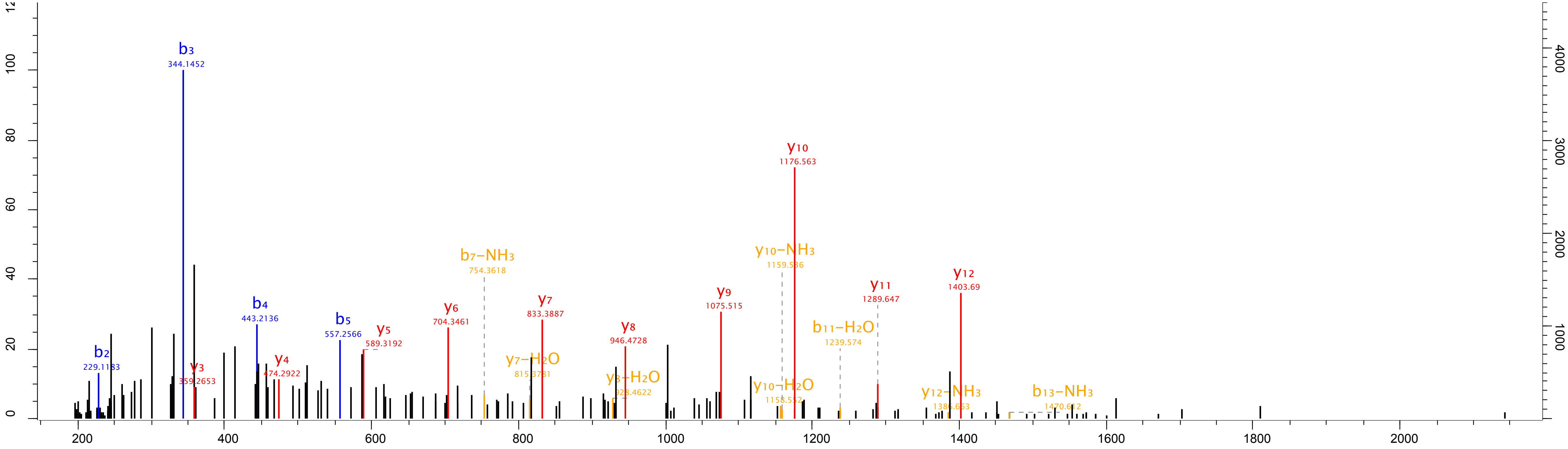

Raw file	Scan	Method	Score	Mass	Gene names
UPS1+500ngY_90minTop17_BC4_01_358	56316	TOF; CID	88.38	1913.9	TSR2

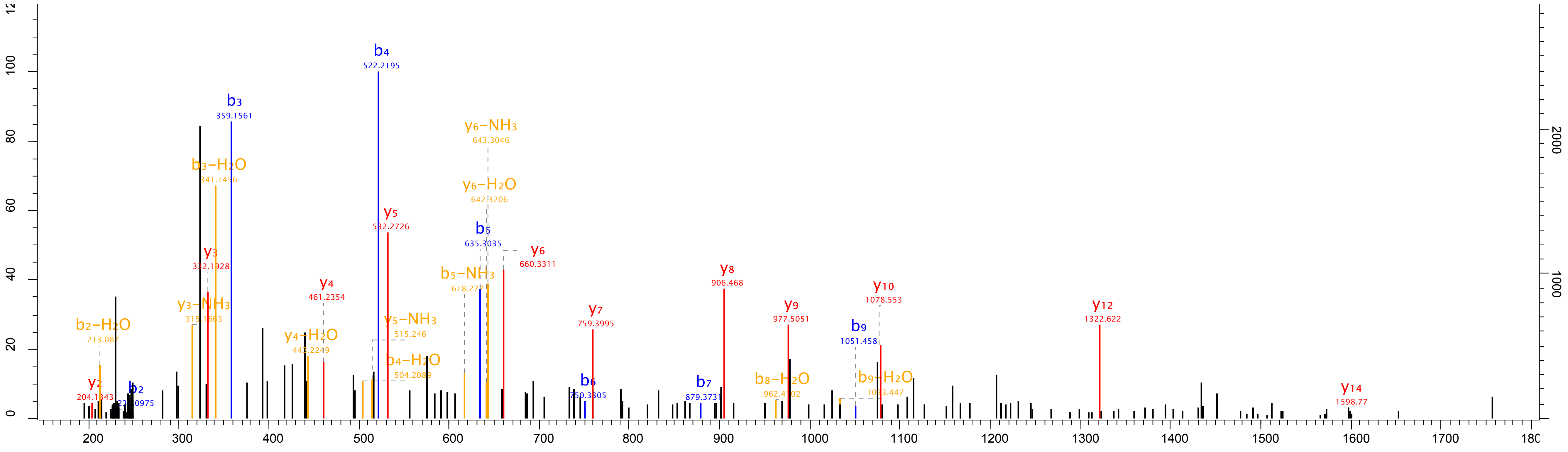

Raw file	Scan	Method	Score	Mass	Gene names
UPS1+500ngY_90minTop17_BC4_01_358	56508	TOF; CID	45.24	2216.99	MET12

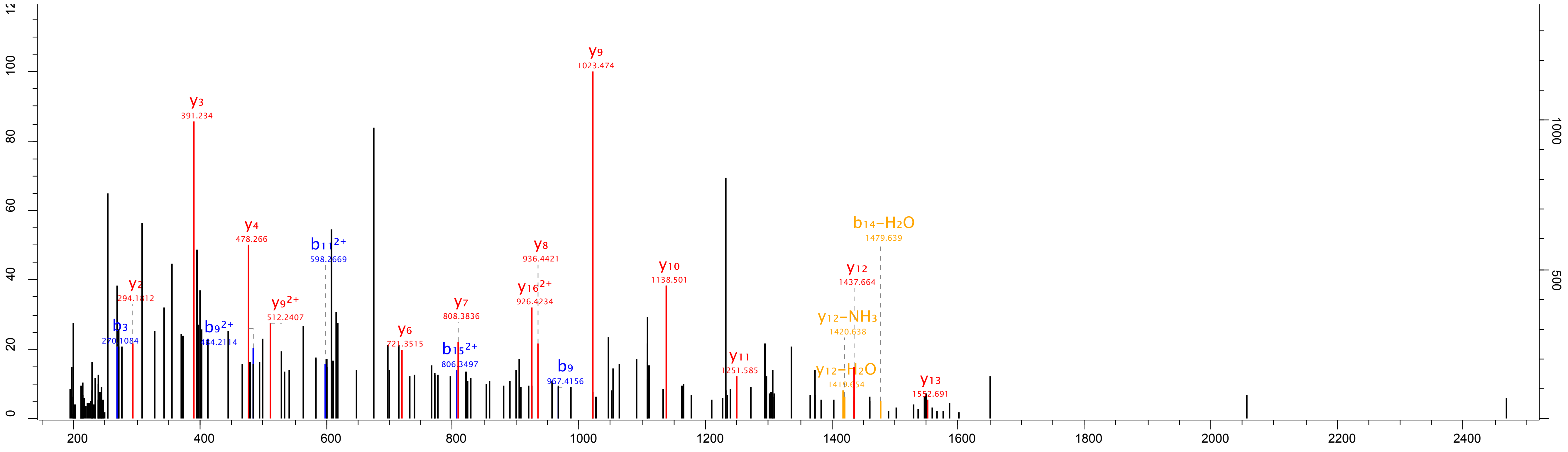

Raw file	Scan	Method	Score	Mass	Gene names
UPS1+500ngY_90minTop17_BC4_01_358	56555	TOF; CID	40.14	2405.03	RPC37

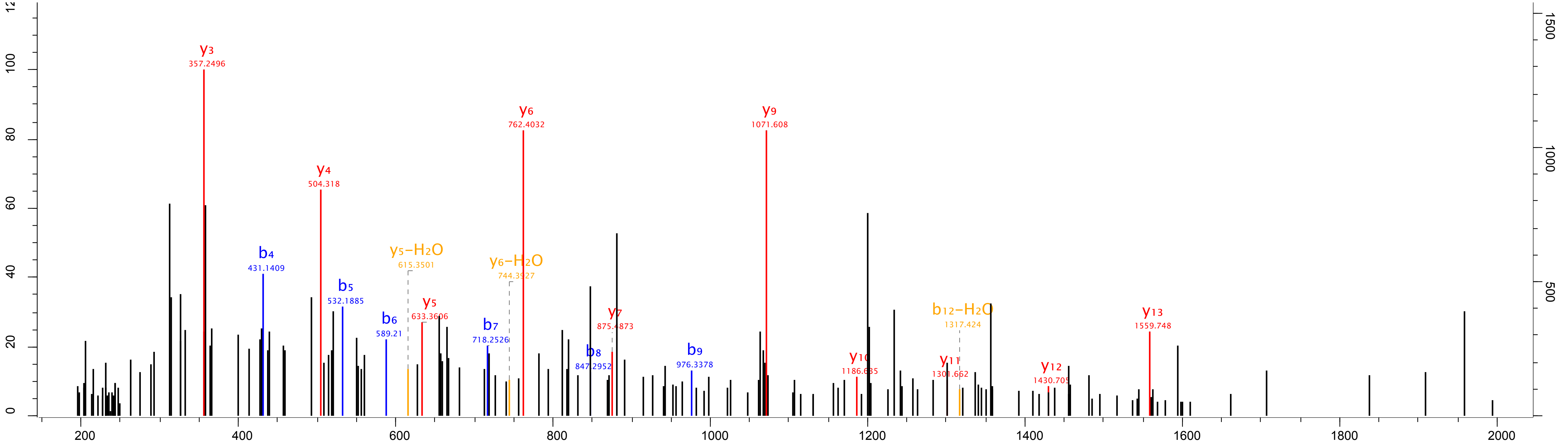

Raw file	Scan	Method	Score	Mass	Gene names
UPS1+500ngY_90minTop17_BC4_01_358	56762	TOF; CID	48.39	1691.83	MIC14

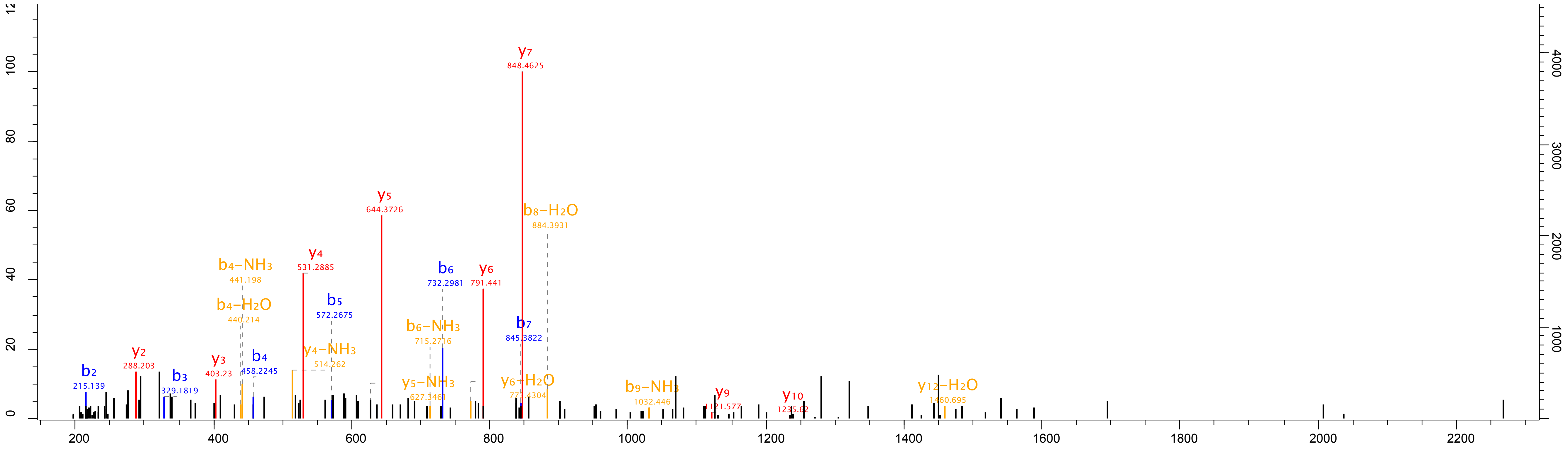

Raw file	Scan	Method	Score	Mass
UPS1+500ngY_90minTop17_BC4_01_358	58250	TOF; CID	56.57	1440.75

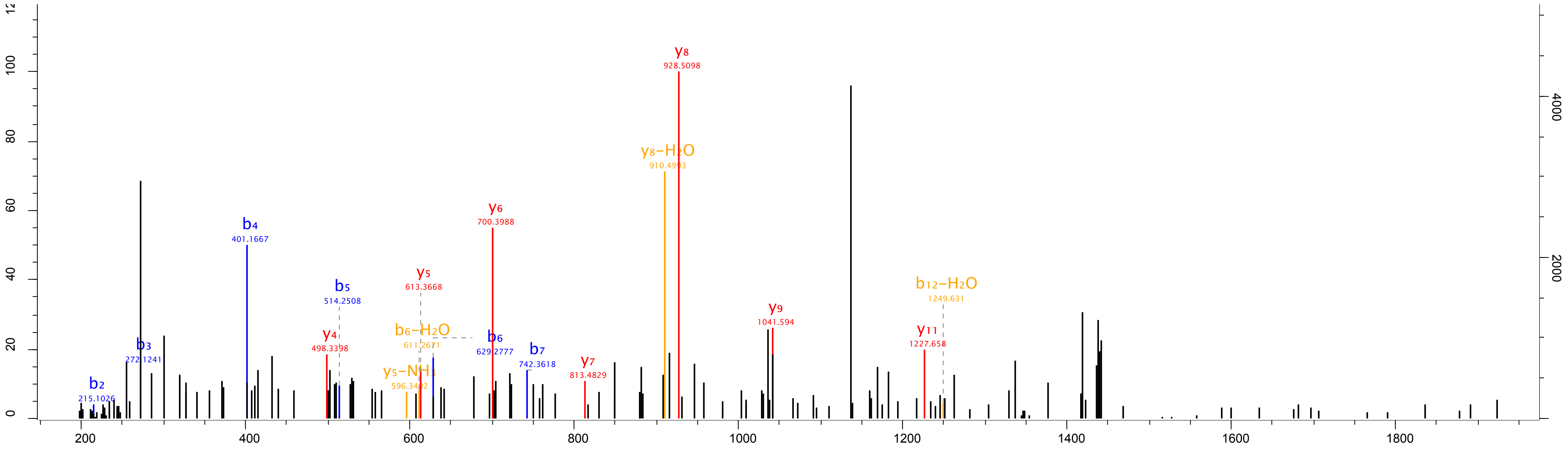

Raw file	Scan	Method	Score	Mass	Gene names
UPS1+500ngY_90minTop17_BC4_01_358	58341	TOF; CID	42.63	2053.02	NUP84

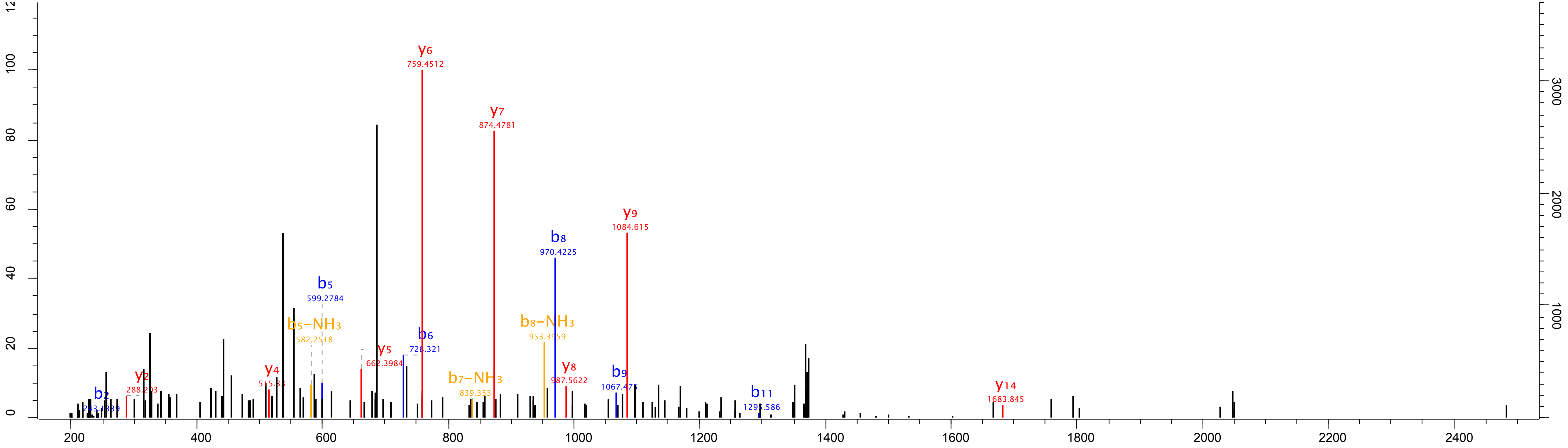

Raw file	Scan	Method	Score	Mass	Gene names
UPS1+500ngY_90minTop17_BC4_01_358	58392	TOF; CID	67.68	1893.02	SNL1

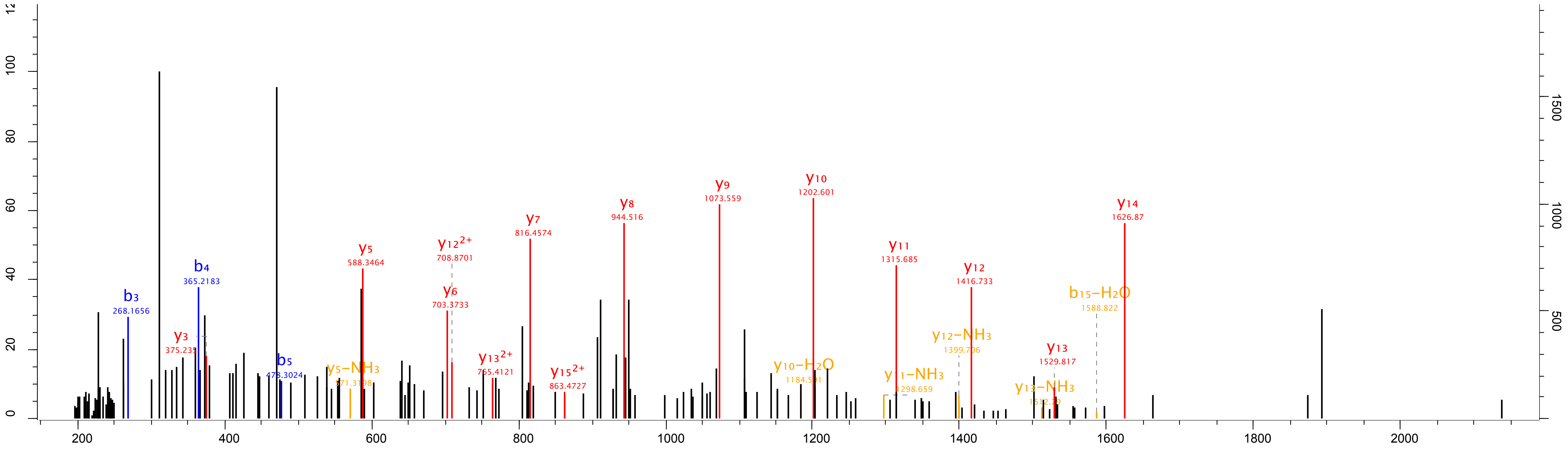

Raw file	Scan	Method	Score	Mass	Gene names
UPS1+500ngY_90minTop17_BC4_01_358	58763	TOF; CID	128.01	1303.71	ERV14

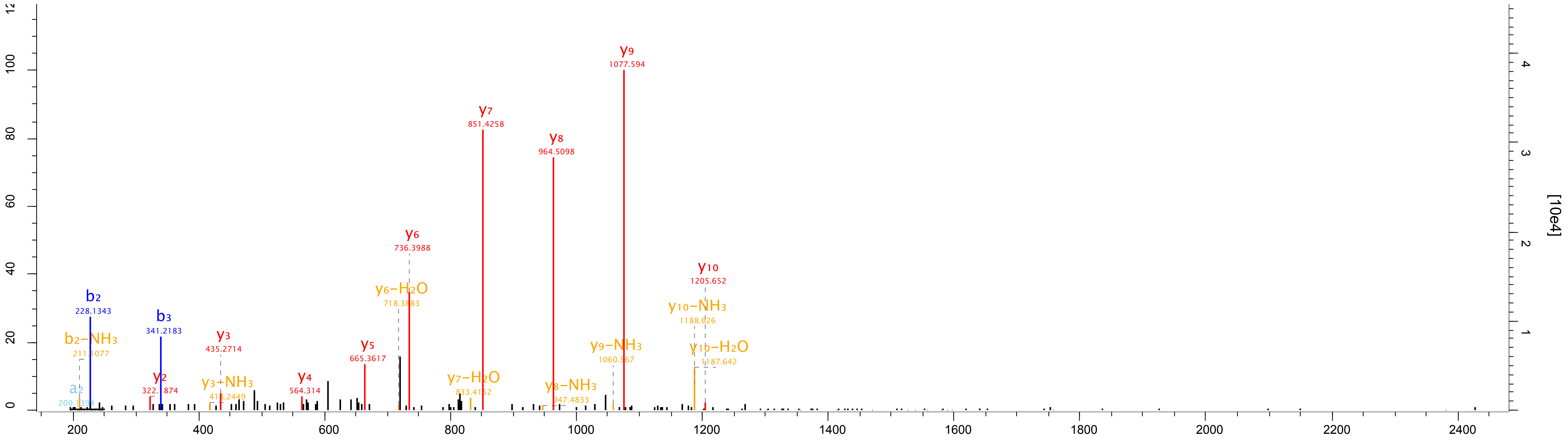

Raw file	Scan	Method	Score	Mass	Gene names
UPS1+500ngY_90minTop17_BC4_01_358	59047	TOF; CID	70.2	1699.94	PAM18

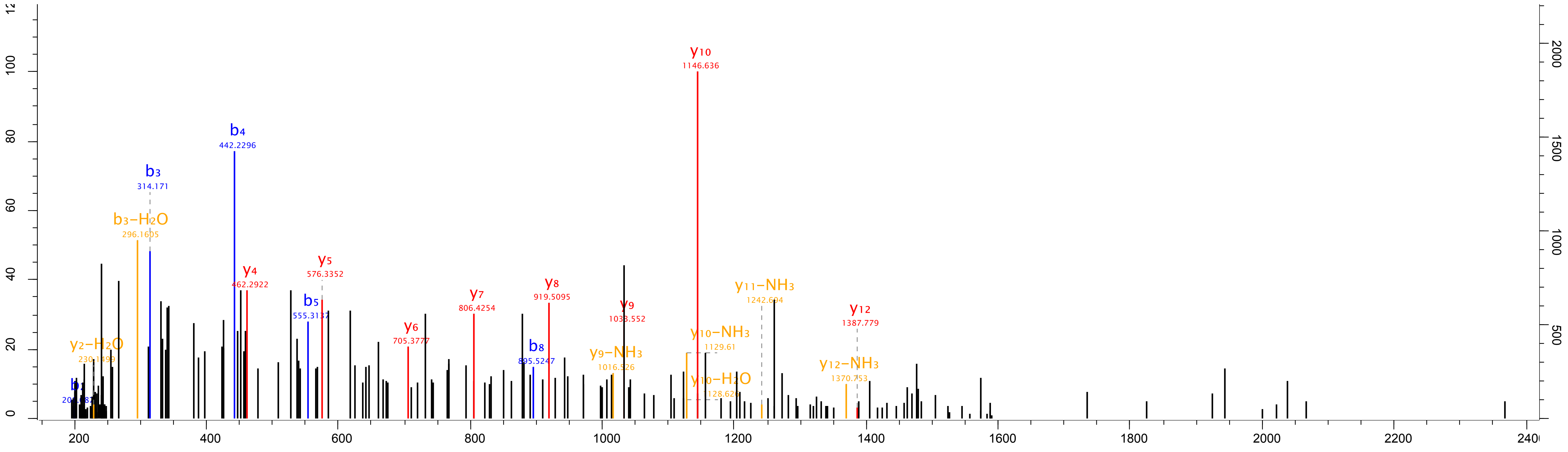

Raw file	Scan	Method	Score	Mass	Gene names
UPS1+500ngY_90minTop17_BC4_01_358	59778	TOF; CID	70.78	2023	ZTA1

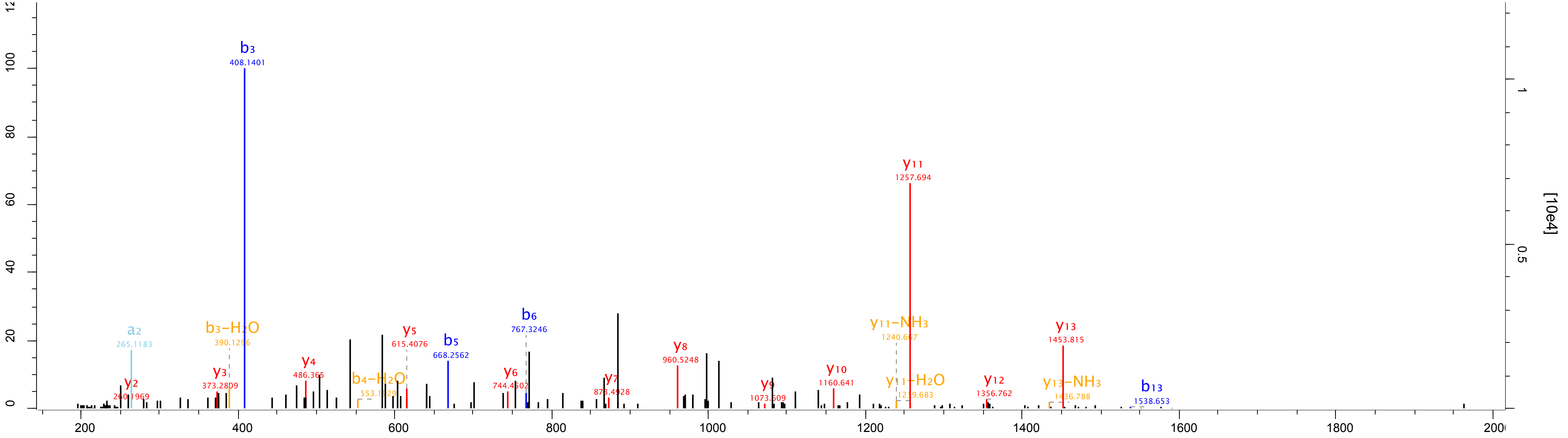

Raw file	Scan	Method	Score	Mass	Gene names
UPS1+500ngY_90minTop17_BC4_01_358	60194	TOF; CID	63.66	1321.69	GIR2

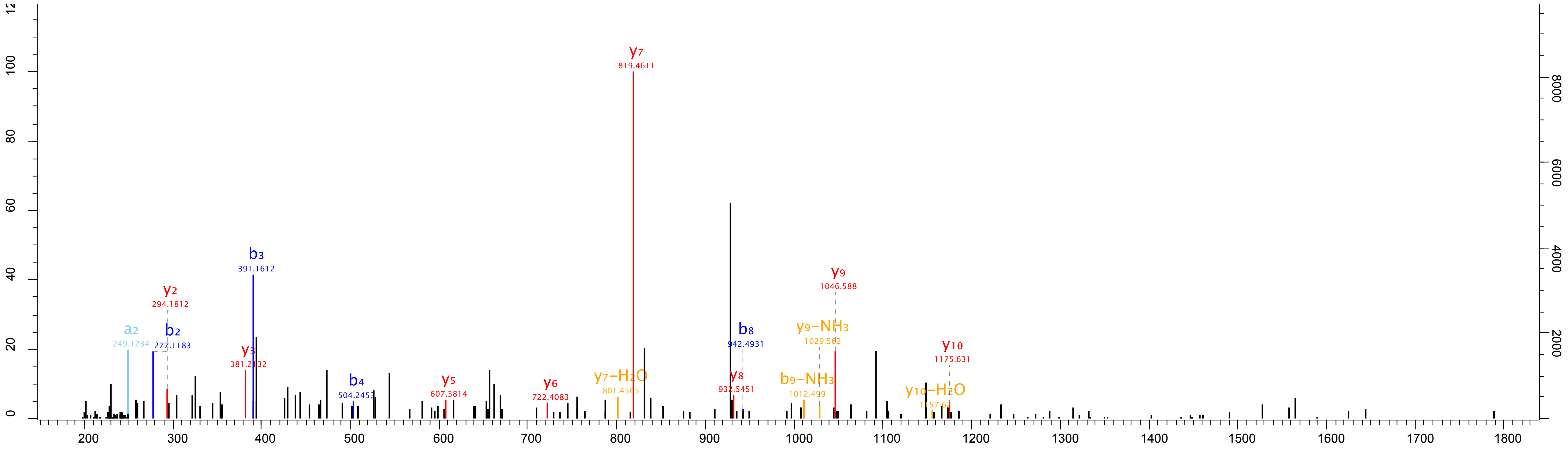

Raw file	Scan	Method	Score	Mass	Gene names
UPS1+500ngY_90minTop17_BC4_01_358	60282	TOF; CID	49.3	1422.72	HAT2

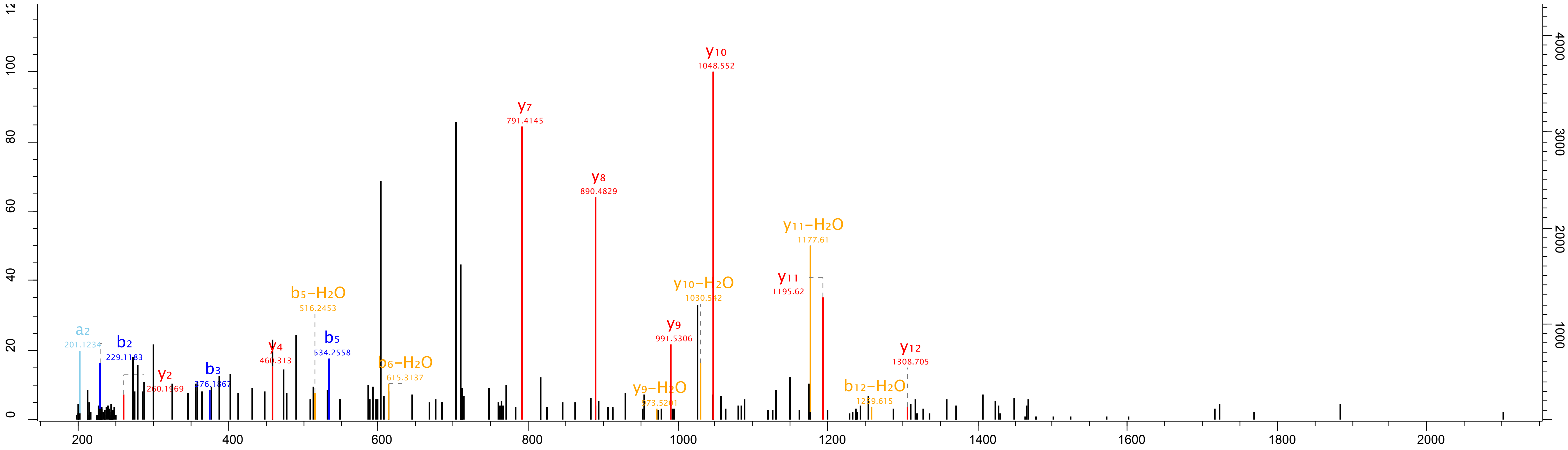

Raw file	Scan	Method	Score	Mass	Gene names
UPS1+500ngY_90minTop17_BC4_01_358	60833	TOF; CID	55.31	1327.77	NOC2

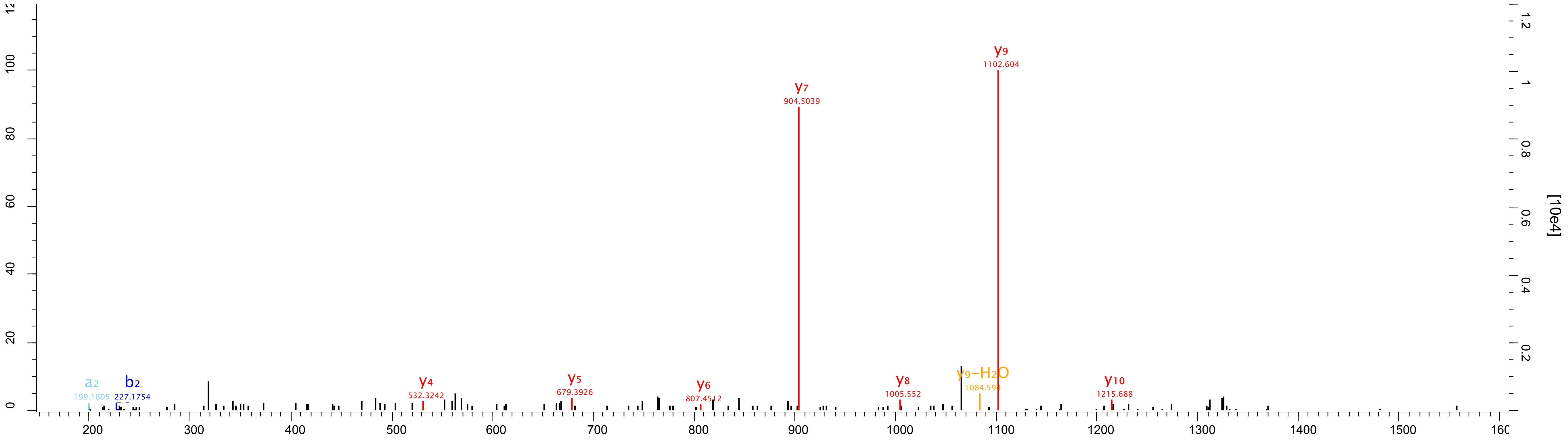

Raw file	Scan	Method	Score	Mass	Gene names
UPS1+500ngY_90minTop17_BC4_01_358	60874	TOF; CID	59.73	1845.97	OSH2

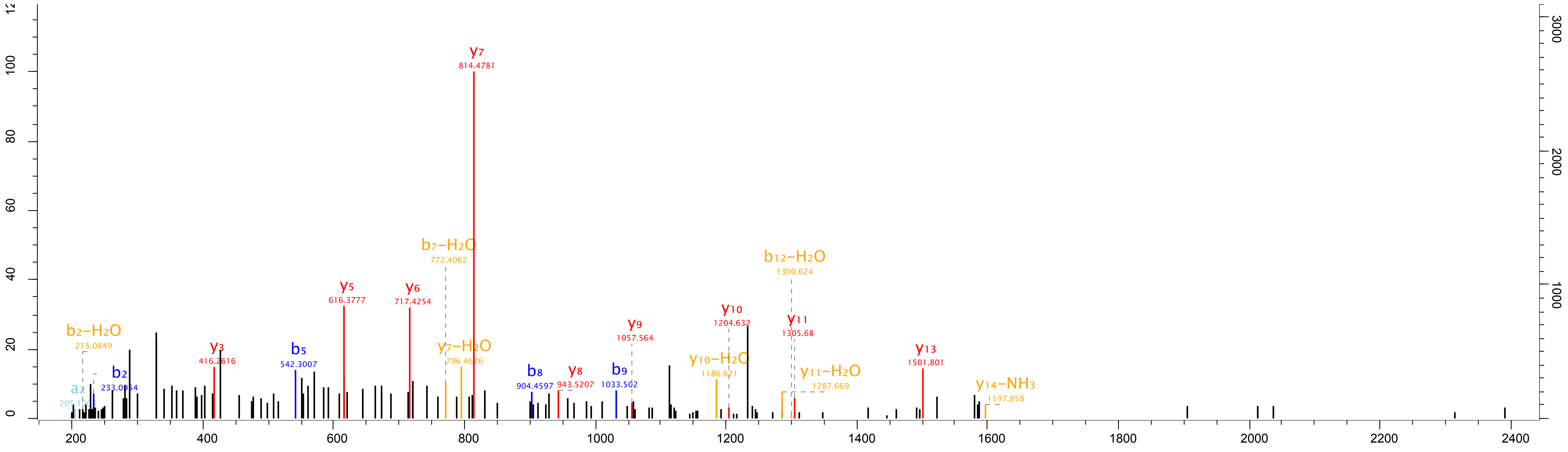

Raw file	Scan	Method	Score	Mass	Gene names
UPS1+500ngY_90minTop17_BC4_01_358	61086	TOF; CID	91.96	1329.74	PHO86

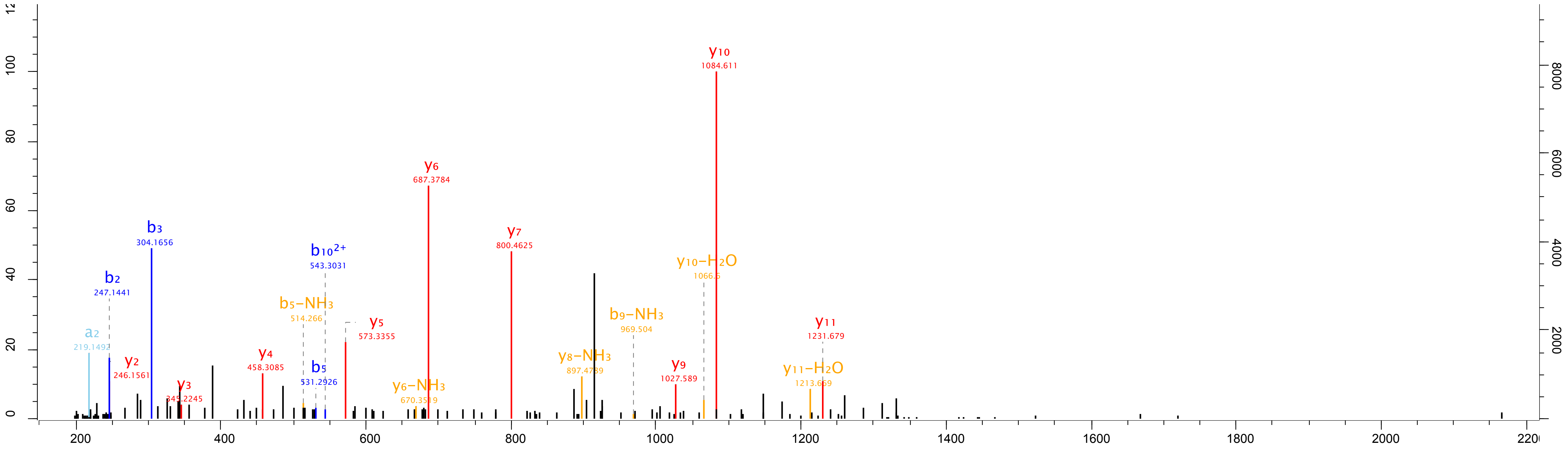

Raw file	Scan	Method	Score	Mass
UPS1+500ngY_90minTop17_BC4_01_358	61122	TOF; CID	44.79	2058.99

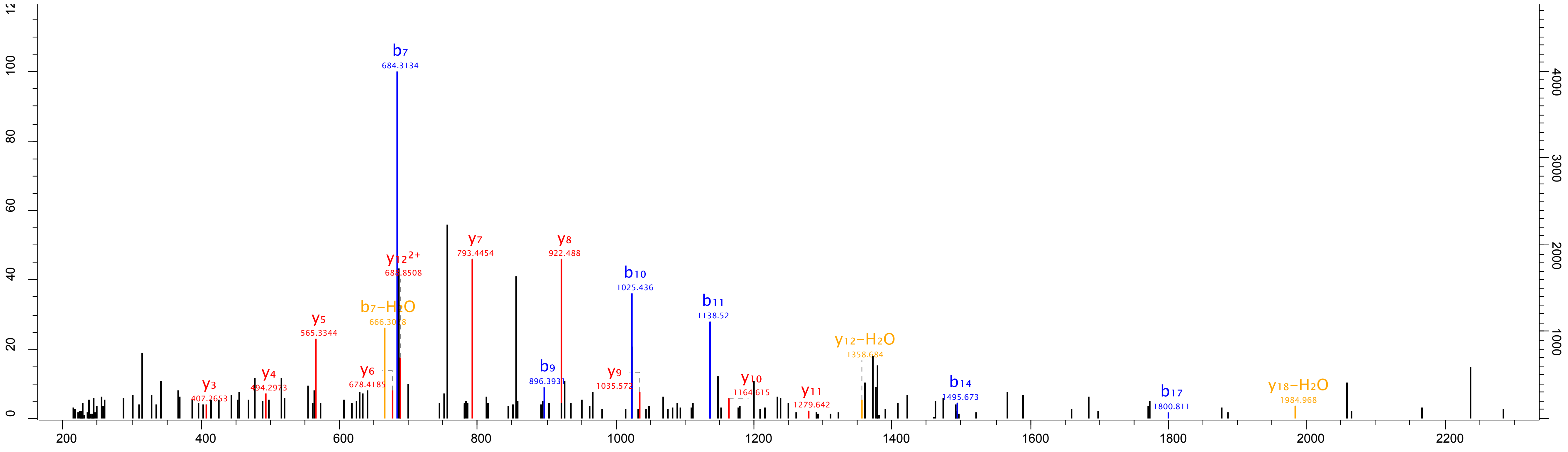

Raw file	Scan	Method	Score	Mass
UPS1+500ngY_90minTop17_BC4_01_358	61131	TOF; CID	44.16	2622.28

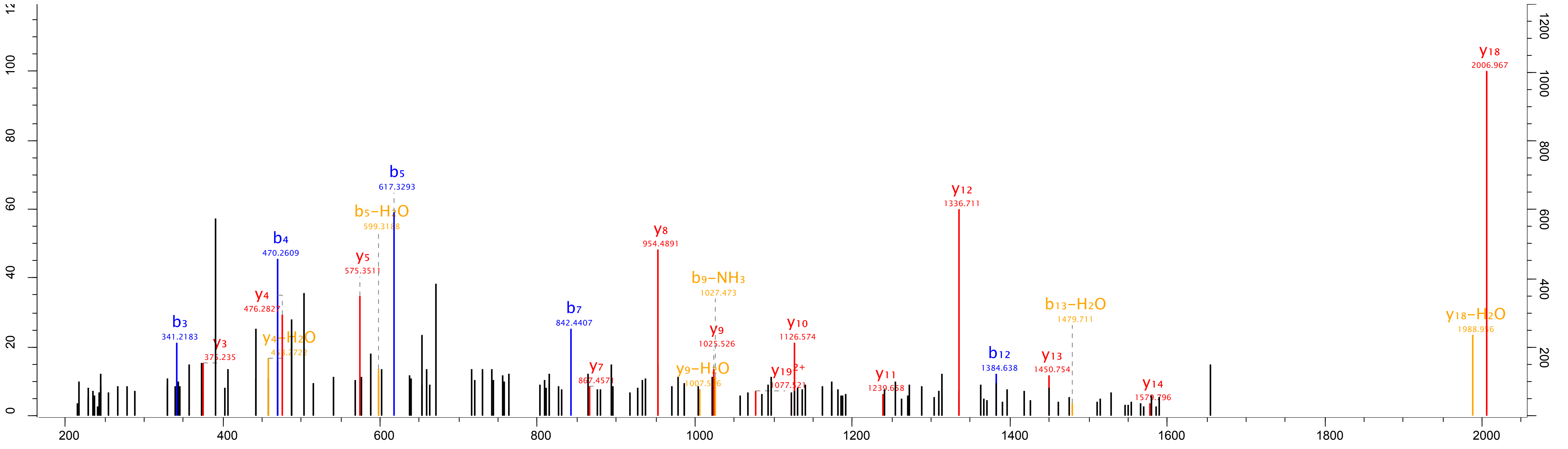

Raw file	Scan	Method	Score	Mass	Gene names
UPS1+500ngY_90minTop17_BC4_01_358	61193	TOF; CID	60.06	2030.02	RIX7

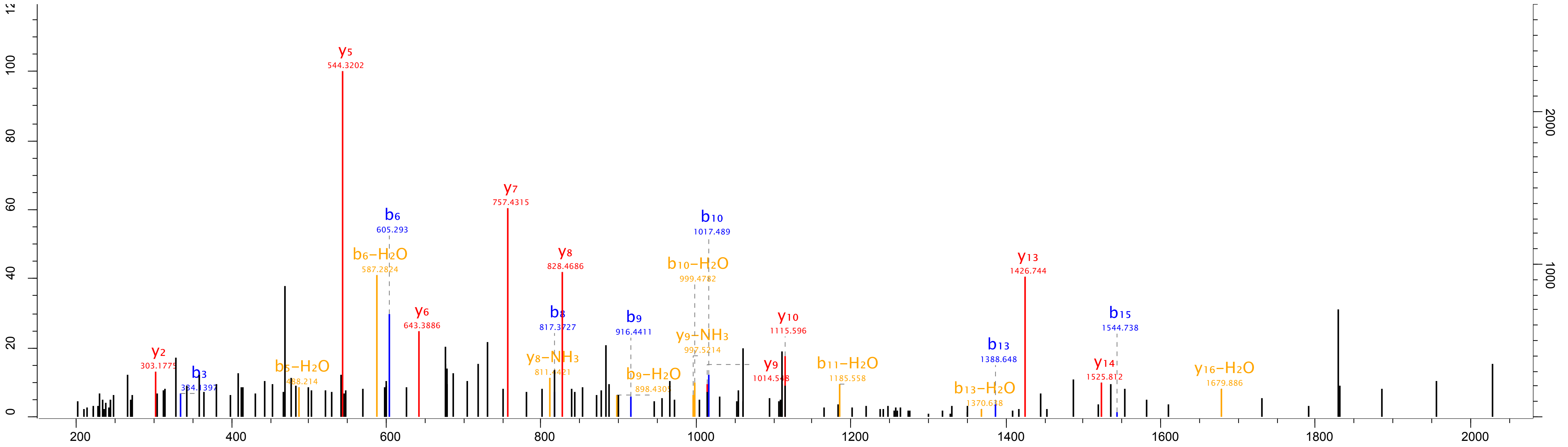

Raw file	Scan	Method	Score	Mass	Gene names
UPS1+500ngY_90minTop17_BC4_01_358	61281	TOF; CID	62.2	2121.05	SRB4

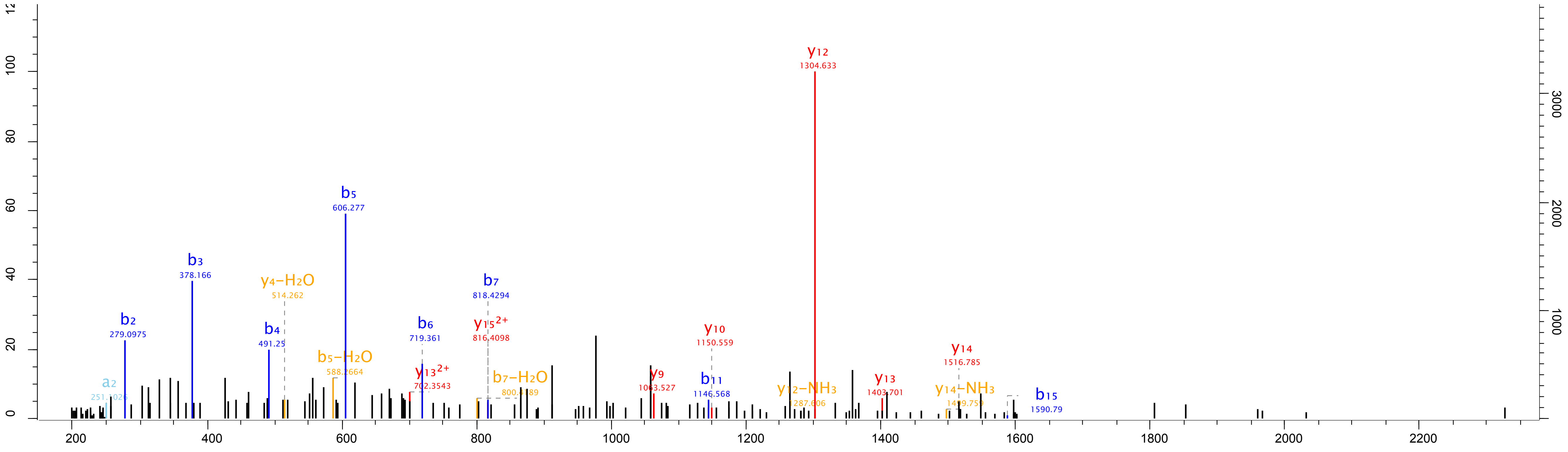

Raw file	Scan	Method	Score	Mass	Gene names
UPS1+500ngY_90minTop17_BC4_01_358	61330	TOF; CID	51.29	1181.68	VPS29

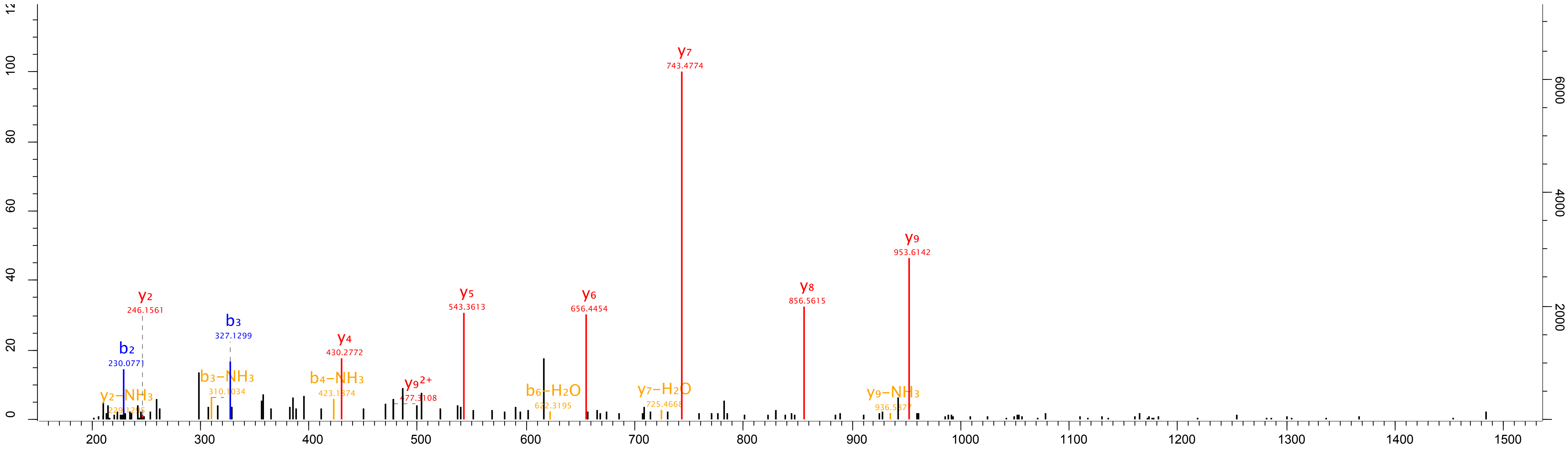

Raw file	Scan	Method	Score	Mass	Gene names
UPS1+500ngY_90minTop17_BC4_01_358	62073	TOF; CID	83.18	1371.67	GCN3

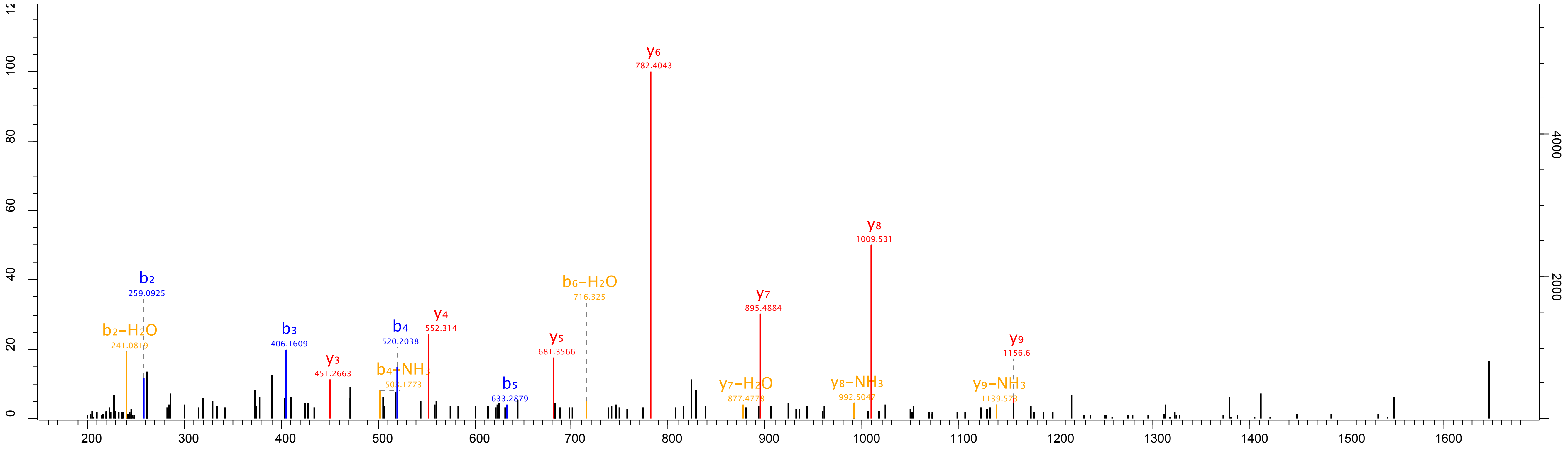

Raw file	Scan	Method	Score	Mass	Gene names
UPS1+500ngY_90minTop17_BC4_01_358	62110	TOF; CID	67.7	1183.66	APT2

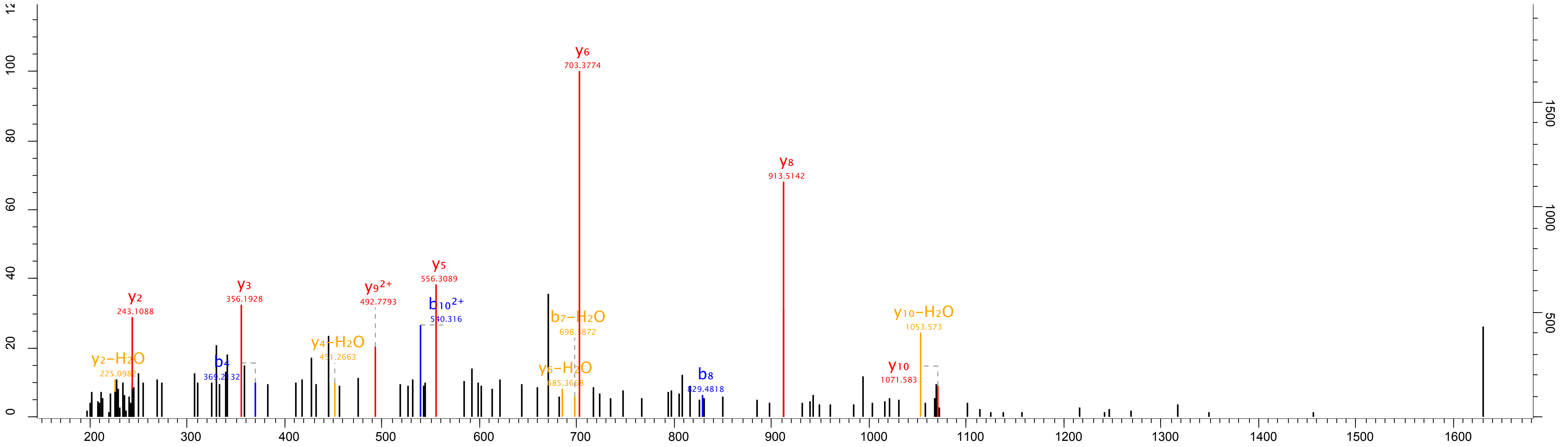

Raw file	Scan	Method	Score	Mass	Gene names
UPS1+500ngY_90minTop17_BC4_01_358	62770	TOF; CID	51.31	2369.07	RCL1

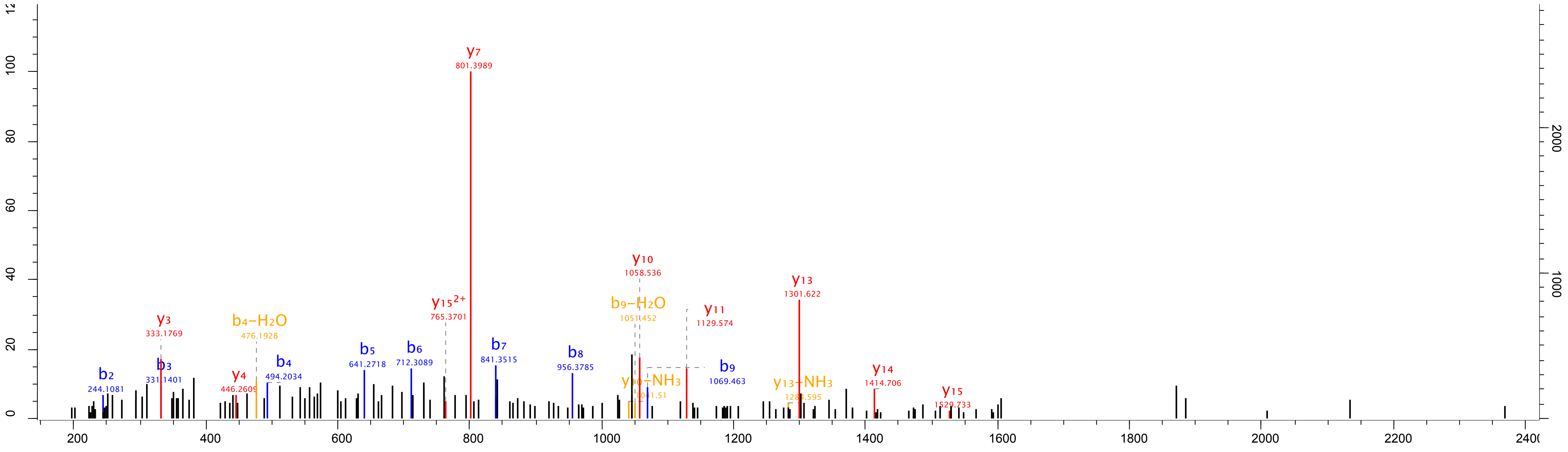

Raw file	Scan	Method	Score	Mass	Gene names
UPS1+500ngY_90minTop17_BC4_01_358	62827	TOF; CID	70.2	1796.92	GIS1

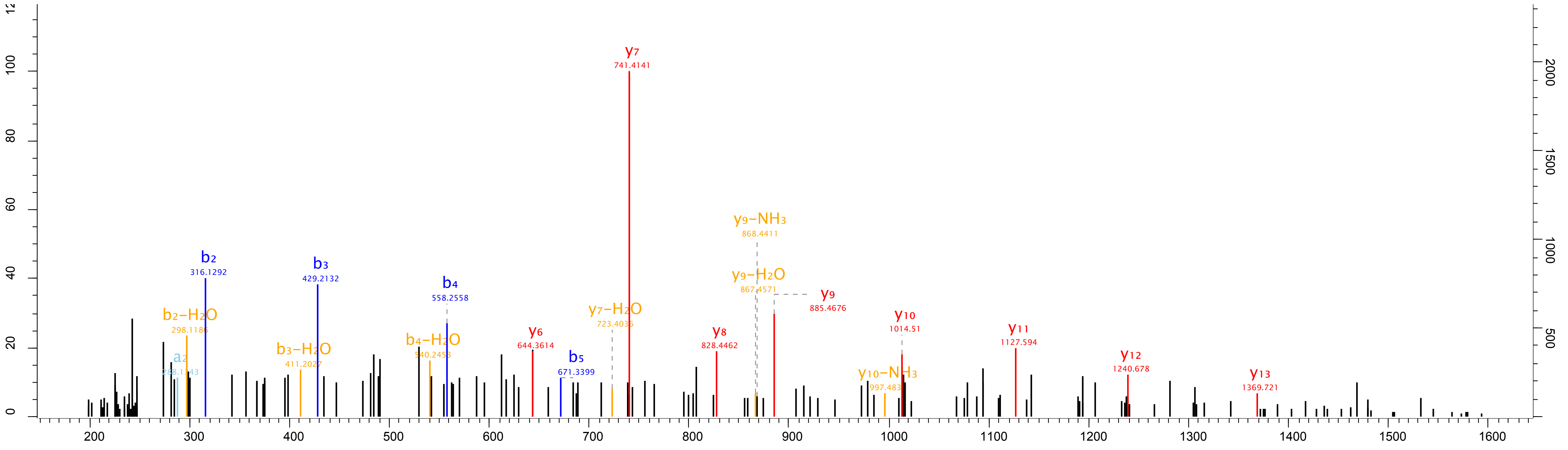

Raw file	Scan	Method	Score	Mass	Gene names
UPS1+500ngY_90minTop17_BC4_01_358	62912	TOF; CID	52.28	3631.66	YIP4

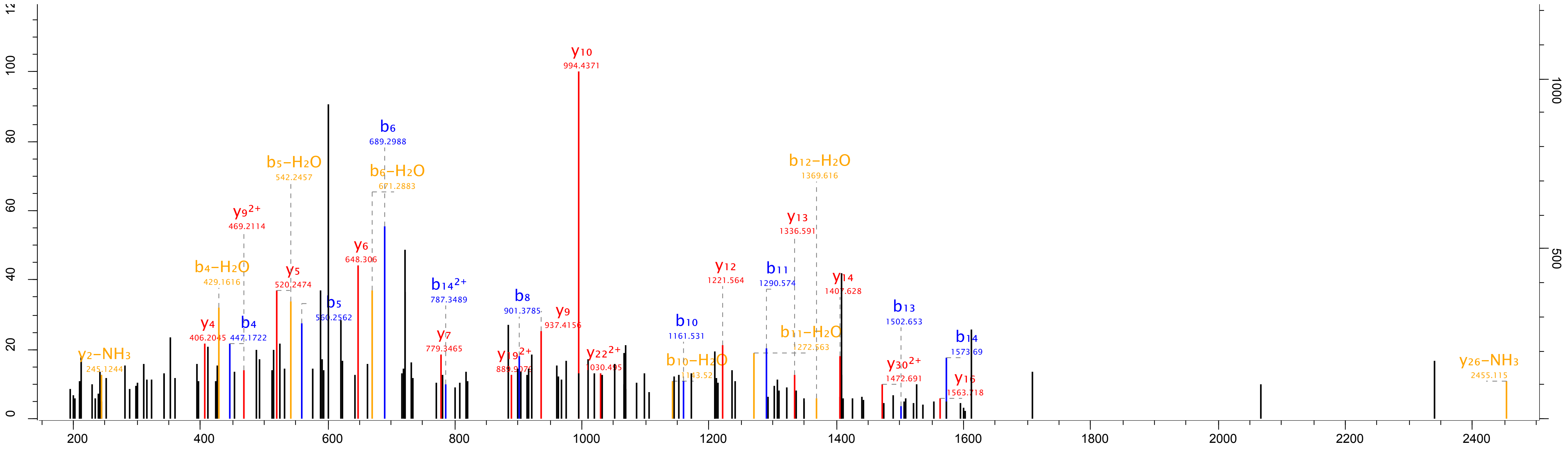

Raw file	Scan	Method	Score	Mass	Gene names
UPS1+500ngY_90minTop17_BC4_01_358	62936	TOF; CID	73.14	2330.97	UGO1

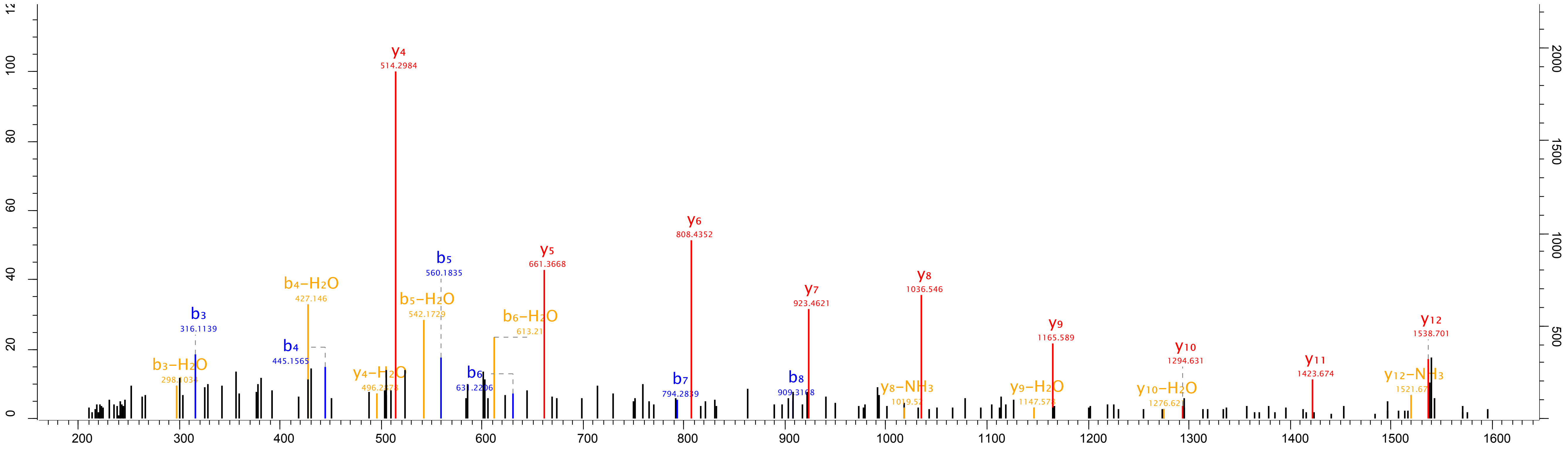

Raw file	Scan	Method	Score	Mass	Gene names
UPS1+500ngY_90minTop17_BC4_01_358	62998	TOF; CID	79.87	1385.78	PSK1

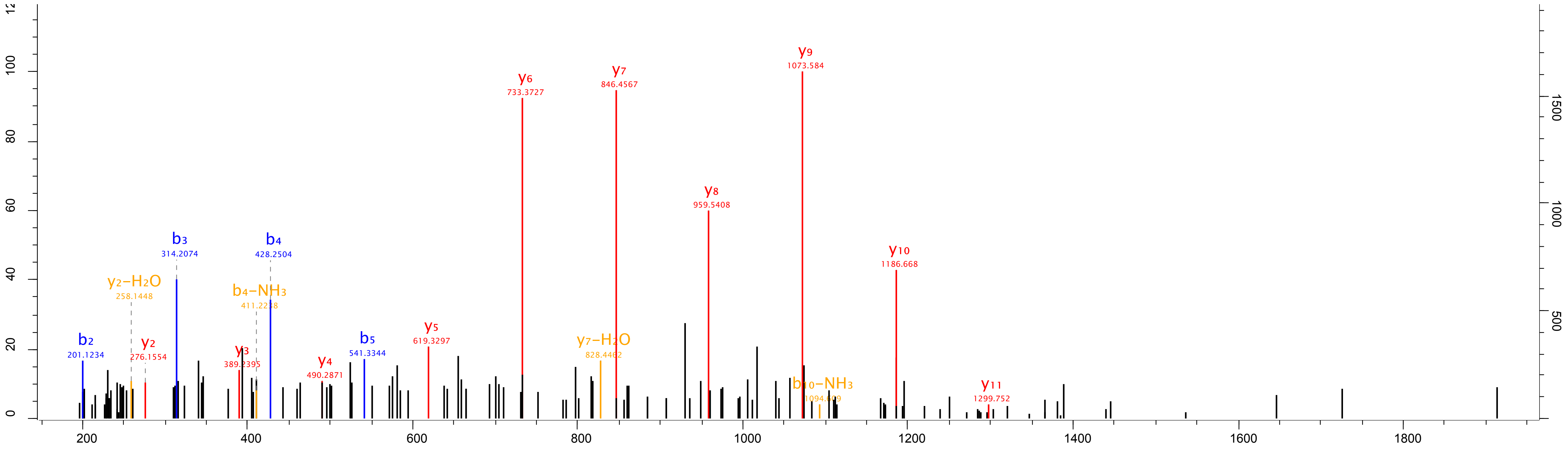

Raw file	Scan	Method	Score	Mass	Gene names
UPS1+500ngY_90minTop17_BC4_01_358	63141	TOF; CID	46.42	2384.15	CTR9

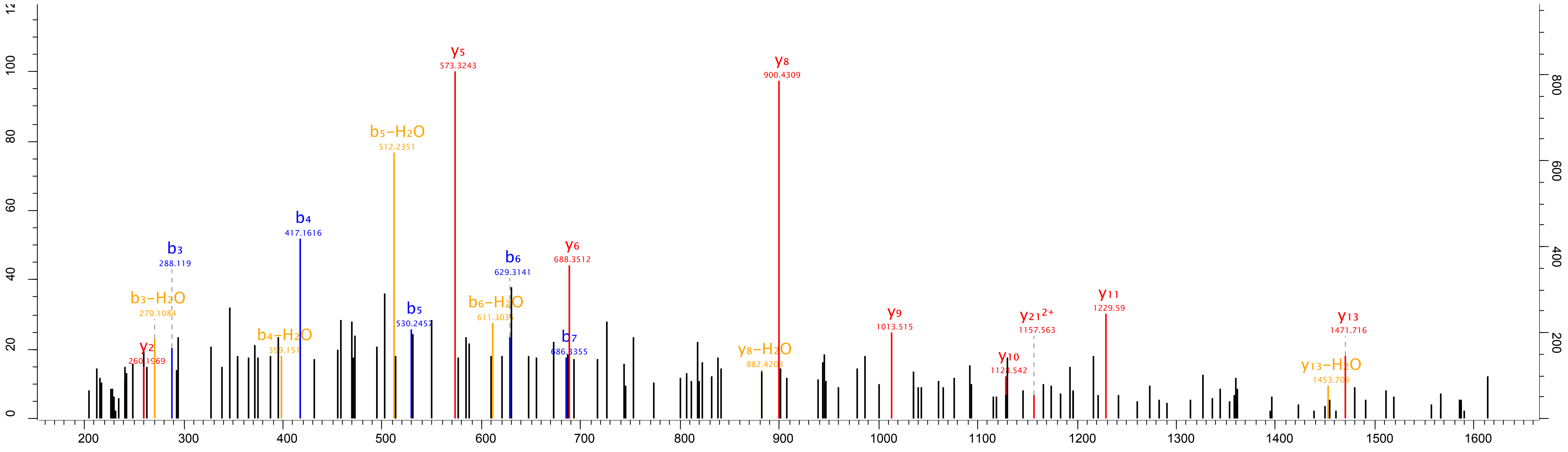

Raw file	Scan	Method	Score	Mass	Gene names
UPS1+500ngY_90minTop17_BC4_01_358	63471	TOF; CID	42.63	2032.93	LSM3

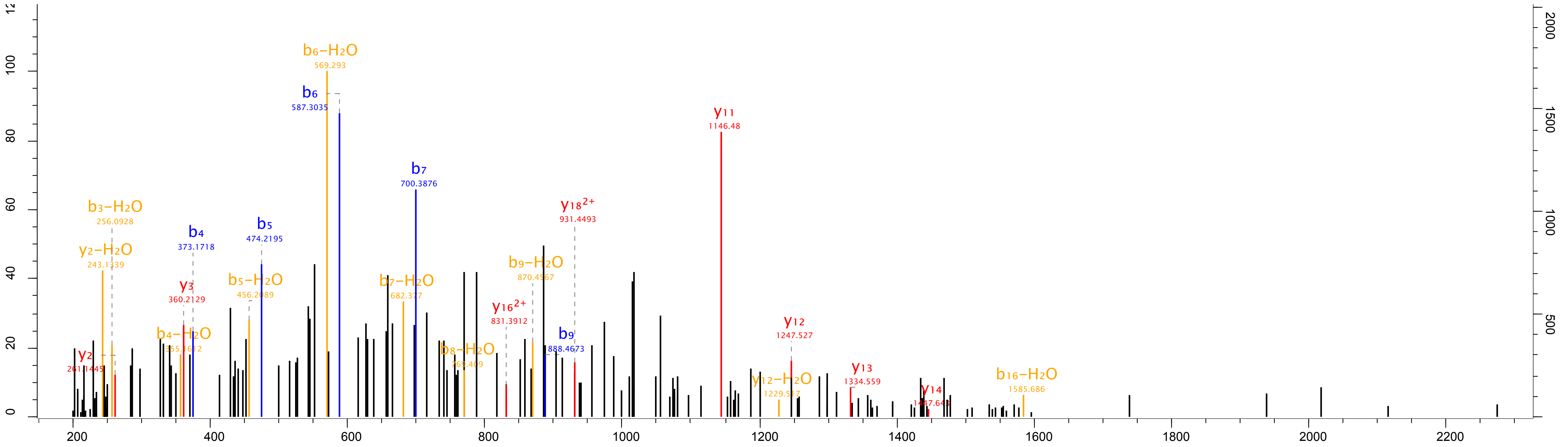

Raw file	Scan	Method	Score	Mass	Gene names
UPS1+500ngY_90minTop17_BC4_01_358	63480	TOF; CID	71.18	1359.7	YMR31

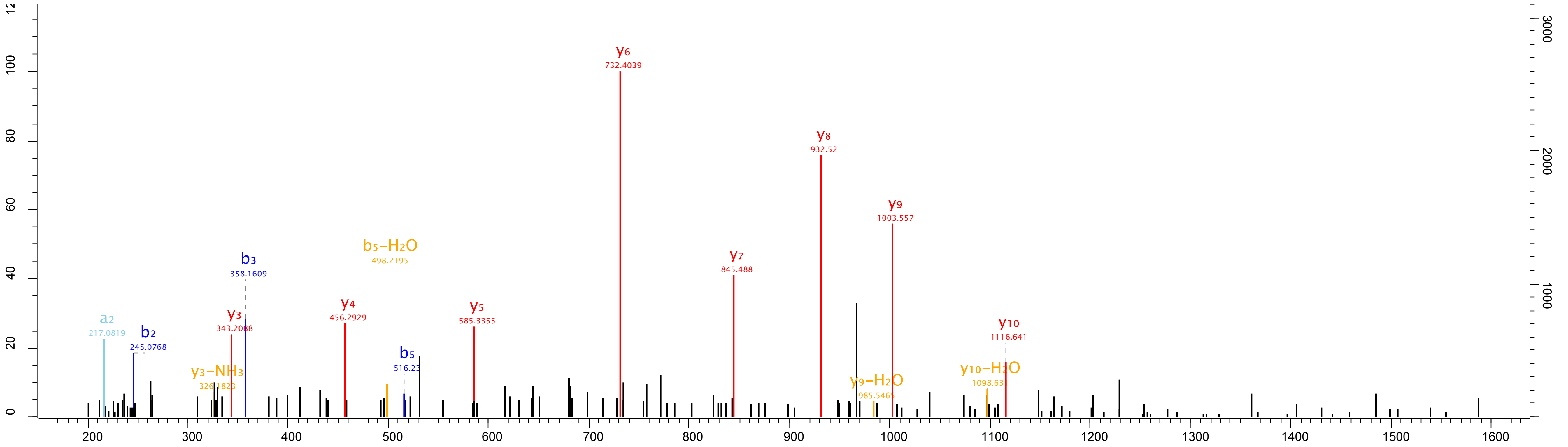

Raw file	Scan	Method	Score	Mass	Gene names
UPS1+500ngY_90minTop17_BC4_01_358	63566	TOF; CID	57.41	2260.02	MVP1

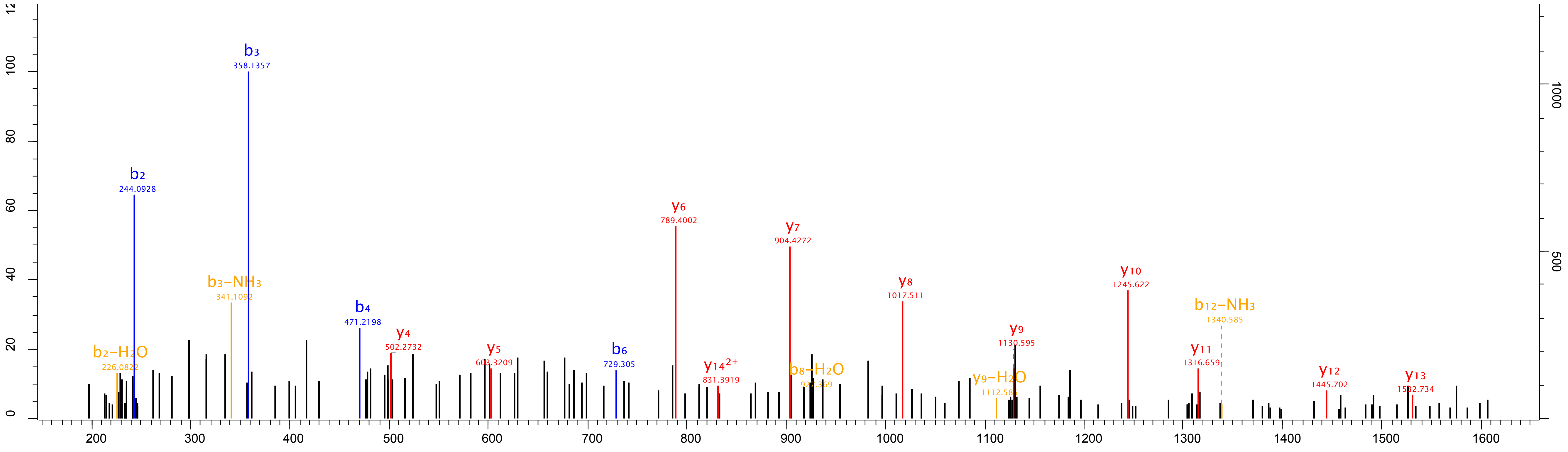

Raw file	Scan	Method	Score	Mass	Gene names
UPS1+500ngY_90minTop17_BC4_01_358	64631	TOF; CID	45.62	1888.98	RXT2

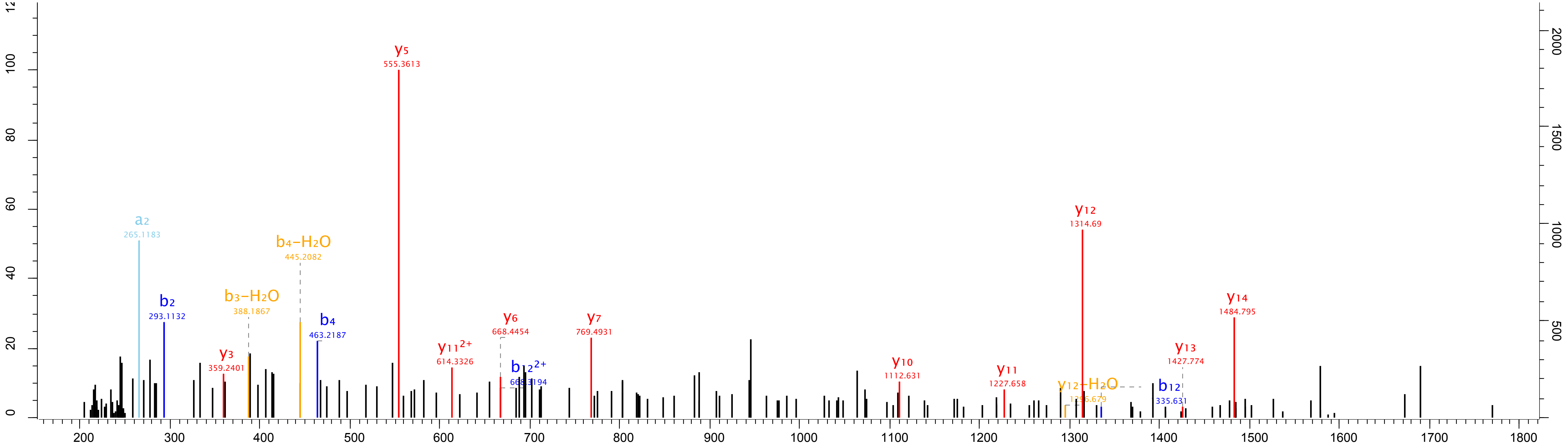

Raw file	Scan	Method	Score	Mass	Gene names
UPS1+500ngY_90minTop17_BC4_01_358	65236	TOF; CID	55.44	1822.9	LAC1

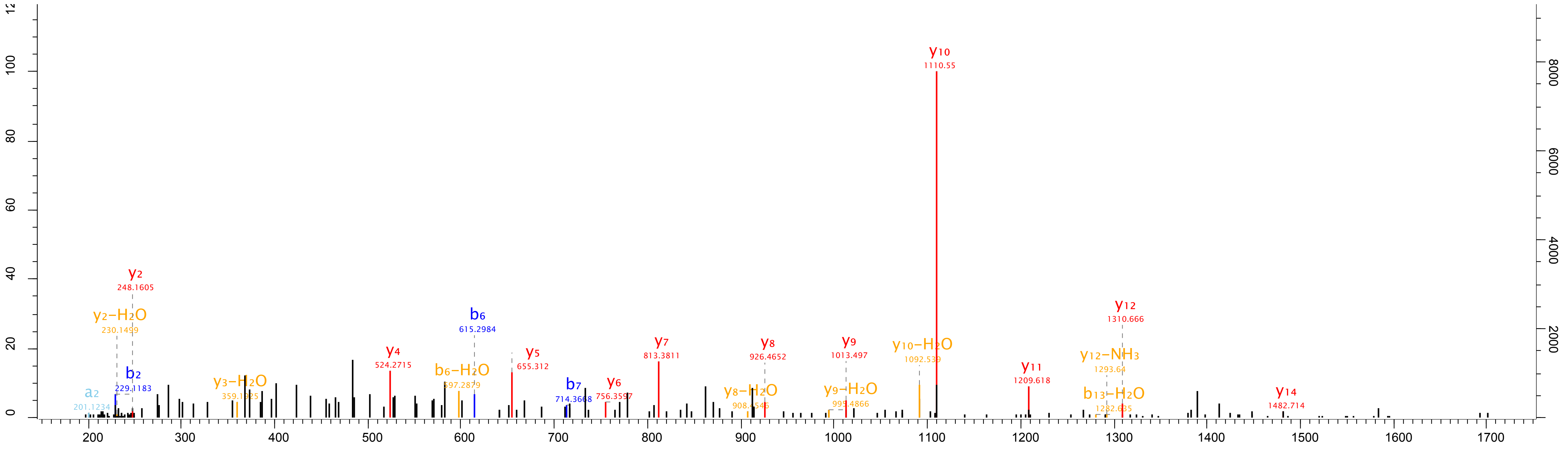

Raw file	Scan	Method	Score	Mass	Gene names
UPS1+500ngY_90minTop17_BC4_01_358	65844	TOF; CID	45.99	2480.17	RPB10

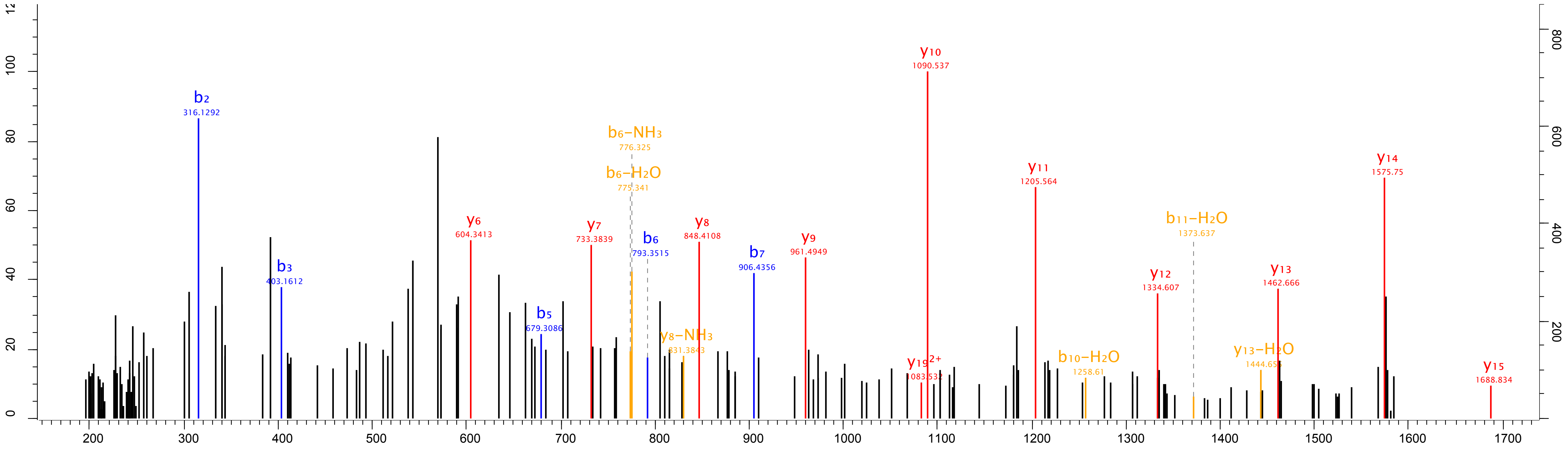

Raw file	Scan	Method	Score	Mass	Gene names
UPS1+500ngY_90minTop17_BC4_01_358	66164	TOF; CID	49.59	1688.88	BUD14

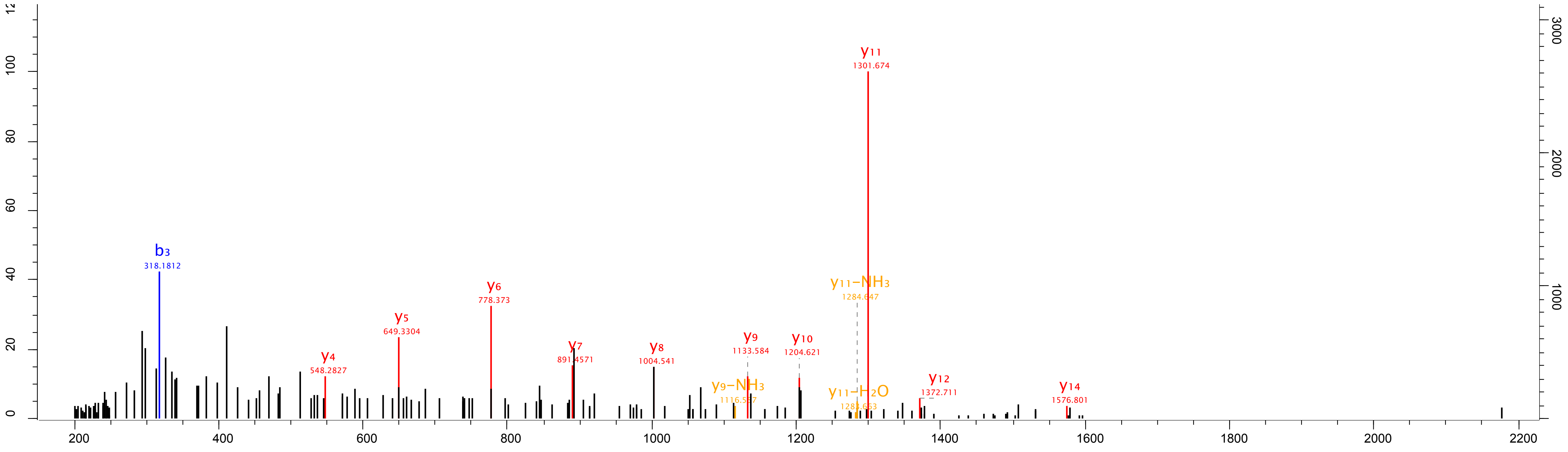

Raw file	Scan	Method	Score	Mass	Gene names
UPS1+500ngY_90minTop17_BC4_01_358	66172	TOF; CID	48.48	1977	CLP1

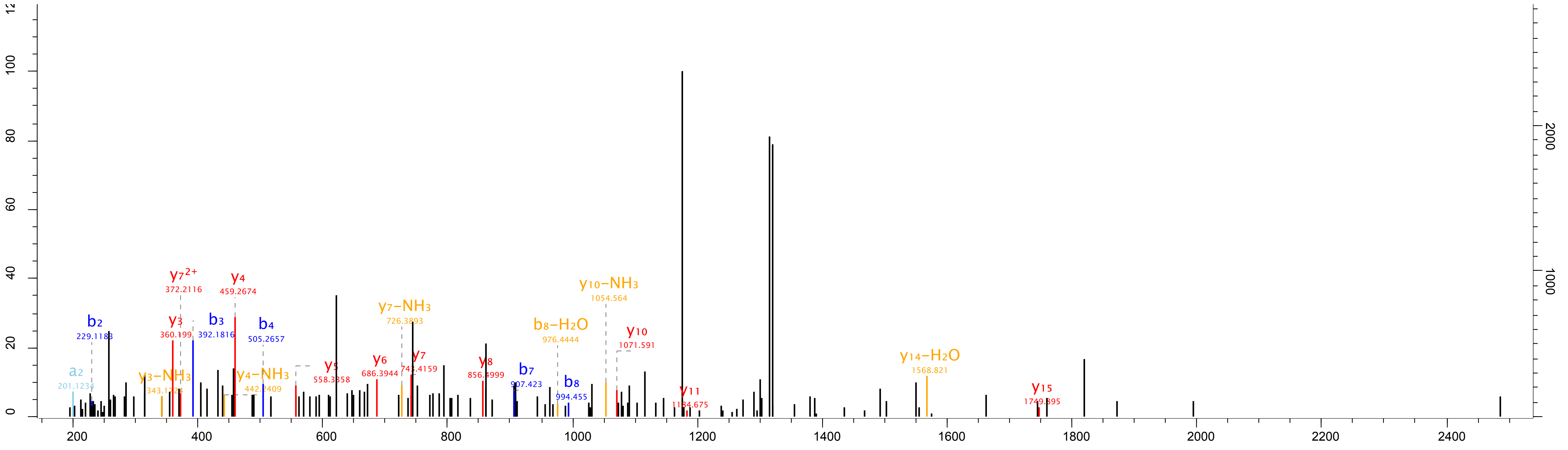

Raw file	Scan	Method	Score	Mass	Gene names
UPS1+500ngY_90minTop17_BC4_01_358	66184	TOF; CID	56.51	1706.84	RIB5

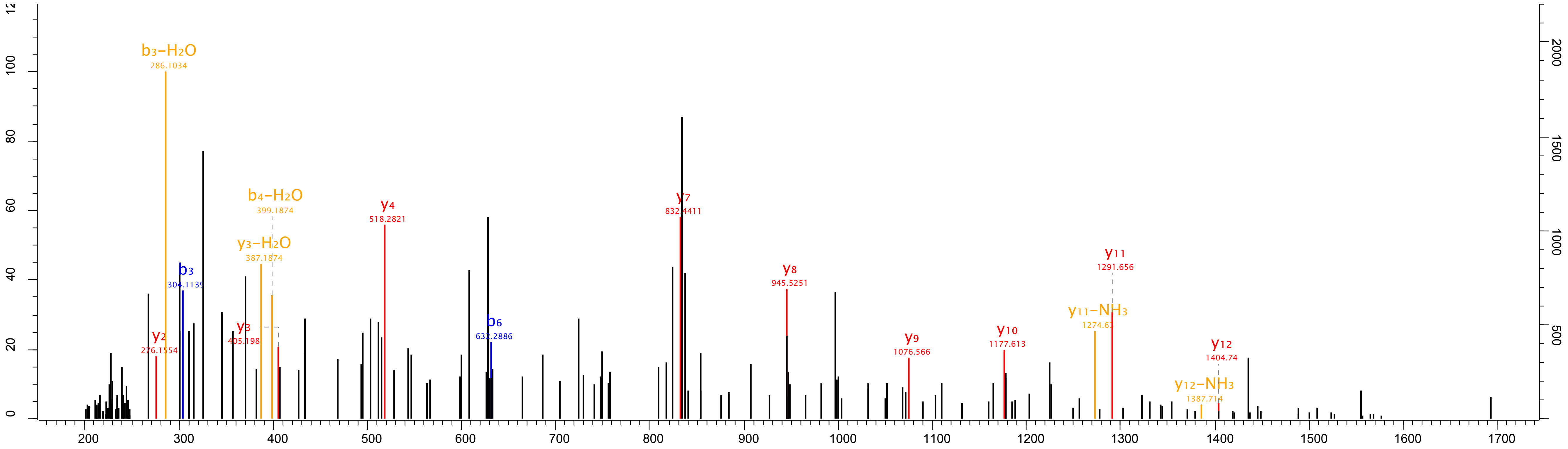

Raw file	Scan	Method	Score	Mass	Gene names
UPS1+500ngY_90minTop17_BC4_01_358	66402	TOF; CID	42.71	2039.98	MTQ2

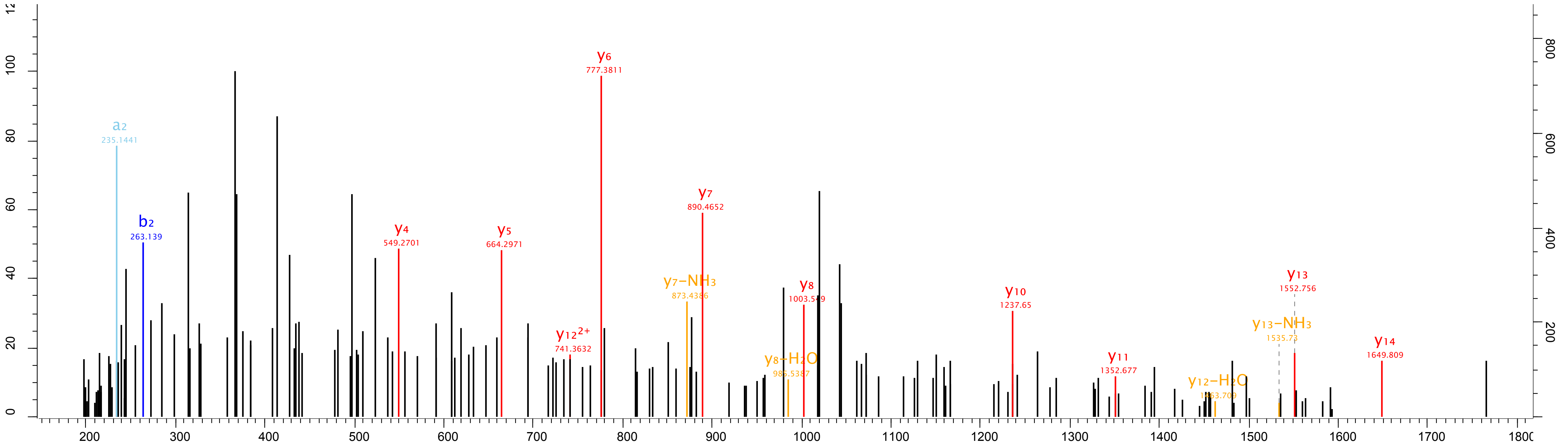

Raw file	Scan	Method	Score	Mass	Gene names
UPS1+500ngY_90minTop17_BC4_01_358	67029	TOF; CID	39.8	2881.47	TAH1

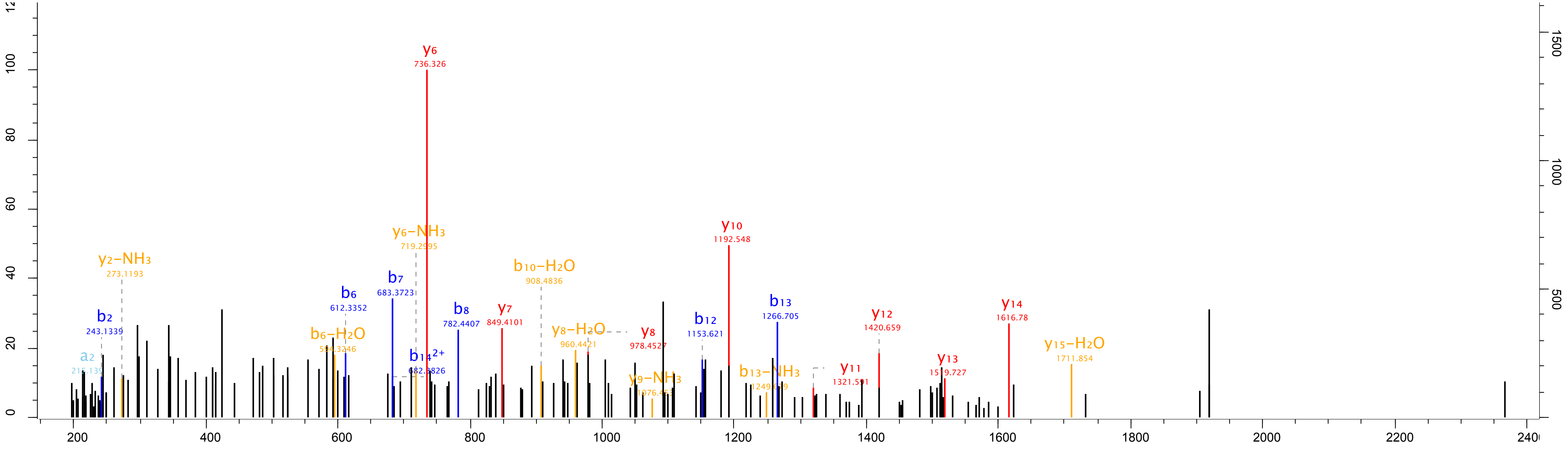

Raw file	Scan	Method	Score	Mass	Gene names
UPS1+500ngY_90minTop17_BC4_01_358	67483	TOF; CID	62.2	1823.92	NOP14

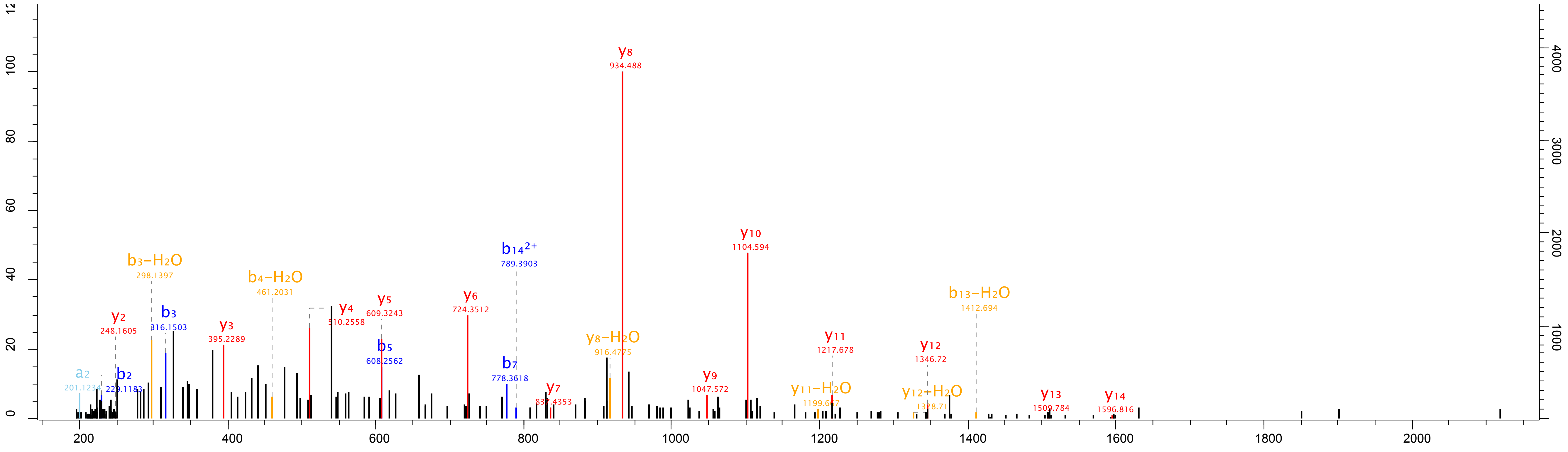

Raw file	Scan	Method	Score	Mass	Gene names
UPS1+500ngY_90minTop17_BC4_01_358	67587	TOF; CID	51.5	1817.88	HTL1

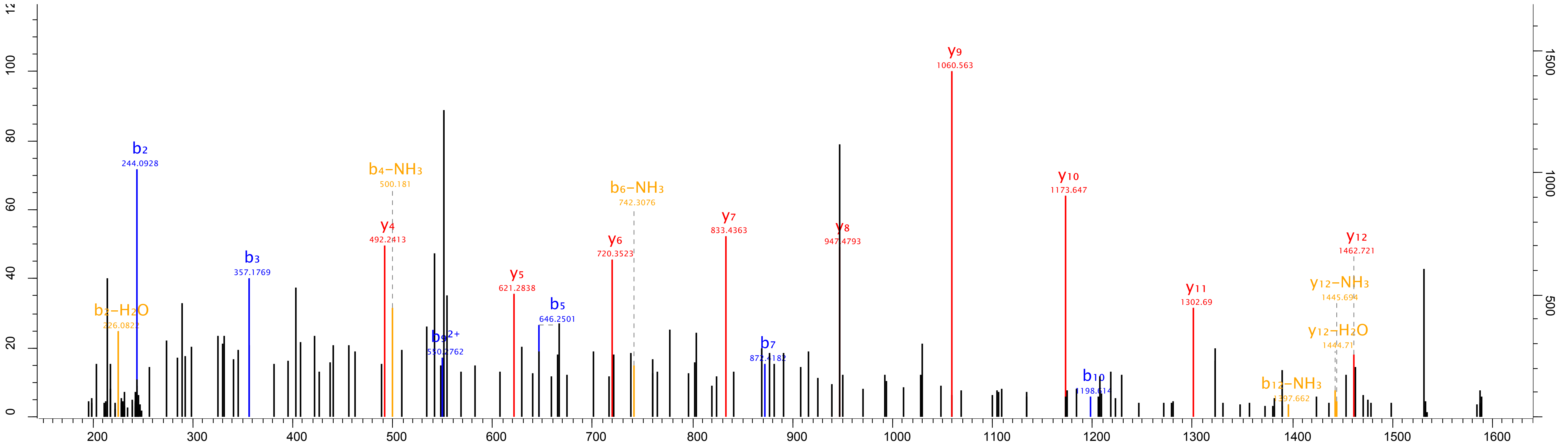

Raw file	Scan	Method	Score	Mass	Gene names
UPS1+500ngY_90minTop17_BC4_01_358	67837	TOF; CID	61.04	2587.29	YPR091C

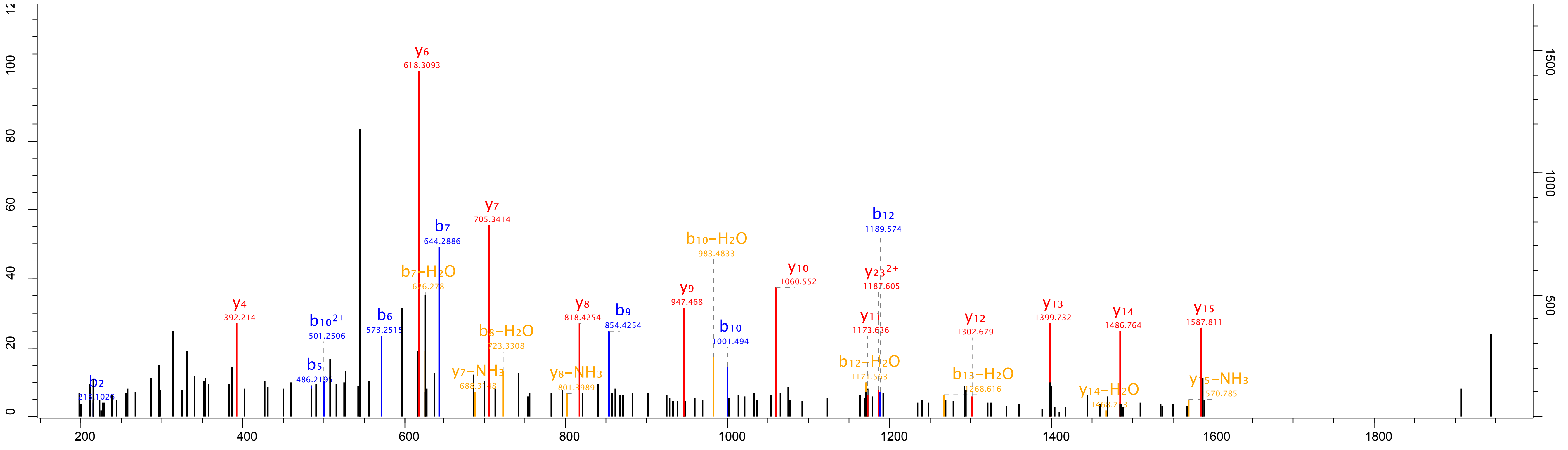

Raw file	Scan	Method	Score	Mass	Gene names
UPS1+500ngY_90minTop17_BC4_01_358	68093	TOF; CID	75.7	3174.42	LSB1

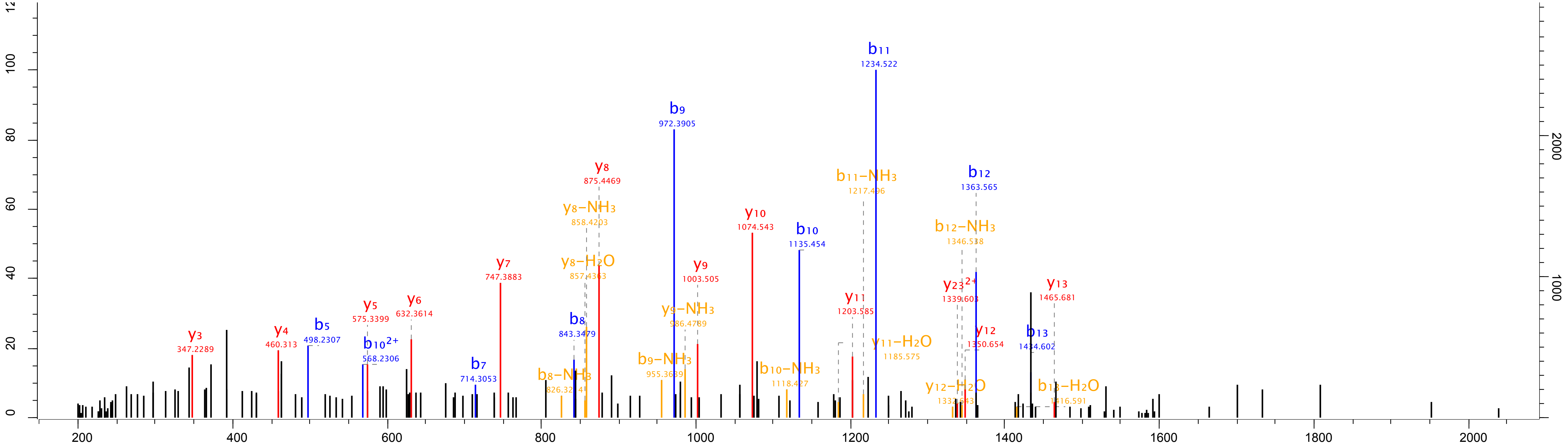

Raw file	Scan	Method	Score	Mass	Gene names
UPS1+500ngY_90minTop17_BC4_01_358	68271	TOF; CID	52.86	1798.87	TRM732

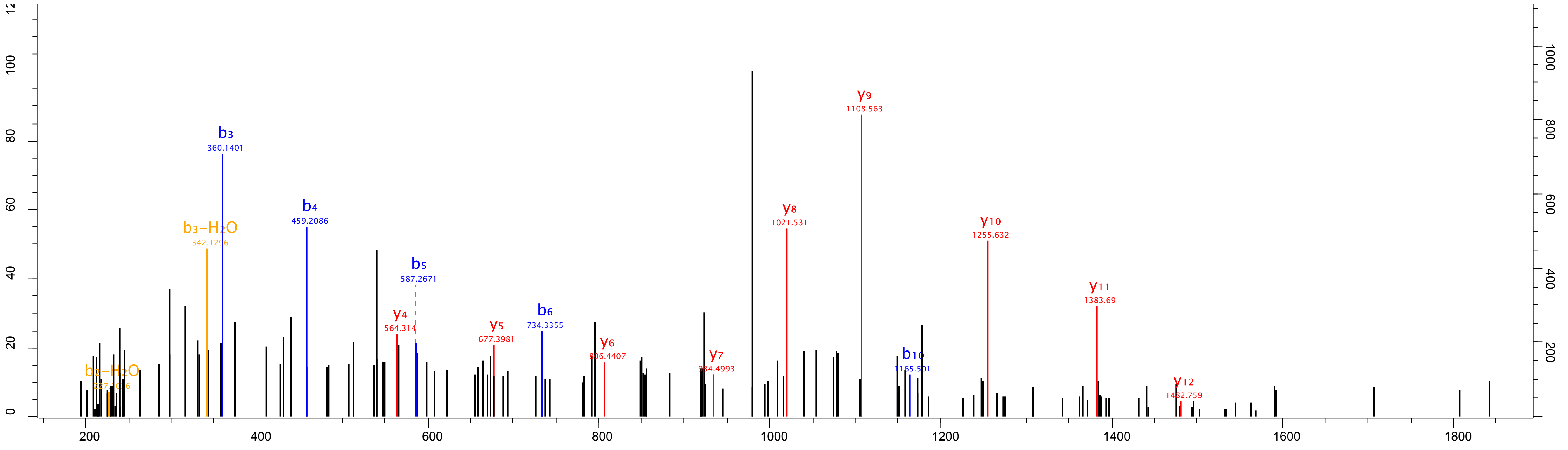

Raw file	Scan	Method	Score	Mass	Gene names
UPS1+500ngY_90minTop17_BC4_01_358	68534	TOF; CID	46.66	2582.38	APE4

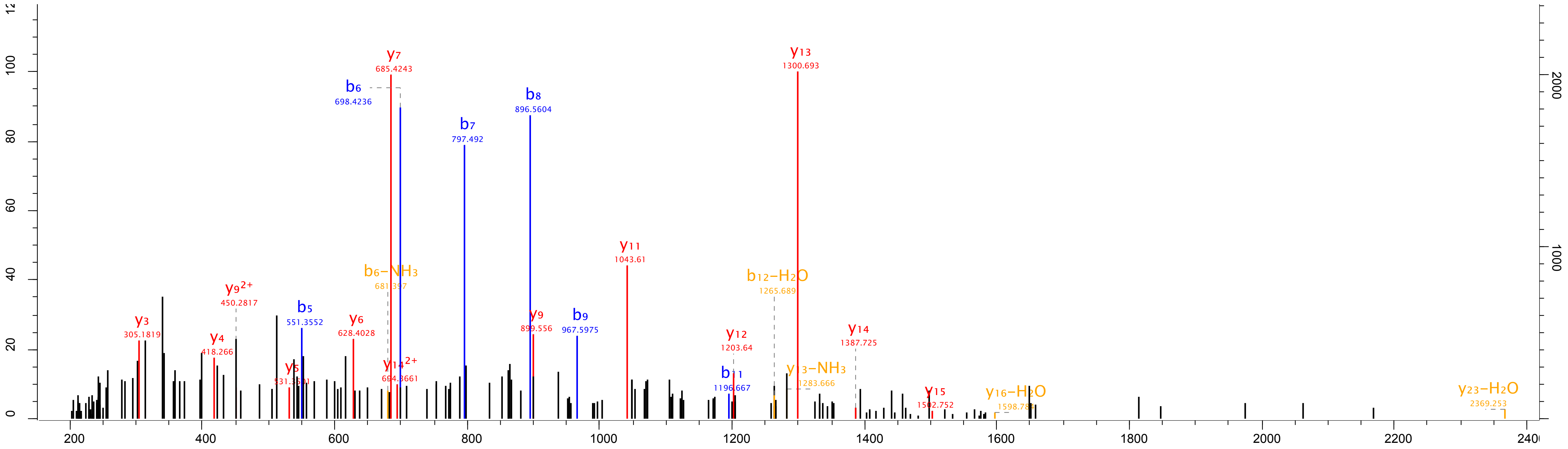

Raw file	Scan	Method	Score	Mass	Gene names
UPS1+500ngY_90minTop17_BC4_01_358	68541	TOF; CID	47.04	1586.85	MSH6

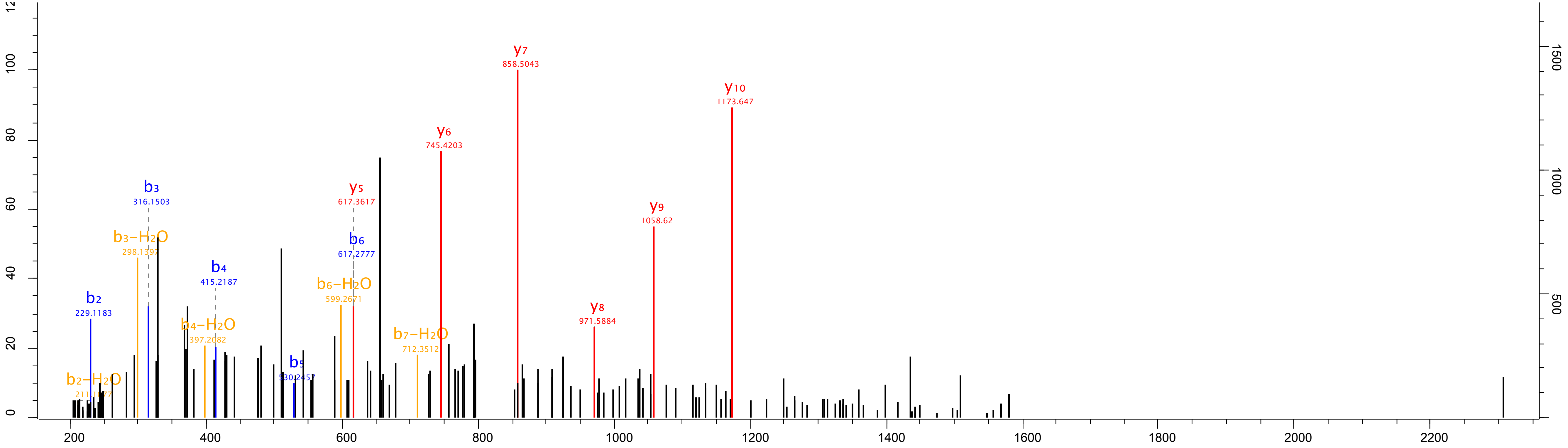

Raw file	Scan	Method	Score	Mass	Gene names
UPS1+500ngY_90minTop17_BC4_01_358	68662	TOF; CID	36.3	3302.48	HMT1

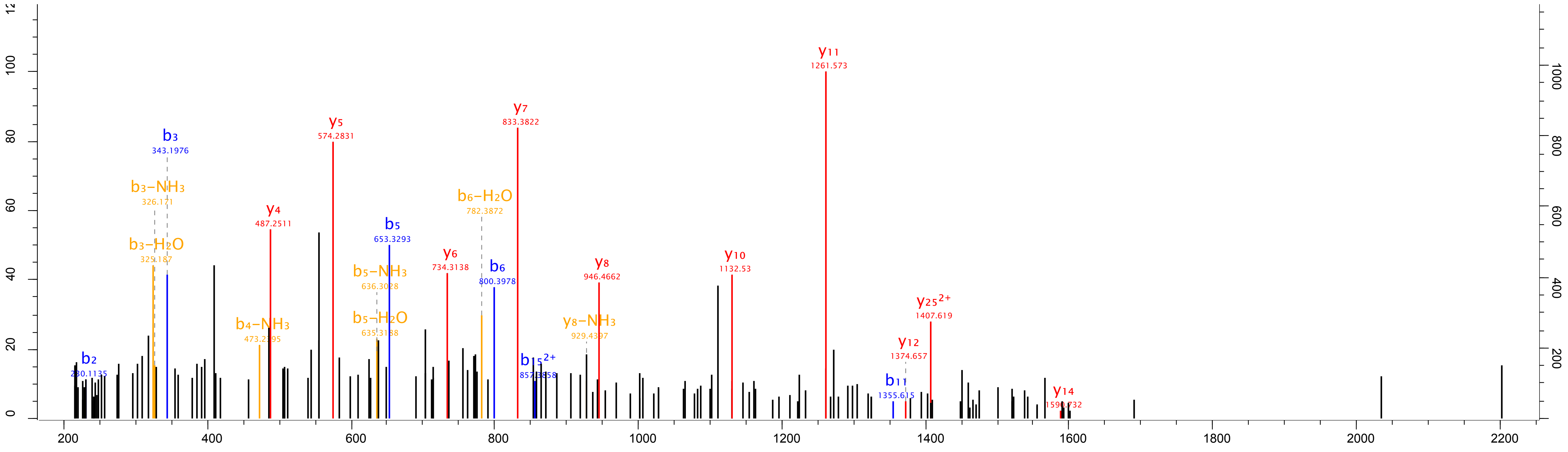

Raw file	Scan	Method	Score	Mass	Gene names
UPS1+500ngY_90minTop17_BC4_01_358	69317	TOF; CID	43.38	2469.21	MNS1

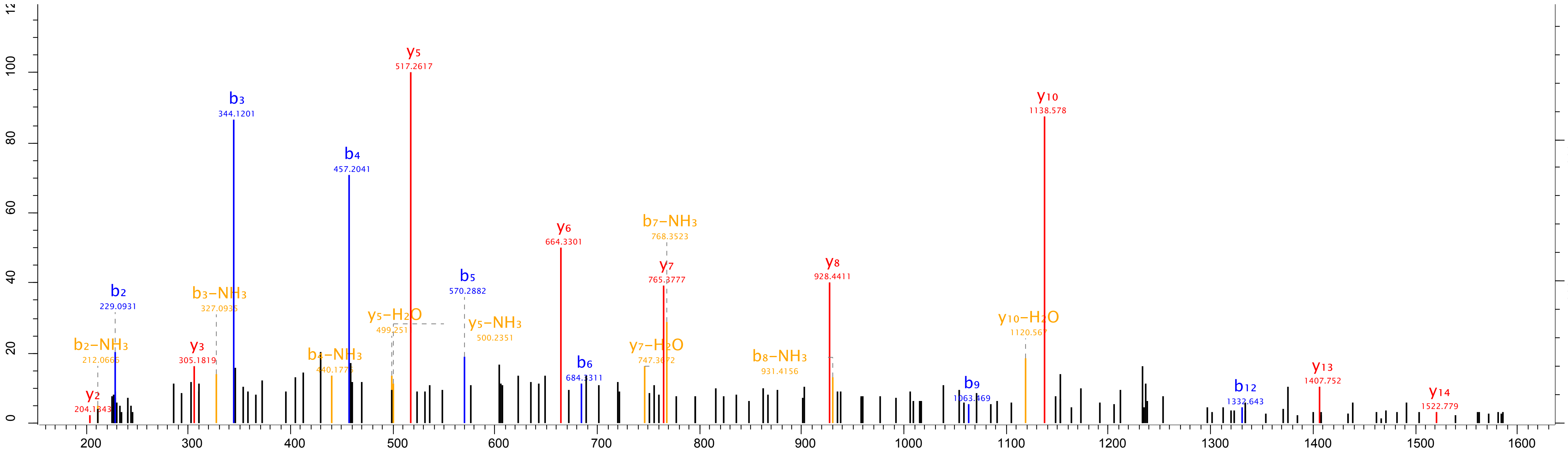

Raw file	Scan	Method	Score	Mass	Gene names
UPS1+500ngY_90minTop17_BC4_01_358	69353	TOF; CID	42.06	2915.54	ATP16

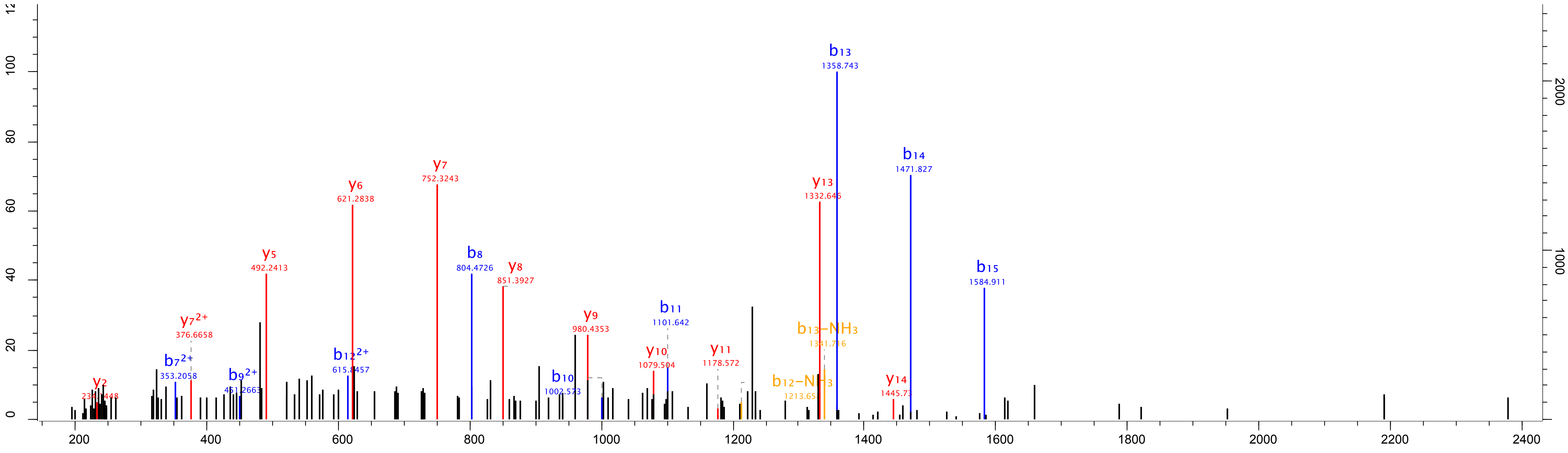

Raw file	Scan	Method	Score	Mass	Gene names
UPS1+500ngY_90minTop17_BC4_01_358	69714	TOF; CID	66.99	1417.74	FDC1

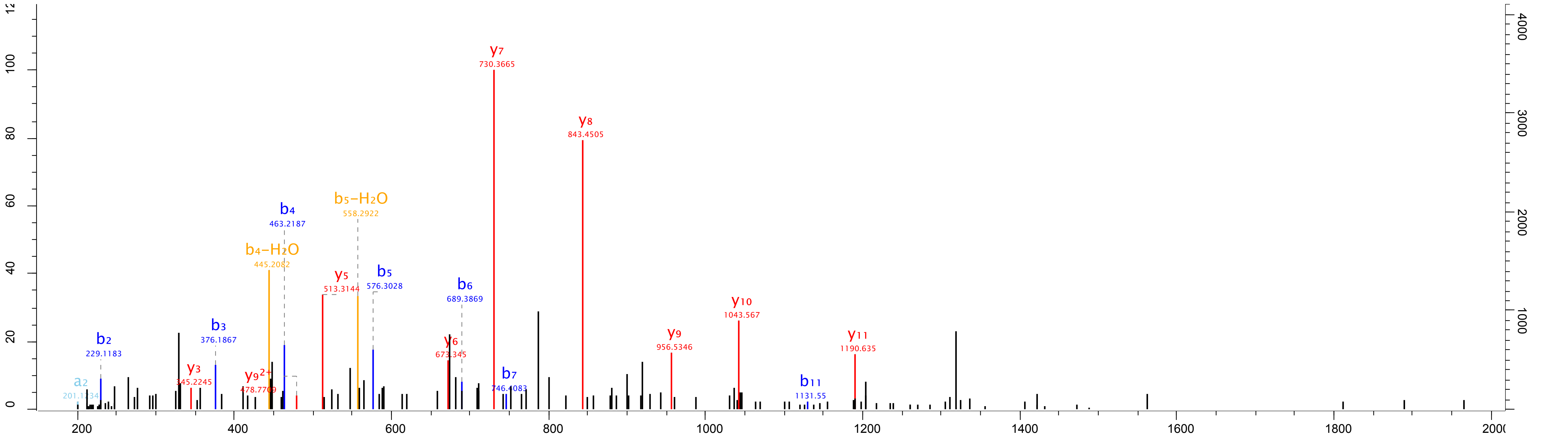

Raw file	Scan	Method	Score	Mass	Gene names
UPS1+500ngY_90minTop17_BC4_01_358	69814	TOF; CID	47.04	1594.8	POM152

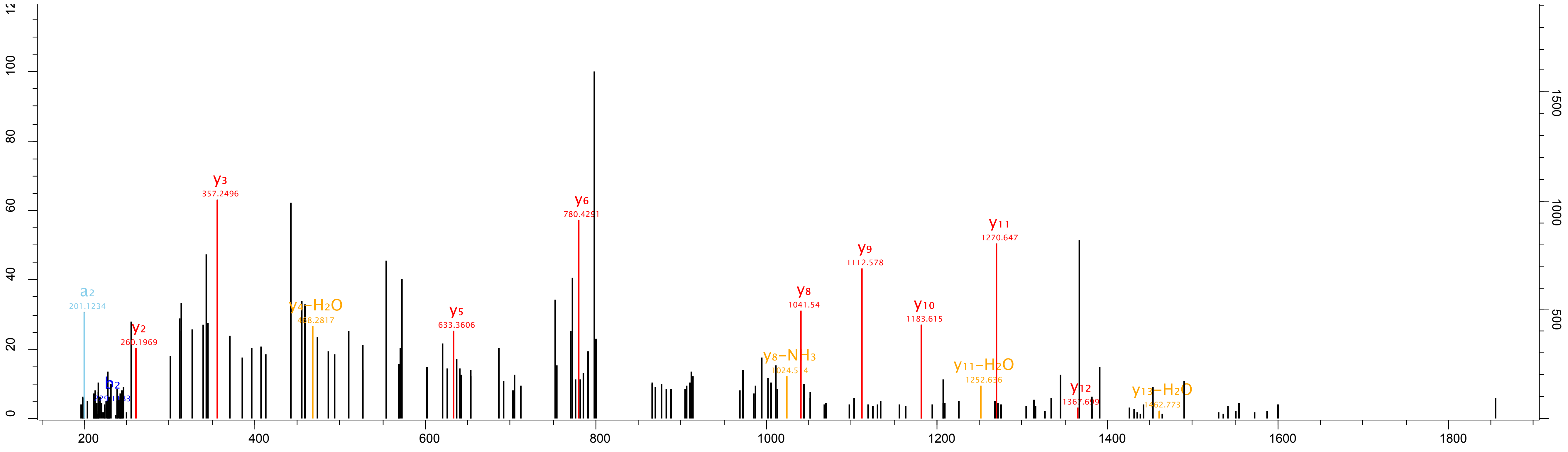

Raw file	Scan	Method	Score	Mass	Gene names
UPS1+500ngY_90minTop17_BC4_01_358	70805	TOF; CID	46.89	1803.9	RSC9

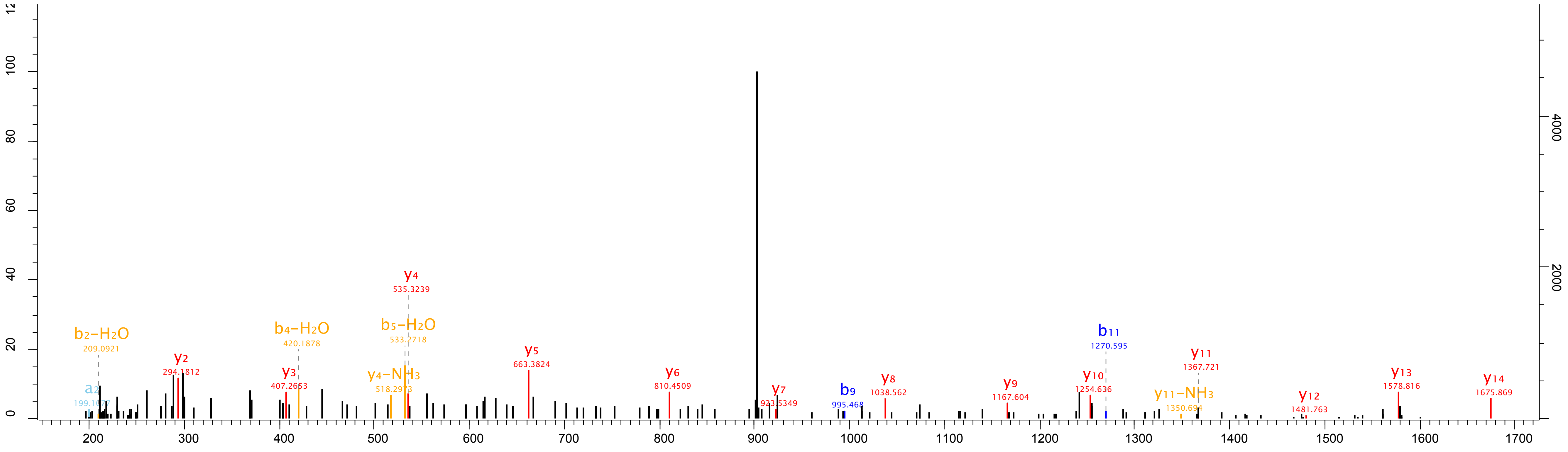

Raw file	Scan	Method	Score	Mass	Gene names
UPS1+500ngY_90minTop17_BC4_01_358	70852	TOF; CID	59.37	2023.12	SMD1

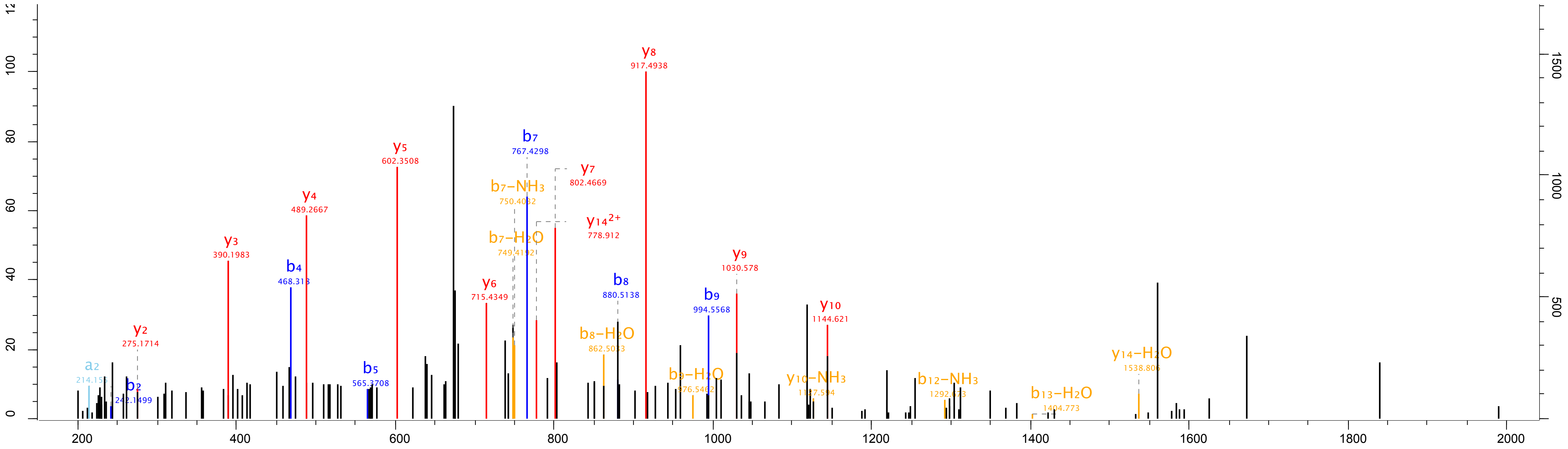

Raw file	Scan	Method	Score	Mass	Gene names
UPS1+500ngY_90minTop17_BC4_01_358	72382	TOF; CID	35.68	2496.13	YVH1

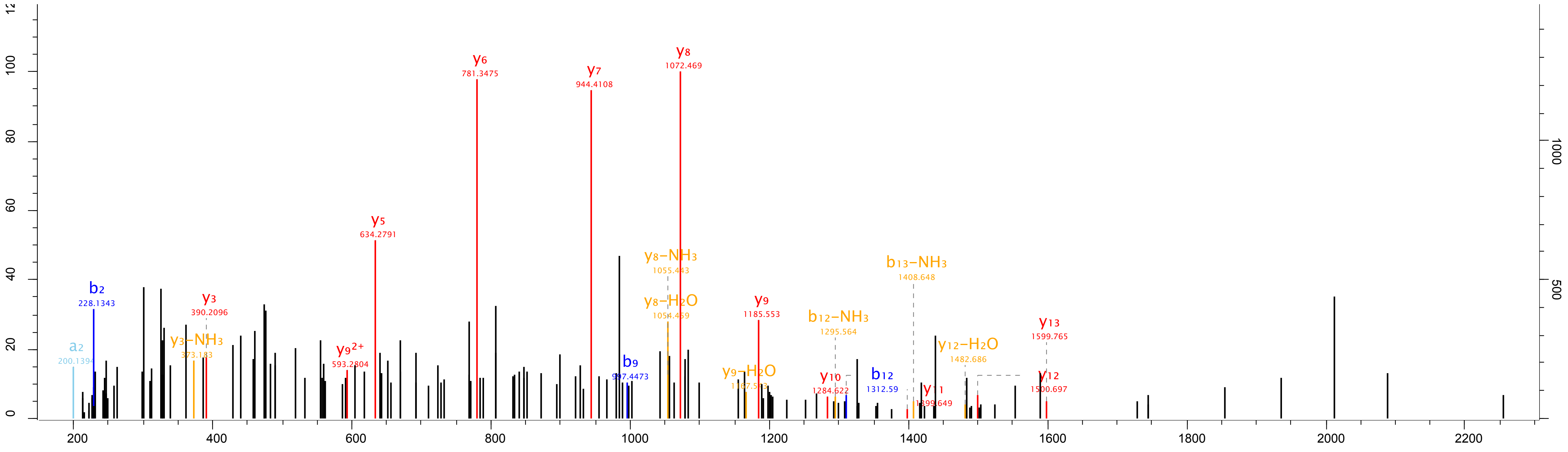

Raw file	Scan	Method	Score	Mass	Gene names
UPS1+500ngY_90minTop17_BC4_01_358	72569	TOF; CID	43.79	1853.04	RKM1

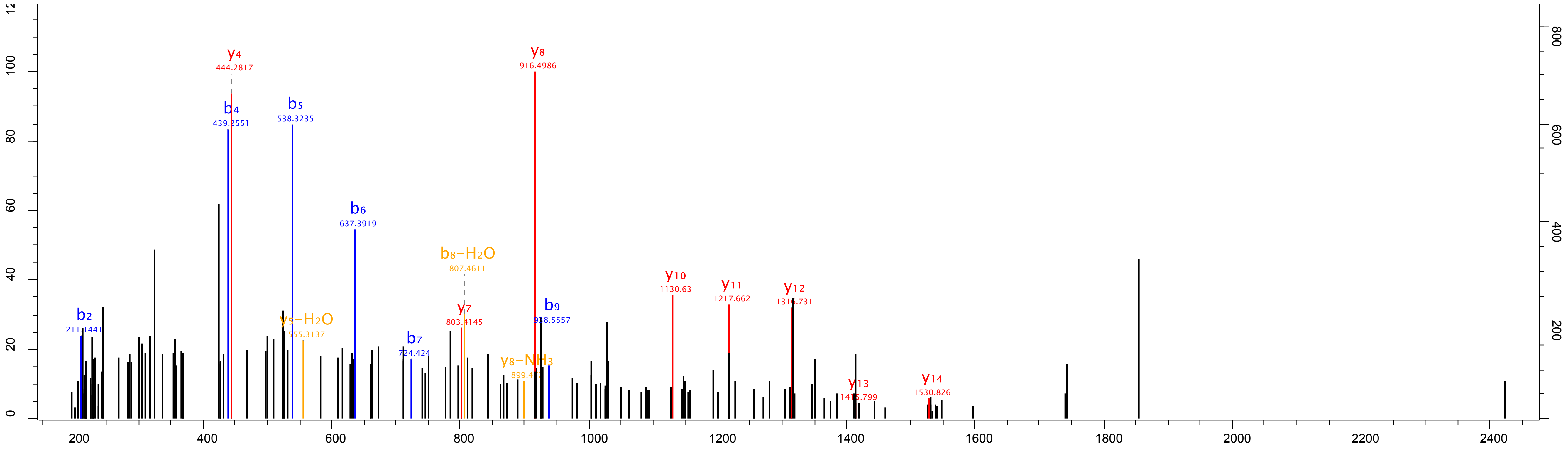

Raw file	Scan	Method	Score	Mass	Gene names
UPS1+500ngY_90minTop17_BC4_01_358	73159	TOF; CID	40.62	1881.92	POF1

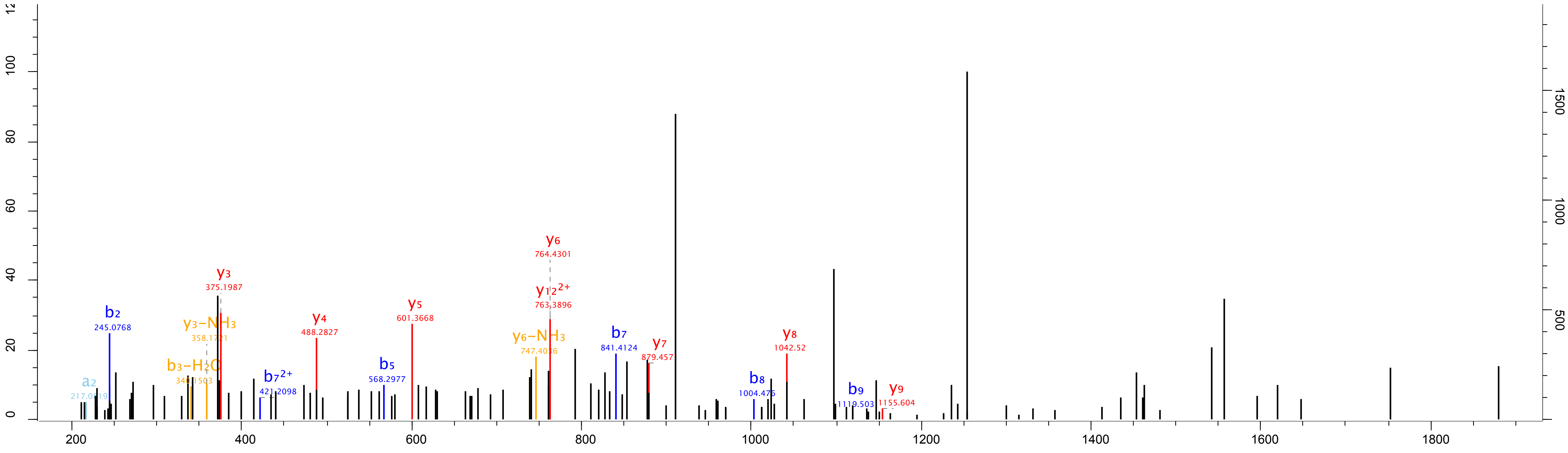

Raw file	Scan	Method	Score	Mass	Gene names
UPS1+500ngY_90minTop17_BC4_01_358	73286	TOF; CID	74.73	2024.99	CDC9

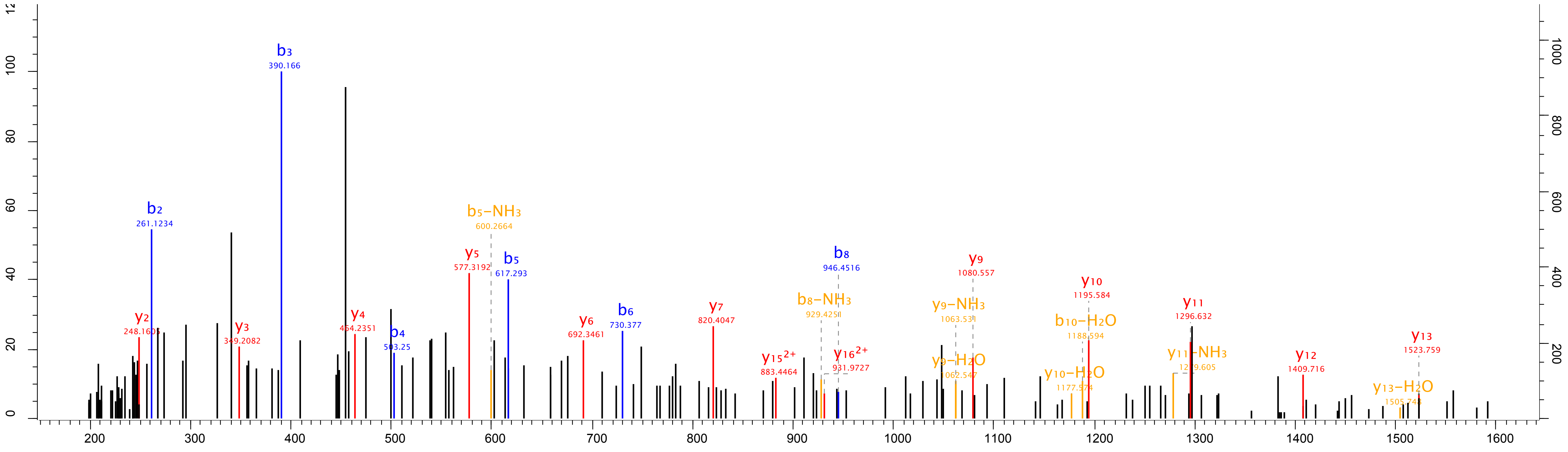

Raw file	Scan	Method	Score	Mass	Gene names
UPS1+500ngY_90minTop17_BC4_01_358	74727	TOF; CID	55.71	2559.34	KAP120

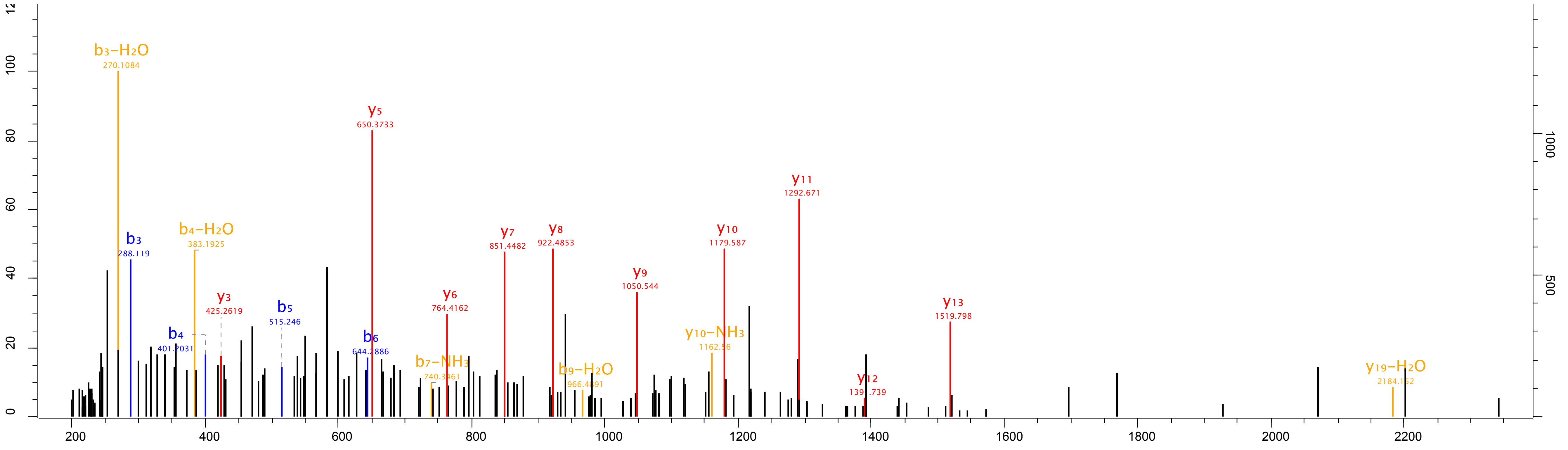

Raw file	Scan	Method	Score	Mass	Gene names
UPS1+500ngY_90minTop17_BC4_01_358	74799	TOF; CID	45	2012.89	CDC123

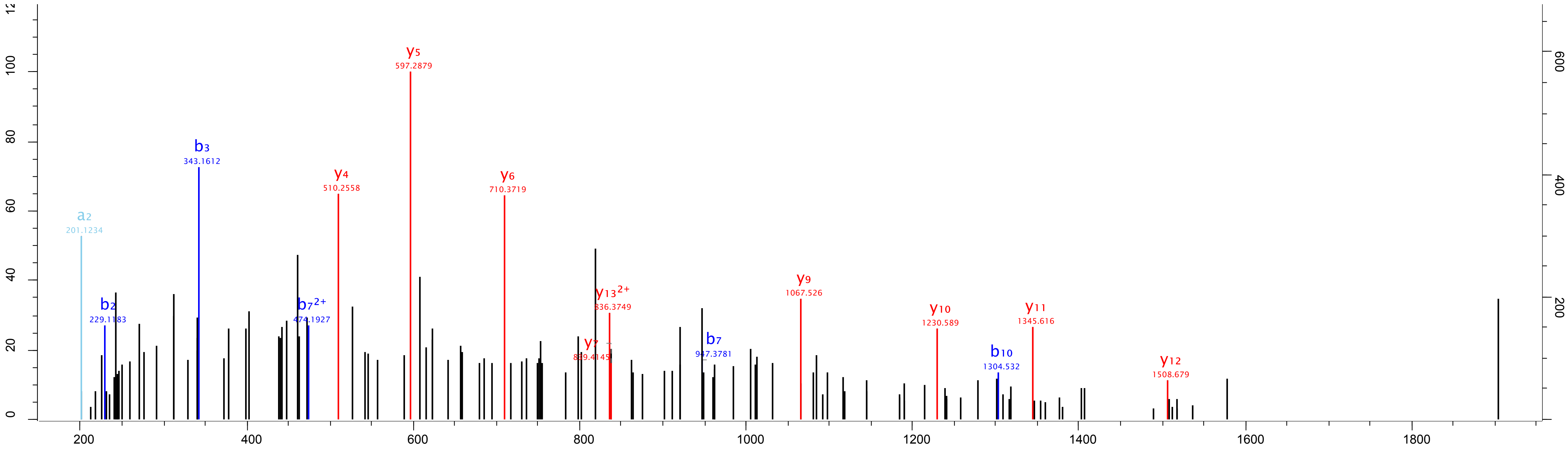

Raw file	Scan	Method	Score	Mass	Gene names
UPS1+500ngY_90minTop17_BC4_01_358	75480	TOF; CID	64.21	3412.62	VPS75

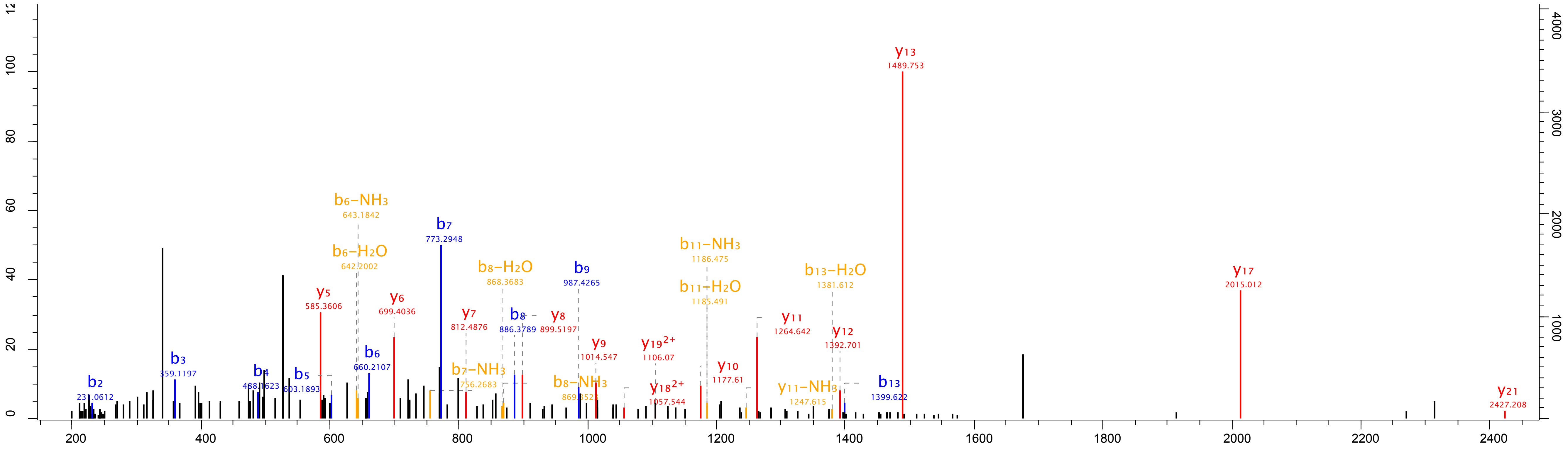

Raw file	Scan	Method	Score	Mass	Gene names
UPS1+500ngY_90minTop17_BC4_01_358	75534	TOF; CID	38.2	3180.49	UBC7

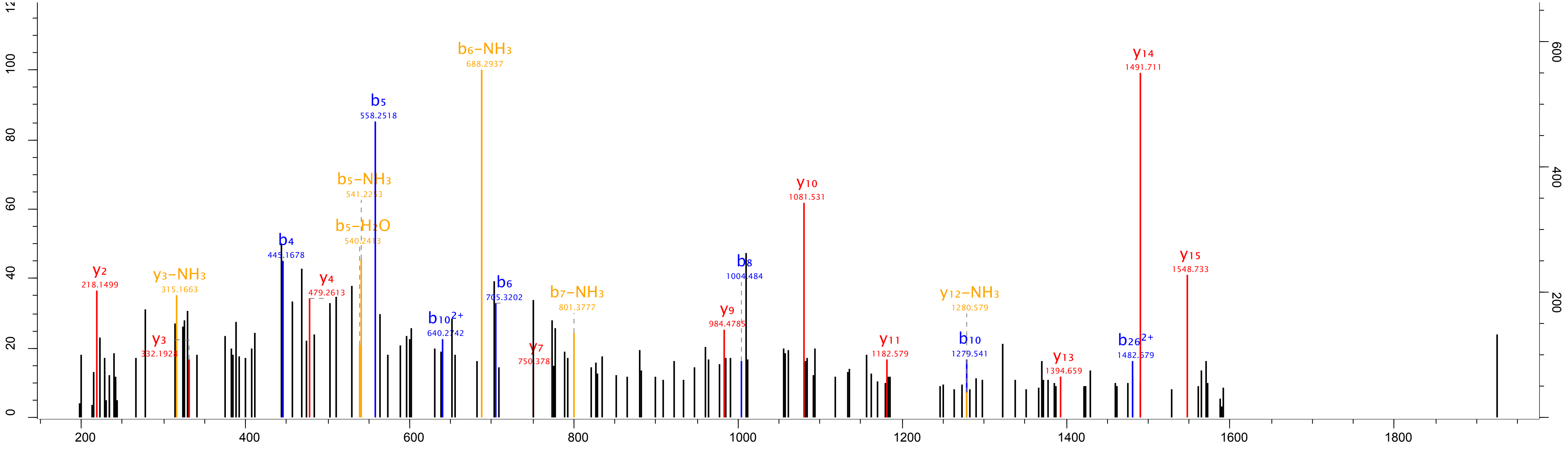

Raw file	Scan	Method	Score	Mass	Gene names
UPS1+500ngY_90minTop17_BC4_01_358	75631	TOF; CID	59.98	2335.16	COA3

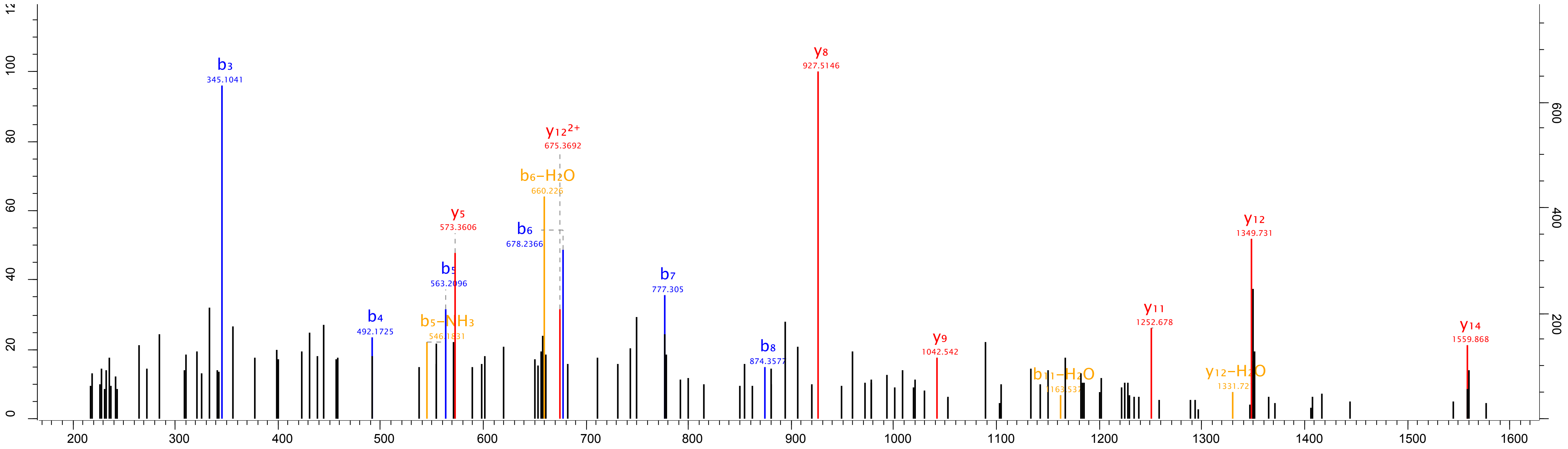

Raw file	Scan	Method	Score	Mass	Gene names
UPS1+500ngY_90minTop17_BC4_01_358	77943	TOF; CID	30.78	2397.17	BZZ1

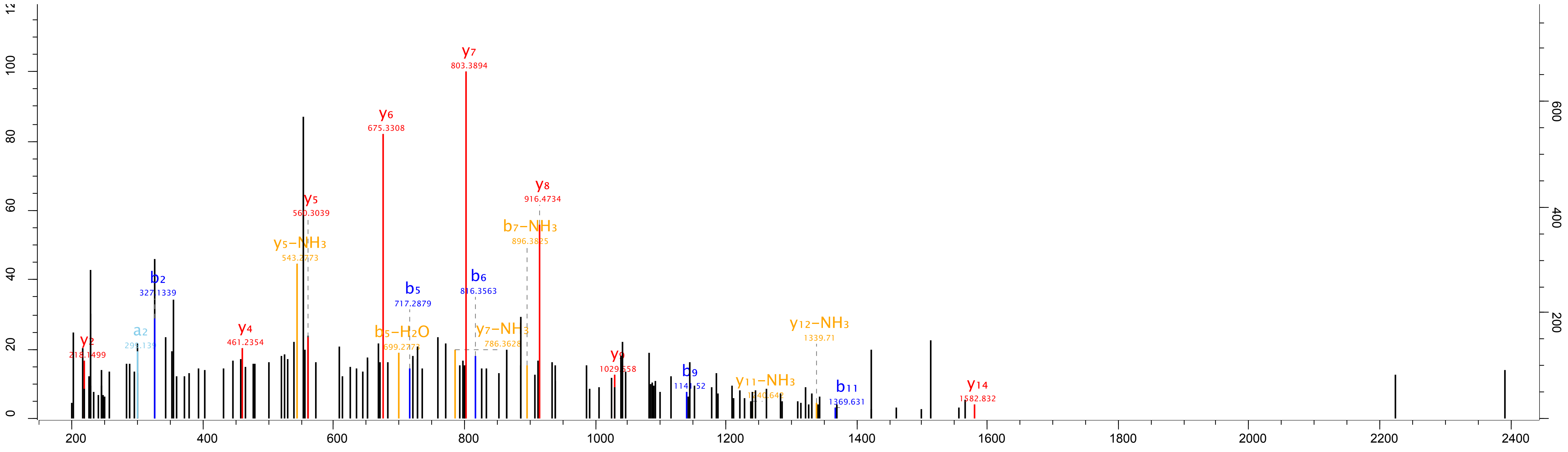

Raw file	Scan	Method	Score	Mass	Gene names
UPS1+500ngY_90minTop17_BC4_01_358	78107	TOF; CID	54.25	2051.03	RRP40

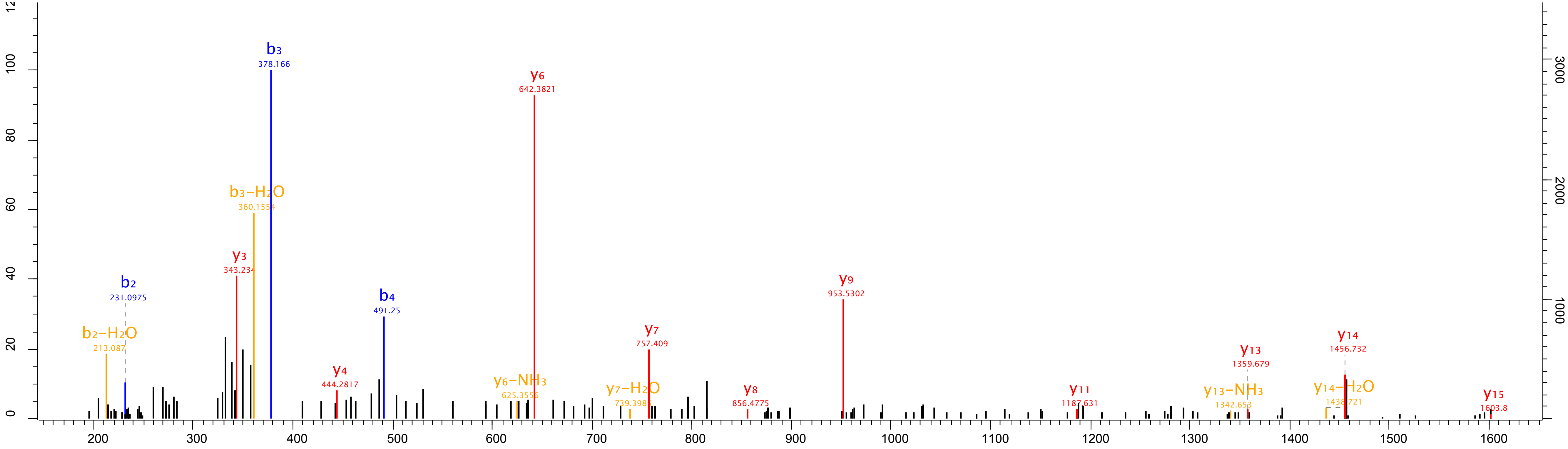

Raw file	Scan	Method	Score	Mass	Gene names
UPS1+500ngY_90minTop17_BC4_01_358	78380	TOF; CID	82.61	2345.27	SEC61

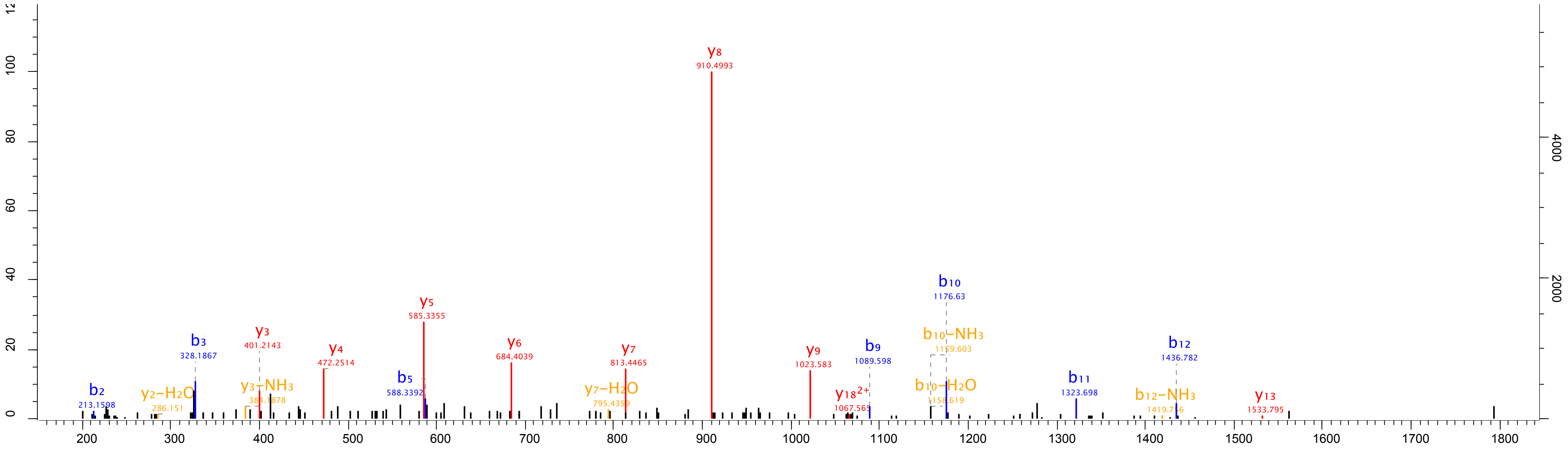

Raw file	Scan	Method	Score	Mass	Gene names
UPS1+500ngY_90minTop17_BC4_01_358	84073	TOF; CID	90.39	2166.26	TSR1

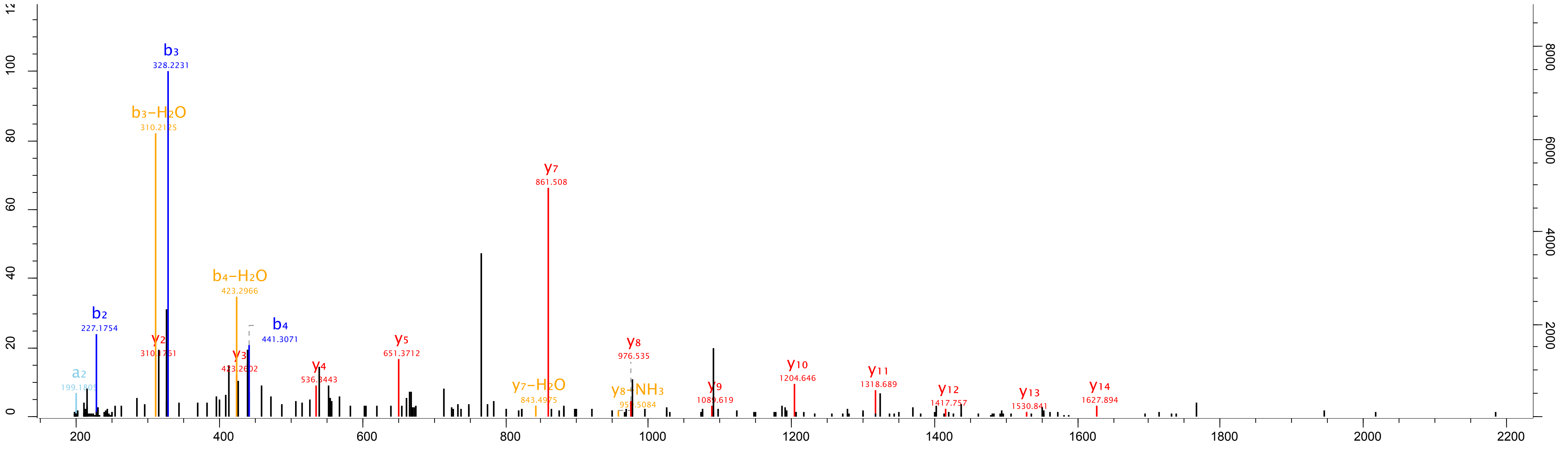

Raw file	Scan	Method	Score	Mass	Gene names
UPS1+500ngY_90minTop17_BC4_01_358	84517	TOF; CID	71.45	1111.57	DID4

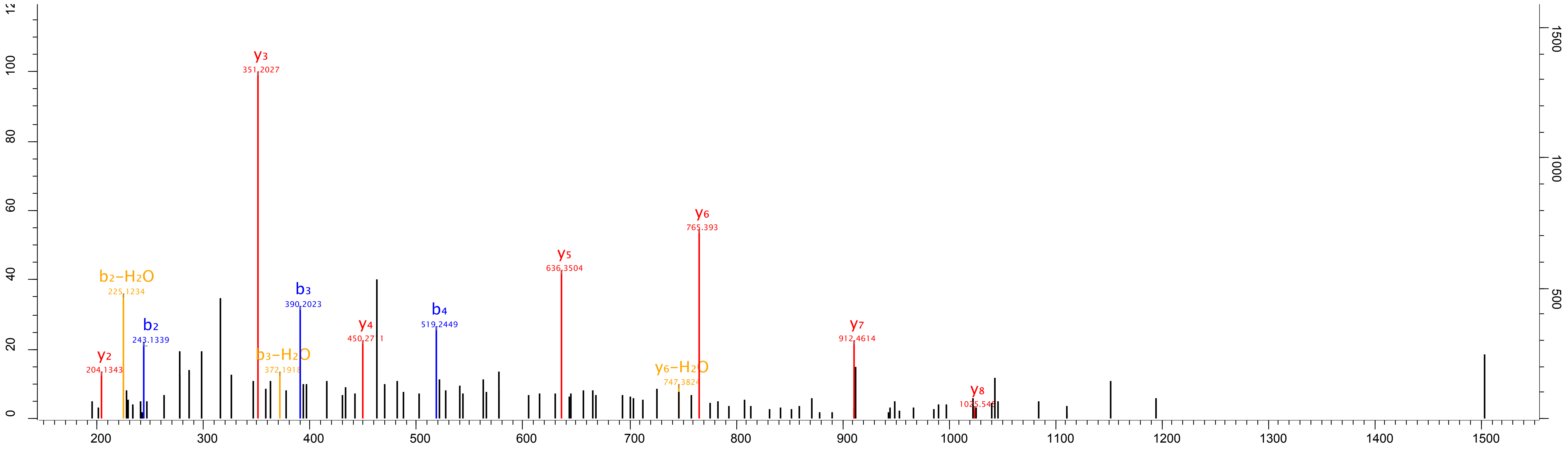

Supplement: Supplemental Data [file supp_M114.047407_mcp.M114.047407-7.pdf]
