# Supplementary material for: The Impact II, a Very High-Resolution Quadrupole Time-of-Flight Instrument (QTOF) for Deep Shotgun Proteomics
Source: Mol Cell Proteomics. 2015 May 19;14(7):2014–29. doi: 10.1074/mcp.M114.047407 (PMC4587313; doi:10.1074/mcp.M114.047407)
Supplement: Supplemental Data [file supp_M114.047407_mcp.M114.047407-8.pdf]

Raw file	Scan	Method	Score	m/z	Gene names
20150306_yeast1_Top_opt_2ug_C1_01_1663	2603	TOF; CID	68.02	422.22	DAM1

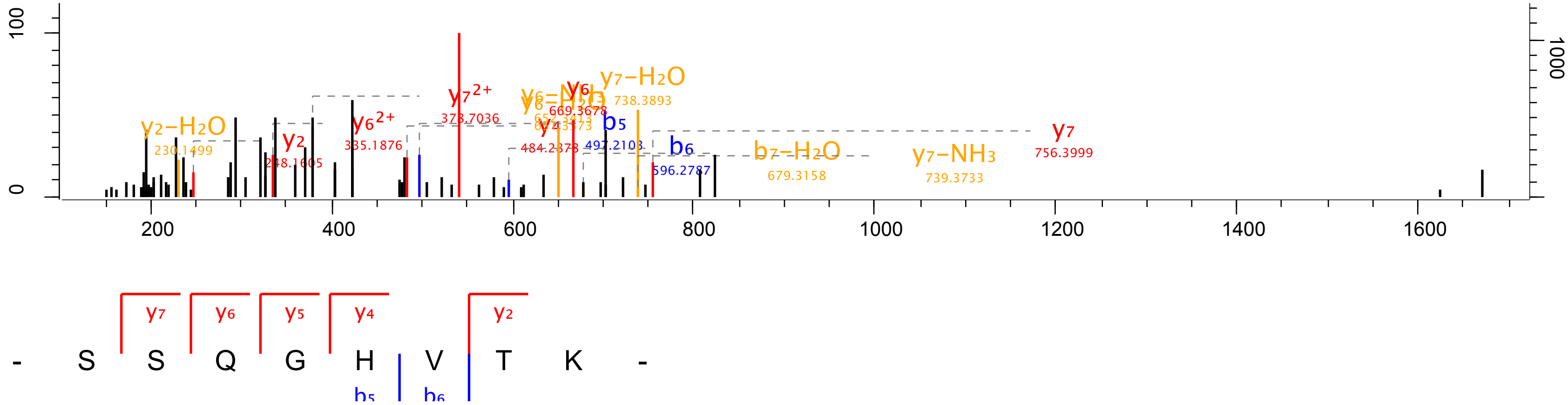

Raw file	Scan	Method	Score	m/z	Gene names
20150306_yeast1_Top_opt_2ug_C1_01_1663	8152	TOF; CID	69.82	379.7	RPS26A

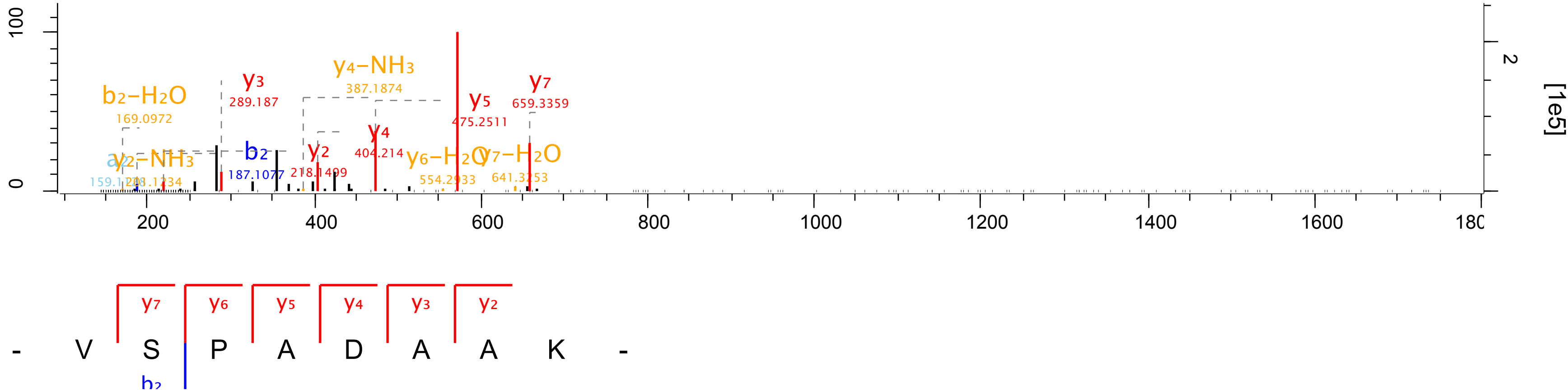

Raw file	Scan	Method	Score	m/z	Gene names
20150306_yeast1_Top_opt_2ug_C1_01_1663	8893	TOF; CID	105.1	549.74	MIX17

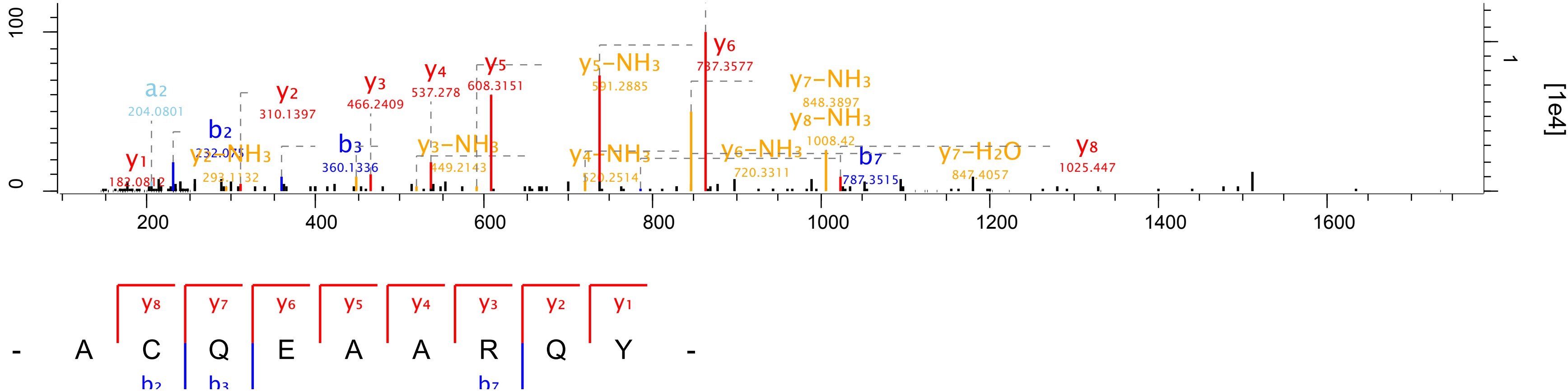

Raw file	Scan	Method	Score	m/z	Gene names
20150306_yeast1_Top_opt_2ug_C1_01_1663	9613	TOF; CID	94.69	648.75	POP8

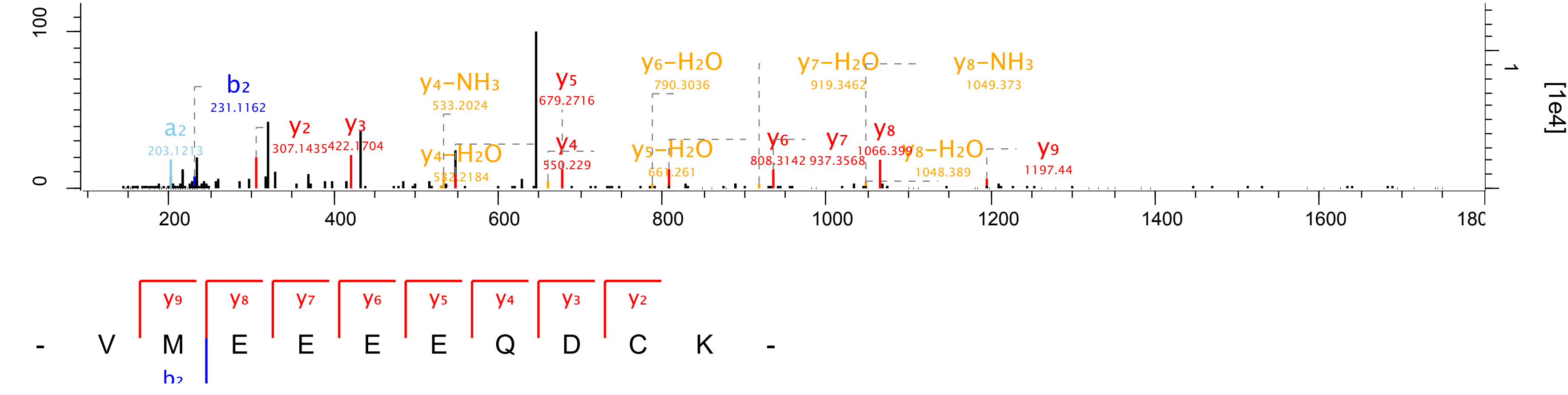

Raw file	Scan	Method	Score	m/z	Gene names
20150306_yeast1_Top_opt_2ug_C1_01_1663	10702	TOF; CID	72.62	564.27	YMD8

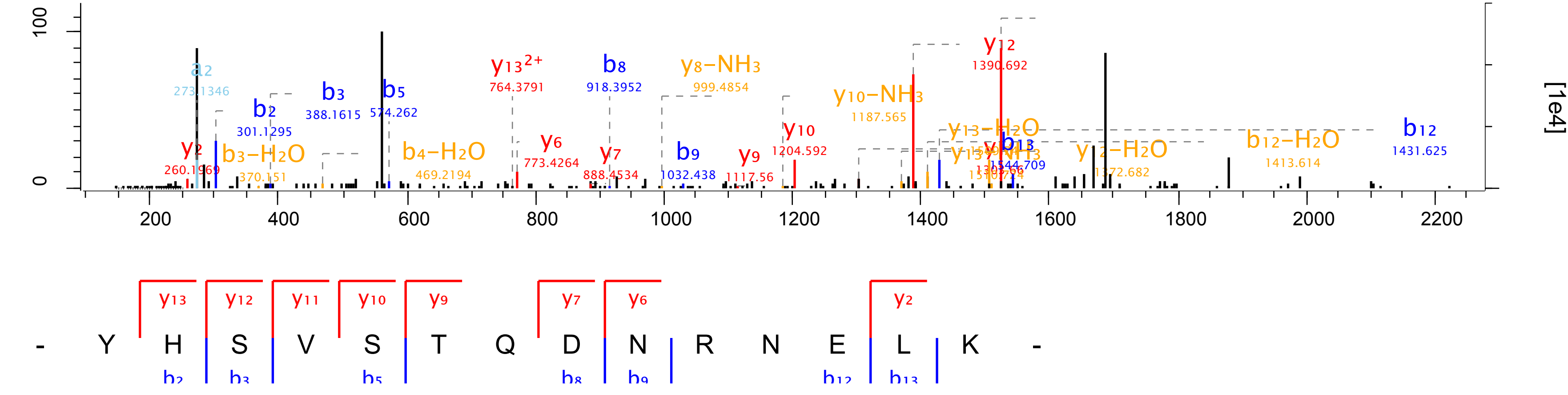

Raw file	Scan	Method	Score	m/z	Gene names
20150306_yeast1_Top_opt_2ug_C1_01_1663	17746	TOF; CID	72.32	705.36	OGG1

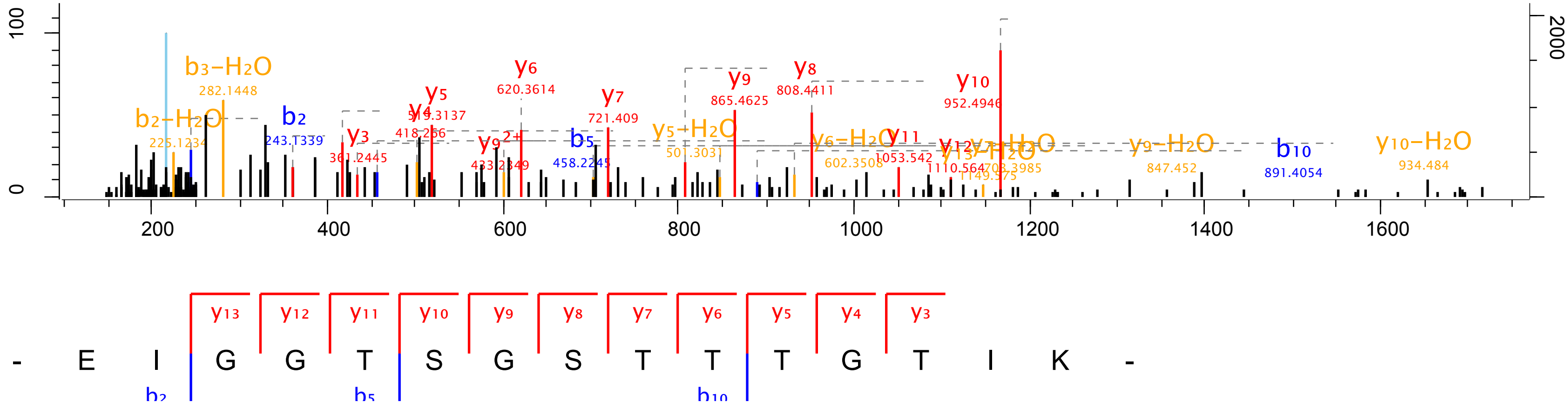

Raw file	Scan	Method	Score	m/z	Gene names
20150306_yeast1_Top_opt_2ug_C1_01_1663	18215	TOF; CID	83.18	580.78	DLS1

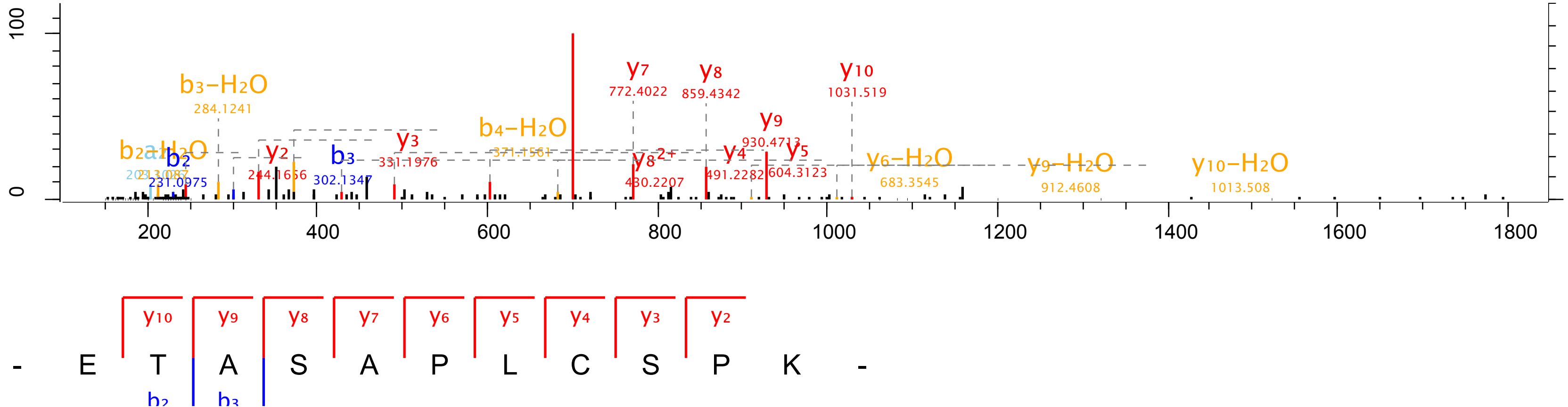

Raw file	Scan	Method	Score	m/z	Gene names
20150306_yeast1_Top_opt_2ug_C1_01_1663	31245	TOF; CID	68.56	513.26	TAT2

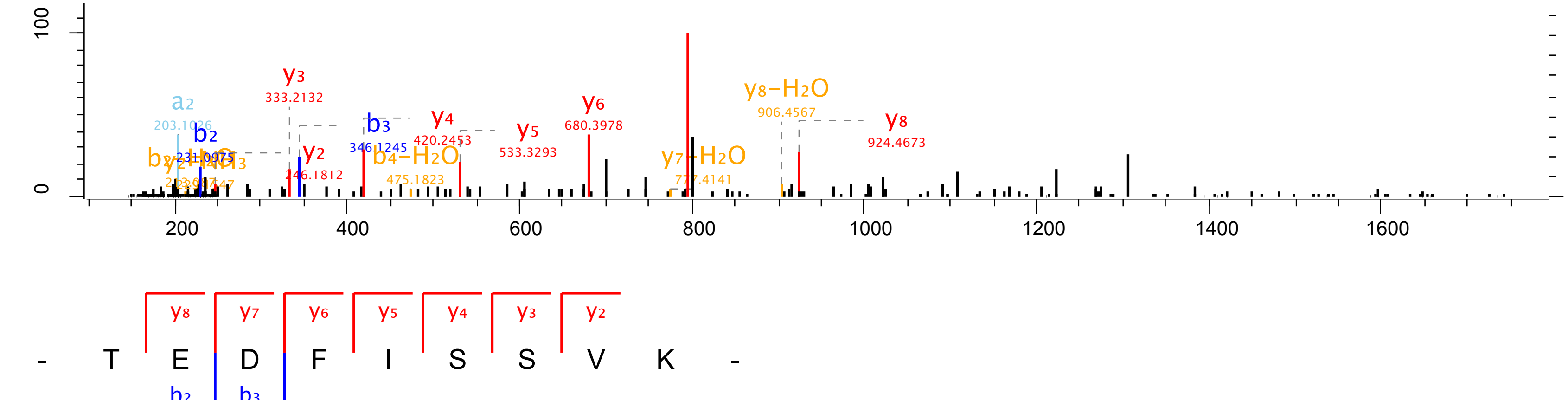

Raw file	Scan	Method	Score	m/z	Gene names
20150306_yeast1_Top_opt_2ug_C1_01_1663	32609	TOF; CID	104.06	513.25	OM14

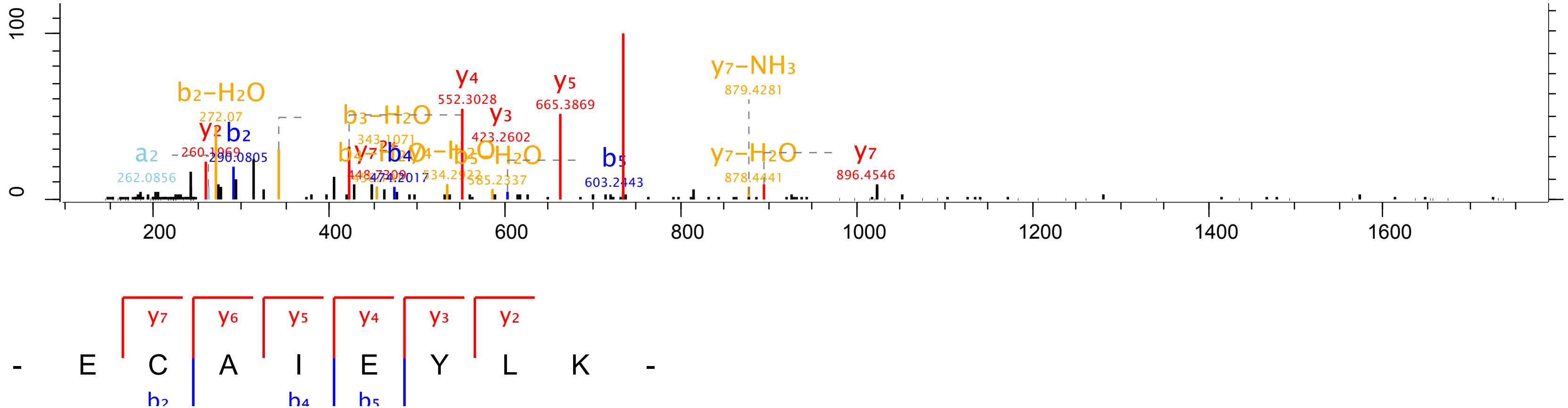

Raw file	Scan	Method	Score	m/z	Gene names
20150306_yeast1_Top_opt_2ug_C1_01_1663	34988	TOF; CID	135.08	1006.48	MGR2

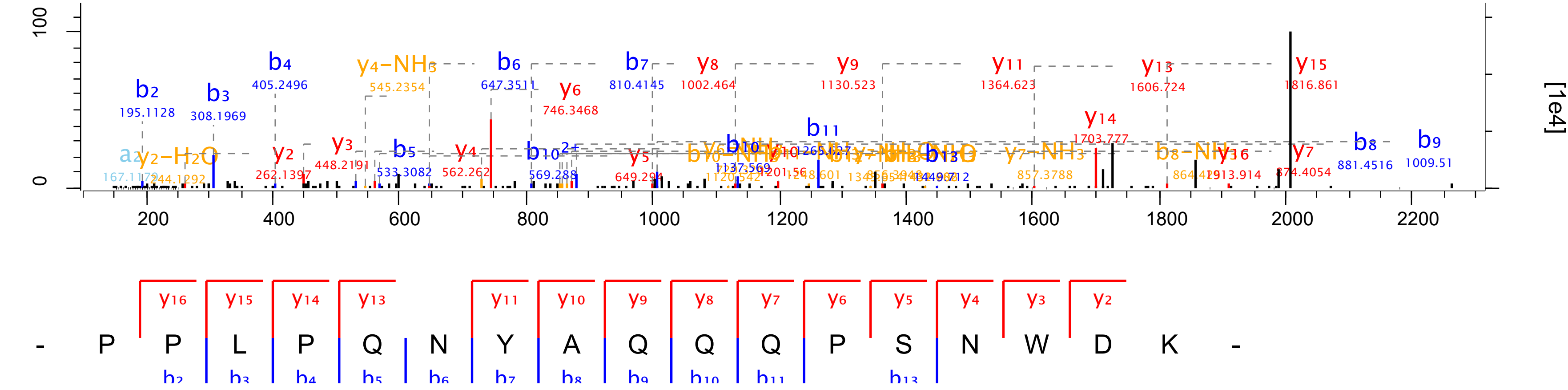

Raw file	Scan	Method	Score	m/z	Gene names
20150306_yeast1_Top_opt_2ug_C1_01_1663	36755	TOF; CID	50.35	458.94	ASE1

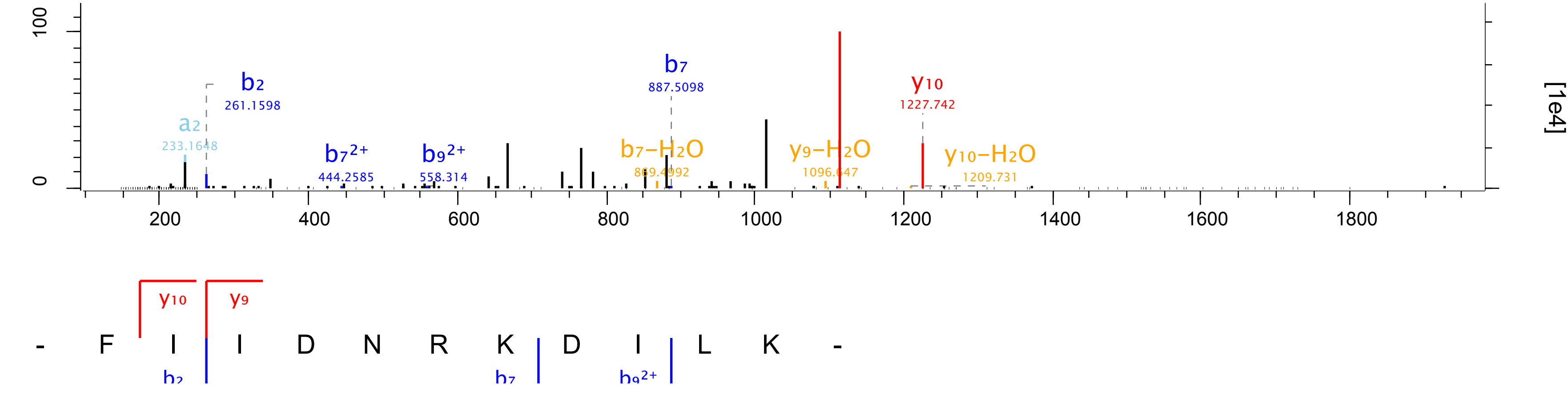

Raw file	Scan	Method	Score	m/z	Gene names
20150306_yeast1_Top_opt_2ug_C1_01_1663	37100	TOF; CID	62.47	458.93	TAD3

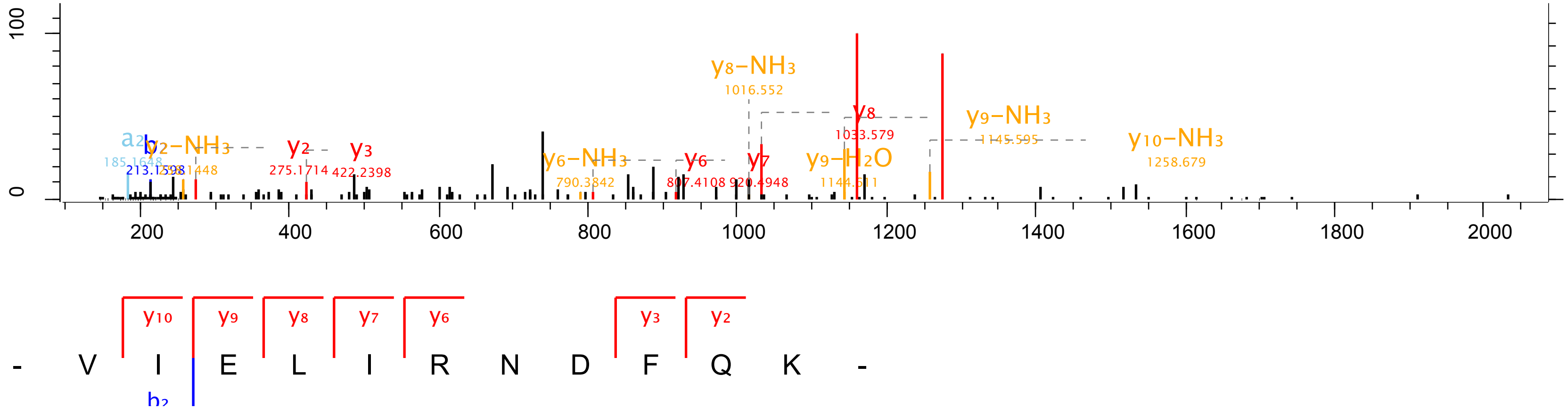

Raw file	Scan	Method	Score	m/z	Gene names
20150306_yeast1_Top_opt_2ug_C1_01_1663	37849	TOF; CID	63.57	450.28	MAM1

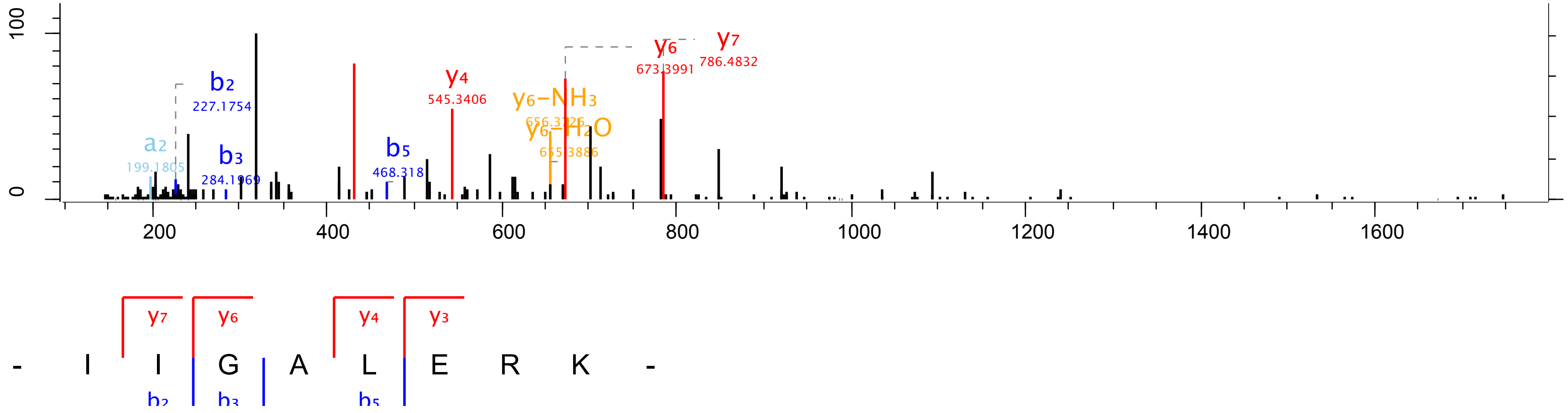

Raw file	Scan	Method	Score	m/z	Gene names
20150306_yeast1_Top_opt_2ug_C1_01_1663	39678	TOF; CID	82.71	513.76	NIS1

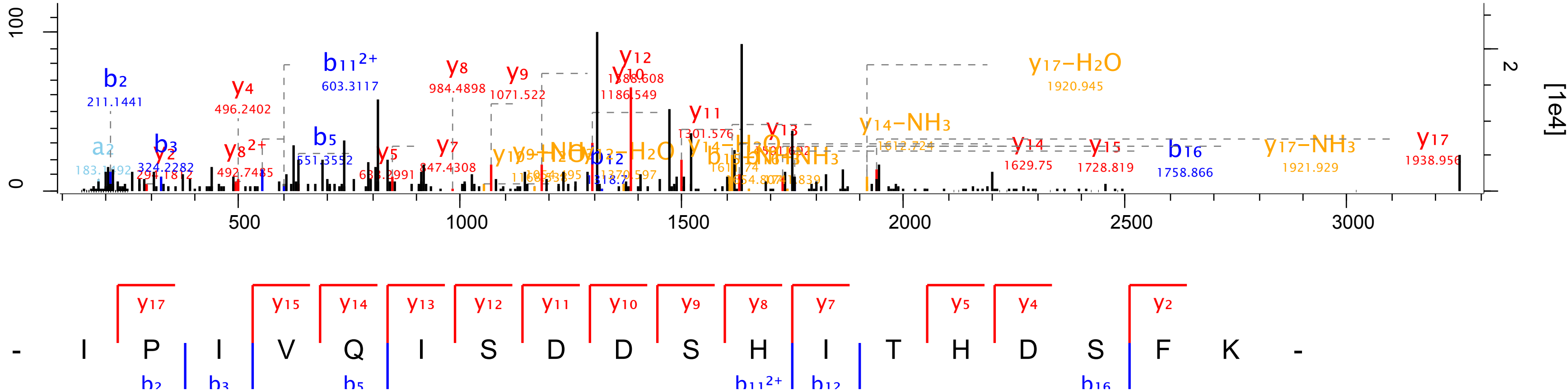

Raw file	Scan	Method	Score	m/z	Gene names
20150306_yeast1_Top_opt_2ug_C1_01_1663	40554	TOF; CID	91.66	400.87	KSH1

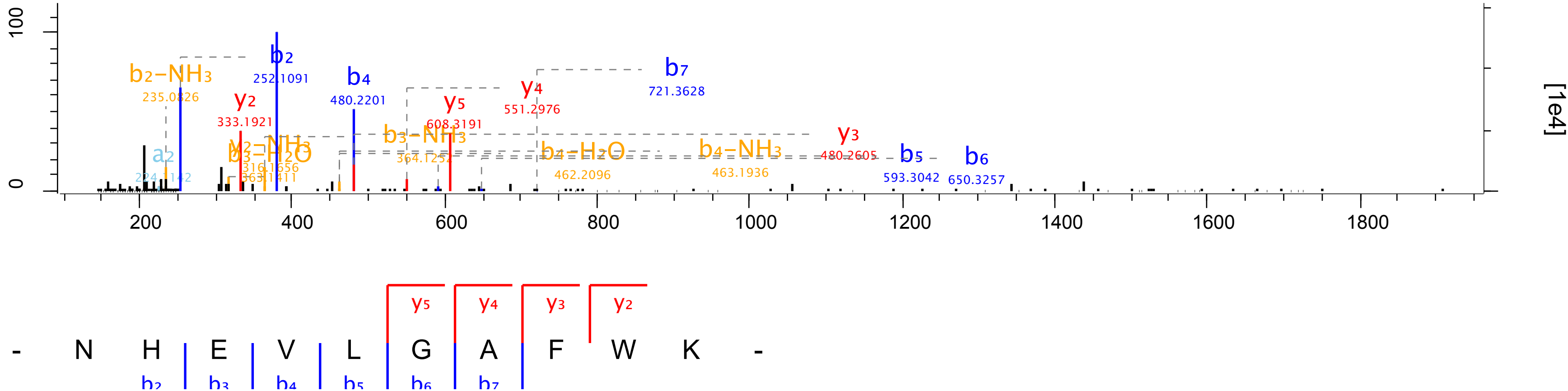

Raw file	Scan	Method	Score	m/z
20150306_yeast1_Top_opt_2ug_C1_01_1663	40784	TOF; CID	56.4	532.8

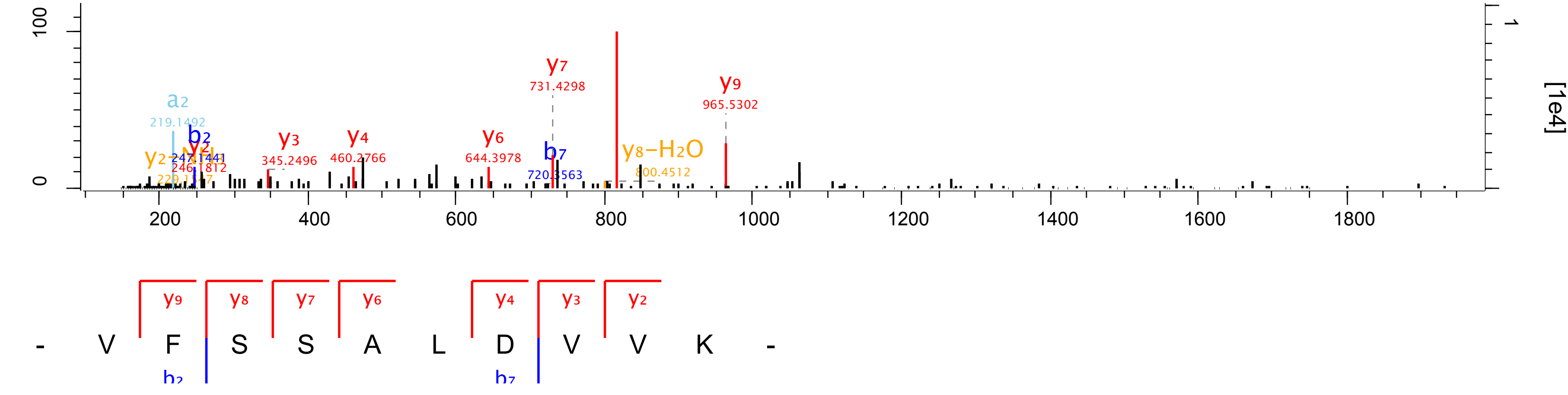

Raw file	Scan	Method	Score	m/z	Gene names
20150306_yeast1_Top_opt_2ug_C1_01_1663	41027	TOF; CID	162.68	477.6	ERV15

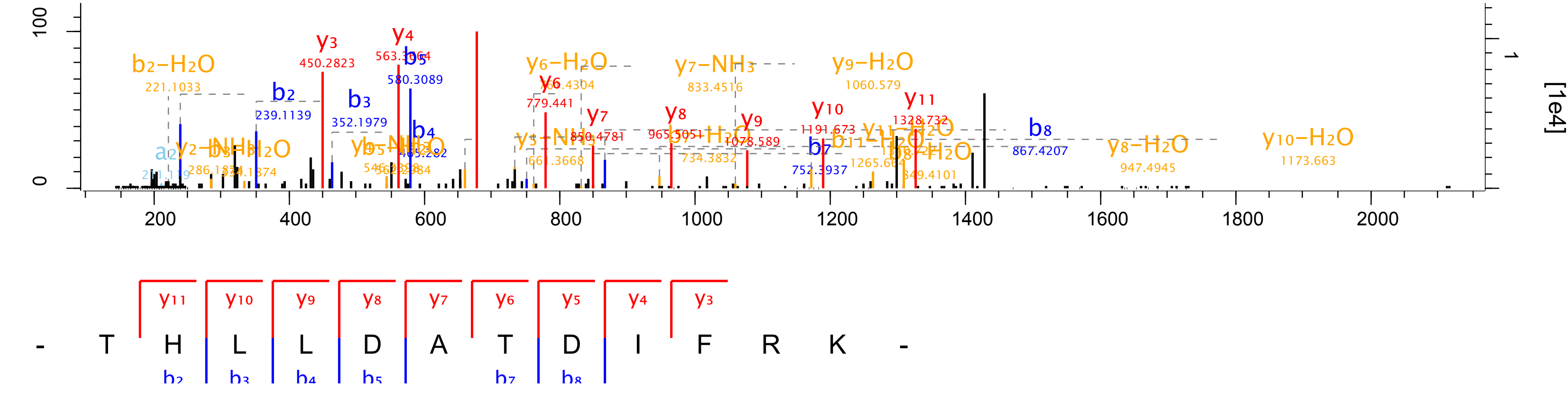

Raw file	Scan	Method	Score	m/z	Gene names
20150306_yeast1_Top_opt_2ug_C1_01_1663	41394	TOF; CID	106.67	645.34	YFH1

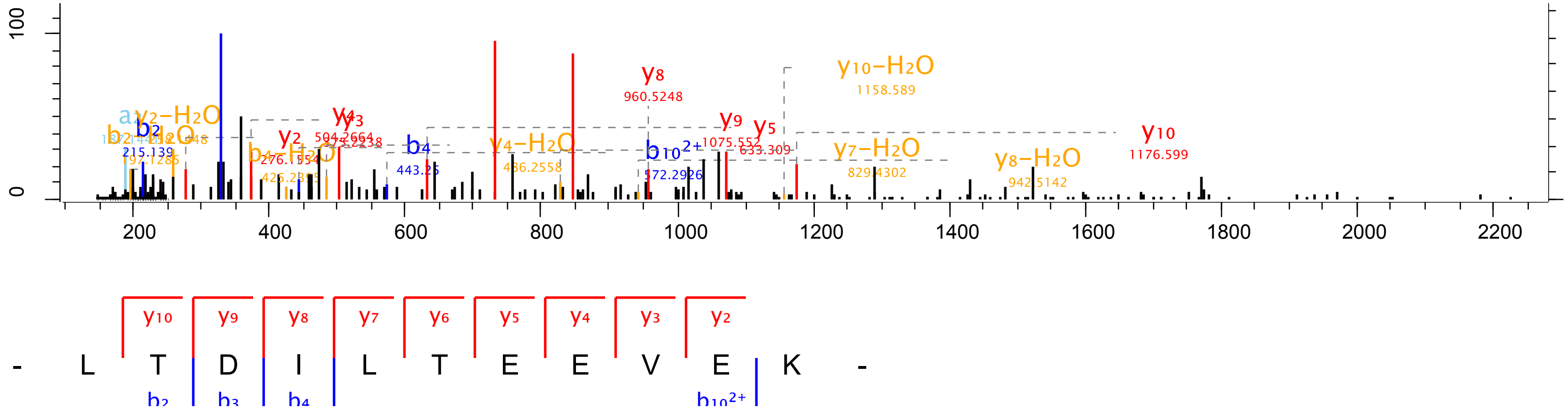

Raw file	Scan	Method	Score	m/z	Gene names
20150306_yeast1_Top_opt_2ug_C1_01_1663	42729	TOF; CID	98.01	439.77	RAM2

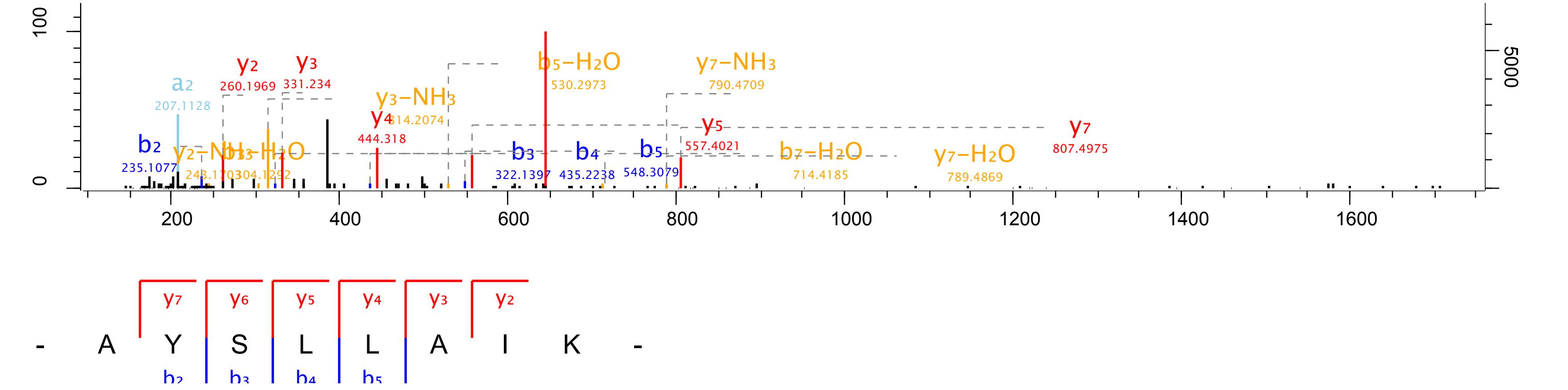

Raw file	Scan	Method	Score	m/z	Gene names
20150306_yeast1_Top_opt_2ug_C1_01_1663	42805	TOF; CID	87.51	645.08	AUR1

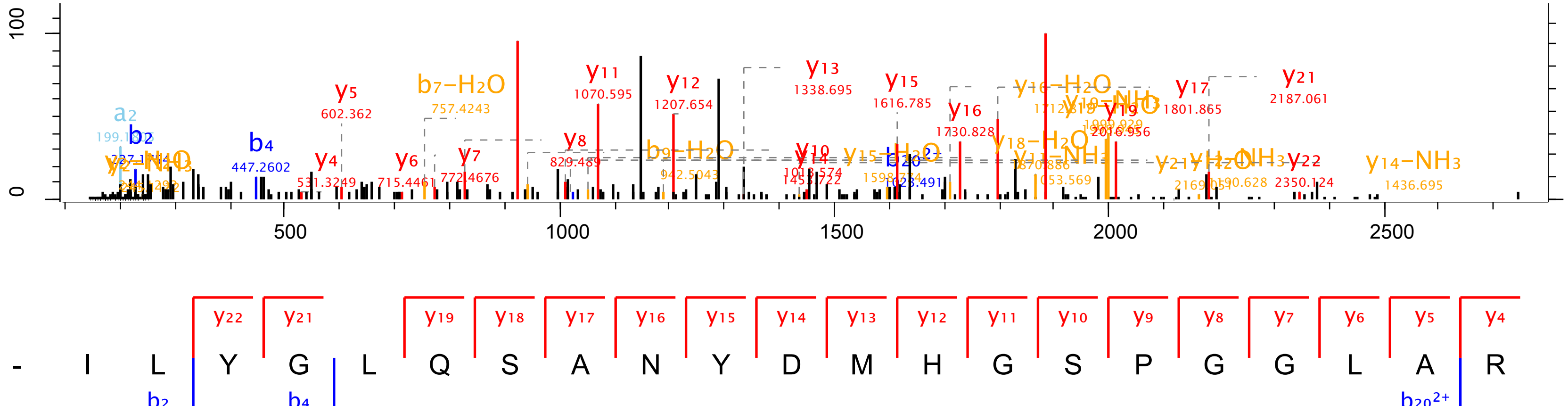

Raw file	Scan	Method	Score	m/z	Gene names
20150306_yeast1_Top_opt_2ug_C1_01_1663	42984	TOF; CID	61.34	548.81	PMT5

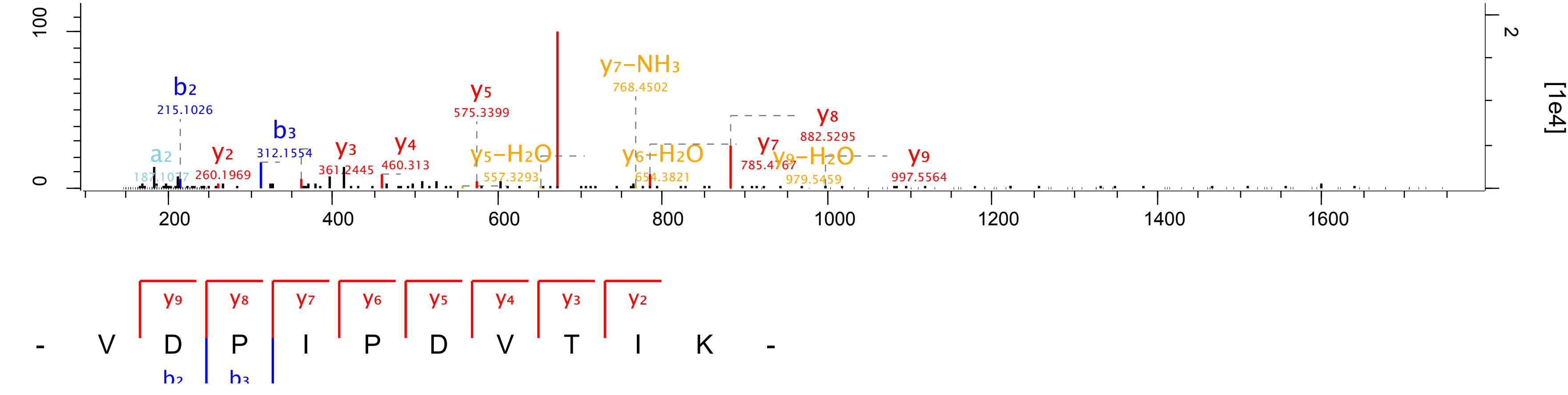

Raw file	Scan	Method	Score	m/z
20150306_yeast1_Top_opt_2ug_C1_01_1663	43550	TOF; CID	81.62	666.32

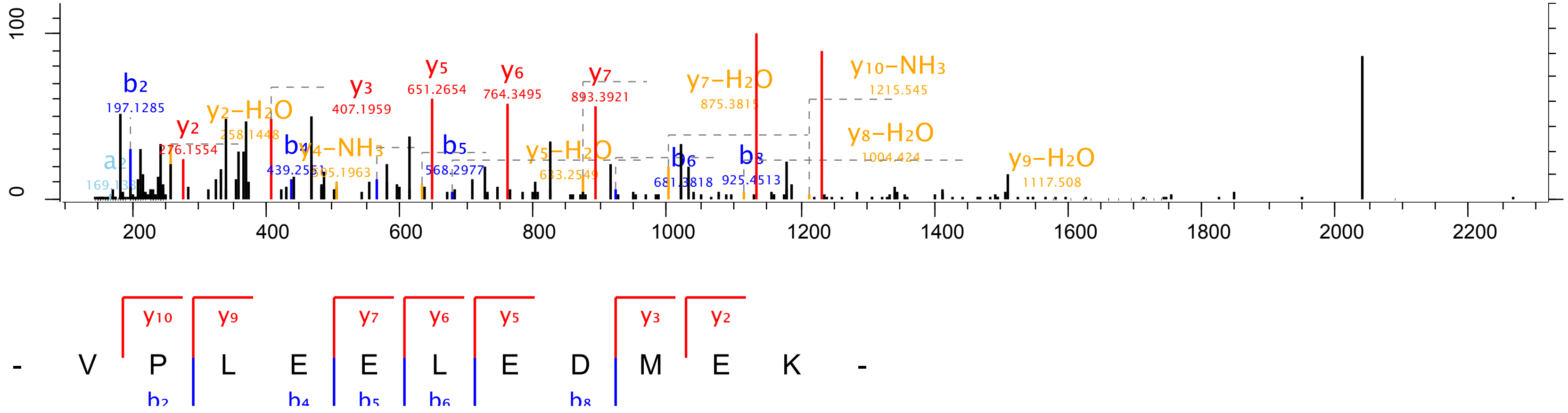

Raw file	Scan	Method	Score	m/z	Gene names
20150306_yeast1_Top_opt_2ug_C1_01_1663	44009	TOF; CID	66.27	469.96	ALG8

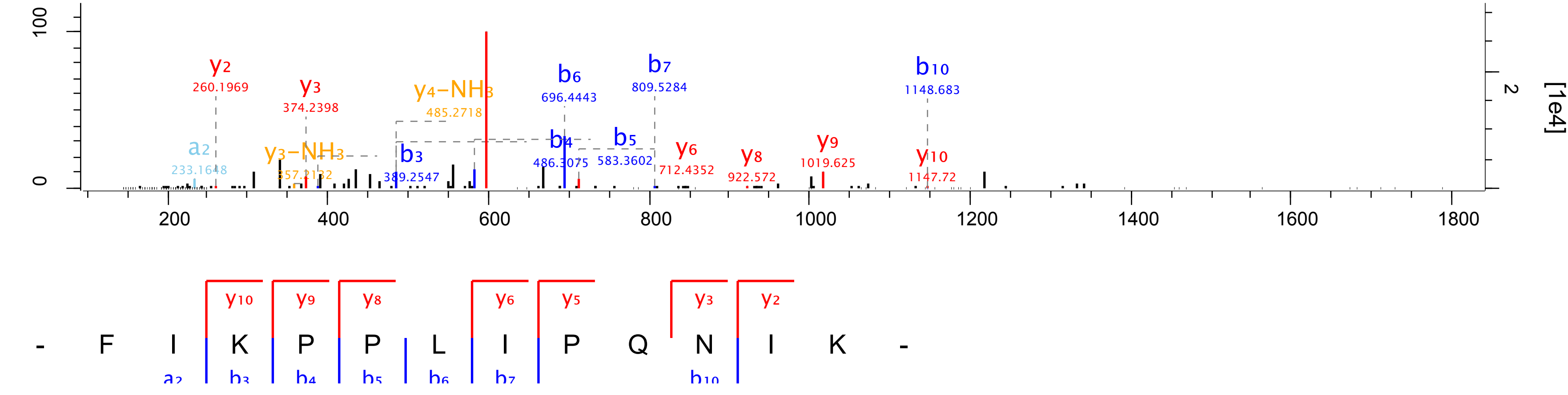

Raw file	Scan	Method	Score	m/z	Gene names
20150306_yeast1_Top_opt_2ug_C1_01_1663	49601	TOF; CID	103.08	604.86	SAN1

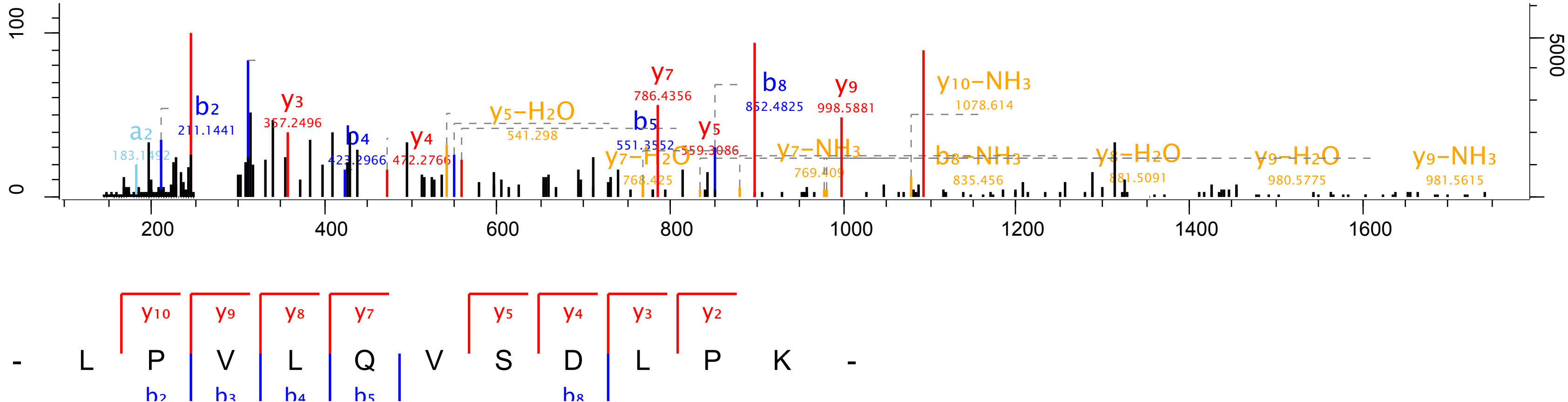

Raw file	Scan	Method	Score	m/z	Gene names
20150306_yeast1_Top_opt_2ug_C1_01_1663	60070	TOF; CID	102.89	917.02	VMA11

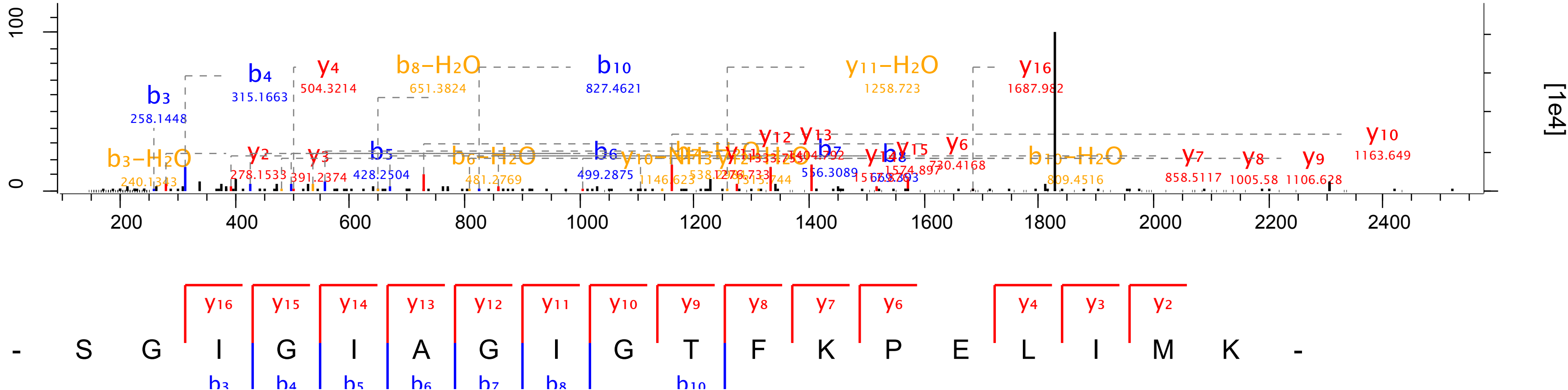

Raw file	Scan	Method	Score	m/z	Gene names
20150306_yeast1_Top_opt_2ug_C1_01_1663	60574	TOF; CID	33.32	1017.82	BAP3

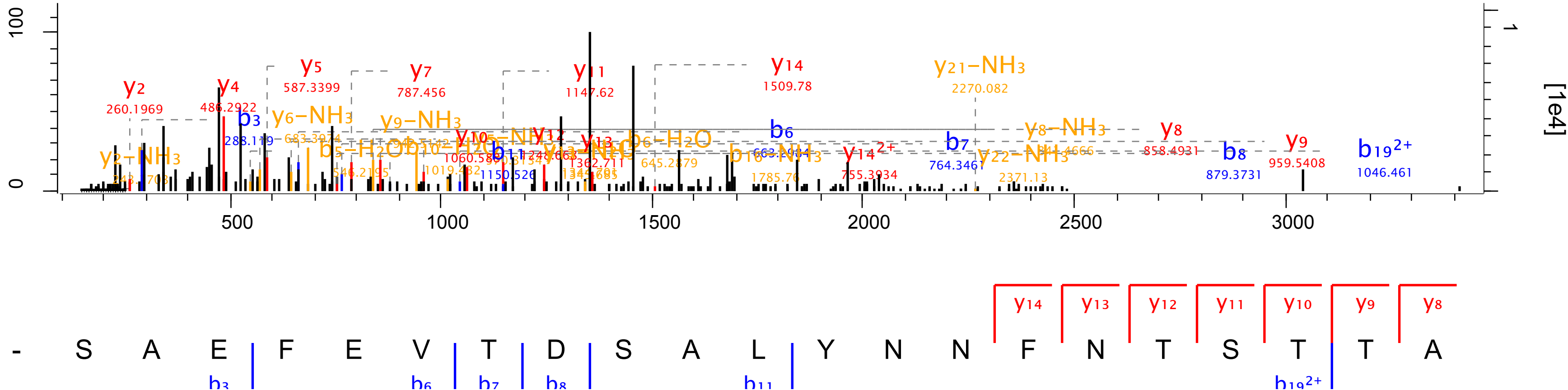

Raw file	Scan	Method	Score	m/z	Gene names
20150306_yeast1_Top_opt_2ug_C1_01_1663	61595	TOF; CID	103.41	1004.03	VMA3

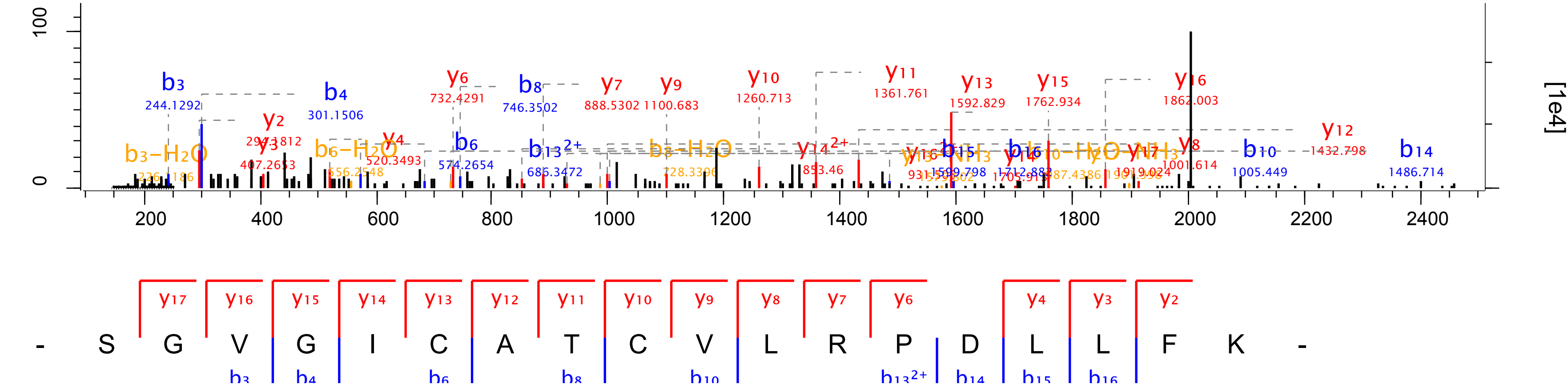

Raw file	Scan	Method	Score	m/z	Gene names
20150306_yeast1_Top_opt_2ug_C1_01_1663	61632	TOF; CID	52.58	709.37	ASI1

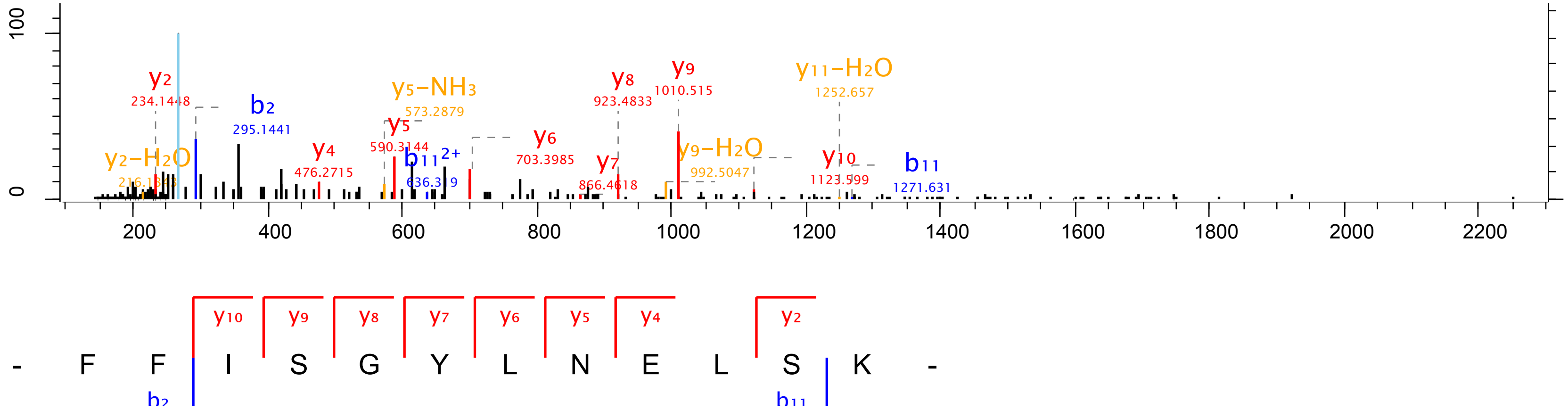

Raw file	Scan	Method	Score	m/z	Gene names
20150306_yeast1_Top_opt_2ug_C1_01_1663	67173	TOF; CID	90.91	1188.54	TRM12

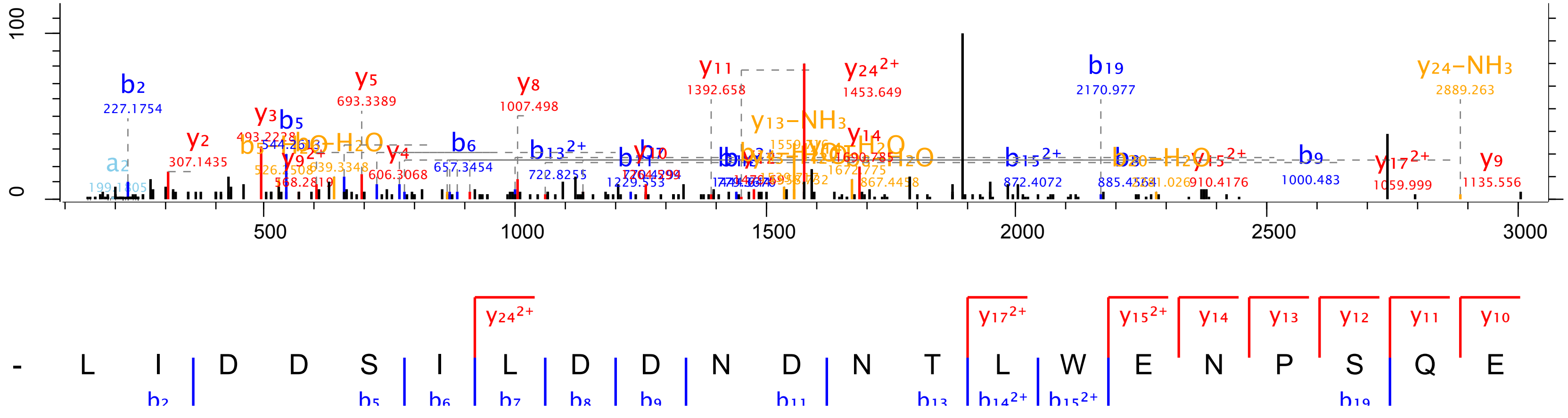

Raw file	Scan	Method	Score	m/z	Gene names
20150306_yeast1_Top_opt_2ug_C1_01_1663	68892	TOF; CID	94.69	683.37	URM1

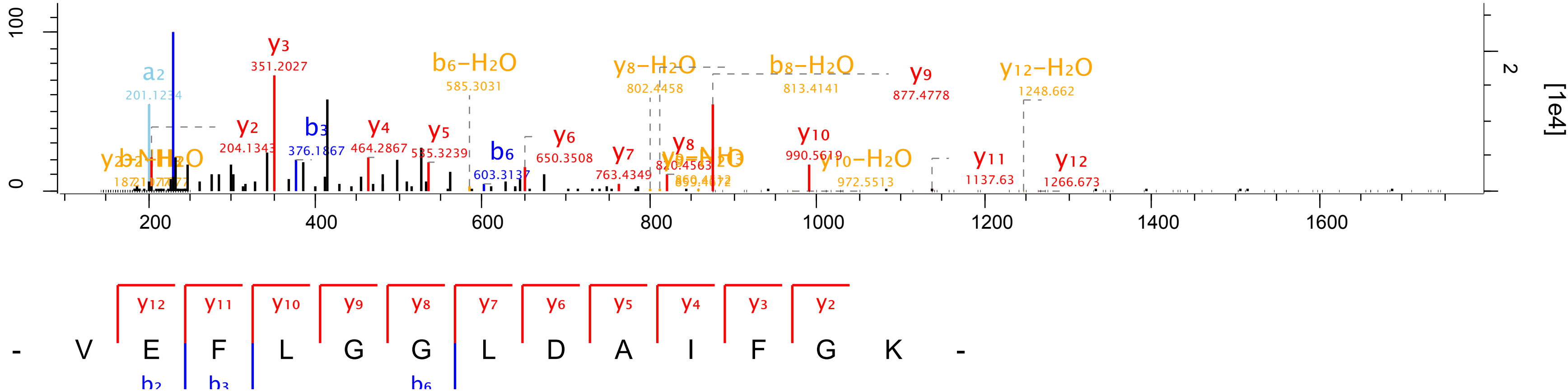

Raw file	Scan	Method	Score	m/z	Gene names
20150306_yeast1_Top_opt_2ug_C1_01_1663	70540	TOF; CID	44.24	765.4	BET5

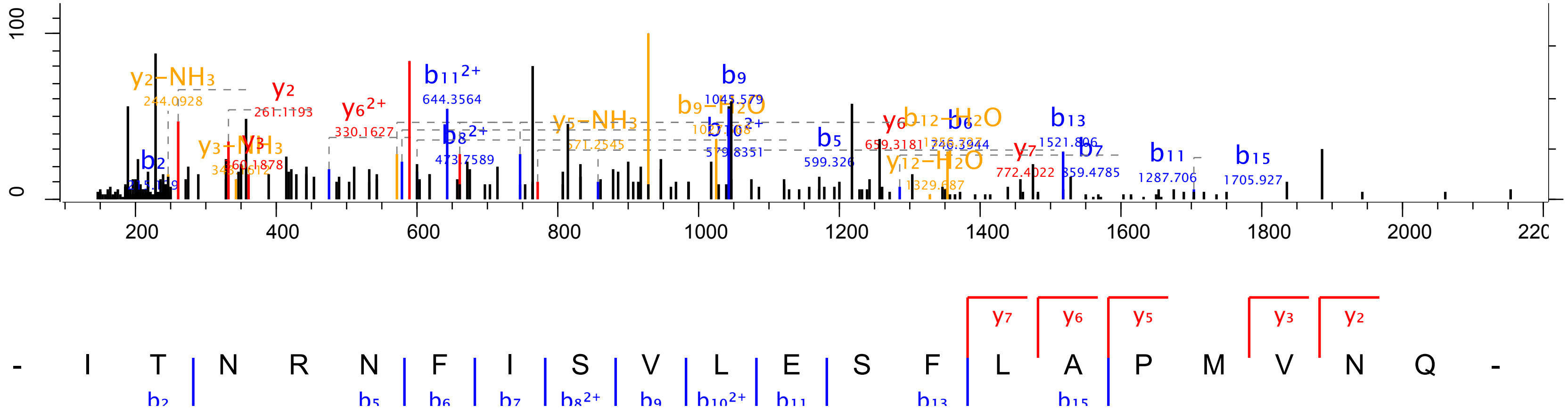

Raw file	Scan	Method	Score	m/z	Gene names
20150306_yeast1_Top_opt_2ug_C1_01_1663	70690	TOF; CID	148.52	836.45	YPR114W

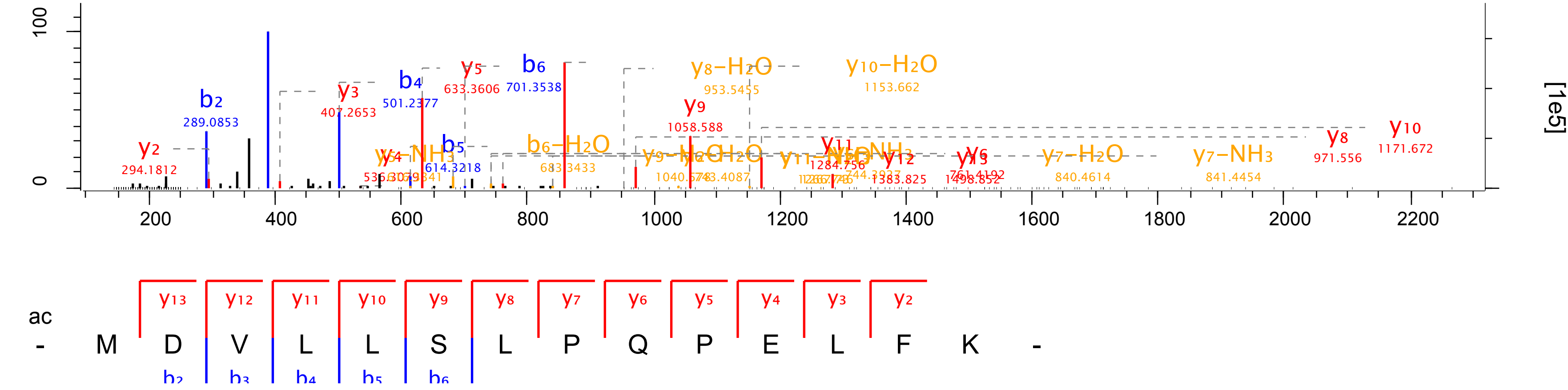

Raw file	Scan	Method	Score	m/z	Gene names
20150306_yeast1_Top_opt_2ug_C1_01_1666	7540	TOF; CID	55.65	366.19	ELG1

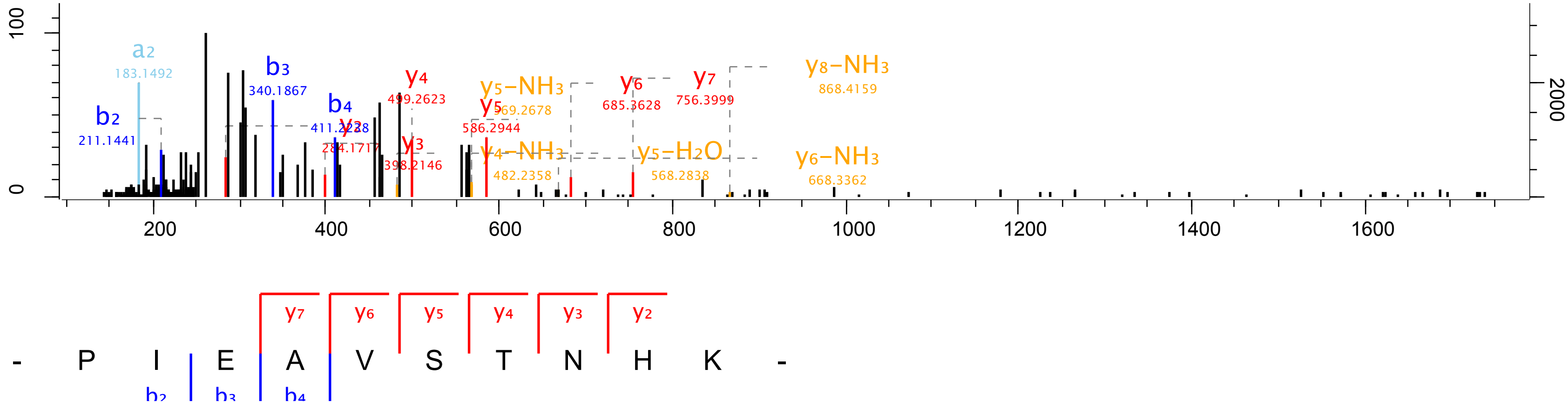

Raw file	Scan	Method	Score	m/z	Gene names
20150306_yeast1_Top_opt_2ug_C1_01_1666	11210	TOF; CID	51.98	413.22	SKP2

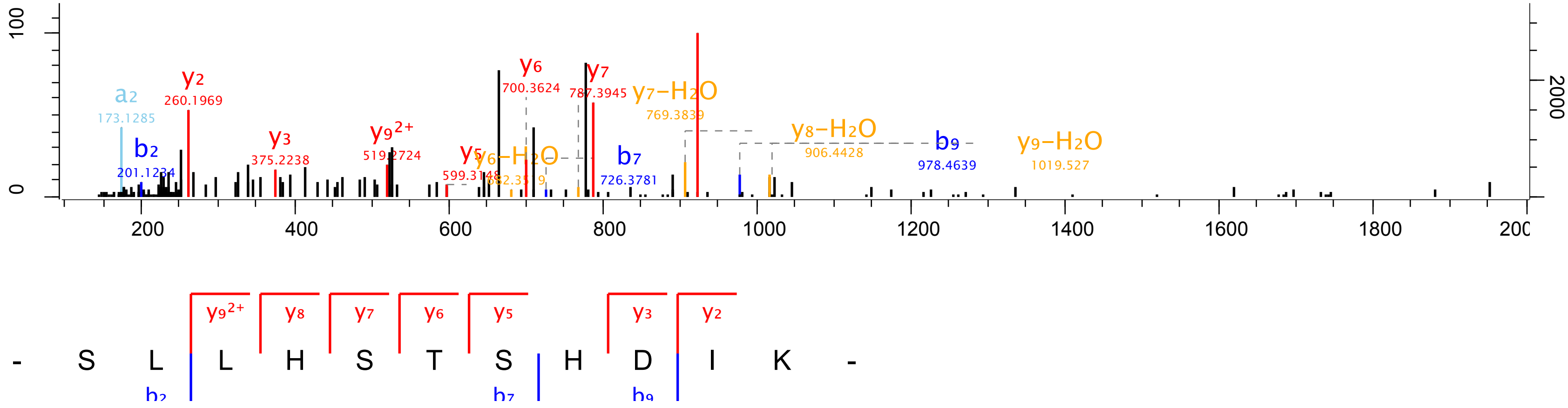

Raw file	Scan	Method	Score	m/z	Gene names
20150306_yeast1_Top_opt_2ug_C1_01_1666	11887	TOF; CID	73.83	597.3	SAS3

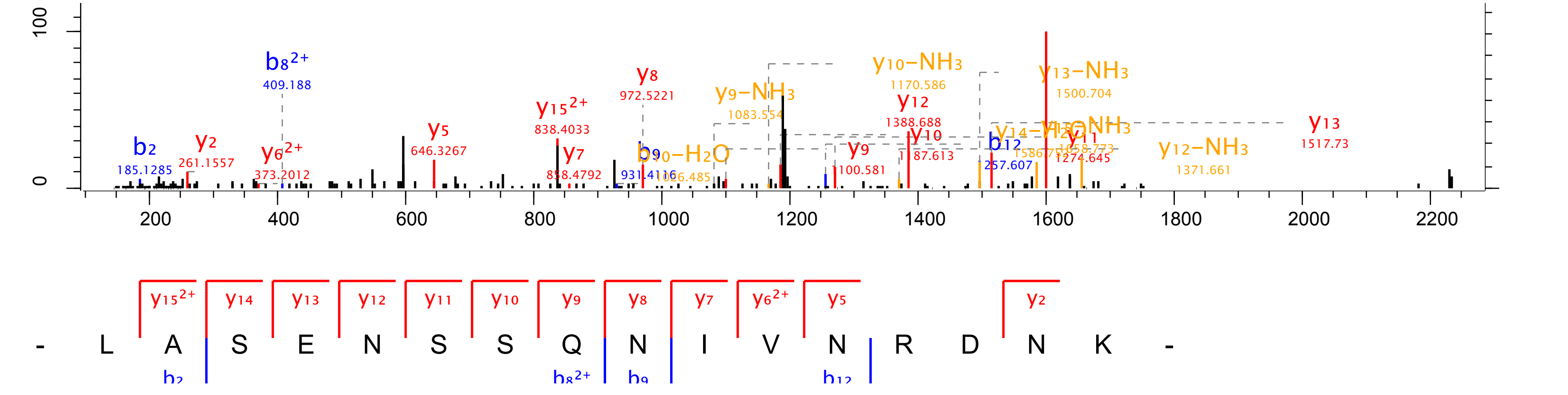

Raw file	Scan	Method	Score	m/z	Gene names
20150306_yeast1_Top_opt_2ug_C1_01_1666	14259	TOF; CID	87.75	365.23	RPS28A;RPS28B

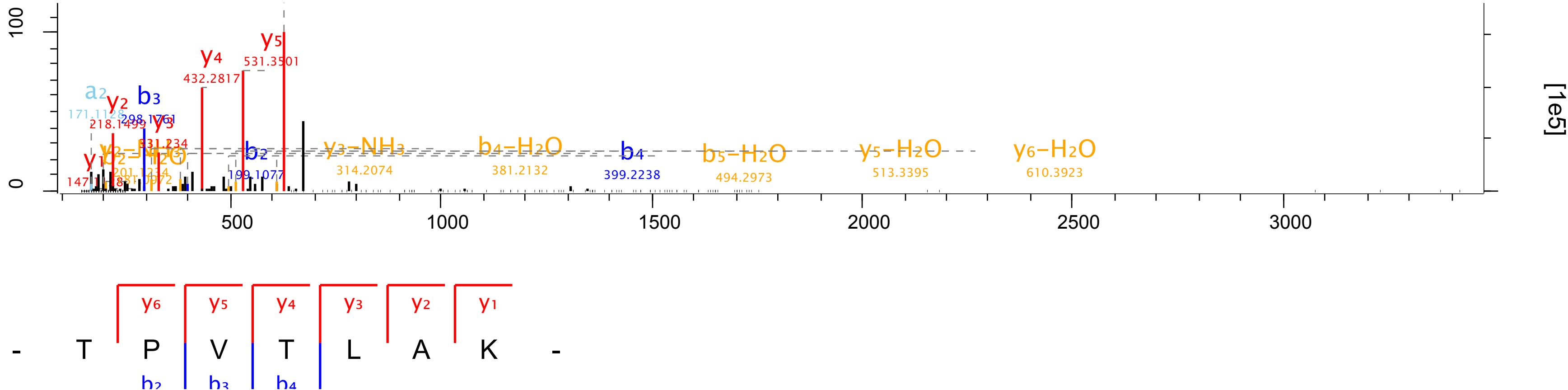

Raw file	Scan	Method	Score	m/z	Gene names
20150306_yeast1_Top_opt_2ug_C1_01_1666	14437	TOF; CID	112.83	543.3	GPI14

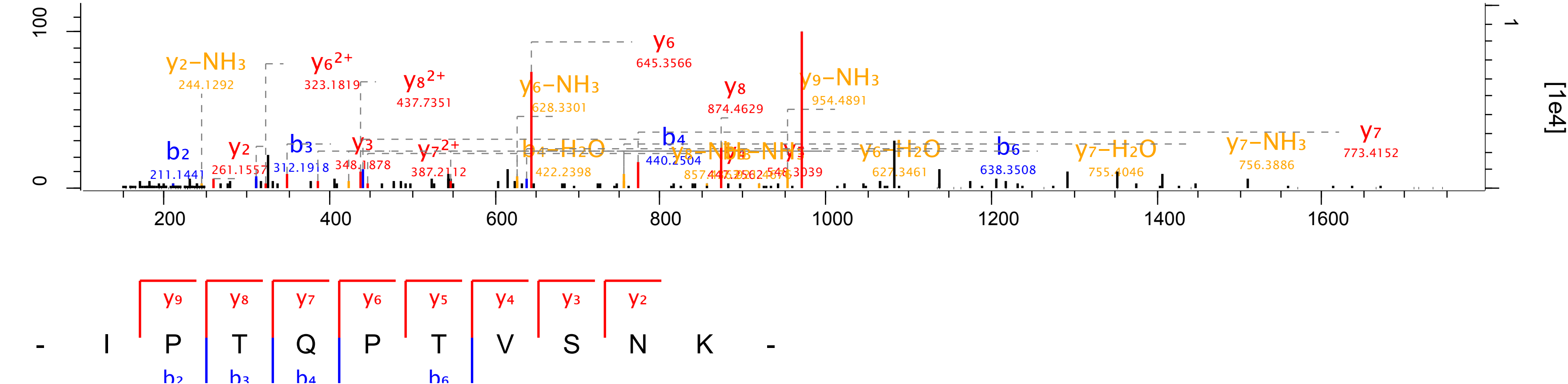

Raw file	Scan	Method	Score	m/z	Gene names
20150306_yeast1_Top_opt_2ug_C1_01_1666	14735	TOF; CID	91.87	502.29	RAD10

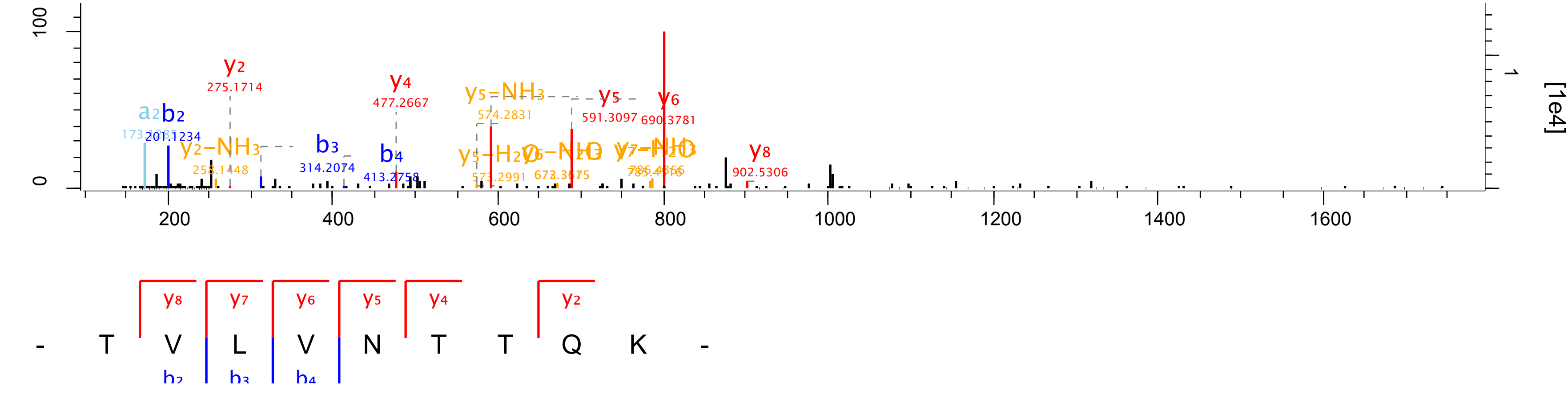

Raw file	Scan	Method	Score	m/z	Gene names
20150306_yeast1_Top_opt_2ug_C1_01_1666	16495	TOF; CID	87.08	548.28	MRPS12

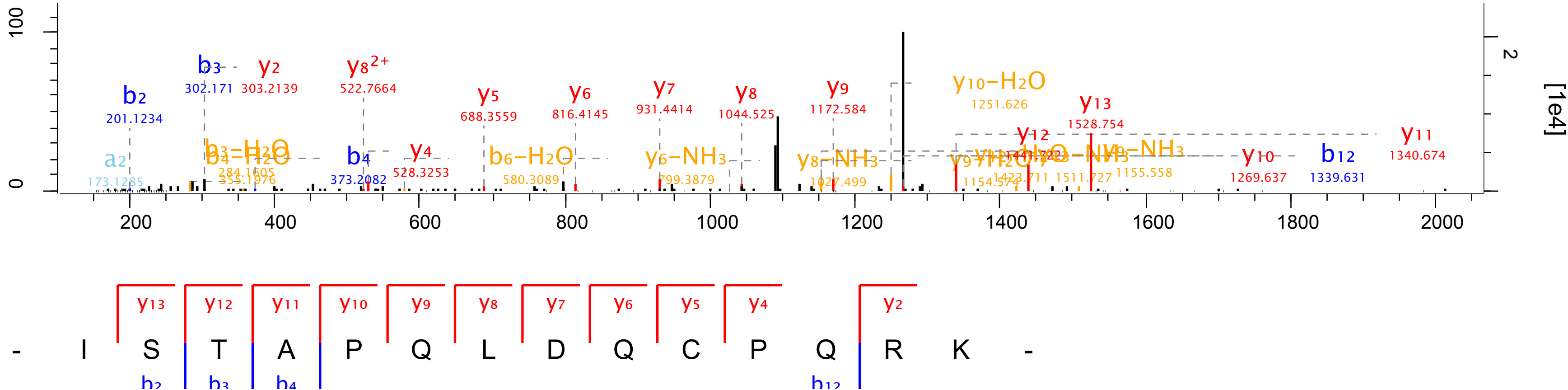

Raw file	Scan	Method	Score	m/z	Gene names
20150306_yeast1_Top_opt_2ug_C1_01_1666	19613	TOF; CID	194.67	856.89	SHR3

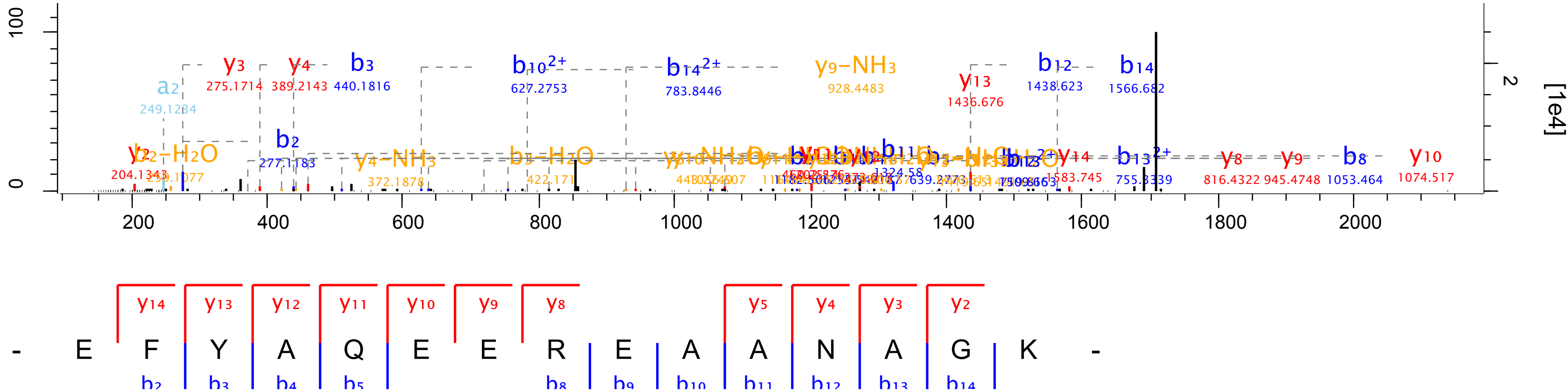

Raw file	Scan	Method	Score	m/z	Gene names
20150306_yeast1_Top_opt_2ug_C1_01_1666	19922	TOF; CID	90.7	663.84	CDC15

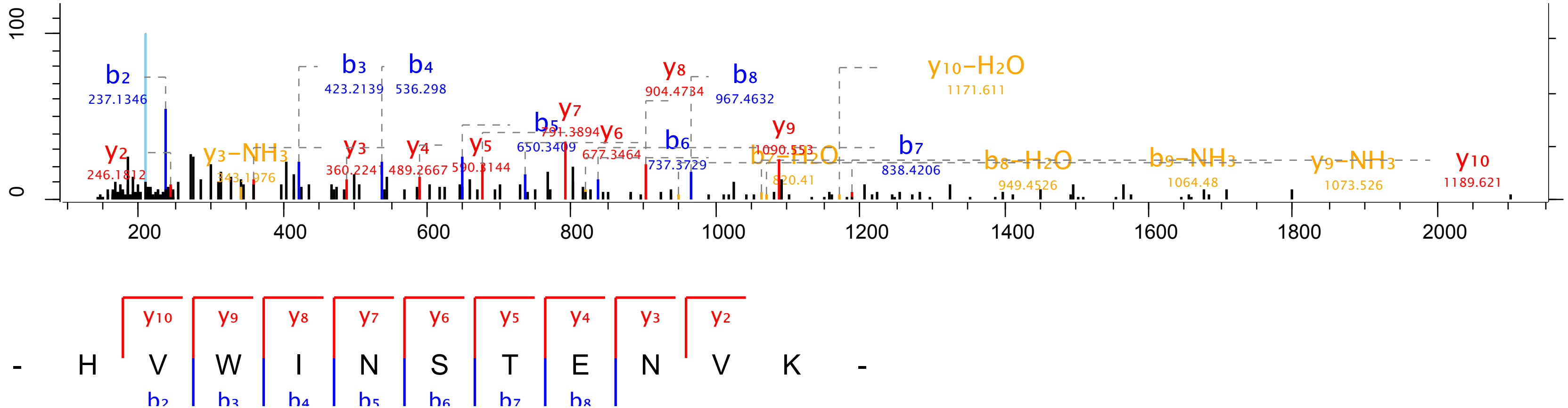

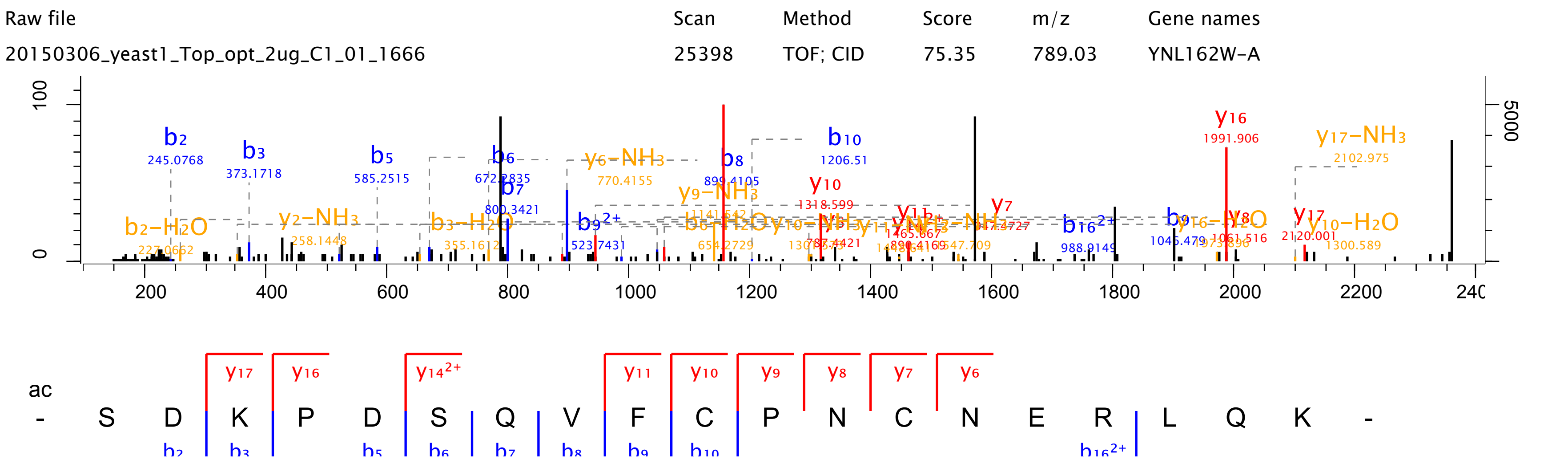
Raw file
20150306_yeast1_Top_opt_2ug_C1_01_1666
Scan
25398
Method
TOF; CID
Score
75.35
m/z
789.03
Gene names
YNL162W-A
100
0
5000
b2 245.0768
b3 373.1718
b5 585.2515
b6 672.2835
b7 800.3421
b8 899.4105
b10 1206.51
b9 2+ 523.7431
b16 2+ 988.9149
y16 1991.906
y17 2120.001
y10 1318.599
y17-NH3 2102.975
y6-NH3 770.4155
y9-NH3 1141.542
y2-NH3 258.1448
b2-H2O 227.0662
b3-H2O 355.1612
y10-H2O 1300.589
200
400
600
800
1000
1200
1400
1600
1800
2000
2200
ac
- S D K P D S Q V F C P N C N E R L Q K -
y17 y16 y14 2+ y11 y10 y9 y8 y7 y6
b2 b3 b5 b6 b7 b8 b9 b10 b16 2+

Raw file	Scan	Method	Score	m/z	Gene names
20150306_yeast1_Top_opt_2ug_C1_01_1666	27340	TOF; CID	81.66	812.87	HLR1

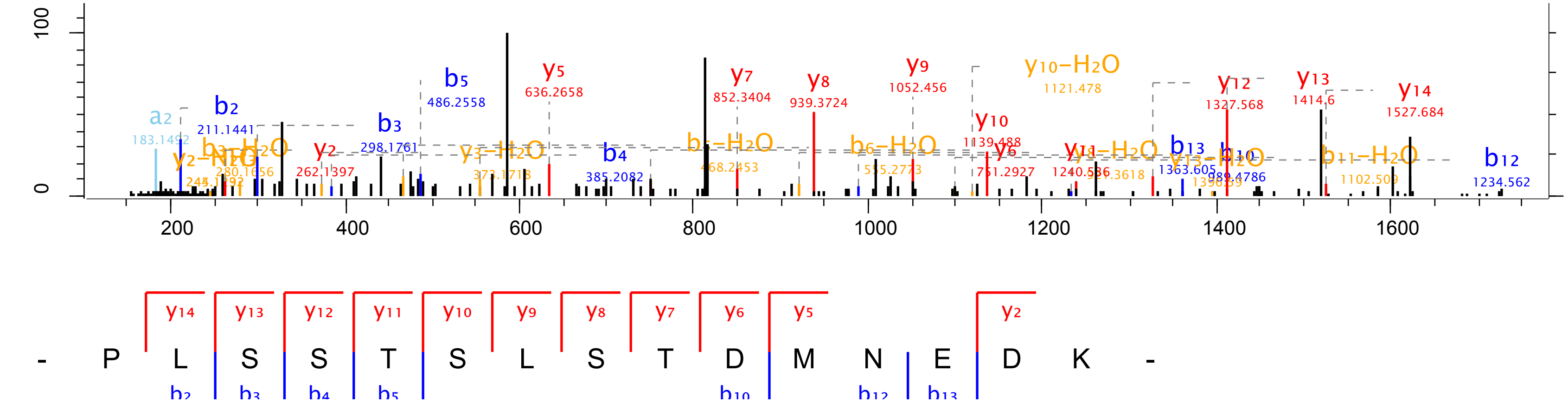

Raw file	Scan	Method	Score	m/z	Gene names
20150306_yeast1_Top_opt_2ug_C1_01_1666	29042	TOF; CID	57.53	536.8	IML3

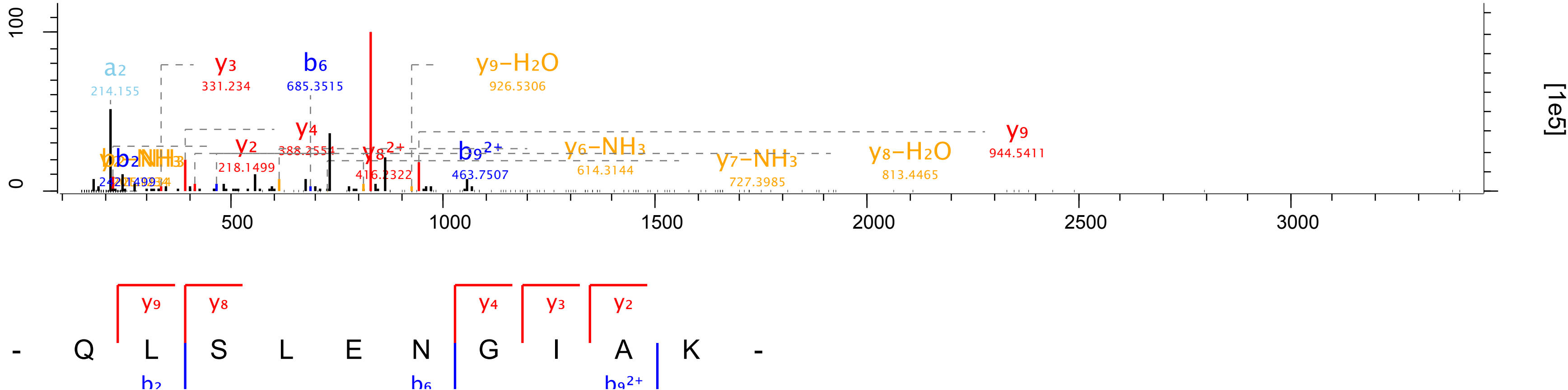

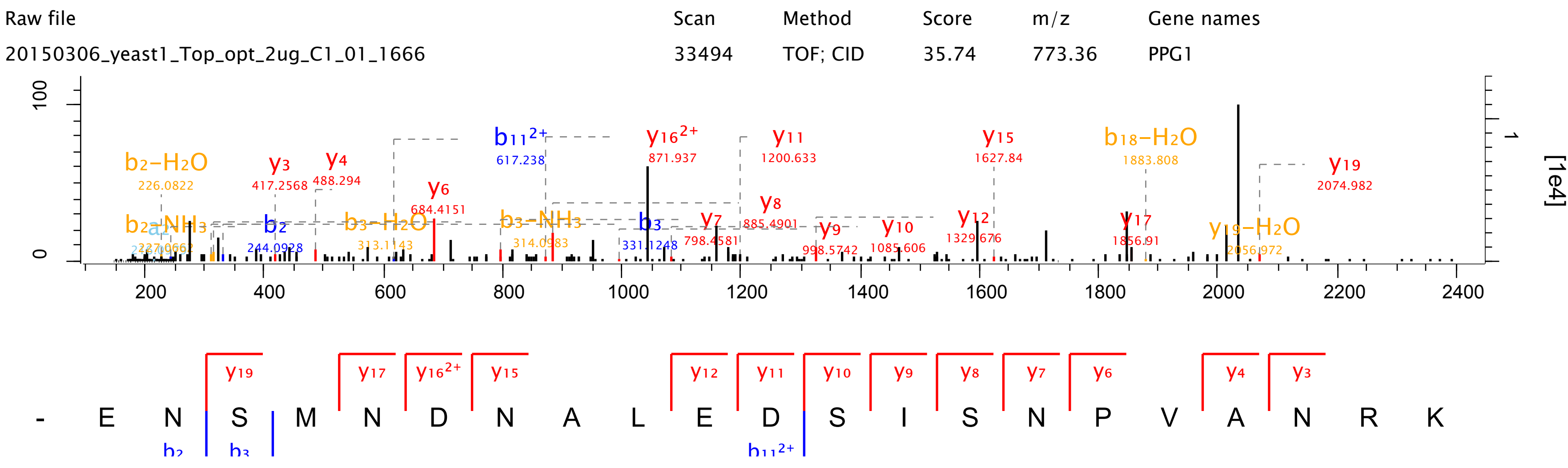
Raw file
20150306_yeast1_Top_opt_2ug_C1_01_1666
Scan
33494
Method
TOF; CID
Score
35.74
m/z
773.36
Gene names
PPG1
100
0
[1e4]
1
b2−H2O 226.0822
y3 417.2568
y4 488.294
b11 2+ 617.238
y6 684.4151
y16 2+ 871.937
y11 1200.633
y8 885.4901
y7 798.4581
y15 1627.84
y12 1329.676
b18−H2O 1883.808
y17 1856.91
y19 2074.982
y19−H2O 2056.972
b2 244.0928
b3−H2O 313.1143
b3−NH3 314.0983
b3 331.1248
y9 998.5742
y10 1085.606
200
400
600
800
1000
1200
1400
1600
1800
2000
2200
2400
- E N S M N D N A L E D S I S N P V A N R K
y19 y17 y16 2+ y15 y12 y11 y10 y9 y8 y7 y6 y4 y3
b2 b3 b11 2+

Raw file	Scan	Method	Score	m/z	Gene names
20150306_yeast1_Top_opt_2ug_C1_01_1666	33787	TOF; CID	81.3	467.27	TCA17

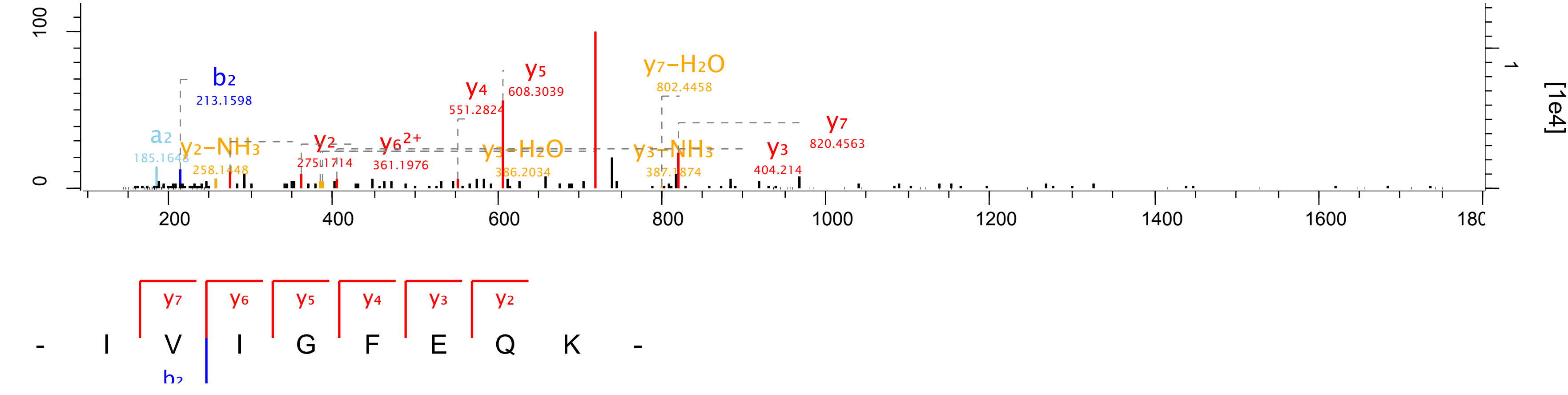

Raw file	Scan	Method	Score	m/z	Gene names
20150306_yeast1_Top_opt_2ug_C1_01_1666	34607	TOF; CID	97.69	535.62	AIF1

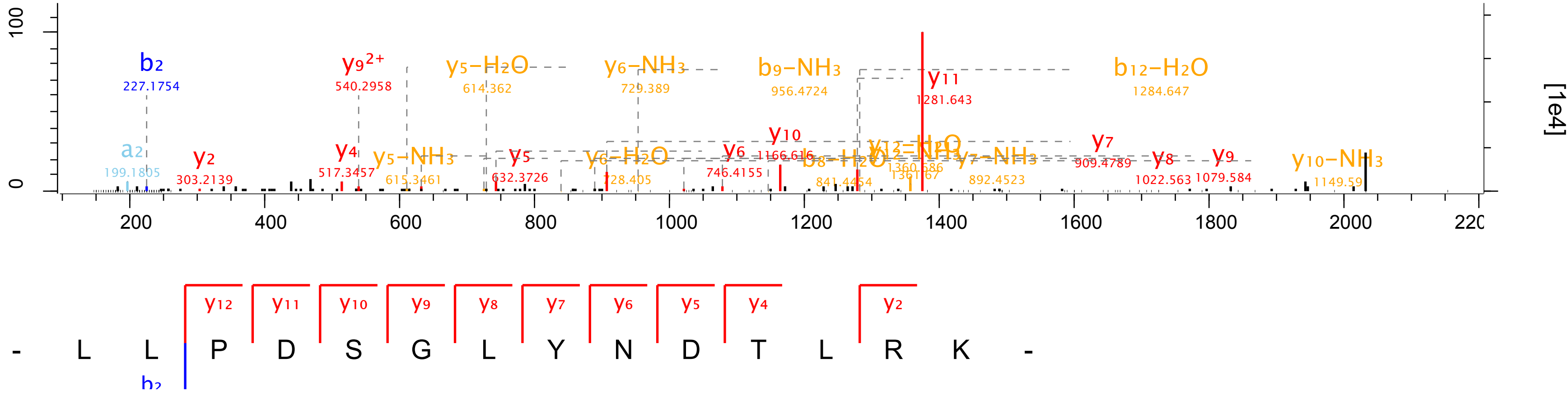

Raw file	Scan	Method	Score	m/z	Gene names
20150306_yeast1_Top_opt_2ug_C1_01_1666	40015	TOF; CID	63.57	488.28	IRC10

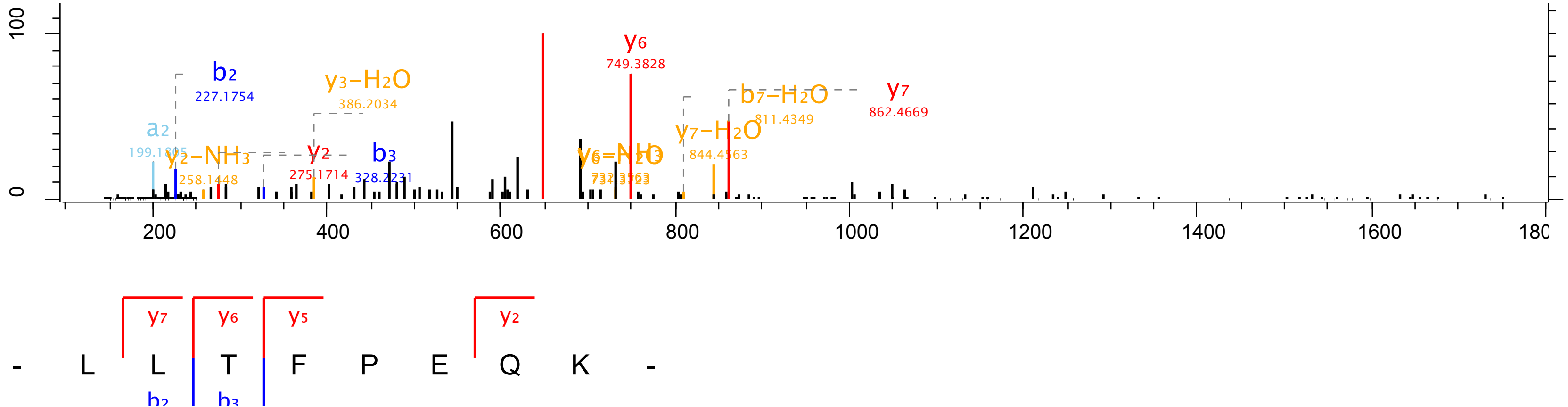

Raw file	Scan	Method	Score	m/z	Gene names
20150306_yeast1_Top_opt_2ug_C1_01_1666	40948	TOF; CID	50.09	760.36	MCM10

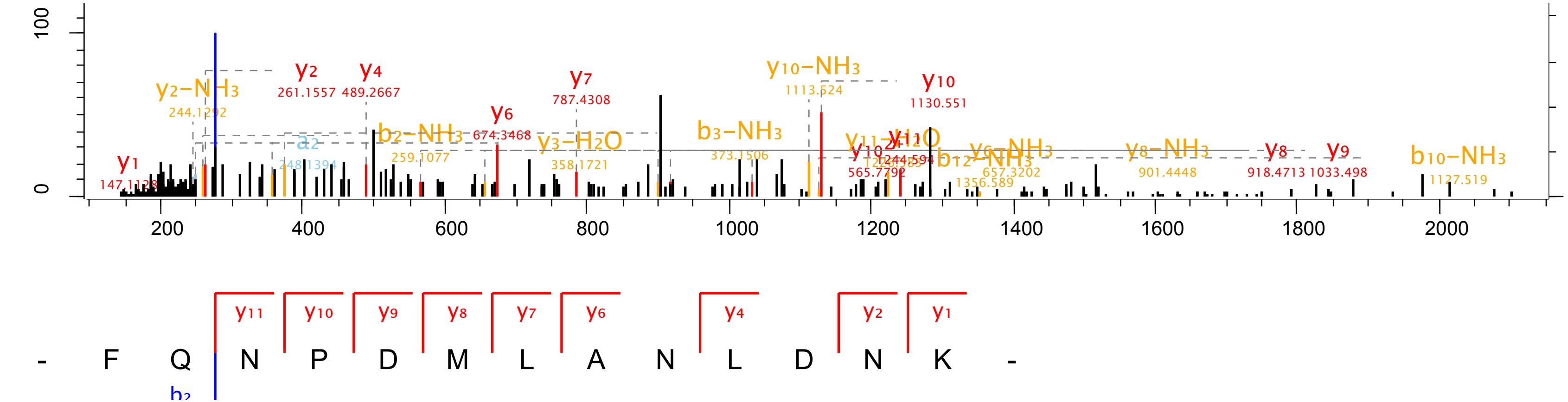

Raw file	Scan	Method	Score	m/z	Gene names
20150306_yeast1_Top_opt_2ug_C1_01_1666	43177	TOF; CID	129.42	518.79	TRS20

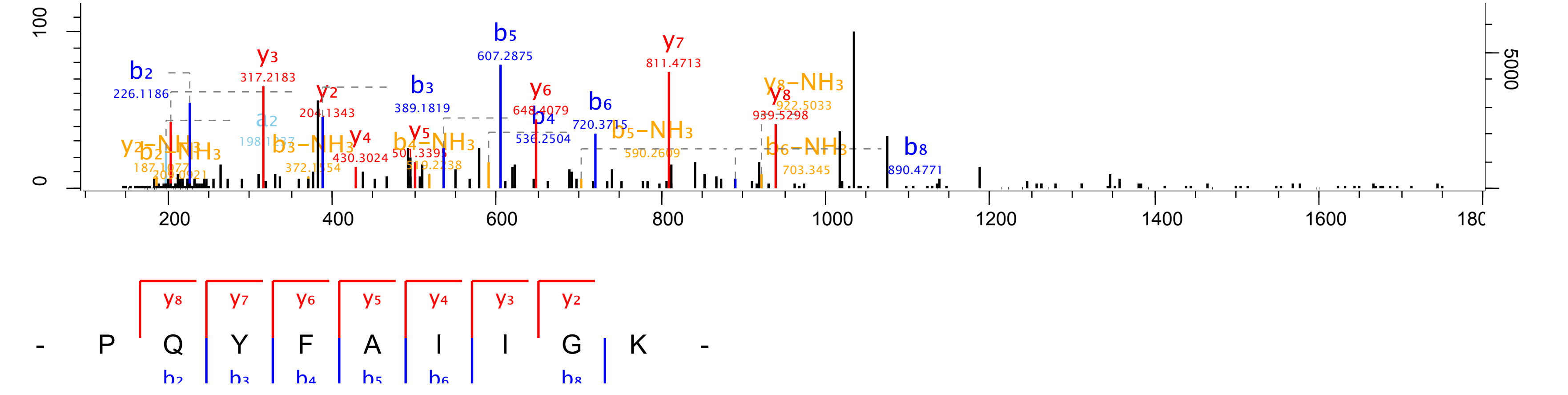

Raw file	Scan	Method	Score	m/z	Gene names
20150306_yeast1_Top_opt_2ug_C1_01_1666	43962	TOF; CID	95.62	551.82	SNM1

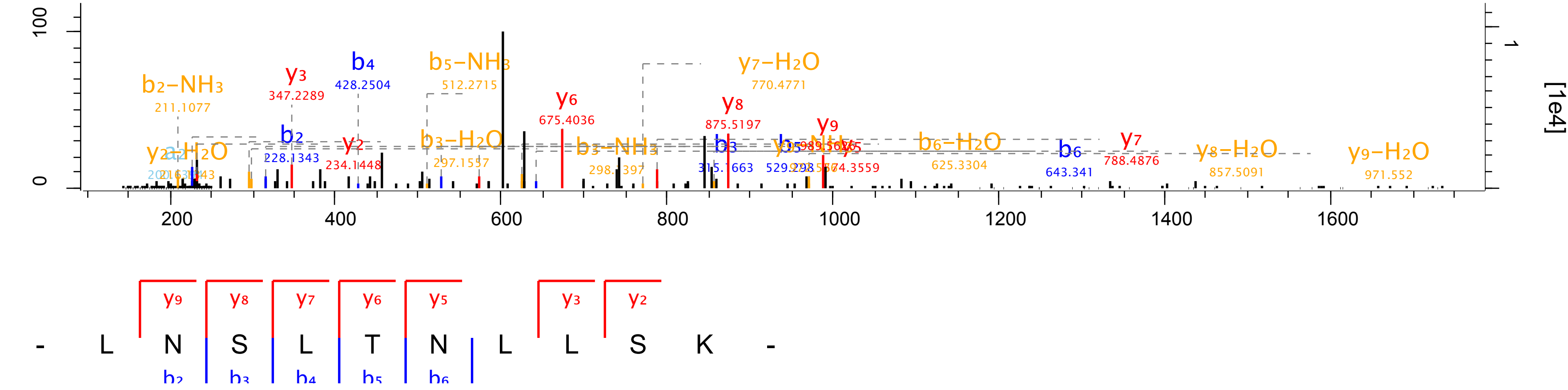

Raw file	Scan	Method	Score	m/z	Gene names
20150306_yeast1_Top_opt_2ug_C1_01_1666	45083	TOF; CID	84.95	767.89	LOT5

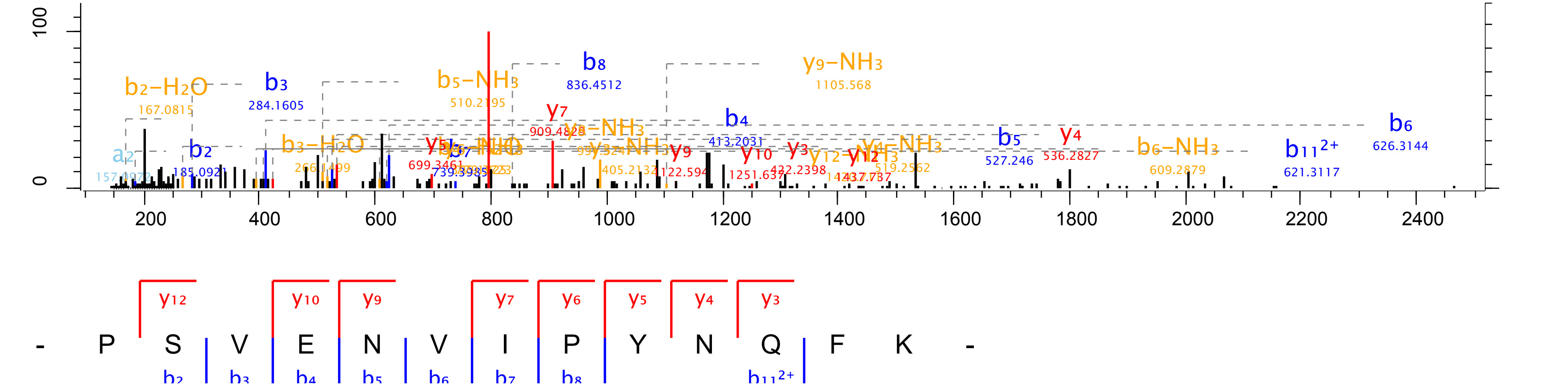

Raw file	Scan	Method	Score	m/z	Gene names
20150306_yeast1_Top_opt_2ug_C1_01_1666	48637	TOF; CID	39.28	1004.49	ROG1

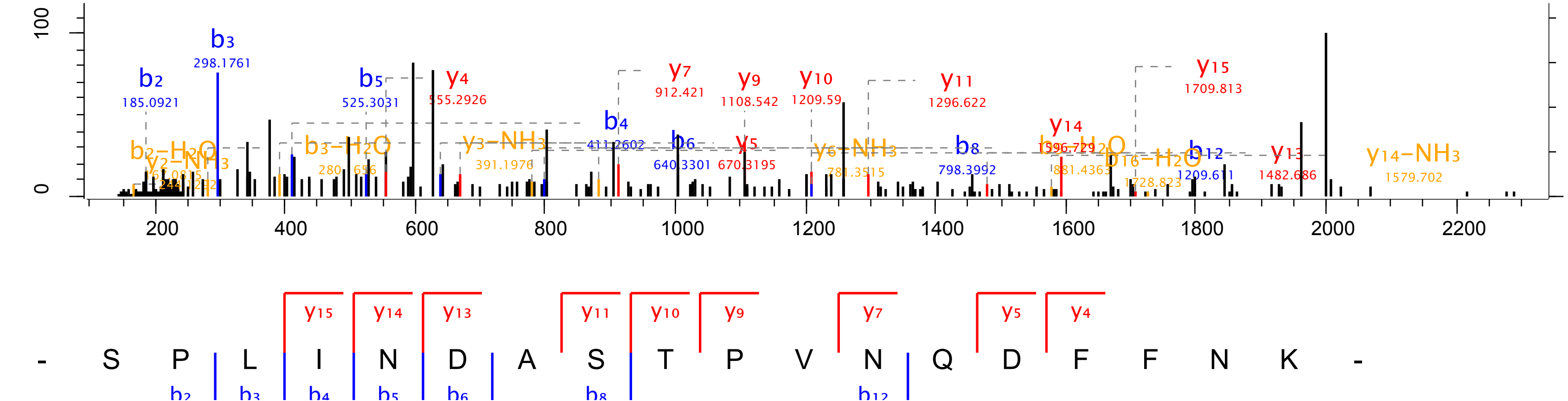

Raw file	Scan	Method	Score	m/z	Gene names
20150306_yeast1_Top_opt_2ug_C1_01_1666	49617	TOF; CID	79.15	568.32	NCE101

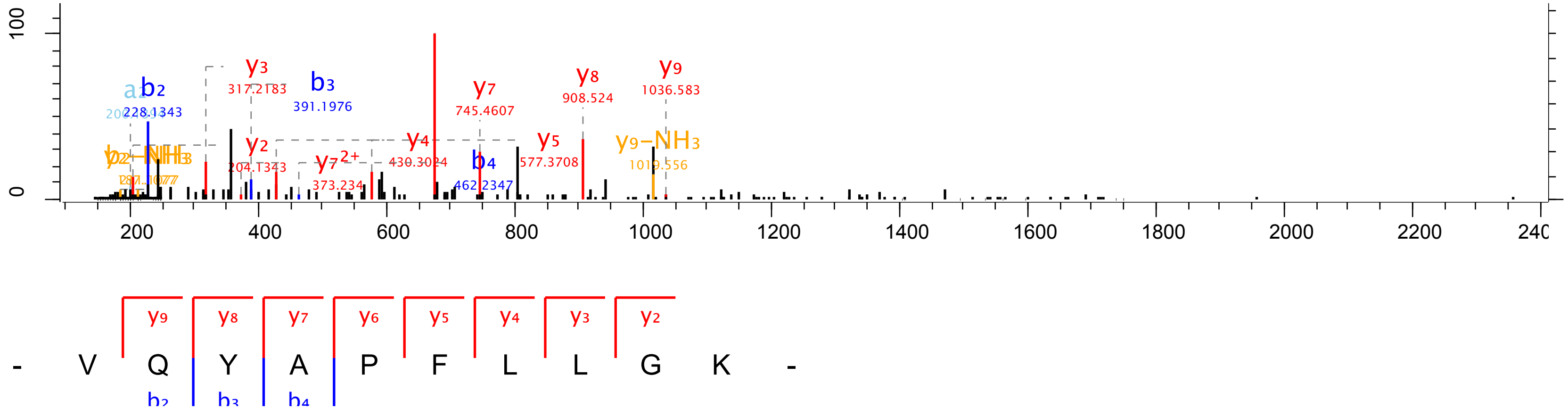

Raw file	Scan	Method	Score	m/z	Gene names
20150306_yeast1_Top_opt_2ug_C1_01_1666	53337	TOF; CID	74.39	704.38	CPT1

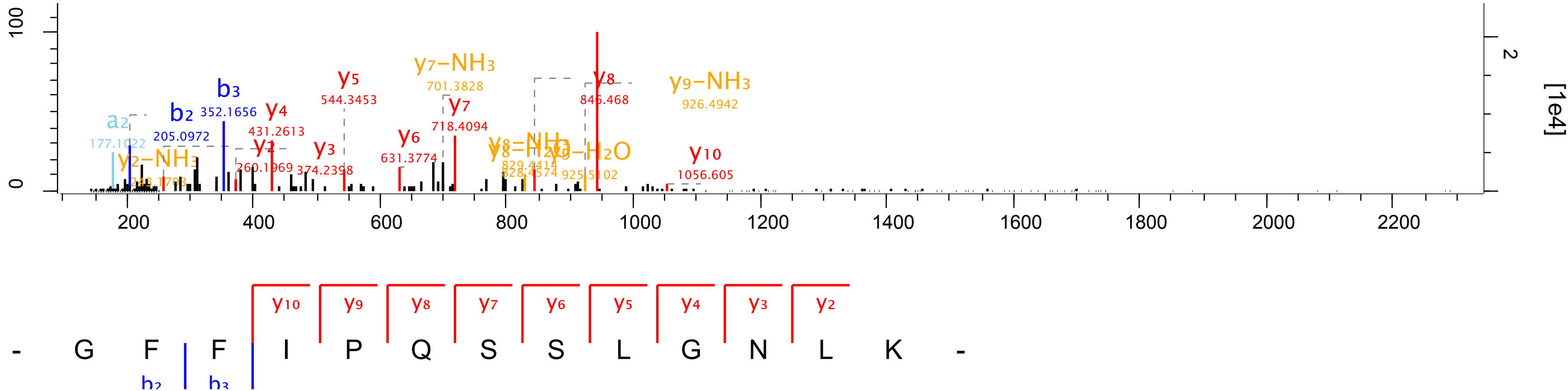

Raw file	Scan	Method	Score	m/z	Gene names
20150306_yeast1_Top_opt_2ug_C1_01_1666	54847	TOF; CID	106.93	669.36	YPR010C-A

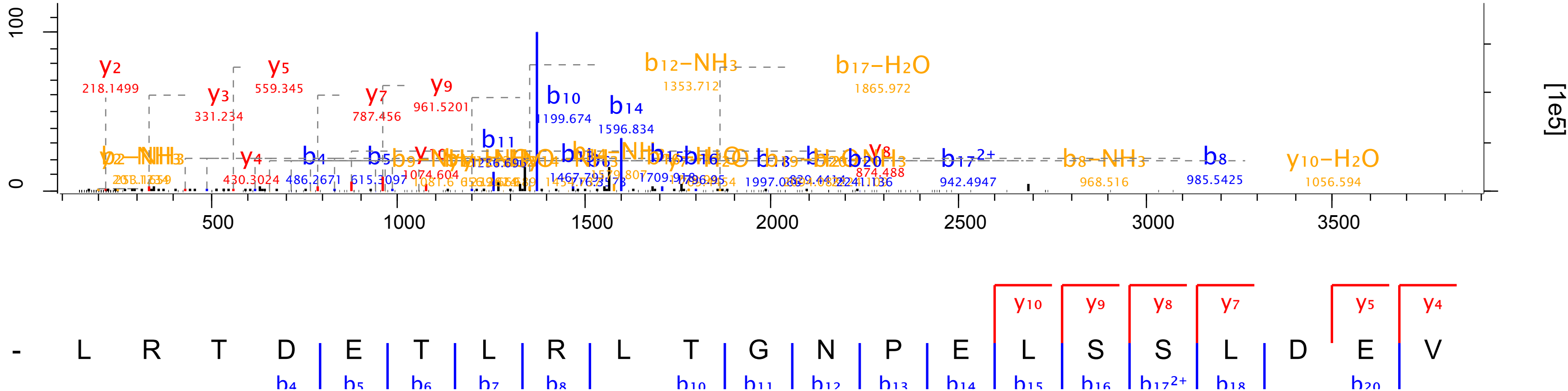

Raw file	Scan	Method	Score	m/z	Gene names
20150306_yeast1_Top_opt_2ug_C1_01_1666	57513	TOF; CID	247.35	804.13	HTA2;HTA1

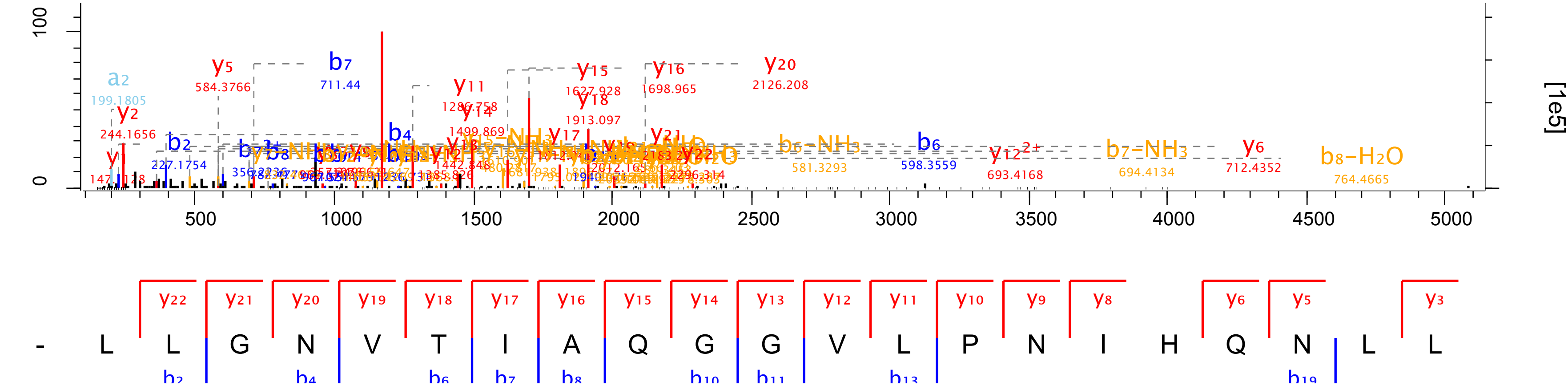

Raw file	Scan	Method	Score	m/z	Gene names
20150306_yeast1_Top_opt_2ug_C1_01_1666	59239	TOF; CID	57.48	782.41	PRM4

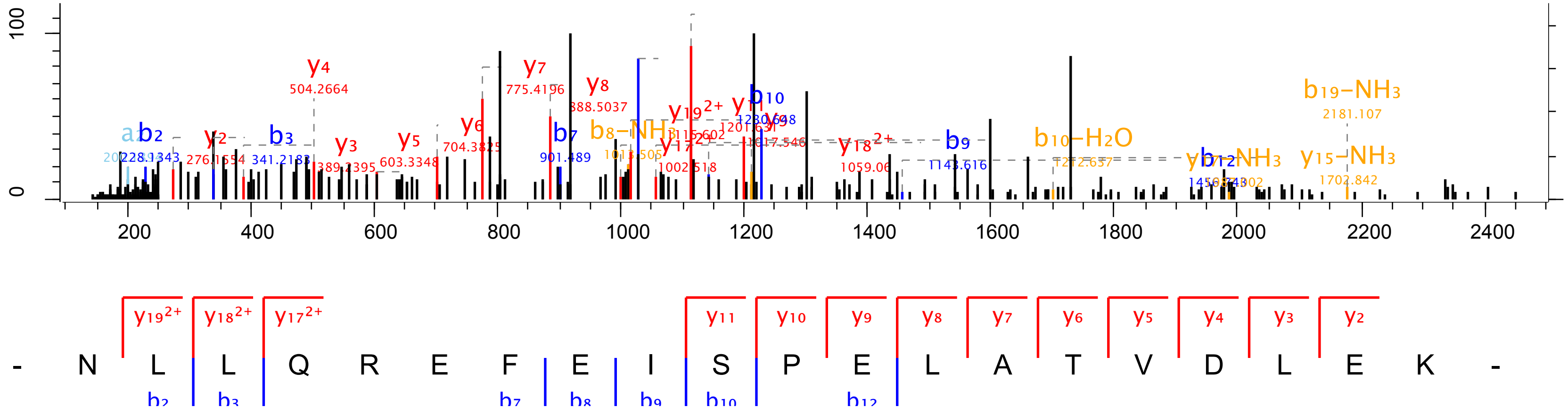

Raw file	Scan	Method	Score	m/z	Gene names
20150306_yeast1_Top_opt_2ug_C1_01_1666	69820	TOF; CID	50.9	729.89	CWP2

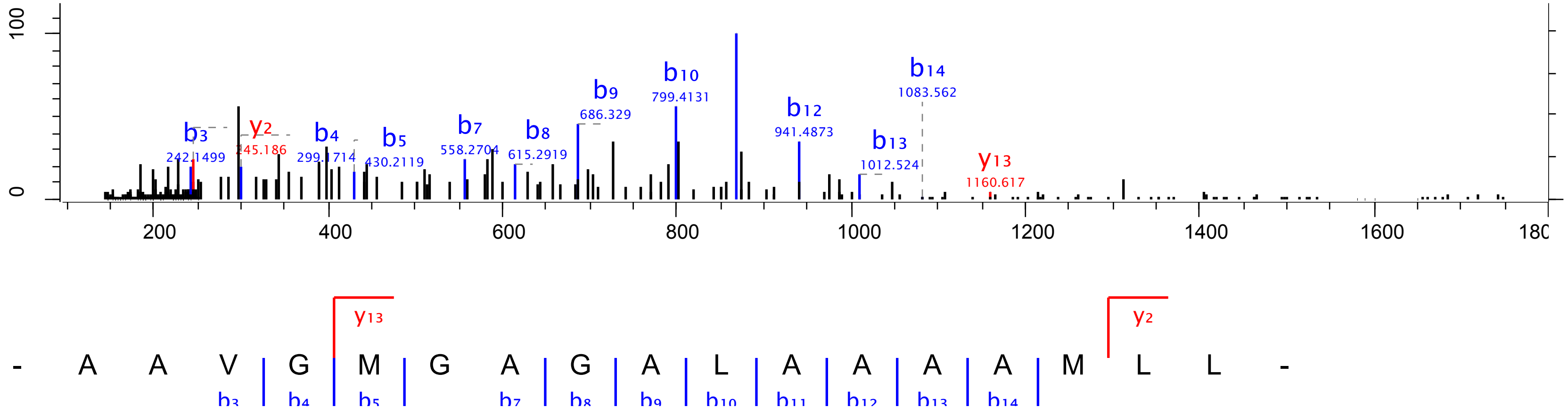

Raw file	Scan	Method	Score	m/z	Gene names
20150306_yeast1_Top_opt_2ug_C1_01_1668	7721	TOF; CID	99	416.72	YDR090C

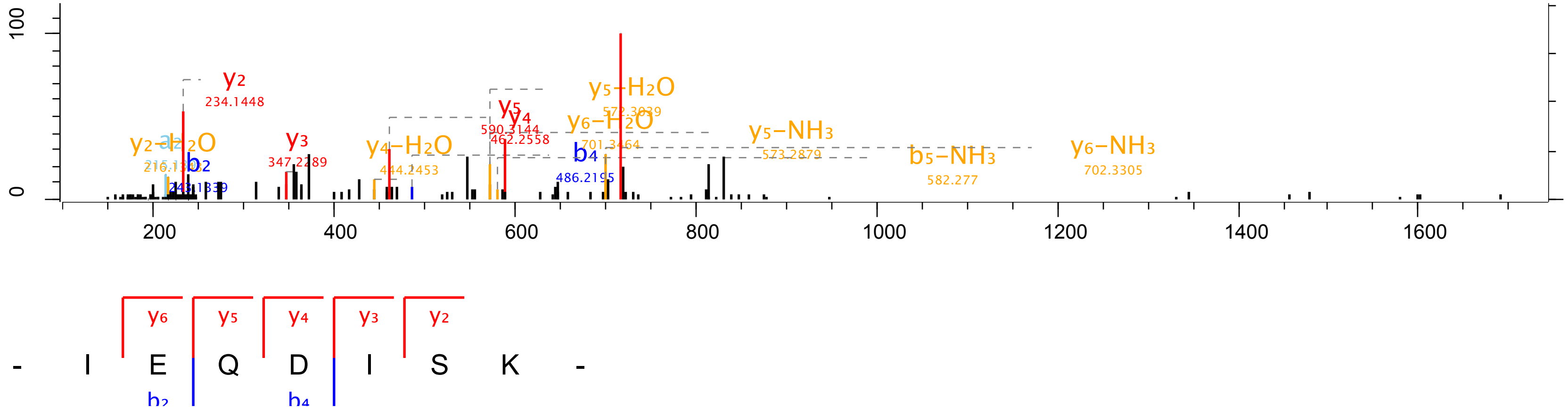

Raw file	Scan	Method	Score	m/z
20150306_yeast1_Top_opt_2ug_C1_01_1668	8865	TOF; CID	71.61	540.25

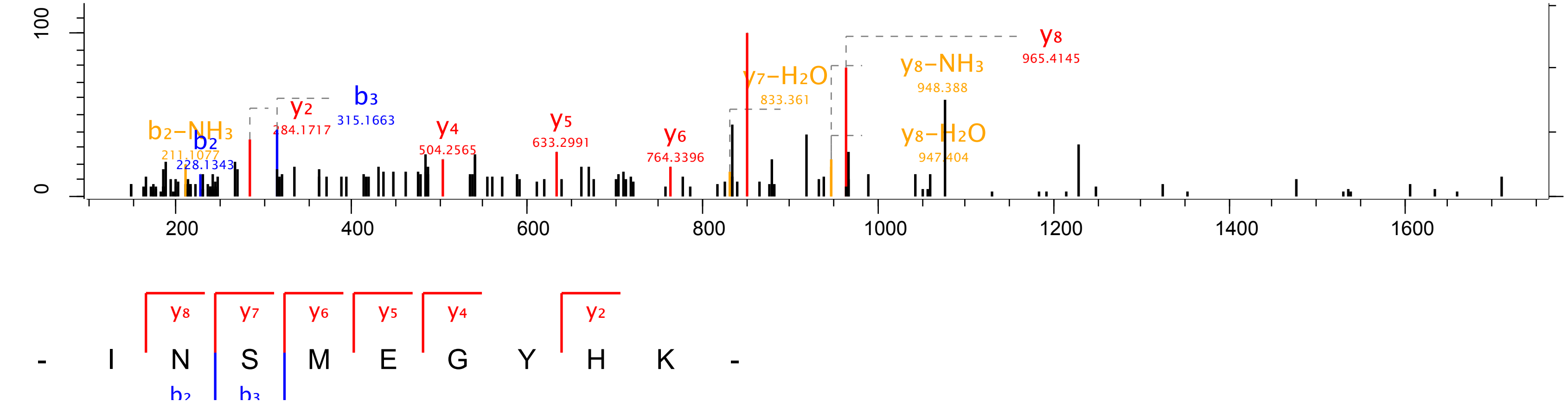

Raw file	Scan	Method	Score	m/z	Gene names
20150306_yeast1_Top_opt_2ug_C1_01_1668	10378	TOF; CID	99.93	731.87	YLR326W

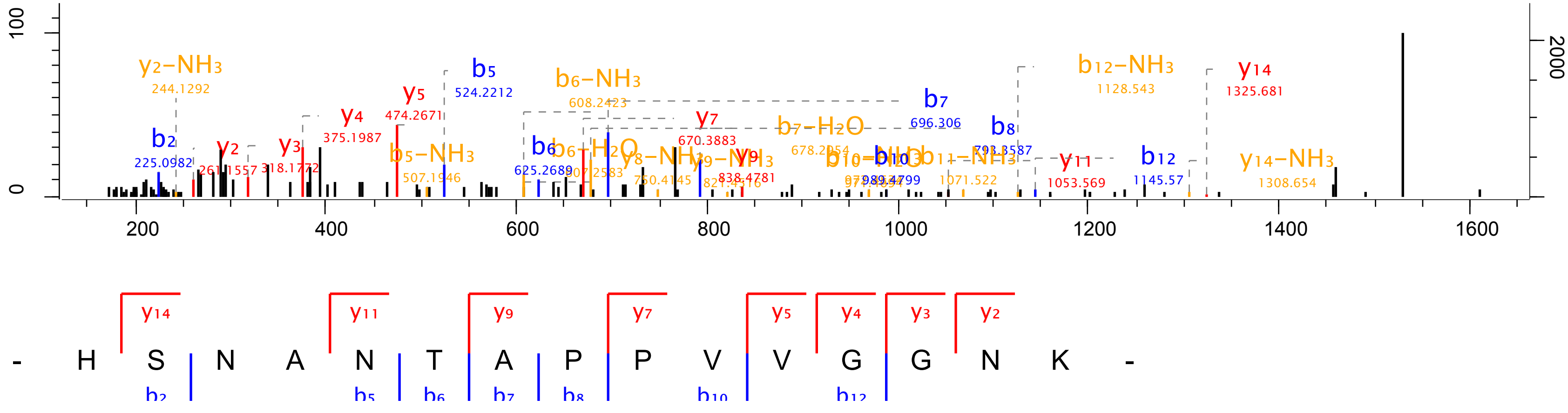

Raw file	Scan	Method	Score	m/z	Gene names
20150306_yeast1_Top_opt_2ug_C1_01_1668	11018	TOF; CID	111.79	625.29	ATO3

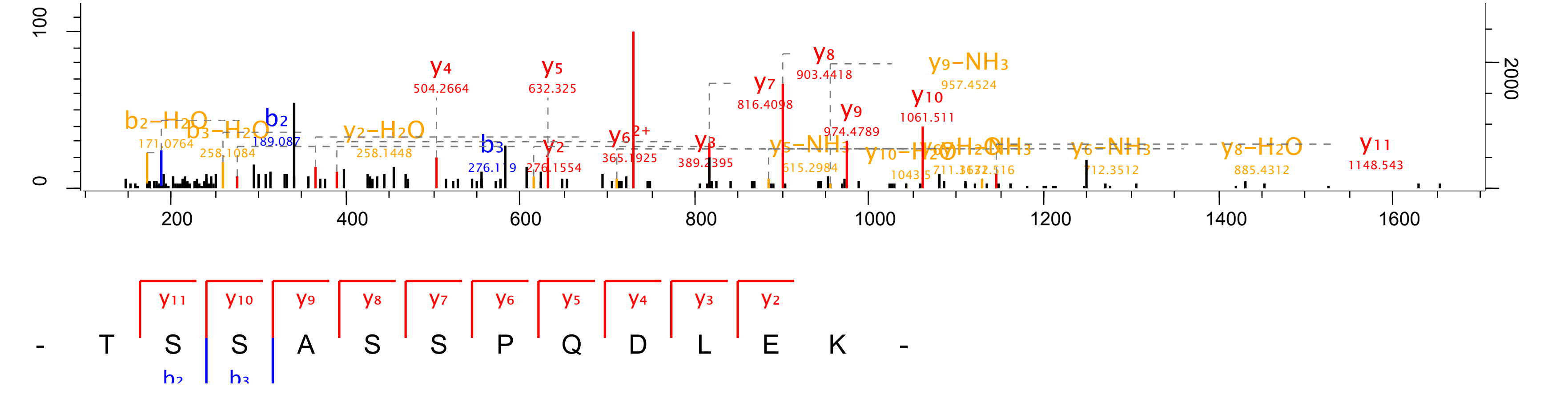

Raw file	Scan	Method	Score	m/z	Gene names
20150306_yeast1_Top_opt_2ug_C1_01_1668	13685	TOF; CID	59.2	544.78	YER077C

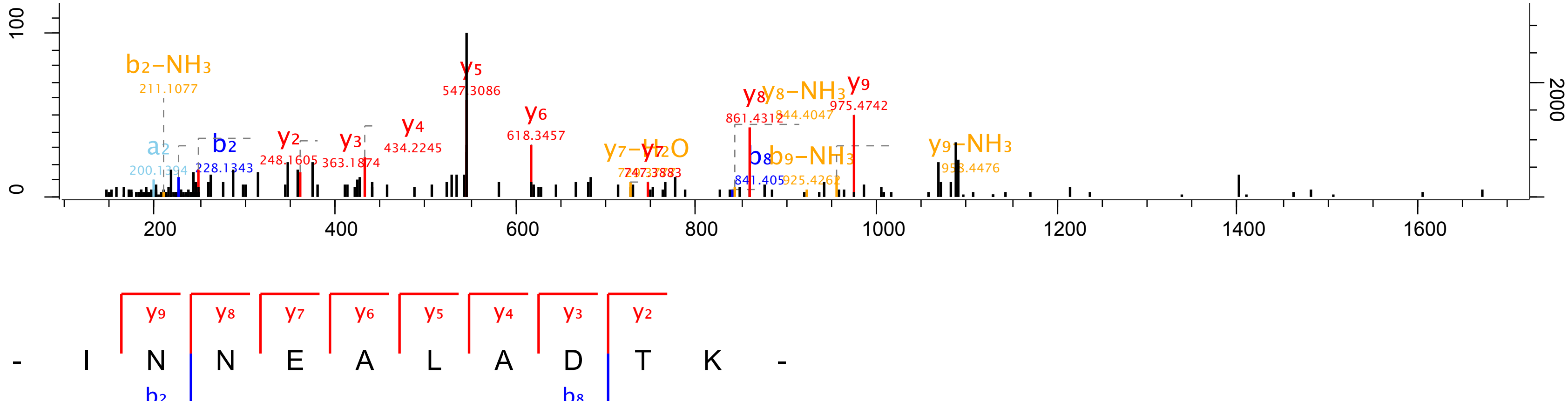

Raw file	Scan	Method	Score	m/z	Gene names
20150306_yeast1_Top_opt_2ug_C1_01_1668	16047	TOF; CID	52.07	635.3	MIH1

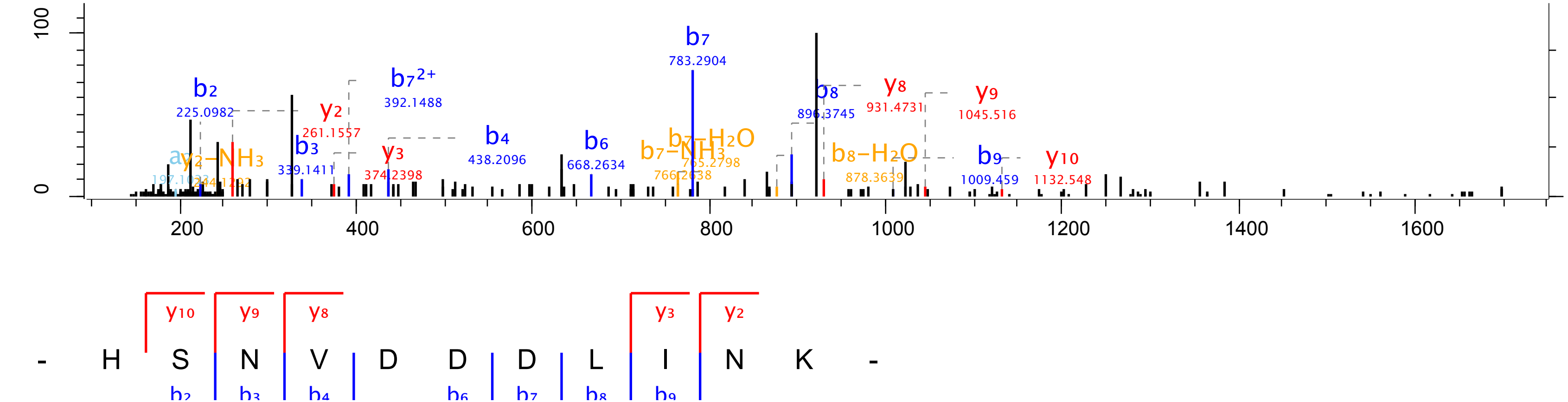

Raw file	Scan	Method	Score	m/z	Gene names
20150306_yeast1_Top_opt_2ug_C1_01_1668	17218	TOF; CID	80.74	634.96	UBR2

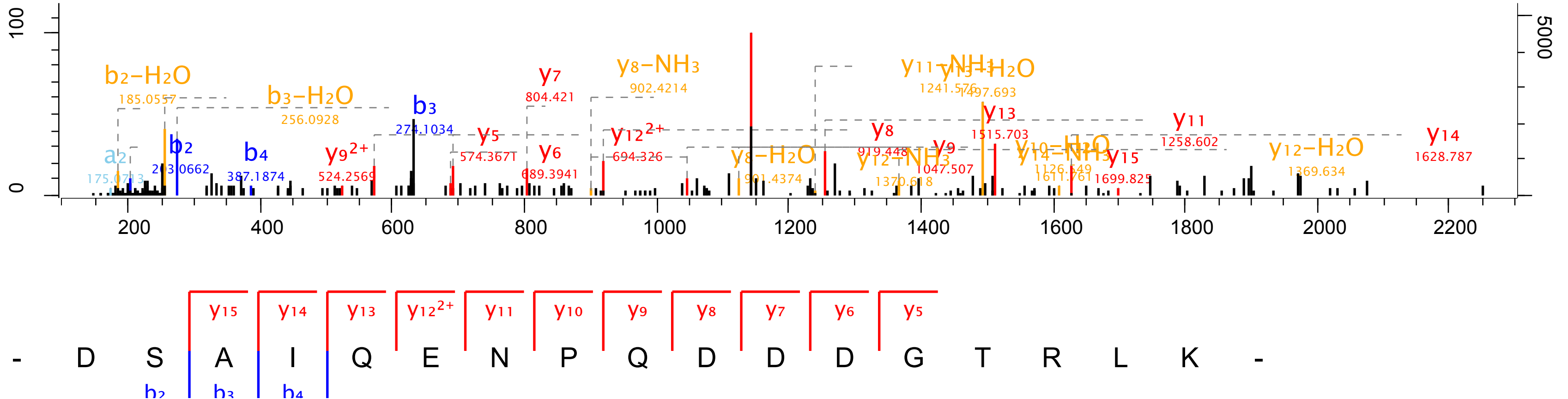

Raw file	Scan	Method	Score	m/z	Gene names
20150306_yeast1_Top_opt_2ug_C1_01_1668	20171	TOF; CID	151.04	916.4	EOS1

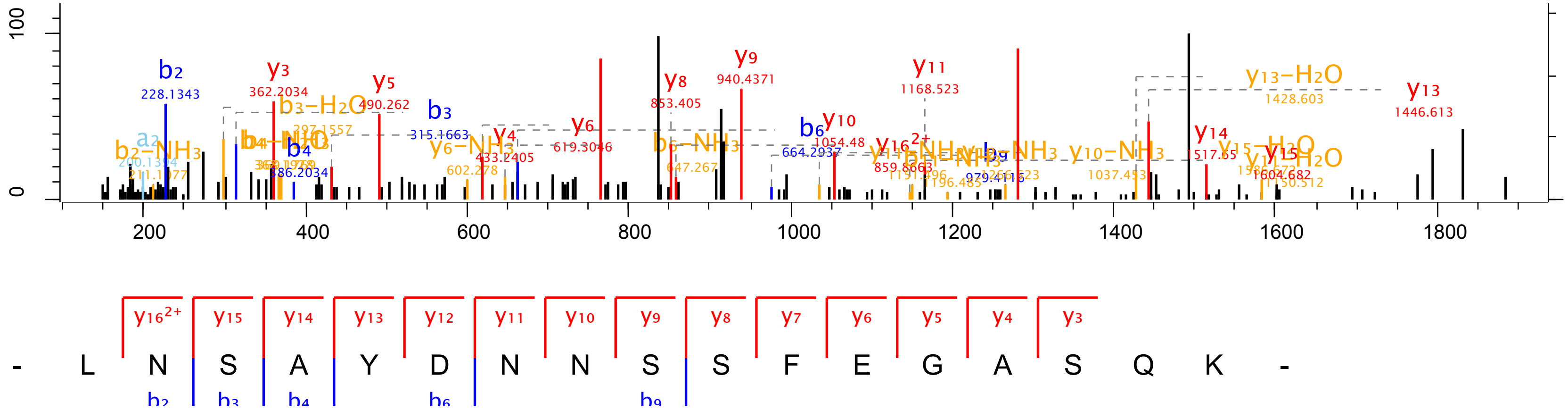

Raw file	Scan	Method	Score	m/z	Gene names
20150306_yeast1_Top_opt_2ug_C1_01_1668	21002	TOF; CID	134.48	376.2	COX9

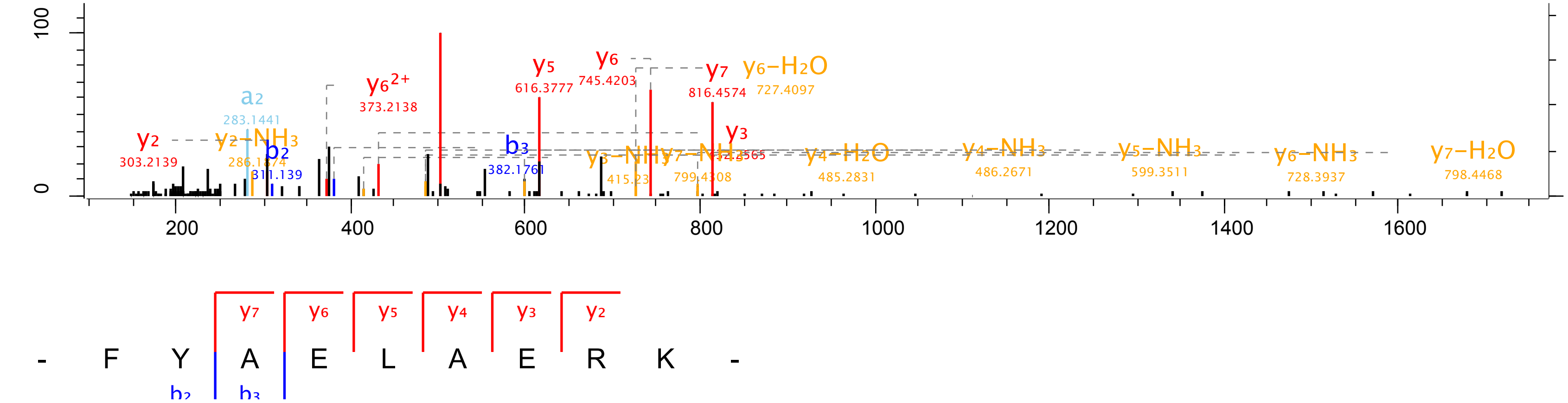

Raw file	Scan	Method	Score	m/z	Gene names
20150306_yeast1_Top_opt_2ug_C1_01_1668	22326	TOF; CID	64.82	556.77	INP54

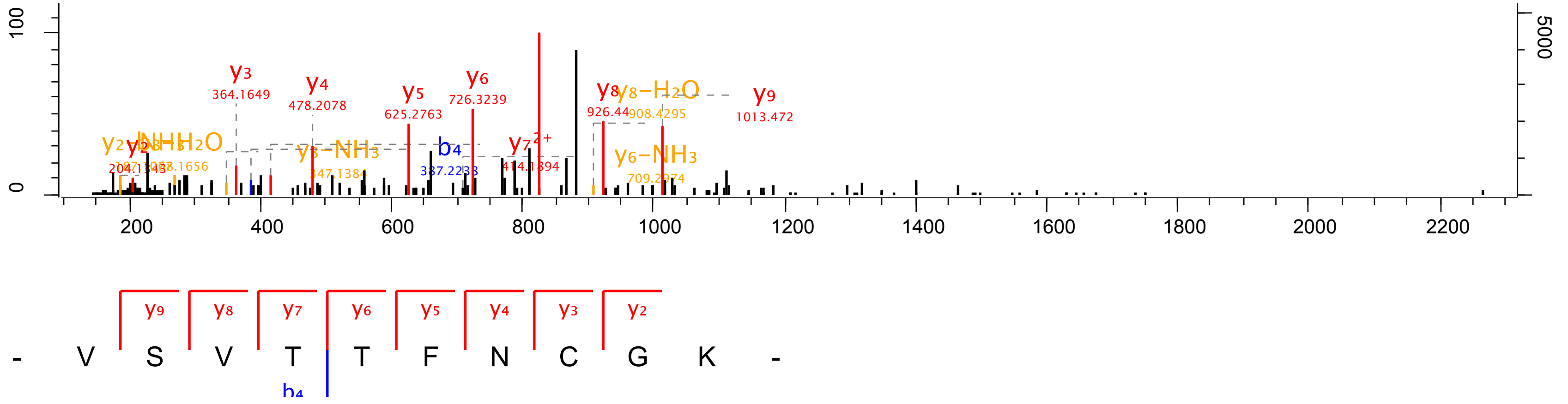

Raw file	Scan	Method	Score	m/z	Gene names
20150306_yeast1_Top_opt_2ug_C1_01_1668	24645	TOF; CID	68.11	628.79	MRPL31

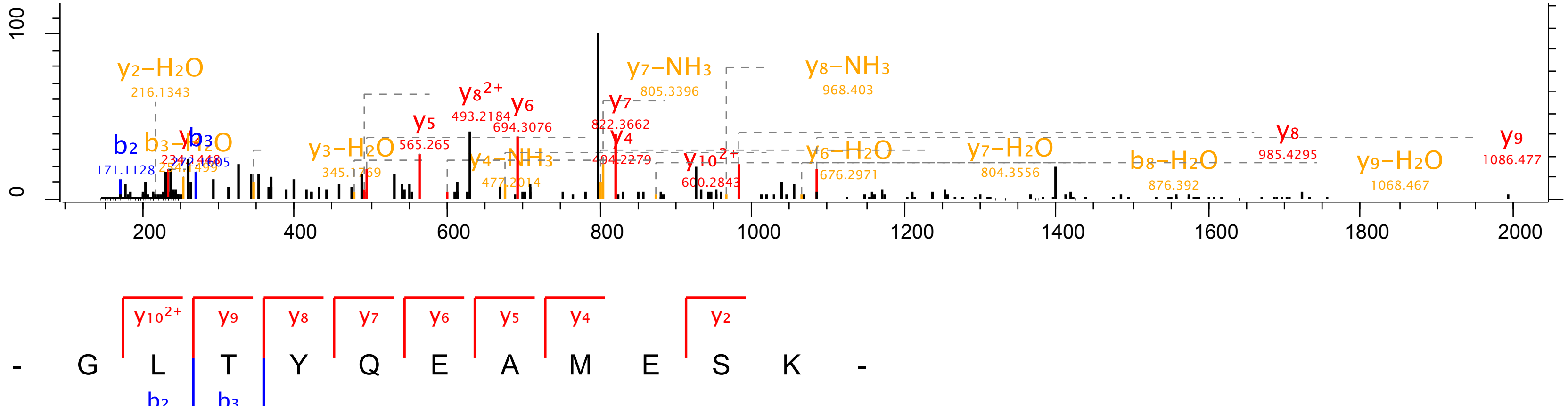

Raw file	Scan	Method	Score	m/z	Gene names
20150306_yeast1_Top_opt_2ug_C1_01_1668	24883	TOF; CID	52.9	685.34	MCH1

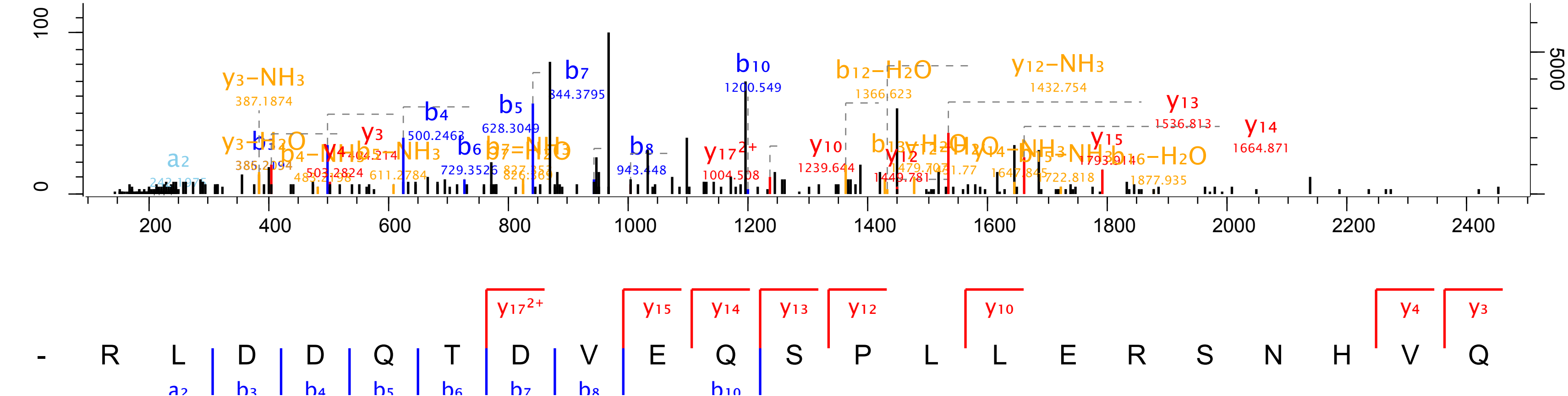

Raw file	Scan	Method	Score	m/z	Gene names
20150306_yeast1_Top_opt_2ug_C1_01_1668	29201	TOF; CID	86.79	470.23	TIM22

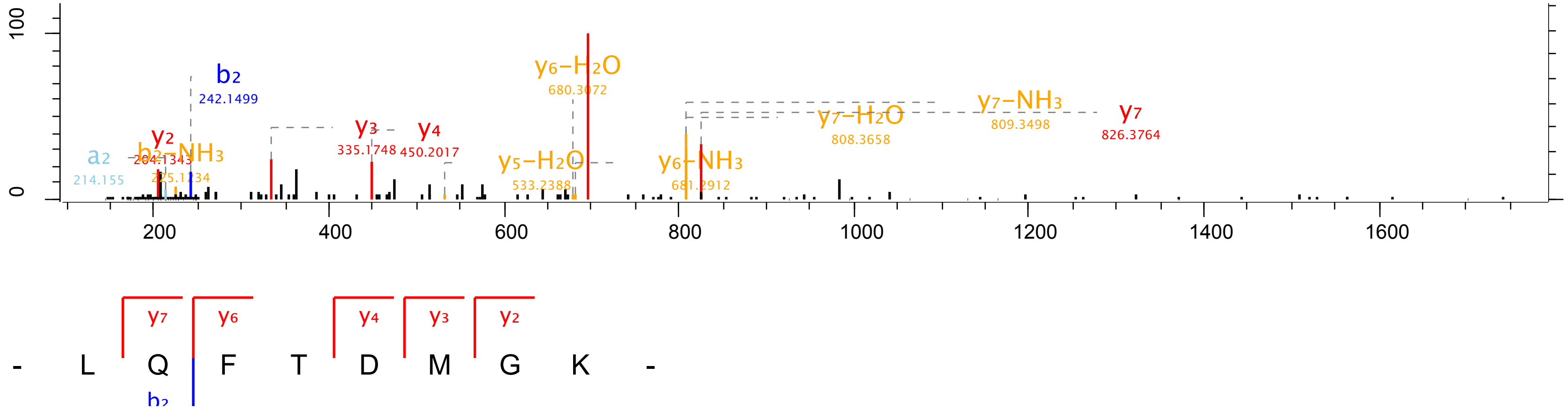

Raw file	Scan	Method	Score	m/z	Gene names
20150306_yeast1_Top_opt_2ug_C1_01_1668	29235	TOF; CID	47.71	651.79	ERP6

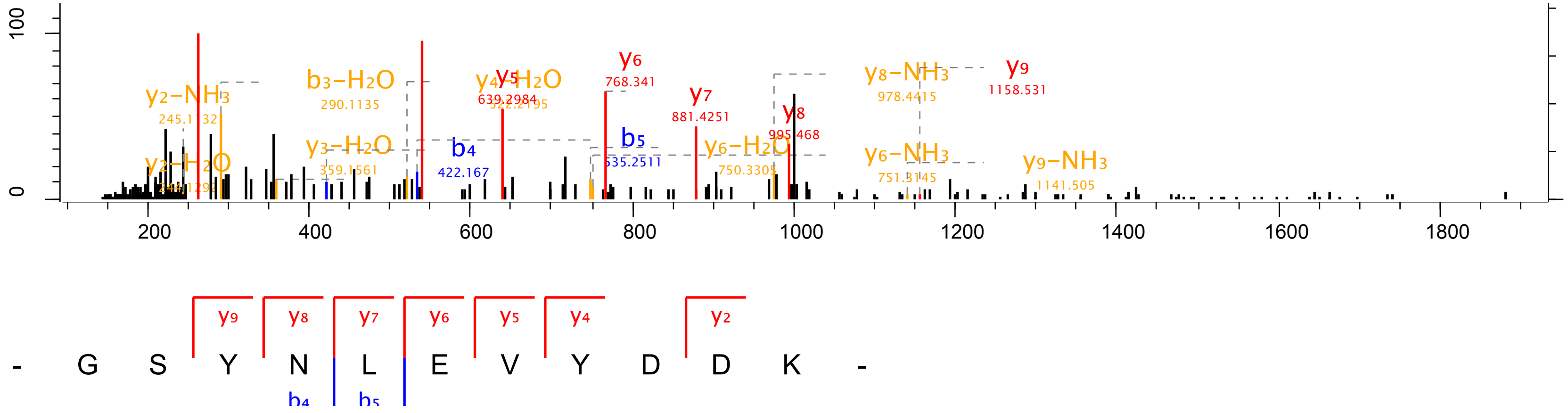

Raw file	Scan	Method	Score	m/z	Gene names
20150306_yeast1_Top_opt_2ug_C1_01_1668	30483	TOF; CID	121.36	556.81	MAK31

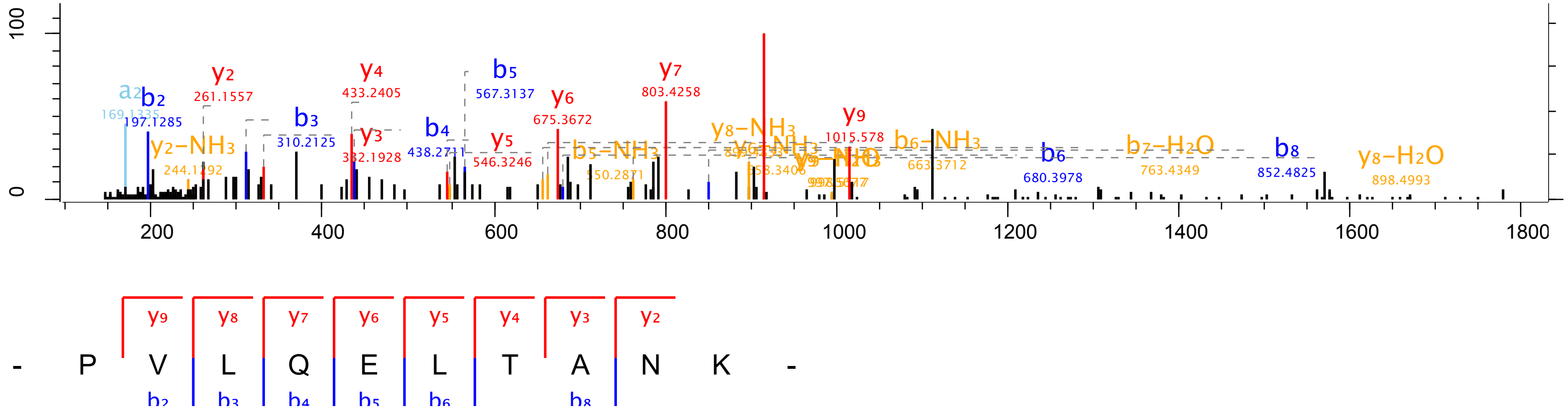

Raw file	Scan	Method	Score	m/z	Gene names
20150306_yeast1_Top_opt_2ug_C1_01_1668	30500	TOF; CID	79.94	793.38	STP22

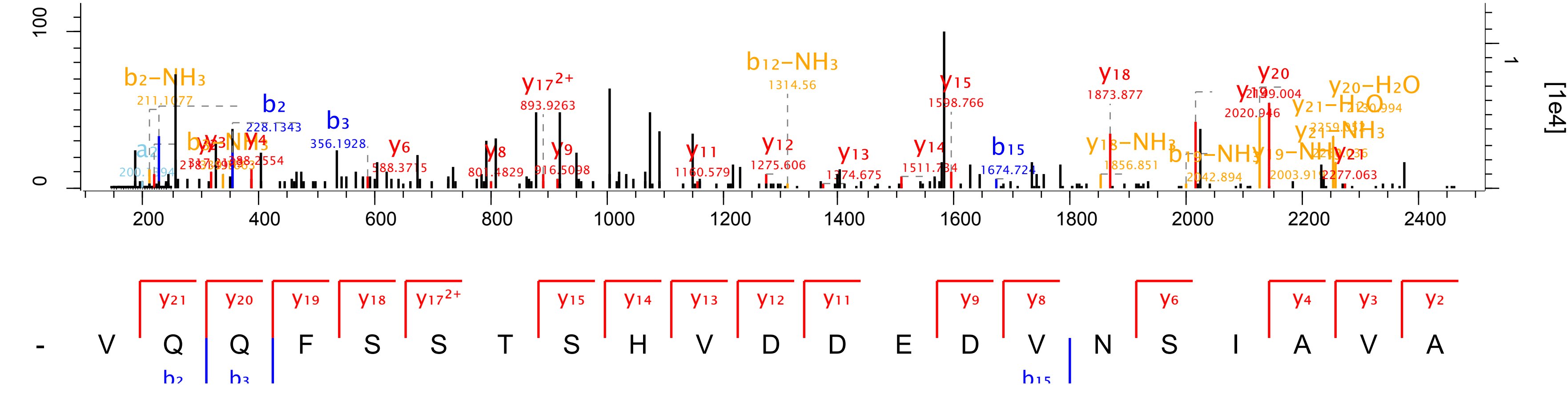

Raw file	Scan	Method	Score	m/z	Gene names
20150306_yeast1_Top_opt_2ug_C1_01_1668	30642	TOF; CID	49.3	374.88	MET8

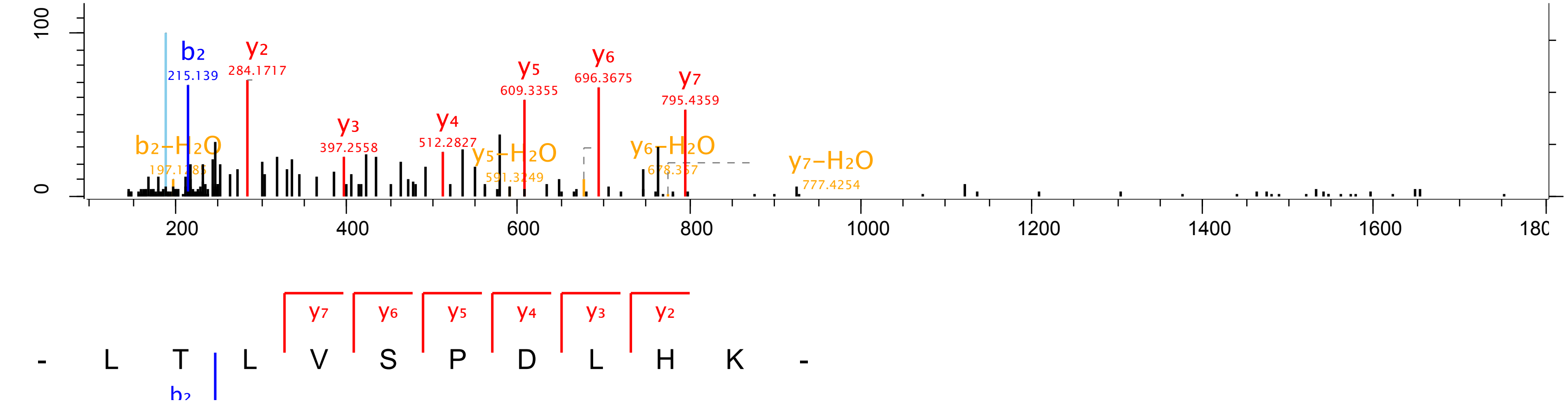

Raw file	Scan	Method	Score	m/z	Gene names
20150306_yeast1_Top_opt_2ug_C1_01_1668	31556	TOF; CID	75.51	541.3	ASI3

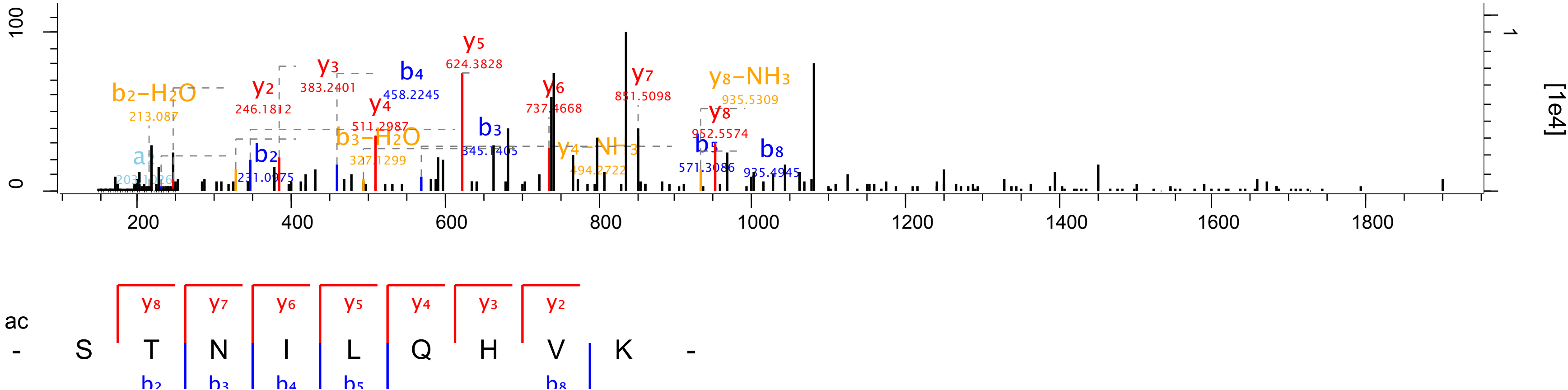

Raw file	Scan	Method	Score	m/z	Gene names
20150306_yeast1_Top_opt_2ug_C1_01_1668	41551	TOF; CID	85.36	886.45	MSB2

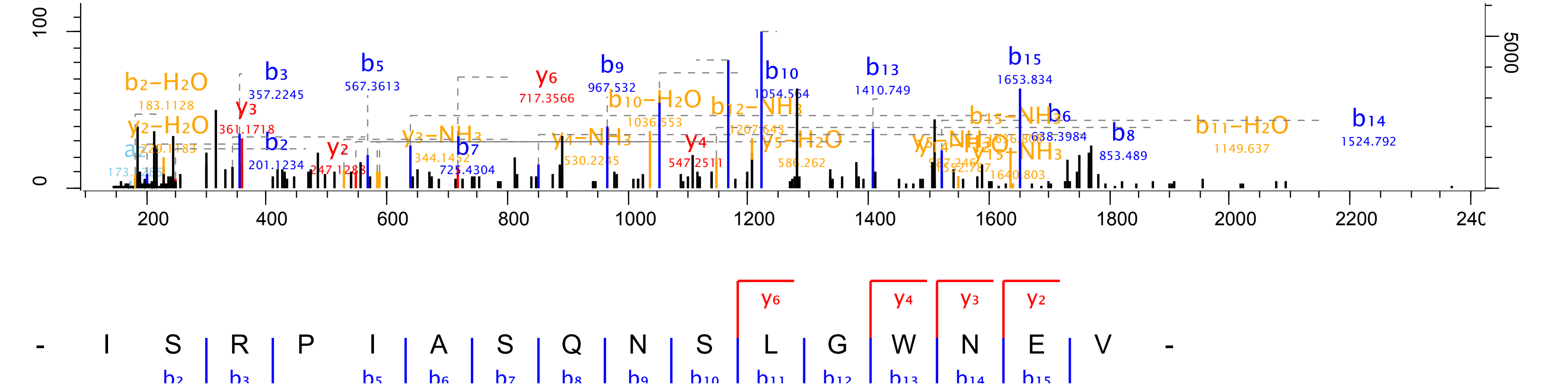

Raw file	Scan	Method	Score	m/z	Gene names
20150306_yeast1_Top_opt_2ug_C1_01_1668	41940	TOF; CID	60.16	693.35	AXL1

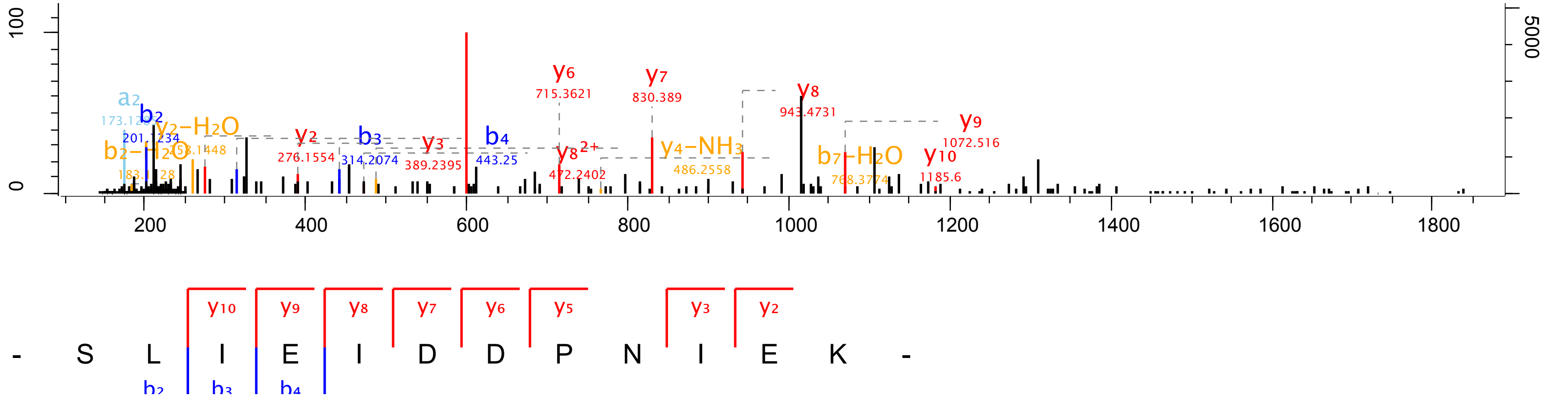

Raw file	Scan	Method	Score	m/z	Gene names
20150306_yeast1_Top_opt_2ug_C1_01_1668	43693	TOF; CID	50.03	933.46	ESC8

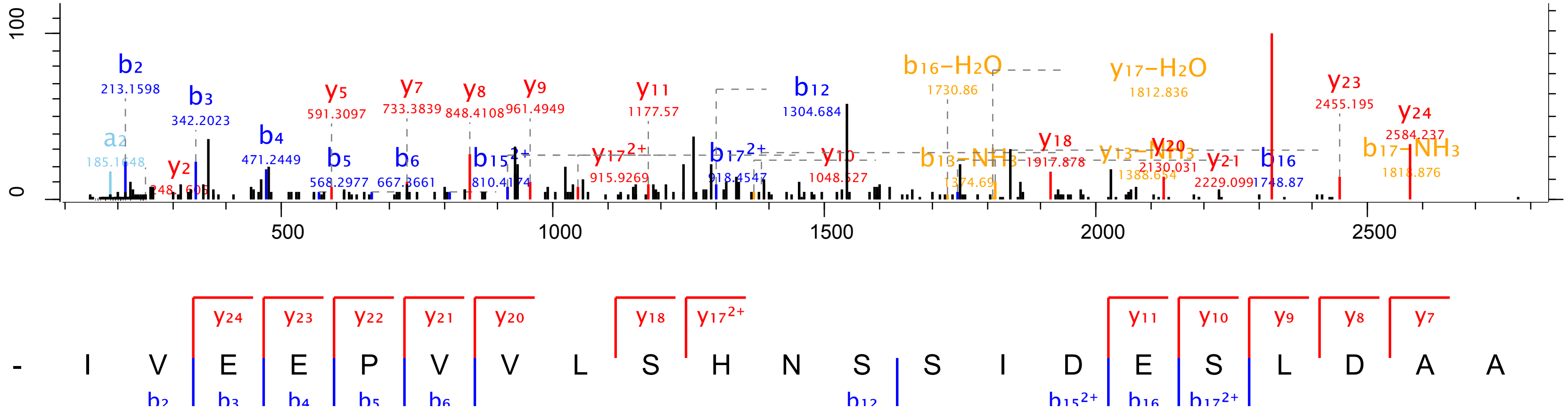

Raw file	Scan	Method	Score	m/z	Gene names
20150306_yeast1_Top_opt_2ug_C1_01_1668	43774	TOF; CID	73.95	665.85	MET2

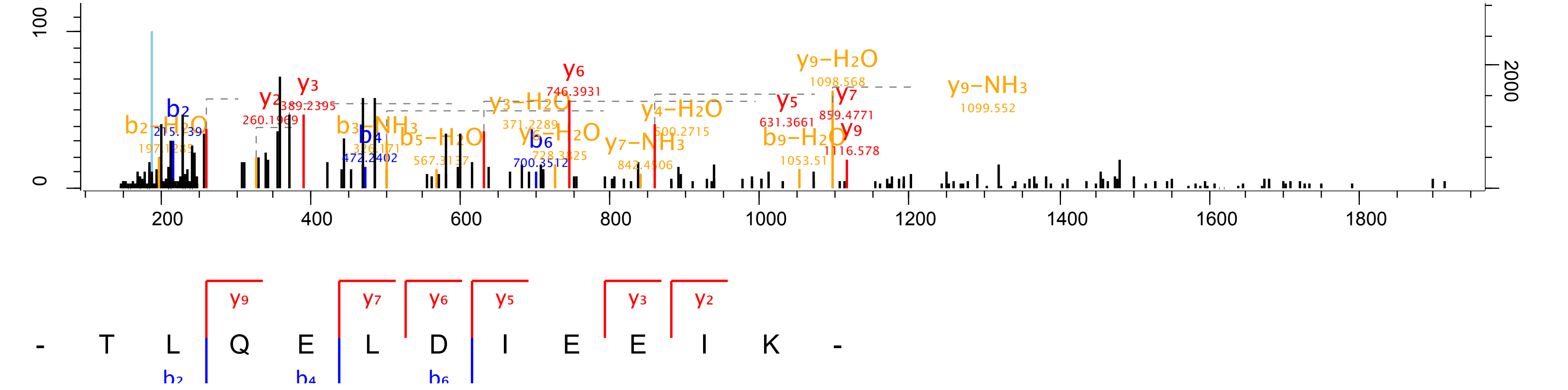

Raw file	Scan	Method	Score	m/z	Gene names
20150306_yeast1_Top_opt_2ug_C1_01_1668	46790	TOF; CID	72.88	671.88	OCA4

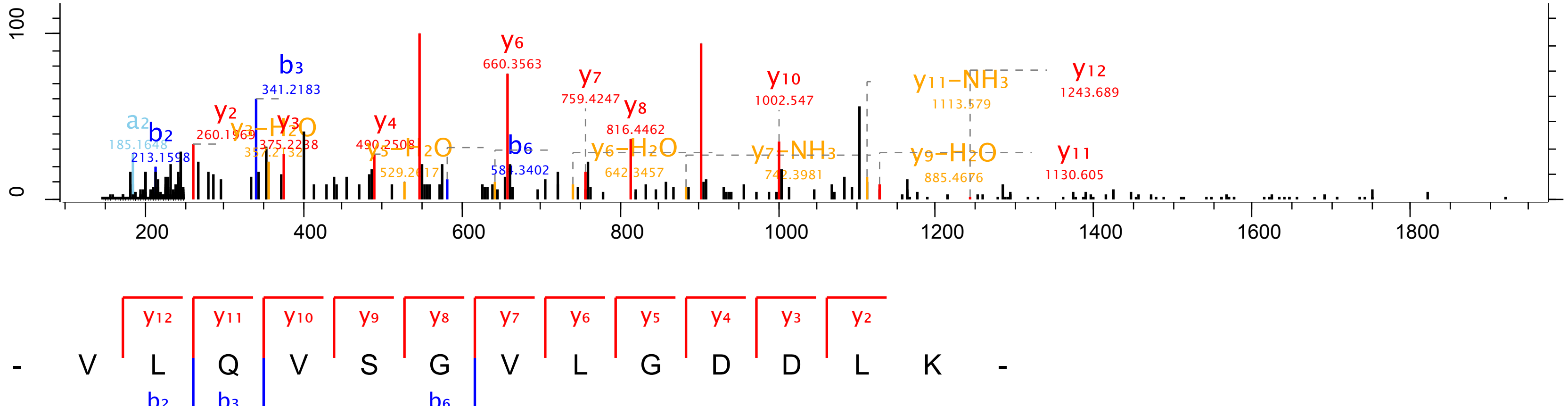

Raw file	Scan	Method	Score	m/z	Gene names
20150306_yeast1_Top_opt_2ug_C1_01_1668	46898	TOF; CID	76.8	1009.14	YPL108W

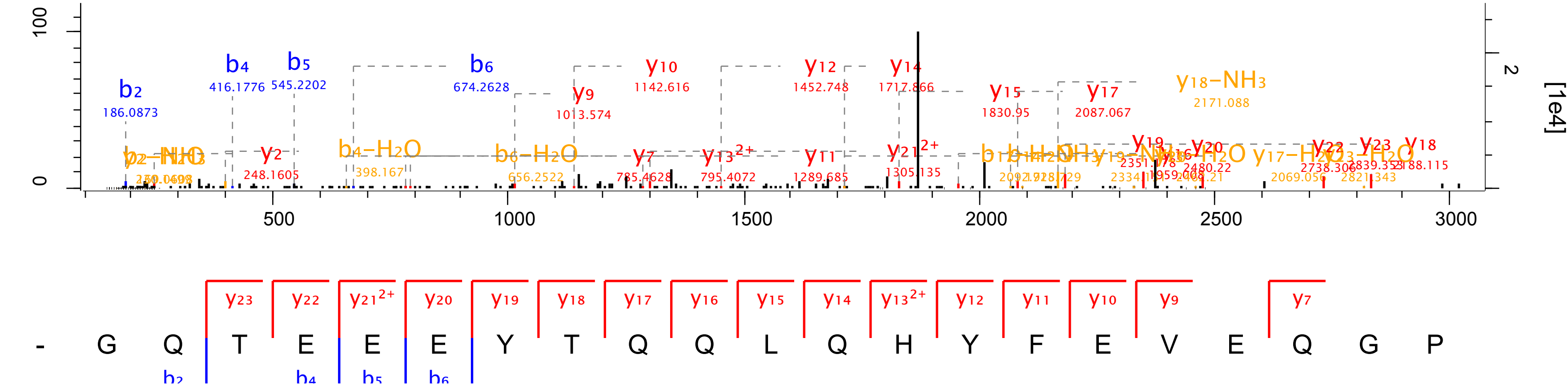

Raw file	Scan	Method	Score	m/z	Gene names
20150306_yeast1_Top_opt_2ug_C1_01_1668	47274	TOF; CID	73.27	729.87	AIM22

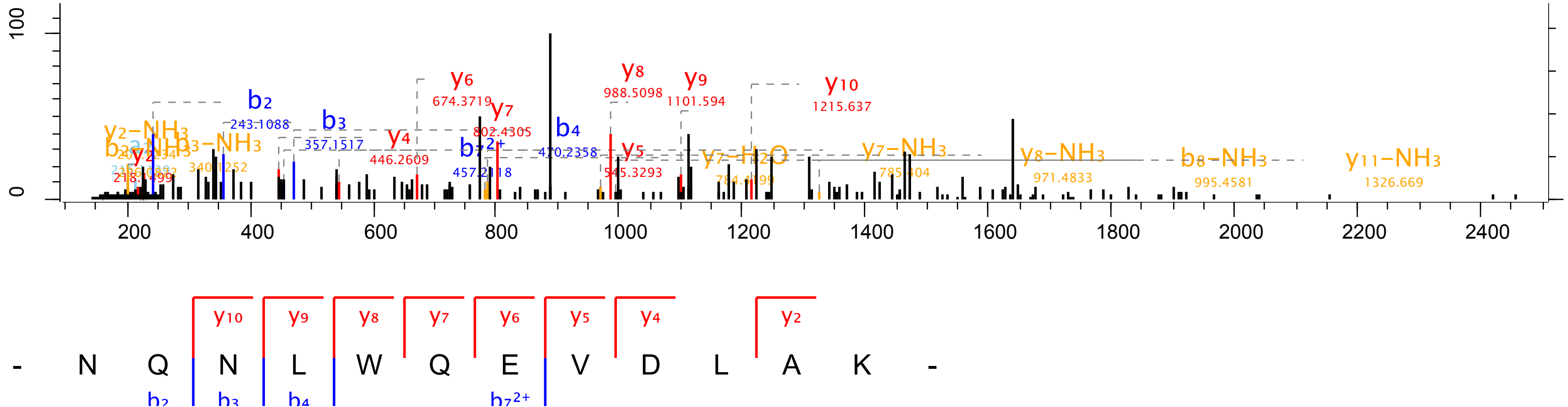

Raw file	Scan	Method	Score	m/z	Gene names
20150306_yeast1_Top_opt_2ug_C1_01_1668	48441	TOF; CID	145.44	1176.07	EMC6

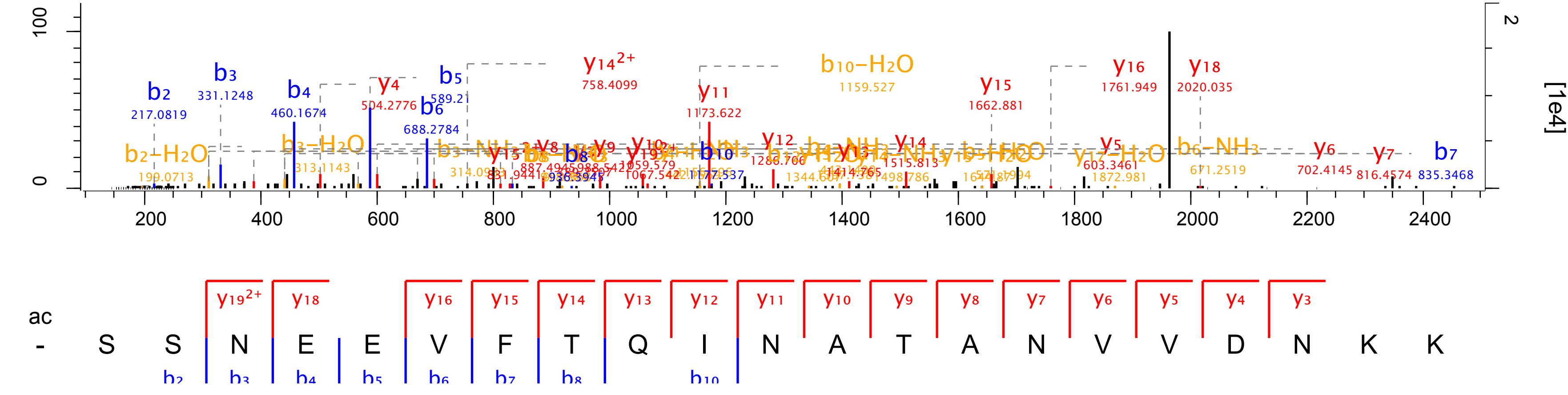

Raw file	Scan	Method	Score	m/z	Gene names
20150306_yeast1_Top_opt_2ug_C1_01_1668	51336	TOF; CID	55.44	686.68	ISC1

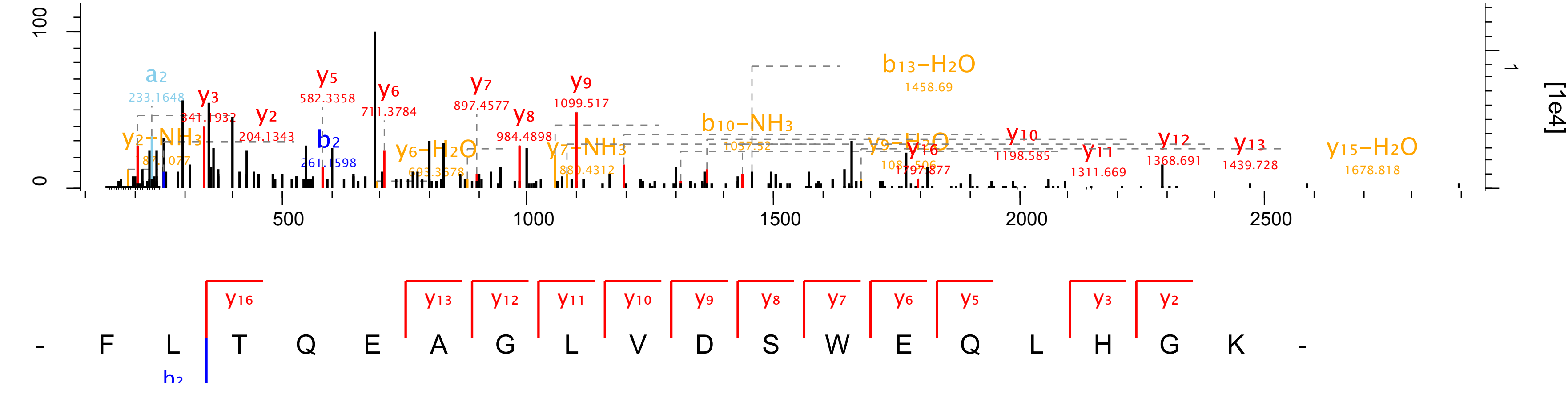

Raw file	Scan	Method	Score	m/z	Gene names
20150306_yeast1_Top_opt_2ug_C1_01_1668	55720	TOF; CID	79.99	525.31	ICE2

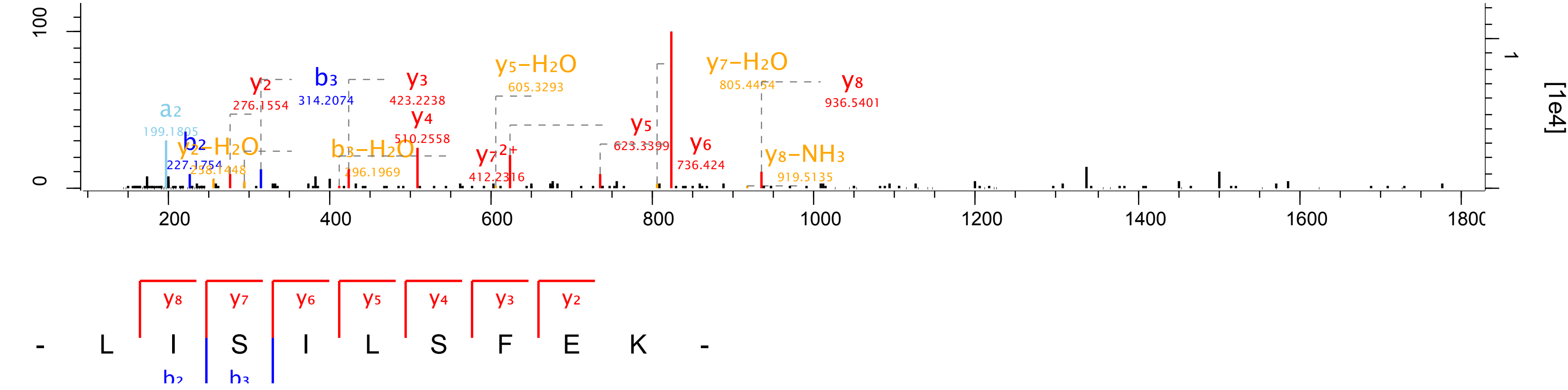

Raw file	Scan	Method	Score	m/z	Gene names
20150306_yeast1_Top_opt_2ug_C1_01_1668	60578	TOF; CID	133.21	756.41	NKP2

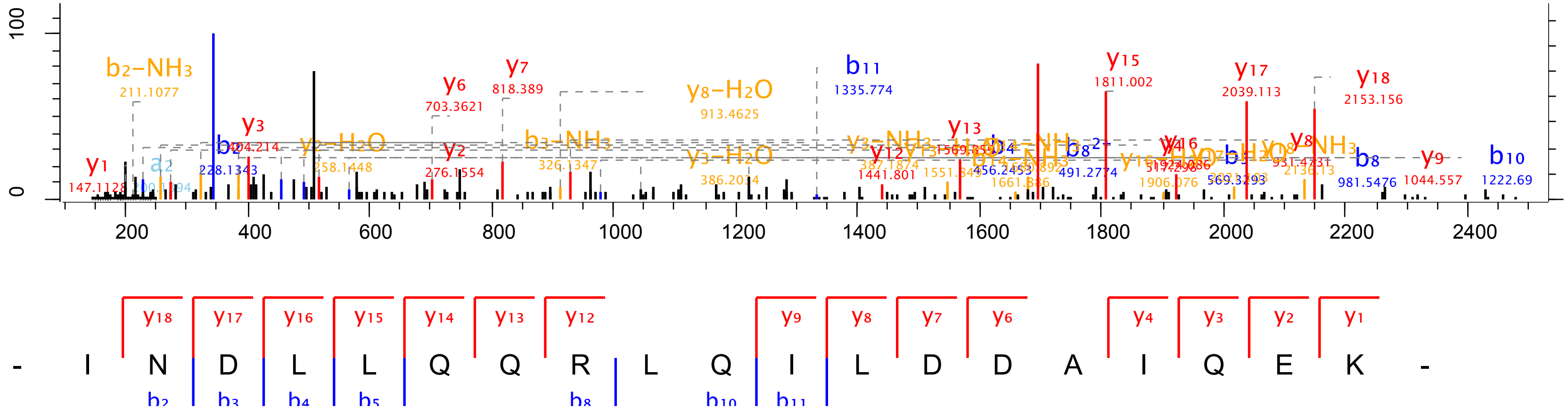

Raw file	Scan	Method	Score	m/z	Gene names
20150306_yeast1_Top_opt_2ug_C1_01_1668	65156	TOF; CID	154.65	568.99	ERV14

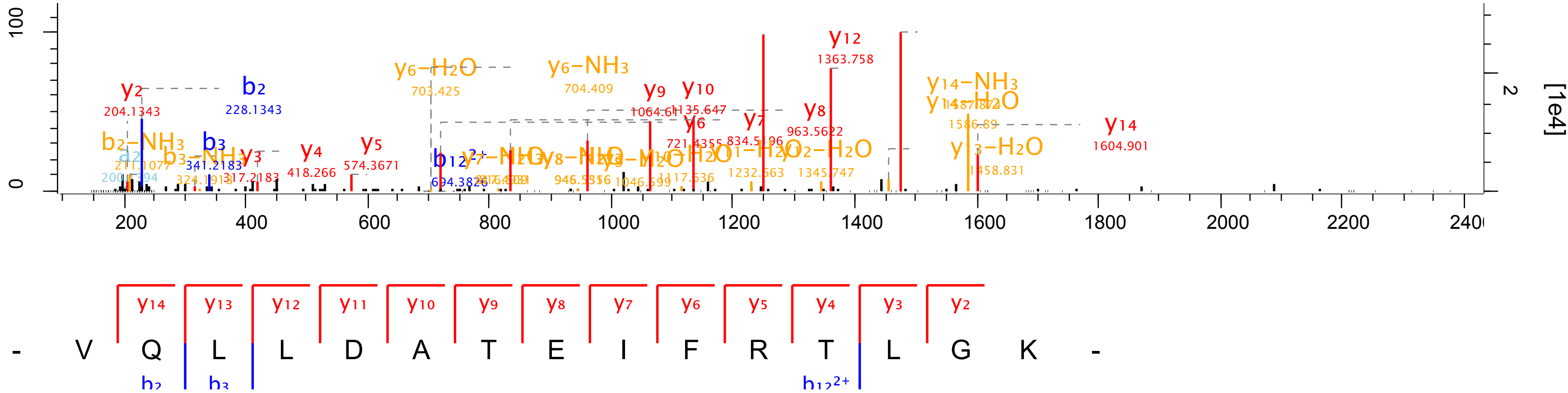

Raw file	Scan	Method	Score	m/z	Gene names
20150306_yeast1_Top_opt_2ug_C1_01_1670	7060	TOF; CID	66.69	474.71	CAN1

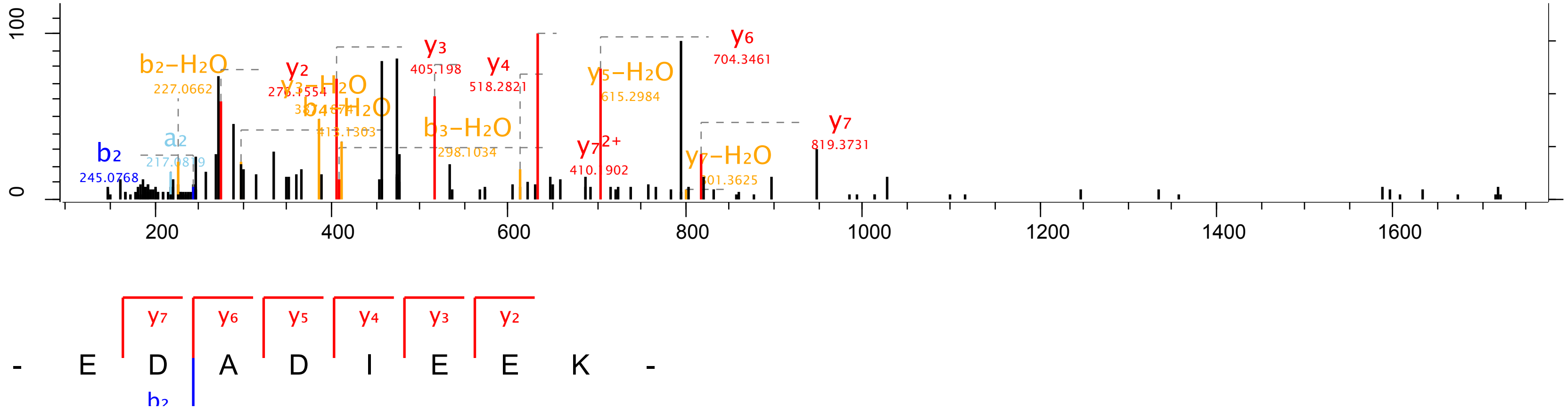

Raw file	Scan	Method	Score	m/z	Gene names
20150306_yeast1_Top_opt_2ug_C1_01_1670	12678	TOF; CID	96.63	634.27	TPN1

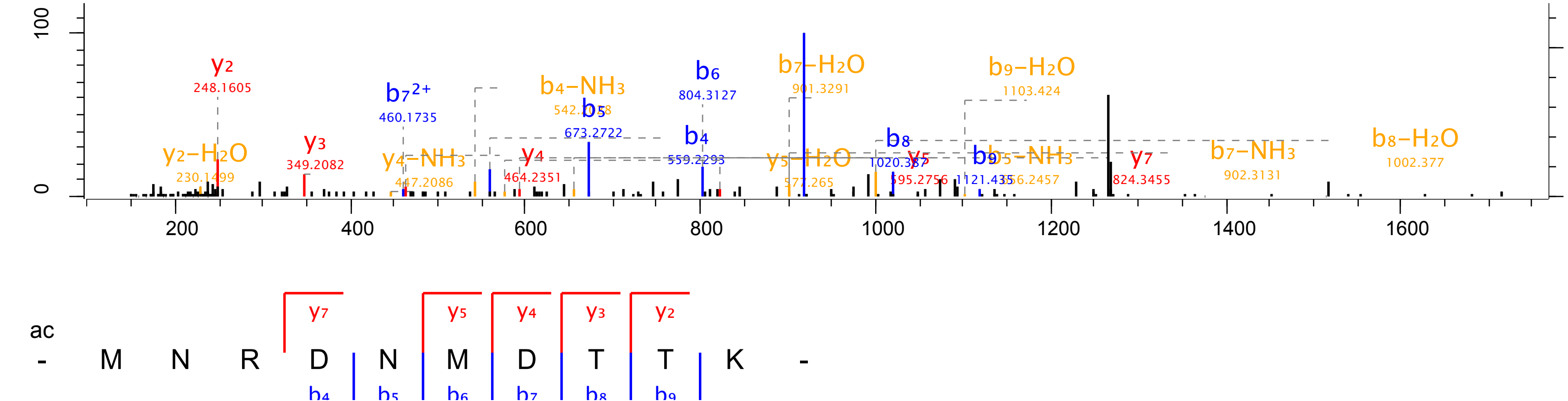

Raw file	Scan	Method	Score	m/z	Gene names
20150306_yeast1_Top_opt_2ug_C1_01_1670	13059	TOF; CID	80.69	651.29	FUN26

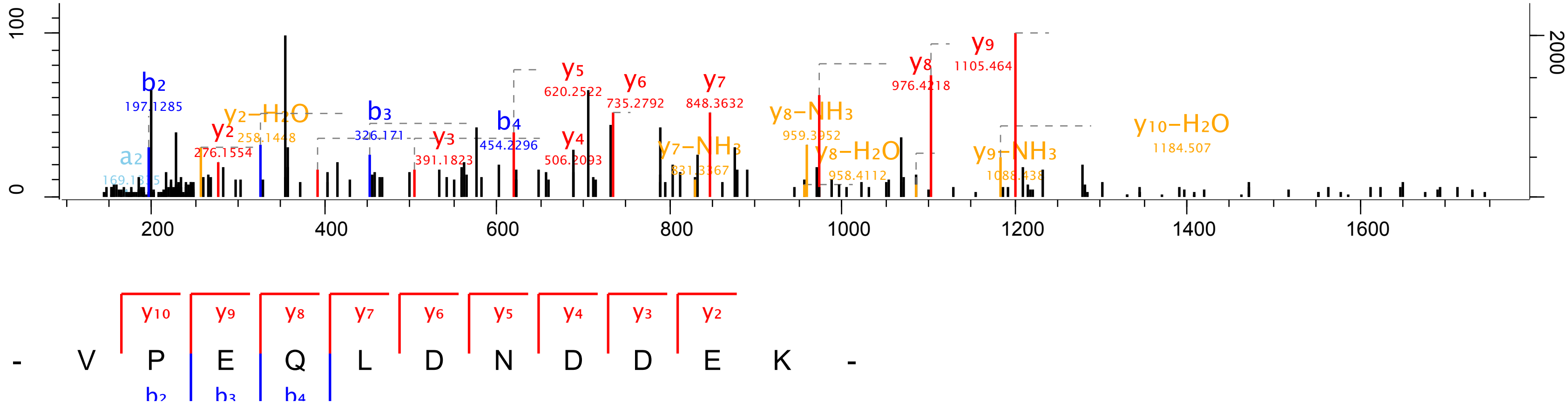

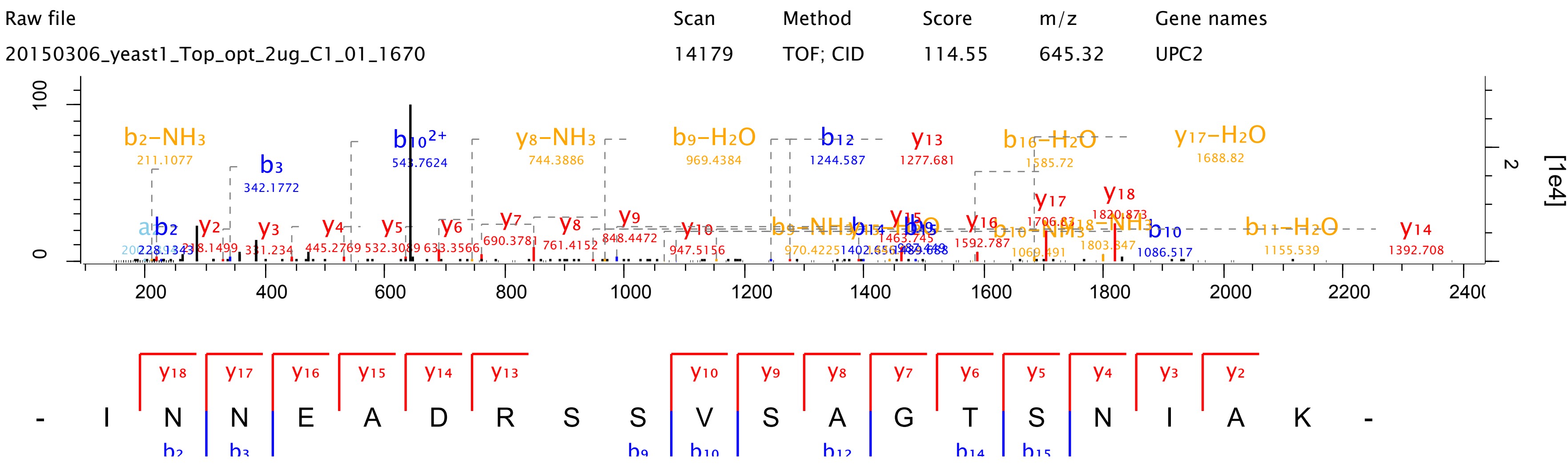
Raw file
20150306_yeast1_Top_opt_2ug_C1_01_1670
Scan
14179
Method
TOF; CID
Score
114.55
m/z
645.32
Gene names
UPC2
- I N N E A D R S S V S A G T S N I A K -

Raw file	Scan	Method	Score	m/z	Gene names
20150306_yeast1_Top_opt_2ug_C1_01_1670	16000	TOF; CID	113.41	439.72	RPS29A

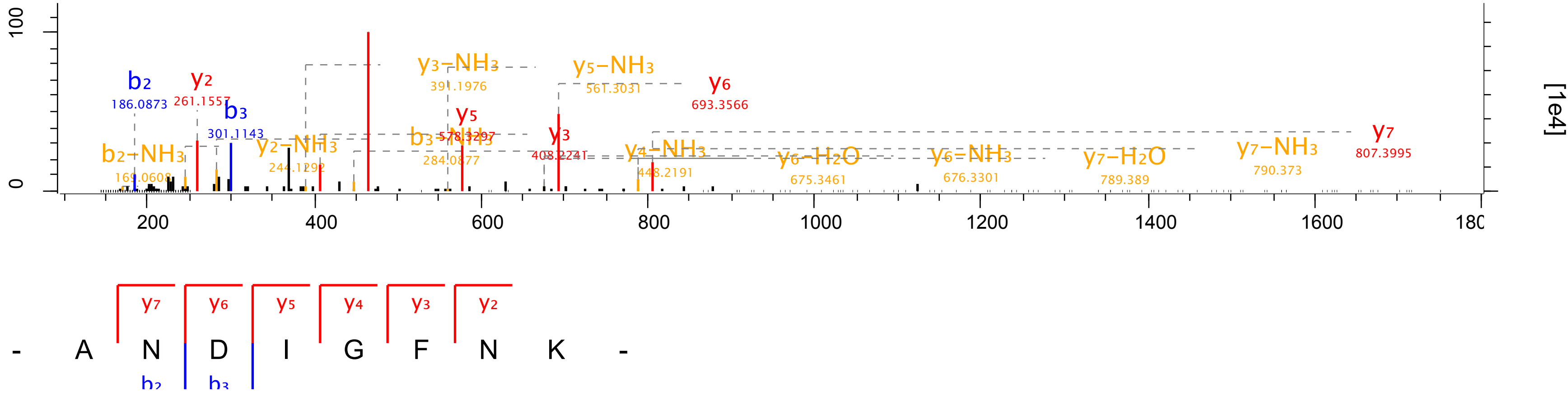

Raw file	Scan	Method	Score	m/z	Gene names
20150306_yeast1_Top_opt_2ug_C1_01_1670	16193	TOF; CID	75.65	531.27	SIP3

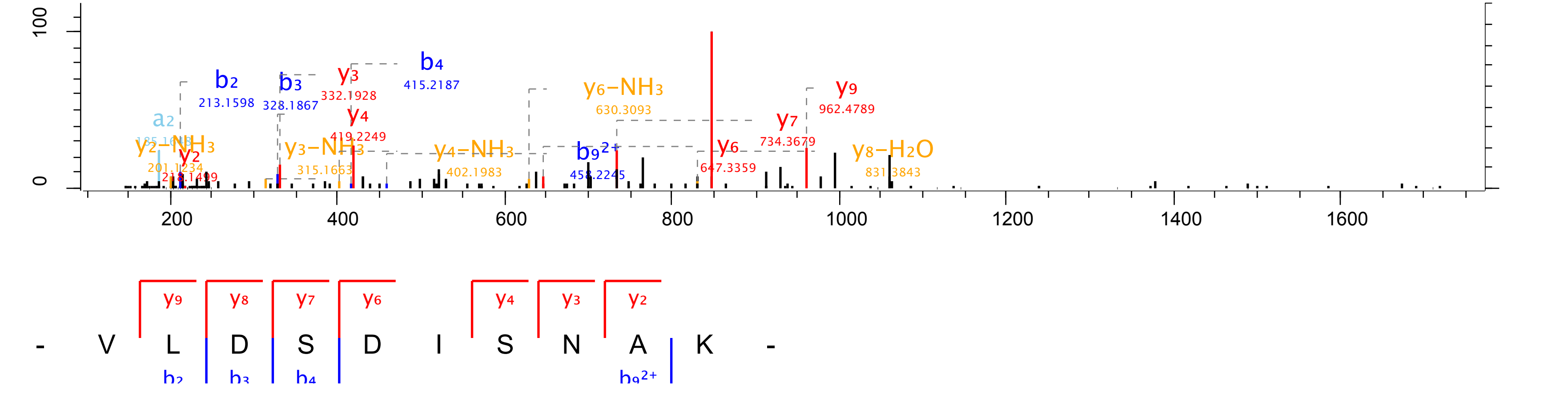

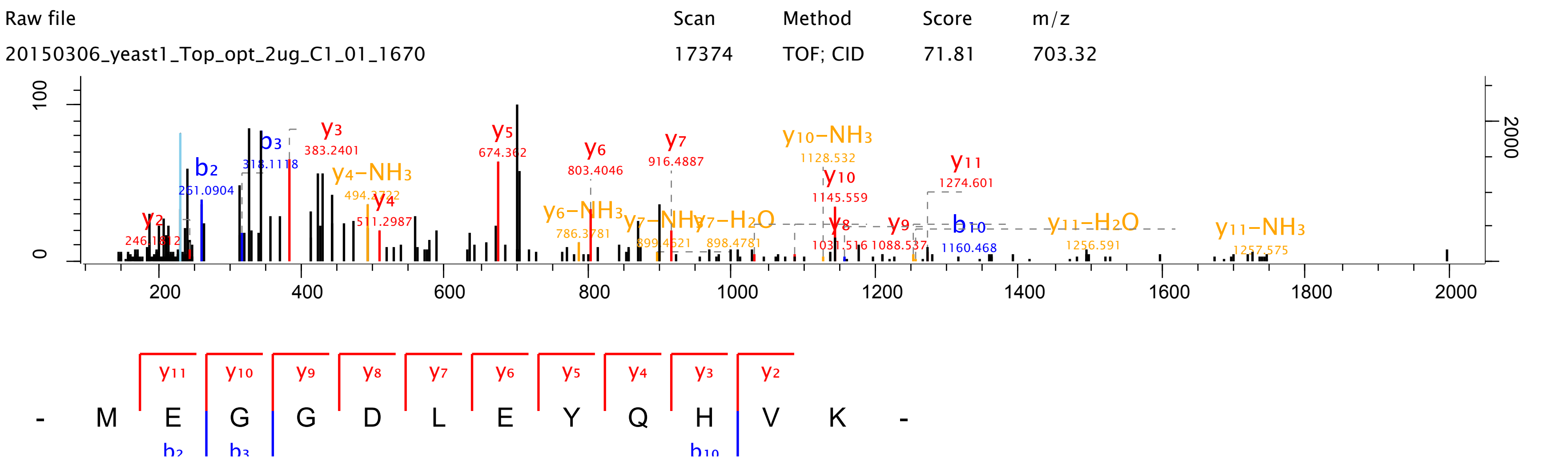

Raw file
20150306_yeast1_Top_opt_2ug_C1_01_1670
Scan
17374
Method
TOF; CID
Score
71.81
m/z
703.32
y2
246.1812
b2
251.0904
b3
318.1118
y3
383.2401
y4−NH3
494.2722
y4
511.2987
y5
674.362
y6−NH3
786.3781
y6
803.4046
y7−NH3
899.4621
y7−H2O
898.4781
y7
916.4887
y8
1031.516
y9
1088.537
y10−NH3
1128.532
y10
1145.559
b10
1160.468
y11
1274.601
y11−H2O
1256.591
y11−NH3
1257.575
- M E G G D L E Y Q H V K -

Raw file	Scan	Method	Score	m/z	Gene names
20150306_yeast1_Top_opt_2ug_C1_01_1670	18656	TOF; CID	69.81	613.3	HMS2

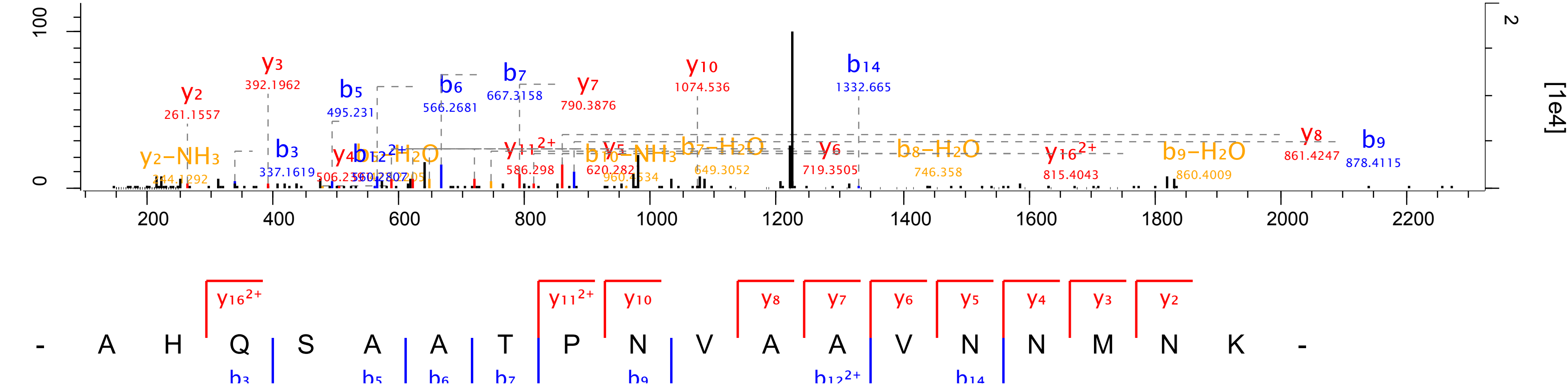

Raw file	Scan	Method	Score	m/z	Gene names
20150306_yeast1_Top_opt_2ug_C1_01_1670	21064	TOF; CID	98.41	587.32	UGO1

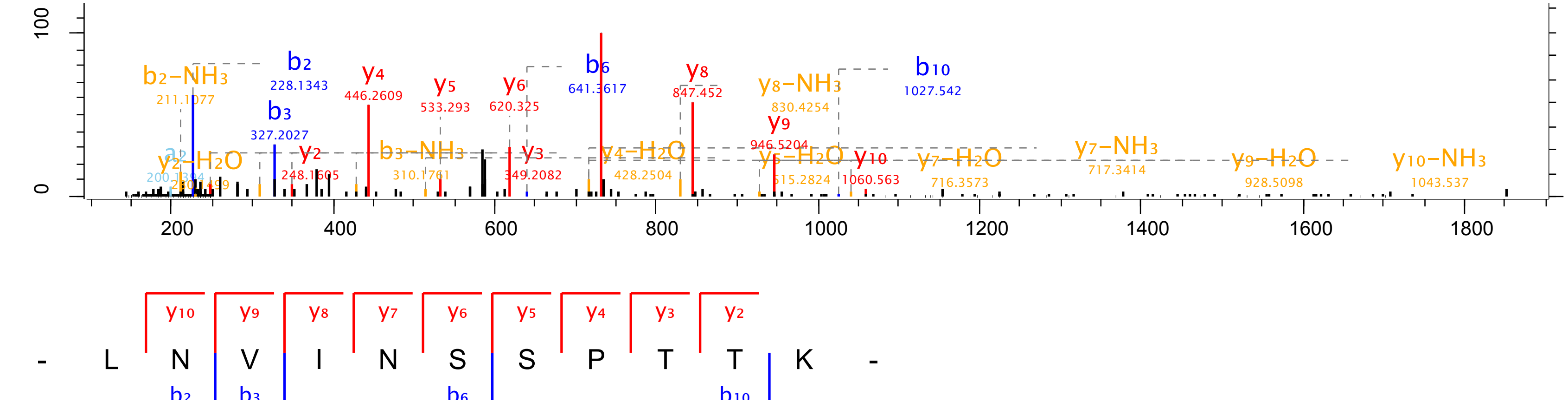

Raw file	Scan	Method	Score	m/z	Gene names
20150306_yeast1_Top_opt_2ug_C1_01_1670	23244	TOF; CID	68.66	481.26	YSC83

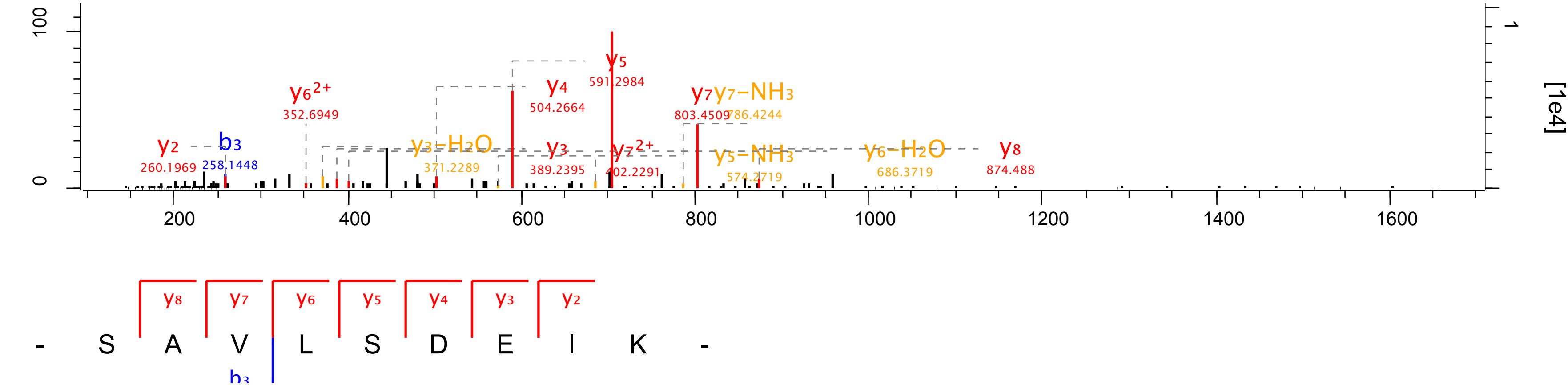

Raw file	Scan	Method	Score	m/z	Gene names
20150306_yeast1_Top_opt_2ug_C1_01_1670	23637	TOF; CID	96.23	433.22	LSM8

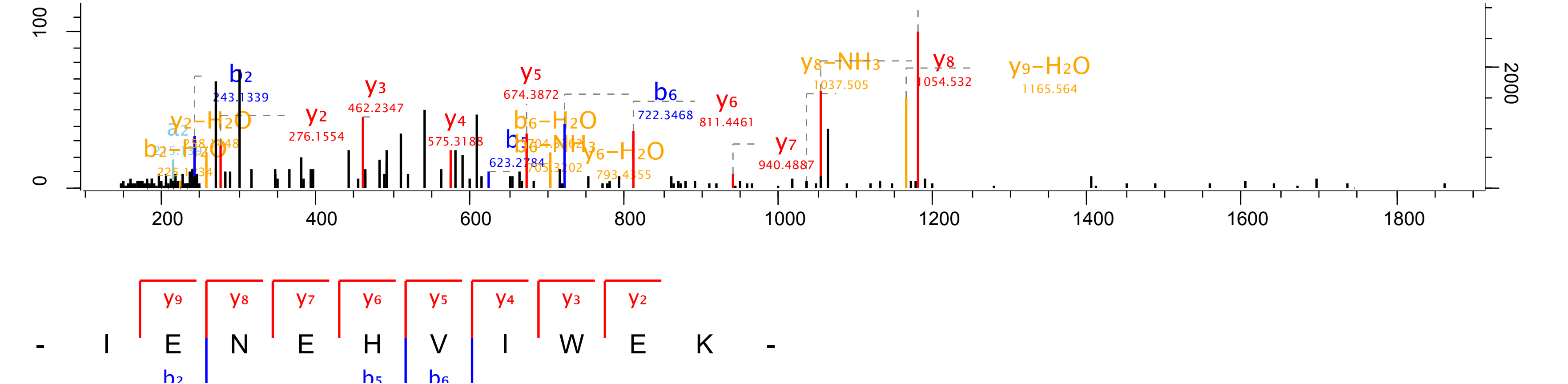

Raw file	Scan	Method	Score	m/z	Gene names
20150306_yeast1_Top_opt_2ug_C1_01_1670	25067	TOF; CID	88.06	618.8	NGR1

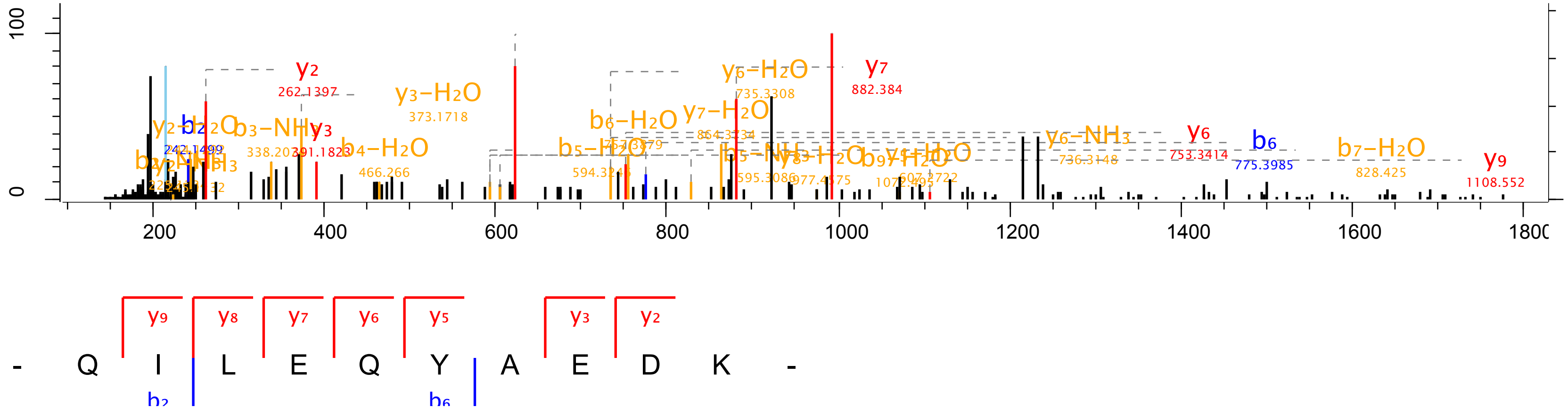

Raw file	Scan	Method	Score	m/z	Gene names
20150306_yeast1_Top_opt_2ug_C1_01_1670	25520	TOF; CID	56.75	823.05	YDR374W-A

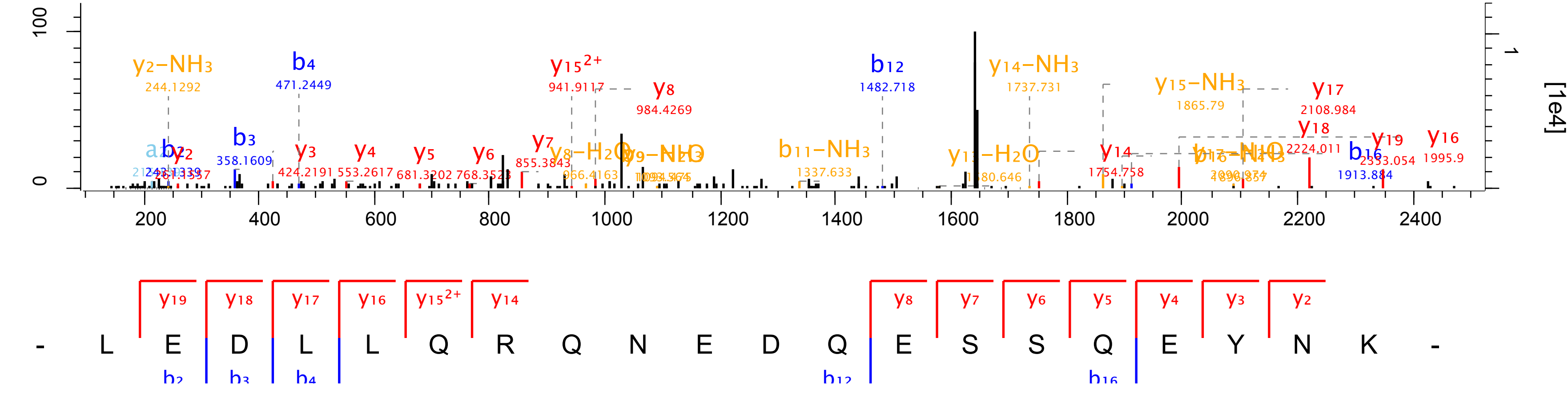

Raw file	Scan	Method	Score	m/z	Gene names
20150306_yeast1_Top_opt_2ug_C1_01_1670	26807	TOF; CID	189.81	610.96	MDM35

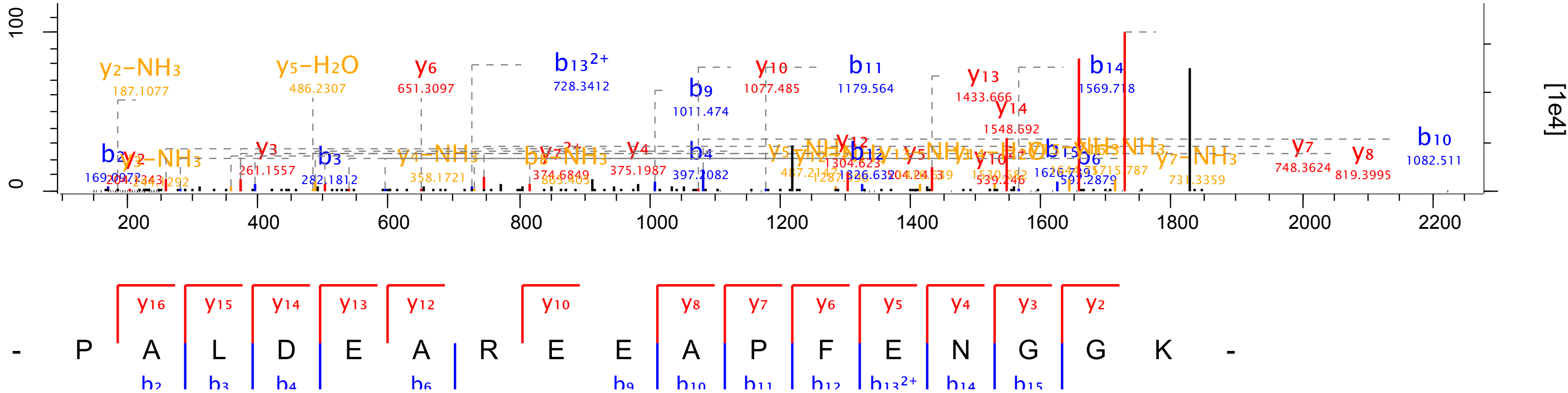

Raw file	Scan	Method	Score	m/z	Gene names
20150306_yeast1_Top_opt_2ug_C1_01_1670	29171	TOF; CID	52.39	589.82	ECM3

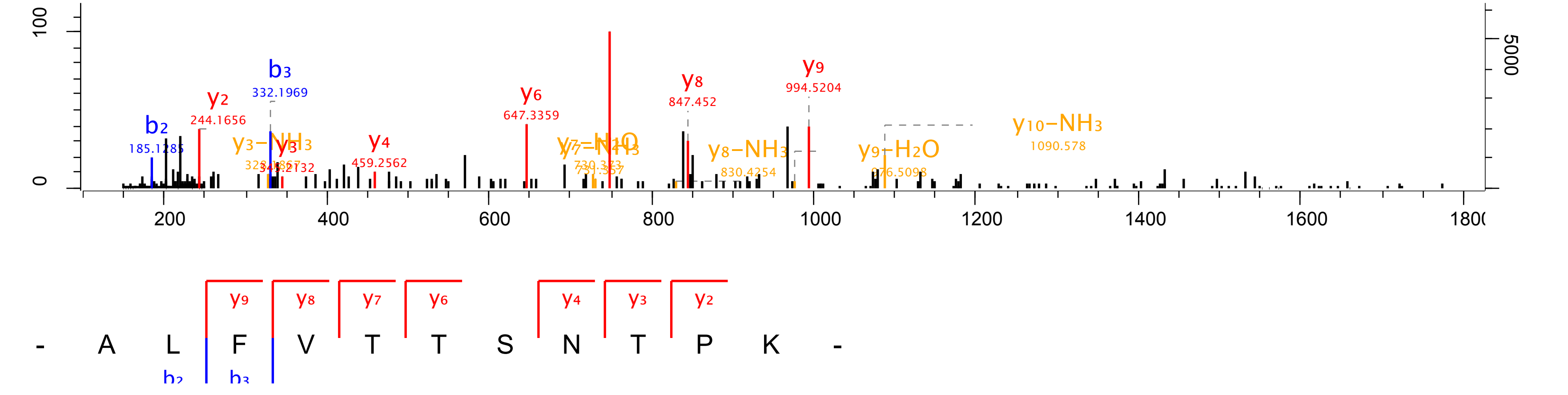

Raw file	Scan	Method	Score	m/z	Gene names
20150306_yeast1_Top_opt_2ug_C1_01_1670	29172	TOF; CID	69.19	572.97	TBS1

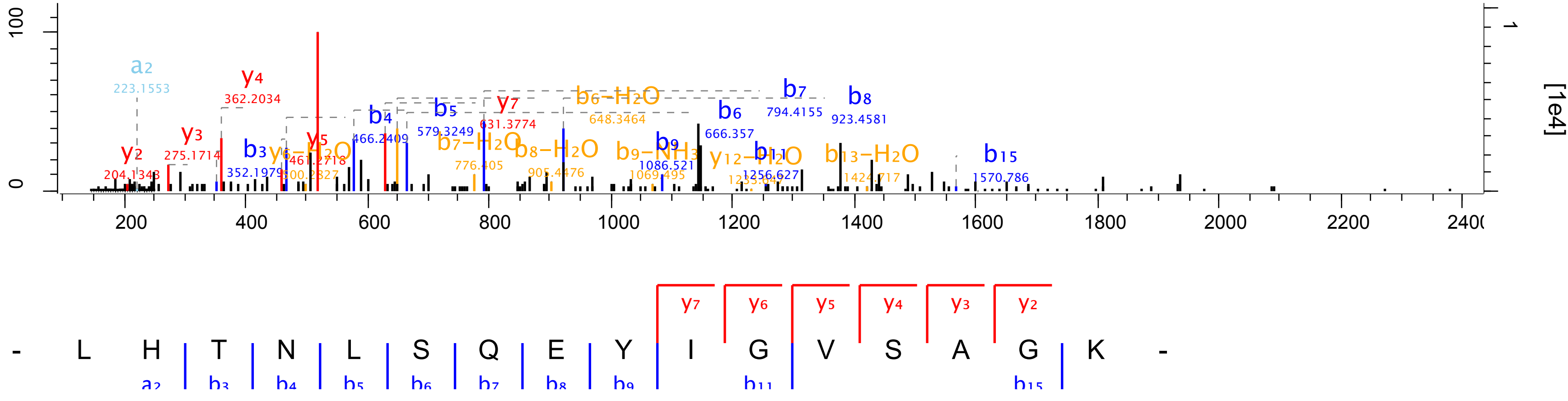

Raw file	Scan	Method	Score	m/z	Gene names
20150306_yeast1_Top_opt_2ug_C1_01_1670	29190	TOF; CID	71.08	593.83	TEC1

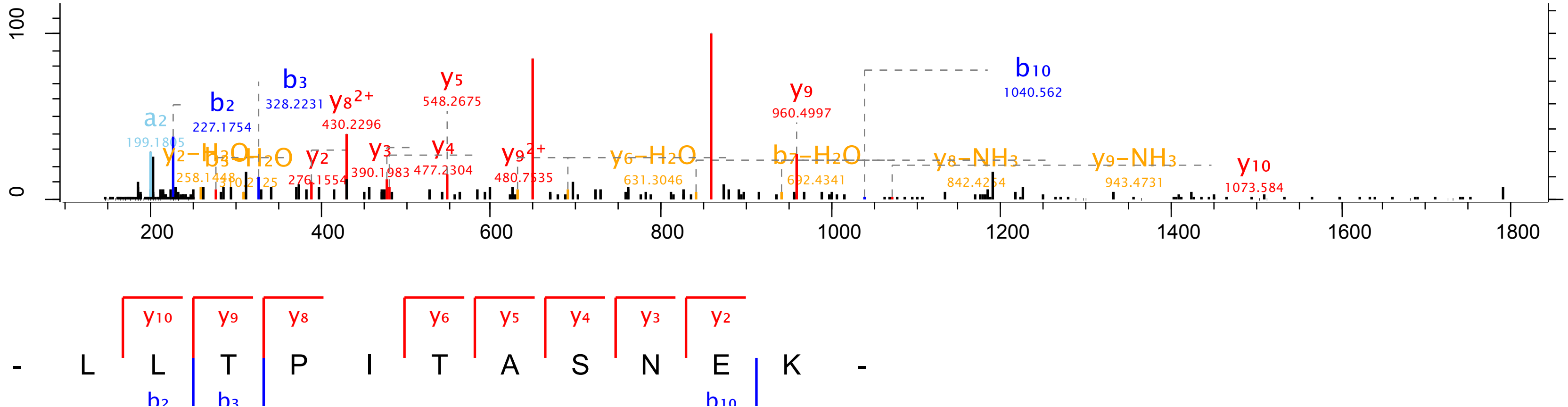

Raw file	Scan	Method	Score	m/z	Gene names
20150306_yeast1_Top_opt_2ug_C1_01_1670	33331	TOF; CID	68.21	436.28	COQ9

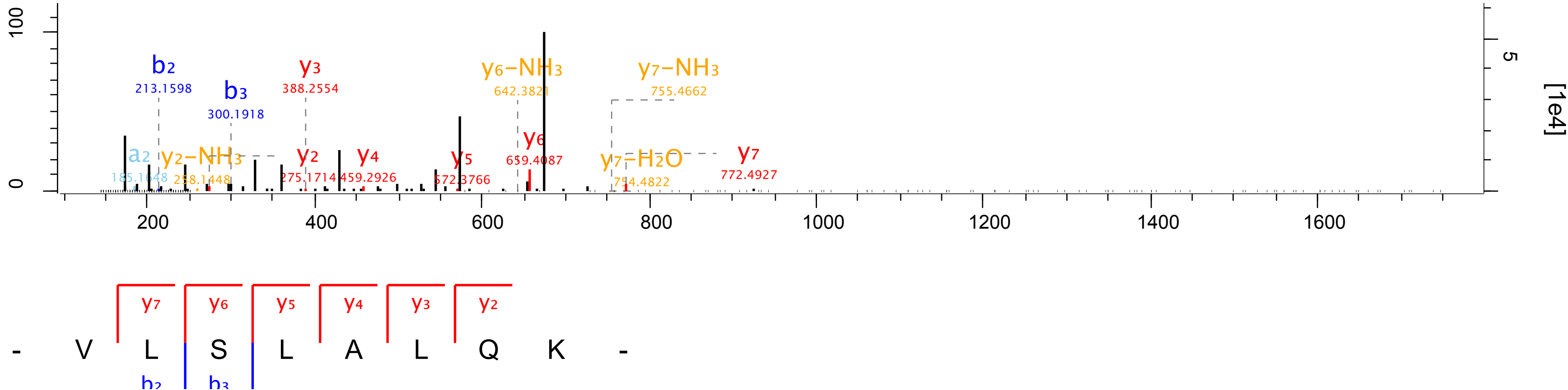

Raw file	Scan	Method	Score	m/z
20150306_yeast1_Top_opt_2ug_C1_01_1670	36251	TOF; CID	107.21	548.76

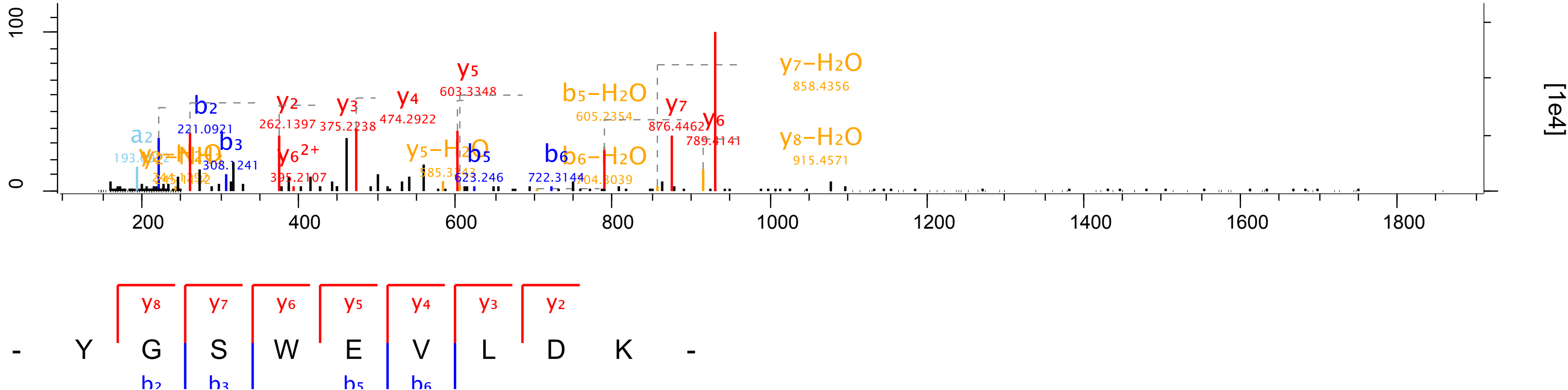

Raw file	Scan	Method	Score	m/z
20150306_yeast1_Top_opt_2ug_C1_01_1670	37795	TOF; CID	63.18	575.28

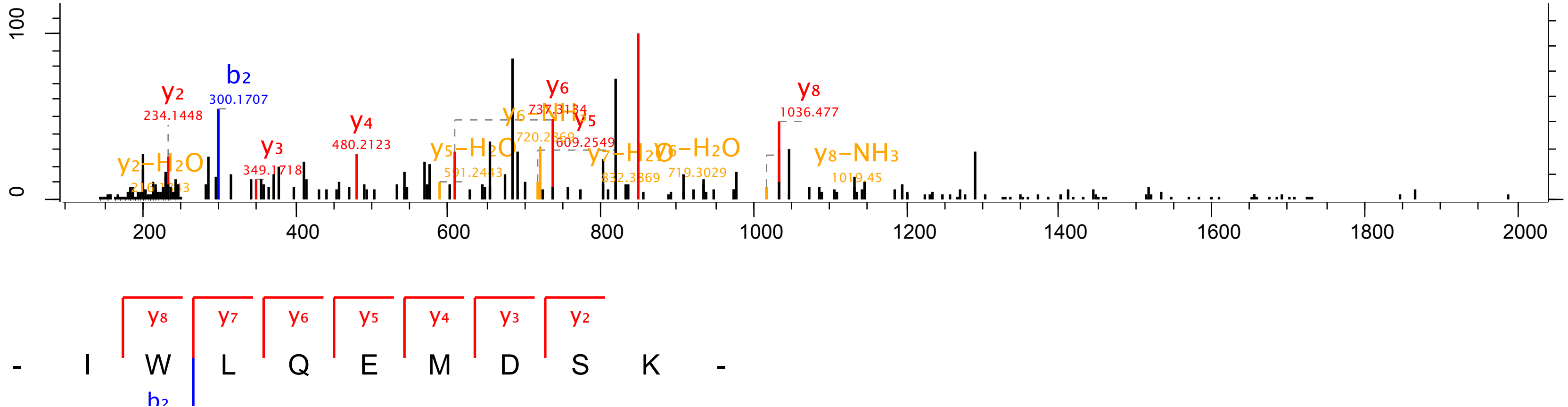

Raw file	Scan	Method	Score	m/z	Gene names
20150306_yeast1_Top_opt_2ug_C1_01_1670	39299	TOF; CID	60.55	610.29	AIR2

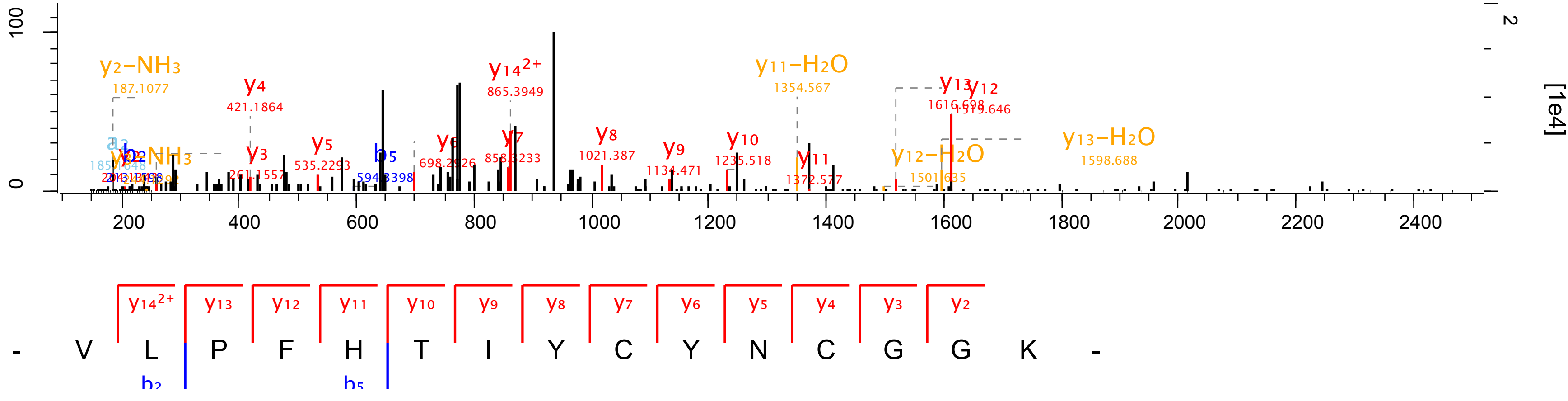

Raw file	Scan	Method	Score	m/z	Gene names
20150306_yeast1_Top_opt_2ug_C1_01_1670	39982	TOF; CID	94.69	400.55	YHR162W;FMP43

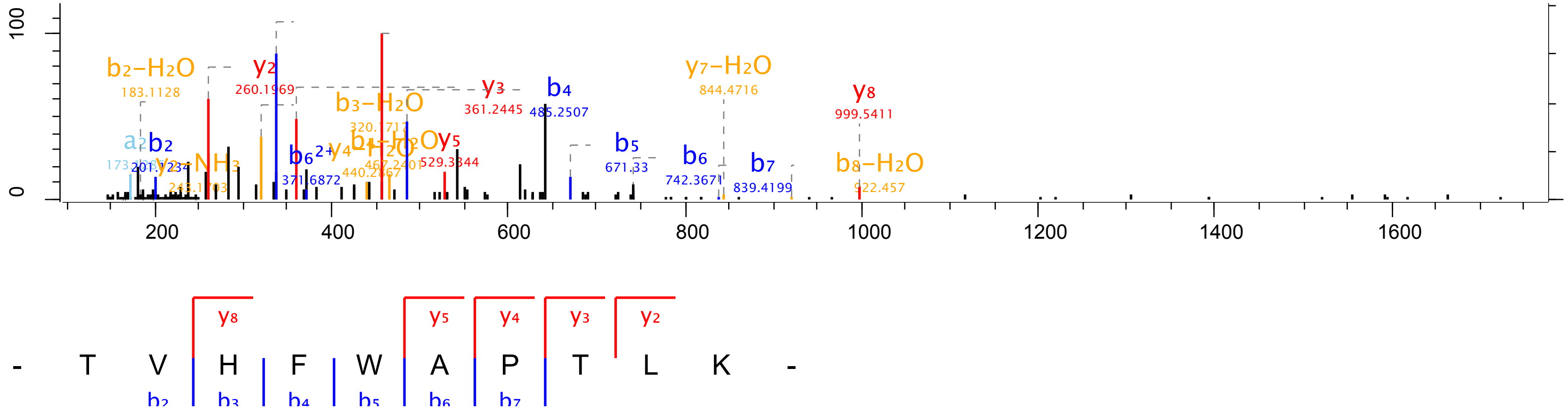

Raw file	Scan	Method	Score	m/z	Gene names
20150306_yeast1_Top_opt_2ug_C1_01_1670	40244	TOF; CID	190.96	985.98	TIF6

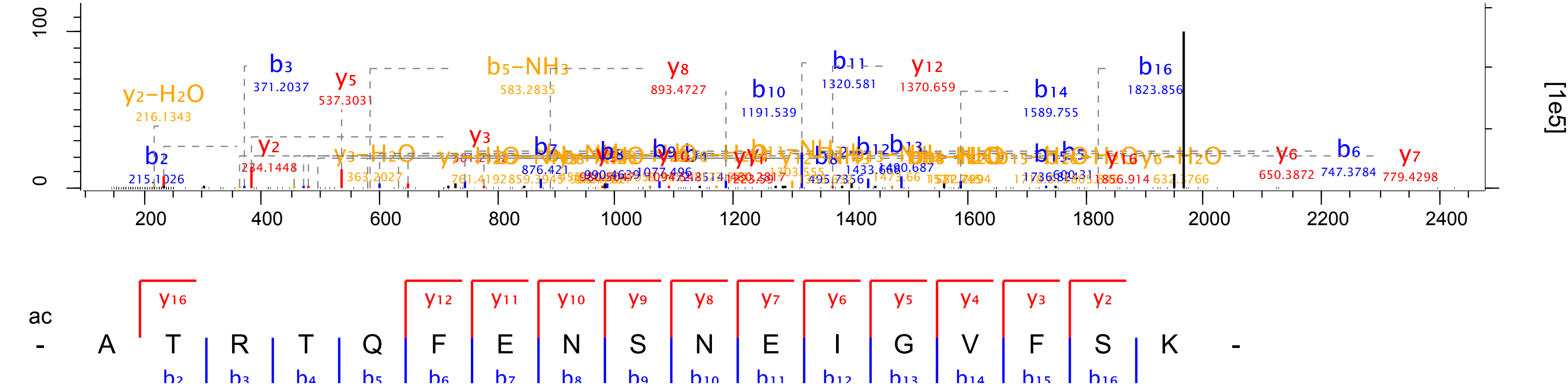

Raw file	Scan	Method	Score	m/z	Gene names
20150306_yeast1_Top_opt_2ug_C1_01_1670	40889	TOF; CID	43.59	857.9	PSD2

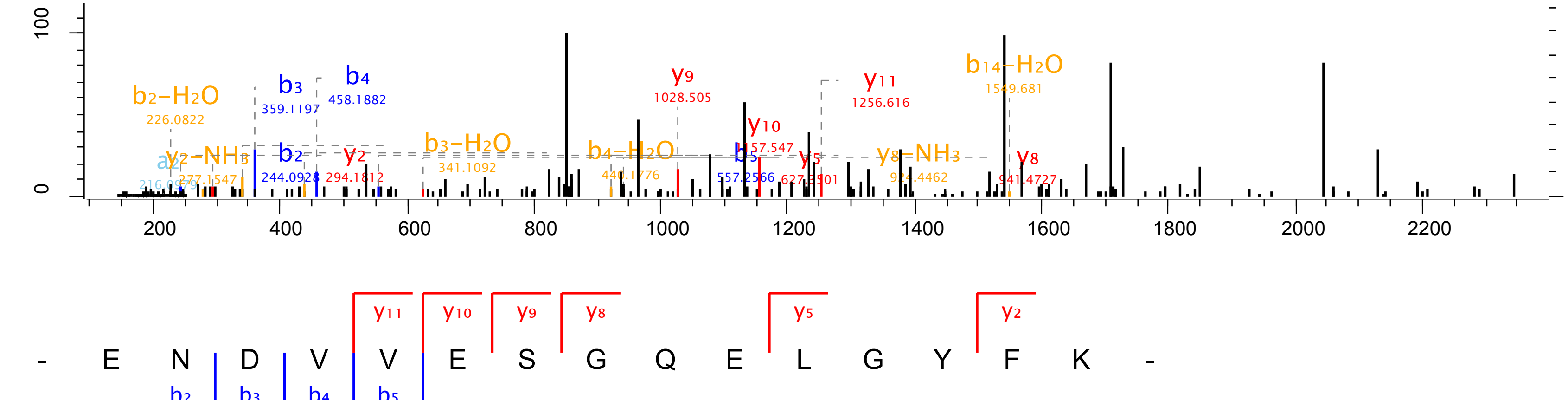

Raw file	Scan	Method	Score	m/z	Gene names
20150306_yeast1_Top_opt_2ug_C1_01_1670	42627	TOF; CID	52.07	658.85	FUS3

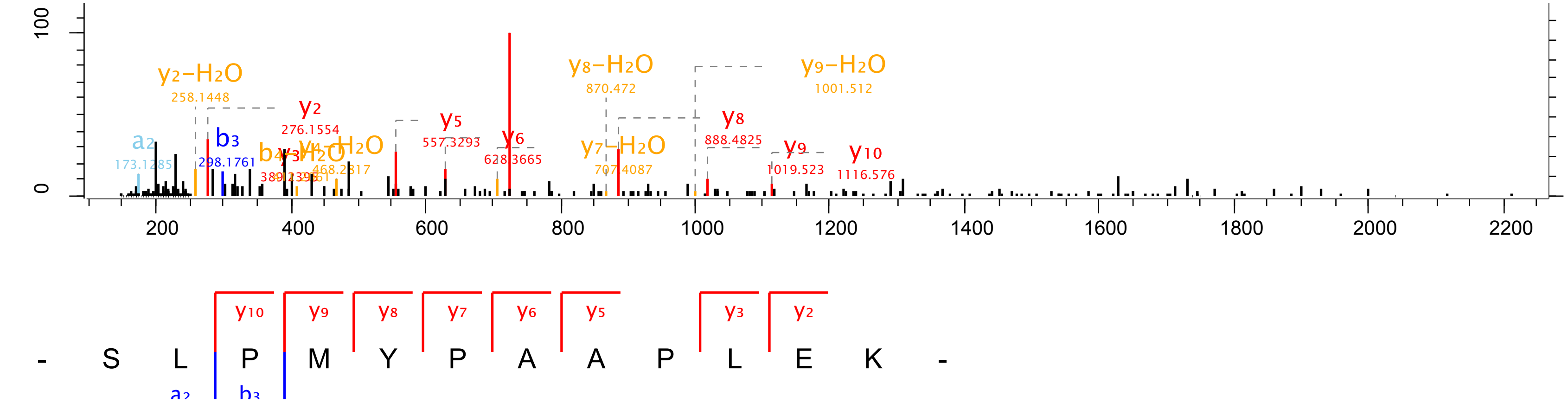

Raw file	Scan	Method	Score	m/z	Gene names
20150306_yeast1_Top_opt_2ug_C1_01_1670	42859	TOF; CID	50.08	736.89	LST4

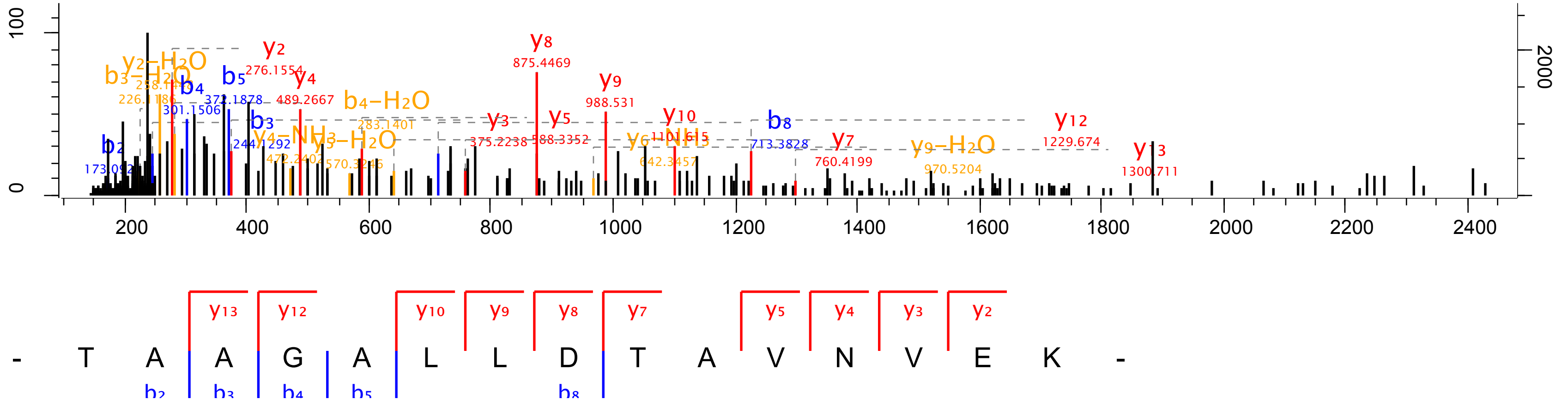

Raw file	Scan	Method	Score	m/z	Gene names
20150306_yeast1_Top_opt_2ug_C1_01_1670	43302	TOF; CID	92.54	626.83	DOT1

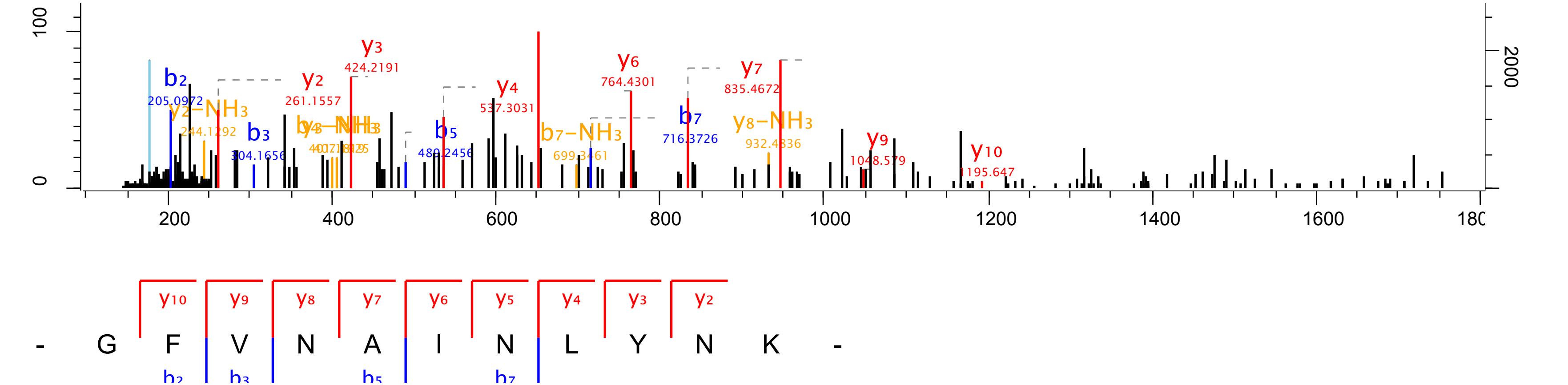

Raw file	Scan	Method	Score	m/z	Gene names
20150306_yeast1_Top_opt_2ug_C1_01_1670	44790	TOF; CID	107.75	851.42	RFX1

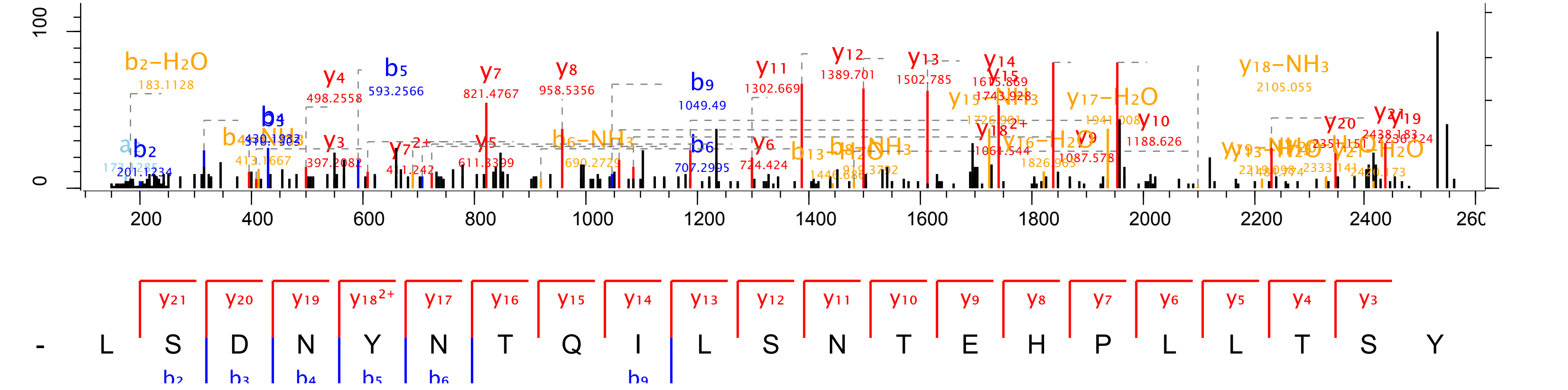

Raw file	Scan	Method	Score	m/z	Gene names
20150306_yeast1_Top_opt_2ug_C1_01_1670	45312	TOF; CID	124.19	891.42	YBR255C-A

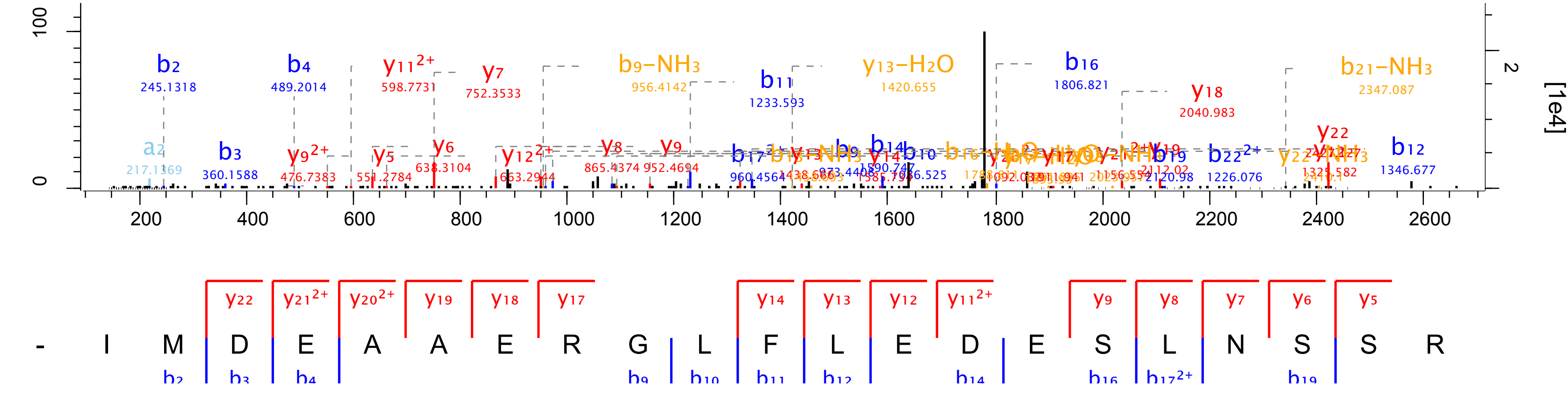

Raw file	Scan	Method	Score	m/z	Gene names
20150306_yeast1_Top_opt_2ug_C1_01_1670	47784	TOF; CID	54.26	590.8	MRL1

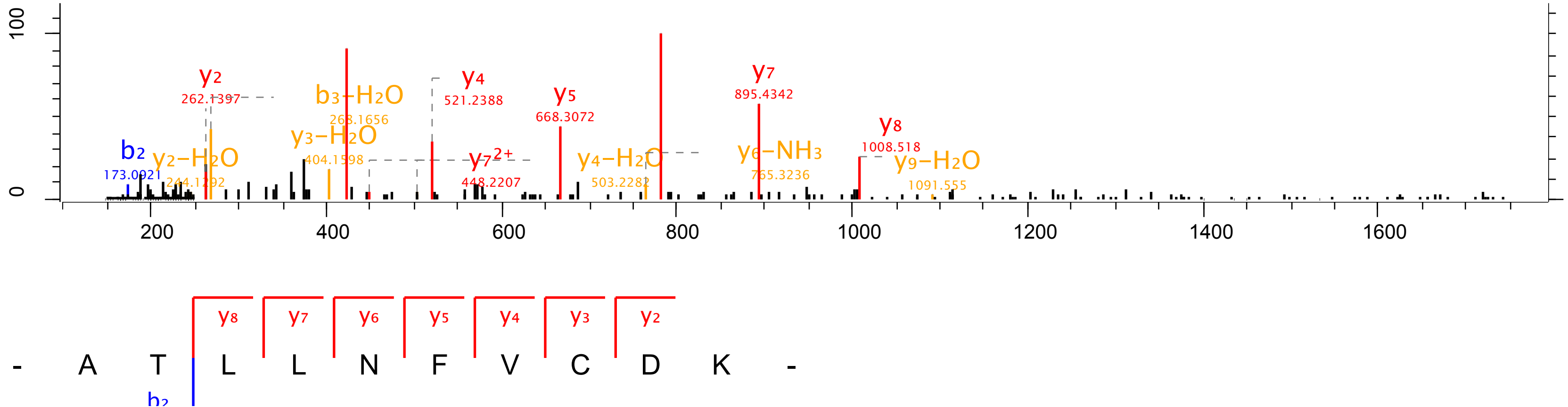

Raw file	Scan	Method	Score	m/z	Gene names
20150306_yeast1_Top_opt_2ug_C1_01_1670	48513	TOF; CID	61.94	730.37	ADF1

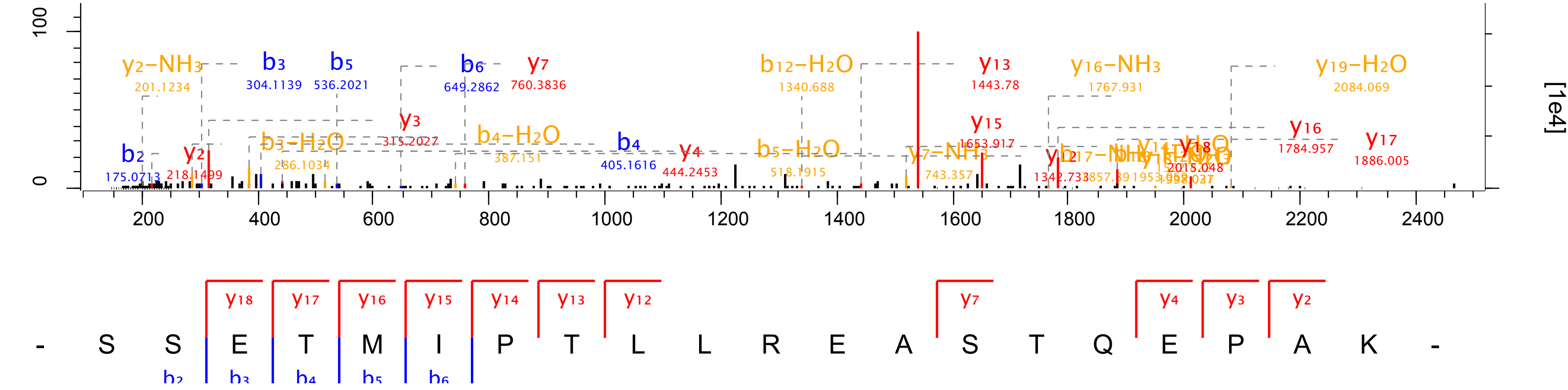

Raw file	Scan	Method	Score	m/z	Gene names
20150306_yeast1_Top_opt_2ug_C1_01_1670	49457	TOF; CID	87.96	745.37	DAS1

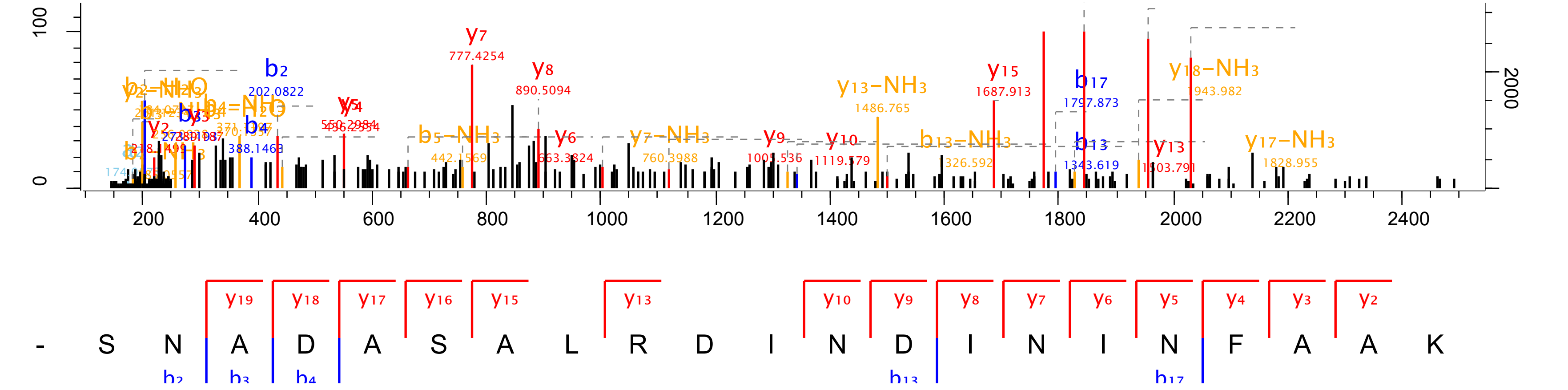

Raw file	Scan	Method	Score	m/z	Gene names
20150306_yeast1_Top_opt_2ug_C1_01_1670	62064	TOF; CID	105.58	627.97	VPS68

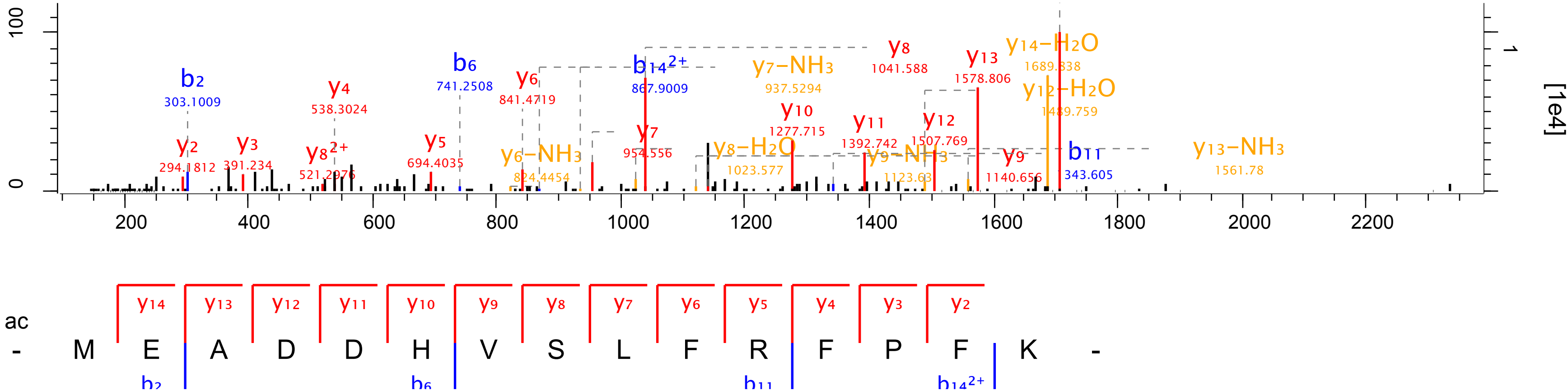

Raw file	Scan	Method	Score	m/z	Gene names
20150306_yeast1_Top_opt_2ug_C1_01_1670	62654	TOF; CID	104.17	735.38	TOM7

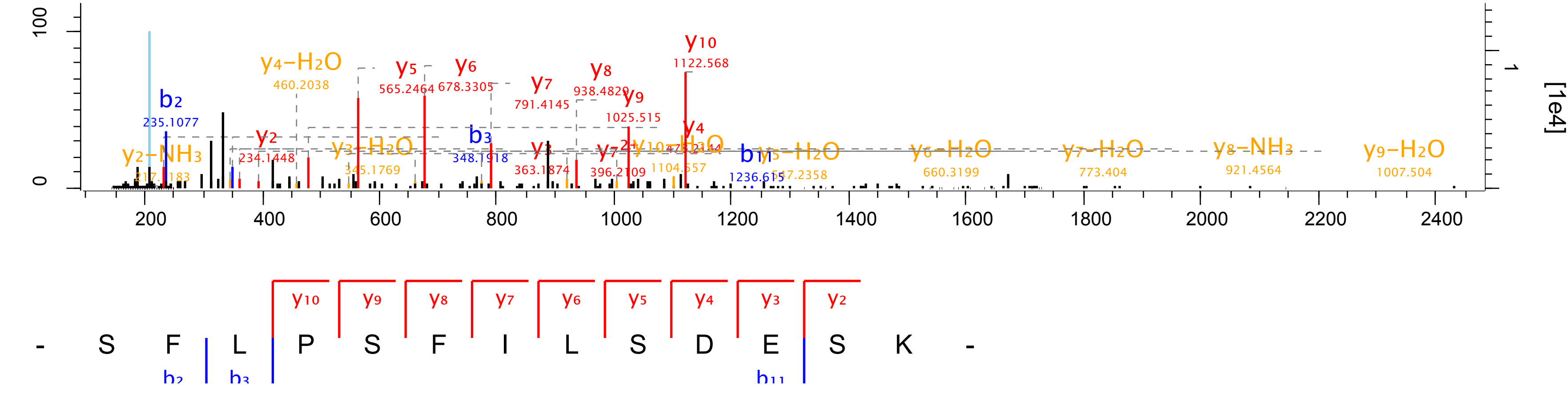

Raw file	Scan	Method	Score	m/z	Gene names
20150306_yeast1_Top_opt_2ug_C1_01_1670	63431	TOF; CID	92.26	1093.54	PPH3

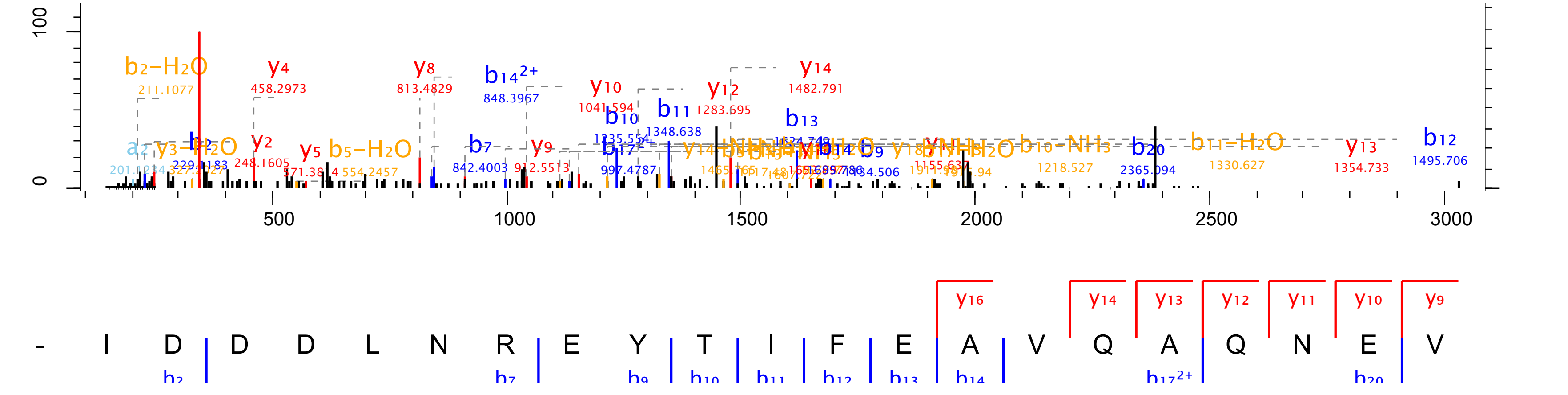

Raw file	Scan	Method	Score	m/z	Gene names
20150306_yeast1_Top_opt_2ug_C1_01_1670	63859	TOF; CID	48.23	1127.57	JJJ2

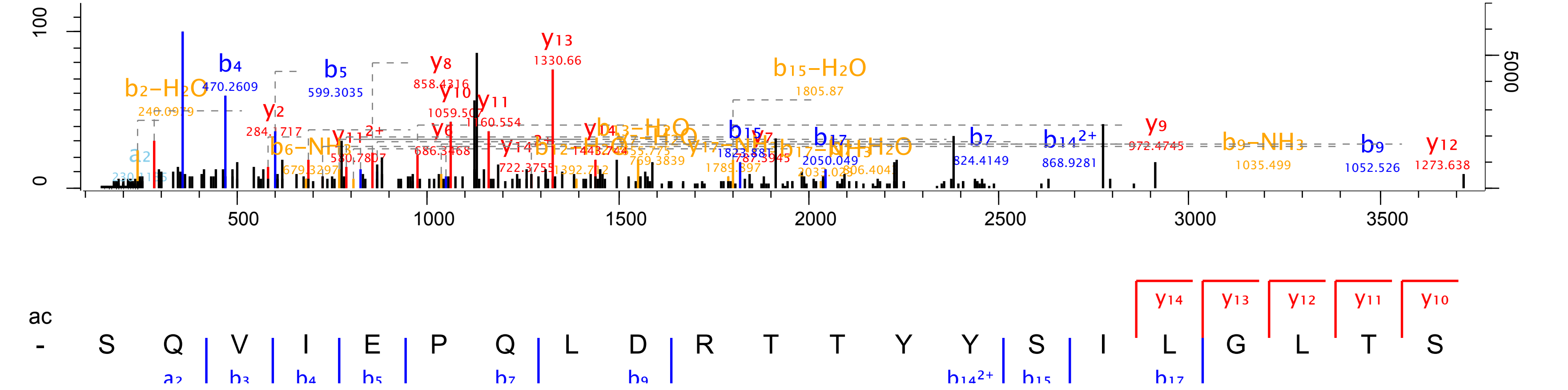

Raw file	Scan	Method	Score	m/z	Gene names
20150306_yeast1_Top_opt_2ug_C1_01_1670	66955	TOF; CID	67.93	1018.51	CIA1

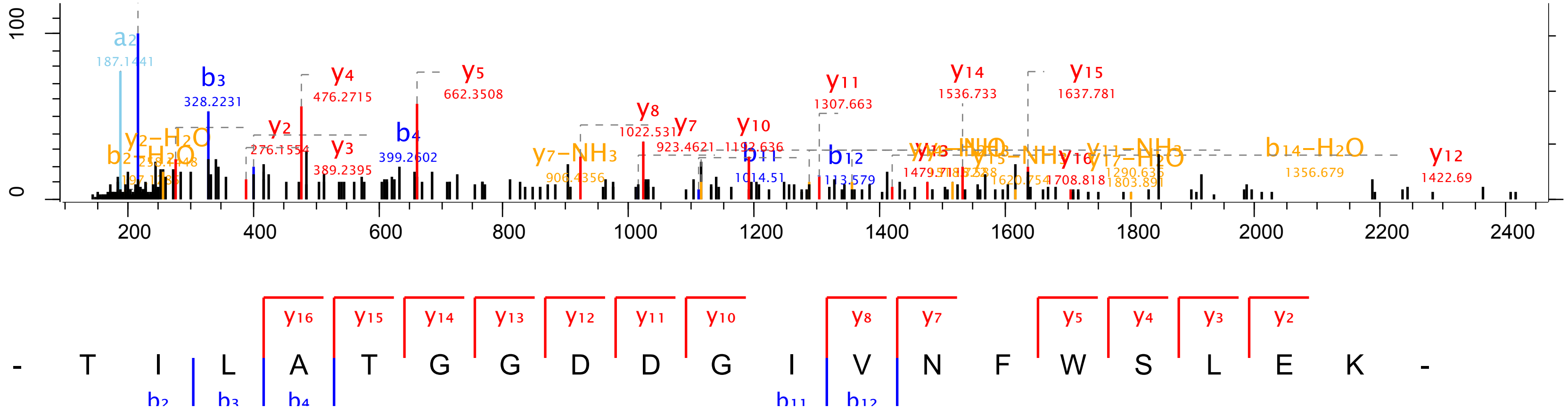

Raw file	Scan	Method	Score	m/z	Gene names
20150306_yeast1_Top_opt_2ug_C1_01_1670	69634	TOF; CID	151.52	754.43	LSM5

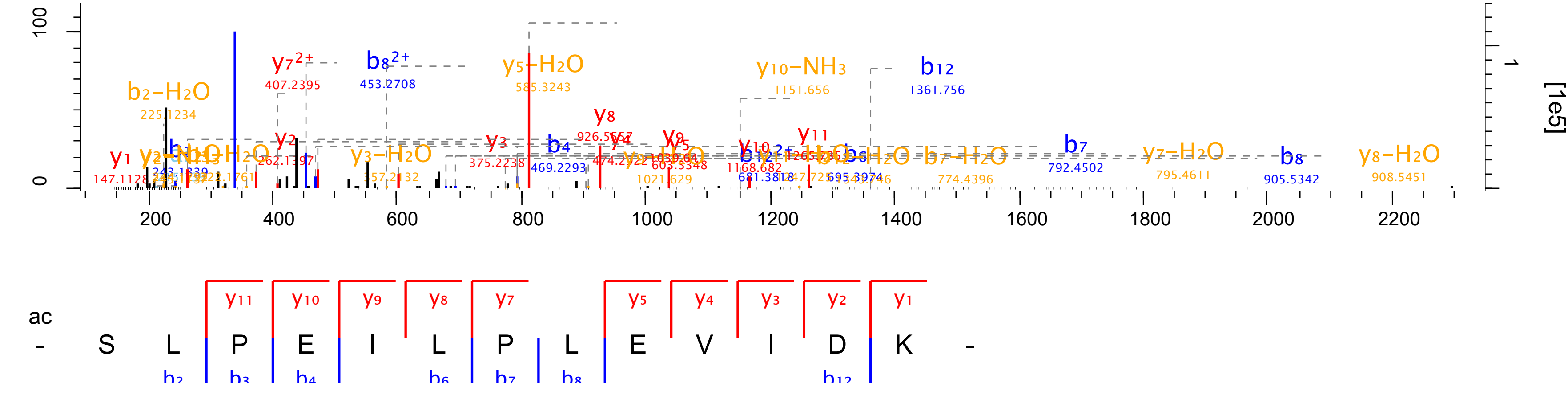

Raw file	Scan	Method	Score	m/z	Gene names
20150306_yeast1_Top_opt_2ug_C1_01_1670	70145	TOF; CID	119.38	1008.54	SPC19

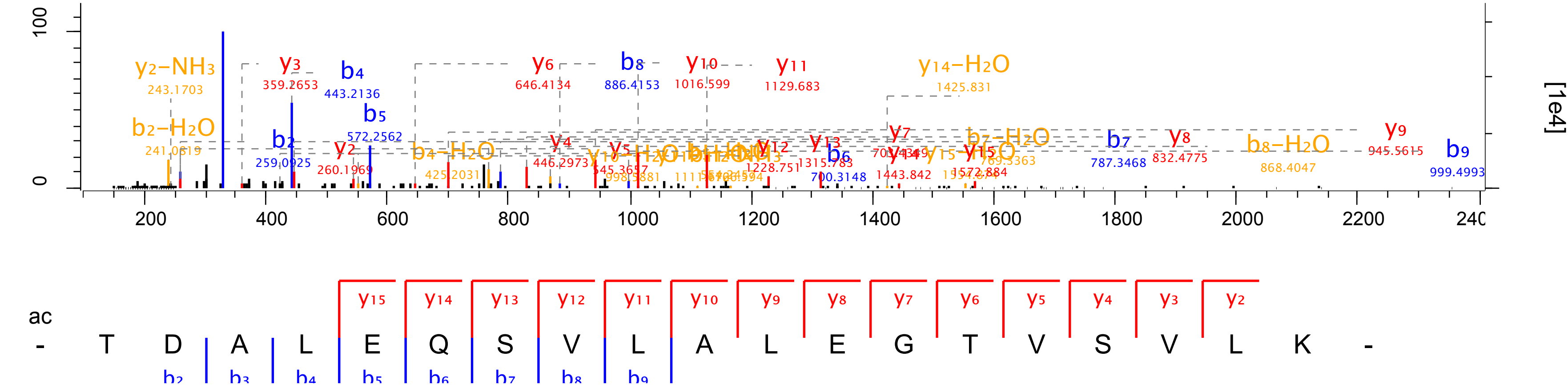

Raw file	Scan	Method	Score	m/z	Gene names
20150306_yeast3_Top_opt_2ug_C3_01_1664	4882	TOF; CID	136.53	640.32	COX14

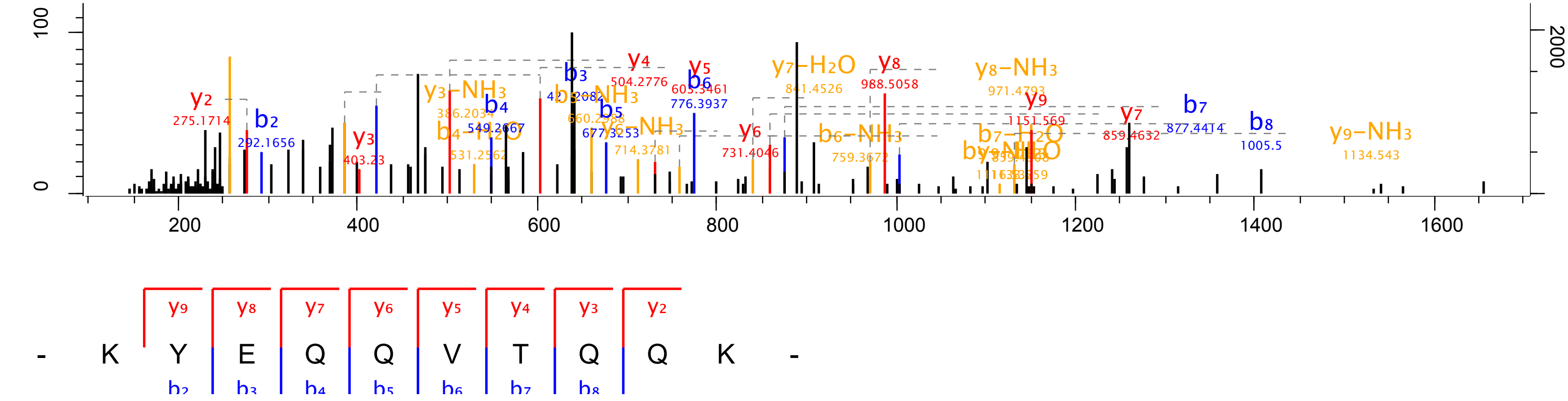

Raw file	Scan	Method	Score	m/z	Gene names
20150306_yeast3_Top_opt_2ug_C3_01_1664	8025	TOF; CID	82.65	576.26	VPS20

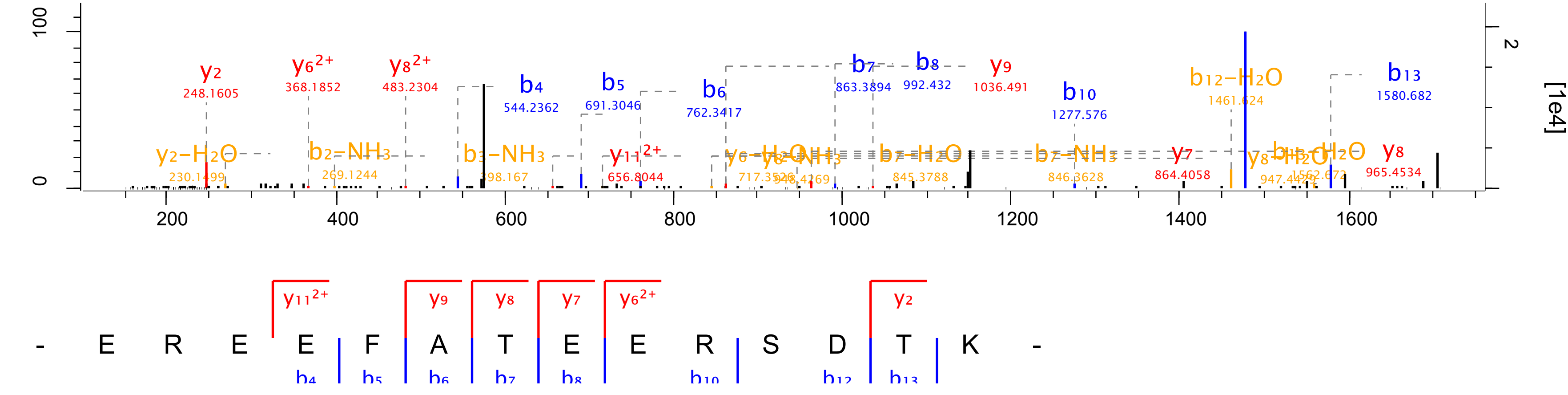

Raw file	Scan	Method	Score	m/z	Gene names
20150306_yeast3_Top_opt_2ug_C3_01_1664	9490	TOF; CID	102.07	549.27	COA3

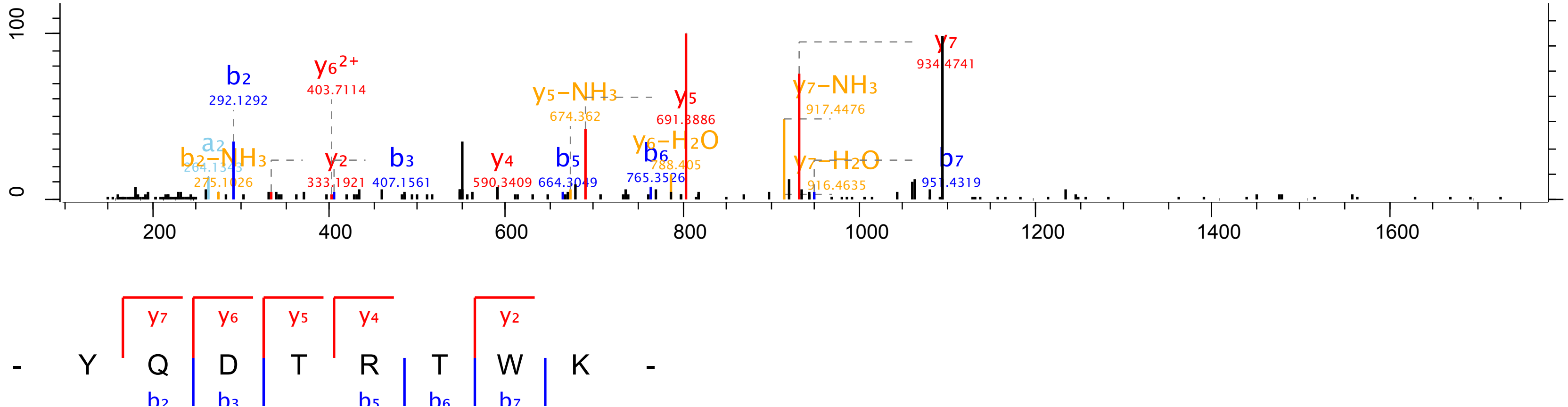

Raw file	Scan	Method	Score	m/z
20150306_yeast3_Top_opt_2ug_C3_01_1664	10617	TOF; CID	94.31	599.77

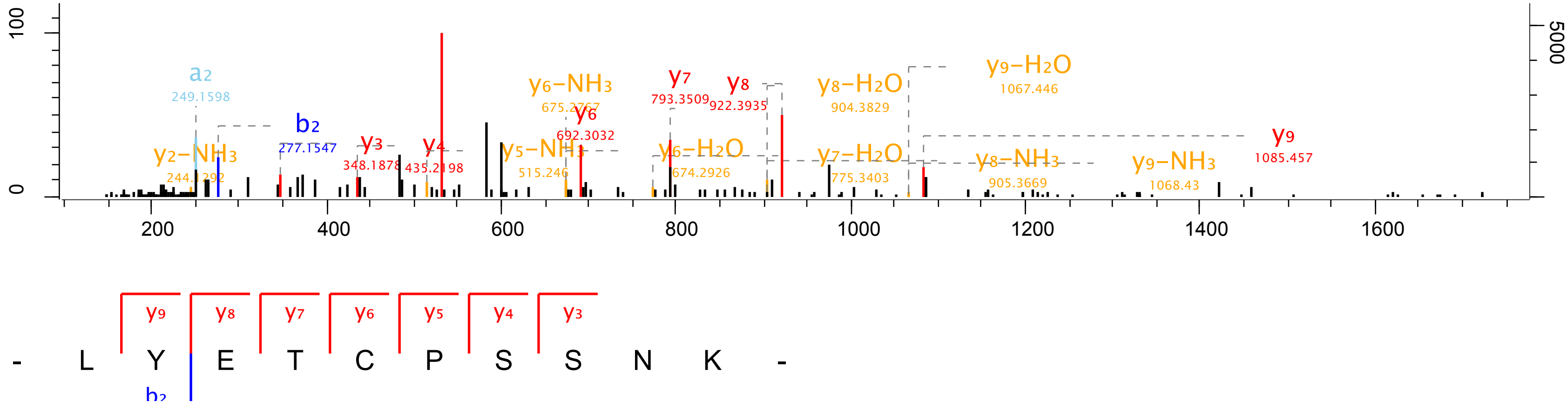

Raw file	Scan	Method	Score	m/z	Gene names
20150306_yeast3_Top_opt_2ug_C3_01_1664	14603	TOF; CID	83.87	543.75	PRY3

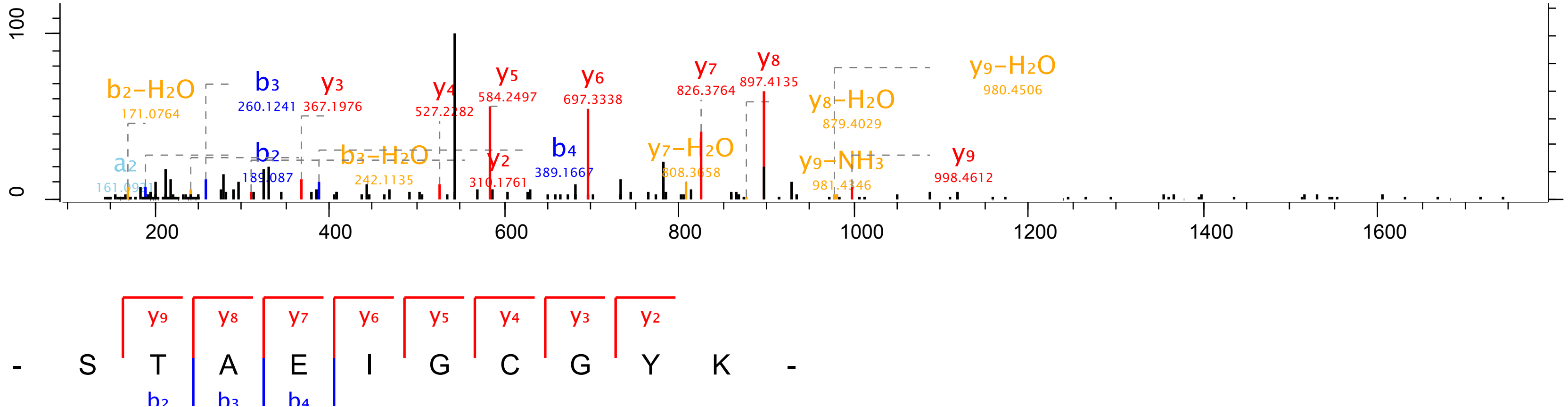

Raw file	Scan	Method	Score	m/z	Gene names
20150306_yeast3_Top_opt_2ug_C3_01_1664	15502	TOF; CID	53.56	495.91	ISY1

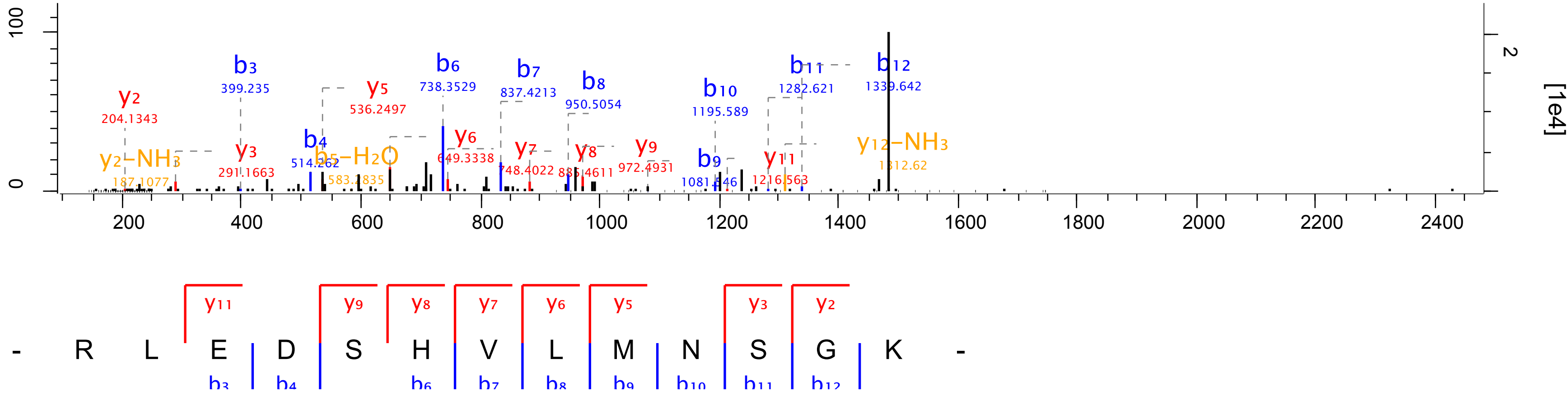

Raw file	Scan	Method	Score	m/z	Gene names
20150306_yeast3_Top_opt_2ug_C3_01_1664	15617	TOF; CID	83.69	525.92	YLR149C

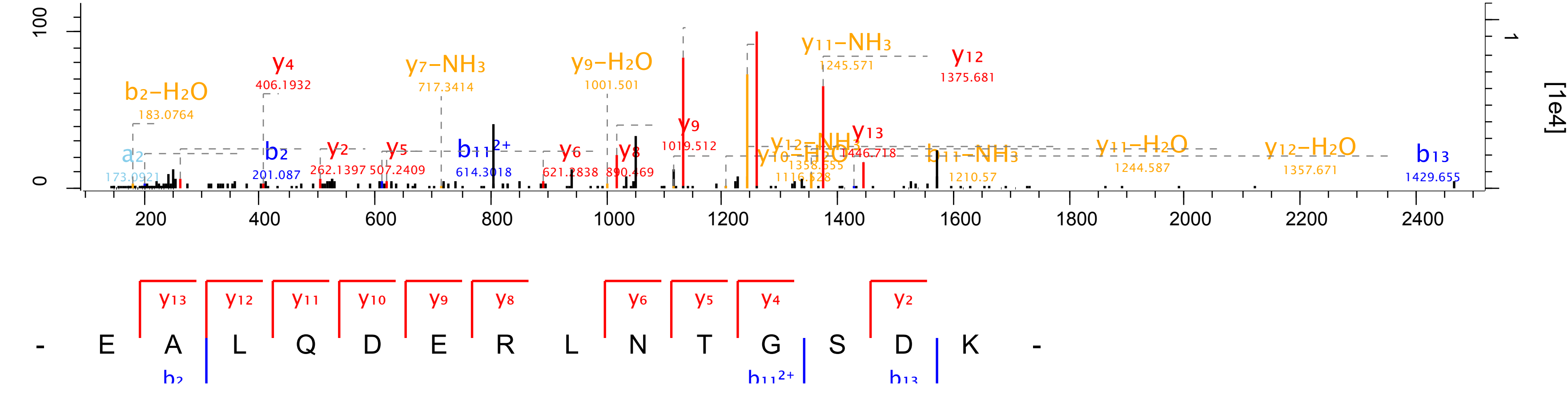

Raw file	Scan	Method	Score	m/z	Gene names
20150306_yeast3_Top_opt_2ug_C3_01_1664	17705	TOF; CID	84.73	701.35	ARG80

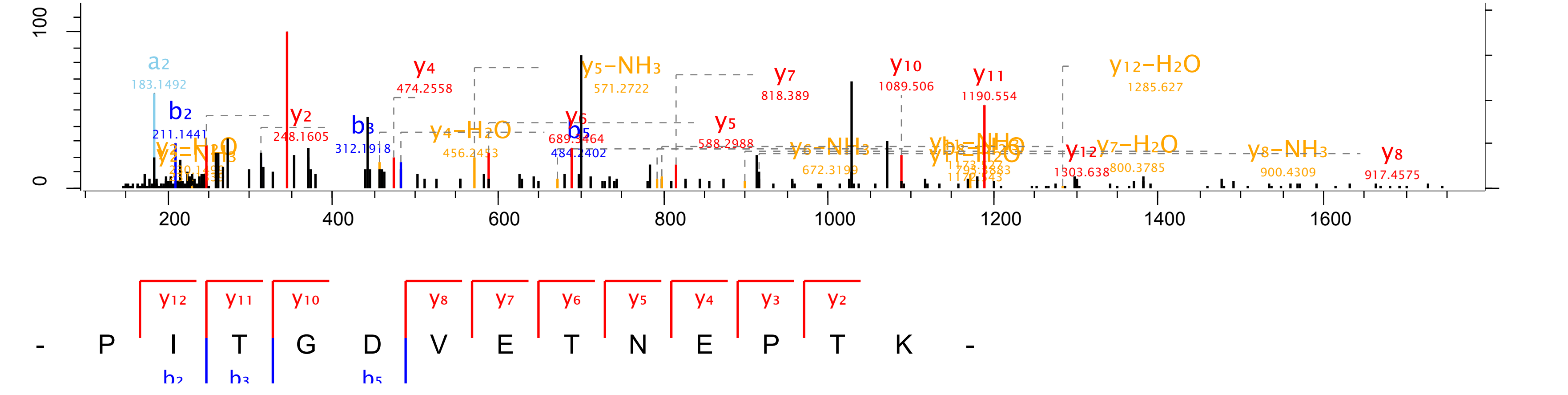

Raw file	Scan	Method	Score	m/z	Gene names
20150306_yeast3_Top_opt_2ug_C3_01_1664	21055	TOF; CID	67.75	634.34	HOR7

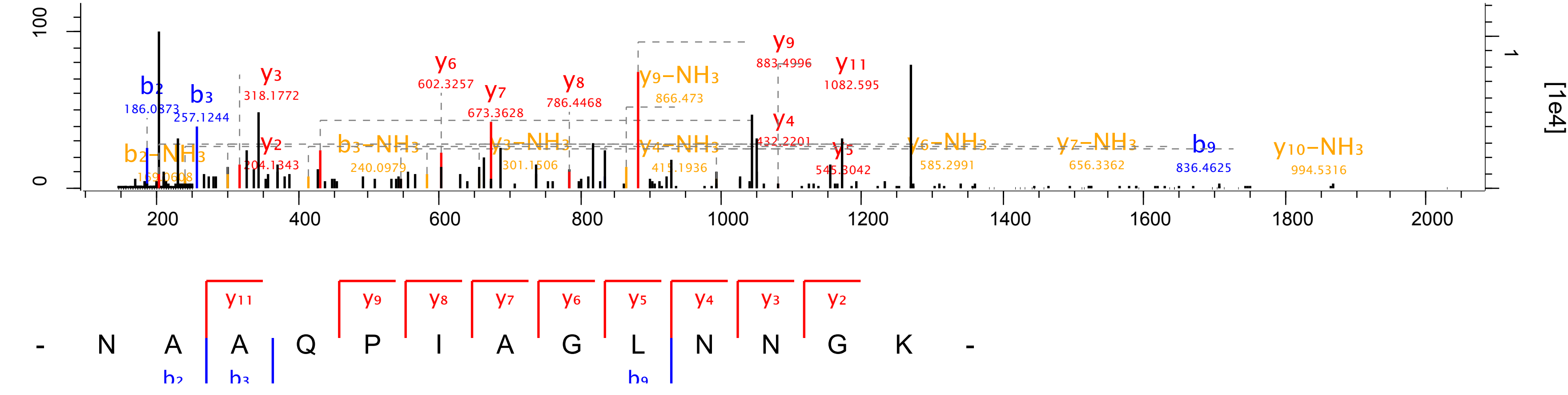

Raw file	Scan	Method	Score	m/z	Gene names
20150306_yeast3_Top_opt_2ug_C3_01_1664	22060	TOF; CID	88.56	514.27	REX3

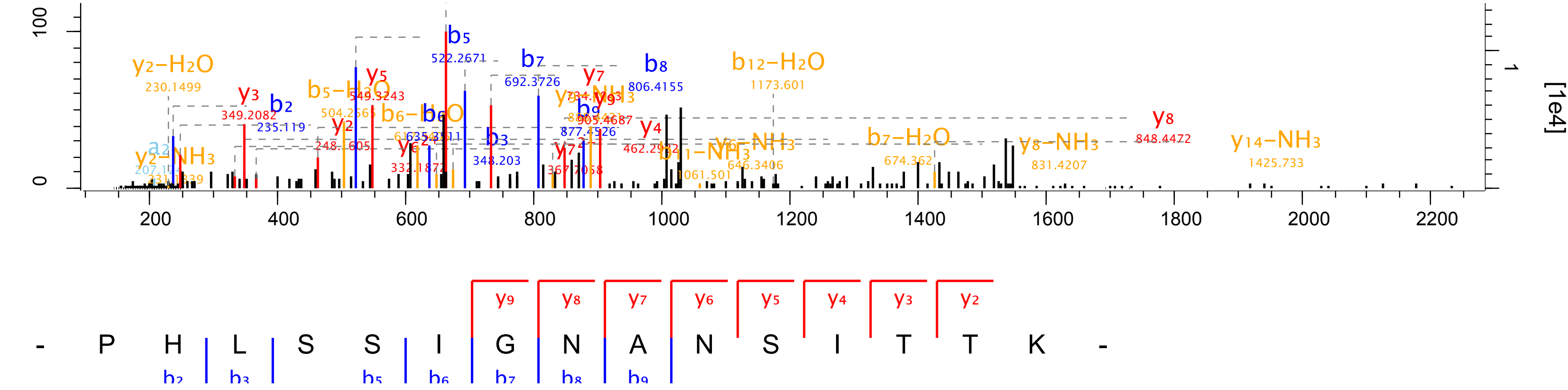

Raw file	Scan	Method	Score	m/z	Gene names
20150306_yeast3_Top_opt_2ug_C3_01_1664	26039	TOF; CID	67.25	485.57	YPL088W

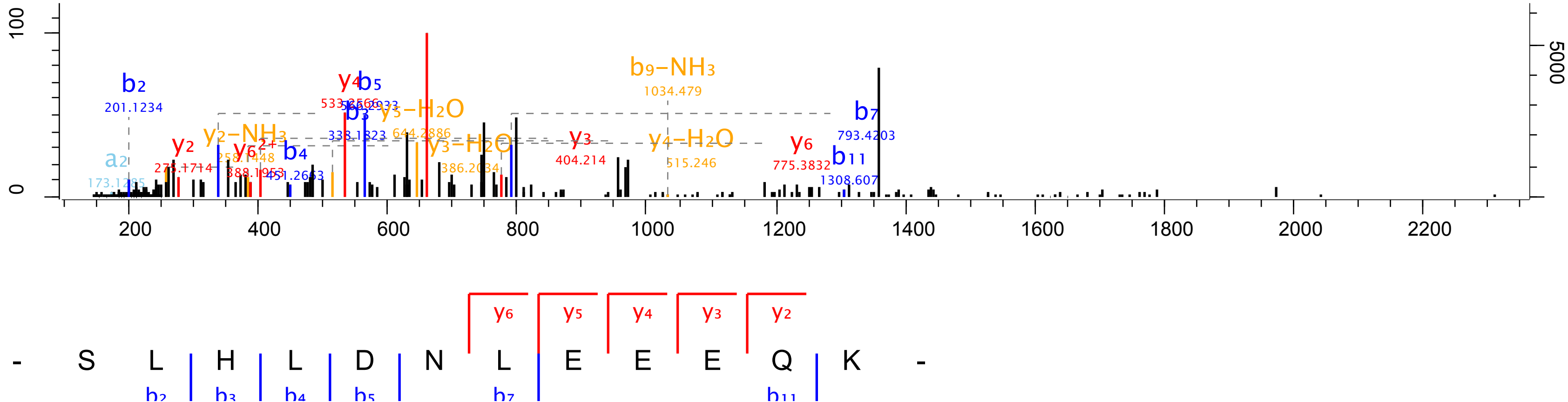

Raw file	Scan	Method	Score	m/z	Gene names
20150306_yeast3_Top_opt_2ug_C3_01_1664	26783	TOF; CID	64.12	448.26	SMD1

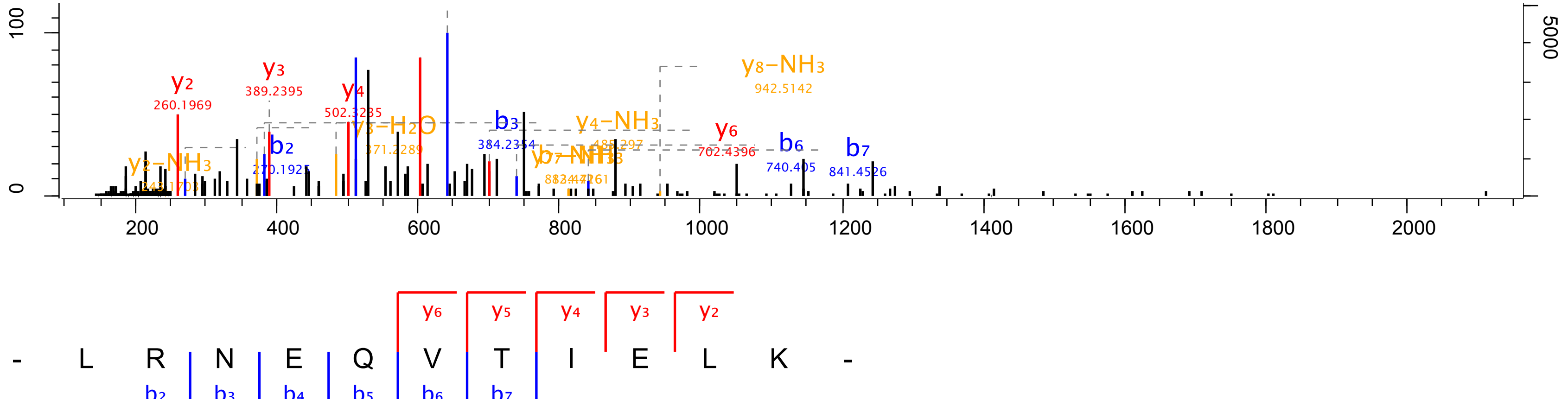

Raw file	Scan	Method	Score	m/z	Gene names
20150306_yeast3_Top_opt_2ug_C3_01_1664	28286	TOF; CID	130.01	971.43	COX17

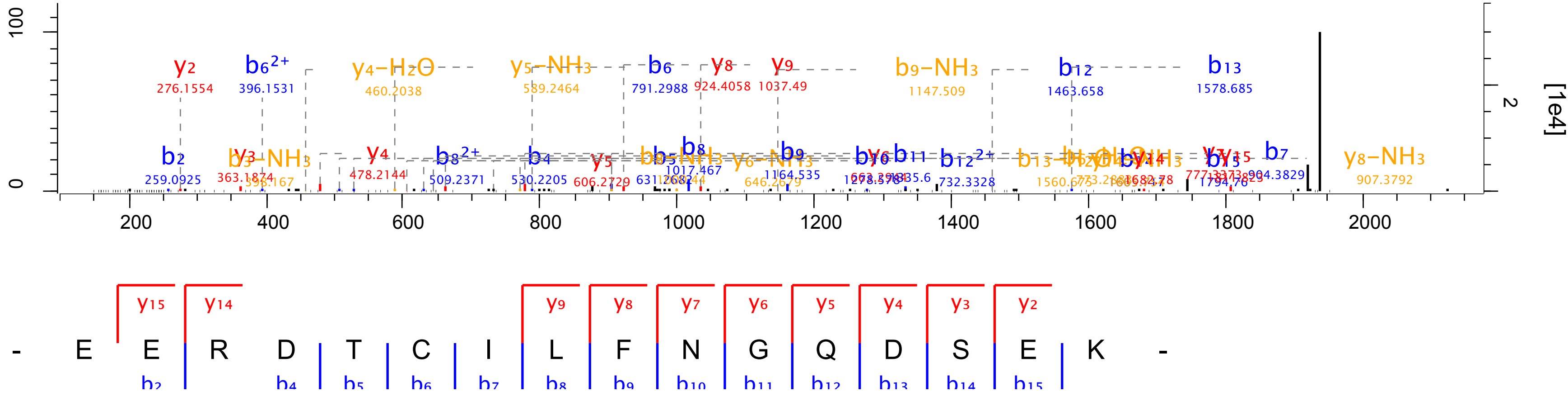

Raw file	Scan	Method	Score	m/z	Gene names
20150306_yeast3_Top_opt_2ug_C3_01_1664	31614	TOF; CID	51.07	815.35	ALF1

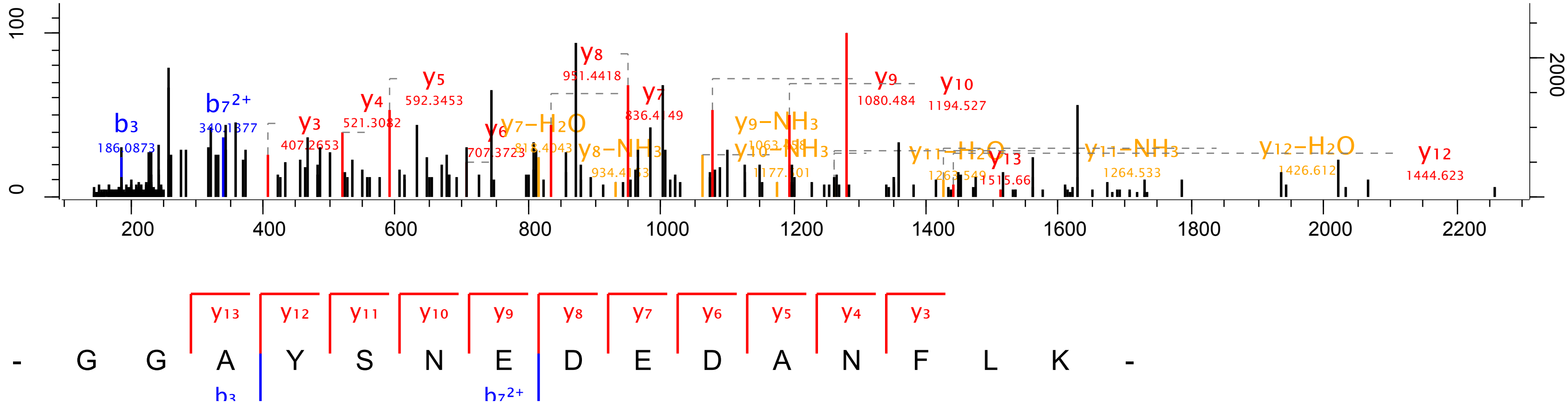

Raw file	Scan	Method	Score	m/z	Gene names
20150306_yeast3_Top_opt_2ug_C3_01_1664	33041	TOF; CID	56.4	601.8	RAD16

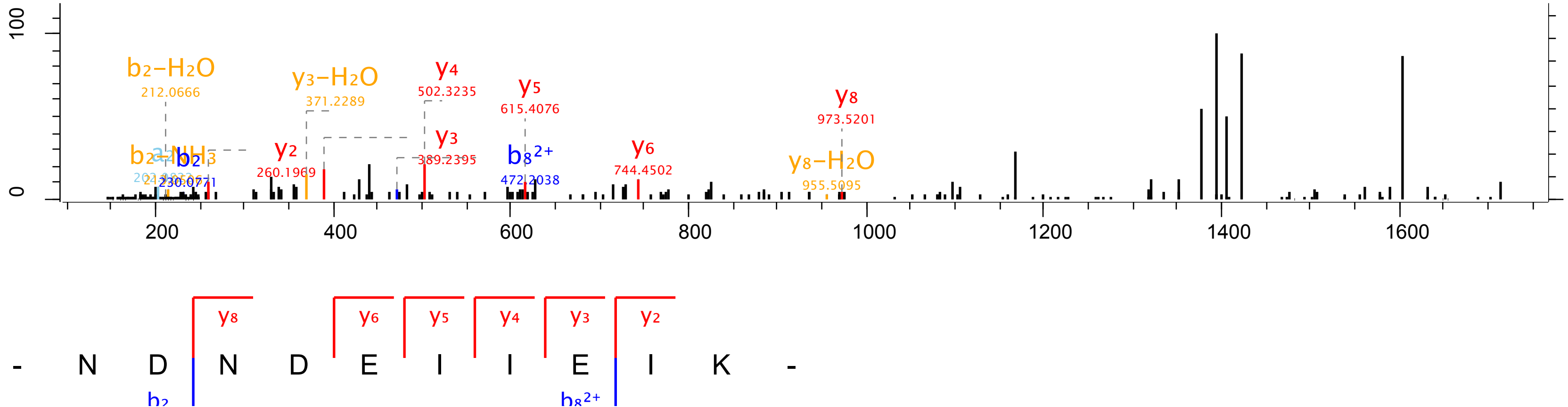

Raw file	Scan	Method	Score	m/z	Gene names
20150306_yeast3_Top_opt_2ug_C3_01_1664	33065	TOF; CID	94.72	586.95	YIL156W-B

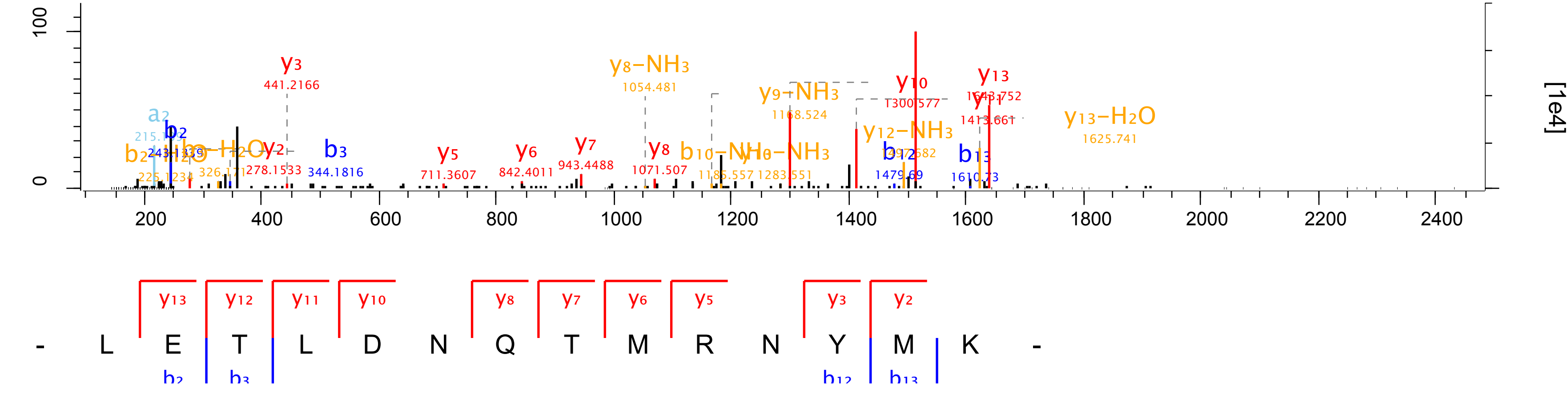

Raw file	Scan	Method	Score	m/z	Gene names
20150306_yeast3_Top_opt_2ug_C3_01_1664	34344	TOF; CID	66.06	781.39	PRP38

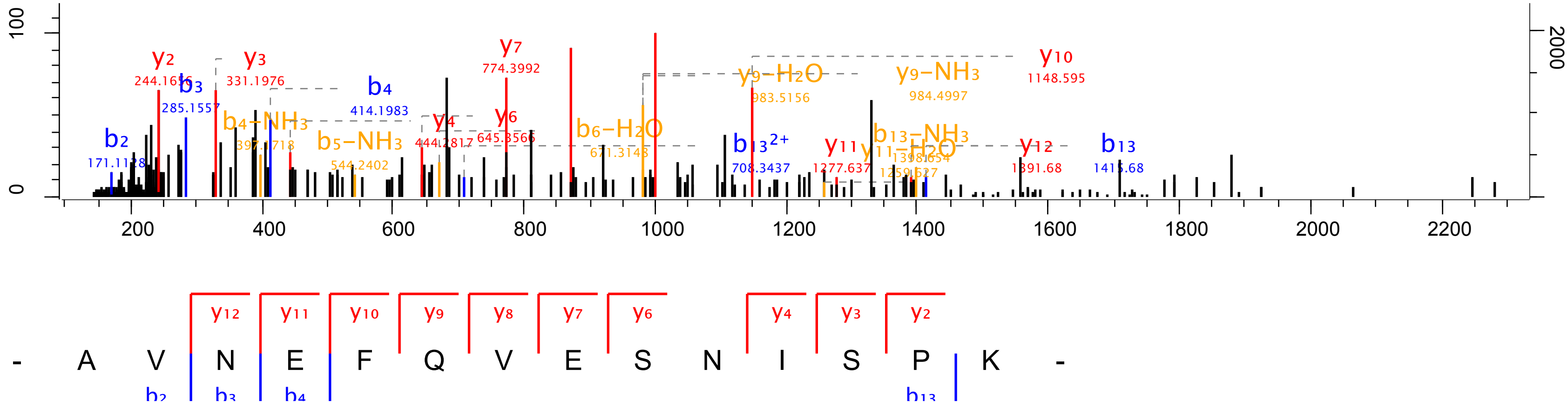

Raw file	Scan	Method	Score	m/z	Gene names
20150306_yeast3_Top_opt_2ug_C3_01_1664	34381	TOF; CID	79.29	767.69	AKR2

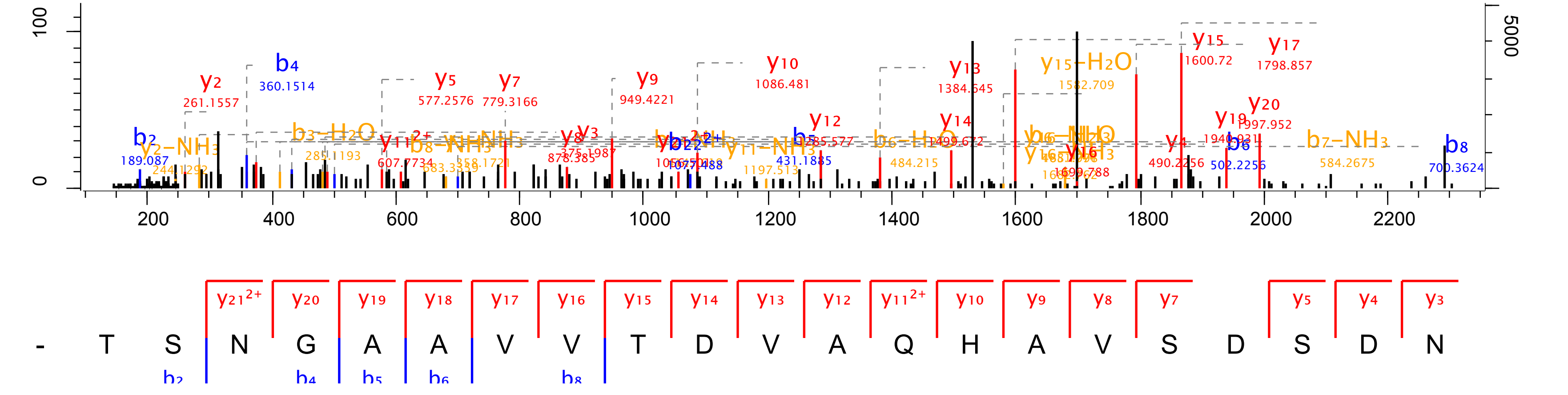

Raw file	Scan	Method	Score	m/z	Gene names
20150306_yeast3_Top_opt_2ug_C3_01_1664	38873	TOF; CID	123.55	1157.99	COX23

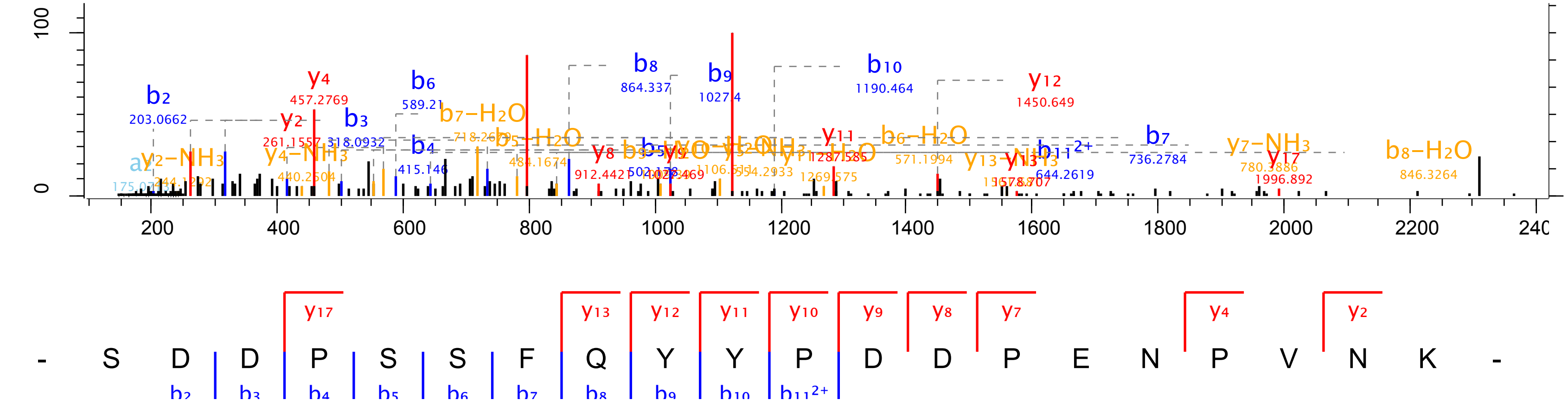

Raw file	Scan	Method	Score	m/z	Gene names
20150306_yeast3_Top_opt_2ug_C3_01_1664	42040	TOF; CID	122.53	998.46	MRPL36

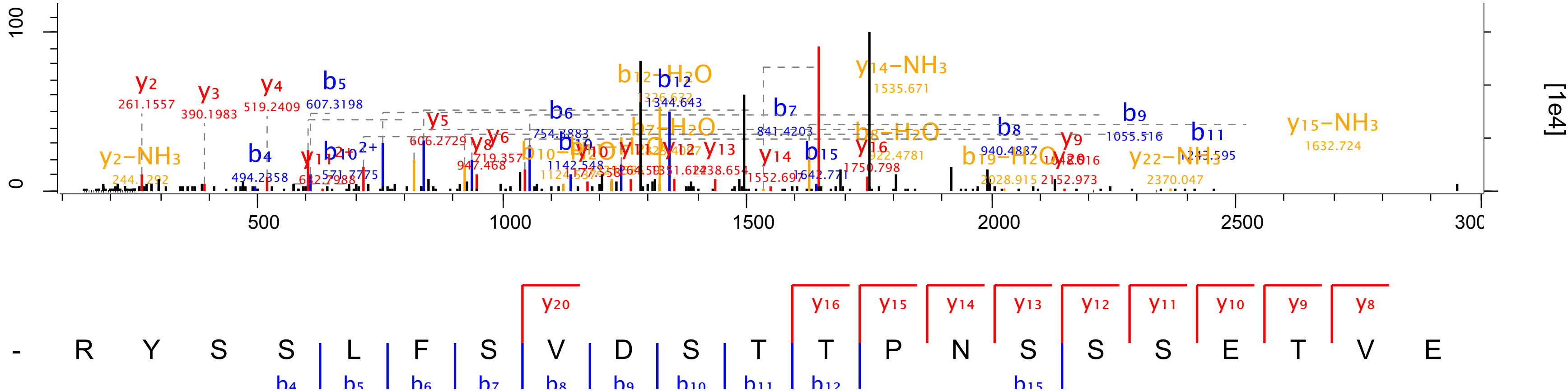

Raw file	Scan	Method	Score	m/z	Gene names
20150306_yeast3_Top_opt_2ug_C3_01_1664	42797	TOF; CID	39.43	528.28	TEA1

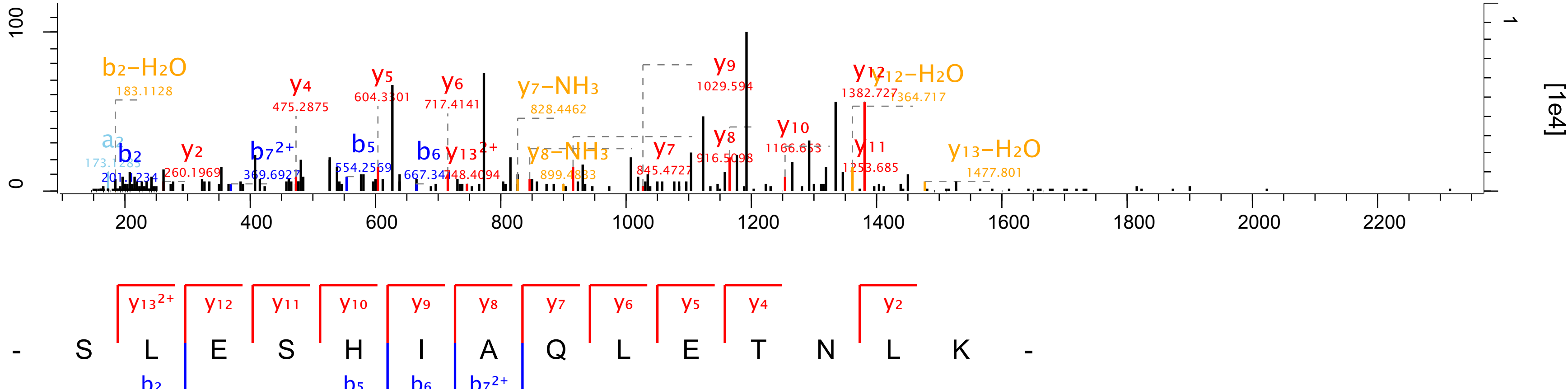

Raw file	Scan	Method	Score	m/z	Gene names
20150306_yeast3_Top_opt_2ug_C3_01_1664	43067	TOF; CID	109.15	517.61	POP2

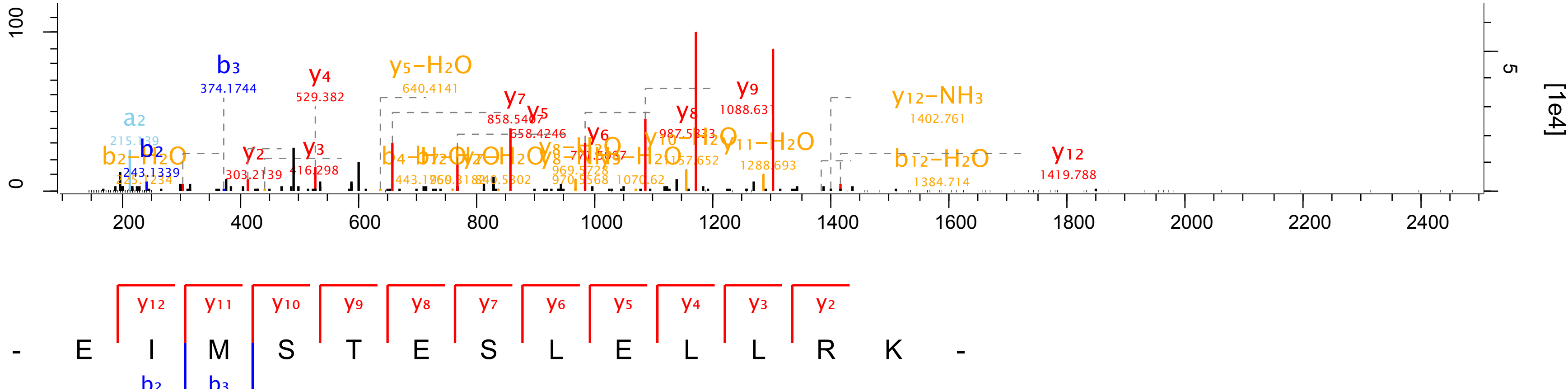

Raw file	Scan	Method	Score	m/z	Gene names
20150306_yeast3_Top_opt_2ug_C3_01_1664	48107	TOF; CID	62.01	694.81	MRPL44

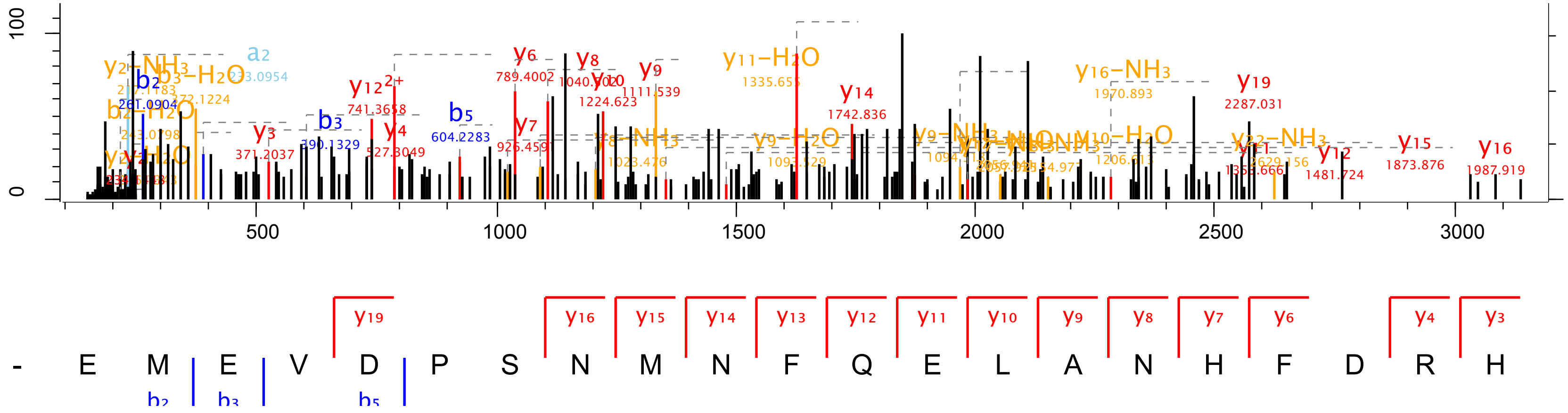

Raw file	Scan	Method	Score	m/z	Gene names
20150306_yeast3_Top_opt_2ug_C3_01_1664	48644	TOF; CID	159.58	511.28	MCM22

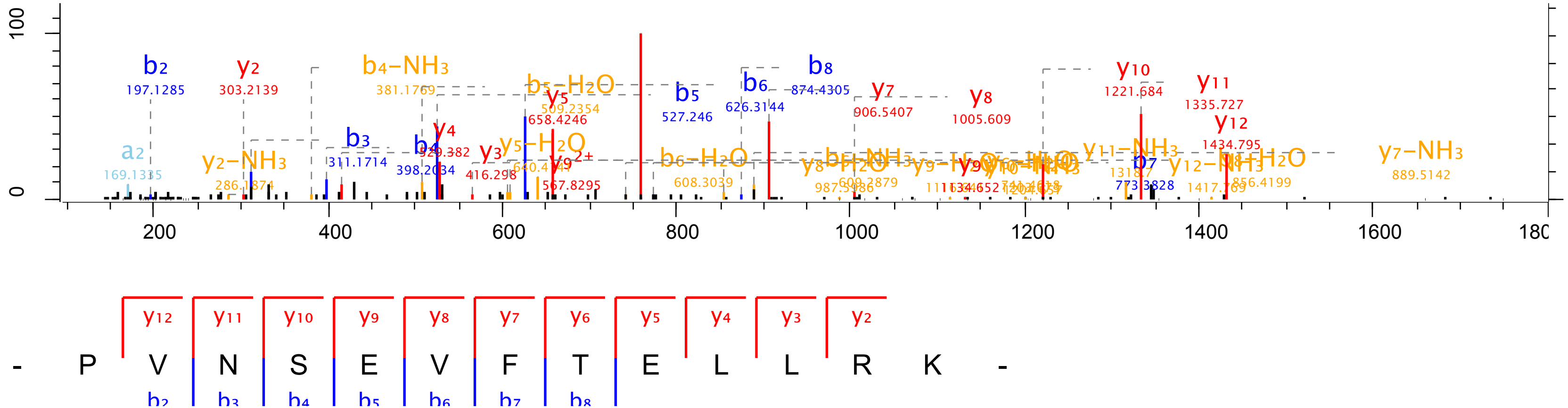

Raw file	Scan	Method	Score	m/z	Gene names
20150306_yeast3_Top_opt_2ug_C3_01_1664	66358	TOF; CID	83.25	1173.6	YDL180W

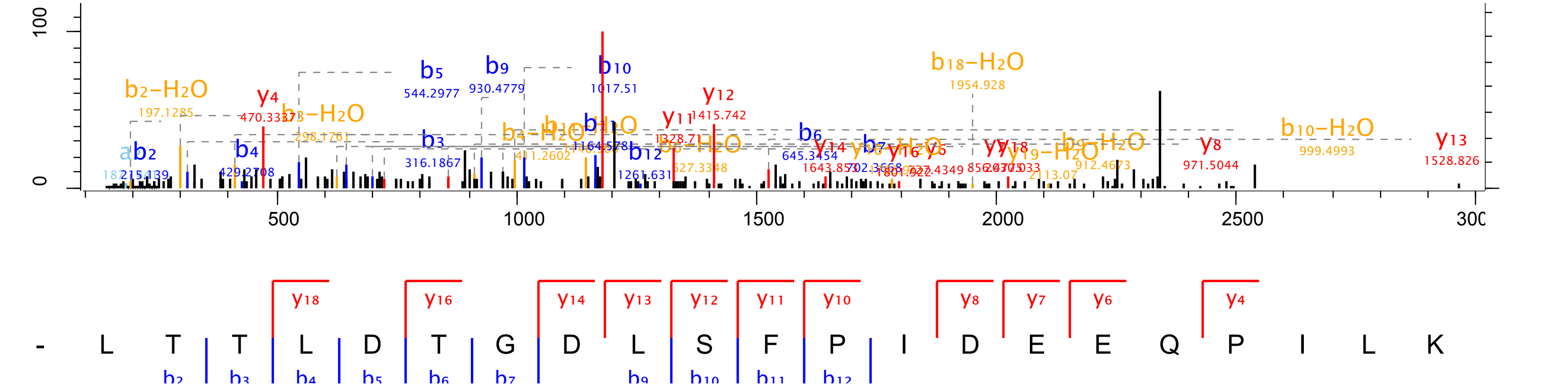

Raw file	Scan	Method	Score	m/z	Gene names
20150306_yeast3_Top_opt_2ug_C3_01_1664	68059	TOF; CID	52.5	762.38	MRP17

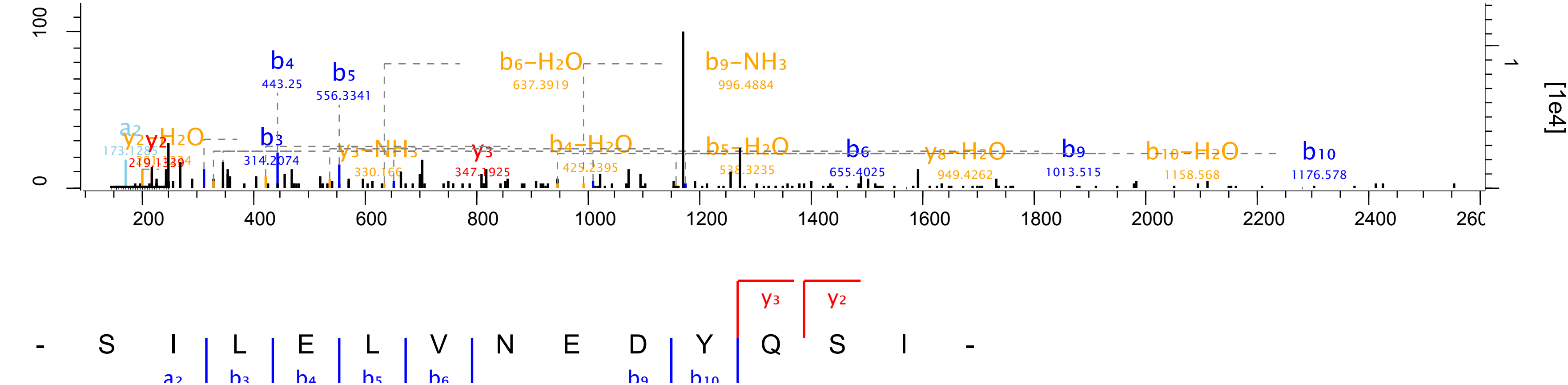

Raw file	Scan	Method	Score	m/z	Gene names
20150306_yeast3_Top_opt_2ug_C3_01_1664	70746	TOF; CID	87.18	689.94	YLR099W-A

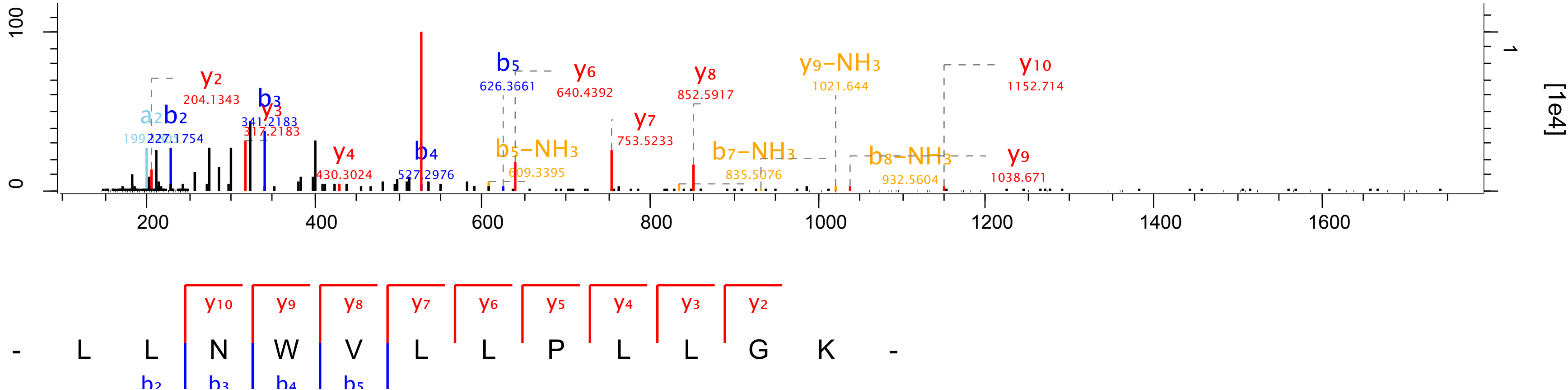

Raw file	Scan	Method	Score	m/z	Gene names
20150306_yeast3_Top_opt_2ug_C3_01_1664	72557	TOF; CID	125.31	870.14	HTZ1

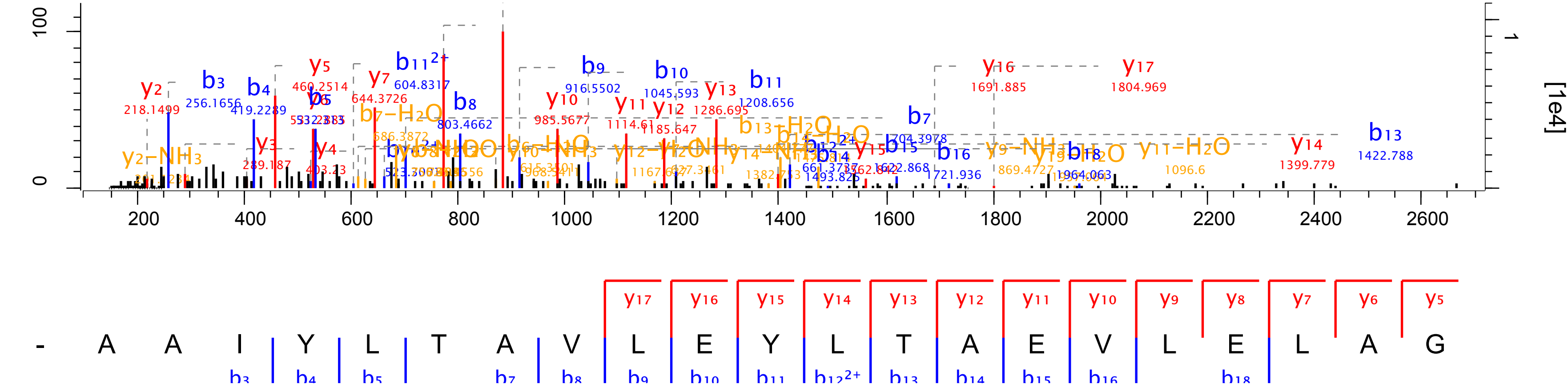

Raw file	Scan	Method	Score	m/z	Gene names
20150306_yeast3_Top_opt_2ug_C3_01_1665	6370	TOF; CID	79.81	424.68	PKR1

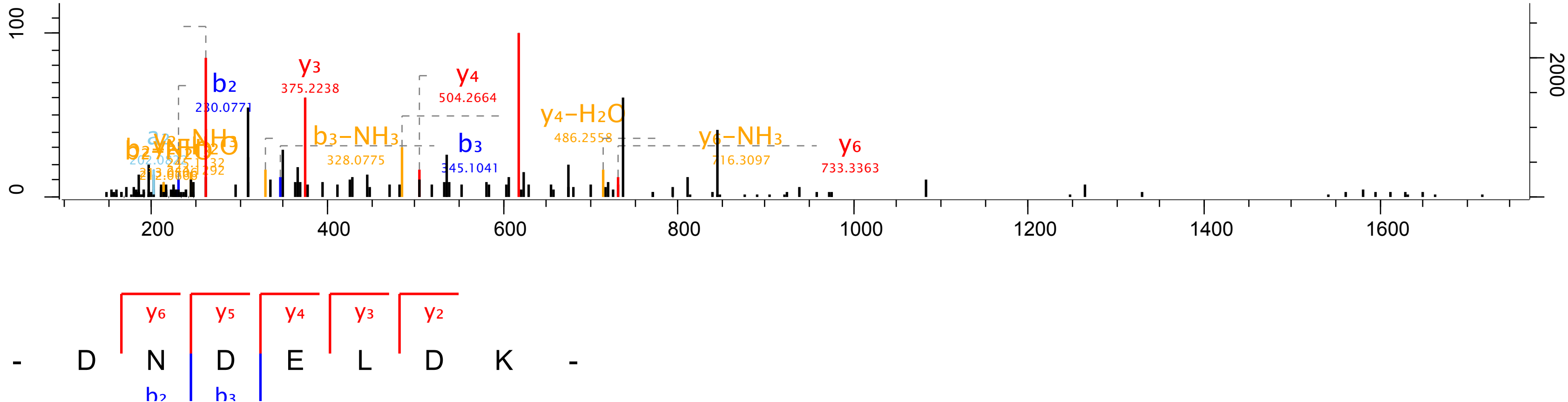

Raw file	Scan	Method	Score	m/z	Gene names
20150306_yeast3_Top_opt_2ug_C3_01_1665	8992	TOF; CID	100.07	661.27	QCR9

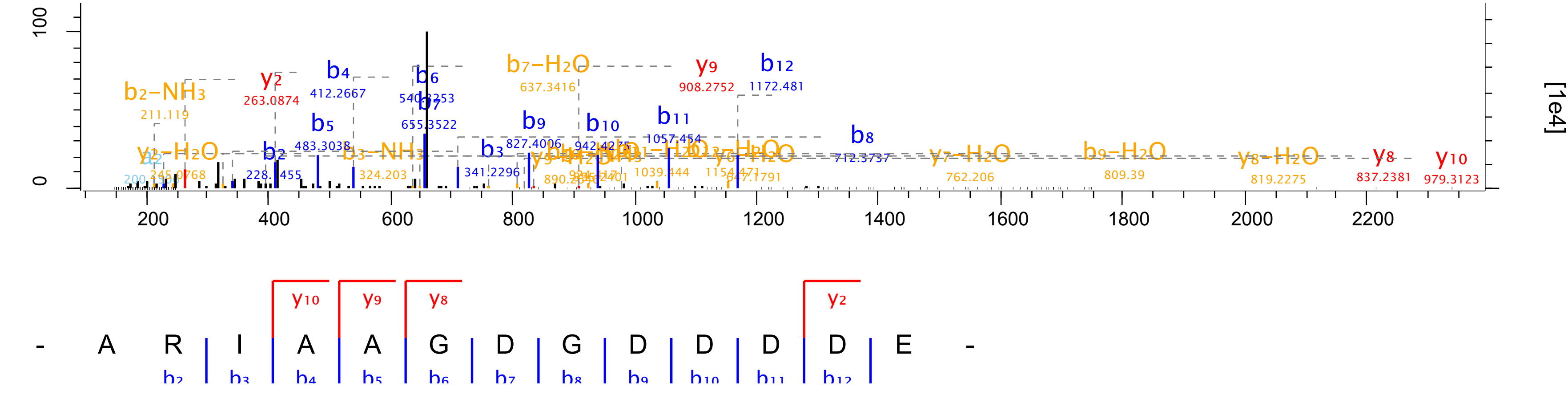

Raw file	Scan	Method	Score	m/z	Gene names
20150306_yeast3_Top_opt_2ug_C3_01_1665	9144	TOF; CID	64.83	590.29	NCA3

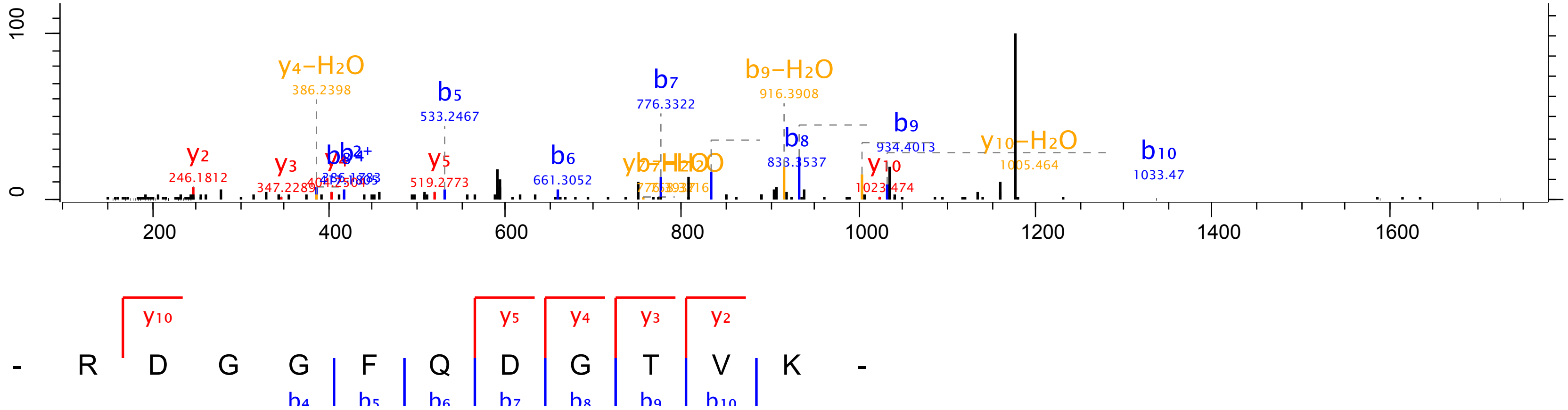

Raw file	Scan	Method	Score	m/z	Gene names
20150306_yeast3_Top_opt_2ug_C3_01_1665	9275	TOF; CID	76.83	707.33	IFM1

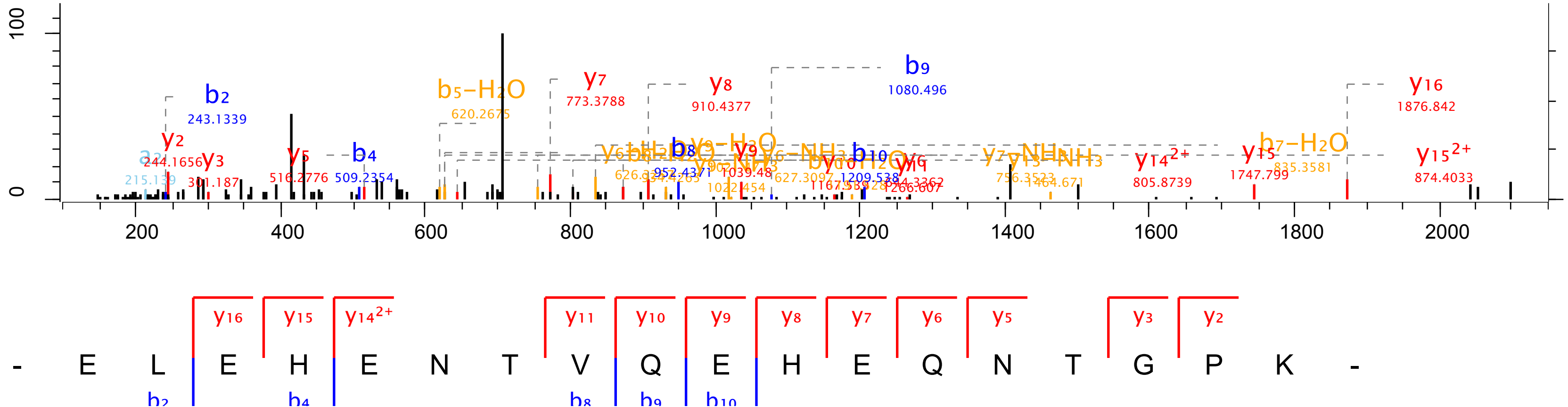

Raw file	Scan	Method	Score	m/z	Gene names
20150306_yeast3_Top_opt_2ug_C3_01_1665	13616	TOF; CID	90.79	664.8	ESA1

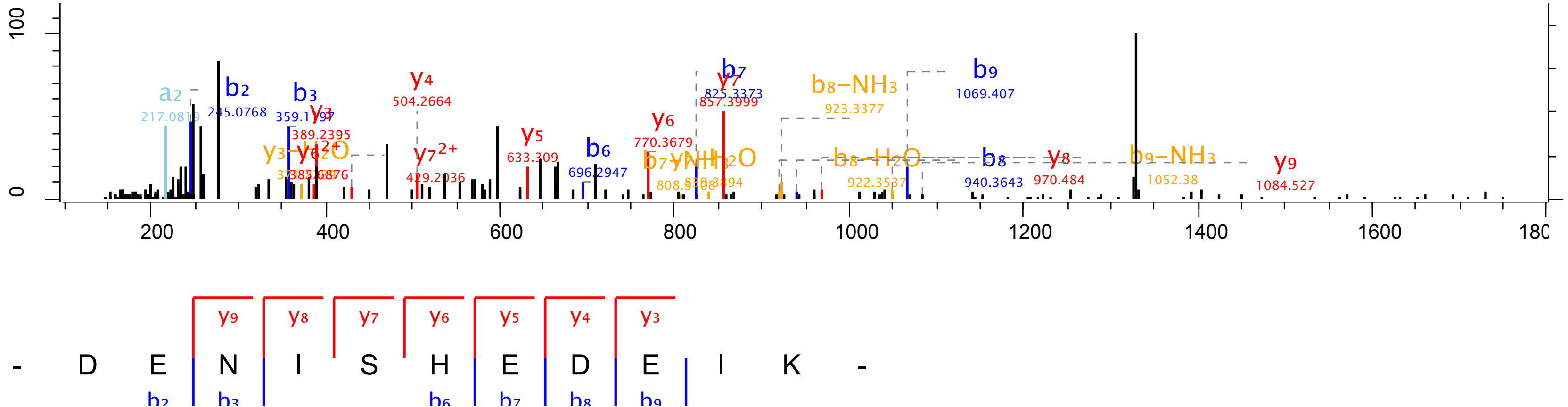

Raw file	Scan	Method	Score	m/z	Gene names
20150306_yeast3_Top_opt_2ug_C3_01_1665	17648	TOF; CID	66.57	524.76	GPI12

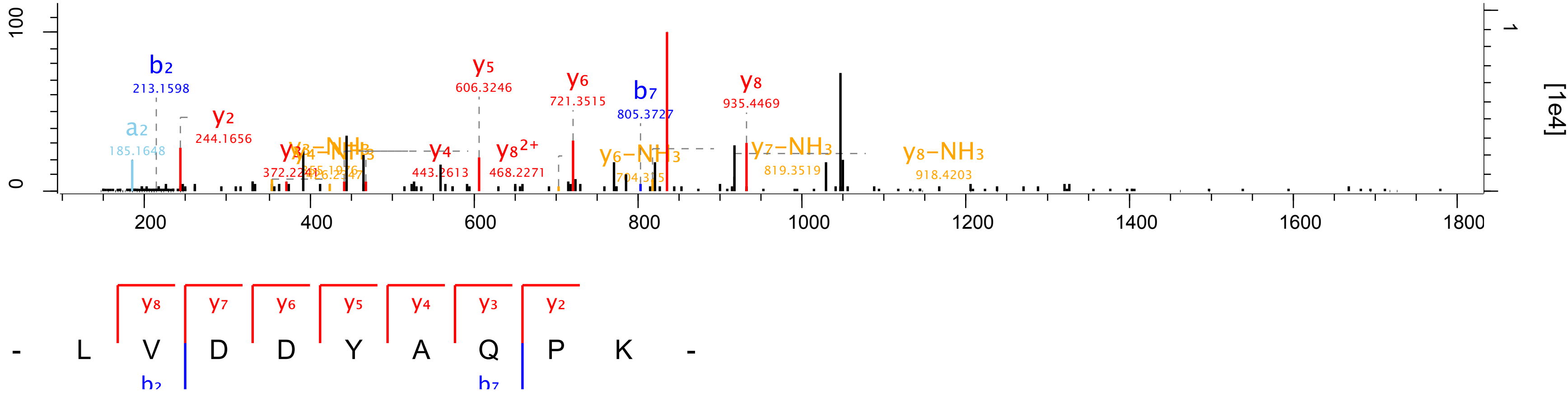

Raw file	Scan	Method	Score	m/z	Gene names
20150306_yeast3_Top_opt_2ug_C3_01_1665	18151	TOF; CID	91.36	555.25	IBD2

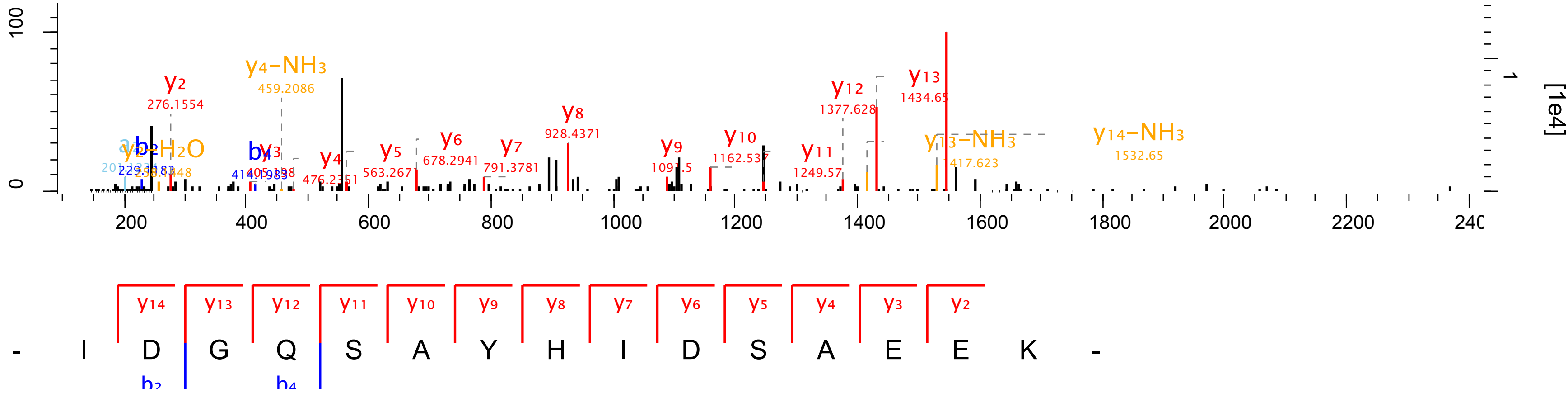

Raw file	Scan	Method	Score	m/z	Gene names
20150306_yeast3_Top_opt_2ug_C3_01_1665	20370	TOF; CID	44.74	808.36	BIT61

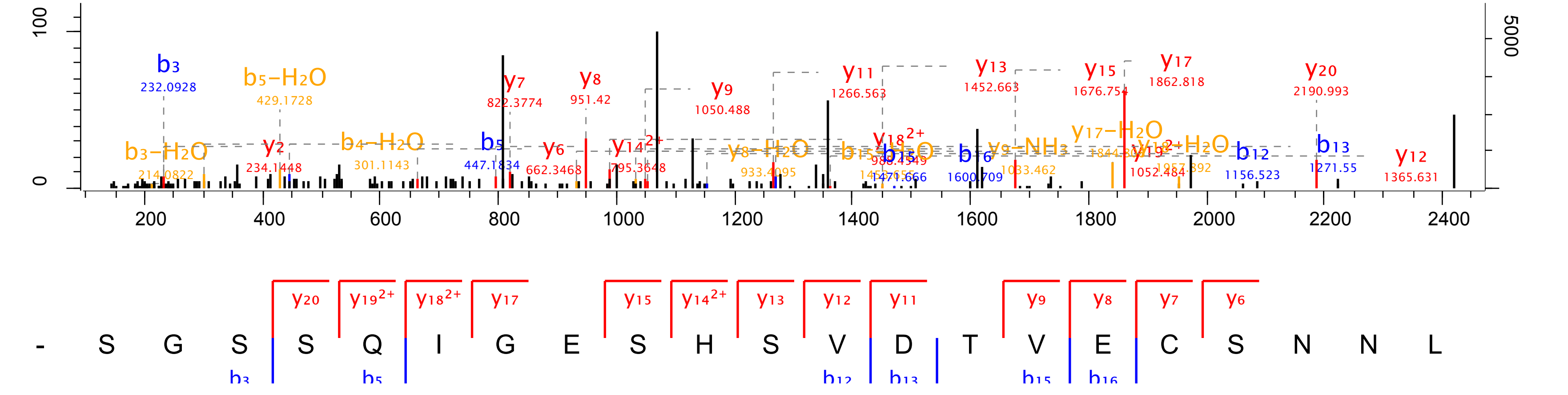

Raw file	Scan	Method	Score	m/z	Gene names
20150306_yeast3_Top_opt_2ug_C3_01_1665	21394	TOF; CID	40.48	500.25	PRP11

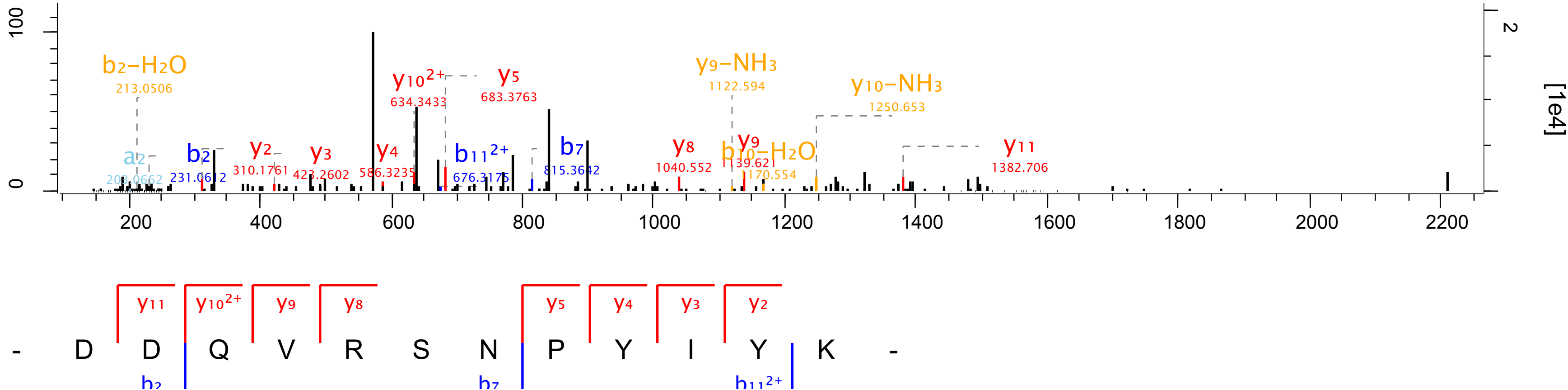

Raw file	Scan	Method	Score	m/z	Gene names
20150306_yeast3_Top_opt_2ug_C3_01_1665	23230	TOF; CID	50.24	763.01	PHO80

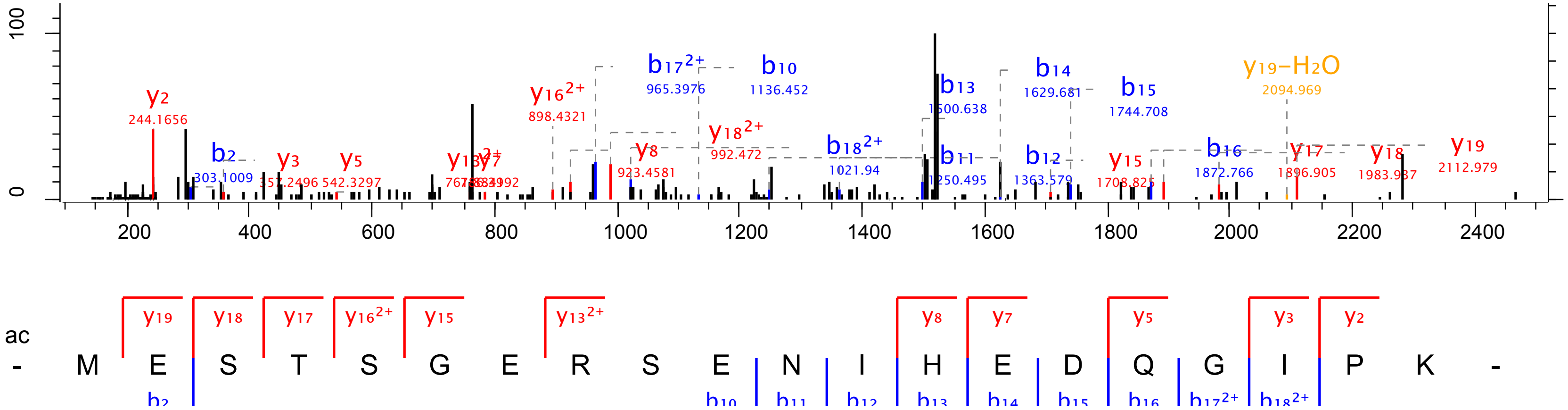

Raw file	Scan	Method	Score	m/z	Gene names
20150306_yeast3_Top_opt_2ug_C3_01_1665	25380	TOF; CID	68.97	669.31	DBF20

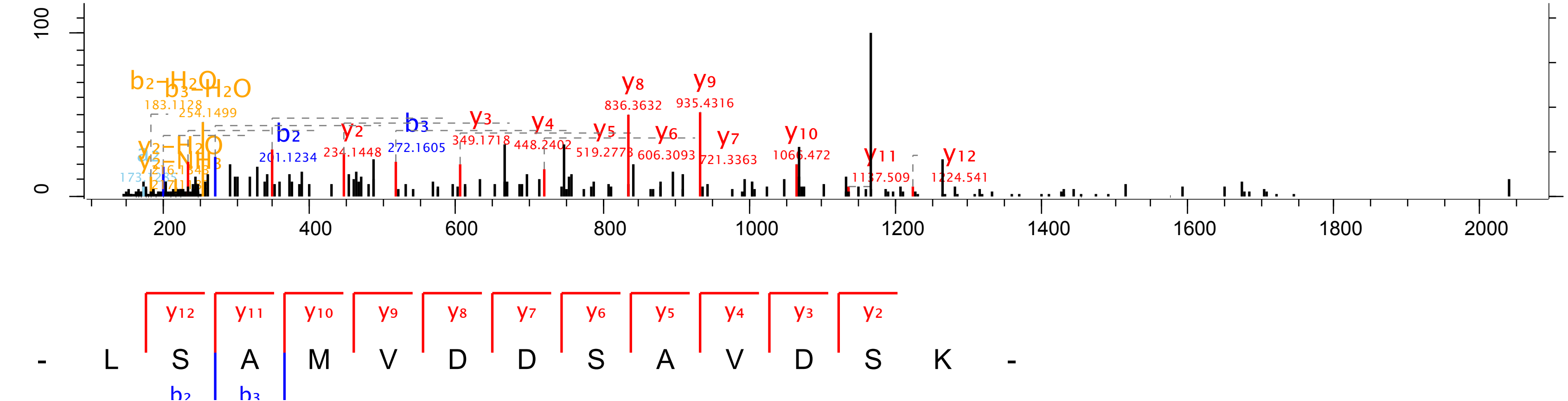

Raw file	Scan	Method	Score	m/z	Gene names
20150306_yeast3_Top_opt_2ug_C3_01_1665	30355	TOF; CID	95.07	911.4	VPS34

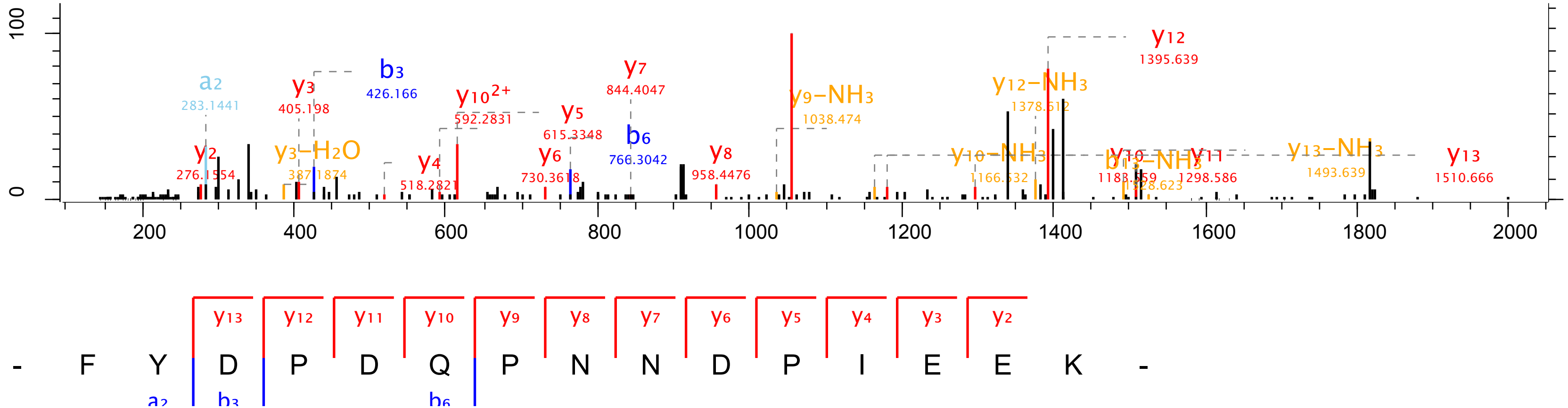

Raw file	Scan	Method	Score	m/z	Gene names
20150306_yeast3_Top_opt_2ug_C3_01_1665	32505	TOF; CID	148.28	596.81	FEN1

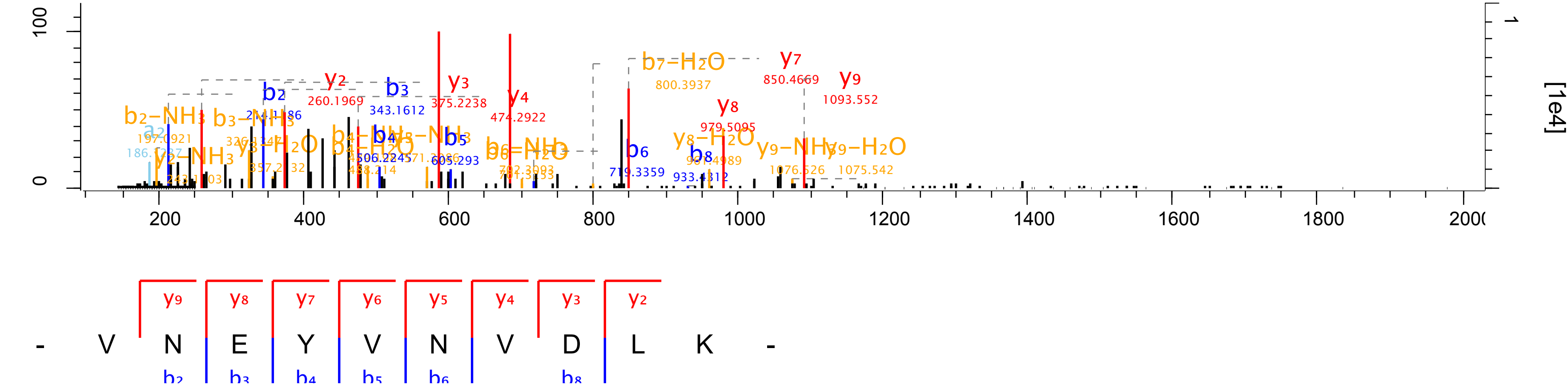

Raw file	Scan	Method	Score	m/z	Gene names
20150306_yeast3_Top_opt_2ug_C3_01_1665	33445	TOF; CID	52.52	688.32	COA4

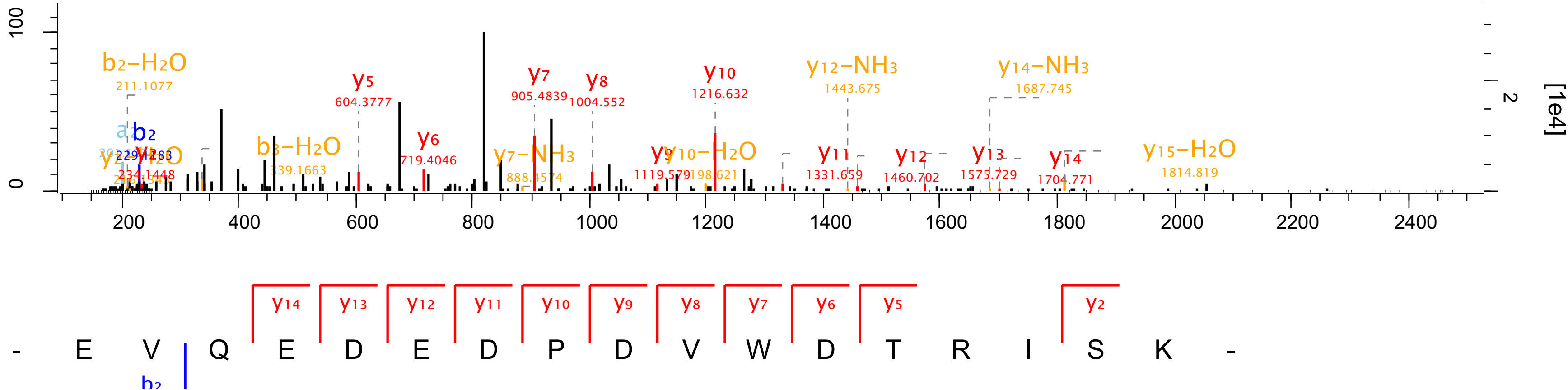

Raw file	Scan	Method	Score	m/z	Gene names
20150306_yeast3_Top_opt_2ug_C3_01_1665	33989	TOF; CID	119.41	838.71	ATG18

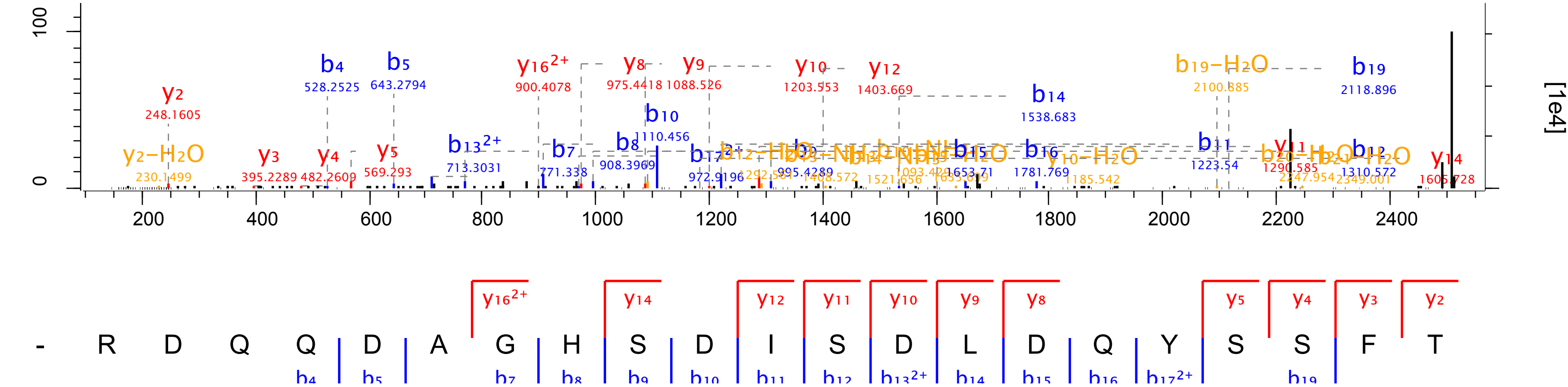

Raw file	Scan	Method	Score	m/z	Gene names
20150306_yeast3_Top_opt_2ug_C3_01_1665	37107	TOF; CID	88.95	577.62	RRN10

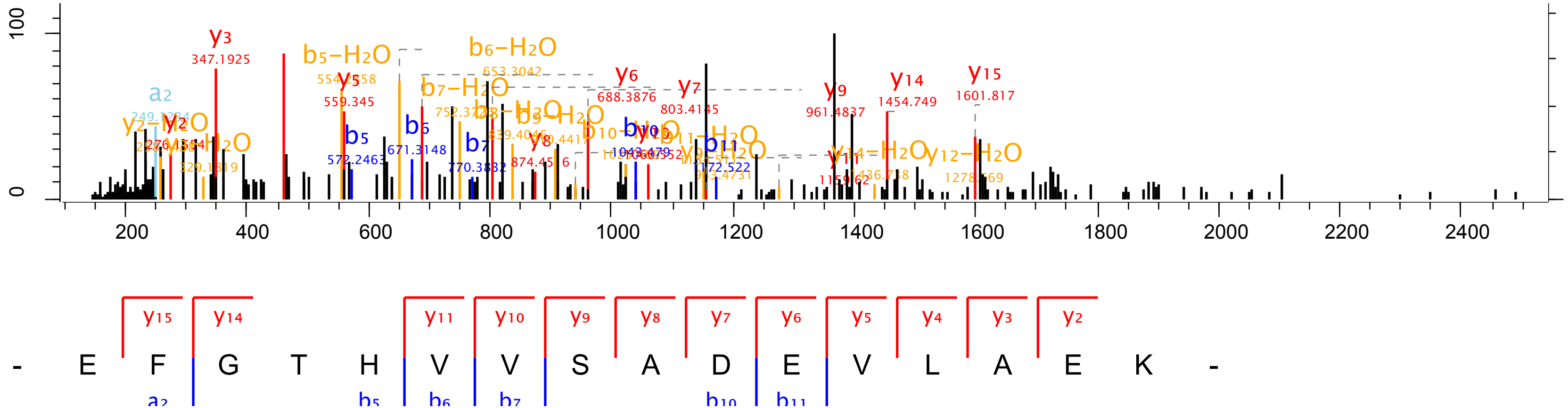

Raw file	Scan	Method	Score	m/z	Gene names
20150306_yeast3_Top_opt_2ug_C3_01_1665	38349	TOF; CID	46.25	686.36	CWC23

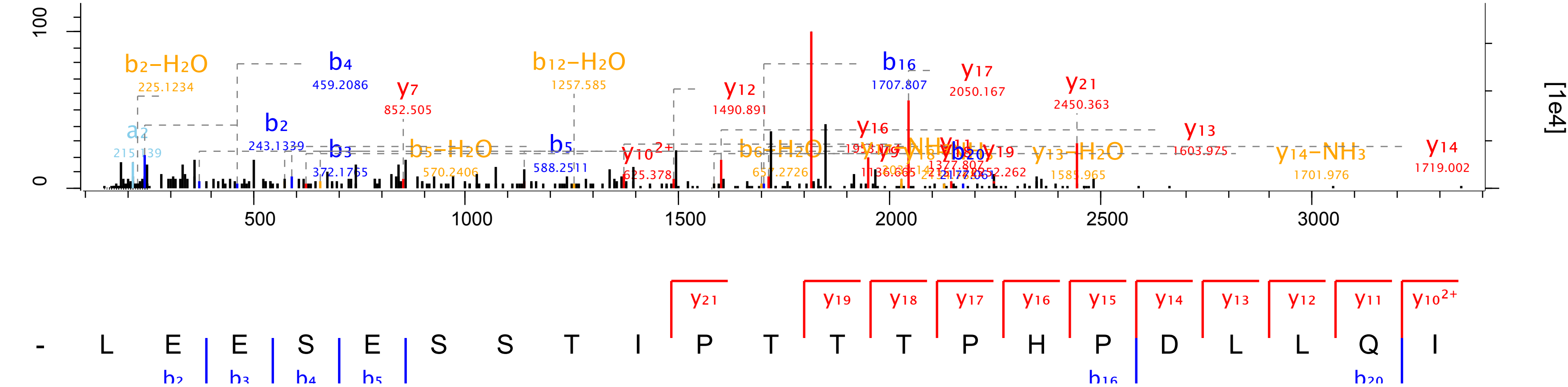

Raw file	Scan	Method	Score	m/z	Gene names
20150306_yeast3_Top_opt_2ug_C3_01_1665	38545	TOF; CID	62.46	408.23	SAF1

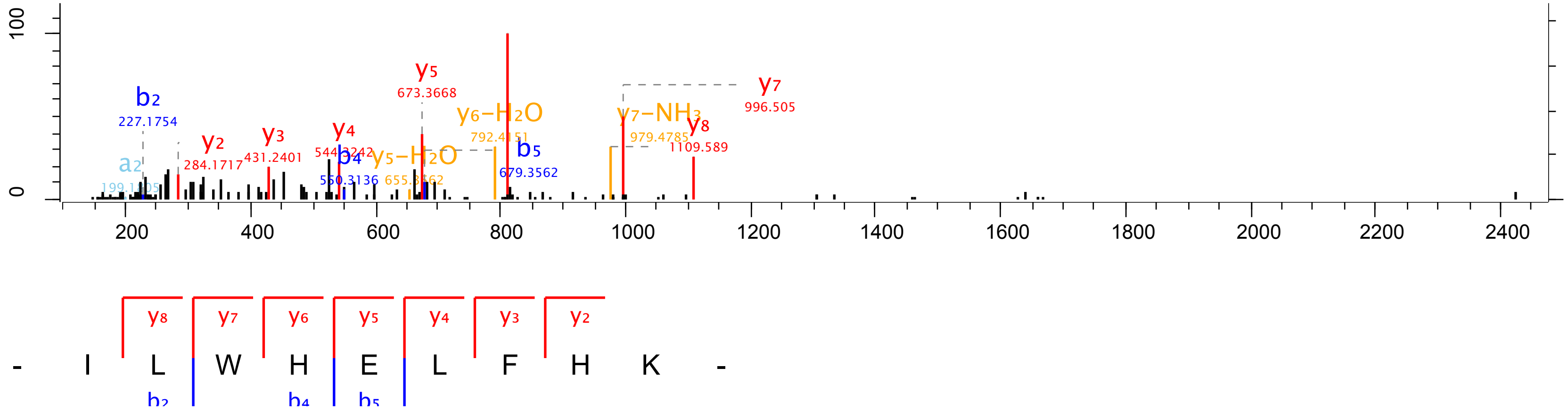

Raw file	Scan	Method	Score	m/z	Gene names
20150306_yeast3_Top_opt_2ug_C3_01_1665	39180	TOF; CID	66.47	1071.99	SFL1

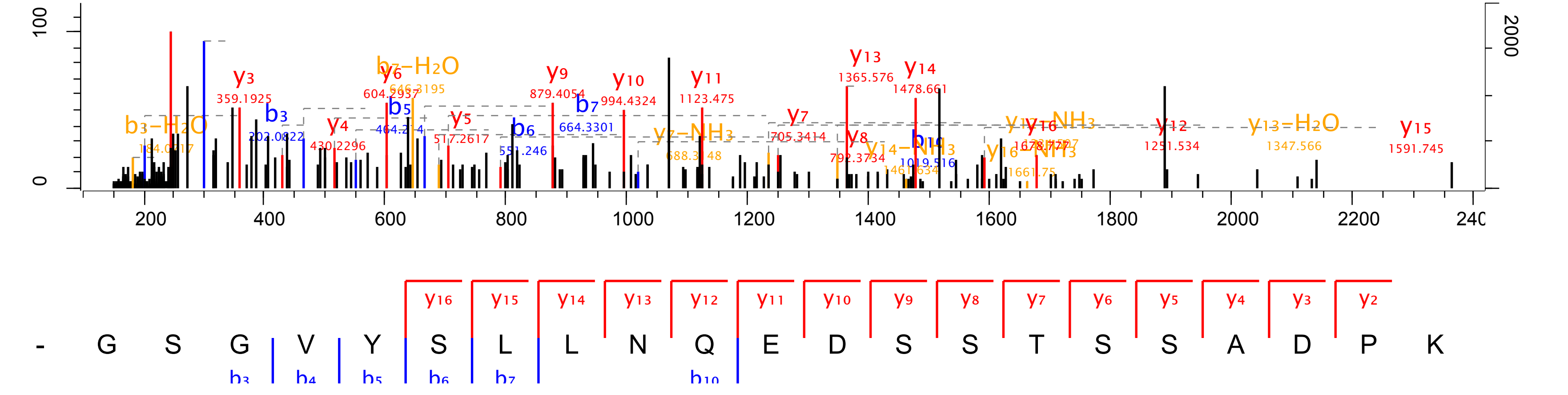

Raw file	Scan	Method	Score	m/z	Gene names
20150306_yeast3_Top_opt_2ug_C3_01_1665	39303	TOF; CID	68.66	547.78	ACS1

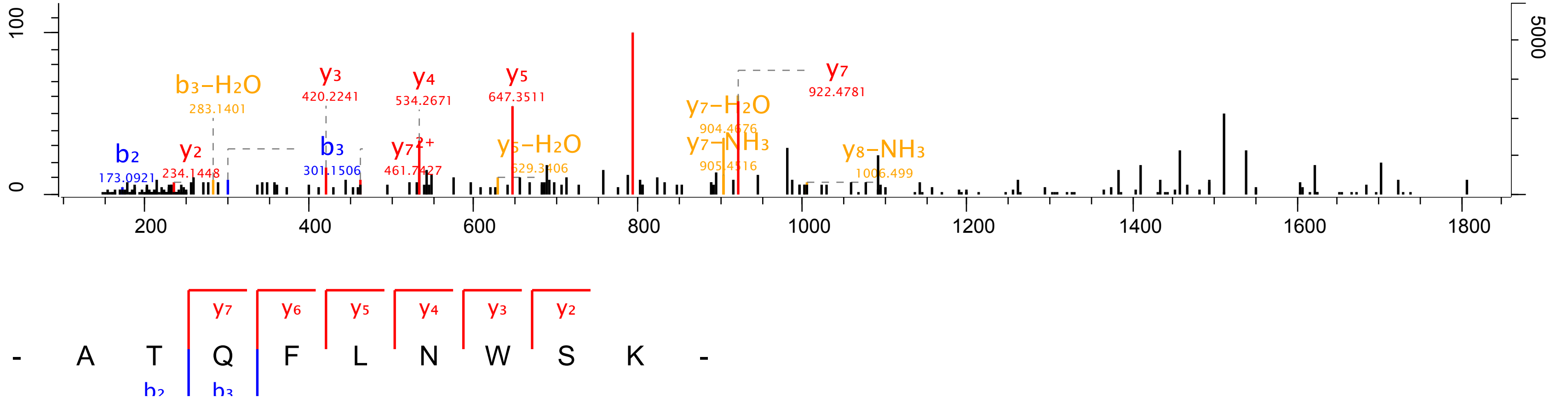

Raw file	Scan	Method	Score	m/z	Gene names
20150306_yeast3_Top_opt_2ug_C3_01_1665	42474	TOF; CID	57.74	1021.82	PTC1

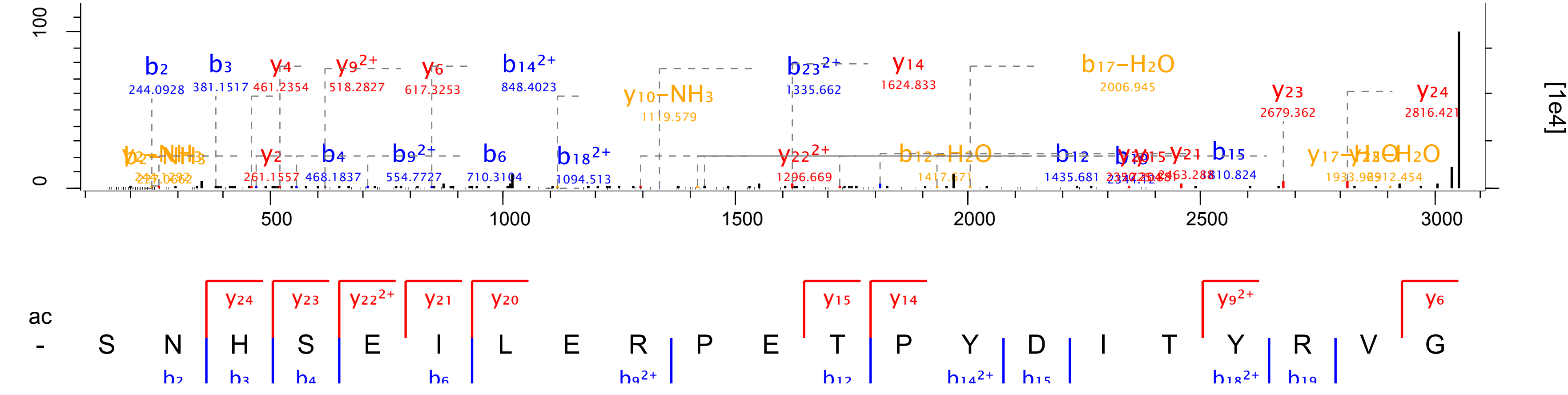

Raw file	Scan	Method	Score	m/z	Gene names
20150306_yeast3_Top_opt_2ug_C3_01_1665	44197	TOF; CID	67.14	759.88	MET31

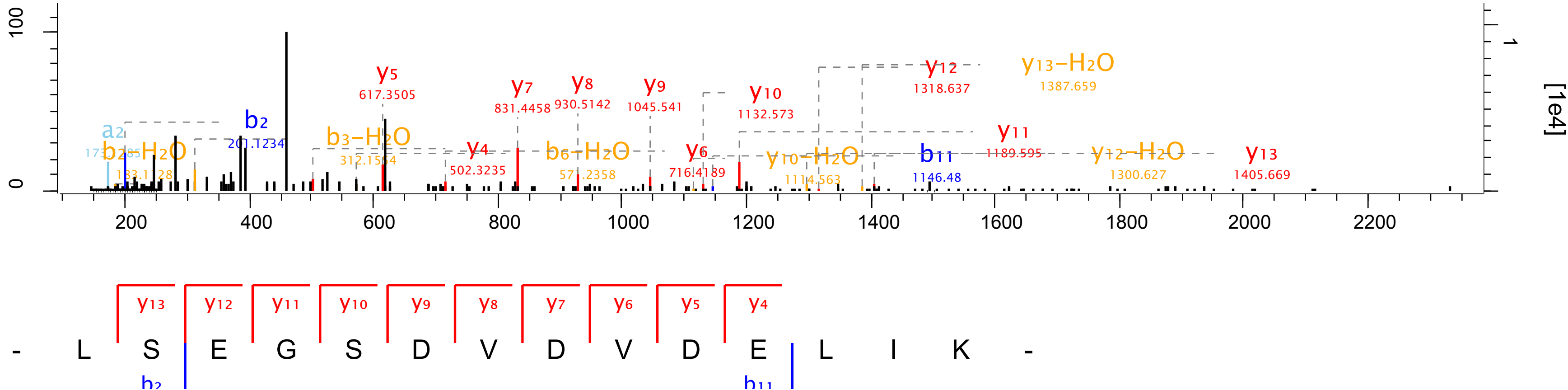

Raw file	Scan	Method	Score	m/z	Gene names
20150306_yeast3_Top_opt_2ug_C3_01_1665	44935	TOF; CID	55.53	841.41	QRI7

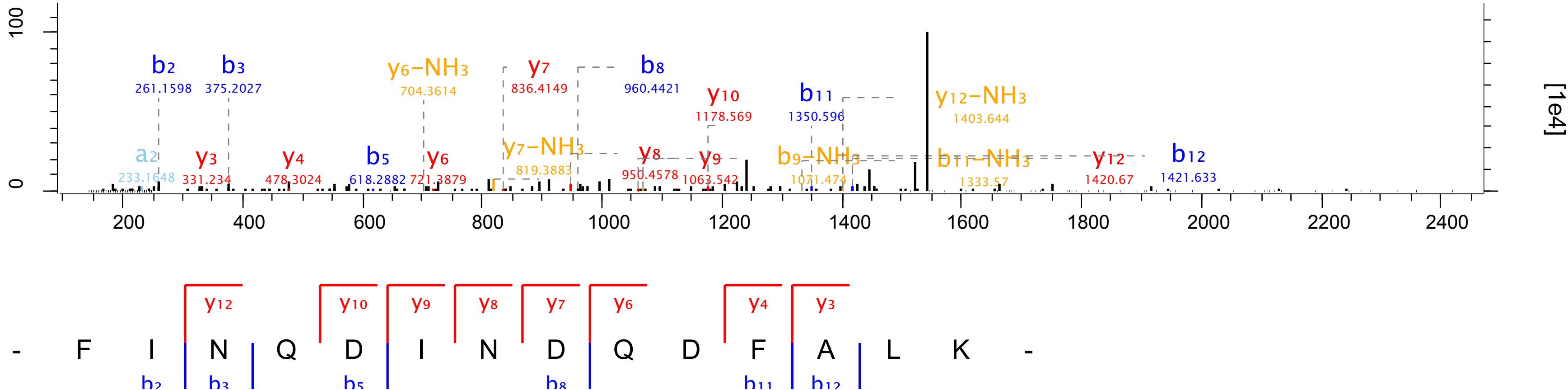

Raw file	Scan	Method	Score	m/z	Gene names
20150306_yeast3_Top_opt_2ug_C3_01_1665	45866	TOF; CID	45.28	498.9	IGD1

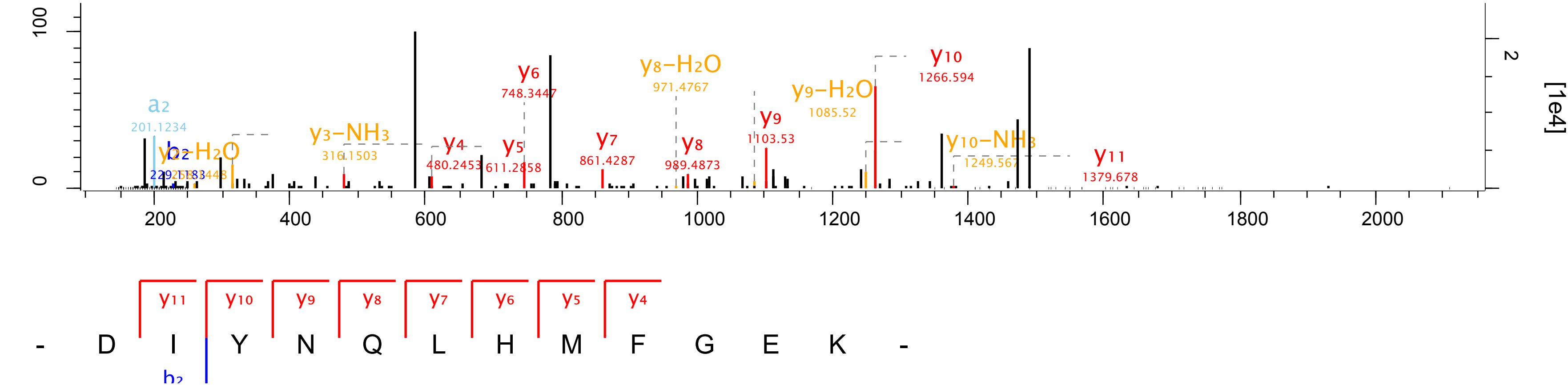

Raw file	Scan	Method	Score	m/z	Gene names
20150306_yeast3_Top_opt_2ug_C3_01_1665	47280	TOF; CID	44.34	717.37	COX16

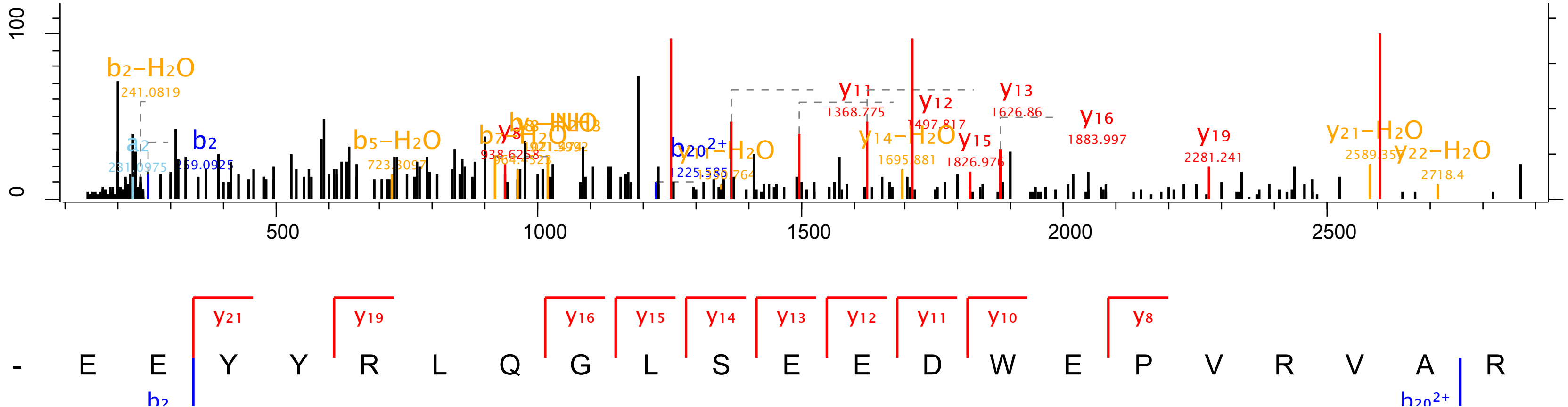

Raw file	Scan	Method	Score	m/z	Gene names
20150306_yeast3_Top_opt_2ug_C3_01_1665	47513	TOF; CID	103.97	553.94	ATG21

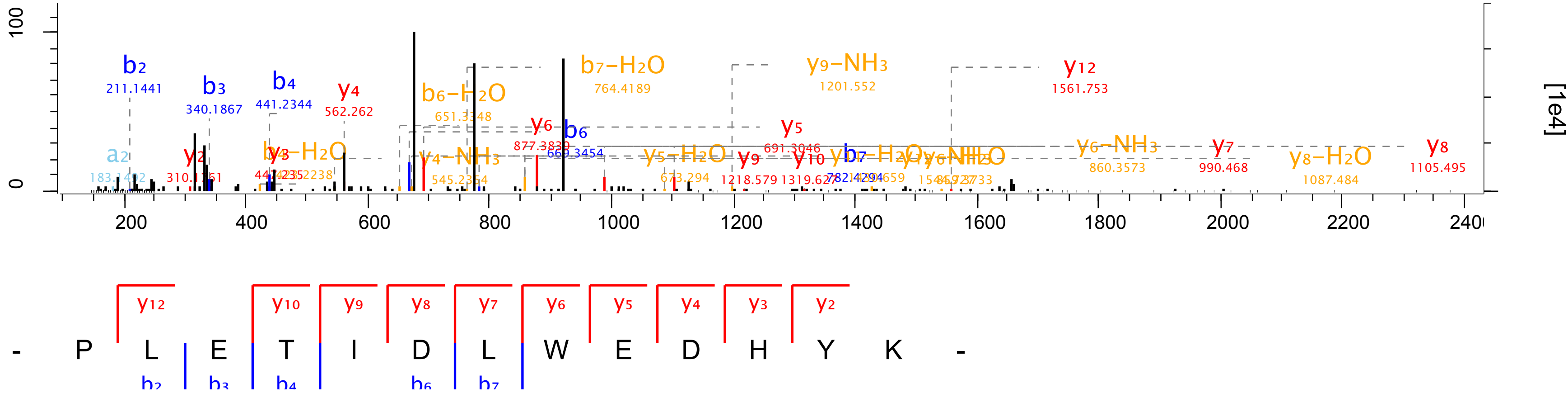

Raw file	Scan	Method	Score	m/z	Gene names
20150306_yeast3_Top_opt_2ug_C3_01_1665	47568	TOF; CID	89.31	808.39	TMA10

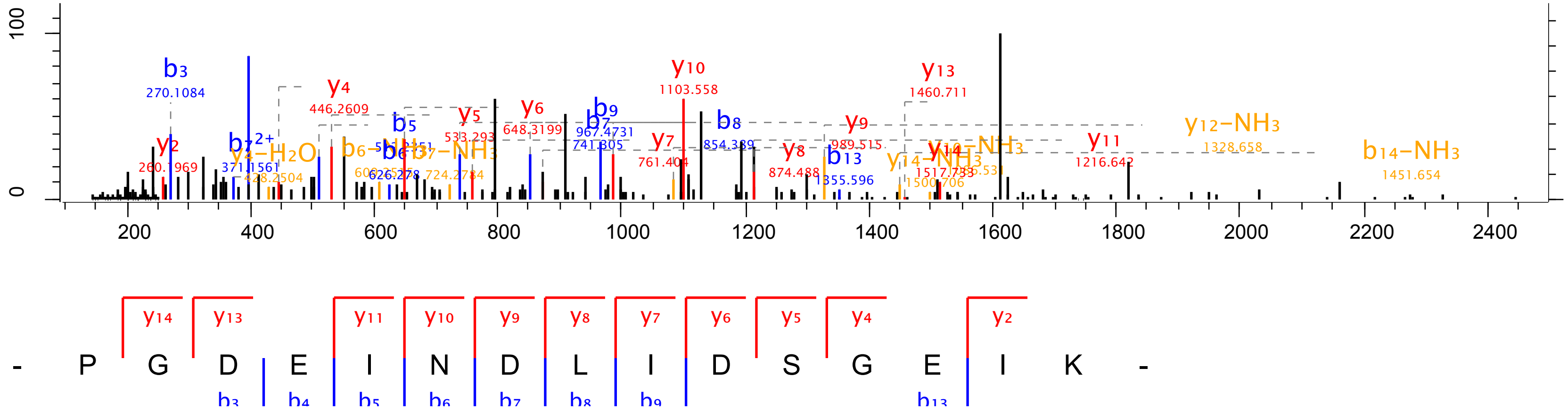

Raw file	Scan	Method	Score	m/z	Gene names
20150306_yeast3_Top_opt_2ug_C3_01_1665	49471	TOF; CID	123.32	1147.6	TPO2

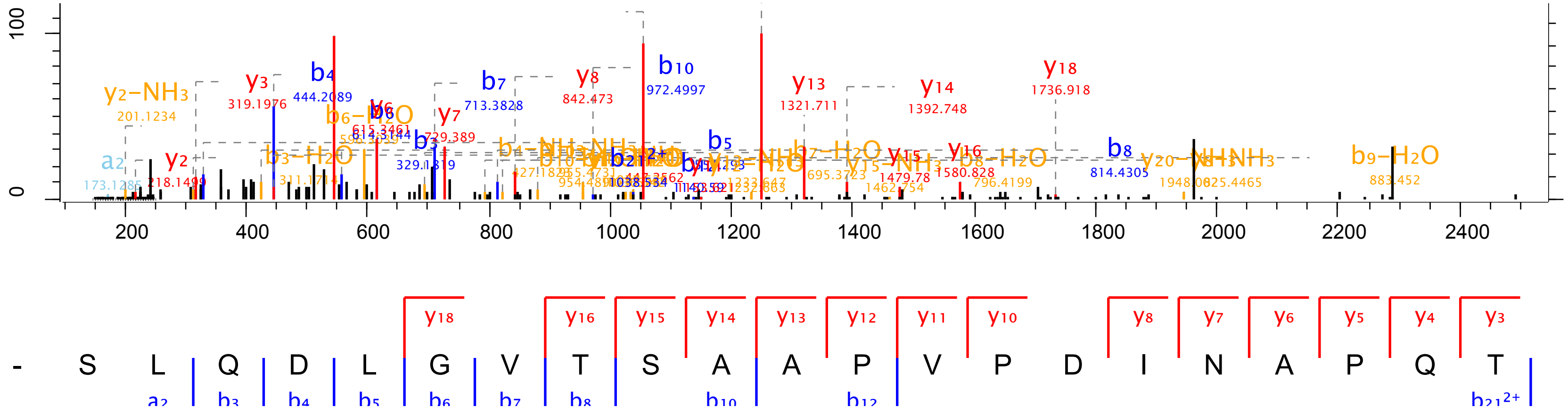

Raw file	Scan	Method	Score	m/z	Gene names
20150306_yeast3_Top_opt_2ug_C3_01_1665	53596	TOF; CID	50.39	891.45	AVO2

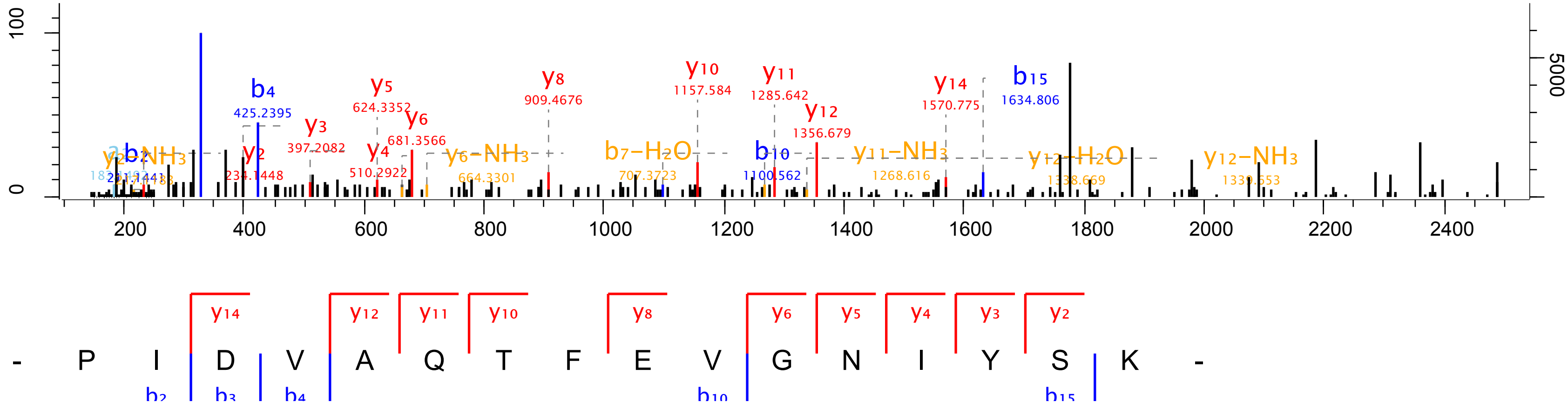

Raw file	Scan	Method	Score	m/z	Gene names
20150306_yeast3_Top_opt_2ug_C3_01_1665	53738	TOF; CID	48.8	1207.54	BXI1

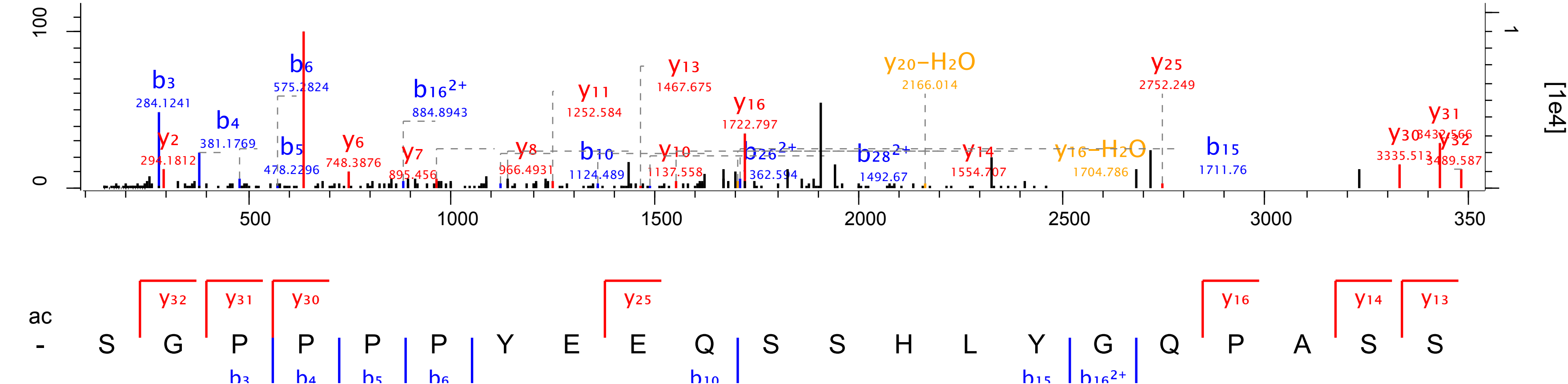

Raw file	Scan	Method	Score	m/z	Gene names
20150306_yeast3_Top_opt_2ug_C3_01_1665	53956	TOF; CID	57.21	1048.82	PCL10

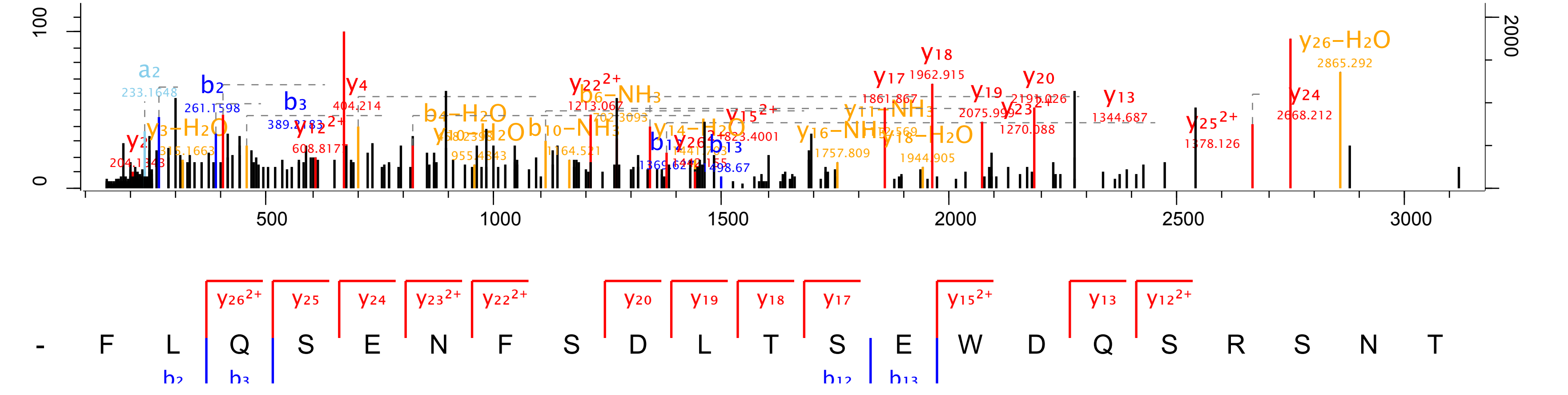

Raw file	Scan	Method	Score	m/z	Gene names
20150306_yeast3_Top_opt_2ug_C3_01_1665	54169	TOF; CID	61.34	562.87	FMS1

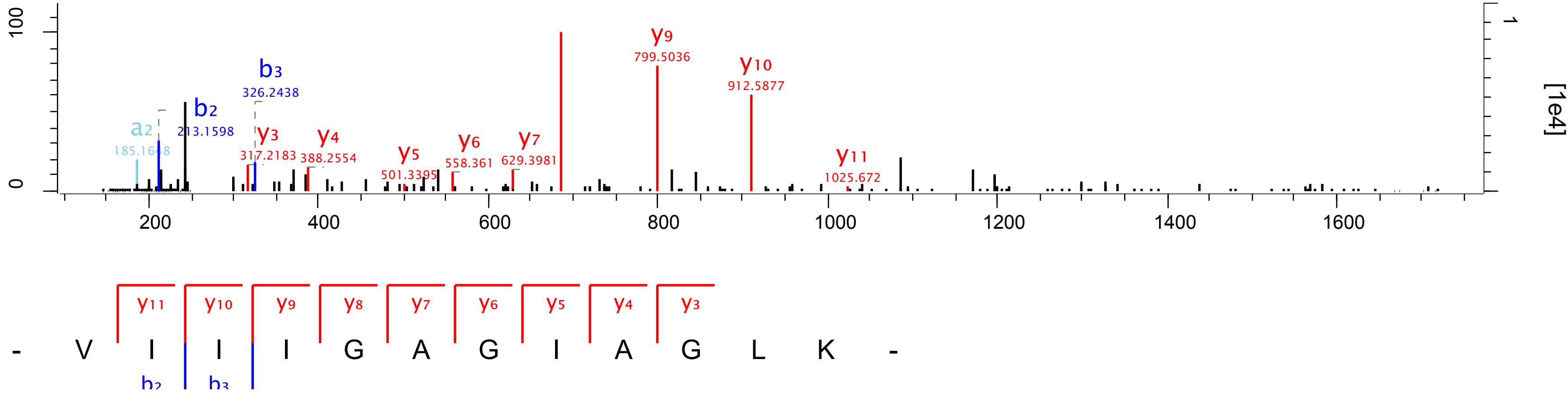

Raw file	Scan	Method	Score	m/z	Gene names
20150306_yeast3_Top_opt_2ug_C3_01_1665	54334	TOF; CID	48.24	831.09	YAH1

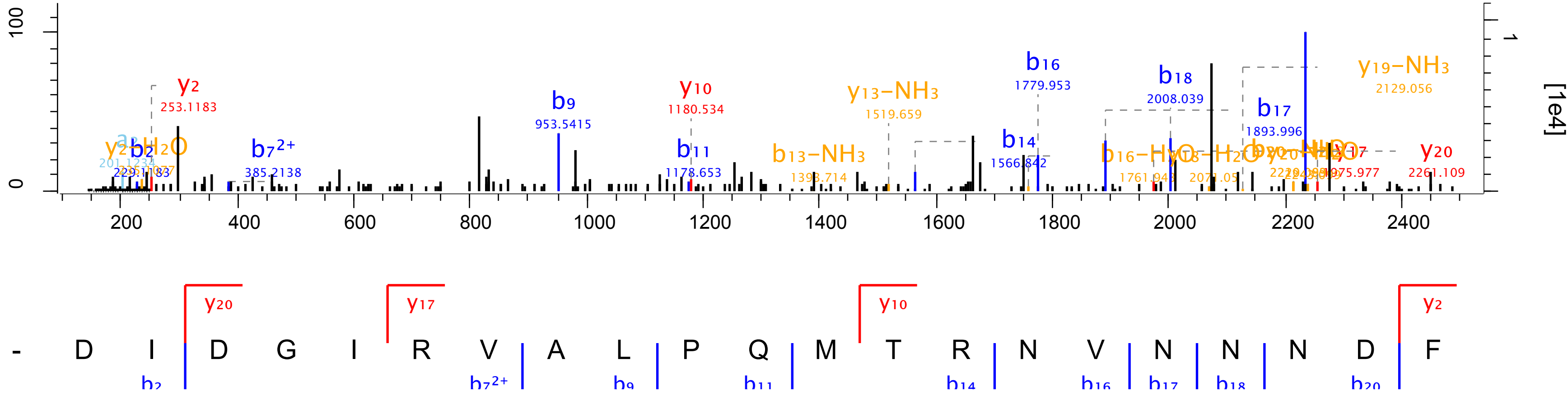

Raw file	Scan	Method	Score	m/z	Gene names
20150306_yeast3_Top_opt_2ug_C3_01_1665	55357	TOF; CID	101.82	599.33	MRPL49

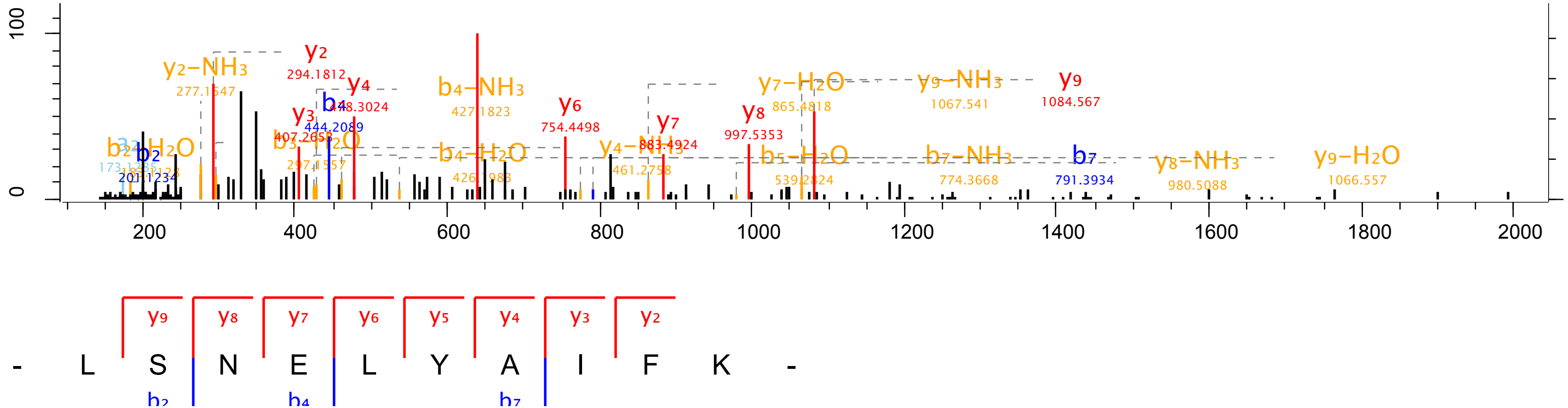

Raw file	Scan	Method	Score	m/z	Gene names
20150306_yeast3_Top_opt_2ug_C3_01_1665	56708	TOF; CID	41.91	684.03	AIM43

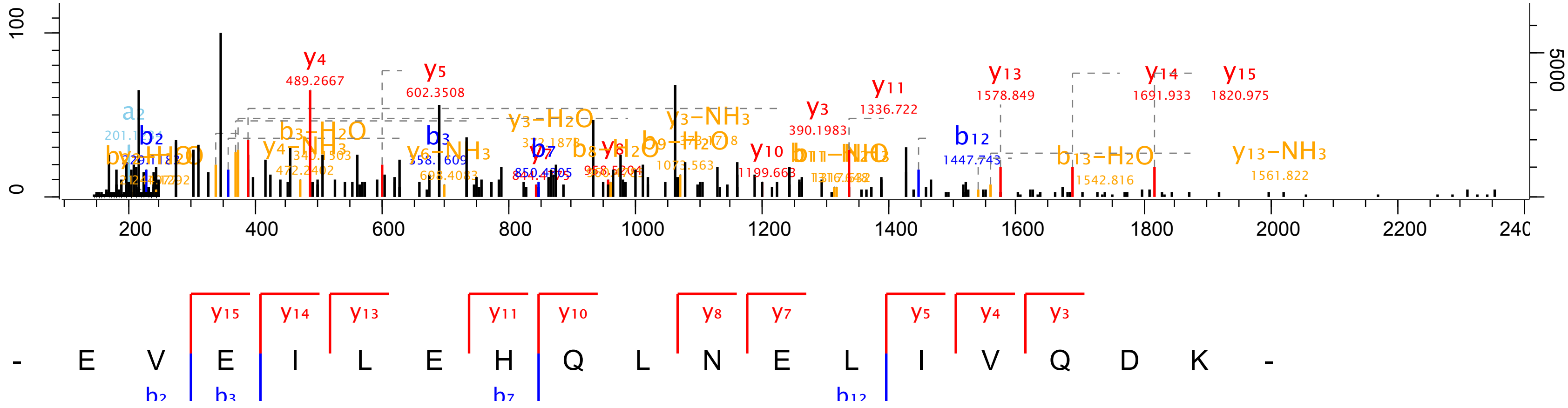

Raw file	Scan	Method	Score	m/z	Gene names
20150306_yeast3_Top_opt_2ug_C3_01_1665	57313	TOF; CID	39.21	853.43	YPC1

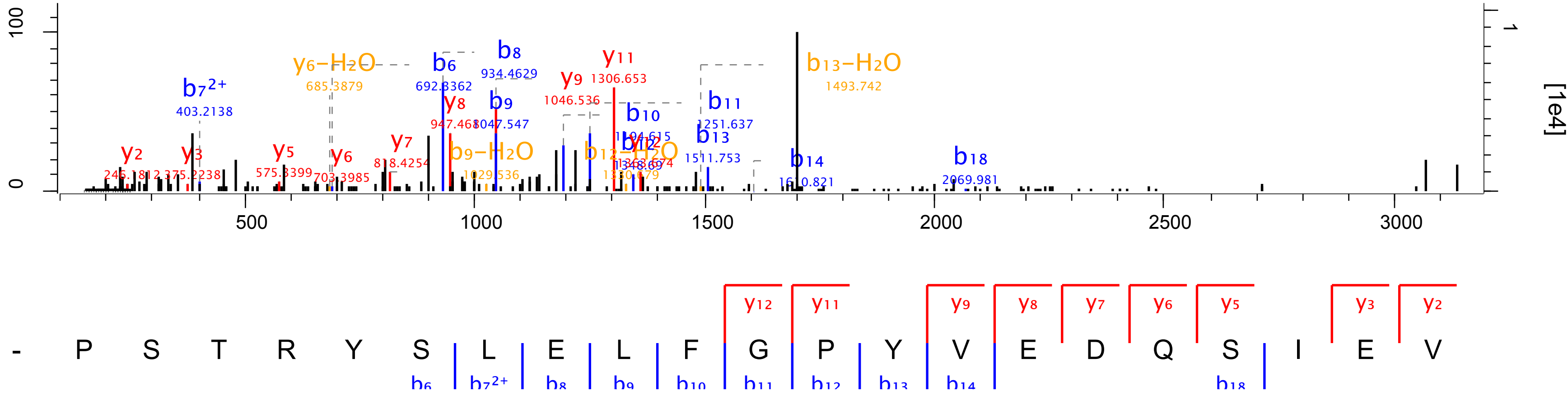

Raw file	Scan	Method	Score	m/z	Gene names
20150306_yeast3_Top_opt_2ug_C3_01_1665	57429	TOF; CID	32.5	912.43	DSS4

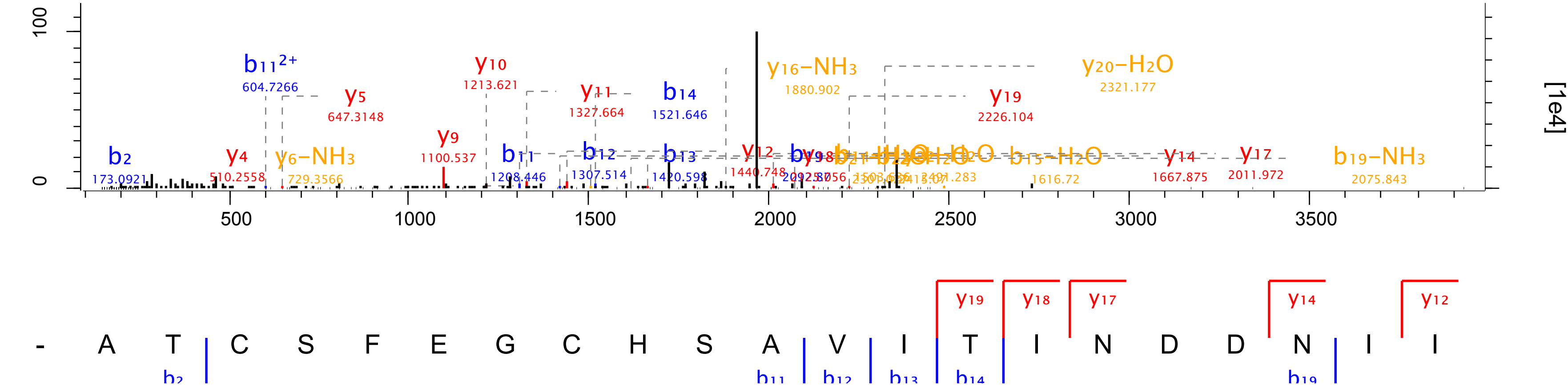

Raw file	Scan	Method	Score	m/z	Gene names
20150306_yeast3_Top_opt_2ug_C3_01_1665	57733	TOF; CID	44.54	798.41	PSF3

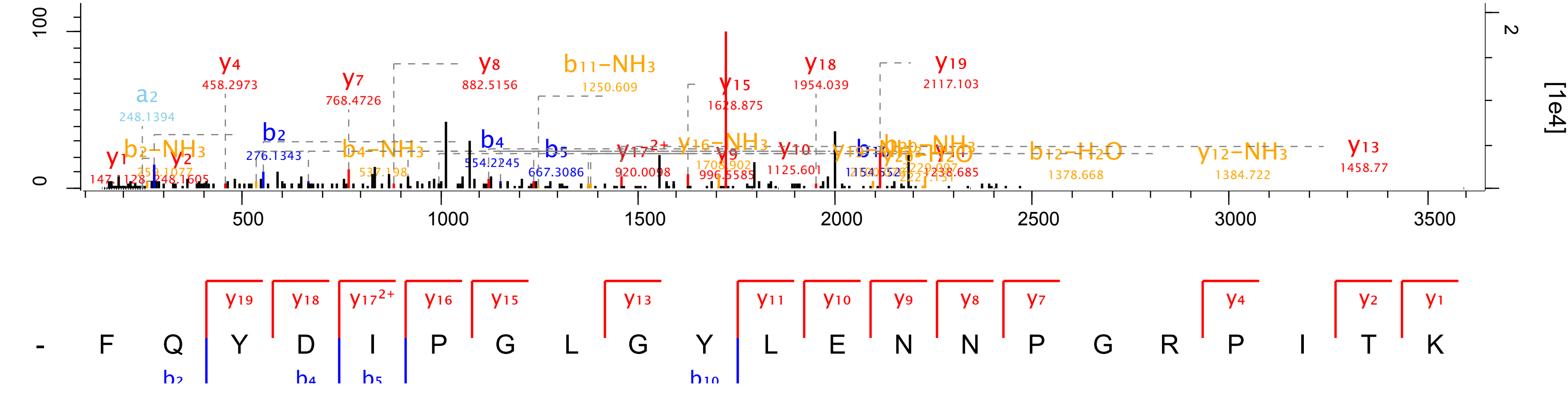

Raw file	Scan	Method	Score	m/z	Gene names
20150306_yeast3_Top_opt_2ug_C3_01_1665	61986	TOF; CID	97.49	1038.97	TOM6

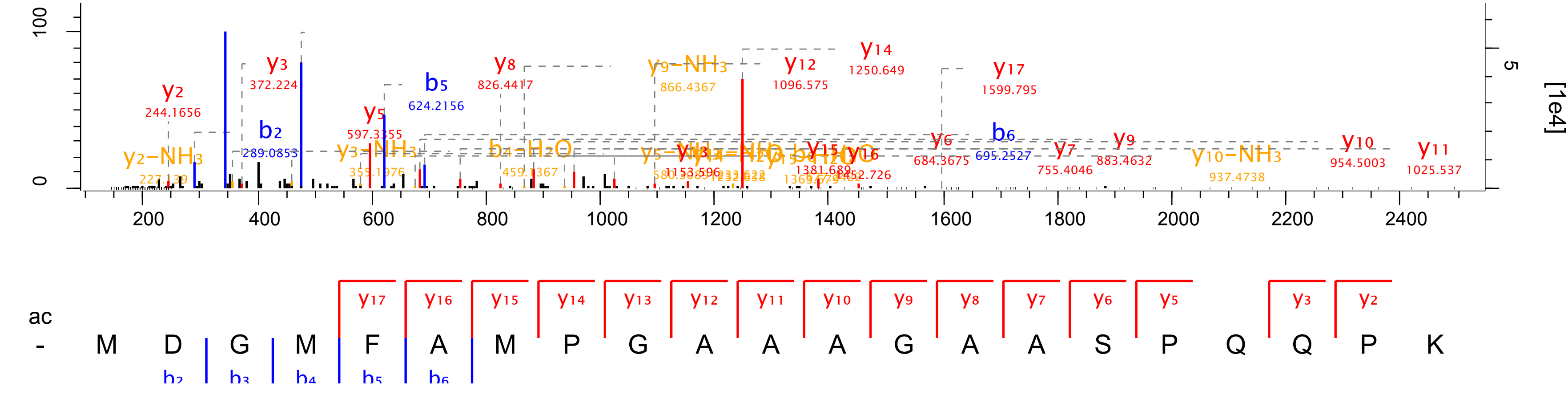

Raw file	Scan	Method	Score	m/z	Gene names
20150306_yeast3_Top_opt_2ug_C3_01_1665	64601	TOF; CID	38.26	899.45	PSF1

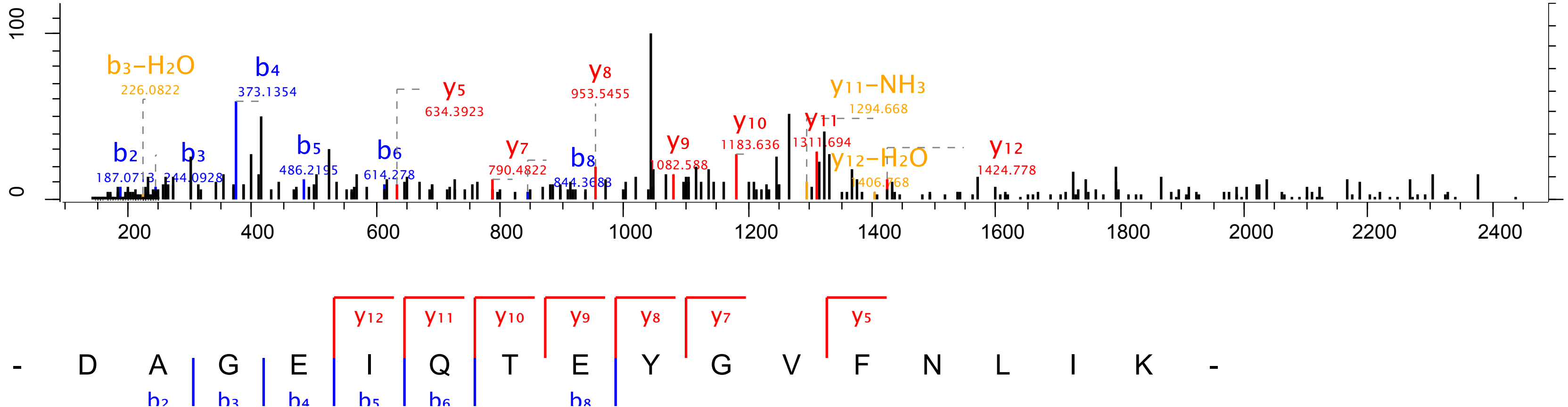

Raw file	Scan	Method	Score	m/z	Gene names
20150306_yeast3_Top_opt_2ug_C3_01_1665	68046	TOF; CID	72.16	937.47	YDC1

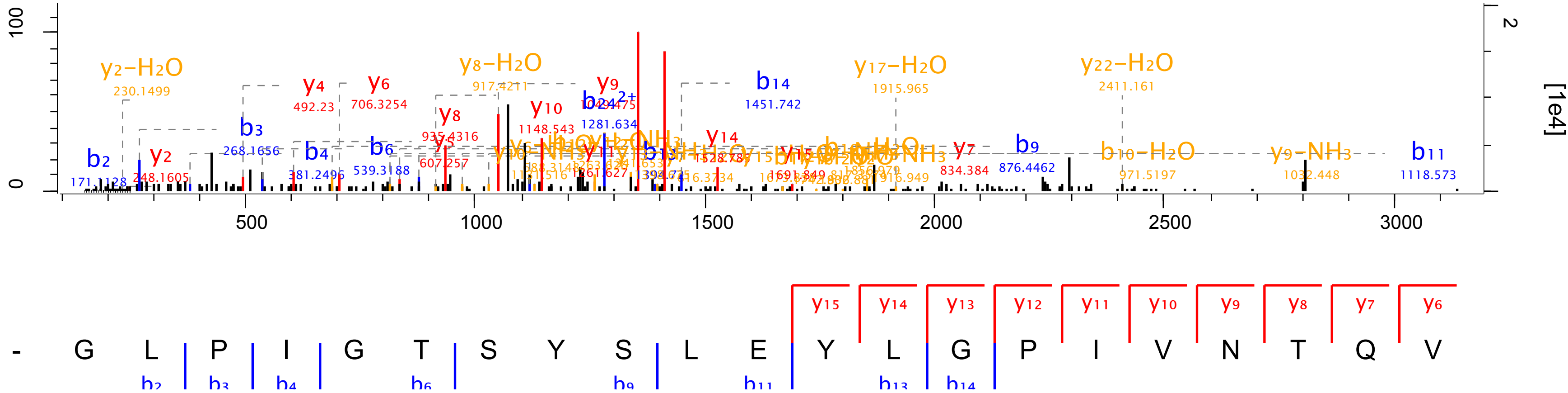

Raw file	Scan	Method	Score	m/z	Gene names
20150306_yeast3_Top_opt_2ug_C3_01_1665	69992	TOF; CID	153.25	1014.55	SRP21

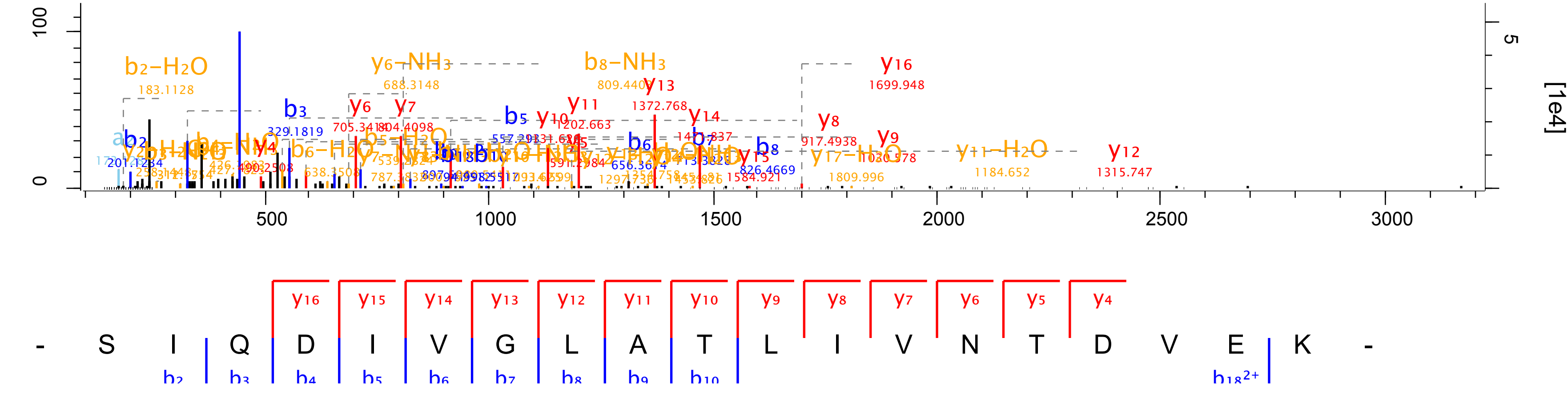

Raw file	Scan	Method	Score	m/z	Gene names
20150306_yeast3_Top_opt_2ug_C3_01_1667	5773	TOF; CID	189.15	981.45	YMR295C

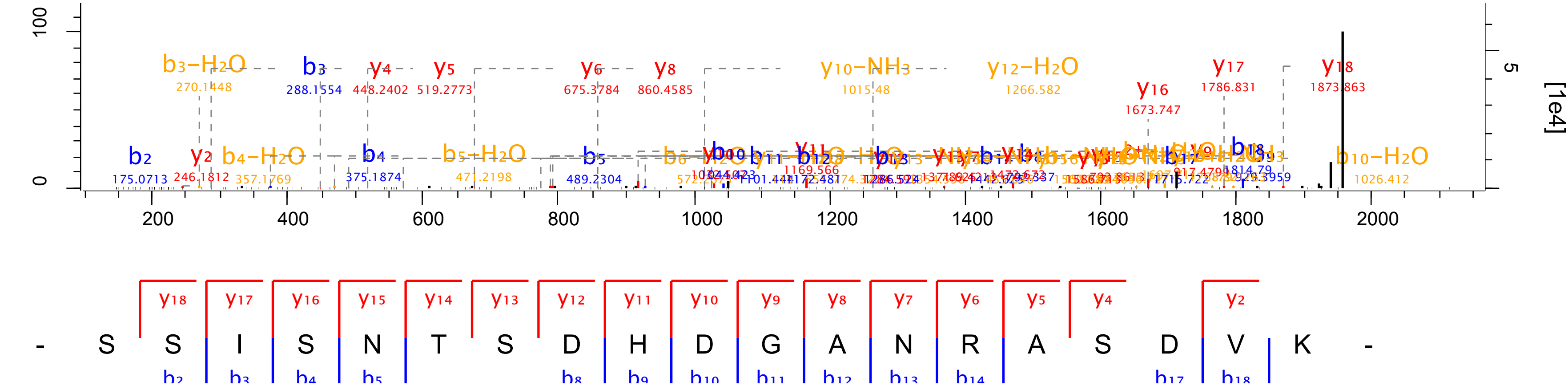

Raw file	Scan	Method	Score	m/z
20150306_yeast3_Top_opt_2ug_C3_01_1667	8095	TOF; CID	45.22	918.38

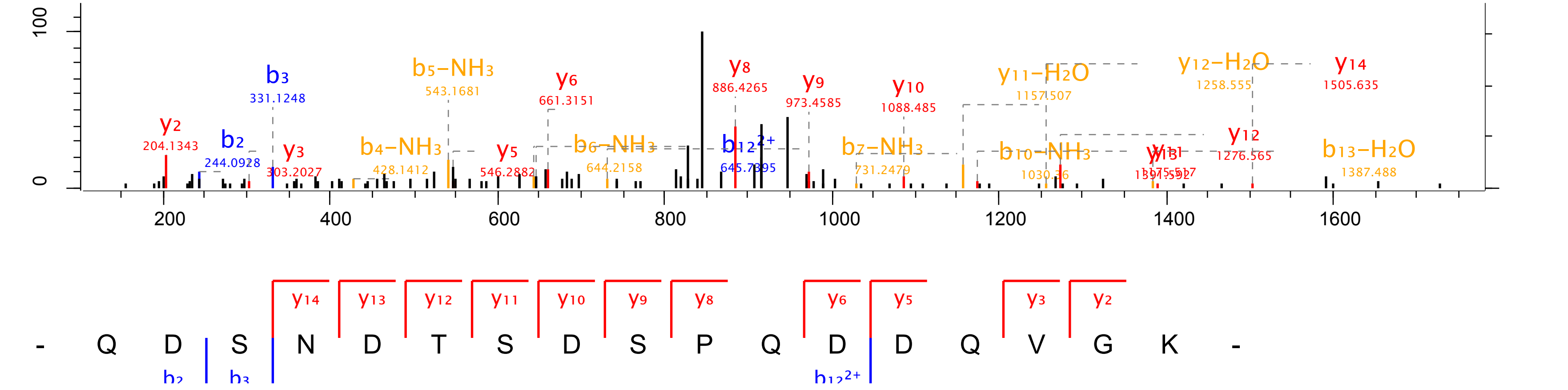

Raw file	Scan	Method	Score	m/z	Gene names
20150306_yeast3_Top_opt_2ug_C3_01_1667	11644	TOF; CID	64.52	527.02	ZRG8

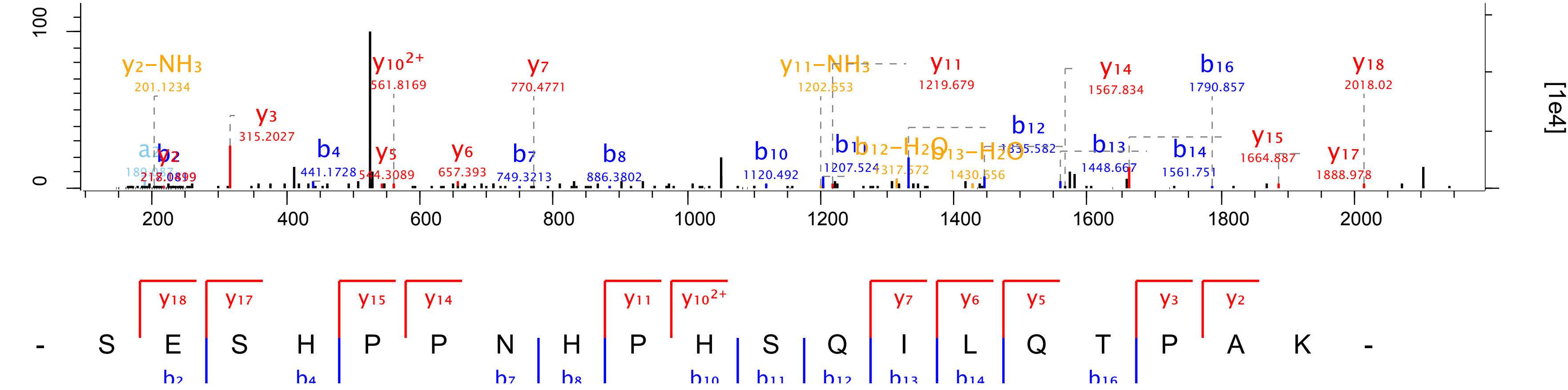

Raw file	Scan	Method	Score	m/z	Gene names
20150306_yeast3_Top_opt_2ug_C3_01_1667	15796	TOF; CID	84.48	651.33	ACE2

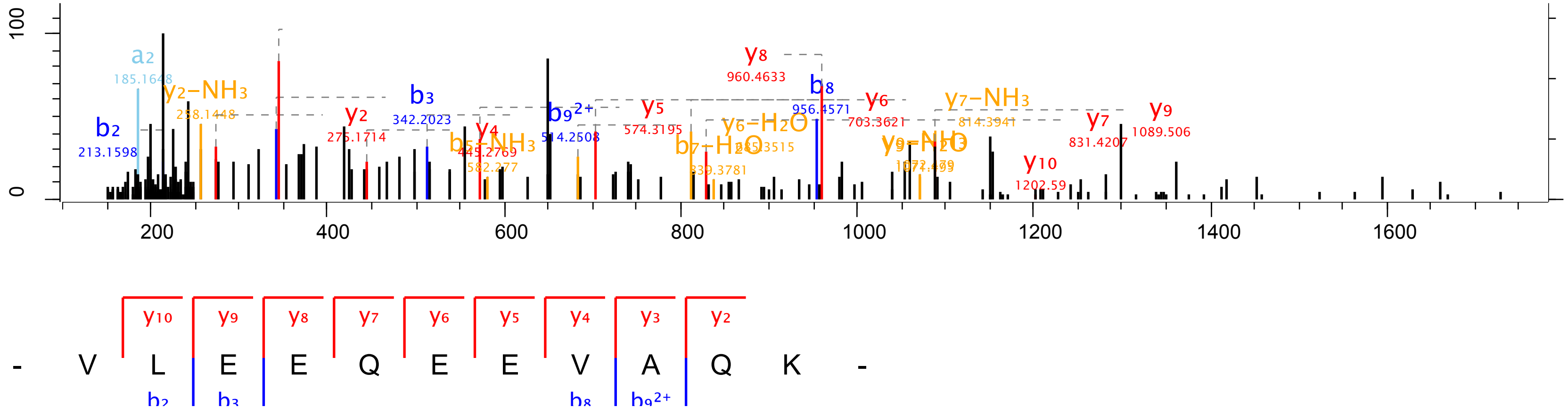

Raw file	Scan	Method	Score	m/z	Gene names
20150306_yeast3_Top_opt_2ug_C3_01_1667	17010	TOF; CID	73.09	585.28	GLN3

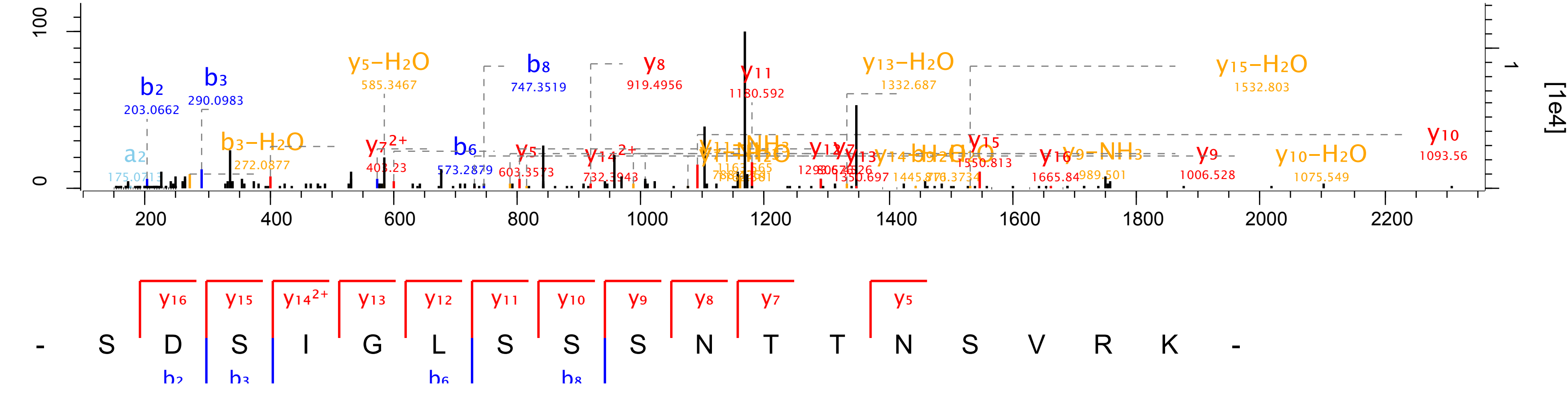

Raw file	Scan	Method	Score	m/z	Gene names
20150306_yeast3_Top_opt_2ug_C3_01_1667	17185	TOF; CID	65.22	712.81	ATG16

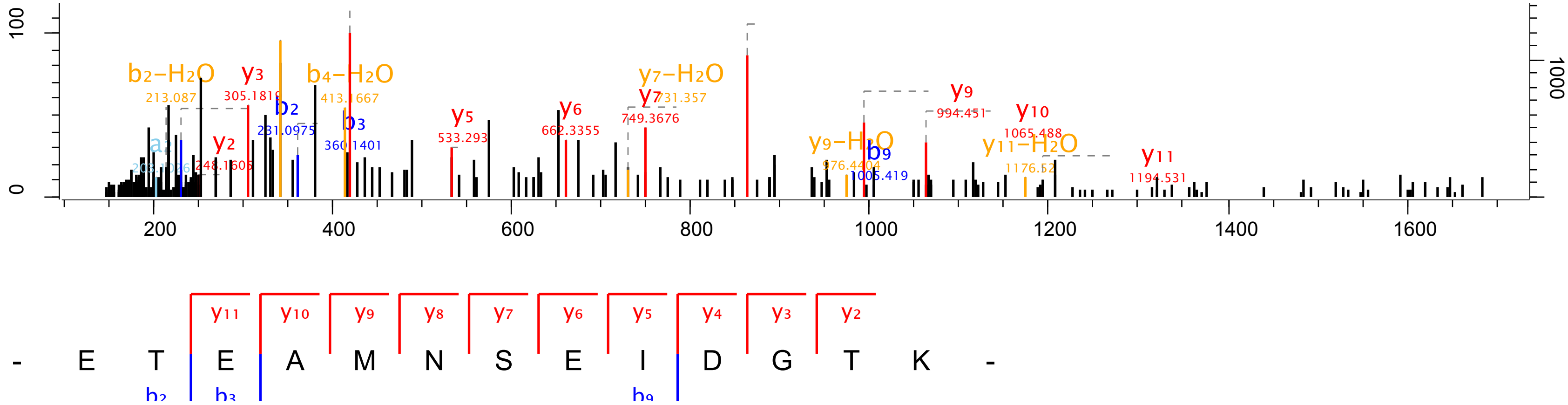

Raw file	Scan	Method	Score	m/z	Gene names
20150306_yeast3_Top_opt_2ug_C3_01_1667	20339	TOF; CID	48.9	609.31	YGR042W

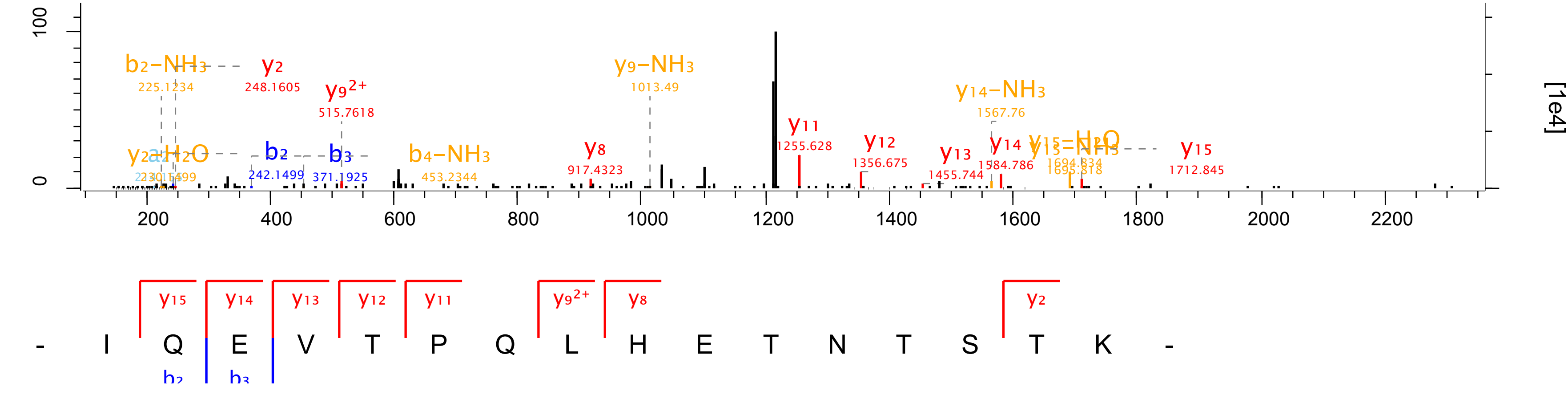

Raw file	Scan	Method	Score	m/z	Gene names
20150306_yeast3_Top_opt_2ug_C3_01_1667	20910	TOF; CID	84.3	700.31	SFK1

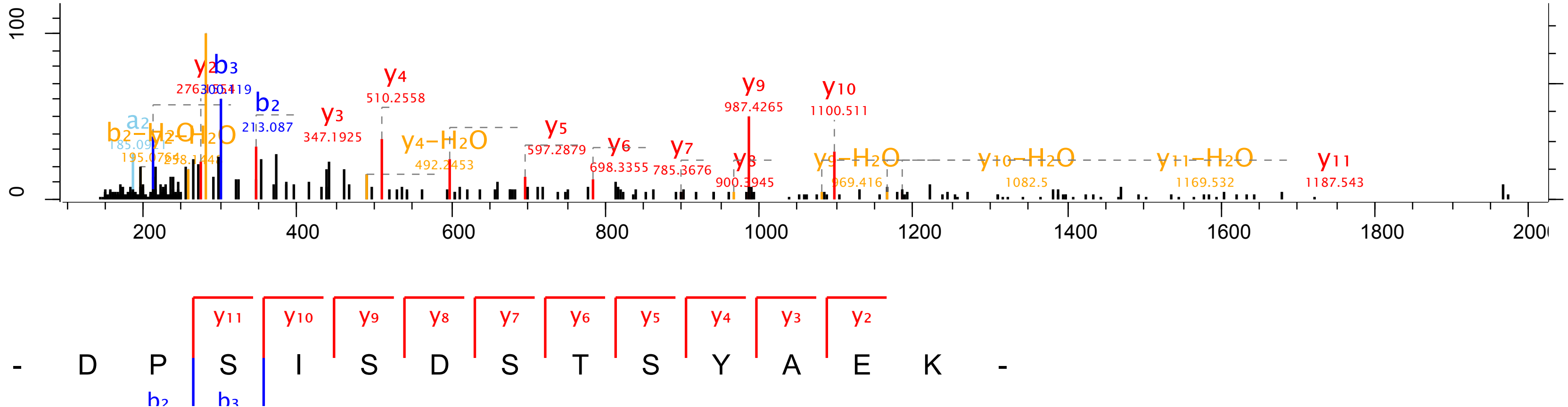

Raw file	Scan	Method	Score	m/z	Gene names
20150306_yeast3_Top_opt_2ug_C3_01_1667	21653	TOF; CID	70.72	743.35	SPT10

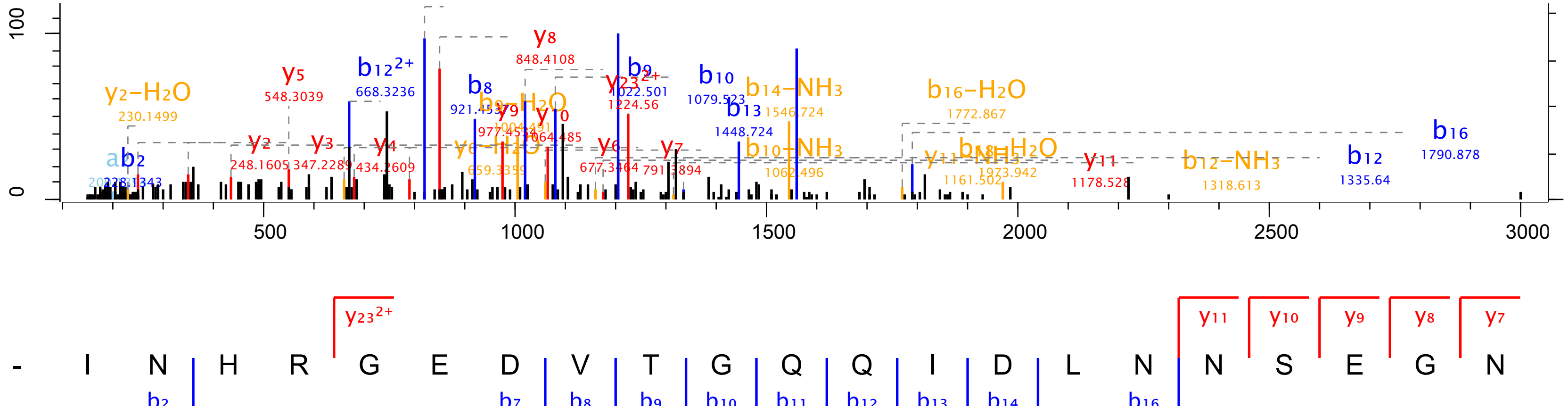

Raw file	Scan	Method	Score	m/z	Gene names
20150306_yeast3_Top_opt_2ug_C3_01_1667	24746	TOF; CID	80.39	812.37	MMT2

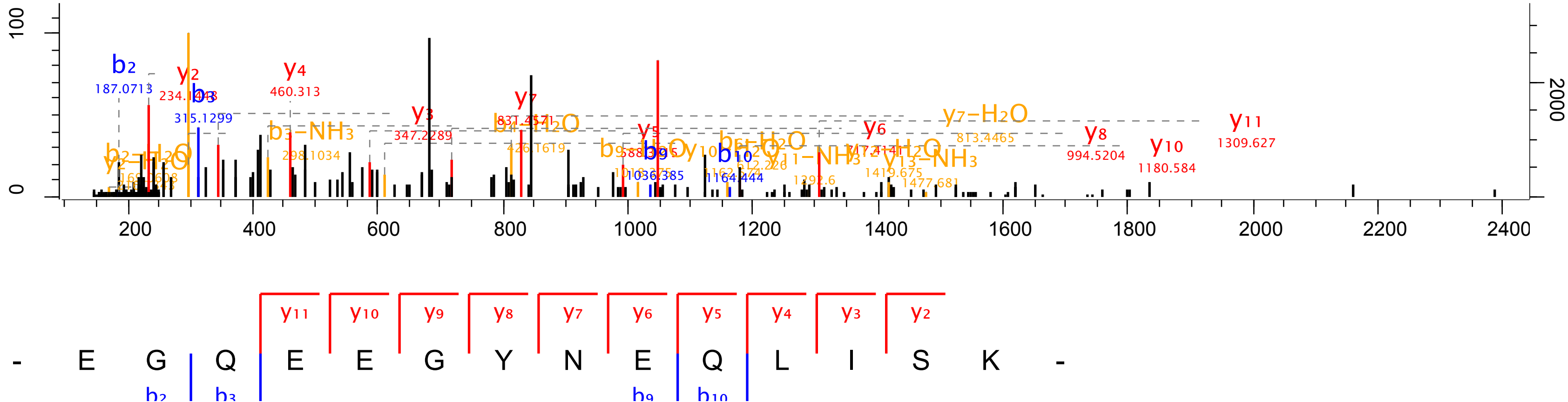

Raw file	Scan	Method	Score	m/z	Gene names
20150306_yeast3_Top_opt_2ug_C3_01_1667	24864	TOF; CID	72.23	476.22	SDH4

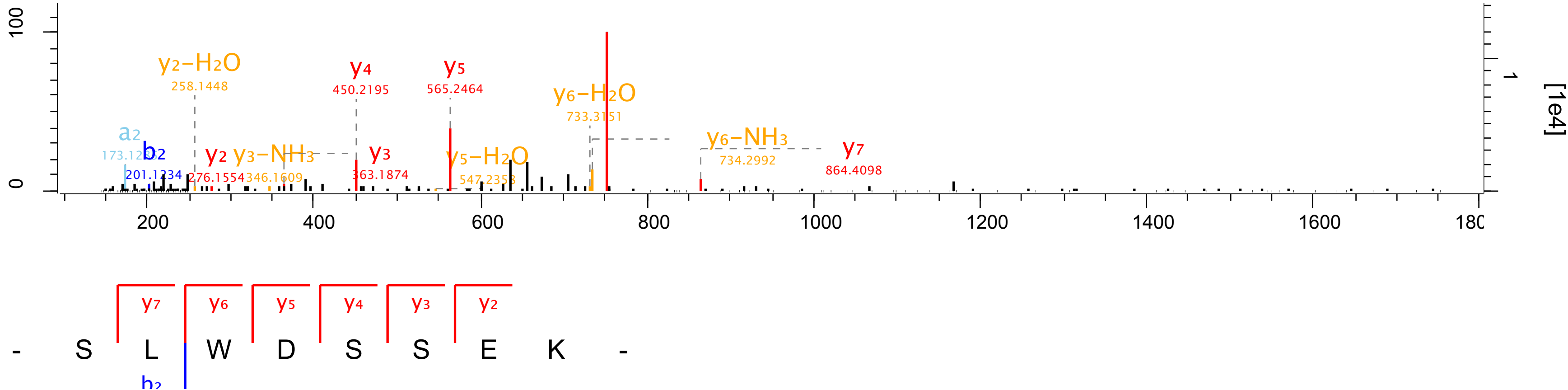

Raw file	Scan	Method	Score	m/z	Gene names
20150306_yeast3_Top_opt_2ug_C3_01_1667	24936	TOF; CID	81.78	487.73	COX19

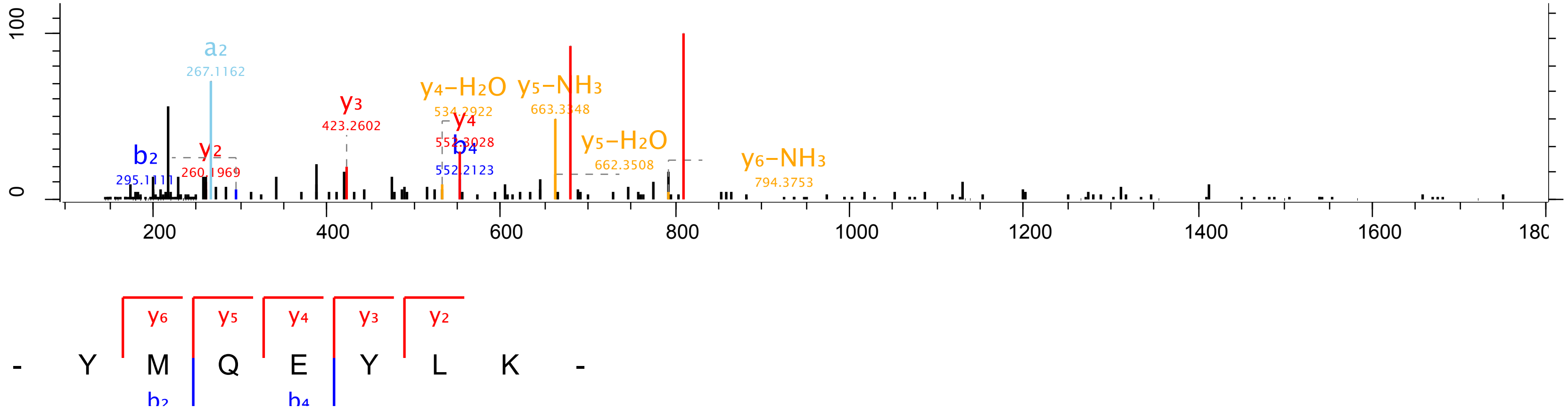

Raw file	Scan	Method	Score	m/z	Gene names
20150306_yeast3_Top_opt_2ug_C3_01_1667	24939	TOF; CID	58.11	765.7	NSE5

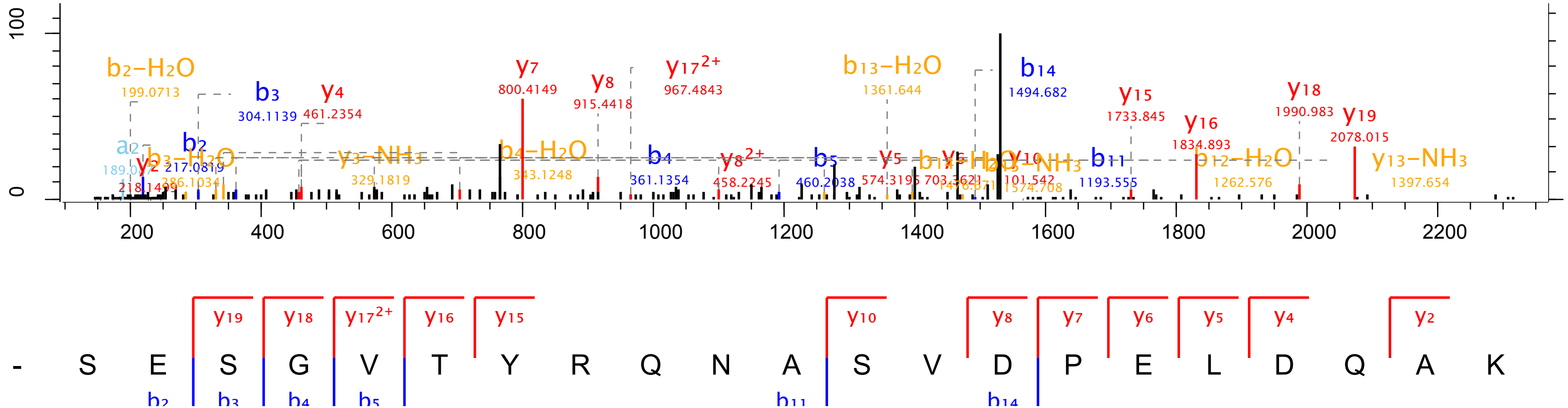

Raw file	Scan	Method	Score	m/z	Gene names
20150306_yeast3_Top_opt_2ug_C3_01_1667	25324	TOF; CID	58.7	579.32	DAL3

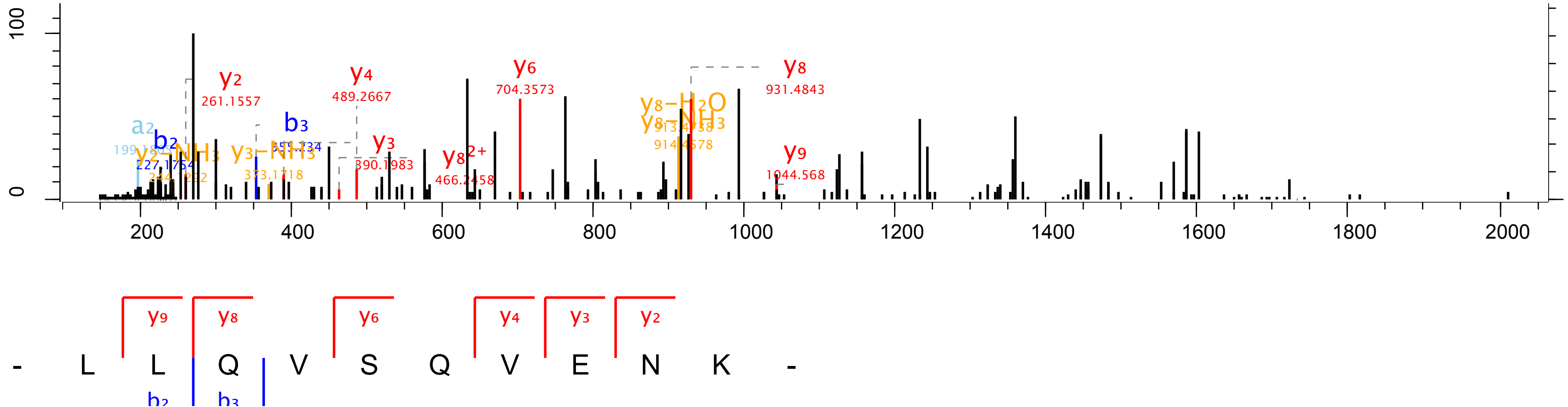

Raw file	Scan	Method	Score	m/z	Gene names
20150306_yeast3_Top_opt_2ug_C3_01_1667	25913	TOF; CID	49.99	687.37	BAG7

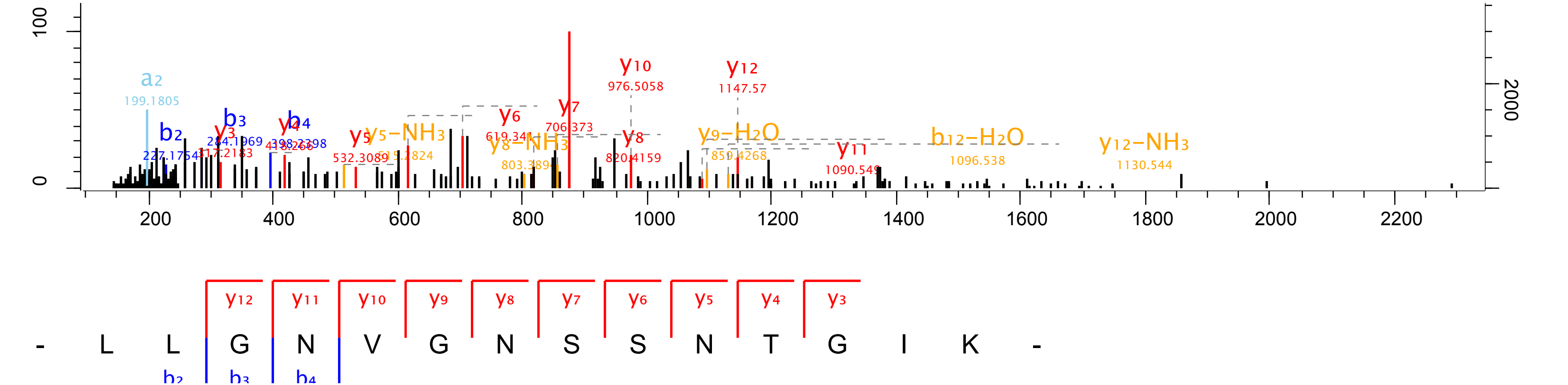

Raw file	Scan	Method	Score	m/z	Gene names
20150306_yeast3_Top_opt_2ug_C3_01_1667	29502	TOF; CID	67.02	714.39	CDD1

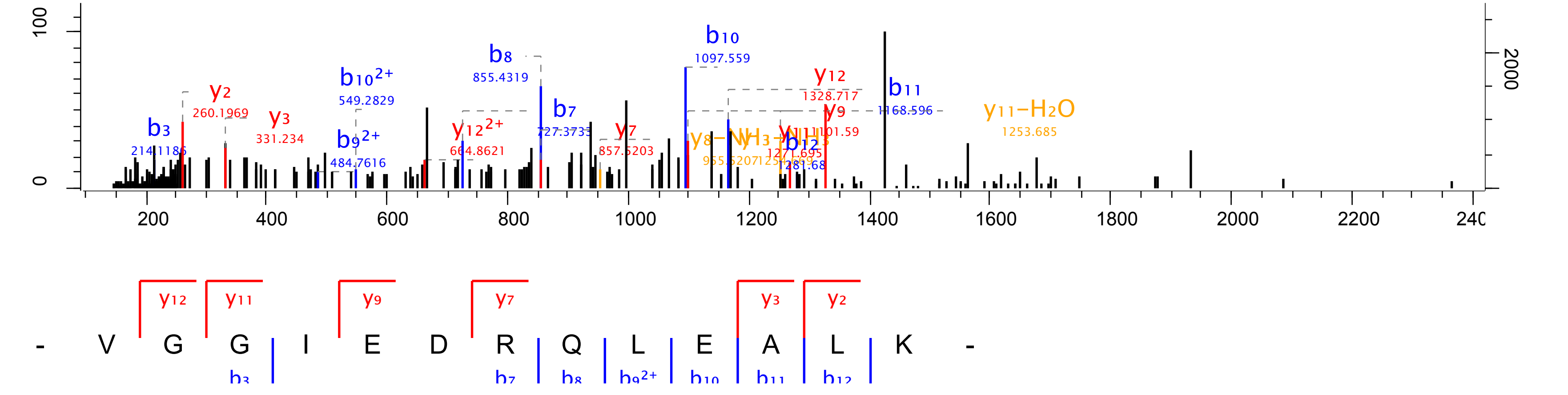

Raw file	Scan	Method	Score	m/z	Gene names
20150306_yeast3_Top_opt_2ug_C3_01_1667	31422	TOF; CID	103.91	612.31	MRPS17

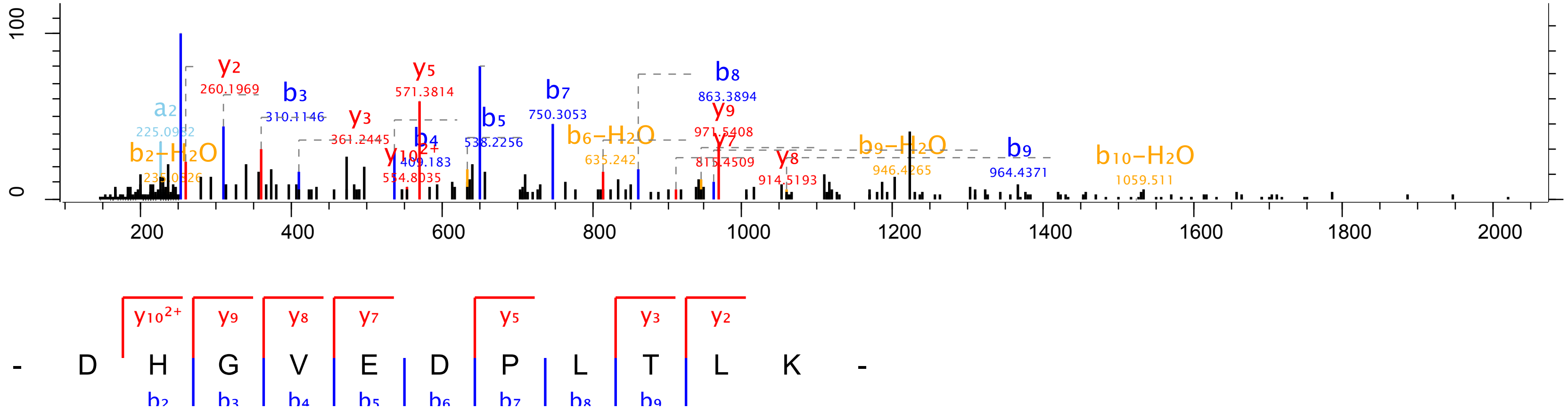

Raw file	Scan	Method	Score	m/z	Gene names
20150306_yeast3_Top_opt_2ug_C3_01_1667	31528	TOF; CID	91.81	527.28	MST27;MST28

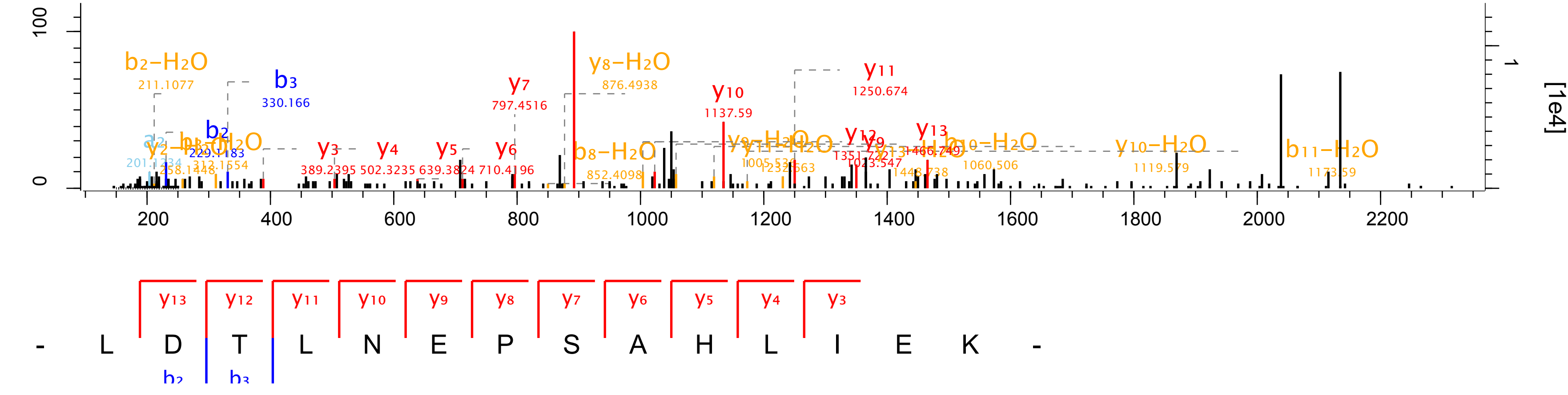

Raw file	Scan	Method	Score	m/z	Gene names
20150306_yeast3_Top_opt_2ug_C3_01_1667	33688	TOF; CID	82.75	354.21	MRPL39

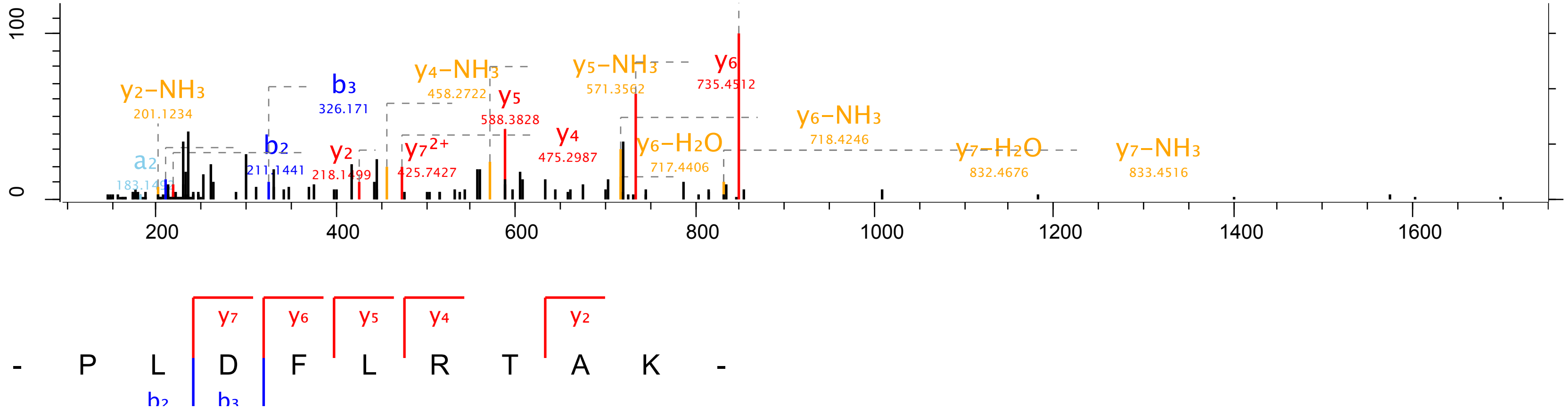

Raw file	Scan	Method	Score	m/z	Gene names
20150306_yeast3_Top_opt_2ug_C3_01_1667	35236	TOF; CID	44.93	772.88	PTP1

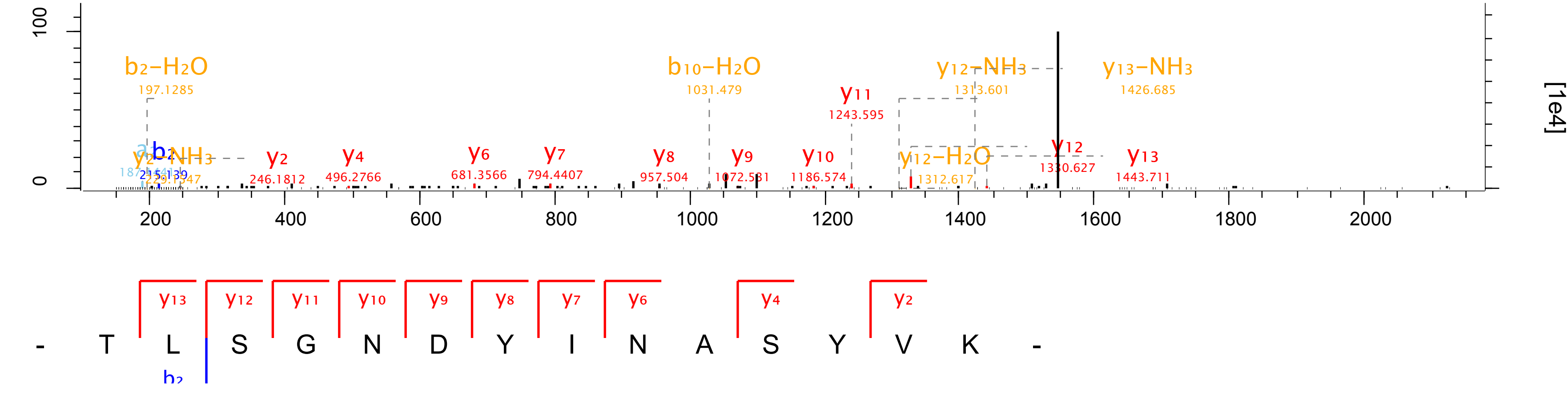

Raw file	Scan	Method	Score	m/z	Gene names
20150306_yeast3_Top_opt_2ug_C3_01_1667	37821	TOF; CID	47.71	643.33	PHS1

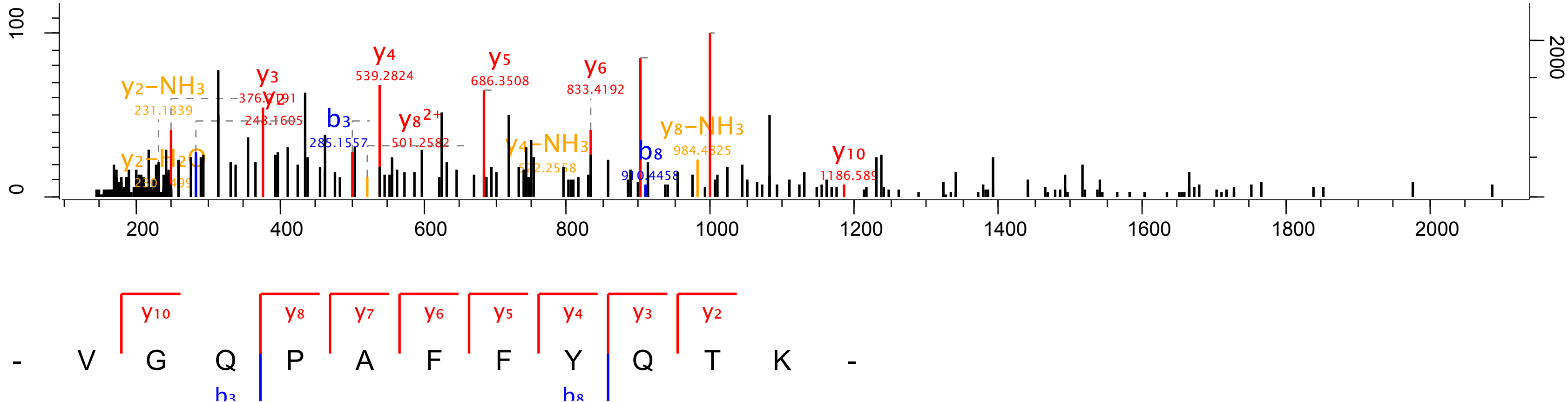

Raw file	Scan	Method	Score	m/z	Gene names
20150306_yeast3_Top_opt_2ug_C3_01_1667	40566	TOF; CID	46.07	645.35	RAD51

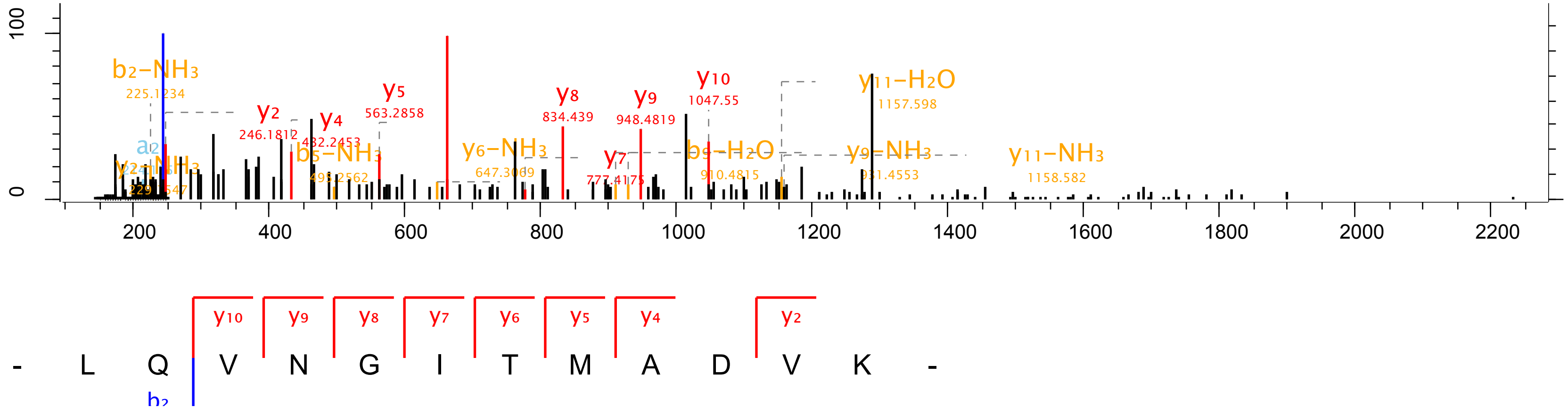

Raw file	Scan	Method	Score	m/z	Gene names
20150306_yeast3_Top_opt_2ug_C3_01_1667	42709	TOF; CID	58.32	491.27	RML2

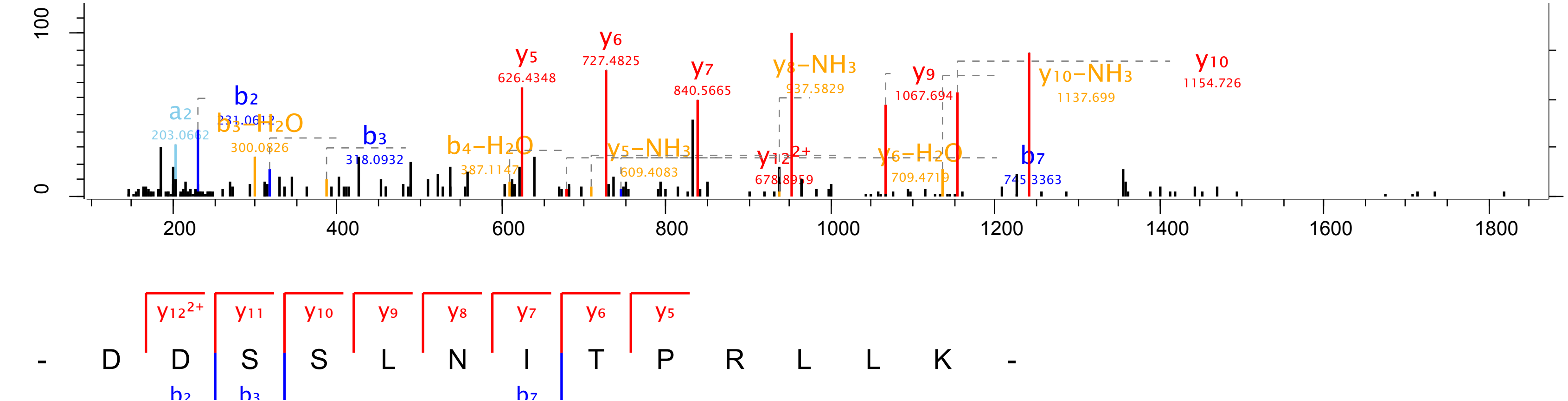

Raw file	Scan	Method	Score	m/z	Gene names
20150306_yeast3_Top_opt_2ug_C3_01_1667	42716	TOF; CID	79.31	591.29	COX8

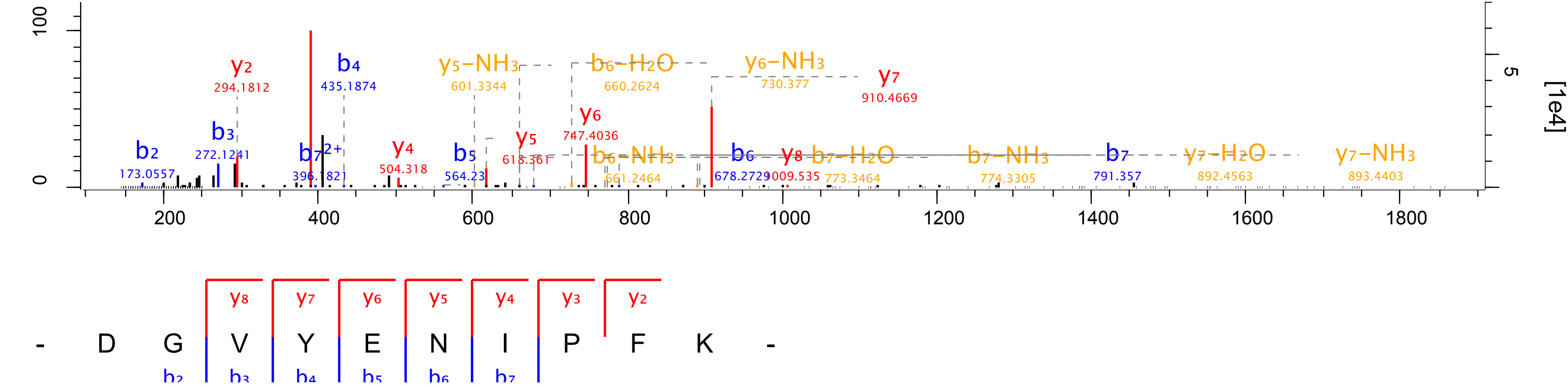

Raw file	Scan	Method	Score	m/z	Gene names
20150306_yeast3_Top_opt_2ug_C3_01_1667	43088	TOF; CID	60.55	729.4	PEX2

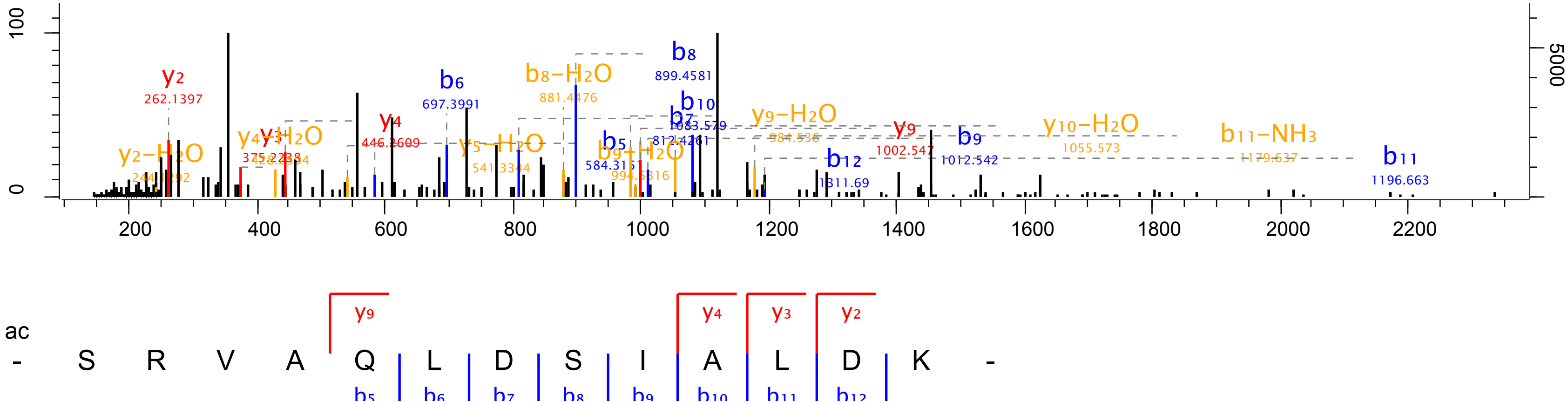

Raw file	Scan	Method	Score	m/z	Gene names
20150306_yeast3_Top_opt_2ug_C3_01_1667	43827	TOF; CID	39.91	699.37	YLL019W-A

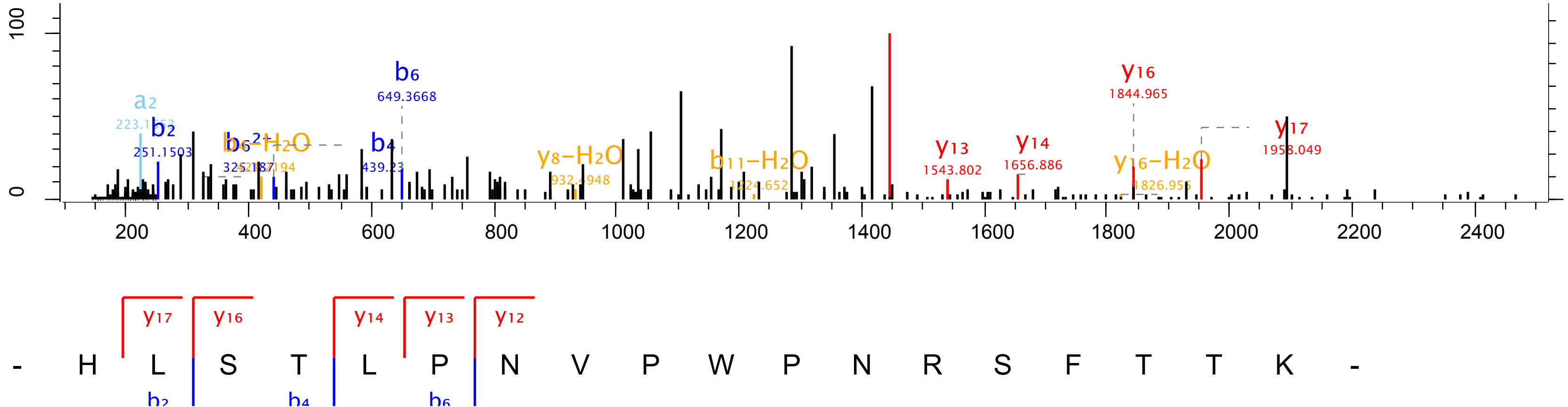

Raw file	Scan	Method	Score	m/z	Gene names
20150306_yeast3_Top_opt_2ug_C3_01_1667	44346	TOF; CID	130.72	575.29	SRL3

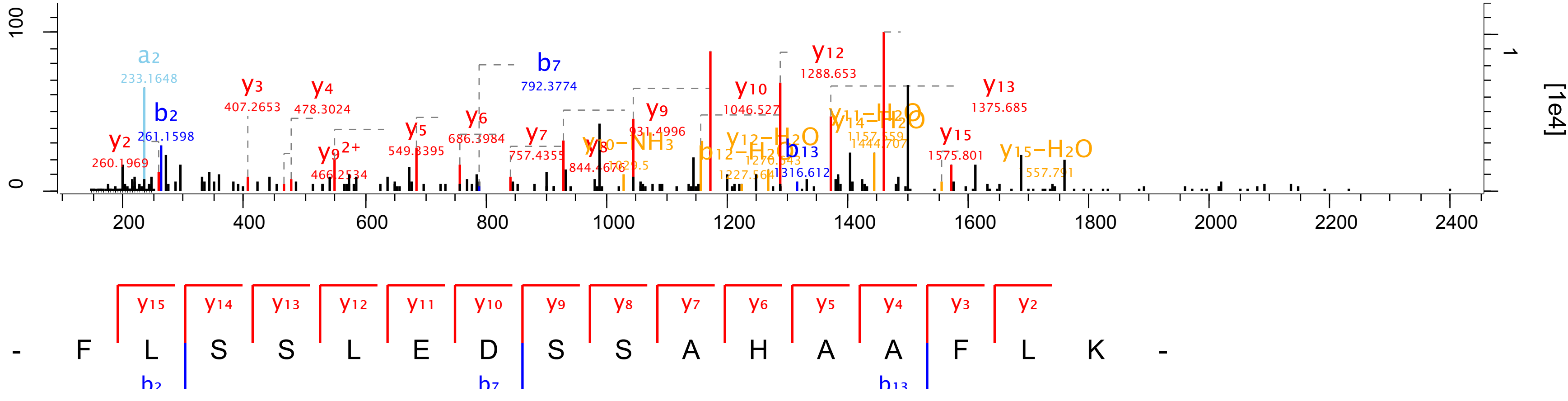

Raw file	Scan	Method	Score	m/z	Gene names
20150306_yeast3_Top_opt_2ug_C3_01_1667	44772	TOF; CID	70.99	799.07	MRPL51

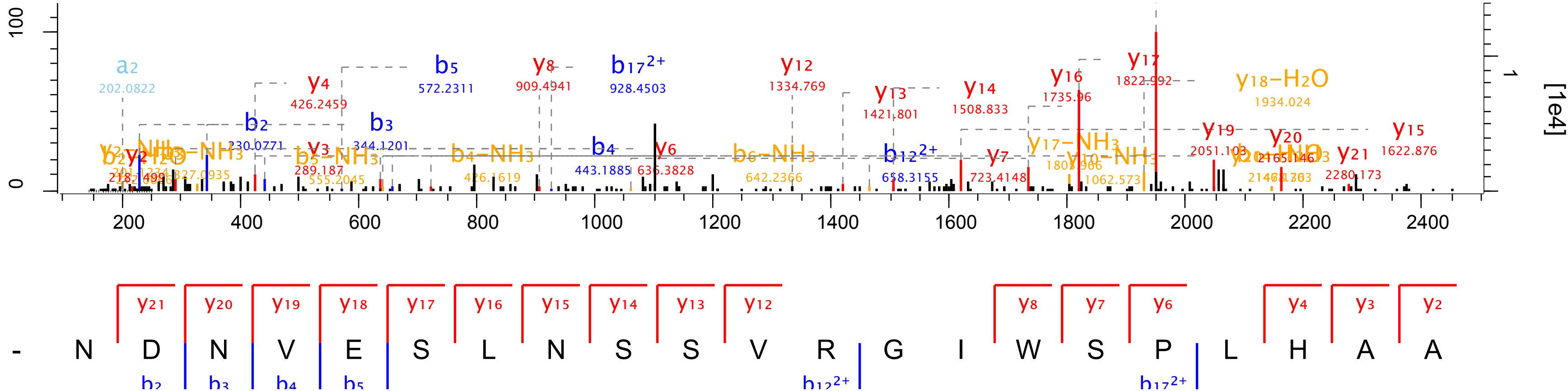

Raw file	Scan	Method	Score	m/z	Gene names
20150306_yeast3_Top_opt_2ug_C3_01_1667	47717	TOF; CID	66.69	495.27	FAR7

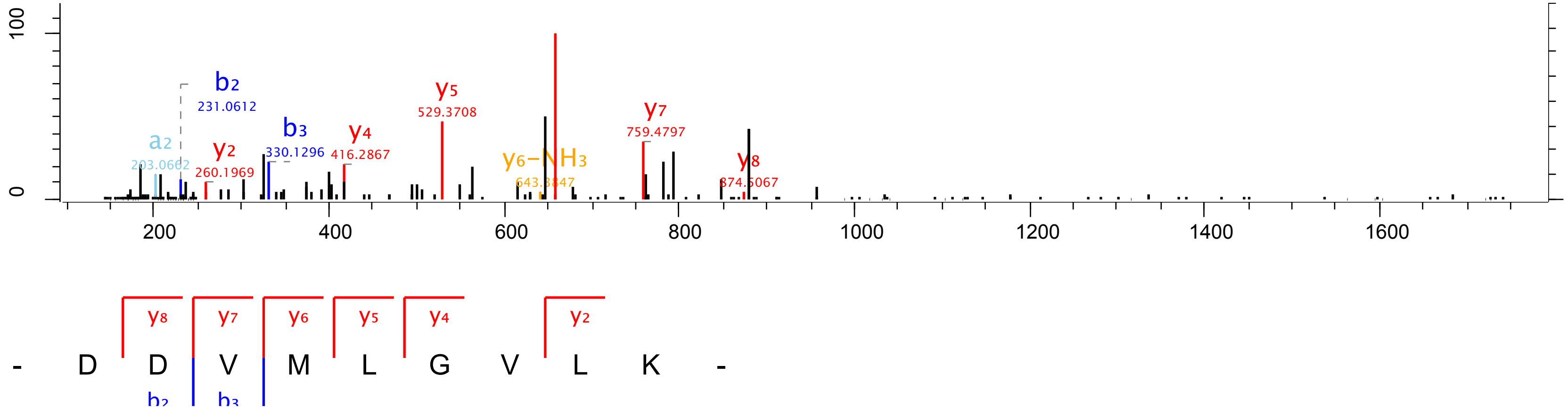

Raw file	Scan	Method	Score	m/z	Gene names
20150306_yeast3_Top_opt_2ug_C3_01_1667	49948	TOF; CID	47.68	667.34	PUS9

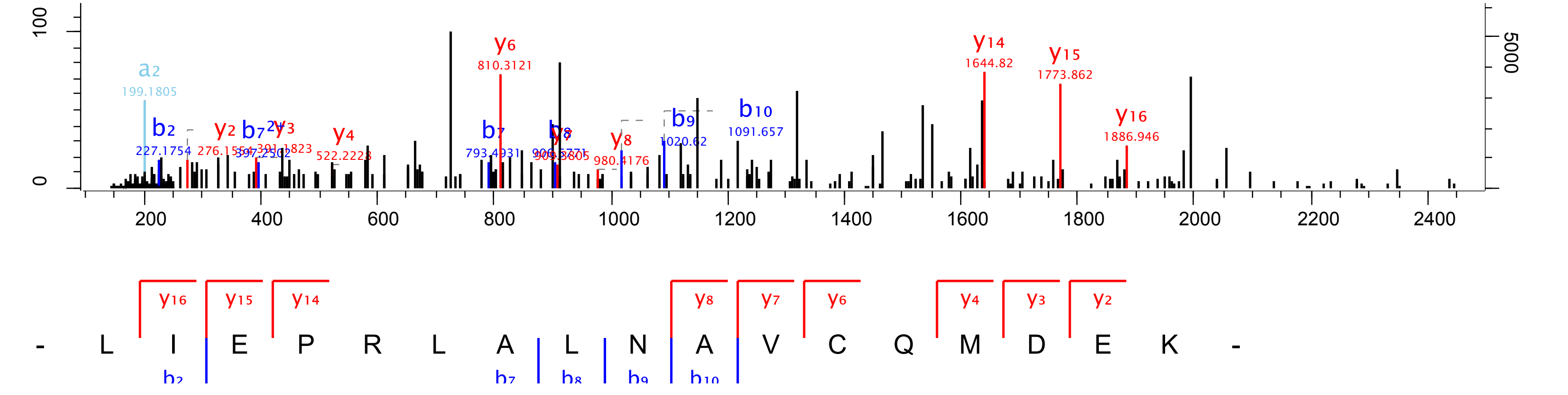

Raw file	Scan	Method	Score	m/z	Gene names
20150306_yeast3_Top_opt_2ug_C3_01_1667	53076	TOF; CID	125.98	906.41	ART5

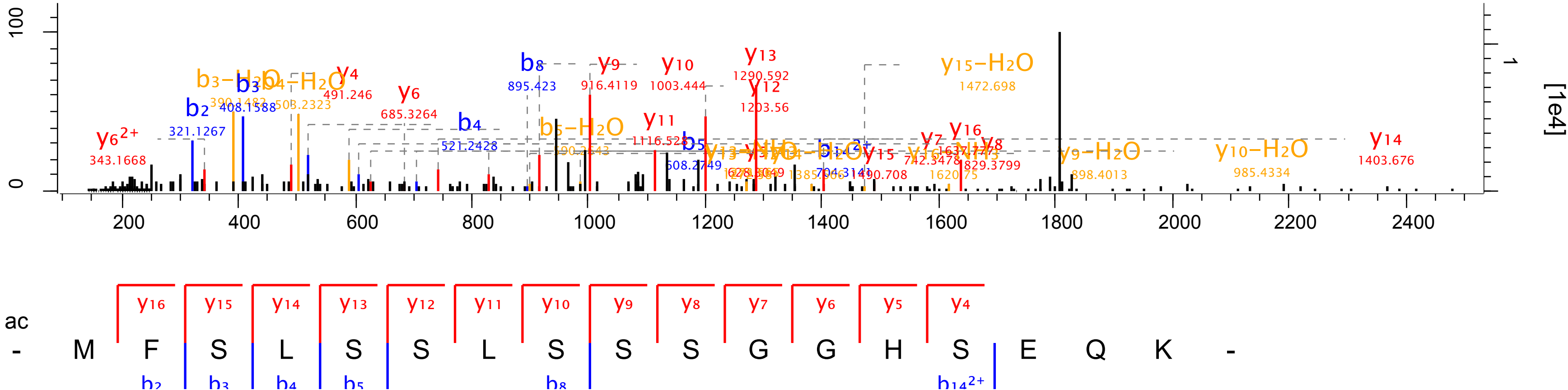

Raw file	Scan	Method	Score	m/z	Gene names
20150306_yeast3_Top_opt_2ug_C3_01_1667	56113	TOF; CID	63.09	1033.12	UMP1

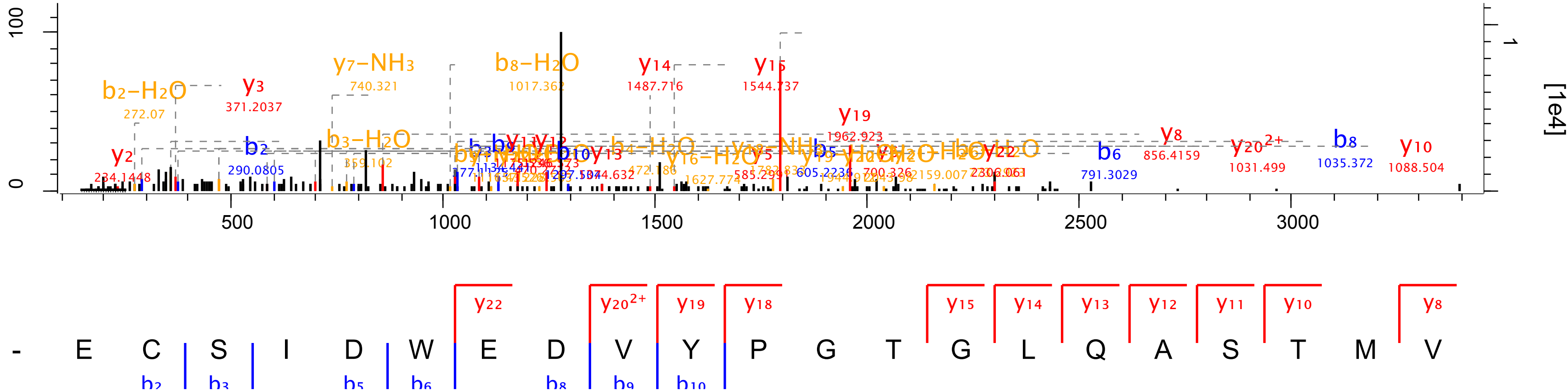

Raw file	Scan	Method	Score	m/z	Gene names
20150306_yeast3_Top_opt_2ug_C3_01_1667	57129	TOF; CID	48.62	1098.03	UBX3

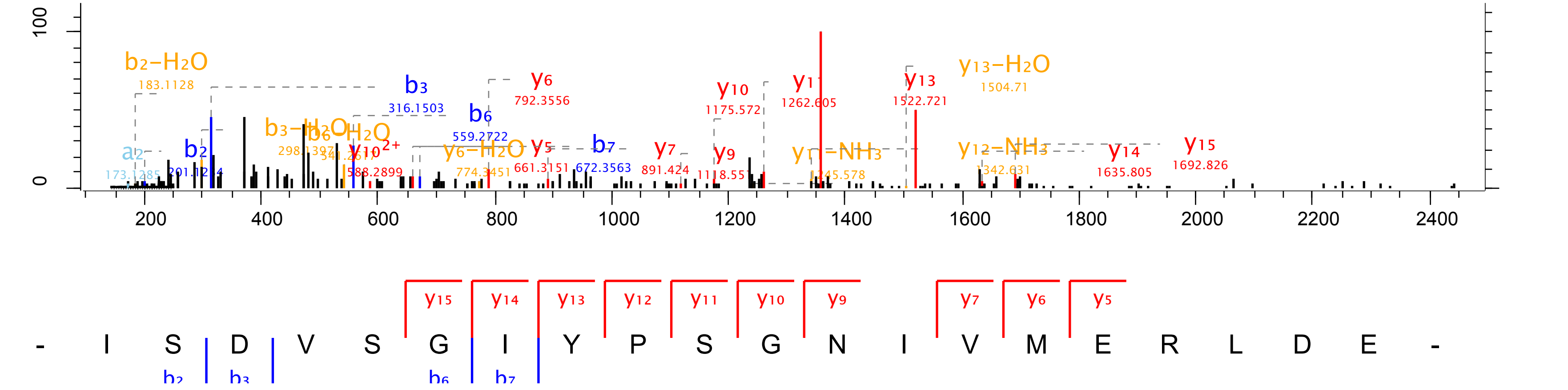

Raw file	Scan	Method	Score	m/z	Gene names
20150306_yeast3_Top_opt_2ug_C3_01_1667	57706	TOF; CID	82.89	870.41	YLR243W

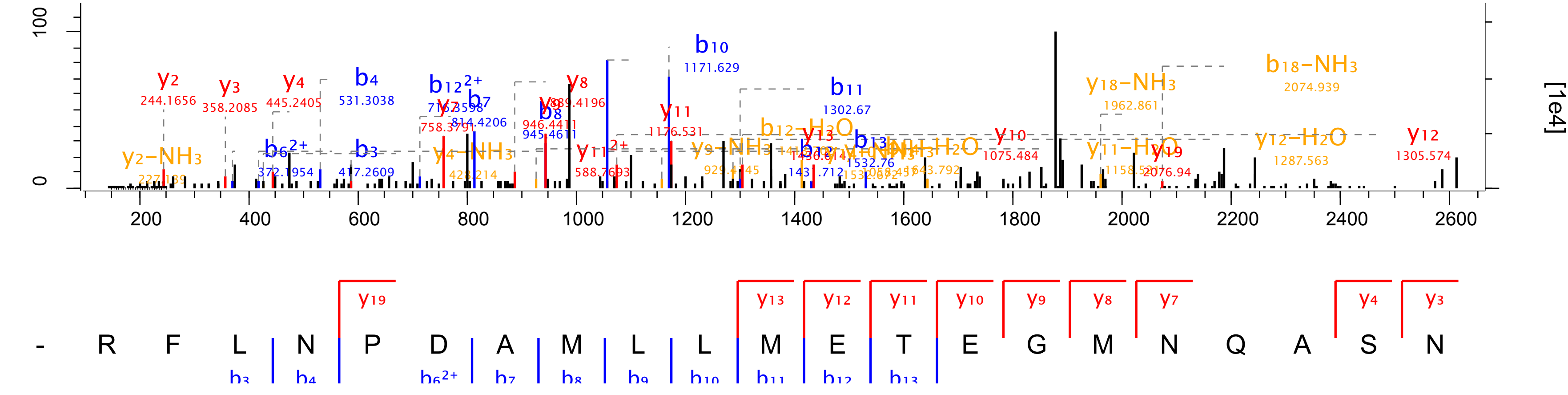

Raw file	Scan	Method	Score	m/z	Gene names
20150306_yeast3_Top_opt_2ug_C3_01_1667	65689	TOF; CID	80.66	1018.51	OSW5

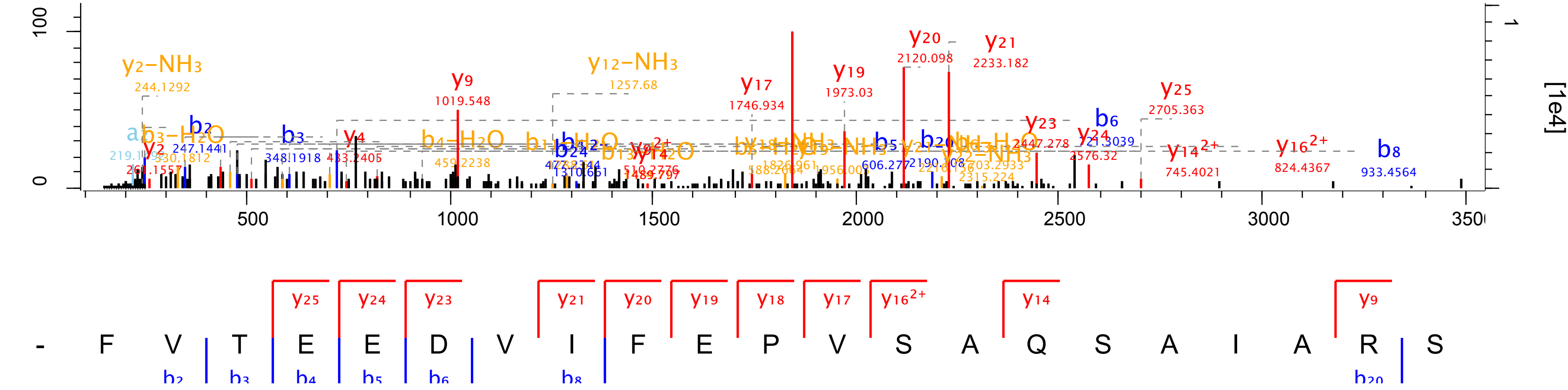

Raw file	Scan	Method	Score	m/z	Gene names
20150306_yeast3_Top_opt_2ug_C3_01_1667	65867	TOF; CID	49.45	659.36	MIC10

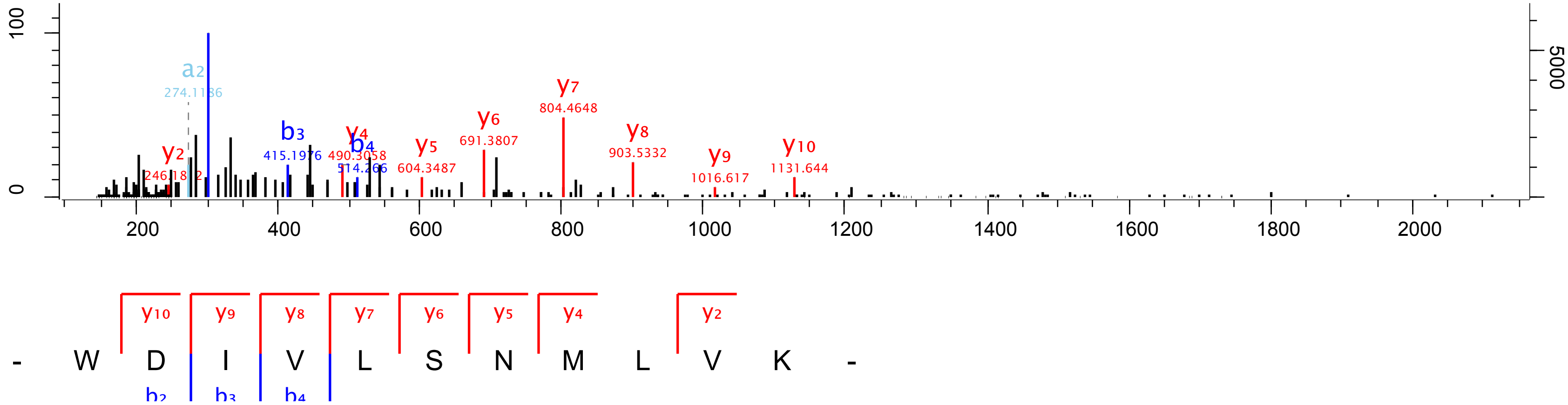

Raw file	Scan	Method	Score	m/z	Gene names
20150306_yeast3_Top_opt_2ug_C3_01_1667	71001	TOF; CID	75.54	849.49	APQ12

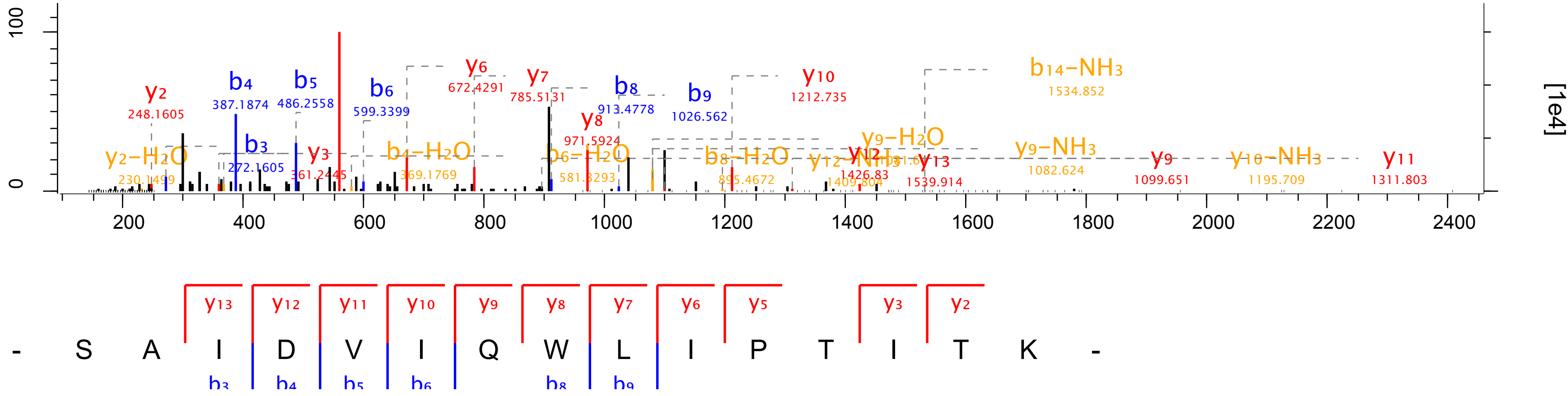

Raw file	Scan	Method	Score	m/z	Gene names
20150306_yeast3_Top_opt_2ug_C3_01_1669	9243	TOF; CID	93.32	581.75	YDR286C

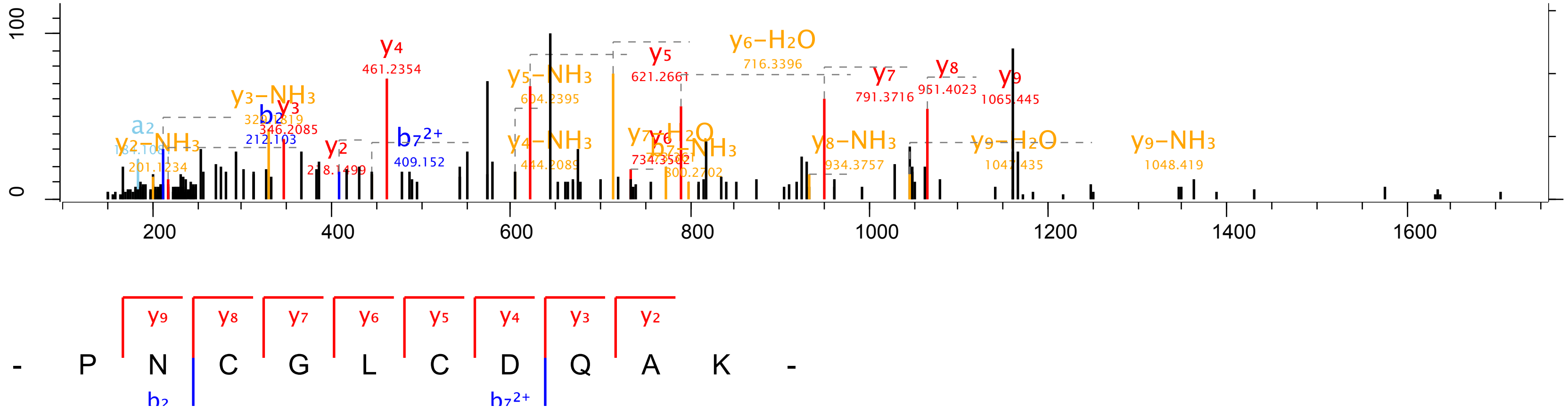

Raw file	Scan	Method	Score	m/z	Gene names
20150306_yeast3_Top_opt_2ug_C3_01_1669	10238	TOF; CID	47.08	611.28	ERT1

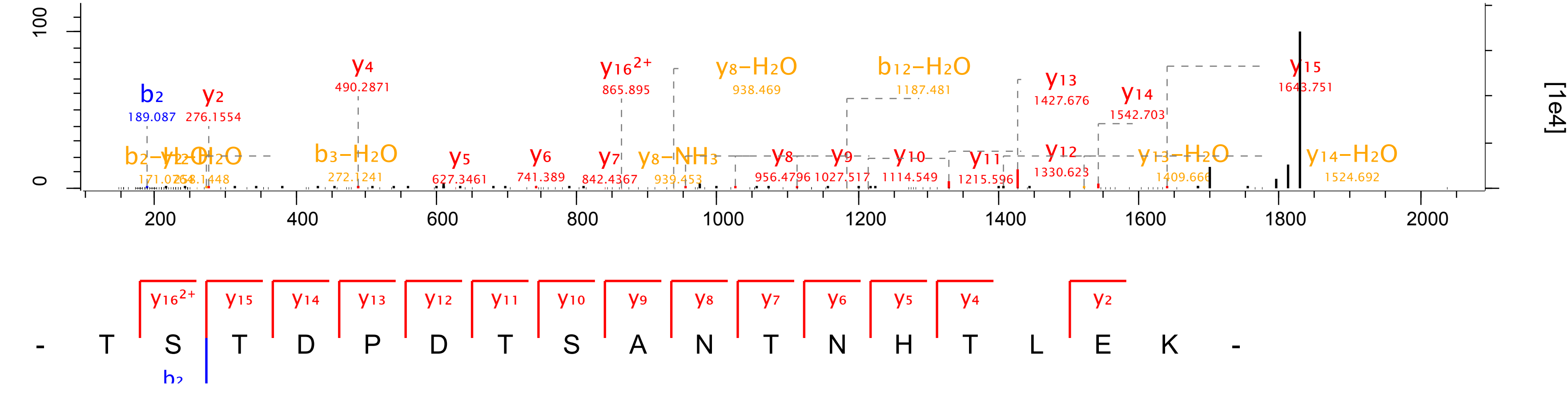

Raw file	Scan	Method	Score	m/z	Gene names
20150306_yeast3_Top_opt_2ug_C3_01_1669	10890	TOF; CID	89.35	493.76	PDH1

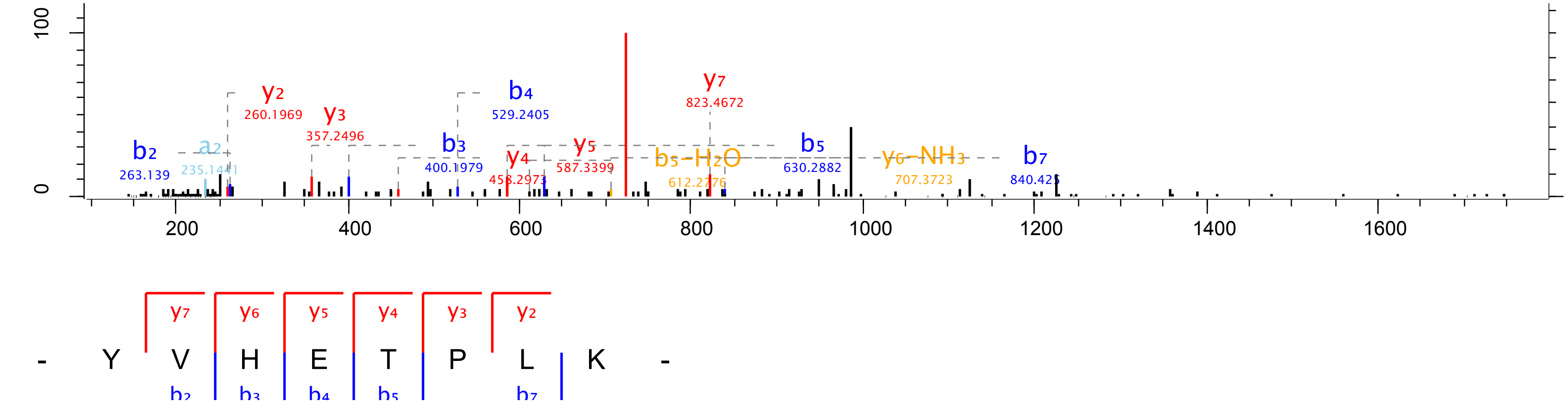

Raw file	Scan	Method	Score	m/z	Gene names
20150306_yeast3_Top_opt_2ug_C3_01_1669	11493	TOF; CID	58.7	526.26	PRY1

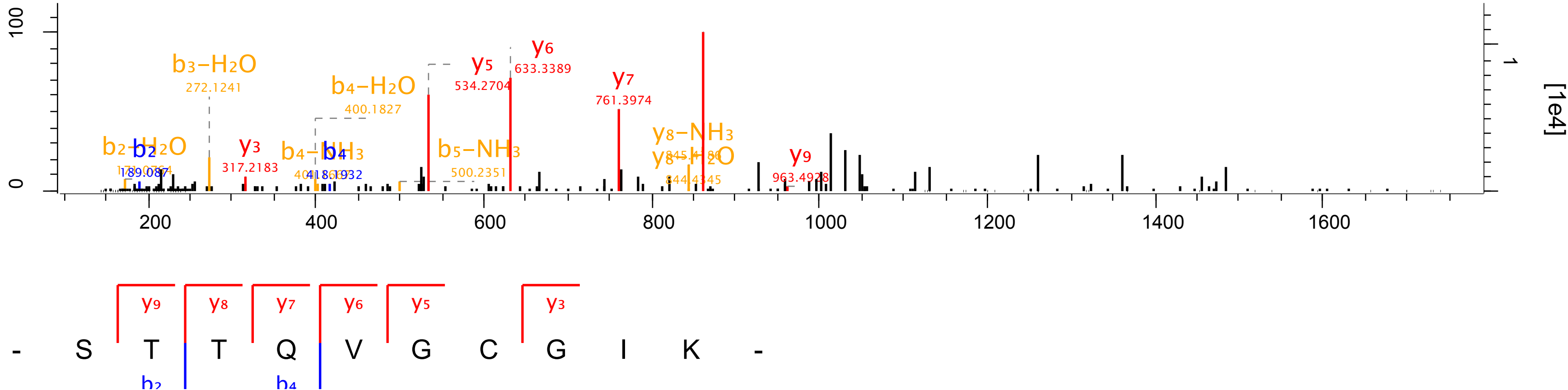

Raw file	Scan	Method	Score	m/z
20150306_yeast3_Top_opt_2ug_C3_01_1669	12842	TOF; CID	55.34	923.89

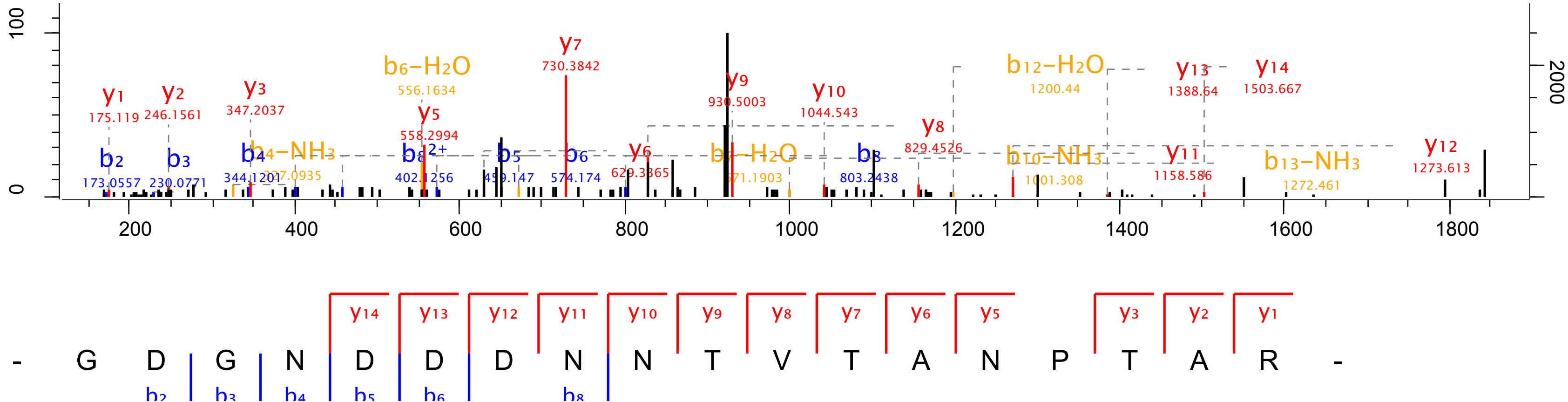

Raw file	Scan	Method	Score	m/z	Gene names
20150306_yeast3_Top_opt_2ug_C3_01_1669	15161	TOF; CID	39.36	886.42	CLB2

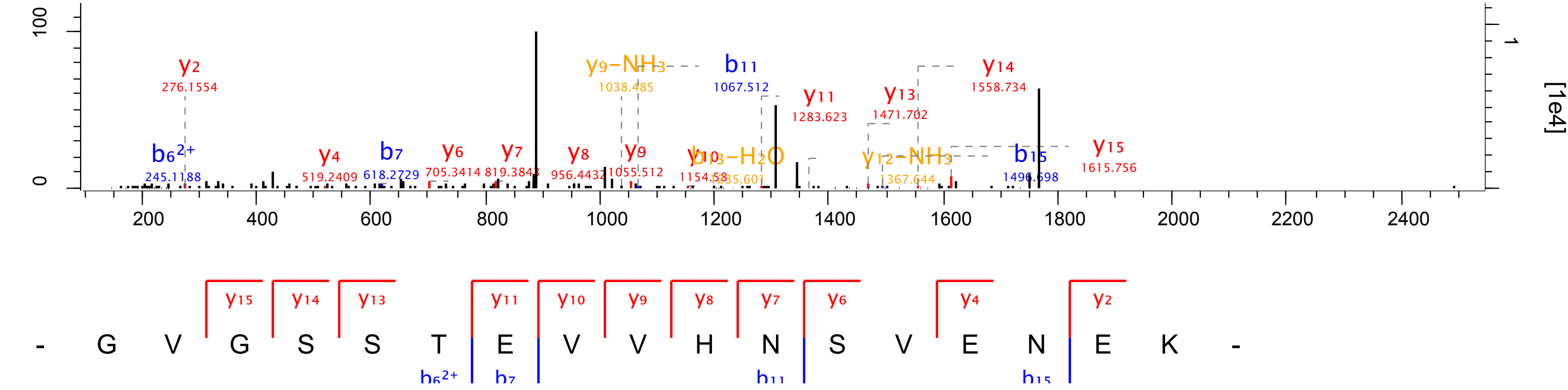

Raw file	Scan	Method	Score	m/z	Gene names
20150306_yeast3_Top_opt_2ug_C3_01_1669	15707	TOF; CID	186.16	492.59	YDL085C-A

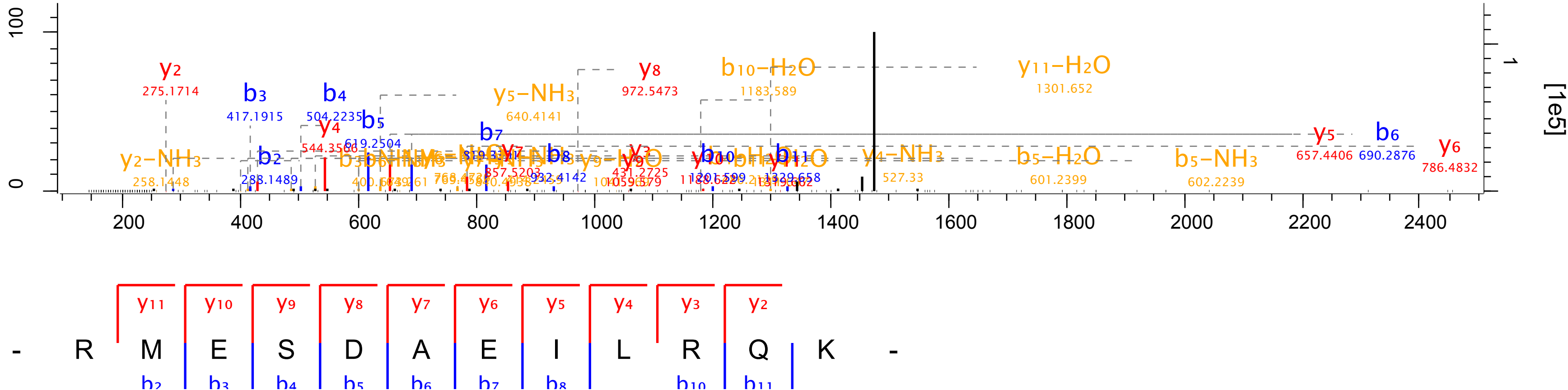

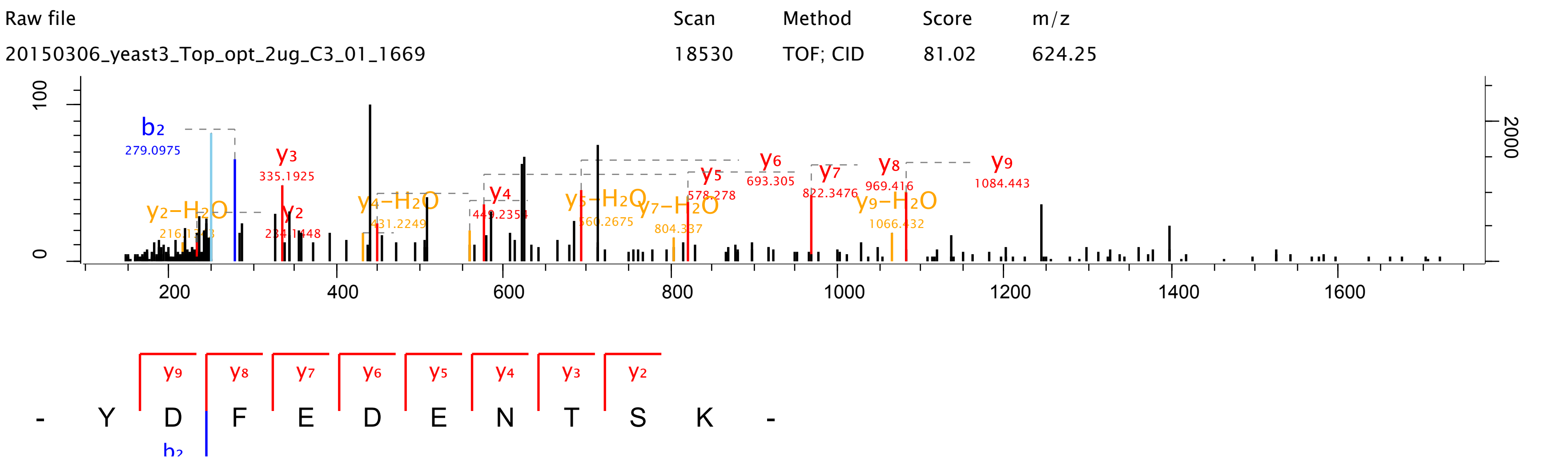
Raw file
20150306_yeast3_Top_opt_2ug_C3_01_1669
Scan
18530
Method
TOF; CID
Score
81.02
m/z
624.25
100
0
2000
b2
279.0975
y3
335.1925
y2
234.1448
y2−H2O
y4−H2O
431.2249
y4
449.2354
y5−H2O
560.2675
y5
578.278
y6
693.305
y7−H2O
804.337
y7
822.3476
y8
969.416
y9−H2O
1066.432
y9
1084.443
200
400
600
800
1000
1200
1400
1600
y9 y8 y7 y6 y5 y4 y3 y2
- Y D F E D E N T S K -
b2

Raw file	Scan	Method	Score	m/z	Gene names
20150306_yeast3_Top_opt_2ug_C3_01_1669	18972	TOF; CID	50.11	578.32	LIF1

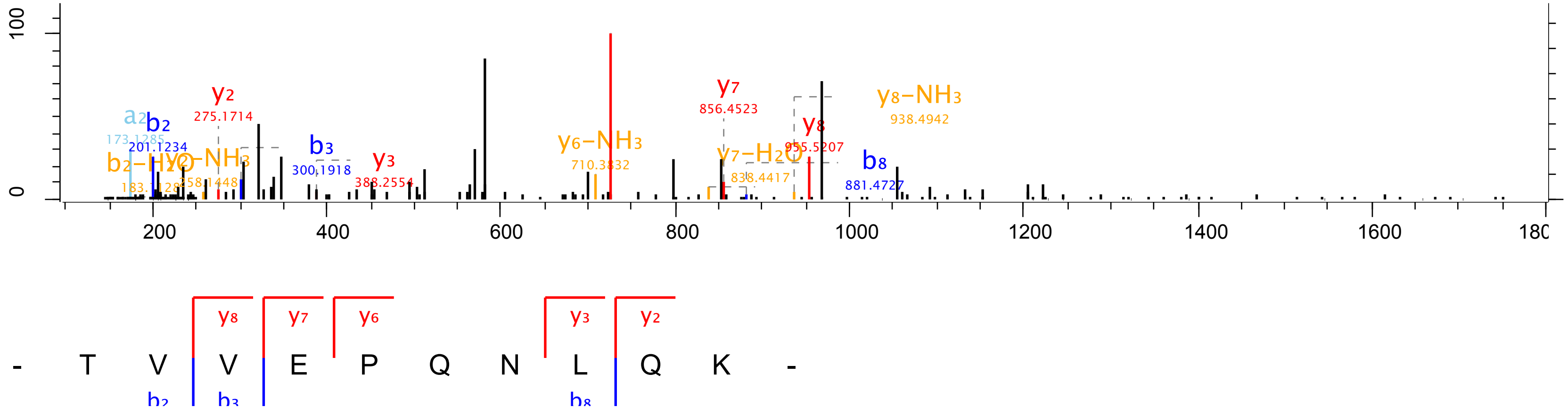

Raw file	Scan	Method	Score	m/z
20150306_yeast3_Top_opt_2ug_C3_01_1669	24236	TOF; CID	54.65	703.33

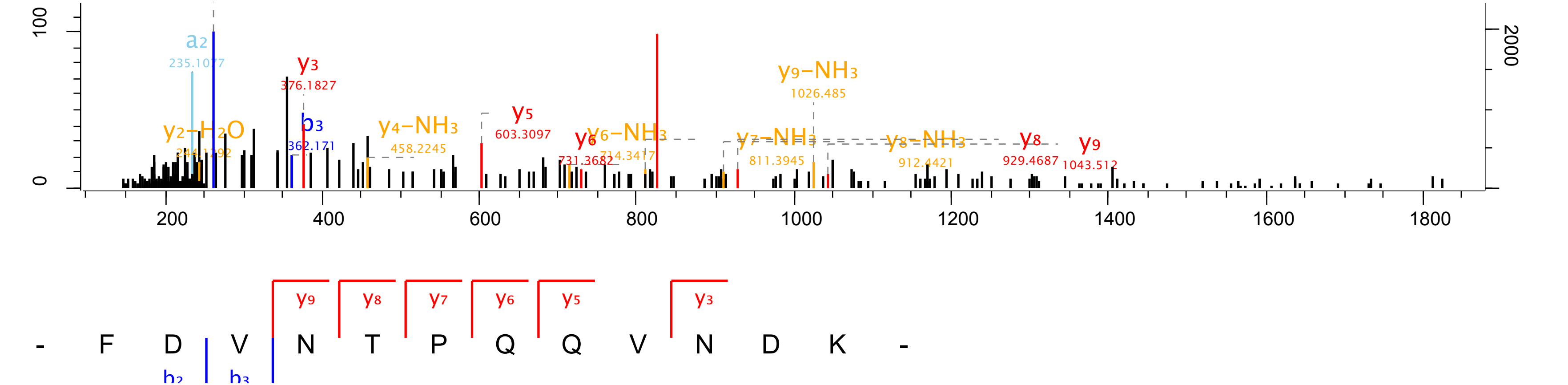

Raw file	Scan	Method	Score	m/z	Gene names
20150306_yeast3_Top_opt_2ug_C3_01_1669	27583	TOF; CID	54.83	994.47	CHS6

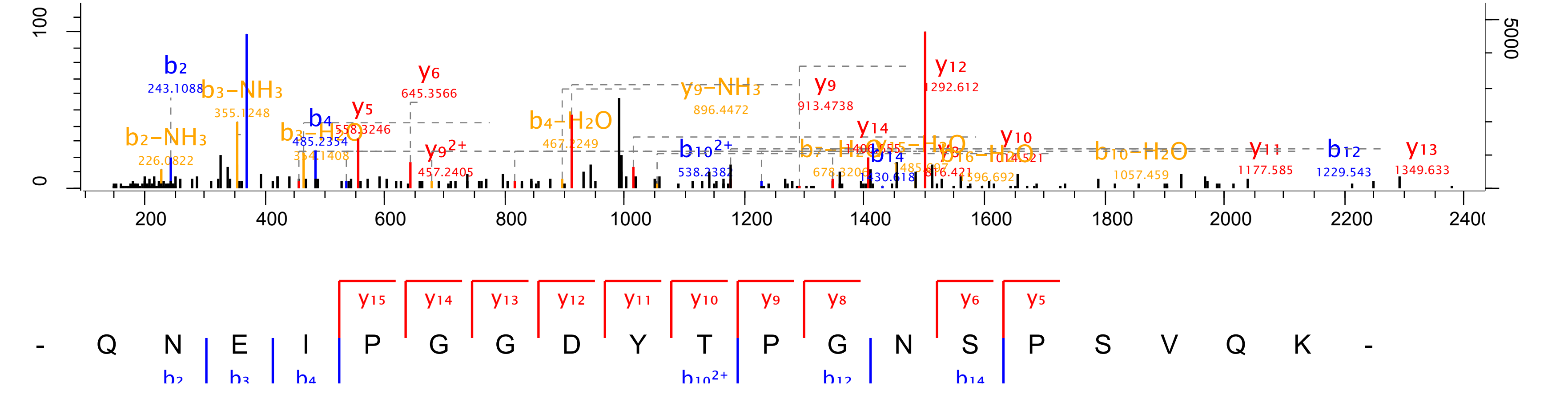

Raw file	Scan	Method	Score	m/z	Gene names
20150306_yeast3_Top_opt_2ug_C3_01_1669	28120	TOF; CID	153.56	893.03	CCW14

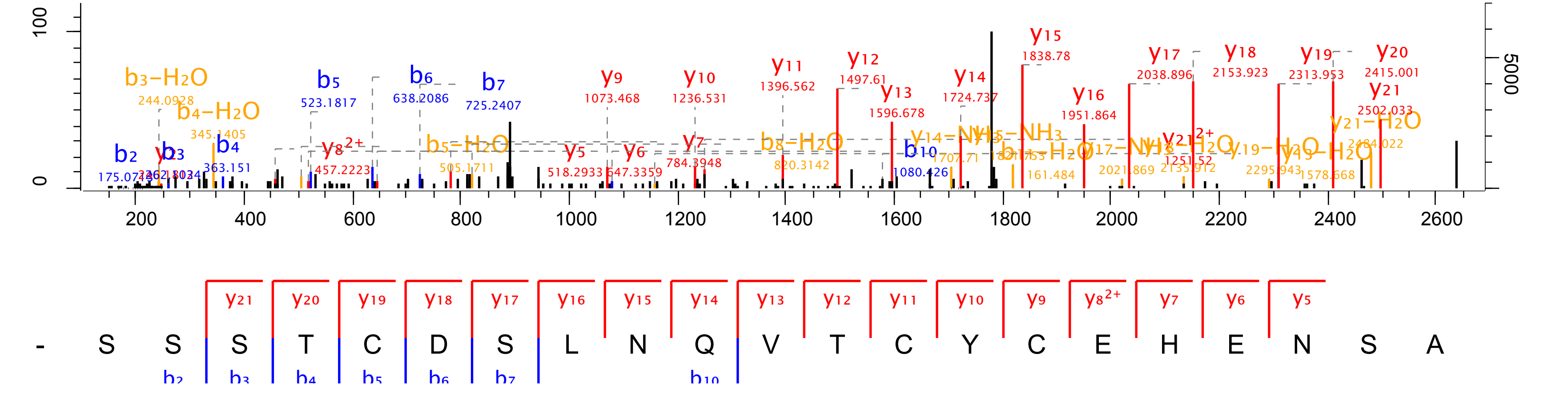

Raw file	Scan	Method	Score	m/z	Gene names
20150306_yeast3_Top_opt_2ug_C3_01_1669	30012	TOF; CID	62.19	671.59	SRB5

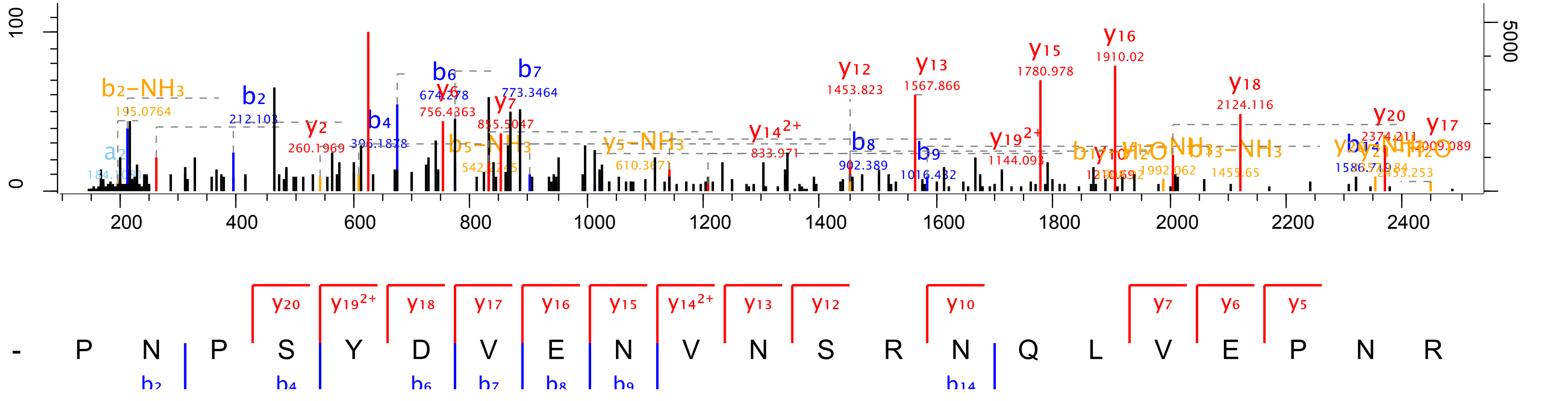

Raw file	Scan	Method	Score	m/z	Gene names
20150306_yeast3_Top_opt_2ug_C3_01_1669	30041	TOF; CID	72.23	479.76	SNT309

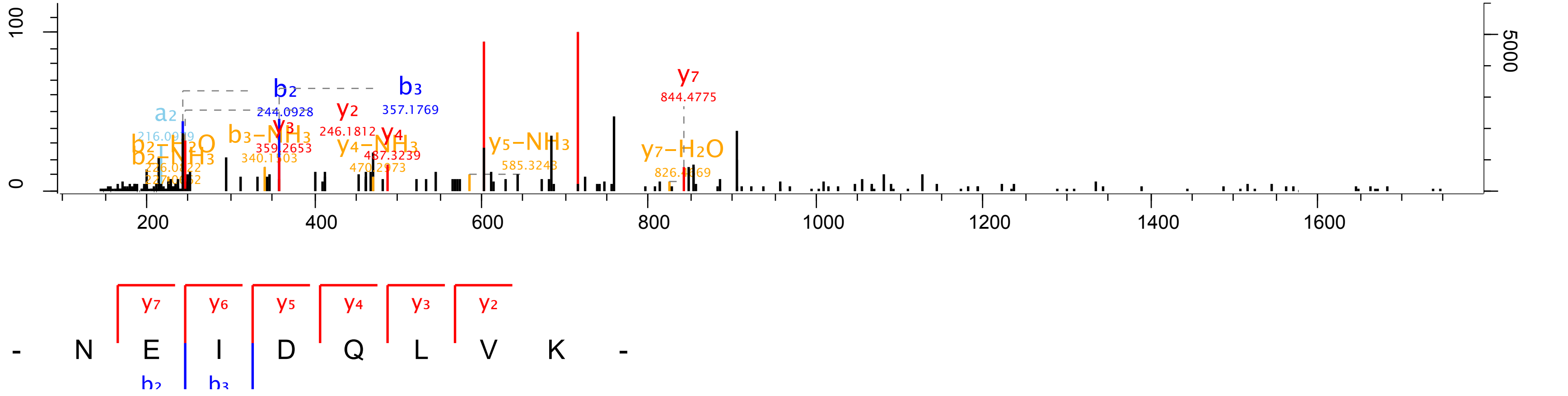

Raw file	Scan	Method	Score	m/z	Gene names
20150306_yeast3_Top_opt_2ug_C3_01_1669	31018	TOF; CID	120.9	1080.43	OMA1

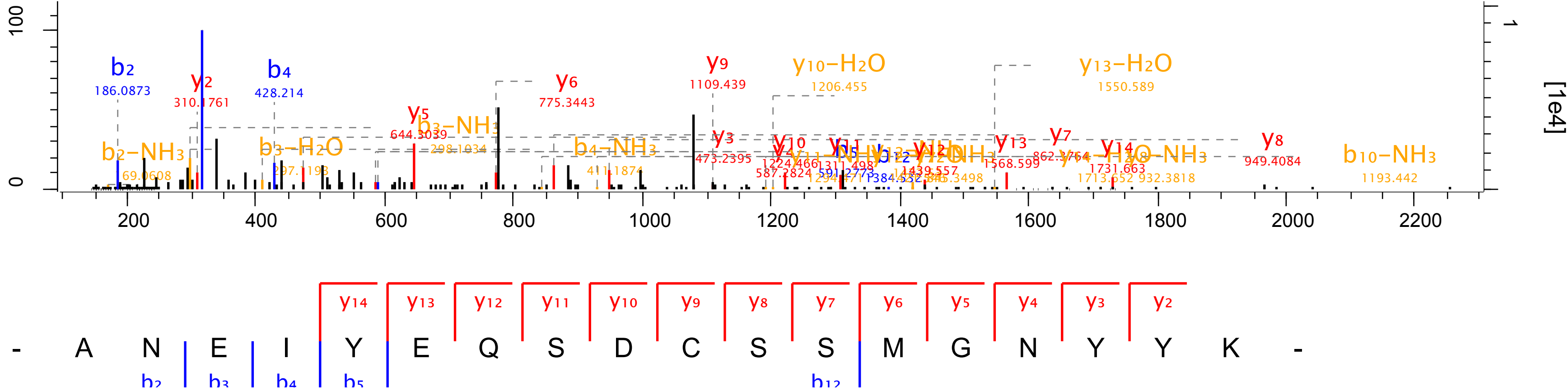

Raw file	Scan	Method	Score	m/z	Gene names
20150306_yeast3_Top_opt_2ug_C3_01_1669	32550	TOF; CID	71.52	563.3	SPP382

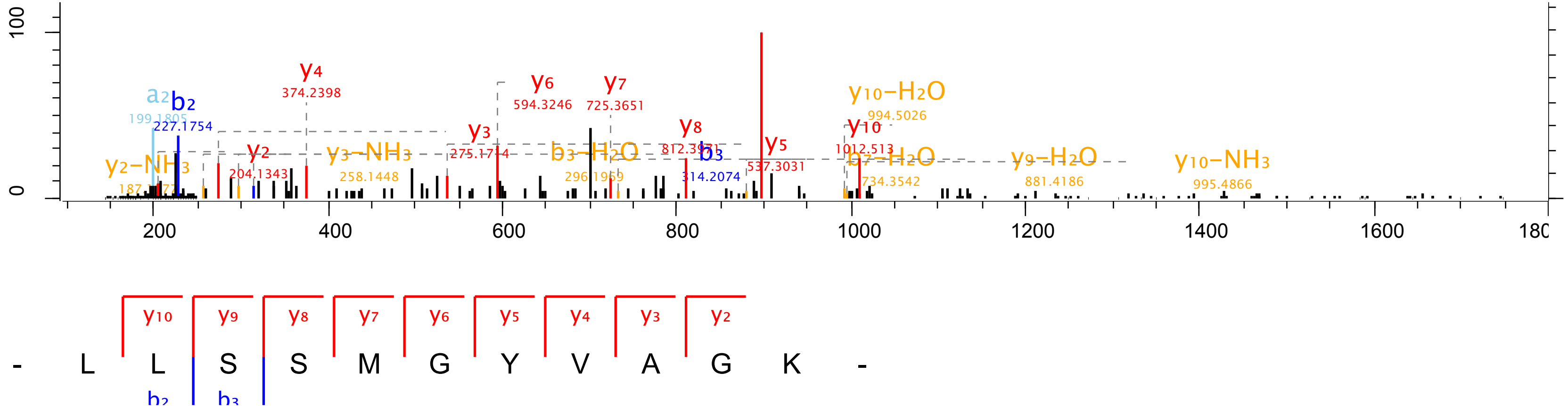

Raw file	Scan	Method	Score	m/z
20150306_yeast3_Top_opt_2ug_C3_01_1669	33216	TOF; CID	52.66	833.38

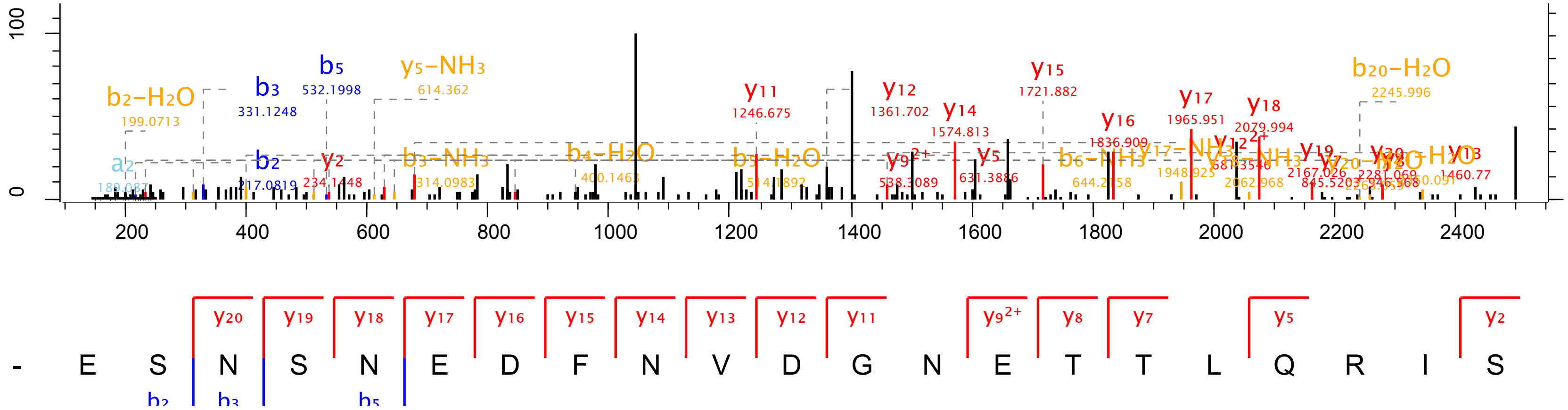

Raw file	Scan	Method	Score	m/z	Gene names
20150306_yeast3_Top_opt_2ug_C3_01_1669	38786	TOF; CID	144.56	748.86	MLF3

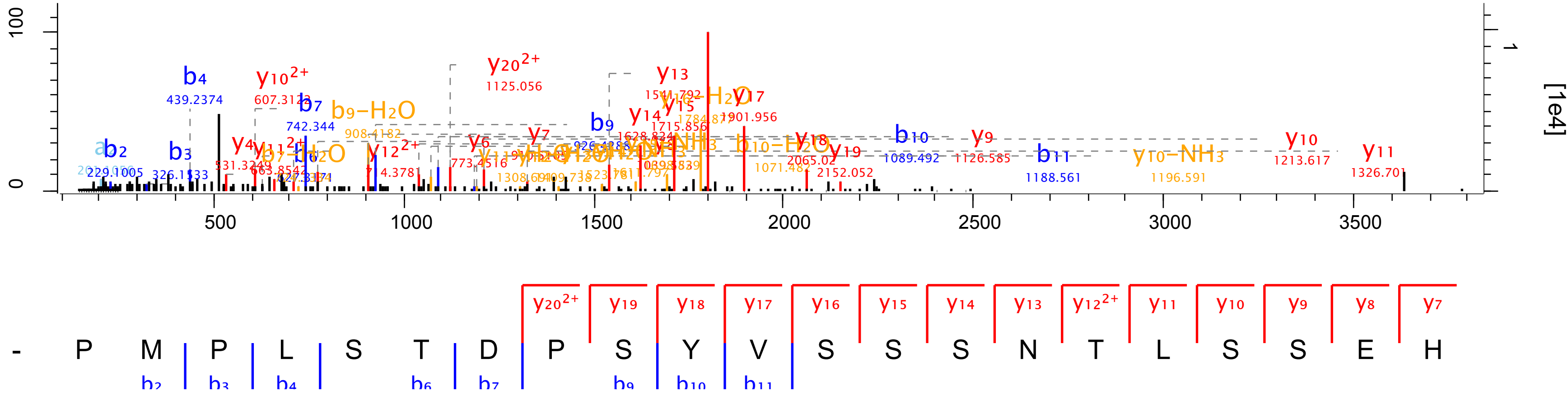

Raw file	Scan	Method	Score	m/z	Gene names
20150306_yeast3_Top_opt_2ug_C3_01_1669	38976	TOF; CID	84.74	451.29	STB4

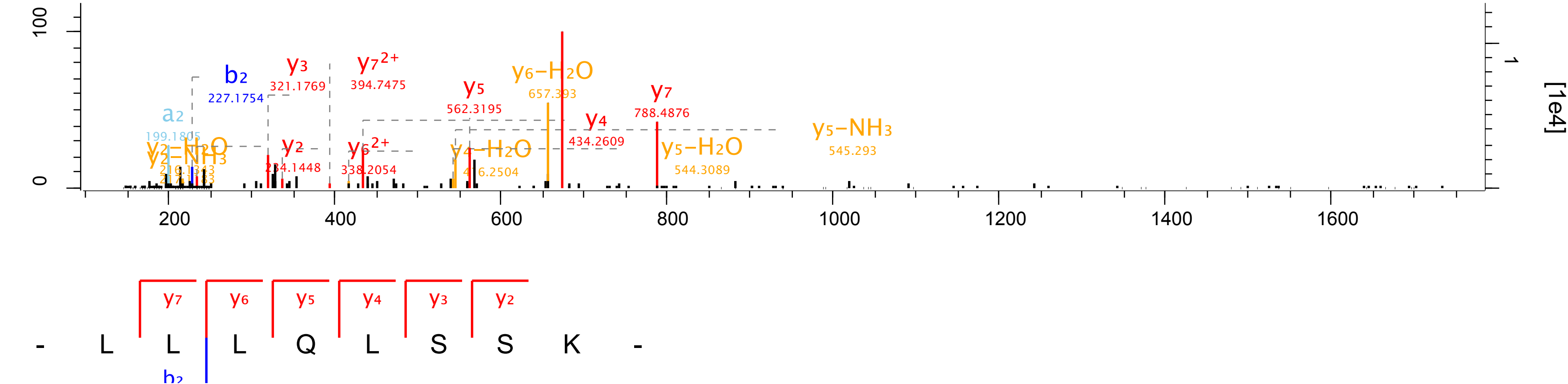

Raw file	Scan	Method	Score	m/z	Gene names
20150306_yeast3_Top_opt_2ug_C3_01_1669	39084	TOF; CID	56.51	819.41	NBP1

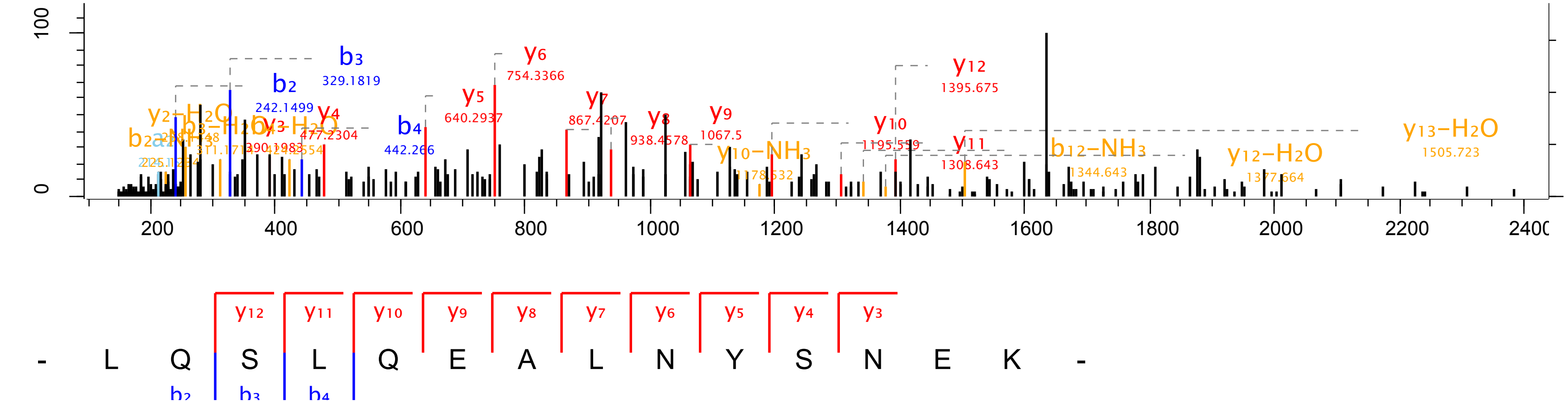

Raw file	Scan	Method	Score	m/z
20150306_yeast3_Top_opt_2ug_C3_01_1669	39547	TOF; CID	58.04	497.62

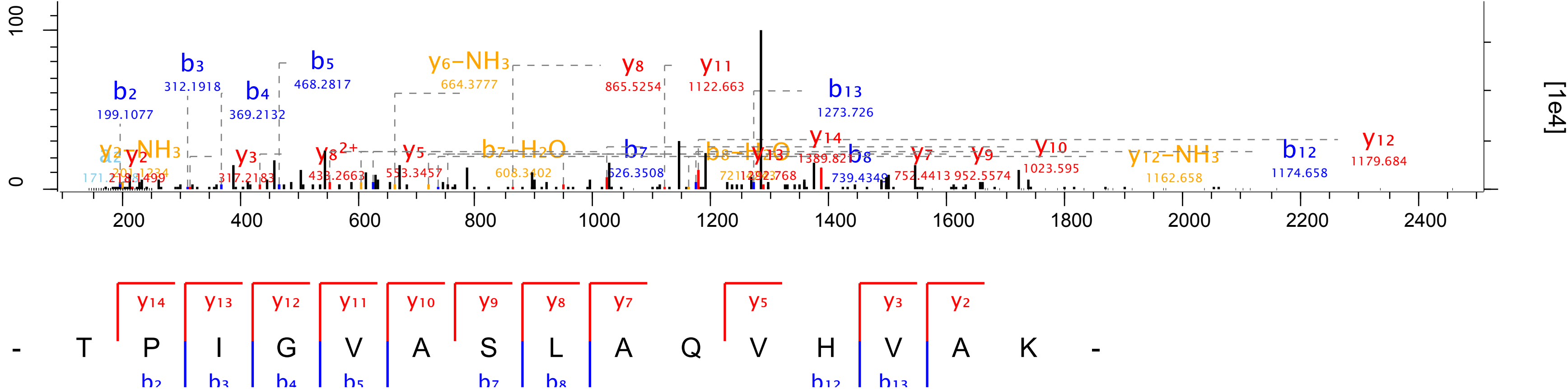

Raw file	Scan	Method	Score	m/z	Gene names
20150306_yeast3_Top_opt_2ug_C3_01_1669	41183	TOF; CID	91.32	443.78	CUL3

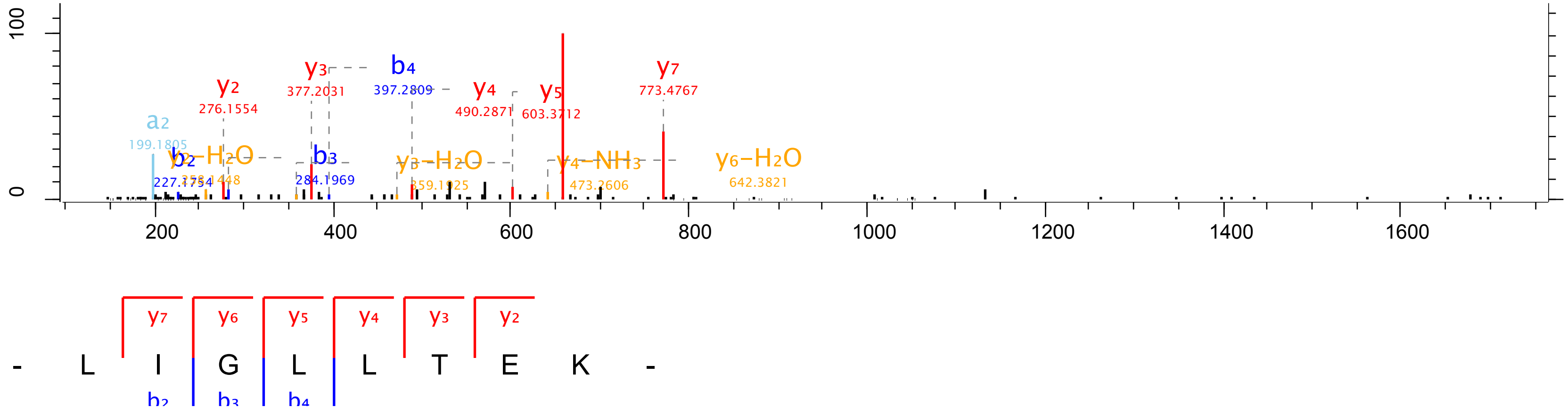

Raw file	Scan	Method	Score	m/z	Gene names
20150306_yeast3_Top_opt_2ug_C3_01_1669	43157	TOF; CID	128.57	466.28	NTF2

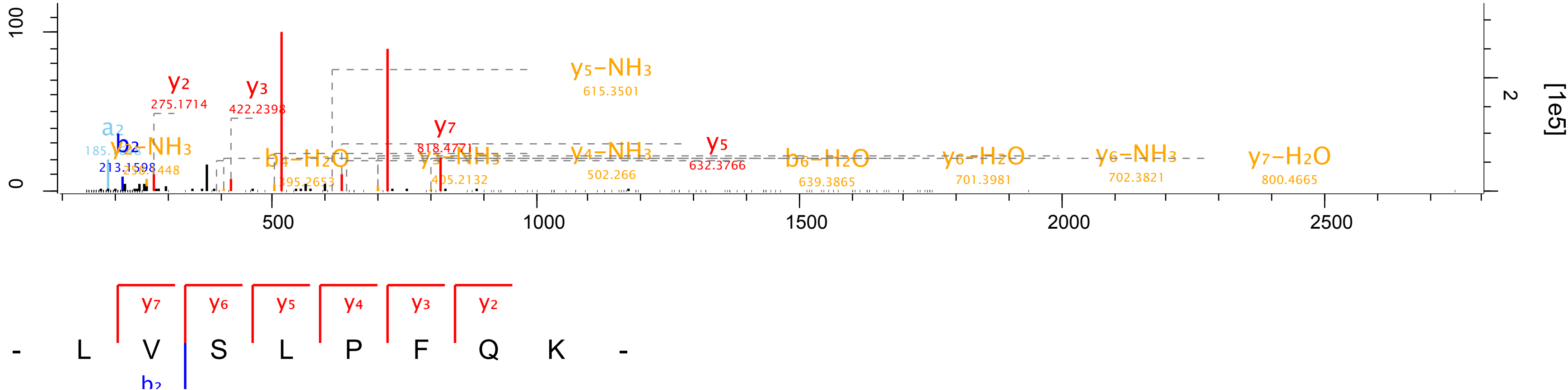

Raw file	Scan	Method	Score	m/z	Gene names
20150306_yeast3_Top_opt_2ug_C3_01_1669	44841	TOF; CID	64.7	716.65	YLR177W

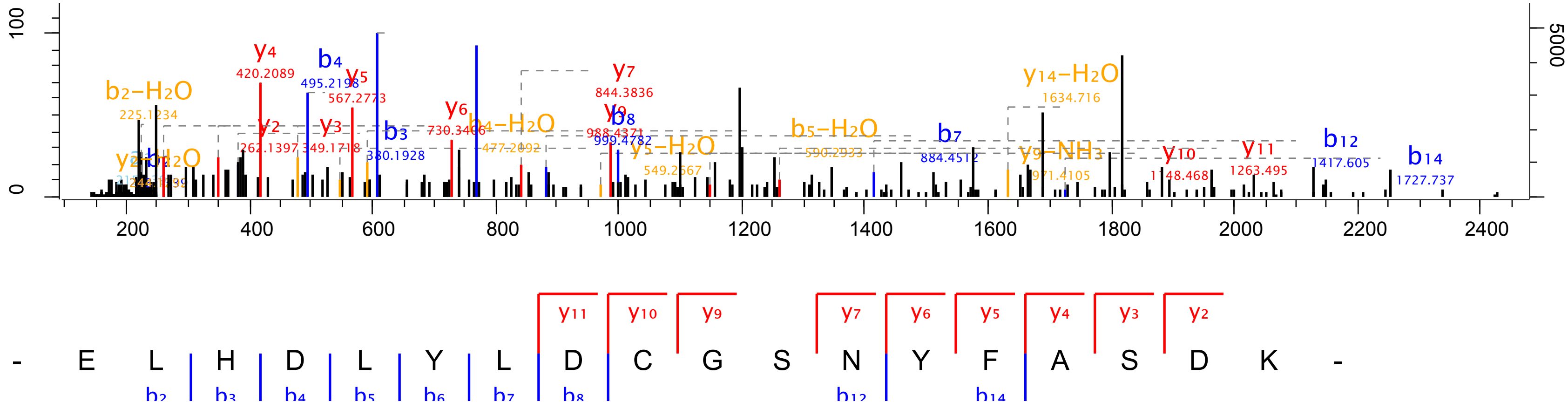

Raw file	Scan	Method	Score	m/z	Gene names
20150306_yeast3_Top_opt_2ug_C3_01_1669	45421	TOF; CID	41.7	746.37	FMP45

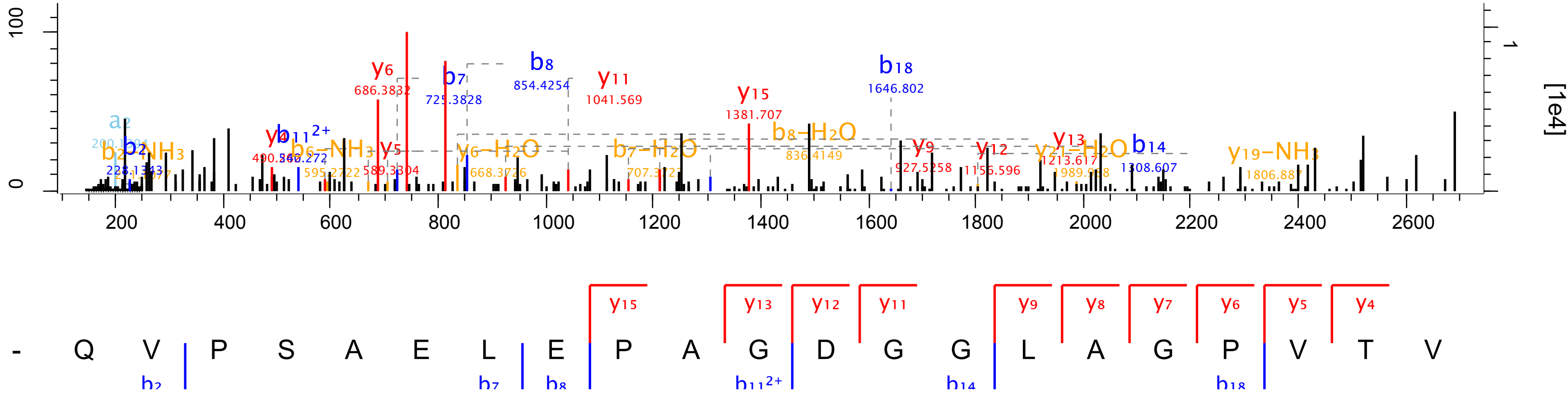

Raw file	Scan	Method	Score	m/z	Gene names
20150306_yeast3_Top_opt_2ug_C3_01_1669	46128	TOF; CID	142.32	923.92	RPC11

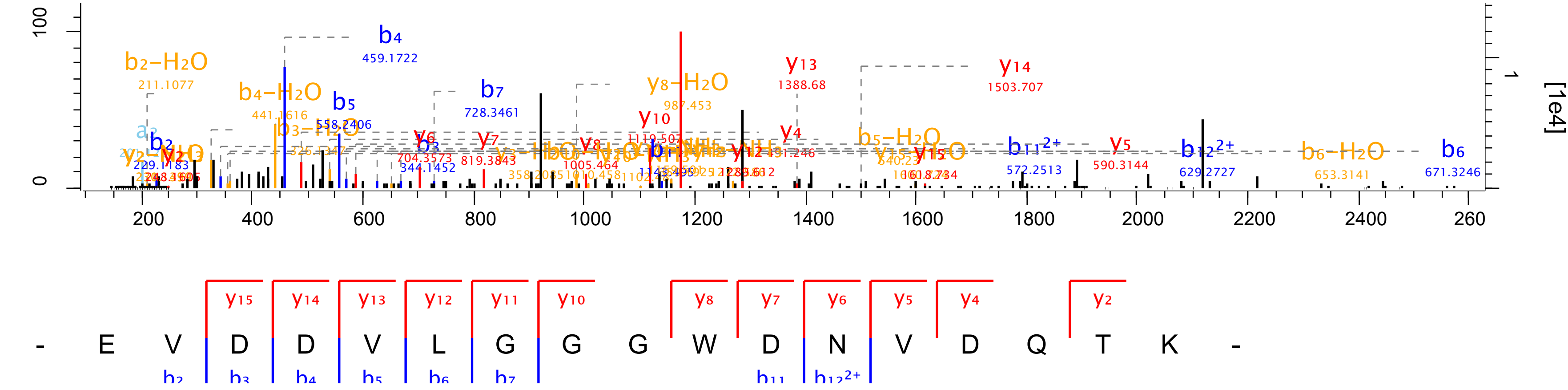

Raw file	Scan	Method	Score	m/z	Gene names
20150306_yeast3_Top_opt_2ug_C3_01_1669	51578	TOF; CID	40.94	754.37	LST7

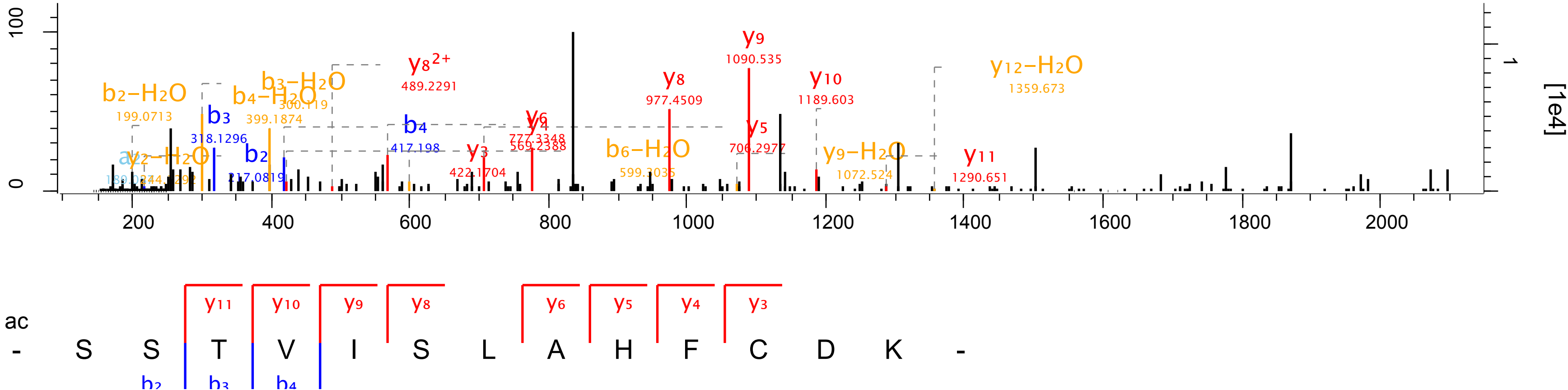

Raw file	Scan	Method	Score	m/z	Gene names
20150306_yeast3_Top_opt_2ug_C3_01_1669	52101	TOF; CID	60.64	655.39	MKK2

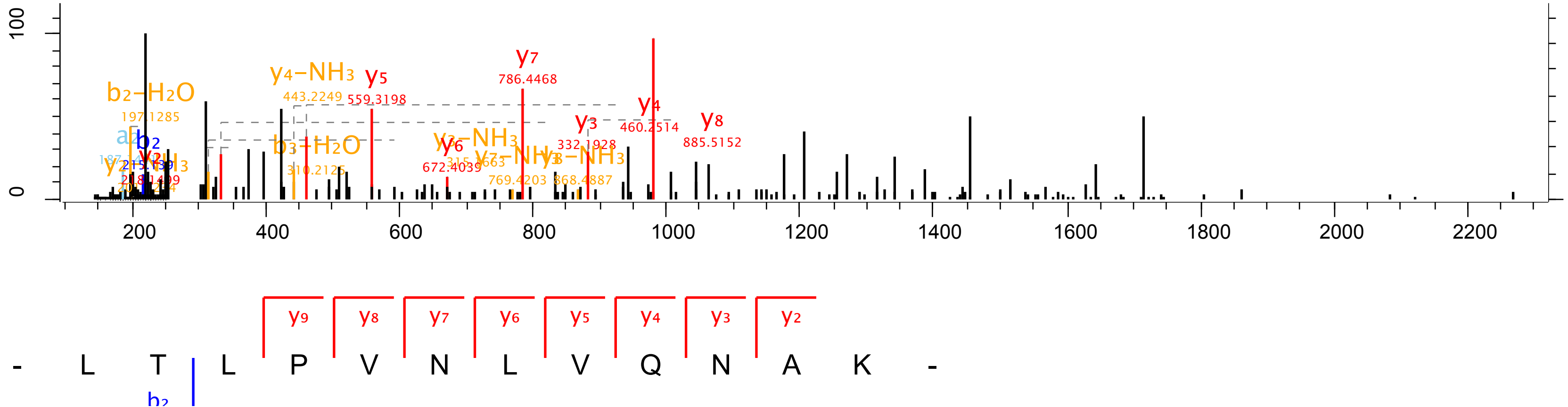

Raw file	Scan	Method	Score	m/z	Gene names
20150306_yeast3_Top_opt_2ug_C3_01_1669	53127	TOF; CID	68.54	646.34	GEM1

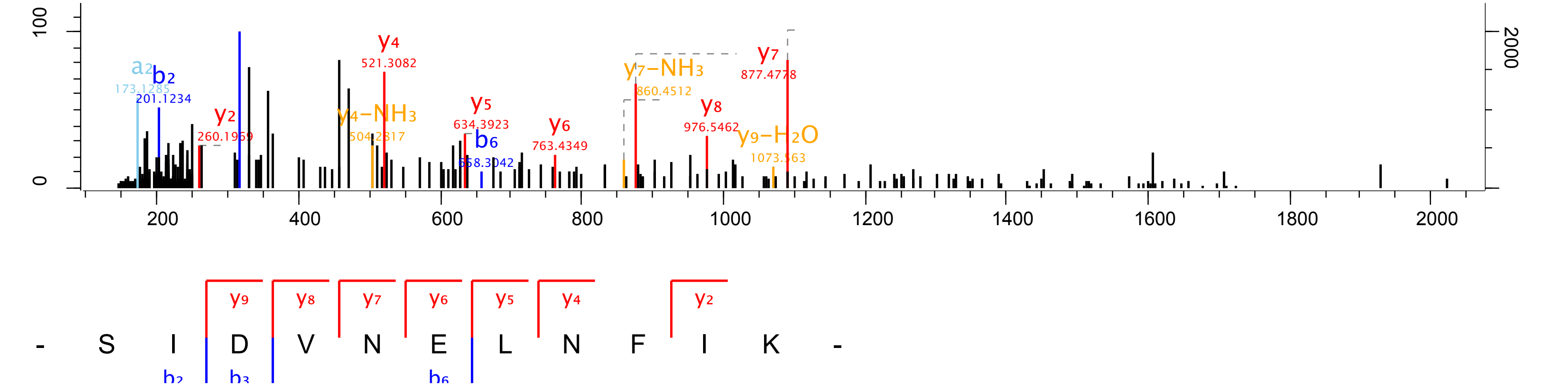

Raw file	Scan	Method	Score	m/z	Gene names
20150306_yeast3_Top_opt_2ug_C3_01_1669	55439	TOF; CID	50.86	1050.16	DUR3

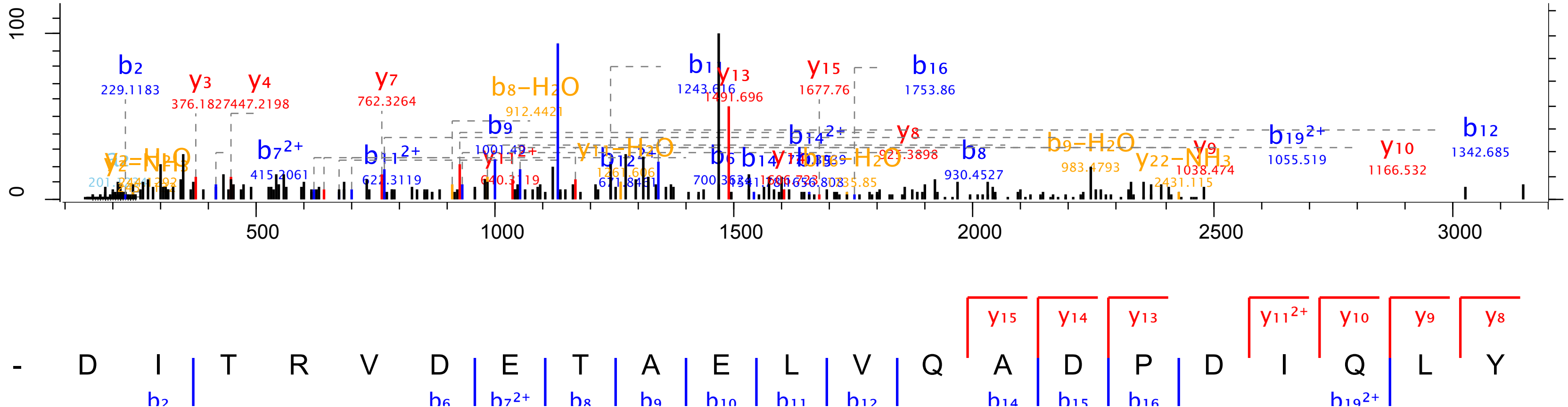

Raw file	Scan	Method	Score	m/z
20150306_yeast3_Top_opt_2ug_C3_01_1669	58481	TOF; CID	96.6	564.35

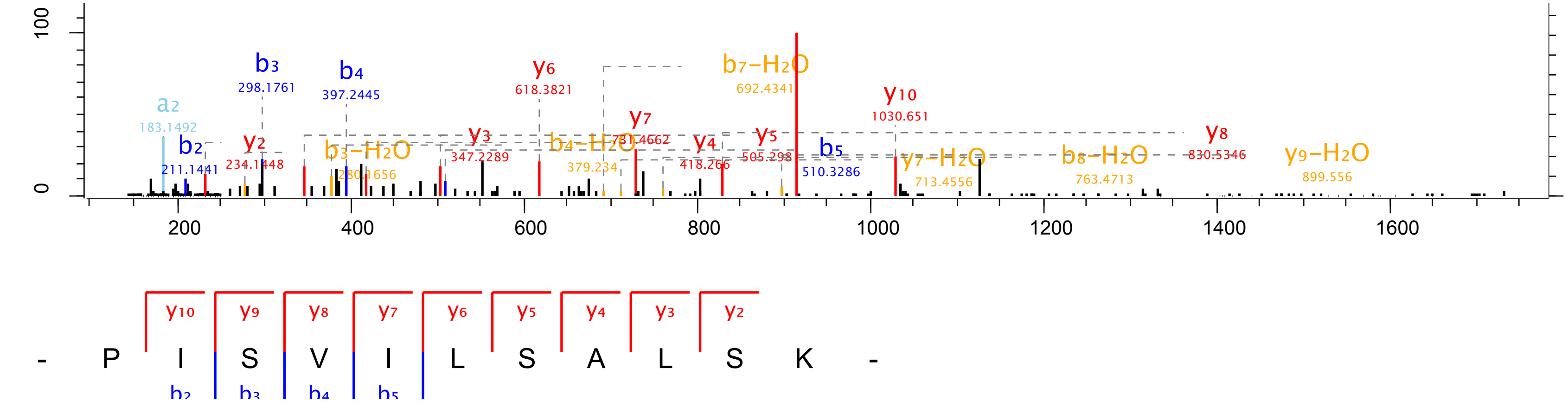

Raw file	Scan	Method	Score	m/z	Gene names
20150306_yeast3_Top_opt_2ug_C3_01_1669	59974	TOF; CID	106.38	637.32	COX1

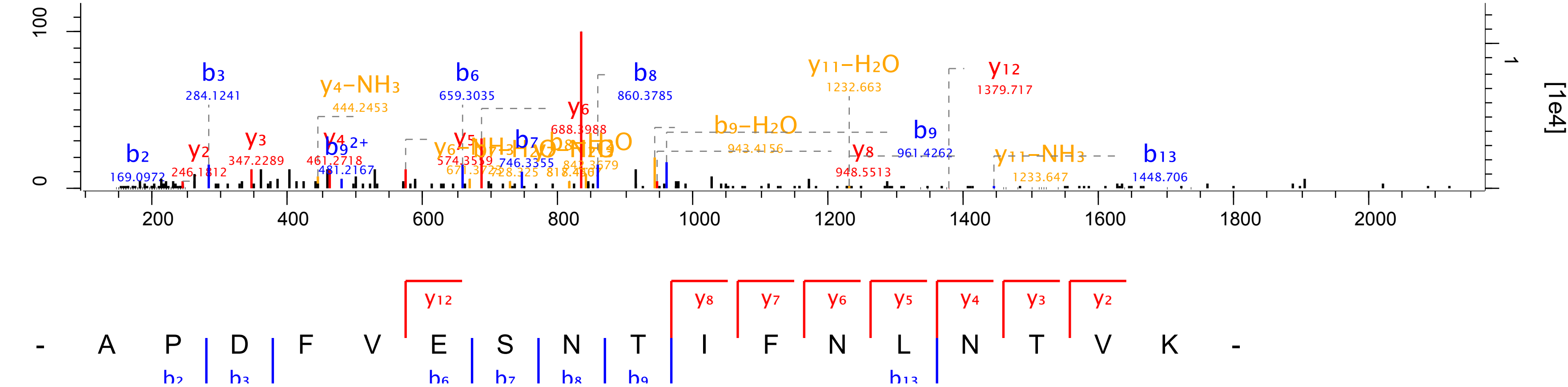

Raw file	Scan	Method	Score	m/z	Gene names
20150306_yeast3_Top_opt_2ug_C3_01_1669	60819	TOF; CID	70.34	814.12	SNF11

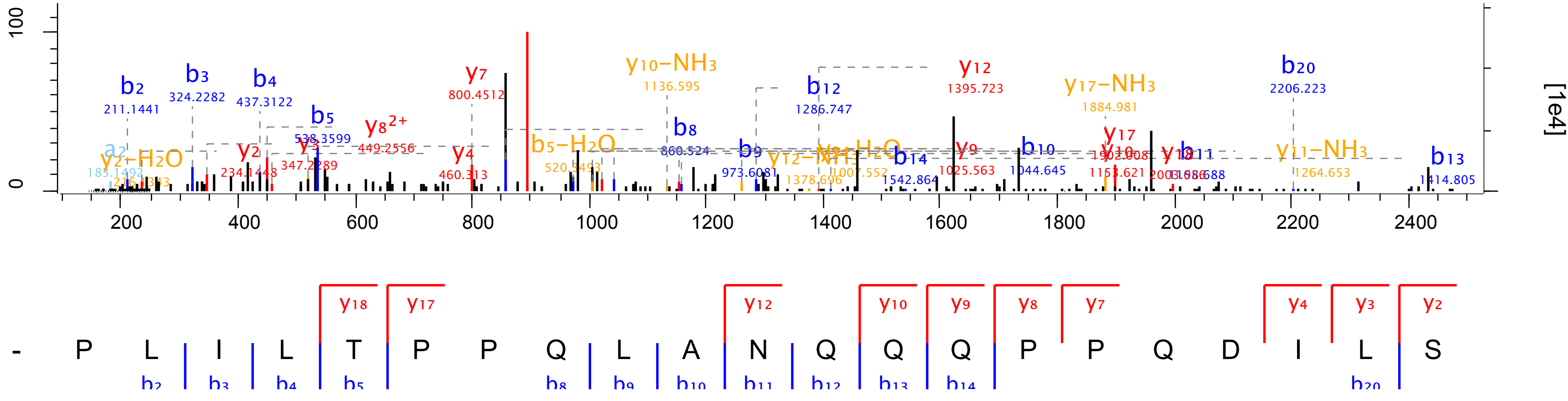

Raw file	Scan	Method	Score	m/z	Gene names
20150306_yeast3_Top_opt_2ug_C3_01_1669	61585	TOF; CID	102.08	710.34	SPC25

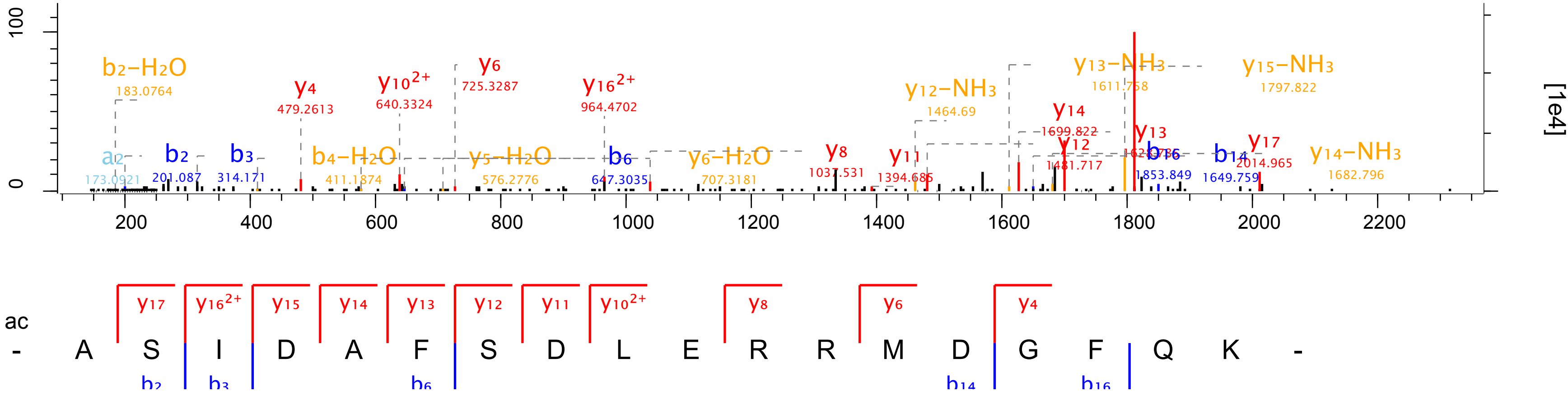

Raw file	Scan	Method	Score	m/z	Gene names
20150306_yeast3_Top_opt_2ug_C3_01_1669	63019	TOF; CID	124.08	811.76	DAD4

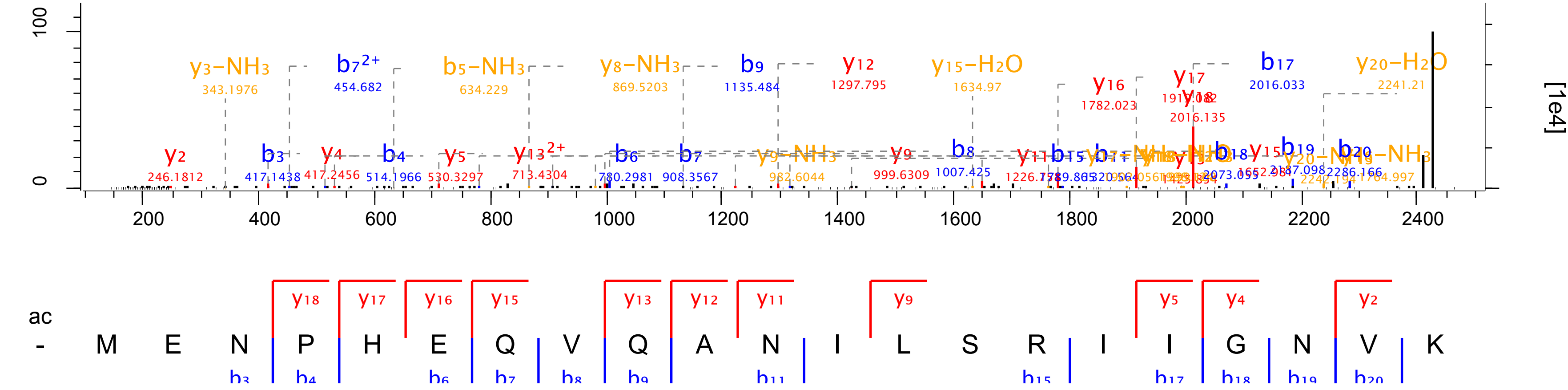

Raw file	Scan	Method	Score	m/z	Gene names
20150306_yeast3_Top_opt_2ug_C3_01_1669	68127	TOF; CID	80.14	731.39	SPS100

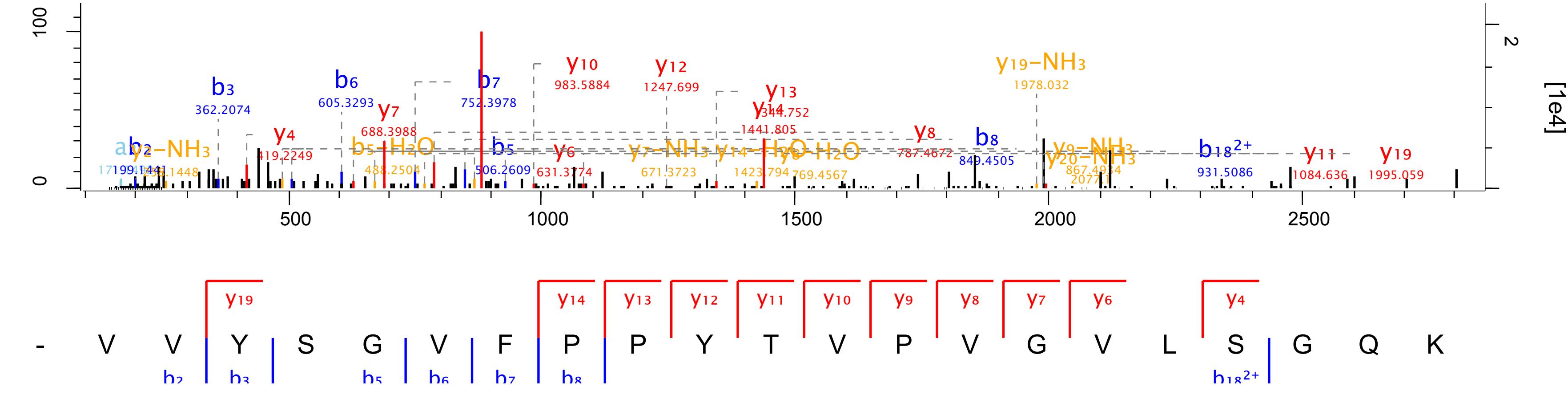

Raw file	Scan	Method	Score	m/z	Gene names
20150306_yeast3_Top_opt_2ug_C3_01_1669	68496	TOF; CID	41.25	1053.52	PEX4

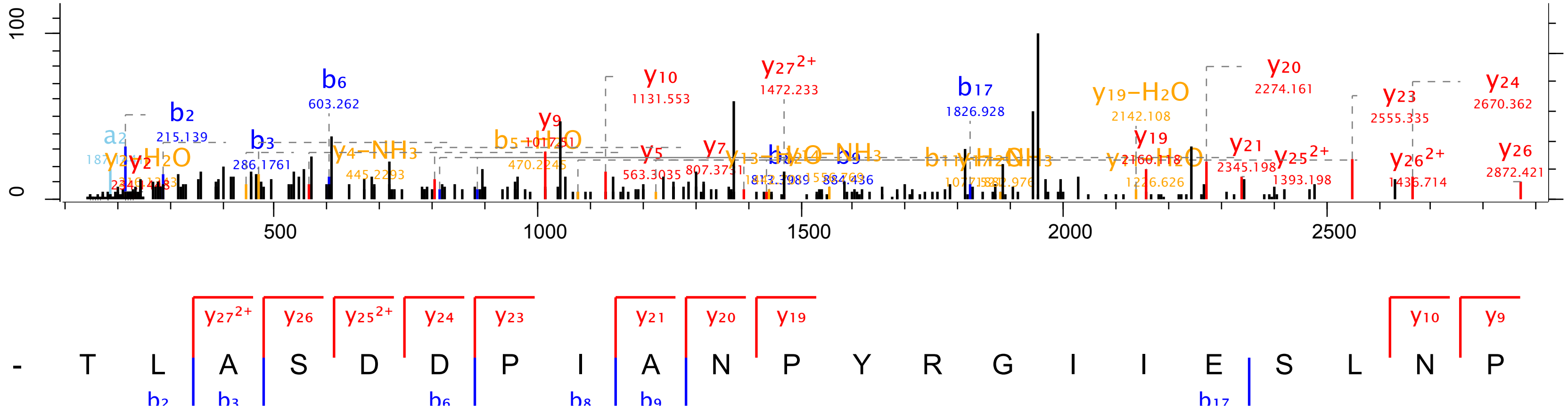

Raw file	Scan	Method	Score	m/z	Gene names
20150306_yeast3_Top_opt_2ug_C3_01_1669	71744	TOF; CID	142.26	729.08	GUP1

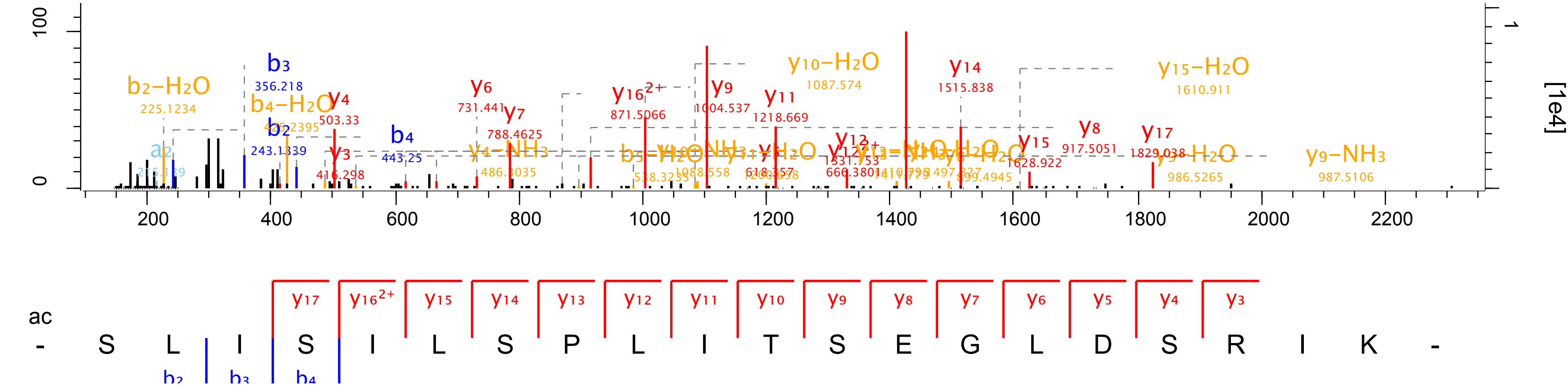

Raw file dentified harge ype Fragmentation ass analyzer odifications xperiment can number etention time on injection time otal ion current ase peak intensity lapsed time ength m/z ass Parent intensity fraction raction of total spectrum ase peak fraction recursor full scan number recursor intensity recursor apex fraction recursor apex offset recursor apex offset time can event number core Intens Comp Factor TCD Comp awOvFtT GC Fill can index S scan index S scan number equence odified sequence roteins S/MS IDs
20150306_yeast1_Top_opt_2ug_C1_01_1663 2 ULTI CID OF Unmodified aploid_01 603 5.6508 21861 59 0 422.22 42.425 0 0 -1 0 0 0 1 8.0161 aN NaN NaN 2218 83 2602 SQGHVTK SSQGHVTK_ 53267 01092
20150306_yeast1_Top_opt_2ug_C1_01_1663 2 ULTI CID OF Unmodified aploid_01 152 16.269 946020 4904 8 379.705 57.396 0 0 1 0 0 0 1 69.8246 aN NaN aN 0 071 1079 151 VSPADAAK VSPADAAK_ 39938 235513
20150306_yeast1_Top_opt_2ug_C1_01_1663 2 ULTI CID OF Unmodified aploid_01 893 17.571 132850 348 9 548.747 095.48 0 0 1 0 0 0 10 105.101 aN NaN aN 0 771 1120 883 ACQEAARQY ACQEAARQY_ 03667 576
20150306_yeast1_Top_opt_2ug_C1_01_1663 2 MULTI ID TOF nmodified aploid_01 613 8.852 128450 753 0 10 48.758 295.5 0 0 -1 0 0 0 12 4.6921 aN NaN NaN 8451 160 9601 MEEEEQDCK VMEEEEQDCK_ 38208 231878
20150306_yeast1_Top_opt_2ug_C1_01_1663 3 ULTI CID OF Unmodified aploid_01 0702 20.748 230900 643 14 564.277 689.81 0 -1 0 0 0 3 72.6151 aN aN NaN 0 479 1221 0699 YHSVSTQDNRNELK YHSVSTQDNRNELK_ 03697 45784
20150306_yeast1_Top_opt_2ug_C1_01_1663 2 ULTI CID OF Unmodified aploid_01 7746 31.86 97611 811 15 705.359 408.7 0 0 -1 0 0 0 72.3155 aN NaN aN 0 16132 612 7737 EIGGTSGSTTTGTIK EIGGTSGSTTTGTIK_ 53397 5474
20150306_yeast1_Top_opt_2ug_C1_01_1663 2 ULTI CID OF Unmodified aploid_01 8215 32.533 88136 994 11 580.785 159.56 0 0 1 0 0 0 10 83.1822 aN aN NaN 0 6575 1638 8205 TASAPLCSPK ETASAPLCSPK_ 40366 56707
20150306_yeast1_Top_opt_2ug_C1_01_1663 2 ULTI CID OF Unmodified aploid_01 1245 51.05 190680 665 9 513.263 024.51 0 0 1 0 0 0 8 68.5575 aN NaN aN 0 28881 362 1237 TEDFISSVK TEDFISSVK_ 38967 08155
20150306_yeast1_Top_opt_2ug_C1_01_1663 2 ULTI CID OF Unmodified aploid_01 2609 52.833 102440 754 8 513.251 024.49 0 0 1 0 0 0 4 104.057 aN NaN aN 0 0169 2438 2605 ECAIEYLK ECAIEYLK_ 38325 8474
20150306_yeast1_Top_opt_2ug_C1_01_1663 2 MULTI ID OFUnmodified aploid_01 4988 56.112 218450 431 17 1005.99 009.96 0 0 1 0 0 0 7 135.077 aN NaN aN 0 32416 570 4981 PPLPQNYAQQQPSNWDK PPLPQNYAQQQPSNWDK_ 02889 66937
20150306_yeast1_Top_opt_2ug_C1_01_1663 3 ULTI CID OF Unmodified aploid_01 6755 58.562 243060 1917 11 458.941 373.8 0 -1 0 0 0 10 50.3534 aN aN NaN 34085 2668 6745 IIDNRKDILK FIIDNRKDILK_ 50275 65898
20150306_yeast1_Top_opt_2ug_C1_01_1663 3 ULTI CID OF Unmodified aploid_01 7100 59.009 134930 882 11 458.93 373.77 0 0 1 0 0 0 13 62.4662 aN aN NaN 0 4411 2687 7087 IELIRNDFQK VIELIRNDFQK_ 9URQ3 228334
20150306_yeast1_Top_opt_2ug_C1_01_1663 2 ULTI CID OF Unmodified aploid_01 7849 60.059 91775 291 8 450.282 98.55 0 0 -1 0 0 0 63.5651 aN NaN aN 0 35118 729 7843 IIGALERK IIGALERK_ 40065 96818
20150306_yeast1_Top_opt_2ug_C1_01_1663 4 ULTI CID OF Unmodified aploid_01 9678 62.568 653400 1571 18 513.766 051.04 0 -1 0 0 0 17 82.7086 aN aN NaN 36846 830 39661 PIVQISDDSHITHDSFK IPIVQISDDSHITHDSFK_ 53939 101715
20150306_yeast1_Top_opt_2ug_C1_01_1663 3 ULTI CID OF Unmodified aploid_01 0554 63.739 126140 0718 10 400.877 199.61 0 -1 0 0 0 11 91.6583 aN aN NaN 37673 879 40543 HEVLGAFWK NHEVLGAFWK_ 8TGJ3 151390
20150306_yeast1_Top_opt_2ug_C1_01_1663 2 ULTI CID OF Unmodified aploid_01 0784 64.036 158060 058 10 532.801 063.59 0 -1 0 0 0 7 56.4044 aN aN NaN 0 7890 2892 0777 FSSALDVVK VFSSALDVVK_ 12198 26431

20150306_yeast1_Top_opt_2ug_C1_01_1663 3 ULTI CID OF Unmodified aploid_01 1027 64.376 219680
352 12 477.264 428.77 0 -1 0 0 0 16 162.684 aN aN NaN 38120 2905 1011
HLLDATDIFRK THLLDATDIFRK_ 38312 11476
20150306_yeast1_Top_opt_2ug_C1_01_1663 2 ULTI CID OF Unmodified aploid_01 1394 64.883 256510
376 11 645.344 288.67 0 -1 0 0 0 5 106.668 aN aN NaN 0 8466 2926 1389
TDILTEEVEK LTDILTEEVEK_ 07540 136943
20150306_yeast1_Top_opt_2ug_C1_01_1663 2 ULTI CID OF Unmodified aploid_01 2729 66.776 47815 073
8 439.771 77.528 0 0 1 0 0 0 8 98.0089 aN NaN aN 0 39727 000 2721
AYSLLAIK AYSLLAIK_ 29703 17795
20150306_yeast1_Top_opt_2ug_C1_01_1663 4 ULTI CID OF Unmodified aploid_01 2805 66.893 361850
774 24 644.829 575.28 0 -1 0 0 0 12 87.5109 aN aN NaN 39799 3004 2793
LYGLQSANYDMHGSPGGLARIDK ILYGLQSANYDMHGSPGGLARIDK_ 36107 00049
20150306_yeast1_Top_opt_2ug_C1_01_1663 2 ULTI CID OF Unmodified aploid_01 2984 67.139 150660
6318 10 548.816 095.62 0 -1 0 0 0 11 61.3438 aN aN NaN 39968 014 42973
DPIPDVTIK VDPIPDVTIK_ 52867 224123
20150306_yeast1_Top_opt_2ug_C1_01_1663 2 ULTI CID OF Unmodified aploid_01 3550 67.931 258250
856 11 666.324 330.63 0 -1 0 0 0 1 81.6249 aN aN NaN 0 0502 3046 3549
PLEELEDMEK VPLEELEDMEK_ 38276 233559
20150306_yeast1_Top_opt_2ug_C1_01_1663 3 ULTI CID OF Unmodified aploid_01 4009 68.602 169880
6653 12 469.963 406.87 0 -1 0 0 0 10 66.2666 aN aN NaN 40936 071 43999
IKPPLIPQNIK FIKPPLIPQNIK_ 40351 5921
20150306_yeast1_Top_opt_2ug_C1_01_1663 2 ULTI CID OF Unmodified aploid_01 9601 76.442 254040
113 11 604.869 207.72 0 -1 0 0 0 4 103.083 aN aN NaN 0 6217 3382 9597
PVLQVSDLPK LPVLQVSDLPK_ 22470 132315
20150306_yeast1_Top_opt_2ug_C1_01_1663 2 ULTI CID OF Unmodified aploid_01 0070 91.668 445930
5753 18 916.526 831.04 0 -1 0 0 0 15 102.895 aN aN NaN 56105 963 60055
GIGIAGIGTFKPELIMK SGIGIAGIGTFKPELIMK_ 32842 190481
20150306_yeast1_Top_opt_2ug_C1_01_1663 3 ULTI CID OF Unmodified aploid_01 0574 92.442 507980
438 28 1017.49 049.45 0 -1 0 0 0 15 33.317 aN aN NaN 0 6581 3991 0559
AEFEVTDSALYNNFNTSTTASLTPEIK SAEFEVTDSALYNNFNTSTTASLTPEIK_ 41815 85552
20150306_yeast1_Top_opt_2ug_C1_01_1663 2 ULTI CID OF Unmodified aploid_01 1595 93.975 477360
077 18 1003.53 005.04 0 0 1 0 0 0 10 103.413 aN aN NaN 0 7545 4048 1585
GVGICATCVLRPDLLFK SGVGICATCVLRPDLLFK_ 25515 90995
20150306_yeast1_Top_opt_2ug_C1_01_1663 2 ULTI CID OF Unmodified aploid_01 1632 94.035 164460
710 12 709.372 416.73 0 -1 0 0 0 11 52.5758 aN aN NaN 57580 4050 1621
FISGYLNELSK FFISGYLNELSK_ 54074 4073
20150306_yeast1_Top_opt_2ug_C1_01_1663 3 ULTI CID OF Unmodified aploid_01 7173 102.86 257970
565 30 1188.22 561.62 0 0 1 0 0 0 8 90.907 aN NaN aN 0 62813 358 7165
LIDDSILDDNDNTLWENPSQEQLNSSIWCK LIDDSILDDNDNTLWENPSQEQLNSSIWCK_ 04235 22754
20150306_yeast1_Top_opt_2ug_C1_01_1663 2 ULTI CID OF Unmodified aploid_01 8892 104.85 338380
2540 13 683.374 364.73 0 -1 0 0 0 17 94.6877 aN aN NaN 64437 453 68875
EFLGGLDAIFGK VEFLGGLDAIFGK_ 40554 24993
20150306_yeast1_Top_opt_2ug_C1_01_1663 3 ULTI CID OF Unmodified aploid_01 0540 106.32 104710
431 20 765.072 292.19 0 -1 0 0 0 9 44.2378 aN aN NaN 0 5993 4545 0531
TNRNFISVLESFLAPMVNQ ITNRNFISVLESFLAPMVNQ_ 03630 06078
20150306_yeast1_Top_opt_2ug_C1_01_1663 2 ULTI CID OF Acetyl (Protein N-term) aploid_01 0690 06.51
1188800 30170 14 836.456 670.9 0 0 -1 0 0 0 5 148.52 aN NaN aN 0 66135 553
0675 MDVLLSLPQPELFK (ac)MDVLLSLPQPELFK_ 06107 142495
20150306_yeast1_Top_opt_2ug_C1_01_1666 3 ULTI CID OF Unmodified aploid_02 540 14.834 100910
030 10 365.863 094.57 0 0 1 0 0 0 14 55.6486 aN aN NaN 0 577 961 526 PIEAVSTNHK
PIEAVSTNHK_ 12050 63948
20150306_yeast1_Top_opt_2ug_C1_01_1666 3 ULTI CID OF Unmodified aploid_02 1210 20.989 70058 775
11 413.221 236.64 0 0 1 0 0 0 12 51.9785 aN aN NaN 0 0043 1165 1198
LLHSTSHDIK SLLHSTSHDIK_ 42843 194801

20150306_yeast1_Top_opt_2ug_C1_01_1666 3 ULTI CID OF Unmodified aploid_02 1887 22.048 129660
985 16 596.967 787.88 0 -1 0 0 0 5 73.8334 aN aN NaN 0 0682 1203 1882
ASENSSQNIVNRDNK LASENSSQNIVNRDNK_ 34218 14883
20150306_yeast1_Top_opt_2ug_C1_01_1666 2 ULTI CID OF Unmodified aploid_02 4259 25.59 3076200
96260 7 365.229 28.444 0 -1 0 0 0 1 87.7543 aN aN NaN 0 2922 1335 4258
PVTLAK_TPVTLAK_ 3E7X9;P0C0X0 16898
20150306_yeast1_Top_opt_2ug_C1_01_1666 2 ULTI CID OF Unmodified aploid_02 4437 25.867 130000
973 10 542.803 083.59 0 -1 0 0 0 17 112.834 aN aN NaN 13091 1344 4420
PTQPTVSNK IPTQPTVSNK_ 47088 02008
20150306_yeast1_Top_opt_2ug_C1_01_1666 2 ULTI CID OF Unmodified aploid_02 4735 26.305 131250
553 9 502.292 002.57 0 0 1 0 0 0 9 91.8667 aN NaN aN 0 3372 1361 4726
TVLVNTTQK TVLVNTTQK_ 06838 20746
20150306_yeast1_Top_opt_2ug_C1_01_1666 3 ULTI CID OF Unmodified aploid_02 6495 29.045 152400
618 14 547.952 640.83 0 -1 0 0 0 5 87.0829 aN aN NaN 0 5034 1459 6490
STAPQLDQCPQRK ISTAPQLDQCPQRK_ 53732 05188
20150306_yeast1_Top_opt_2ug_C1_01_1666 2 ULTI CID OF Unmodified aploid_02 9613 33.761 135690
0851 15 856.898 711.78 0 -1 0 0 0 9 194.667 aN aN NaN 17979 1632 9604
FYAQEEREAANAGK EFYAQEEREAANAGK_ 02774 43092
20150306_yeast1_Top_opt_2ug_C1_01_1666 2 ULTI CID OF Unmodified aploid_02 9922 34.219 103980
103 11 663.844 325.67 0 -1 0 0 0 12 90.697 aN aN NaN 0 8271 1649 9910 VWINSTENVK
HVWINSTENVK_ 27636 89255
20150306_yeast1_Top_opt_2ug_C1_01_1666 3 ULTI CID OF Acetyl (Protein N-term) aploid_02 5398 2.331
142120 205 0 9 788.698 363.07 0 0 -1 0 0 0 6 75.3492 aN NaN aN 0 23443 953 5382
SDKPDSQVFCPNCNERLQK (ac)SDKPDSQVFCPNCNERLQK_ 3E7A8 87240
20150306_yeast1_Top_opt_2ug_C1_01_1666 2 ULTI CID OF Unmodified aploid_02 7340 45.141 142070
610 15 812.876 623.74 0 0 1 0 0 0 14 81.6559 aN aN NaN 0 5277 2061 7326
LSSTSLSTDMNEDK PLSSTSLSTDMNEDK_ 04429 165946
20150306_yeast1_Top_opt_2ug_C1_01_1666 2 ULTI CID OF Unmodified aploid_02 9042 47.508 5436500
16810 10 36.803 071.59 0 0 -1 0 0 0 6 7.5317 NaN aN NaN 26884 156 29036
LSLENGIAK QLSLENGIAK_ 38265 175064
20150306_yeast1_Top_opt_2ug_C1_01_1666 3 ULTI CID OF Unmodified aploid_02 3494 53.961 247670
955 21 773.363 317.07 0 -1 0 0 0 12 35.7362 aN aN NaN 31089 2403 3482
NSMNDNALEDSISNPVANRK ENSMNDNALEDSISNPVANRK_ 32838 2167
20150306_yeast1_Top_opt_2ug_C1_01_1666 2 ULTI CID OF Unmodified aploid_02 3787 54.408 121800
825 8 467.271 32.528 0 0 1 0 0 0 17 81.2963 aN aN NaN 0 1366 2419 3770 VIGFEQK
IVIGFEQK_ 32613 07160
20150306_yeast1_Top_opt_2ug_C1_01_1666 3 ULTI CID OF Unmodified aploid_02 4607 55.534 235220
1788 14 535.627 603.86 0 -1 0 0 0 9 97.6896 aN aN NaN 0 2140 2465 4598
LPDSGLYNDTLRK LLPDSGLYNDTLRK_ 52923 26989
20150306_yeast1_Top_opt_2ug_C1_01_1666 2 ULTI CID OF Unmodified aploid_02 0015 63.098 135880
738 8 488.28 74.546 0 0 1 0 0 0 17 63.5651 aN NaN aN 0 7248 2765 9998
LLTFPEQK LLTFPEQK_ 08118 28160
20150306_yeast1_Top_opt_2ug_C1_01_1666 2 ULTI CID OF Unmodified aploid_02 0948 64.46 248820
628 13 760.366 518.72 0 0 1 0 0 0 14 50.0878 aN NaN aN 0 8129 2817 0934
FQNPDMLANLDNK FQNPDMLANLDNK_ 32354 9191
20150306_yeast1_Top_opt_2ug_C1_01_1666 2 ULTI CID OF Unmodified aploid_02 3177 67.877 172840
682 9 518.795 035.58 0 0 1 0 0 0 11 129.421 aN aN NaN 0 0234 2941 3166 QYFAIIGK
PQYFAIIGK_ 38334 67358
20150306_yeast1_Top_opt_2ug_C1_01_1666 2 ULTI CID OF Unmodified aploid_02 3962 69.016 168760
803 10 551.827 101.64 0 -1 0 0 0 4 95.6181 aN aN NaN 0 0975 2985 3958 NSLTNLLSK
LNSLTNLLSK_ 40993 30723
20150306_yeast1_Top_opt_2ug_C1_01_1666 0 EAK CID OF Unmodified aploid_02 5083 70.64 208660
853 13 767.897 aN 0 0 -1 0 0 0 9 4.9536 NaN aN NaN 42034 047 45074
SVENVIPYNQFK PSVENVIPYNQFK_ 34234 68369

20150306_yeast1_Top_opt_2ug_C1_01_1666 2 ULTI CID OF Unmodified aploid_02 8637 75.682 256800
904 18 1004 005.98 0 0 -1 0 0 0 7 39.276 aN NaN aN 0 45391 244 8620
SPLINDASTPVNQDFFNK SPLINDASTPVNQDFFNK_ 53118 97902
20150306_yeast1_Top_opt_2ug_C1_01_1666 2 ULTI CID OF Unmodified aploid_02 9617 77.045 151710
105 10 568.329 134.64 0 -1 0 0 0 7 79.1482 aN aN NaN 0 6316 3299 9610
QYAPFLLGK VQYAPFLLGK_ 02820 34479
20150306_yeast1_Top_opt_2ug_C1_01_1666 2 ULTI CID OF Unmodified aploid_02 3337 82.673 422880
1180 13 704.385 406.75 0 -1 0 0 0 1 74.3925 aN aN NaN 49829 3506 3336
FFIPQSSLGNLK GFFIPQSSLGNLK_ 17898 4831
20150306_yeast1_Top_opt_2ug_C1_01_1666 4 ULTI CID OF Unmodified aploid_02 4847 84.824 1042100
3802 24 68.363 2669.42 0 -1 0 0 0 17 06.93 NaN aN NaN 51256 589 54830
RTDETLRLTGNPELSSLDEVLAK LRTDETLRLTGNPELSSLDEVLAK_ 5Z2X5 34044
20150306_yeast1_Top_opt_2ug_C1_01_1666 3 ULTI CID OF Unmodified aploid_02 7513 88.648 4933500
52300 23 03.804 408.39 0 0 -1 0 0 0 1 47.354 NaN aN NaN 53773 738 57512
LGNVTIAQGGVLPNIHQNLLPK LLGNVTIAQGGVLPNIHQNLLPK_ 04912;P04911 26215
20150306_yeast1_Top_opt_2ug_C1_01_1666 3 ULTI CID OF Unmodified aploid_02 9239 91.178 379230
260 20 782.083 343.23 0 0 1 0 0 0 17 57.4836 aN aN NaN 0 5404 3833 9222
LLQREFEISPELATVDLEK NLLQREFEISPELATVDLEK_ 12498 53734
20150306_yeast1_Top_opt_2ug_C1_01_1666 0 EAK CID OF Unmodified aploid_02 9820 106.1 177610
180 0 7 729.896 aN 0 0 -1 0 0 0 14 0.8968 NaN aN NaN 65397 421 69806
AVGMGAGALAAAAMLL AAVGMGAGALAAAAMLL_ 43497 336
20150306_yeast1_Top_opt_2ug_C1_01_1668 2 ULTI CID OF Unmodified aploid_03 721 14.899 56612 774
7 416.725 31.435 0 0 -1 0 0 0 98.9966 aN NaN aN 0 6842 77 7716 EQDISK
IEQDISK_ 03193 3549
20150306_yeast1_Top_opt_2ug_C1_01_1668 2 ULTI CID OF Unmodified aploid_03 865 16.865 46139 001
0 539.756 077.5 0 0 -1 0 0 0 15 1.6136 aN NaN aN 0 7923 40 8850 NSMEGYHK
INSMEGYHK_ 53947 01079
20150306_yeast1_Top_opt_2ug_C1_01_1668 2 ULTI CID OF Unmodified aploid_03 0378 19.374 41872 032
15 731.873 461.73 0 0 1 0 0 0 16 99.927 aN NaN aN 0 352 1024 0362
HSNANTAPPVVGGNK HSNANTAPPVVGGNK_ 06170 8496
20150306_yeast1_Top_opt_2ug_C1_01_1668 2 ULTI CID OF Unmodified aploid_03 1018 20.398 52011 751
12 625.299 248.58 0 0 1 0 0 0 8 111.793 aN NaN aN 0 956 1060 1010
TSSASSPQDLEK TSSASSPQDLEK_ 12359 218820
20150306_yeast1_Top_opt_2ug_C1_01_1668 2 ULTI CID OF Unmodified aploid_03 3685 24.552 82469 759
10 544.783 087.55 0 0 1 0 0 0 11 59.1984 aN aN NaN 0 2475 1208 3674
NNEALADTK INNEALADTK_ 40050 00903
20150306_yeast1_Top_opt_2ug_C1_01_1668 2 ULTI CID OF Unmodified aploid_03 6047 28.232 96584 720
11 635.309 268.6 0 0 1 0 0 0 15 52.0715 aN NaN aN 0 4706 1339 6032
HSNVDDDLINK HSNVDDDLINK_ 23748 88555
20150306_yeast1_Top_opt_2ug_C1_01_1668 3 ULTI CID OF Unmodified aploid_03 7218 29.973 147240
796 17 634.633 900.88 0 -1 0 0 0 16 80.7384 aN aN NaN 15812 1404 7202
SAIQENPQDDDGTRLK DSAIQENPQDDDGTRLK_ 07963 2119
20150306_yeast1_Top_opt_2ug_C1_01_1668 2 ULTI CID OF Unmodified aploid_03 0171 34.388 57768 55
17 916.41 830.81 0 0 -1 0 0 0 7 151.044 aN NaN aN 0 18601 568 0154
LNSAYDNNSSFEGASQK LNSAYDNNSSFEGASQK_ 53938 30669
20150306_yeast1_Top_opt_2ug_C1_01_1668 3 ULTI CID OF Unmodified aploid_03 1002 35.624 54055 586
9 376.2 125.58 0 0 -1 0 0 0 134.478 aN NaN aN 0 19385 615 21000 YAELAERK
FYAELAERK_ 07255 72058
20150306_yeast1_Top_opt_2ug_C1_01_1668 2 ULTI CID OF Unmodified aploid_03 2326 37.568 140060
568 10 556.776 111.54 0 -1 0 0 0 12 64.82 aN NaN aN 0 20636 1688 22314
VSVTTFNCGK _VSVTTFNCGK_ Q08227 235917
20150306_yeast1_Top_opt_2ug_C1_01_1668 + 2 MULTI CID TOF Unmodified haploid_03 24645
41.033 1 222820 6749 0 11 628.796 1255.58 0 0 0 -1 0 0 0 0 9 68.1074
NaN NaN NaN 0 22826 1817 24636 GLTYQEAMESK _GLTYQEAMESK_ P14063 78074

20150306_yeast1_Top_opt_2ug_C1_01_1668 + 4 MULTI CID TOF Unmodified haploid_03 24883
41.404 1 213790 3149 0 23 684.841 2735.34 0 0 0 -1 0 0 0 0 13 52.898
NaN NaN NaN 0 23051 1830 24870 RLDDQTDVEQSPLLERSNHVQEK
RLDDQTDVEQSPLLERSNHVQEK Q07376 181802
20150306_yeast1_Top_opt_2ug_C1_01_1668 + 2 MULTI CID TOF Unmodified haploid_03 29201
47.546 1 87748 5050 0 8 470.237 938.46 0 0 0 -1 0 0 0 0 11 86.7939
NaN NaN NaN 0 27129 2070 29190 LQFTDMGK _LQFTDMGK_ Q12328 132834
20150306_yeast1_Top_opt_2ug_C1_01_1668 + 2 MULTI CID TOF Unmodified haploid_03 29235
47.588 1 170780 2646 0 11 651.796 1301.58 0 0 0 -1 0 0 0 0 9 47.7121
NaN NaN NaN 0 27161 2072 29226 GSYNLEVYDDK _GSYNLEVYDDK_ P53198 81152
20150306_yeast1_Top_opt_2ug_C1_01_1668 + 0 PEAK CID TOF Unmodified haploid_03 30483
49.358 1 152710 2725 0 10 556.819 NaN 0 0 0 -1 0 0 0 0 15 121.36 NaN
NaN NaN 0 28340 2141 30468 PVLQELTANK _PVLQELTANK_ P23059 169549
20150306_yeast1_Top_opt_2ug_C1_01_1668 + 3 MULTI CID TOF Unmodified haploid_03 30500
49.386 1 441500 6692 0 22 792.716 2375.13 0 0 0 -1 0 0 0 0 14 79.9422
NaN NaN NaN 0 28356 2142 30486 VQQFSSTSHVDDEDVNSIAVAK
VQQFSSTSHVDDEDVNSIAVAK P25604 234326
20150306_yeast1_Top_opt_2ug_C1_01_1668 + 3 MULTI CID TOF Unmodified haploid_03 30642
49.591 1 77160 3147 0 10 374.89 1121.65 0 0 0 -1 0 0 0 0 12 49.3004
NaN NaN NaN 0 28490 2150 30630 LTLVSPDLHK _LTLVSPDLHK_ P15807 137998
20150306_yeast1_Top_opt_2ug_C1_01_1668 + 2 MULTI CID TOF Acetyl (Protein N-term) haploid_03
31556 50.941 1 360040 6438 0 9 541.302 1080.59 0 0 0 -1 0 0 0 0 8
75.509 NaN NaN NaN 0 29353 2201 31548 STNILQHVK _(ac)STNILQHVK_ P53983 202641
20150306_yeast1_Top_opt_2ug_C1_01_1668 + 2 MULTI CID TOF Unmodified haploid_03 41551
65.122 1 312340 2246 0 16 885.957 1769.9 0 0 0 -1 0 0 0 0 13 85.3625
NaN NaN NaN 0 38793 2756 41538 ISRPIASQNSLGWNEV _ISRPIASQNSLGWNEV_ P32334
105034
20150306_yeast1_Top_opt_2ug_C1_01_1668 + 2 MULTI CID TOF Unmodified haploid_03 41940
65.697 1 166590 3206 0 12 693.361 1384.71 0 0 0 -1 0 0 0 0 6 60.1571
NaN NaN NaN 0 39160 2778 41934 SLIEIDDPNIEK _SLIEIDDPNIEK_ P40851 194659
20150306_yeast1_Top_opt_2ug_C1_01_1668 + 3 MULTI CID TOF Unmodified haploid_03 43693
68.242 1 287860 2766 0 26 932.802 2795.38 0 0 0 -1 0 0 0 0 13 50.0291
NaN NaN NaN 0 40816 2875 43680 IVEEPVVLSHNSSIDESLDAATQNTK
IVEEPVVLSHNSSIDESLDAATQNTK Q08119 106920
20150306_yeast1_Top_opt_2ug_C1_01_1668 + 2 MULTI CID TOF Unmodified haploid_03 43774
68.368 1 160740 2556 0 11 665.858 1329.7 0 0 0 -1 0 0 0 0 4 73.9513
NaN NaN NaN 0 40892 2880 43770 TLQELDIEEIK _TLQELDIEEIK_ P08465 214588
20150306_yeast1_Top_opt_2ug_C1_01_1668 + 0 PEAK CID TOF Unmodified haploid_03 46790
72.649 1 355040 4765 0 13 671.882 NaN 0 0 0 -1 0 0 0 0 14 72.8801 NaN
NaN NaN 0 43741 3047 46776 VLQVSGVLGDDLK _VLQVSGVLGDDLK_ P25366 231224
20150306_yeast1_Top_opt_2ug_C1_01_1668 + 3 MULTI CID TOF Unmodified haploid_03 46898
72.798 1 326850 7806 0 25 1008.82 3023.43 0 0 0 -1 0 0 0 0 14 76.7993
NaN NaN NaN 0 43843 3053 46884 GQTEEEYTQQLQHYFEVEQGPVRTK
GQTEEEYTQQLQHYFEVEQGPVRTK Q02872 79748
20150306_yeast1_Top_opt_2ug_C1_01_1668 + 2 MULTI CID TOF Unmodified haploid_03 47274
73.301 1 210930 1681 0 12 729.37 1456.73 0 0 0 -1 0 0 0 0 12 73.2733
NaN NaN NaN 0 44198 3074 47262 NQNLWQEVDLAK _NQNLWQEVDLAK_ P47051 156952
20150306_yeast1_Top_opt_2ug_C1_01_1668 + 2 MULTI CID TOF Acetyl (Protein N-term) haploid_03
48441 75.102 1 306660 6800 0 21 1175.58 2349.14 0 0 0 -1 0 0 0 0 9
145.441 NaN NaN NaN 0 45300 3139 48432 SSNEEVFTQINATANVVDNKK
(ac)SSNEEVFTQINATANVVDNKK Q12431 200882
20150306_yeast1_Top_opt_2ug_C1_01_1668 + 3 MULTI CID TOF Unmodified haploid_03 51336
79.338 1 529080 9671 0 18 686.682 2057.02 0 0 0 -1 0 0 0 0 6 55.4367
NaN NaN NaN 0 48034 3300 51330 FLTQEAGLVDSWEQLHGK _FLTQEAGLVDSWEQLHGK_

P40015 67406
20150306_yeast1_Top_opt_2ug_C1_01_1668 + 2 MULTI CID TOF Unmodified haploid_03 55720
85.773 1 92899 7418 0 9 525.315 1048.62 0 0 0 -1 0 0 0 0 16 79.9855
NaN NaN NaN 0 52175 3543 55704 LISILSFEK _LISILSFEK_ P40499 124708
20150306_yeast1_Top_opt_2ug_C1_01_1668 + 3 MULTI CID TOF Unmodified haploid_03 60578
92.923 1 267050 5095 0 19 756.086 2265.23 0 0 0 -1 0 0 0 0 14 133.208
NaN NaN NaN 0 56763 3813 60564 INDLLQQRLQILDDAIQEK _INDLLQQRLQILDDAIQEK_
Q06162 100515
20150306_yeast1_Top_opt_2ug_C1_01_1668 + 3 MULTI CID TOF Unmodified haploid_03 65156
99.939 1 334910 12291 0 15 568.661 1702.96 0 0 0 -1 0 0 0 0 2 154.651
NaN NaN NaN 0 61086 4068 65154 VQLLDATEIFRTLGK _VQLLDATEIFRTLGK_ P53173
234257
20150306_yeast1_Top_opt_2ug_C1_01_1670 + 2 MULTI CID TOF Unmodified haploid_04 7060
13.96 1 52526 1354 0 8 474.713 947.411 0 0 0 -1 0 0 0 0 7 66.6919
NaN NaN NaN 0 6137 921 7053 EDADIEEK _EDADIEEK_ P04817 38630
20150306_yeast1_Top_opt_2ug_C1_01_1670 + 2 MULTI CID TOF Acetyl (Protein N-term) haploid_04
12678 23.122 1 82486 3813 0 10 634.274 1266.53 0 0 0 -1 0 0 0 0 9
105.524 NaN NaN NaN 0 11443 1233 12669 MNRDNMDTTK _(ac)MNRDNMDTTK_ P53099
145312
20150306_yeast1_Top_opt_2ug_C1_01_1670 + 2 MULTI CID TOF Unmodified haploid_04 13059
23.692 1 112930 1761 0 11 651.299 1300.58 0 0 0 -1 0 0 0 0 12 80.6901
NaN NaN NaN 0 11803 1254 13047 VPEQLDNDDEK _VPEQLDNDDEK_ P31381 233330
20150306_yeast1_Top_opt_2ug_C1_01_1670 + 3 MULTI CID TOF Unmodified haploid_04 14179
25.371 1 199910 20305 0 19 645.324 1932.95 0 0 0 -1 0 0 0 0 16 114.554
NaN NaN NaN 0 12861 1316 14163 INNEADRSSVSAGTSNIAK _INNEADRSSVSAGTSNIAK_
Q12151 100893
20150306_yeast1_Top_opt_2ug_C1_01_1670 + 2 MULTI CID TOF Unmodified haploid_04 16000
28.137 1 574080 53509 0 8 439.722 877.429 0 0 0 -1 0 0 0 0 1 113.412
NaN NaN NaN 0 14580 1418 15999 ANDIGFNK _ANDIGFNK_ P41057 10427
20150306_yeast1_Top_opt_2ug_C1_01_1670 + 2 MULTI CID TOF Unmodified haploid_04 16193
28.452 1 110260 5362 0 10 531.275 1060.54 0 0 0 -1 0 0 0 0 14 75.6522
NaN NaN NaN 0 14763 1428 16179 VLDSDISNAK _VLDSDISNAK_ P38717 230005
20150306_yeast1_Top_opt_2ug_C1_01_1670 + 2 MULTI CID TOF Unmodified haploid_04 17374
30.201 1 106410 1825 0 12 703.325 1404.64 0 0 0 -1 0 0 0 0 7 71.8061
NaN NaN NaN 0 15878 1494 17367 MEGGDLEYQHVK _MEGGDLEYQHVK_ P25361 142742
20150306_yeast1_Top_opt_2ug_C1_01_1670 + 3 MULTI CID TOF Unmodified haploid_04 18656
32.274 1 164980 7662 0 18 613.304 1836.89 0 0 0 -1 0 0 0 0 11 69.8052
NaN NaN NaN 0 17089 1565 18645 AHQSAATPNVAAVNNMNK _AHQSAATPNVAAVNNMNK_
P47175 5986
20150306_yeast1_Top_opt_2ug_C1_01_1670 + 2 MULTI CID TOF Unmodified haploid_04 21064
35.881 1 125020 5153 0 11 587.326 1172.64 0 0 0 -1 0 0 0 0 7 98.4072
NaN NaN NaN 0 19363 1699 21057 LNVINSSPTTK _LNVINSSPTTK_ Q03327 130883
20150306_yeast1_Top_opt_2ug_C1_01_1670 + 2 MULTI CID TOF Unmodified haploid_04 23244
39.08 1 96819 6477 0 9 481.262 960.51 0 0 0 -1 0 0 0 0 9 68.657
NaN NaN NaN 0 21422 1820 23235 SAVLSDEIK _SAVLSDEIK_ P32792 186525
20150306_yeast1_Top_opt_2ug_C1_01_1670 + 3 MULTI CID TOF Unmodified haploid_04 23637
39.657 1 75314 1914 0 10 432.893 1295.66 0 0 0 -1 0 0 0 0 6 96.2294
NaN NaN NaN 0 21793 1842 23631 IENEHVIWEK _IENEHVIWEK_ P47093 93356
20150306_yeast1_Top_opt_2ug_C1_01_1670 + 2 MULTI CID TOF Unmodified haploid_04 25067
41.777 1 178580 2847 0 10 618.81 1235.6 0 0 0 -1 0 0 0 0 14 88.0565
NaN NaN NaN 0 23144 1921 25053 QILEQYAEDK _QILEQYAEDK_ P32831 173645
20150306_yeast1_Top_opt_2ug_C1_01_1670 + 3 MULTI CID TOF Unmodified haploid_04 25520
42.427 1 158050 4677 0 20 822.718 2465.13 0 0 0 -1 0 0 0 0 17 56.7483
NaN NaN NaN 0 23572 1946 25503 LEDLLQRQNEDQESSQEYNK _LEDLLQRQNEDQESSQEYNK_

Q2V2P8 117682
20150306_yeast1_Top_opt_2ug_C1_01_1670 + 3 MULTI CID TOF Unmodified haploid_04 26807
44.307 1 172980 8128 0 17 610.627 1828.86 0 0 0 -1 0 0 0 0 8 189.809
NaN NaN NaN 0 24787 2018 26799 PALDEAREEAPFENGGK _PALDEAREEAPFENGGK_
O60200 160923
20150306_yeast1_Top_opt_2ug_C1_01_1670 + 2 MULTI CID TOF Unmodified haploid_04 29171
47.782 1 177740 4091 0 11 589.822 1177.63 0 0 0 -1 0 0 0 0 14 52.3906
NaN NaN NaN 0 27020 2149 29157 ALFVTTSNTPK _ALFVTTSNTPK_ Q99252 8366
20150306_yeast1_Top_opt_2ug_C1_01_1670 + 3 MULTI CID TOF Unmodified haploid_04 29172
47.784 1 247140 7083 0 16 572.969 1715.89 0 0 0 -1 0 0 0 0 15 69.1882
NaN NaN NaN 0 27021 2149 29157 LHTNLSQEYIGVSAGK _LHTNLSQEYIGVSAGK_ P38114
122563
20150306_yeast1_Top_opt_2ug_C1_01_1670 + 2 MULTI CID TOF Unmodified haploid_04 29190
47.815 1 180040 5103 0 11 593.836 1185.66 0 0 0 -1 0 0 0 0 15 71.0845
NaN NaN NaN 0 27038 2150 29175 LLTPITASNEK _LLTPITASNEK_ P18412 128279
20150306_yeast1_Top_opt_2ug_C1_01_1670 + 2 MULTI CID TOF Unmodified haploid_04 33331
53.699 1 344060 41412 0 8 436.284 870.553 0 0 0 -1 0 0 0 0 16 68.2141
NaN NaN NaN 0 30949 2380 33315 VLSLALQK _VLSLALQK_ Q05779 231373
20150306_yeast1_Top_opt_2ug_C1_01_1670 + 2 MULTI CID TOF Unmodified haploid_04 36251
57.94 1 204070 10319 0 9 548.769 1095.52 0 0 0 -1 0 0 0 0 2 107.205
NaN NaN NaN 0 33706 2543 36249 YGSWEVLDK _YGSWEVLDK_ P53066 245367
20150306_yeast1_Top_opt_2ug_C1_01_1670 + 2 MULTI CID TOF Unmodified haploid_04 37795
60.03 1 290380 5075 0 9 575.287 1148.56 0 0 0 -1 0 0 0 0 16 63.1842
NaN NaN NaN 0 35165 2628 37779 IWLQEMDSK _IWLQEMDSK_ Q03823 108163
20150306_yeast1_Top_opt_2ug_C1_01_1670 + 3 MULTI CID TOF Unmodified haploid_04 39299
62.142 1 503920 12233 0 15 610.291 1827.85 0 0 0 -1 0 0 0 0 8 60.5499
NaN NaN NaN 0 36585 2712 39291 VLPFHTIYCYNCGGK _VLPFHTIYCYNCGGK_ Q12476
231010
20150306_yeast1_Top_opt_2ug_C1_01_1670 + 3 MULTI CID TOF Unmodified haploid_04 39982
63.105 1 59576 2773 0 10 400.558 1198.65 0 0 0 -1 0 0 0 0 7 94.6921
NaN NaN NaN 0 37230 2750 39975 TVHFWAPTLK _TVHFWAPTLK_ P38857;P53311 220472
20150306_yeast1_Top_opt_2ug_C1_01_1670 + 2 MULTI CID TOF Acetyl (Protein N-term) haploid_04
40244 63.475 1 599980 48281 0 17 985.484 1968.95 0 0 0 -1 0 0 0 0 17
190.958 NaN NaN NaN 0 37478 2764 40227 ATRTQFENSNEIGVFSK
(ac)ATRTQFENSNEIGVFSK Q12522 15024
20150306_yeast1_Top_opt_2ug_C1_01_1670 + 2 MULTI CID TOF Unmodified haploid_04 40889
64.478 1 276300 3921 0 15 857.4 1712.79 0 0 0 -1 0 0 0 0 14 43.5923
NaN NaN NaN 0 38087 2800 40875 ENDVVESGQELGYFK _ENDVVESGQELGYFK_ P53037
51112
20150306_yeast1_Top_opt_2ug_C1_01_1670 + 2 MULTI CID TOF Unmodified haploid_04 42627
67.115 1 239980 5667 0 12 658.851 1315.69 0 0 0 -1 0 0 0 0 6 52.0715
NaN NaN NaN 0 39728 2897 42621 SLPMYPAAPLEK _SLPMYPAAPLEK_ P16892 195166
20150306_yeast1_Top_opt_2ug_C1_01_1670 + 2 MULTI CID TOF Unmodified haploid_04 42859
67.478 1 253820 2249 0 15 736.897 1471.78 0 0 0 -1 0 0 0 0 4 50.0775
NaN NaN NaN 0 39947 2910 42855 TAAGALLDTAVNVEK _TAAGALLDTAVNVEK_ P34239
205489
20150306_yeast1_Top_opt_2ug_C1_01_1670 + 2 MULTI CID TOF Unmodified haploid_04 43302
68.14 1 198460 2002 0 11 626.839 1251.66 0 0 0 -1 0 0 0 0 15 92.5385
NaN NaN NaN 0 40366 2934 43287 GFVNAINLYNK _GFVNAINLYNK_ Q04089 75331
20150306_yeast1_Top_opt_2ug_C1_01_1670 + 3 MULTI CID TOF Unmodified haploid_04 44790
70.167 1 228330 1714 0 22 851.095 2550.26 0 0 0 -1 0 0 0 0 9 107.747
NaN NaN NaN 0 41771 3017 44781 LSDNYNTQILSNTEHPLLTSYK
LSDNYNTQILSNTEHPLLTSYK P48743 134511
20150306_yeast1_Top_opt_2ug_C1_01_1670 + 3 MULTI CID TOF Unmodified haploid_04 45312

70.892 1 287290 10506 0 24 891.091 2670.25 0 0 0 -1 0 0 0 0 9 124.195
NaN NaN NaN 0 42264 3046 45303 IMDEAAERGLFLEDESLNSSRSTT
IMDEAAERGLFLEDESLNSSRSTT Q3E776 100107
20150306_yeast1_Top_opt_2ug_C1_01_1670 + 2 MULTI CID TOF Unmodified haploid_04 47784
74.387 1 215020 5504 0 10 590.806 1179.6 0 0 0 -1 0 0 0 0 15 54.2593
NaN NaN NaN 0 44599 3183 47769 ATLLNFVCDK _ATLLNFVCDK_ Q06815 14775
20150306_yeast1_Top_opt_2ug_C1_01_1670 + 3 MULTI CID TOF Unmodified haploid_04 48513
75.495 1 231270 6456 0 20 730.373 2188.1 0 0 0 -1 0 0 0 0 6 61.9415
NaN NaN NaN 0 45287 3224 48507 SSETMIPTLLREASTQEPAK _SSETMIPTLLREASTQEPAK_
Q2V2Q1 200396
20150306_yeast1_Top_opt_2ug_C1_01_1670 + 3 MULTI CID TOF Unmodified haploid_04 49457
76.812 1 277200 1513 0 21 745.041 2232.1 0 0 0 -1 0 0 0 0 14 87.9633
NaN NaN NaN 0 46179 3276 49443 SNADASALRDINDININFAAK
SNADASALRDINDININFAAK P47005 196252
20150306_yeast1_Top_opt_2ug_C1_01_1670 + 3 MULTI CID TOF Acetyl (Protein N-term) haploid_04
62064 95.45 1 158030 4495 0 15 627.638 1879.89 0 0 0 -1 0 0 0 0 3
105.582 NaN NaN NaN 0 58085 3977 62061 MEADDHVSLFRFPFK _(ac)MEADDHVSLFRFPFK_
Q12016 142586
20150306_yeast1_Top_opt_2ug_C1_01_1670 + 2 MULTI CID TOF Unmodified haploid_04 62654
96.395 1 264690 11349 0 13 735.38 1468.74 0 0 0 -1 0 0 0 0 17 104.174
NaN NaN NaN 0 58643 4009 62637 SFLPSFILSDESK _SFLPSFILSDESK_ P53507 189749
20150306_yeast1_Top_opt_2ug_C1_01_1670 + 3 MULTI CID TOF Unmodified haploid_04 63431
97.66 1 297870 6170 0 29 1092.88 3275.63 0 0 0 -1 0 0 0 0 2 92.2584
NaN NaN NaN 0 59376 4053 63429 IDDDLNREYTIFEAVQAQNEVGNAIIPTK
IDDDLNREYTIFEAVQAQNEVGNAIIPTK P32345 91495
20150306_yeast1_Top_opt_2ug_C1_01_1670 + 3 MULTI CID TOF Acetyl (Protein N-term) haploid_04
63859 98.344 1 397860 4812 0 30 1127.24 3378.7 0 0 0 -1 0 0 0 0 16
48.2263 NaN NaN NaN 0 59781 4076 63843 SQVIEPQLDRTTYYSILGLTSNATSSEVHK
(ac)SQVIEPQLDRTTYYSILGLTSNATSSEVHK P46997 199256
20150306_yeast1_Top_opt_2ug_C1_01_1670 + 2 MULTI CID TOF Unmodified haploid_04 66955
103.16 1 283930 3050 0 19 1018.52 2035.02 0 0 0 -1 0 0 0 0 16 67.928
NaN NaN NaN 0 62705 4248 66939 TILATGGDDGIVNFWSLEK _TILATGGDDGIVNFWSLEK_
Q05583 212199
20150306_yeast1_Top_opt_2ug_C1_01_1670 + 2 MULTI CID TOF Acetyl (Protein N-term) haploid_04
69634 105.93 1 859110 93502 0 13 754.434 1506.85 0 0 0 -1 0 0 0 0 13
151.52 NaN NaN NaN 0 65235 4397 69621 SLPEILPLEVIDK _(ac)SLPEILPLEVIDK_ P40089
195110
20150306_yeast1_Top_opt_2ug_C1_01_1670 + 2 MULTI CID TOF Acetyl (Protein N-term) haploid_04
70145 106.48 1 226620 12489 0 19 1008.05 2014.08 0 0 0 -1 0 0 0 0 2
119.377 NaN NaN NaN 0 65717 4426 70143 TDALEQSVLALEGTVSVLK
(ac)TDALEQSVLALEGTVSVLK Q03954 207411
20150306_yeast3_Top_opt_2ug_C3_01_1664 + 2 MULTI CID TOF Unmodified diploid_01 4882
10.379 1 111740 1332 0 10 640.335 1278.65 0 0 0 -1 0 0 0 0 10 136.53
NaN NaN NaN 0 4089 791 4872 KYEQQVTQQK _KYEQQVTQQK_ P39103 113178
20150306_yeast3_Top_opt_2ug_C3_01_1664 + 3 MULTI CID TOF Unmodified diploid_01 8025
15.355 1 157860 12913 0 14 576.268 1725.78 0 0 0 -1 0 0 0 0 3 82.6513
NaN NaN NaN 0 7057 966 8022 EREEFATEERSDTK _EREEFATEERSDTK_ Q04272 54385
20150306_yeast3_Top_opt_2ug_C3_01_1664 + 2 MULTI CID TOF Unmodified diploid_01 9490
17.775 1 106140 4657 0 8 549.272 1096.53 0 0 0 -1 0 0 0 0 10 102.067
NaN NaN NaN 0 8441 1047 9480 YQDTRTWK _YQDTRTWK_ Q3E7B2 248490
20150306_yeast3_Top_opt_2ug_C3_01_1664 + 2 MULTI CID TOF Unmodified diploid_01 10617
19.604 1 104710 3504 0 10 599.774 1197.53 0 0 0 -1 0 0 0 0 3 94.3092
NaN NaN NaN 0 9505 1110 10614 LYETCPSSNK _LYETCPSSNK_ P42840 141177
20150306_yeast3_Top_opt_2ug_C3_01_1664 + 2 MULTI CID TOF Unmodified diploid_01 14603

25.576 1 106330 4790 0 10 543.251 1084.49 0 0 0 -1 0 0 0 0 11 83.8686
NaN NaN NaN 0 13270 1331 14592 STAEIGCGYK _STAEIGCGYK_ P47033 201623
20150306_yeast3_Top_opt_2ug_C3_01_1664 + 3 MULTI CID TOF Unmodified diploid_01 15502
26.867 1 196390 8933 0 13 495.917 1484.73 0 0 0 -1 0 0 0 0 10 53.5645
NaN NaN NaN 0 14119 1381 15492 RLEDSHVLMNSGK _RLEDSHVLMNSGK_ P21374 181884
20150306_yeast3_Top_opt_2ug_C3_01_1664 + 3 MULTI CID TOF Unmodified diploid_01 15617
27.022 1 154550 5551 0 14 525.924 1574.75 0 0 0 -1 0 0 0 0 17 83.6916
NaN NaN NaN 0 14228 1387 15600 EALQDERLNTGSDK _EALQDERLNTGSDK_ Q99296
37390
20150306_yeast3_Top_opt_2ug_C3_01_1664 + 2 MULTI CID TOF Unmodified diploid_01 17705
30.127 1 129120 3078 0 13 700.85 1399.69 0 0 0 -1 0 0 0 0 17 84.7337
NaN NaN NaN 0 16200 1503 17688 PITGDVETNEPTK _PITGDVETNEPTK_ P07249 164468
20150306_yeast3_Top_opt_2ug_C3_01_1664 + 2 MULTI CID TOF Unmodified diploid_01 21055
35.078 1 323240 10283 0 13 634.339 1266.66 0 0 0 -1 0 0 0 0 1 67.7494
NaN NaN NaN 0 19363 1690 21054 NAAQPIAGLNNGK _NAAQPIAGLNNGK_ Q05827 147301
20150306_yeast3_Top_opt_2ug_C3_01_1664 + 3 MULTI CID TOF Unmodified diploid_01 22060
36.567 1 412710 7837 0 15 513.943 1538.81 0 0 0 -1 0 0 0 0 16 88.5614
NaN NaN NaN 0 20313 1745 22044 PHLSSIGNANSITTK _PHLSSIGNANSITTK_ Q12090
163634
20150306_yeast3_Top_opt_2ug_C3_01_1664 + 3 MULTI CID TOF Unmodified diploid_01 26039
42.255 1 169940 4086 0 12 485.578 1453.71 0 0 0 -1 0 0 0 0 17 67.2517
NaN NaN NaN 0 24071 1966 26022 SLHLDNLEEEQK _SLHLDNLEEEQK_ Q02895 194580
20150306_yeast3_Top_opt_2ug_C3_01_1664 + 3 MULTI CID TOF Unmodified diploid_01 26783
43.257 1 143060 3355 0 11 448.263 1341.77 0 0 0 -1 0 0 0 0 5 64.1212
NaN NaN NaN 0 24773 2008 26778 LRNEQVTIELK _LRNEQVTIELK_ Q02260 133896
20150306_yeast3_Top_opt_2ug_C3_01_1664 + 2 MULTI CID TOF Unmodified diploid_01 28286
45.369 1 151720 11099 0 16 970.938 1939.86 0 0 0 -1 0 0 0 0 14 130.01
NaN NaN NaN 0 26193 2091 28272 EERDTCILFNGQDSEK _EERDTCILFNGQDSEK_ Q12287
41487
20150306_yeast3_Top_opt_2ug_C3_01_1664 + 2 MULTI CID TOF Unmodified diploid_01 31614
49.965 1 233700 1499 0 15 815.359 1628.7 0 0 0 -1 0 0 0 0 12 51.0726
NaN NaN NaN 0 29336 2276 31602 GGAYSNEDEDANFLK _GGAYSNEDEDANFLK_ P53904
75389
20150306_yeast3_Top_opt_2ug_C3_01_1664 + 2 MULTI CID TOF Unmodified diploid_01 33041
51.959 1 196490 3036 0 10 601.799 1201.58 0 0 0 -1 0 0 0 0 17 56.4044
NaN NaN NaN 0 30684 2355 33024 NDNDEIIEIK _NDNDEIIEIK_ P31244 148689
20150306_yeast3_Top_opt_2ug_C3_01_1664 + 3 MULTI CID TOF Unmodified diploid_01 33065
51.996 1 444100 14092 0 14 586.284 1755.83 0 0 0 -1 0 0 0 0 5 94.7168
NaN NaN NaN 0 30706 2357 33060 LETLDNQTMRNYMK _LETLDNQTMRNYMK_ Q2V2P4
119277
20150306_yeast3_Top_opt_2ug_C3_01_1664 + 2 MULTI CID TOF Unmodified diploid_01 34344
53.776 1 187240 1547 0 14 781.396 1560.78 0 0 0 -1 0 0 0 0 6 66.0588
NaN NaN NaN 0 31914 2428 34338 AVNEFQVESNISPK _AVNEFQVESNISPK_ Q00723 16366
20150306_yeast3_Top_opt_2ug_C3_01_1664 + 3 MULTI CID TOF Unmodified diploid_01 34381
53.819 1 245740 2072 0 23 767.363 2299.07 0 0 0 -1 0 0 0 0 7 79.2876
NaN NaN NaN 0 31949 2430 34374 TSNGAAVVTDVAQHAVSDSDNNK
TSNGAAVVTDVAQHAVSDSDNNK Q12013 218519
20150306_yeast3_Top_opt_2ug_C3_01_1664 + 2 MULTI CID TOF Unmodified diploid_01 38873
60.044 1 217220 3583 0 20 1157.49 2312.97 0 0 0 -1 0 0 0 0 17 123.552
NaN NaN NaN 0 36192 2679 38856 SDDPSSFQYYPDDPENPVNK _SDDPSSFQYYPDDPENPVNK_
P38824 186866
20150306_yeast3_Top_opt_2ug_C3_01_1664 + 3 MULTI CID TOF Unmodified diploid_01 42040
64.48 1 401380 6502 0 27 998.467 2992.38 0 0 0 -1 0 0 0 0 16 122.531
NaN NaN NaN 0 39183 2855 42024 RYSSLFSVDSTTPNSSSETVELSEENK

RYSSLFSVDSTTPNSSSETVELSEENK P36531 185311
20150306_yeast3_Top_opt_2ug_C3_01_1664 + 3 MULTI CID TOF Unmodified diploid_01 42797
65.637 1 240290 3850 0 14 528.284 1581.83 0 0 0 -1 0 0 0 0 17 39.4267
NaN NaN NaN 0 39898 2897 42780 SLESHIAQLETNLK _SLESHIAQLETNLK_ P47988 194218
20150306_yeast3_Top_opt_2ug_C3_01_1664 + 3 MULTI CID TOF Unmodified diploid_01 43067
66.012 1 642370 27793 0 13 516.949 1547.82 0 0 0 -1 0 0 0 0 17 109.145
NaN NaN NaN 0 40153 2912 43050 EIMSTESLELLRK _EIMSTESLELLRK_ P39008 46207
20150306_yeast3_Top_opt_2ug_C3_01_1664 + 4 MULTI CID TOF Unmodified diploid_01 48107
73.041 1 512920 2744 0 23 694.563 2774.22 0 0 0 -1 0 0 0 0 17 62.0054
NaN NaN NaN 0 44913 3192 48090 EMEVDPSNMNFQELANHFDRHSK
EMEVDPSNMNFQELANHFDRHSK P19956 50591
20150306_yeast3_Top_opt_2ug_C3_01_1664 + 0 PEAK CID TOF Unmodified diploid_01 48644
73.777 1 93851 3212 0 13 511.287 NaN 0 0 0 -1 0 0 0 0 14 159.576 NaN
NaN NaN 0 45420 3222 48630 PVNSEVFTELLRK _PVNSEVFTELLRK_ P47167 169640
20150306_yeast3_Top_opt_2ug_C3_01_1664 + 2 MULTI CID TOF Unmodified diploid_01 66358
100.01 1 347020 3814 0 21 1173.11 2344.2 0 0 0 -1 0 0 0 0 16 83.2506
NaN NaN NaN 0 62150 4206 66342 LTTLDTGDLSFPIDEEQPILK _LTTLDTGDLSFPIDEEQPILK_
Q12301 138440
20150306_yeast3_Top_opt_2ug_C3_01_1664 + 2 MULTI CID TOF Unmodified diploid_01 68059
102.63 1 265550 3150 0 13 761.891 1521.77 0 0 0 -1 0 0 0 0 7 52.4955
NaN NaN NaN 0 63756 4301 68052 SILELVNEDYQSI _SILELVNEDYQSI_ P28778 192613
20150306_yeast3_Top_opt_2ug_C3_01_1664 + 2 MULTI CID TOF Unmodified diploid_01 70746
105.59 1 142800 7530 0 12 689.944 1377.87 0 0 0 -1 0 0 0 0 12 87.1843
NaN NaN NaN 0 66294 4450 70734 LLNWVLLPLLGK _LLNWVLLPLLGK_ Q3E798 126944
20150306_yeast3_Top_opt_2ug_C3_01_1664 + 3 MULTI CID TOF Unmodified diploid_01 72557
107.91 1 291120 6265 0 25 869.815 2606.42 0 0 0 -1 0 0 0 0 5 125.311
NaN NaN NaN 0 68004 4551 72552 AAIYLTAVLEYLTAEVLELAGNAAK
AAIYLTAVLEYLTAEVLELAGNAAK Q12692 766
20150306_yeast3_Top_opt_2ug_C3_01_1665 + 2 MULTI CID TOF Unmodified diploid_02 6370
12.468 1 54872 1918 0 7 424.685 847.356 0 0 0 -1 0 0 0 0 3 79.8094
NaN NaN NaN 0 5585 783 6367 DNDELDK _DNDELDK_ Q03880 29665
20150306_yeast3_Top_opt_2ug_C3_01_1665 + 2 MULTI CID TOF Unmodified diploid_02 8992
16.912 1 562160 49607 0 13 660.271 1318.53 0 0 0 -1 0 0 0 0 15 100.072
NaN NaN NaN 0 8062 928 8977 ARIAAGDGDDDDE _ARIAAGDGDDDDE_ P22289 12843
20150306_yeast3_Top_opt_2ug_C3_01_1665 + 2 MULTI CID TOF Unmodified diploid_02 9144
17.164 1 78180 4117 0 11 590.289 1178.56 0 0 0 -1 0 0 0 0 5 64.8267
NaN NaN NaN 0 8205 937 9139 RDGGFQDGTVK _RDGGFQDGTVK_ P46955 179287
20150306_yeast3_Top_opt_2ug_C3_01_1665 + 3 MULTI CID TOF Unmodified diploid_02 9275
17.378 1 51702 1775 0 18 706.995 2117.96 0 0 0 -1 0 0 0 0 10 76.8261
NaN NaN NaN 0 8329 944 9265 ELEHENTVQEHEQNTGPK _ELEHENTVQEHEQNTGPK_ P25038
47886
20150306_yeast3_Top_opt_2ug_C3_01_1665 + 2 MULTI CID TOF Unmodified diploid_02 13616
24.155 1 118790 3720 0 11 664.804 1327.59 0 0 0 -1 0 0 0 0 13 90.7928
NaN NaN NaN 0 12429 1185 13603 DENISHEDEIK _DENISHEDEIK_ Q08649 22237
20150306_yeast3_Top_opt_2ug_C3_01_1665 + 2 MULTI CID TOF Unmodified diploid_02 17648
30.122 1 150890 6612 0 9 524.769 1047.52 0 0 0 -1 0 0 0 0 13 66.5676
NaN NaN NaN 0 16237 1409 17635 LVDDYAQPK _LVDDYAQPK_ P23797 138757
20150306_yeast3_Top_opt_2ug_C3_01_1665 + 3 MULTI CID TOF Unmodified diploid_02 18151
30.821 1 220420 7931 0 15 554.925 1661.75 0 0 0 -1 0 0 0 0 12 91.3551
NaN NaN NaN 0 16712 1437 18139 IDGQSAYHIDSAEEK _IDGQSAYHIDSAEEK_ P53892
91819
20150306_yeast3_Top_opt_2ug_C3_01_1665 + 3 MULTI CID TOF Unmodified diploid_02 20370
34.243 1 106660 2402 0 23 808.032 2421.08 0 0 0 -1 0 0 0 0 17 44.7397
NaN NaN NaN 0 18808 1560 20353 SGSSQIGESHSVDTVECSNNLSK

SGSSQIGESHSVDTVECSNNLSK P47041 190844
20150306_yeast3_Top_opt_2ug_C3_01_1665 + 3 MULTI CID TOF Unmodified diploid_02 21394
35.793 1 283080 13322 0 12 499.917 1496.73 0 0 0 -1 0 0 0 0 15 40.4755
NaN NaN NaN 0 19775 1617 21379 DDQVRSNPYIYK _DDQVRSNPYIYK_ Q07350 21288
20150306_yeast3_Top_opt_2ug_C3_01_1665 + 3 MULTI CID TOF Acetyl (Protein N-term) diploid_02
23230 38.504 1 178590 3445 0 20 762.682 2285.02 0 0 0 -1 0 0 0 0 15
50.2365 NaN NaN NaN 0 21509 1719 23215 MESTSGERSENIHEDQGIPK
(ac)MESTSGERSENIHEDQGIPK P20052 143005
20150306_yeast3_Top_opt_2ug_C3_01_1665 + 2 MULTI CID TOF Unmodified diploid_02 25380
41.642 1 143040 1461 0 13 669.316 1336.62 0 0 0 -1 0 0 0 0 5 68.9722
NaN NaN NaN 0 23539 1839 25375 LSAMVDDSAVDSK _LSAMVDDSAVDSK_ P32328 134228
20150306_yeast3_Top_opt_2ug_C3_01_1665 + 2 MULTI CID TOF Unmodified diploid_02 30355
48.66 1 192650 4602 0 15 910.902 1819.79 0 0 0 -1 0 0 0 0 12 95.0671
NaN NaN NaN 0 28238 2115 30343 FYDPDQPNNDPIEEK _FYDPDQPNNDPIEEK_ P22543
72105
20150306_yeast3_Top_opt_2ug_C3_01_1665 + 2 MULTI CID TOF Unmodified diploid_02 32505
51.774 1 245350 6966 0 10 596.814 1191.61 0 0 0 -1 0 0 0 0 2 148.28
NaN NaN NaN 0 30268 2235 32503 VNEYVNVDLK _VNEYVNVDLK_ P25358 232332
20150306_yeast3_Top_opt_2ug_C3_01_1665 + 3 MULTI CID TOF Unmodified diploid_02 33445
53.114 1 646590 20741 0 17 687.653 2059.94 0 0 0 -1 0 0 0 0 6 52.5197
NaN NaN NaN 0 31156 2287 33439 EVQEDEDPDVWDTRISK _EVQEDEDPDVWDTRISK_
Q05809 58963
20150306_yeast3_Top_opt_2ug_C3_01_1665 + 3 MULTI CID TOF Unmodified diploid_02 33989
53.851 1 248910 11783 0 22 838.38 2512.12 0 0 0 -1 0 0 0 0 10 119.412
NaN NaN NaN 0 31670 2317 33979 RDQQDAGHSDISDLDQYSSFTK
RDQQDAGHSDISDLDQYSSFTK P43601 179425
20150306_yeast3_Top_opt_2ug_C3_01_1665 + 3 MULTI CID TOF Unmodified diploid_02 37107
58.269 1 280800 2783 0 16 577.627 1729.86 0 0 0 -1 0 0 0 0 14 88.9514
NaN NaN NaN 0 34615 2490 37093 EFGTHVVSADEVLAEK _EFGTHVVSADEVLAEK_ P38204
42344
20150306_yeast3_Top_opt_2ug_C3_01_1665 + 5 MULTI CID TOF Unmodified diploid_02 38349
59.985 1 547800 5722 0 30 685.966 3424.79 0 0 0 -1 0 0 0 0 14 46.2517
NaN NaN NaN 0 35788 2559 38335 LEESESSTIPTTTPHPDLLQIQRHGELLRK
LEESESSTIPTTTPHPDLLQIQRHGELLRK P52868 118085
20150306_yeast3_Top_opt_2ug_C3_01_1665 + 3 MULTI CID TOF Unmodified diploid_02 38545
60.232 1 49372 1445 0 9 408.23 1221.67 0 0 0 -1 0 0 0 0 12 62.4631
NaN NaN NaN 0 35973 2570 38533 ILWHELFHK _ILWHELFHK_ P38352 99984
20150306_yeast3_Top_opt_2ug_C3_01_1665 + 2 MULTI CID TOF Unmodified diploid_02 39180
61.102 1 178920 1685 0 21 1071.5 2140.98 0 0 0 -1 0 0 0 0 17 66.4672
NaN NaN NaN 0 36573 2605 39163 GSGVYSLLNQEDSSTSSADPK
GSGVYSLLNQEDSSTSSADPK P20134 80491
20150306_yeast3_Top_opt_2ug_C3_01_1665 + 2 MULTI CID TOF Unmodified diploid_02 39303
61.287 1 144530 2876 0 9 547.786 1093.56 0 0 0 -1 0 0 0 0 14 68.657
NaN NaN NaN 0 36689 2612 39289 ATQFLNWSK _ATQFLNWSK_ Q01574 14997
20150306_yeast3_Top_opt_2ug_C3_01_1665 + 3 MULTI CID TOF Acetyl (Protein N-term) diploid_02
42474 65.951 1 222080 8908 0 26 1020.51 3058.5 0 0 0 -1 0 0 0 0 17
57.7376 NaN NaN NaN 0 39684 2788 42457 SNHSEILERPETPYDITYRVGVAENK
(ac)SNHSEILERPETPYDITYRVGVAENK P35182 196729
20150306_yeast3_Top_opt_2ug_C3_01_1665 + 2 MULTI CID TOF Unmodified diploid_02 44197
68.529 1 286920 8023 0 14 759.882 1517.75 0 0 0 -1 0 0 0 0 12 67.136
NaN NaN NaN 0 41311 2884 44185 LSEGSDVDVDELIK _LSEGSDVDVDELIK_ Q03081 134647
20150306_yeast3_Top_opt_2ug_C3_01_1665 + 2 MULTI CID TOF Unmodified diploid_02 44935
69.579 1 746150 38070 0 14 840.914 1679.81 0 0 0 -1 0 0 0 0 12 55.5313
NaN NaN NaN 0 42008 2925 44923 FINQDINDQDFALK _FINQDINDQDFALK_ P43122 65994

20150306_yeast3_Top_opt_2ug_C3_01_1665 + 3 MULTI CID TOF Unmodified diploid_02 45866
70.842 1 330840 15102 0 12 498.904 1493.69 0 0 0 -1 0 0 0 0 7 45.2798
NaN NaN NaN 0 42887 2977 45859 DIYNQLHMFGEK _DIYNQLHMFGEK_ P43598 26609
20150306_yeast3_Top_opt_2ug_C3_01_1665 + 4 MULTI CID TOF Unmodified diploid_02 47280
72.782 1 378370 2623 0 23 717.119 2864.45 0 0 0 -1 0 0 0 0 17 44.3413
NaN NaN NaN 0 44223 3055 47263 EEYYRLQGLSEEDWEPVRVARLK
EEYYRLQGLSEEDWEPVRVARLK P47081 42122
20150306_yeast3_Top_opt_2ug_C3_01_1665 + 3 MULTI CID TOF Unmodified diploid_02 47513
73.11 1 452410 25932 0 13 553.609 1657.81 0 0 0 -1 0 0 0 0 16 103.975
NaN NaN NaN 0 44443 3068 47497 PLETIDLWEDHYK _PLETIDLWEDHYK_ Q02887 164966
20150306_yeast3_Top_opt_2ug_C3_01_1665 + 2 MULTI CID TOF Unmodified diploid_02 47568
73.178 1 300600 4687 0 15 807.896 1613.78 0 0 0 -1 0 0 0 0 17 89.3114
NaN NaN NaN 0 44495 3071 47551 PGDEINDLIDSGEIK _PGDEINDLIDSGEIK_ Q06177 162578
20150306_yeast3_Top_opt_2ug_C3_01_1665 + 2 MULTI CID TOF Unmodified diploid_02 49471
75.996 1 253300 6208 0 23 1147.11 2292.2 0 0 0 -1 0 0 0 0 12 123.322
NaN NaN NaN 0 46292 3177 49459 SLQDLGVTSAAPVPDINAPQTAK
SLQDLGVTSAAPVPDINAPQTAK P53283 195290
20150306_yeast3_Top_opt_2ug_C3_01_1665 + 2 MULTI CID TOF Unmodified diploid_02 53596
82.238 1 298540 4652 0 16 890.954 1779.89 0 0 0 -1 0 0 0 0 15 50.3923
NaN NaN NaN 0 50188 3406 53581 PIDVAQTFEVGNIYSK _PIDVAQTFEVGNIYSK_ Q04749
163947
20150306_yeast3_Top_opt_2ug_C3_01_1665 + 3 MULTI CID TOF Acetyl (Protein N-term) diploid_02
53738 82.435 1 197010 5835 0 33 1206.88 3617.63 0 0 0 -1 0 0 0 0 13
48.798 NaN NaN NaN 0 50322 3414 53725 SGPPPPYEEQSSHLYGQPASSQDGNAFIPEDFK
(ac)SGPPPPYEEQSSHLYGQPASSQDGNAFIPEDFK P48558 190713
20150306_yeast3_Top_opt_2ug_C3_01_1665 + 3 MULTI CID TOF Unmodified diploid_02 53956
82.76 1 235950 1446 0 28 1048.49 3142.46 0 0 0 -1 0 0 0 0 15 57.2098
NaN NaN NaN 0 50528 3426 53941 FLQSENFSDLTSEWDQSRSNTPGLAEGK
FLQSENFSDLTSEWDQSRSNTPGLAEGK P53124 67188
20150306_yeast3_Top_opt_2ug_C3_01_1665 + 2 MULTI CID TOF Unmodified diploid_02 54169
83.056 1 144170 6160 0 12 562.874 1123.73 0 0 0 -1 0 0 0 0 12 61.3438
NaN NaN NaN 0 50729 3438 54157 VIIIGAGIAGLK _VIIIGAGIAGLK_P50264 228621
20150306_yeast3_Top_opt_2ug_C3_01_1665 + 3 MULTI CID TOF Unmodified diploid_02 54334
83.291 1 348930 3203 0 22 830.414 2488.22 0 0 0 -1 0 0 0 0 15 48.2444
NaN NaN NaN 0 50885 3447 54319 DIDGIRVALPQMTRNVNNNDFS
DIDGIRVALPQMTRNVNNNDFS Q12184 25084
20150306_yeast3_Top_opt_2ug_C3_01_1665 + 2 MULTI CID TOF Unmodified diploid_02 55357
84.717 1 140140 2972 0 10 599.329 1196.64 0 0 0 -1 0 0 0 0 12 101.817
NaN NaN NaN 0 51851 3504 55345 LSNELYAIFK _LSNELYAIFK_ P40858 135676
20150306_yeast3_Top_opt_2ug_C3_01_1665 + 3 MULTI CID TOF Unmodified diploid_02 56708
86.616 1 359390 4695 0 17 683.7 2048.08 0 0 0 -1 0 0 0 0 13 41.9121
NaN NaN NaN 0 53127 3579 56695 EVEILEHQLNELIVQDK _EVEILEHQLNELIVQDK_ Q02888
58121
20150306_yeast3_Top_opt_2ug_C3_01_1665 + 3 MULTI CID TOF Unmodified diploid_02 57313
87.51 1 270010 3633 0 22 853.099 2556.28 0 0 0 -1 0 0 0 0 6 39.2097
NaN NaN NaN 0 53698 3613 57307 PSTRYSLELFGPYVEDQSIEVK
PSTRYSLELFGPYVEDQSIEVK P38298 168344
20150306_yeast3_Top_opt_2ug_C3_01_1665 + 4 MULTI CID TOF Unmodified diploid_02 57429
87.671 1 708240 20405 0 32 912.18 3644.69 0 0 0 -1 0 0 0 0 14 32.4955
NaN NaN NaN 0 53808 3619 57415 ATCSFEGCHSAVITINDDNIINLPEQVHSEFK
ATCSFEGCHSAVITINDDNIINLPEQVHSEFK P32601 14344
20150306_yeast3_Top_opt_2ug_C3_01_1665 + 3 MULTI CID TOF Unmodified diploid_02 57733
88.125 1 419000 8174 0 21 798.081 2391.22 0 0 0 -1 0 0 0 0 12 44.5409
NaN NaN NaN 0 54095 3636 57721 FQYDIPGLGYLENNPGRPITK _FQYDIPGLGYLENNPGRPITK_

Q12146	69319
20150306_yeast3_Top_opt_2ug_C3_01_1665	+	2	MULTI	CID	TOF	Acetyl (Protein N-term)	diploid_02
61986	94.421	1	782090	45787	0	21	1038.47	2074.93	0	0	0	-1	0	0	0	0	17
97.4899	NaN	NaN	NaN	0	58112	3872	61969	MDGMFAMPGAAAGAASPQQPK
(ac)MDGMFAMPGAAAGAASPQQPK	P33448	142088
20150306_yeast3_Top_opt_2ug_C3_01_1665	+	2	MULTI	CID	TOF	Unmodified	diploid_02	64601
98.597	1	467310	5554	0	16	898.955	1795.9	0	0	0	-1	0	0	0	0	4	38.2606
NaN	NaN	NaN	0	60581	4018	64597	DAGEIQTEYGVFNLIK	_DAGEIQTEYGVFNLIK_	Q12488
19524
20150306_yeast3_Top_opt_2ug_C3_01_1665	+	3	MULTI	CID	TOF	Unmodified	diploid_02	68046
104.01	1	617310	6690	0	26	937.145	2808.41	0	0	0	-1	0	0	0	0	11	72.1583
NaN	NaN	NaN	0	63835	4209	68035	GLPIGTSYSLEYLGPIVNTQVDDETK
GLPIGTSYSLEYLGPIVNTQVDDETK	Q02896	77833
20150306_yeast3_Top_opt_2ug_C3_01_1665	+	2	MULTI	CID	TOF	Unmodified	diploid_02	69992
105.64	1	902020	39189	0	19	1014.56	2027.11	0	0	0	-1	0	0	0	0	13	153.25
NaN	NaN	NaN	0	65673	4317	69979	SIQDIVGLATLIVNTDVEK	_SIQDIVGLATLIVNTDVEK_
P32342	193089
20150306_yeast3_Top_opt_2ug_C3_01_1667	+	2	MULTI	CID	TOF	Unmodified	diploid_03	5773
11.621	1	184310	21349	0	19	980.952	1959.89	0	0	0	-1	0	0	0	0	12	189.149
NaN	NaN	NaN	0	4995	776	5761	SSISNTSDHDGANRASDVK	_SSISNTSDHDGANRASDVK_
Q03559	200647
20150306_yeast3_Top_opt_2ug_C3_01_1667	+	2	MULTI	CID	TOF	Unmodified	diploid_03	8095
15.401	1	20600	1186	0	17	918.38	1834.75	0	0	0	-1	0	0	0	0	12	45.2206
NaN	NaN	NaN	0	7188	905	8083	QDSNDTSDSPQDDQVGK	_QDSNDTSDSPQDDQVGK_	Q03687
171784
20150306_yeast3_Top_opt_2ug_C3_01_1667	+	4	MULTI	CID	TOF	Unmodified	diploid_03	11644
21.171	1	136420	11362	0	19	527.018	2104.04	0	0	0	-1	0	0	0	0	15	64.5215
NaN	NaN	NaN	0	10540	1102	11629	SESHPPNHPHSQILQTPAK	_SESHPPNHPHSQILQTPAK_
P40021	189023
20150306_yeast3_Top_opt_2ug_C3_01_1667	+	2	MULTI	CID	TOF	Unmodified	diploid_03	15796
27.248	1	99108	1374	0	11	651.332	1300.65	0	0	0	-1	0	0	0	0	9	84.4793
NaN	NaN	NaN	0	14461	1333	15787	VLEEQEEVAQK	_VLEEQEEVAQK_	P21192	230133
20150306_yeast3_Top_opt_2ug_C3_01_1667	+	3	MULTI	CID	TOF	Unmodified	diploid_03	17010
29.067	1	168290	5018	0	17	584.96	1751.86	0	0	0	-1	0	0	0	0	17	73.0929
NaN	NaN	NaN	0	15608	1400	16993	SDSIGLSSSNTTNSVRK	_SDSIGLSSSNTTNSVRK_	P18494
187551
20150306_yeast3_Top_opt_2ug_C3_01_1667	+	2	MULTI	CID	TOF	Unmodified	diploid_03	17185
29.316	1	102520	1009	0	13	712.817	1423.62	0	0	0	-1	0	0	0	0	12	81.6249
NaN	NaN	NaN	0	15773	1410	17173	ETEAMNSEIDGTK	_ETEAMNSEIDGTK_	Q03818	56796
20150306_yeast3_Top_opt_2ug_C3_01_1667	+	3	MULTI	CID	TOF	Unmodified	diploid_03	20339
33.919	1	181190	9255	0	16	609.311	1824.91	0	0	0	-1	0	0	0	0	16	48.8985
NaN	NaN	NaN	0	18752	1585	20323	IQEVTPQLHETNTSTK	_IQEVTPQLHETNTSTK_	P53227
102532
20150306_yeast3_Top_opt_2ug_C3_01_1667	+	2	MULTI	CID	TOF	Unmodified	diploid_03	20910
34.753	1	112090	2487	0	13	700.316	1398.62	0	0	0	-1	0	0	0	0	11	84.2973
NaN	NaN	NaN	0	19291	1617	20899	DPSISDSTSYAEK	_DPSISDSTSYAEK_	P35735	30605
20150306_yeast3_Top_opt_2ug_C3_01_1667	+	4	MULTI	CID	TOF	Unmodified	diploid_03	21653
35.868	1	181920	1906	0	27	742.855	2967.39	0	0	0	-1	0	0	0	0	16	70.7178
NaN	NaN	NaN	0	19993	1658	21637	INHRGEDVTGQQIDLNNSEGNENSVTK
INHRGEDVTGQQIDLNNSEGNENSVTK	P35208	100793
20150306_yeast3_Top_opt_2ug_C3_01_1667	+	2	MULTI	CID	TOF	Unmodified	diploid_03	24746
40.301	1	161780	2842	0	14	812.379	1622.74	0	0	0	-1	0	0	0	0	13	80.3867
NaN	NaN	NaN	0	22914	1830	24733	EGQEEGYNEQLISK	_EGQEEGYNEQLISK_	Q08970	43838
20150306_yeast3_Top_opt_2ug_C3_01_1667	+	2	MULTI	CID	TOF	Unmodified	diploid_03	24864

40.461 1 106160 8377 0 8 476.225 950.436 0 0 0 -1 0 0 0 0 5 72.23
NaN NaN NaN 0 23025 1837 24859 SLWDSSEK _SLWDSSEK_ P37298 195898
20150306_yeast3_Top_opt_2ug_C3_01_1667 + 2 MULTI CID TOF Unmodified diploid_03 24936
40.558 1 129760 4316 0 7 487.737 973.46 0 0 0 -1 0 0 0 0 5 81.7839
NaN NaN NaN 0 23093 1841 24931 YMQEYLK _YMQEYLK_Q3E731 247761
20150306_yeast3_Top_opt_2ug_C3_01_1667 + 3 MULTI CID TOF Unmodified diploid_03 24939
40.562 1 136080 3410 0 21 765.368 2293.08 0 0 0 -1 0 0 0 0 8 58.107
NaN NaN NaN 0 23096 1841 24931 SESGVTYRQNASVDPELDQAK
SESGVTYRQNASVDPELDQAK Q03718 189022
20150306_yeast3_Top_opt_2ug_C3_01_1667 + 2 MULTI CID TOF Unmodified diploid_03 25324
41.111 1 328380 5790 0 10 579.328 1156.64 0 0 0 -1 0 0 0 0 15 58.6993
NaN NaN NaN 0 23460 1862 25309 LLQVSQVENK _LLQVSQVENK_ P32459 127753
20150306_yeast3_Top_opt_2ug_C3_01_1667 + 2 MULTI CID TOF Unmodified diploid_03 25913
41.922 1 183180 1809 0 14 687.37 1372.73 0 0 0 -1 0 0 0 0 10 49.9877
NaN NaN NaN 0 24016 1895 25903 LLGNVGNSSNTGIK _LLGNVGNSSNTGIK_ Q12128
126206
20150306_yeast3_Top_opt_2ug_C3_01_1667 + 2 MULTI CID TOF Unmodified diploid_03 29502
46.805 1 177440 1219 0 13 714.395 1426.78 0 0 0 -1 0 0 0 0 17 67.0193
NaN NaN NaN 0 27406 2094 29485 VGGIEDRQLEALK _VGGIEDRQLEALK_ Q06549 226981
20150306_yeast3_Top_opt_2ug_C3_01_1667 + 2 MULTI CID TOF Unmodified diploid_03 31422
49.523 1 323740 6058 0 11 612.314 1222.61 0 0 0 -1 0 0 0 0 11 103.909
NaN NaN NaN 0 29219 2201 31411 DHGVEDPLTLK _DHGVEDPLTLK_ Q03246 24601
20150306_yeast3_Top_opt_2ug_C3_01_1667 + 3 MULTI CID TOF Unmodified diploid_03 31528
49.687 1 305630 4814 0 14 527.282 1578.82 0 0 0 -1 0 0 0 0 9 91.812
NaN NaN NaN 0 29319 2207 31519 LDTLNEPSAHLIEK _LDTLNEPSAHLIEK_ P39552;P53176
117194
20150306_yeast3_Top_opt_2ug_C3_01_1667 + 3 MULTI CID TOF Unmodified diploid_03 33688
52.737 1 47723 1868 0 9 354.21 1059.61 0 0 0 -1 0 0 0 0 9 82.7505
NaN NaN NaN 0 31359 2327 33679 PLDFLRTAK _PLDFLRTAK_ P36533 164757
20150306_yeast3_Top_opt_2ug_C3_01_1667 + 2 MULTI CID TOF Unmodified diploid_03 35236
54.866 1 410370 38063 0 14 772.884 1543.75 0 0 0 -1 0 0 0 0 9 44.9254
NaN NaN NaN 0 32821 2413 35227 TLSGNDYINASYVK _TLSGNDYINASYVK_ P25044
214804
20150306_yeast3_Top_opt_2ug_C3_01_1667 + 2 MULTI CID TOF Unmodified diploid_03 37821
58.459 1 189050 1509 0 11 643.331 1284.65 0 0 0 -1 0 0 0 0 2 47.7121
NaN NaN NaN 0 35262 2557 37819 VGQPAFFYQTK _VGQPAFFYQTK_ P40857 227346
20150306_yeast3_Top_opt_2ug_C3_01_1667 + 2 MULTI CID TOF Unmodified diploid_03 40566
62.135 1 427990 6691 0 12 644.851 1287.69 0 0 0 -1 0 0 0 0 11 46.0691
NaN NaN NaN 0 37855 2709 40555 LQVNGITMADVK _LQVNGITMADVK_ P25454 133458
20150306_yeast3_Top_opt_2ug_C3_01_1667 + 3 MULTI CID TOF Unmodified diploid_03 42709
65.2 1 99331 2007 0 13 491.276 1470.81 0 0 0 -1 0 0 0 0 12 58.32 NaN
NaN NaN 0 39879 2828 42697 DDSSLNITPRLLK _DDSSLNITPRLLK_ P32611 21336
20150306_yeast3_Top_opt_2ug_C3_01_1667 + 2 MULTI CID TOF Unmodified diploid_03 42716
65.213 1 456950 47980 0 10 591.296 1180.58 0 0 0 -1 0 0 0 0 1 79.3106
NaN NaN NaN 0 39885 2829 42715 DGVYENIPFK _DGVYENIPFK_ P04039 24430
20150306_yeast3_Top_opt_2ug_C3_01_1667 + 2 MULTI CID TOF Acetyl (Protein N-term) diploid_03
43088 65.756 1 272590 4391 0 13 729.402 1456.79 0 0 0 -1 0 0 0 0 13
60.5499 NaN NaN NaN 0 40237 2849 43075 SRVAQLDSIALDK _(ac)SRVAQLDSIALDK_
P32800 199950
20150306_yeast3_Top_opt_2ug_C3_01_1667 + 3 MULTI CID TOF Unmodified diploid_03 43827
66.889 1 394860 3409 0 18 699.037 2094.09 0 0 0 -1 0 0 0 0 14 39.91
NaN NaN NaN 0 40935 2890 43813 HLSTLPNVPWPNRSFTTK _HLSTLPNVPWPNRSFTTK_
Q8TGM887085
20150306_yeast3_Top_opt_2ug_C3_01_1667 + 3 MULTI CID TOF Unmodified diploid_03 44346

67.659 1 337320 6521 0 16 574.961 1721.86 0 0 0 -1 0 0 0 0 11 130.717
NaN NaN NaN 0 41425 2919 44335 FLSSLEDSSAHAAFLK _FLSSLEDSSAHAAFLK_ P36167
67341
20150306_yeast3_Top_opt_2ug_C3_01_1667 + 3 MULTI CID TOF Unmodified diploid_03 44772
68.273 1 286210 4525 0 22 798.743 2393.21 0 0 0 -1 0 0 0 0 5 70.9935
NaN NaN NaN 0 41827 2943 44767 NDNVESLNSSVRGIWSPLHAAK
NDNVESLNSSVRGIWSPLHAAK Q06090 148781
20150306_yeast3_Top_opt_2ug_C3_01_1667 + 2 MULTI CID TOF Unmodified diploid_03 47717
72.32 1 113780 4910 0 9 495.271 988.528 0 0 0 -1 0 0 0 0 16 66.6919
NaN NaN NaN 0 44609 3106 47701 DDVMLGVLK _DDVMLGVLK_ P43592 21429
20150306_yeast3_Top_opt_2ug_C3_01_1667 + 3 MULTI CID TOF Unmodified diploid_03 49948
75.47 1 407960 3084 0 17 667.345 1999.01 0 0 0 -1 0 0 0 0 15 47.6798
NaN NaN NaN 0 46716 3230 49933 LIEPRLALNAVCQMDEK _LIEPRLALNAVCQMDEK_
Q12069 123353
20150306_yeast3_Top_opt_2ug_C3_01_1667 + 2 MULTI CID TOF Acetyl (Protein N-term) diploid_03
53076 80.047 1 411070 4607 0 17 905.92 1809.82 0 0 0 -1 0 0 0 0 11
125.975 NaN NaN NaN 0 49670 3404 53065 MFSLSSLSSSGGHSEQK
(ac)MFSLSSLSSSGGHSEQK P53244 143312
20150306_yeast3_Top_opt_2ug_C3_01_1667 + 3 MULTI CID TOF Unmodified diploid_03 56113
84.456 1 362090 4069 0 28 1032.79 3095.35 0 0 0 -1 0 0 0 0 6 63.0948
NaN NaN NaN 0 52538 3573 56107 ECSIDWEDVYPGTGLQASTMVGDDVHSK
ECSIDWEDVYPGTGLQASTMVGDDVHSK P38293 38614
20150306_yeast3_Top_opt_2ug_C3_01_1667 + 2 MULTI CID TOF Unmodified diploid_03 57129
85.901 1 375510 3630 0 20 1097.54 2193.06 0 0 0 -1 0 0 0 0 14 48.6162
NaN NaN NaN 0 53498 3629 57115 ISDVSGIYPSGNIVMERLDE _ISDVSGIYPSGNIVMERLDE_
Q12229 103865
20150306_yeast3_Top_opt_2ug_C3_01_1667 + 3 MULTI CID TOF Unmodified diploid_03 57706
86.731 1 567980 4850 0 23 869.75 2606.23 0 0 0 -1 0 0 0 0 15 82.8854
NaN NaN NaN 0 54043 3661 57691 RFLNPDAMLLMETEGMNQASNPK
RFLNPDAMLLMETEGMNQASNPK Q06543 180217
20150306_yeast3_Top_opt_2ug_C3_01_1667 + 3 MULTI CID TOF Unmodified diploid_03 65689
98.657 1 488430 3370 0 28 1018.19 3051.53 0 0 0 -1 0 0 0 0 6 80.6641
NaN NaN NaN 0 61582 4105 65683 FVTEEDVIFEPVSAQSAIARSLETTANK
FVTEEDVIFEPVSAQSAIARSLETTANK P40219 71825
20150306_yeast3_Top_opt_2ug_C3_01_1667 + 2 MULTI CID TOF Unmodified diploid_03 65867
98.919 1 147240 4845 0 11 659.363 1316.71 0 0 0 -1 0 0 0 0 4 49.4481
NaN NaN NaN 0 61750 4115 65863 WDIVLSNMLVK _WDIVLSNMLVK_ Q96VH5 240452
20150306_yeast3_Top_opt_2ug_C3_01_1667 + 2 MULTI CID TOF Unmodified diploid_03 71001
105.68 1 465660 24392 0 15 849.492 1696.97 0 0 0 -1 0 0 0 0 8 75.5351
NaN NaN NaN 0 66599 4400 70993 SAIDVIQWLIPTITK _SAIDVIQWLIPTITK_ P40532 185743
20150306_yeast3_Top_opt_2ug_C3_01_1669 + 2 MULTI CID TOF Unmodified diploid_04 9243
17.177 1 76281 958 0 10 581.753 1161.49 0 0 0 -1 0 0 0 0 17 93.3246 NaN
NaN NaN 0 8313 928 9226 PNCGLCDQAK _PNCGLCDQAK_ Q05530 166585
20150306_yeast3_Top_opt_2ug_C3_01_1669 + 3 MULTI CID TOF Unmodified diploid_04 10238
18.725 1 139660 15054 0 17 611.287 1830.84 0 0 0 -1 0 0 0 0 4 47.0818
NaN NaN NaN 0 9252 984 10234 TSTDPDTSANTNHTLEK _TSTDPDTSANTNHTLEK_ P38140
218933
20150306_yeast3_Top_opt_2ug_C3_01_1669 + 2 MULTI CID TOF Unmodified diploid_04 10890
19.787 1 102560 3395 0 8 493.772 985.529 0 0 0 -1 0 0 0 0 8 89.3546
NaN NaN NaN 0 9868 1020 10882 YVHETPLK _YVHETPLK_ Q12428 250130
20150306_yeast3_Top_opt_2ug_C3_01_1669 + 2 MULTI CID TOF Unmodified diploid_04 11493
20.73 1 196050 5414 0 10 525.766 1049.52 0 0 0 -1 0 0 0 0 17 58.6986
NaN NaN NaN 0 10438 1053 11476 STTQVGCGIK _STTQVGCGIK_ P47032 203040
20150306_yeast3_Top_opt_2ug_C3_01_1669 + 2 MULTI CID TOF Unmodified diploid_04 12842

22.732 1 47733 2130 0 18 923.896 1845.78 0 0 0 -1 0 0 0 0 16 55.3373
NaN NaN NaN 0 11712 1128 12826 GDGNDDDNNTVTANPTAR _GDGNDDDNNTVTANPTAR_
P47087 73343
20150306_yeast3_Top_opt_2ug_C3_01_1669 + 2 MULTI CID TOF Unmodified diploid_04 15161
26.124 1 91000 9231 0 17 886.425 1770.84 0 0 0 -1 0 0 0 0 13 39.364
NaN NaN NaN 0 13902 1257 15148 GVGSSTEVVHNSVENEK _GVGSSTEVVHNSVENEK_
P24869 82555
20150306_yeast3_Top_opt_2ug_C3_01_1669 + 3 MULTI CID TOF Unmodified diploid_04 15707
26.936 1 439810 49918 0 12 492.593 1474.76 0 0 0 -1 0 0 0 0 1 186.159
NaN NaN NaN 0 14417 1288 15706 RMESDAEILRQK _RMESDAEILRQK_ Q3E7B7 182641
20150306_yeast3_Top_opt_2ug_C3_01_1669 + 2 MULTI CID TOF Unmodified diploid_04 18530
31.04 1 126220 1606 0 10 624.257 1246.5 0 0 0 -1 0 0 0 0 16 81.0165
NaN NaN NaN 0 17084 1444 18514 YDFEDENTSK _YDFEDENTSK_ Q03795 243031
20150306_yeast3_Top_opt_2ug_C3_01_1669 + 2 MULTI CID TOF Unmodified diploid_04 18972
31.708 1 131510 5588 0 10 578.32 1154.63 0 0 0 -1 0 0 0 0 8 50.1076
NaN NaN NaN 0 17501 1469 18964 TVVEPQNLQK _TVVEPQNLQK_ P53150 221423
20150306_yeast3_Top_opt_2ug_C3_01_1669 + 2 MULTI CID TOF Unmodified diploid_04 24236
39.214 1 136760 1660 0 12 702.841 1403.67 0 0 0 -1 0 0 0 0 16 54.6504
NaN NaN NaN 0 22473 1761 24220 FDVNTPQQVNDK _FDVNTPQQVNDK_ P47090 62935
20150306_yeast3_Top_opt_2ug_C3_01_1669 + 2 MULTI CID TOF Unmodified diploid_04 27583
44.021 1 150300 3665 0 19 994.474 1986.93 0 0 0 -1 0 0 0 0 15 54.8342
NaN NaN NaN 0 25634 1947 27568 QNEIPGGDYTPGNSPSVQK _QNEIPGGDYTPGNSPSVQK_
P40955 175362
20150306_yeast3_Top_opt_2ug_C3_01_1669 + 3 MULTI CID TOF Unmodified diploid_04 28120
44.762 1 188220 3406 0 23 892.704 2675.09 0 0 0 -1 0 0 0 0 12 153.562
NaN NaN NaN 0 26141 1977 28108 SSSTCDSLNQVTCYCEHENSAVK
SSSTCDSLNQVTCYCEHENSAVK O13547 201351
20150306_yeast3_Top_opt_2ug_C3_01_1669 + 4 MULTI CID TOF Unmodified diploid_04 30012
47.337 1 297360 3421 0 23 671.344 2681.35 0 0 0 -1 0 0 0 0 14 62.193
NaN NaN NaN 0 27928 2082 29998 PNPSYDVENVNSRNQLVEPNRIK
PNPSYDVENVNSRNQLVEPNRIK P32585 166749
20150306_yeast3_Top_opt_2ug_C3_01_1669 + 2 MULTI CID TOF Unmodified diploid_04 30041
47.379 1 160250 3752 0 8 479.765 957.516 0 0 0 -1 0 0 0 0 7 72.23
NaN NaN NaN 0 27955 2084 30034 NEIDQLVK _NEIDQLVK_ Q06091 149445
20150306_yeast3_Top_opt_2ug_C3_01_1669 + 2 MULTI CID TOF Unmodified diploid_04 31018
48.693 1 184090 7330 0 18 1079.94 2157.86 0 0 0 -1 0 0 0 0 12 120.9
NaN NaN NaN 0 28878 2138 31006 ANEIYEQSDCSSMGNYYK _ANEIYEQSDCSSMGNYYK_
P36163 10482
20150306_yeast3_Top_opt_2ug_C3_01_1669 + 2 MULTI CID TOF Unmodified diploid_04 32550
50.862 1 194490 4480 0 11 563.301 1124.59 0 0 0 -1 0 0 0 0 14 71.5242
NaN NaN NaN 0 30325 2223 32536 LLSSMGYVAGK _LLSSMGYVAGK_ Q06411 128053
20150306_yeast3_Top_opt_2ug_C3_01_1669 + 3 MULTI CID TOF Unmodified diploid_04 33216
51.783 1 245260 2465 0 22 833.054 2496.14 0 0 0 -1 0 0 0 0 14 52.6567
NaN NaN NaN 0 30954 2260 33202 ESNSNEDFNVDGNETTLQRISK
ESNSNEDFNVDGNETTLQRISK Q04835 55986
20150306_yeast3_Top_opt_2ug_C3_01_1669 + 4 MULTI CID TOF Unmodified diploid_04 38786
59.546 1 259440 3921 0 27 748.367 2989.44 0 0 0 -1 0 0 0 0 4 144.563
NaN NaN NaN 0 36214 2570 38782 PMPLSTDPSYVSSSNTLSSEHELRVEK
PMPLSTDPSYVSSSNTLSSEHELRVEK P32047 166363
20150306_yeast3_Top_opt_2ug_C3_01_1669 + 2 MULTI CID TOF Unmodified diploid_04 38976
59.802 1 127040 8038 0 8 451.288 900.562 0 0 0 -1 0 0 0 0 14 84.7431
NaN NaN NaN 0 36394 2580 38962 LLLQLSSK _LLLQLSSK_ P50104 126632
20150306_yeast3_Top_opt_2ug_C3_01_1669 + 2 MULTI CID TOF Unmodified diploid_04 39084
59.937 1 307770 1639 0 14 818.911 1635.81 0 0 0 -1 0 0 0 0 14 56.514

NaN NaN NaN 0 36496 2586 39070 LQSLQEALNYSNEK _LQSLQEALNYSNEK_ P52919
133242
20150306_yeast3_Top_opt_2ug_C3_01_1669 + 3 MULTI CID TOF Unmodified diploid_04 39547
60.559 1 455030 7446 0 15 497.626 1489.86 0 0 0 -1 0 0 0 0 9 58.0449
NaN NaN NaN 0 36933 2612 39538 TPIGVASLAQVHVAK _TPIGVASLAQVHVAK_ Q06567
216508
20150306_yeast3_Top_opt_2ug_C3_01_1669 + 2 MULTI CID TOF Unmodified diploid_04 41183
62.801 1 67583 6498 0 8 443.783 885.551 0 0 0 -1 0 0 0 0 7 91.318
NaN NaN NaN 0 38478 2703 41176 LIGLLTEK _LIGLLTEK_ P53202 123495
20150306_yeast3_Top_opt_2ug_C3_01_1669 + 2 MULTI CID TOF Unmodified diploid_04 43157
65.601 1 1385500 221500 0 8 466.284 930.554 0 0 0 -1 0 0 0 0 1 128.566
NaN NaN NaN 0 40342 2813 43156 LVSLPFQK _LVSLPFQK_ P33331 140099
20150306_yeast3_Top_opt_2ug_C3_01_1669 + 3 MULTI CID TOF Unmodified diploid_04 44841
68.052 1 389650 3593 0 18 716.32 2145.94 0 0 0 -1 0 0 0 0 11 64.7047
NaN NaN NaN 0 41933 2906 44830 ELHDLYLDCGSNYFASDK _ELHDLYLDCGSNYFASDK_
Q06251 48492
20150306_yeast3_Top_opt_2ug_C3_01_1669 + 3 MULTI CID TOF Unmodified diploid_04 45421
68.849 1 484600 7312 0 23 745.711 2234.11 0 0 0 -1 0 0 0 0 15 41.703
NaN NaN NaN 0 42481 2938 45406 QVPSAELEPAGDGGLAGPVTVRD
QVPSAELEPAGDGGLAGPVTVRD Q07651 178002
20150306_yeast3_Top_opt_2ug_C3_01_1669 + 2 MULTI CID TOF Unmodified diploid_04 46128
69.835 1 339930 7726 0 17 923.927 1845.84 0 0 0 -1 0 0 0 0 2 142.324
NaN NaN NaN 0 43148 2978 46126 EVDDVLGGGWDNVDQTK _EVDDVLGGGWDNVDQTK_
Q04307 57885
20150306_yeast3_Top_opt_2ug_C3_01_1669 + 2 MULTI CID TOF Acetyl (Protein N-term) diploid_04
51578 77.521 1 373580 5237 0 13 753.87 1505.73 0 0 0 -1 0 0 0 0 16
40.9413 NaN NaN NaN 0 48296 3280 51562 SSTVISLAHFCDK _(ac)SSTVISLAHFCDK_
P53237 201492
20150306_yeast3_Top_opt_2ug_C3_01_1669 + 2 MULTI CID TOF Unmodified diploid_04 52101
78.348 1 294470 6391 0 12 655.395 1308.78 0 0 0 -1 0 0 0 0 17 60.6413
NaN NaN NaN 0 48790 3309 52084 LTLPVNLVQNAK _LTLPVNLVQNAK_ P32491 137972
20150306_yeast3_Top_opt_2ug_C3_01_1669 + 2 MULTI CID TOF Unmodified diploid_04 53127
79.882 1 180940 1993 0 11 646.347 1290.68 0 0 0 -1 0 0 0 0 17 68.5357
NaN NaN NaN 0 49759 3366 53110 SIDVNELNFIK _SIDVNELNFIK_ P39722 192204
20150306_yeast3_Top_opt_2ug_C3_01_1669 + 3 MULTI CID TOF Unmodified diploid_04 55439
83.111 1 414580 2940 0 28 1049.83 3146.48 0 0 0 -1 0 0 0 0 7 50.8649
NaN NaN NaN 0 51942 3495 55432 DITRVDETAELVQADPDIQLYDAEANDK
DITRVDETAELVQADPDIQLYDAEANDK P33413 26406
20150306_yeast3_Top_opt_2ug_C3_01_1669 + 2 MULTI CID TOF Unmodified diploid_04 58481
87.369 1 162480 5909 0 11 564.354 1126.69 0 0 0 -1 0 0 0 0 7 96.6043
NaN NaN NaN 0 54815 3664 58474 PISVILSALSK _PISVILSALSK_ P47085 164454
20150306_yeast3_Top_opt_2ug_C3_01_1669 + 3 MULTI CID TOF Unmodified diploid_04 59974
89.53 1 175860 7336 0 17 636.996 1907.97 0 0 0 -1 0 0 0 0 6 106.377
NaN NaN NaN 0 56225 3747 59968 APDFVESNTIFNLNTVK _APDFVESNTIFNLNTVK_ P00401
11072
20150306_yeast3_Top_opt_2ug_C3_01_1669 + 3 MULTI CID TOF Unmodified diploid_04 60819
90.777 1 413430 8493 0 22 813.791 2438.35 0 0 0 -1 0 0 0 0 5 70.3446
NaN NaN NaN 0 57023 3794 60814 PLILTPPQLANQQQPPQDILSK
PLILTPPQLANQQQPPQDILSK P38956 165272
20150306_yeast3_Top_opt_2ug_C3_01_1669 + 3 MULTI CID TOF Acetyl (Protein N-term) diploid_04
61585 91.879 1 182380 5134 0 18 710.01 2127.01 0 0 0 -1 0 0 0 0 15
102.085 NaN NaN NaN 0 57747 3836 61570 ASIDAFSDLERRMDGFQK
(ac)ASIDAFSDLERRMDGFQK P40014 13677
20150306_yeast3_Top_opt_2ug_C3_01_1669 + 3 MULTI CID TOF Acetyl (Protein N-term) diploid_04

63019 94.027 1 323090 12773 0 21 811.427 2431.26 0 0 0 -1 0 0 0 0 9
124.076 NaN NaN NaN 0 59101 3916 63010 MENPHEQVQANILSRIIGNVK
(ac)MENPHEQVQANILSRIIGNVK P69851 142824
20150306_yeast3_Top_opt_2ug_C3_01_1669 + 3 MULTI CID TOF Unmodified diploid_04 68127
101.82 1 471690 10132 0 21 731.737 2192.19 0 0 0 -1 0 0 0 0 5 80.1418
NaN NaN NaN 0 63925 4200 68122 VVYSGVFPPYTVPVGVLSGQK
VVYSGVFPPYTVPVGVLSGQK P13130 239284
20150306_yeast3_Top_opt_2ug_C3_01_1669 + 3 MULTI CID TOF Unmodified diploid_04 68496
102.39 1 364690 3024 0 29 1053.2 3156.58 0 0 0 -1 0 0 0 0 14 41.2471
NaN NaN NaN 0 64274 4220 68482 TLASDDPIANPYRGIIESLNPIDETDLSK
TLASDDPIANPYRGIIESLNPIDETDLSK P29340 213318
20150306_yeast3_Top_opt_2ug_C3_01_1669 + 3 MULTI CID TOF Acetyl (Protein N-term) diploid_04
71744 105.94 1 129910 4806 0 20 728.755 2183.24 0 0 0 -1 0 0 0 0 4
142.258 NaN NaN NaN 0 67341 4401 71740 SLISILSPLITSEGLDSRIK
(ac)SLISILSPLITSEGLDSRIK P53154 194699
