# Supplementary material for: The Impact II, a Very High-Resolution Quadrupole Time-of-Flight Instrument (QTOF) for Deep Shotgun Proteomics
Source: Mol Cell Proteomics. 2015 May 19;14(7):2014–29. doi: 10.1074/mcp.M114.047407 (PMC4587313; doi:10.1074/mcp.M114.047407)

Raw file	Scan	Method	Score	m/z	Gene names
20150307_Hepa1_Top_opt_D1_01_1671	24207	TOF; CID	89.23	688.34	Procr

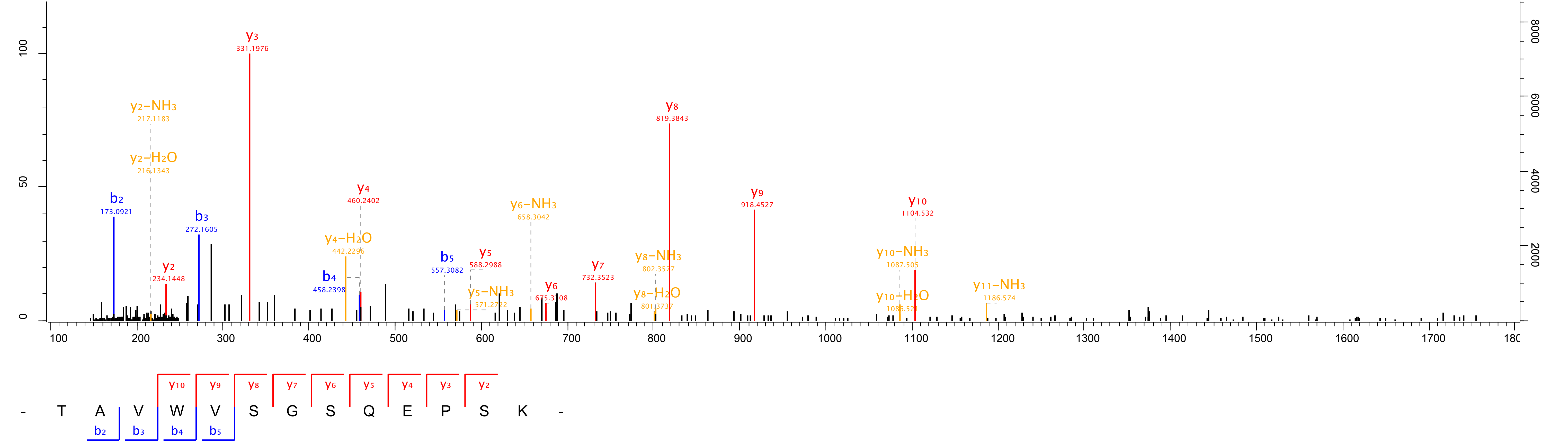

Raw file	Scan	Method	Score	m/z	Gene names
20150307_Hepa1_Top_opt_D1_01_1671	28943	TOF; CID	83.42	682.65	Chchd7

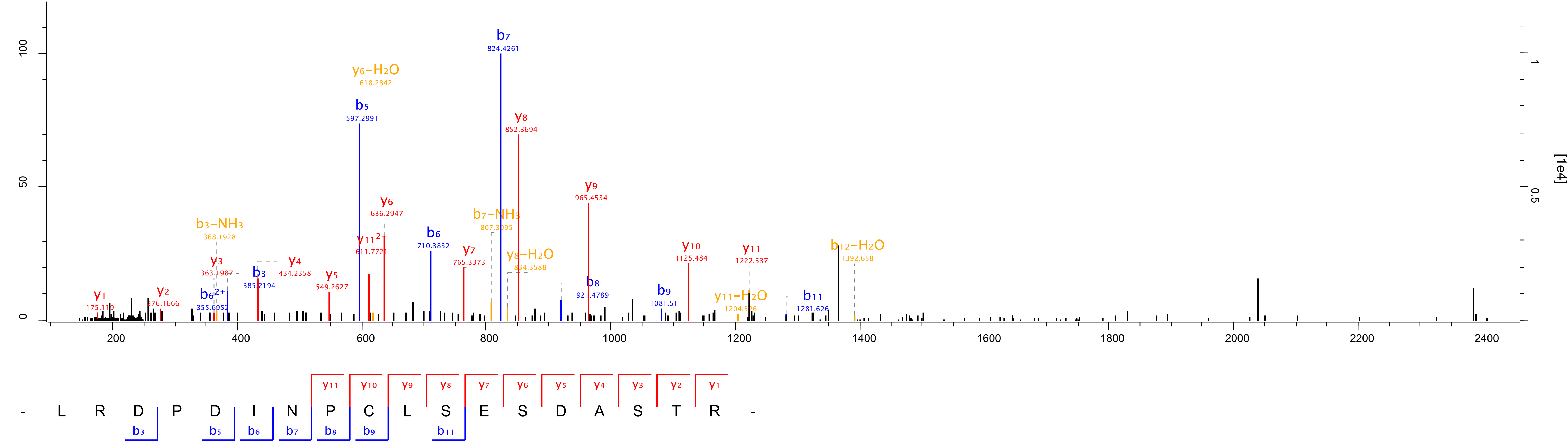

Raw file	Scan	Method	Score	m/z	Gene names
20150307_Hepa1_Top_opt_D1_01_1671	40501	TOF; CID	82.48	891.08	Igfbp1

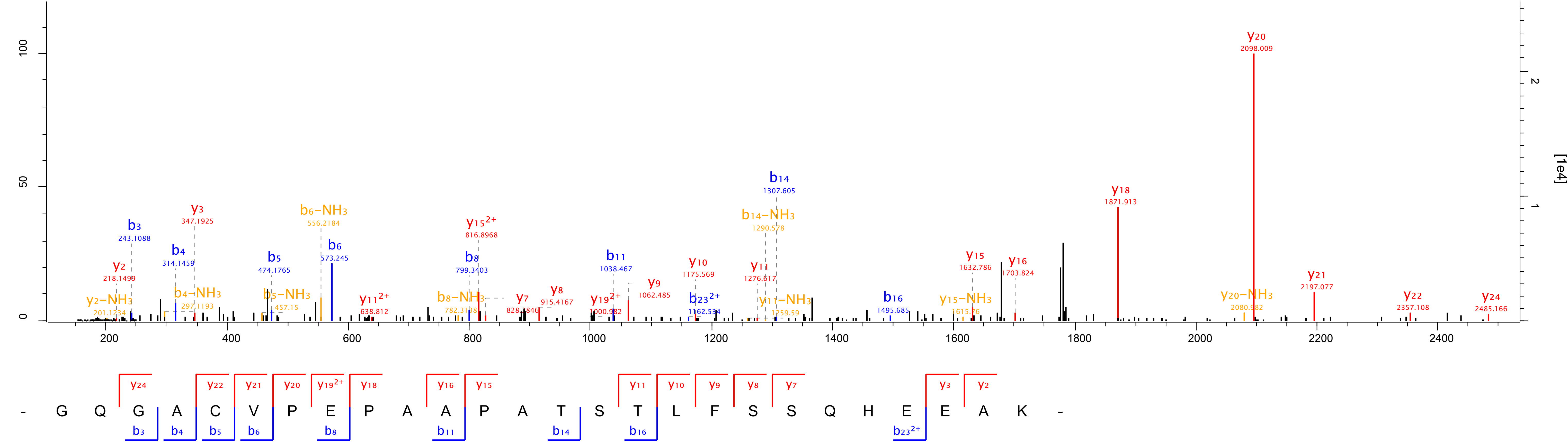

Raw file	Scan	Method	Score	m/z	Gene names
20150307_Hepa1_Top_opt_D1_01_1671	45109	TOF; CID	62.69	638.33	Myd88

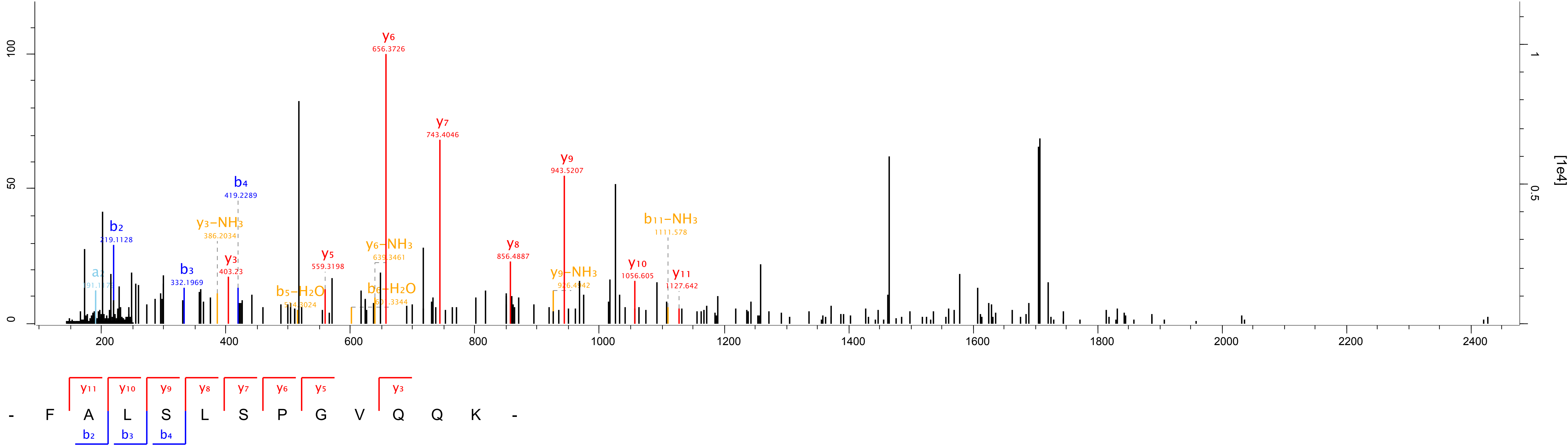

Raw file	Scan	Method	Score	m/z	Gene names
20150307_Hepa1_Top_opt_D1_01_1671	46372	TOF; CID	150.22	881.4	Ly6e

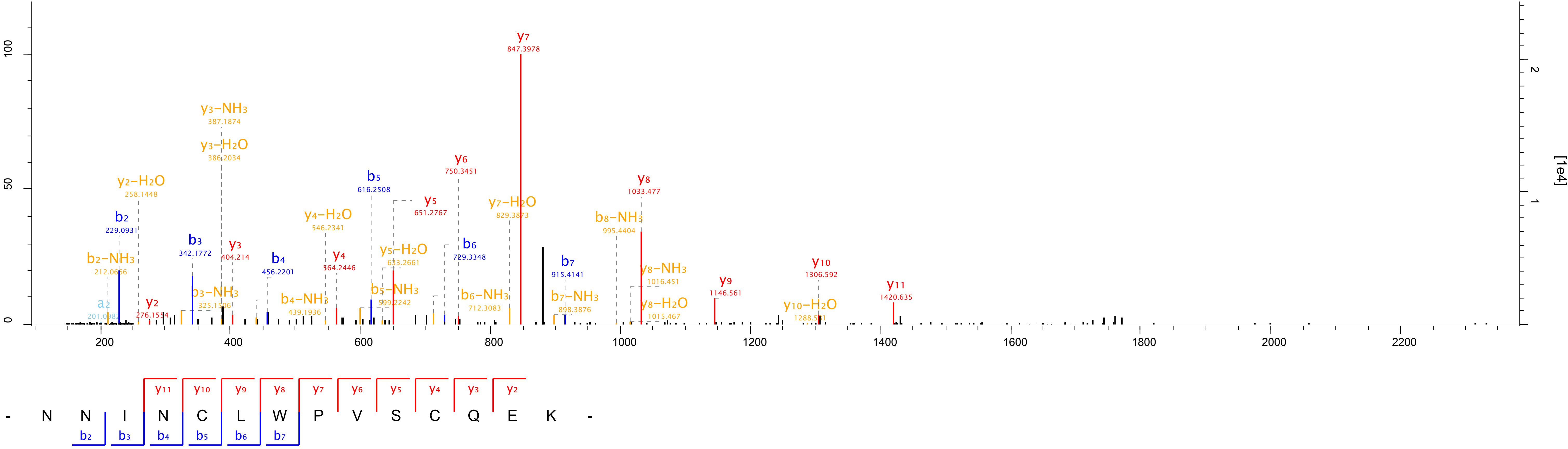

Raw file	Scan	Method	Score	m/z	Gene names
20150307_Hepa1_Top_opt_D1_01_1671	47165	TOF; CID	50.3	619.31	Lgals3bp

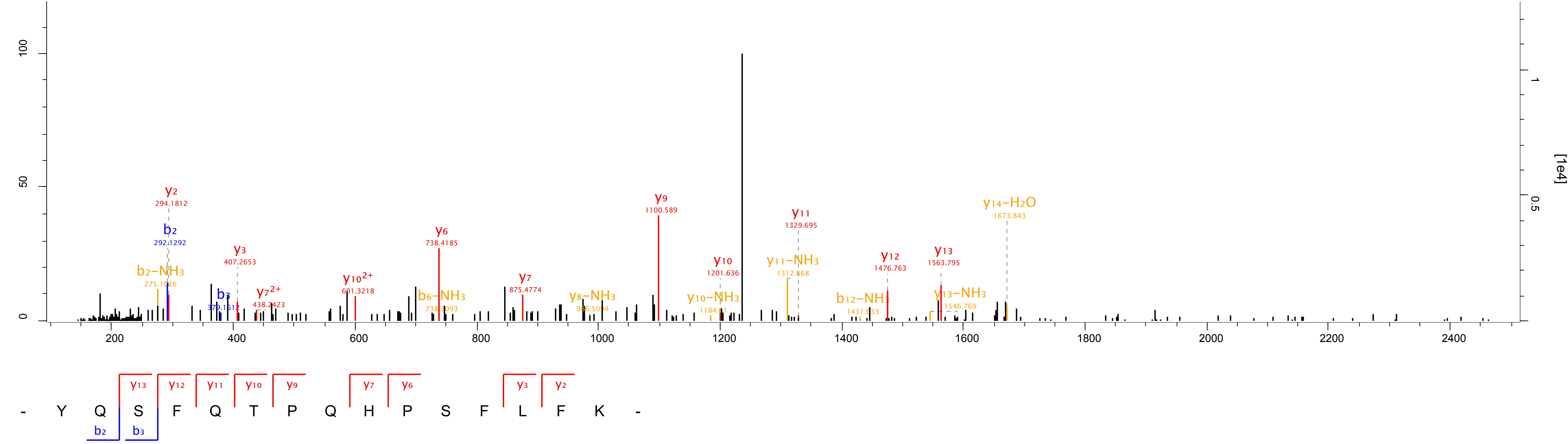

Raw file	Scan	Method	Score	m/z	Gene names
20150307_Hepa1_Top_opt_D1_01_1671	50486	TOF; CID	45.21	559.6	Asb7

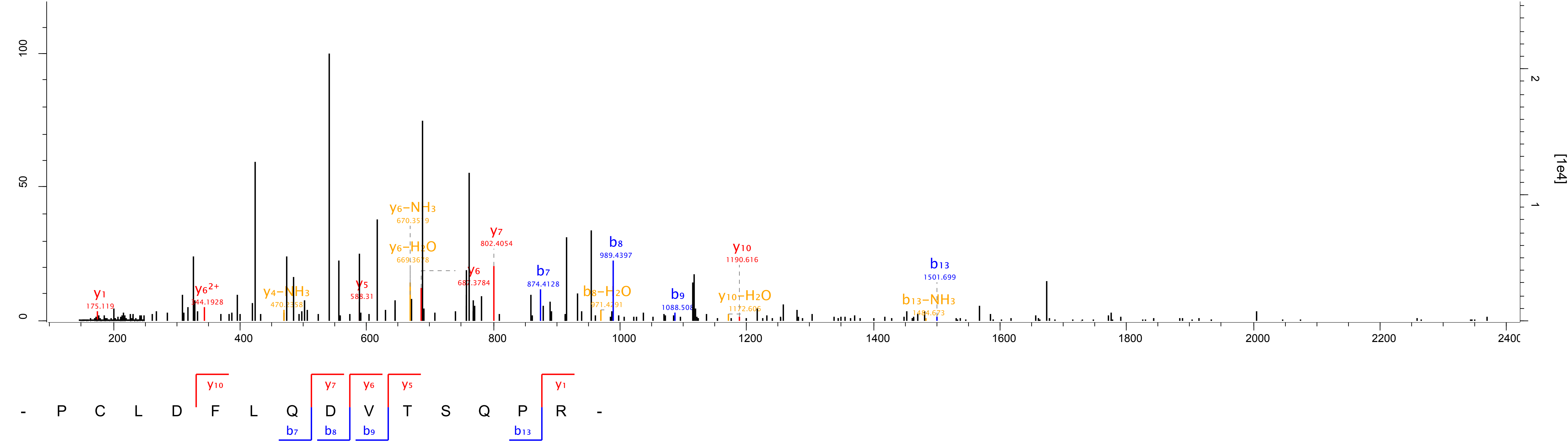

Raw file	Scan	Method	Score	m/z	Gene names
20150307_Hepa1_Top_opt_D1_01_1671	58244	TOF; CID	76.3	781.37	Stc1

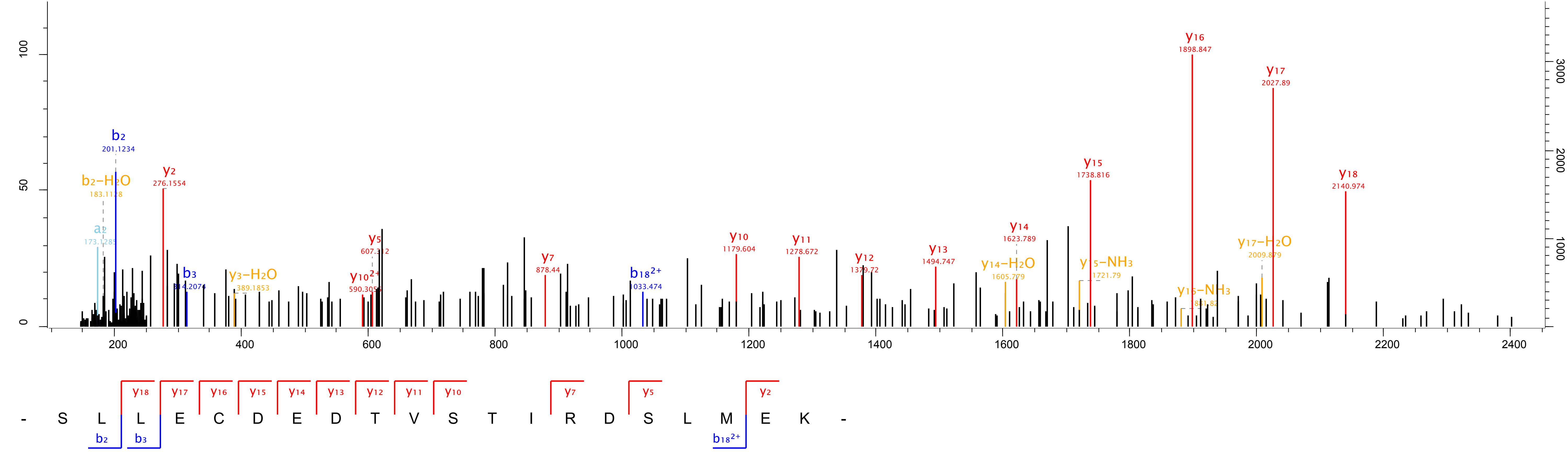

Raw file	Scan	Method	Score	m/z	Gene names
20150307_Hepa1_Top_opt_D1_01_1671	69497	TOF; CID	91.4	698.38	Scoc

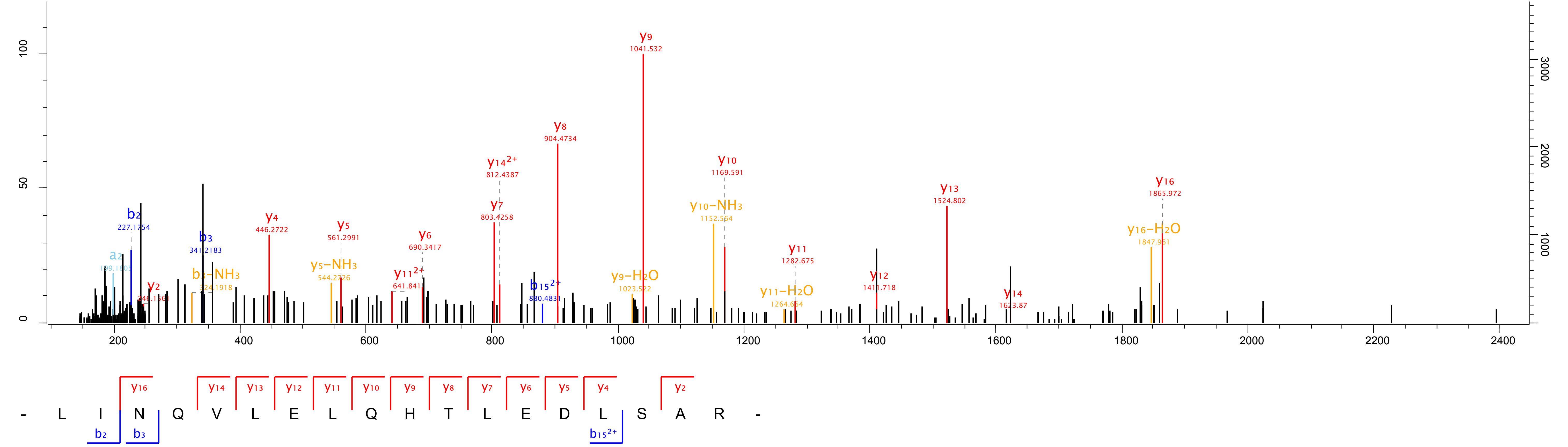

Raw file	Scan	Method	Score	m/z	Gene names
20150307_Hepa1_Top_opt_D1_01_1683	5405	TOF; CID	72.32	825.37	Papd5

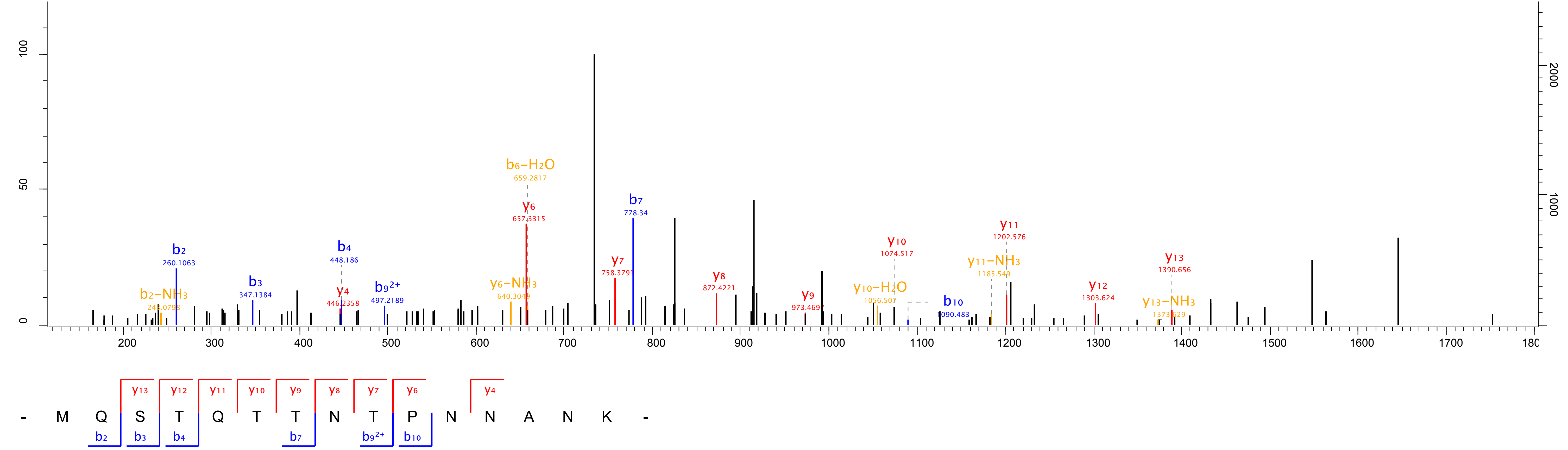

Raw file	Scan	Method	Score	m/z	Gene names
20150307_Hepa1_Top_opt_D1_01_1683	8227	TOF; CID	54.25	565.6	Fam76b

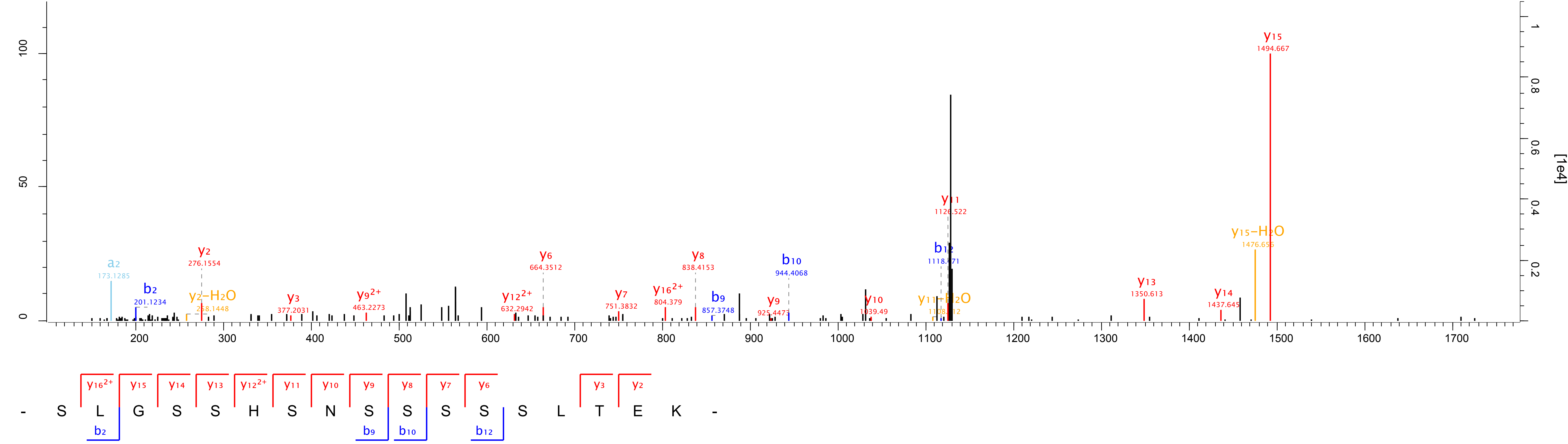

Raw file	Scan	Method	Score	m/z	Gene names
20150307_Hepa1_Top_opt_D1_01_1683	13304	TOF; CID	73.44	625.26	Coa6

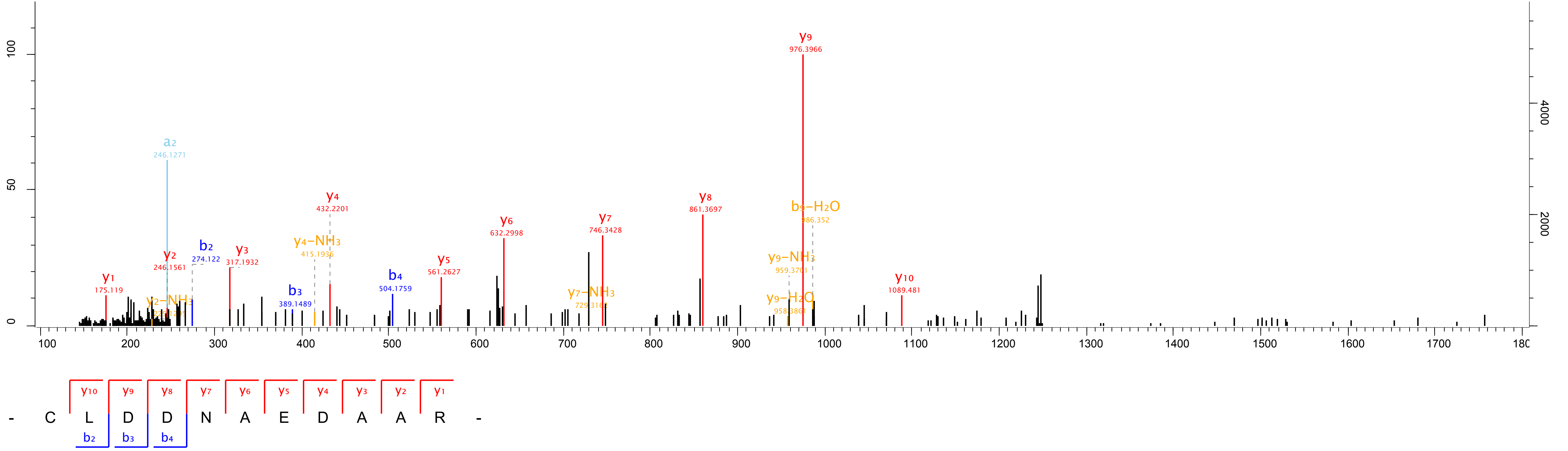

Raw file	Scan	Method	Score	m/z	Gene names
20150307_Hepa1_Top_opt_D1_01_1683	22974	TOF; CID	54.34	635.32	Dnajb2

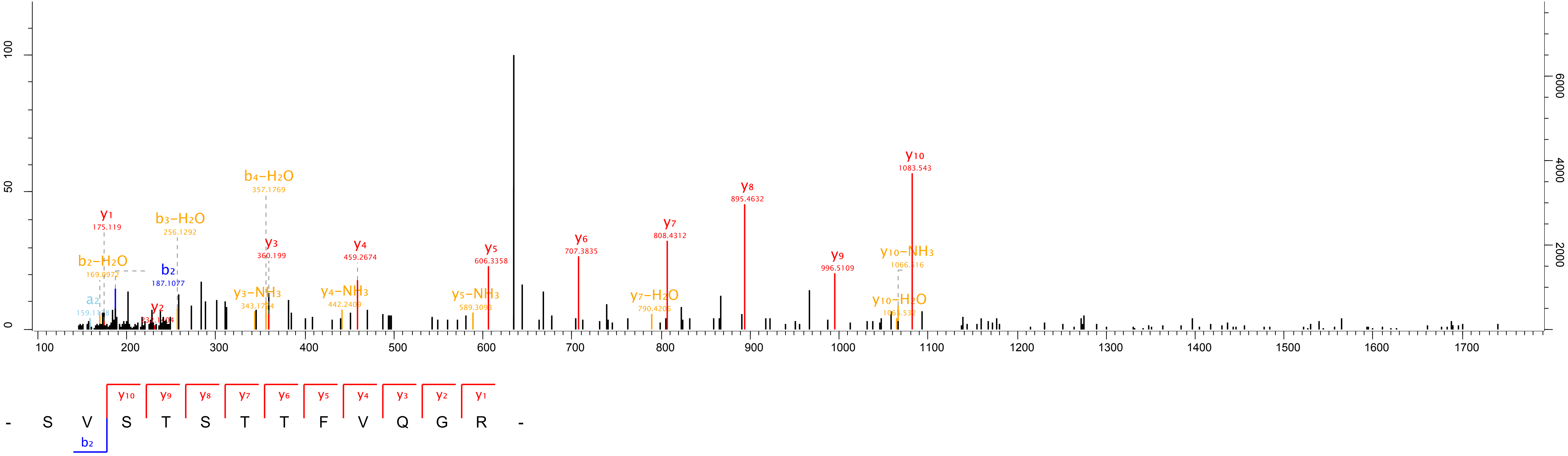

Raw file	Scan	Method	Score	m/z	Gene names
20150307_Hepa1_Top_opt_D1_01_1683	31253	TOF; CID	77.28	705.33	Vkorc1

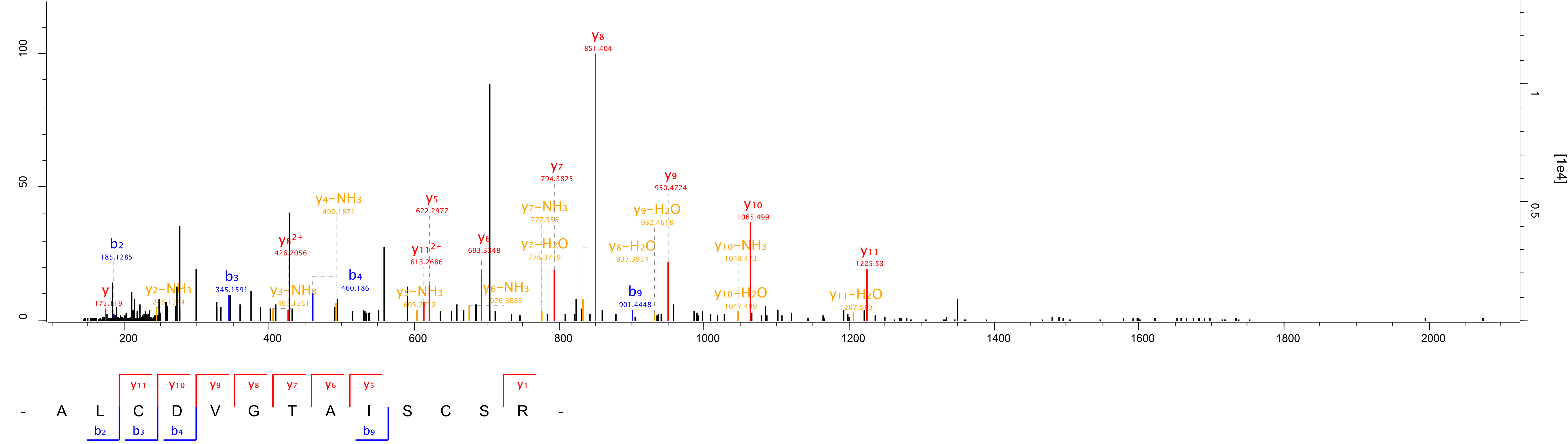

Raw file	Scan	Method	Score	m/z	Gene names
20150307_Hepa1_Top_opt_D1_01_1683	35469	TOF; CID	161.11	476.24	Mapk15

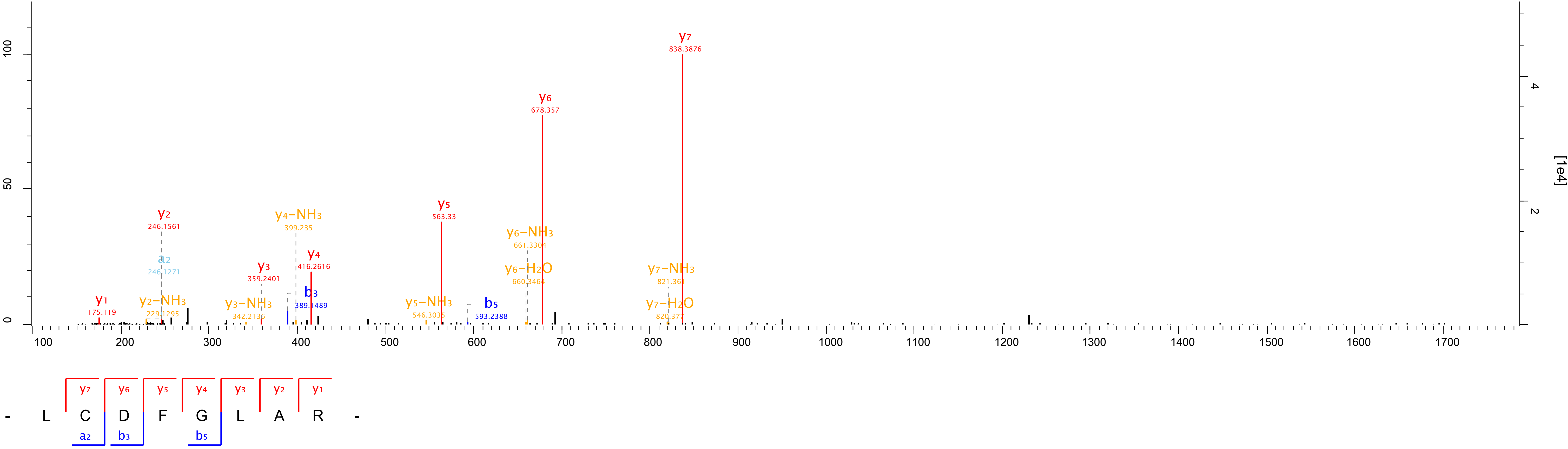

Raw file	Scan	Method	Score	m/z	Gene names
20150307_Hepa1_Top_opt_D1_01_1683	46496	TOF; CID	75.68	1052.49	Lifr

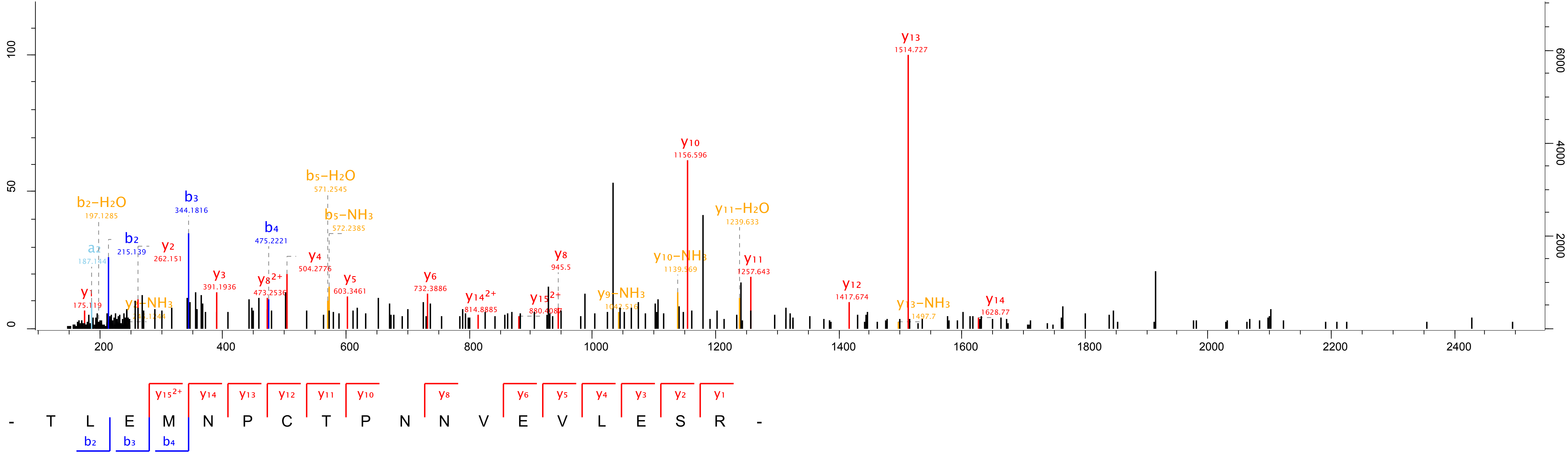

Raw file	Scan	Method	Score	m/z	Gene names
20150307_Hepa1_Top_opt_D1_01_1683	47202	TOF; CID	84.75	782.41	N6amt1

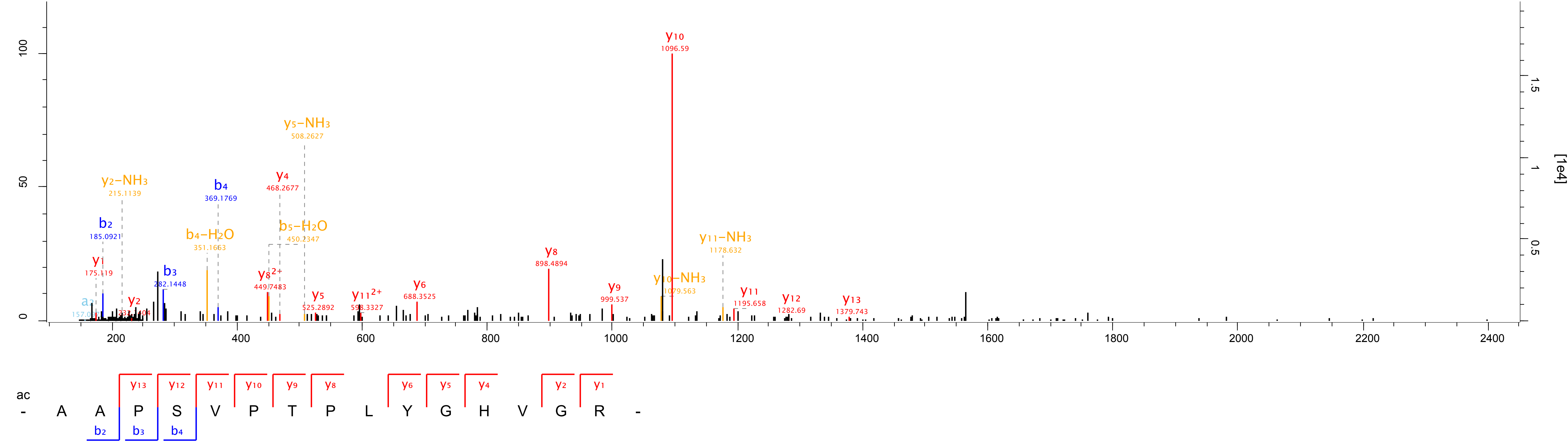

Raw file	Scan	Method	Score	m/z	Gene names
20150307_Hepa1_Top_opt_D1_01_1683	54707	TOF; CID	120.7	594.33	Mboat7

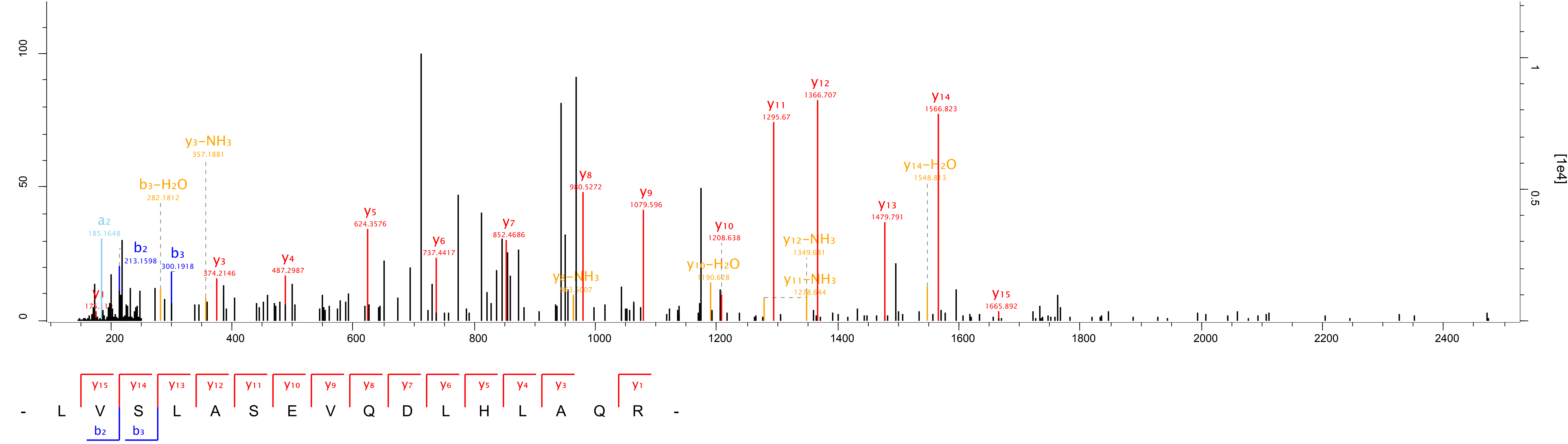

Raw file	Scan	Method	Score	m/z	Gene names
20150307_Hepa1_Top_opt_D1_01_1683	61410	TOF; CID	72.3	911.12	Rpp25l

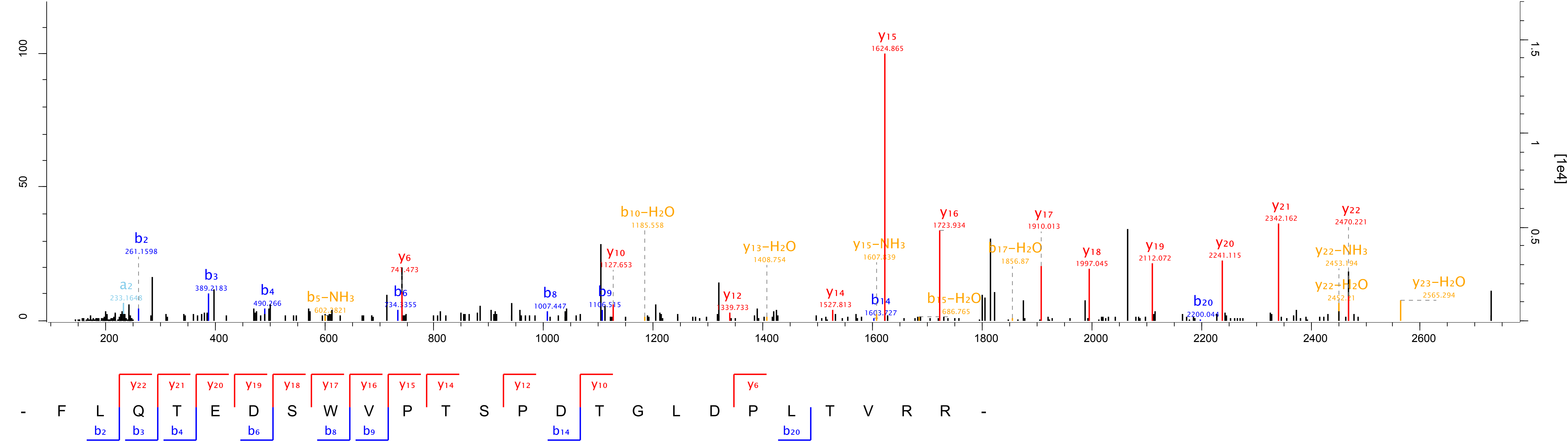

Raw file	Scan	Method	Score	m/z	Gene names
20150307_Hepa1_Top_opt_D1_01_1683	63543	TOF; CID	94.28	1084.01	Ly6c2;Ly6c1

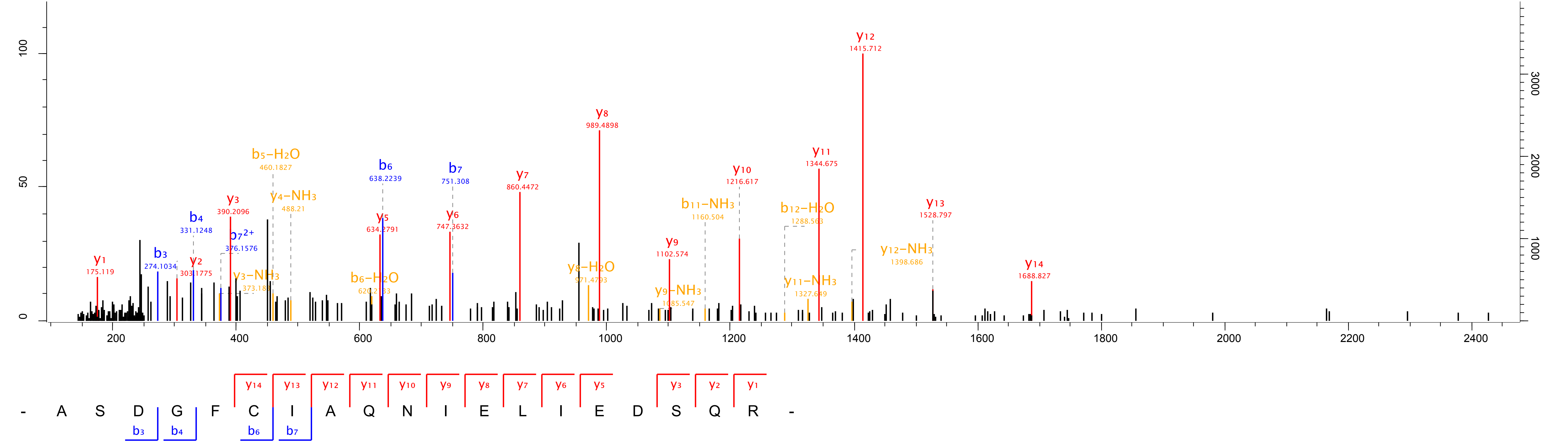

Raw file	Scan	Method	Score	m/z	Gene names
20150307_Hepa1_Top_opt_D1_01_1683	64066	TOF; CID	69.03	660.36	Ptpra

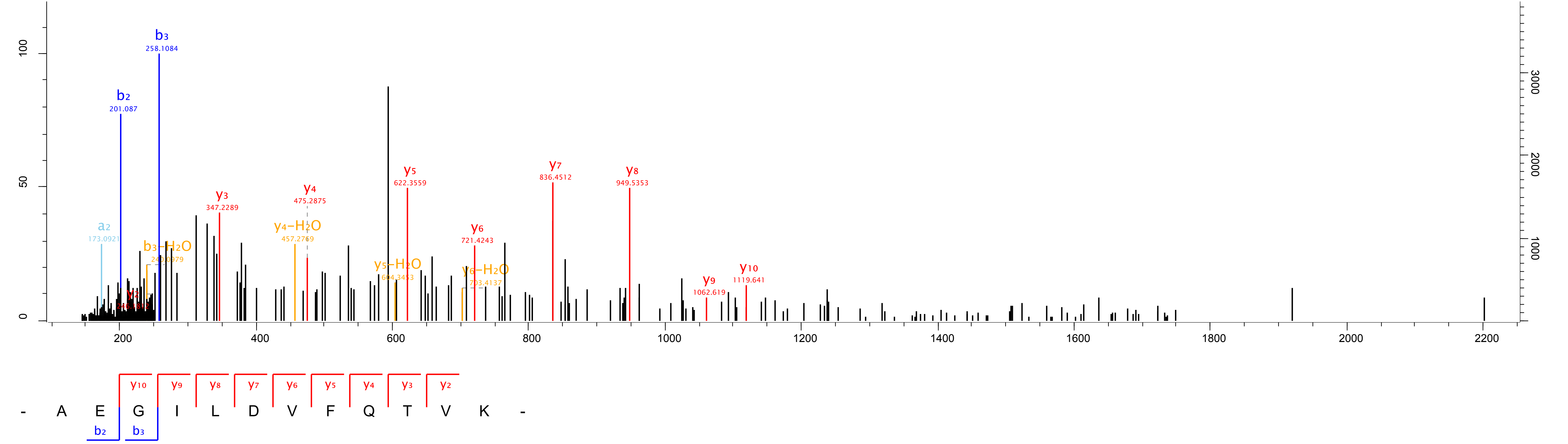

Raw file	Scan	Method	Score	m/z	Gene names
20150307_Hepa1_Top_opt_D1_01_1683	64551	TOF; CID	47.69	687.4	Hpgds

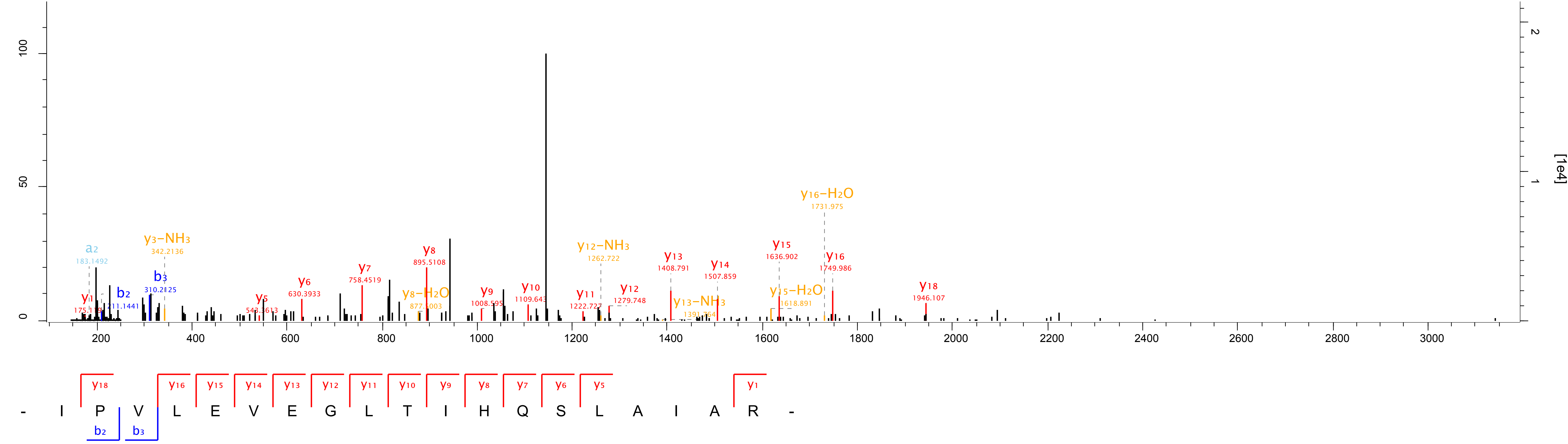

Raw file	Scan	Method	Score	m/z	Gene names
20150307_Hepa1_Top_opt_D1_01_1683	64751	TOF; CID	38.27	869.44	Als2

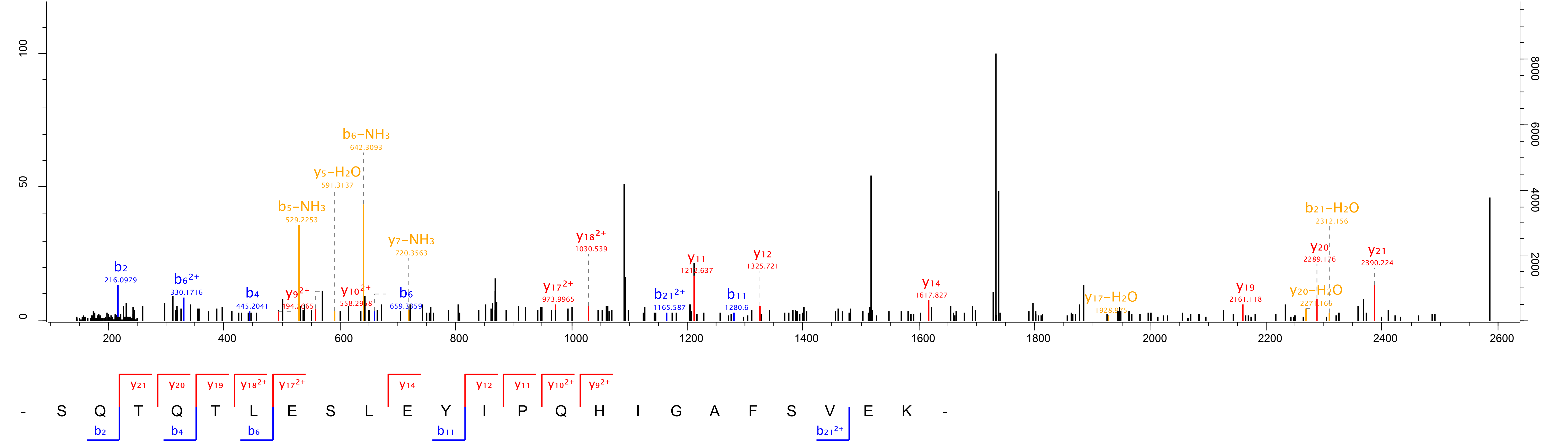

Raw file	Scan	Method	Score	m/z	Gene names
20150307_Hepa1_Top_opt_D1_01_1683	65230	TOF; CID	91.4	672.05	Tsen54

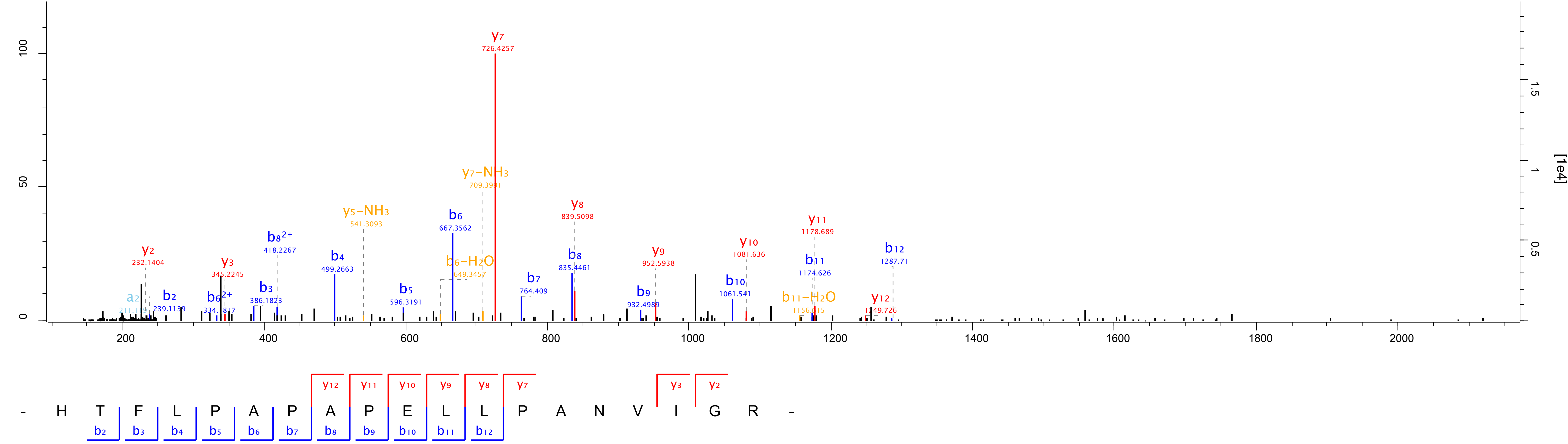

Raw file	Scan	Method	Score	m/z	Gene names
20150307_Hepa1_Top_opt_D1_01_1683	65233	TOF; CID	34.99	973.47	Ndufa3

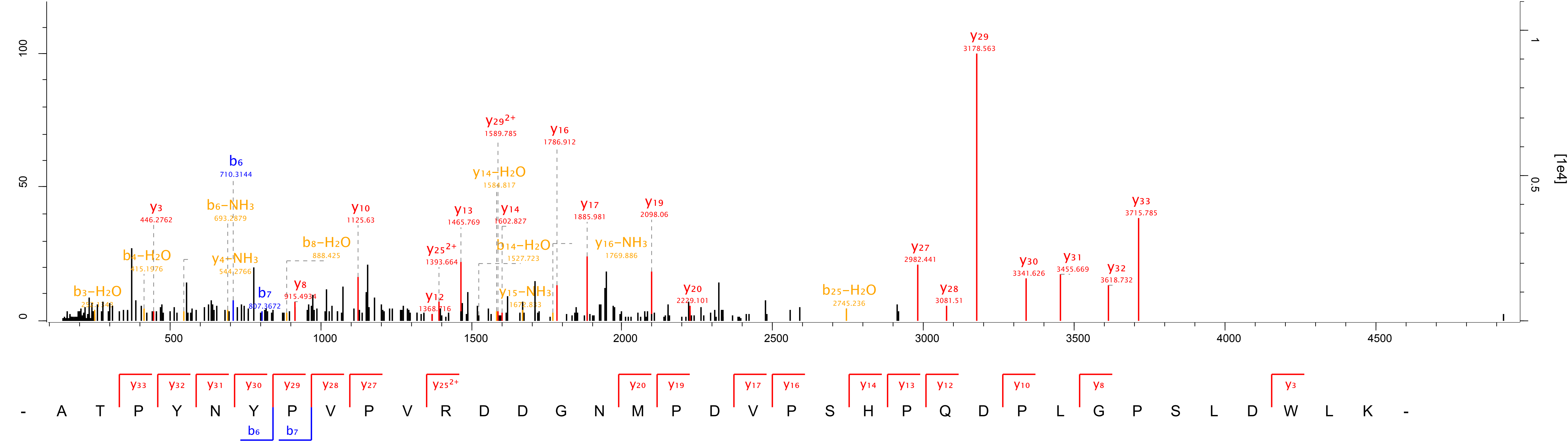

Raw file	Scan	Method	Score	m/z	Gene names
20150307_Hepa1_Top_opt_D1_01_1683	66257	TOF; CID	86.49	969.47	Enpep

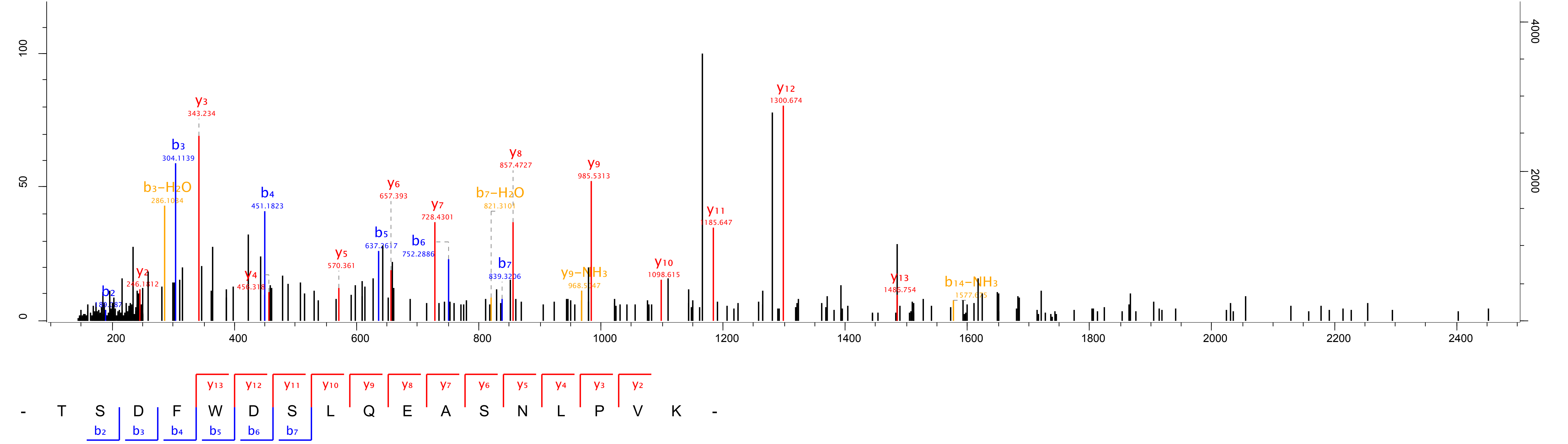

Raw file	Scan	Method	Score	m/z	Gene names
20150307_Hepa1_Top_opt_D1_01_1683	67304	TOF; CID	56.82	960.47	Bcar3

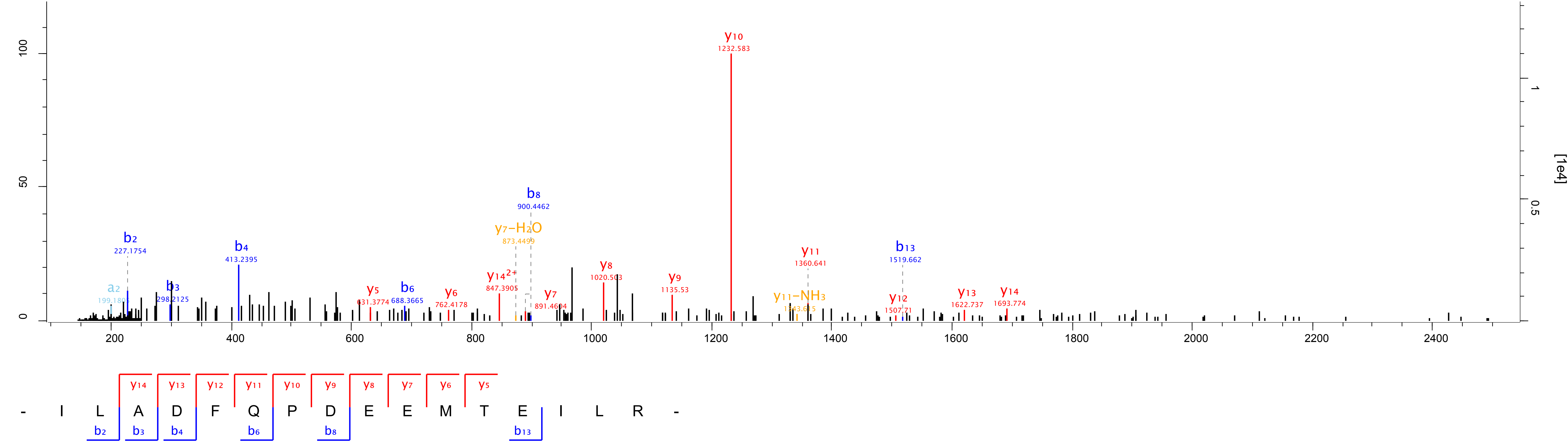

Raw file	Scan	Method	Score	m/z	Gene names
20150307_Hepa1_Top_opt_D1_01_1683	68708	TOF; CID	62.06	843.98	Slc25a30;Slc25a14

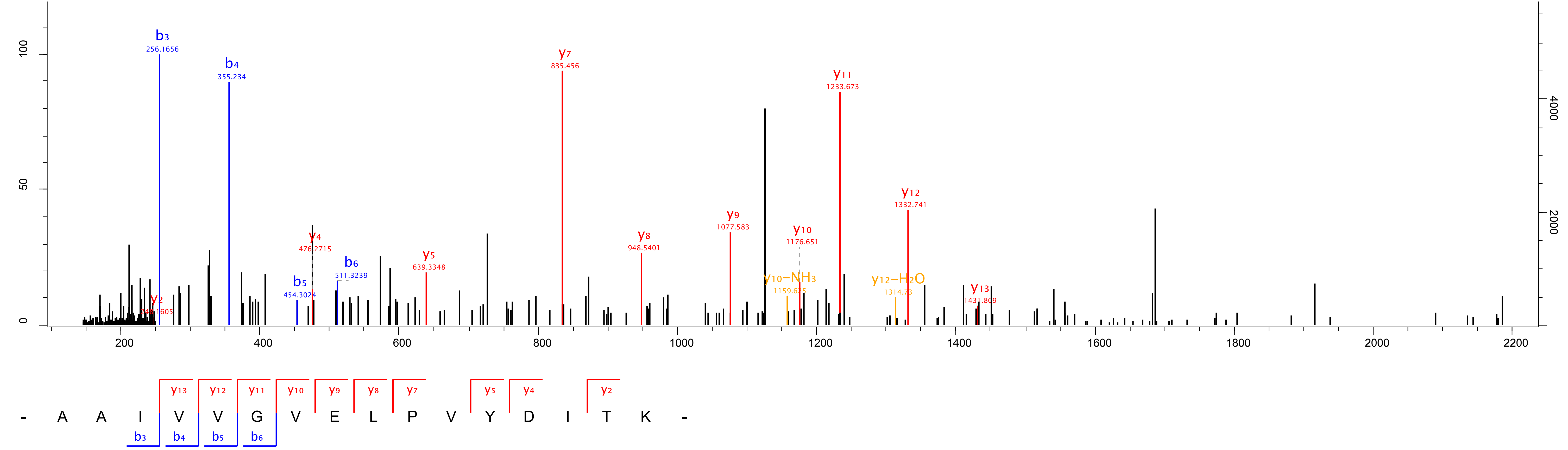

Raw file	Scan	Method	Score	m/z	Gene names
20150307_Hepa1_Top_opt_D1_01_1683	68775	TOF; CID	52.17	837.41	Mmp13

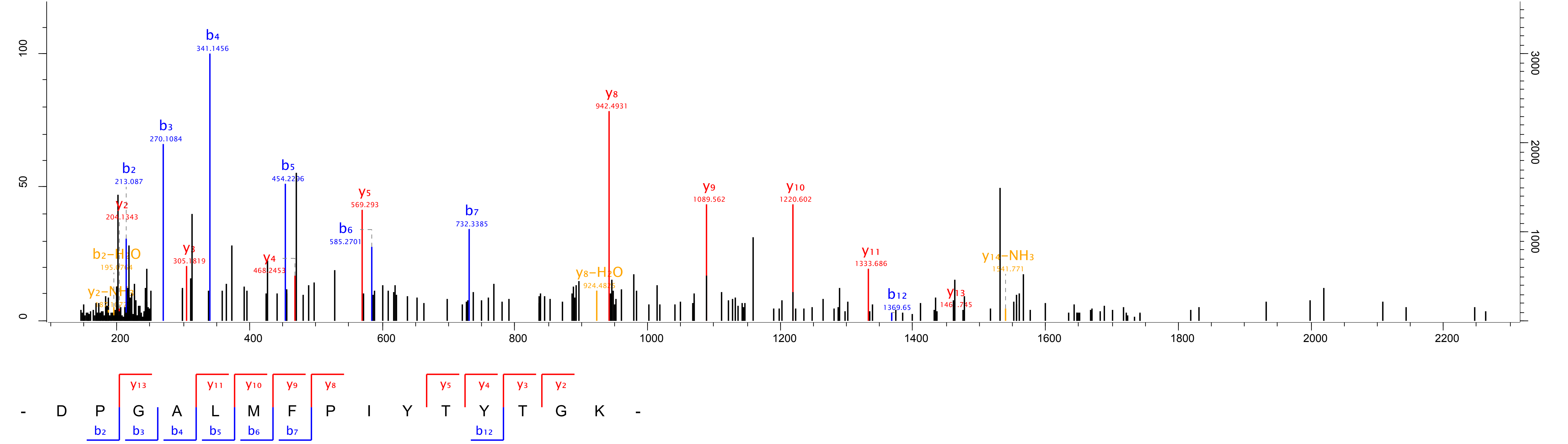

Raw file	Scan	Method	Score	m/z	Gene names
20150307_Hepa1_Top_opt_D1_01_1683	70733	TOF; CID	100.28	805.74	Ly6a

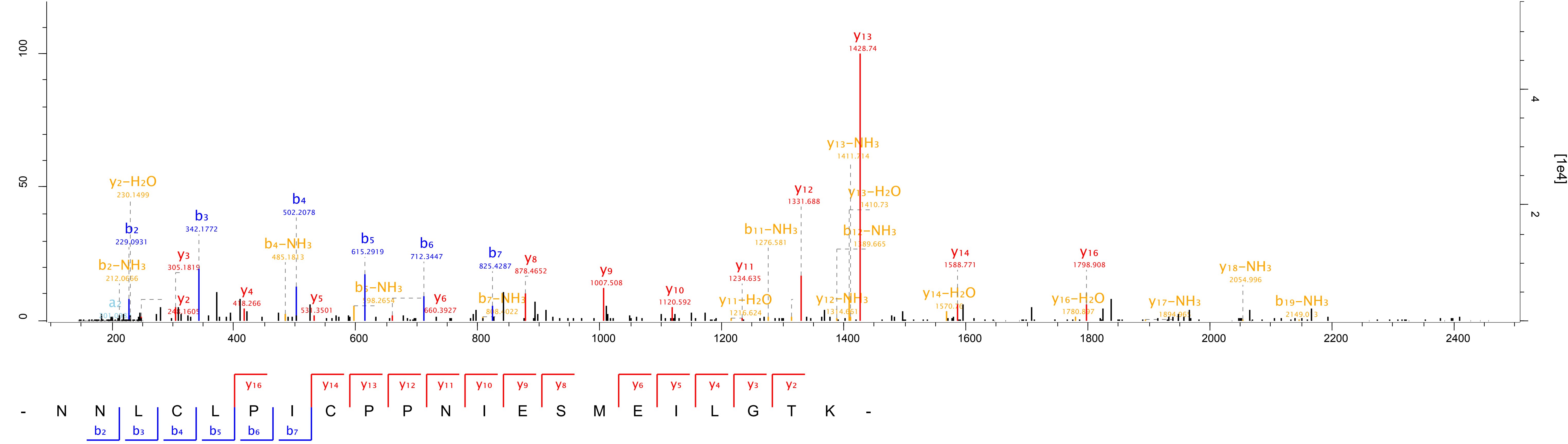

Raw file	Scan	Method	Score	m/z	Gene names
20150307_Hepa1_Top_opt_D1_01_1683	71086	TOF; CID	65.02	1042.54	Gpnmb

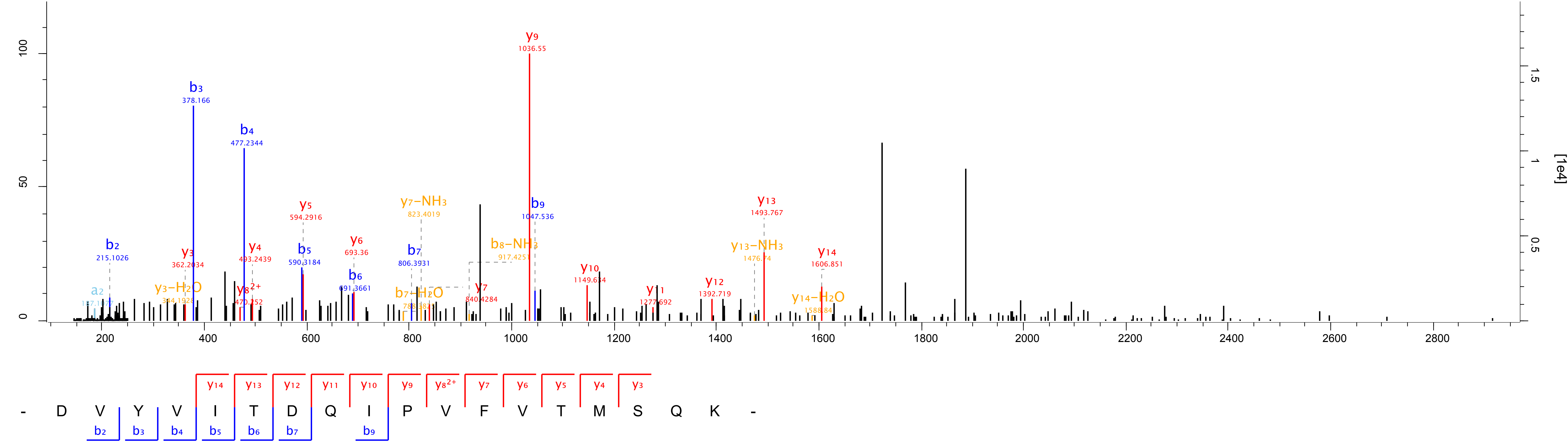

Raw file	Scan	Method	Score	m/z	Gene names
20150307_Hepa1_Top_opt_D1_01_1683	71898	TOF; CID	62.03	948.48	Polr2f

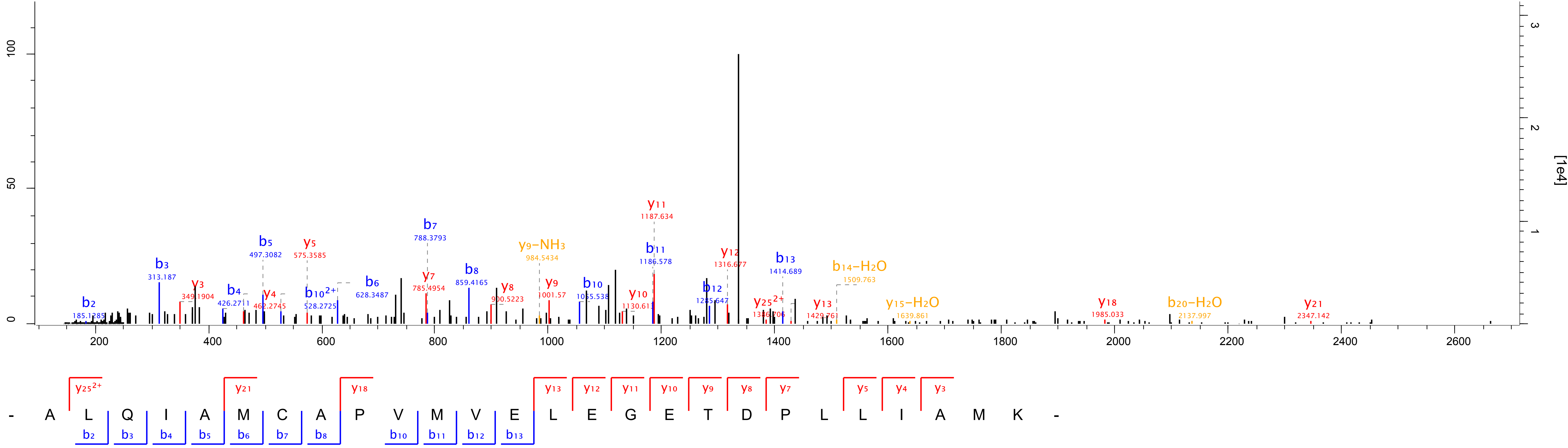

Raw file	Scan	Method	Score	m/z	Gene names
20150307_Hepa1_Top_opt_D1_01_1683	72128	TOF; CID	68.64	693.35	Ninj1

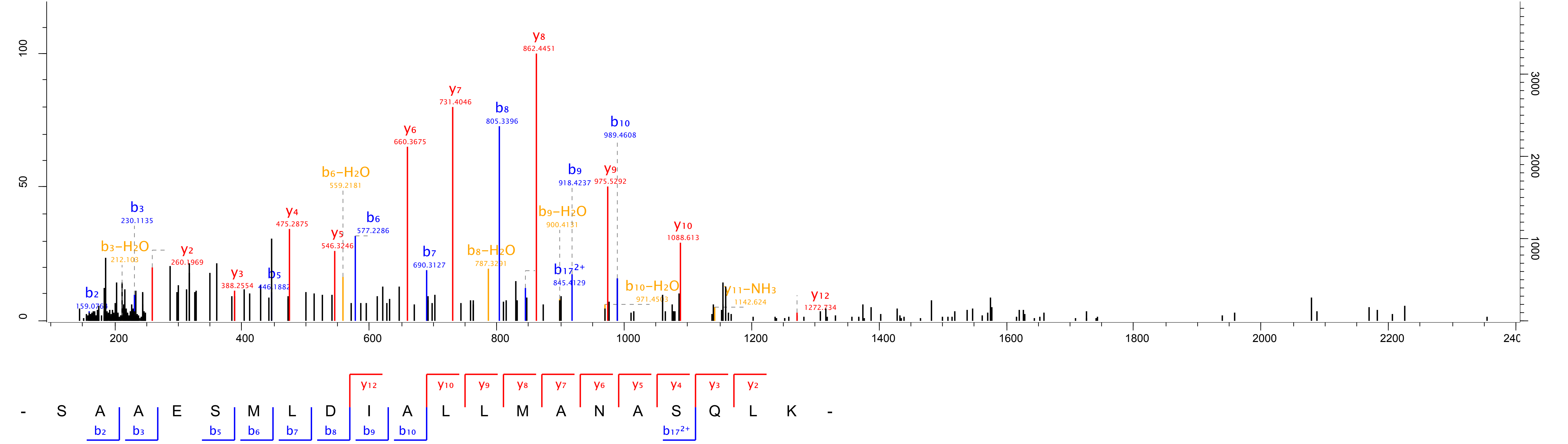

Raw file	Scan	Method	Score	m/z	Gene names
20150307_Hepa2_Top_opt_D2_01_1673	6252	TOF; CID	73.5	605.74	Fst

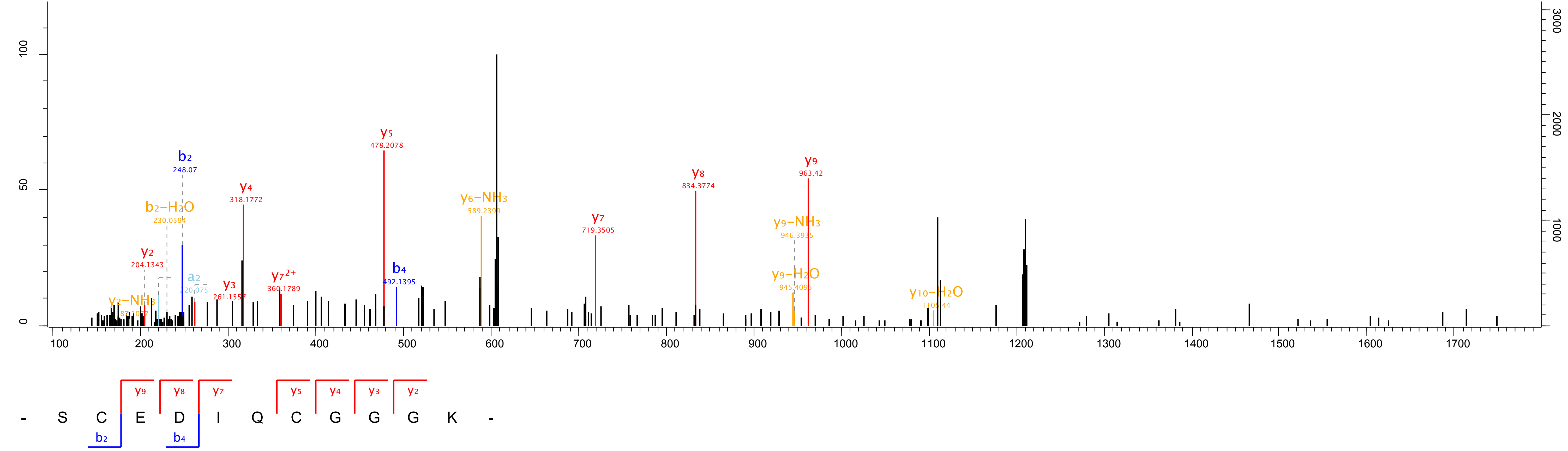

Raw file	Scan	Method	Score	m/z	Gene names
20150307_Hepa2_Top_opt_D2_01_1673	10356	TOF; CID	83.95	541.74	Sprr2k;Sprr2b;Sprr2g;Sprr2d;Sprr2h

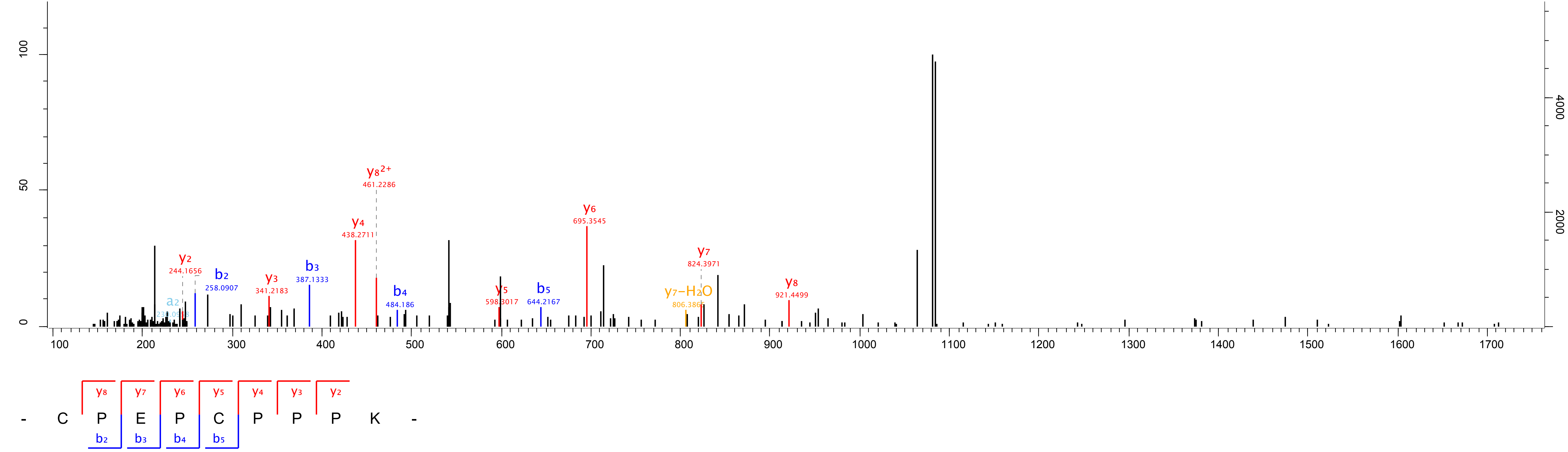

Raw file	Scan	Method	Score	m/z	Gene names
20150307_Hepa2_Top_opt_D2_01_1673	13469	TOF; CID	67.42	576.28	Gm17296

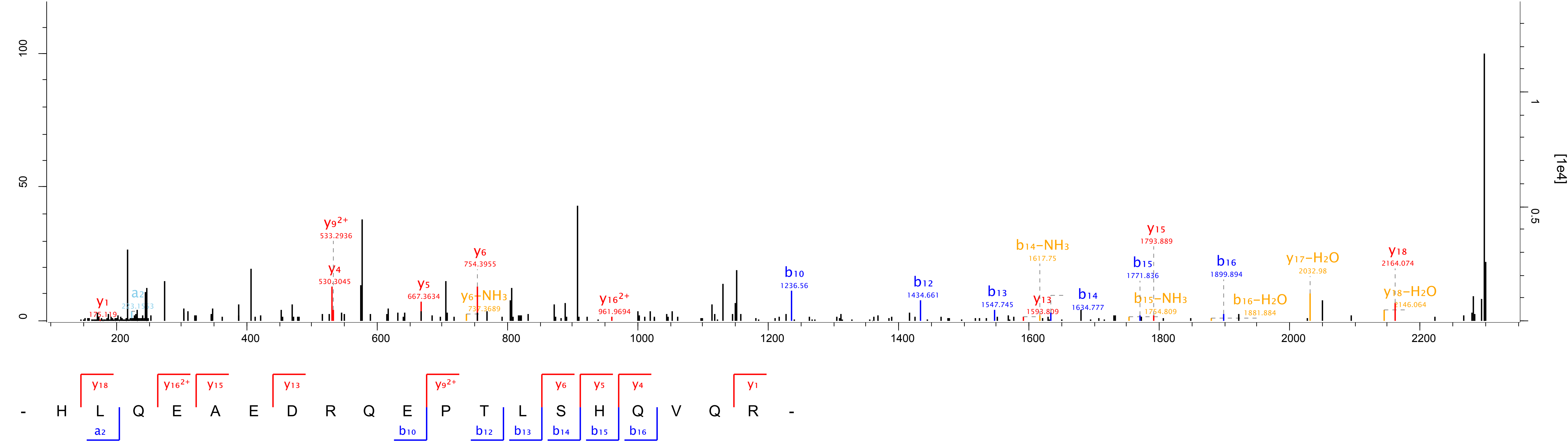

Raw file	Scan	Method	Score	m/z	Gene names
20150307_Hepa2_Top_opt_D2_01_1673	13493	TOF; CID	85.92	647.3	Polr3e

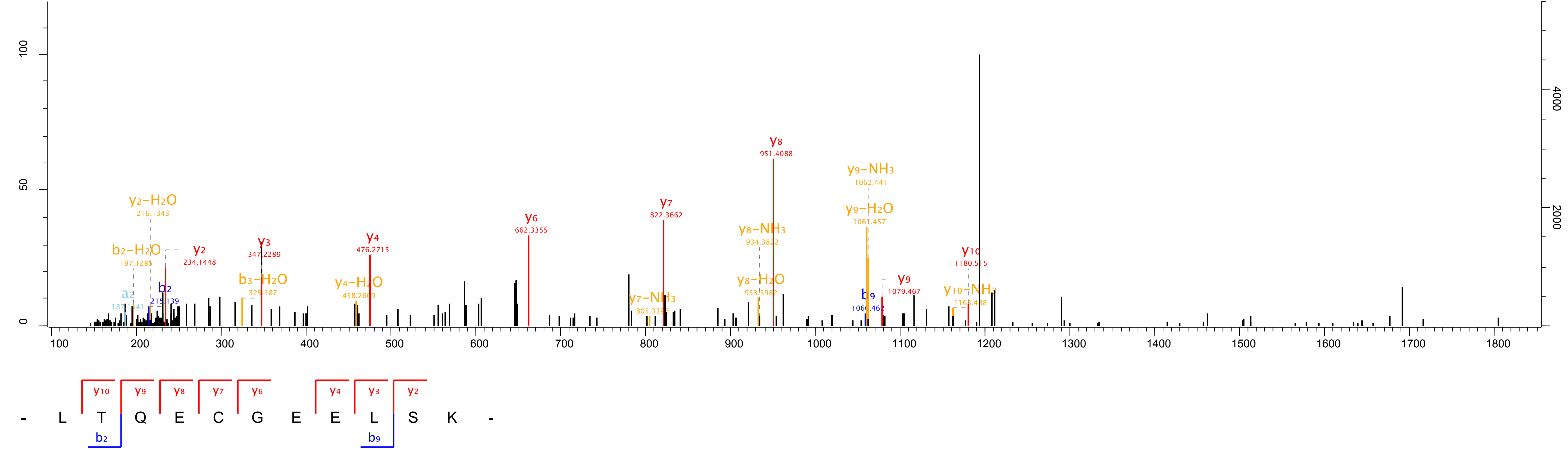

Raw file	Scan	Method	Score	m/z	Gene names
20150307_Hepa2_Top_opt_D2_01_1673	18476	TOF; CID	83.5	664.79	Mrpl32

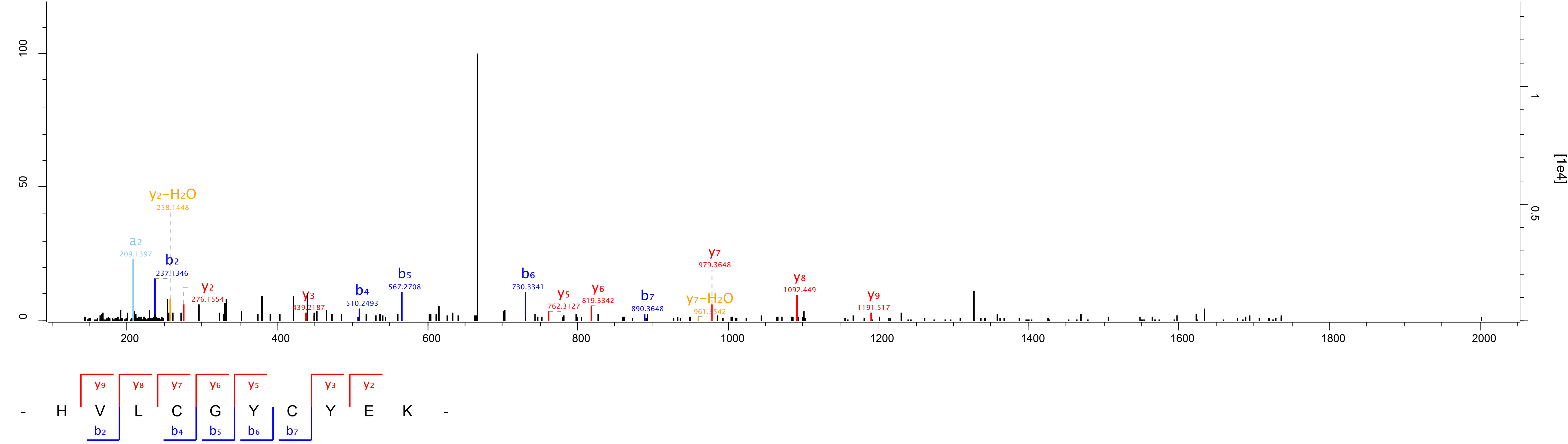

Raw file	Scan	Method	Score	m/z	Gene names
20150307_Hepa2_Top_opt_D2_01_1673	18658	TOF; CID	73.5	551.27	Fam168a

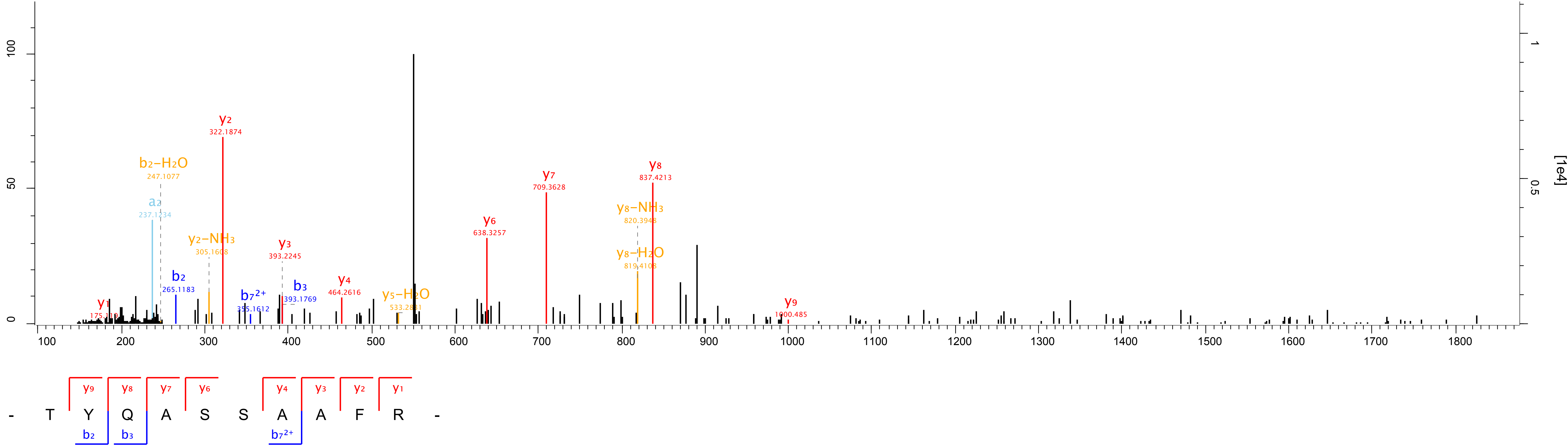

Raw file	Scan	Method	Score	m/z	Gene names
20150307_Hepa2_Top_opt_D2_01_1673	20411	TOF; CID	44.93	579.96	Cdh16

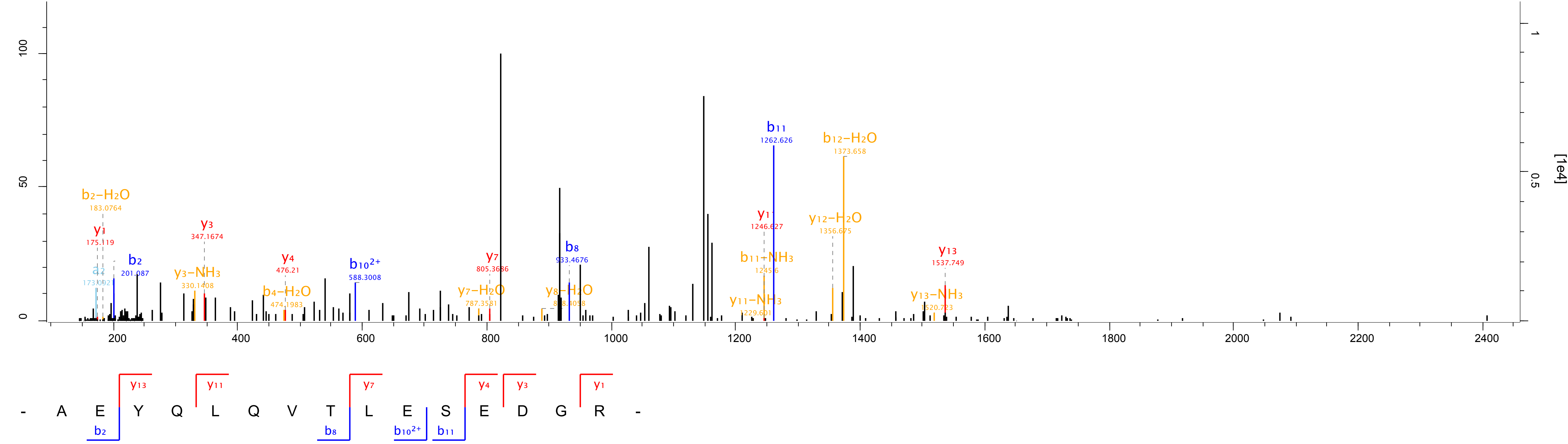

Raw file	Scan	Method	Score	m/z	Gene names
20150307_Hepa2_Top_opt_D2_01_1673	23176	TOF; CID	60.55	634.33	Ndfip2

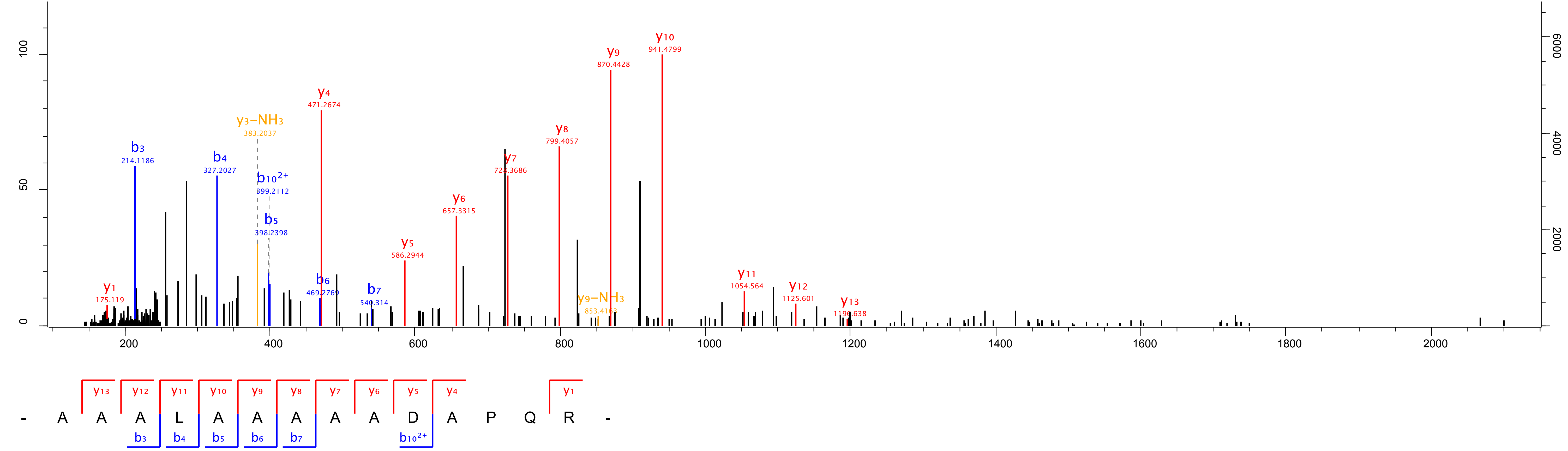

Raw file	Scan	Method	Score	m/z	Gene names
20150307_Hepa2_Top_opt_D2_01_1673	30900	TOF; CID	130.29	834.4	Ormdl2;Ormdl1

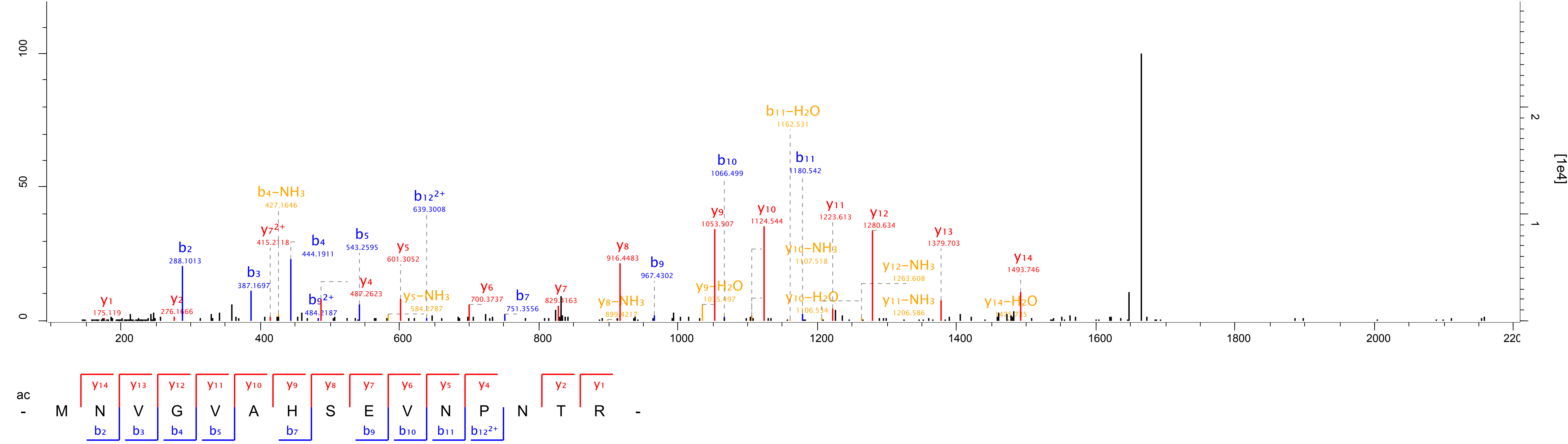

Raw file	Scan	Method	Score	m/z	Gene names
20150307_Hepa2_Top_opt_D2_01_1673	31870	TOF; CID	72.43	634.77	Slc11a2

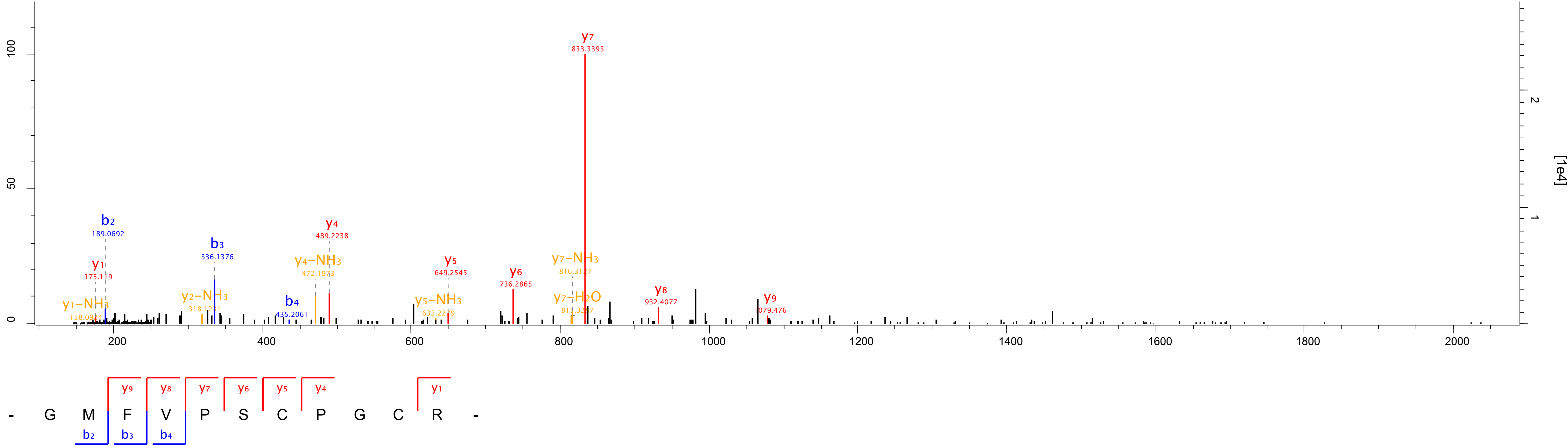

Raw file	Scan	Method	Score	m/z	Gene names
20150307_Hepa2_Top_opt_D2_01_1673	33326	TOF; CID	73.88	706.31	Mgst1

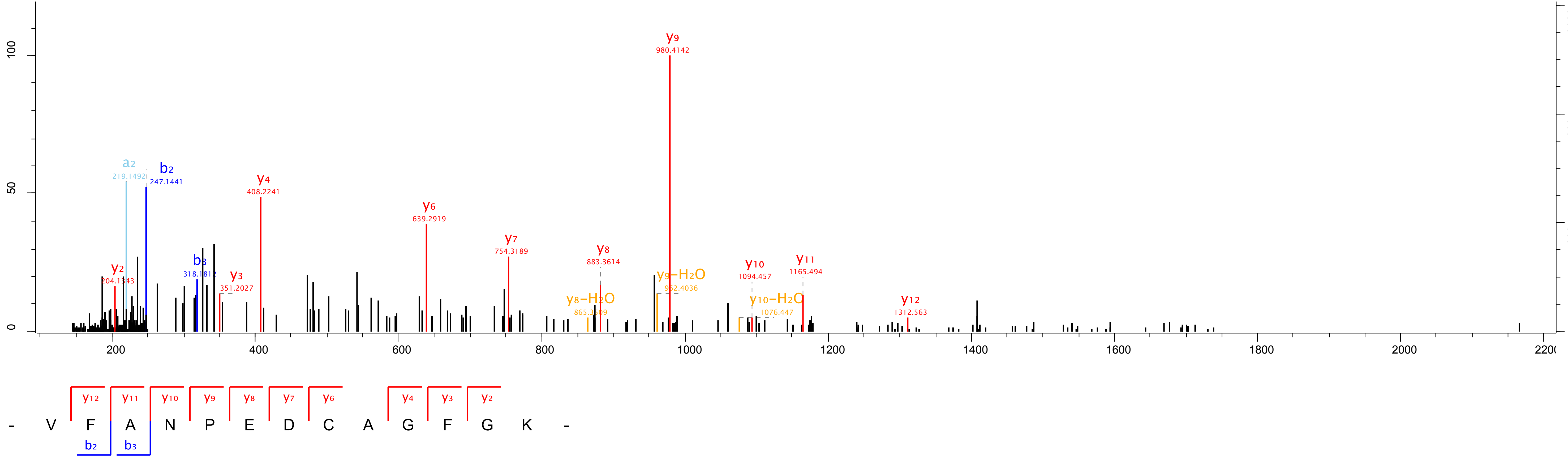

Raw file	Scan	Method	Score	m/z	Gene names
20150307_Hepa2_Top_opt_D2_01_1673	37446	TOF; CID	61.65	736.39	St6gal1

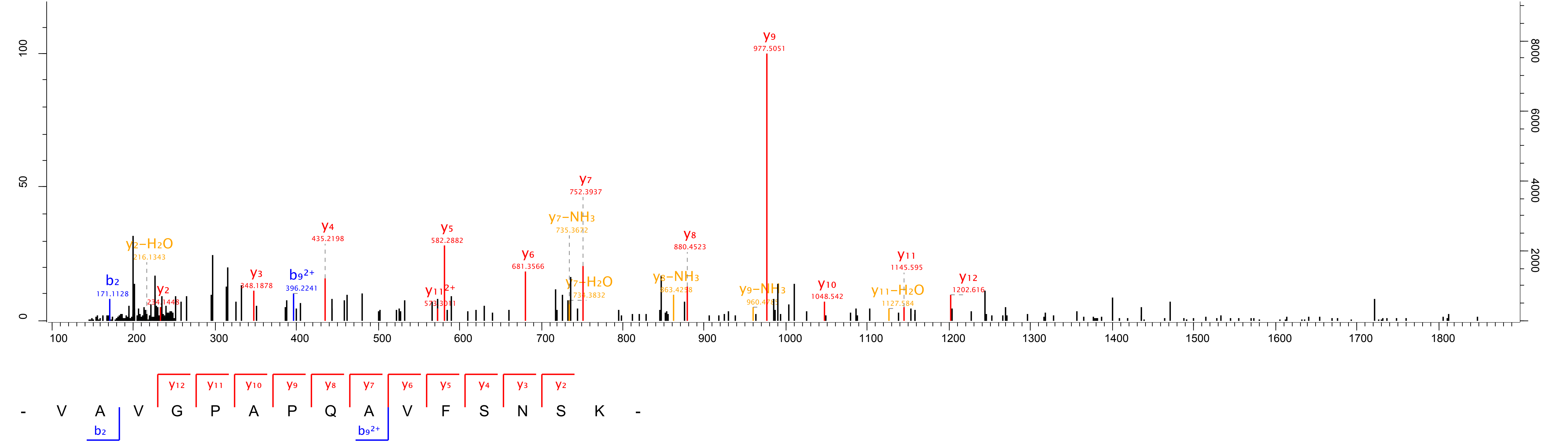

Raw file	Scan	Method	Score	m/z	Gene names
20150307_Hepa2_Top_opt_D2_01_1673	38798	TOF; CID	78.46	578.81	Dnajc22

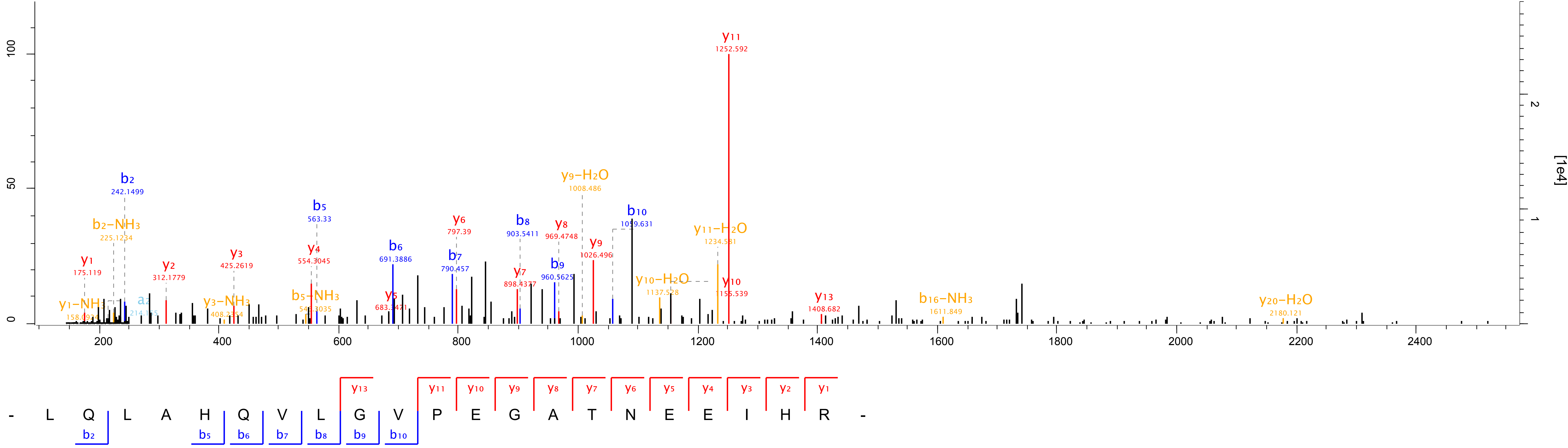

Raw file	Scan	Method	Score	m/z	Gene names
20150307_Hepa2_Top_opt_D2_01_1673	42094	TOF; CID	62.83	965.79	Trim44

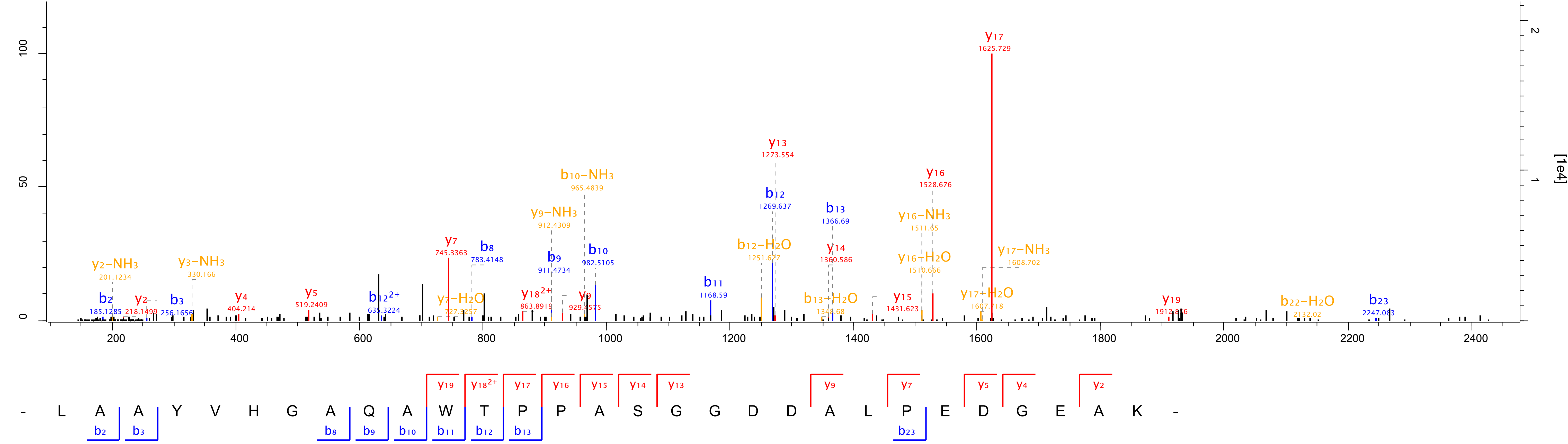

Raw file	Scan	Method	Score	m/z	Gene names
20150307_Hepa2_Top_opt_D2_01_1673	43113	TOF; CID	56.78	942.1	Polr3h

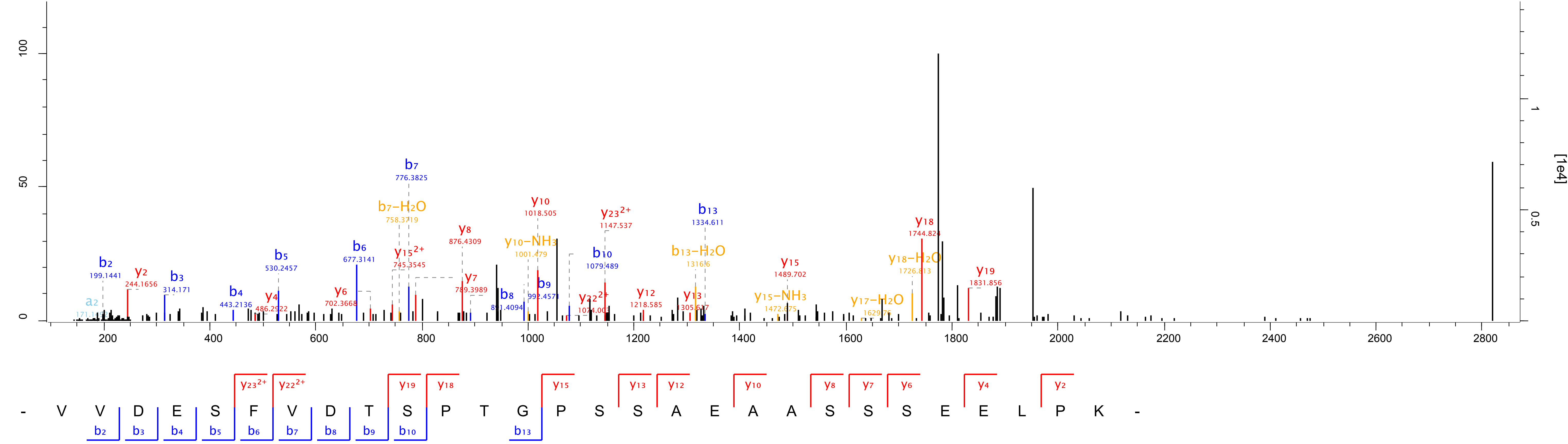

Raw file	Scan	Method	Score	m/z	Gene names
20150307_Hepa2_Top_opt_D2_01_1673	44228	TOF; CID	57.29	587.34	Slc30a6

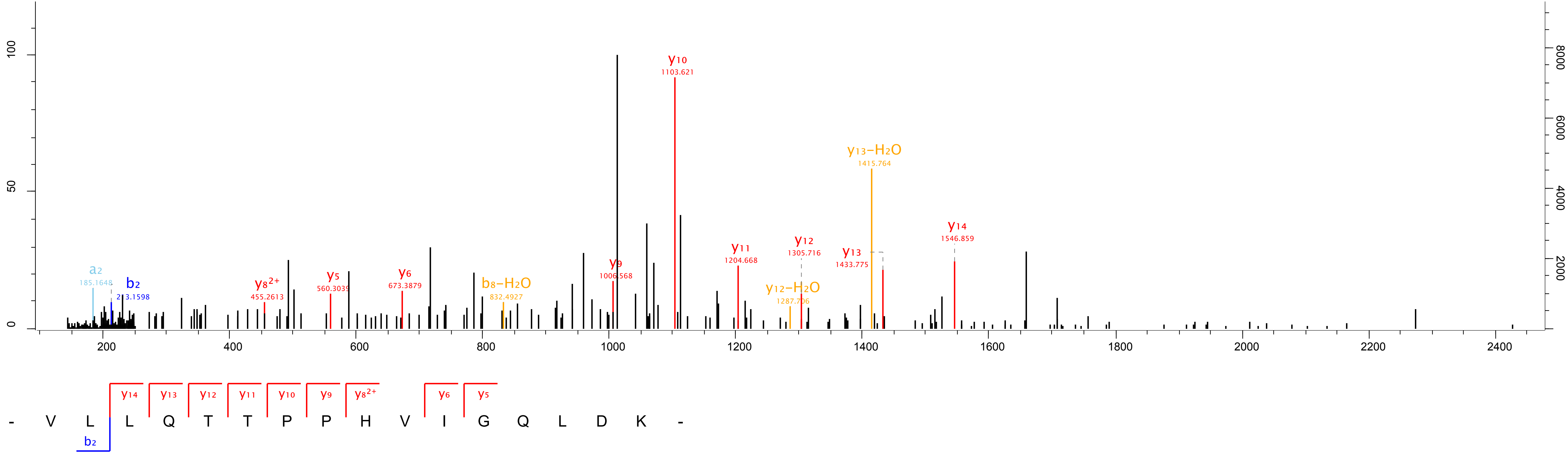

Raw file	Scan	Method	Score	m/z	Gene names
20150307_Hepa2_Top_opt_D2_01_1673	44592	TOF; CID	49.66	780.15	Hyal2

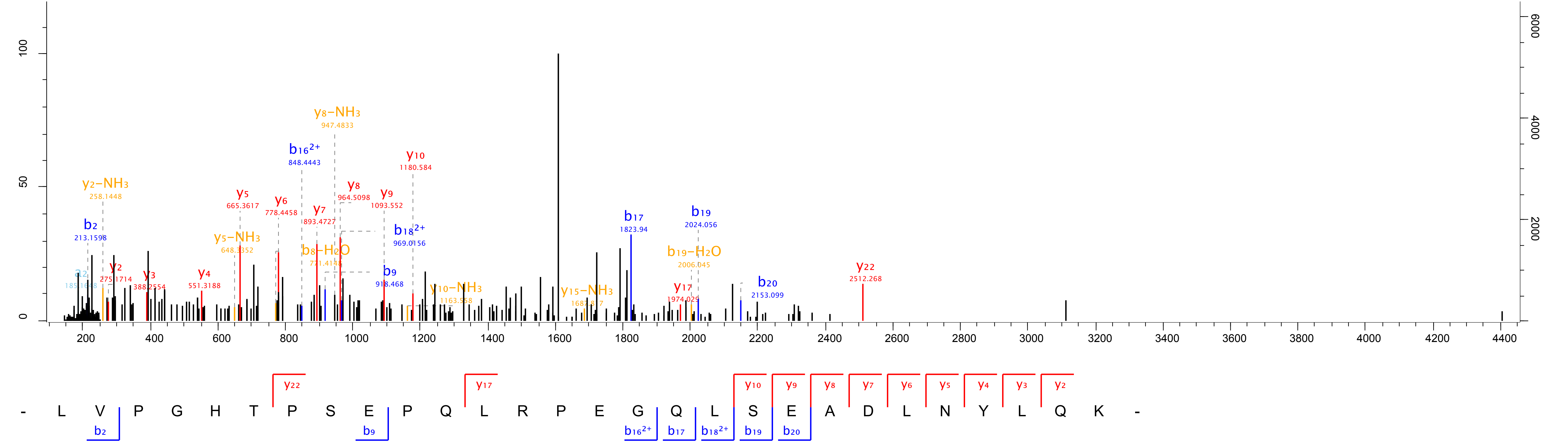

Raw file	Scan	Method	Score	m/z	Gene names
20150307_Hepa2_Top_opt_D2_01_1673	47037	TOF; CID	69.07	902.46	Phrf1

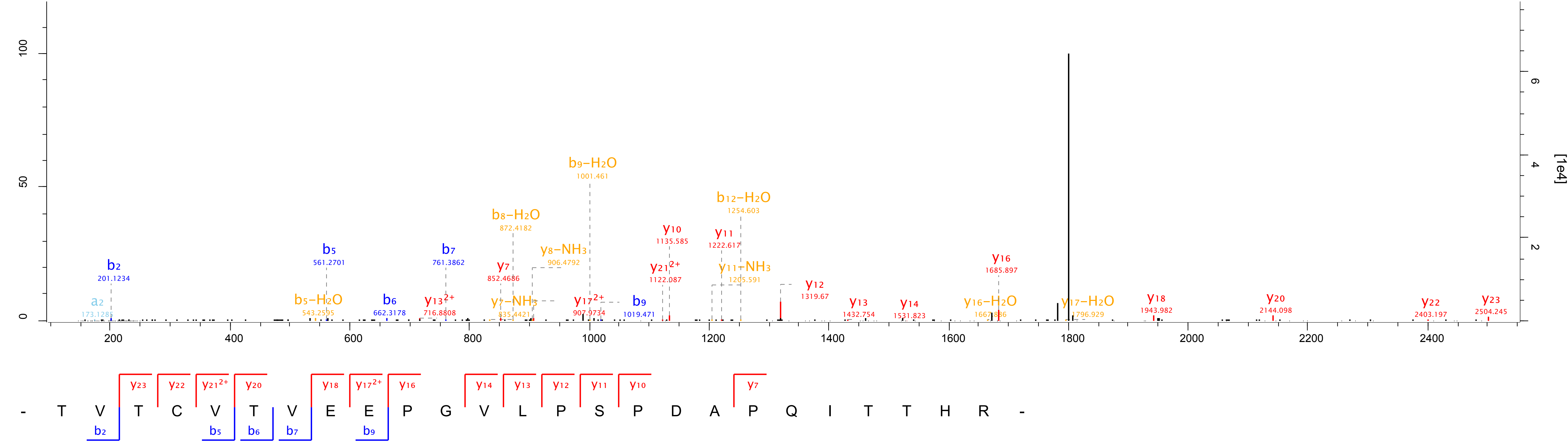

Raw file	Scan	Method	Score	m/z	Gene names
20150307_Hepa2_Top_opt_D2_01_1673	50610	TOF; CID	51.03	828.42	Nme7

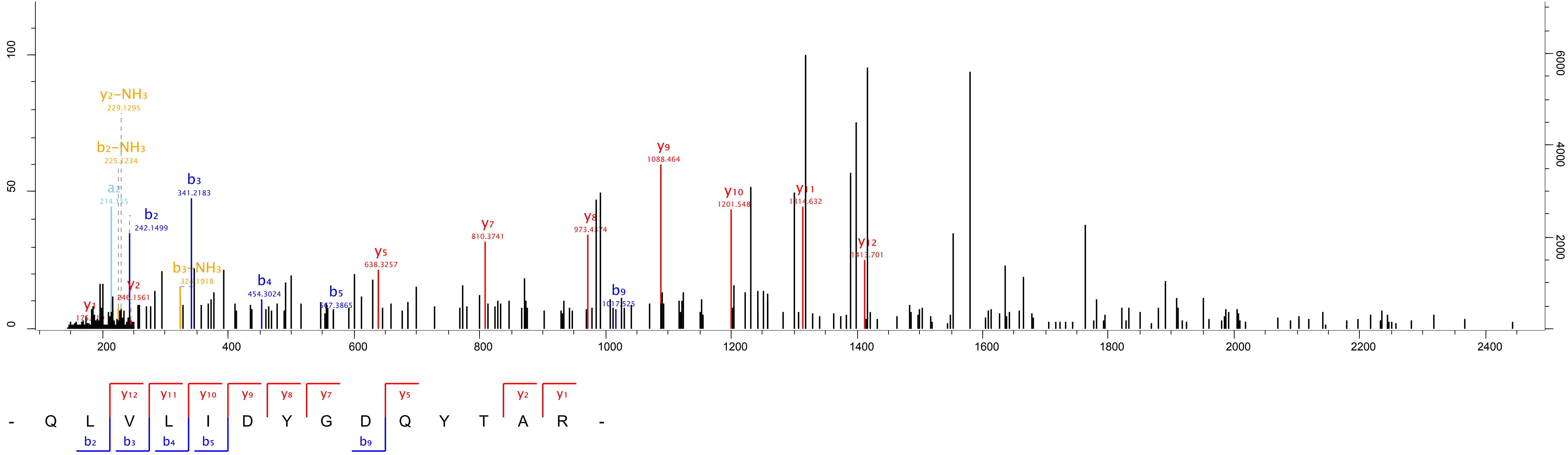

Raw file	Scan	Method	Score	m/z	Gene names
20150307_Hepa2_Top_opt_D2_01_1673	52325	TOF; CID	51.77	748.9	Mgst2

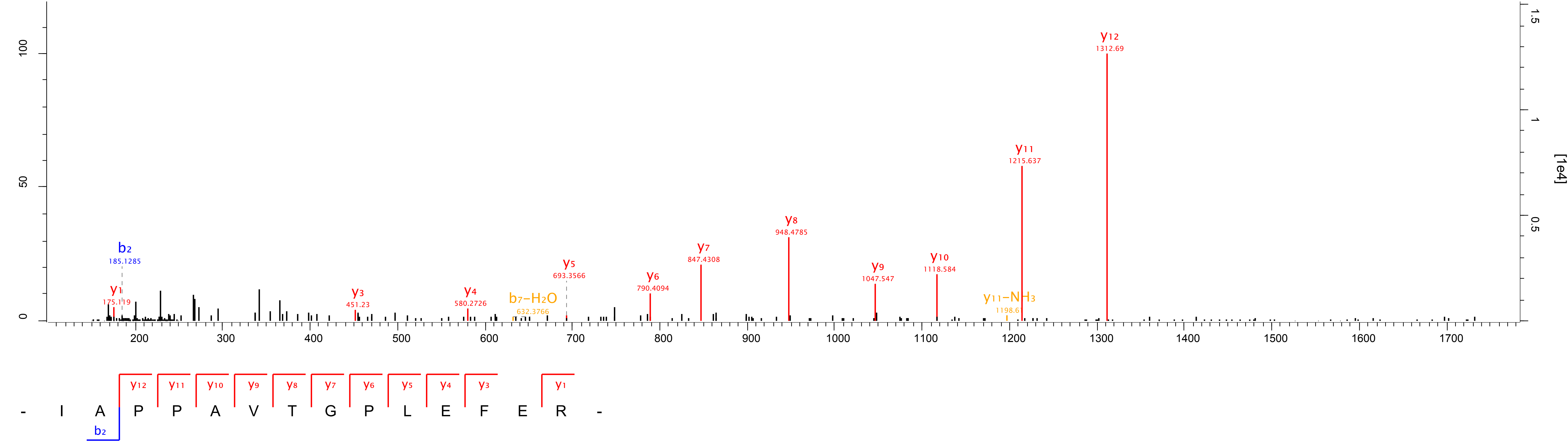

Raw file	Scan	Method	Score	m/z	Gene names
20150307_Hepa2_Top_opt_D2_01_1673	52801	TOF; CID	57.21	799.38	Ndfip1

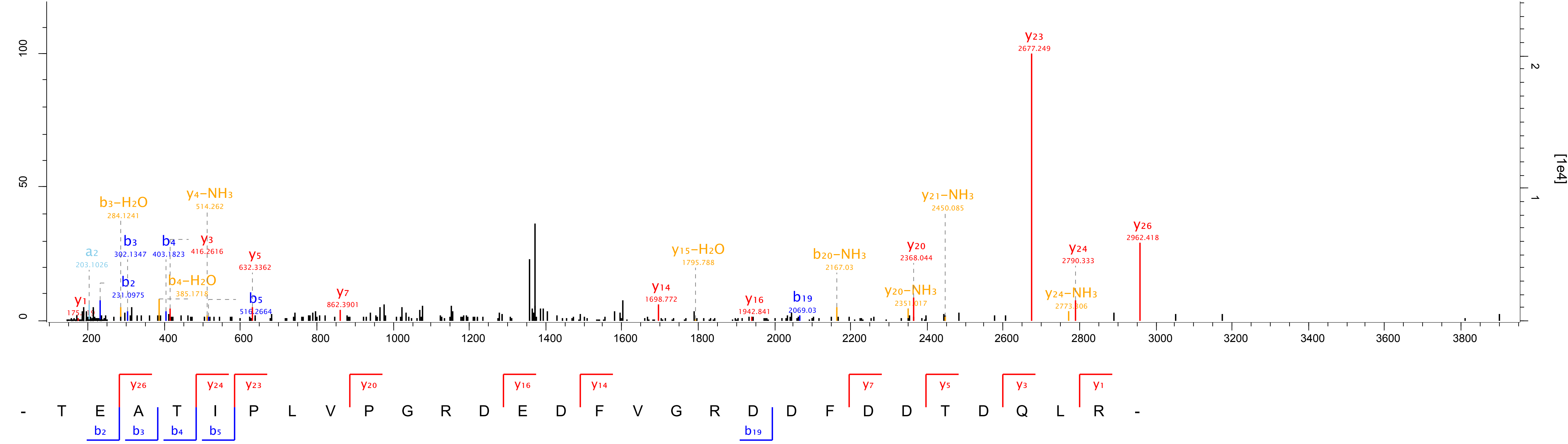

Raw file	Scan	Method	Score	m/z	Gene names
20150307_Hepa2_Top_opt_D2_01_1673	54029	TOF; CID	56.12	659.35	Ngly1

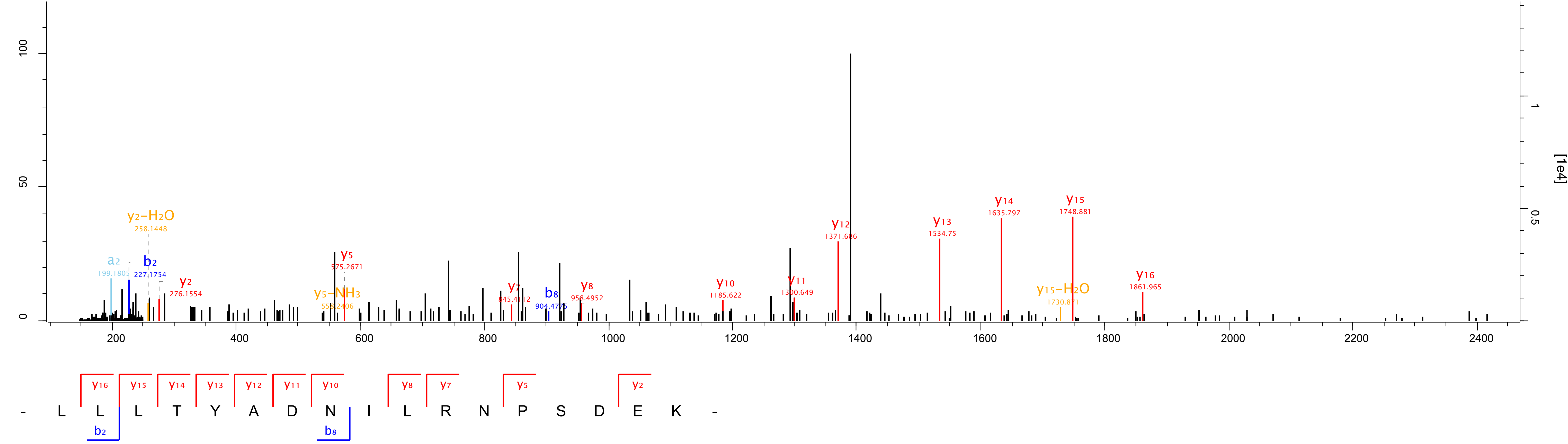

Raw file	Scan	Method	Score	m/z	Gene names
20150307_Hepa2_Top_opt_D2_01_1673	58469	TOF; CID	131.45	971.51	Cdca3

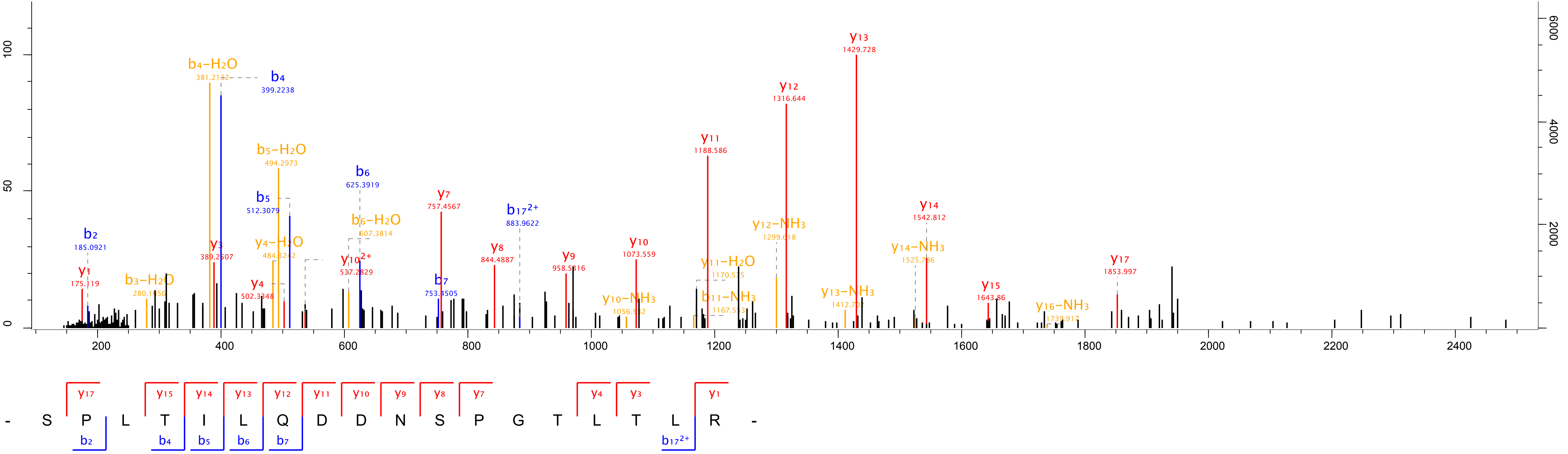

Raw file	Scan	Method	Score	m/z	Gene names
20150307_Hepa2_Top_opt_D2_01_1673	59212	TOF; CID	47.92	917.46	Nthl1

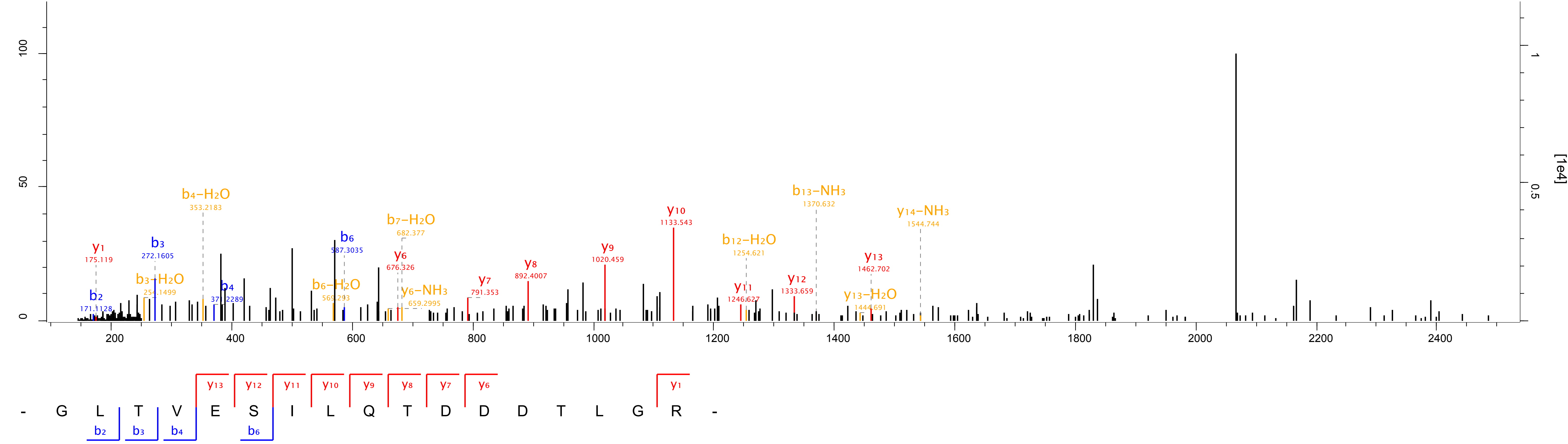

Raw file	Scan	Method	Score	m/z	Gene names
20150307_Hepa2_Top_opt_D2_01_1673	59859	TOF; CID	51.77	783.41	Gemin8

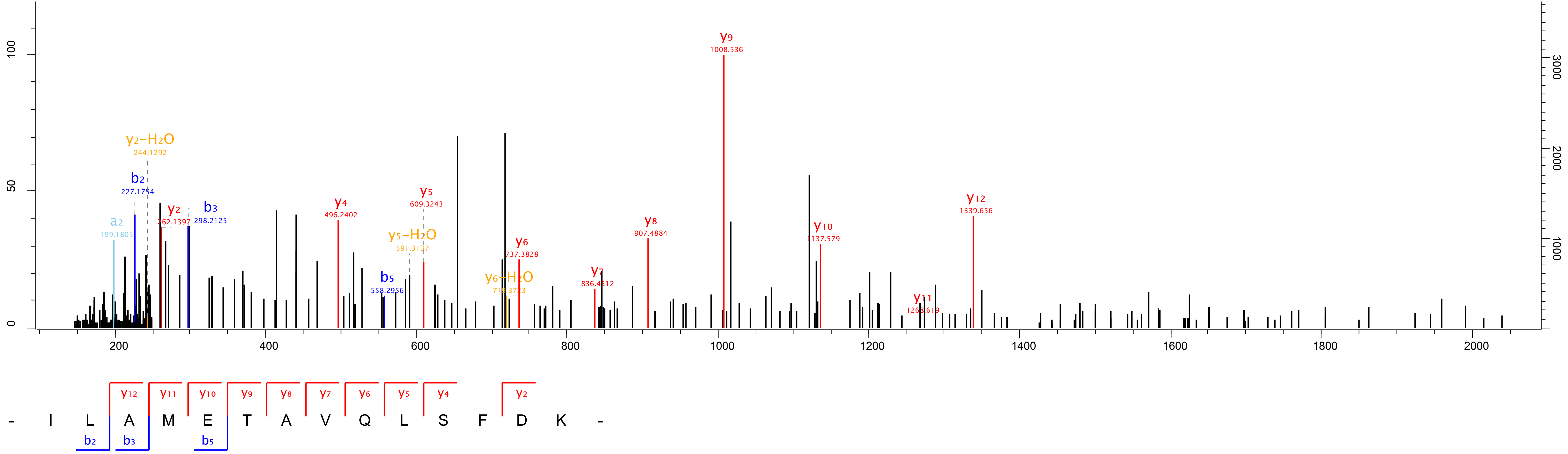

Raw file	Scan	Method	Score	m/z	Gene names
20150307_Hepa2_Top_opt_D2_01_1673	61370	TOF; CID	82.26	567.33	Rp2

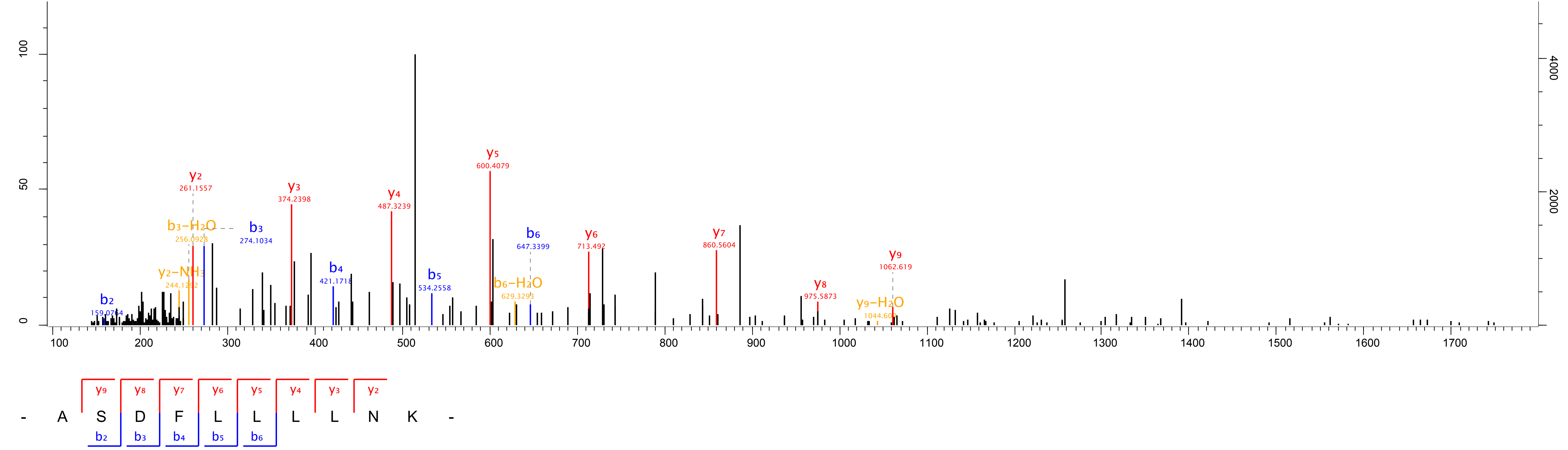

Raw file	Scan	Method	Score	m/z	Gene names
20150307_Hepa2_Top_opt_D2_01_1673	63388	TOF; CID	33.49	858.43	Rnpc3

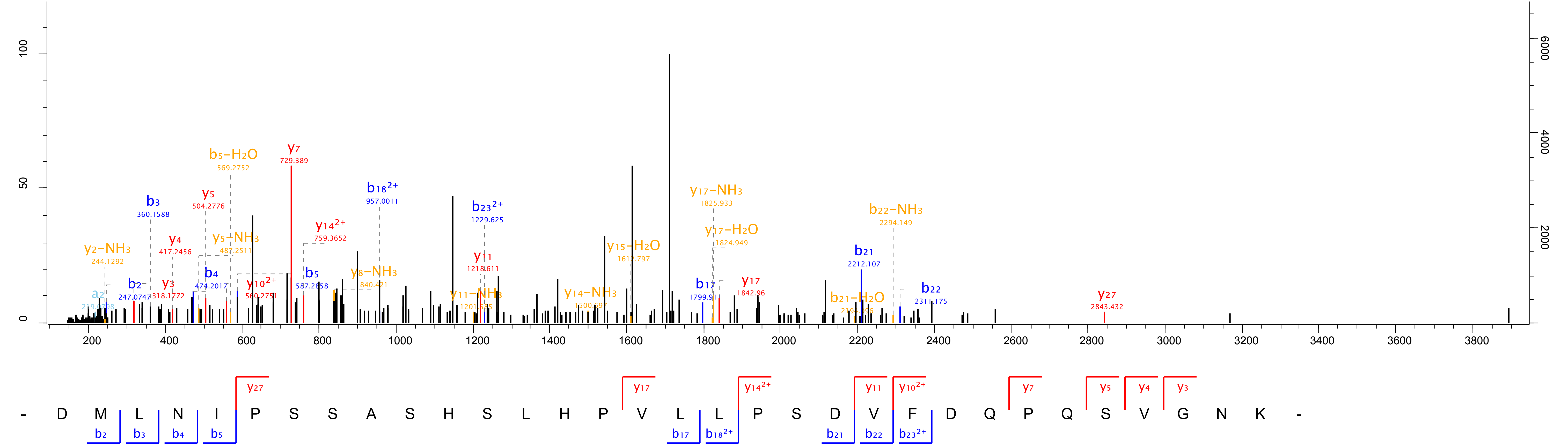

Raw file	Scan	Method	Score	m/z	Gene names
20150307_Hepa2_Top_opt_D2_01_1673	63495	TOF; CID	76.15	967.49	Cox11

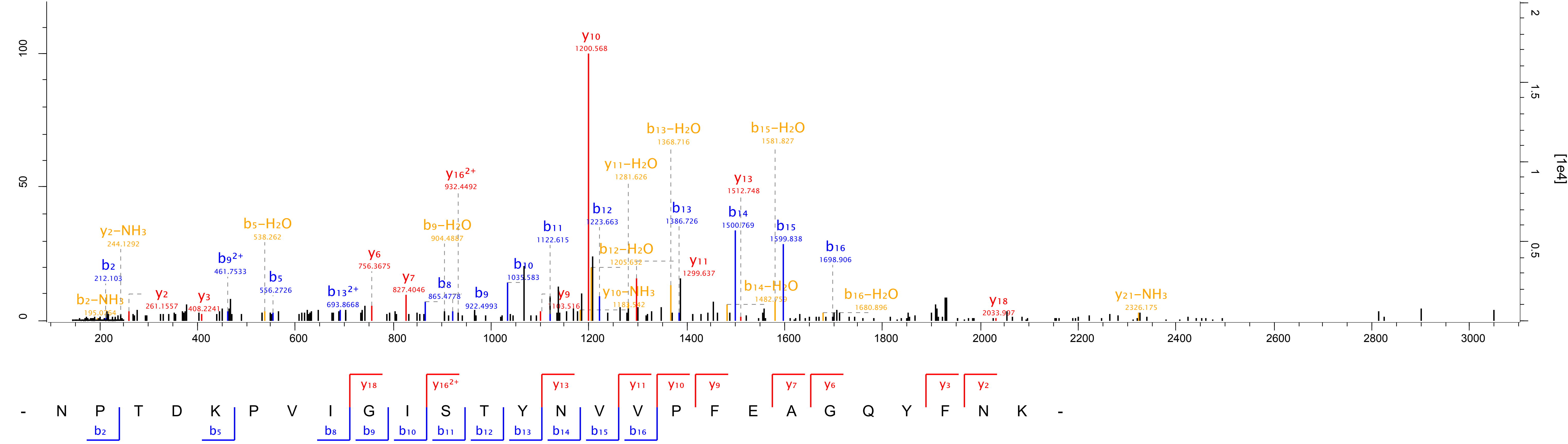

Raw file	Scan	Method	Score	m/z	Gene names
20150307_Hepa2_Top_opt_D2_01_1673	63700	TOF; CID	104.2	866.47	Tp53rk;Trp53rk;2810408M09Rik

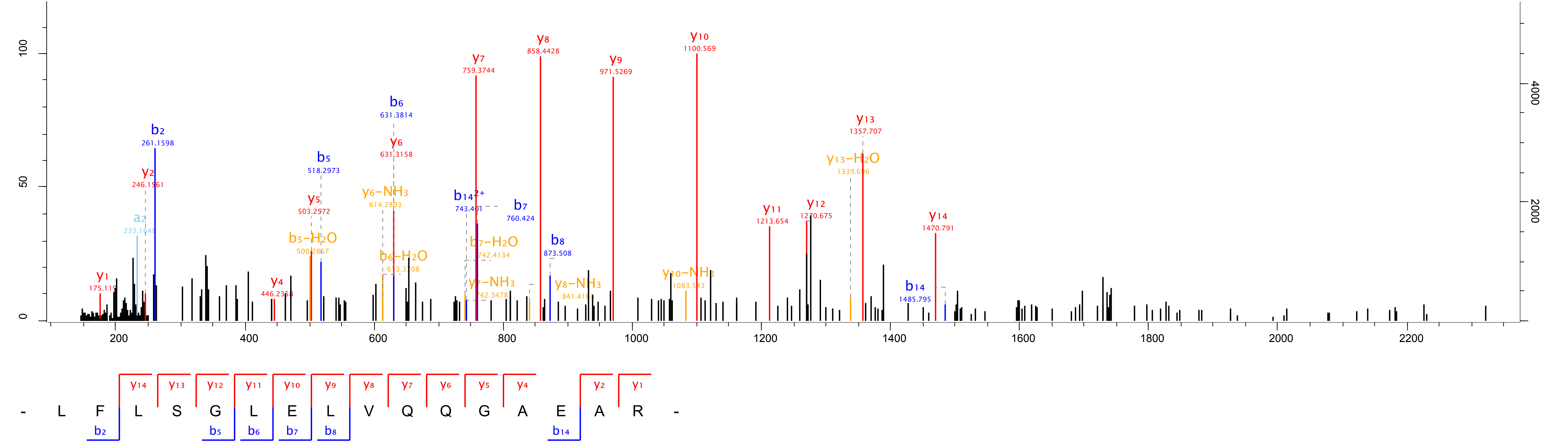

Raw file	Scan	Method	Score	m/z	Gene names
20150307_Hepa2_Top_opt_D2_01_1673	64371	TOF; CID	70.84	695.36	Mcee

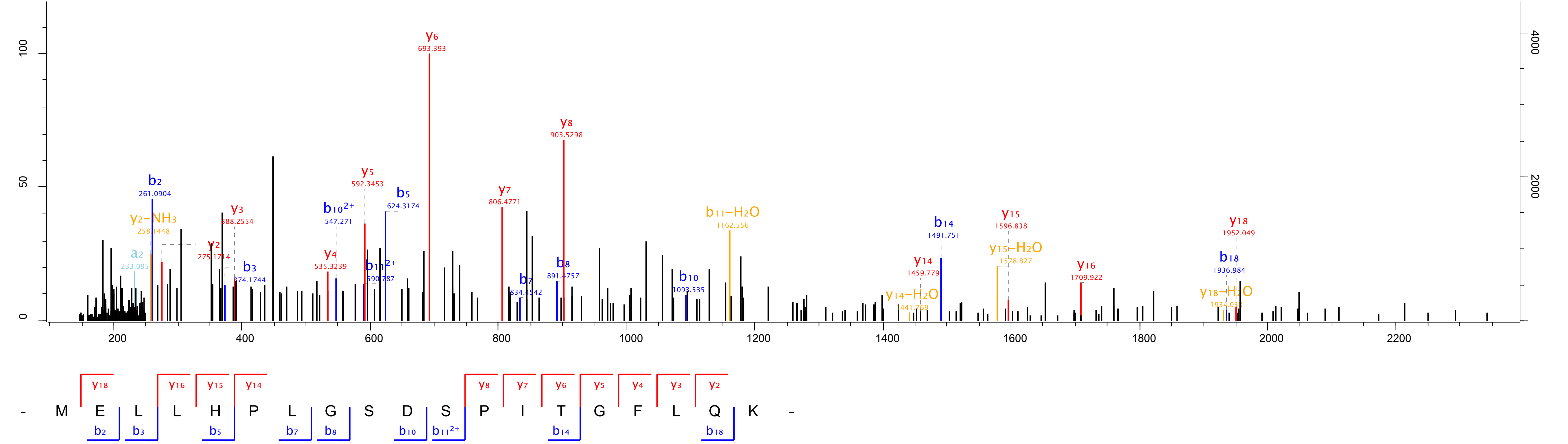

Raw file	Scan	Method	Score	m/z	Gene names
20150307_Hepa2_Top_opt_D2_01_1673	65602	TOF; CID	57.15	884.45	Adat3

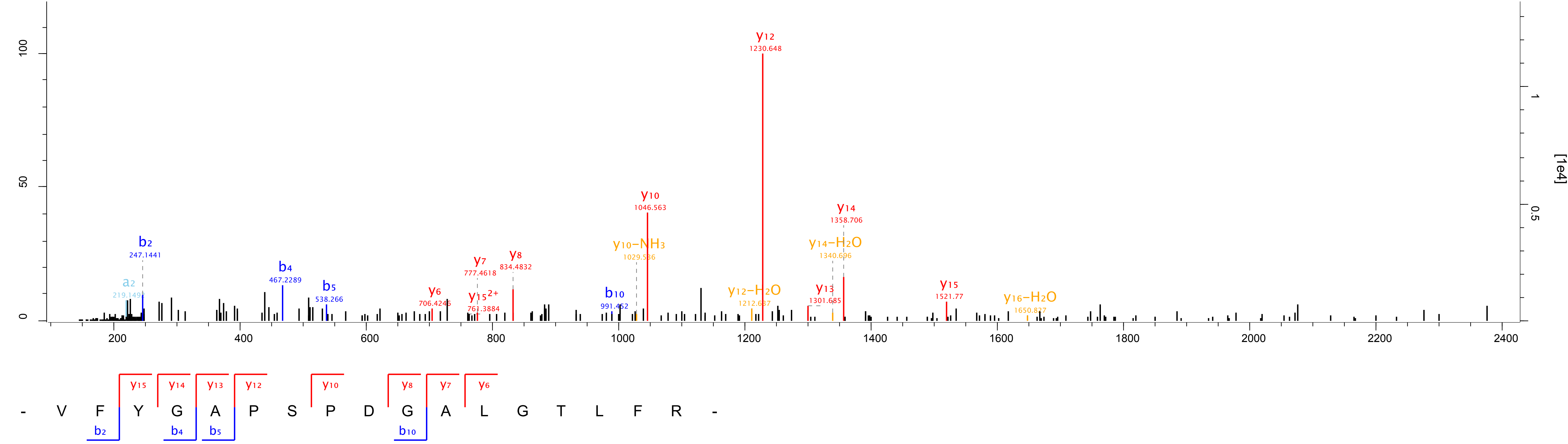

Raw file	Scan	Method	Score	m/z	Gene names
20150307_Hepa2_Top_opt_D2_01_1673	67454	TOF; CID	52.81	706.38	Mtnd1

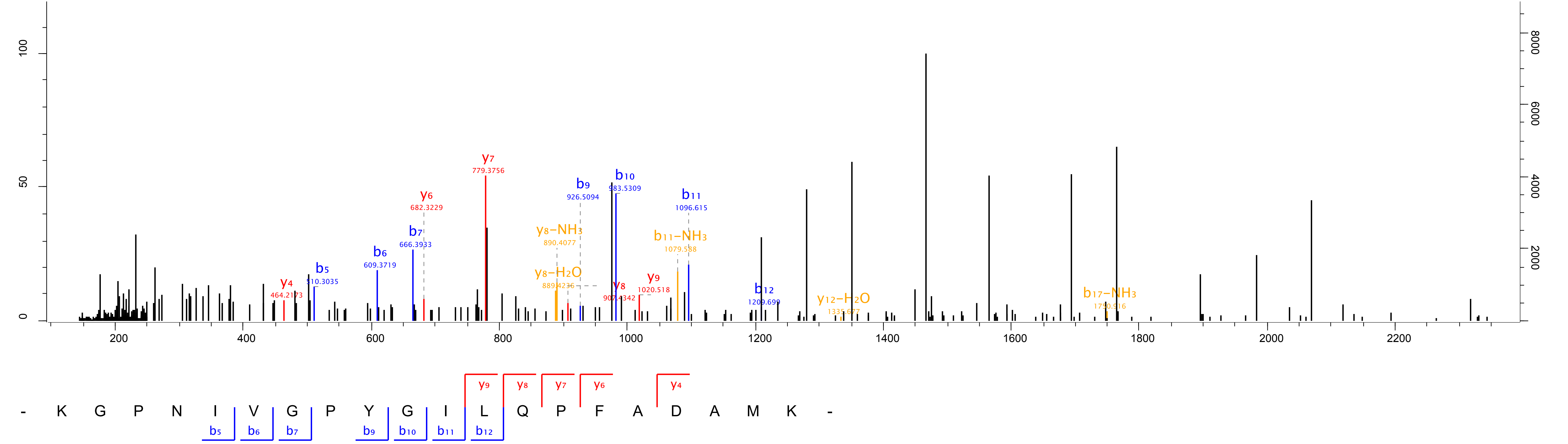

Raw file	Scan	Method	Score	m/z	Gene names
20150307_Hepa2_Top_opt_D2_01_1673	67886	TOF; CID	37.53	865.45	Med28

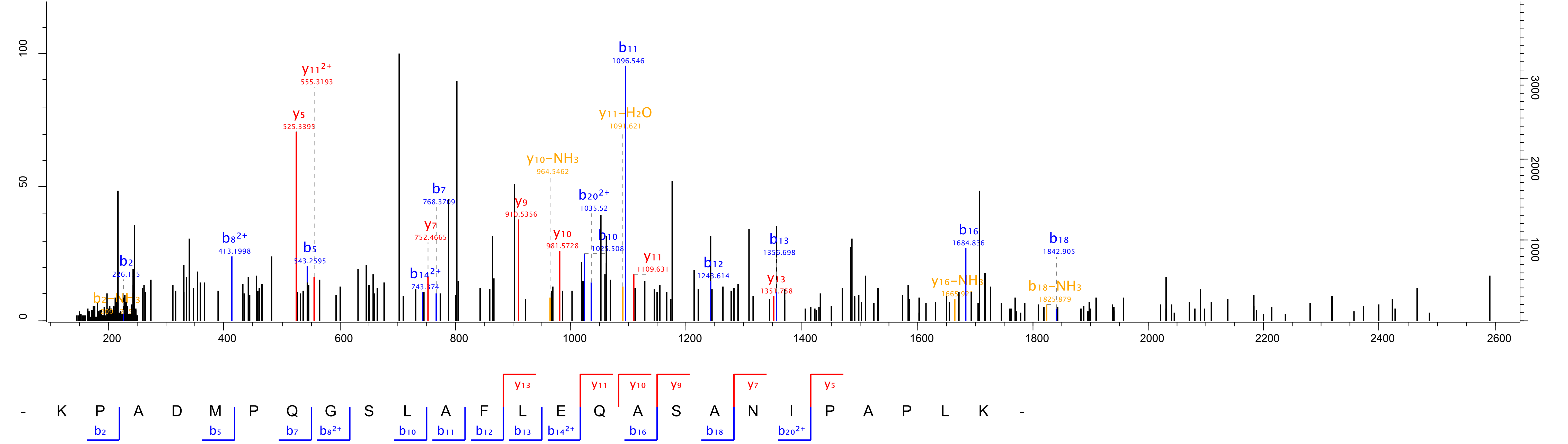

Raw file	Scan	Method	Score	m/z
20150307_Hepa2_Top_opt_D2_01_1686	7820	TOF; CID	78.34	635.28

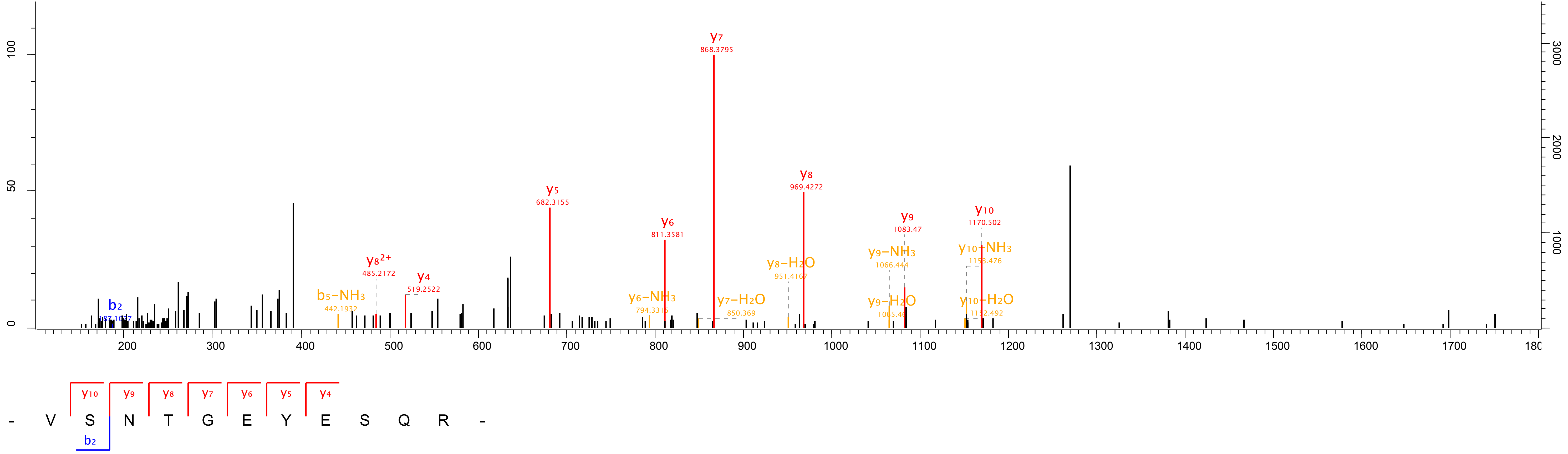

Raw file	Scan	Method	Score	m/z	Gene names
20150307_Hepa2_Top_opt_D2_01_1686	10335	TOF; CID	86.9	538.76	Med22

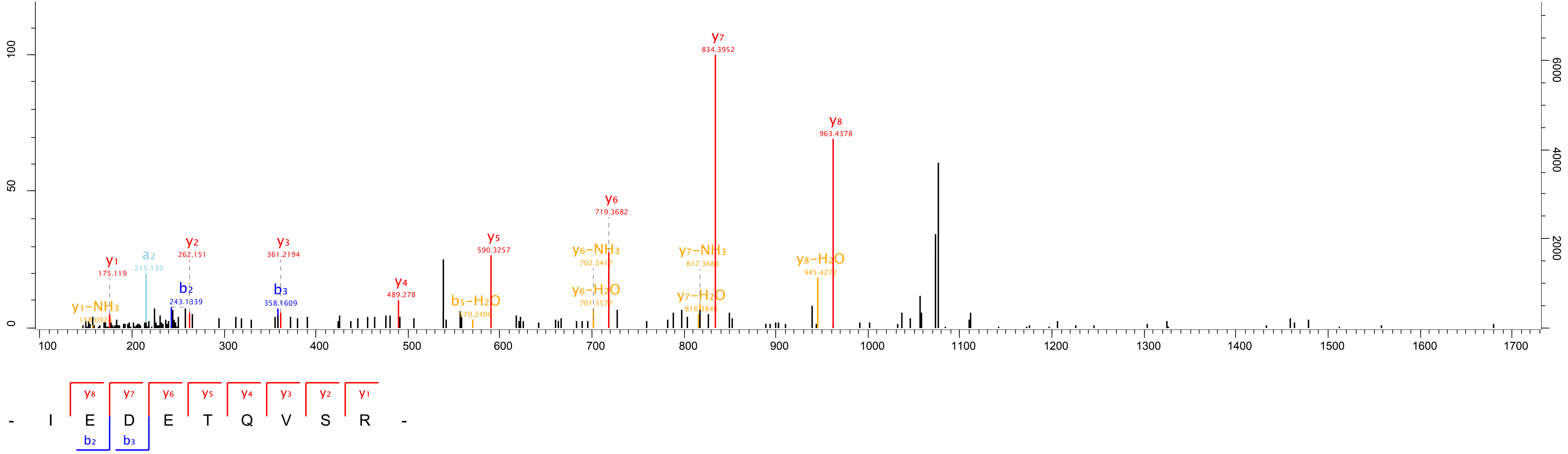

Raw file	Scan	Method	Score	m/z	Gene names
20150307_Hepa2_Top_opt_D2_01_1686	13137	TOF; CID	76.34	860.41	Sgol2

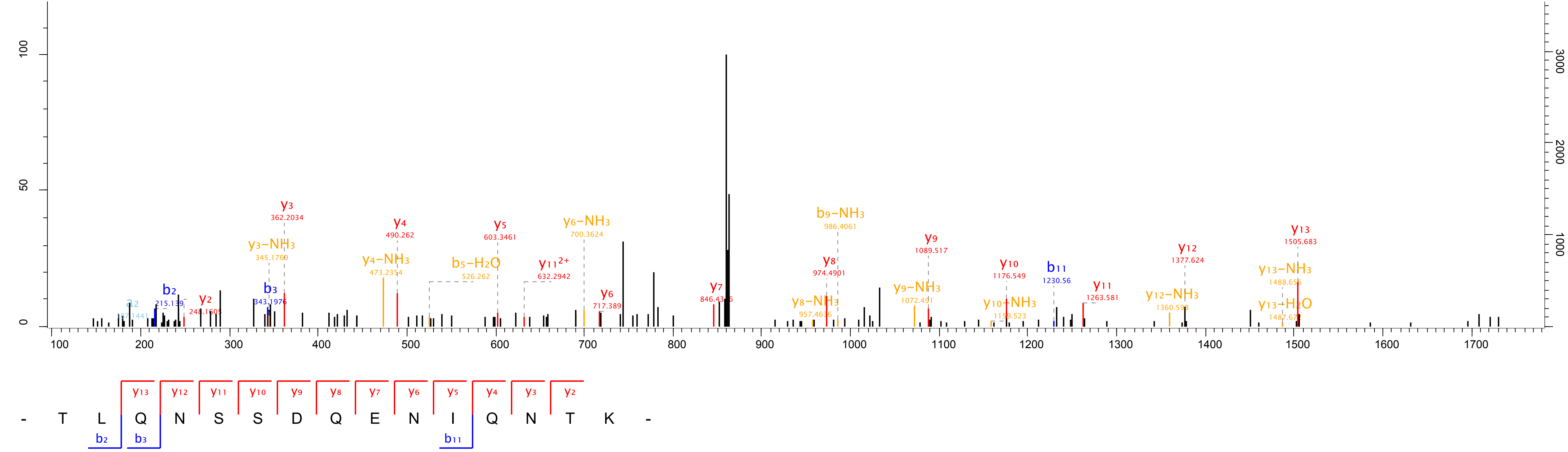

Raw file	Scan	Method	Score	m/z
20150307_Hepa2_Top_opt_D2_01_1686	15685	TOF; CID	78.14	510.27

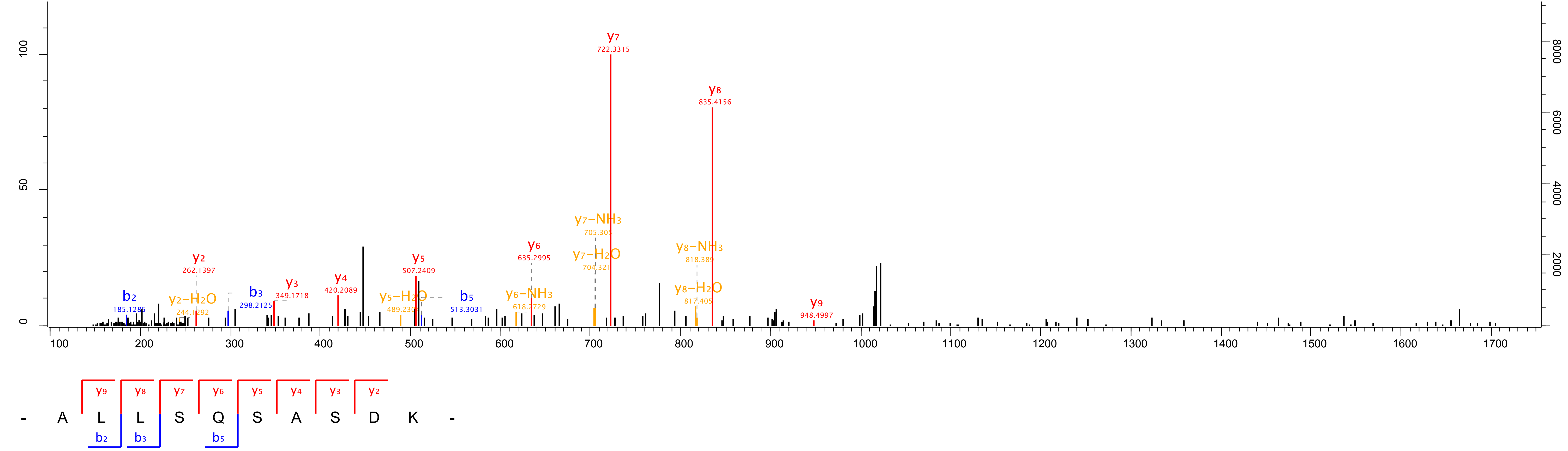

Raw file	Scan	Method	Score	m/z	Gene names
20150307_Hepa2_Top_opt_D2_01_1686	17330	TOF; CID	68.48	548.27	Tdp2

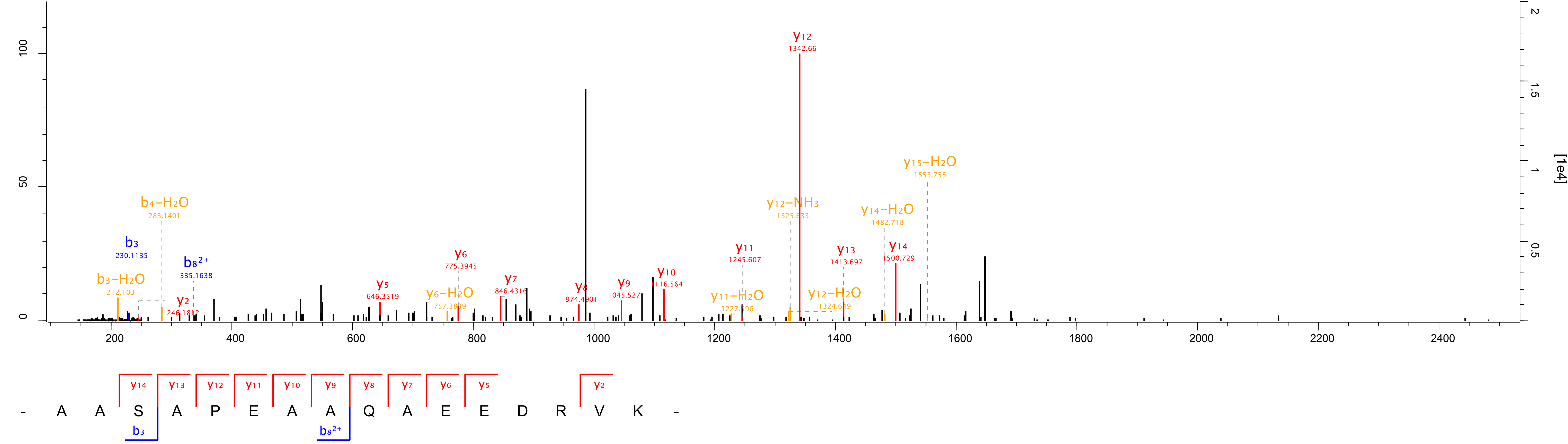

Raw file	Scan	Method	Score	m/z	Gene names
20150307_Hepa2_Top_opt_D2_01_1686	18805	TOF; CID	104.2	810.9	2310022A10Rik

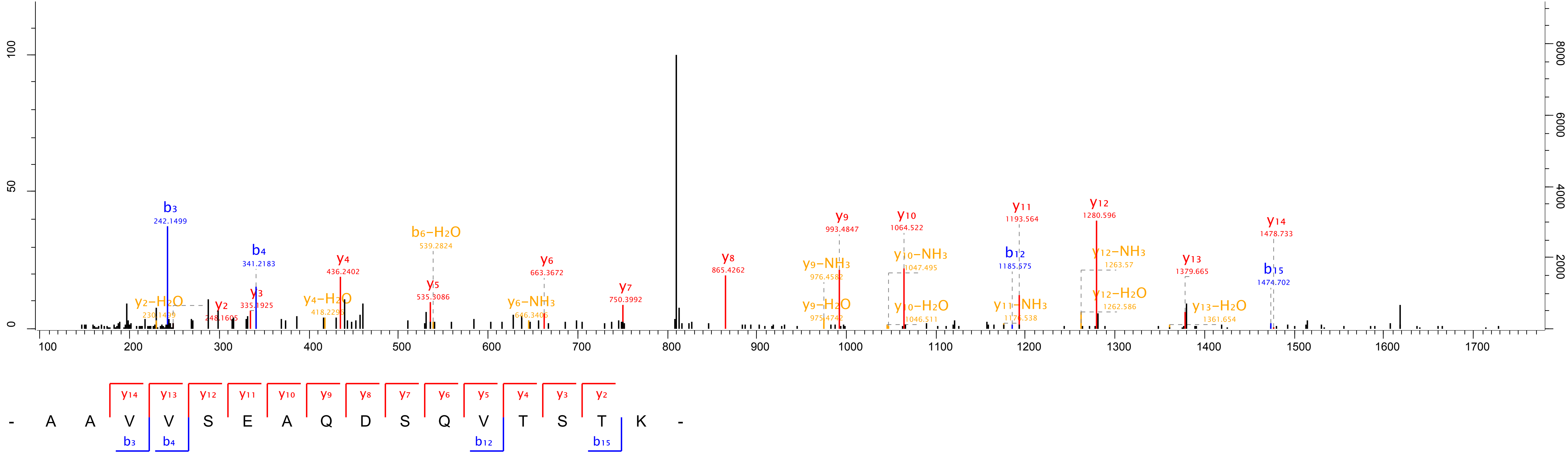

Raw file	Scan	Method	Score	m/z	Gene names
20150307_Hepa2_Top_opt_D2_01_1686	21715	TOF; CID	79.66	599.82	Psmb10

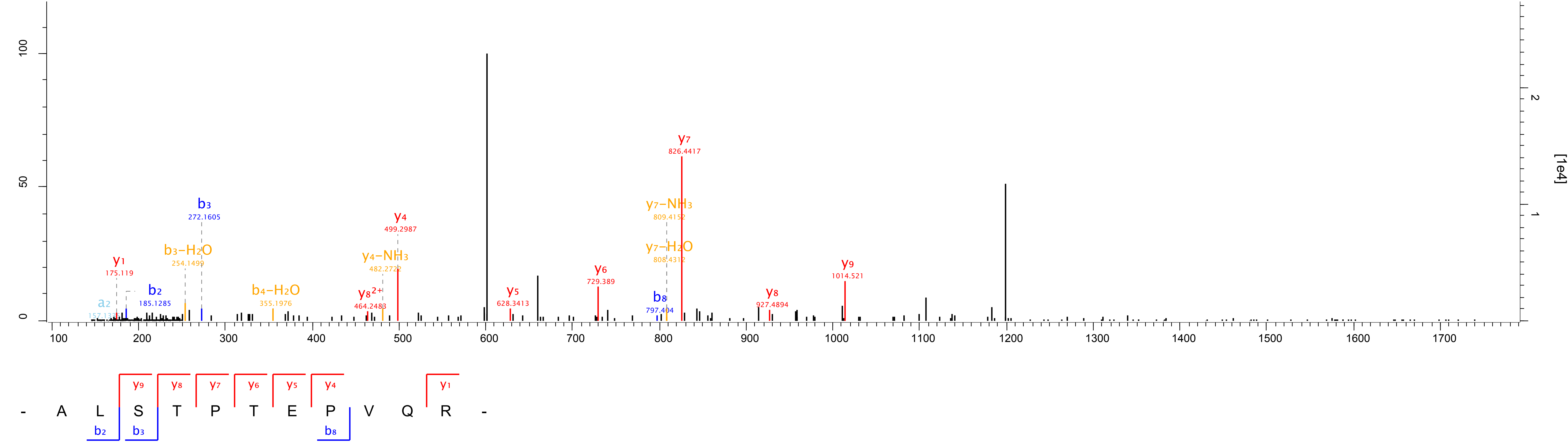

Raw file	Scan	Method	Score	m/z	Gene names
20150307_Hepa2_Top_opt_D2_01_1686	26563	TOF; CID	60.26	453.24	Nsun4

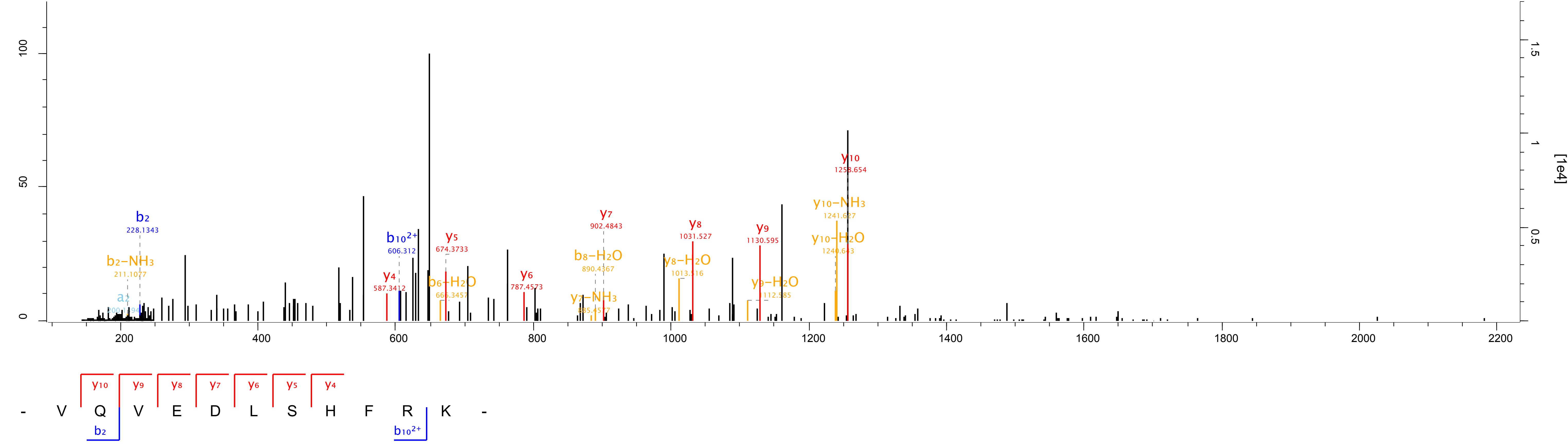

Raw file	Scan	Method	Score	m/z	Gene names
20150307_Hepa2_Top_opt_D2_01_1686	30992	TOF; CID	76.82	569.64	Klhl42

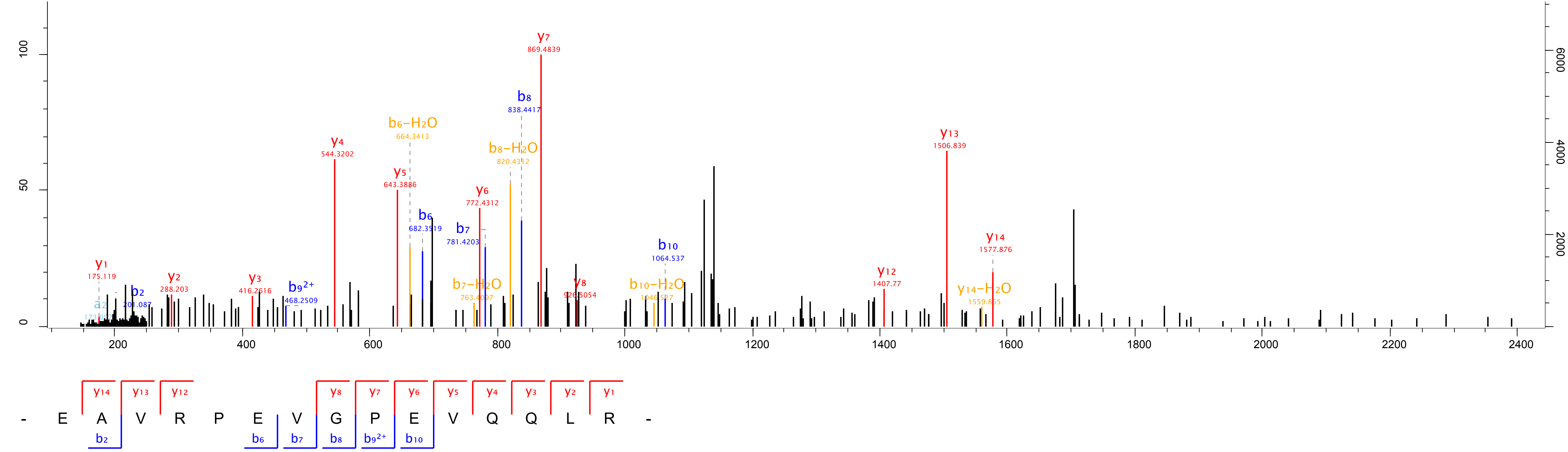

Raw file	Scan	Method	Score	m/z	Gene names
20150307_Hepa2_Top_opt_D2_01_1686	39518	TOF; CID	50.3	777.39	Suv39h1

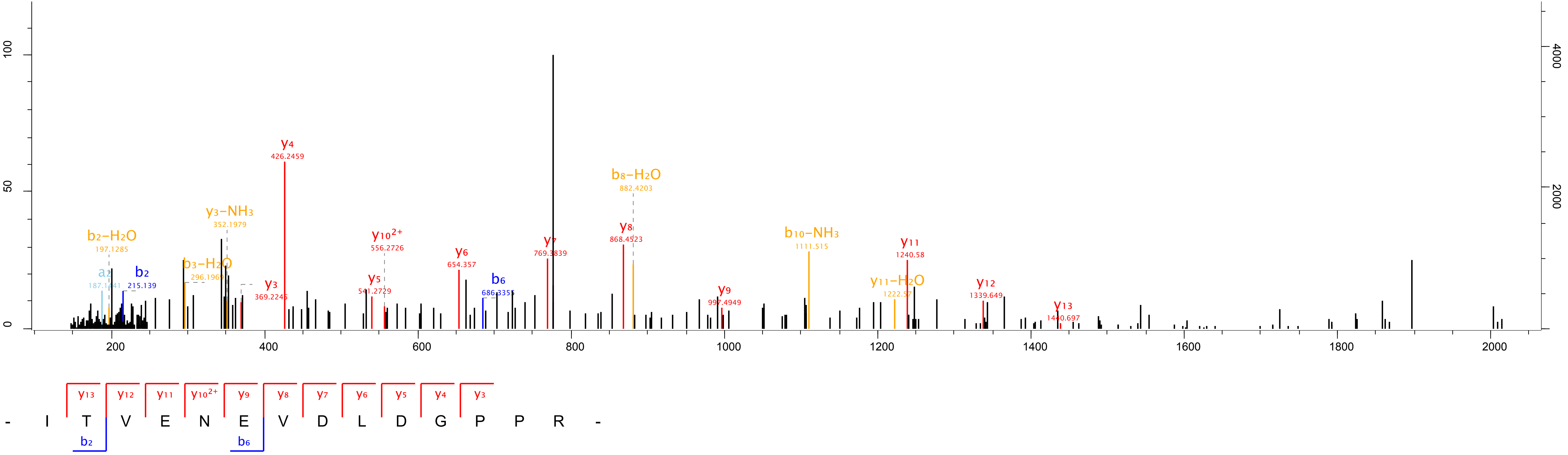

Raw file	Scan	Method	Score	m/z	Gene names
20150307_Hepa2_Top_opt_D2_01_1686	40103	TOF; CID	43.03	438.74	Grik5

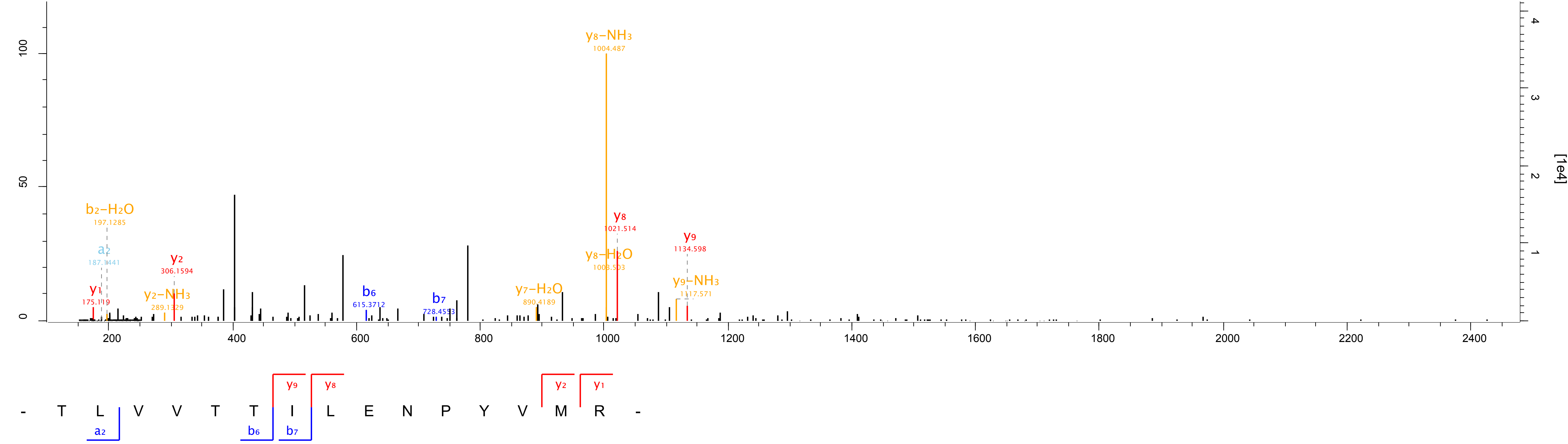

Raw file	Scan	Method	Score	m/z	Gene names
20150307_Hepa2_Top_opt_D2_01_1686	42293	TOF; CID	66.99	503.26	Slc35e1

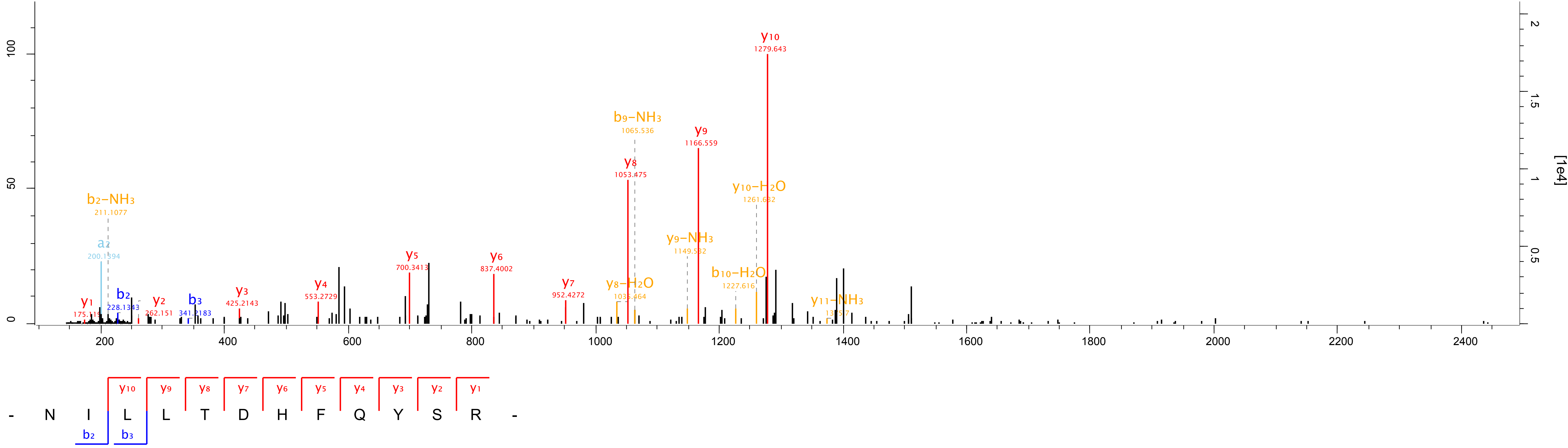

Raw file	Scan	Method	Score	m/z	Gene names
20150307_Hepa2_Top_opt_D2_01_1686	47373	TOF; CID	48.22	596.31	Vps72

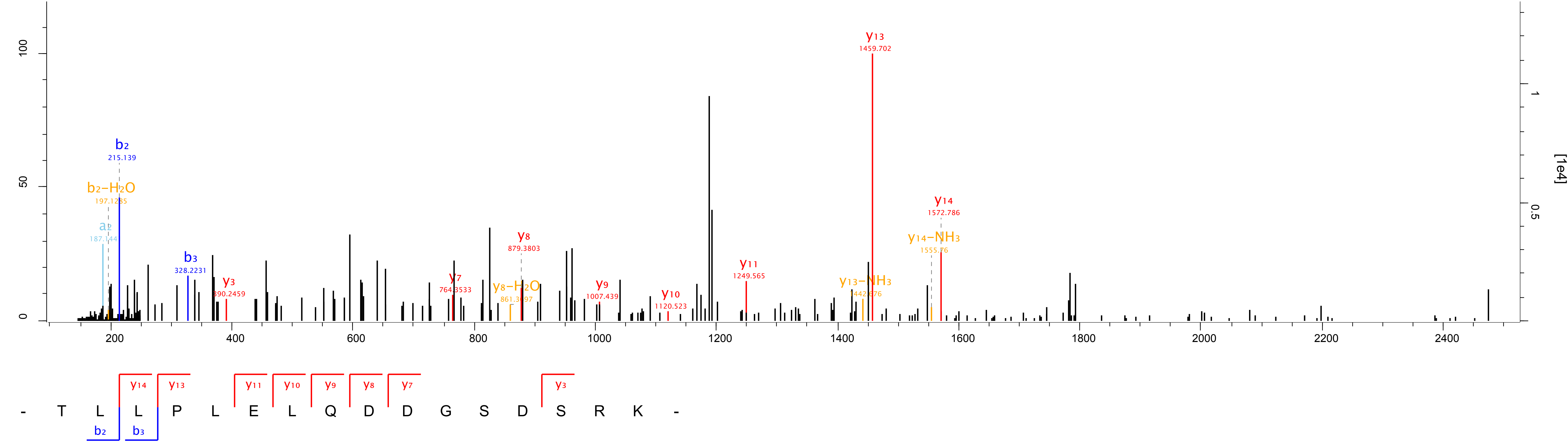

Raw file	Scan	Method	Score	m/z	Gene names
20150307_Hepa2_Top_opt_D2_01_1686	47636	TOF; CID	109.72	1008.46	Rhbdd1

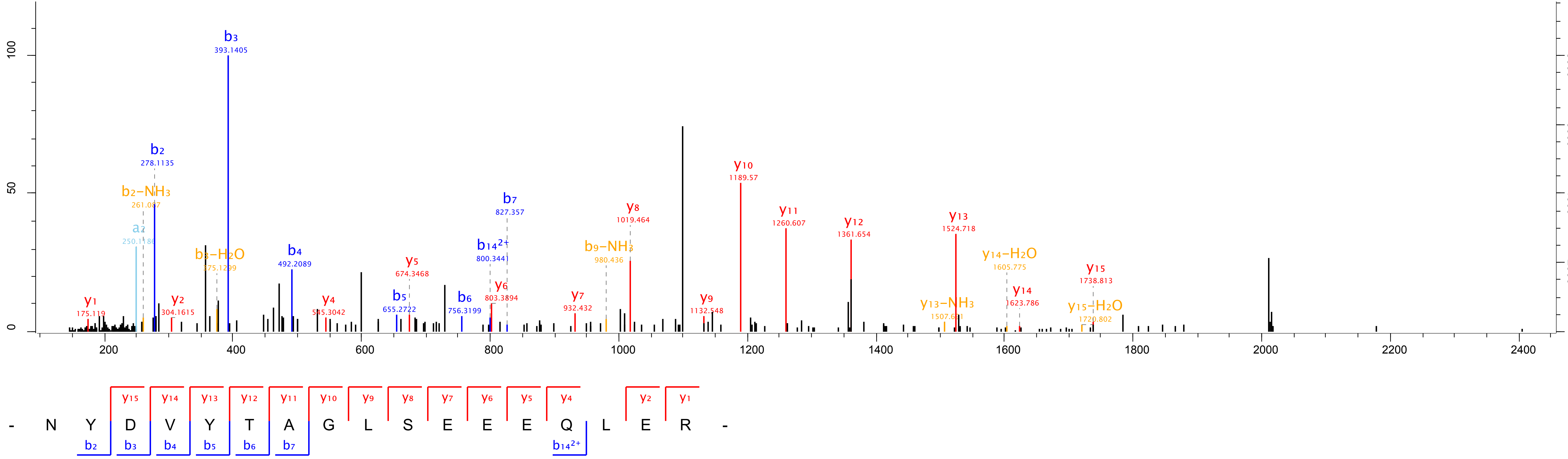

Raw file	Scan	Method	Score	m/z	Gene names
20150307_Hepa2_Top_opt_D2_01_1686	47760	TOF; CID	65.92	725.35	Stard4

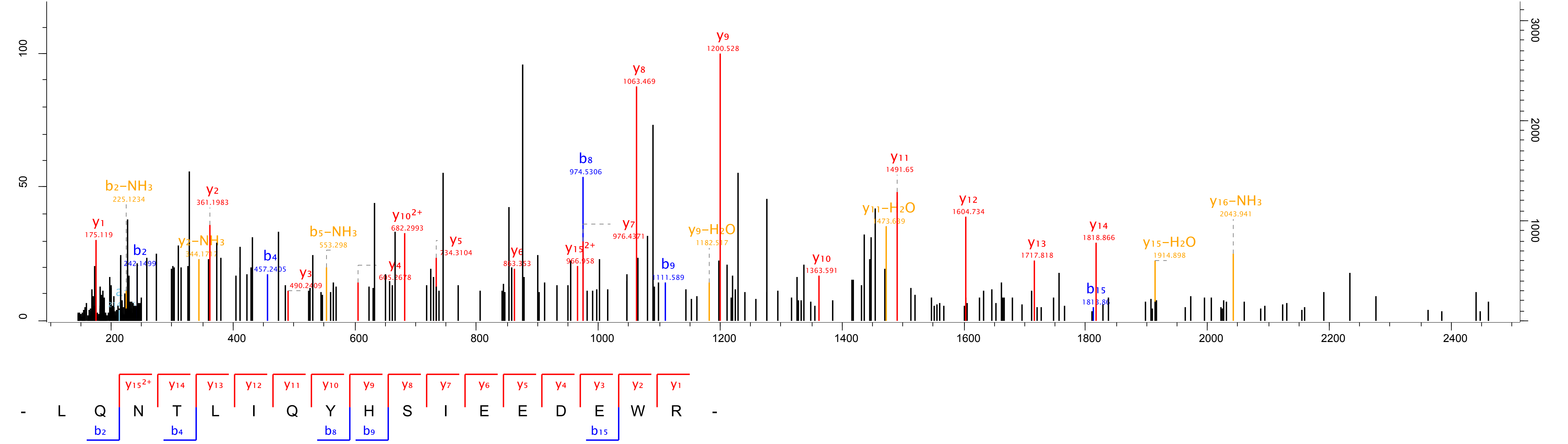

Raw file	Scan	Method	Score	m/z	Gene names
20150307_Hepa2_Top_opt_D2_01_1686	47804	TOF; CID	77.51	894.46	Bicc1

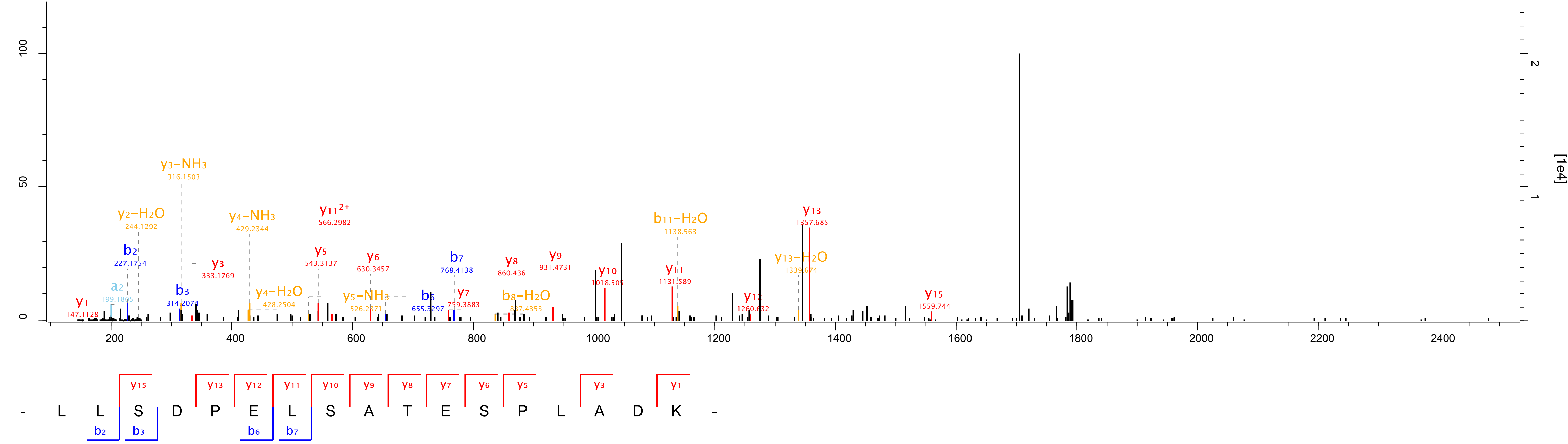

Raw file	Scan	Method	Score	m/z	Gene names
20150307_Hepa2_Top_opt_D2_01_1686	51692	TOF; CID	70.45	764.39	Tmppe

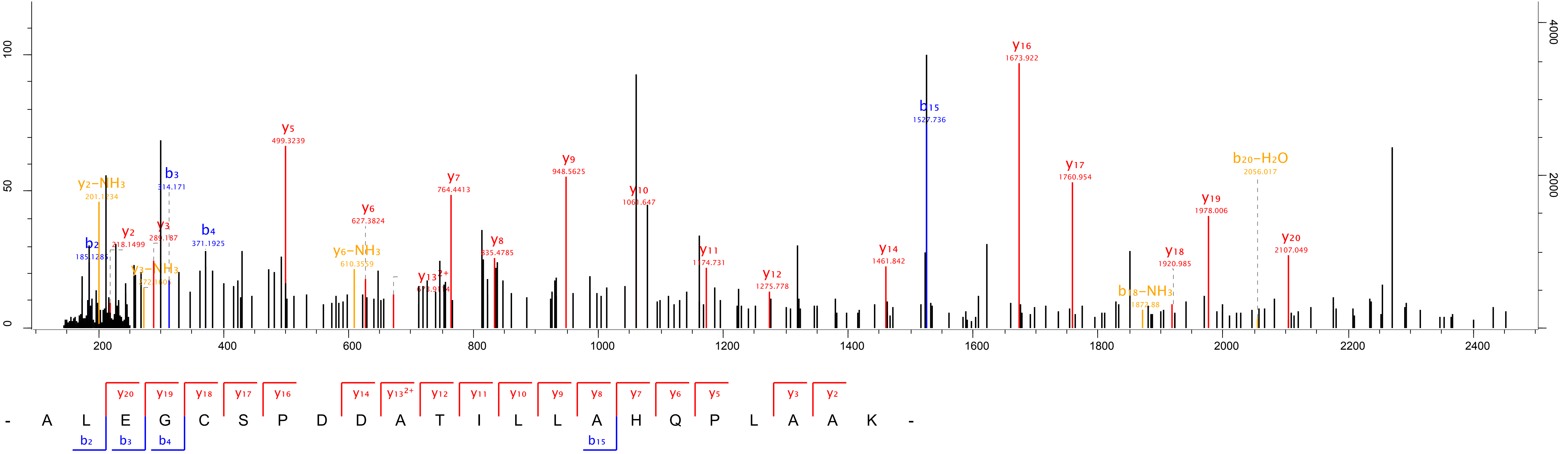

Raw file	Scan	Method	Score	m/z	Gene names
20150307_Hepa2_Top_opt_D2_01_1686	53274	TOF; CID	76.23	595.83	Ggact

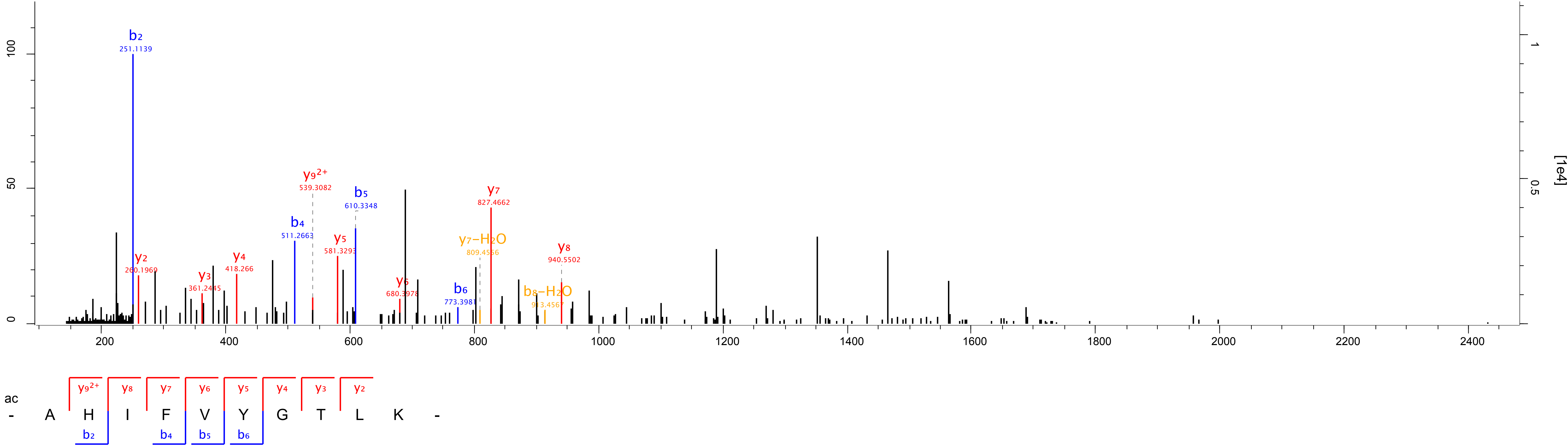

Raw file	Scan	Method	Score	m/z	Gene names
20150307_Hepa2_Top_opt_D2_01_1686	60047	TOF; CID	122.53	1135.85	Derl2

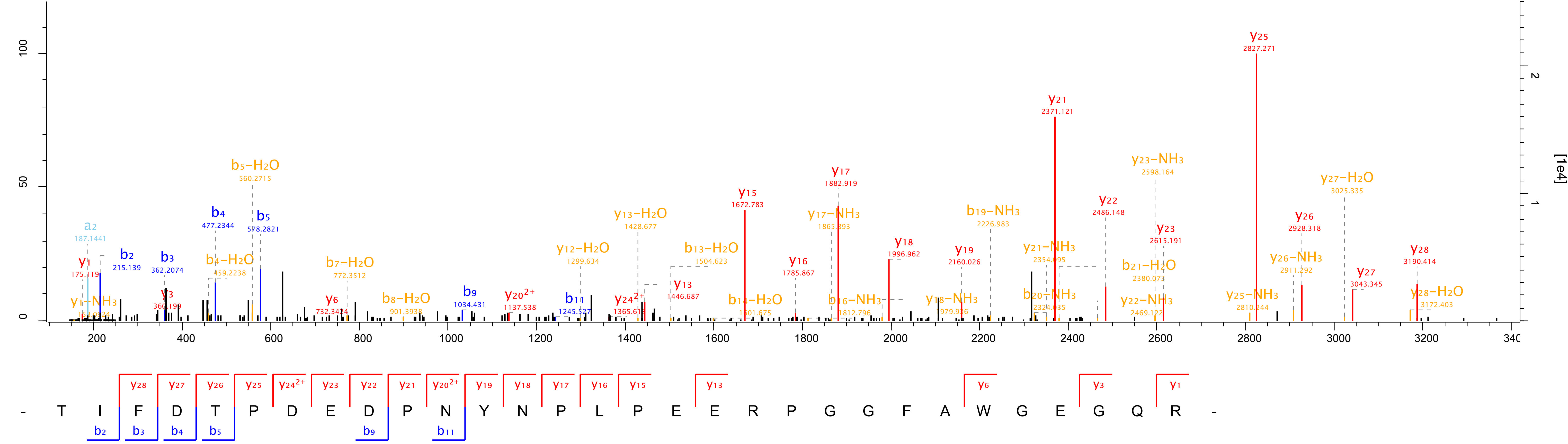

Raw file	Scan	Method	Score	m/z	Gene names
20150307_Hepa2_Top_opt_D2_01_1686	61589	TOF; CID	77.47	762.4	Mthfsd

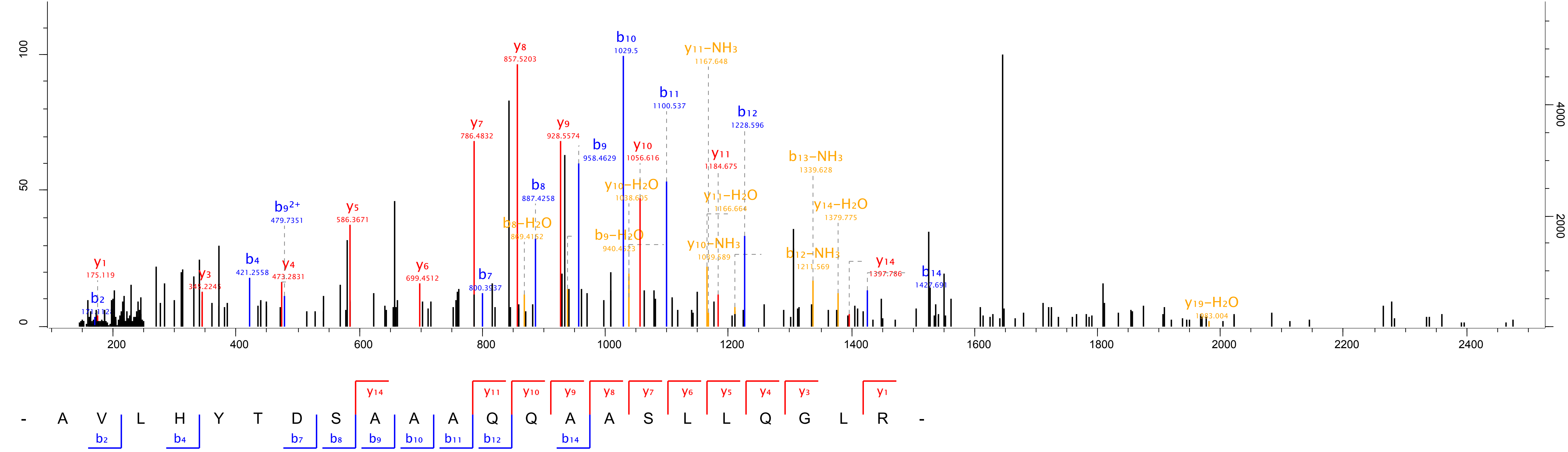

Raw file	Scan	Method	Score	m/z	Gene names
20150307_Hepa2_Top_opt_D2_01_1686	62047	TOF; CID	135.38	754.69	Cnih4

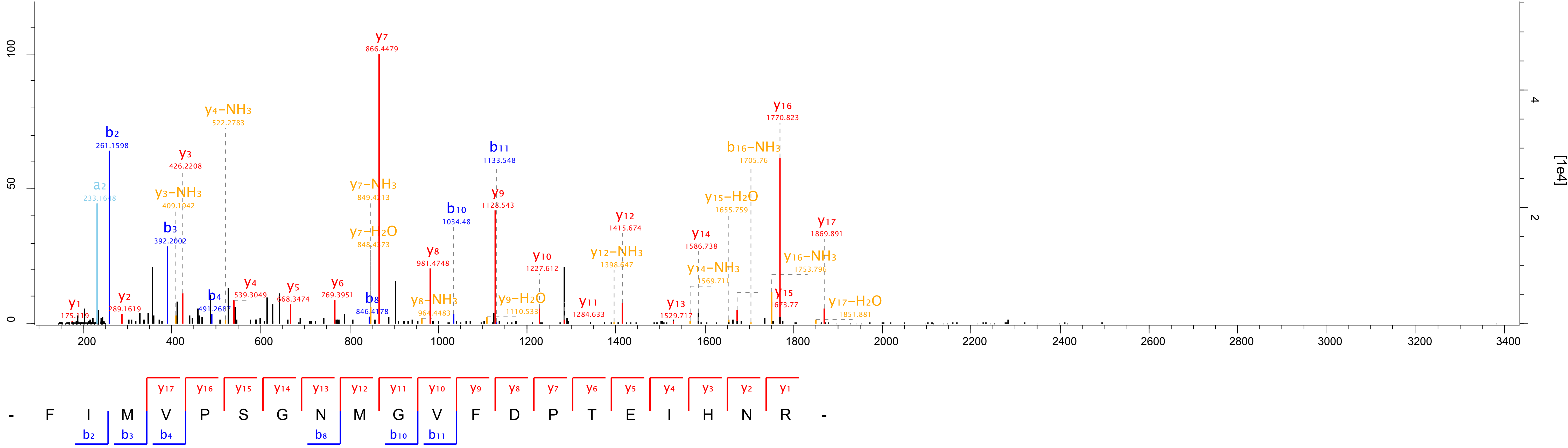

Raw file	Scan	Method	Score	m/z	Gene names
20150307_Hepa2_Top_opt_D2_01_1686	62069	TOF; CID	88.39	642.32	Slc4a4

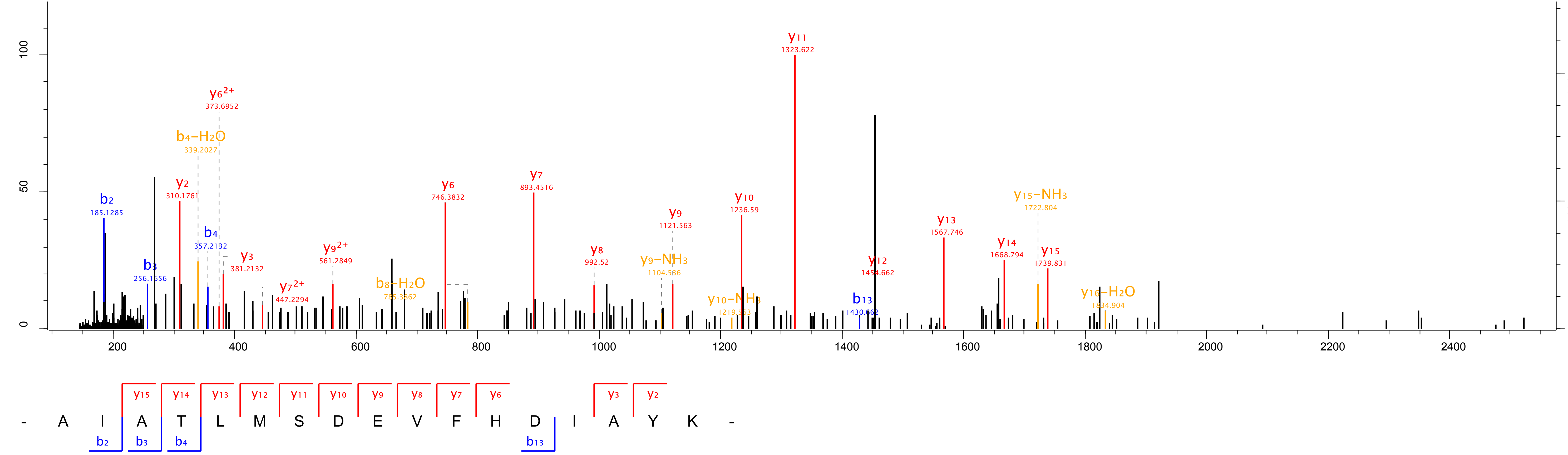

Raw file	Scan	Method	Score	m/z	Gene names
20150307_Hepa2_Top_opt_D2_01_1686	62533	TOF; CID	66.66	590.35	Ryr1

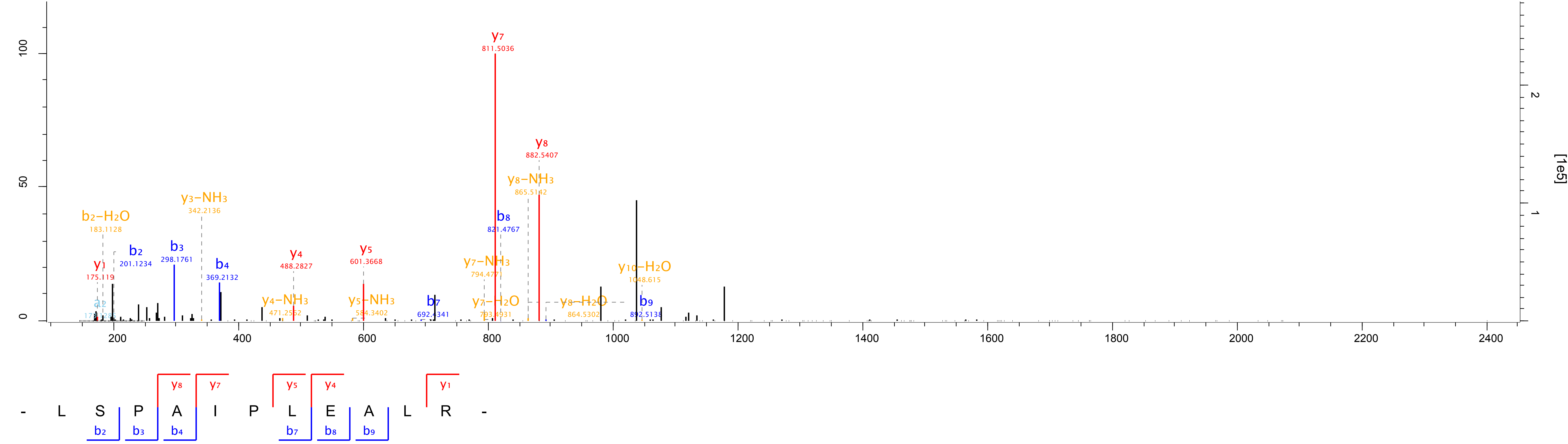

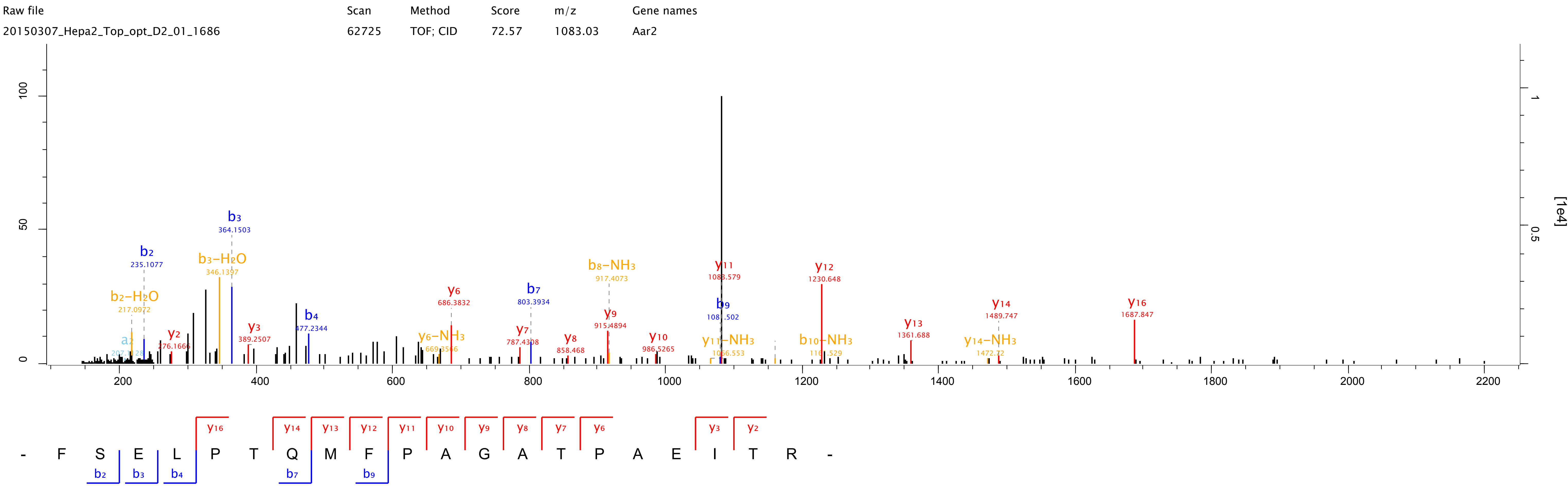

Raw file
20150307_Hepa2_Top_opt_D2_01_1686
Scan
62725
Method
TOF; CID
Score
72.57
m/z
1083.03
Gene names
Aar2
100
50
0
1
0.5
[1e4]
200
400
600
800
1000
1200
1400
1600
1800
2000
2200
b2 235.1077
b3 364.1503
b4 477.2344
b7 803.3934
b9
b2-H2O 217.0972
b3-H2O 346.1397
b8-NH3 917.4073
y6-NH3
y11-NH3
b10-NH3
y14-NH3
y2 276.1666
y3 389.2507
y6 686.3832
y7
y8 858.468
y9
y10 986.5265
y11
y12 1230.648
y13 1361.688
y14 1489.747
y16 1687.847
- F S E L P T Q M F P A G A T P A E I T R -
y16 y14 y13 y12 y11 y10 y9 y8 y7 y6 y3 y2
b2 b3 b4 b7 b9

Raw file	Scan	Method	Score	m/z	Gene names
20150307_Hepa2_Top_opt_D2_01_1686	64601	TOF; CID	50.3	792.44	Slc7a11

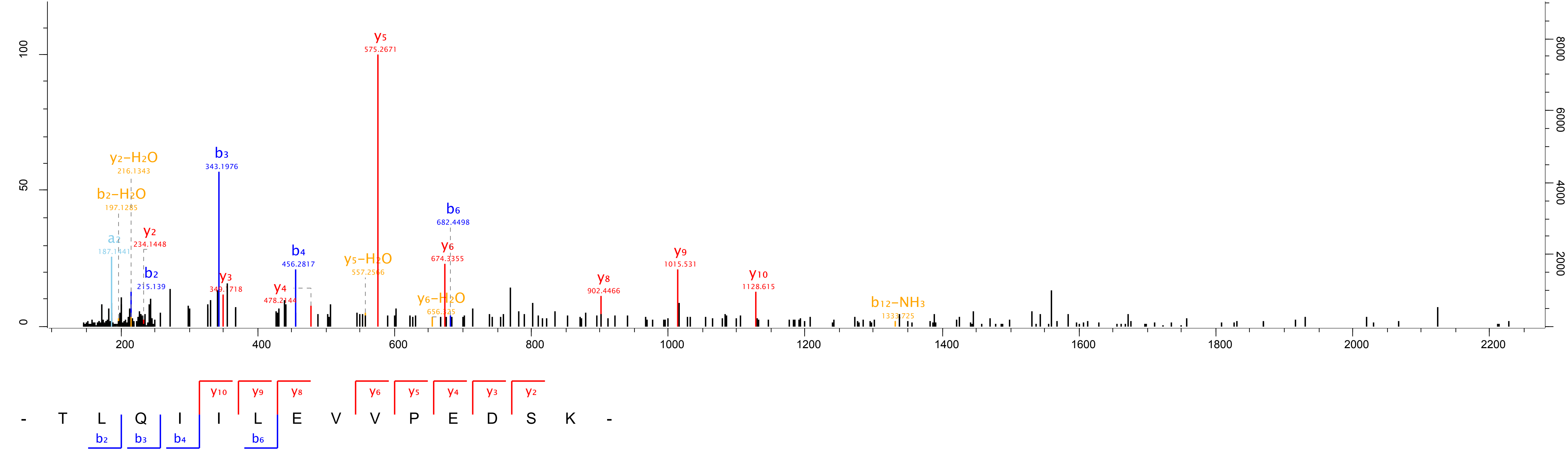

Raw file	Scan	Method	Score	m/z	Gene names
20150307_Hepa2_Top_opt_D2_01_1686	66815	TOF; CID	90.15	521.83	Pik3ca

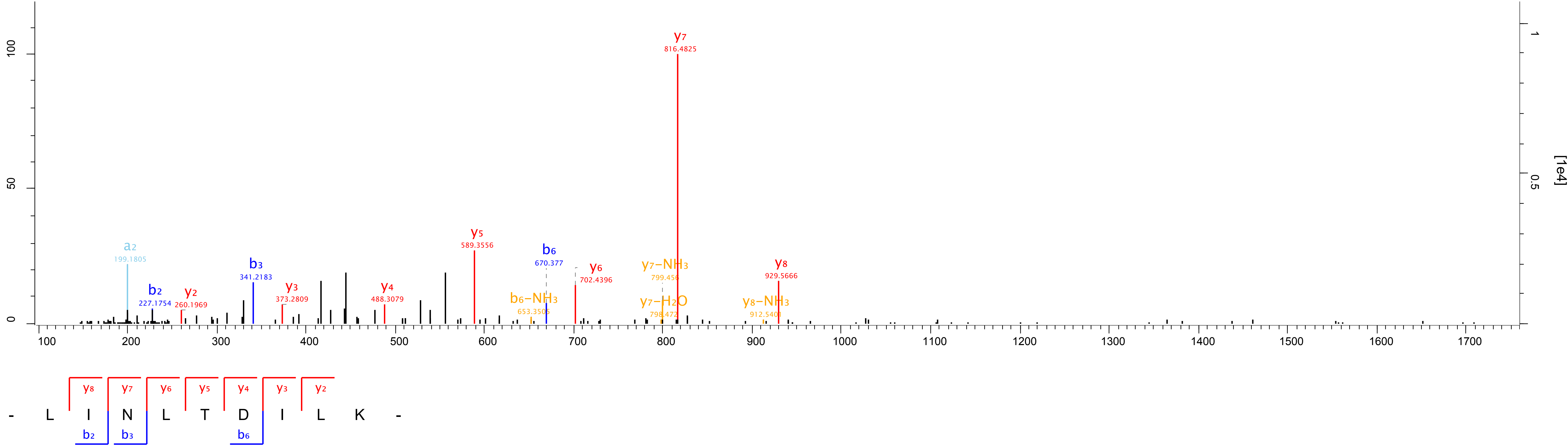

Raw file	Scan	Method	Score	m/z	Gene names
20150307_Hepa2_Top_opt_D2_01_1686	69259	TOF; CID	54.28	835.41	Cdca7

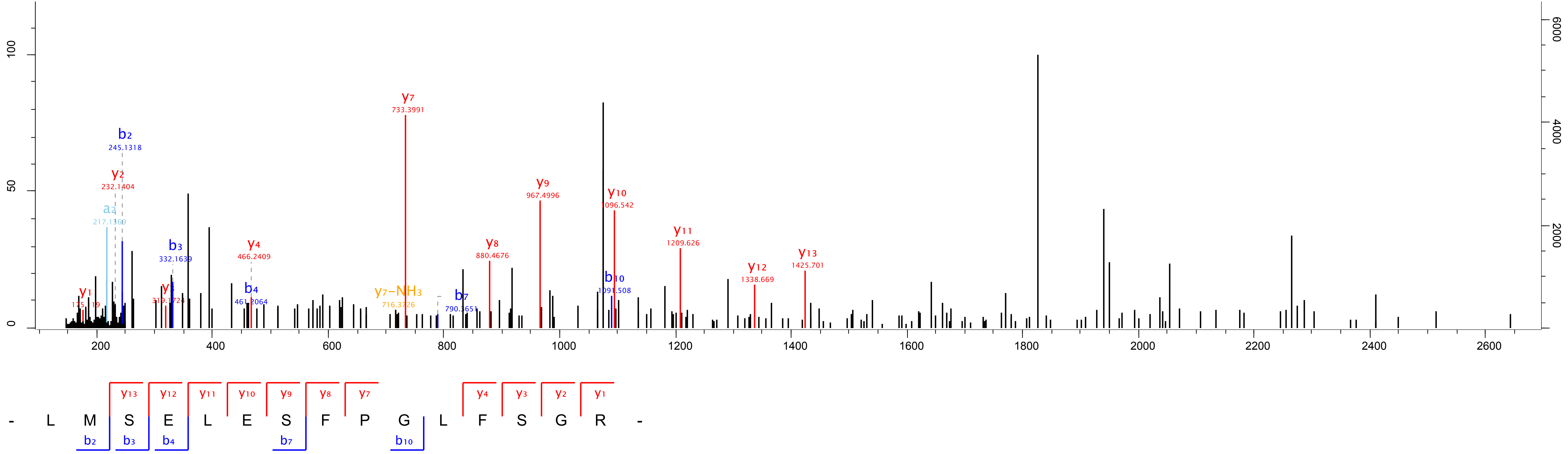

Raw file	Scan	Method	Score	m/z	Gene names
20150307_Hepa2_Top_opt_D2_01_1686	70480	TOF; CID	61.96	1051.24	Tmem184b

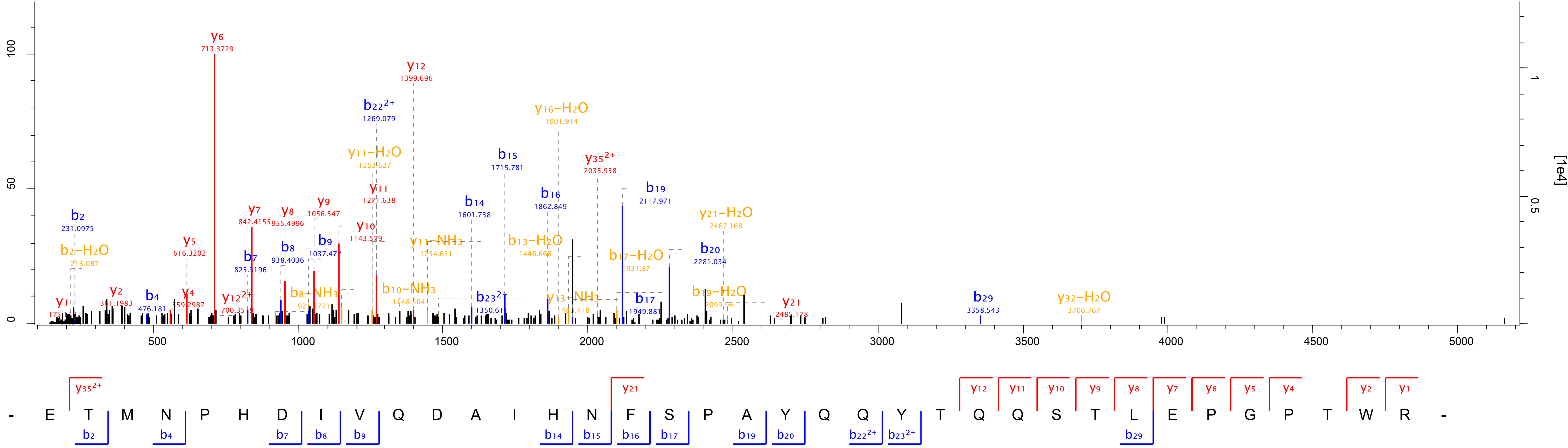

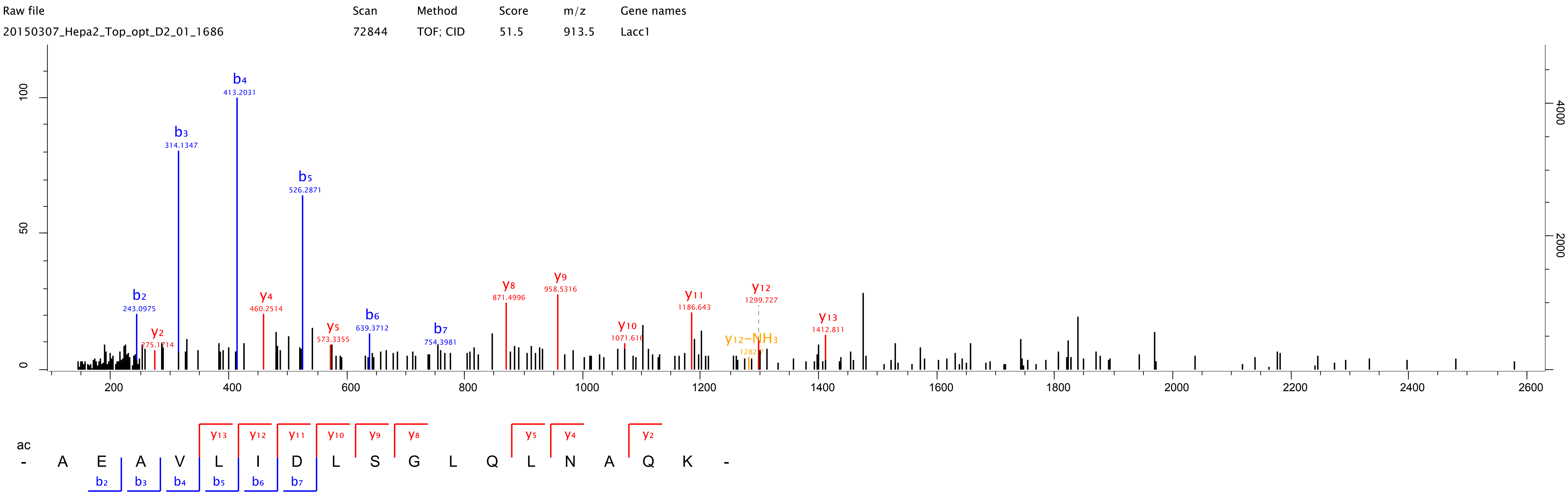

Raw file
20150307_Hepa2_Top_opt_D2_01_1686
Scan
72844
Method
TOF; CID
Score
51.5
m/z
913.5
Gene names
Lacc1
b2 243.0975
b3 314.1347
b4 413.2031
b5 526.2871
b6 639.3712
b7 754.3981
y2 275.1714
y4 460.2514
y5 573.3355
y8 871.4996
y9 958.5316
y10 1071.616
y11 1186.643
y12 1299.727
y12−NH3
y13 1412.811
0
50
100
2000
4000
200
400
600
800
1000
1200
1400
1600
1800
2000
2200
2400
2600
ac
- A E A V L I D L S G L Q L N A Q K -
y13 y12 y11 y10 y9 y8 y5 y4 y2
b2 b3 b4 b5 b6 b7

Raw file	Scan	Method	Score	m/z	Gene names
20150307_Hepa3_Top_opt_D3_01_1675	8702	TOF; CID	133.16	481.58	Smim4

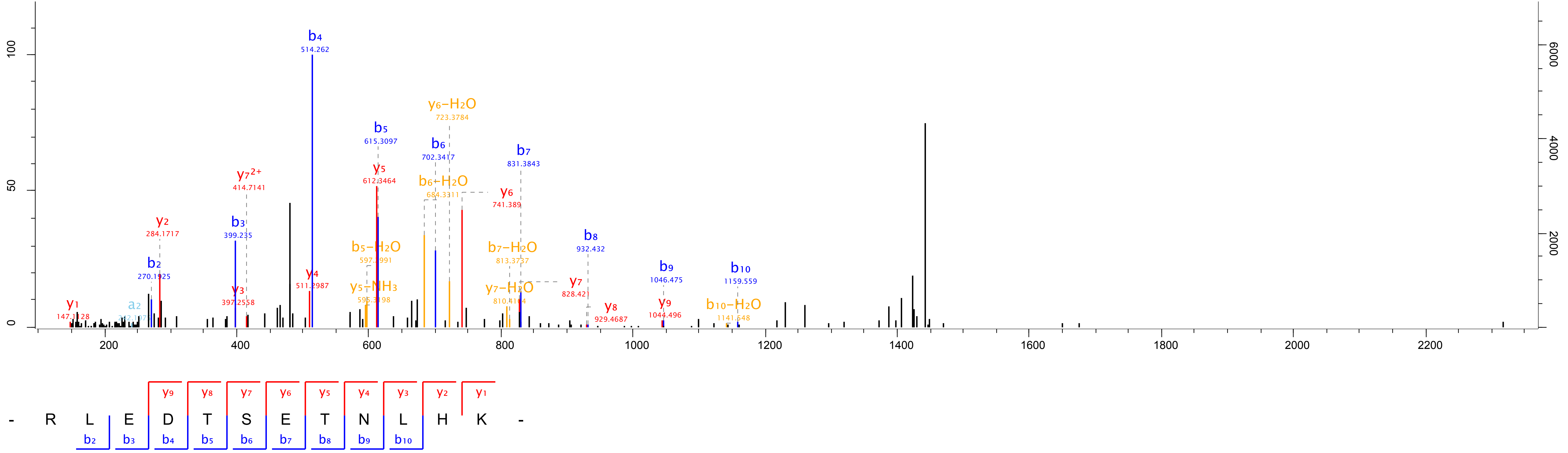

Raw file	Scan	Method	Score	m/z	Gene names
20150307_Hepa3_Top_opt_D3_01_1675	20342	TOF; CID	61.11	482.58	Foxred1

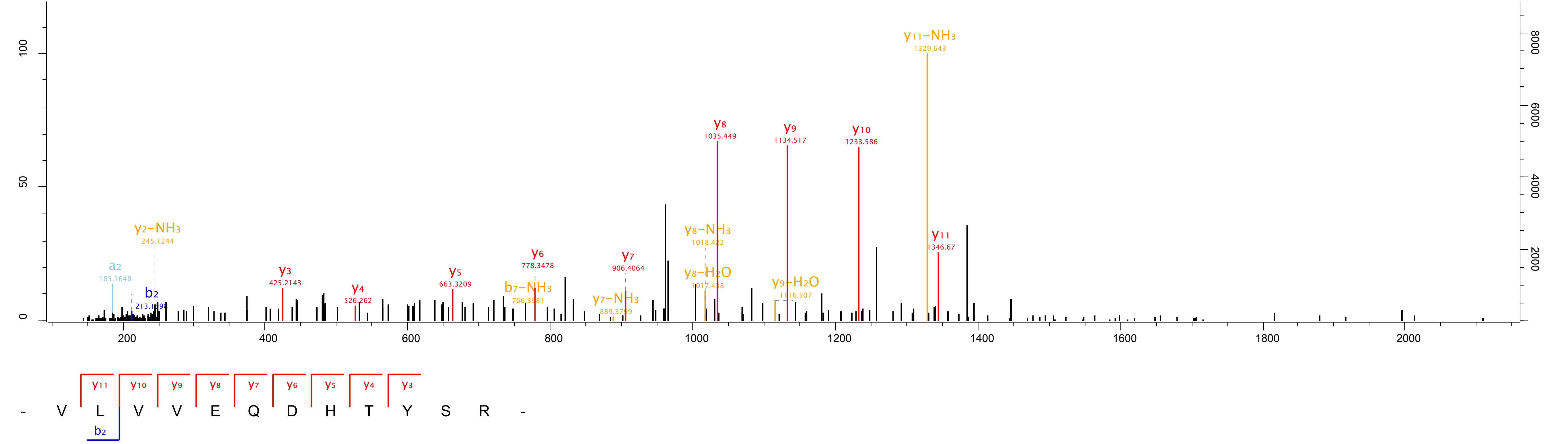

Raw file	Scan	Method	Score	m/z	Gene names
20150307_Hepa3_Top_opt_D3_01_1675	22570	TOF; CID	58.32	683.83	Esam

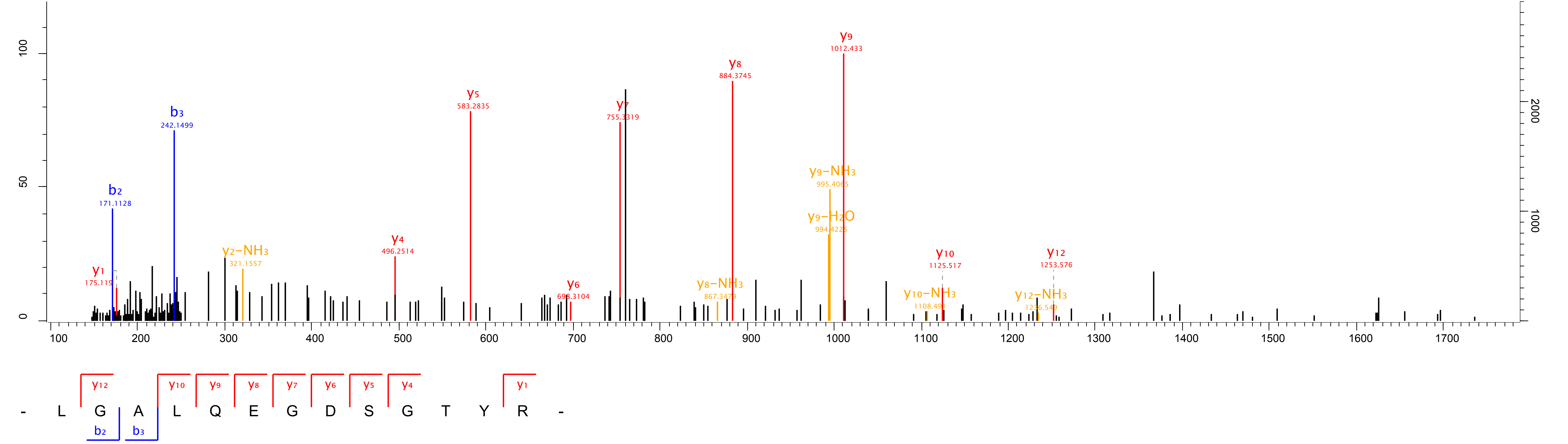

Raw file	Scan	Method	Score	m/z
20150307_Hepa3_Top_opt_D3_01_1675	27988	TOF; CID	89.62	640.3

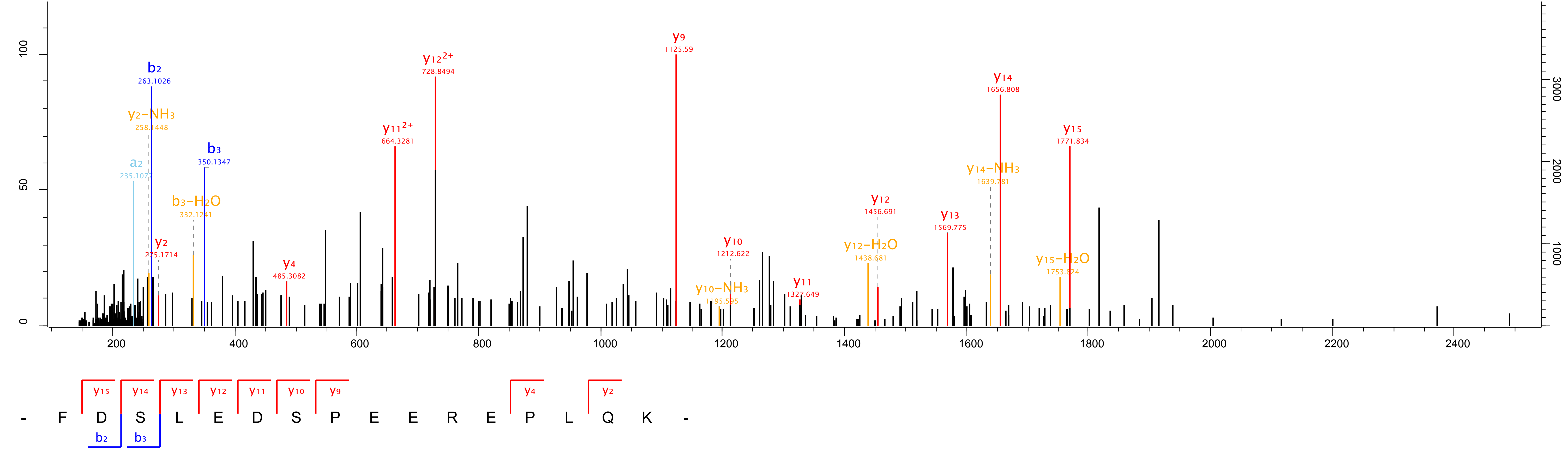

Raw file	Scan	Method	Score	m/z	Gene names
20150307_Hepa3_Top_opt_D3_01_1675	37532	TOF; CID	46.84	678.3	Cd59a

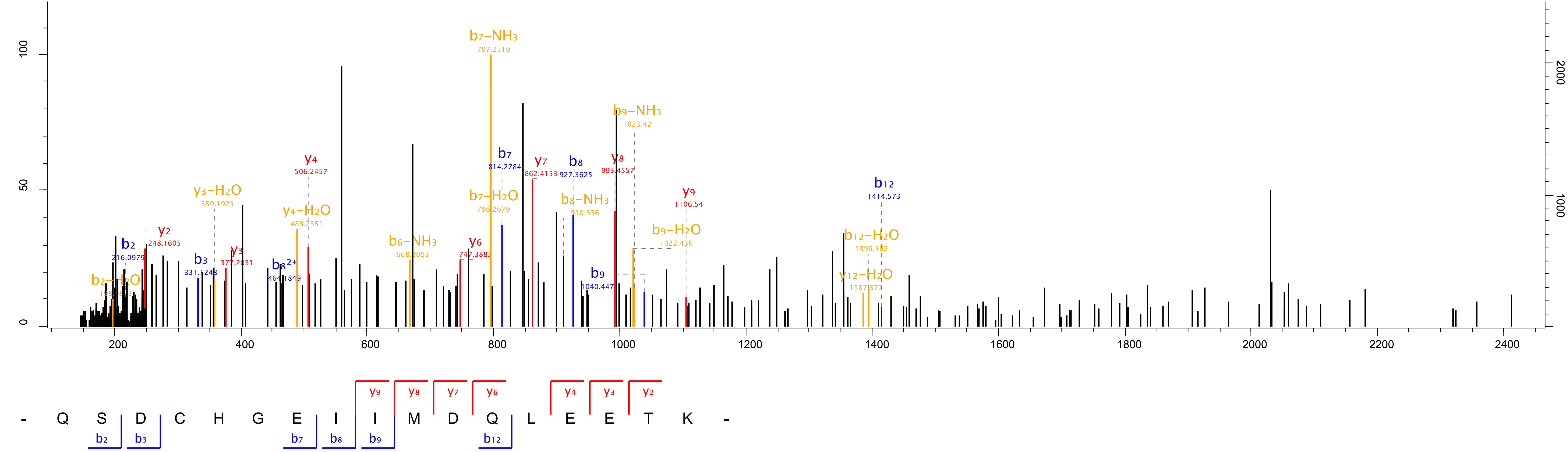

Raw file	Scan	Method	Score	m/z	Gene names
20150307_Hepa3_Top_opt_D3_01_1675	42656	TOF; CID	47.09	734.65	Elof1

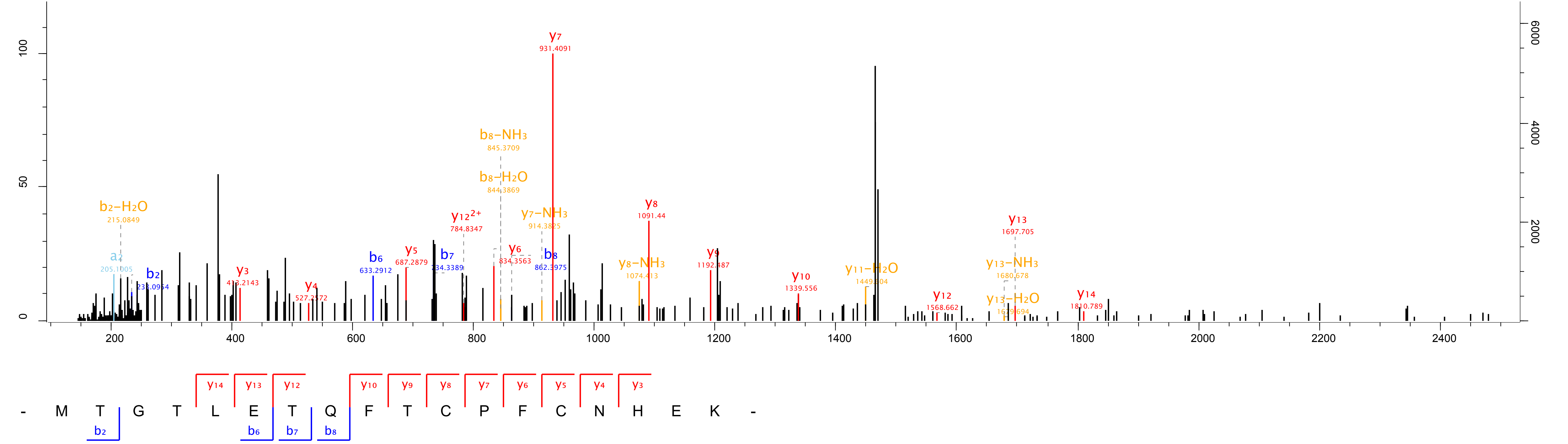

Raw file	Scan	Method	Score	m/z	Gene names
20150307_Hepa3_Top_opt_D3_01_1675	46744	TOF; CID	71.56	730.41	Zcchc10

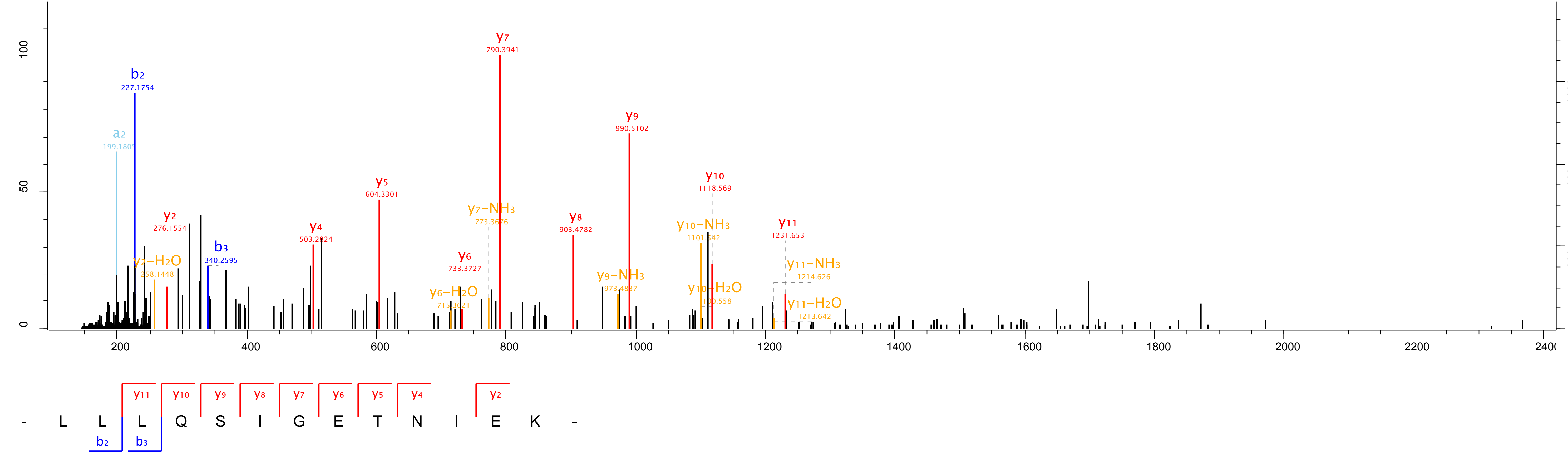

Raw file	Scan	Method	Score	m/z	Gene names
20150307_Hepa3_Top_opt_D3_01_1675	49767	TOF; CID	62.2	624.01	Echdc3

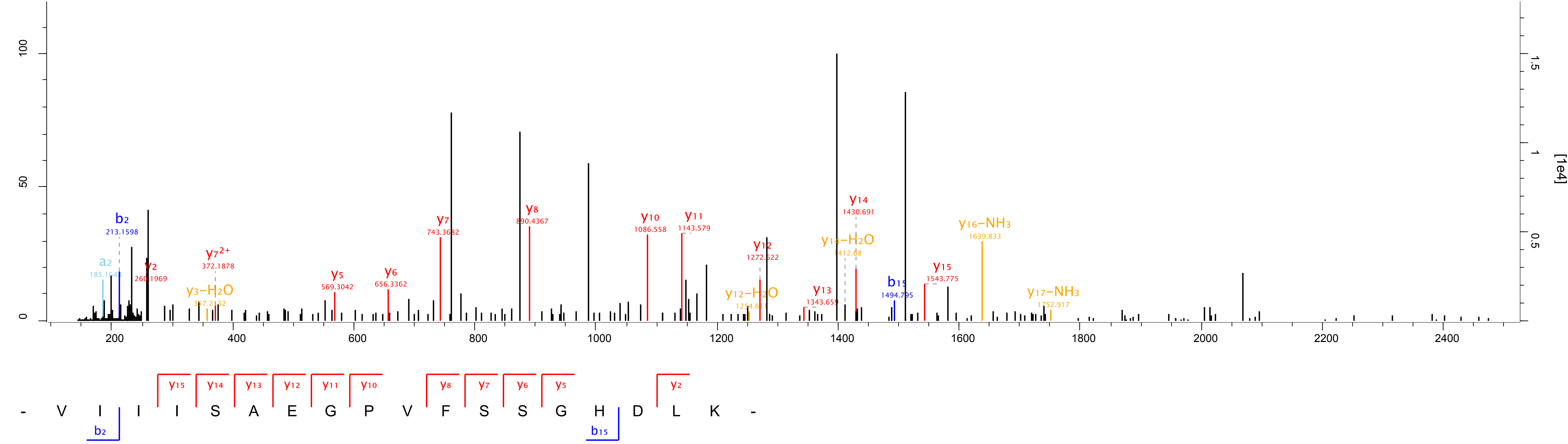

Raw file	Scan	Method	Score	m/z	Gene names
20150307_Hepa3_Top_opt_D3_01_1675	49832	TOF; CID	80.51	578.68	Tmem11

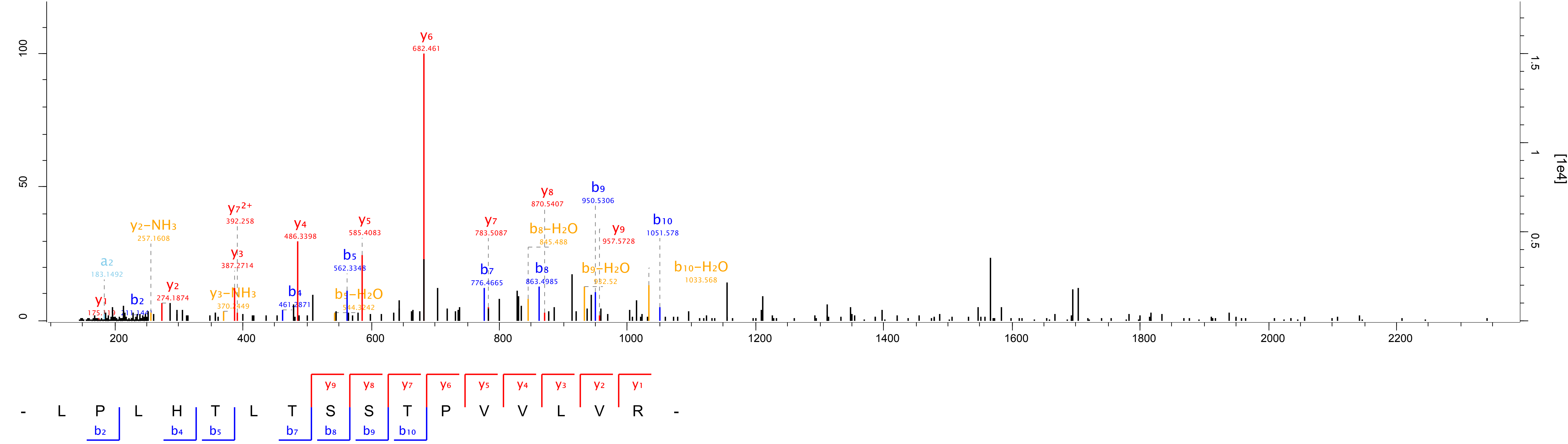

Raw file	Scan	Method	Score	m/z	Gene names
20150307_Hepa3_Top_opt_D3_01_1675	51464	TOF; CID	49.66	1059.51	Gm20604

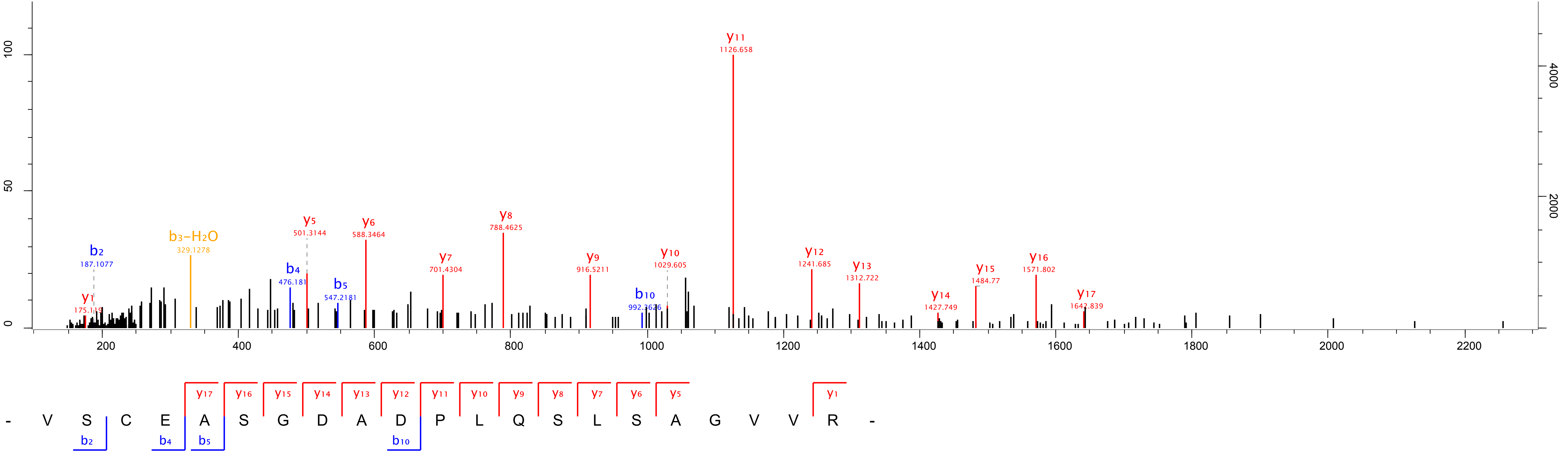

Raw file	Scan	Method	Score	m/z	Gene names
20150307_Hepa3_Top_opt_D3_01_1675	54737	TOF; CID	78.95	675.32	Fosl1

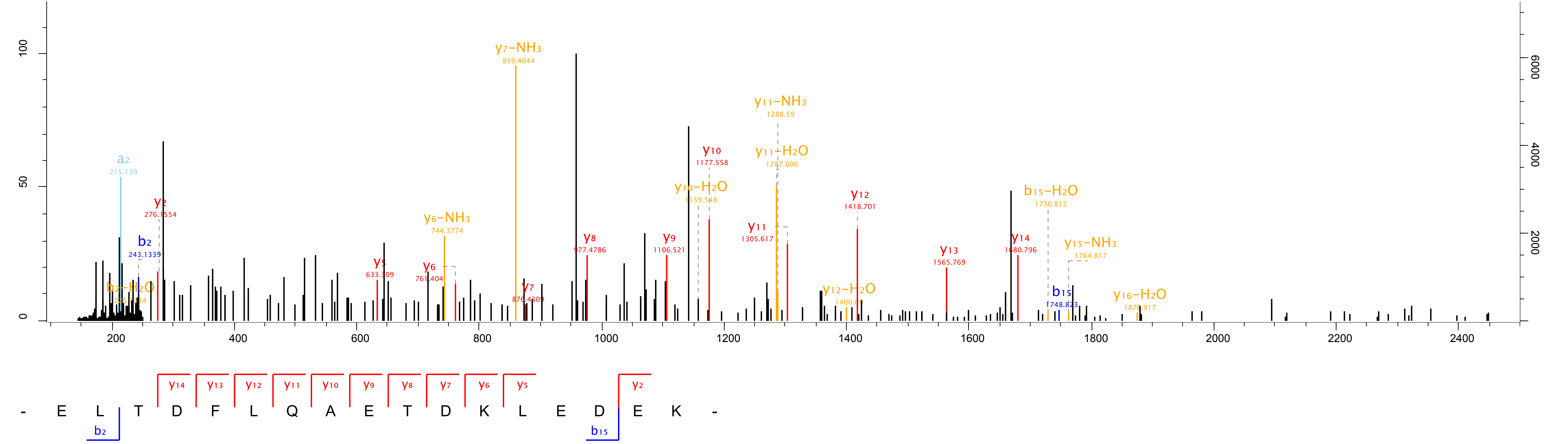

Raw file	Scan	Method	Score	m/z	Gene names
20150307_Hepa3_Top_opt_D3_01_1675	56508	TOF; CID	57.29	813.45	Plac8

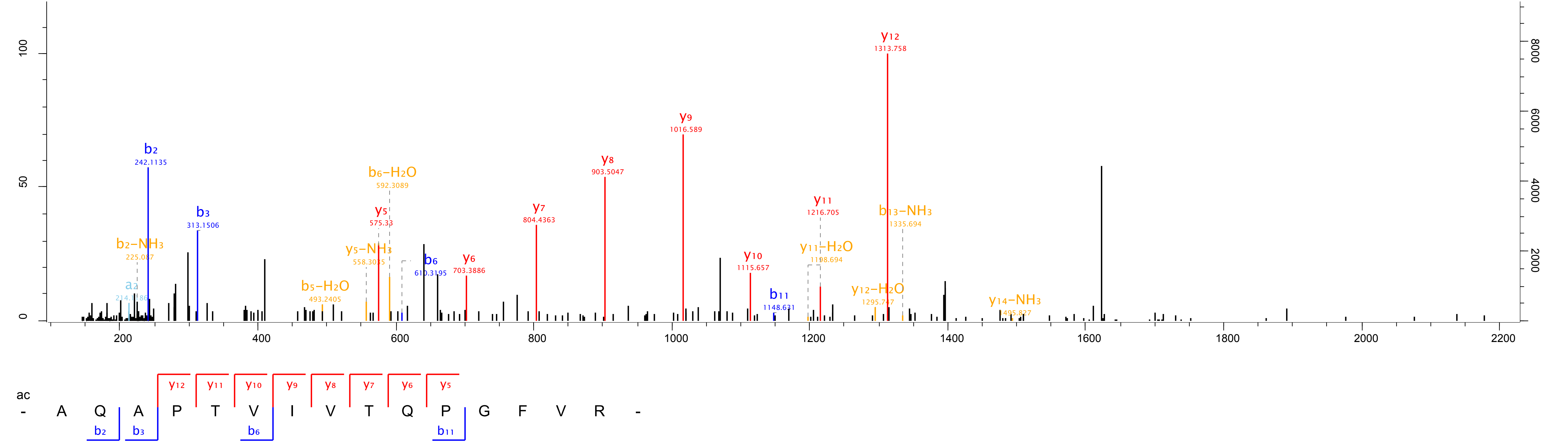

Raw file	Scan	Method	Score	m/z	Gene names
20150307_Hepa3_Top_opt_D3_01_1675	58653	TOF; CID	45.35	1038.5	Tnfrsf12a

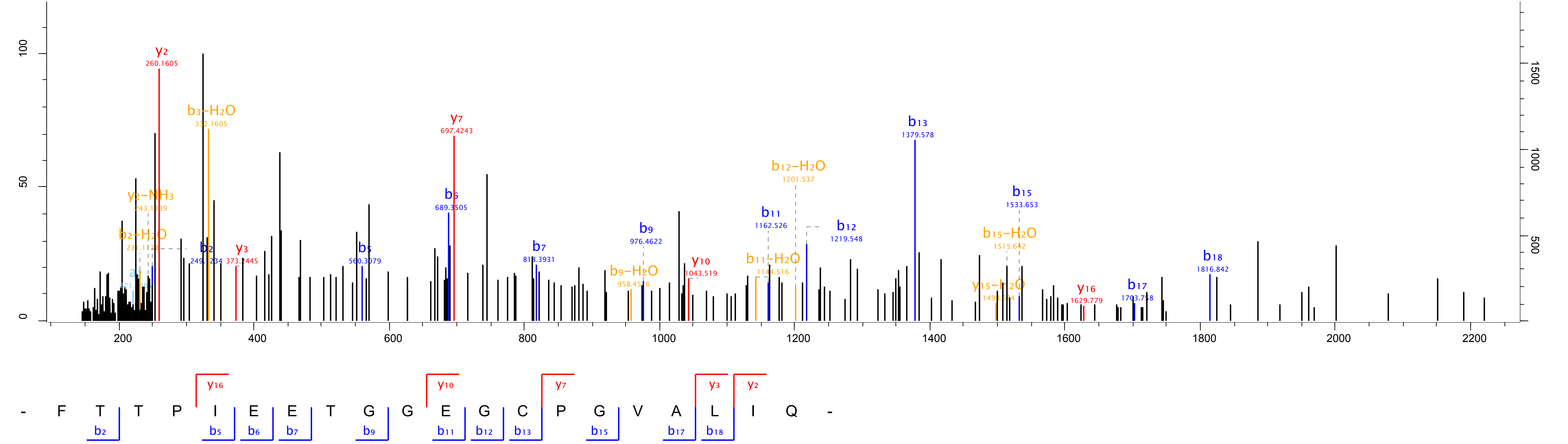

Raw file	Scan	Method	Score	m/z	Gene names
20150307_Hepa3_Top_opt_D3_01_1675	69382	TOF; CID	184.48	597.37	Kdelr2

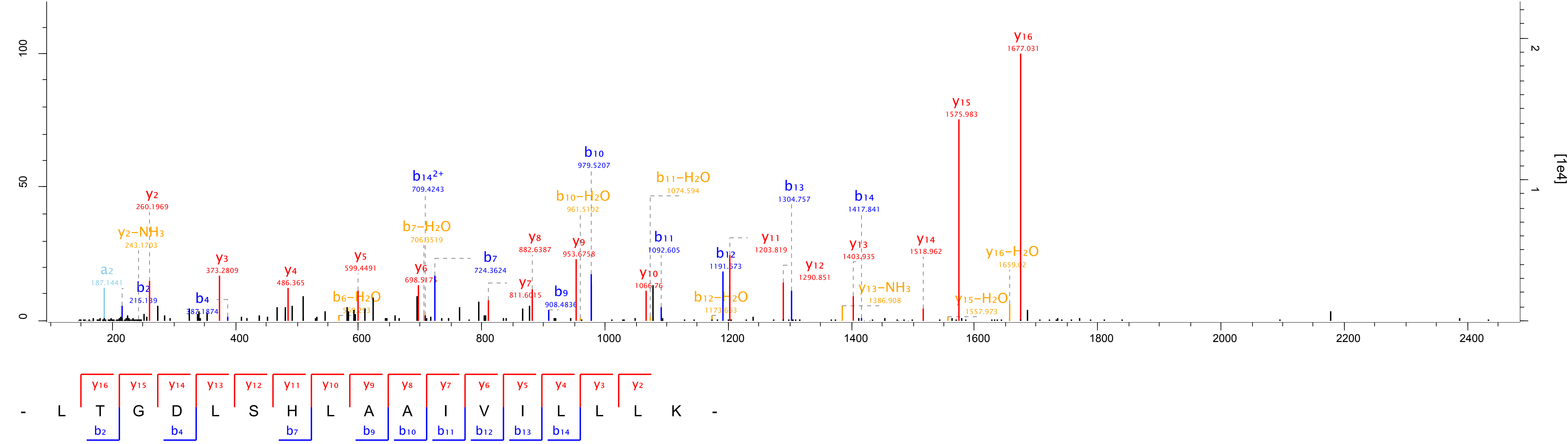

Raw file	Scan	Method	Score	m/z	Gene names
20150307_Hepa3_Top_opt_D3_01_1675	69431	TOF; CID	90.3	1132.59	Ssr2

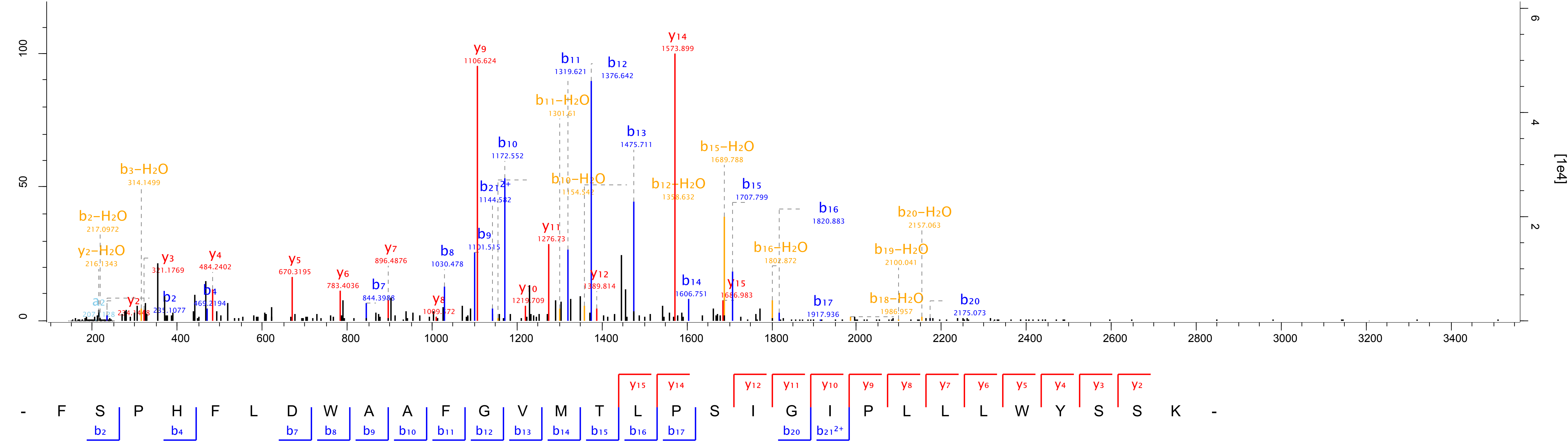

Raw file	Scan	Method	Score	m/z	Gene names
20150307_Hepa3_Top_opt_D3_01_1675	69437	TOF; CID	94.18	1294.63	Cox8a

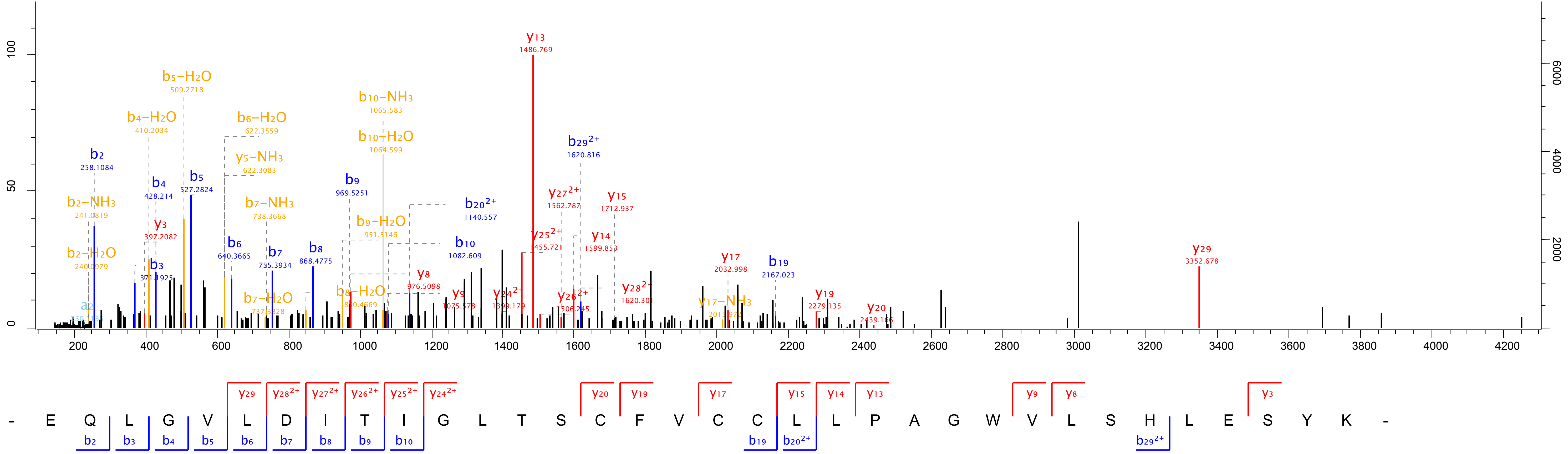

Raw file	Scan	Method	Score	m/z	Gene names
20150307_Hepa3_Top_opt_D3_01_1689	6594	TOF; CID	166.69	555.27	Mfsd1

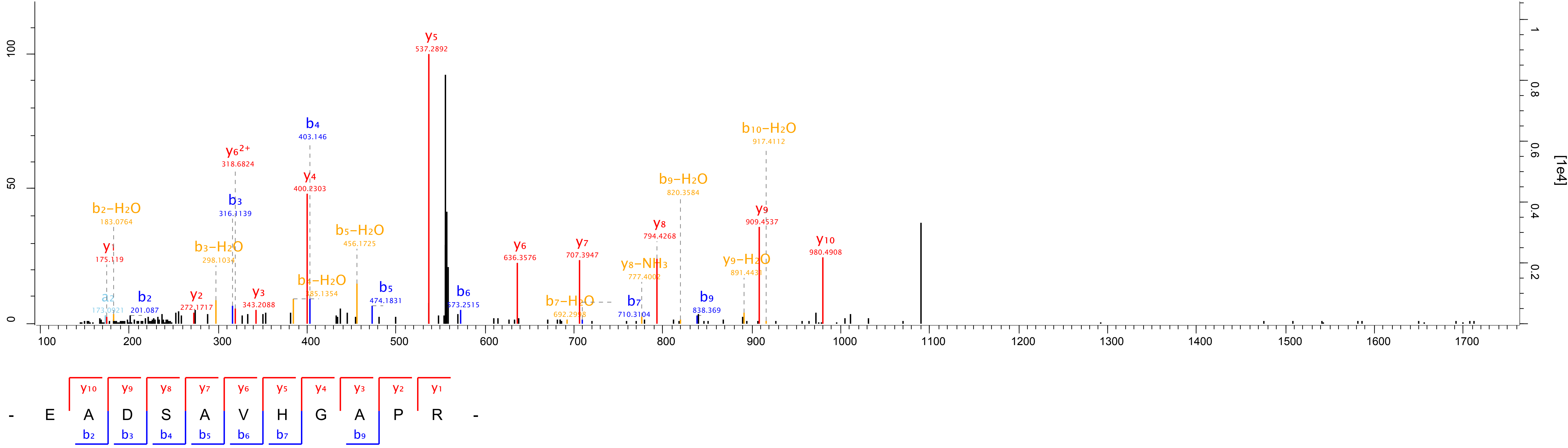

Raw file	Scan	Method	Score	m/z	Gene names
20150307_Hepa3_Top_opt_D3_01_1689	11454	TOF; CID	83.87	544.27	Egln1

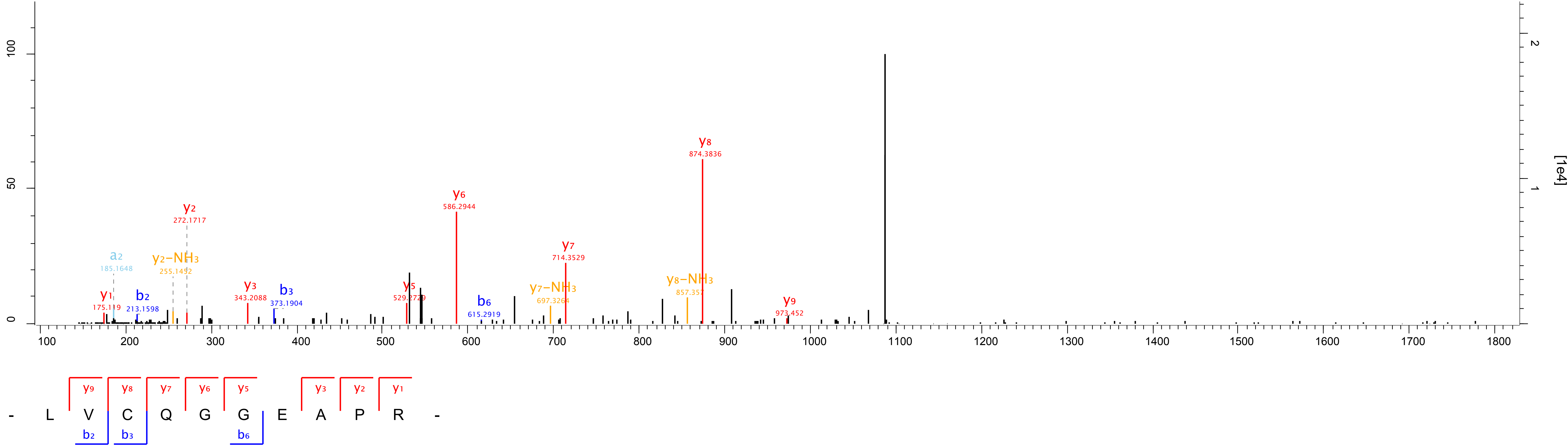

Raw file	Scan	Method	Score	m/z	Gene names
20150307_Hepa3_Top_opt_D3_01_1689	11714	TOF; CID	74.61	695.32	Tspan2

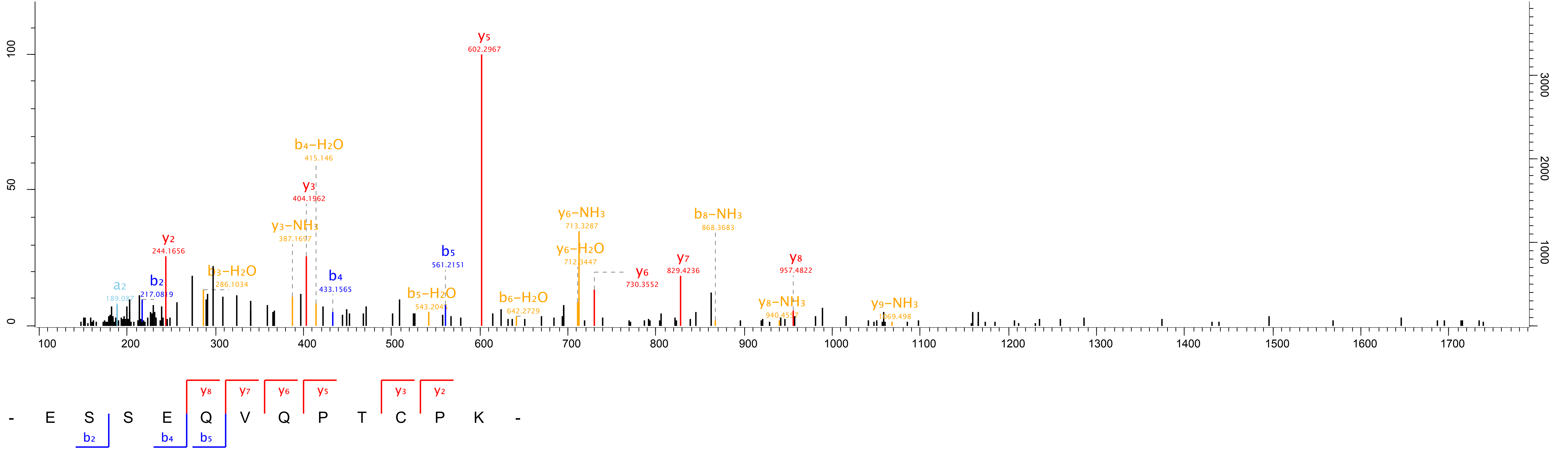

Raw file	Scan	Method	Score	m/z	Gene names
20150307_Hepa3_Top_opt_D3_01_1689	14054	TOF; CID	84.17	638.3	Nudt1

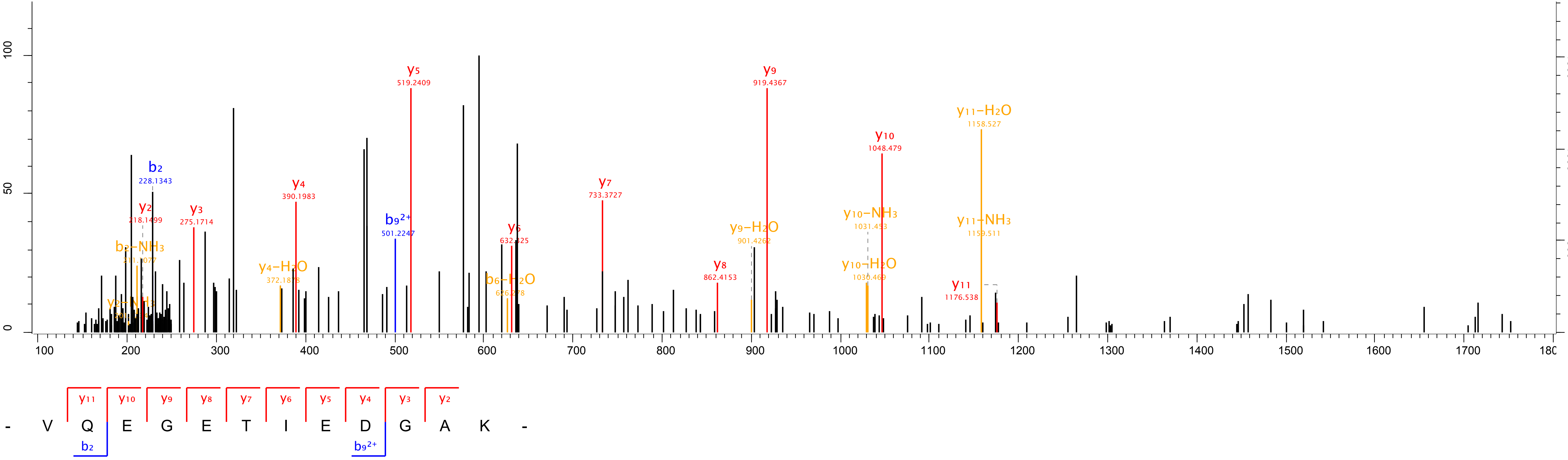

Raw file	Scan	Method	Score	m/z	Gene names
20150307_Hepa3_Top_opt_D3_01_1689	18676	TOF; CID	40.72	685.33	Kmt2d

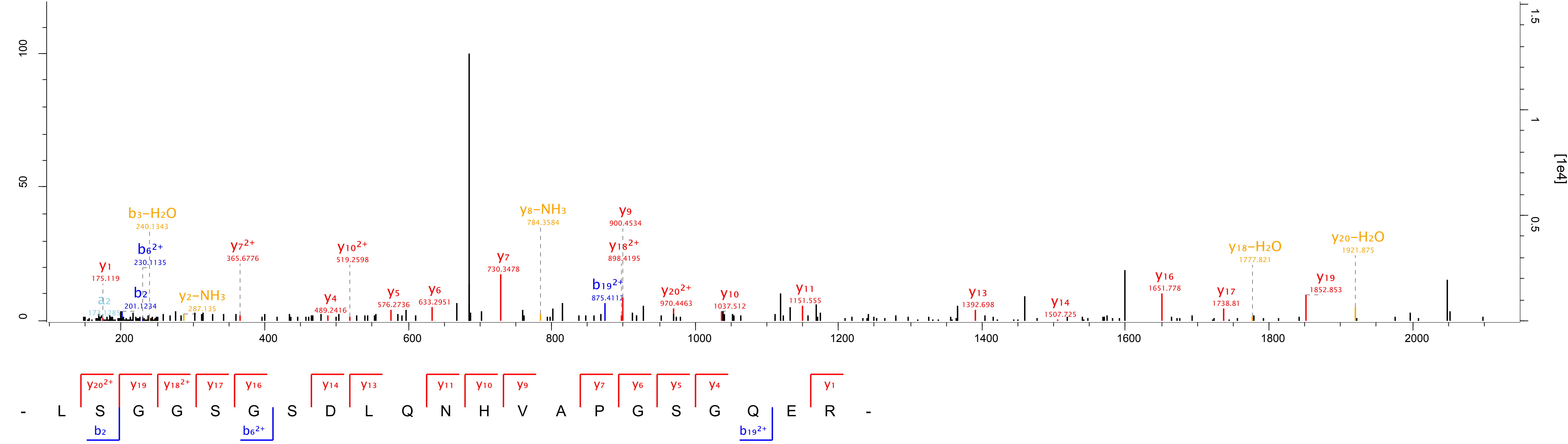

Raw file	Scan	Method	Score	m/z	Gene names
20150307_Hepa3_Top_opt_D3_01_1689	20387	TOF; CID	56.57	658.84	Banp

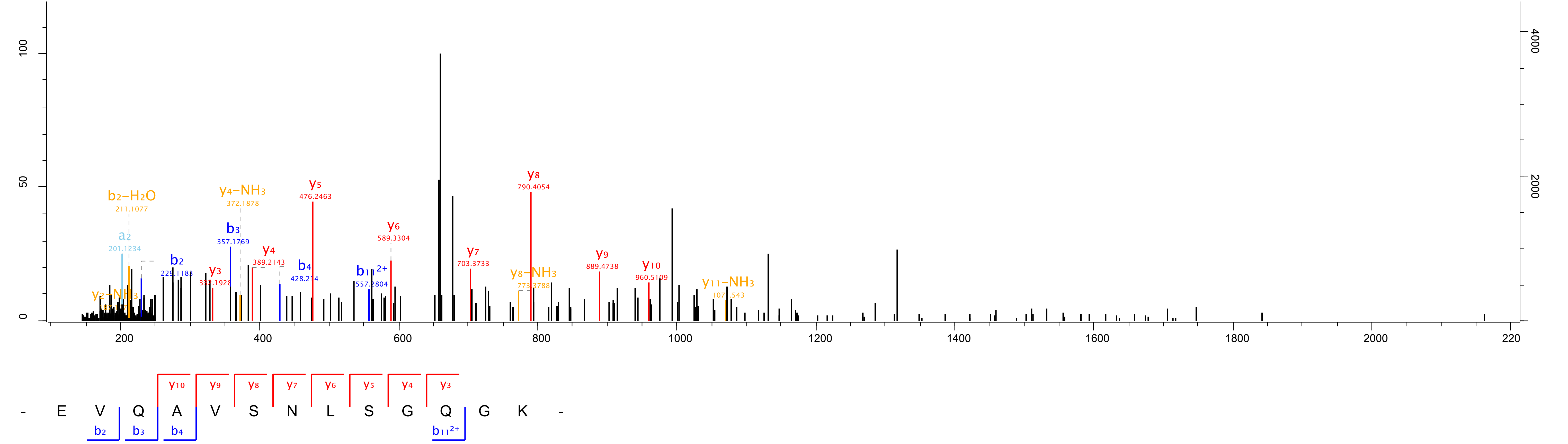

Raw file	Scan	Method	Score	m/z	Gene names
20150307_Hepa3_Top_opt_D3_01_1689	45529	TOF; CID	42.56	692	Cd99

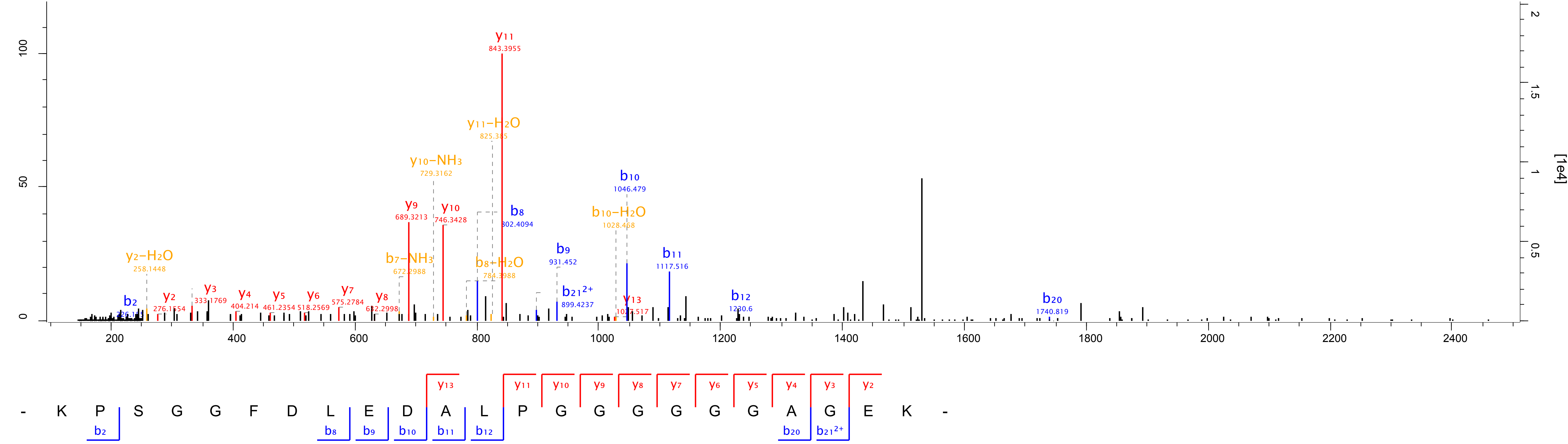

Raw file	Scan	Method	Score	m/z	Gene names
20150307_Hepa3_Top_opt_D3_01_1689	48369	TOF; CID	72.89	738.88	Fitm2

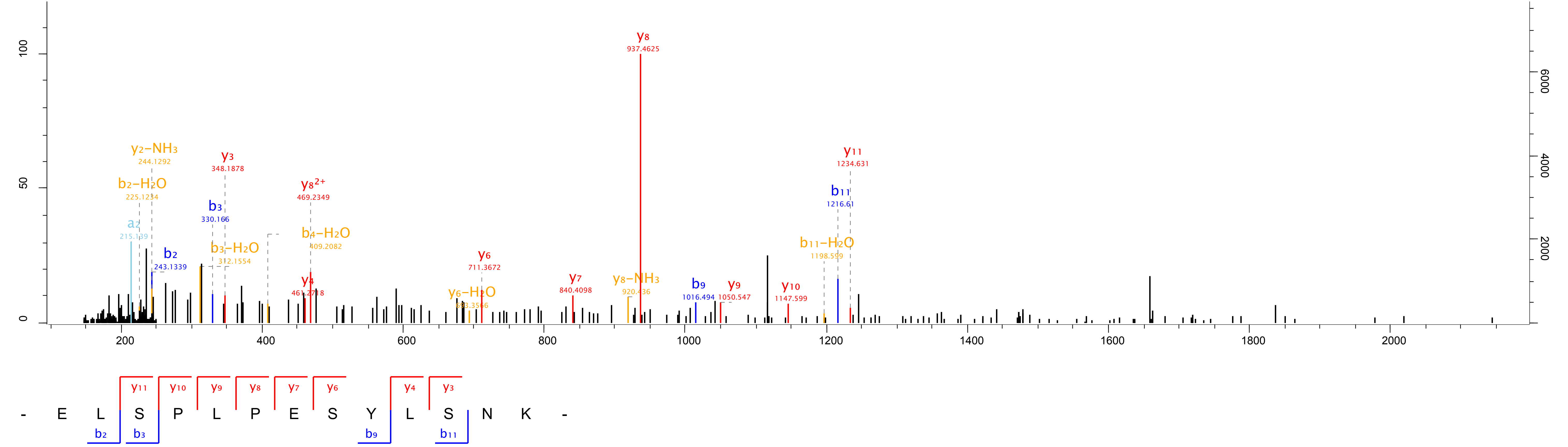

Raw file	Scan	Method	Score	m/z	Gene names
20150307_Hepa3_Top_opt_D3_01_1689	49165	TOF; CID	99.44	504.72	Tmem258

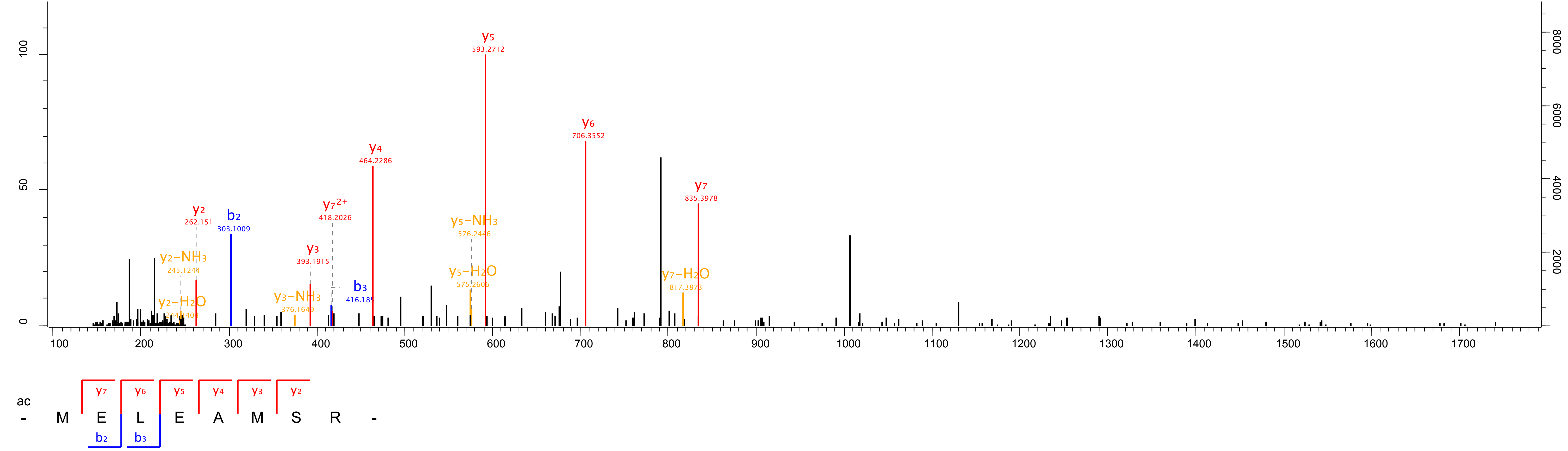

Raw file	Scan	Method	Score	m/z	Gene names
20150307_Hepa3_Top_opt_D3_01_1689	51662	TOF; CID	66.22	775.89	Mbd1

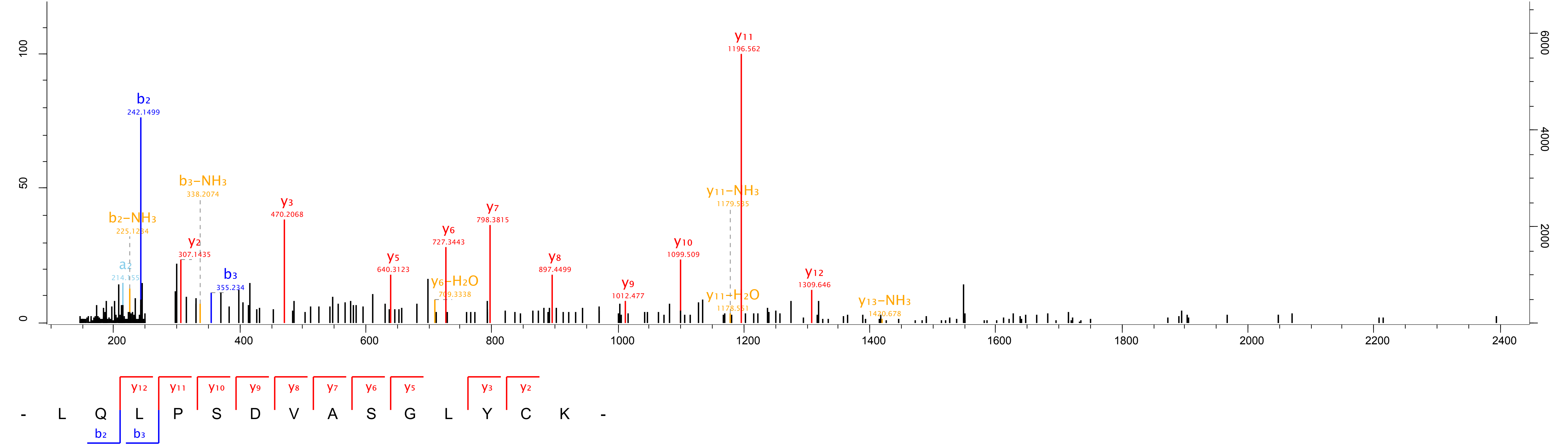

Raw file	Scan	Method	Score	m/z	Gene names
20150307_Hepa3_Top_opt_D3_01_1689	54824	TOF; CID	124.51	876.42	Pdzd11

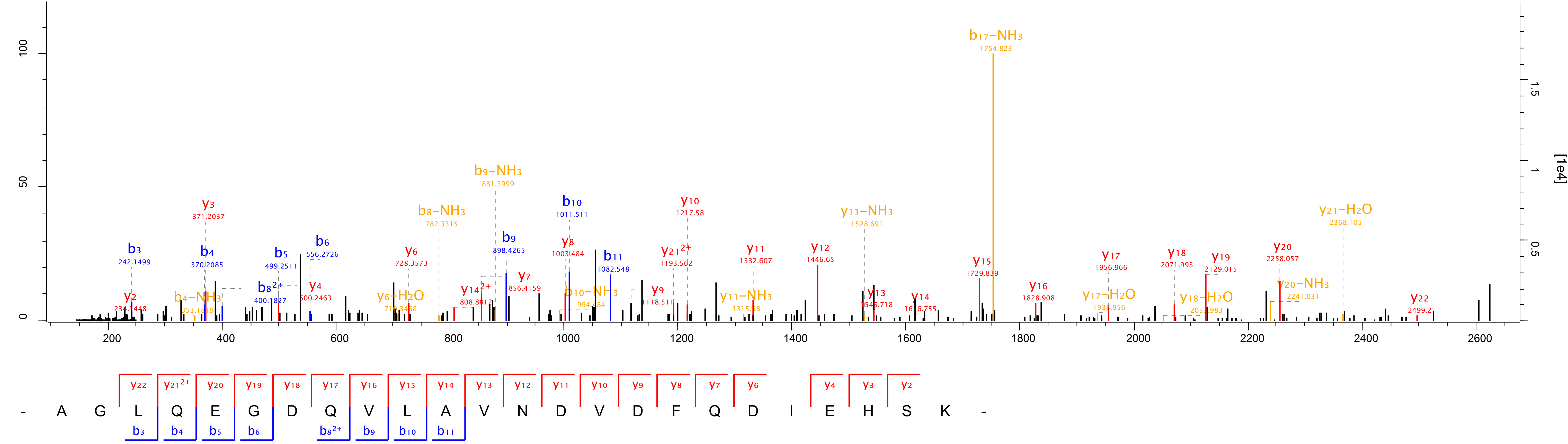

Raw file	Scan	Method	Score	m/z	Gene names
20150307_Hepa3_Top_opt_D3_01_1689	54983	TOF; CID	80.17	471.8	Rhebl1

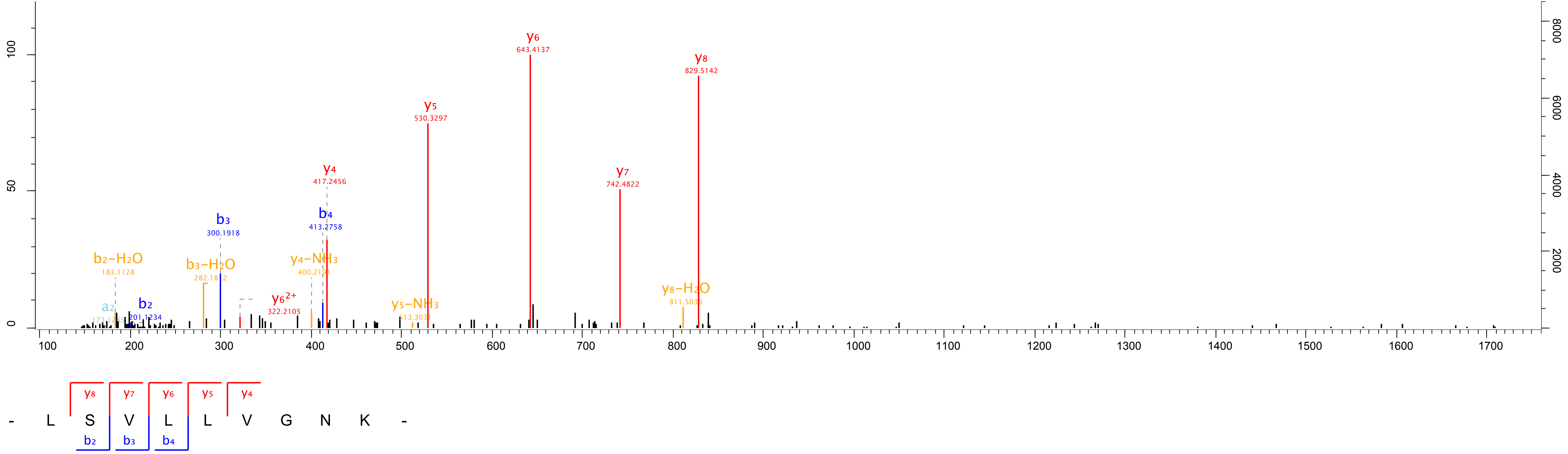

Raw file	Scan	Method	Score	m/z	Gene names
20150307_Hepa3_Top_opt_D3_01_1689	55158	TOF; CID	45.88	848.41	Rnf13

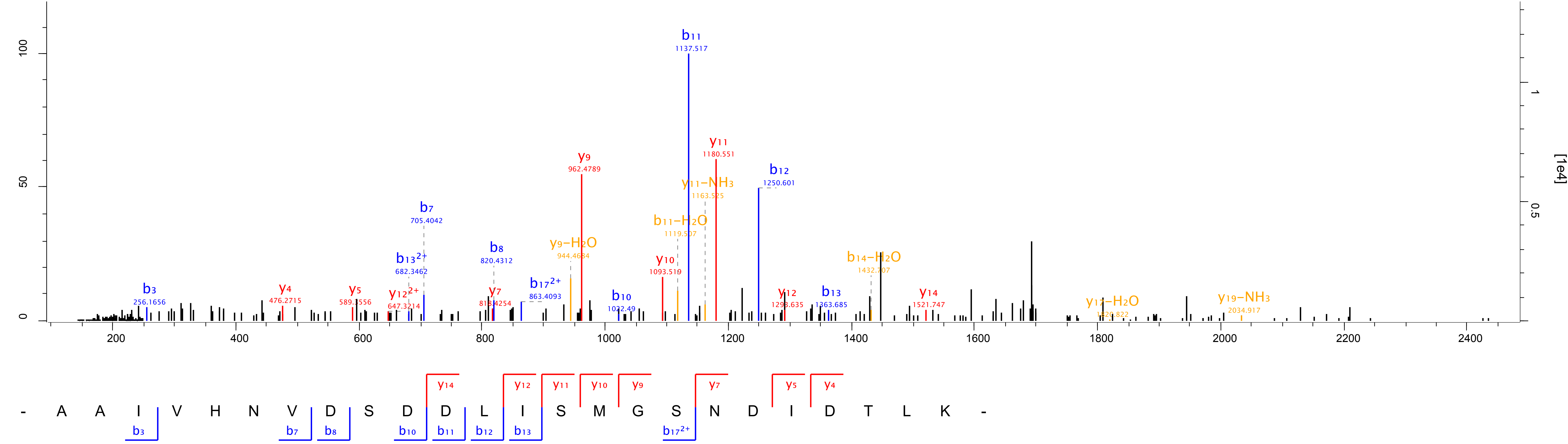

Raw file	Scan	Method	Score	m/z	Gene names
20150307_Hepa3_Top_opt_D3_01_1689	57246	TOF; CID	147.56	854.91	Ssr3

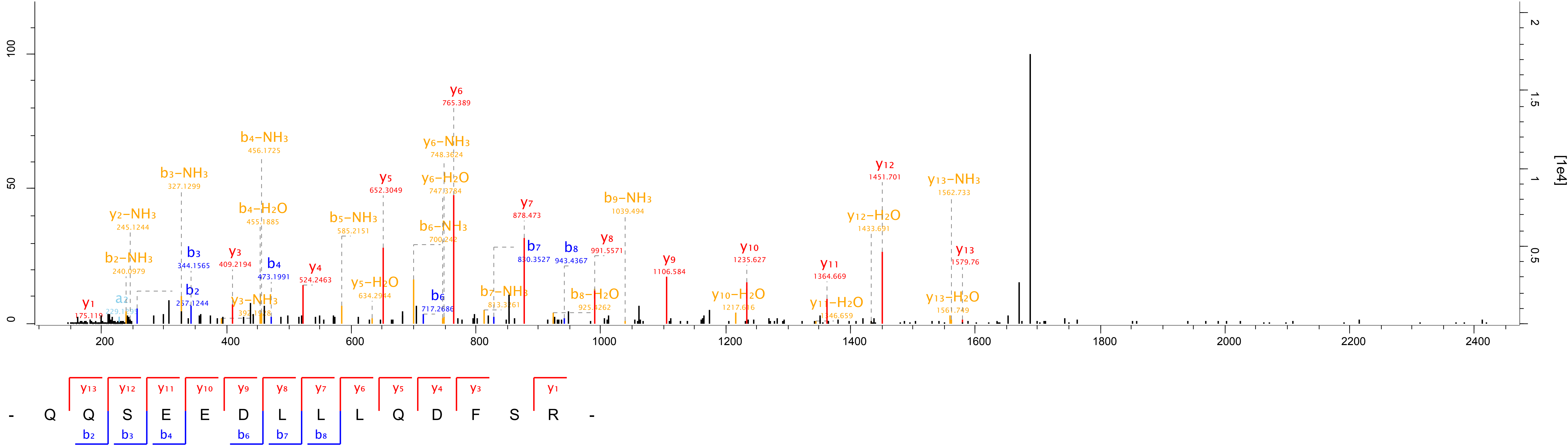

Raw file	Scan	Method	Score	m/z	Gene names
20150307_Hepa3_Top_opt_D3_01_1689	58866	TOF; CID	76.73	842.4	Trappc2;0610009B22Rik

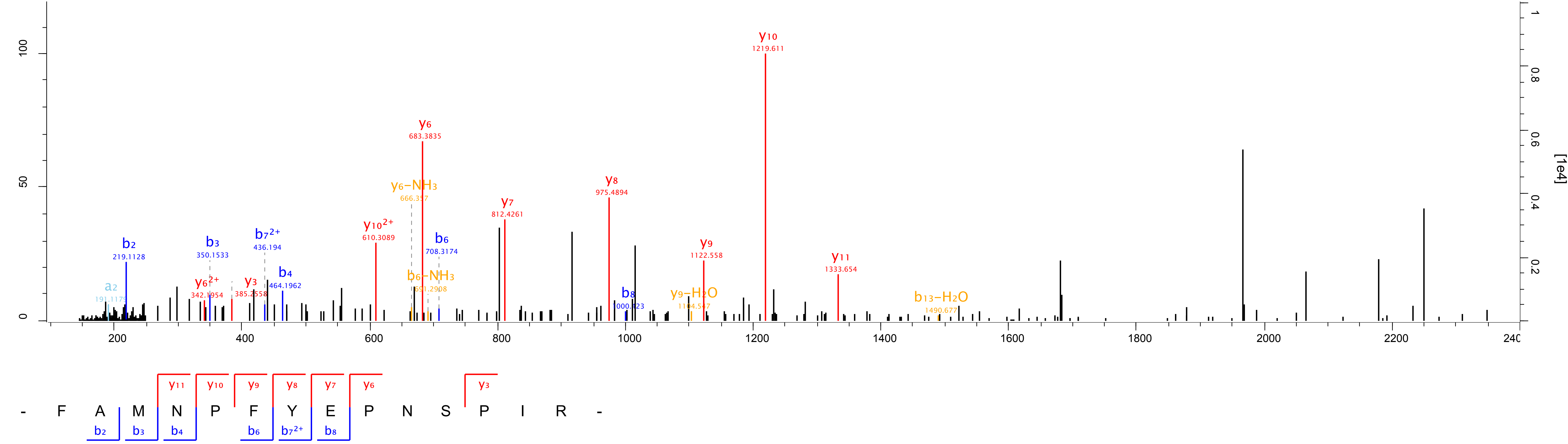

Raw file	Scan	Method	Score	m/z	Gene names
20150307_Hepa3_Top_opt_D3_01_1689	61263	TOF; CID	79.49	648.37	Tmem186

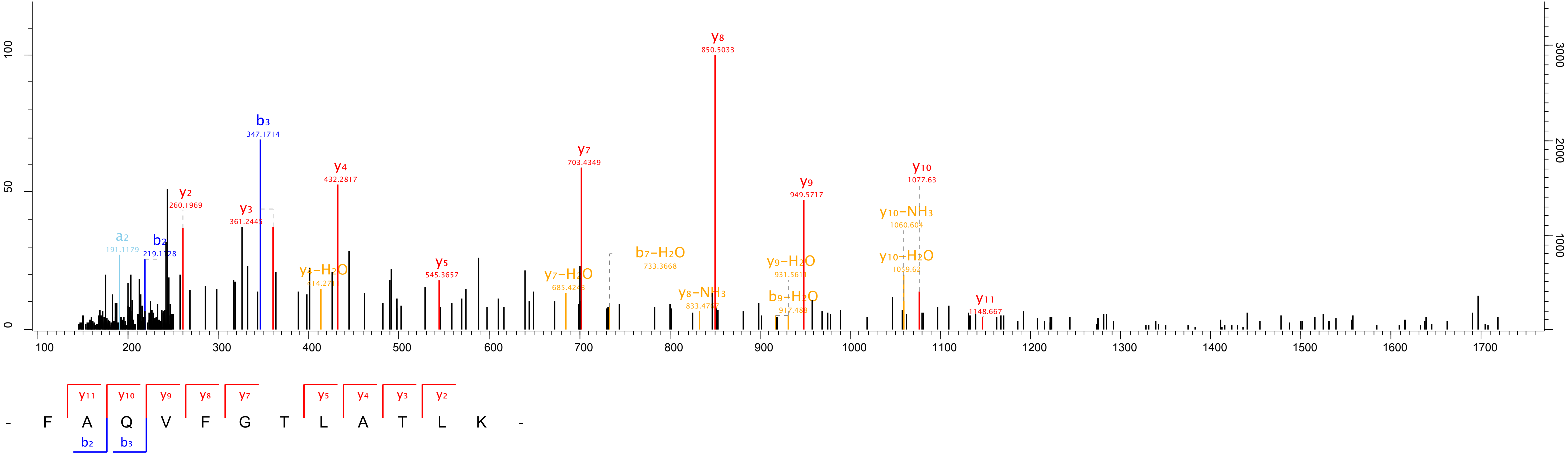

Raw file	Scan	Method	Score	m/z	Gene names
20150307_Hepa3_Top_opt_D3_01_1689	63337	TOF; CID	58.04	699.39	Lpcat4

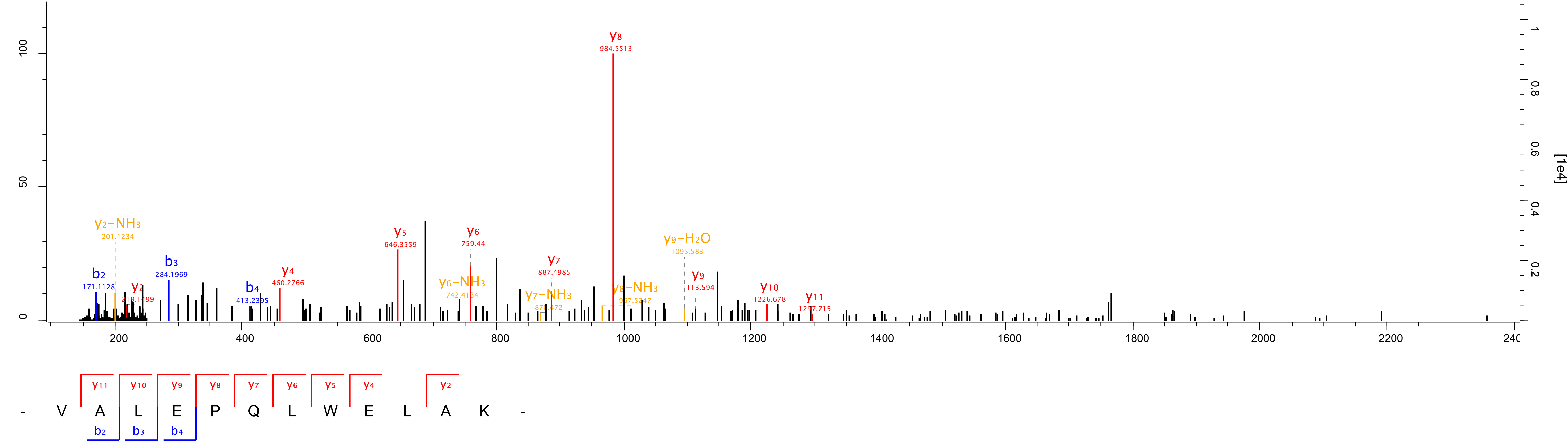

Raw file	Scan	Method	Score	m/z	Gene names
20150307_Hepa3_Top_opt_D3_01_1689	63733	TOF; CID	64.52	850.47	Golga7

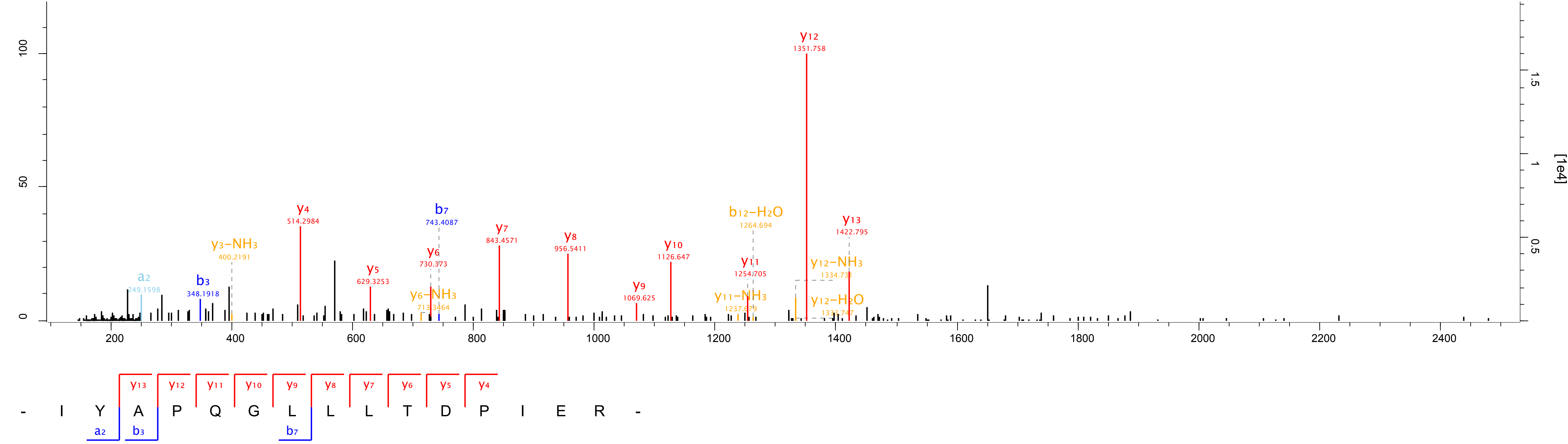

Raw file	Scan	Method	Score	m/z	Gene names
20150307_Hepa3_Top_opt_D3_01_1689	68743	TOF; CID	54.76	794.74	Nlrx1

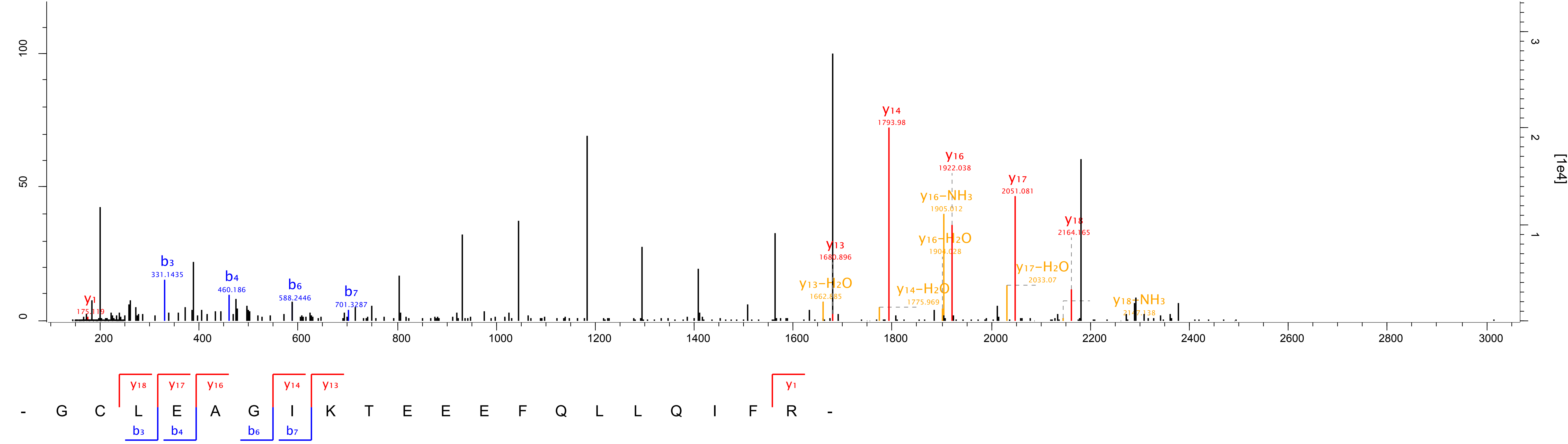

Raw file	Scan	Method	Score	m/z	Gene names
20150307_Hepa3_Top_opt_D3_01_1689	69371	TOF; CID	38.38	824.42	Crx

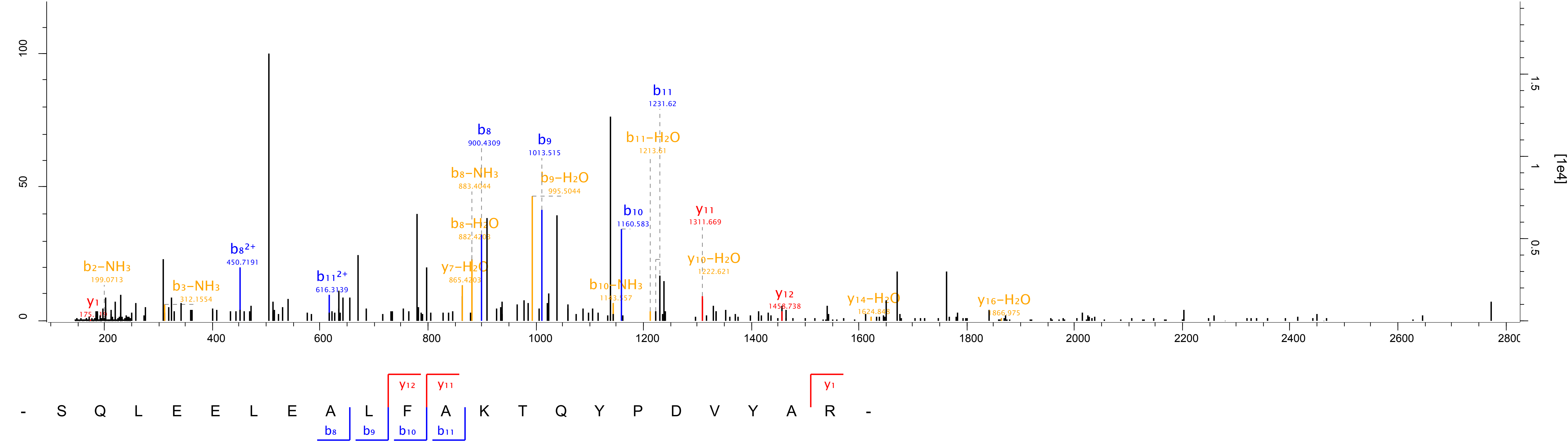

Raw file	Scan	Method	Score	m/z	Gene names
20150307_MEF1_Top_opt_E1_01_1677	38213	TOF; CID	39.9	646.97	Icmt

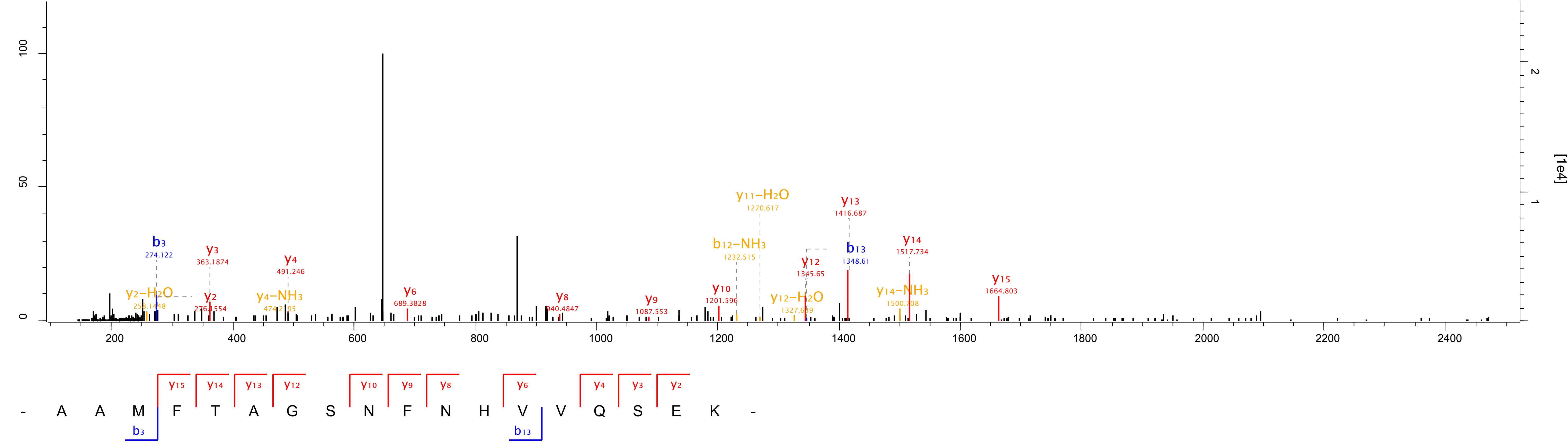

Raw file	Scan	Method	Score	m/z	Gene names
20150307_MEF1_Top_opt_E1_01_1677	42047	TOF; CID	73.39	510.6	Mrpl57

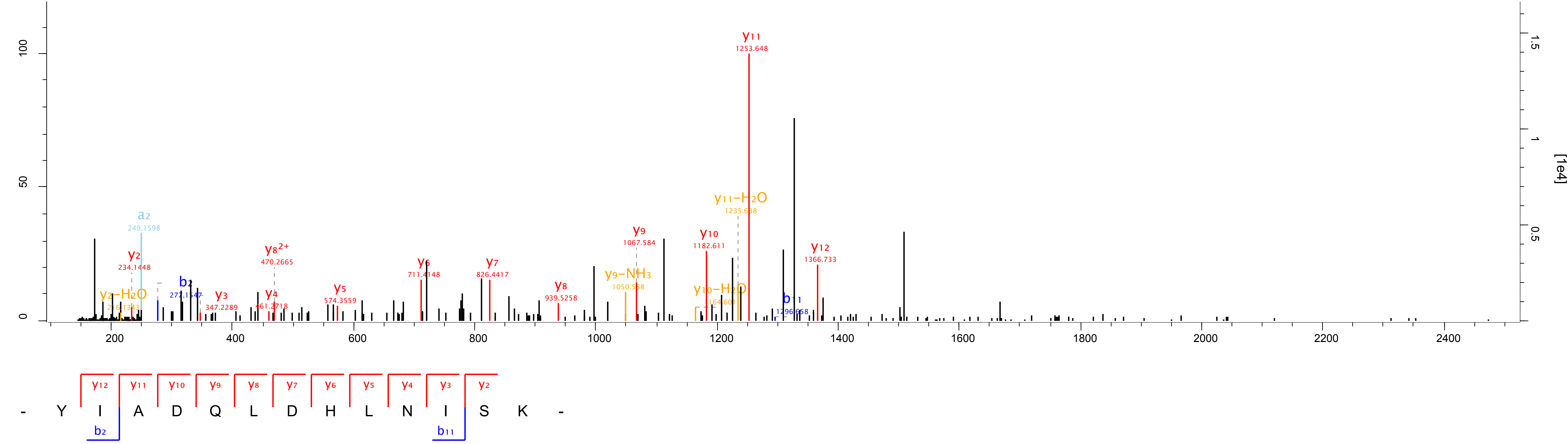

Raw file	Scan	Method	Score	m/z	Gene names
20150307_MEF1_Top_opt_E1_01_1677	45288	TOF; CID	93.23	524.27	Ripk2

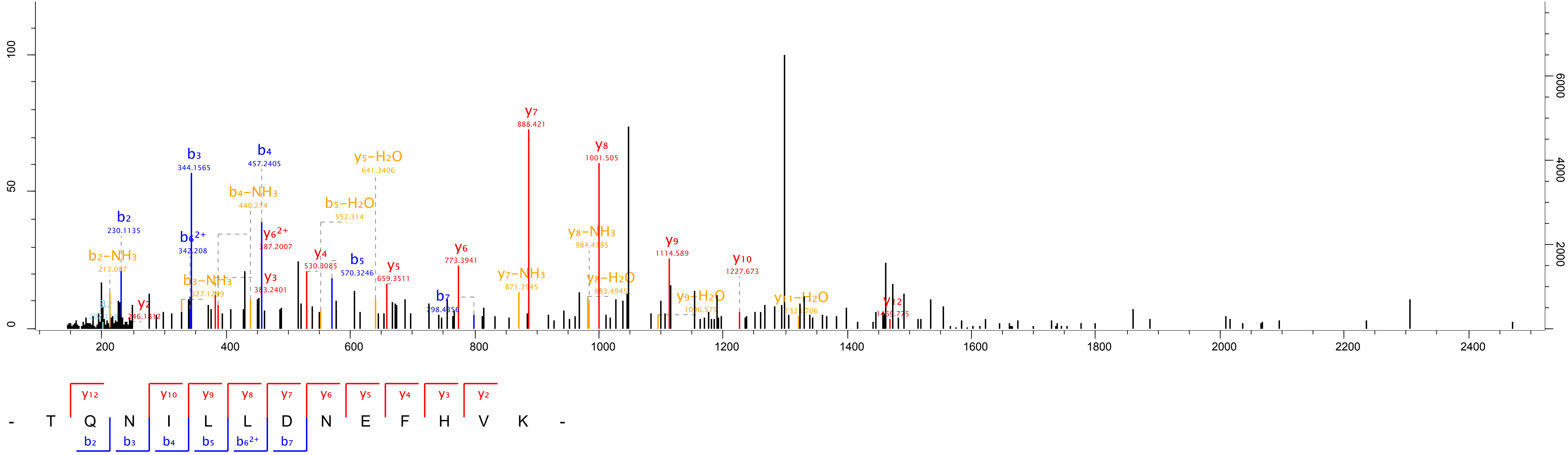

Raw file	Scan	Method	Score	m/z	Gene names
20150307_MEF1_Top_opt_E1_01_1677	51804	TOF; CID	59.71	909.45	Tsr3

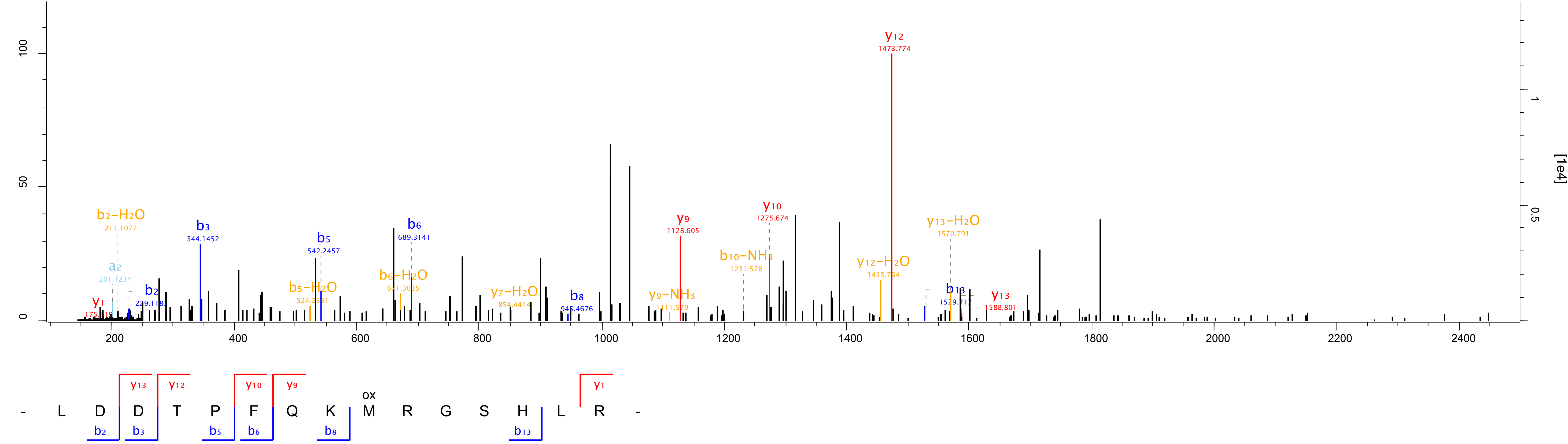

Raw file	Scan	Method	Score	m/z	Gene names
20150307_MEF1_Top_opt_E1_01_1677	57749	TOF; CID	123.03	874.44	Tmem256

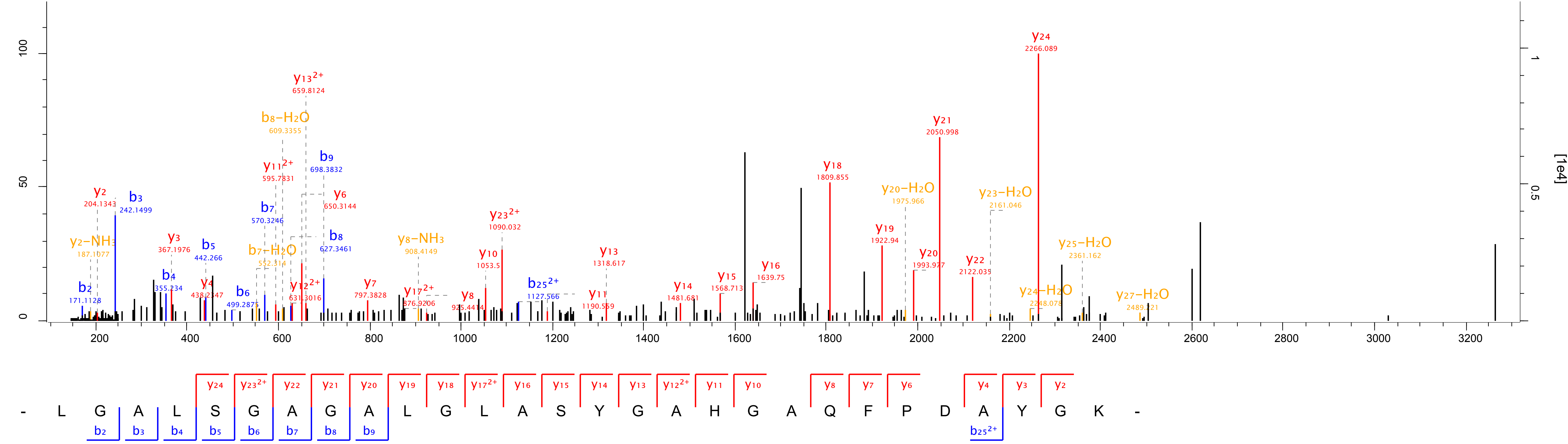

Raw file	Scan	Method	Score	m/z	Gene names
20150307_MEF1_Top_opt_E1_01_1677	60484	TOF; CID	41.26	717.38	0610031J06Rik

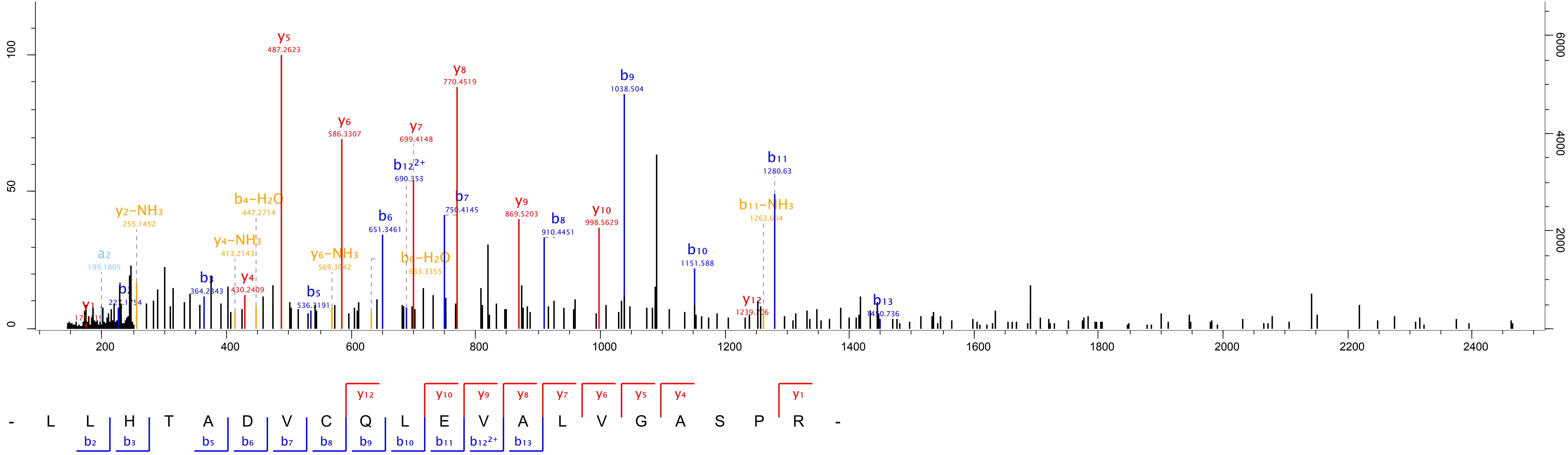

Raw file	Scan	Method	Score	m/z	Gene names
20150307_MEF1_Top_opt_E1_01_1677	60809	TOF; CID	92.15	483.29	Igfbp7

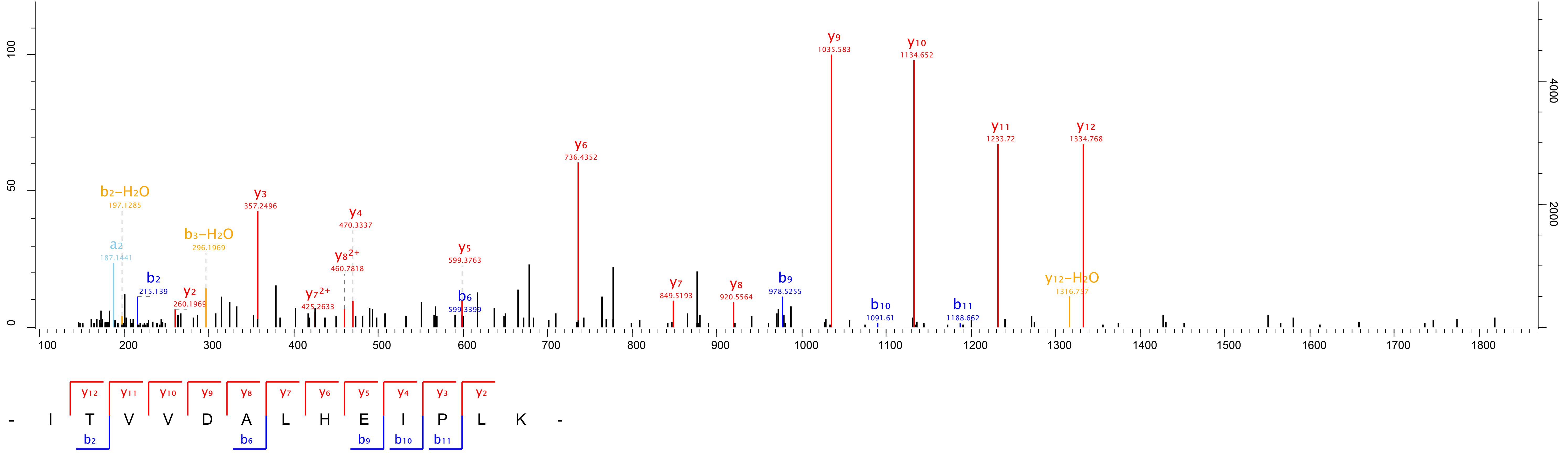

Raw file	Scan	Method	Score	m/z	Gene names
20150307_MEF1_Top_opt_E1_01_1677	65338	TOF; CID	84.89	960.45	Aebp1

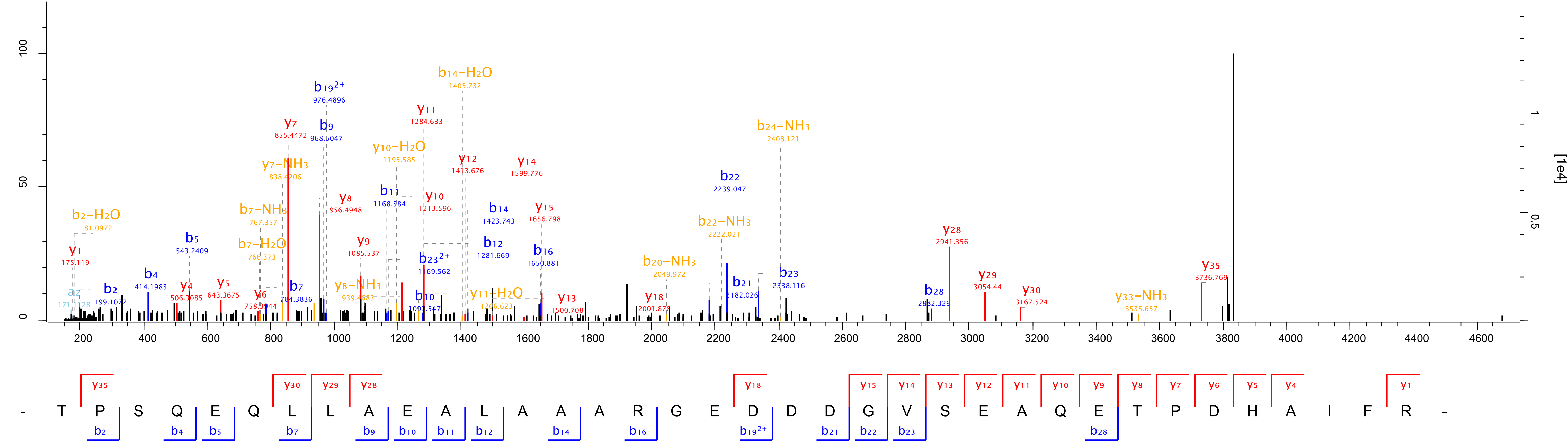

Raw file	Scan	Method	Score	m/z	Gene names
20150307_MEF1_Top_opt_E1_01_1677	65612	TOF; CID	81.02	857.43	Rasa4

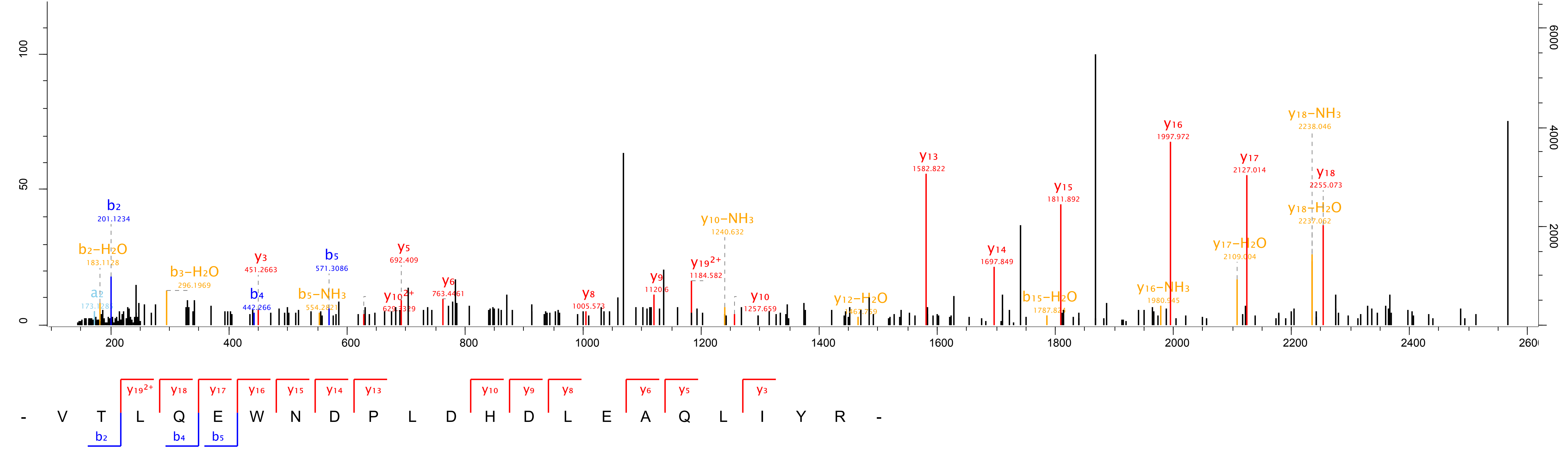

Raw file	Scan	Method	Score	m/z	Gene names
20150307_MEF1_Top_opt_E1_01_1677	66367	TOF; CID	88.6	631.87	Casp6

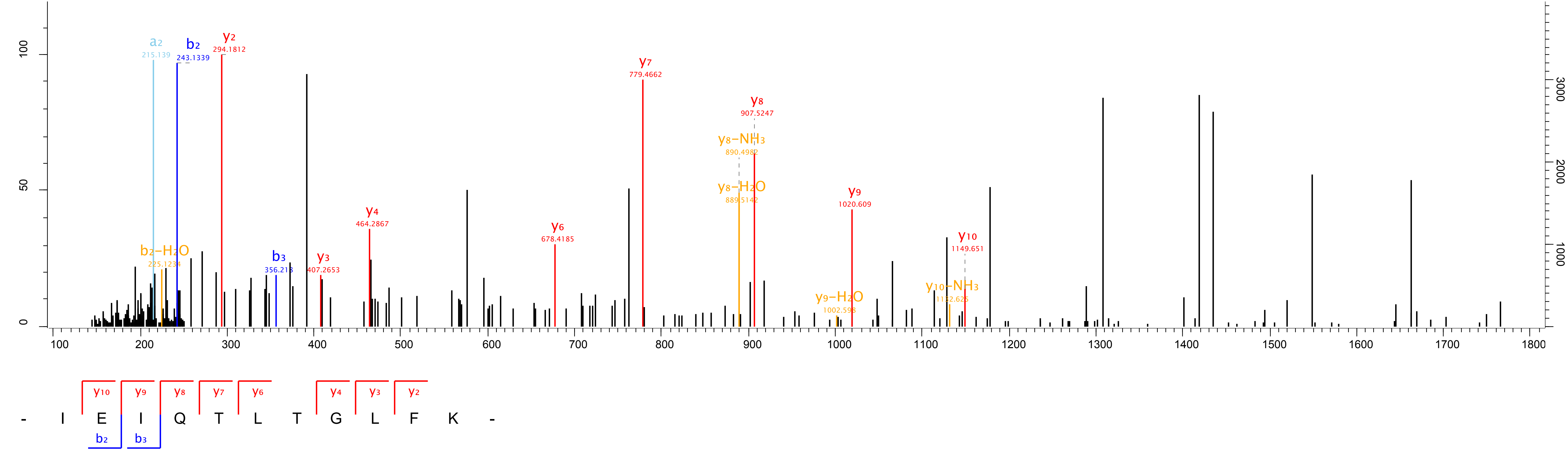

Raw file	Scan	Method	Score	m/z	Gene names
20150307_MEF1_Top_opt_E1_01_1677	68185	TOF; CID	53.03	785.39	Tm9sf1

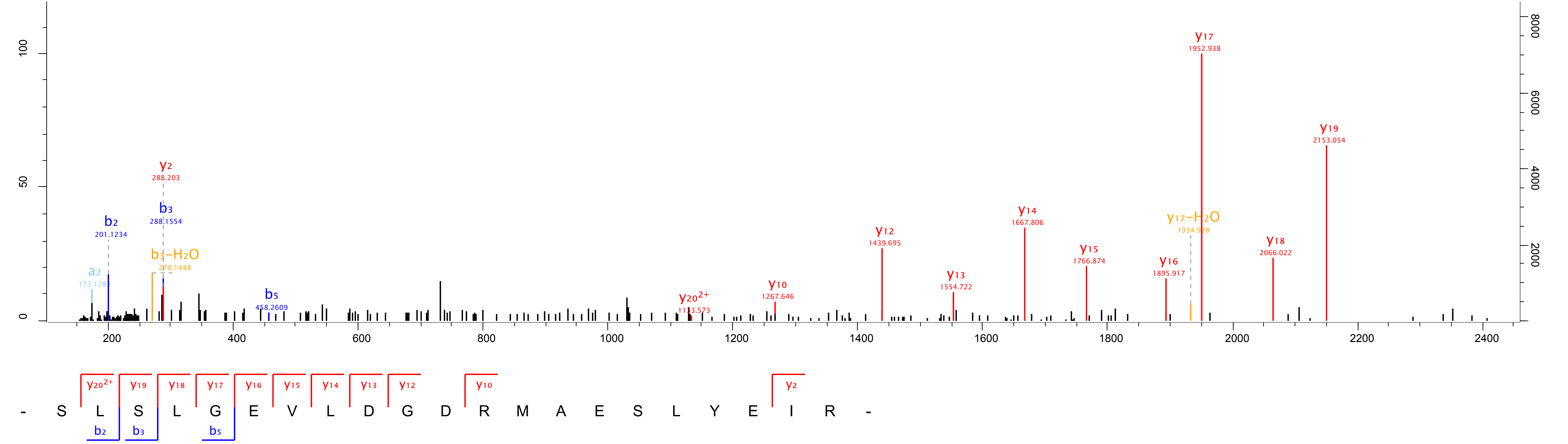

Raw file	Scan	Method	Score	m/z	Gene names
20150307_MEF1_Top_opt_E1_01_1684	8950	TOF; CID	71.61	364.51	Tm4sf1

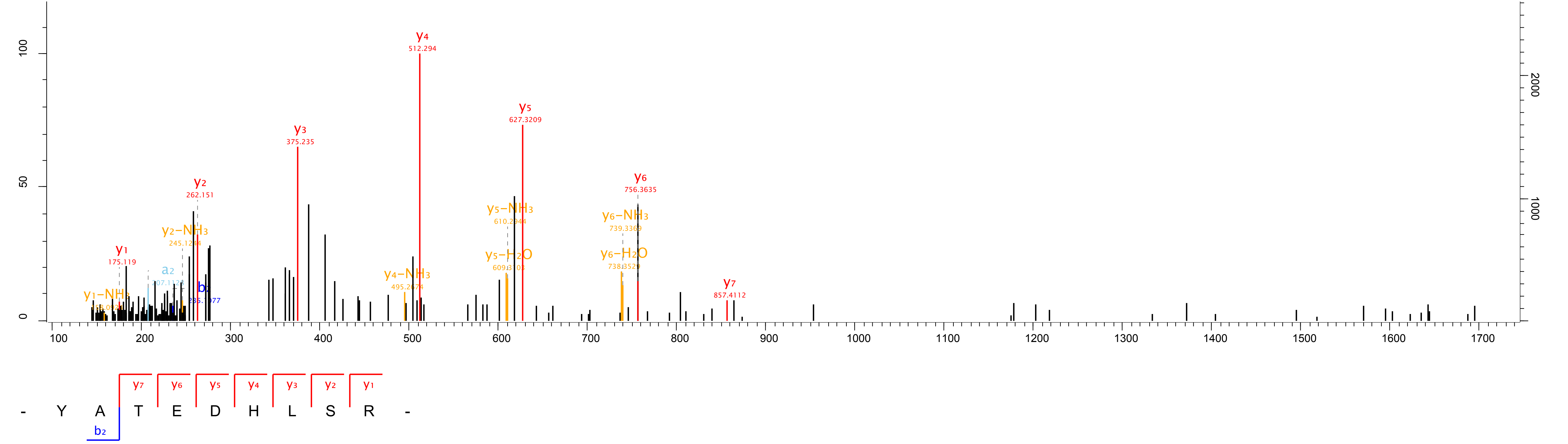

Raw file	Scan	Method	Score	m/z	Gene names
20150307_MEF1_Top_opt_E1_01_1684	21987	TOF; CID	60.26	461.23	Gsdma3

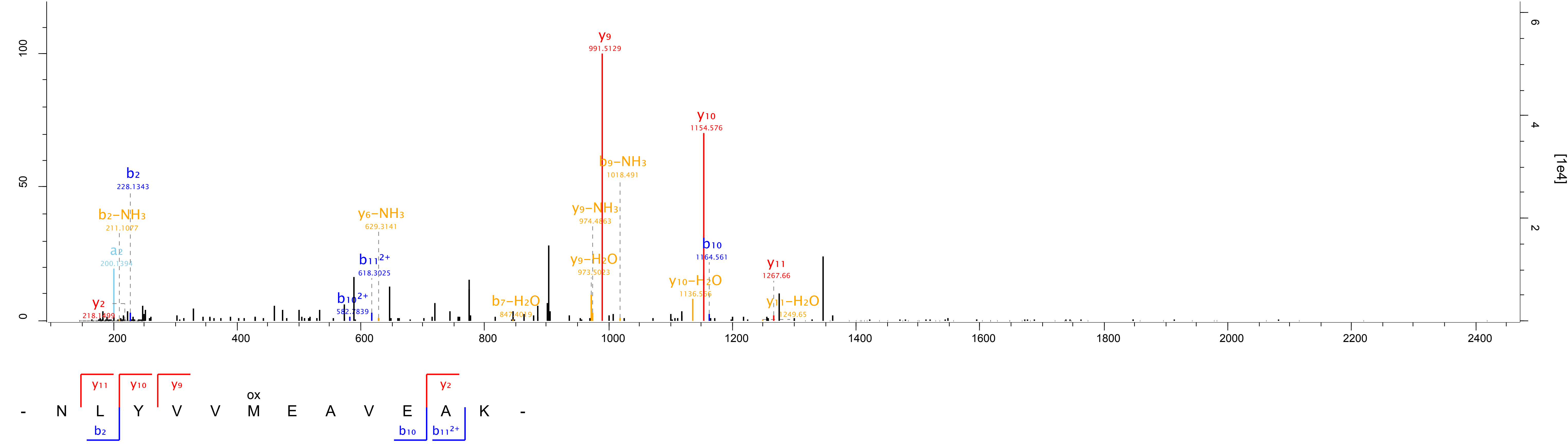

Raw file	Scan	Method	Score	m/z	Gene names
20150307_MEF1_Top_opt_E1_01_1684	47497	TOF; CID	100.04	880.99	Krtcap2

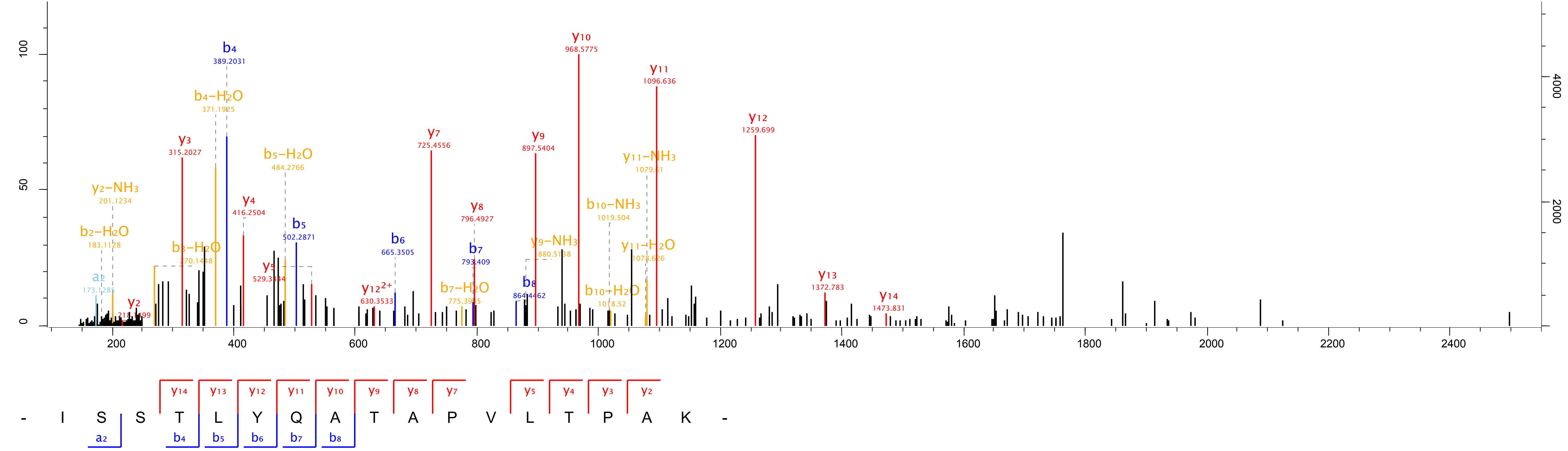

Raw file	Scan	Method	Score	m/z	Gene names
20150307_MEF1_Top_opt_E1_01_1684	51804	TOF; CID	35.81	750.08	Pcdh19

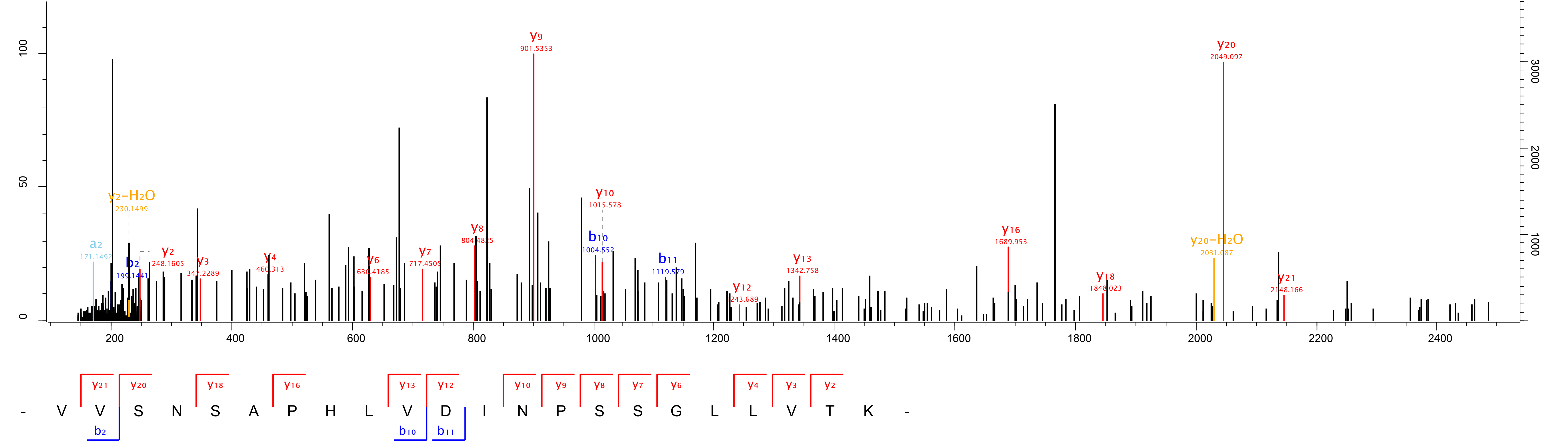

Raw file	Scan	Method	Score	m/z	Gene names
20150307_MEF1_Top_opt_E1_01_1684	54948	TOF; CID	158.37	882.77	Mapk1ip1l

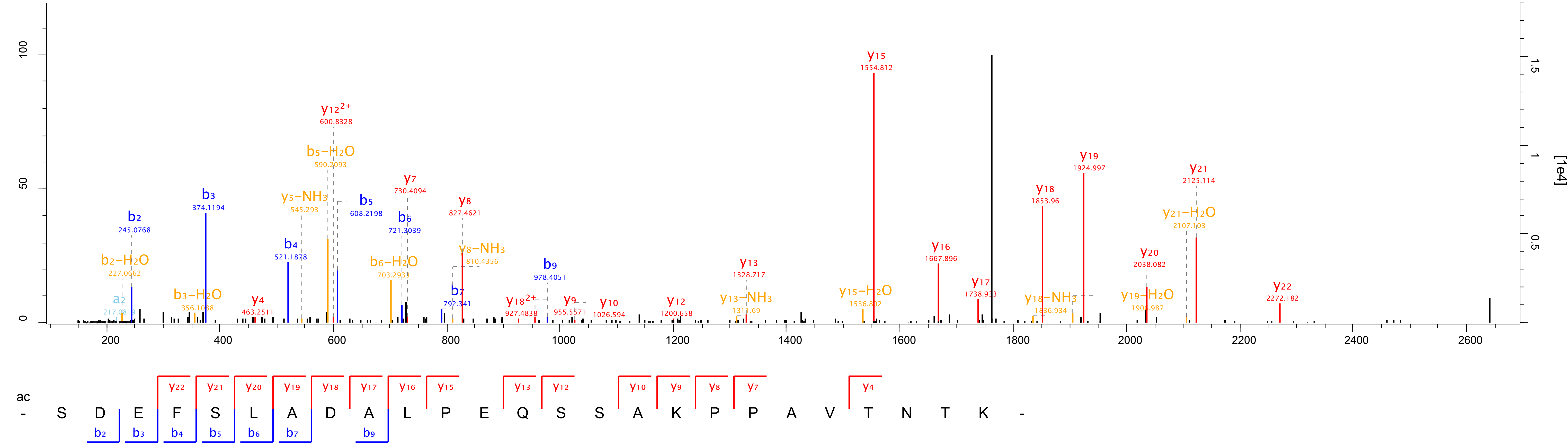

Raw file	Scan	Method	Score	m/z	Gene names
20150307_MEF1_Top_opt_E1_01_1684	62588	TOF; CID	52.01	815.4	Anapc16

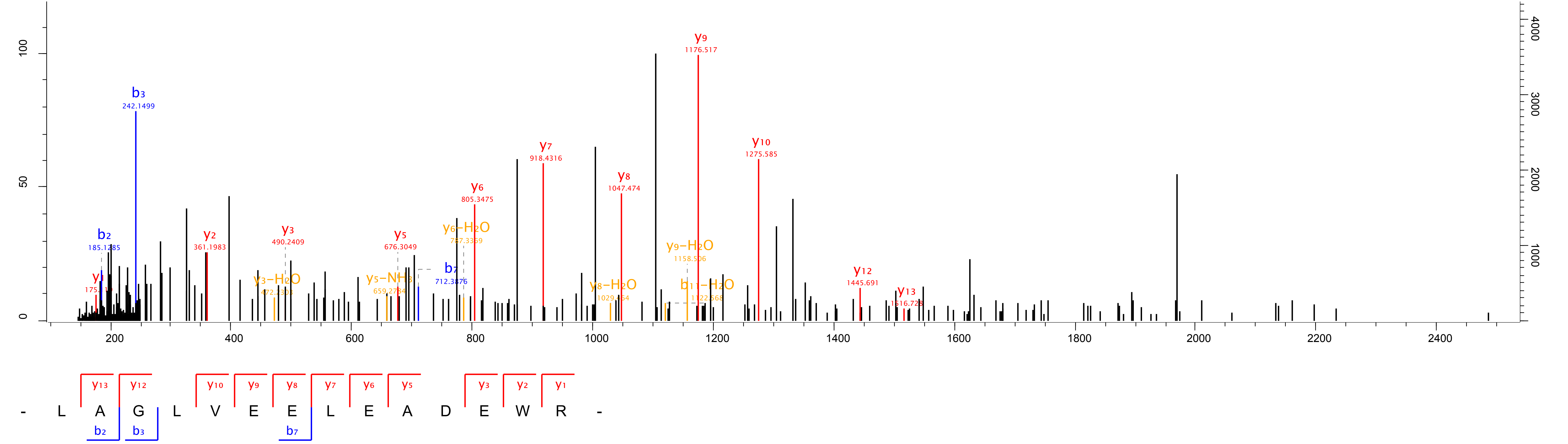

Raw file	Scan	Method	Score	m/z	Gene names
20150307_MEF1_Top_opt_E1_01_1684	63743	TOF; CID	79.68	737.03	Eri3

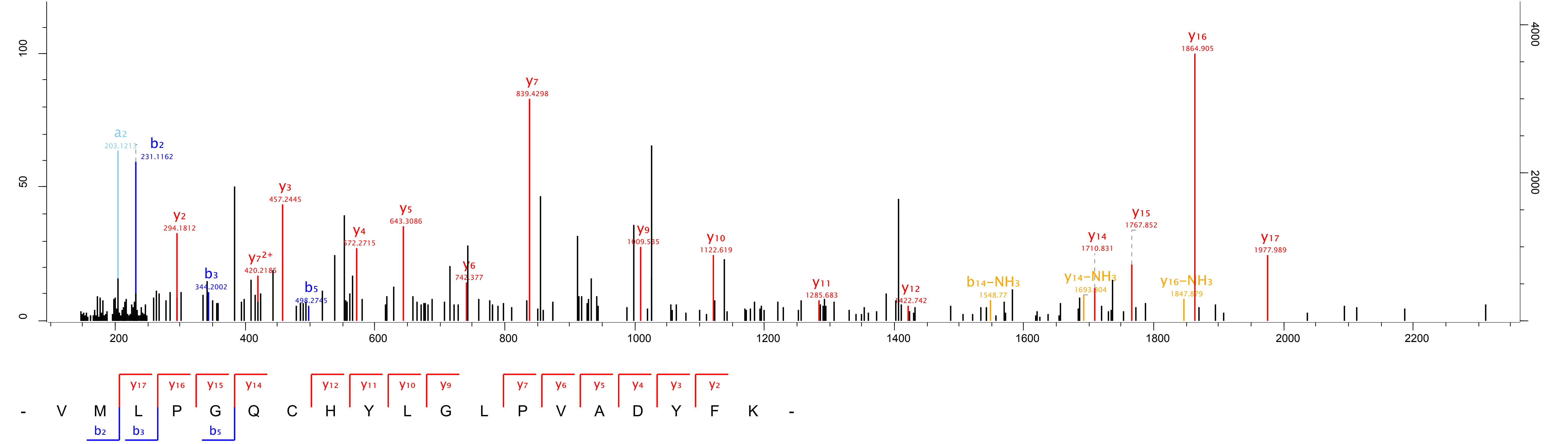

Raw file	Scan	Method	Score	m/z	Gene names
20150307_MEF1_Top_opt_E1_01_1684	66442	TOF; CID	42.73	1161.24	Mrpl52

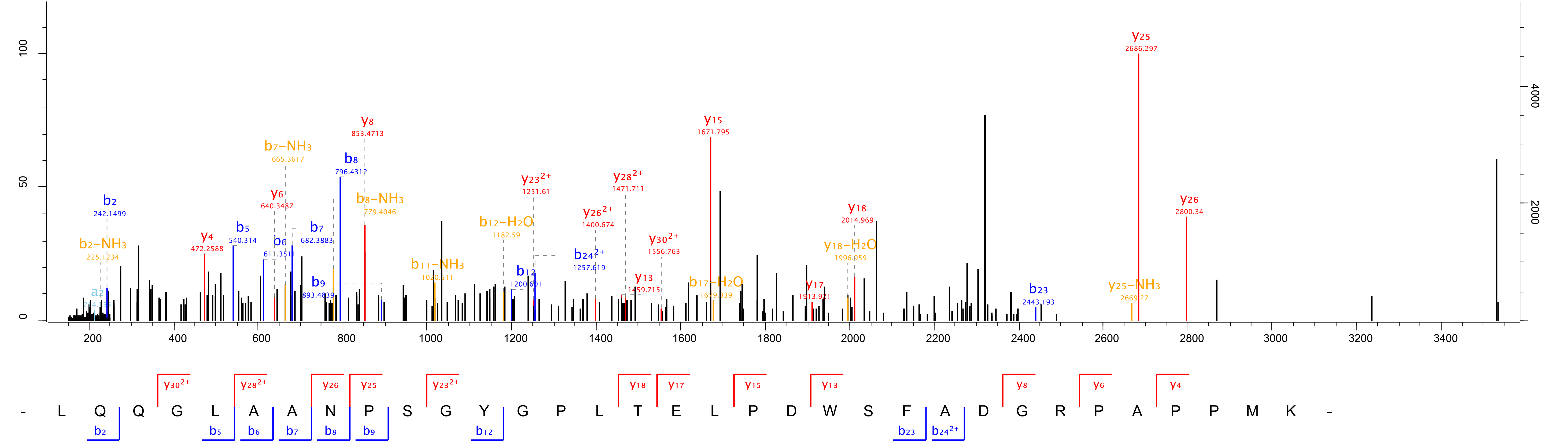

Raw file	Scan	Method	Score	m/z	Gene names
20150307_MEF2_Top_opt_E2_01_1679	15449	TOF; CID	40.24	486.23	1700034J04Rik

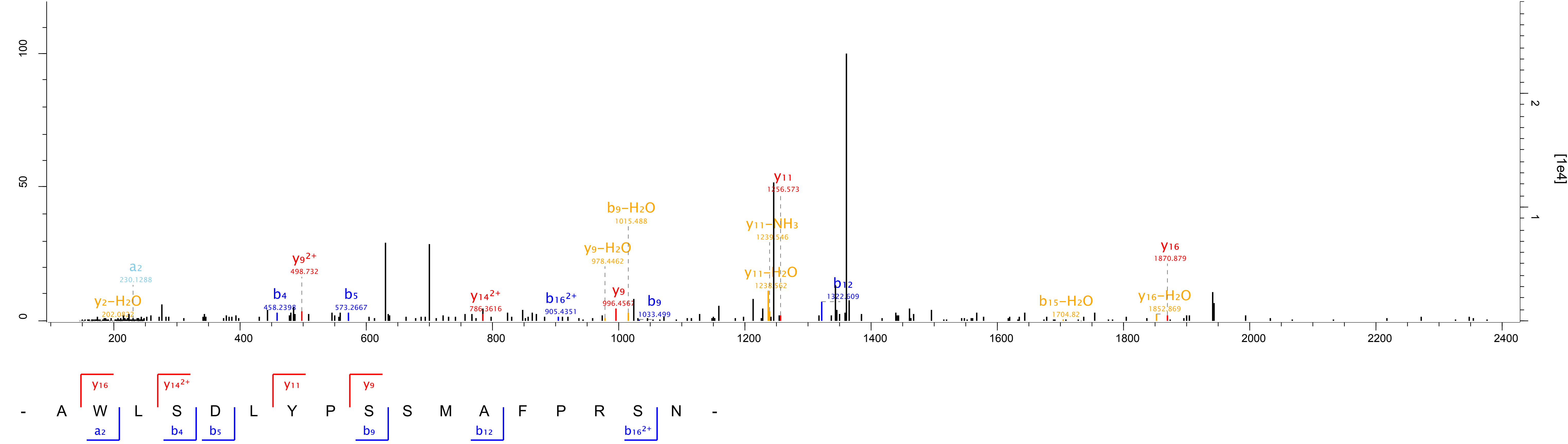

Raw file	Scan	Method	Score	m/z	Gene names
20150307_MEF2_Top_opt_E2_01_1679	48023	TOF; CID	81.68	659.37	Mrps33

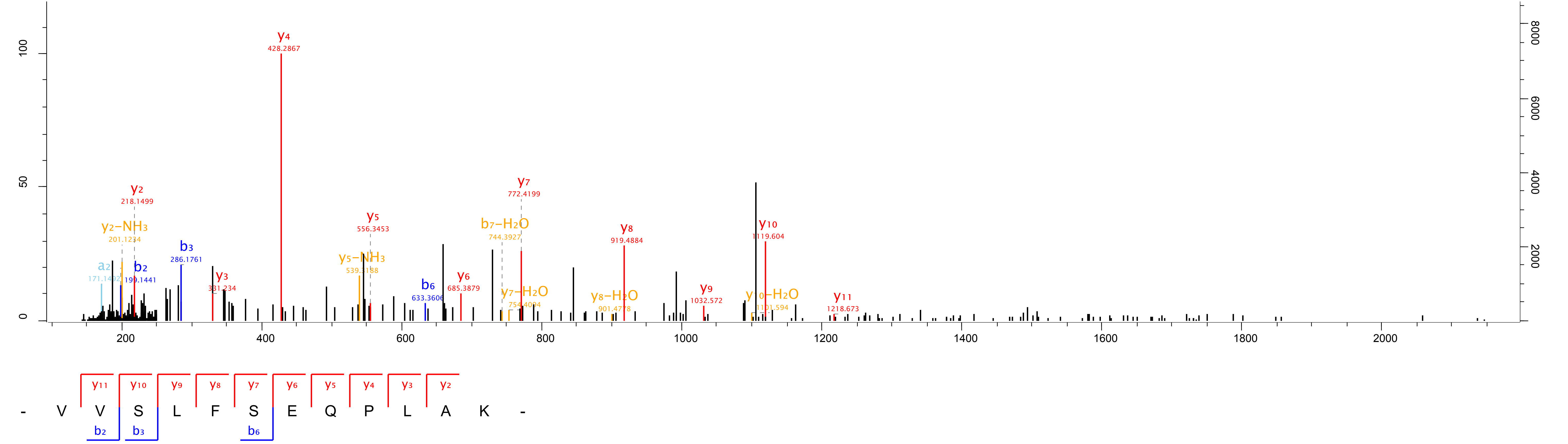

Raw file	Scan	Method	Score	m/z	Gene names
20150307_MEF2_Top_opt_E2_01_1679	61671	TOF; CID	58.09	820.42	Ctdnep1

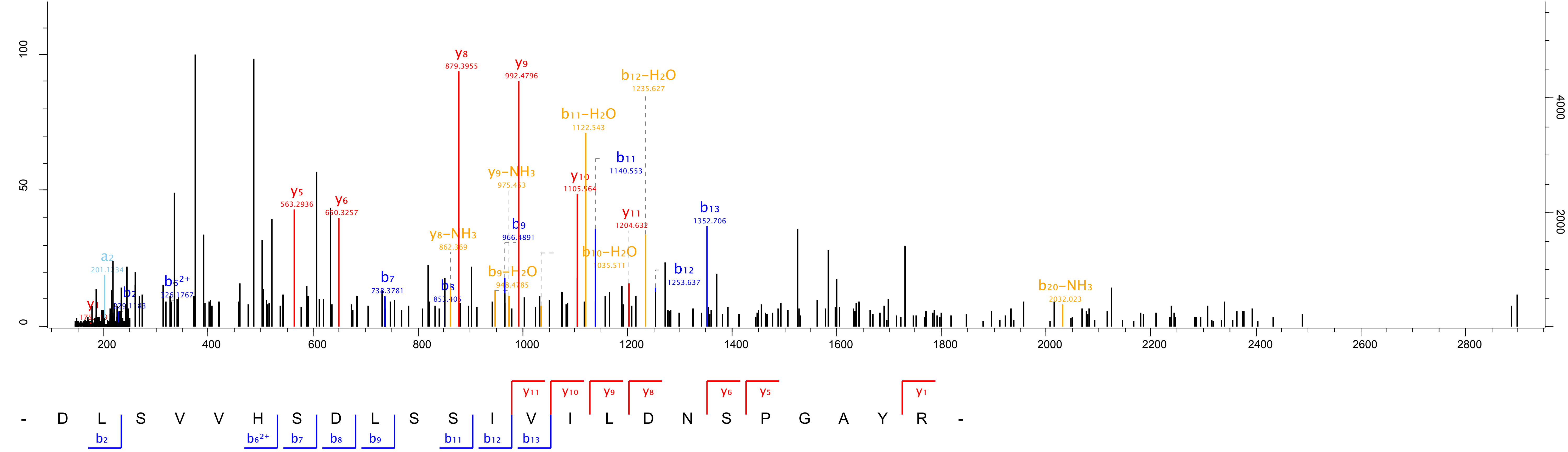

Raw file	Scan	Method	Score	m/z	Gene names
20150307_MEF2_Top_opt_E2_01_1687	9922	TOF; CID	79.88	622.97	Cgref1

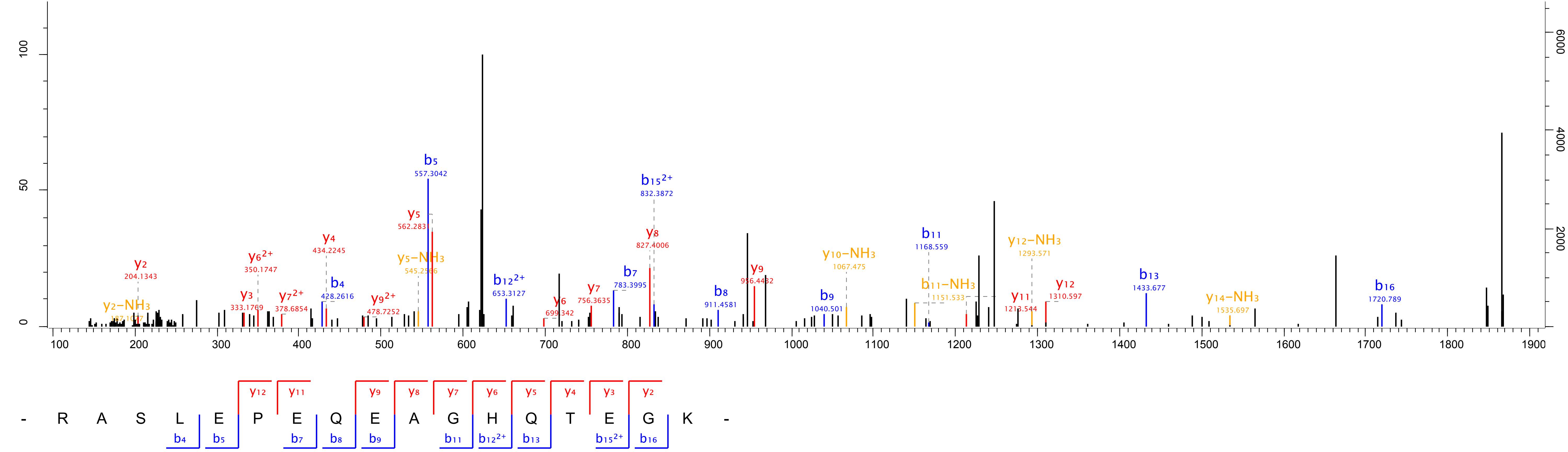

Raw file	Scan	Method	Score	m/z	Gene names
20150307_MEF2_Top_opt_E2_01_1687	13121	TOF; CID	83.14	609.31	Armcx2

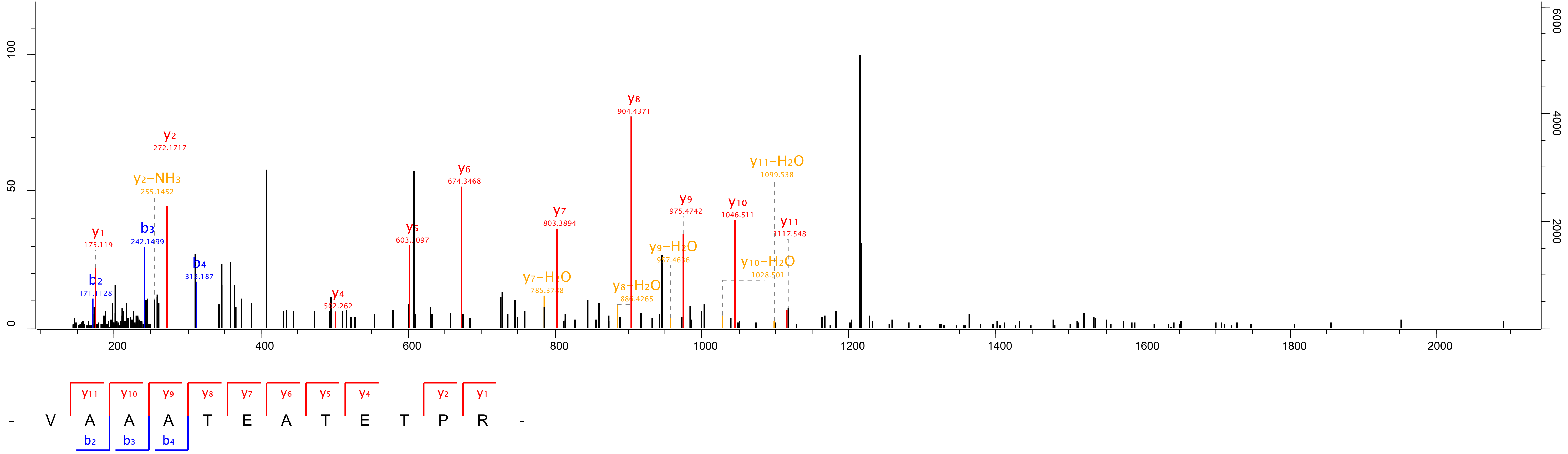

Raw file	Scan	Method	Score	m/z	Gene names
20150307_MEF2_Top_opt_E2_01_1687	14173	TOF; CID	48.42	483.5	Wfikkn1

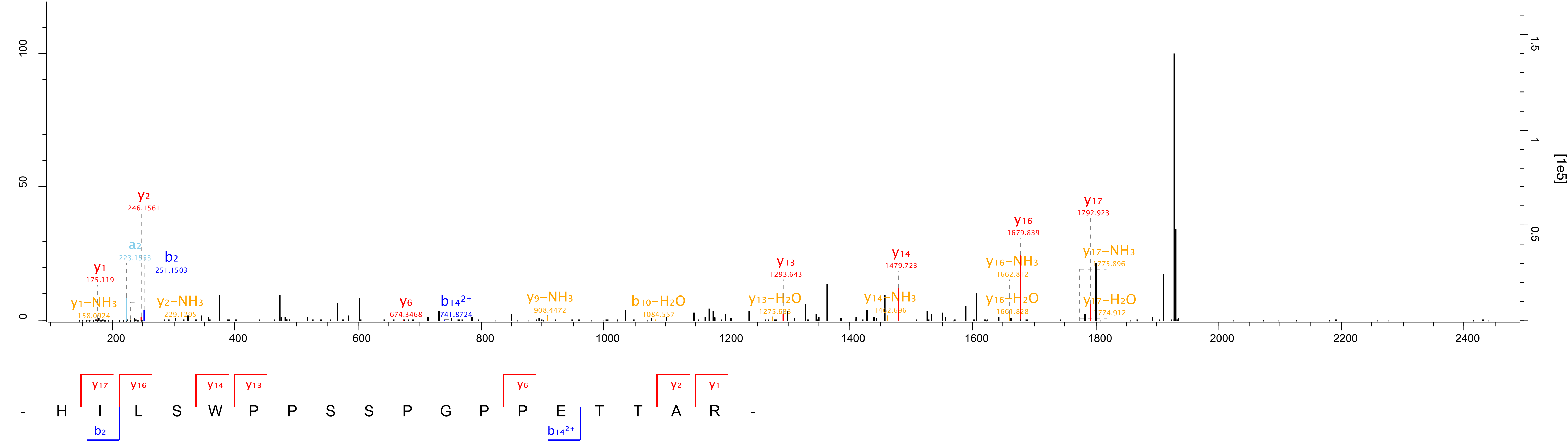

Raw file	Scan	Method	Score	m/z	Gene names
20150307_MEF2_Top_opt_E2_01_1687	14537	TOF; CID	65.84	641.29	Ndufv3

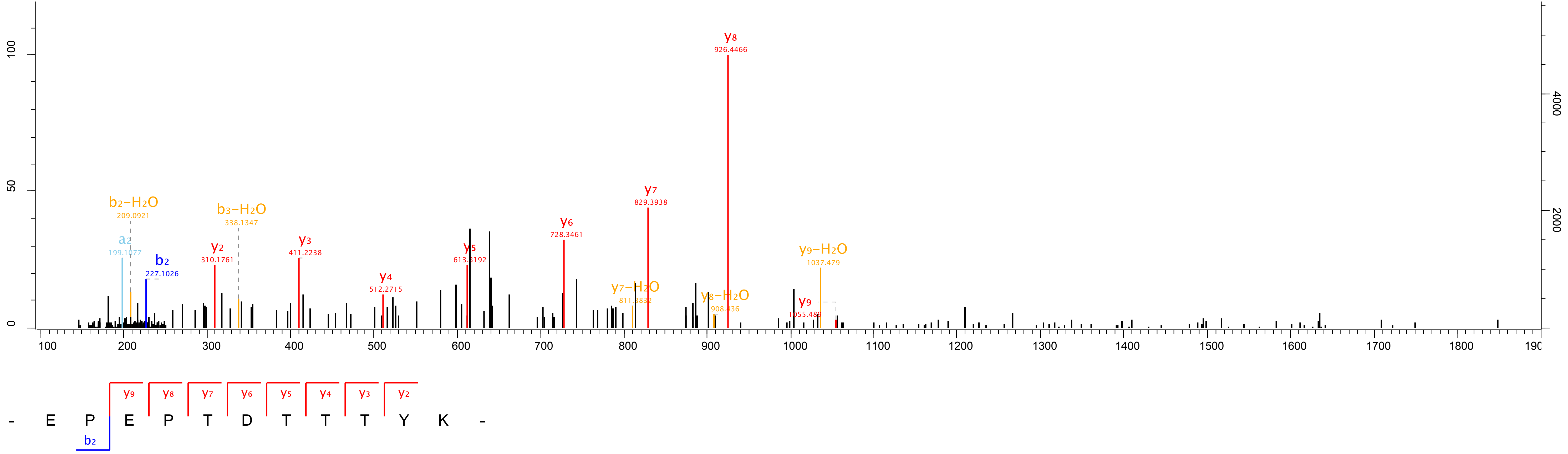

Raw file	Scan	Method	Score	m/z	Gene names
20150307_MEF2_Top_opt_E2_01_1687	15716	TOF; CID	79.15	568.78	Dyrk1a;Dyrk1b

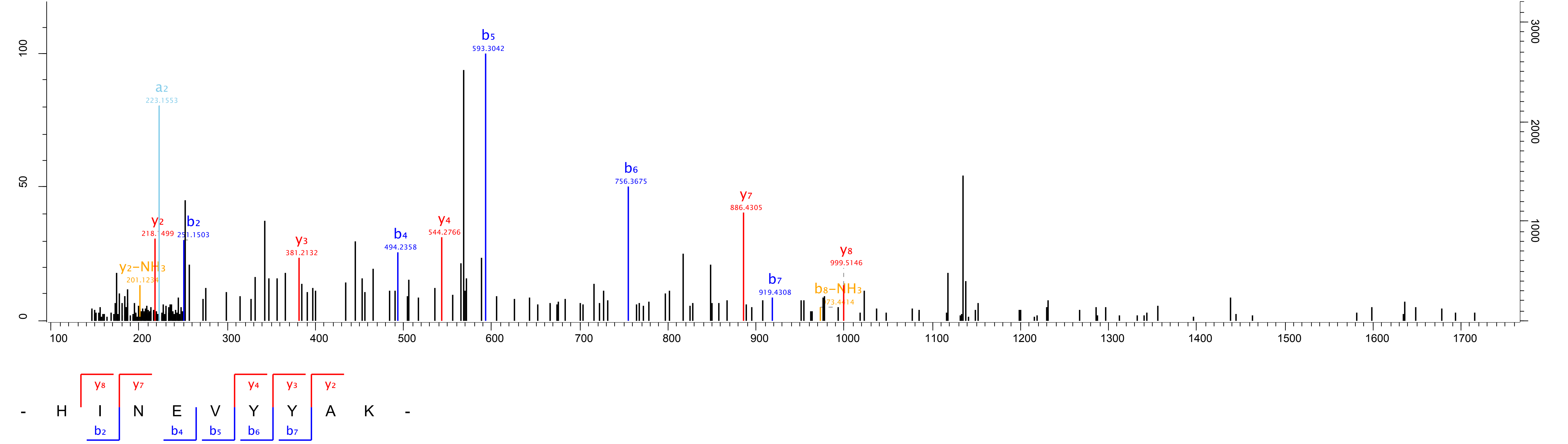

Raw file	Scan	Method	Score	m/z	Gene names
20150307_MEF2_Top_opt_E2_01_1687	37855	TOF; CID	50.9	786.42	Endod1

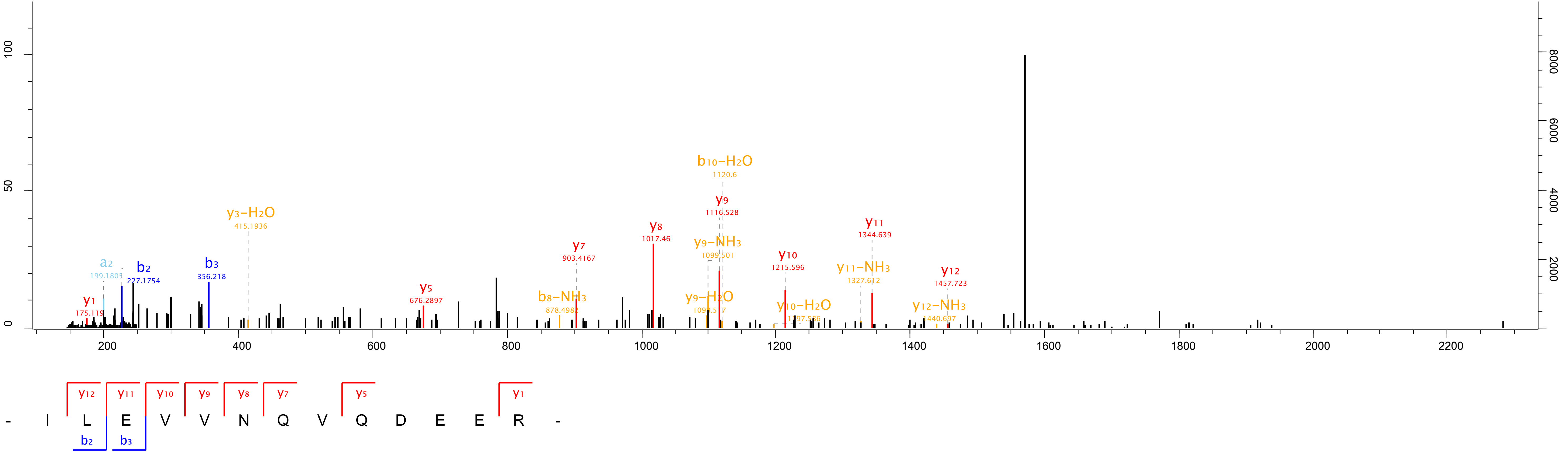

Raw file	Scan	Method	Score	m/z	Gene names
20150307_MEF2_Top_opt_E2_01_1687	39813	TOF; CID	60.97	577.29	Akt1s1

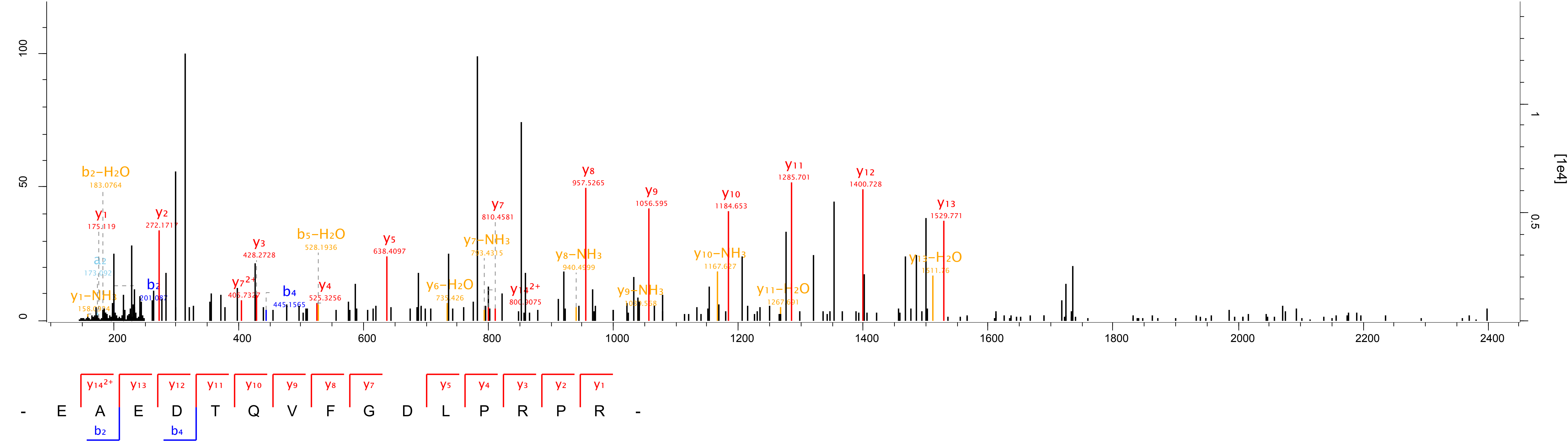

Raw file	Scan	Method	Score	m/z	Gene names
20150307_MEF2_Top_opt_E2_01_1687	41202	TOF; CID	56.96	600.86	Gmeb2

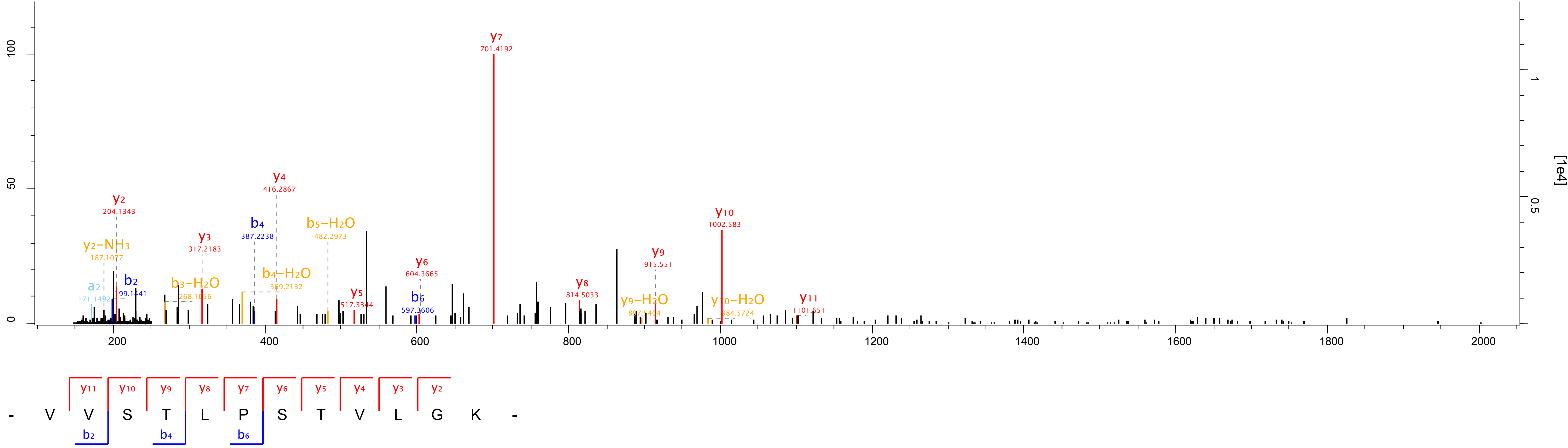

Raw file	Scan	Method	Score	m/z	Gene names
20150307_MEF2_Top_opt_E2_01_1687	46351	TOF; CID	43.95	579.29	Napepld

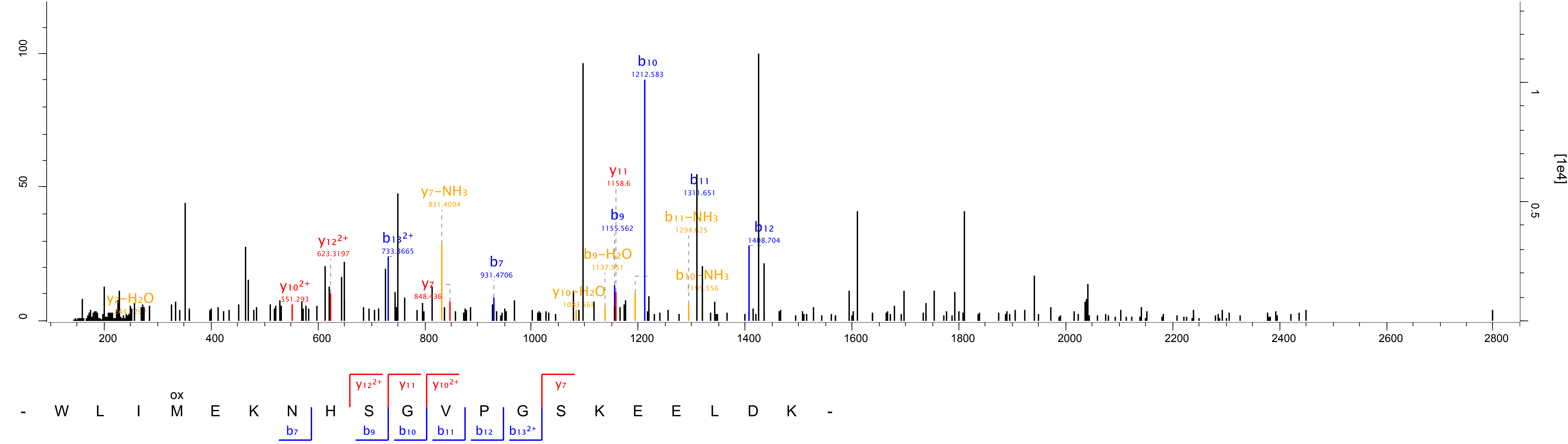

Raw file	Scan	Method	Score	m/z	Gene names
20150307_MEF2_Top_opt_E2_01_1687	55276	TOF; CID	100.62	882.44	Rpe

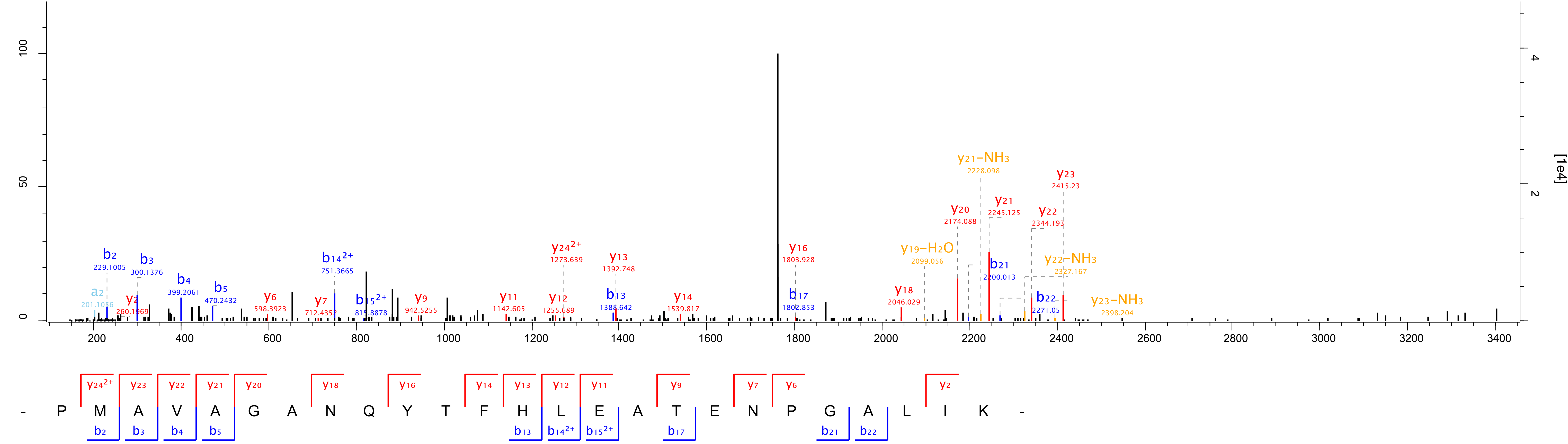

Raw file	Scan	Method	Score	m/z	Gene names
20150307_MEF2_Top_opt_E2_01_1687	58576	TOF; CID	118.31	940.46	Desi2

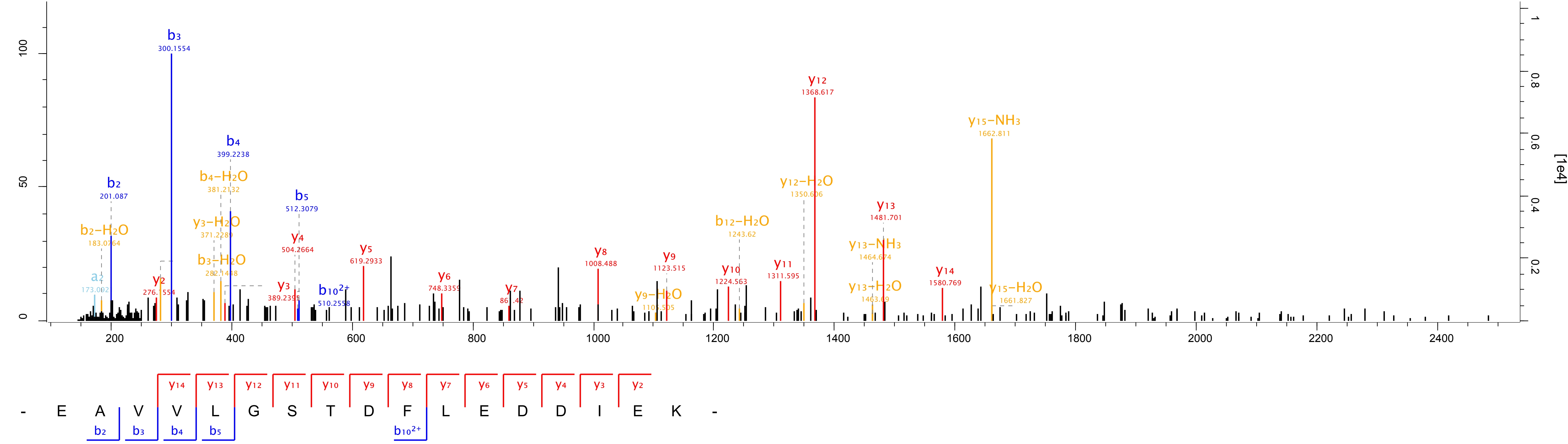

Raw file	Scan	Method	Score	m/z	Gene names
20150307_MEF2_Top_opt_E2_01_1687	59996	TOF; CID	49.66	829.08	Fdx1l

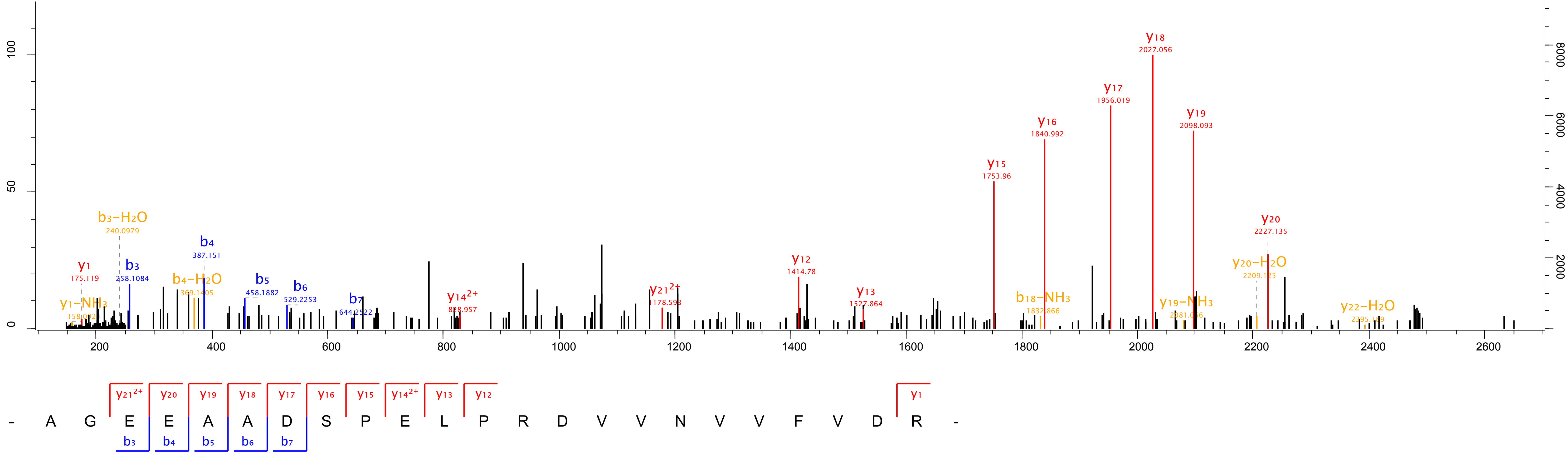

Raw file	Scan	Method	Score	m/z	Gene names
20150307_MEF2_Top_opt_E2_01_1687	65933	TOF; CID	69.77	740.38	Txndc15

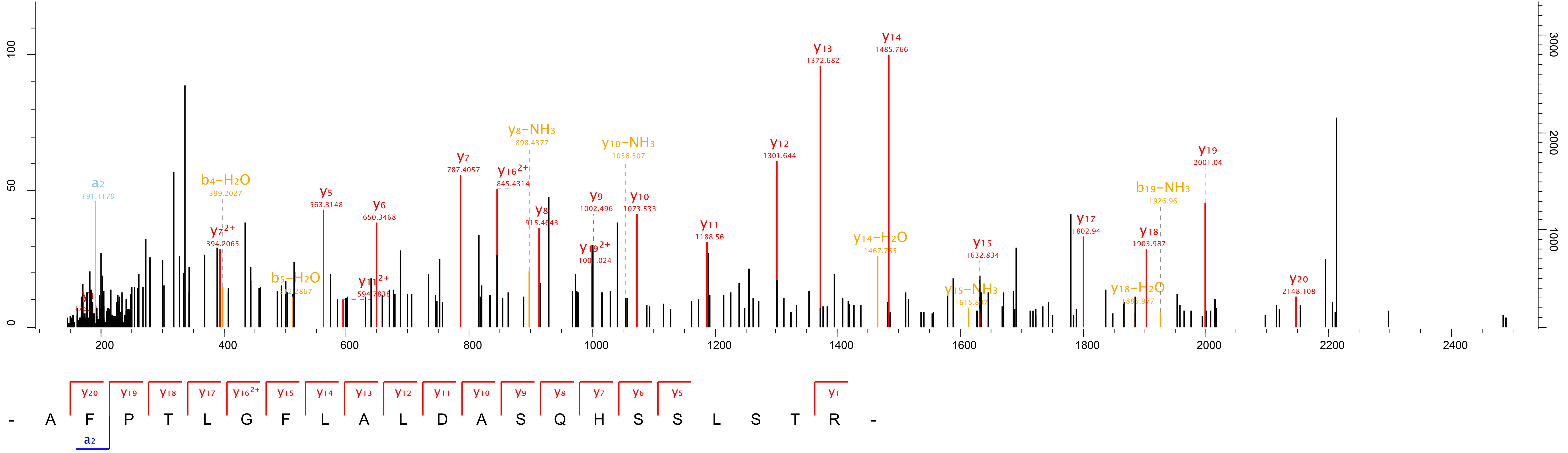

Raw file	Scan	Method	Score	m/z	Gene names
20150307_MEF2_Top_opt_E2_01_1687	70556	TOF; CID	85.47	814.95	Grk5;Grk6

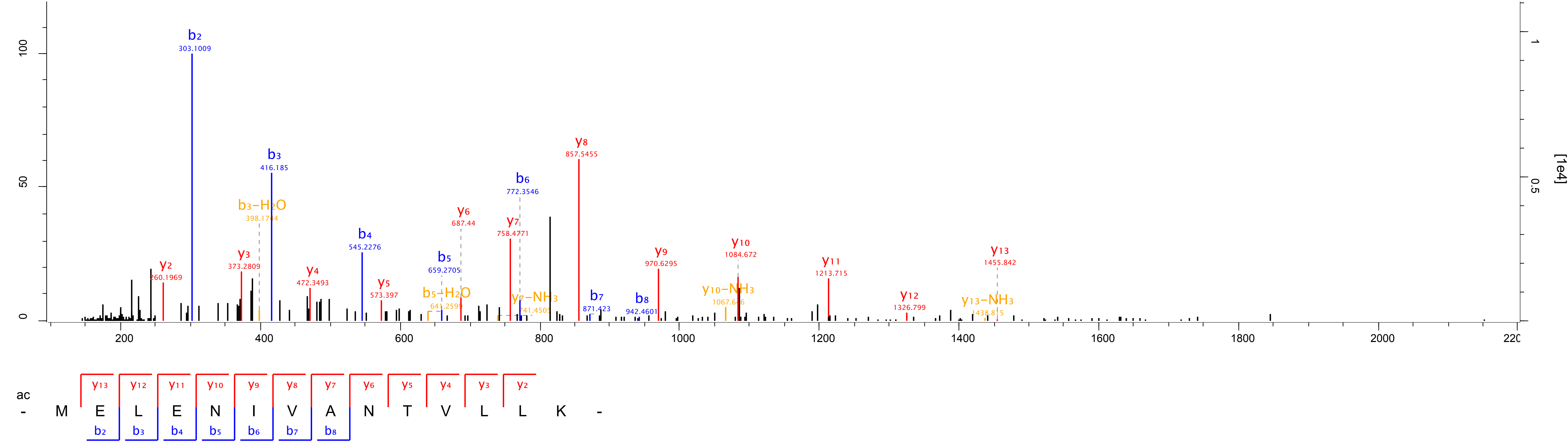

Raw file	Scan	Method	Score	m/z	Gene names
20150307_MEF3_Top_opt_E3_01_1681	10911	TOF; CID	58.7	406.21	Lrrcc1

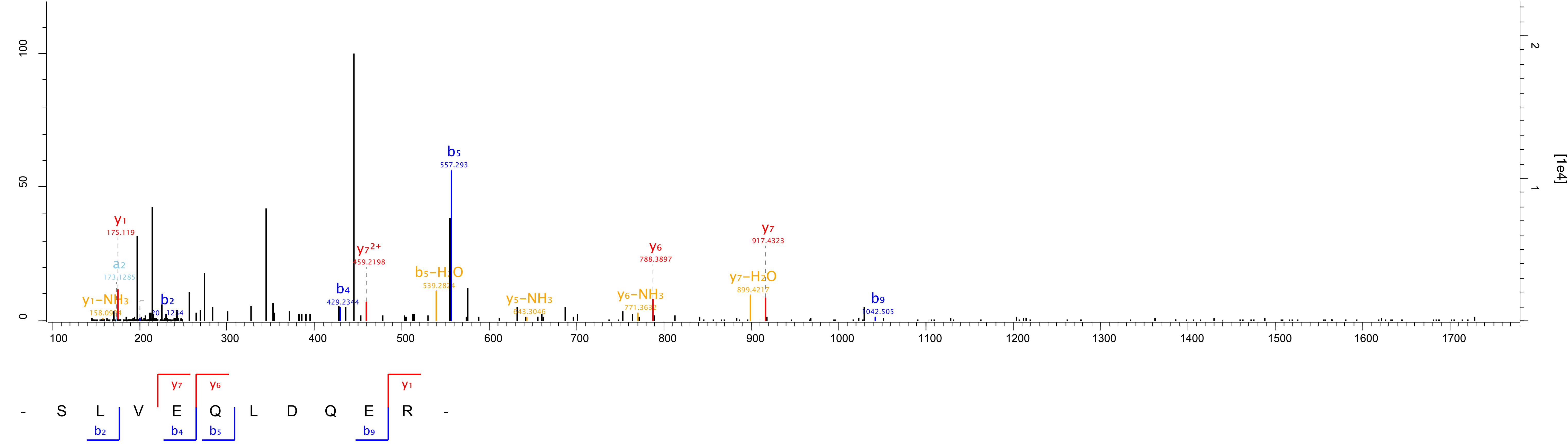

Raw file	Scan	Method	Score	m/z	Gene names
20150307_MEF3_Top_opt_E3_01_1681	12589	TOF; CID	83.87	407.2	Tmed3

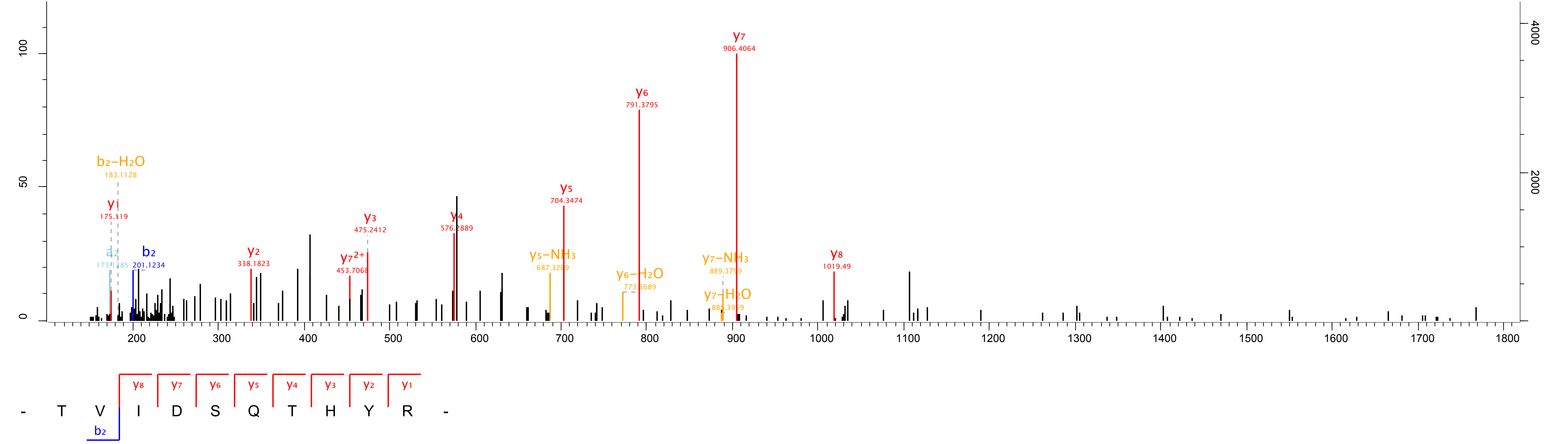

Raw file	Scan	Method	Score	m/z	Gene names
20150307_MEF3_Top_opt_E3_01_1681	16487	TOF; CID	49.3	609.92	Gpr55

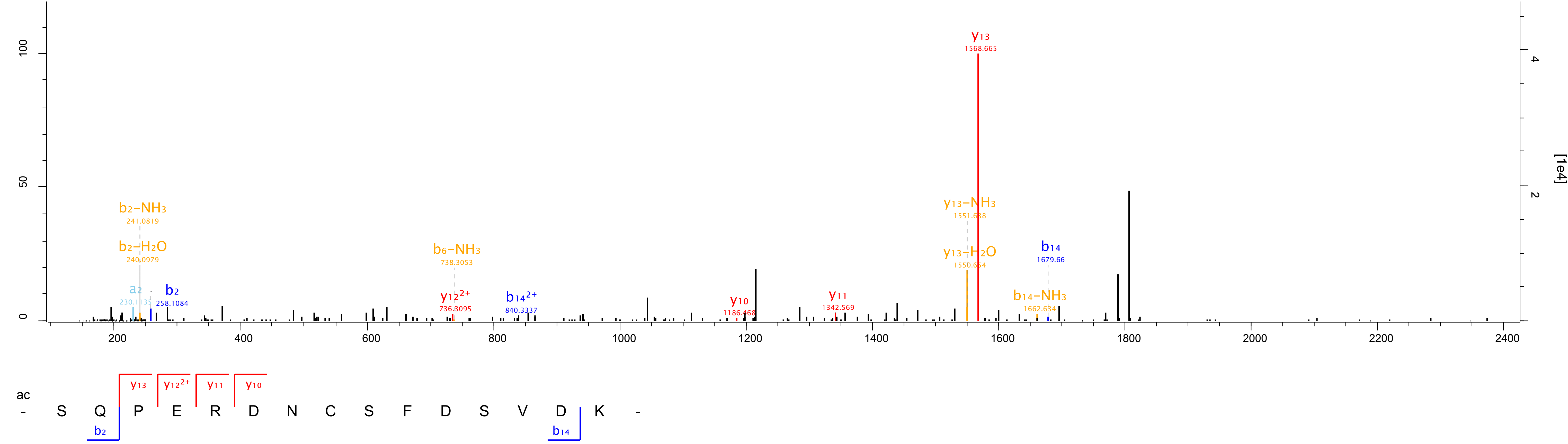

Raw file	Scan	Method	Score	m/z
20150307_MEF3_Top_opt_E3_01_1681	19049	TOF; CID	64.27	465.22

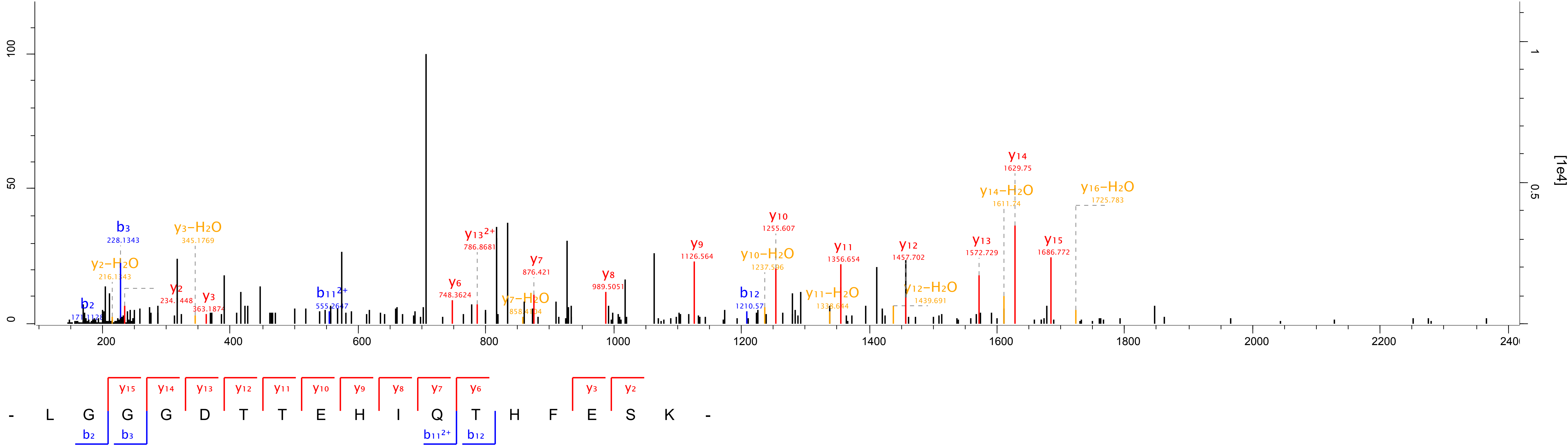

Raw file	Scan	Method	Score	m/z	Gene names
20150307_MEF3_Top_opt_E3_01_1681	25118	TOF; CID	46.25	642.36	Tfap4

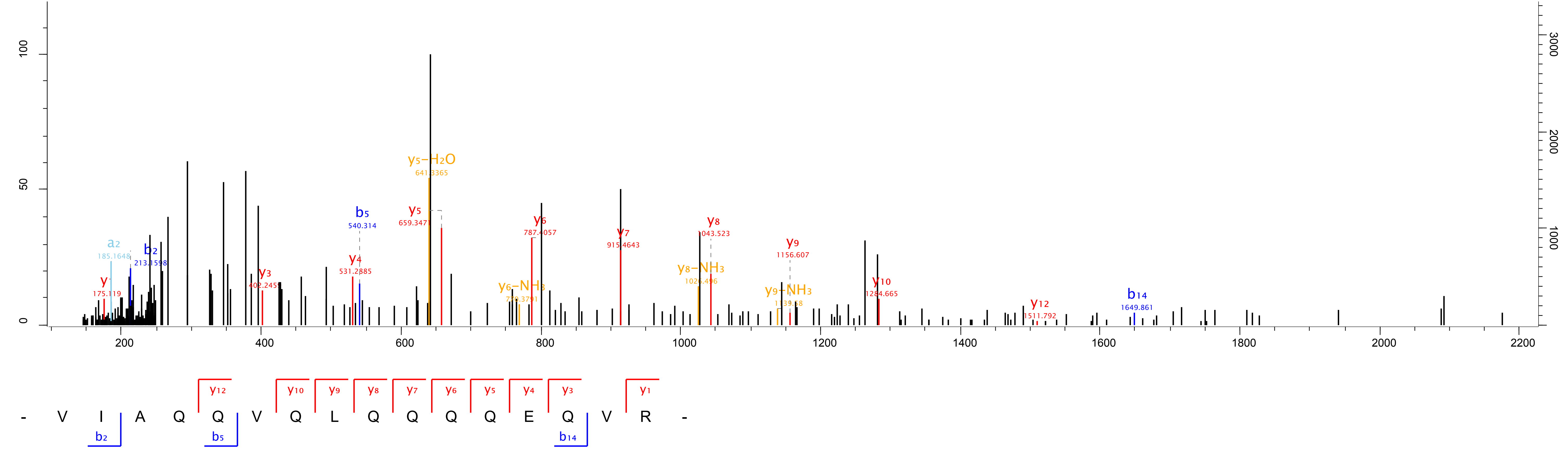

Raw file	Scan	Method	Score	m/z	Gene names
20150307_MEF3_Top_opt_E3_01_1681	50096	TOF; CID	45.28	981.95	Cdc42se2

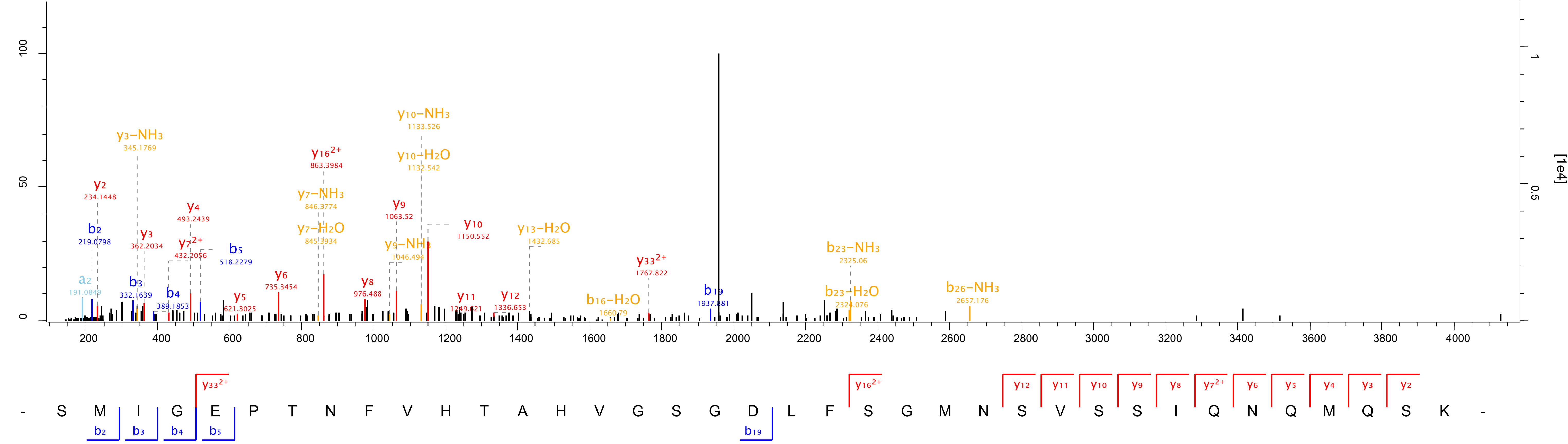

Raw file	Scan	Method	Score	m/z	Gene names
20150307_MEF3_Top_opt_E3_01_1681	58007	TOF; CID	31.17	730.36	Med16

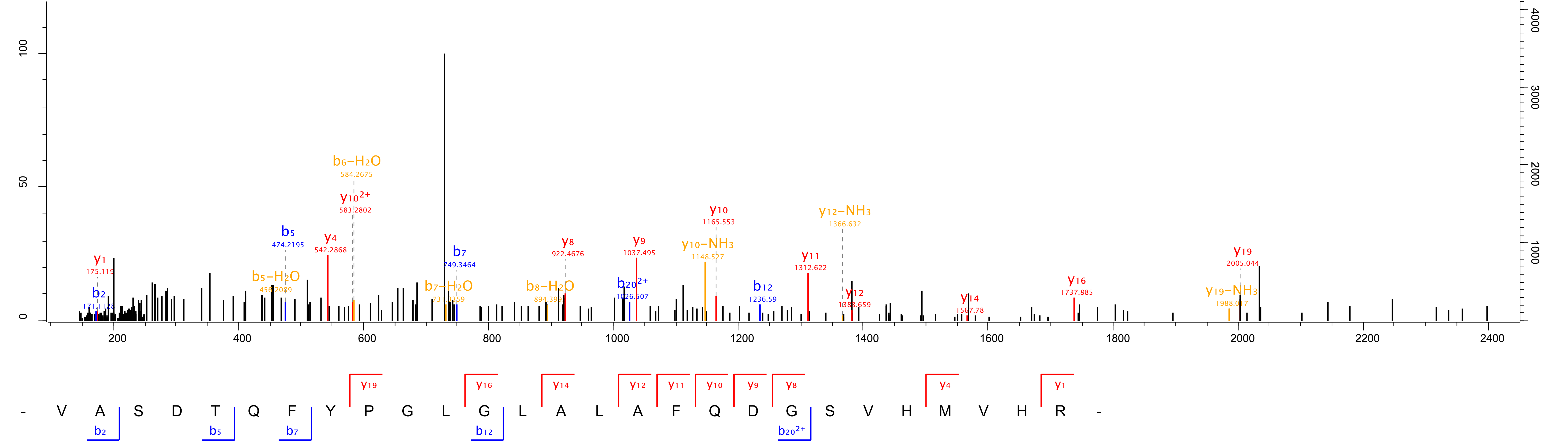

Raw file	Scan	Method	Score	m/z	Gene names
20150307_MEF3_Top_opt_E3_01_1690	13882	TOF; CID	69.26	666.81	Tmf1

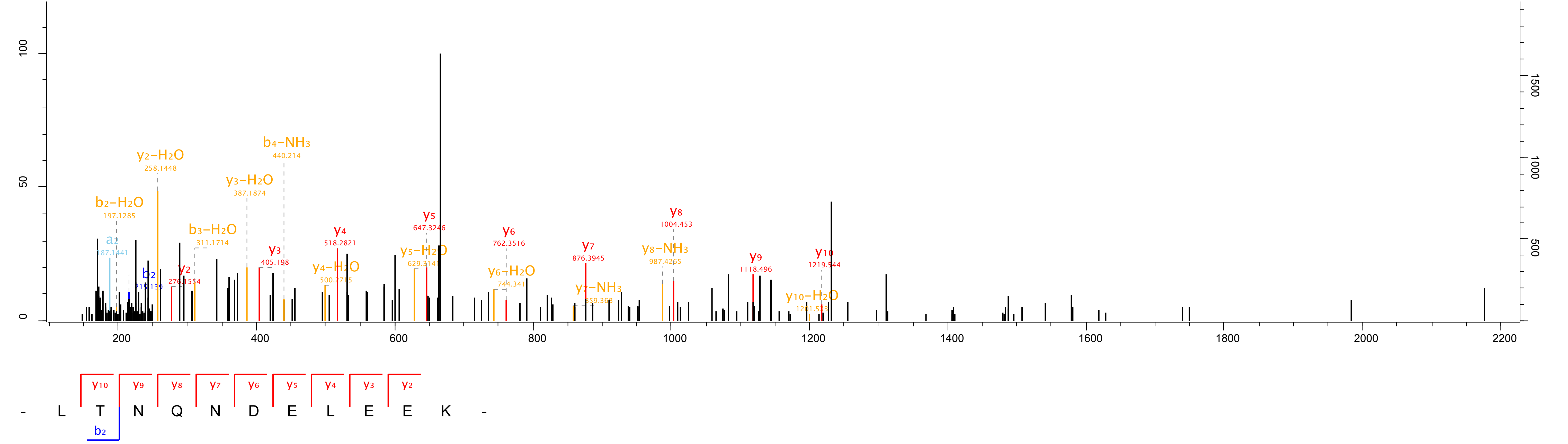

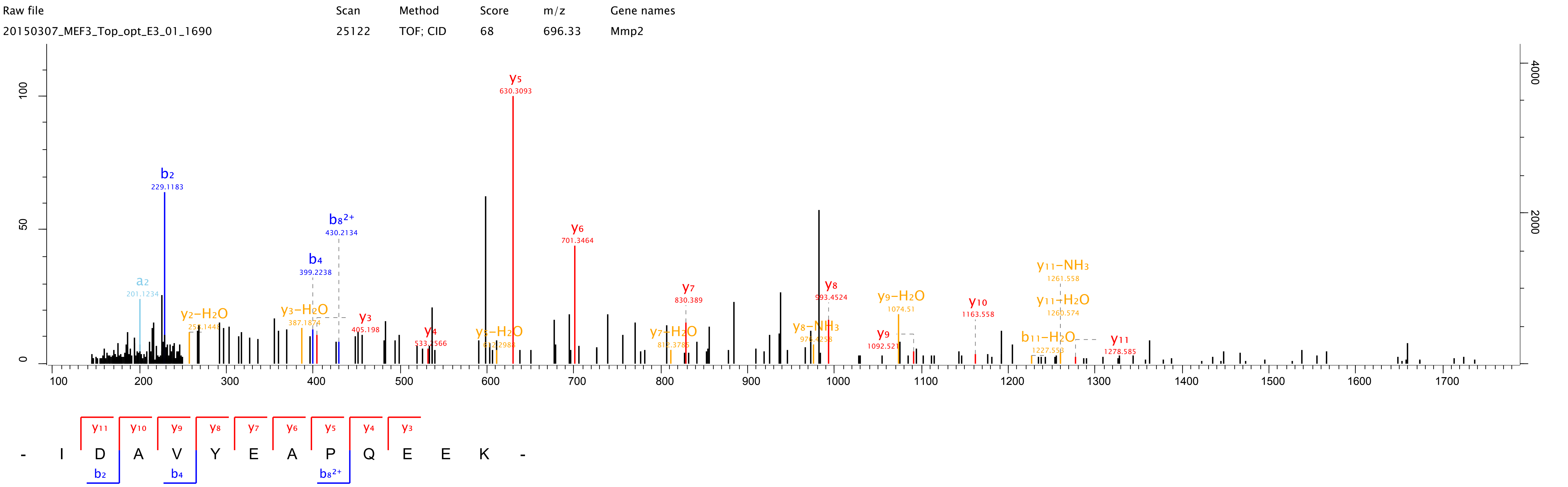

Raw file
20150307_MEF3_Top_opt_E3_01_1690
Scan
25122
Method
TOF; CID
Score
68
m/z
696.33
Gene names
Mmp2
y5
630.3093
b2
229.1183
b8 2+
430.2134
b4
399.2238
a2
201.1234
y6
701.3464
y2−H2O
y3−H2O
387.1874
y3
405.198
y4
y7
830.389
y8
993.4524
y9−H2O
1074.51
y10
1163.558
y11−NH3
1261.558
y11−H2O
y9
1092.521
b11−H2O
1227.553
y11
1278.585
- I D A V Y E A P Q E E K -

Raw file	Scan	Method	Score	m/z	Gene names
20150307_MEF3_Top_opt_E3_01_1690	27887	TOF; CID	78.33	791.37	Sumf1

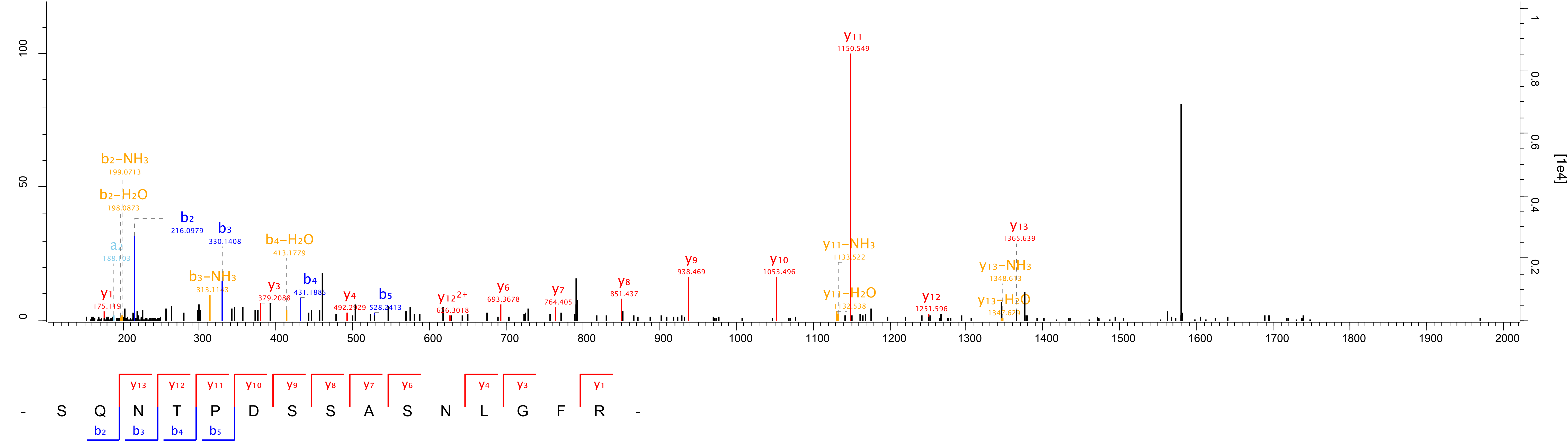

Raw file	Scan	Method	Score	m/z	Gene names
20150307_MEF3_Top_opt_E3_01_1690	35500	TOF; CID	73.5	404.22	Orc6

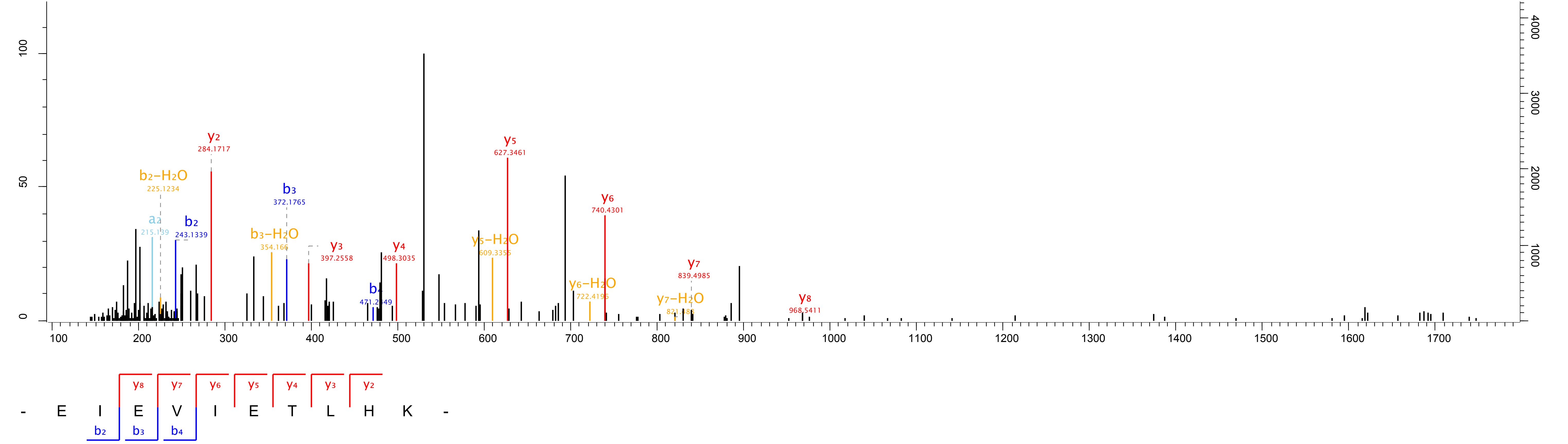

Raw file	Scan	Method	Score	m/z	Gene names
20150307_MEF3_Top_opt_E3_01_1690	36627	TOF; CID	68.66	602.32	Rnf180

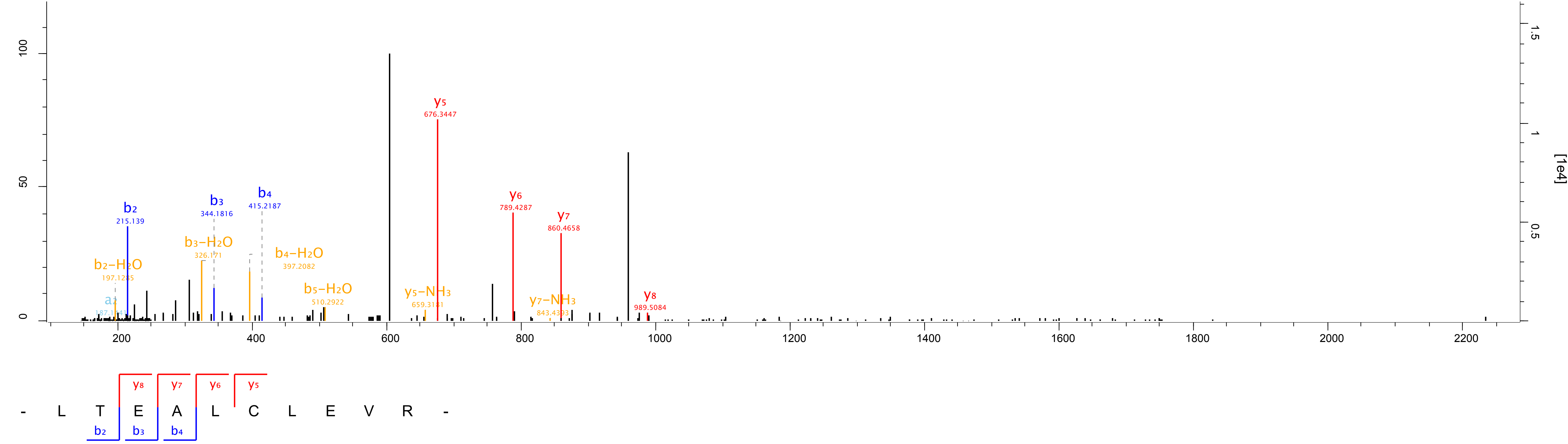

Raw file	Scan	Method	Score	m/z	Gene names
20150307_MEF3_Top_opt_E3_01_1690	37133	TOF; CID	68.22	373.87	Olfr622

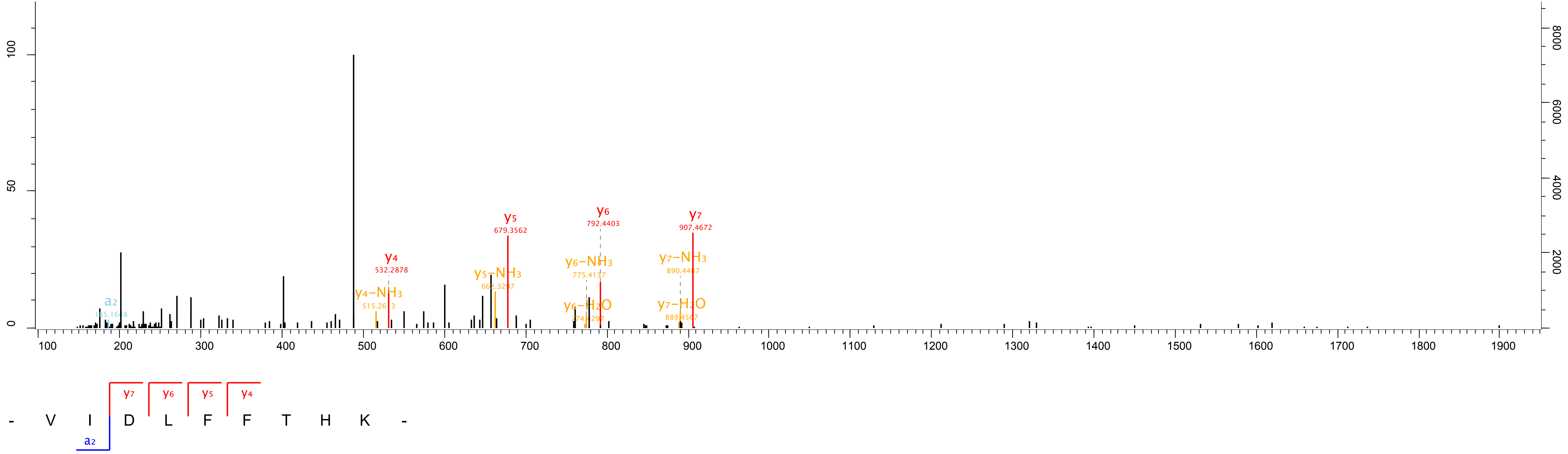

Raw file	Scan	Method	Score	m/z	Gene names
20150307_MEF3_Top_opt_E3_01_1690	37917	TOF; CID	53.3	688.36	Kifap3

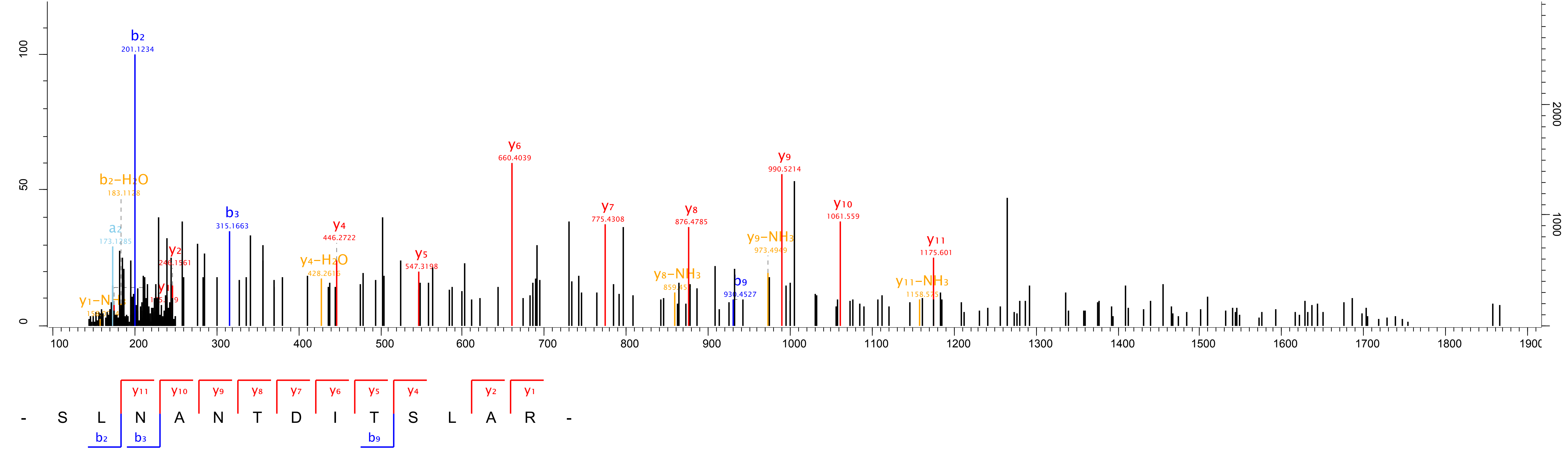

Raw file	Scan	Method	Score	m/z	Gene names
20150307_MEF3_Top_opt_E3_01_1690	47348	TOF; CID	102.08	623	Rcbtb2

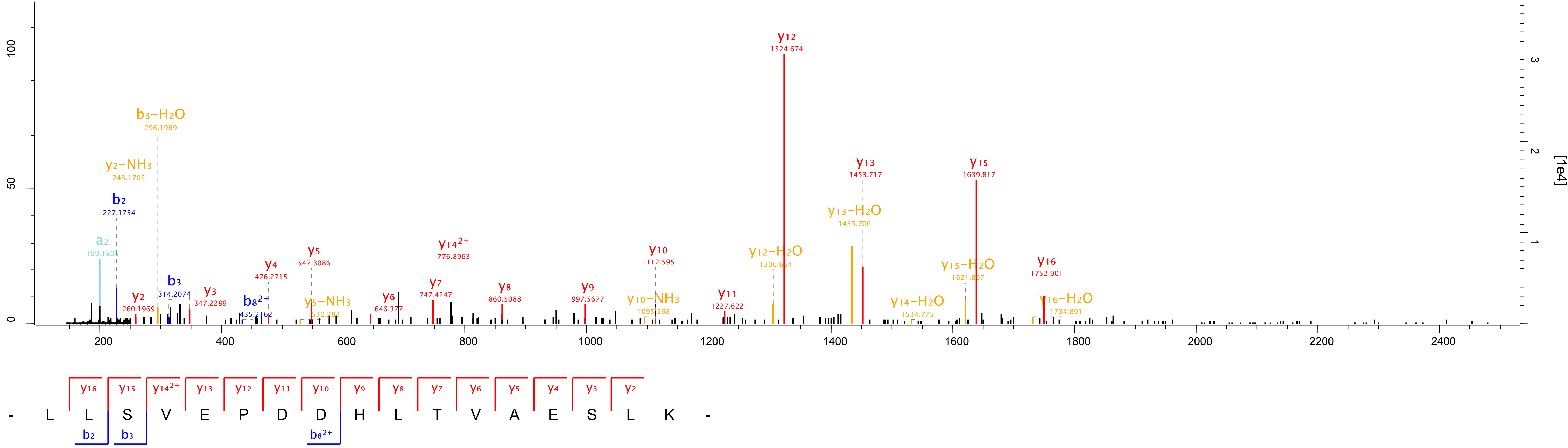

Raw file	Scan	Method	Score	m/z	Gene names
20150307_MEF3_Top_opt_E3_01_1690	55704	TOF; CID	48.29	965.47	Med19

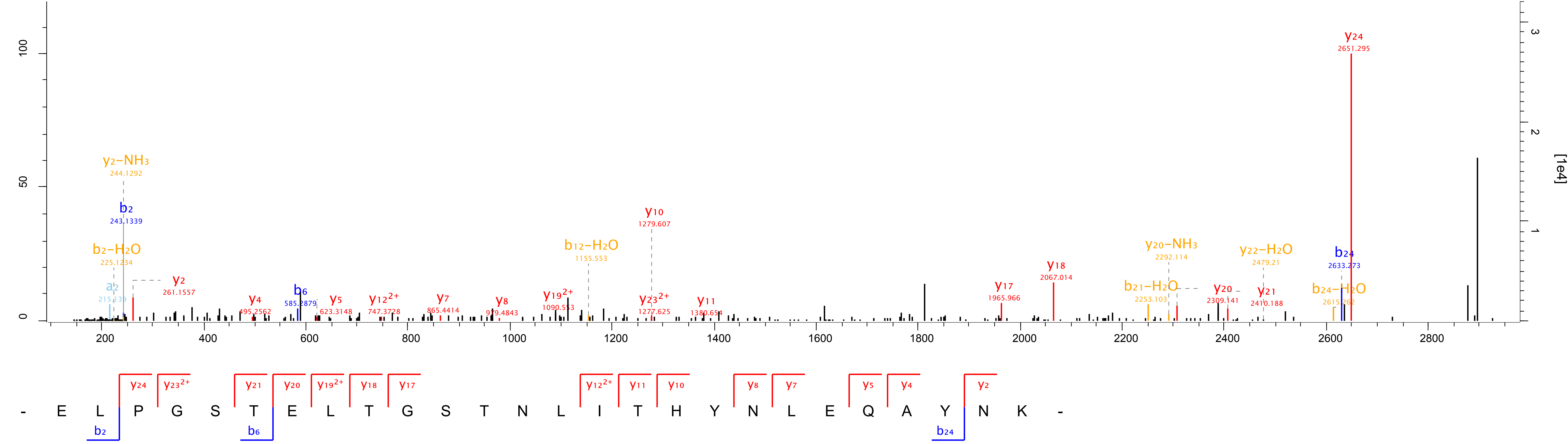

Raw file	Scan	Method	Score	m/z	Gene names
20150307_MEF3_Top_opt_E3_01_1690	55963	TOF; CID	61.34	811.77	Znrd1

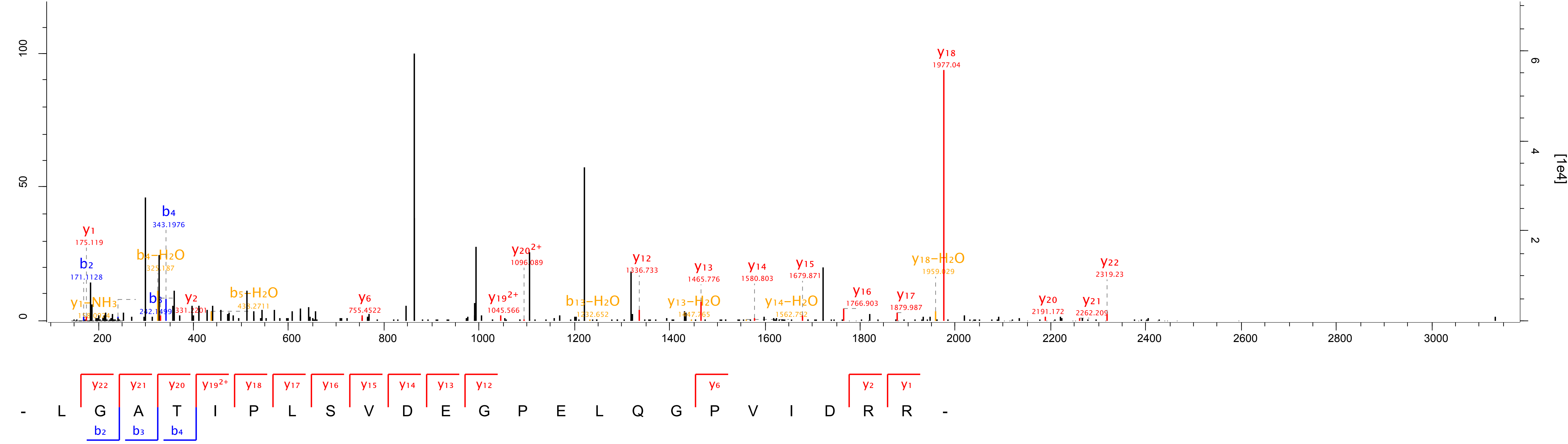

Raw file	Scan	Method	Score	m/z	Gene names
20150307_MEF3_Top_opt_E3_01_1690	62757	TOF; CID	50.18	943.46	Glce

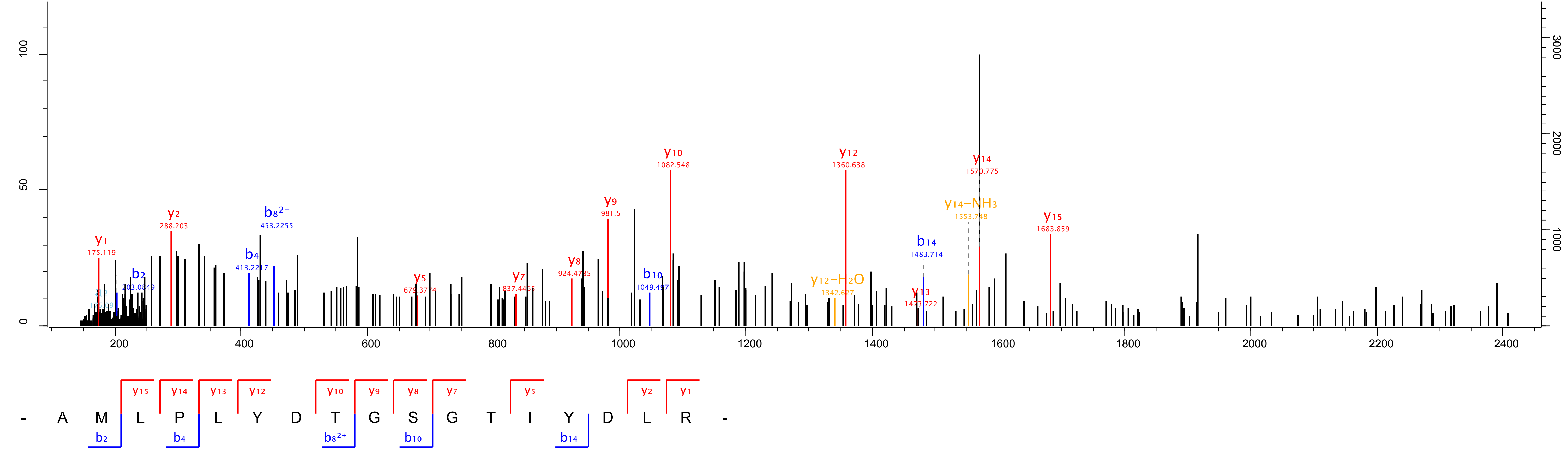

Raw file	Scan	Method	Score	m/z	Gene names
20150307_MEF3_Top_opt_E3_01_1690	64017	TOF; CID	76.21	756.37	Hoxd13

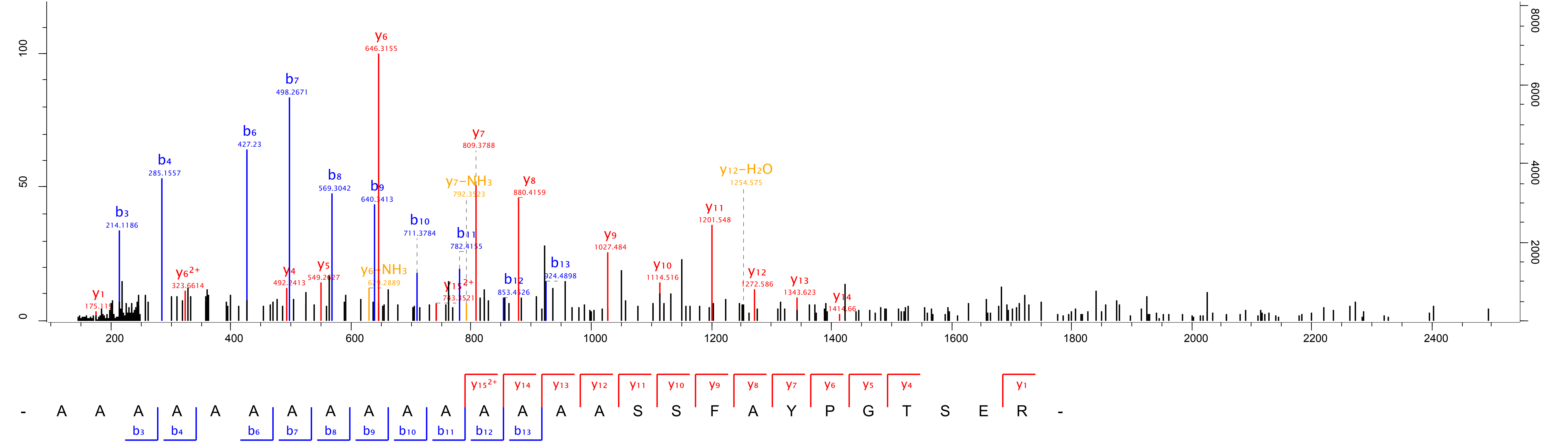

Raw file	Scan	Method	Score	m/z	Gene names
20150307_NSC1_Top_opt_F1_01_1678	6806	TOF; CID	59.71	788.83	Tceal8

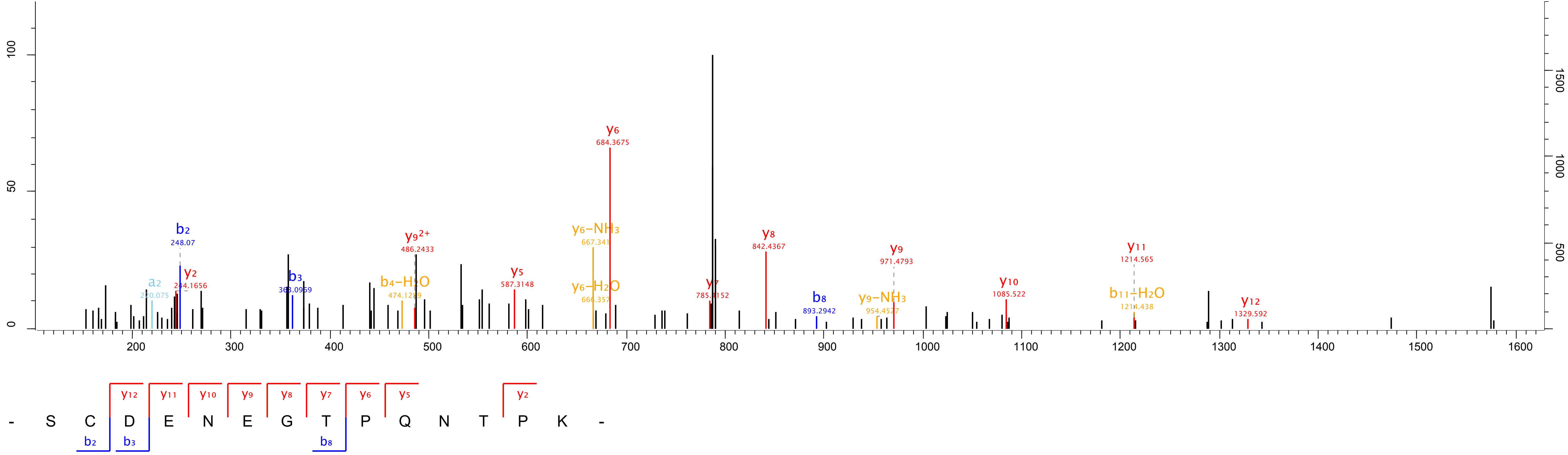

Raw file	Scan	Method	Score	m/z	Gene names
20150307_NSC1_Top_opt_F1_01_1678	9914	TOF; CID	80.44	551.78	Tdrd12

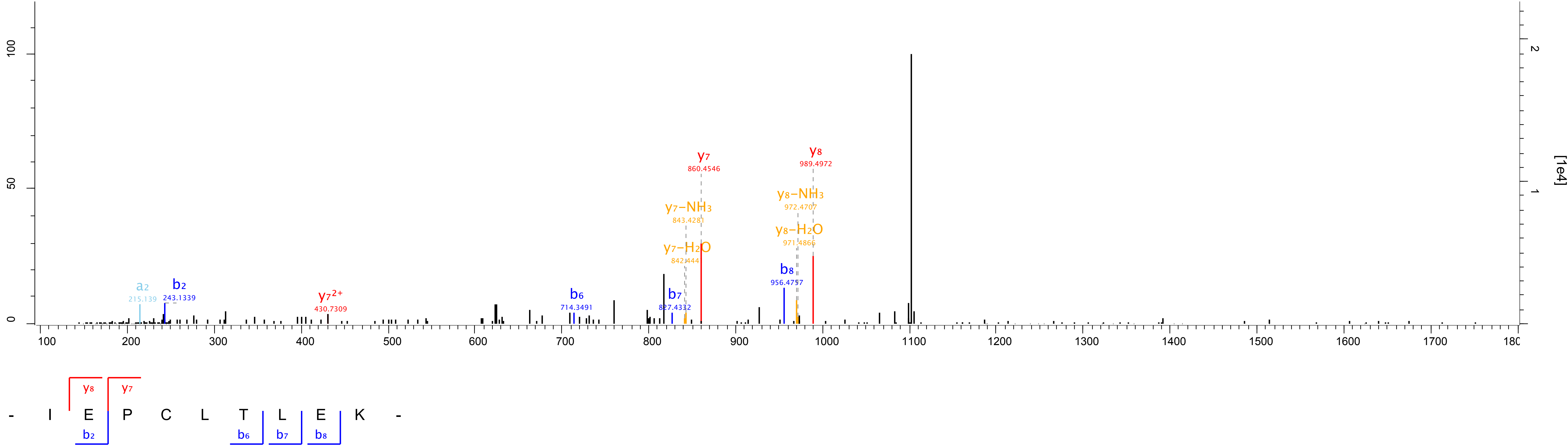

Raw file	Scan	Method	Score	m/z	Gene names
20150307_NSC1_Top_opt_F1_01_1678	10494	TOF; CID	66.06	572.78	Nipa1

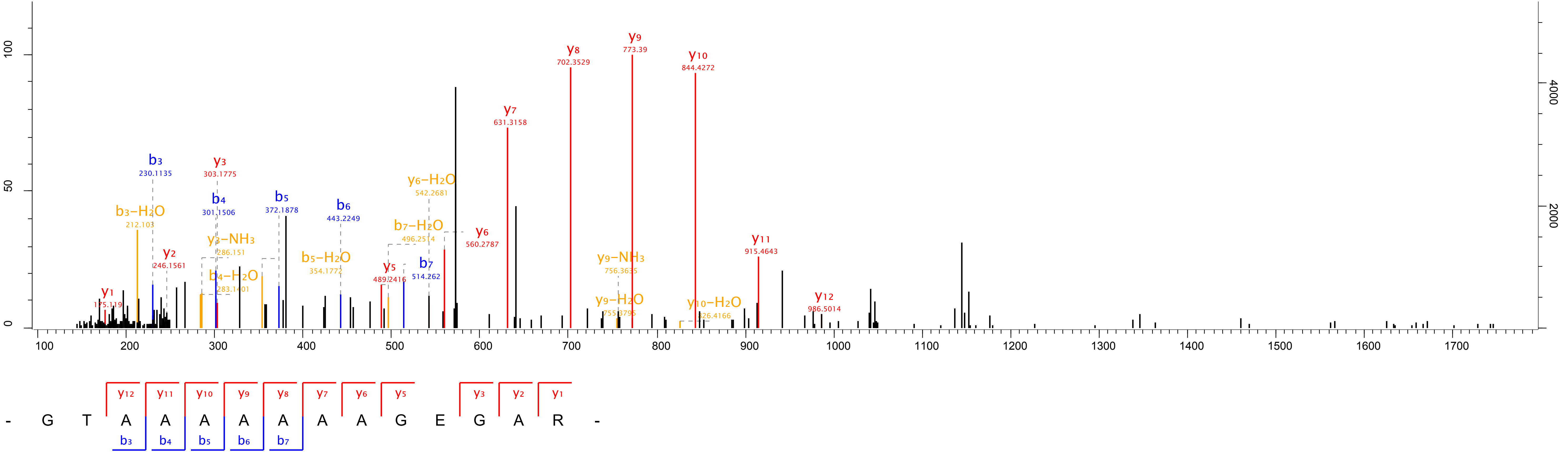

Raw file	Scan	Method	Score	m/z	Gene names
20150307_NSC1_Top_opt_F1_01_1678	10696	TOF; CID	47.65	884.71	Fiz1

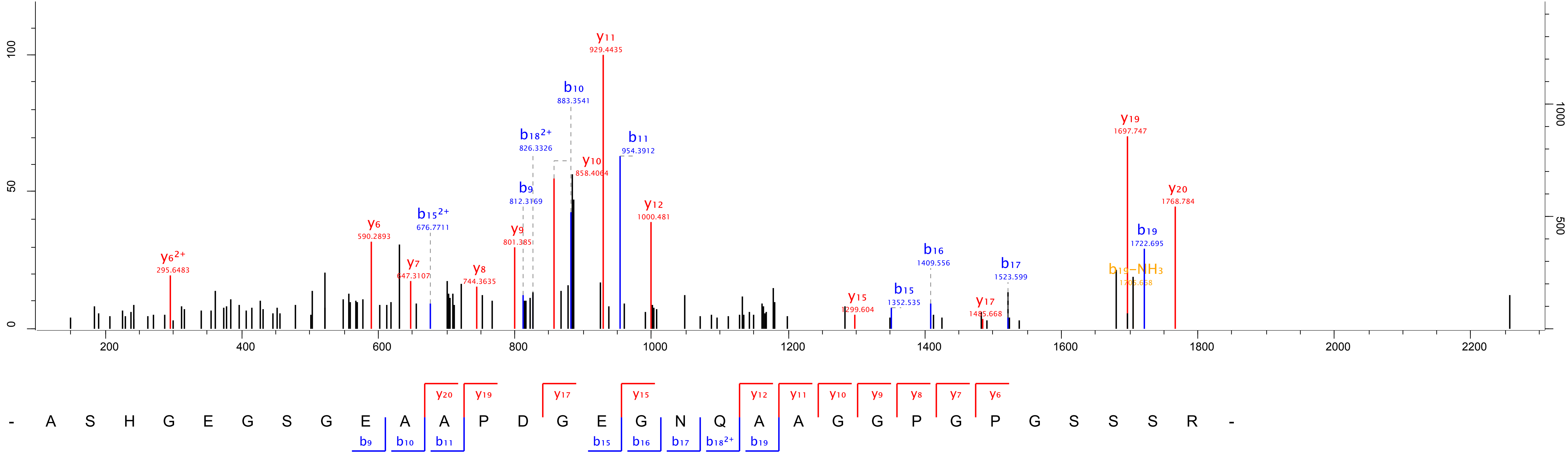

Raw file	Scan	Method	Score	m/z	Gene names
20150307_NSC1_Top_opt_F1_01_1678	14329	TOF; CID	100.39	511.26	Dolpp1

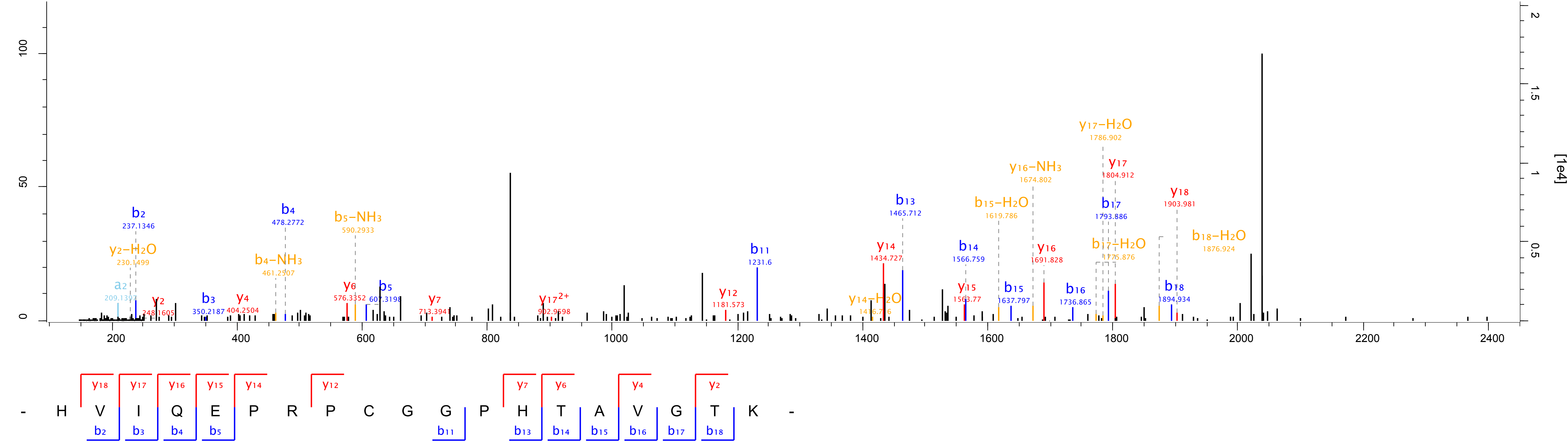

Raw file	Scan	Method	Score	m/z	Gene names
20150307_NSC1_Top_opt_F1_01_1678	17315	TOF; CID	84.82	633.29	Astn1

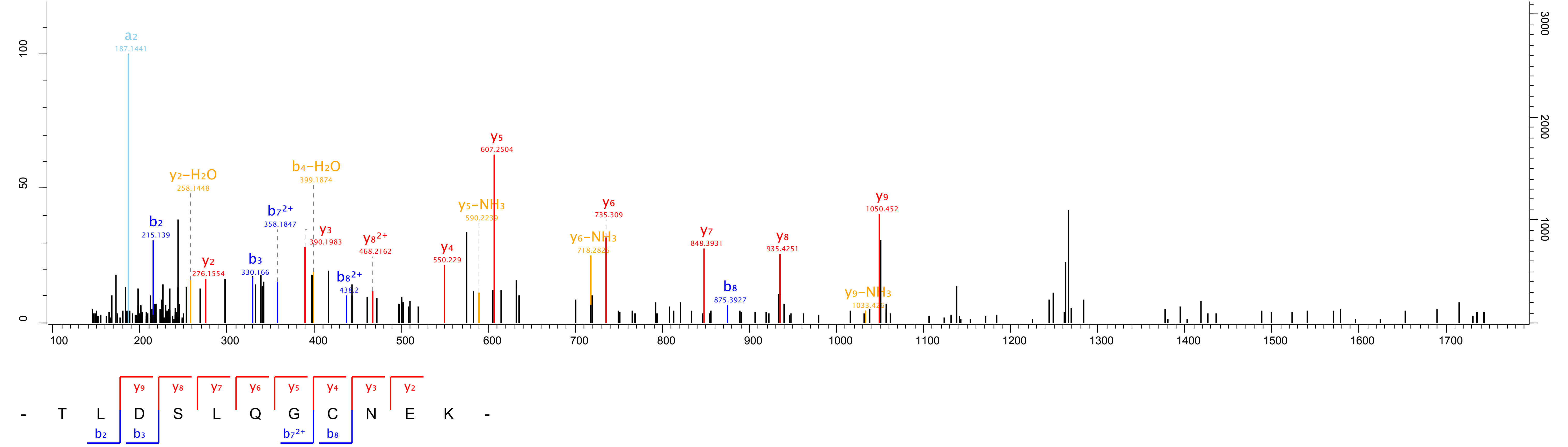

Raw file	Scan	Method	Score	m/z	Gene names
20150307_NSC1_Top_opt_F1_01_1678	19391	TOF; CID	41.51	695.34	Traf3ip2

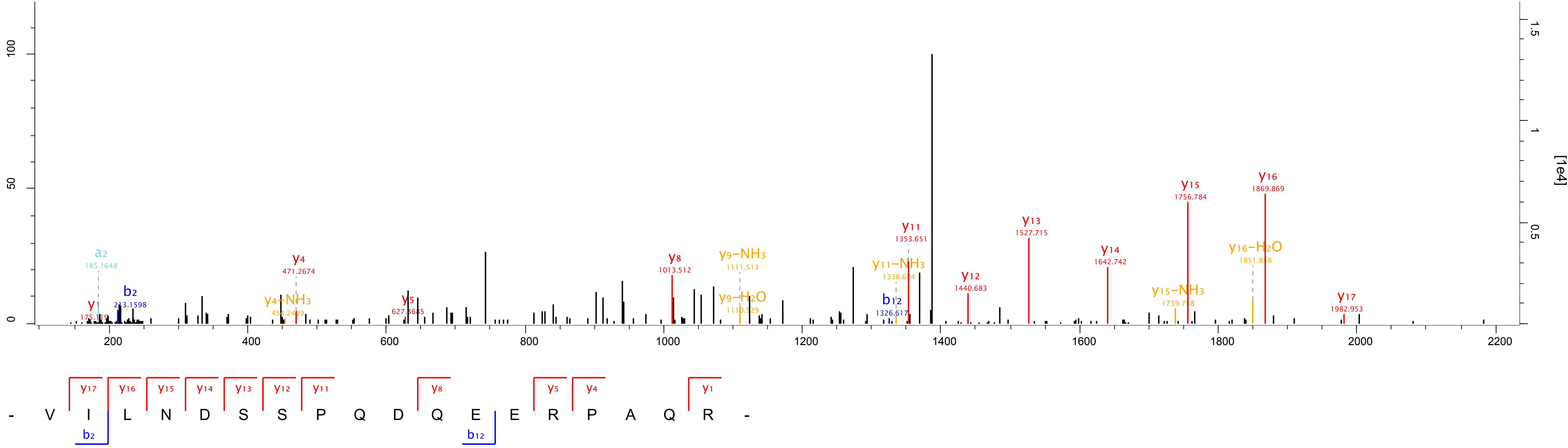

Raw file	Scan	Method	Score	m/z	Gene names
20150307_NSC1_Top_opt_F1_01_1678	21887	TOF; CID	110.35	746.85	Cdyl

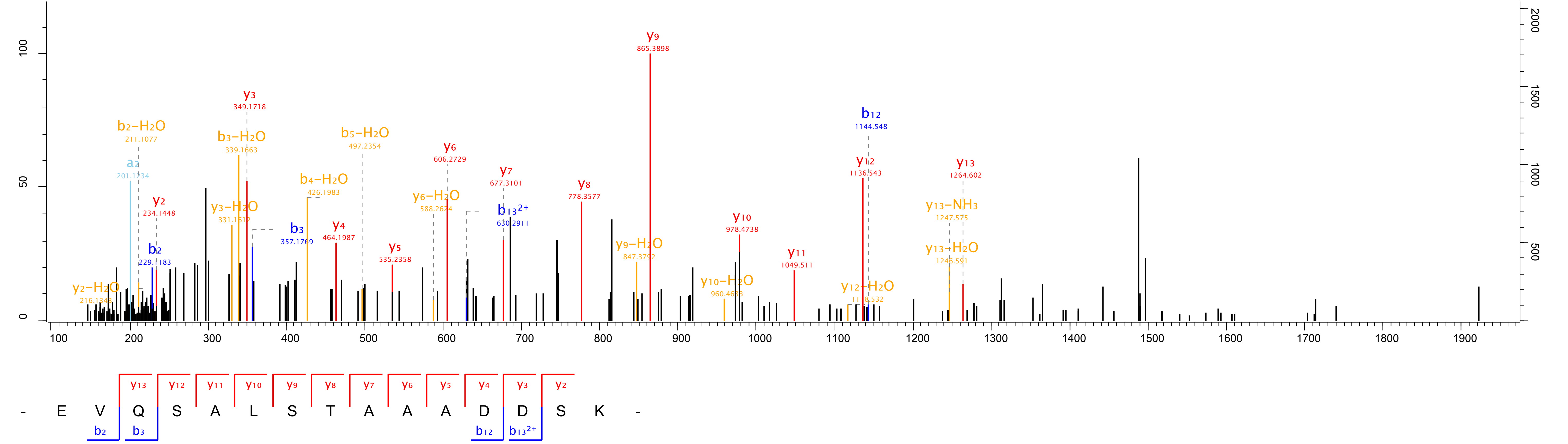

Raw file	Scan	Method	Score	m/z	Gene names
20150307_NSC1_Top_opt_F1_01_1678	27146	TOF; CID	51.03	673.35	R3hdm1

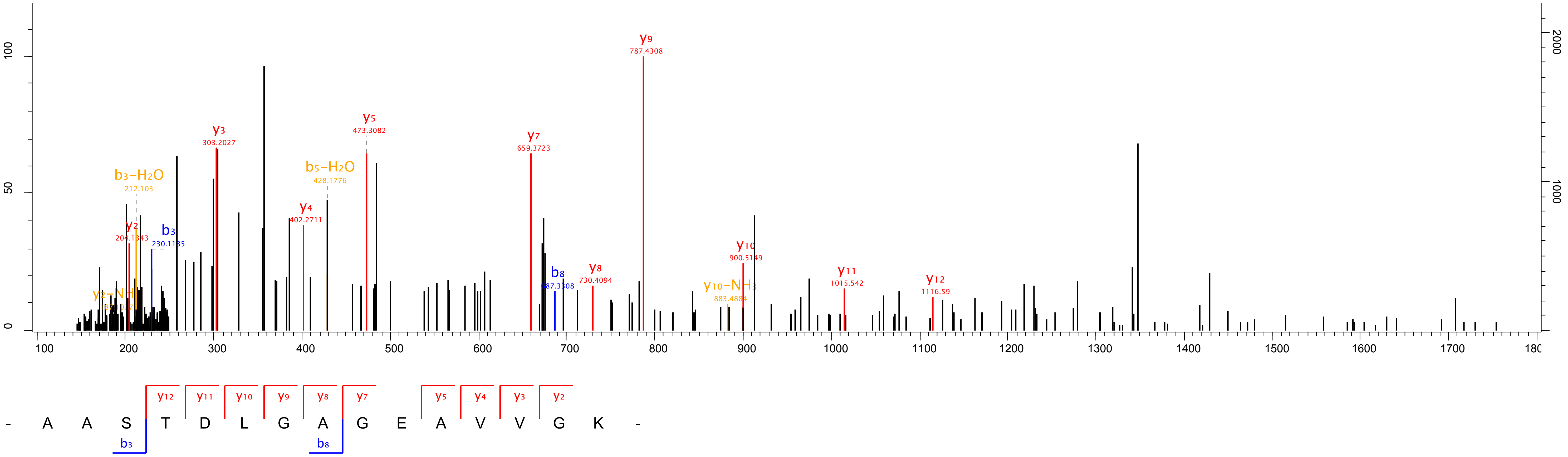

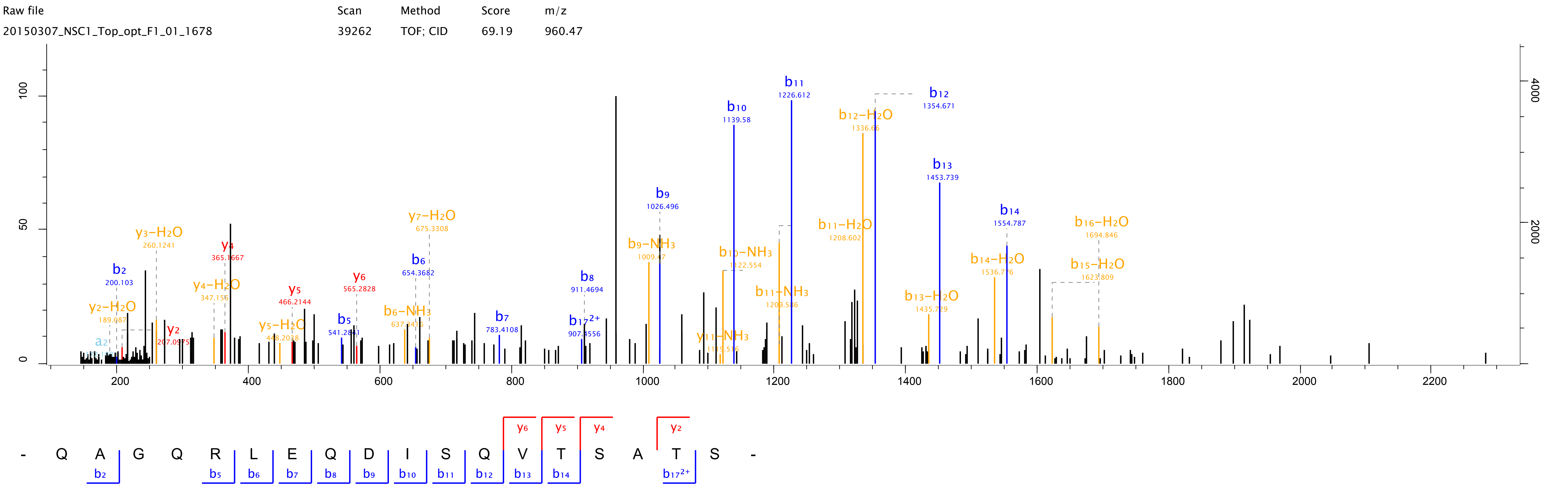

Raw file
20150307_NSC1_Top_opt_F1_01_1678
Scan
39262
Method
TOF; CID
Score
69.19
m/z
960.47
b2 200.103
y2−H2O 189.087
y2 207.0975
y3−H2O 260.1241
y4−H2O 347.156
y4 365.1667
y5−H2O 448.2038
y5 466.2144
b5 541.2841
y6 565.2828
b6−NH3 637.3476
b6 654.3682
y7−H2O 675.3308
b7 783.4108
b17 2+ 907.4556
b8 911.4694
b9−NH3 1009.47
b9 1026.496
y11−NH3 1115.516
b10−NH3 1122.554
b10 1139.58
b11−H2O 1208.602
b11−NH3 1209.586
b11 1226.612
b12−H2O 1336.65
b12 1354.671
b13−H2O 1435.729
b13 1453.739
b14−H2O 1536.776
b14 1554.787
b15−H2O 1623.809
b16−H2O 1694.846
0
50
100
2000
4000
200
400
600
800
1000
1200
1400
1600
1800
2000
2200
- Q A G Q R L E Q D I S Q V T S A T S -
y6 y5 y4 y2
b2 b5 b6 b7 b8 b9 b10 b11 b12 b13 b14 b17 2+

Raw file	Scan	Method	Score	m/z	Gene names
20150307_NSC1_Top_opt_F1_01_1678	40040	TOF; CID	76.22	598.32	Mtatp8;mt-Atp8

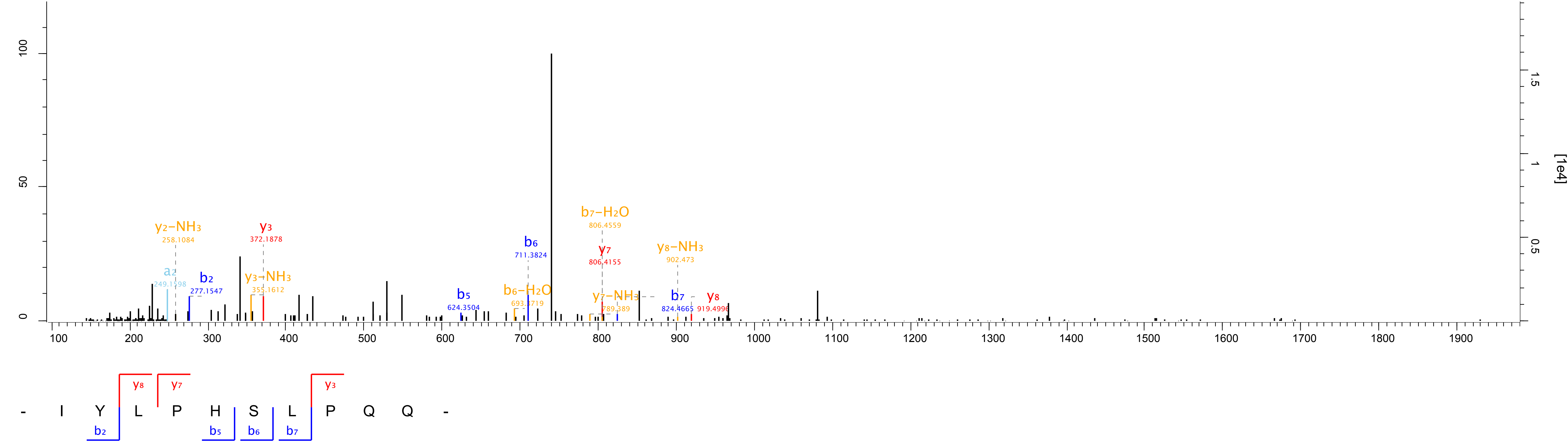

Raw file	Scan	Method	Score	m/z	Gene names
20150307_NSC1_Top_opt_F1_01_1678	41055	TOF; CID	80.74	949.41	Slc36a1

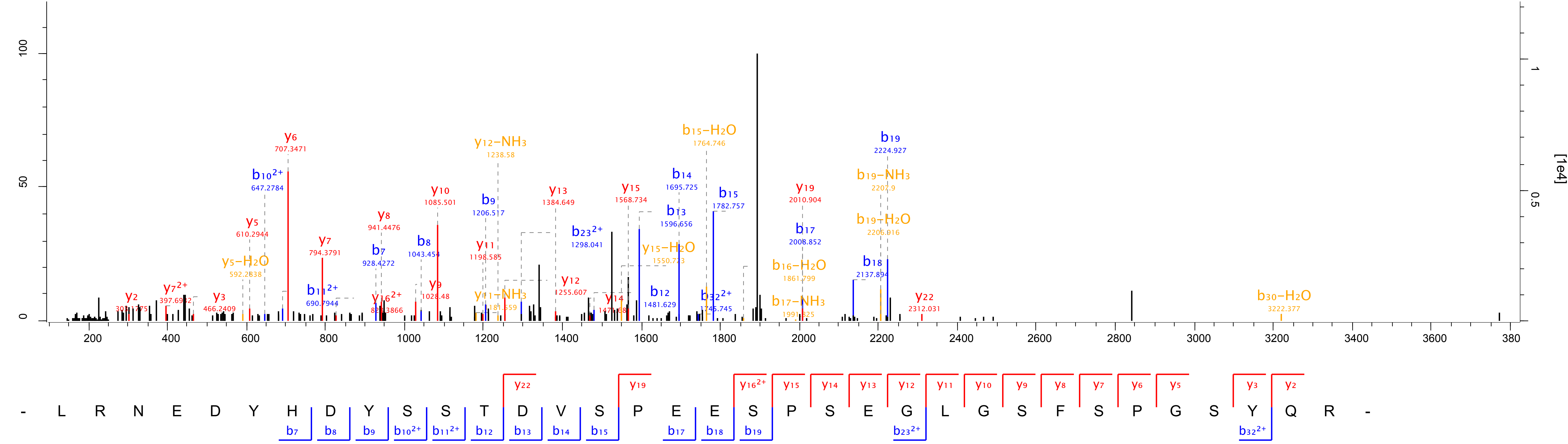

Raw file	Scan	Method	Score	m/z	Gene names
20150307_NSC1_Top_opt_F1_01_1678	43340	TOF; CID	105.06	809.39	Ccdc97

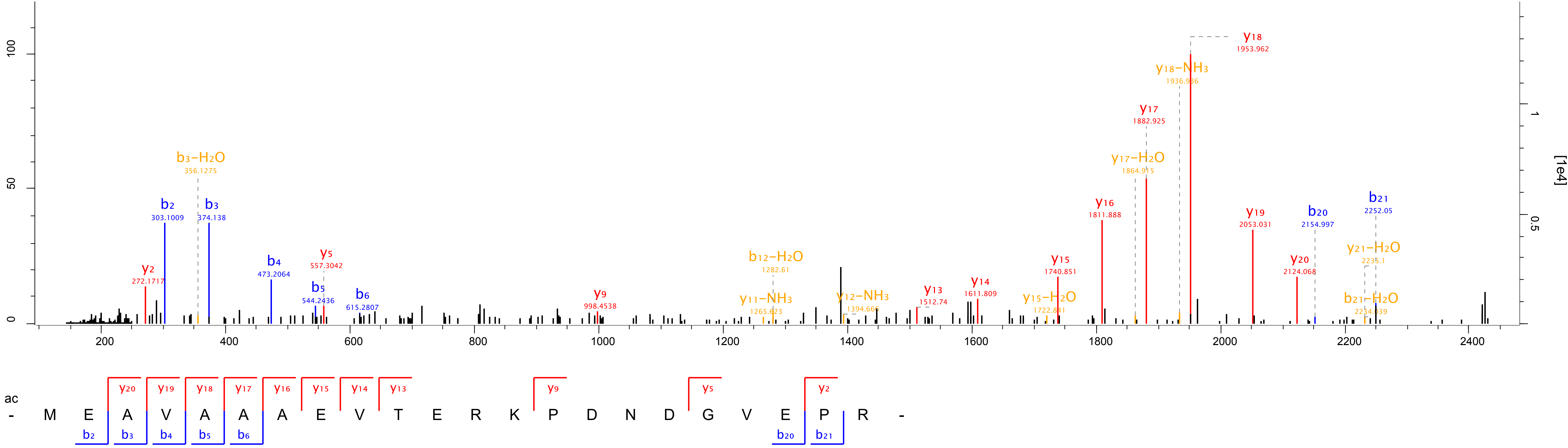

Raw file	Scan	Method	Score	m/z	Gene names
20150307_NSC1_Top_opt_F1_01_1678	46955	TOF; CID	72.2	583.33	Mab21l2;Mab21l1

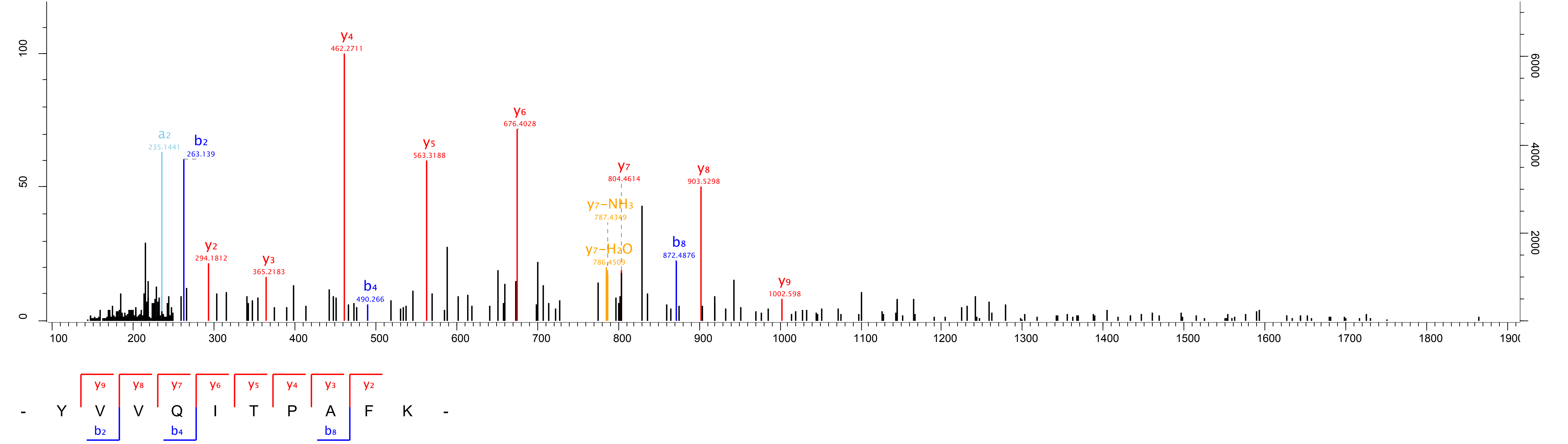

Raw file	Scan	Method	Score	m/z	Gene names
20150307_NSC1_Top_opt_F1_01_1678	50279	TOF; CID	58.69	687.37	Hmgxb4

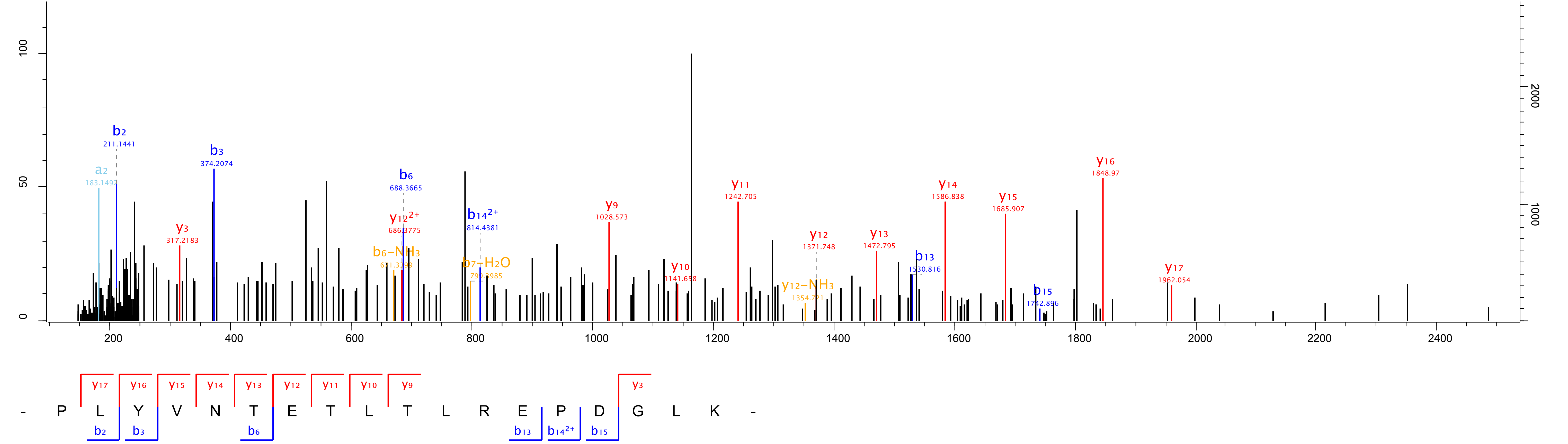

Raw file	Scan	Method	Score	m/z	Gene names
20150307_NSC1_Top_opt_F1_01_1678	50405	TOF; CID	71.6	915.46	Nadk

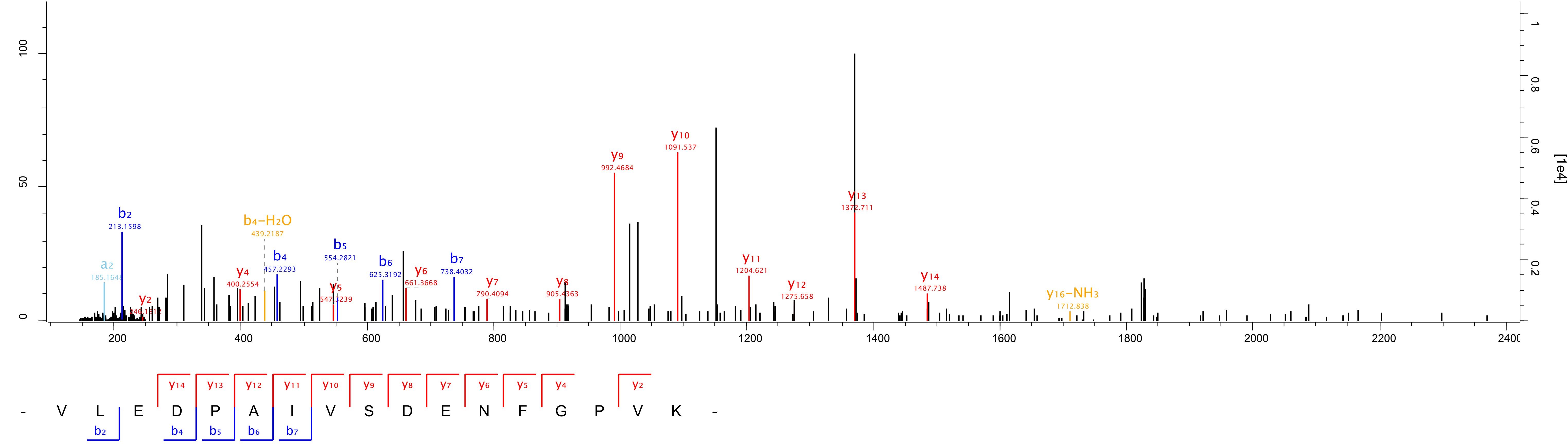

Raw file	Scan	Method	Score	m/z	Gene names
20150307_NSC1_Top_opt_F1_01_1678	52349	TOF; CID	55.44	991.5	Ube2w

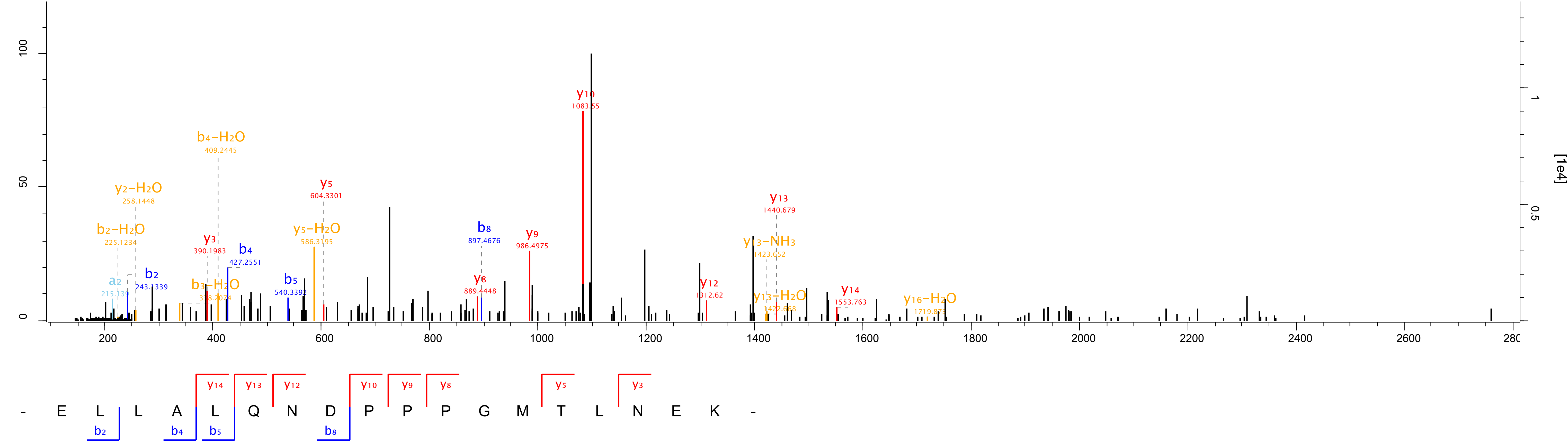

Raw file	Scan	Method	Score	m/z	Gene names
20150307_NSC1_Top_opt_F1_01_1678	52580	TOF; CID	62.36	541.3	Mterf2

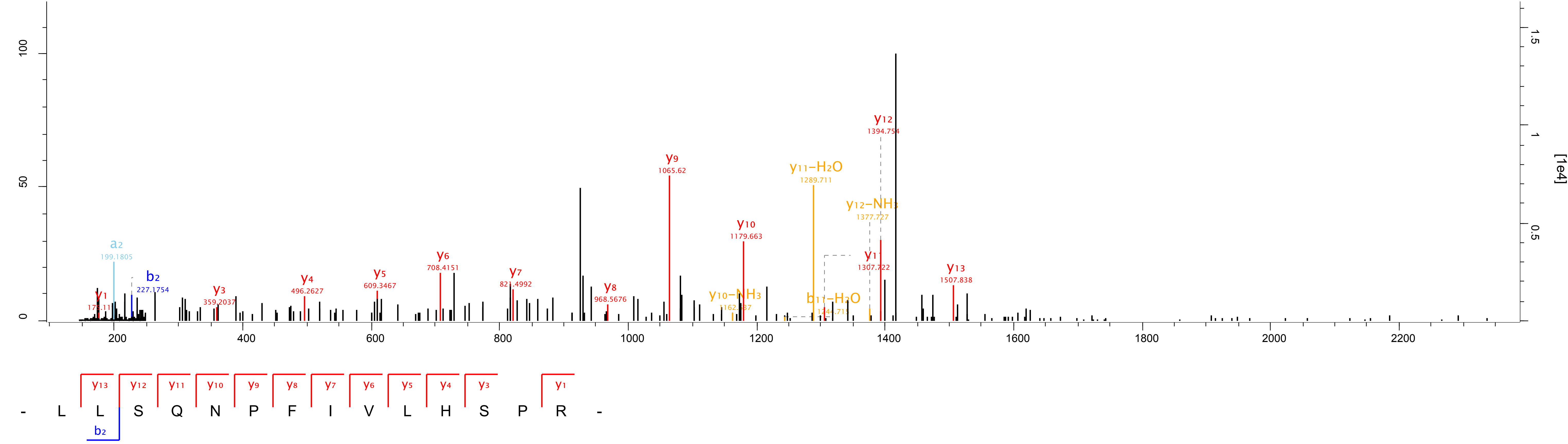

Raw file	Scan	Method	Score	m/z	Gene names
20150307_NSC1_Top_opt_F1_01_1678	52835	TOF; CID	61.03	1108.51	Cebpg

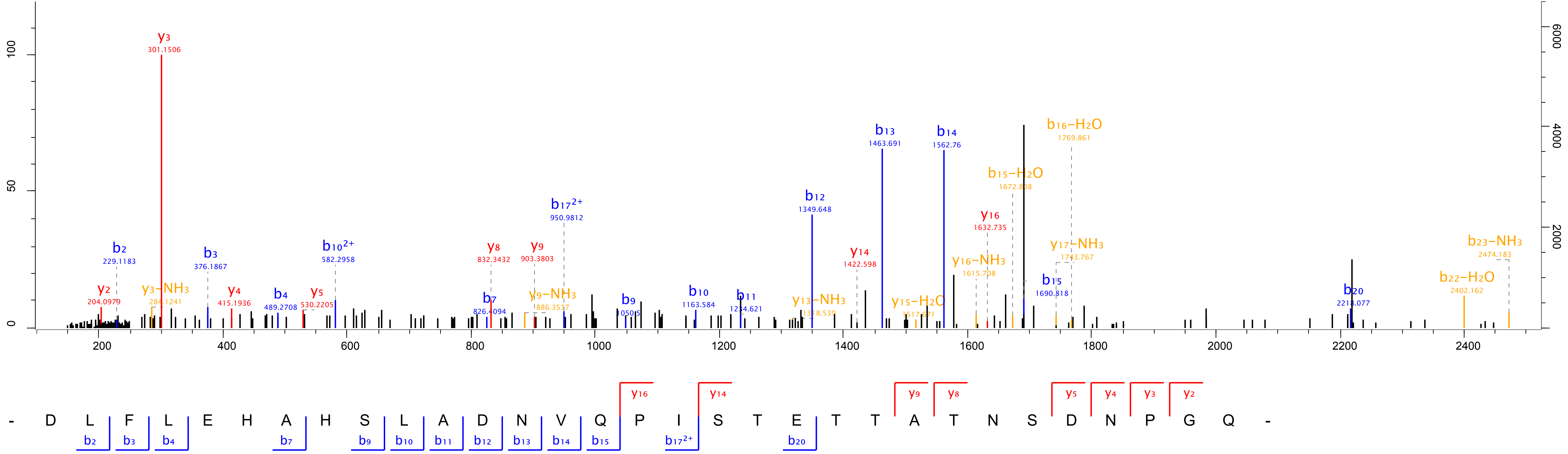

Raw file	Scan	Method	Score	m/z	Gene names
20150307_NSC1_Top_opt_F1_01_1678	54856	TOF; CID	101.74	1016.99	Hdhd2;Ier3ip1

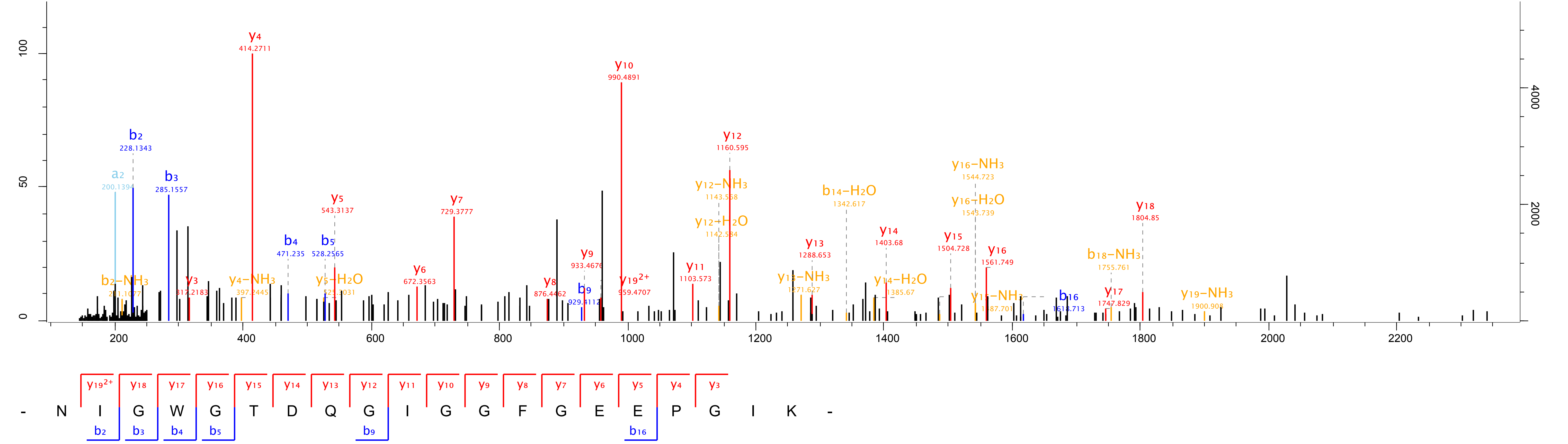

Raw file	Scan	Method	Score	m/z	Gene names
20150307_NSC1_Top_opt_F1_01_1678	55459	TOF; CID	49.12	882.45	Sema4b

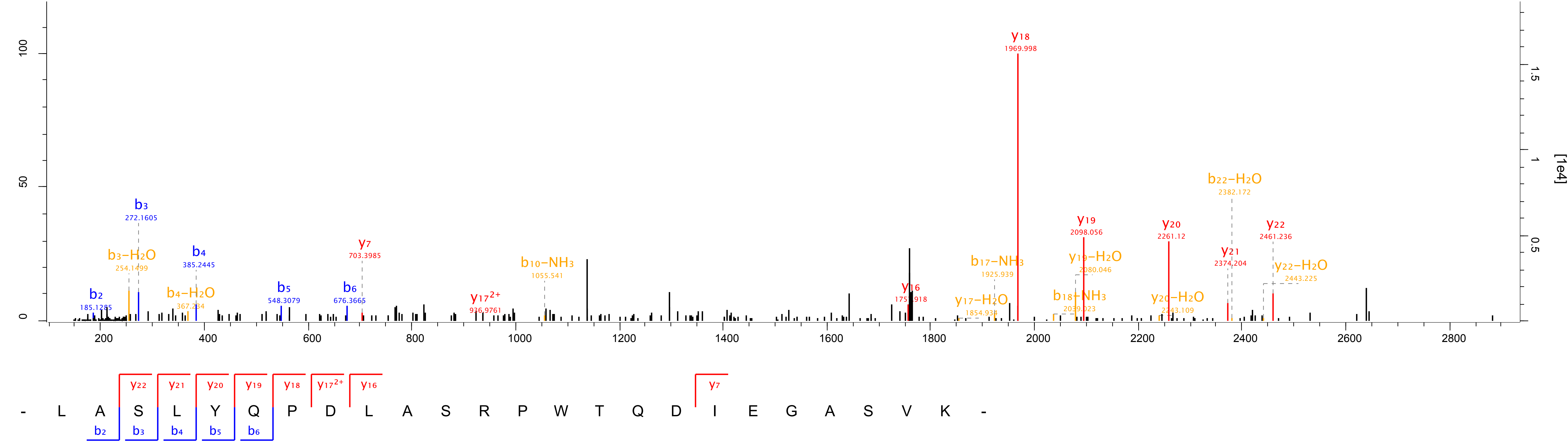

Raw file	Scan	Method	Score	m/z	Gene names
20150307_NSC1_Top_opt_F1_01_1678	55765	TOF; CID	47.81	867.42	Tmem206

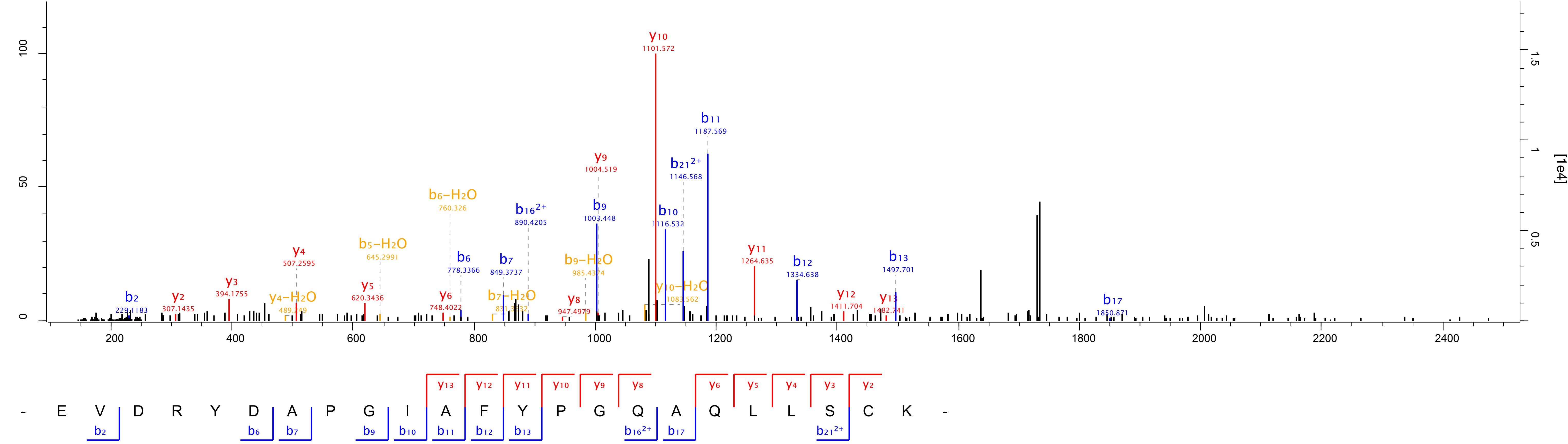

Raw file	Scan	Method	Score	m/z	Gene names
20150307_NSC1_Top_opt_F1_01_1678	56985	TOF; CID	46.89	621.99	Pcif1

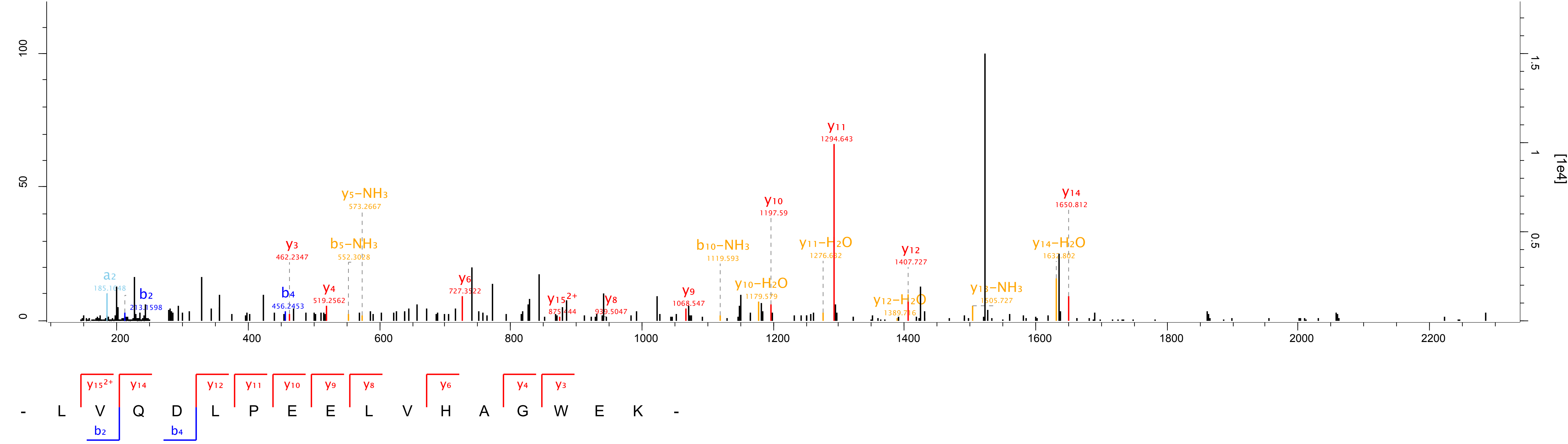

Raw file	Scan	Method	Score	m/z	Gene names
20150307_NSC1_Top_opt_F1_01_1678	61347	TOF; CID	68.68	715.34	Tmem55b

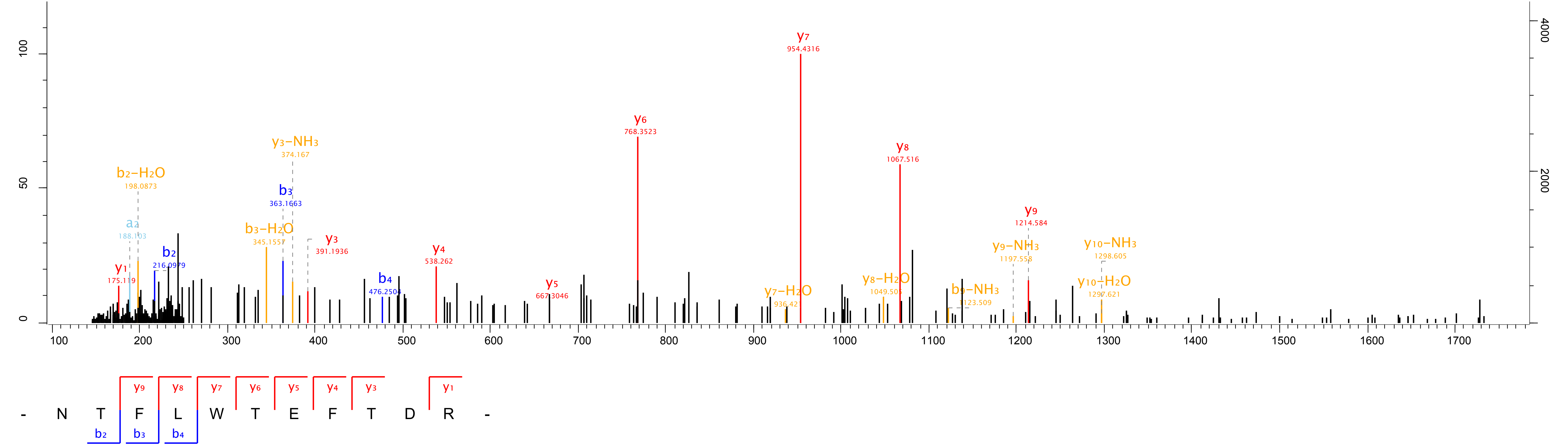

Raw file	Scan	Method	Score	m/z	Gene names
20150307_NSC1_Top_opt_F1_01_1678	61742	TOF; CID	83.65	531.32	Stx16

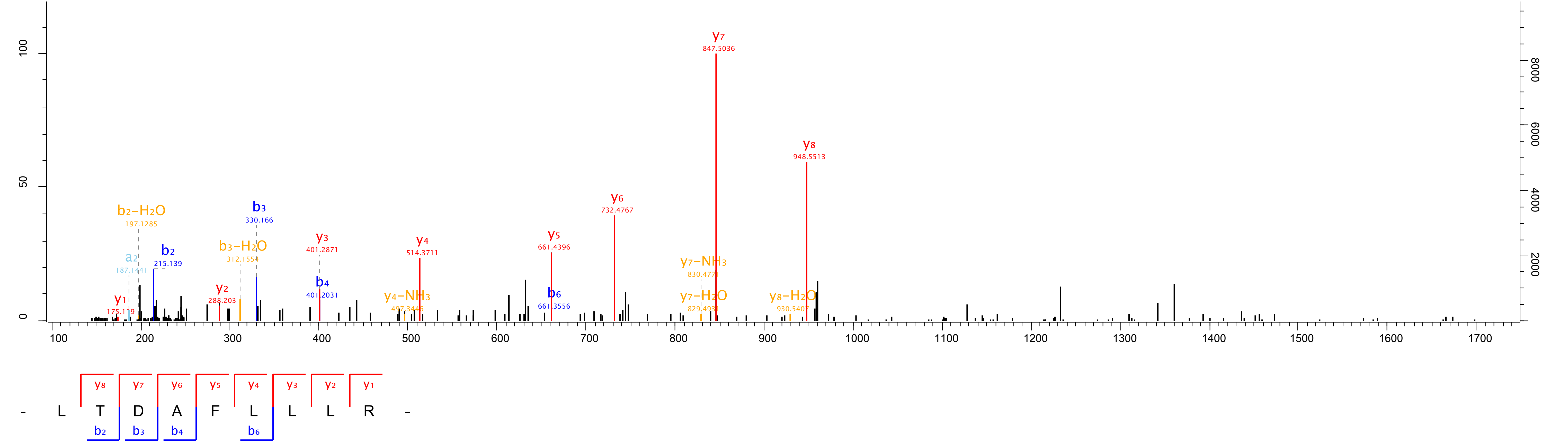

Raw file	Scan	Method	Score	m/z	Gene names
20150307_NSC1_Top_opt_F1_01_1678	62123	TOF; CID	75.44	1038.86	Mrpl2

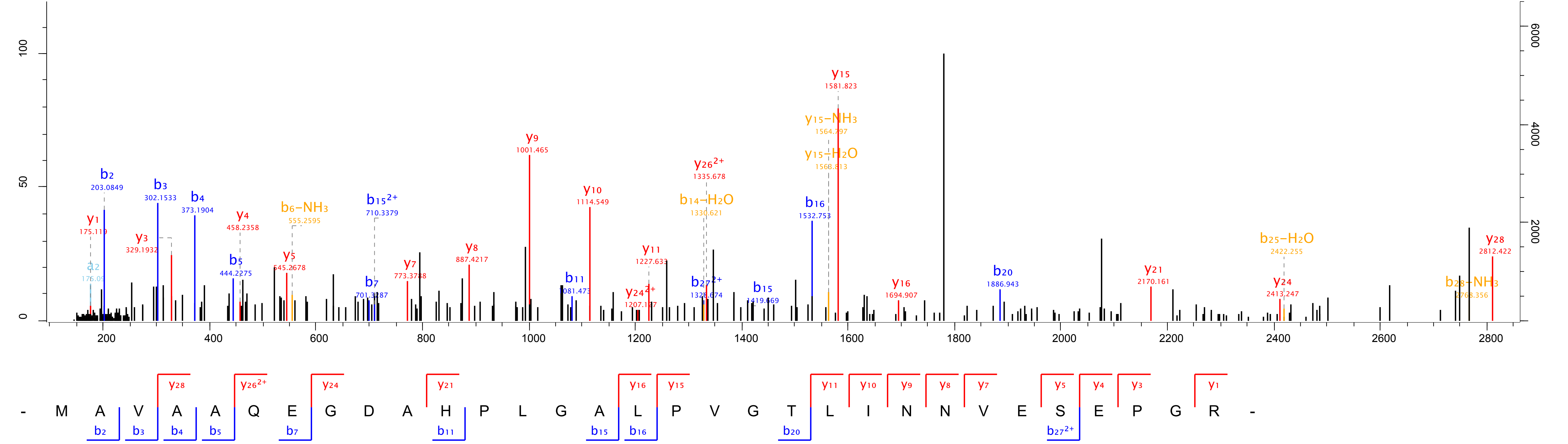

Raw file	Scan	Method	Score	m/z	Gene names
20150307_NSC1_Top_opt_F1_01_1678	62249	TOF; CID	63.38	884.4	Chchd10

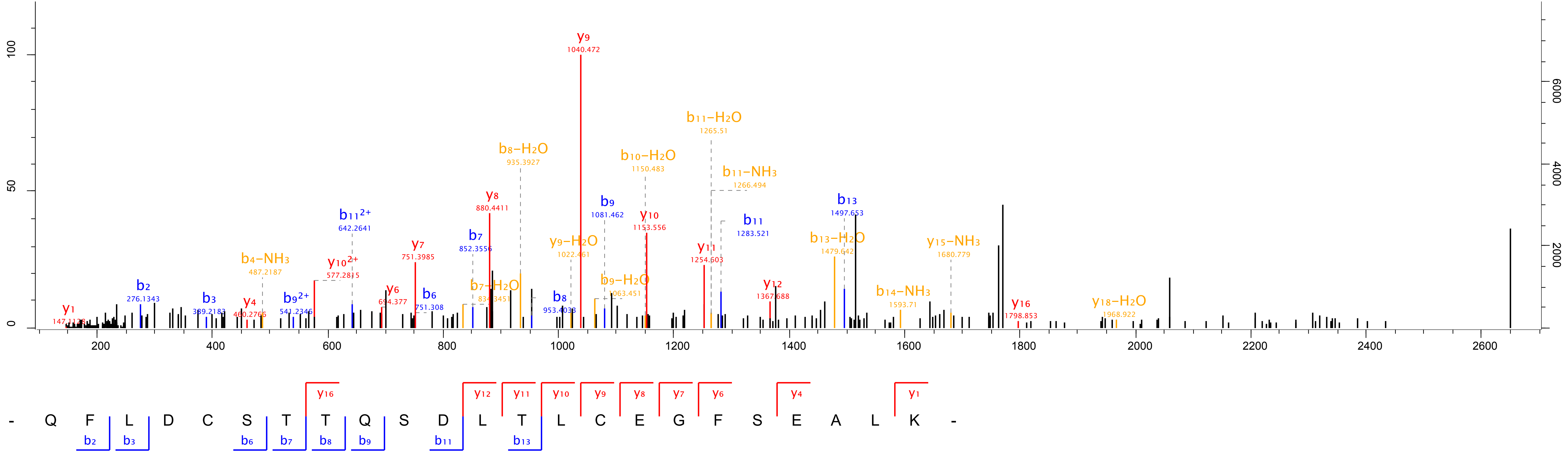

Raw file	Scan	Method	Score	m/z	Gene names
20150307_NSC1_Top_opt_F1_01_1678	66028	TOF; CID	78.98	868.1	Xpr1

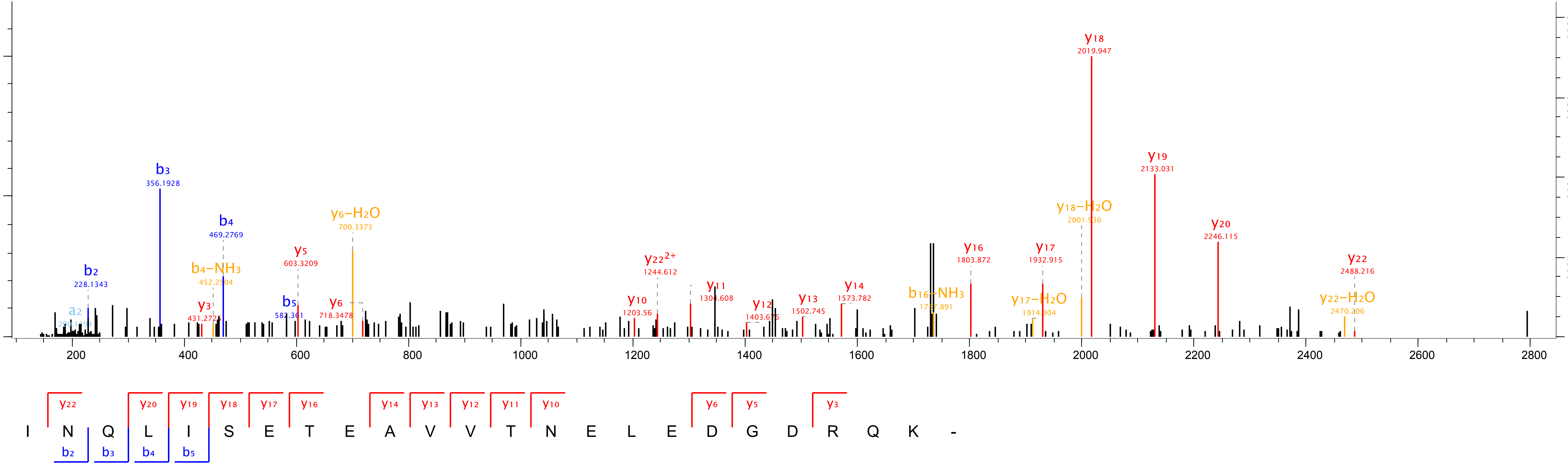

Raw file	Scan	Method	Score	m/z	Gene names
20150307_NSC1_Top_opt_F1_01_1678	69983	TOF; CID	108.66	809.9	Elovl1

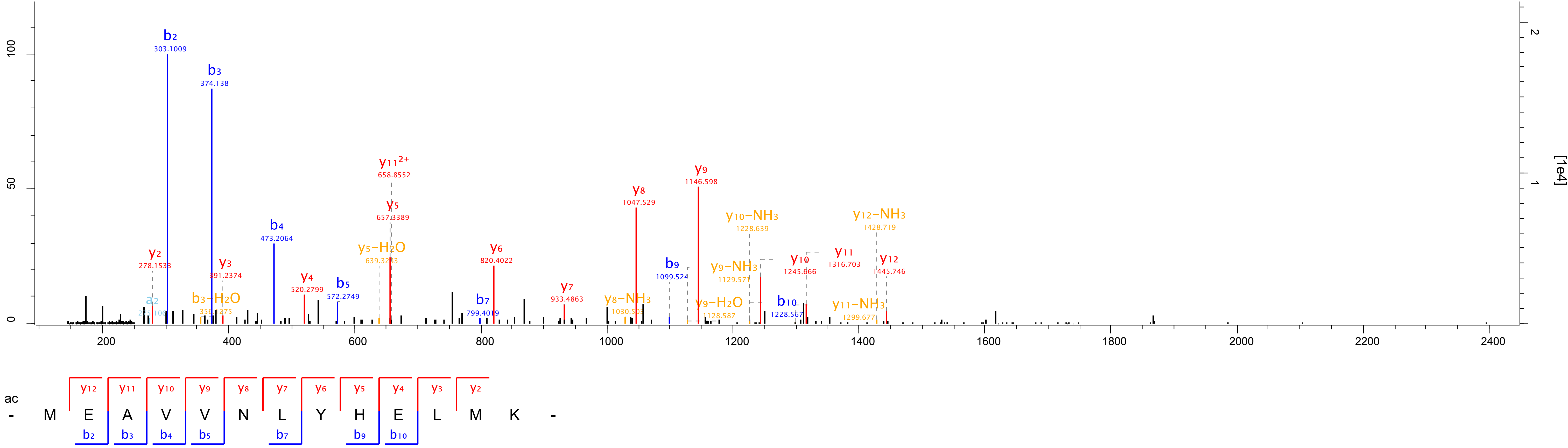

Raw file	Scan	Method	Score	m/z	Gene names
20150307_NSC1_Top_opt_F1_01_1685	10683	TOF; CID	55.58	433.54	Sf1

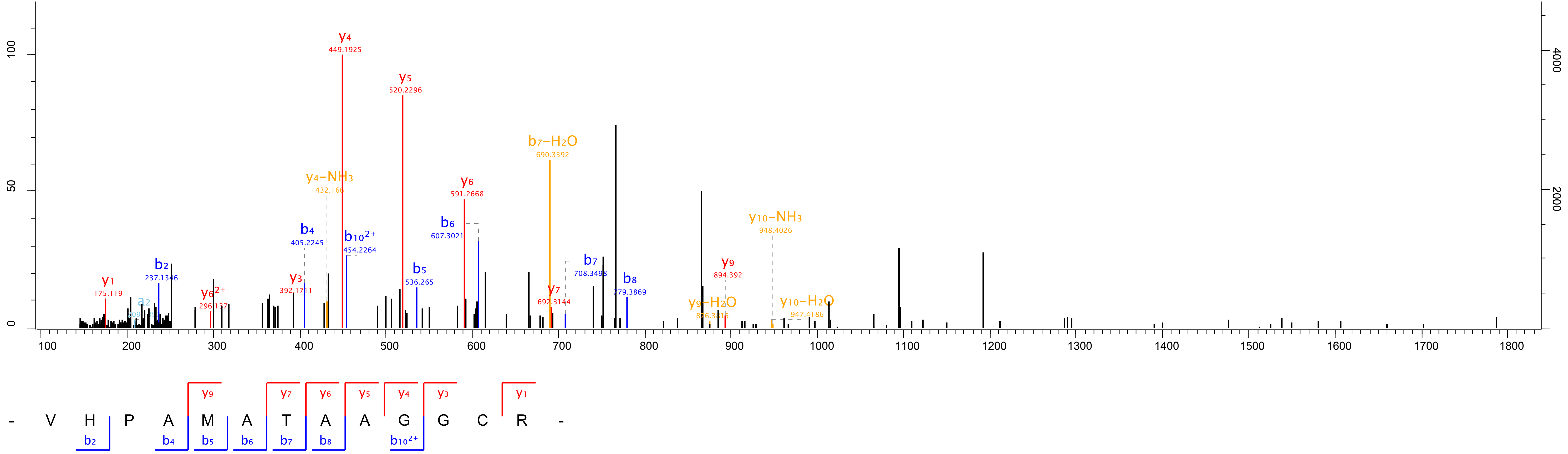

Raw file	Scan	Method	Score	m/z	Gene names
20150307_NSC1_Top_opt_F1_01_1685	13401	TOF; CID	54.9	590.28	Tmem56

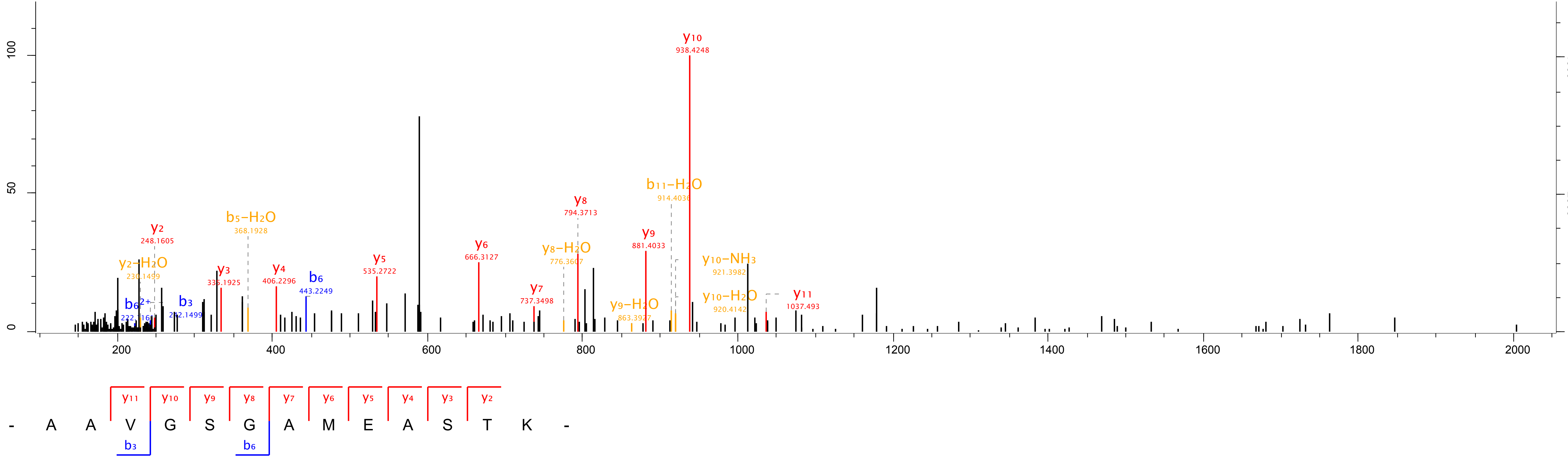

Raw file	Scan	Method	Score	m/z	Gene names
20150307_NSC1_Top_opt_F1_01_1685	15799	TOF; CID	100.28	839.41	Crls1

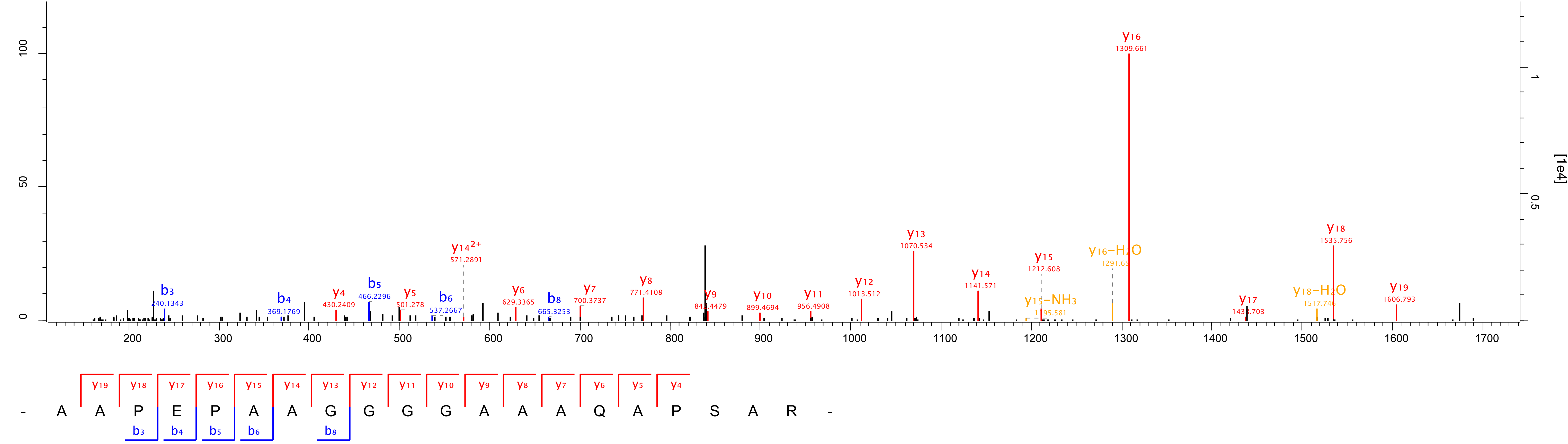

Raw file	Scan	Method	Score	m/z	Gene names
20150307_NSC1_Top_opt_F1_01_1685	20861	TOF; CID	41.49	738.35	Cdr2

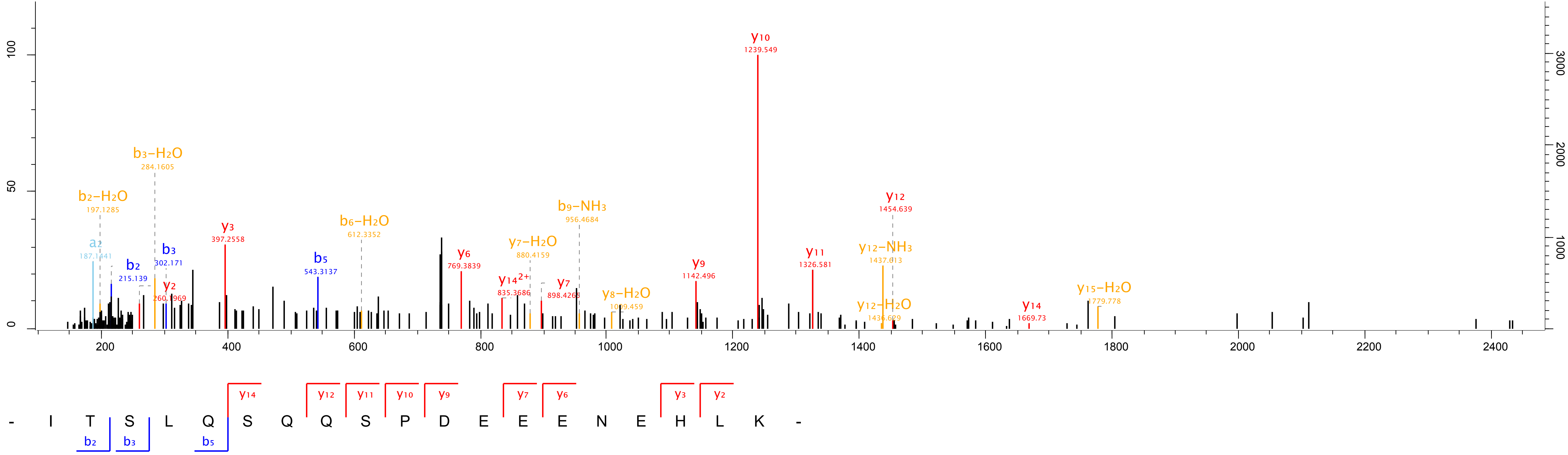

Raw file	Scan	Method	Score	m/z	Gene names
20150307_NSC1_Top_opt_F1_01_1685	24586	TOF; CID	93.16	672.36	Sh3bp5l

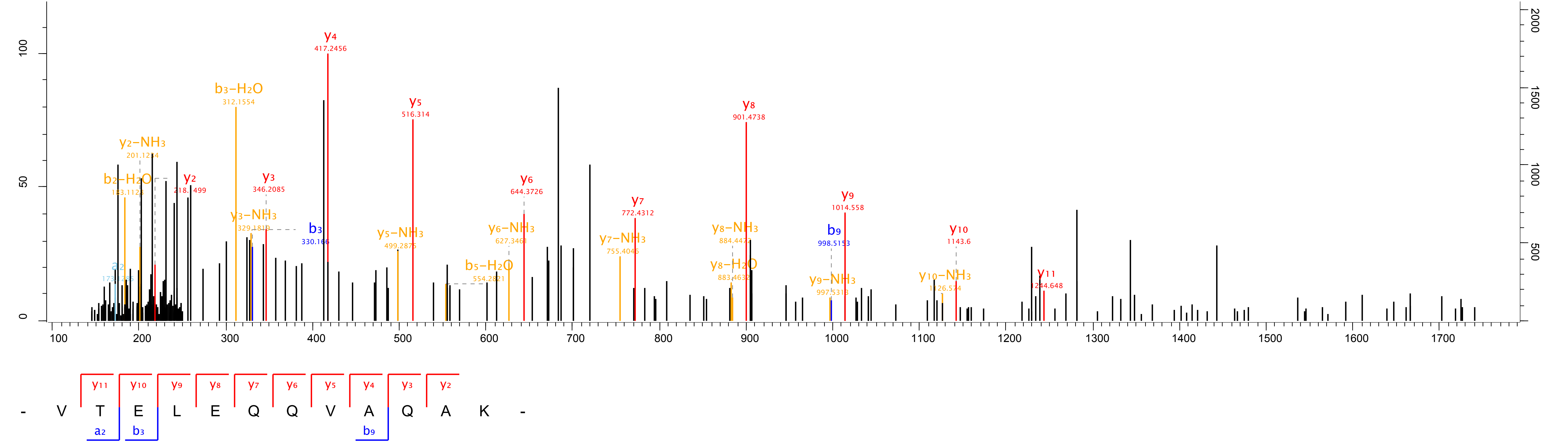

Raw file	Scan	Method	Score	m/z	Gene names
20150307_NSC1_Top_opt_F1_01_1685	31535	TOF; CID	43.95	661.32	Atrnl1

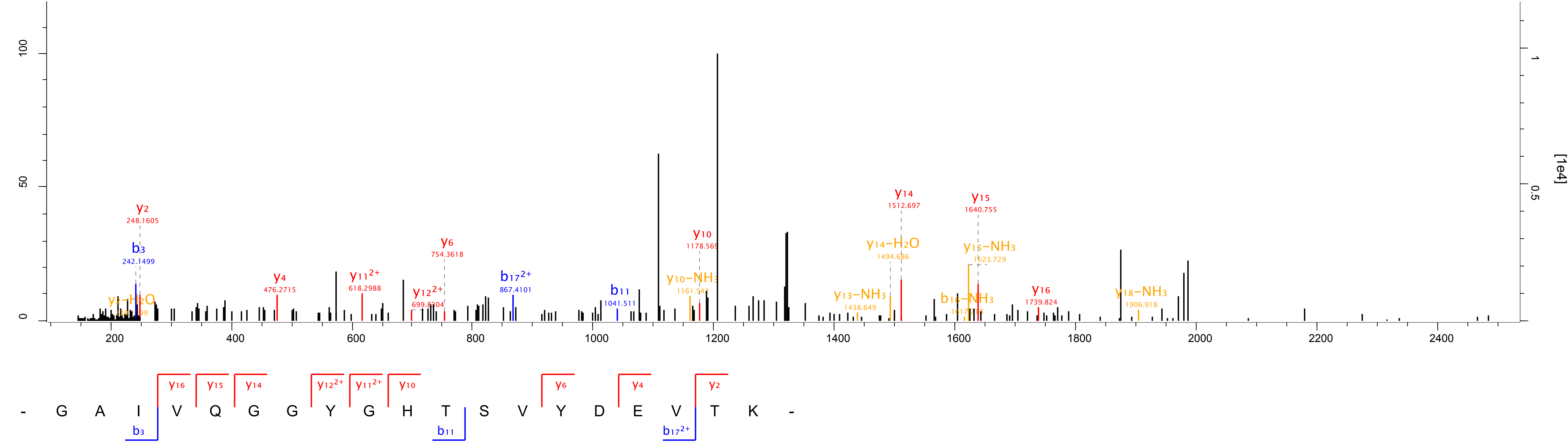

Raw file	Scan	Method	Score	m/z	Gene names
20150307_NSC1_Top_opt_F1_01_1685	32424	TOF; CID	58.98	577.95	Bet1l

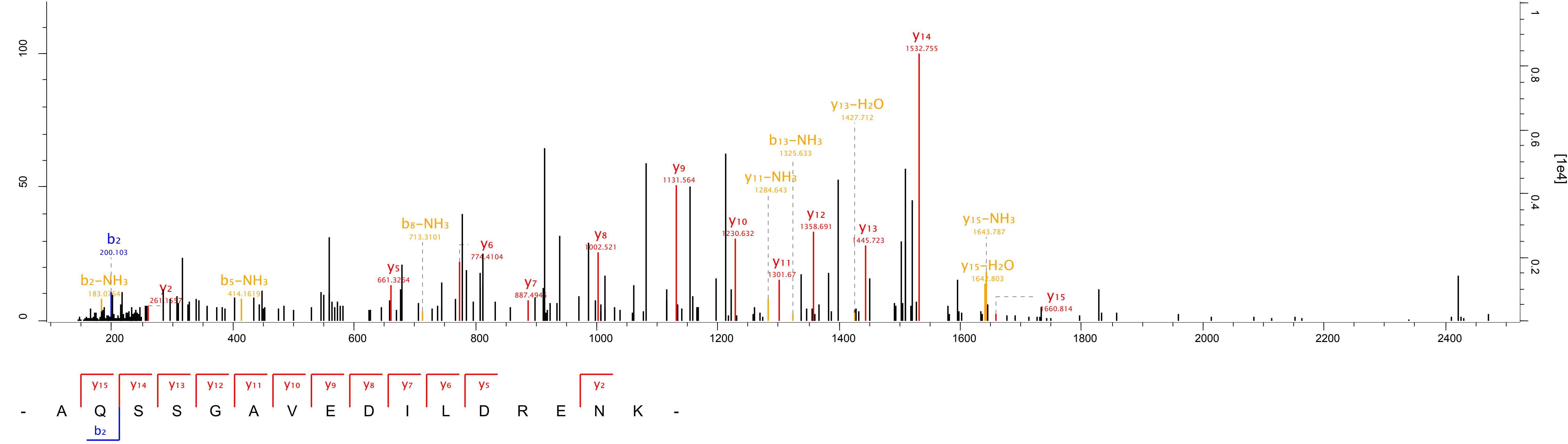

Raw file	Scan	Method	Score	m/z	Gene names
20150307_NSC1_Top_opt_F1_01_1685	34103	TOF; CID	91.48	872.41	Scyl3

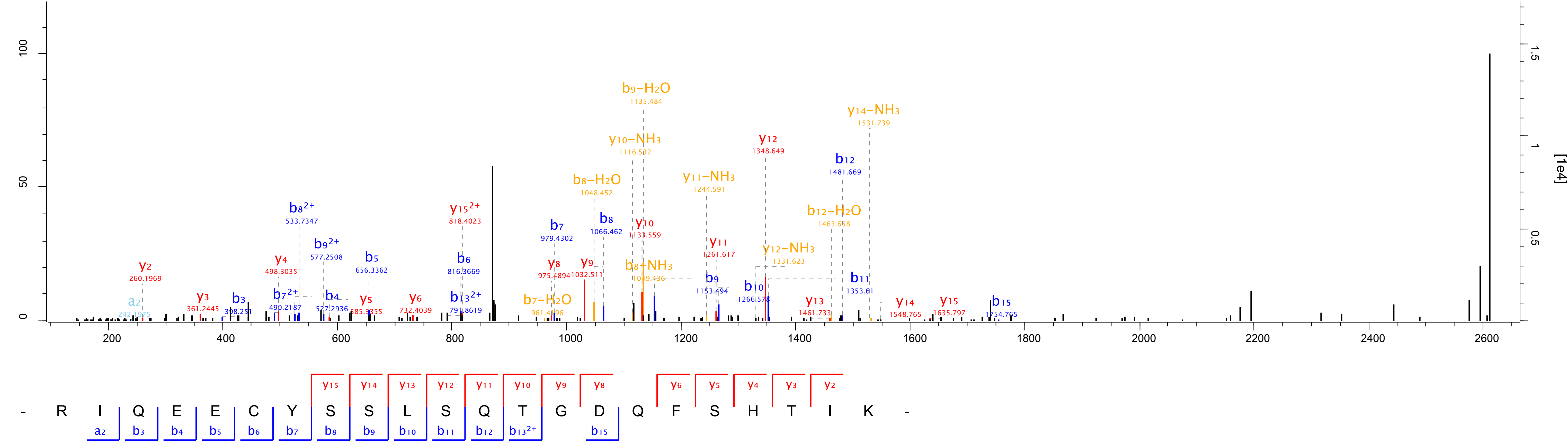

Raw file	Scan	Method	Score	m/z	Gene names
20150307_NSC1_Top_opt_F1_01_1685	35132	TOF; CID	53.45	614.34	Adarb1

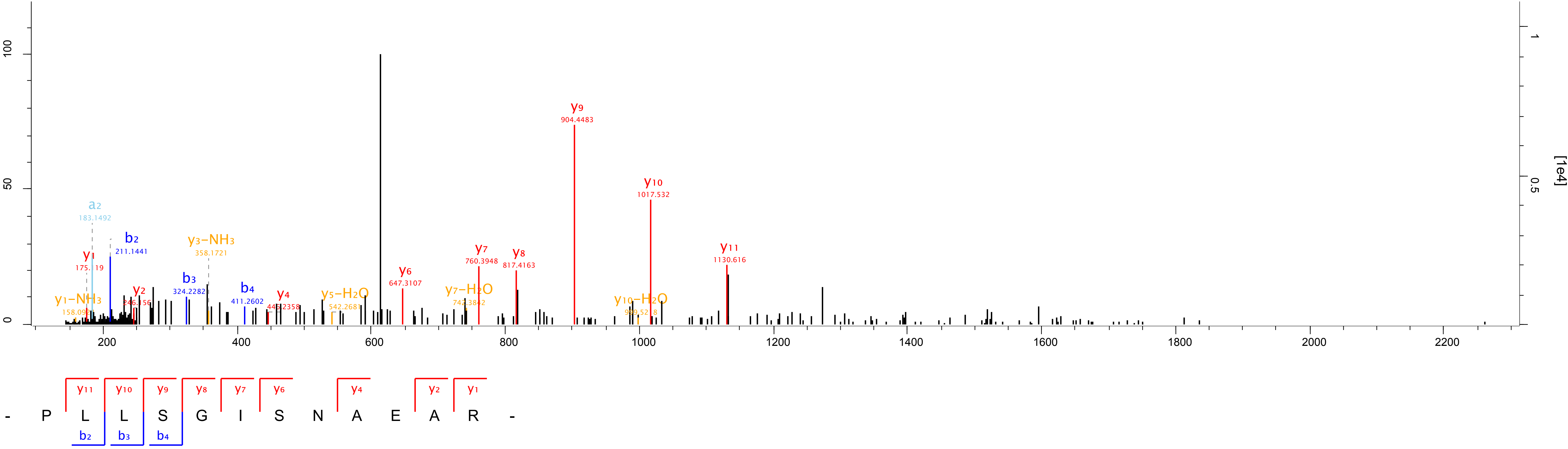

Raw file	Scan	Method	Score	m/z	Gene names
20150307_NSC1_Top_opt_F1_01_1685	35978	TOF; CID	52.9	846.4	Pja2

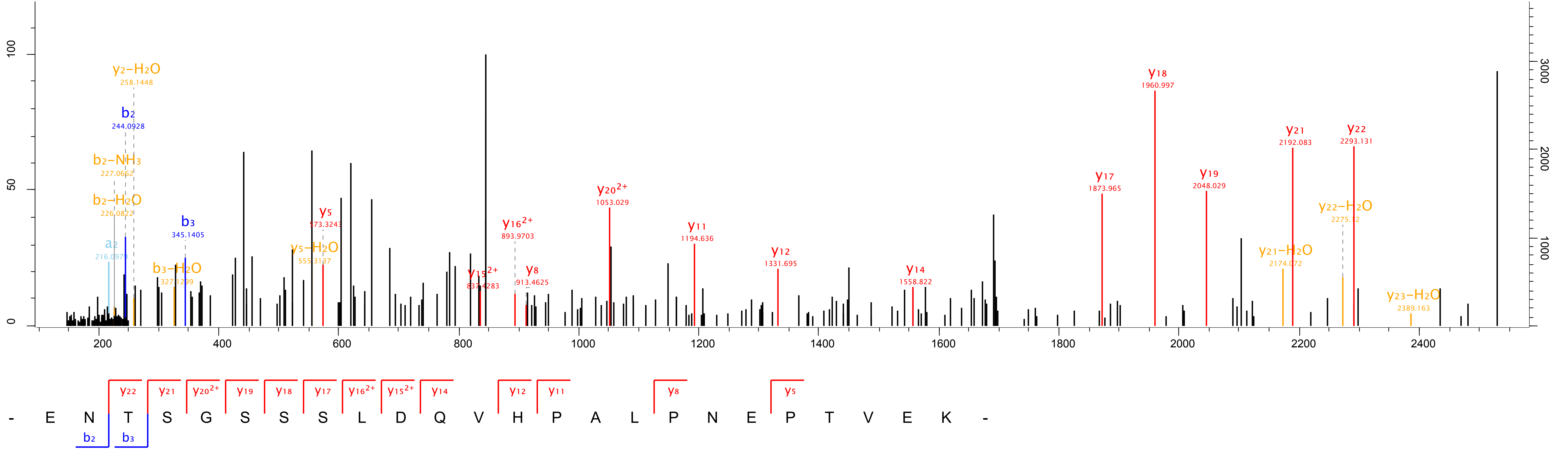

Raw file	Scan	Method	Score	m/z	Gene names
20150307_NSC1_Top_opt_F1_01_1685	39876	TOF; CID	81.97	915.42	Ccsmst1

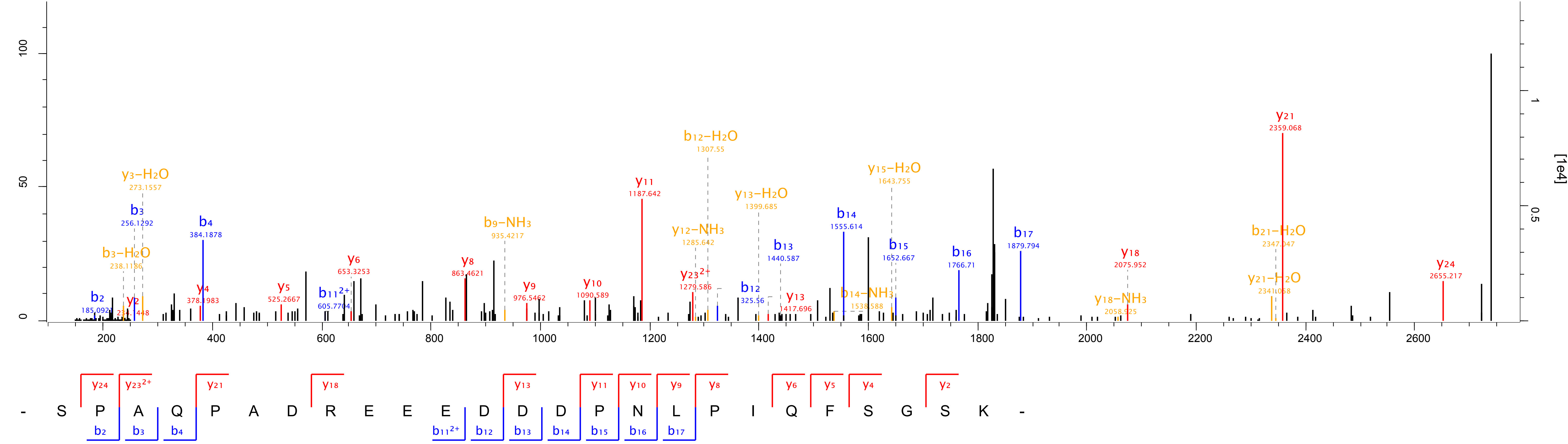

Raw file	Scan	Method	Score	m/z	Gene names
20150307_NSC1_Top_opt_F1_01_1685	41578	TOF; CID	71.03	458.56	Mrpl51

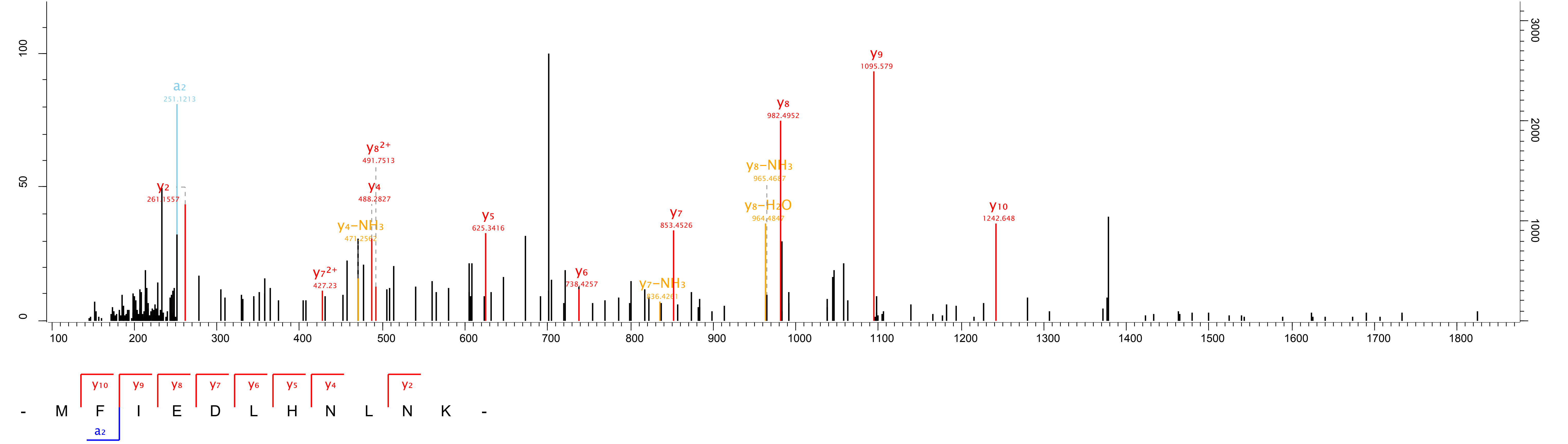

Raw file	Scan	Method	Score	m/z	Gene names
20150307_NSC1_Top_opt_F1_01_1685	42510	TOF; CID	61.34	559.29	Lamtor4

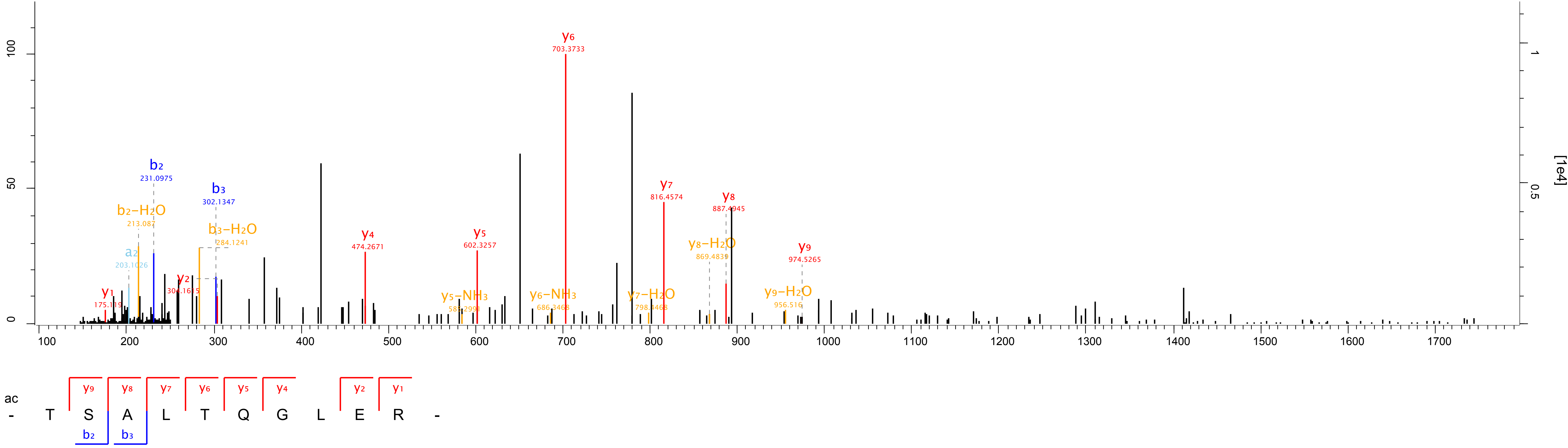

Raw file	Scan	Method	Score	m/z	Gene names
20150307_NSC1_Top_opt_F1_01_1685	44871	TOF; CID	54.56	771.07	Aagab

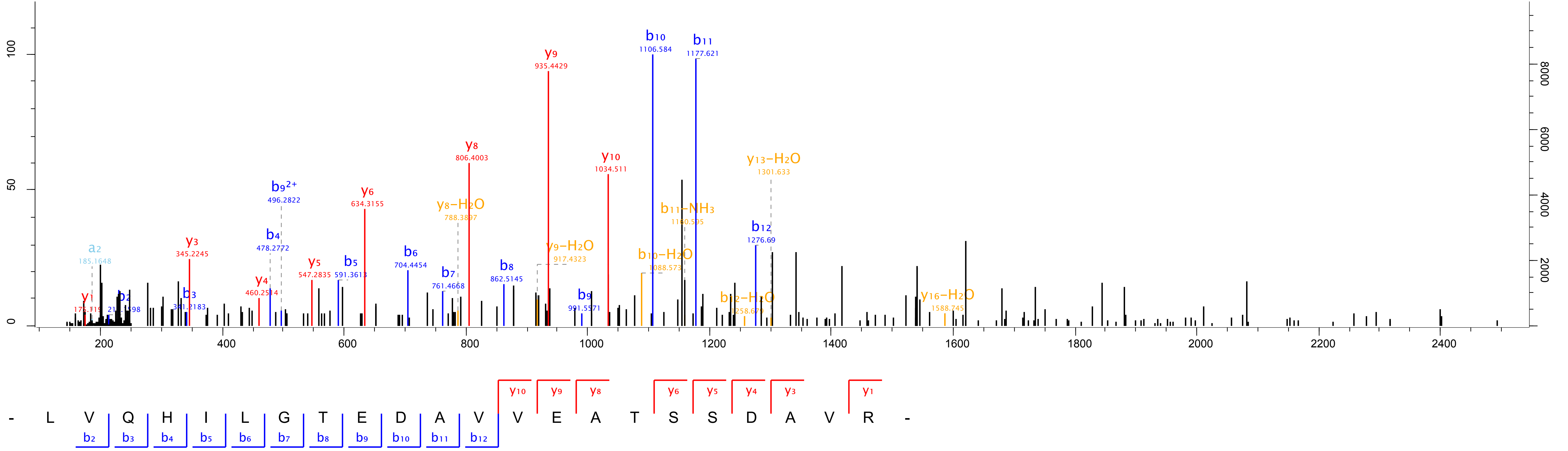

Raw file	Scan	Method	Score	m/z	Gene names
20150307_NSC1_Top_opt_F1_01_1685	49980	TOF; CID	55.2	689.36	Tarbp2

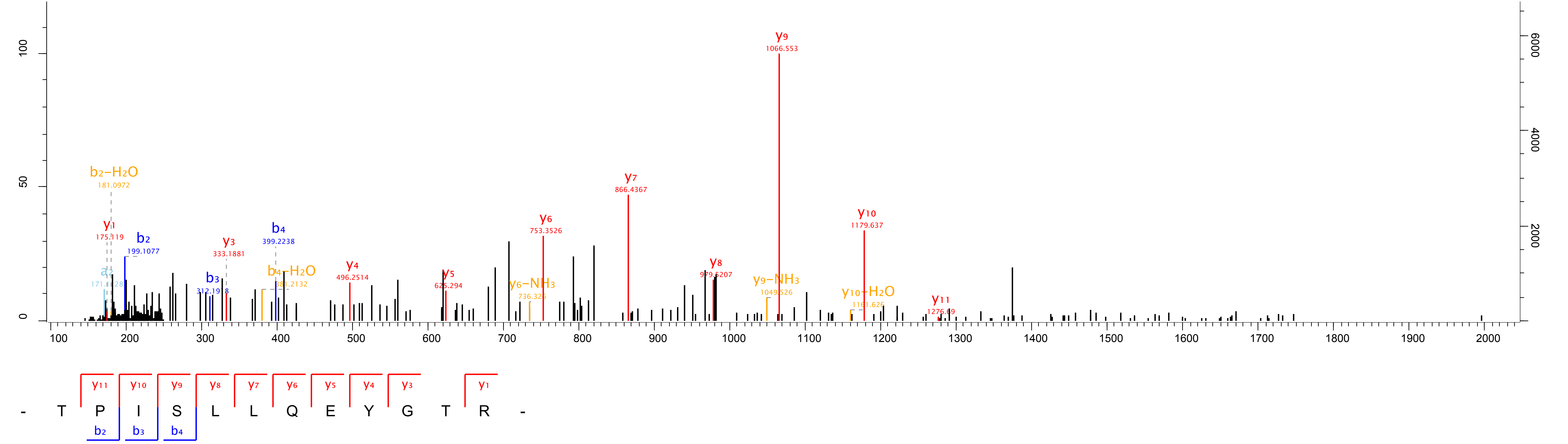

Raw file	Scan	Method	Score	m/z	Gene names
20150307_NSC1_Top_opt_F1_01_1685	52937	TOF; CID	54.09	836.4	Tfb2m;Gm1818

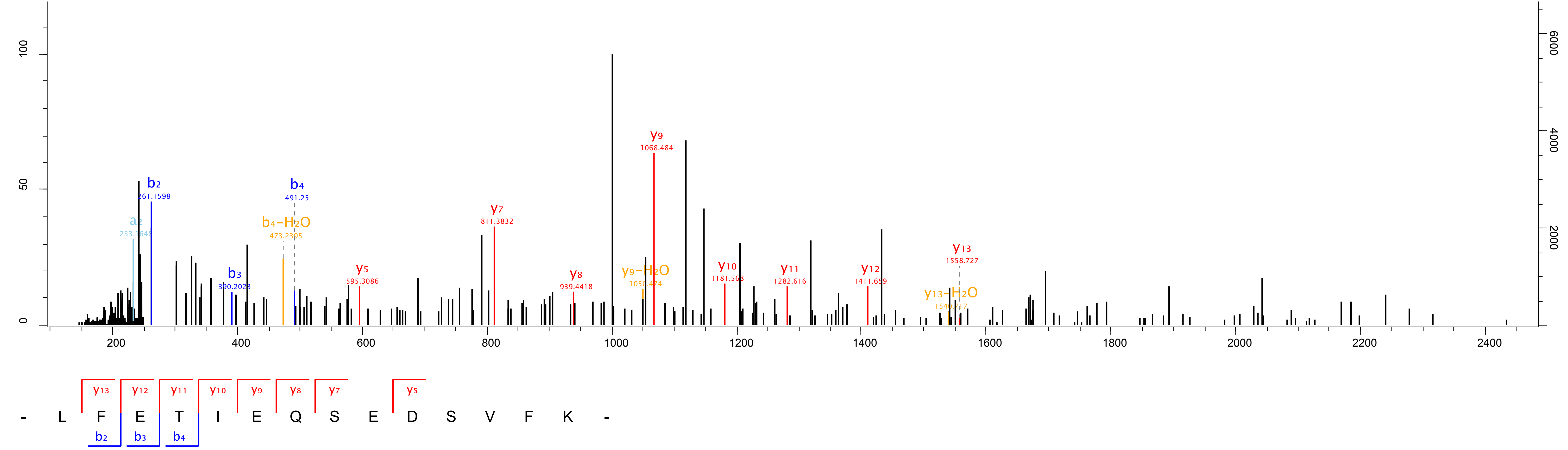

Raw file	Scan	Method	Score	m/z	Gene names
20150307_NSC1_Top_opt_F1_01_1685	53359	TOF; CID	83.2	705.39	Rundc3a

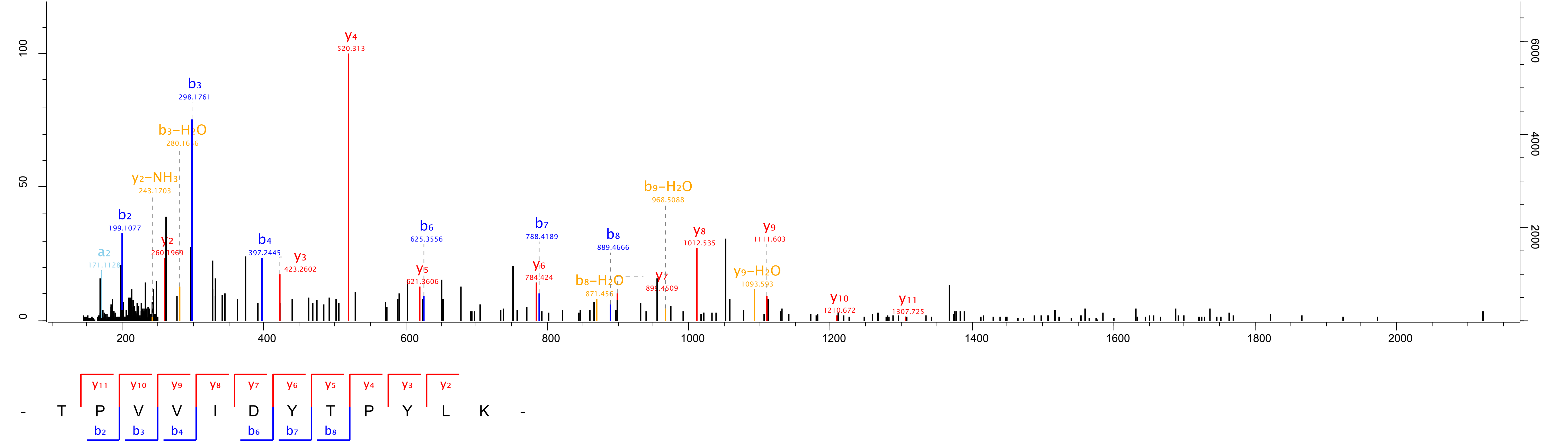

Raw file	Scan	Method	Score	m/z	Gene names
20150307_NSC1_Top_opt_F1_01_1685	56442	TOF; CID	64.45	918.47	Pex10

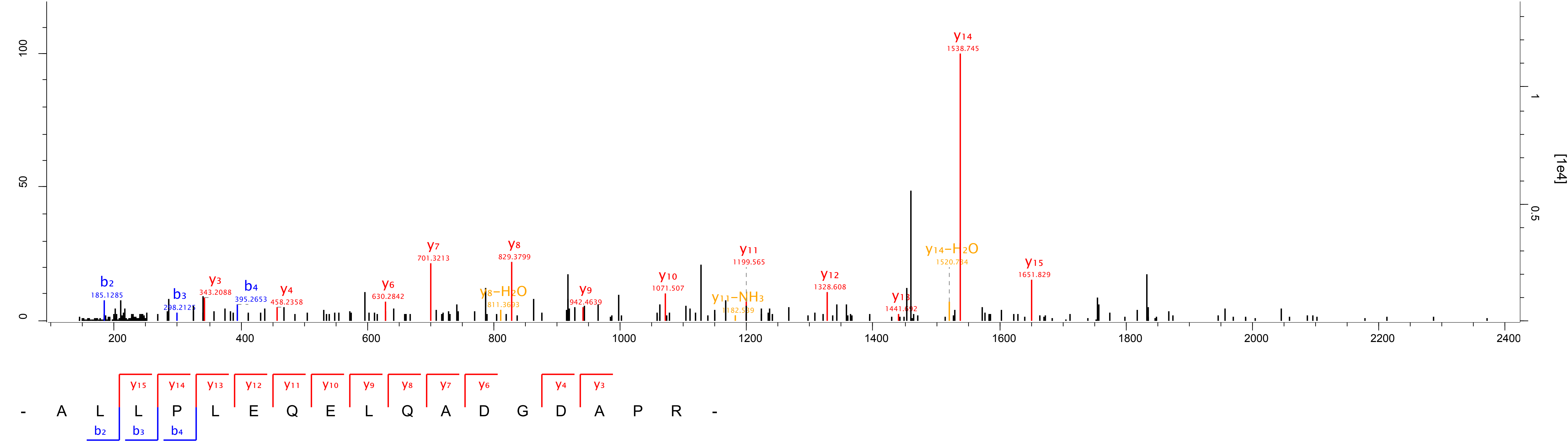

Raw file	Scan	Method	Score	m/z	Gene names
20150307_NSC1_Top_opt_F1_01_1685	57125	TOF; CID	112.26	967.74	Tmem208

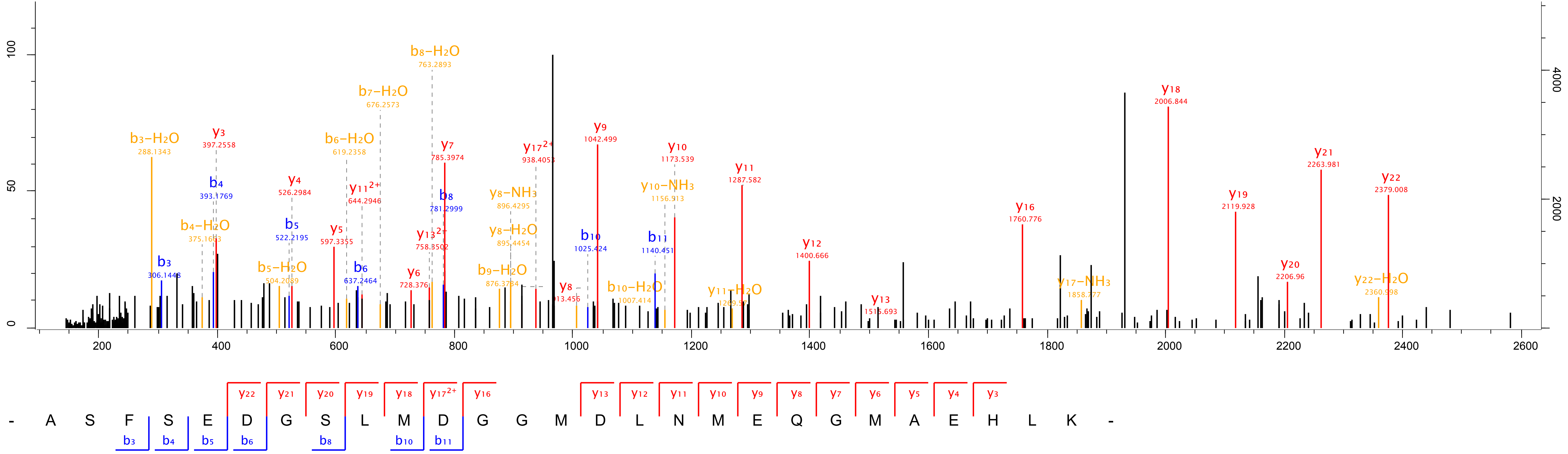

Raw file	Scan	Method	Score	m/z	Gene names
20150307_NSC1_Top_opt_F1_01_1685	58294	TOF; CID	57.48	1163.53	Zfyve27

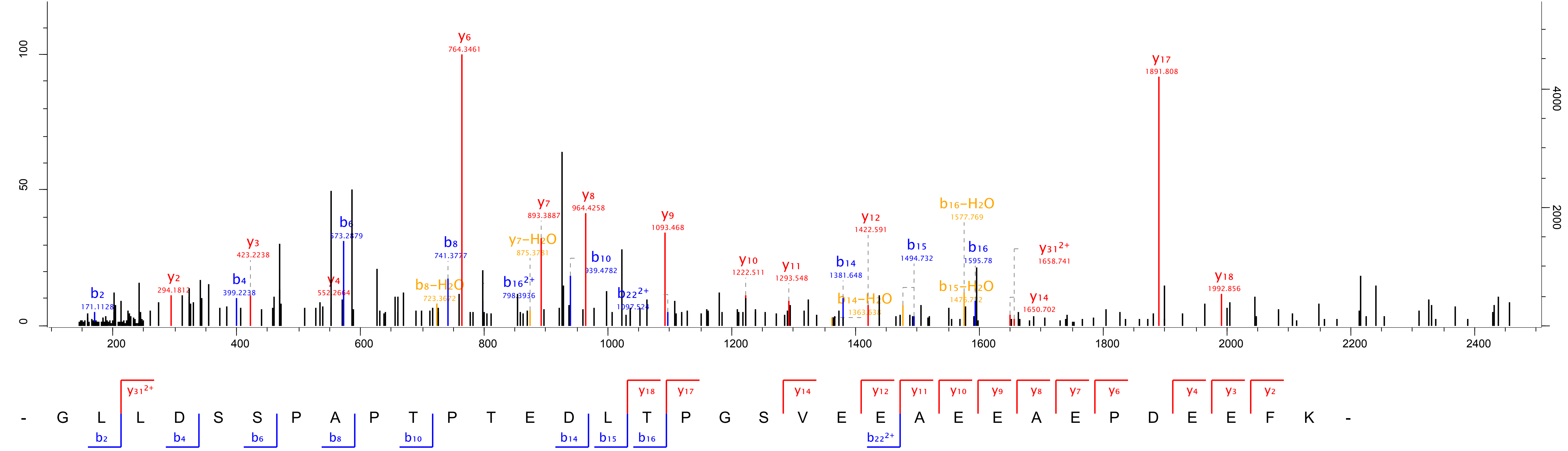

Raw file	Scan	Method	Score	m/z	Gene names
20150307_NSC1_Top_opt_F1_01_1685	58332	TOF; CID	113.47	913.44	Isca1;AK157302

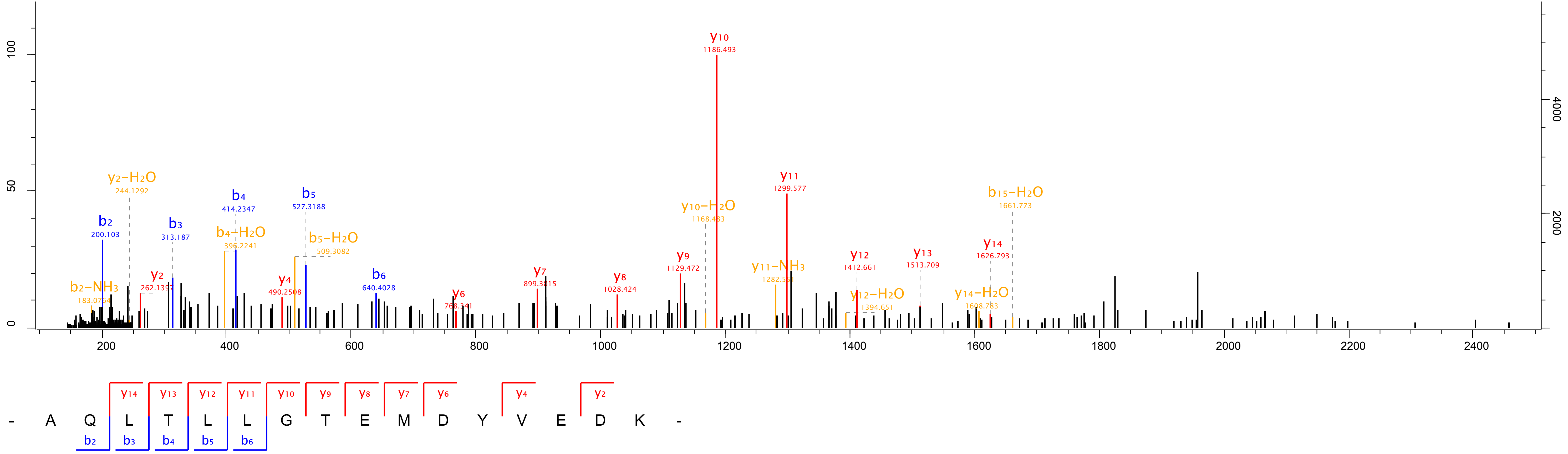

Raw file	Scan	Method	Score	m/z	Gene names
20150307_NSC1_Top_opt_F1_01_1685	61453	TOF; CID	120.86	714.89	Ostc

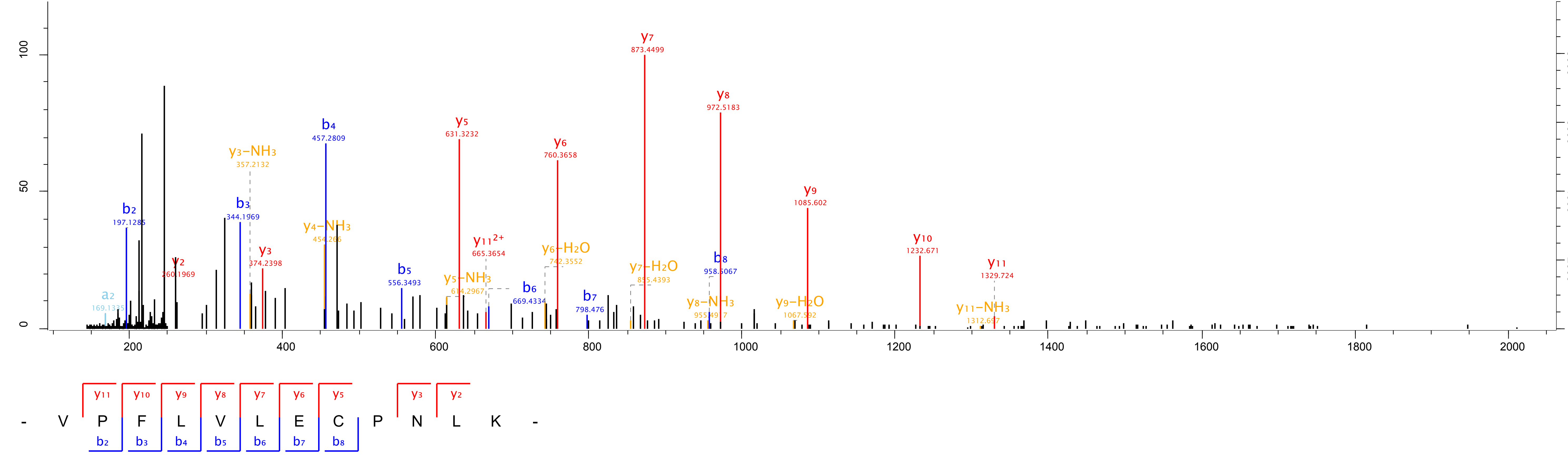

Raw file	Scan	Method	Score	m/z
20150307_NSC1_Top_opt_F1_01_1685	61809	TOF; CID	62.29	783.41

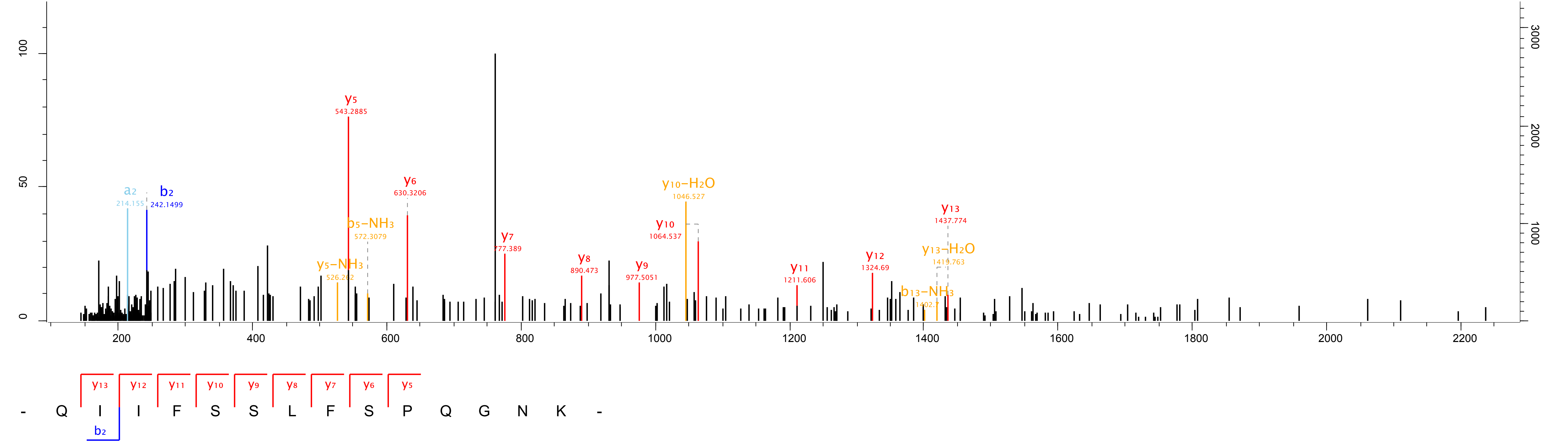

Raw file	Scan	Method	Score	m/z	Gene names
20150307_NSC1_Top_opt_F1_01_1685	63012	TOF; CID	61.19	895.45	Taf5

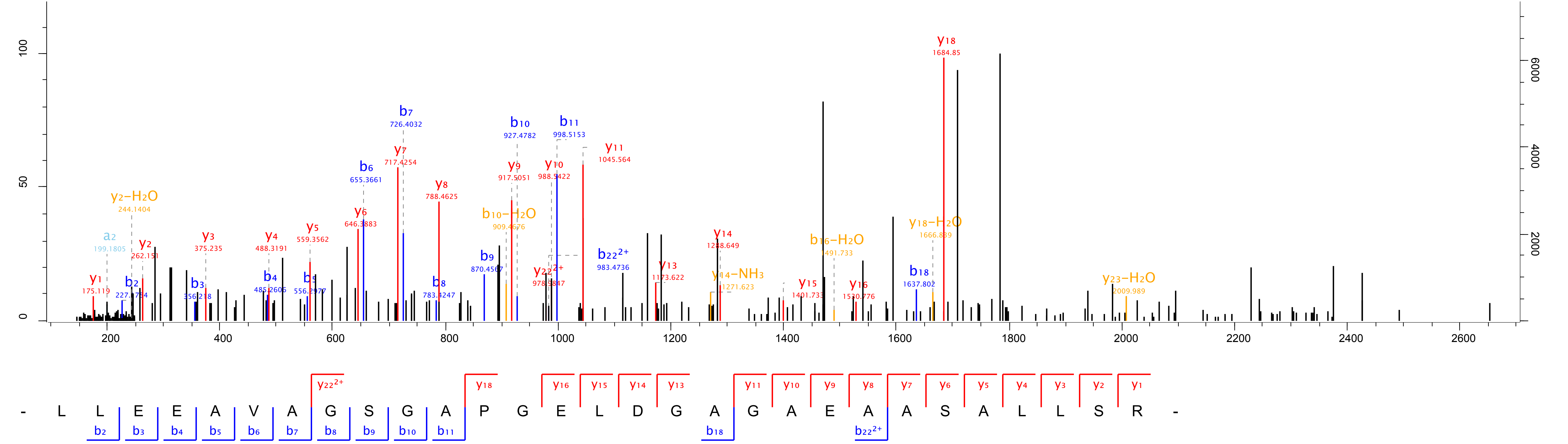

Raw file	Scan	Method	Score	m/z	Gene names
20150307_NSC1_Top_opt_F1_01_1685	69185	TOF; CID	63.7	971.12	Snrpe

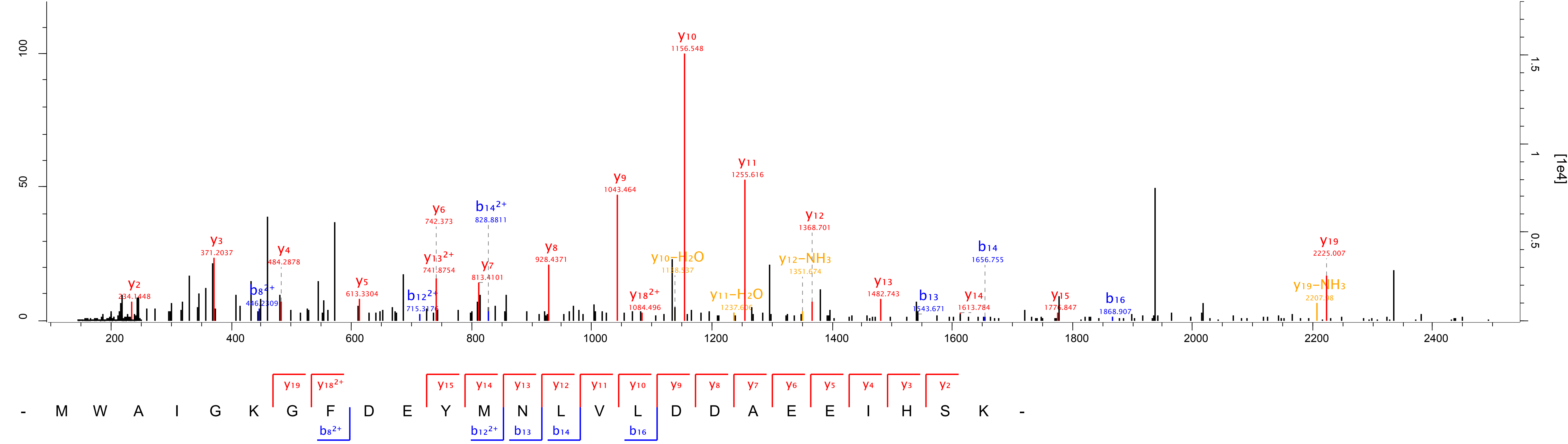

Raw file	Scan	Method	Score	m/z	Gene names
20150307_NSC2_Top_opt_F2_01_1688	13790	TOF; CID	74.51	736.37	Cbx6

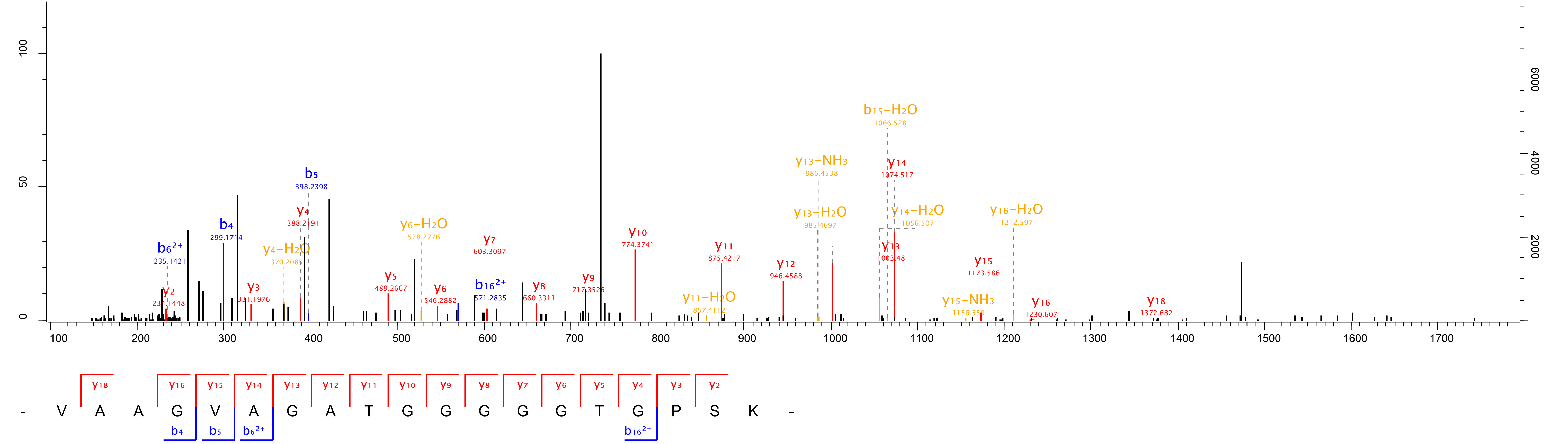

Raw file	Scan	Method	Score	m/z	Gene names
20150307_NSC2_Top_opt_F2_01_1688	20810	TOF; CID	69.03	660.82	Prkg1

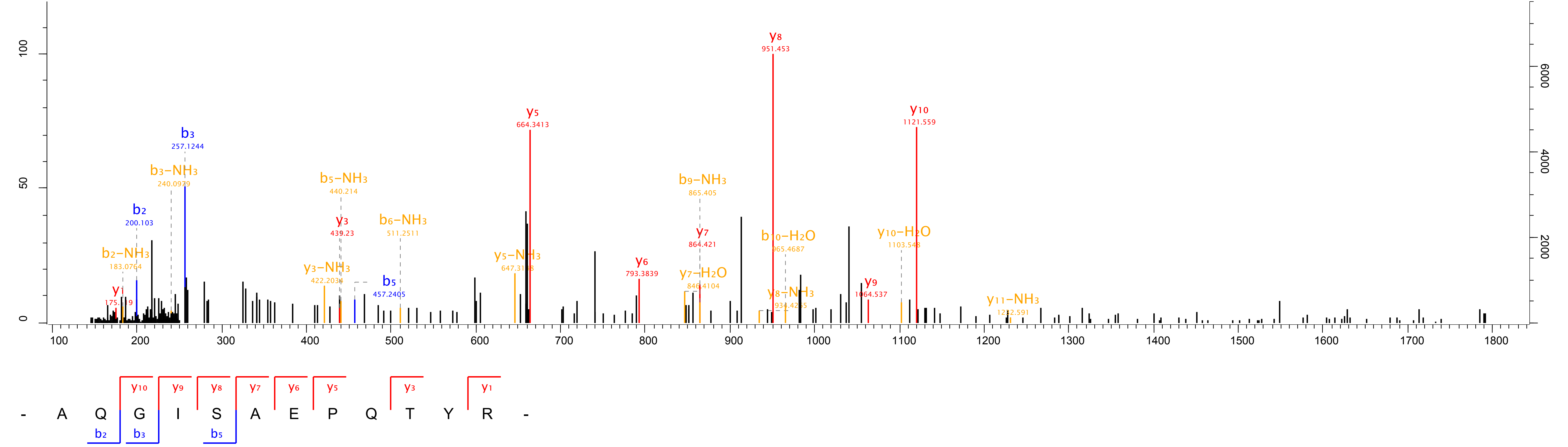

Raw file	Scan	Method	Score	m/z	Gene names
20150307_NSC2_Top_opt_F2_01_1688	26007	TOF; CID	70.54	468.27	Znf423;Zfp423

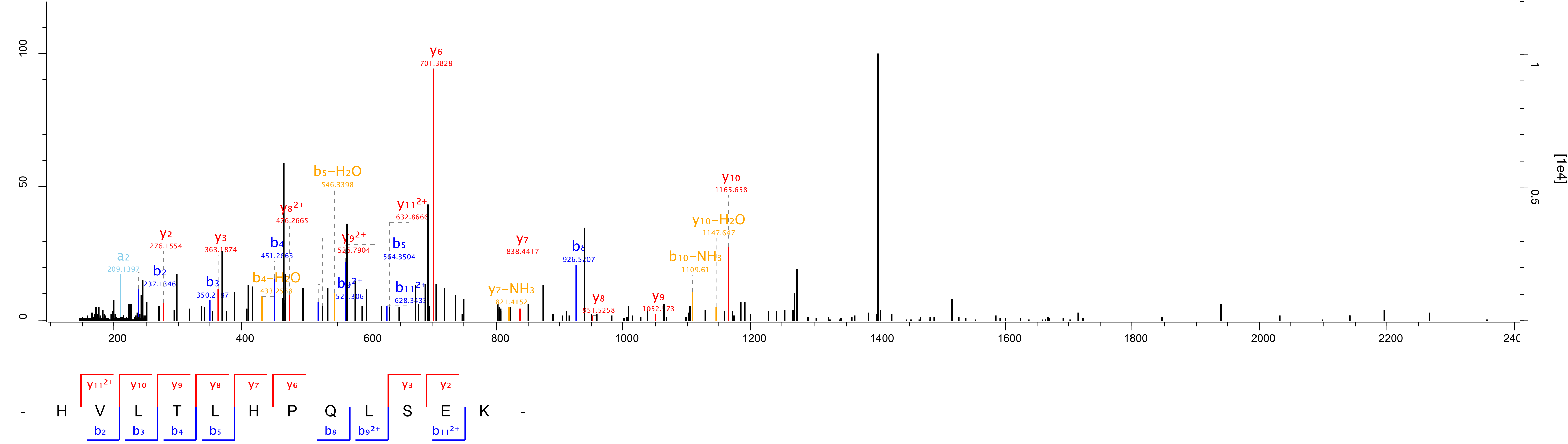

Raw file	Scan	Method	Score	m/z	Gene names
20150307_NSC2_Top_opt_F2_01_1688	28982	TOF; CID	66.83	580.3	Slain2

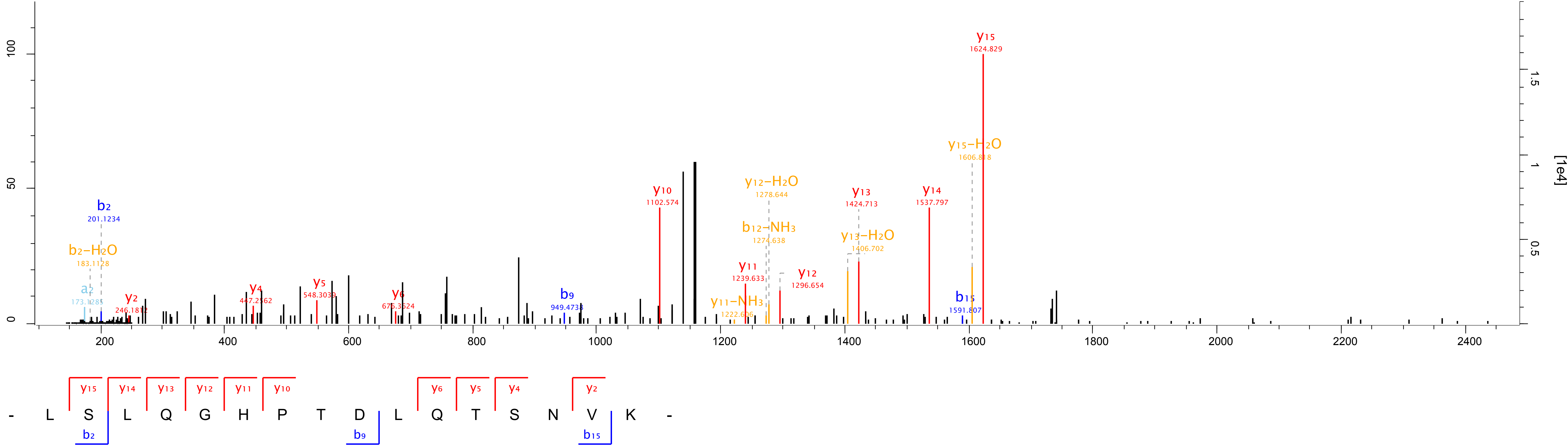

Raw file	Scan	Method	Score	m/z	Gene names
20150307_NSC2_Top_opt_F2_01_1688	29734	TOF; CID	51.32	938.8	Usp30

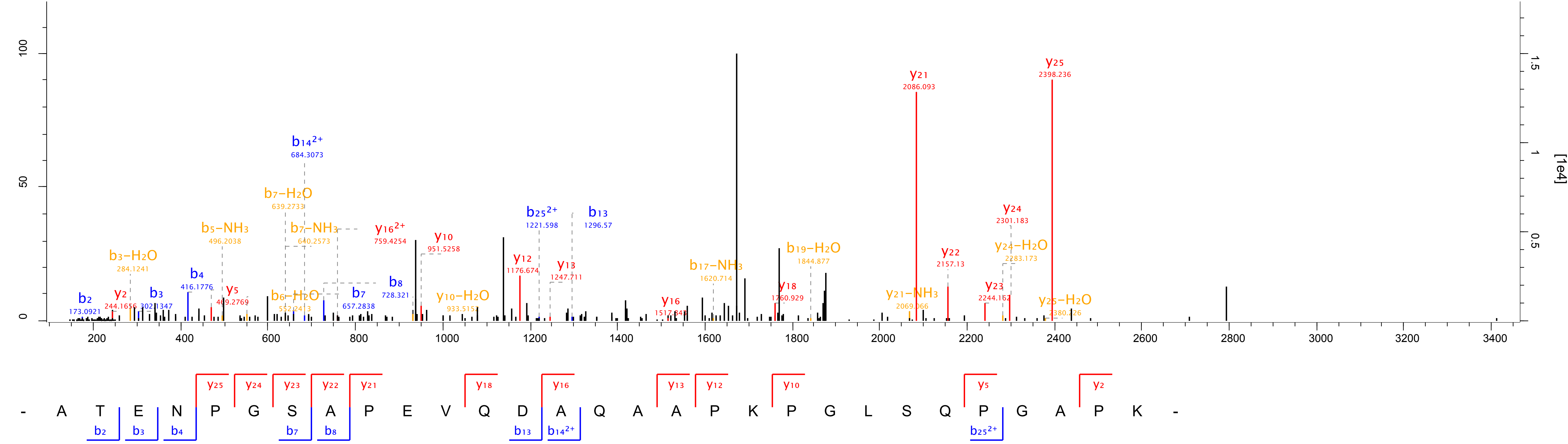

Raw file	Scan	Method	Score	m/z	Gene names
20150307_NSC2_Top_opt_F2_01_1688	38331	TOF; CID	70.26	764.9	Hddc3

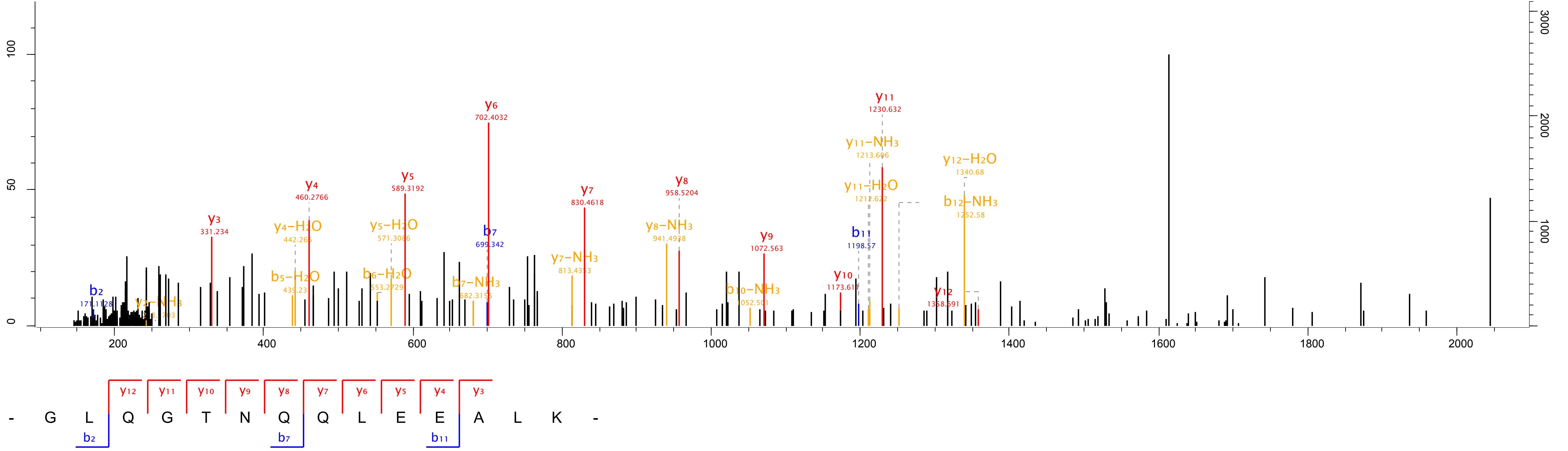

Raw file	Scan	Method	Score	m/z	Gene names
20150307_NSC2_Top_opt_F2_01_1688	44883	TOF; CID	100.93	676.83	Zdhhc6

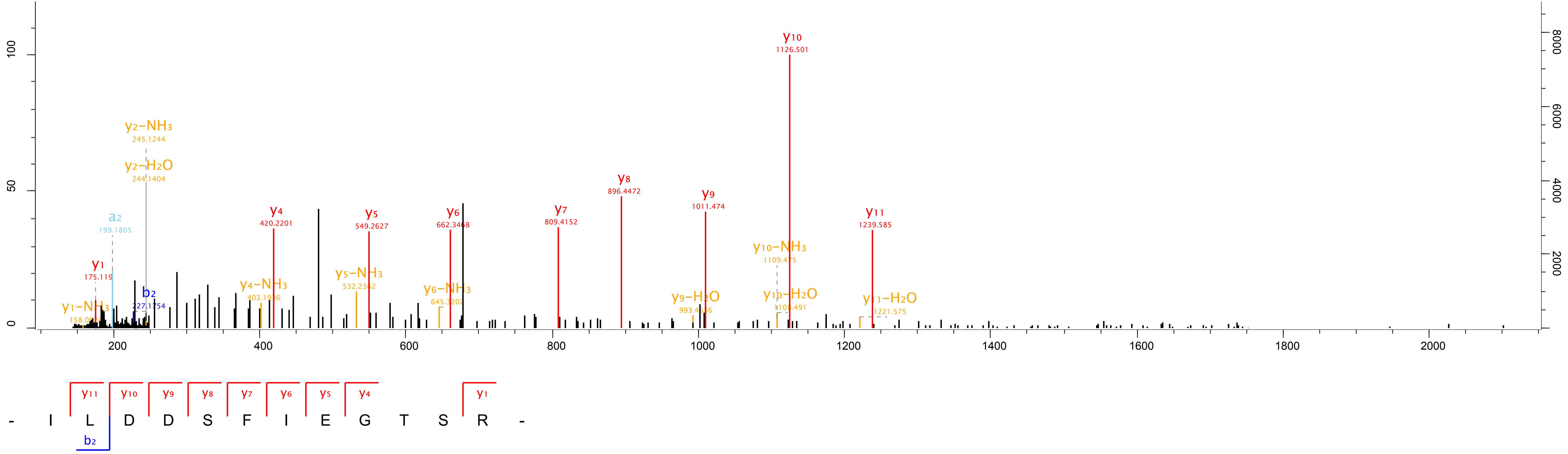

Raw file	Scan	Method	Score	m/z
20150307_NSC2_Top_opt_F2_01_1688	45486	TOF; CID	50.87	699.68

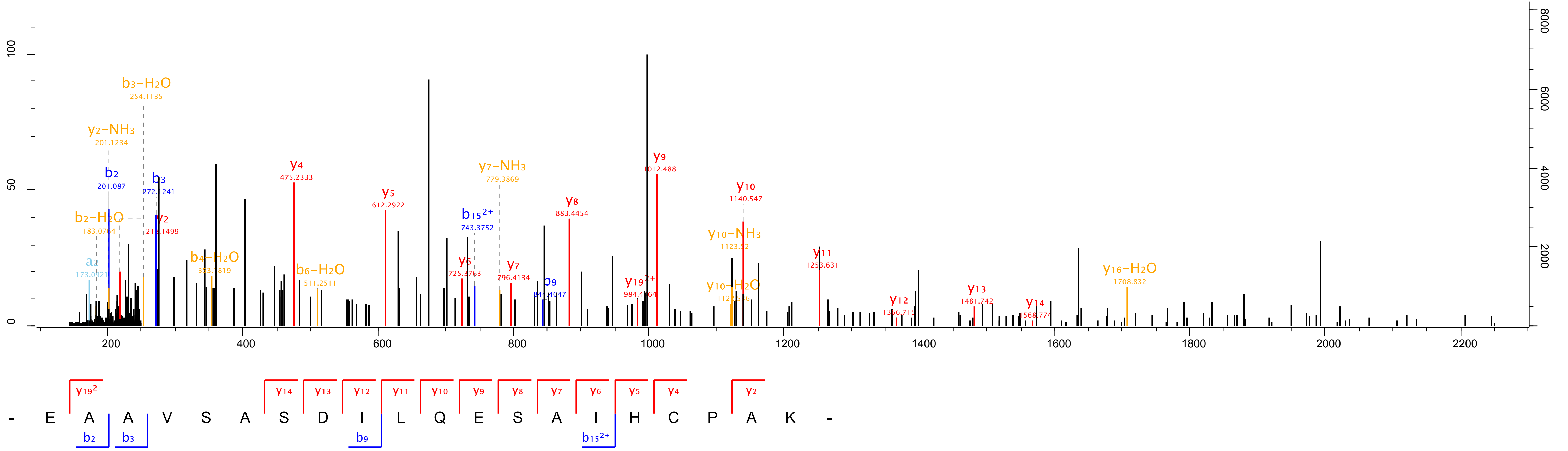

Raw file	Scan	Method	Score	m/z	Gene names
20150307_NSC2_Top_opt_F2_01_1688	48560	TOF; CID	65.72	512.33	Rrad

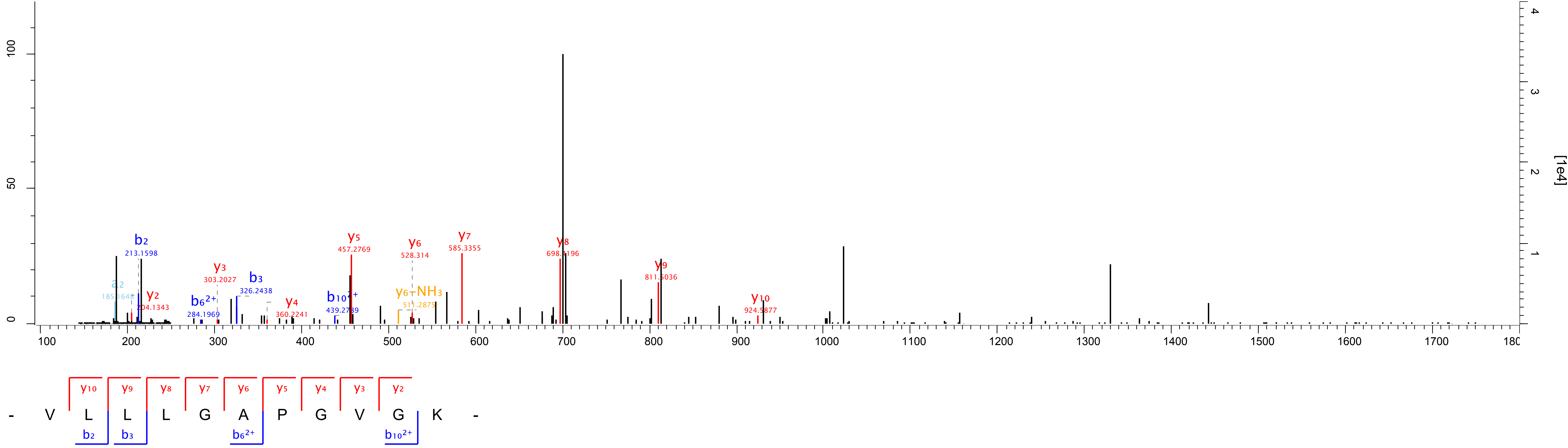

Raw file	Scan	Method	Score	m/z	Gene names
20150307_NSC2_Top_opt_F2_01_1688	50739	TOF; CID	79.65	718.41	Timm22

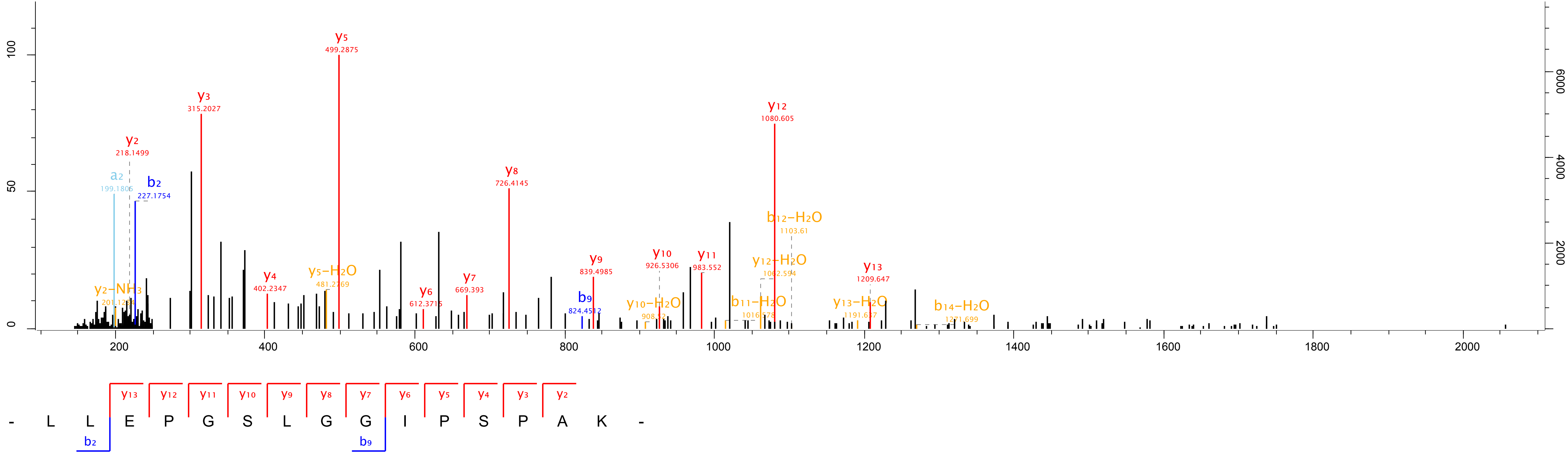

Raw file	Scan	Method	Score	m/z	Gene names
20150307_NSC2_Top_opt_F2_01_1688	50868	TOF; CID	59.23	785.4	Thsd7a

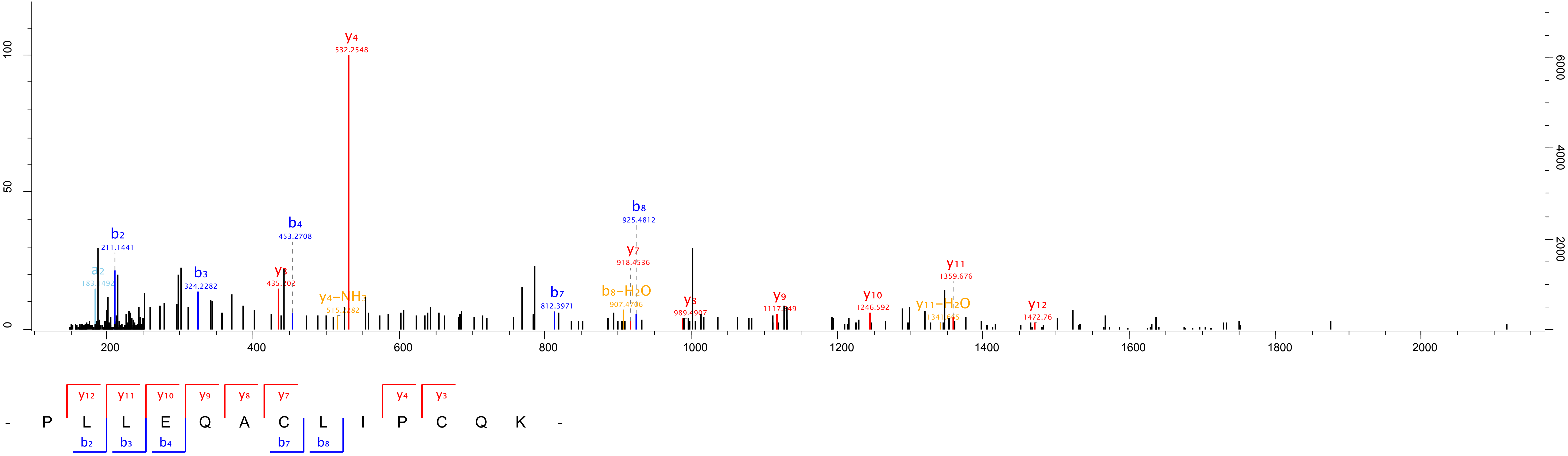

Raw file	Scan	Method	Score	m/z	Gene names
20150307_NSC2_Top_opt_F2_01_1688	53066	TOF; CID	49.43	986.46	Wdr47

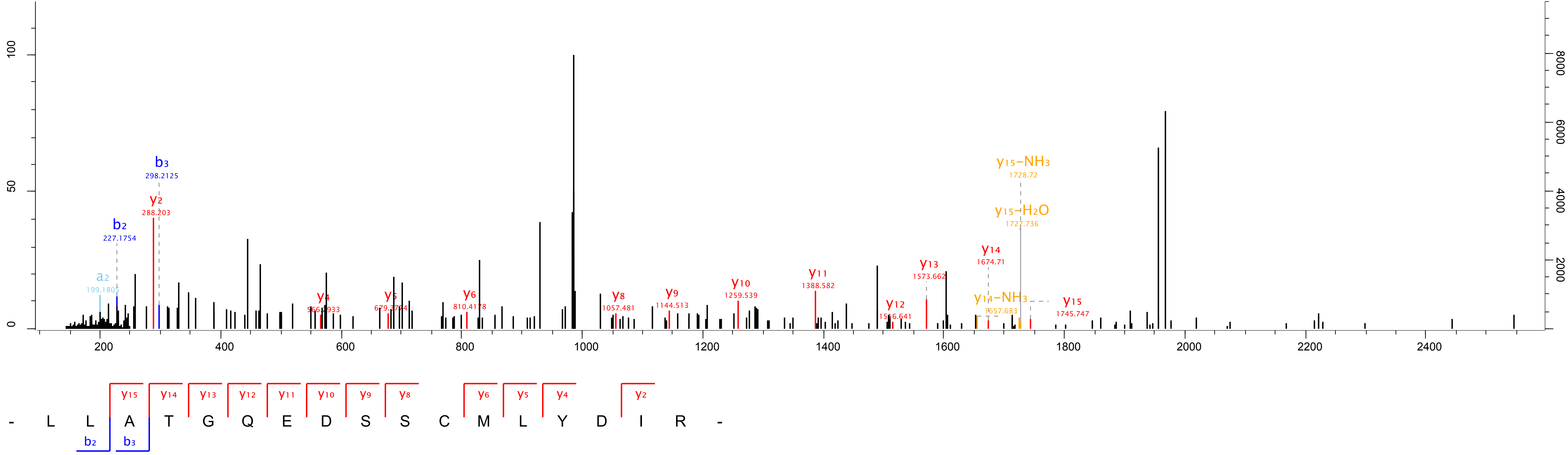

Raw file	Scan	Method	Score	m/z	Gene names
20150307_NSC2_Top_opt_F2_01_1688	53083	TOF; CID	77.19	595.34	Exd2

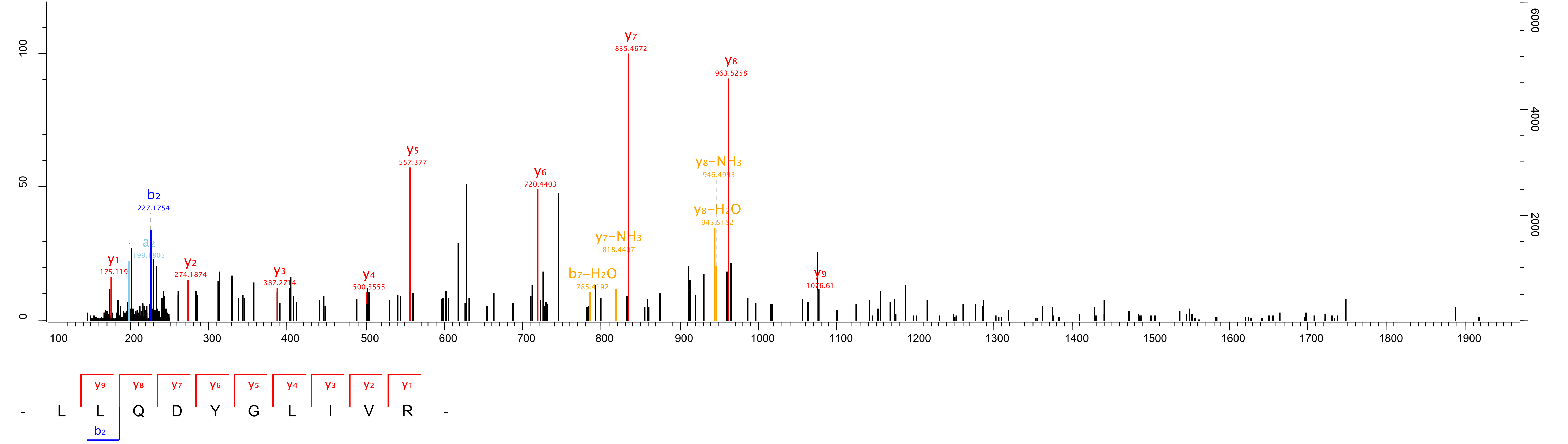

Raw file	Scan	Method	Score	m/z	Gene names
20150307_NSC2_Top_opt_F2_01_1688	55017	TOF; CID	58.27	566.29	Chchd5

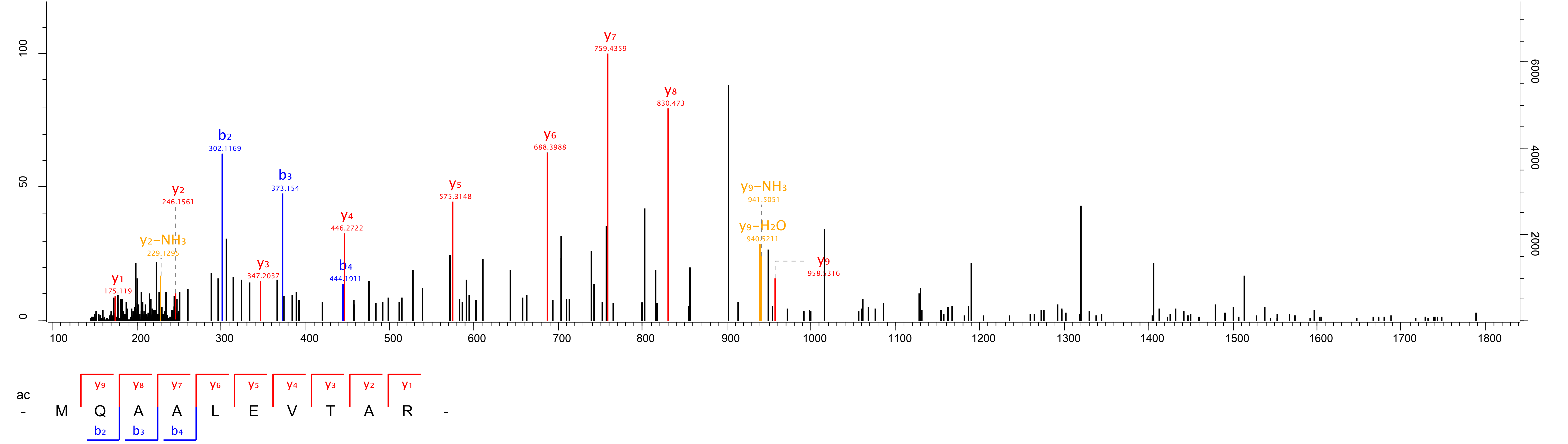

Raw file	Scan	Method	Score	m/z	Gene names
20150307_NSC2_Top_opt_F2_01_1688	57313	TOF; CID	87.52	813.4	Mettl7a2Higd1c;Mettl7a1;UbiE2;Mettl7a2;UbiE-YGHL1(HIG1-4)

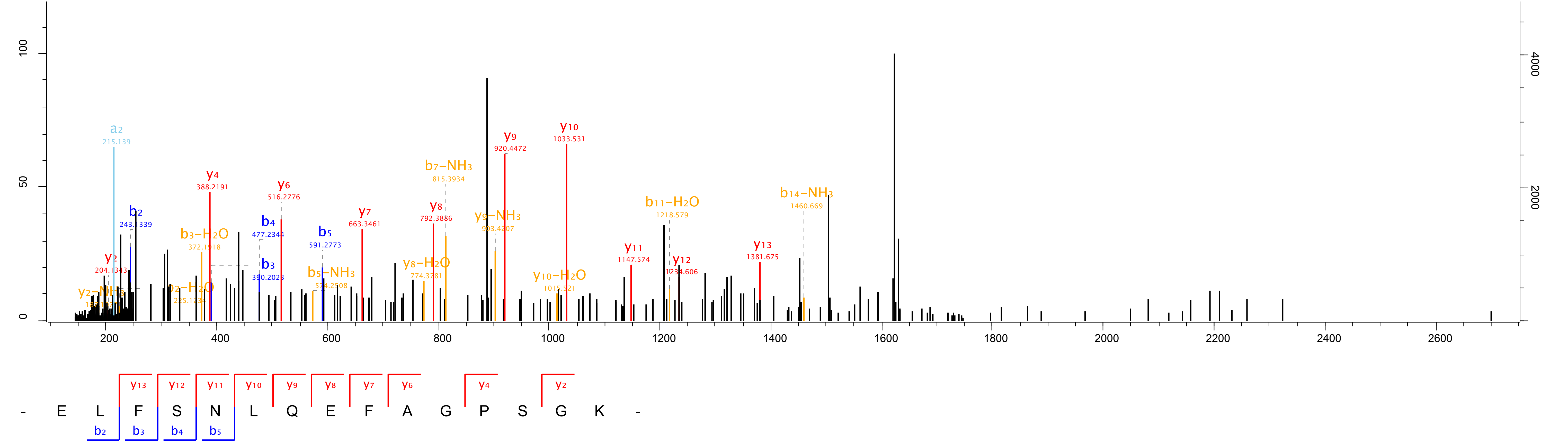

Raw file	Scan	Method	Score	m/z	Gene names
20150307_NSC2_Top_opt_F2_01_1688	58928	TOF; CID	96.82	713.39	Med27

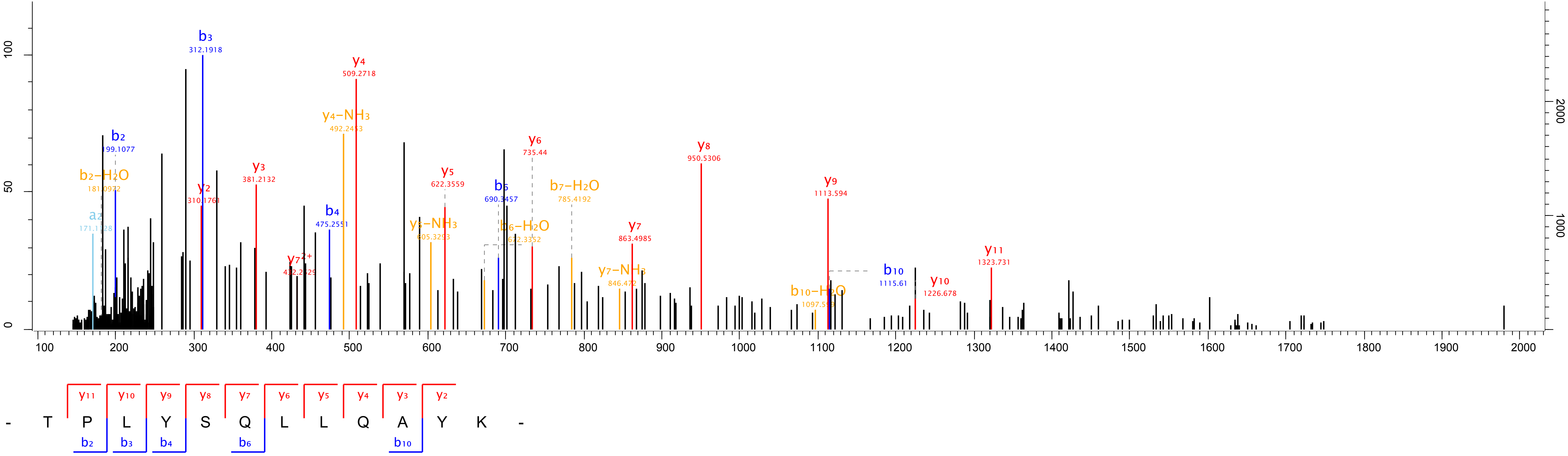

Raw file	Scan	Method	Score	m/z	Gene names
20150307_NSC2_Top_opt_F2_01_1688	70592	TOF; CID	30.7	963.48	Snrk

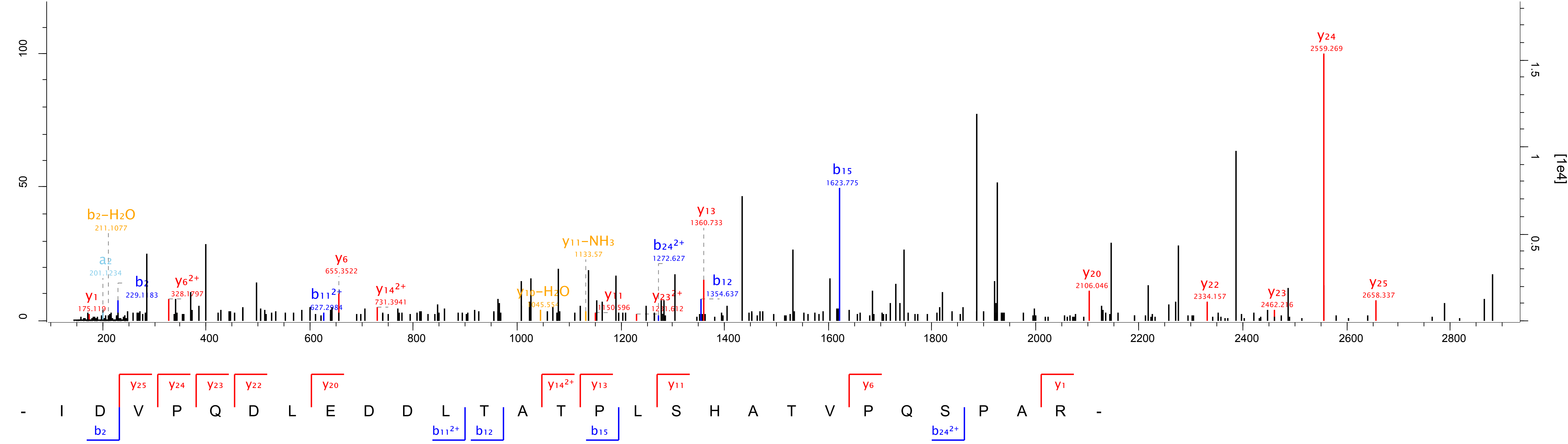

Raw file	Scan	Method	Score	m/z	Gene names
20150307_NSC2_Top_opt_F2_01_1688	76458	TOF; CID	53.48	957.48	Nbea

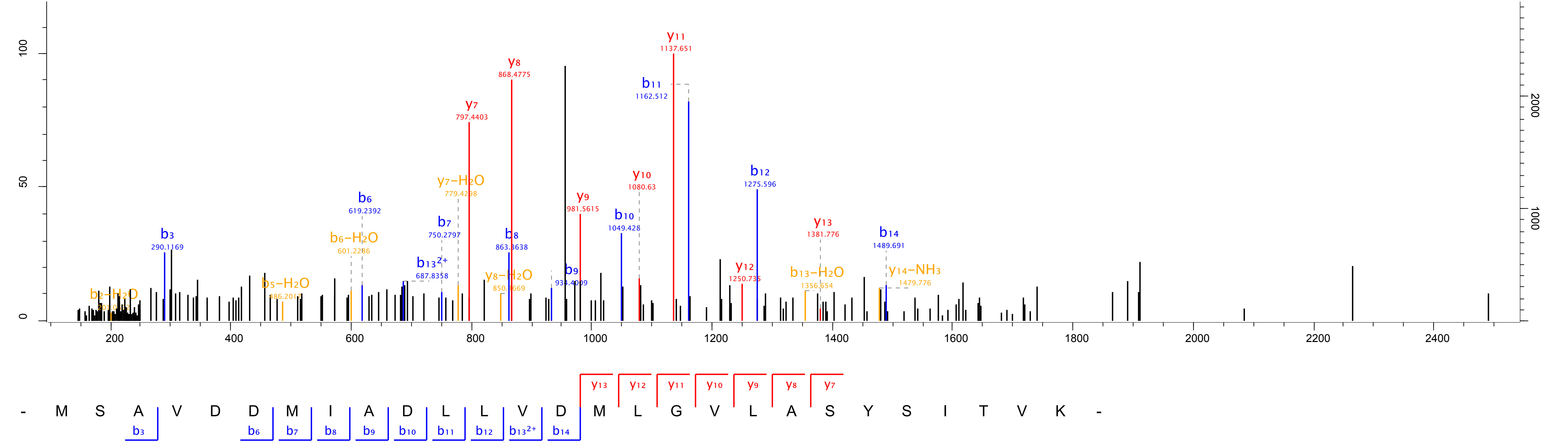

Raw file	Scan	Method	Score	m/z	Gene names
20150307_NSC3_Top_opt_F3_01_1682	5024	TOF; CID	125.54	738.24	Mt3

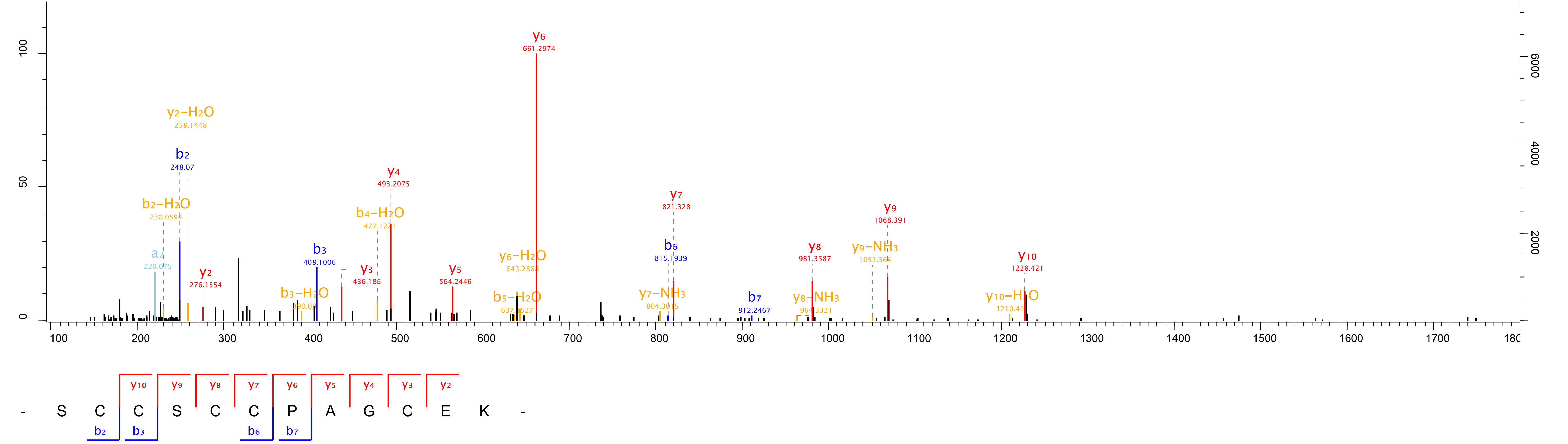

Raw file	Scan	Method	Score	m/z	Gene names
20150307_NSC3_Top_opt_F3_01_1682	5313	TOF; CID	87.18	603.26	Mgarp

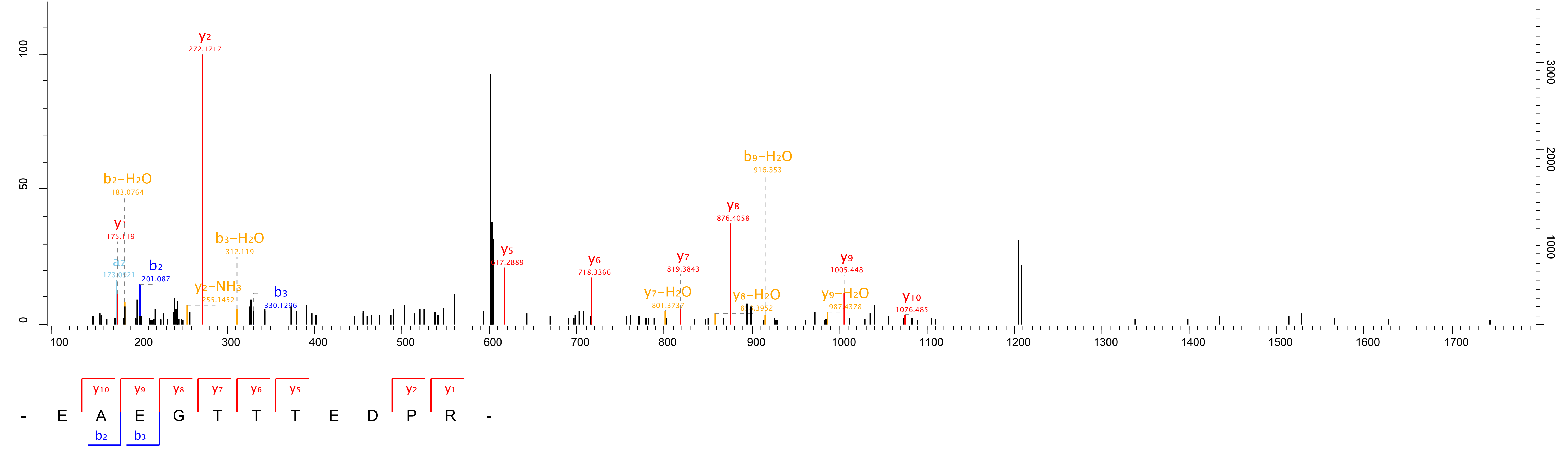

Raw file	Scan	Method	Score	m/z	Gene names
20150307_NSC3_Top_opt_F3_01_1682	15987	TOF; CID	82.26	599.27	Ttf2

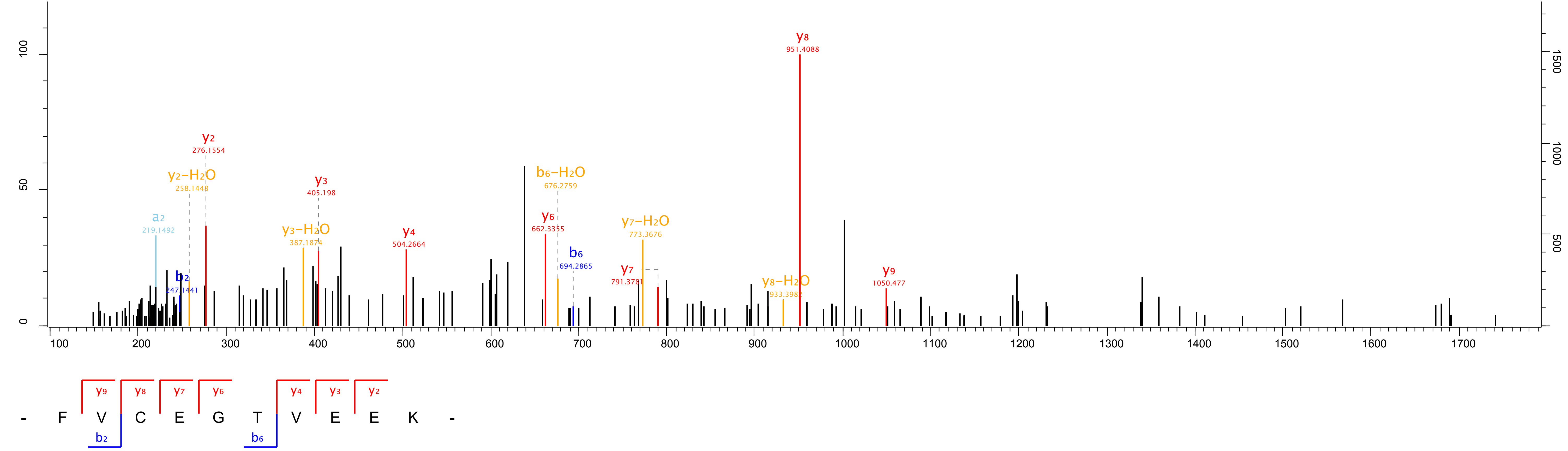

Raw file	Scan	Method	Score	m/z	Gene names
20150307_NSC3_Top_opt_F3_01_1682	27952	TOF; CID	54.65	747.85	Gpm6a

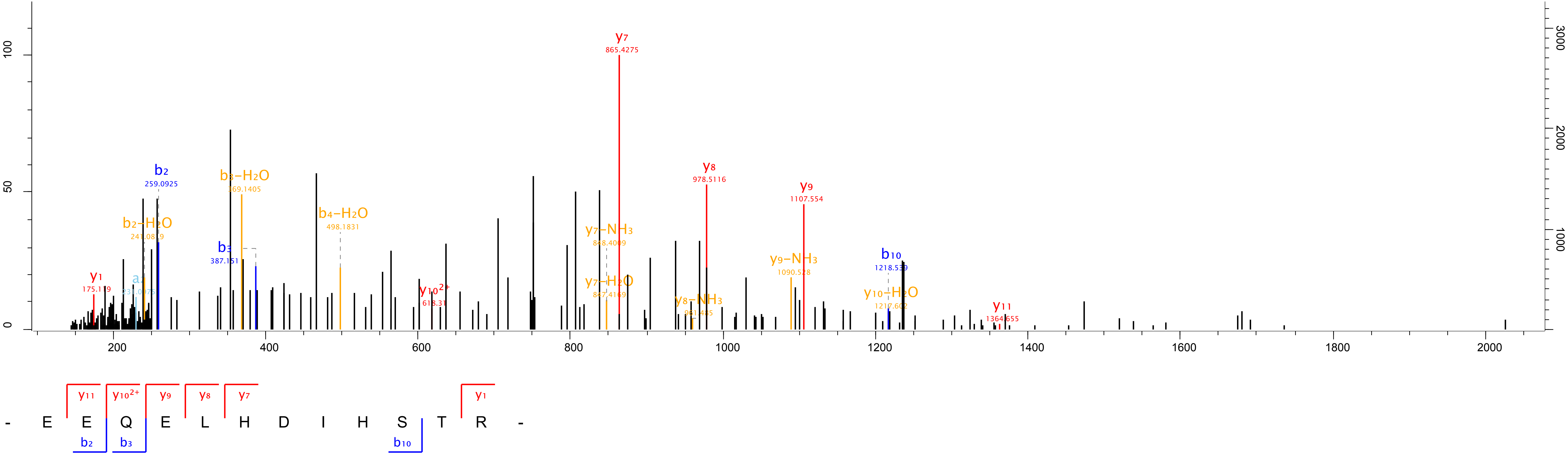

Raw file	Scan	Method	Score	m/z	Gene names
20150307_NSC3_Top_opt_F3_01_1682	30030	TOF; CID	62.69	730.91	Ccdc108

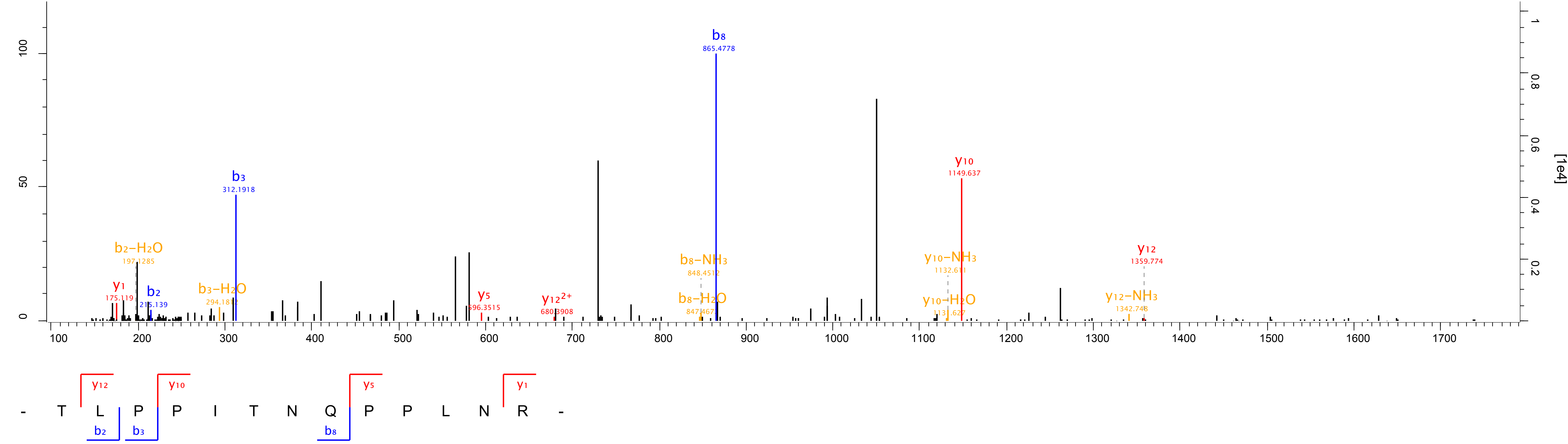

Raw file	Scan	Method	Score	m/z	Gene names
20150307_NSC3_Top_opt_F3_01_1682	38932	TOF; CID	91.27	627.37	Hic2

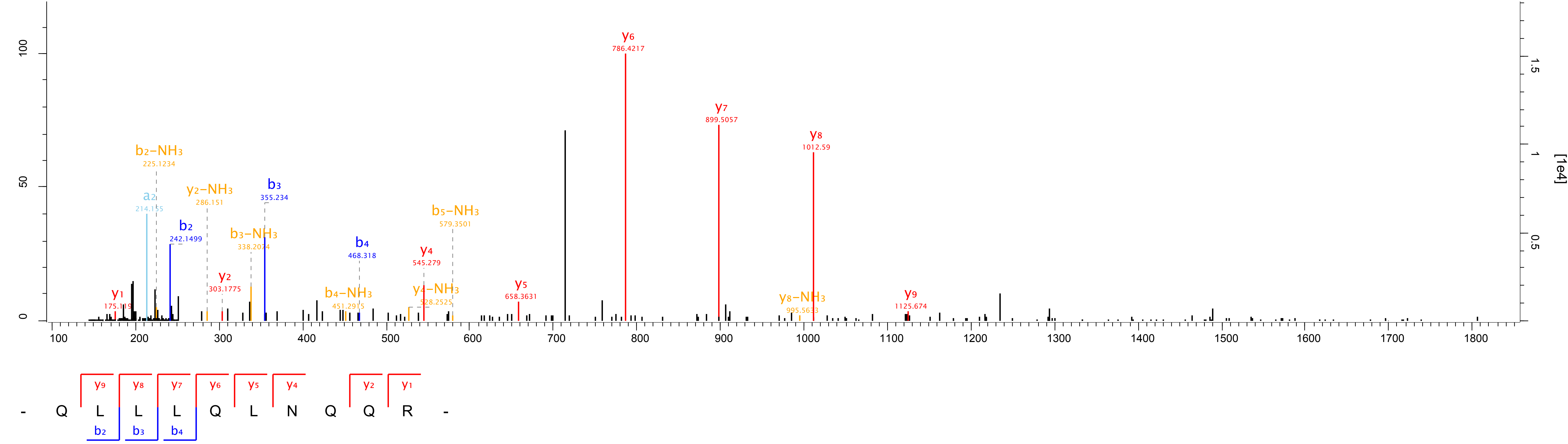

Raw file	Scan	Method	Score	m/z	Gene names
20150307_NSC3_Top_opt_F3_01_1682	58073	TOF; CID	111.57	1120.63	Cox6a1

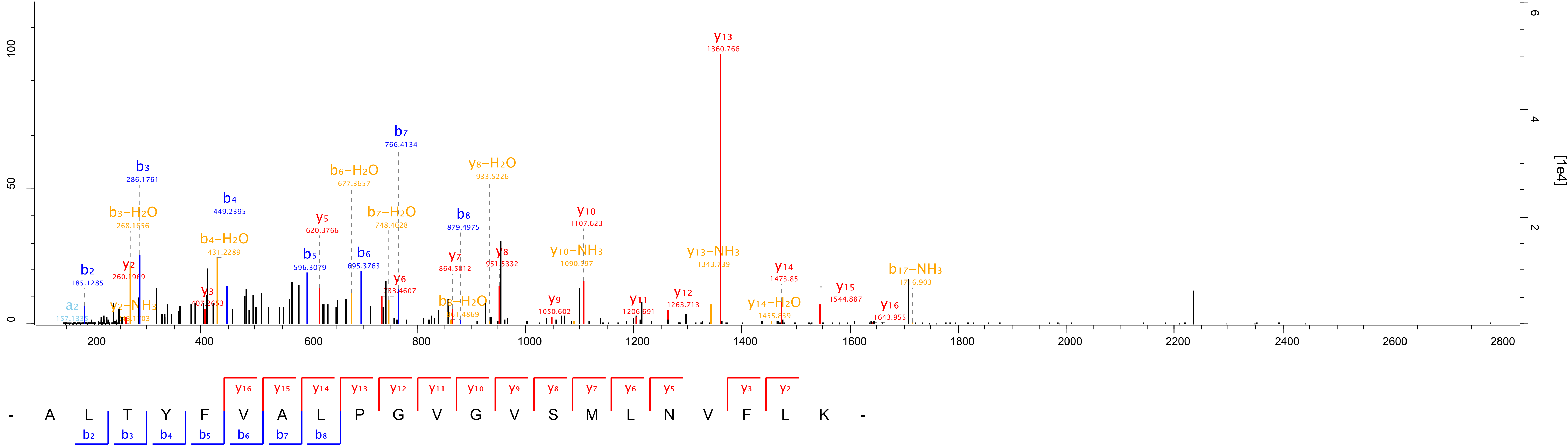

Raw file	Scan	Method	Score	m/z	Gene names
20150307_NSC3_Top_opt_F3_01_1691	9876	TOF; CID	77.06	596.29	Pms2

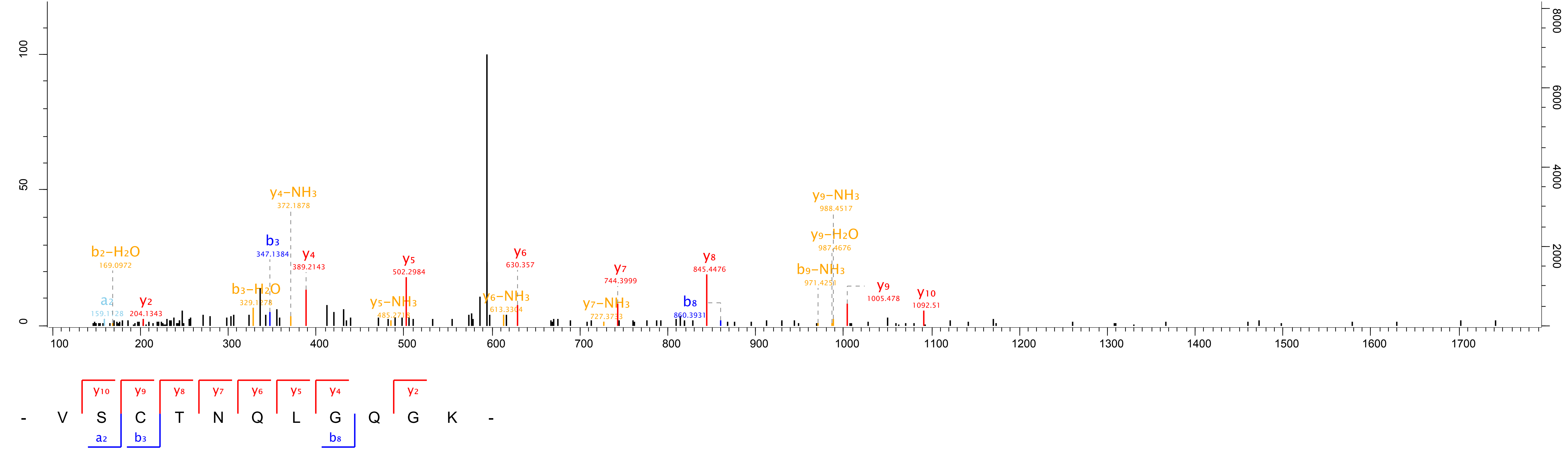

Raw file	Scan	Method	Score	m/z	Gene names
20150307_NSC3_Top_opt_F3_01_1691	10794	TOF; CID	48.04	440.23	Aes

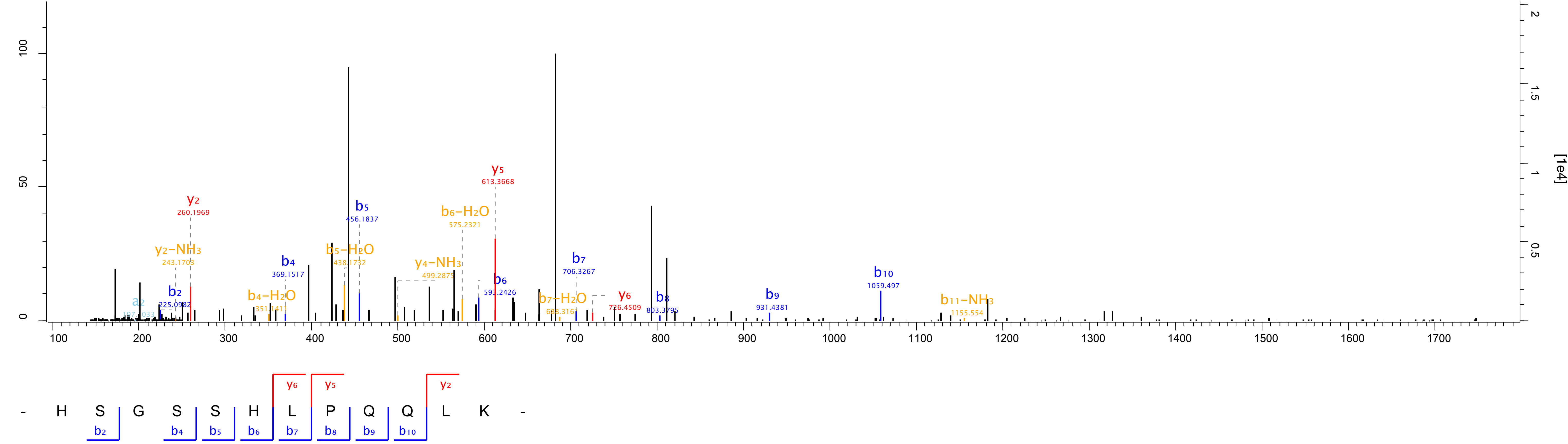

Raw file	Scan	Method	Score	m/z	Gene names
20150307_NSC3_Top_opt_F3_01_1691	11972	TOF; CID	71.35	685.83	Syngr3

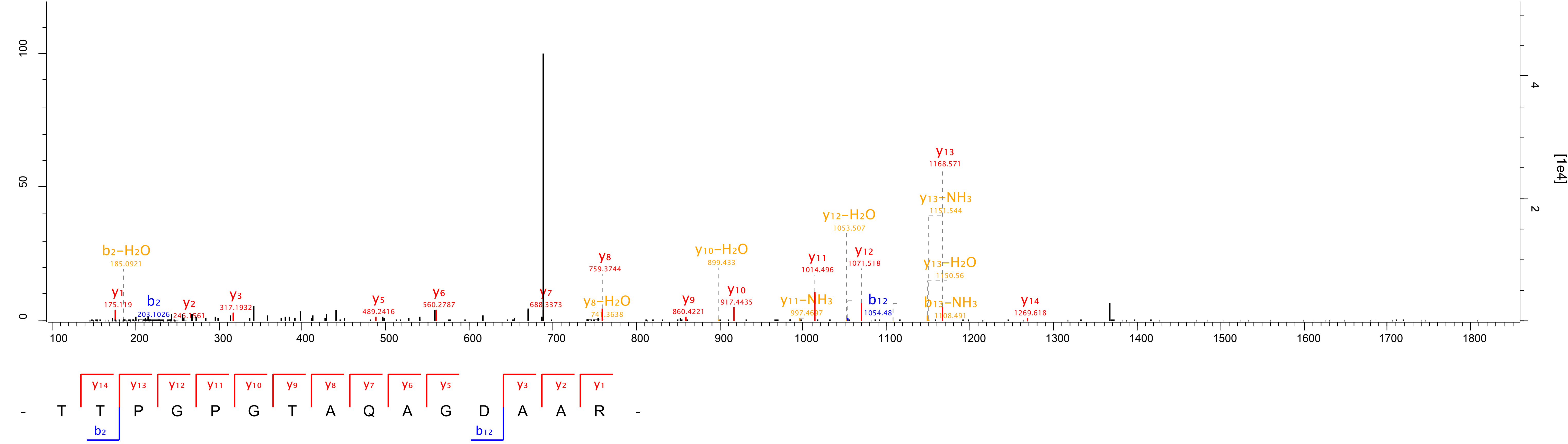

Raw file	Scan	Method	Score	m/z	Gene names
20150307_NSC3_Top_opt_F3_01_1691	14993	TOF; CID	51.09	634.31	Nek3

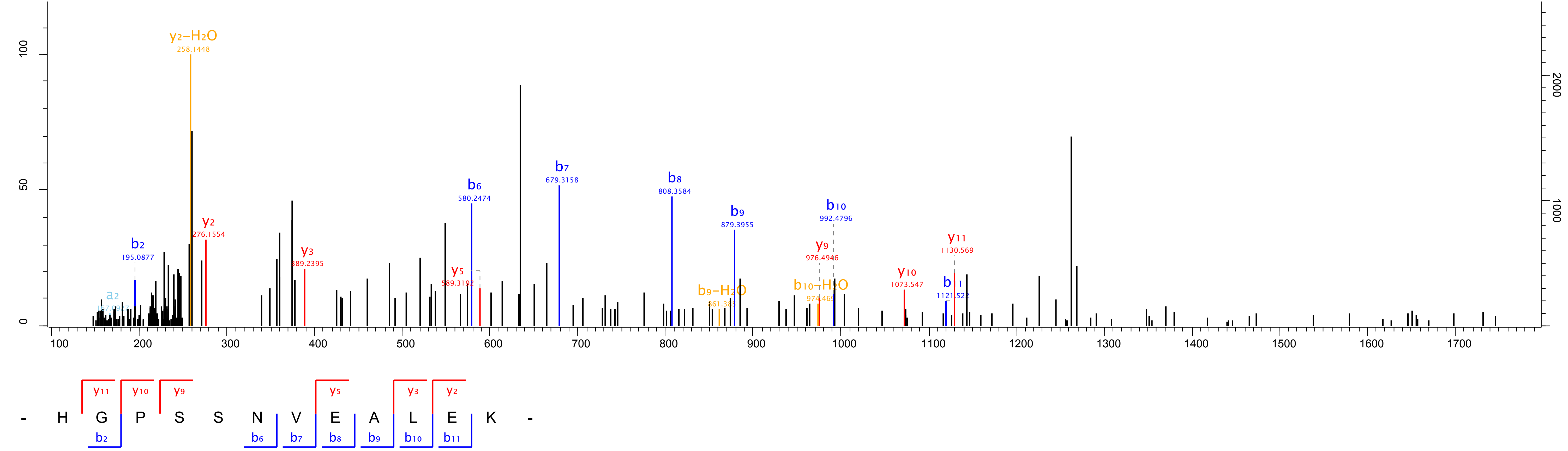

Raw file	Scan	Method	Score	m/z	Gene names
20150307_NSC3_Top_opt_F3_01_1691	21400	TOF; CID	110.57	794.4	Dtd2

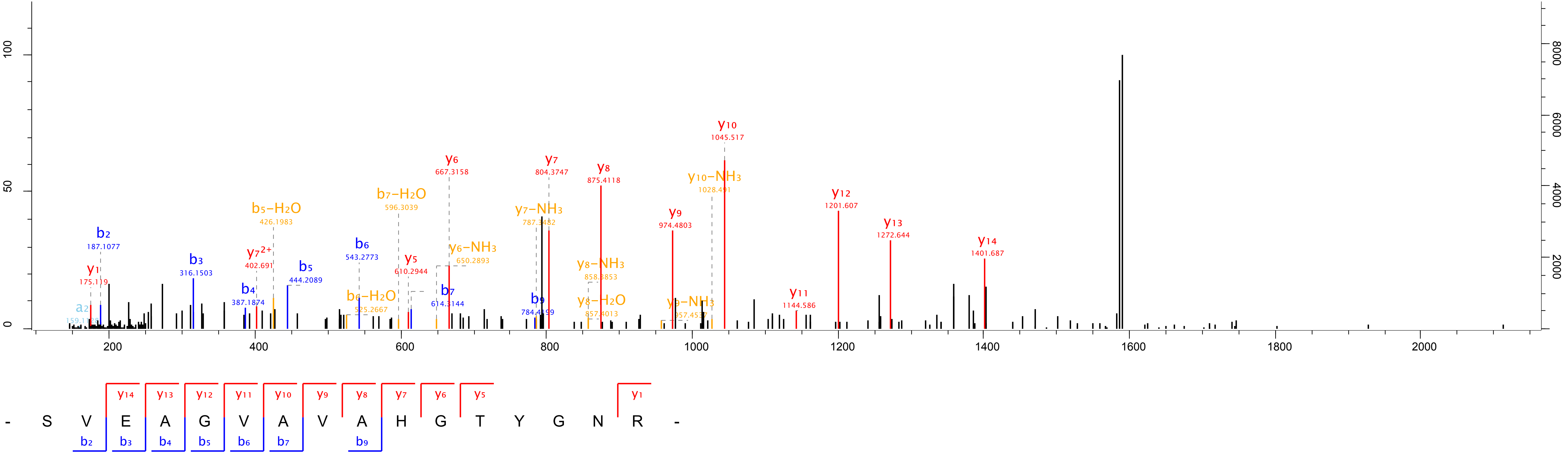

Raw file	Scan	Method	Score	m/z	Gene names
20150307_NSC3_Top_opt_F3_01_1691	25670	TOF; CID	54.51	675.34	Zgpat

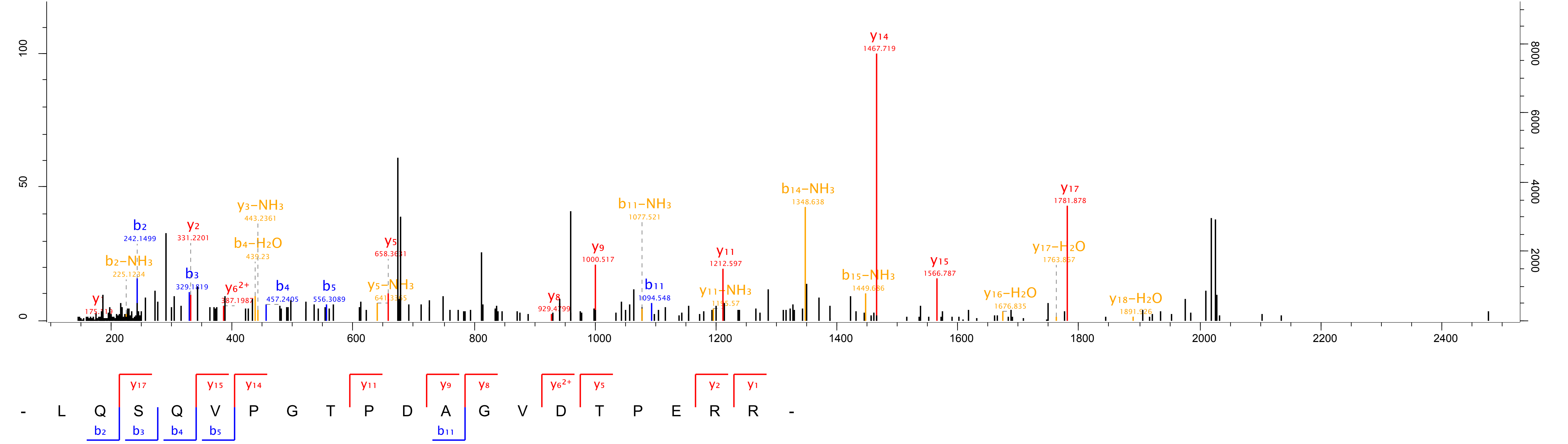

Raw file	Scan	Method	Score	m/z	Gene names
20150307_NSC3_Top_opt_F3_01_1691	40929	TOF; CID	47.09	685.32	Pcp4;Igsf5

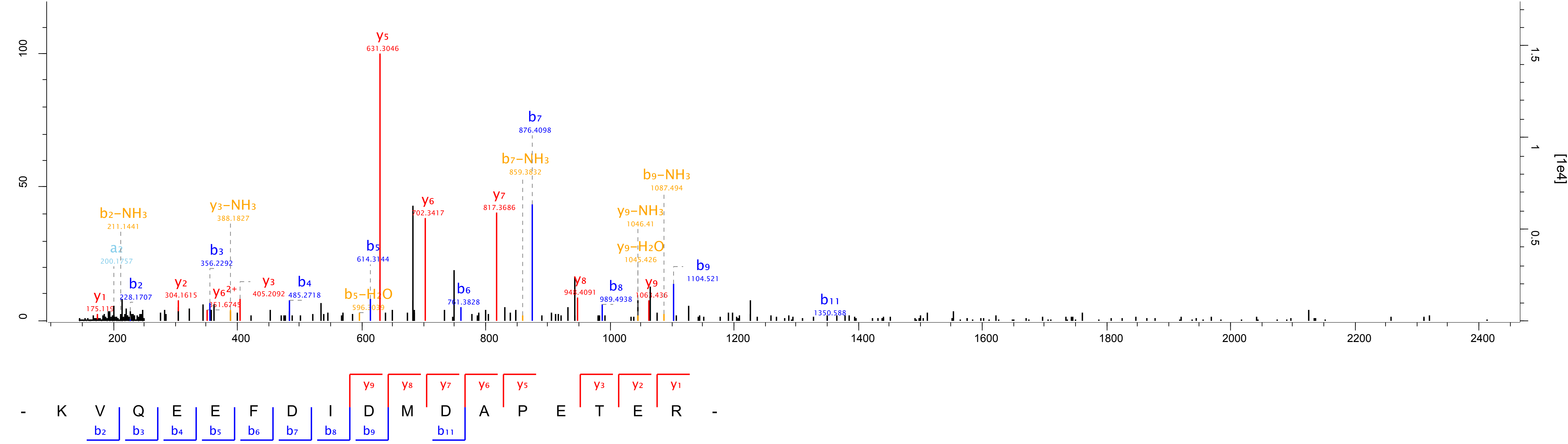

Raw file	Scan	Method	Score	m/z	Gene names
20150307_NSC3_Top_opt_F3_01_1691	47062	TOF; CID	45	921.98	Stab2

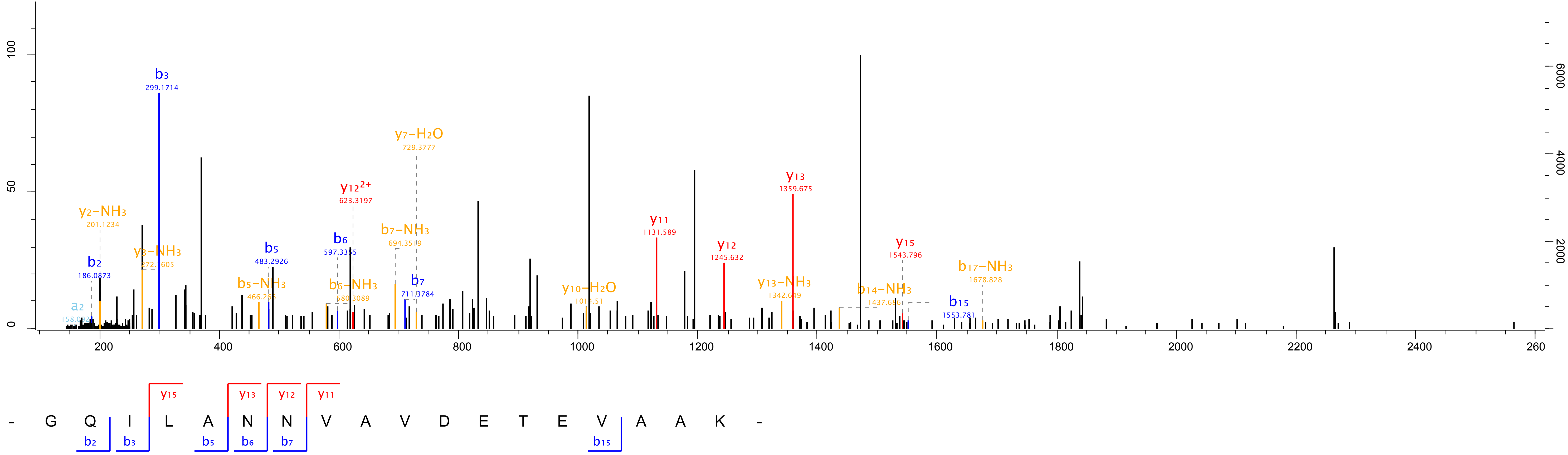

Raw file	Scan	Method	Score	m/z	Gene names
20150307_NSC3_Top_opt_F3_01_1691	48875	TOF; CID	117.79	700.85	Spcs1

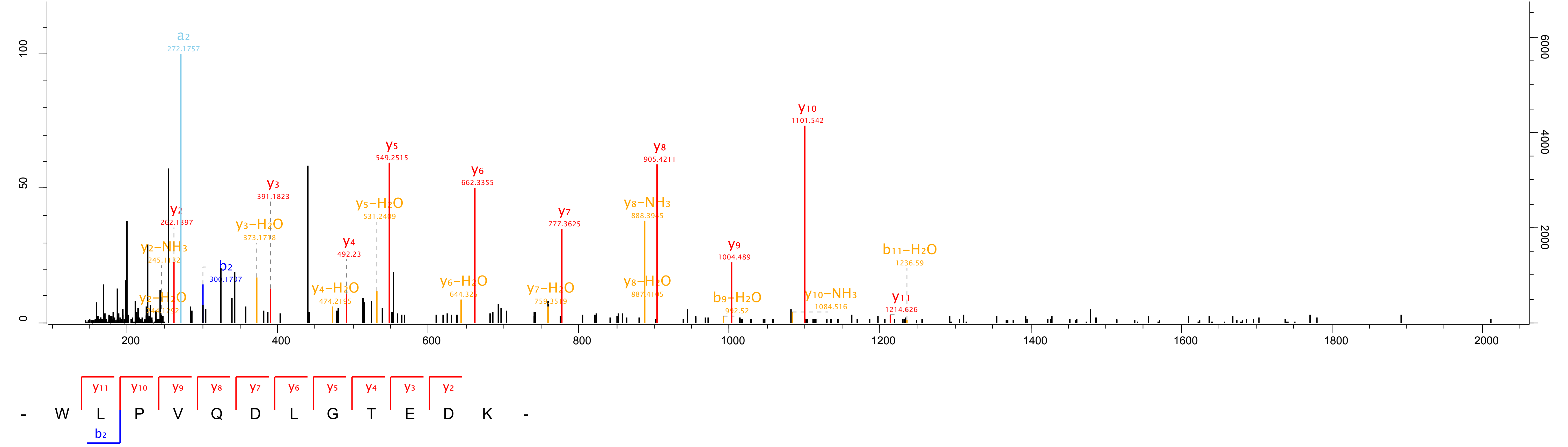

Raw file	Scan	Method	Score	m/z	Gene names
20150307_NSC3_Top_opt_F3_01_1691	56151	TOF; CID	65.18	652.36	Glrx

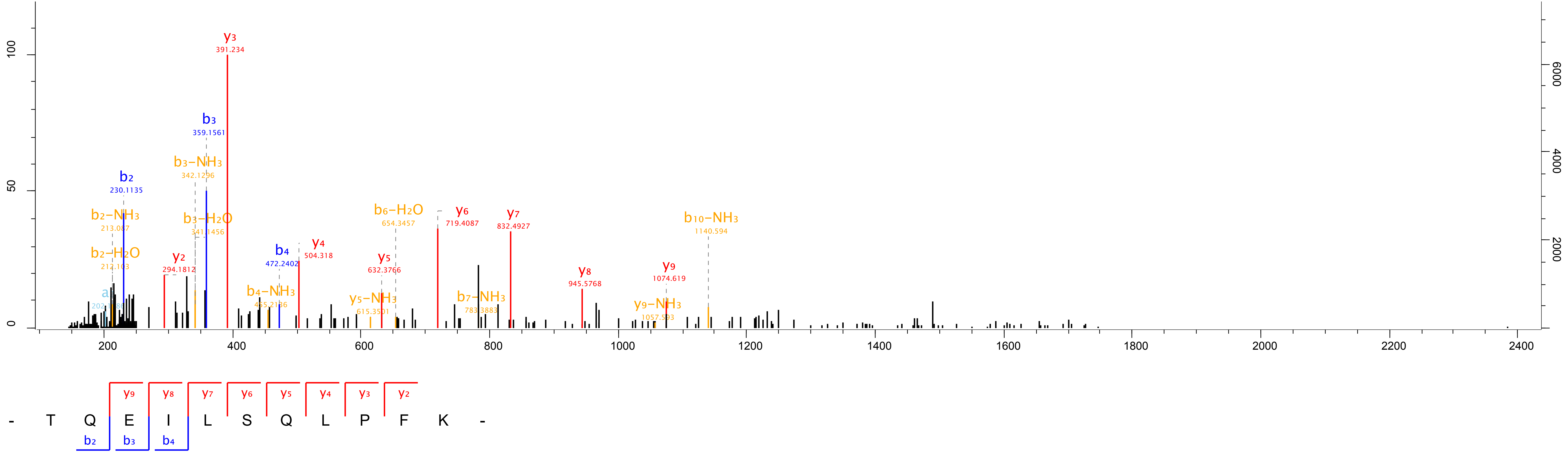

Raw file	Scan	Method	Score	m/z	Gene names
20150307_NSC3_Top_opt_F3_01_1691	60872	TOF; CID	58.69	662.7	Senp8

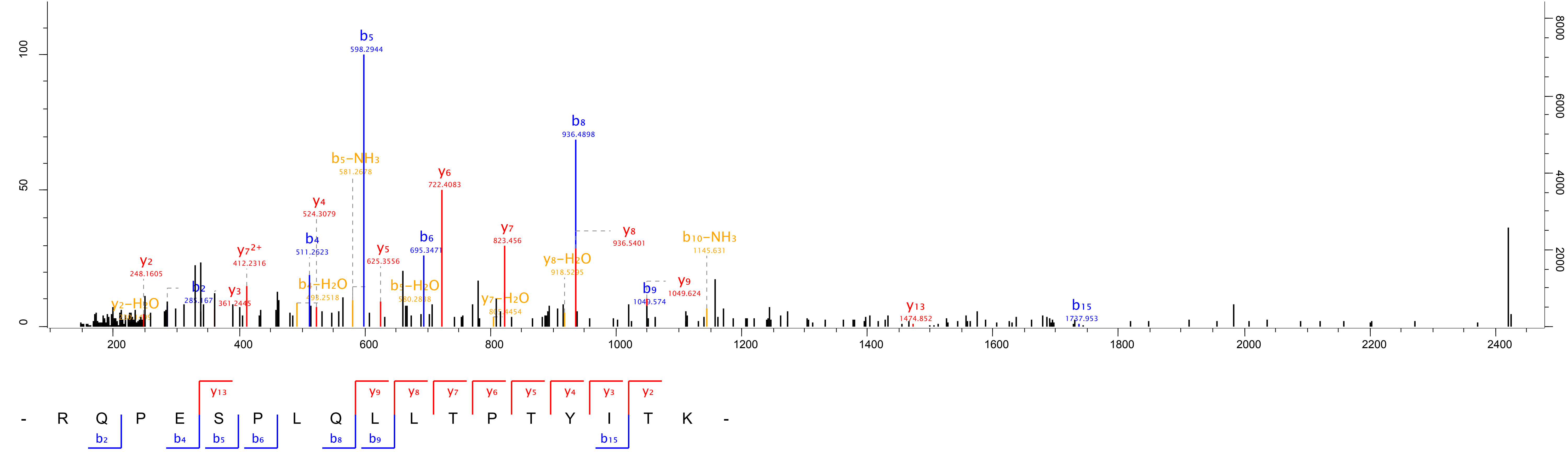

Raw file	Scan	Method	Score	m/z	Gene names
20150307_NSC3_Top_opt_F3_01_1691	60876	TOF; CID	29.67	1008.49	Blzf1

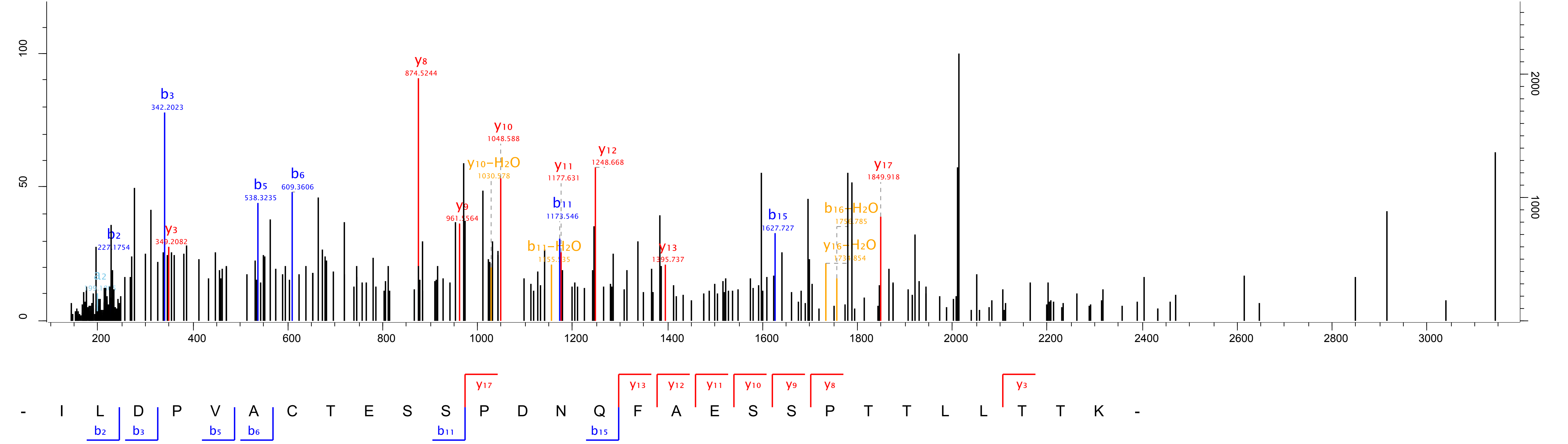

Raw file	Scan	Method	Score	m/z
20150307_NSC3_Top_opt_F3_01_1691	61194	TOF; CID	60.73	852.41

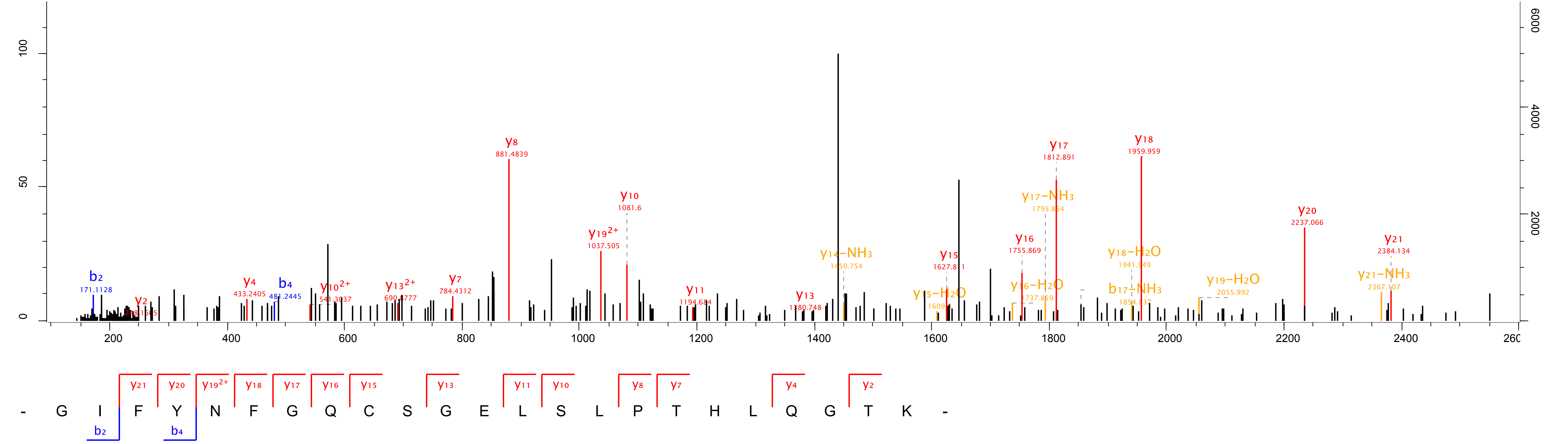

Raw file	Scan	Method	Score	m/z	Gene names
20150307_NSC3_Top_opt_F3_01_1691	62835	TOF; CID	73.92	923.45	Naaa

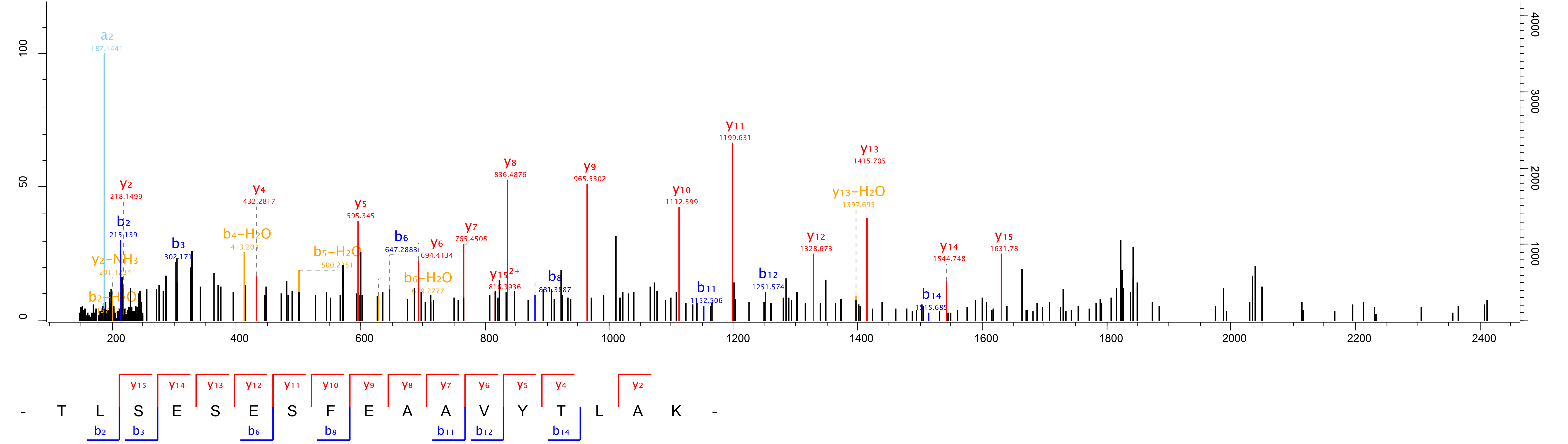

Raw file	Scan	Method	Score	m/z	Gene names
20150307_NSC3_Top_opt_F3_01_1691	63973	TOF; CID	47.59	1121.9	Zeb1

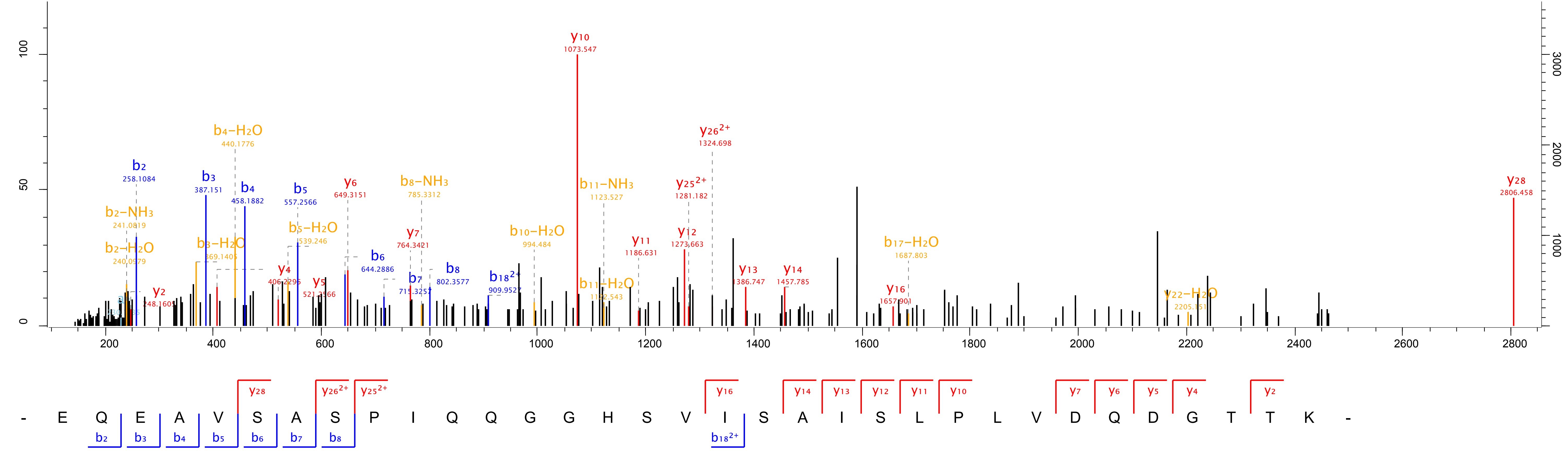

Raw file	Scan	Method	Score	m/z	Gene names
20150307_NSC3_Top_opt_F3_01_1691	65141	TOF; CID	51.09	961.48	Dus4l

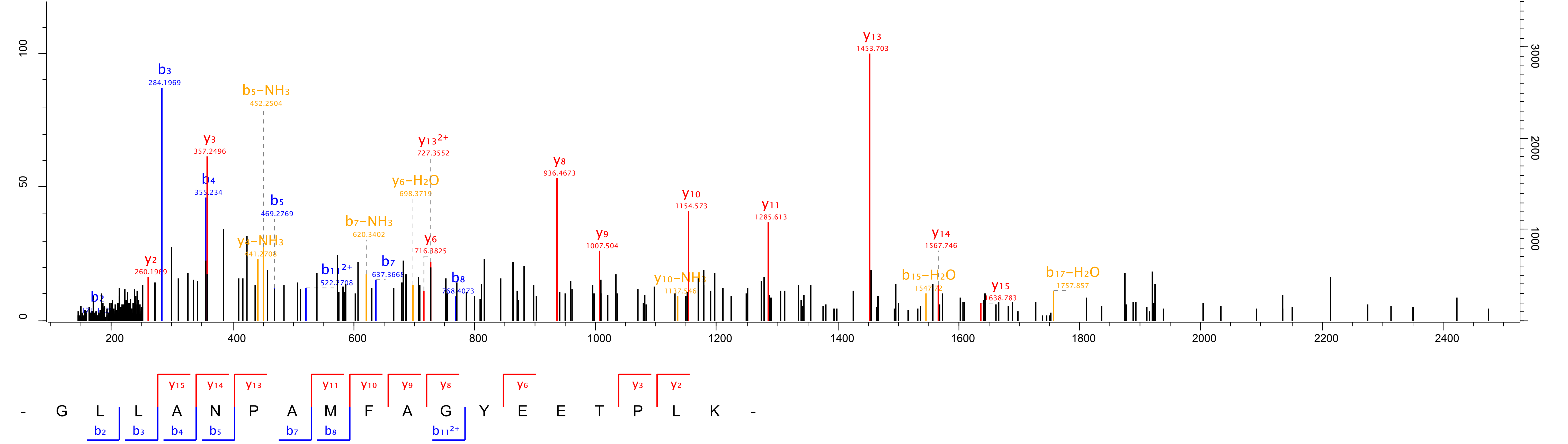

Raw file	Scan	Method	Score	m/z	Gene names
20150307_NSC3_Top_opt_F3_01_1691	65496	TOF; CID	90.91	559.33	Trim23

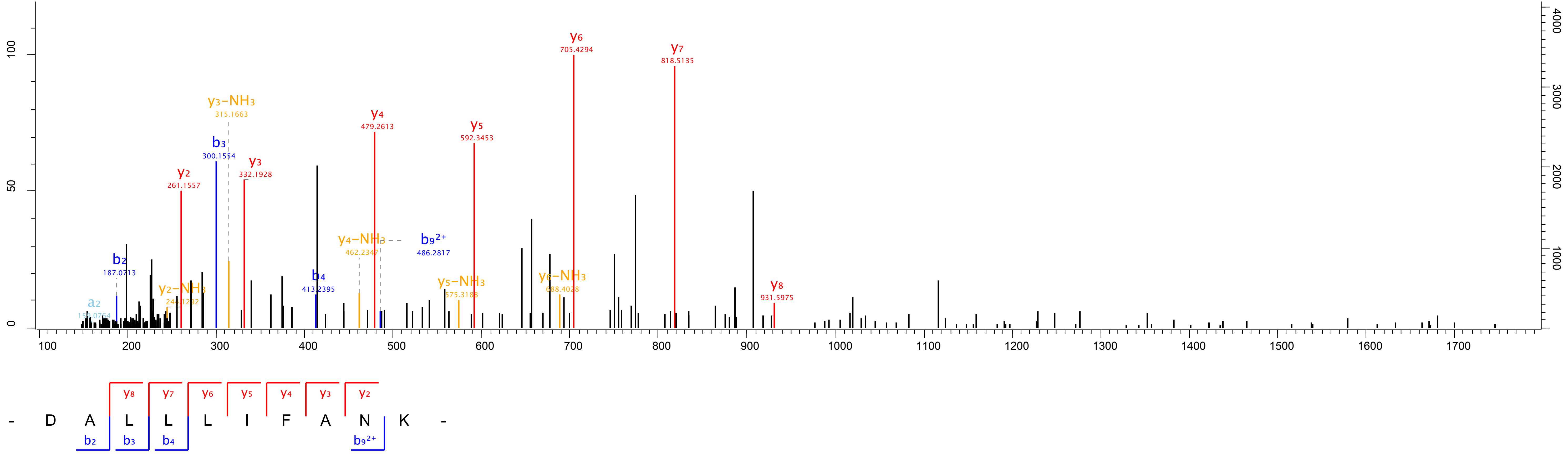

Raw file	Scan	Method	Score	m/z	Gene names
20150307_NSC3_Top_opt_F3_01_1691	67746	TOF; CID	53.03	958.03	Cbr4

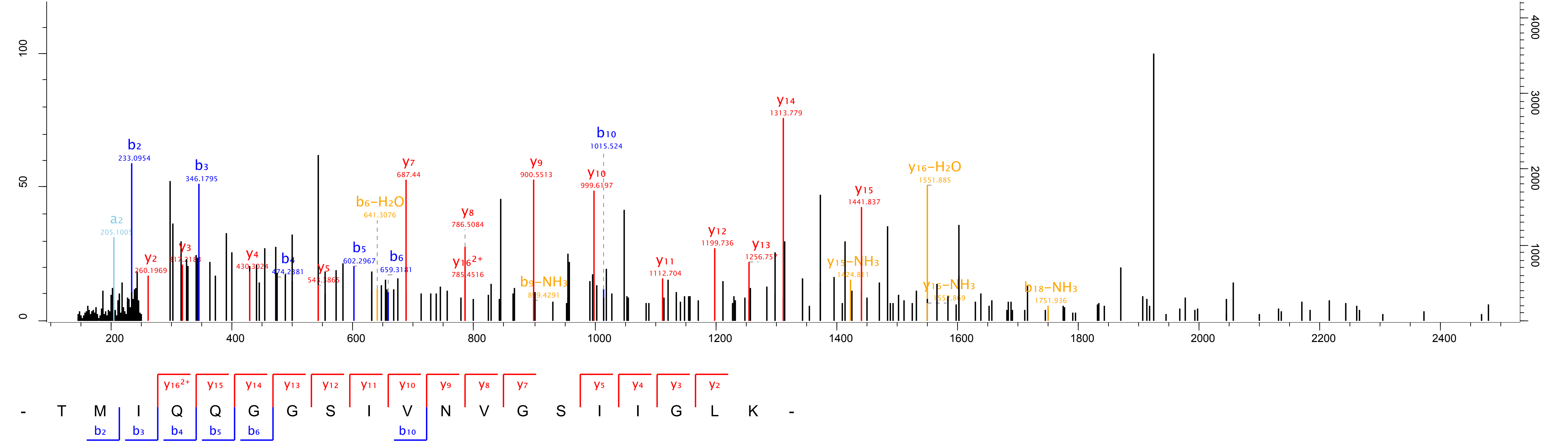

Raw file	Scan	Method	Score	m/z	Gene names
20150307_NSC3_Top_opt_F3_01_1691	67877	TOF; CID	53.89	1064.49	Agtrap

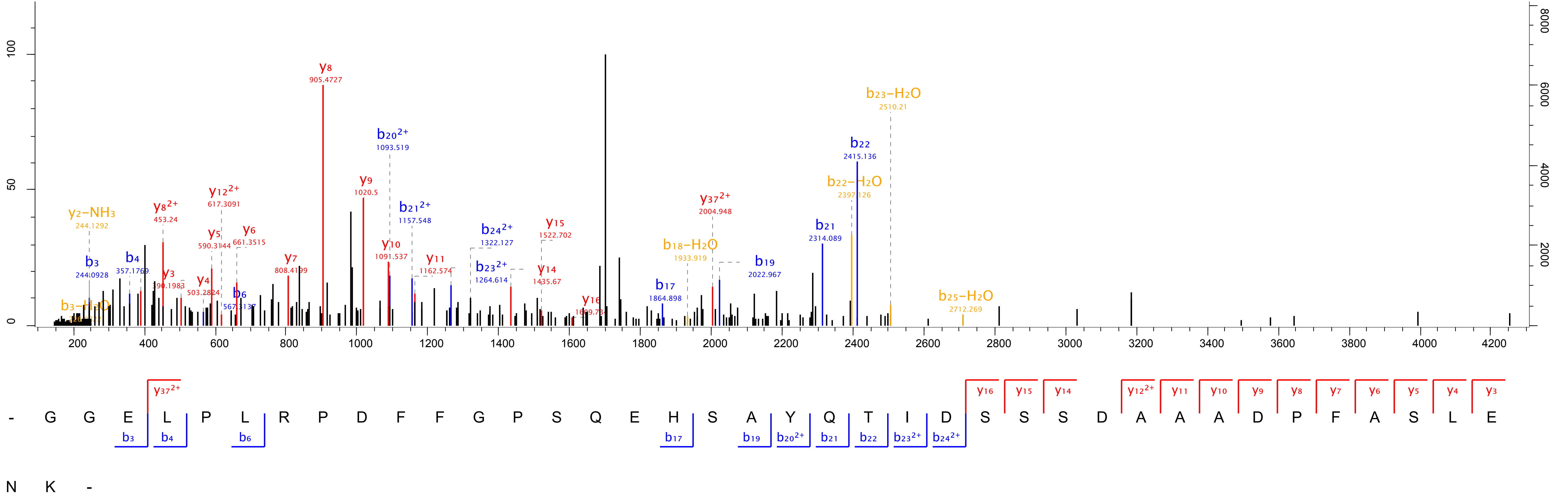

Raw file	Scan	Method	Score	m/z	Gene names
20150307_NSC3_Top_opt_F3_01_1691	70975	TOF; CID	52.46	743.4	Mzt2

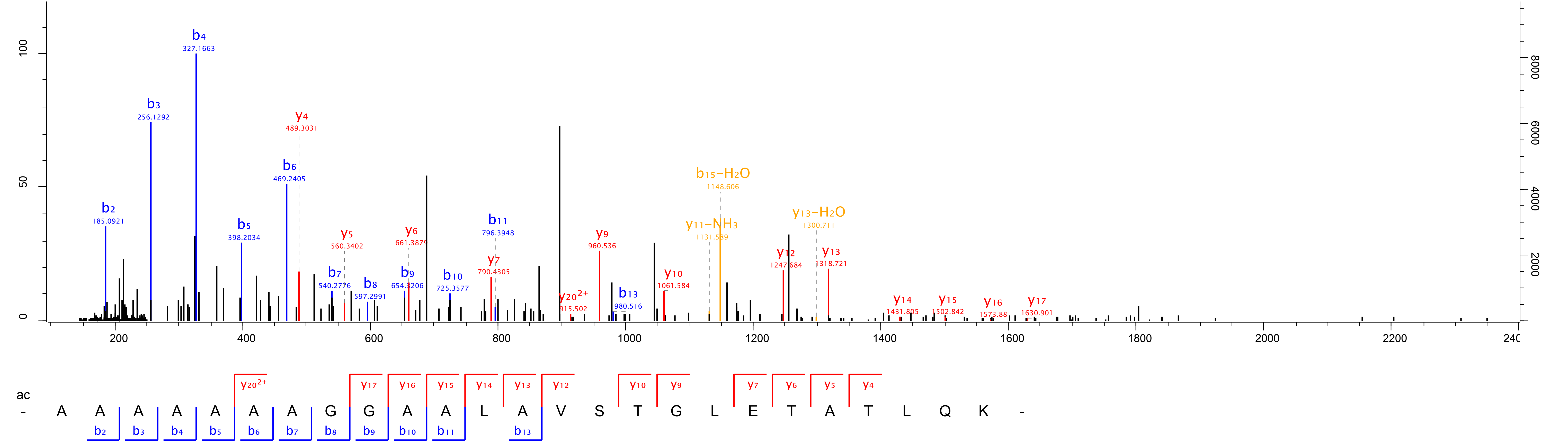

Raw file	Scan	Method	Score	m/z	Gene names
20150307_NSC3_Top_opt_F3_01_1691	71540	TOF; CID	183.7	989.49	G6pc3

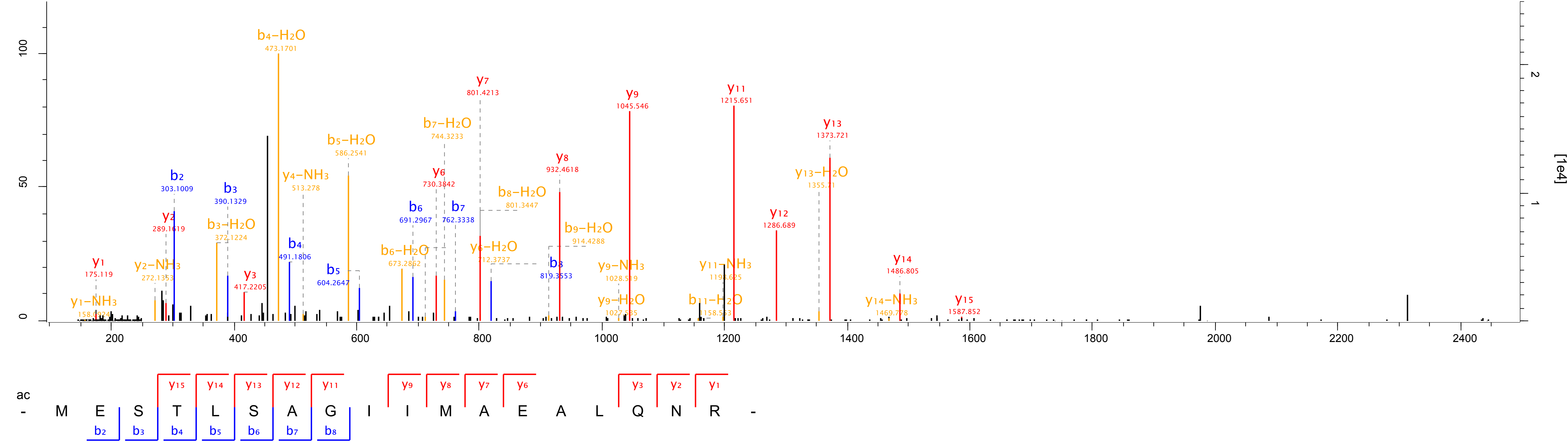

Supplement: Supplemental Data [file supp_M114.047407_mcp.M114.047407-9.pdf]
