# Supplementary material for: The Impact II, a Very High-Resolution Quadrupole Time-of-Flight Instrument (QTOF) for Deep Shotgun Proteomics
Source: Mol Cell Proteomics. 2015 May 19;14(7):2014–29. doi: 10.1074/mcp.M114.047407 (PMC4587313; doi:10.1074/mcp.M114.047407)

Raw file	Scan	Method	Score	m/z	Gene names
20140918_fract1_dyn_5ul_D1_01_367	5371	TOF; CID	131.06	330.19	HMGN4

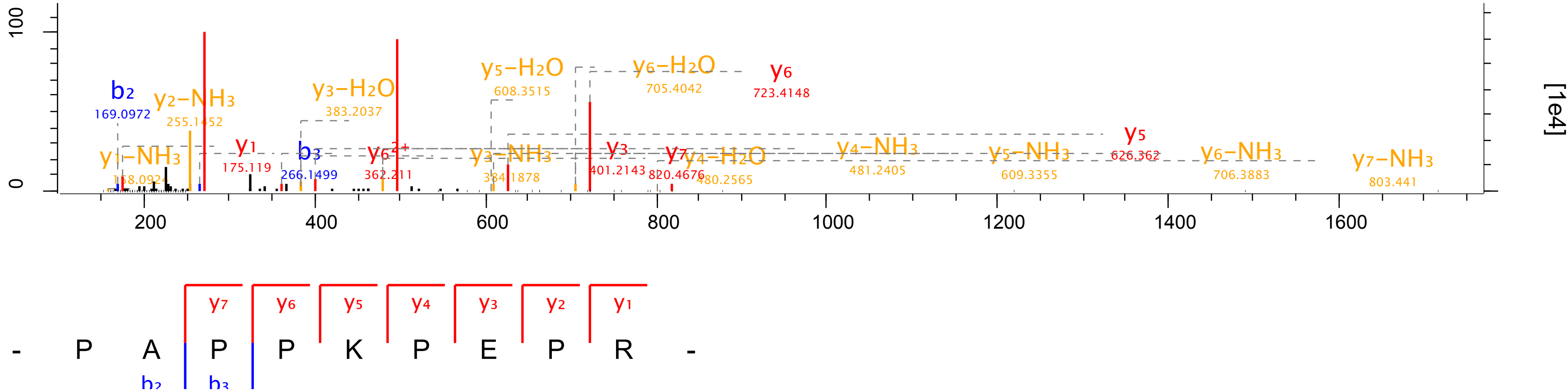

Raw file	Scan	Method	Score	m/z	Gene names
20140918_fract1_dyn_5ul_D1_01_367	11637	TOF; CID	120.9	393.73	BBC3

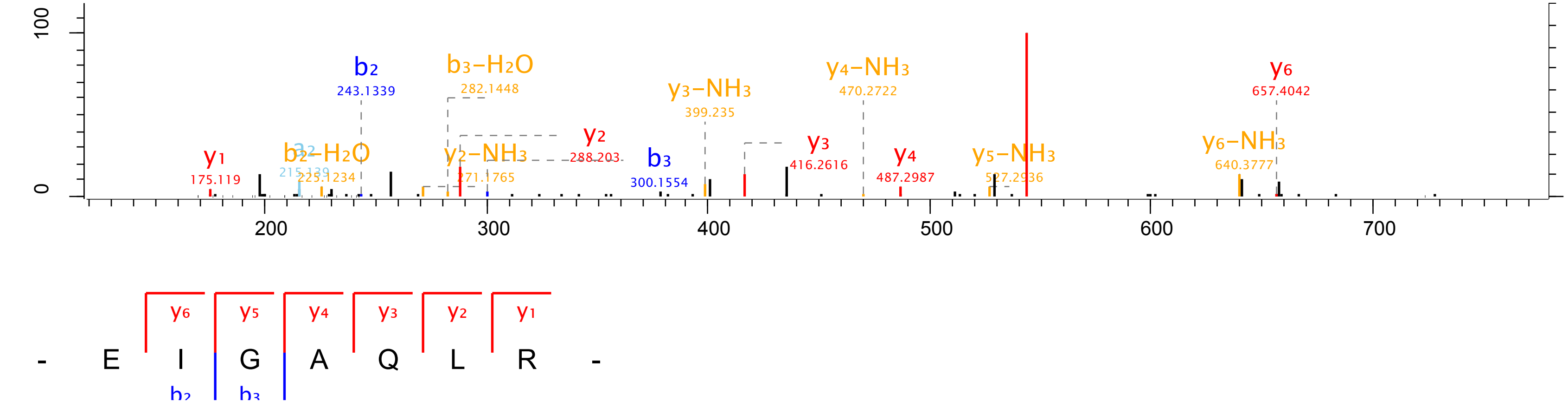

Raw file	Scan	Method	Score	m/z	Gene names
20140918_fract1_dyn_5ul_D1_01_367	12432	TOF; CID	125.75	652.32	USH1C

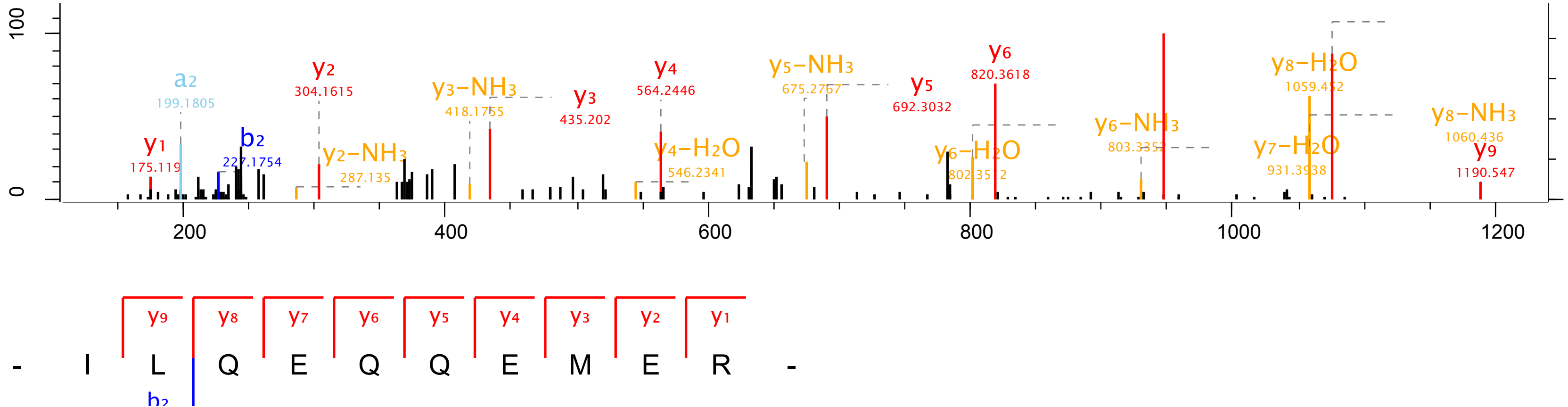

Raw file	Scan	Method	Score	m/z	Gene names
20140918_fract1_dyn_5ul_D1_01_367	13249	TOF; CID	155.84	552.78	VAMP8

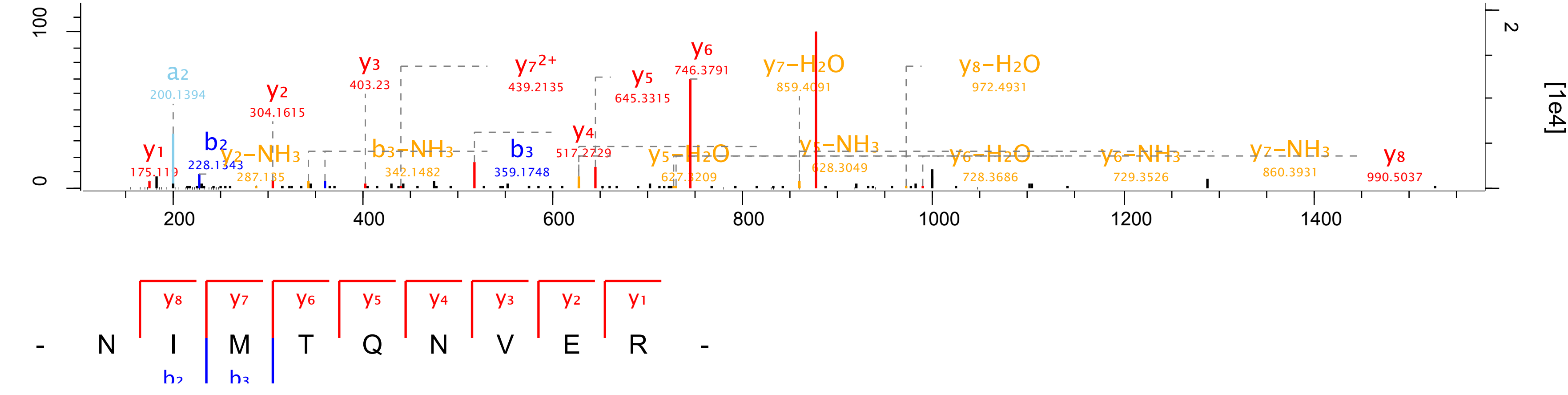

Raw file	Scan	Method	Score	m/z	Gene names
20140918_fract1_dyn_5ul_D1_01_367	14036	TOF; CID	87.78	599.84	SDC4

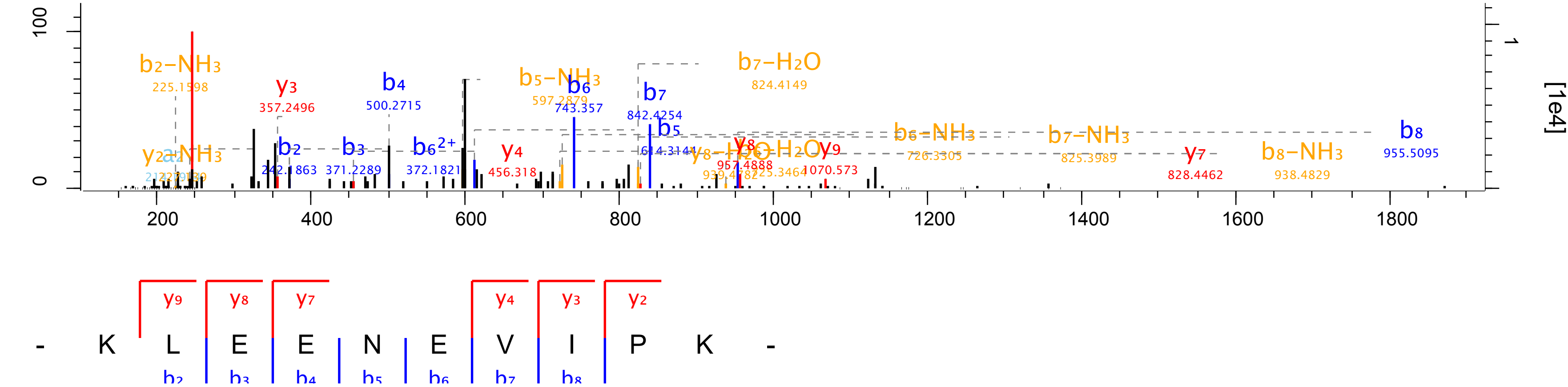

Raw file	Scan	Method	Score	m/z	Gene names
20140918_fract1_dyn_5ul_D1_01_367	14149	TOF; CID	91.31	538.29	PTMS

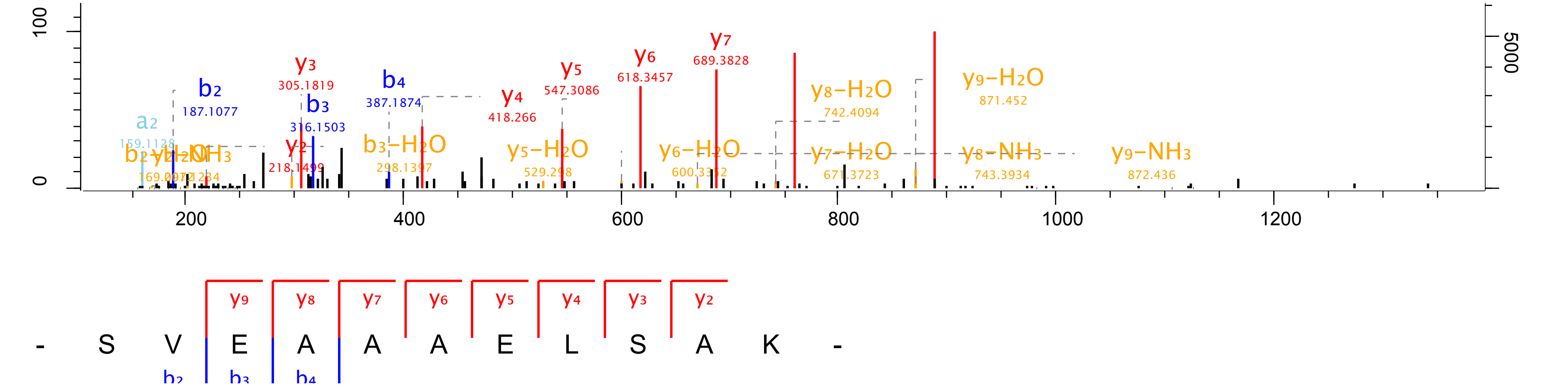

Raw file	Scan	Method	Score	m/z	Gene names
20140918_fract1_dyn_5ul_D1_01_367	17331	TOF; CID	67.9	673.33	NEK3

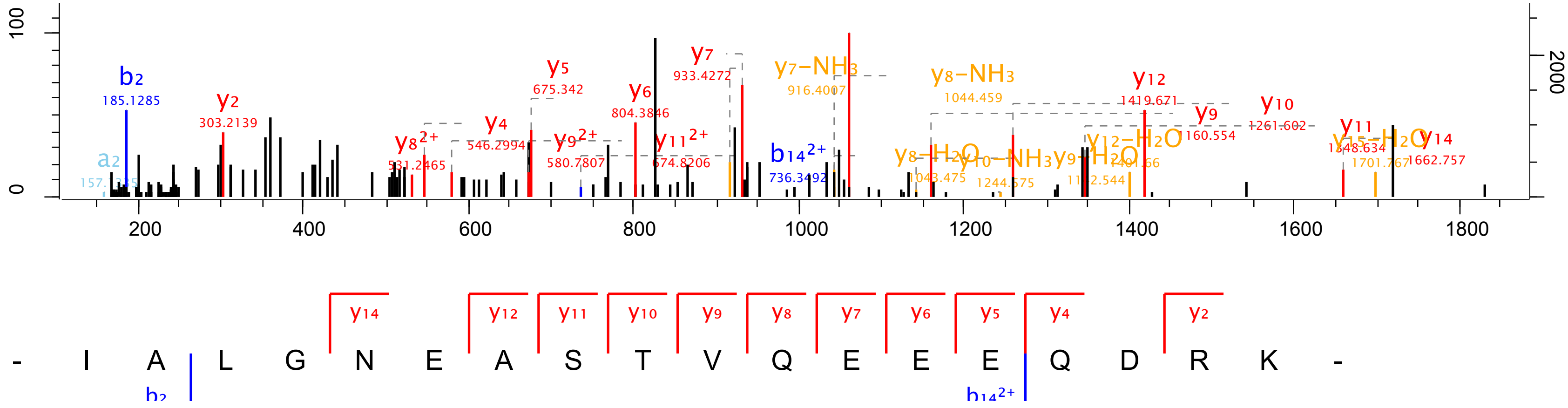

Raw file	Scan	Method	Score	m/z	Gene names
20140918_fract1_dyn_5ul_D1_01_367	18973	TOF; CID	83.75	312.54	ZNF668

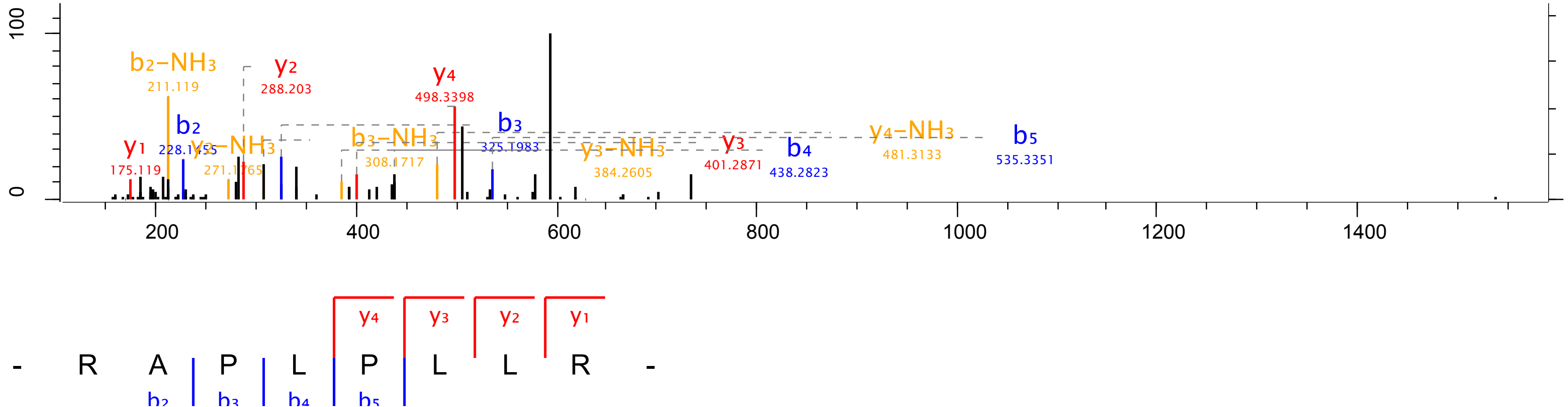

Raw file	Scan	Method	Score	m/z	Gene names
20140918_fract1_dyn_5ul_D1_01_367	19764	TOF; CID	58.71	576.26	GCA

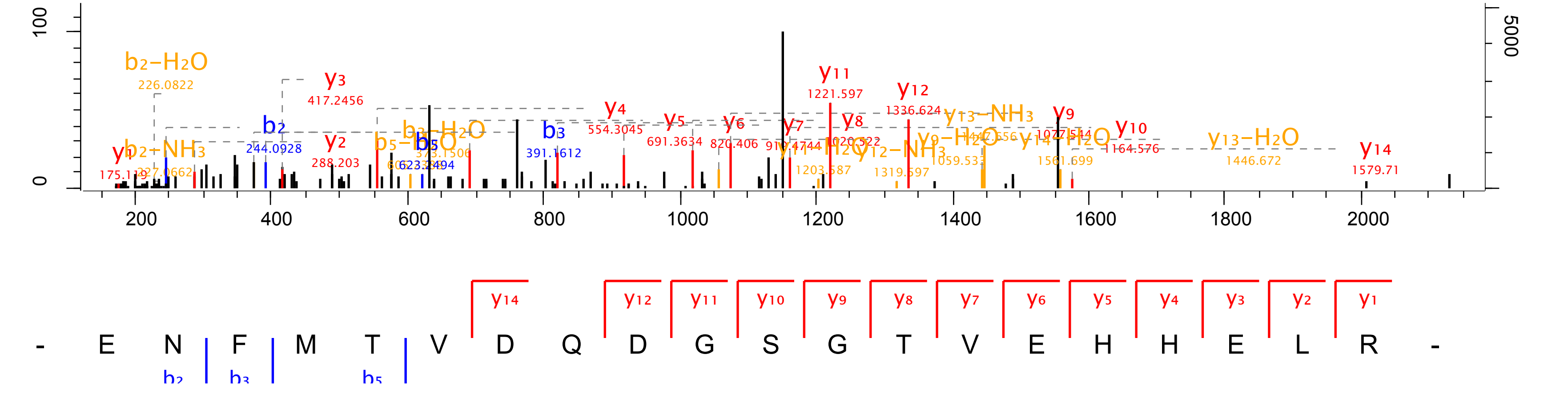

Raw file	Scan	Method	Score	m/z	Gene names
20140918_fract1_dyn_5ul_D1_01_367	22779	TOF; CID	104.01	527.77	S100A3

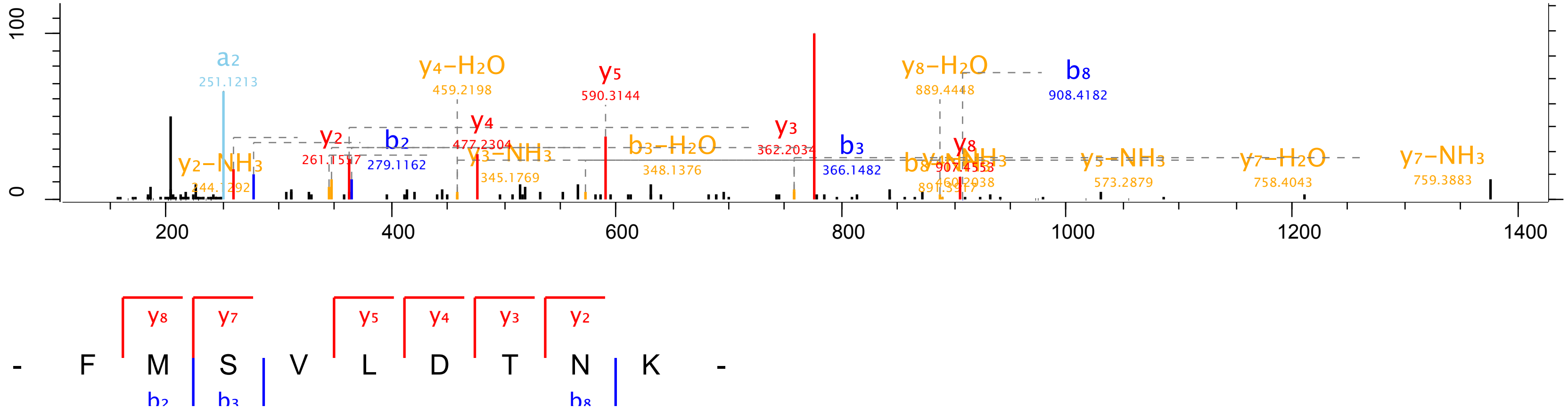

Raw file	Scan	Method	Score	m/z	Gene names
20140918_fract1_dyn_5ul_D1_01_367	25911	TOF; CID	106.4	597.31	FBXL17

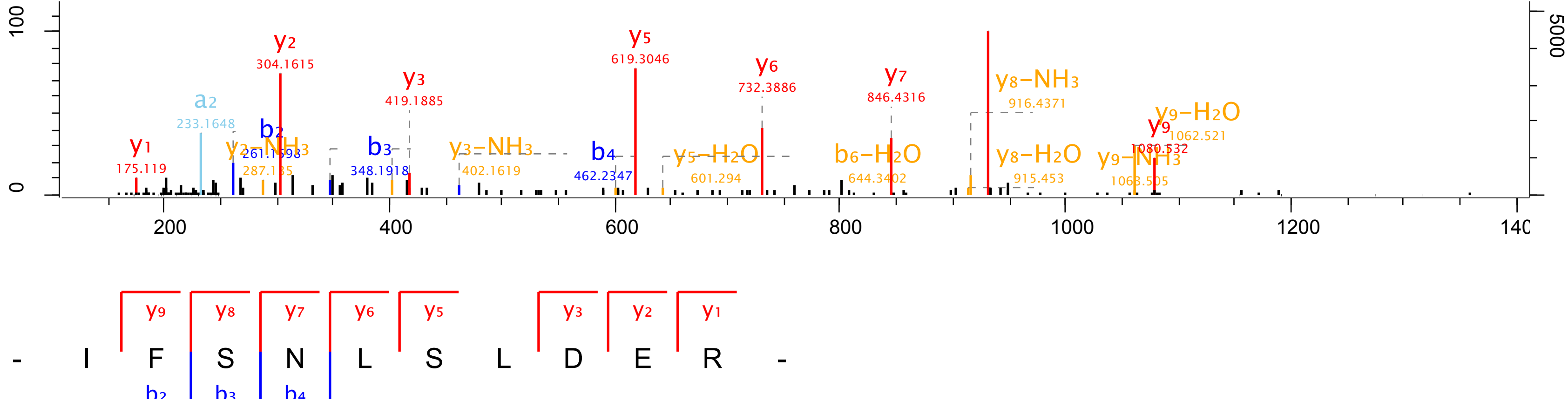

Raw file	Scan	Method	Score	m/z	Gene names
20140918_fract1_dyn_5ul_D1_01_367	25930	TOF; CID	171.46	853.39	TMEM179B

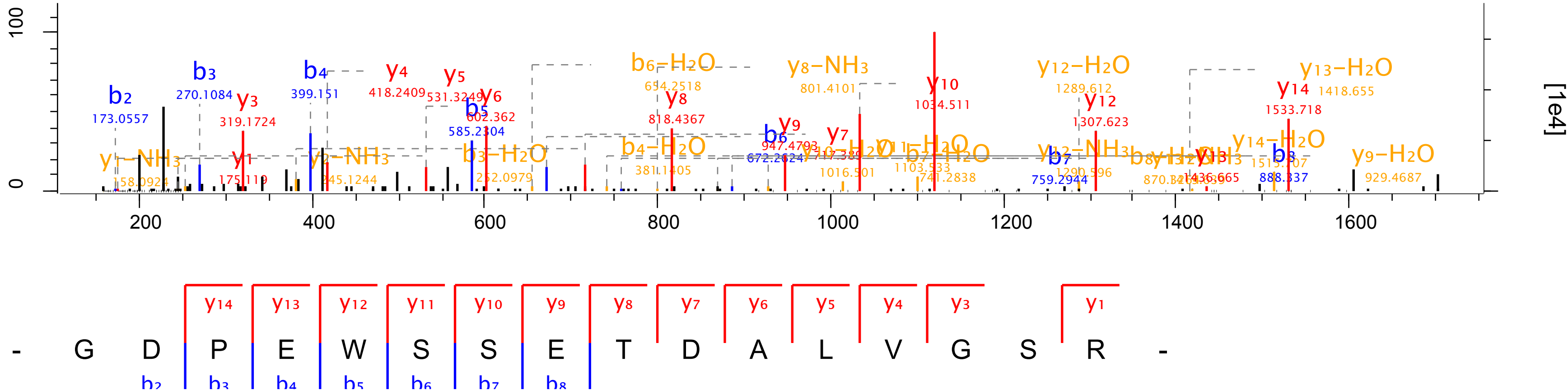

Raw file	Scan	Method	Score	m/z	Gene names
20140918_fract1_dyn_5ul_D1_01_367	27369	TOF; CID	68.49	556.82	HMGN5

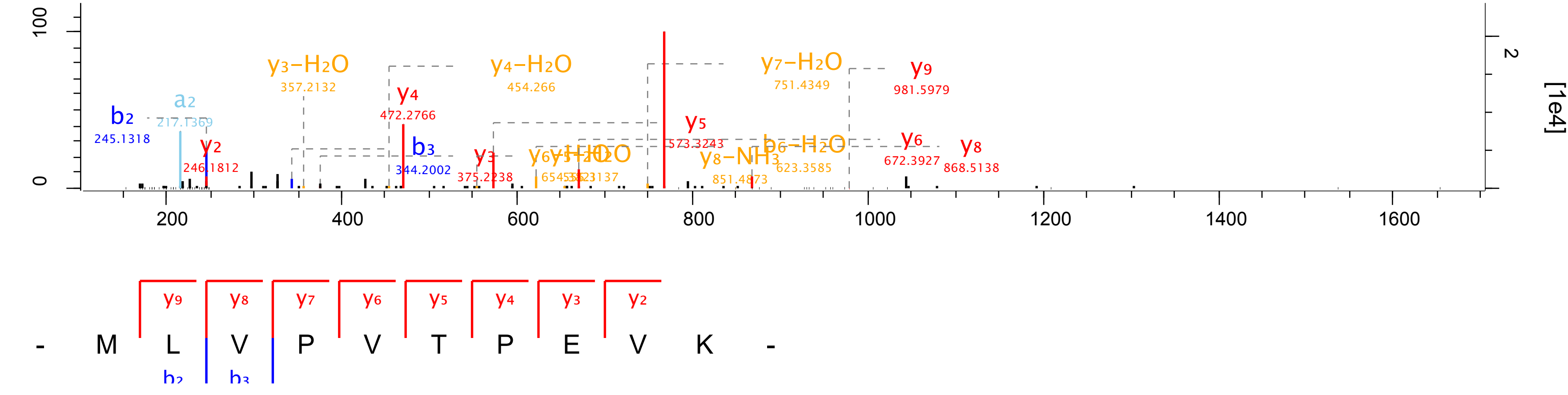

Raw file	Scan	Method	Score	m/z	Gene names
20140918_fract1_dyn_5ul_D1_01_367	27688	TOF; CID	115.78	640.34	METTL23

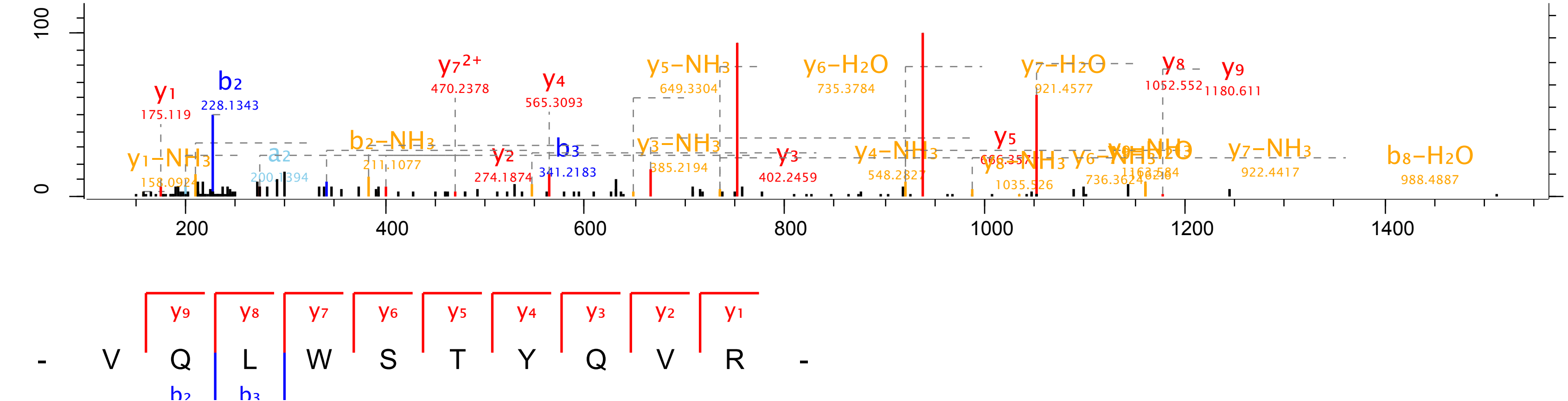

Raw file	Scan	Method	Score	m/z	Gene names
20140918_fract1_dyn_5ul_D1_01_367	28710	TOF; CID	56.57	567.32	NPHP3

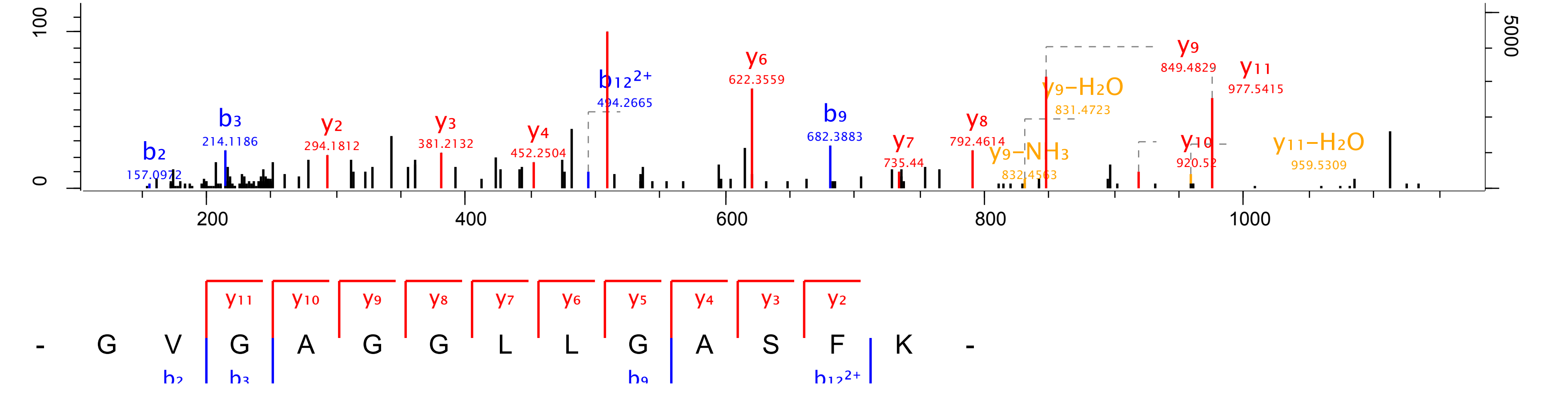

Raw file	Scan	Method	Score	m/z	Gene names
20140918_fract1_dyn_5ul_D1_01_367	29299	TOF; CID	95.27	469.25	CHAC1

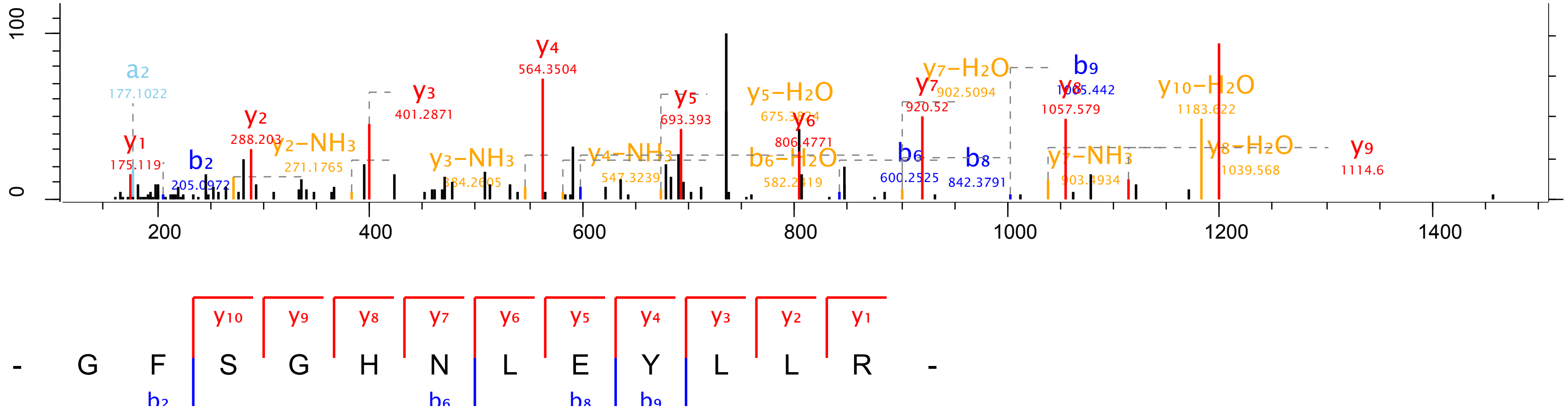

Raw file	Scan	Method	Score	m/z	Gene names
20140918_fract1_dyn_5ul_D1_01_367	30752	TOF; CID	106.68	552.31	CYB5D2

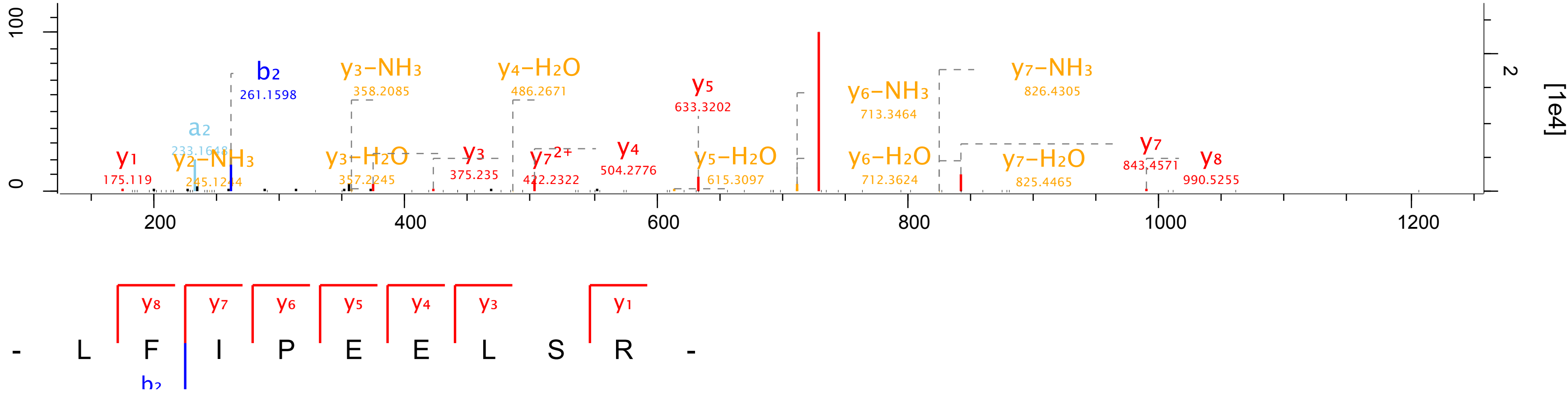

Raw file	Scan	Method	Score	m/z	Gene names
20140918_fract1_dyn_5ul_D1_01_367	31299	TOF; CID	54.67	998.52	GLYCTK

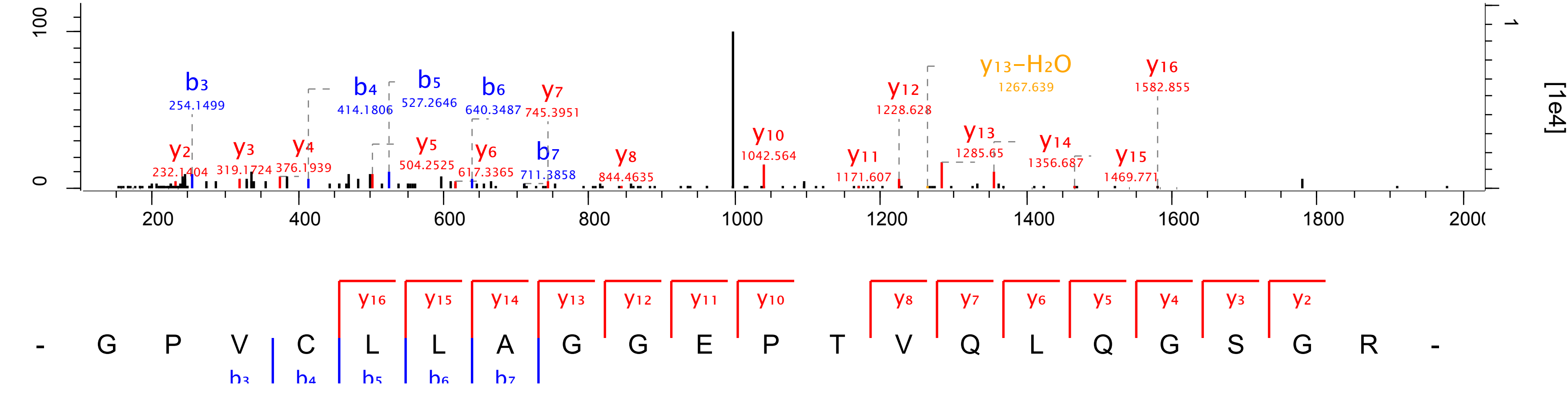

Raw file	Scan	Method	Score	m/z	Gene names
20140918_fract1_dyn_5ul_D1_01_367	31857	TOF; CID	119.65	872.47	BTF3L4

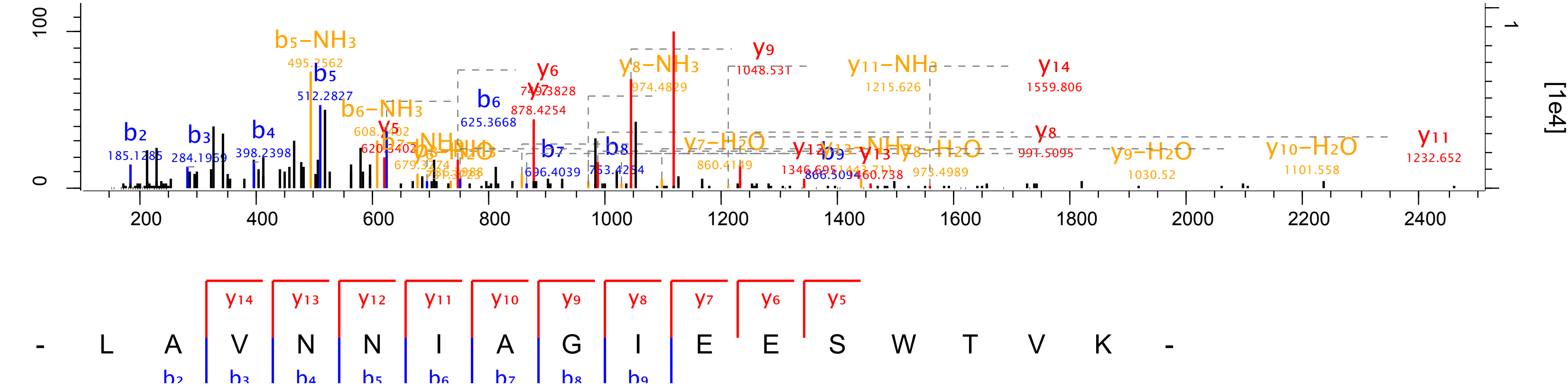

Raw file	Scan	Method	Score	m/z	Gene names
20140918_fract1_dyn_5ul_D1_01_367	31947	TOF; CID	52.81	1047	KMT2E

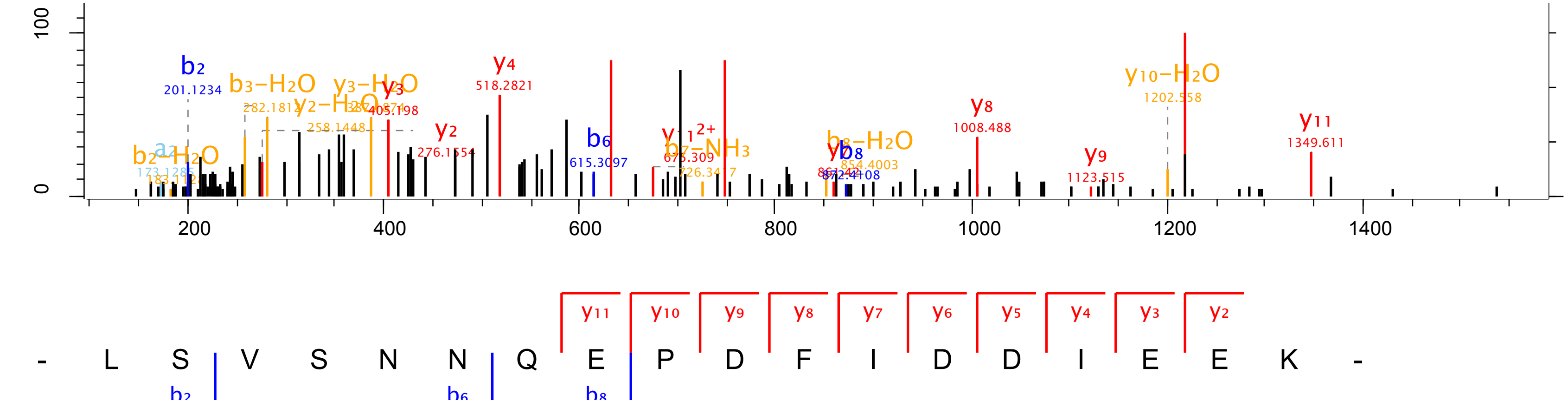

Raw file	Scan	Method	Score	m/z	Gene names
20140918_fract1_dyn_5ul_D1_01_367	33339	TOF; CID	47.56	772.74	PLLP

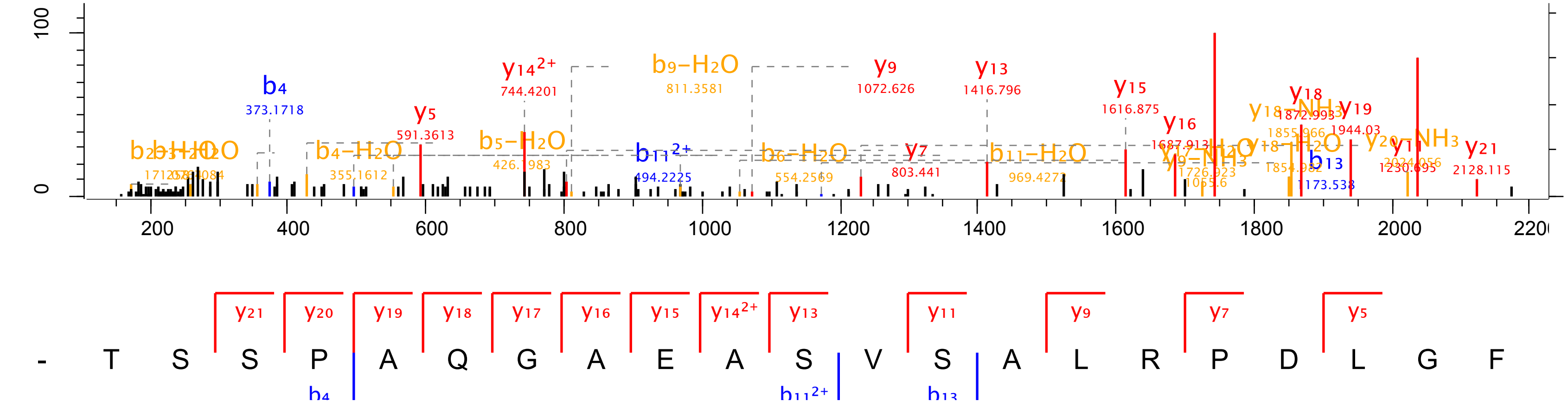

Raw file	Scan	Method	Score	m/z	Gene names
20140918_fract1_dyn_5ul_D1_01_367	38702	TOF; CID	92.27	613.65	TMEM234

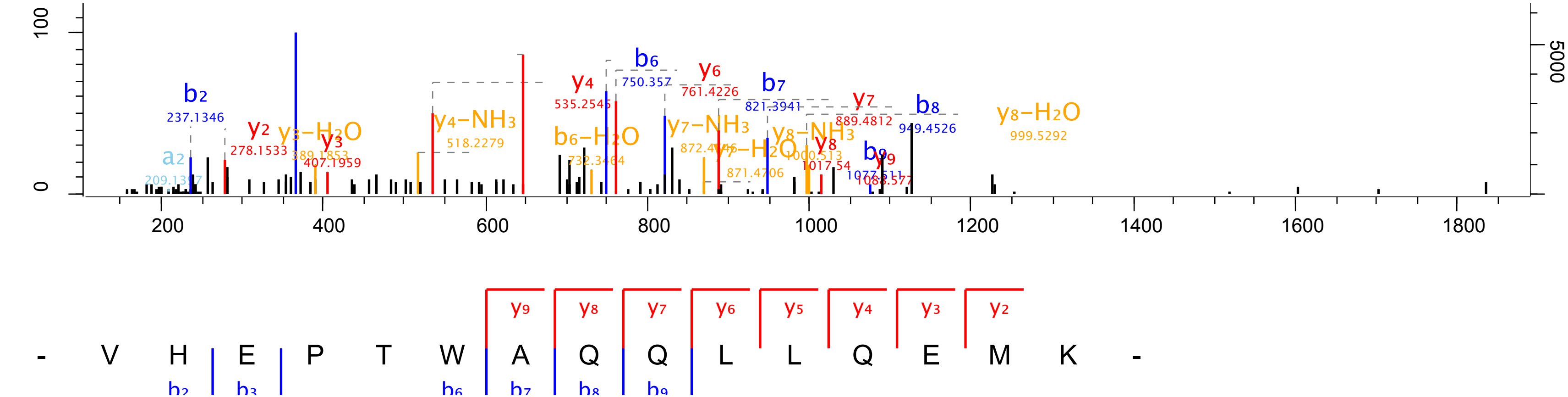

Raw file	Scan	Method	Score	m/z	Gene names
20140918_fract1_dyn_5ul_D1_01_367	38950	TOF; CID	98.06	1026.5	ZNF410

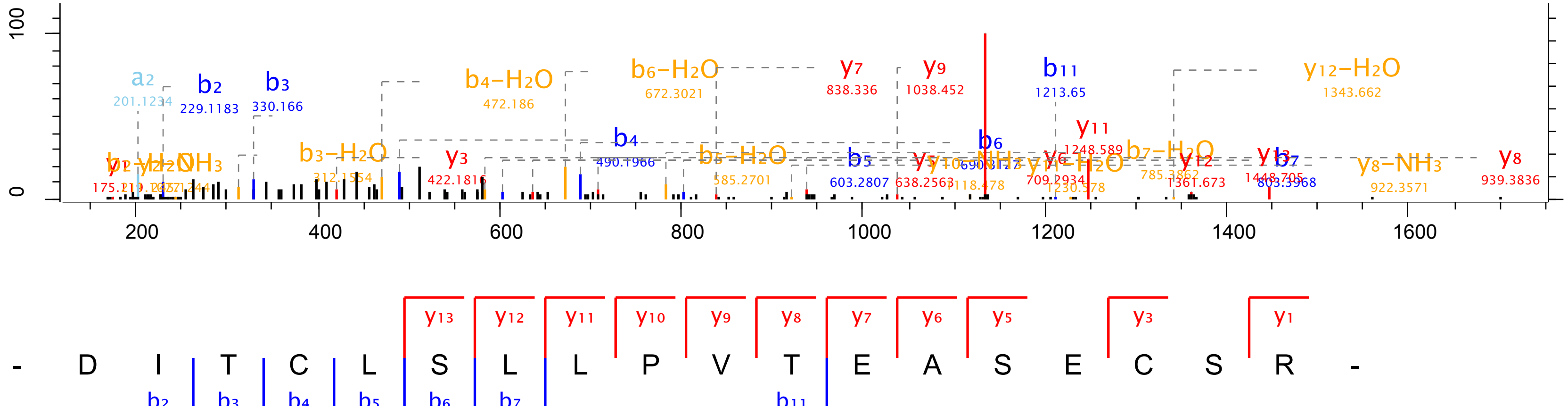

Raw file	Scan	Method	Score	m/z	Gene names
20140918_fract2_dyn_5ul_D2_01_369	5194	TOF; CID	123.99	349.2	E4F1

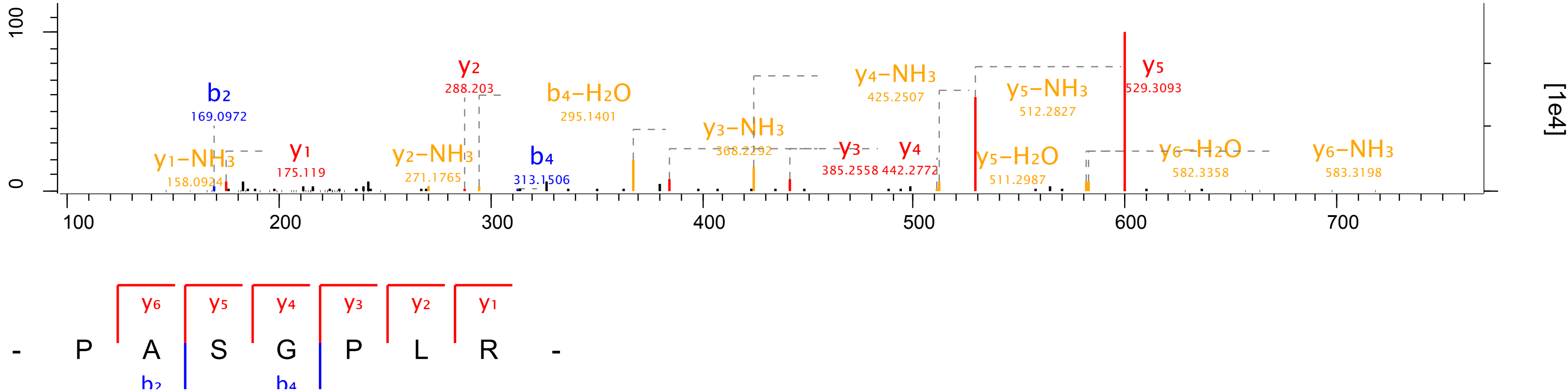

Raw file	Scan	Method	Score	m/z	Gene names
20140918_fract2_dyn_5ul_D2_01_369	7094	TOF; CID	56.53	615.32	CD164

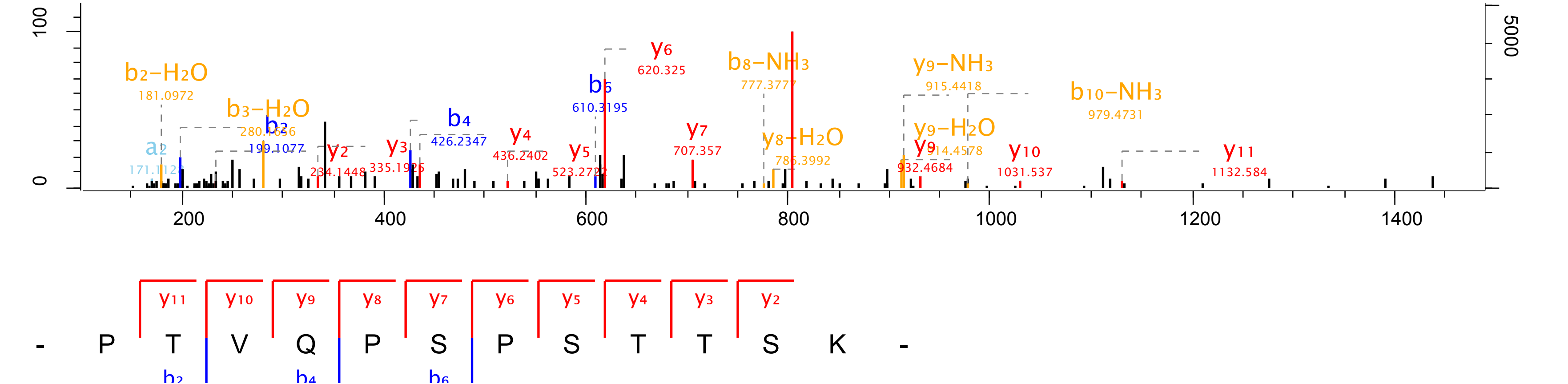

Raw file	Scan	Method	Score	m/z	Gene names
20140918_fract2_dyn_5ul_D2_01_369	8659	TOF; CID	60.17	478.76	EPM2A

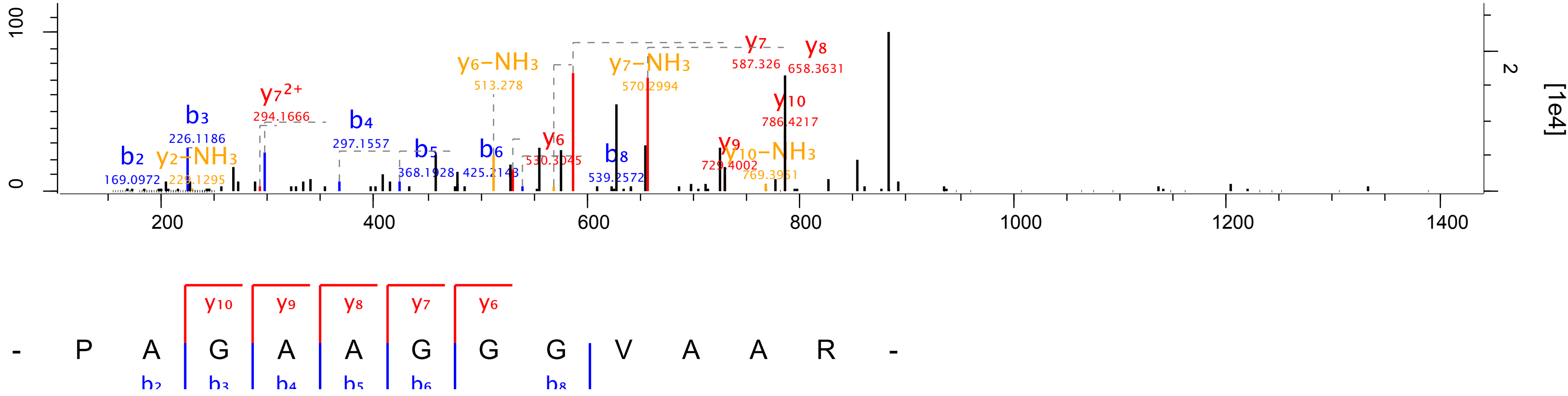

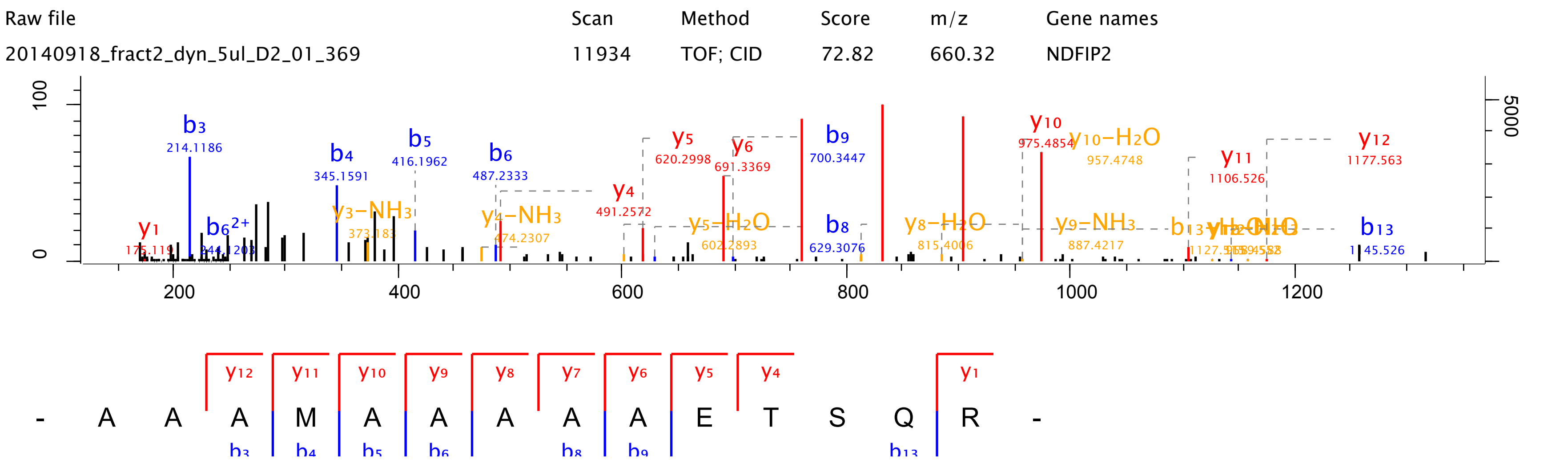

Raw file
20140918_fract2_dyn_5ul_D2_01_369
Scan
11934
Method
TOF; CID
Score
72.82
m/z
660.32
Gene names
NDFIP2
100
0
5000
y1 175.119
b3 214.1186
b6 2+ 244.1203
b4 345.1591
y3−NH3 373.183
b5 416.1962
y4−NH3 474.2307
b6 487.2333
y4 491.2572
y5 620.2998
y5−H2O 602.2893
y6 691.3369
b9 700.3447
b8 629.3076
y8−H2O 815.4006
y10 975.4854
y10−H2O 957.4748
y9−NH3 887.4217
y11 1106.526
b13 1145.526
y12 1177.563
200
400
600
800
1000
1200
- A A A M A A A A A E T S Q R -
y12 y11 y10 y9 y8 y7 y6 y5 y4 y1
b3 b4 b5 b6 b8 b9 b13

Raw file	Scan	Method	Score	m/z	Gene names
20140918_fract2_dyn_5ul_D2_01_369	12204	TOF; CID	117.2	552.56	GNG12

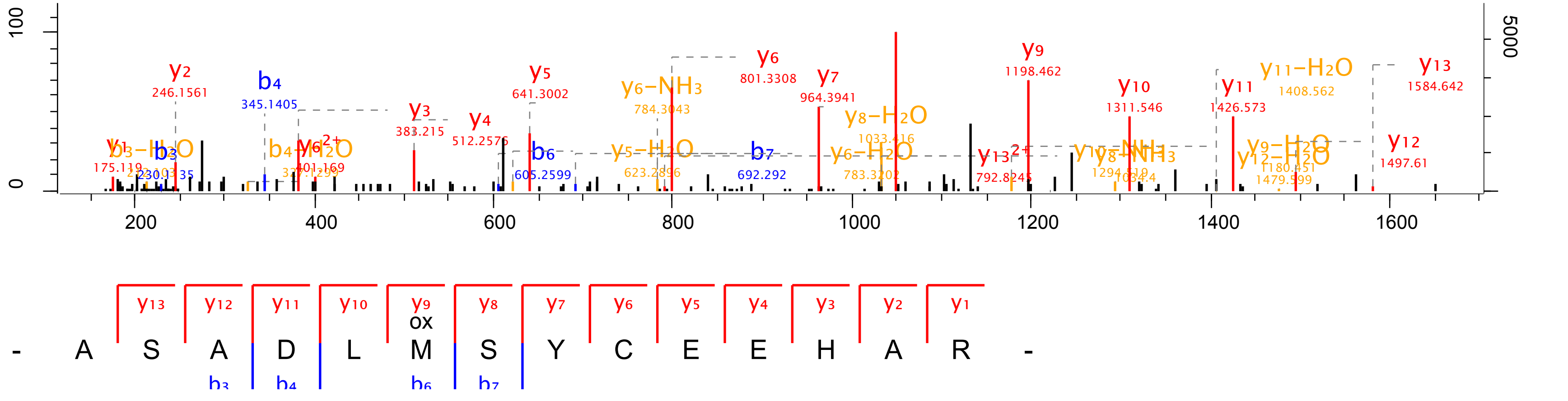

Raw file	Scan	Method	Score	m/z	Gene names
20140918_fract2_dyn_5ul_D2_01_369	12868	TOF; CID	202.56	683.36	CYSTM1

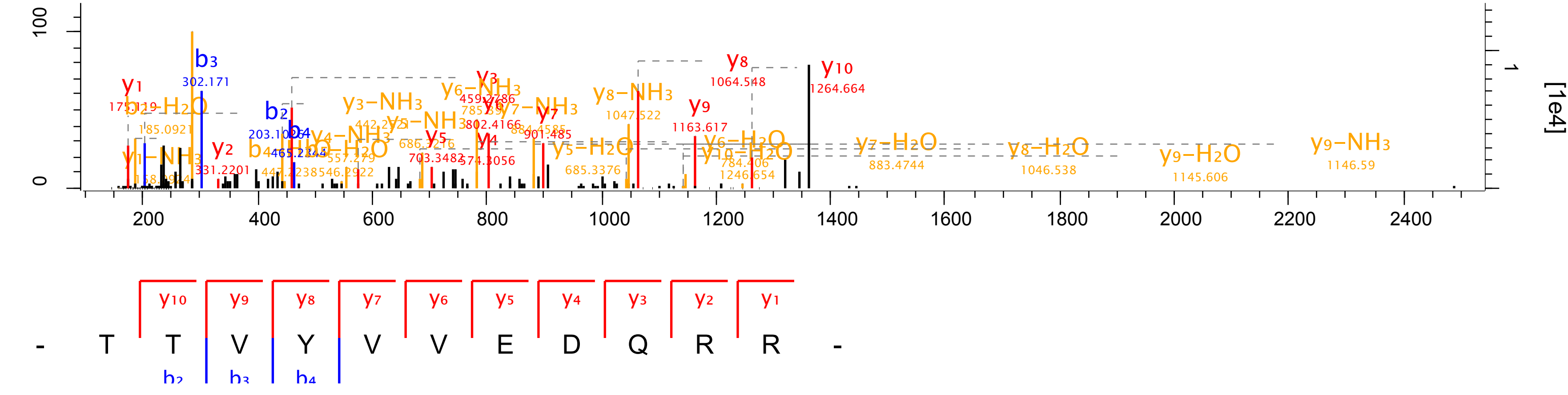

Raw file	Scan	Method	Score	m/z	Gene names
20140918_fract2_dyn_5ul_D2_01_369	13095	TOF; CID	83.86	502.25	C18orf32

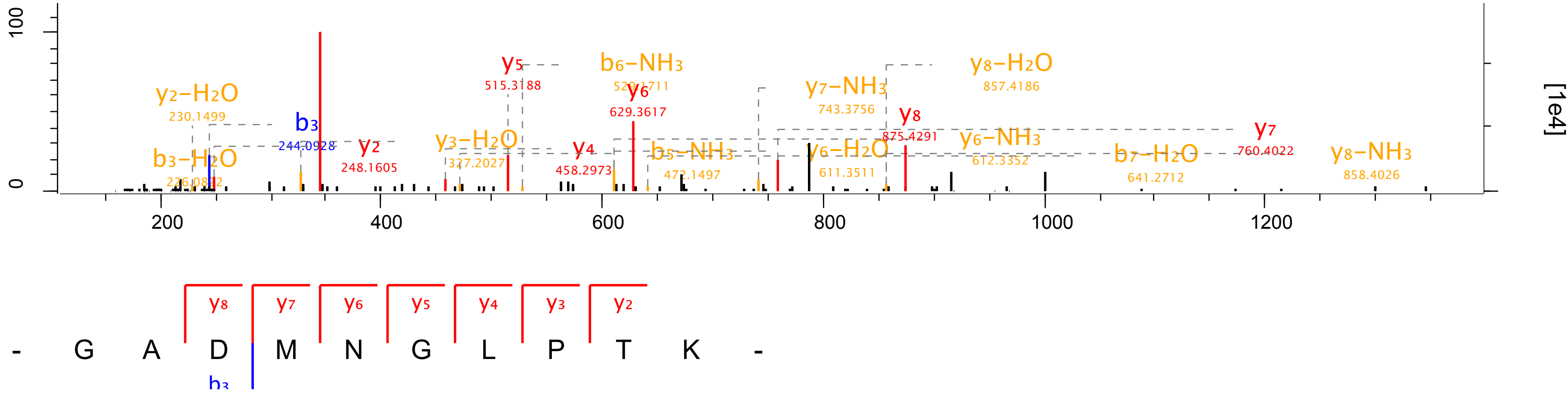

Raw file	Scan	Method	Score	m/z	Gene names
20140918_fract2_dyn_5ul_D2_01_369	13430	TOF; CID	130.56	720.31	LEPROTL1

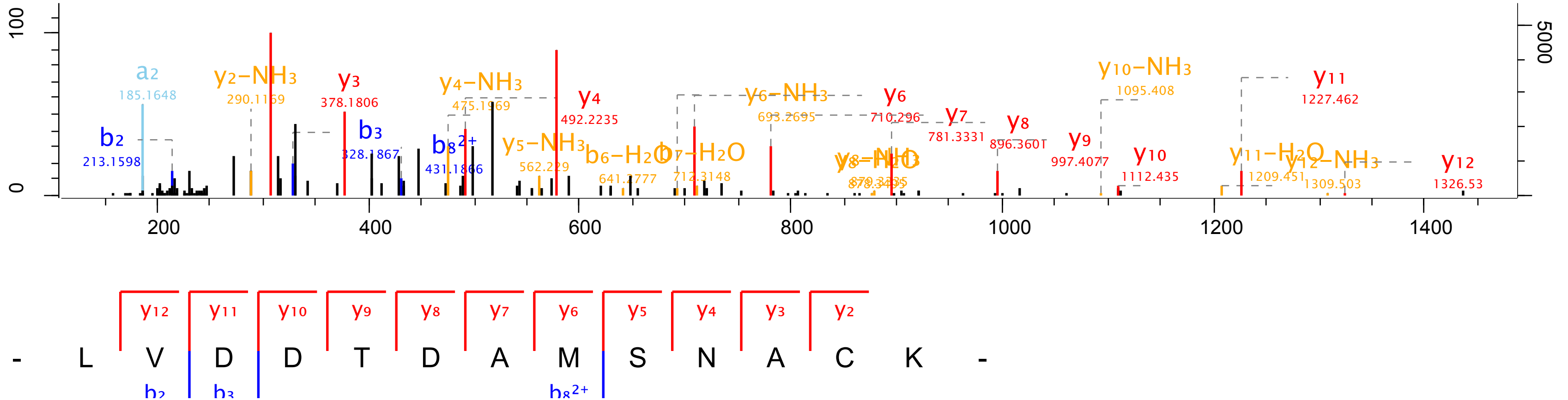

Raw file	Scan	Method	Score	m/z	Gene names
20140918_fract2_dyn_5ul_D2_01_369	13600	TOF; CID	100.76	833.05	C11orf48

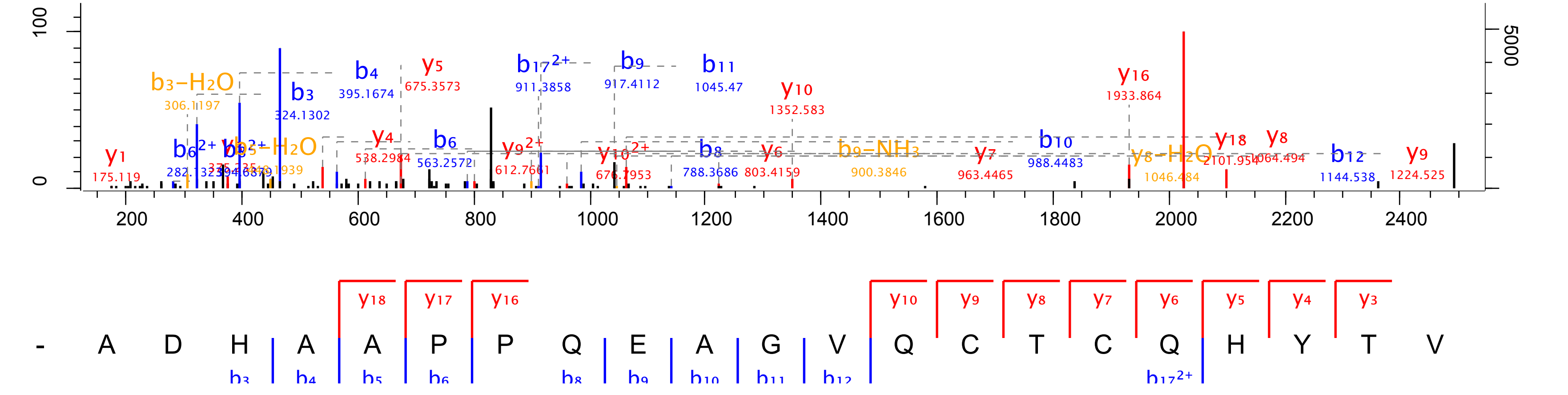

Raw file	Scan	Method	Score	m/z	Gene names
20140918_fract2_dyn_5ul_D2_01_369	14879	TOF; CID	99.14	493.76	UTS2

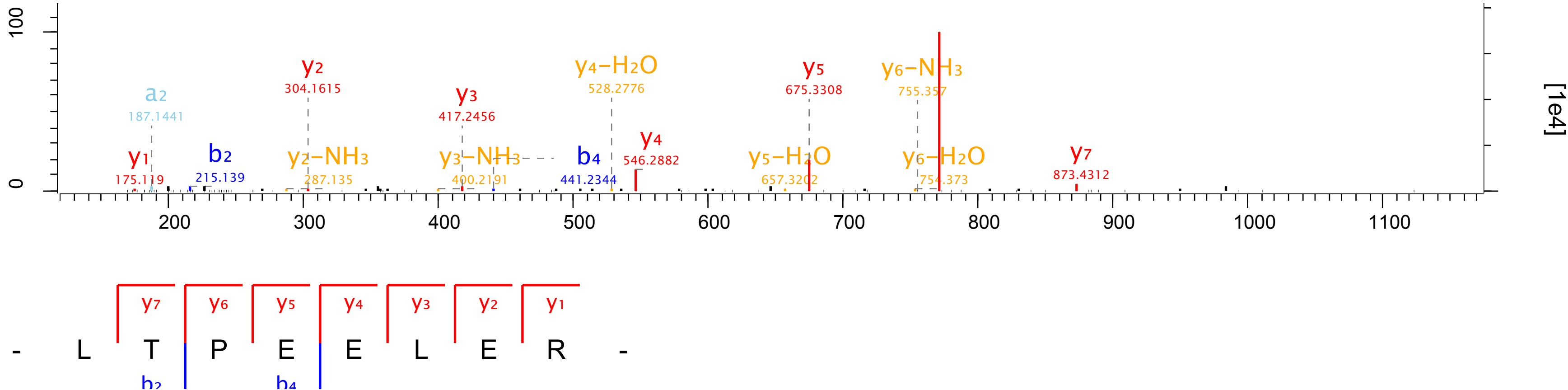

Raw file	Scan	Method	Score	m/z
20140918_fract2_dyn_5ul_D2_01_369	16447	TOF; CID	61.83	609.8

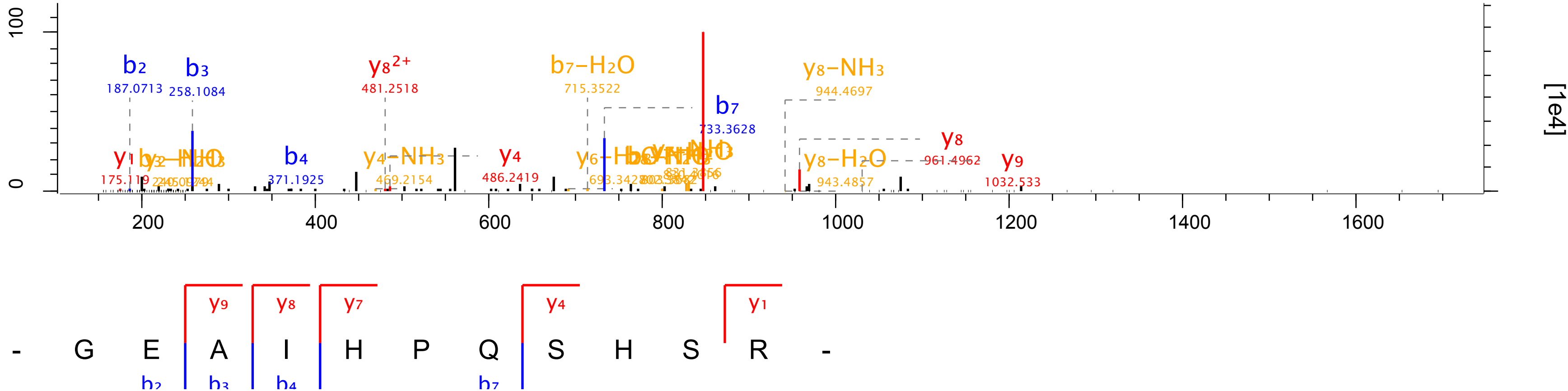

Raw file	Scan	Method	Score	m/z	Gene names
20140918_fract2_dyn_5ul_D2_01_369	18483	TOF; CID	101.65	509.26	PPP2R2A

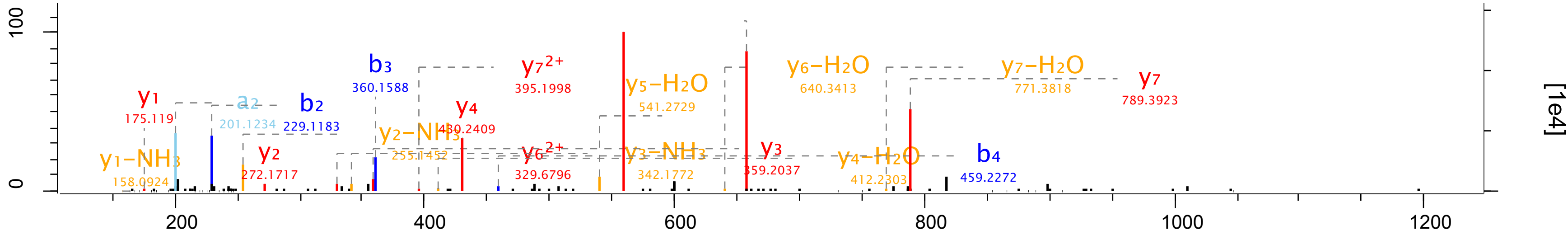

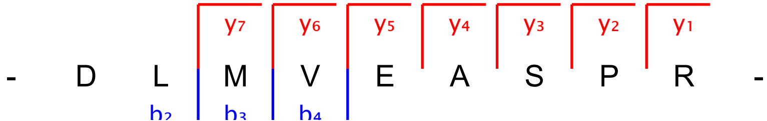

Raw file	Scan	Method	Score	m/z	Gene names
20140918_fract2_dyn_5ul_D2_01_369	21330	TOF; CID	125.97	766.84	SNX16

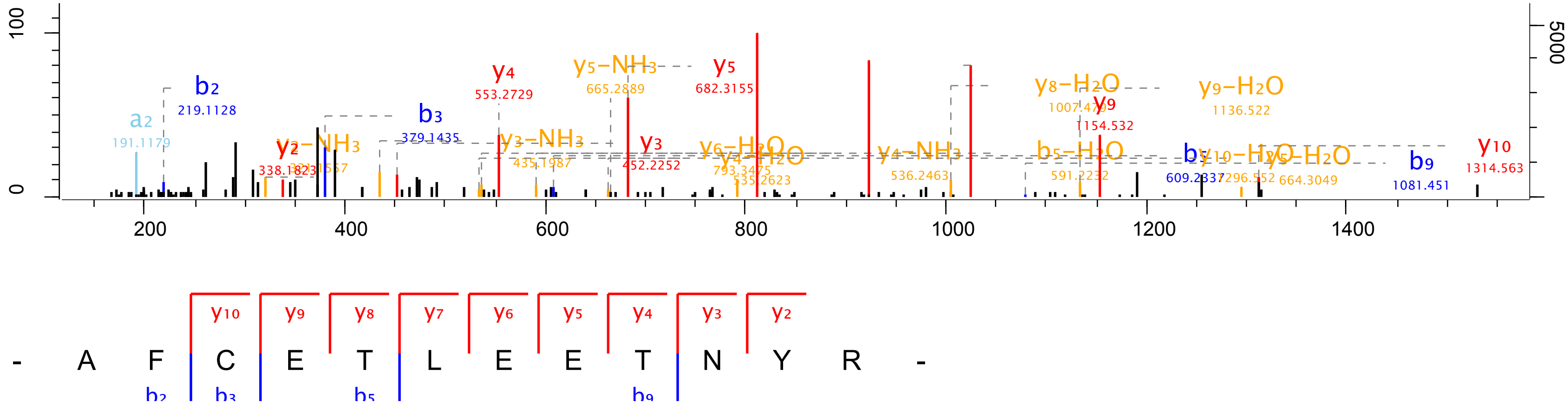

Raw file	Scan	Method	Score	m/z	Gene names
20140918_fract2_dyn_5ul_D2_01_369	23515	TOF; CID	65.37	674.32	BATF3

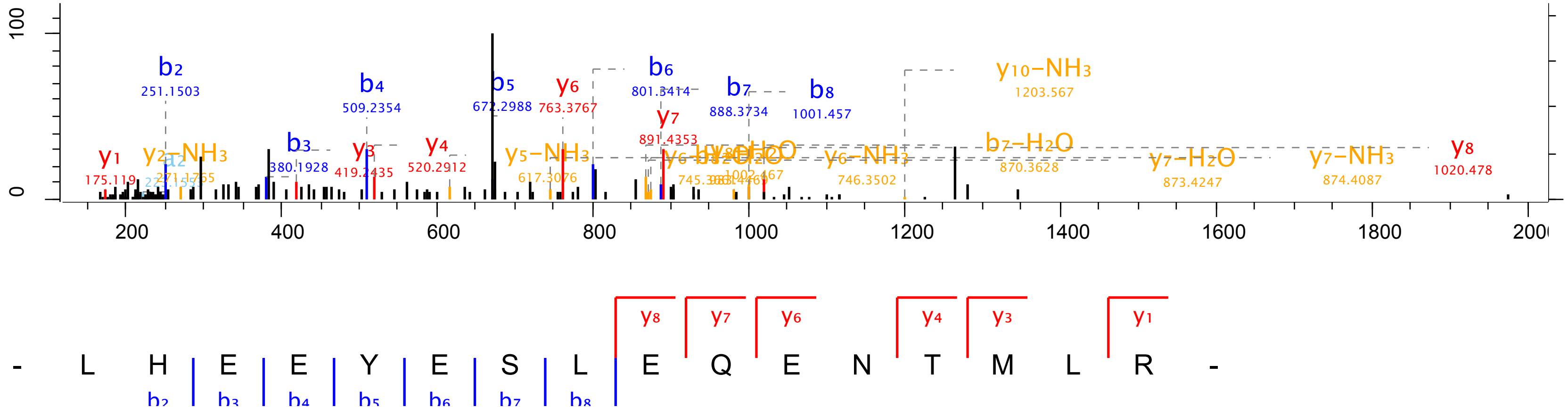

Raw file	Scan	Method	Score	m/z	Gene names
20140918_fract2_dyn_5ul_D2_01_369	24119	TOF; CID	72.32	532.95	METRN

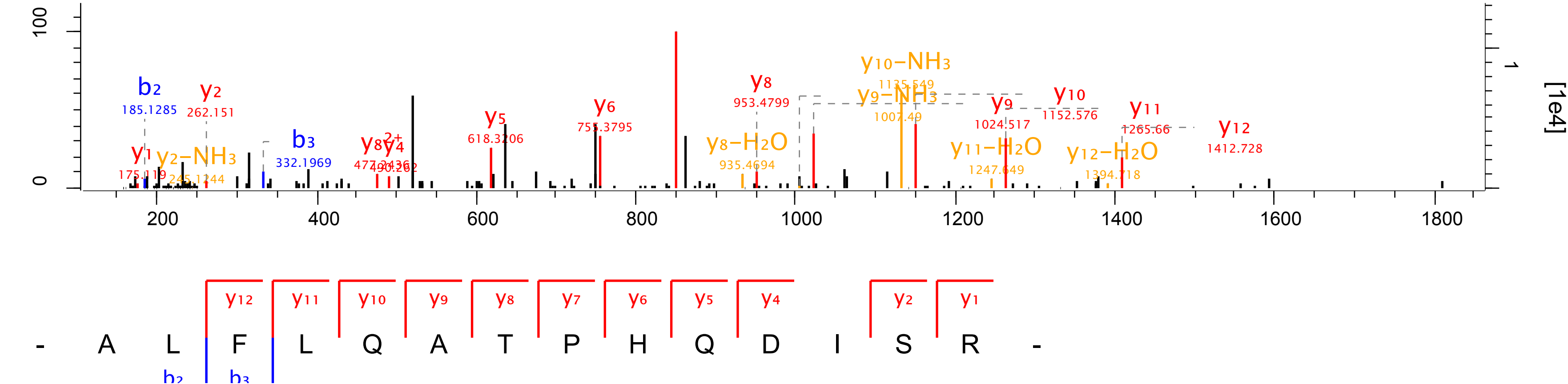

Raw file	Scan	Method	Score	m/z	Gene names
20140918_fract2_dyn_5ul_D2_01_369	26625	TOF; CID	95.18	756.86	FAM73A

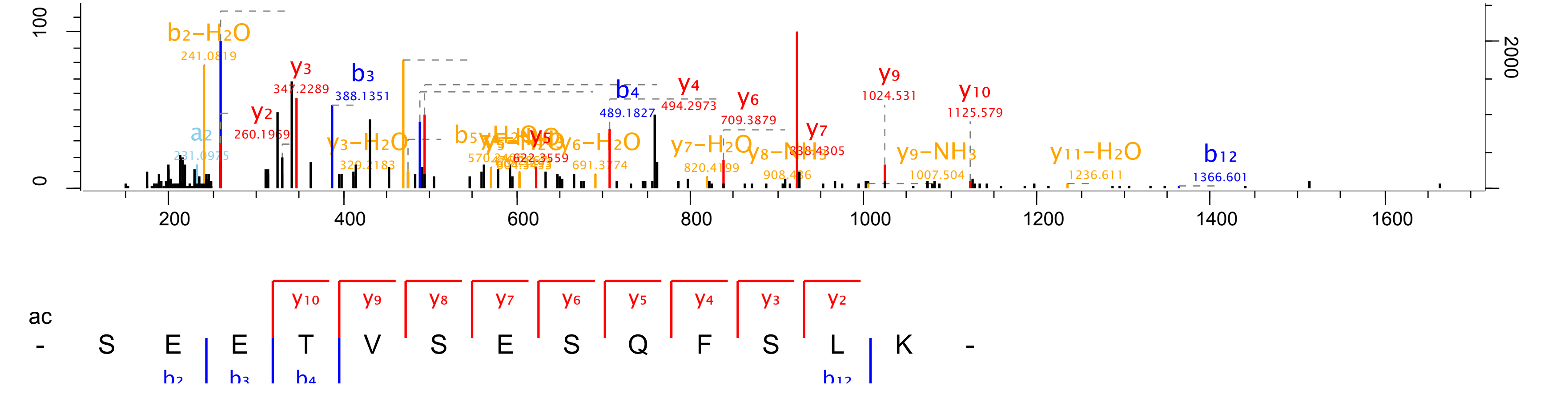

Raw file	Scan	Method	Score	m/z	Gene names
20140918_fract2_dyn_5ul_D2_01_369	29883	TOF; CID	94.66	429.77	JPH4

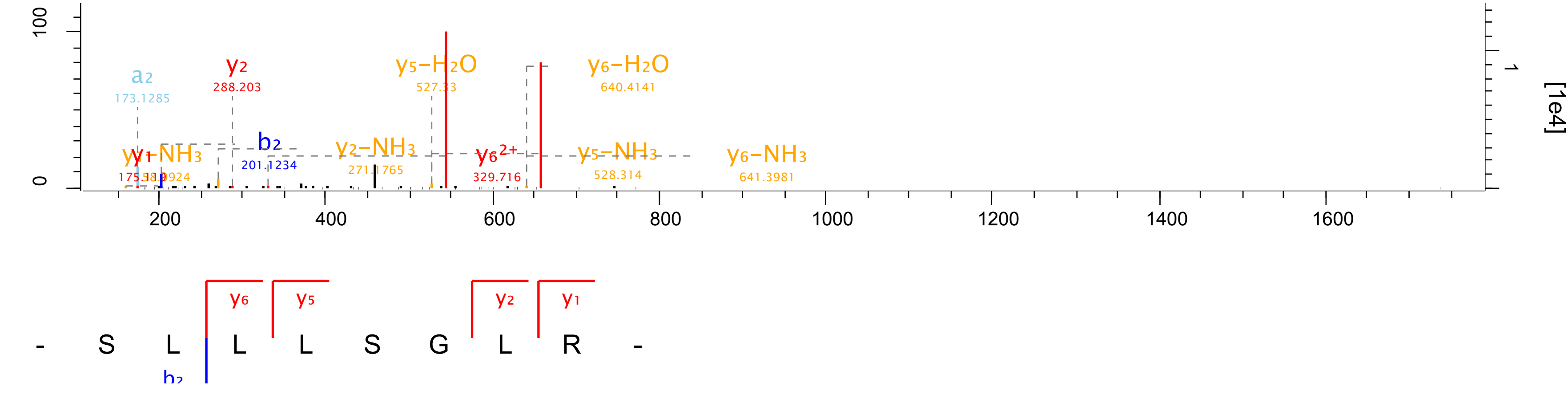

Raw file	Scan	Method	Score	m/z	Gene names
20140918_fract2_dyn_5ul_D2_01_369	34270	TOF; CID	148.44	1066.07	MED10

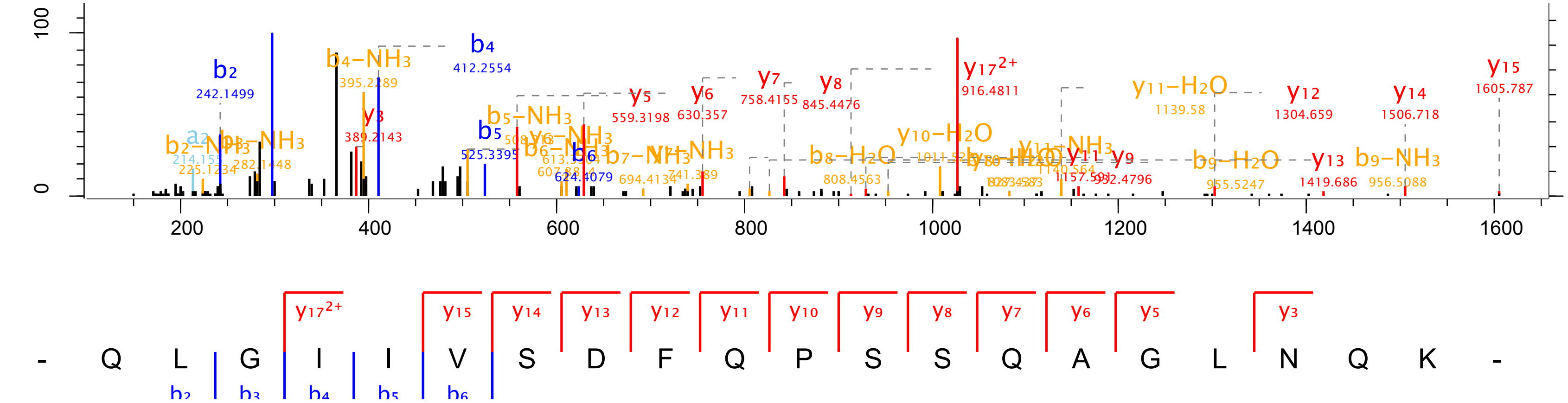

Raw file	Scan	Method	Score	m/z	Gene names
20140918_fract2_dyn_5ul_D2_01_369	35585	TOF; CID	106.68	543.82	NABP1

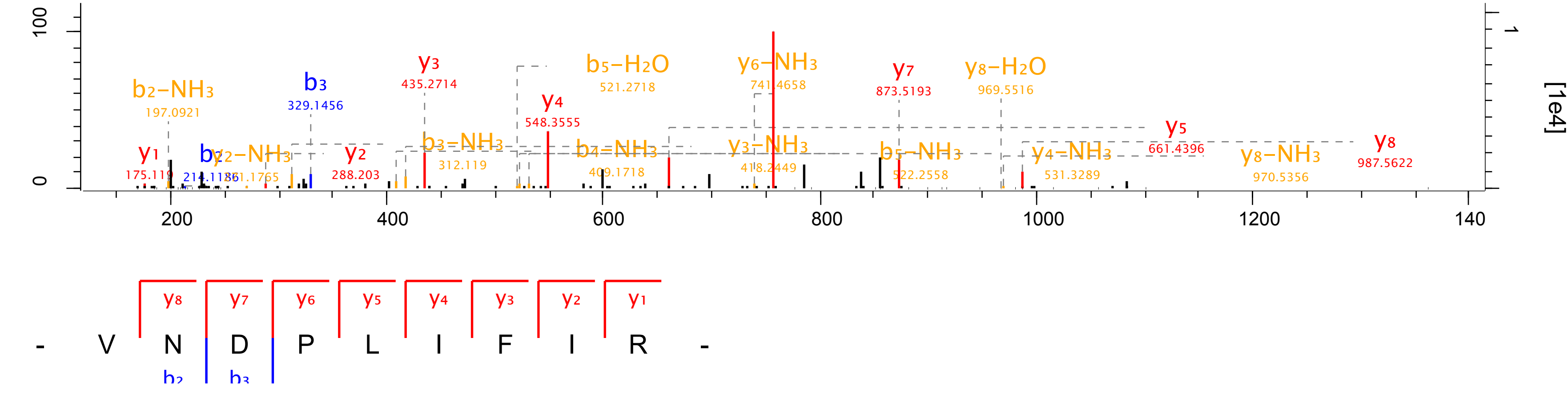

Raw file	Scan	Method	Score	m/z	Gene names
20140918_fract2_dyn_5ul_D2_01_369	35927	TOF; CID	88.18	530.3	PHLDA1

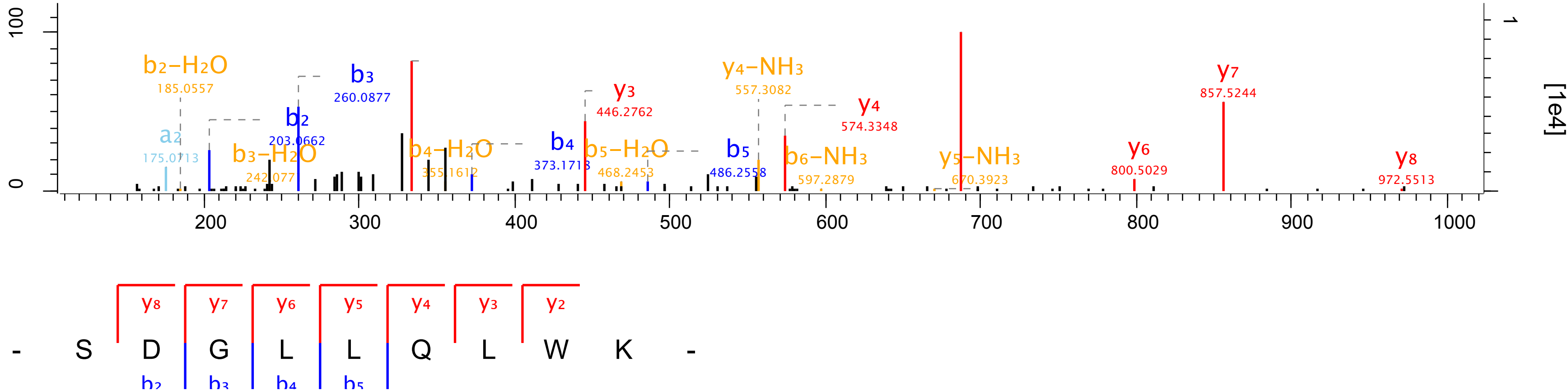

Raw file	Scan	Method	Score	m/z	Gene names
20140918_fract2_dyn_5ul_D2_01_369	41437	TOF; CID	147.64	1019.55	LSM5

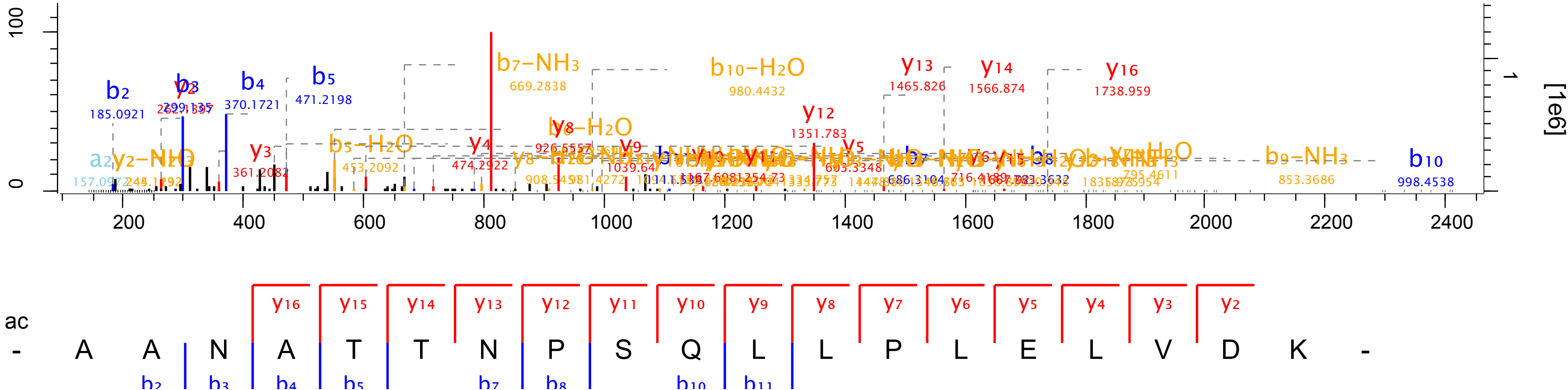

Raw file	Scan	Method	Score	m/z	Gene names
20140918_fract3_dyn_5ul_D3_01_370	8848	TOF; CID	85.74	601.29	C1orf53

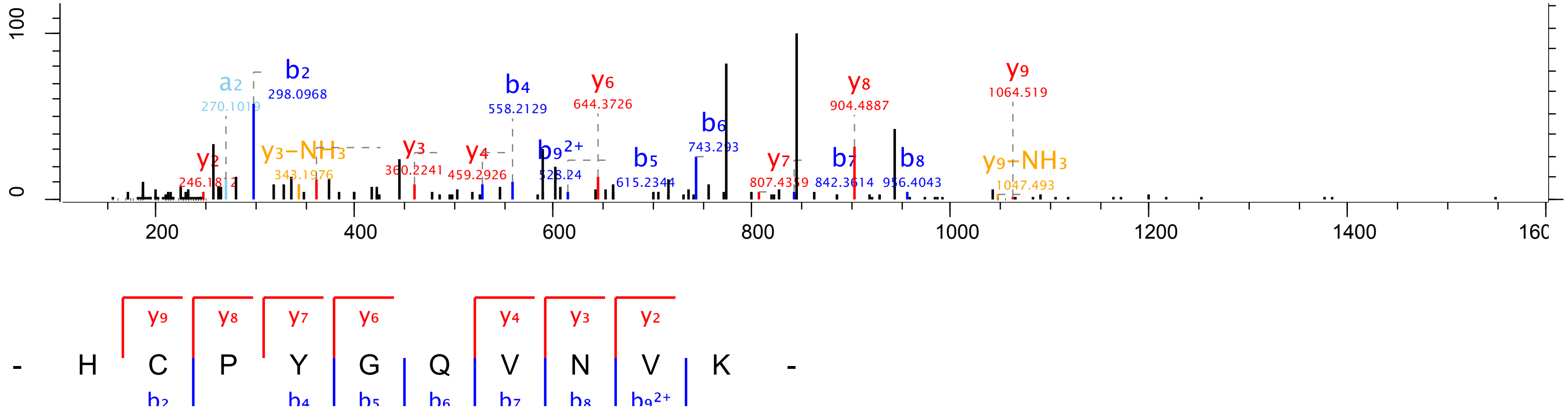

Raw file	Scan	Method	Score	m/z	Gene names
20140918_fract3_dyn_5ul_D3_01_370	11157	TOF; CID	99.44	483.74	C10orf54

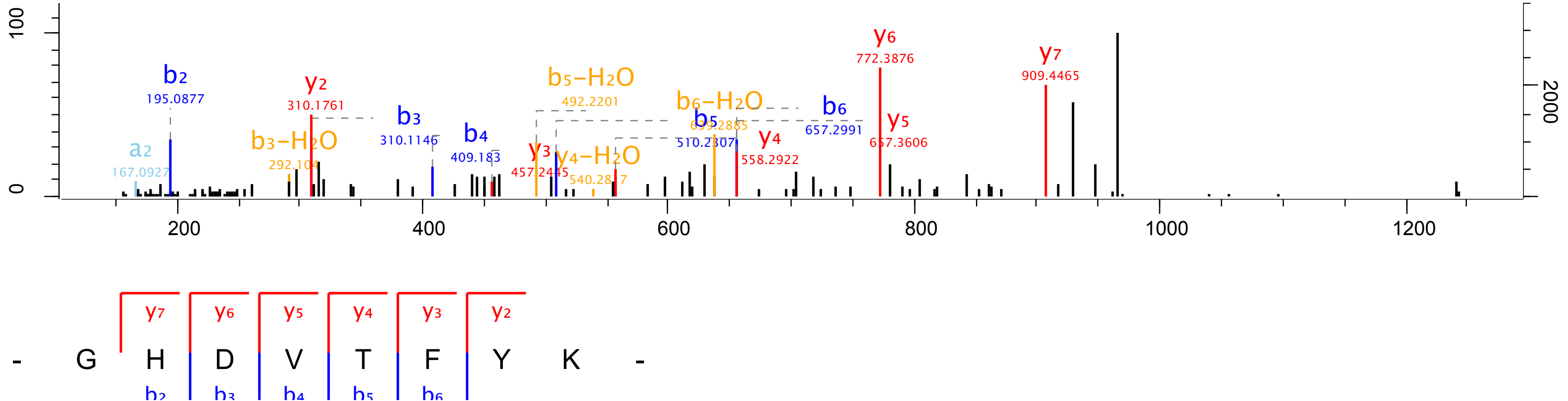

Raw file	Scan	Method	Score	m/z	Gene names
20140918_fract3_dyn_5ul_D3_01_370	12643	TOF; CID	39.39	627.31	CYBRD1

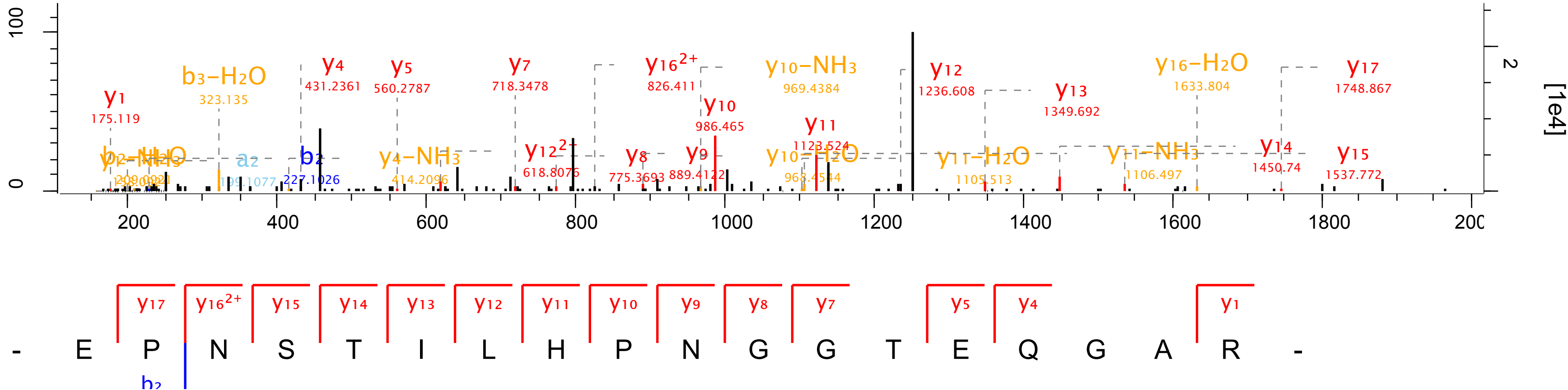

Raw file	Scan	Method	Score	m/z	Gene names
20140918_fract3_dyn_5ul_D3_01_370	12893	TOF; CID	63.08	582.78	CCDC151

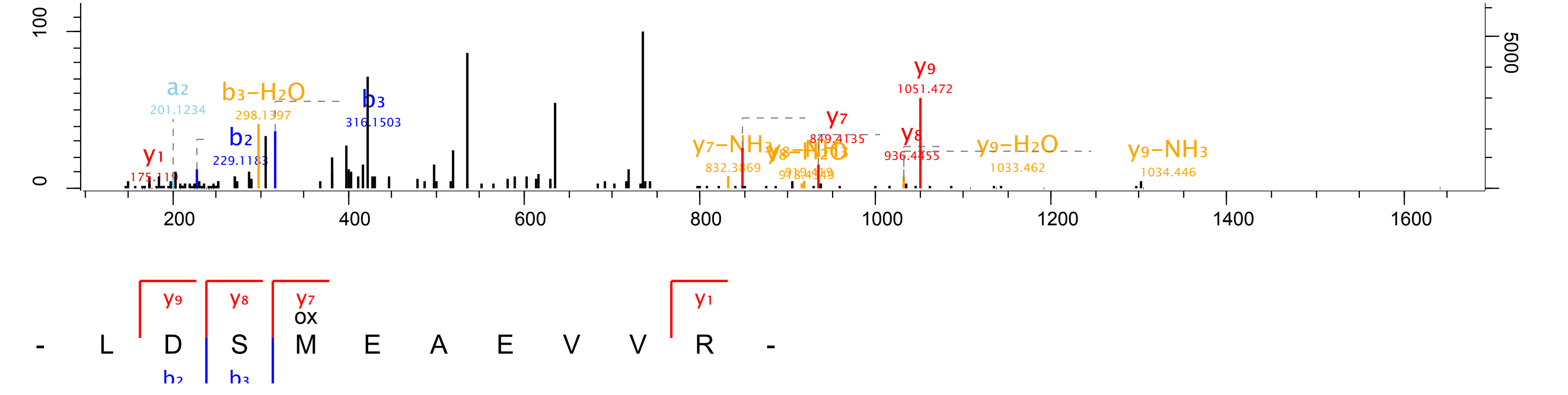

Raw file	Scan	Method	Score	m/z	Gene names
20140918_fract3_dyn_5ul_D3_01_370	14690	TOF; CID	114.76	630.83	TFDP2

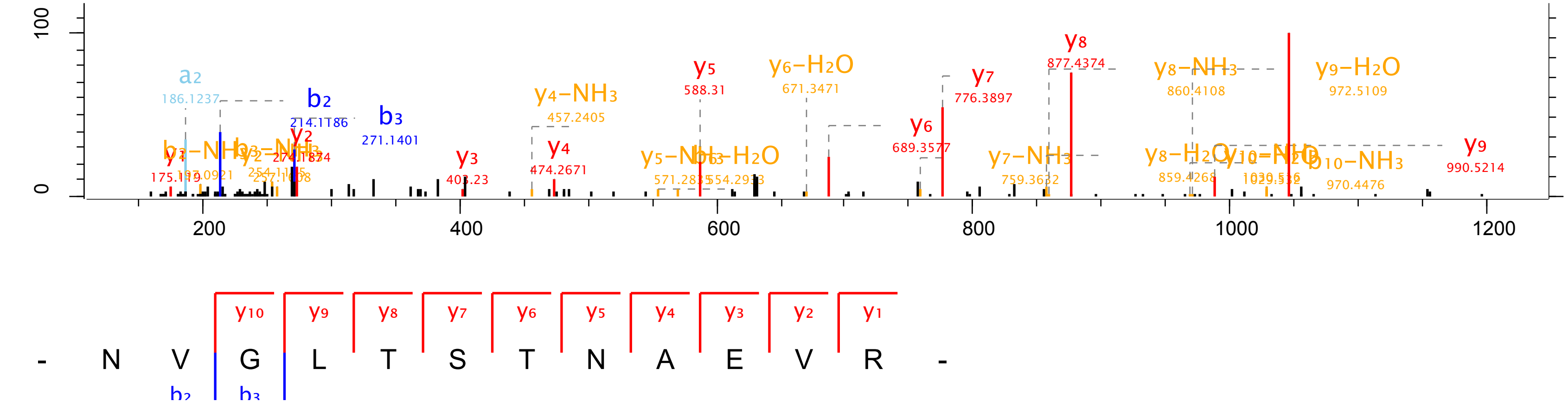

Raw file	Scan	Method	Score	m/z	Gene names
20140918_fract3_dyn_5ul_D3_01_370	15091	TOF; CID	78.9	469.58	TMEM208

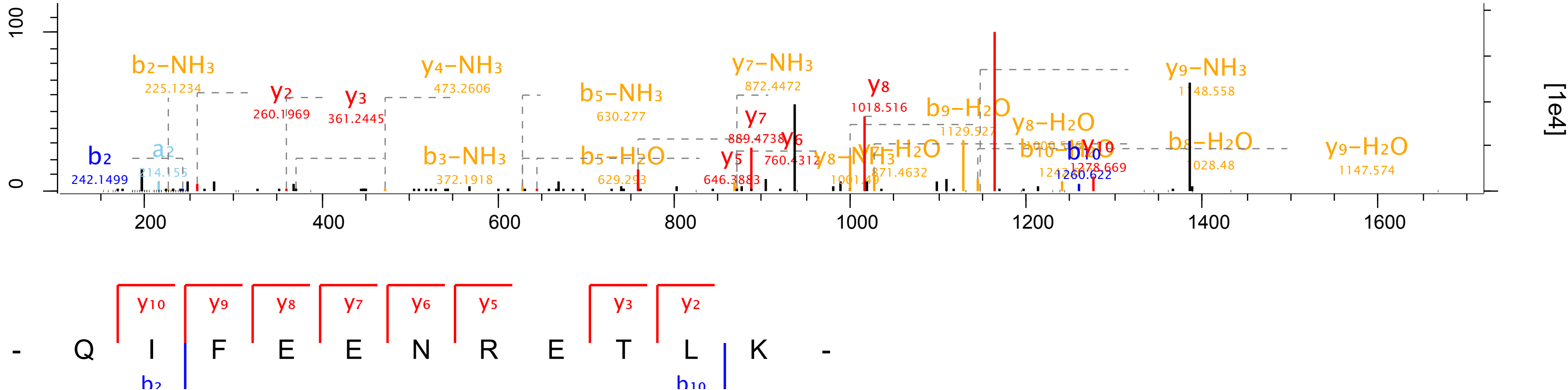

Raw file	Scan	Method	Score	m/z	Gene names
20140918_fract3_dyn_5ul_D3_01_370	16503	TOF; CID	112.64	592.8	MFSD1

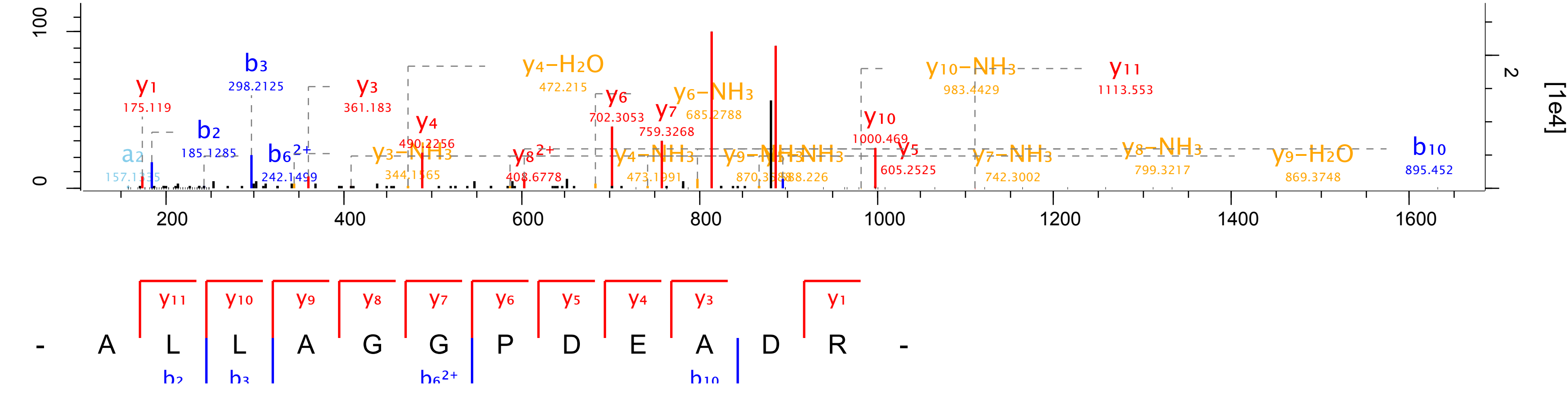

Raw file	Scan	Method	Score	m/z	Gene names
20140918_fract3_dyn_5ul_D3_01_370	17243	TOF; CID	109.71	483.72	TMEM258

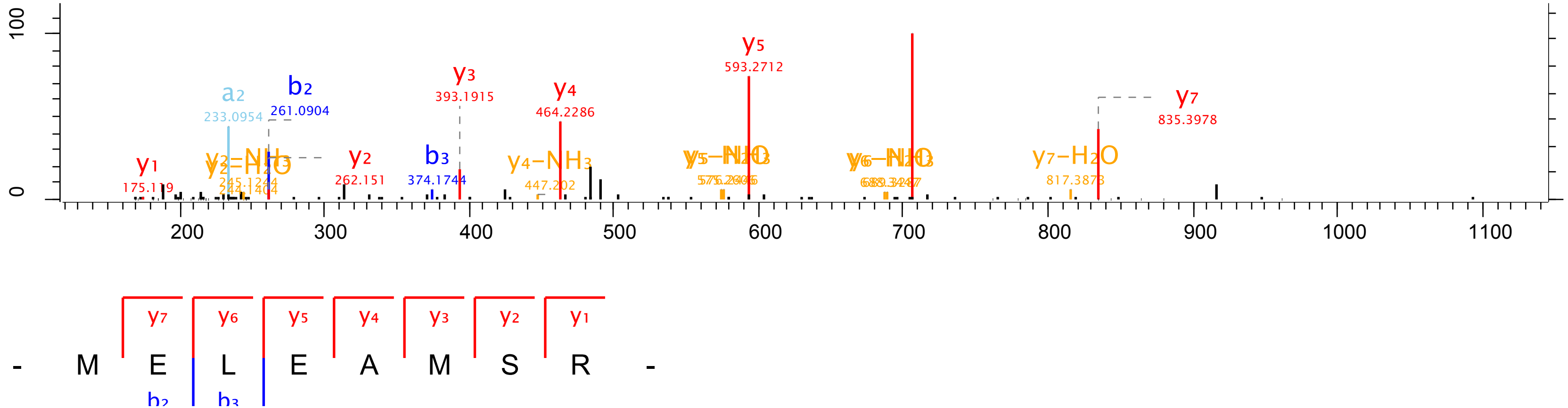

Raw file	Scan	Method	Score	m/z	Gene names
20140918_fract3_dyn_5ul_D3_01_370	18788	TOF; CID	136.81	858.93	APOC3

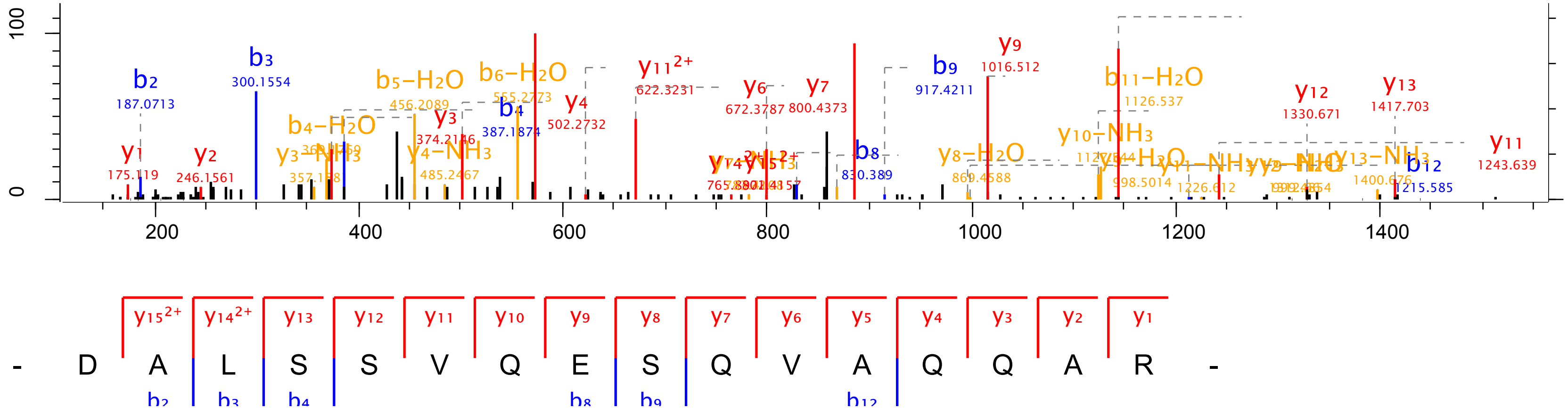

Raw file	Scan	Method	Score	m/z	Gene names
20140918_fract3_dyn_5ul_D3_01_370	24078	TOF; CID	76.23	546.3	SIAH2

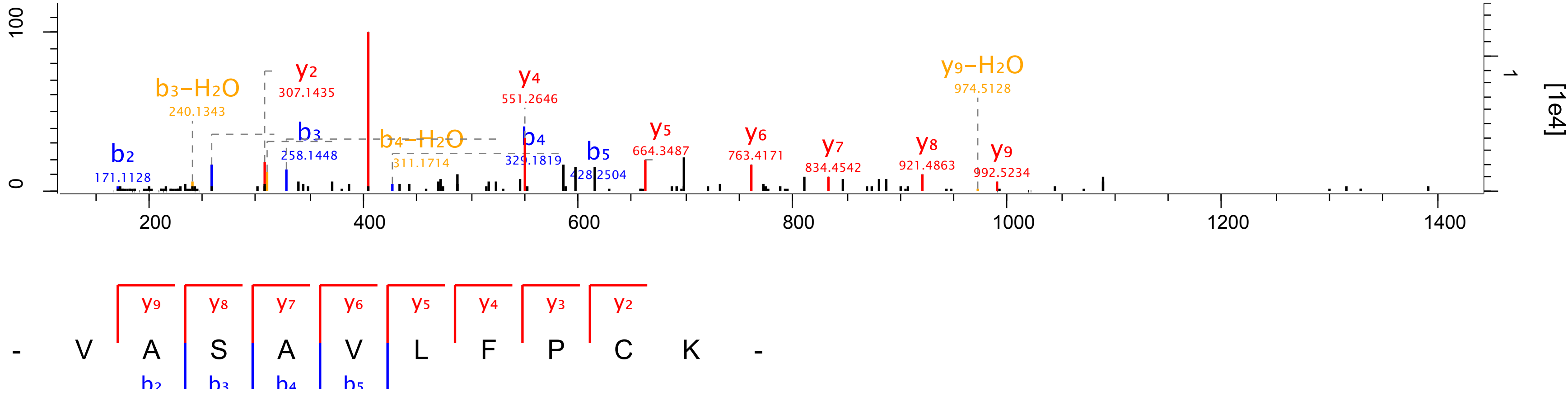

Raw file	Scan	Method	Score	m/z	Gene names
20140918_fract3_dyn_5ul_D3_01_370	24381	TOF; CID	93.65	553.32	WBP5

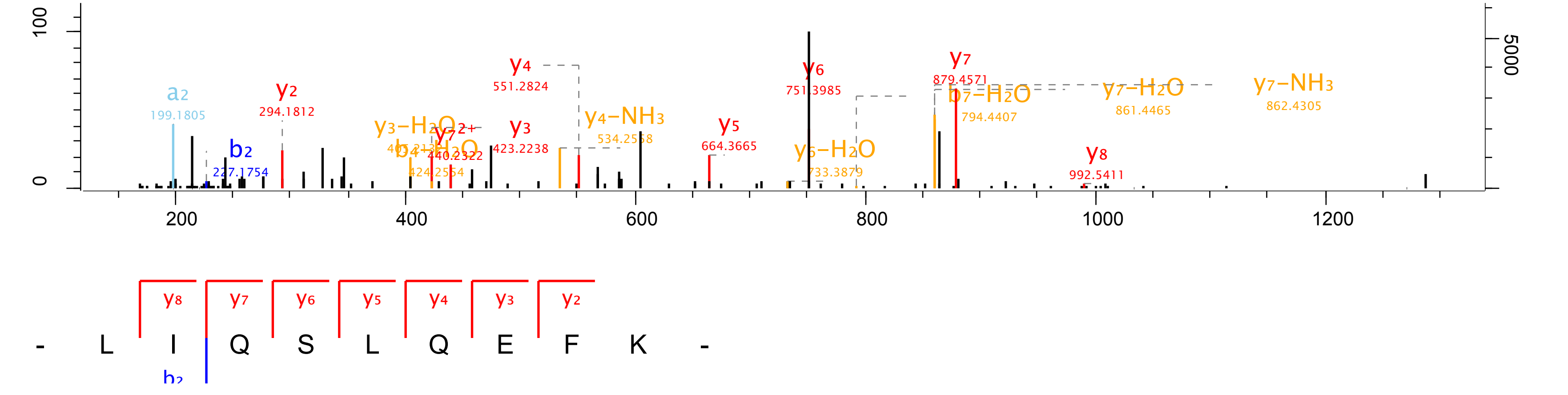

Raw file	Scan	Method	Score	m/z	Gene names
20140918_fract3_dyn_5ul_D3_01_370	26934	TOF; CID	78.95	578.3	MBLAC1

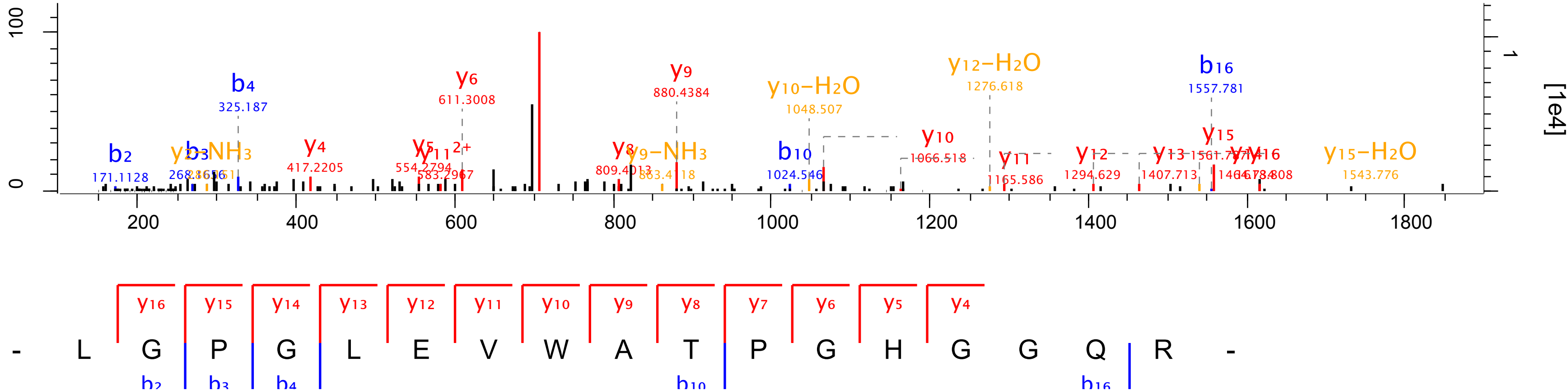

Raw file	Scan	Method	Score	m/z	Gene names
20140918_fract3_dyn_5ul_D3_01_370	27345	TOF; CID	89.3	586.84	KATNAL2

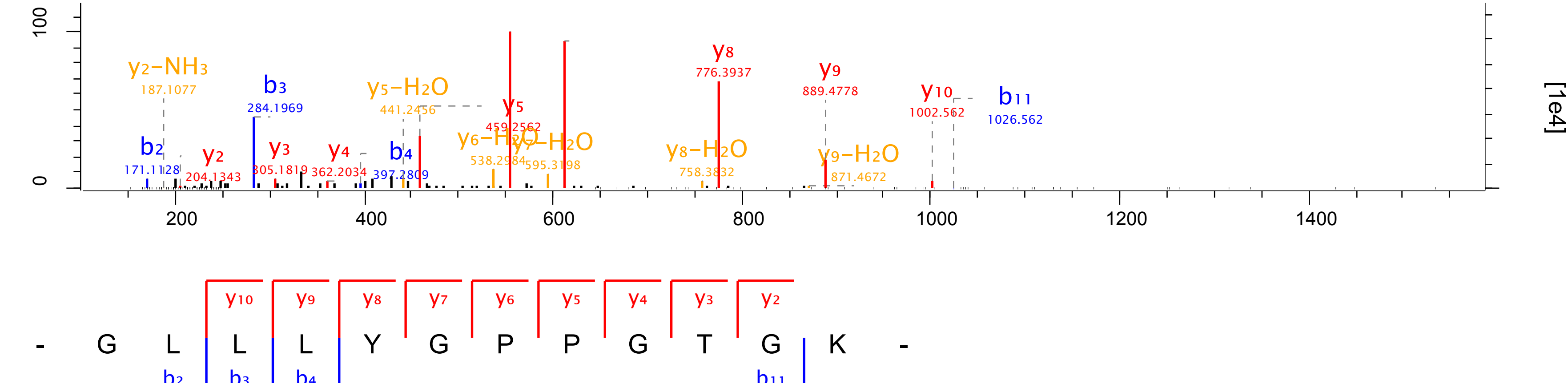

Raw file	Scan	Method	Score	m/z	Gene names
20140918_fract3_dyn_5ul_D3_01_370	28210	TOF; CID	84.17	727.38	NUDT14

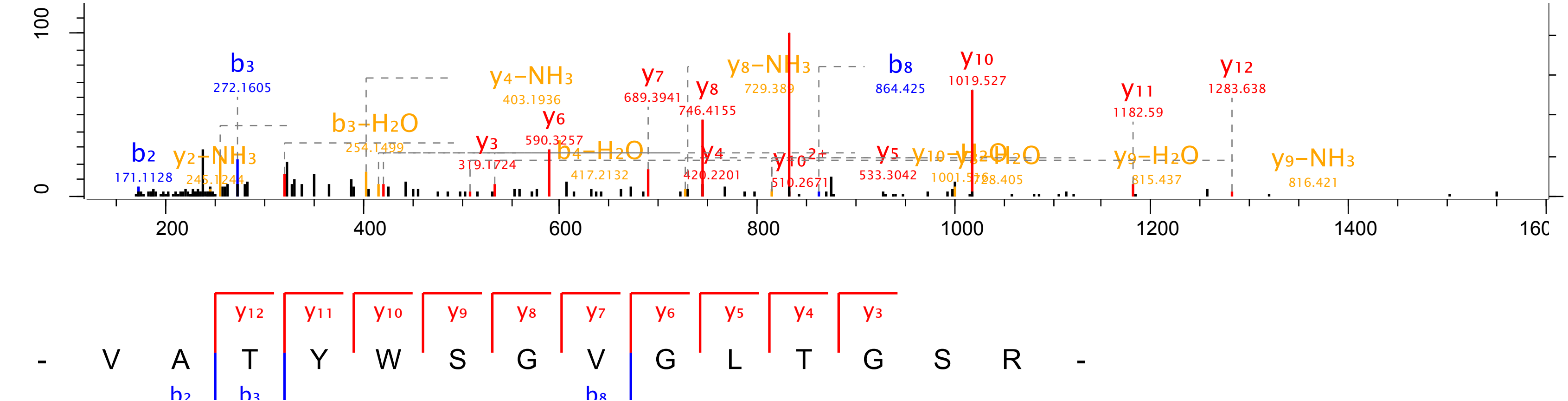

Raw file	Scan	Method	Score	m/z	Gene names
20140918_fract3_dyn_5ul_D3_01_370	32988	TOF; CID	84.65	996.92	CLDN7

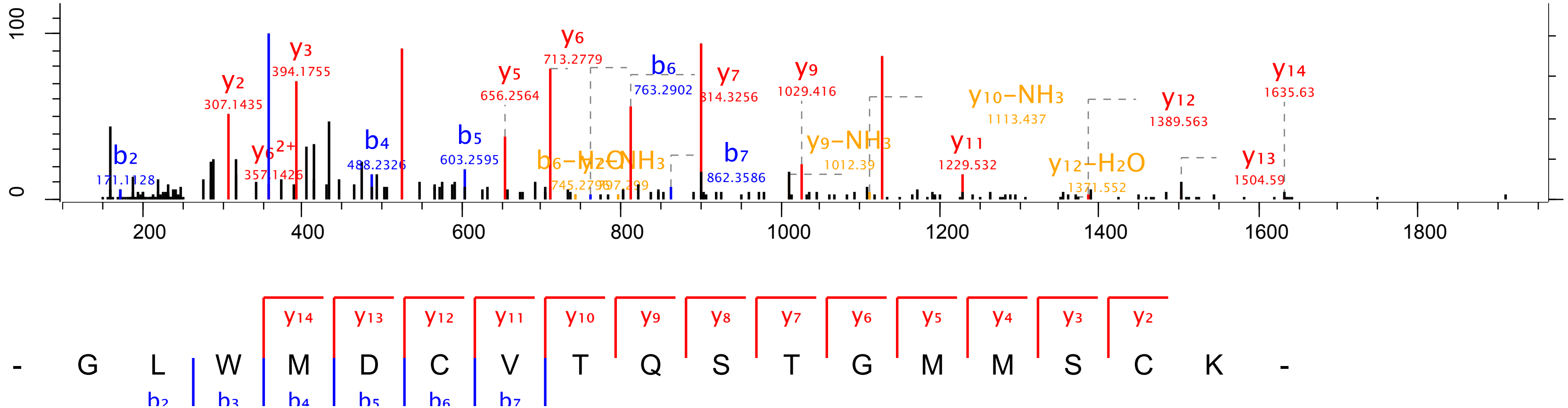

Raw file	Scan	Method	Score	m/z	Gene names
20140918_fract3_dyn_5ul_D3_01_370	33706	TOF; CID	99.07	854.42	SSR3

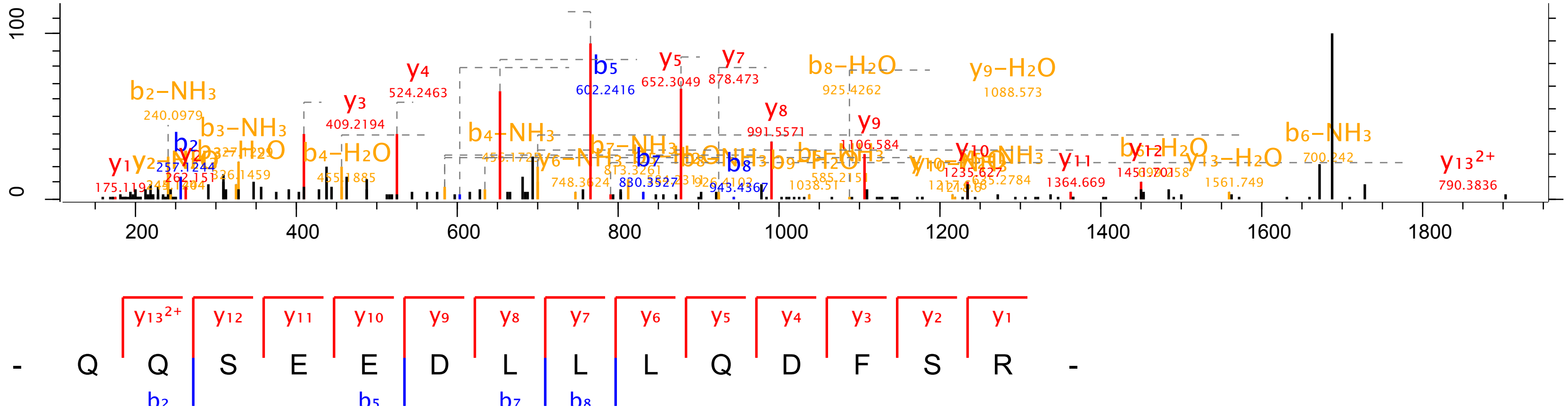

Raw file	Scan	Method	Score	m/z	Gene names
20140918_fract3_dyn_5ul_D3_01_370	34784	TOF; CID	71.67	1169.23	TAF11

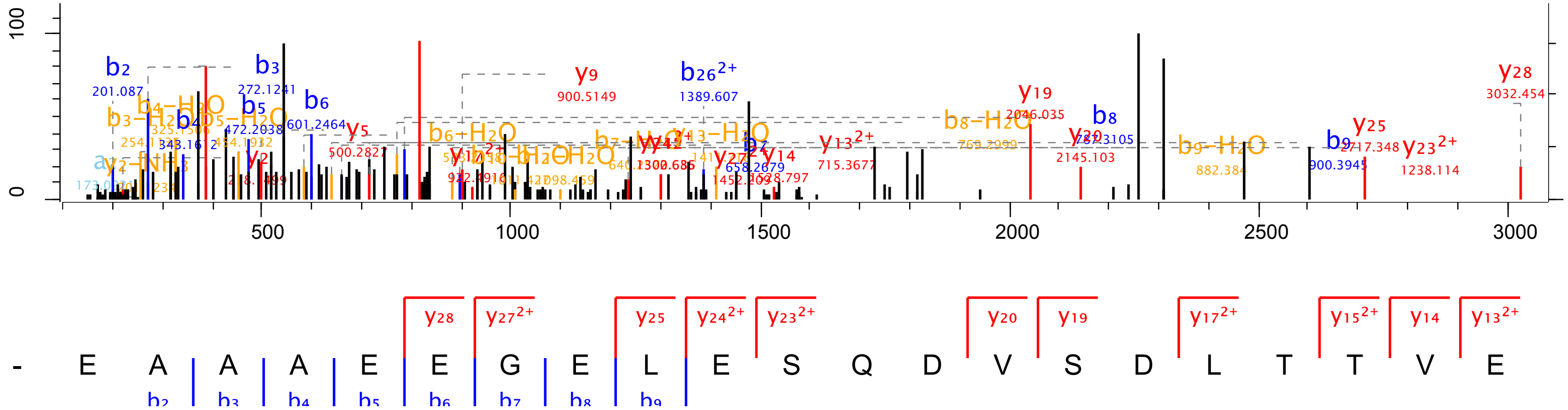

Raw file	Scan	Method	Score	m/z	Gene names
20140918_fract3_dyn_5ul_D3_01_370	35999	TOF; CID	113.71	568.8	MT-ND2

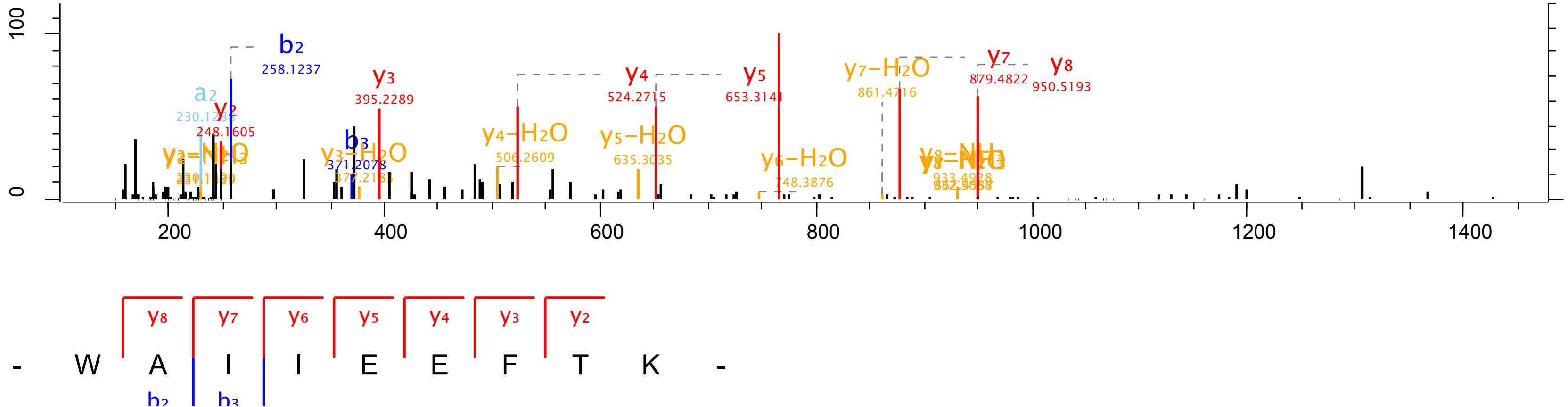

Raw file	Scan	Method	Score	m/z	Gene names
20140918_fract3_dyn_5ul_D3_01_370	39602	TOF; CID	89.62	858.4	SLC29A2

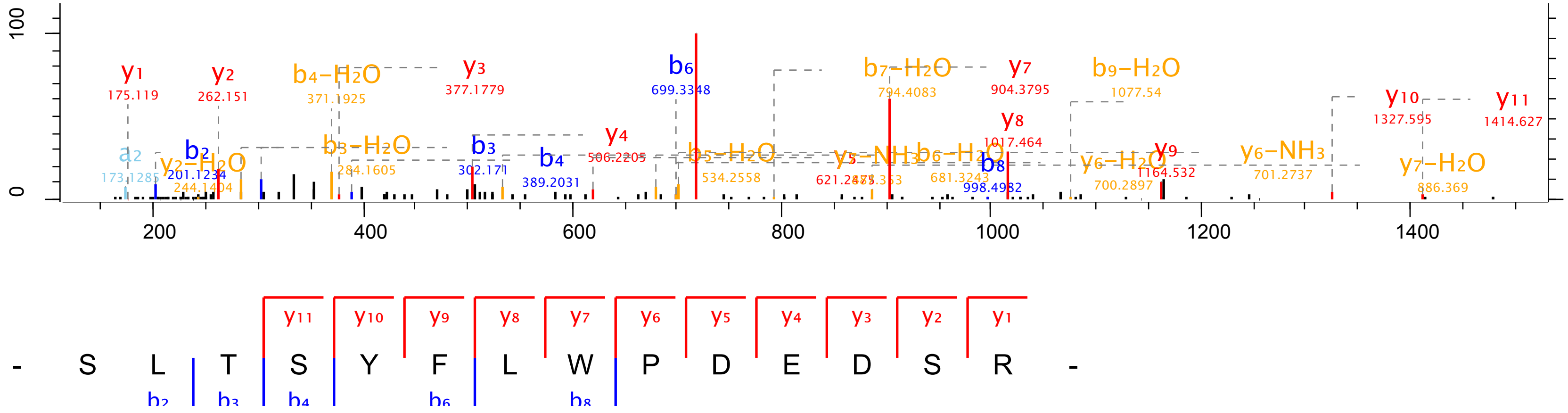

Raw file	Scan	Method	Score	m/z	Gene names
20140918_fract4_dyn_5ul_D4_01_371	5976	TOF; CID	83.86	564.78	TET2

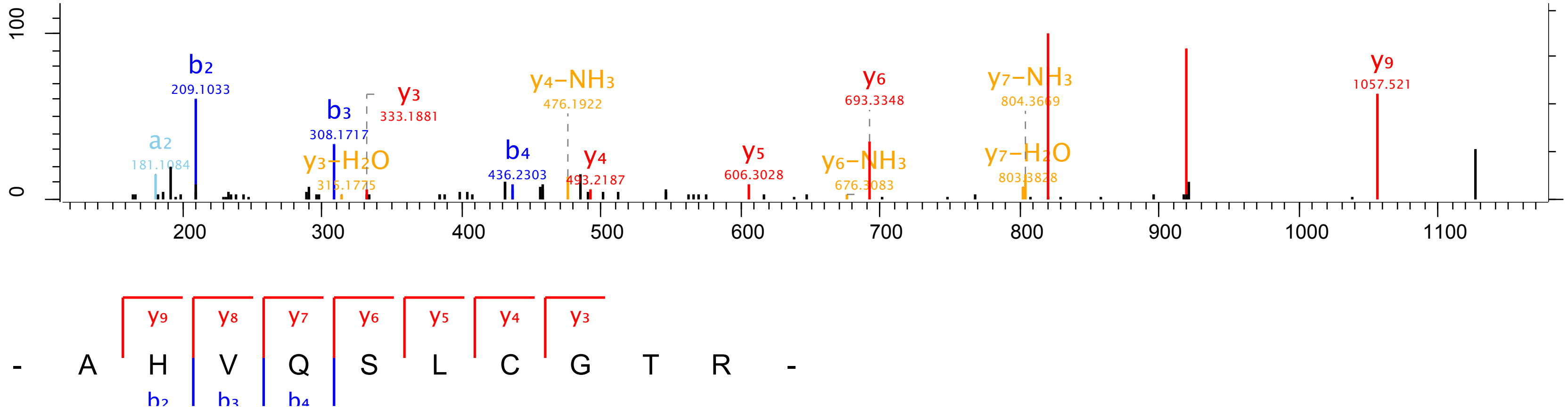

Raw file	Scan	Method	Score	m/z	Gene names
20140918_fract4_dyn_5ul_D4_01_371	7729	TOF; CID	109.48	476.77	ENTPD7

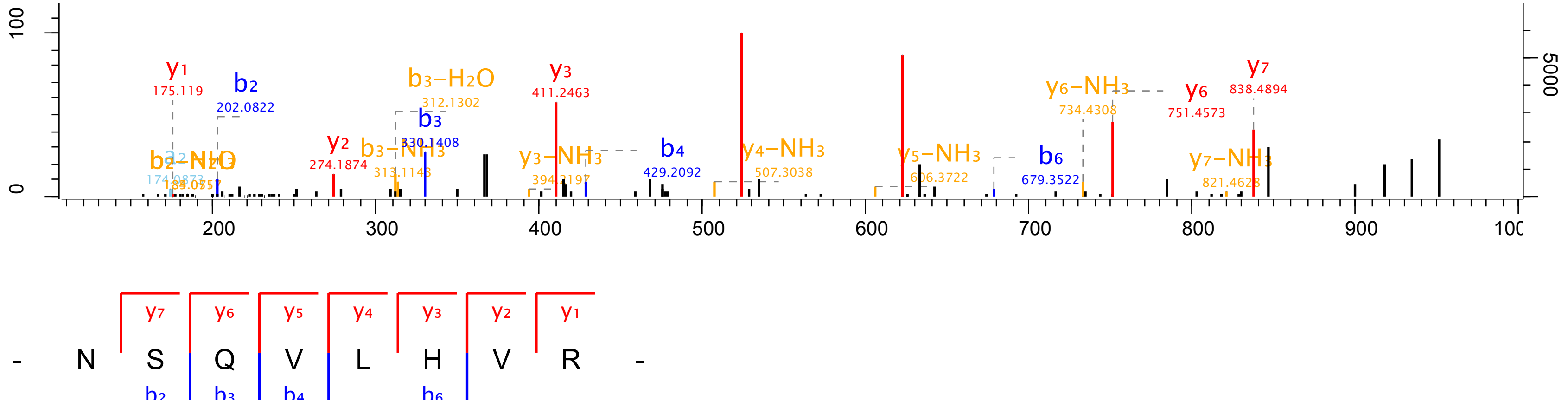

Raw file	Scan	Method	Score	m/z	Gene names
20140918_fract4_dyn_5ul_D4_01_371	8161	TOF; CID	100.11	735.37	RWDD3

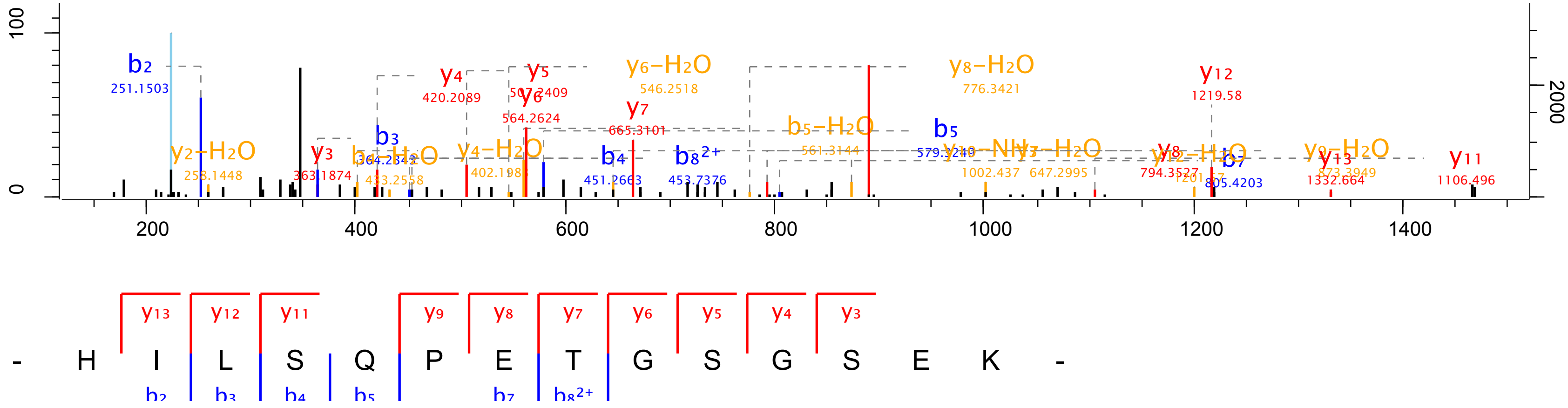

Raw file	Scan	Method	Score	m/z	Gene names
20140918_fract4_dyn_5ul_D4_01_371	8232	TOF; CID	124.89	454.23	KIAA2026

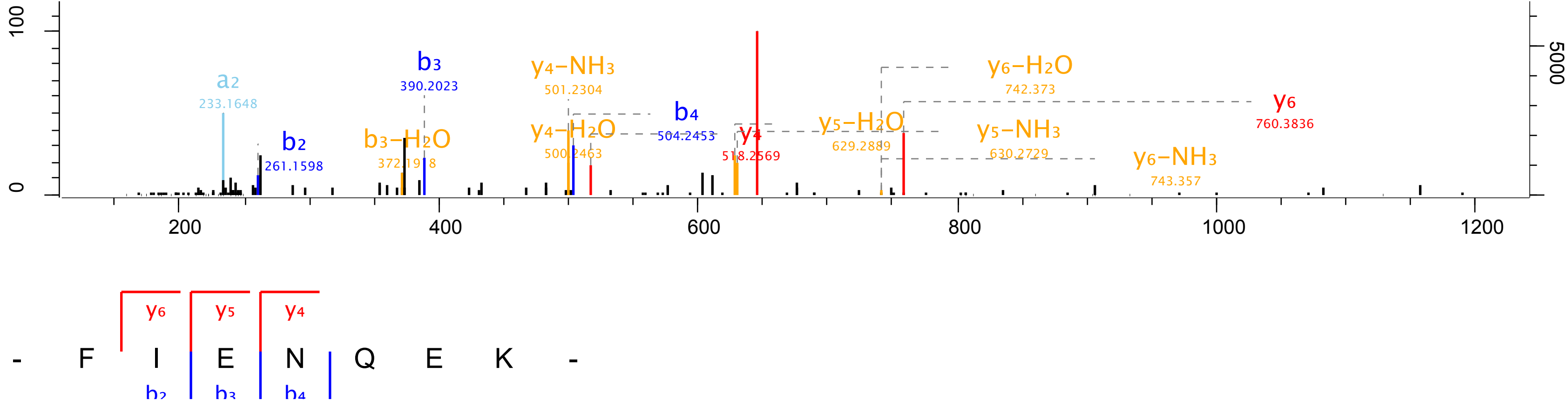

Raw file	Scan	Method	Score	m/z	Gene names
20140918_fract4_dyn_5ul_D4_01_371	8826	TOF; CID	90.79	464.54	DPM3

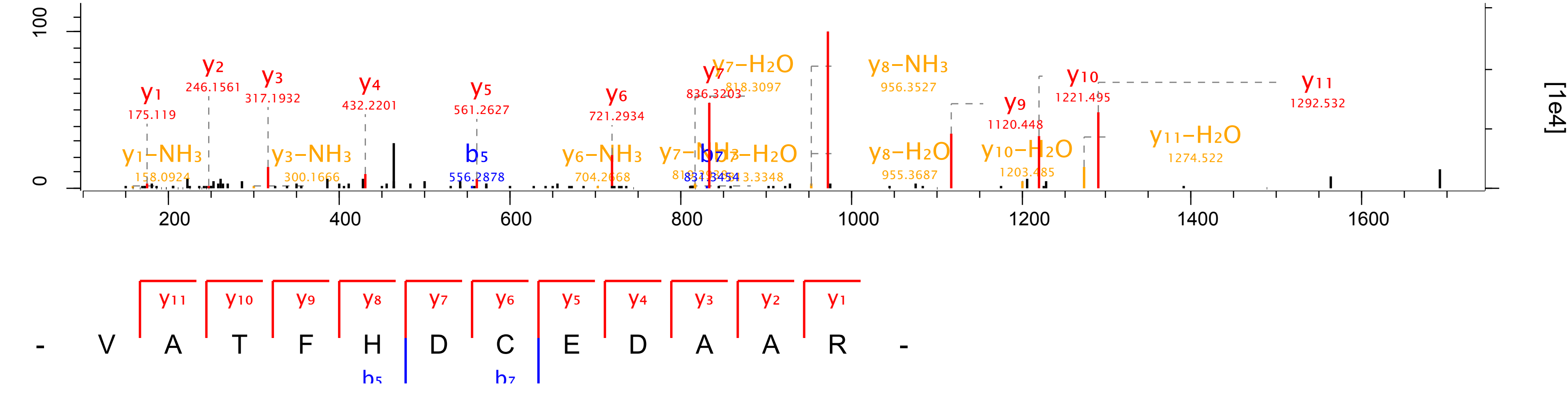

Raw file	Scan	Method	Score	m/z	Gene names
20140918_fract4_dyn_5ul_D4_01_371	11863	TOF; CID	45.62	684.85	ZXDA;ZXDB

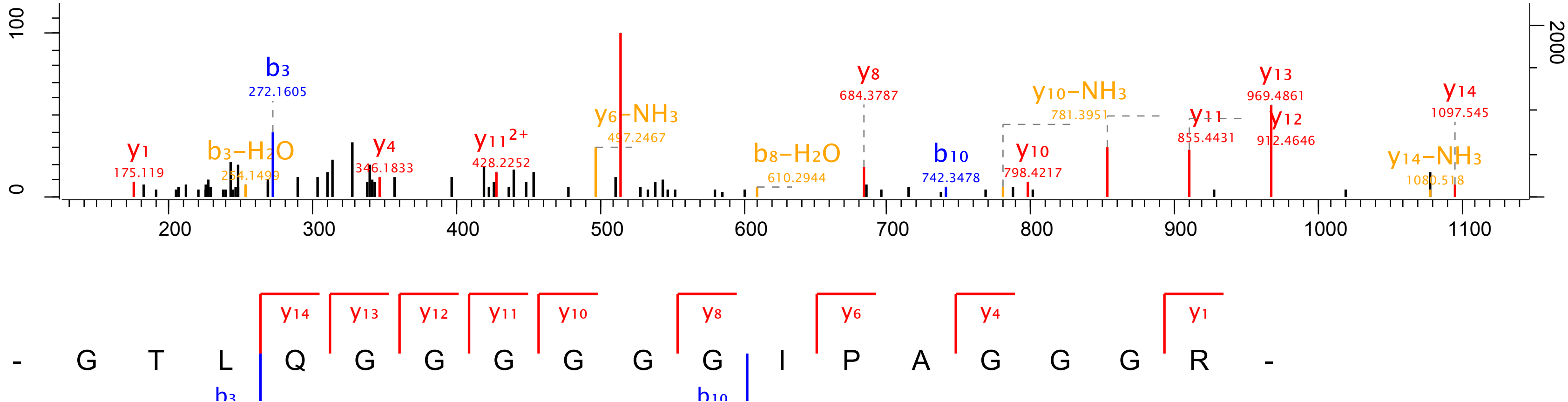

Raw file	Scan	Method	Score	m/z	Gene names
20140918_fract4_dyn_5ul_D4_01_371	13366	TOF; CID	151.98	459.56	CYB561D2

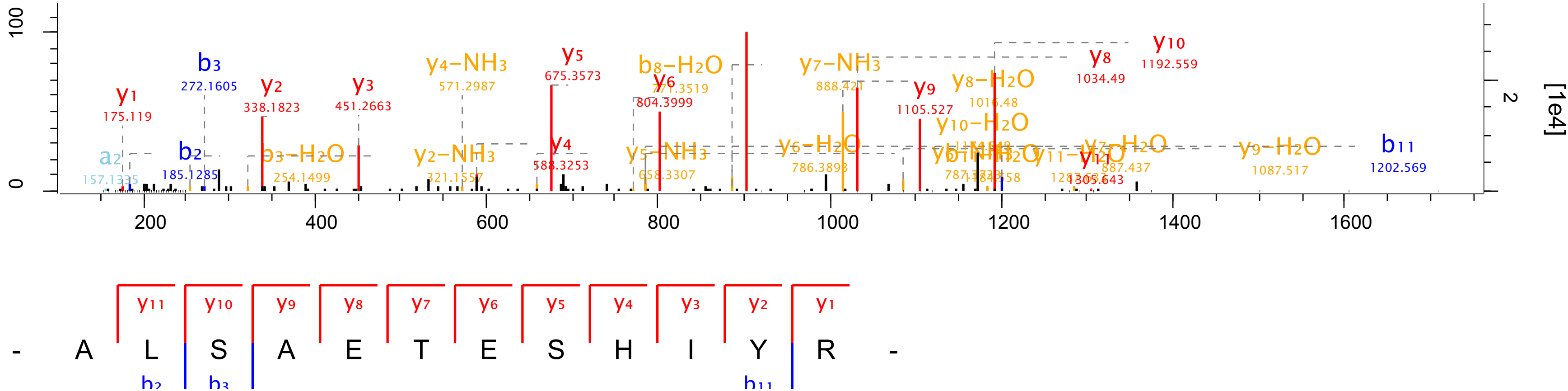

Raw file	Scan	Method	Score	m/z	Gene names
20140918_fract4_dyn_5ul_D4_01_371	13525	TOF; CID	93.84	577.32	PLEKHG4;CYB561A3

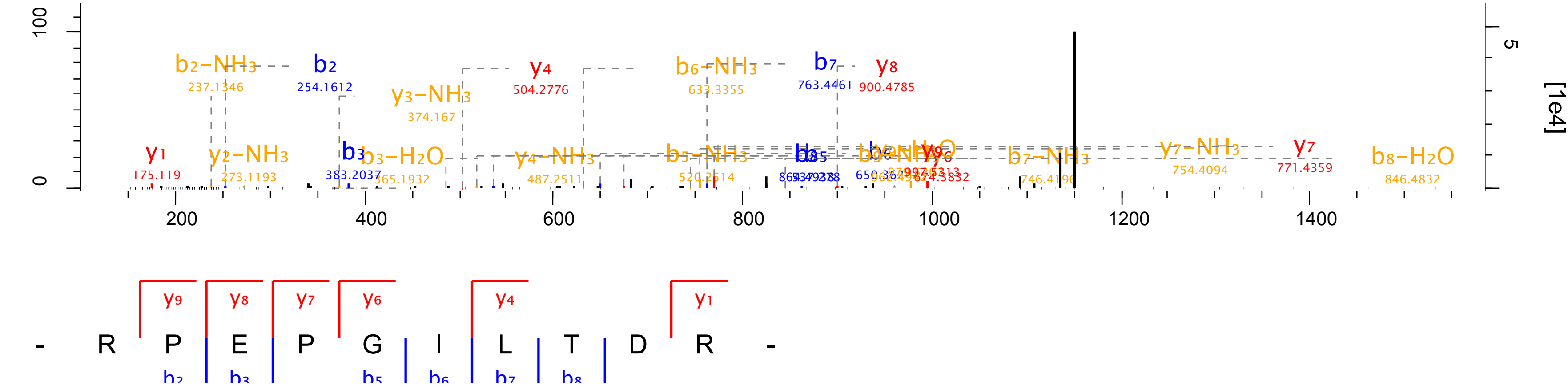

Raw file	Scan	Method	Score	m/z	Gene names
20140918_fract4_dyn_5ul_D4_01_371	17049	TOF; CID	58.32	779.34	ATG14

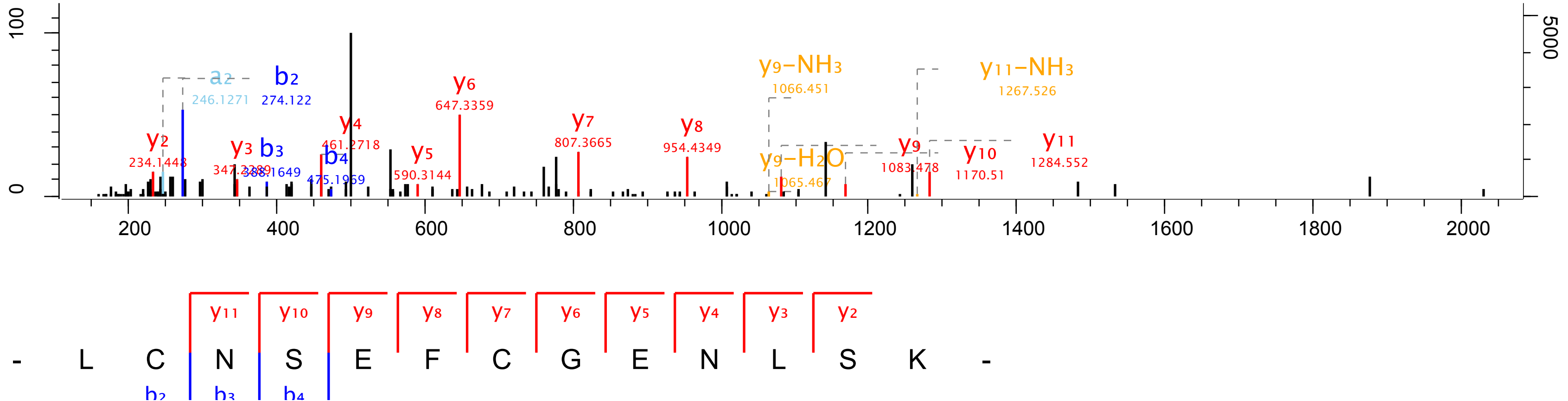

Raw file	Scan	Method	Score	m/z	Gene names
20140918_fract4_dyn_5ul_D4_01_371	19402	TOF; CID	53.56	734.85	CYLC1

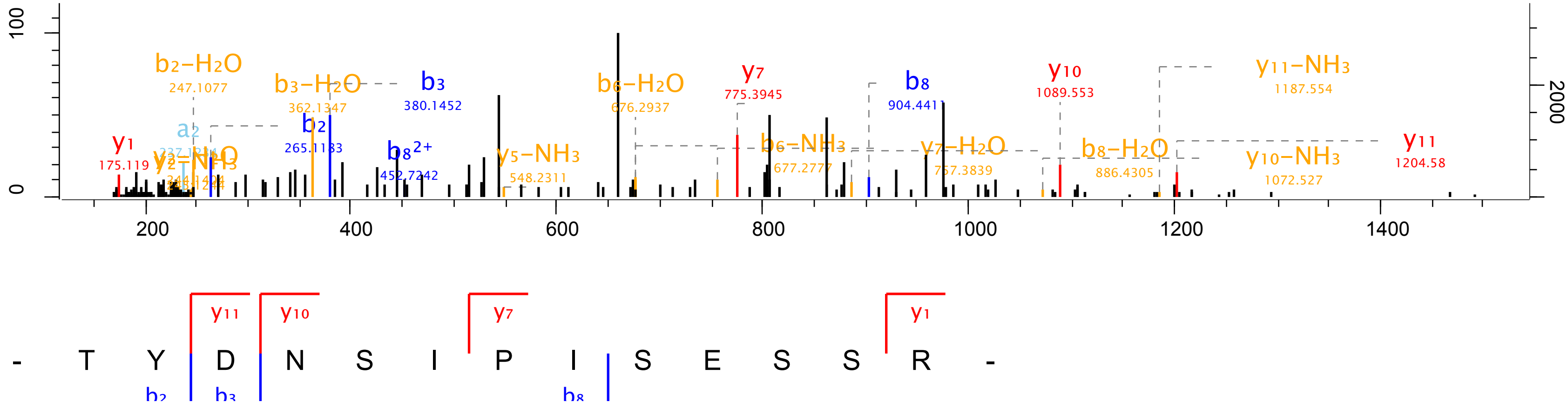

Raw file	Scan	Method	Score	m/z	Gene names
20140918_fract4_dyn_5ul_D4_01_371	19940	TOF; CID	64.1	650.82	SLC36A4

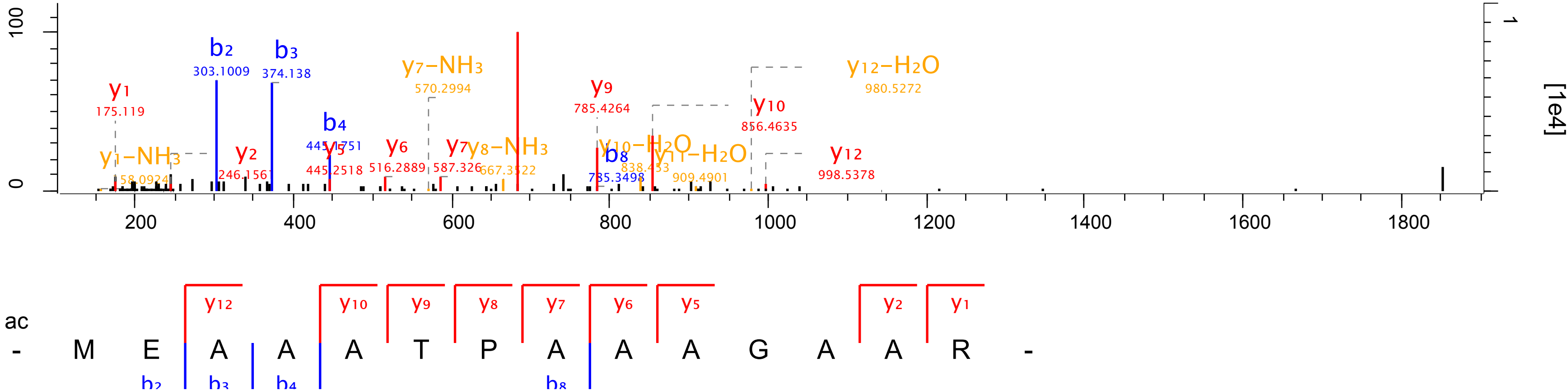

Raw file	Scan	Method	Score	m/z	Gene names
20140918_fract4_dyn_5ul_D4_01_371	21822	TOF; CID	117.48	610.32	FAM110A

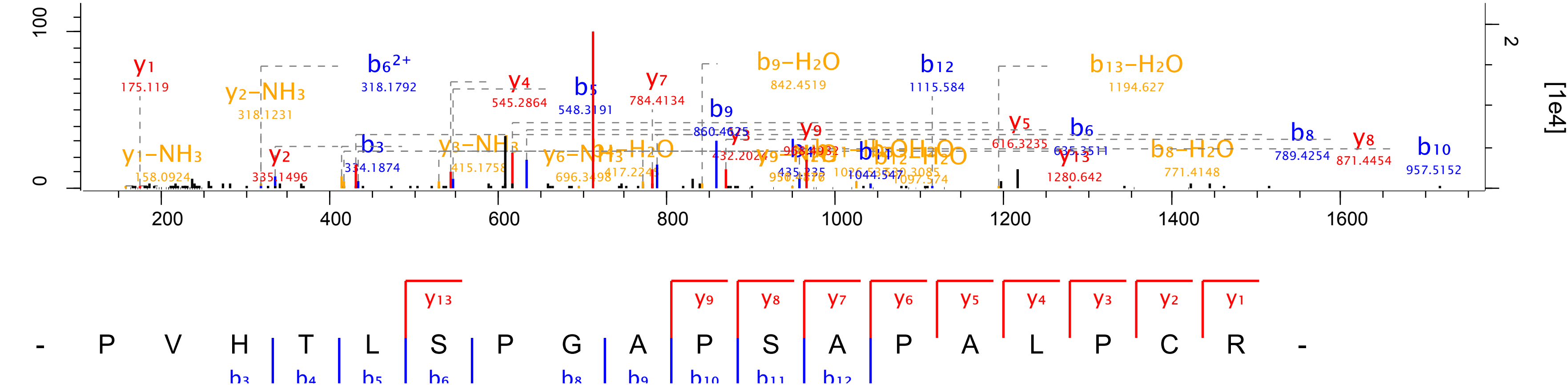

Raw file	Scan	Method	Score	m/z	Gene names
20140918_fract4_dyn_5ul_D4_01_371	22016	TOF; CID	119.87	532.62	SOCS3

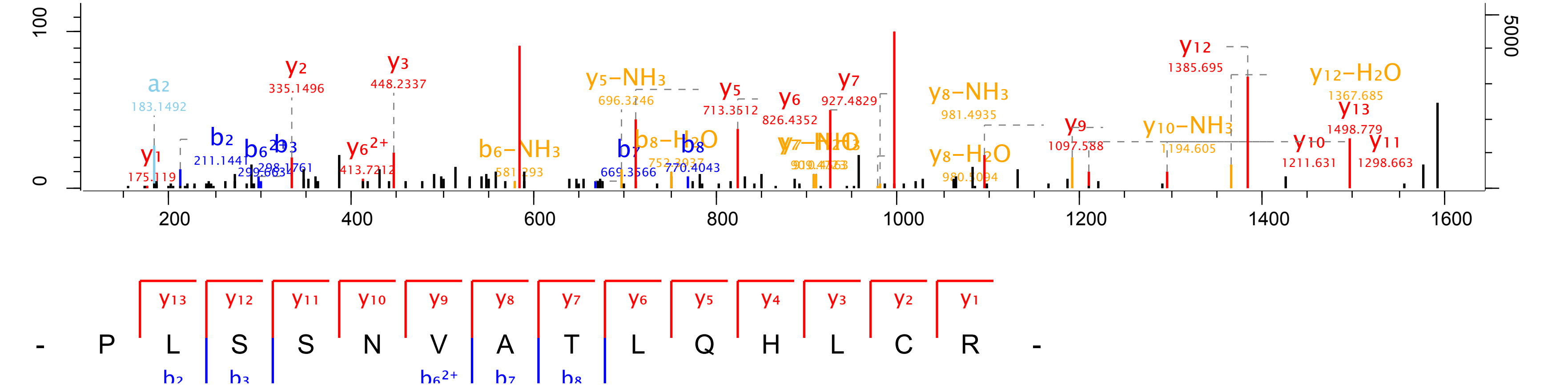

Raw file	Scan	Method	Score	m/z	Gene names
20140918_fract4_dyn_5ul_D4_01_371	24299	TOF; CID	100.74	420.23	SMDT1

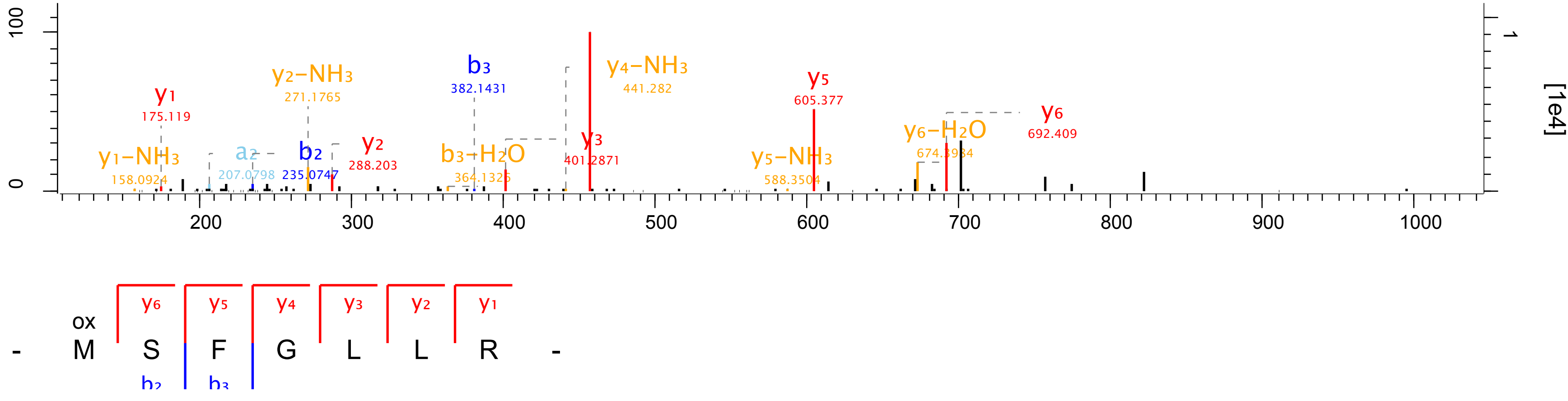

Raw file	Scan	Method	Score	m/z	Gene names
20140918_fract4_dyn_5ul_D4_01_371	26213	TOF; CID	141.55	959.96	DSCR3

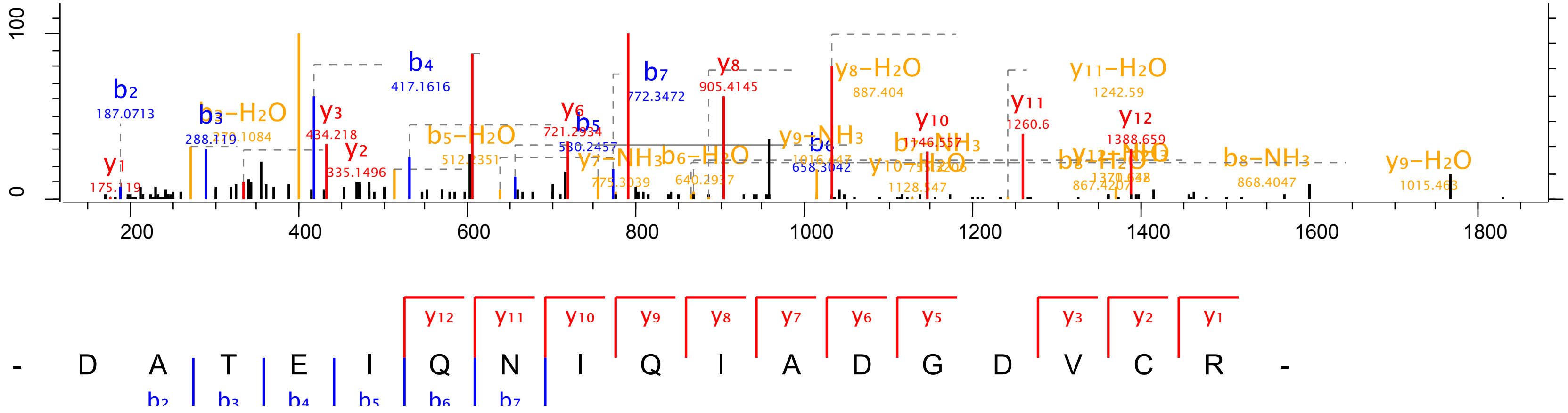

Raw file	Scan	Method	Score	m/z	Gene names
20140918_fract4_dyn_5ul_D4_01_371	27437	TOF; CID	77.75	855.39	METTL7B

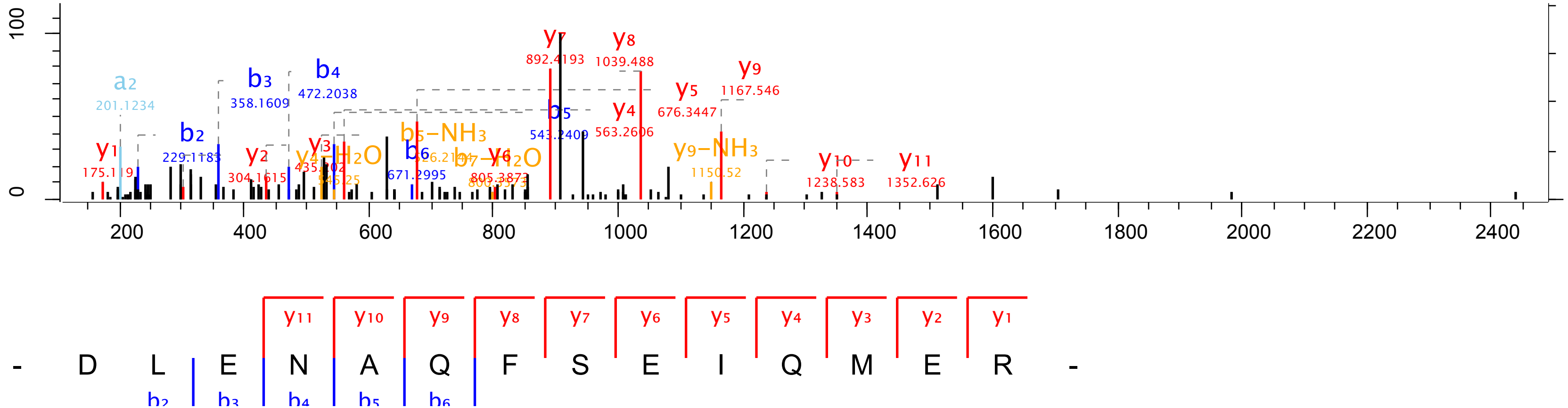

Raw file	Scan	Method	Score	m/z	Gene names
20140918_fract4_dyn_5ul_D4_01_371	30094	TOF; CID	155.92	723.7	LMBRD1

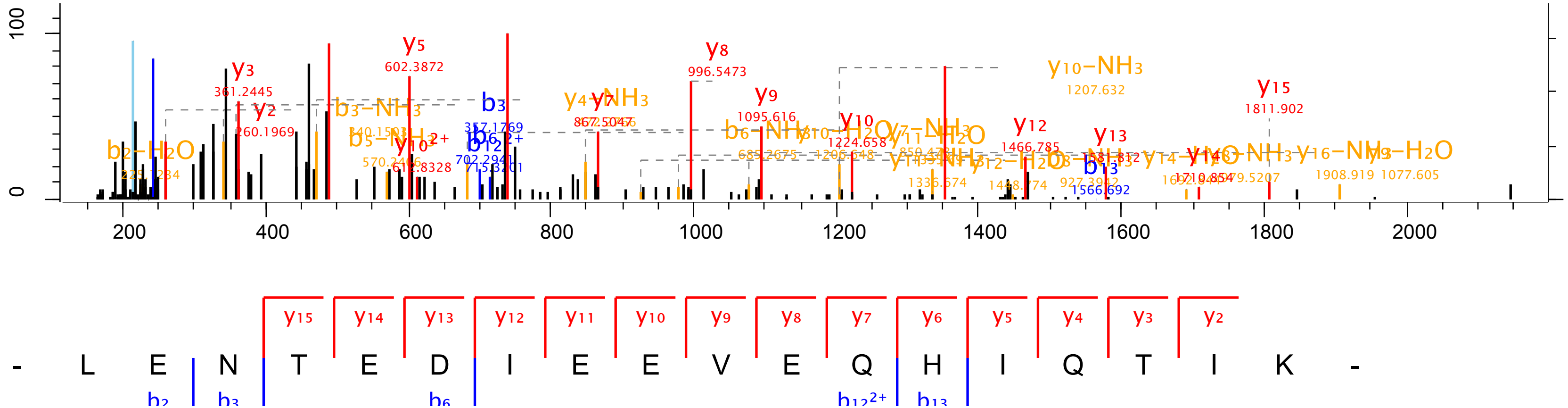

Raw file	Scan	Method	Score	m/z	Gene names
20140918_fract4_dyn_5ul_D4_01_371	30714	TOF; CID	68.04	740.89	TMEM219

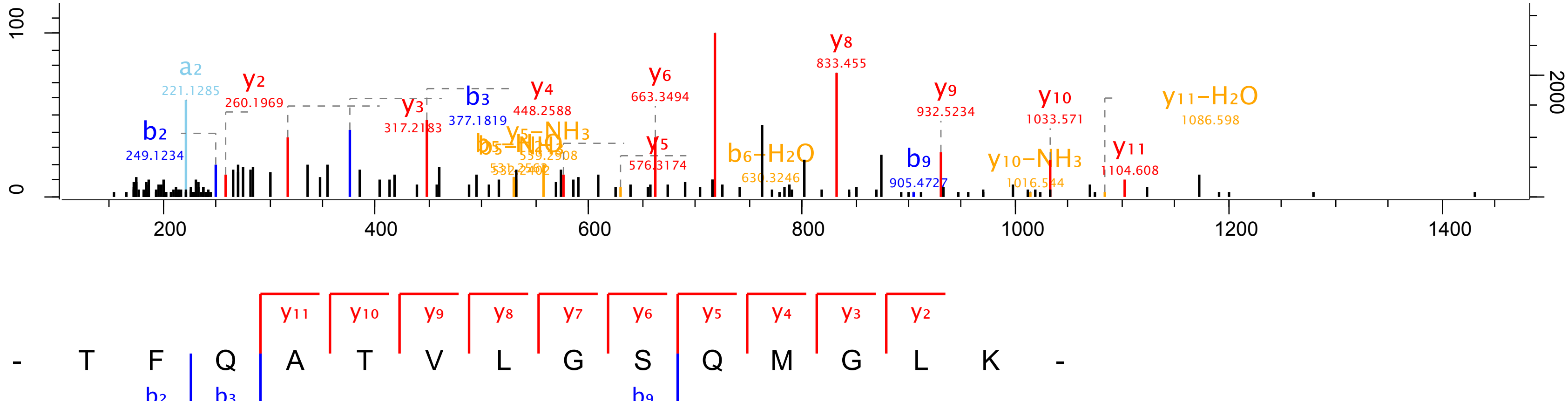

Raw file	Scan	Method	Score	m/z	Gene names
20140918_fract4_dyn_5ul_D4_01_371	32344	TOF; CID	66.83	668.65	PERP

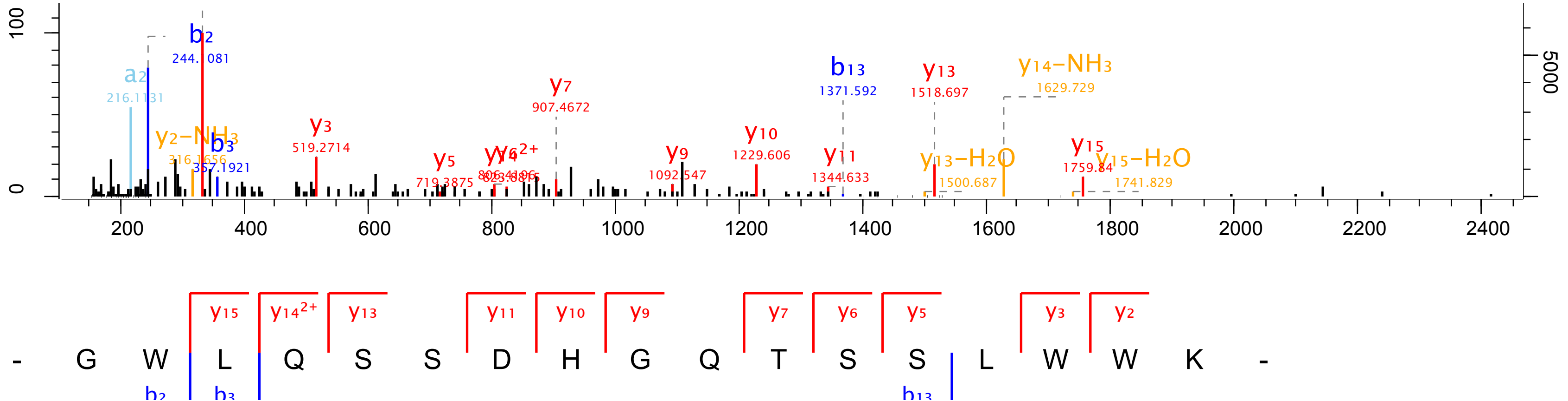

Raw file	Scan	Method	Score	m/z	Gene names
20140918_fract4_dyn_5ul_D4_01_371	37863	TOF; CID	70.27	773.91	DEPDC1B

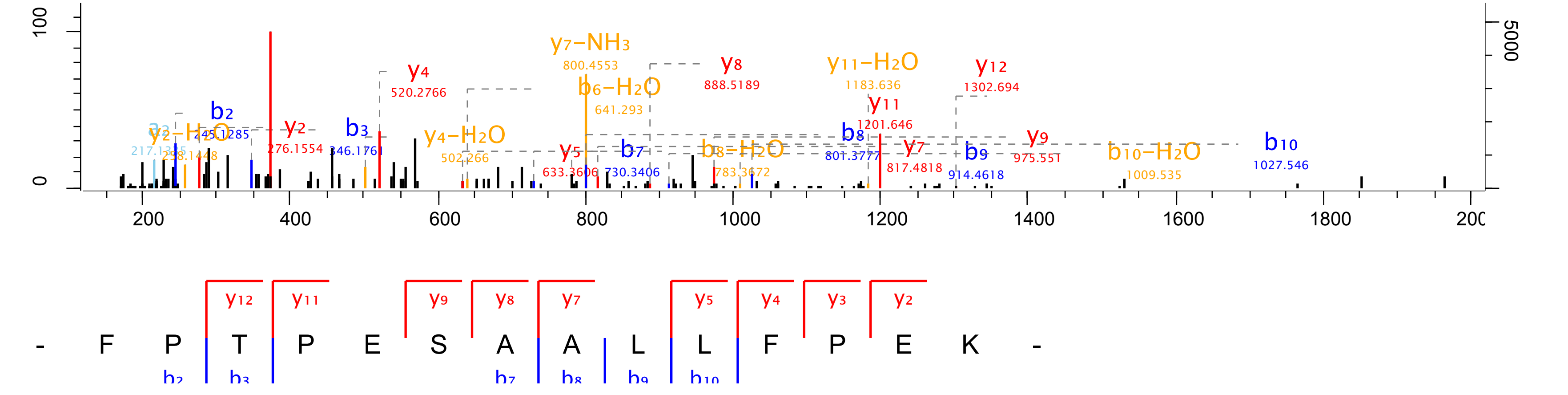

Raw file	Scan	Method	Score	m/z	Gene names
20140918_fract4_dyn_5ul_D4_01_371	38454	TOF; CID	141.58	797.94	CLDN12

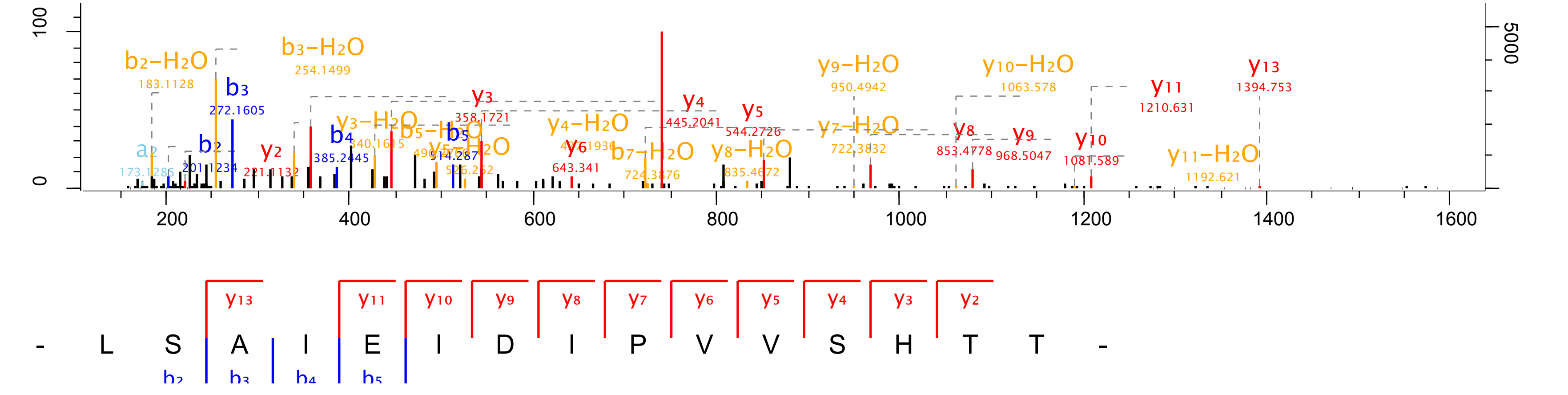

Raw file	Scan	Method	Score	m/z	Gene names
20140918_fract4_dyn_5ul_D4_01_371	39129	TOF; CID	68.24	971.02	GINM1

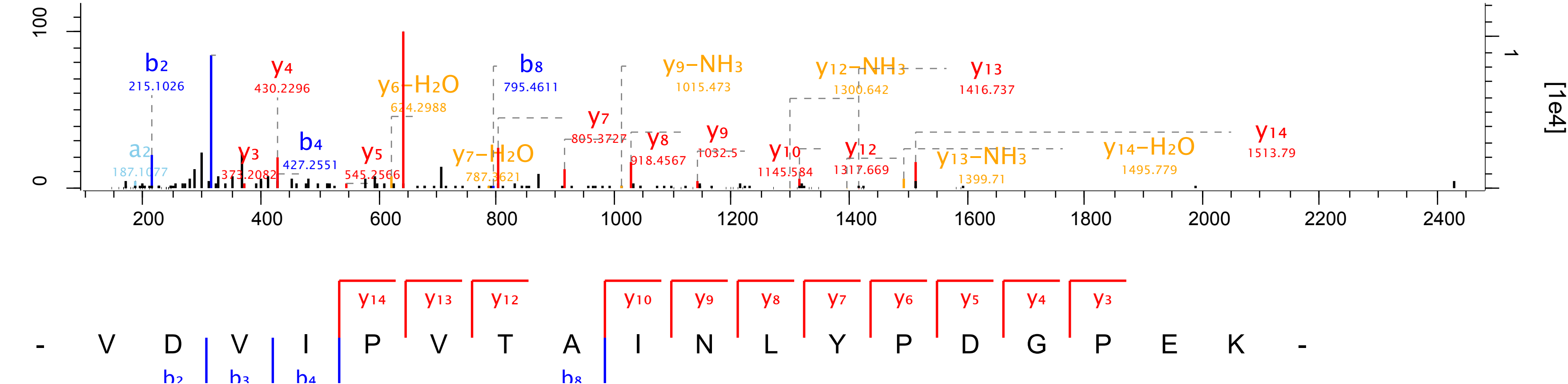

Raw file	Scan	Method	Score	m/z	Gene names
20140918_fract5_dyn_5ul_D5_01_372	5109	TOF; CID	110.53	421.21	FAM104A

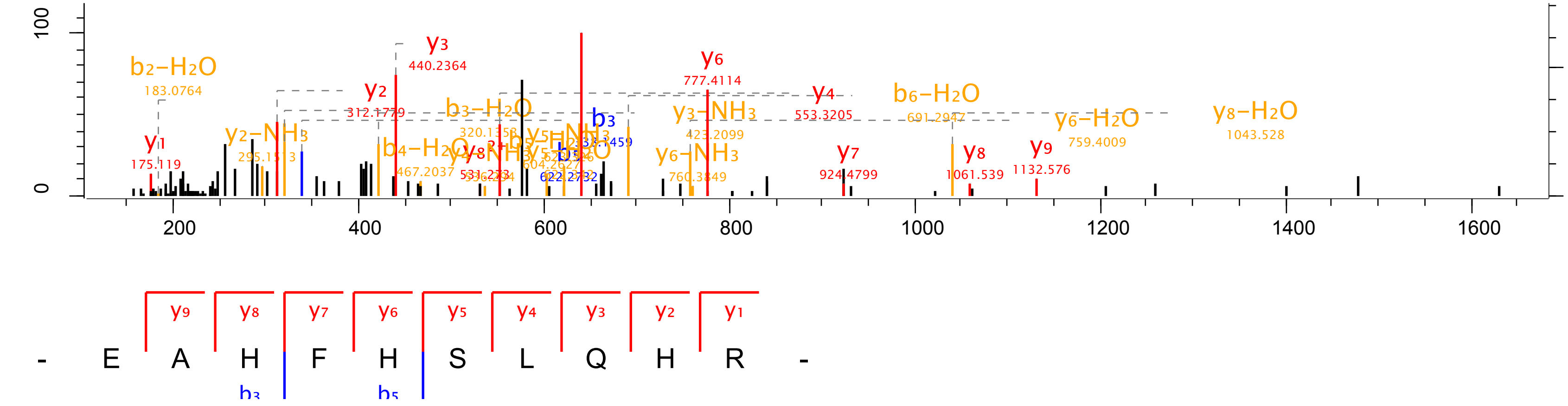

Raw file	Scan	Method	Score	m/z	Gene names
20140918_fract5_dyn_5ul_D5_01_372	7293	TOF; CID	89.83	563.76	CRELD2

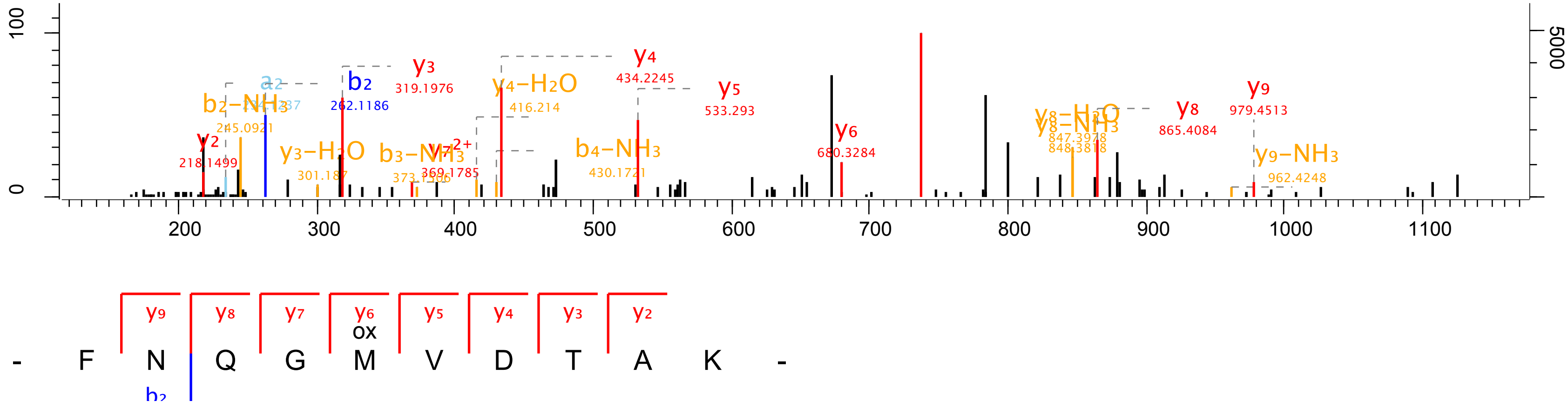

Raw file	Scan	Method	Score	m/z	Gene names
20140918_fract5_dyn_5ul_D5_01_372	11876	TOF; CID	79.47	408.21	TMEM187

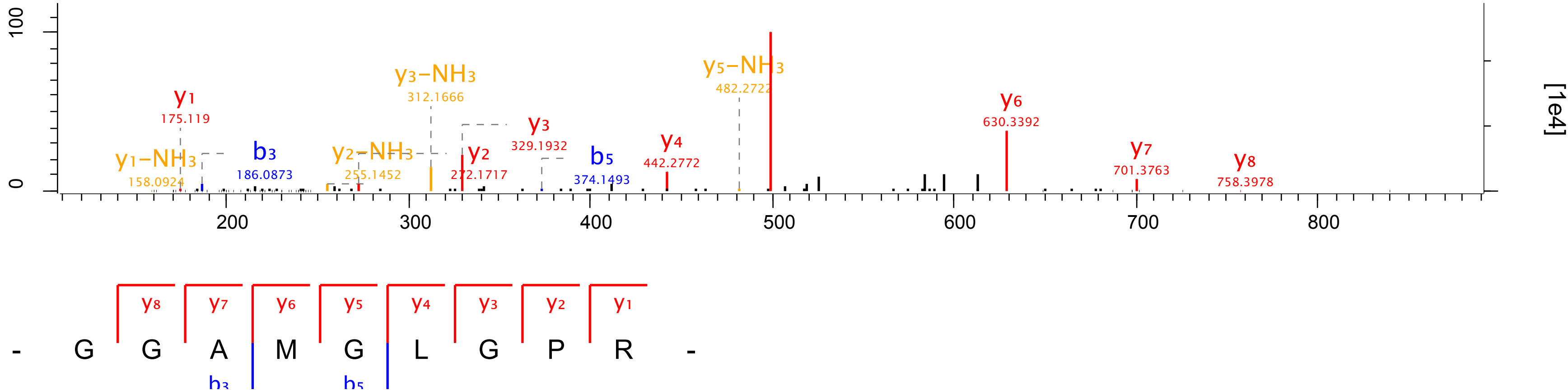

Raw file	Scan	Method	Score	m/z	Gene names
20140918_fract5_dyn_5ul_D5_01_372	13299	TOF; CID	105.13	641.31	C17orf89

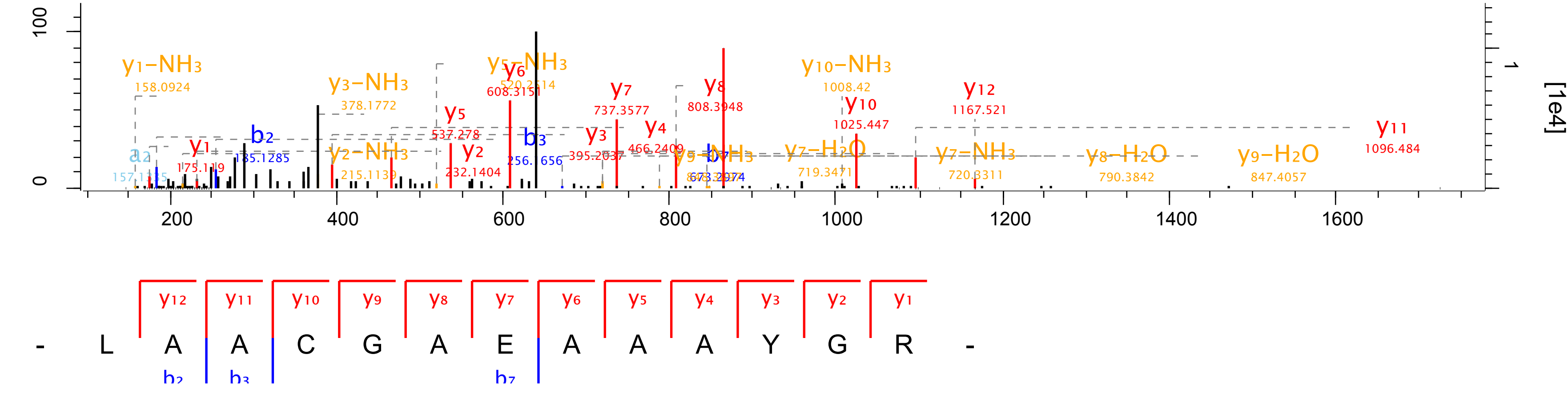

Raw file	Scan	Method	Score	m/z	Gene names
20140918_fract5_dyn_5ul_D5_01_372	14338	TOF; CID	64.82	660.81	RAB19

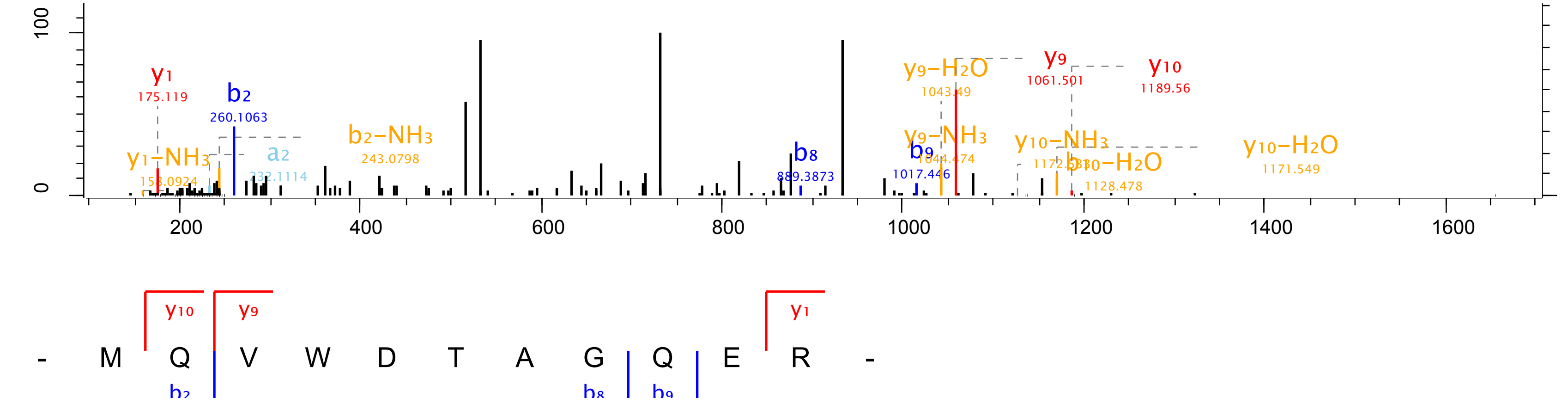

Raw file	Scan	Method	Score	m/z	Gene names
20140918_fract5_dyn_5ul_D5_01_372	14907	TOF; CID	94.02	510.62	IFI27L2

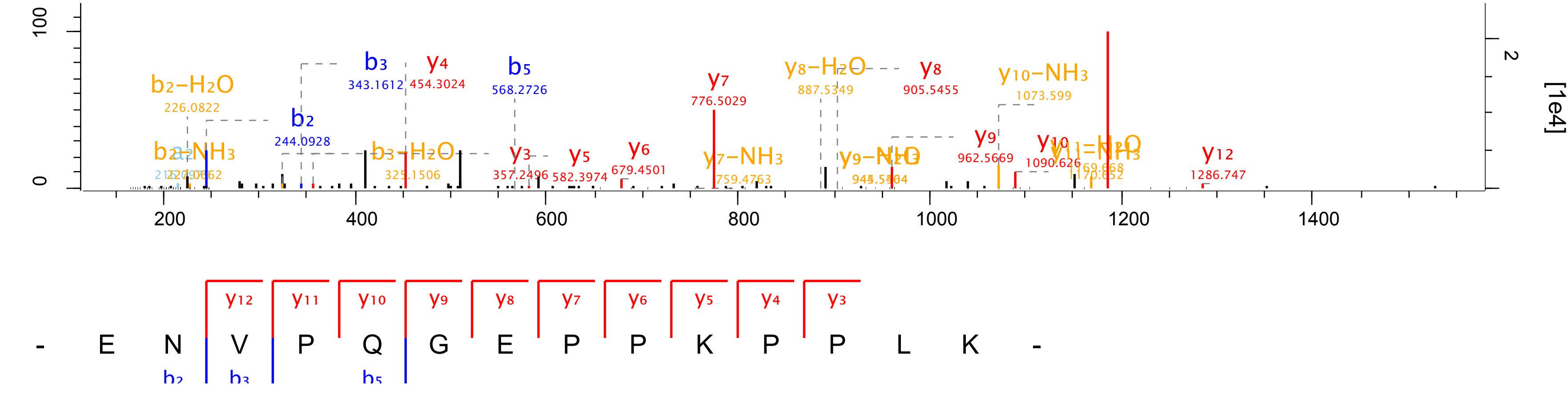

Raw file	Scan	Method	Score	m/z	Gene names
20140918_fract5_dyn_5ul_D5_01_372	19805	TOF; CID	55.72	746.39	C20orf24

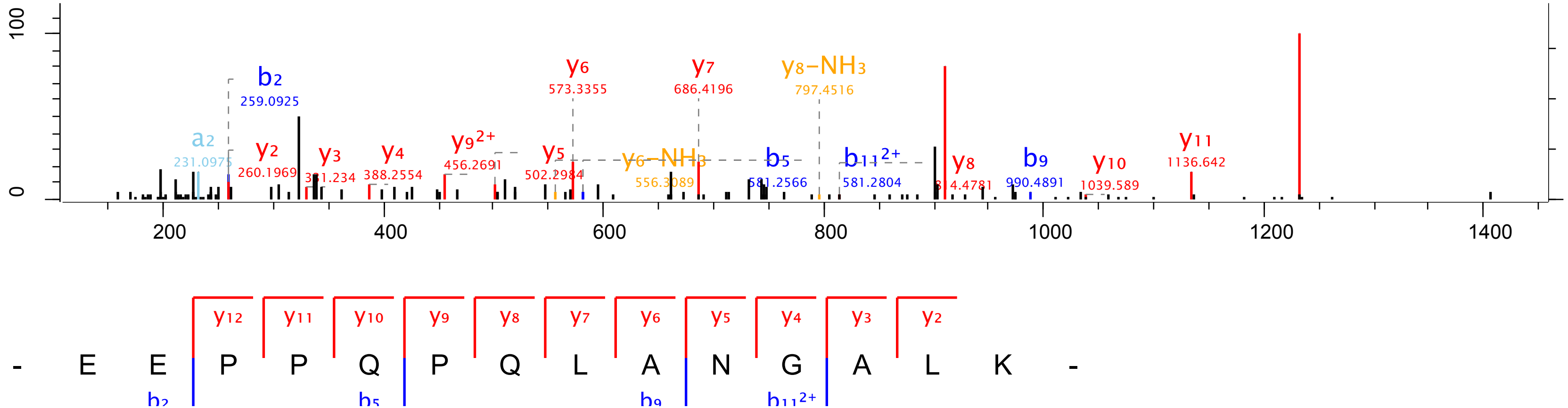

Raw file	Scan	Method	Score	m/z	Gene names
20140918_fract5_dyn_5ul_D5_01_372	19851	TOF; CID	99.71	860.4	DPH3

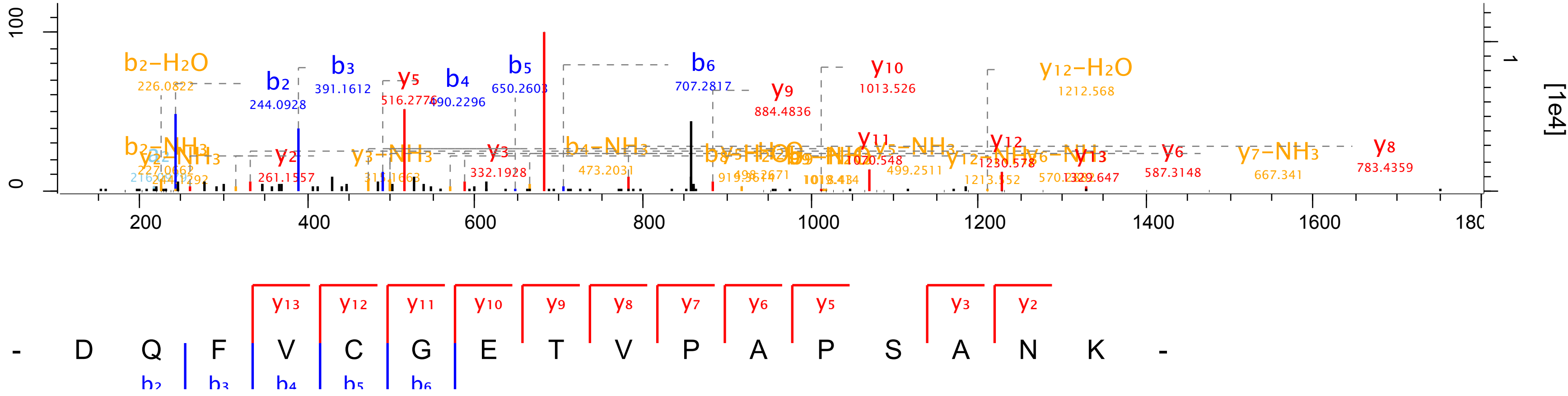

Raw file	Scan	Method	Score	m/z	Gene names
20140918_fract5_dyn_5ul_D5_01_372	22314	TOF; CID	124.59	451.25	BTBD16

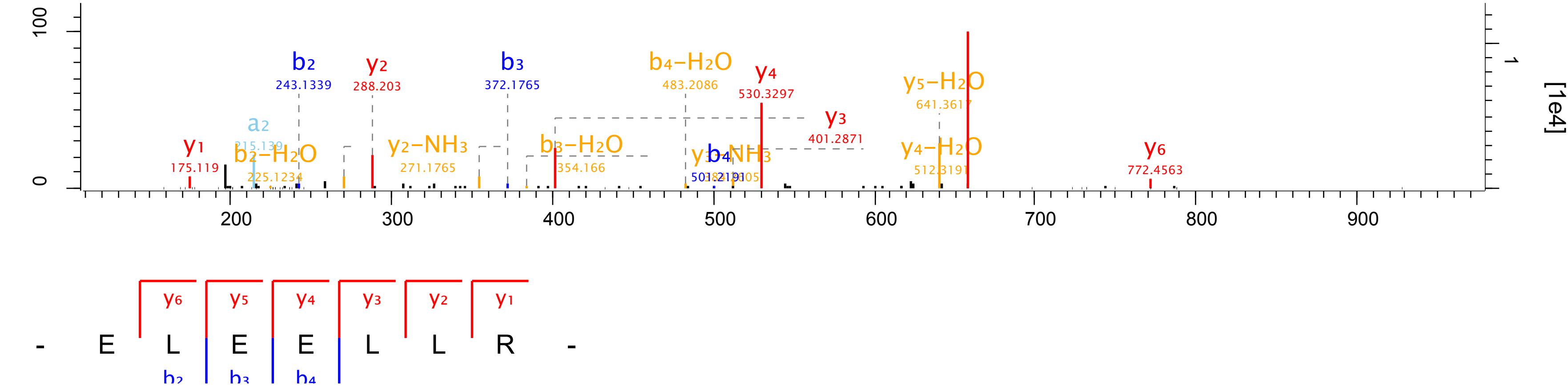

Raw file	Scan	Method	Score	m/z	Gene names
20140918_fract5_dyn_5ul_D5_01_372	26055	TOF; CID	133.13	633.32	DNAH14

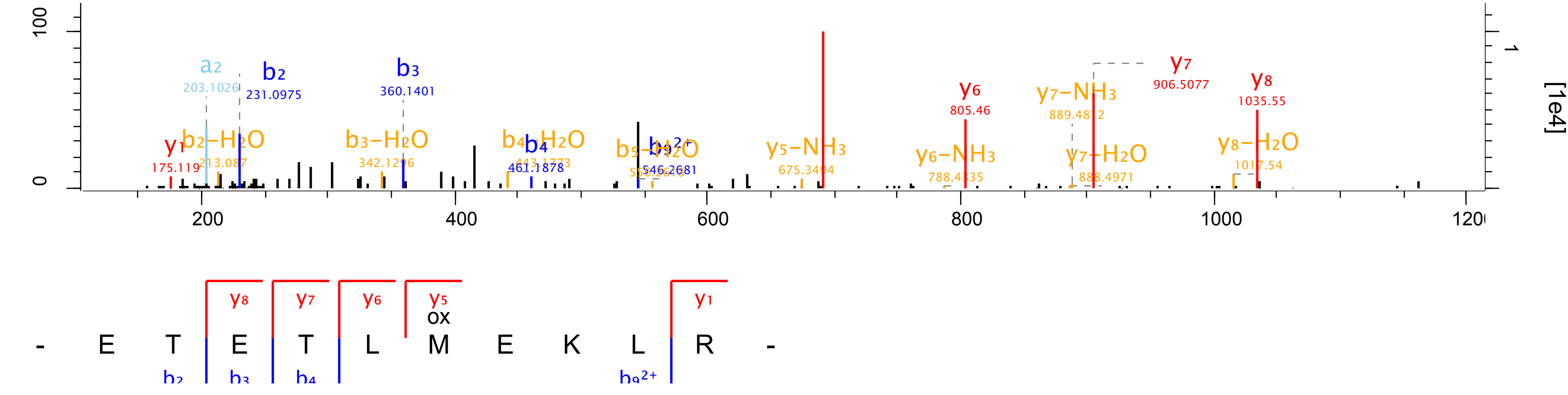

Raw file	Scan	Method	Score	m/z	Gene names
20140918_fract5_dyn_5ul_D5_01_372	26579	TOF; CID	101.28	943.97	MKKS

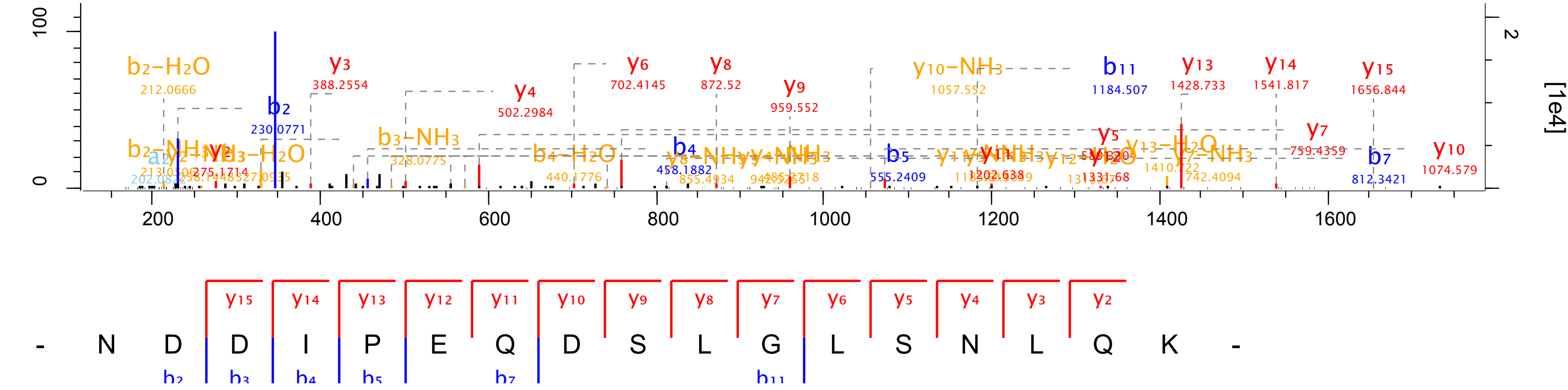

Raw file	Scan	Method	Score	m/z	Gene names
20140918_fract5_dyn_5ul_D5_01_372	27130	TOF; CID	66.99	614.37	MDM1;MAP2K6

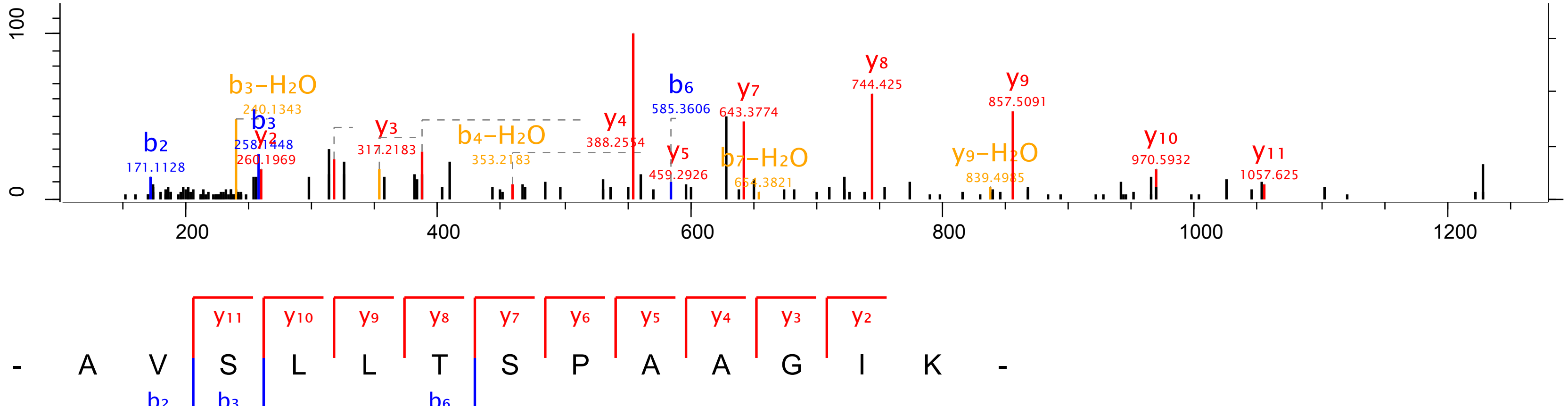

Raw file	Scan	Method	Score	m/z	Gene names
20140918_fract5_dyn_5ul_D5_01_372	28736	TOF; CID	81.14	802.06	CD68

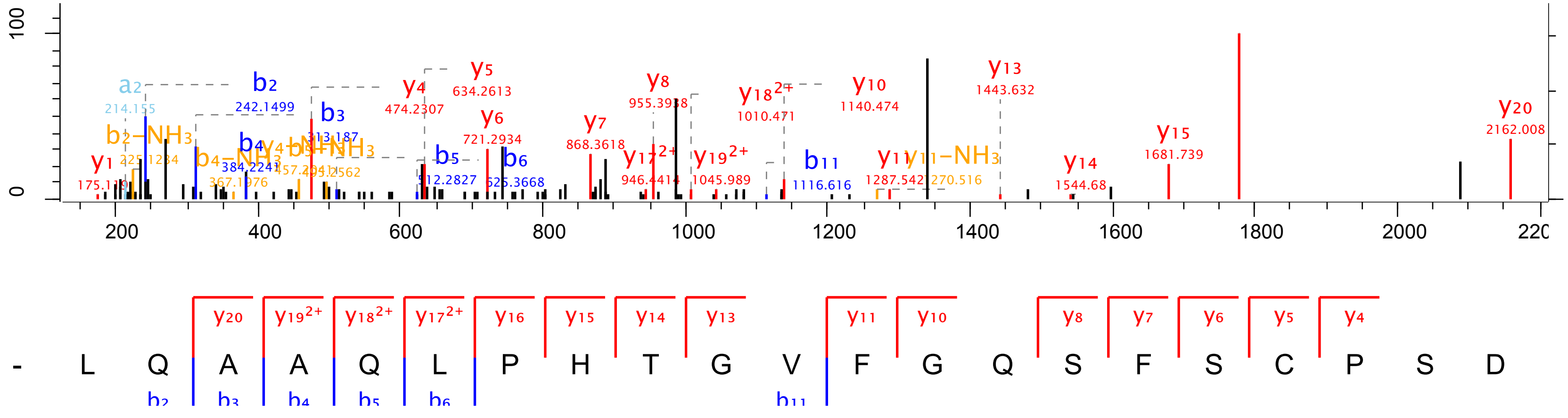

Raw file	Scan	Method	Score	m/z	Gene names
20140918_fract5_dyn_5ul_D5_01_372	30562	TOF; CID	100.02	735.03	UBALD1

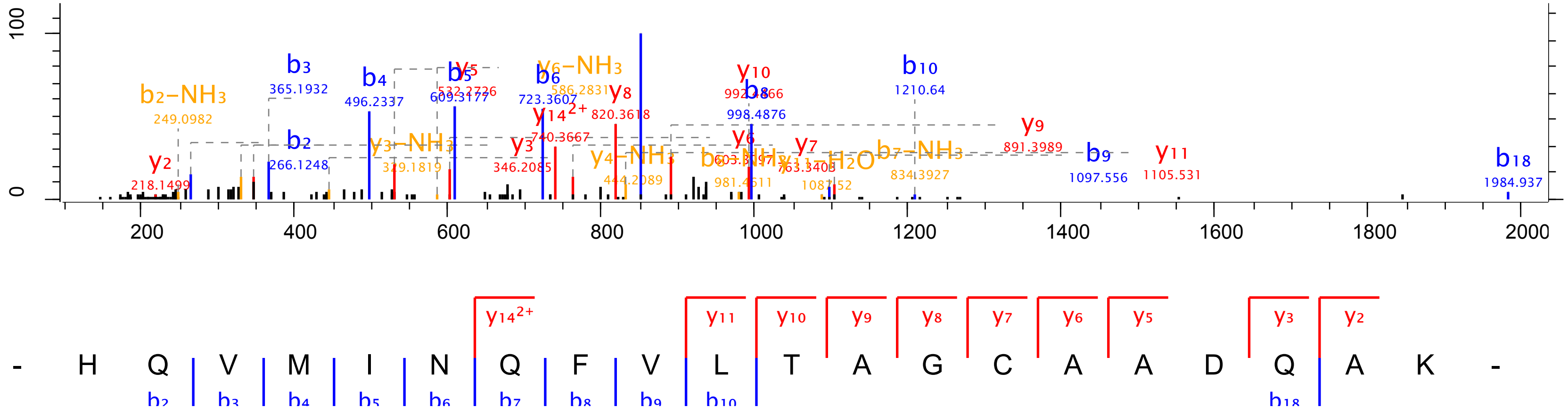

Raw file	Scan	Method	Score	m/z	Gene names
20140918_fract5_dyn_5ul_D5_01_372	31188	TOF; CID	93.37	581.33	ZDHHC12

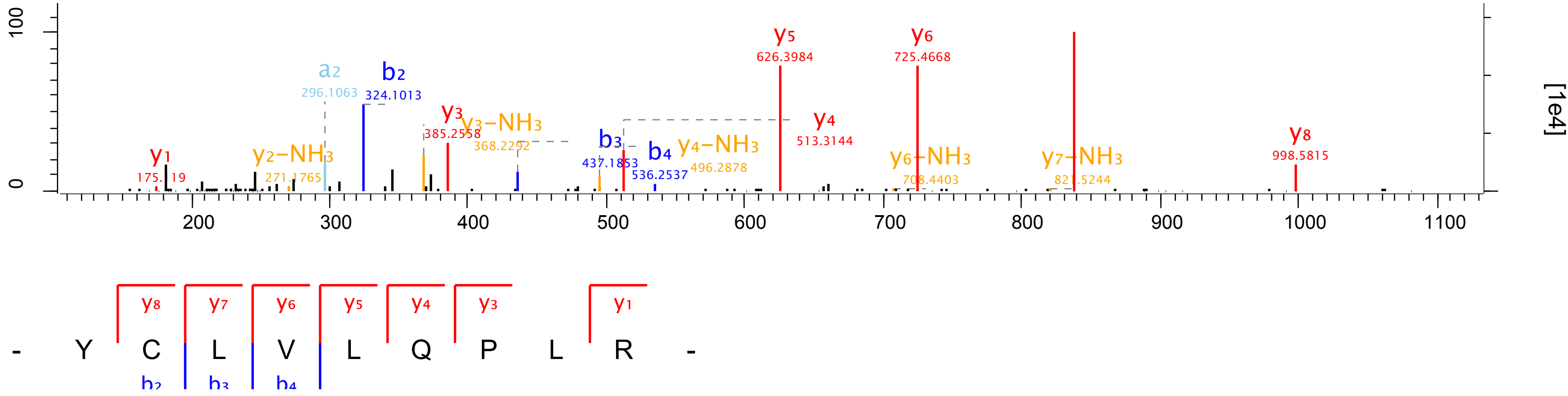

Raw file	Scan	Method	Score	m/z	Gene names
20140918_fract5_dyn_5ul_D5_01_372	31204	TOF; CID	129.38	626.32	OTUB2

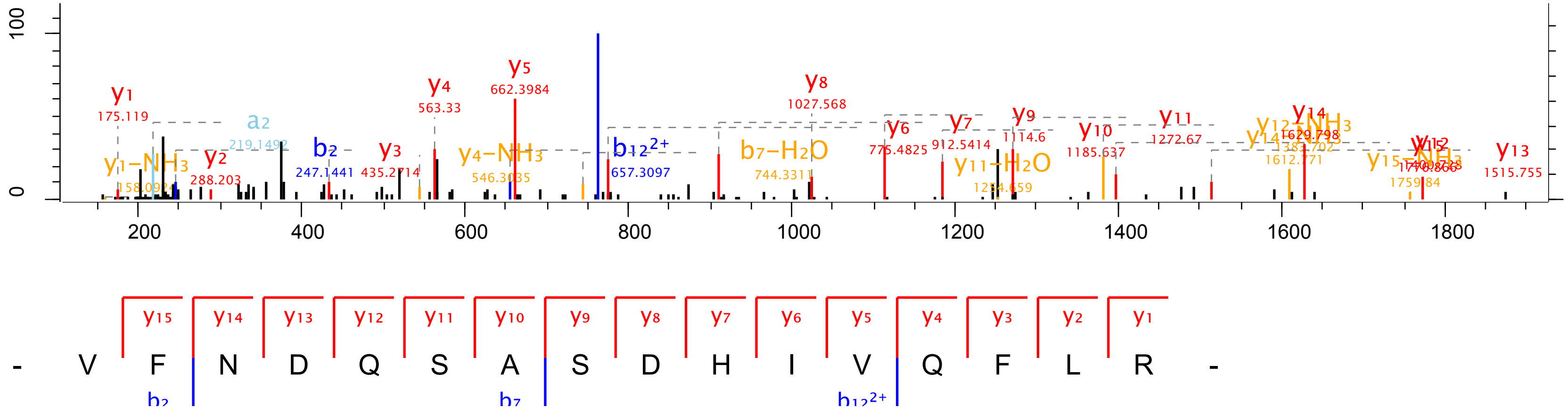

Raw file	Scan	Method	Score	m/z	Gene names
20140918_fract5_dyn_5ul_D5_01_372	32271	TOF; CID	119.53	649.82	TEN1

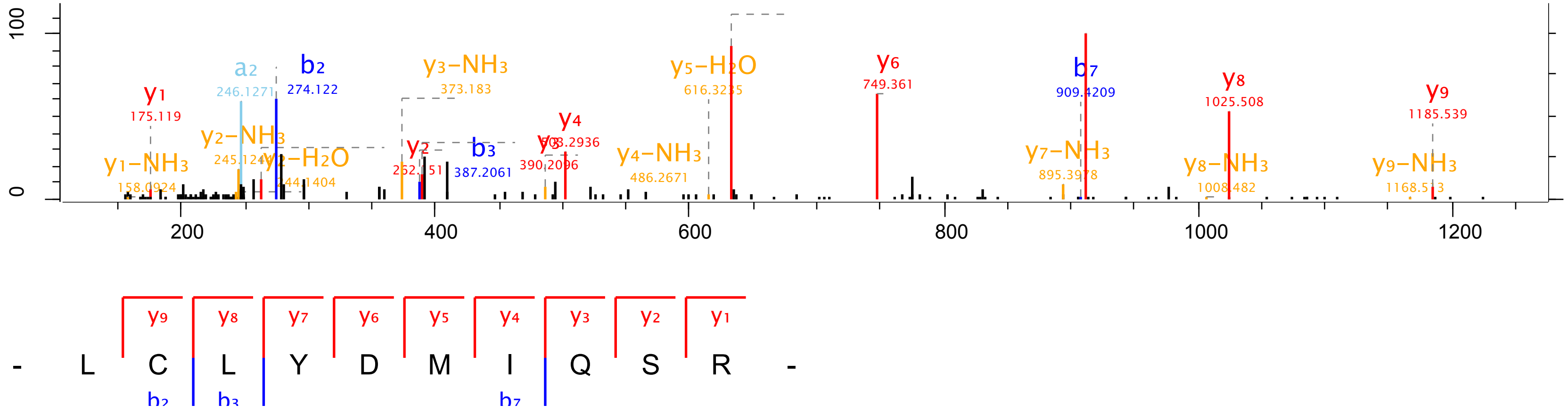

Raw file	Scan	Method	Score	m/z	Gene names
20140918_fract5_dyn_5ul_D5_01_372	32920	TOF; CID	88.28	950.95	APOBEC3C

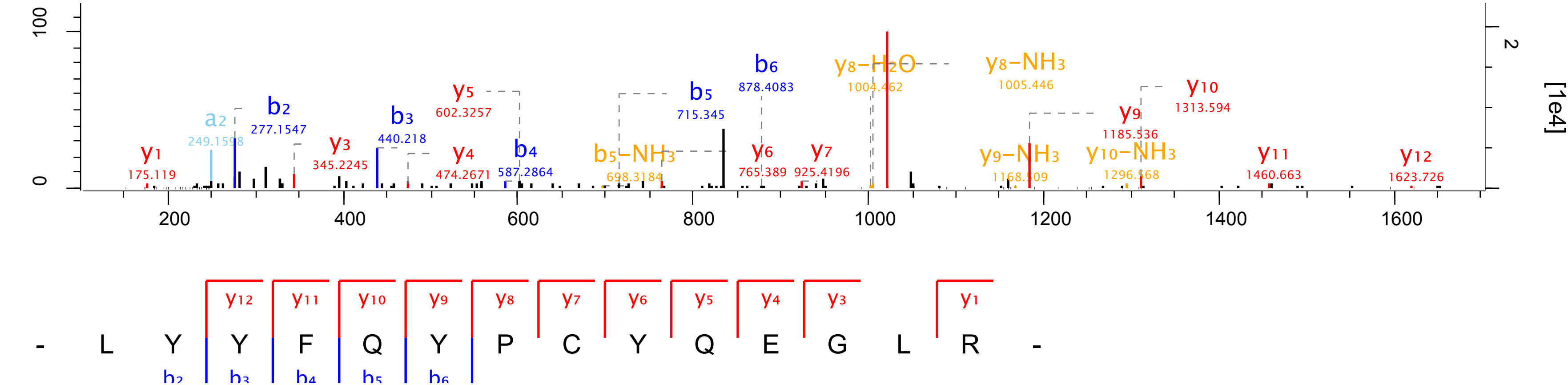

Raw file	Scan	Method	Score	m/z	Gene names
20140918_fract5_dyn_5ul_D5_01_372	33628	TOF; CID	64.1	773.43	PIGL

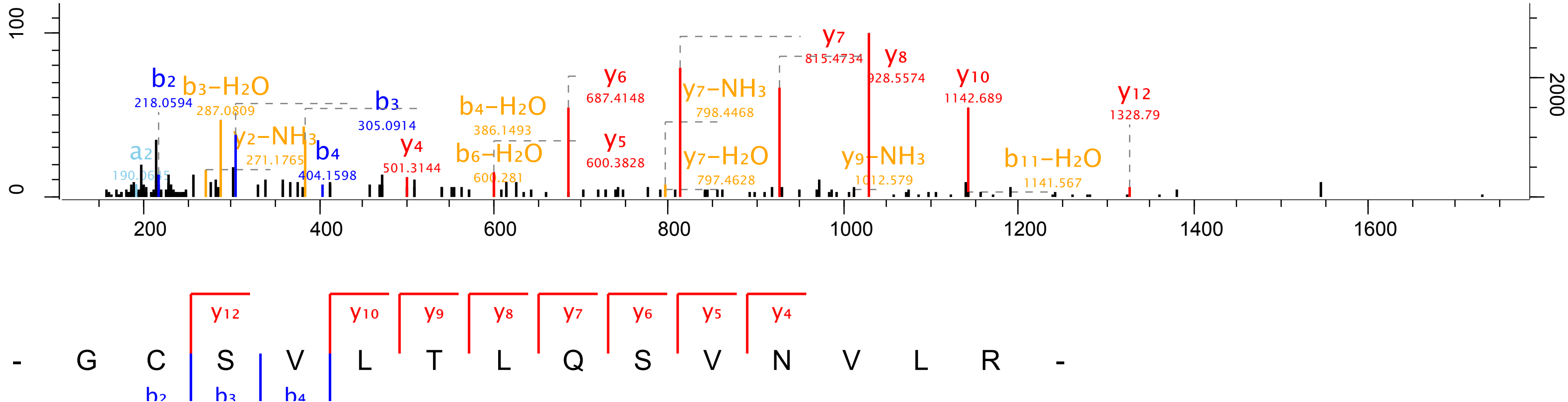

Raw file	Scan	Method	Score	m/z	Gene names
20140918_fract5_dyn_5ul_D5_01_372	34434	TOF; CID	59.23	659.36	CDC14B

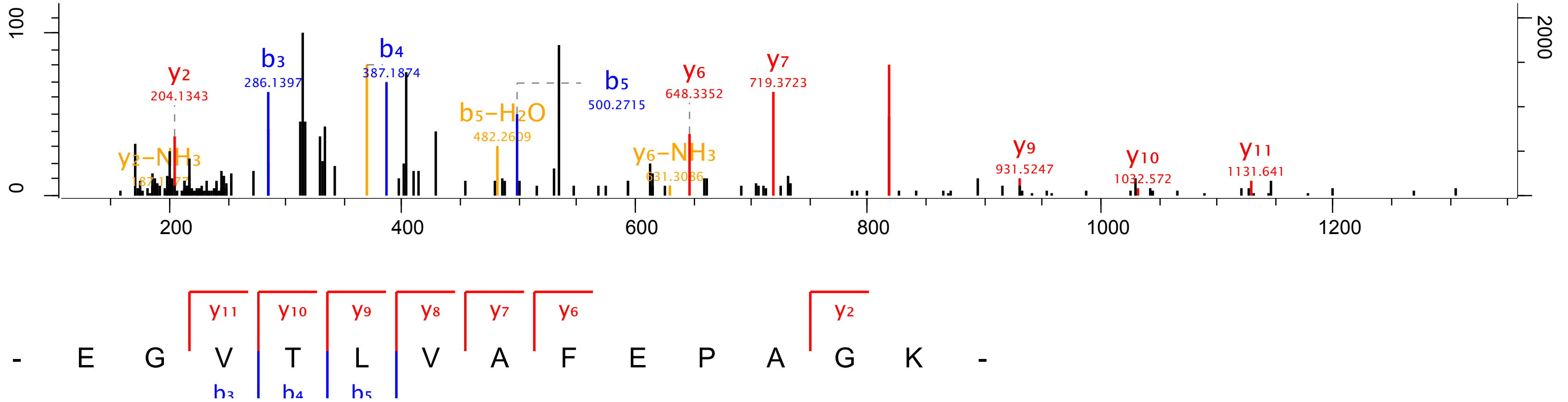

Raw file	Scan	Method	Score	m/z	Gene names
20140918_fract5_dyn_5ul_D5_01_372	34980	TOF; CID	104.22	576.86	SPIN4

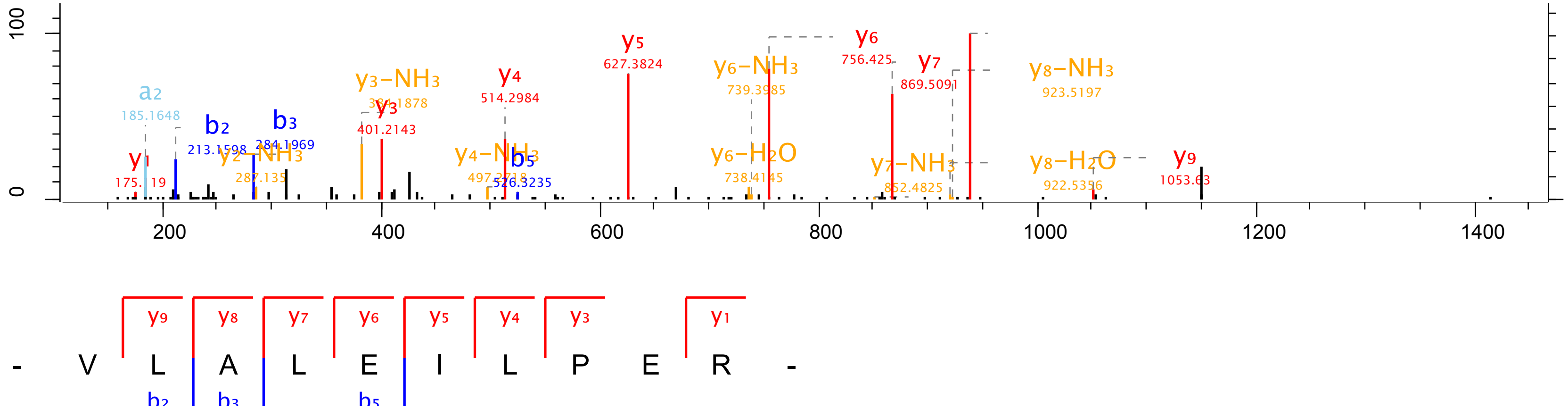

Raw file	Scan	Method	Score	m/z	Gene names
20140918_fract5_dyn_5ul_D5_01_372	36322	TOF; CID	68.24	651.32	BCL2

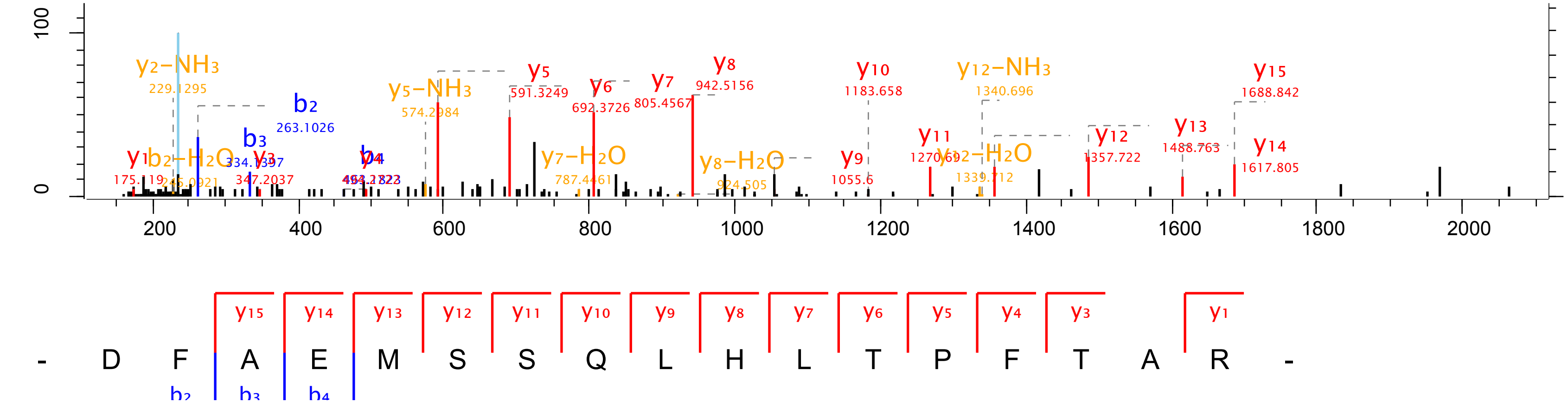

Raw file	Scan	Method	Score	m/z	Gene names
20140918_fract5_dyn_5ul_D5_01_372	37423	TOF; CID	54.09	557.29	IFT46

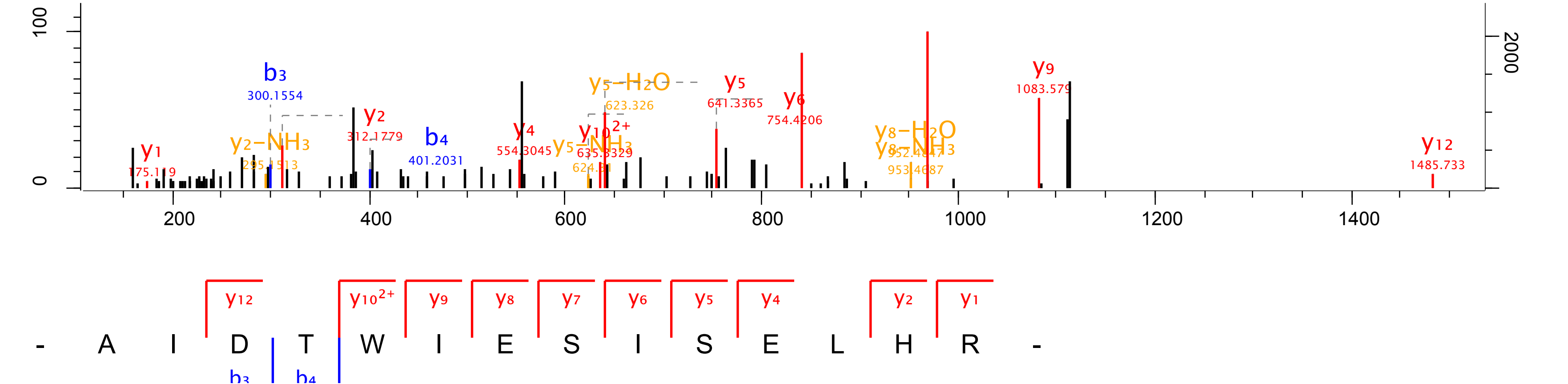

Raw file	Scan	Method	Score	m/z	Gene names
20140918_fract5_dyn_5ul_D5_01_372	37450	TOF; CID	89.23	750.91	TMEM128

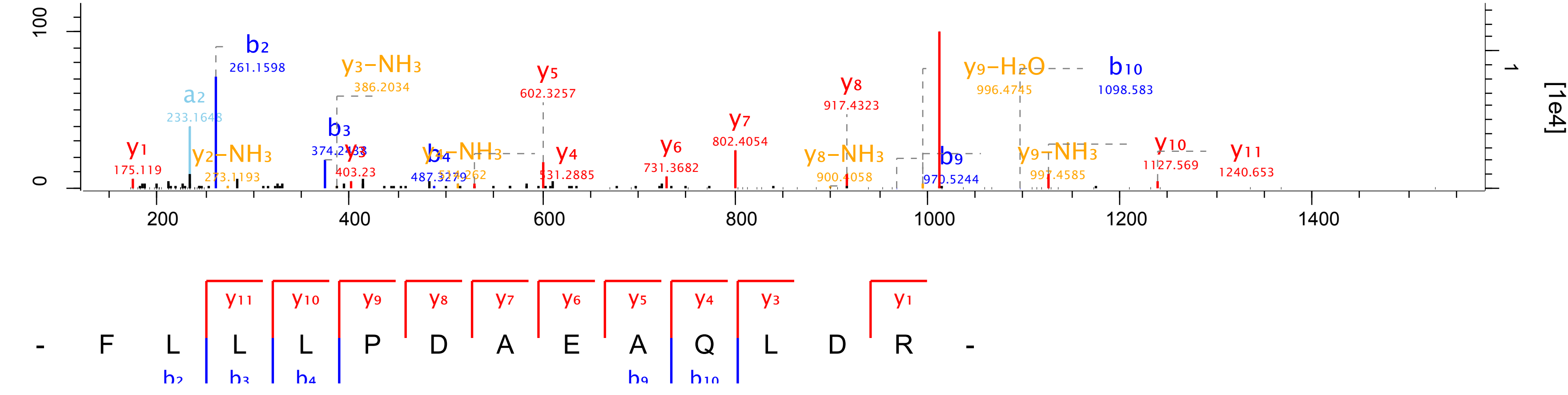

Raw file	Scan	Method	Score	m/z	Gene names
20140918_fract5_dyn_5ul_D5_01_372	38197	TOF; CID	110.9	1119.89	MRPL54

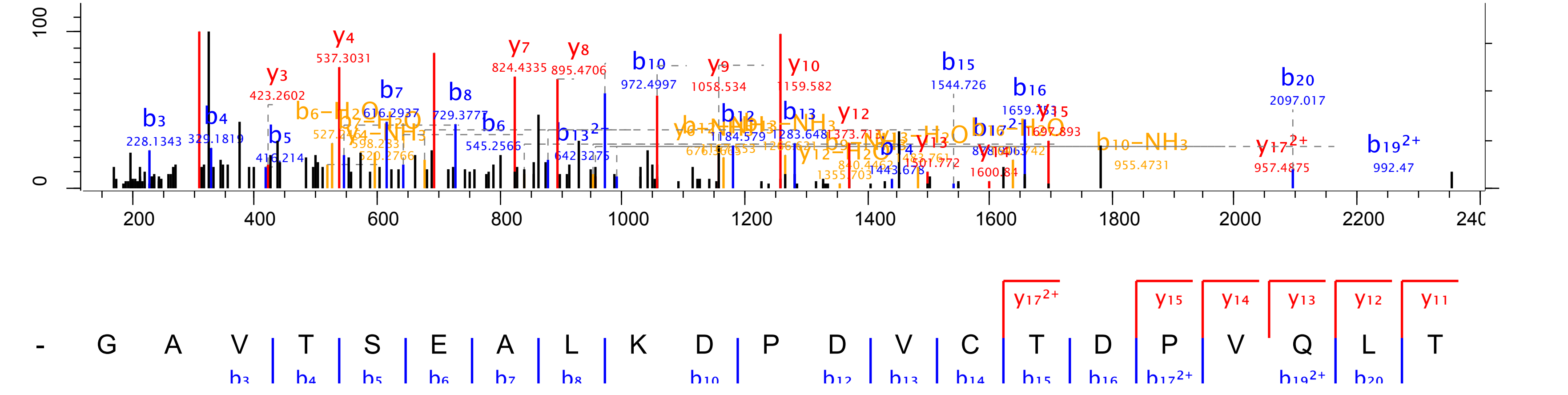

Raw file	Scan	Method	Score	m/z	Gene names
20140918_fract6_dyn_5ul_D6_01_373	5072	TOF; CID	144.09	594.3	NR1H3

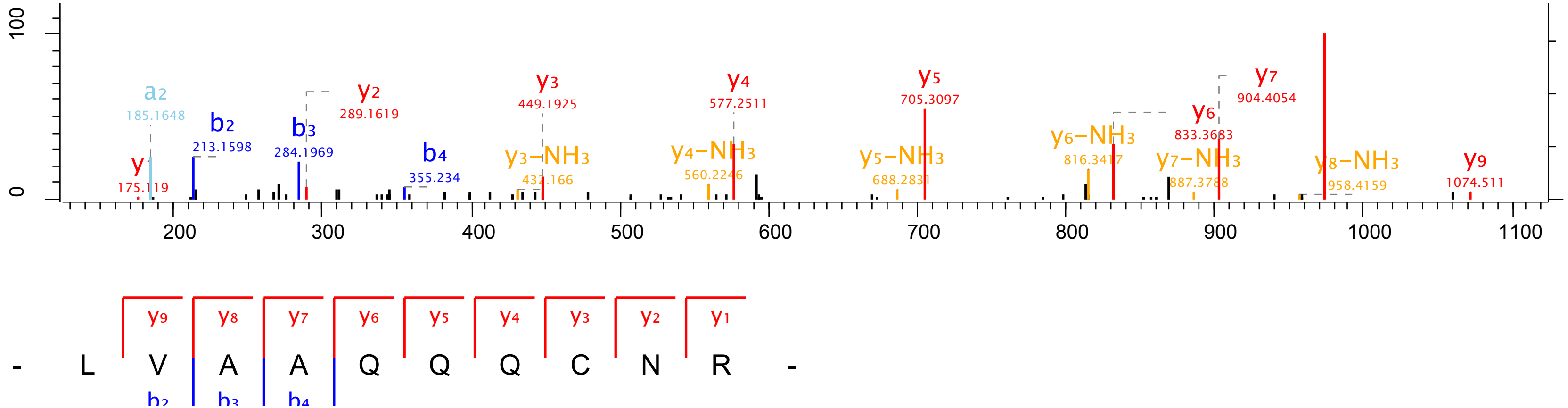

Raw file	Scan	Method	Score	m/z	Gene names
20140918_fract6_dyn_5ul_D6_01_373	7562	TOF; CID	91.07	566.3	ZKSCAN4

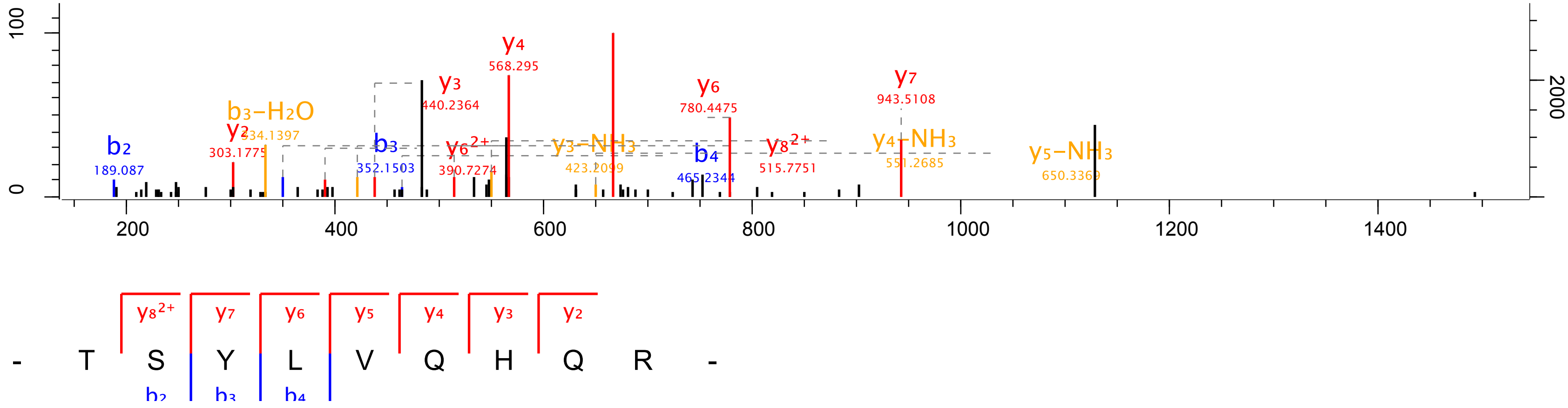

Raw file	Scan	Method	Score	m/z	Gene names
20140918_fract6_dyn_5ul_D6_01_373	8867	TOF; CID	111.63	625.82	STXBP6

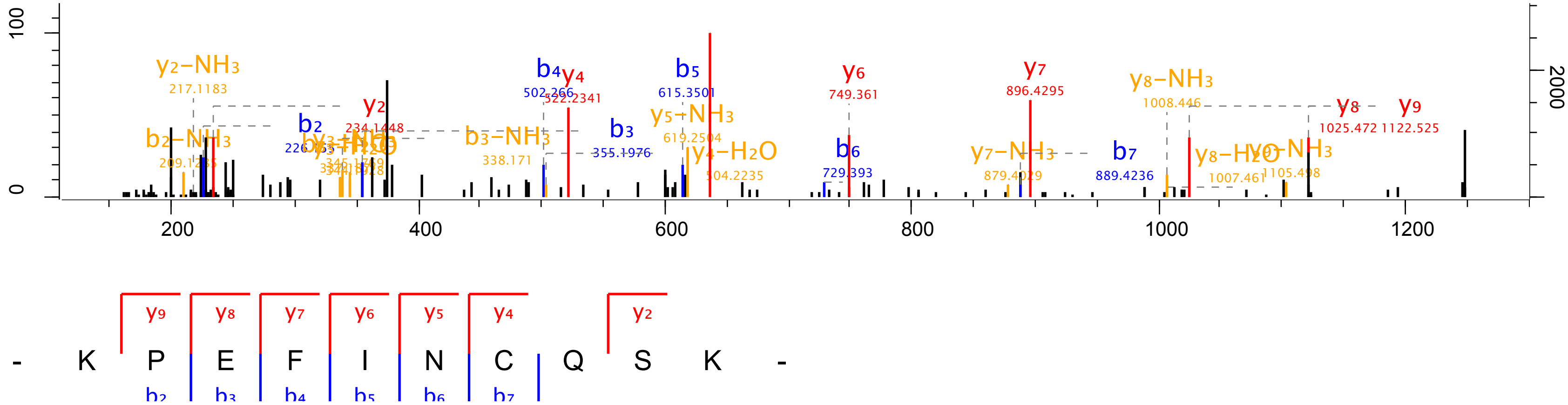

Raw file	Scan	Method	Score	m/z	Gene names
20140918_fract6_dyn_5ul_D6_01_373	10923	TOF; CID	105.95	617.33	HDGFRP2

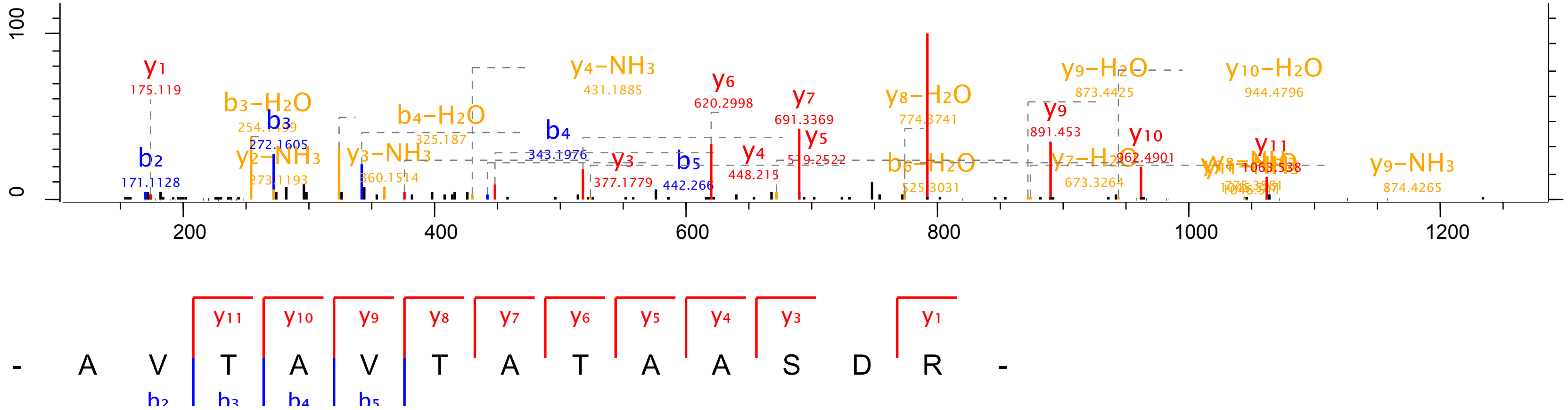

Raw file	Scan	Method	Score	m/z	Gene names
20140918_fract6_dyn_5ul_D6_01_373	13691	TOF; CID	133.42	607.83	SLC25A14

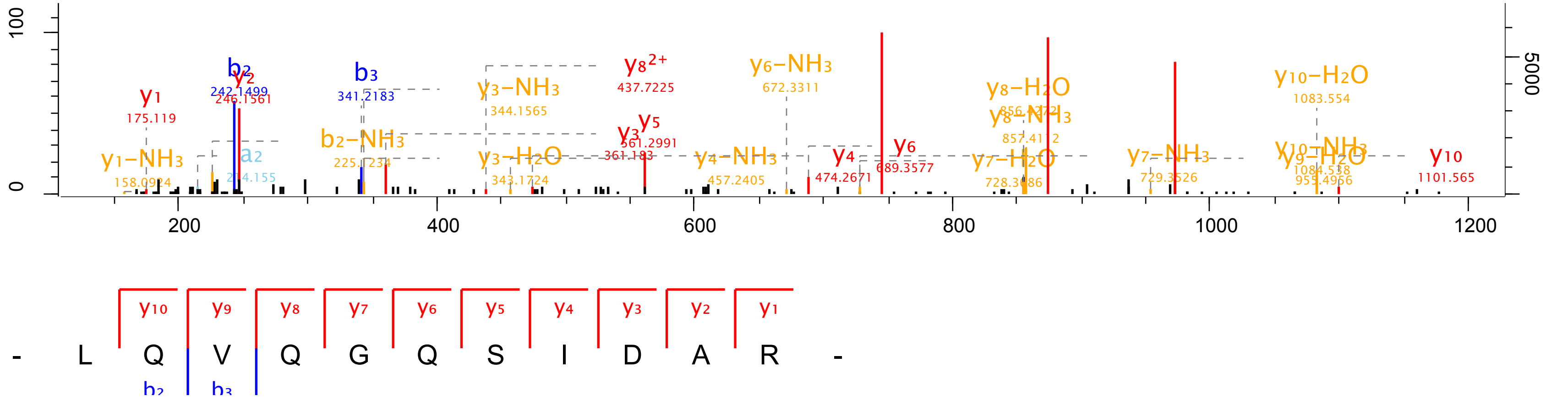

Raw file	Scan	Method	Score	m/z	Gene names
20140918_fract6_dyn_5ul_D6_01_373	15296	TOF; CID	143.5	638.29	CKS1B

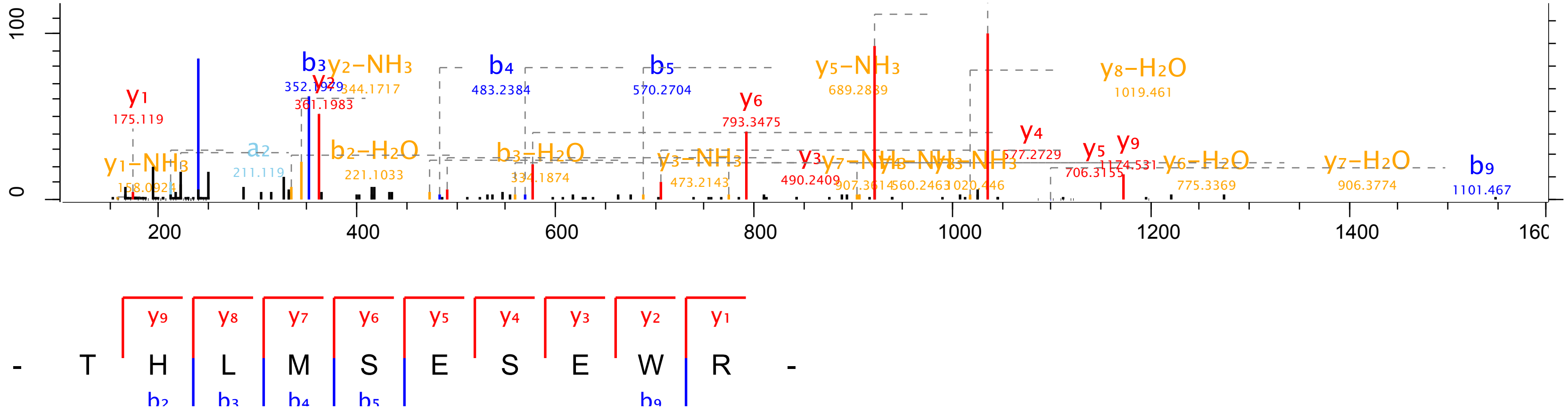

Raw file	Scan	Method	Score	m/z	Gene names
20140918_fract6_dyn_5ul_D6_01_373	17547	TOF; CID	145.69	843.39	ELOVL6

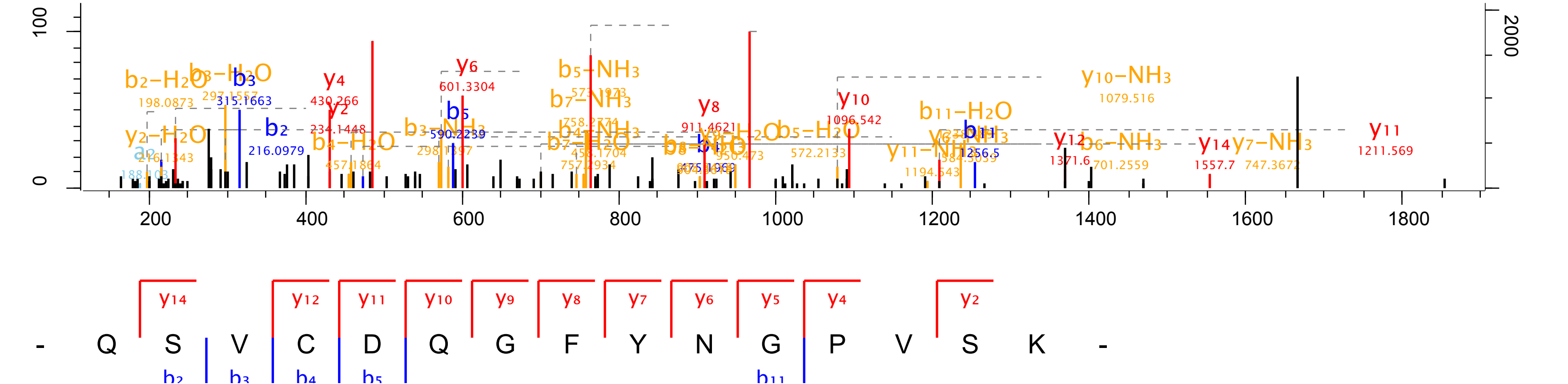

Raw file	Scan	Method	Score	m/z	Gene names
20140918_fract6_dyn_5ul_D6_01_373	19067	TOF; CID	103.13	604.31	TMEM44

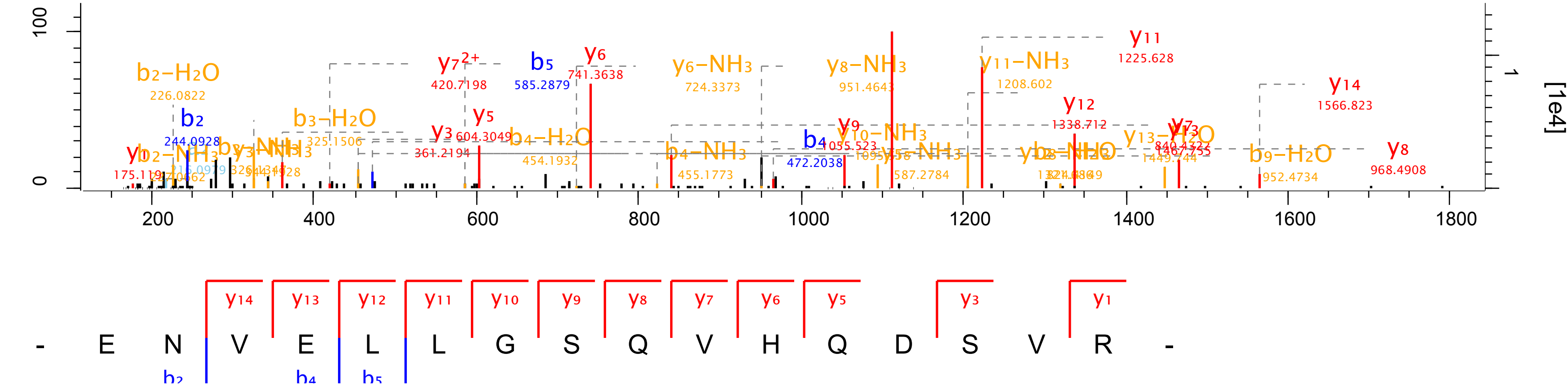

Raw file	Scan	Method	Score	m/z	Gene names
20140918_fract6_dyn_5ul_D6_01_373	19730	TOF; CID	75.09	700.84	JOSD2

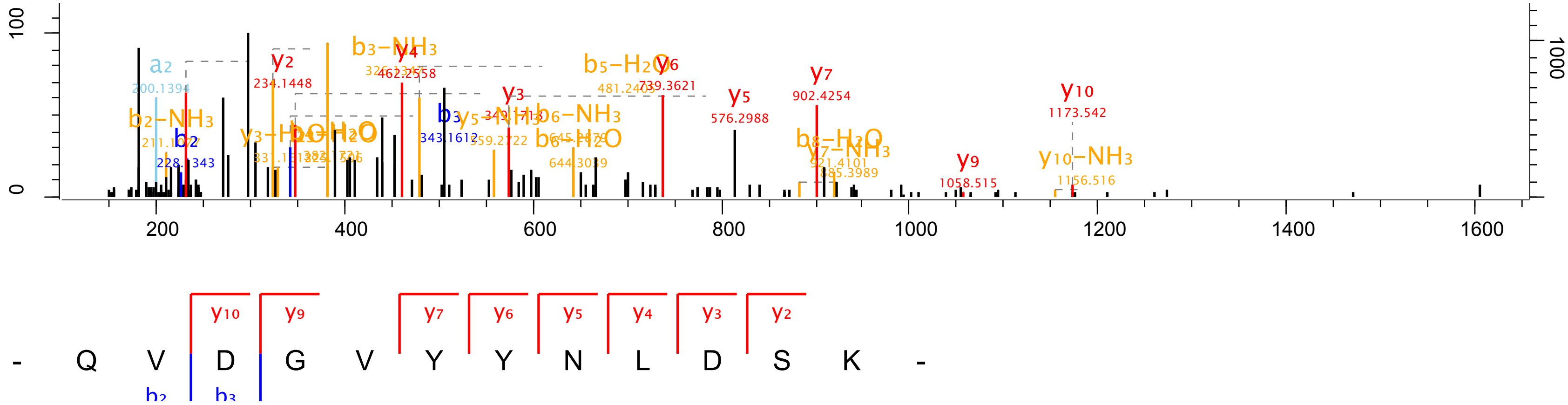

Raw file	Scan	Method	Score	m/z	Gene names
20140918_fract6_dyn_5ul_D6_01_373	20609	TOF; CID	81.66	579.61	CYB561

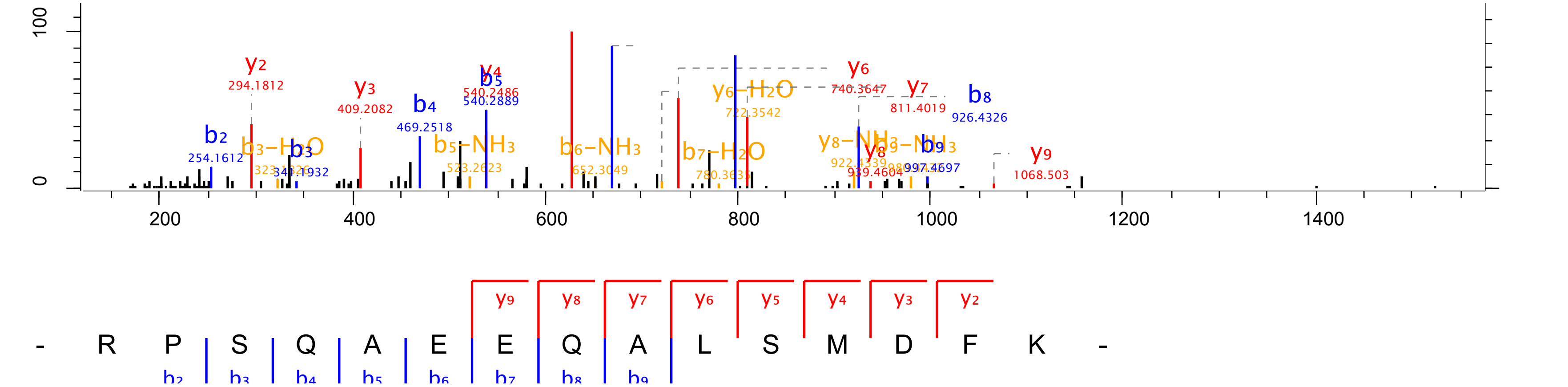

Raw file	Scan	Method	Score	m/z	Gene names
20140918_fract6_dyn_5ul_D6_01_373	21525	TOF; CID	54.87	1137.52	MDM2

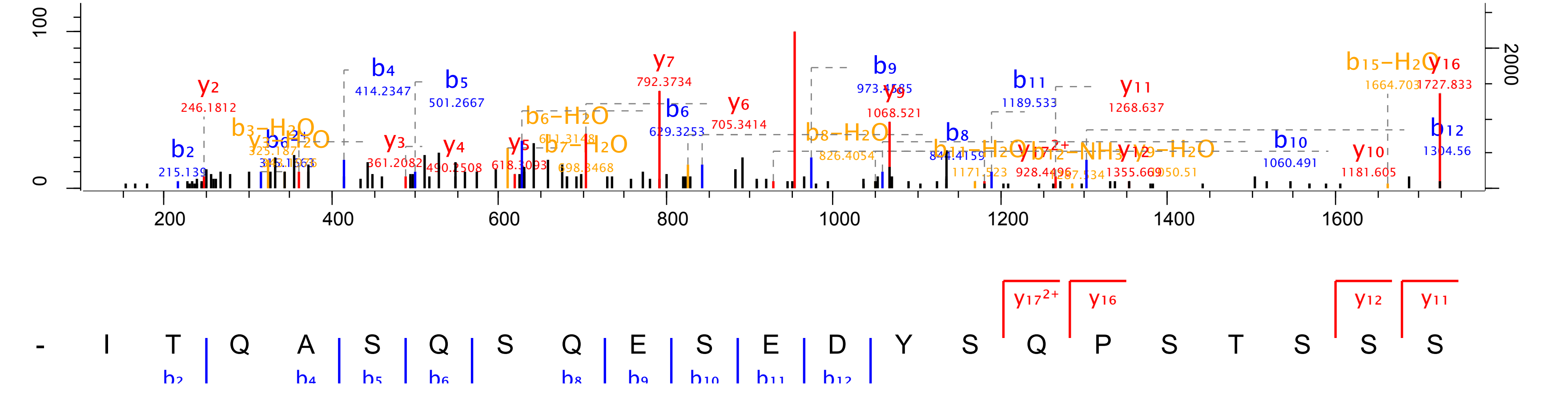

Raw file	Scan	Method	Score	m/z	Gene names
20140918_fract6_dyn_5ul_D6_01_373	22016	TOF; CID	102.52	548.28	SOCS2

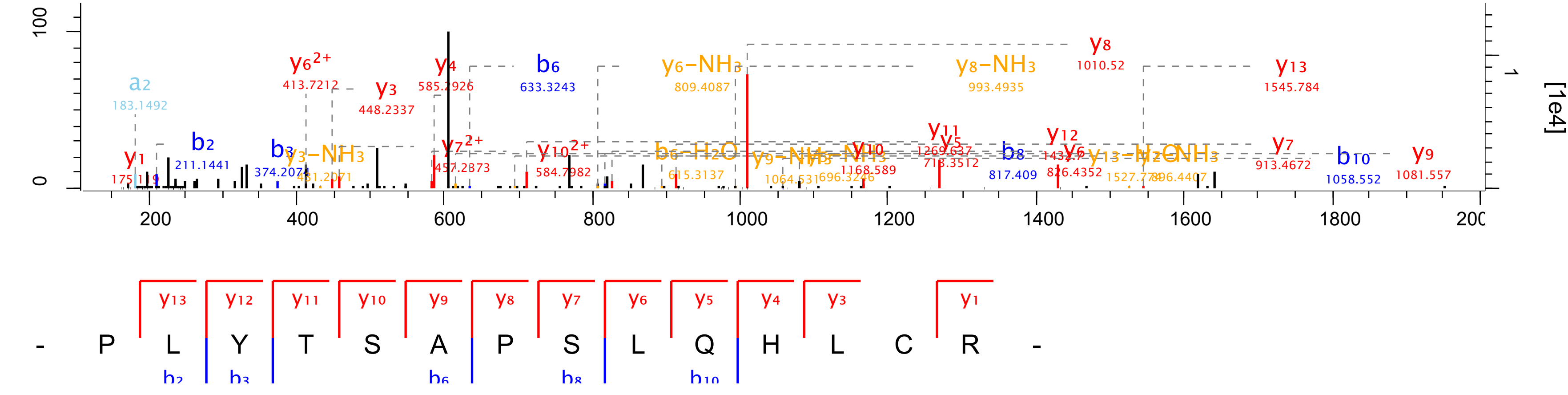

Raw file	Scan	Method	Score	m/z	Gene names
20140918_fract6_dyn_5ul_D6_01_373	22979	TOF; CID	152	949.44	SLC18B1

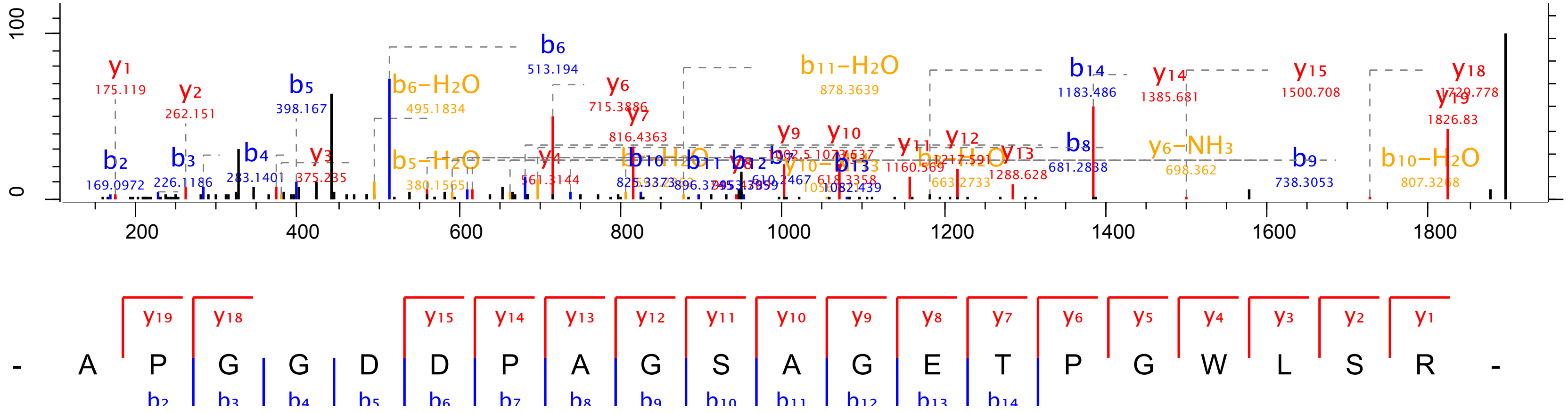

Raw file	Scan	Method	Score	m/z	Gene names
20140918_fract6_dyn_5ul_D6_01_373	22980	TOF; CID	93.16	499.31	ZBTB26

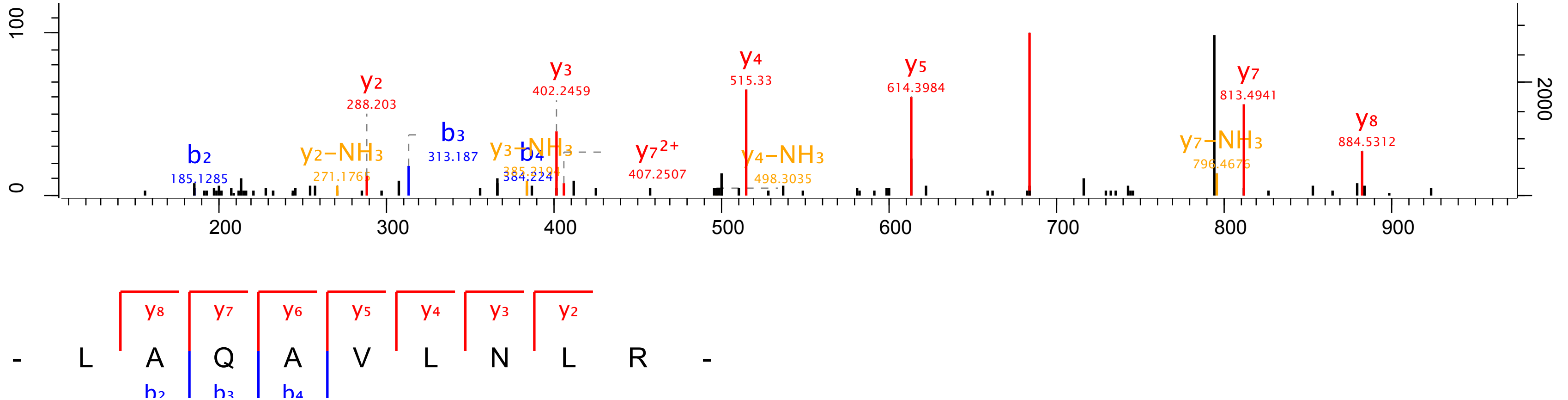

Raw file	Scan	Method	Score	m/z	Gene names
20140918_fract6_dyn_5ul_D6_01_373	25453	TOF; CID	87.67	617.64	HNRNPH1

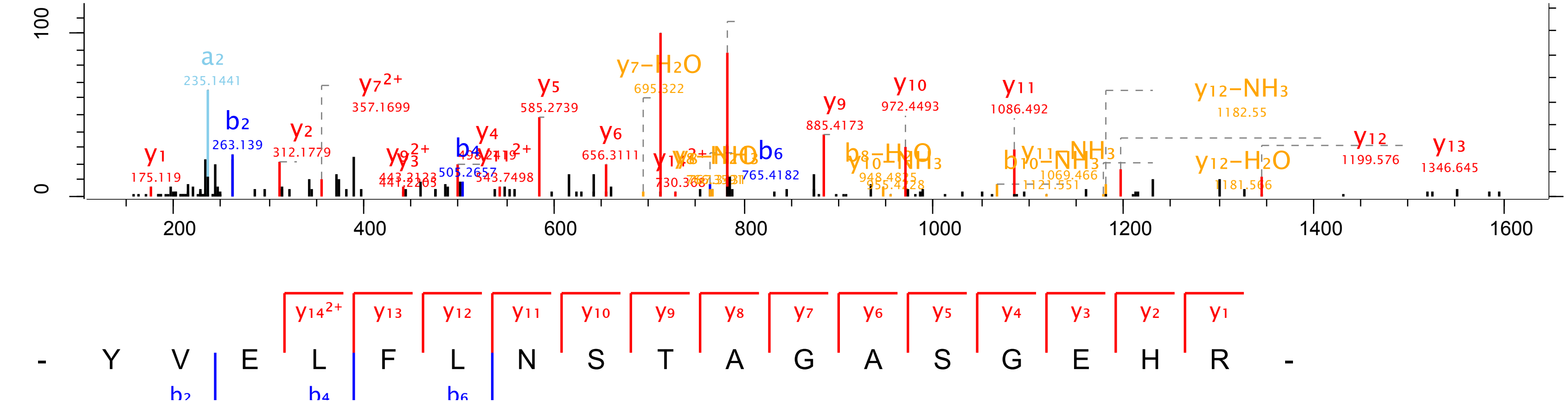

Raw file	Scan	Method	Score	m/z	Gene names
20140918_fract6_dyn_5ul_D6_01_373	27376	TOF; CID	51.64	556.29	FAM210B

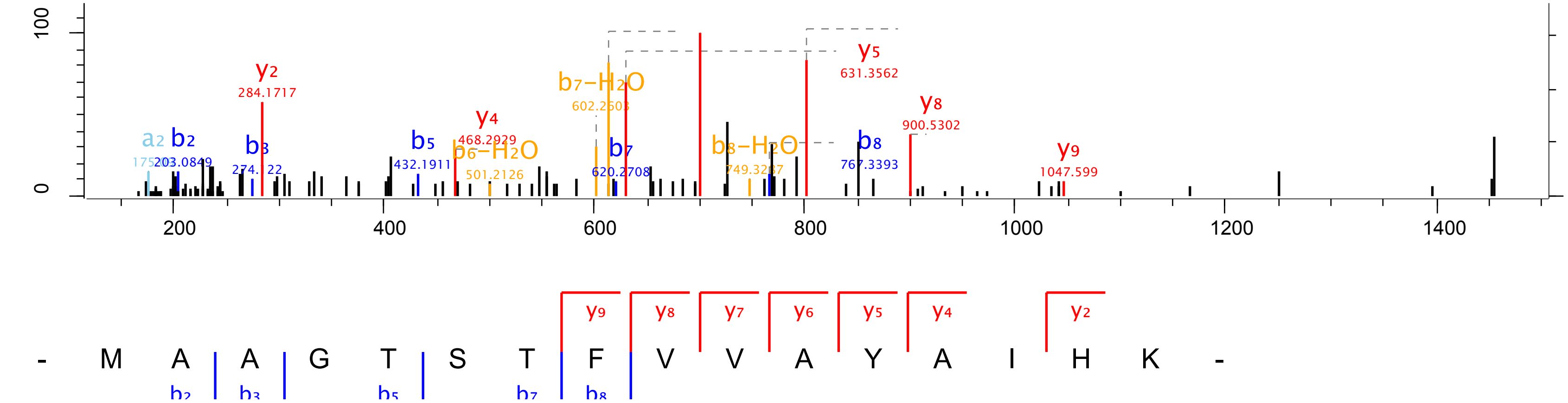

Raw file	Scan	Method	Score	m/z	Gene names
20140918_fract6_dyn_5ul_D6_01_373	29014	TOF; CID	120.15	587.34	GLTSCR1L

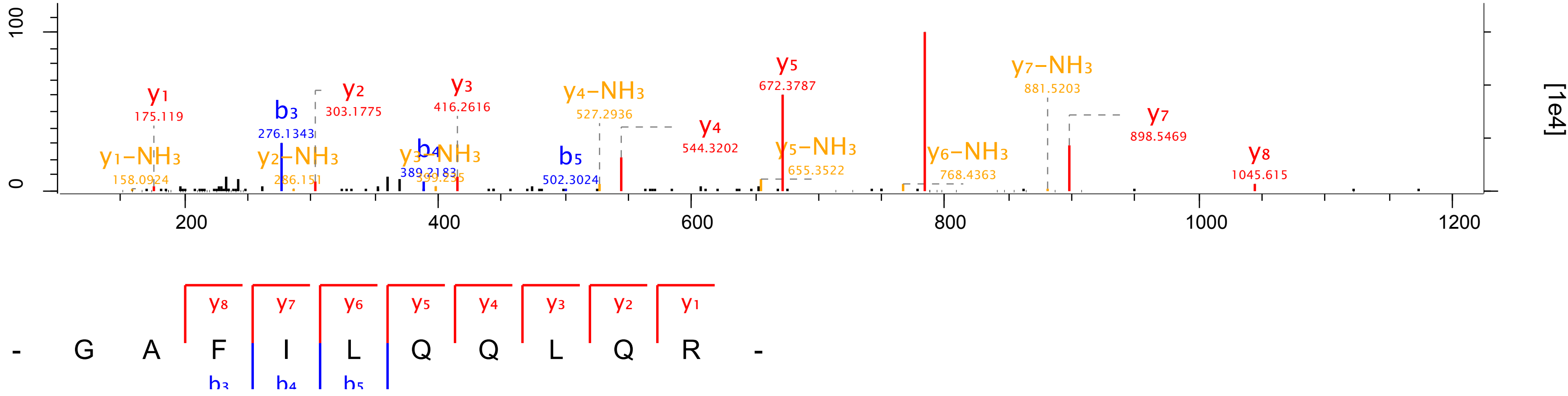

Raw file	Scan	Method	Score	m/z	Gene names
20140918_fract6_dyn_5ul_D6_01_373	31731	TOF; CID	66.77	908.11	CITED2

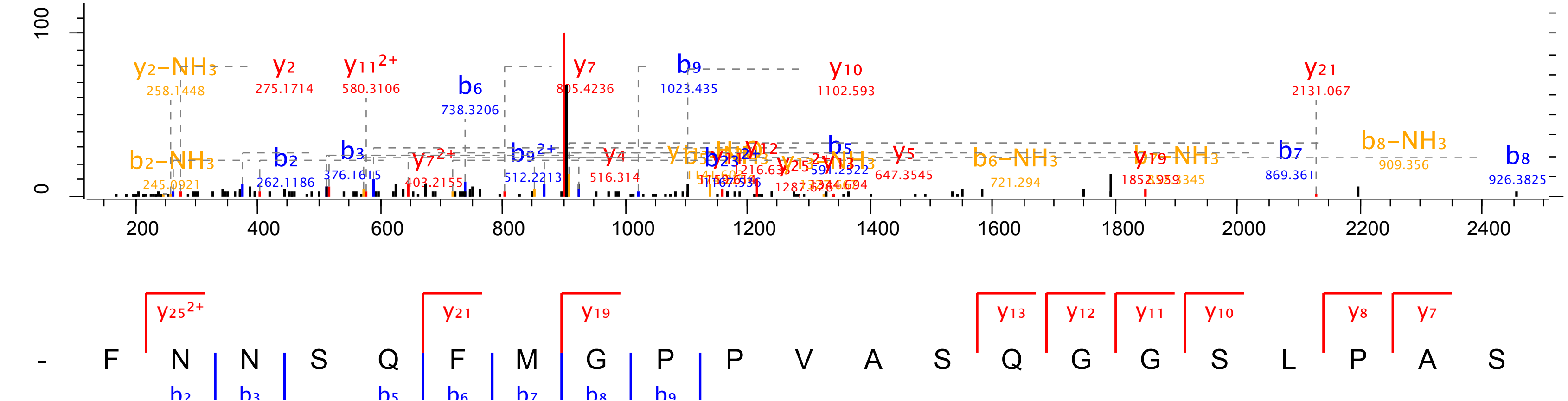

Raw file	Scan	Method	Score	m/z	Gene names
20140918_fract6_dyn_5ul_D6_01_373	38208	TOF; CID	57.24	1235.13	TMEM51

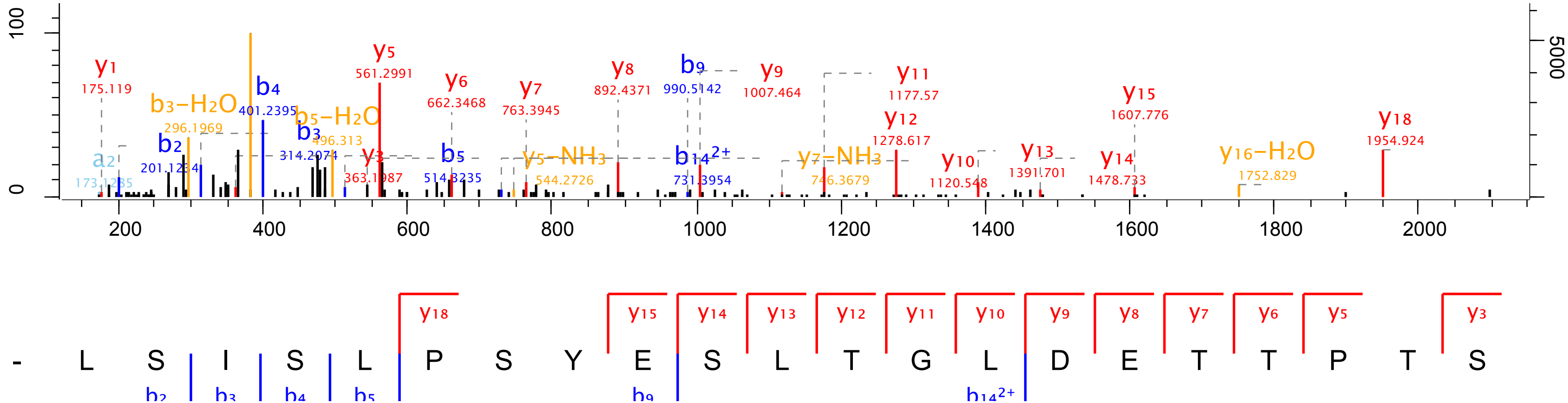

Raw file	Scan	Method	Score	m/z	Gene names
20140918_fract7_dyn_5ul_D7_01_393	8162	TOF; CID	77.89	629.33	CXCR4

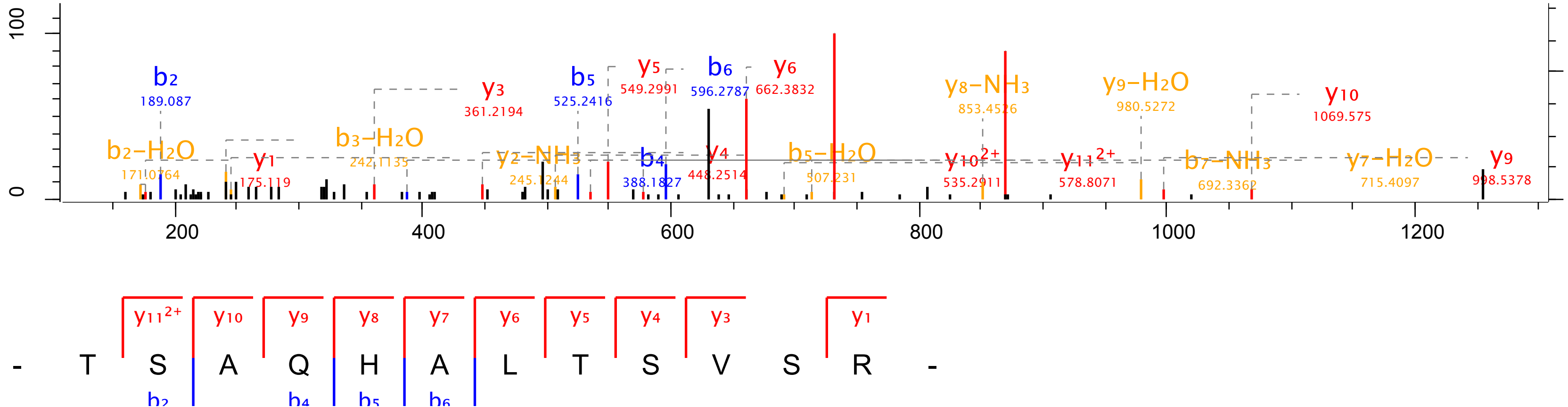

Raw file	Scan	Method	Score	m/z	Gene names
20140918_fract7_dyn_5ul_D7_01_393	9069	TOF; CID	92.86	704.83	TBPL1

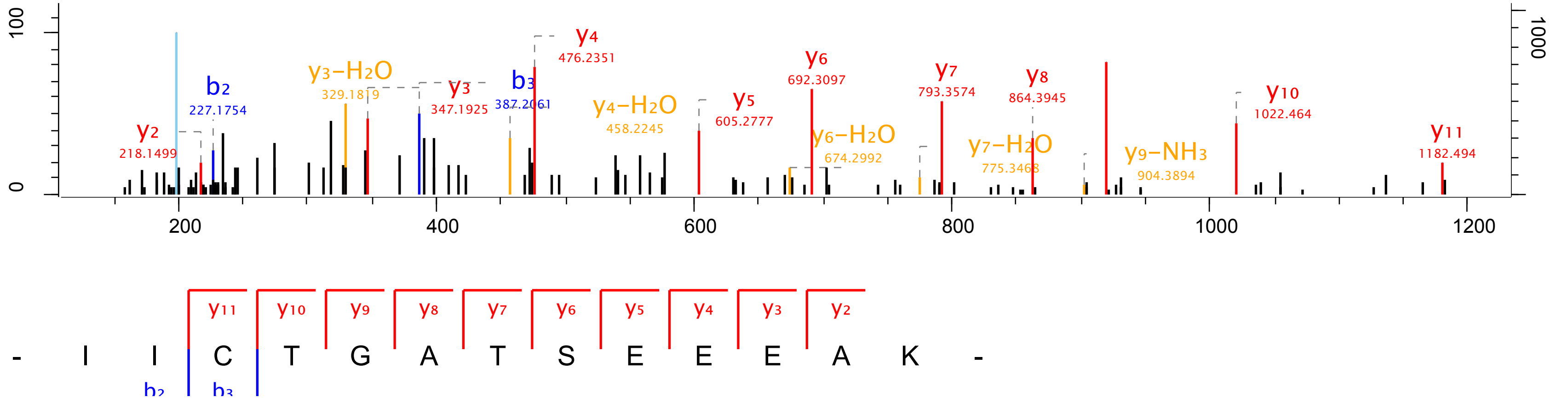

Raw file	Scan	Method	Score	m/z
20140918_fract7_dyn_5ul_D7_01_393	10109	TOF; CID	86.9	574.31

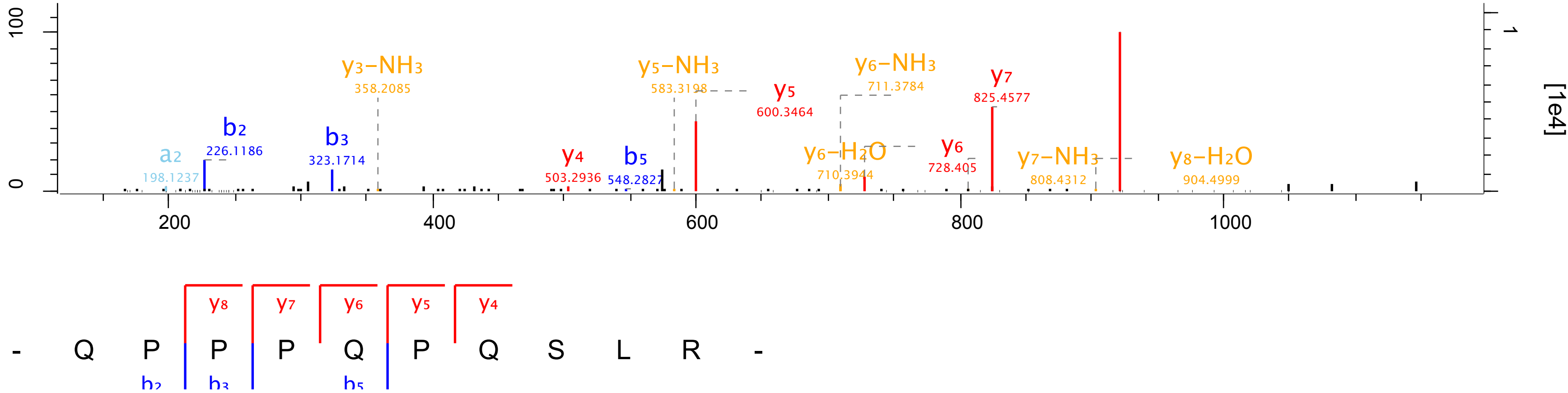

Raw file	Scan	Method	Score	m/z	Gene names
20140918_fract7_dyn_5ul_D7_01_393	10418	TOF; CID	164.97	513.78	SMIM12

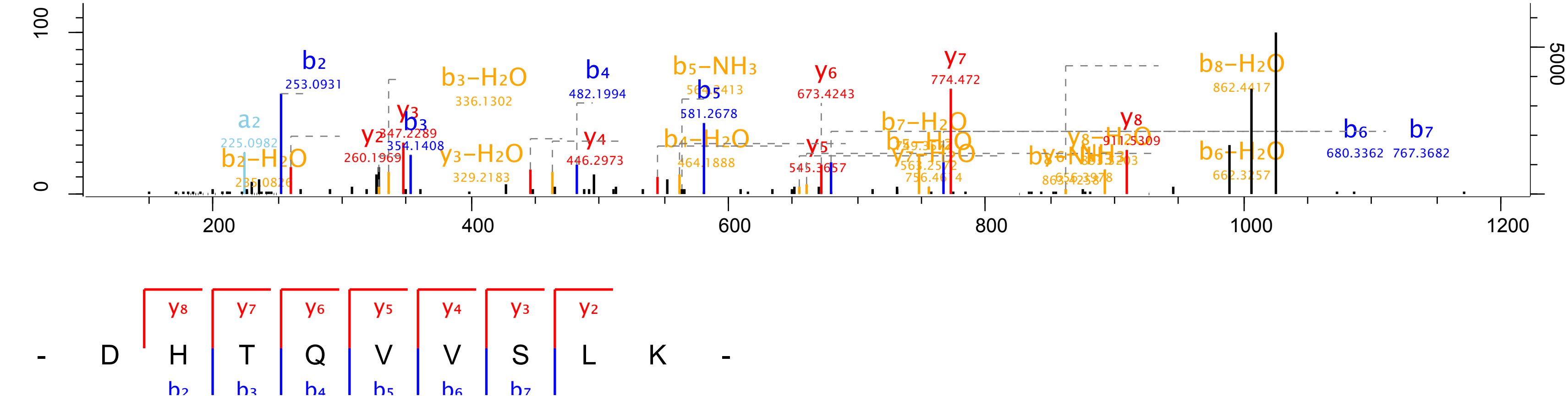

Raw file	Scan	Method	Score	m/z	Gene names
20140918_fract7_dyn_5ul_D7_01_393	11128	TOF; CID	50.9	491.22	JAG2

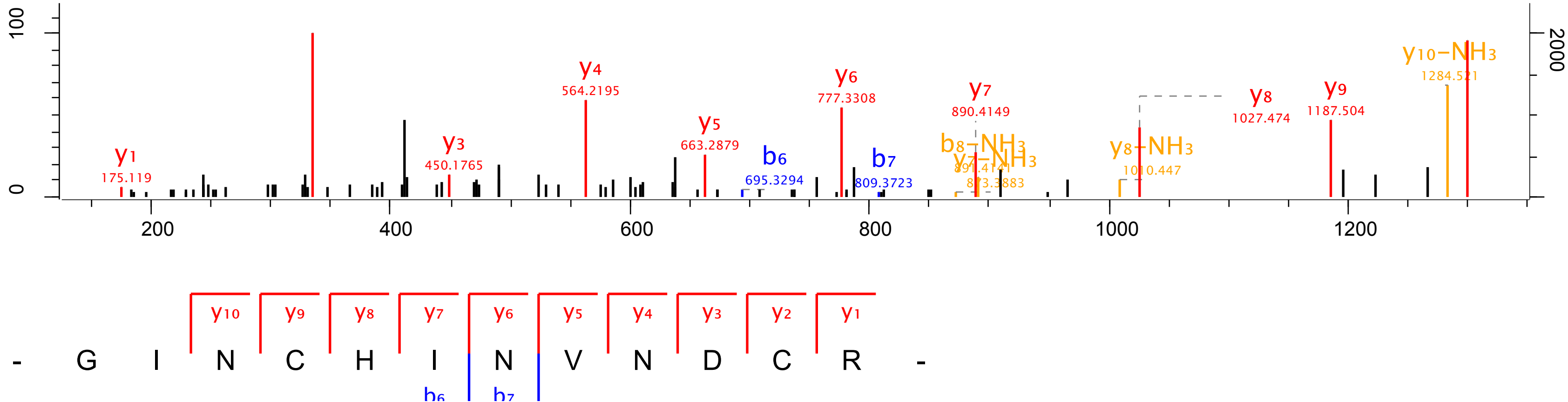

Raw file	Scan	Method	Score	m/z	Gene names
20140918_fract7_dyn_5ul_D7_01_393	12131	TOF; CID	40.97	690.35	DMTF1

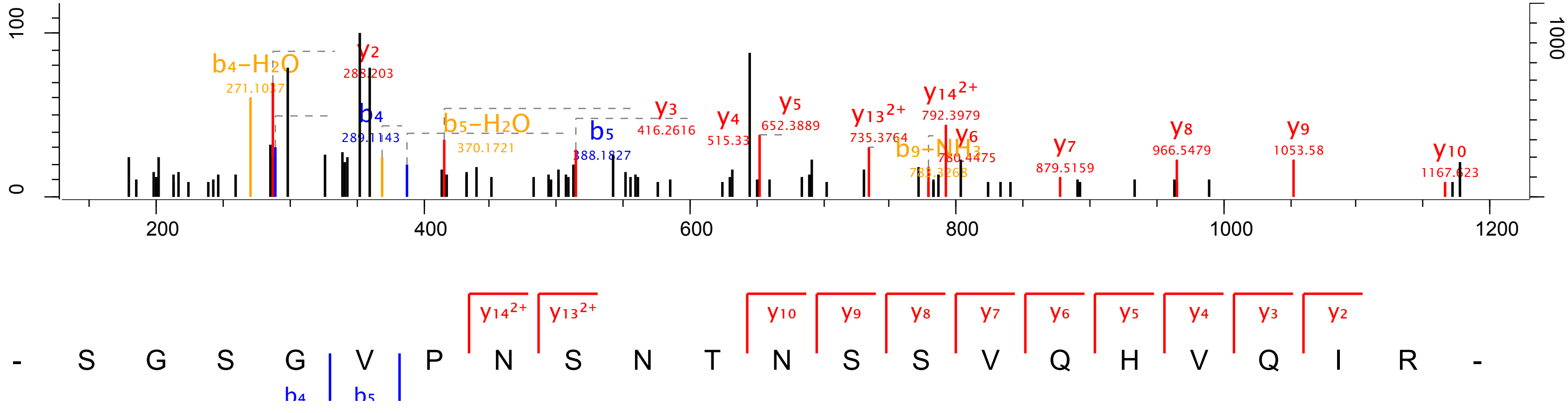

Raw file	Scan	Method	Score	m/z	Gene names
20140918_fract7_dyn_5ul_D7_01_393	12235	TOF; CID	73.44	621.84	TAF12

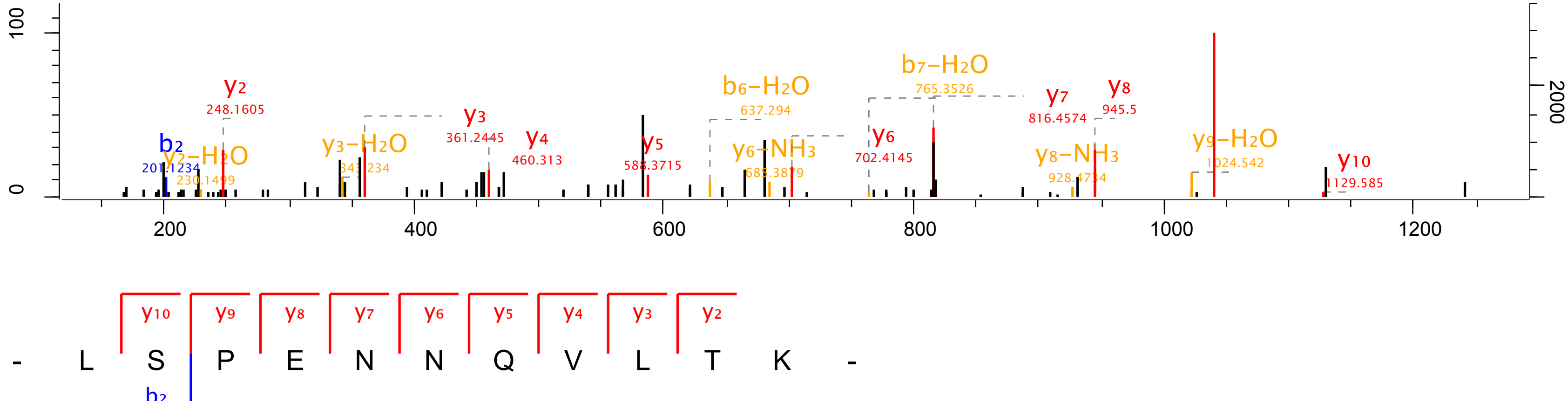

Raw file	Scan	Method	Score	m/z	Gene names
20140918_fract7_dyn_5ul_D7_01_393	14172	TOF; CID	127.84	454.74	KCNIP3

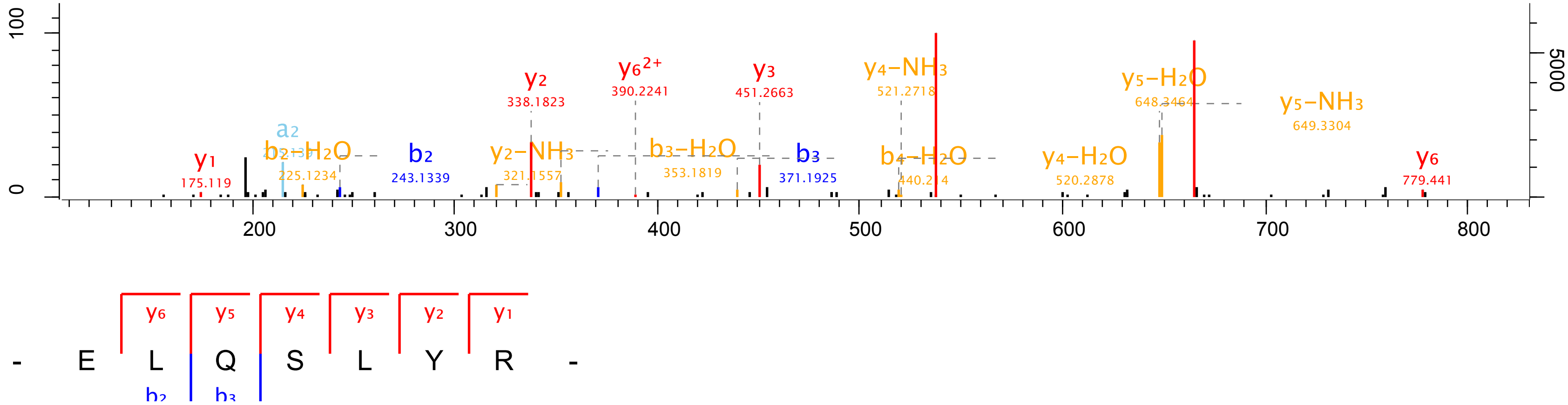

Raw file	Scan	Method	Score	m/z	Gene names
20140918_fract7_dyn_5ul_D7_01_393	17342	TOF; CID	94.31	598.29	PIGC

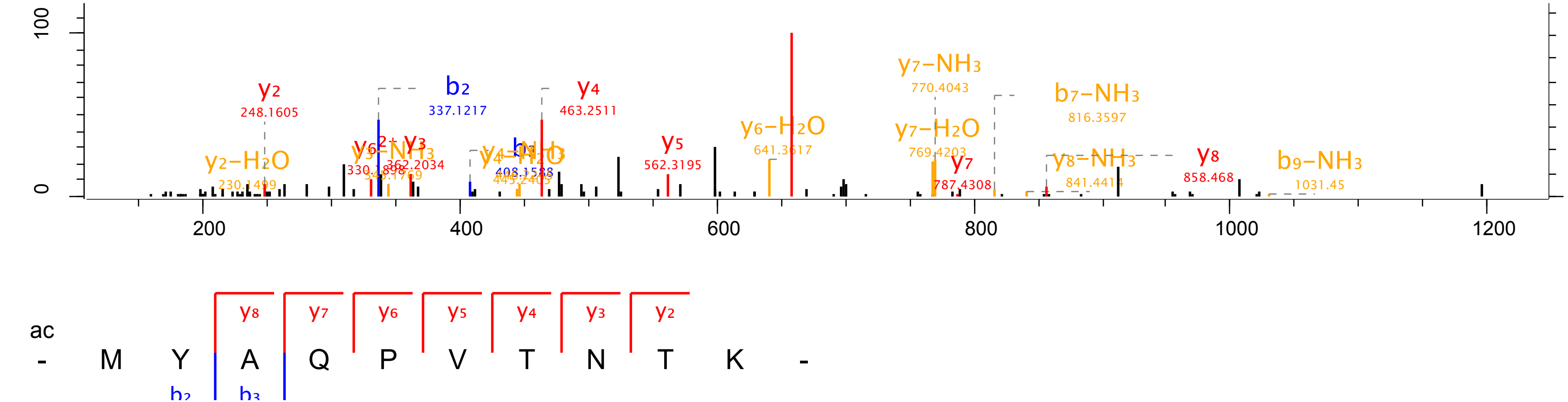

Raw file	Scan	Method	Score	m/z	Gene names
20140918_fract7_dyn_5ul_D7_01_393	17482	TOF; CID	93.56	585.83	DAGLA

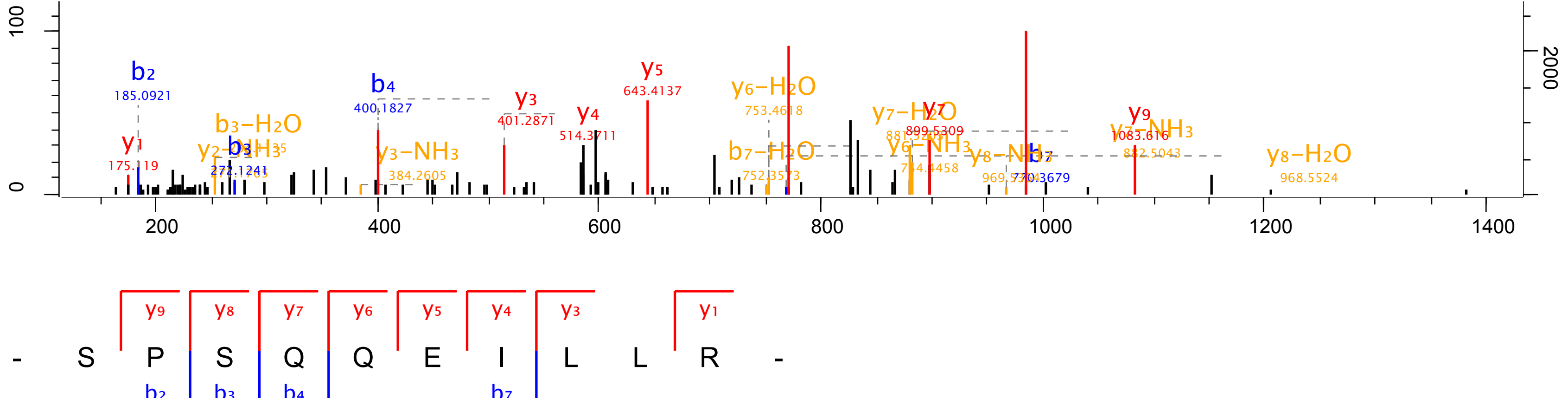

Raw file	Scan	Method	Score	m/z	Gene names
20140918_fract7_dyn_5ul_D7_01_393	19337	TOF; CID	73.93	799.01	PGAP3

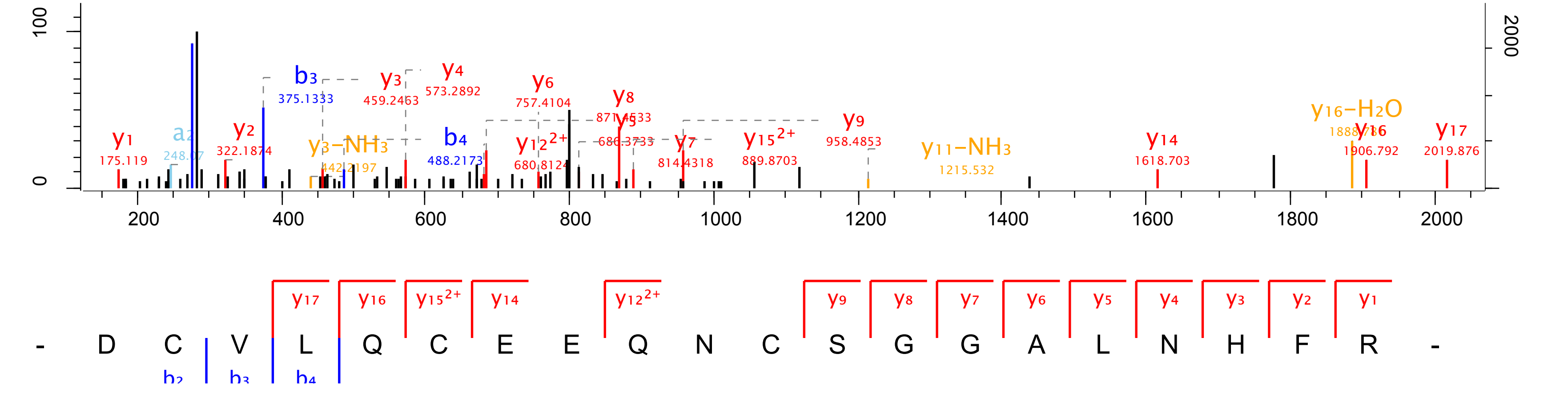

Raw file	Scan	Method	Score	m/z	Gene names
20140918_fract7_dyn_5ul_D7_01_393	22550	TOF; CID	81.7	800.37	CTNNBIP1

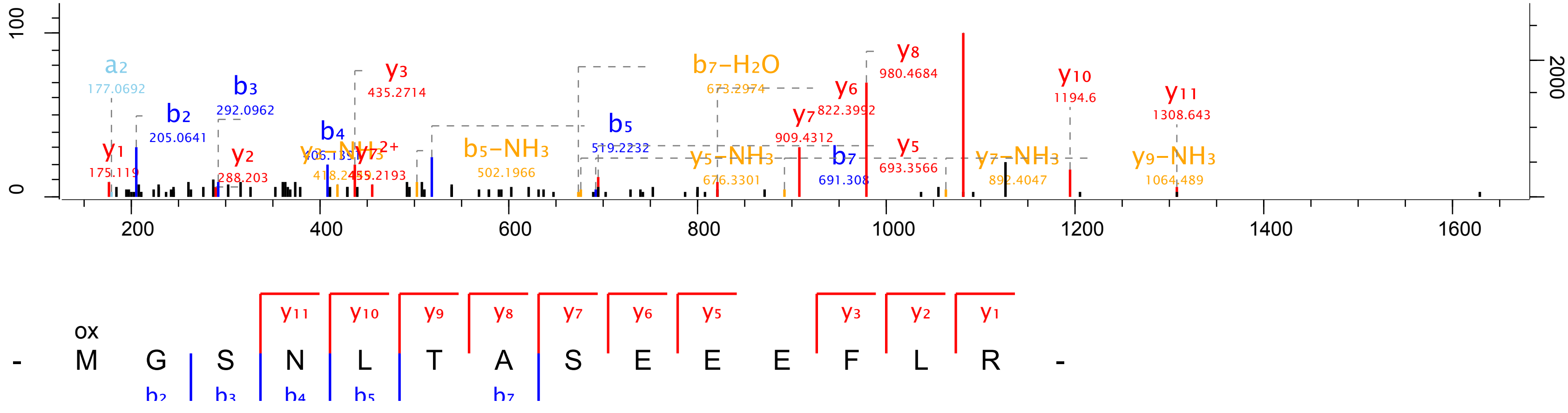

Raw file	Scan	Method	Score	m/z	Gene names
20140918_fract7_dyn_5ul_D7_01_393	22807	TOF; CID	78.96	692.86	PLEKHO2

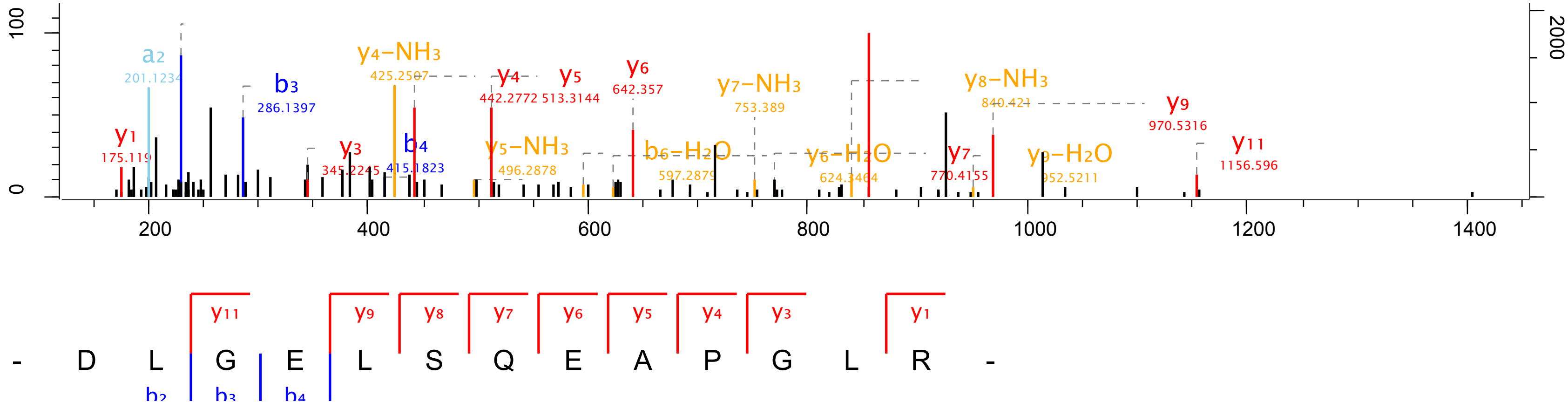

Raw file	Scan	Method	Score	m/z	Gene names
20140918_fract7_dyn_5ul_D7_01_393	23603	TOF; CID	75.74	490.76	LAPTM4A

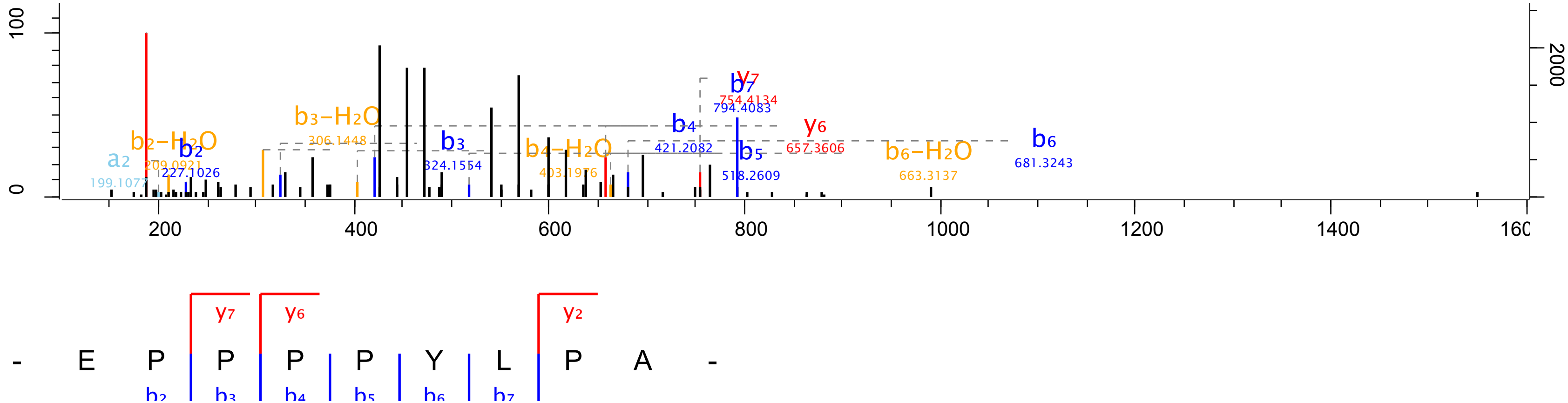

Raw file	Scan	Method	Score	m/z	Gene names
20140918_fract7_dyn_5ul_D7_01_393	24511	TOF; CID	90.83	603.31	TAF13

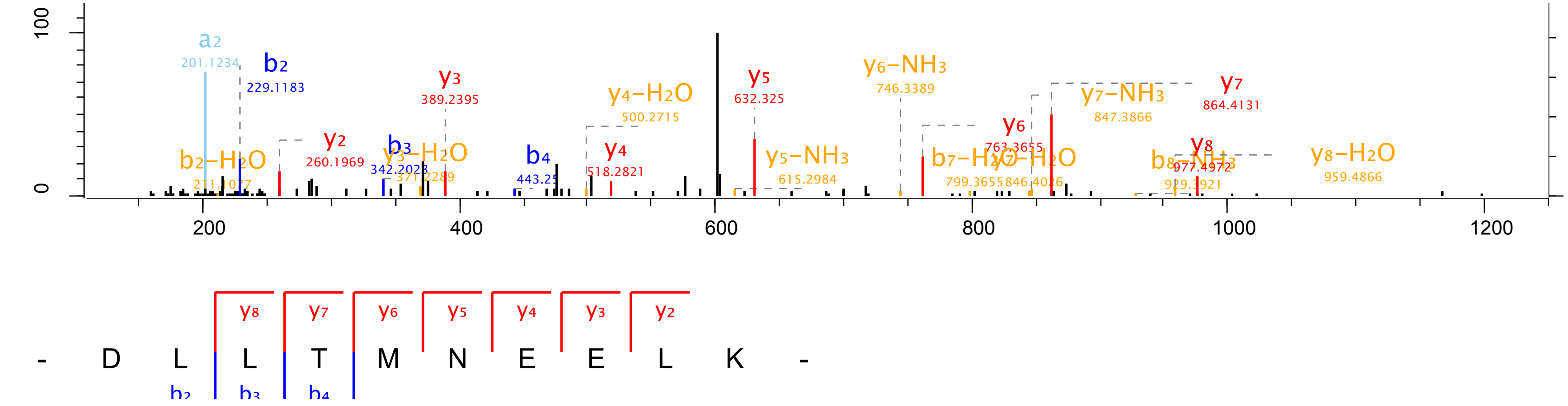

Raw file	Scan	Method	Score	m/z	Gene names
20140918_fract7_dyn_5ul_D7_01_393	26476	TOF; CID	100.45	946.97	PAGE1

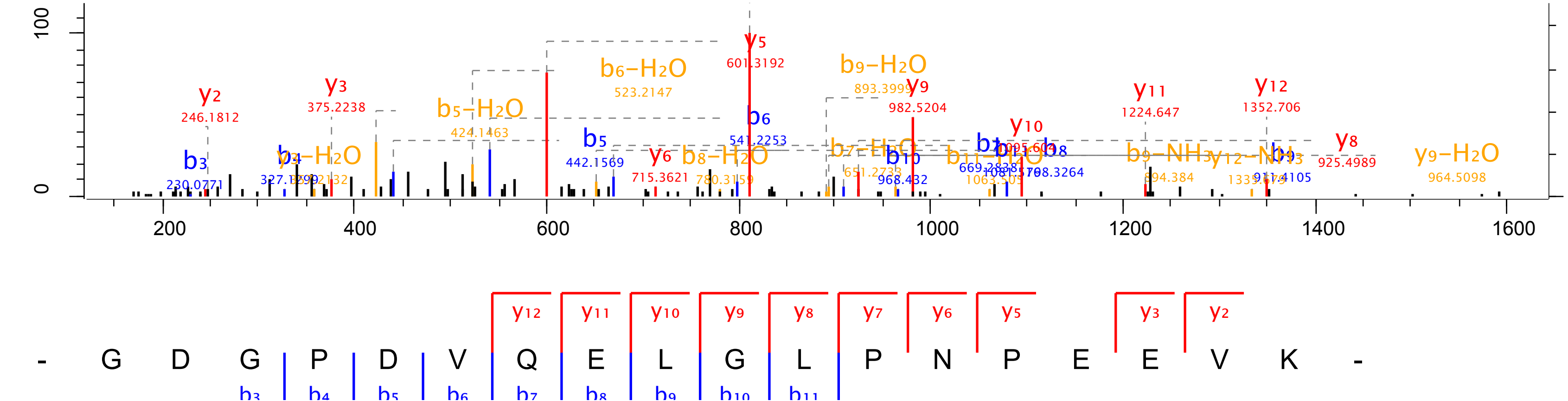

Raw file	Scan	Method	Score	m/z	Gene names
20140918_fract7_dyn_5ul_D7_01_393	26855	TOF; CID	52.53	778.4	TBC1D16

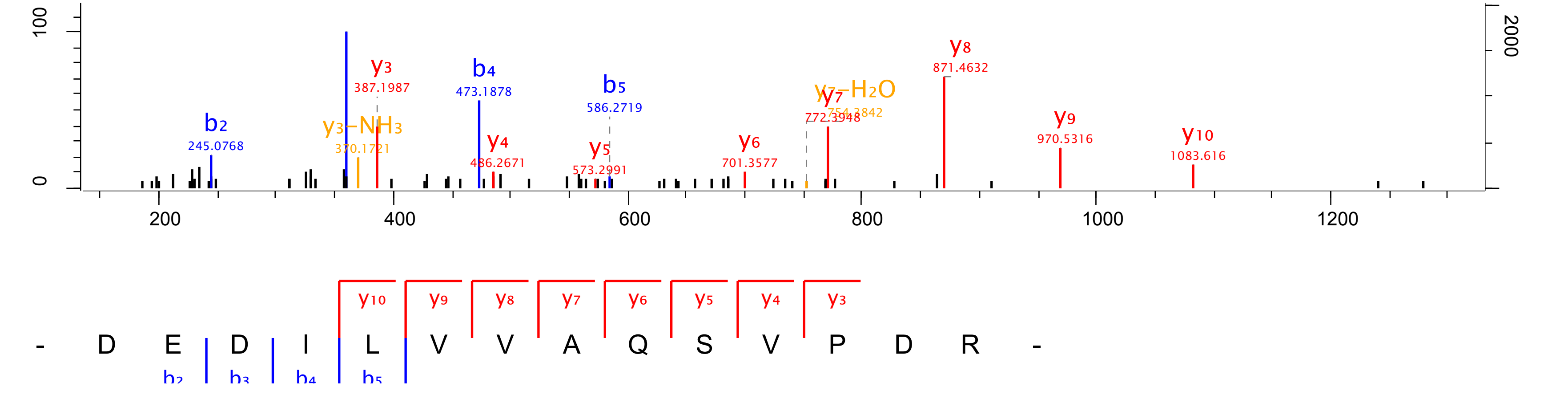

Raw file	Scan	Method	Score	m/z	Gene names
20140918_fract7_dyn_5ul_D7_01_393	27285	TOF; CID	89.36	837.41	GPANK1

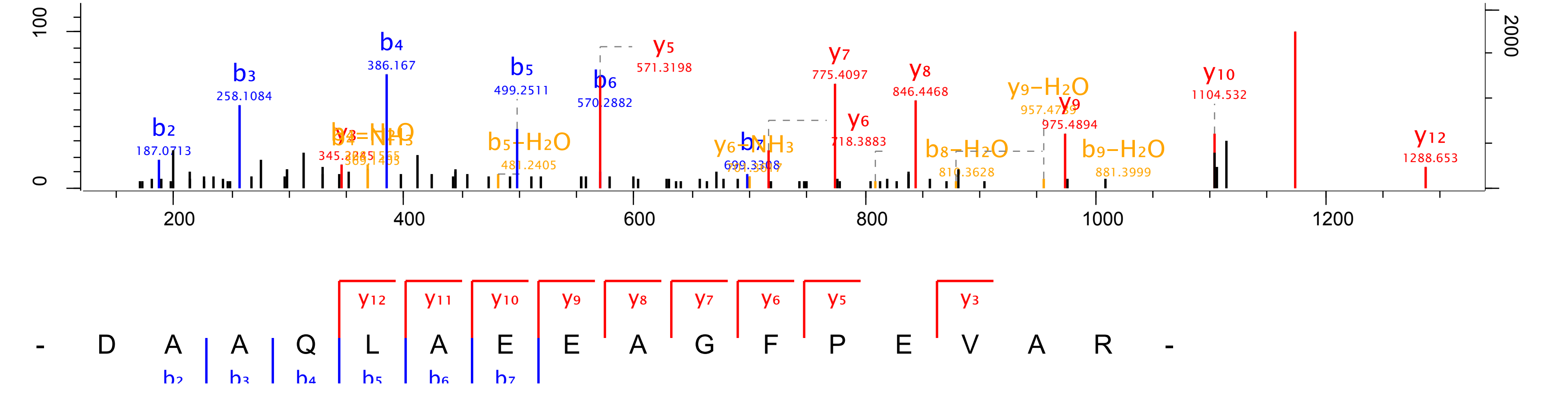

Raw file	Scan	Method	Score	m/z	Gene names
20140918_fract8_dyn_5ul_D8_01_394	6123	TOF; CID	58.1	481.24	ZSCAN21

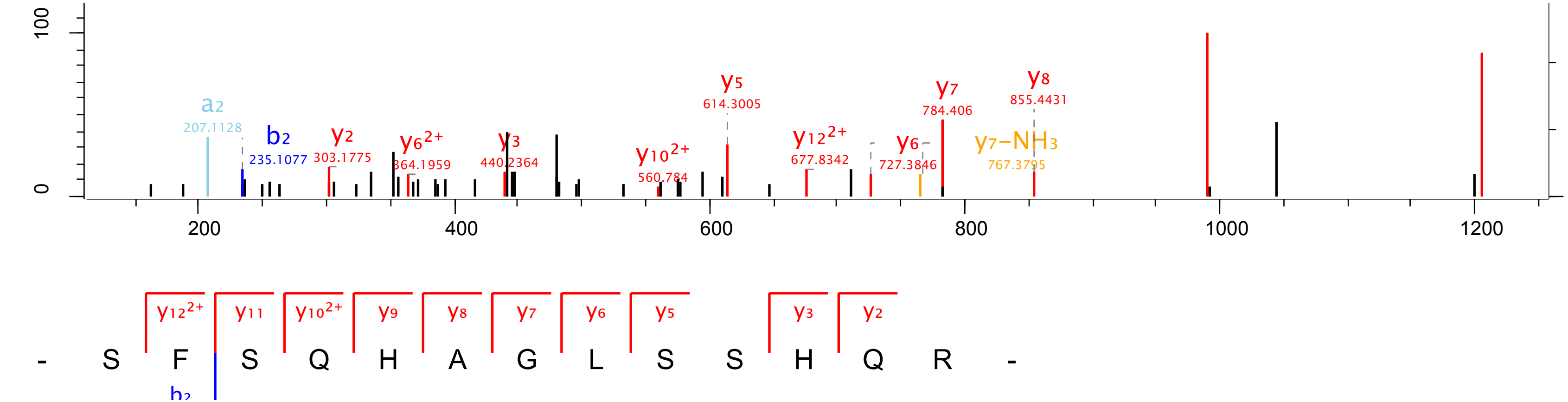

Raw file	Scan	Method	Score	m/z	Gene names
20140918_fract8_dyn_5ul_D8_01_394	9728	TOF; CID	69.19	613.31	THRB

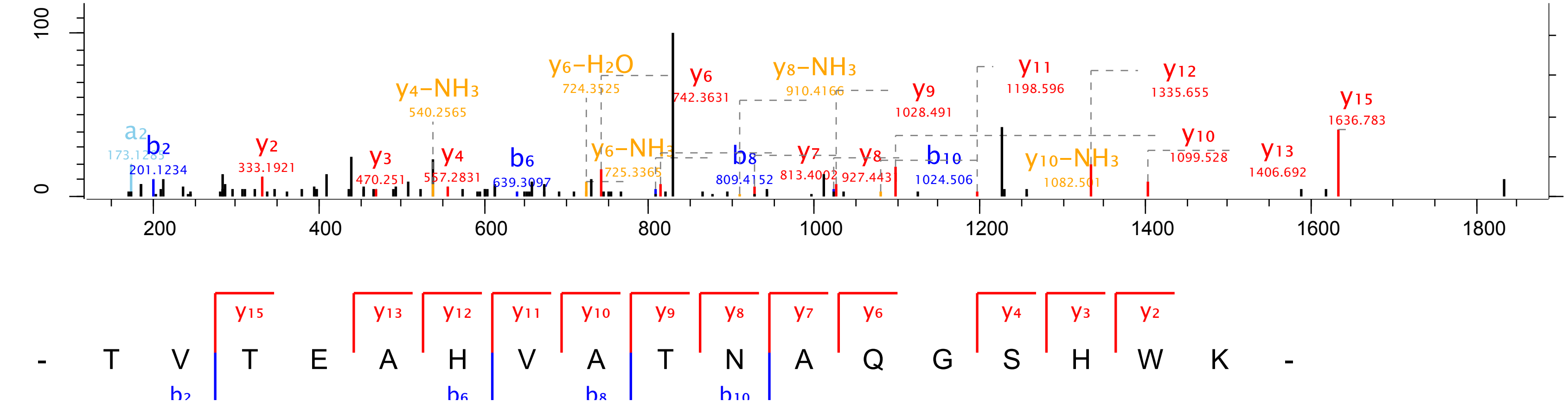

Raw file	Scan	Method	Score	m/z	Gene names
20140918_fract8_dyn_5ul_D8_01_394	10605	TOF; CID	98.52	436.76	RTN1

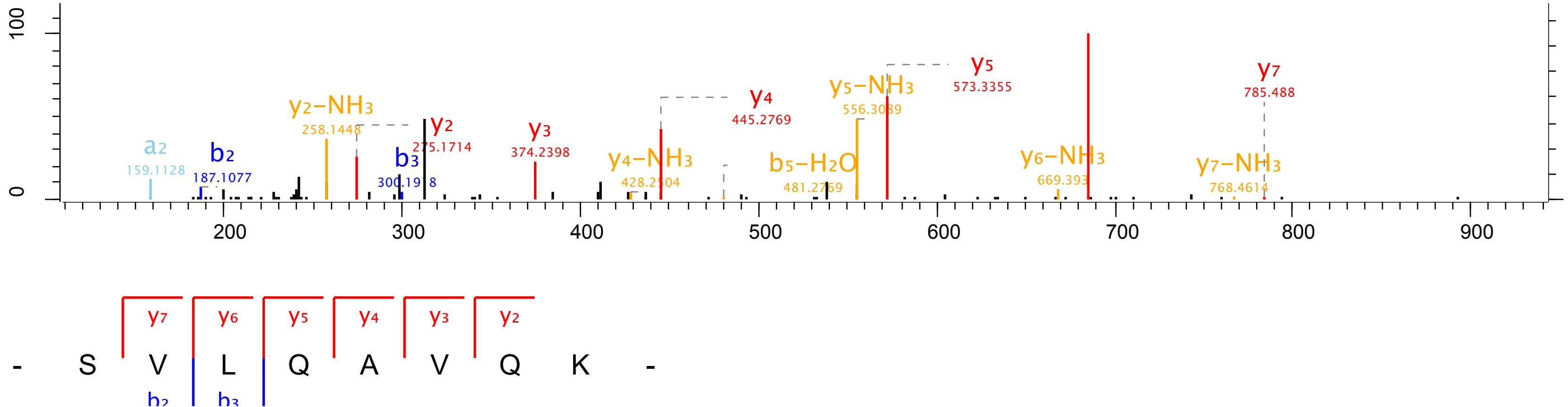

Raw file	Scan	Method	Score	m/z	Gene names
20140918_fract8_dyn_5ul_D8_01_394	14620	TOF; CID	91.97	875.42	TMEM181

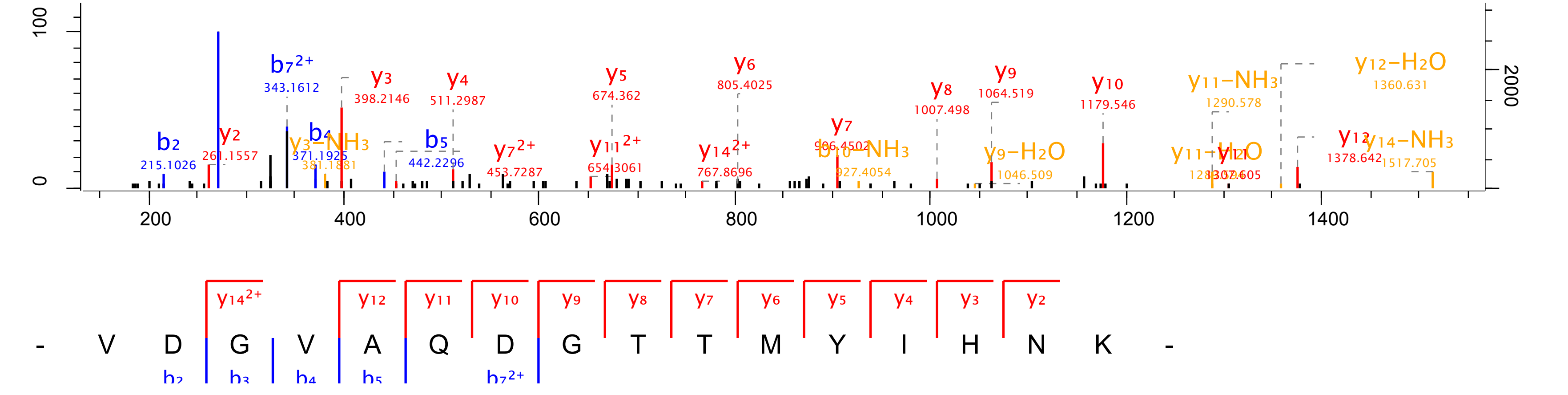

Raw file	Scan	Method	Score	m/z	Gene names
20140918_fract8_dyn_5ul_D8_01_394	16871	TOF; CID	75.57	439.91	PIGP

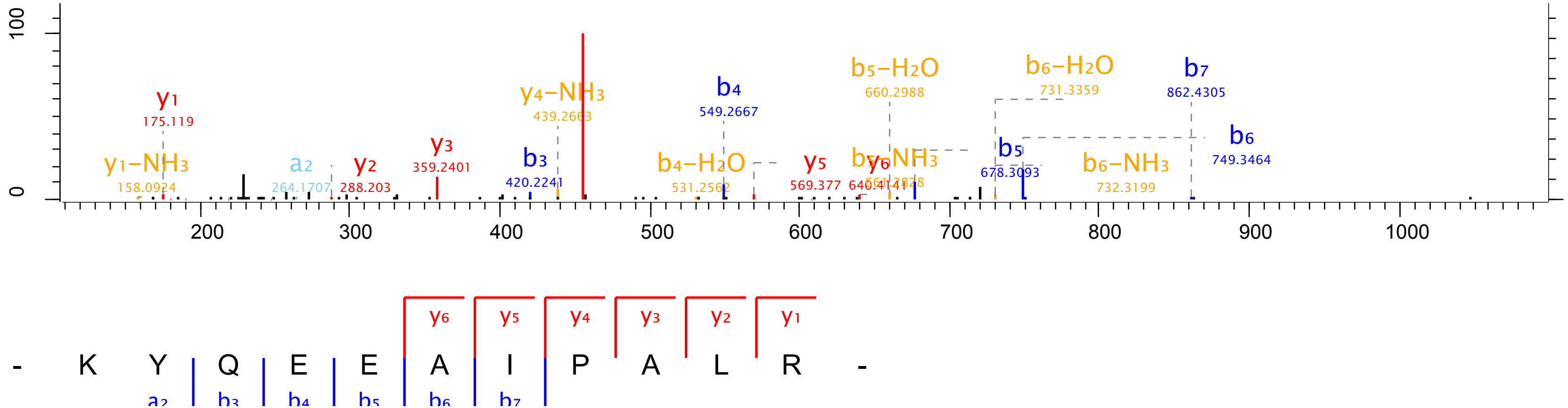

Raw file	Scan	Method	Score	m/z	Gene names
20140918_fract8_dyn_5ul_D8_01_394	16974	TOF; CID	93.44	566.29	C6orf57

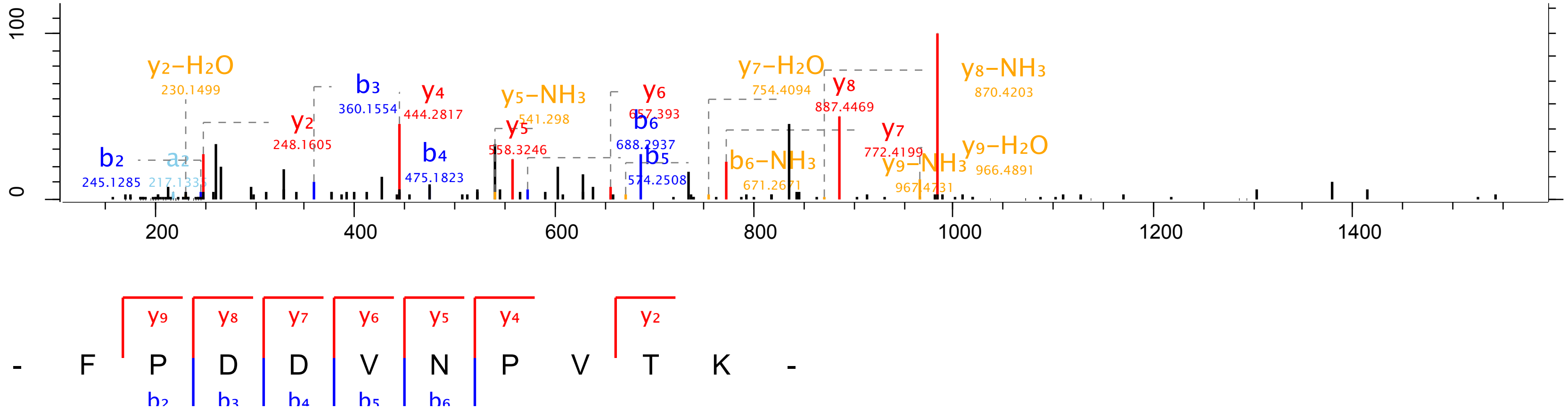

Raw file	Scan	Method	Score	m/z	Gene names
20140918_fract8_dyn_5ul_D8_01_394	17254	TOF; CID	90.15	517.27	CGRRF1

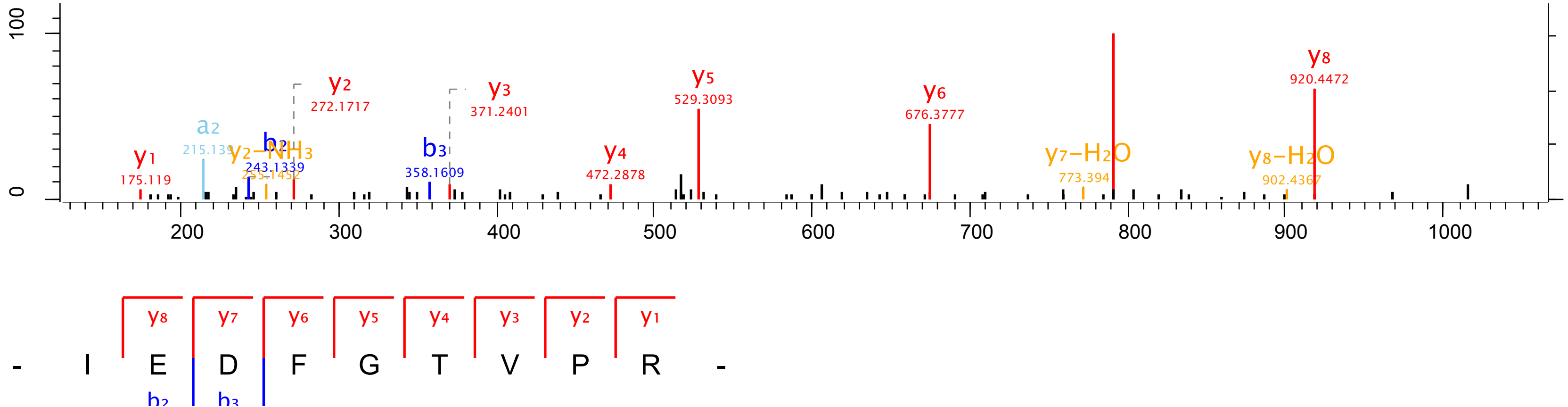

Raw file	Scan	Method	Score	m/z	Gene names
20140918_fract8_dyn_5ul_D8_01_394	19851	TOF; CID	106.36	456.76	LOXHD1

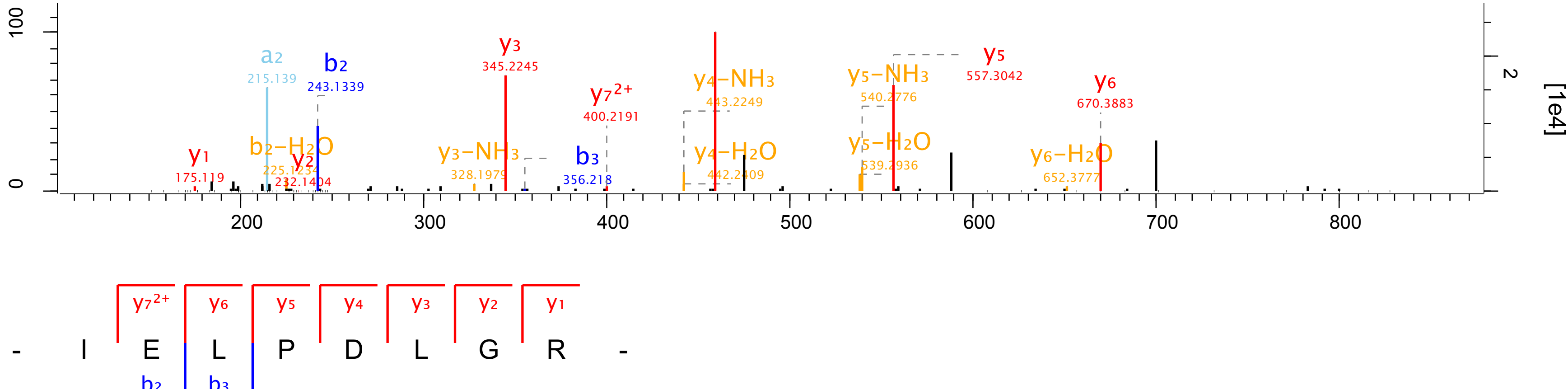

Raw file	Scan	Method	Score	m/z	Gene names
20140918_fract8_dyn_5ul_D8_01_394	22152	TOF; CID	57.11	670.36	CSTA

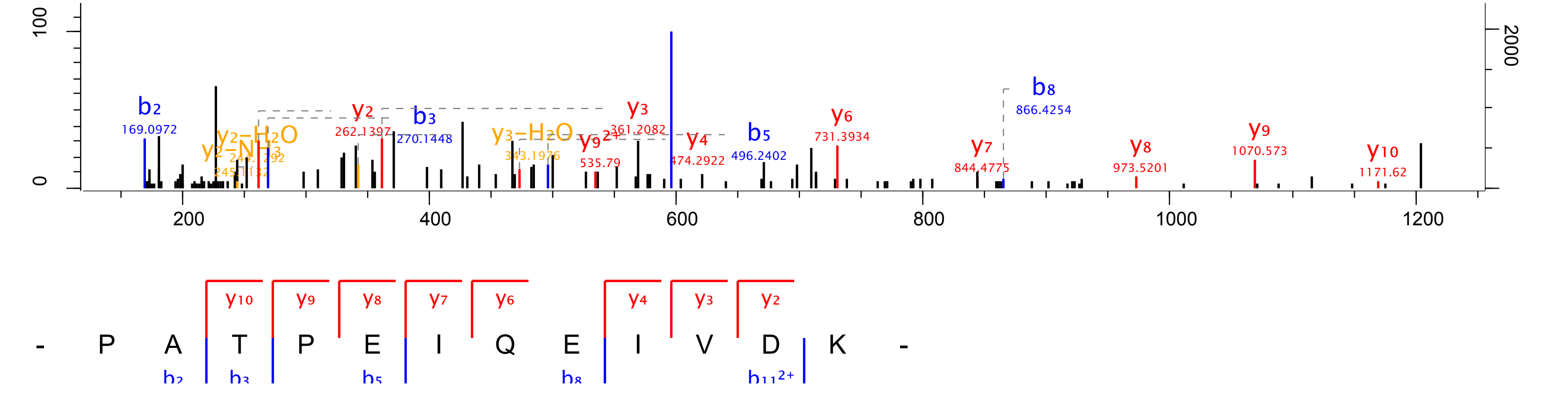

Raw file	Scan	Method	Score	m/z	Gene names
20140918_fract8_dyn_5ul_D8_01_394	23088	TOF; CID	57.53	789.41	CELSR1

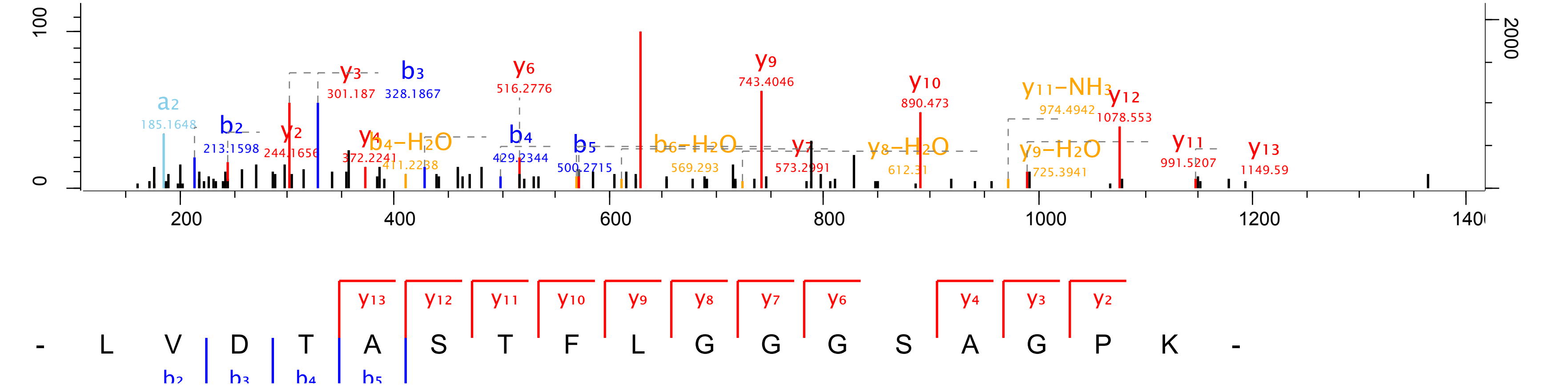

Raw file	Scan	Method	Score	m/z	Gene names
20140918_fract8_dyn_5ul_D8_01_394	23593	TOF; CID	76.28	595.28	RPS3

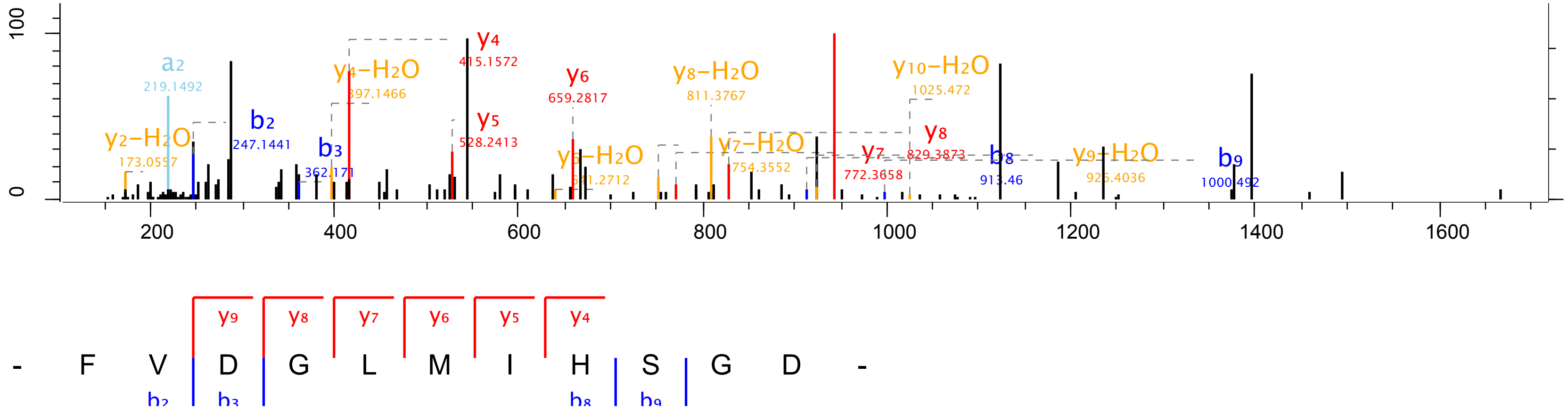

Raw file	Scan	Method	Score	m/z	Gene names
20140918_fract8_dyn_5ul_D8_01_394	23664	TOF; CID	52.32	676.35	ZNF202

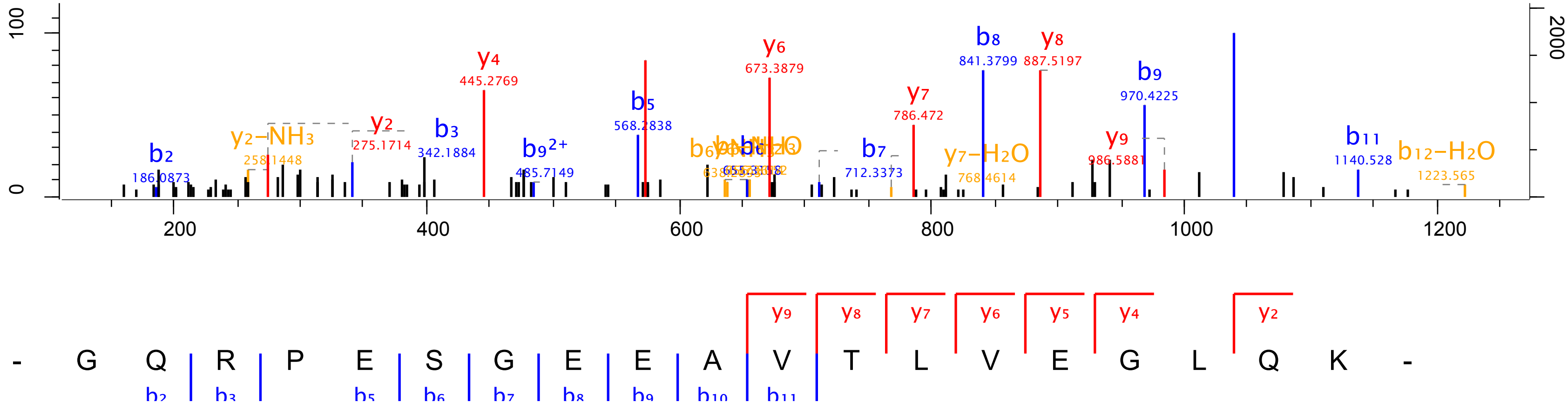

Raw file	Scan	Method	Score	m/z	Gene names
20140918_fract8_dyn_5ul_D8_01_394	27365	TOF; CID	88.98	1173.05	PKIG

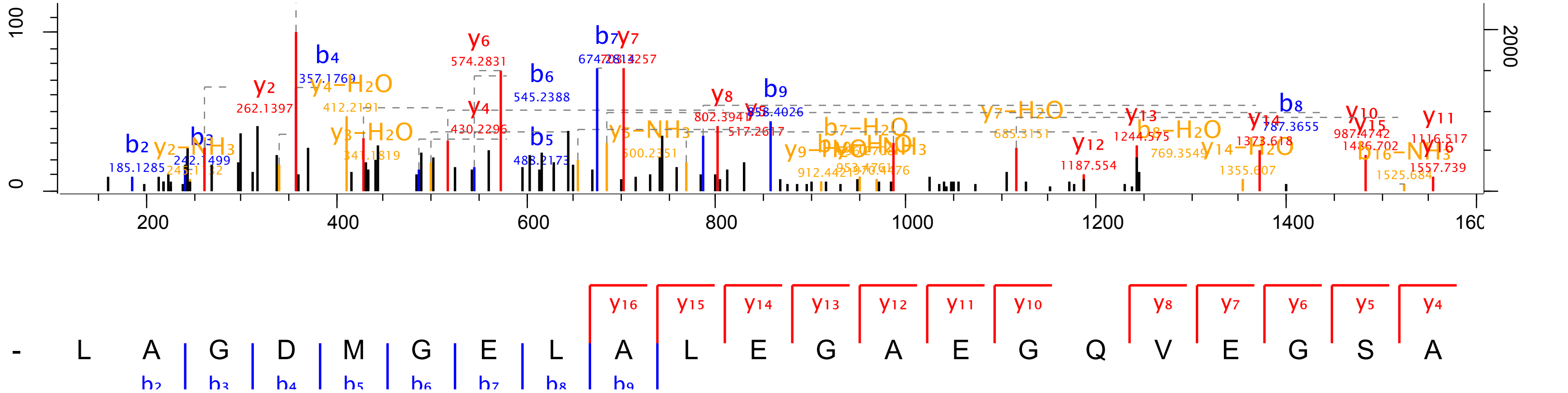

Raw file	Scan	Method	Score	m/z	Gene names
20140918_fract8_dyn_5ul_D8_01_394	30967	TOF; CID	88.6	606.37	FAAP24;C19orf40

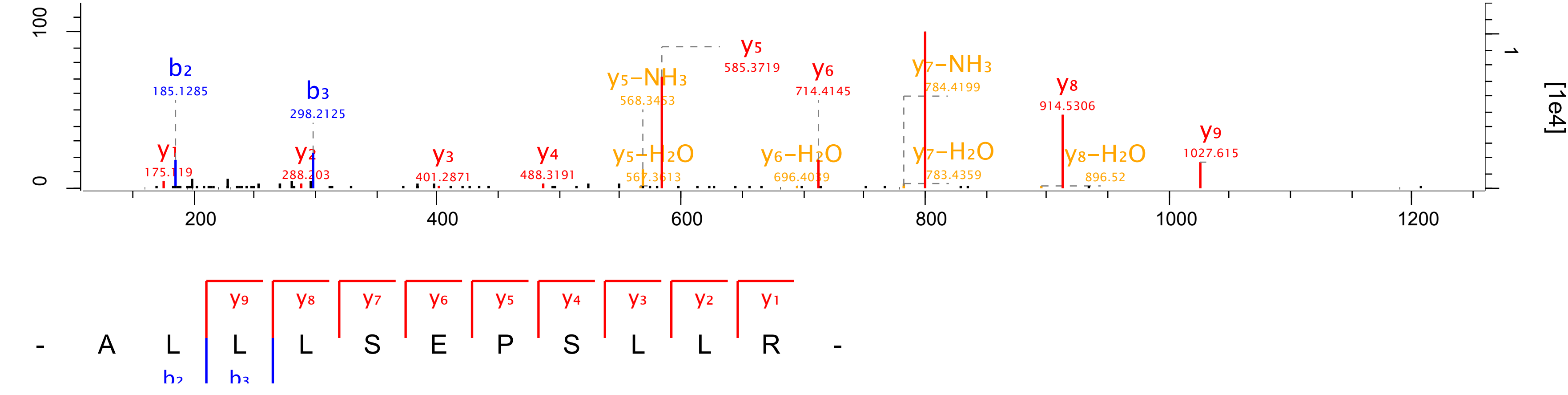

Raw file	Scan	Method	Score	m/z	Gene names
20140918_fract9_dyn_5ul_E1_01_377	10193	TOF; CID	189.7	475.79	SMC1B

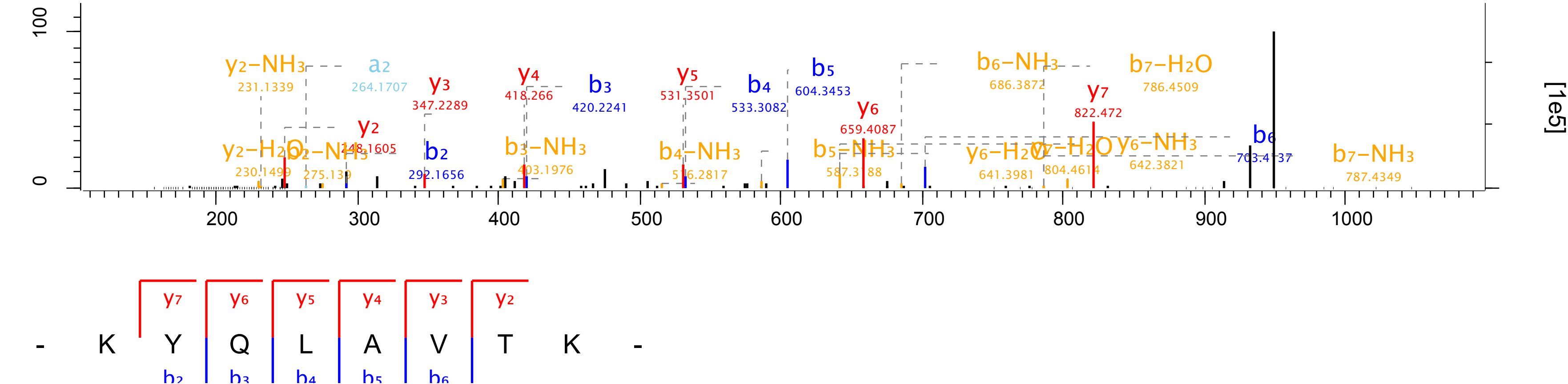

Raw file	Scan	Method	Score	m/z	Gene names
20140918_fract9_dyn_5ul_E1_01_377	14012	TOF; CID	110.38	600.83	SPATA24

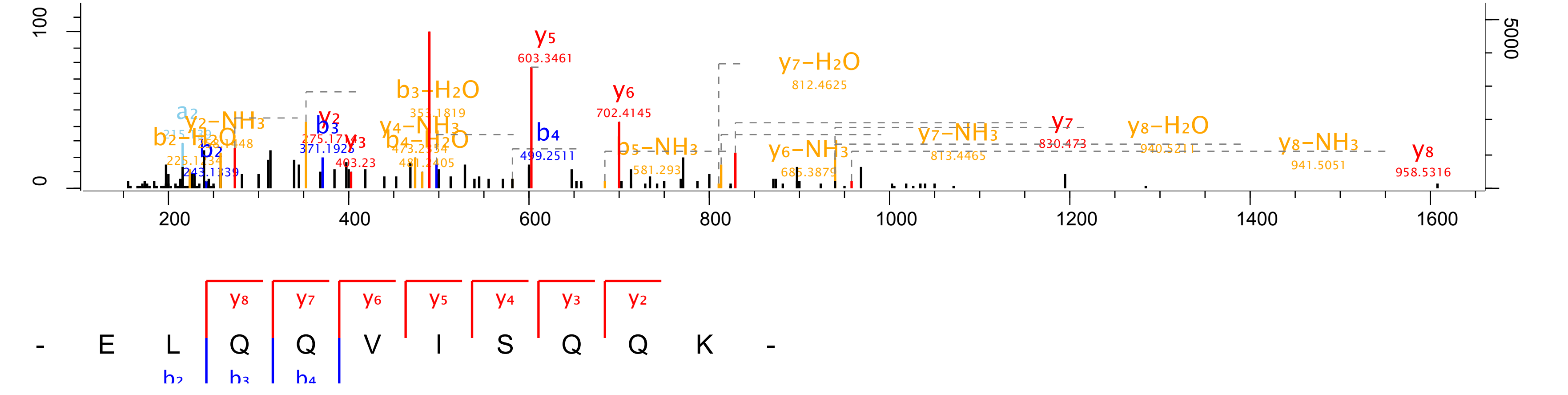

Raw file	Scan	Method	Score	m/z	Gene names
20140918_fract9_dyn_5ul_E1_01_377	15473	TOF; CID	41.91	599.55	COL16A1

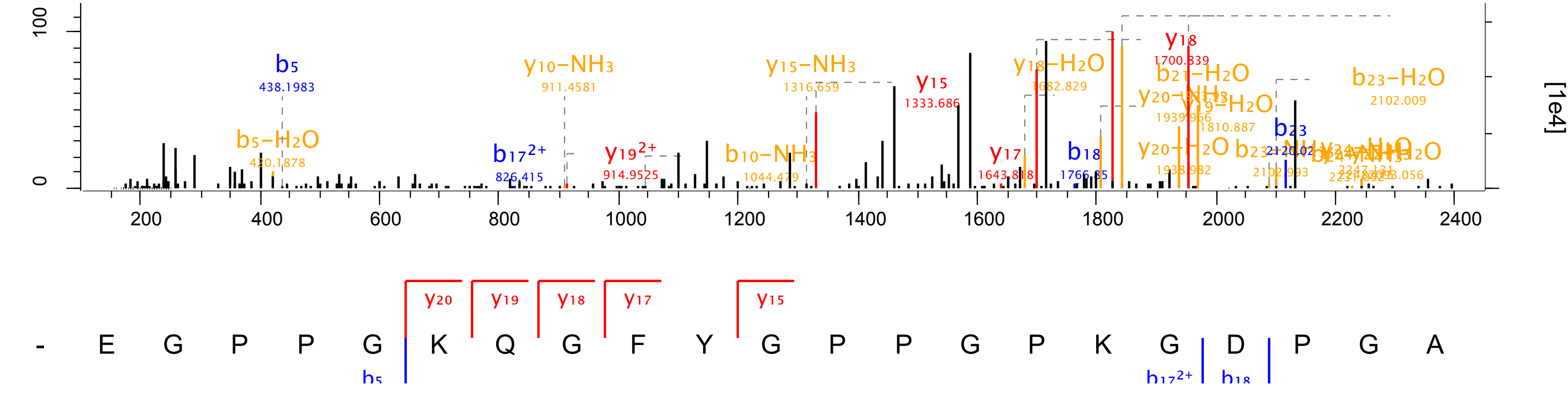

Raw file	Scan	Method	Score	m/z	Gene names
20140918_fract9_dyn_5ul_E1_01_377	16756	TOF; CID	54.95	564.61	PRDM4

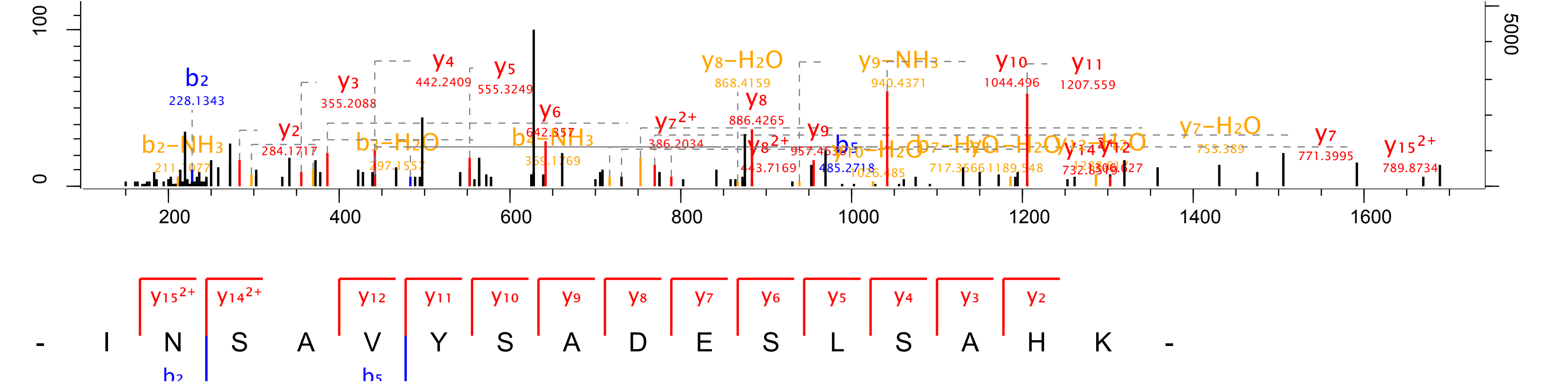

Raw file	Scan	Method	Score	m/z	Gene names
20140918_fract9_dyn_5ul_E1_01_377	18256	TOF; CID	94.31	513.27	YIPF4

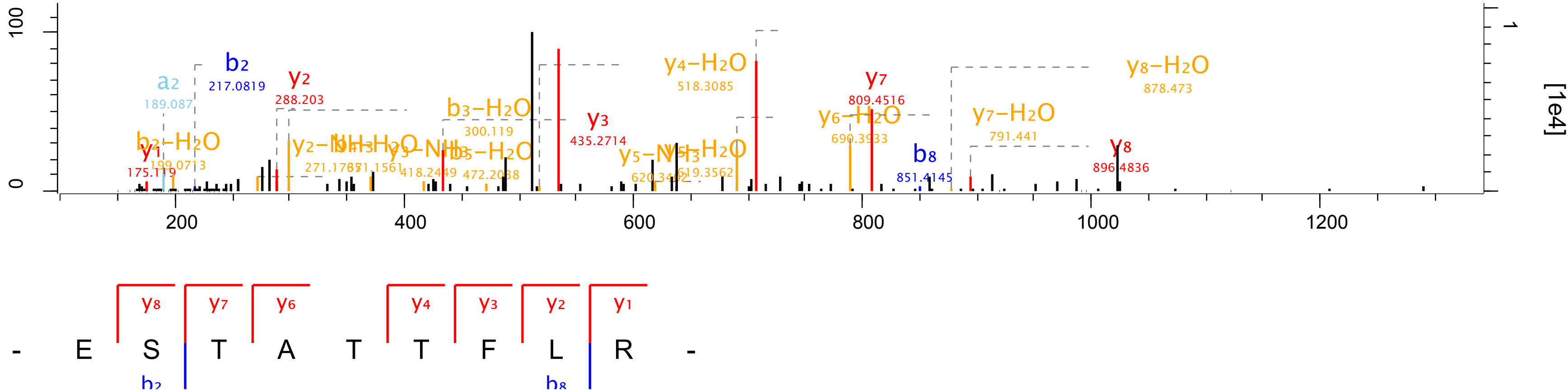

Raw file	Scan	Method	Score	m/z	Gene names
20140918_fract9_dyn_5ul_E1_01_377	19258	TOF; CID	61.24	627.3	TTC31

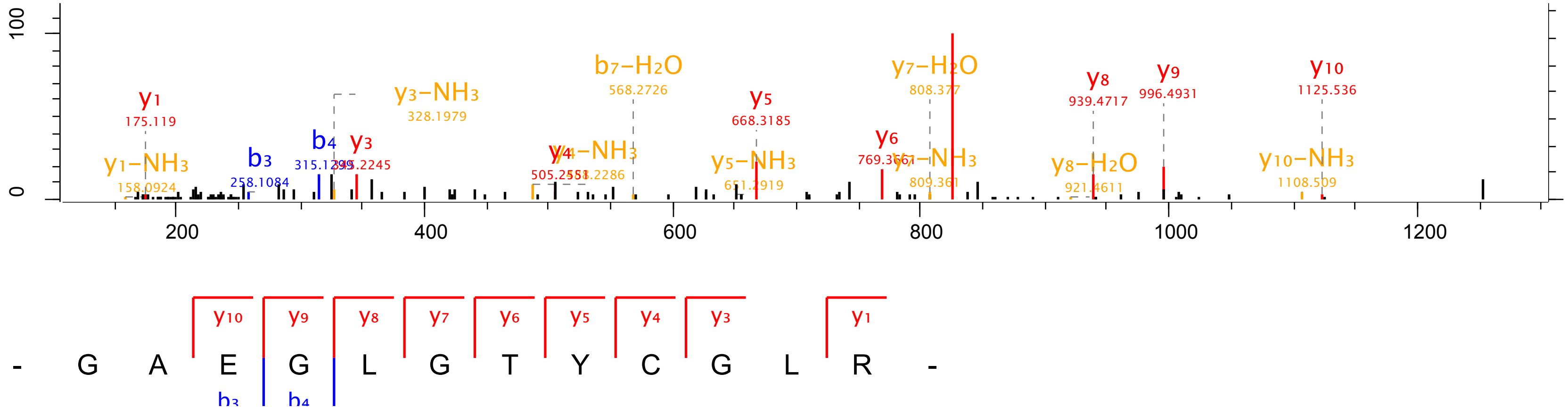

Raw file	Scan	Method	Score	m/z	Gene names
20140918_fract9_dyn_5ul_E1_01_377	19503	TOF; CID	91.87	538.77	TNIP3

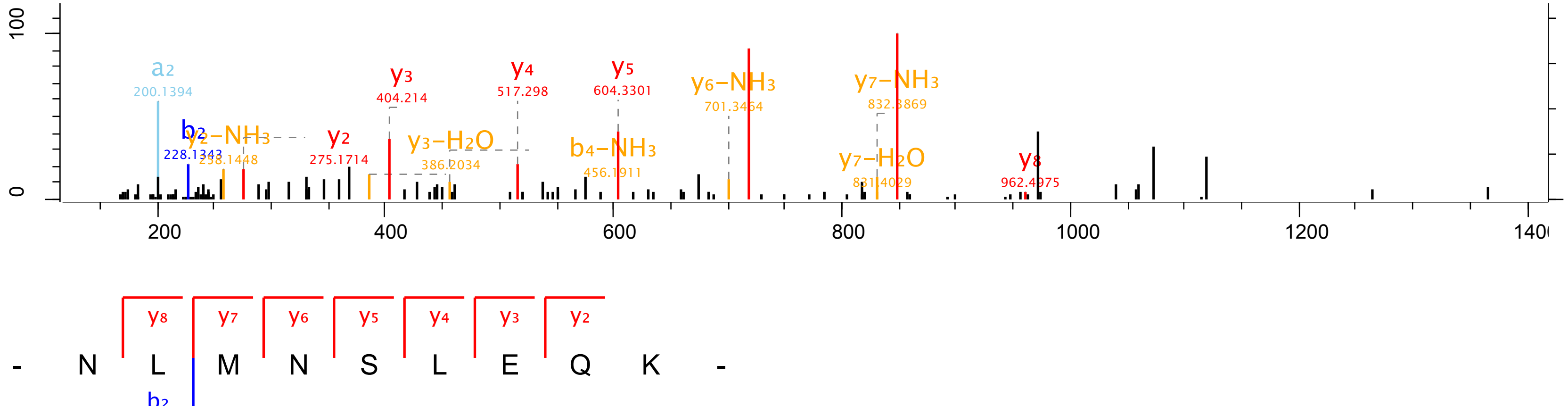

Raw file	Scan	Method	Score	m/z	Gene names
20140918_fract9_dyn_5ul_E1_01_377	20242	TOF; CID	126.07	525.76	SLC35A1

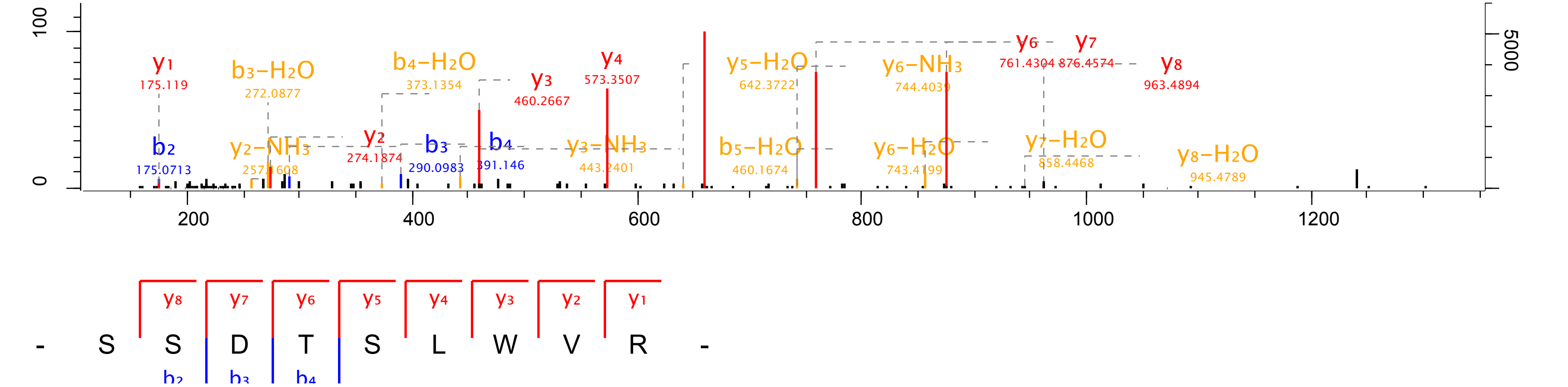

Raw file	Scan	Method	Score	m/z	Gene names
20140918_fract9_dyn_5ul_E1_01_377	20847	TOF; CID	70.94	572.77	ELF3

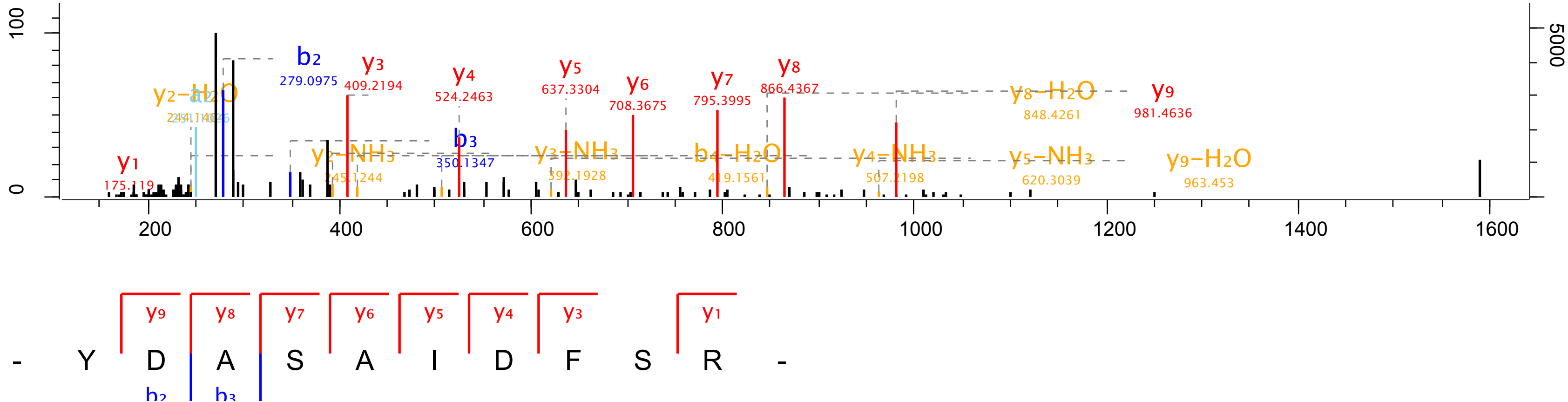

Raw file	Scan	Method	Score	m/z	Gene names
20140918_fract9_dyn_5ul_E1_01_377	21888	TOF; CID	72.29	698.36	TRAIP

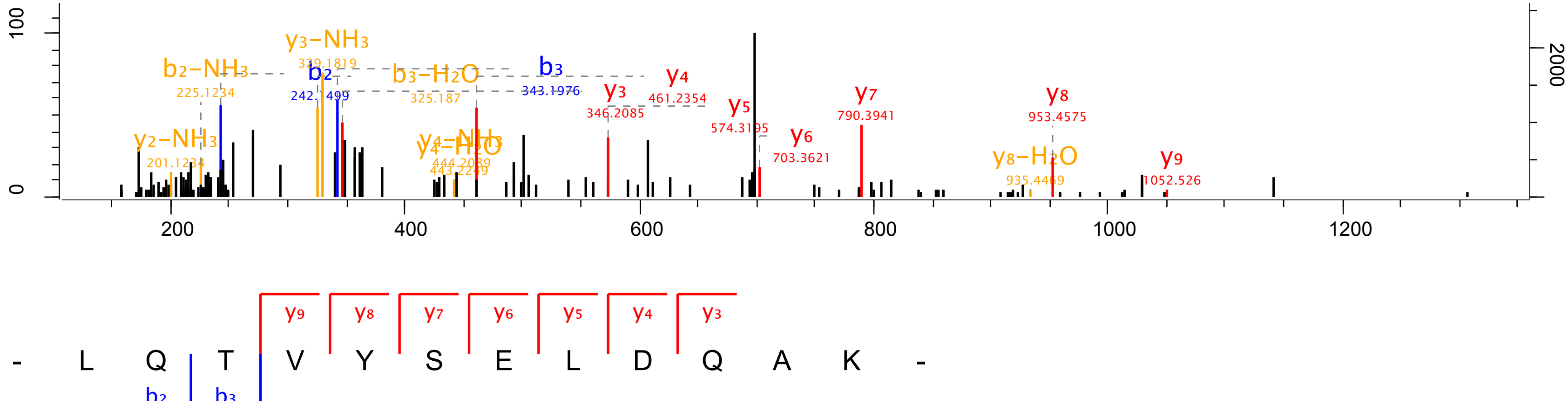

Raw file	Scan	Method	Score	m/z	Gene names
20140918_fract9_dyn_5ul_E1_01_377	21957	TOF; CID	99.92	1195.56	PRRG1

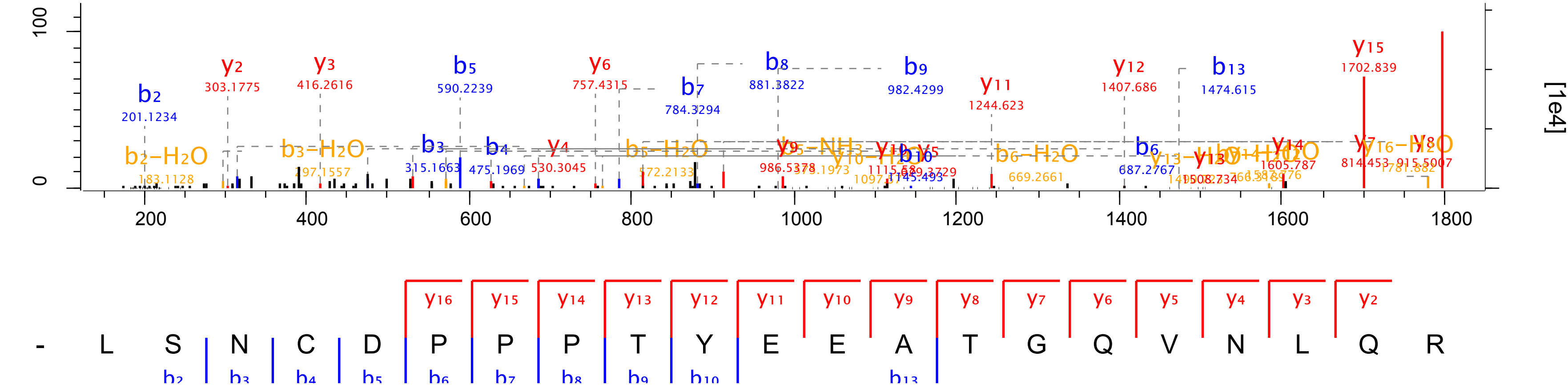

Raw file	Scan	Method	Score	m/z	Gene names
20140918_fract9_dyn_5ul_E1_01_377	26611	TOF; CID	159.14	889.45	SERINC1

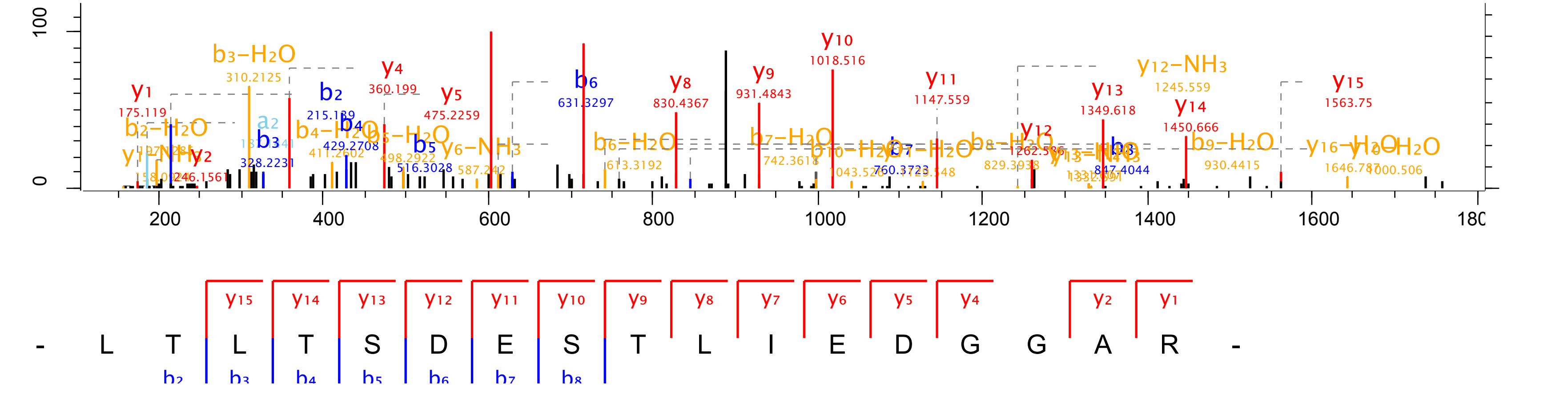

Raw file	Scan	Method	Score	m/z	Gene names
20140918_fract9_dyn_5ul_E1_01_377	29824	TOF; CID	59.23	827.4	SYCP1

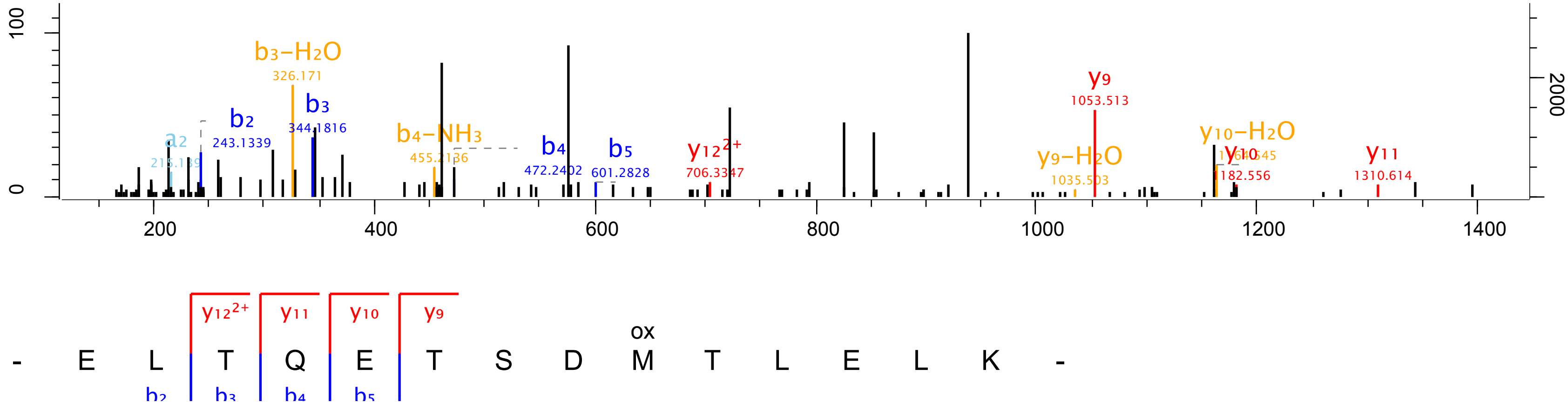

Raw file	Scan	Method	Score	m/z	Gene names
20140918_fract9_dyn_5ul_E1_01_377	30984	TOF; CID	90.05	624.86	RITA1

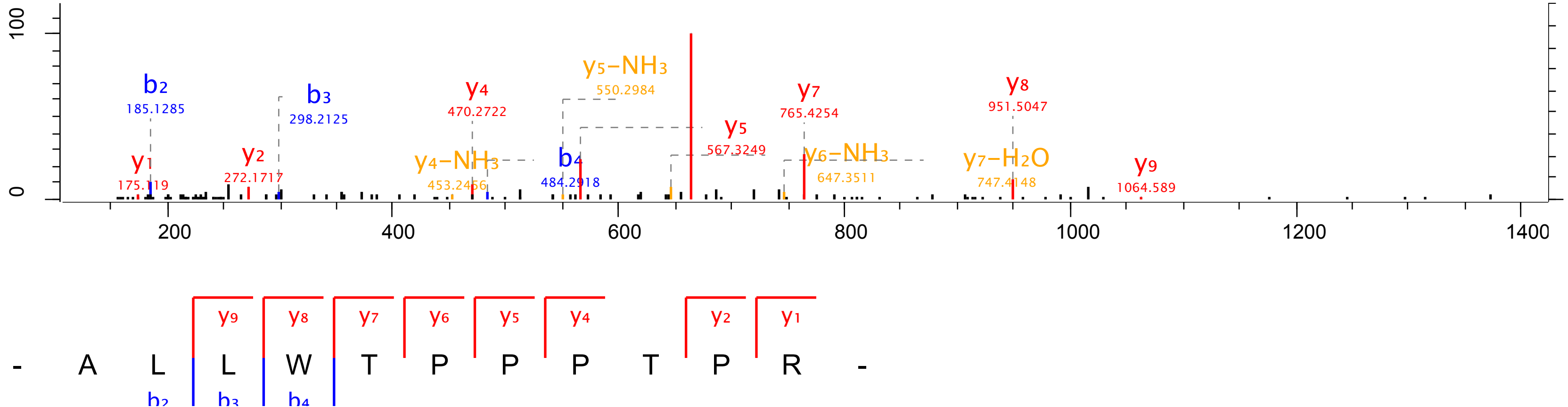

Raw file	Scan	Method	Score	m/z	Gene names
20140918_fract9_dyn_5ul_E1_01_377	34039	TOF; CID	46.27	1196.09	PEX2

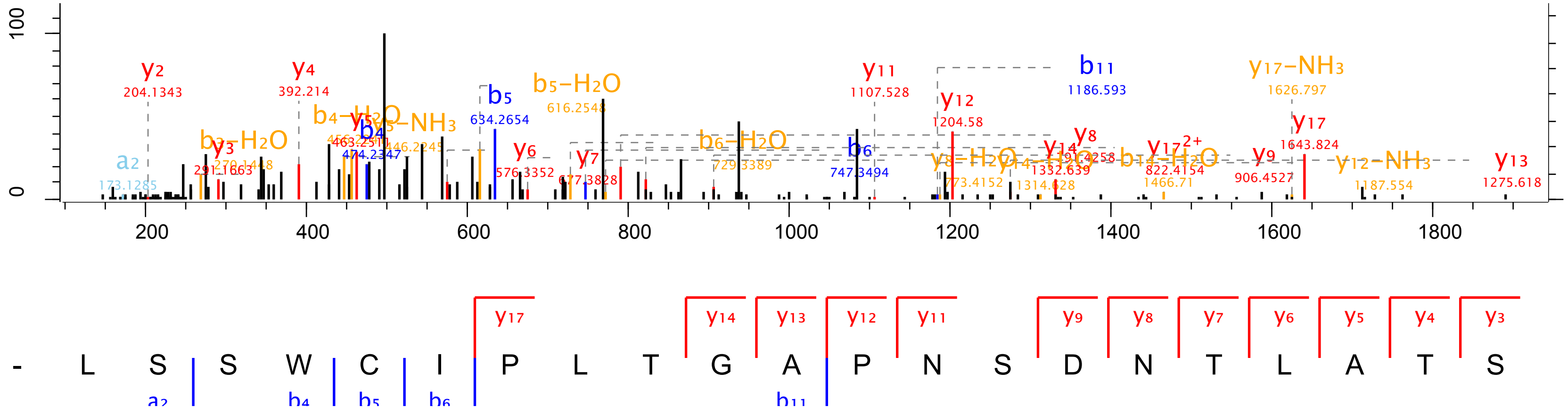

Raw file	Scan	Method	Score	m/z	Gene names
20140918_fract9_dyn_5ul_E1_01_377	34107	TOF; CID	128.72	760.03	CNIH4

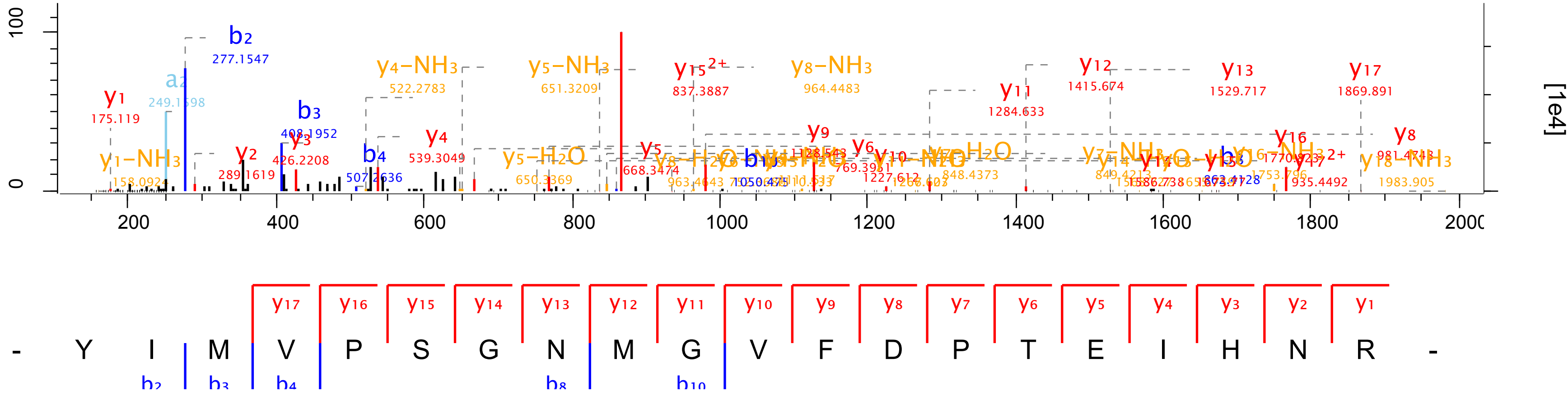

Raw file	Scan	Method	Score	m/z	Gene names
20140918_fract9_dyn_5ul_E1_01_377	38027	TOF; CID	61.13	673.02	PLAGL2

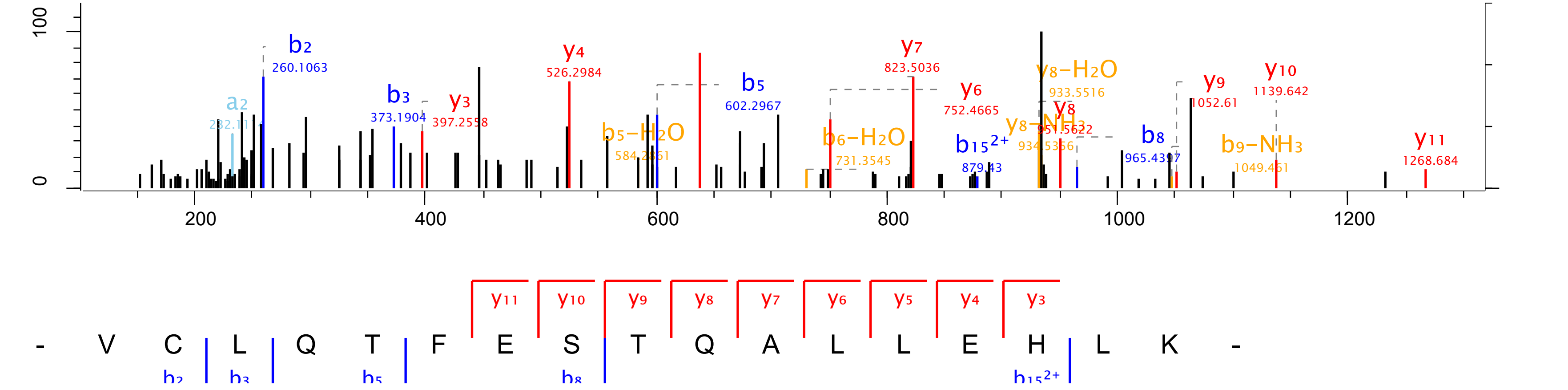

Raw file	Scan	Method	Score	m/z	Gene names
20140918_fract9_dyn_5ul_E1_01_377	38516	TOF; CID	52.86	1017.54	ZFAND2B

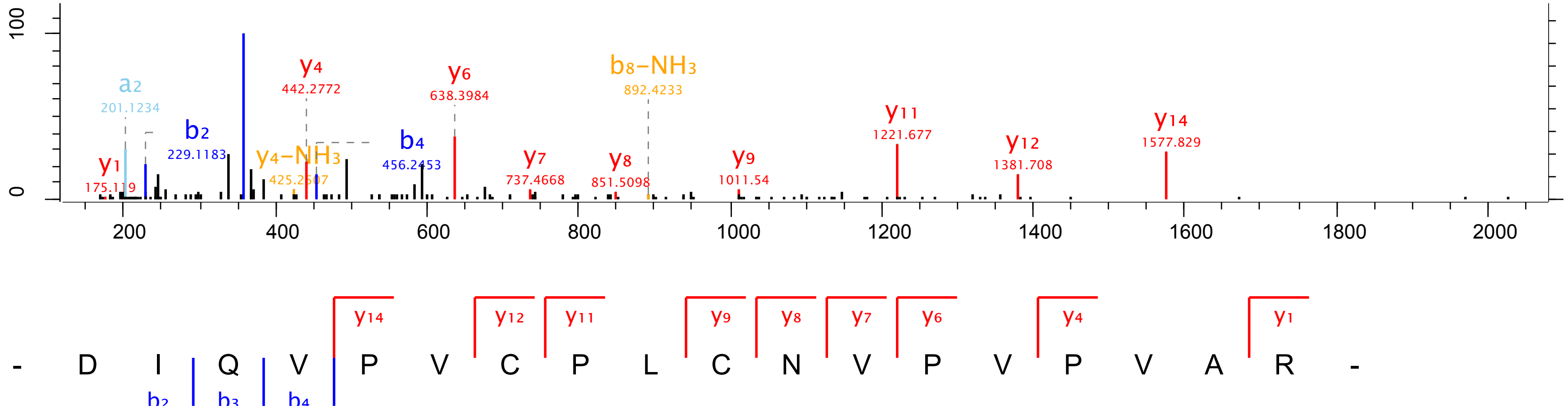

Raw file	Scan	Method	Score	m/z	Gene names
20140918_fract9_dyn_5ul_E1_01_377	38574	TOF; CID	75.55	1065.54	KDELR1

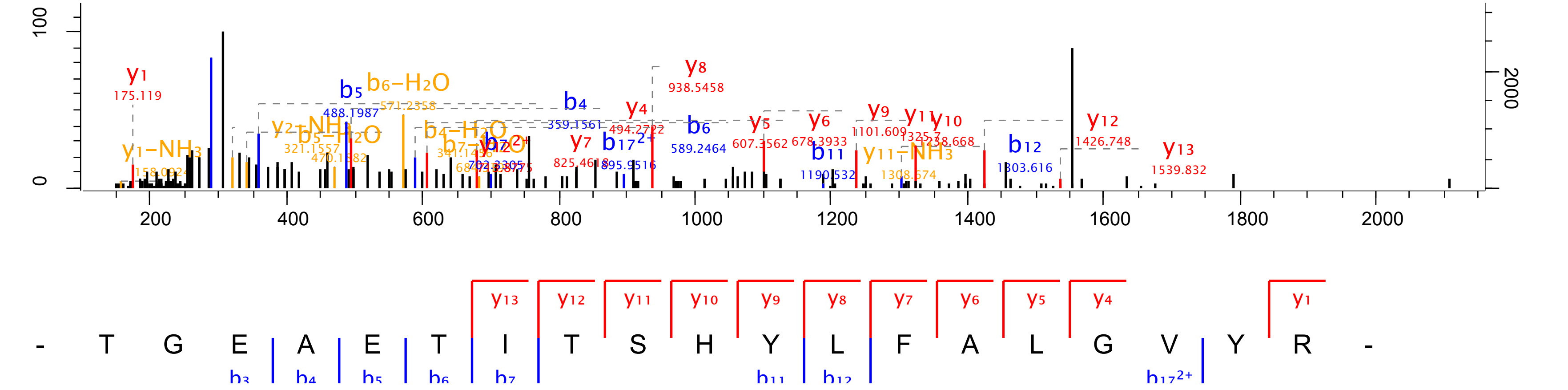

Raw file	Scan	Method	Score	m/z	Gene names
20140918_fract10_dyn_5ul_E2_01_378	6158	TOF; CID	75.32	465.23	CRAT

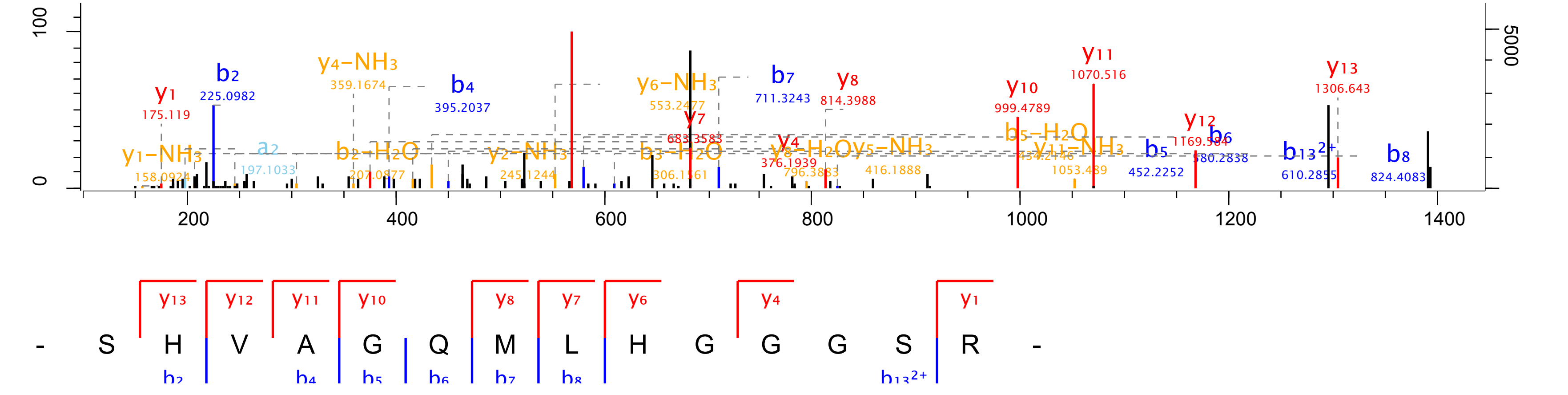

Raw file	Scan	Method	Score	m/z
20140918_fract10_dyn_5ul_E2_01_378	6940	TOF; CID	49.77	581.28

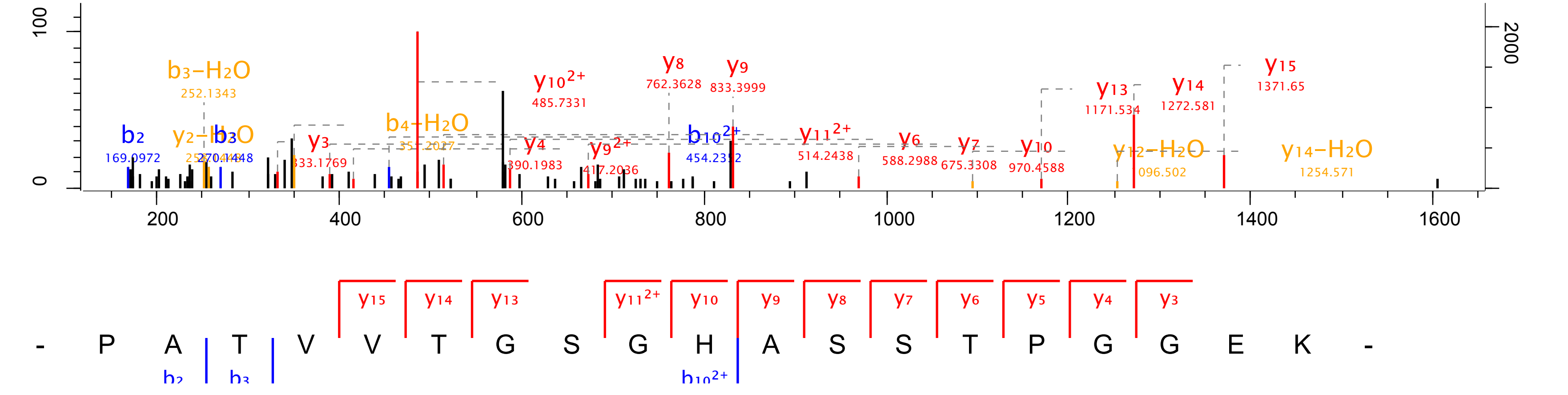

Raw file	Scan	Method	Score	m/z	Gene names
20140918_fract10_dyn_5ul_E2_01_378	7502	TOF; CID	102.97	446.23	NINJ1

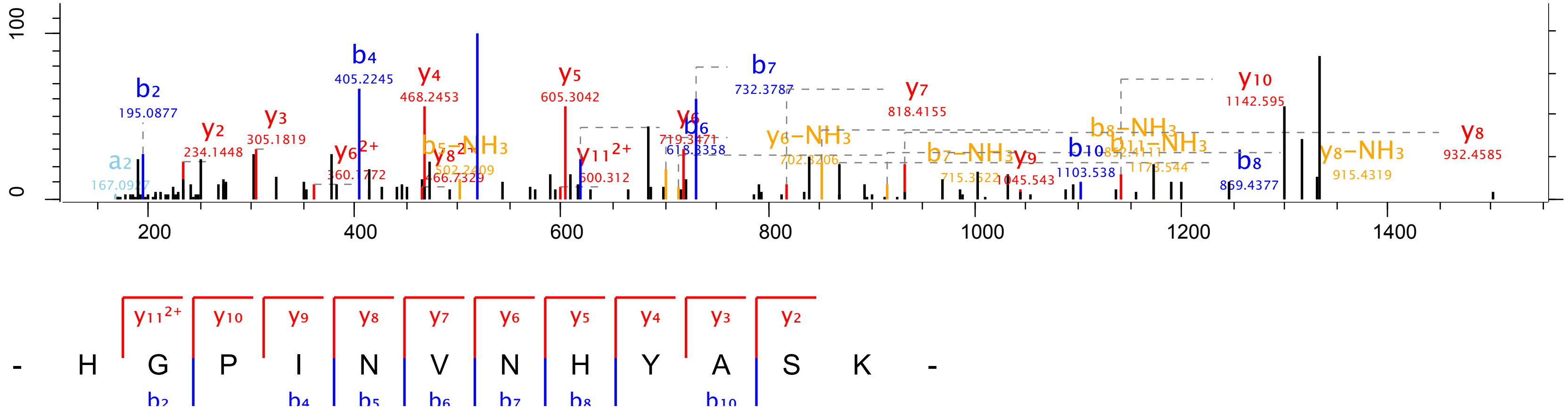

Raw file	Scan	Method	Score	m/z	Gene names
20140918_fract10_dyn_5ul_E2_01_378	7973	TOF; CID	94.12	505.25	ZNF566;TMEM92

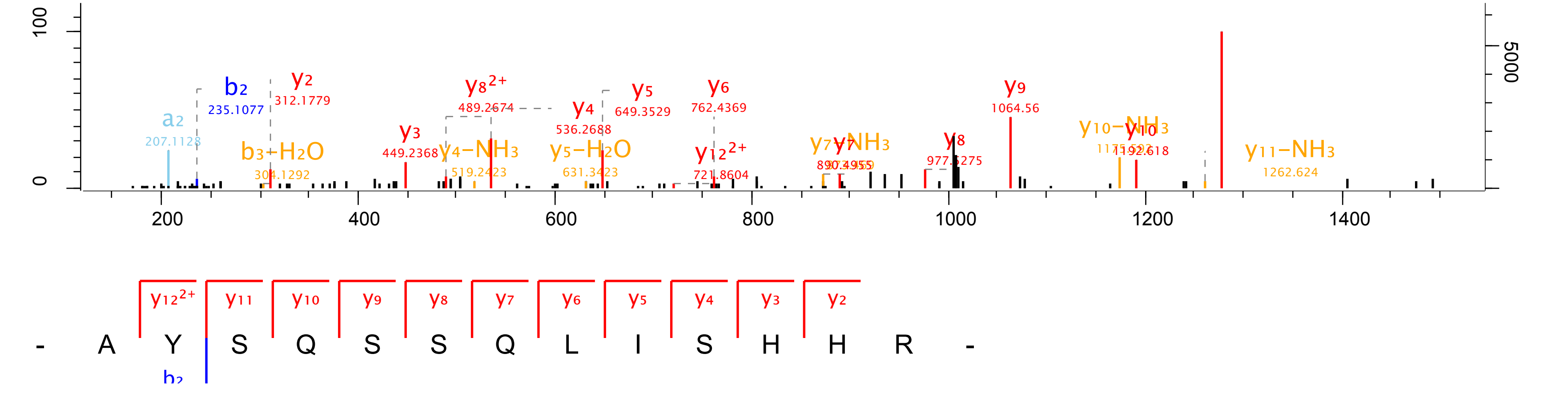

Raw file	Scan	Method	Score	m/z	Gene names
20140918_fract10_dyn_5ul_E2_01_378	10331	TOF; CID	87.57	353.44	C15orf41

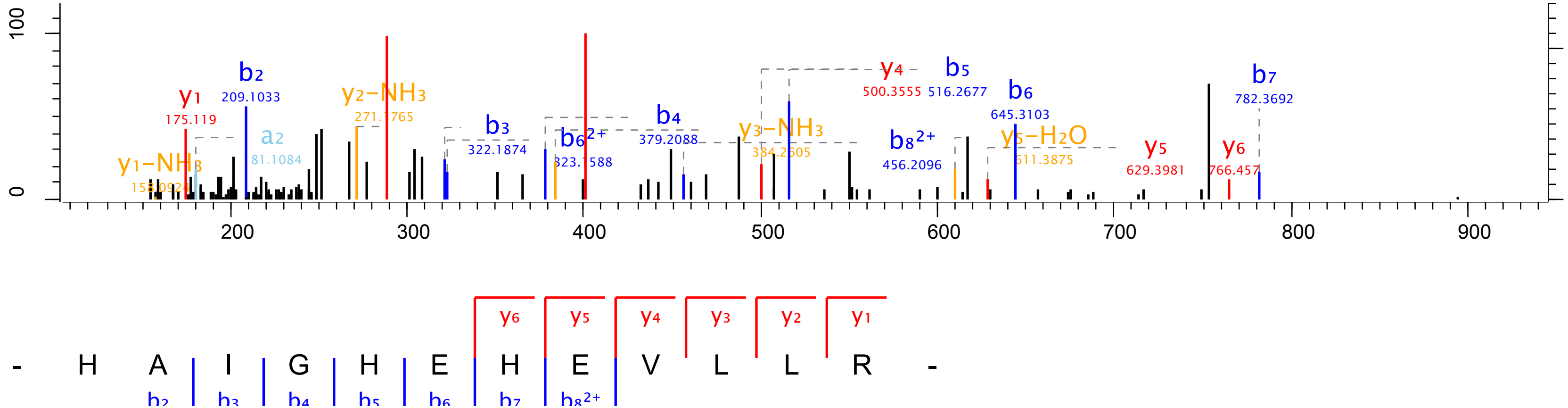

Raw file	Scan	Method	Score	m/z	Gene names
20140918_fract10_dyn_5ul_E2_01_378	10459	TOF; CID	125.74	545.32	PRICKLE2

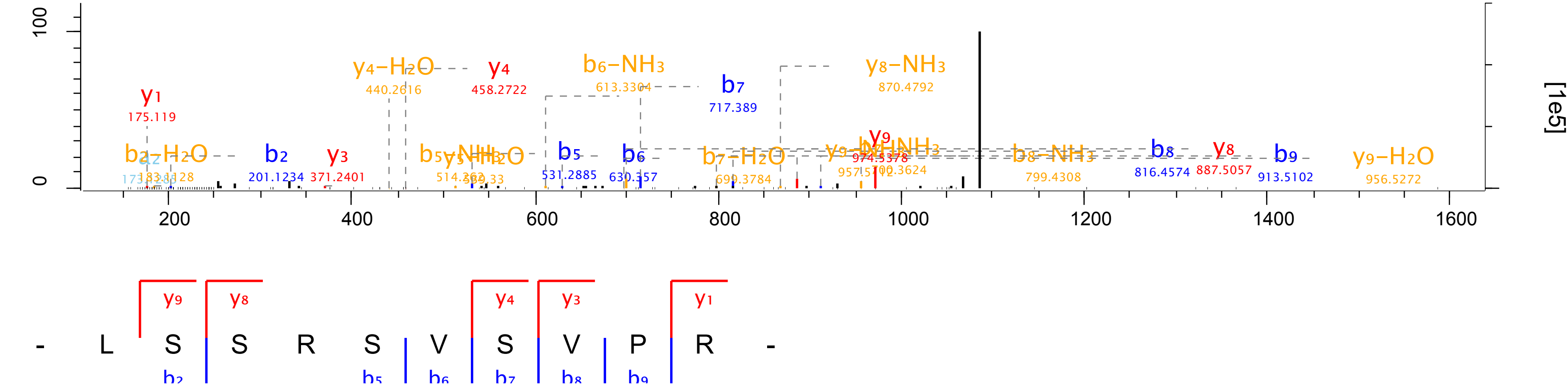

Raw file	Scan	Method	Score	m/z	Gene names
20140918_fract10_dyn_5ul_E2_01_378	13201	TOF; CID	78.82	529.32	RPGRIP1

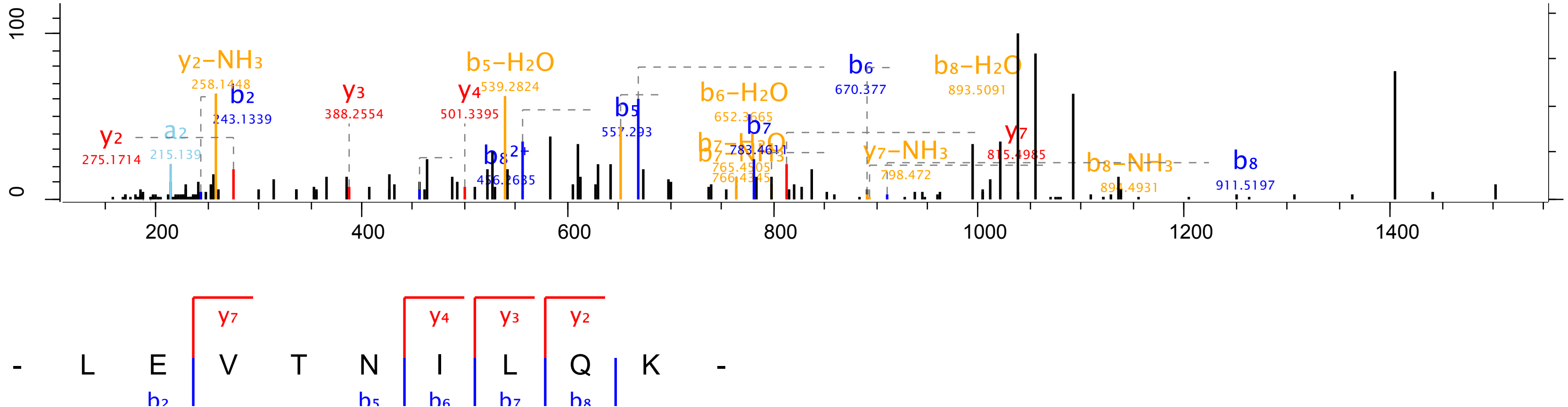

Raw file	Scan	Method	Score	m/z	Gene names
20140918_fract10_dyn_5ul_E2_01_378	17242	TOF; CID	57.17	432.23	NDUFC1

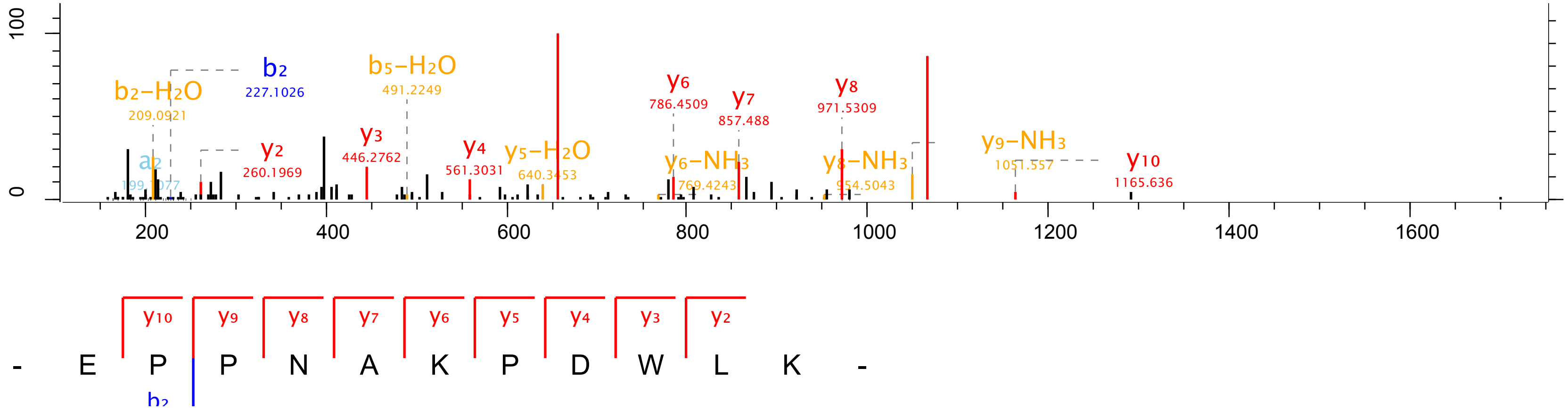

Raw file	Scan	Method	Score	m/z	Gene names
20140918_fract10_dyn_5ul_E2_01_378	17727	TOF; CID	47.55	628.31	METAP1D

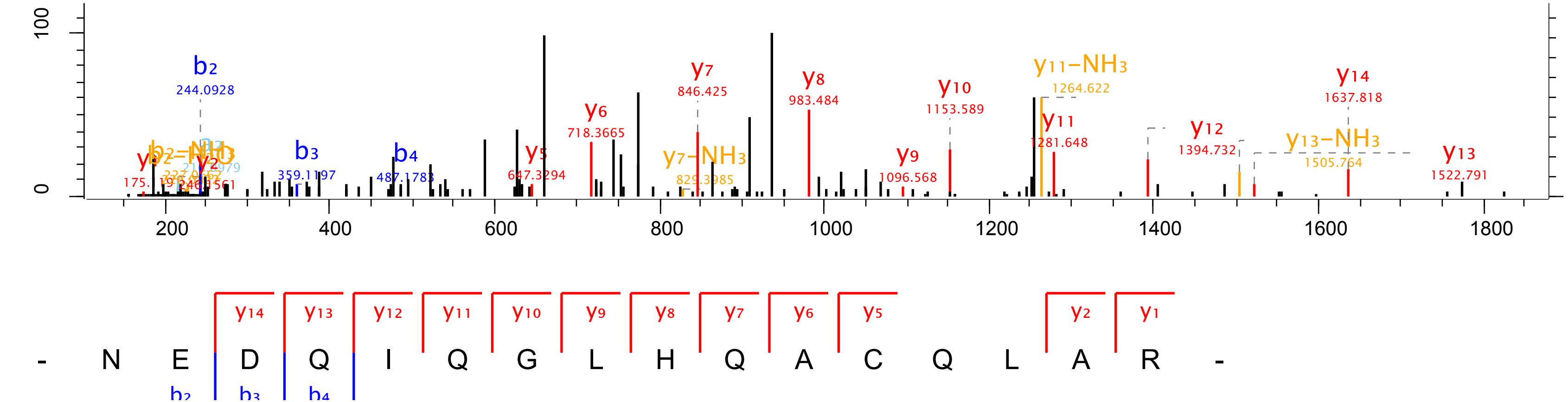

Raw file	Scan	Method	Score	m/z	Gene names
20140918_fract10_dyn_5ul_E2_01_378	20538	TOF; CID	66.27	421.23	GNG11

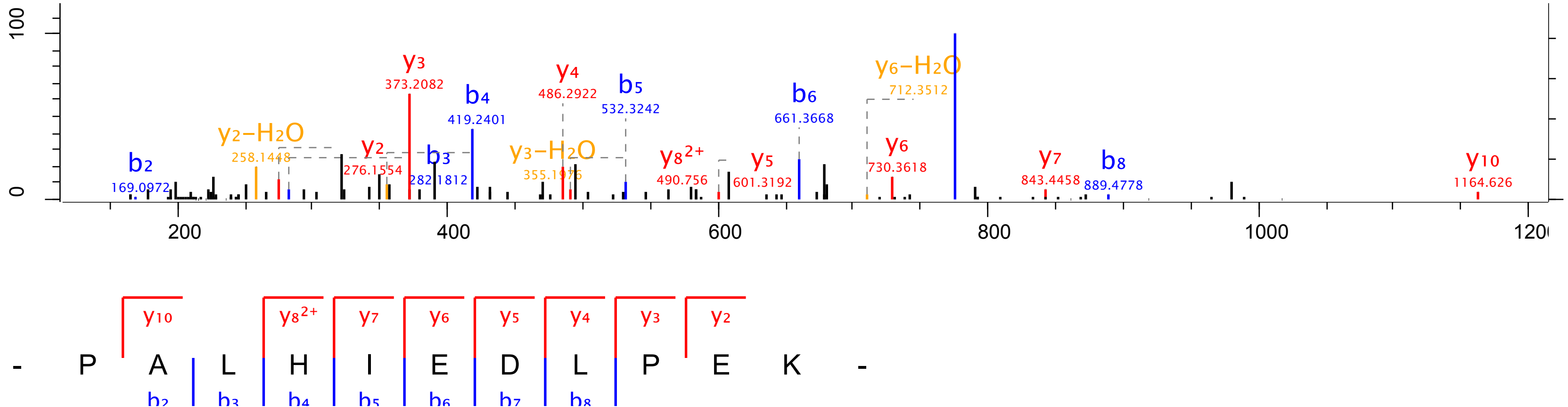

Raw file	Scan	Method	Score	m/z	Gene names
20140918_fract10_dyn_5ul_E2_01_378	26597	TOF; CID	119.57	414.27	C2orf72

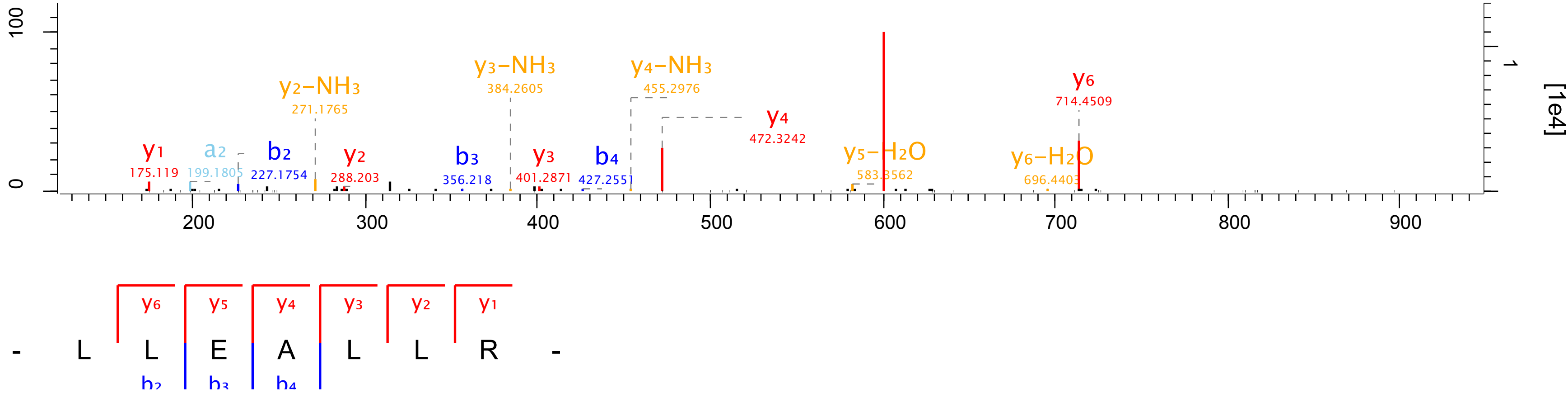

Raw file	Scan	Method	Score	m/z	Gene names
20140918_fract10_dyn_5ul_E2_01_378	26649	TOF; CID	43.33	979.49	OSBPL8

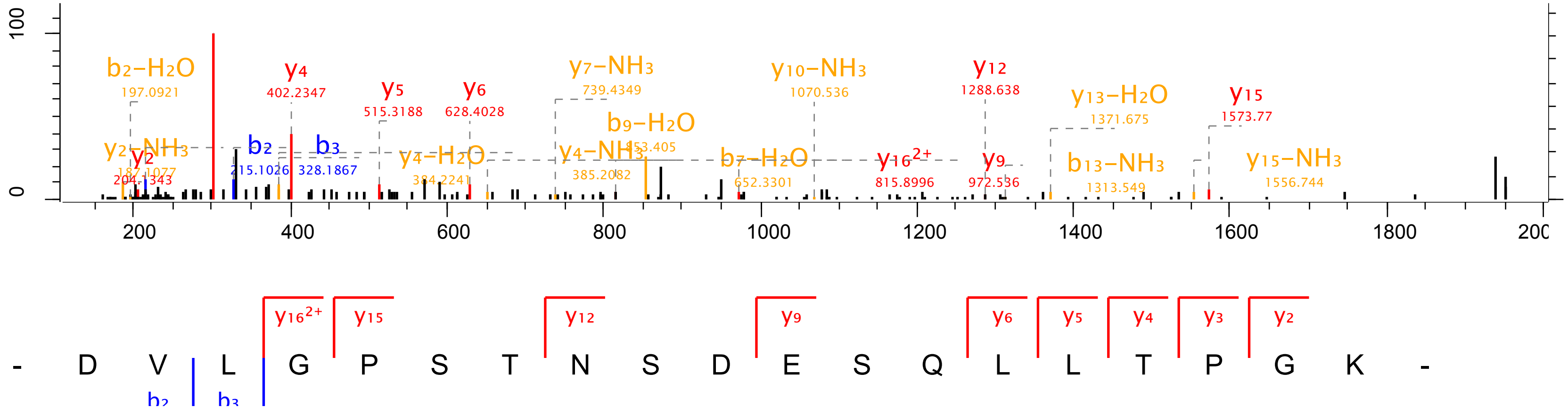

Raw file	Scan	Method	Score	m/z	Gene names
20140918_fract10_dyn_5ul_E2_01_378	30767	TOF; CID	120.9	865.99	KRTCAP2

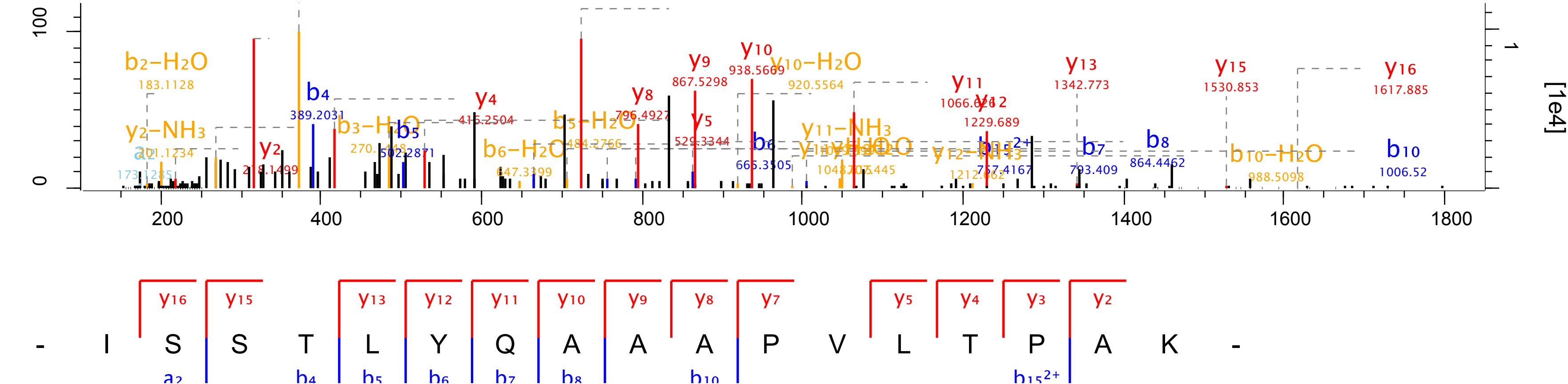

Raw file	Scan	Method	Score	m/z	Gene names
20140918_fract10_dyn_5ul_E2_01_378	33088	TOF; CID	216	928.44	MAPK1IP1L

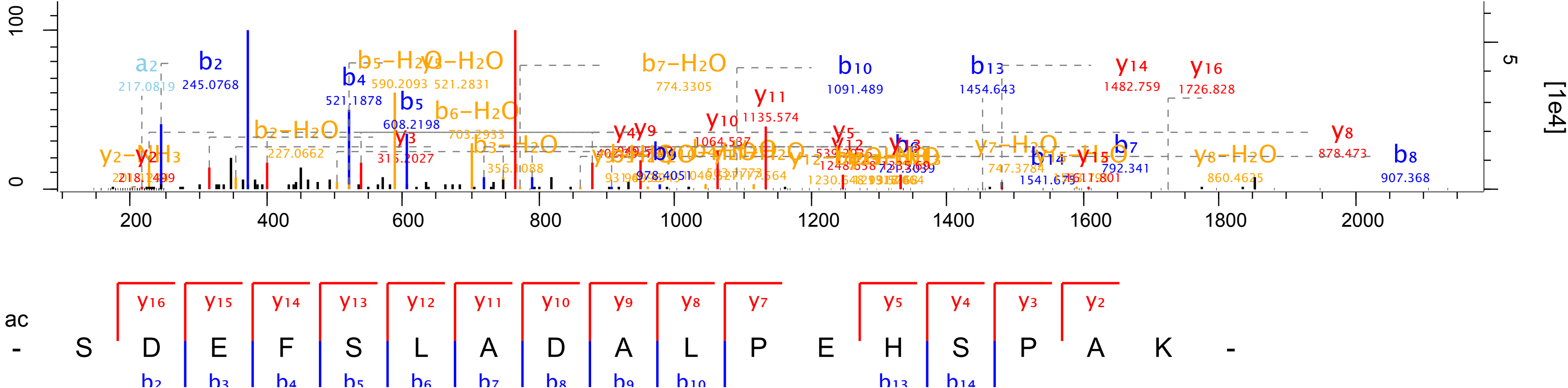

Raw file	Scan	Method	Score	m/z	Gene names
20140918_fract10_dyn_5ul_E2_01_378	34653	TOF; CID	137.89	814.92	CCDC167

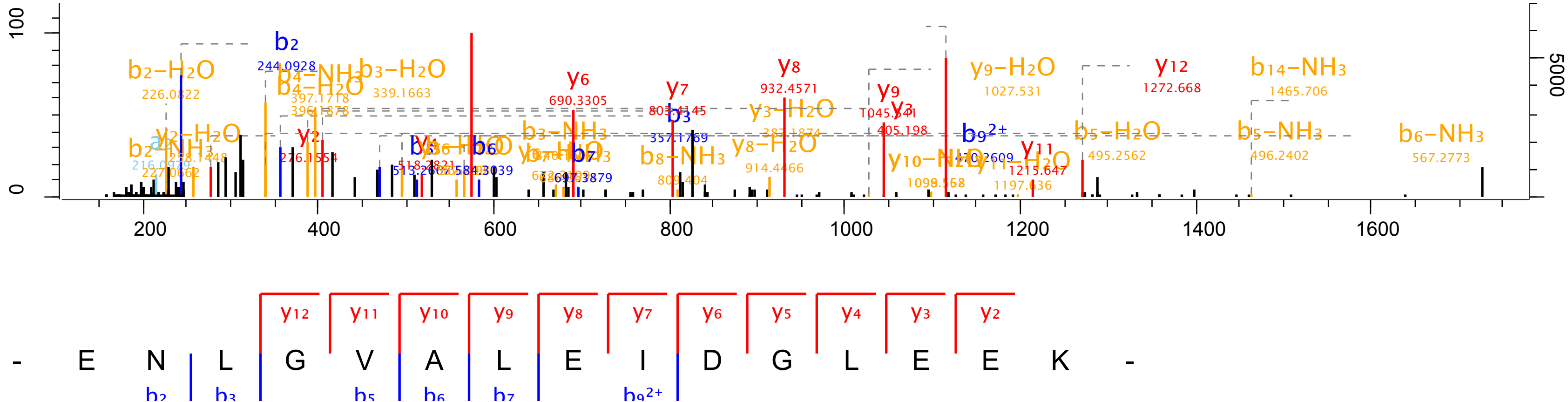

Raw file	Scan	Method	Score	m/z	Gene names
20140918_fract11_dyn_5ul_E3_01_379	6911	TOF; CID	113.24	544.91	ZNF302

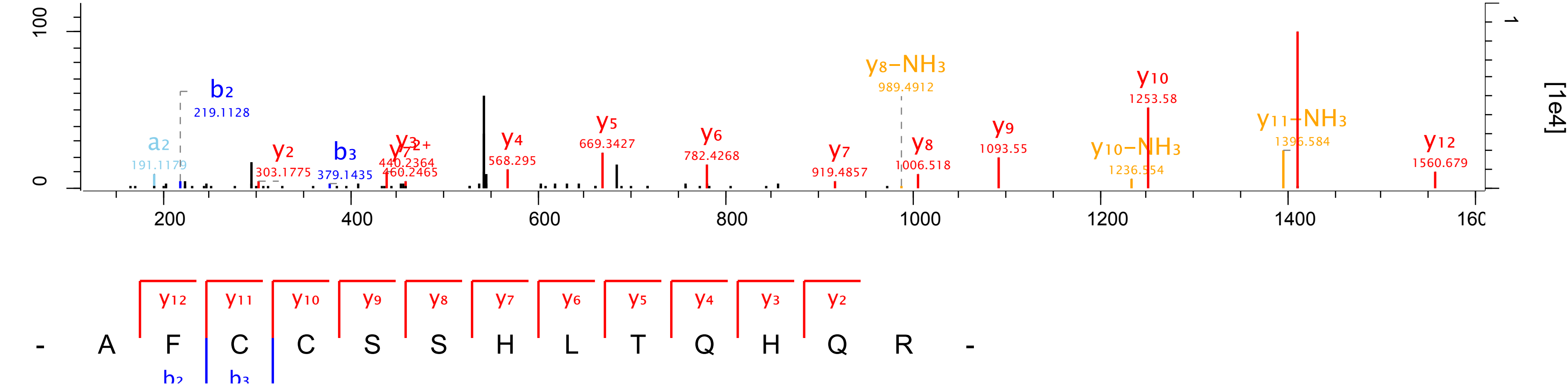

Raw file	Scan	Method	Score	m/z	Gene names
20140918_fract11_dyn_5ul_E3_01_379	7039	TOF; CID	80.39	526.62	ZNF251

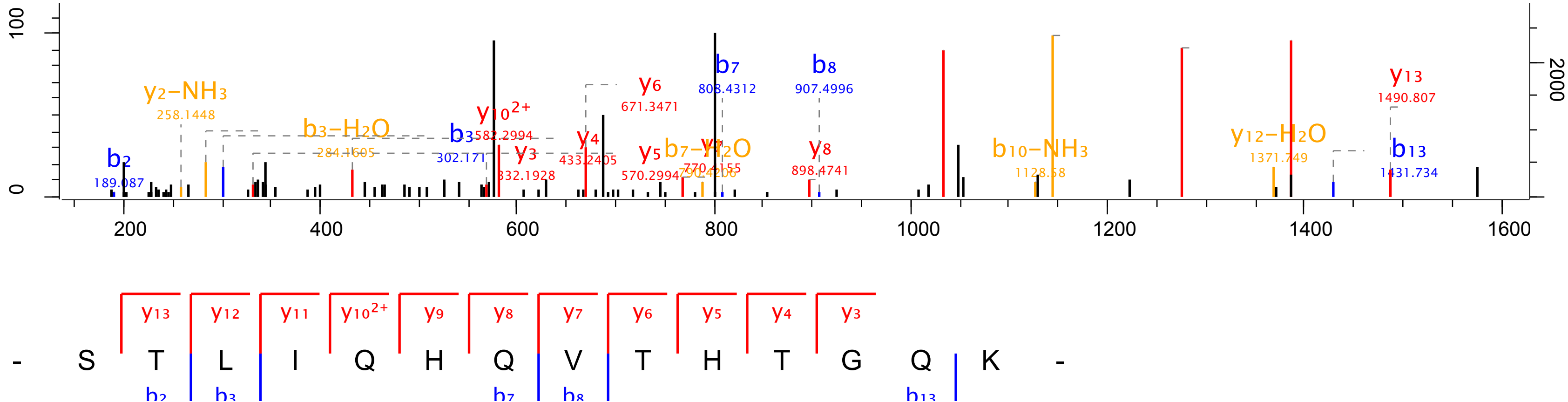

Raw file	Scan	Method	Score	m/z	Gene names
20140918_fract11_dyn_5ul_E3_01_379	9261	TOF; CID	106.38	465.24	TMEM41A

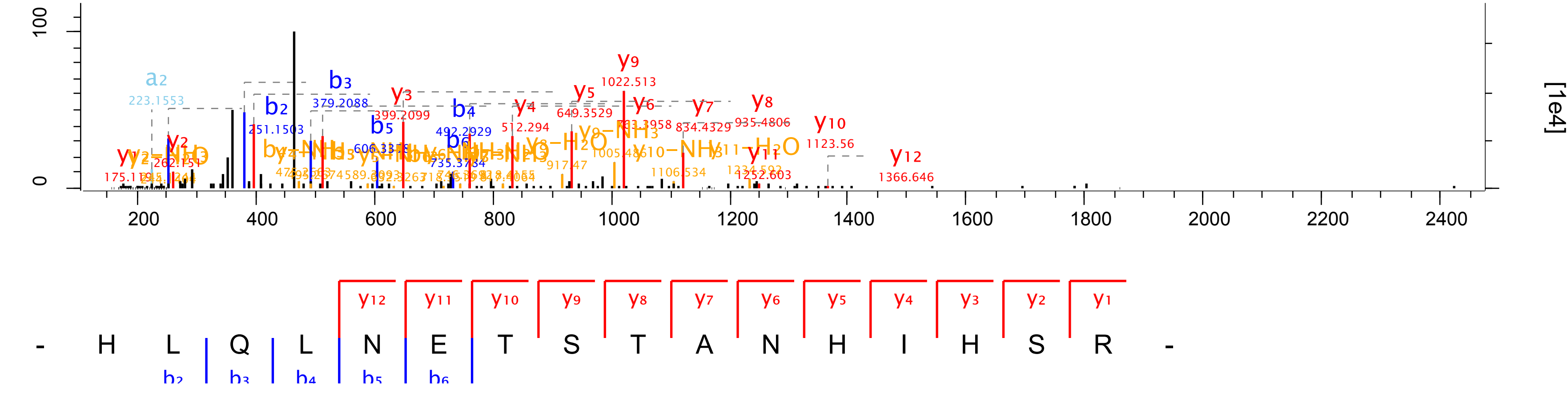

Raw file	Scan	Method	Score	m/z	Gene names
20140918_fract11_dyn_5ul_E3_01_379	9894	TOF; CID	74.53	368.21	SPATA24

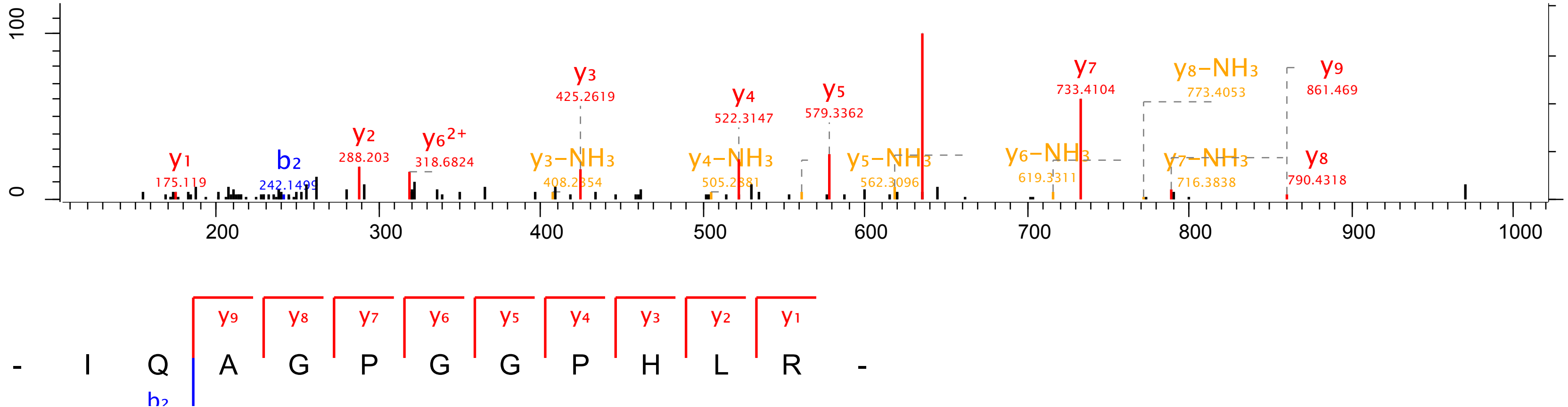

Raw file	Scan	Method	Score	m/z	Gene names
20140918_fract11_dyn_5ul_E3_01_379	16771	TOF; CID	133.05	617.83	SUMO2;SUMO3;SUMO4

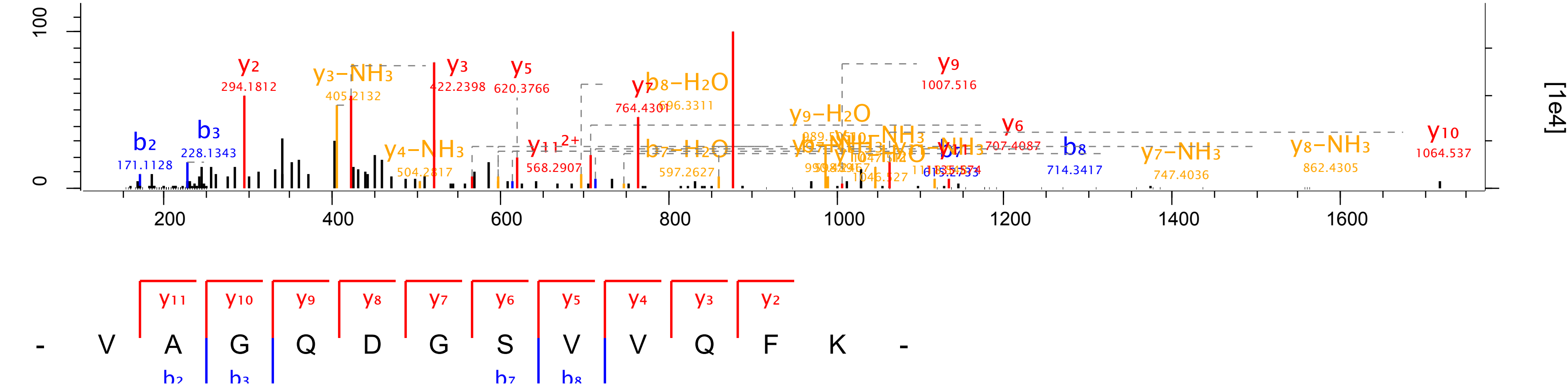

Raw file	Scan	Method	Score	m/z	Gene names
20140918_fract11_dyn_5ul_E3_01_379	16787	TOF; CID	38.38	700.35	MIDN

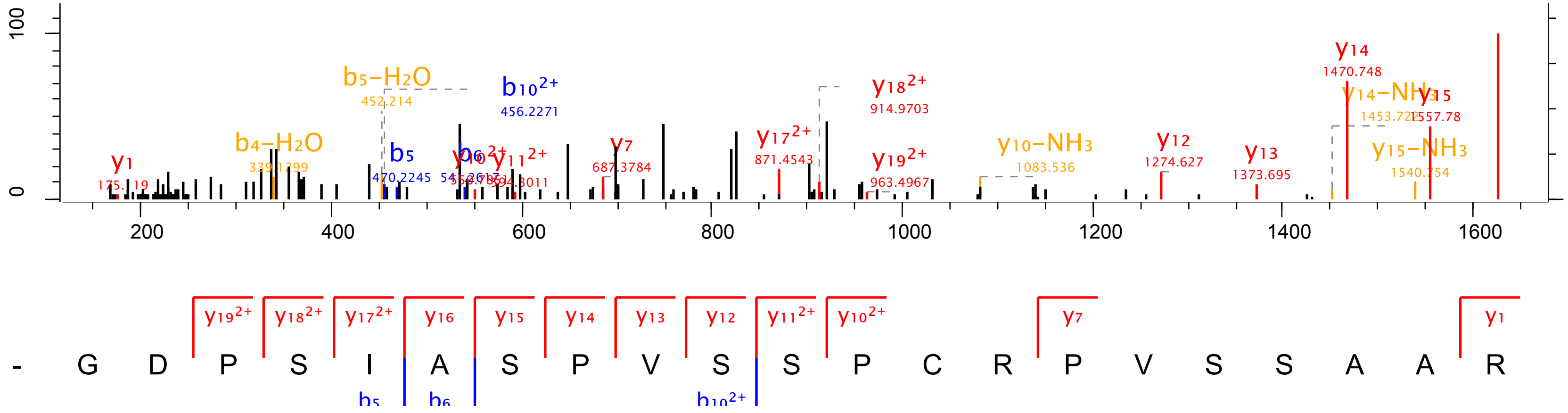

Raw file	Scan	Method	Score	m/z	Gene names
20140918_fract11_dyn_5ul_E3_01_379	20114	TOF; CID	74.85	581.6	RCHY1

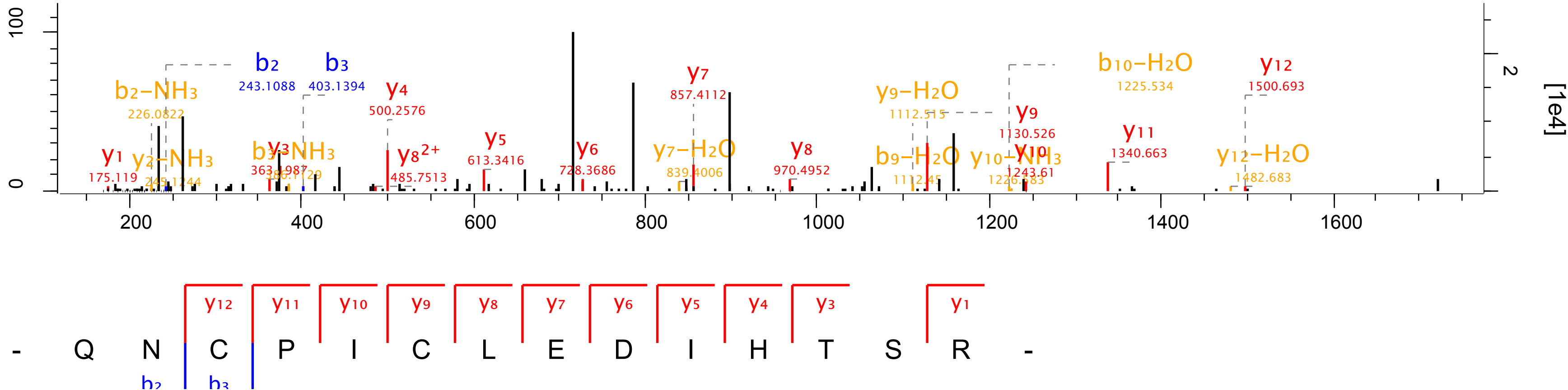

Raw file	Scan	Method	Score	m/z	Gene names
20140918_fract11_dyn_5ul_E3_01_379	20243	TOF; CID	61.41	513.28	MYEOV2

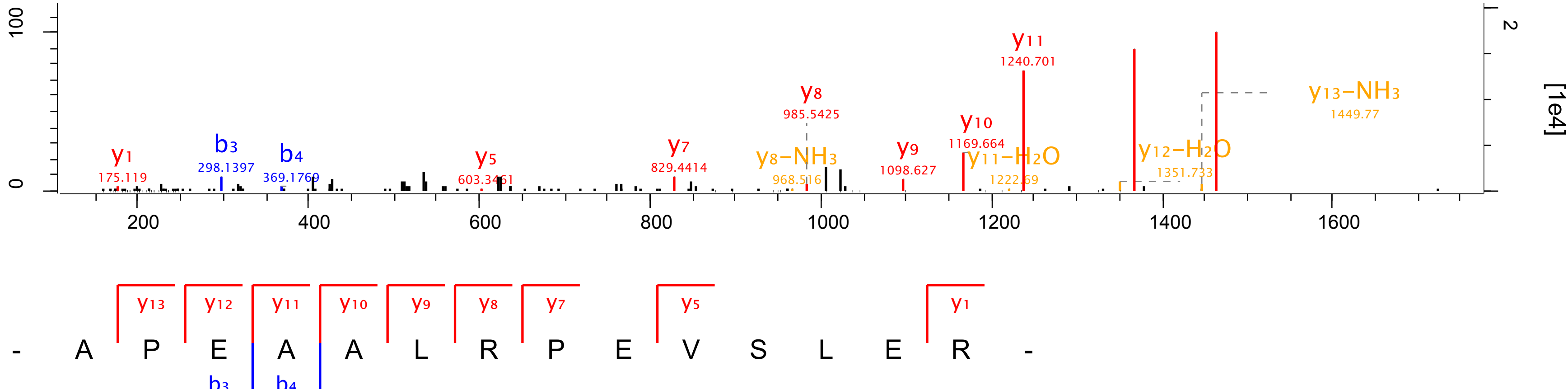

Raw file	Scan	Method	Score	m/z	Gene names
20140918_fract11_dyn_5ul_E3_01_379	21398	TOF; CID	132.25	608.33	SVIP

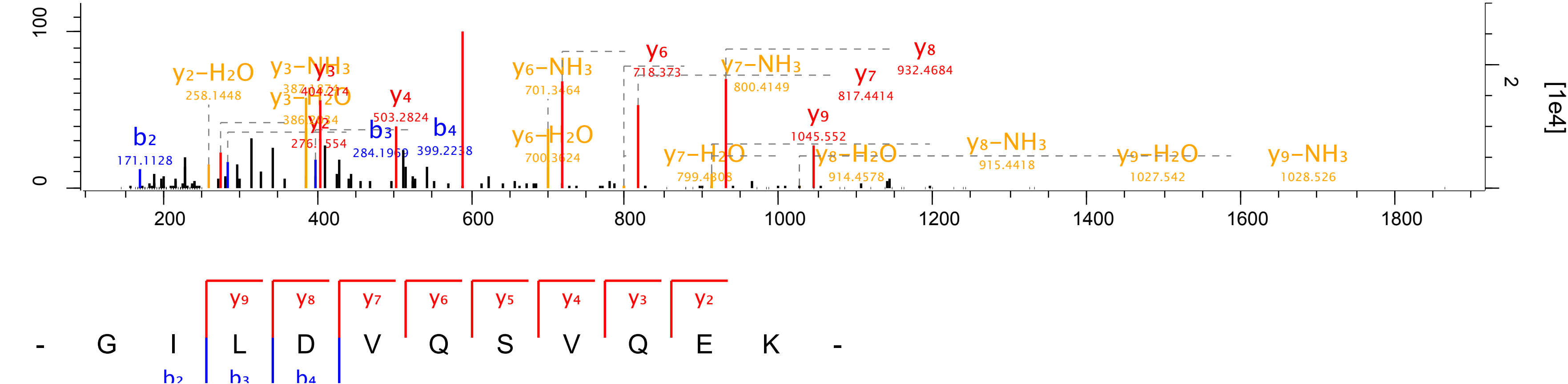

Raw file	Scan	Method	Score	m/z	Gene names
20140918_fract11_dyn_5ul_E3_01_379	24909	TOF; CID	83.82	857.86	MT-ND3

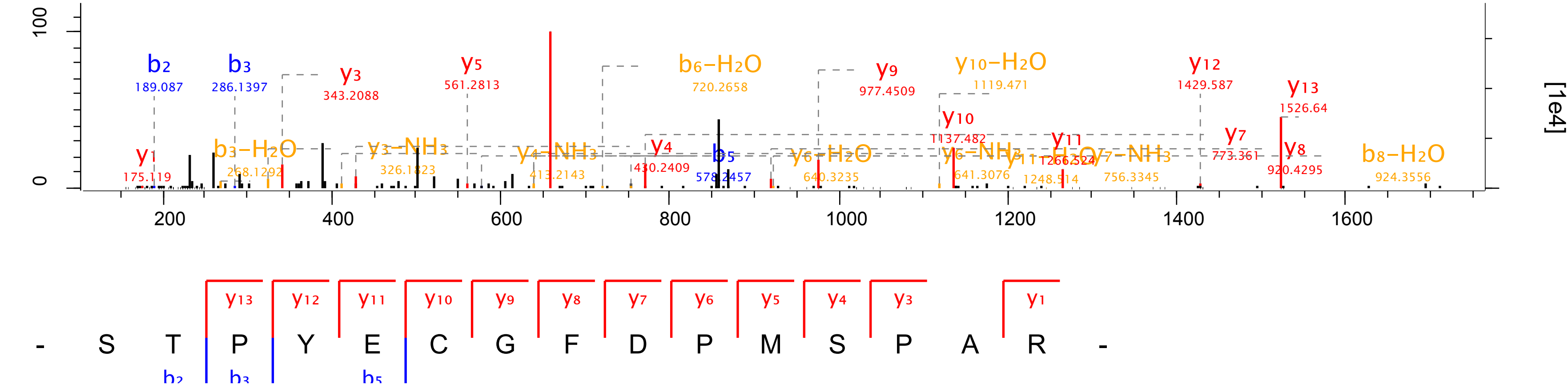

Raw file	Scan	Method	Score	m/z	Gene names
20140918_fract11_dyn_5ul_E3_01_379	25227	TOF; CID	125.45	914.41	RNF5

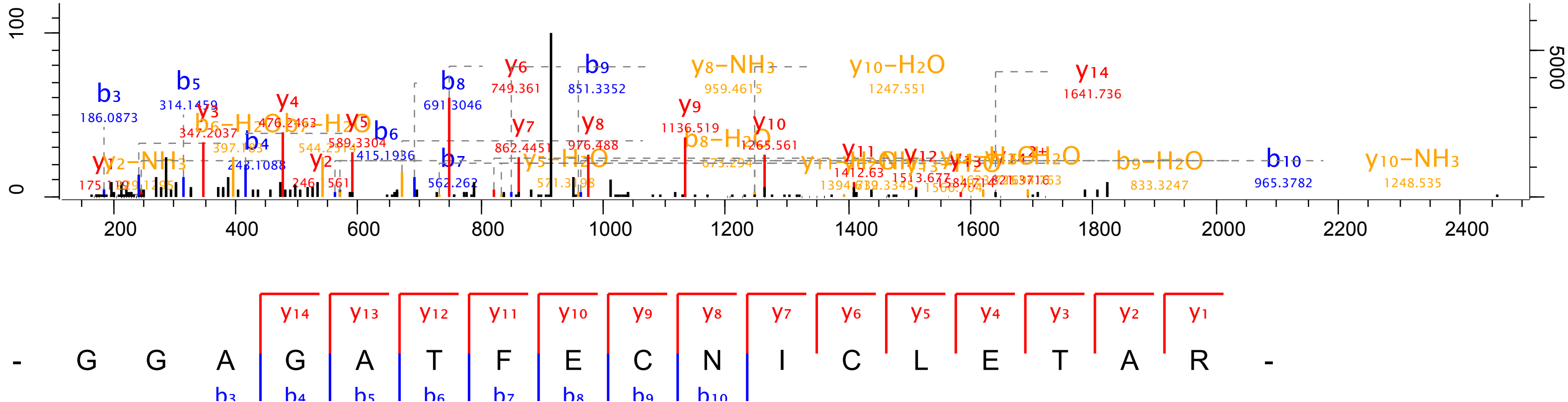

Raw file	Scan	Method	Score	m/z	Gene names
20140918_fract11_dyn_5ul_E3_01_379	25906	TOF; CID	85.47	664.98	DCUN1D2

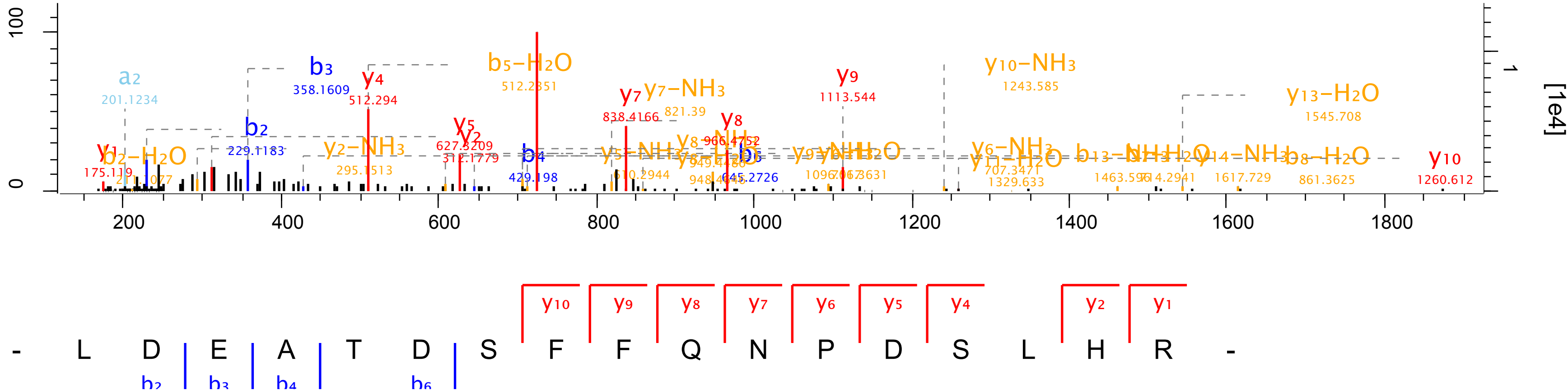

Raw file	Scan	Method	Score	m/z	Gene names
20140918_fract11_dyn_5ul_E3_01_379	26489	TOF; CID	121.52	497.28	MT-ND1

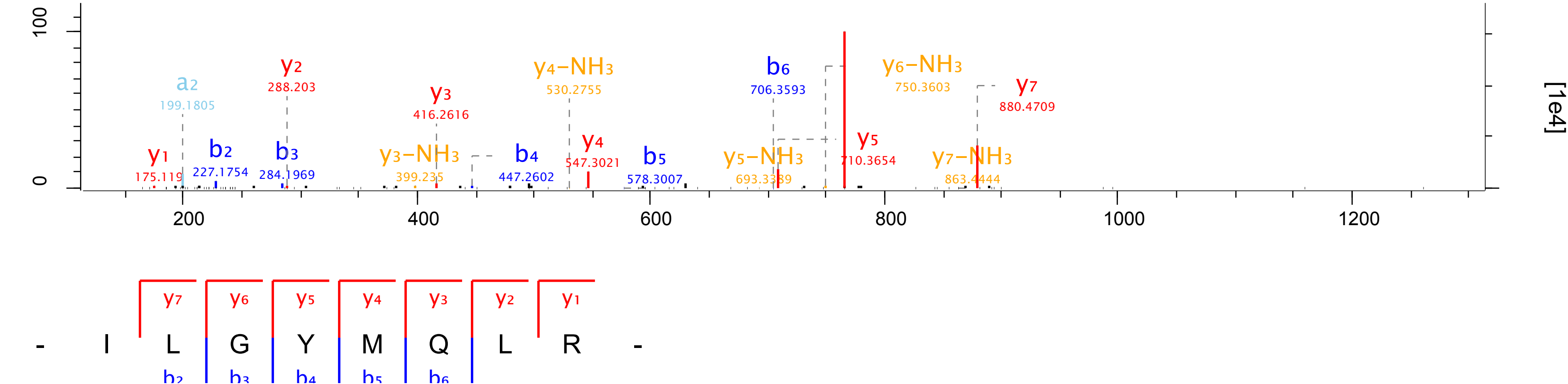

Raw file	Scan	Method	Score	m/z	Gene names
20140918_fract11_dyn_5ul_E3_01_379	27824	TOF; CID	75.74	363.88	UVSSA

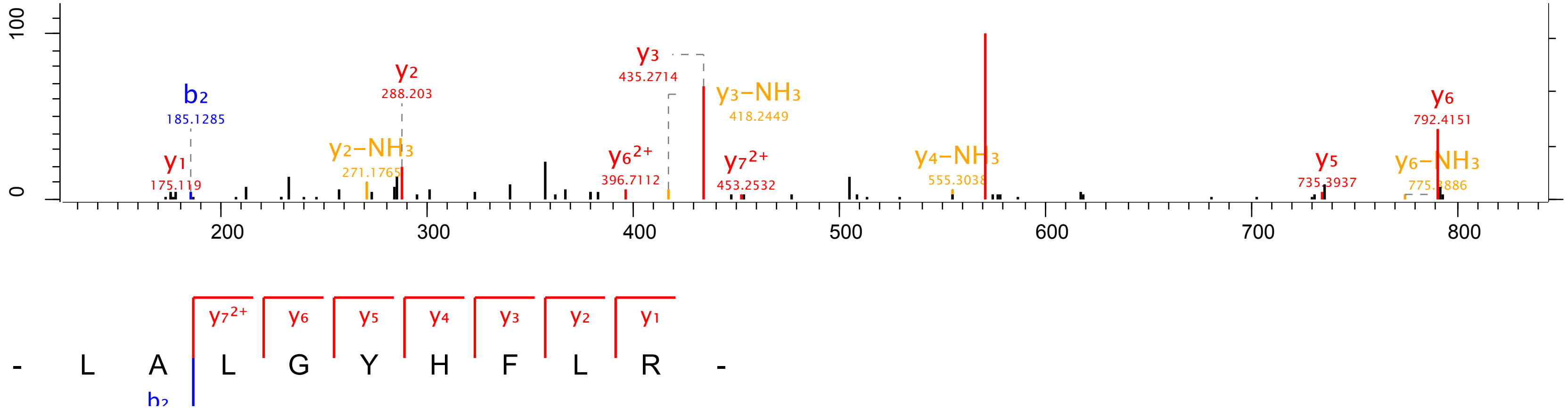

Raw file	Scan	Method	Score	m/z	Gene names
20140918_fract11_dyn_5ul_E3_01_379	28758	TOF; CID	119.87	734.84	AGPAT2

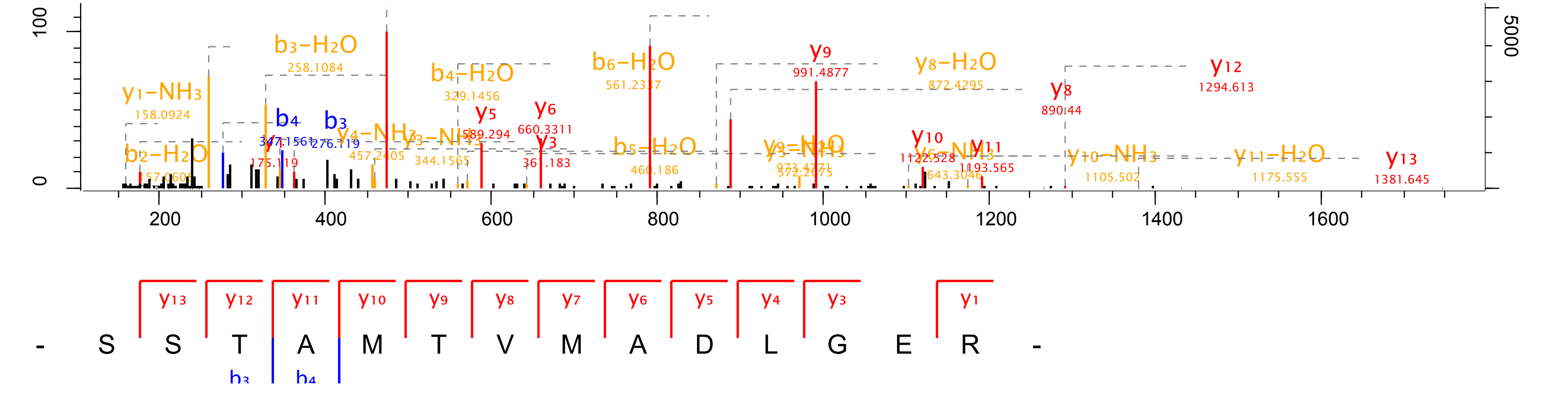

Raw file	Scan	Method	Score	m/z	Gene names
20140918_fract11_dyn_5ul_E3_01_379	29796	TOF; CID	120.46	452.77	MYLK

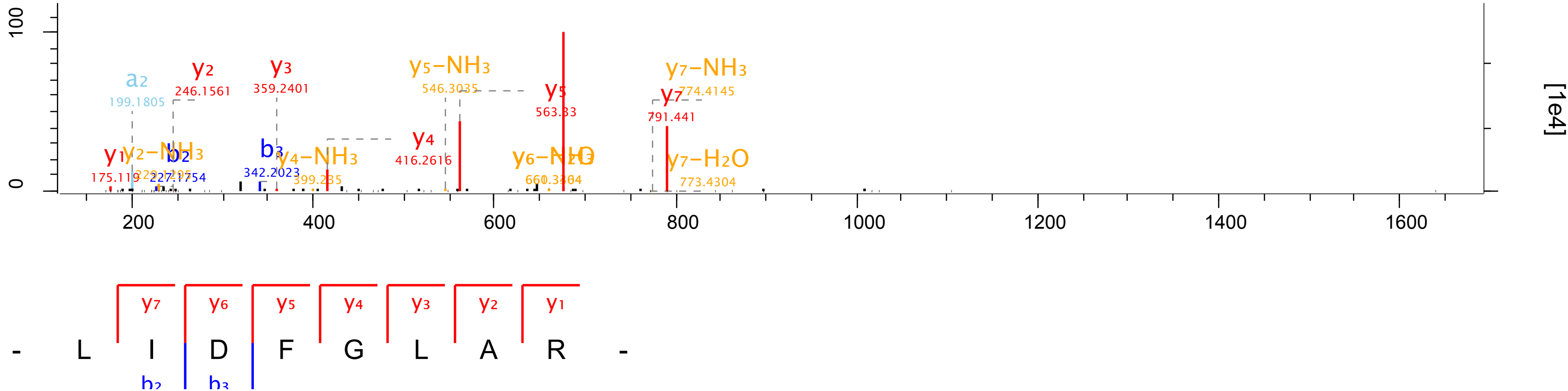

Raw file	Scan	Method	Score	m/z	Gene names
20140918_fract11_dyn_5ul_E3_01_379	32811	TOF; CID	69.72	493.28	TOR1AIP2

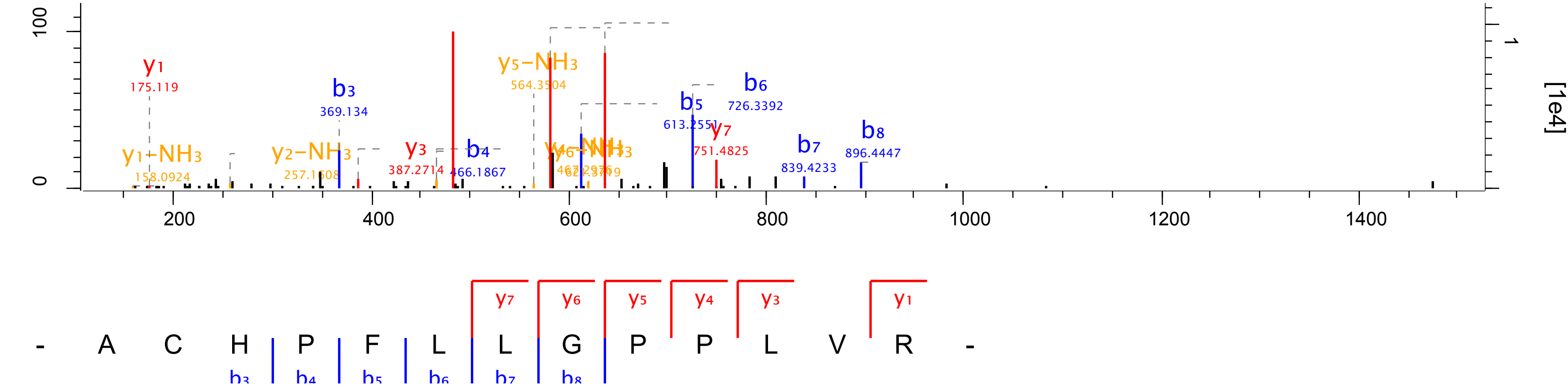

Raw file	Scan	Method	Score	m/z	Gene names
20140918_fract11_dyn_5ul_E3_01_379	33243	TOF; CID	120.77	441.6	SNX24

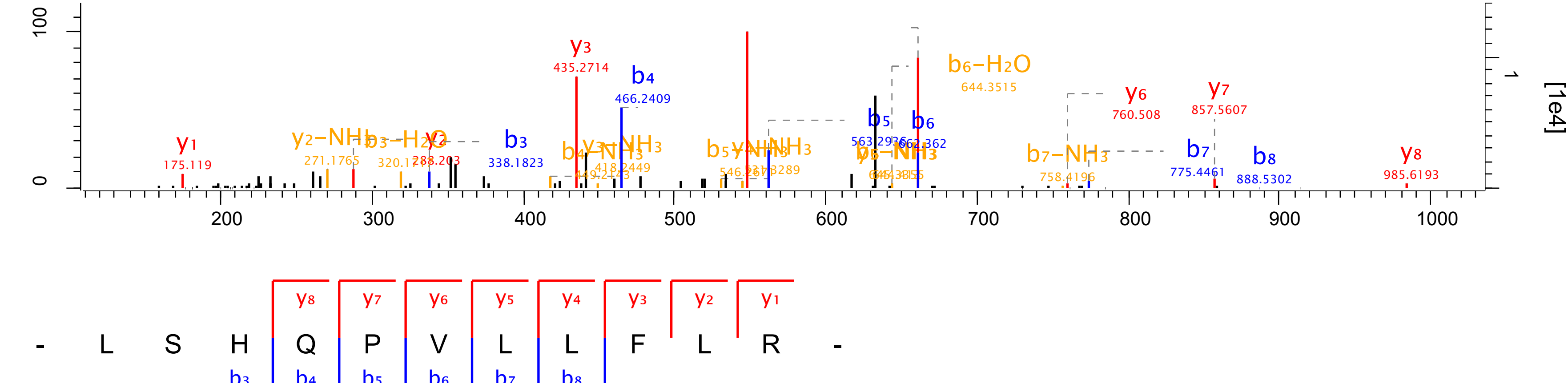

Raw file	Scan	Method	Score	m/z	Gene names
20140918_fract11_dyn_5ul_E3_01_379	34360	TOF; CID	132.32	629.84	GTF2A1;HIST1H4F

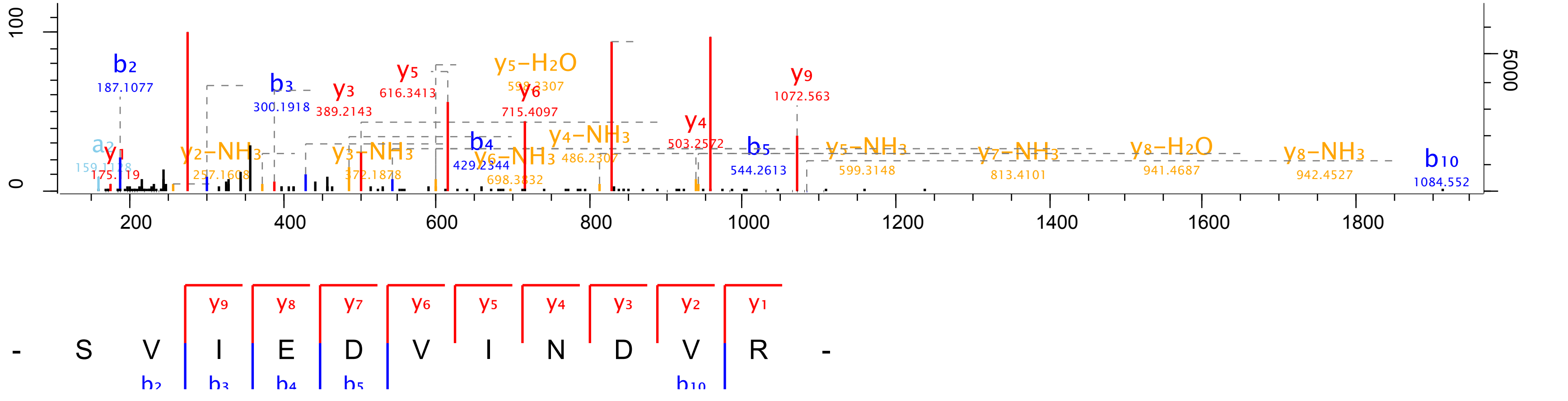

Raw file	Scan	Method	Score	m/z	Gene names
20140918_fract11_dyn_5ul_E3_01_379	34473	TOF; CID	66.06	574.82	AGBL2

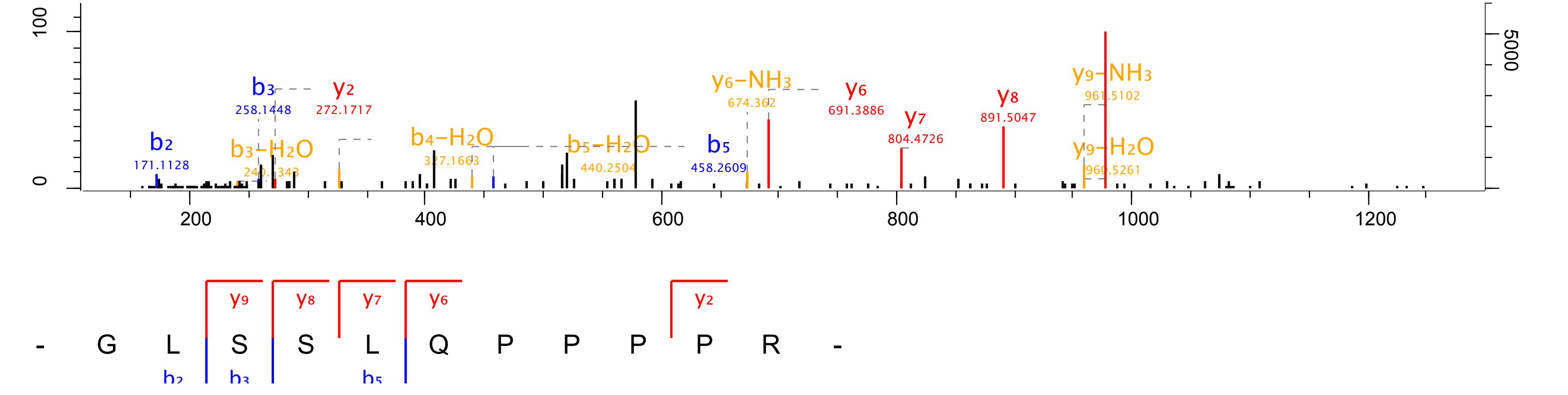

Raw file	Scan	Method	Score	m/z	Gene names
20140918_fract11_dyn_5ul_E3_01_379	36638	TOF; CID	139.88	560.32	KDELR3

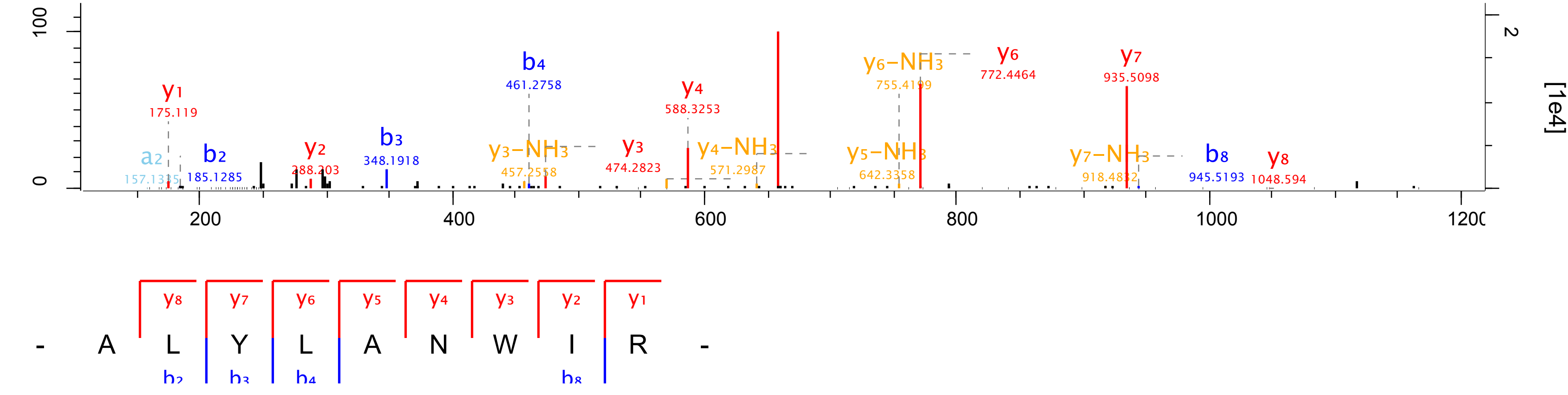

Raw file	Scan	Method	Score	m/z	Gene names
20140918_fract11_dyn_5ul_E3_01_379	37635	TOF; CID	105.72	1351.63	SMAGP

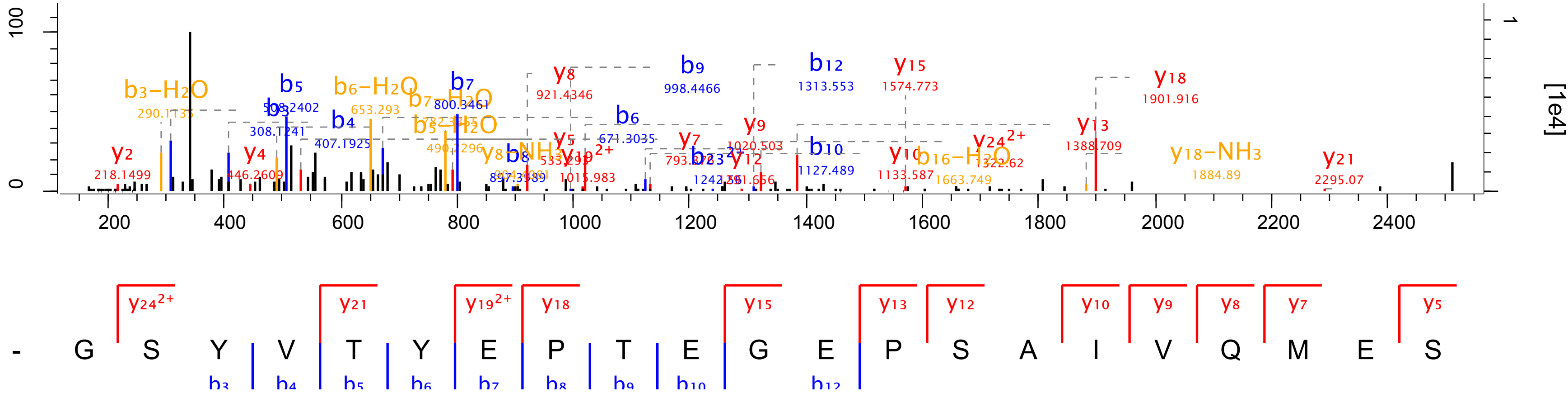

Raw file	Scan	Method	Score	m/z	Gene names
20140918_fract11_dyn_5ul_E3_01_379	38389	TOF; CID	128.03	610.3	CMTM6

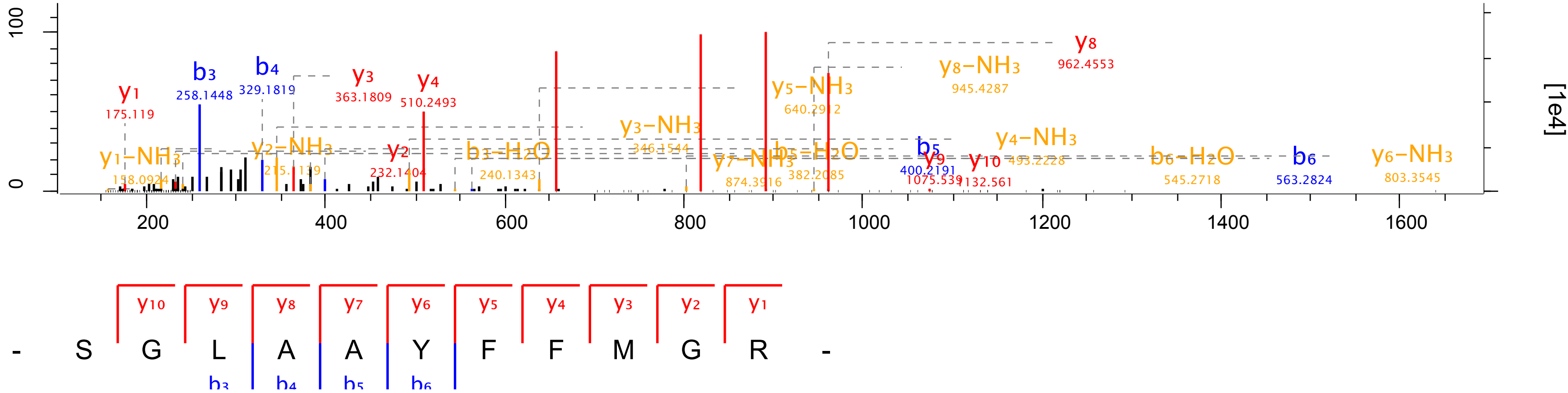

Raw file	Scan	Method	Score	m/z	Gene names
20140918_fract12_dyn_5ul_E4_01_380	6672	TOF; CID	87.64	499.22	ZNF580

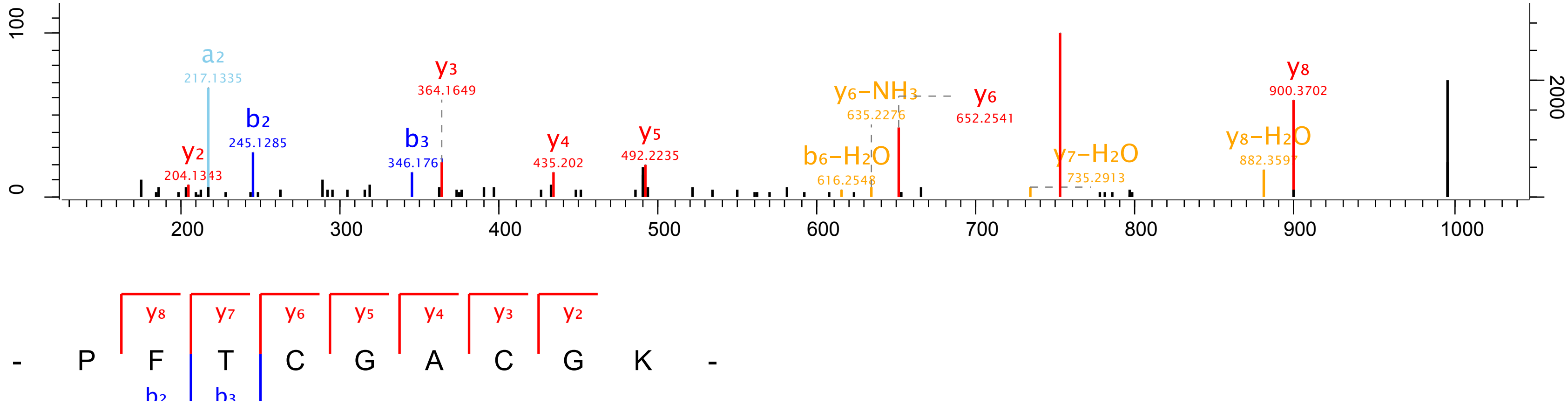

Raw file	Scan	Method	Score	m/z	Gene names
20140918_fract12_dyn_5ul_E4_01_380	11045	TOF; CID	64.22	705.87	SAMD10

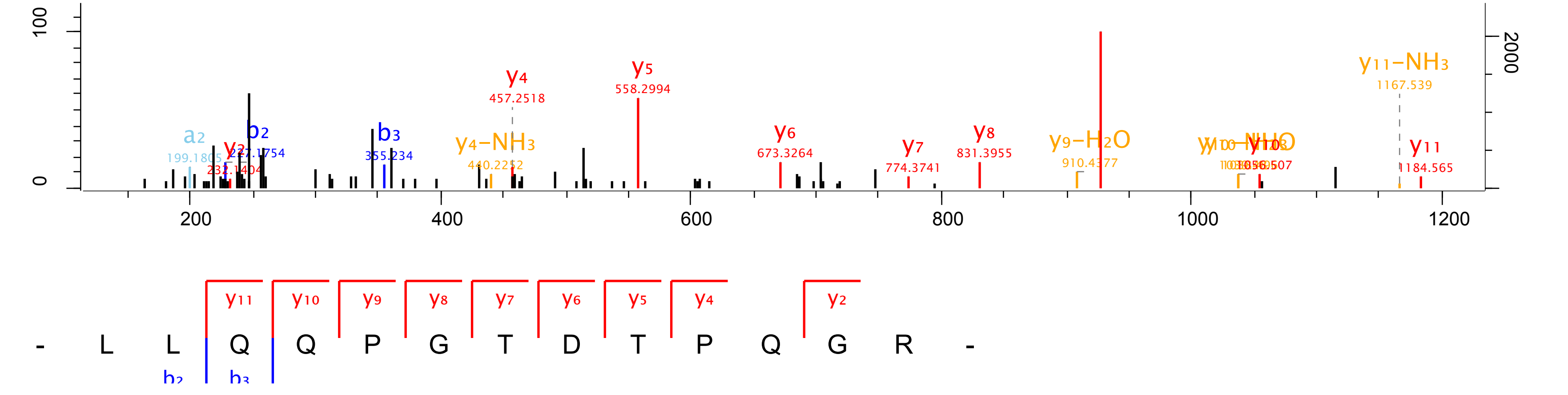

Raw file	Scan	Method	Score	m/z	Gene names
20140918_fract12_dyn_5ul_E4_01_380	14311	TOF; CID	53.56	799.41	DMWD

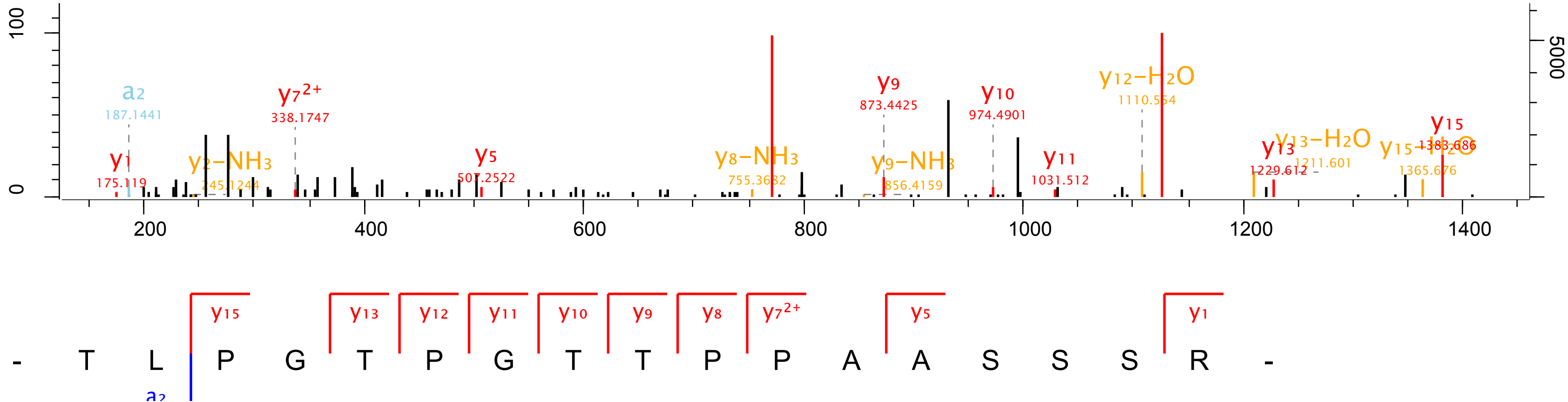

Raw file	Scan	Method	Score	m/z	Gene names
20140918_fract12_dyn_5ul_E4_01_380	14421	TOF; CID	124.42	587.31	SULT1E1

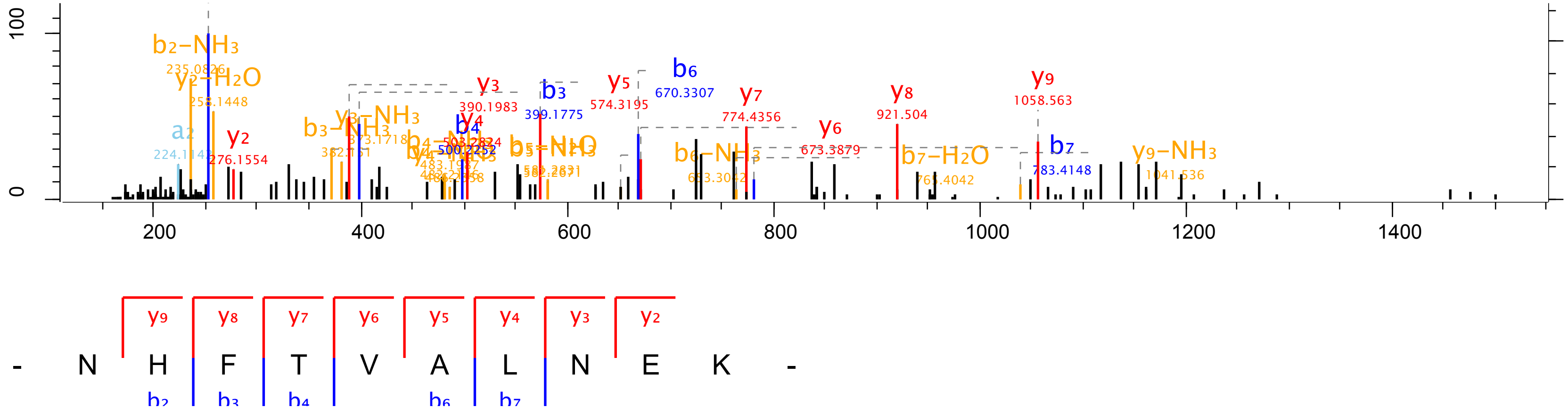

Raw file	Scan	Method	Score	m/z	Gene names
20140918_fract12_dyn_5ul_E4_01_380	14666	TOF; CID	62.47	558.31	GJA8

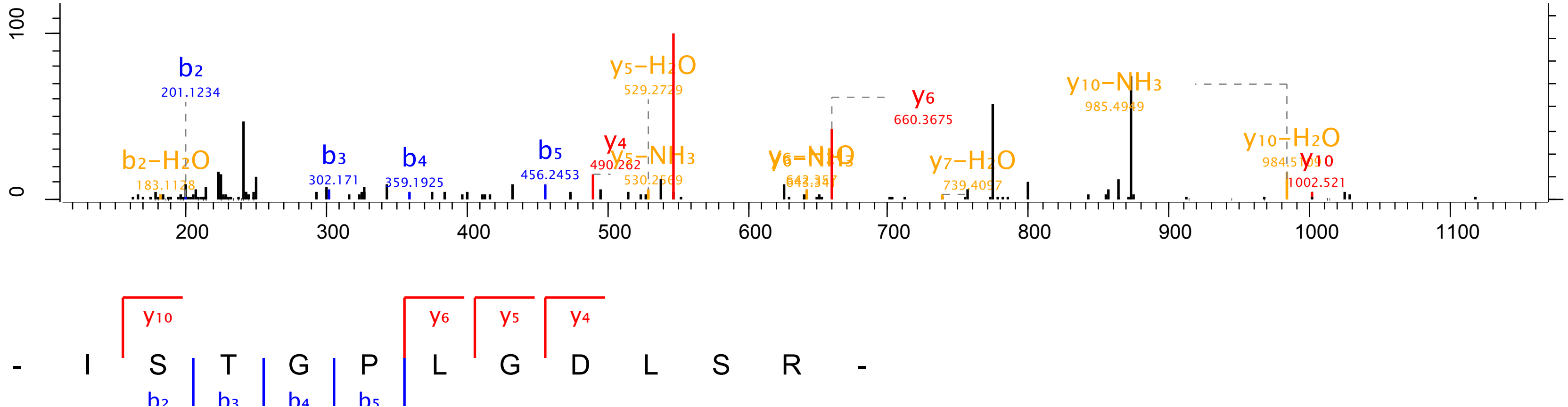

Raw file	Scan	Method	Score	m/z	Gene names
20140918_fract12_dyn_5ul_E4_01_380	20560	TOF; CID	77.64	655.82	CLEC2B

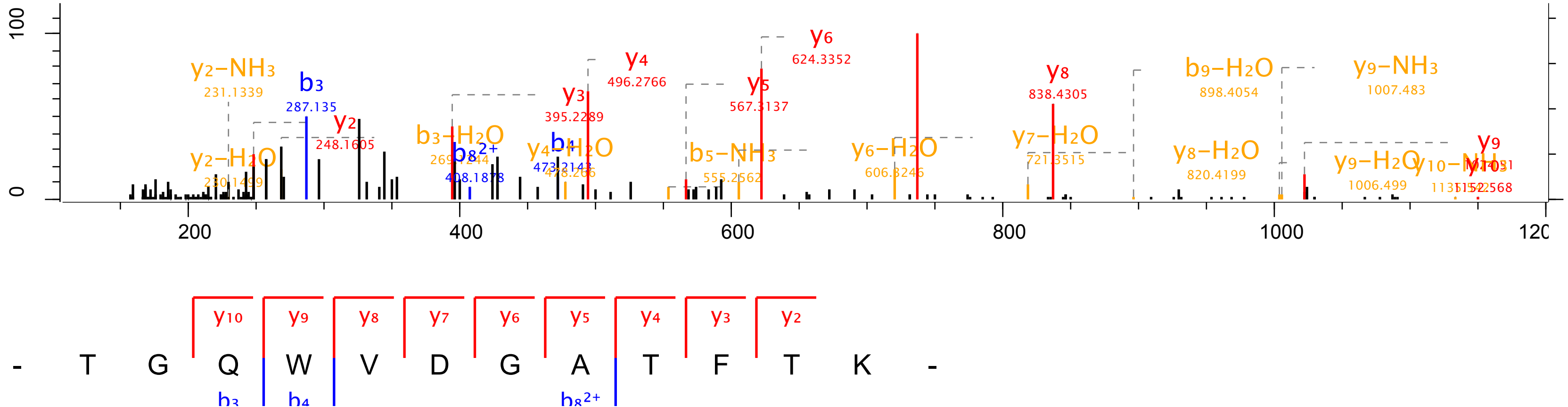

Raw file	Scan	Method	Score	m/z	Gene names
20140918_fract12_dyn_5ul_E4_01_380	21996	TOF; CID	89.35	524.25	BTN2A2

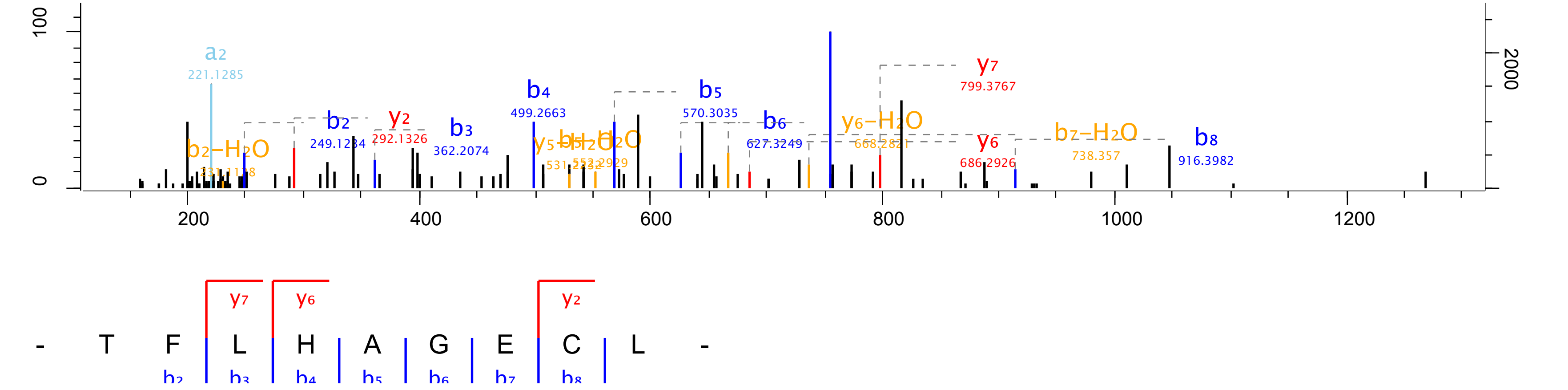

Raw file	Scan	Method	Score	m/z	Gene names
20140918_fract12_dyn_5ul_E4_01_380	22288	TOF; CID	49.14	953.1	C6orf120

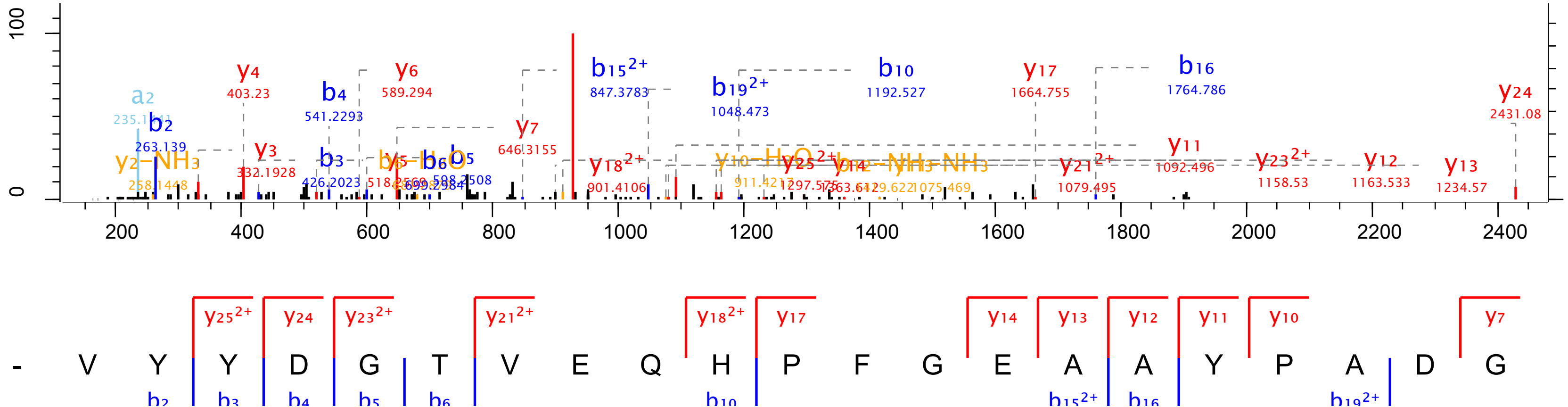

Raw file	Scan	Method	Score	m/z	Gene names
20140918_fract12_dyn_5ul_E4_01_380	24532	TOF; CID	71.56	535.6	C12orf49

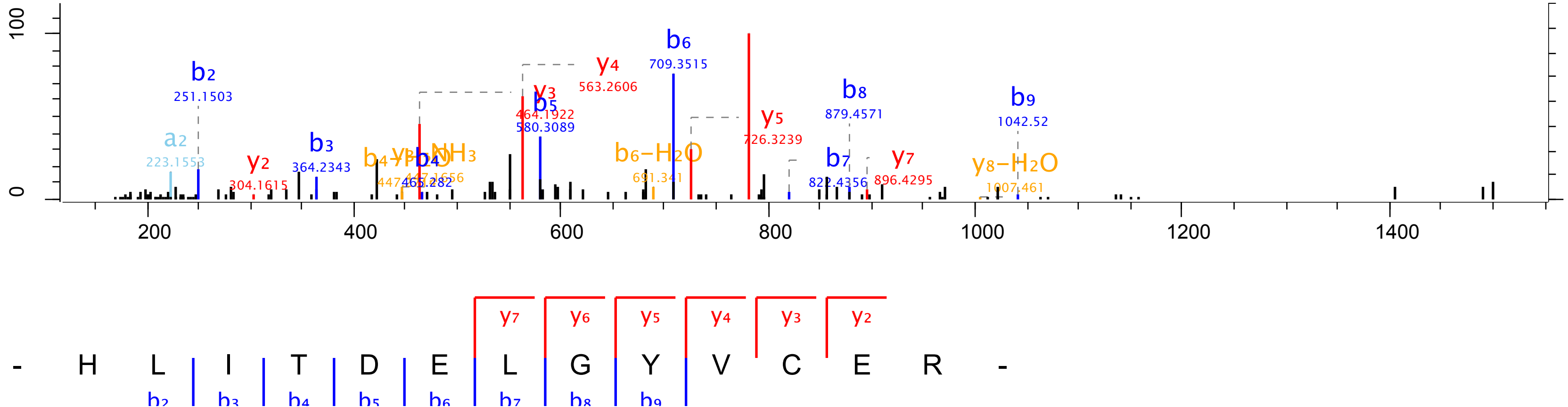

Raw file	Scan	Method	Score	m/z	Gene names
20140918_fract12_dyn_5ul_E4_01_380	26404	TOF; CID	90.79	486.95	TIGD1

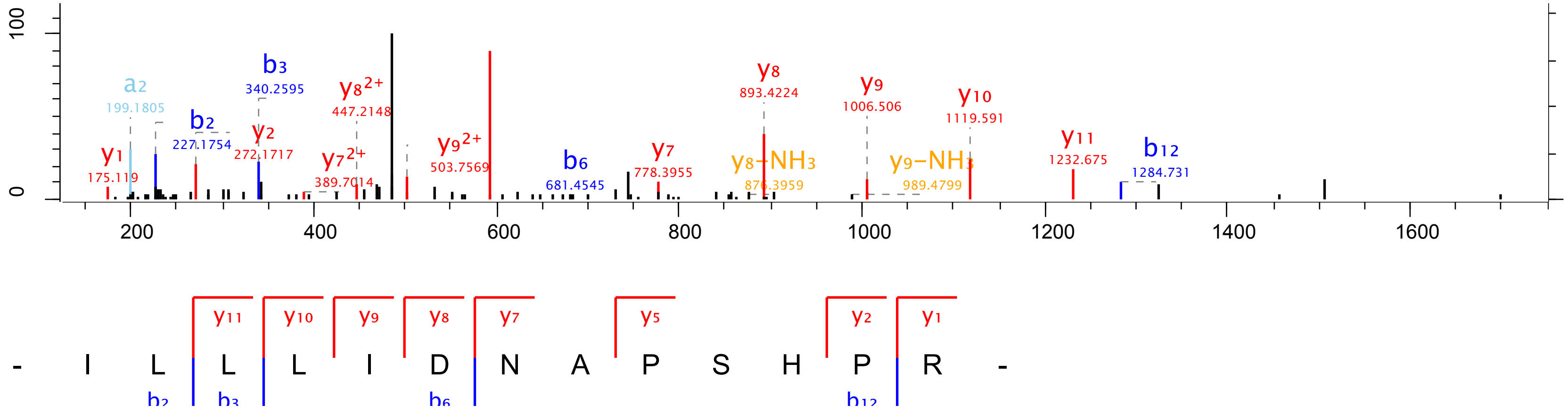

Raw file	Scan	Method	Score	m/z	Gene names
20140918_fract12_dyn_5ul_E4_01_380	26677	TOF; CID	55.66	653.31	HDAC10

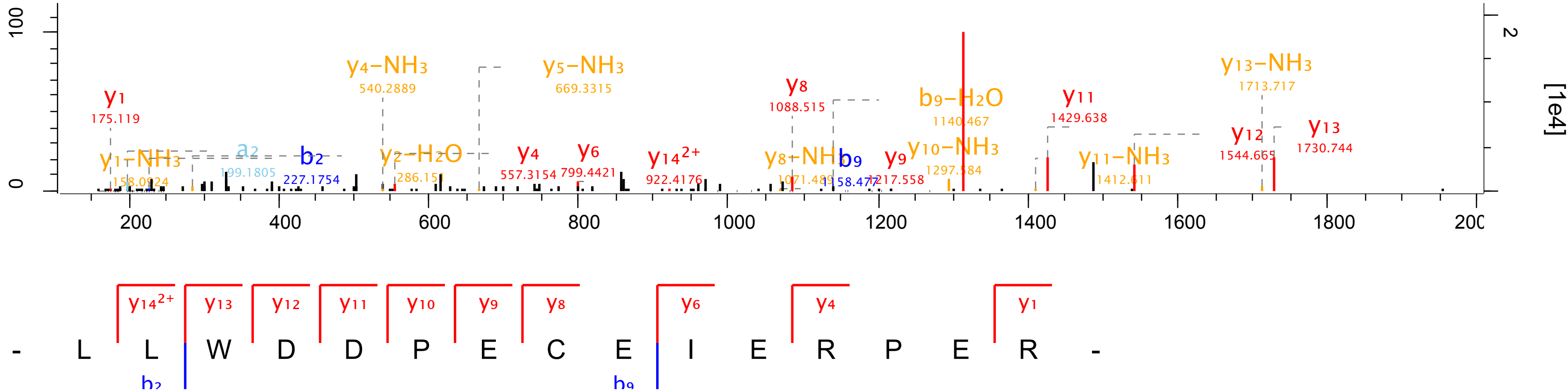

Raw file	Scan	Method	Score	m/z	Gene names
20140918_fract12_dyn_5ul_E4_01_380	27276	TOF; CID	128.01	667.83	LRRC8B

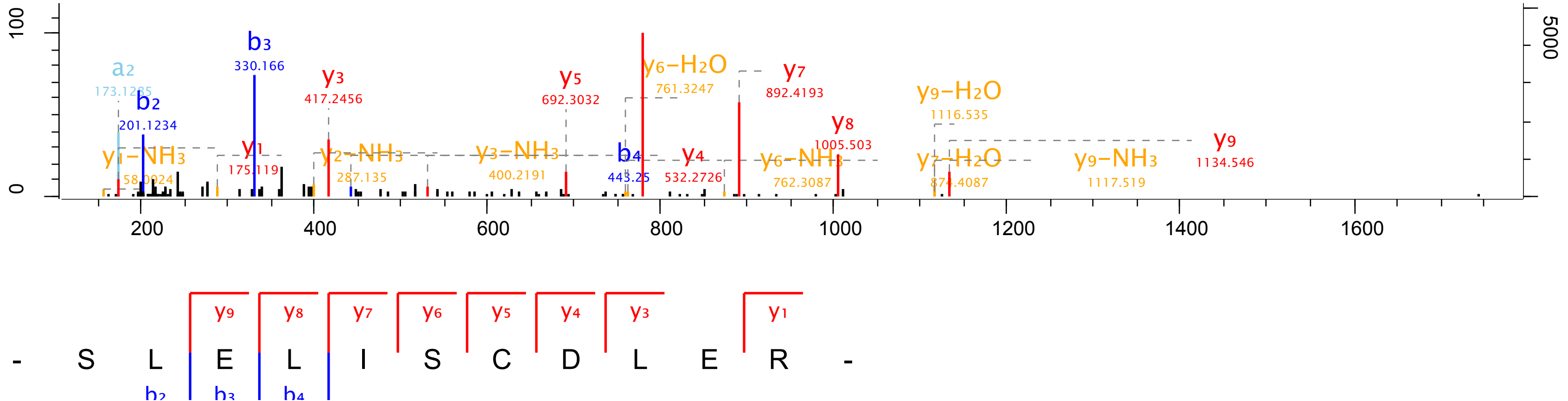

Raw file	Scan	Method	Score	m/z	Gene names
20140918_fract12_dyn_5ul_E4_01_380	28706	TOF; CID	101.33	710.84	SLC39A13

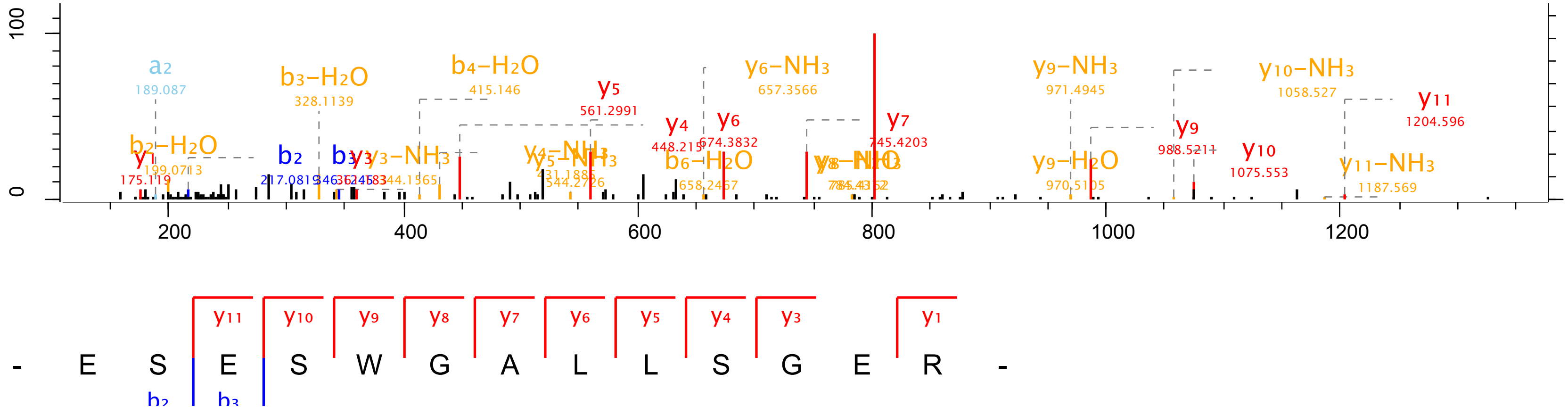

Raw file	Scan	Method	Score	m/z	Gene names
20140918_fract12_dyn_5ul_E4_01_380	29259	TOF; CID	122.13	516.24	B9D1

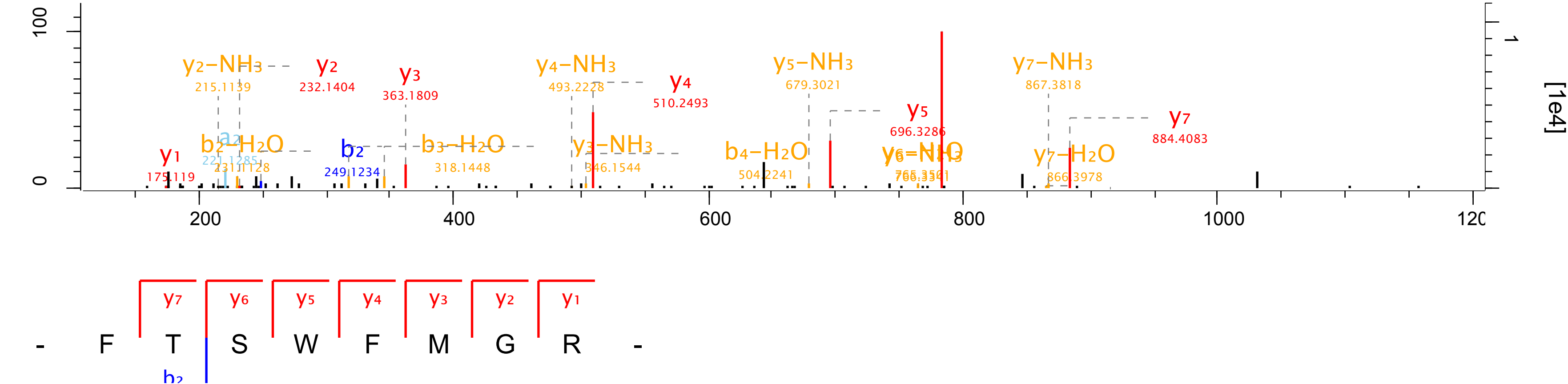

Raw file	Scan	Method	Score	m/z	Gene names
20140918_fract12_dyn_5ul_E4_01_380	29316	TOF; CID	79.66	622.37	MT-ATP6

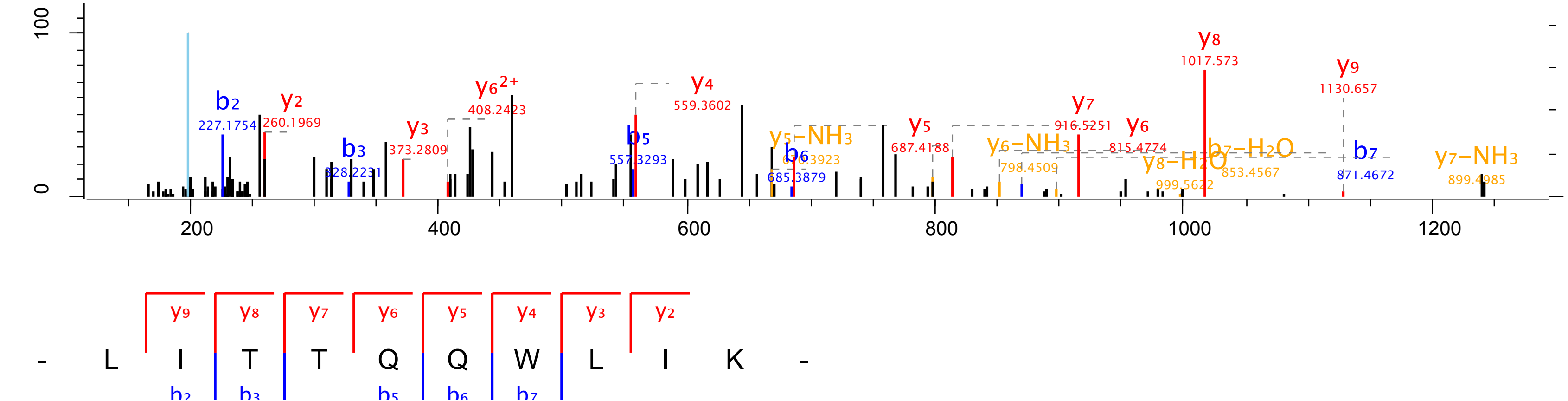

Raw file	Scan	Method	Score	m/z	Gene names
20140918_fract12_dyn_5ul_E4_01_380	29656	TOF; CID	93.37	1247.65	CDH19

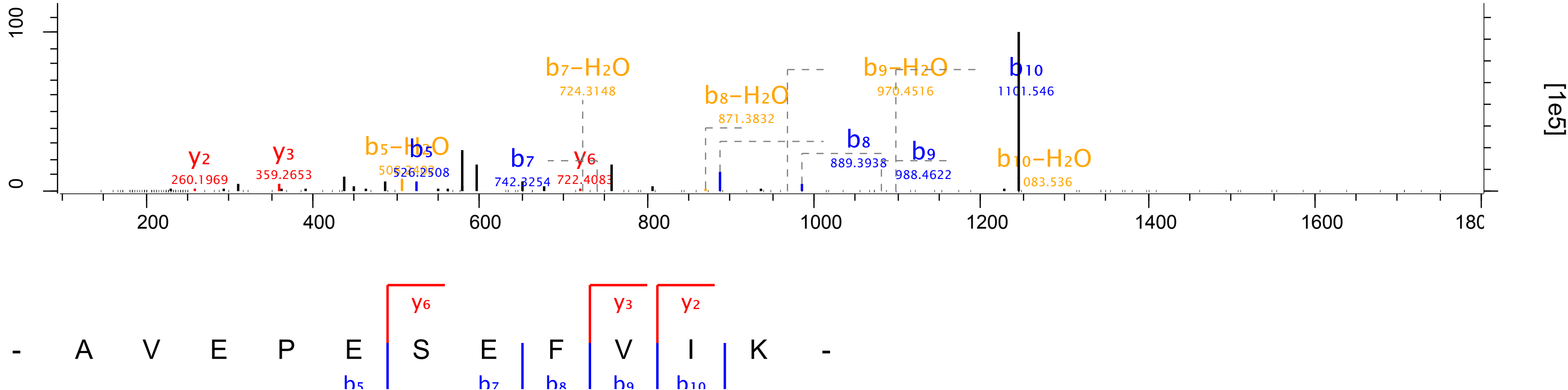

Raw file	Scan	Method	Score	m/z	Gene names
20140918_fract12_dyn_5ul_E4_01_380	30218	TOF; CID	84.33	879.1	RARA

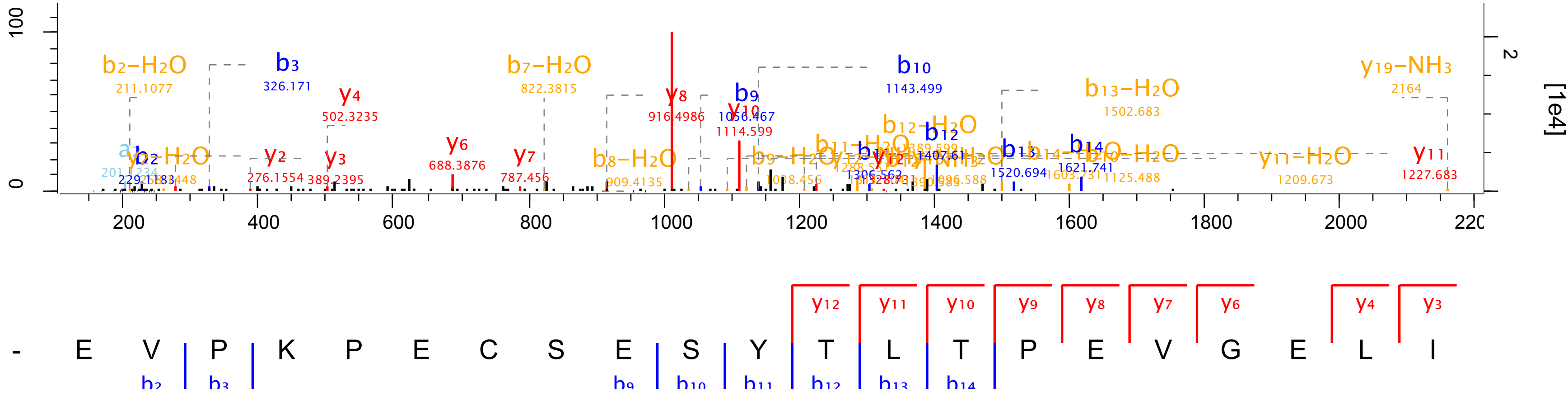

Raw file	Scan	Method	Score	m/z	Gene names
20140918_fract12_dyn_5ul_E4_01_380	32309	TOF; CID	104.88	531.29	DUSP19

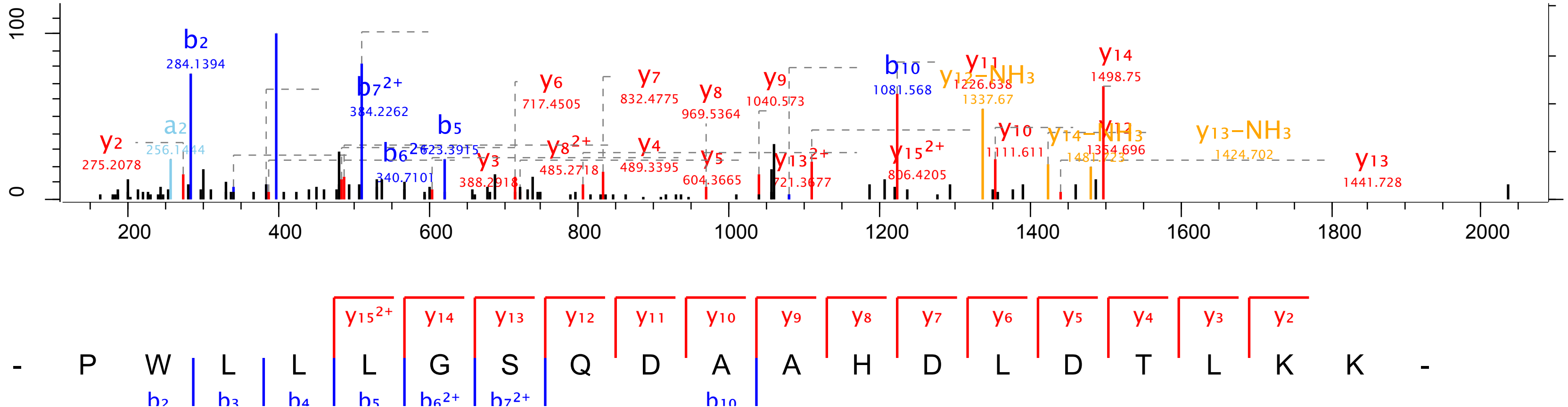

Raw file	Scan	Method	Score	m/z	Gene names
20140918_fract12_dyn_5ul_E4_01_380	35495	TOF; CID	96.46	574.65	ZMYND19

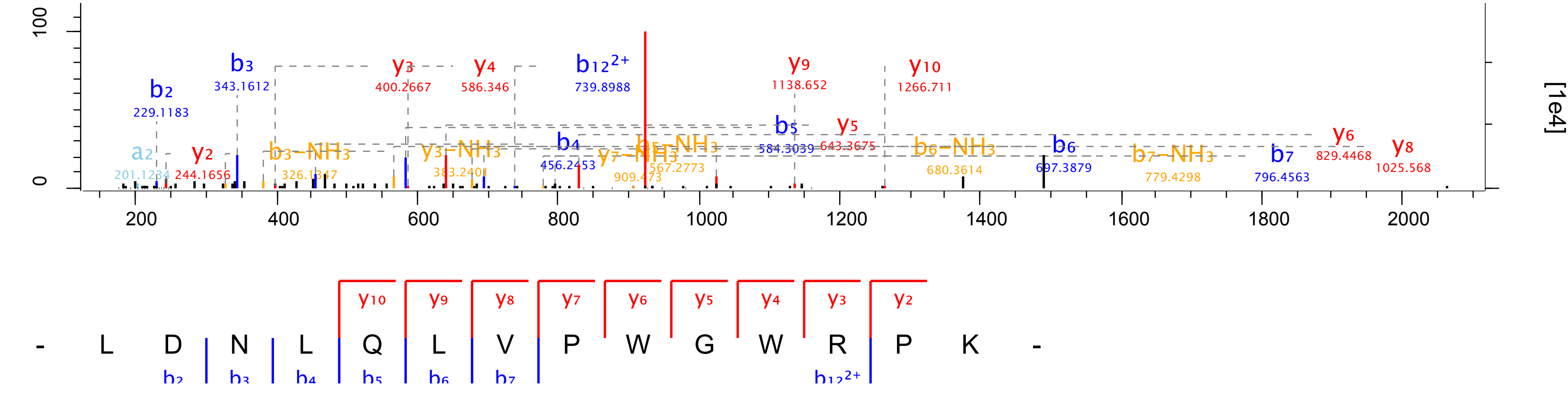

Raw file	Scan	Method	Score	m/z	Gene names
20140918_fract12_dyn_5ul_E4_01_380	35864	TOF; CID	134.29	666.09	SLC39A8

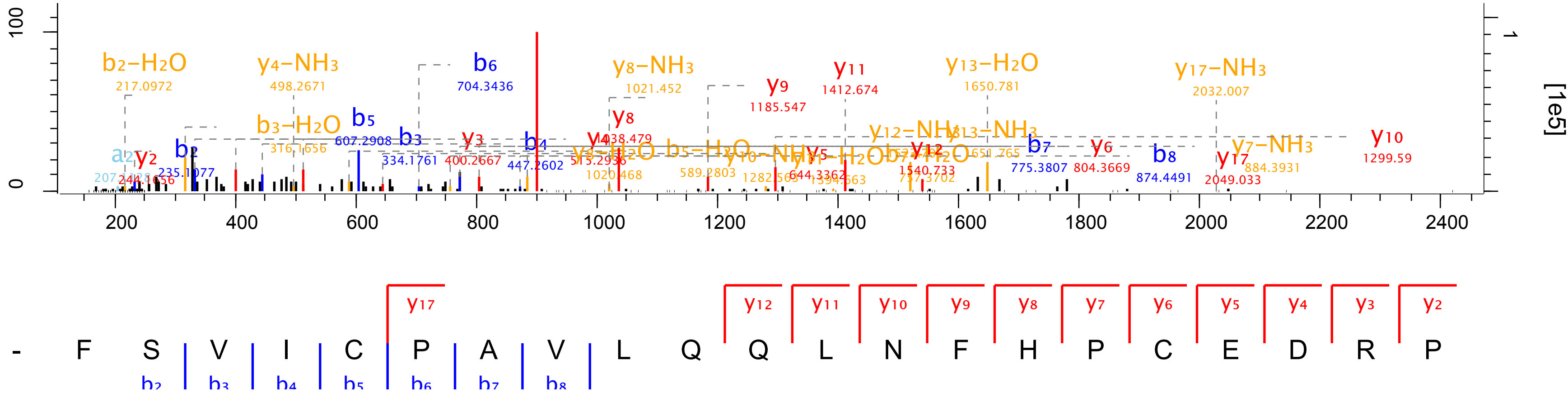

Raw file	Scan	Method	Score	m/z	Gene names
20140918_fract13_dyn_5ul_E5_01_381	22797	TOF; CID	63.49	972.43	LYPD3

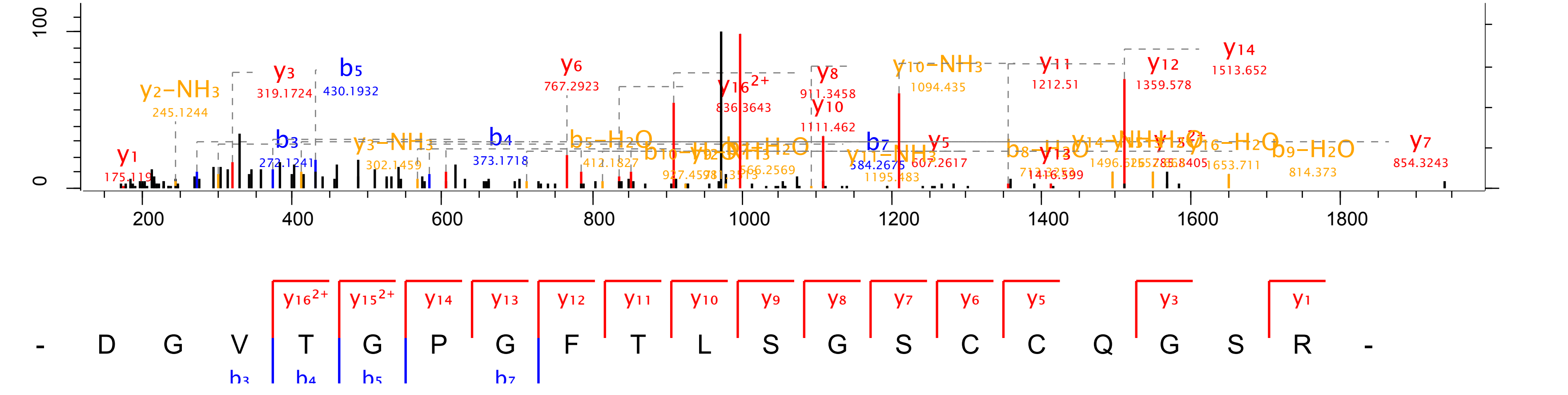

Raw file	Scan	Method	Score	m/z	Gene names
20140918_fract13_dyn_5ul_E5_01_381	24186	TOF; CID	116.24	1031.46	TM2D2

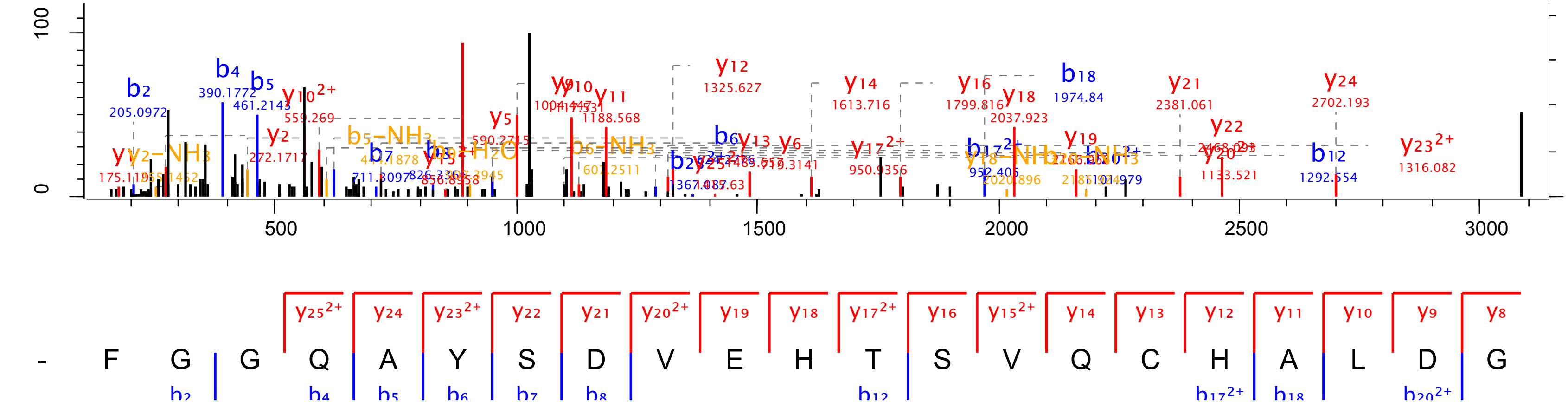

Raw file	Scan	Method	Score	m/z	Gene names
20140918_fract13_dyn_5ul_E5_01_381	24961	TOF; CID	100.73	747.37	C19orf54

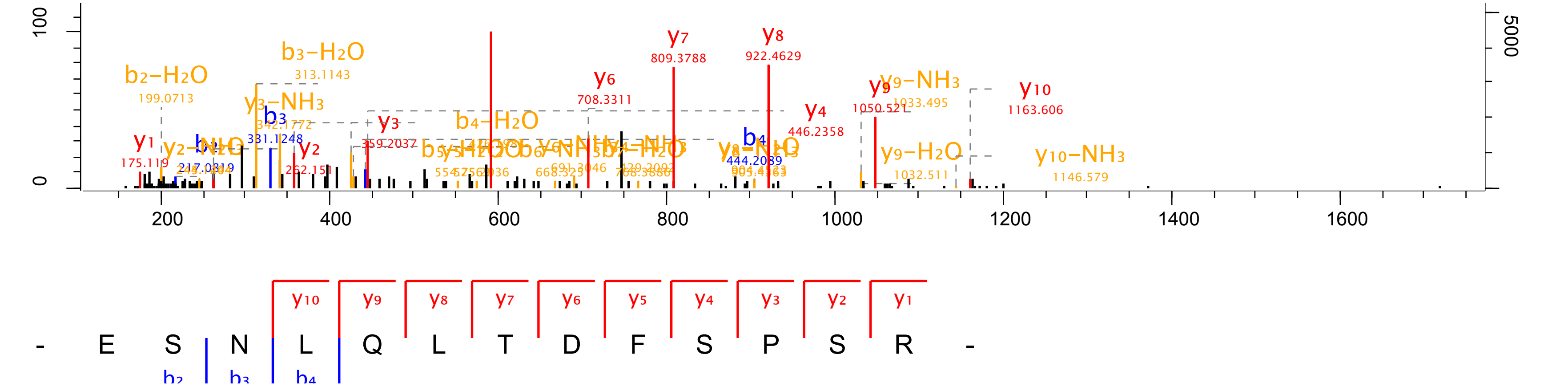

Raw file	Scan	Method	Score	m/z	Gene names
20140918_fract13_dyn_5ul_E5_01_381	25017	TOF; CID	99.41	876.08	NABP2

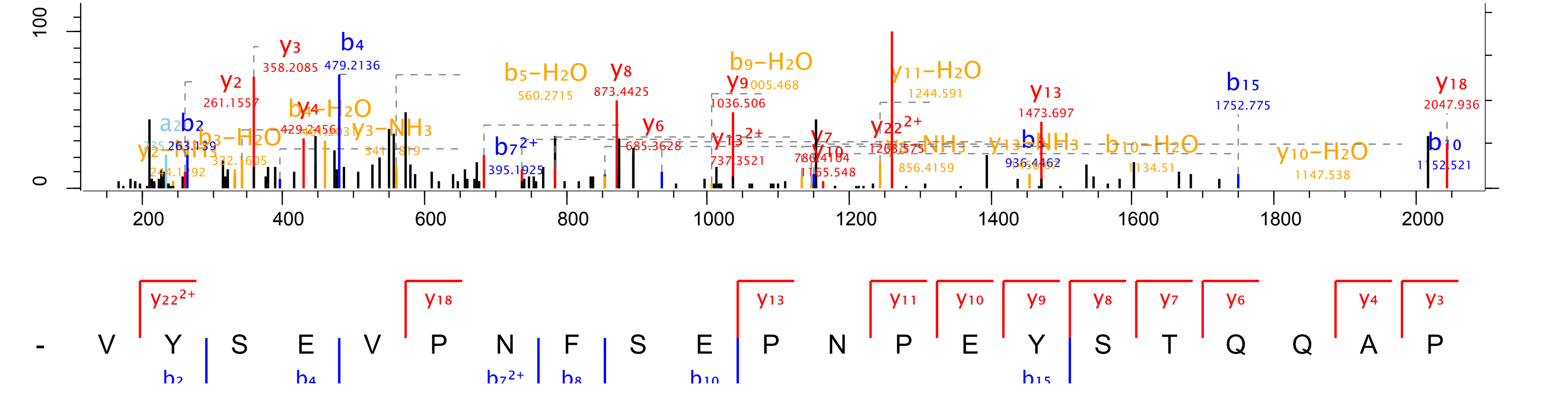

Raw file	Scan	Method	Score	m/z	Gene names
20140918_fract13_dyn_5ul_E5_01_381	26313	TOF; CID	77.66	516.78	RGS20

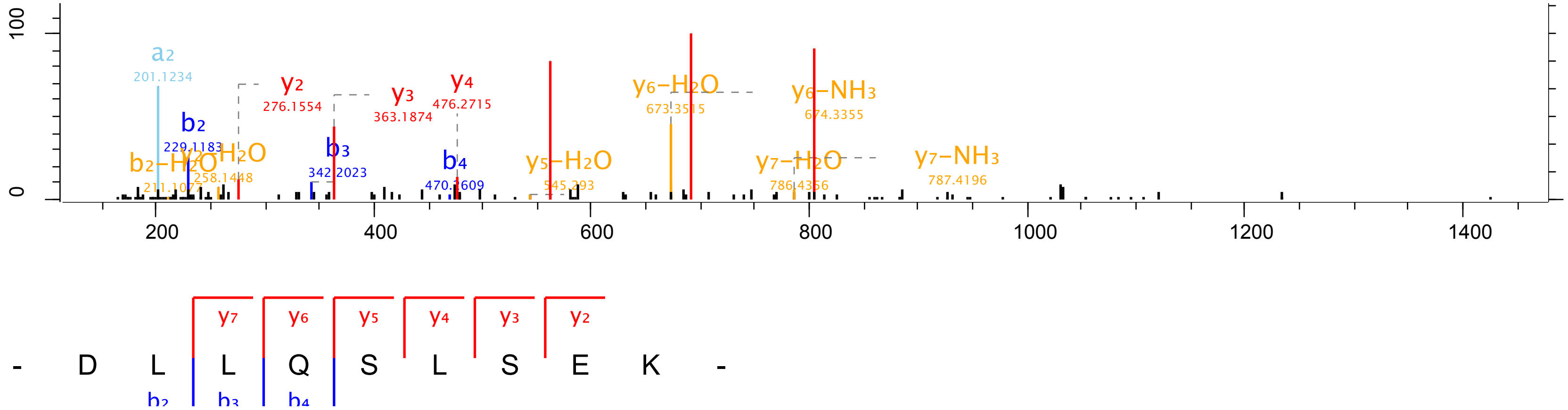

Raw file	Scan	Method	Score	m/z	Gene names
20140918_fract13_dyn_5ul_E5_01_381	27792	TOF; CID	73.08	580.31	GADD45A

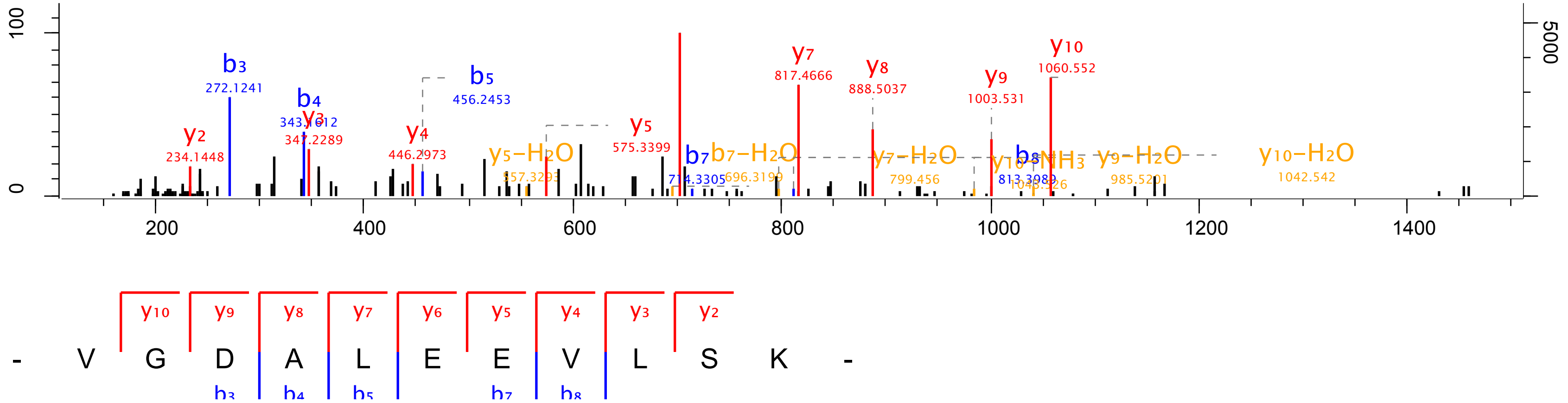

Raw file	Scan	Method	Score	m/z	Gene names
20140918_fract13_dyn_5ul_E5_01_381	32232	TOF; CID	88.43	605.02	ZFPM1

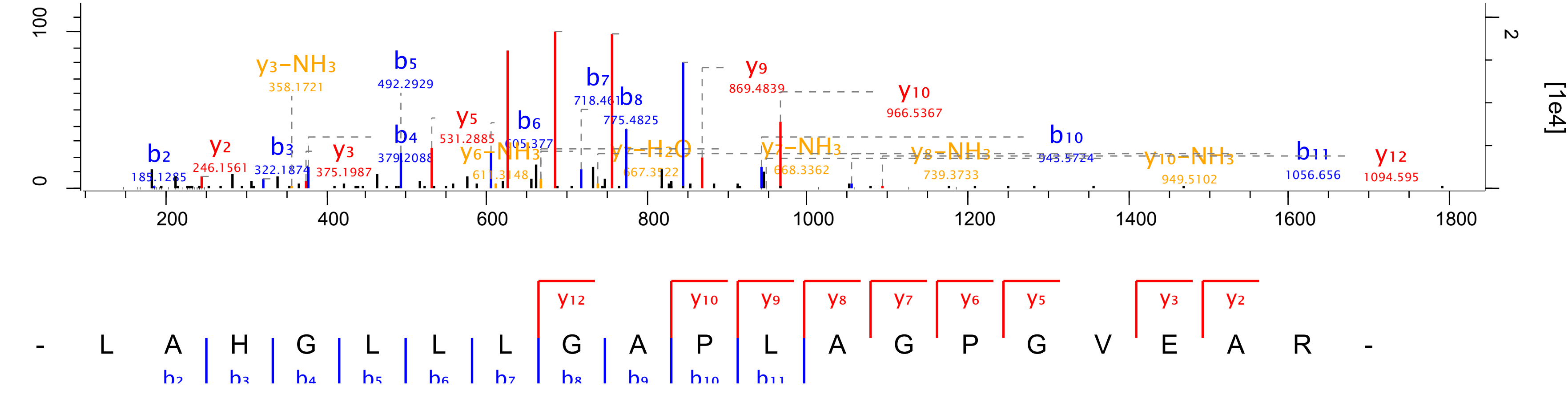

Raw file	Scan	Method	Score	m/z	Gene names
20140918_fract13_dyn_5ul_E5_01_381	33194	TOF; CID	74.99	745.38	PPARGC1B

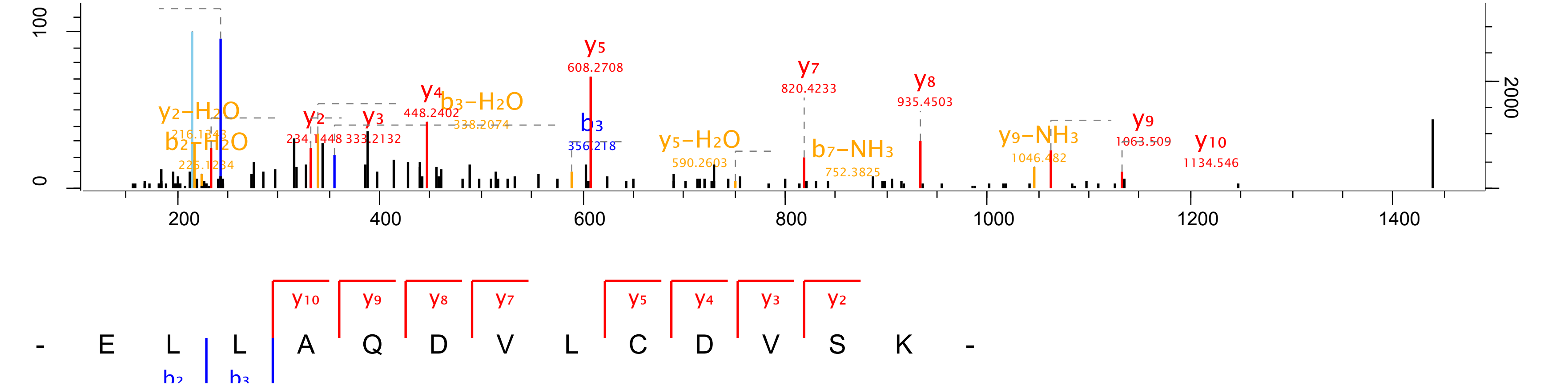

Raw file	Scan	Method	Score	m/z	Gene names
20140918_fract13_dyn_5ul_E5_01_381	33269	TOF; CID	72.89	707.41	MRI

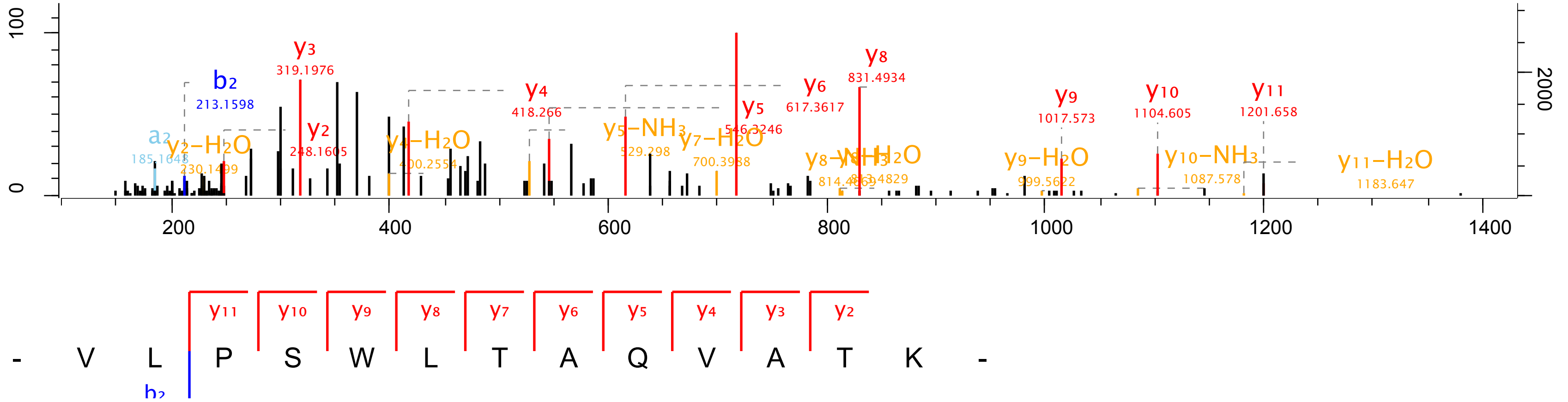

Raw file	Scan	Method	Score	m/z	Gene names
20140918_fract13_dyn_5ul_E5_01_381	35481	TOF; CID	73.5	603.35	IP6K2

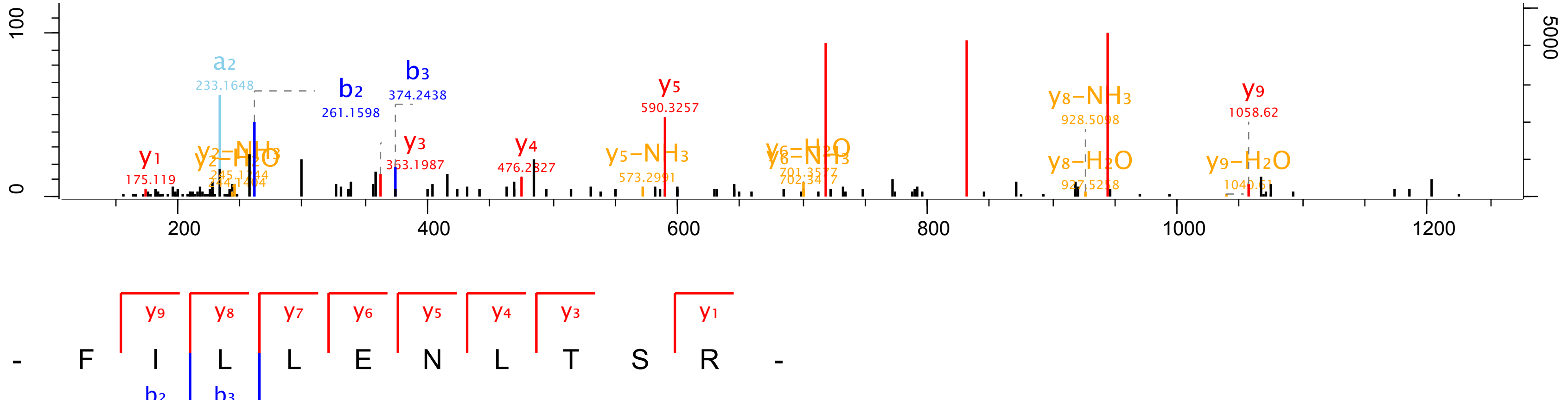

Raw file	Scan	Method	Score	m/z	Gene names
20140918_fract13_dyn_5ul_E5_01_381	36102	TOF; CID	34.41	1036.49	PHLPP2

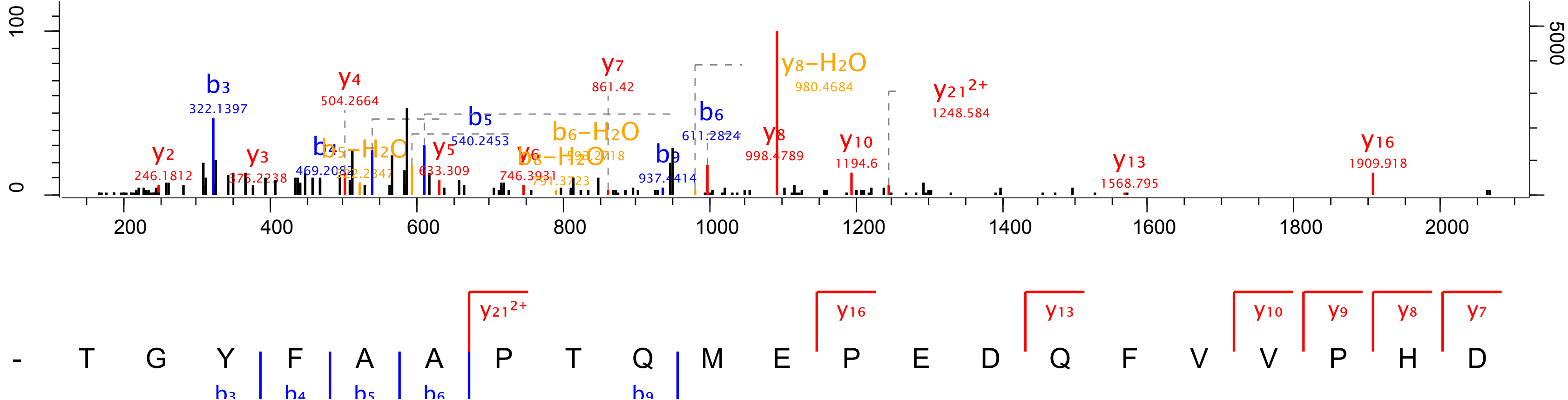

Raw file	Scan	Method	Score	m/z	Gene names
20140918_fract13_dyn_5ul_E5_01_381	37516	TOF; CID	63.68	880.42	TPM4

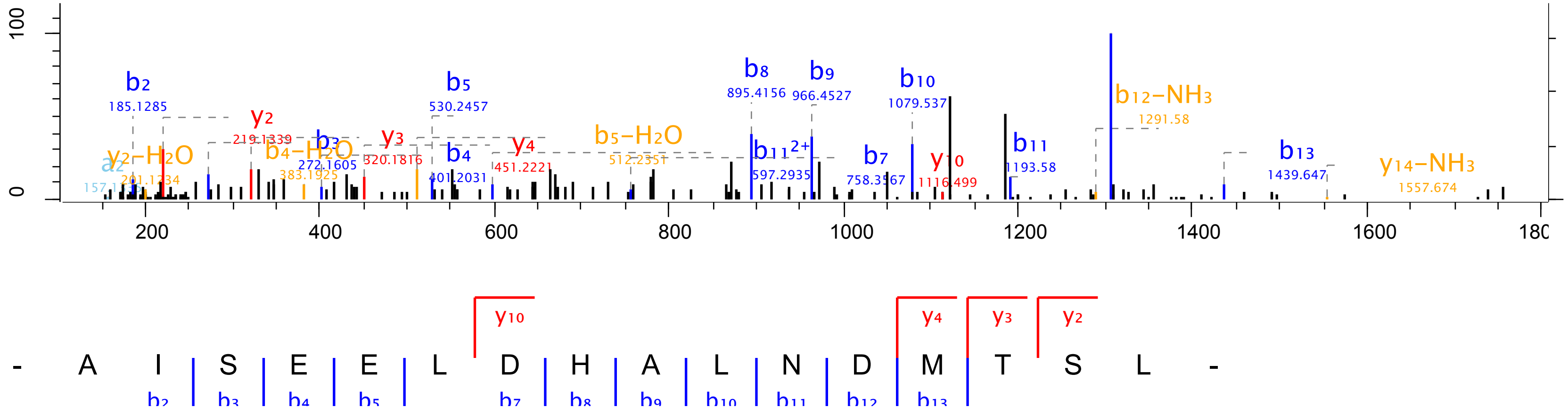

Raw file	Scan	Method	Score	m/z	Gene names
20140918_fract14_dyn_5ul_E6_01_382	9962	TOF; CID	51.5	735.88	PPP1R26

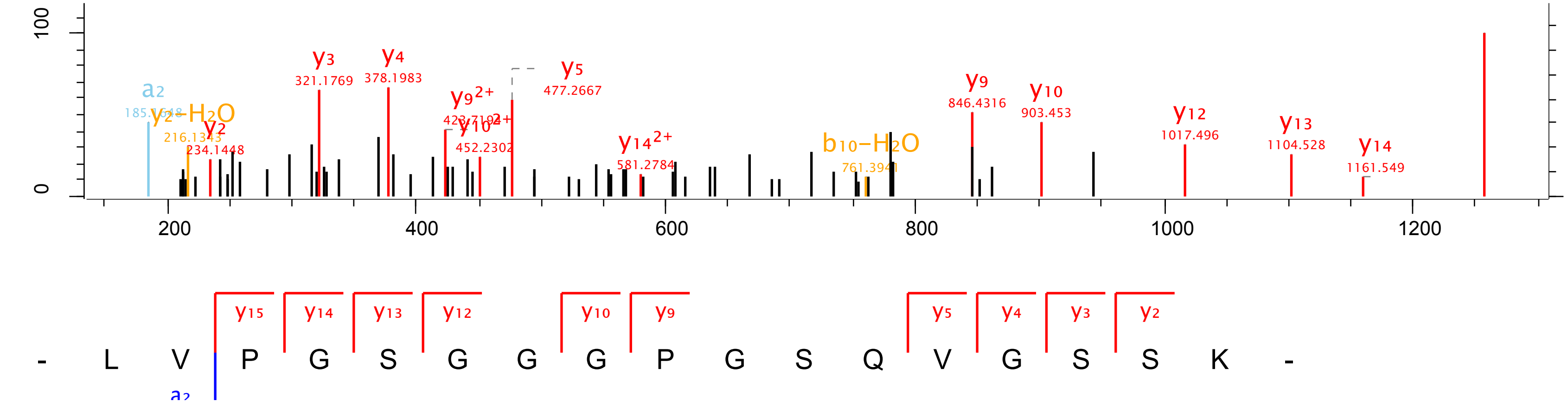

Raw file	Scan	Method	Score	m/z	Gene names
20140918_fract14_dyn_5ul_E6_01_382	10509	TOF; CID	42.95	543.27	BNIP3L

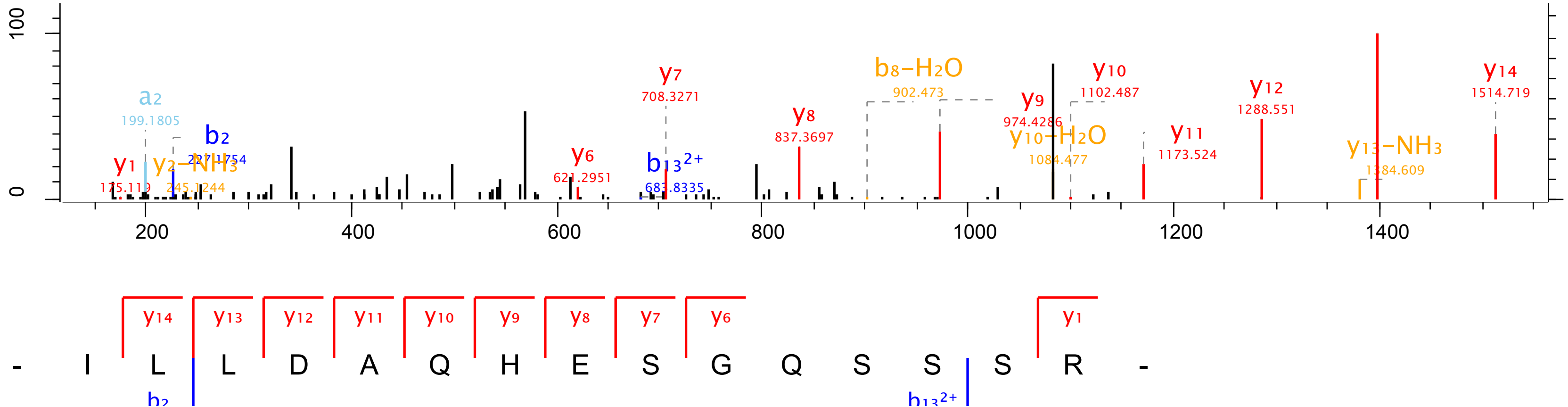

Raw file	Scan	Method	Score	m/z	Gene names
20140918_fract14_dyn_5ul_E6_01_382	13466	TOF; CID	103.69	430.92	DPY19L3

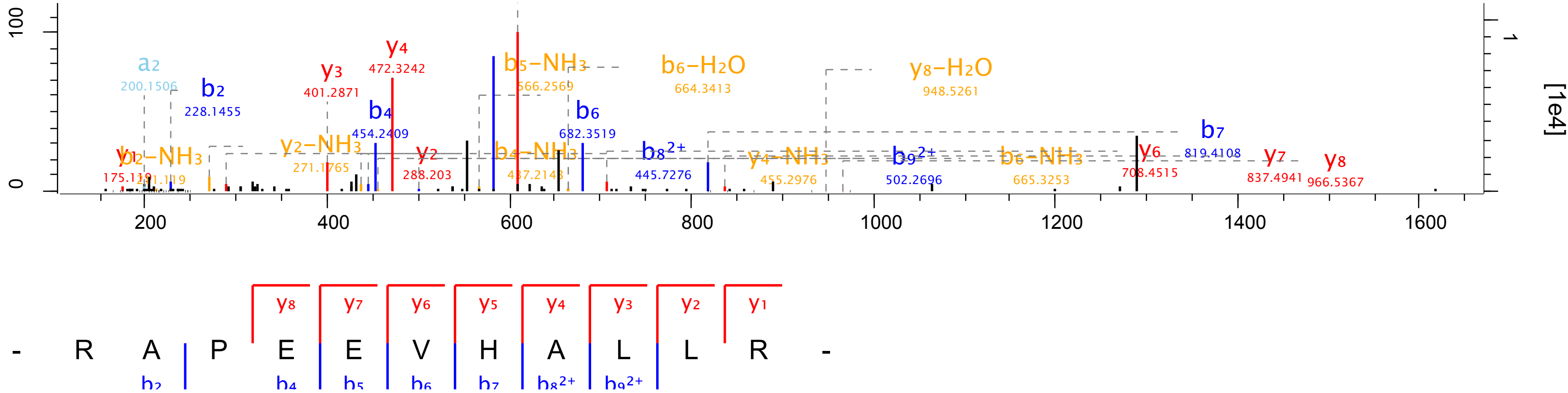

Raw file	Scan	Method	Score	m/z	Gene names
20140918_fract14_dyn_5ul_E6_01_382	16840	TOF; CID	113.76	764.88	LIN37

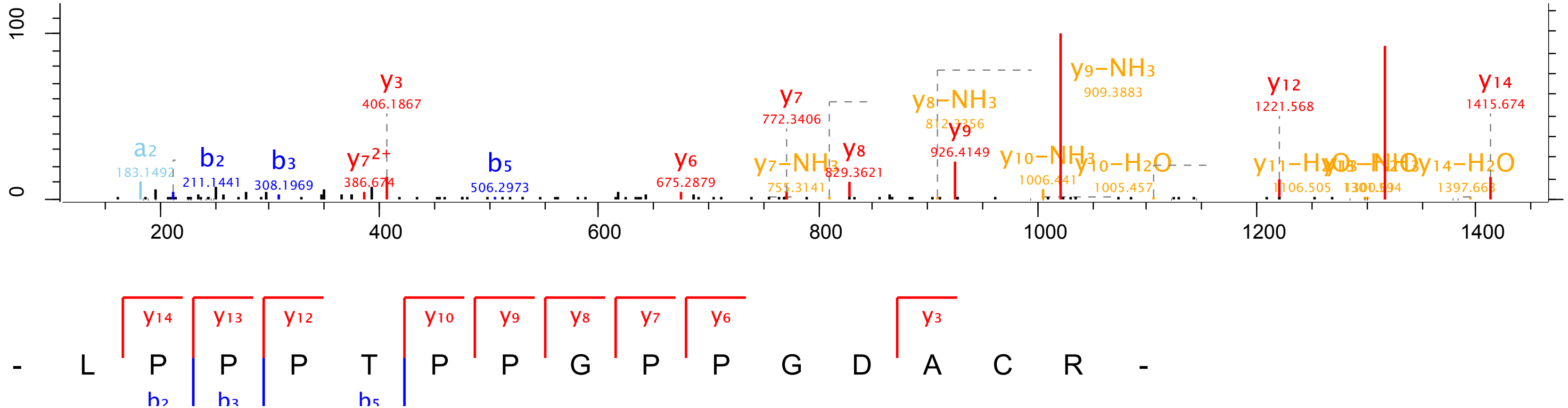

Raw file	Scan	Method	Score	m/z	Gene names
20140918_fract14_dyn_5ul_E6_01_382	17177	TOF; CID	90.7	619.82	PHF19

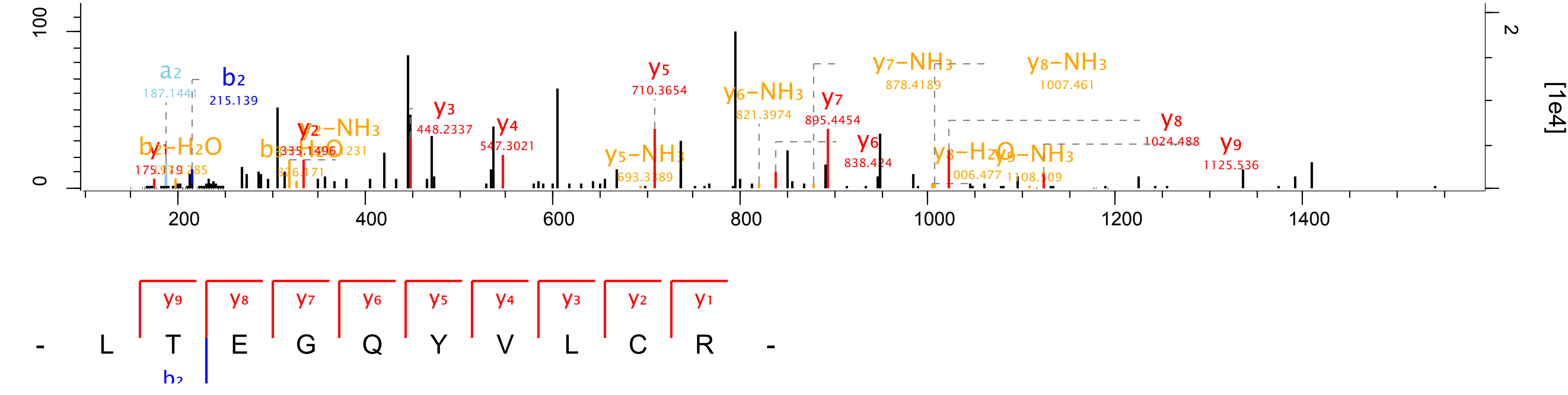

Raw file	Scan	Method	Score	m/z	Gene names
20140918_fract14_dyn_5ul_E6_01_382	20987	TOF; CID	69.72	588.31	ZDHHC14

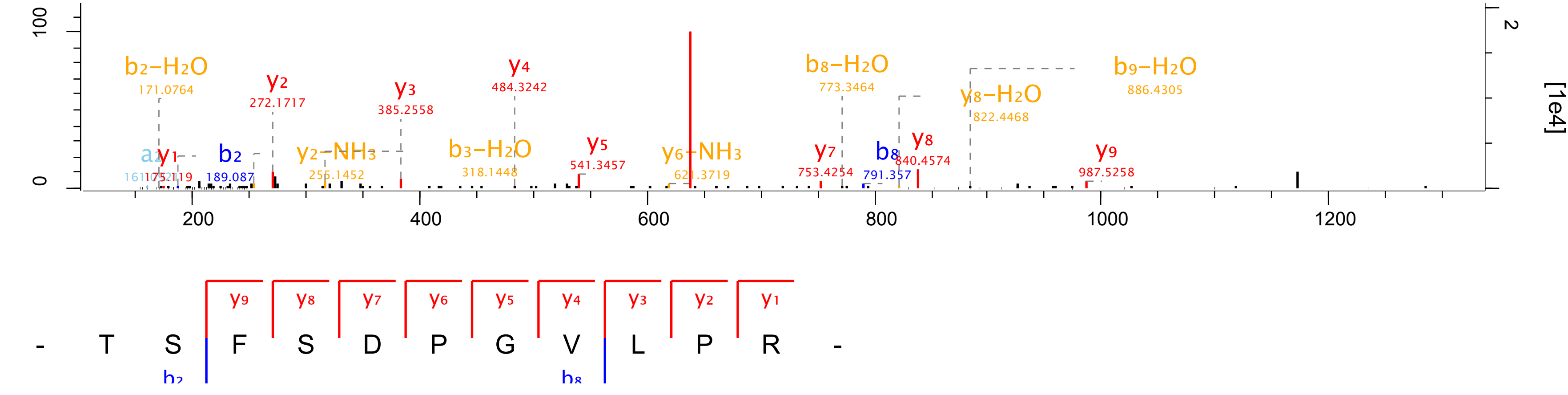

Raw file	Scan	Method	Score	m/z	Gene names
20140918_fract14_dyn_5ul_E6_01_382	23391	TOF; CID	66.39	743.84	OTX1

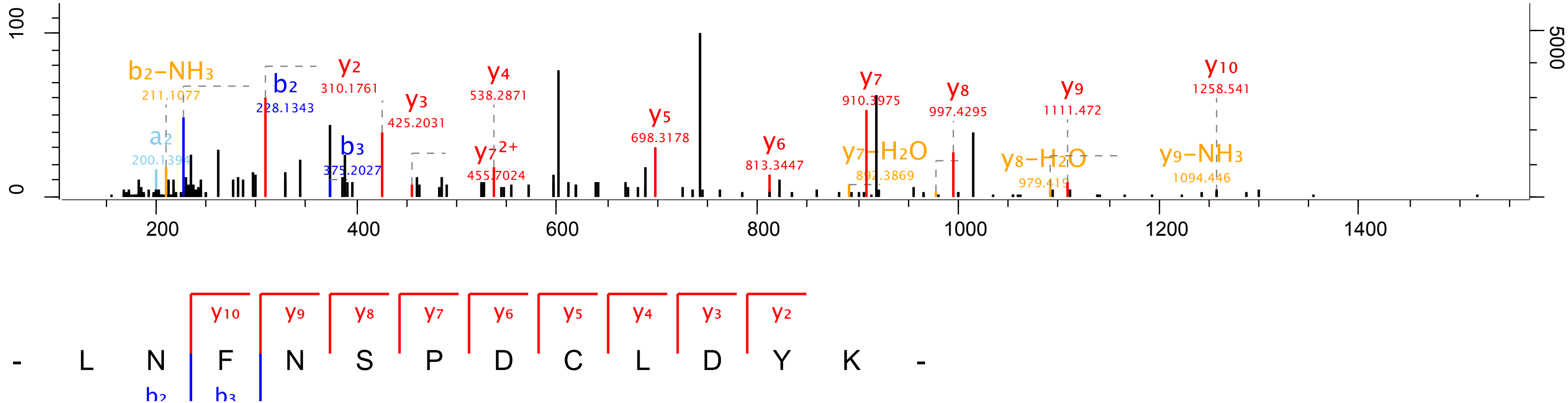

Raw file	Scan	Method	Score	m/z	Gene names
20140918_fract14_dyn_5ul_E6_01_382	25573	TOF; CID	163.45	929.48	HIATL1

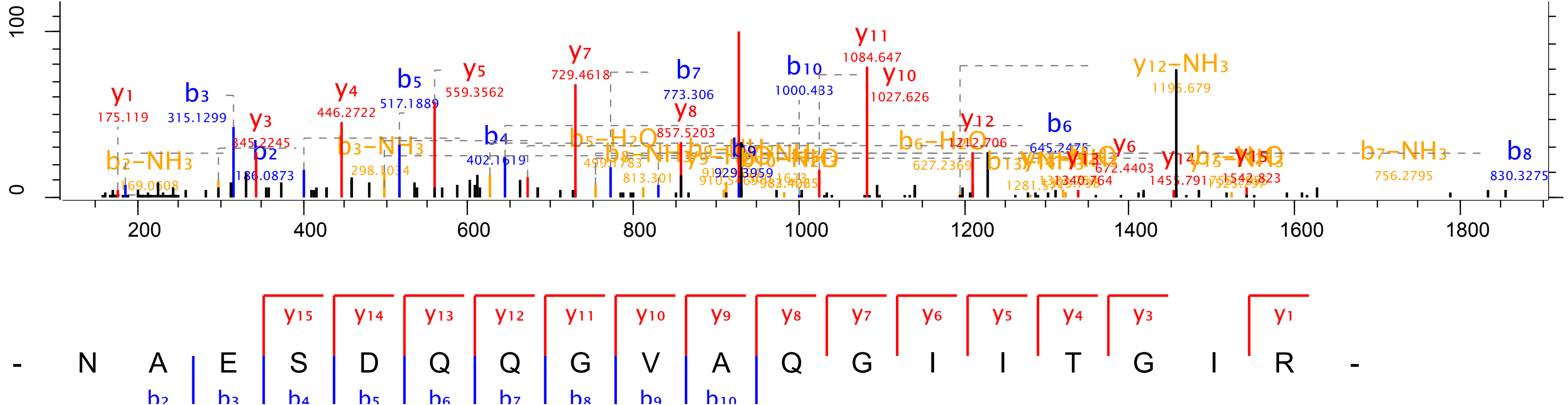

Raw file	Scan	Method	Score	m/z	Gene names
20140918_fract14_dyn_5ul_E6_01_382	26190	TOF; CID	88.02	478.56	WDR17

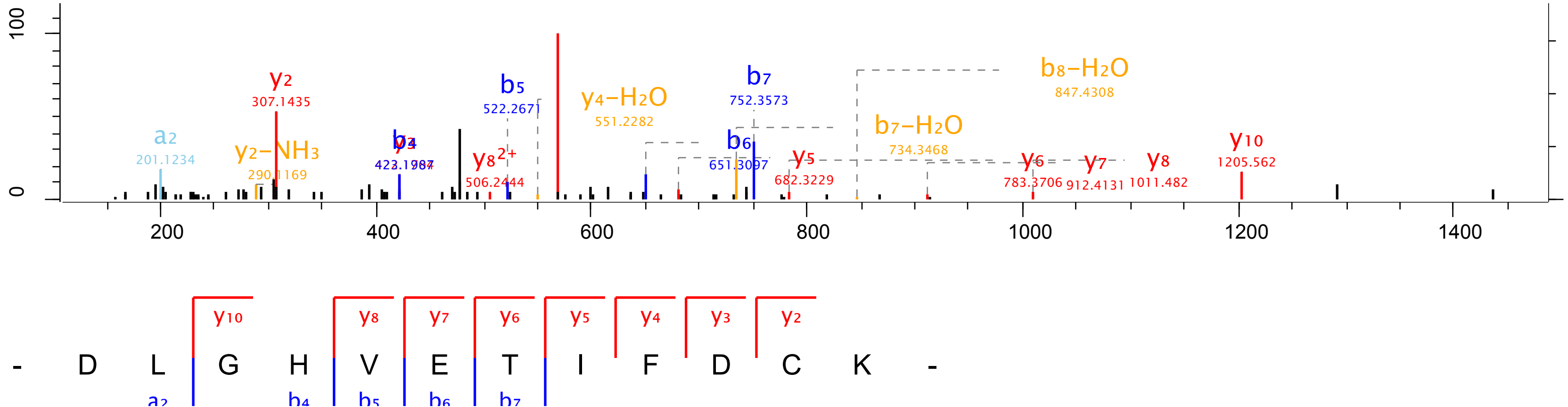

Raw file	Scan	Method	Score	m/z	Gene names
20140918_fract14_dyn_5ul_E6_01_382	26406	TOF; CID	89.23	672.36	SPATA13

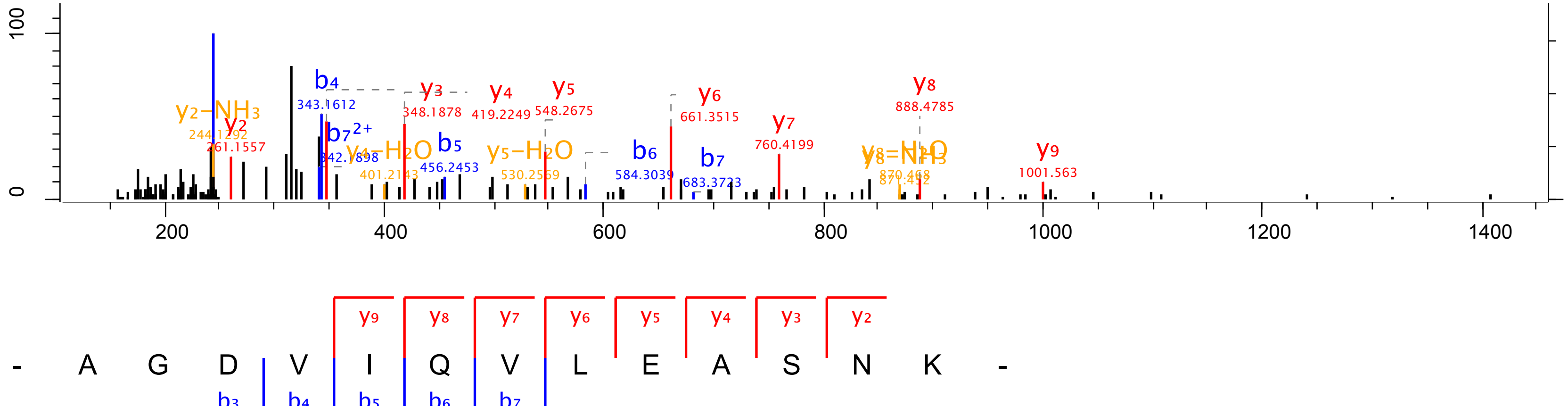

Raw file	Scan	Method	Score	m/z	Gene names
20140918_fract14_dyn_5ul_E6_01_382	27979	TOF; CID	116.24	731.87	TSPAN15

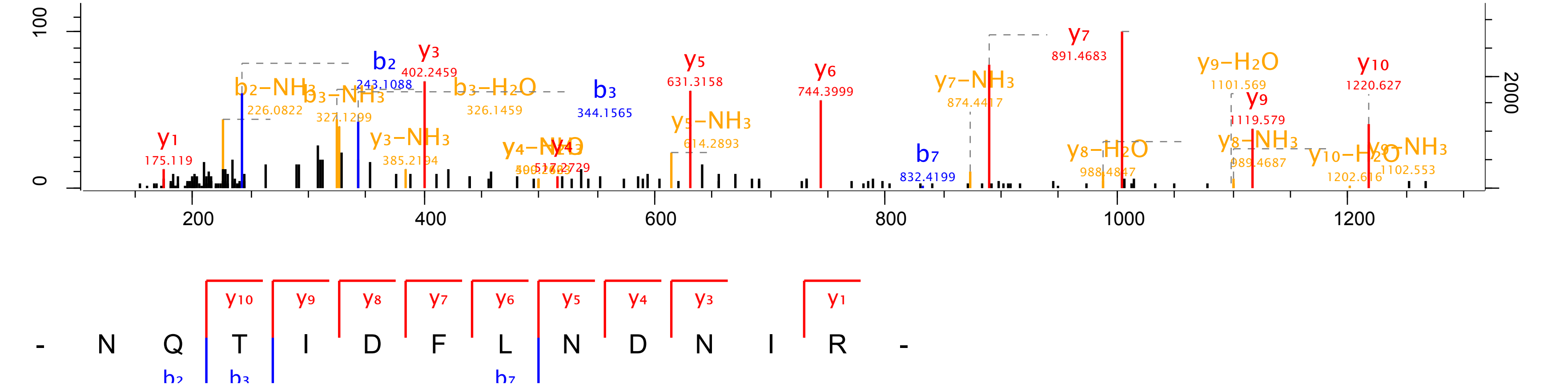

Raw file	Scan	Method	Score	m/z	Gene names
20140918_fract14_dyn_5ul_E6_01_382	28753	TOF; CID	123.5	575.3	JOSD1

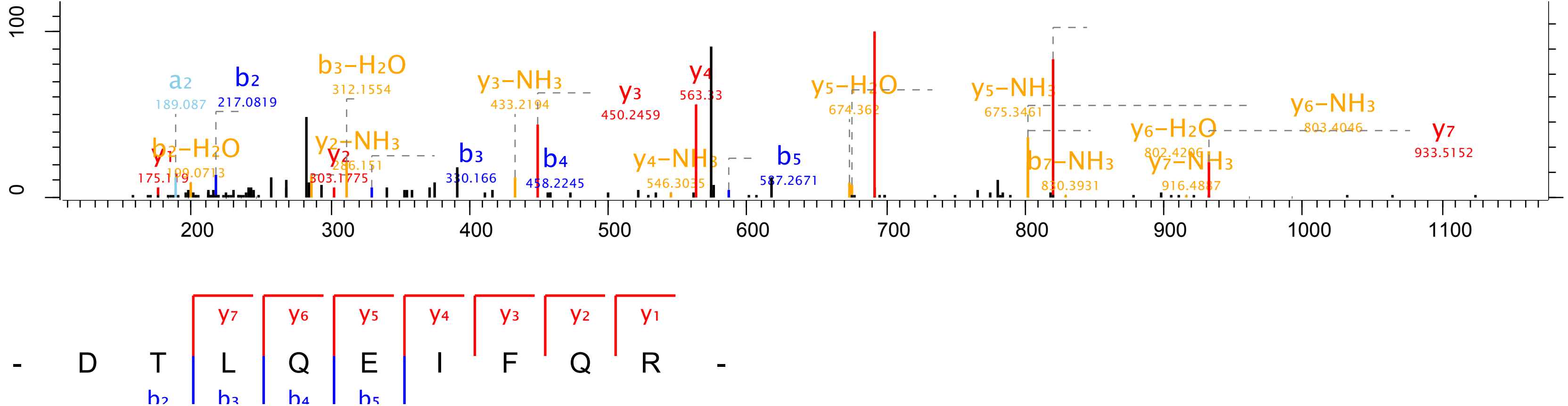

Raw file	Scan	Method	Score	m/z	Gene names
20140918_fract14_dyn_5ul_E6_01_382	32306	TOF; CID	94.49	856.38	MSANTD4

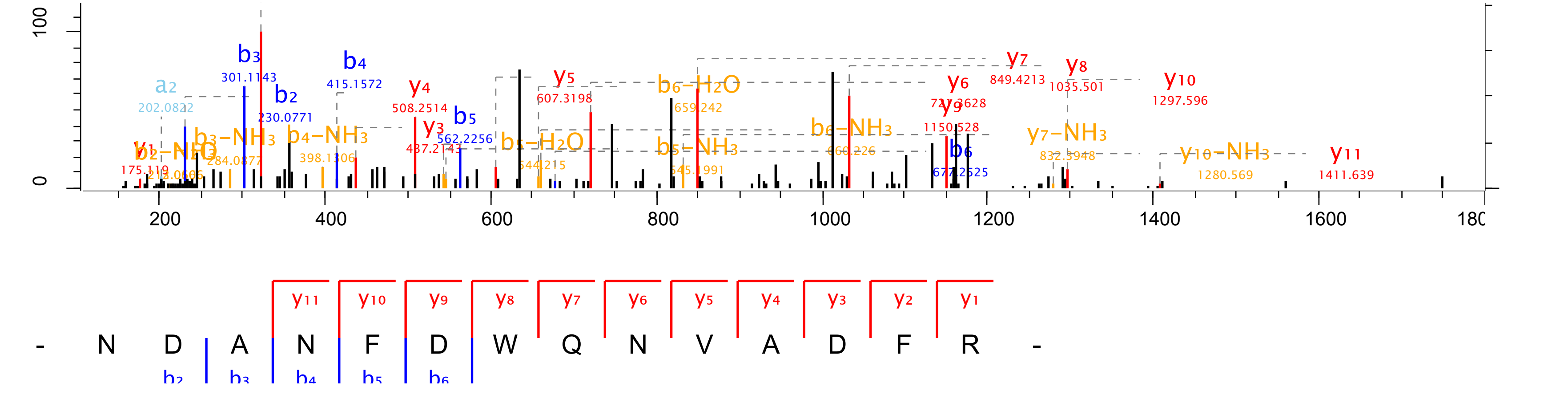

Raw file	Scan	Method	Score	m/z	Gene names
20140918_fract14_dyn_5ul_E6_01_382	34095	TOF; CID	37.88	730.02	PTPRH

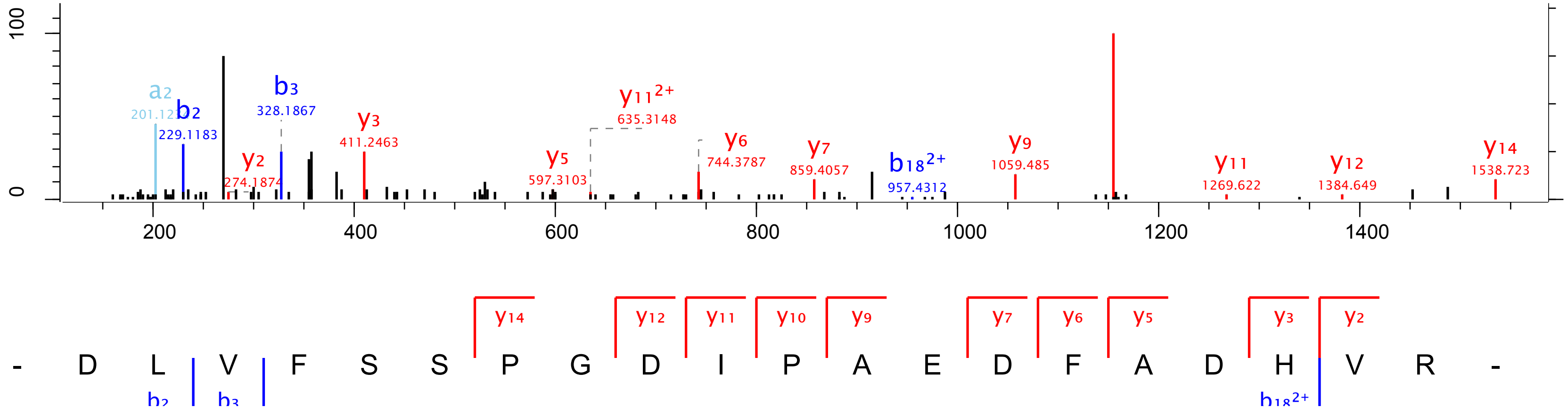

Raw file	Scan	Method	Score	m/z	Gene names
20140918_fract14_dyn_5ul_E6_01_382	38899	TOF; CID	73.07	907.8	HIGD2A

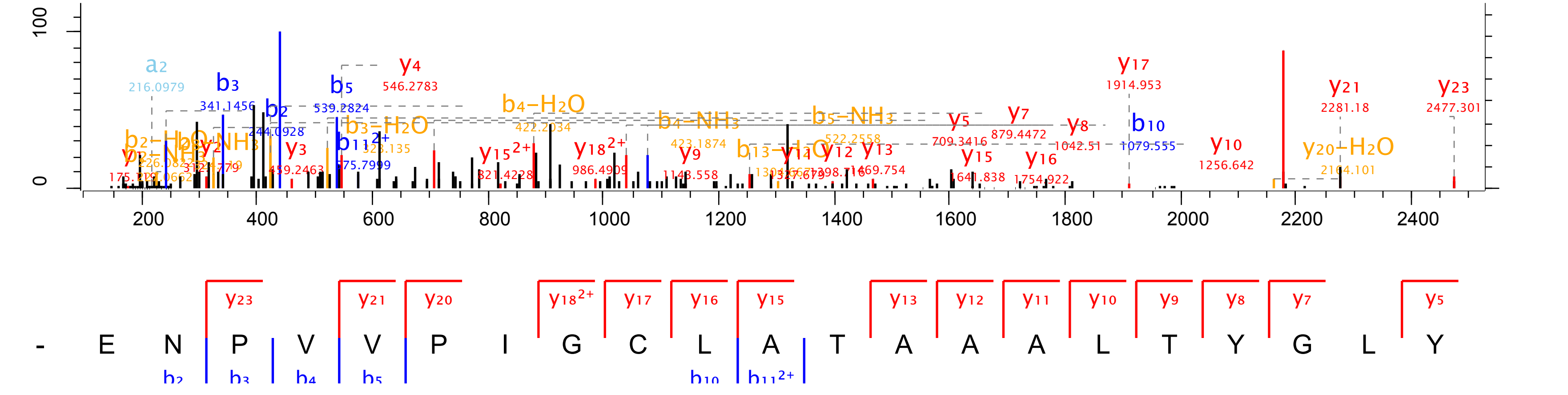

Raw file	Scan	Method	Score	m/z	Gene names
20140918_fract15_dyn_5ul_E7_01_383	7412	TOF; CID	73.08	417.89	SPATA33

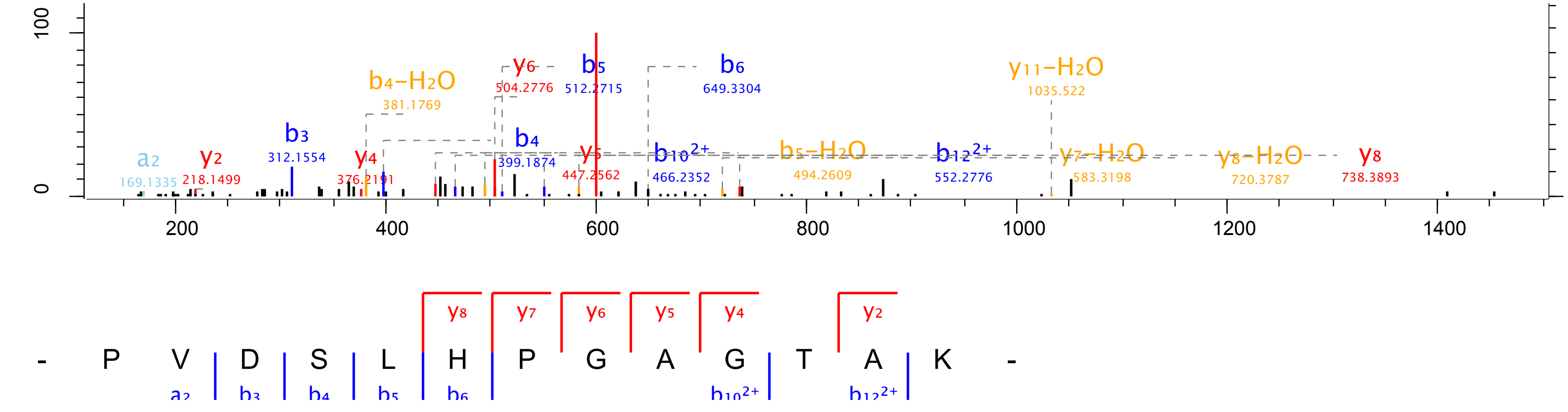

Raw file	Scan	Method	Score	m/z	Gene names
20140918_fract15_dyn_5ul_E7_01_383	10912	TOF; CID	109.72	613.83	TMC7

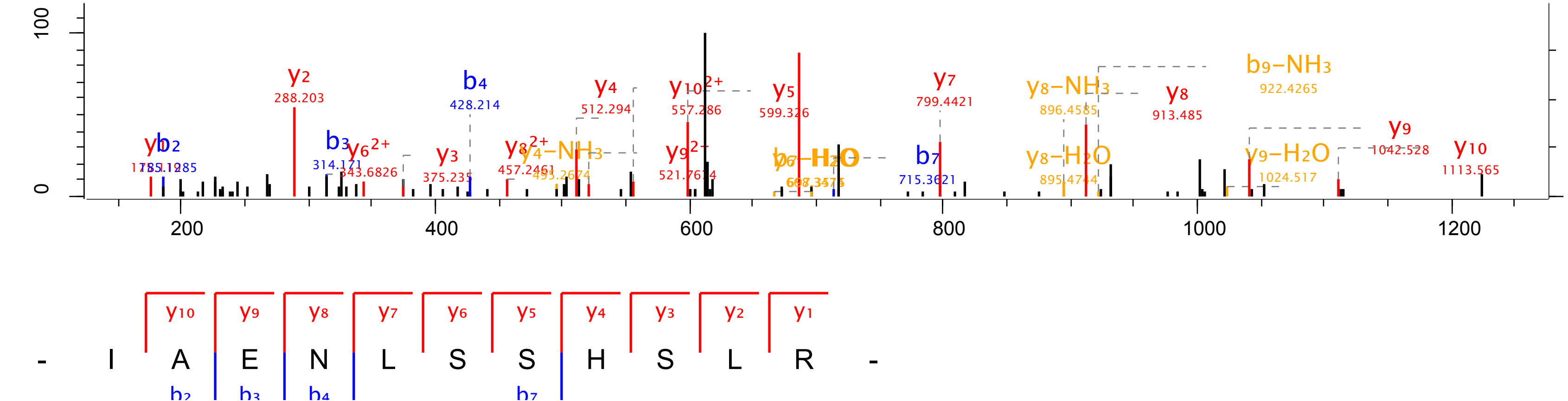

Raw file	Scan	Method	Score	m/z	Gene names
20140918_fract15_dyn_5ul_E7_01_383	11143	TOF; CID	92.61	413.89	BNIP3

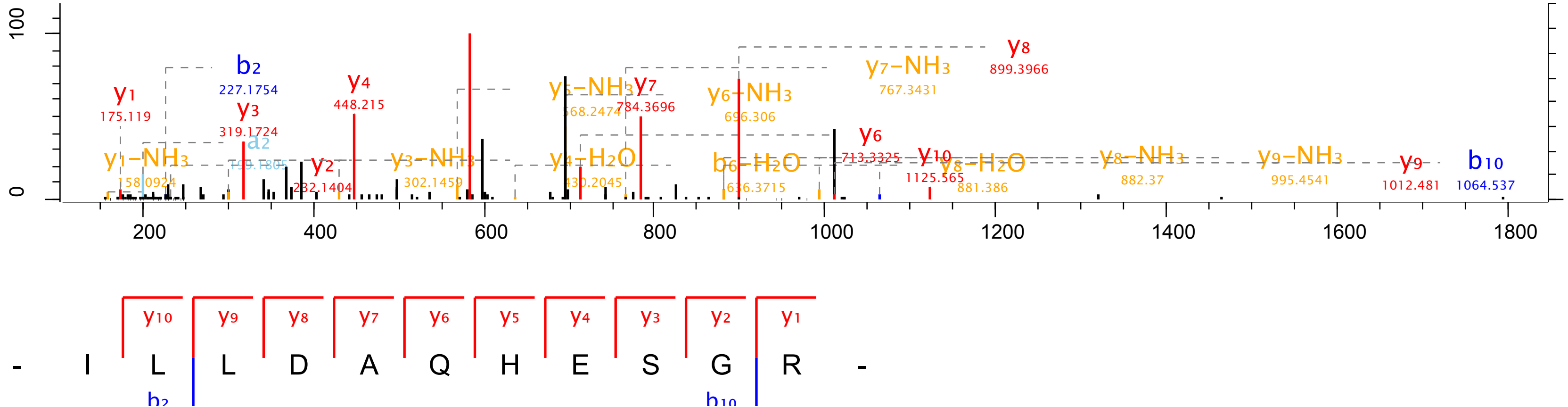

Raw file	Scan	Method	Score	m/z	Gene names
20140918_fract15_dyn_5ul_E7_01_383	13650	TOF; CID	50.35	411.87	FBP1;FBP2

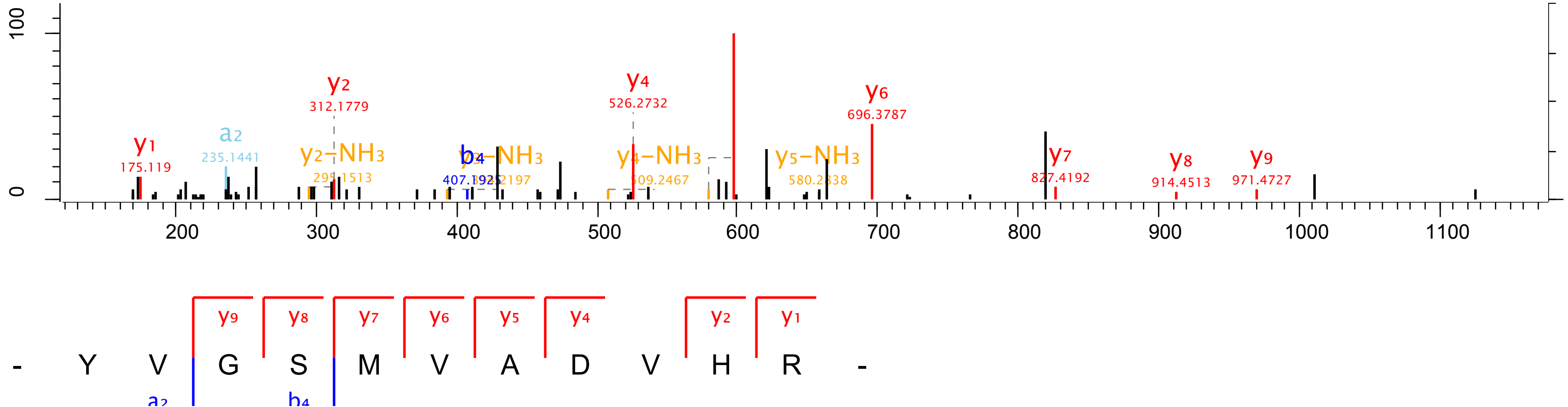

Raw file	Scan	Method	Score	m/z	Gene names
20140918_fract15_dyn_5ul_E7_01_383	14342	TOF; CID	128.59	817.93	FRAT2

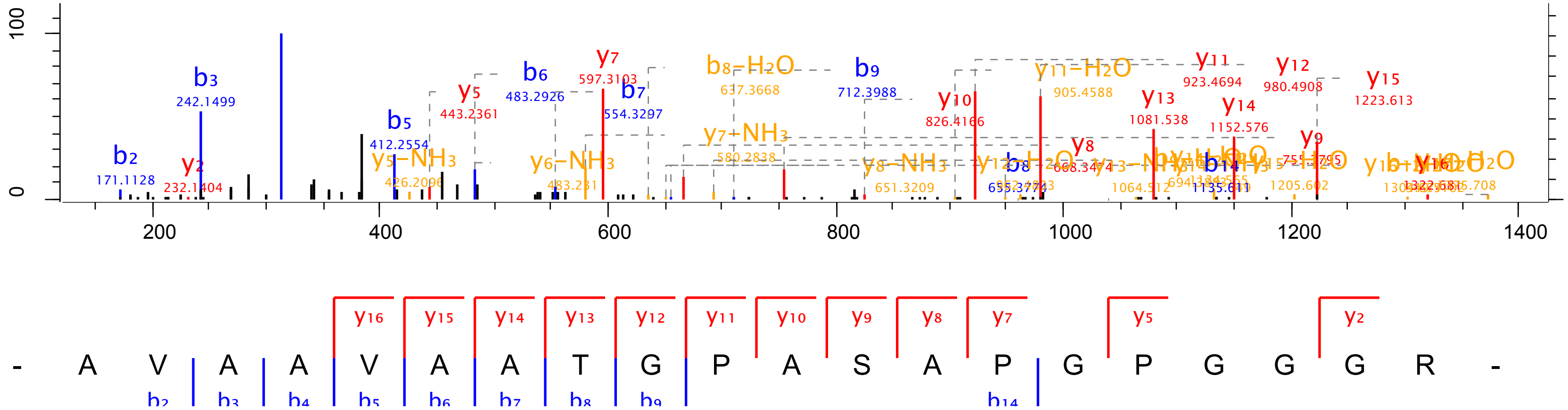

Raw file	Scan	Method	Score	m/z	Gene names
20140918_fract15_dyn_5ul_E7_01_383	14478	TOF; CID	68.85	521.3	CCDC17

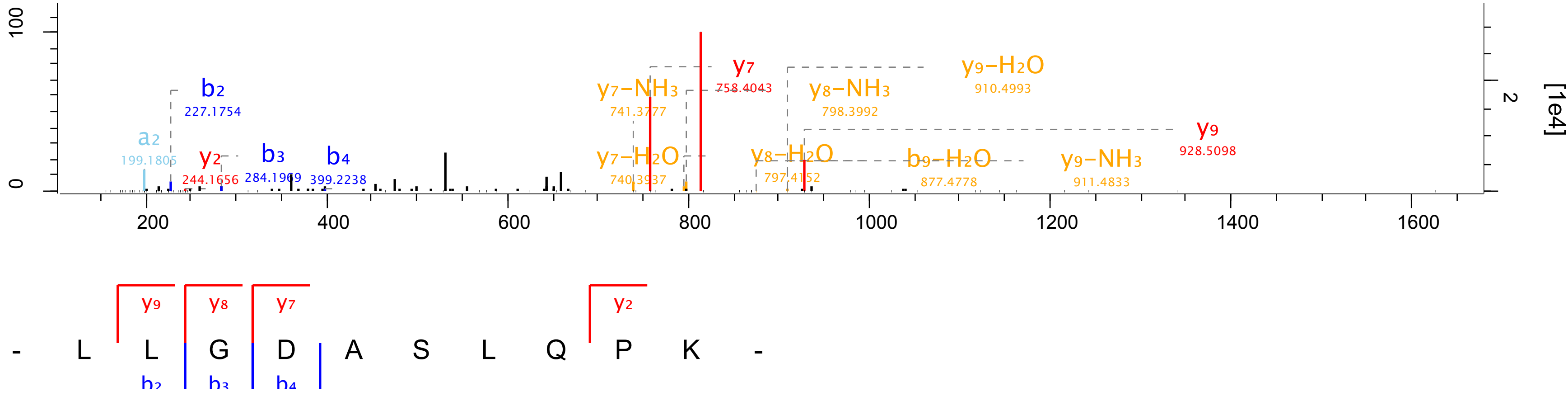

Raw file	Scan	Method	Score	m/z	Gene names
20140918_fract15_dyn_5ul_E7_01_383	20484	TOF; CID	74.44	1043.03	ZFP36

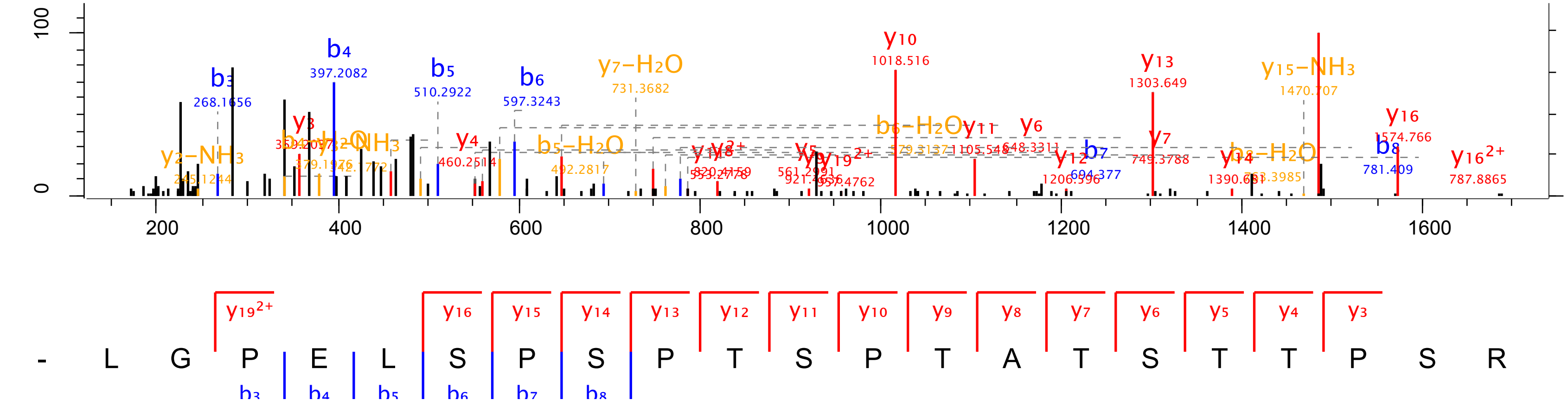

Raw file	Scan	Method	Score	m/z	Gene names
20140918_fract15_dyn_5ul_E7_01_383	20885	TOF; CID	124.59	401.24	CDO1

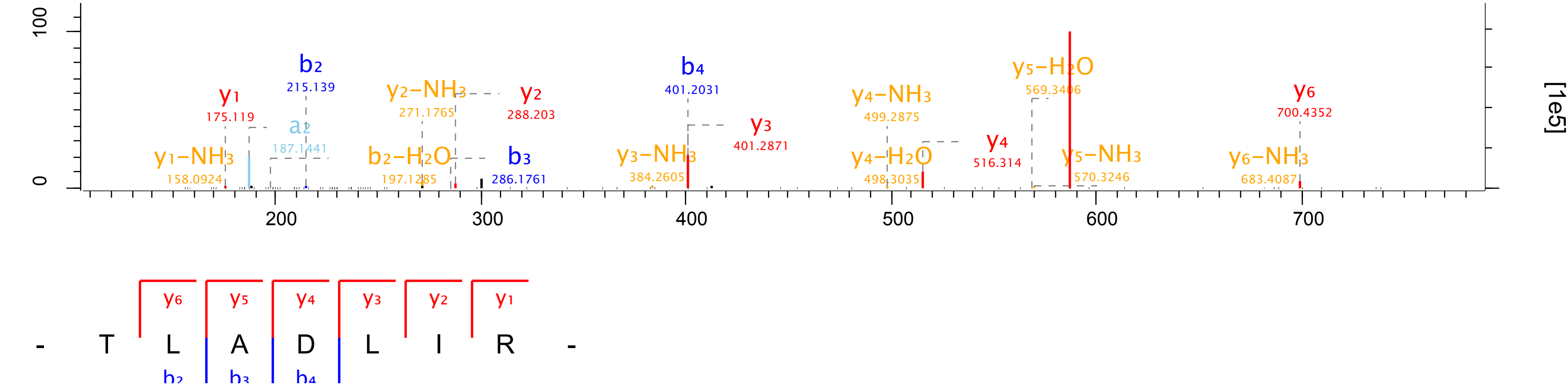

Raw file	Scan	Method	Score	m/z	Gene names
20140918_fract15_dyn_5ul_E7_01_383	21284	TOF; CID	37.88	672.34	TMEM243

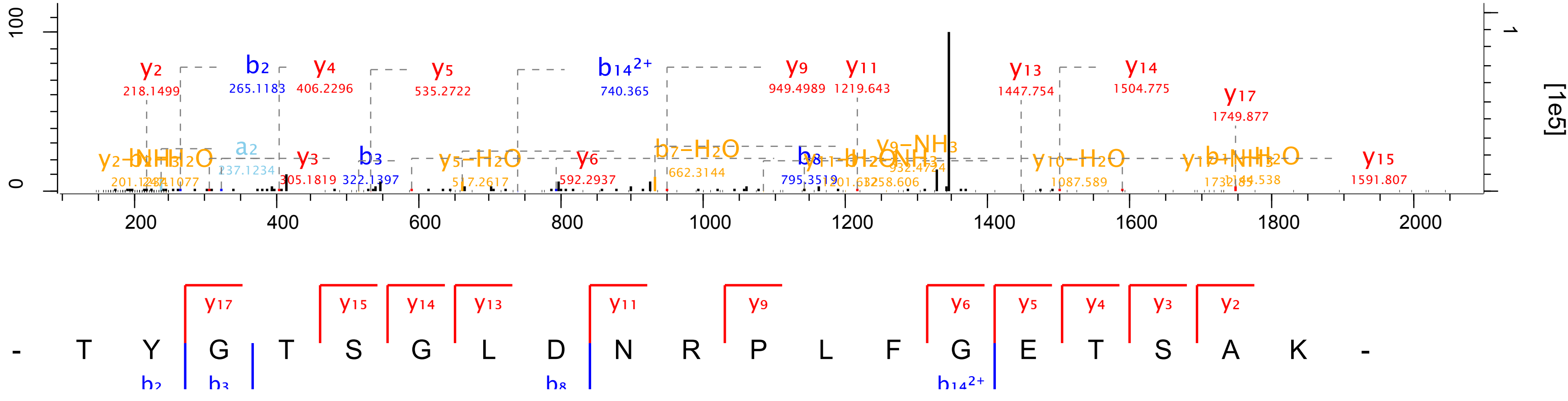

Raw file	Scan	Method	Score	m/z	Gene names
20140918_fract15_dyn_5ul_E7_01_383	21517	TOF; CID	118.67	524.28	COX7C

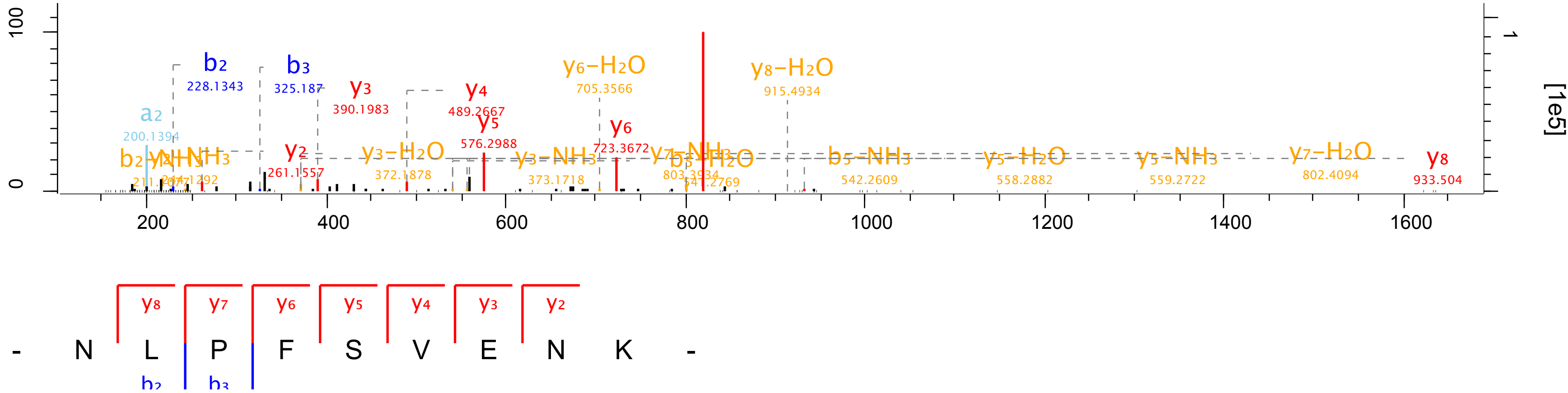

Raw file	Scan	Method	Score	m/z	Gene names
20140918_fract15_dyn_5ul_E7_01_383	28146	TOF; CID	66.06	629.85	C10orf90

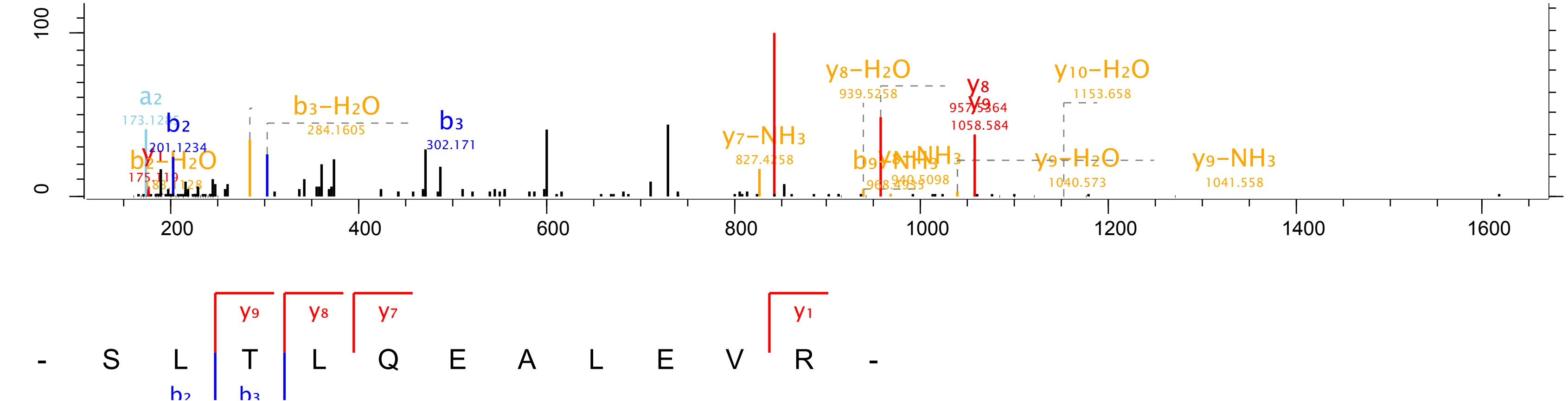

Raw file	Scan	Method	Score	m/z	Gene names
20140918_fract15_dyn_5ul_E7_01_383	35442	TOF; CID	179.74	935.51	VAMP1

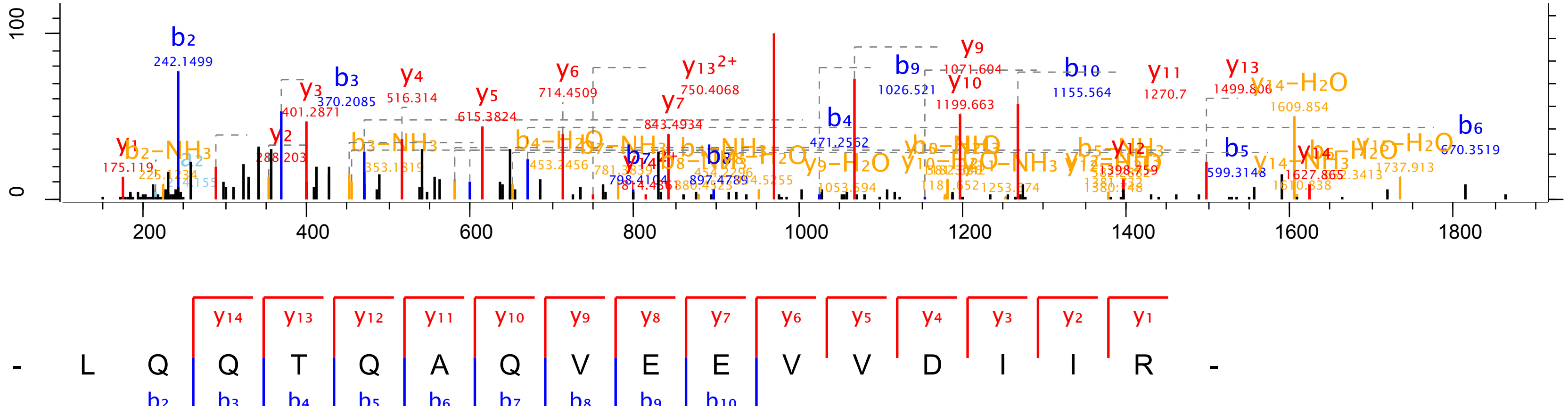

Raw file	Scan	Method	Score	m/z	Gene names
20140918_fract15_dyn_5ul_E7_01_383	36900	TOF; CID	125.74	615.86	ORAOV1

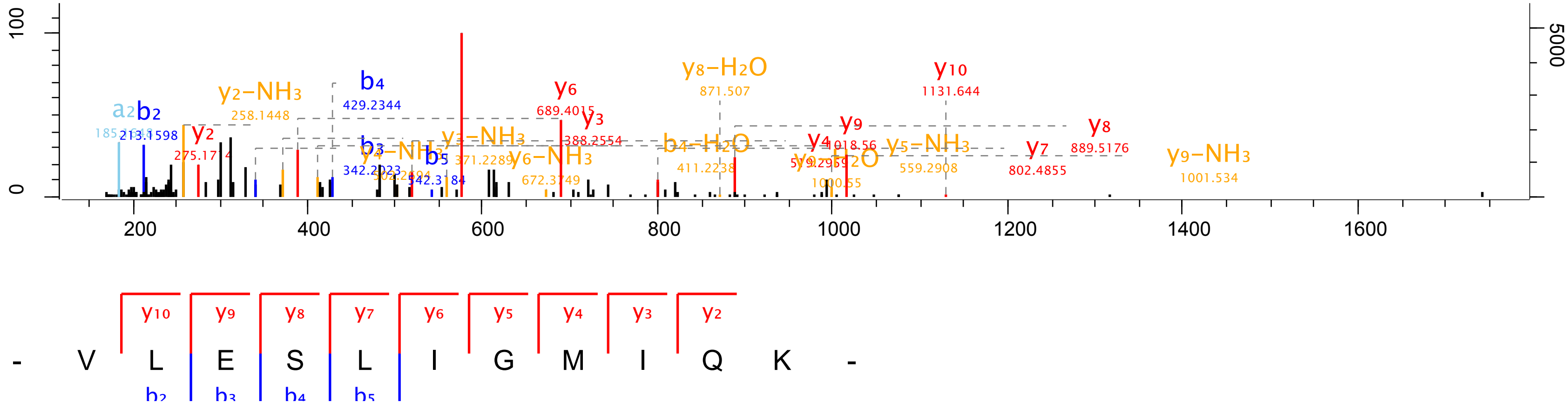

Raw file	Scan	Method	Score	m/z	Gene names
20140918_fract15_dyn_5ul_E7_01_383	37782	TOF; CID	85.99	908.43	EPT1

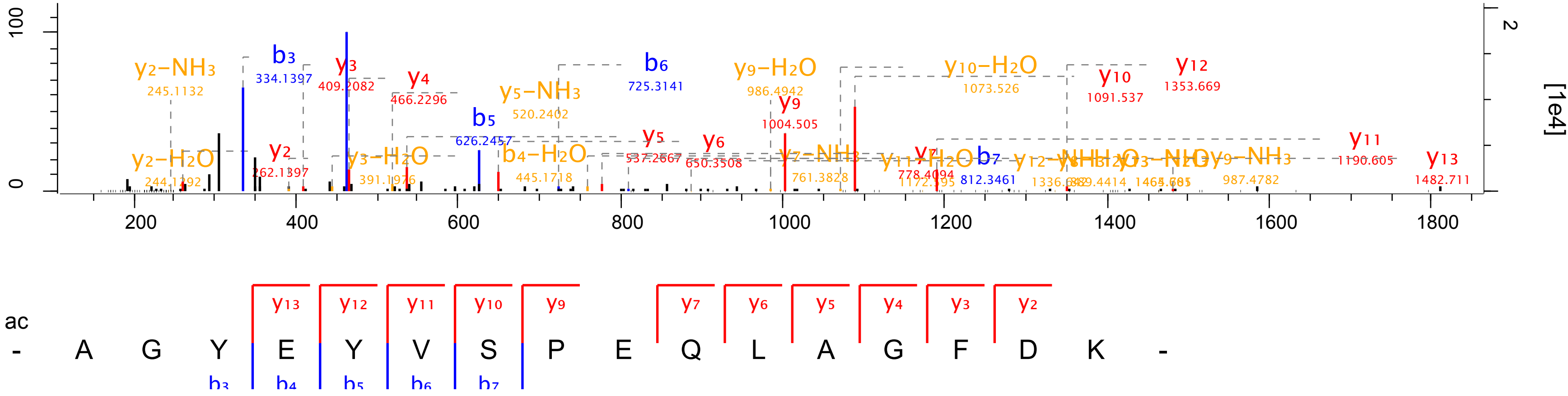

Raw file	Scan	Method	Score	m/z	Gene names
20140918_fract16_dyn_5ul_E8_01_384	7490	TOF; CID	67.65	551.28	RCBTB1

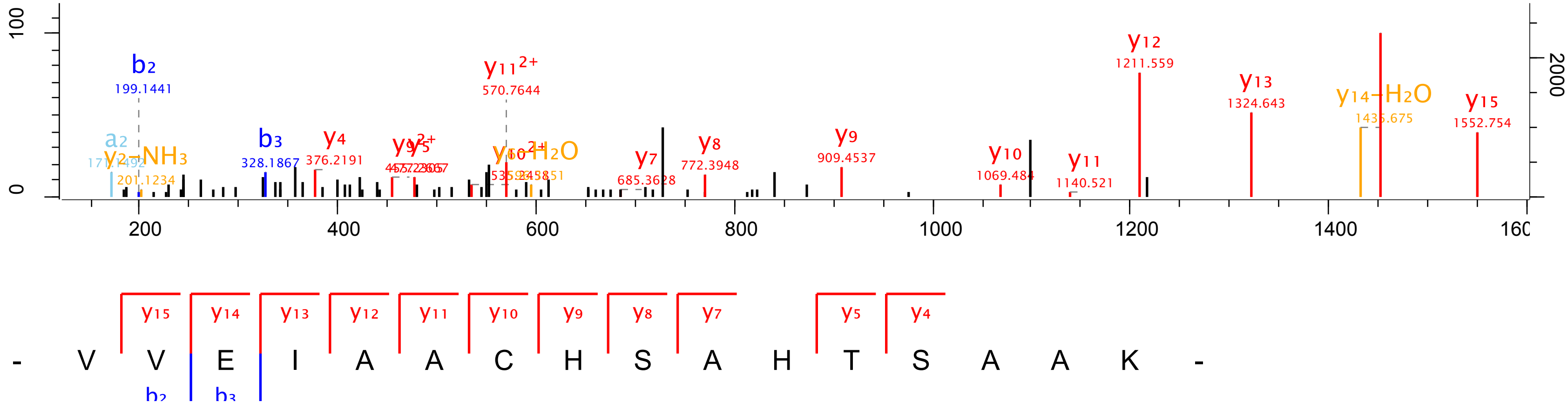

Raw file	Scan	Method	Score	m/z	Gene names
20140918_fract16_dyn_5ul_E8_01_384	10407	TOF; CID	108.72	637.81	FUNDC1

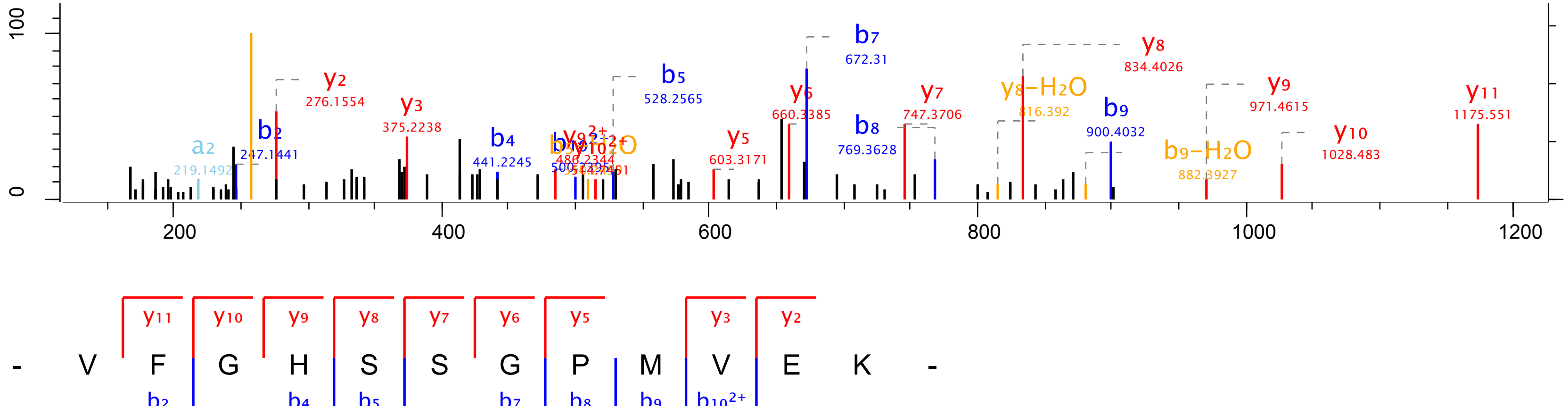

Raw file	Scan	Method	Score	m/z	Gene names
20140918_fract16_dyn_5ul_E8_01_384	15801	TOF; CID	130.41	631.79	CRLS1

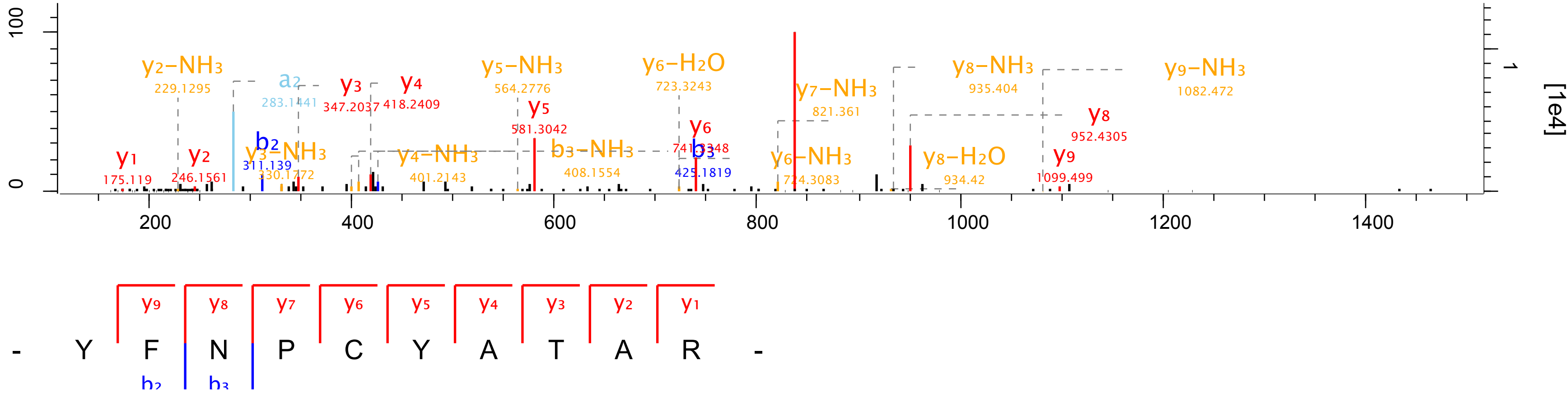

Raw file	Scan	Method	Score	m/z	Gene names
20140918_fract16_dyn_5ul_E8_01_384	19188	TOF; CID	147.26	705.36	TMEM69

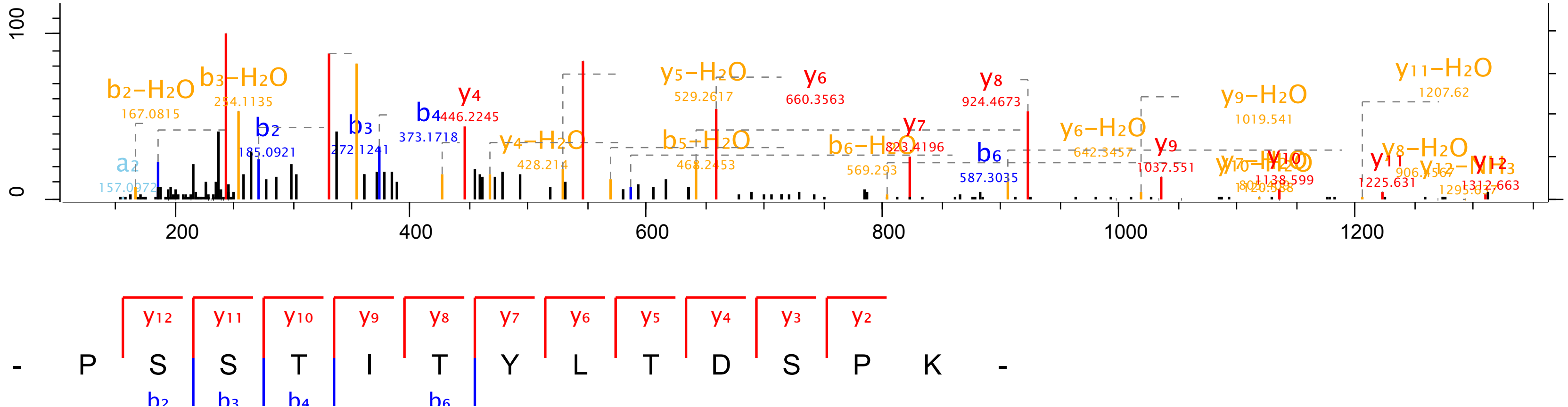

Raw file	Scan	Method	Score	m/z	Gene names
20140918_fract16_dyn_5ul_E8_01_384	20600	TOF; CID	122.69	643.3	TC2N

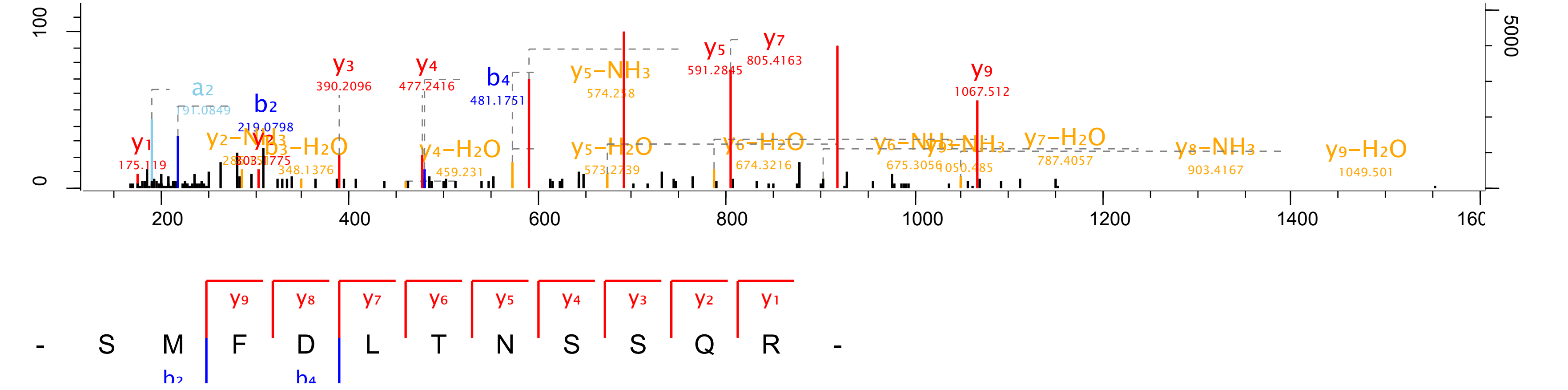

Raw file	Scan	Method	Score	m/z	Gene names
20140918_fract16_dyn_5ul_E8_01_384	21868	TOF; CID	99.82	517.78	TMEM18

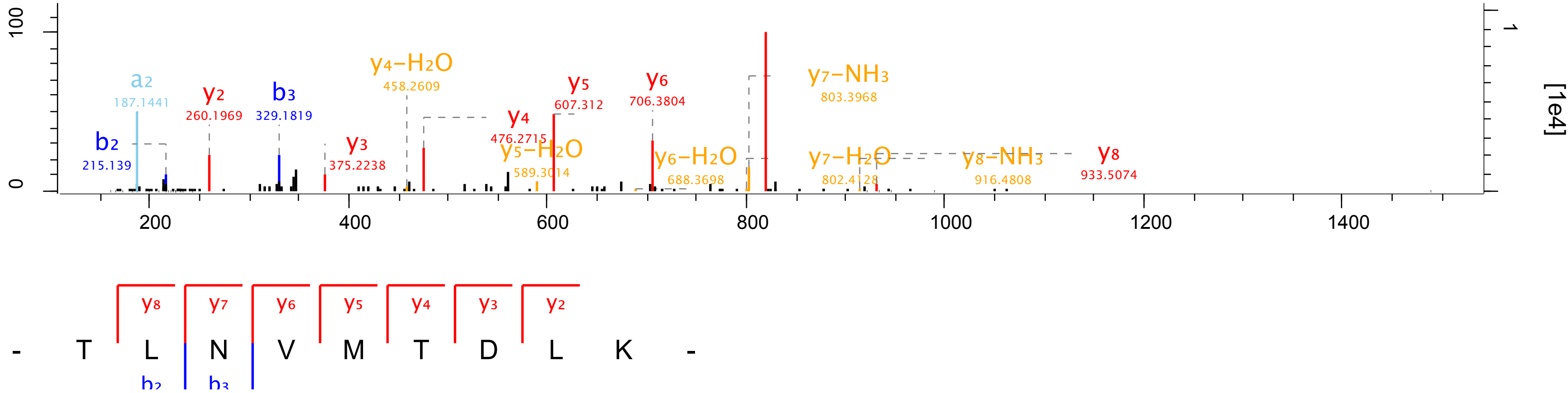

Raw file	Scan	Method	Score	m/z	Gene names
20140918_fract16_dyn_5ul_E8_01_384	23783	TOF; CID	85.94	919.96	TMEM184A

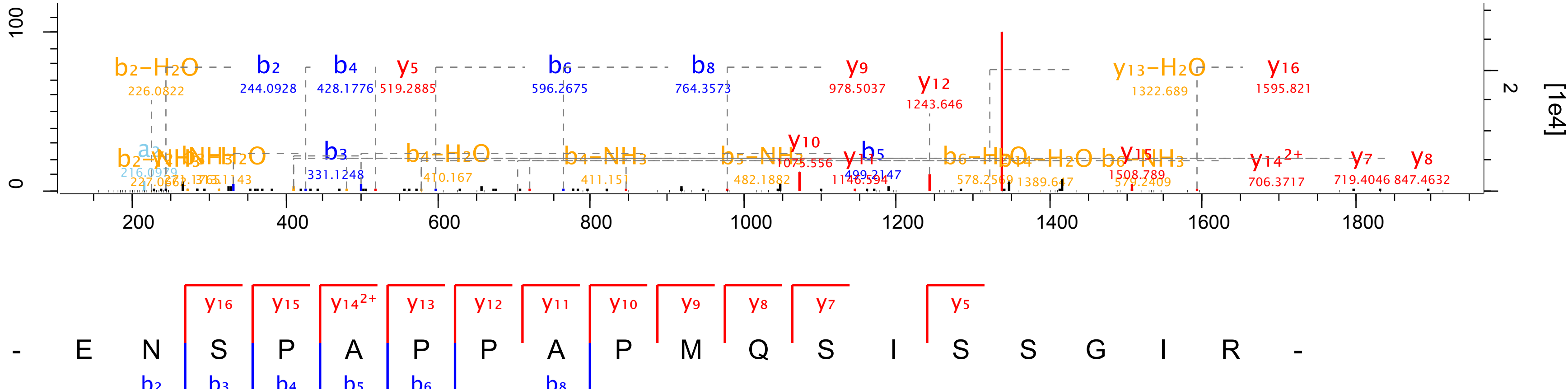

Raw file	Scan	Method	Score	m/z	Gene names
20140918_fract16_dyn_5ul_E8_01_384	25775	TOF; CID	74.96	648.83	GSKIP

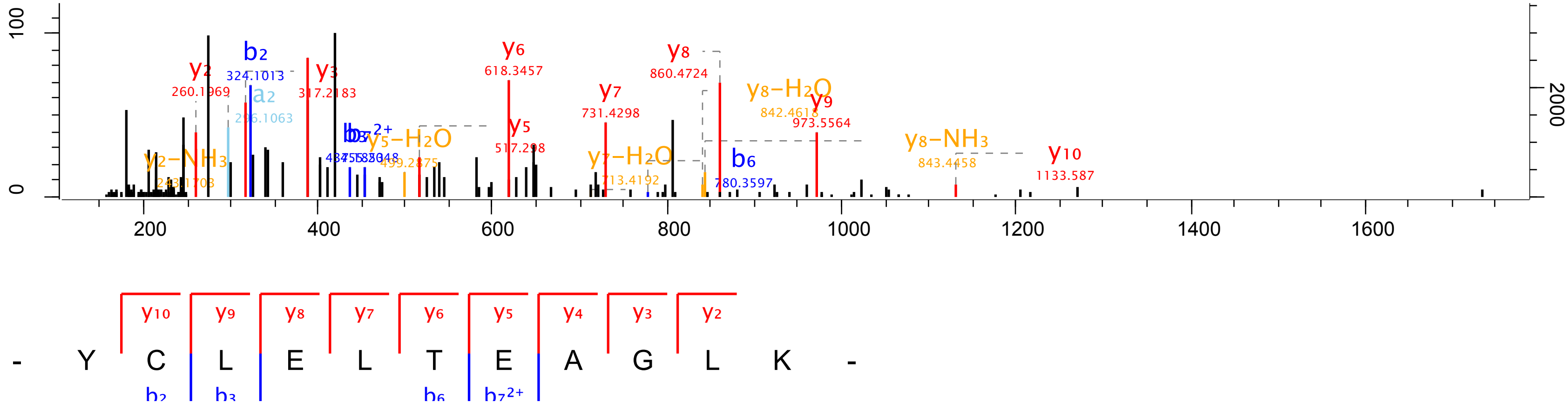

Raw file	Scan	Method	Score	m/z	Gene names
20140918_fract16_dyn_5ul_E8_01_384	32586	TOF; CID	70.26	851.43	GTF3C6

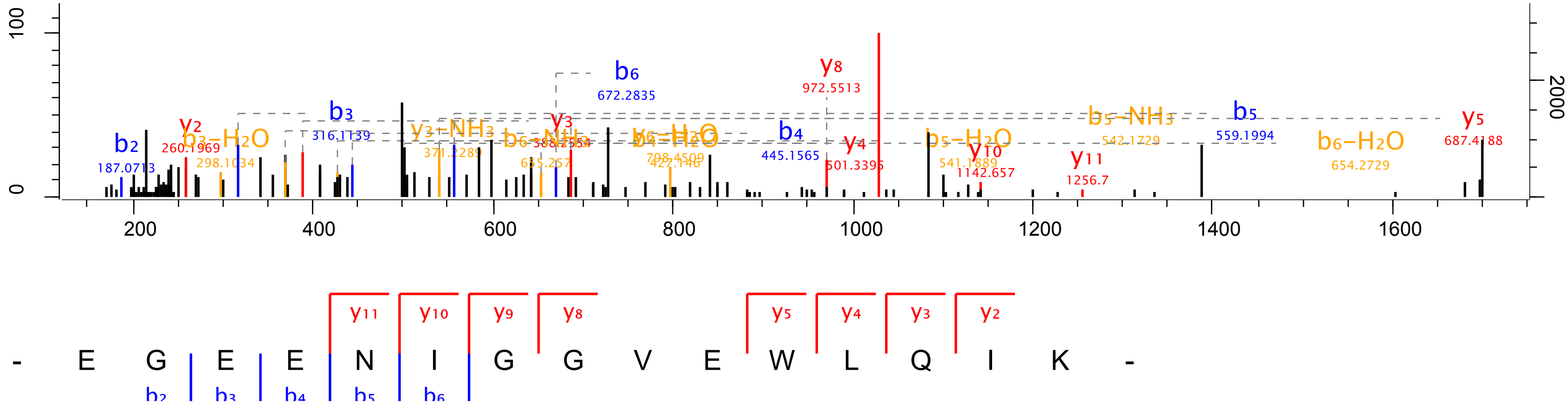

Raw file	Scan	Method	Score	m/z	Gene names
20140918_fract16_dyn_5ul_E8_01_384	36809	TOF; CID	225.13	664	ABRACL

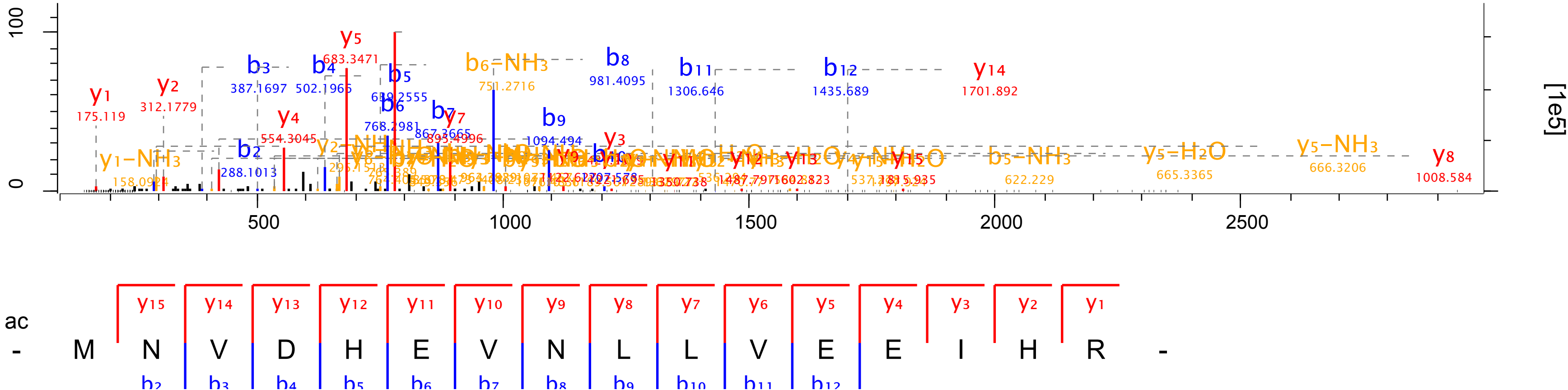

Raw file	Scan	Method	Score	m/z	Gene names
20140918_fract17_dyn_5ul_F1_01_385	11497	TOF; CID	166.04	485.91	SELT

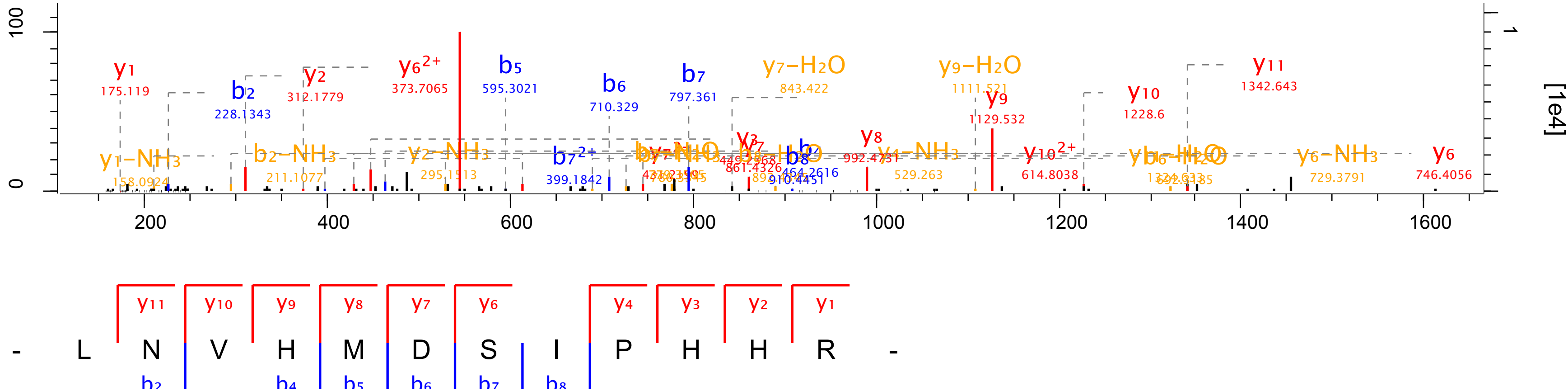

Raw file	Scan	Method	Score	m/z	Gene names
20140918_fract17_dyn_5ul_F1_01_385	13023	TOF; CID	53.3	512.62	PNMA1

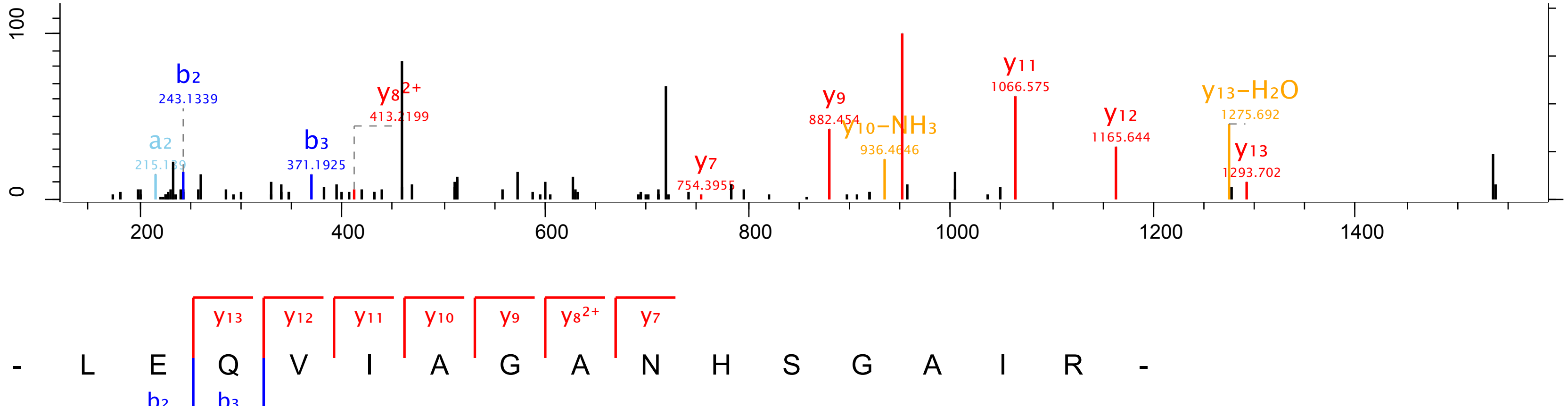

Raw file	Scan	Method	Score	m/z	Gene names
20140918_fract17_dyn_5ul_F1_01_385	19598	TOF; CID	71.45	576.32	ADAM12

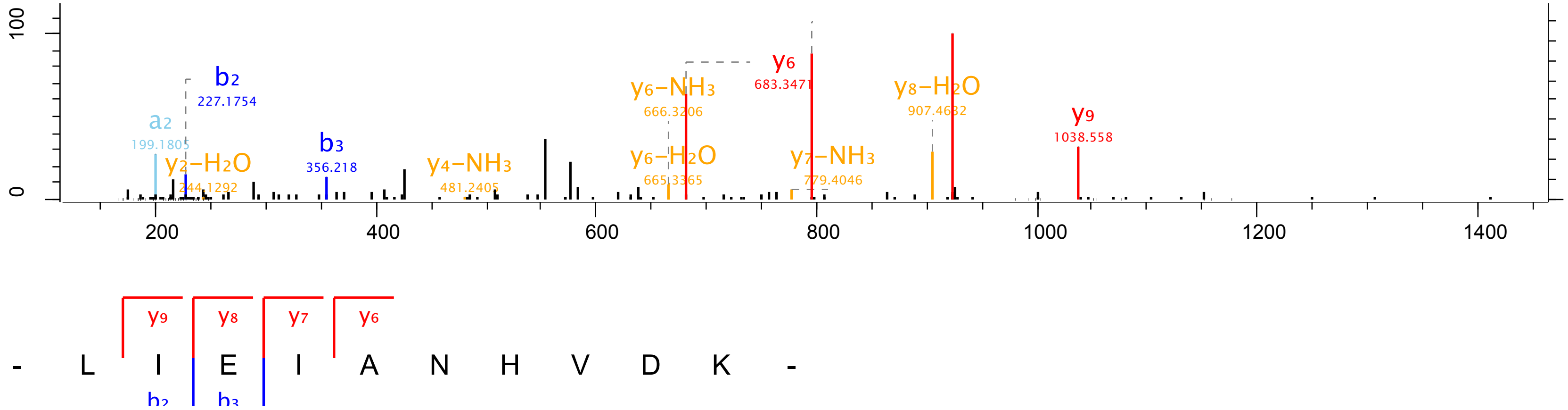

Raw file	Scan	Method	Score	m/z	Gene names
20140918_fract17_dyn_5ul_F1_01_385	20317	TOF; CID	34.51	813.9	BAD

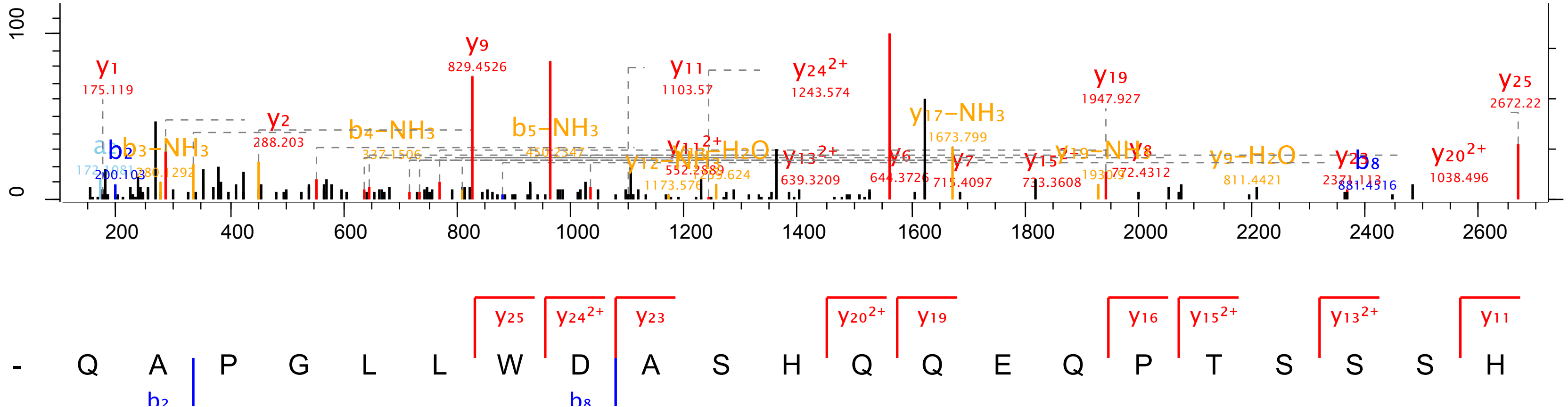

Raw file	Scan	Method	Score	m/z	Gene names
20140918_fract17_dyn_5ul_F1_01_385	20475	TOF; CID	68.58	563.64	ELF4

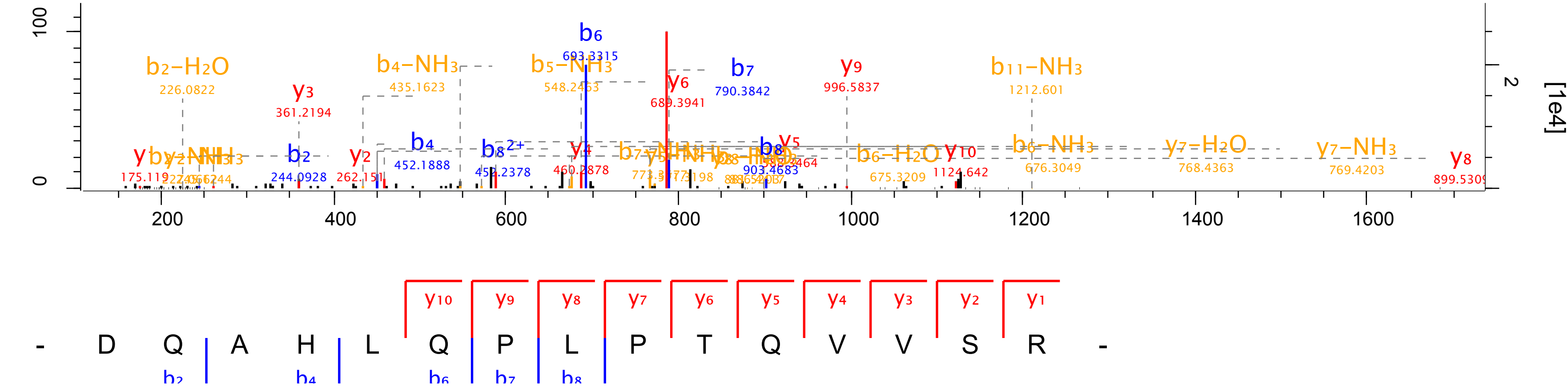

Raw file	Scan	Method	Score	m/z	Gene names
20140918_fract17_dyn_5ul_F1_01_385	20713	TOF; CID	87.45	834.92	SRF

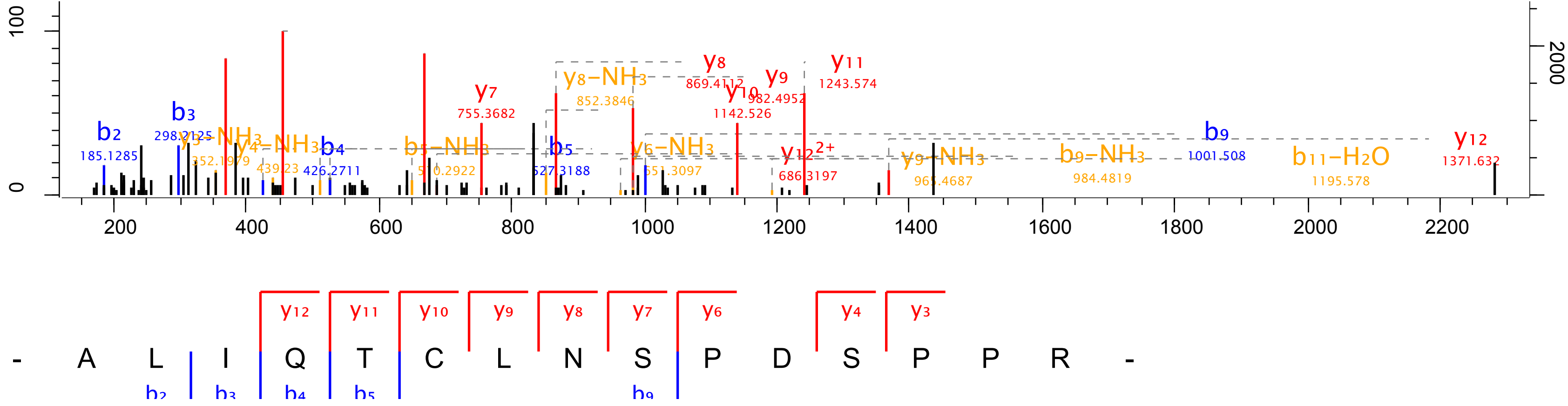

Raw file	Scan	Method	Score	m/z	Gene names
20140918_fract17_dyn_5ul_F1_01_385	21011	TOF; CID	107.06	574.31	C18orf25

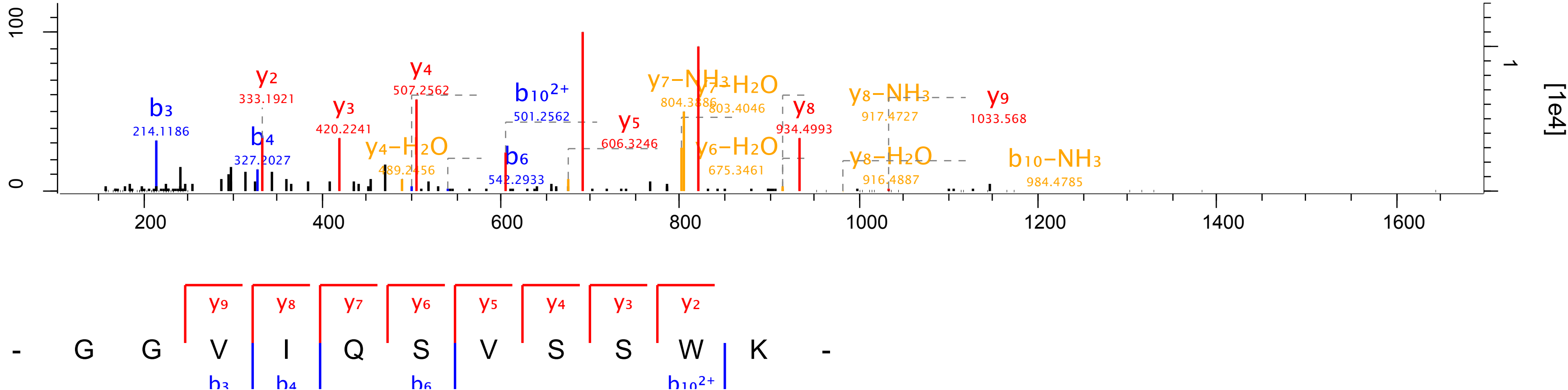

Raw file	Scan	Method	Score	m/z	Gene names
20140918_fract17_dyn_5ul_F1_01_385	21637	TOF; CID	76.33	637.84	FICD

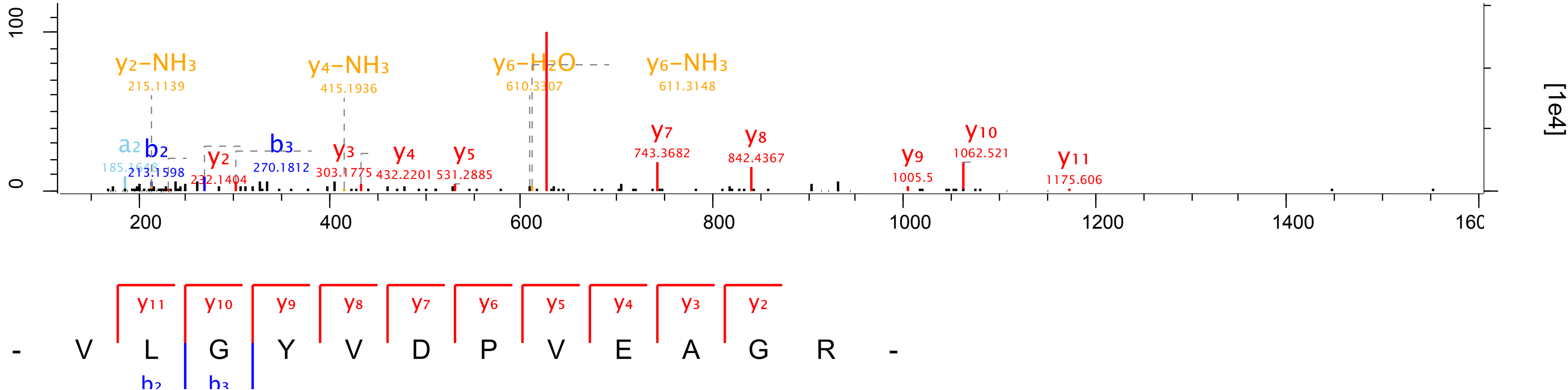

Raw file	Scan	Method	Score	m/z	Gene names
20140918_fract17_dyn_5ul_F1_01_385	23864	TOF; CID	83.36	518.28	LSM10

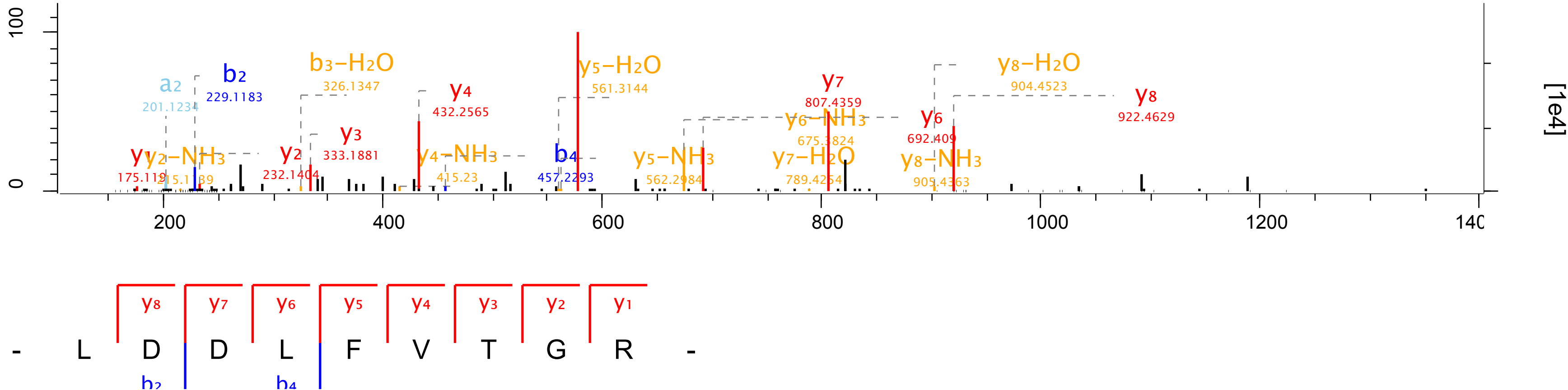

Raw file	Scan	Method	Score	m/z	Gene names
20140918_fract17_dyn_5ul_F1_01_385	23963	TOF; CID	86.95	813.07	LHFPL2

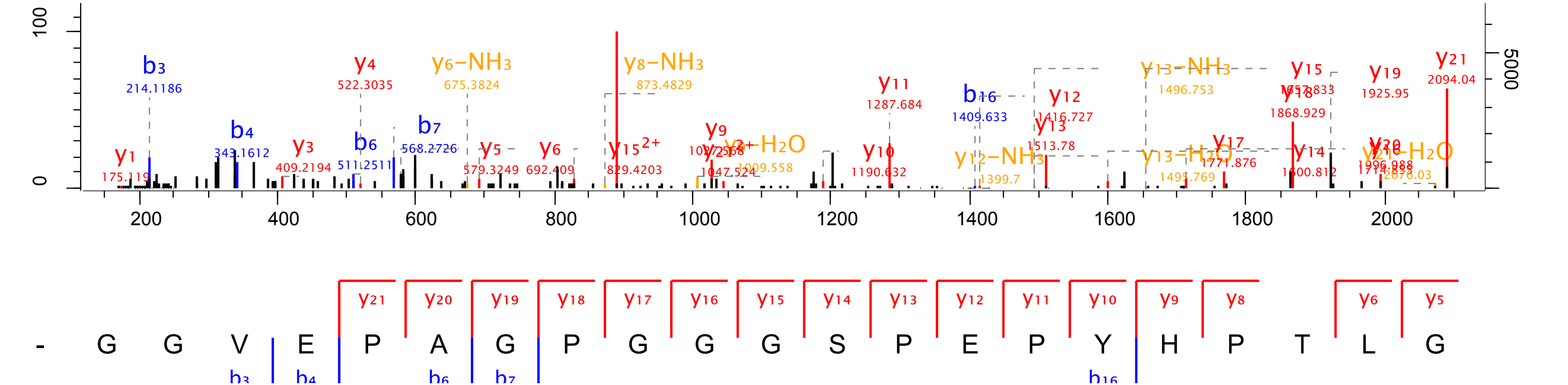

Raw file	Scan	Method	Score	m/z	Gene names
20140918_fract17_dyn_5ul_F1_01_385	24002	TOF; CID	73.44	587.82	SYNGR3

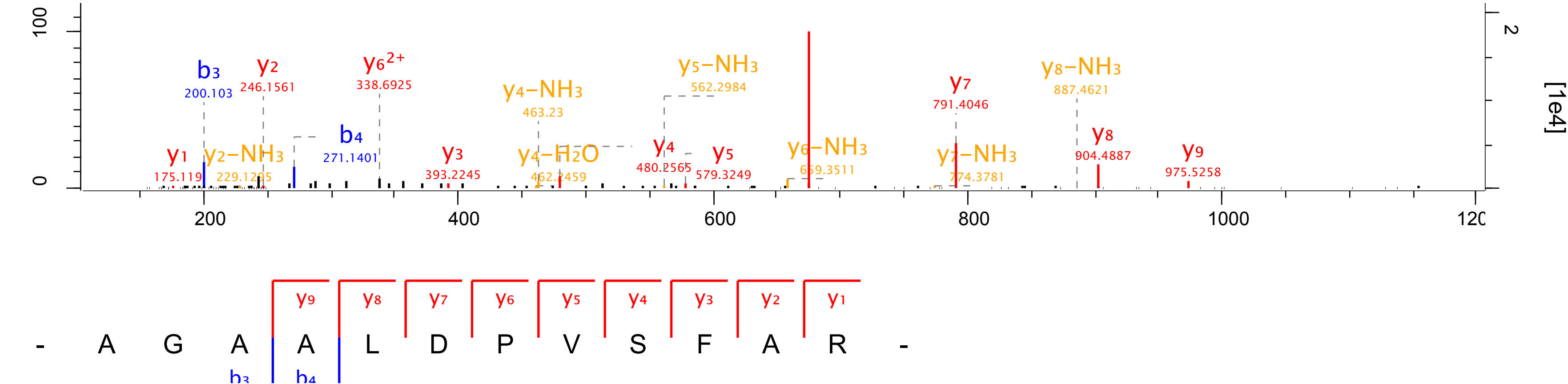

Raw file	Scan	Method	Score	m/z	Gene names
20140918_fract17_dyn_5ul_F1_01_385	24990	TOF; CID	93.65	502.27	PGM5

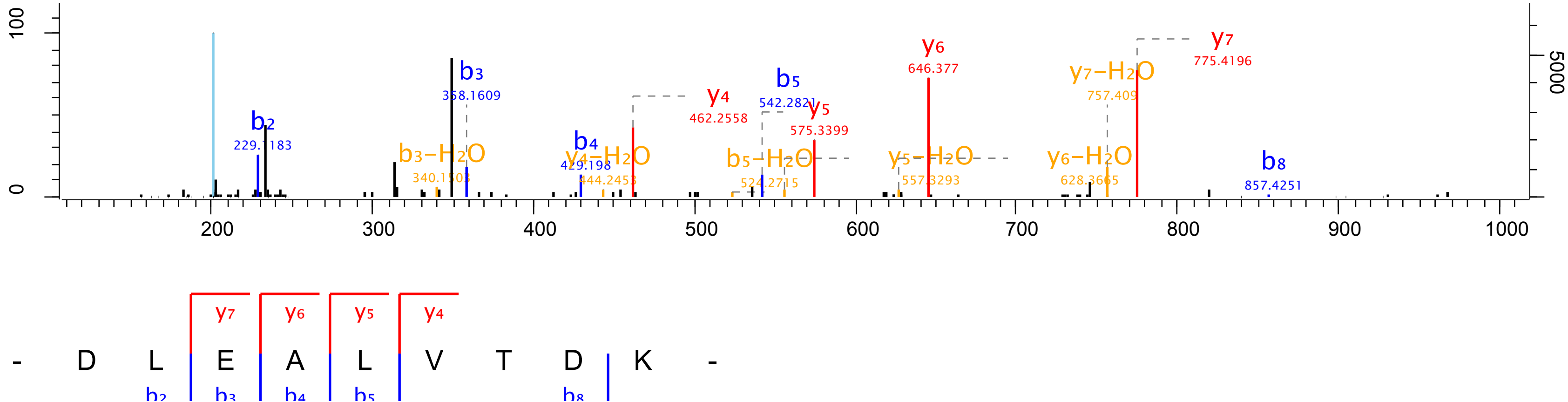

Raw file	Scan	Method	Score	m/z	Gene names
20140918_fract17_dyn_5ul_F1_01_385	25591	TOF; CID	89.75	415.75	APBA1

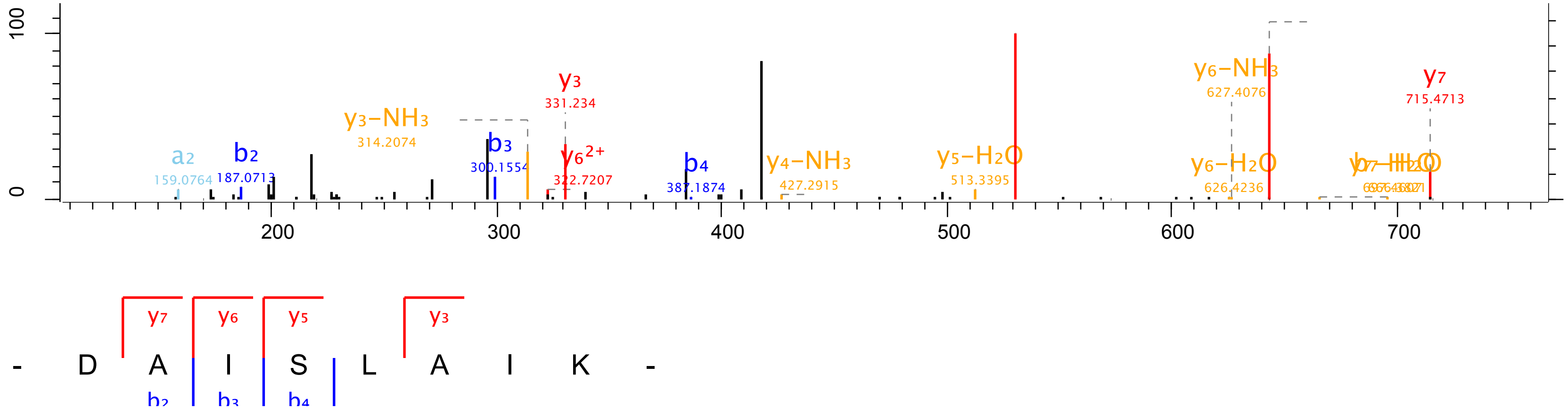

Raw file	Scan	Method	Score	m/z	Gene names
20140918_fract17_dyn_5ul_F1_01_385	27872	TOF; CID	65.37	862.46	PSENEN

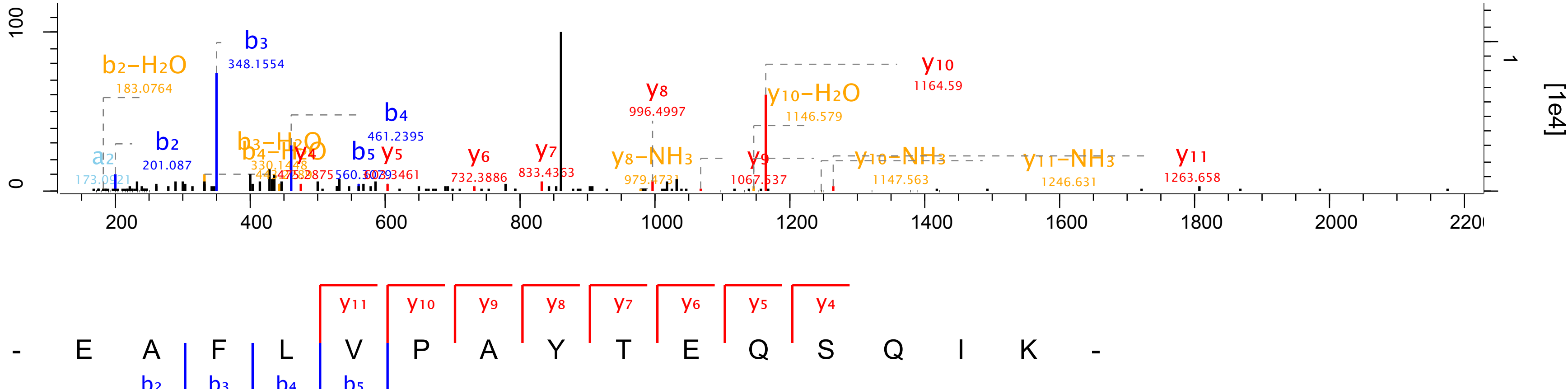

Raw file	Scan	Method	Score	m/z	Gene names
20140918_fract17_dyn_5ul_F1_01_385	28637	TOF; CID	76.3	1074.52	GGACT

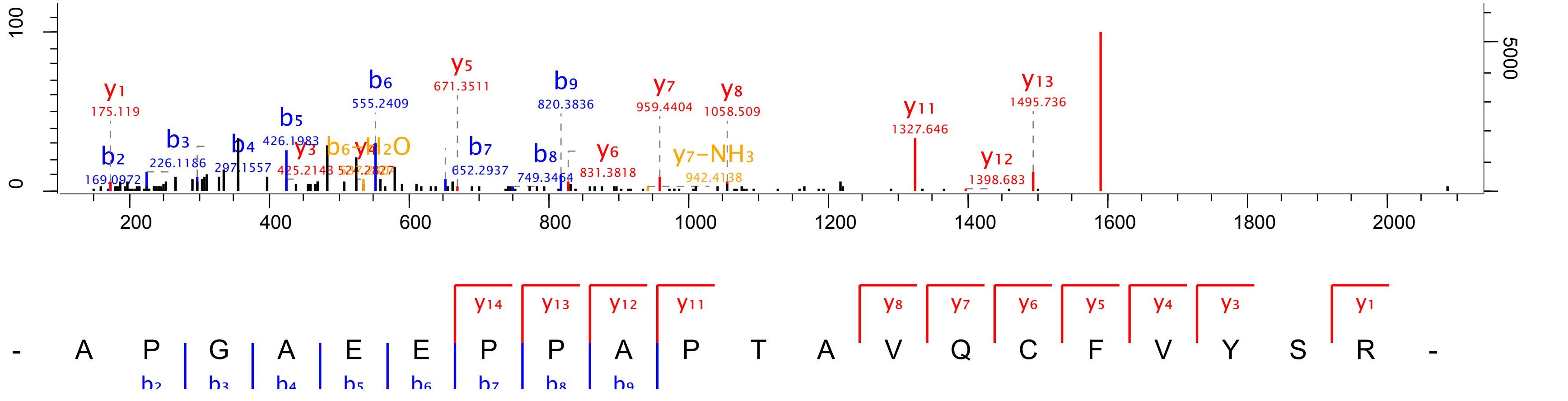

Raw file	Scan	Method	Score	m/z
20140918_fract17_dyn_5ul_F1_01_385	32221	TOF; CID	44.93	963.44

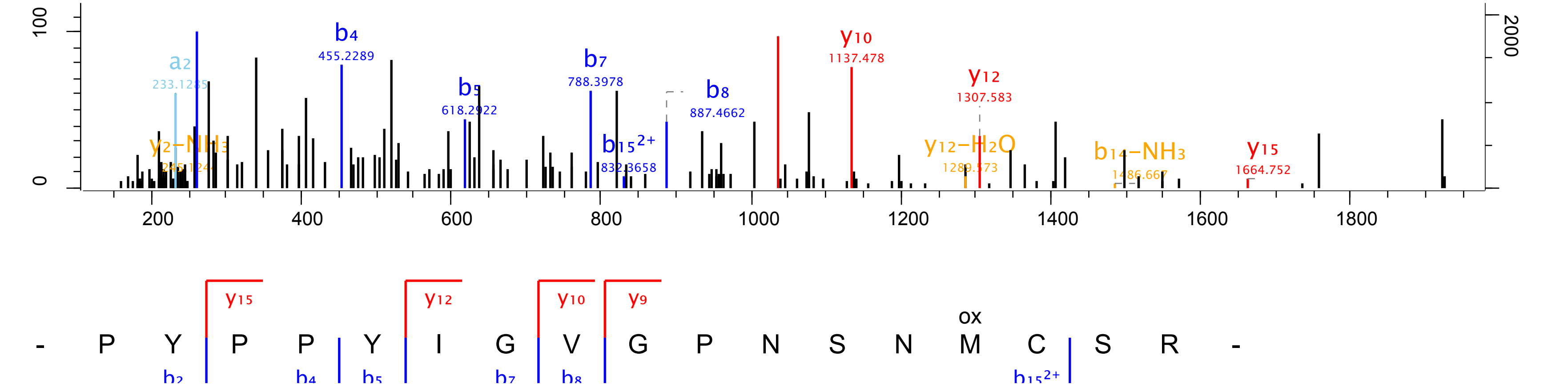

Raw file	Scan	Method	Score	m/z	Gene names
20140918_fract17_dyn_5ul_F1_01_385	34561	TOF; CID	160.43	1007.98	TMEM106C

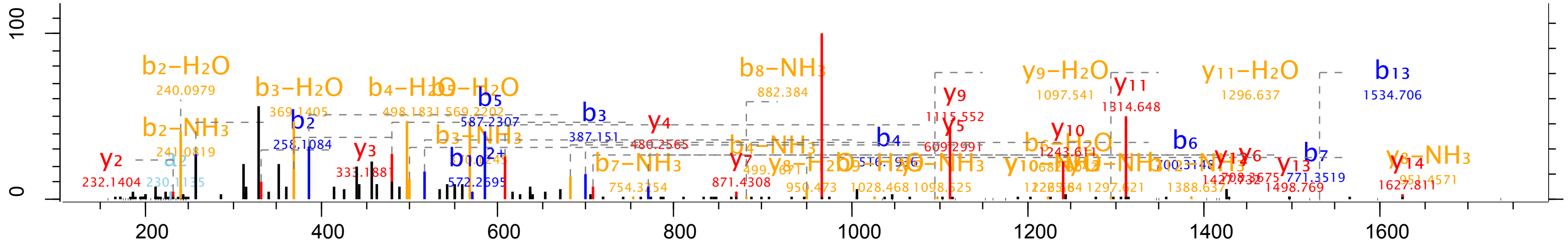

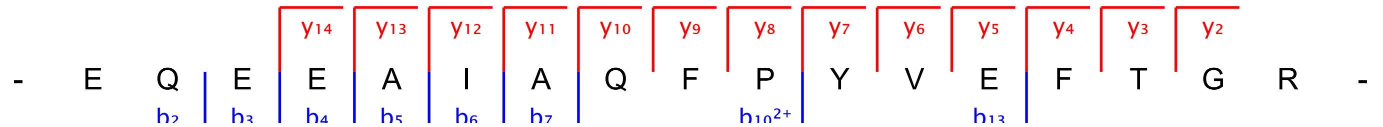

Raw file	Scan	Method	Score	m/z	Gene names
20140918_fract18_dyn_5ul_F2_01_386	9805	TOF; CID	91.96	457.24	ZFP62

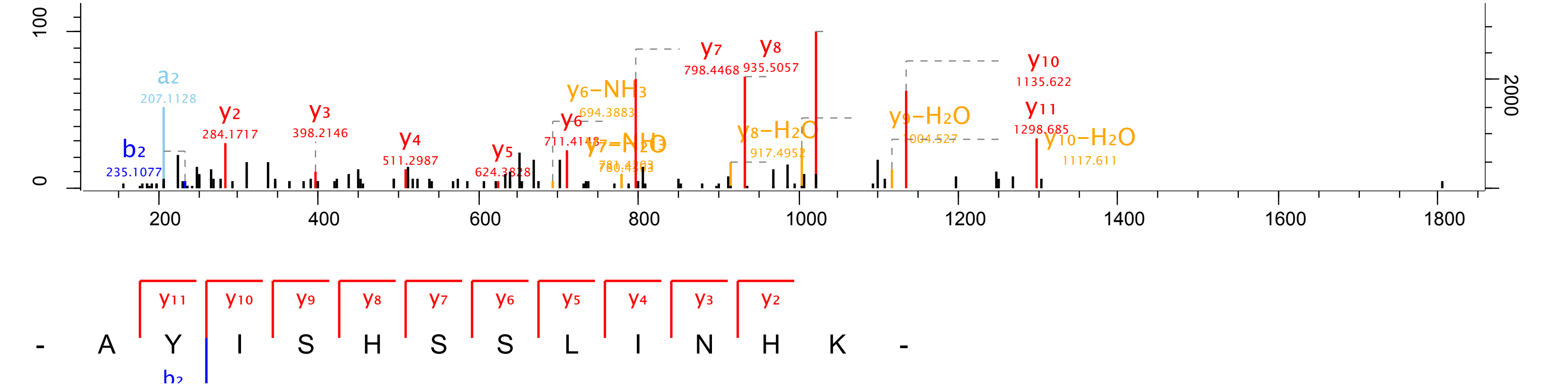

Raw file	Scan	Method	Score	m/z	Gene names
20140918_fract18_dyn_5ul_F2_01_386	10641	TOF; CID	70.94	583.3	PRDM15;ZNF298

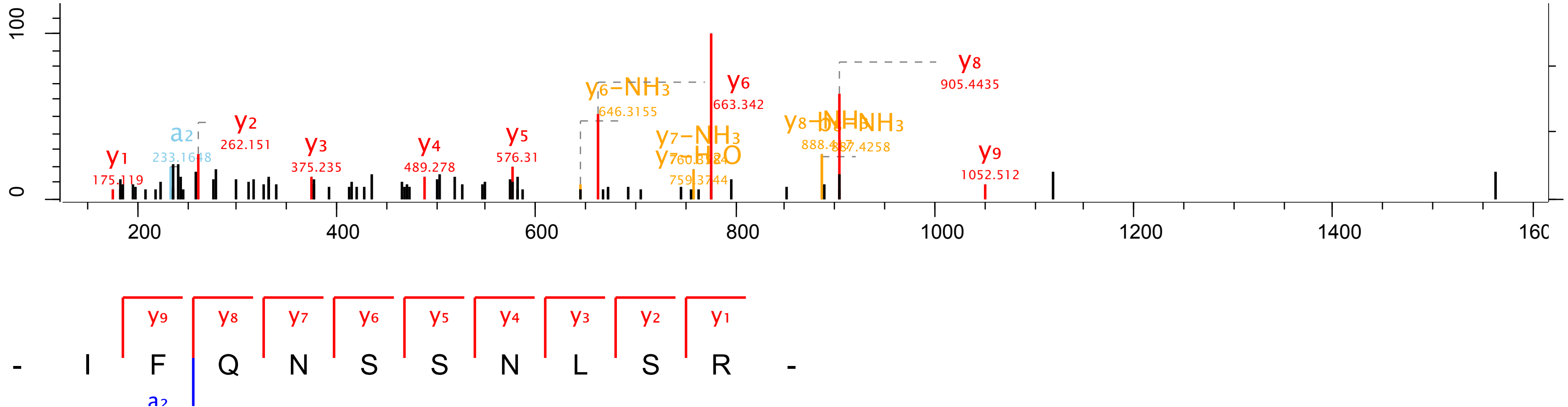

Raw file	Scan	Method	Score	m/z	Gene names
20140918_fract18_dyn_5ul_F2_01_386	12792	TOF; CID	120.65	407.24	MYO1G

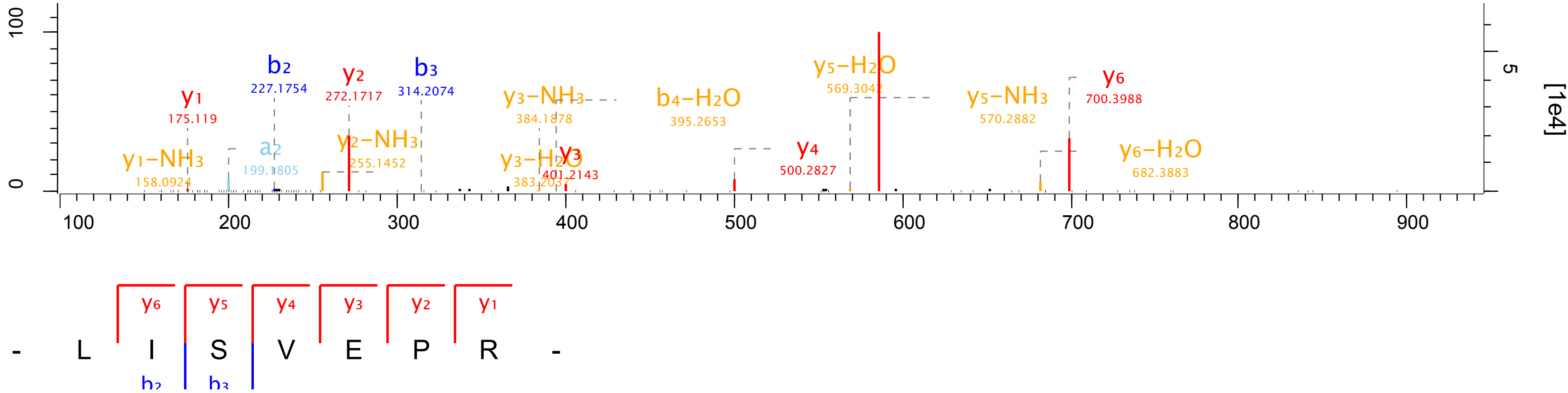

Raw file	Scan	Method	Score	m/z	Gene names
20140918_fract18_dyn_5ul_F2_01_386	15134	TOF; CID	73.83	558.61	BCL2L11

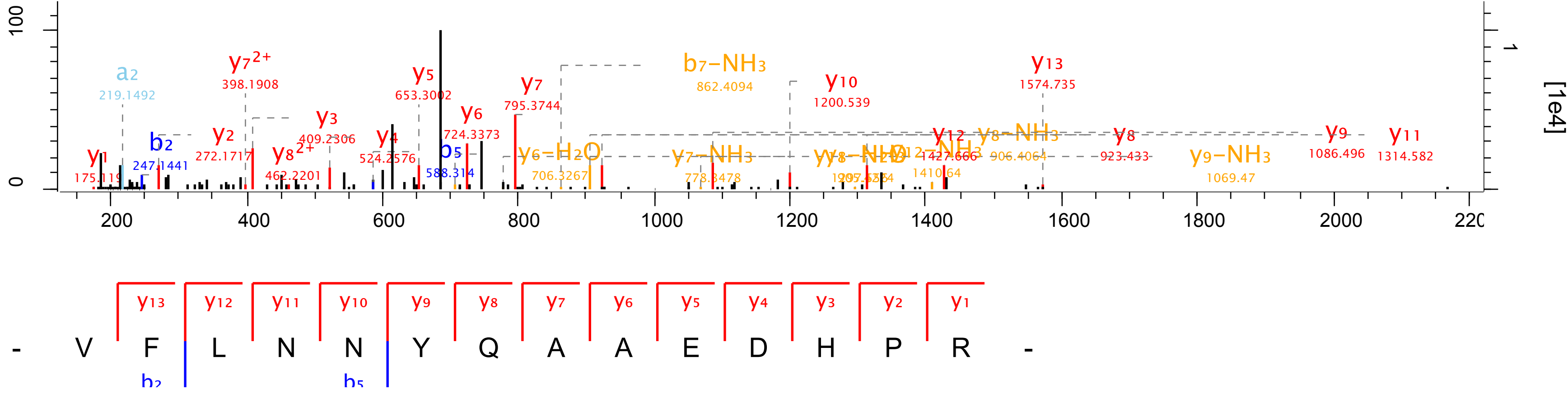

Raw file	Scan	Method	Score	m/z	Gene names
20140918_fract18_dyn_5ul_F2_01_386	15567	TOF; CID	56.49	736.4	TTC8

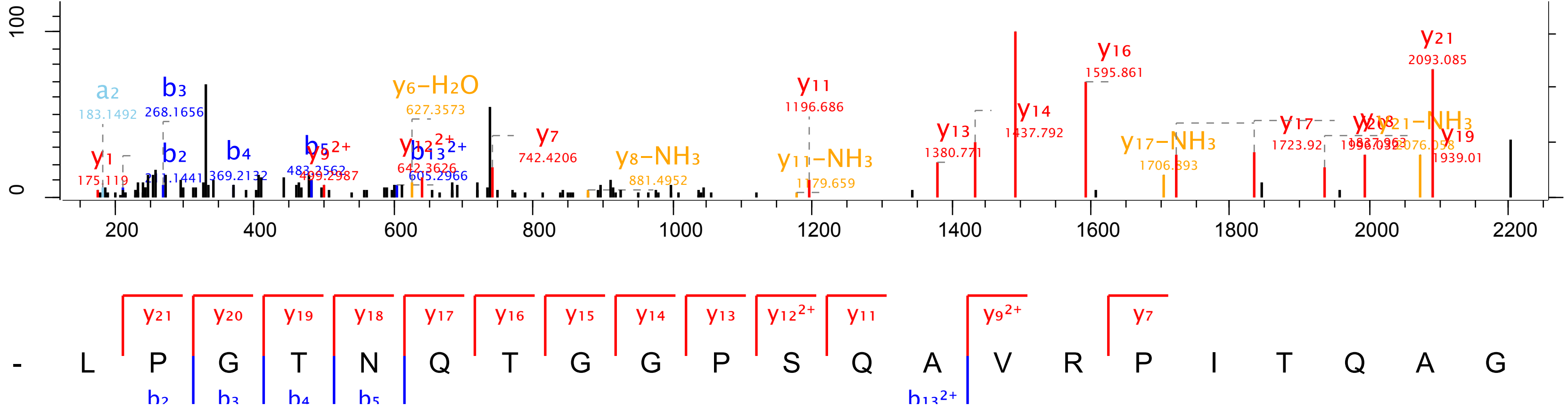

Raw file	Scan	Method	Score	m/z	Gene names
20140918_fract18_dyn_5ul_F2_01_386	17566	TOF; CID	93.42	552.28	HECA

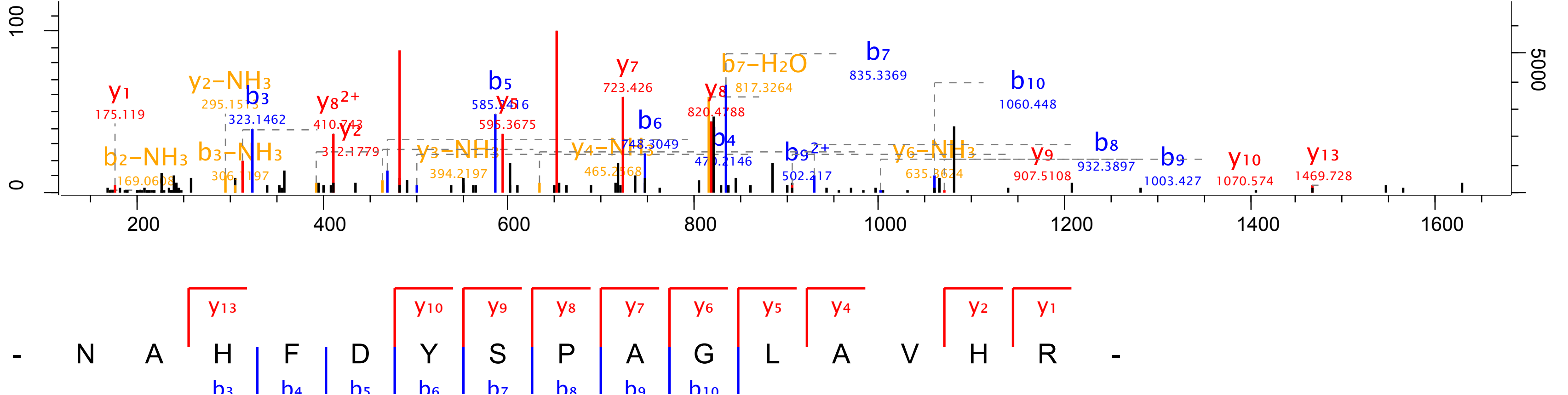

Raw file	Scan	Method	Score	m/z	Gene names
20140918_fract18_dyn_5ul_F2_01_386	18760	TOF; CID	81.92	553.8	C19orf43

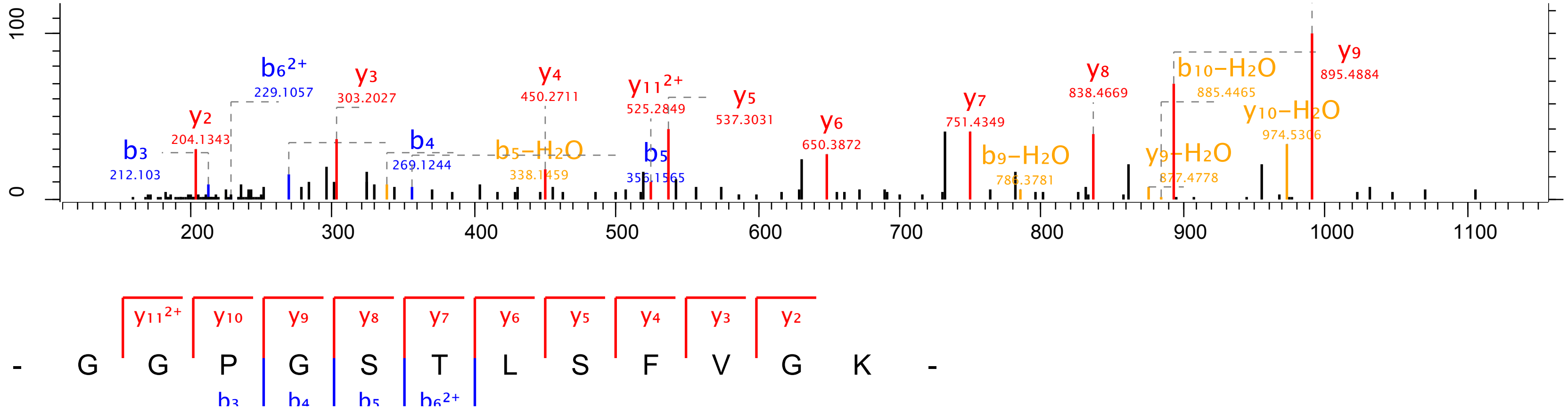

Raw file	Scan	Method	Score	m/z	Gene names
20140918_fract18_dyn_5ul_F2_01_386	19576	TOF; CID	72.85	436.91	LAMB2

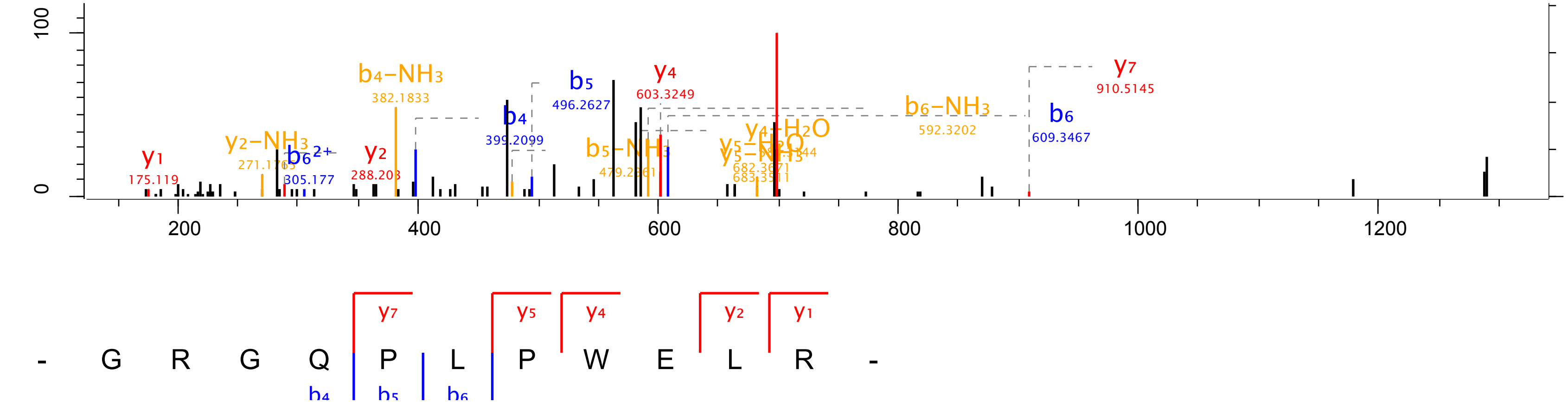

Raw file	Scan	Method	Score	m/z	Gene names
20140918_fract18_dyn_5ul_F2_01_386	21444	TOF; CID	63.38	868.38	KREMEN2

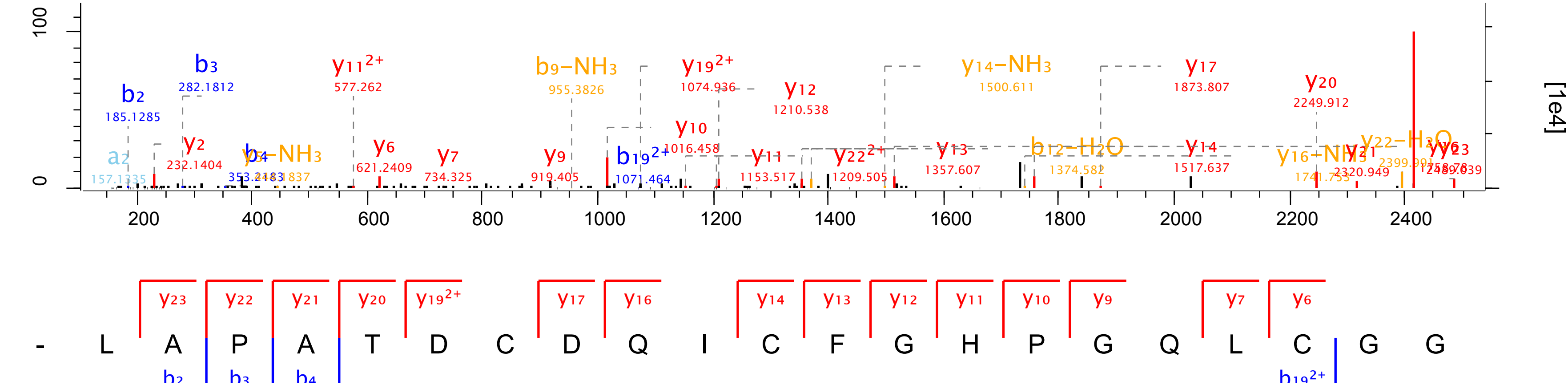

Raw file	Scan	Method	Score	m/z	Gene names
20140918_fract18_dyn_5ul_F2_01_386	22470	TOF; CID	48.91	948.95	FAM168B

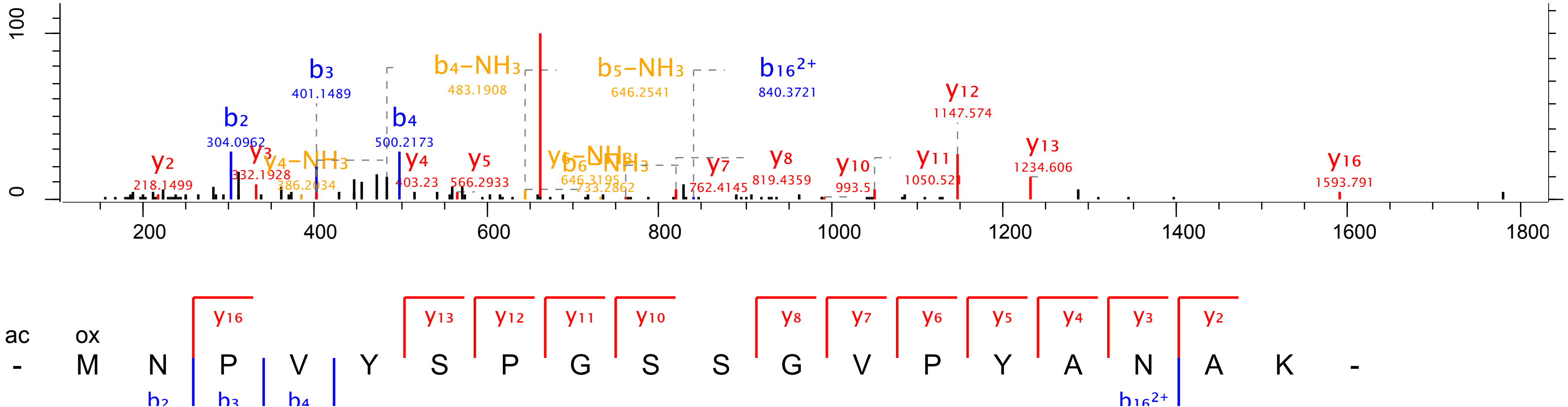

Raw file	Scan	Method	Score	m/z	Gene names
20140918_fract18_dyn_5ul_F2_01_386	23050	TOF; CID	108.09	506.24	PLGRKT

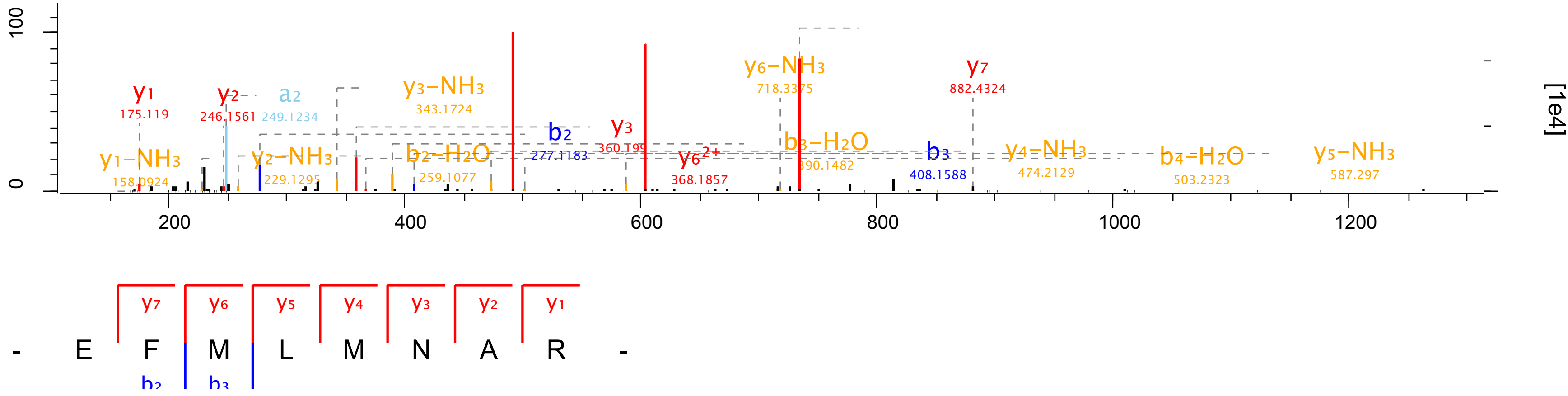

Raw file	Scan	Method	Score	m/z	Gene names
20140918_fract18_dyn_5ul_F2_01_386	23633	TOF; CID	90.71	567.31	TSPAN9

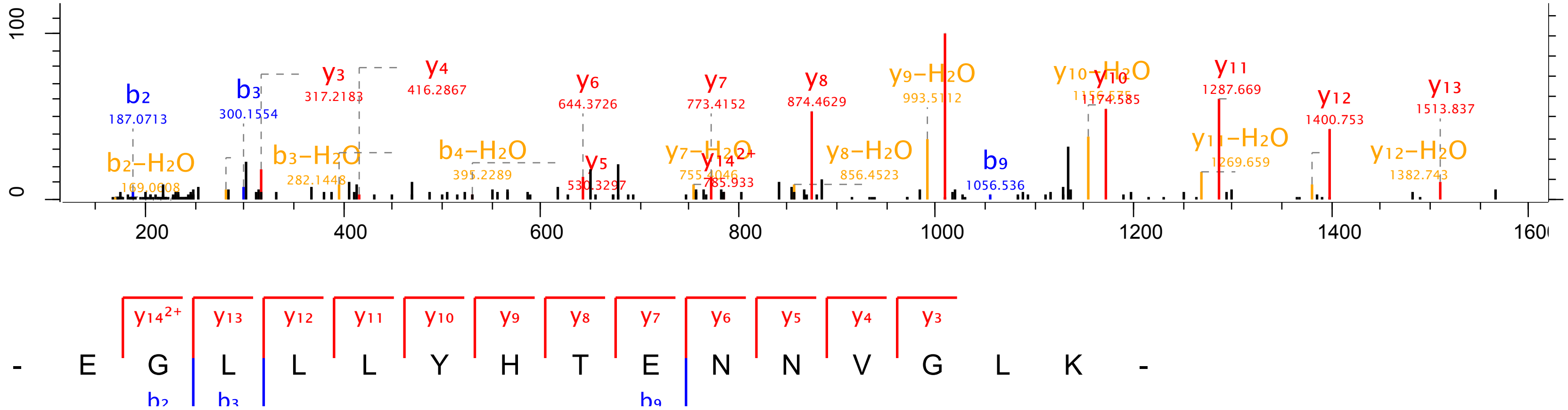

Raw file	Scan	Method	Score	m/z	Gene names
20140918_fract18_dyn_5ul_F2_01_386	25409	TOF; CID	61.48	925.46	PPDPF

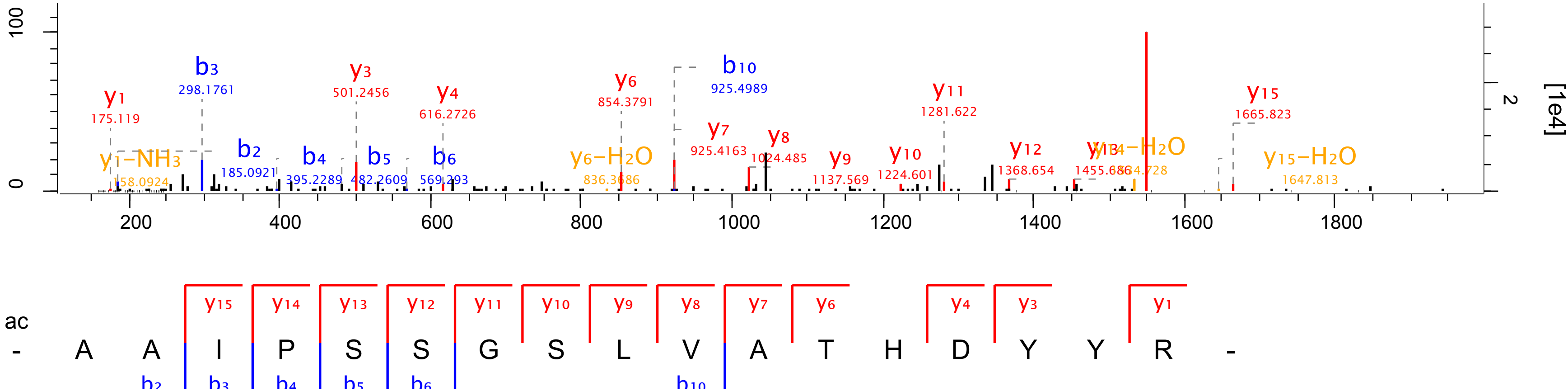

Raw file	Scan	Method	Score	m/z	Gene names
20140918_fract18_dyn_5ul_F2_01_386	27114	TOF; CID	49.34	676.88	BARX1

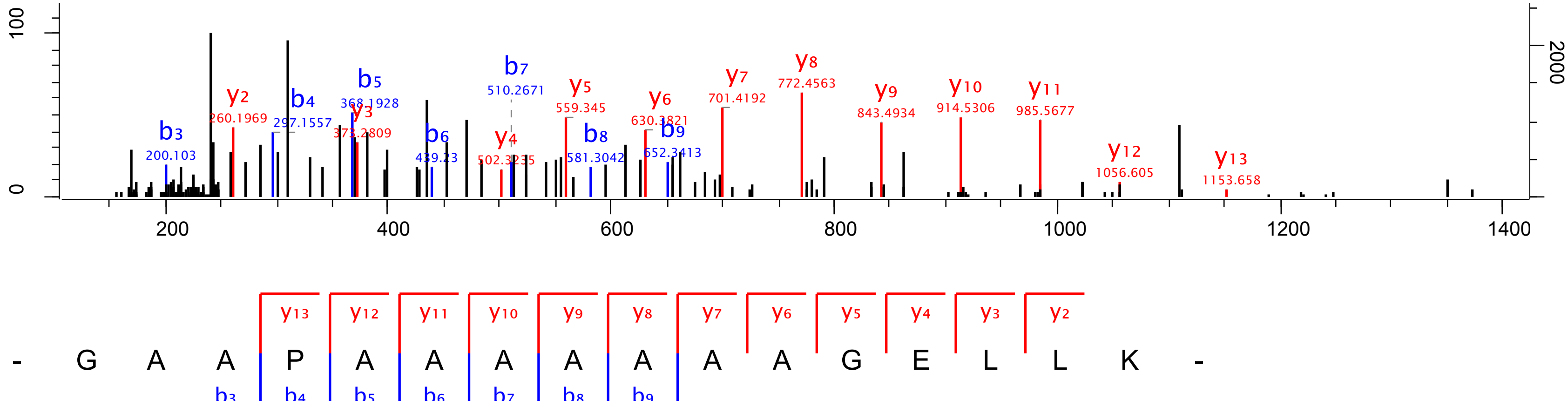

Raw file	Scan	Method	Score	m/z	Gene names
20140918_fract18_dyn_5ul_F2_01_386	27291	TOF; CID	55.55	830.4	C11orf74

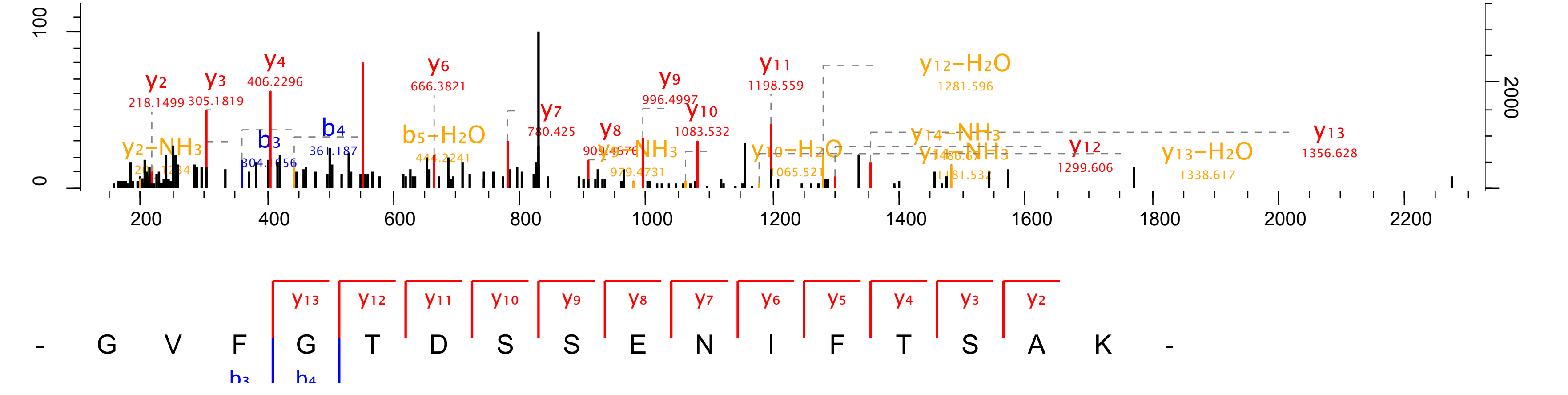

Raw file	Scan	Method	Score	m/z	Gene names
20140918_fract18_dyn_5ul_F2_01_386	29698	TOF; CID	103.42	1101.17	CEBPG

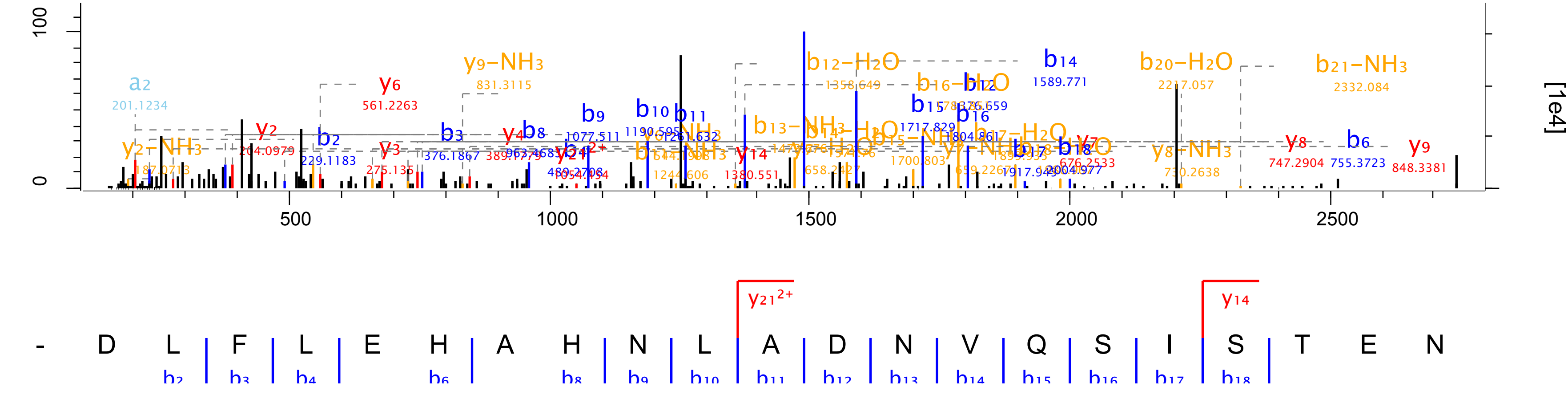

Raw file	Scan	Method	Score	m/z	Gene names
20140918_fract18_dyn_5ul_F2_01_386	29705	TOF; CID	28.43	688.34	EIF4EBP1

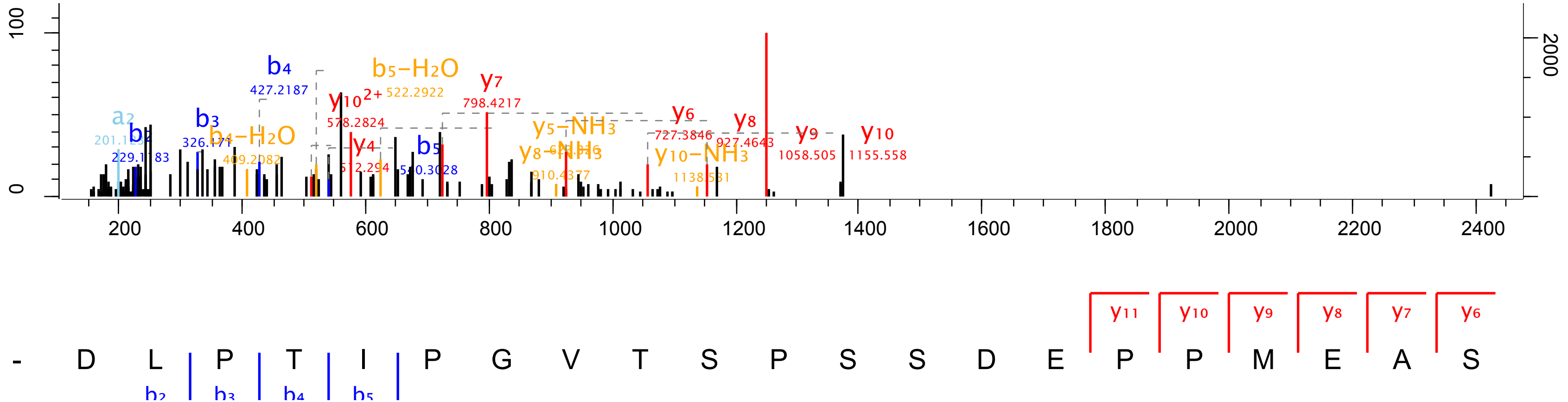

Raw file	Scan	Method	Score	m/z	Gene names
20140918_fract18_dyn_5ul_F2_01_386	30818	TOF; CID	70.94	633.81	BACE1

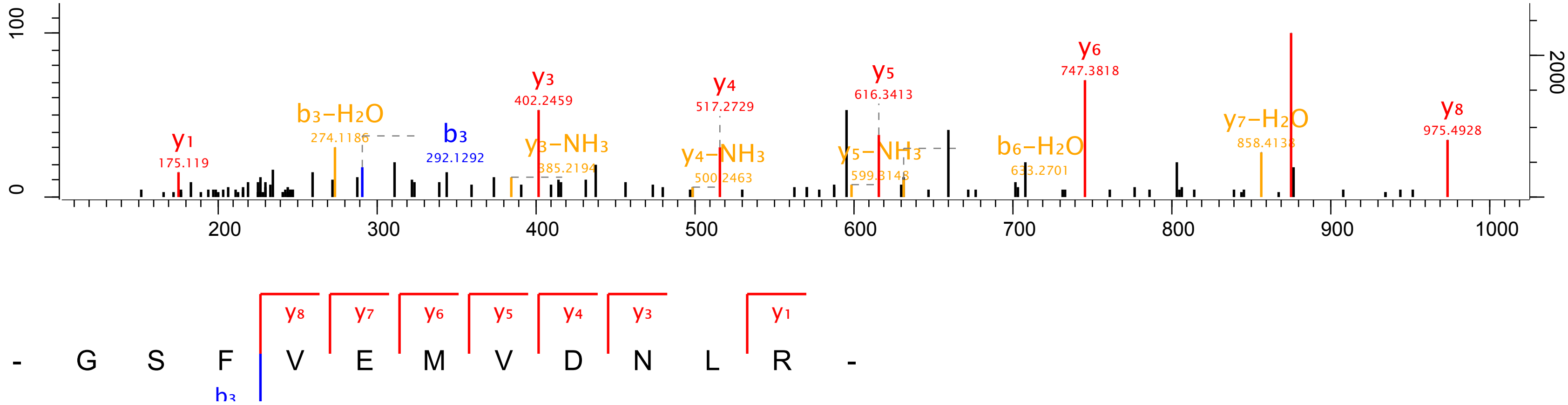

Raw file	Scan	Method	Score	m/z	Gene names
20140918_fract18_dyn_5ul_F2_01_386	32742	TOF; CID	85.56	968	EFHC1

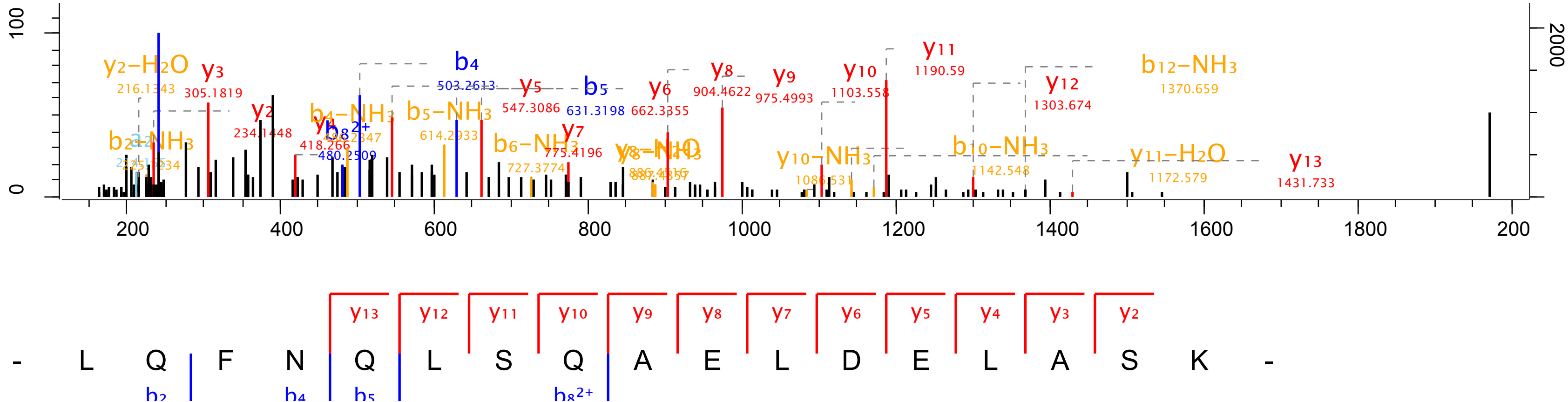

Raw file	Scan	Method	Score	m/z	Gene names
20140918_fract18_dyn_5ul_F2_01_386	34286	TOF; CID	70.2	808.43	NAA60

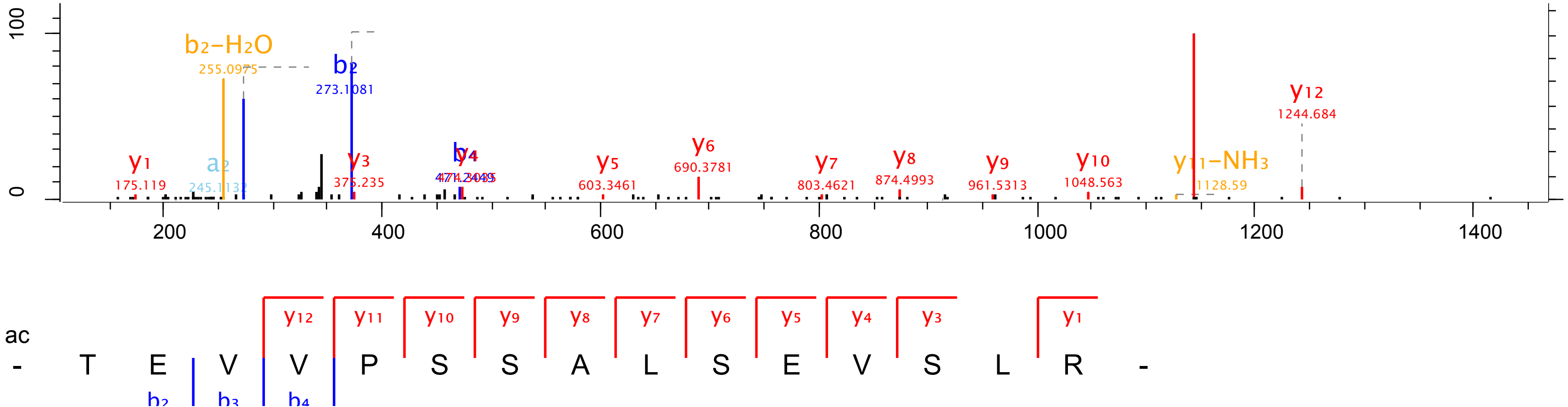

Raw file	Scan	Method	Score	m/z	Gene names
20140918_fract18_dyn_5ul_F2_01_386	34809	TOF; CID	76.12	748.38	PTPN14

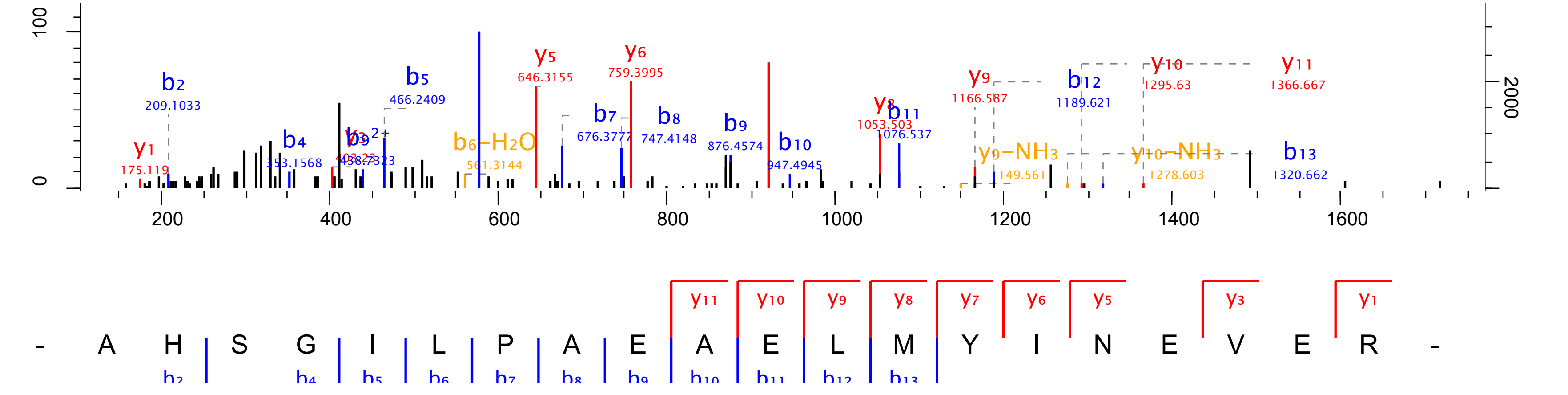

Raw file	Scan	Method	Score	m/z	Gene names
20140918_fract18_dyn_5ul_F2_01_386	35229	TOF; CID	77.78	742.89	AURKAIP1

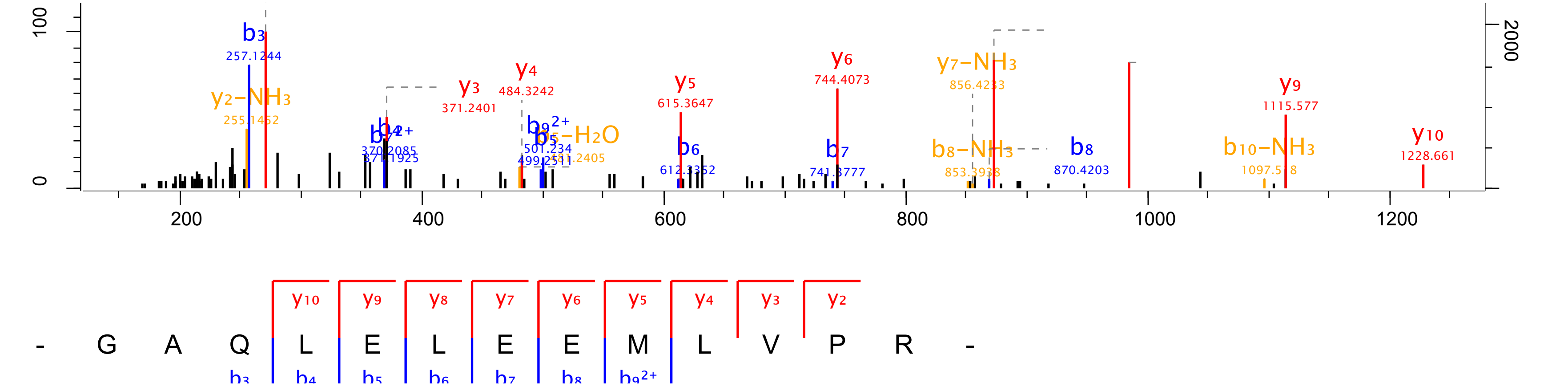

Raw file	Scan	Method	Score	m/z	Gene names
20140918_fract18_dyn_5ul_F2_01_386	36870	TOF; CID	76.97	1015.17	ADIRF

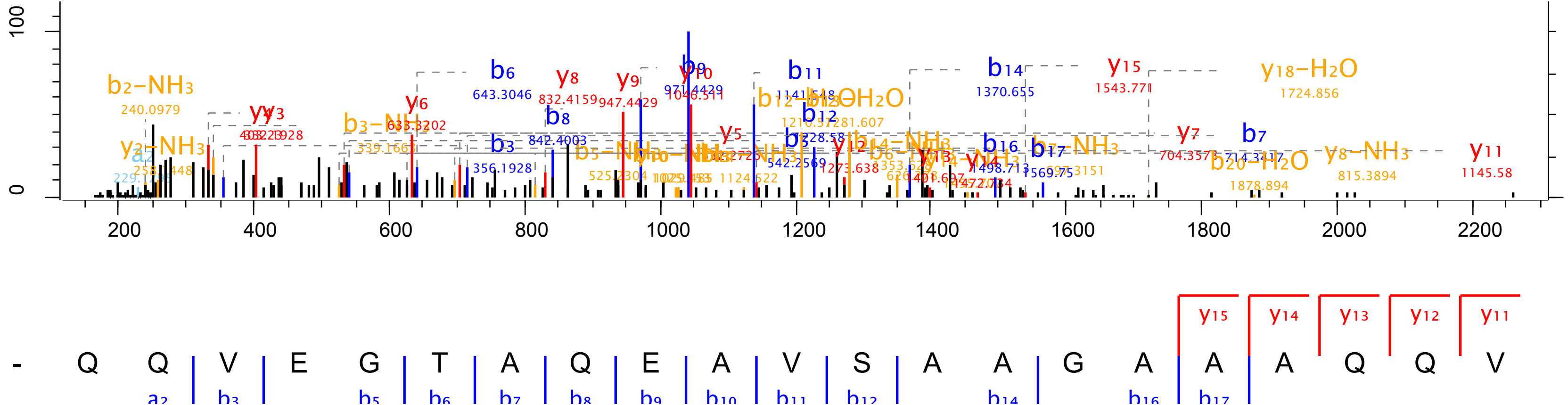

Raw file	Scan	Method	Score	m/z	Gene names
20140918_fract19_dyn_5ul_F3_01_387	15790	TOF; CID	88.5	499.8	DNAJB14

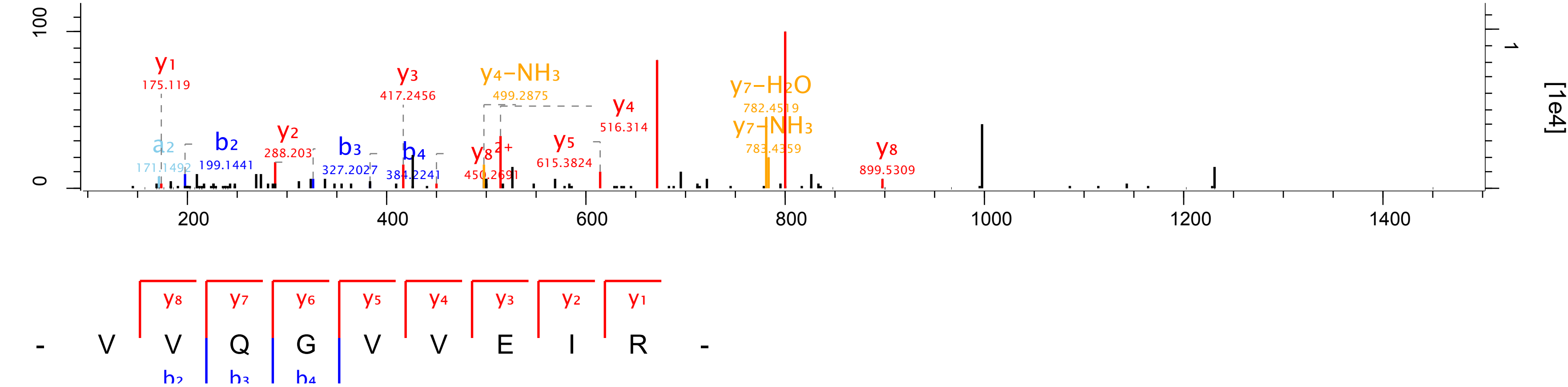

Raw file	Scan	Method	Score	m/z
20140918_fract19_dyn_5ul_F3_01_387	17028	TOF; CID	90.79	631.82

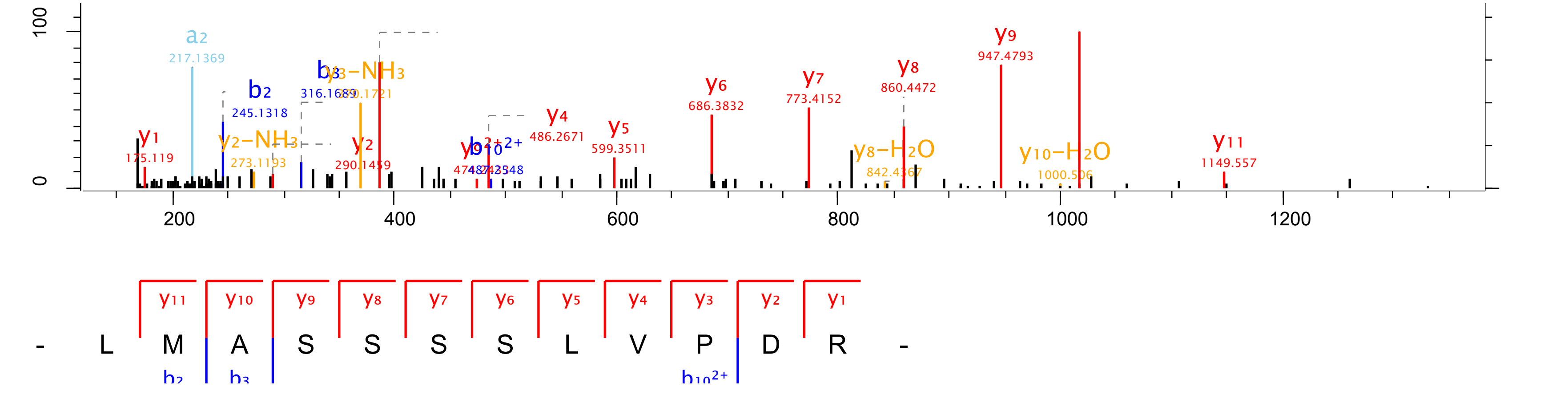

Raw file	Scan	Method	Score	m/z	Gene names
20140918_fract19_dyn_5ul_F3_01_387	18389	TOF; CID	147.52	476.24	MAPK15

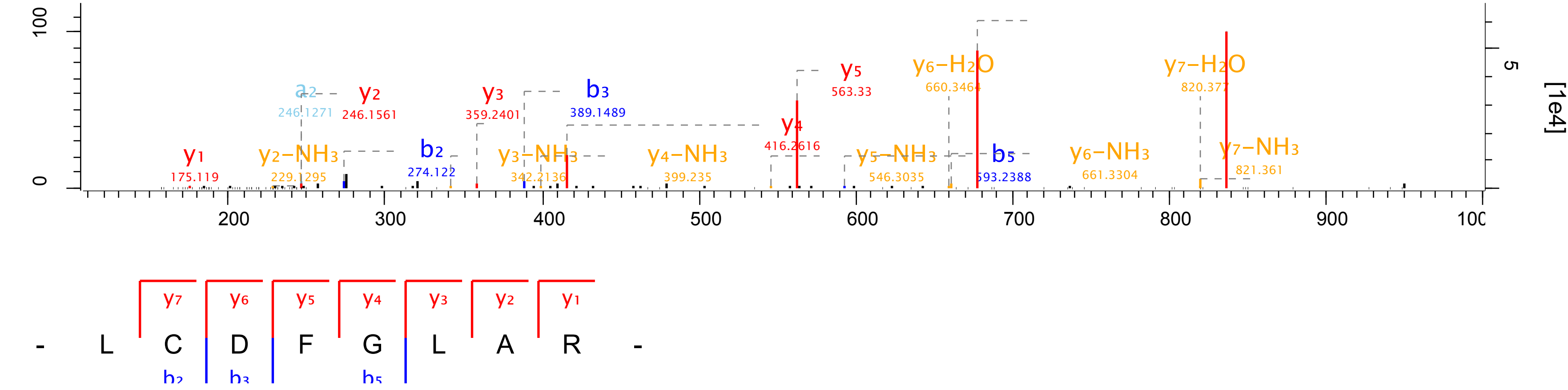

Raw file	Scan	Method	Score	m/z	Gene names
20140918_fract19_dyn_5ul_F3_01_387	19285	TOF; CID	95.41	731.36	ZDHHC24

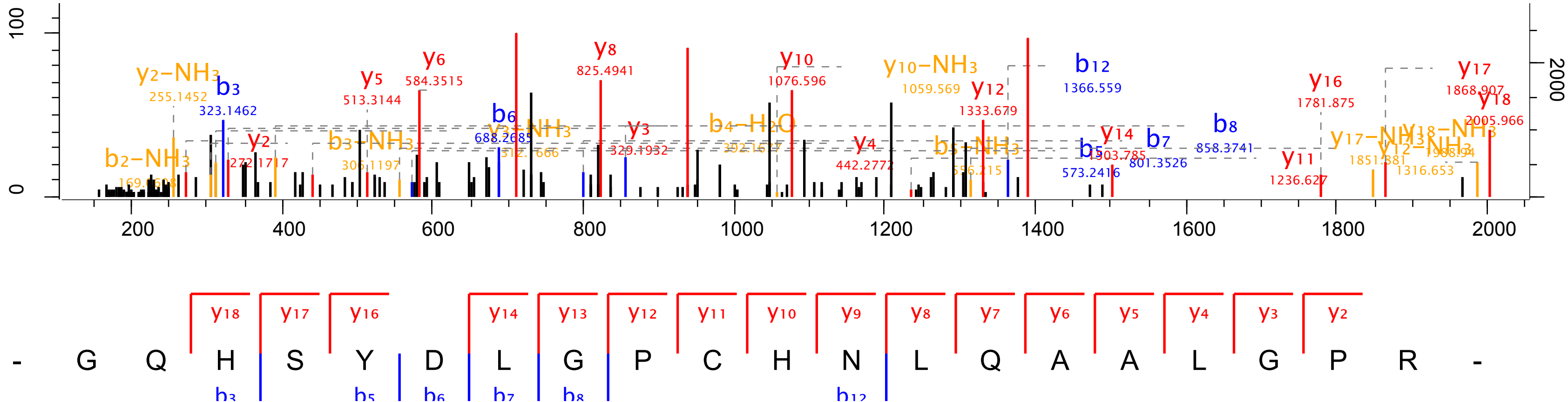

Raw file	Scan	Method	Score	m/z	Gene names
20140918_fract19_dyn_5ul_F3_01_387	19929	TOF; CID	78.15	559.76	ZNF77

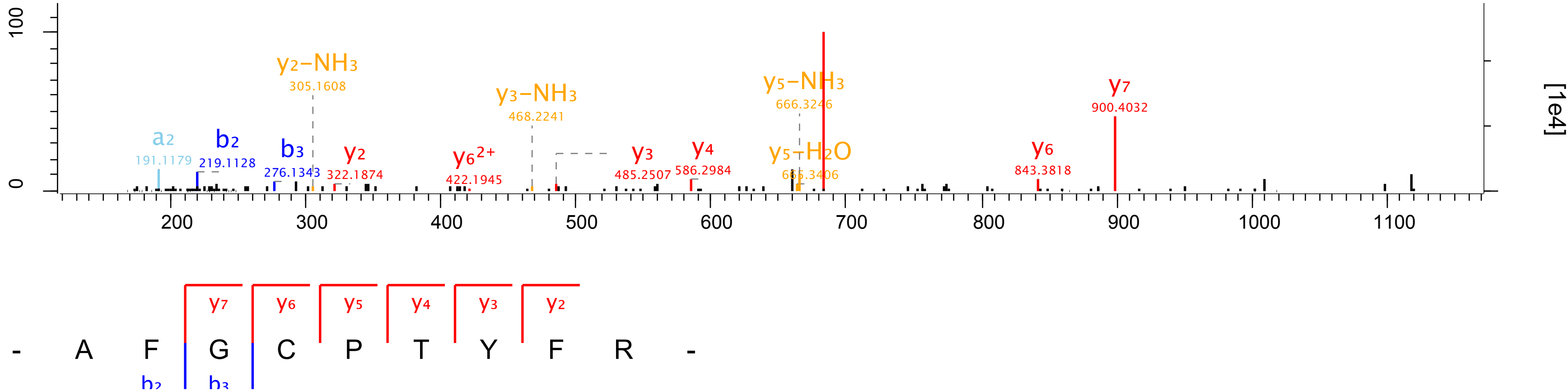

Raw file	Scan	Method	Score	m/z	Gene names
20140918_fract19_dyn_5ul_F3_01_387	24337	TOF; CID	79.09	771.36	FBXO17

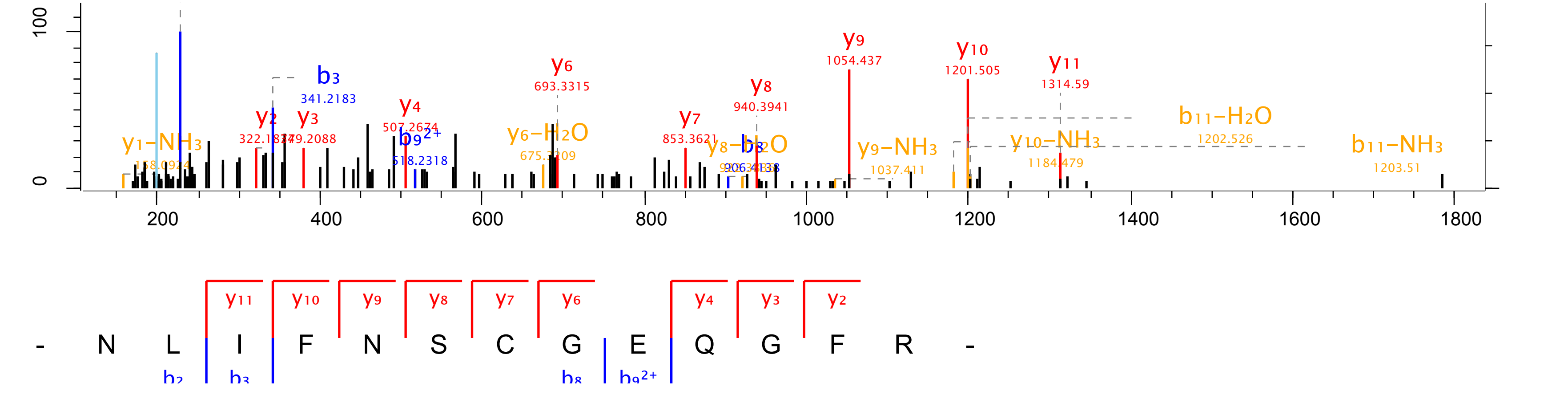

Raw file	Scan	Method	Score	m/z	Gene names
20140918_fract19_dyn_5ul_F3_01_387	34988	TOF; CID	83.42	1091.52	CDKN2AIPNL

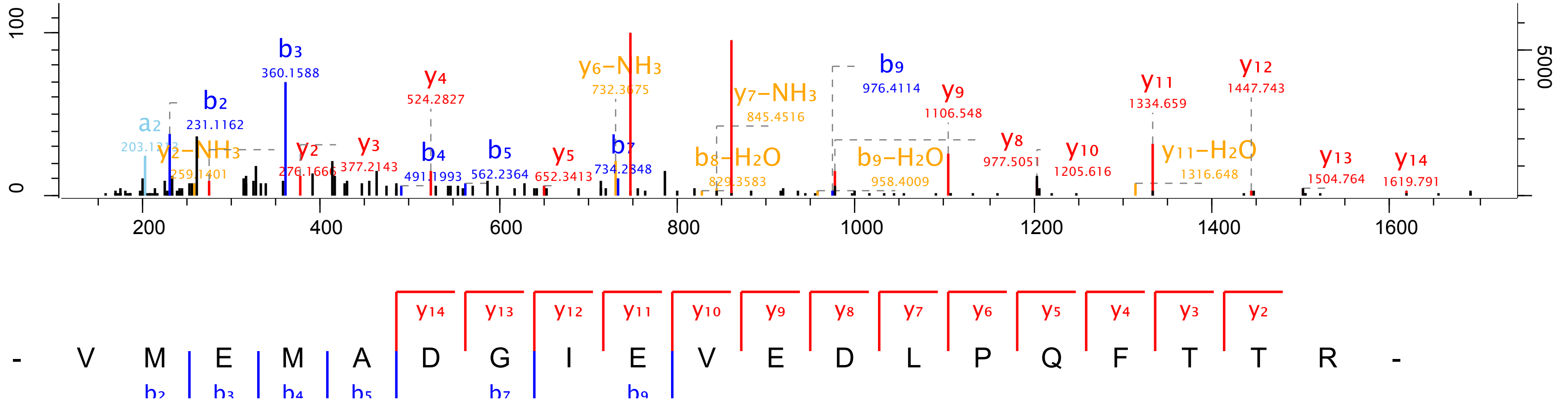

Raw file	Scan	Method	Score	m/z	Gene names
20140918_fract19_dyn_5ul_F3_01_387	37851	TOF; CID	75.76	565.02	SMIM11

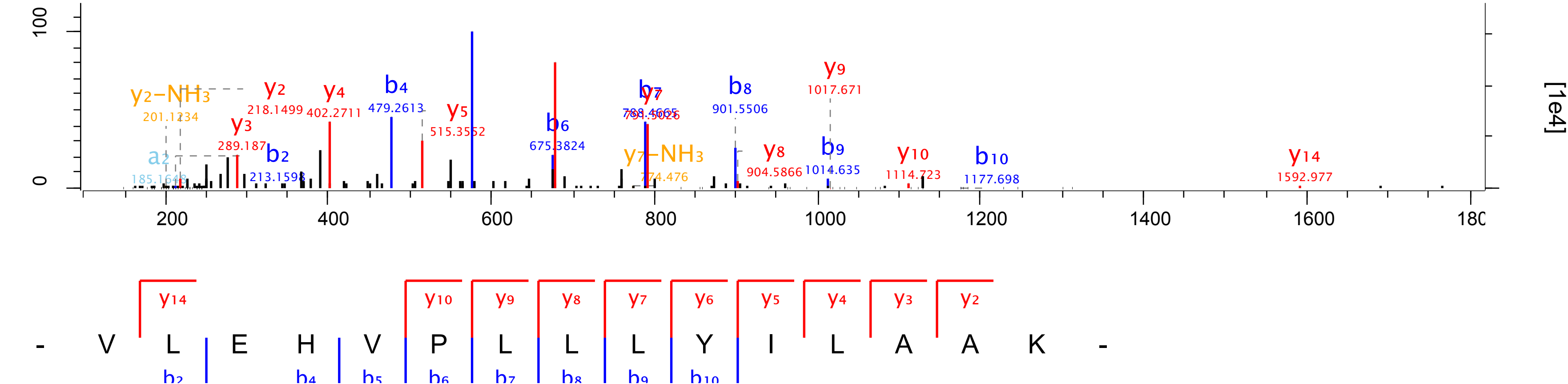

Raw file	Scan	Method	Score	m/z	Gene names
20140918_fract20_dyn_5ul_F4_01_388	10698	TOF; CID	108.09	495.28	ARID3B

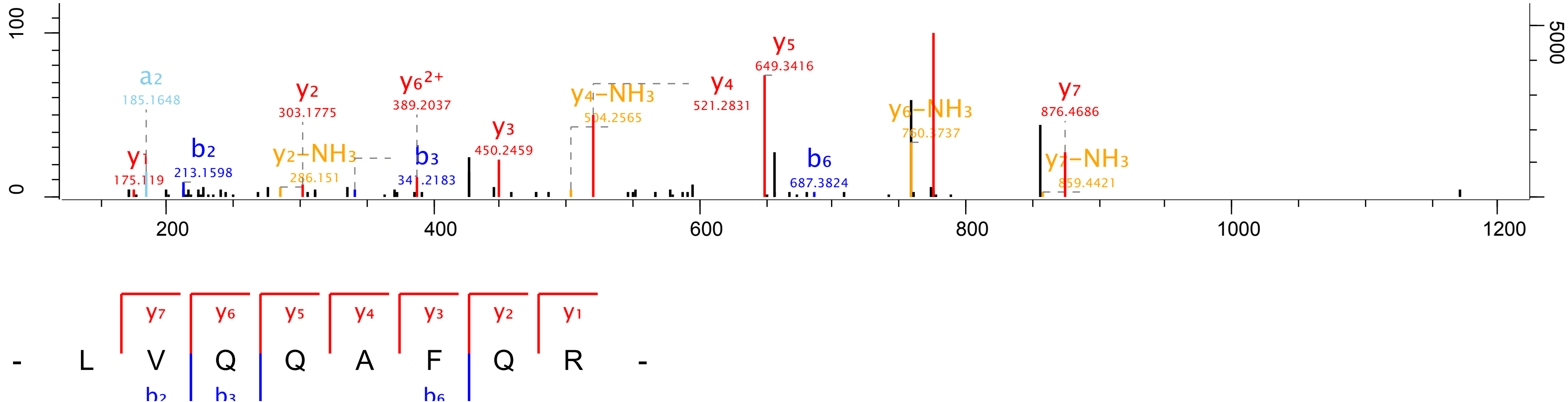

Raw file	Scan	Method	Score	m/z	Gene names
20140918_fract20_dyn_5ul_F4_01_388	16067	TOF; CID	93.18	505.78	NAIF1

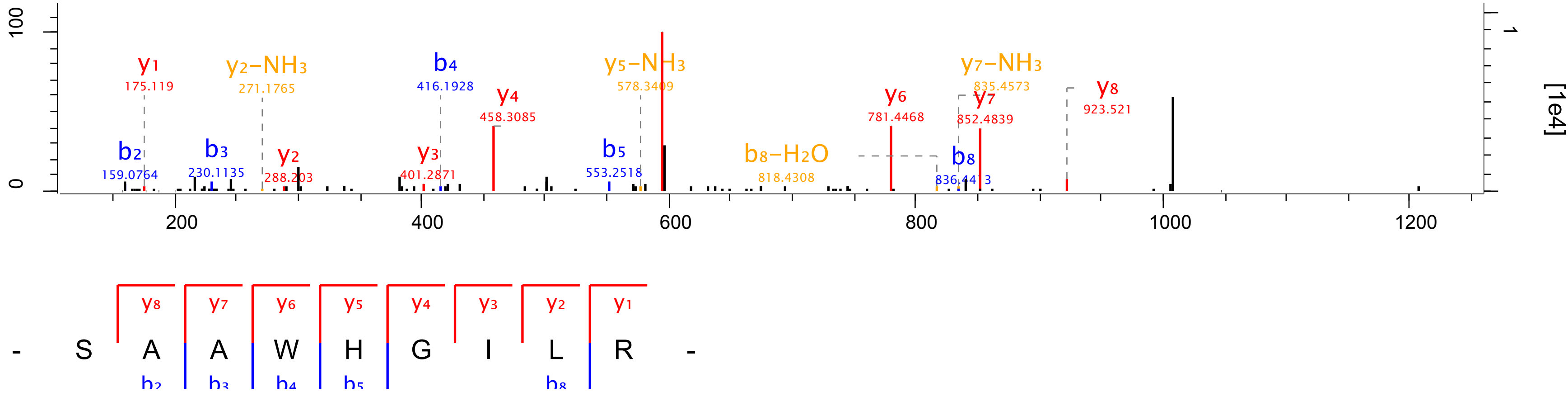

Raw file	Scan	Method	Score	m/z	Gene names
20140918_fract20_dyn_5ul_F4_01_388	17487	TOF; CID	30.94	624.83	ZNF831

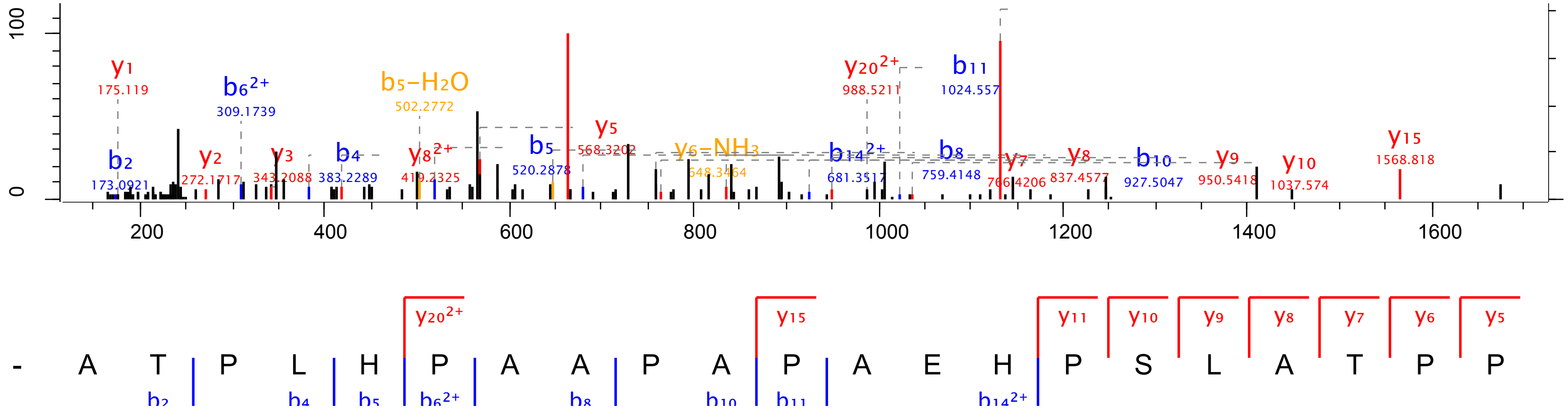

Raw file	Scan	Method	Score	m/z	Gene names
20140918_fract20_dyn_5ul_F4_01_388	20434	TOF; CID	90.71	476.26	TPCN2

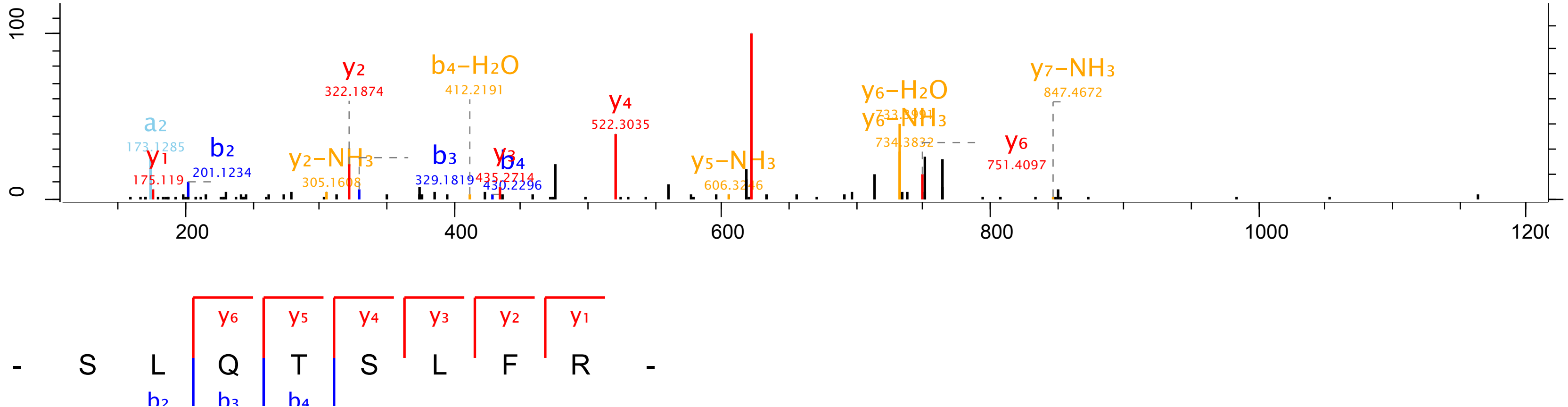

Raw file	Scan	Method	Score	m/z	Gene names
20140918_fract20_dyn_5ul_F4_01_388	21256	TOF; CID	90.83	614.86	C2orf68

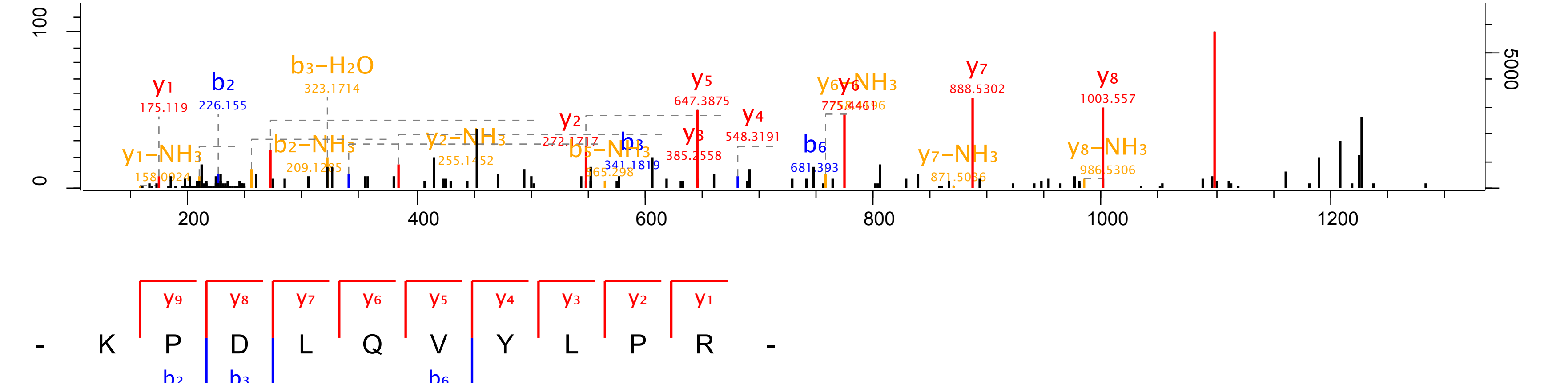

Raw file	Scan	Method	Score	m/z	Gene names
20140918_fract20_dyn_5ul_F4_01_388	23570	TOF; CID	65.18	680.31	C16orf87

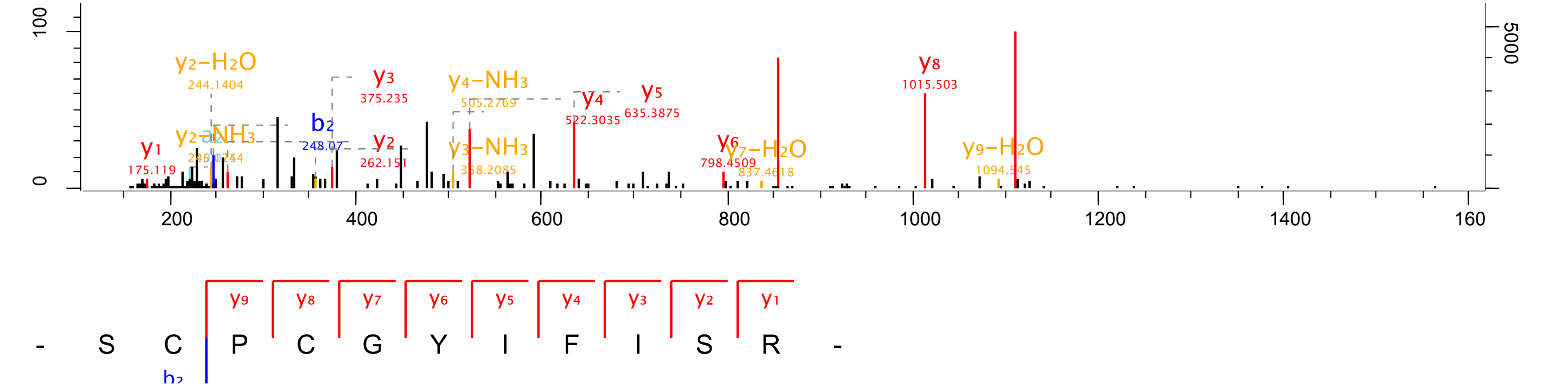

Raw file	Scan	Method	Score	m/z	Gene names
20140918_fract20_dyn_5ul_F4_01_388	27036	TOF; CID	91.59	718.05	DTD2

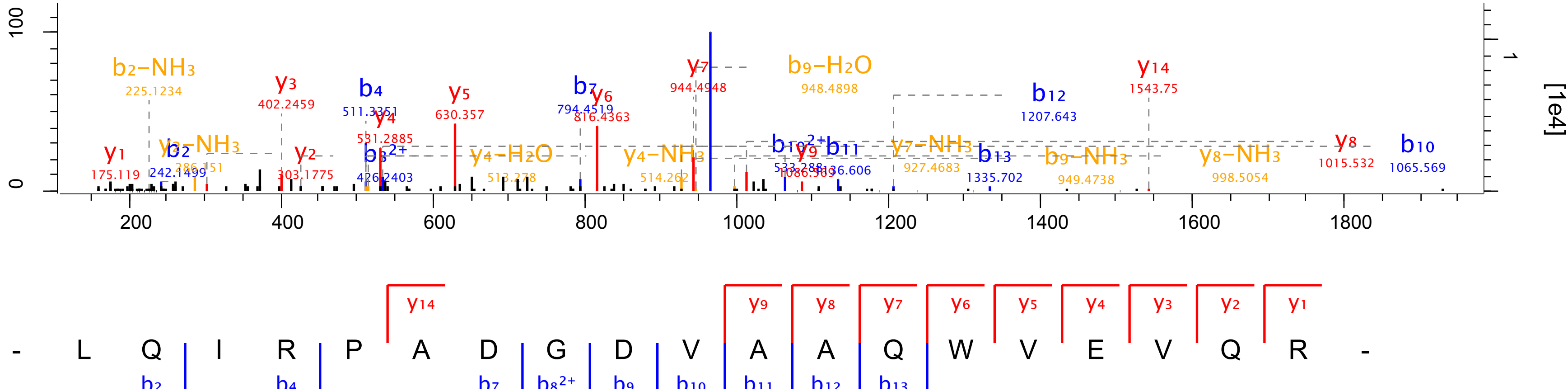

Raw file	Scan	Method	Score	m/z	Gene names
20140918_fract20_dyn_5ul_F4_01_388	27422	TOF; CID	94.12	493.24	CCDC82

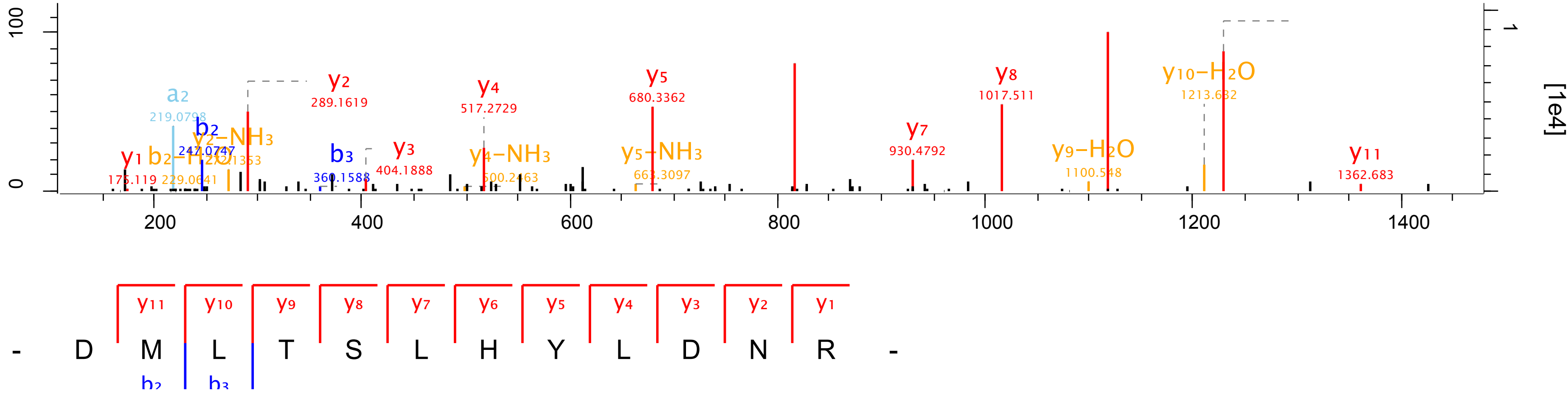

Raw file	Scan	Method	Score	m/z	Gene names
20140918_fract20_dyn_5ul_F4_01_388	30172	TOF; CID	95.57	622.31	C1QTNF6

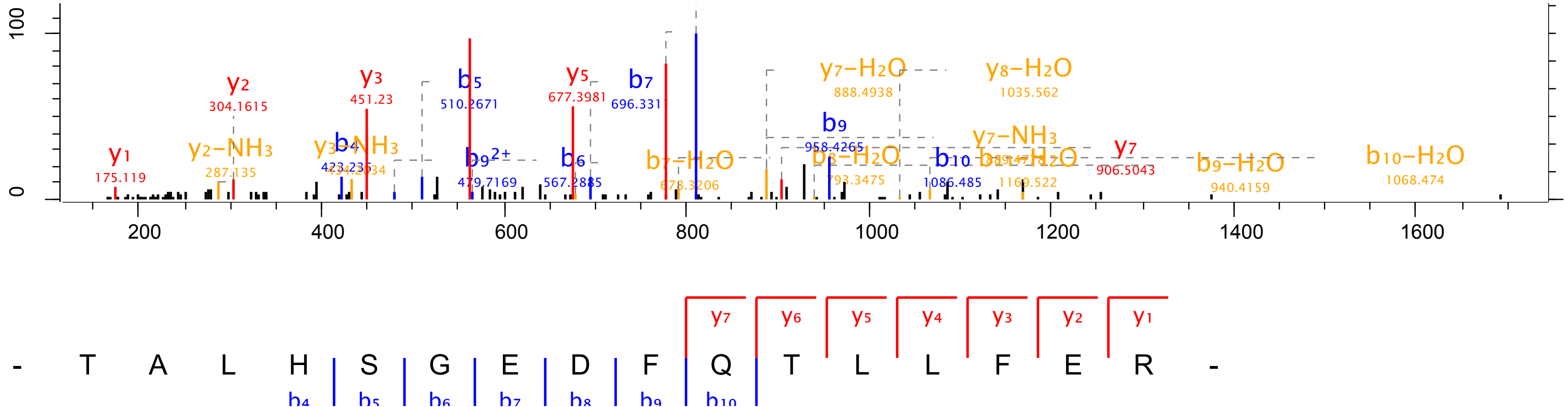

Raw file	Scan	Method	Score	m/z	Gene names
20140918_fract20_dyn_5ul_F4_01_388	35695	TOF; CID	41.51	1073.57	ULK4

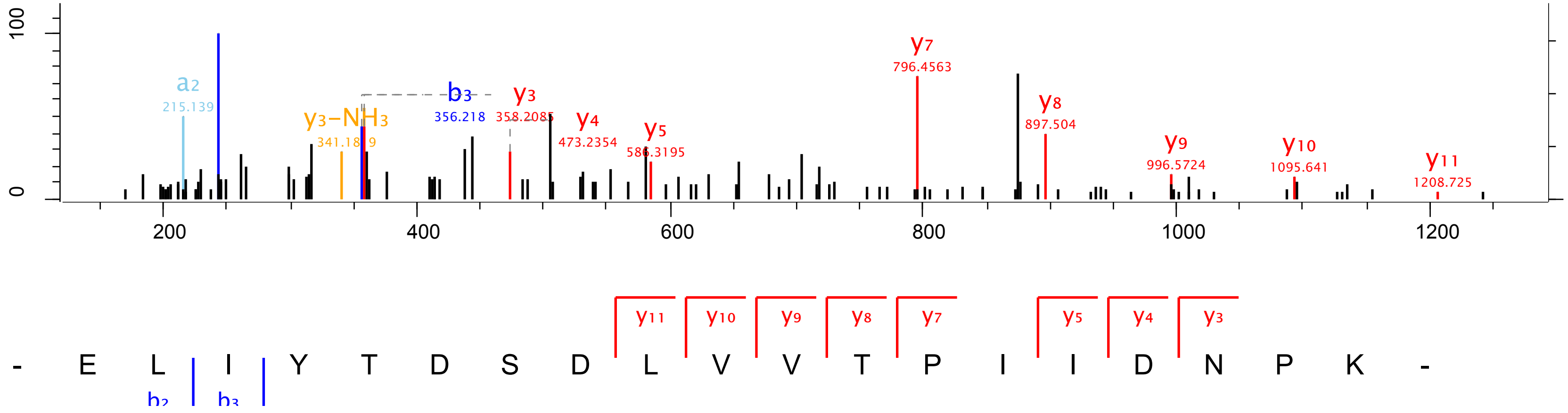

Raw file	Scan	Method	Score	m/z	Gene names
20140918_fract21_dyn_5ul_F5_01_389	19644	TOF; CID	65.04	627.36	C17orf97

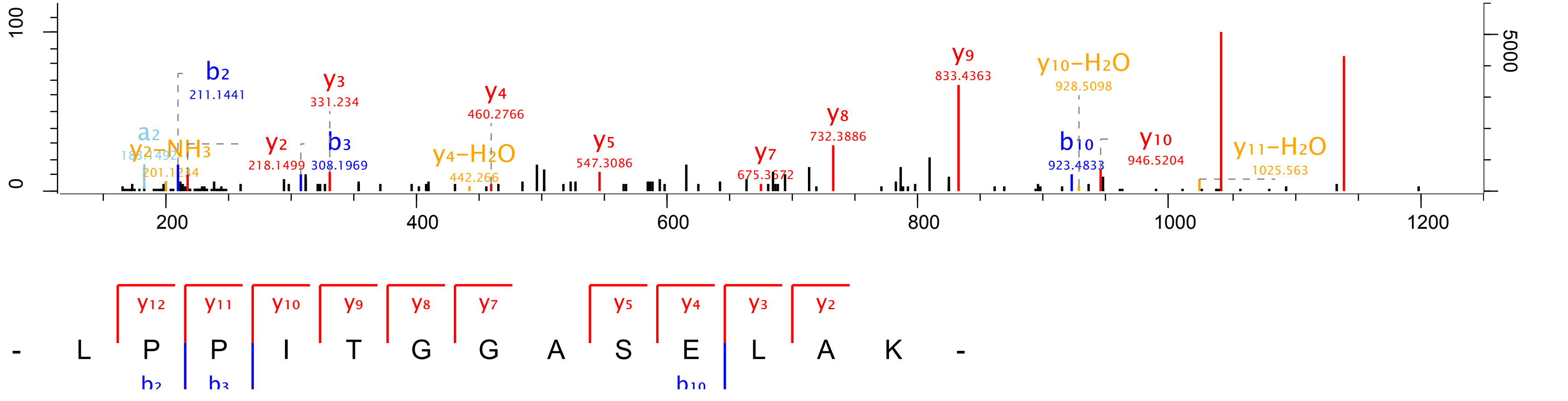

Raw file	Scan	Method	Score	m/z	Gene names
20140918_fract21_dyn_5ul_F5_01_389	19673	TOF; CID	39.68	771.05	RPUSD1

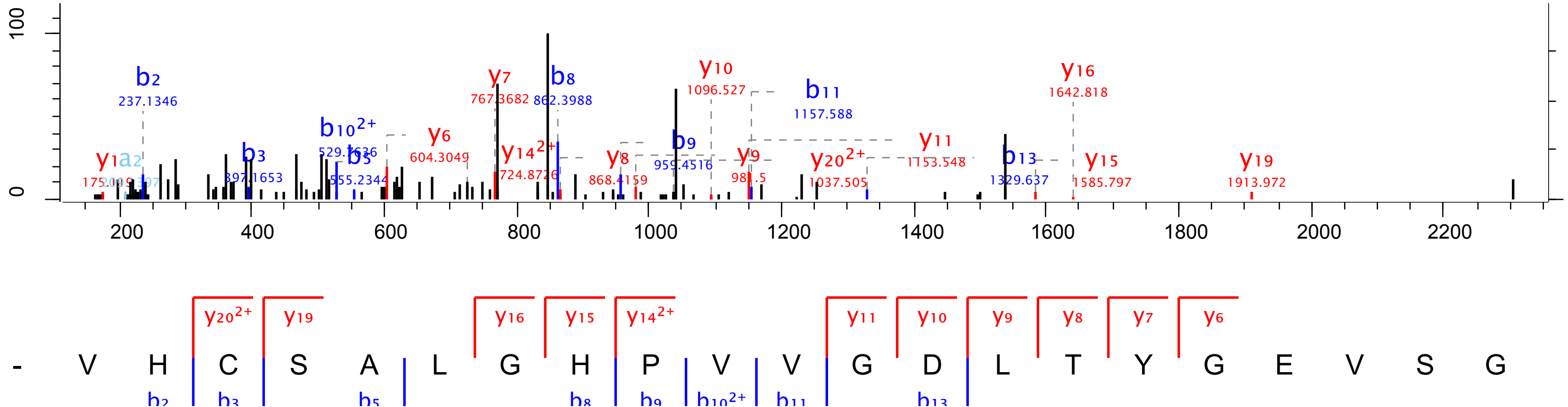

Raw file	Scan	Method	Score	m/z	Gene names
20140918_fract21_dyn_5ul_F5_01_389	20694	TOF; CID	51.03	749.39	TMEM67

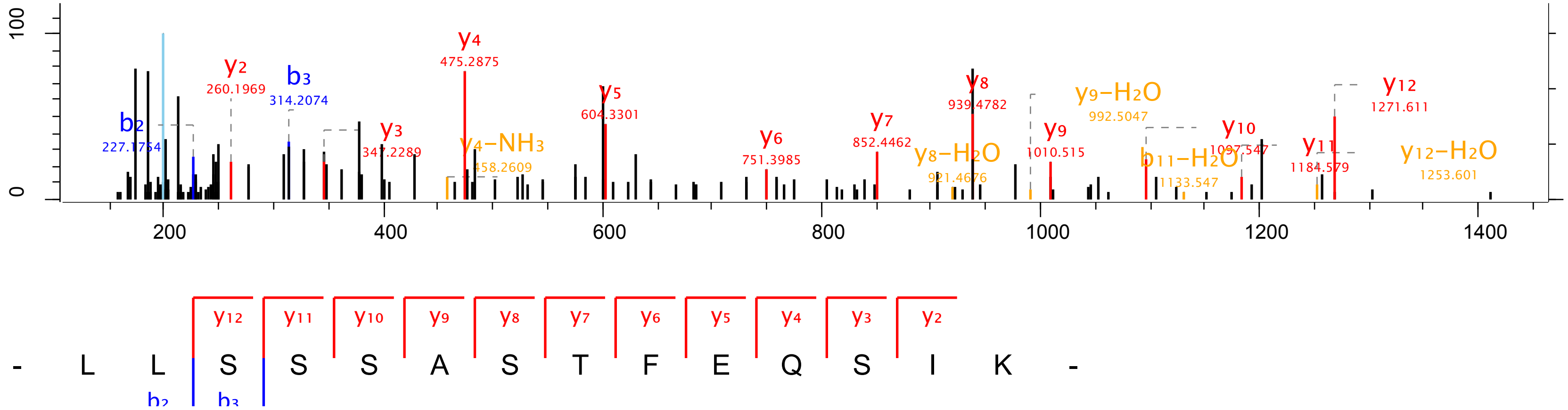

Raw file	Scan	Method	Score	m/z	Gene names
20140918_fract21_dyn_5ul_F5_01_389	22241	TOF; CID	69.77	999.01	LMO4

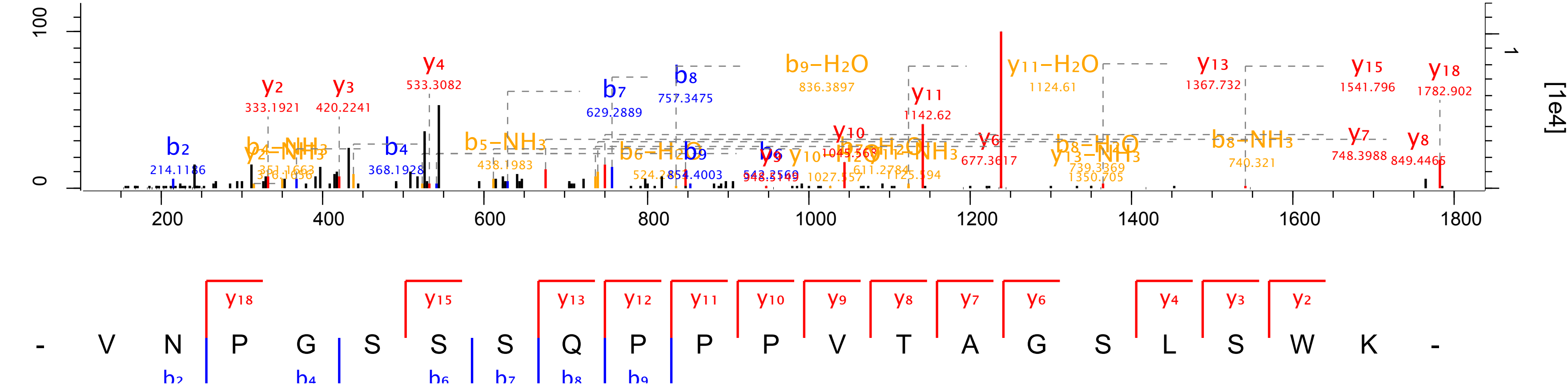

Raw file	Scan	Method	Score	m/z	Gene names
20140918_fract21_dyn_5ul_F5_01_389	23432	TOF; CID	91.81	544.29	FN1

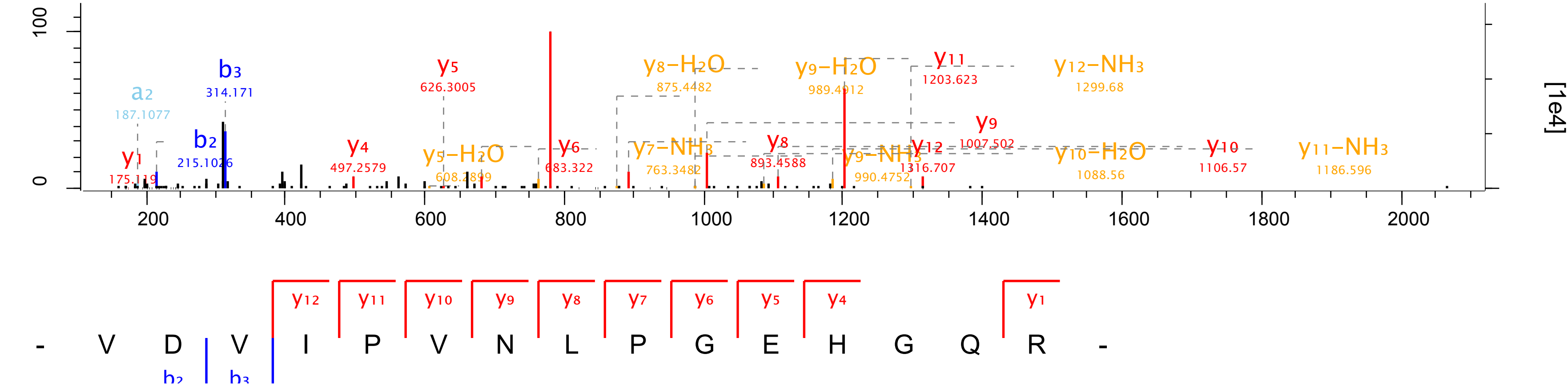

Raw file	Scan	Method	Score	m/z	Gene names
20140918_fract21_dyn_5ul_F5_01_389	24025	TOF; CID	52.48	741.03	ZBTB8A

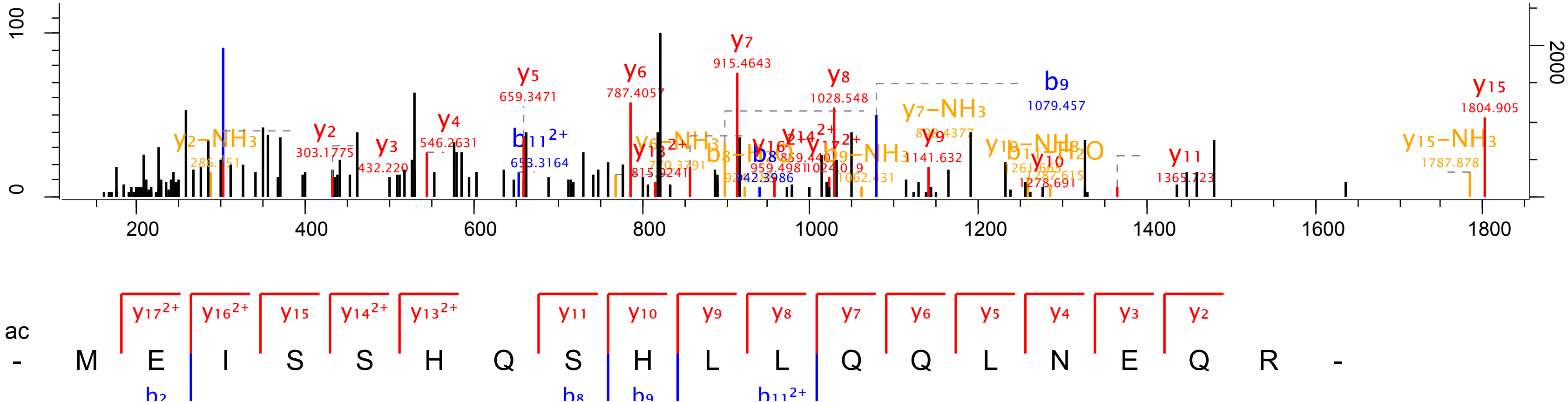

Raw file	Scan	Method	Score	m/z	Gene names
20140918_fract21_dyn_5ul_F5_01_389	24342	TOF; CID	109.11	486.29	LGI1

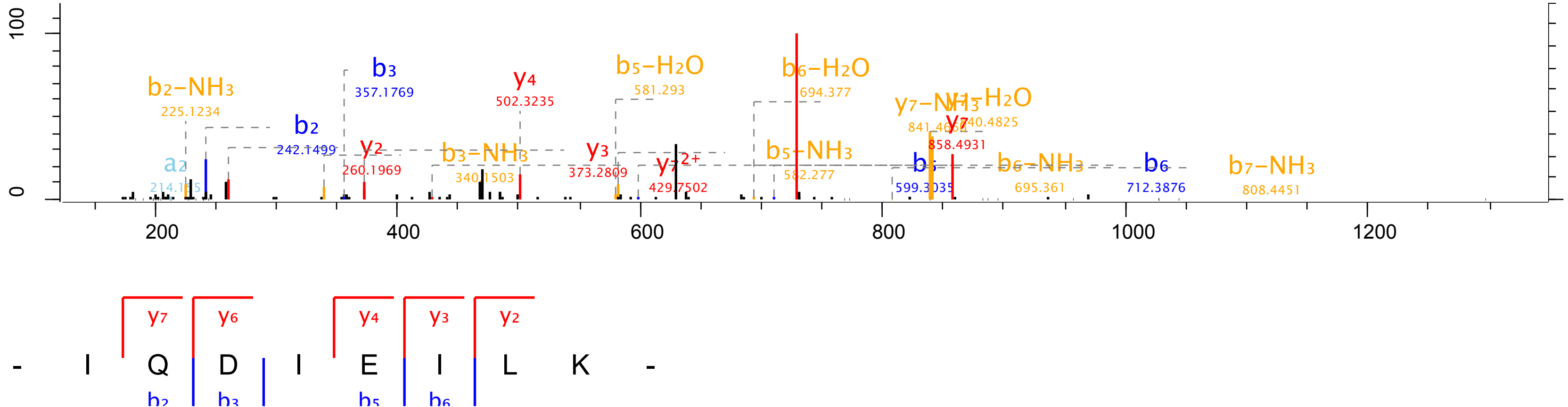

Raw file	Scan	Method	Score	m/z	Gene names
20140918_fract21_dyn_5ul_F5_01_389	24816	TOF; CID	43.76	501.6	HEMK1

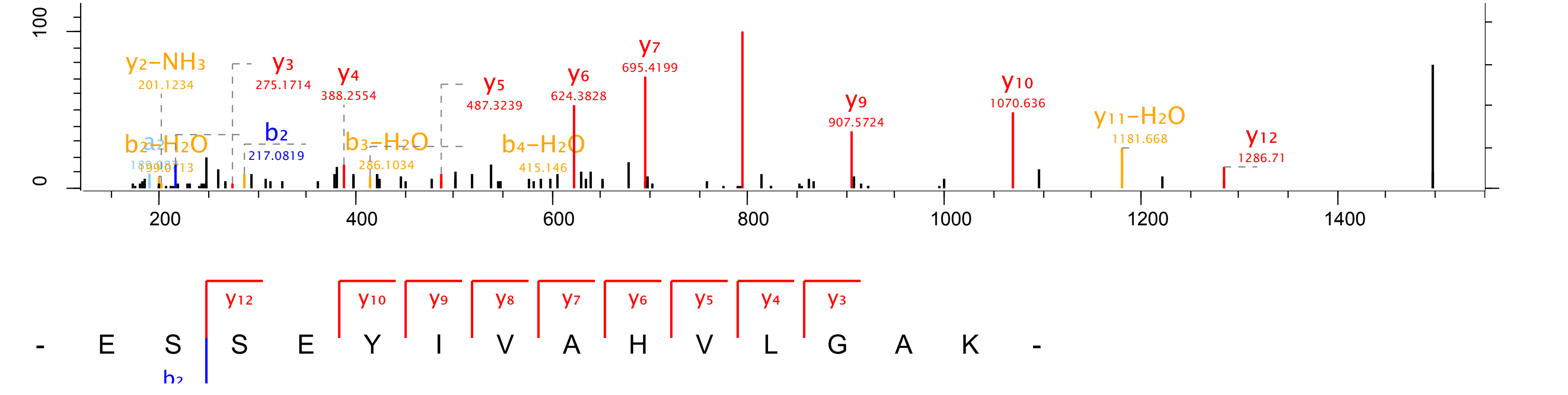

Raw file	Scan	Method	Score	m/z	Gene names
20140918_fract21_dyn_5ul_F5_01_389	27728	TOF; CID	117.93	731.88	SNAPC5

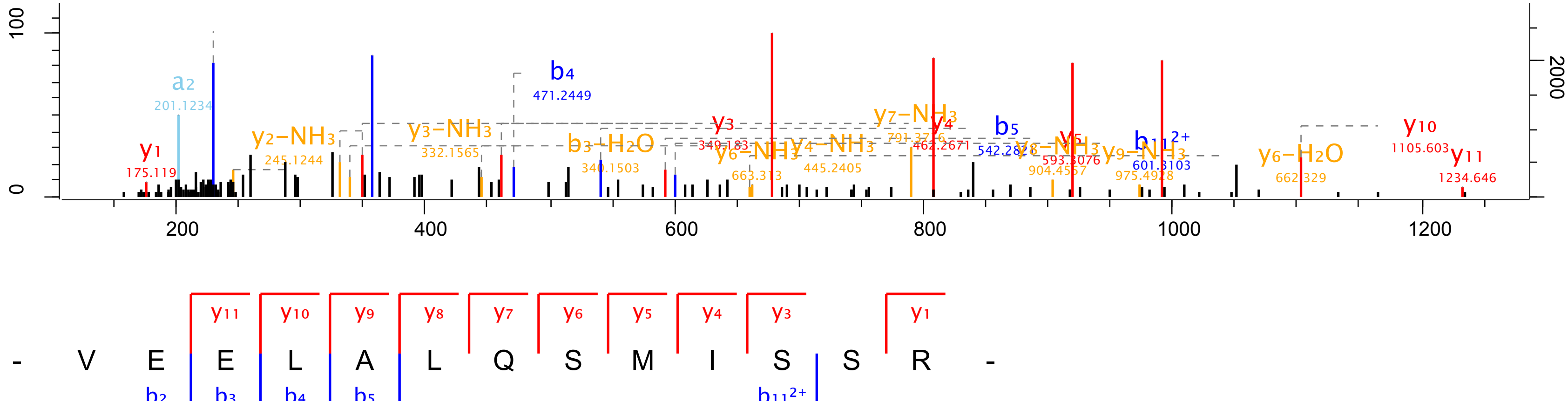

Raw file	Scan	Method	Score	m/z	Gene names
20140918_fract22_dyn_5ul_F6_01_390	12436	TOF; CID	115.29	608.33	TRAF5

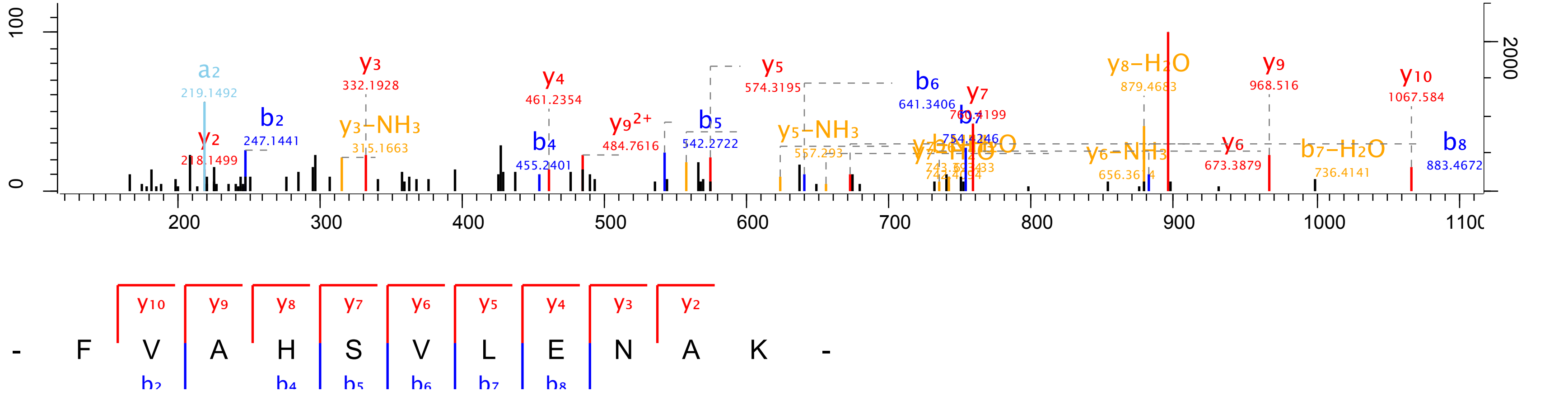

Raw file	Scan	Method	Score	m/z	Gene names
20140918_fract22_dyn_5ul_F6_01_390	12713	TOF; CID	54.95	604.31	HORMAD1

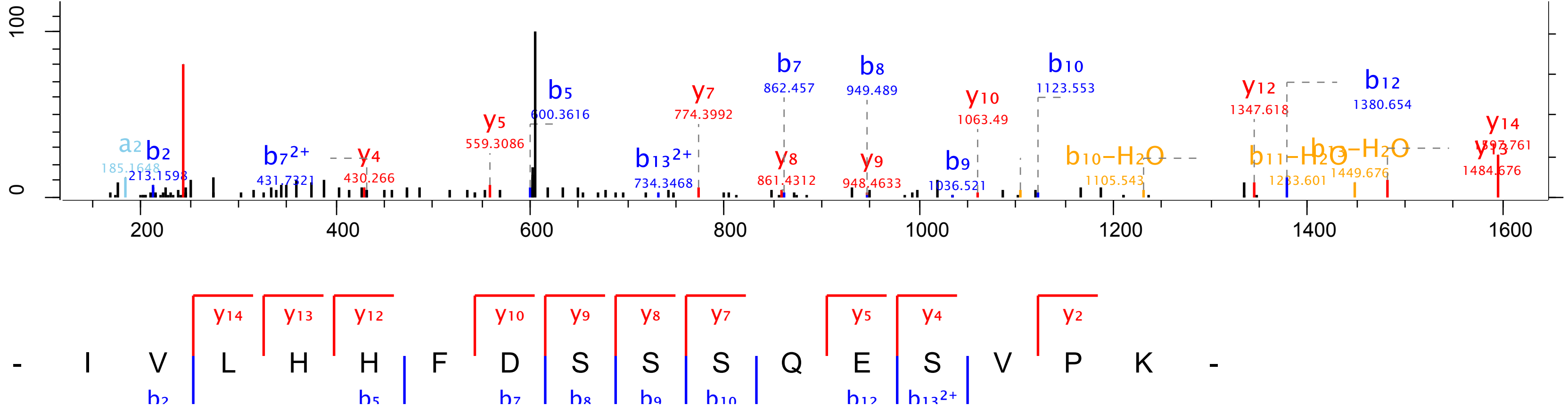

Raw file	Scan	Method	Score	m/z	Gene names
20140918_fract22_dyn_5ul_F6_01_390	13537	TOF; CID	70.94	496.77	NPTXR

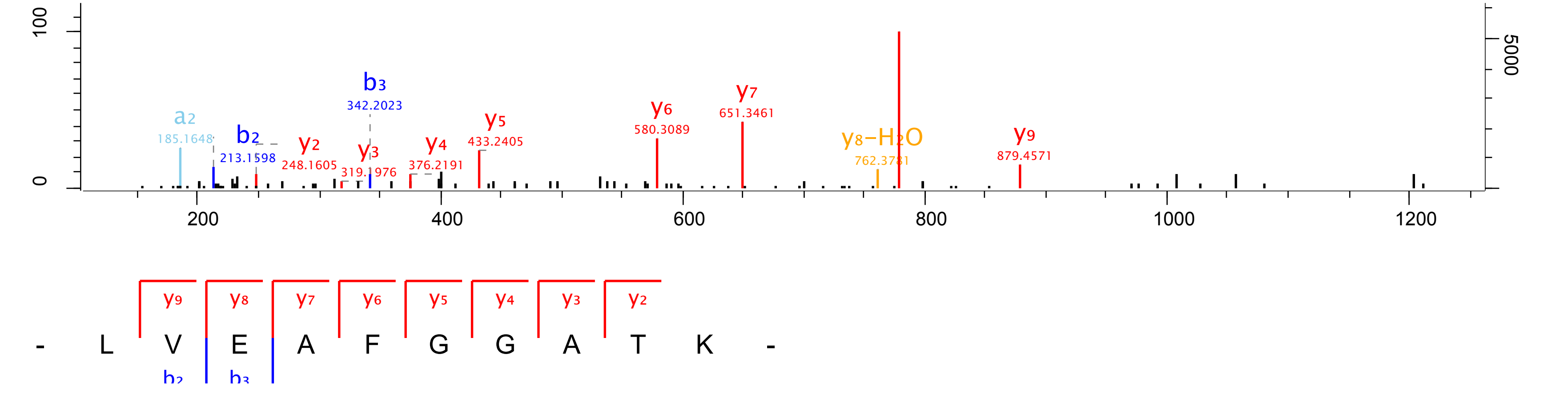

Raw file	Scan	Method	Score	m/z	Gene names
20140918_fract22_dyn_5ul_F6_01_390	20955	TOF; CID	73.93	491.6	ZNF121

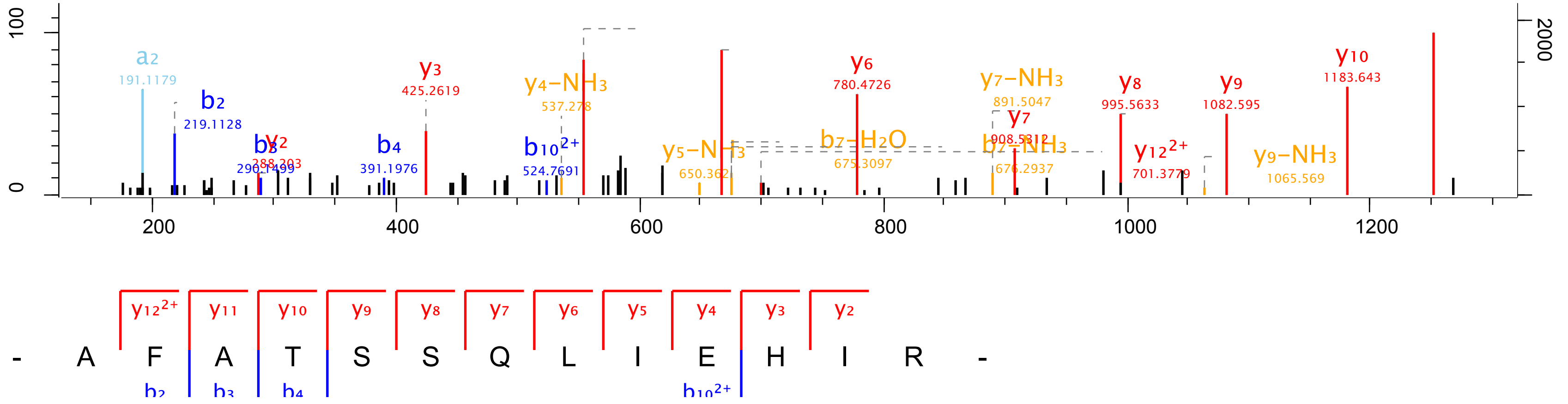

Raw file	Scan	Method	Score	m/z	Gene names
20140918_fract22_dyn_5ul_F6_01_390	21998	TOF; CID	90.79	509.28	NLK

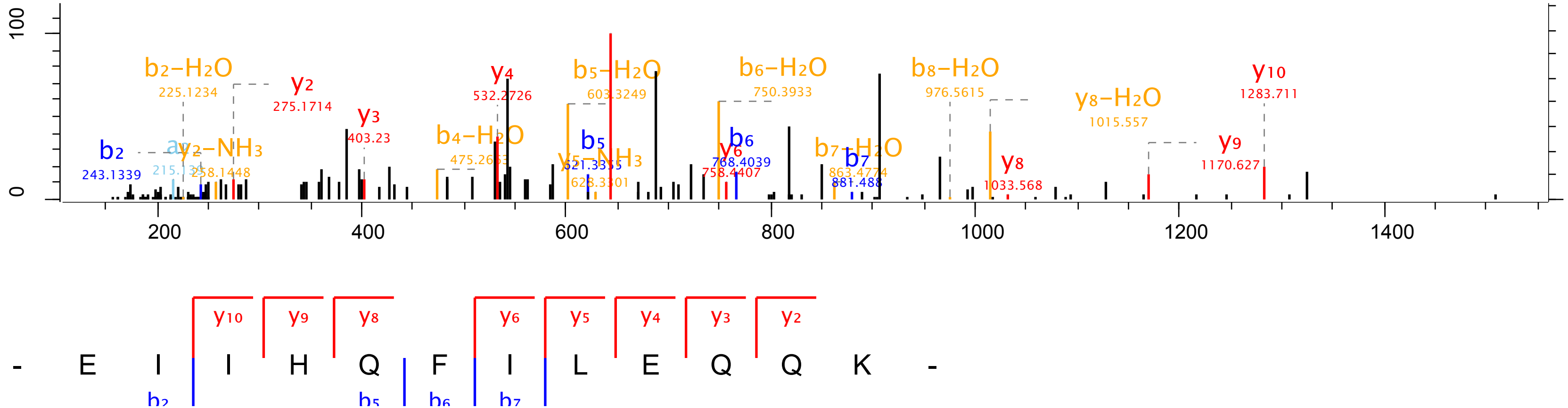

Raw file	Scan	Method	Score	m/z	Gene names
20140918_fract22_dyn_5ul_F6_01_390	22556	TOF; CID	84.38	1101.48	ELOF1

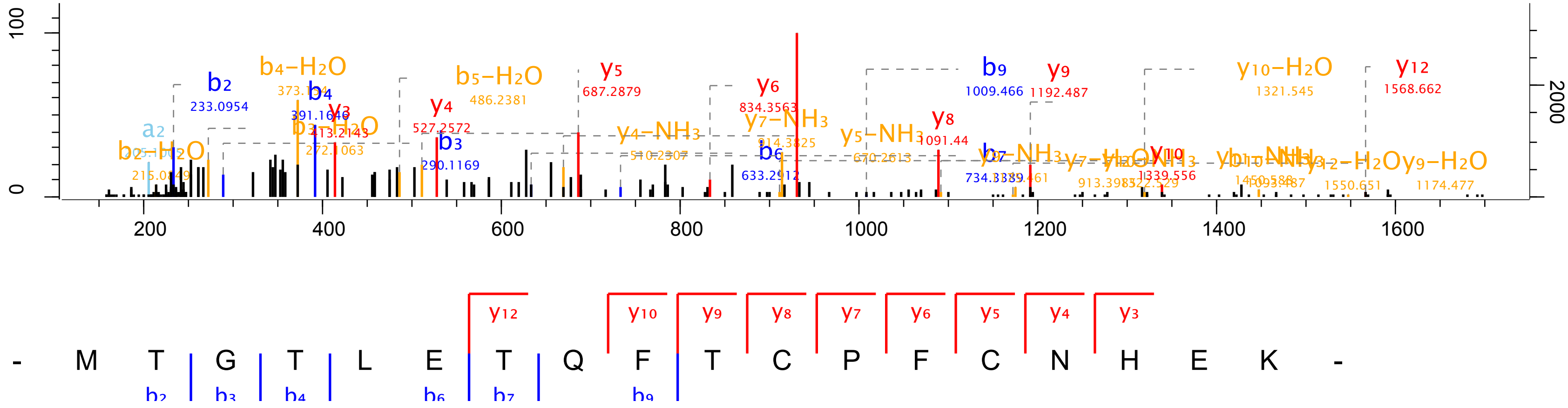

Raw file	Scan	Method	Score	m/z	Gene names
20140918_fract22_dyn_5ul_F6_01_390	28205	TOF; CID	43.26	654.34	NATD1

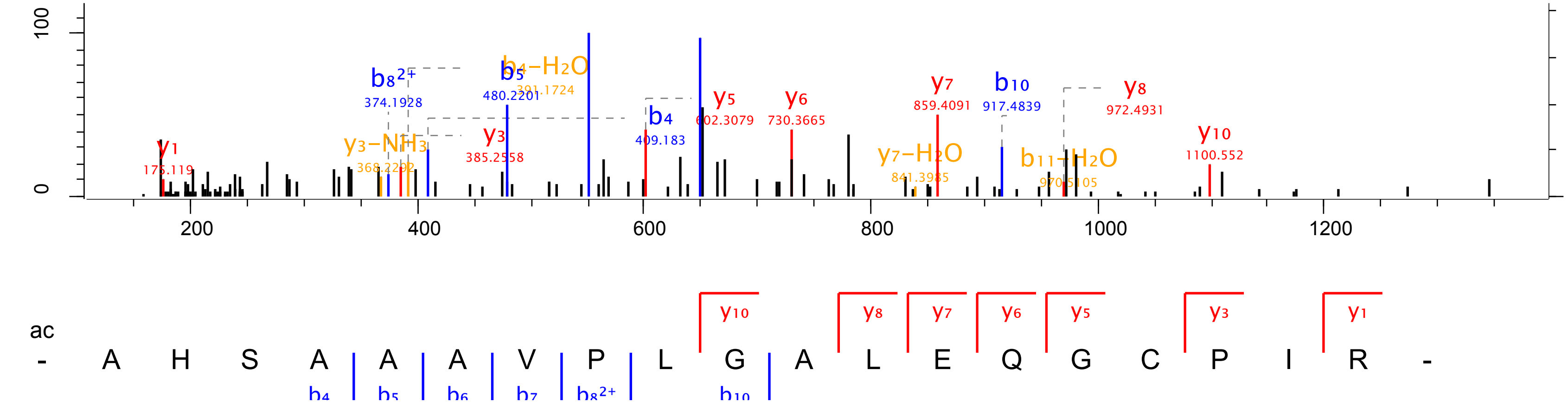

Raw file	Scan	Method	Score	m/z	Gene names
20140918_fract22_dyn_5ul_F6_01_390	29716	TOF; CID	97.46	751.4	NFYB

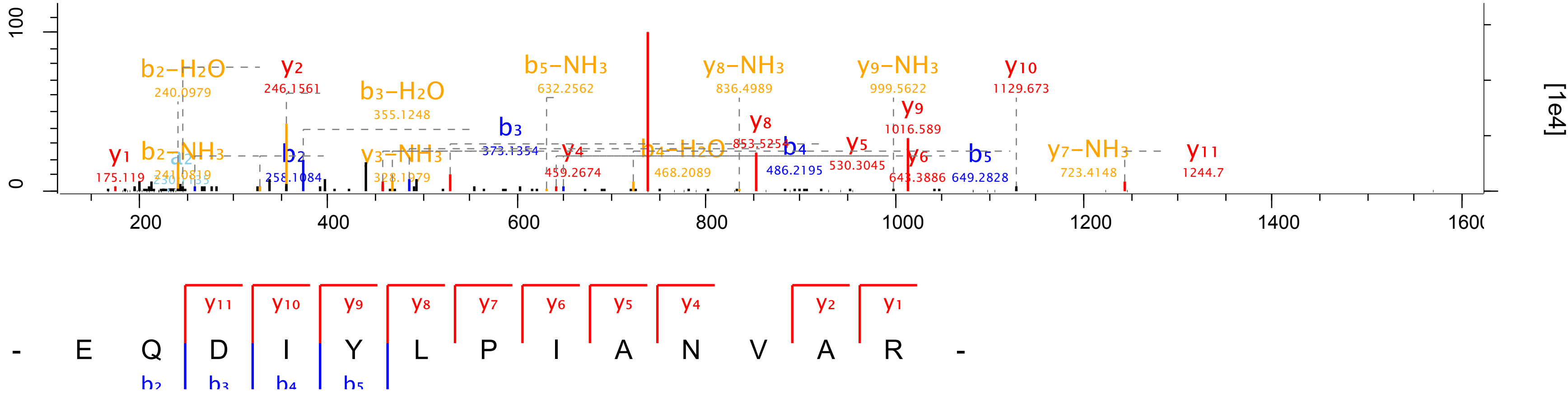

Raw file	Scan	Method	Score	m/z	Gene names
20140918_fract22_dyn_5ul_F6_01_390	32616	TOF; CID	102.52	813.9	TSPAN1

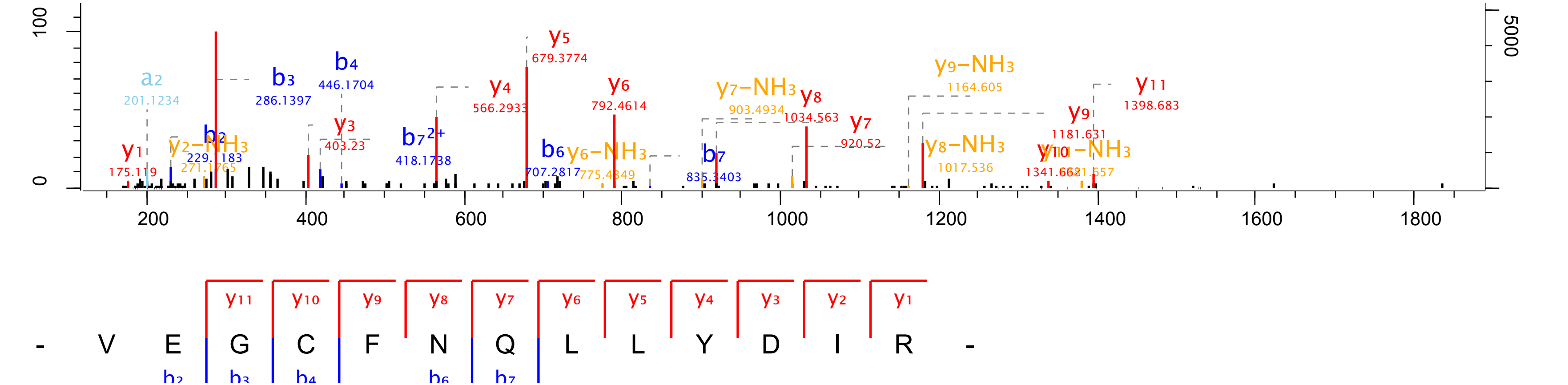

Raw file	Scan	Method	Score	m/z	Gene names
20140918_fract22_dyn_5ul_F6_01_390	32988	TOF; CID	103.9	1191.07	BTBD3

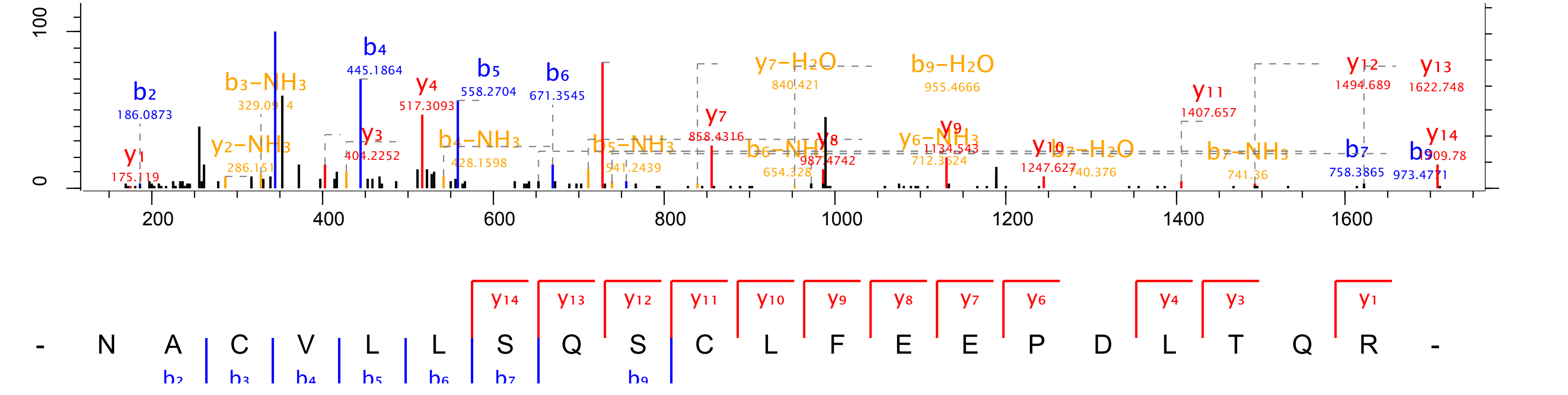

Raw file	Scan	Method	Score	m/z	Gene names
20140918_fract22_dyn_5ul_F6_01_390	33263	TOF; CID	95.41	1162.62	PAQR7

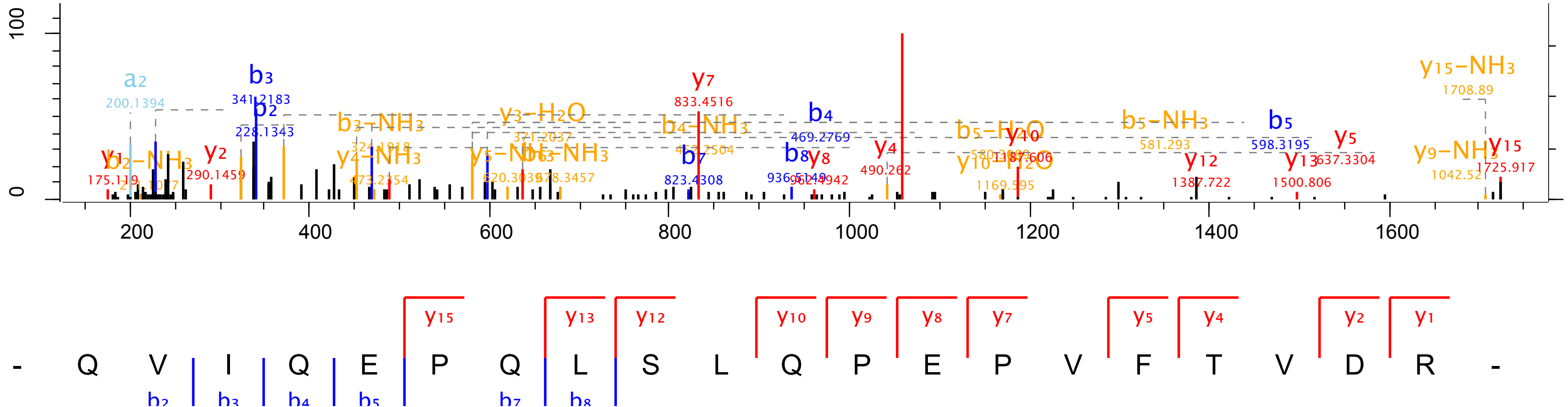

Raw file	Scan	Method	Score	m/z	Gene names
20140918_fract22_dyn_5ul_F6_01_390	33578	TOF; CID	105.35	1123.84	NABP2

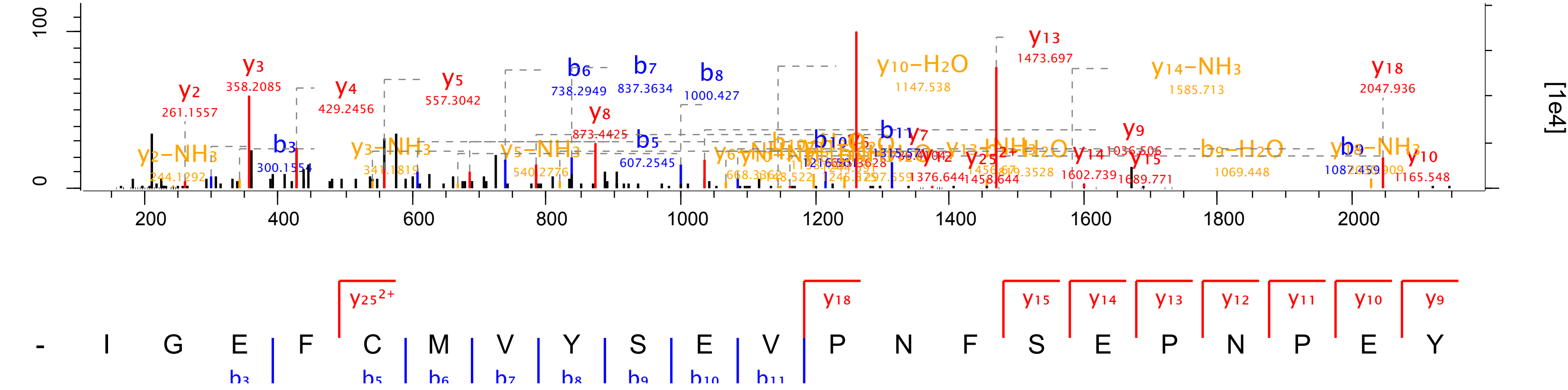

Raw file	Scan	Method	Score	m/z	Gene names
20140918_fract23_dyn_5ul_F7_01_391	9225	TOF; CID	46.88	473.23	ZSCAN32

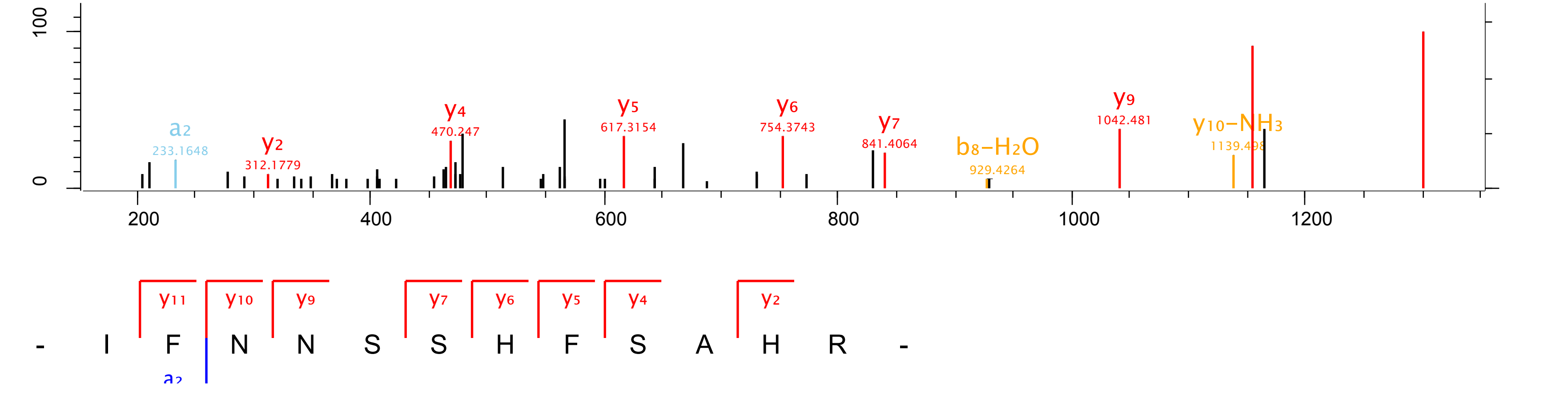

Raw file	Scan	Method	Score	m/z	Gene names
20140918_fract23_dyn_5ul_F7_01_391	12980	TOF; CID	111.04	452.74	HSD17B13

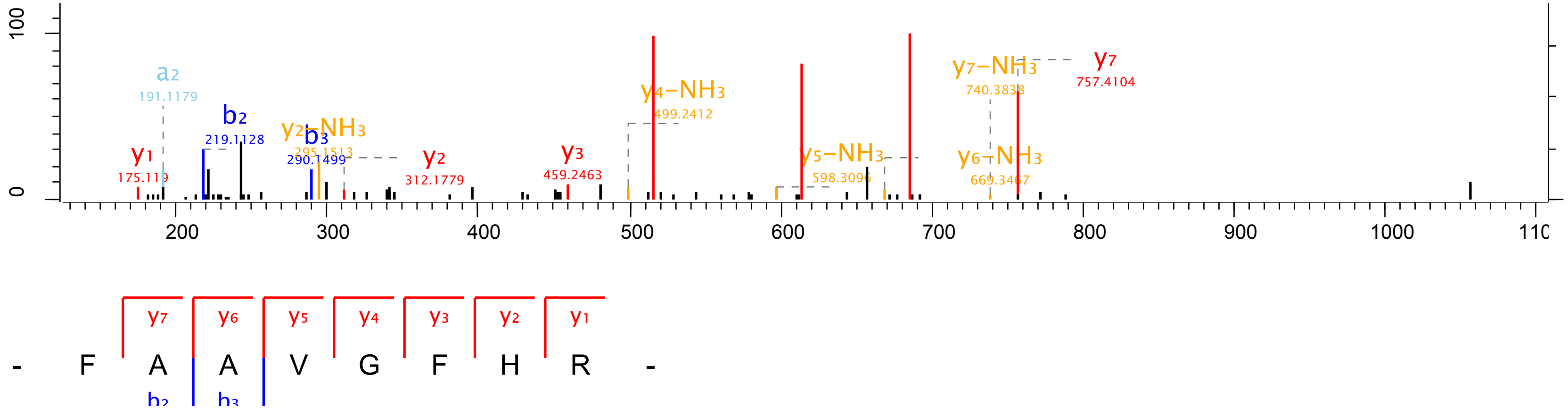

Raw file	Scan	Method	Score	m/z	Gene names
20140918_fract23_dyn_5ul_F7_01_391	14436	TOF; CID	96.74	625.86	FKRP

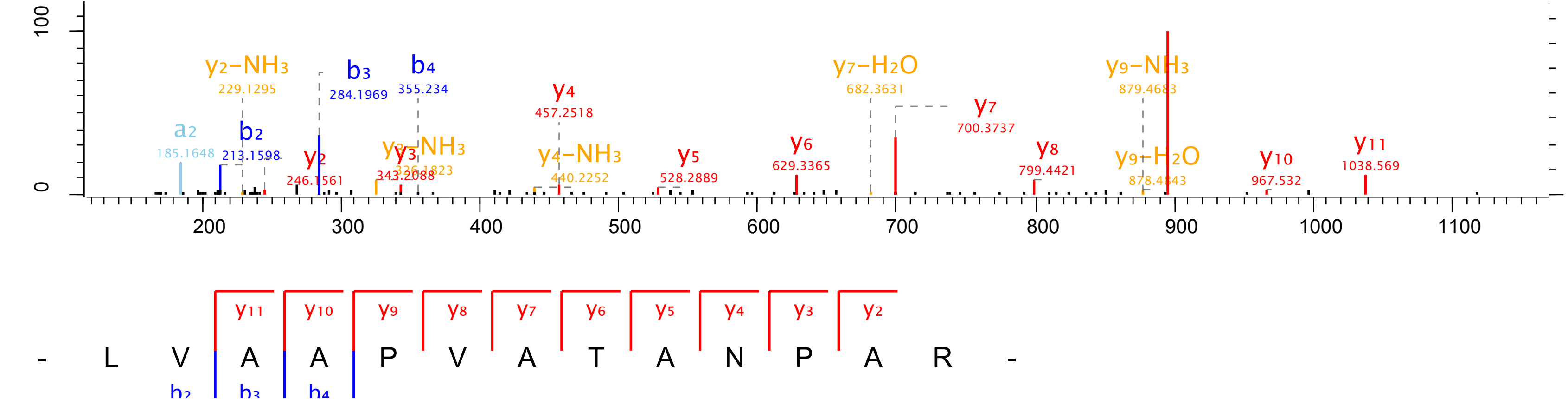

Raw file	Scan	Method	Score	m/z	Gene names
20140918_fract23_dyn_5ul_F7_01_391	14601	TOF; CID	147.26	654.82	KCNK1

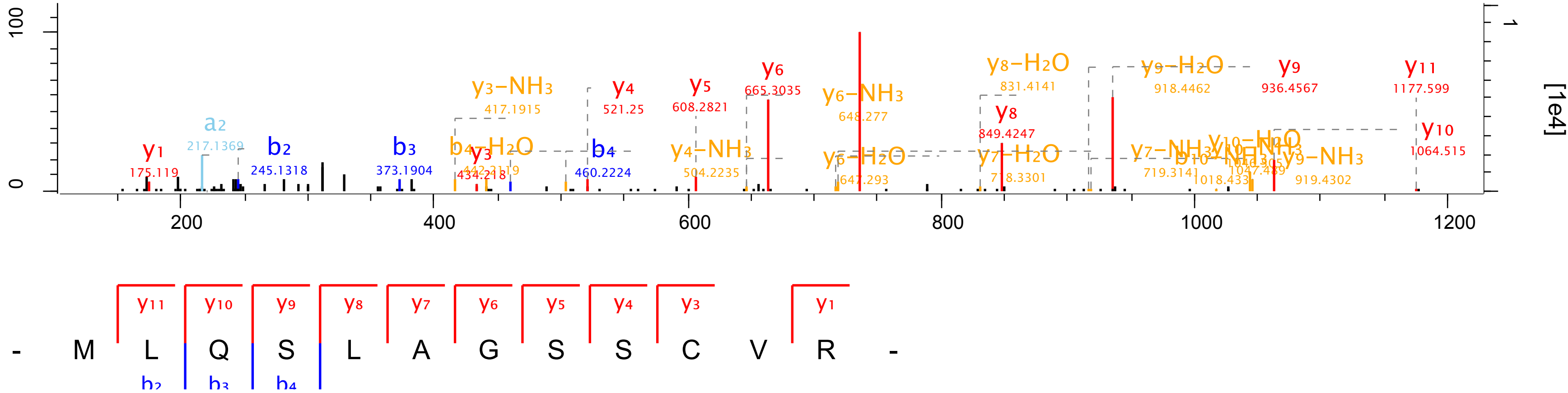

Raw file	Scan	Method	Score	m/z	Gene names
20140918_fract23_dyn_5ul_F7_01_391	16629	TOF; CID	84.51	569.32	ZNF219

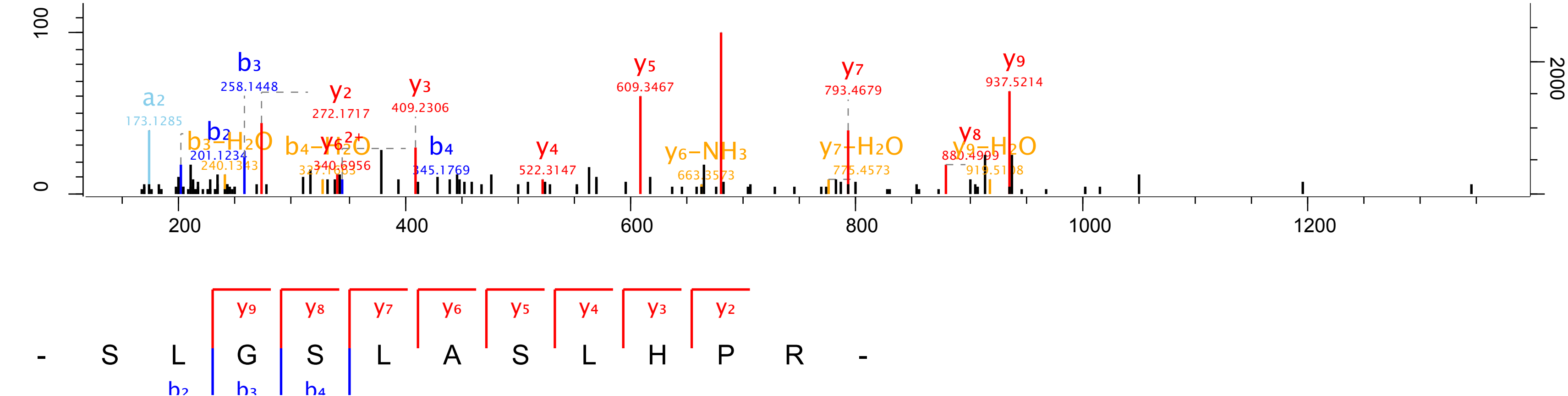

Raw file	Scan	Method	Score	m/z	Gene names
20140918_fract23_dyn_5ul_F7_01_391	18077	TOF; CID	76.07	734.85	STYXL1

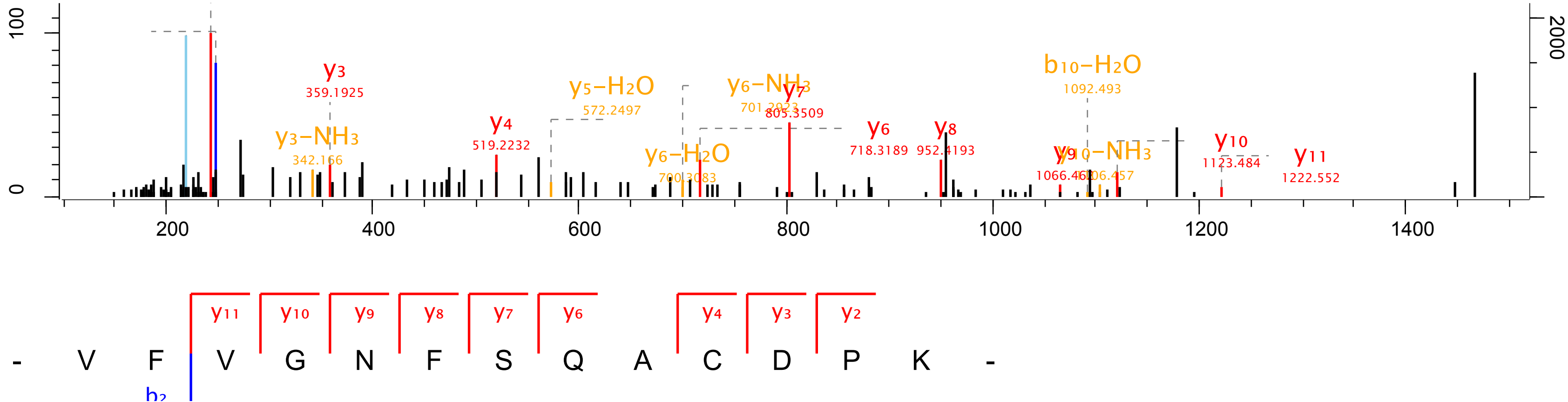

Raw file	Scan	Method	Score	m/z	Gene names
20140918_fract23_dyn_5ul_F7_01_391	18445	TOF; CID	119.39	672.33	XK

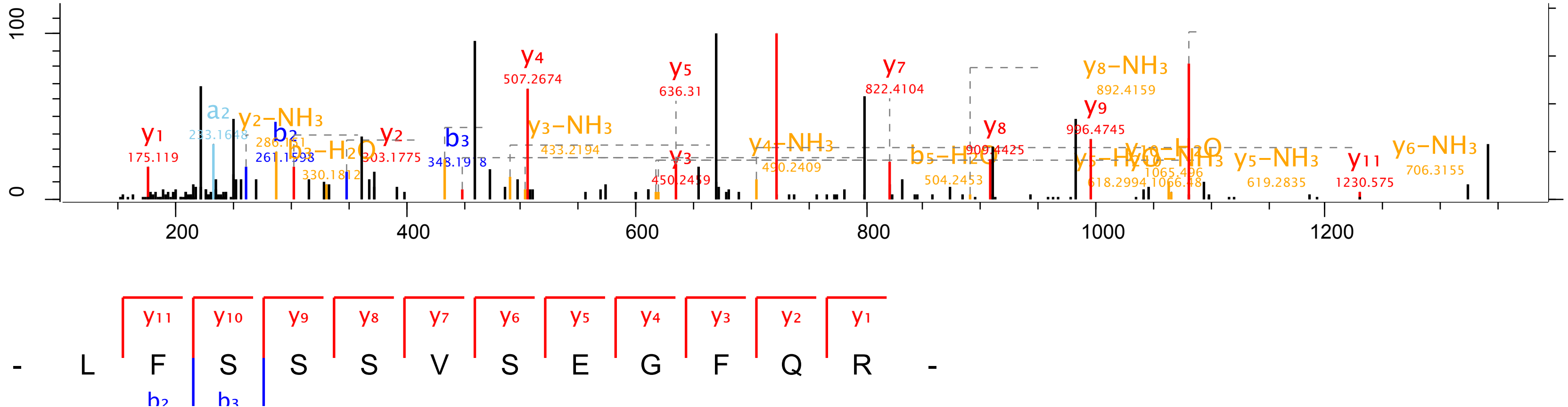

Raw file	Scan	Method	Score	m/z	Gene names
20140918_fract23_dyn_5ul_F7_01_391	18516	TOF; CID	69.09	599.3	C9orf9

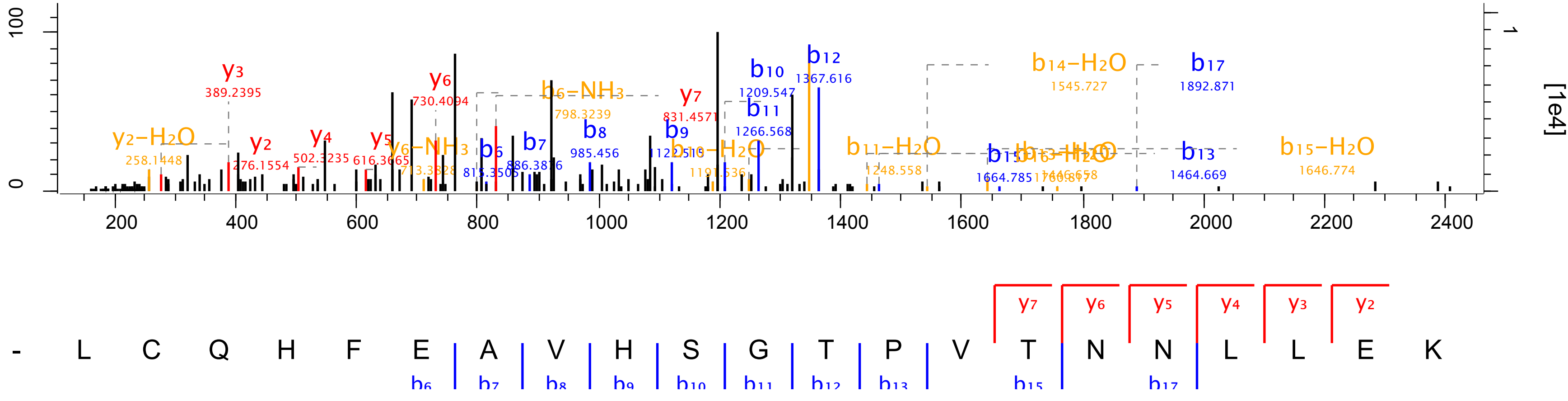

Raw file	Scan	Method	Score	m/z	Gene names
20140918_fract23_dyn_5ul_F7_01_391	19632	TOF; CID	90.76	670.38	FAM178A

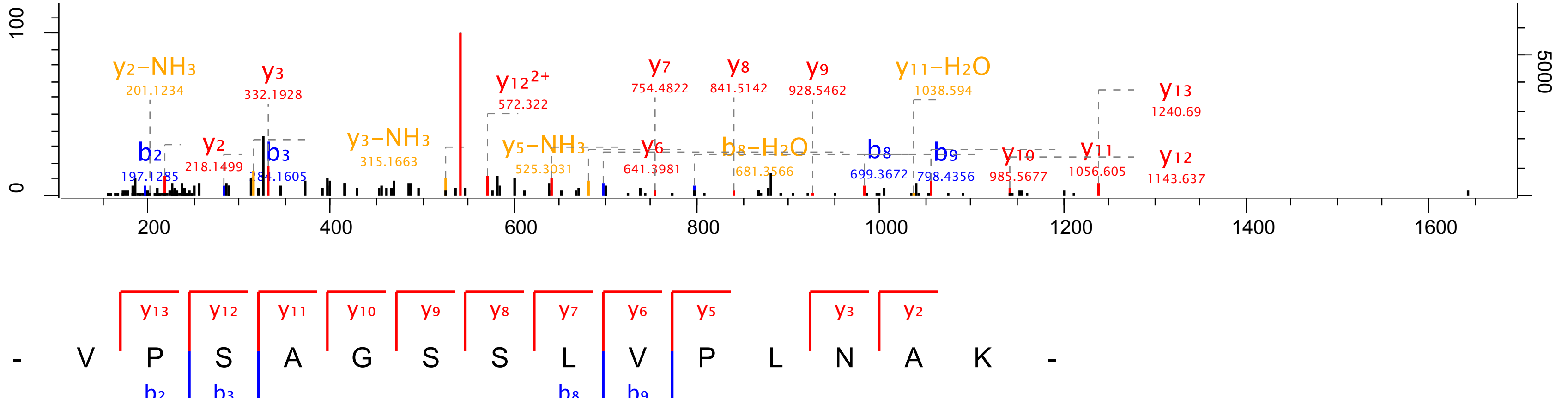

Raw file	Scan	Method	Score	m/z	Gene names
20140918_fract23_dyn_5ul_F7_01_391	20512	TOF; CID	63.62	657.84	EVA1C

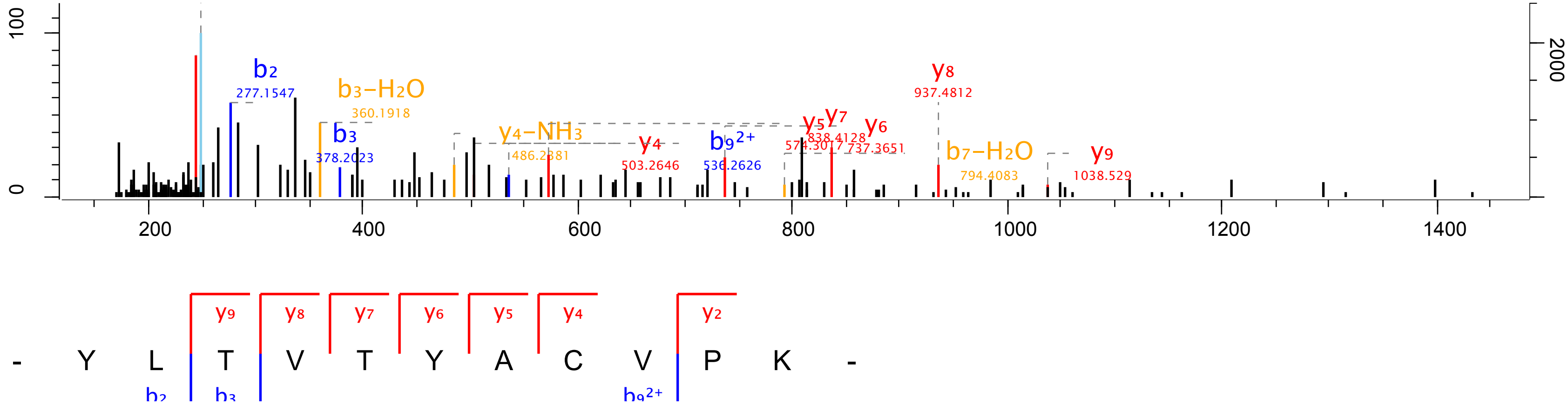

Raw file	Scan	Method	Score	m/z	Gene names
20140918_fract23_dyn_5ul_F7_01_391	22685	TOF; CID	117.03	849.38	PLP2

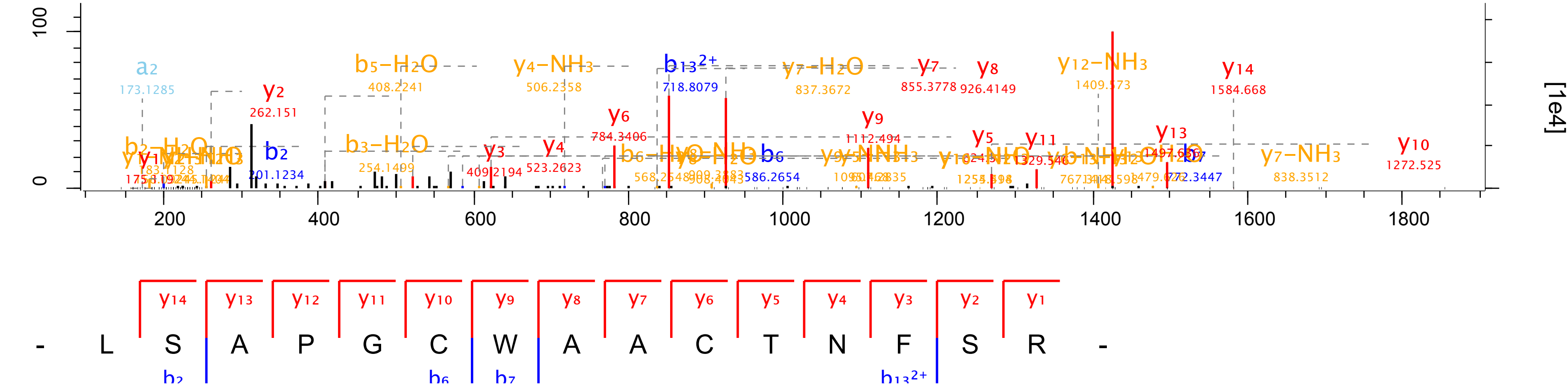

Raw file	Scan	Method	Score	m/z	Gene names
20140918_fract23_dyn_5ul_F7_01_391	22745	TOF; CID	113.24	518.28	PARD3B

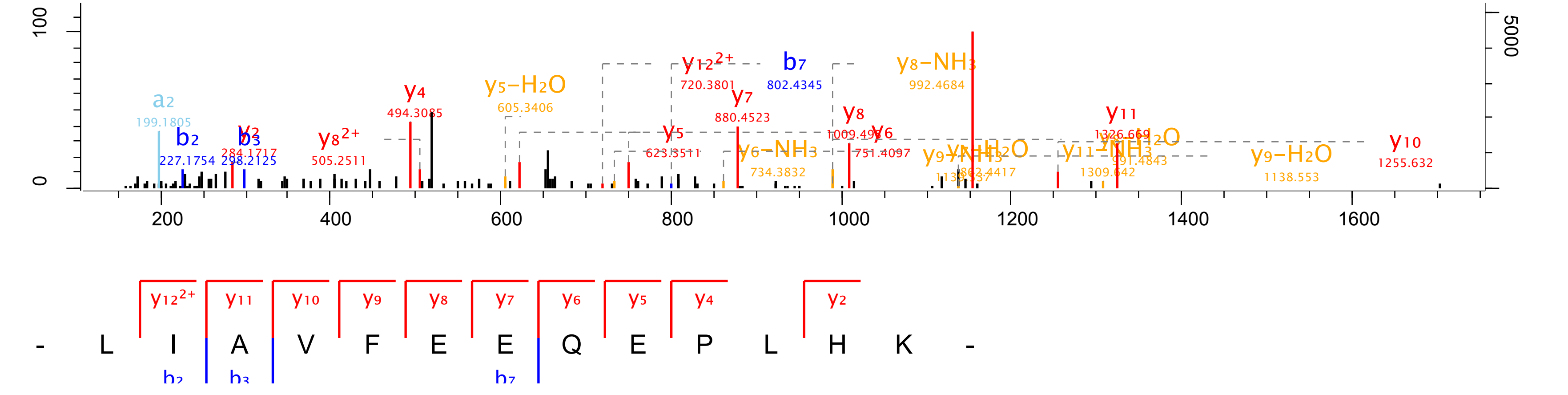

Raw file	Scan	Method	Score	m/z	Gene names
20140918_fract23_dyn_5ul_F7_01_391	25240	TOF; CID	61.11	460.93	ANKRA2

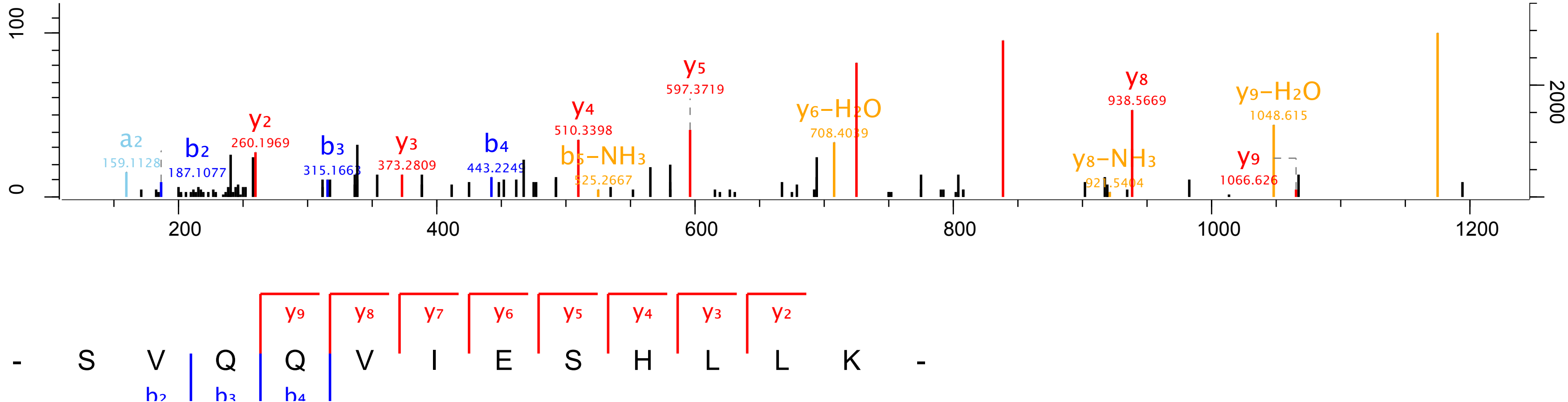

Raw file	Scan	Method	Score	m/z	Gene names
20140918_fract23_dyn_5ul_F7_01_391	25691	TOF; CID	32.51	754.85	INO80D

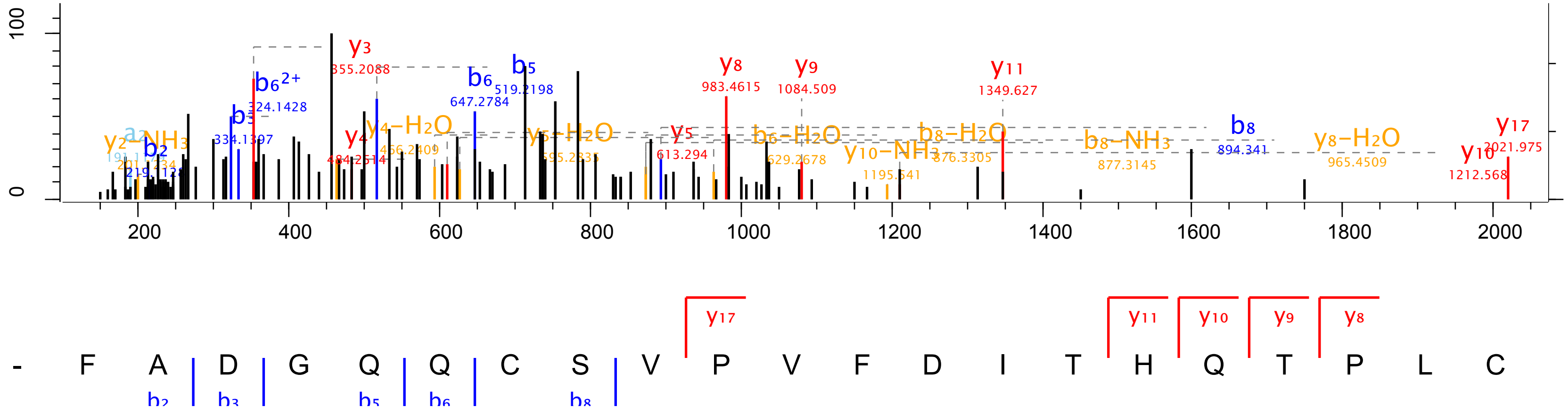

Raw file	Scan	Method	Score	m/z	Gene names
20140918_fract23_dyn_5ul_F7_01_391	26852	TOF; CID	81.68	710.89	NFE2L1

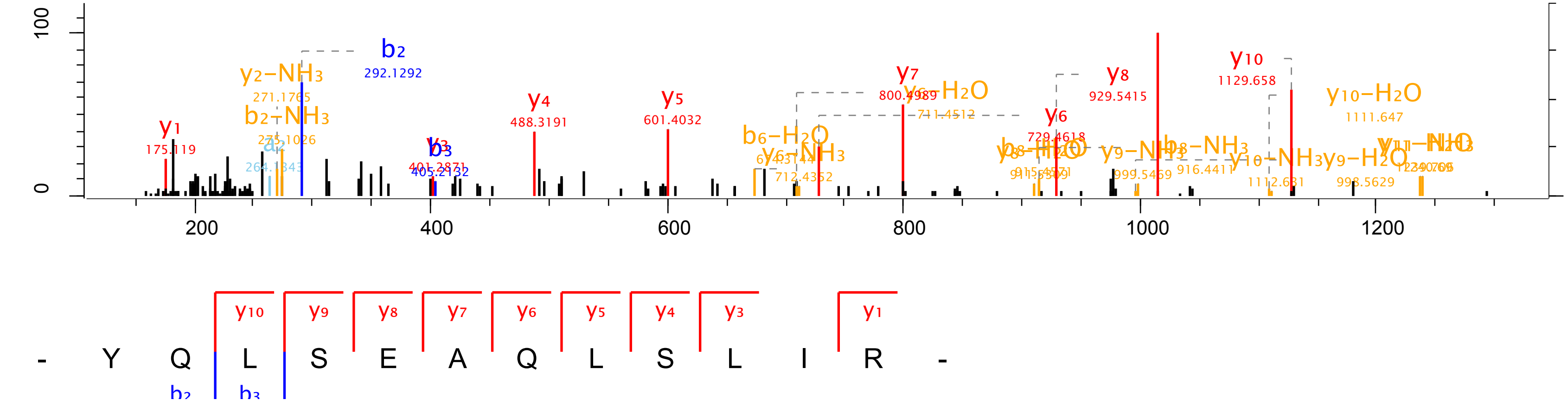

Raw file	Scan	Method	Score	m/z	Gene names
20140918_fract23_dyn_5ul_F7_01_391	27715	TOF; CID	125.23	948.52	ITM2C

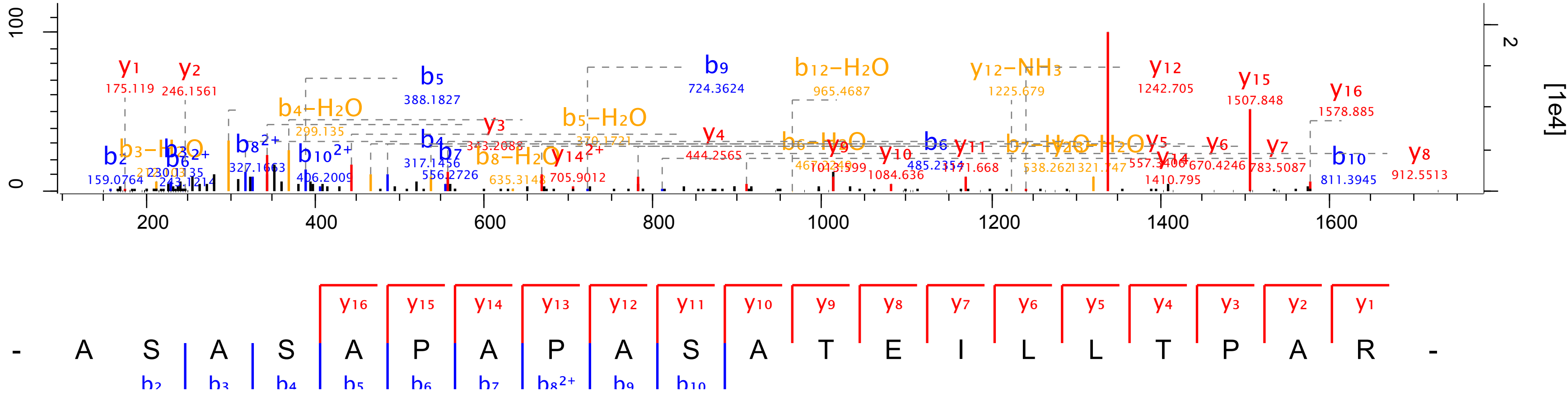

Raw file	Scan	Method	Score	m/z	Gene names
20140918_fract23_dyn_5ul_F7_01_391	30129	TOF; CID	69.7	908.73	TSPAN4

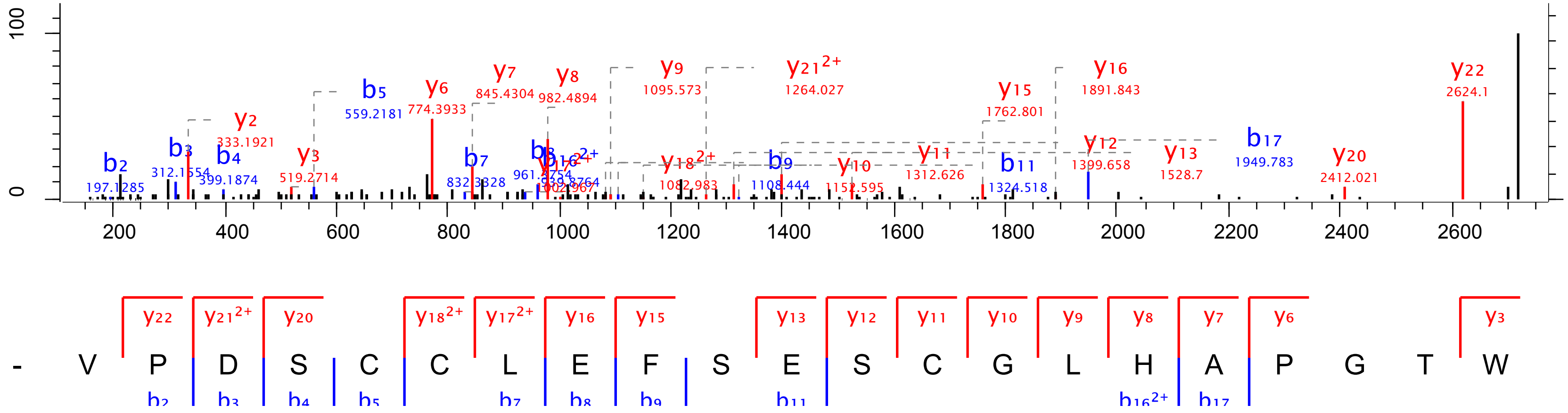

Raw file	Scan	Method	Score	m/z	Gene names
20140918_fract23_dyn_5ul_F7_01_391	33281	TOF; CID	109.1	928.51	PET117

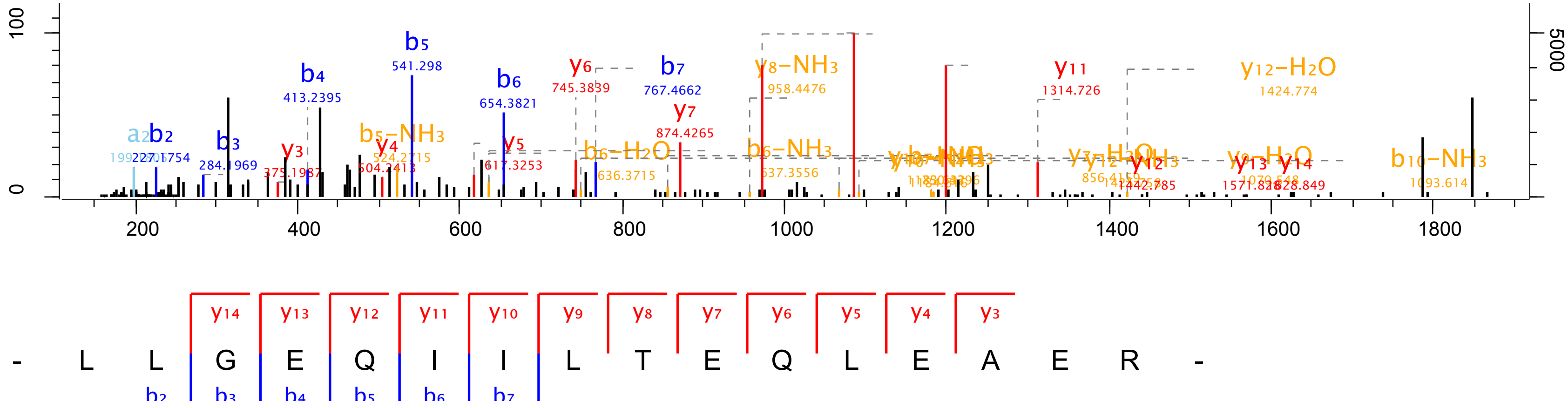

Raw file	Scan	Method	Score	m/z	Gene names
20140918_fract23_dyn_5ul_F7_01_391	33865	TOF; CID	99.4	961.13	SFT2D1

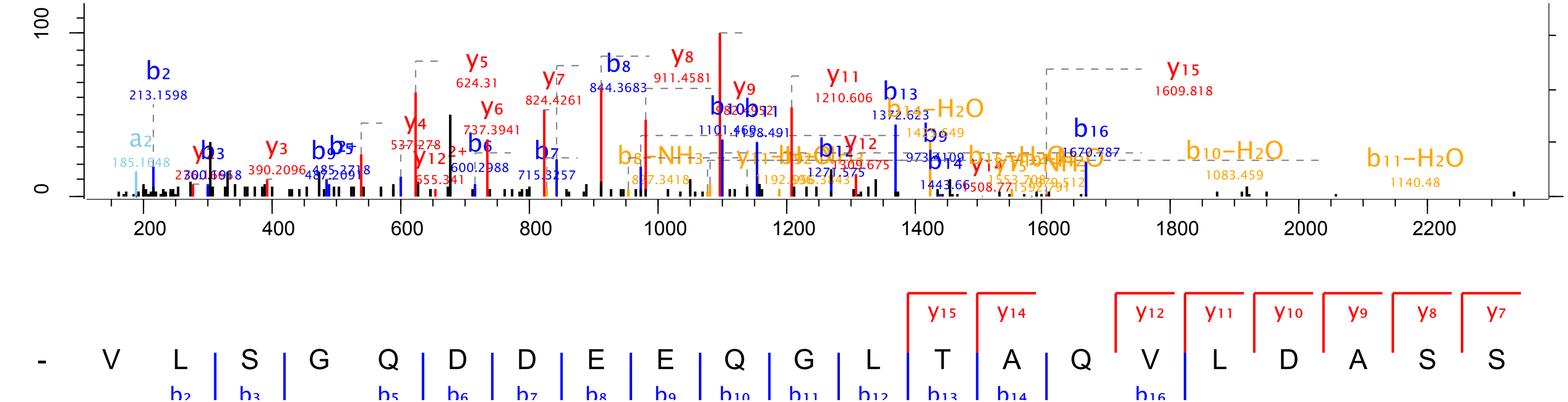

Raw file	Scan	Method	Score	m/z	Gene names
20140918_fract23_dyn_5ul_F7_01_391	34833	TOF; CID	122.74	491.24	TMEM50B

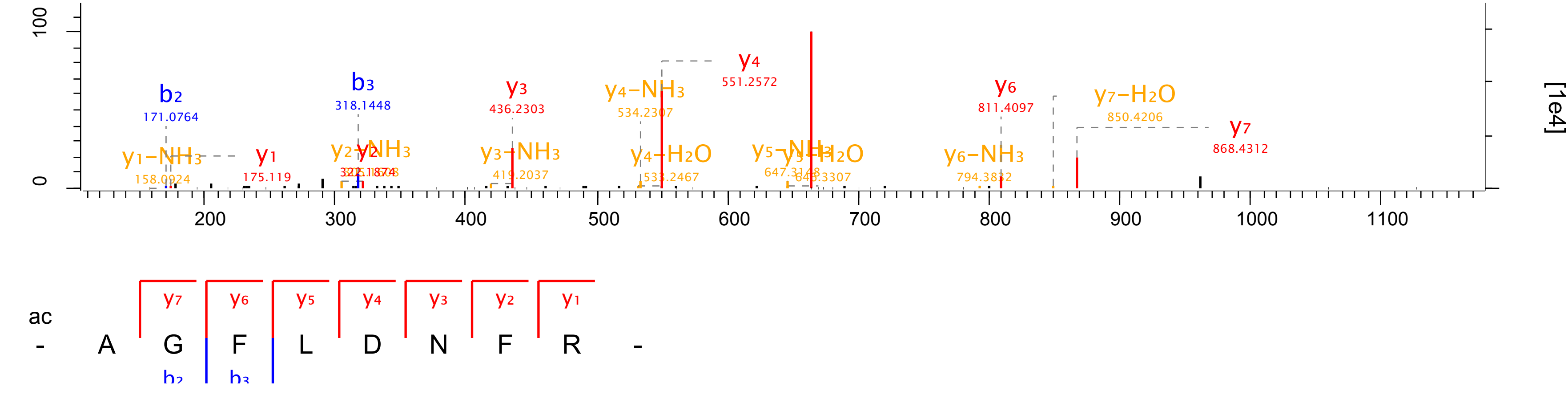

Raw file	Scan	Method	Score	m/z	Gene names
20140918_fract23_dyn_5ul_F7_01_391	35008	TOF; CID	65.24	698.88	PRKD2

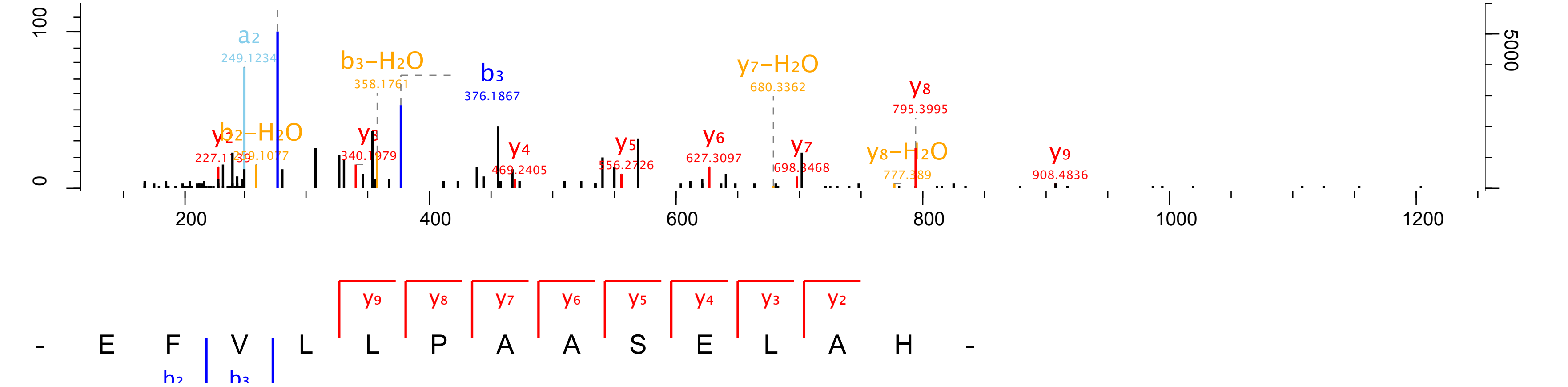

Raw file	Scan	Method	Score	m/z	Gene names
20140918_fract24_dyn_5ul_F8_01_392	10309	TOF; CID	78.32	527.62	ZNF34

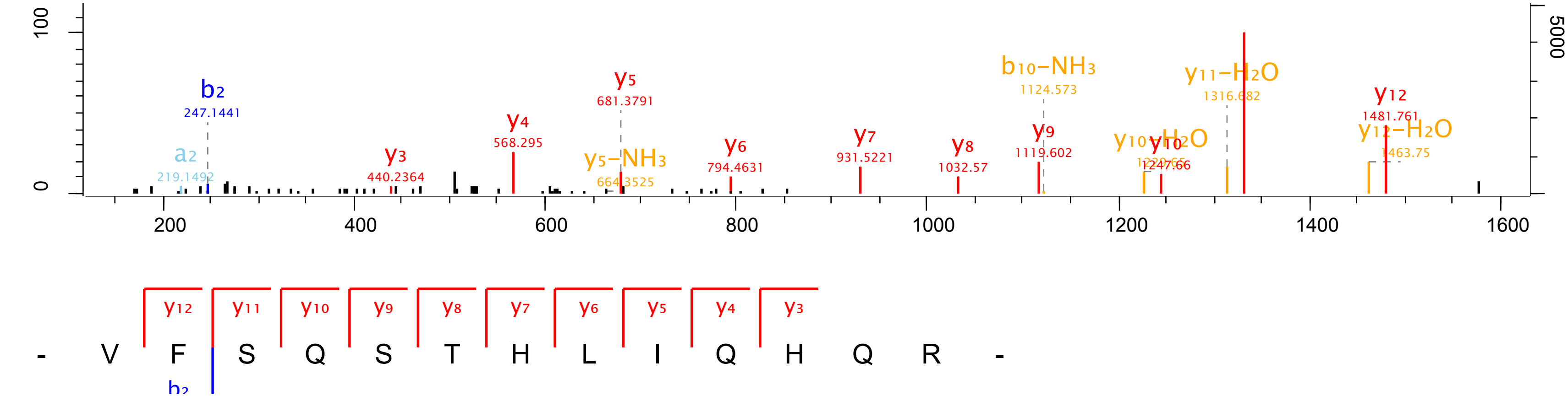

Raw file	Scan	Method	Score	m/z	Gene names
20140918_fract24_dyn_5ul_F8_01_392	13618	TOF; CID	89.35	528.81	TTC7B

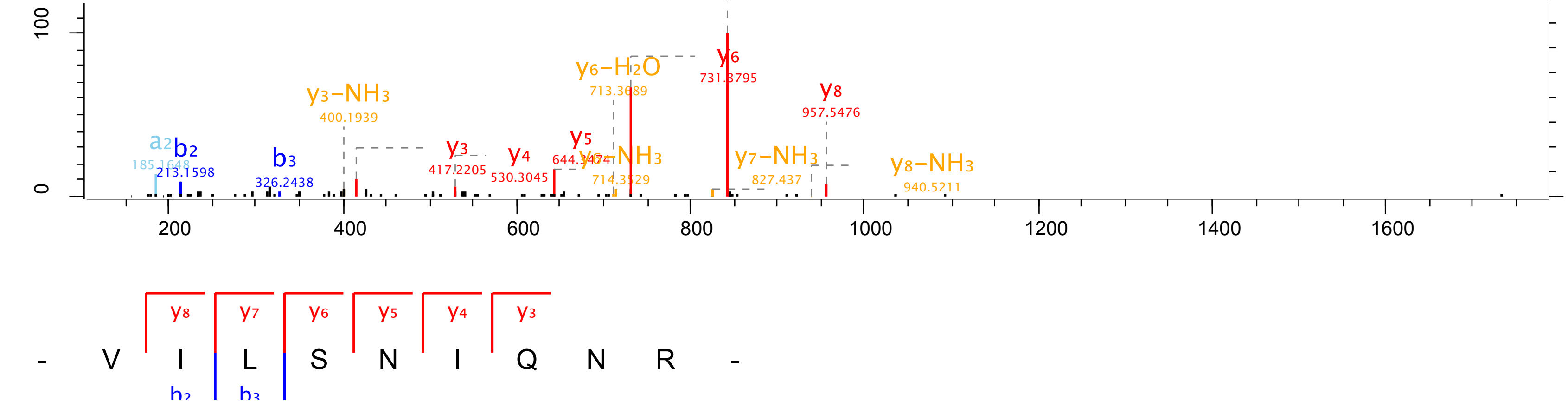

Raw file	Scan	Method	Score	m/z	Gene names
20140918_fract24_dyn_5ul_F8_01_392	17669	TOF; CID	66.64	758.04	UBXN2B

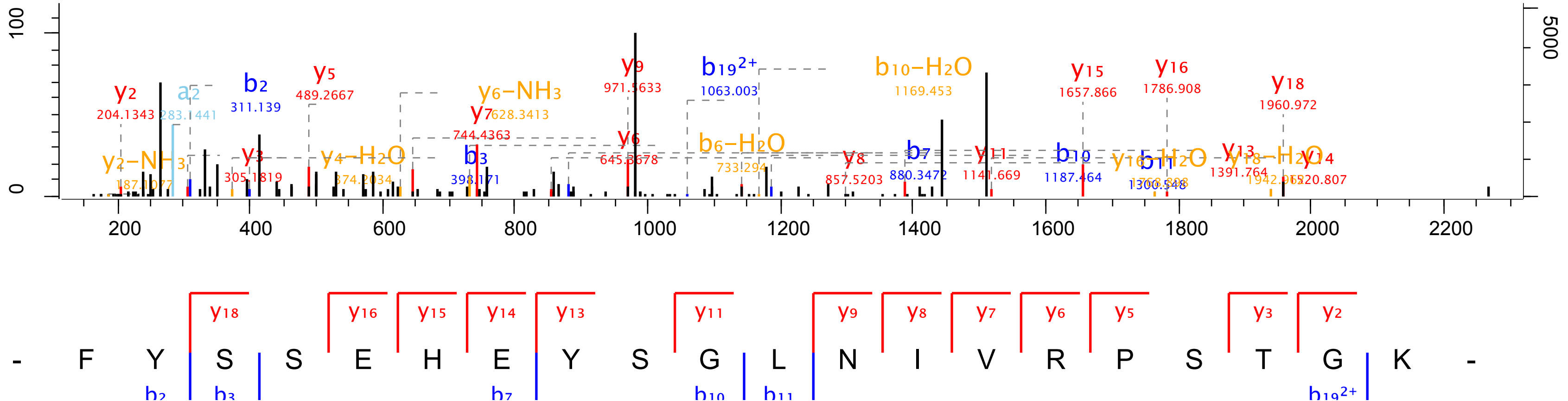

Raw file	Scan	Method	Score	m/z	Gene names
20140918_fract24_dyn_5ul_F8_01_392	17720	TOF; CID	77.37	738.91	F3

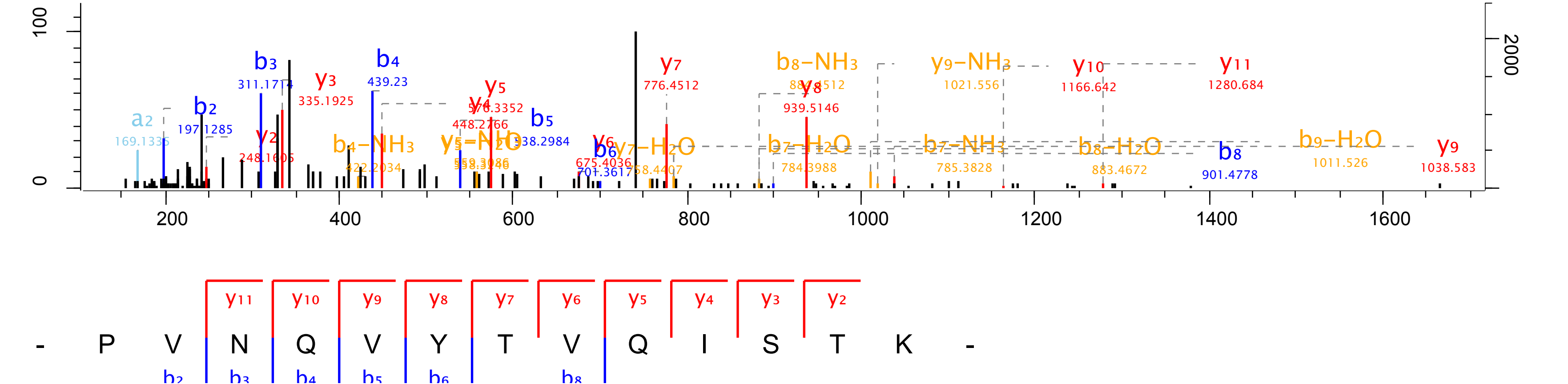

Raw file	Scan	Method	Score	m/z	Gene names
20140918_fract24_dyn_5ul_F8_01_392	17863	TOF; CID	93.18	482.27	ZDHHC16

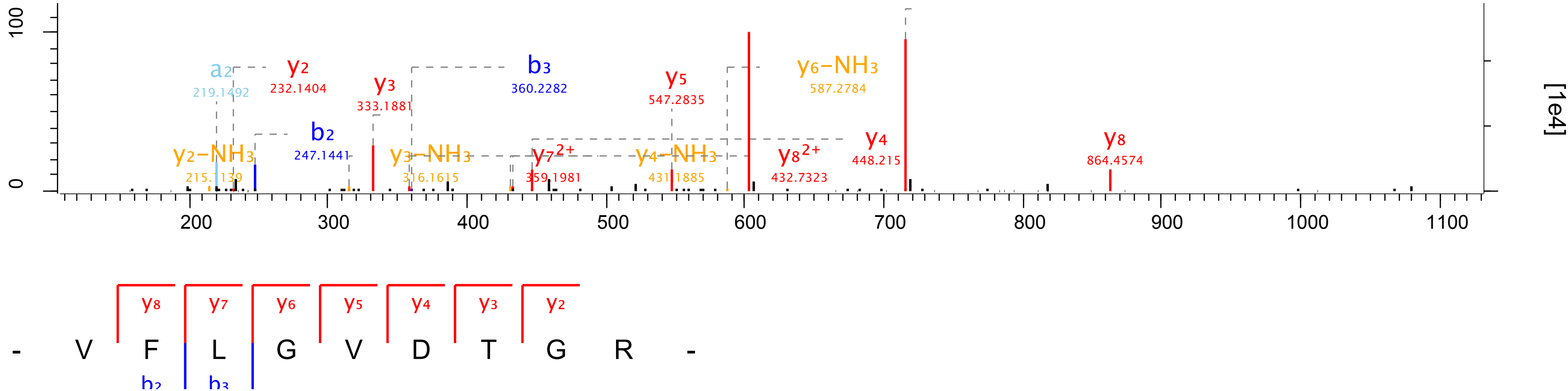

Raw file	Scan	Method	Score	m/z	Gene names
20140918_fract24_dyn_5ul_F8_01_392	18933	TOF; CID	58.27	553.3	EXPH5

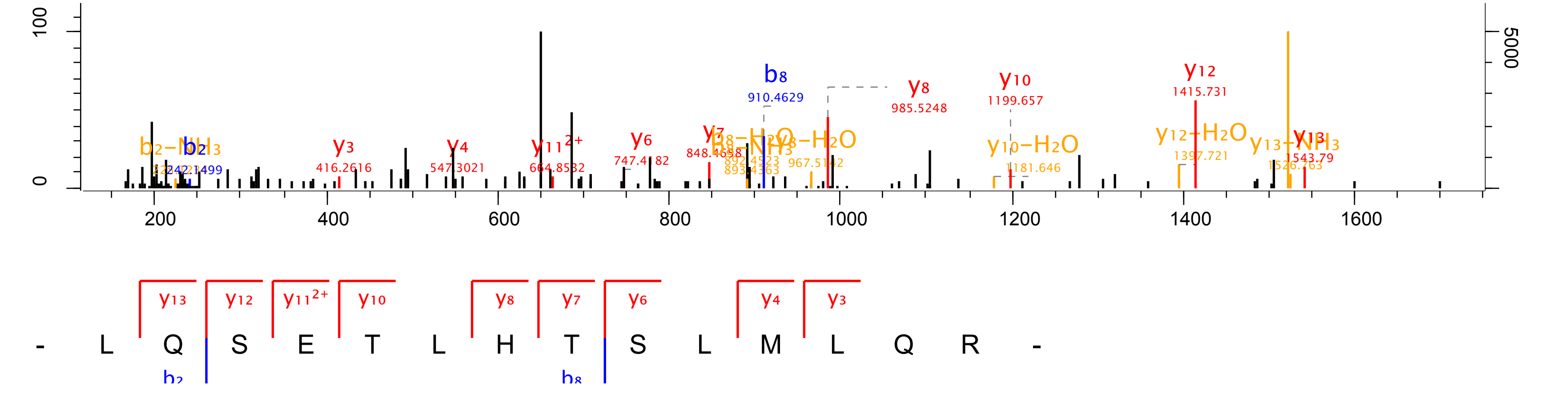

Raw file	Scan	Method	Score	m/z	Gene names
20140918_fract24_dyn_5ul_F8_01_392	19046	TOF; CID	66.27	638.32	PIGB

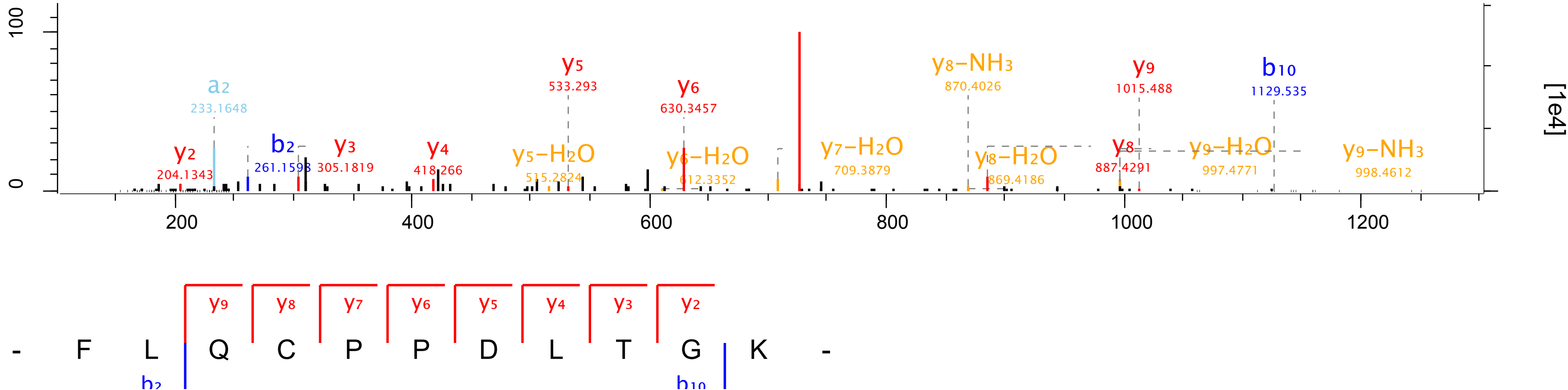

Raw file	Scan	Method	Score	m/z	Gene names
20140918_fract24_dyn_5ul_F8_01_392	19146	TOF; CID	84.36	643.35	FBXO5

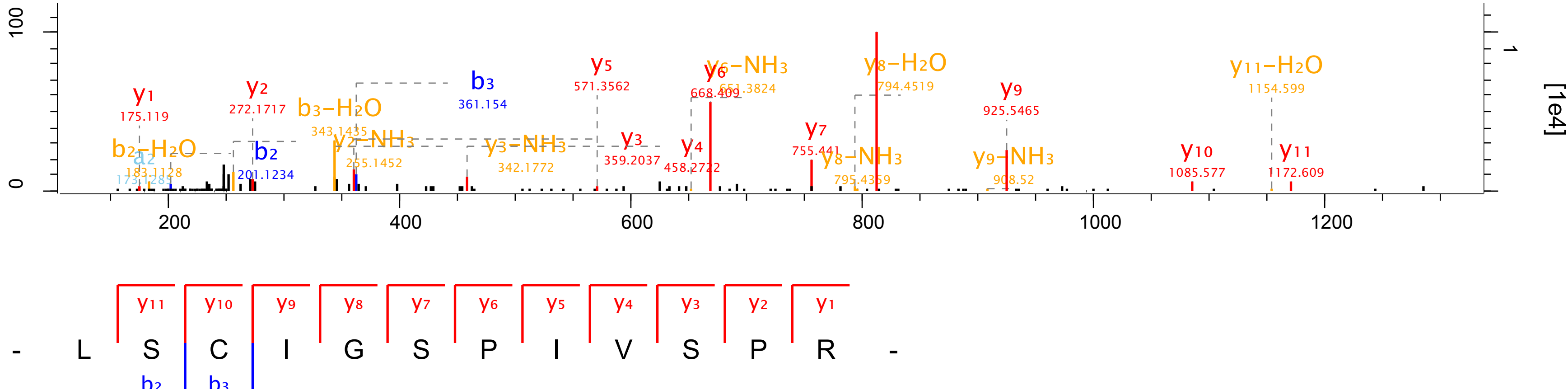

Raw file	Scan	Method	Score	m/z	Gene names
20140918_fract24_dyn_5ul_F8_01_392	20157	TOF; CID	42.12	762.34	PEG10

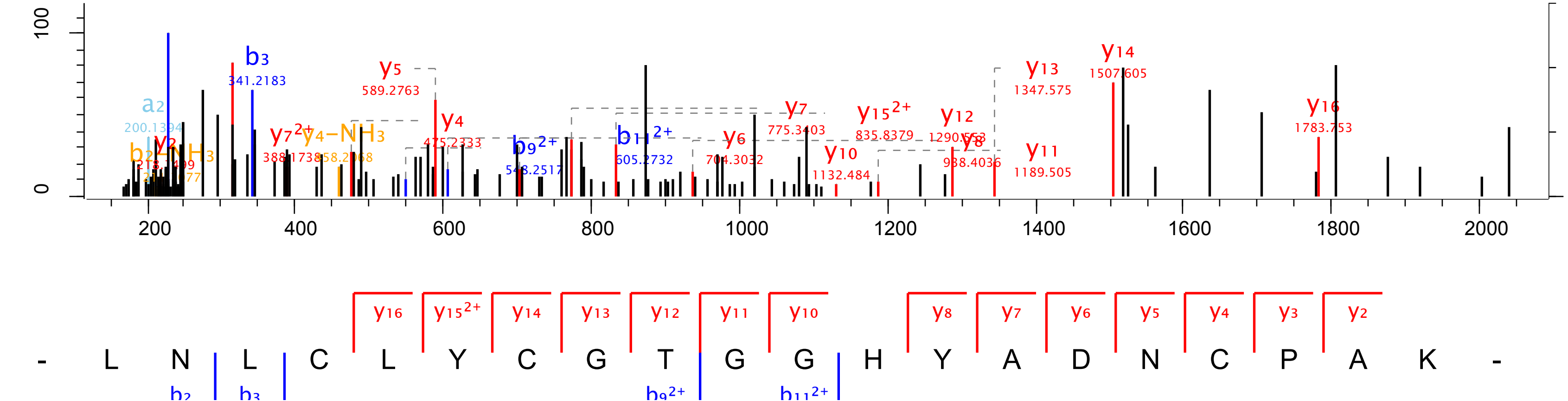

Raw file	Scan	Method	Score	m/z	Gene names
20140918_fract24_dyn_5ul_F8_01_392	20417	TOF; CID	40.97	553.32	FAM189B

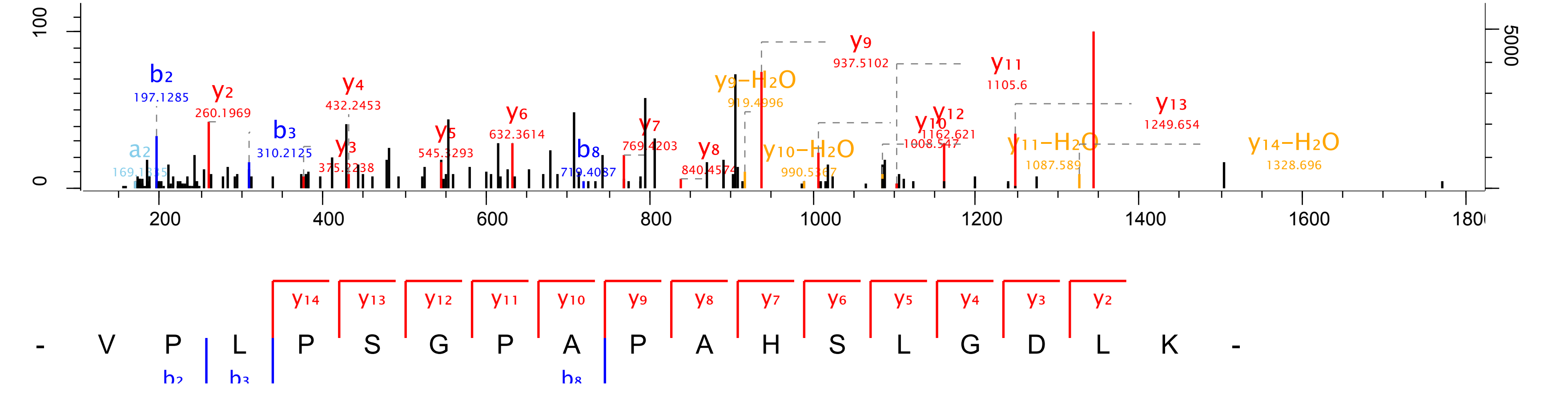

Raw file	Scan	Method	Score	m/z	Gene names
20140918_fract24_dyn_5ul_F8_01_392	23998	TOF; CID	119.53	575.32	BTG1

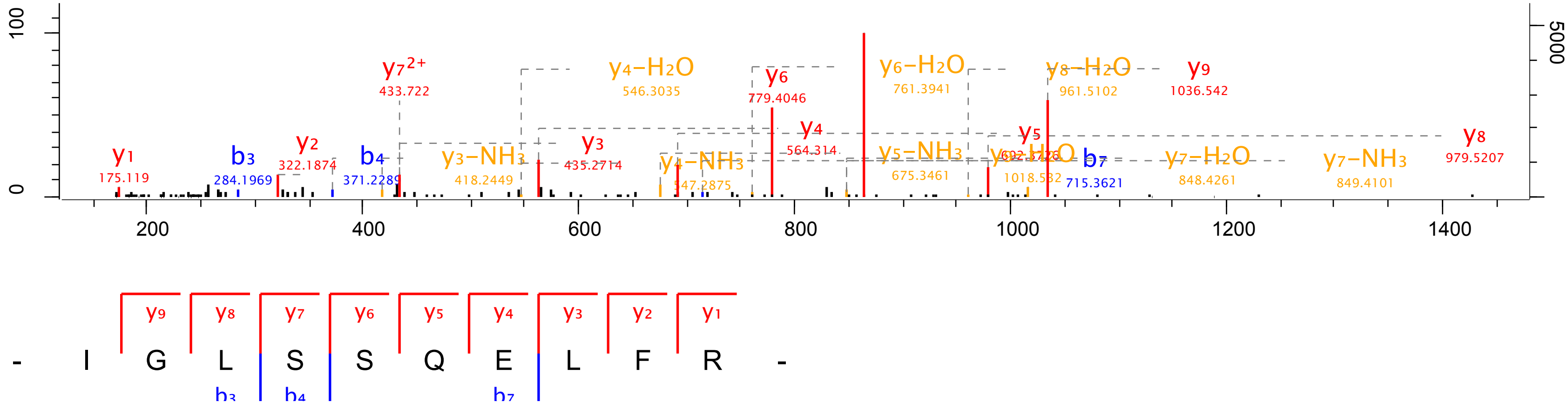

Raw file	Scan	Method	Score	m/z	Gene names
20140918_fract24_dyn_5ul_F8_01_392	29116	TOF; CID	139.58	512.29	FAM118A

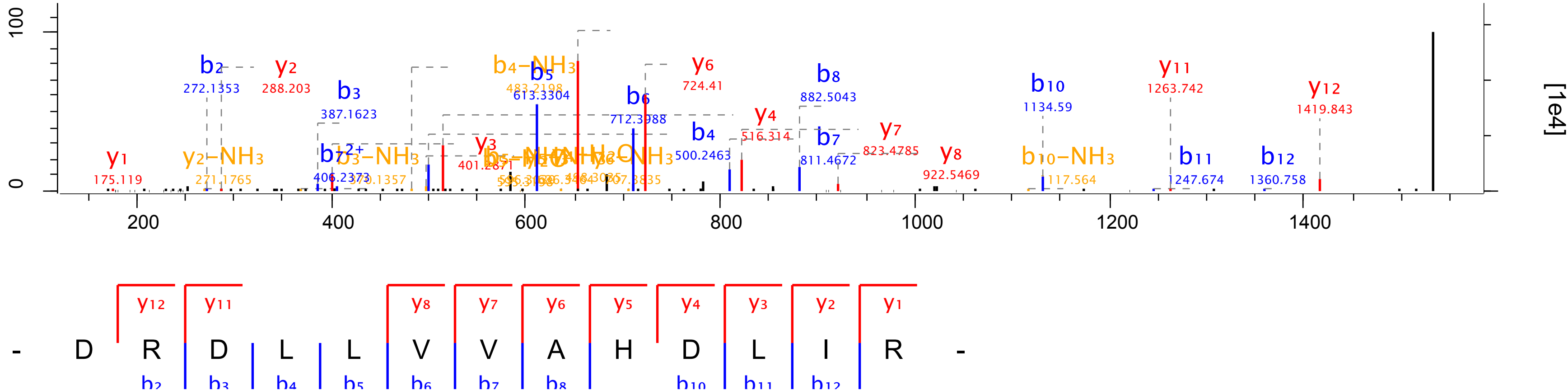

Raw file	Scan	Method	Score	m/z	Gene names
20140918_fract24_dyn_5ul_F8_01_392	31489	TOF; CID	147.73	614.8	ZBTB8OS

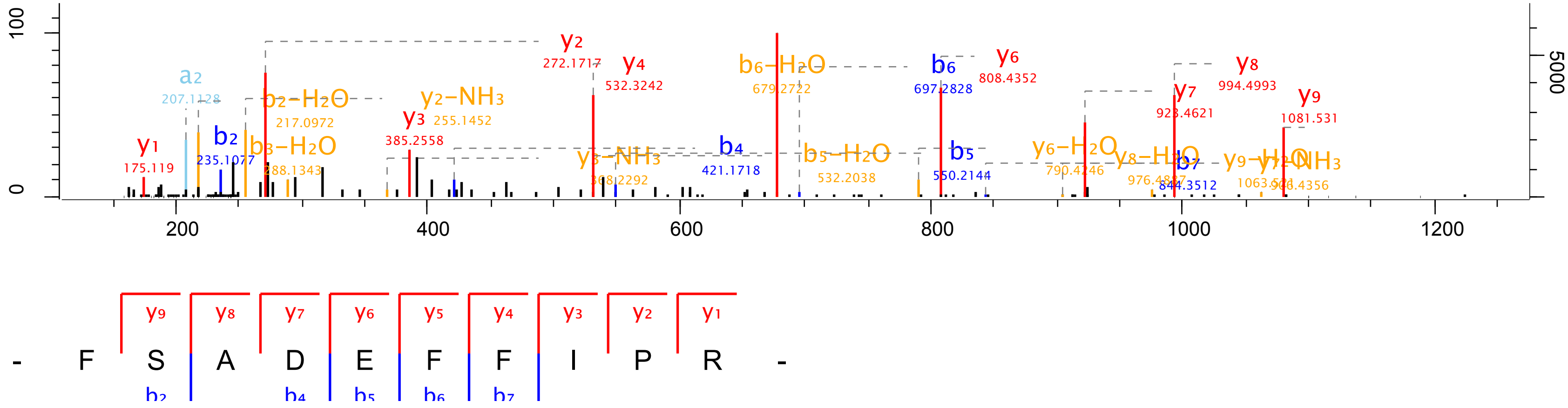

Raw file	Scan	Method	Score	m/z	Gene names
20140918_fract24_dyn_5ul_F8_01_392	33403	TOF; CID	50.3	824.43	POLI

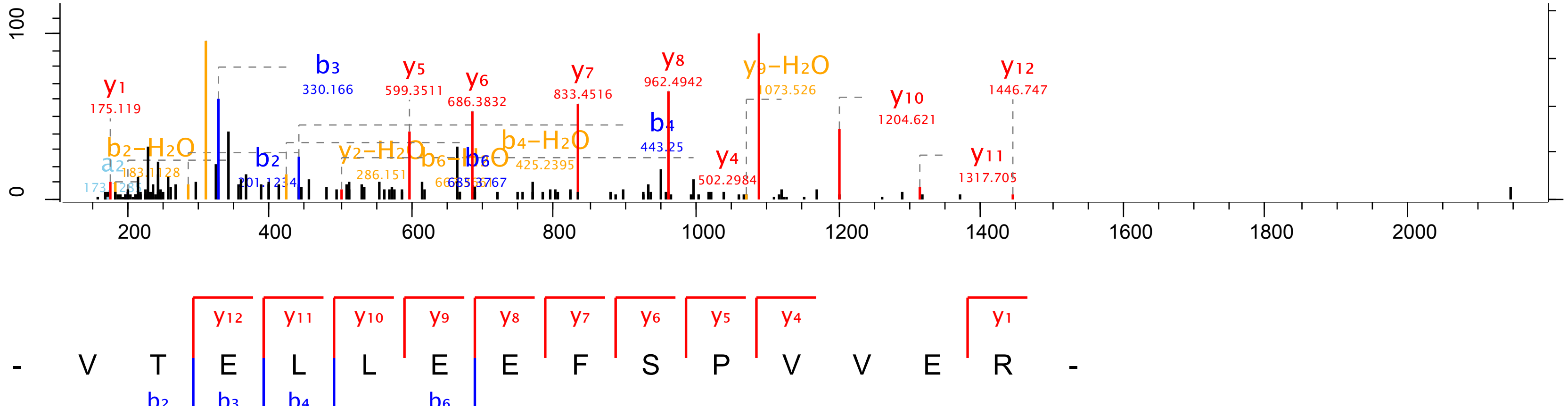

Raw file	Scan	Method	Score	m/z	Gene names
20140918_fract24_dyn_5ul_F8_01_392	34044	TOF; CID	83.23	711.86	SMIM15

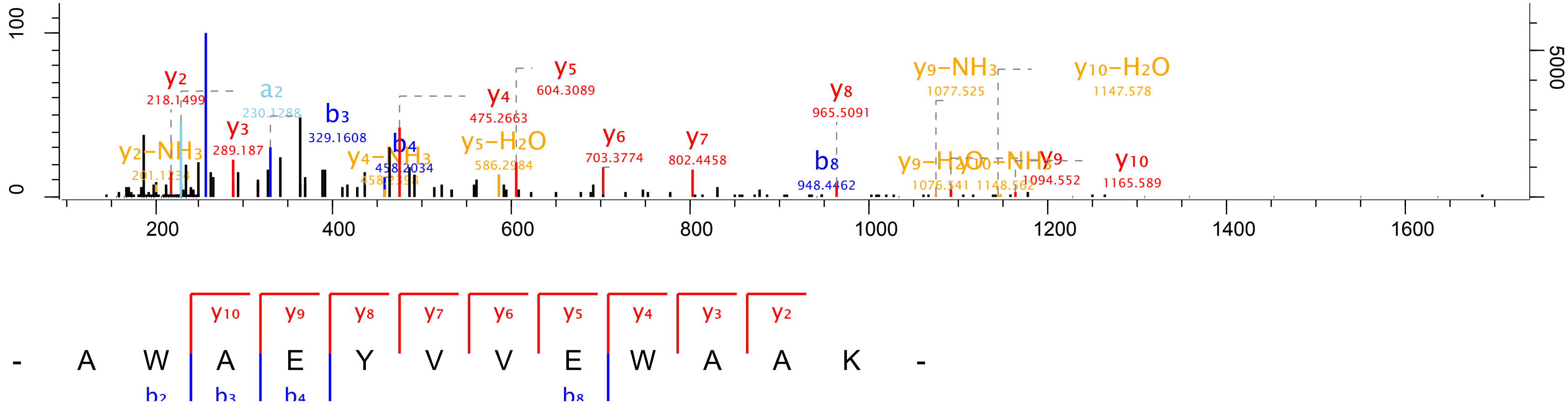

Supplement: Supplemental Data [file supp_M114.047407_mcp.M114.047407-10.pdf]
