# Supplementary material for: The Impact II, a Very High-Resolution Quadrupole Time-of-Flight Instrument (QTOF) for Deep Shotgun Proteomics
Source: Mol Cell Proteomics. 2015 May 19;14(7):2014–29. doi: 10.1074/mcp.M114.047407 (PMC4587313; doi:10.1074/mcp.M114.047407)

Raw file	Scan	Method	Score	m/z	Gene names
20140925_fract1_dyn_5ul_B1_01_436	3761	TOF; CID	88.5	538.76	TM4SF1

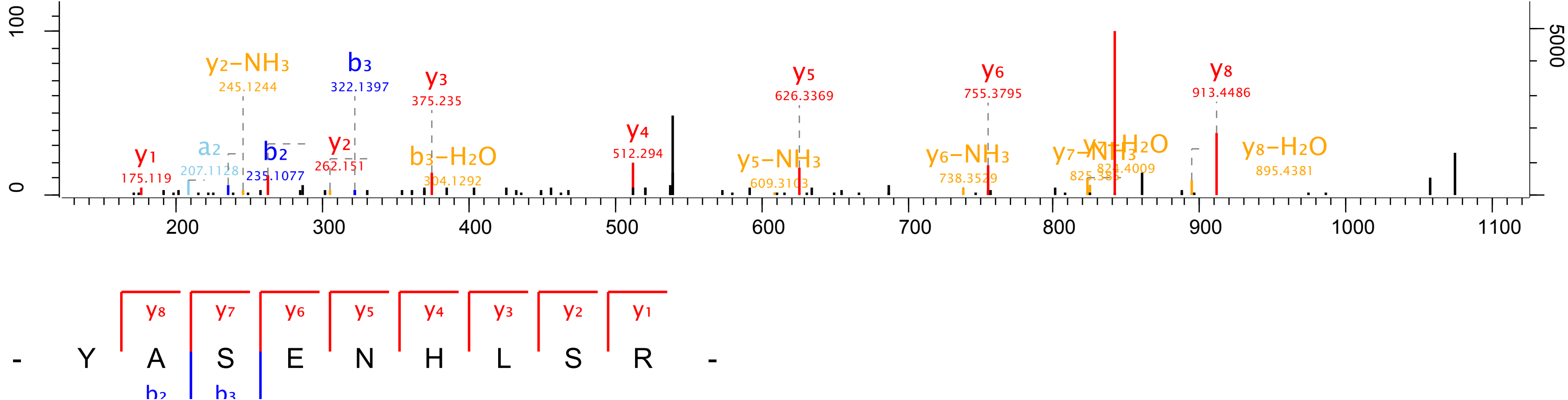

Raw file	Scan	Method	Score	m/z	Gene names
20140925_fract1_dyn_5ul_B1_01_436	9153	TOF; CID	38.88	690.65	SETD5

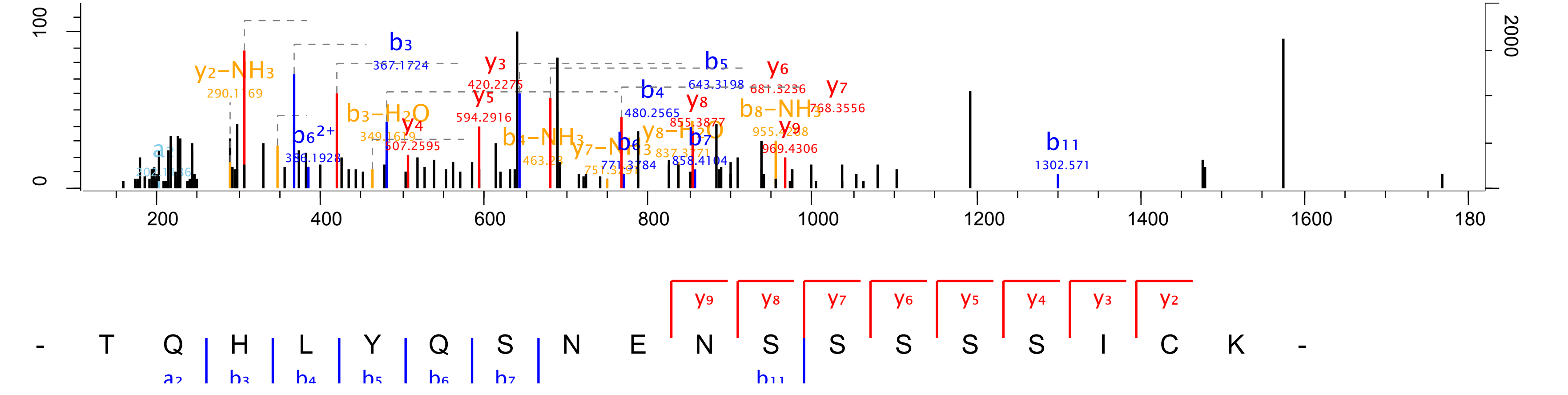

Raw file	Scan	Method	Score	m/z	Gene names
20140925_fract1_dyn_5ul_B1_01_436	11407	TOF; CID	44.51	483.88	KLHL5

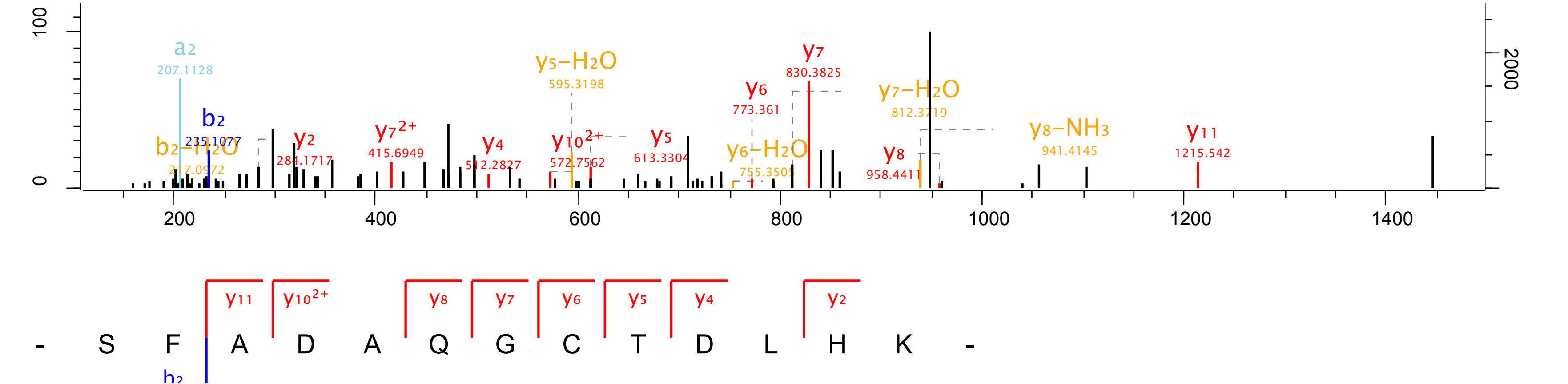

Raw file	Scan	Method	Score	m/z	Gene names
20140925_fract1_dyn_5ul_B1_01_436	11708	TOF; CID	48.71	590.62	PRDM4

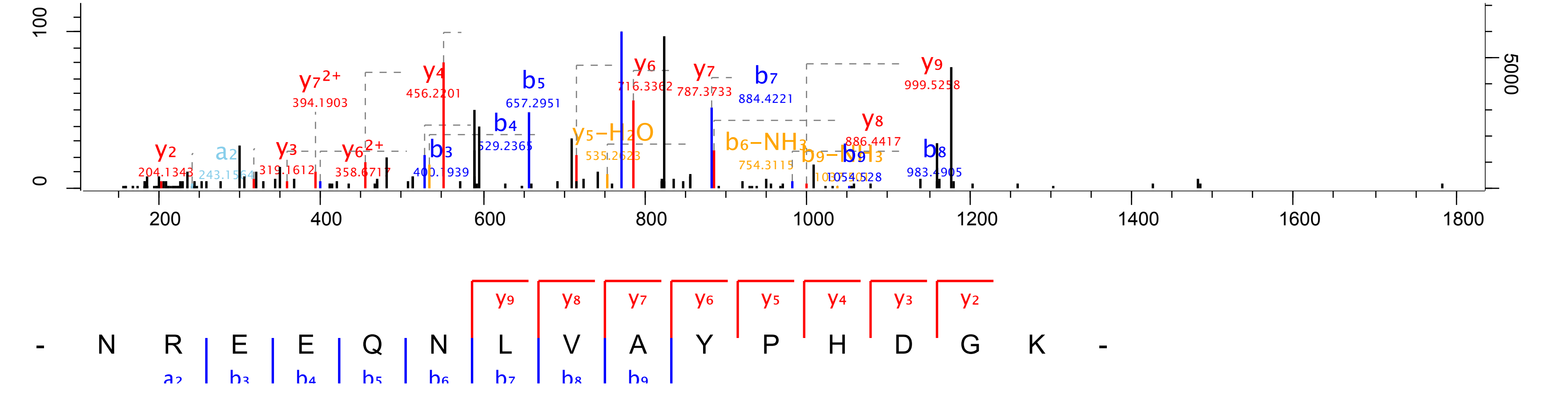

Raw file	Scan	Method	Score	m/z	Gene names
20140925_fract1_dyn_5ul_B1_01_436	12547	TOF; CID	59.23	632.32	HERC6

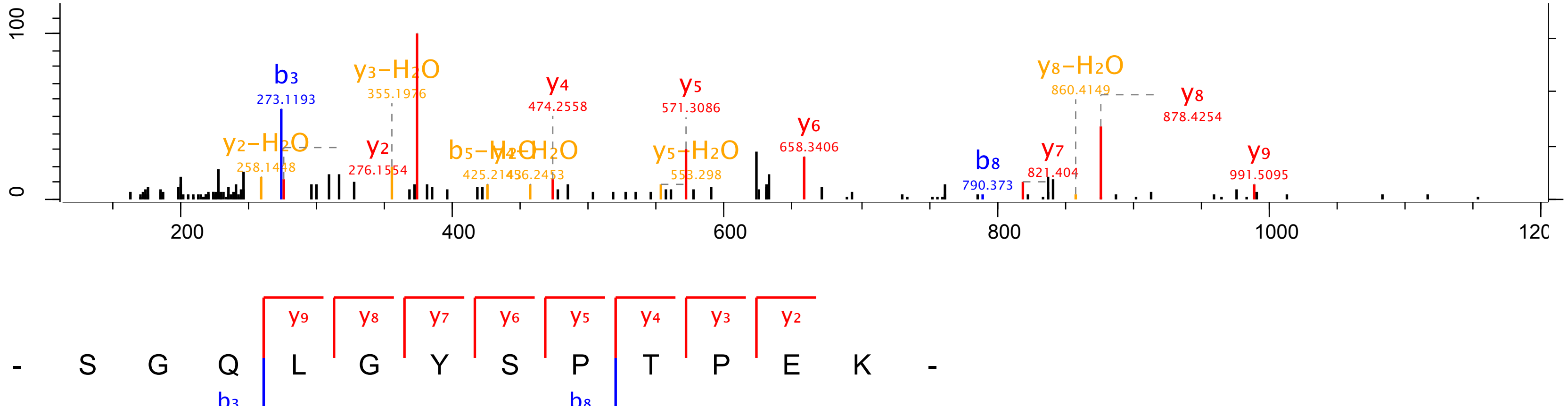

Raw file	Scan	Method	Score	m/z	Gene names
20140925_fract1_dyn_5ul_B1_01_436	17592	TOF; CID	81.8	726.36	SCNN1B

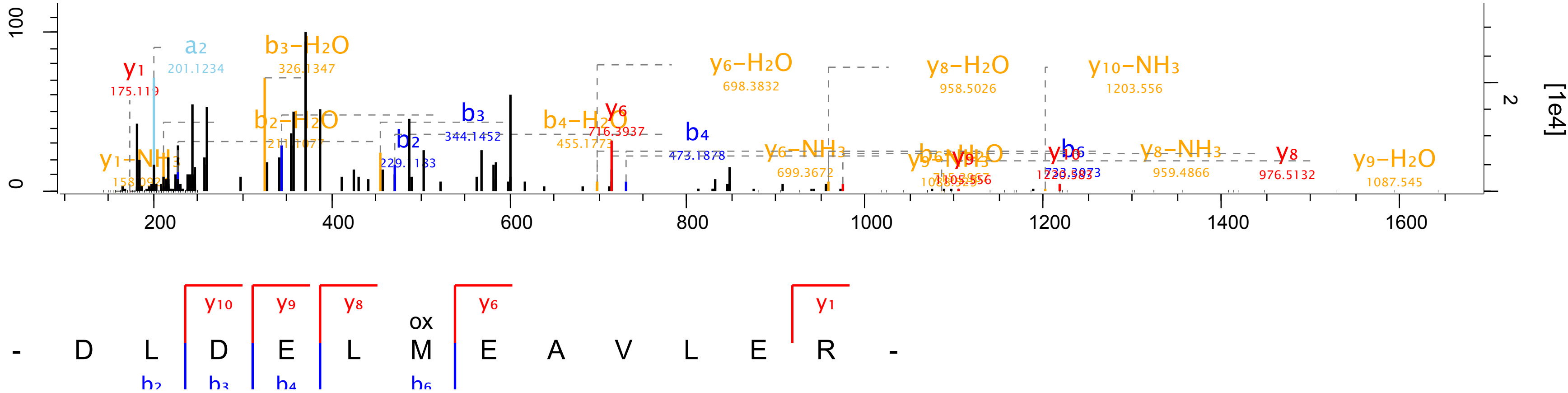

Raw file	Scan	Method	Score	m/z	Gene names
20140925_fract1_dyn_5ul_B1_01_436	18212	TOF; CID	77.06	312.54	ZNF668

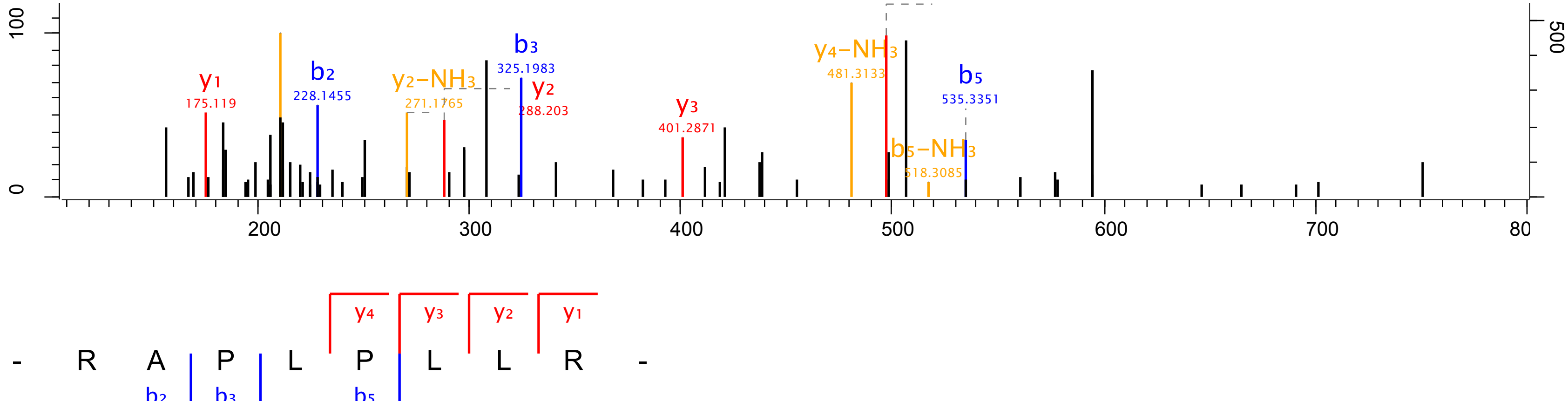

Raw file	Scan	Method	Score	m/z	Gene names
20140925_fract1_dyn_5ul_B1_01_436	21807	TOF; CID	82.29	545.82	CDCA4

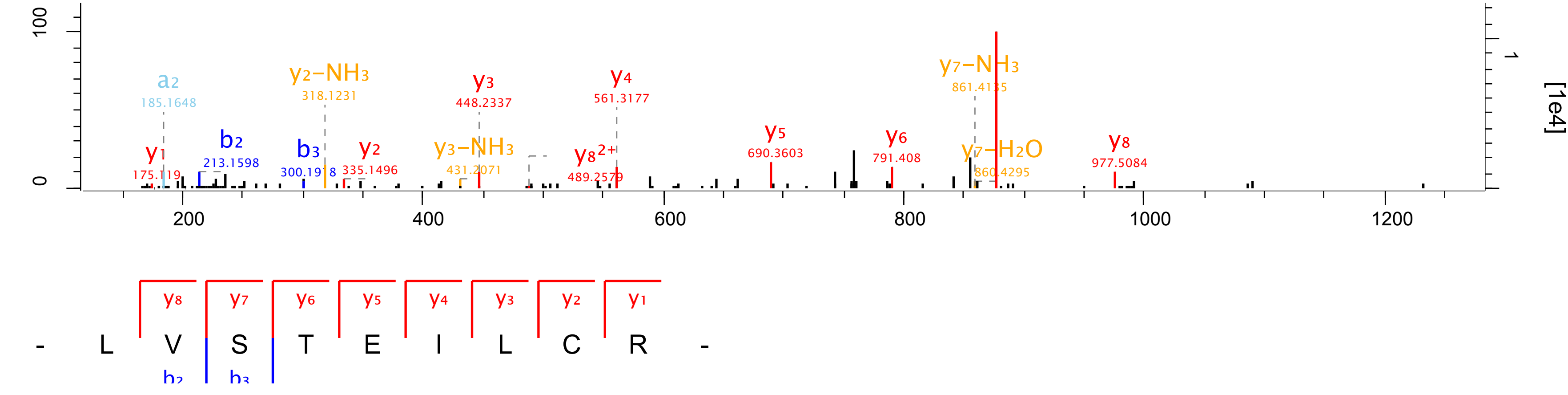

Raw file	Scan	Method	Score	m/z	Gene names
20140925_fract1_dyn_5ul_B1_01_436	22467	TOF; CID	77.42	445.75	SLC35A3

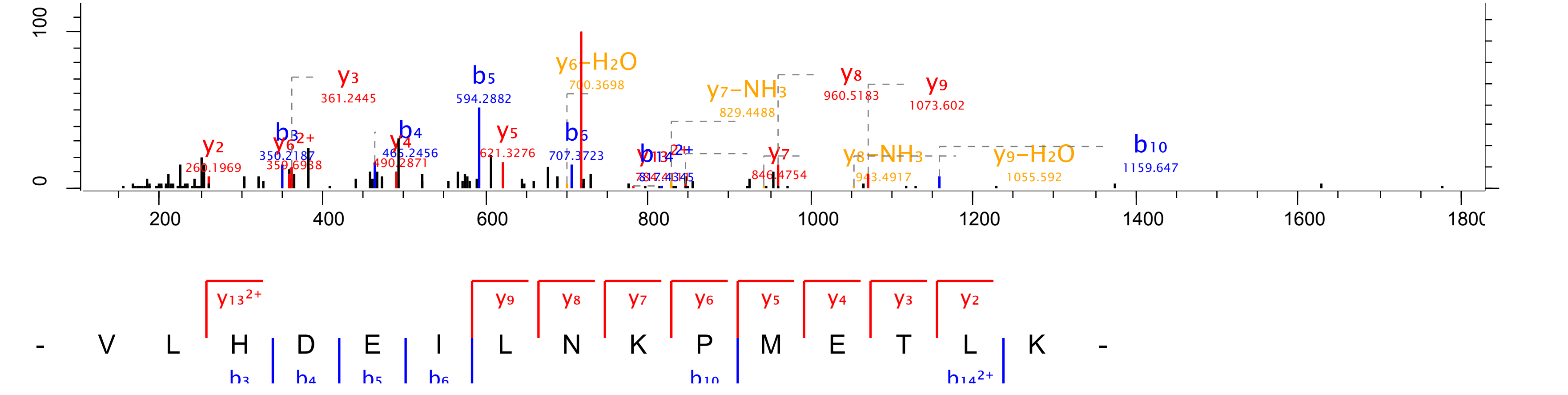

Raw file	Scan	Method	Score	m/z	Gene names
20140925_fract1_dyn_5ul_B1_01_436	25443	TOF; CID	68.28	796.39	SRRD

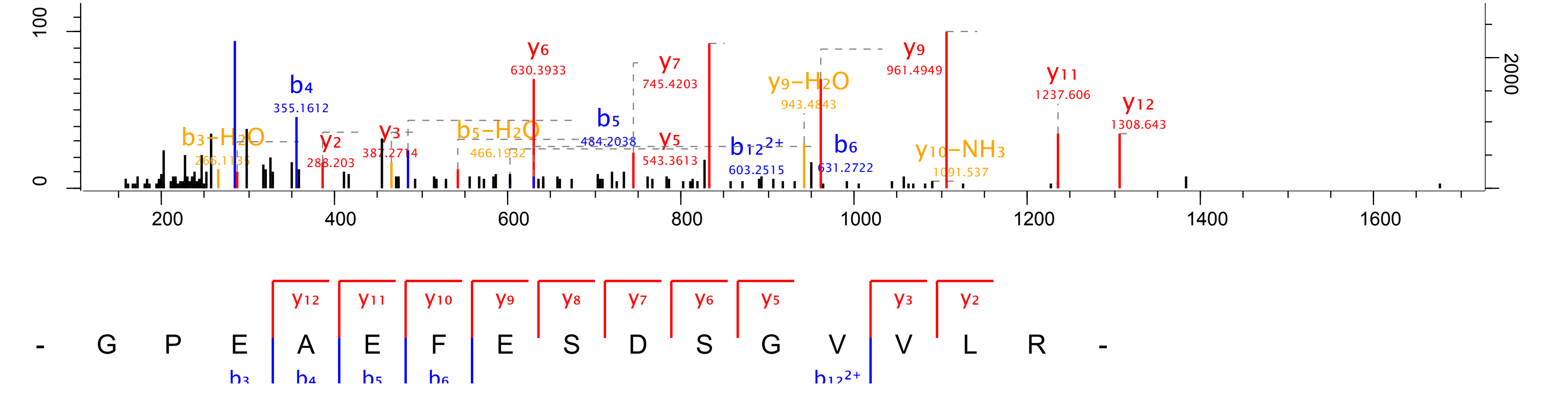

Raw file	Scan	Method	Score	m/z	Gene names
20140925_fract1_dyn_5ul_B1_01_436	27361	TOF; CID	47.77	499.29	SPATA31C2;SPATA31C1

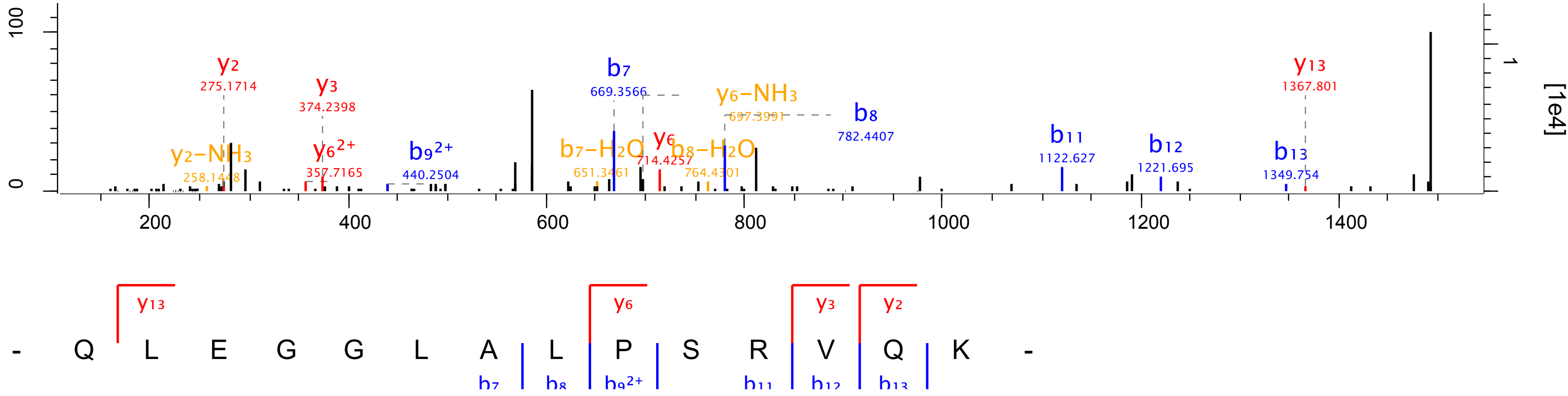

Raw file	Scan	Method	Score	m/z	Gene names
20140925_fract1_dyn_5ul_B1_01_436	28512	TOF; CID	65.29	469.25	CHAC1

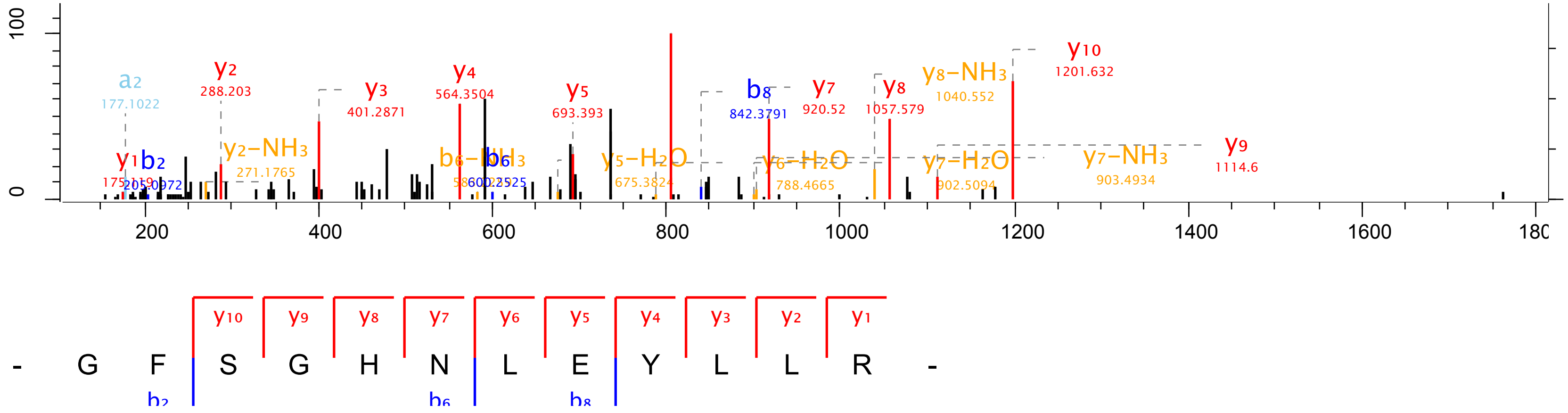

Raw file	Scan	Method	Score	m/z	Gene names
20140925_fract1_dyn_5ul_B1_01_436	29388	TOF; CID	66.57	790.96	ARL4A

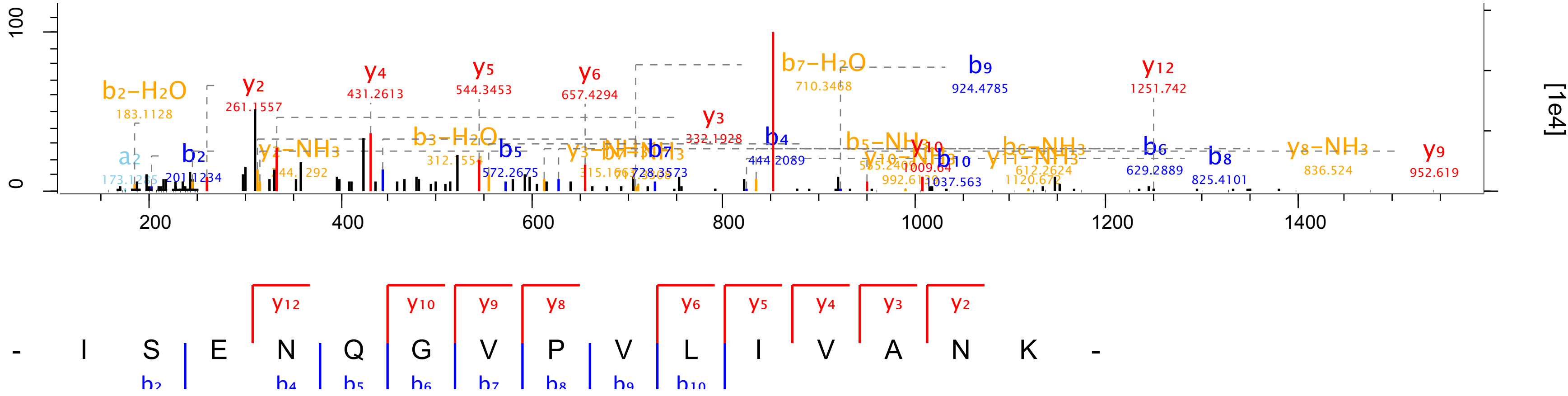

Raw file	Scan	Method	Score	m/z	Gene names
20140925_fract1_dyn_5ul_B1_01_436	30244	TOF; CID	41.62	567.3	TECTA

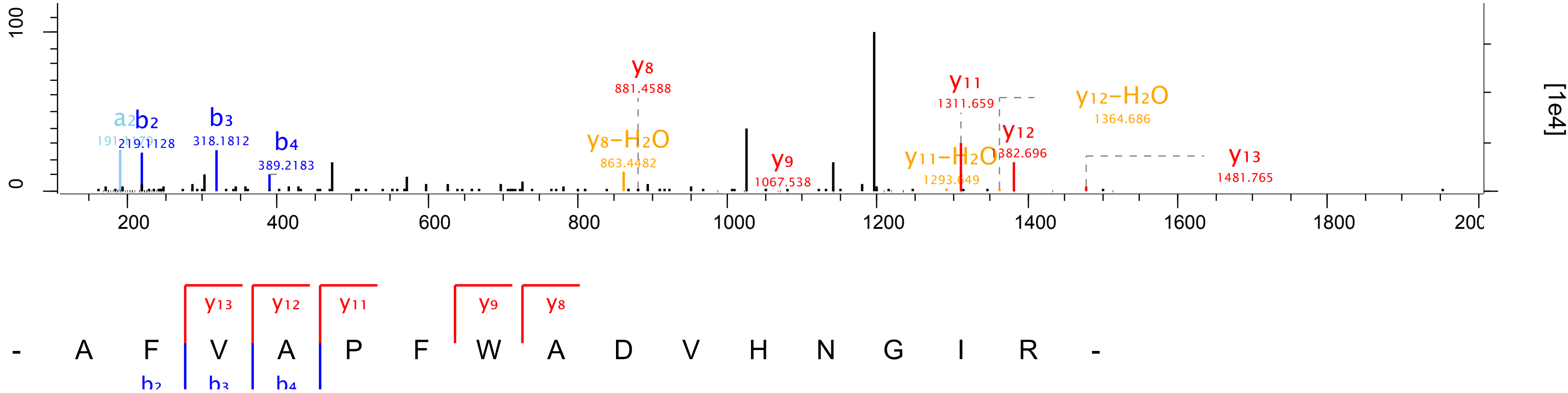

Raw file	Scan	Method	Score	m/z	Gene names
20140925_fract1_dyn_5ul_B1_01_436	30678	TOF; CID	61.28	999.02	GLYCTK

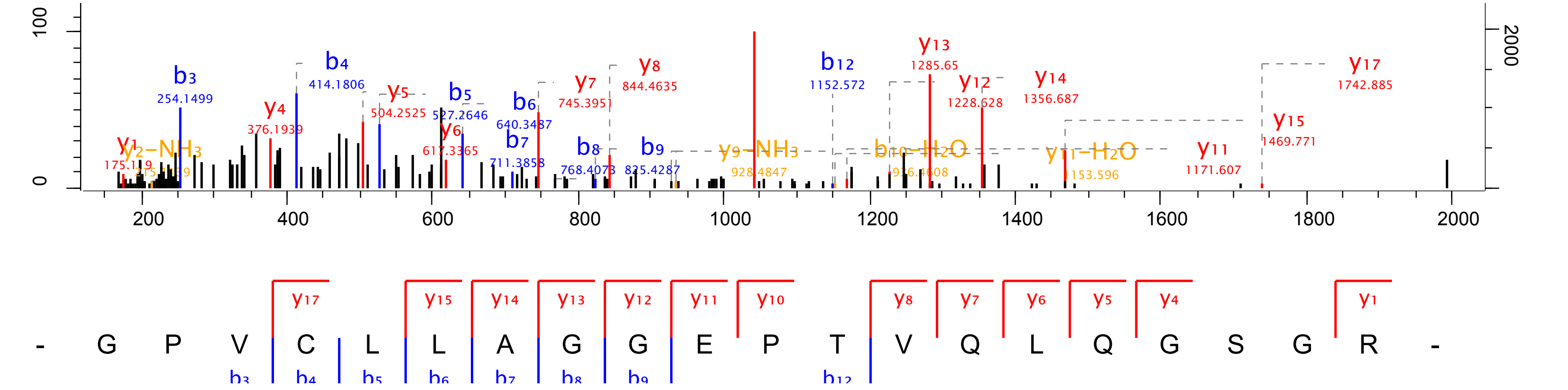

Raw file	Scan	Method	Score	m/z	Gene names
20140925_fract1_dyn_5ul_B1_01_436	32676	TOF; CID	44.34	772.74	PLLP

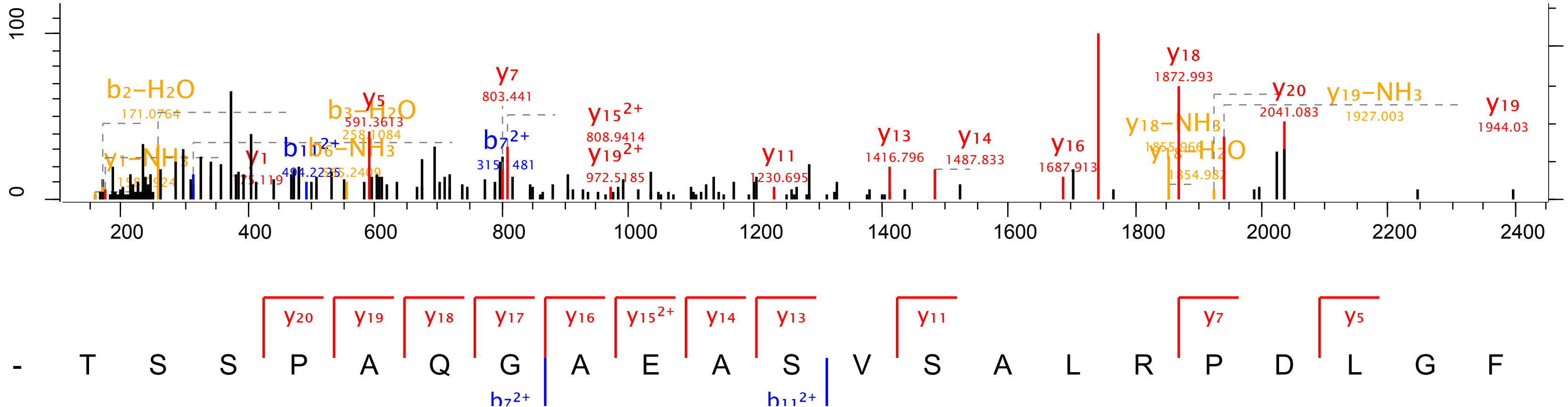

Raw file	Scan	Method	Score	m/z	Gene names
20140925_fract1_dyn_5ul_B1_01_436	34114	TOF; CID	126.91	575.32	STK19

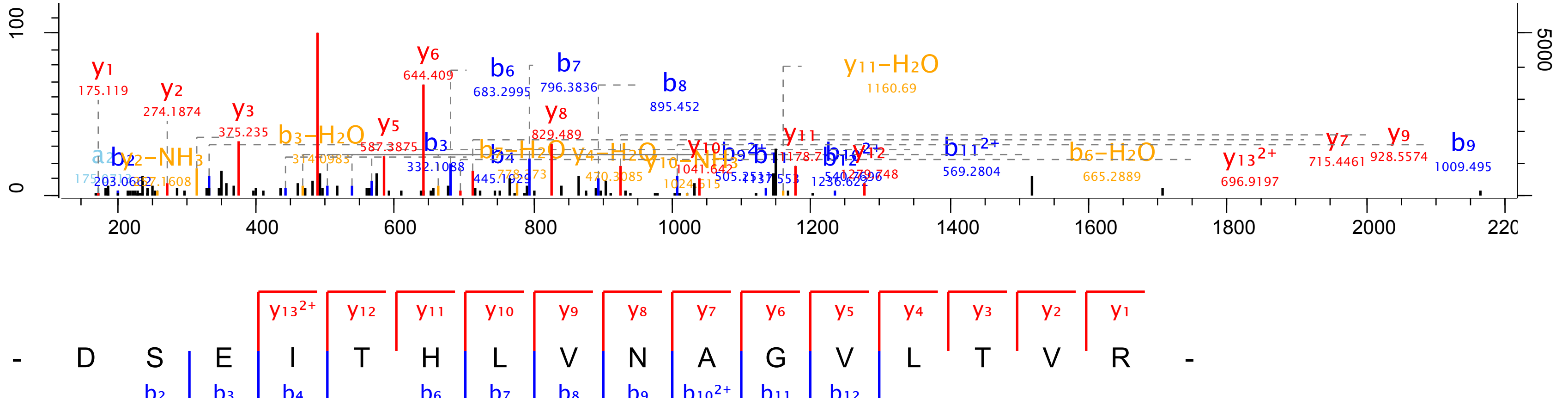

Raw file	Scan	Method	Score	m/z	Gene names
20140925_fract1_dyn_5ul_B1_01_436	37990	TOF; CID	101.3	613.31	TMEM234

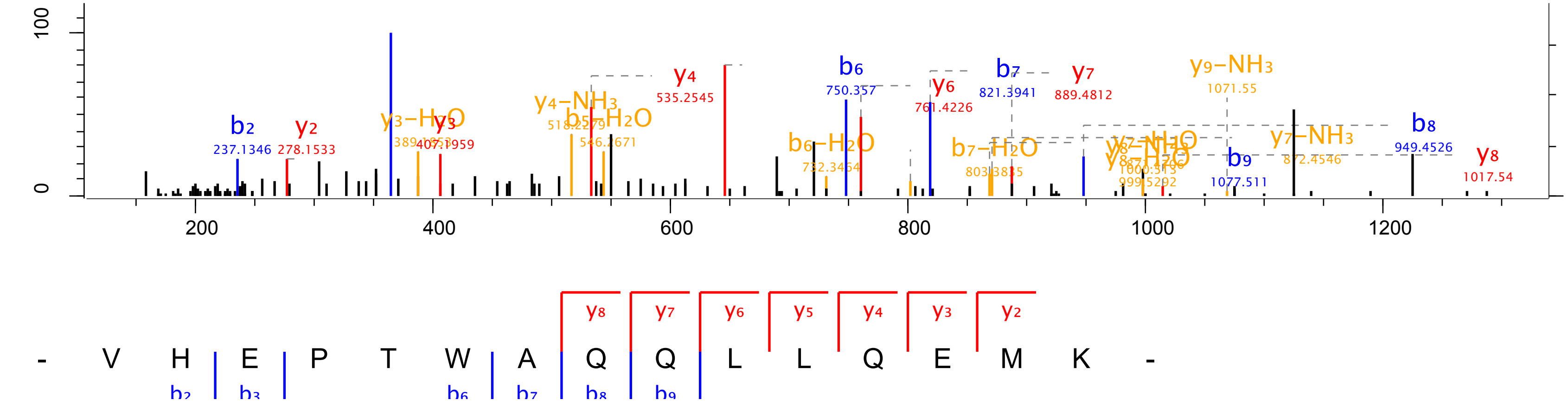

Raw file	Scan	Method	Score	m/z	Gene names
20140925_fract1_dyn_5ul_B1_01_436	38064	TOF; CID	95.2	943.47	ZBTB3

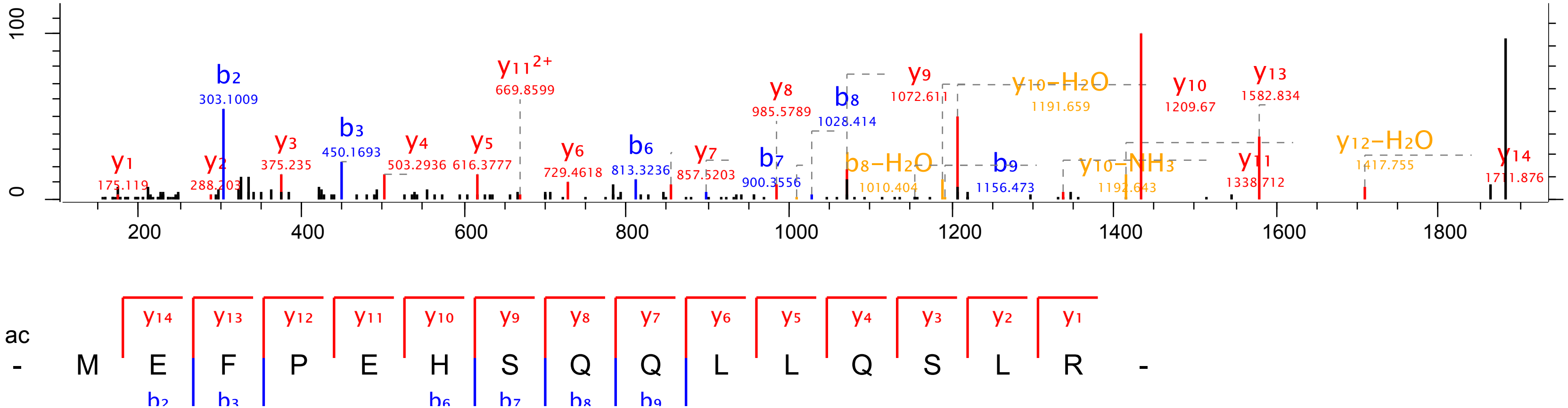

Raw file	Scan	Method	Score	m/z	Gene names
20140925_fract1_dyn_5ul_B1_01_436	39762	TOF; CID	83.37	967.99	CLIC5;CLIC6

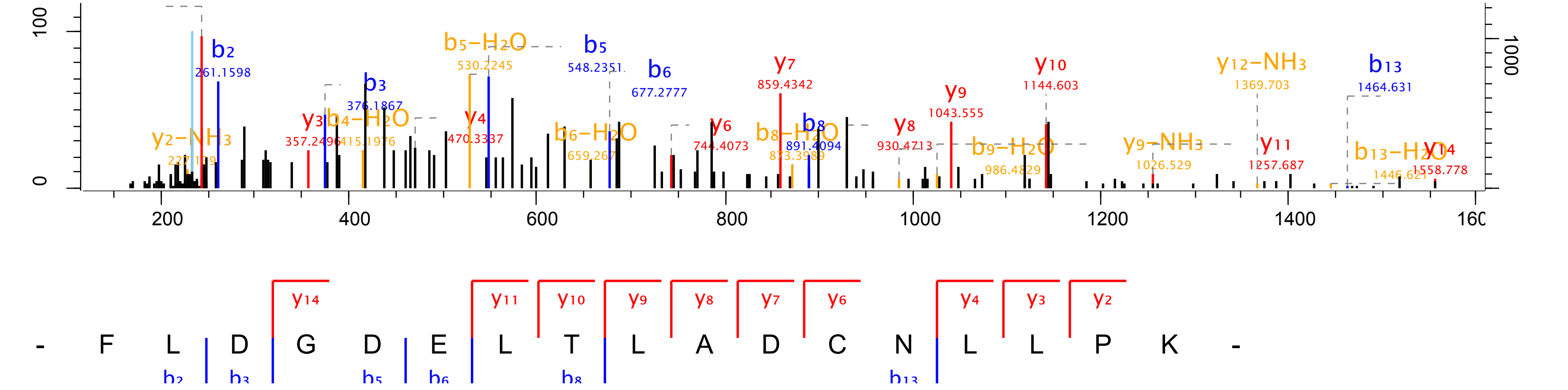

Raw file	Scan	Method	Score	m/z	Gene names
20140925_fract2_dyn_5ul_B2_01_437	3804	TOF; CID	76.23	390.53	BBIP1

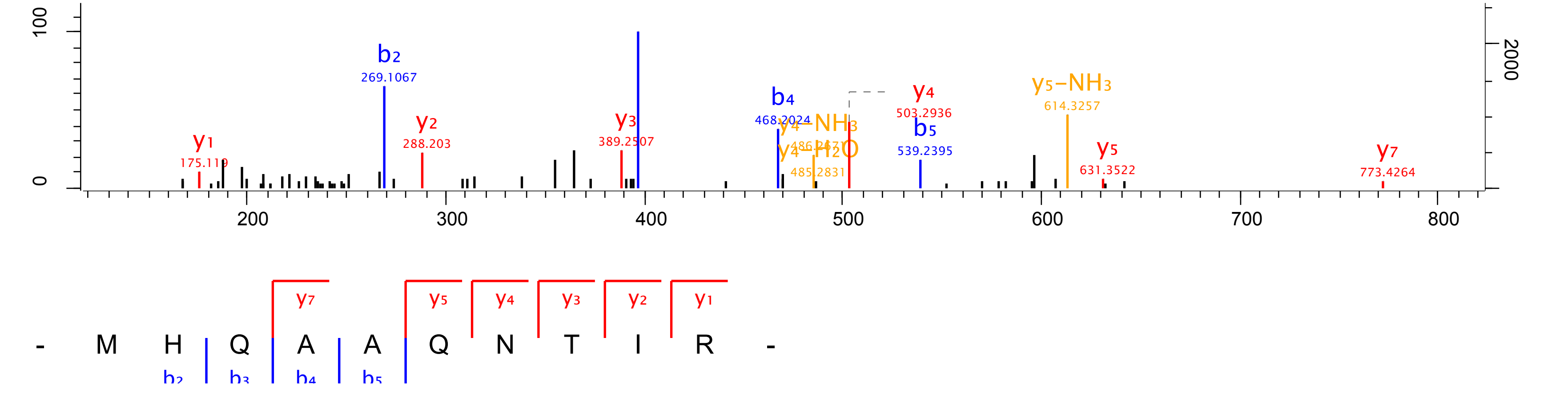

Raw file	Scan	Method	Score	m/z	Gene names
20140925_fract2_dyn_5ul_B2_01_437	4899	TOF; CID	119.21	330.19	HMGN4

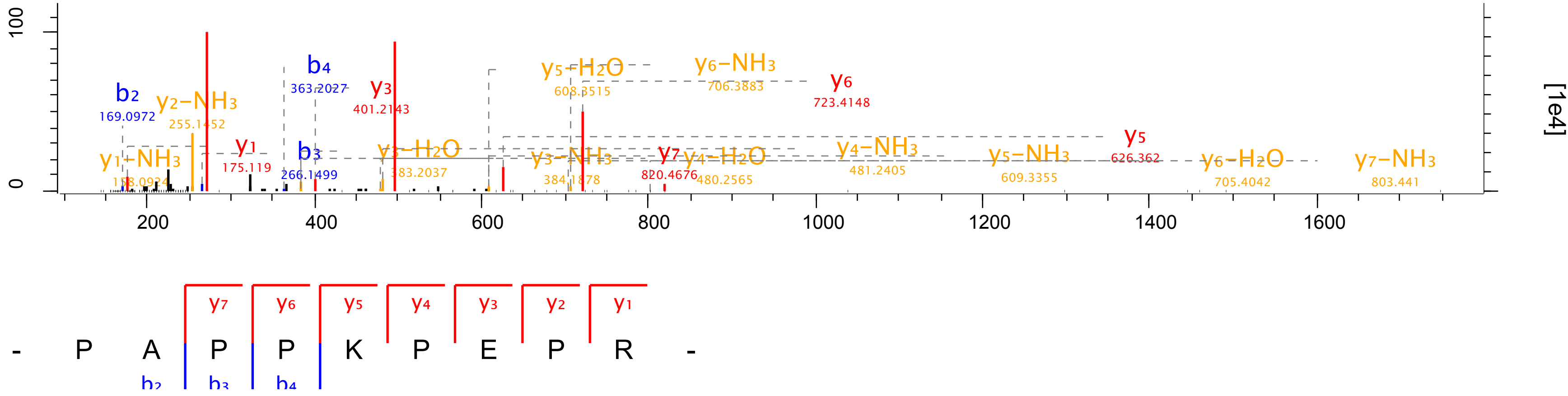

Raw file	Scan	Method	Score	m/z	Gene names
20140925_fract2_dyn_5ul_B2_01_437	4992	TOF; CID	67.33	556.26	ZNF485

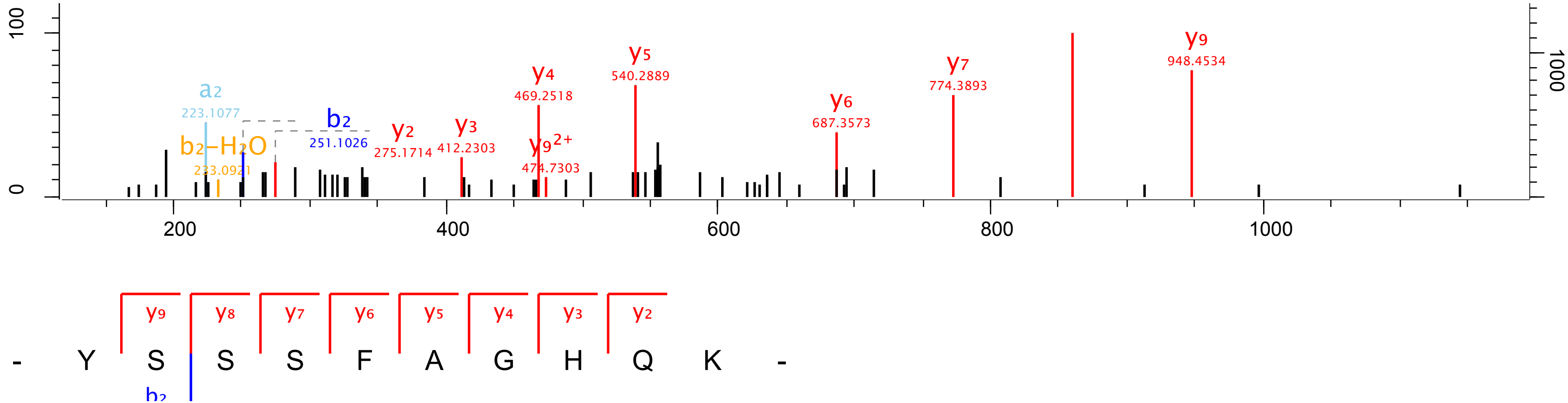

Raw file	Scan	Method	Score	m/z	Gene names
20140925_fract2_dyn_5ul_B2_01_437	7133	TOF; CID	73.67	510.75	C18orf32

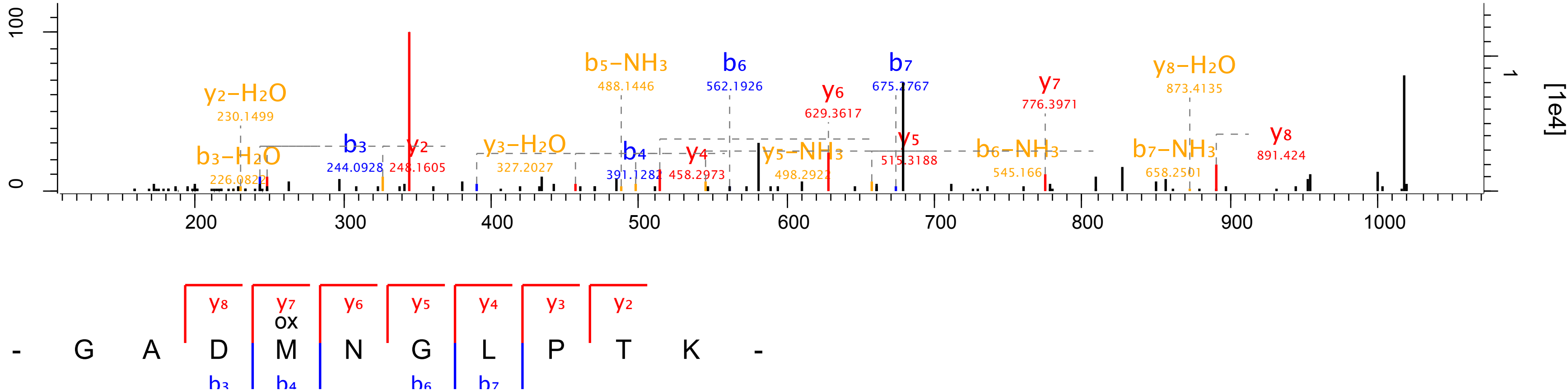

Raw file	Scan	Method	Score	m/z	Gene names
20140925_fract2_dyn_5ul_B2_01_437	9624	TOF; CID	79.47	323.51	CHIC1

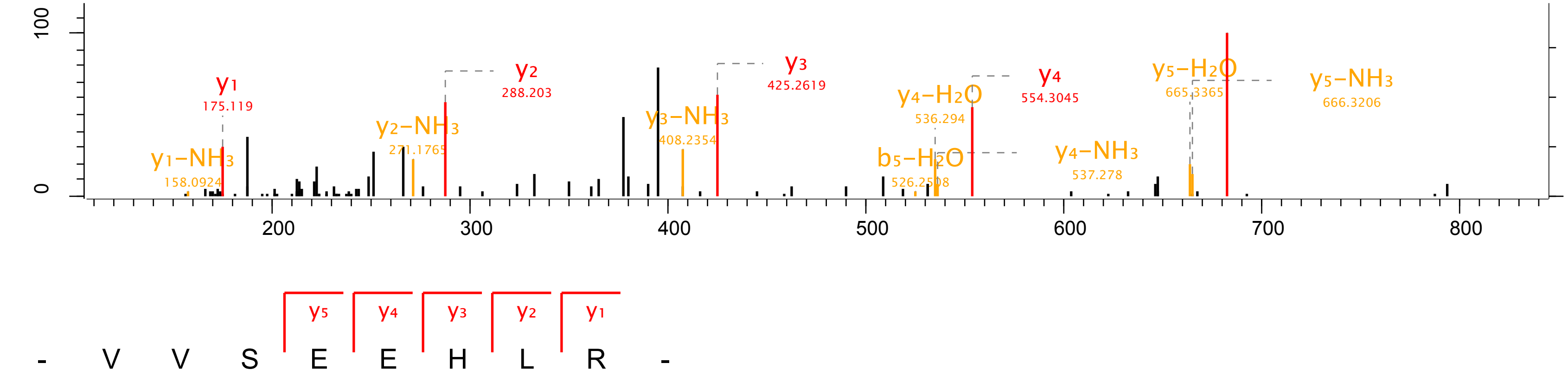

Raw file	Scan	Method	Score	m/z	Gene names
20140925_fract2_dyn_5ul_B2_01_437	11066	TOF; CID	79.64	660.32	NDFIP2

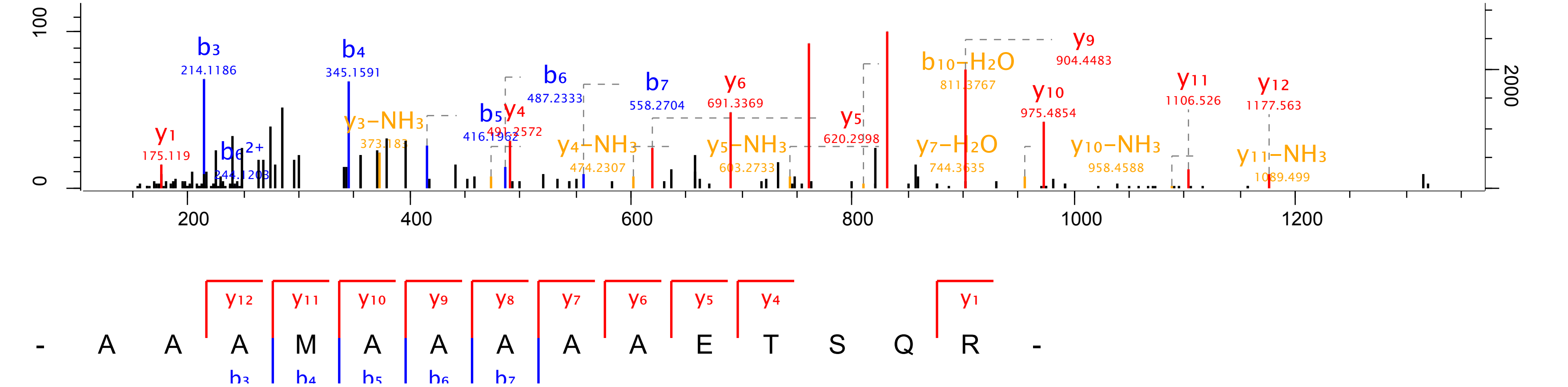

Raw file	Scan	Method	Score	m/z	Gene names
20140925_fract2_dyn_5ul_B2_01_437	12490	TOF; CID	72.89	720.31	LEPROTL1

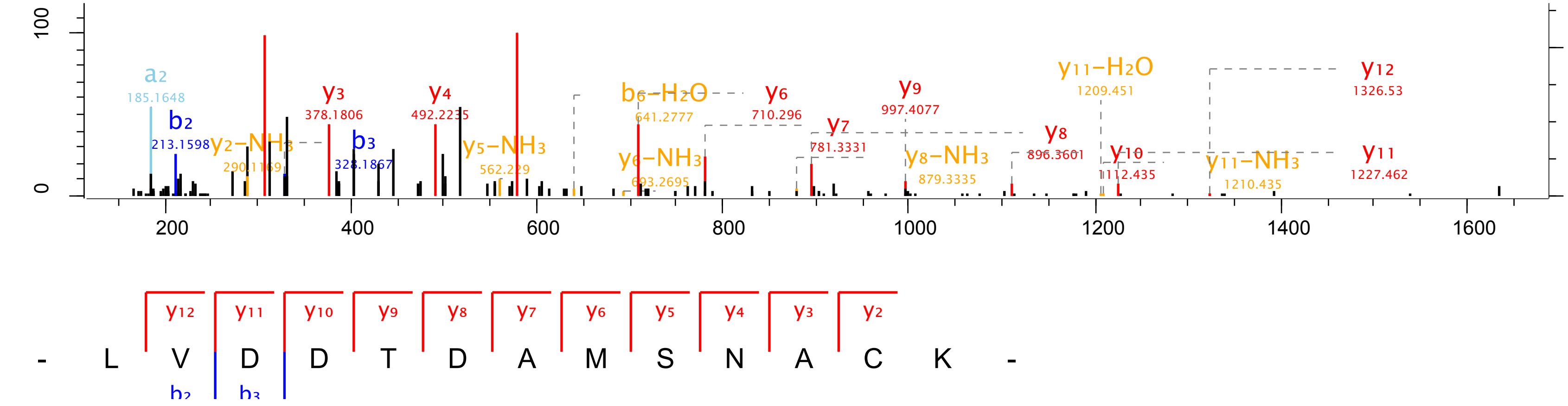

Raw file	Scan	Method	Score	m/z	Gene names
20140925_fract2_dyn_5ul_B2_01_437	13182	TOF; CID	54.9	735.84	ZNF276

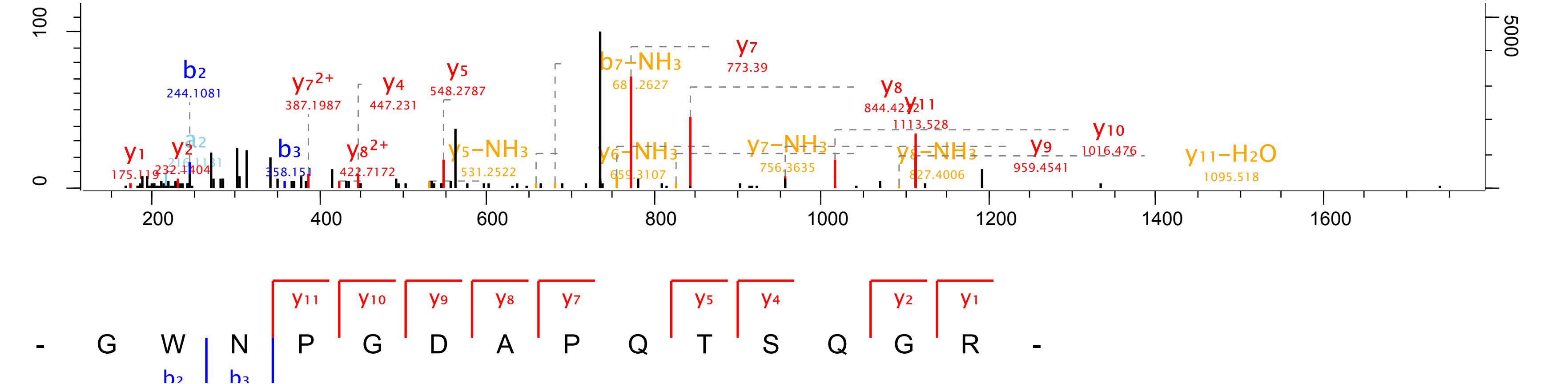

Raw file	Scan	Method	Score	m/z
20140925_fract2_dyn_5ul_B2_01_437	15478	TOF; CID	72.2	609.8

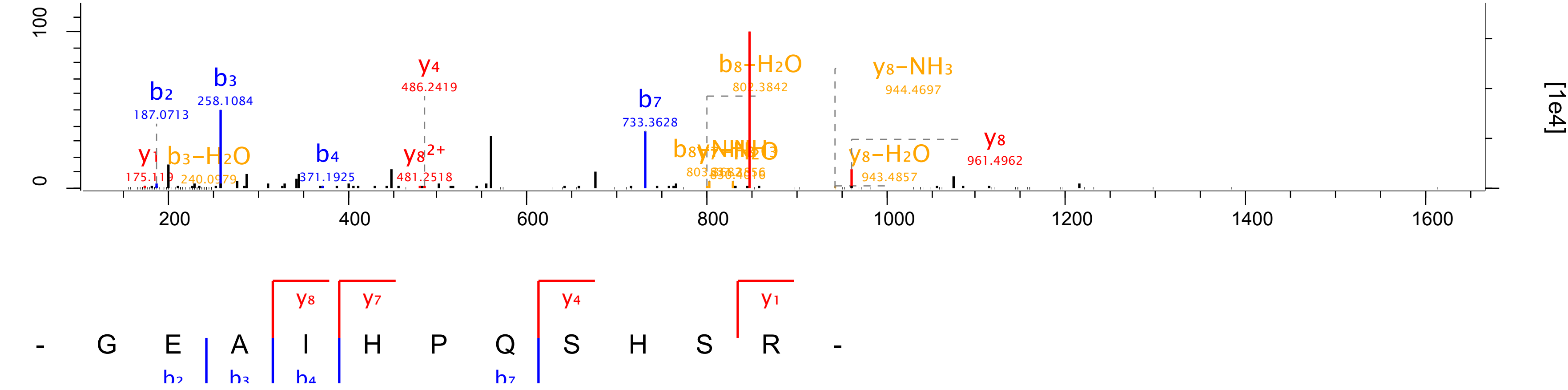

Raw file	Scan	Method	Score	m/z	Gene names
20140925_fract2_dyn_5ul_B2_01_437	18205	TOF; CID	120.63	858.93	APOC3

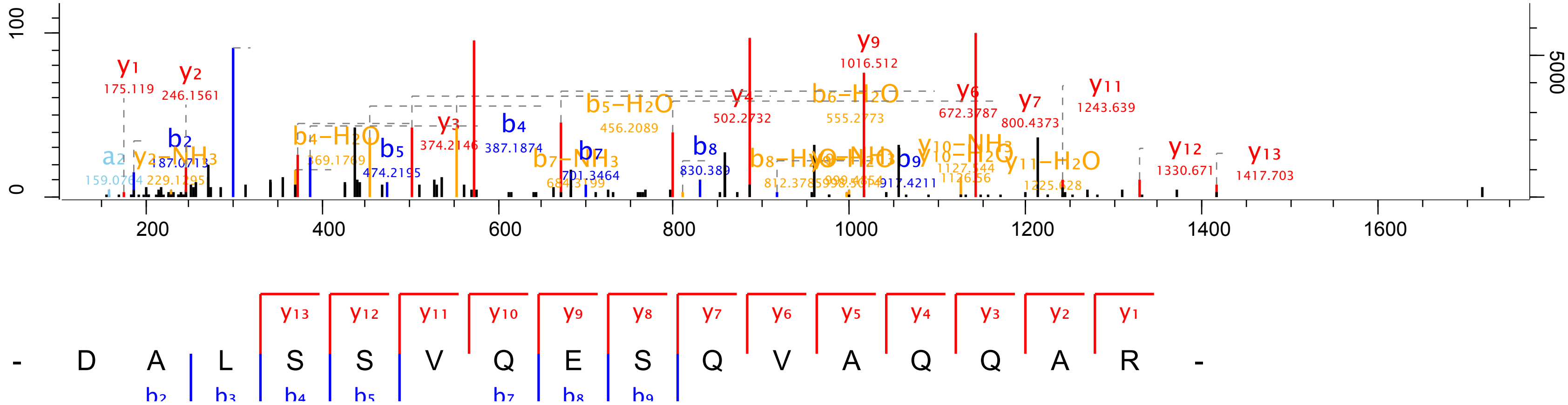

Raw file	Scan	Method	Score	m/z	Gene names
20140925_fract2_dyn_5ul_B2_01_437	22456	TOF; CID	118.52	674.31	BATF3

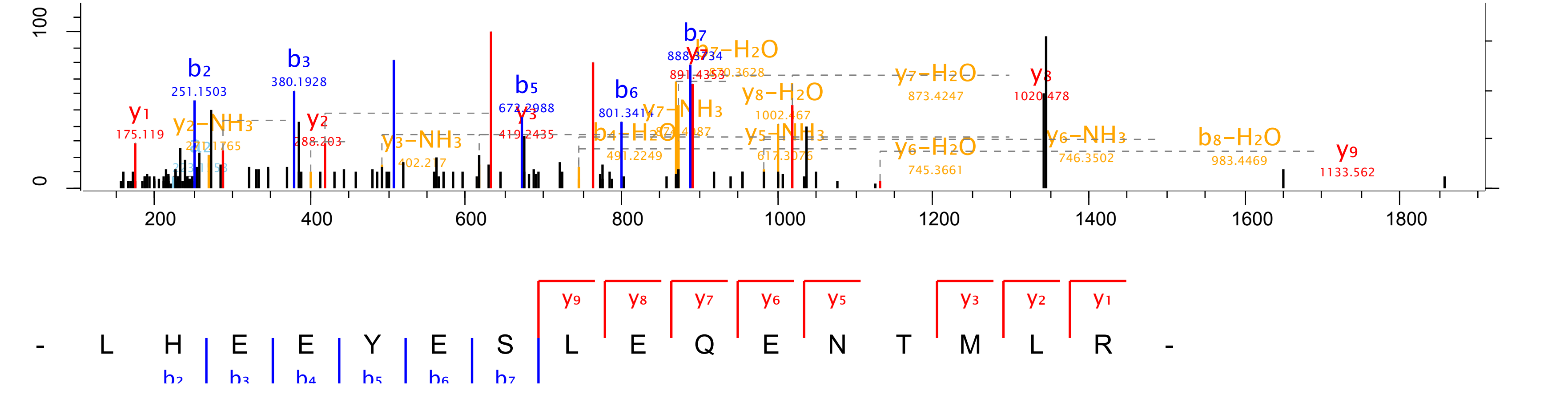

Raw file	Scan	Method	Score	m/z	Gene names
20140925_fract2_dyn_5ul_B2_01_437	25530	TOF; CID	101.32	756.86	FAM73A

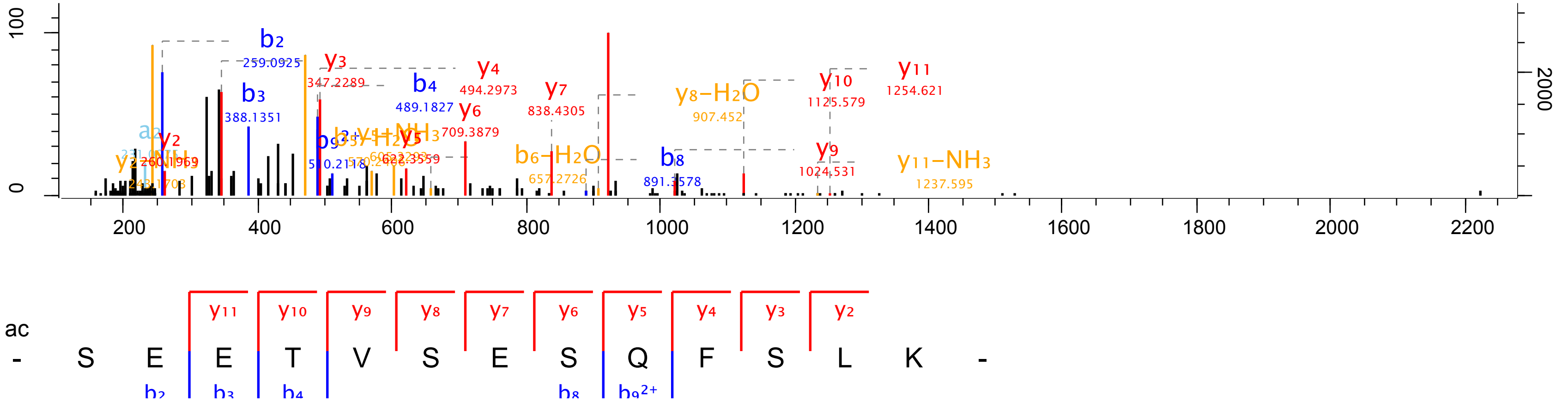

Raw file	Scan	Method	Score	m/z	Gene names
20140925_fract2_dyn_5ul_B2_01_437	26853	TOF; CID	116.63	505.27	MRPL57

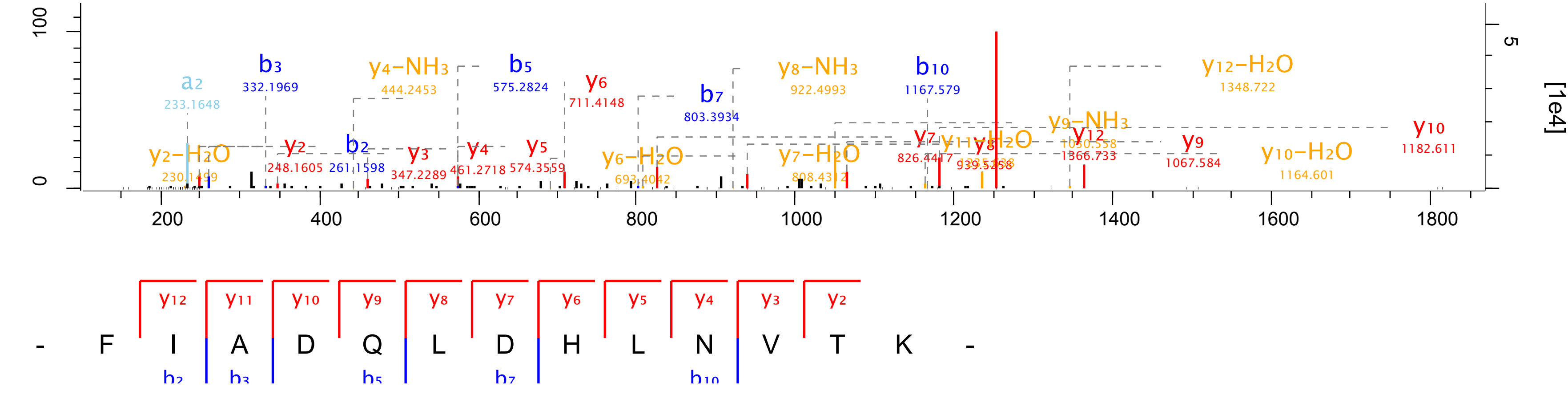

Raw file	Scan	Method	Score	m/z	Gene names
20140925_fract2_dyn_5ul_B2_01_437	29916	TOF; CID	101.05	983.44	PPP1R16A

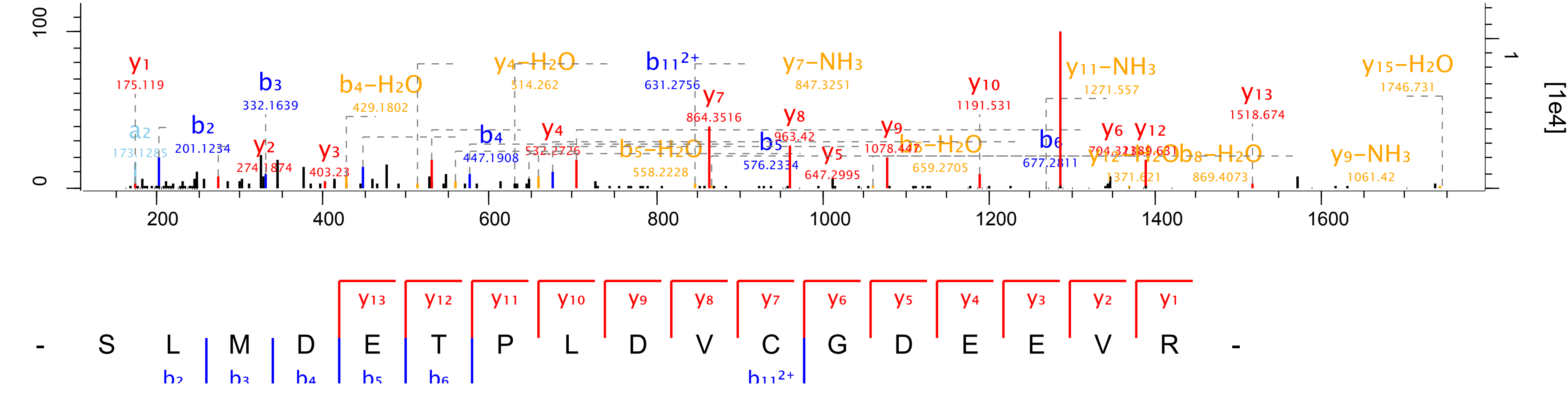

Raw file	Scan	Method	Score	m/z	Gene names
20140925_fract2_dyn_5ul_B2_01_437	30628	TOF; CID	110.41	496.76	SEPT3

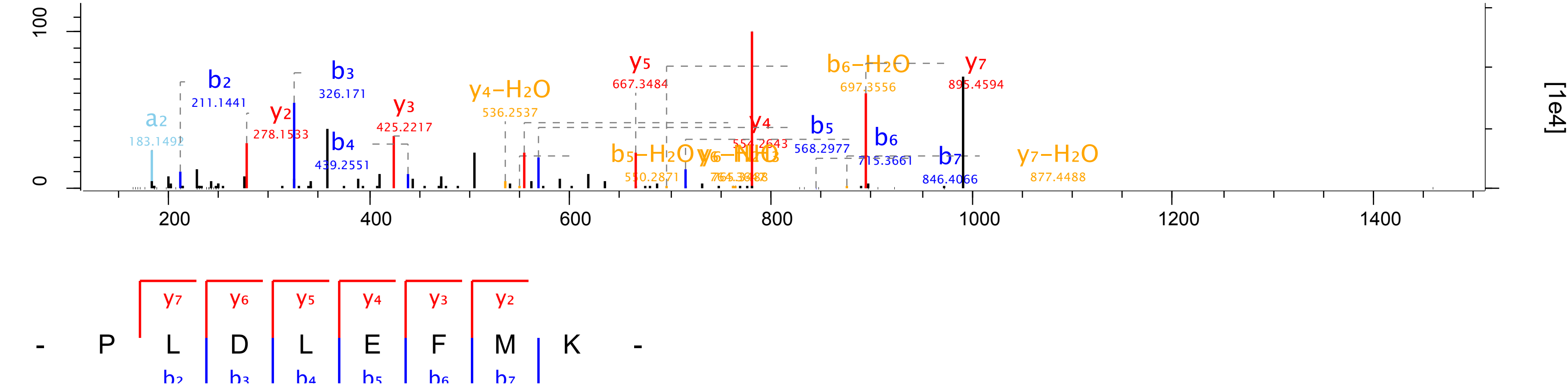

Raw file	Scan	Method	Score	m/z	Gene names
20140925_fract2_dyn_5ul_B2_01_437	30785	TOF; CID	99.44	534.25	HYAL1

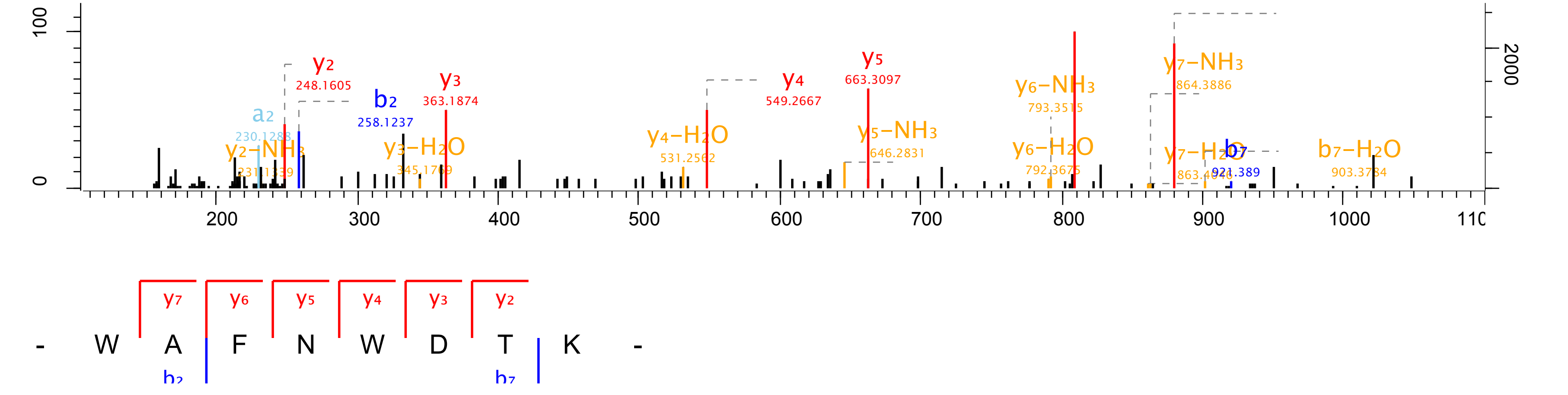

Raw file	Scan	Method	Score	m/z	Gene names
20140925_fract2_dyn_5ul_B2_01_437	31476	TOF; CID	74.3	508.62	GABPB1

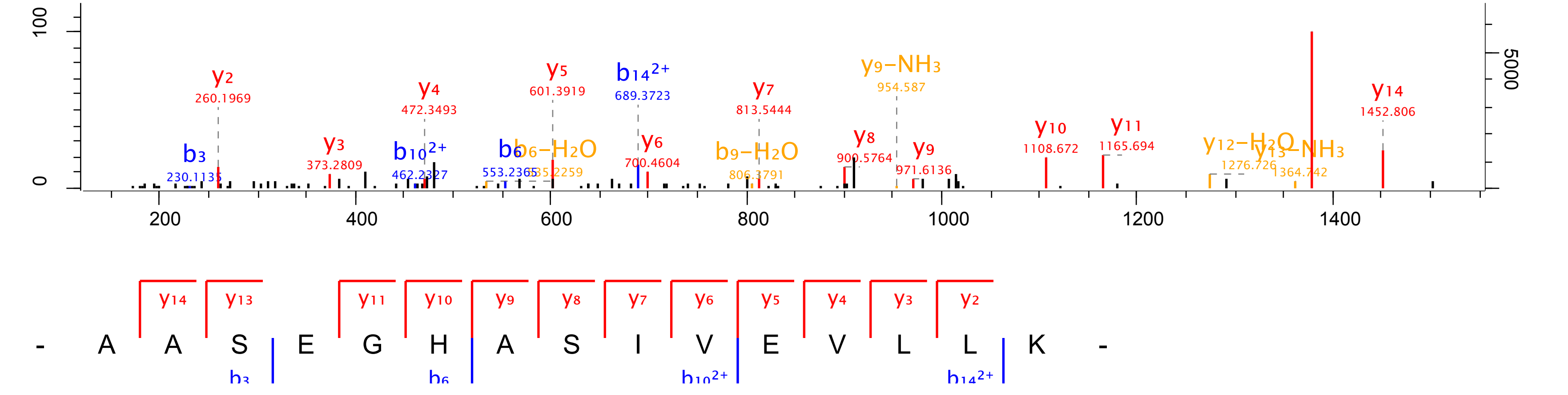

Raw file	Scan	Method	Score	m/z	Gene names
20140925_fract2_dyn_5ul_B2_01_437	33699	TOF; CID	97.22	583.3	TMEM138

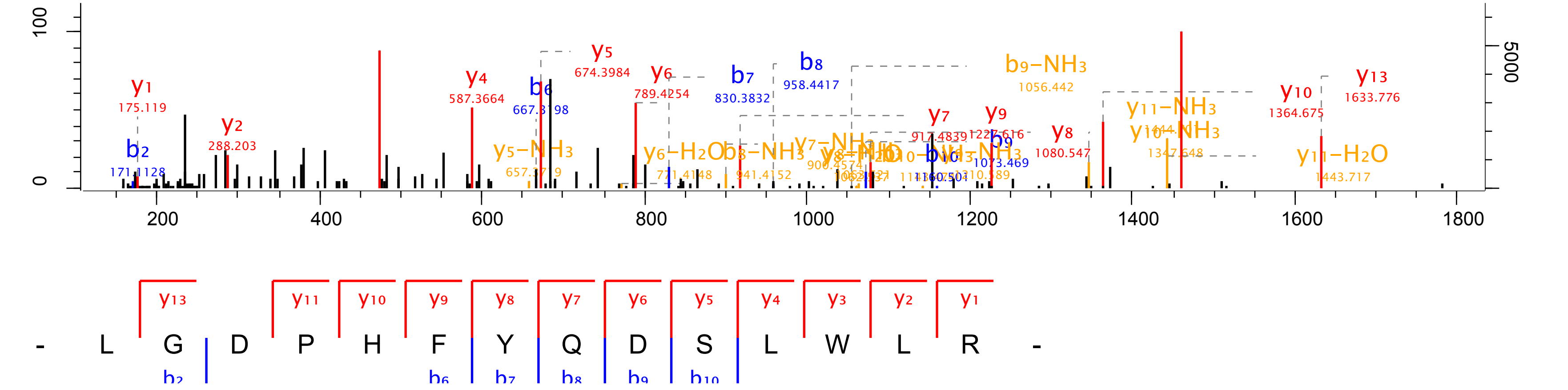

Raw file	Scan	Method	Score	m/z	Gene names
20140925_fract2_dyn_5ul_B2_01_437	36475	TOF; CID	29.84	984.49	HOXC5

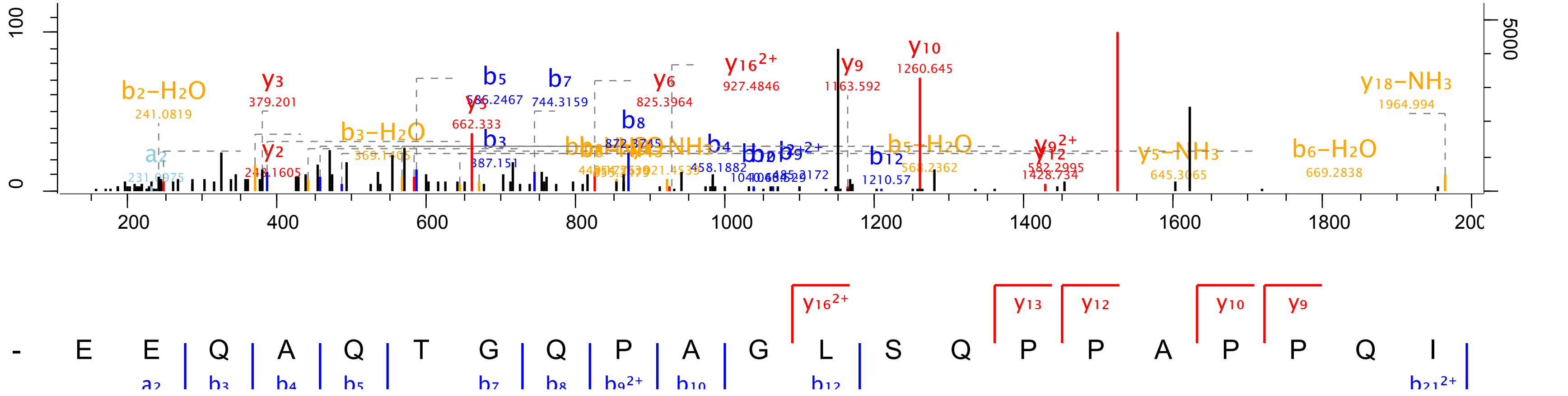

Raw file	Scan	Method	Score	m/z	Gene names
20140925_fract2_dyn_5ul_B2_01_437	36693	TOF; CID	74.46	580.84	ZBED9

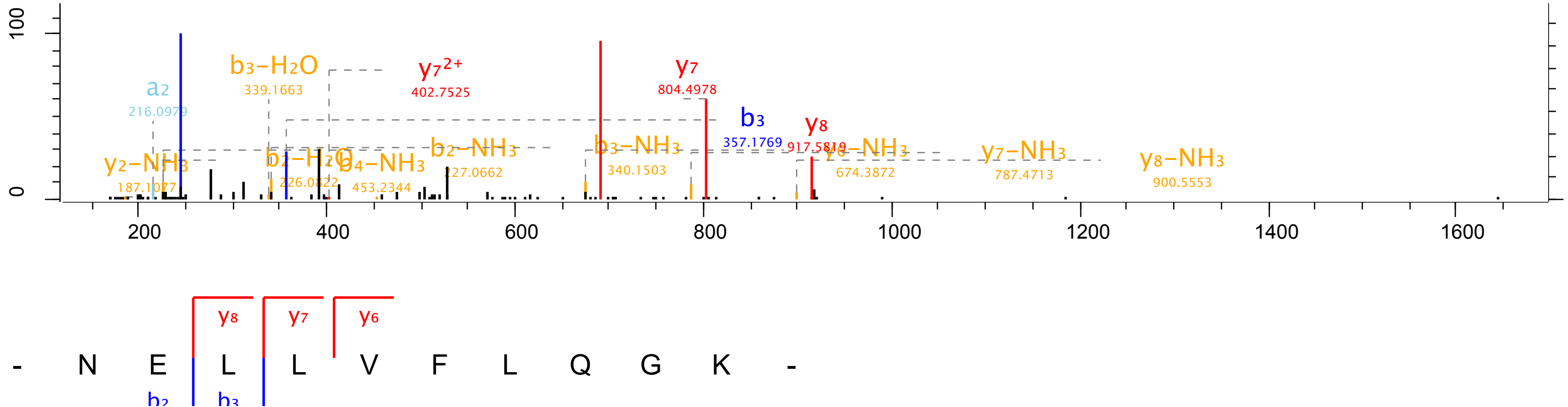

Raw file	Scan	Method	Score	m/z	Gene names
20140925_fract2_dyn_5ul_B2_01_437	37413	TOF; CID	73.92	972.02	SLC10A7

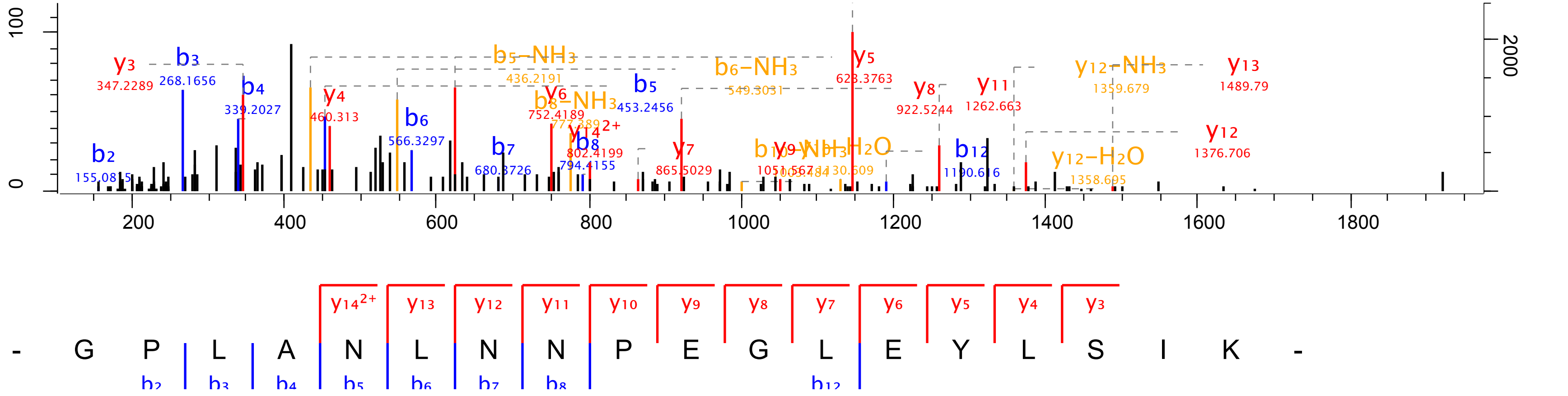

Raw file	Scan	Method	Score	m/z	Gene names
20140925_fract2_dyn_5ul_B2_01_437	41459	TOF; CID	47.69	1020.5	LSM5

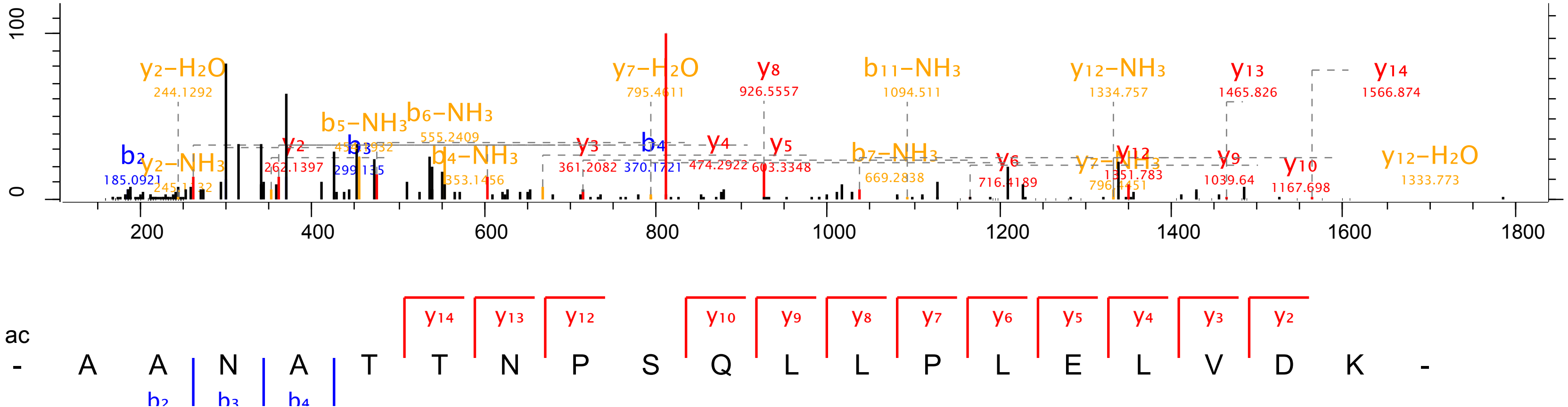

Raw file	Scan	Method	Score	m/z	Gene names
20140925_fract3_dyn_5ul_B3_01_438	2670	TOF; CID	101.39	452.21	MT-CO3

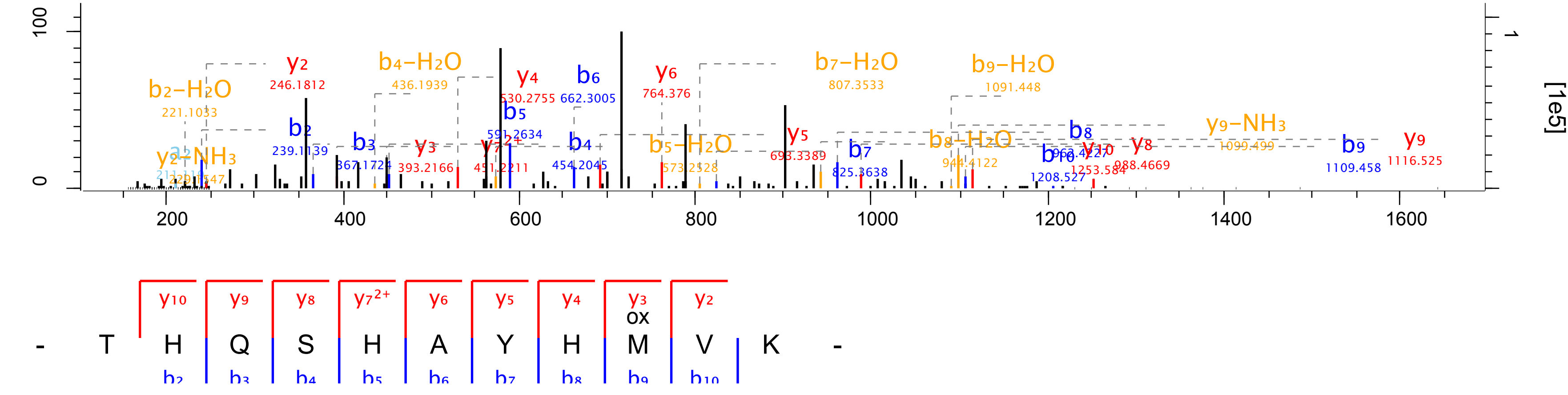

Raw file	Scan	Method	Score	m/z	Gene names
20140925_fract3_dyn_5ul_B3_01_438	8033	TOF; CID	88.06	521.79	HIPK3

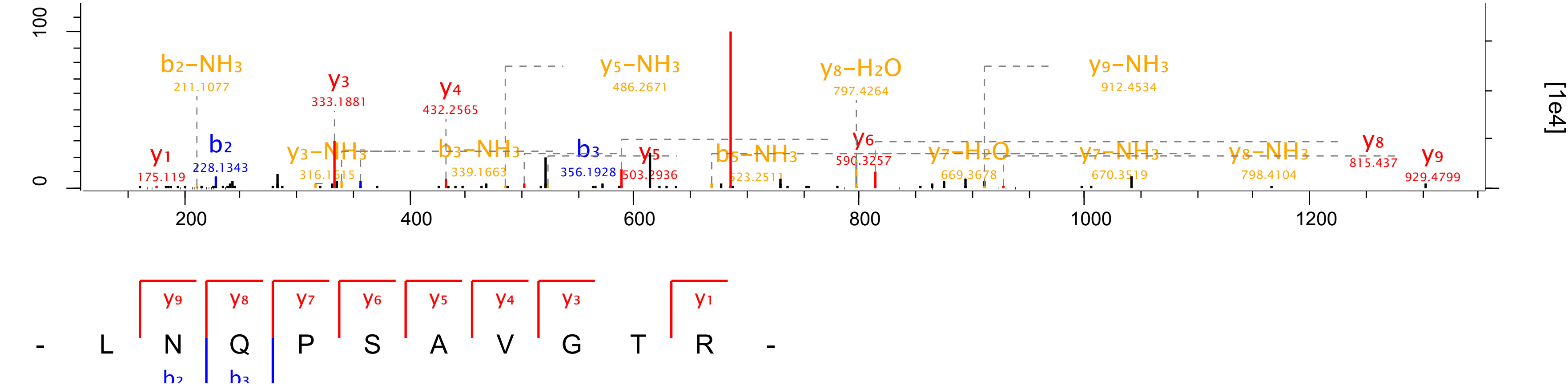

Raw file	Scan	Method	Score	m/z	Gene names
20140925_fract3_dyn_5ul_B3_01_438	12301	TOF; CID	61.78	582.78	CCDC151

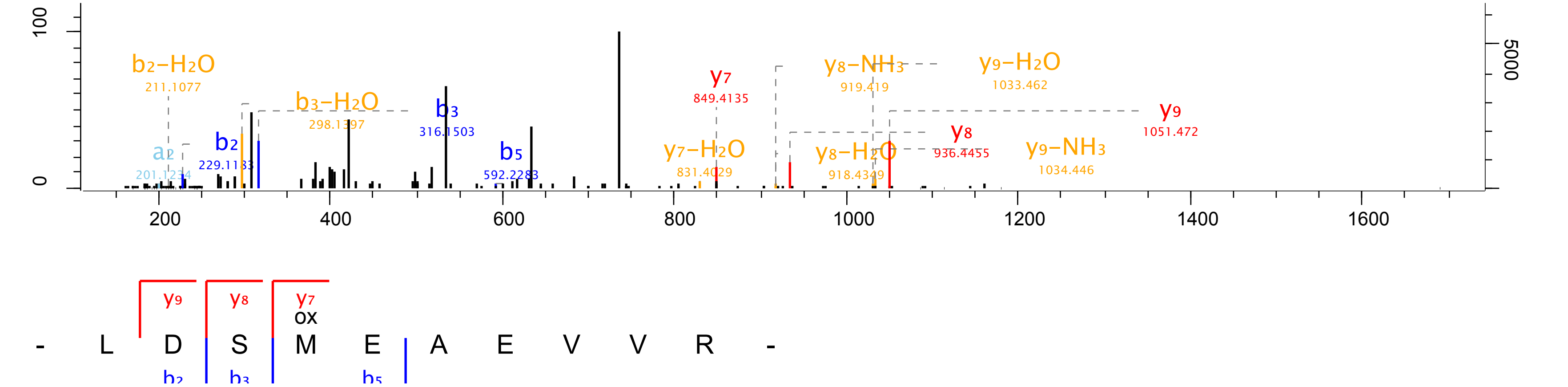

Raw file	Scan	Method	Score	m/z	Gene names
20140925_fract3_dyn_5ul_B3_01_438	12772	TOF; CID	91.09	499.72	PIGH

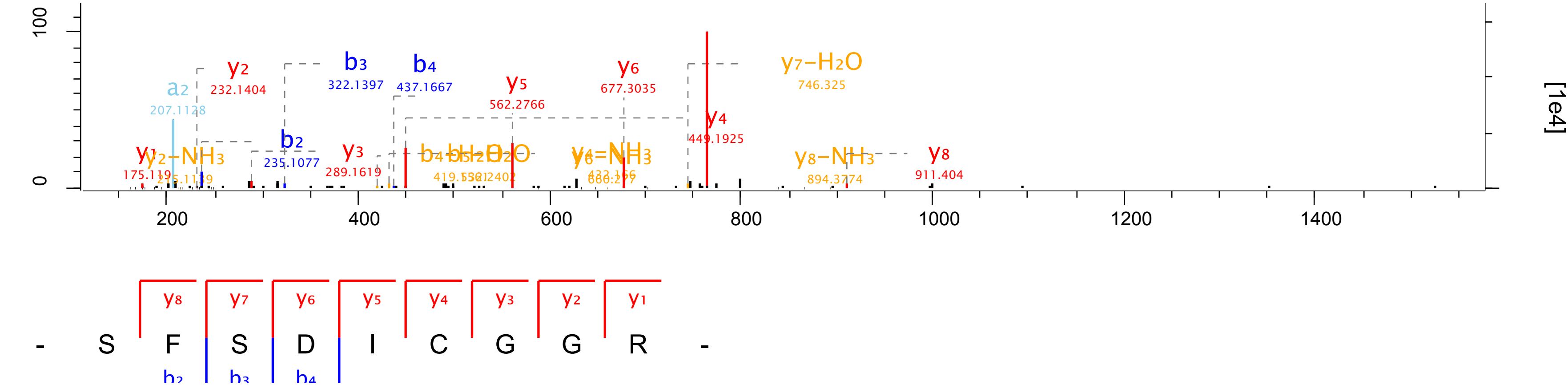

Raw file	Scan	Method	Score	m/z	Gene names
20140925_fract3_dyn_5ul_B3_01_438	14078	TOF; CID	122.33	630.83	TFDP2

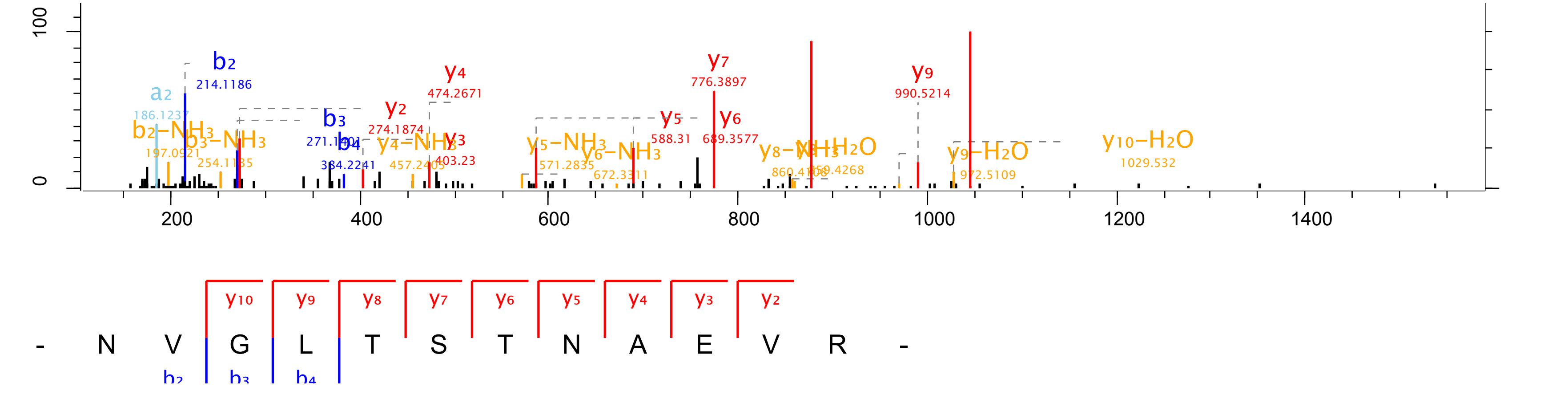

Raw file	Scan	Method	Score	m/z	Gene names
20140925_fract3_dyn_5ul_B3_01_438	17414	TOF; CID	120.57	692.83	SLPI

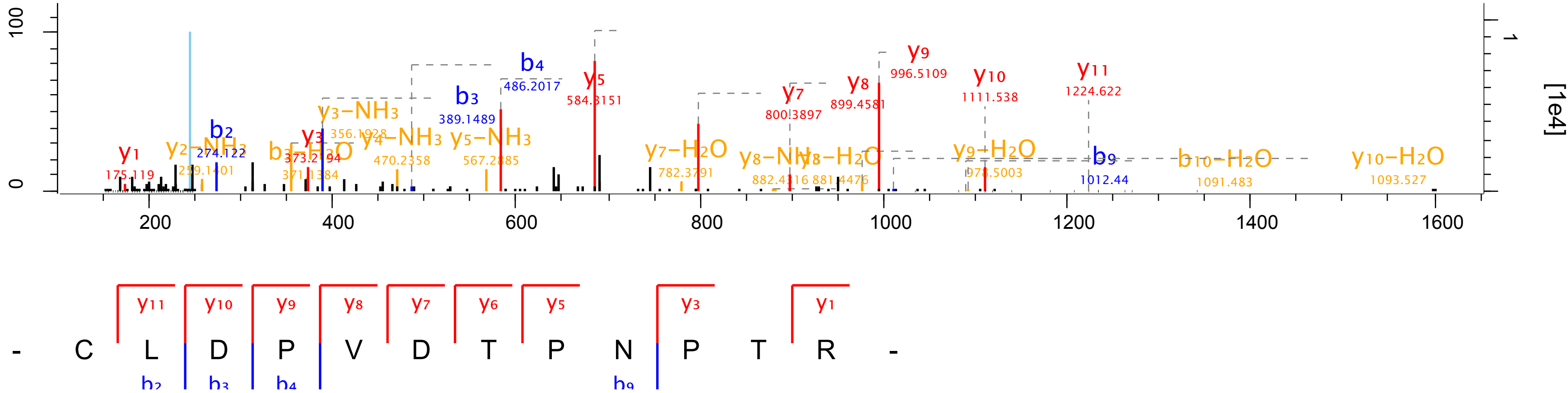

Raw file	Scan	Method	Score	m/z	Gene names
20140925_fract3_dyn_5ul_B3_01_438	17637	TOF; CID	33.32	778.38	FXYD5

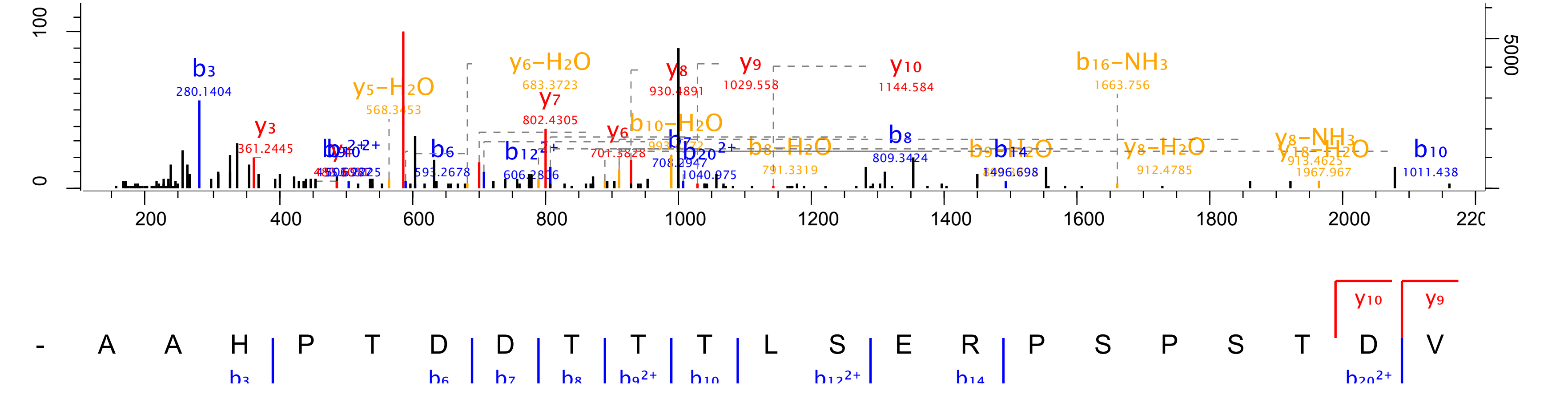

Raw file	Scan	Method	Score	m/z	Gene names
20140925_fract3_dyn_5ul_B3_01_438	21328	TOF; CID	69.75	764.63	SMIM14

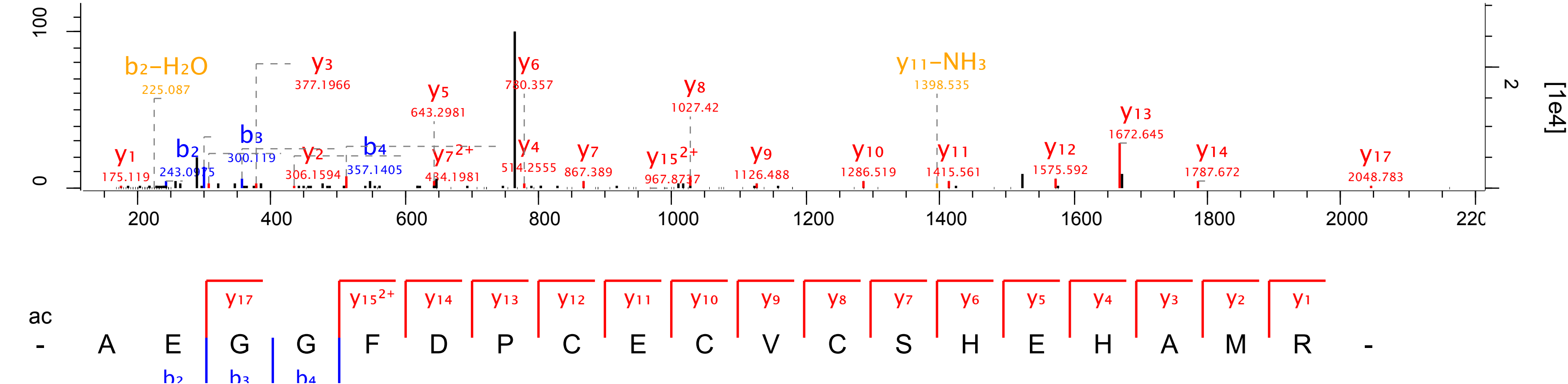

Raw file	Scan	Method	Score	m/z	Gene names
20140925_fract3_dyn_5ul_B3_01_438	23720	TOF; CID	91.87	553.32	WBP5

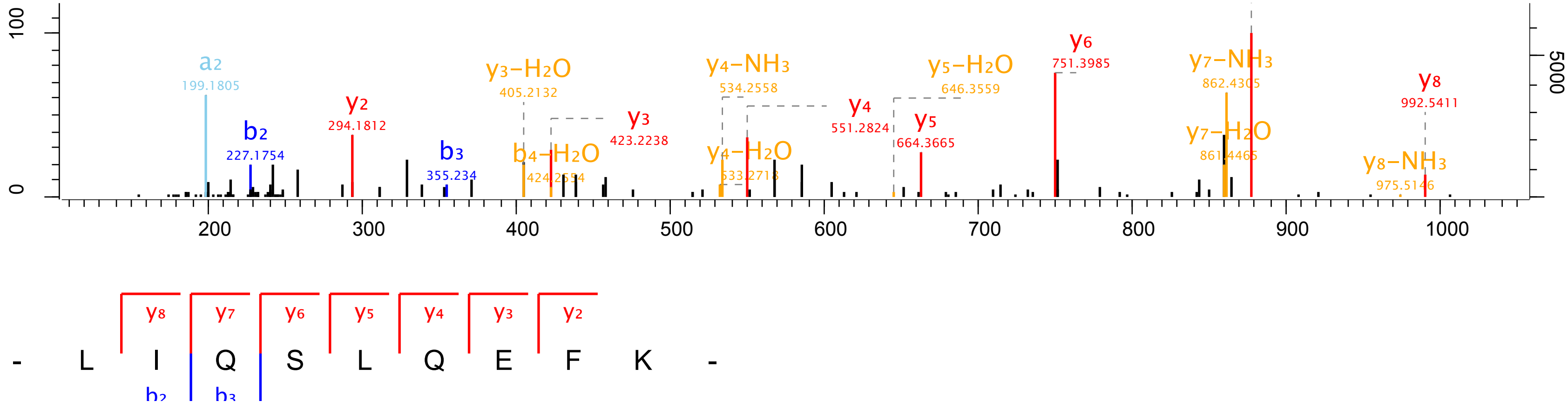

Raw file	Scan	Method	Score	m/z	Gene names
20140925_fract3_dyn_5ul_B3_01_438	26331	TOF; CID	114.27	578.3	MBLAC1

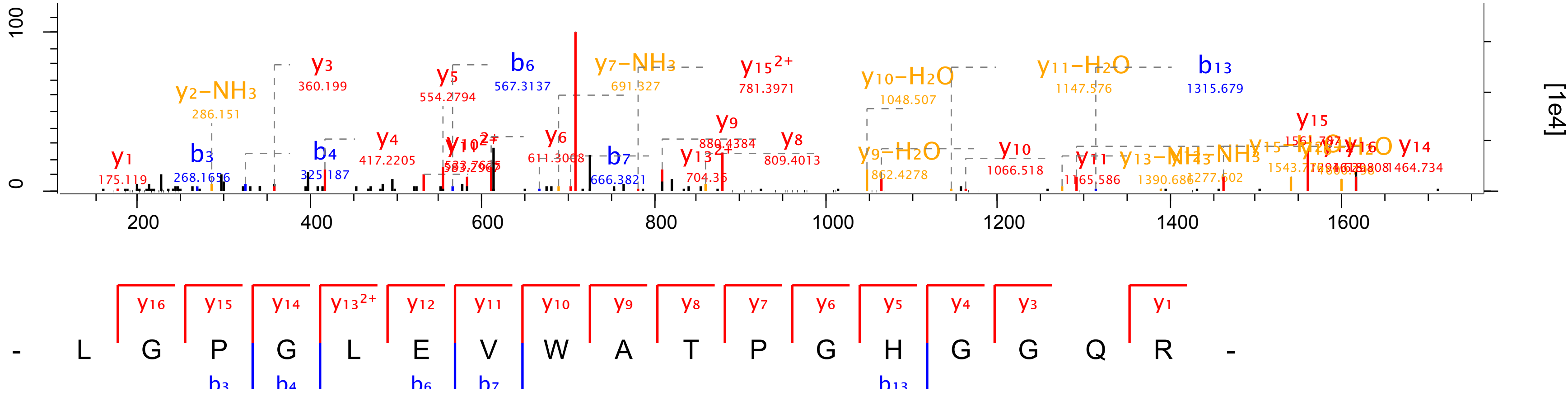

Raw file	Scan	Method	Score	m/z	Gene names
20140925_fract3_dyn_5ul_B3_01_438	27630	TOF; CID	62.29	727.38	NUDT14

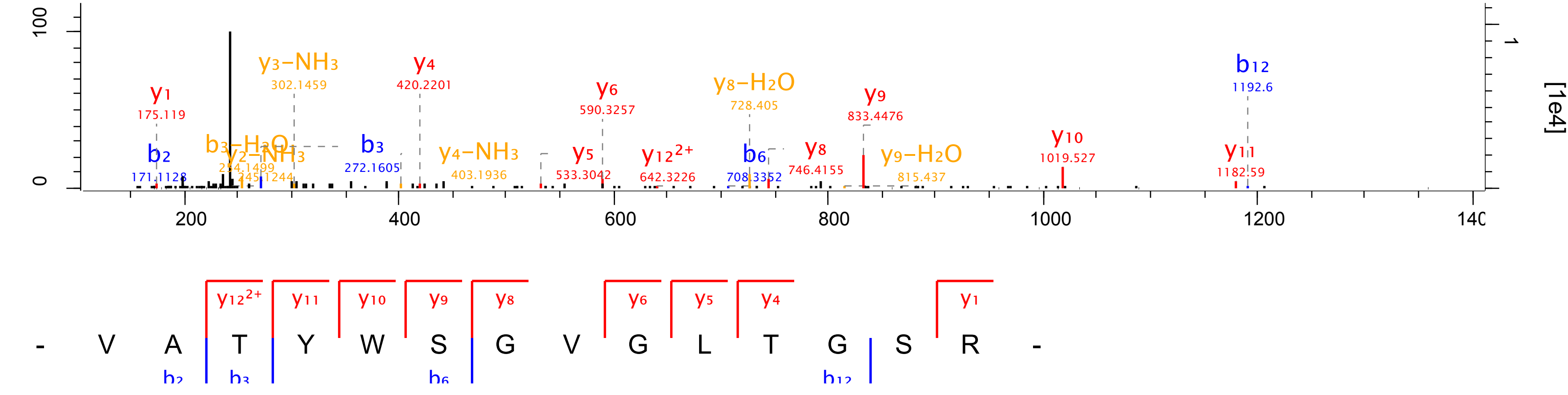

Raw file	Scan	Method	Score	m/z	Gene names
20140925_fract3_dyn_5ul_B3_01_438	28767	TOF; CID	91.32	585.28	SNX10

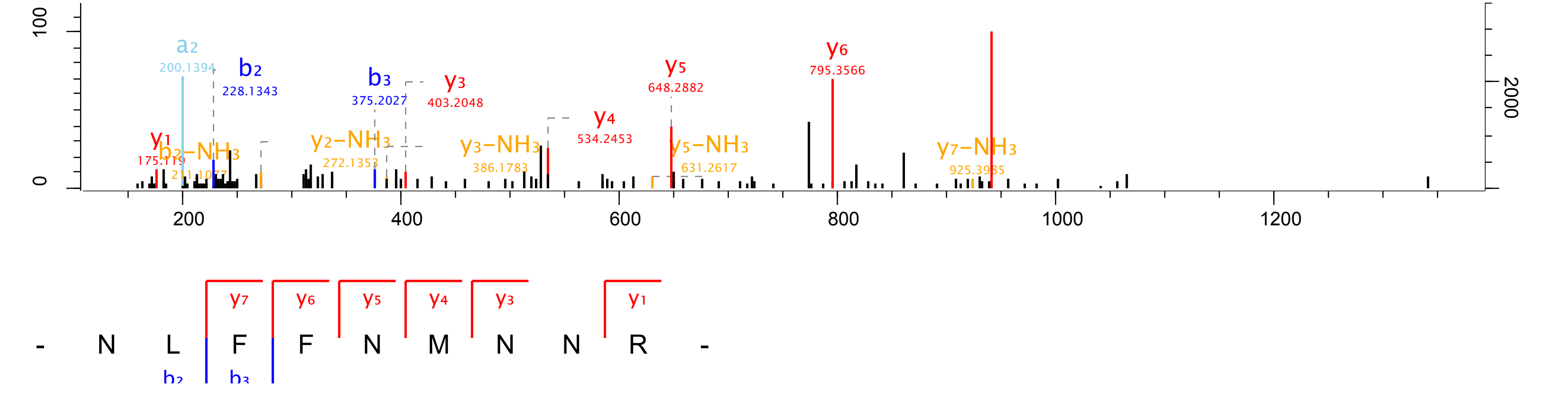

Raw file	Scan	Method	Score	m/z	Gene names
20140925_fract3_dyn_5ul_B3_01_438	28783	TOF; CID	90.23	766.88	C2orf76

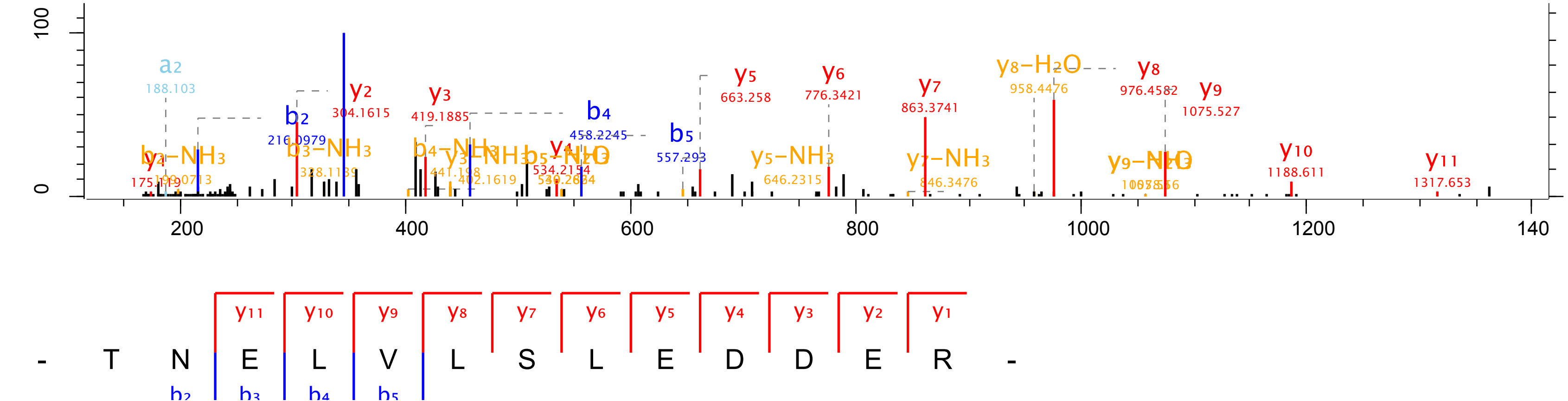

Raw file	Scan	Method	Score	m/z	Gene names
20140925_fract3_dyn_5ul_B3_01_438	30492	TOF; CID	93.35	576.34	MAL2

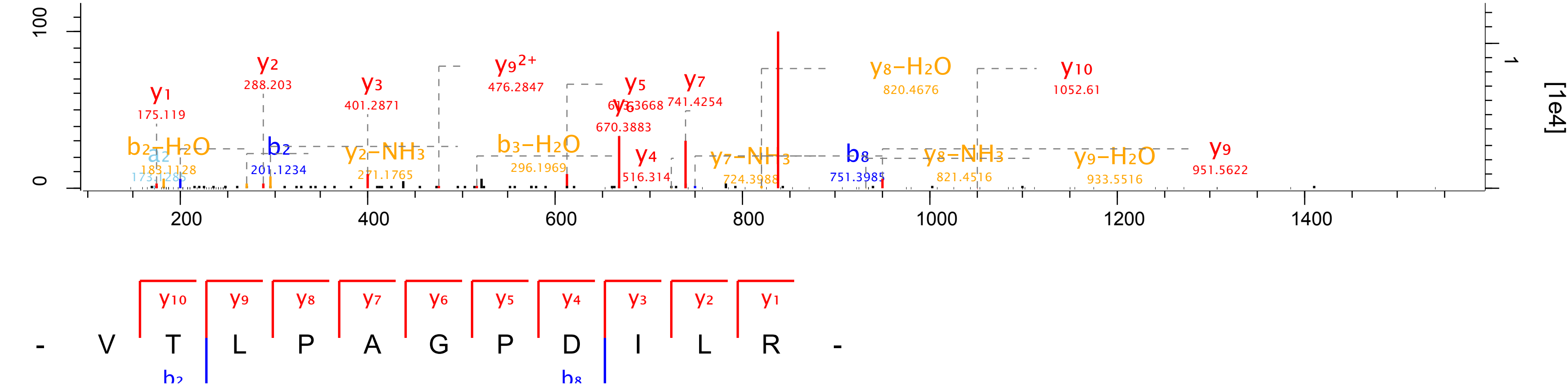

Raw file	Scan	Method	Score	m/z	Gene names
20140925_fract3_dyn_5ul_B3_01_438	32375	TOF; CID	92.26	996.91	CLDN7

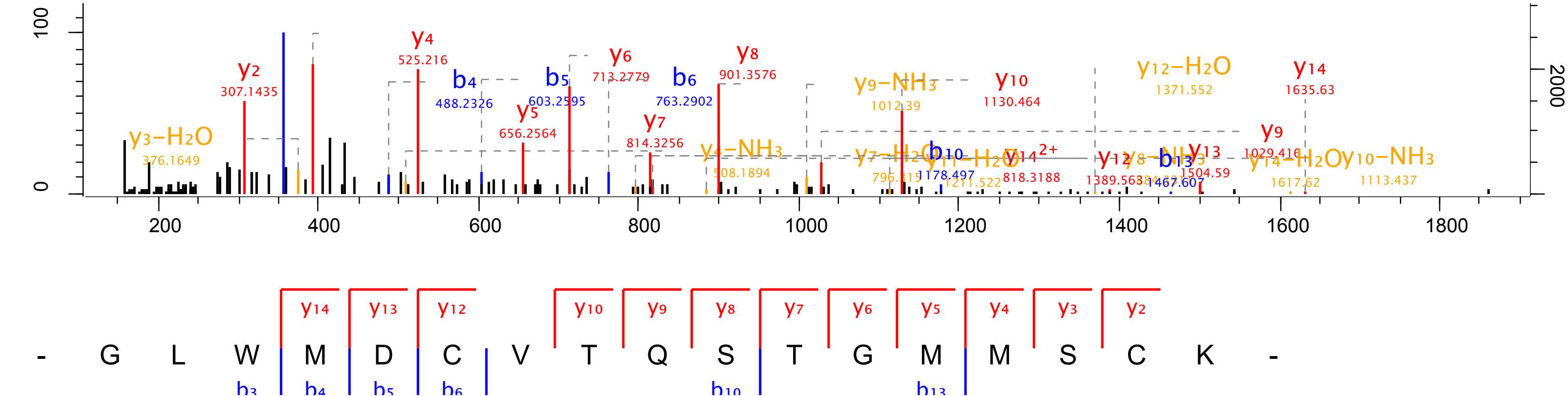

Raw file	Scan	Method	Score	m/z	Gene names
20140925_fract3_dyn_5ul_B3_01_438	34833	TOF; CID	37.29	717.02	ULBP3

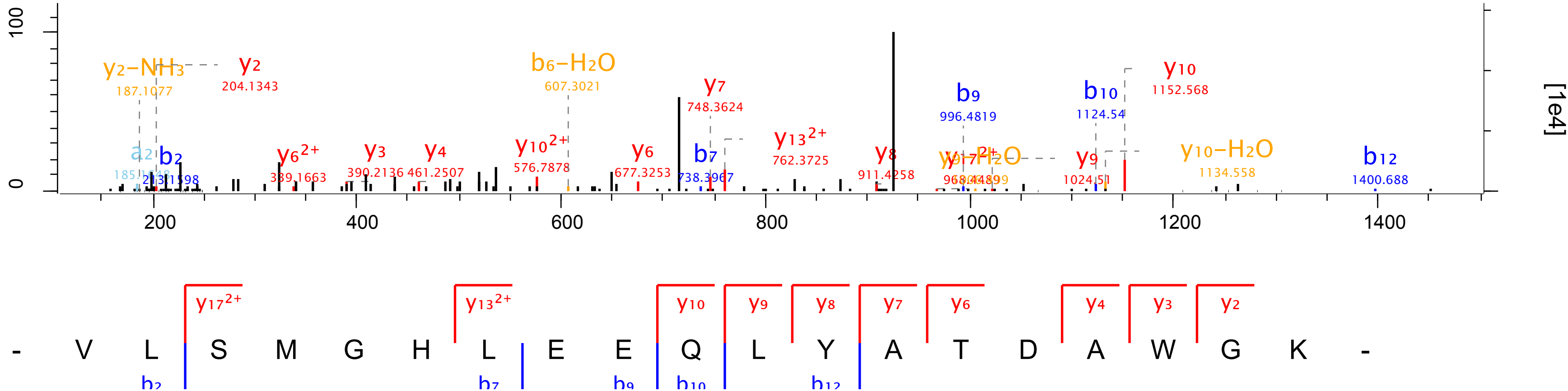

Raw file	Scan	Method	Score	m/z	Gene names
20140925_fract3_dyn_5ul_B3_01_438	35387	TOF; CID	103.55	568.8	MT-ND2

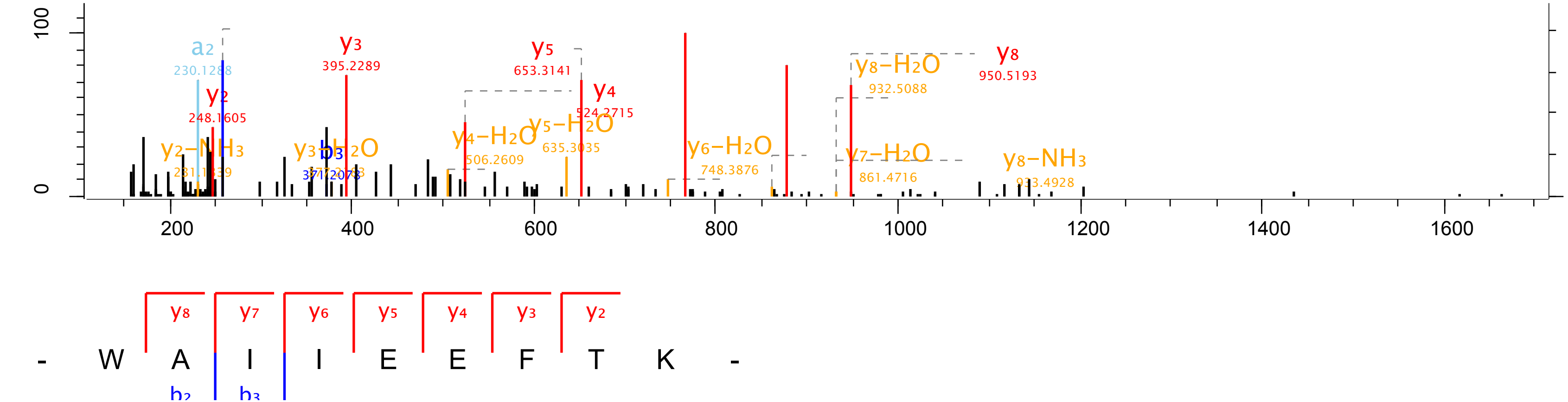

Raw file	Scan	Method	Score	m/z	Gene names
20140925_fract3_dyn_5ul_B3_01_438	37800	TOF; CID	43.33	1050.54	CLN8

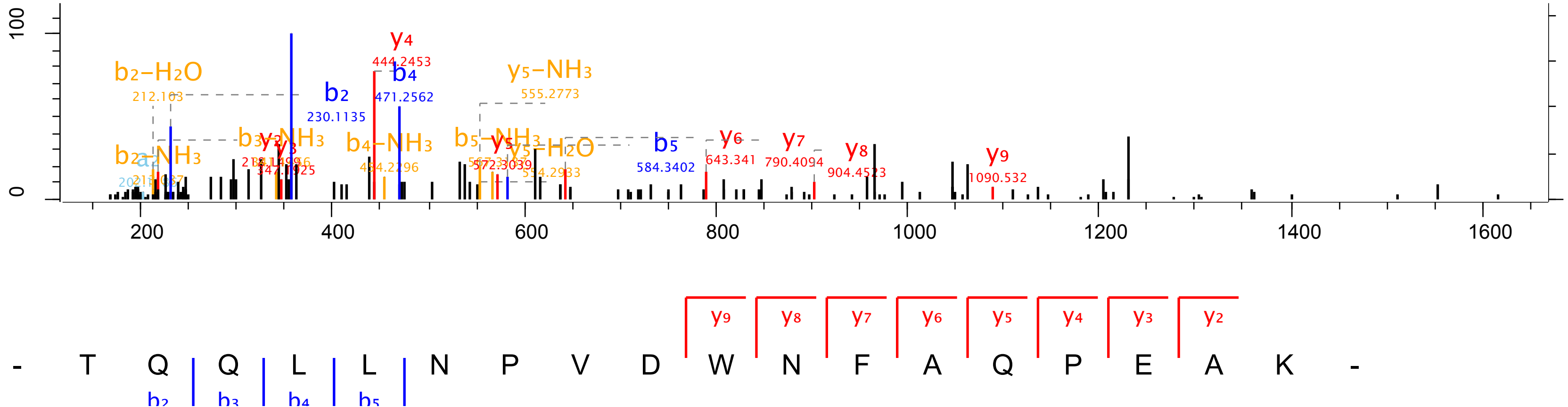

Raw file	Scan	Method	Score	m/z	Gene names
20140925_fract3_dyn_5ul_B3_01_438	38323	TOF; CID	58.1	776.9	TPRA1

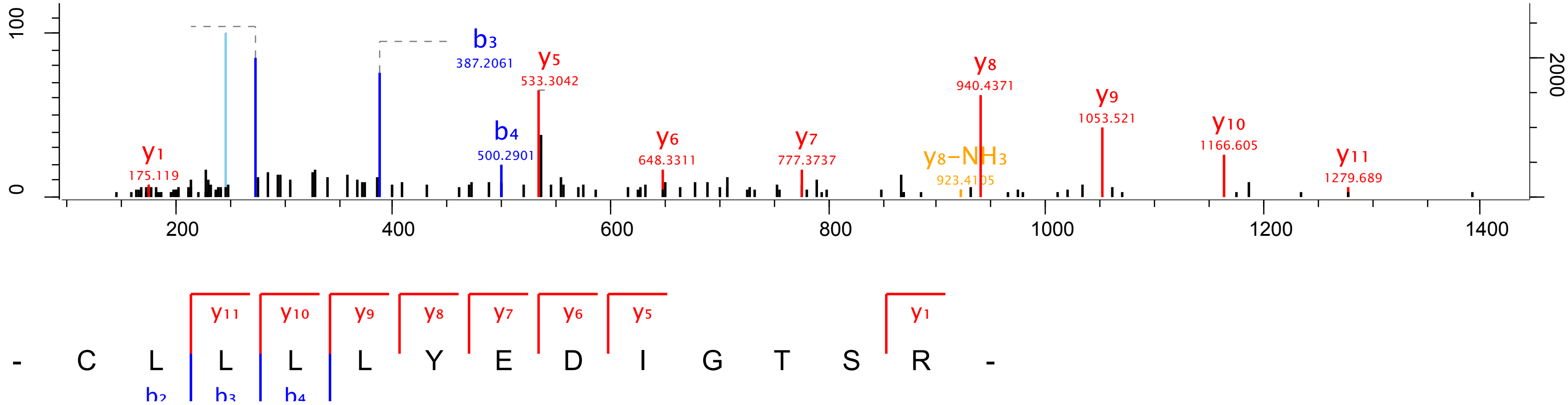

Raw file	Scan	Method	Score	m/z	Gene names
20140925_fract4_dyn_5ul_B4_01_439	3443	TOF; CID	69.93	541.27	KIAA1958

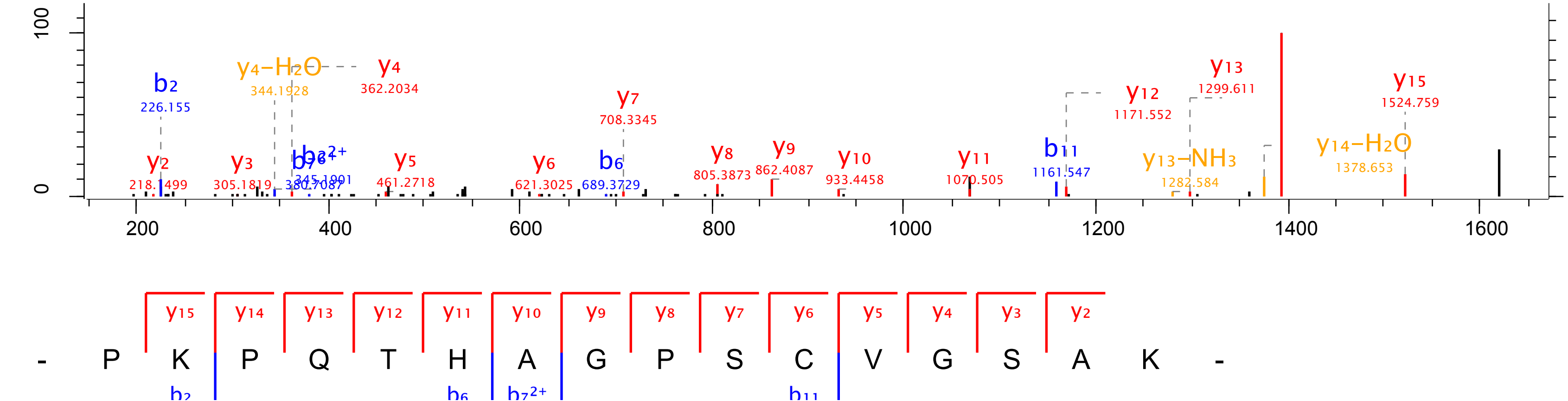

Raw file	Scan	Method	Score	m/z	Gene names
20140925_fract4_dyn_5ul_B4_01_439	3804	TOF; CID	116.37	438.2	SMOC2

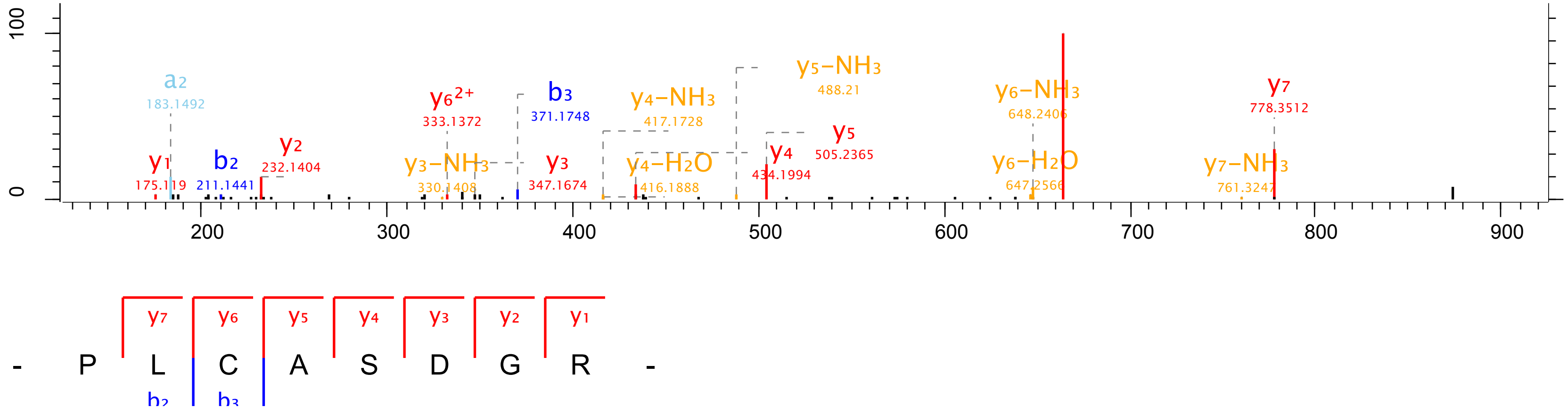

Raw file	Scan	Method	Score	m/z	Gene names
20140925_fract4_dyn_5ul_B4_01_439	5237	TOF; CID	74.84	564.78	TET2

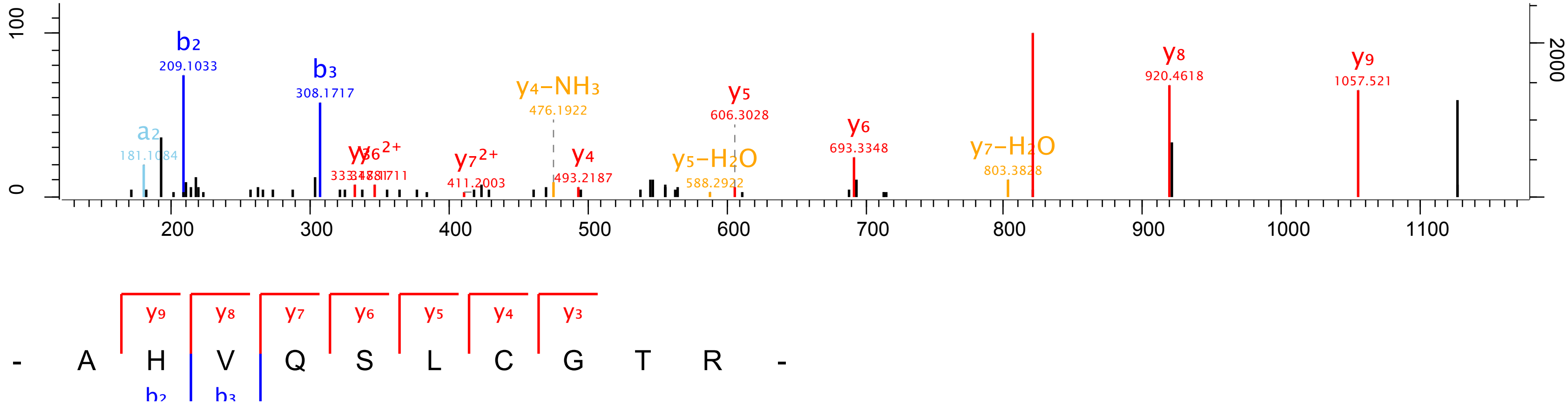

Raw file	Scan	Method	Score	m/z	Gene names
20140925_fract4_dyn_5ul_B4_01_439	7060	TOF; CID	87.18	601.28	ZBTB1

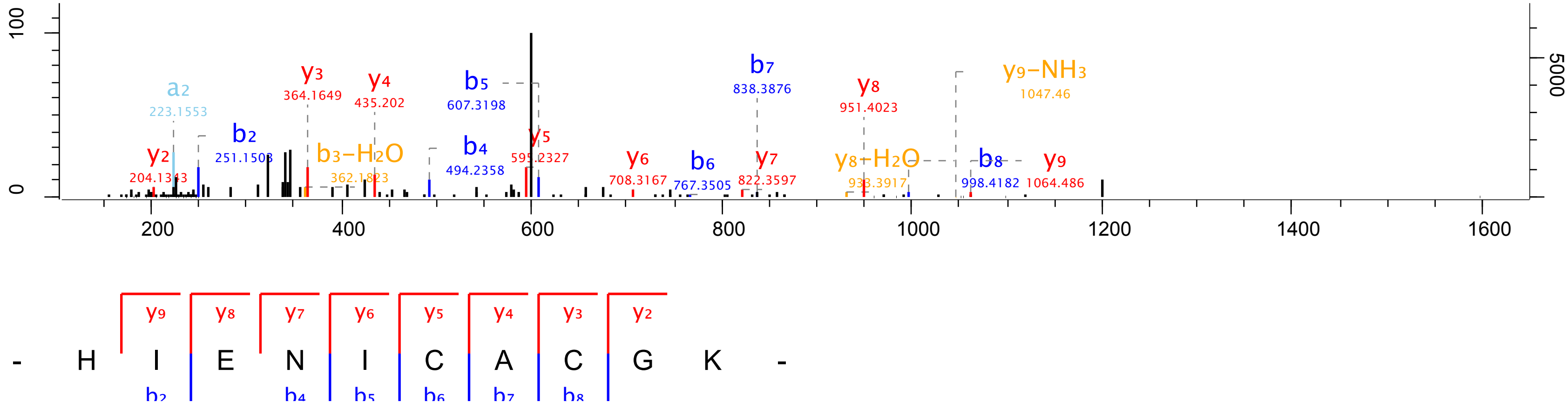

Raw file	Scan	Method	Score	m/z	Gene names
20140925_fract4_dyn_5ul_B4_01_439	7355	TOF; CID	54.9	735.37	RWDD3

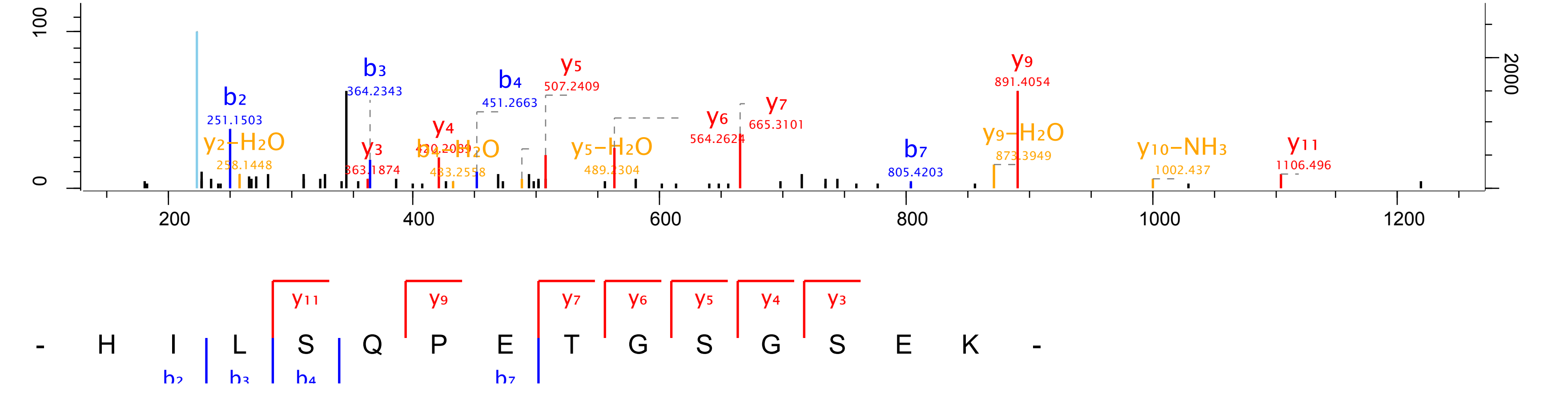

Raw file	Scan	Method	Score	m/z	Gene names
20140925_fract4_dyn_5ul_B4_01_439	7838	TOF; CID	78.26	571.25	ZFP3

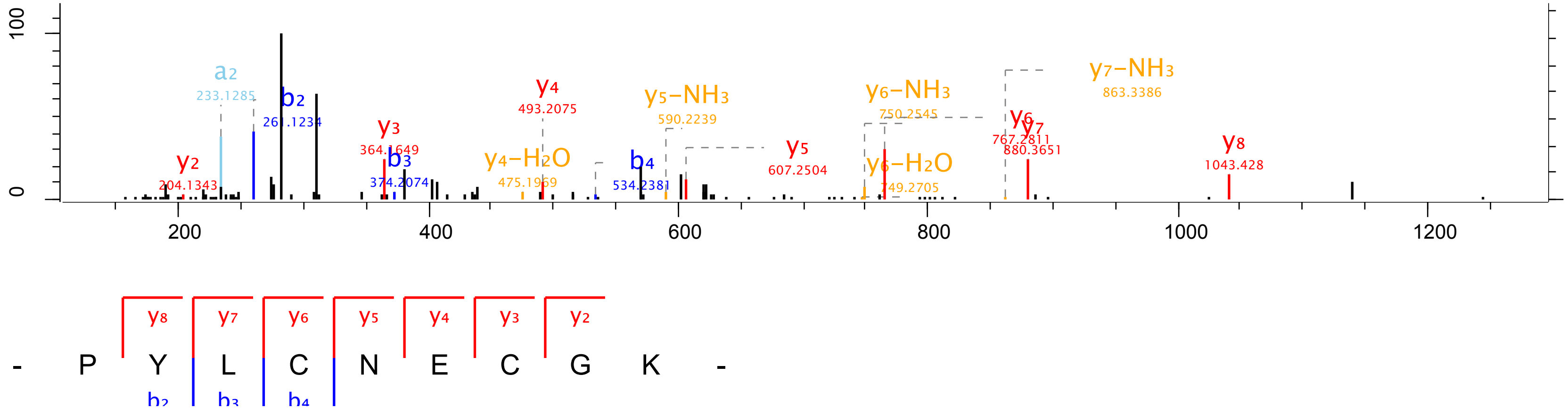

Raw file	Scan	Method	Score	m/z	Gene names
20140925_fract4_dyn_5ul_B4_01_439	8052	TOF; CID	54.34	464.54	DPM3

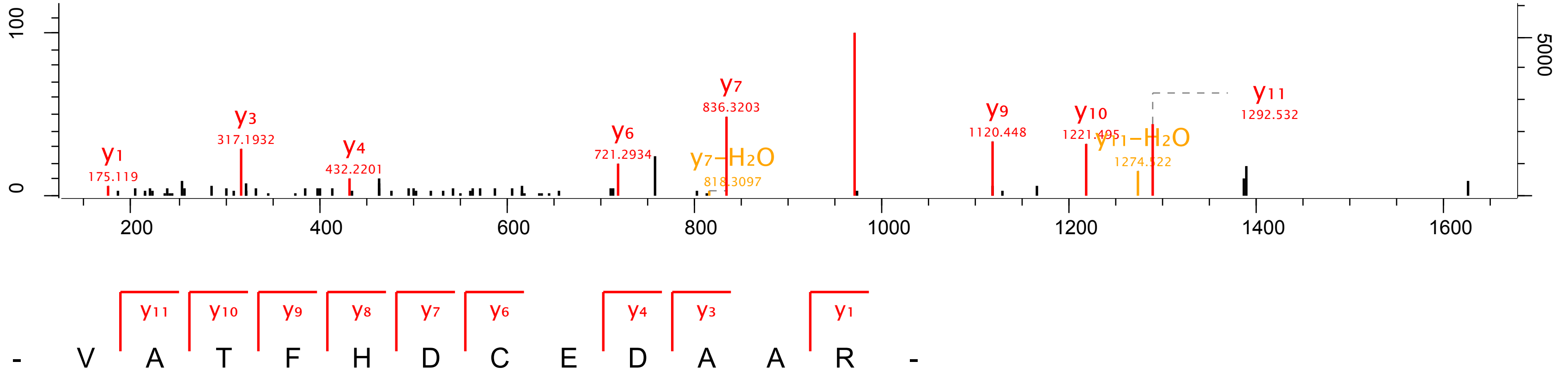

Raw file	Scan	Method	Score	m/z	Gene names
20140925_fract4_dyn_5ul_B4_01_439	12391	TOF; CID	142.08	459.56	CYB561D2

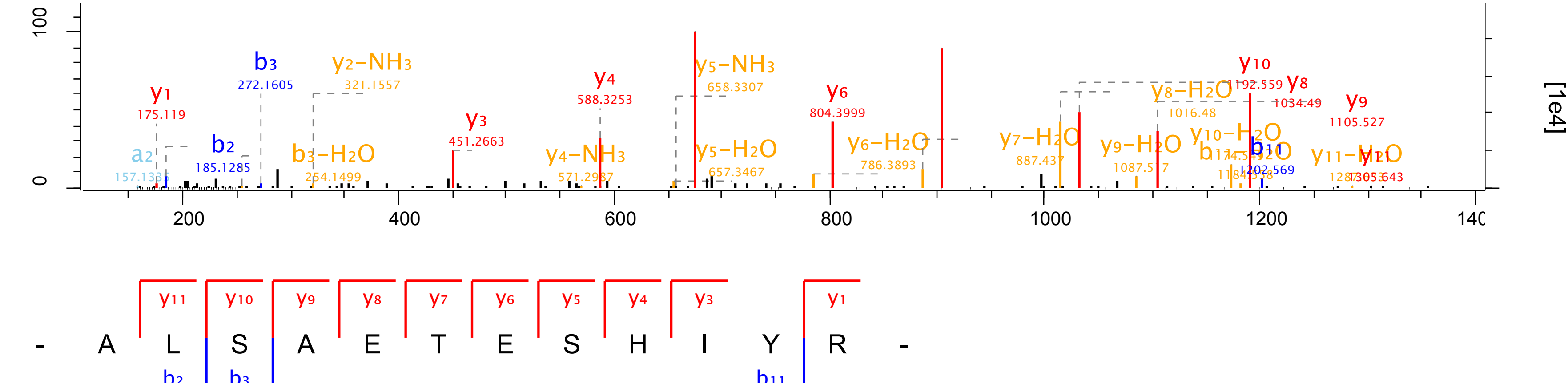

Raw file	Scan	Method	Score	m/z	Gene names
20140925_fract4_dyn_5ul_B4_01_439	12557	TOF; CID	144.1	577.32	PLEKHG4;CYB561A3

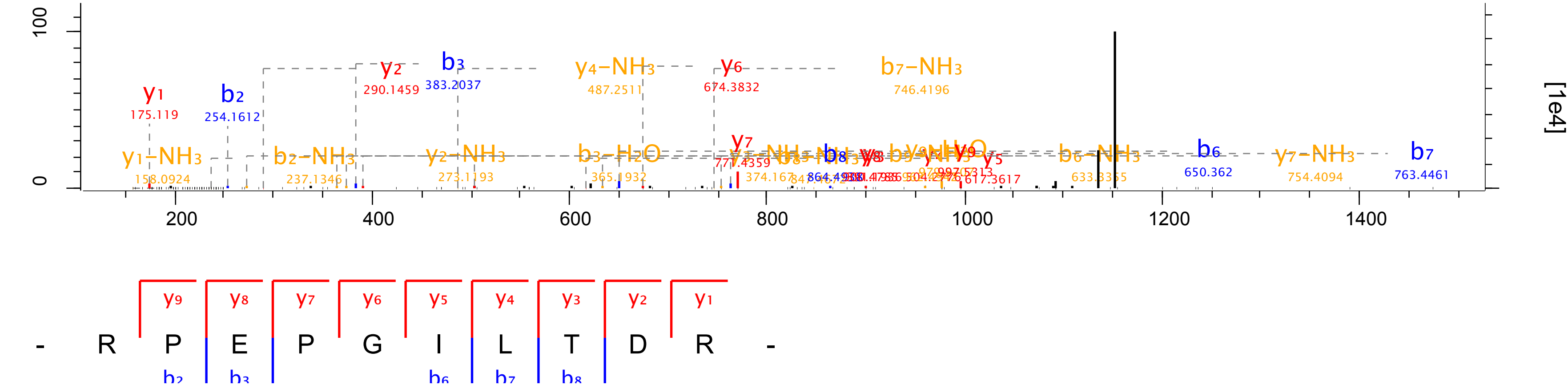

Raw file	Scan	Method	Score	m/z	Gene names
20140925_fract4_dyn_5ul_B4_01_439	17334	TOF; CID	85.81	658.84	ZMYM6NB

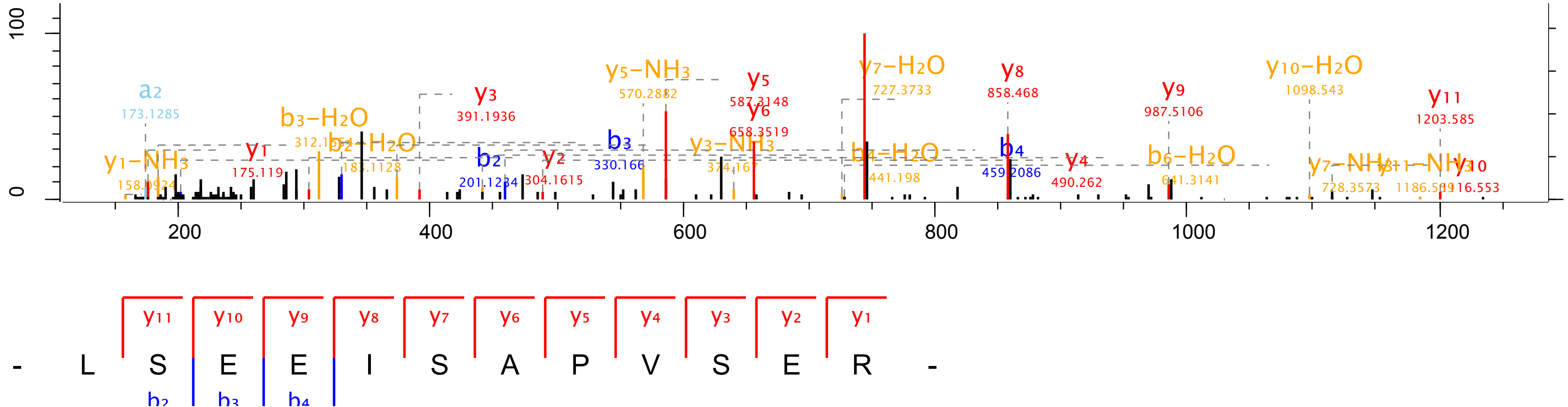

Raw file	Scan	Method	Score	m/z	Gene names
20140925_fract4_dyn_5ul_B4_01_439	19479	TOF; CID	79.15	495.79	PORCN

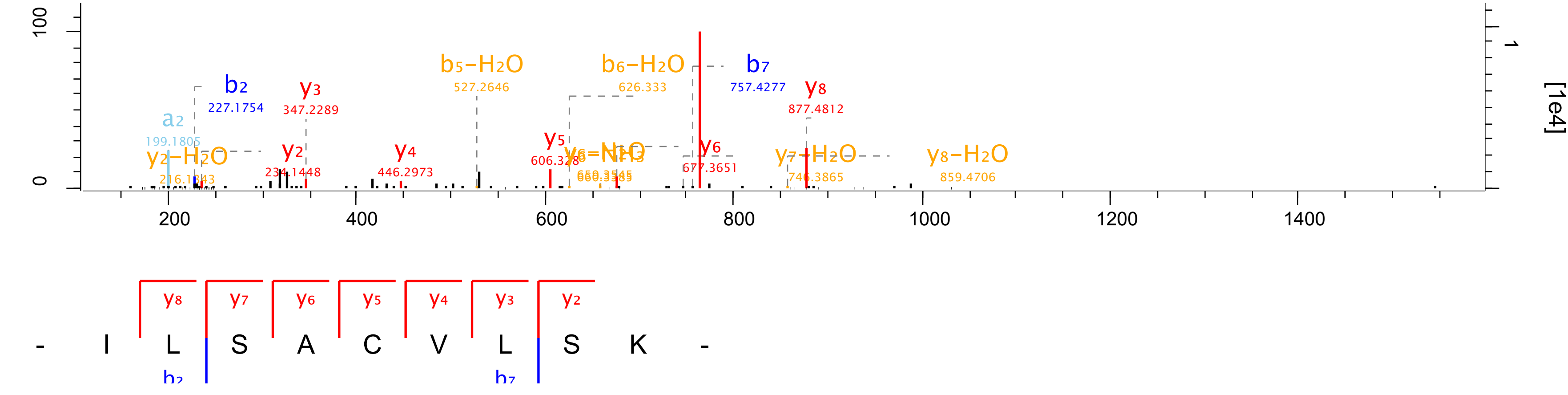

Raw file	Scan	Method	Score	m/z	Gene names
20140925_fract4_dyn_5ul_B4_01_439	20342	TOF; CID	70.2	600.31	C8orf37

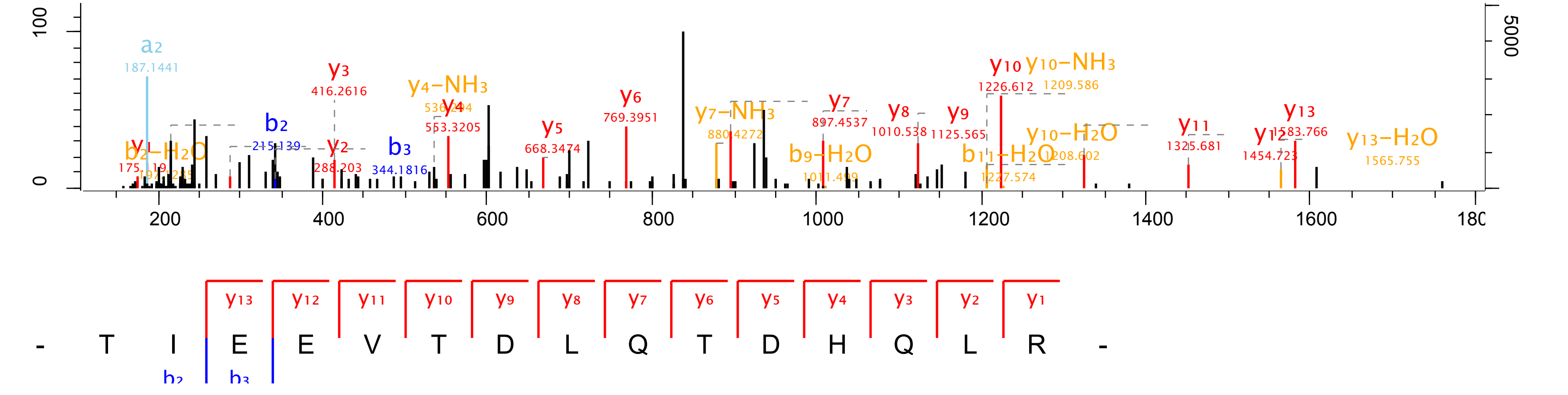

Raw file	Scan	Method	Score	m/z	Gene names
20140925_fract4_dyn_5ul_B4_01_439	21878	TOF; CID	100.22	668.33	DGCR6L

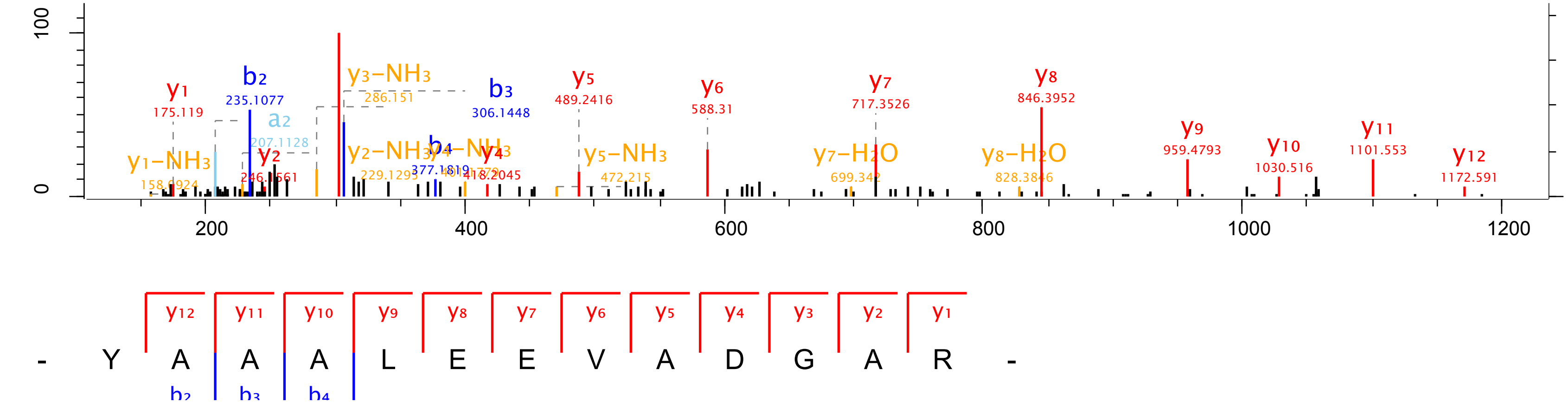

Raw file	Scan	Method	Score	m/z	Gene names
20140925_fract4_dyn_5ul_B4_01_439	24369	TOF; CID	85.21	547.27	RGMA

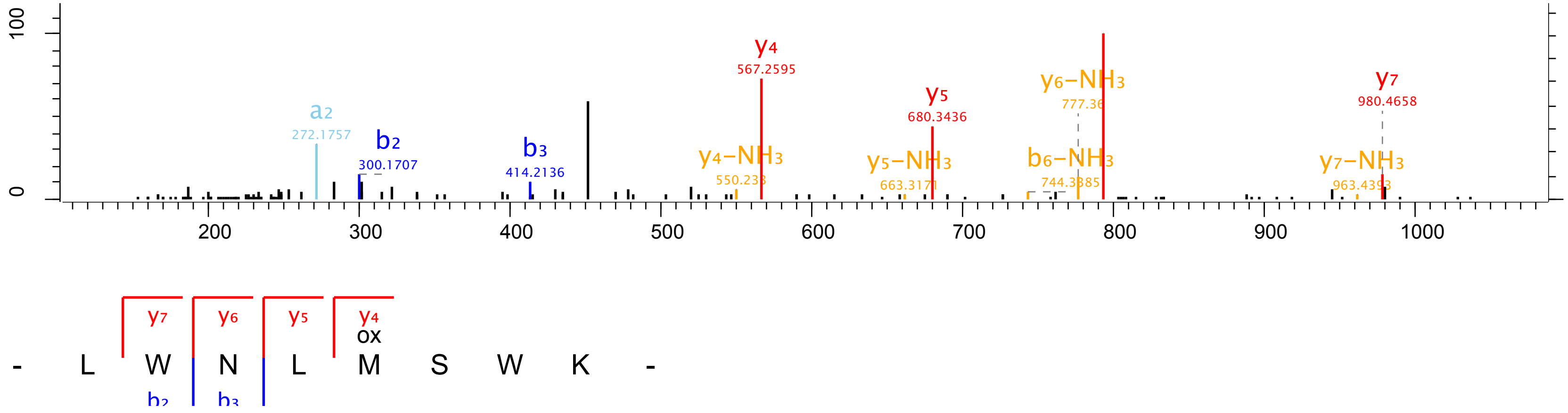

Raw file	Scan	Method	Score	m/z	Gene names
20140925_fract4_dyn_5ul_B4_01_439	26254	TOF; CID	38.99	1133.2	TECPR1

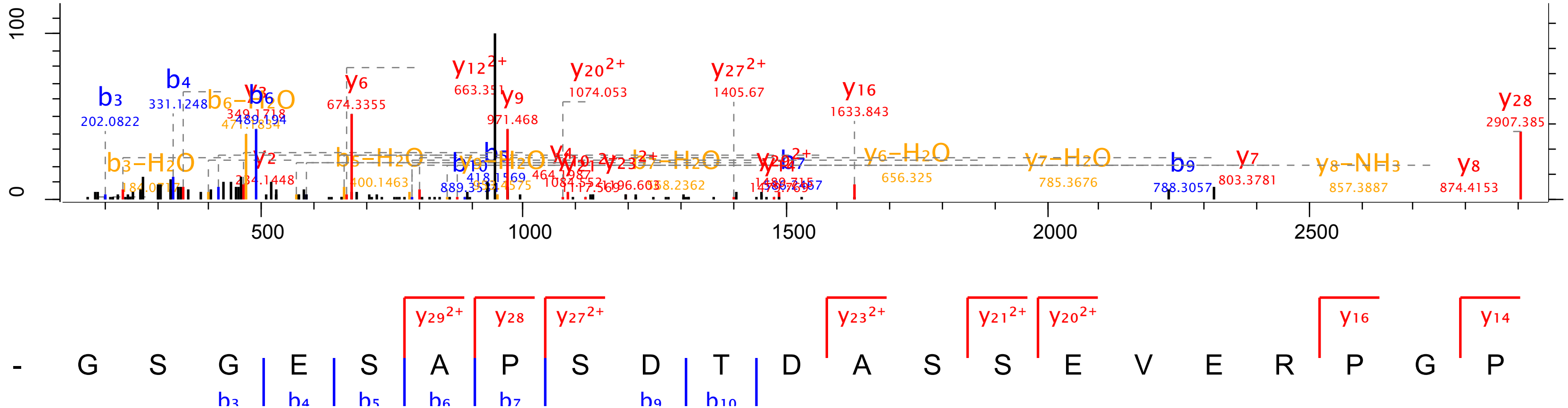

Raw file	Scan	Method	Score	m/z	Gene names
20140925_fract4_dyn_5ul_B4_01_439	29259	TOF; CID	112.16	1085.04	LMBRD1

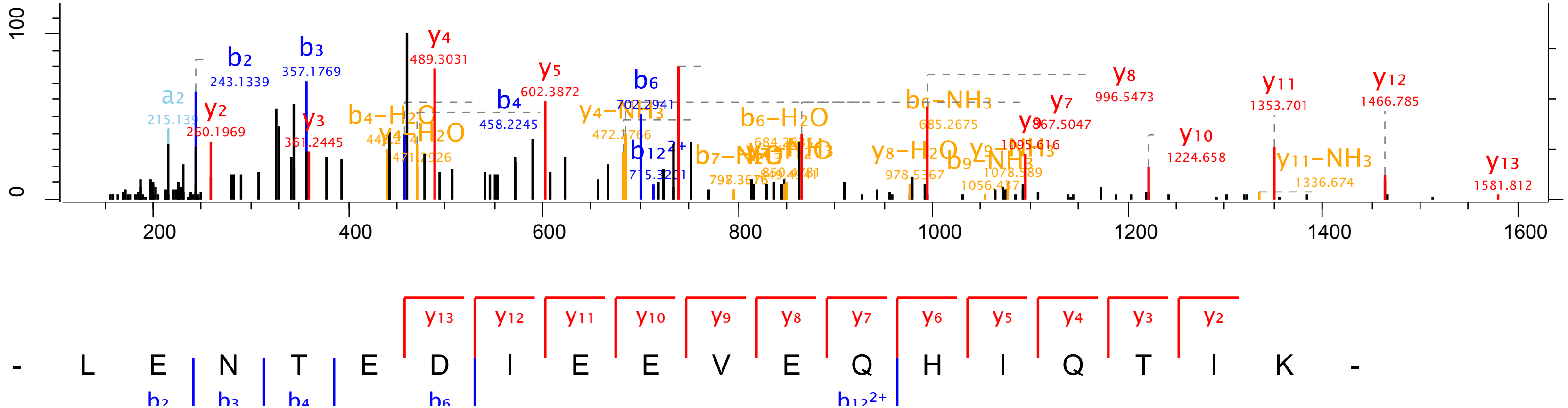

Raw file	Scan	Method	Score	m/z	Gene names
20140925_fract4_dyn_5ul_B4_01_439	29369	TOF; CID	77.66	572.32	BOK

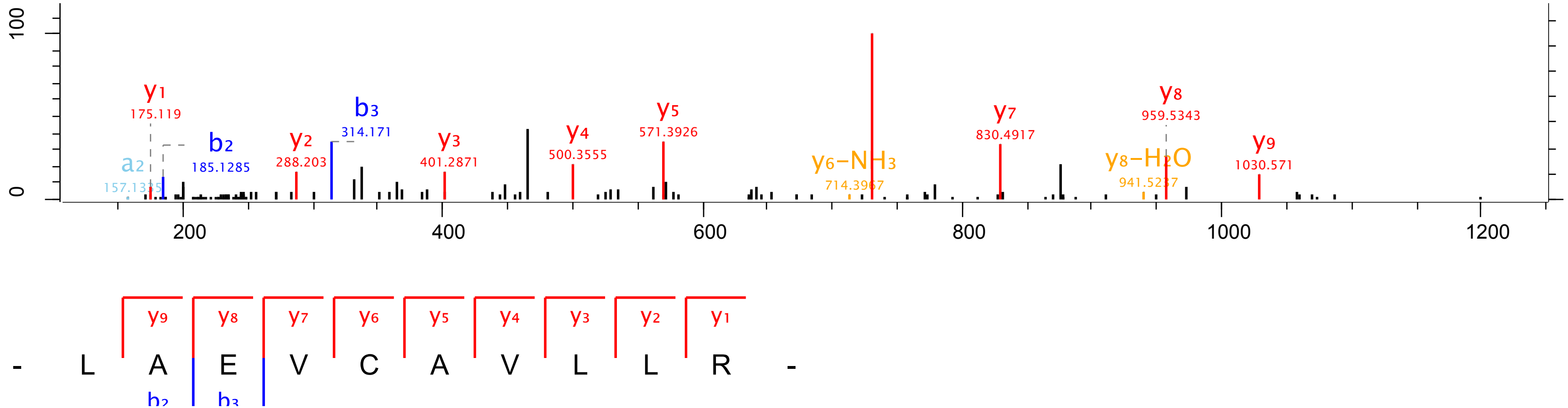

Raw file	Scan	Method	Score	m/z	Gene names
20140925_fract4_dyn_5ul_B4_01_439	31196	TOF; CID	39.48	668.32	PERP

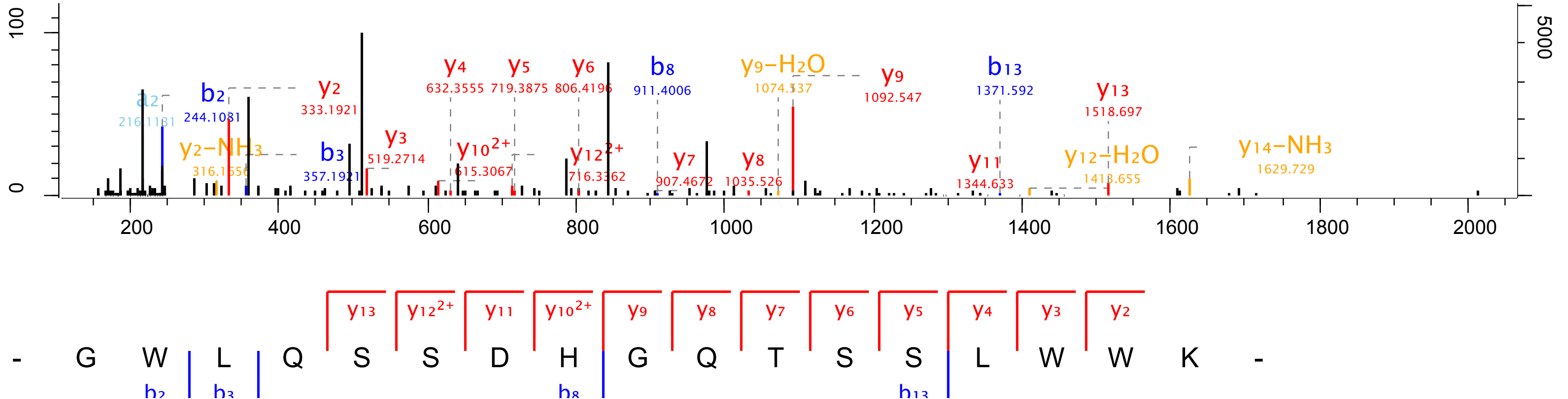

Raw file	Scan	Method	Score	m/z	Gene names
20140925_fract4_dyn_5ul_B4_01_439	31561	TOF; CID	101.56	717.43	EMC9

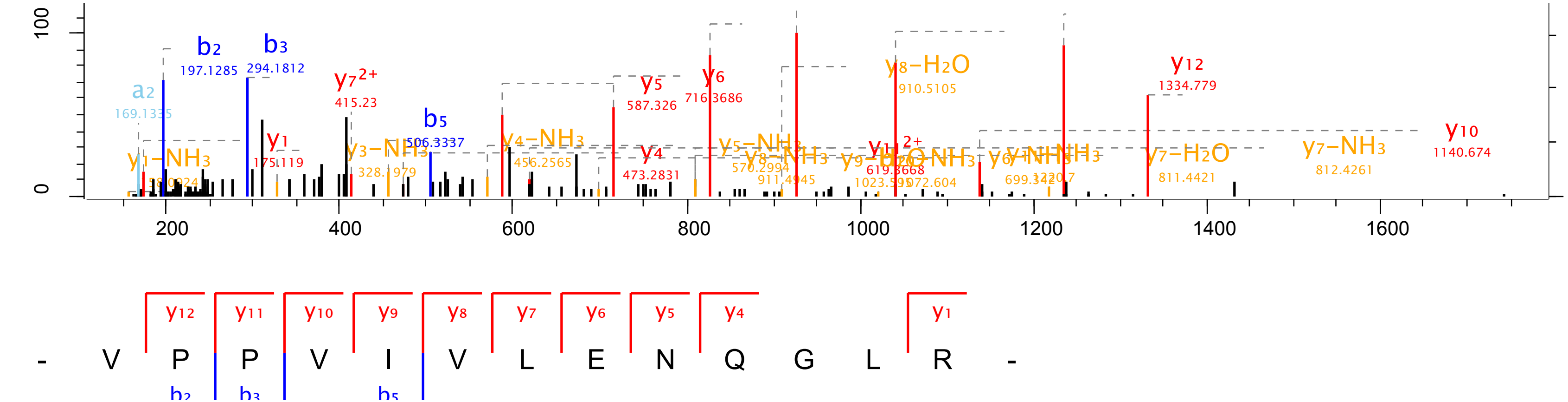

Raw file	Scan	Method	Score	m/z	Gene names
20140925_fract4_dyn_5ul_B4_01_439	32642	TOF; CID	244.37	854.42	SSR3

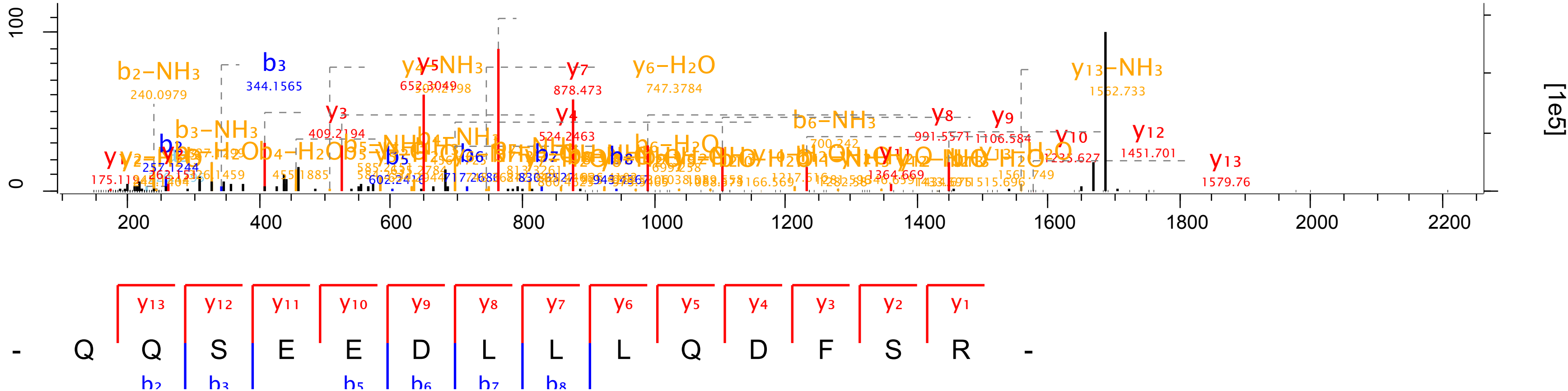

Raw file	Scan	Method	Score	m/z	Gene names
20140925_fract4_dyn_5ul_B4_01_439	35372	TOF; CID	61.26	833.45	CES3

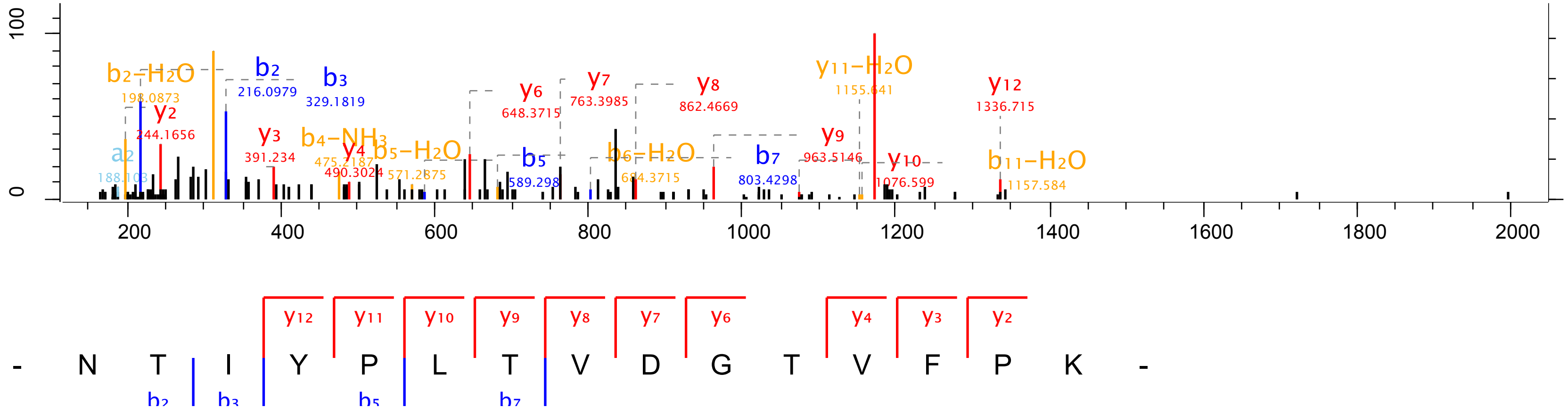

Raw file	Scan	Method	Score	m/z	Gene names
20140925_fract4_dyn_5ul_B4_01_439	35606	TOF; CID	104.17	744.37	VCPKMT

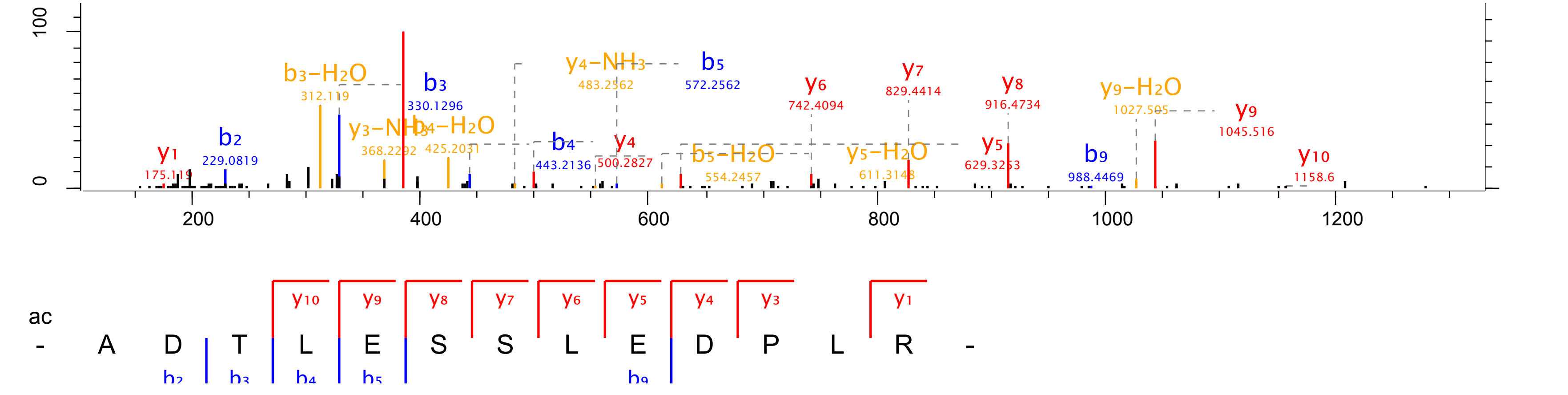

Raw file	Scan	Method	Score	m/z	Gene names
20140925_fract4_dyn_5ul_B4_01_439	36201	TOF; CID	65.72	917.44	CNBP

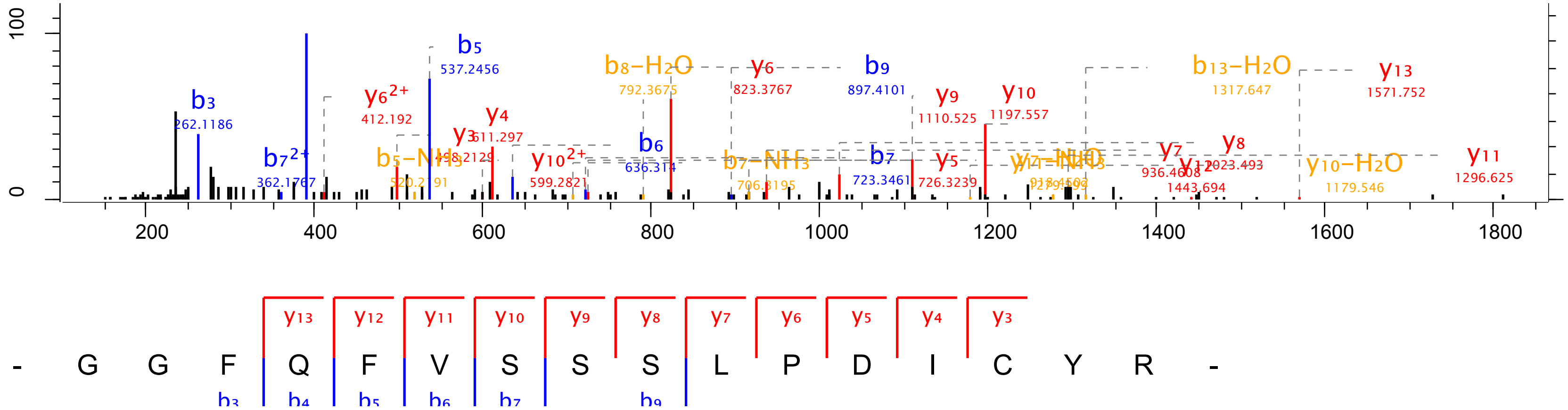

Raw file	Scan	Method	Score	m/z	Gene names
20140925_fract4_dyn_5ul_B4_01_439	37504	TOF; CID	101.32	797.94	CLDN12

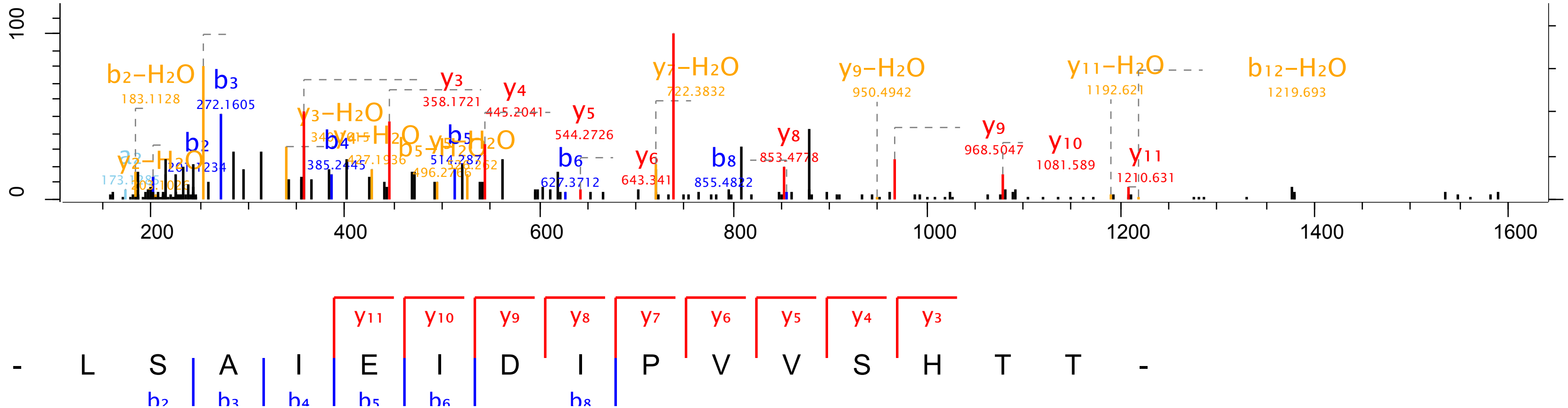

Raw file	Scan	Method	Score	m/z	Gene names
20140925_fract4_dyn_5ul_B4_01_439	37880	TOF; CID	43.41	797.38	B9D2

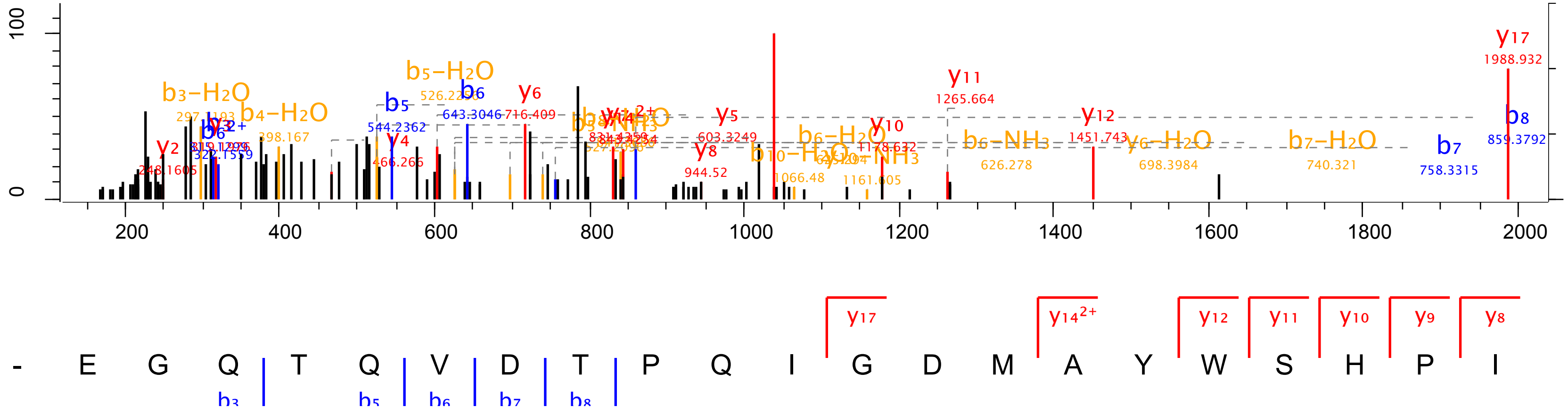

Raw file	Scan	Method	Score	m/z	Gene names
20140925_fract5_dyn_5ul_B5_01_440	7057	TOF; CID	183.43	489.27	COX8A

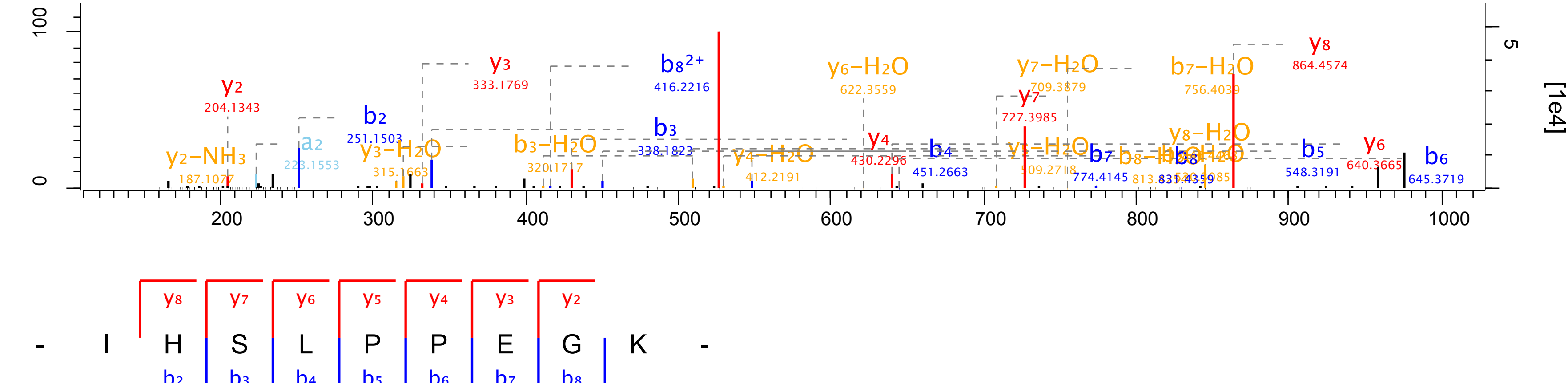

Raw file	Scan	Method	Score	m/z	Gene names
20140925_fract5_dyn_5ul_B5_01_440	10987	TOF; CID	94.11	408.21	TMEM187

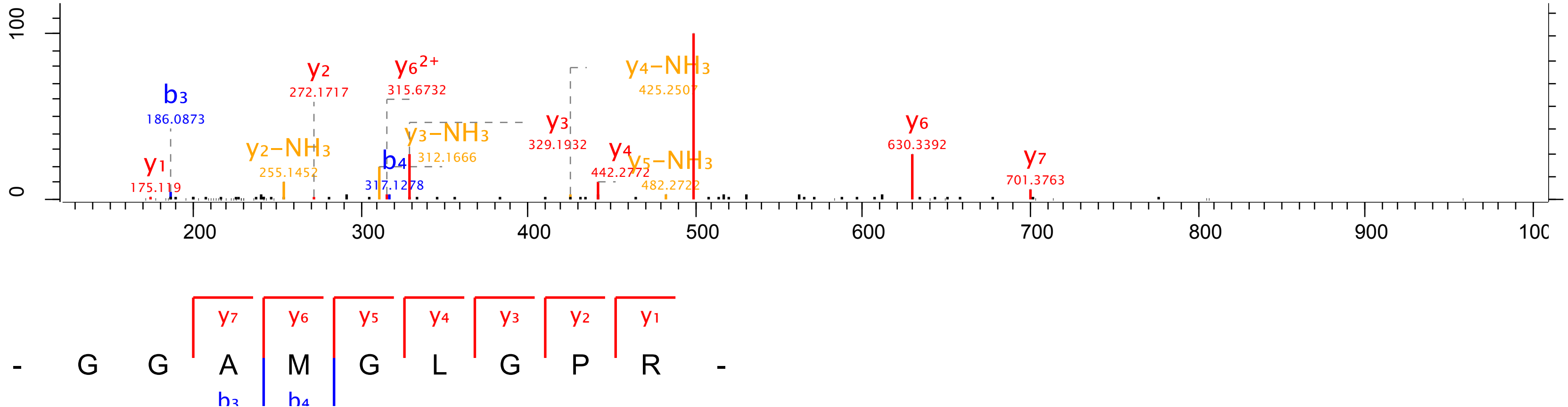

Raw file	Scan	Method	Score	m/z	Gene names
20140925_fract5_dyn_5ul_B5_01_440	12457	TOF; CID	114.72	640.8	C17orf89

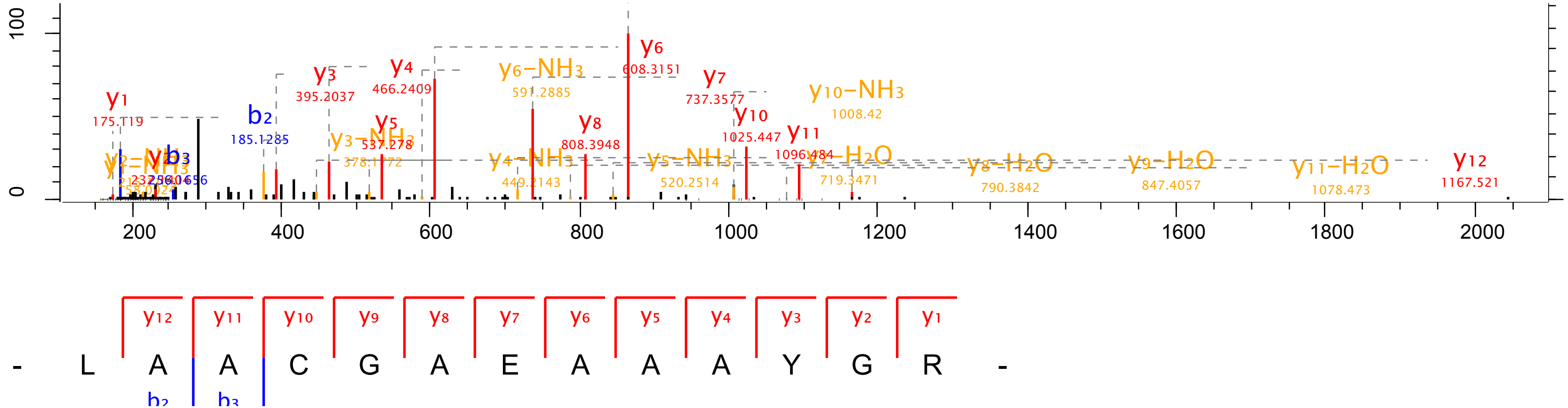

Raw file	Scan	Method	Score	m/z	Gene names
20140925_fract5_dyn_5ul_B5_01_440	14538	TOF; CID	142.43	425.86	CKS1B

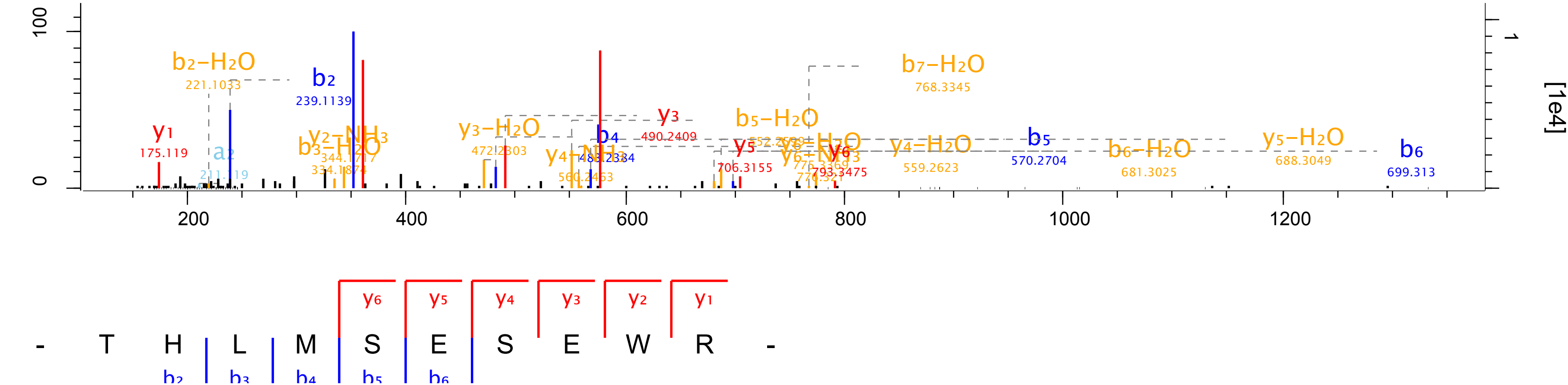

Raw file	Scan	Method	Score	m/z	Gene names
20140925_fract5_dyn_5ul_B5_01_440	16153	TOF; CID	46.51	932.47	JOSD2

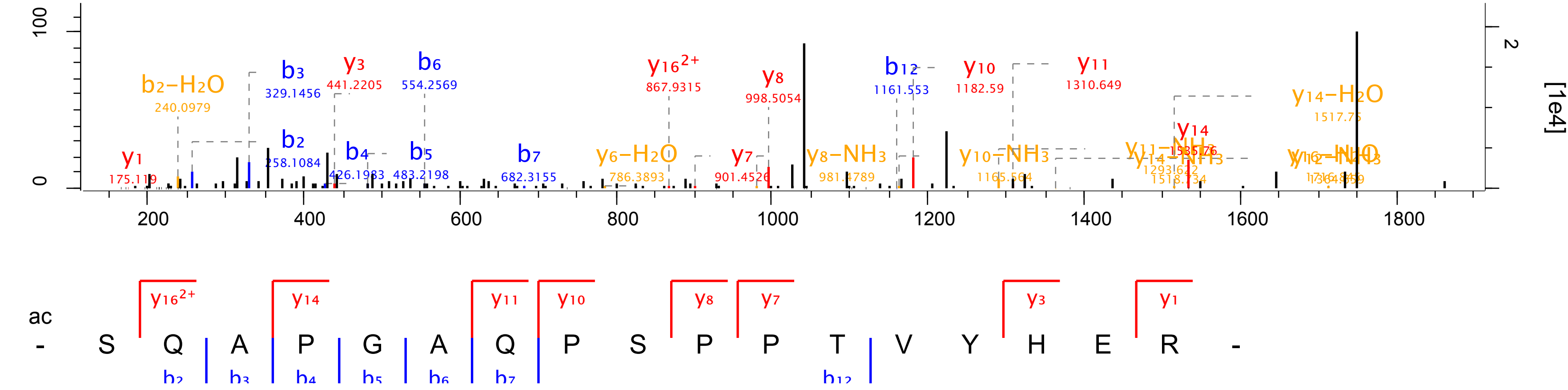

Raw file	Scan	Method	Score	m/z	Gene names
20140925_fract5_dyn_5ul_B5_01_440	16866	TOF; CID	57.41	834.91	FAM174A

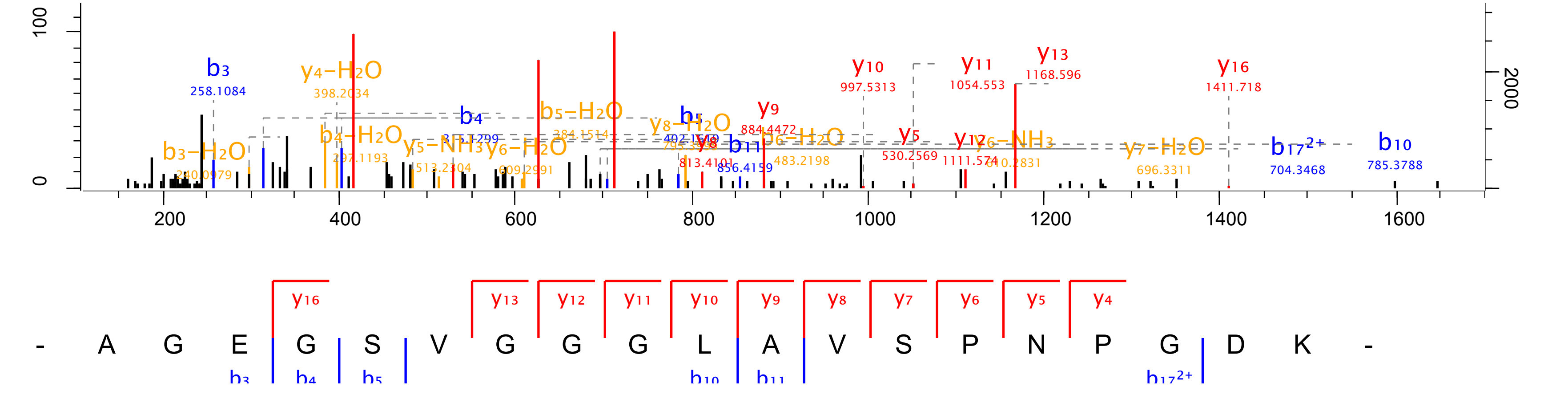

Raw file	Scan	Method	Score	m/z	Gene names
20140925_fract5_dyn_5ul_B5_01_440	18901	TOF; CID	89.51	807.38	CENPO

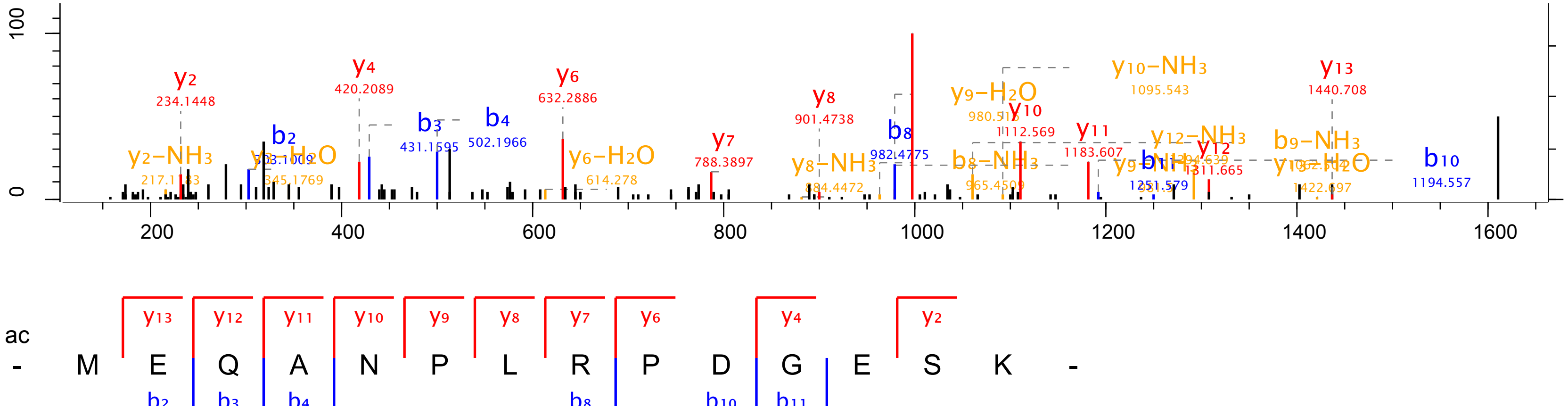

Raw file	Scan	Method	Score	m/z	Gene names
20140925_fract5_dyn_5ul_B5_01_440	20273	TOF; CID	86.14	512.73	TMEM258

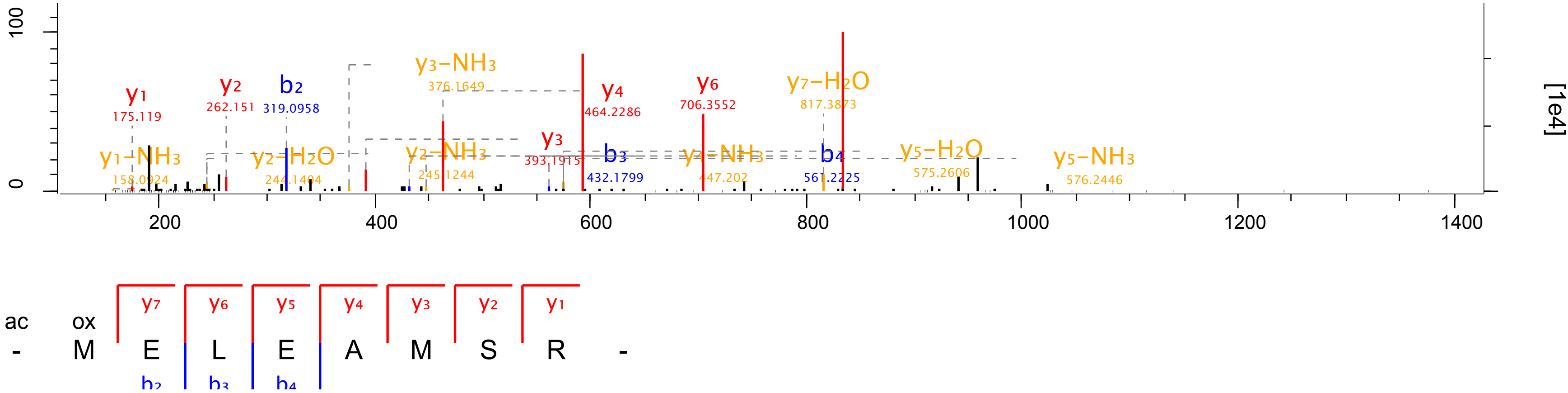

Raw file	Scan	Method	Score	m/z	Gene names
20140925_fract5_dyn_5ul_B5_01_440	20511	TOF; CID	116.78	579.95	CYB561

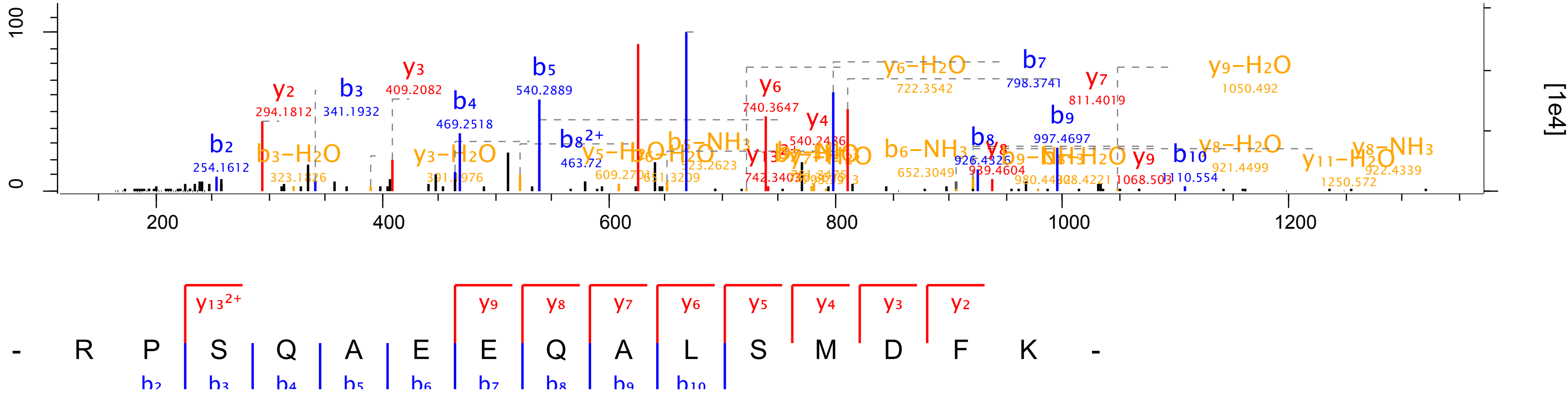

Raw file	Scan	Method	Score	m/z	Gene names
20140925_fract5_dyn_5ul_B5_01_440	23273	TOF; CID	46.07	463.89	TERF1

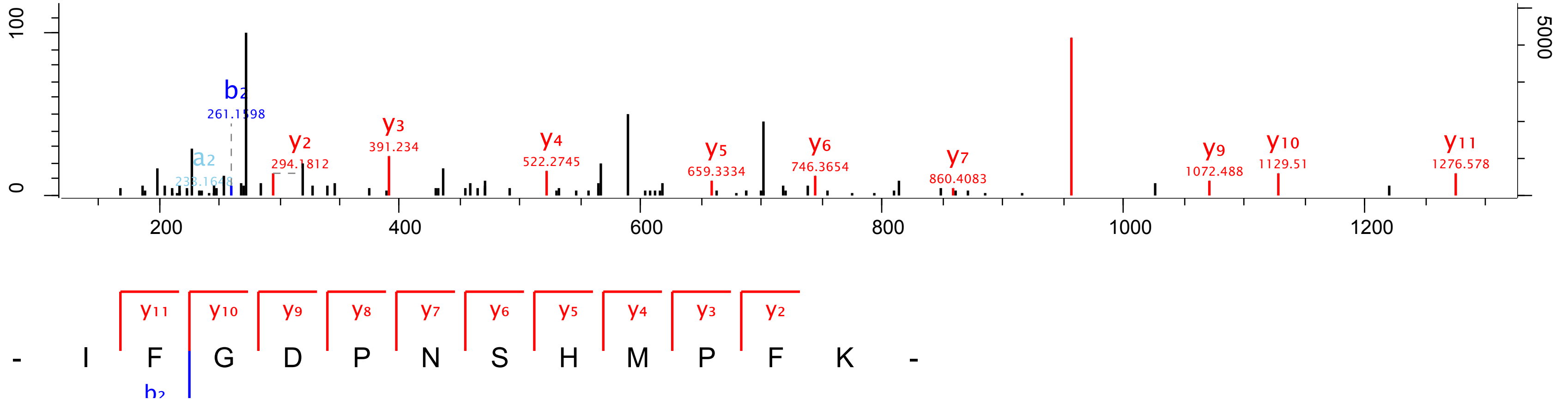

Raw file	Scan	Method	Score	m/z	Gene names
20140925_fract5_dyn_5ul_B5_01_440	23820	TOF; CID	77.06	722.87	SAMD12

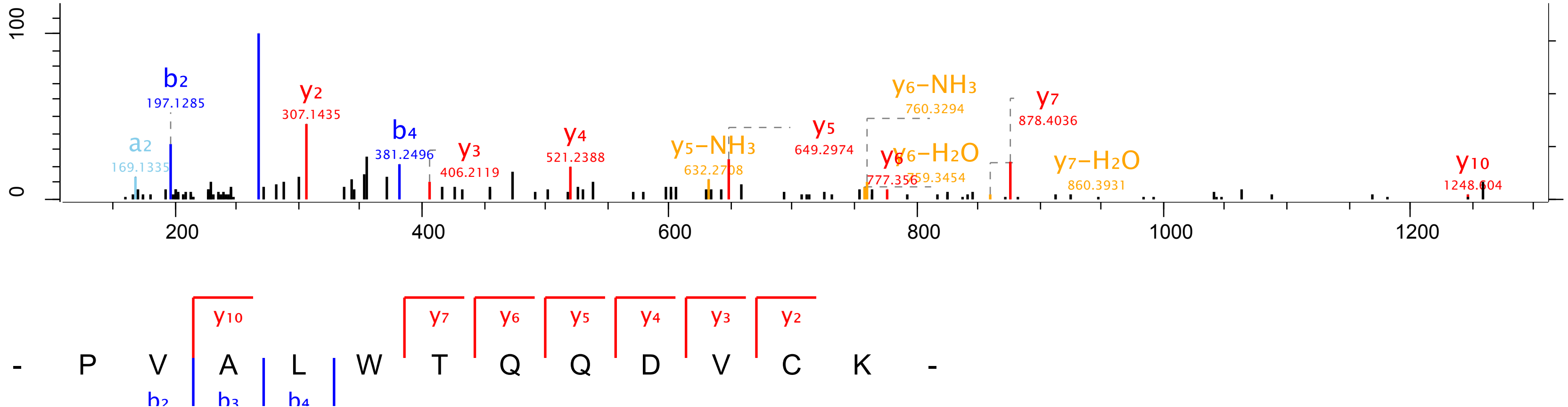

Raw file	Scan	Method	Score	m/z	Gene names
20140925_fract5_dyn_5ul_B5_01_440	25198	TOF; CID	66	693.36	SLC22A3

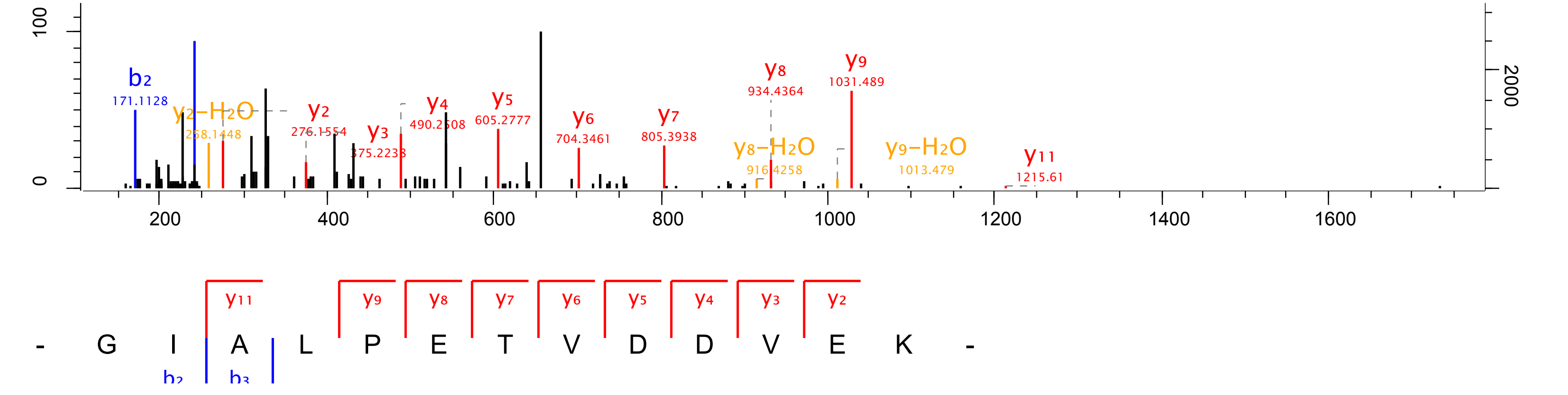

Raw file	Scan	Method	Score	m/z	Gene names
20140925_fract5_dyn_5ul_B5_01_440	25642	TOF; CID	114.96	943.96	MKKS

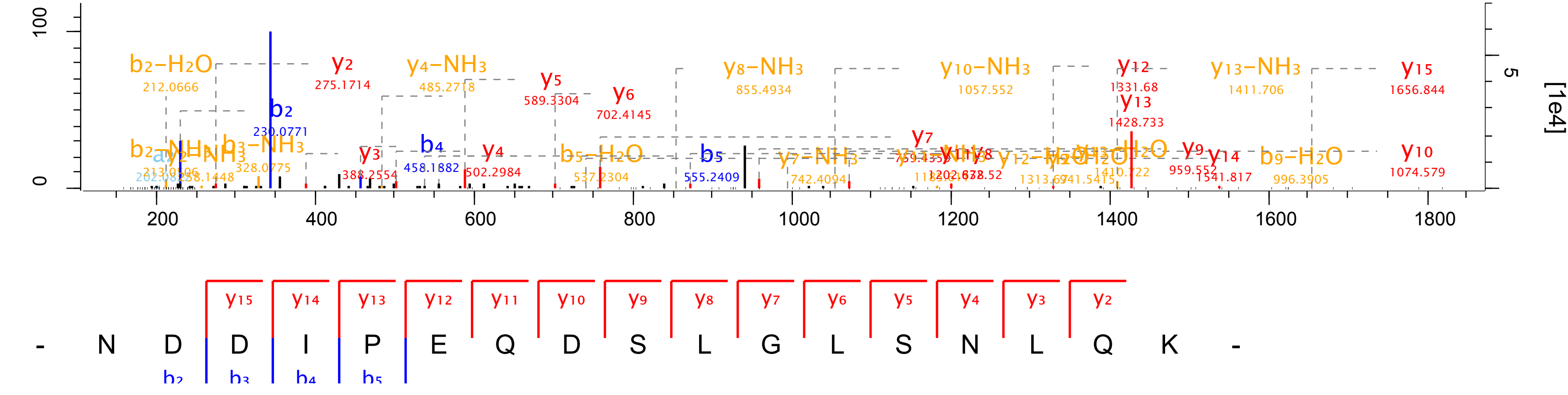

Raw file	Scan	Method	Score	m/z	Gene names
20140925_fract5_dyn_5ul_B5_01_440	29617	TOF; CID	58.89	687.86	FAM89B

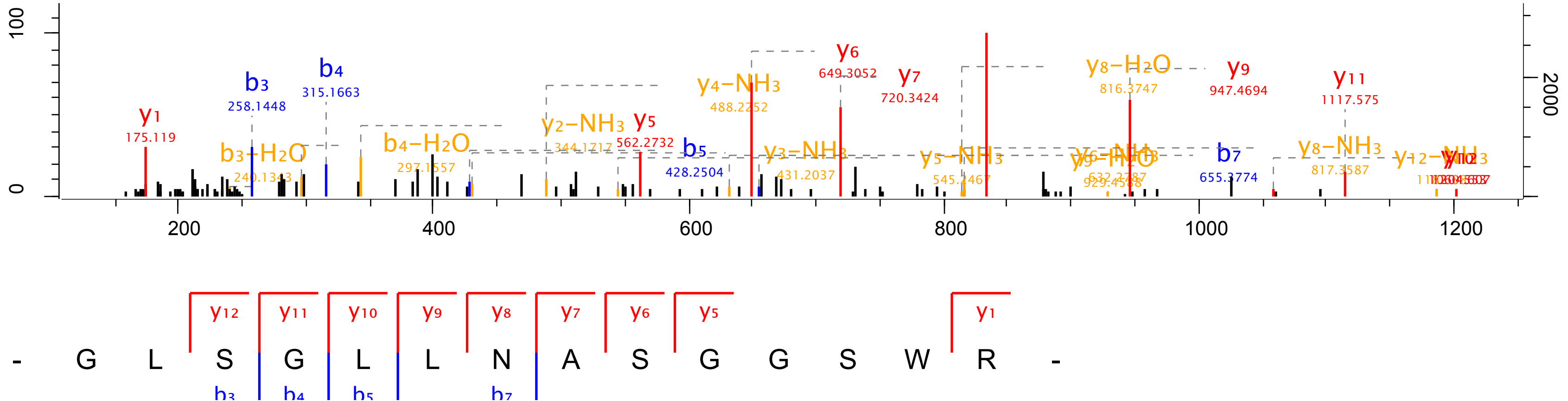

Raw file	Scan	Method	Score	m/z	Gene names
20140925_fract5_dyn_5ul_B5_01_440	29669	TOF; CID	91.59	735.03	UBALD1

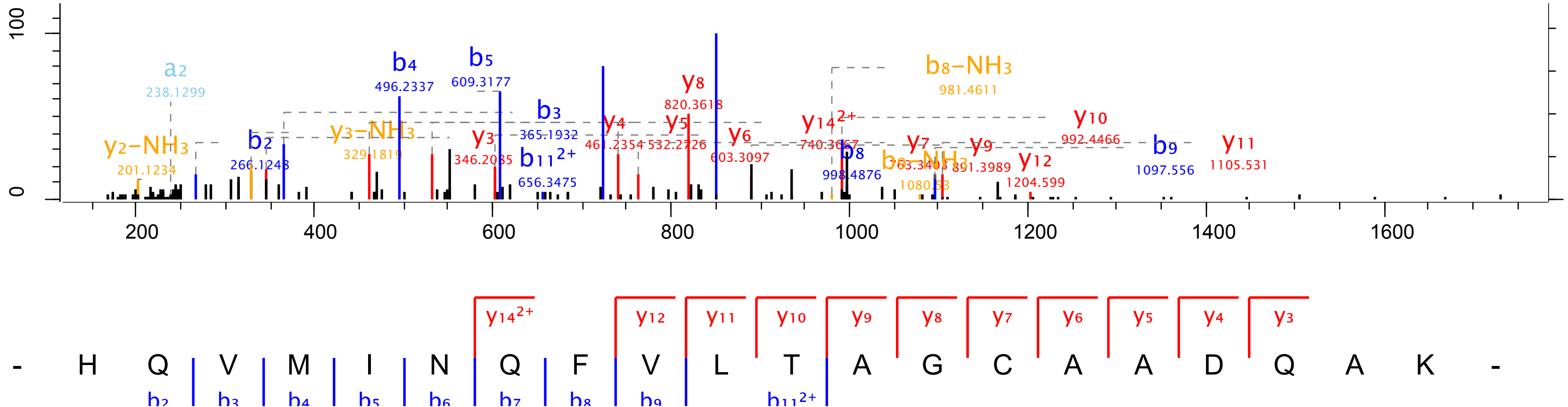

Raw file	Scan	Method	Score	m/z	Gene names
20140925_fract5_dyn_5ul_B5_01_440	30227	TOF; CID	112.17	626.32	OTUB2

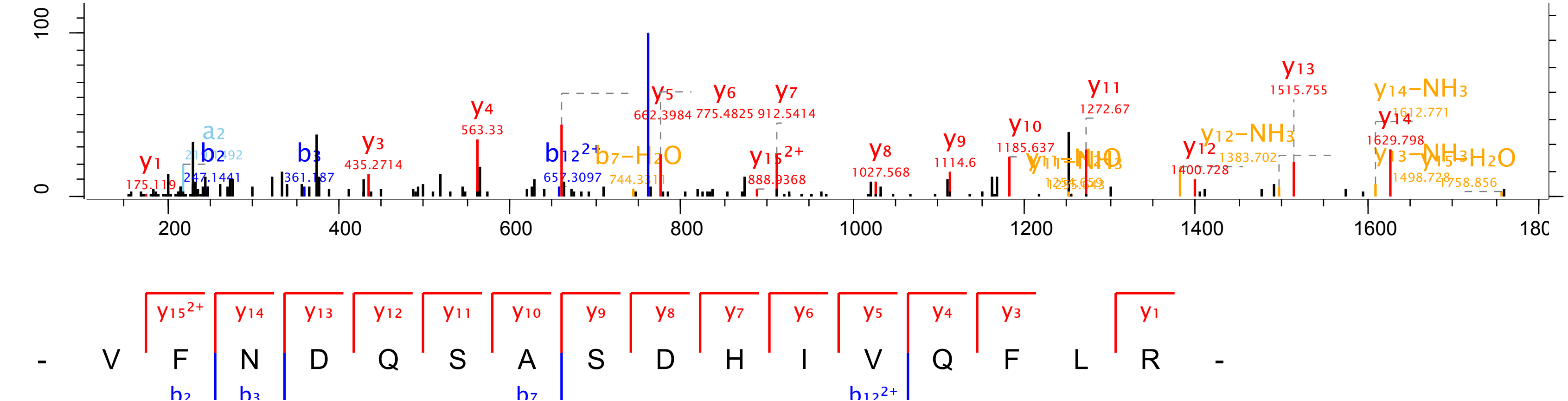

Raw file	Scan	Method	Score	m/z	Gene names
20140925_fract5_dyn_5ul_B5_01_440	31940	TOF; CID	52.18	725.35	PRR3

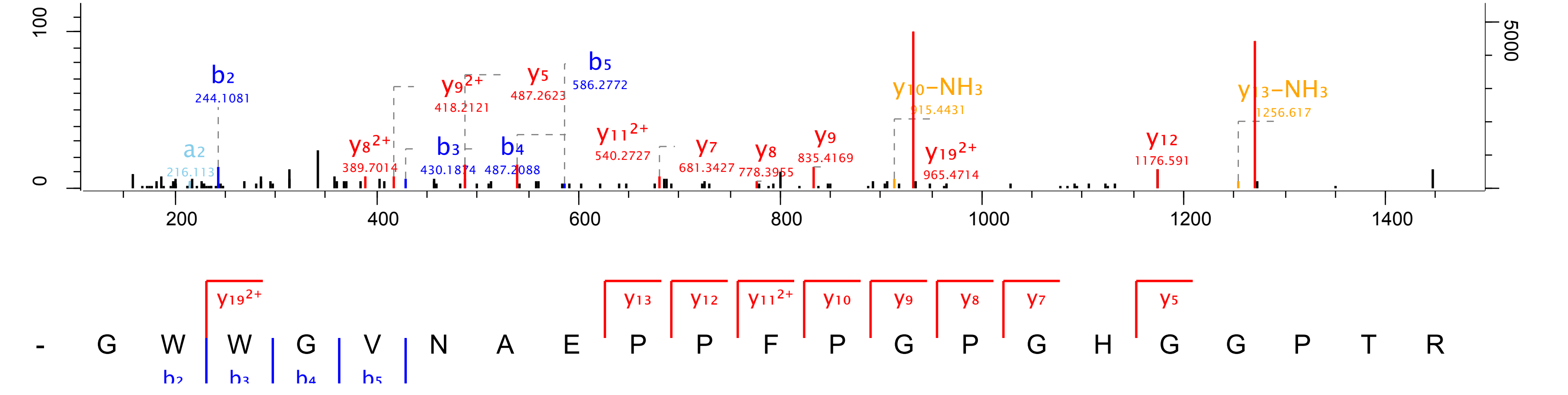

Raw file	Scan	Method	Score	m/z	Gene names
20140925_fract5_dyn_5ul_B5_01_440	32102	TOF; CID	41.21	1213.6	JAG1

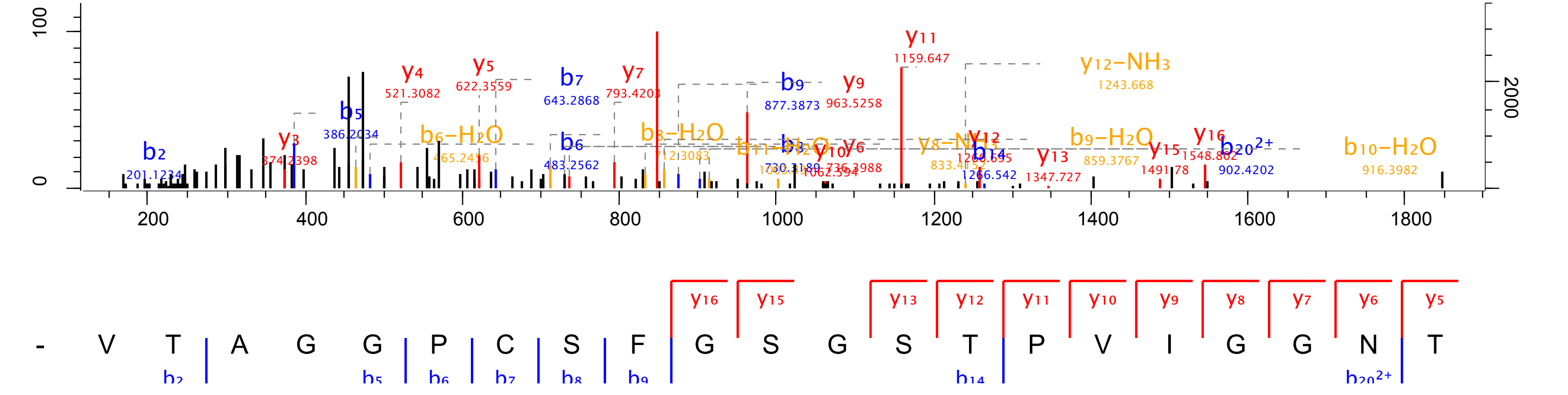

Raw file	Scan	Method	Score	m/z	Gene names
20140925_fract5_dyn_5ul_B5_01_440	34064	TOF; CID	117.7	576.85	SPIN4

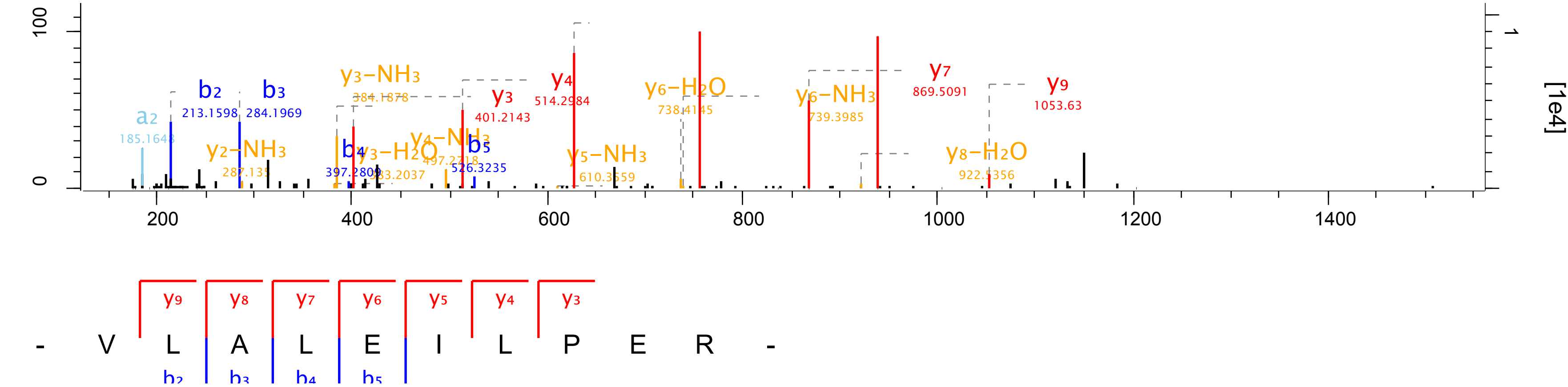

Raw file	Scan	Method	Score	m/z	Gene names
20140925_fract5_dyn_5ul_B5_01_440	35034	TOF; CID	70.41	650.32	RNF141

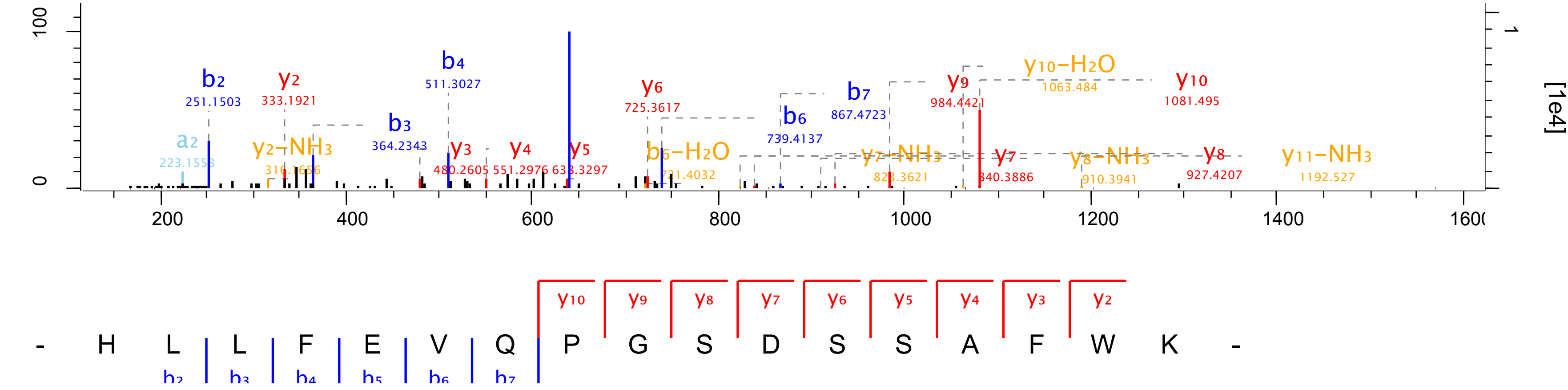

Raw file	Scan	Method	Score	m/z	Gene names
20140925_fract5_dyn_5ul_B5_01_440	35562	TOF; CID	69.98	651.32	BCL2

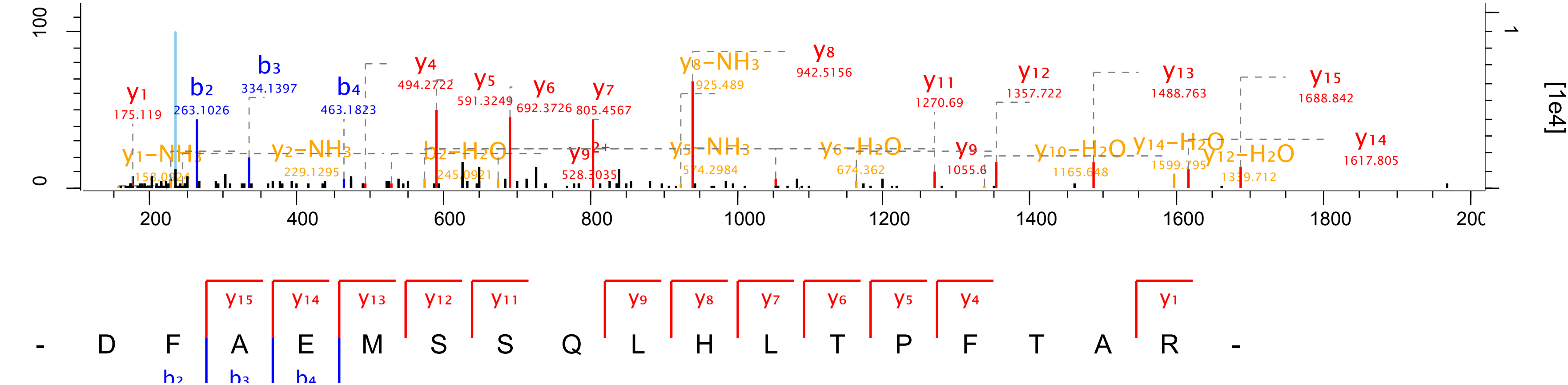

Raw file	Scan	Method	Score	m/z	Gene names
20140925_fract6_dyn_5ul_B6_01_441	3737	TOF; CID	53.45	675.29	HMGN1

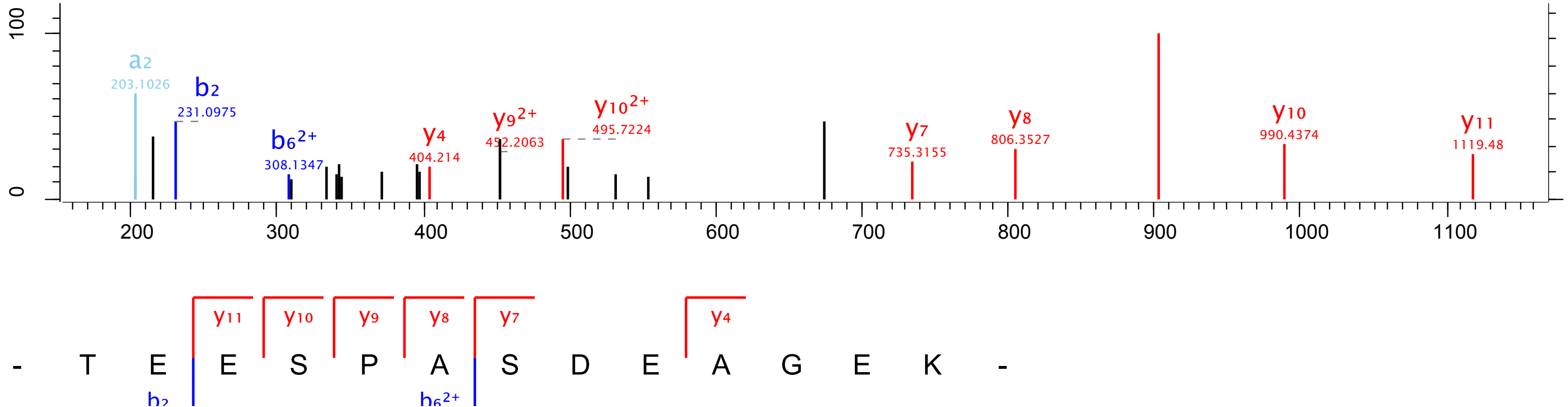

Raw file	Scan	Method	Score	m/z	Gene names
20140925_fract6_dyn_5ul_B6_01_441	4453	TOF; CID	157.91	594.3	NR1H3

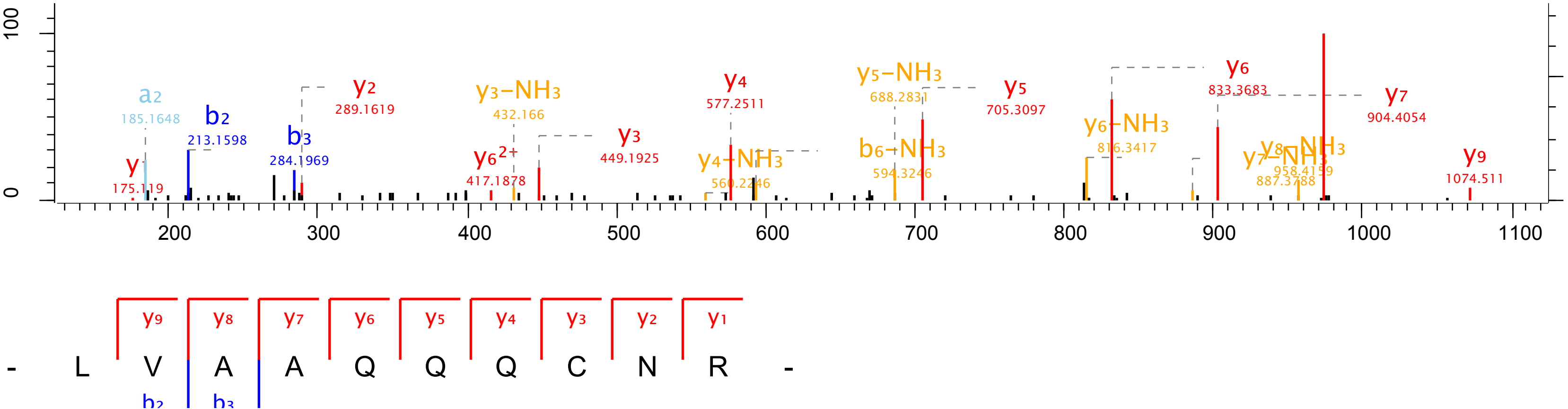

Raw file	Scan	Method	Score	m/z	Gene names
20140925_fract6_dyn_5ul_B6_01_441	7012	TOF; CID	97.28	527.29	ZNF229

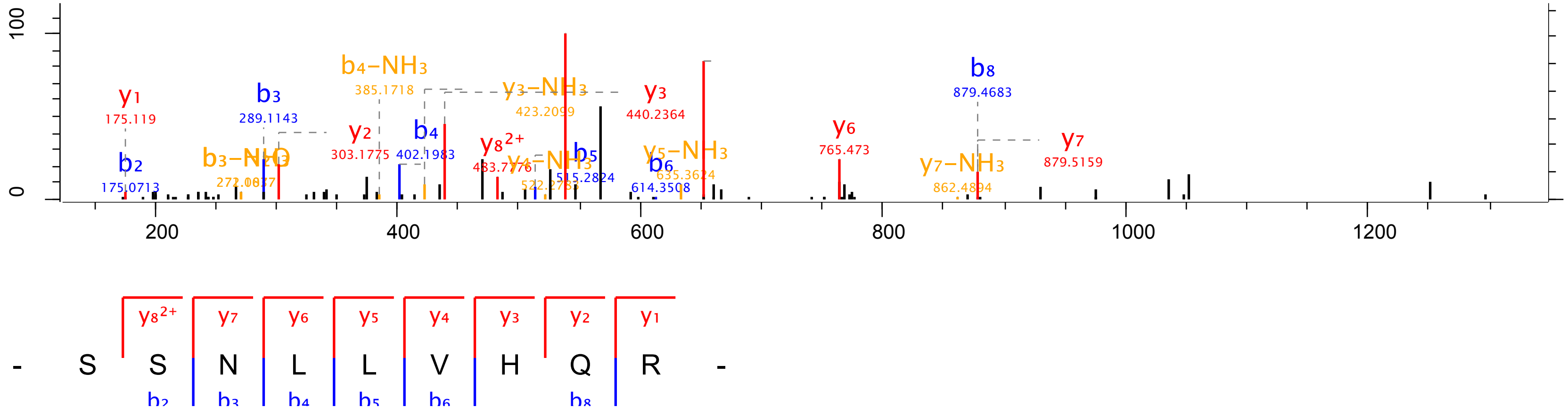

Raw file	Scan	Method	Score	m/z	Gene names
20140925_fract6_dyn_5ul_B6_01_441	10157	TOF; CID	118.76	617.32	HDGFRP2

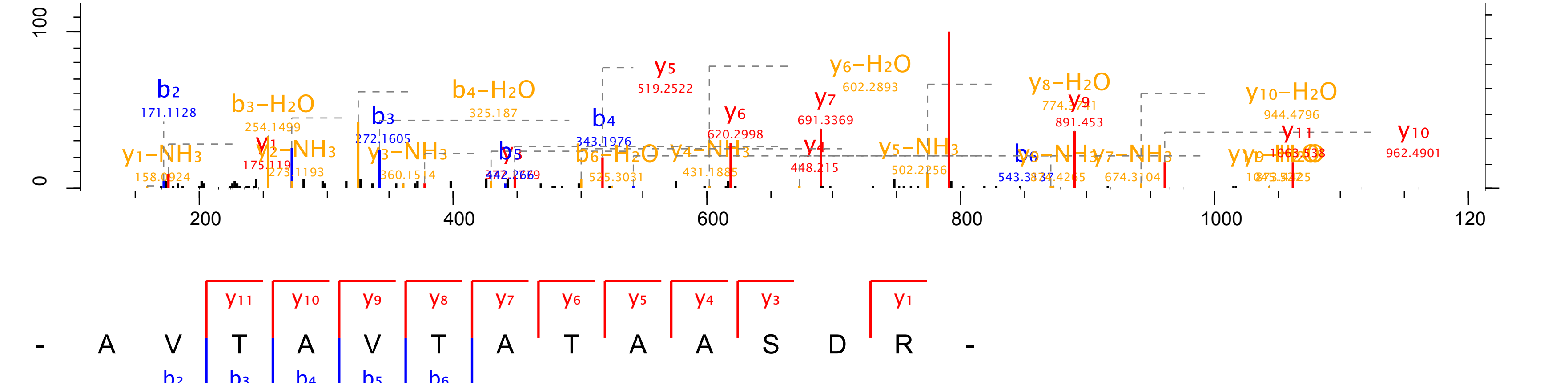

Raw file	Scan	Method	Score	m/z	Gene names
20140925_fract6_dyn_5ul_B6_01_441	13025	TOF; CID	95.2	804.39	GPRC5C

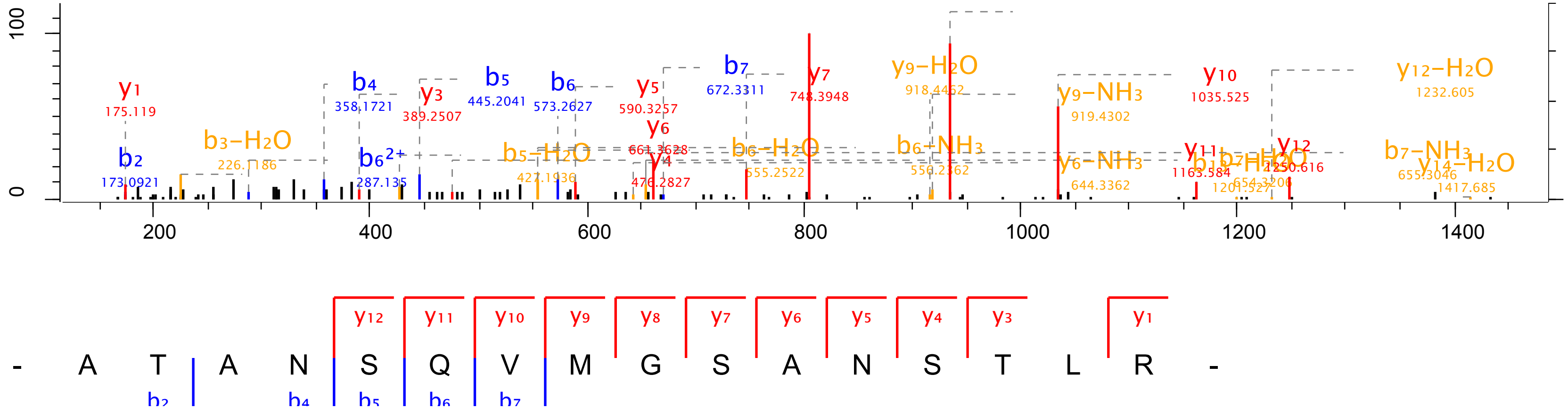

Raw file	Scan	Method	Score	m/z	Gene names
20140925_fract6_dyn_5ul_B6_01_441	14027	TOF; CID	137.21	835.4	SNAP25

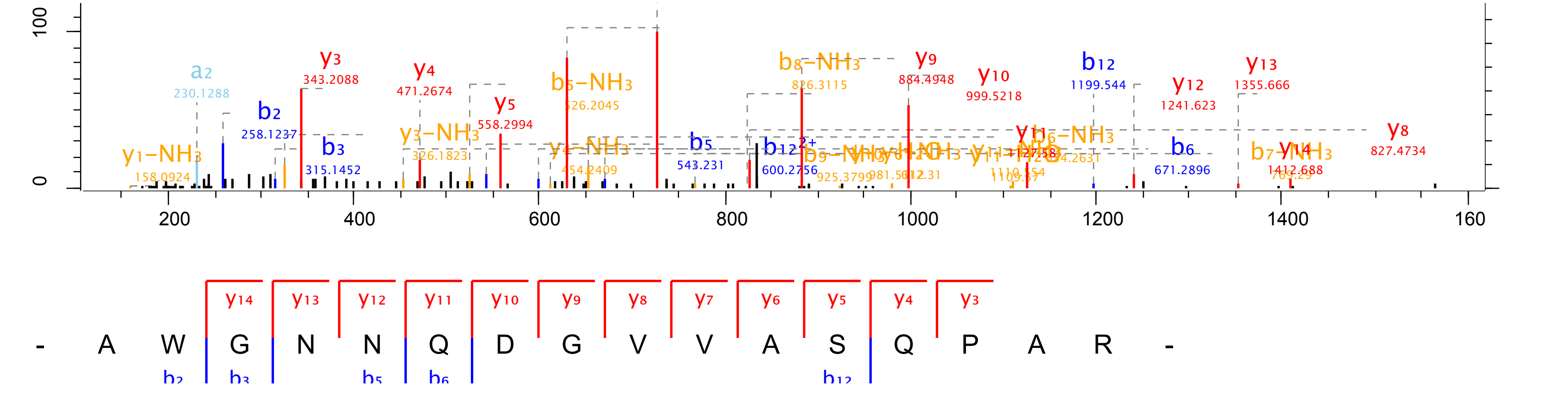

Raw file	Scan	Method	Score	m/z	Gene names
20140925_fract6_dyn_5ul_B6_01_441	16625	TOF; CID	118.19	843.38	ELOVL6

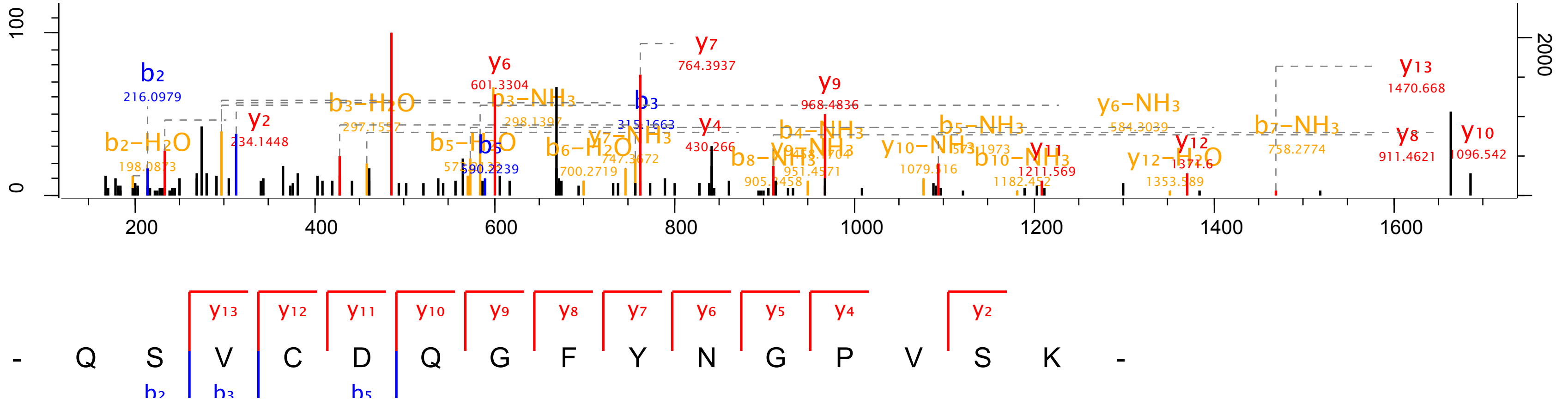

Raw file	Scan	Method	Score	m/z	Gene names
20140925_fract6_dyn_5ul_B6_01_441	16864	TOF; CID	104.7	560.27	TRIM38

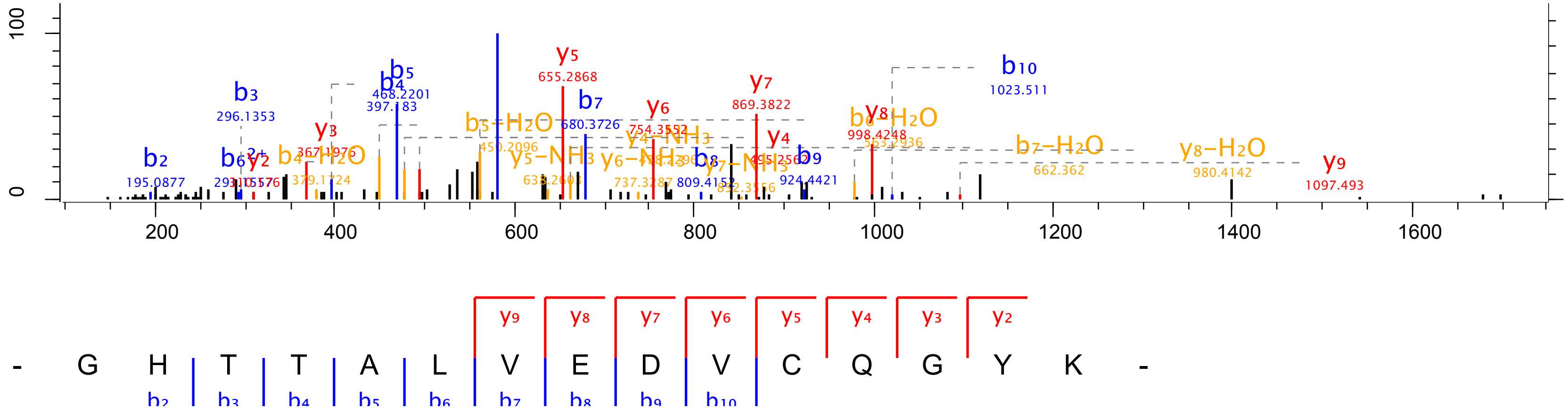

Raw file	Scan	Method	Score	m/z	Gene names
20140925_fract6_dyn_5ul_B6_01_441	17095	TOF; CID	69.92	498.24	FBXO46

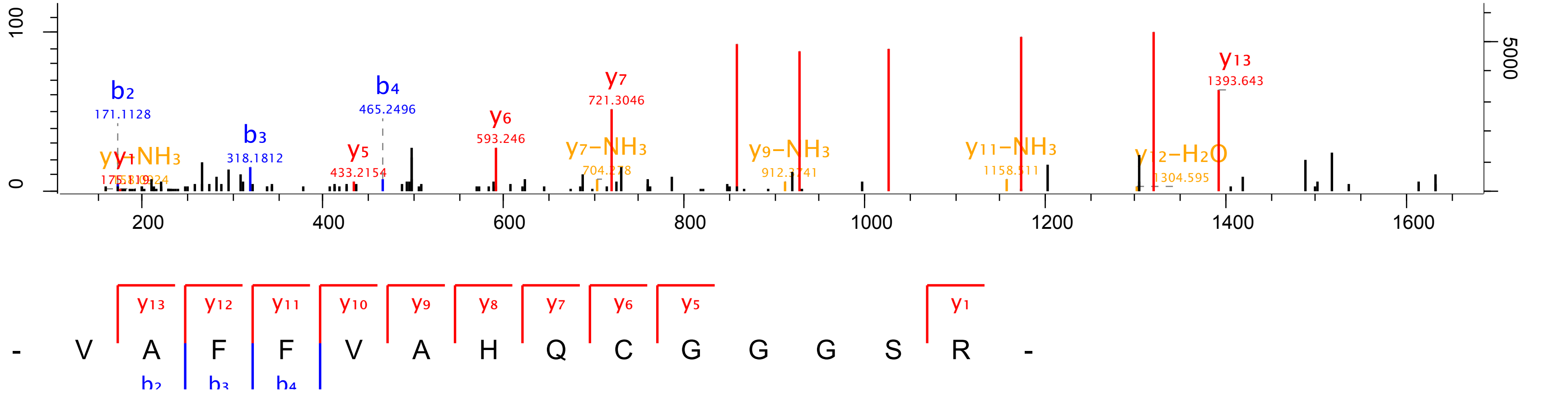

Raw file	Scan	Method	Score	m/z	Gene names
20140925_fract6_dyn_5ul_B6_01_441	17611	TOF; CID	139.39	578.82	FAM109A

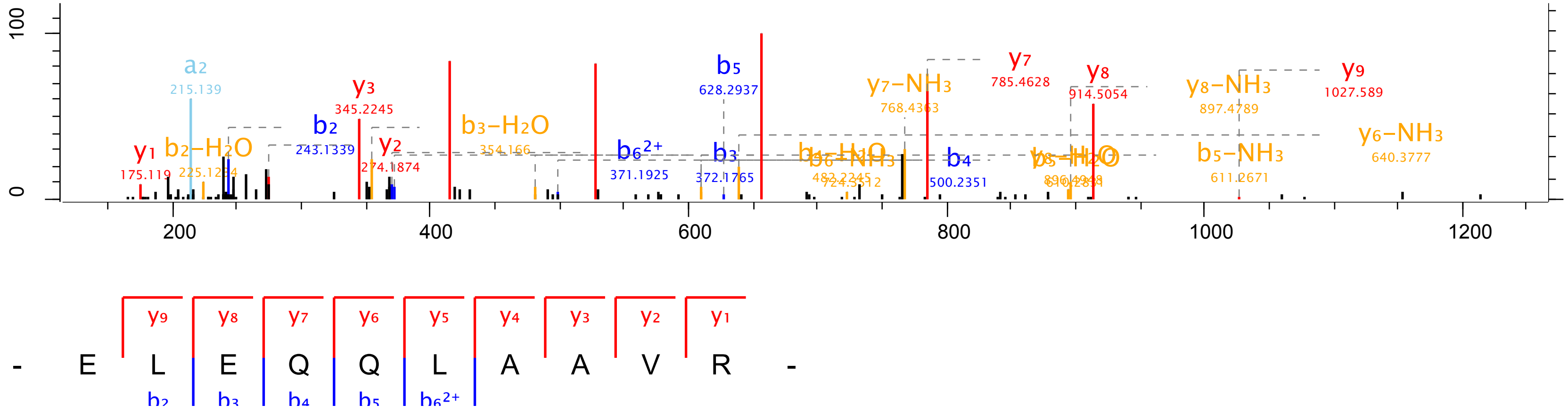

Raw file	Scan	Method	Score	m/z	Gene names
20140925_fract6_dyn_5ul_B6_01_441	20019	TOF; CID	65.84	718.32	ATG10

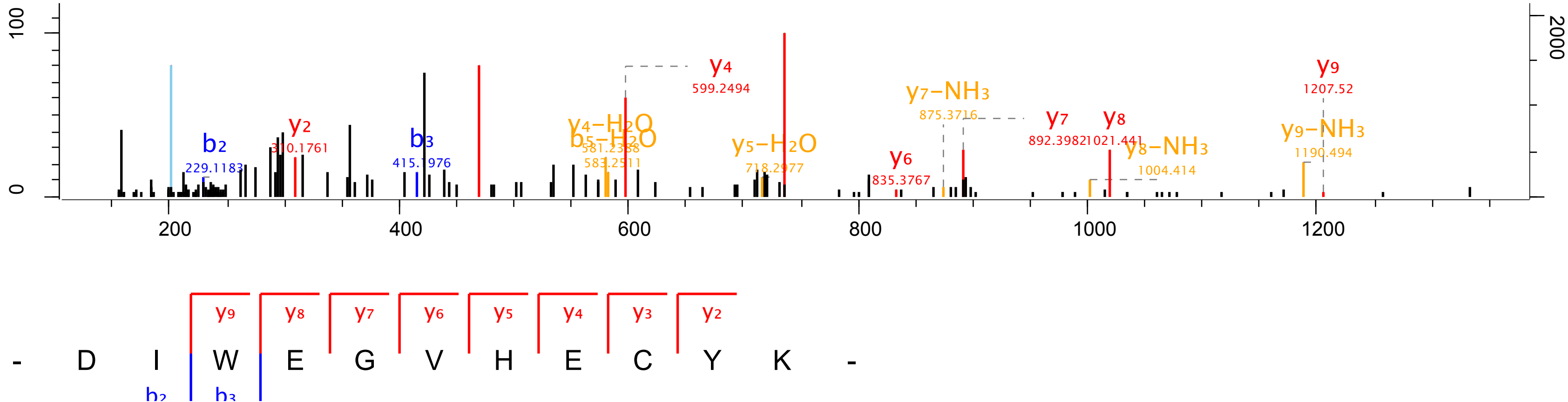

Raw file	Scan	Method	Score	m/z	Gene names
20140925_fract6_dyn_5ul_B6_01_441	20736	TOF; CID	30.92	1137.52	MDM2

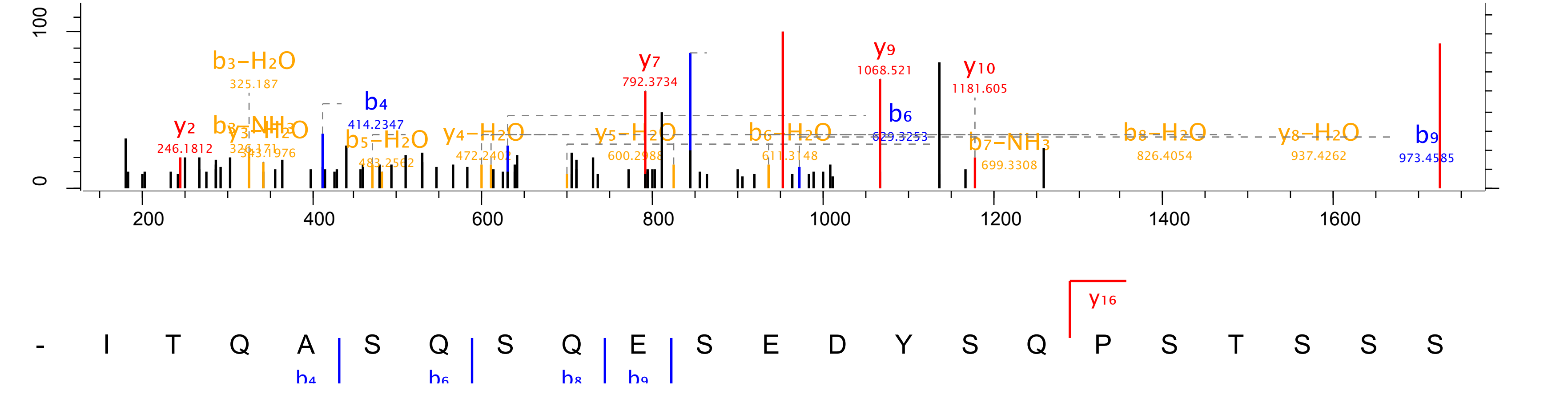

Raw file	Scan	Method	Score	m/z	Gene names
20140925_fract6_dyn_5ul_B6_01_441	21185	TOF; CID	121.95	591.31	BTBD7

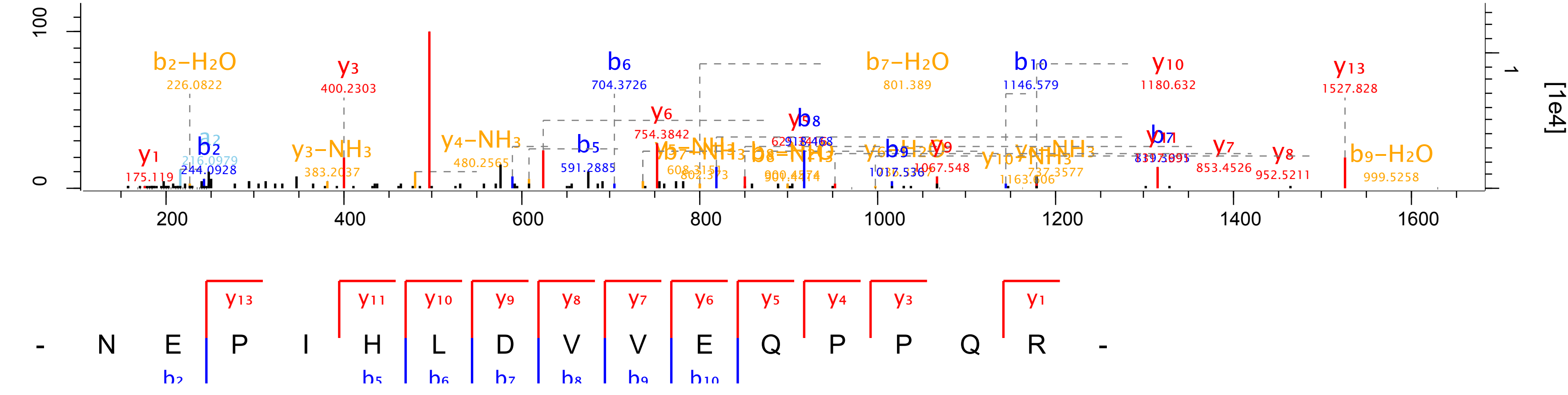

Raw file	Scan	Method	Score	m/z	Gene names
20140925_fract6_dyn_5ul_B6_01_441	22628	TOF; CID	86.8	461.23	ZKSCAN5

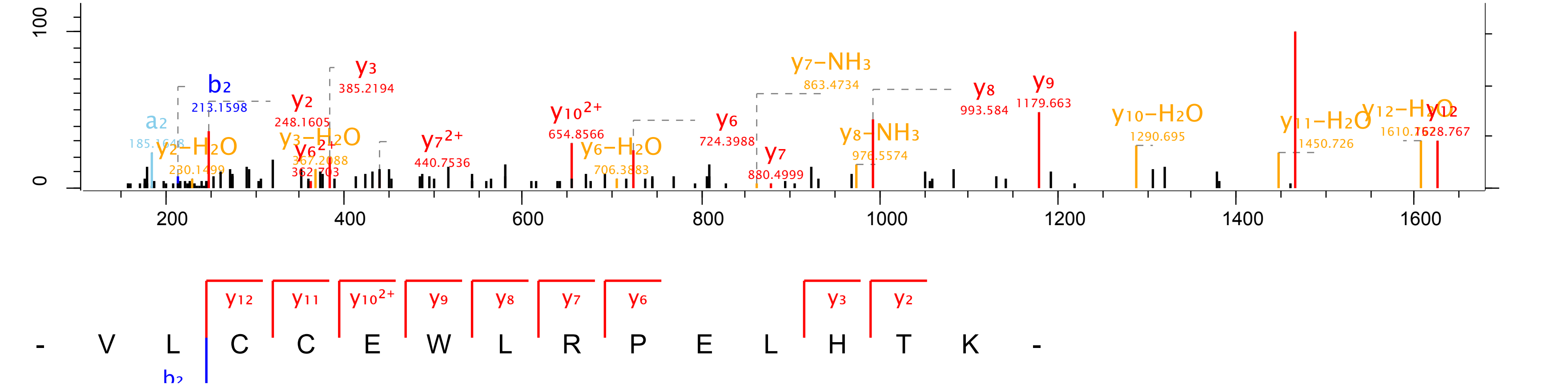

Raw file	Scan	Method	Score	m/z	Gene names
20140925_fract6_dyn_5ul_B6_01_441	24882	TOF; CID	64.12	586.84	KATNAL2

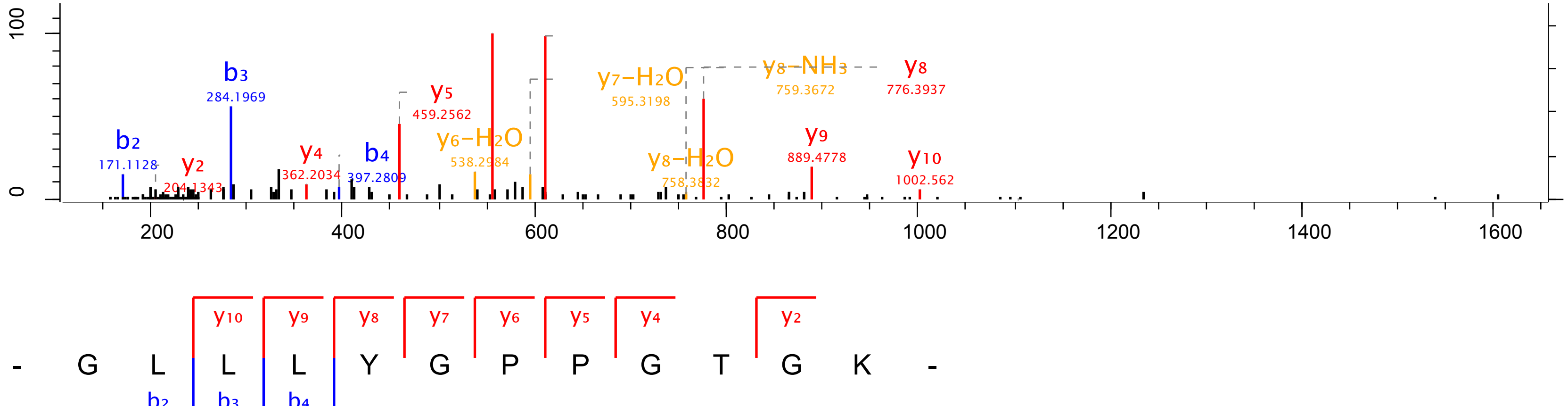

Raw file	Scan	Method	Score	m/z	Gene names
20140925_fract6_dyn_5ul_B6_01_441	26241	TOF; CID	109.48	541.8	PRELID1

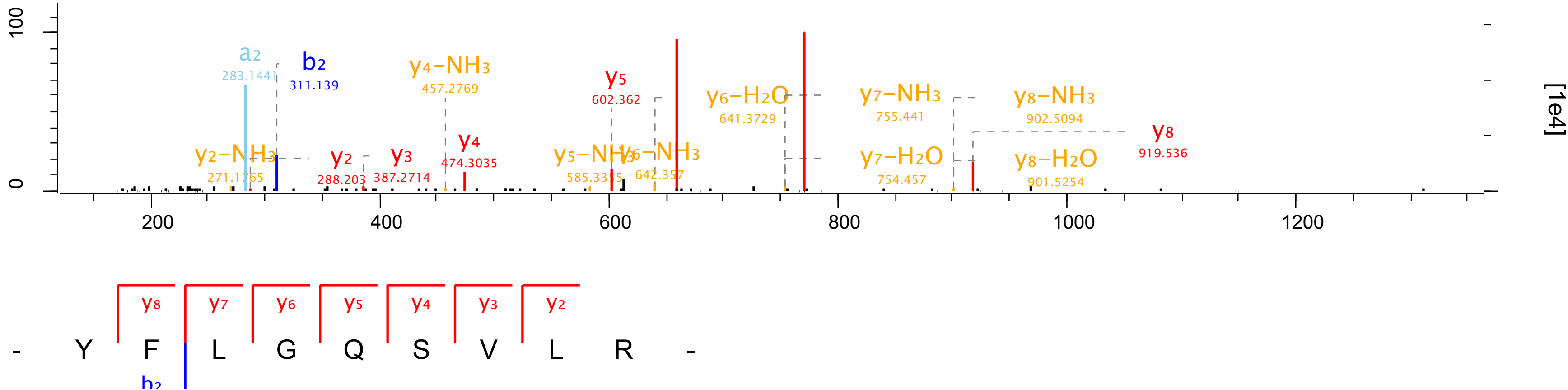

Raw file	Scan	Method	Score	m/z	Gene names
20140925_fract6_dyn_5ul_B6_01_441	26604	TOF; CID	73.83	556.29	FAM210B

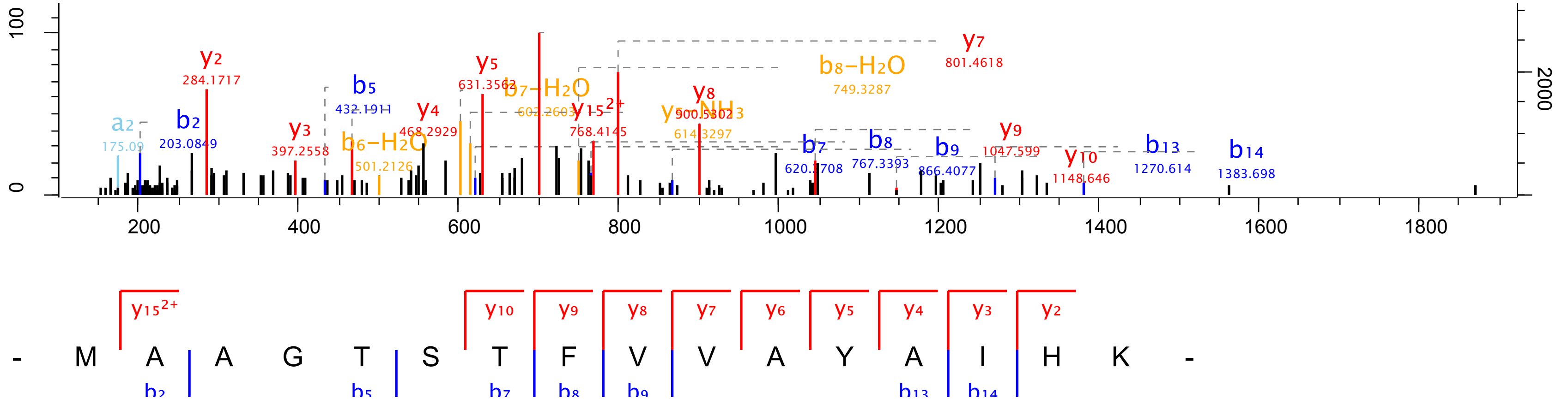

Raw file	Scan	Method	Score	m/z	Gene names
20140925_fract6_dyn_5ul_B6_01_441	28115	TOF; CID	83.95	587.34	GLTSCR1L

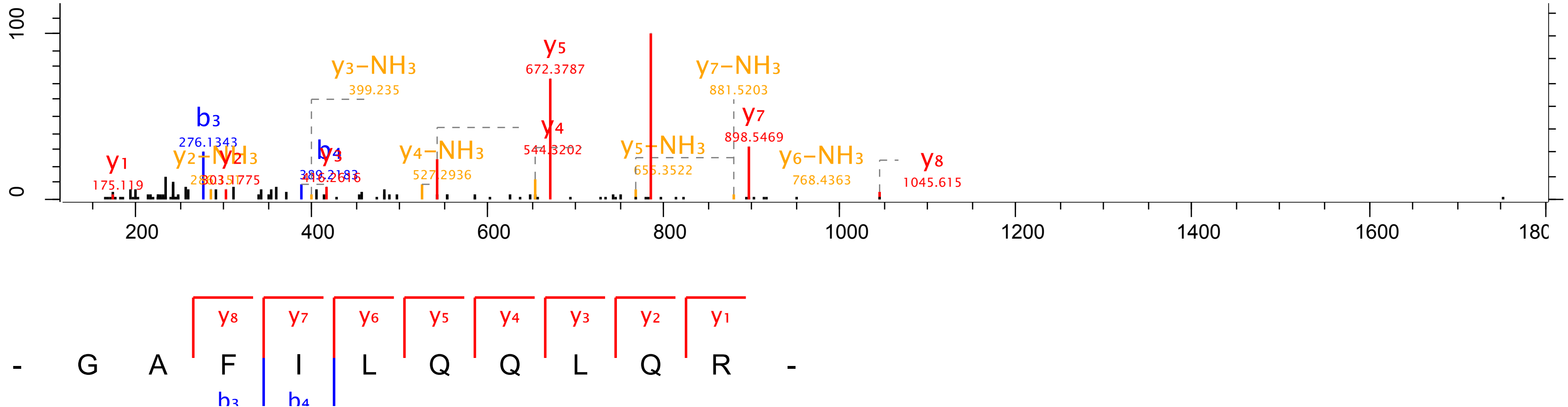

Raw file	Scan	Method	Score	m/z	Gene names
20140925_fract6_dyn_5ul_B6_01_441	29609	TOF; CID	107.82	569.29	CENPP

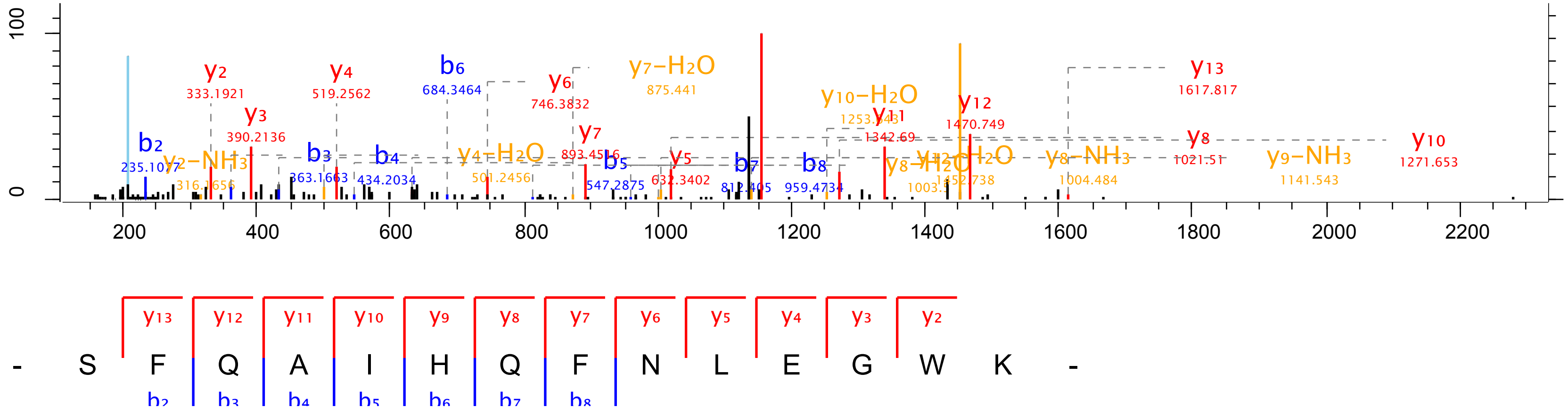

Raw file	Scan	Method	Score	m/z	Gene names
20140925_fract6_dyn_5ul_B6_01_441	29925	TOF; CID	67.23	725.03	UBALD2

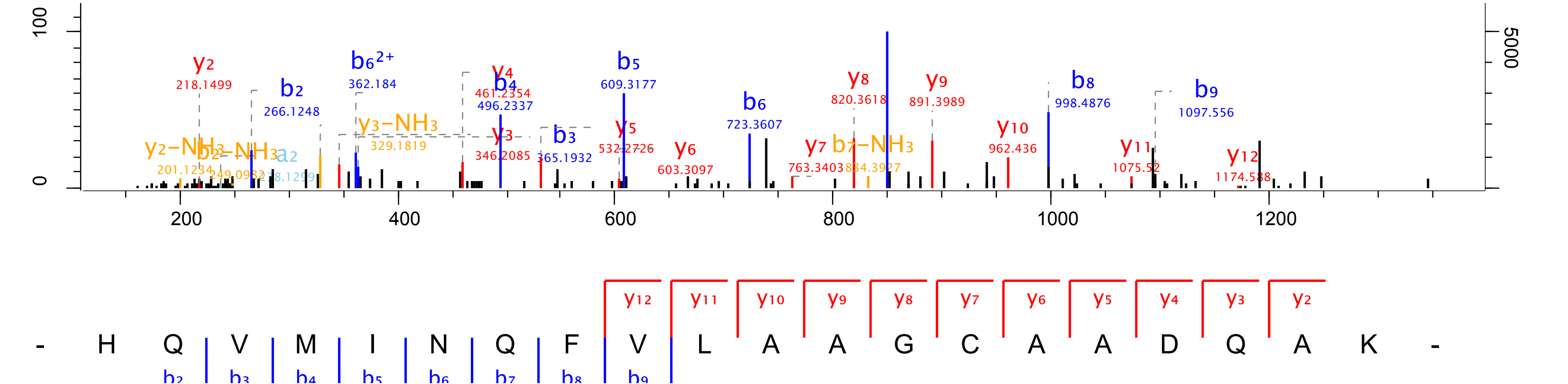

Raw file	Scan	Method	Score	m/z	Gene names
20140925_fract6_dyn_5ul_B6_01_441	32154	TOF; CID	55.84	834.43	TFR2

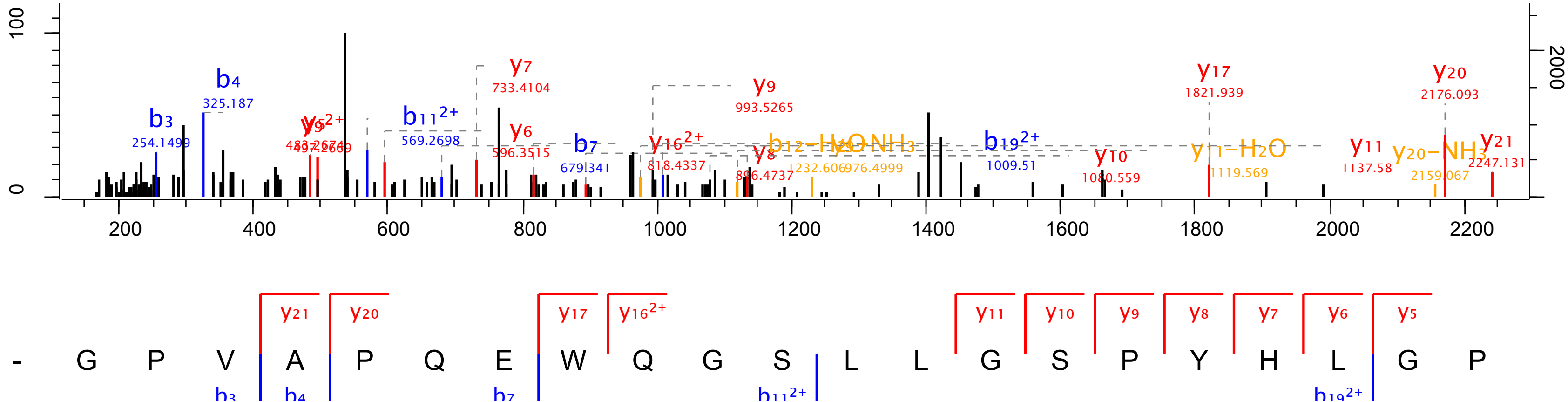

Raw file	Scan	Method	Score	m/z	Gene names
20140925_fract6_dyn_5ul_B6_01_441	33623	TOF; CID	52.43	1021.04	HIPK2;HIPK1

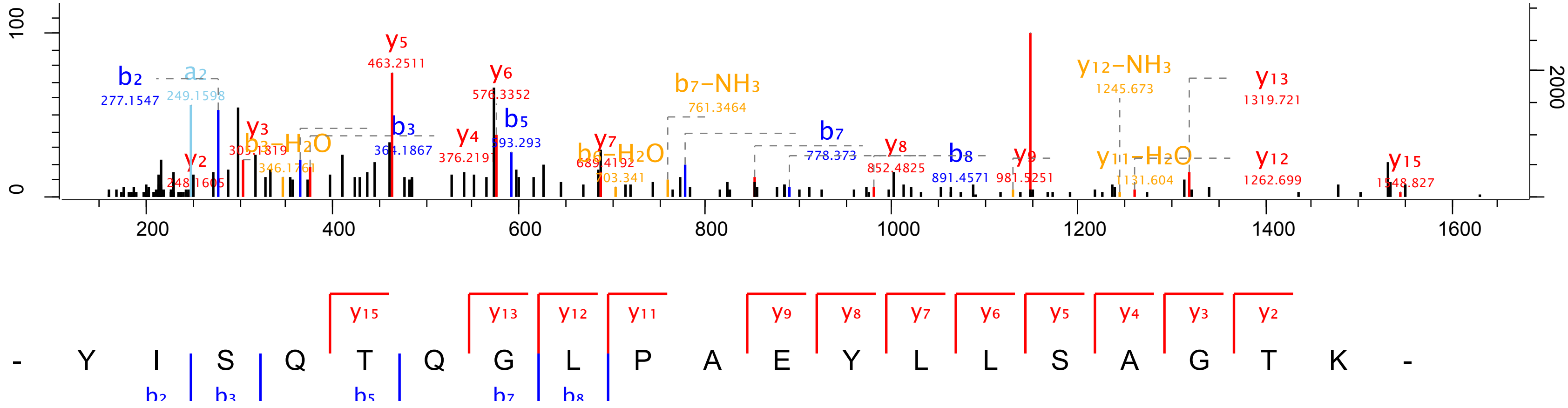

Raw file	Scan	Method	Score	m/z	Gene names
20140925_fract6_dyn_5ul_B6_01_441	34441	TOF; CID	60.54	866.47	TTC32

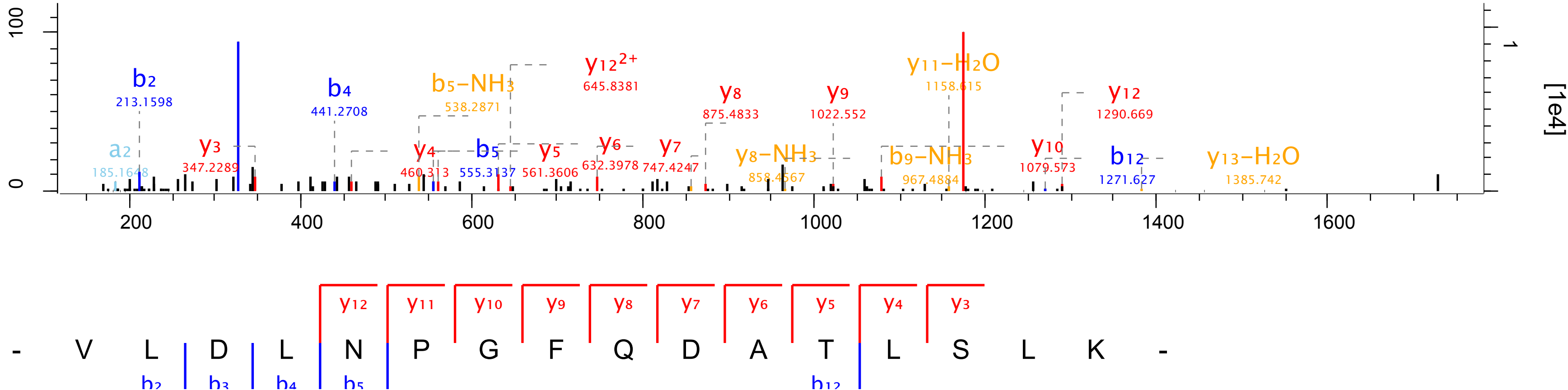

Raw file	Scan	Method	Score	m/z	Gene names
20140925_fract6_dyn_5ul_B6_01_441	38112	TOF; CID	31.55	809.42	DOS;C19orf26

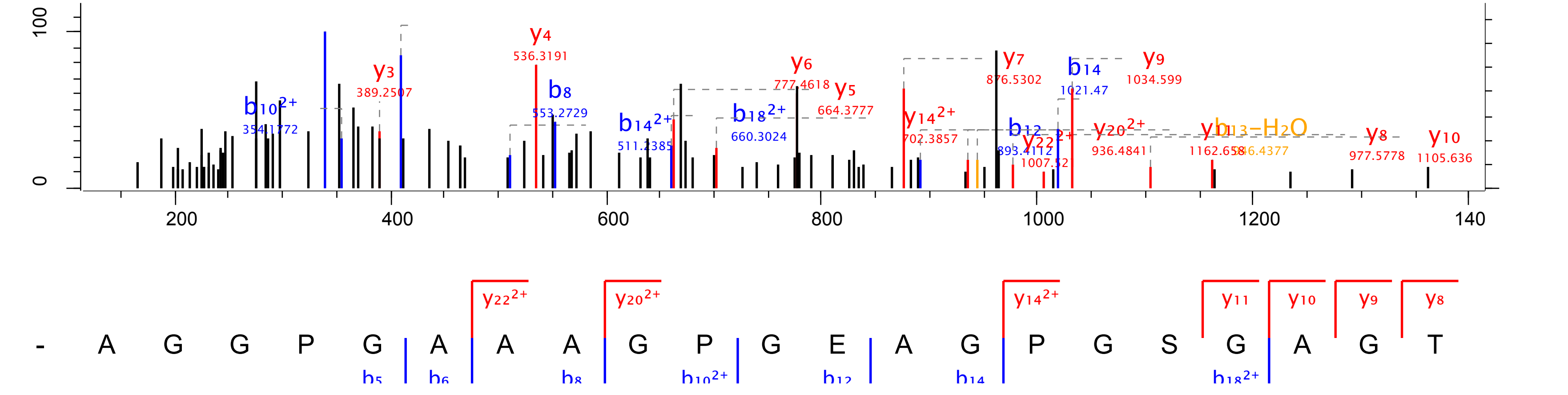

Raw file	Scan	Method	Score	m/z	Gene names
20140925_fract7_dyn_5ul_B7_01_442	5466	TOF; CID	43.03	506.59	STOX2;GDAP1

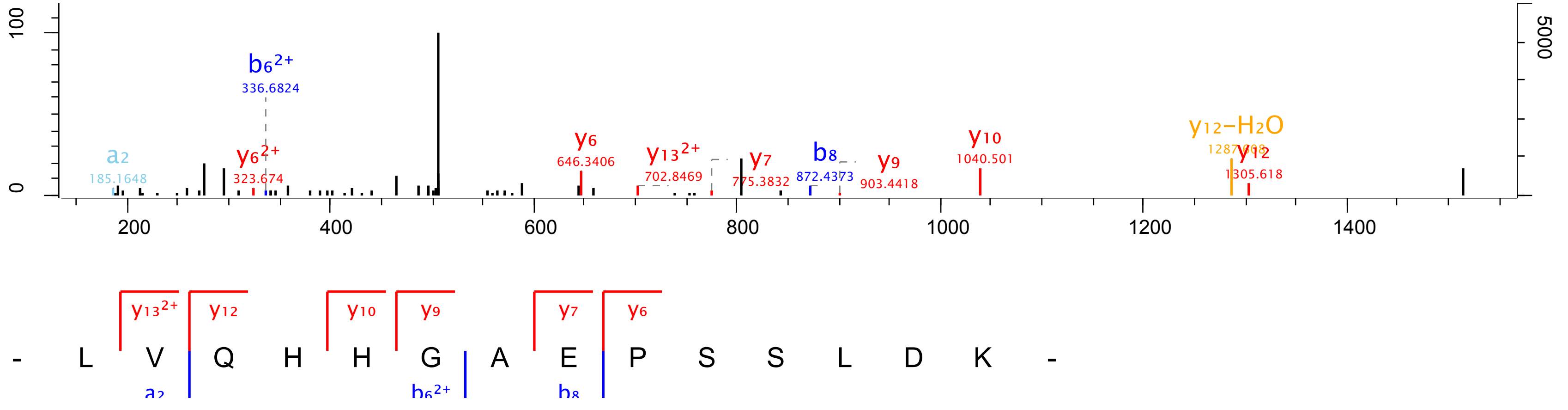

Raw file	Scan	Method	Score	m/z	Gene names
20140925_fract7_dyn_5ul_B7_01_442	7569	TOF; CID	89.43	629.33	CXCR4

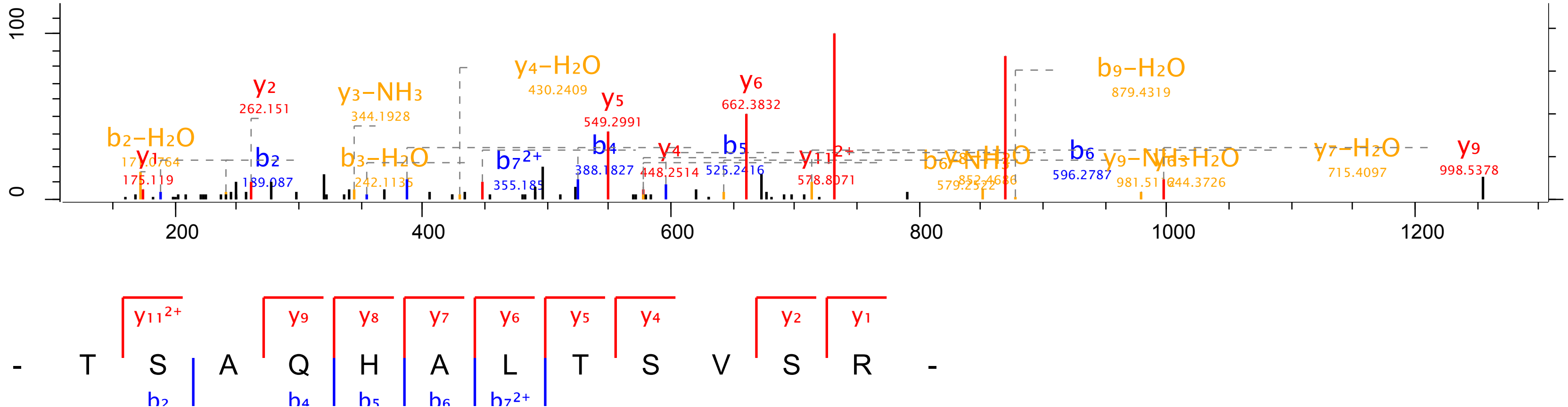

Raw file	Scan	Method	Score	m/z	Gene names
20140925_fract7_dyn_5ul_B7_01_442	8519	TOF; CID	92.86	705.84	TBPL1

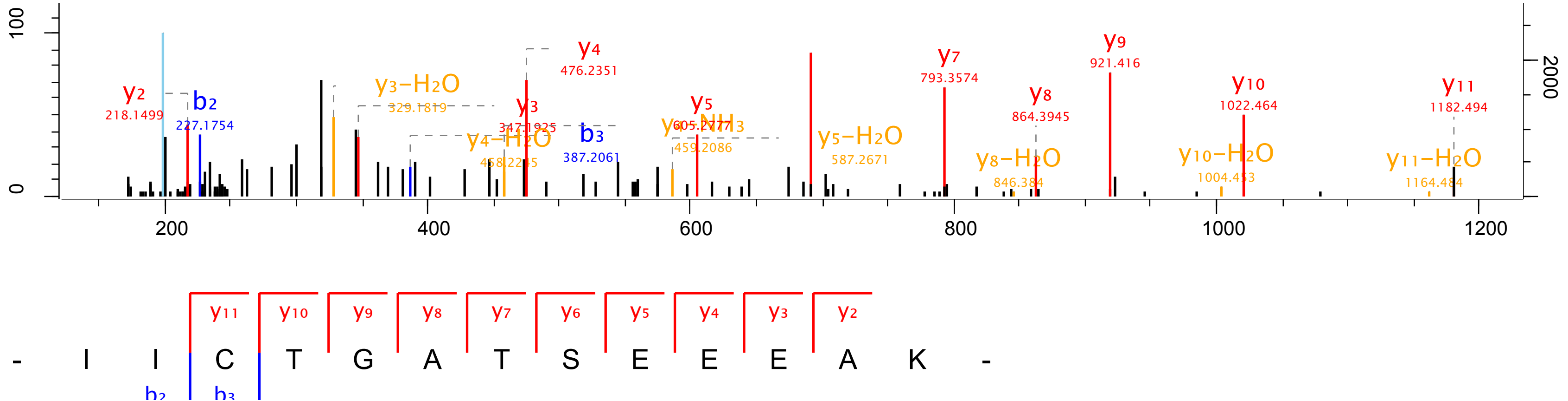

Raw file	Scan	Method	Score	m/z
20140925_fract7_dyn_5ul_B7_01_442	11359	TOF; CID	58.1	608.79

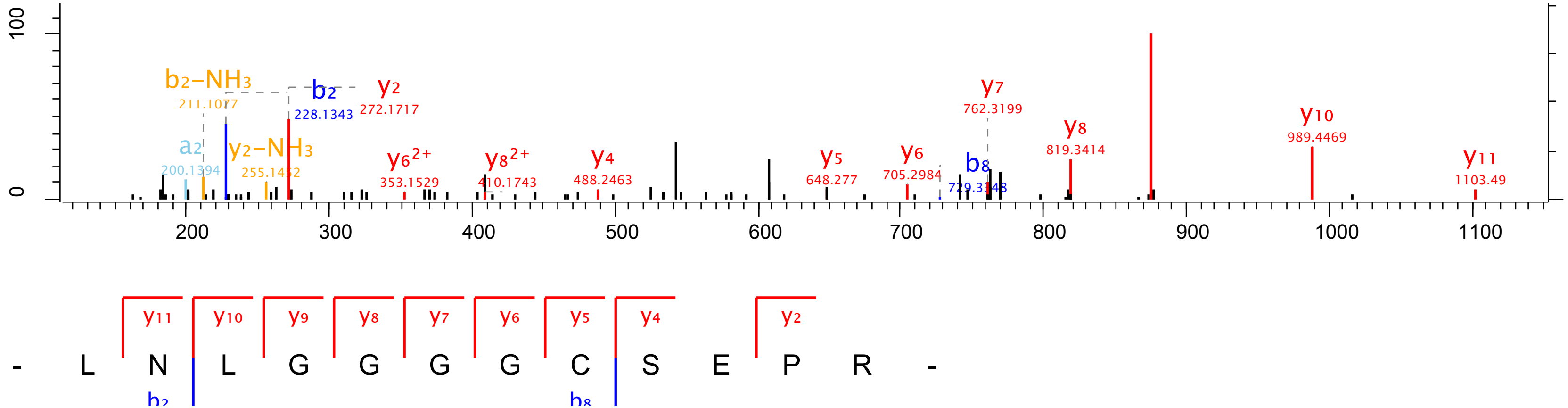

Raw file	Scan	Method	Score	m/z	Gene names
20140925_fract7_dyn_5ul_B7_01_442	11706	TOF; CID	66.15	690.34	DMTF1

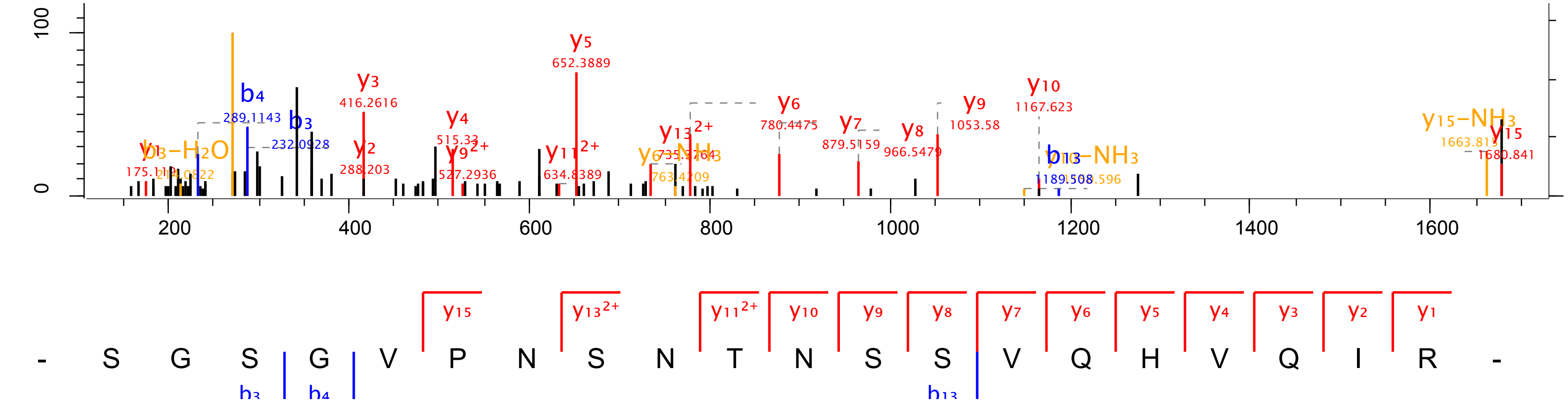

Raw file	Scan	Method	Score	m/z	Gene names
20140925_fract7_dyn_5ul_B7_01_442	12226	TOF; CID	76.24	836.05	TMEM39B

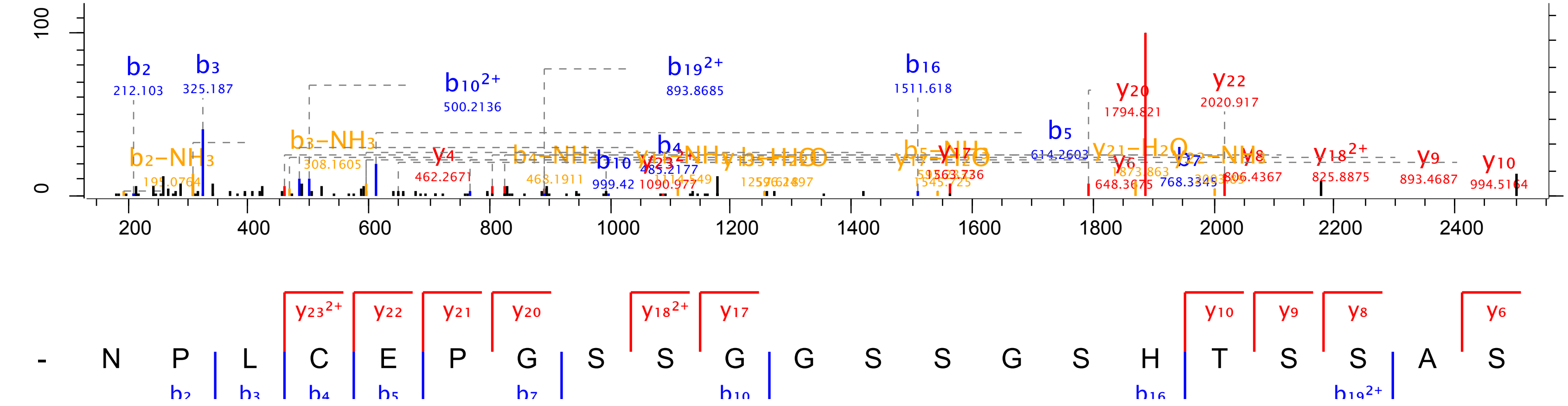

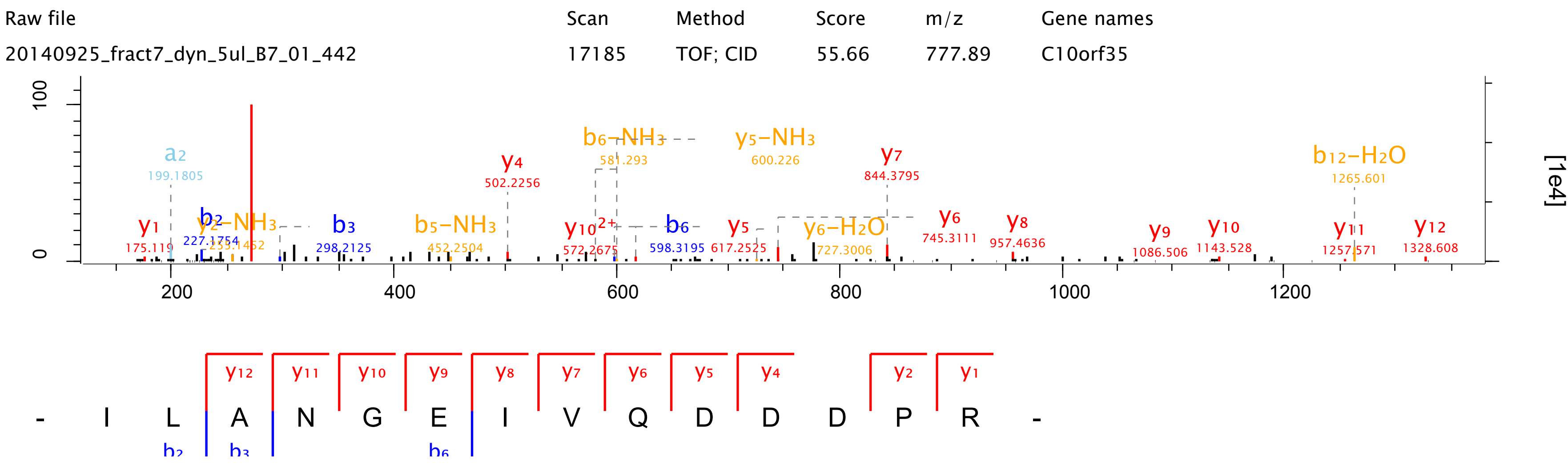

Raw file
20140925_fract7_dyn_5ul_B7_01_442
Scan
17185
Method
TOF; CID
Score
55.66
m/z
777.89
Gene names
C10orf35
[1e4]
a2 199.1805
y1 175.119
b2 227.1754
y2−NH3 253.1452
b3 298.2125
b5−NH3 452.2504
y4 502.2256
y10 2+ 572.2675
b6−NH3 581.293
b6 598.3195
y5−NH3 600.226
y5 617.2525
y6−H2O 727.3006
y6 745.3111
y7 844.3795
y8 957.4636
y9 1086.506
y10 1143.528
y11 1257.571
b12−H2O 1265.601
y12 1328.608
- I L A N G E I V Q D D D P R -

Raw file	Scan	Method	Score	m/z	Gene names
20140925_fract7_dyn_5ul_B7_01_442	17433	TOF; CID	49.93	452.9	UBXN2A

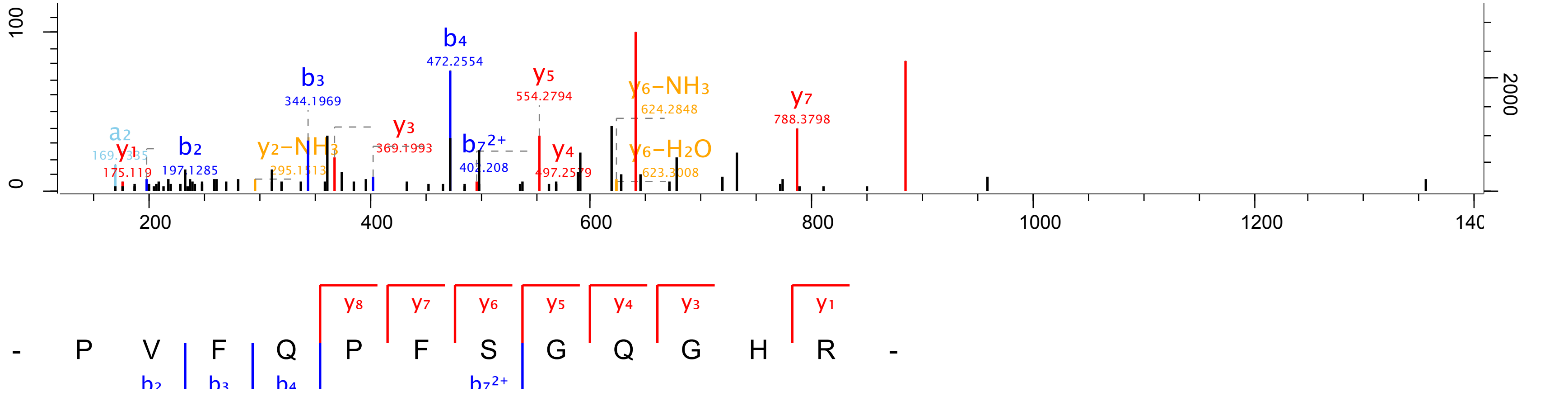

Raw file	Scan	Method	Score	m/z	Gene names
20140925_fract7_dyn_5ul_B7_01_442	18863	TOF; CID	88.08	794.39	GPNMB

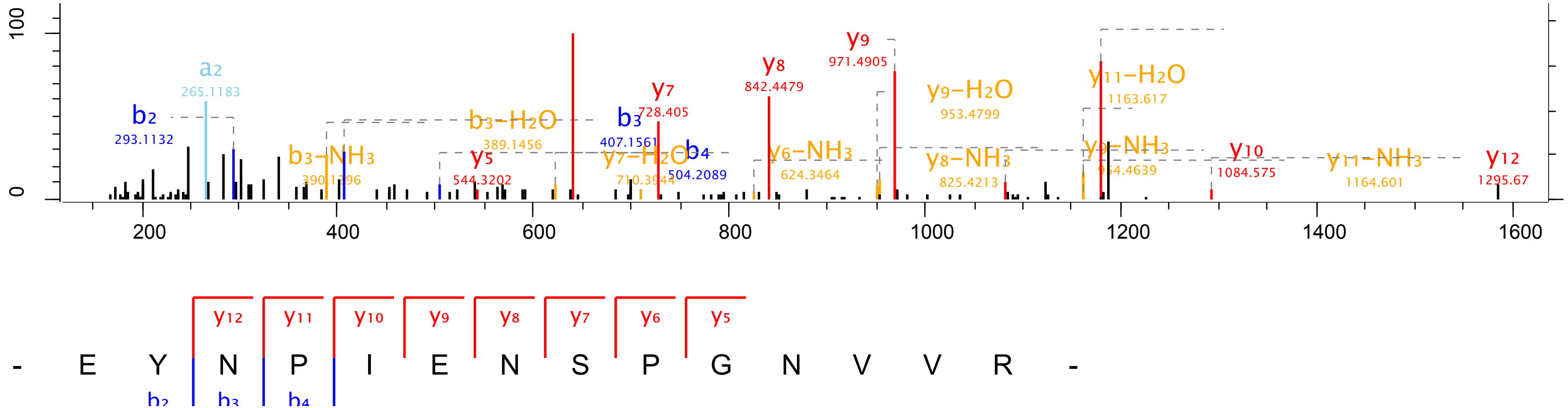

Raw file	Scan	Method	Score	m/z	Gene names
20140925_fract7_dyn_5ul_B7_01_442	19373	TOF; CID	42.71	678.33	ODF2L

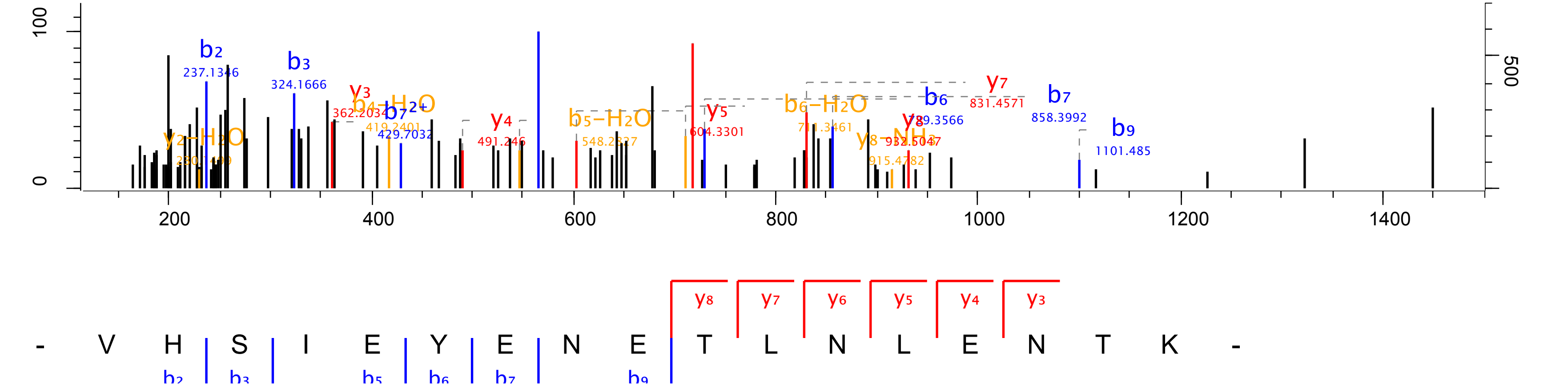

Raw file	Scan	Method	Score	m/z	Gene names
20140925_fract7_dyn_5ul_B7_01_442	19862	TOF; CID	69.82	616.31	ZDHHC8

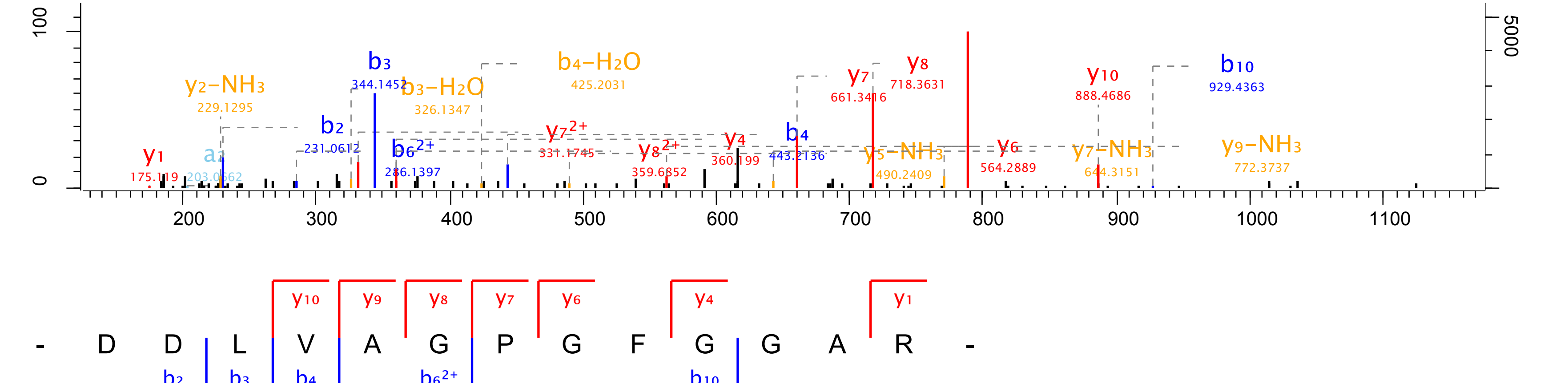

Raw file	Scan	Method	Score	m/z	Gene names
20140925_fract7_dyn_5ul_B7_01_442	21649	TOF; CID	50.94	1098.01	BRPF3

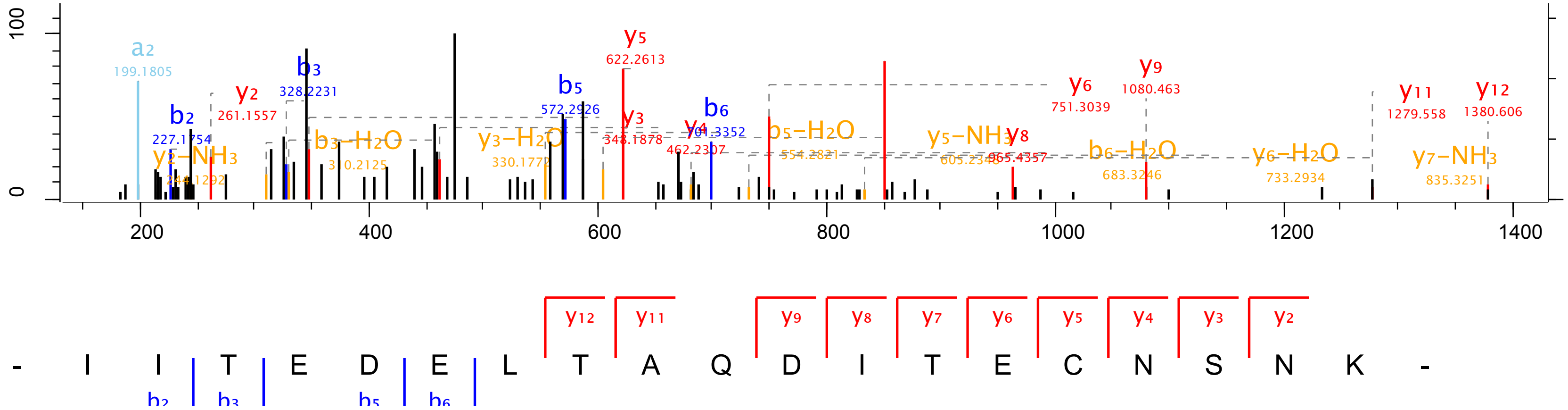

Raw file	Scan	Method	Score	m/z	Gene names
20140925_fract7_dyn_5ul_B7_01_442	22742	TOF; CID	113.76	800.37	CTNNBIP1

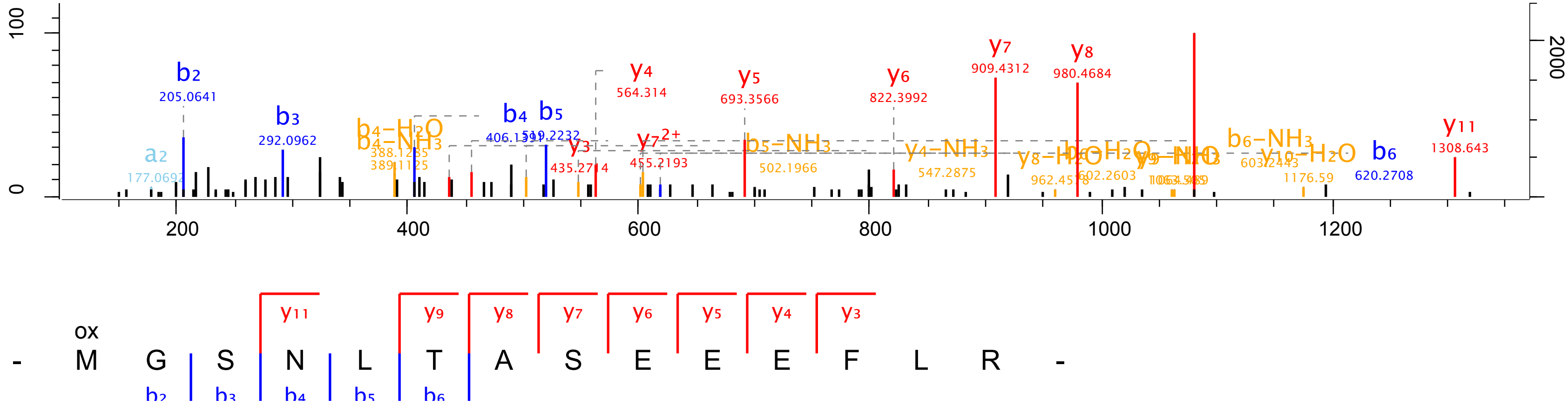

Raw file	Scan	Method	Score	m/z	Gene names
20140925_fract7_dyn_5ul_B7_01_442	23880	TOF; CID	105.53	617.97	HNRNPH1

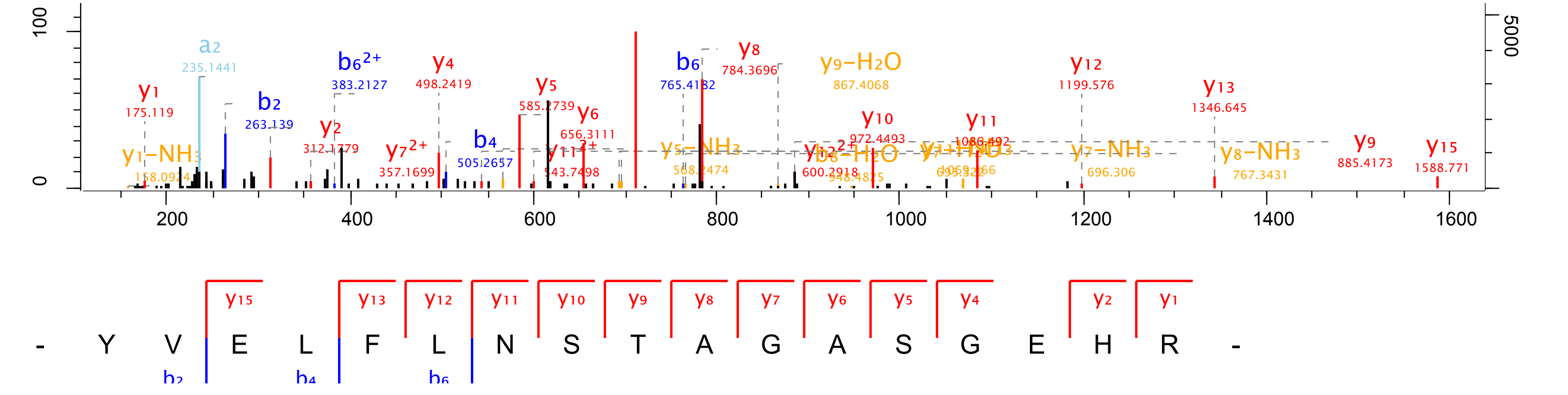

Raw file	Scan	Method	Score	m/z	Gene names
20140925_fract7_dyn_5ul_B7_01_442	23891	TOF; CID	74.46	574.84	ANKEF1

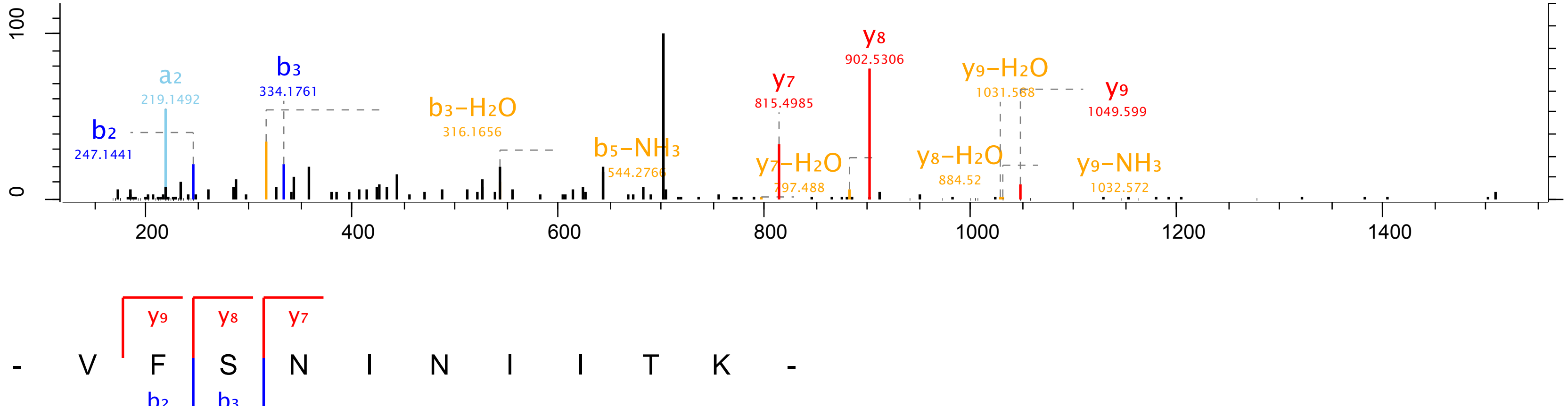

Raw file	Scan	Method	Score	m/z	Gene names
20140925_fract7_dyn_5ul_B7_01_442	24744	TOF; CID	105.98	603.31	TAF13

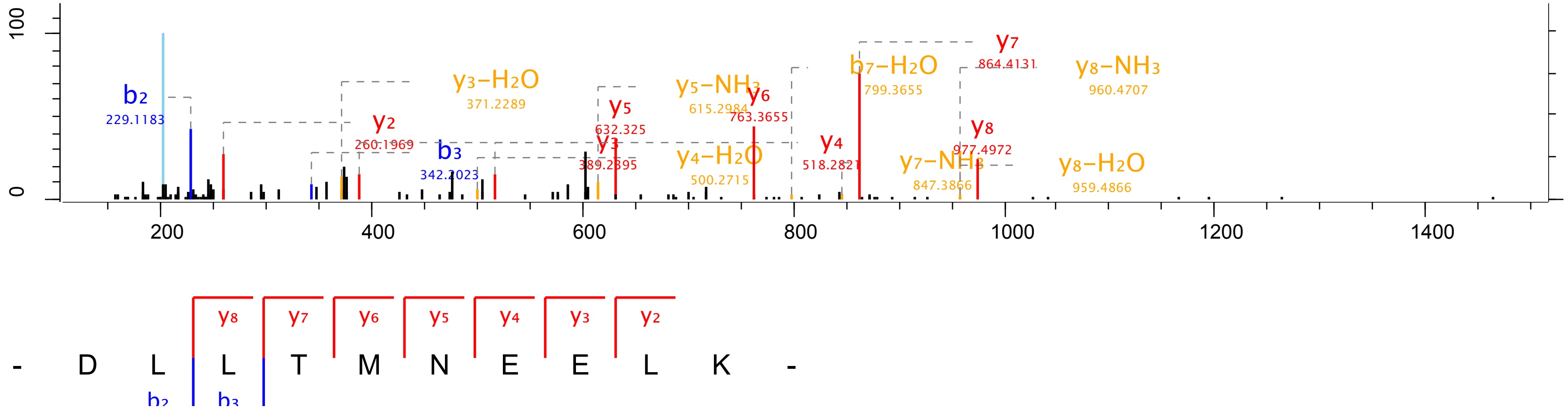

Raw file	Scan	Method	Score	m/z	Gene names
20140925_fract7_dyn_5ul_B7_01_442	24899	TOF; CID	56.87	878.92	INPP5A

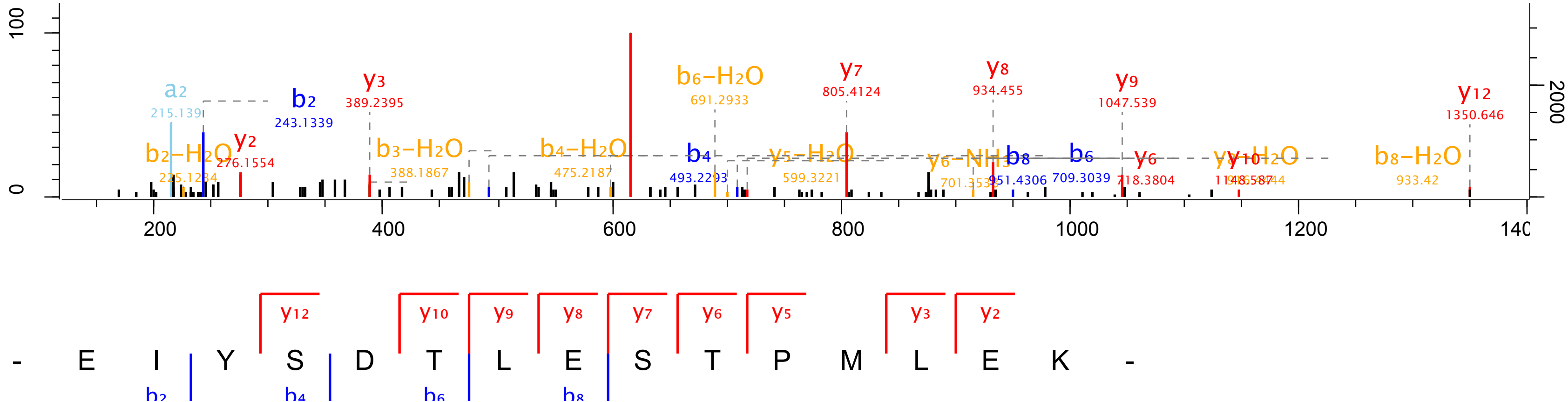

Raw file	Scan	Method	Score	m/z	Gene names
20140925_fract7_dyn_5ul_B7_01_442	32048	TOF; CID	91.4	996	PTTG1IP

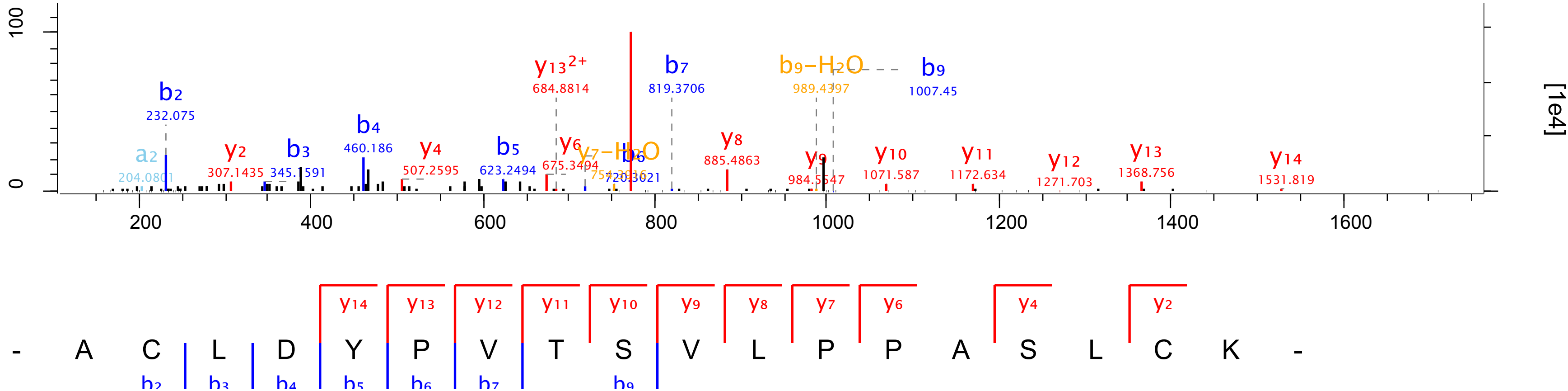

Raw file	Scan	Method	Score	m/z	Gene names
20140925_fract7_dyn_5ul_B7_01_442	32461	TOF; CID	75.3	868.78	ICAM5

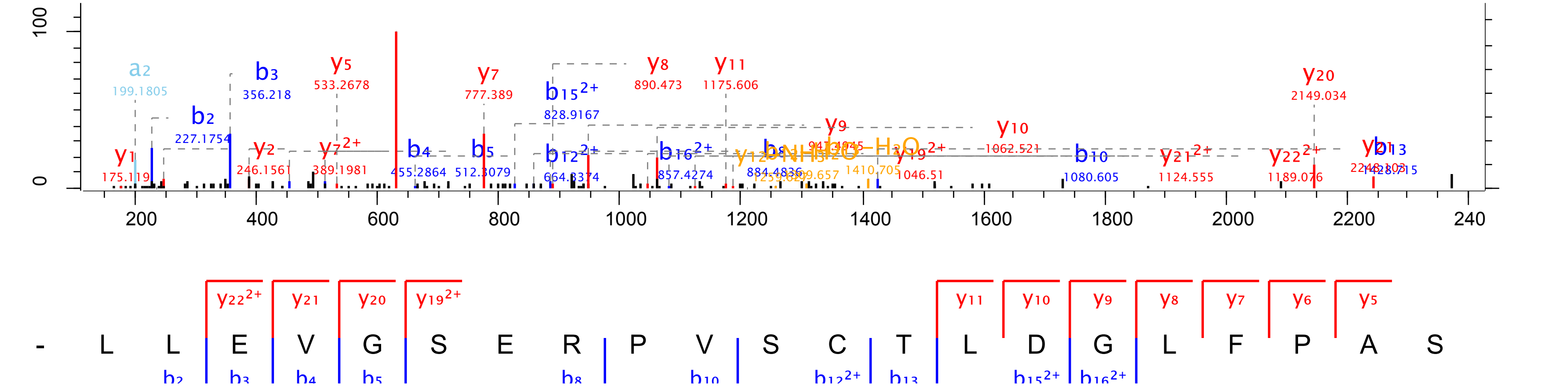

Raw file	Scan	Method	Score	m/z	Gene names
20140925_fract7_dyn_5ul_B7_01_442	34350	TOF; CID	63.69	891.99	PPP1R3F

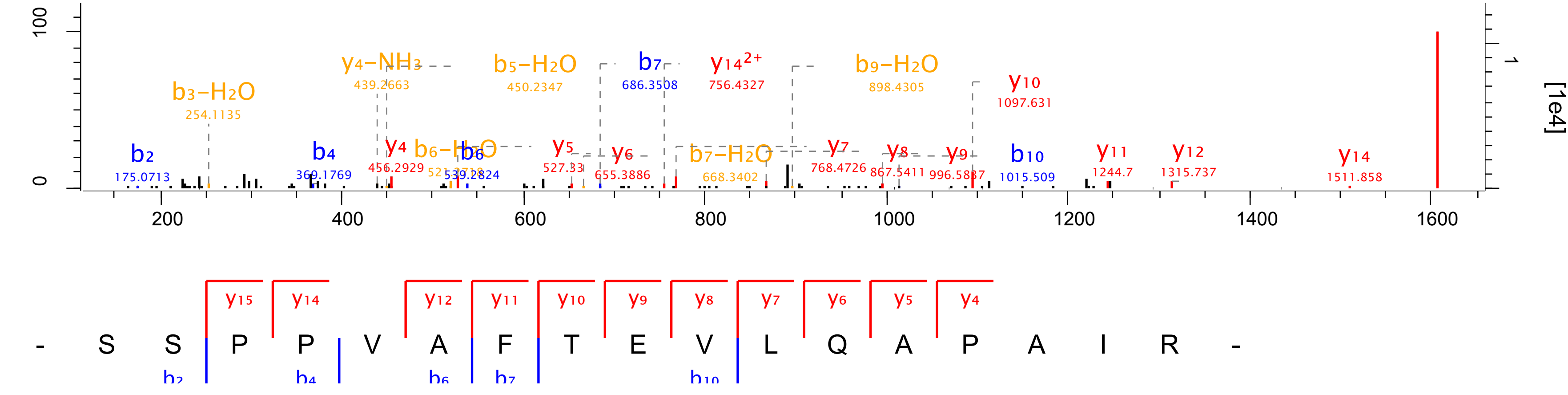

Raw file	Scan	Method	Score	m/z	Gene names
20140925_fract7_dyn_5ul_B7_01_442	35244	TOF; CID	71.08	886.4	HOXC11

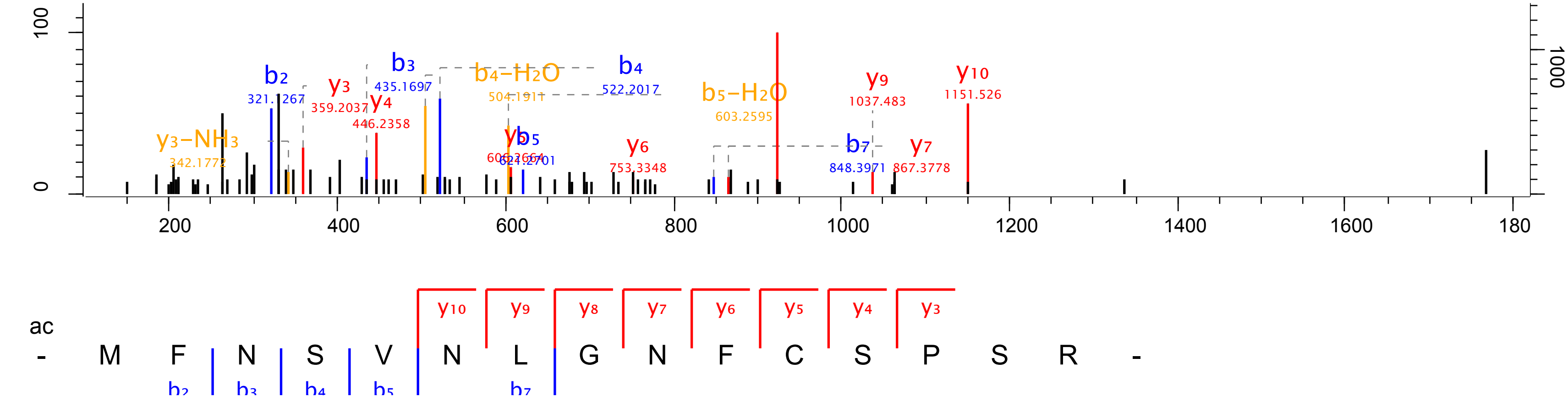

Raw file	Scan	Method	Score	m/z	Gene names
20140925_fract7_dyn_5ul_B7_01_442	35325	TOF; CID	56.82	839.42	ZRANB3

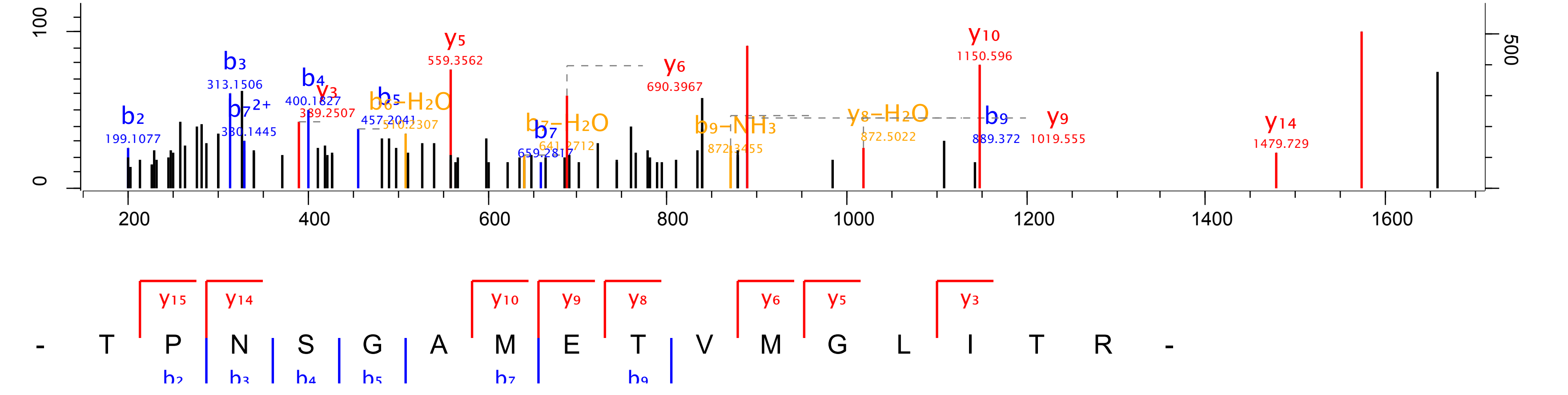

Raw file	Scan	Method	Score	m/z	Gene names
20140925_fract8_dyn_5ul_B8_01_443	5102	TOF; CID	79.47	313.85	ZNF212

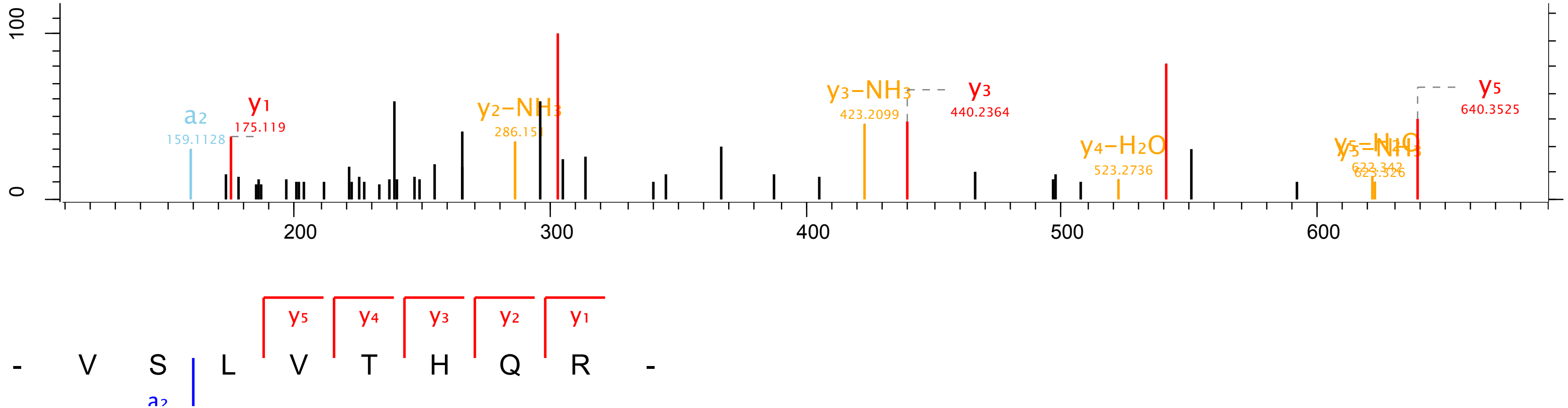

Raw file	Scan	Method	Score	m/z	Gene names
20140925_fract8_dyn_5ul_B8_01_443	9002	TOF; CID	101.05	612.97	THRB

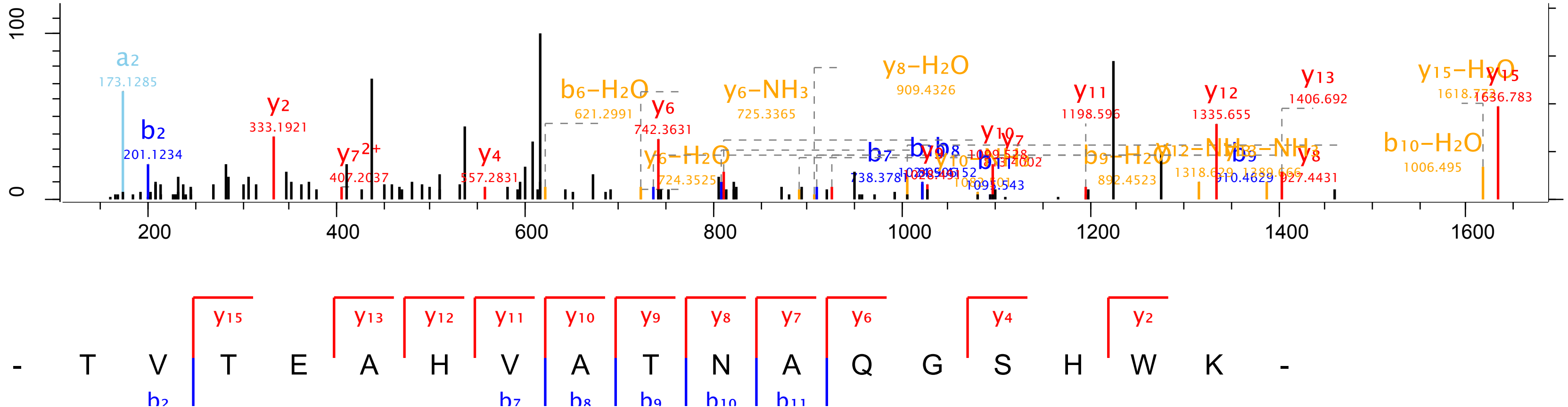

Raw file	Scan	Method	Score	m/z
20140925_fract8_dyn_5ul_B8_01_443	9549	TOF; CID	103.26	574.32

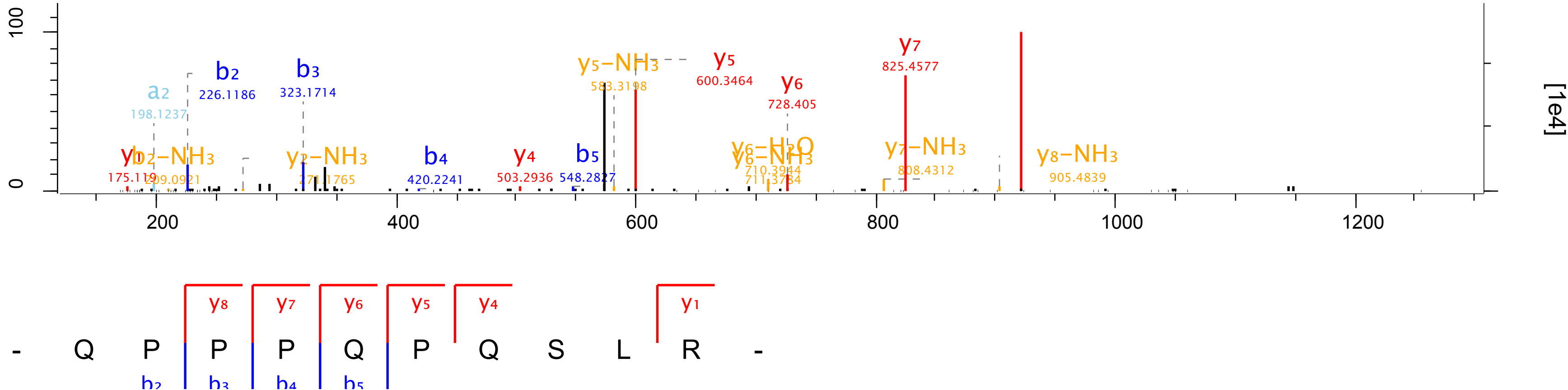

Raw file	Scan	Method	Score	m/z	Gene names
20140925_fract8_dyn_5ul_B8_01_443	12927	TOF; CID	79.12	339.16	AQP3

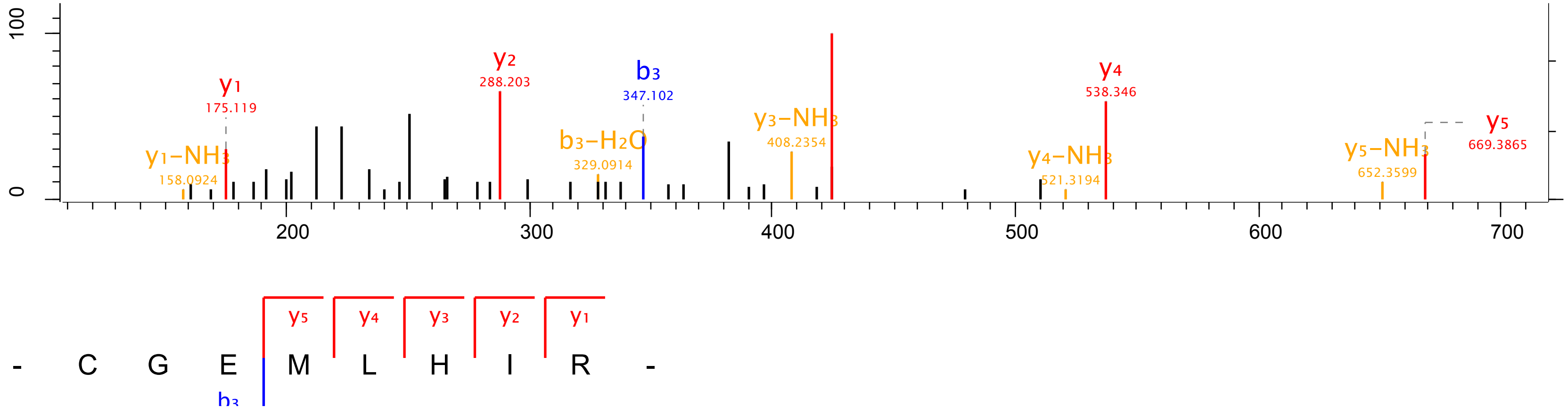

Raw file	Scan	Method	Score	m/z	Gene names
20140925_fract8_dyn_5ul_B8_01_443	13166	TOF; CID	97.73	578.79	TMEM254

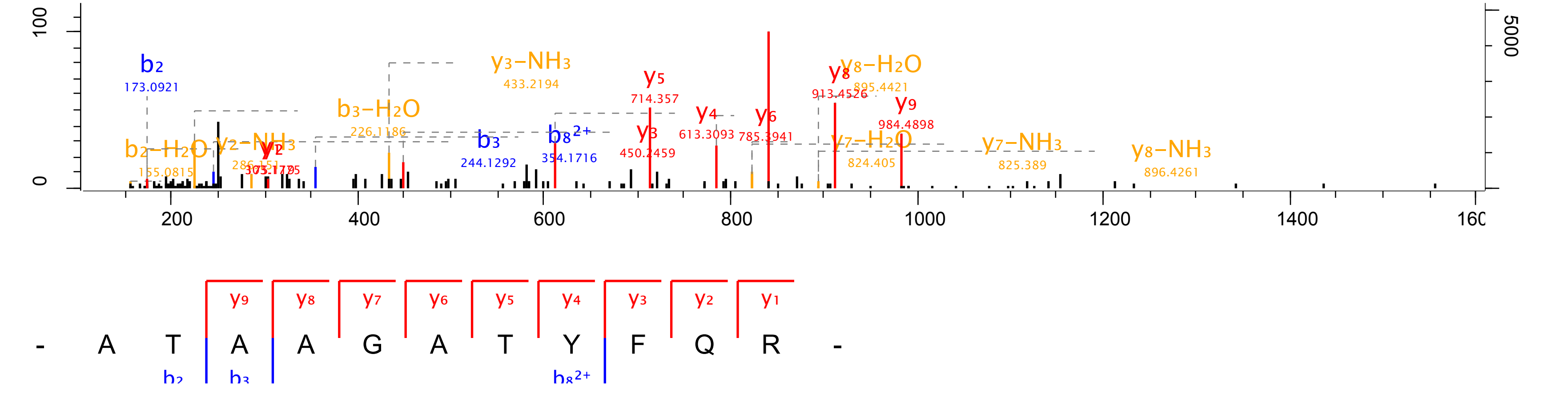

Raw file	Scan	Method	Score	m/z	Gene names
20140925_fract8_dyn_5ul_B8_01_443	14497	TOF; CID	107.82	874.92	TMEM181

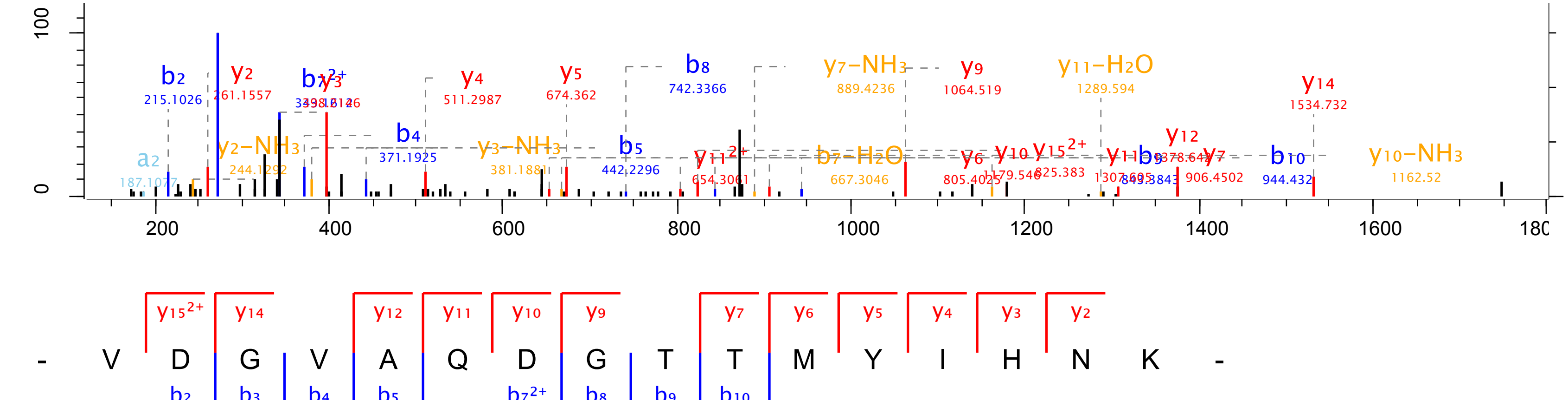

Raw file	Scan	Method	Score	m/z	Gene names
20140925_fract8_dyn_5ul_B8_01_443	16430	TOF; CID	74.13	560.28	UBTD1

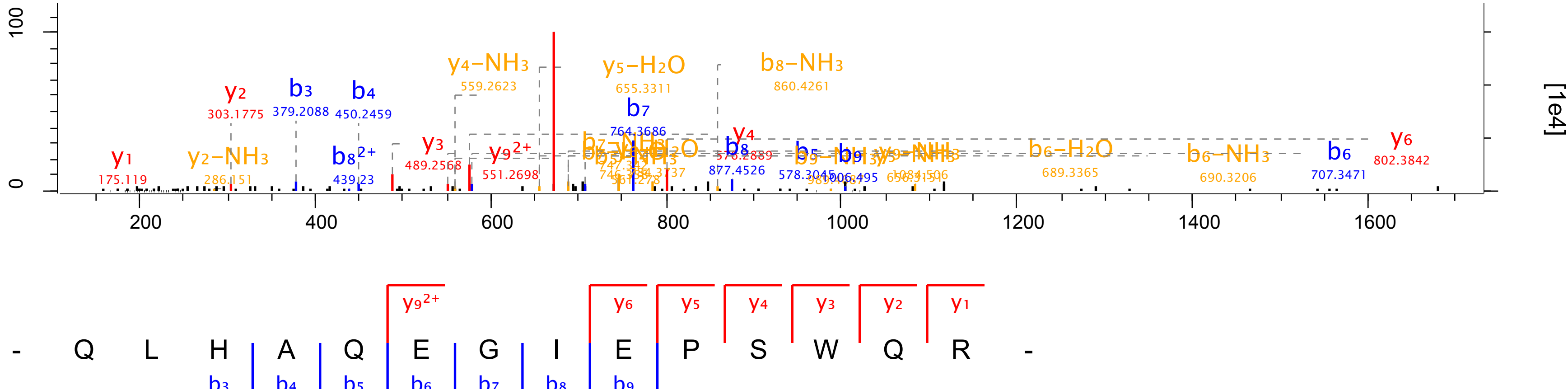

Raw file	Scan	Method	Score	m/z	Gene names
20140925_fract8_dyn_5ul_B8_01_443	16846	TOF; CID	91.7	439.91	PIGP

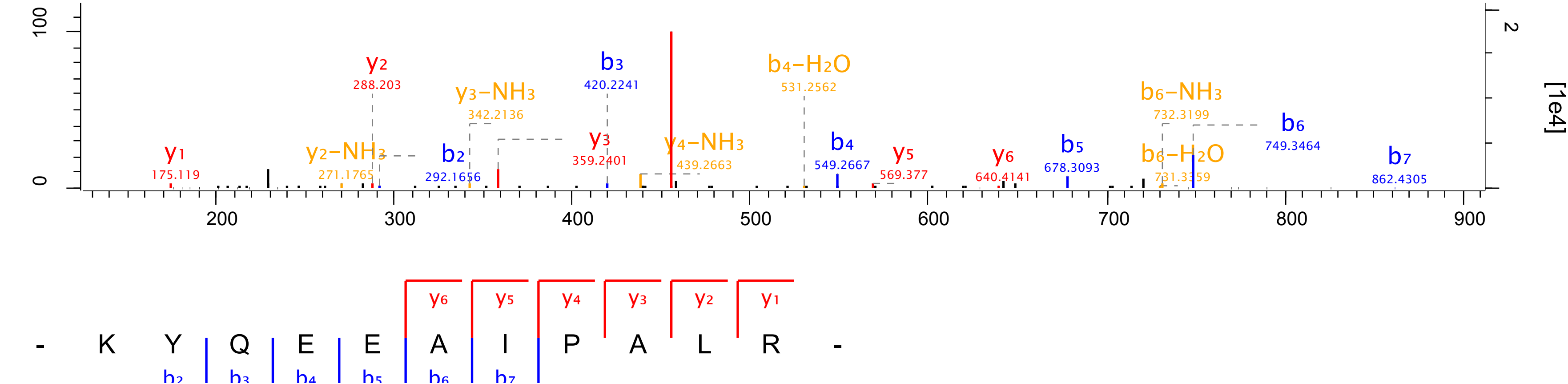

Raw file	Scan	Method	Score	m/z	Gene names
20140925_fract8_dyn_5ul_B8_01_443	16943	TOF; CID	71.9	566.29	C6orf57

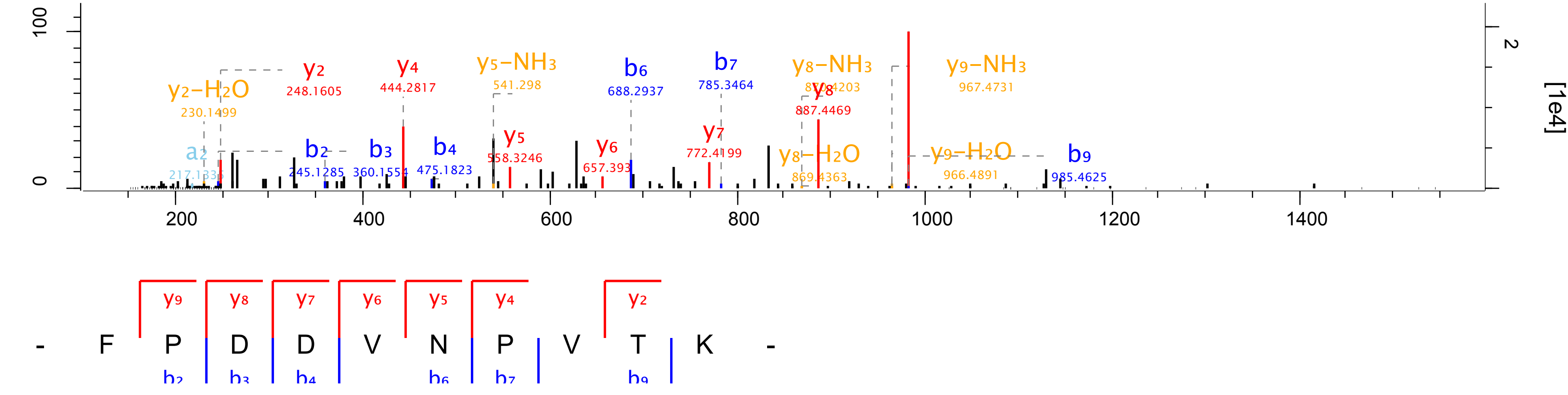

Raw file	Scan	Method	Score	m/z	Gene names
20140925_fract8_dyn_5ul_B8_01_443	19351	TOF; CID	44.83	804.38	HOXA13

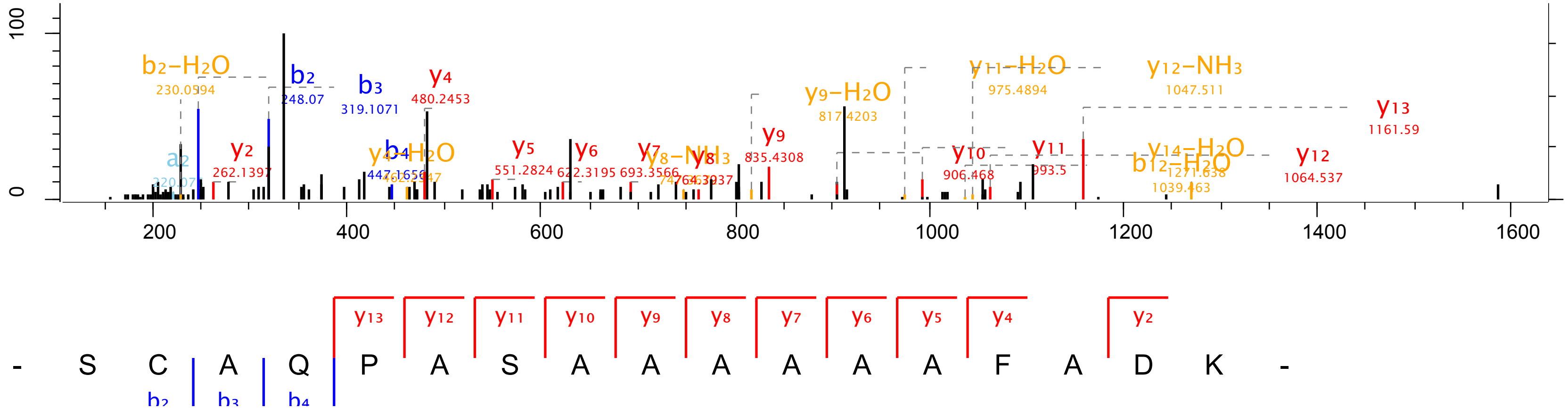

Raw file	Scan	Method	Score	m/z	Gene names
20140925_fract8_dyn_5ul_B8_01_443	21321	TOF; CID	111.94	639.35	ALG14

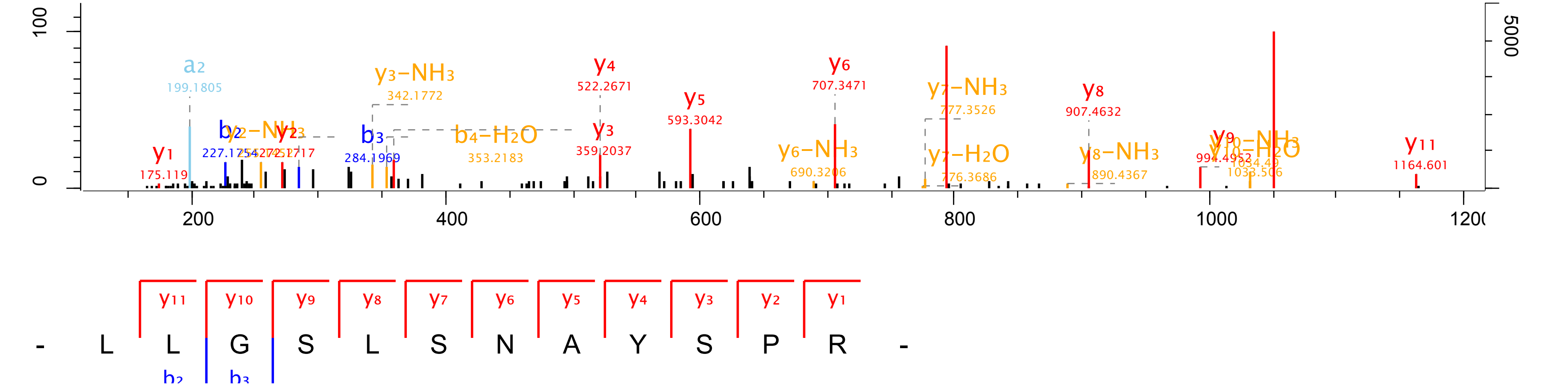

Raw file	Scan	Method	Score	m/z	Gene names
20140925_fract8_dyn_5ul_B8_01_443	22789	TOF; CID	53.87	670.36	CSTA

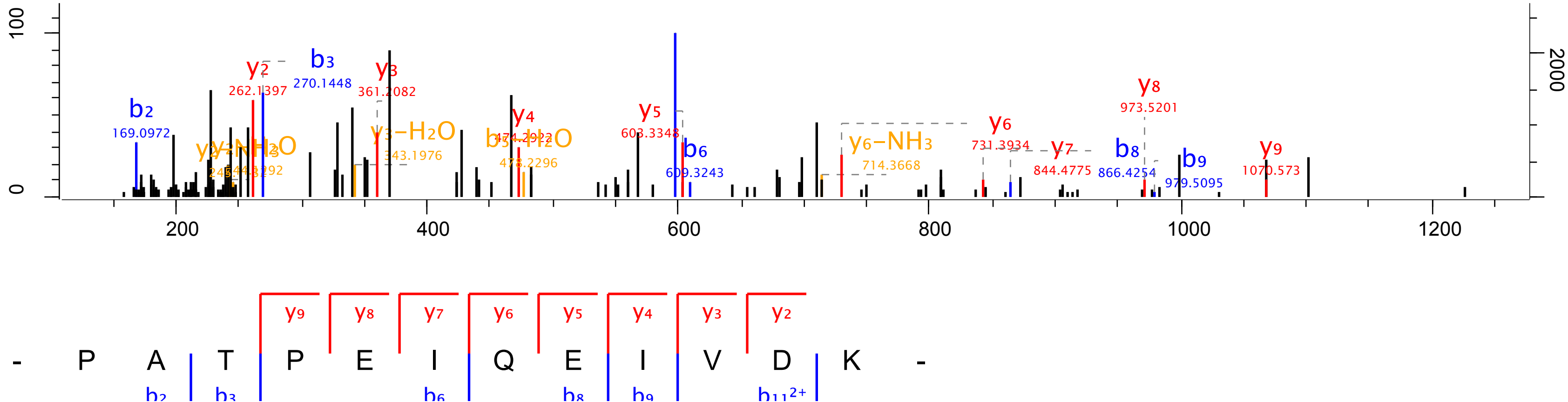

Raw file	Scan	Method	Score	m/z	Gene names
20140925_fract8_dyn_5ul_B8_01_443	23769	TOF; CID	56.82	789.41	CELSR1

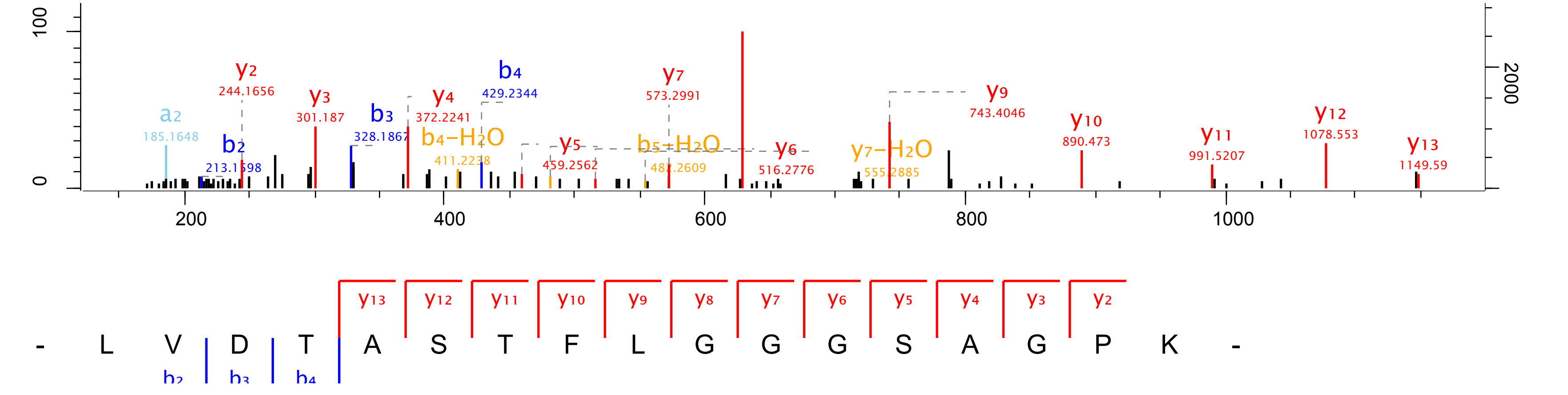

Raw file	Scan	Method	Score	m/z	Gene names
20140925_fract8_dyn_5ul_B8_01_443	23826	TOF; CID	45.42	798.38	ATXN7L2

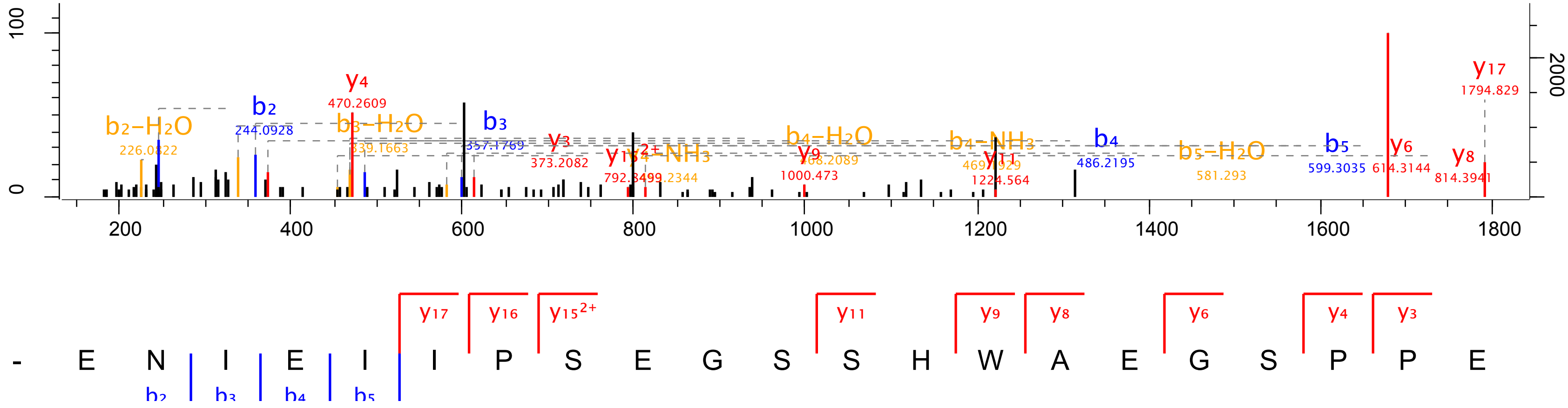

Raw file	Scan	Method	Score	m/z	Gene names
20140925_fract8_dyn_5ul_B8_01_443	24478	TOF; CID	75.82	658.85	PLAC1

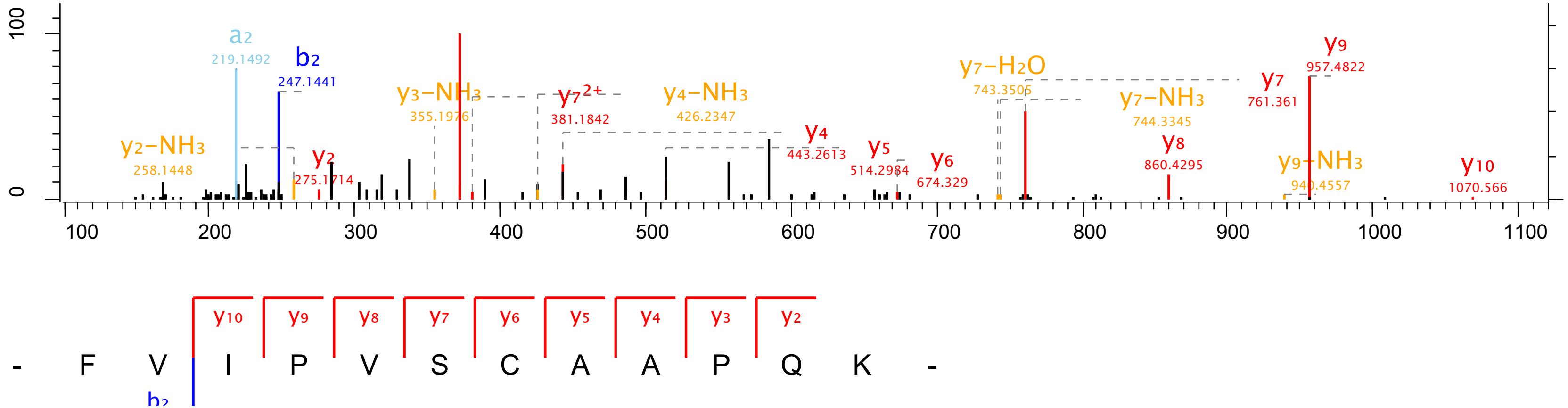

Raw file	Scan	Method	Score	m/z	Gene names
20140925_fract8_dyn_5ul_B8_01_443	25227	TOF; CID	51.59	578.98	DCLRE1B

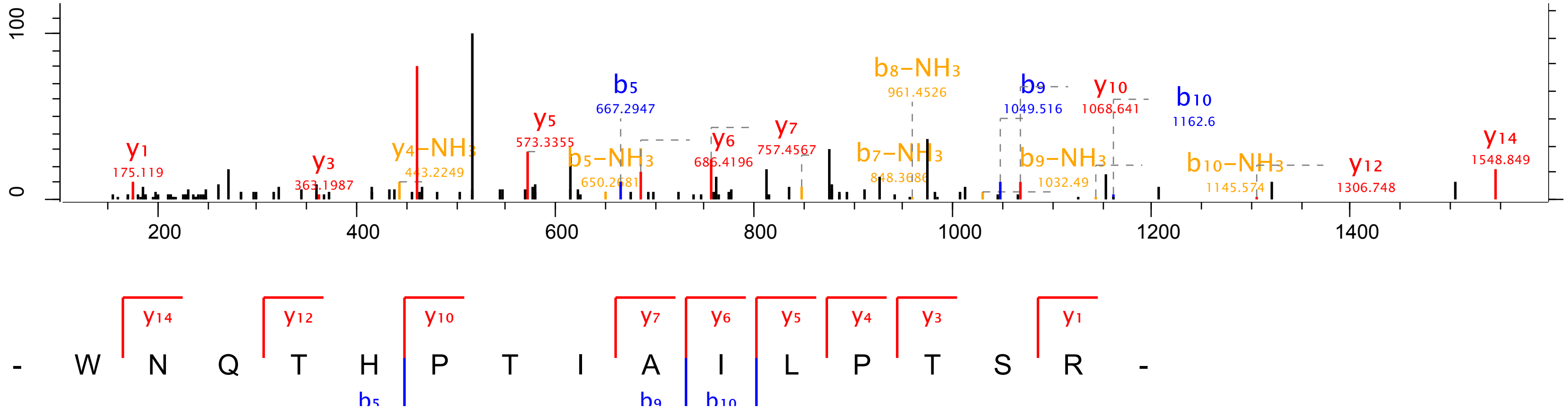

Raw file	Scan	Method	Score	m/z	Gene names
20140925_fract8_dyn_5ul_B8_01_443	28629	TOF; CID	110.57	1173.06	PKIG

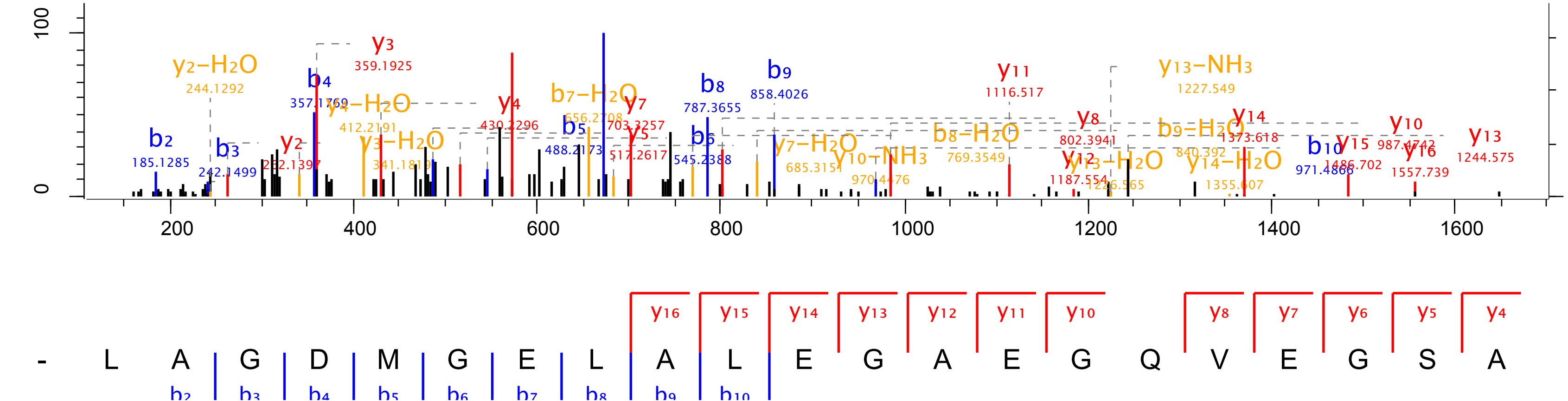

Raw file	Scan	Method	Score	m/z	Gene names
20140925_fract8_dyn_5ul_B8_01_443	30412	TOF; CID	64.27	869.43	MFSD8

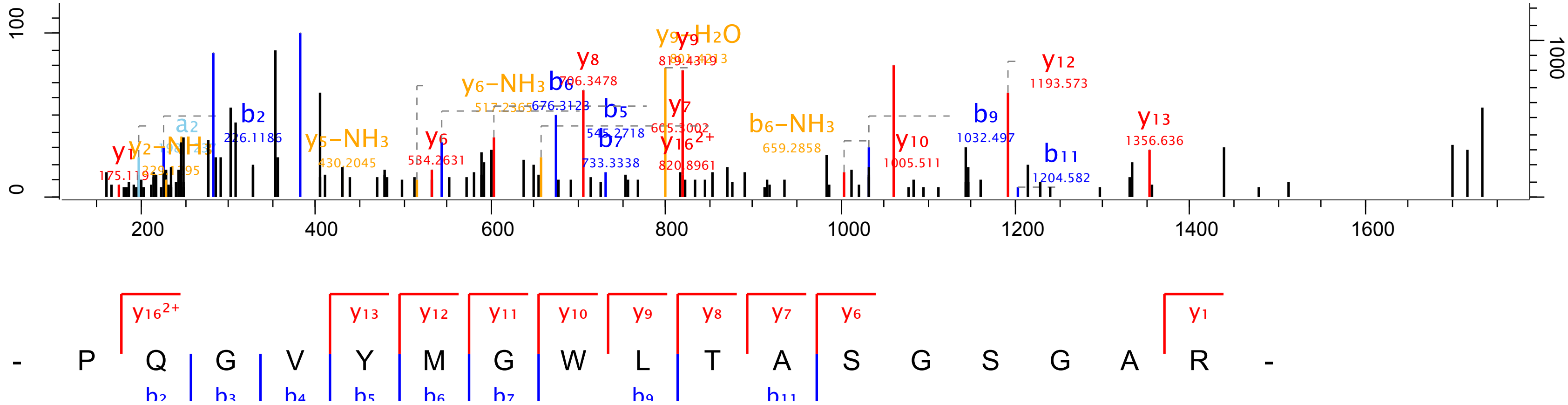

Raw file	Scan	Method	Score	m/z	Gene names
20140925_fract8_dyn_5ul_B8_01_443	32575	TOF; CID	103.31	606.37	FAAP24;C19orf40

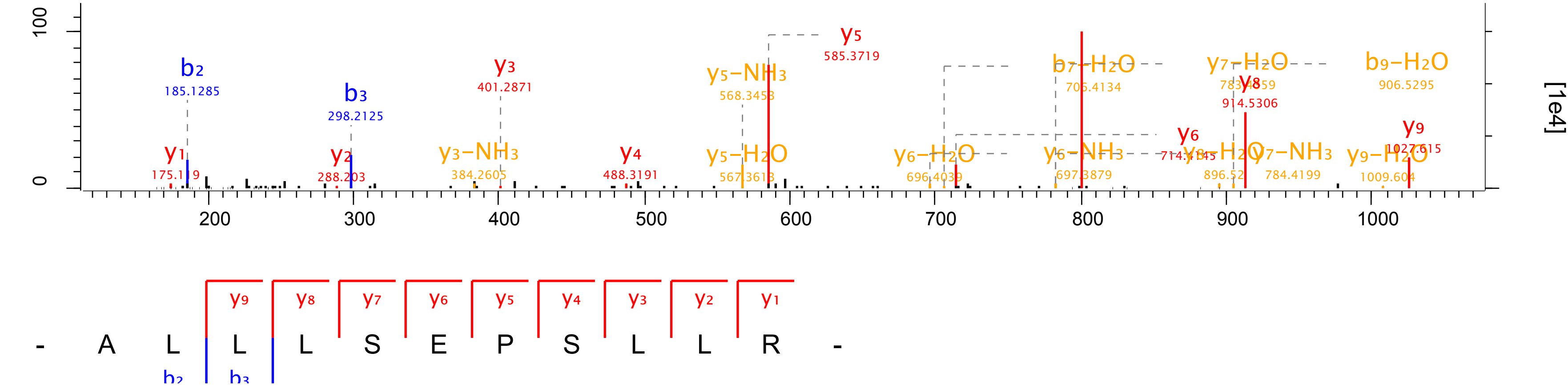

Raw file	Scan	Method	Score	m/z	Gene names
20140925_fract9_dyn_5ul_C1_01_444	3856	TOF; CID	59.23	453.58	SPOCK1

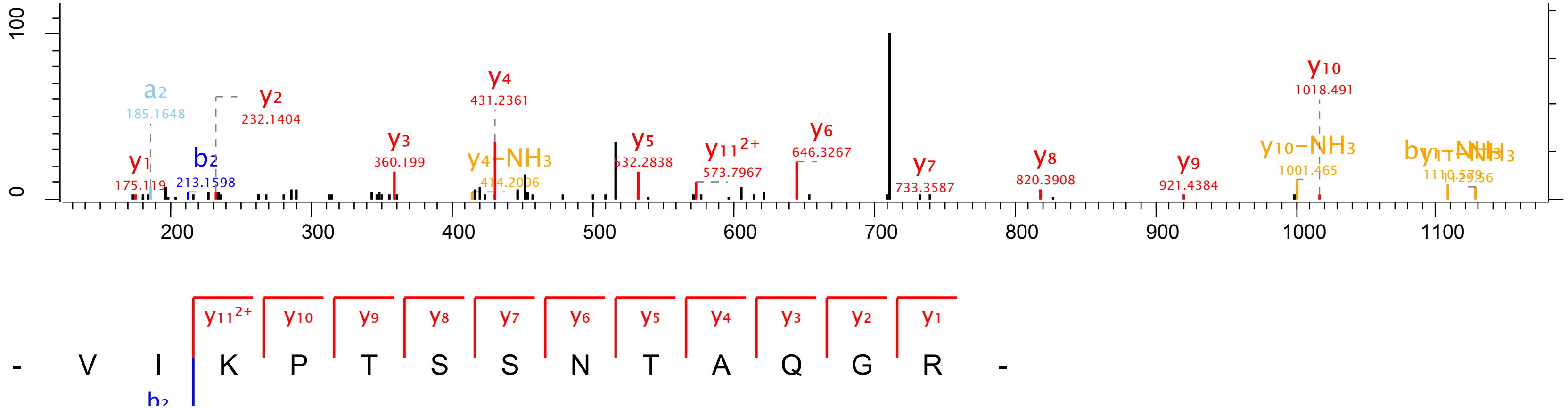

Raw file	Scan	Method	Score	m/z	Gene names
20140925_fract9_dyn_5ul_C1_01_444	6056	TOF; CID	114.28	443.23	MRPS18C

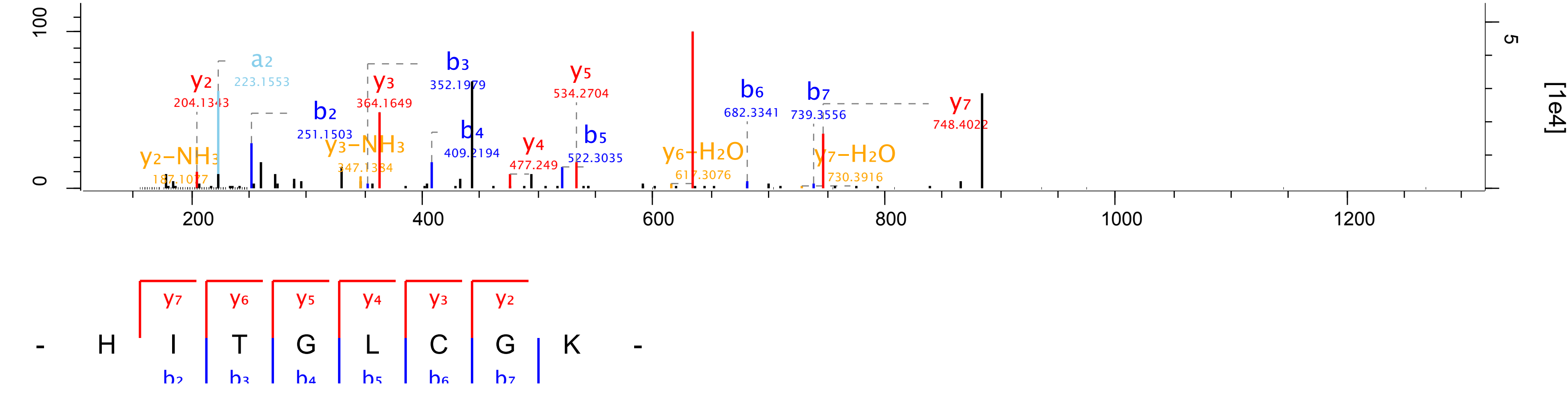

Raw file	Scan	Method	Score	m/z	Gene names
20140925_fract9_dyn_5ul_C1_01_444	7437	TOF; CID	80.69	472.77	ZNF236

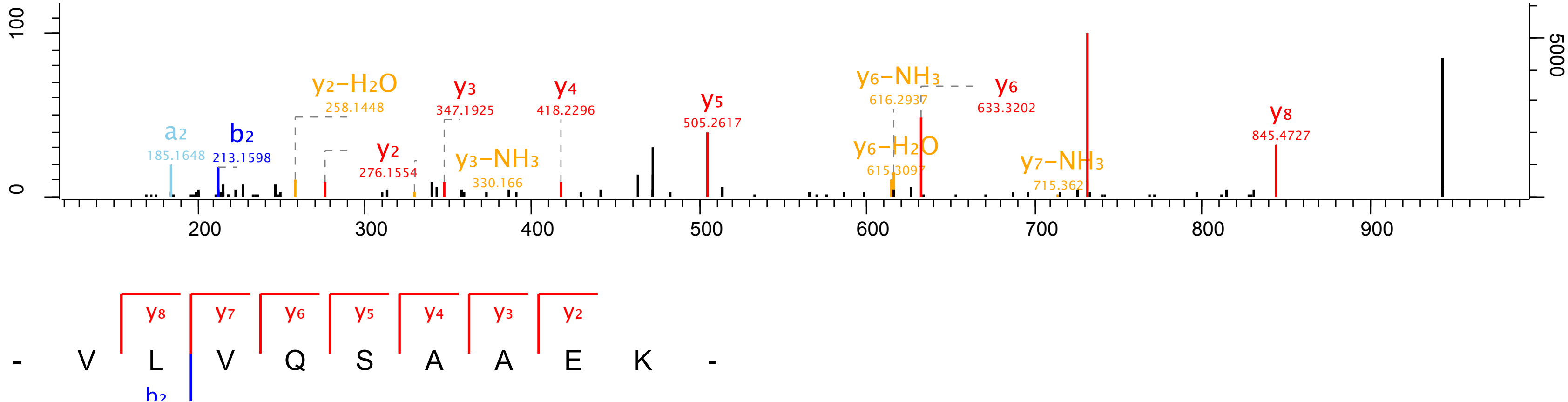

Raw file	Scan	Method	Score	m/z	Gene names
20140925_fract9_dyn_5ul_C1_01_444	11579	TOF; CID	108.47	438.18	CITED2

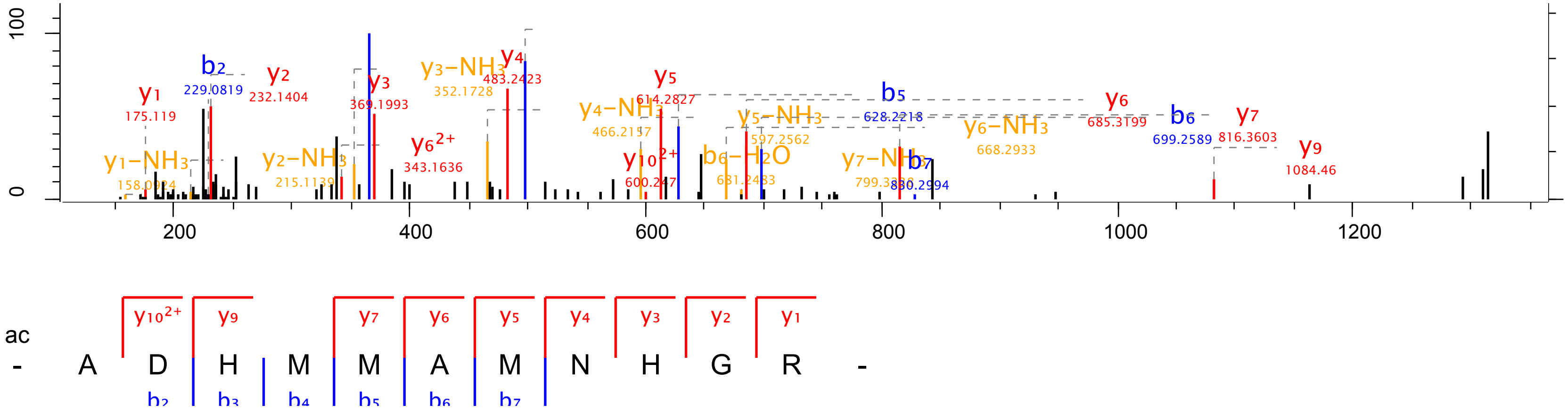

Raw file	Scan	Method	Score	m/z	Gene names
20140925_fract9_dyn_5ul_C1_01_444	17719	TOF; CID	72.29	627.3	TTC31

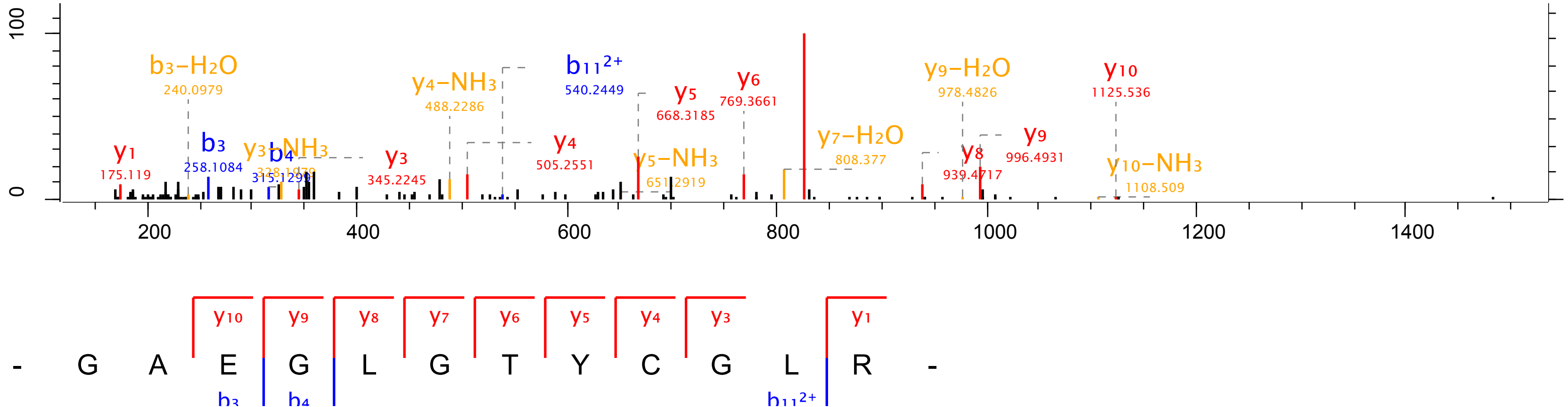

Raw file	Scan	Method	Score	m/z	Gene names
20140925_fract9_dyn_5ul_C1_01_444	24095	TOF; CID	81.35	889.45	SERINC1

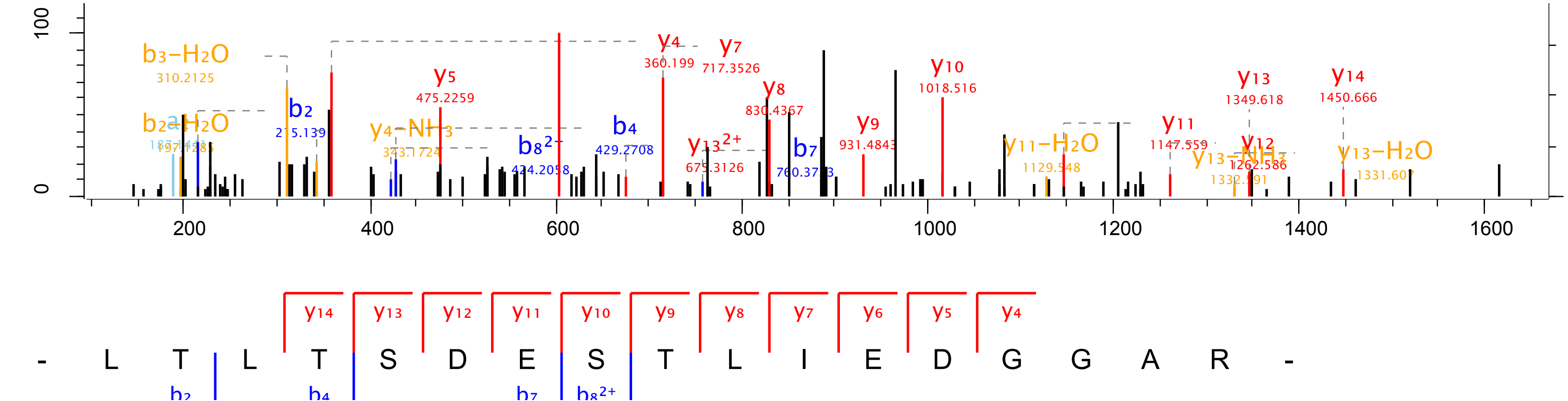

Raw file	Scan	Method	Score	m/z	Gene names
20140925_fract9_dyn_5ul_C1_01_444	24322	TOF; CID	82.51	958.16	OTUD1

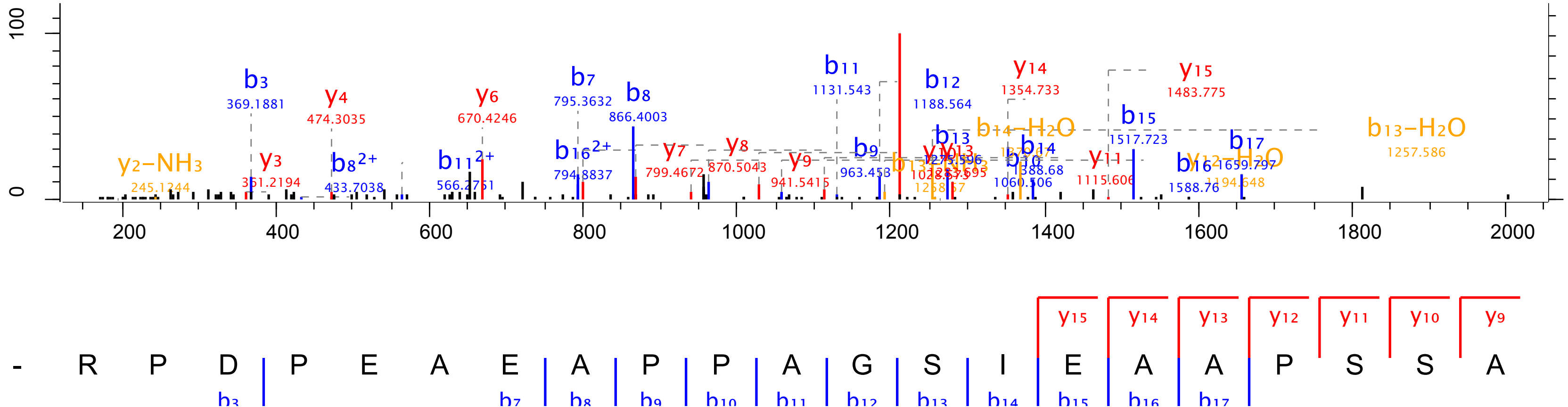

Raw file	Scan	Method	Score	m/z	Gene names
20140925_fract9_dyn_5ul_C1_01_444	25436	TOF; CID	102.07	502.27	PGM5

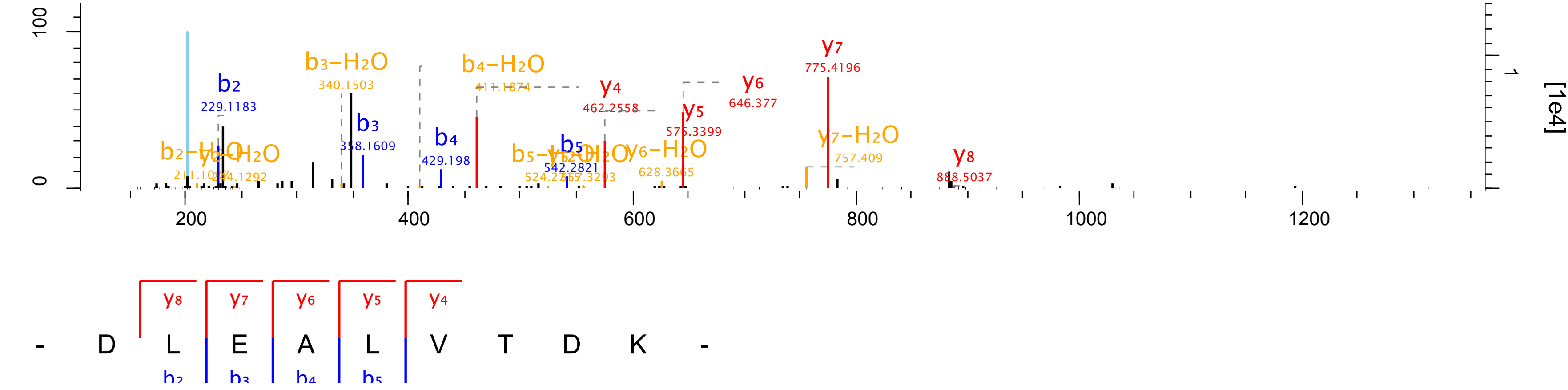

Raw file	Scan	Method	Score	m/z	Gene names
20140925_fract9_dyn_5ul_C1_01_444	25470	TOF; CID	65.37	749.36	MT-ND6

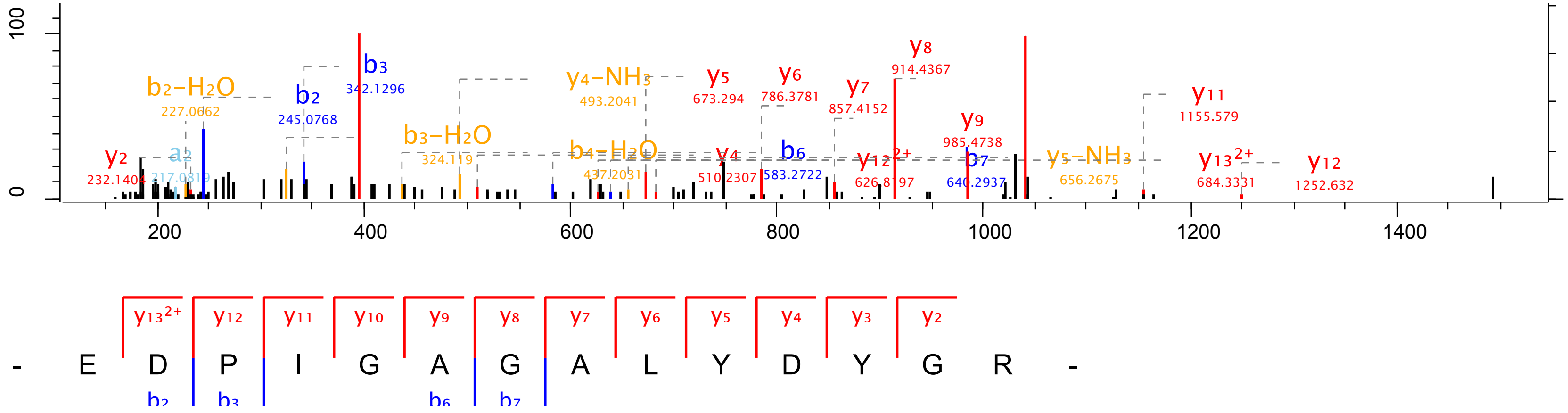

Raw file	Scan	Method	Score	m/z	Gene names
20140925_fract9_dyn_5ul_C1_01_444	27368	TOF; CID	86.37	901.45	C21orf2

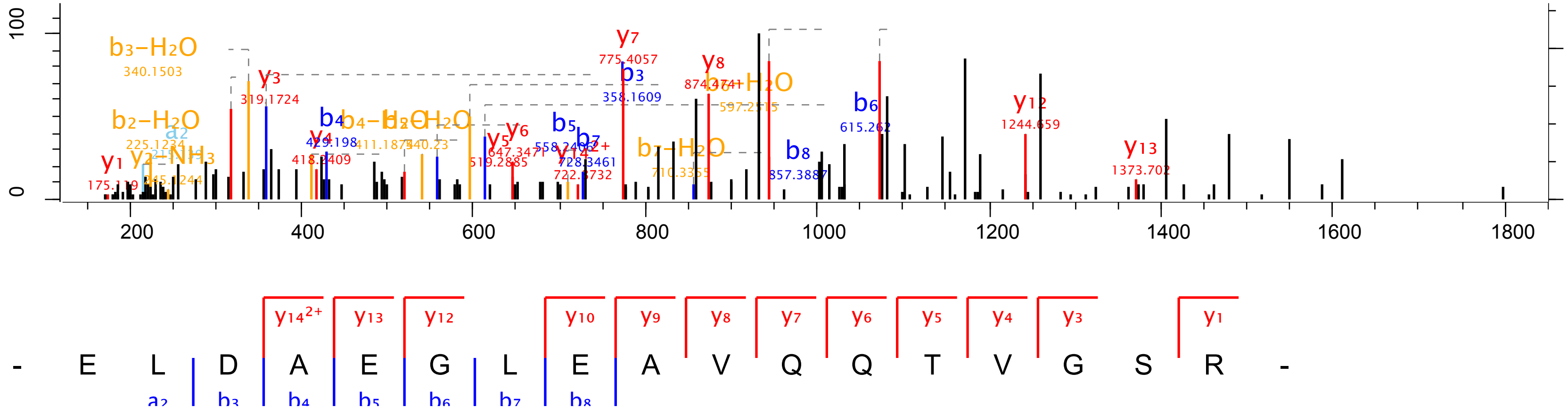

Raw file	Scan	Method	Score	m/z	Gene names
20140925_fract9_dyn_5ul_C1_01_444	29165	TOF; CID	87.36	1207.54	CMTM4

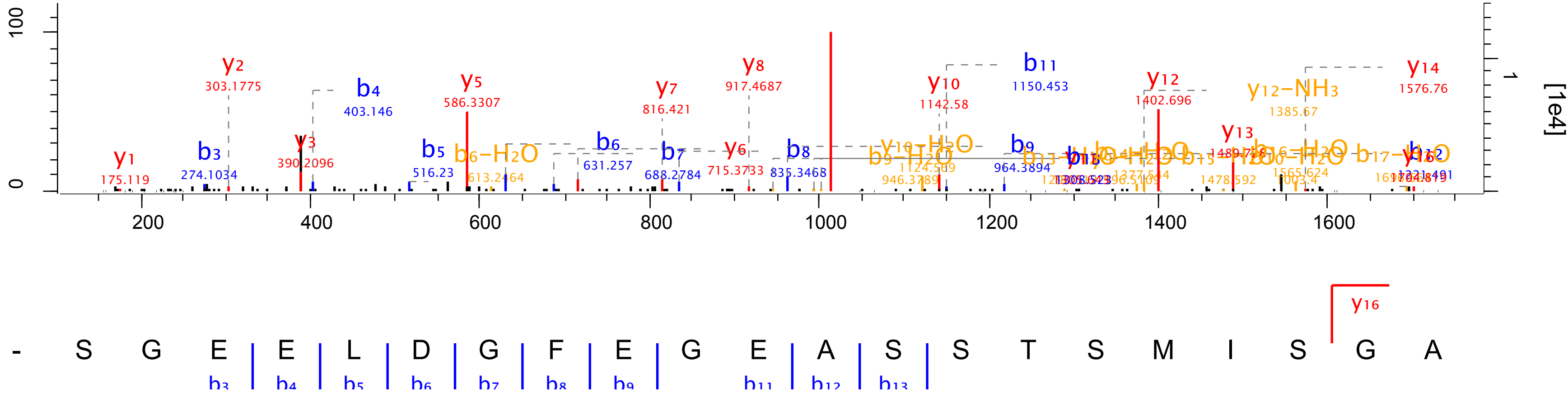

Raw file	Scan	Method	Score	m/z	Gene names
20140925_fract9_dyn_5ul_C1_01_444	30623	TOF; CID	102.59	765.36	CNIH4

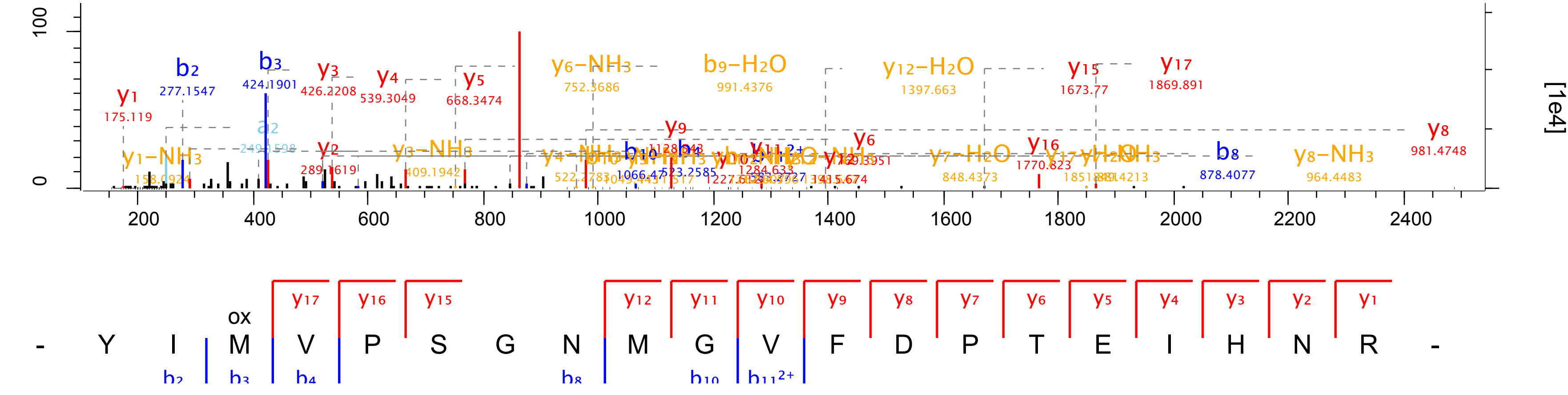

Raw file	Scan	Method	Score	m/z	Gene names
20140925_fract9_dyn_5ul_C1_01_444	30647	TOF; CID	57.48	638.32	G2E3

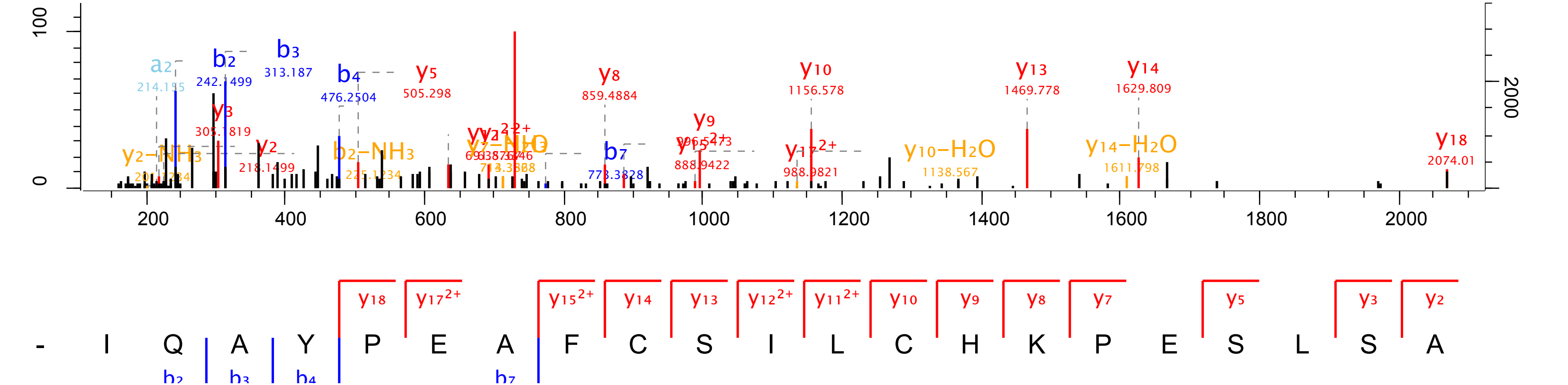

Raw file	Scan	Method	Score	m/z	Gene names
20140925_fract9_dyn_5ul_C1_01_444	33274	TOF; CID	77.75	922.45	HYAL2

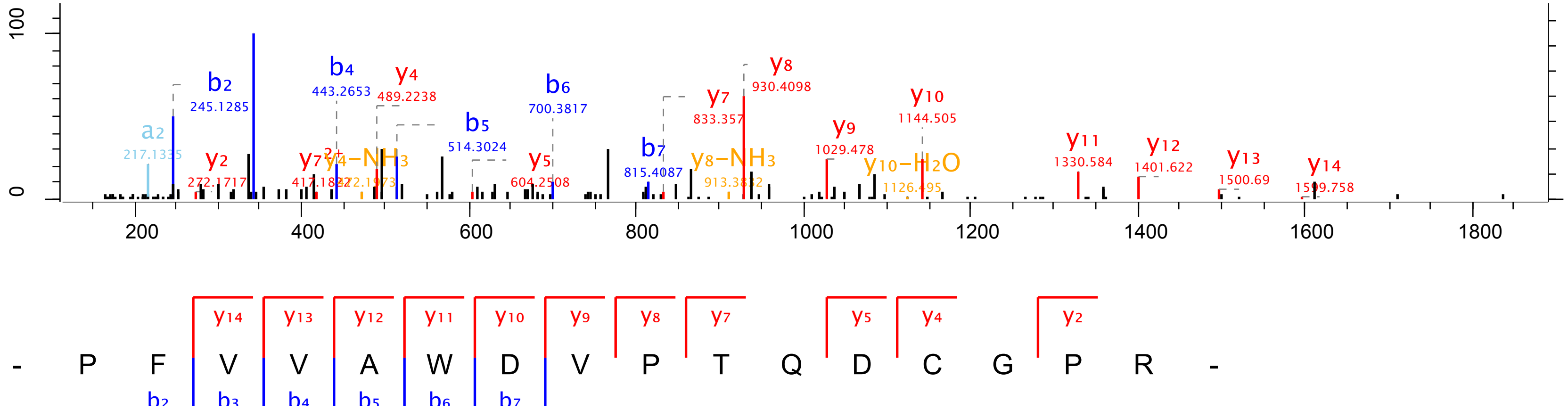

Raw file	Scan	Method	Score	m/z	Gene names
20140925_fract9_dyn_5ul_C1_01_444	36826	TOF; CID	128.06	835.4	IL32

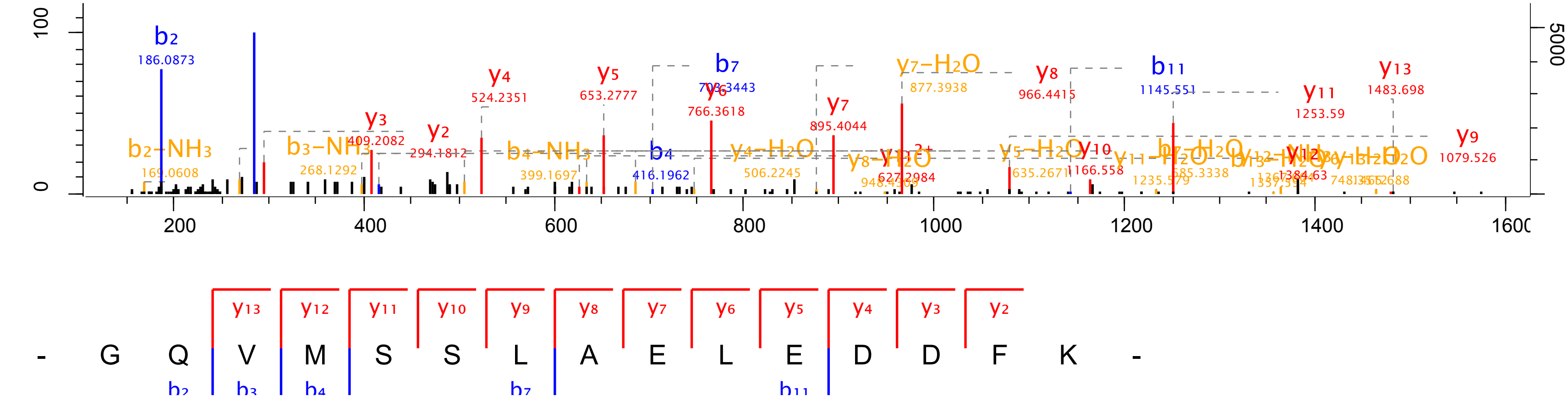

Raw file	Scan	Method	Score	m/z	Gene names
20140925_fract9_dyn_5ul_C1_01_444	37645	TOF; CID	104.88	1065.55	KDELR1

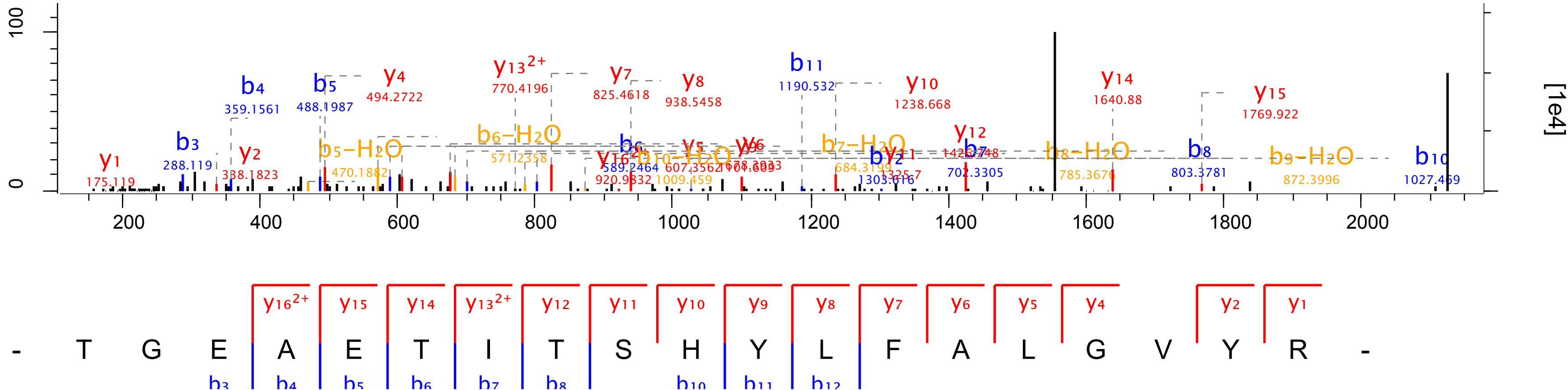

Raw file	Scan	Method	Score	m/z	Gene names
20140925_fract9_dyn_5ul_C1_01_444	37770	TOF; CID	79.84	1017.54	ZFAND2B

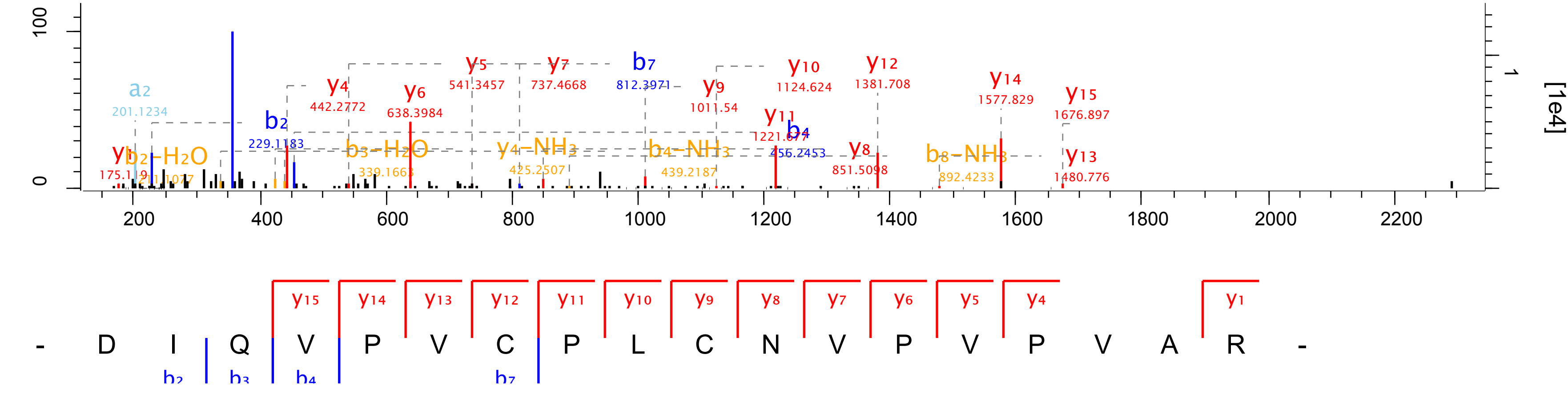

Raw file	Scan	Method	Score	m/z	Gene names
20140925_fract9_dyn_5ul_C1_01_444	37924	TOF; CID	61.94	1018.48	SCHIP1;IQCJ-SCHIP1

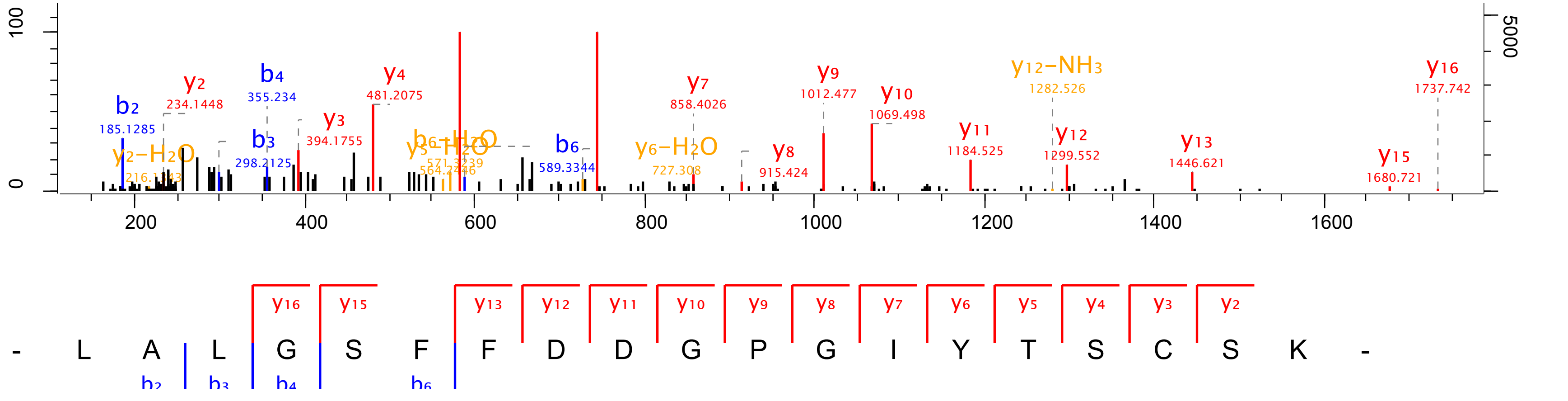

Raw file	Scan	Method	Score	m/z	Gene names
20140925_fract9_dyn_5ul_C1_01_444	38245	TOF; CID	49.83	969.45	CDC42SE1

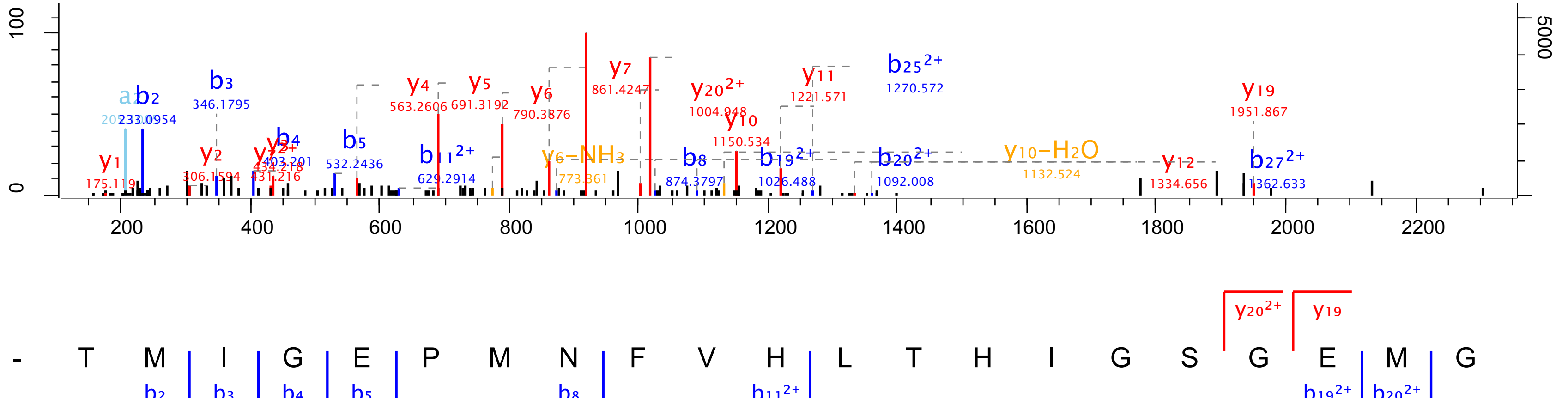

Raw file	Scan	Method	Score	m/z	Gene names
20140925_fract9_dyn_5ul_C1_01_444	40227	TOF; CID	86.21	522.28	PXDNL

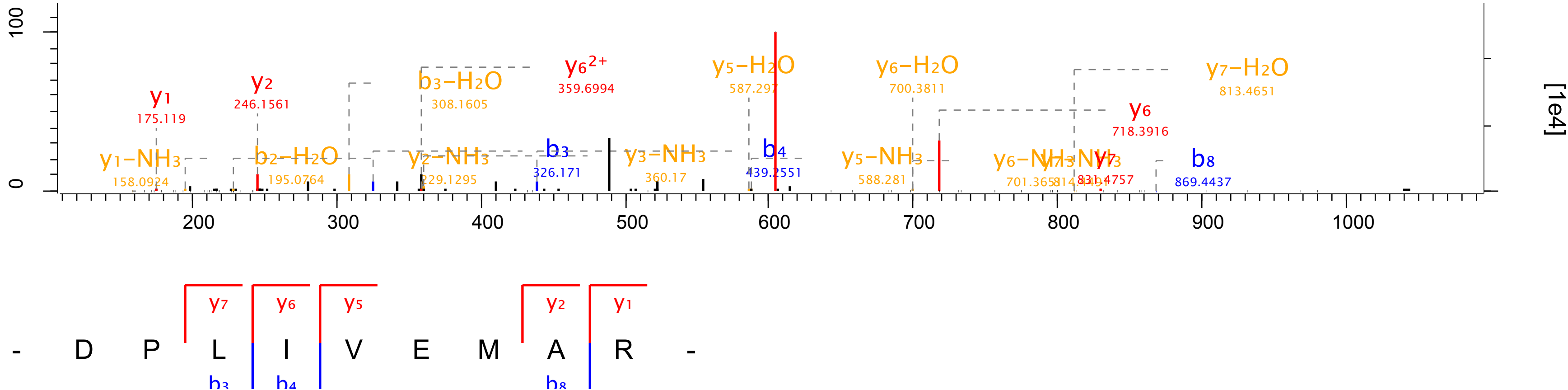

Raw file	Scan	Method	Score	m/z	Gene names
20140925_fract10_dyn_5ul_C2_01_445	5310	TOF; CID	65.37	465.23	CRAT

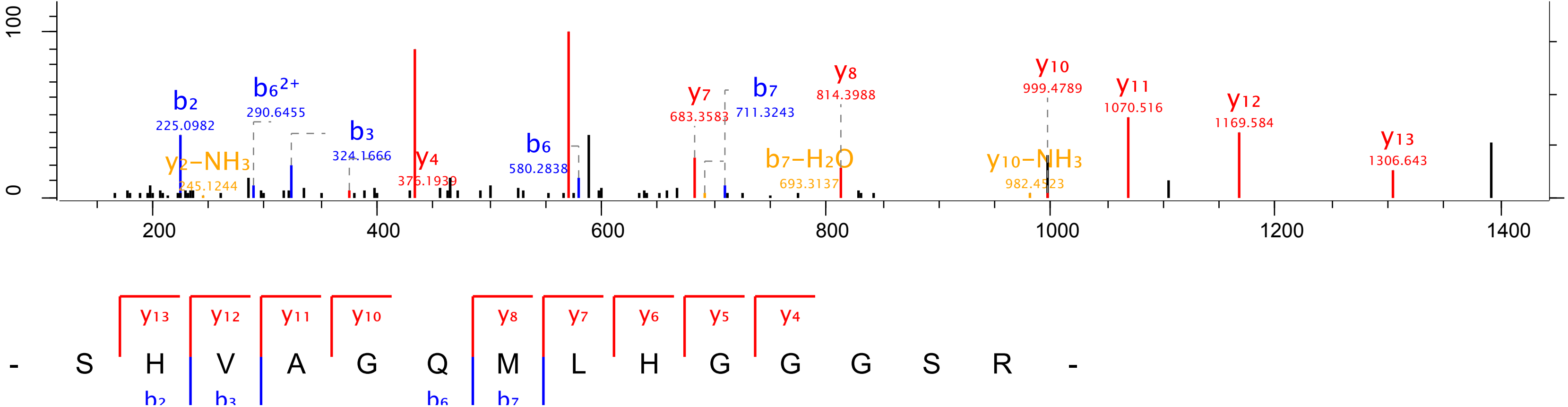

Raw file	Scan	Method	Score	m/z	Gene names
20140925_fract10_dyn_5ul_C2_01_445	6793	TOF; CID	196.27	446.23	NINJ1

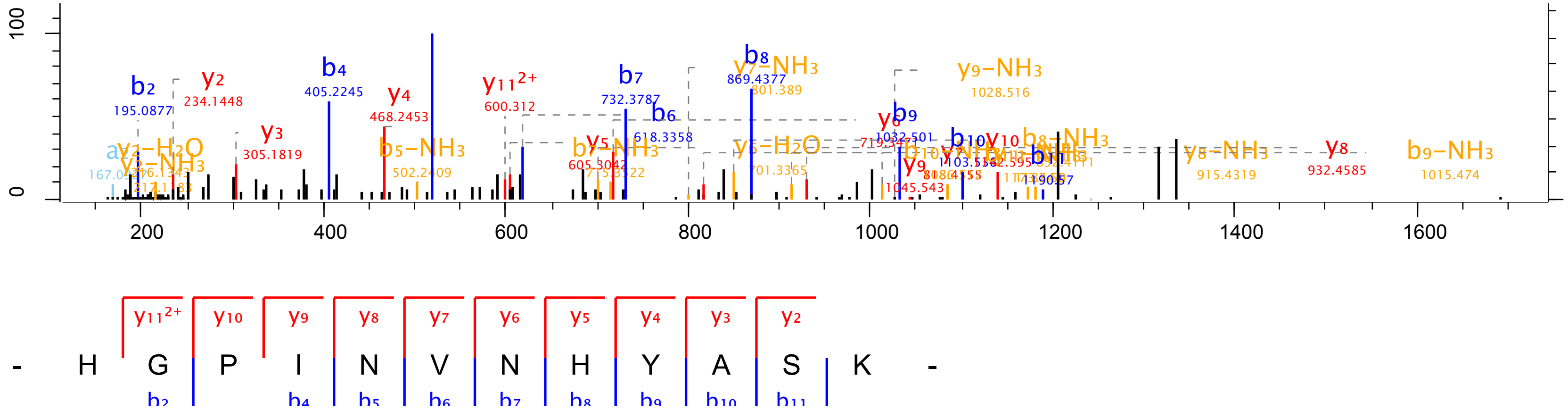

Raw file	Scan	Method	Score	m/z	Gene names
20140925_fract10_dyn_5ul_C2_01_445	7750	TOF; CID	47.77	501.26	PRRG4

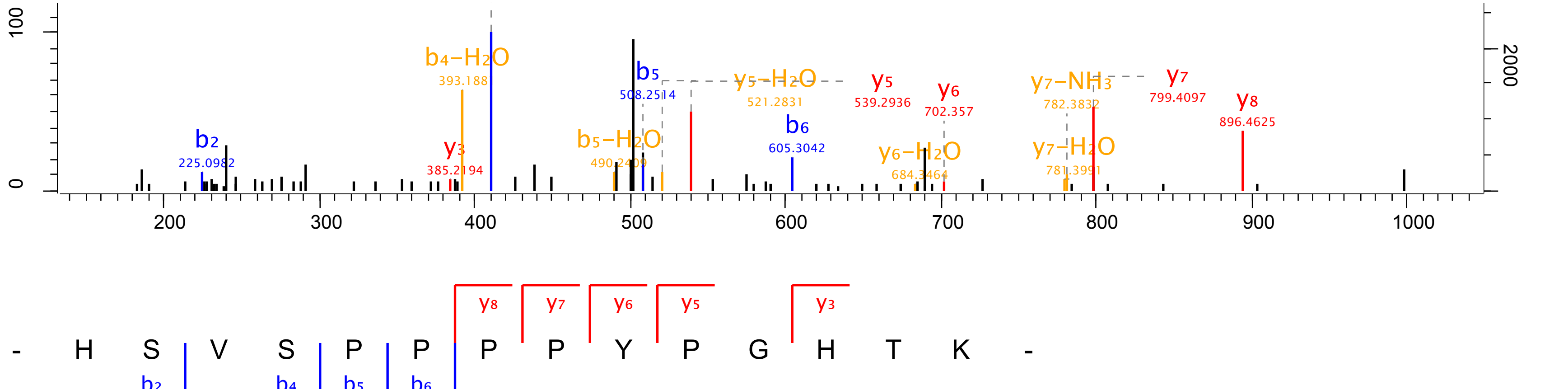

Raw file	Scan	Method	Score	m/z	Gene names
20140925_fract10_dyn_5ul_C2_01_445	12153	TOF; CID	93.6	529.32	RPGRIP1

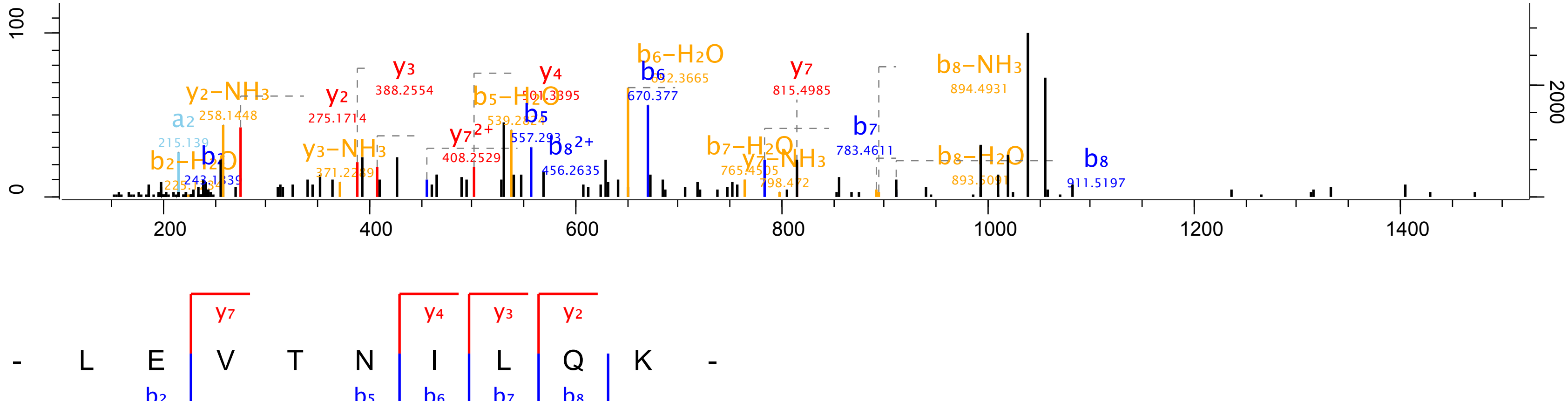

Raw file	Scan	Method	Score	m/z	Gene names
20140925_fract10_dyn_5ul_C2_01_445	14434	TOF; CID	125.16	497.26	AKR1D1

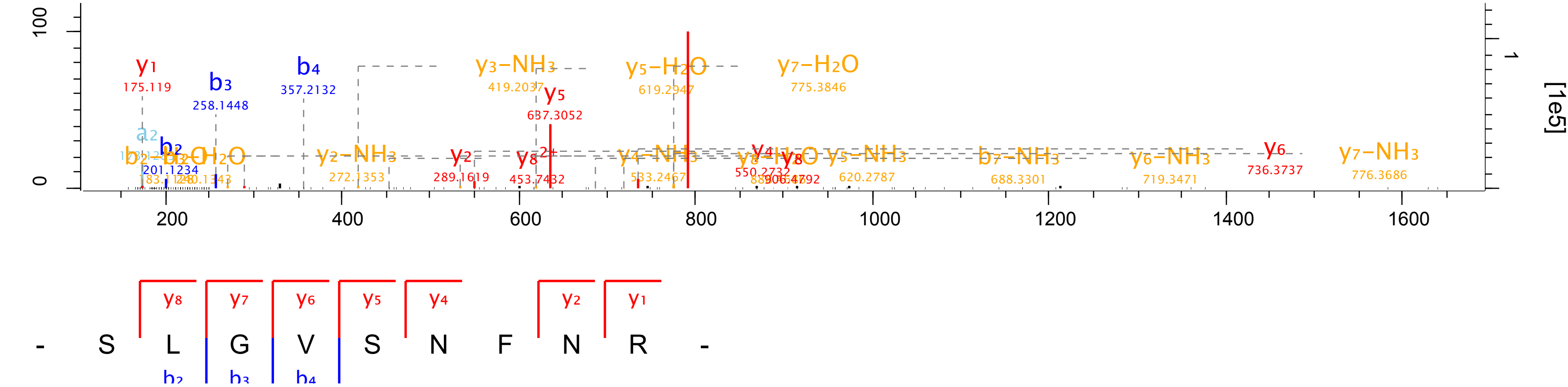

Raw file	Scan	Method	Score	m/z	Gene names
20140925_fract10_dyn_5ul_C2_01_445	16455	TOF; CID	75.82	432.56	NDUFC1

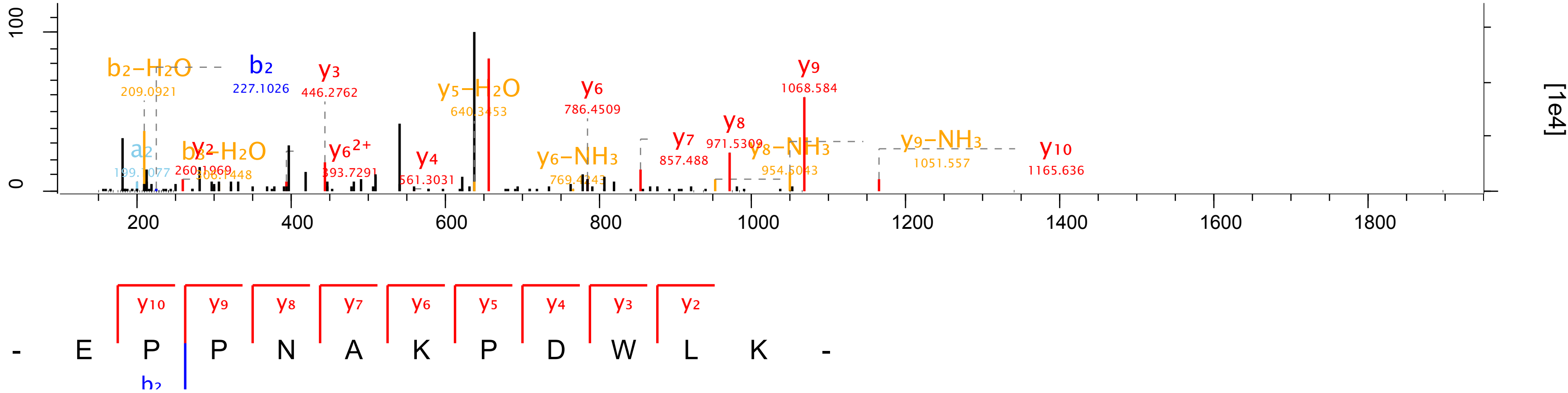

Raw file	Scan	Method	Score	m/z	Gene names
20140925_fract10_dyn_5ul_C2_01_445	17301	TOF; CID	79.34	551.61	YIPF6

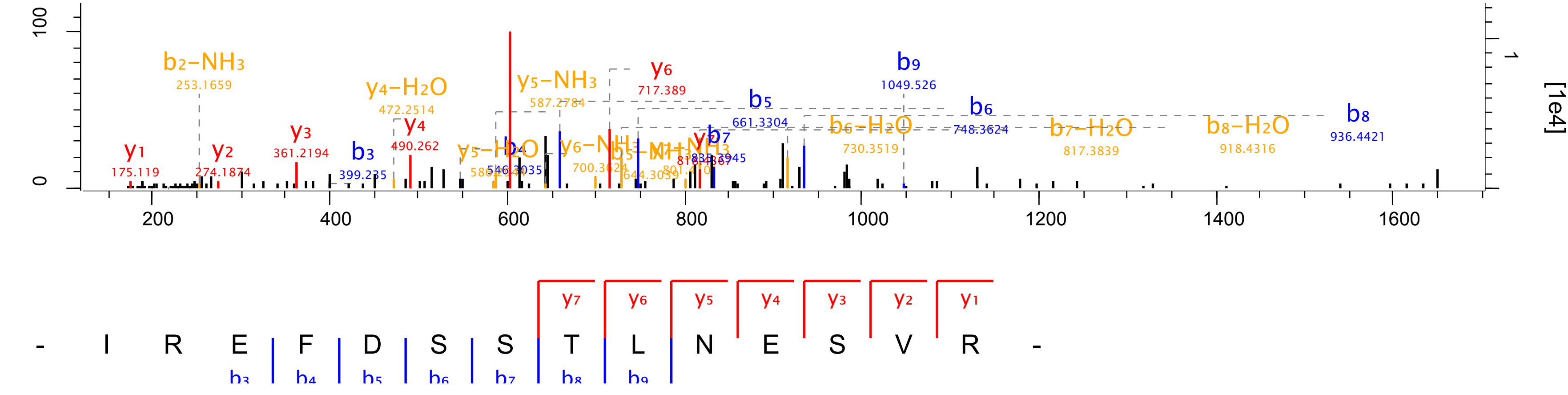

Raw file	Scan	Method	Score	m/z	Gene names
20140925_fract10_dyn_5ul_C2_01_445	19493	TOF; CID	73.44	421.23	GNG11

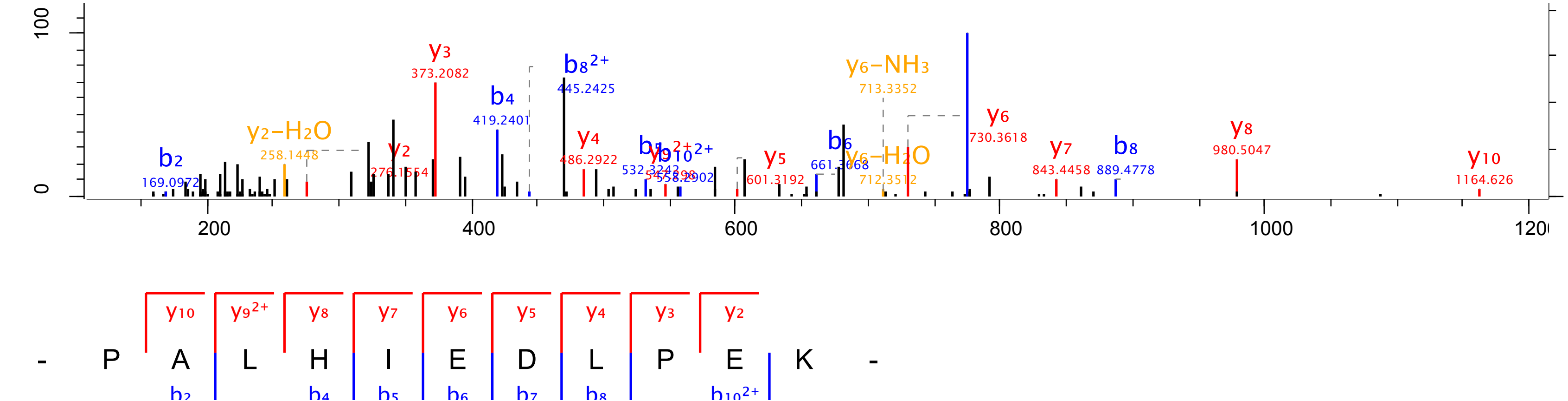

Raw file	Scan	Method	Score	m/z	Gene names
20140925_fract10_dyn_5ul_C2_01_445	25602	TOF; CID	95.26	710.37	TMEM184C

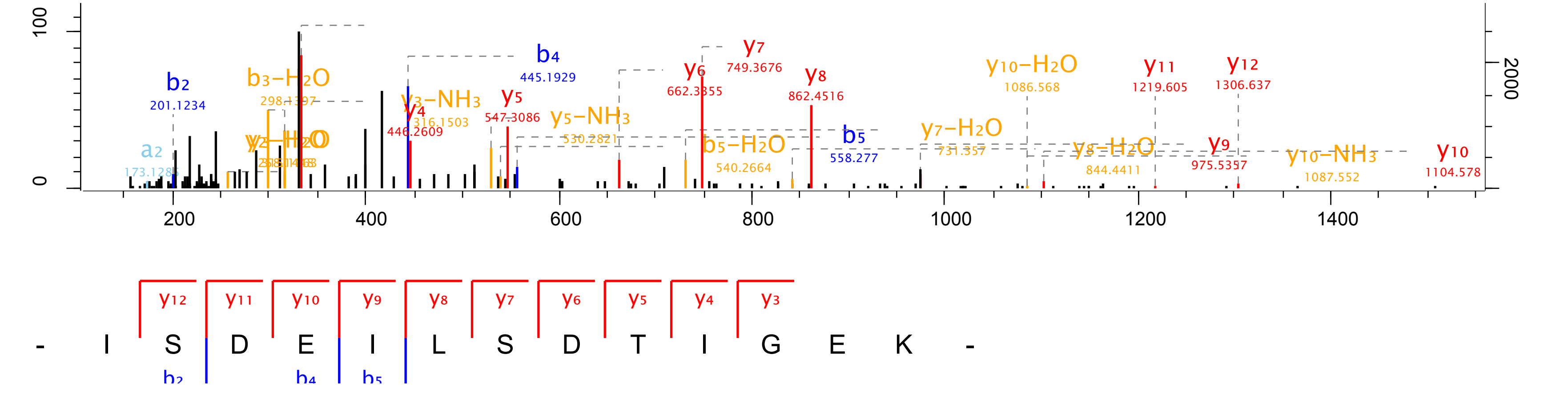

Raw file	Scan	Method	Score	m/z	Gene names
20140925_fract10_dyn_5ul_C2_01_445	28508	TOF; CID	84.61	550.28	GRPEL2

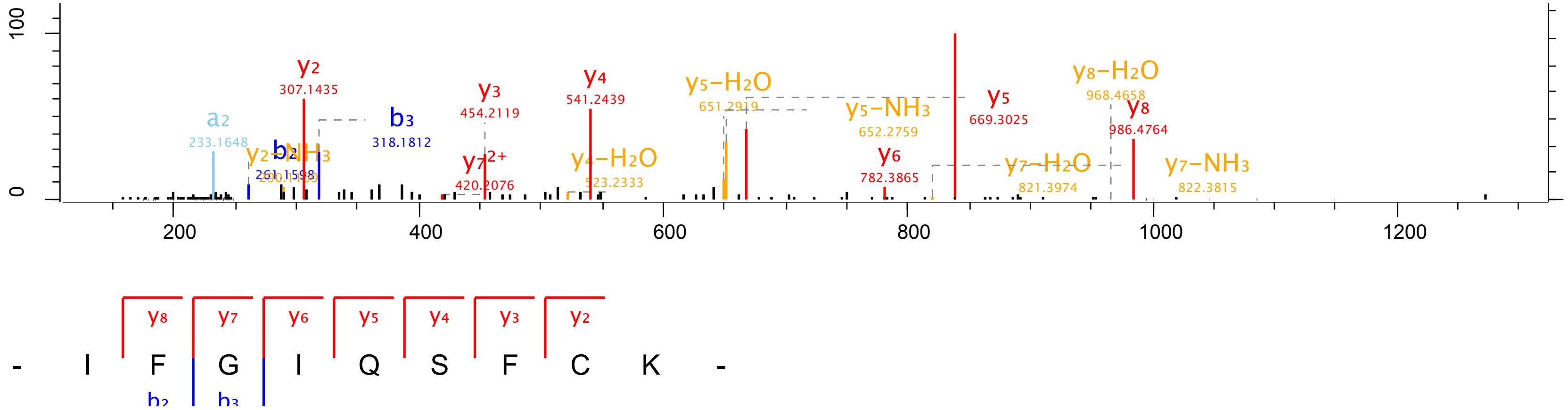

Raw file	Scan	Method	Score	m/z	Gene names
20140925_fract10_dyn_5ul_C2_01_445	29586	TOF; CID	98.06	865.99	KRTCAP2

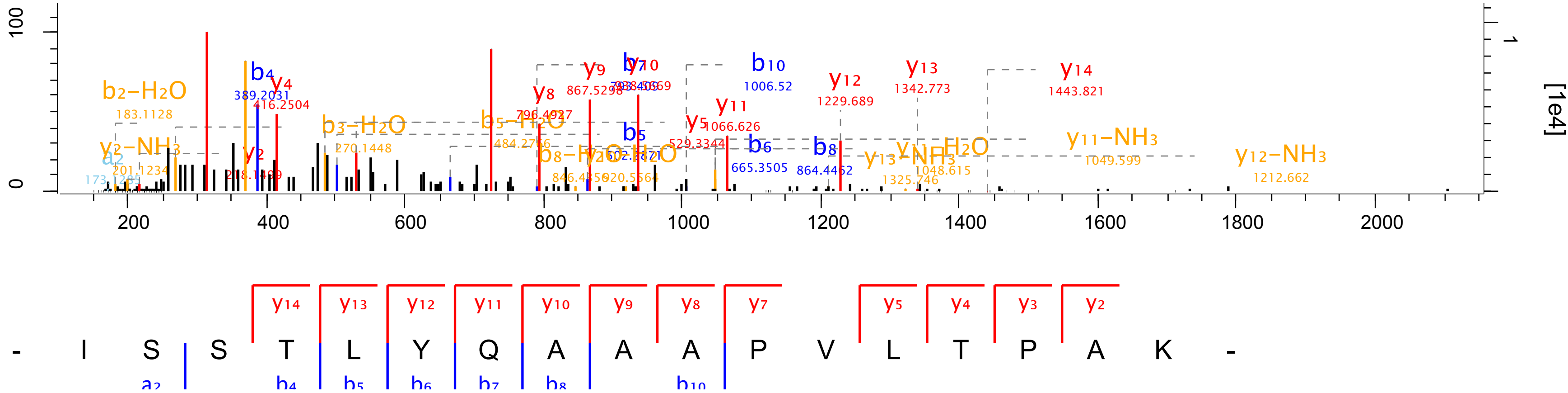

Raw file	Scan	Method	Score	m/z	Gene names
20140925_fract10_dyn_5ul_C2_01_445	33269	TOF; CID	147.62	629.83	GTF2A1;HIST1H4F

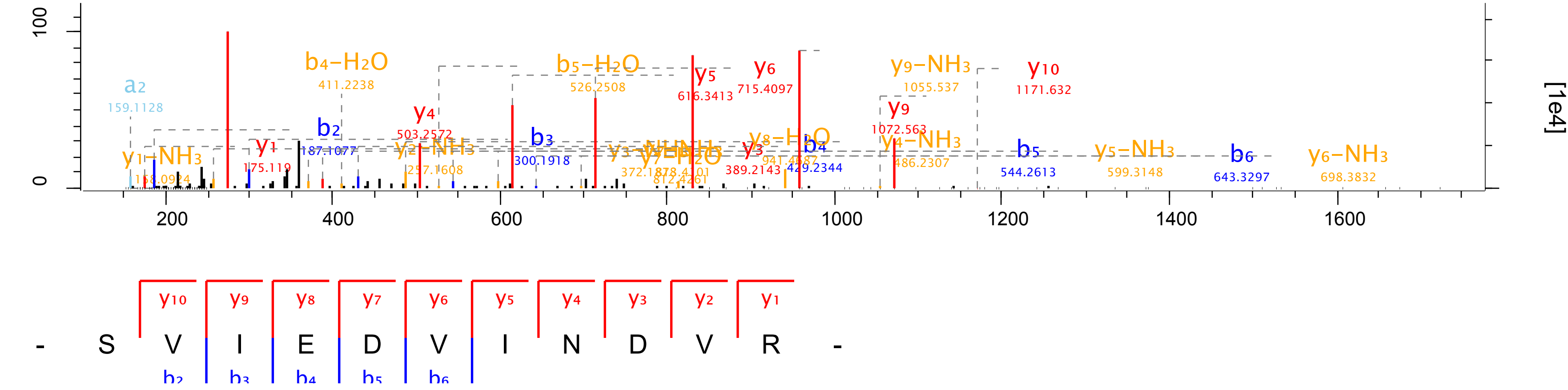

Raw file	Scan	Method	Score	m/z	Gene names
20140925_fract10_dyn_5ul_C2_01_445	33457	TOF; CID	128.06	814.92	CCDC167

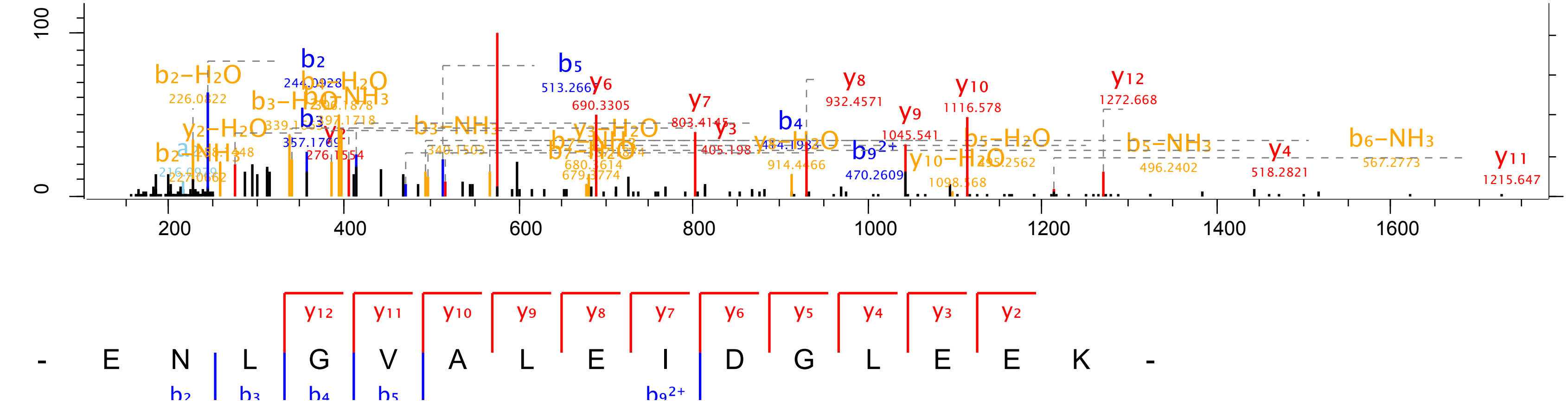

Raw file	Scan	Method	Score	m/z	Gene names
20140925_fract10_dyn_5ul_C2_01_445	36125	TOF; CID	58.08	499.76	TRAM2

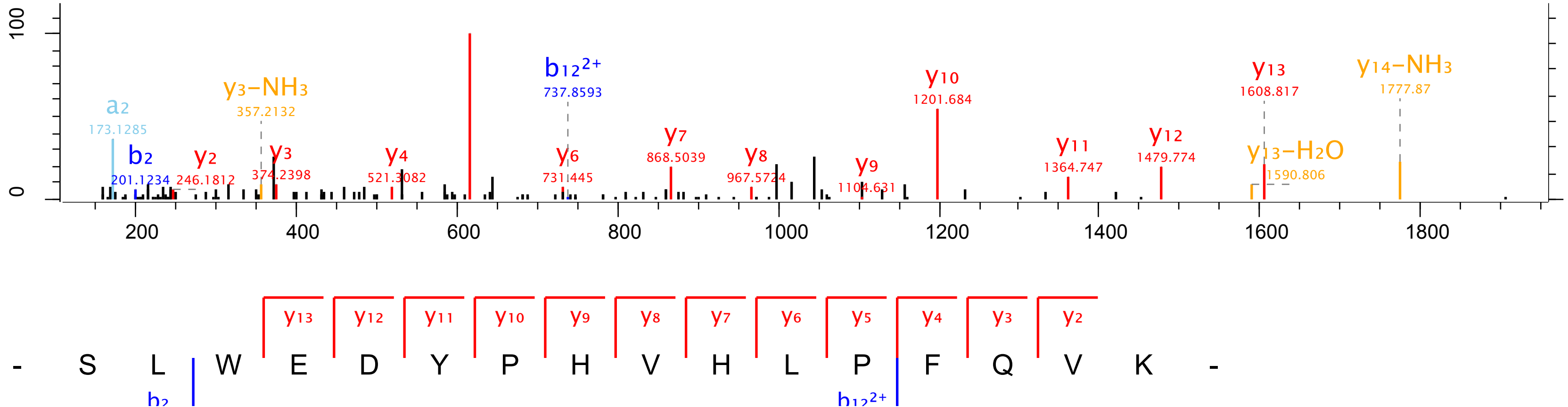

Raw file	Scan	Method	Score	m/z	Gene names
20140925_fract10_dyn_5ul_C2_01_445	40949	TOF; CID	106.28	1099.53	CNIH1

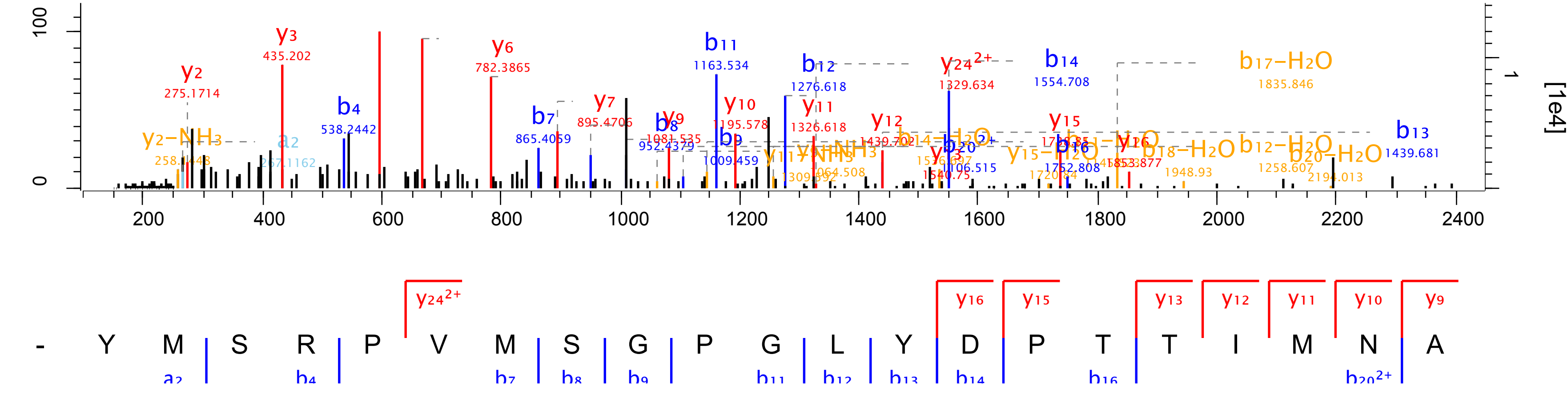

Raw file	Scan	Method	Score	m/z	Gene names
20140925_fract11_dyn_5ul_C3_01_446	6238	TOF; CID	44.97	526.62	ZNF251

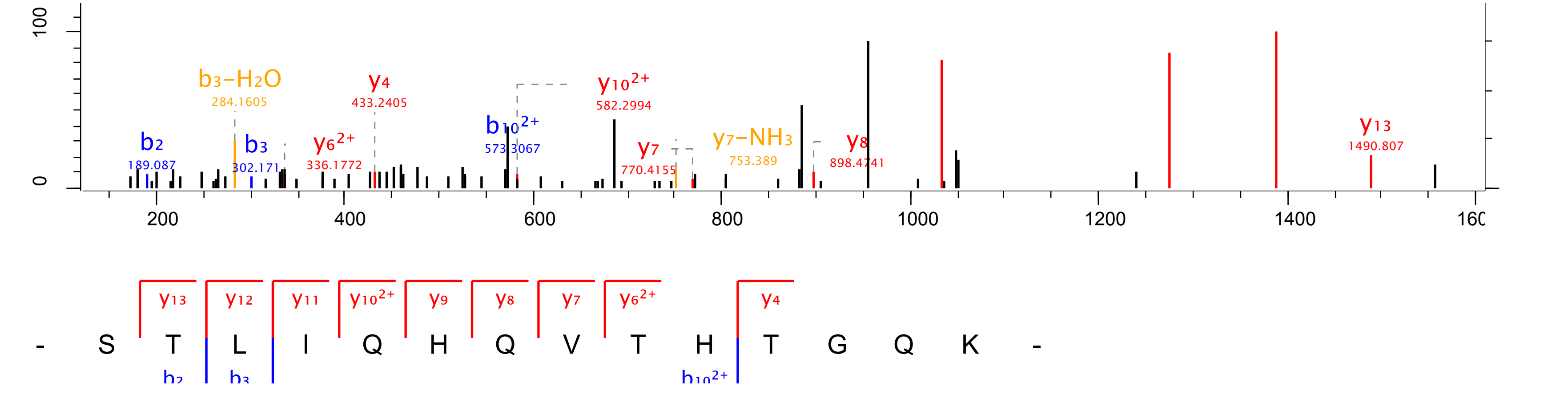

Raw file	Scan	Method	Score	m/z	Gene names
20140925_fract11_dyn_5ul_C3_01_446	8495	TOF; CID	125.5	465.49	TMEM41A

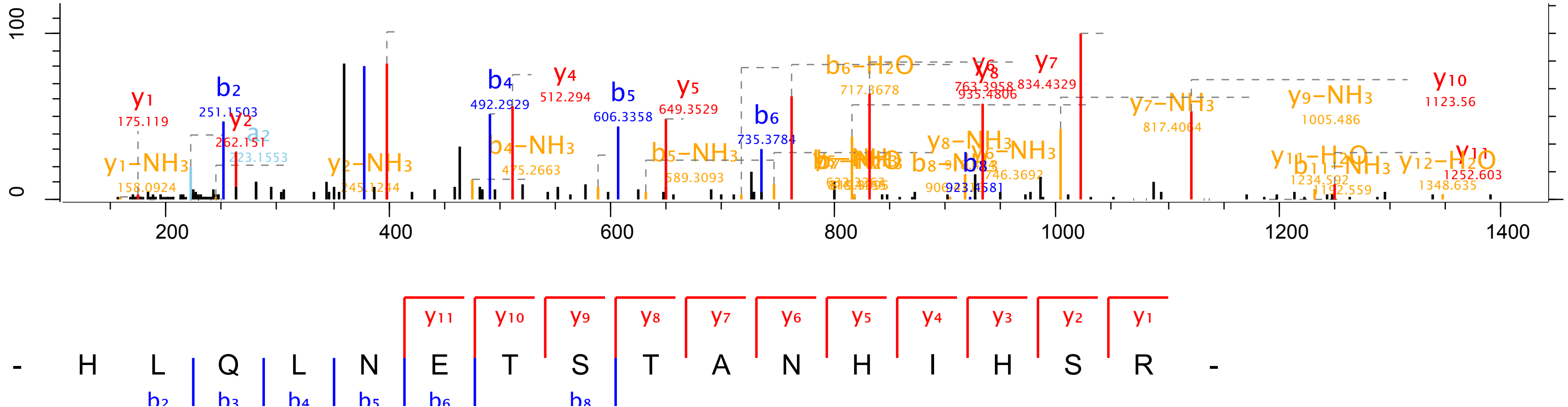

Raw file	Scan	Method	Score	m/z	Gene names
20140925_fract11_dyn_5ul_C3_01_446	9106	TOF; CID	69.81	371.53	R3HDM4

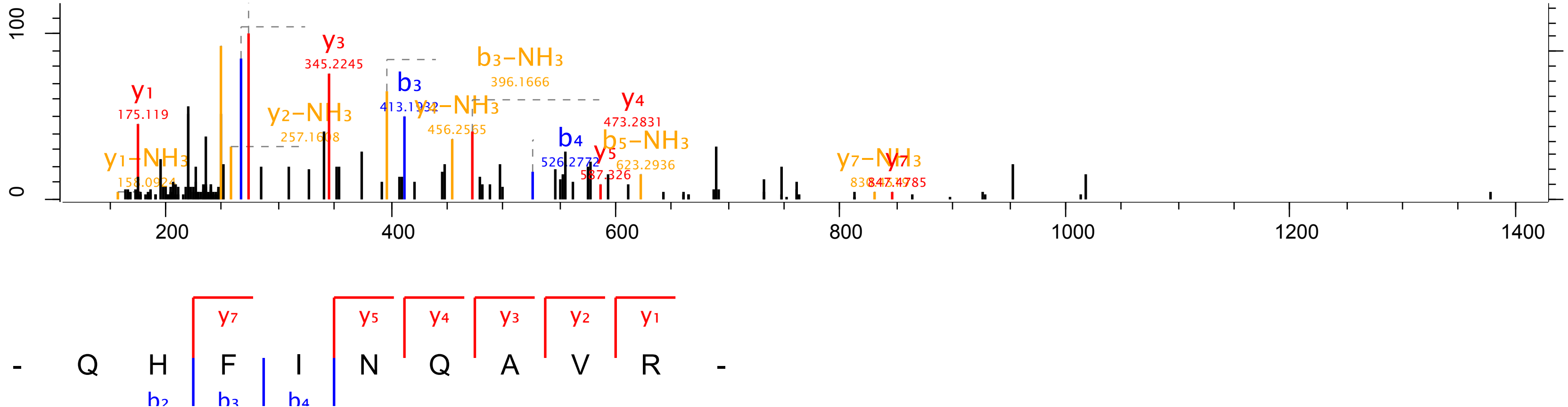

Raw file	Scan	Method	Score	m/z	Gene names
20140925_fract11_dyn_5ul_C3_01_446	12344	TOF; CID	98.18	691.66	FAM156A

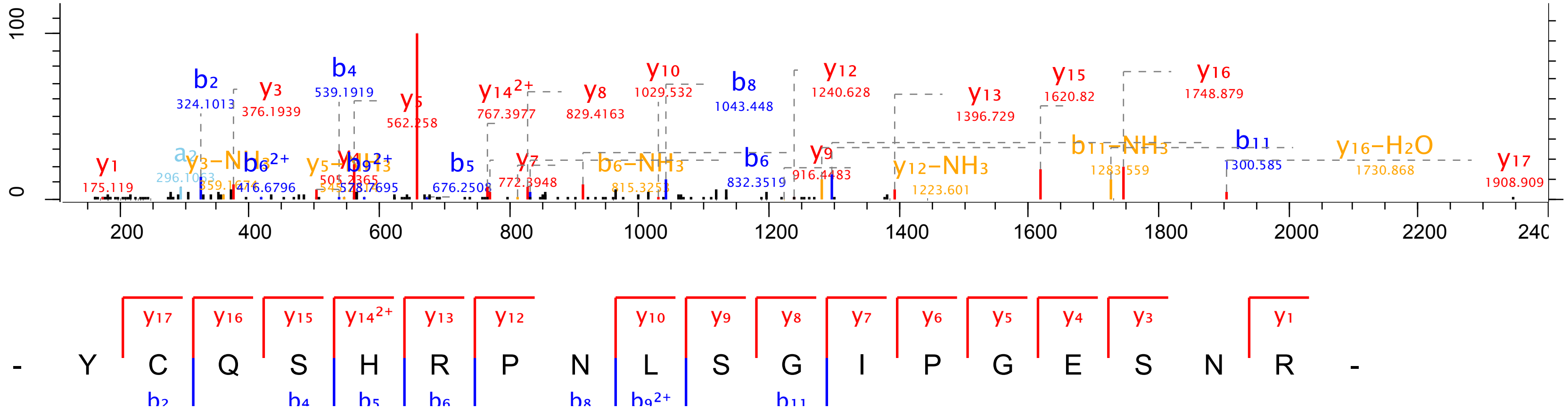

Raw file	Scan	Method	Score	m/z	Gene names
20140925_fract11_dyn_5ul_C3_01_446	15350	TOF; CID	53.45	487.59	SLC25A27

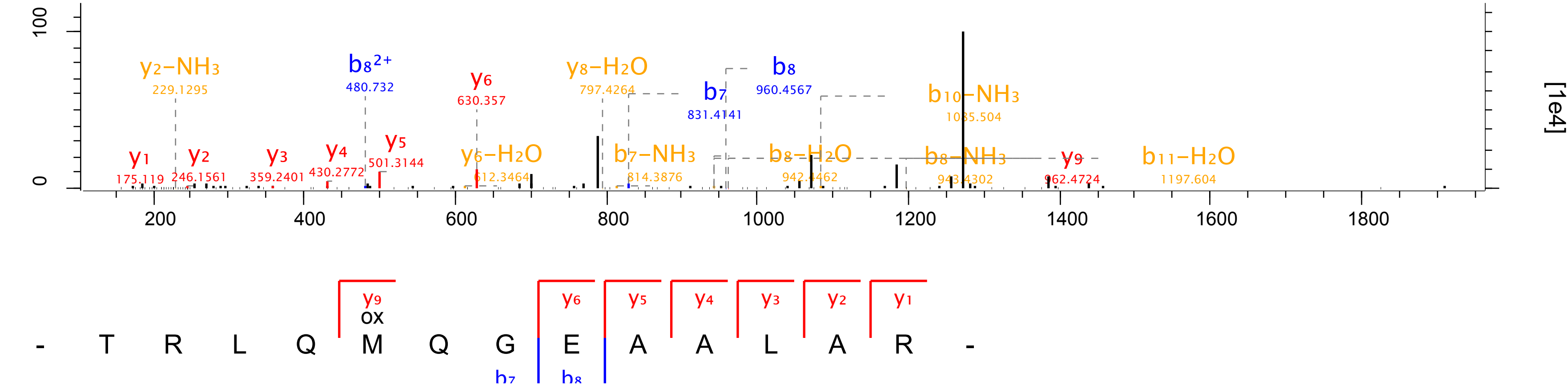

Raw file	Scan	Method	Score	m/z	Gene names
20140925_fract11_dyn_5ul_C3_01_446	15726	TOF; CID	81.53	479.27	SLIT1

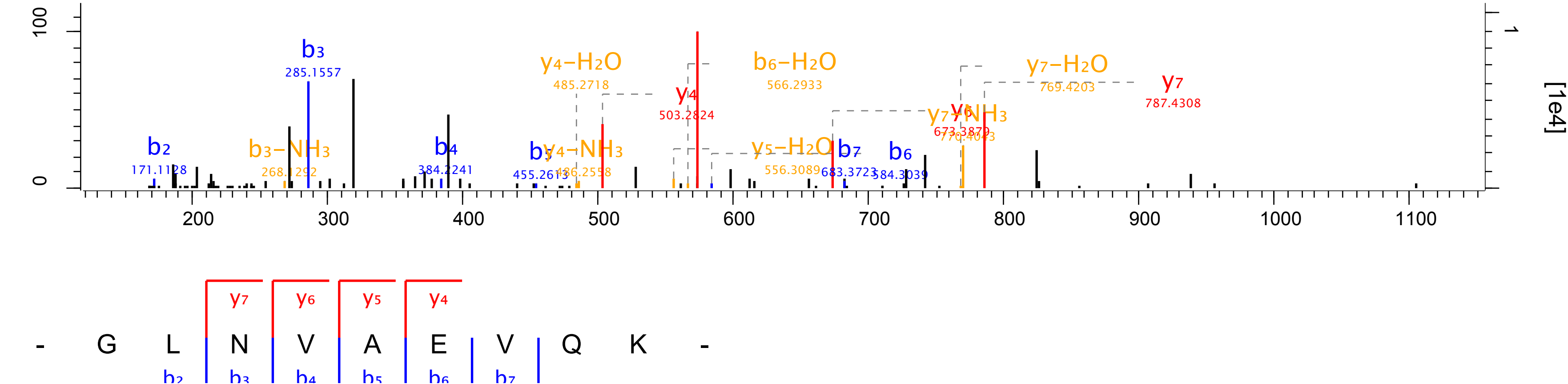

Raw file	Scan	Method	Score	m/z	Gene names
20140925_fract11_dyn_5ul_C3_01_446	16053	TOF; CID	168.85	617.83	SUMO2;SUMO3;SUMO4

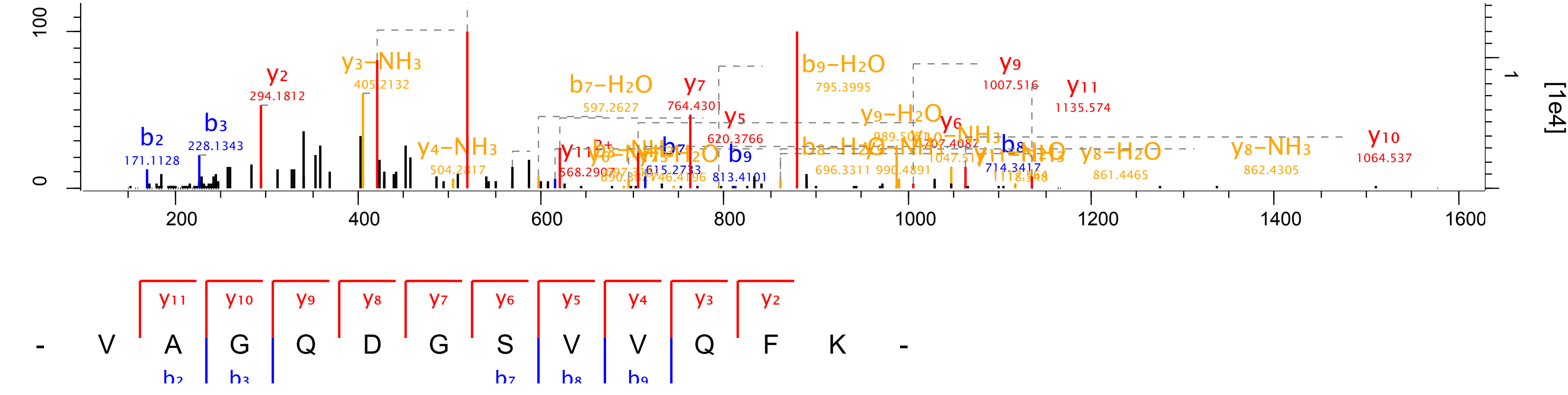

Raw file	Scan	Method	Score	m/z	Gene names
20140925_fract11_dyn_5ul_C3_01_446	19613	TOF; CID	79.68	666.34	ENKD1

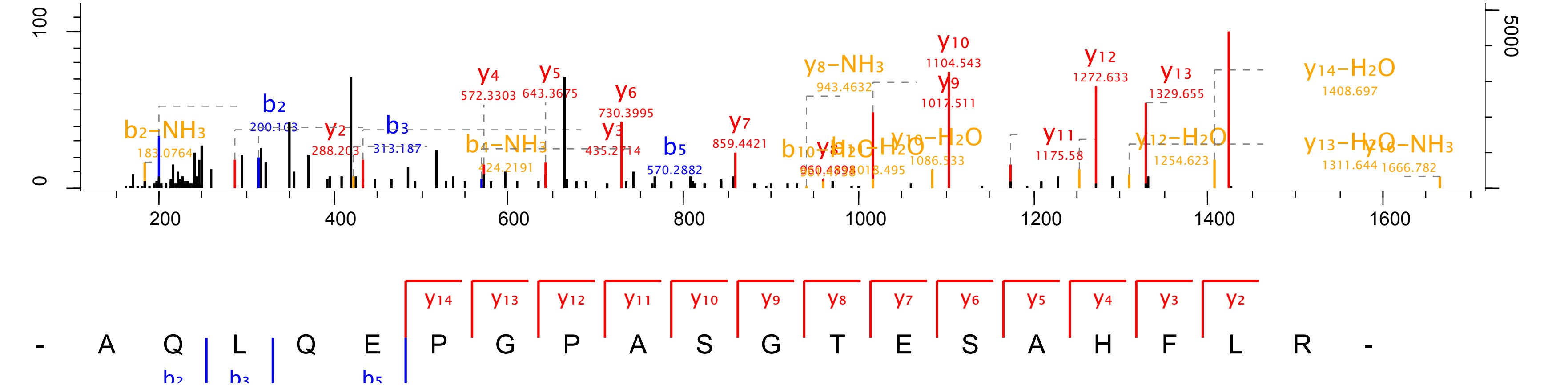

Raw file	Scan	Method	Score	m/z	Gene names
20140925_fract11_dyn_5ul_C3_01_446	23347	TOF; CID	58.08	786.4	DACH1

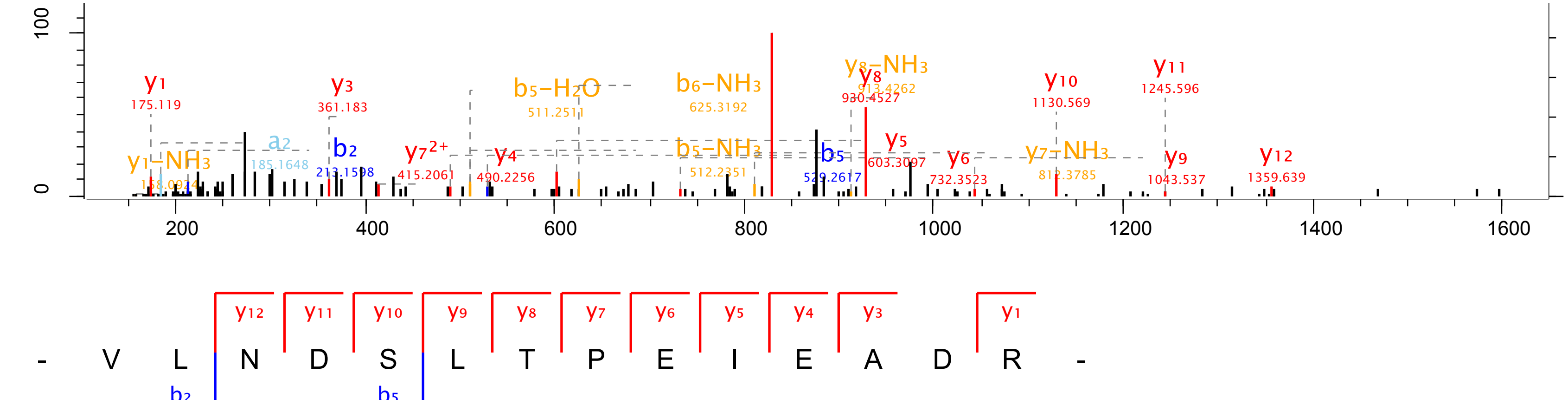

Raw file	Scan	Method	Score	m/z	Gene names
20140925_fract11_dyn_5ul_C3_01_446	23959	TOF; CID	57.15	857.86	MT-ND3

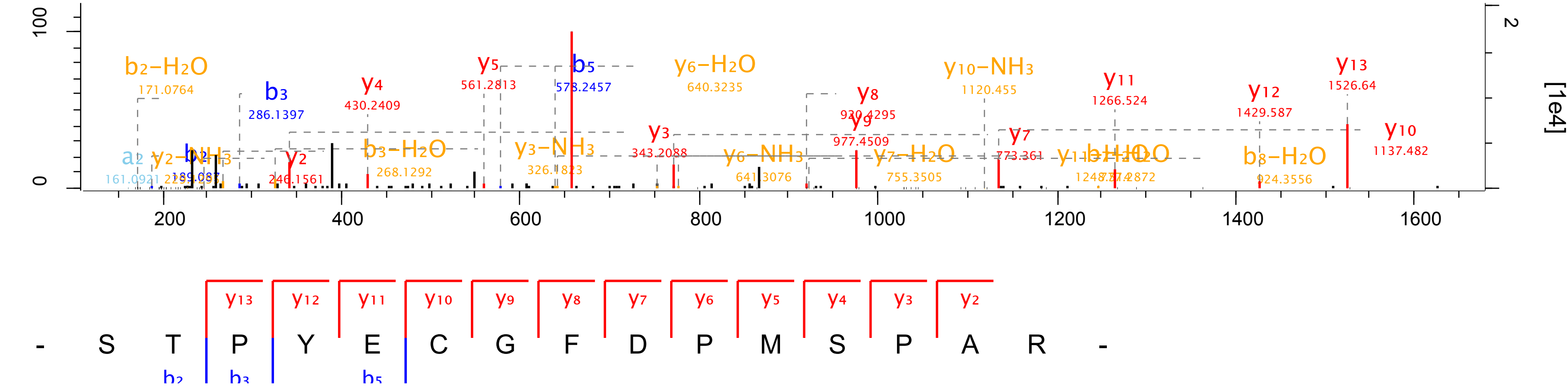

Raw file	Scan	Method	Score	m/z	Gene names
20140925_fract11_dyn_5ul_C3_01_446	25013	TOF; CID	75.38	664.98	DCUN1D2

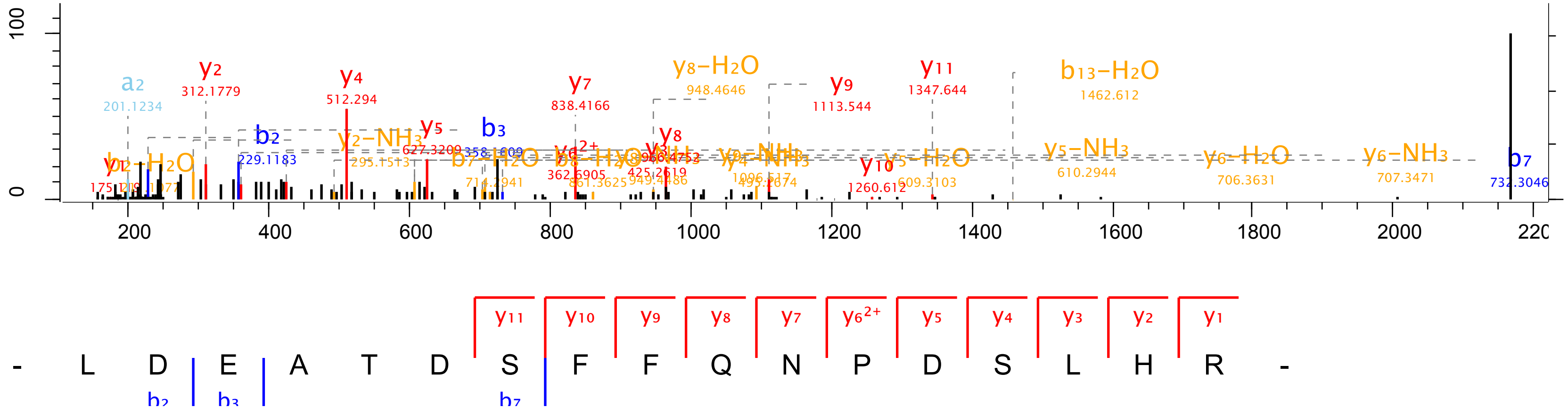

Raw file	Scan	Method	Score	m/z	Gene names
20140925_fract11_dyn_5ul_C3_01_446	27615	TOF; CID	70.94	648.33	PEX2

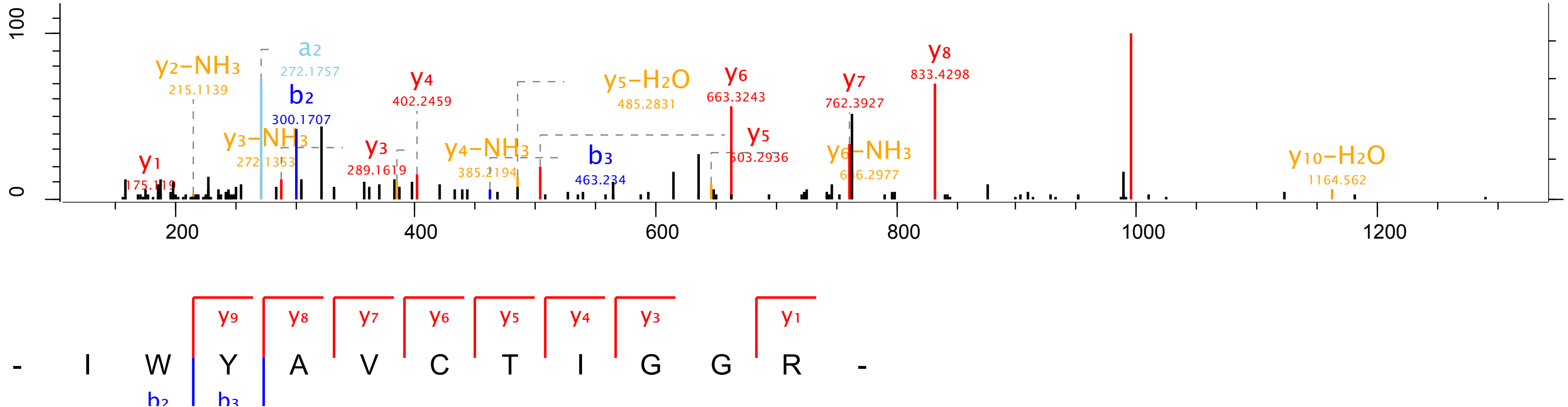

Raw file	Scan	Method	Score	m/z	Gene names
20140925_fract11_dyn_5ul_C3_01_446	28160	TOF; CID	90.71	597.99	DGCR2

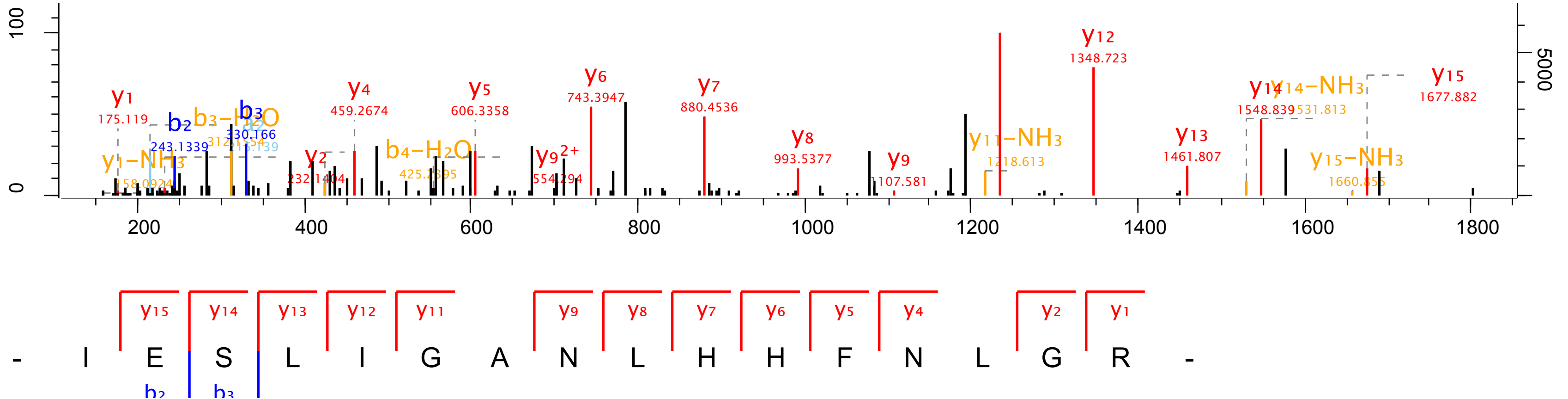

Raw file	Scan	Method	Score	m/z	Gene names
20140925_fract11_dyn_5ul_C3_01_446	28225	TOF; CID	57.53	404.23	SLITRK5;SLITRK6

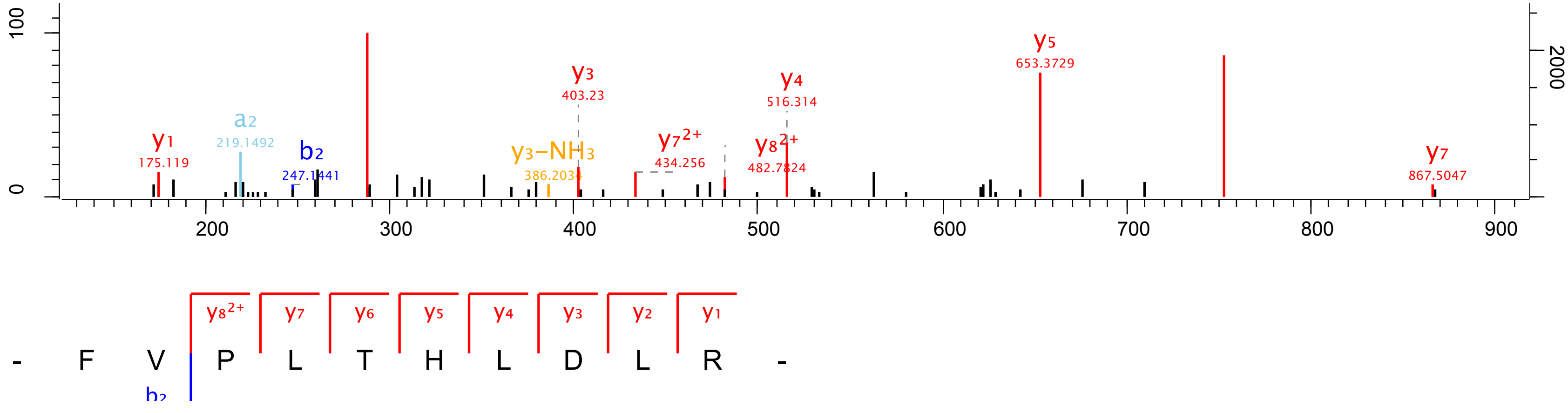

Raw file	Scan	Method	Score	m/z	Gene names
20140925_fract11_dyn_5ul_C3_01_446	29914	TOF; CID	114.97	623.38	MT-ATP6

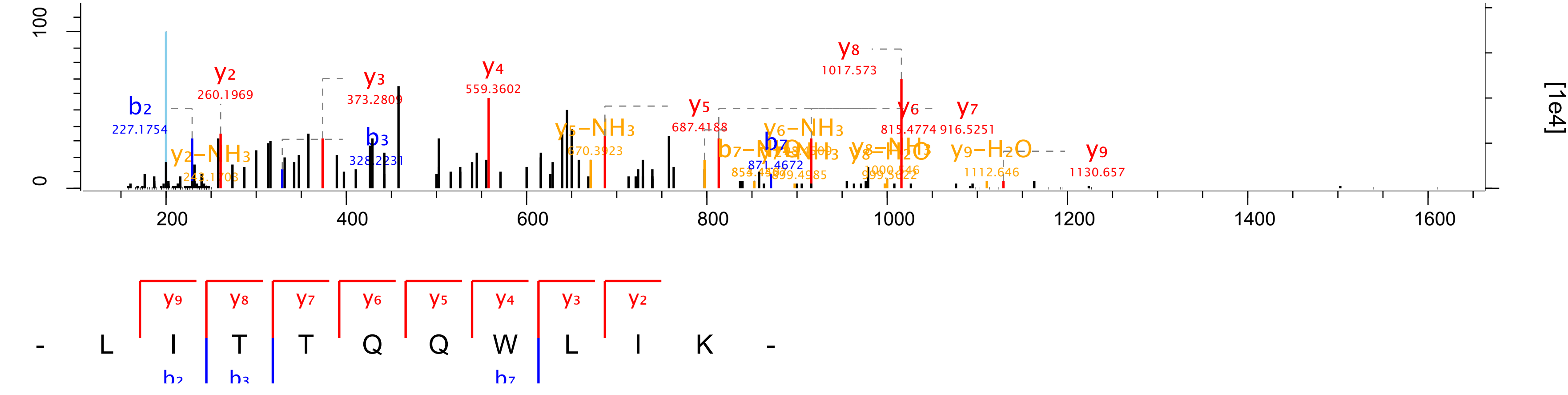

Raw file	Scan	Method	Score	m/z	Gene names
20140925_fract11_dyn_5ul_C3_01_446	31134	TOF; CID	101.05	982.05	RNF166

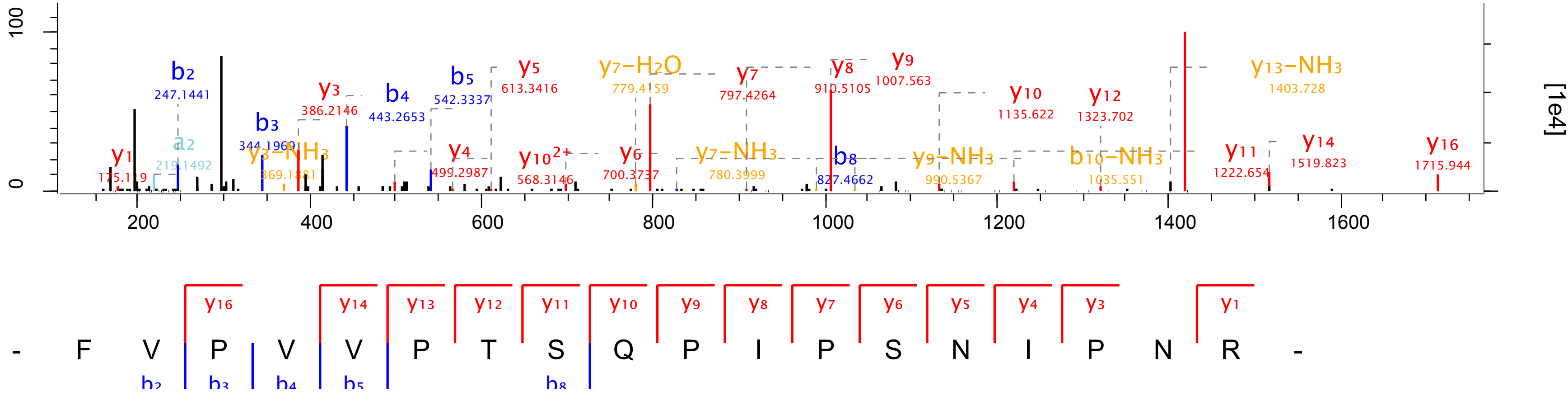

Raw file	Scan	Method	Score	m/z	Gene names
20140925_fract11_dyn_5ul_C3_01_446	32157	TOF; CID	101.39	441.6	SNX24

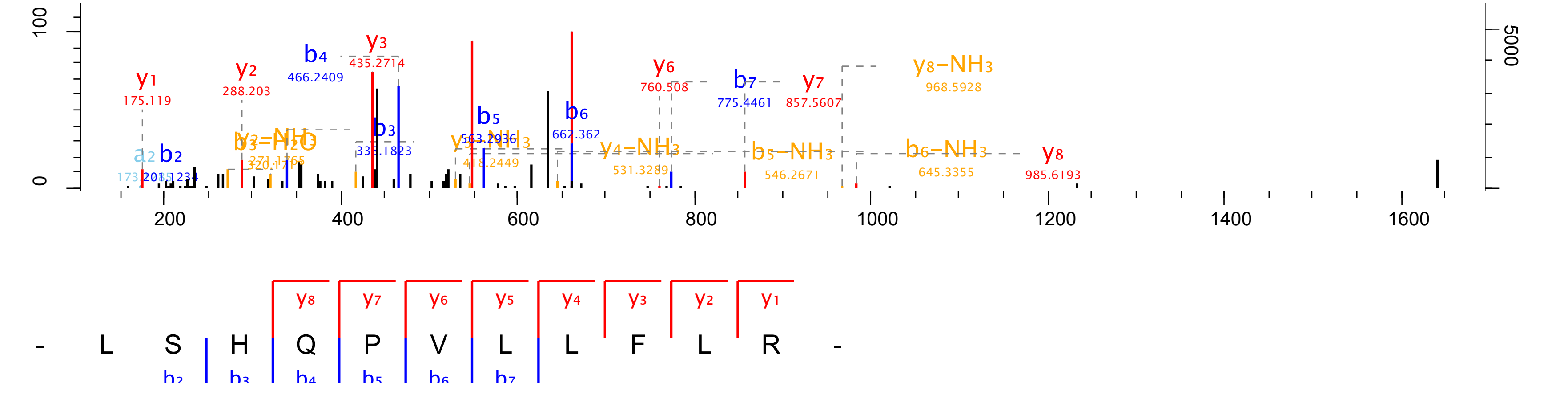

Raw file	Scan	Method	Score	m/z	Gene names
20140925_fract11_dyn_5ul_C3_01_446	36697	TOF; CID	106.12	1351.64	SMAGP

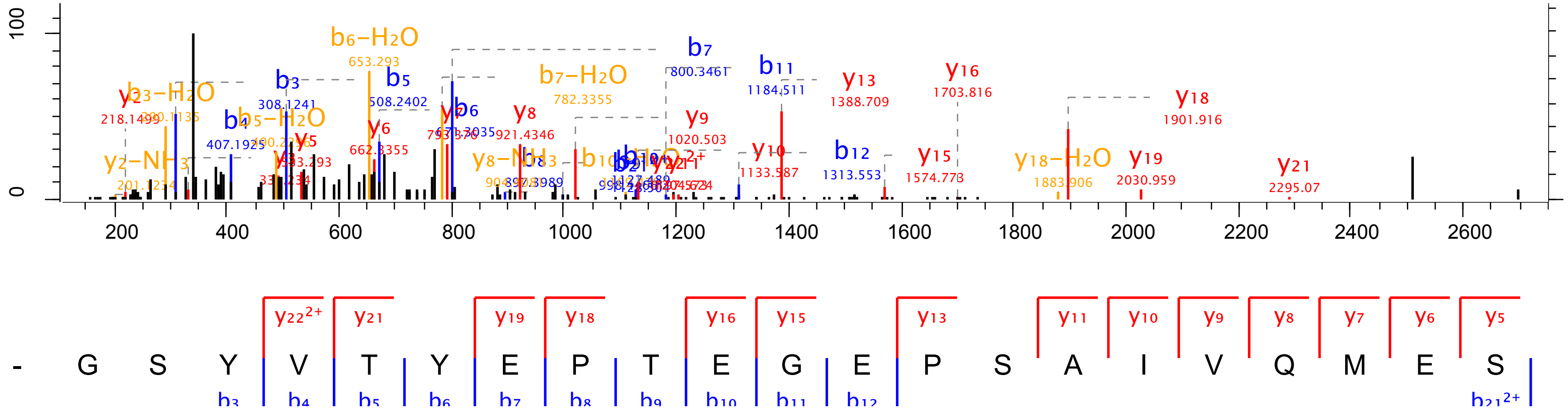

Raw file	Scan	Method	Score	m/z	Gene names
20140925_fract11_dyn_5ul_C3_01_446	37178	TOF; CID	80.92	588.34	SLC35B3

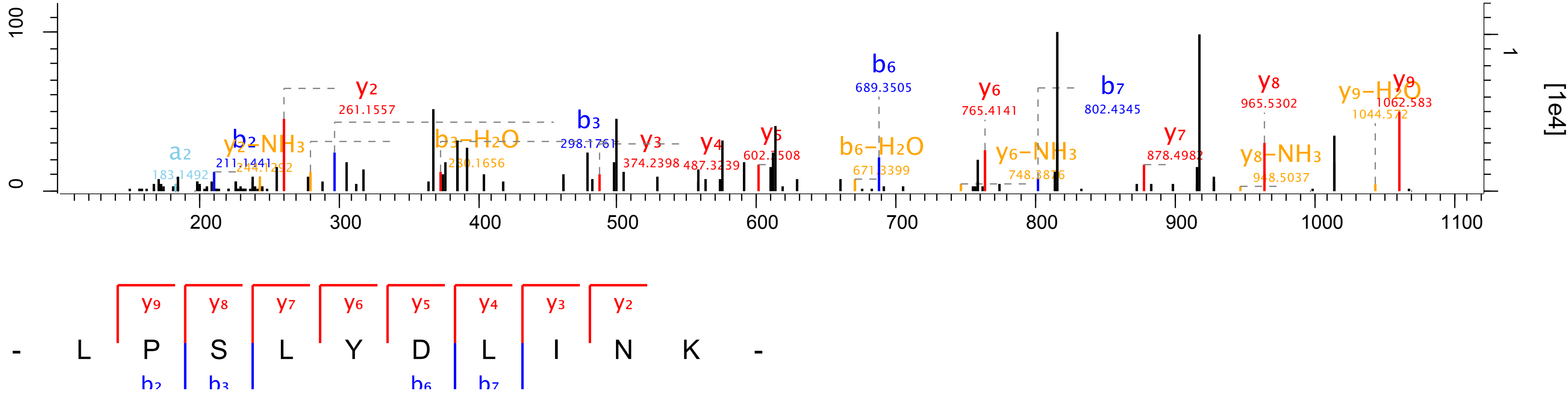

Raw file	Scan	Method	Score	m/z	Gene names
20140925_fract11_dyn_5ul_C3_01_446	37227	TOF; CID	113.93	610.3	CMTM6

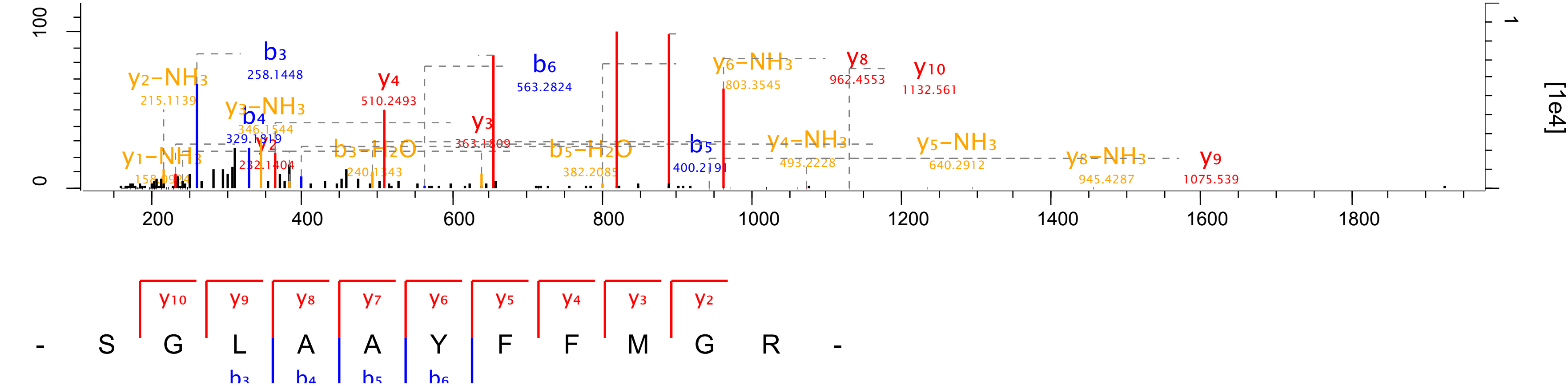

Raw file	Scan	Method	Score	m/z	Gene names
20140925_fract12_dyn_5ul_C4_01_447	9556	TOF; CID	101.6	497.3	RWDD2A

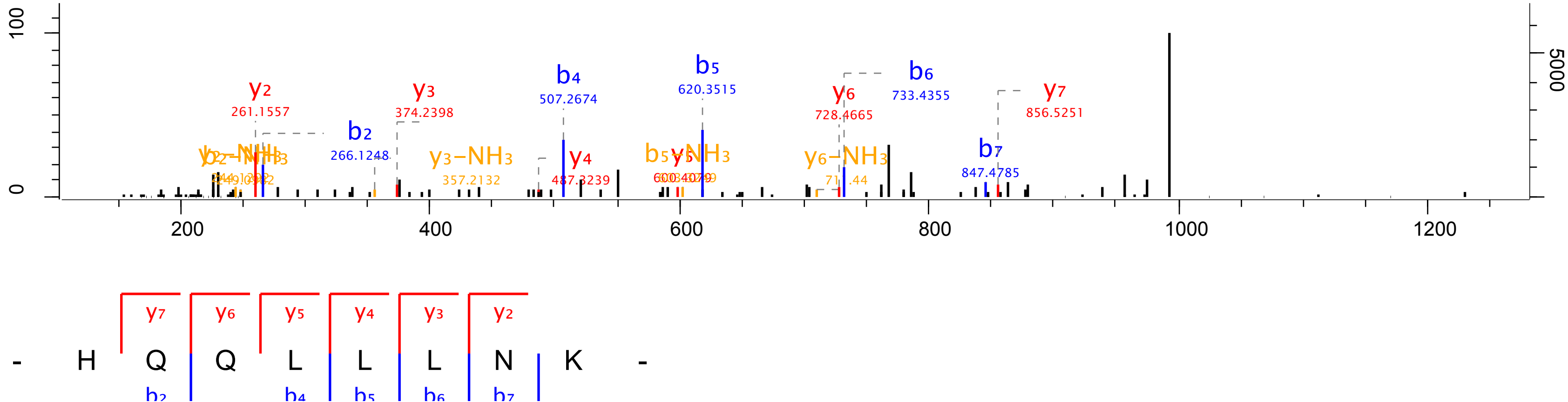

Raw file	Scan	Method	Score	m/z	Gene names
20140925_fract12_dyn_5ul_C4_01_447	10892	TOF; CID	67.38	543.8	SLC26A11

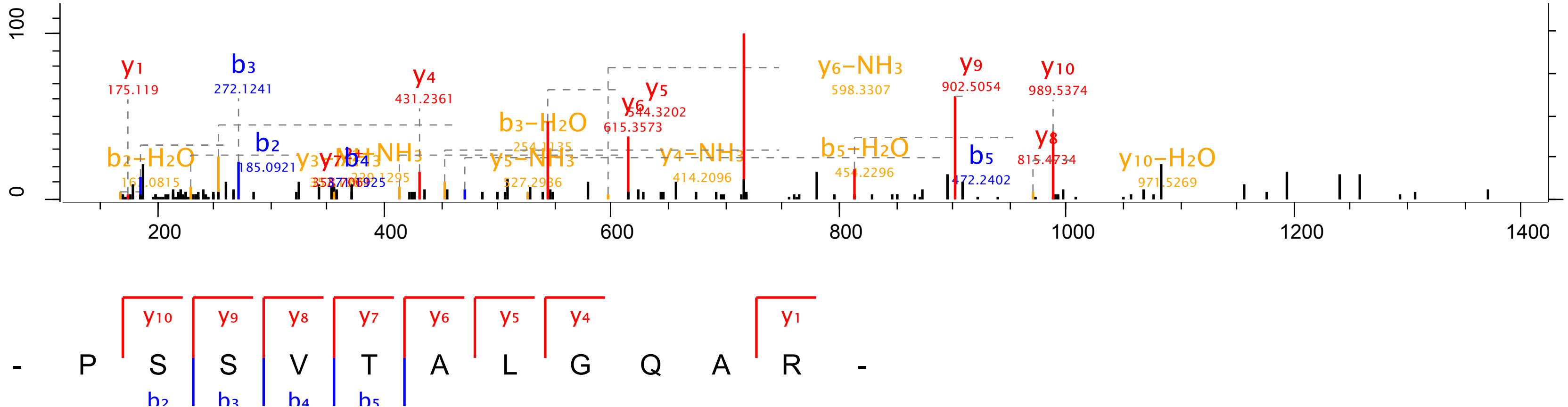

Raw file	Scan	Method	Score	m/z	Gene names
20140925_fract12_dyn_5ul_C4_01_447	17730	TOF; CID	83.21	480.77	FBXW11

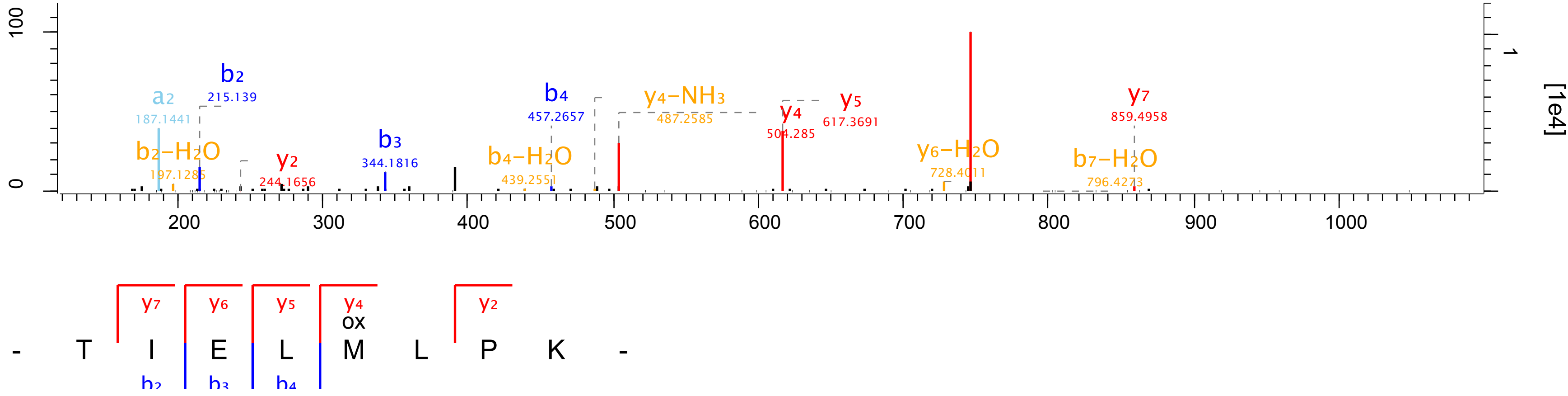

Raw file	Scan	Method	Score	m/z	Gene names
20140925_fract12_dyn_5ul_C4_01_447	18858	TOF; CID	32.88	938.45	CYSRT1

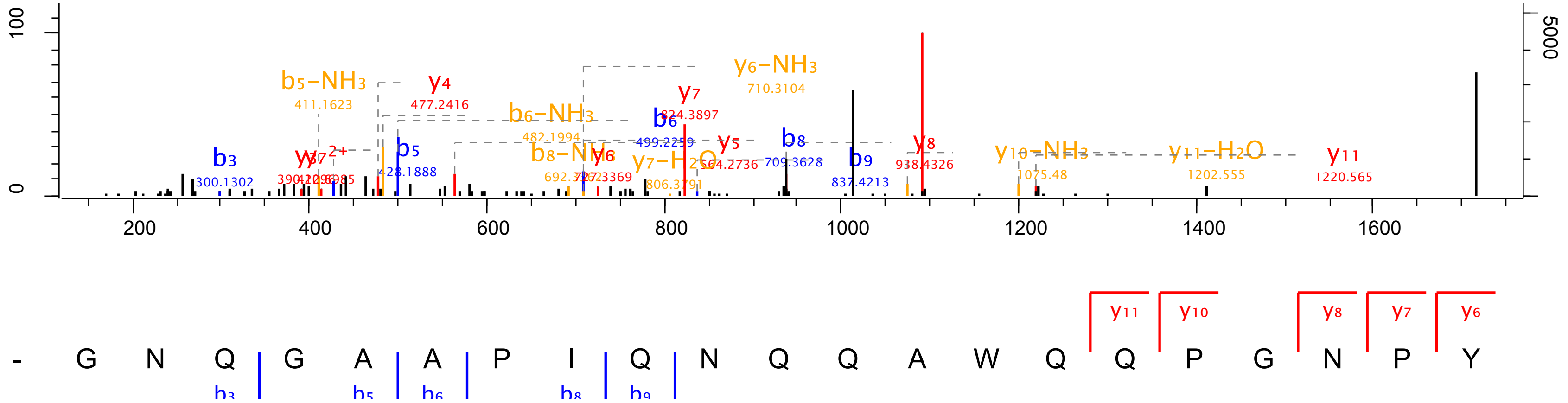

Raw file	Scan	Method	Score	m/z	Gene names
20140925_fract12_dyn_5ul_C4_01_447	20140	TOF; CID	95.57	765.31	SLC35F6

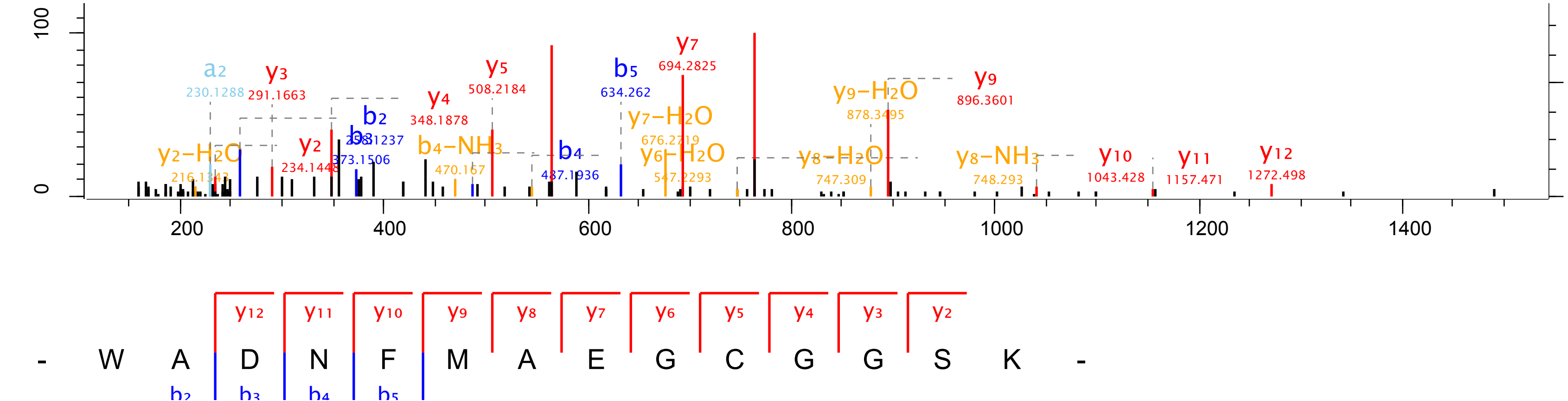

Raw file	Scan	Method	Score	m/z	Gene names
20140925_fract12_dyn_5ul_C4_01_447	22540	TOF; CID	55.26	746.37	FRMD4B

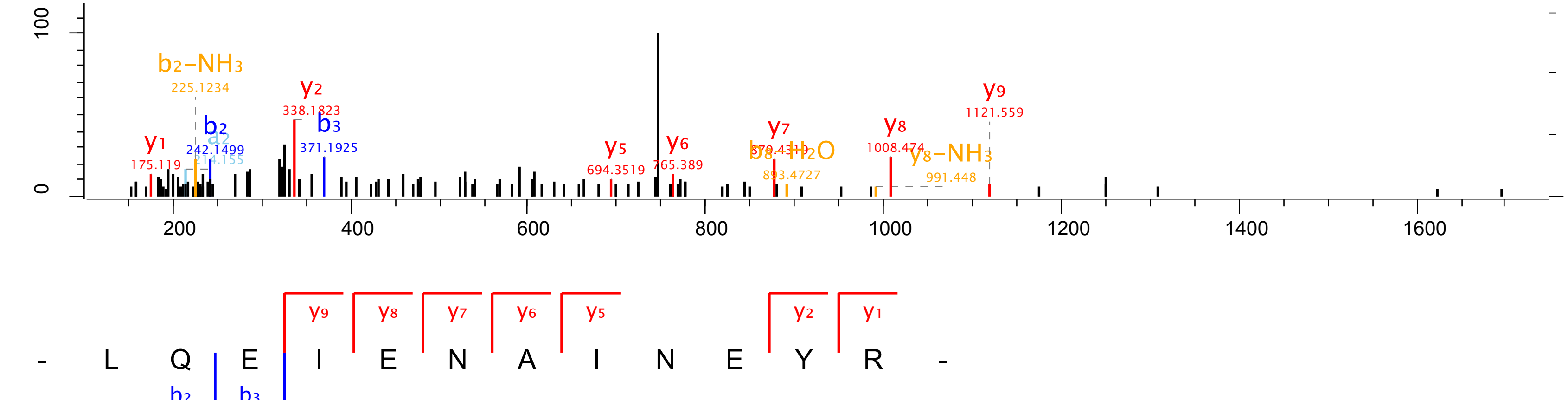

Raw file	Scan	Method	Score	m/z	Gene names
20140925_fract12_dyn_5ul_C4_01_447	24858	TOF; CID	45.56	984.13	DOK1

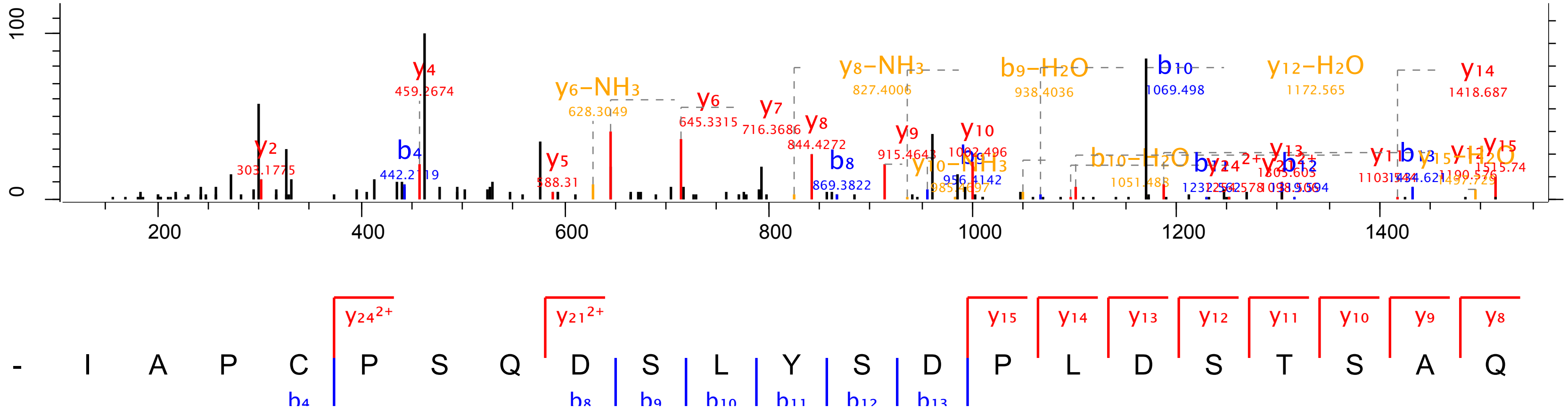

Raw file	Scan	Method	Score	m/z	Gene names
20140925_fract12_dyn_5ul_C4_01_447	24983	TOF; CID	68.85	572.31	ICA1

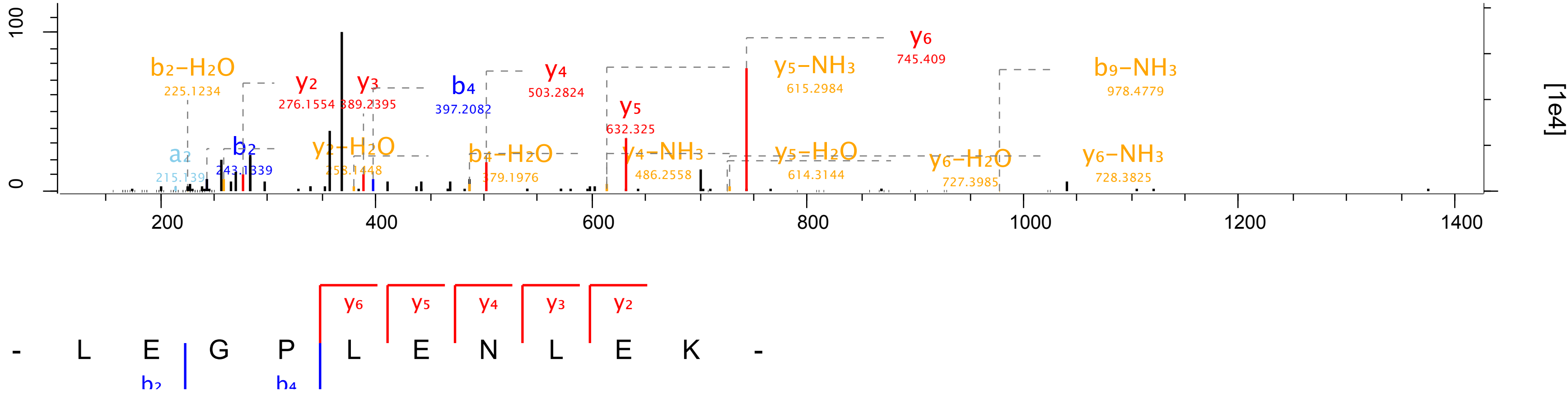

Raw file	Scan	Method	Score	m/z	Gene names
20140925_fract12_dyn_5ul_C4_01_447	25295	TOF; CID	61.11	486.95	TIGD1

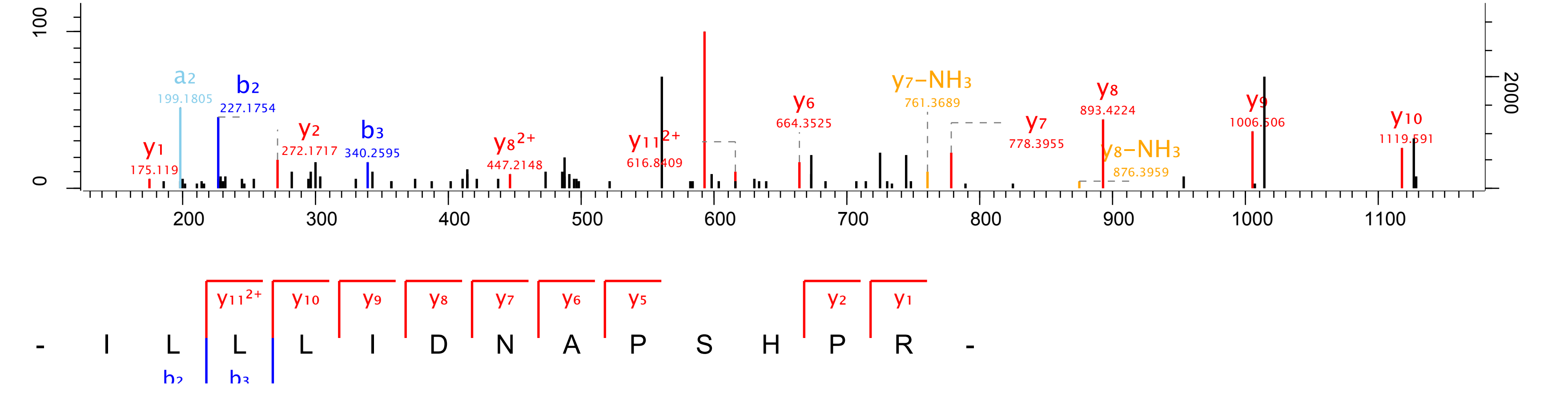

Raw file	Scan	Method	Score	m/z	Gene names
20140925_fract12_dyn_5ul_C4_01_447	29198	TOF; CID	79.69	879.44	RARA

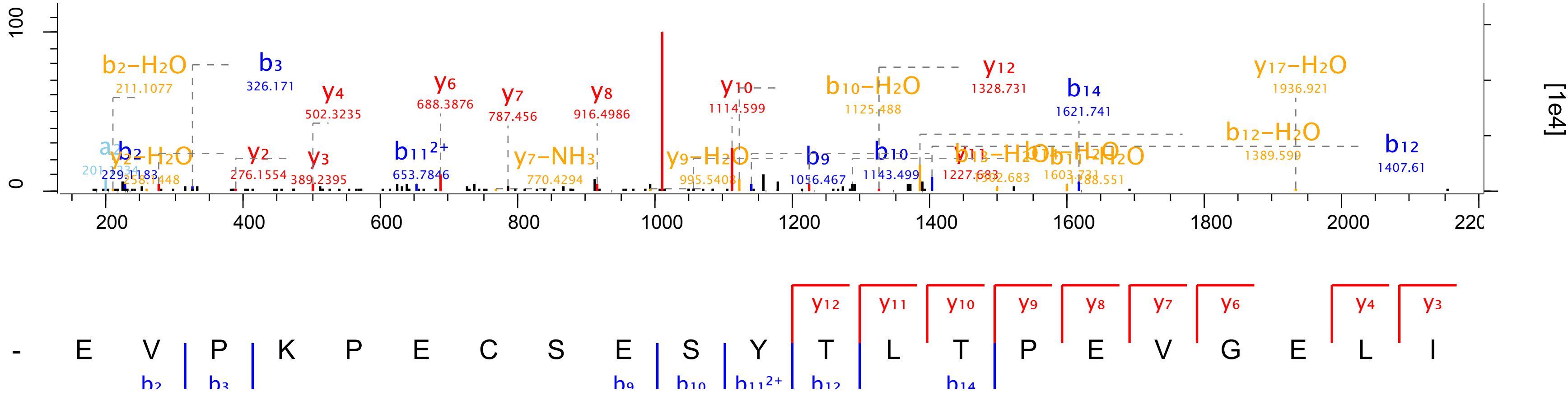

Raw file	Scan	Method	Score	m/z	Gene names
20140925_fract12_dyn_5ul_C4_01_447	30367	TOF; CID	69.72	492.94	TOR1AIP2

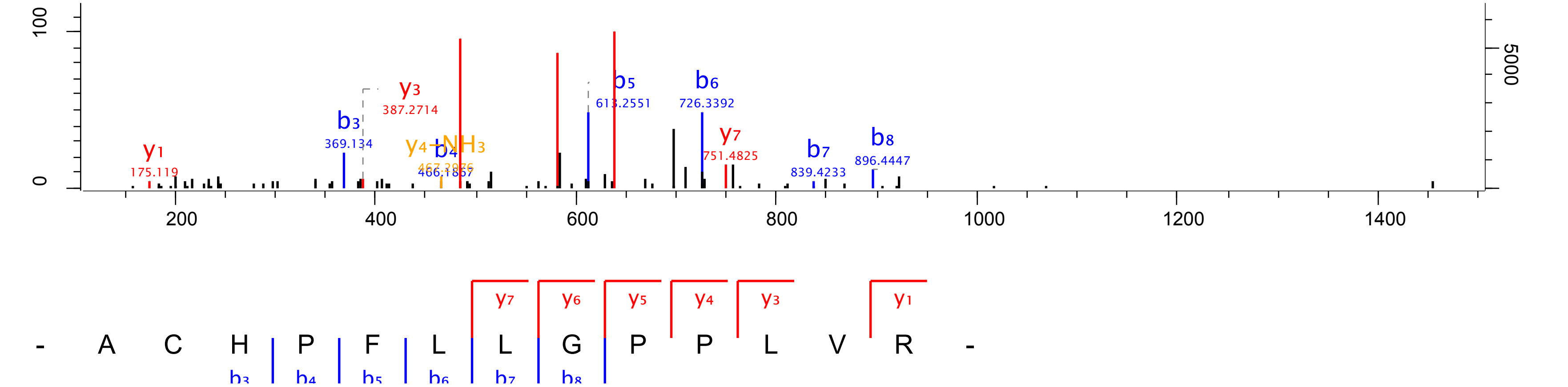

Raw file	Scan	Method	Score	m/z	Gene names
20140925_fract12_dyn_5ul_C4_01_447	31046	TOF; CID	77.22	531.29	DUSP19

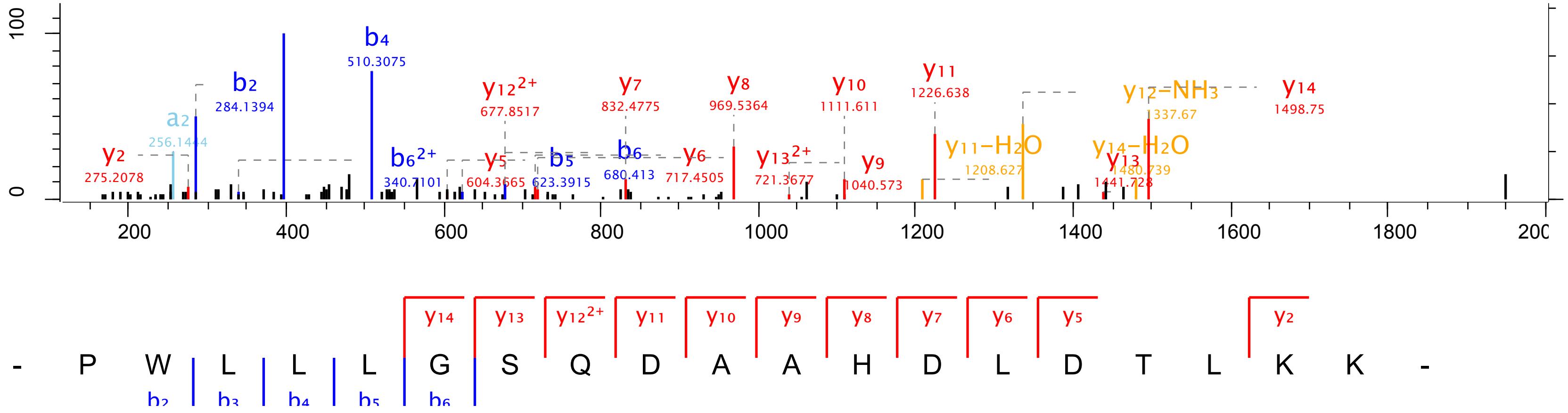

Raw file	Scan	Method	Score	m/z	Gene names
20140925_fract12_dyn_5ul_C4_01_447	31107	TOF; CID	118.51	510.62	TNFSF13

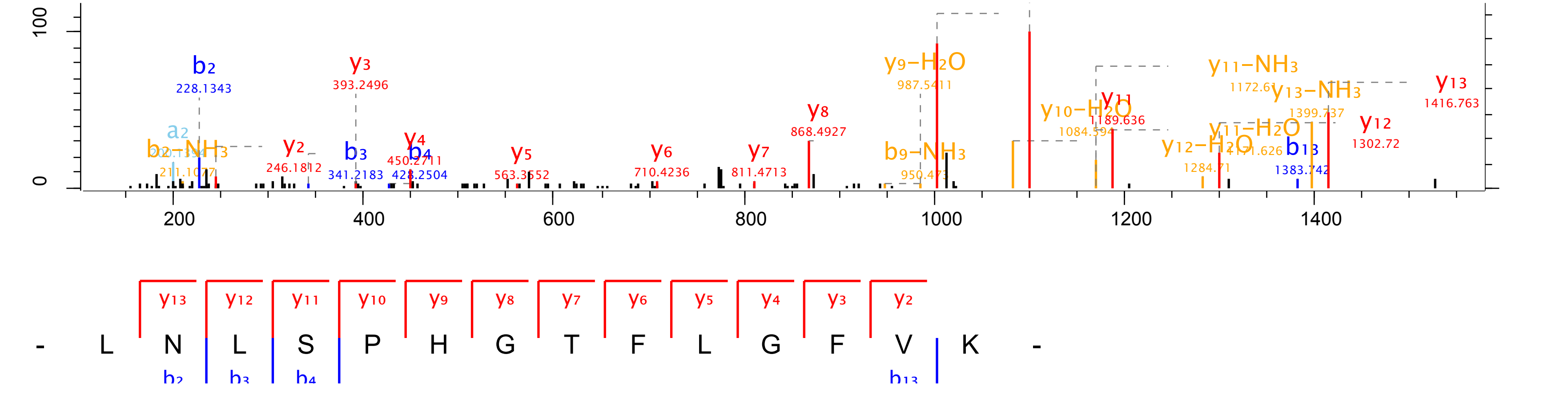

Raw file	Scan	Method	Score	m/z	Gene names
20140925_fract12_dyn_5ul_C4_01_447	34863	TOF; CID	86.56	886.44	SLC39A8

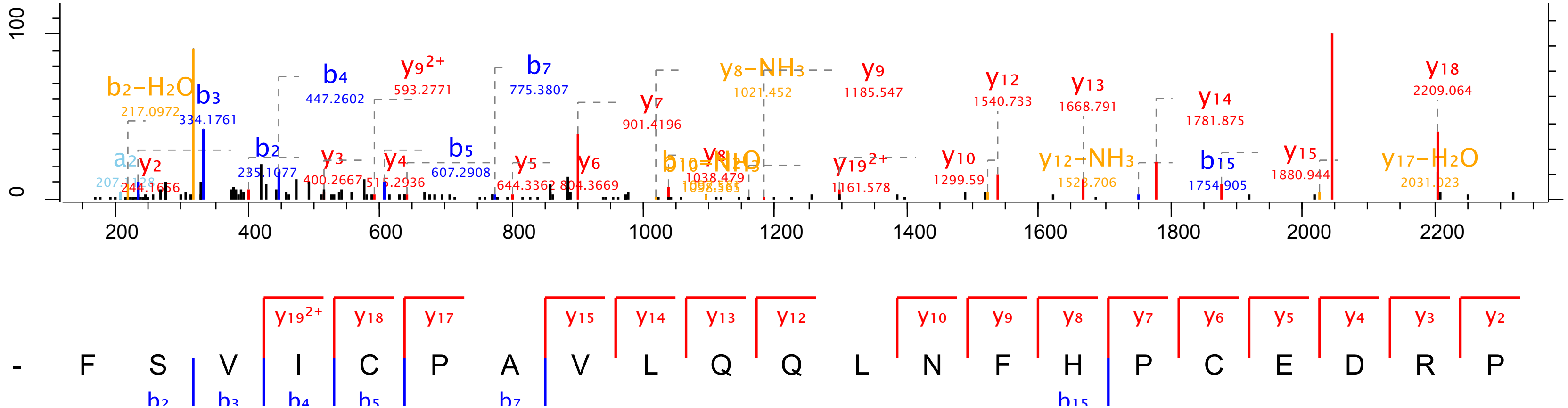

Raw file	Scan	Method	Score	m/z	Gene names
20140925_fract12_dyn_5ul_C4_01_447	34891	TOF; CID	39.96	539.64	CARD8

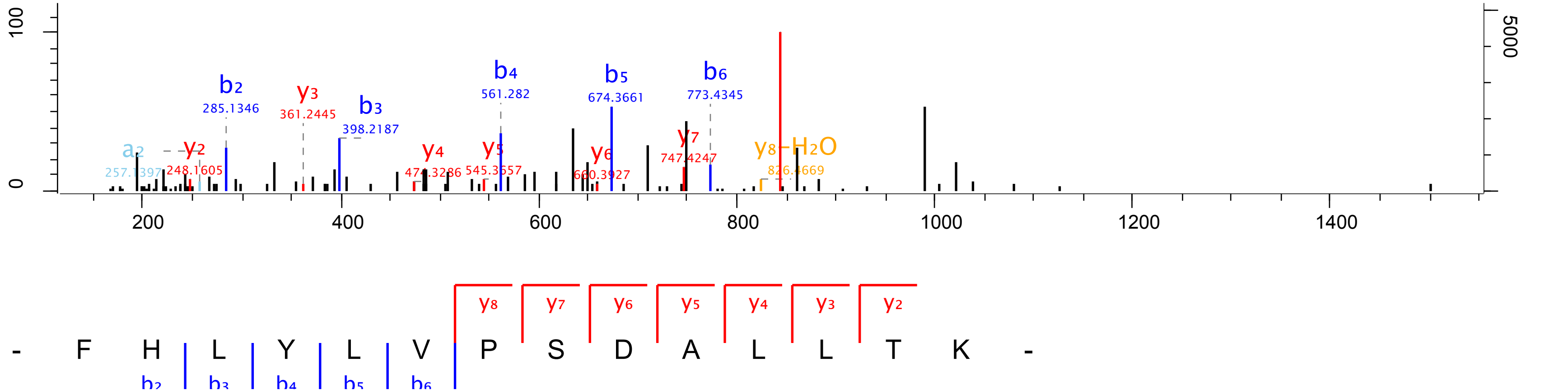

Raw file	Scan	Method	Score	m/z	Gene names
20140925_fract12_dyn_5ul_C4_01_447	36572	TOF; CID	70.06	608.83	PCDH15

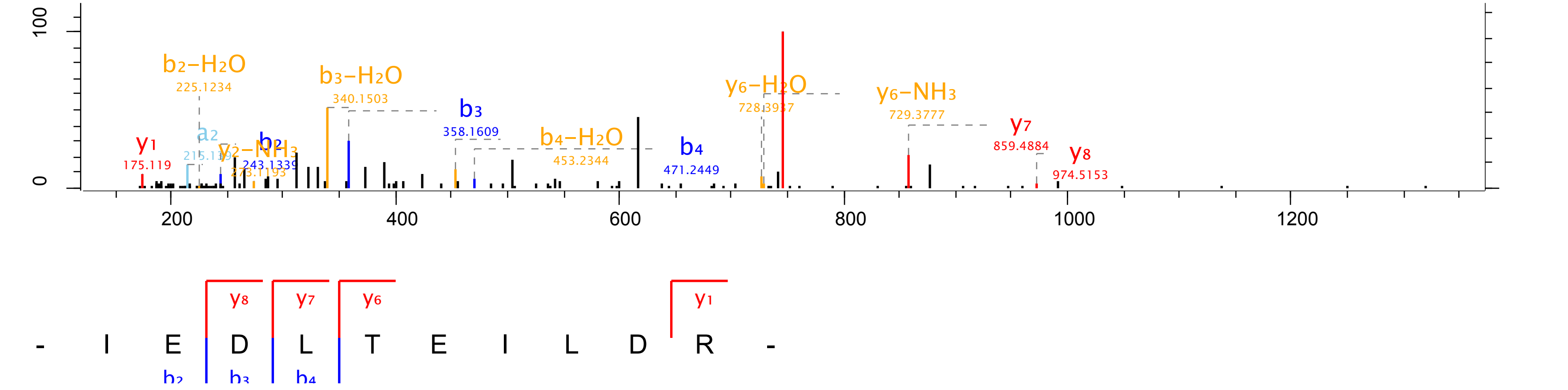

Raw file	Scan	Method	Score	m/z	Gene names
20140925_fract12_dyn_5ul_C4_01_447	37046	TOF; CID	36.13	728.38	S100A12

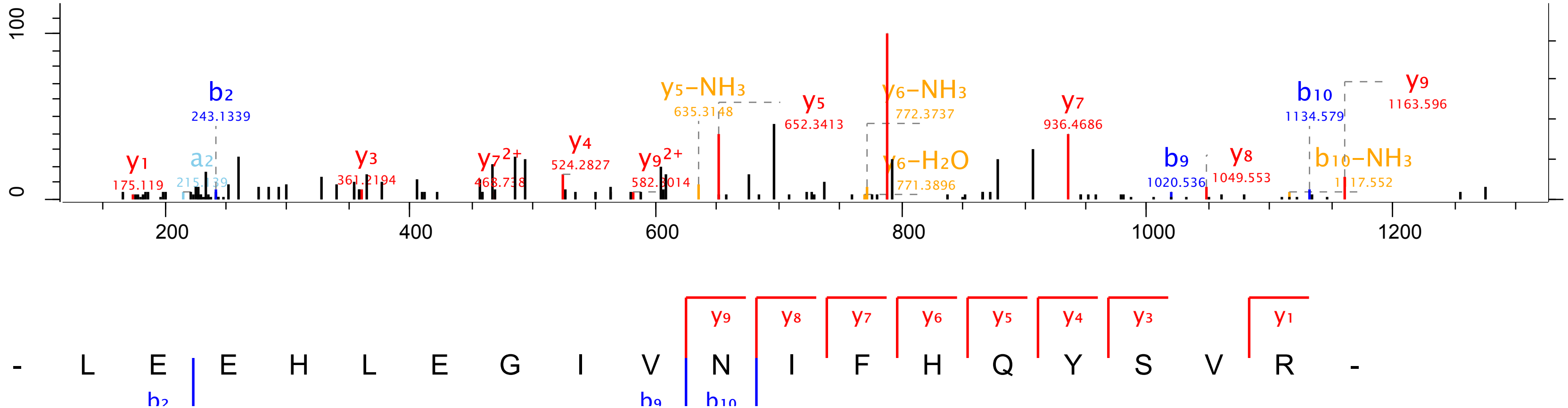

Raw file	Scan	Method	Score	m/z	Gene names
20140925_fract12_dyn_5ul_C4_01_447	37375	TOF; CID	115.68	821.45	ATP6V0D2

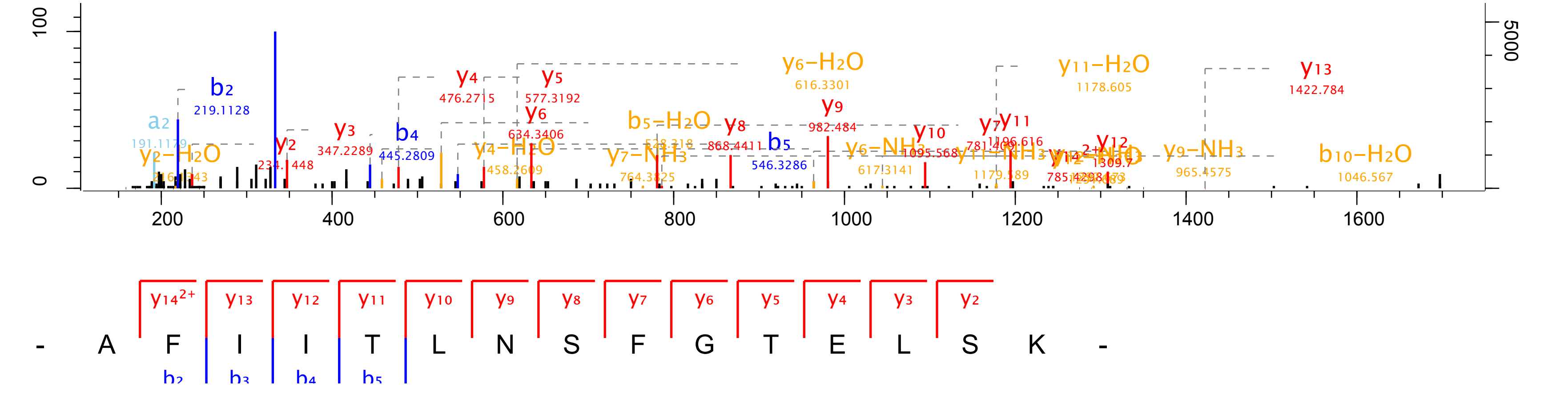

Raw file	Scan	Method	Score	m/z	Gene names
20140925_fract12_dyn_5ul_C4_01_447	38790	TOF; CID	145.46	603.83	ANAPC15

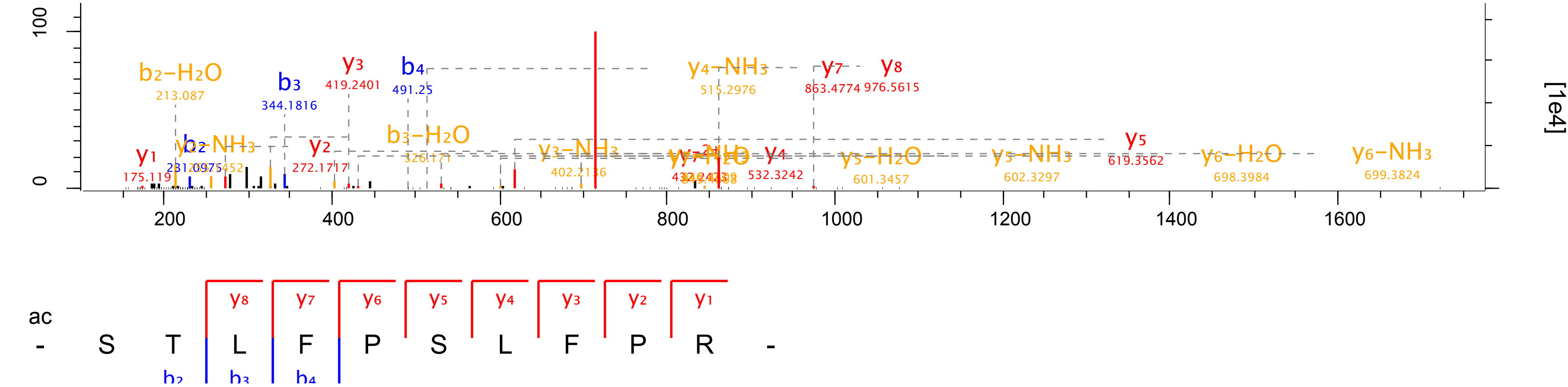

Raw file	Scan	Method	Score	m/z	Gene names
20140925_fract13_dyn_5ul_C5_01_448	8066	TOF; CID	107.65	463.92	ARNTL2

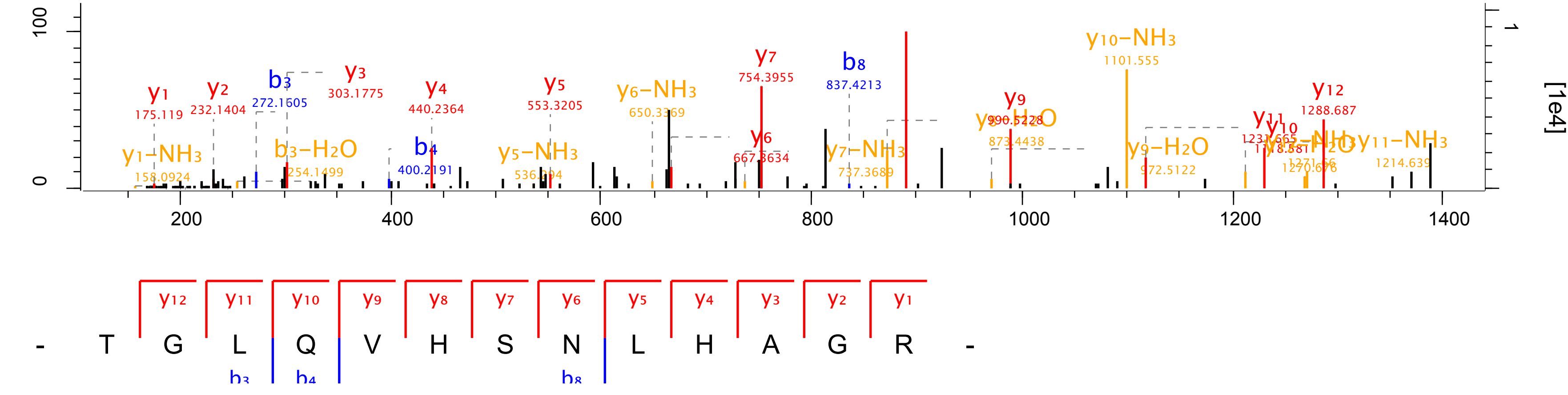

Raw file	Scan	Method	Score	m/z	Gene names
20140925_fract13_dyn_5ul_C5_01_448	10820	TOF; CID	99.3	464.27	GTF2A2

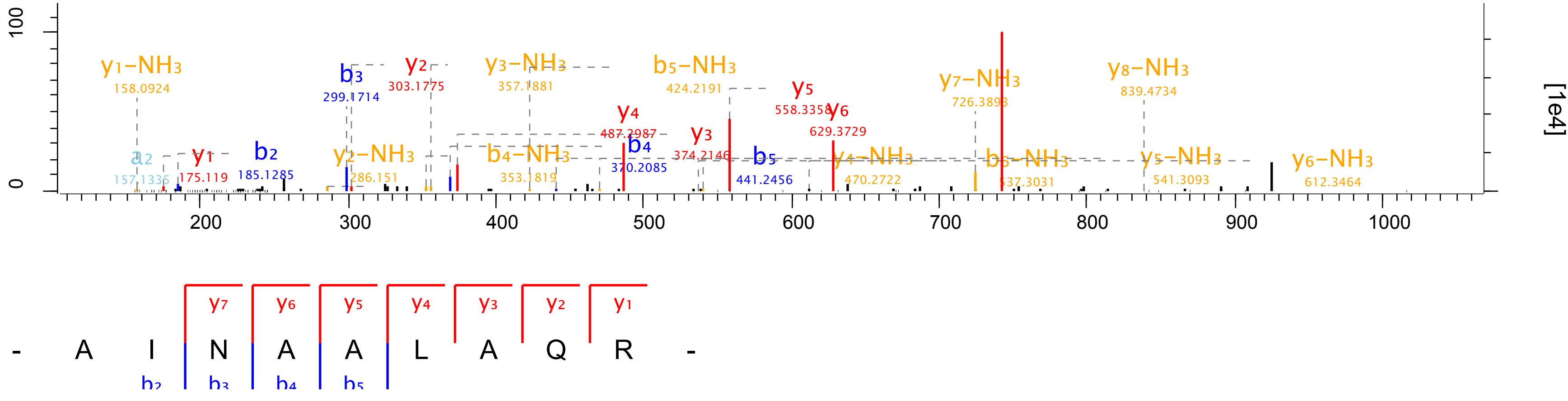

Raw file	Scan	Method	Score	m/z	Gene names
20140925_fract13_dyn_5ul_C5_01_448	13203	TOF; CID	83.21	432.26	FOSL1

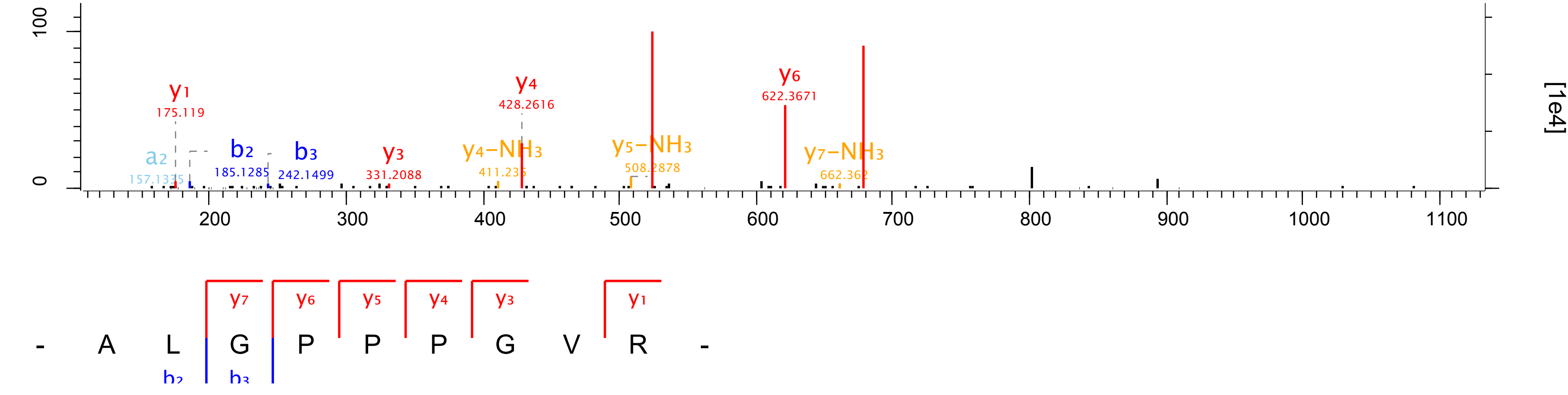

Raw file	Scan	Method	Score	m/z	Gene names
20140925_fract13_dyn_5ul_C5_01_448	19649	TOF; CID	73.83	713.36	YIF1A

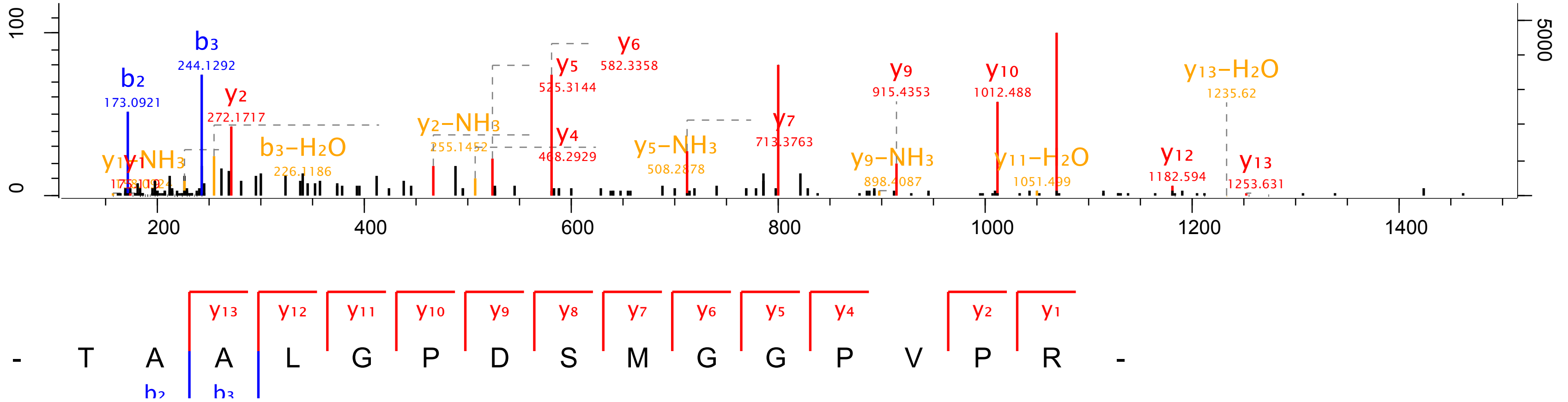

Raw file	Scan	Method	Score	m/z	Gene names
20140925_fract13_dyn_5ul_C5_01_448	20213	TOF; CID	65.42	536.02	IRAK2

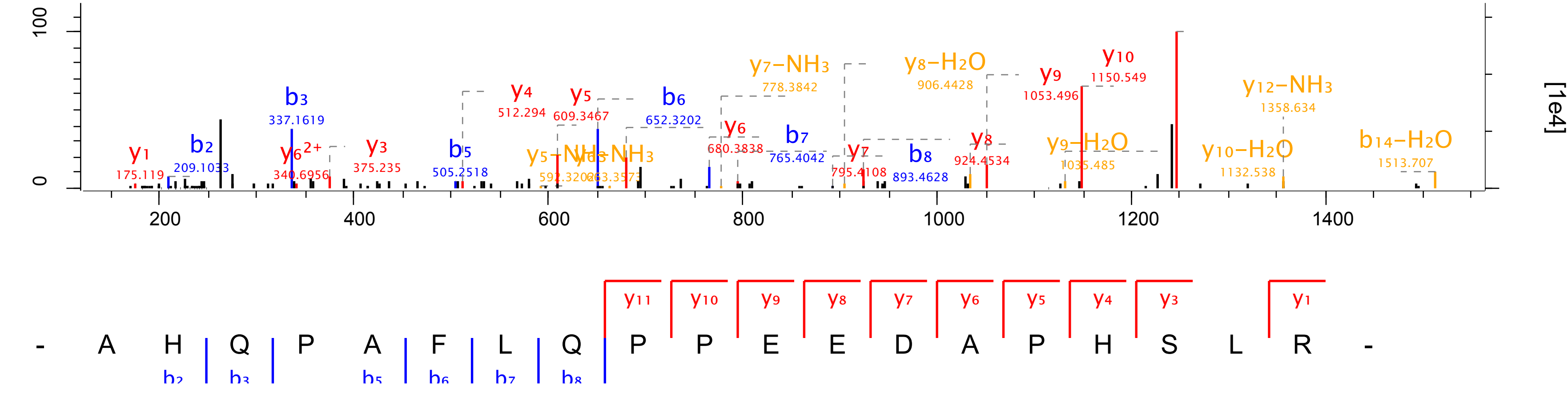

Raw file	Scan	Method	Score	m/z	Gene names
20140925_fract13_dyn_5ul_C5_01_448	21856	TOF; CID	52.87	971.93	LYPD3

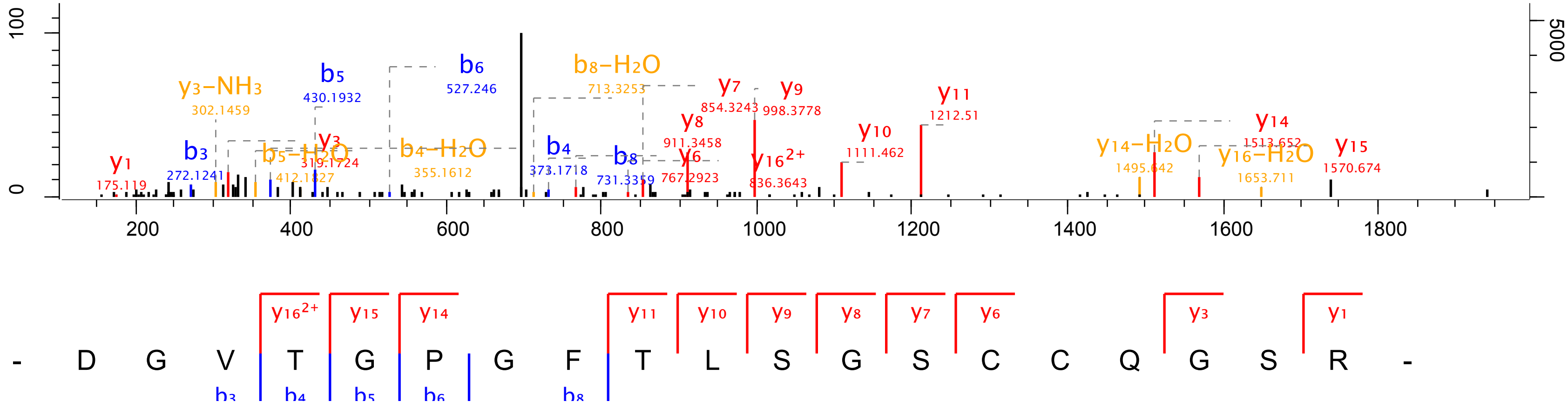

Raw file	Scan	Method	Score	m/z	Gene names
20140925_fract13_dyn_5ul_C5_01_448	22877	TOF; CID	73.39	767.89	PREP

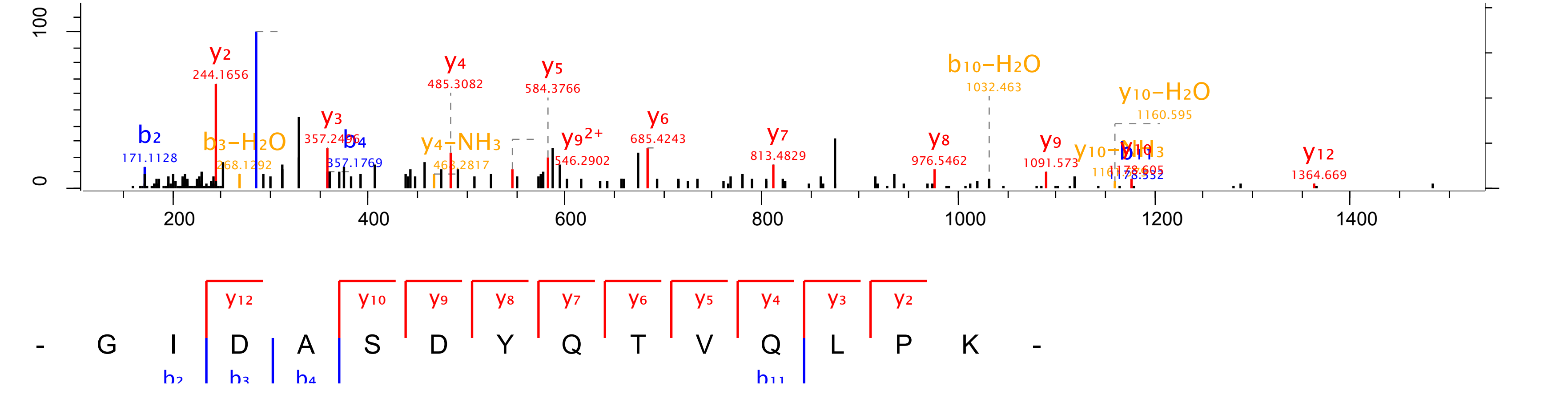

Raw file	Scan	Method	Score	m/z	Gene names
20140925_fract13_dyn_5ul_C5_01_448	22975	TOF; CID	100.76	811.39	ZDHHC2

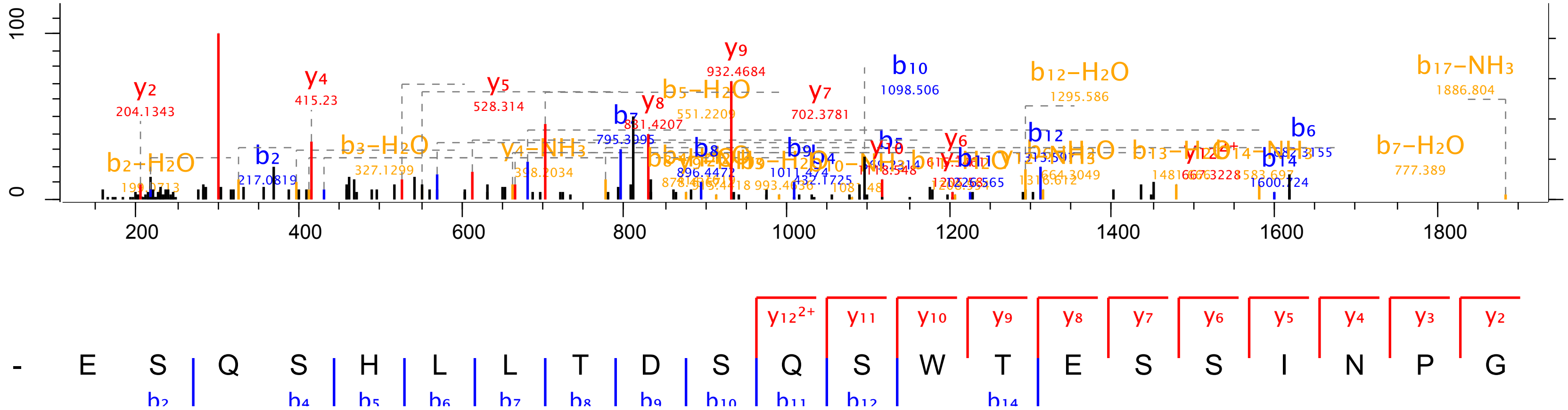

Raw file	Scan	Method	Score	m/z	Gene names
20140925_fract13_dyn_5ul_C5_01_448	23106	TOF; CID	56.37	773.85	TM2D2

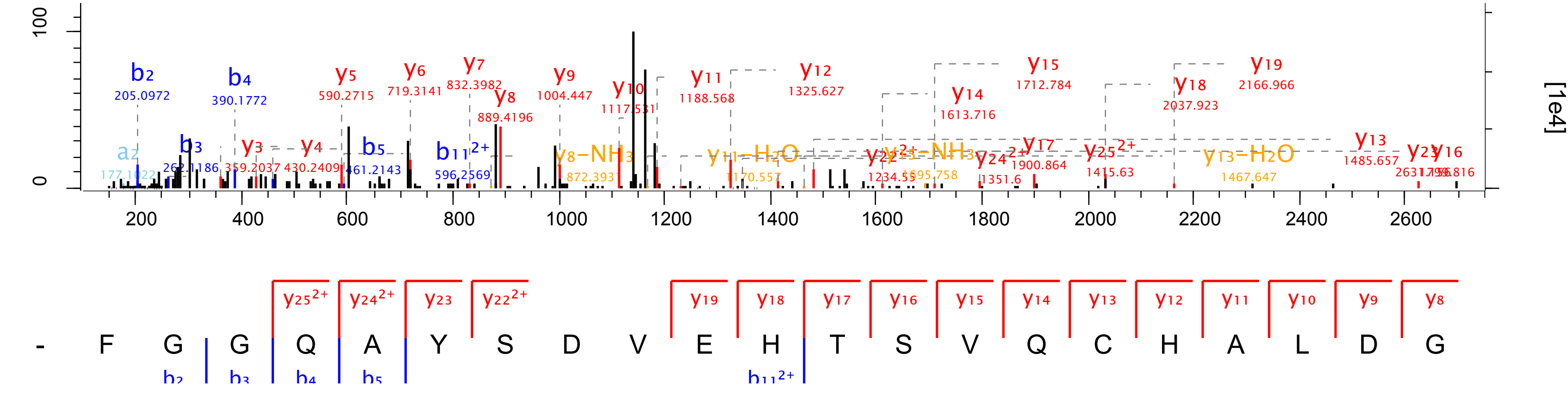

Raw file	Scan	Method	Score	m/z	Gene names
20140925_fract13_dyn_5ul_C5_01_448	26681	TOF; CID	61.11	580.32	GADD45A

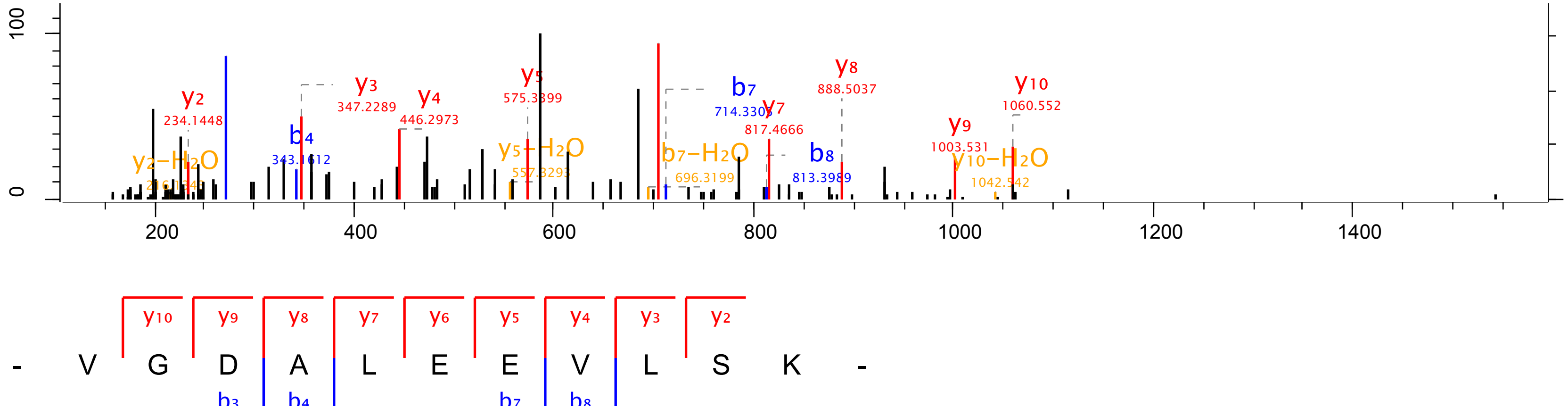

Raw file	Scan	Method	Score	m/z	Gene names
20140925_fract13_dyn_5ul_C5_01_448	28201	TOF; CID	110.6	1001.49	PSEN1

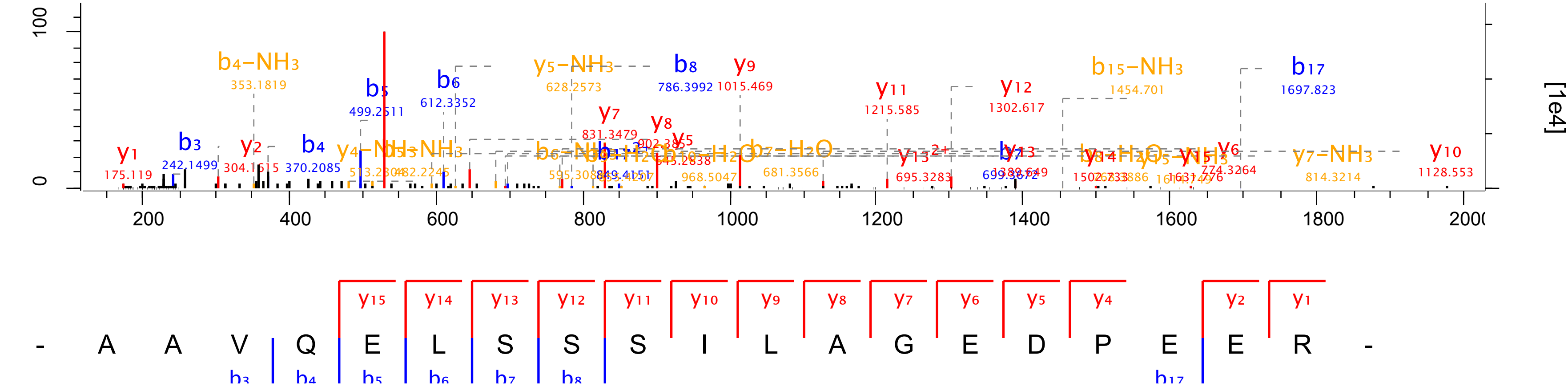

Raw file	Scan	Method	Score	m/z	Gene names
20140925_fract13_dyn_5ul_C5_01_448	32763	TOF; CID	111.94	512.94	TMCO3

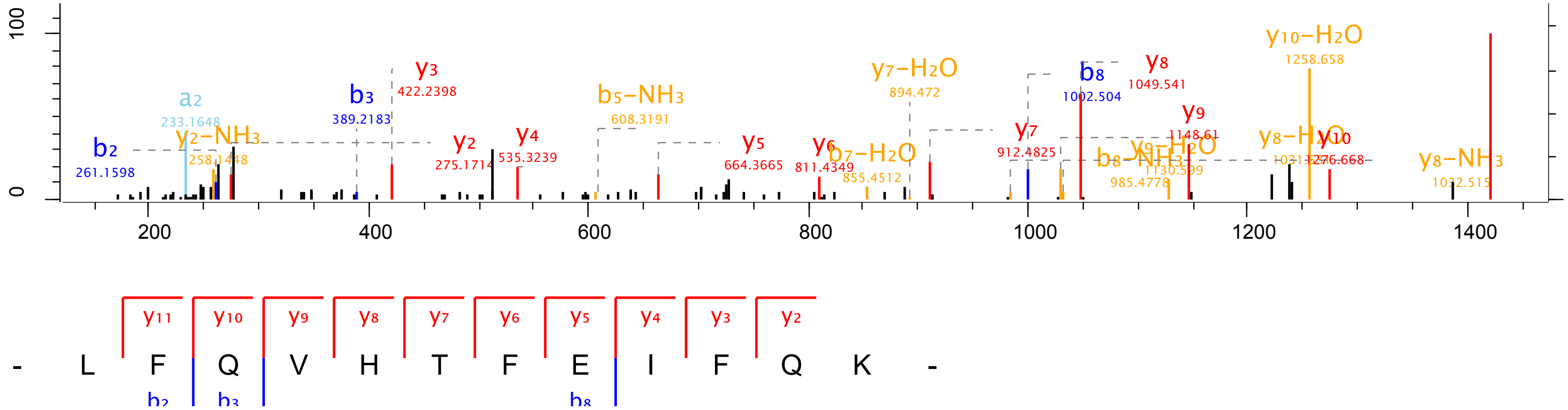

Raw file	Scan	Method	Score	m/z	Gene names
20140925_fract13_dyn_5ul_C5_01_448	33950	TOF; CID	57.61	1025.83	CERK

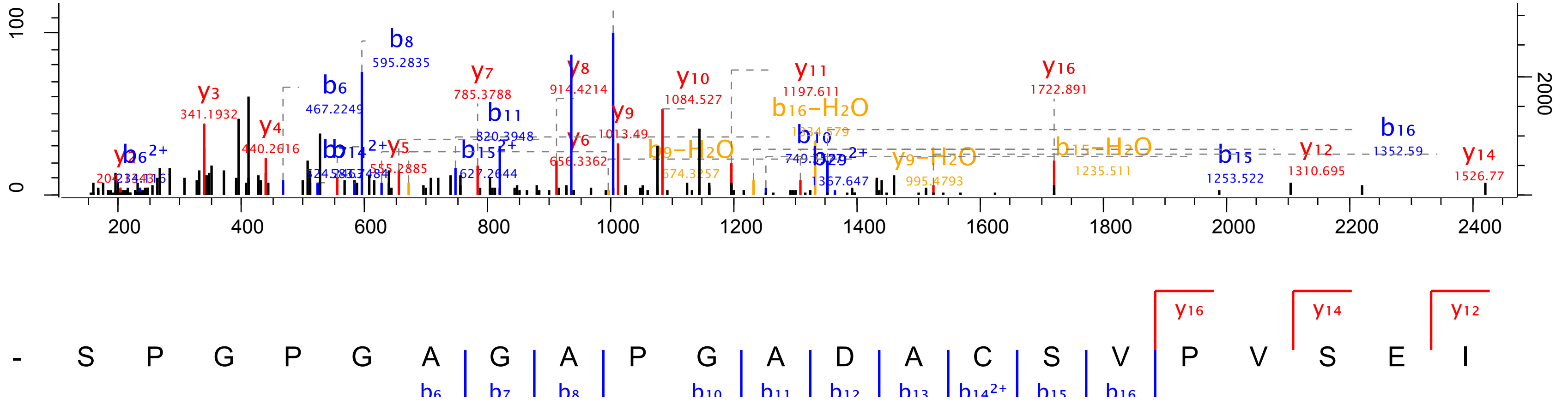

Raw file	Scan	Method	Score	m/z	Gene names
20140925_fract13_dyn_5ul_C5_01_448	34168	TOF; CID	57.11	499.94	METTL4

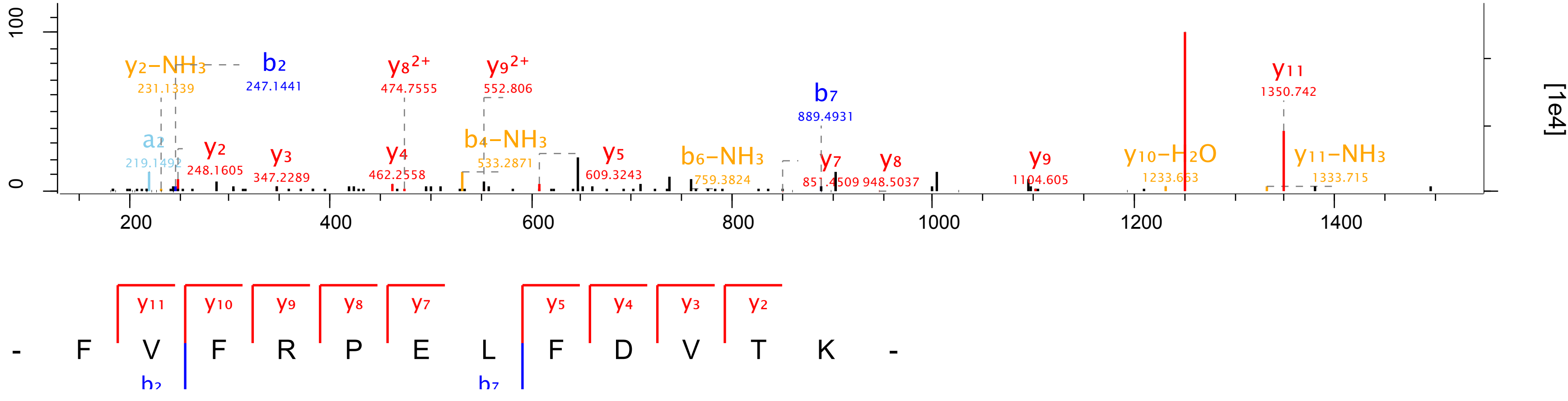

Raw file	Scan	Method	Score	m/z	Gene names
20140925_fract13_dyn_5ul_C5_01_448	36002	TOF; CID	51.94	799.92	SLC8B1

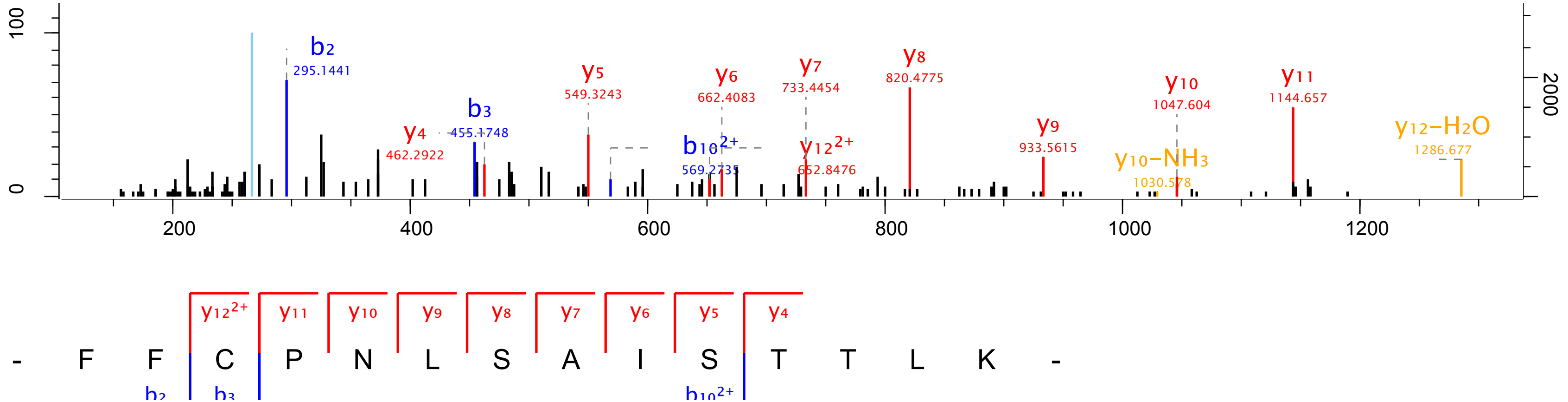

Raw file	Scan	Method	Score	m/z	Gene names
20140925_fract13_dyn_5ul_C5_01_448	36368	TOF; CID	68.75	879.92	TPM4

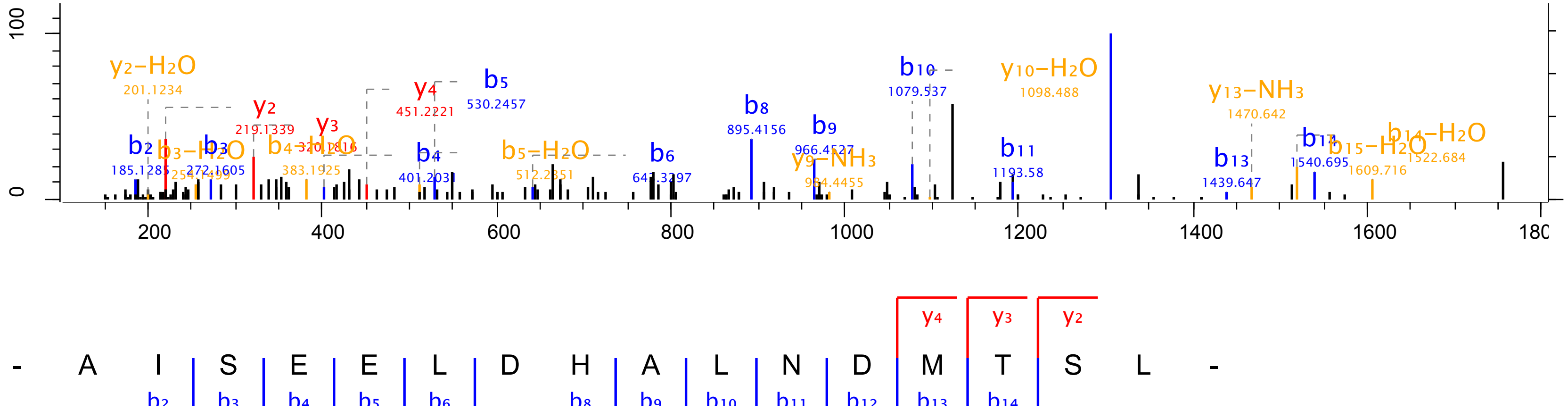

Raw file	Scan	Method	Score	m/z	Gene names
20140925_fract14_dyn_5ul_C6_01_449	8085	TOF; CID	68	521.27	GDF11

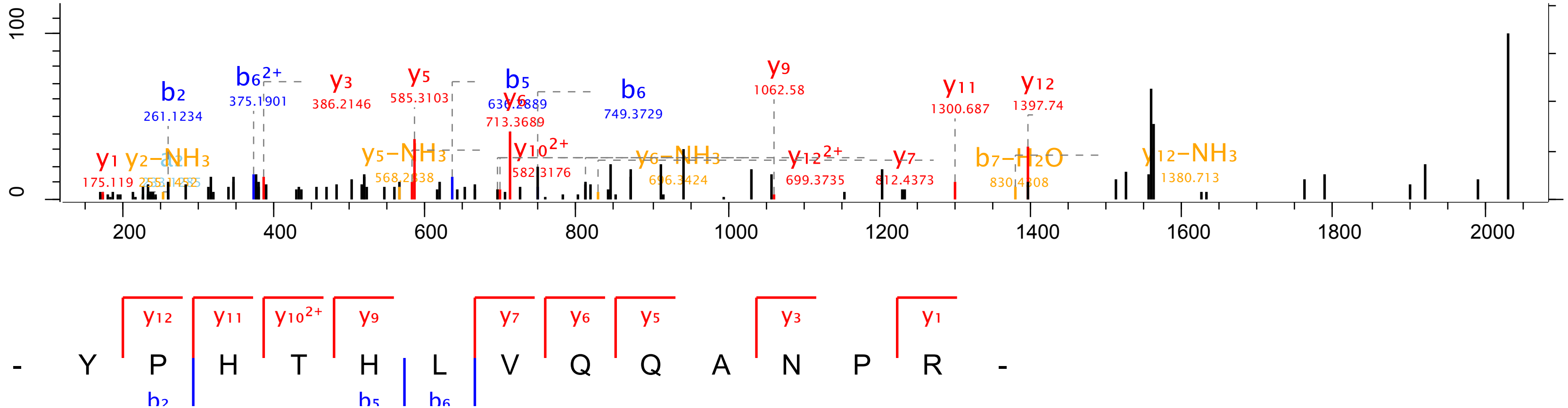

Raw file	Scan	Method	Score	m/z	Gene names
20140925_fract14_dyn_5ul_C6_01_449	9765	TOF; CID	48.9	814.41	BNIP3L

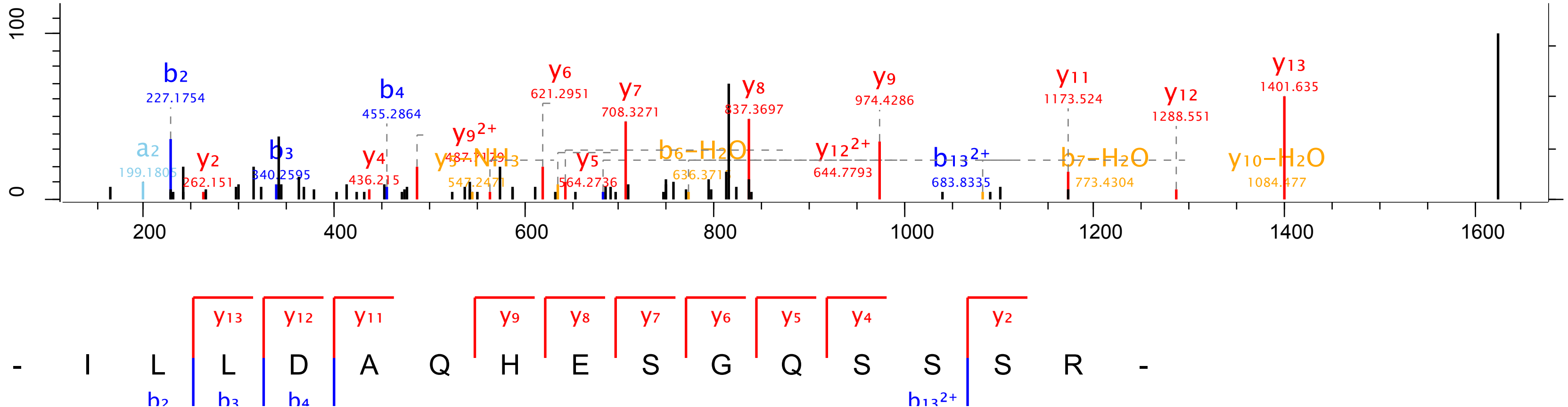

Raw file	Scan	Method	Score	m/z	Gene names
20140925_fract14_dyn_5ul_C6_01_449	11023	TOF; CID	102.51	467.26	GARS

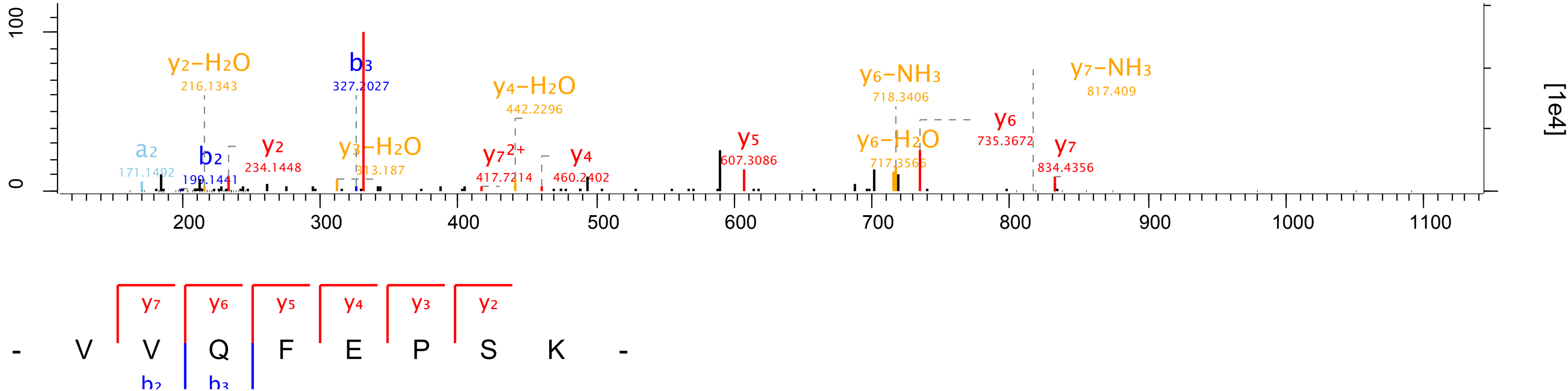

Raw file	Scan	Method	Score	m/z	Gene names
20140925_fract14_dyn_5ul_C6_01_449	12538	TOF; CID	98.9	430.91	DPY19L3

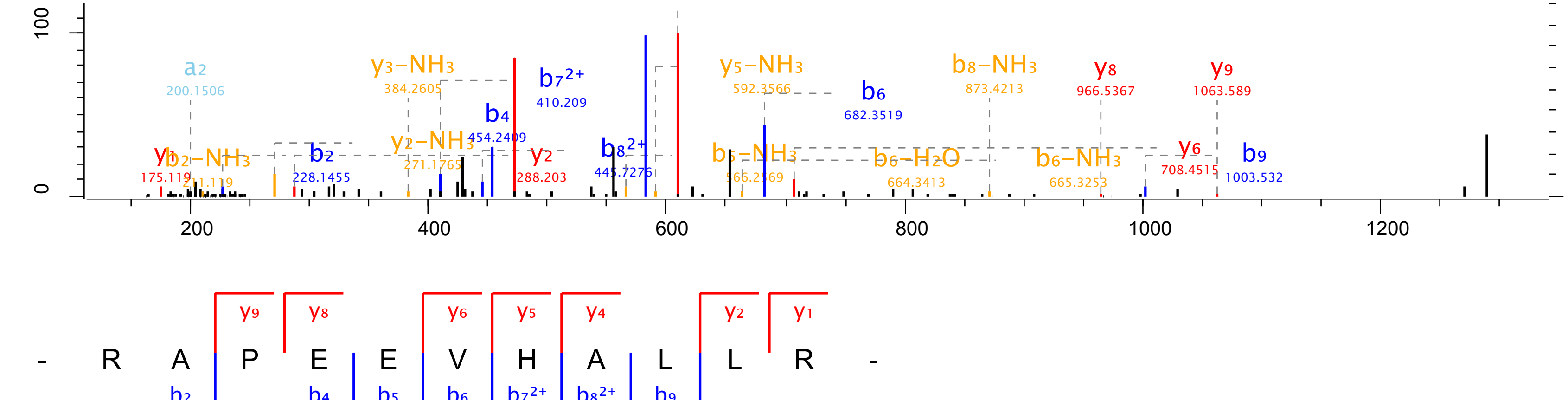

Raw file	Scan	Method	Score	m/z	Gene names
20140925_fract14_dyn_5ul_C6_01_449	15984	TOF; CID	59.71	540.28	SLC25A36

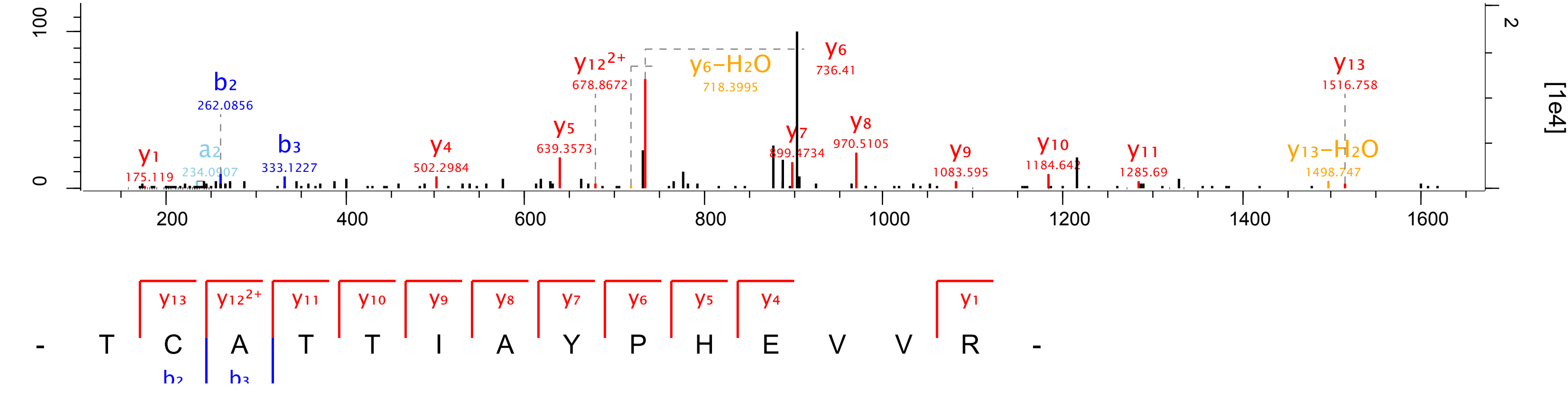

Raw file	Scan	Method	Score	m/z	Gene names
20140925_fract14_dyn_5ul_C6_01_449	16427	TOF; CID	123.75	619.81	PHF19

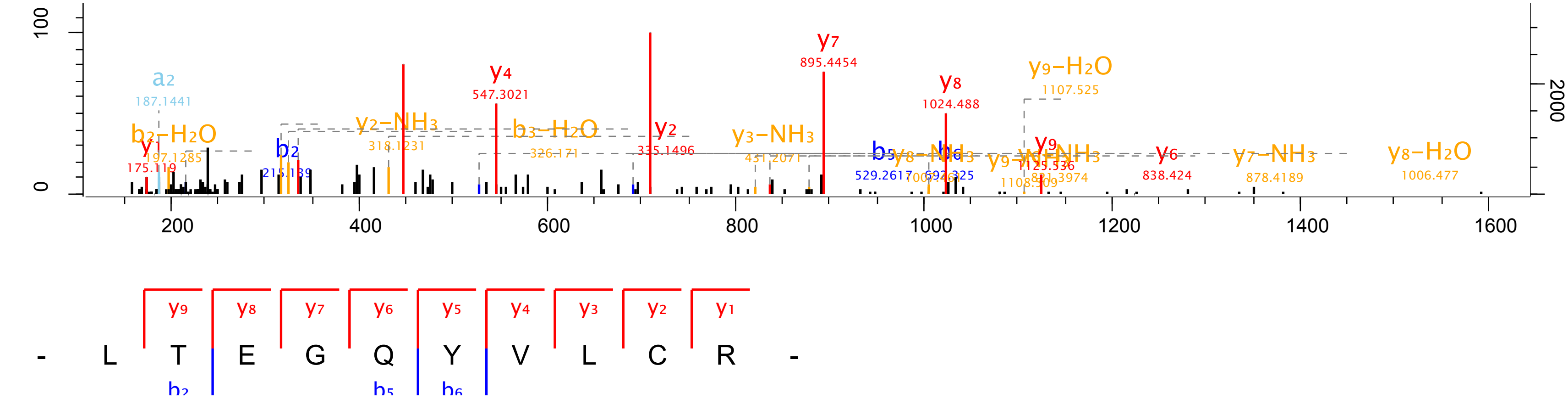

Raw file	Scan	Method	Score	m/z	Gene names
20140925_fract14_dyn_5ul_C6_01_449	18034	TOF; CID	140.14	587.78	MAX

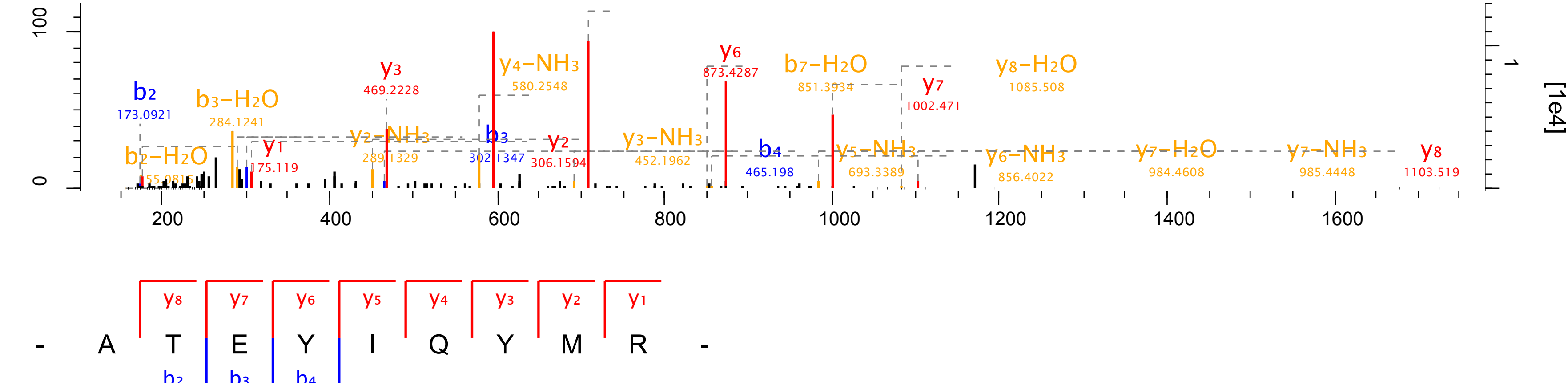

Raw file	Scan	Method	Score	m/z	Gene names
20140925_fract14_dyn_5ul_C6_01_449	22789	TOF; CID	112.44	786.41	NIPA1

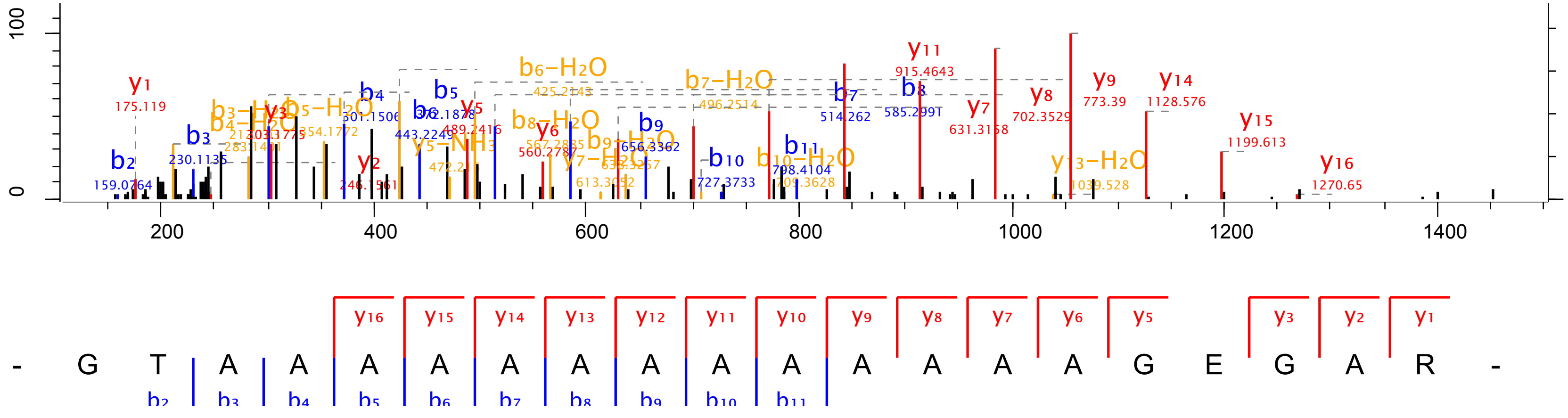

Raw file	Scan	Method	Score	m/z	Gene names
20140925_fract14_dyn_5ul_C6_01_449	23576	TOF; CID	129.82	627.31	KIAA1161

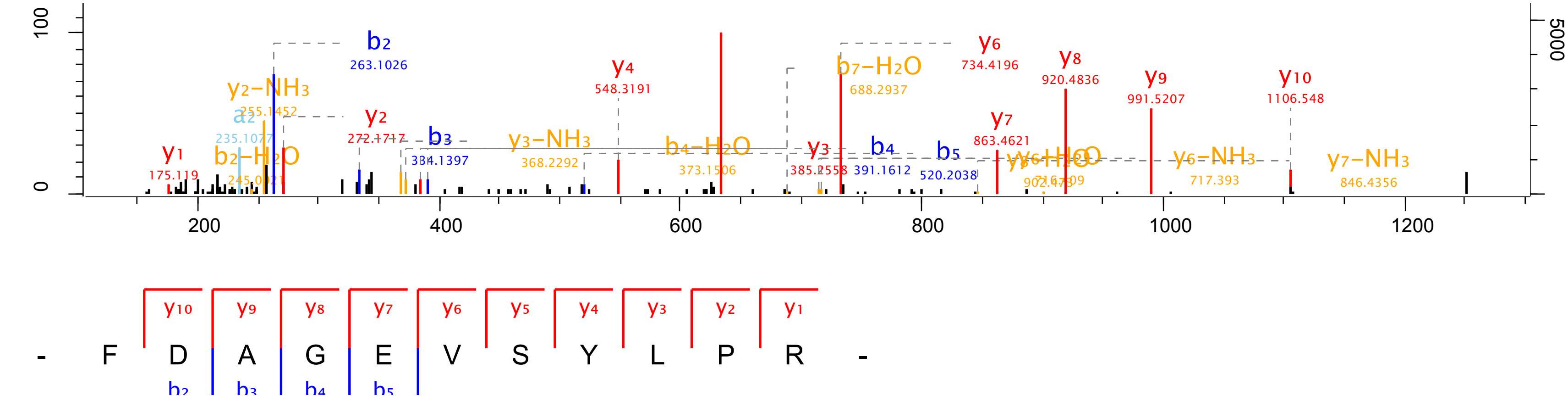

Raw file	Scan	Method	Score	m/z	Gene names
20140925_fract14_dyn_5ul_C6_01_449	24817	TOF; CID	160.47	929.48	HIATL1

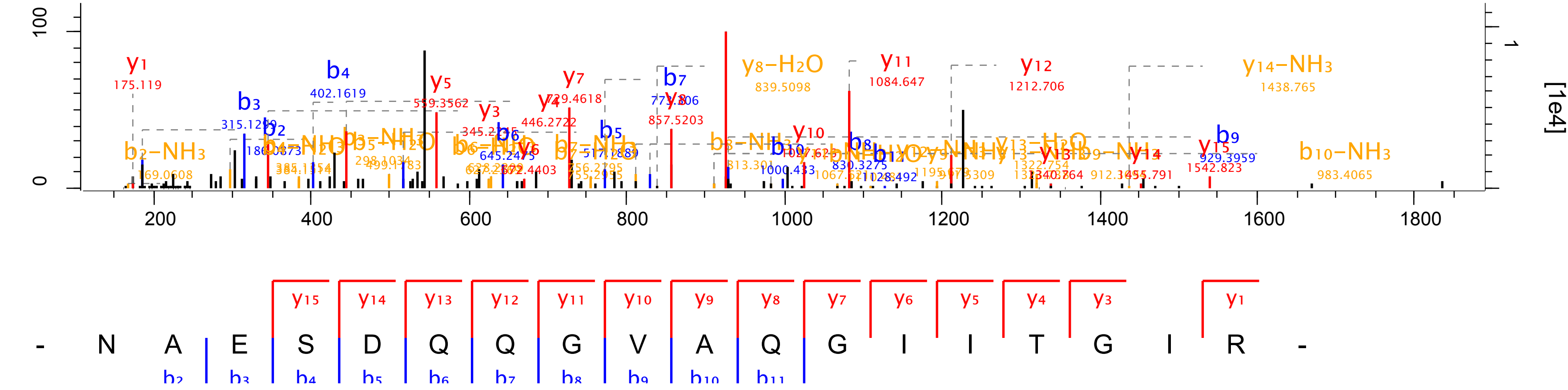

Raw file	Scan	Method	Score	m/z	Gene names
20140925_fract14_dyn_5ul_C6_01_449	25068	TOF; CID	104.42	546.29	D2HGDH

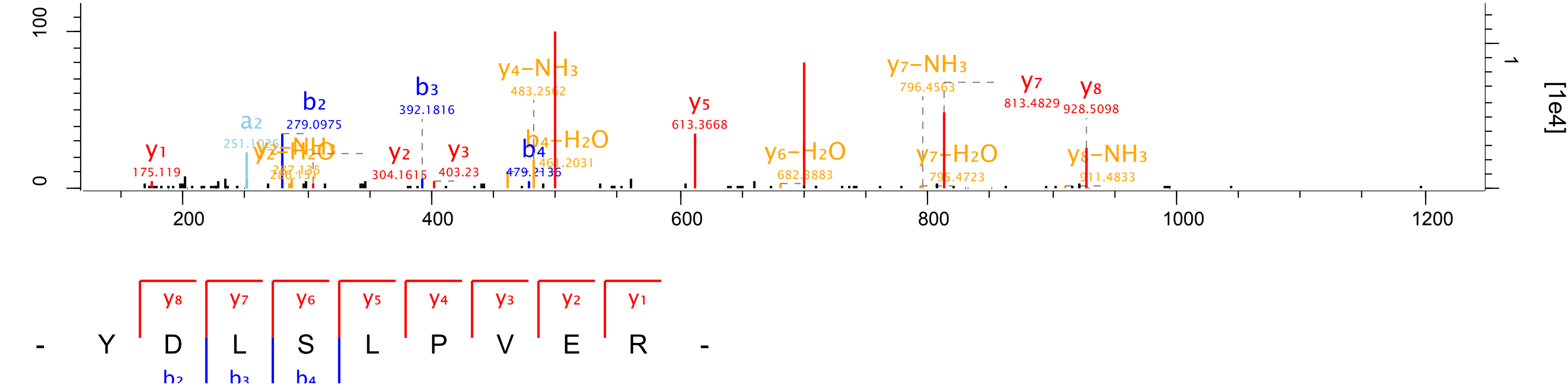

Raw file	Scan	Method	Score	m/z	Gene names
20140925_fract14_dyn_5ul_C6_01_449	25542	TOF; CID	66.07	655	IFT172

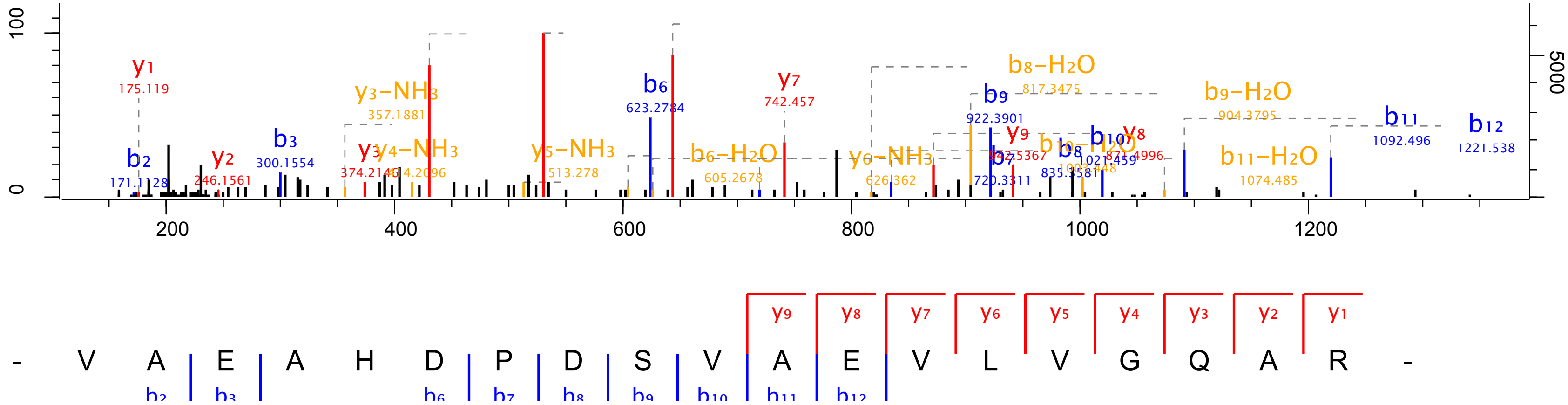

Raw file	Scan	Method	Score	m/z	Gene names
20140925_fract14_dyn_5ul_C6_01_449	25600	TOF; CID	74.99	672.36	SPATA13

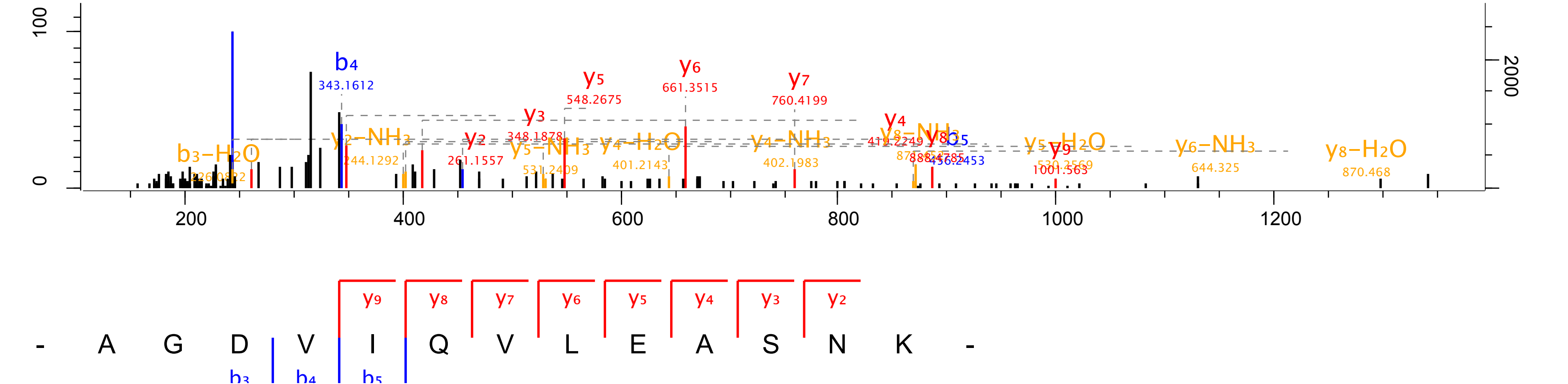

Raw file	Scan	Method	Score	m/z	Gene names
20140925_fract14_dyn_5ul_C6_01_449	27929	TOF; CID	125.82	575.3	JOSD1

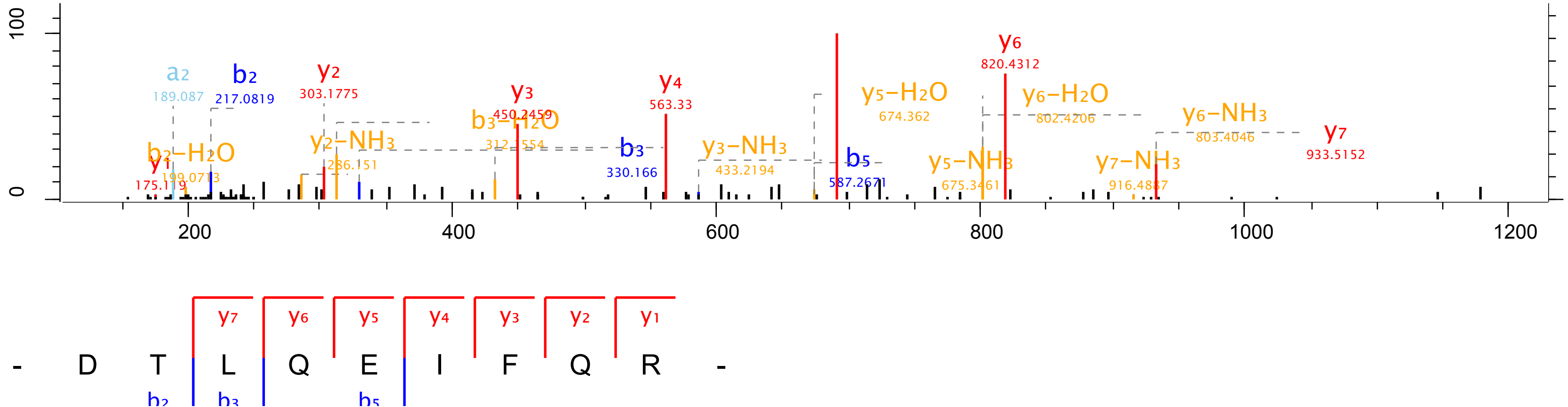

Raw file	Scan	Method	Score	m/z	Gene names
20140925_fract14_dyn_5ul_C6_01_449	30669	TOF; CID	87.2	813.9	PAG1

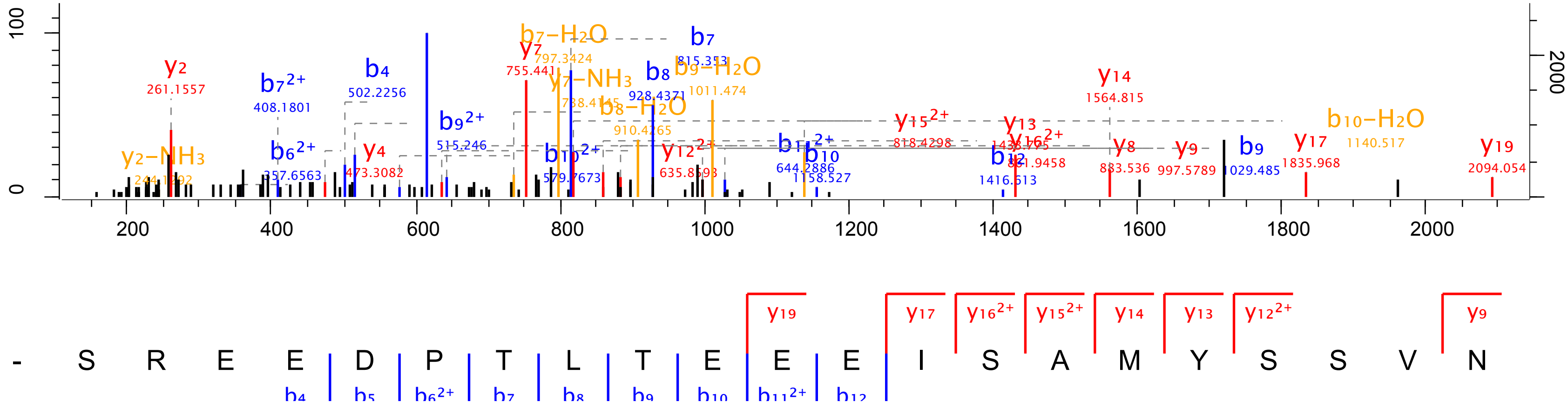

Raw file	Scan	Method	Score	m/z	Gene names
20140925_fract14_dyn_5ul_C6_01_449	31563	TOF; CID	106.14	856.38	MSANTD4

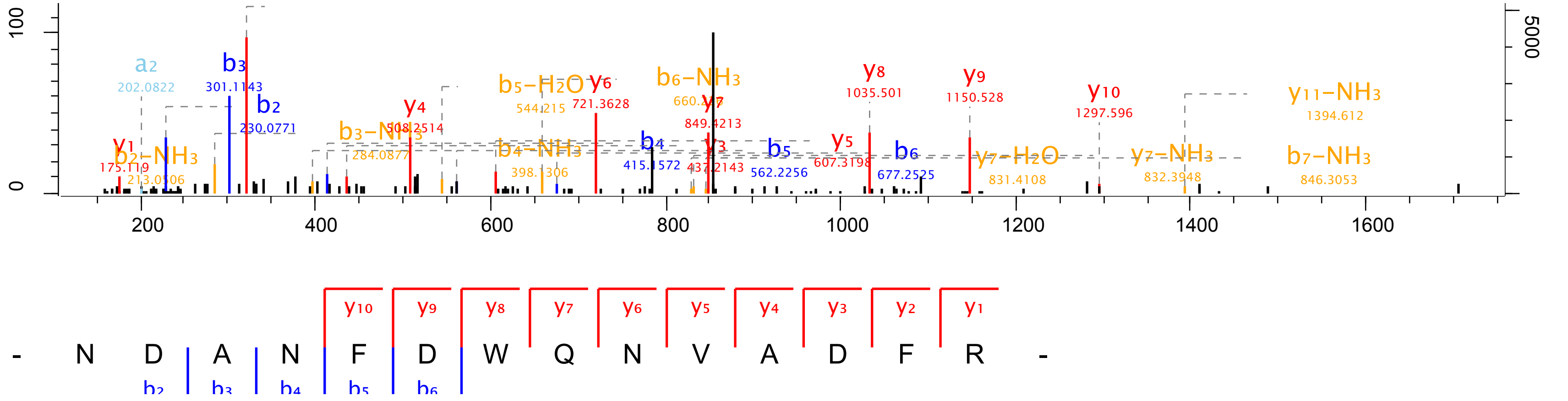

Raw file	Scan	Method	Score	m/z	Gene names
20140925_fract14_dyn_5ul_C6_01_449	33308	TOF; CID	66.81	646.35	NFE2L2

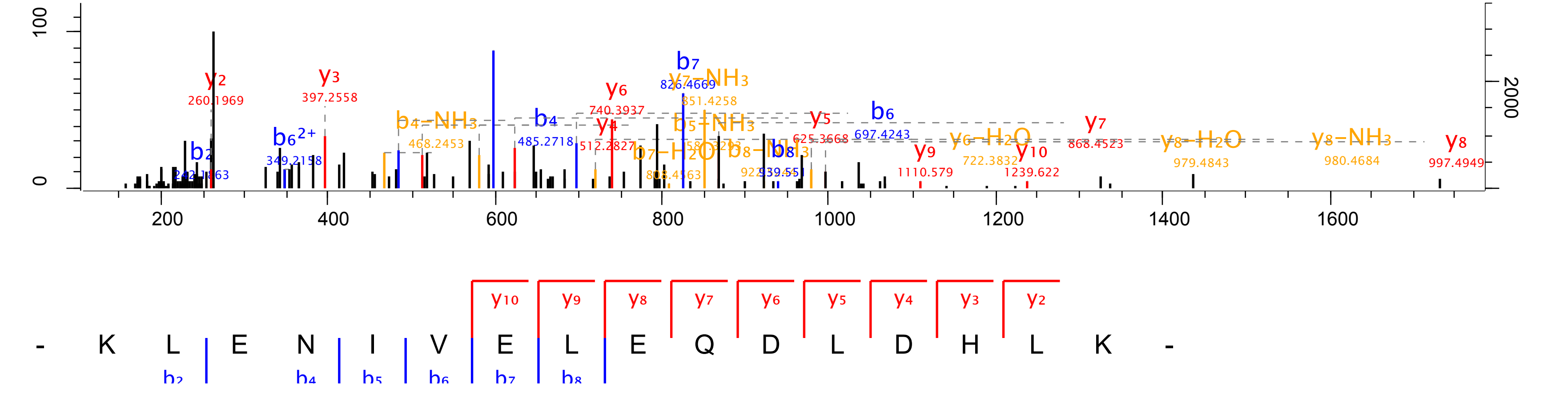

Raw file	Scan	Method	Score	m/z	Gene names
20140925_fract14_dyn_5ul_C6_01_449	37146	TOF; CID	94.77	588.36	KREMEN2

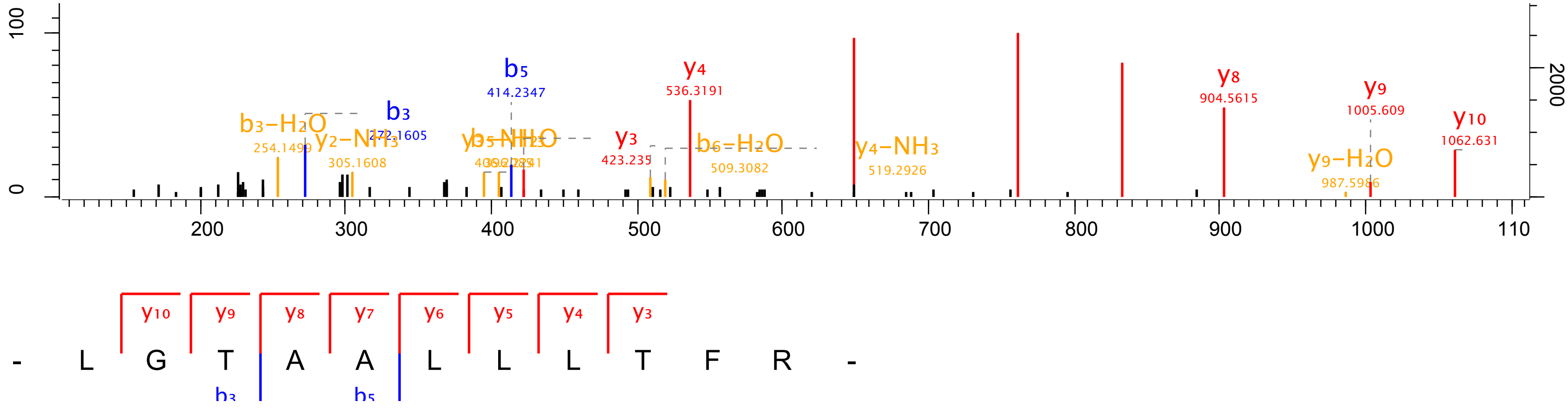

Raw file	Scan	Method	Score	m/z	Gene names
20140925_fract14_dyn_5ul_C6_01_449	38602	TOF; CID	128.74	620.02	GIPC2

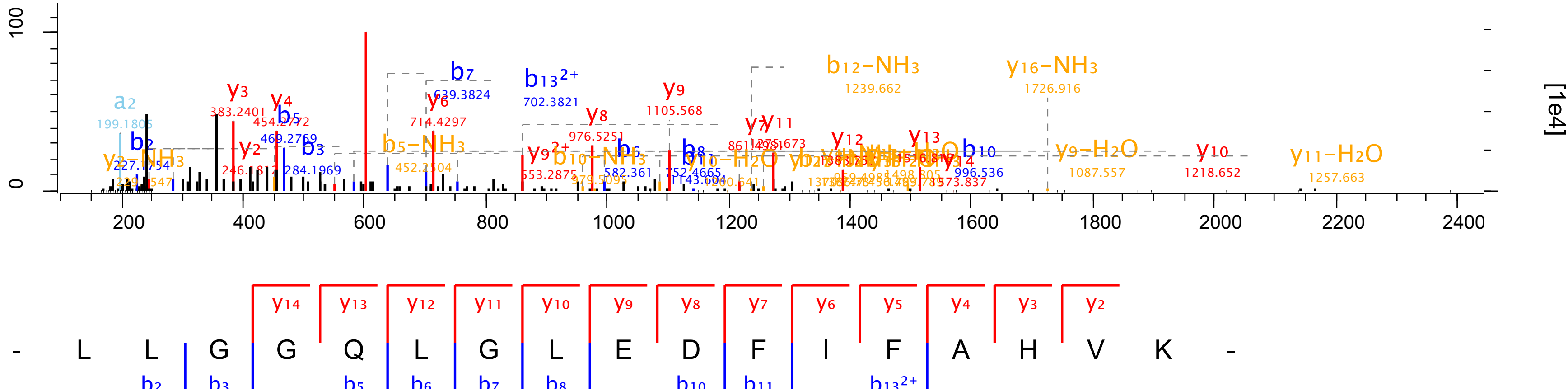

Raw file	Scan	Method	Score	m/z	Gene names
20140925_fract15_dyn_5ul_C7_01_450	5331	TOF; CID	78.55	519.94	ZNF250

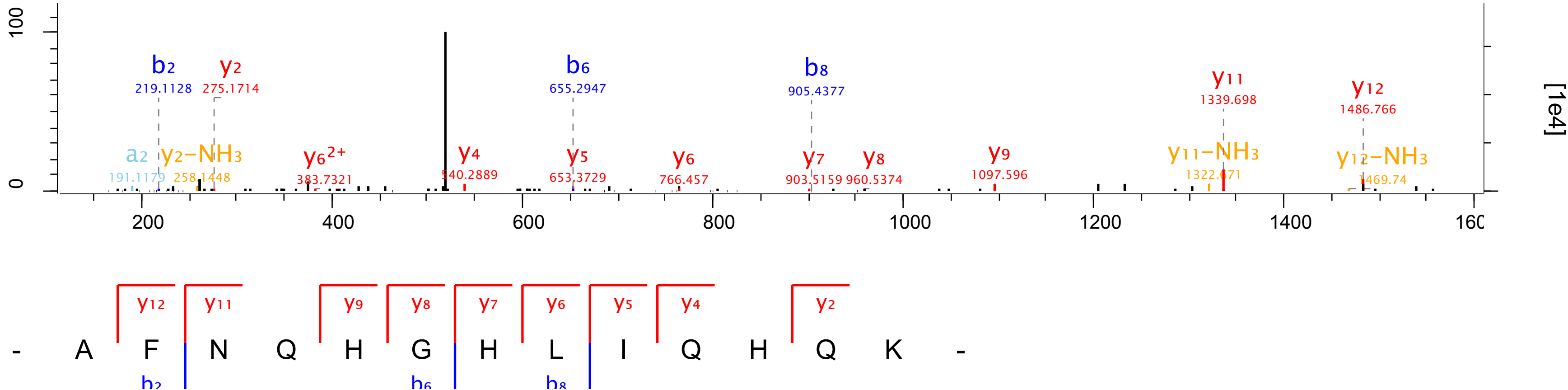

Raw file	Scan	Method	Score	m/z	Gene names
20140925_fract15_dyn_5ul_C7_01_450	6724	TOF; CID	72.68	417.22	SPATA33

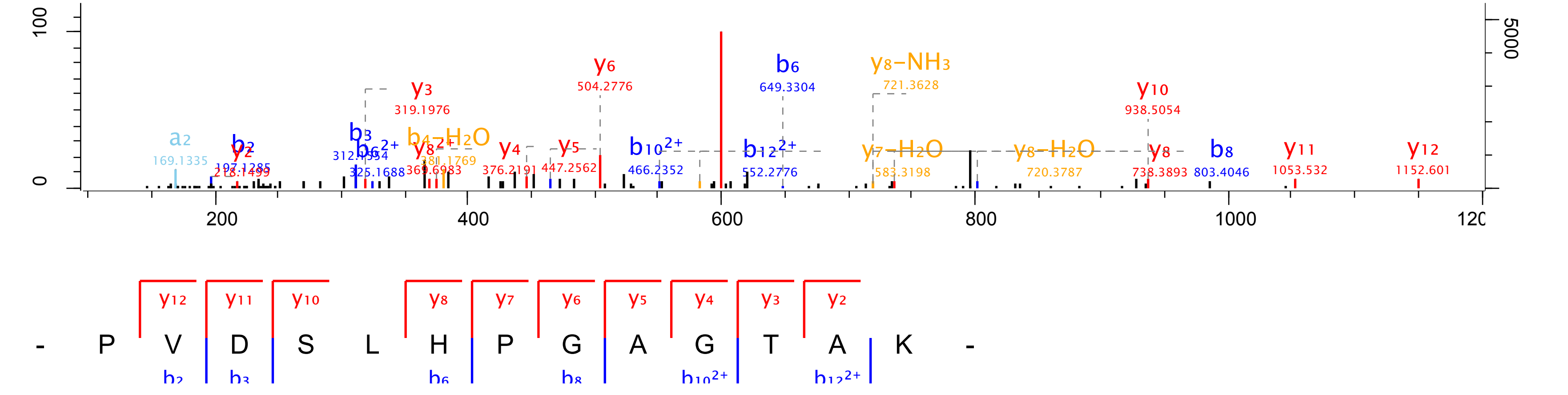

Raw file	Scan	Method	Score	m/z	Gene names
20140925_fract15_dyn_5ul_C7_01_450	10271	TOF; CID	71.08	613.83	TMC7

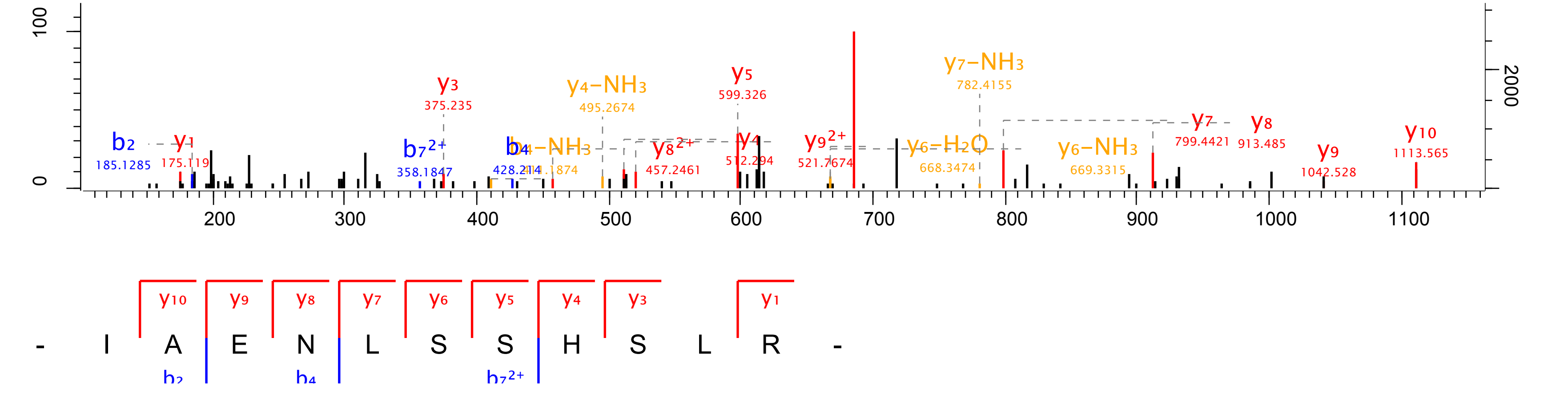

Raw file	Scan	Method	Score	m/z	Gene names
20140925_fract15_dyn_5ul_C7_01_450	10433	TOF; CID	130.01	413.55	BNIP3

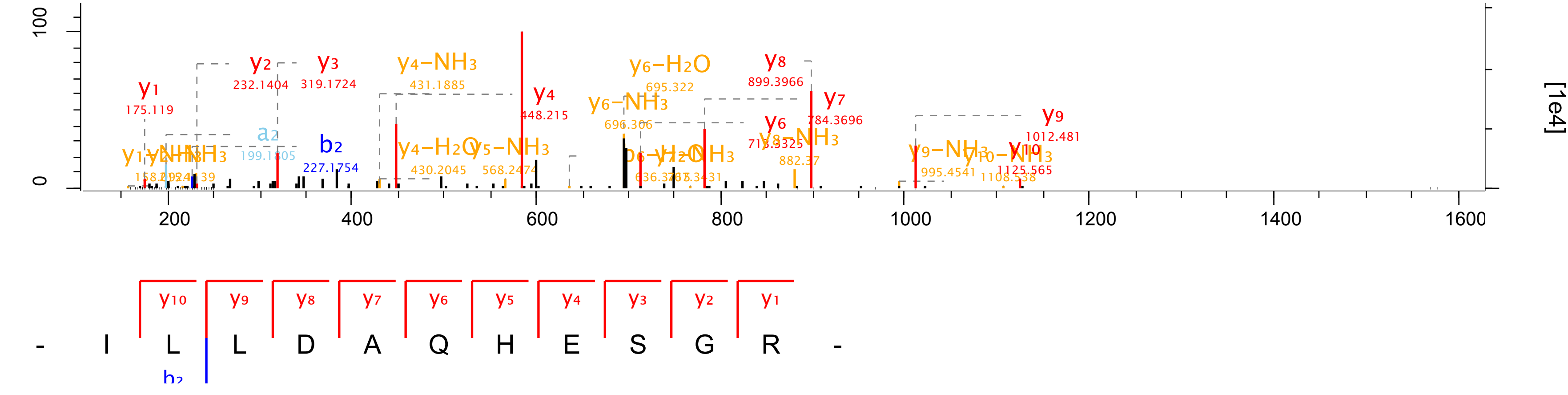

Raw file	Scan	Method	Score	m/z	Gene names
20140925_fract15_dyn_5ul_C7_01_450	13702	TOF; CID	67.42	817.94	FRAT2

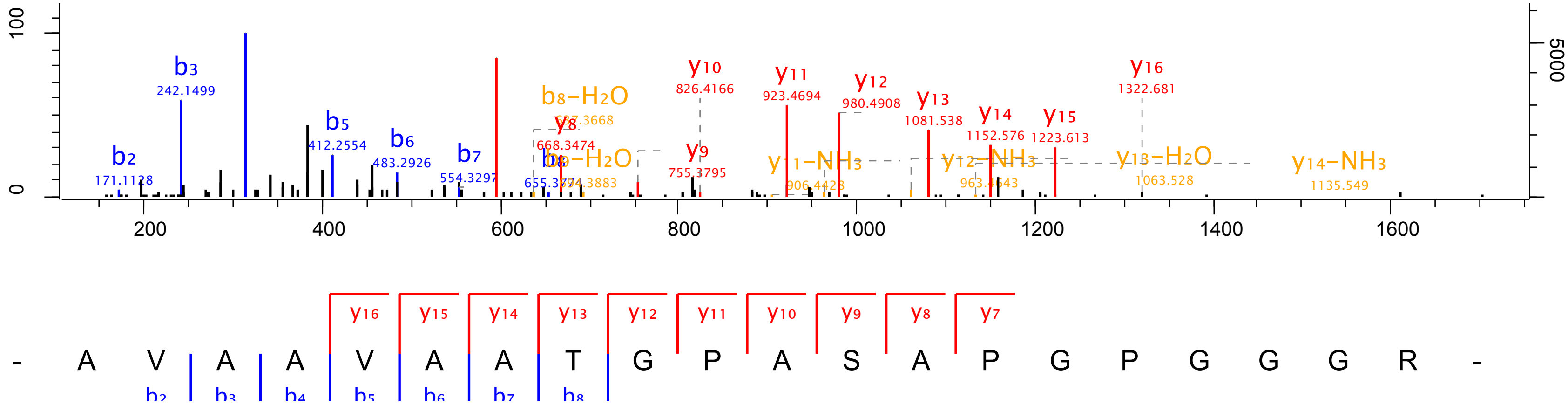

Raw file	Scan	Method	Score	m/z	Gene names
20140925_fract15_dyn_5ul_C7_01_450	14929	TOF; CID	57.79	416.24	GLDN

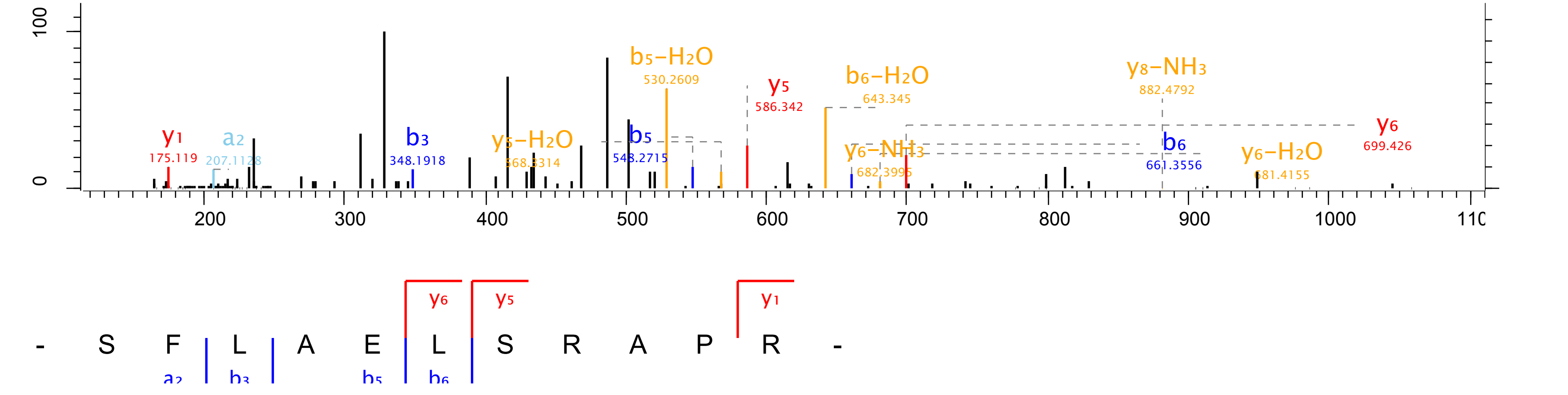

Raw file	Scan	Method	Score	m/z	Gene names
20140925_fract15_dyn_5ul_C7_01_450	16618	TOF; CID	138.42	676.85	SELK

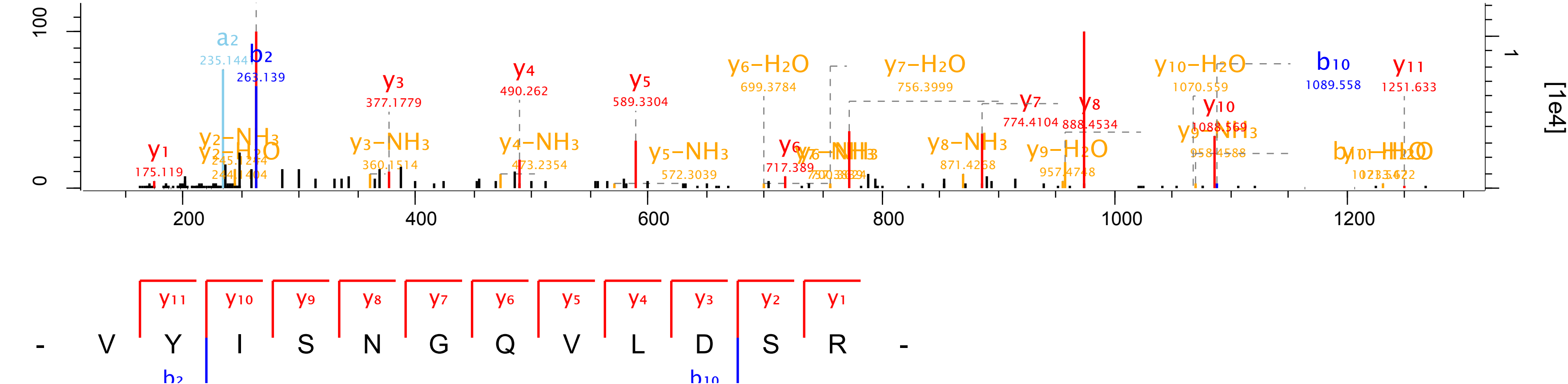

Raw file	Scan	Method	Score	m/z	Gene names
20140925_fract15_dyn_5ul_C7_01_450	20476	TOF; CID	56.04	595.81	TENM4

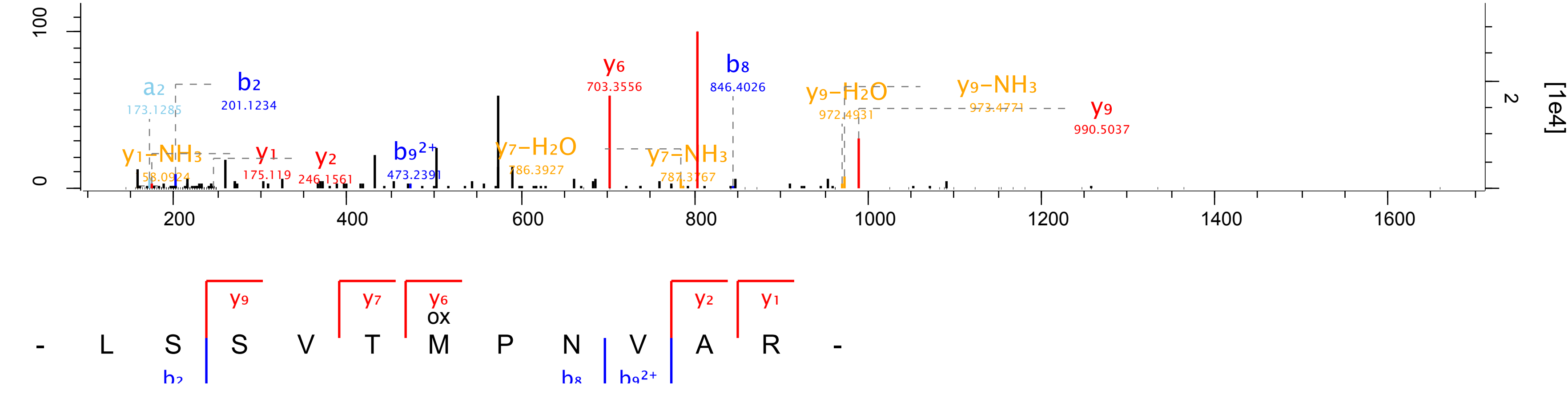

Raw file	Scan	Method	Score	m/z	Gene names
20140925_fract15_dyn_5ul_C7_01_450	20604	TOF; CID	93.1	524.79	COX7C

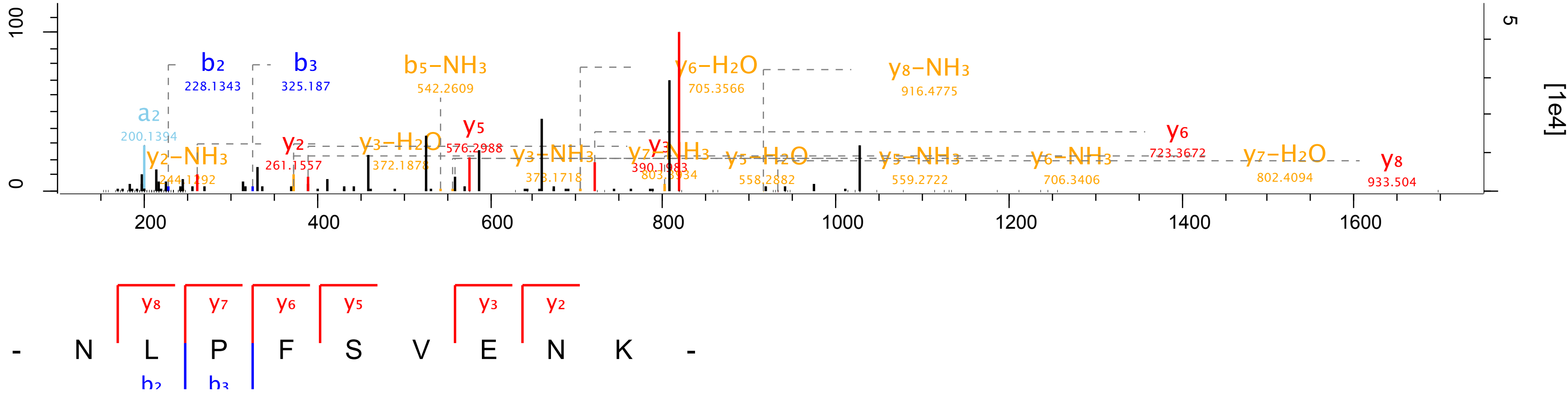

Raw file	Scan	Method	Score	m/z	Gene names
20140925_fract15_dyn_5ul_C7_01_450	27155	TOF; CID	96.14	629.85	C10orf90

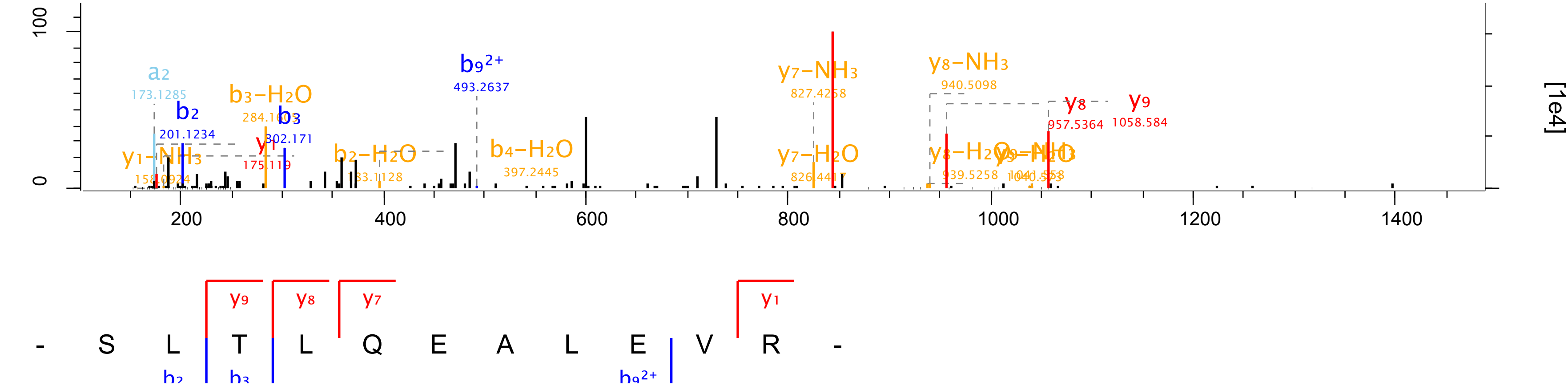

Raw file	Scan	Method	Score	m/z	Gene names
20140925_fract15_dyn_5ul_C7_01_450	29844	TOF; CID	58.47	1009.43	FAM199X

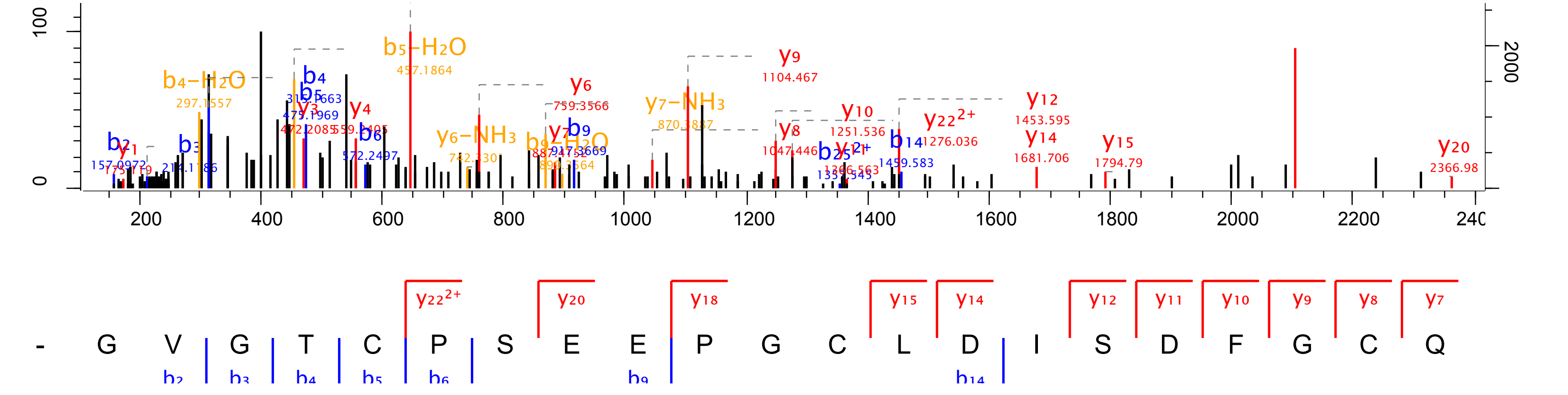

Raw file	Scan	Method	Score	m/z	Gene names
20140925_fract15_dyn_5ul_C7_01_450	34494	TOF; CID	99.04	935.51	VAMP1

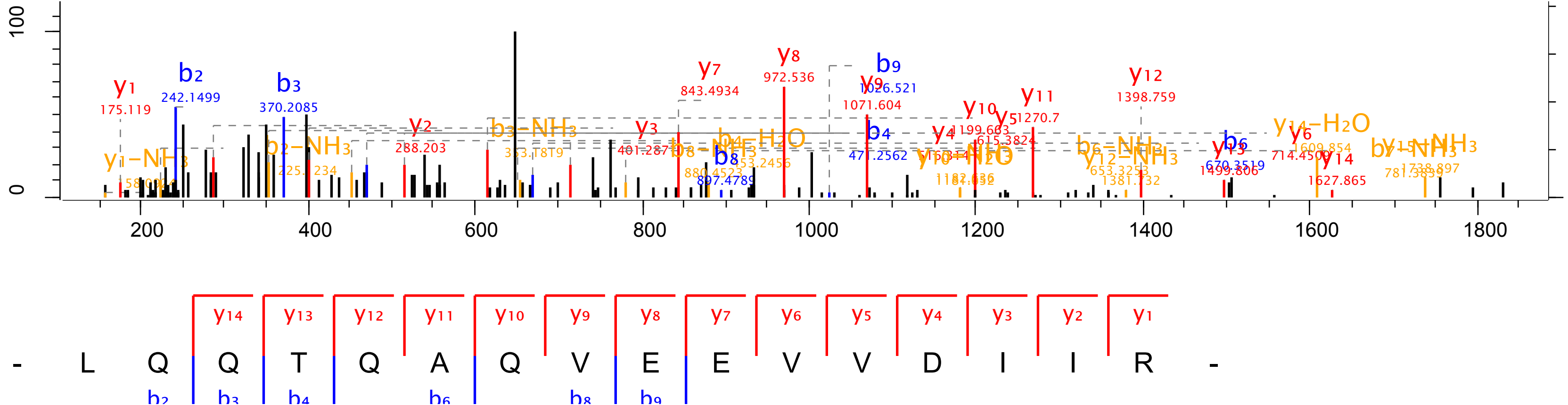

Raw file	Scan	Method	Score	m/z	Gene names
20140925_fract15_dyn_5ul_C7_01_450	35763	TOF; CID	90.15	615.86	ORAOV1

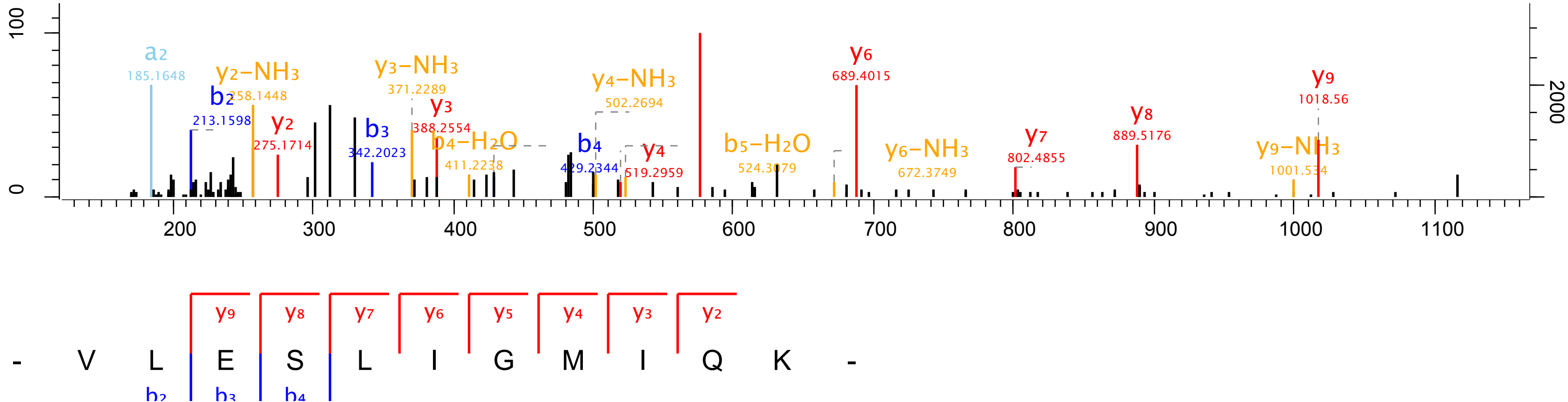

Raw file	Scan	Method	Score	m/z	Gene names
20140925_fract15_dyn_5ul_C7_01_450	36672	TOF; CID	143.46	908.43	EPT1

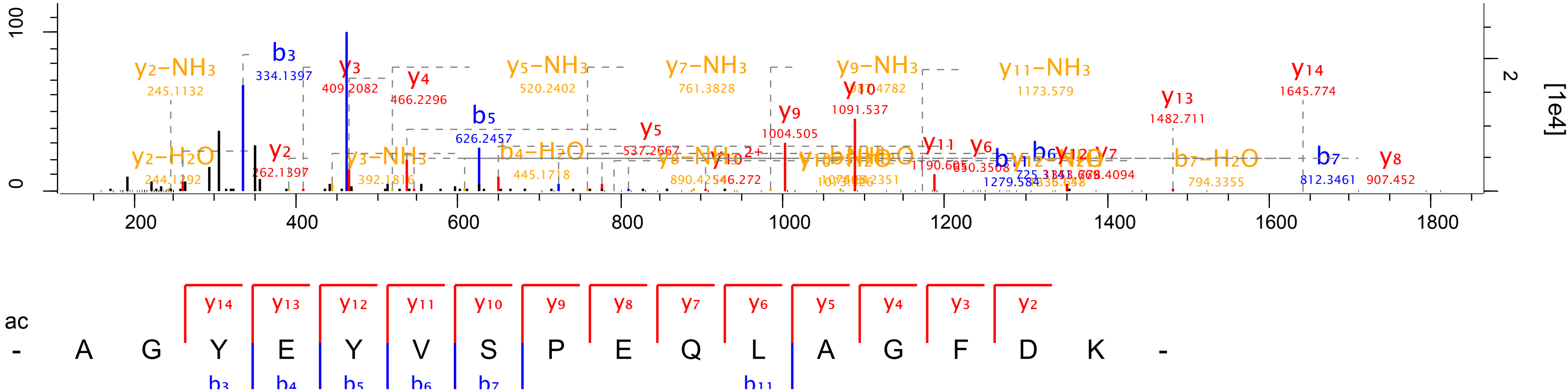

Raw file	Scan	Method	Score	m/z	Gene names
20140925_fract15_dyn_5ul_C7_01_450	38479	TOF; CID	64.22	753.4	UHMK1

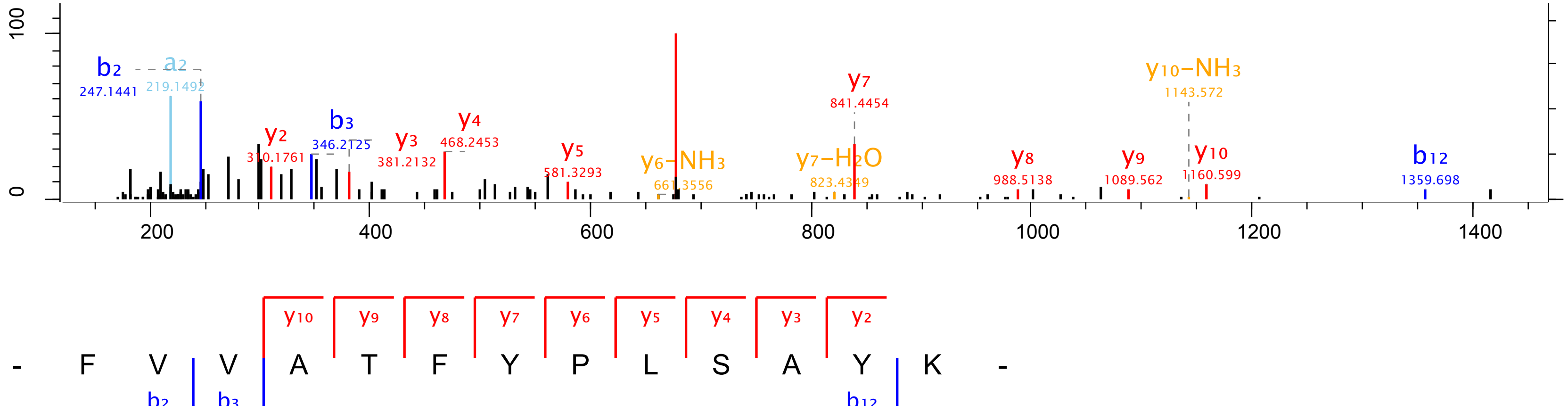

Raw file	Scan	Method	Score	m/z	Gene names
20140925_fract16_dyn_5ul_C8_01_451	9528	TOF; CID	85.52	637.81	FUNDC1

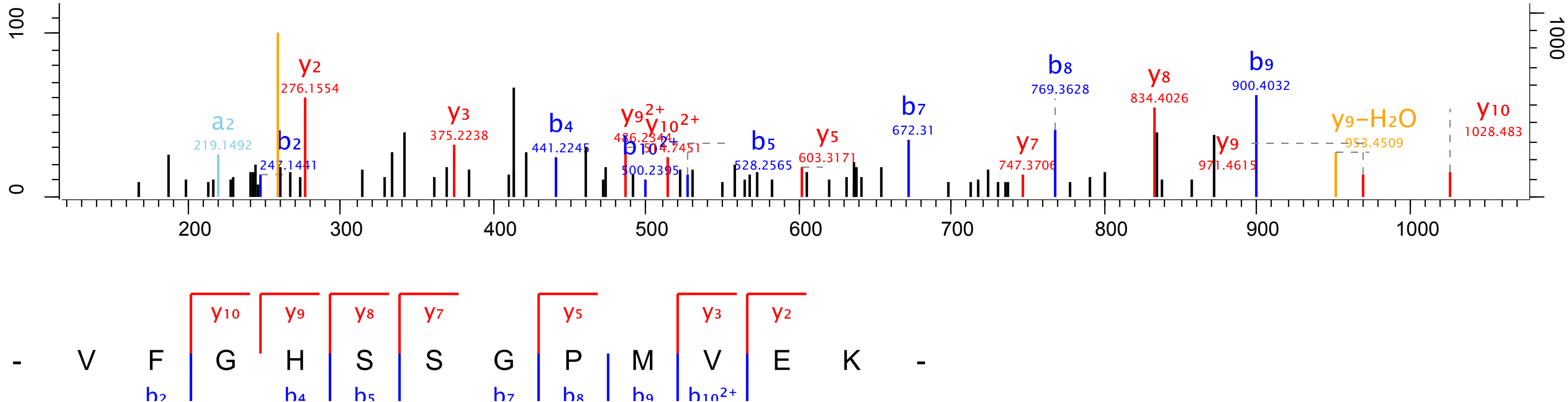

Raw file	Scan	Method	Score	m/z	Gene names
20140925_fract16_dyn_5ul_C8_01_451	14904	TOF; CID	113.93	631.79	CRLS1

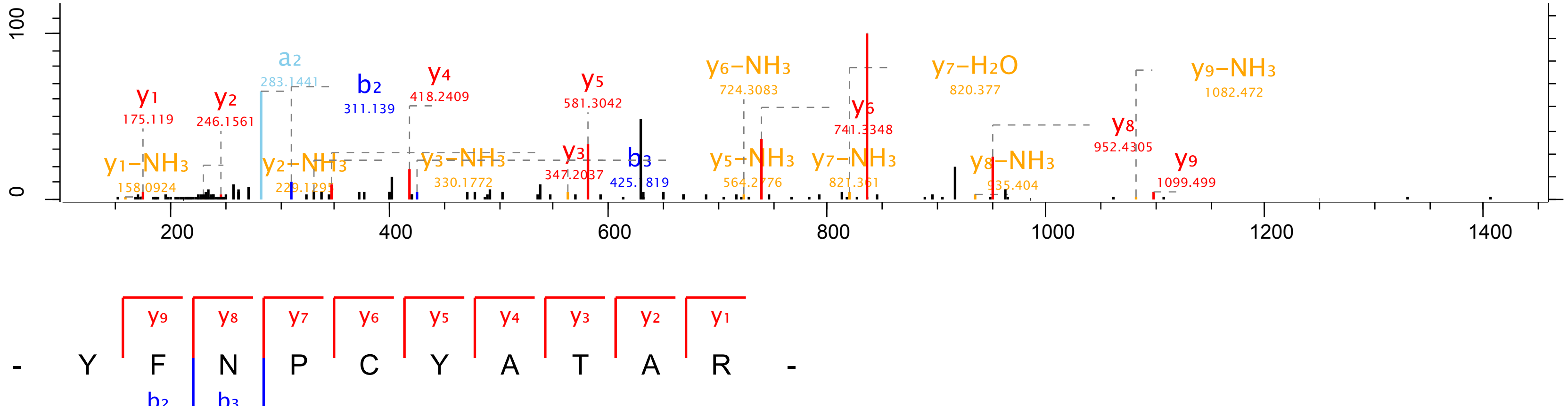

Raw file	Scan	Method	Score	m/z
20140925_fract16_dyn_5ul_C8_01_451	15773	TOF; CID	70.54	601.83

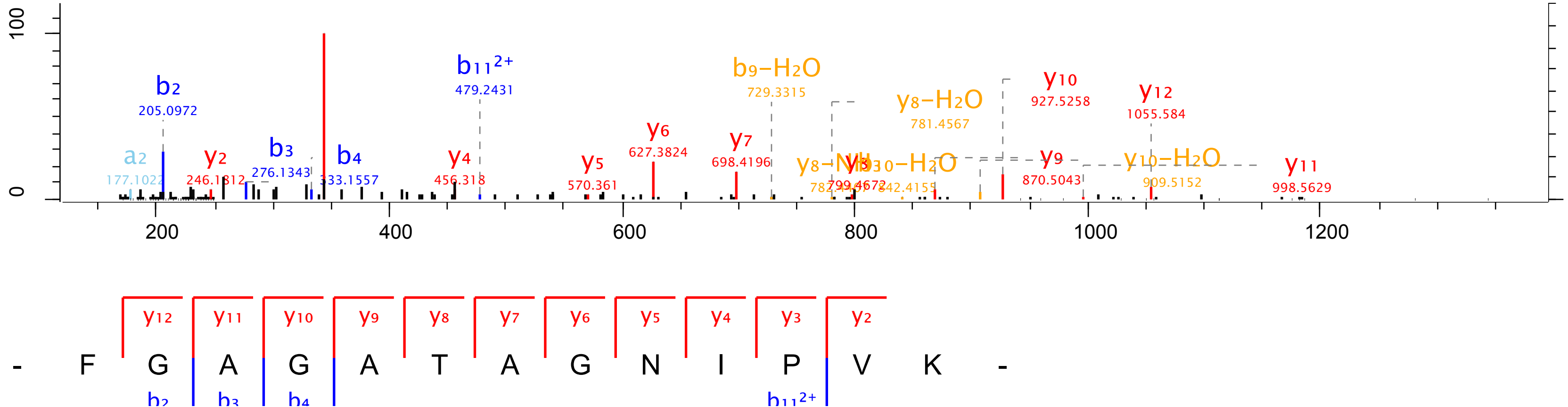

Raw file	Scan	Method	Score	m/z	Gene names
20140925_fract16_dyn_5ul_C8_01_451	18234	TOF; CID	73.44	553.8	C19orf43

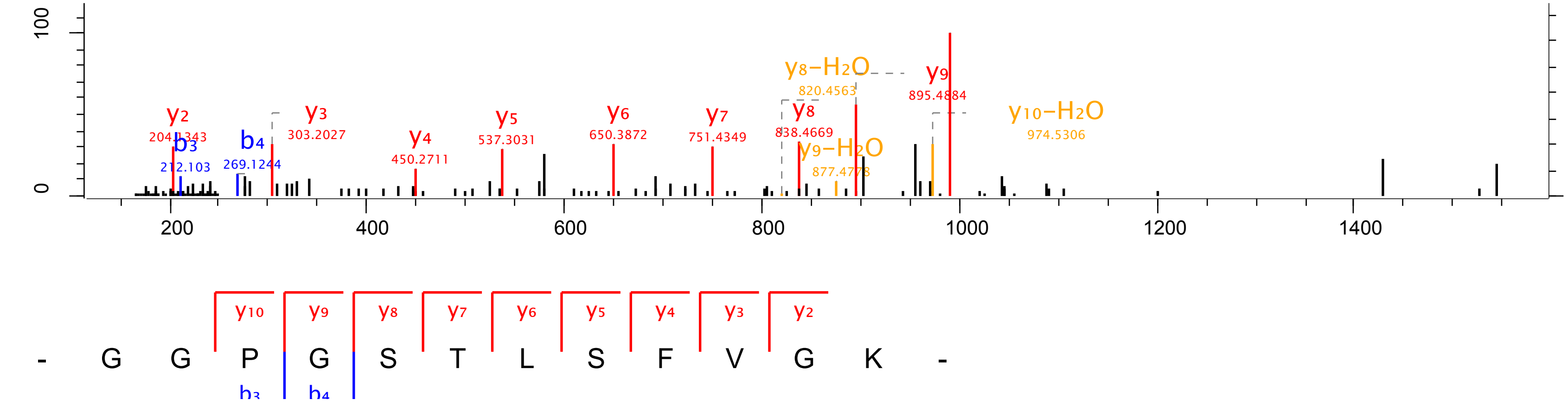

Raw file	Scan	Method	Score	m/z	Gene names
20140925_fract16_dyn_5ul_C8_01_451	18592	TOF; CID	81.57	1043.03	ZFP36

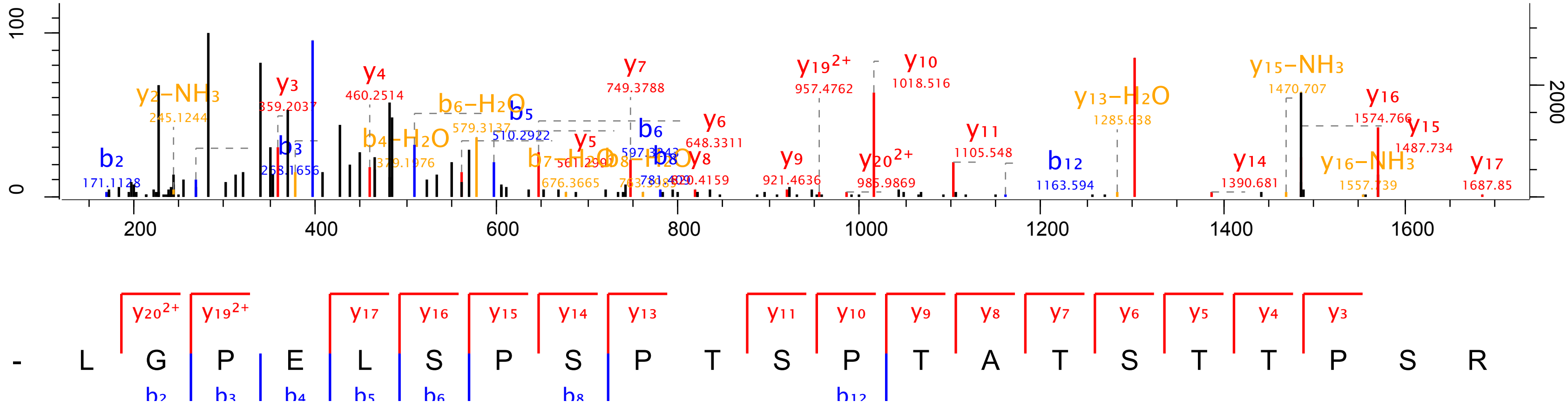

Raw file	Scan	Method	Score	m/z	Gene names
20140925_fract16_dyn_5ul_C8_01_451	19513	TOF; CID	93.37	672.33	TMEM243

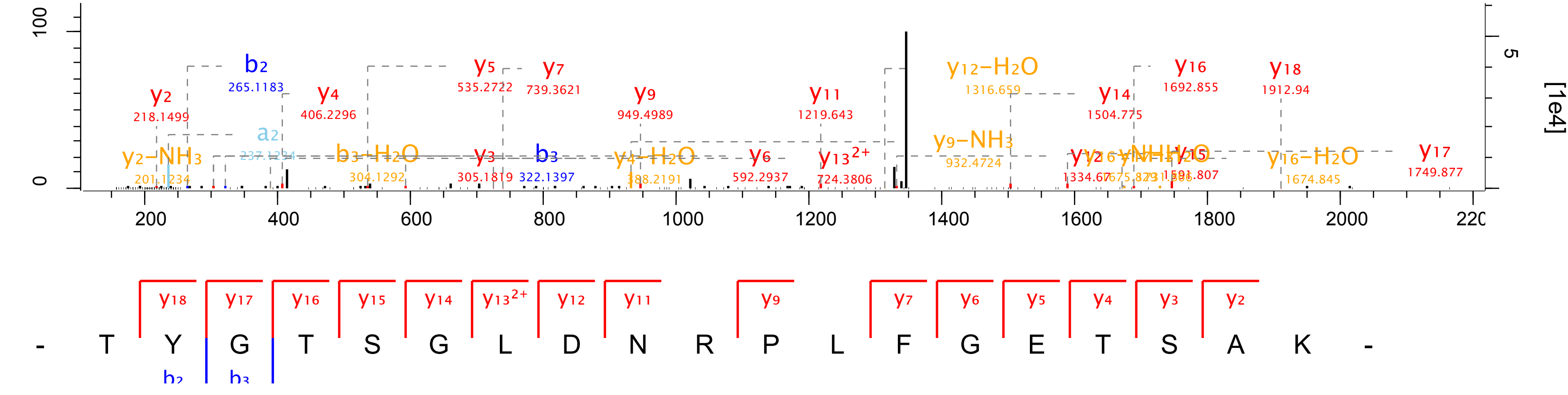

Raw file	Scan	Method	Score	m/z	Gene names
20140925_fract16_dyn_5ul_C8_01_451	20857	TOF; CID	74.84	517.78	TMEM18

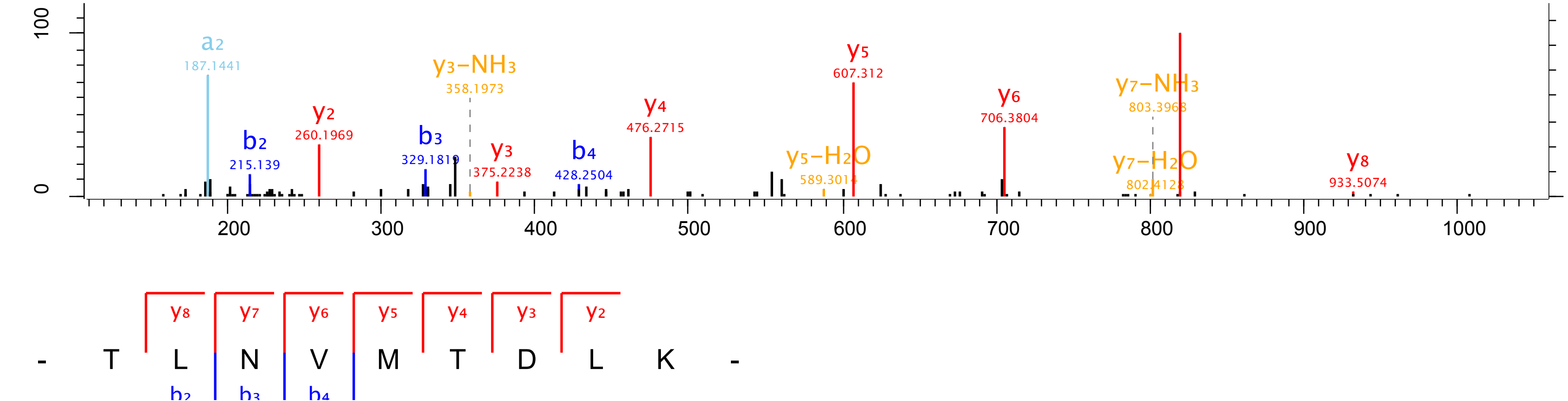

Raw file	Scan	Method	Score	m/z	Gene names
20140925_fract16_dyn_5ul_C8_01_451	24987	TOF; CID	63.06	730.38	DMBT1

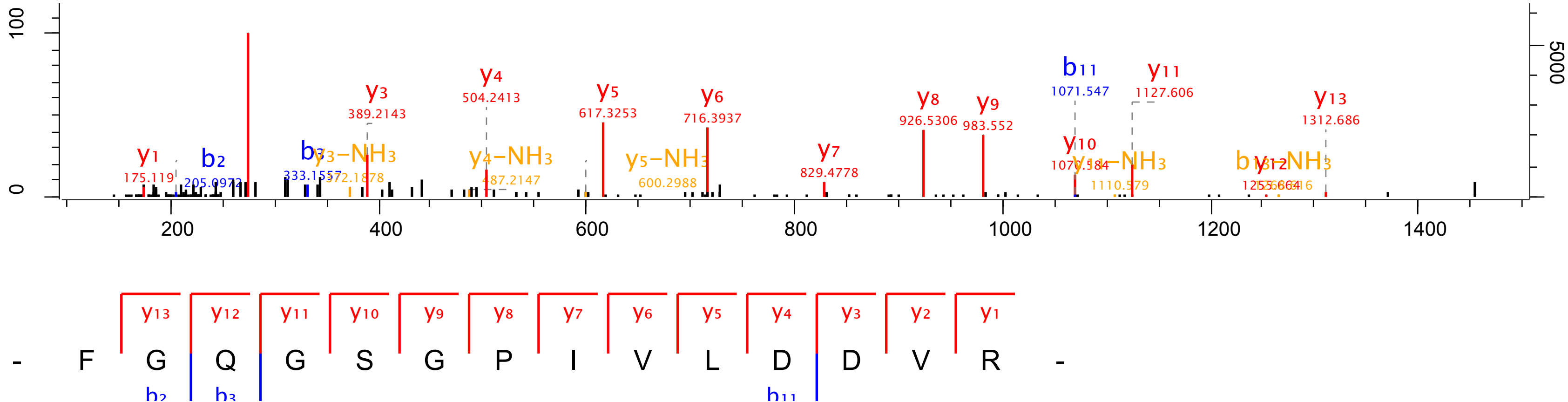

Raw file	Scan	Method	Score	m/z	Gene names
20140925_fract16_dyn_5ul_C8_01_451	25322	TOF; CID	92.27	900.41	RAET1E

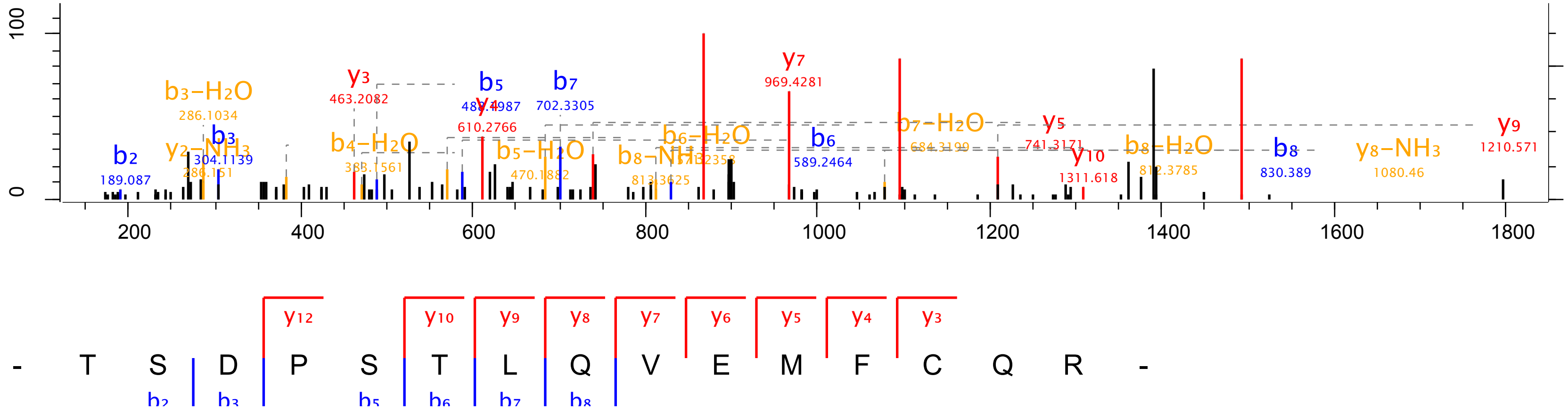

Raw file	Scan	Method	Score	m/z	Gene names
20140925_fract16_dyn_5ul_C8_01_451	30967	TOF; CID	109.25	945.77	TMEM222

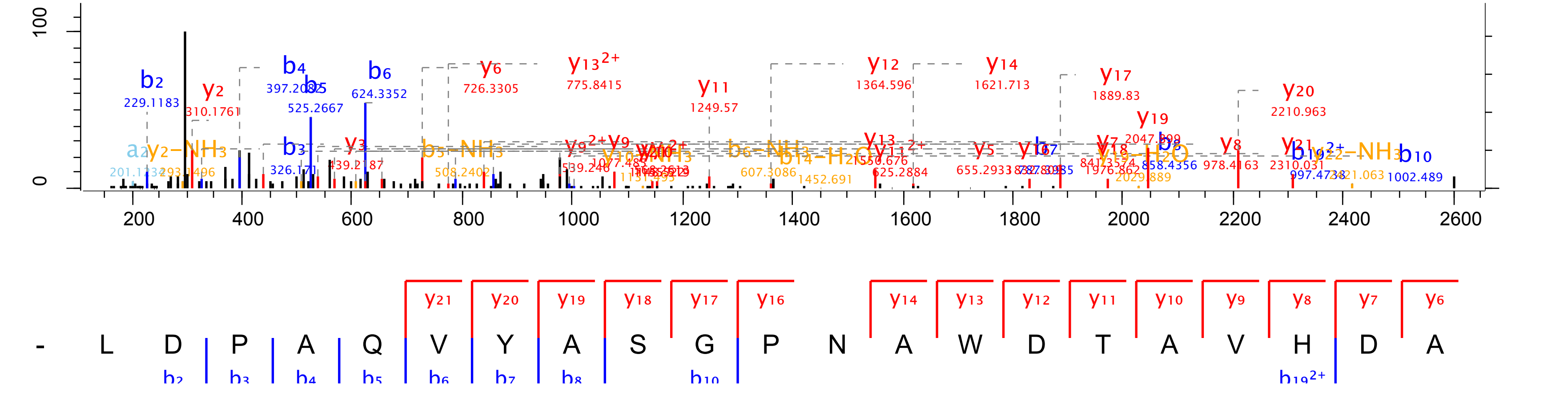

Raw file	Scan	Method	Score	m/z	Gene names
20140925_fract16_dyn_5ul_C8_01_451	32372	TOF; CID	66.99	647.91	MAD2L1BP

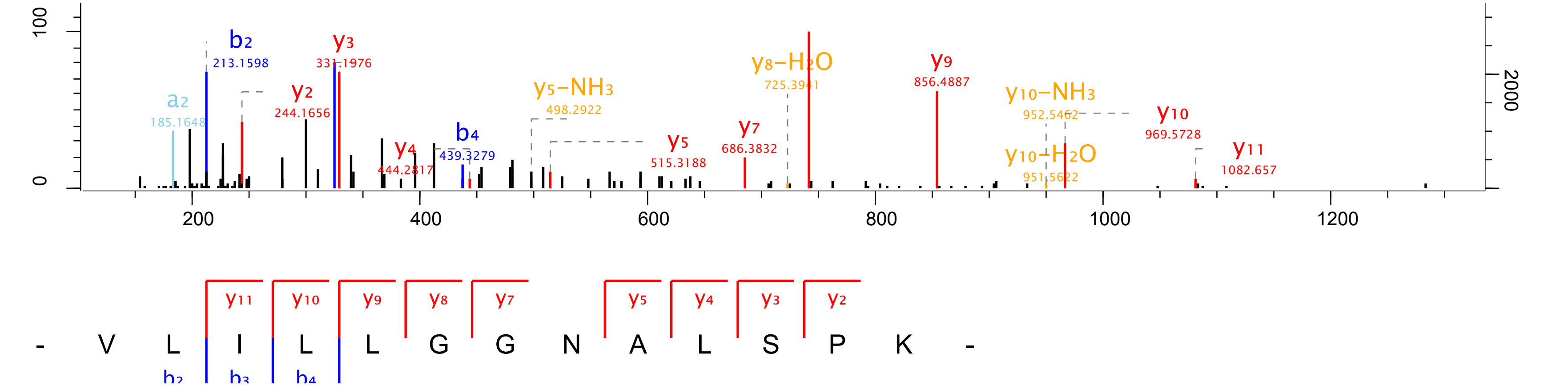

Raw file	Scan	Method	Score	m/z	Gene names
20140925_fract16_dyn_5ul_C8_01_451	36437	TOF; CID	138.91	664	ABRACL

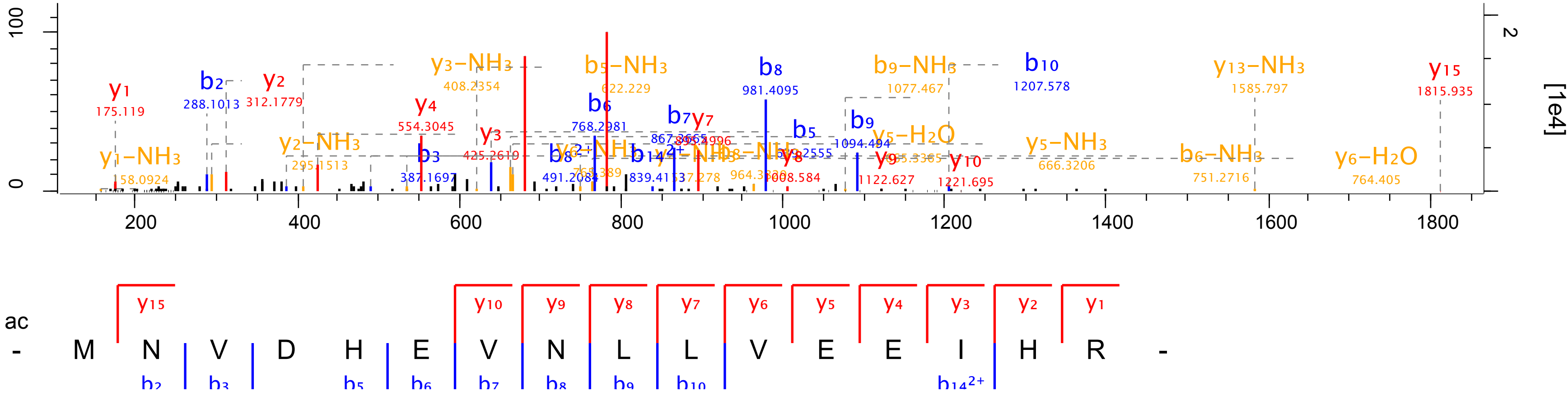

Raw file	Scan	Method	Score	m/z	Gene names
20140925_fract16_dyn_5ul_C8_01_451	37296	TOF; CID	70.11	907.81	HIGD2A

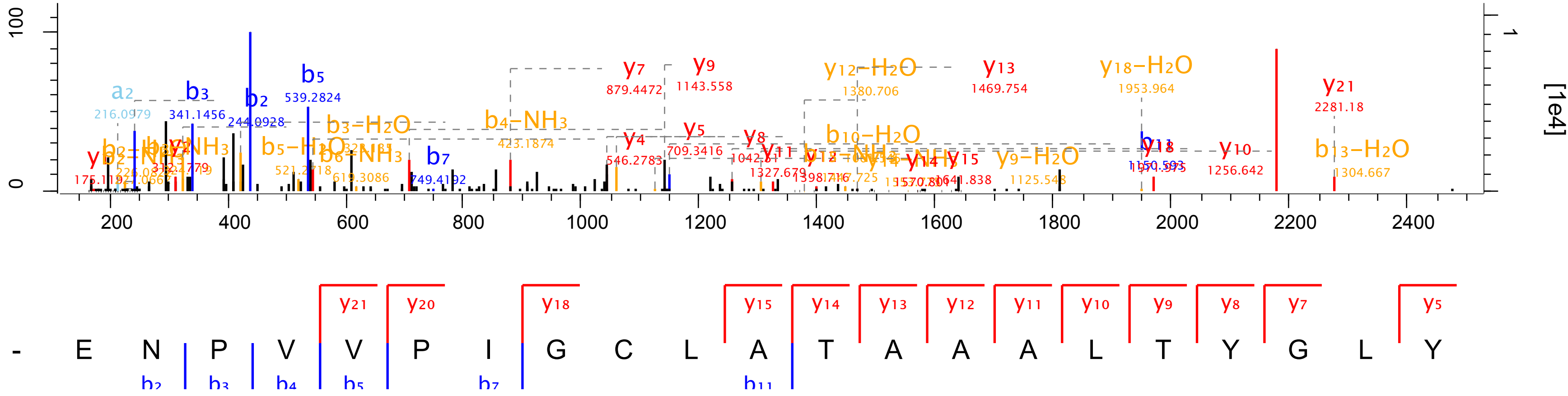

Raw file	Scan	Method	Score	m/z	Gene names
20140925_fract17_dyn_5ul_H1_01_452	9066	TOF; CID	46.28	513.27	RBL1

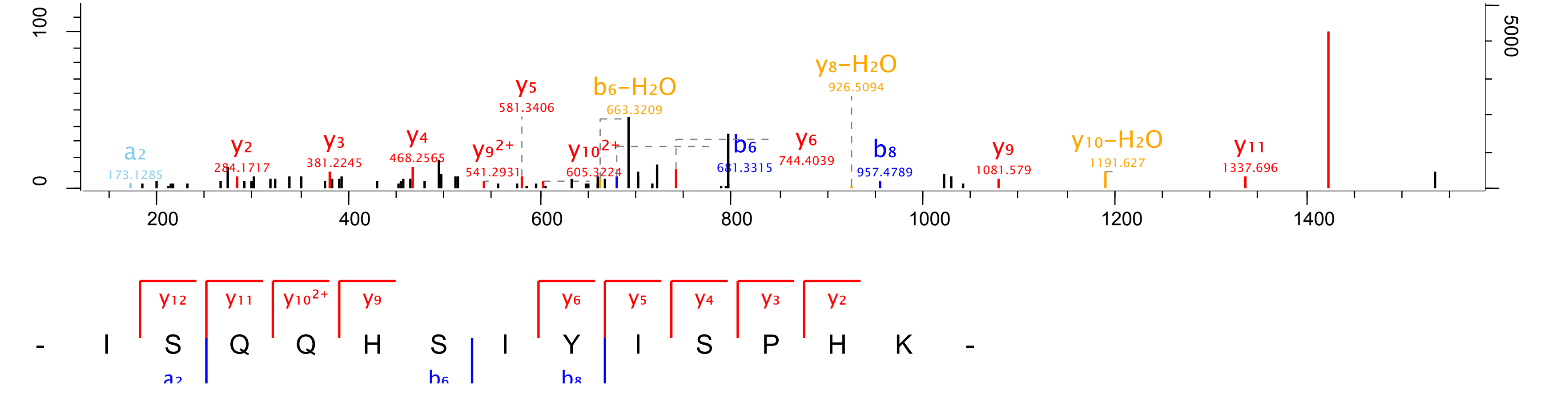

Raw file	Scan	Method	Score	m/z	Gene names
20140925_fract17_dyn_5ul_H1_01_452	9341	TOF; CID	120.65	429.74	SLC46A1

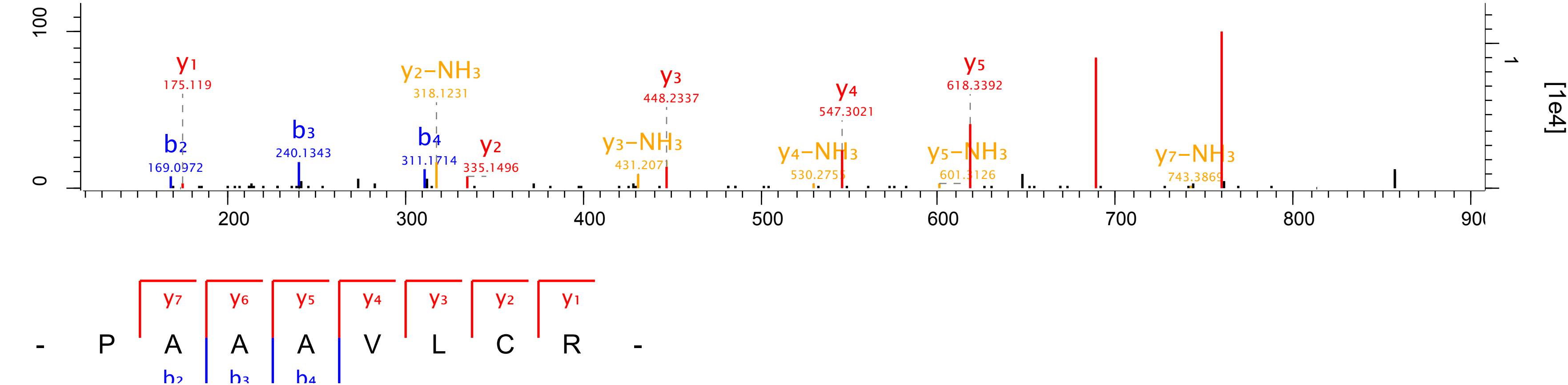

Raw file	Scan	Method	Score	m/z	Gene names
20140925_fract17_dyn_5ul_H1_01_452	12167	TOF; CID	96.67	512.61	PNMA1

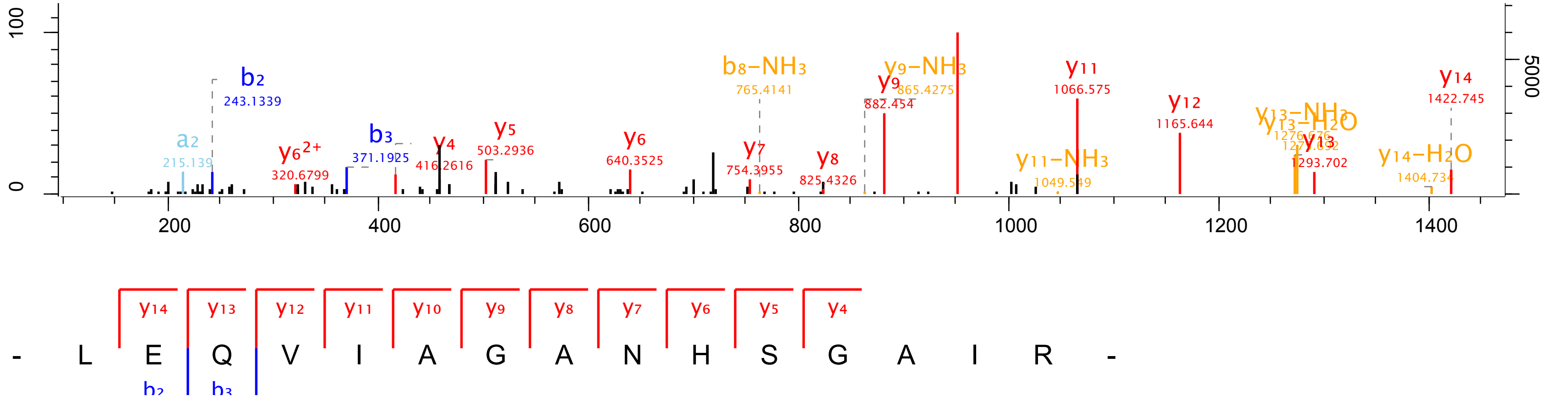

Raw file	Scan	Method	Score	m/z	Gene names
20140925_fract17_dyn_5ul_H1_01_452	13149	TOF; CID	86.46	602.81	OTUD3

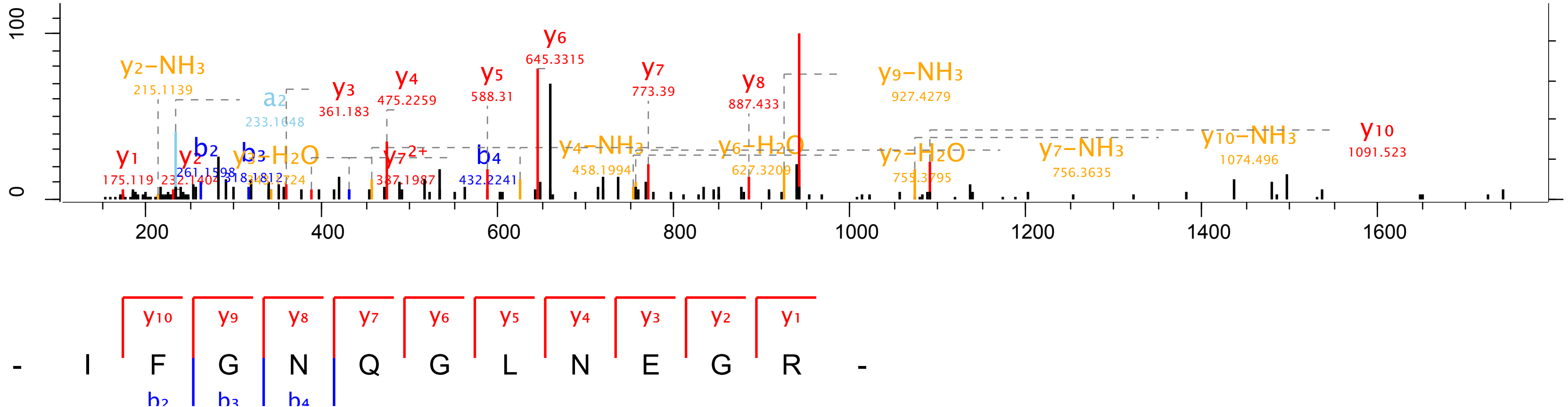

Raw file	Scan	Method	Score	m/z	Gene names
20140925_fract17_dyn_5ul_H1_01_452	18506	TOF; CID	104.81	705.36	TMEM69

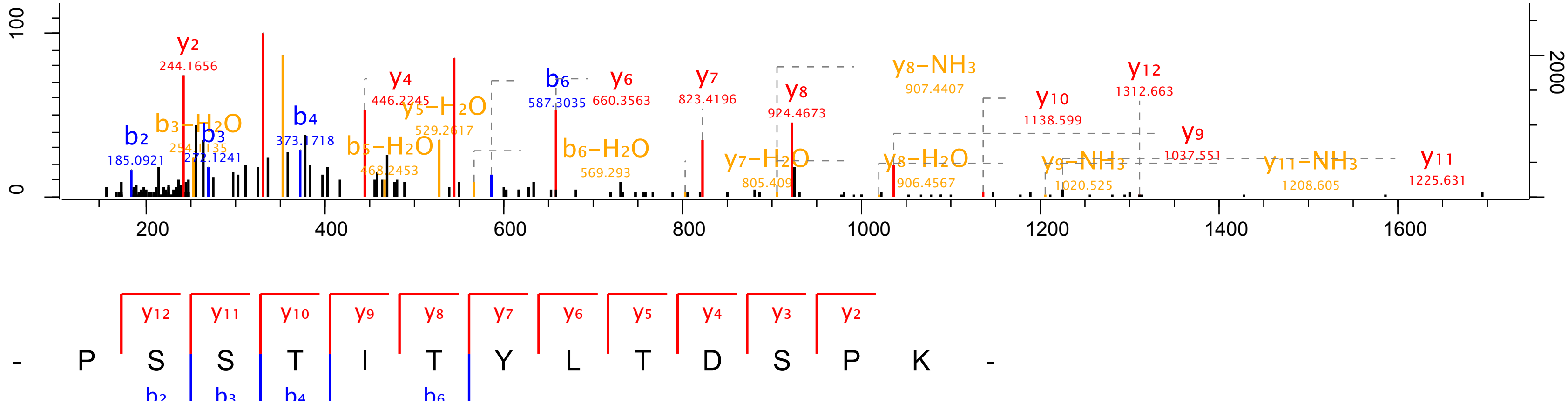

Raw file	Scan	Method	Score	m/z	Gene names
20140925_fract17_dyn_5ul_H1_01_452	19014	TOF; CID	109.47	685.87	KDM6A

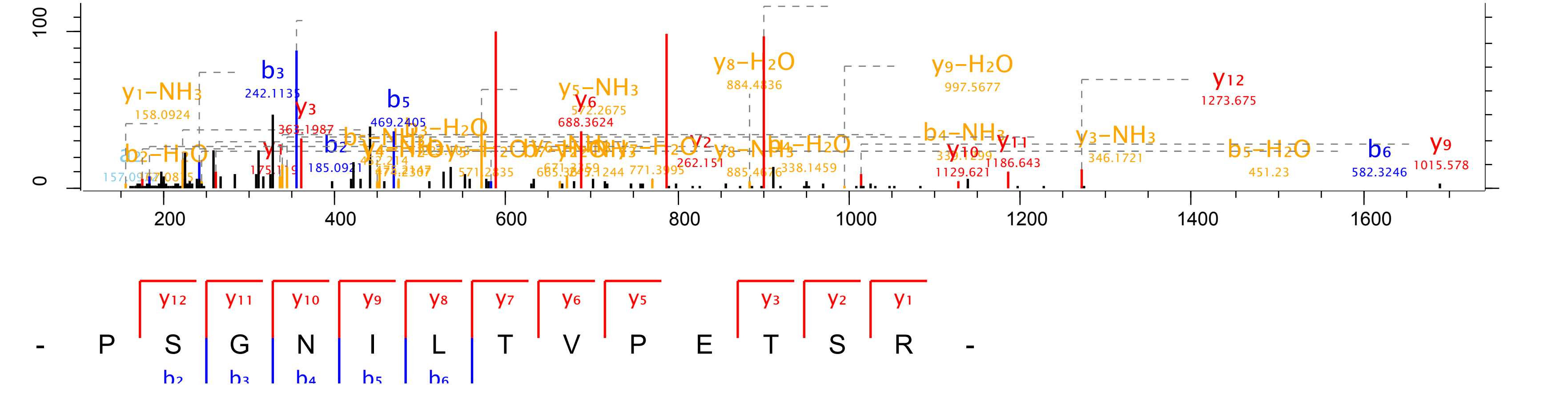

Raw file	Scan	Method	Score	m/z	Gene names
20140925_fract17_dyn_5ul_H1_01_452	19441	TOF; CID	42.71	813.9	BAD

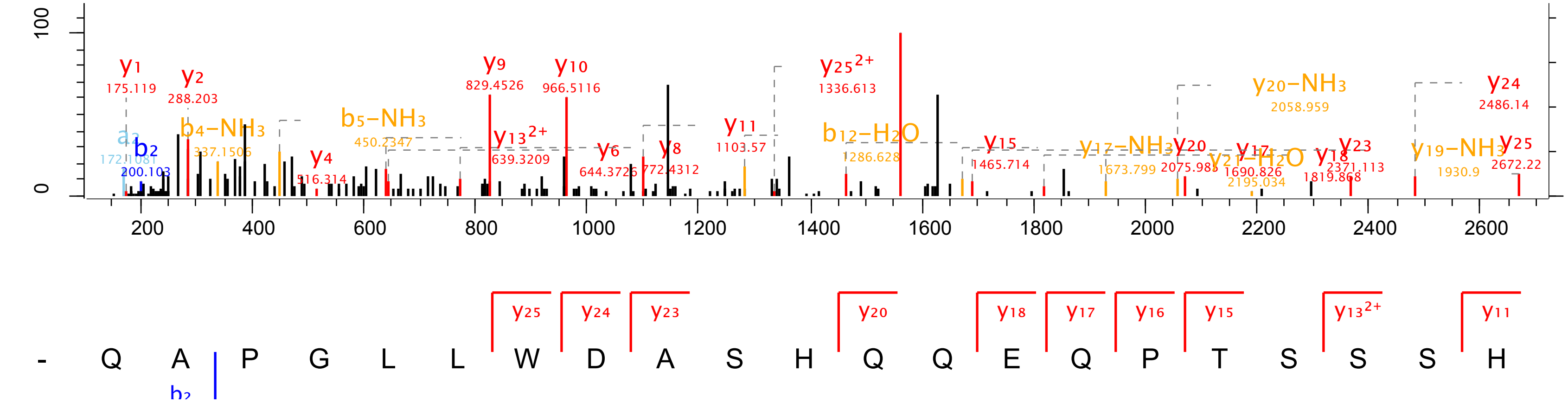

Raw file	Scan	Method	Score	m/z	Gene names
20140925_fract17_dyn_5ul_H1_01_452	20775	TOF; CID	104.39	632.31	SPA17

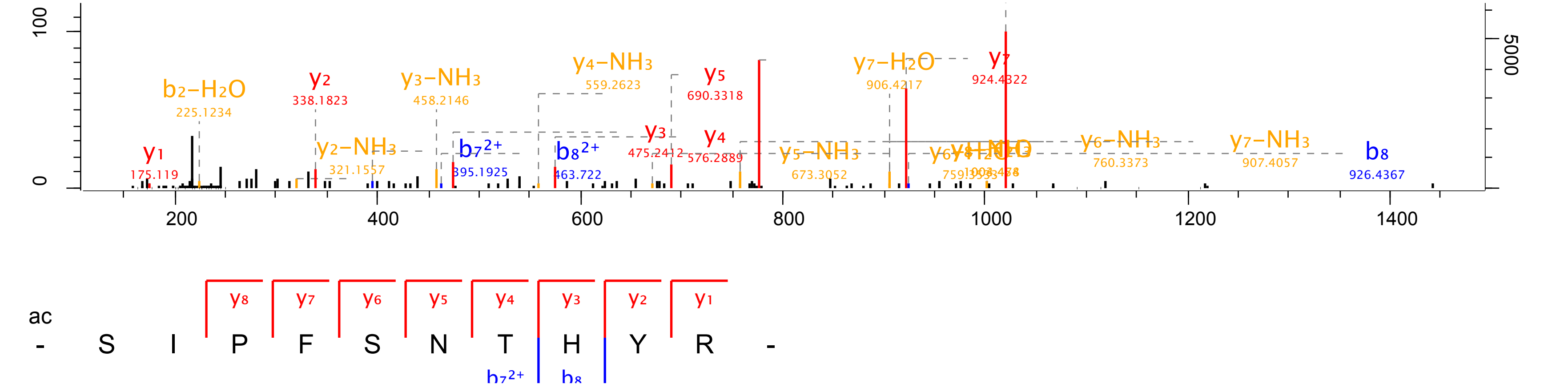

Raw file	Scan	Method	Score	m/z	Gene names
20140925_fract17_dyn_5ul_H1_01_452	23179	TOF; CID	80.69	587.82	SYNGR3

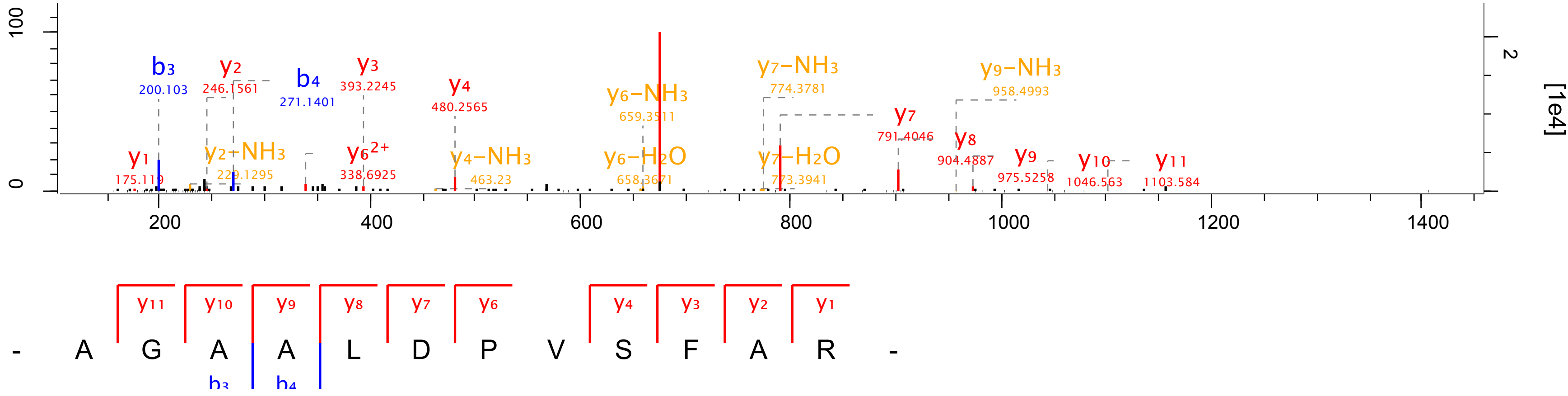

Raw file	Scan	Method	Score	m/z	Gene names
20140925_fract17_dyn_5ul_H1_01_452	23260	TOF; CID	99.45	813.07	LHFPL2

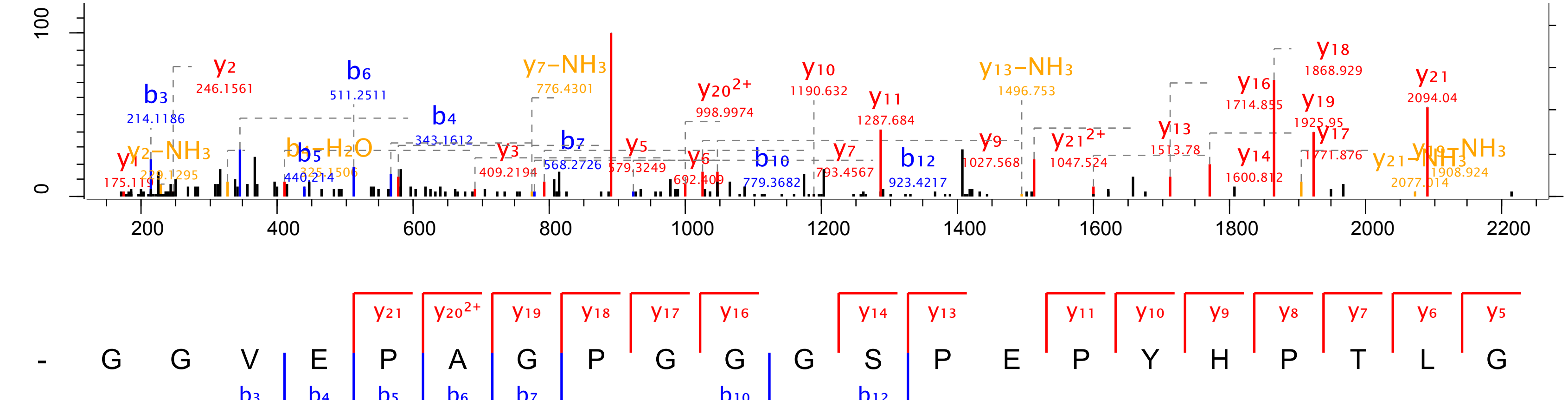

Raw file	Scan	Method	Score	m/z	Gene names
20140925_fract17_dyn_5ul_H1_01_452	27140	TOF; CID	60.55	862.45	PSENEN

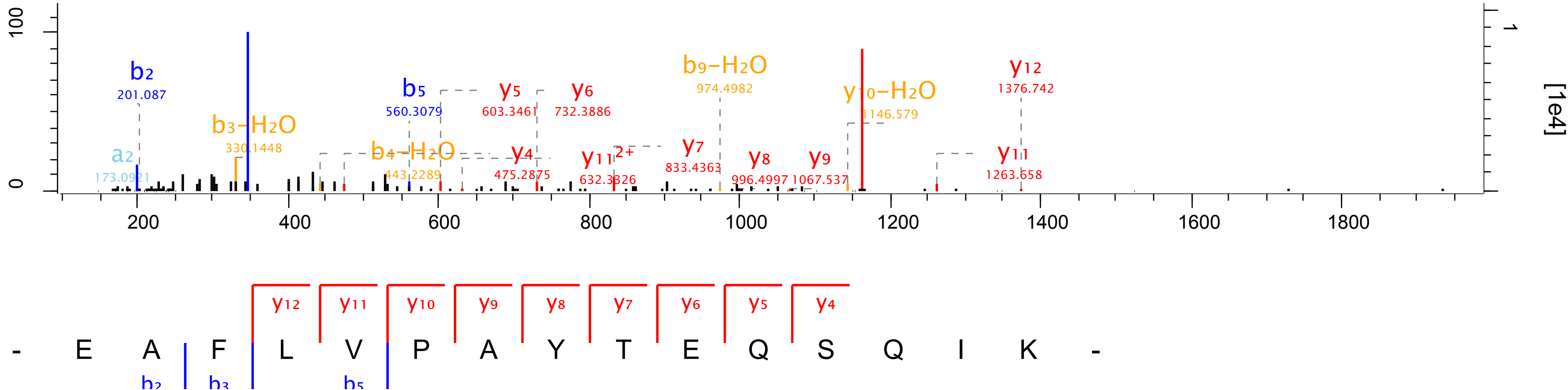

Raw file	Scan	Method	Score	m/z	Gene names
20140925_fract17_dyn_5ul_H1_01_452	29034	TOF; CID	54.76	991.01	UBE2W

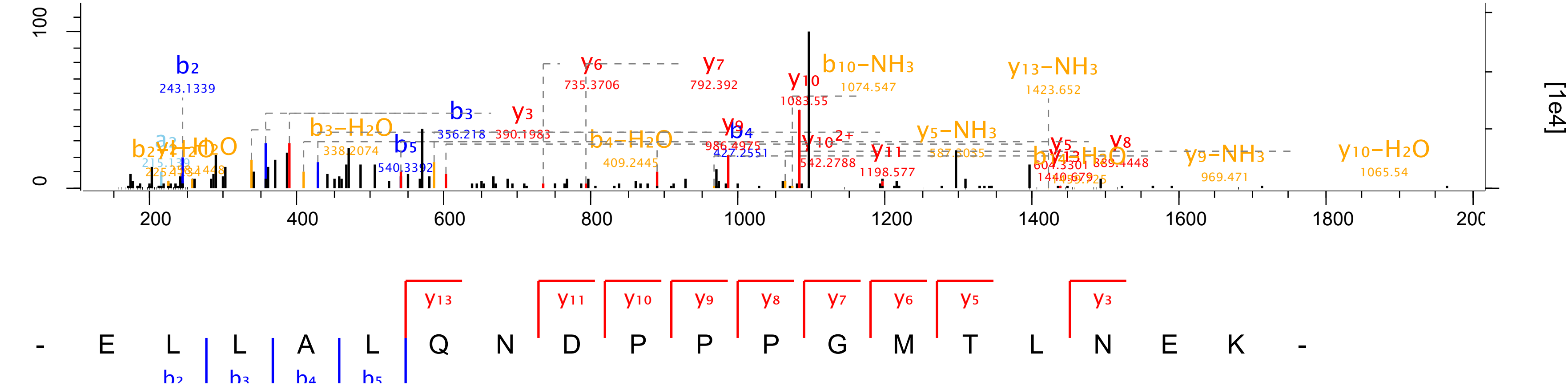

Raw file	Scan	Method	Score	m/z	Gene names
20140925_fract17_dyn_5ul_H1_01_452	32819	TOF; CID	79.84	1015.45	NPL

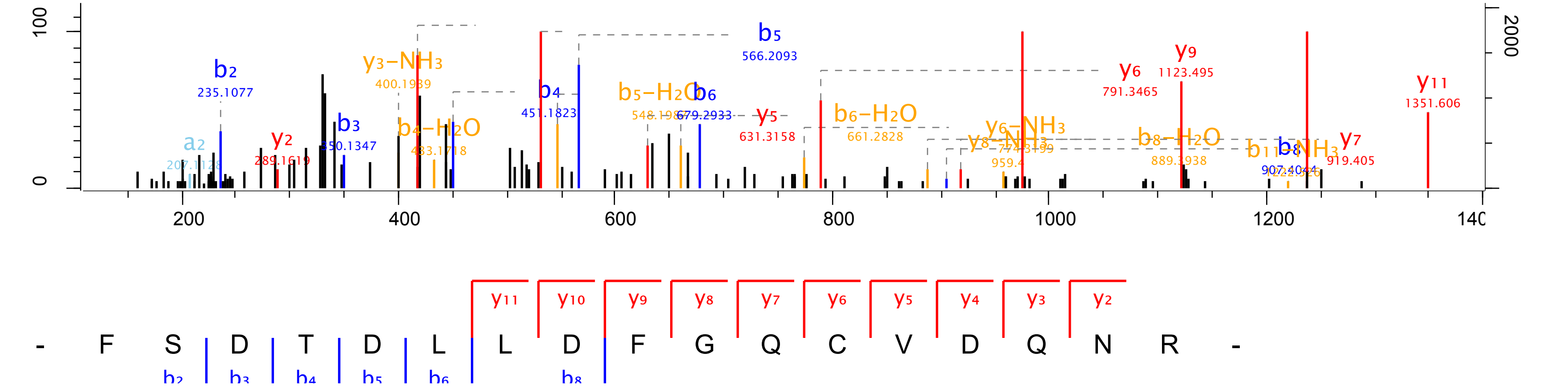

Raw file	Scan	Method	Score	m/z	Gene names
20140925_fract17_dyn_5ul_H1_01_452	34843	TOF; CID	41.48	1146.88	SWI5

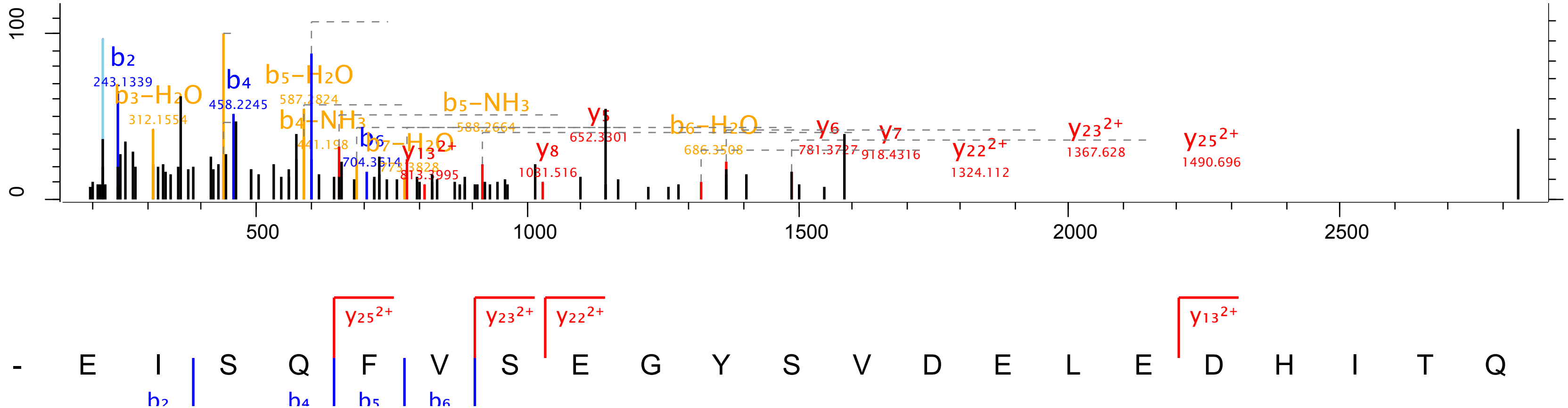

Raw file	Scan	Method	Score	m/z	Gene names
20140925_fract17_dyn_5ul_H1_01_452	36344	TOF; CID	39.83	743.39	ZNF512B

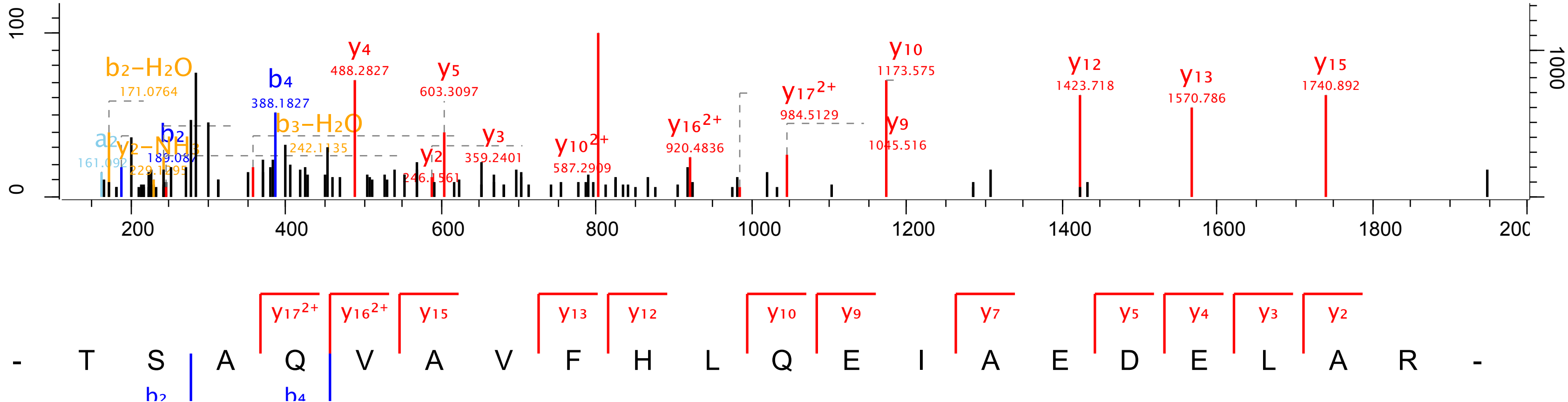

Raw file	Scan	Method	Score	m/z	Gene names
20140925_fract18_dyn_5ul_H2_01_453	5019	TOF; CID	72.29	436.25	ZNF707

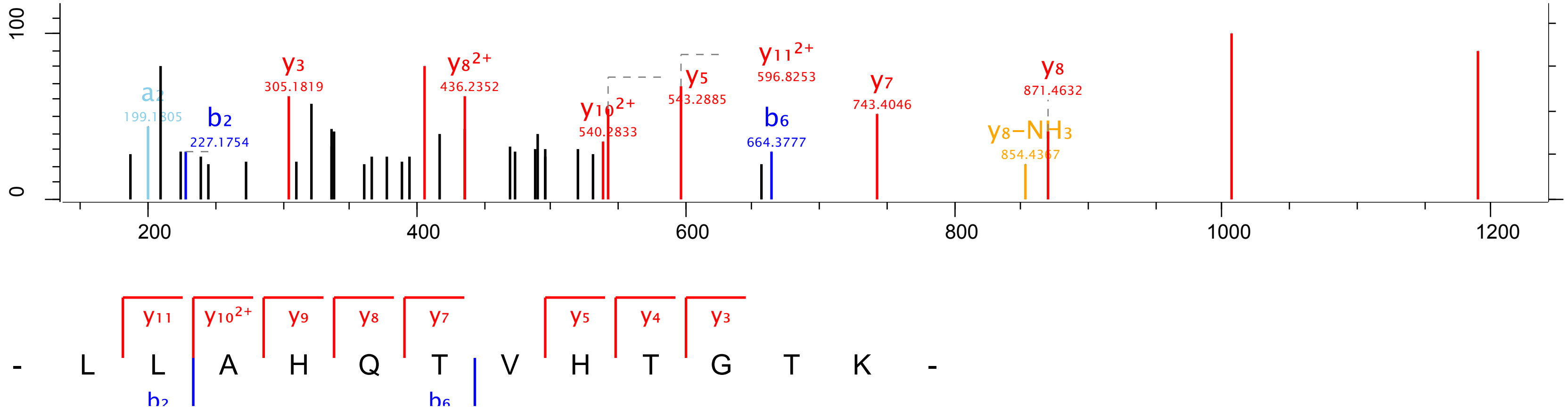

Raw file	Scan	Method	Score	m/z	Gene names
20140925_fract18_dyn_5ul_H2_01_453	5714	TOF; CID	55.26	483.92	ZXDB

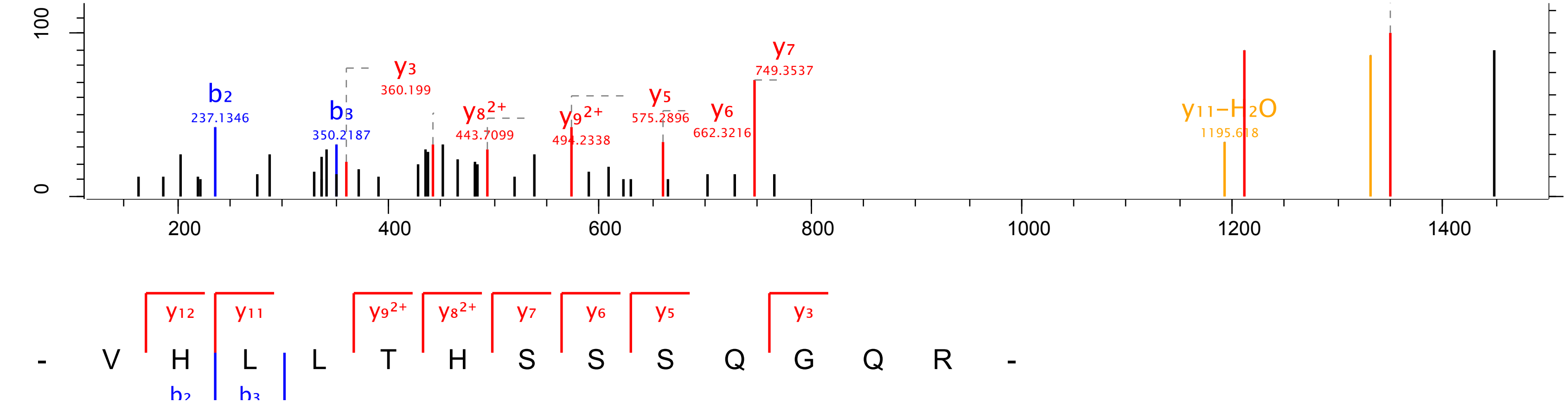

Raw file	Scan	Method	Score	m/z	Gene names
20140925_fract18_dyn_5ul_H2_01_453	6526	TOF; CID	84.51	655.83	RNF167

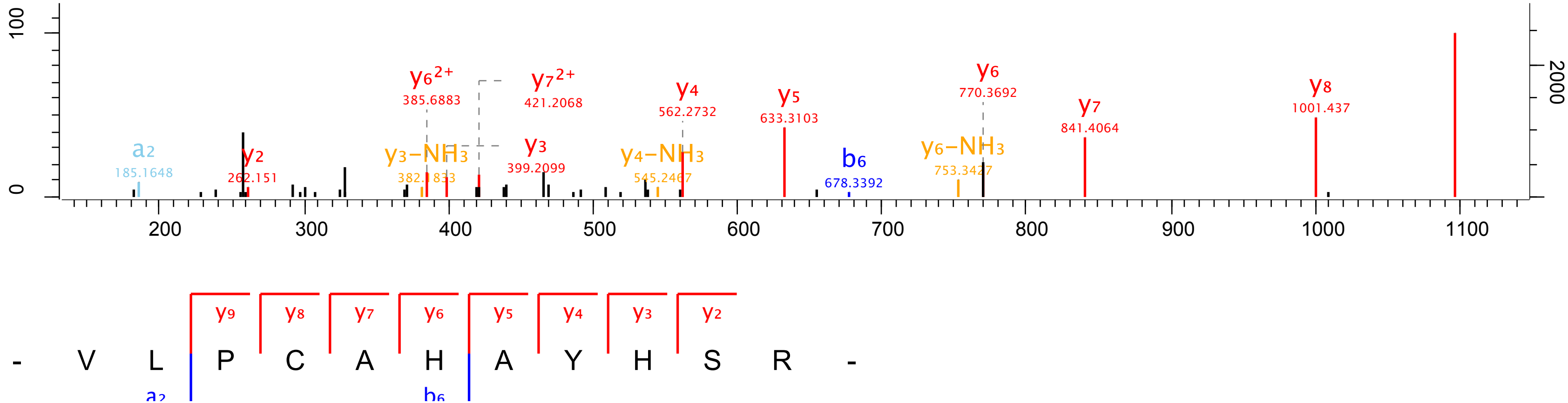

Raw file	Scan	Method	Score	m/z	Gene names
20140925_fract18_dyn_5ul_H2_01_453	8990	TOF; CID	123.67	457.24	ZFP62

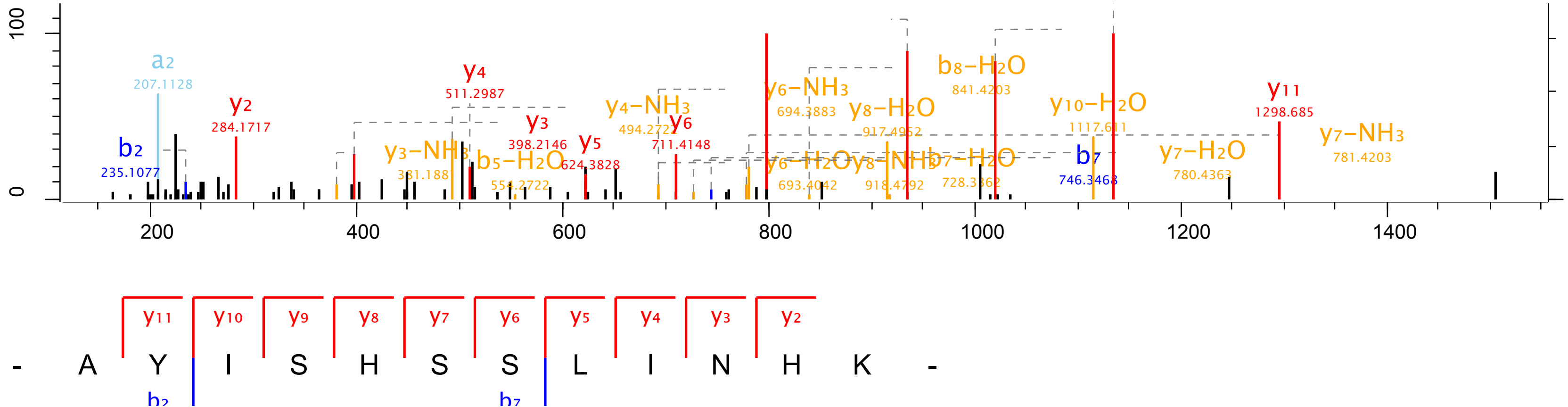

Raw file	Scan	Method	Score	m/z	Gene names
20140925_fract18_dyn_5ul_H2_01_453	11106	TOF; CID	63.32	362.2	RTN4R

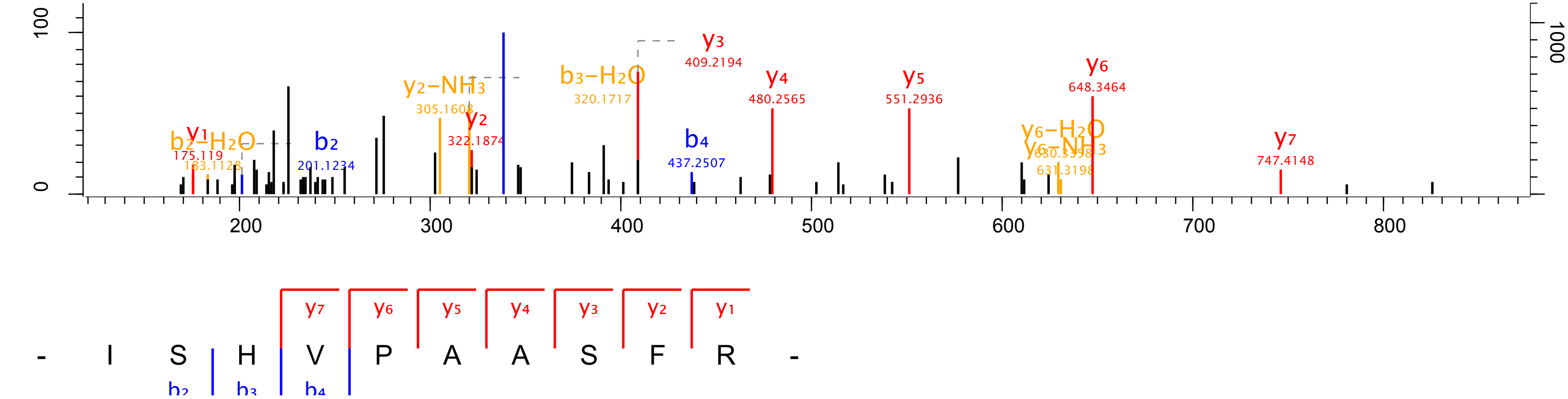

Raw file	Scan	Method	Score	m/z	Gene names
20140925_fract18_dyn_5ul_H2_01_453	12108	TOF; CID	89.19	621.31	C14orf119

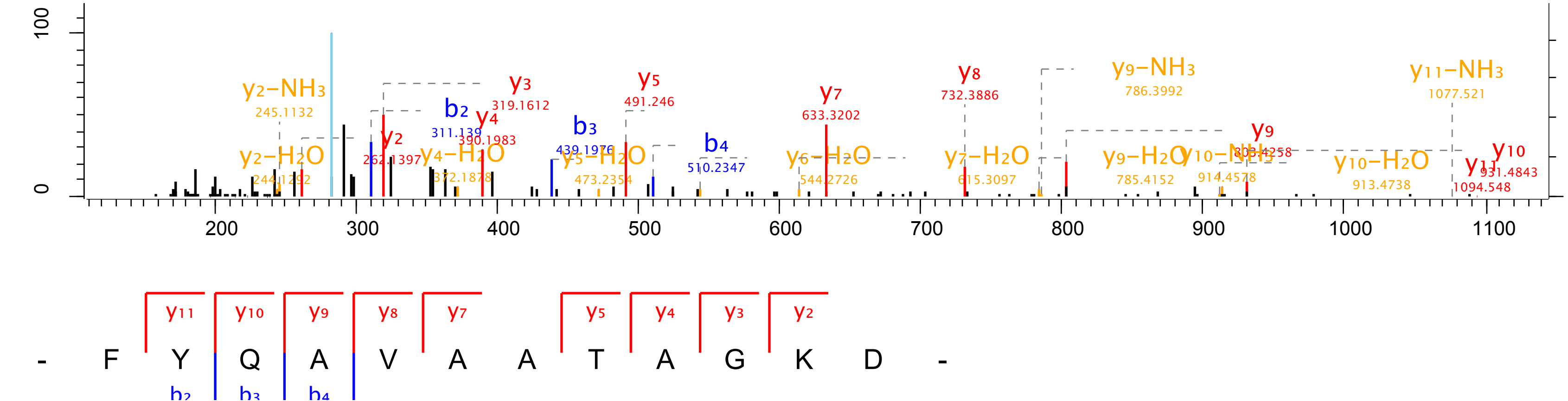

Raw file	Scan	Method	Score	m/z	Gene names
20140925_fract18_dyn_5ul_H2_01_453	14821	TOF; CID	44.86	736.06	TTC8

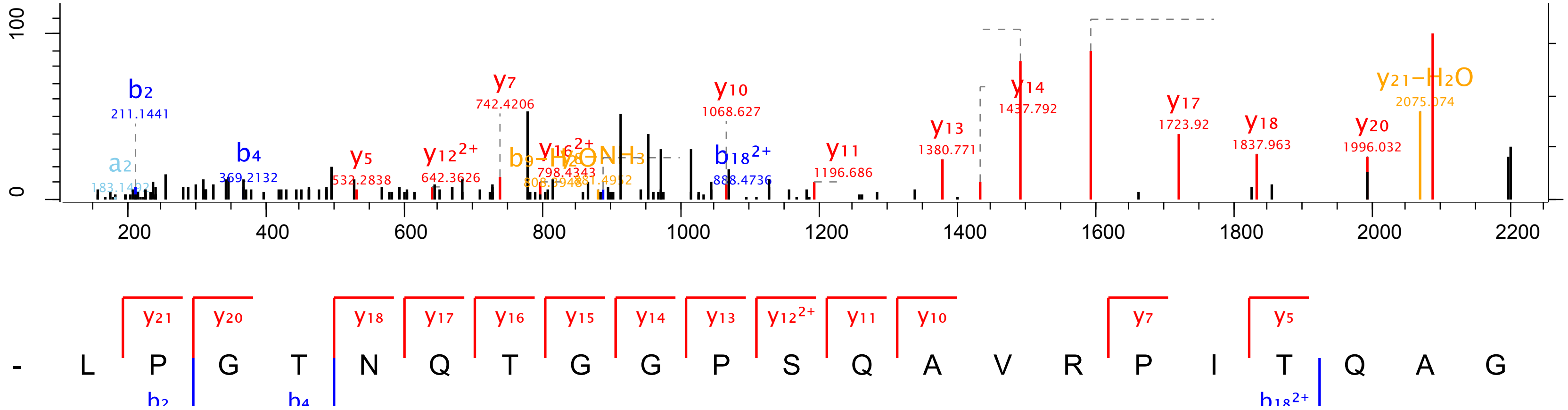

Raw file	Scan	Method	Score	m/z	Gene names
20140925_fract18_dyn_5ul_H2_01_453	15116	TOF; CID	111.12	781.86	PEMT

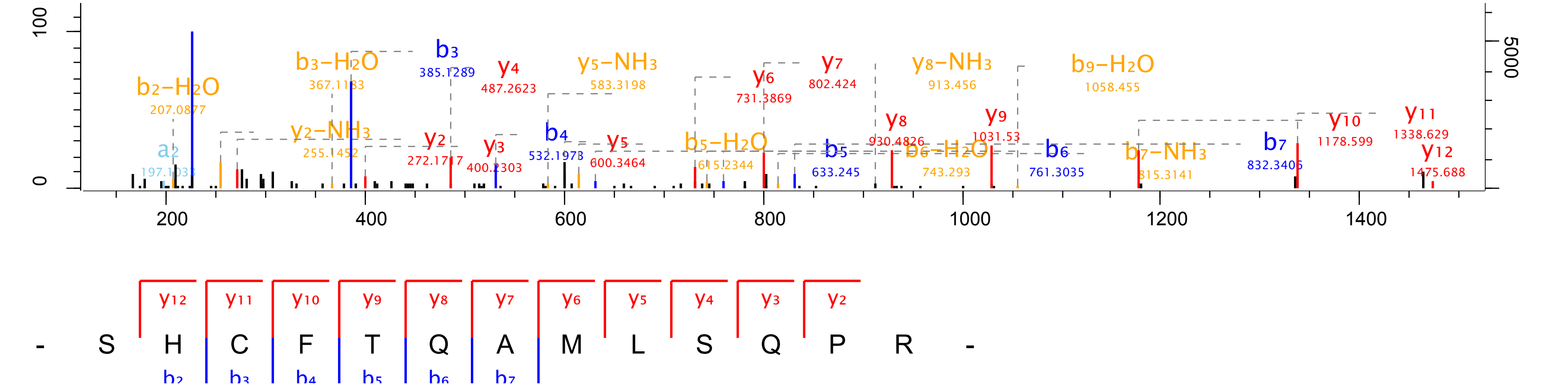

Raw file	Scan	Method	Score	m/z	Gene names
20140925_fract18_dyn_5ul_H2_01_453	15973	TOF; CID	81.3	365.55	ATAT1

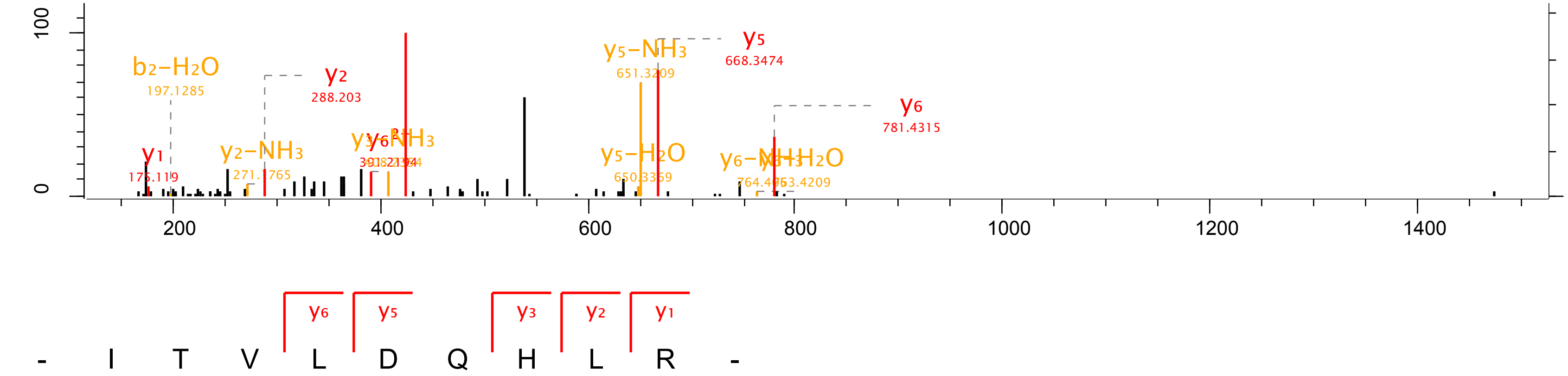

Raw file	Scan	Method	Score	m/z	Gene names
20140925_fract18_dyn_5ul_H2_01_453	17699	TOF; CID	157.99	436.58	RPL39;RPL39P5

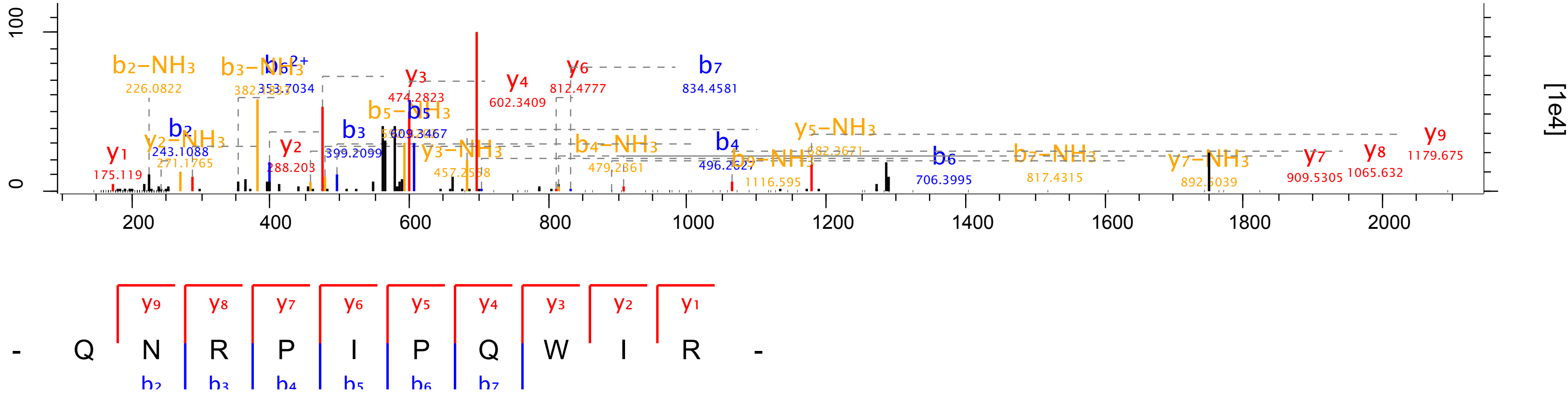

Raw file	Scan	Method	Score	m/z	Gene names
20140925_fract18_dyn_5ul_H2_01_453	22779	TOF; CID	78.08	810.91	MFSD1

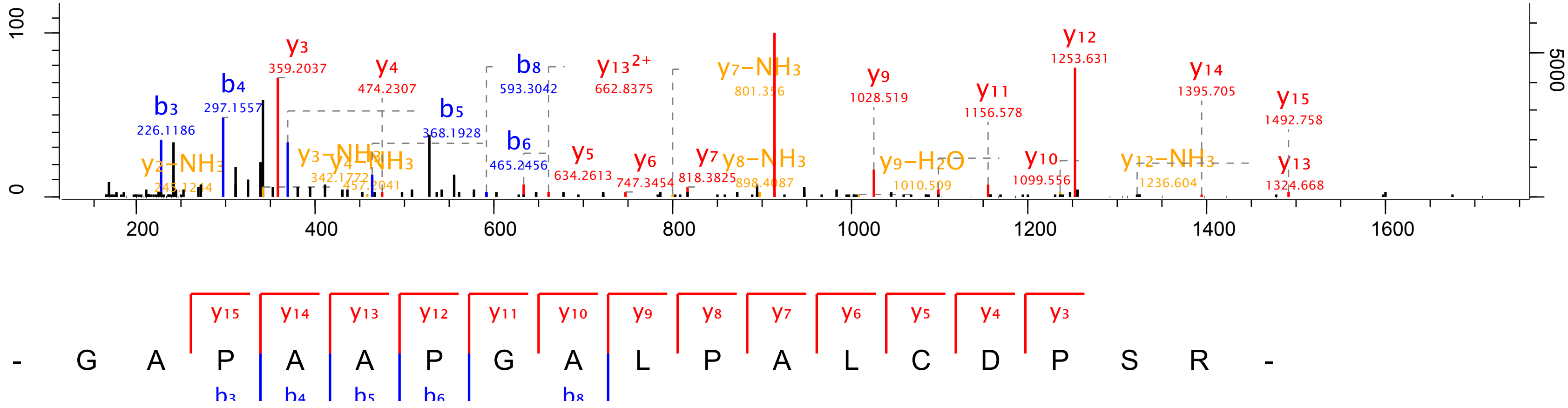

Raw file	Scan	Method	Score	m/z	Gene names
20140925_fract18_dyn_5ul_H2_01_453	23605	TOF; CID	106.3	788.38	SMIM10

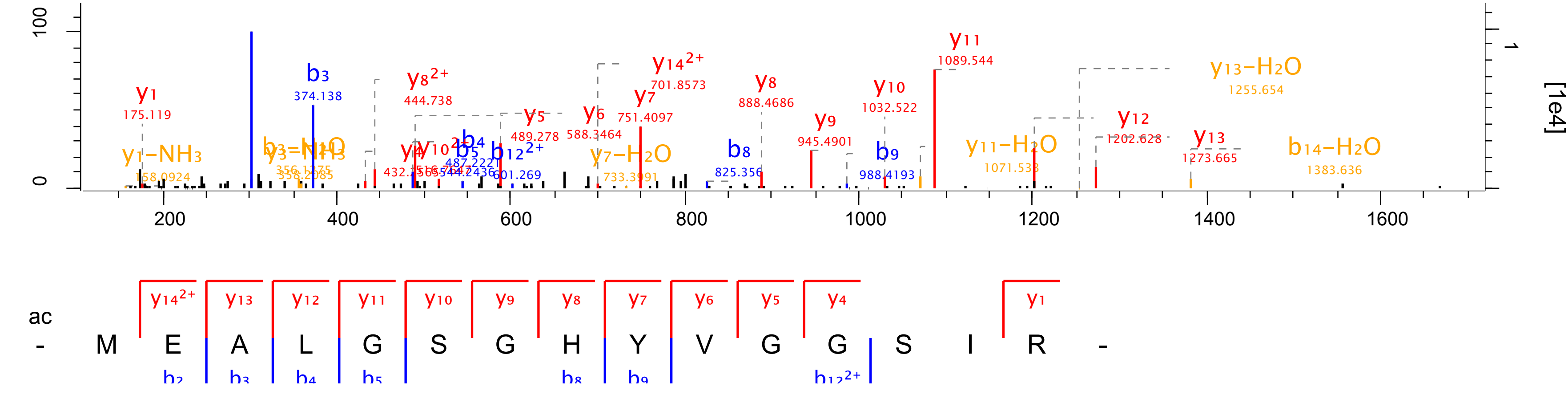

Raw file	Scan	Method	Score	m/z	Gene names
20140925_fract18_dyn_5ul_H2_01_453	24761	TOF; CID	55.44	925.46	PPDPF

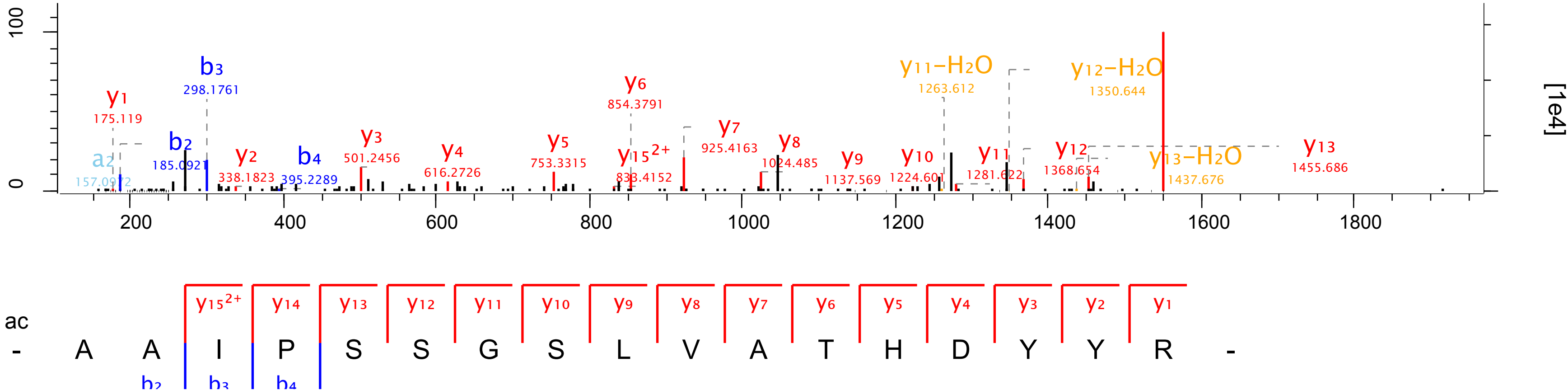

Raw file	Scan	Method	Score	m/z	Gene names
20140925_fract18_dyn_5ul_H2_01_453	26144	TOF; CID	106.28	941.45	FAM168B

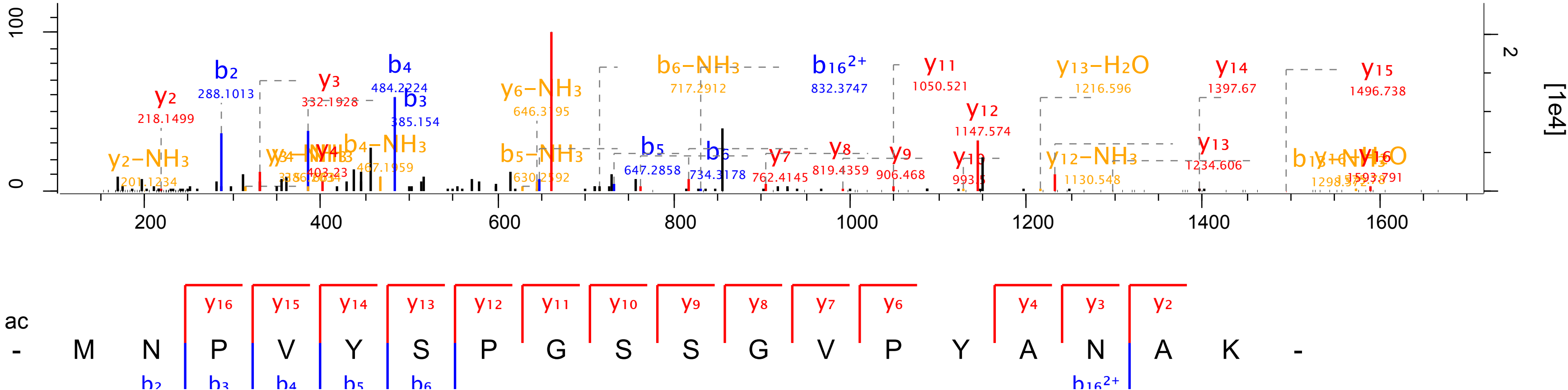

Raw file	Scan	Method	Score	m/z
20140925_fract18_dyn_5ul_H2_01_453	26801	TOF; CID	55.44	809.4

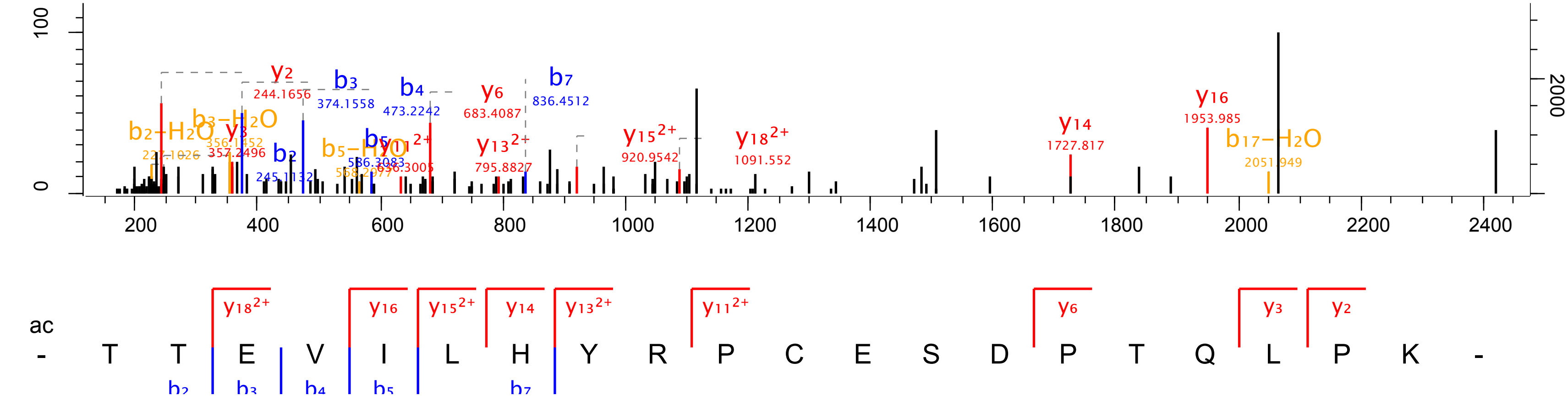

Raw file	Scan	Method	Score	m/z	Gene names
20140925_fract18_dyn_5ul_H2_01_453	27740	TOF; CID	61.42	702.37	VPS37C

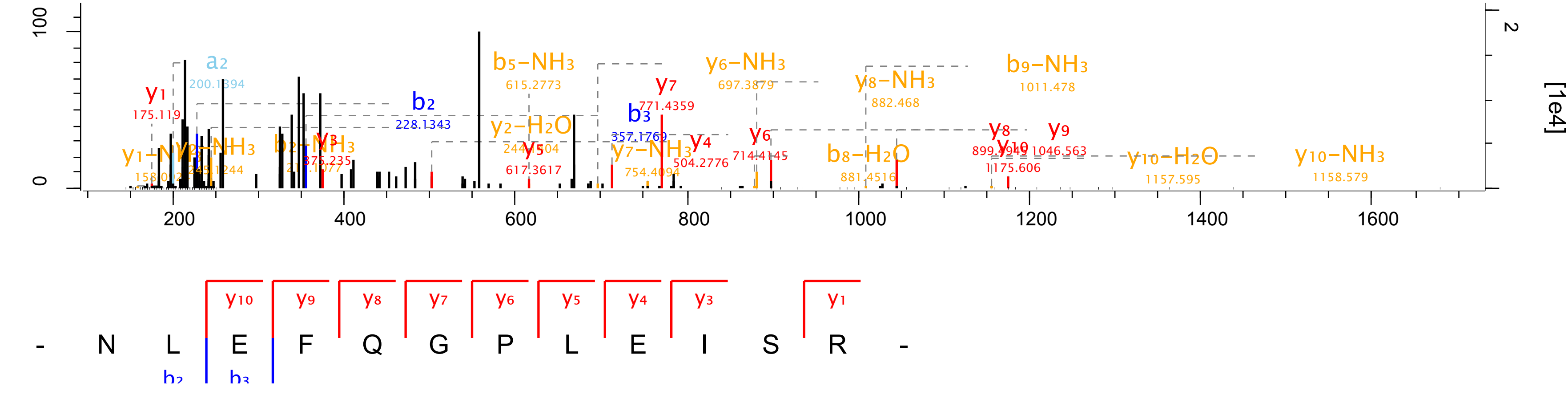

Raw file	Scan	Method	Score	m/z	Gene names
20140925_fract18_dyn_5ul_H2_01_453	28949	TOF; CID	81.77	1100.51	CEBPG

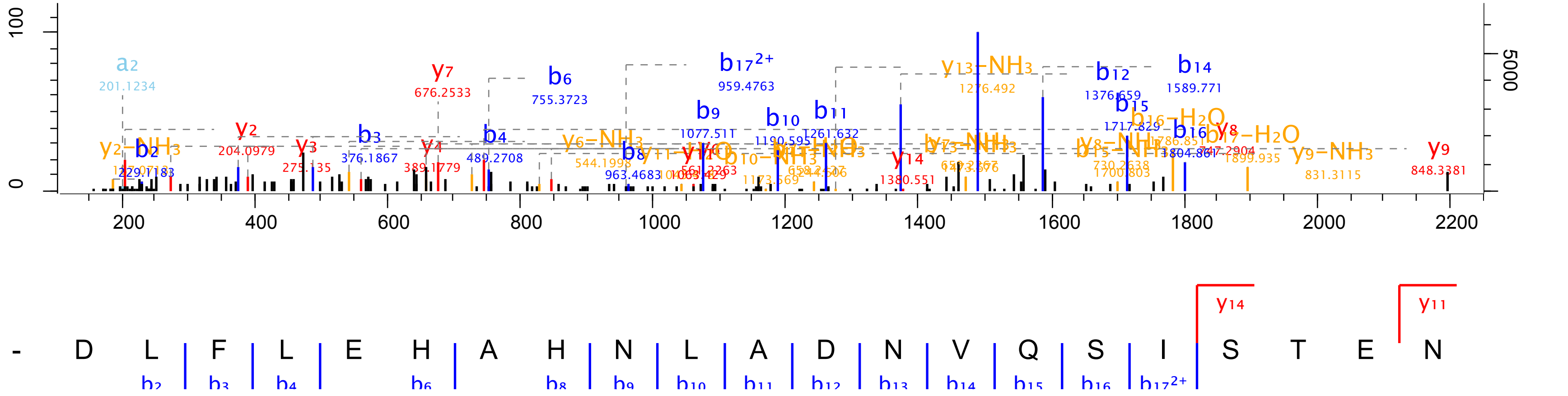

Raw file	Scan	Method	Score	m/z	Gene names
20140925_fract18_dyn_5ul_H2_01_453	30058	TOF; CID	62.47	633.81	BACE1

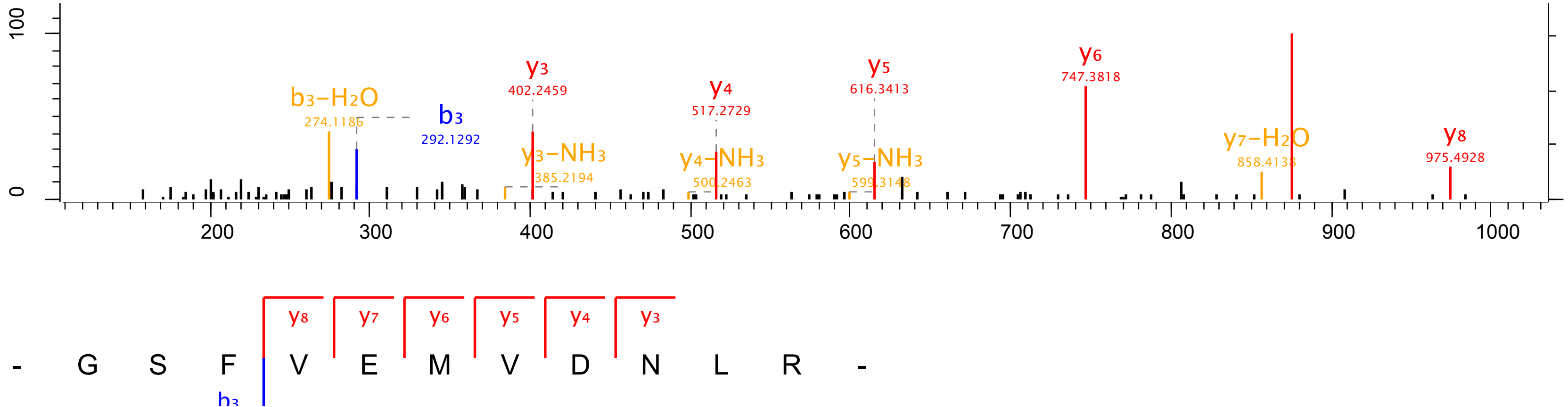

Raw file	Scan	Method	Score	m/z	Gene names
20140925_fract18_dyn_5ul_H2_01_453	32290	TOF; CID	77.22	968	EFHC1

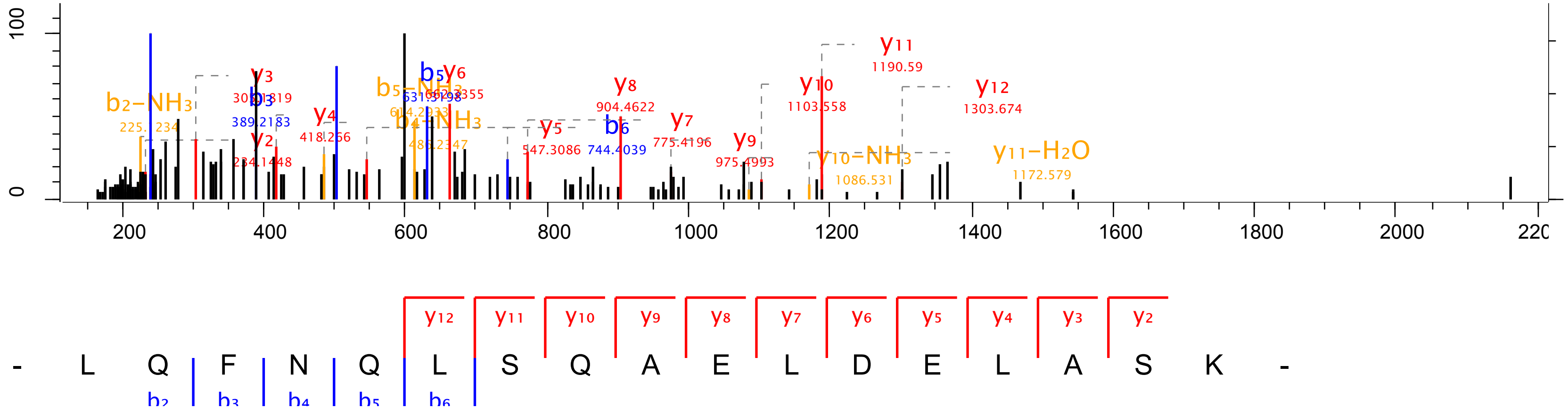

Raw file	Scan	Method	Score	m/z	Gene names
20140925_fract18_dyn_5ul_H2_01_453	33602	TOF; CID	77.75	808.43	NAA60

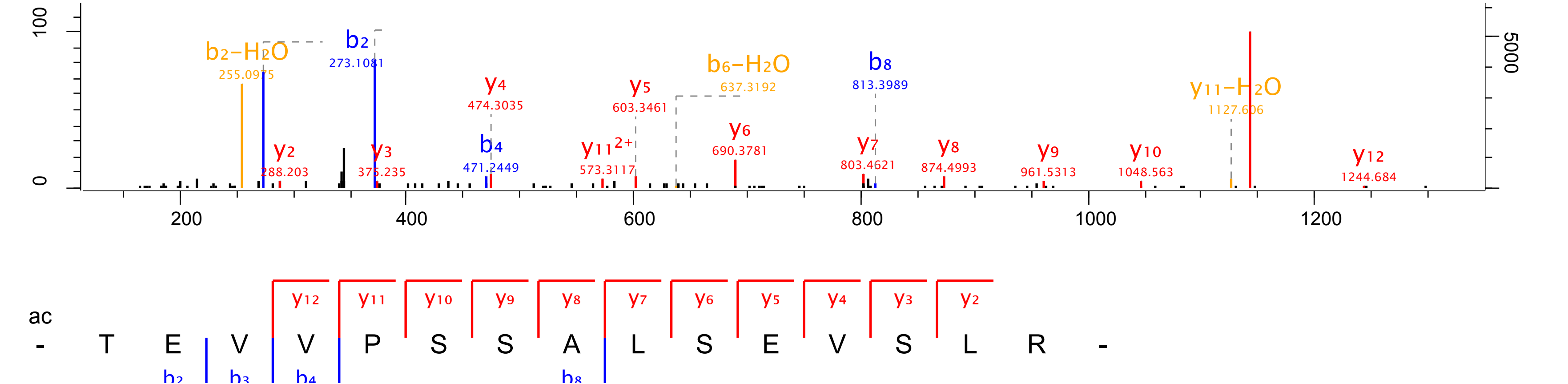

Raw file	Scan	Method	Score	m/z	Gene names
20140925_fract19_dyn_5ul_H3_01_454	6187	TOF; CID	79.12	348.87	ZNF12

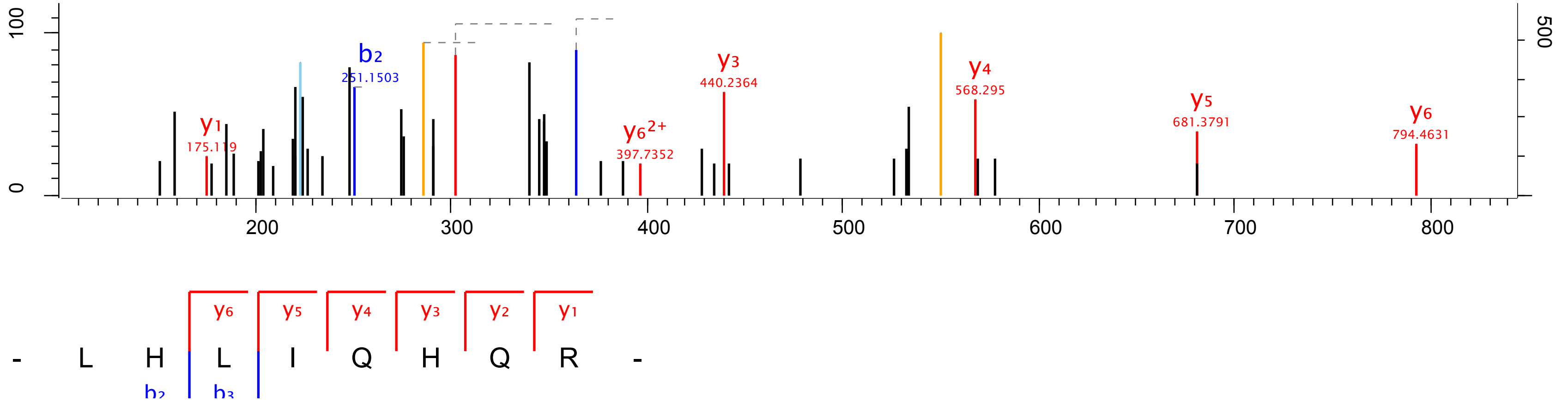

Raw file	Scan	Method	Score	m/z	Gene names
20140925_fract19_dyn_5ul_H3_01_454	9959	TOF; CID	49.45	431.23	C6orf136

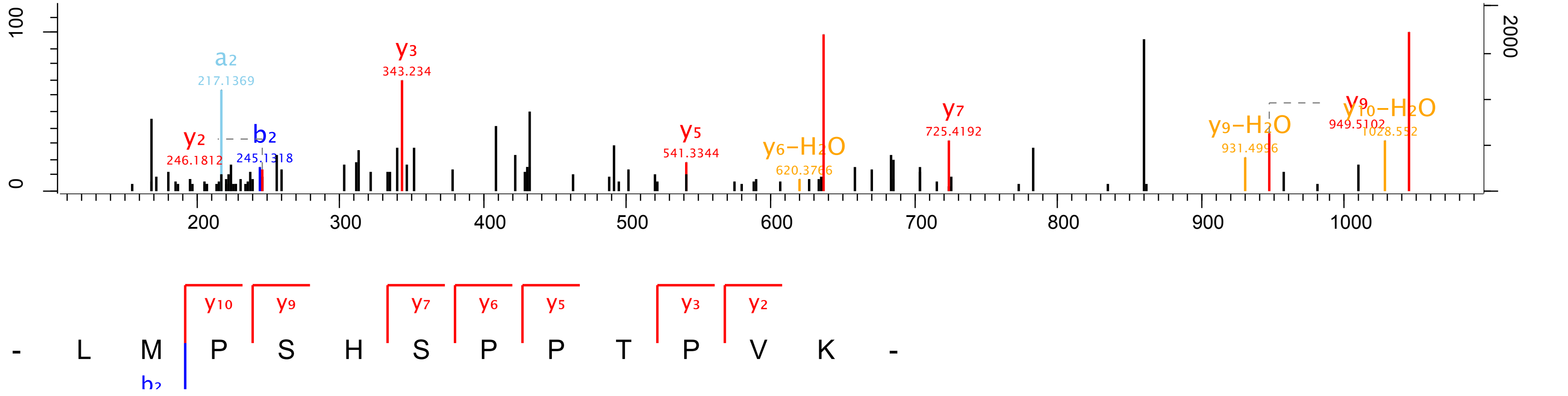

Raw file	Scan	Method	Score	m/z	Gene names
20140925_fract19_dyn_5ul_H3_01_454	14659	TOF; CID	49.19	514.27	TMEM53

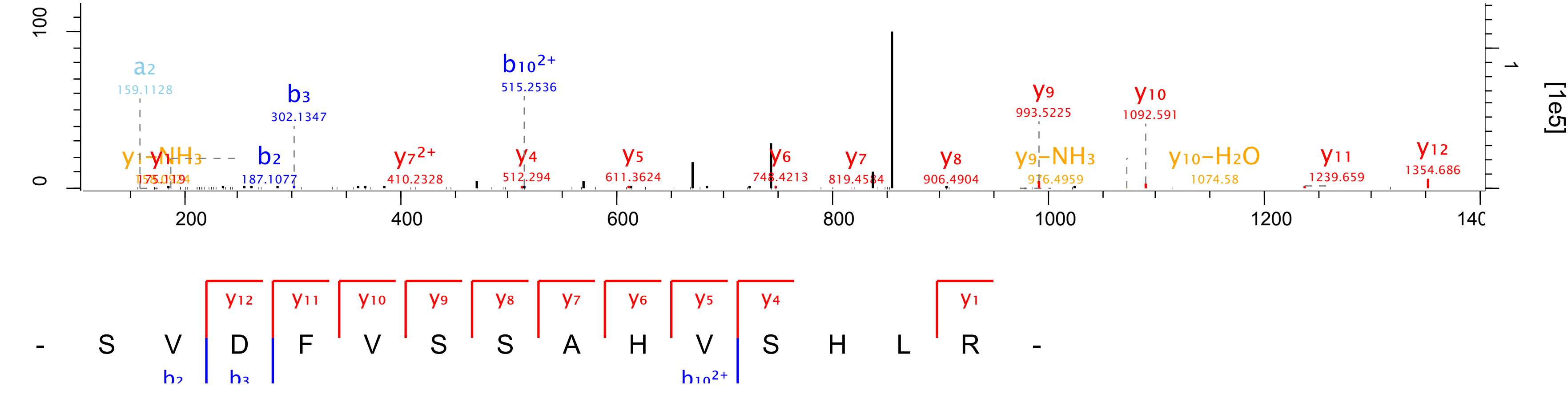

Raw file	Scan	Method	Score	m/z	Gene names
20140925_fract19_dyn_5ul_H3_01_454	15075	TOF; CID	90.15	499.8	DNAJB14

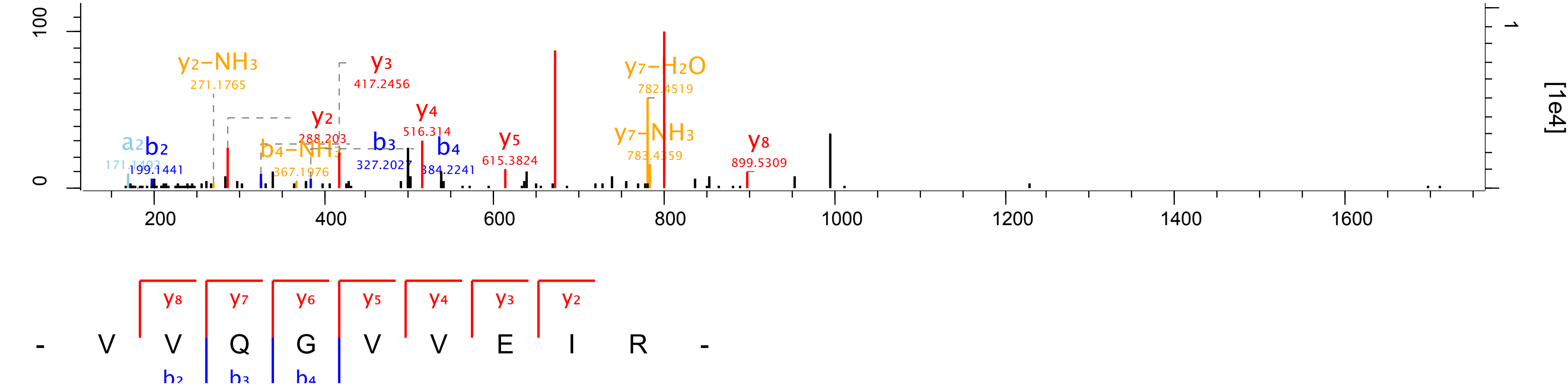

Raw file	Scan	Method	Score	m/z	Gene names
20140925_fract19_dyn_5ul_H3_01_454	17579	TOF; CID	152.47	476.24	MAPK15

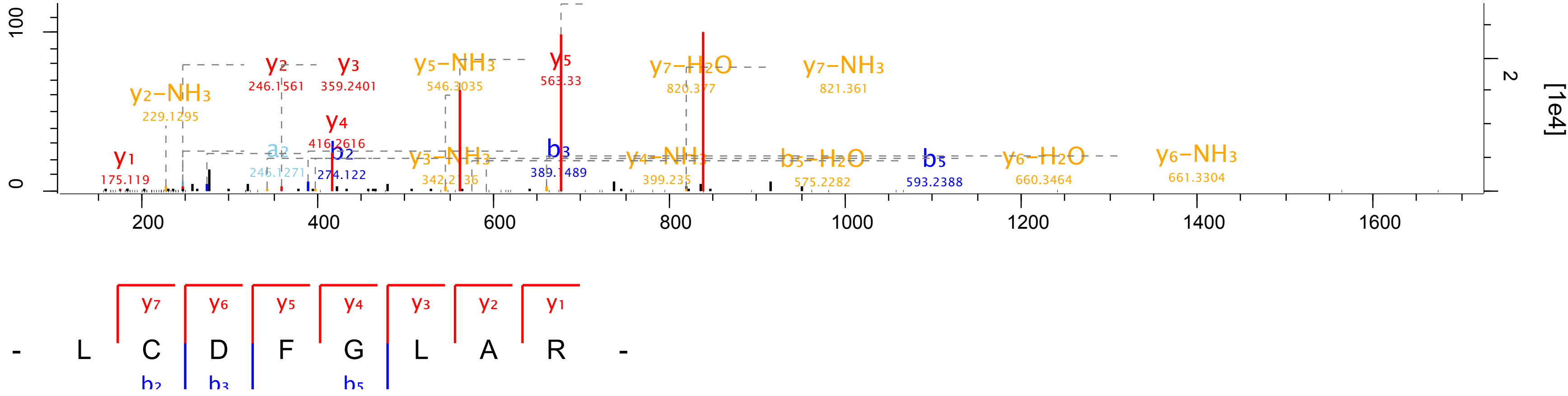

Raw file	Scan	Method	Score	m/z	Gene names
20140925_fract19_dyn_5ul_H3_01_454	18392	TOF; CID	130.04	510.78	MAP2K5

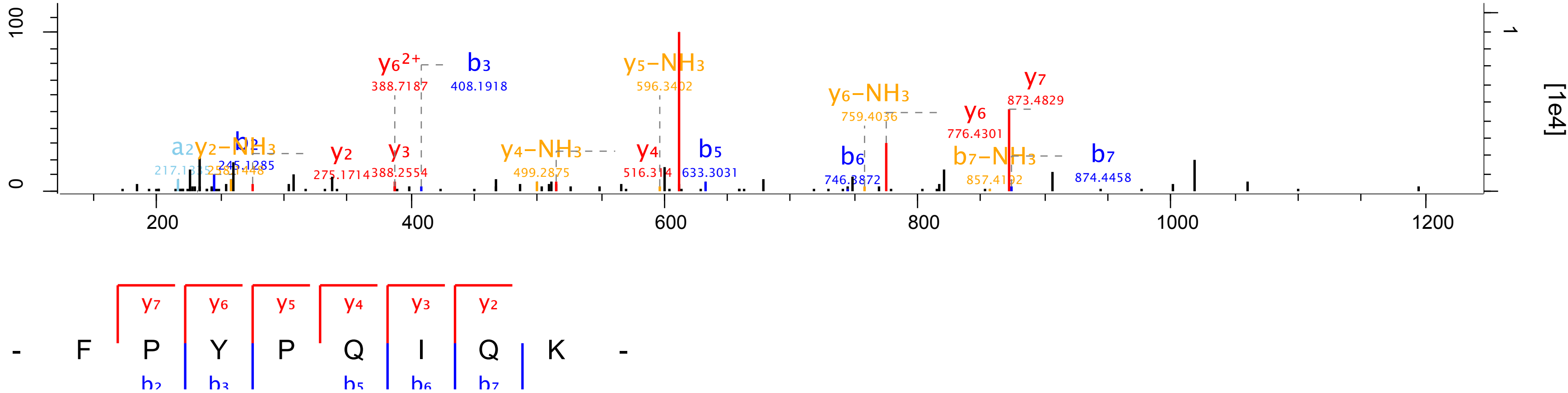

Raw file	Scan	Method	Score	m/z	Gene names
20140925_fract19_dyn_5ul_H3_01_454	18582	TOF; CID	93.5	548.52	ZDHHC24

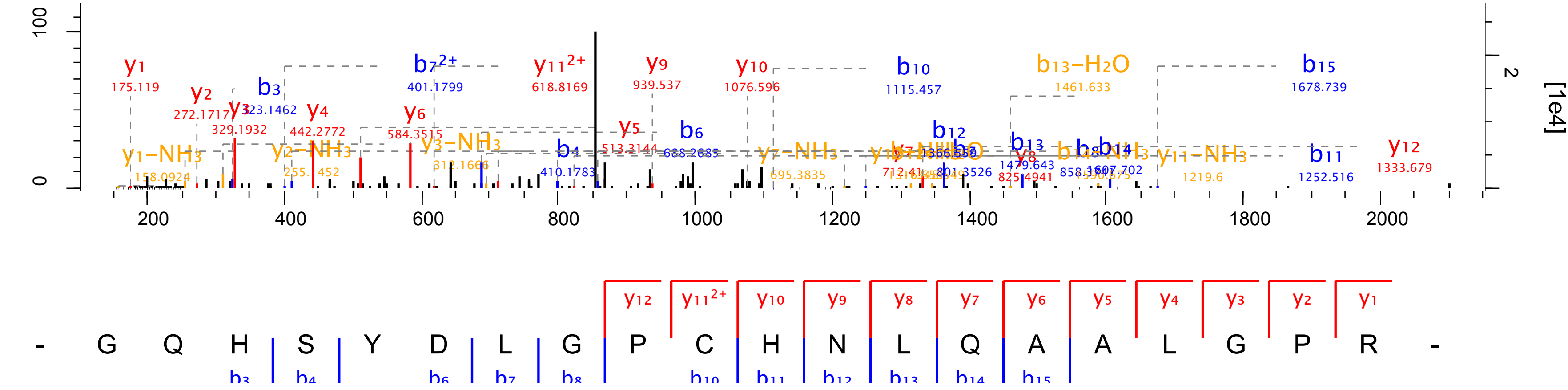

Raw file	Scan	Method	Score	m/z	Gene names
20140925_fract19_dyn_5ul_H3_01_454	20773	TOF; CID	63.47	462.25	EFCAB7

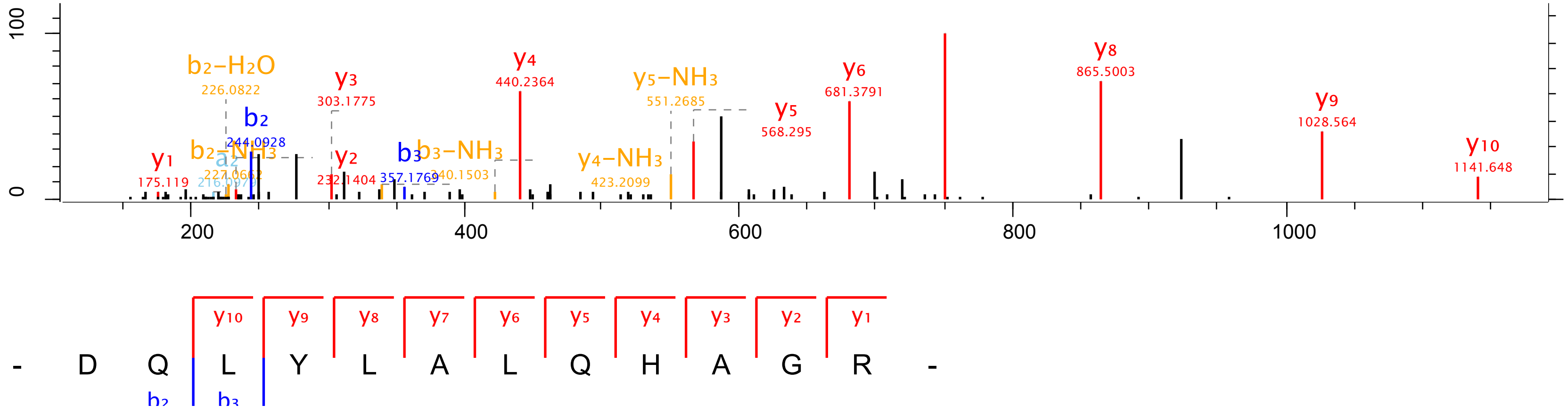

Raw file	Scan	Method	Score	m/z	Gene names
20140925_fract19_dyn_5ul_H3_01_454	23464	TOF; CID	73.93	771.36	FBXO17

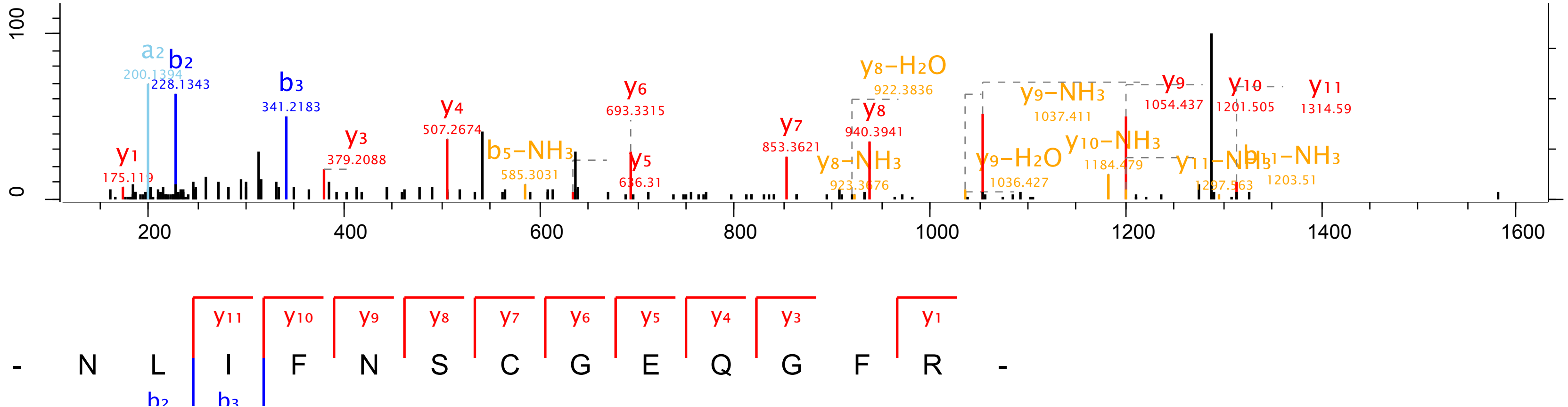

Raw file	Scan	Method	Score	m/z	Gene names
20140925_fract19_dyn_5ul_H3_01_454	24558	TOF; CID	74.53	538.29	S100P

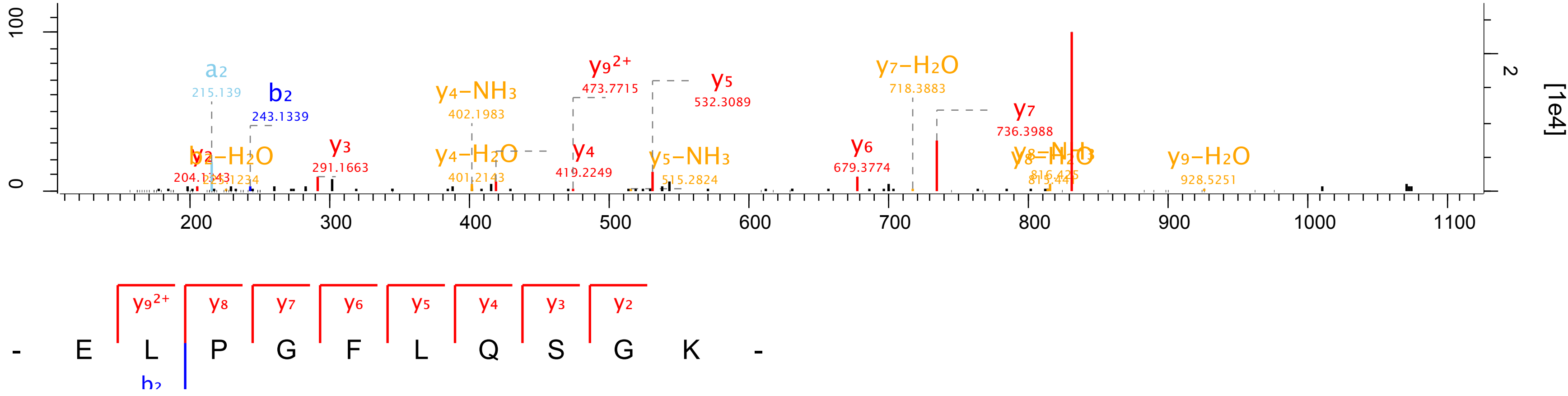

Raw file	Scan	Method	Score	m/z	Gene names
20140925_fract19_dyn_5ul_H3_01_454	26432	TOF; CID	42.64	712.7	SDC3

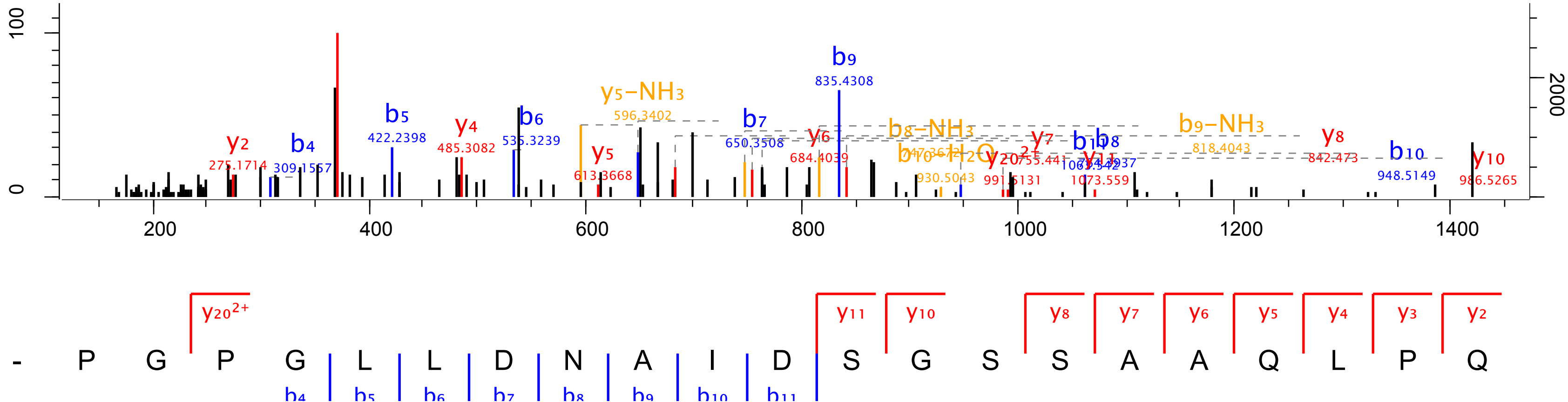

Raw file	Scan	Method	Score	m/z	Gene names
20140925_fract19_dyn_5ul_H3_01_454	33397	TOF; CID	51.23	718.7	BCL2L11

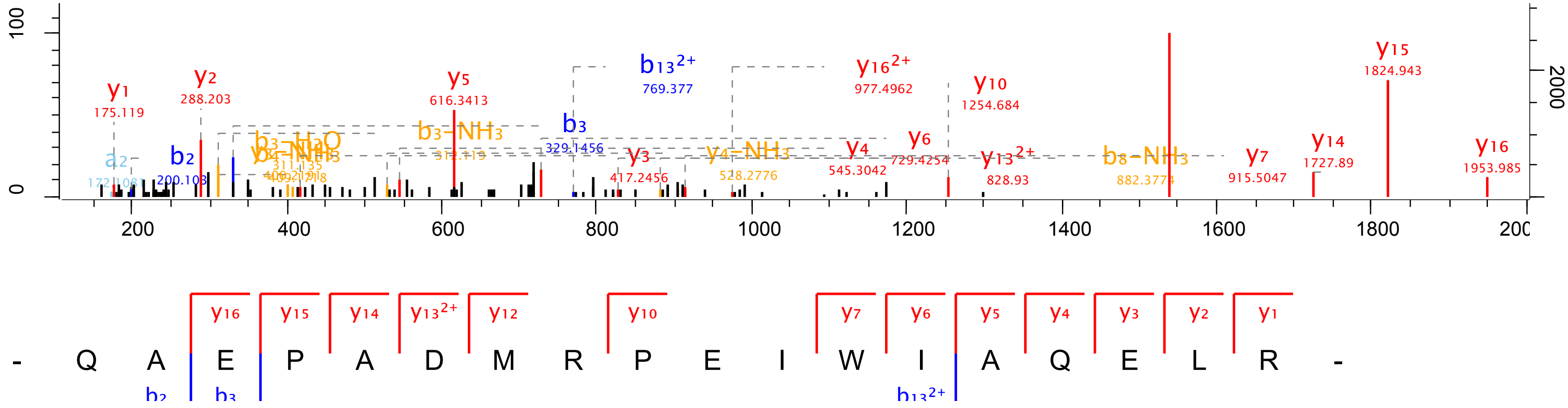

Raw file	Scan	Method	Score	m/z	Gene names
20140925_fract19_dyn_5ul_H3_01_454	33877	TOF; CID	125.31	1319.1	CHCHD10

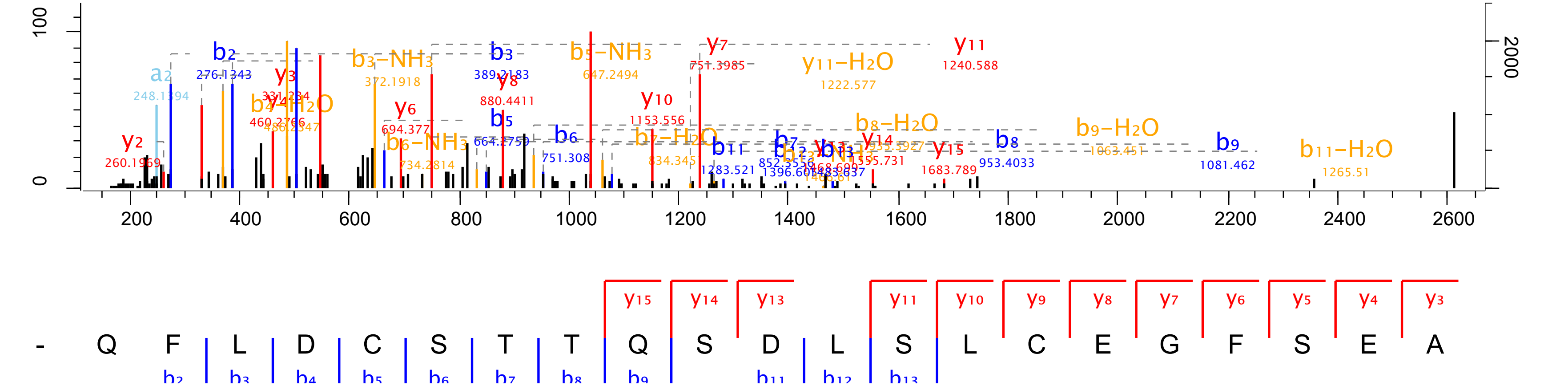

Raw file	Scan	Method	Score	m/z	Gene names
20140925_fract19_dyn_5ul_H3_01_454	34248	TOF; CID	61.94	1091.52	CDKN2AIPNL

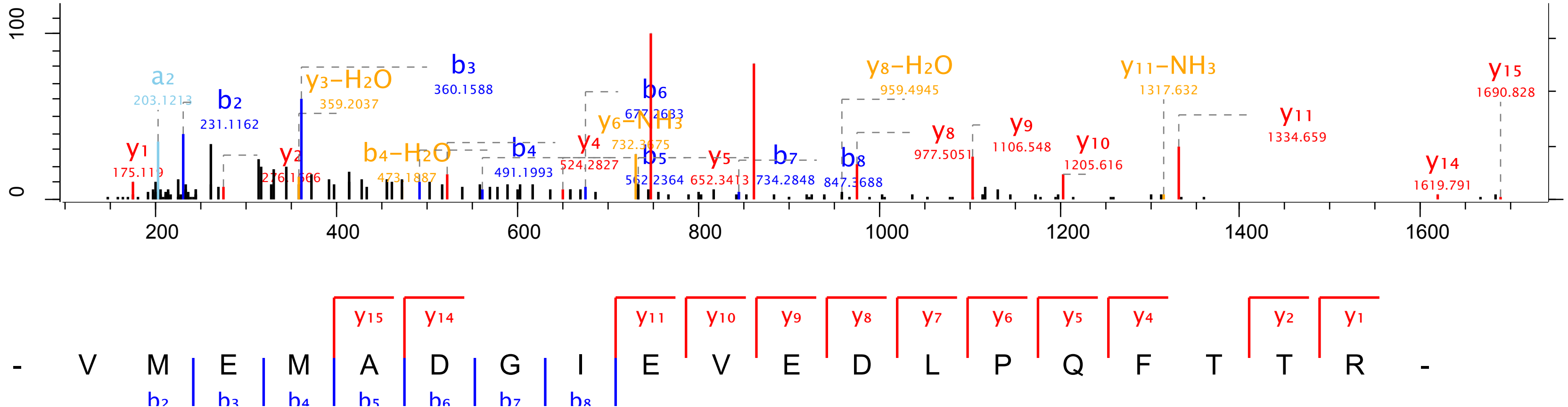

Raw file	Scan	Method	Score	m/z	Gene names
20140925_fract20_dyn_5ul_H4_01_455	8917	TOF; CID	80.83	437.25	KIAA1328

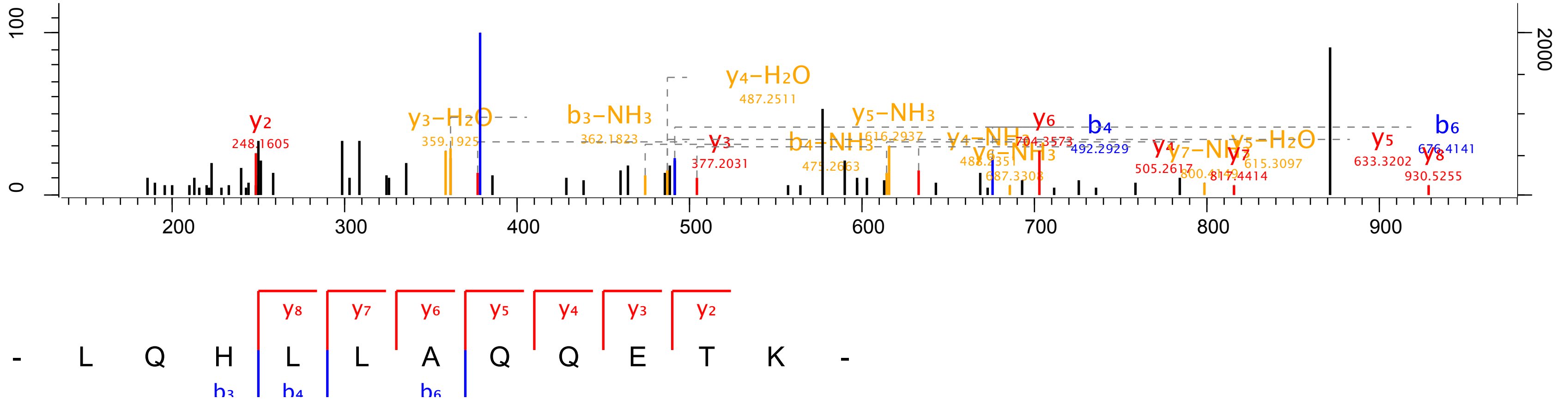

Raw file	Scan	Method	Score	m/z	Gene names
20140925_fract20_dyn_5ul_H4_01_455	9852	TOF; CID	97.8	495.28	ARID3B

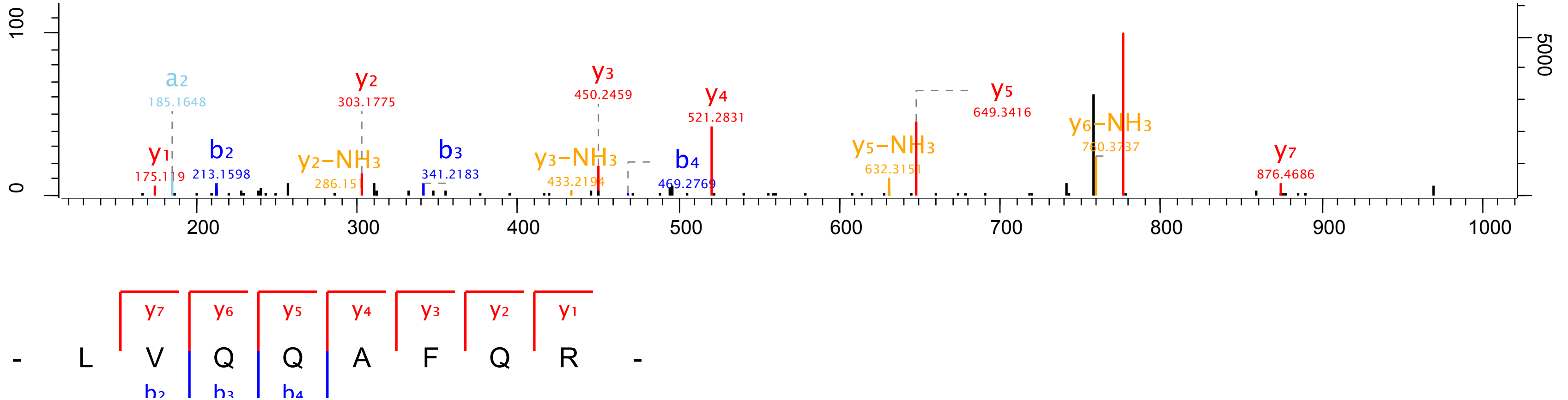

Raw file	Scan	Method	Score	m/z	Gene names
20140925_fract20_dyn_5ul_H4_01_455	13873	TOF; CID	78.32	486.27	MSRB3

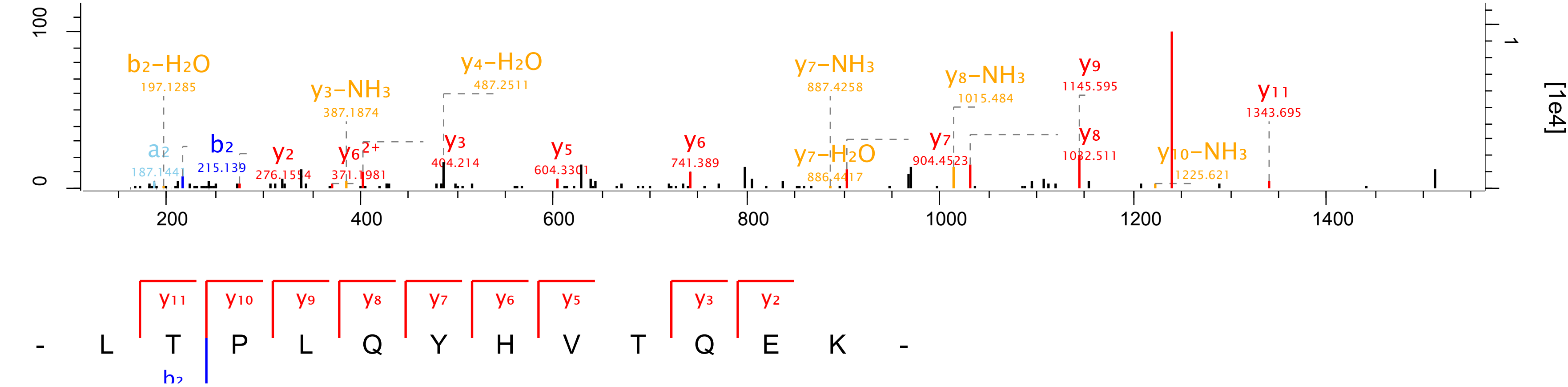

Raw file	Scan	Method	Score	m/z	Gene names
20140925_fract20_dyn_5ul_H4_01_455	15079	TOF; CID	84	505.78	NAIF1

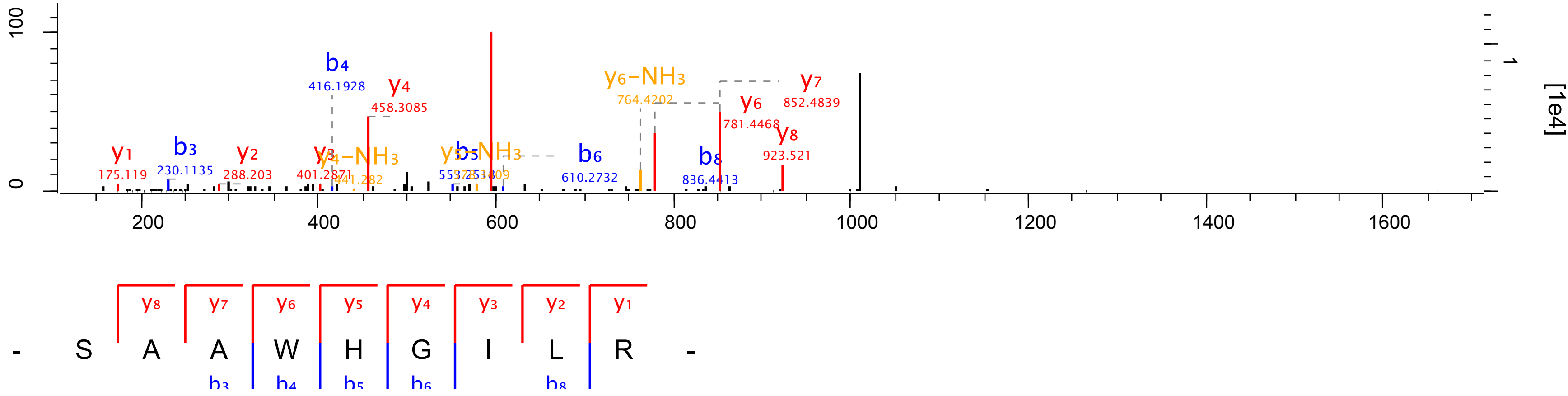

Raw file	Scan	Method	Score	m/z	Gene names
20140925_fract20_dyn_5ul_H4_01_455	16399	TOF; CID	46.89	610.33	SLC22A20

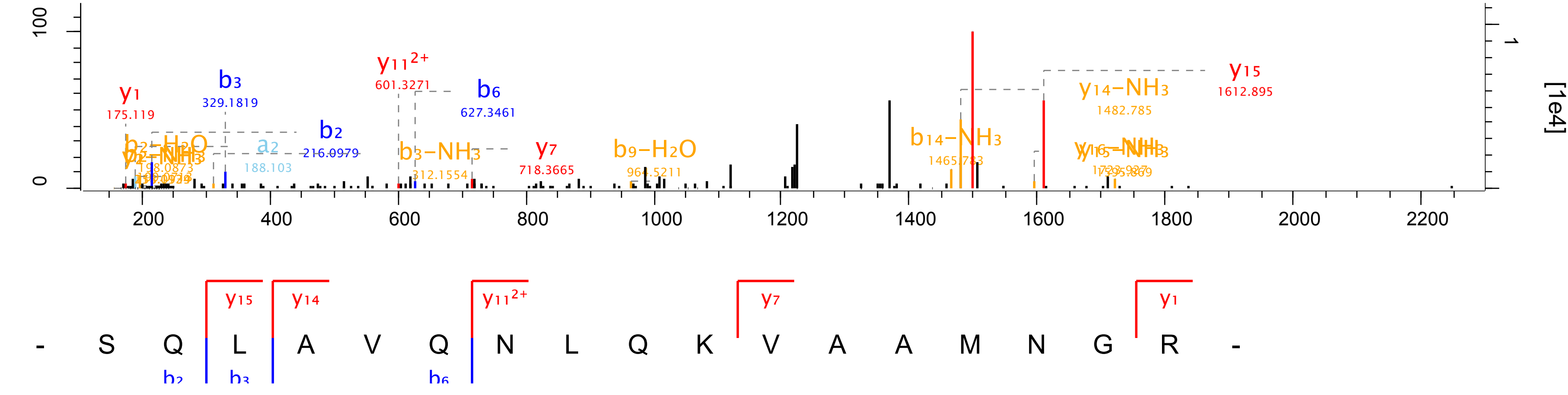

Raw file	Scan	Method	Score	m/z	Gene names
20140925_fract20_dyn_5ul_H4_01_455	17586	TOF; CID	168.05	932.93	ROMO1

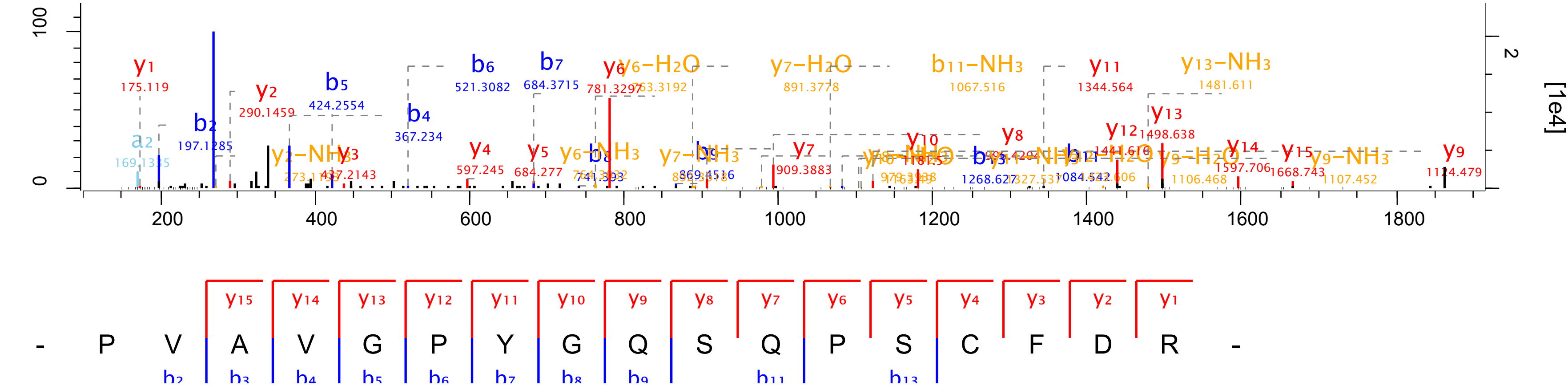

Raw file	Scan	Method	Score	m/z	Gene names
20140925_fract20_dyn_5ul_H4_01_455	21001	TOF; CID	69.81	395.88	CCDC65

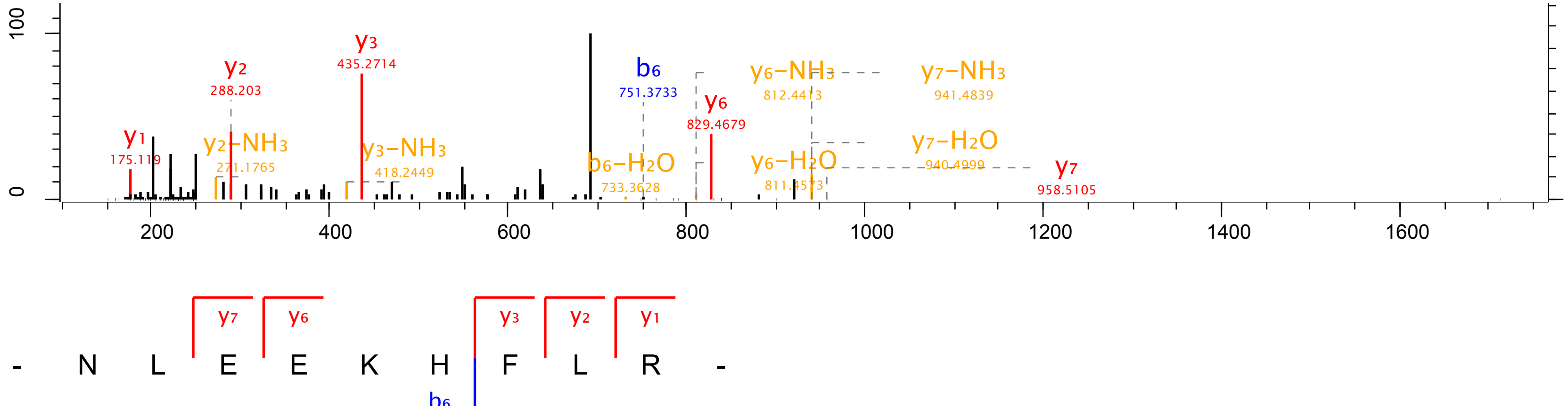

Raw file	Scan	Method	Score	m/z	Gene names
20140925_fract20_dyn_5ul_H4_01_455	23305	TOF; CID	48.9	867.95	FAM102A

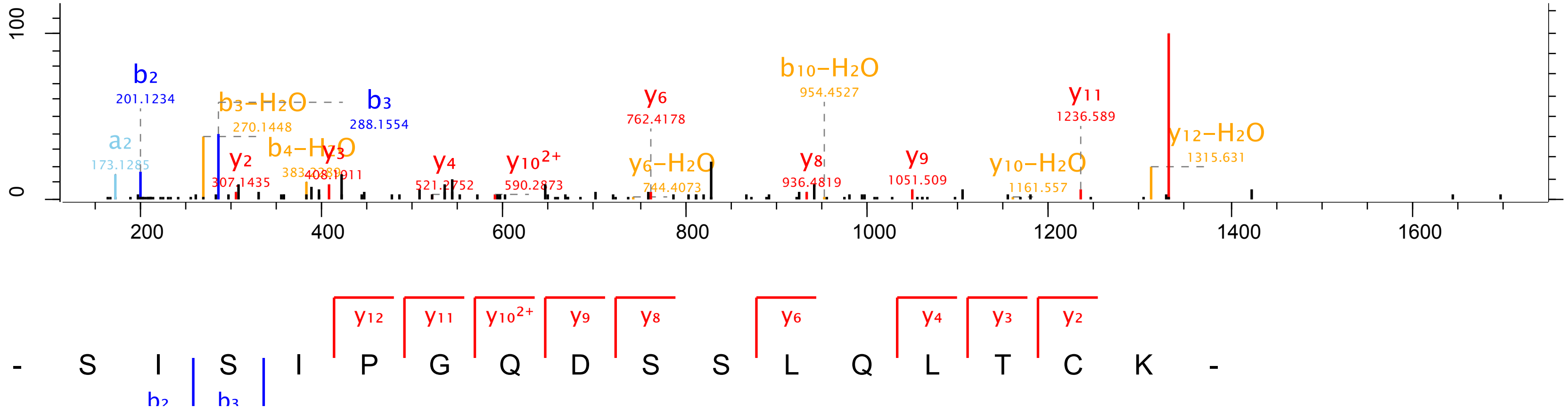

Raw file	Scan	Method	Score	m/z	Gene names
20140925_fract20_dyn_5ul_H4_01_455	27402	TOF; CID	99.94	732.38	SNAPC5

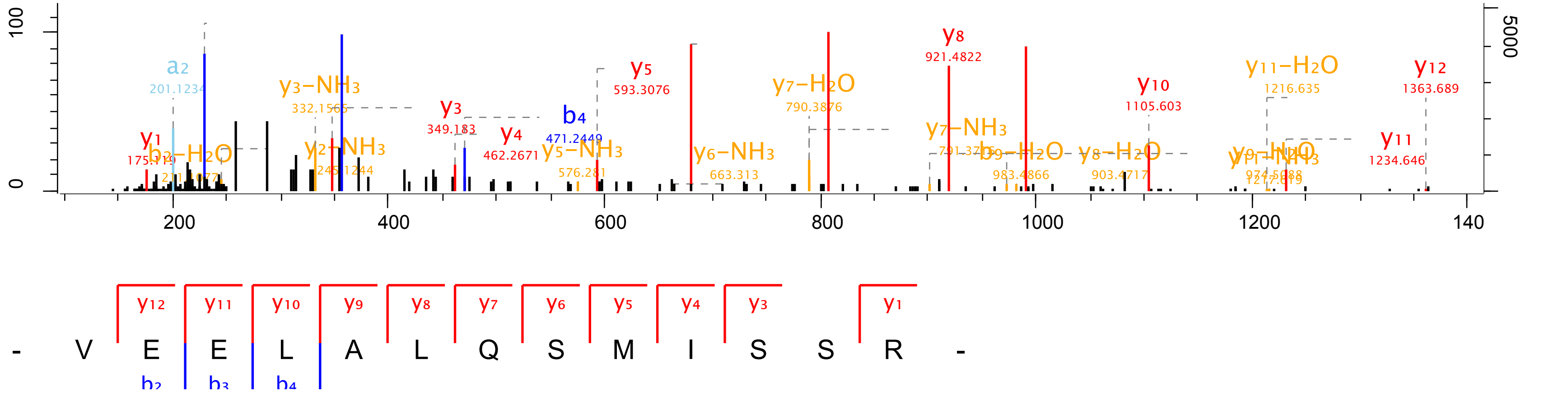

Raw file	Scan	Method	Score	m/z	Gene names
20140925_fract20_dyn_5ul_H4_01_455	27466	TOF; CID	90.26	979.98	FAM168A

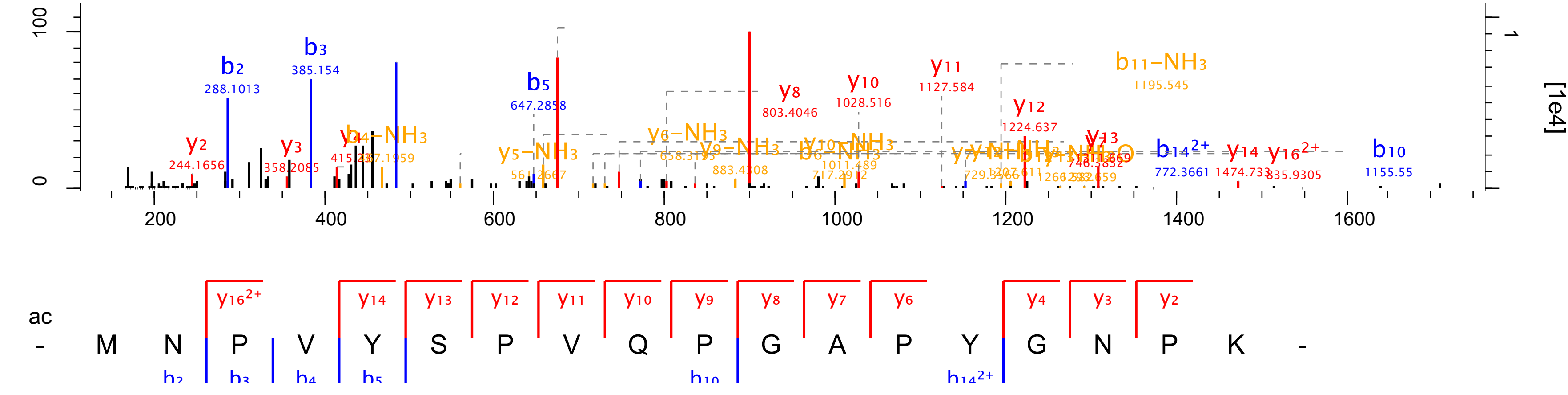

Raw file	Scan	Method	Score	m/z	Gene names
20140925_fract20_dyn_5ul_H4_01_455	29308	TOF; CID	137.39	817.42	SLC26A6

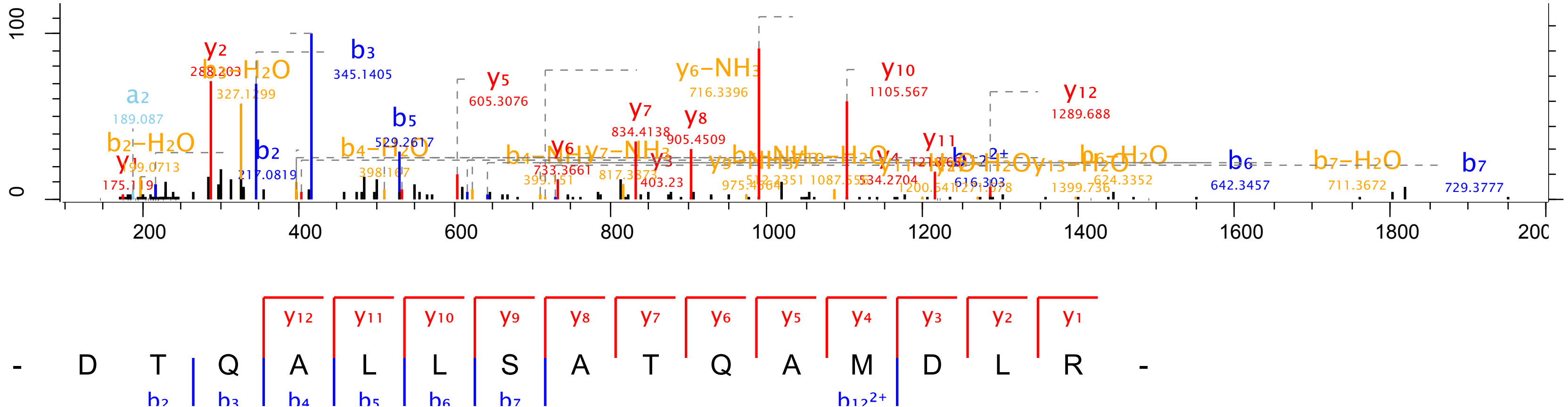

Raw file	Scan	Method	Score	m/z	Gene names
20140925_fract20_dyn_5ul_H4_01_455	31205	TOF; CID	68	673.36	CD82

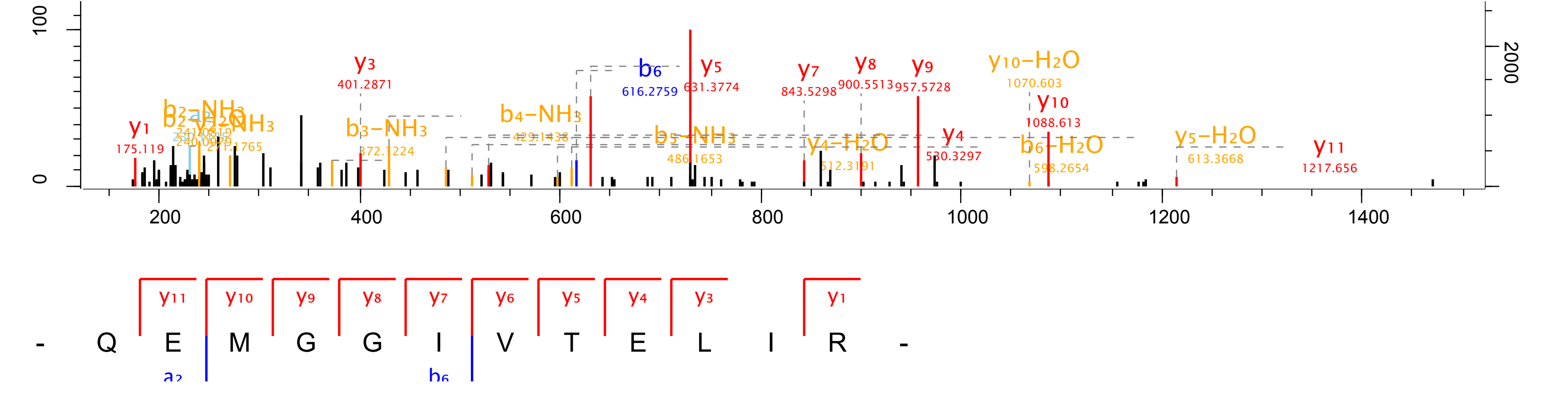

Raw file	Scan	Method	Score	m/z	Gene names
20140925_fract20_dyn_5ul_H4_01_455	31520	TOF; CID	48.97	1230.11	FAM219A

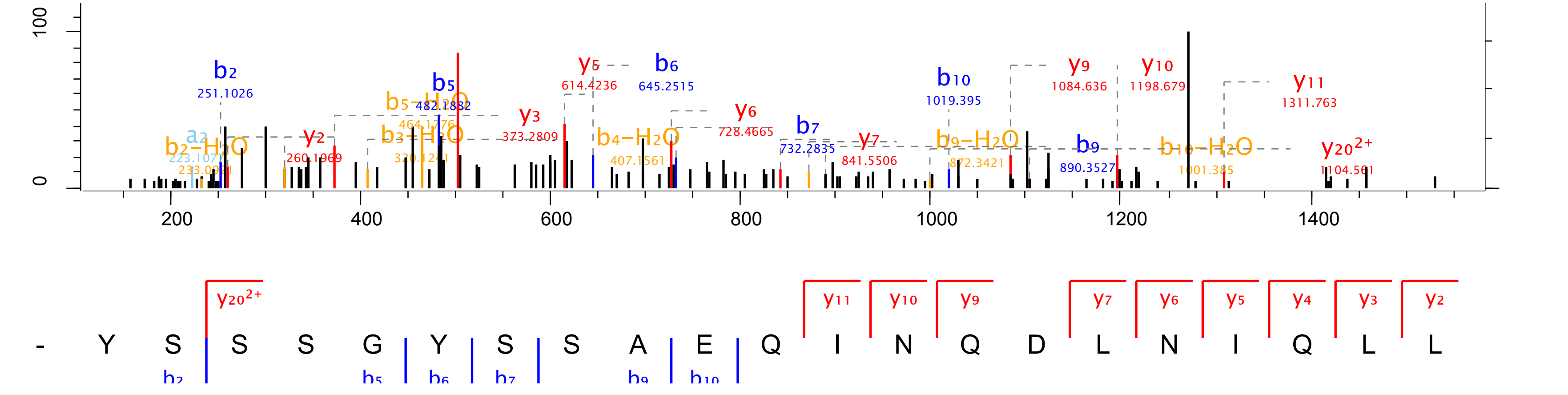

Raw file	Scan	Method	Score	m/z	Gene names
20140925_fract20_dyn_5ul_H4_01_455	34894	TOF; CID	40.97	1073.57	ULK4

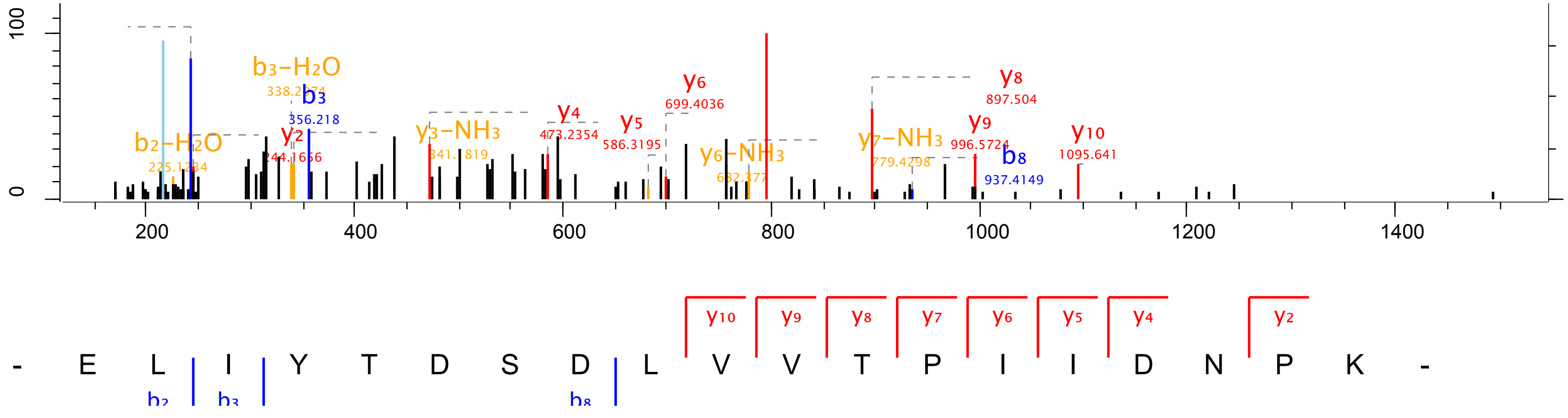

Raw file	Scan	Method	Score	m/z	Gene names
20140925_fract20_dyn_5ul_H4_01_455	37382	TOF; CID	70.89	564.69	SMIM11

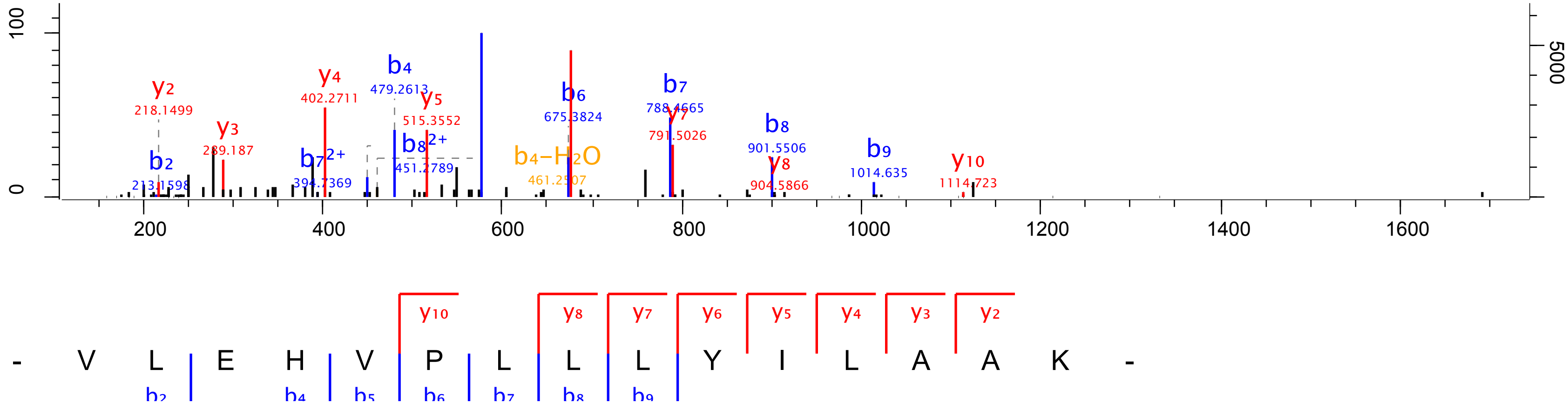

Raw file	Scan	Method	Score	m/z	Gene names
20140925_fract21_dyn_5ul_H5_01_456	16339	TOF; CID	115.71	565.34	NIPA2

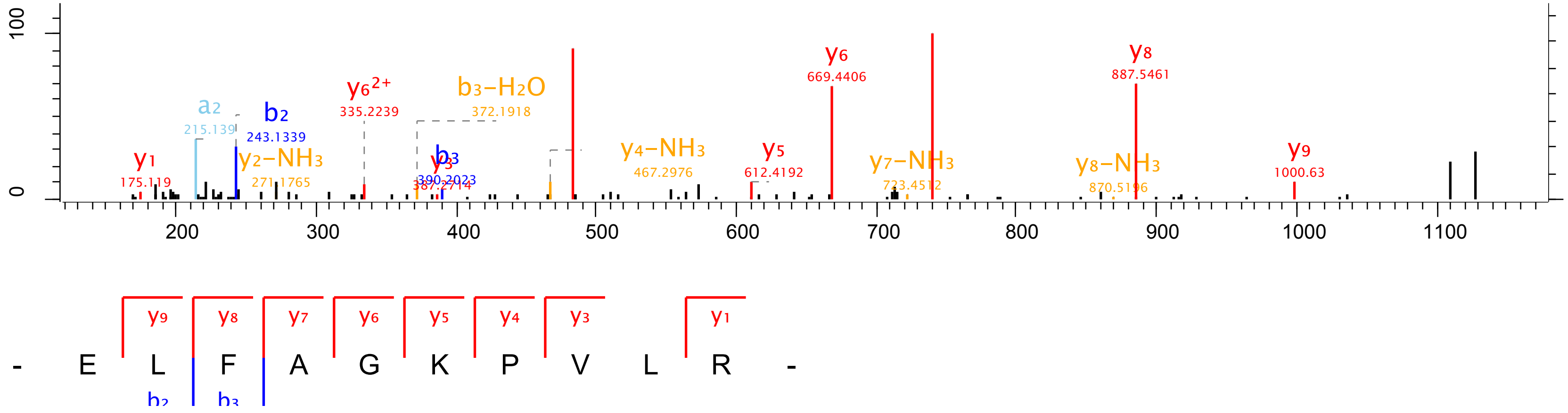

Raw file	Scan	Method	Score	m/z	Gene names
20140925_fract21_dyn_5ul_H5_01_456	17882	TOF; CID	94.59	735.37	AVPI1

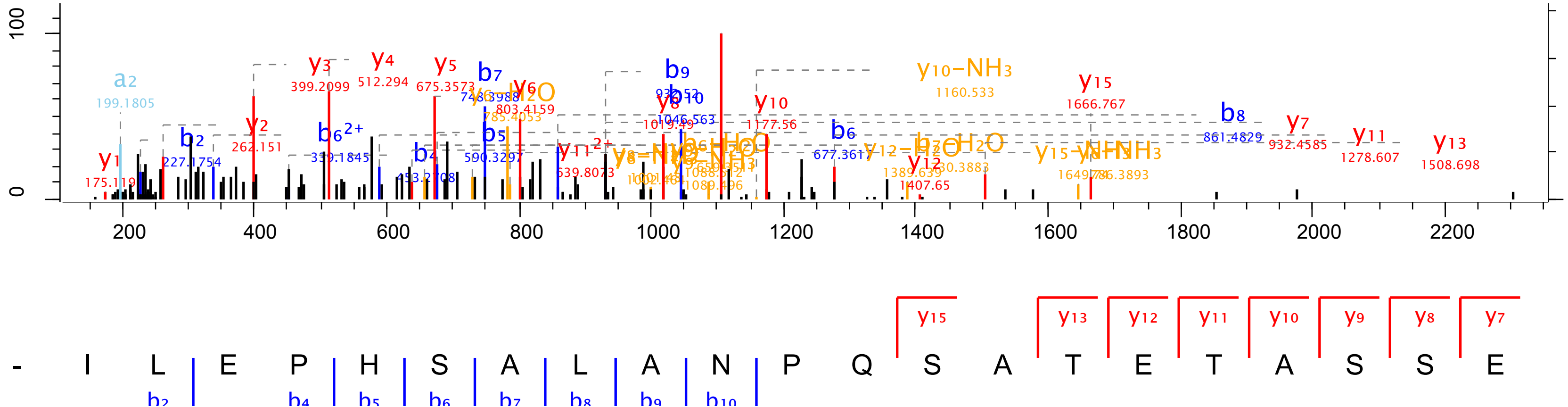

Raw file	Scan	Method	Score	m/z	Gene names
20140925_fract21_dyn_5ul_H5_01_456	20846	TOF; CID	59.71	500.93	USP40

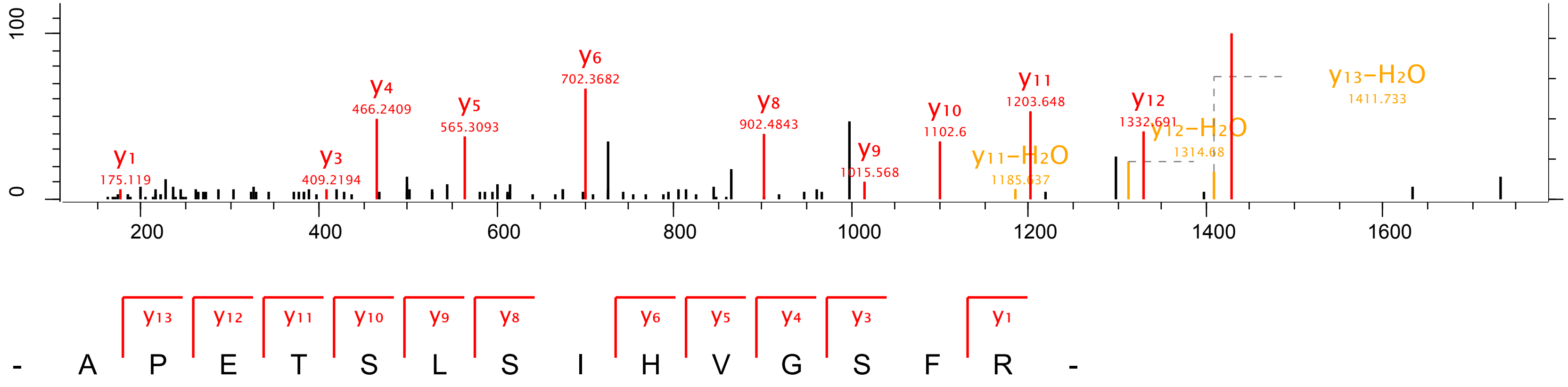

Raw file	Scan	Method	Score	m/z	Gene names
20140925_fract21_dyn_5ul_H5_01_456	23175	TOF; CID	42.12	741.03	ZBTB8A

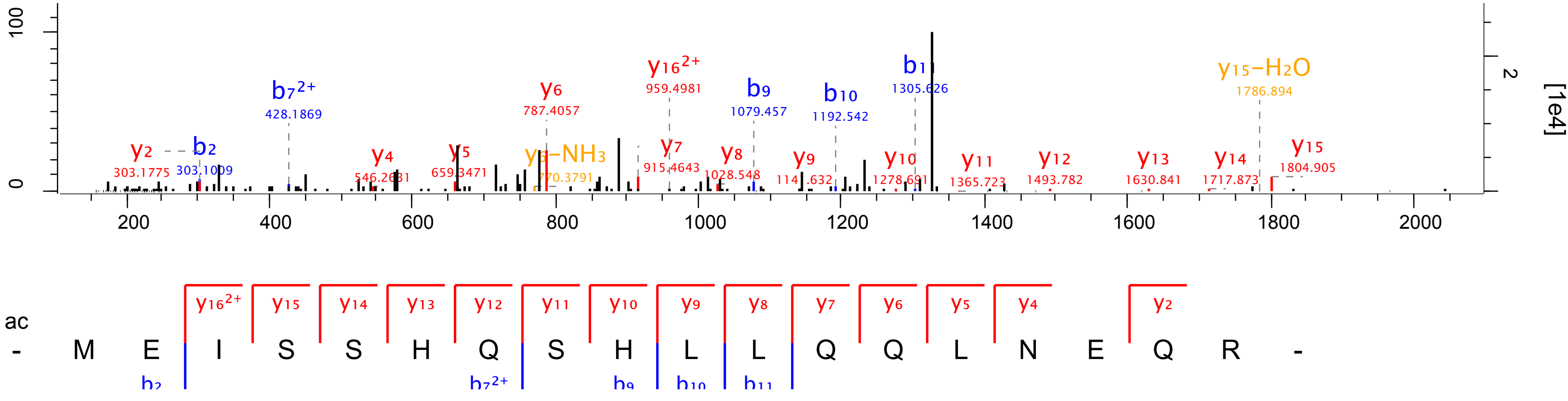

Raw file	Scan	Method	Score	m/z	Gene names
20140925_fract21_dyn_5ul_H5_01_456	23785	TOF; CID	63.62	458.92	CHRNA7

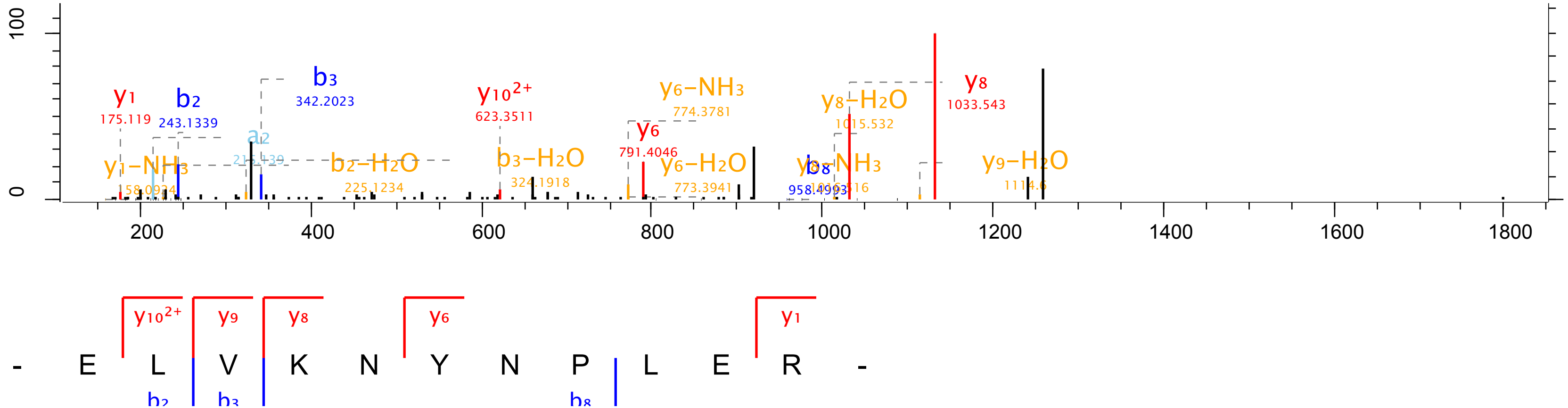

Raw file	Scan	Method	Score	m/z	Gene names
20140925_fract21_dyn_5ul_H5_01_456	24059	TOF; CID	91.62	501.6	HEMK1

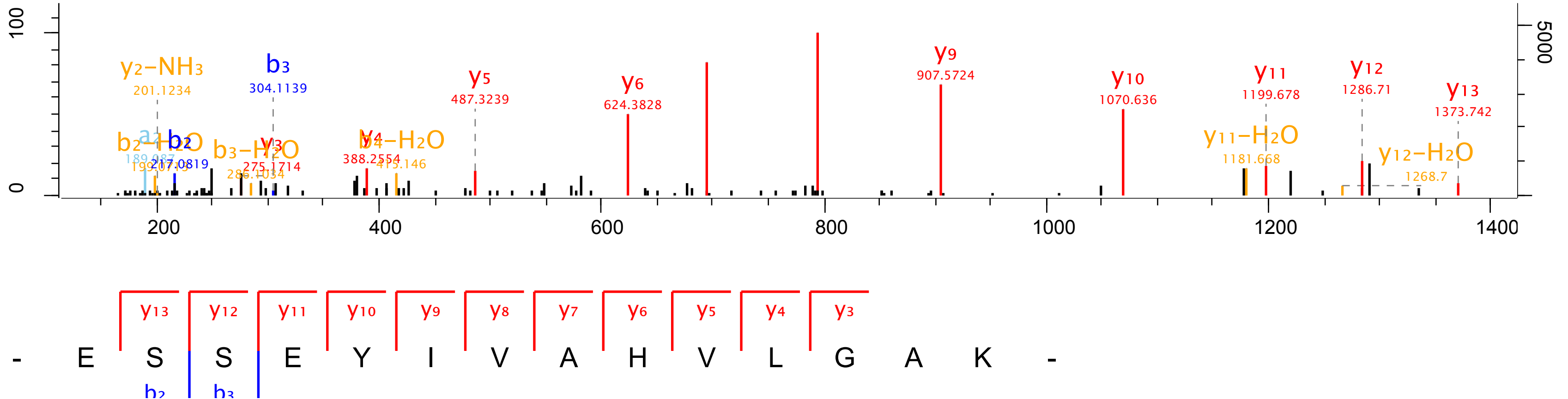

Raw file	Scan	Method	Score	m/z	Gene names
20140925_fract21_dyn_5ul_H5_01_456	26508	TOF; CID	61.11	833.41	IL10RB

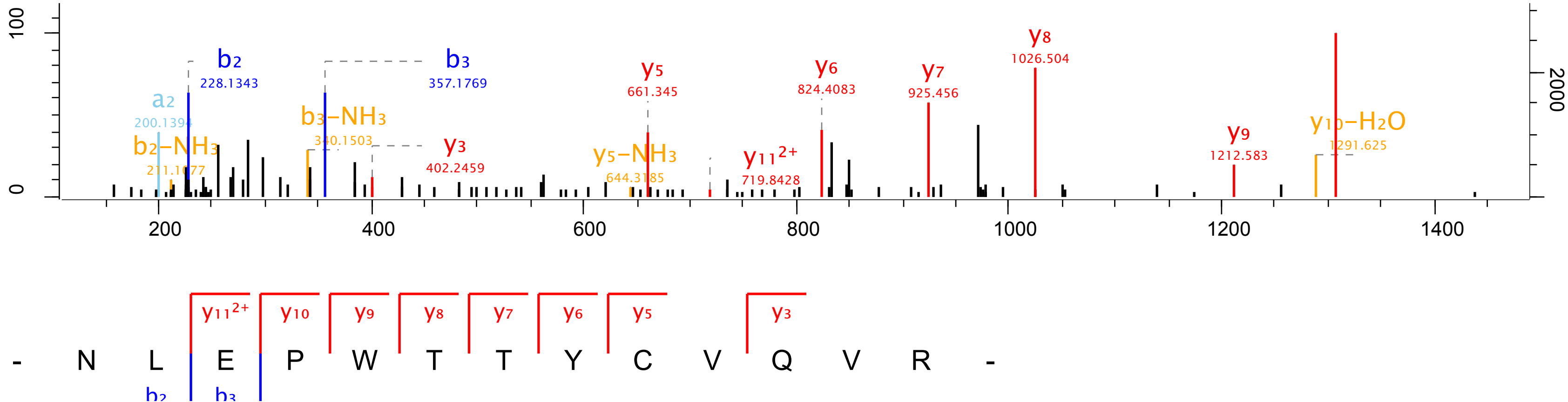

Raw file	Scan	Method	Score	m/z	Gene names
20140925_fract21_dyn_5ul_H5_01_456	27714	TOF; CID	81.02	704.83	OAF

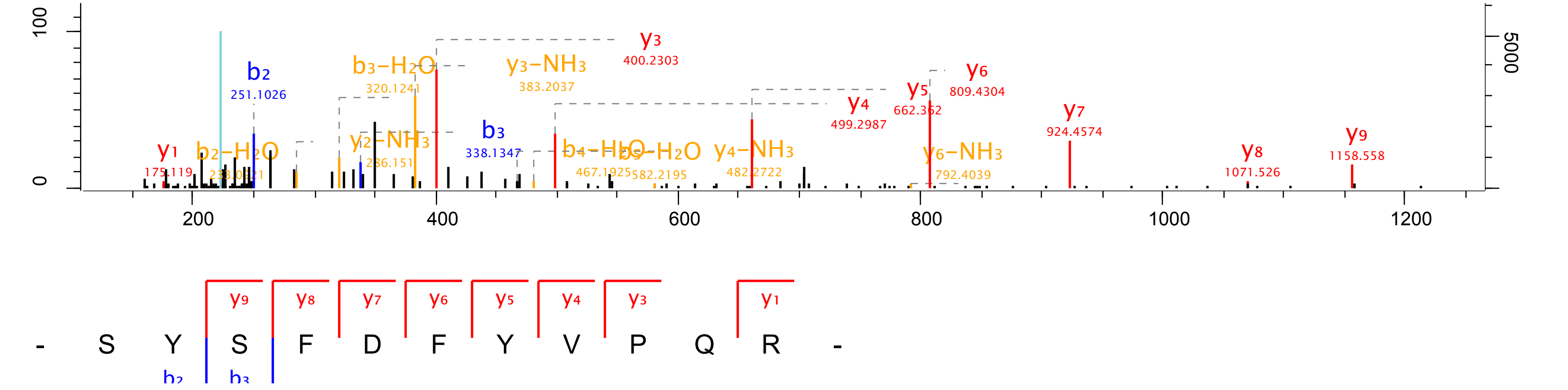

Raw file	Scan	Method	Score	m/z	Gene names
20140925_fract21_dyn_5ul_H5_01_456	28121	TOF; CID	43.86	585.3	TWSG1

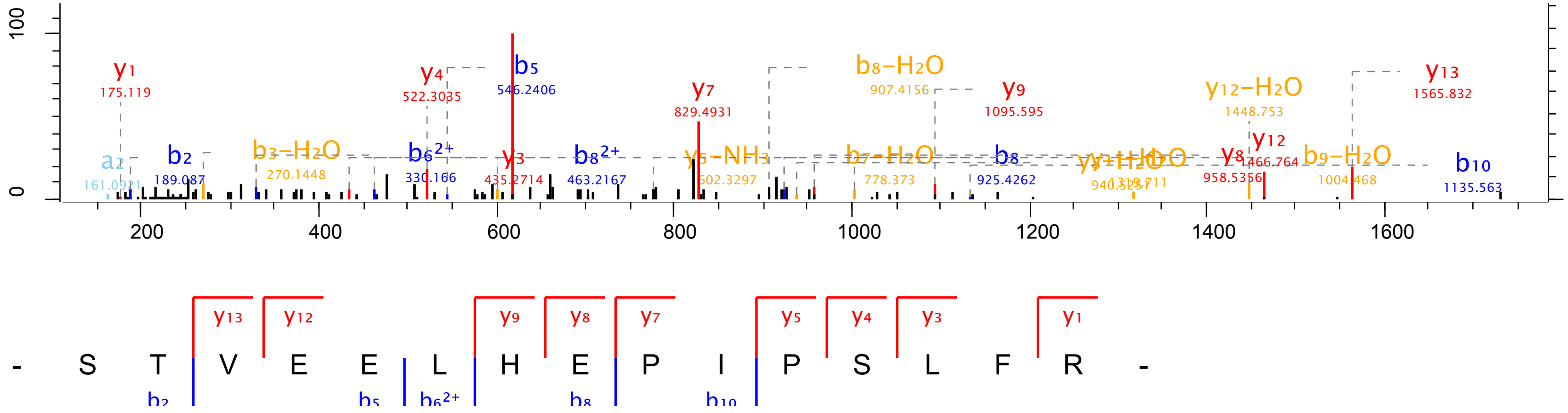

Raw file	Scan	Method	Score	m/z	Gene names
20140925_fract22_dyn_5ul_H6_01_457	13222	TOF; CID	70.91	513.26	ZNF740

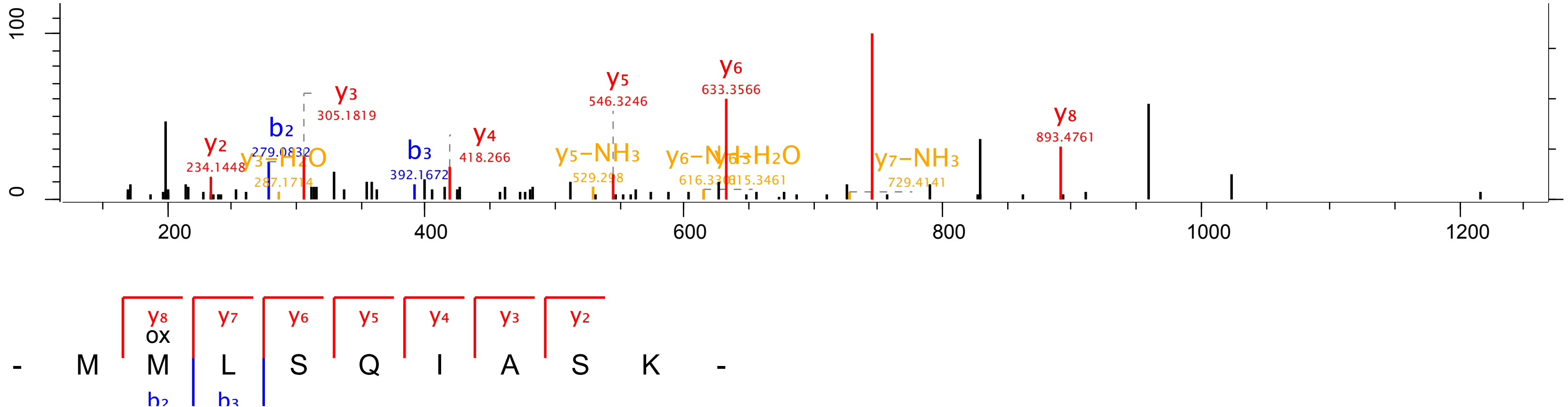

Raw file	Scan	Method	Score	m/z	Gene names
20140925_fract22_dyn_5ul_H6_01_457	15401	TOF; CID	128.38	504.33	SNRNP35

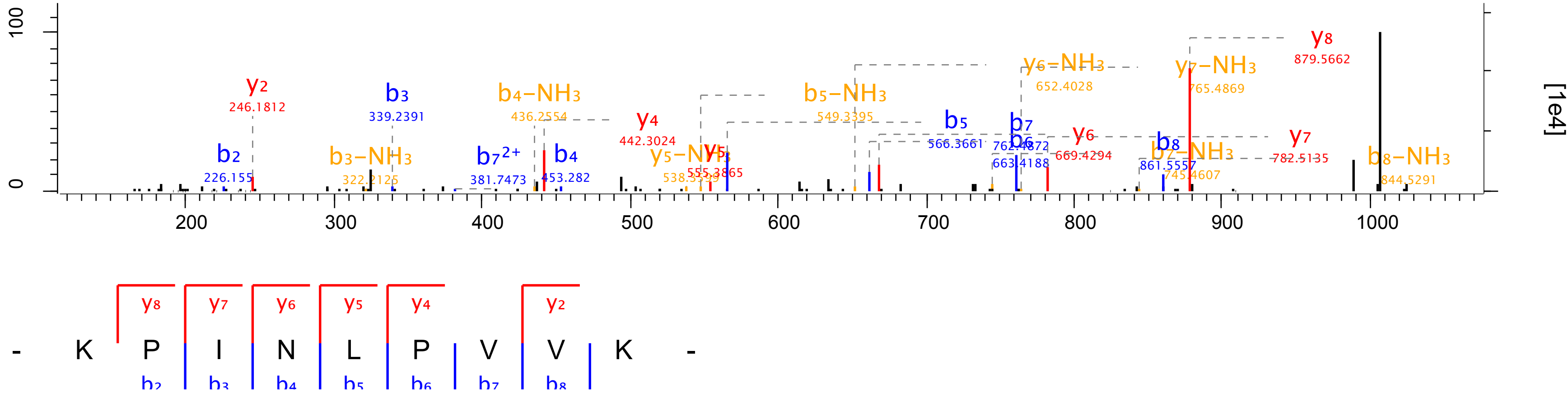

Raw file	Scan	Method	Score	m/z	Gene names
20140925_fract22_dyn_5ul_H6_01_457	16966	TOF; CID	105.52	575.55	SLC39A3

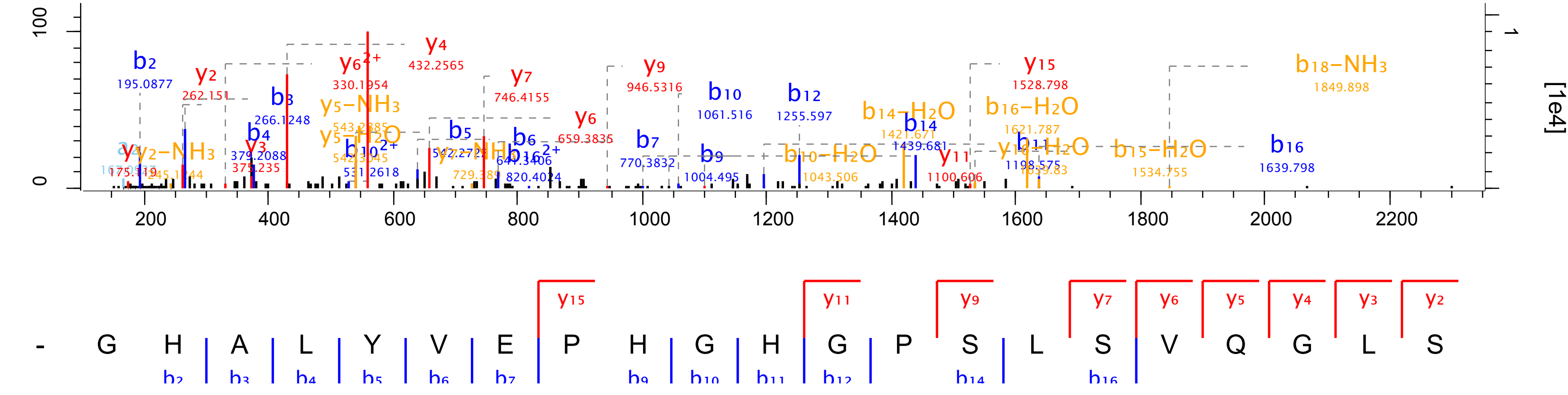

Raw file	Scan	Method	Score	m/z	Gene names
20140925_fract22_dyn_5ul_H6_01_457	17733	TOF; CID	92.25	672.33	XK

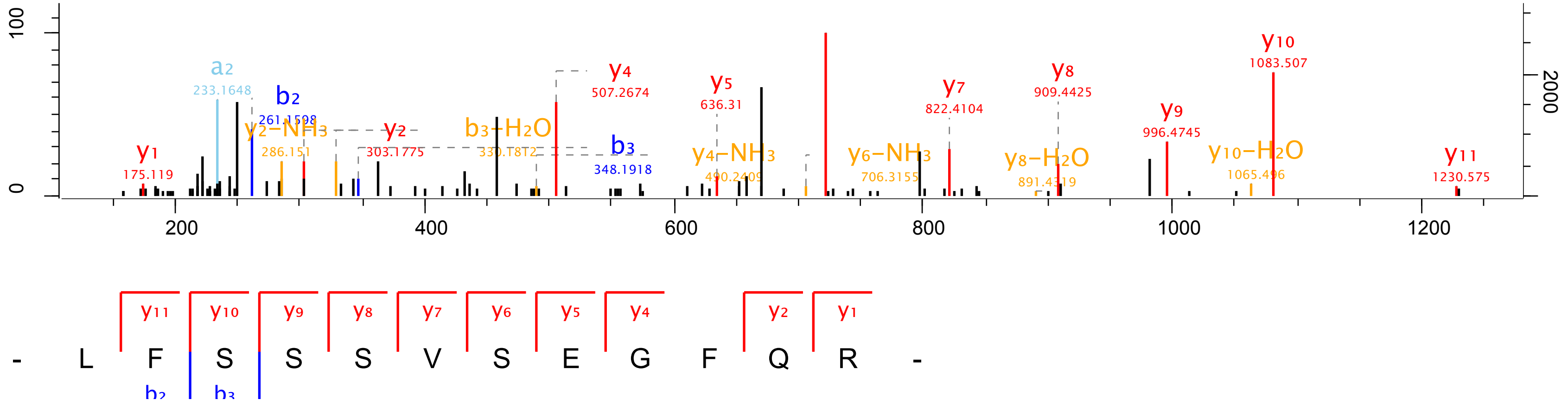

Raw file	Scan	Method	Score	m/z	Gene names
20140925_fract22_dyn_5ul_H6_01_457	21078	TOF; CID	120.55	509.28	NLK

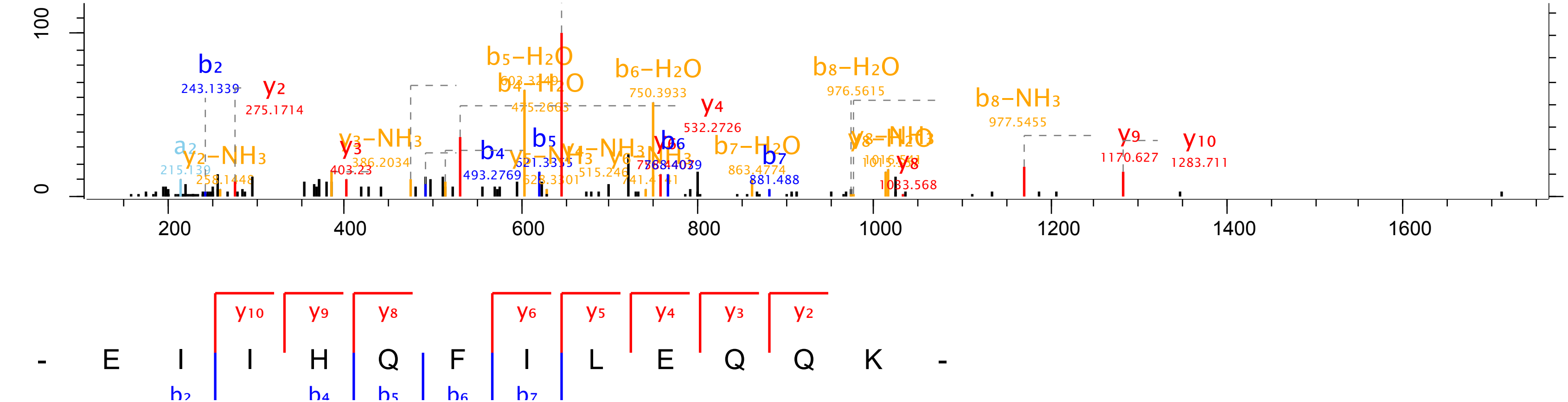

Raw file	Scan	Method	Score	m/z	Gene names
20140925_fract22_dyn_5ul_H6_01_457	21715	TOF; CID	97.21	1101.48	ELOF1

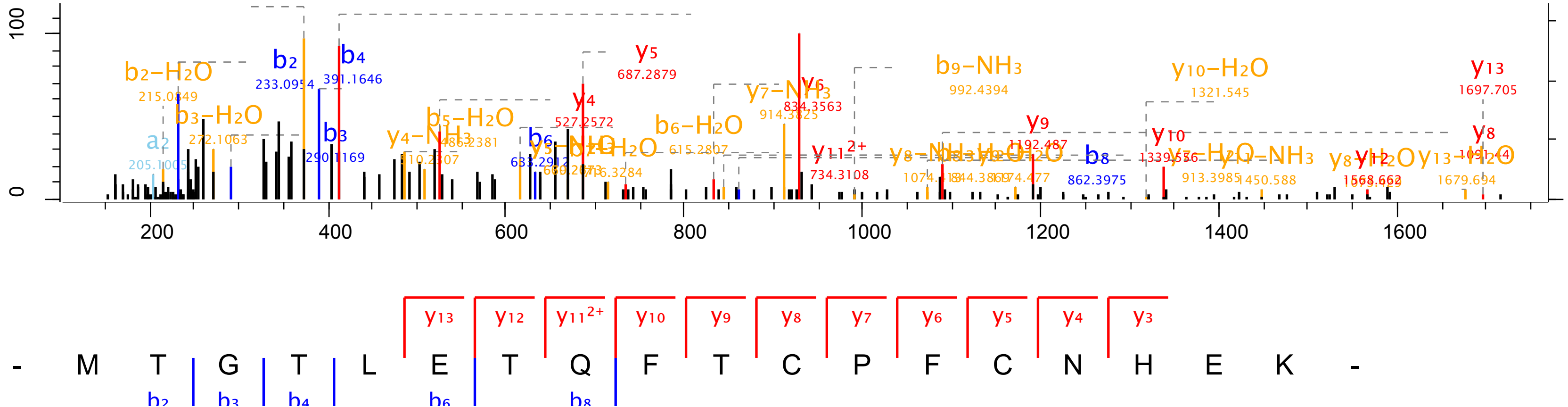

Raw file	Scan	Method	Score	m/z	Gene names
20140925_fract22_dyn_5ul_H6_01_457	28654	TOF; CID	73.23	498.8	ZNF114

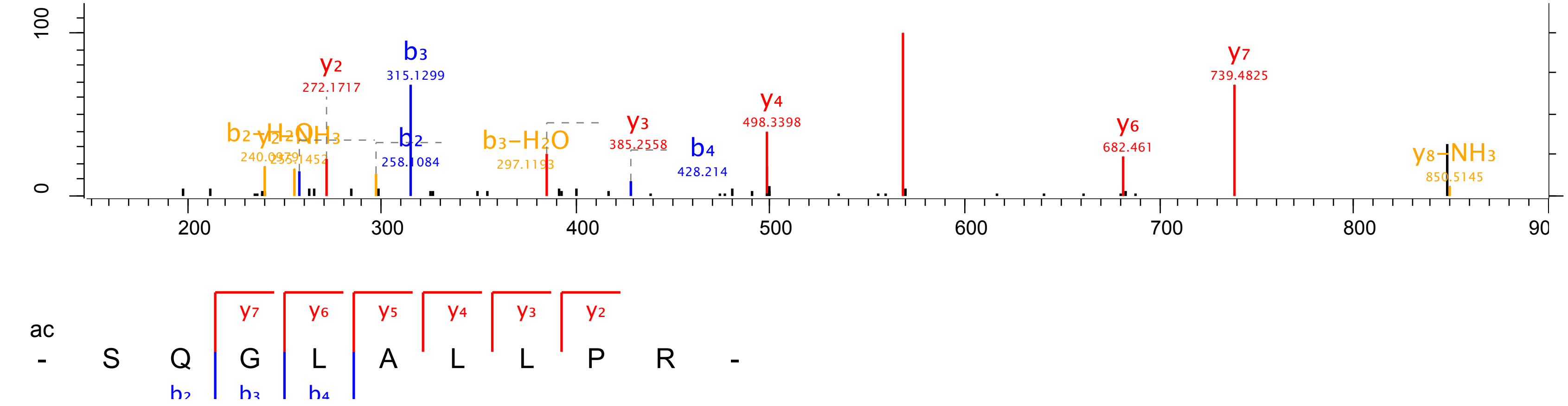

Raw file	Scan	Method	Score	m/z	Gene names
20140925_fract22_dyn_5ul_H6_01_457	31286	TOF; CID	93.37	570.82	FIGN

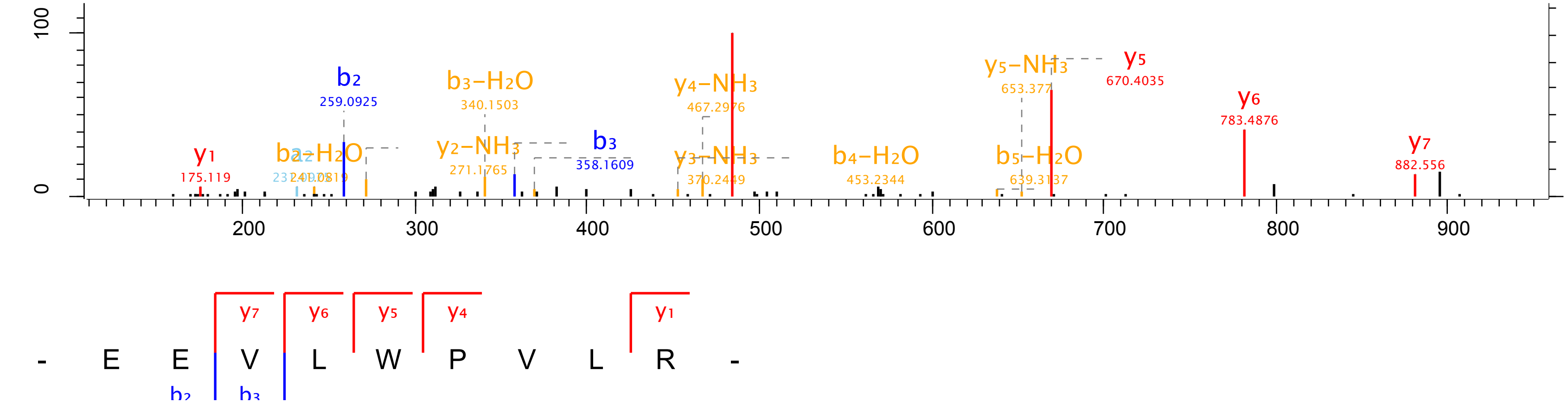

Raw file	Scan	Method	Score	m/z	Gene names
20140925_fract22_dyn_5ul_H6_01_457	31800	TOF; CID	64.24	814.39	TSPAN1

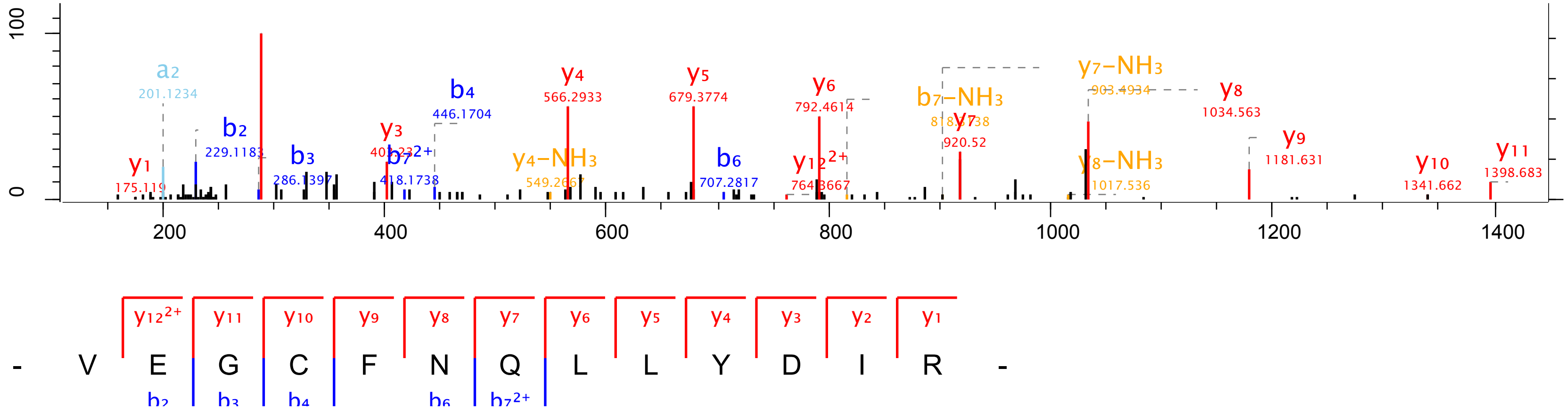

Raw file	Scan	Method	Score	m/z	Gene names
20140925_fract23_dyn_5ul_H7_01_458	10320	TOF; CID	76.23	450.27	SOX4

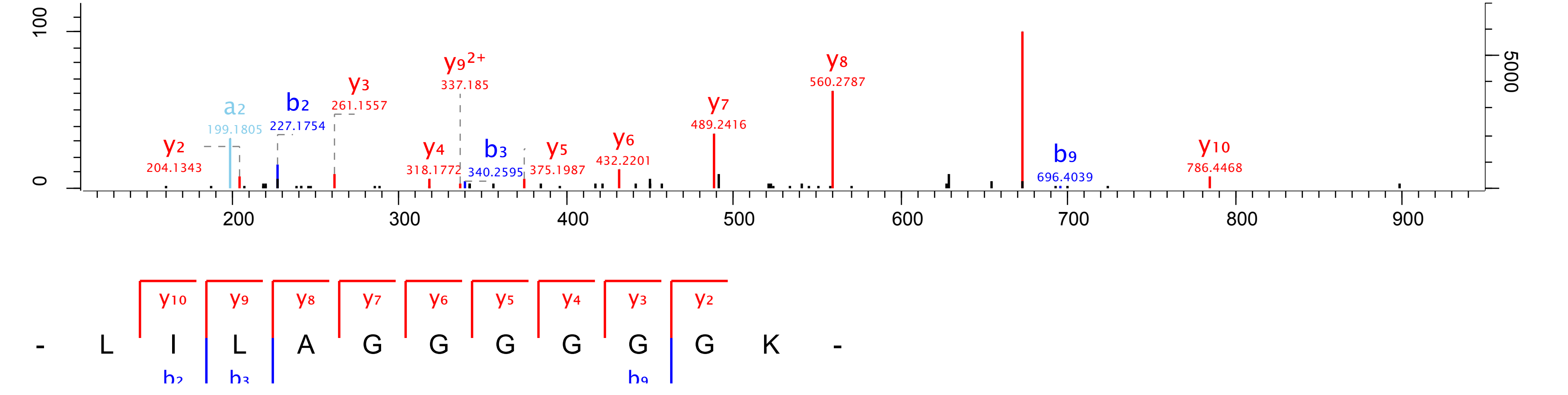

Raw file	Scan	Method	Score	m/z	Gene names
20140925_fract23_dyn_5ul_H7_01_458	13516	TOF; CID	93.62	625.87	FKRP

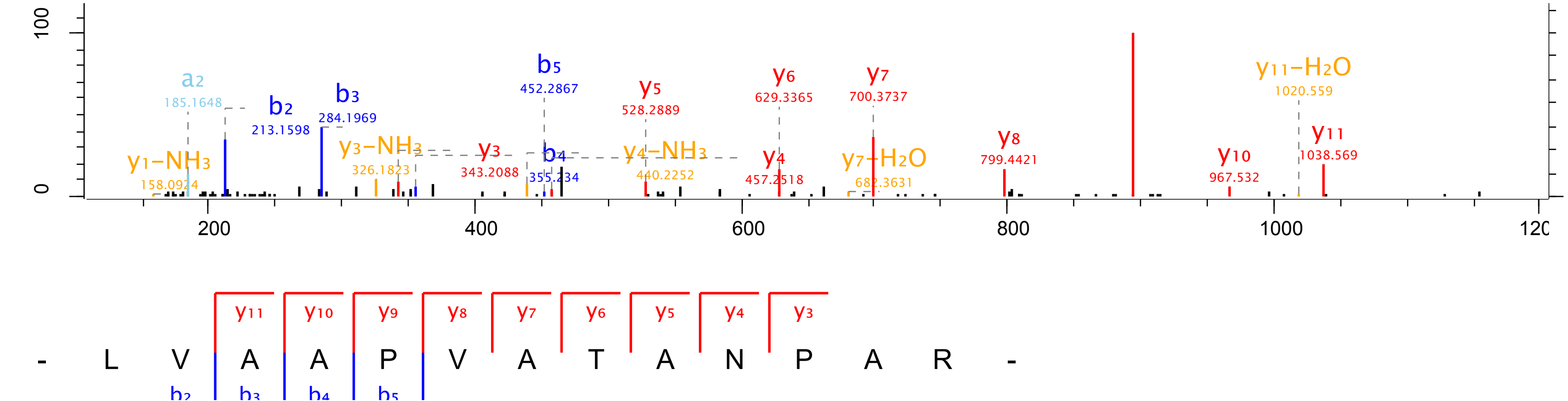

Raw file	Scan	Method	Score	m/z	Gene names
20140925_fract23_dyn_5ul_H7_01_458	18044	TOF; CID	58.1	448.56	B9D1

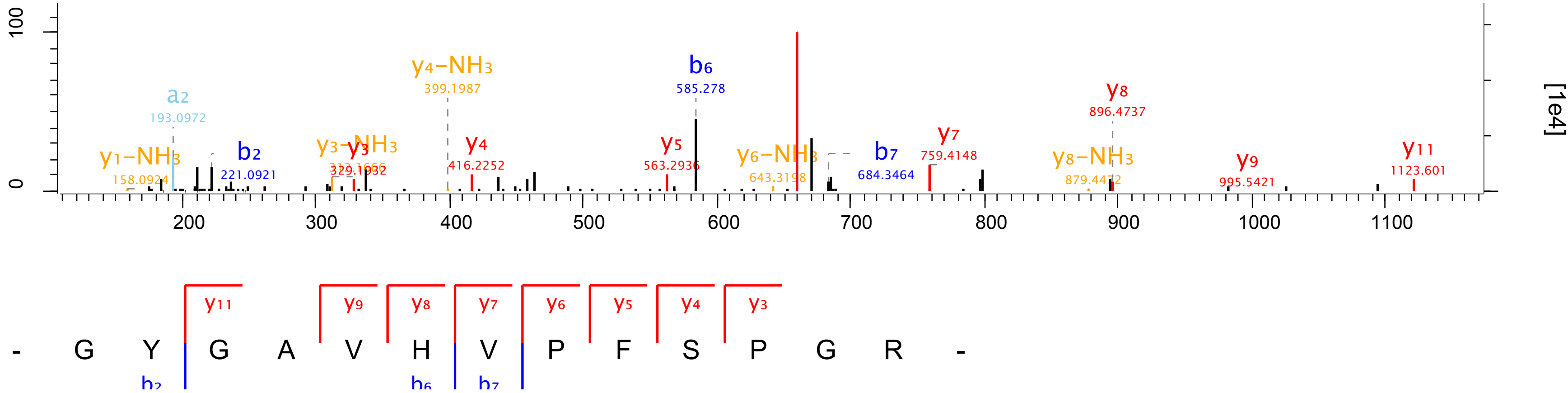

Raw file	Scan	Method	Score	m/z	Gene names
20140925_fract23_dyn_5ul_H7_01_458	19417	TOF; CID	66.22	554.93	MANEAL

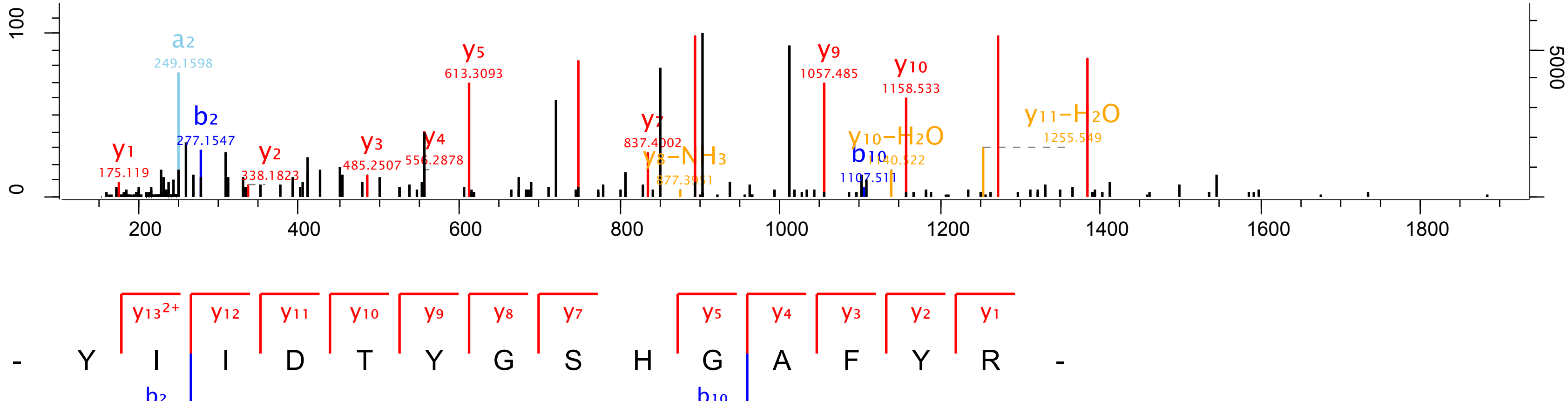

Raw file	Scan	Method	Score	m/z	Gene names
20140925_fract23_dyn_5ul_H7_01_458	20113	TOF; CID	62.68	800.44	ZCCHC10

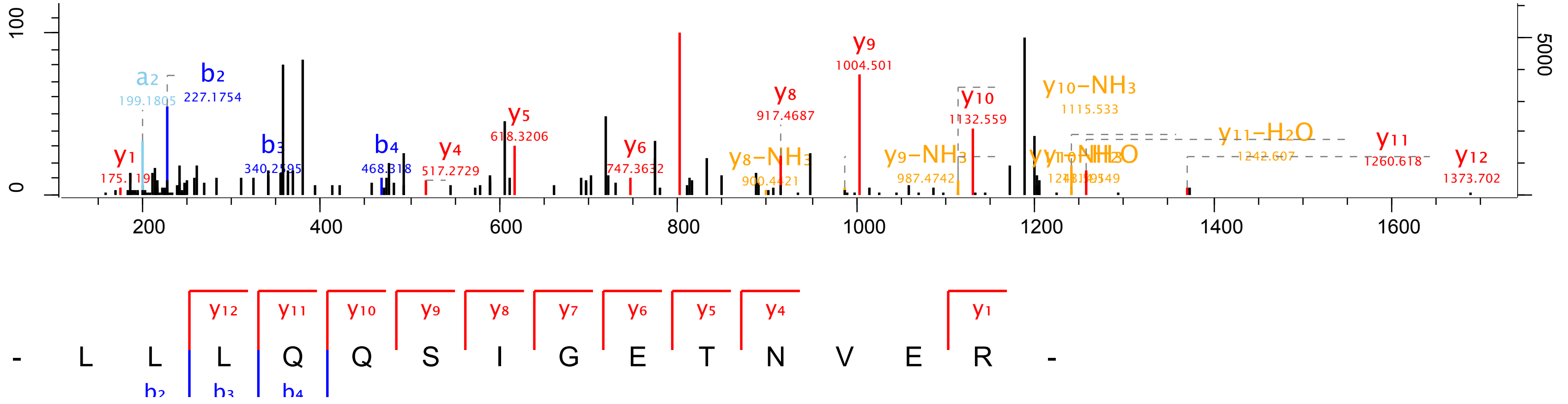

Raw file	Scan	Method	Score	m/z	Gene names
20140925_fract23_dyn_5ul_H7_01_458	20707	TOF; CID	101.62	874.44	BRI3

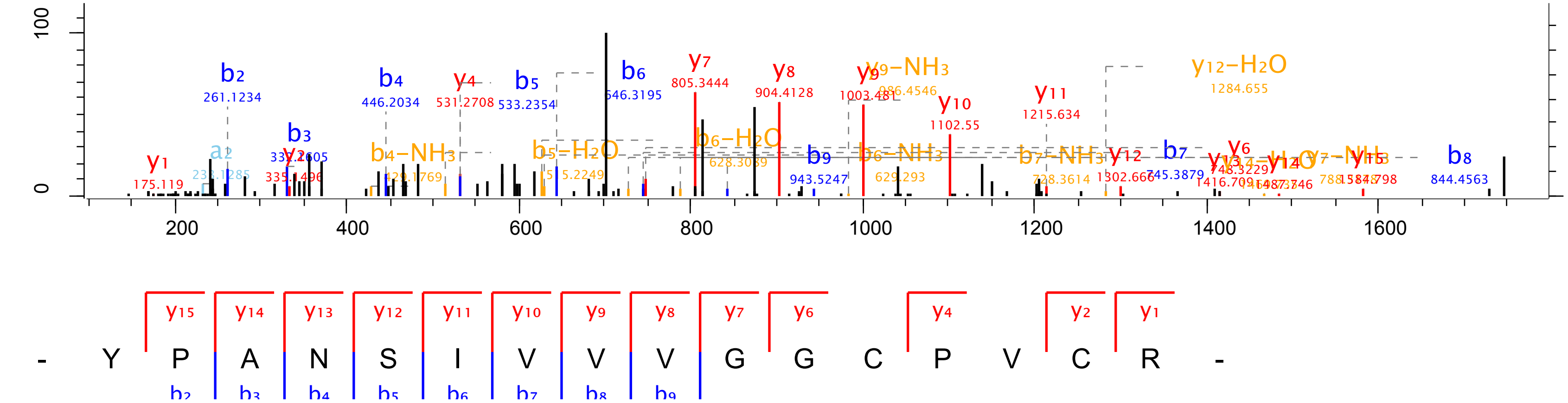

Raw file	Scan	Method	Score	m/z	Gene names
20140925_fract23_dyn_5ul_H7_01_458	21573	TOF; CID	79.15	492.26	ITGB6

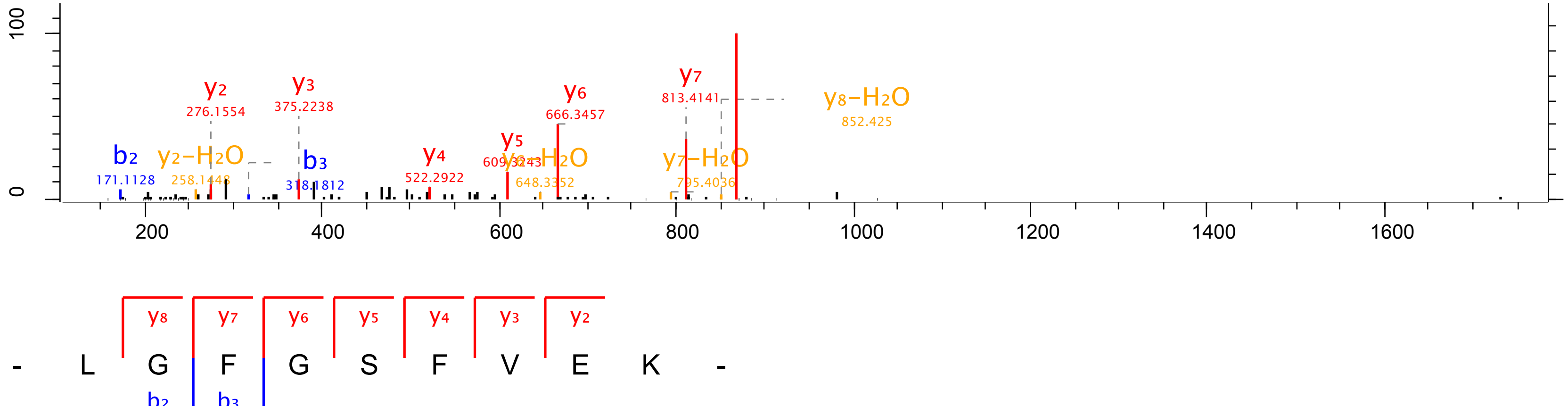

Raw file	Scan	Method	Score	m/z	Gene names
20140925_fract23_dyn_5ul_H7_01_458	26978	TOF; CID	41.85	810.1	ZBTB3

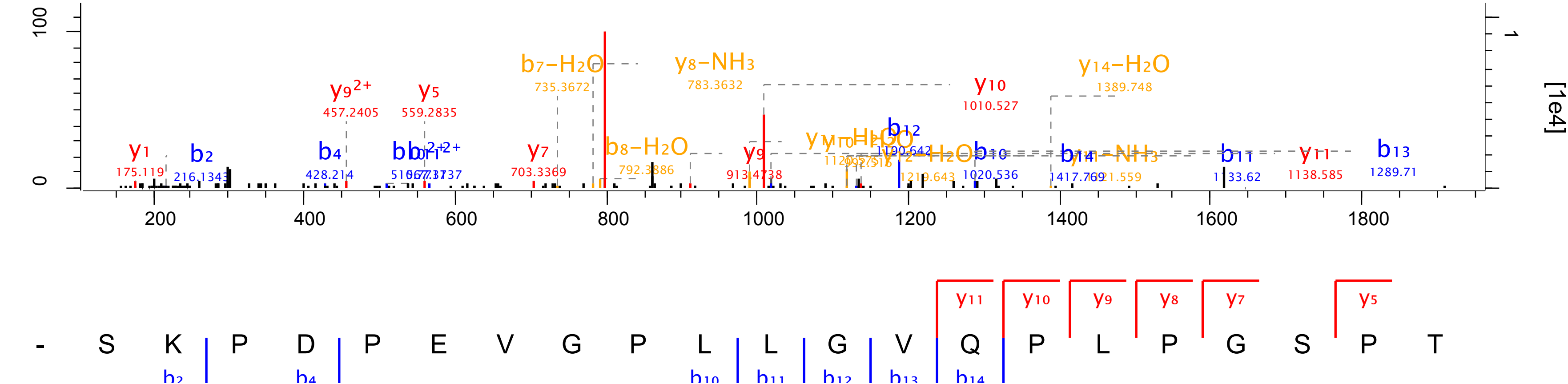

Raw file	Scan	Method	Score	m/z	Gene names
20140925_fract23_dyn_5ul_H7_01_458	28773	TOF; CID	43.7	1072.54	METTL12

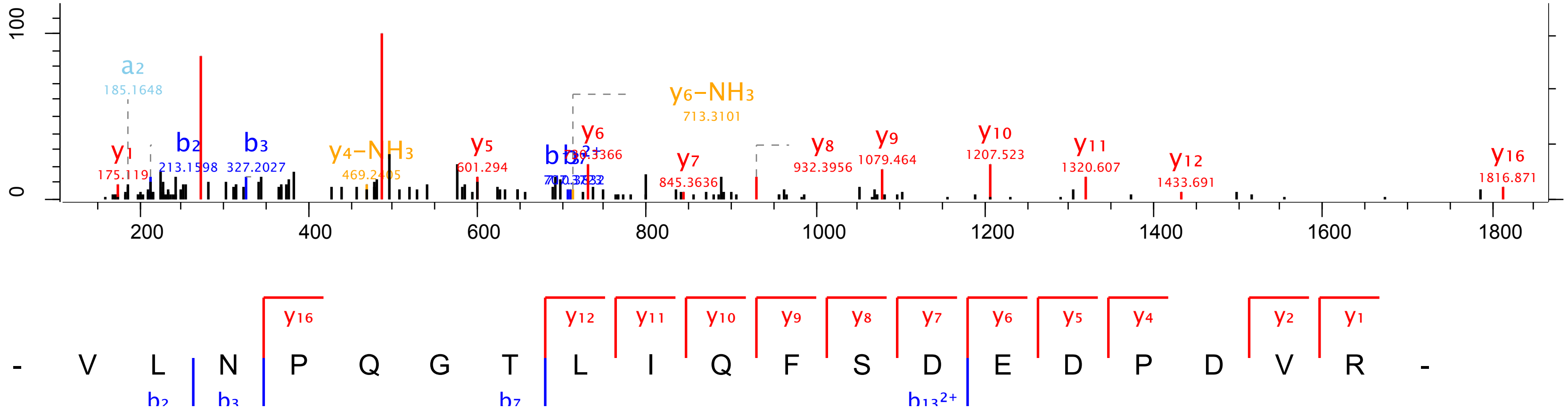

Raw file	Scan	Method	Score	m/z	Gene names
20140925_fract23_dyn_5ul_H7_01_458	33082	TOF; CID	107.75	961.13	SFT2D1

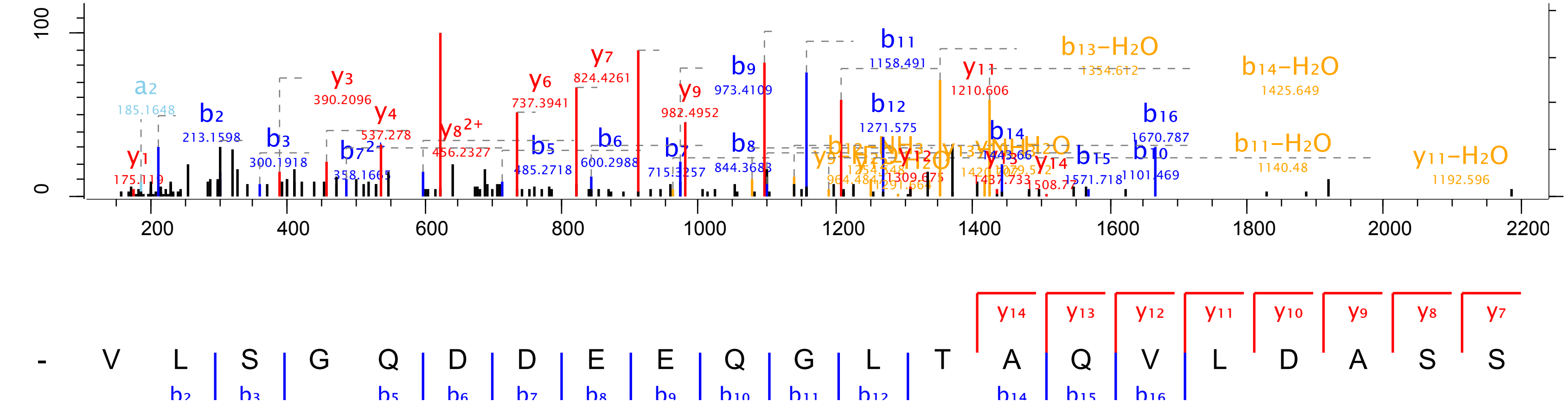

Raw file	Scan	Method	Score	m/z	Gene names
20140925_fract23_dyn_5ul_H7_01_458	34170	TOF; CID	57.23	698.88	PRKD2

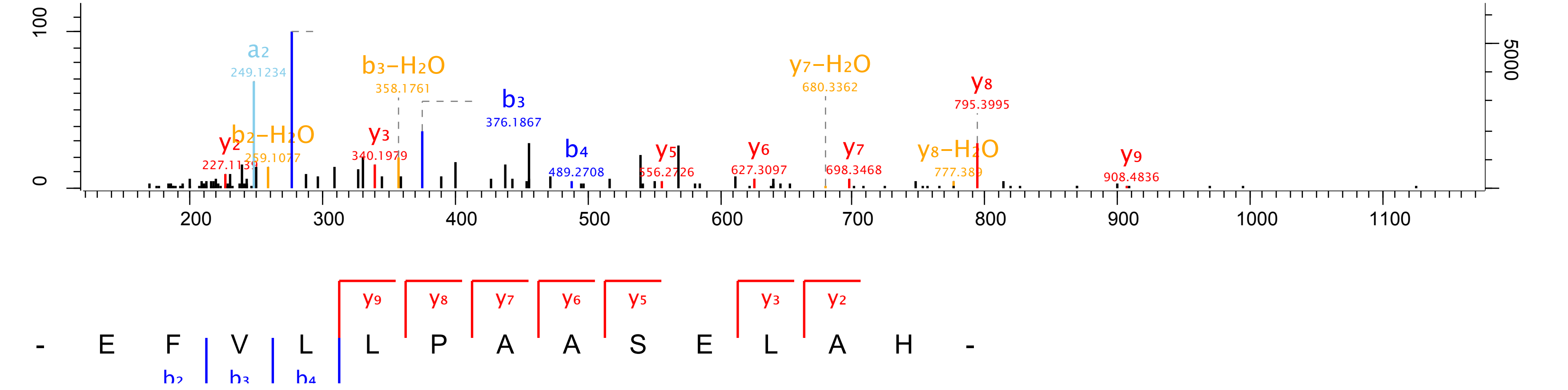

Raw file	Scan	Method	Score	m/z	Gene names
20140925_fract24_dyn_5ul_H8_01_459	9206	TOF; CID	74.79	570.63	RCBTB2

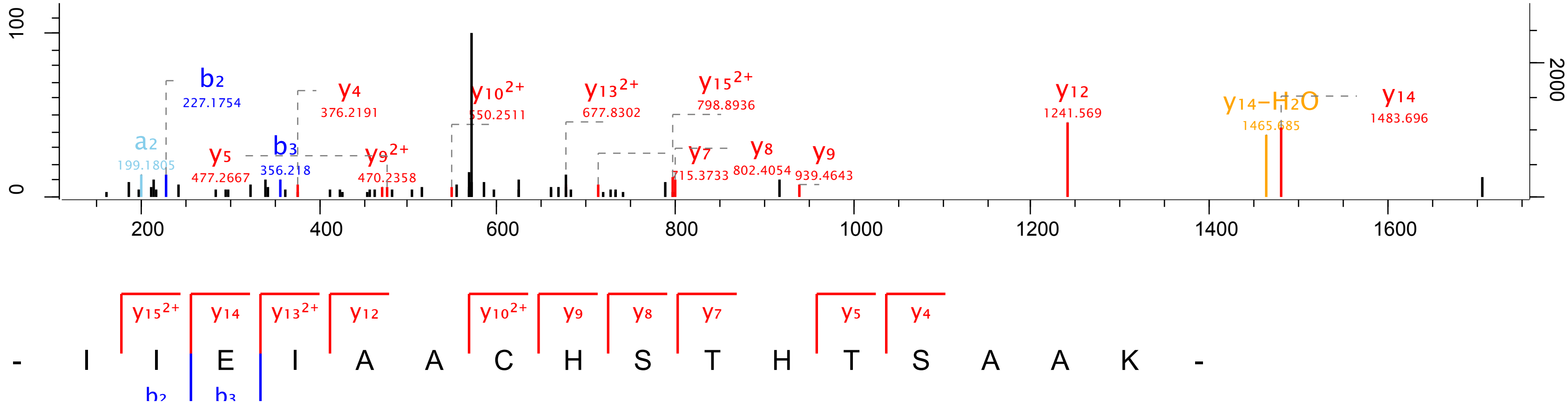

Raw file	Scan	Method	Score	m/z	Gene names
20140925_fract24_dyn_5ul_H8_01_459	9393	TOF; CID	77.66	523.78	SLC25A23

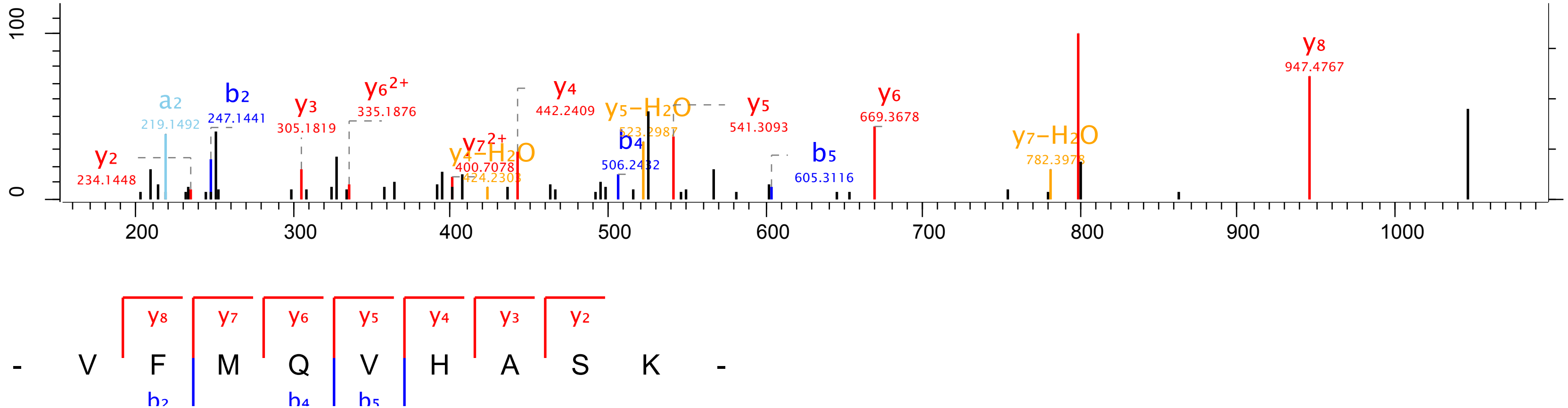

Raw file	Scan	Method	Score	m/z	Gene names
20140925_fract24_dyn_5ul_H8_01_459	12749	TOF; CID	76.06	528.81	TTC7B

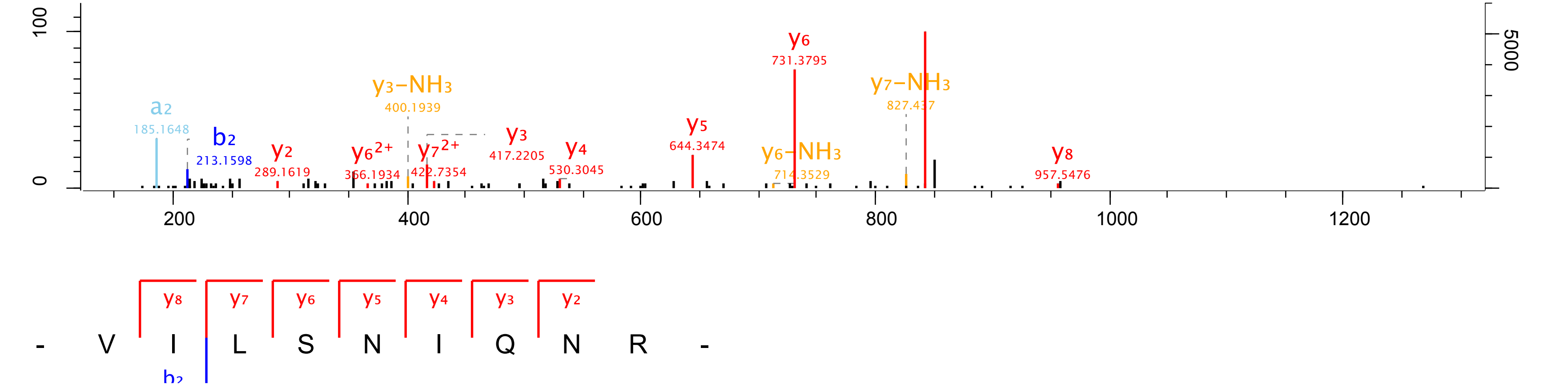

Raw file	Scan	Method	Score	m/z	Gene names
20140925_fract24_dyn_5ul_H8_01_459	15262	TOF; CID	61.34	399.22	WDR90

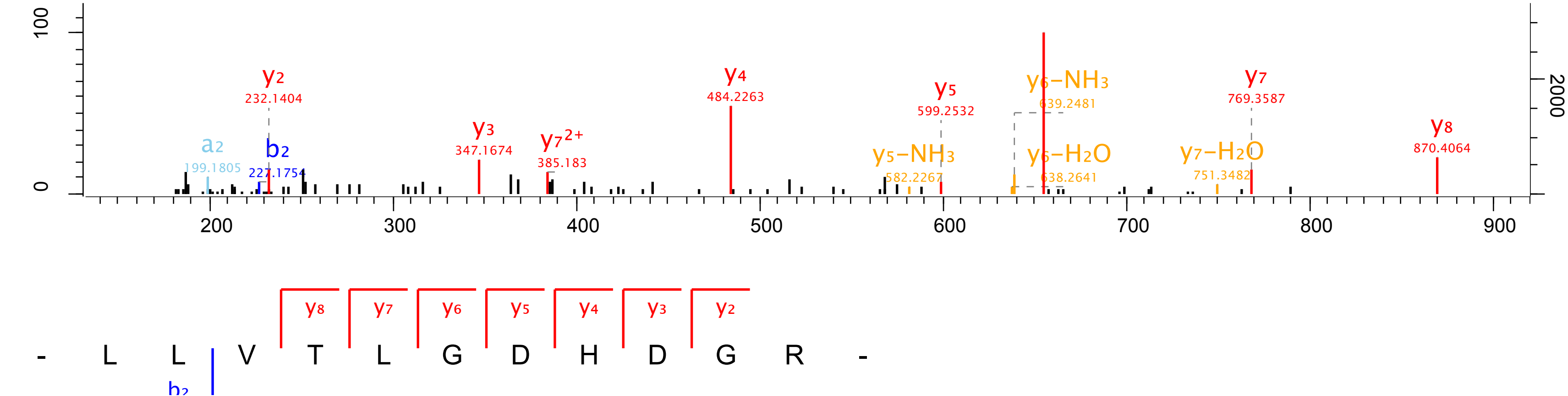

Raw file	Scan	Method	Score	m/z	Gene names
20140925_fract24_dyn_5ul_H8_01_459	16548	TOF; CID	97.8	428.76	STRC

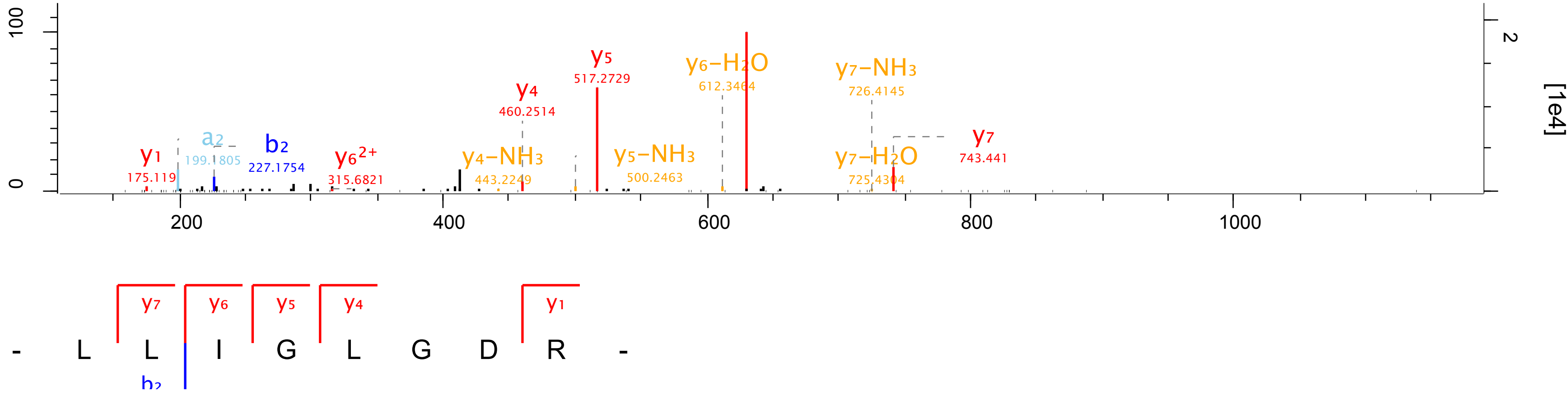

Raw file	Scan	Method	Score	m/z	Gene names
20140925_fract24_dyn_5ul_H8_01_459	19885	TOF; CID	53.45	552.98	FAM189B

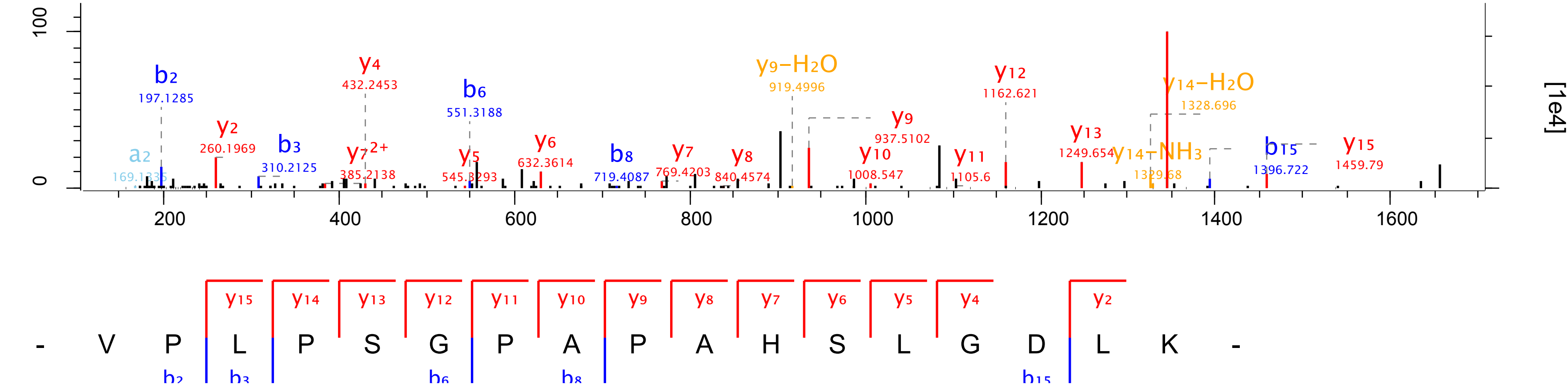

Raw file	Scan	Method	Score	m/z	Gene names
20140925_fract24_dyn_5ul_H8_01_459	27364	TOF; CID	68.48	891.44	TAF11

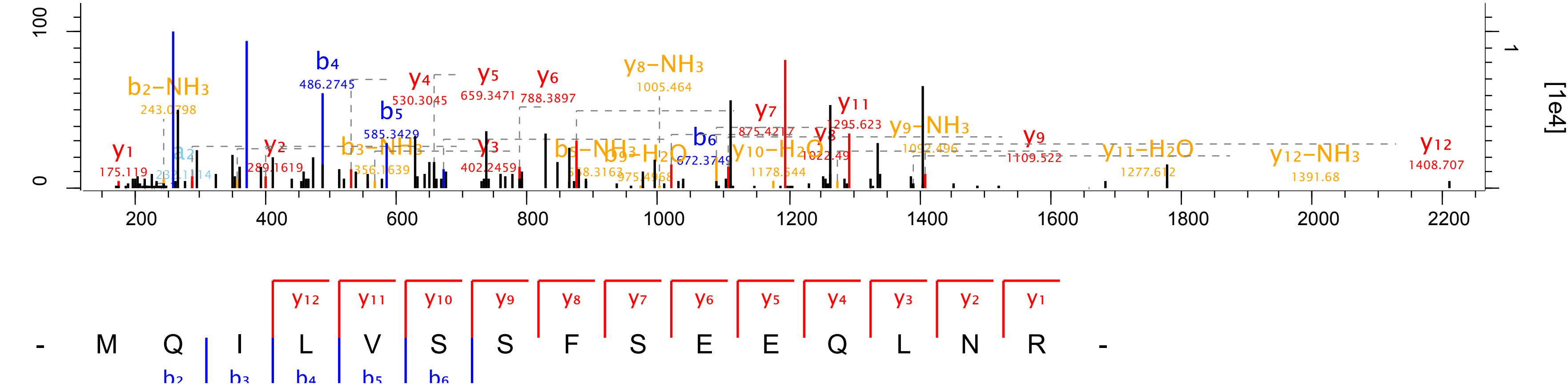

Raw file	Scan	Method	Score	m/z	Gene names
20140925_fract24_dyn_5ul_H8_01_459	28153	TOF; CID	62.34	693.38	IFT88

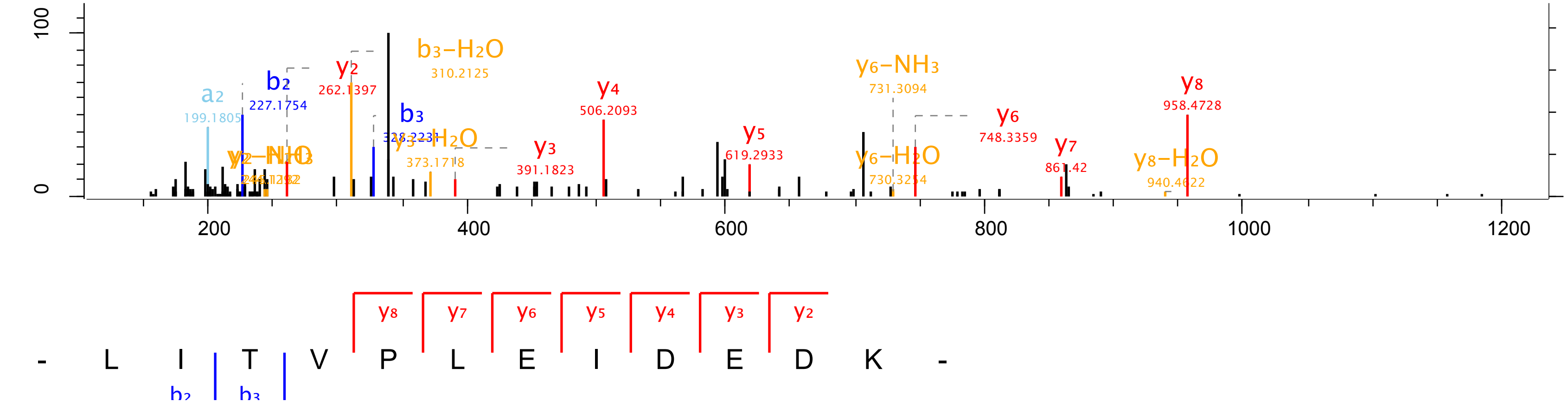

Raw file	Scan	Method	Score	m/z	Gene names
20140925_fract24_dyn_5ul_H8_01_459	28568	TOF; CID	88.71	512.29	FAM118A

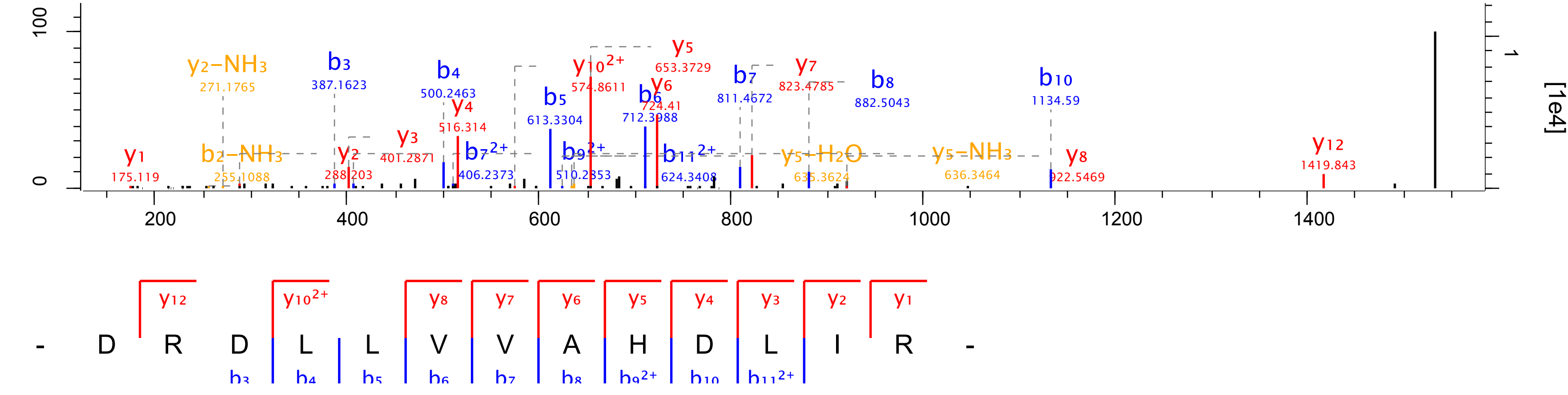

Raw file	Scan	Method	Score	m/z	Gene names
20140925_fract24_dyn_5ul_H8_01_459	34509	TOF; CID	55.75	823.44	NCKAP5

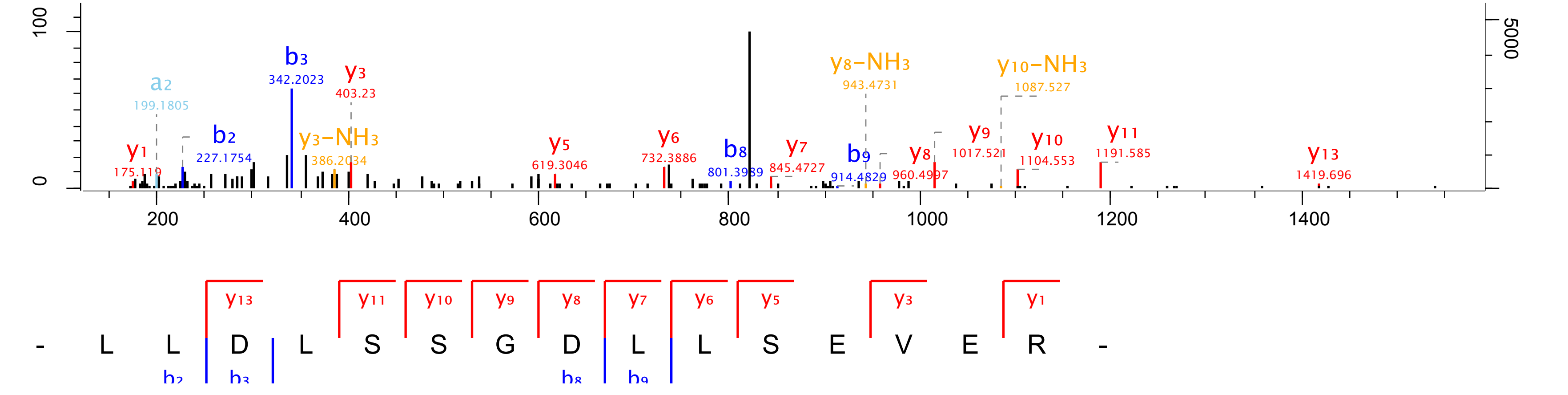

Supplement: Supplemental Data [file supp_M114.047407_mcp.M114.047407-11.pdf]
