# Supplementary material for: The Impact II, a Very High-Resolution Quadrupole Time-of-Flight Instrument (QTOF) for Deep Shotgun Proteomics
Source: Mol Cell Proteomics. 2015 May 19;14(7):2014–29. doi: 10.1074/mcp.M114.047407 (PMC4587313; doi:10.1074/mcp.M114.047407)

Raw file	Scan	Method	Score	m/z	Gene names
20141014_fract1_dyn_5ul_E1_01_580	6102	TOF; CID	107.69	330.19	HMGN4

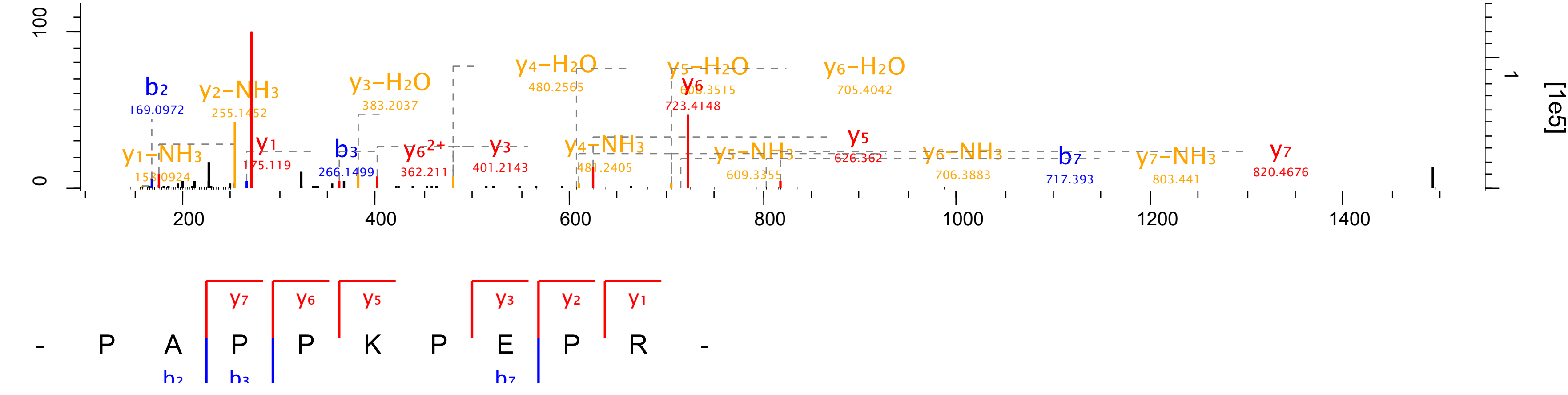

Raw file	Scan	Method	Score	m/z	Gene names
20141014_fract1_dyn_5ul_E1_01_580	6441	TOF; CID	90.05	377.2	NDUFB2

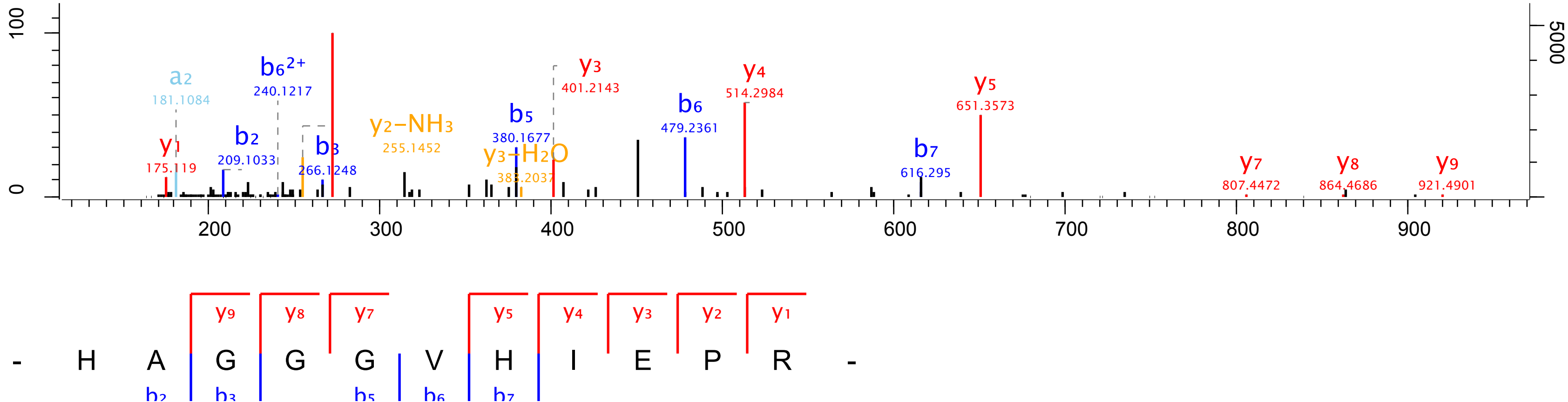

Raw file	Scan	Method	Score	m/z	Gene names
20141014_fract1_dyn_5ul_E1_01_580	8834	TOF; CID	70.09	447.55	BTBD7

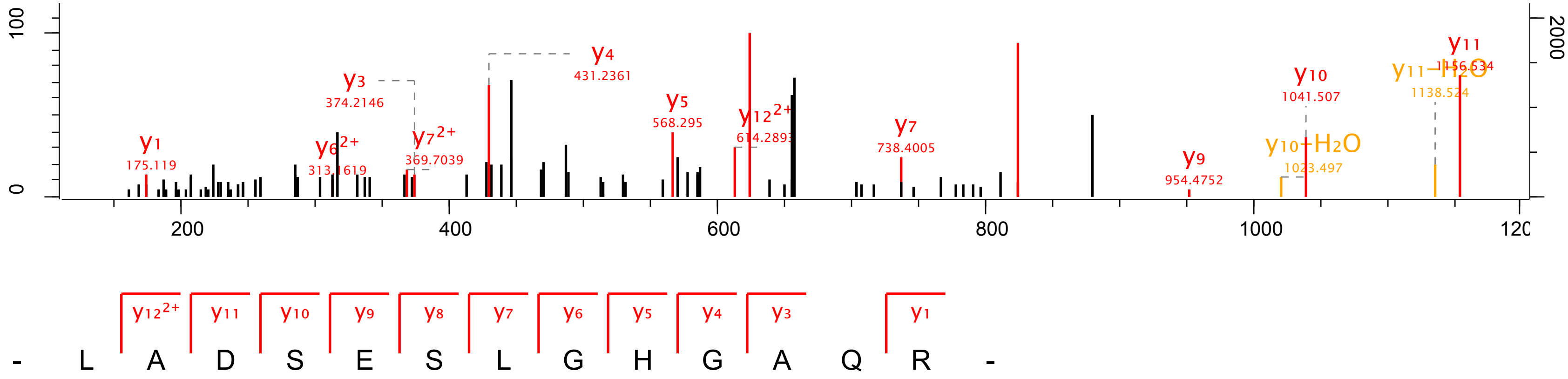

Raw file	Scan	Method	Score	m/z	Gene names
20141014_fract1_dyn_5ul_E1_01_580	9299	TOF; CID	66.55	560.28	SPSB3

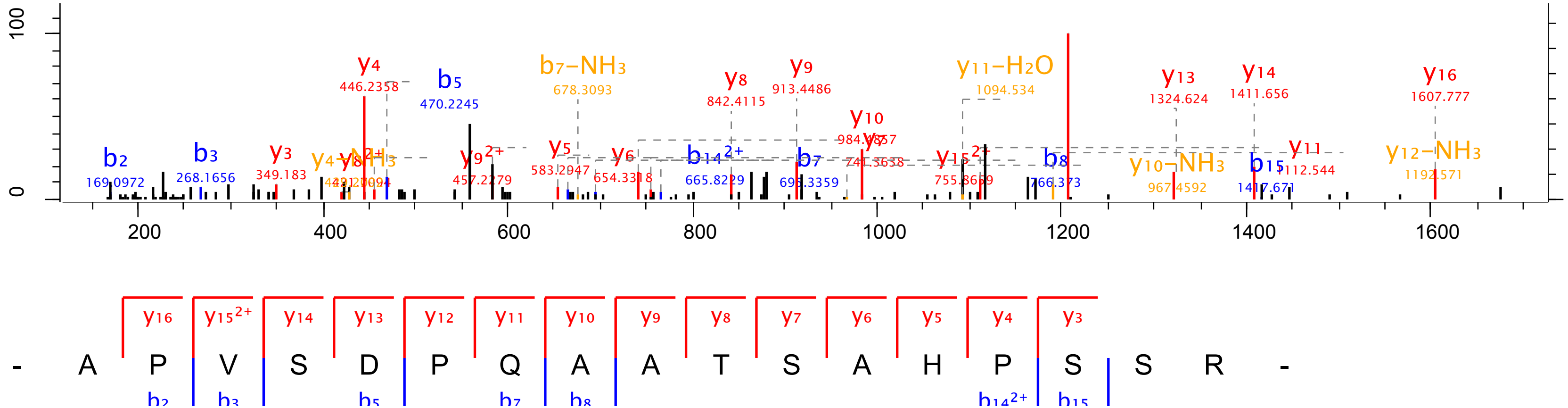

Raw file	Scan	Method	Score	m/z	Gene names
20141014_fract1_dyn_5ul_E1_01_580	13546	TOF; CID	96.74	702.33	TNFRSF10D

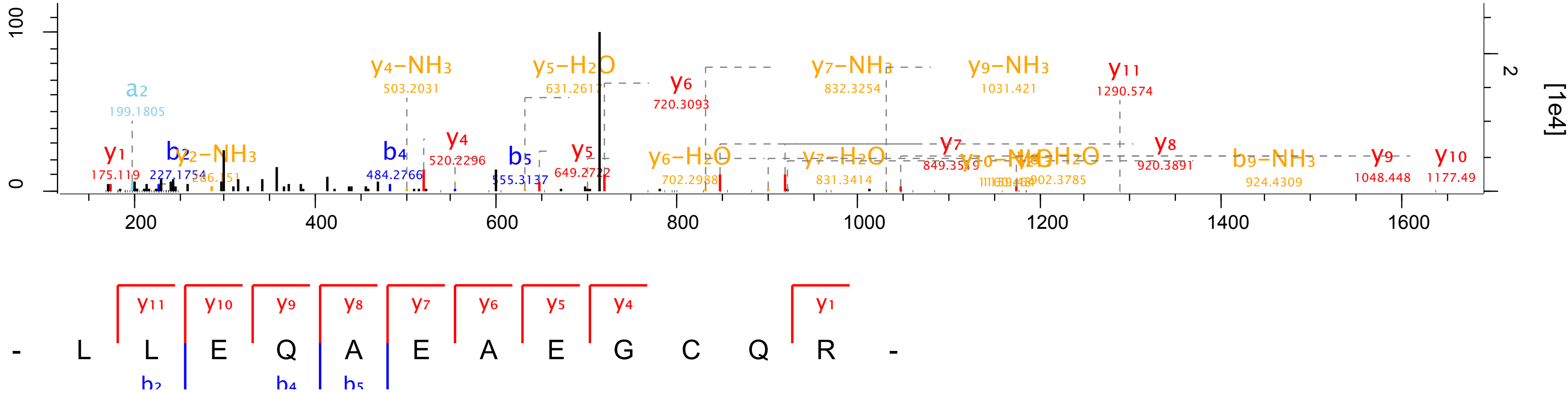

Raw file	Scan	Method	Score	m/z	Gene names
20141014_fract1_dyn_5ul_E1_01_580	14845	TOF; CID	65.18	538.28	PTMS

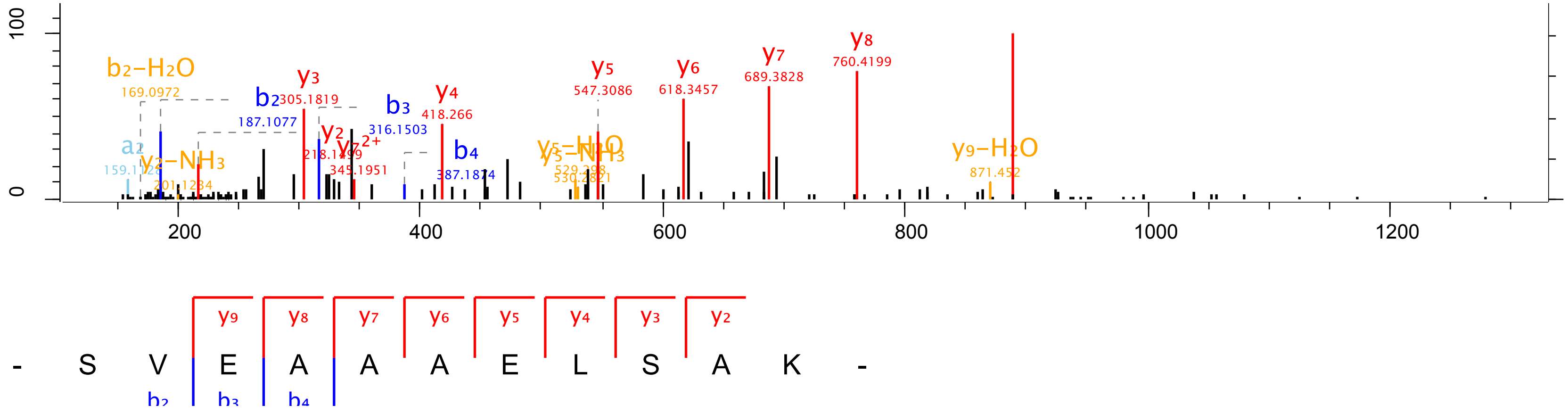

Raw file	Scan	Method	Score	m/z	Gene names
20141014_fract1_dyn_5ul_E1_01_580	21407	TOF; CID	67.08	566.8	RABL2B;RABL2A

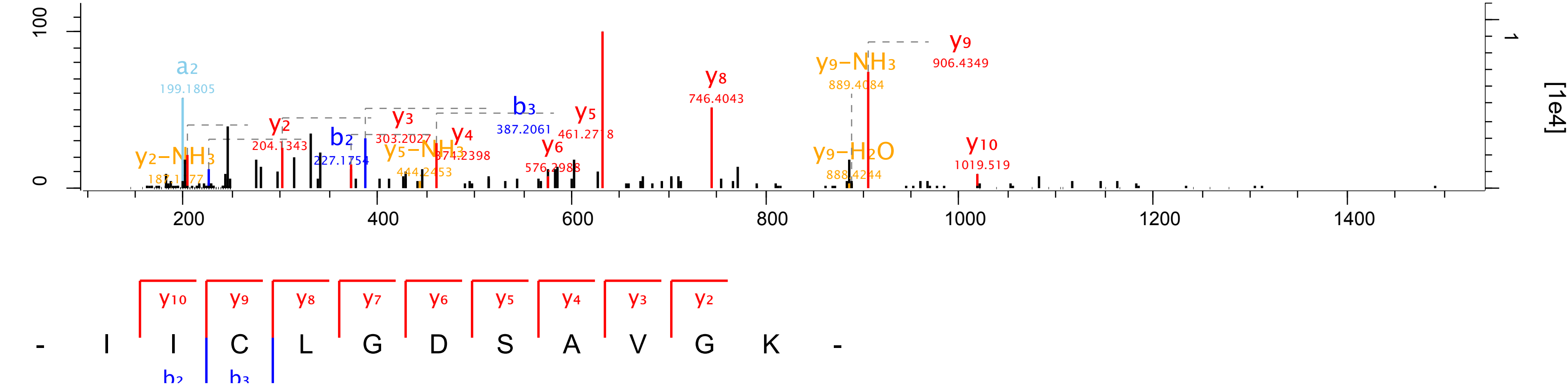

Raw file	Scan	Method	Score	m/z	Gene names
20141014_fract1_dyn_5ul_E1_01_580	22474	TOF; CID	69.43	593.99	SLC35A3

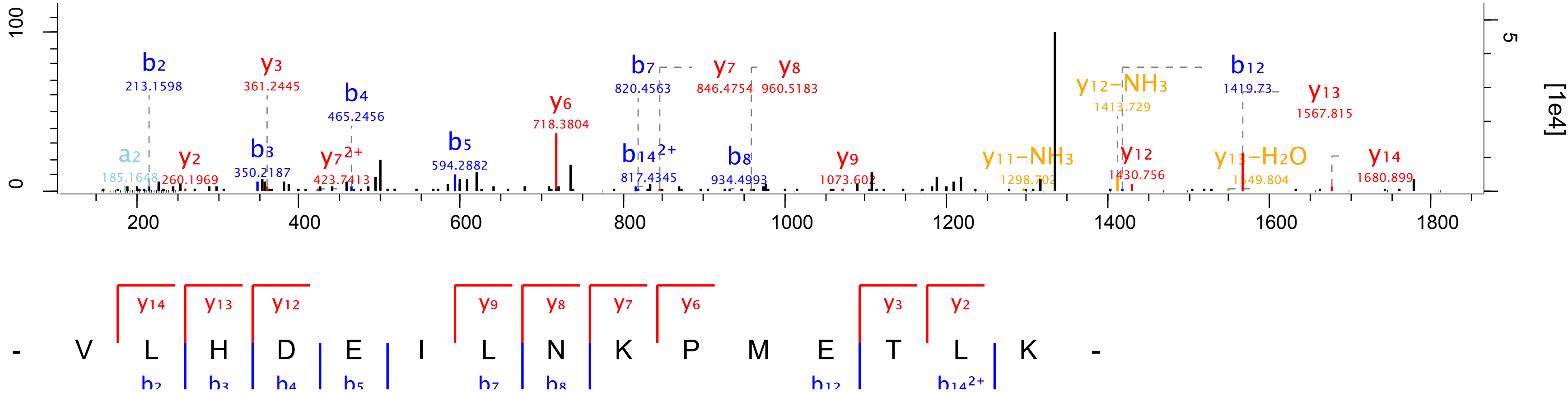

Raw file	Scan	Method	Score	m/z	Gene names
20141014_fract1_dyn_5ul_E1_01_580	25904	TOF; CID	68.54	597.31	FBXL17

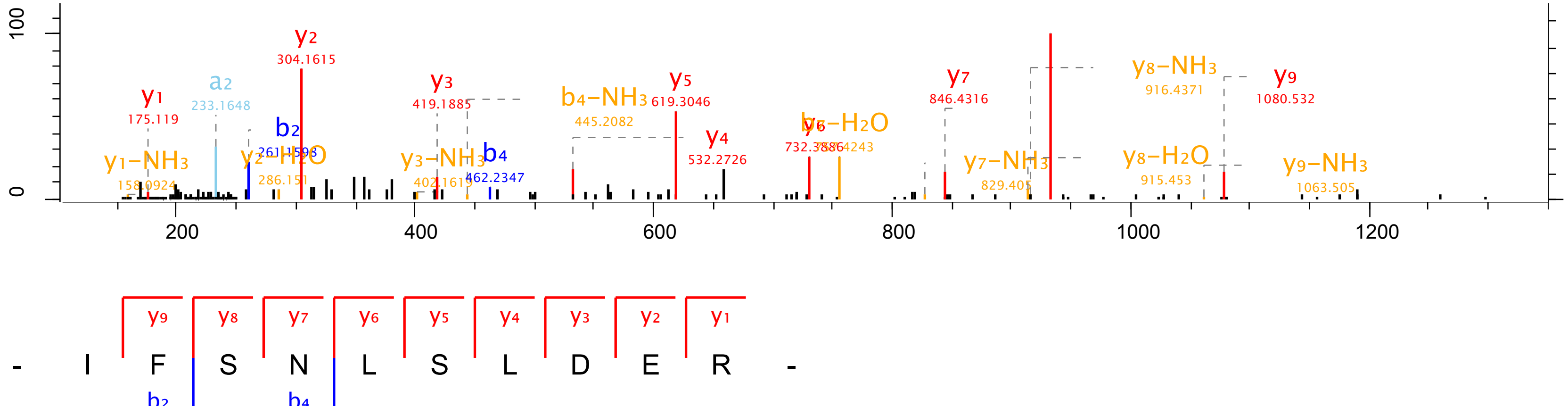

Raw file	Scan	Method	Score	m/z	Gene names
20141014_fract1_dyn_5ul_E1_01_580	27070	TOF; CID	80.24	699.87	DEPTOR

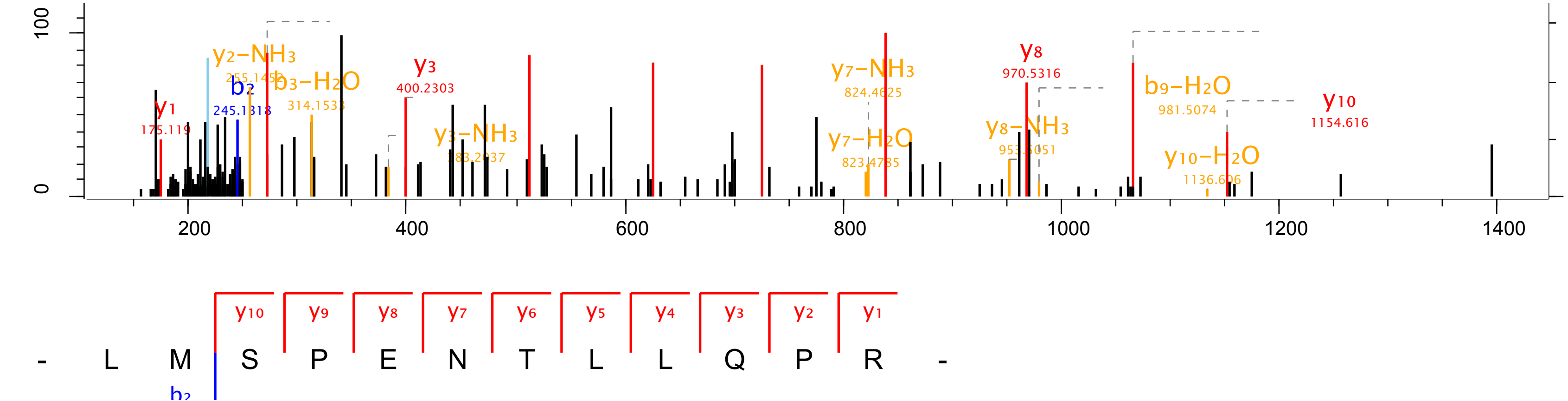

Raw file	Scan	Method	Score	m/z	Gene names
20141014_fract1_dyn_5ul_E1_01_580	27783	TOF; CID	48.9	984.49	HOXA11

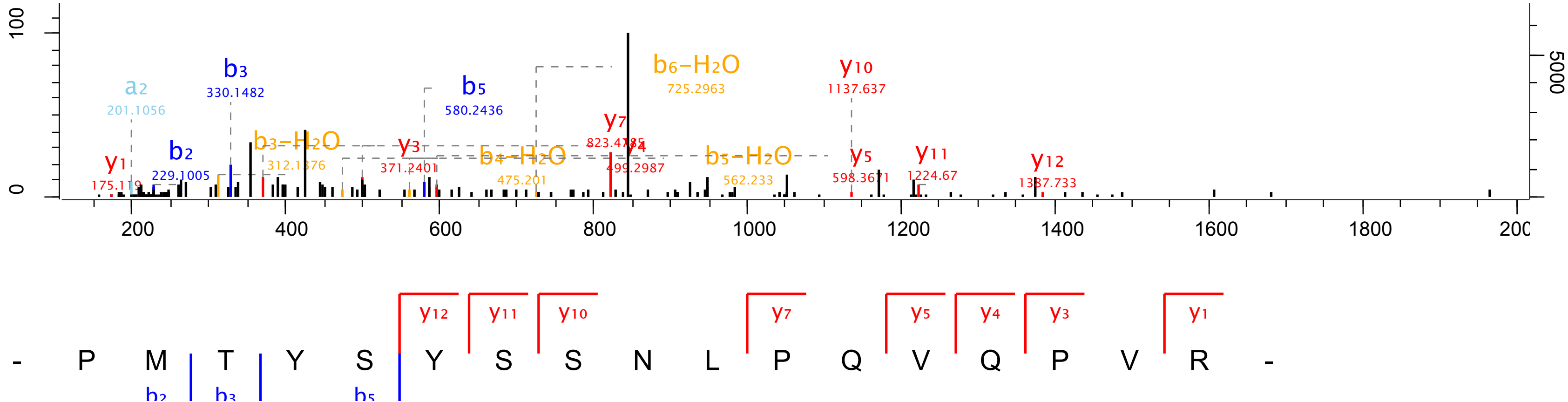

Raw file	Scan	Method	Score	m/z	Gene names
20141014_fract1_dyn_5ul_E1_01_580	33597	TOF; CID	51.73	723.38	SIVA1;GRM6

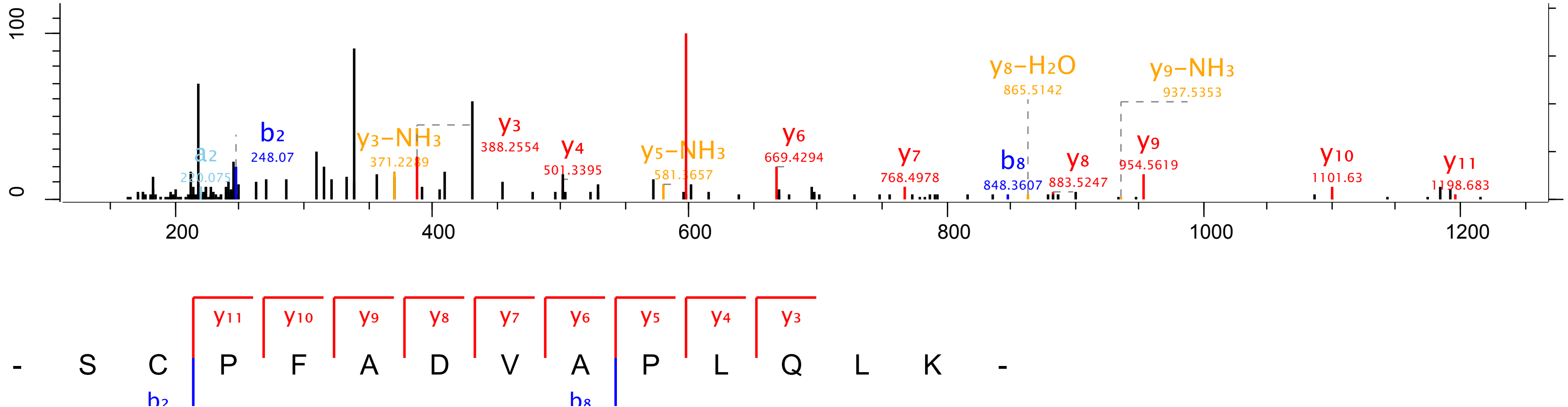

Raw file	Scan	Method	Score	m/z	Gene names
20141014_fract1_dyn_5ul_E1_01_580	34618	TOF; CID	78.6	906.98	RIMKLB

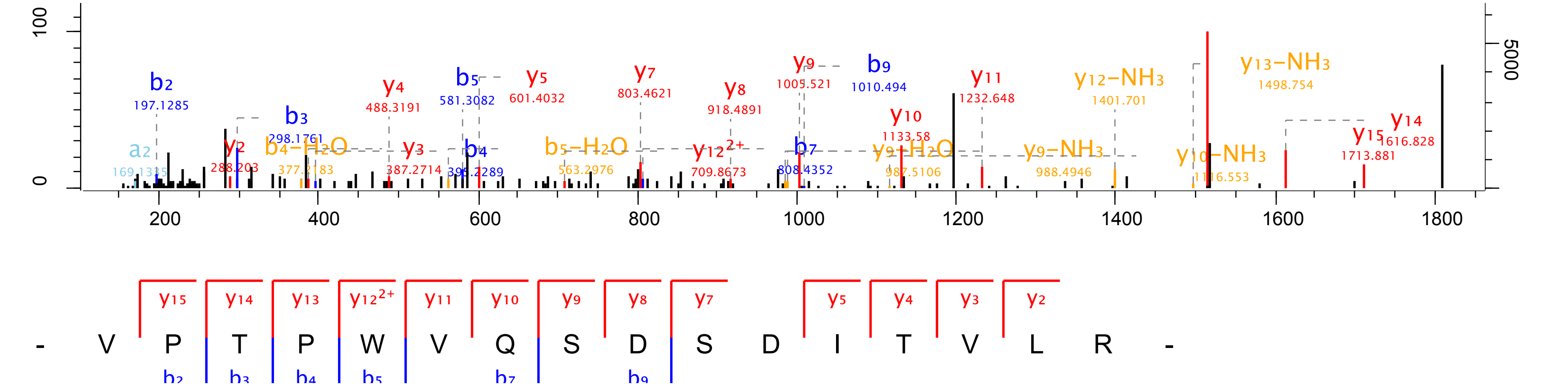

Raw file	Scan	Method	Score	m/z	Gene names
20141014_fract1_dyn_5ul_E1_01_580	35665	TOF; CID	80.69	520.31	SAMD11

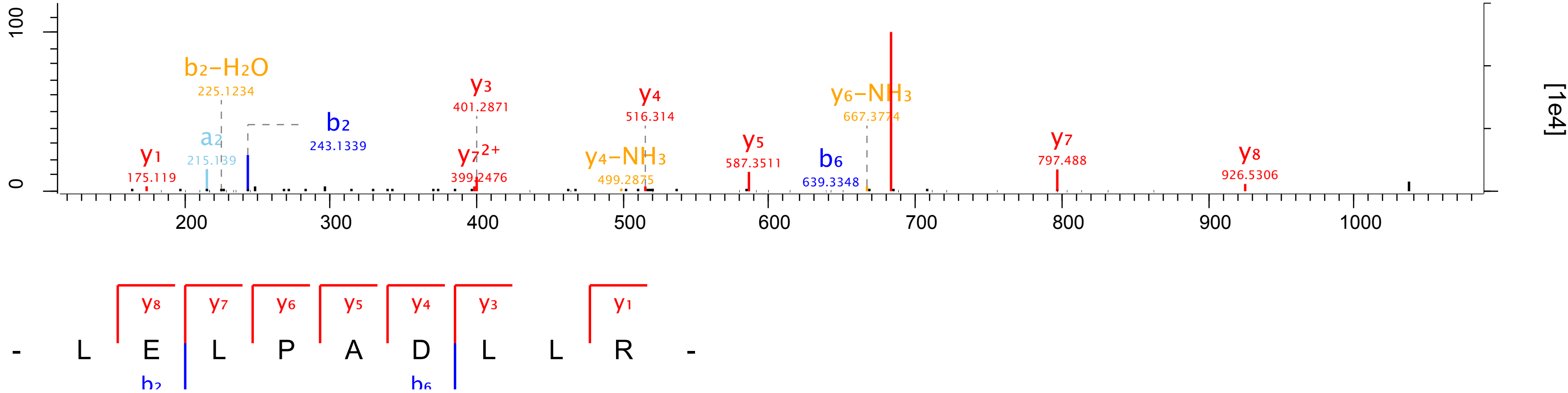

Raw file	Scan	Method	Score	m/z	Gene names
20141014_fract2_dyn_5ul_E2_01_581	3355	TOF; CID	81.1	390.53	BBIP1

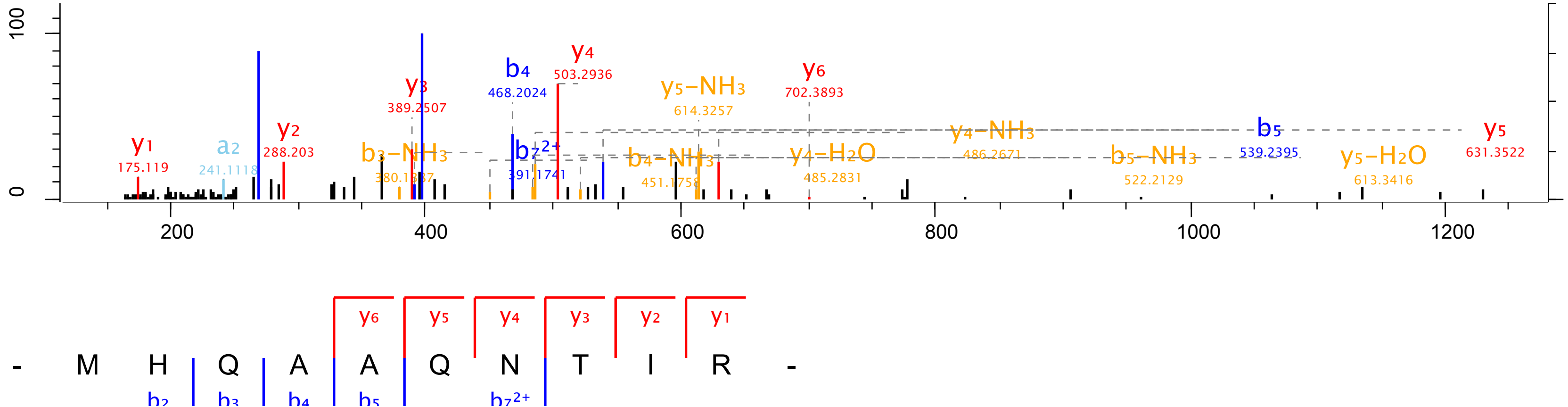

Raw file	Scan	Method	Score	m/z	Gene names
20141014_fract2_dyn_5ul_E2_01_581	5353	TOF; CID	104.44	534.72	SIAH1

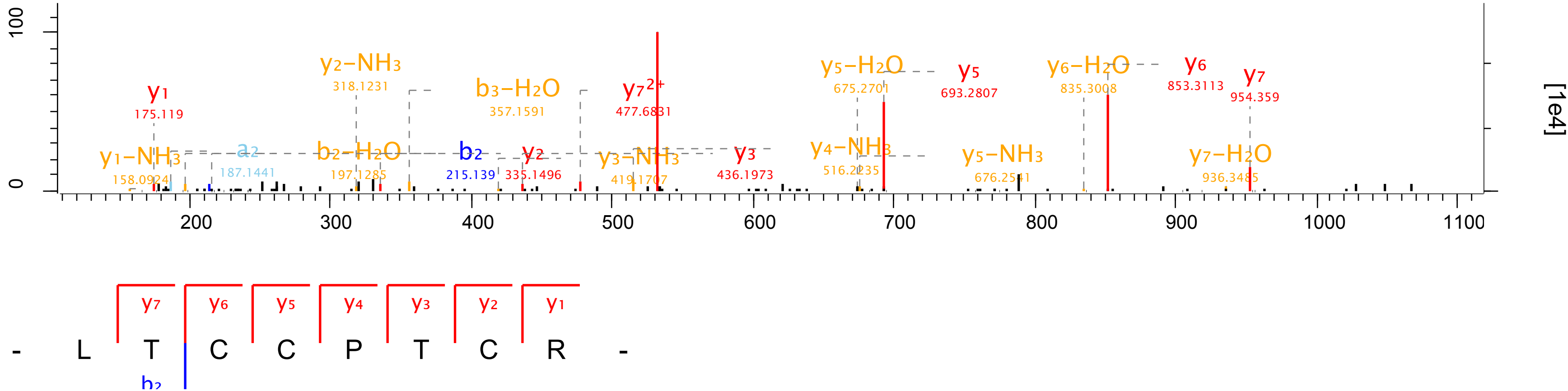

Raw file	Scan	Method	Score	m/z	Gene names
20141014_fract2_dyn_5ul_E2_01_581	5967	TOF; CID	86.5	615.32	CD164

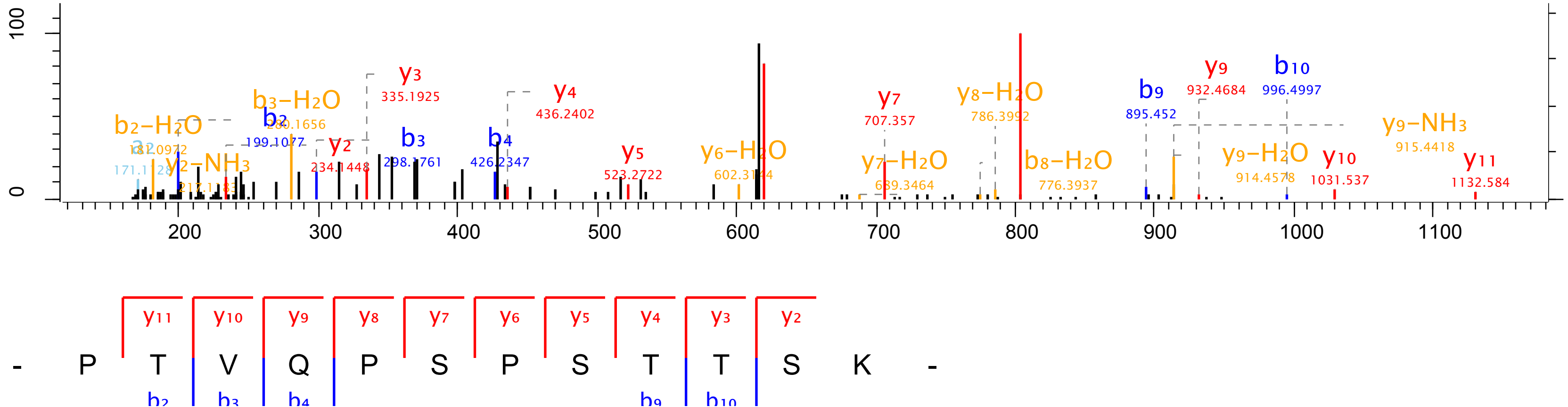

Raw file	Scan	Method	Score	m/z	Gene names
20141014_fract2_dyn_5ul_E2_01_581	7794	TOF; CID	127.46	509.26	VPS9D1

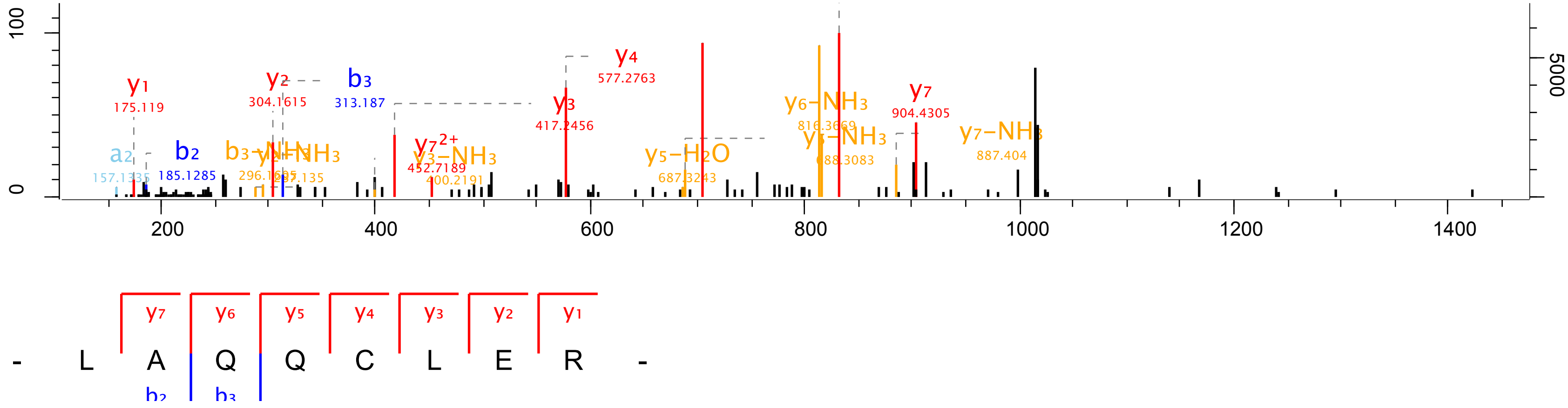

Raw file	Scan	Method	Score	m/z	Gene names
20141014_fract2_dyn_5ul_E2_01_581	8678	TOF; CID	52.25	482.24	ZNF165

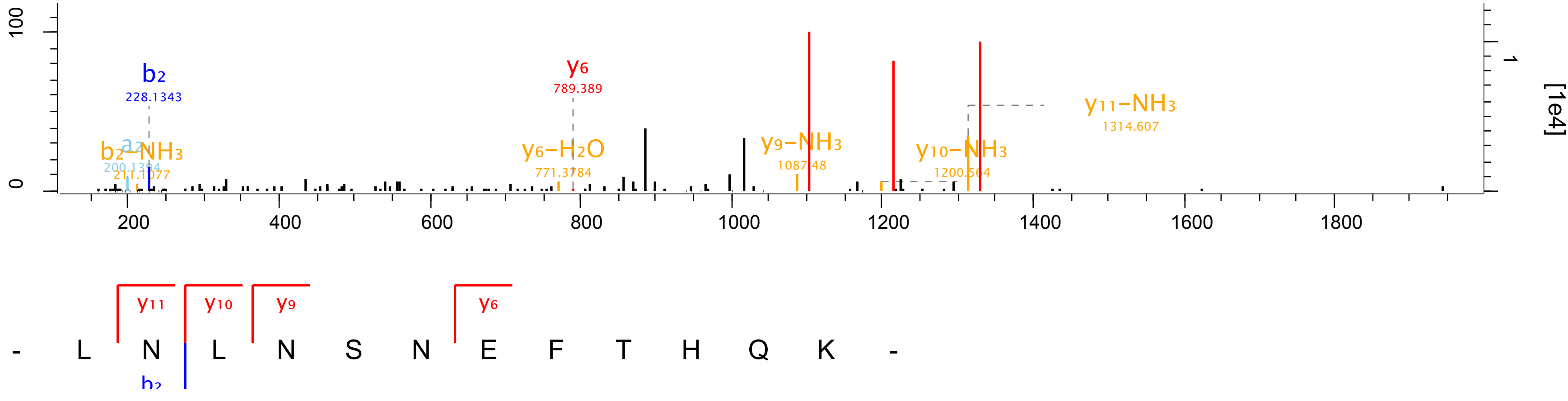

Raw file	Scan	Method	Score	m/z	Gene names
20141014_fract2_dyn_5ul_E2_01_581	10721	TOF; CID	54.9	660.32	NDFIP2

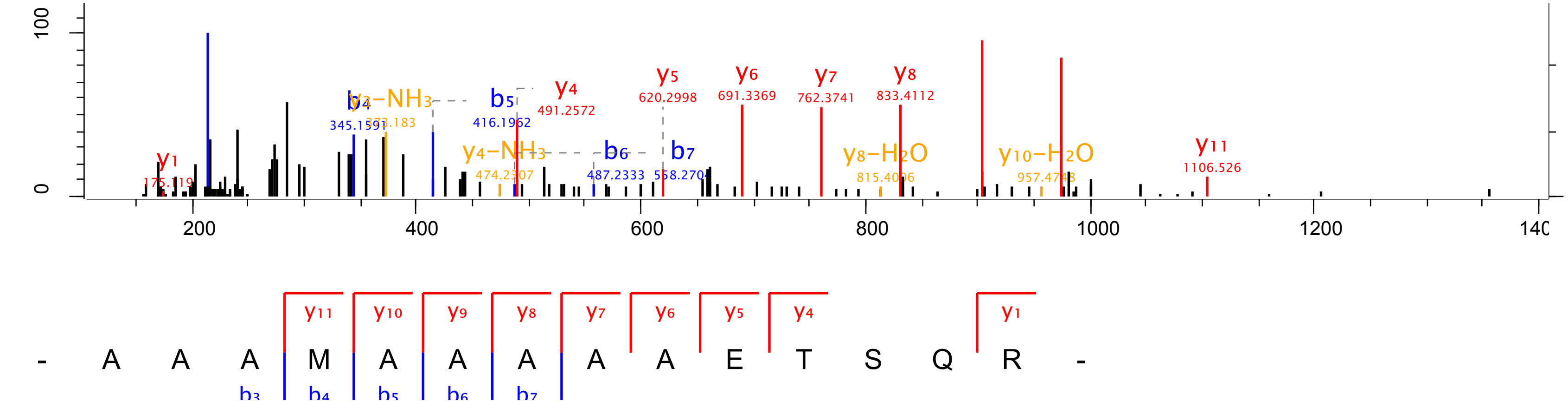

Raw file	Scan	Method	Score	m/z	Gene names
20141014_fract2_dyn_5ul_E2_01_581	11931	TOF; CID	87.18	502.25	C18orf32

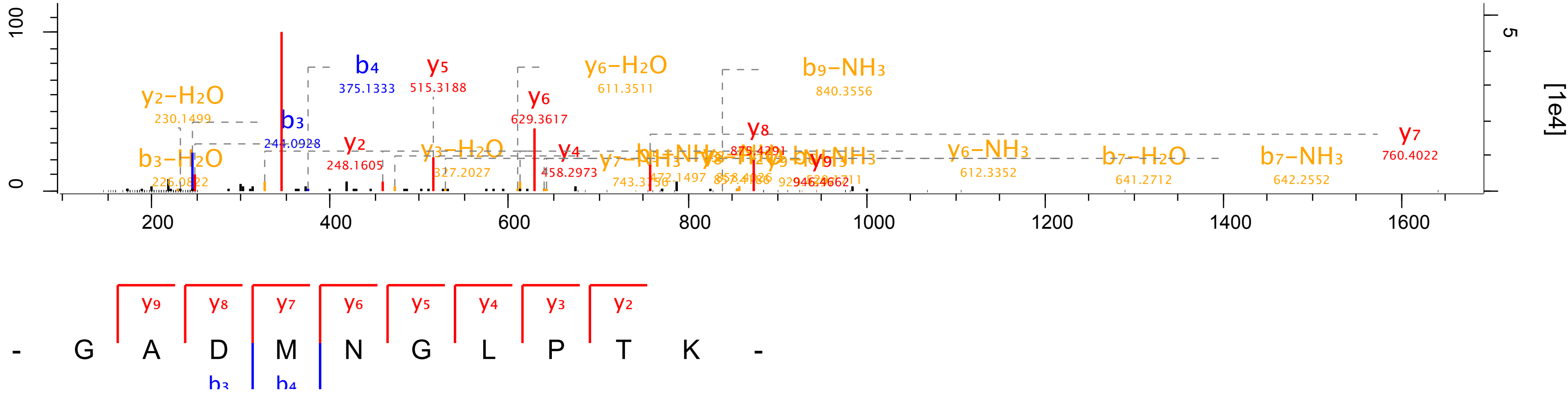

Raw file	Scan	Method	Score	m/z	Gene names
20141014_fract2_dyn_5ul_E2_01_581	12068	TOF; CID	95.52	552.78	VAMP8

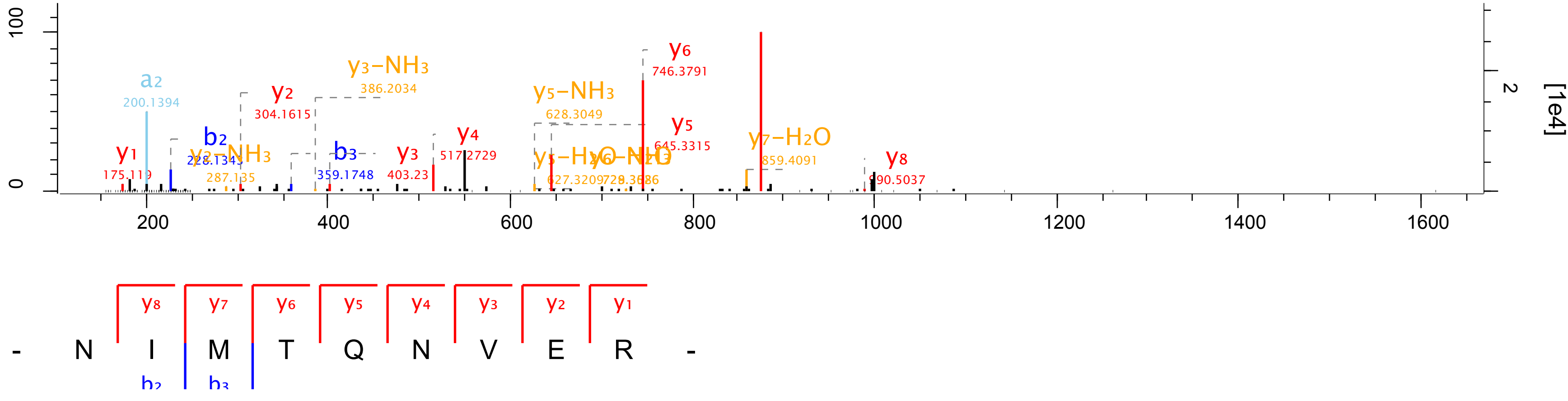

Raw file	Scan	Method	Score	m/z	Gene names
20141014_fract2_dyn_5ul_E2_01_581	12220	TOF; CID	113.51	625.03	C11orf48

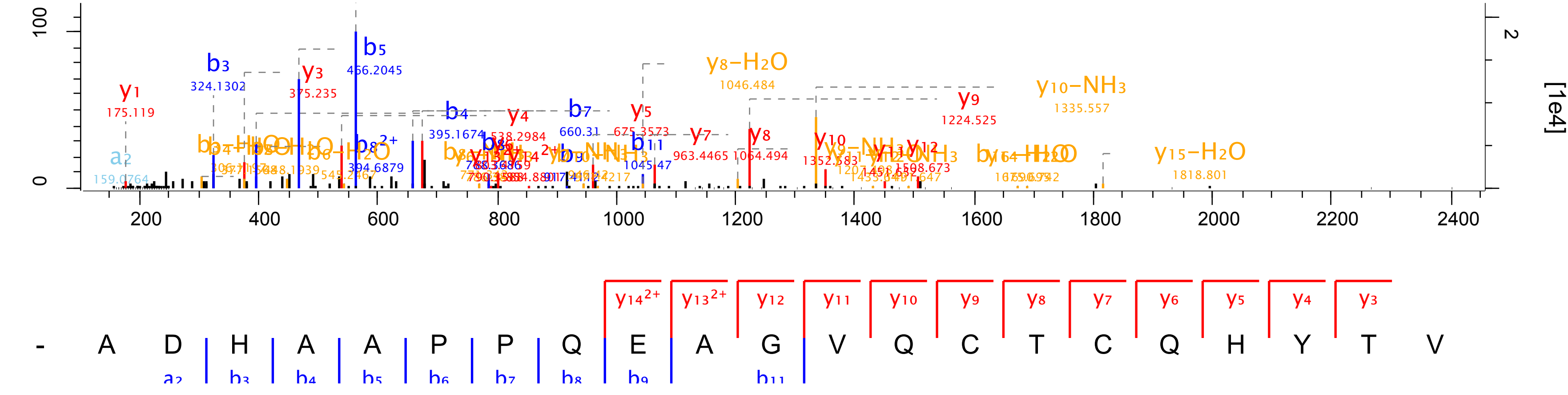

Raw file	Scan	Method	Score	m/z	Gene names
20141014_fract2_dyn_5ul_E2_01_581	12521	TOF; CID	94.12	720.31	LEPROTL1

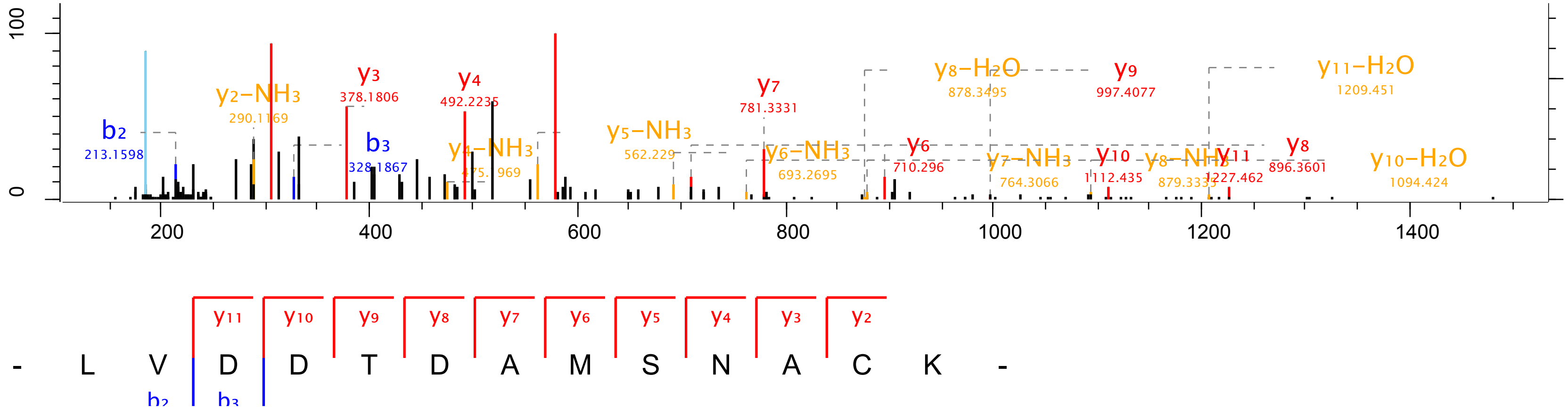

Raw file	Scan	Method	Score	m/z	Gene names
20141014_fract2_dyn_5ul_E2_01_581	14642	TOF; CID	116.19	739.35	ZNHIT1

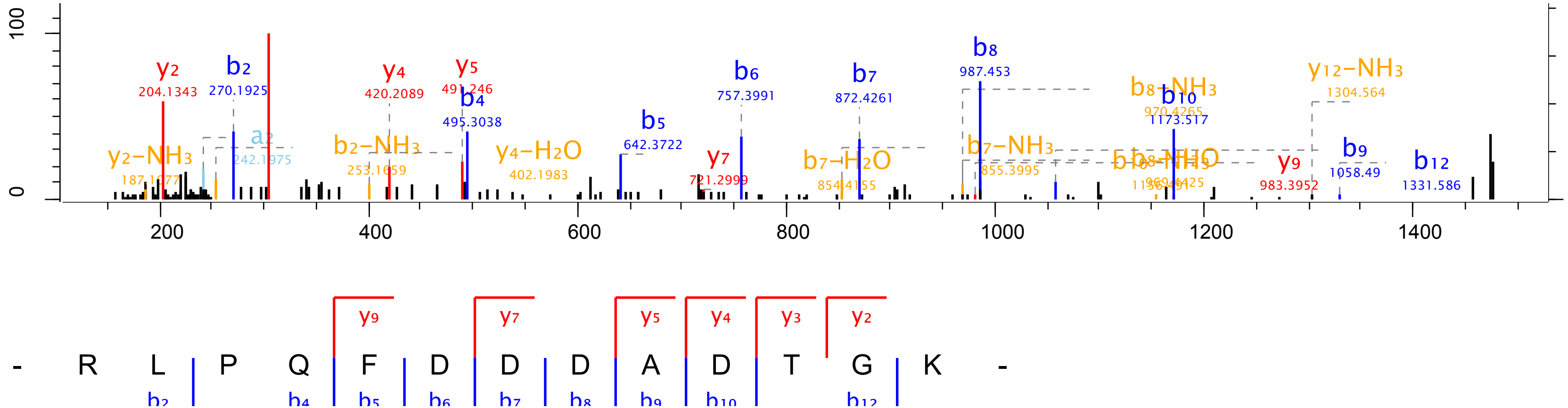

Raw file	Scan	Method	Score	m/z	Gene names
20141014_fract2_dyn_5ul_E2_01_581	18284	TOF; CID	64.54	641.97	ITPKA

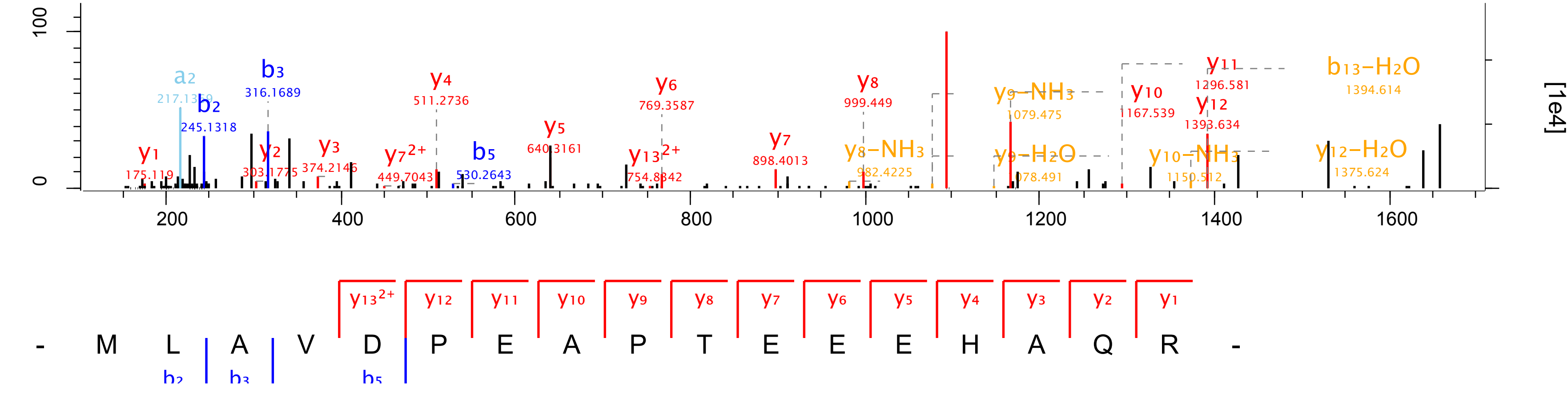

Raw file	Scan	Method	Score	m/z	Gene names
20141014_fract2_dyn_5ul_E2_01_581	18570	TOF; CID	105.63	859.93	APOC3

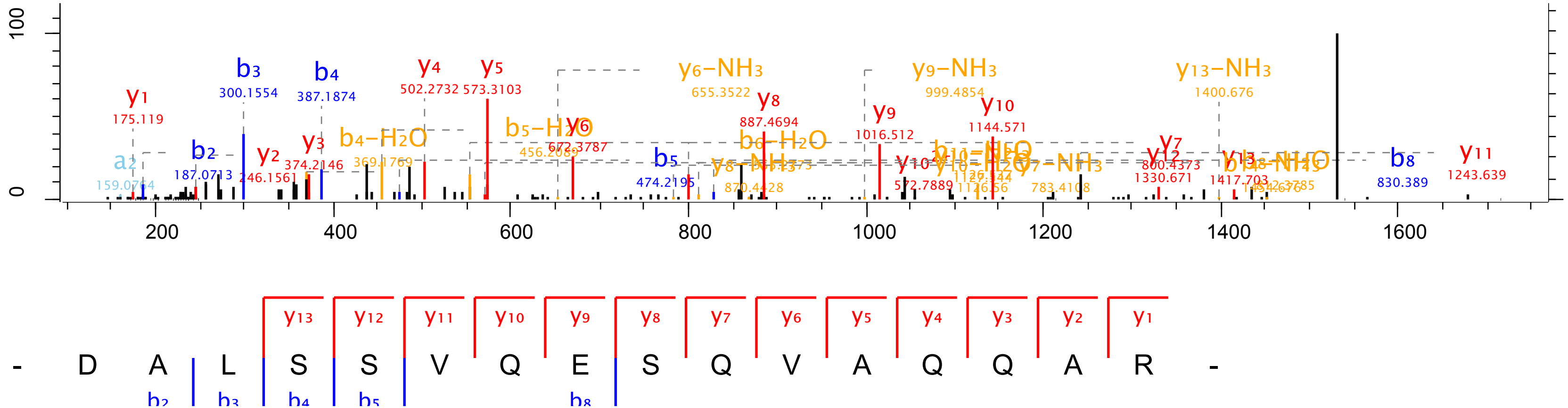

Raw file	Scan	Method	Score	m/z	Gene names
20141014_fract2_dyn_5ul_E2_01_581	26560	TOF; CID	73.93	756.86	FAM73A

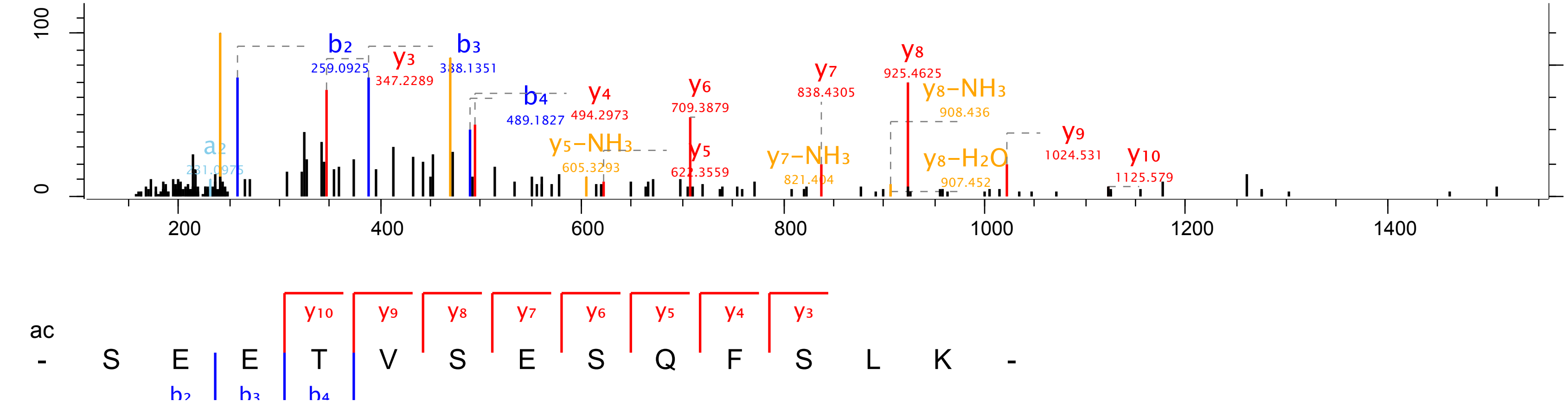

Raw file	Scan	Method	Score	m/z	Gene names
20141014_fract2_dyn_5ul_E2_01_581	26737	TOF; CID	133.48	757.4	MRPL57

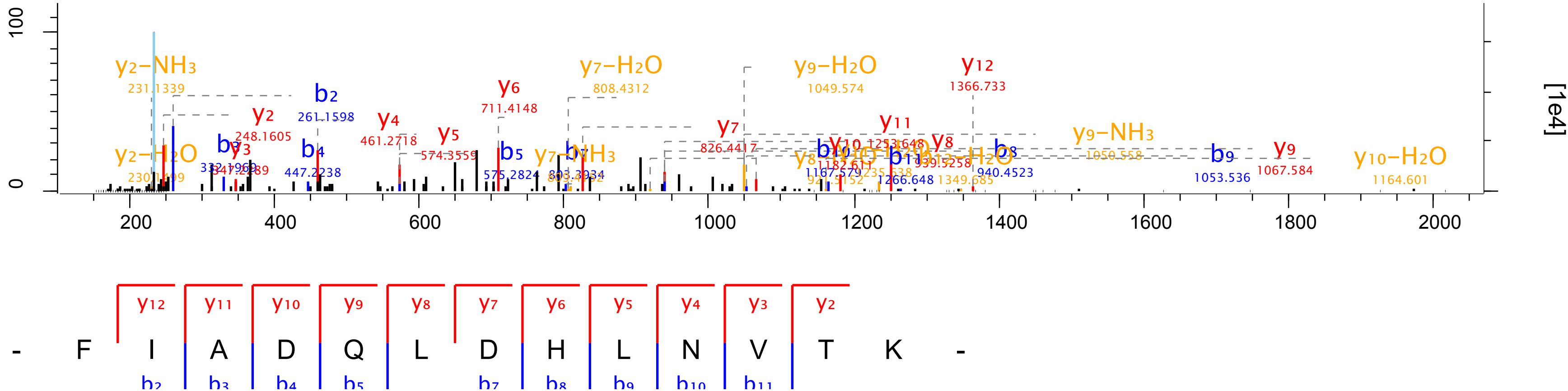

Raw file	Scan	Method	Score	m/z	Gene names
20141014_fract2_dyn_5ul_E2_01_581	27754	TOF; CID	49.3	615.36	EFNB1

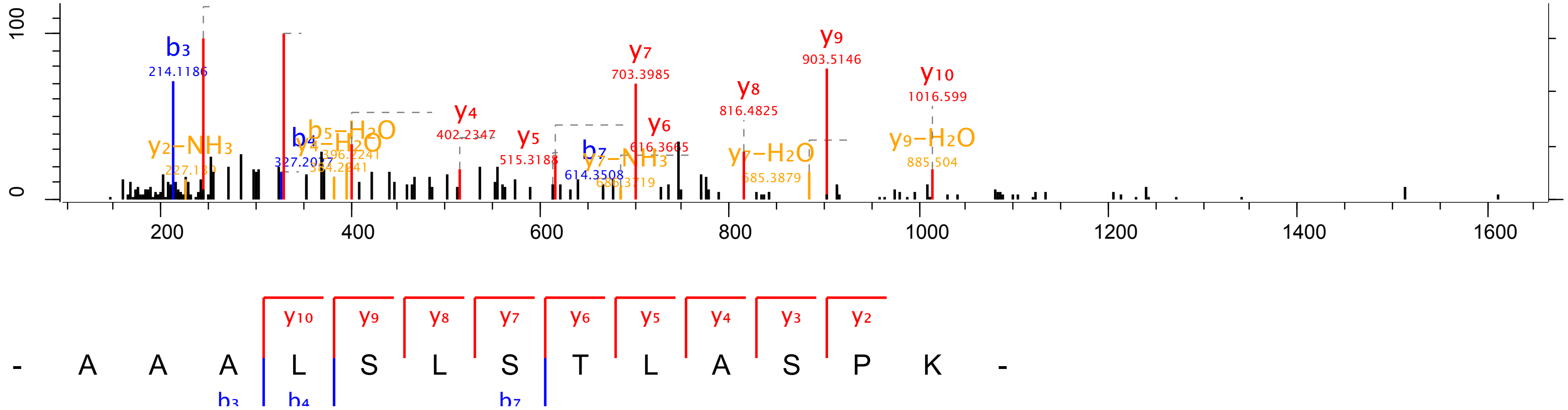

Raw file	Scan	Method	Score	m/z	Gene names
20141014_fract2_dyn_5ul_E2_01_581	28473	TOF; CID	94.49	565.29	BTG2

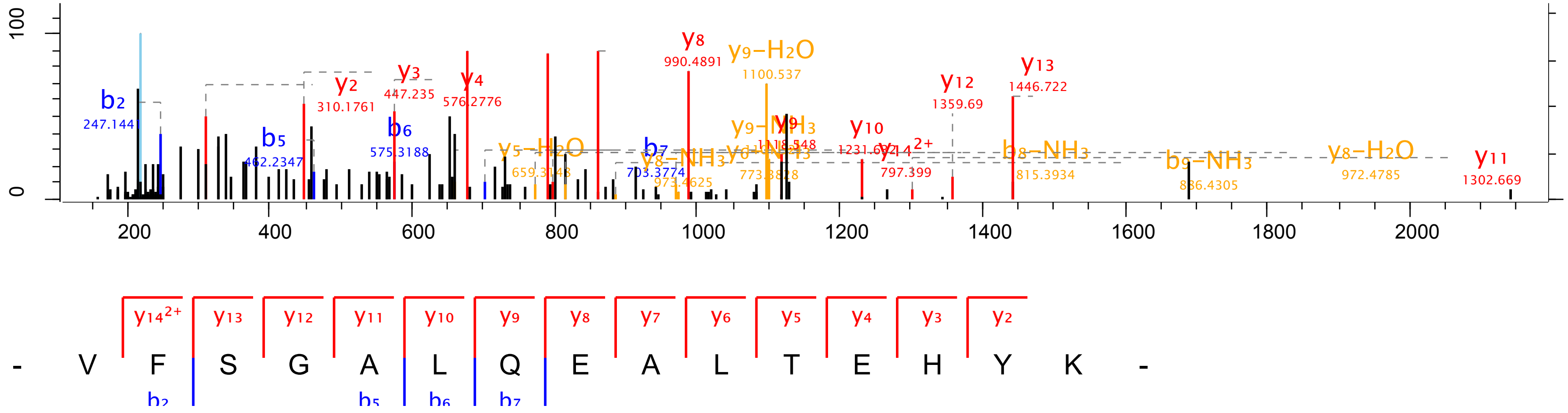

Raw file	Scan	Method	Score	m/z	Gene names
20141014_fract2_dyn_5ul_E2_01_581	30966	TOF; CID	109.11	496.76	SEPT3

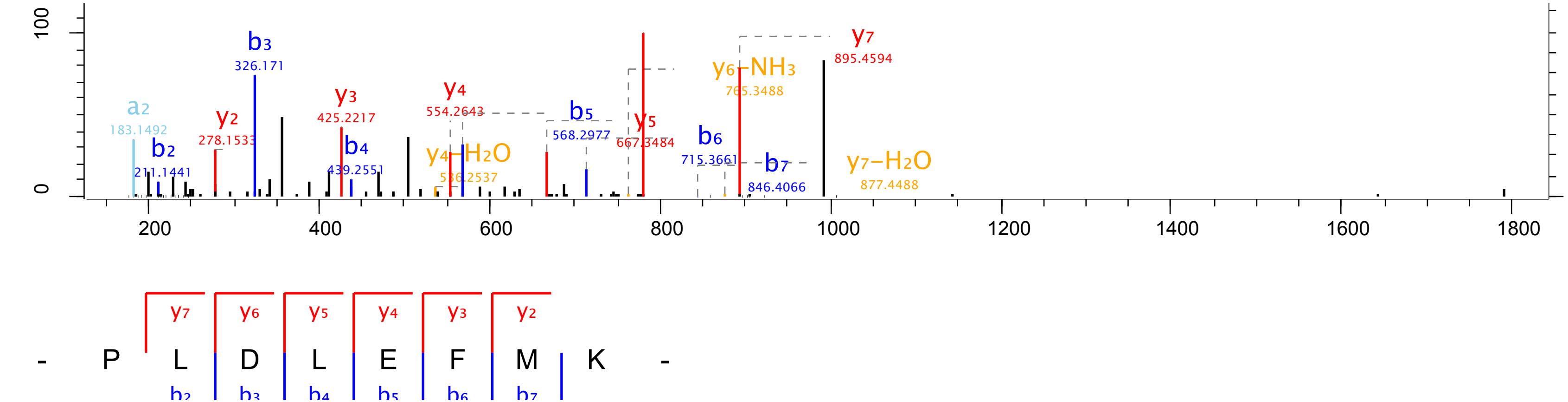

Raw file	Scan	Method	Score	m/z	Gene names
20141014_fract2_dyn_5ul_E2_01_581	33792	TOF; CID	136.83	582.95	TMEM138

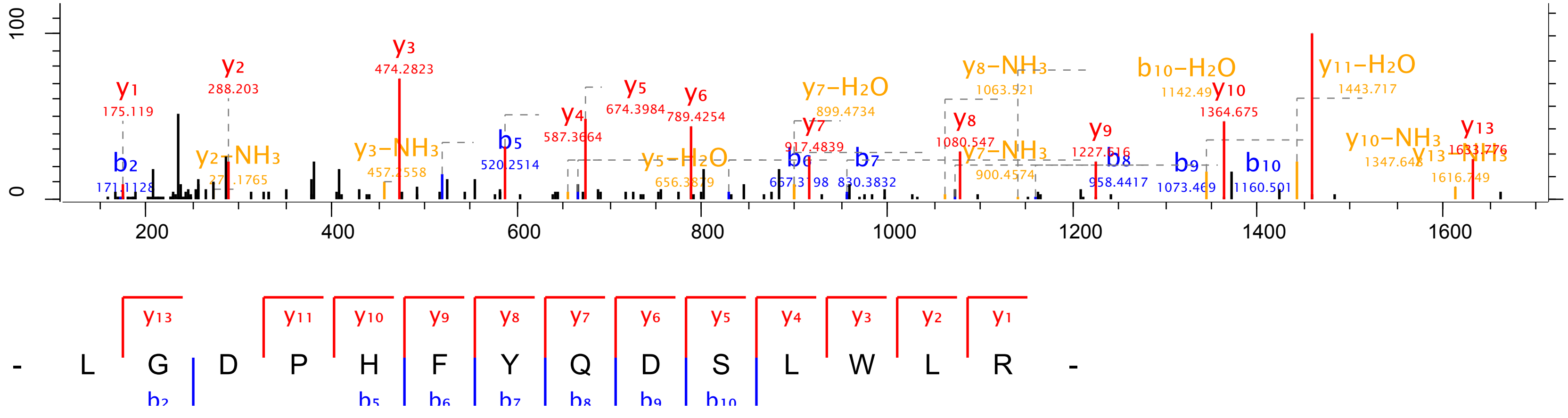

Raw file	Scan	Method	Score	m/z	Gene names
20141014_fract2_dyn_5ul_E2_01_581	35148	TOF; CID	90.15	530.29	PHLDA1

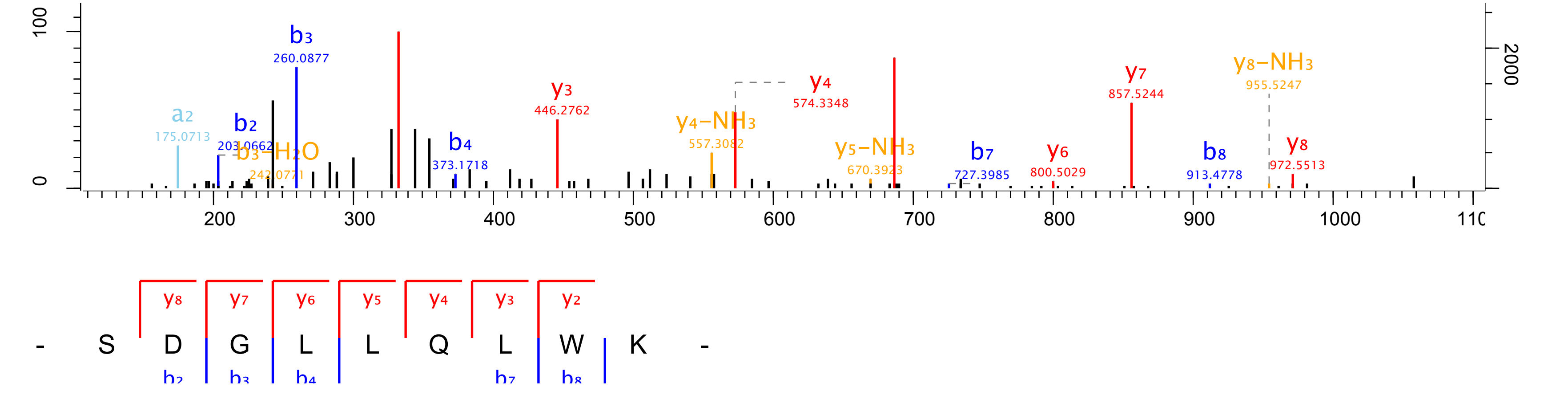

Raw file	Scan	Method	Score	m/z	Gene names
20141014_fract2_dyn_5ul_E2_01_581	37360	TOF; CID	82.65	516.97	STK17B

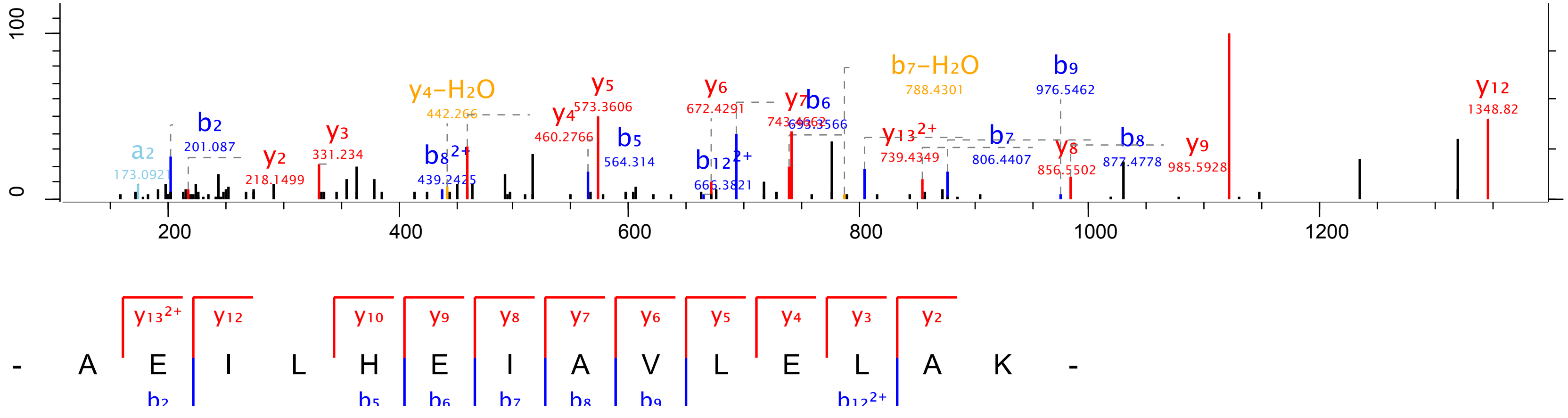

Raw file	Scan	Method	Score	m/z	Gene names
20141014_fract2_dyn_5ul_E2_01_581	38373	TOF; CID	142.43	628.32	SLC2A6

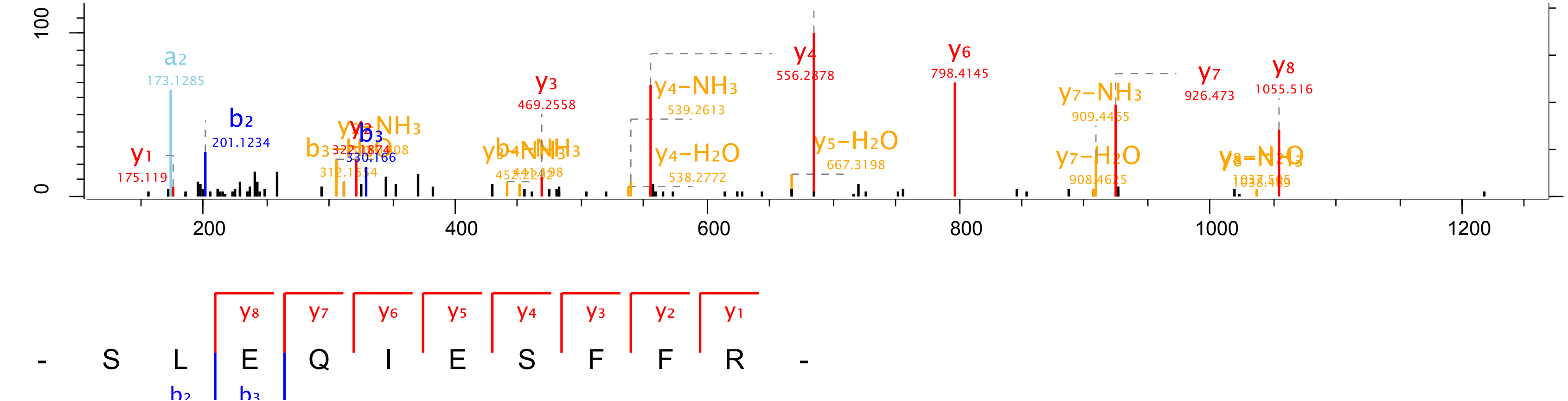

Raw file	Scan	Method	Score	m/z	Gene names
20141014_fract2_dyn_5ul_E2_01_581	41523	TOF; CID	54.96	1068.02	FKTN

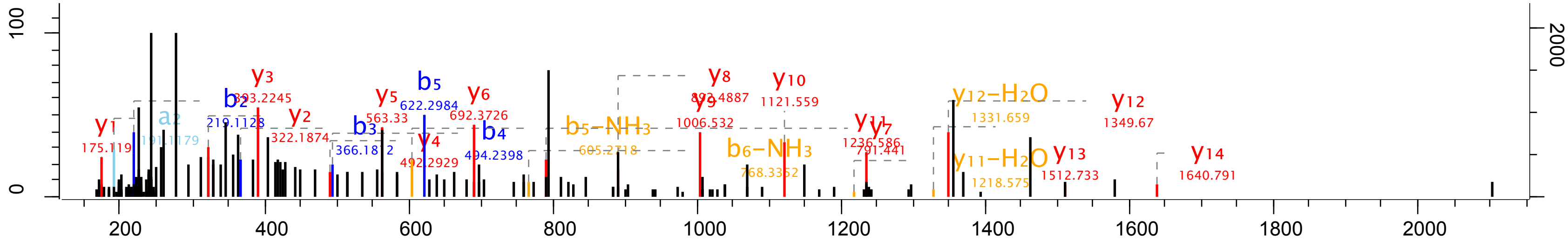

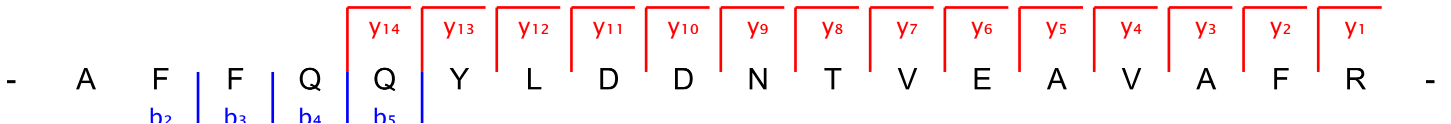

Raw file	Scan	Method	Score	m/z	Gene names
20141014_fract2_dyn_5ul_E2_01_581	41524	TOF; CID	43.73	1040.48	FUOM

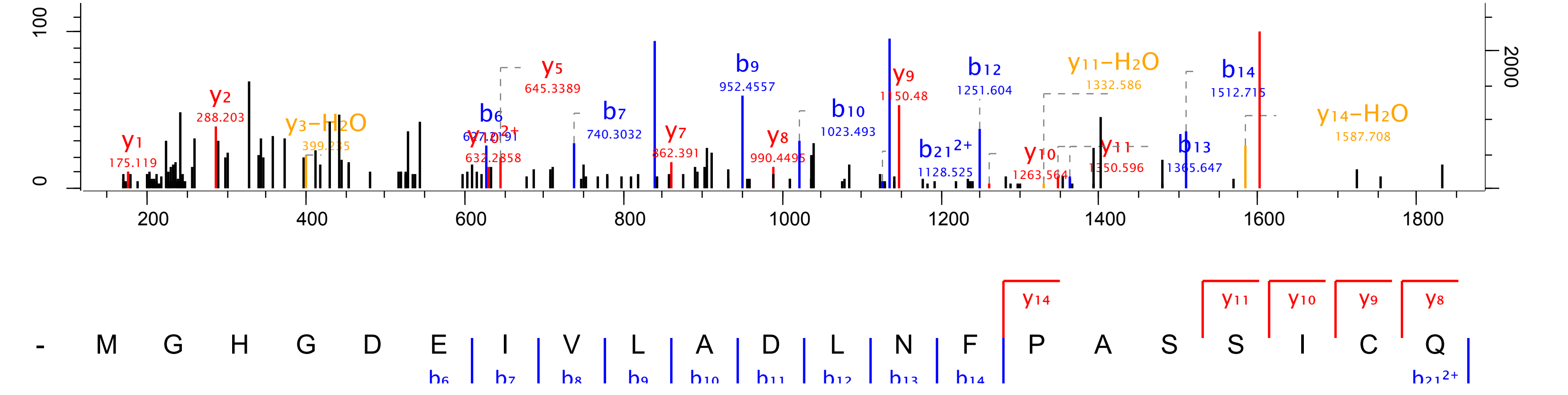

Raw file	Scan	Method	Score	m/z	Gene names
20141014_fract3_dyn_5ul_E3_01_582	7727	TOF; CID	104.01	601.79	C1orf53

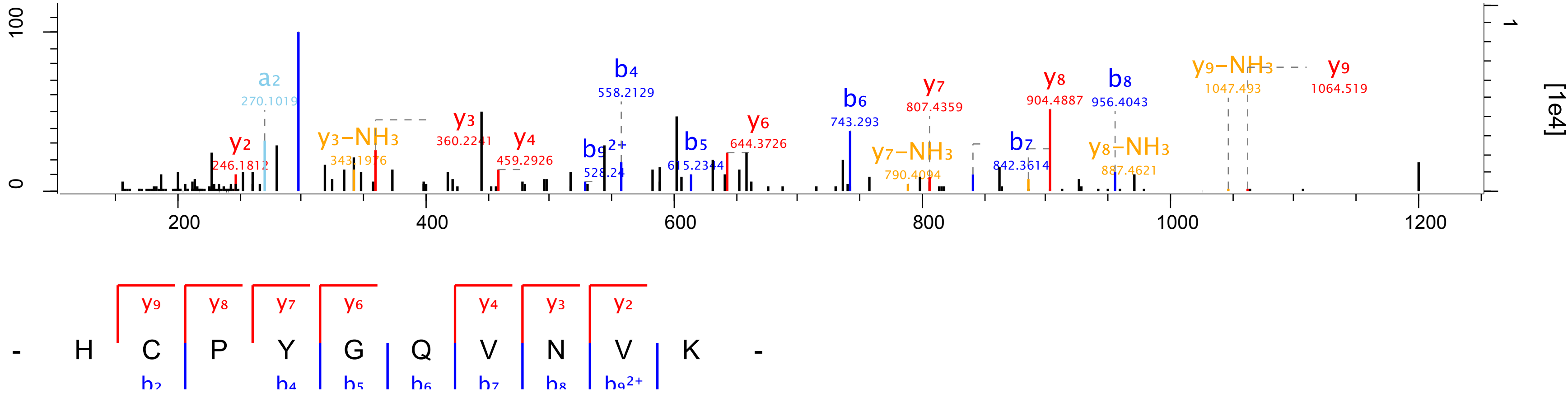

Raw file	Scan	Method	Score	m/z	Gene names
20141014_fract3_dyn_5ul_E3_01_582	11647	TOF; CID	84.16	673.36	ZNF784

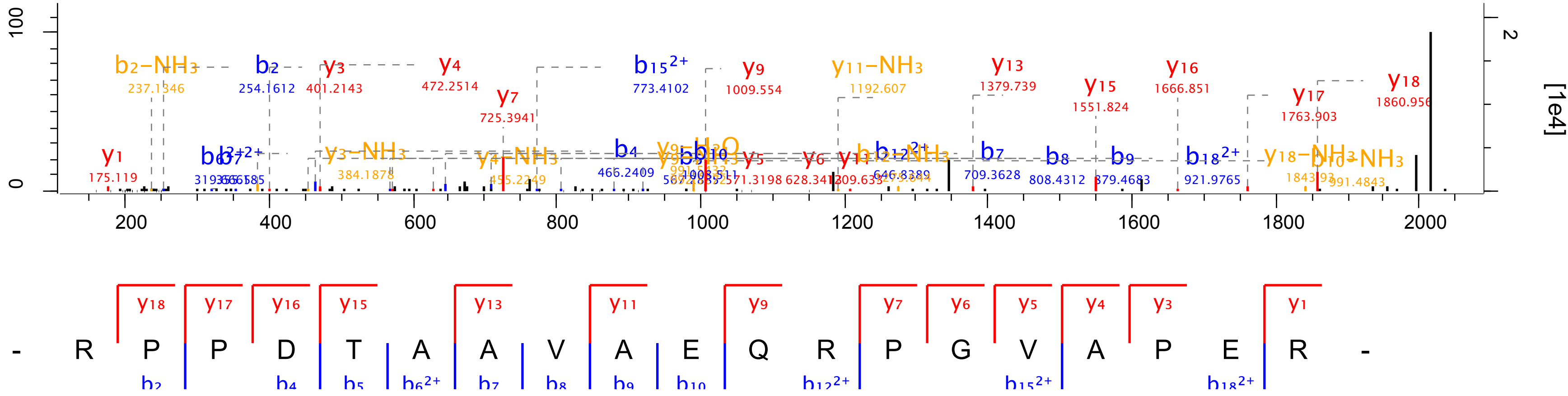

Raw file	Scan	Method	Score	m/z	Gene names
20141014_fract3_dyn_5ul_E3_01_582	12784	TOF; CID	55.72	634.33	ADCK5

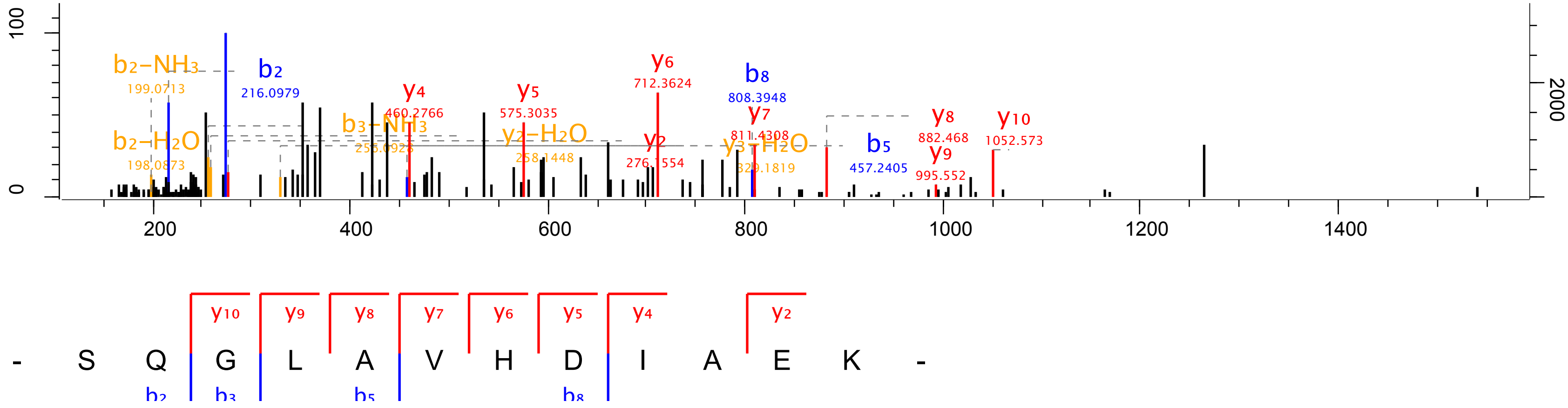

Raw file	Scan	Method	Score	m/z	Gene names
20141014_fract3_dyn_5ul_E3_01_582	13752	TOF; CID	49.22	696.34	KLHL24

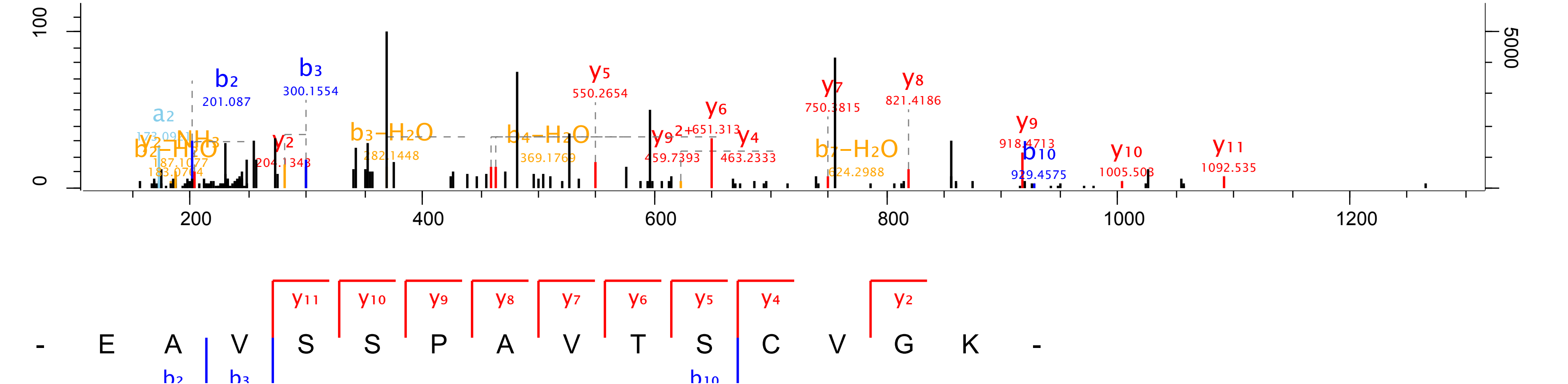

Raw file	Scan	Method	Score	m/z	Gene names
20141014_fract3_dyn_5ul_E3_01_582	13989	TOF; CID	54.81	698.35	IFT43

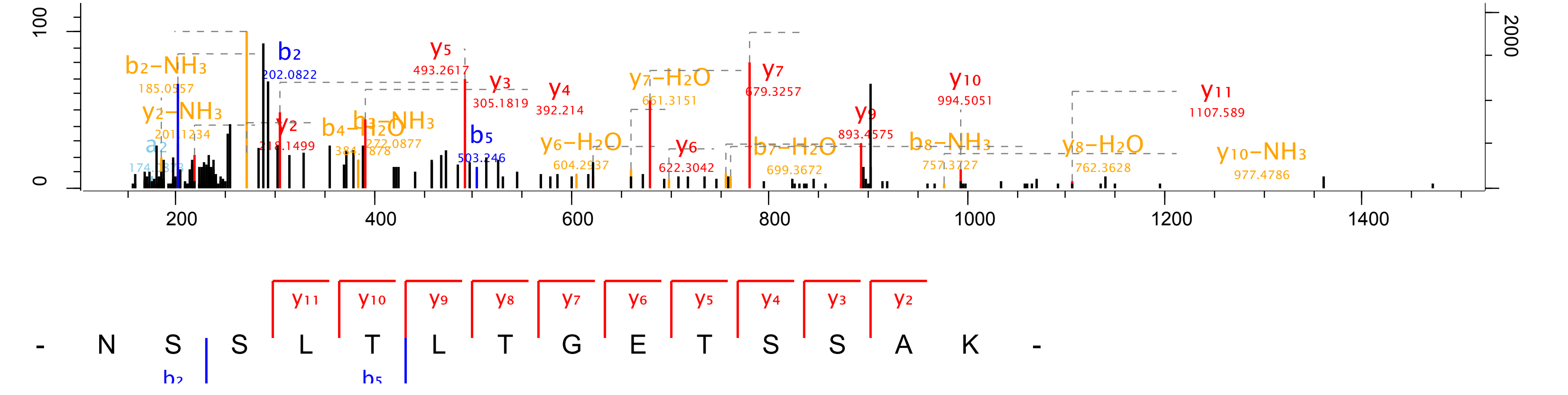

Raw file	Scan	Method	Score	m/z	Gene names
20141014_fract3_dyn_5ul_E3_01_582	14124	TOF; CID	183.65	526.75	C14orf1

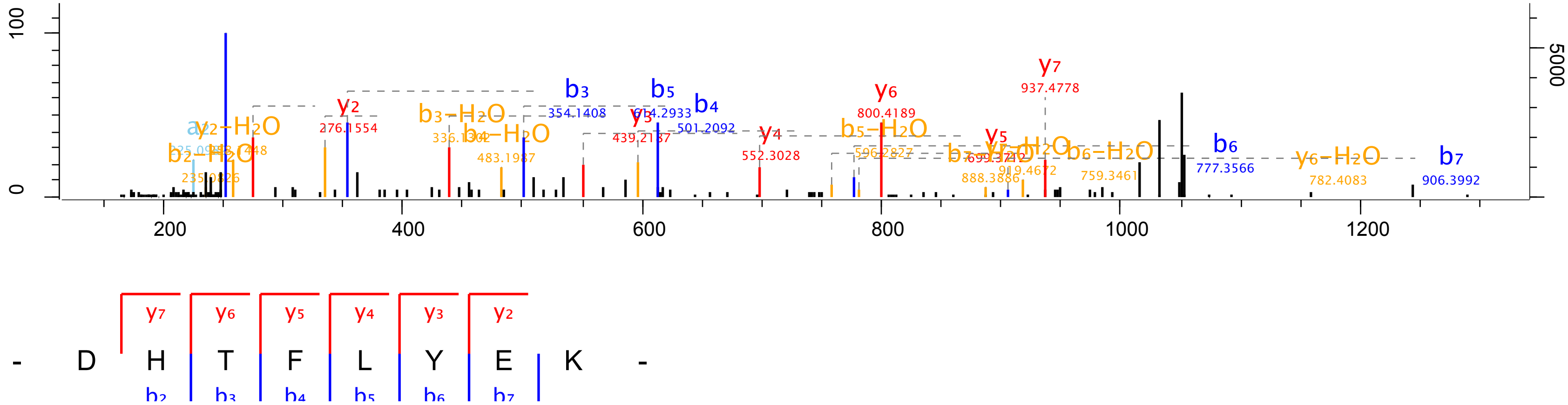

Raw file	Scan	Method	Score	m/z	Gene names
20141014_fract3_dyn_5ul_E3_01_582	15692	TOF; CID	90.7	592.8	MFSD1

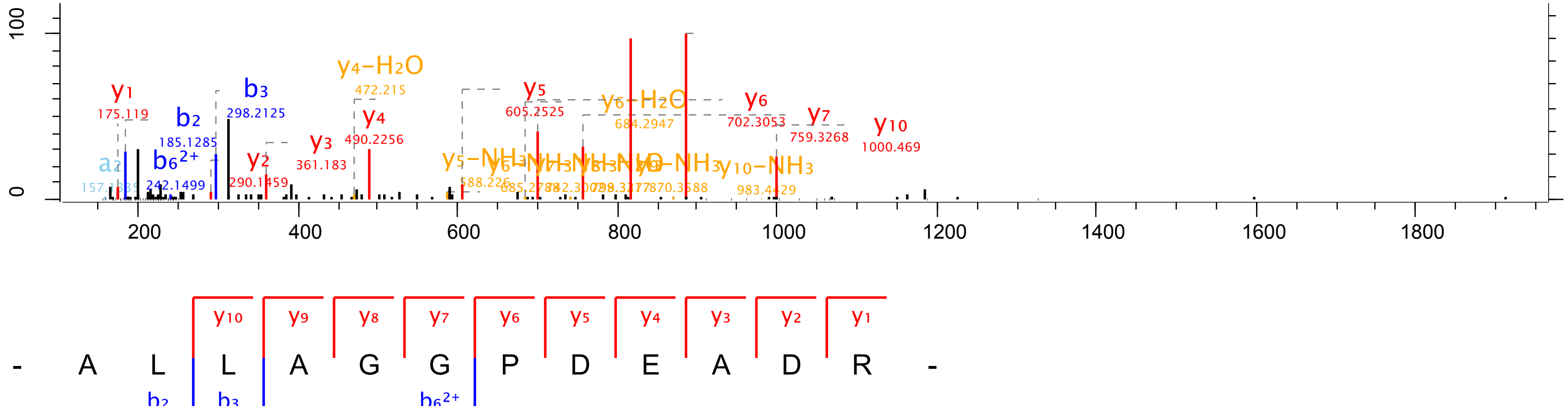

Raw file	Scan	Method	Score	m/z	Gene names
20141014_fract3_dyn_5ul_E3_01_582	17790	TOF; CID	71.18	658.84	ZMYM6NB

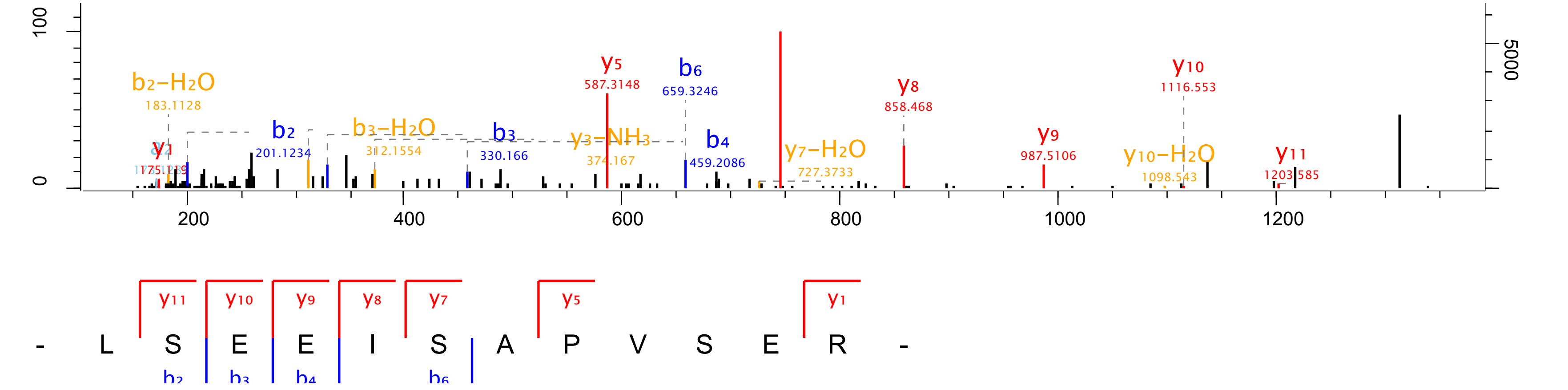

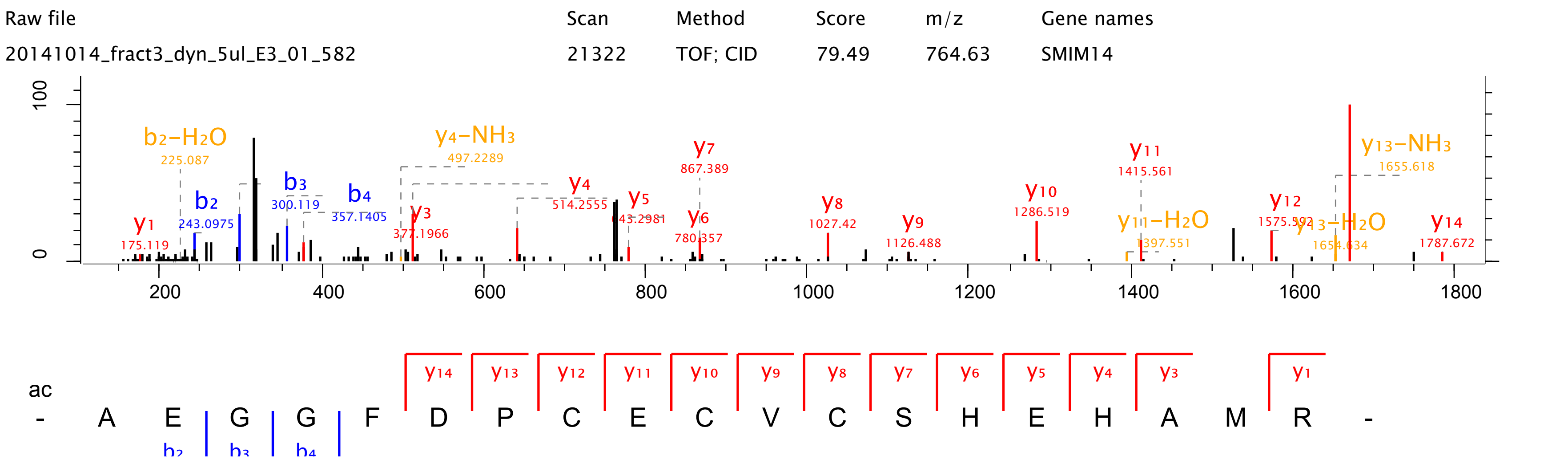

Raw file
20141014_fract3_dyn_5ul_E3_01_582
Scan
21322
Method
TOF; CID
Score
79.49
m/z
764.63
Gene names
SMIM14
100
0
b2-H2O
225.087
b2
243.0975
b3
300.119
b4
357.1405
y1
175.119
y3
377.1966
y4-NH3
497.2289
y4
514.2555
y5
643.2981
y6
780.357
y7
867.389
y8
1027.42
y9
1126.488
y10
1286.519
y11
1415.561
y11-H2O
1397.551
y12
1575.592
y13-H2O
1654.634
y13-NH3
1655.618
y14
1787.672
200
400
600
800
1000
1200
1400
1600
1800
ac
- A E G G F D P C E C V C S H E H A M R -
y14 y13 y12 y11 y10 y9 y8 y7 y6 y5 y4 y3 y1
b2 b3 b4

Raw file	Scan	Method	Score	m/z	Gene names
20141014_fract3_dyn_5ul_E3_01_582	22524	TOF; CID	81.62	446.6	HMGN5

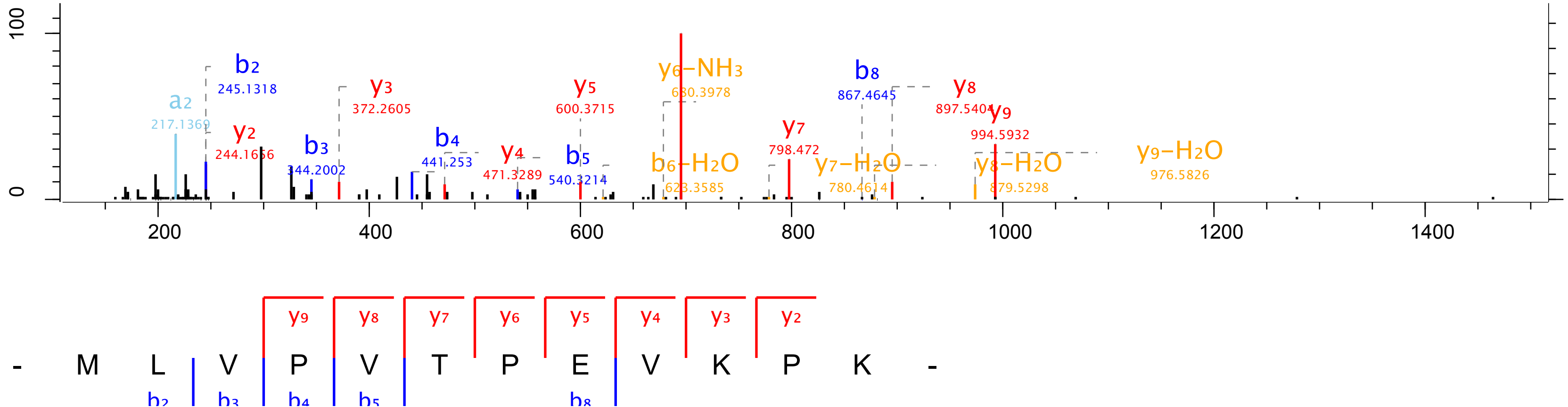

Raw file	Scan	Method	Score	m/z	Gene names
20141014_fract3_dyn_5ul_E3_01_582	24106	TOF; CID	186.81	507.59	CDC26

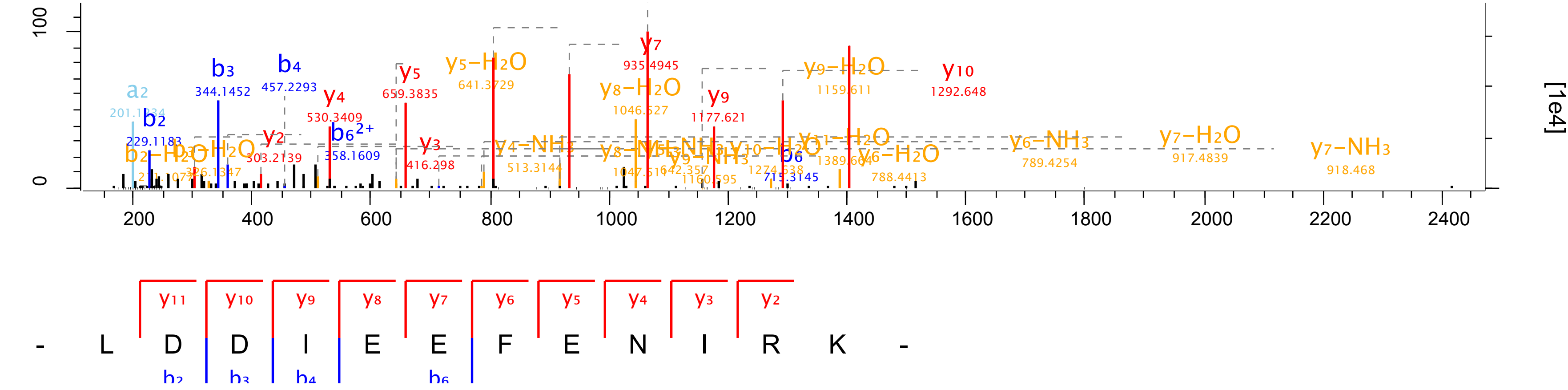

Raw file	Scan	Method	Score	m/z	Gene names
20141014_fract3_dyn_5ul_E3_01_582	25926	TOF; CID	51.64	669.37	GREB1

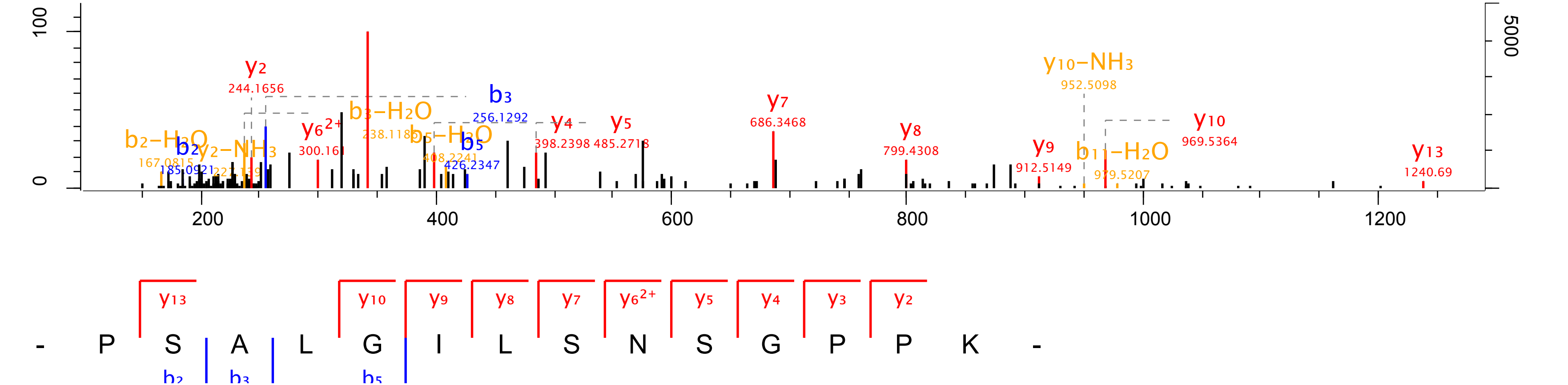

Raw file	Scan	Method	Score	m/z	Gene names
20141014_fract3_dyn_5ul_E3_01_582	27220	TOF; CID	81.64	1040.48	TCTEX1D2

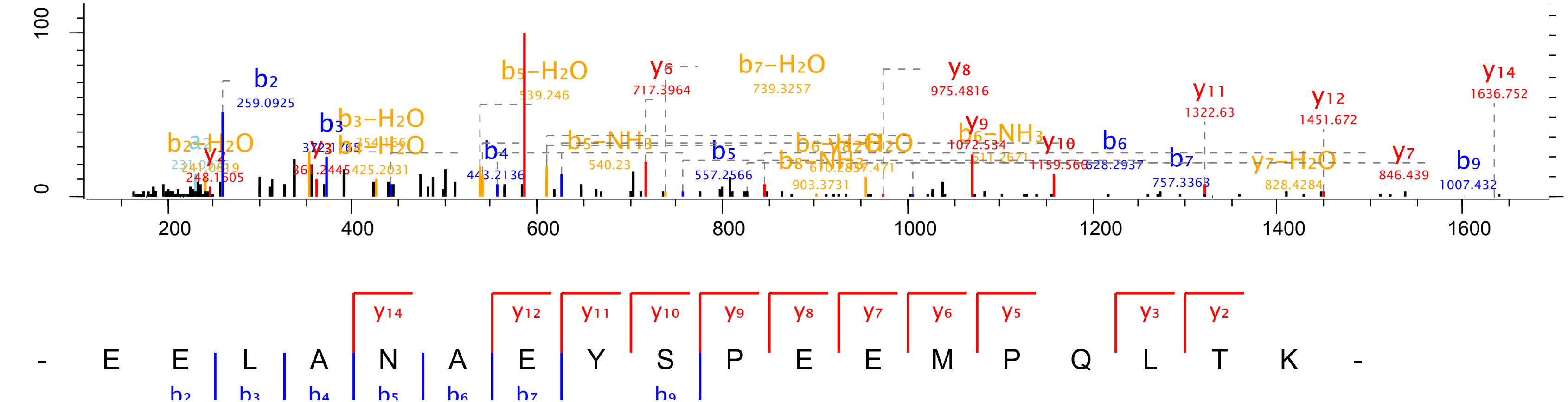

Raw file	Scan	Method	Score	m/z	Gene names
20141014_fract3_dyn_5ul_E3_01_582	28058	TOF; CID	58.32	487.59	TMEM231

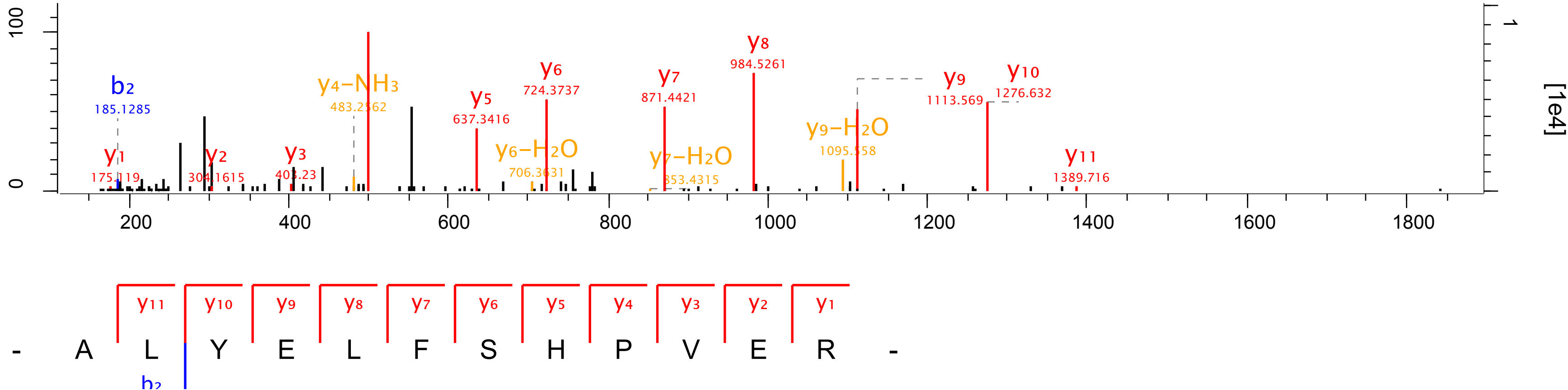

Raw file	Scan	Method	Score	m/z	Gene names
20141014_fract3_dyn_5ul_E3_01_582	28977	TOF; CID	72.68	609.33	SMIM10

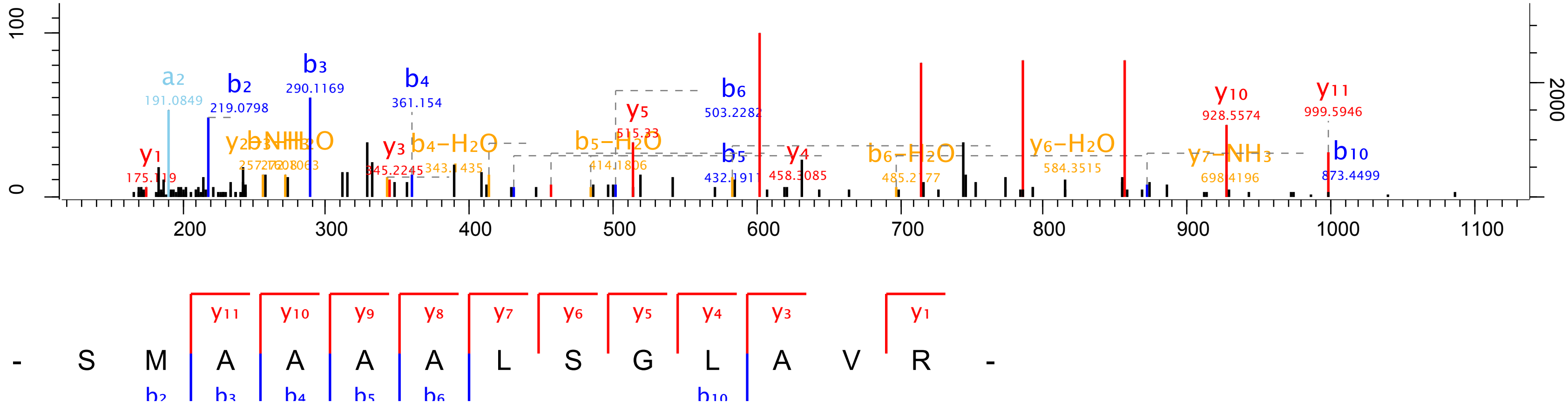

Raw file	Scan	Method	Score	m/z	Gene names
20141014_fract3_dyn_5ul_E3_01_582	30250	TOF; CID	97.16	627.38	MRPL34

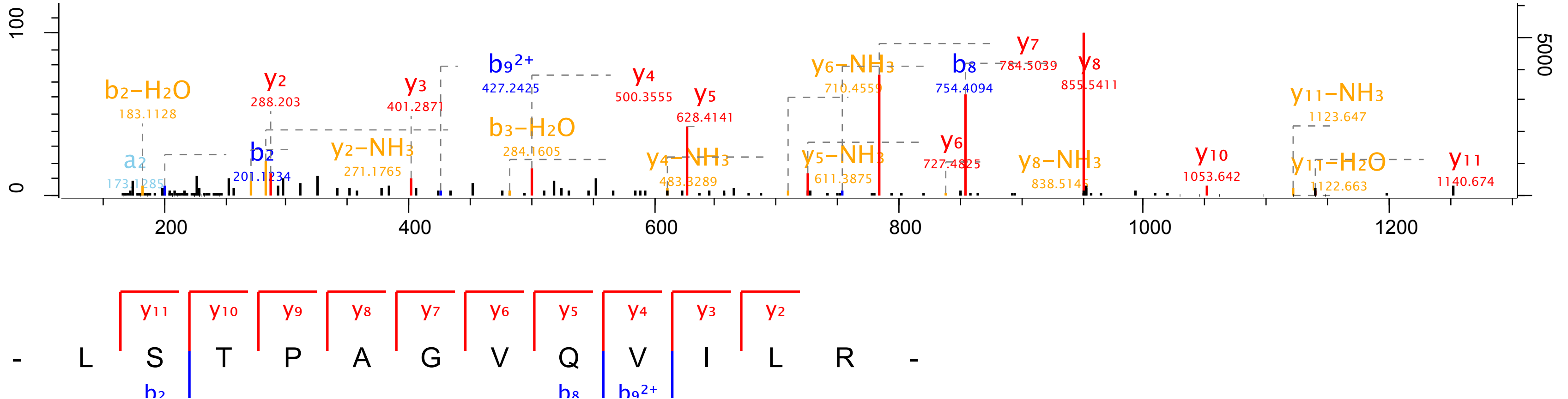

Raw file	Scan	Method	Score	m/z	Gene names
20141014_fract3_dyn_5ul_E3_01_582	30675	TOF; CID	50.09	695.39	CCDC71L

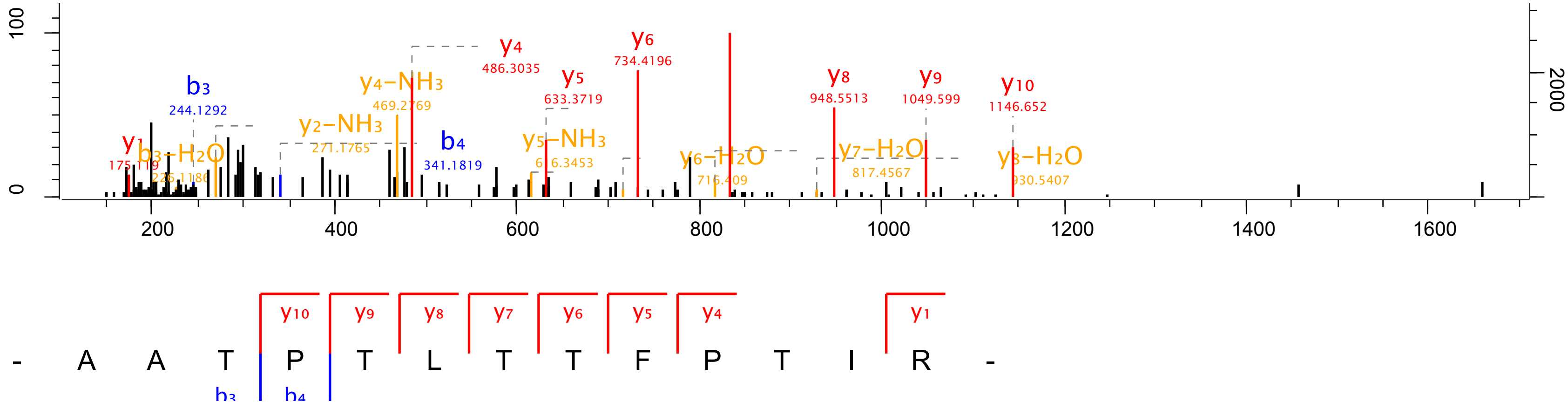

Raw file	Scan	Method	Score	m/z	Gene names
20141014_fract3_dyn_5ul_E3_01_582	38024	TOF; CID	65.04	777.4	TPRA1

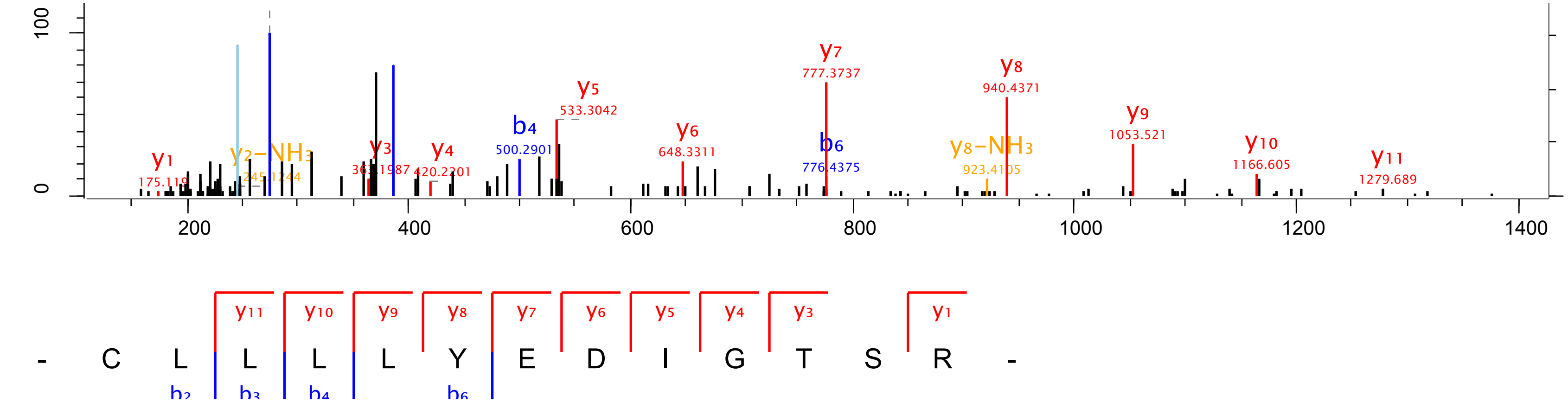

Raw file	Scan	Method	Score	m/z	Gene names
20141014_fract3_dyn_5ul_E3_01_582	38068	TOF; CID	86.8	798.44	CLDN12

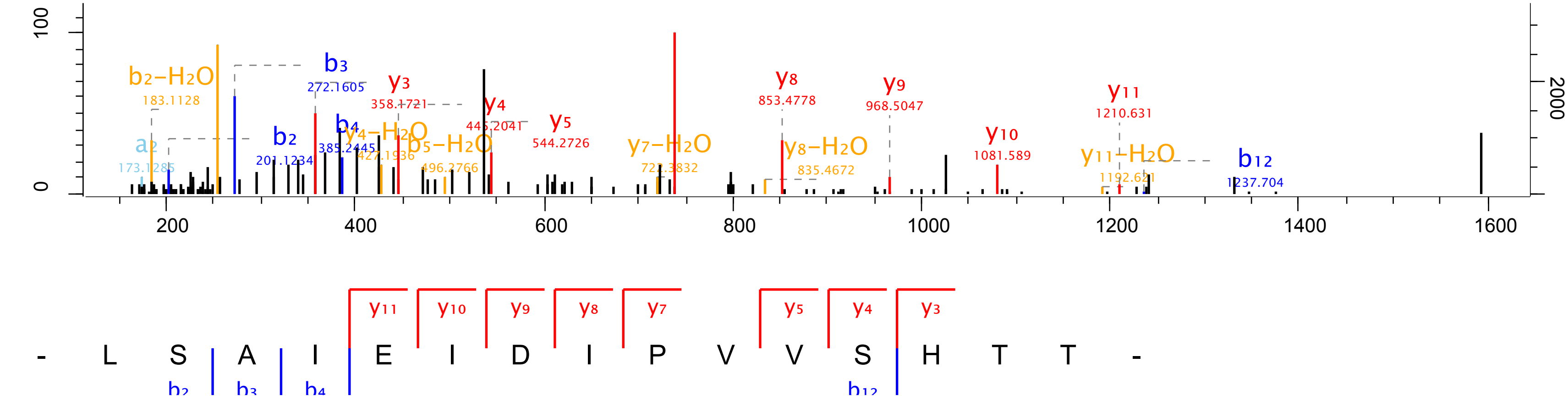

Raw file	Scan	Method	Score	m/z	Gene names
20141014_fract4_dyn_5ul_E4_01_583	3789	TOF; CID	111.01	438.2	SMOC2

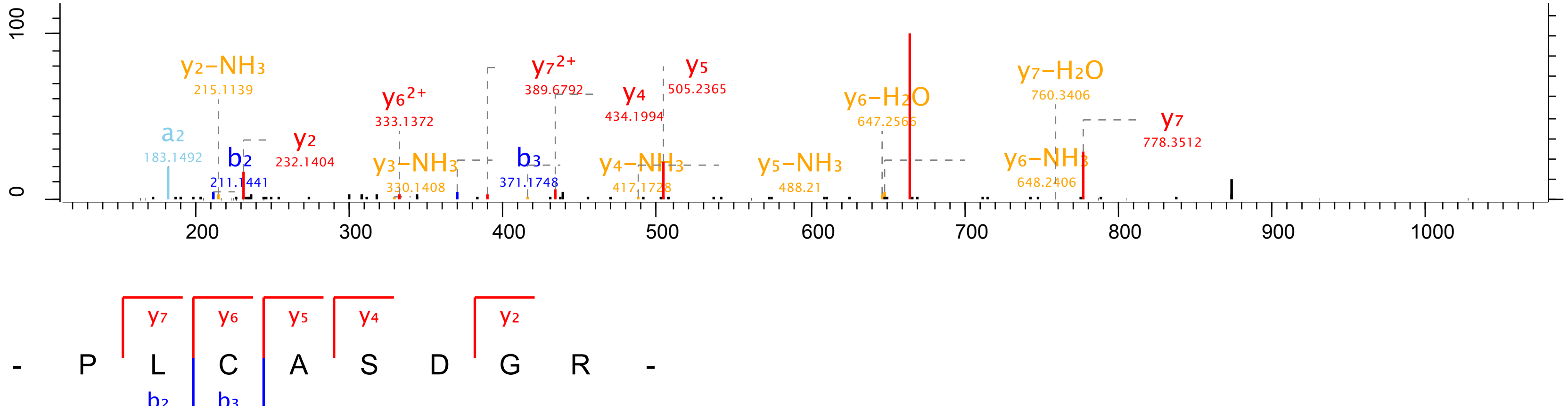

Raw file	Scan	Method	Score	m/z	Gene names
20141014_fract4_dyn_5ul_E4_01_583	4789	TOF; CID	132.09	416.74	TP53INP2

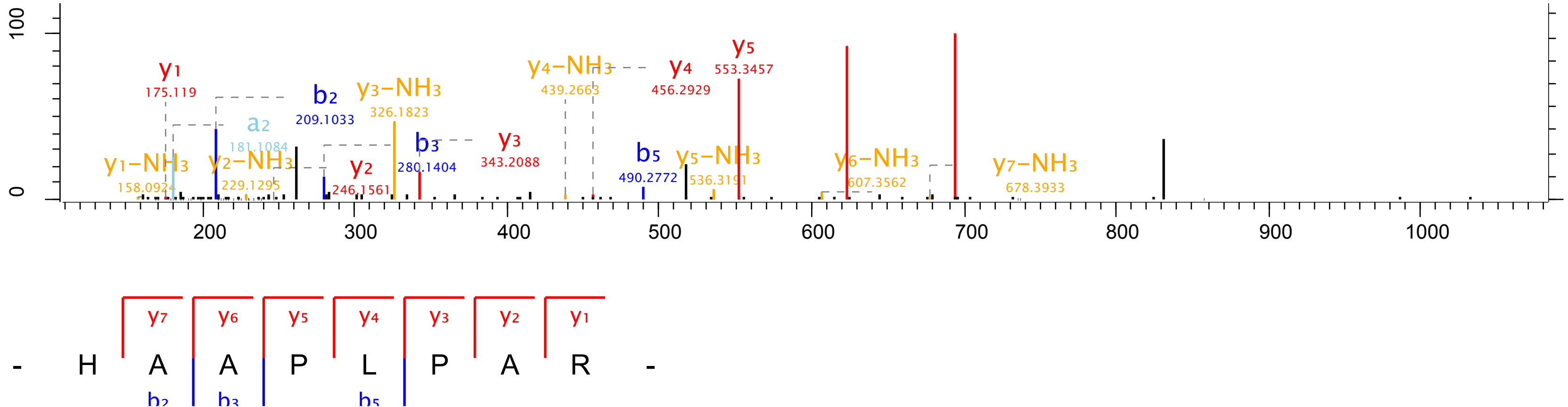

Raw file	Scan	Method	Score	m/z	Gene names
20141014_fract4_dyn_5ul_E4_01_583	8664	TOF; CID	130.77	406.19	C1D

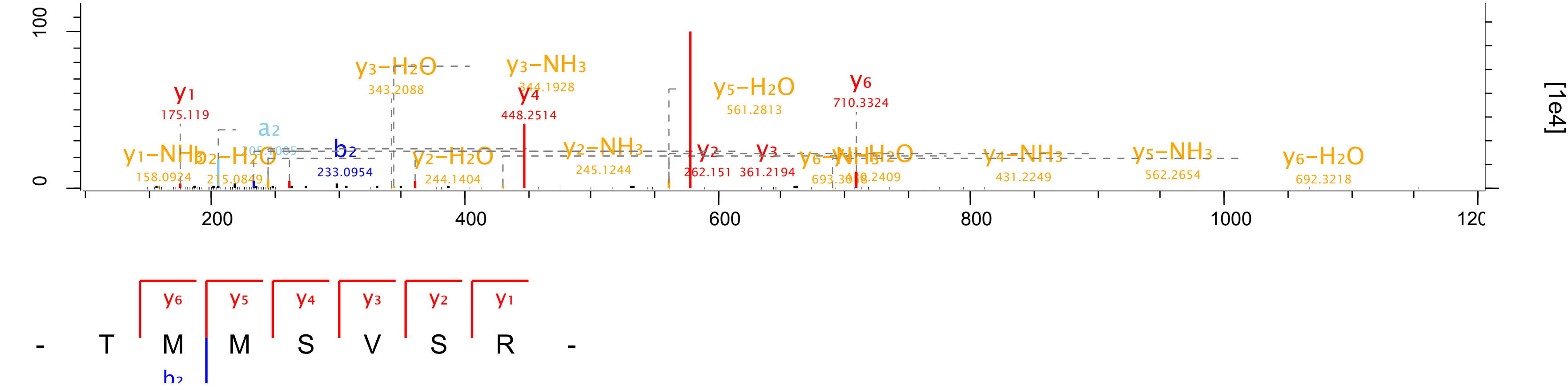

Raw file	Scan	Method	Score	m/z	Gene names
20141014_fract4_dyn_5ul_E4_01_583	9921	TOF; CID	85.35	626.26	ZNF652;ZBTB47

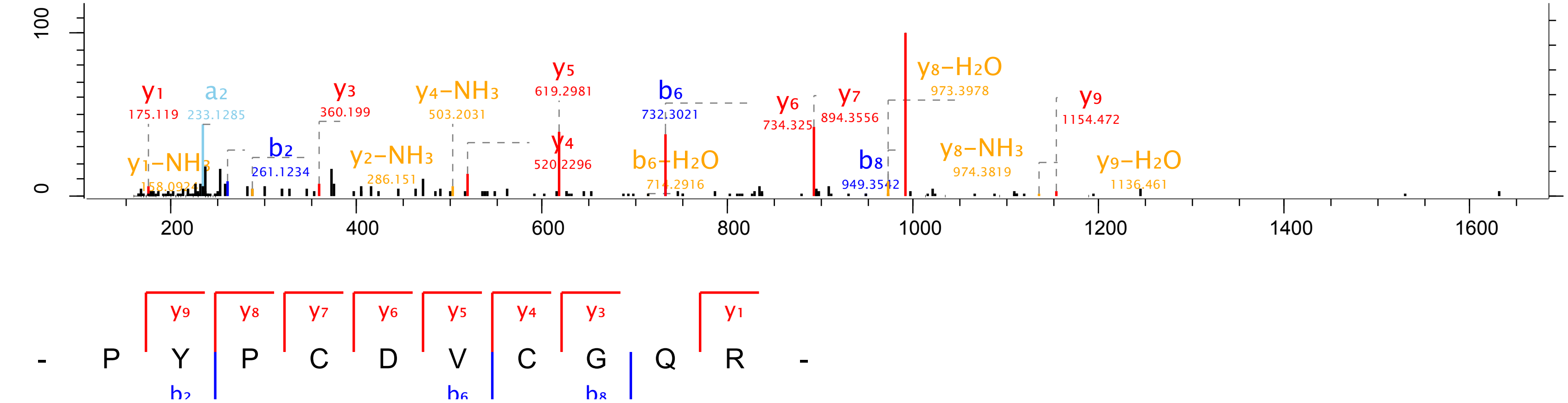

Raw file	Scan	Method	Score	m/z	Gene names
20141014_fract4_dyn_5ul_E4_01_583	17515	TOF; CID	160.81	657.3	DYNLT3

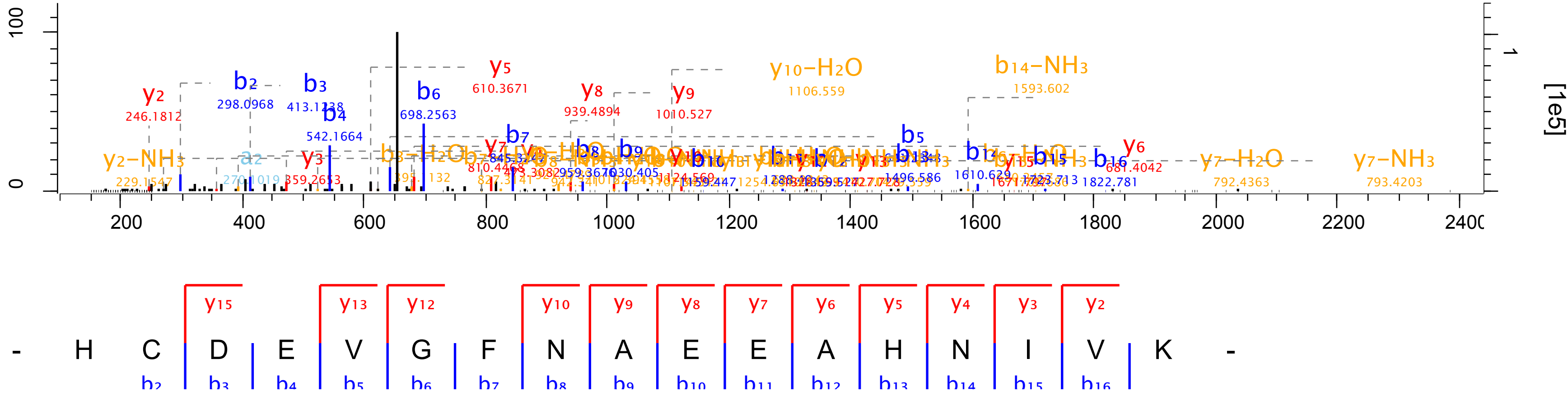

Raw file	Scan	Method	Score	m/z	Gene names
20141014_fract4_dyn_5ul_E4_01_583	19196	TOF; CID	91.28	650.82	SLC36A4

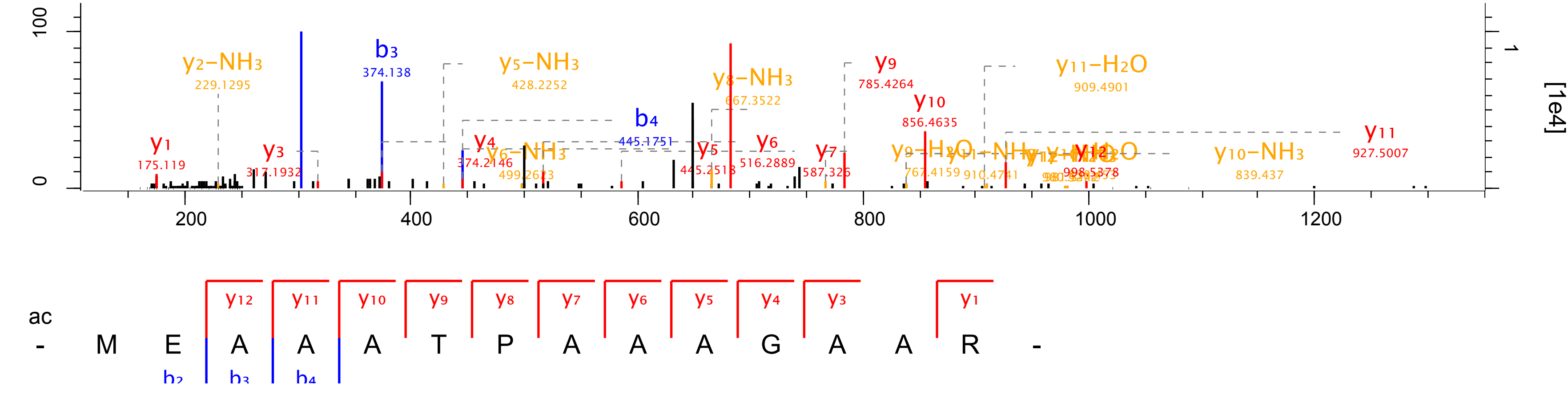

Raw file	Scan	Method	Score	m/z	Gene names
20141014_fract4_dyn_5ul_E4_01_583	20269	TOF; CID	57.43	787.32	FHL2

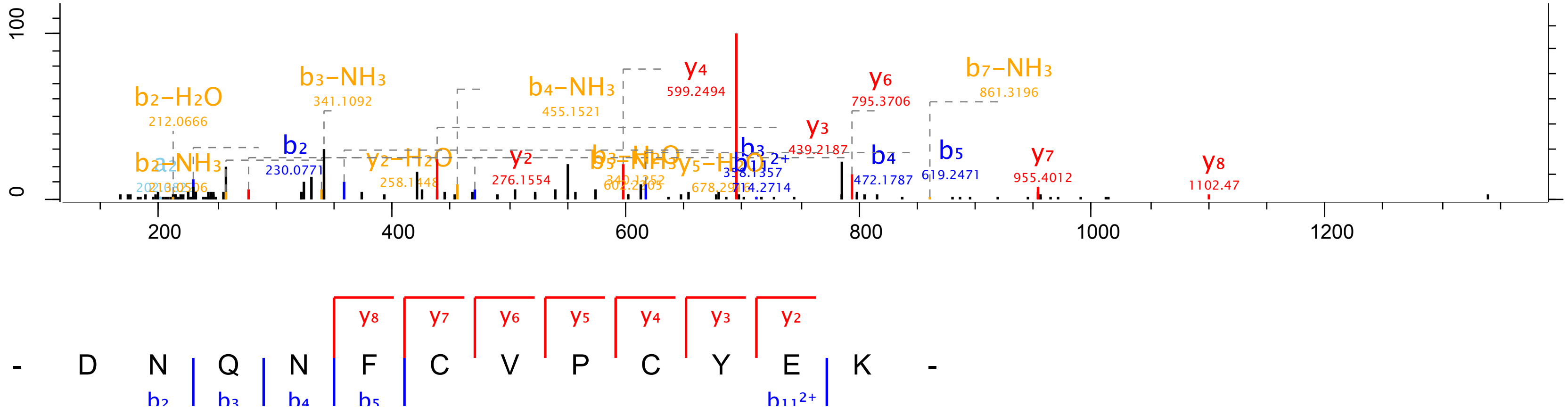

Raw file	Scan	Method	Score	m/z	Gene names
20141014_fract4_dyn_5ul_E4_01_583	20370	TOF; CID	109.83	532.61	SOCS3

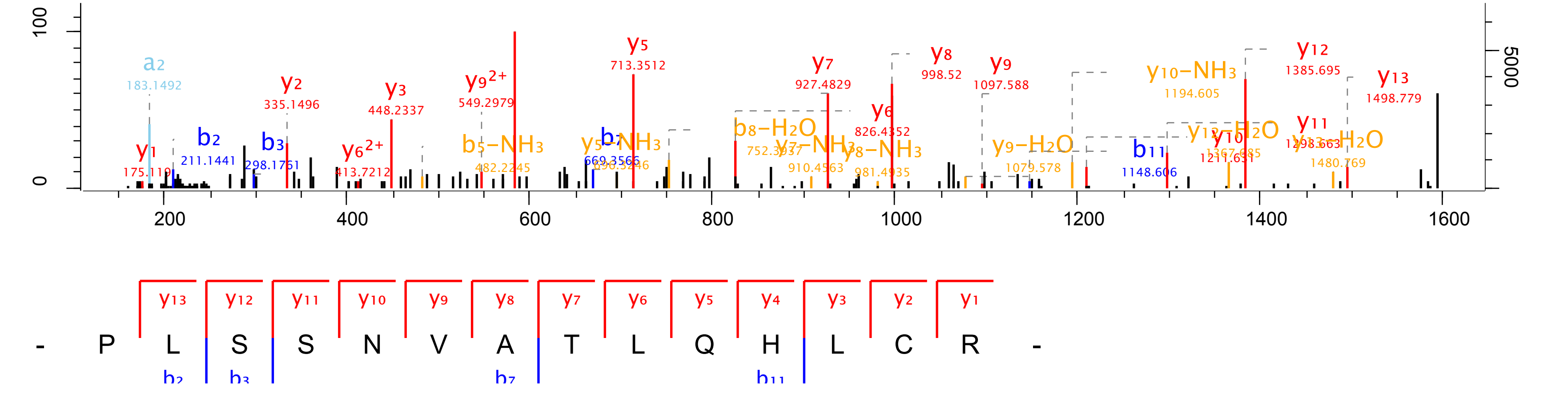

Raw file	Scan	Method	Score	m/z	Gene names
20141014_fract4_dyn_5ul_E4_01_583	20560	TOF; CID	111.52	709.85	CYB561D2

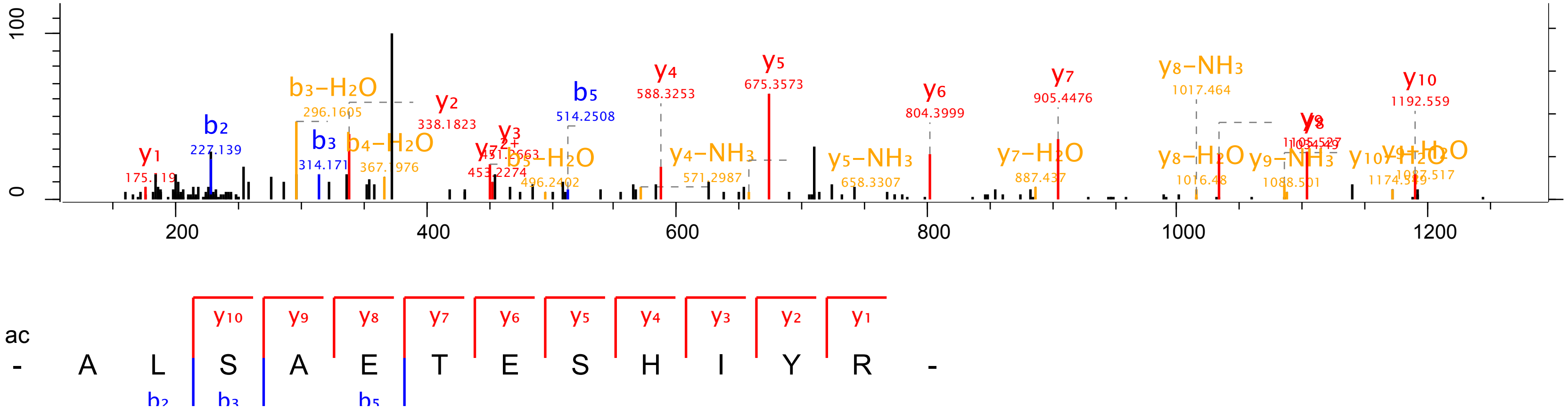

Raw file	Scan	Method	Score	m/z	Gene names
20141014_fract4_dyn_5ul_E4_01_583	24861	TOF; CID	64.24	814.89	C4orf3

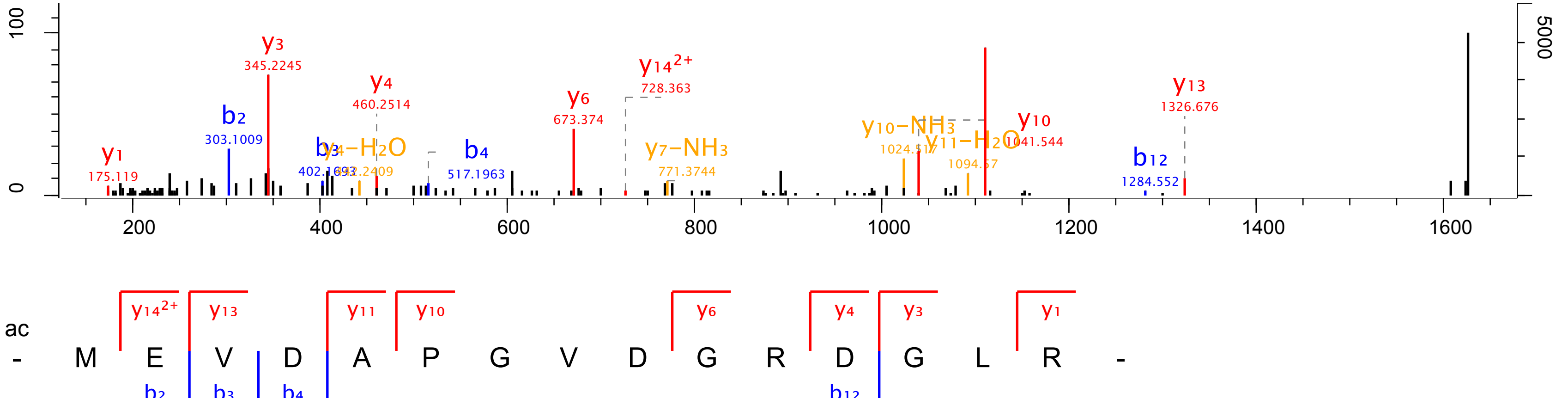

Raw file	Scan	Method	Score	m/z	Gene names
20141014_fract4_dyn_5ul_E4_01_583	25014	TOF; CID	59.91	668.35	SYTL1

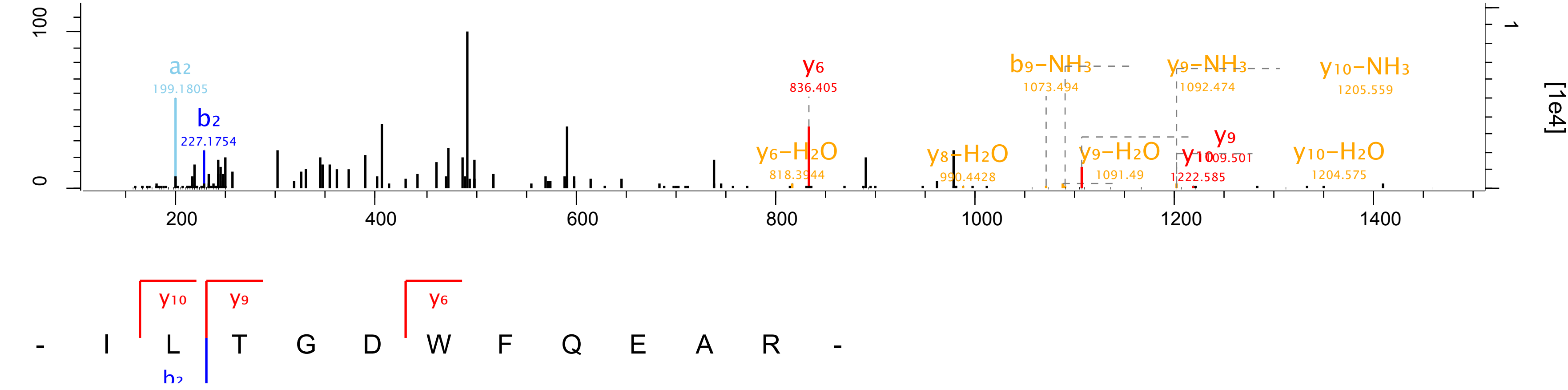

Raw file	Scan	Method	Score	m/z	Gene names
20141014_fract4_dyn_5ul_E4_01_583	25540	TOF; CID	65.37	785.87	HGSNAT

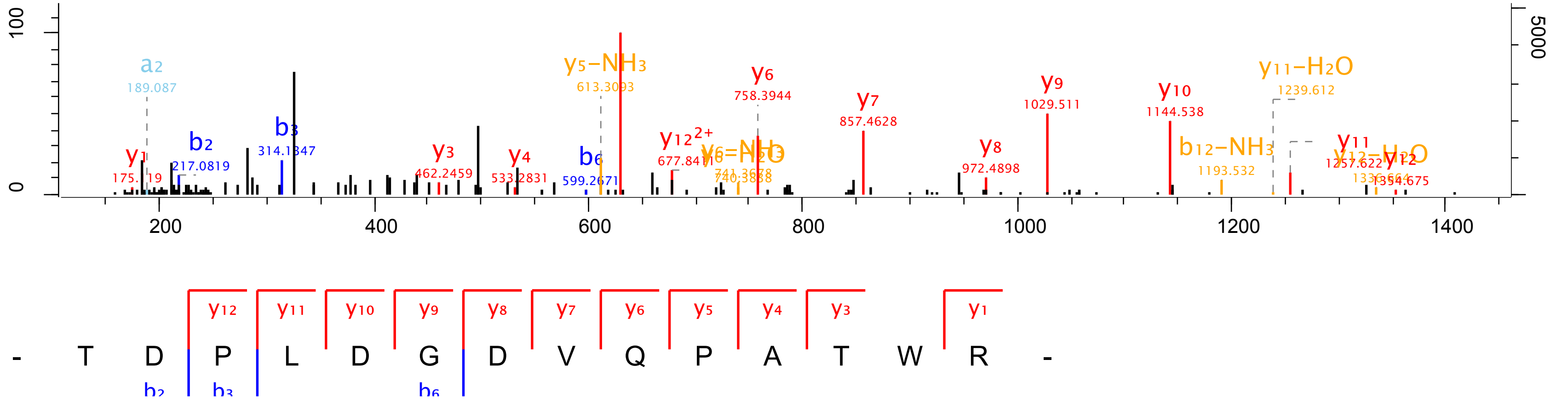

Raw file	Scan	Method	Score	m/z	Gene names
20141014_fract4_dyn_5ul_E4_01_583	26021	TOF; CID	60.16	586.84	KATNAL2

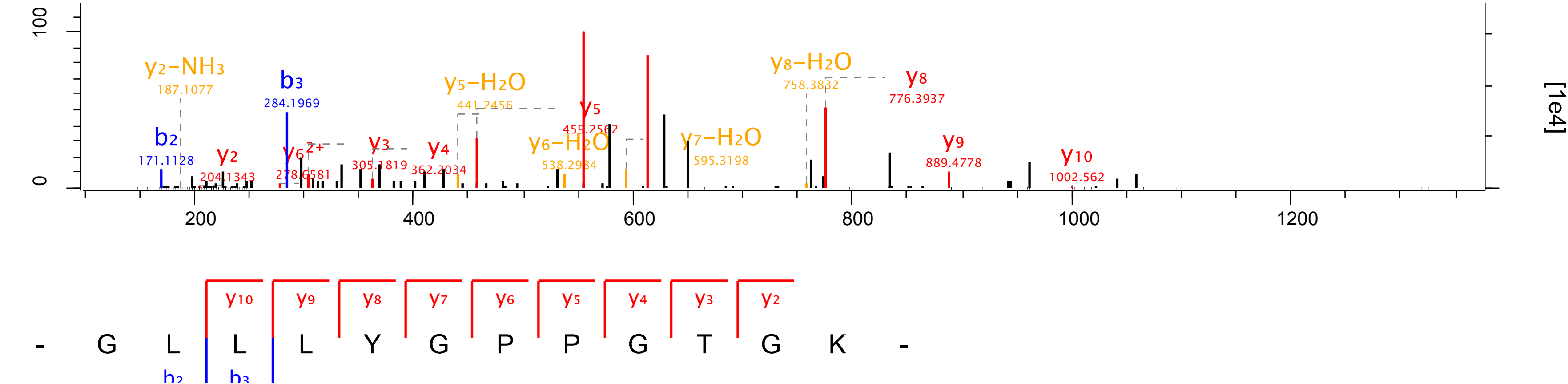

Raw file	Scan	Method	Score	m/z	Gene names
20141014_fract4_dyn_5ul_E4_01_583	26163	TOF; CID	57.97	755.91	KLF10

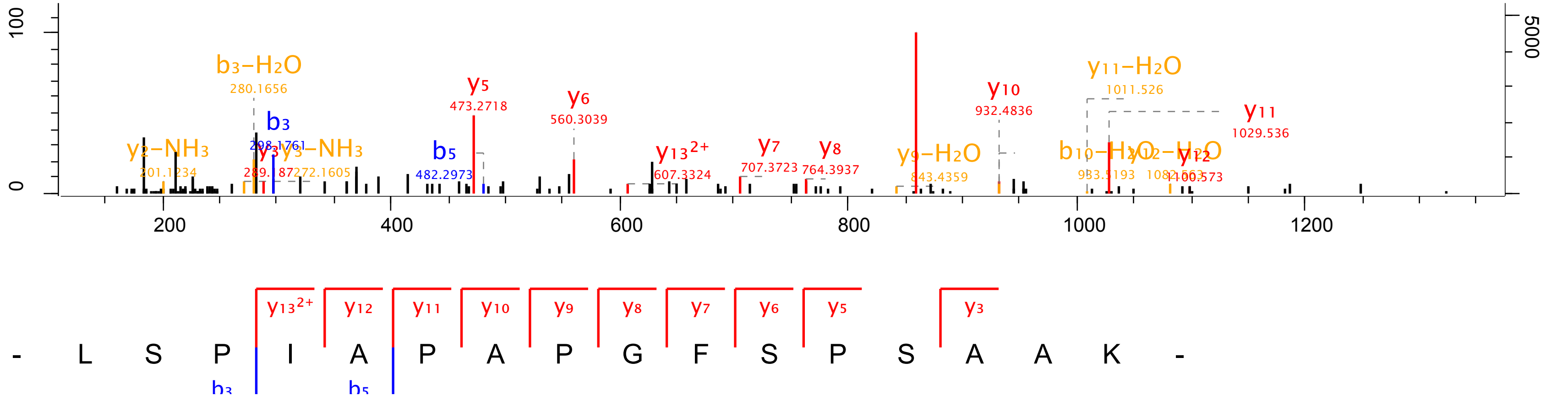

Raw file	Scan	Method	Score	m/z	Gene names
20141014_fract4_dyn_5ul_E4_01_583	26226	TOF; CID	27.53	1132.86	TECPR1

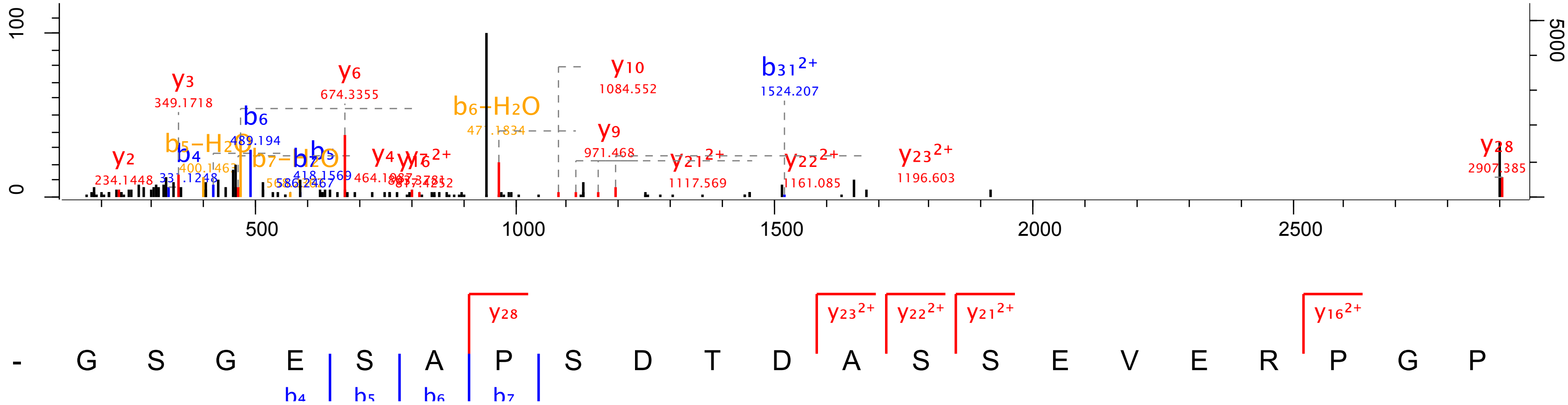

Raw file	Scan	Method	Score	m/z	Gene names
20141014_fract4_dyn_5ul_E4_01_583	26734	TOF; CID	78.32	721.36	CHURC1-FNTB;CHURC1

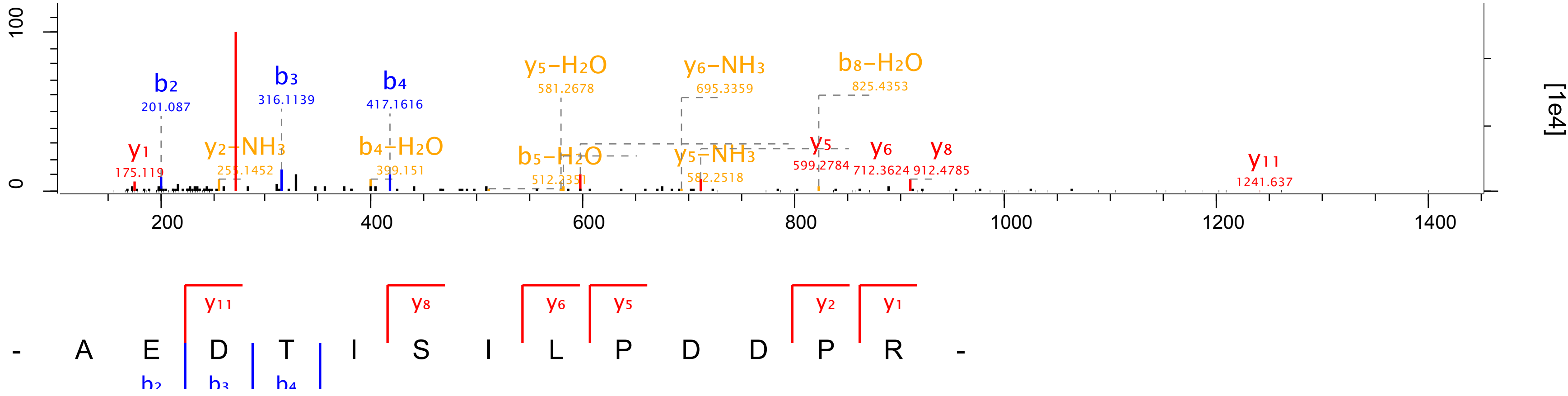

Raw file	Scan	Method	Score	m/z	Gene names
20141014_fract4_dyn_5ul_E4_01_583	26770	TOF; CID	52.53	855.39	METTL7B

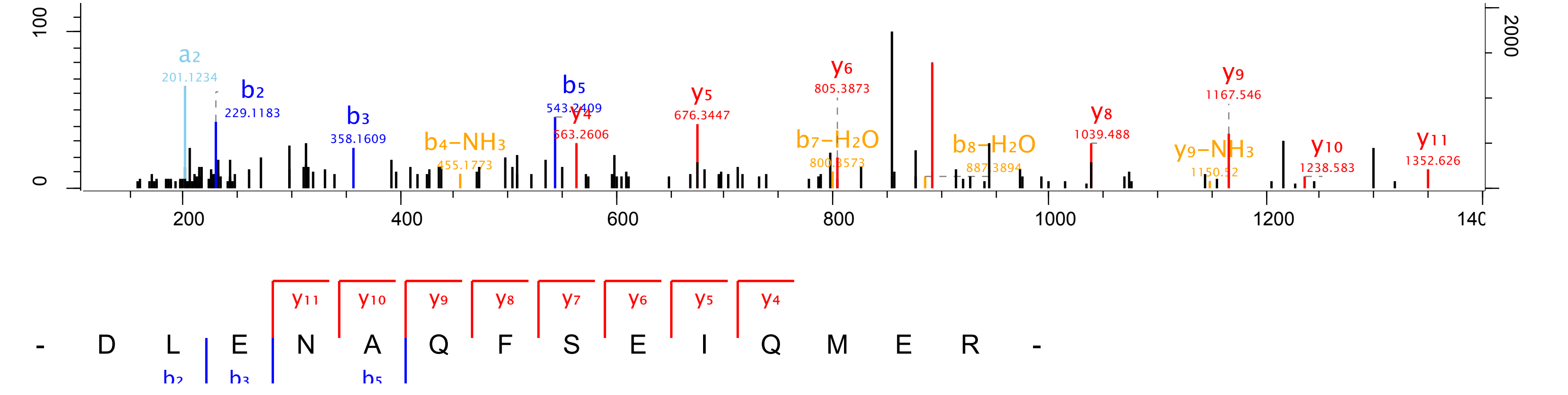

Raw file	Scan	Method	Score	m/z	Gene names
20141014_fract4_dyn_5ul_E4_01_583	27431	TOF; CID	51.09	609.32	SETD6

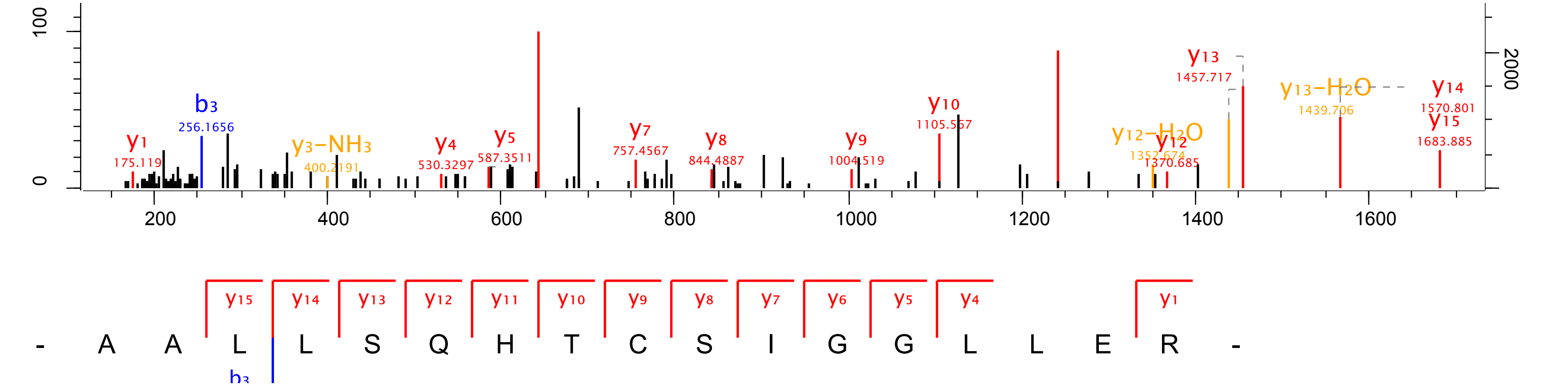

Raw file	Scan	Method	Score	m/z	Gene names
20141014_fract4_dyn_5ul_E4_01_583	28844	TOF; CID	87.43	858.42	CLUAP1

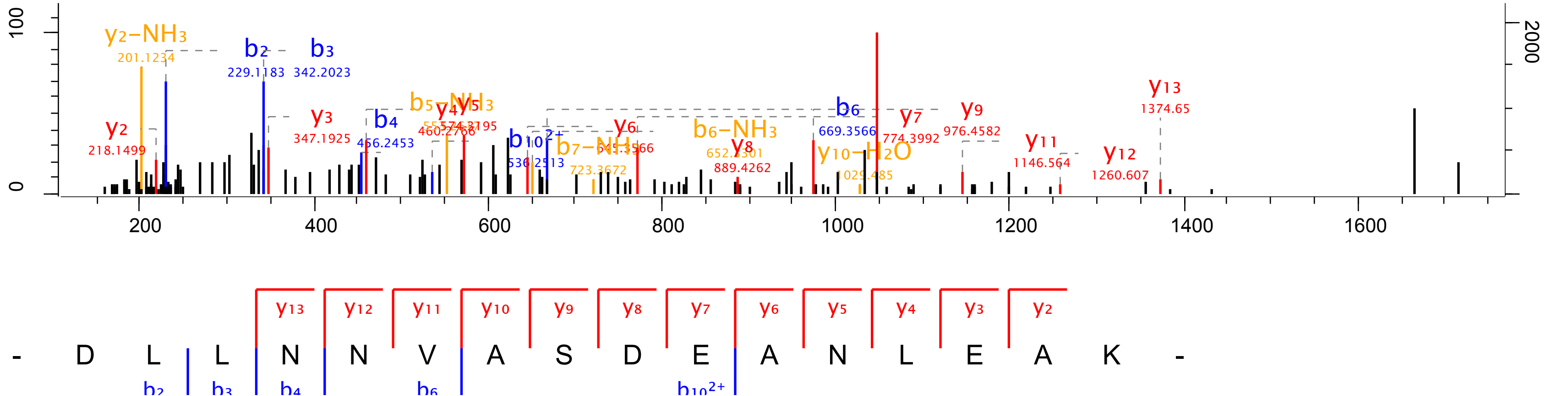

Raw file	Scan	Method	Score	m/z	Gene names
20141014_fract4_dyn_5ul_E4_01_583	29058	TOF; CID	86.18	572.33	BOK

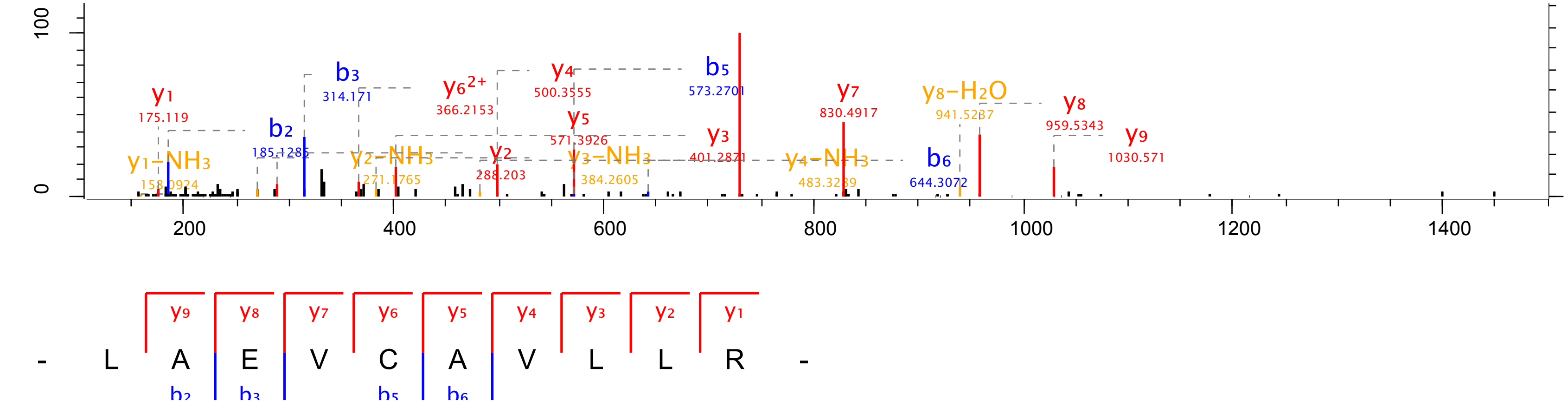

Raw file	Scan	Method	Score	m/z	Gene names
20141014_fract4_dyn_5ul_E4_01_583	29096	TOF; CID	99.09	735.03	UBALD1

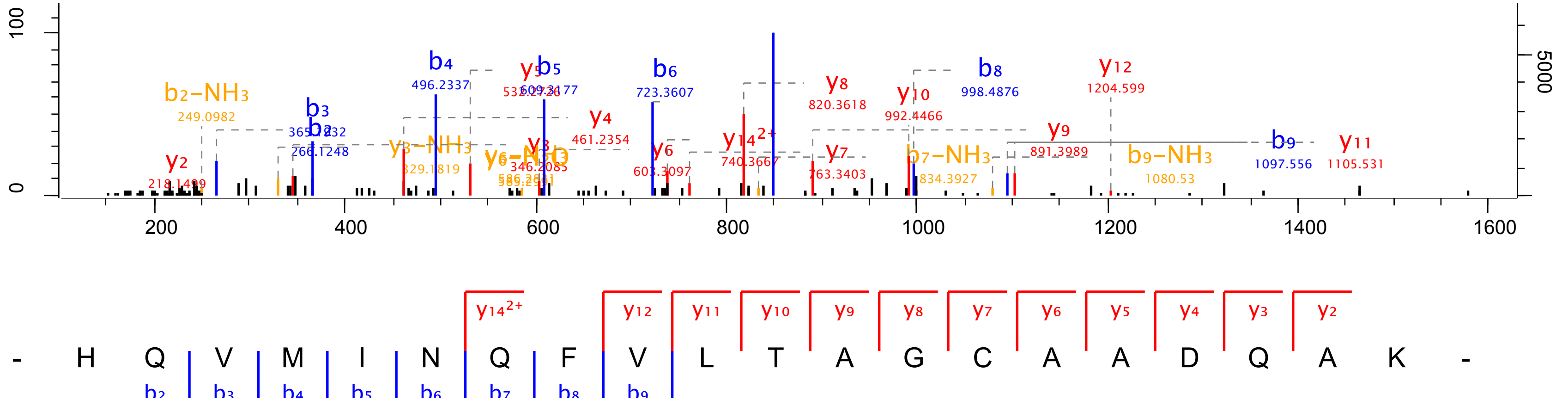

Raw file	Scan	Method	Score	m/z	Gene names
20141014_fract4_dyn_5ul_E4_01_583	29929	TOF; CID	103.43	623.86	SLC22A18

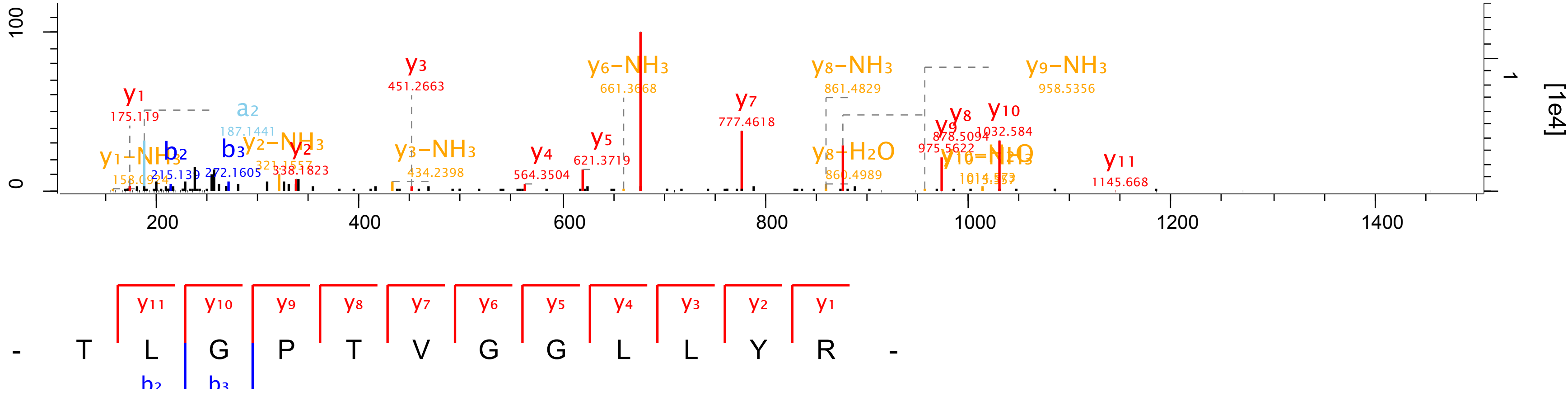

Raw file	Scan	Method	Score	m/z	Gene names
20141014_fract4_dyn_5ul_E4_01_583	30589	TOF; CID	53.56	668.31	PERP

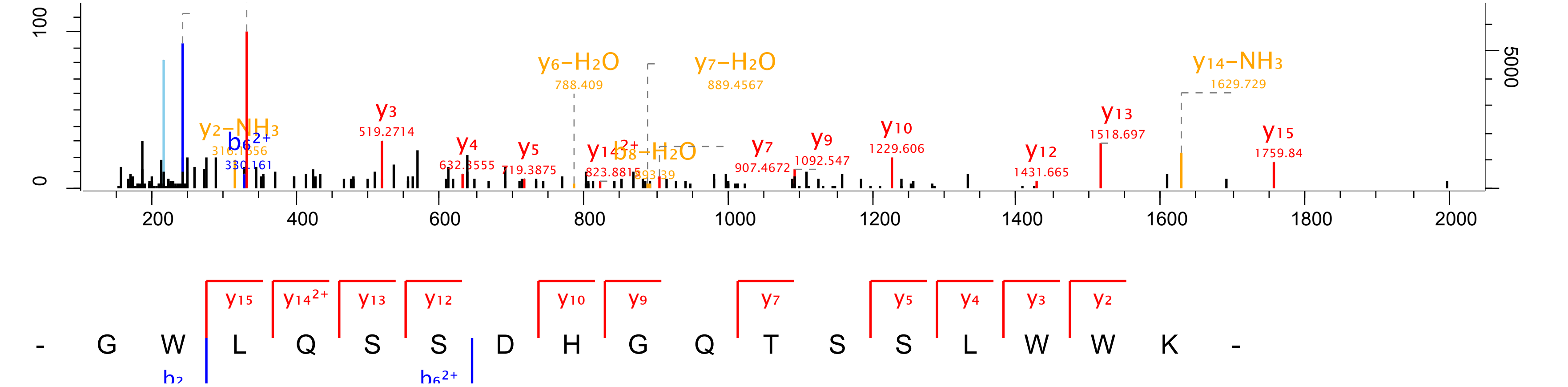

Raw file	Scan	Method	Score	m/z	Gene names
20141014_fract4_dyn_5ul_E4_01_583	32830	TOF; CID	146.37	854.41	SSR3

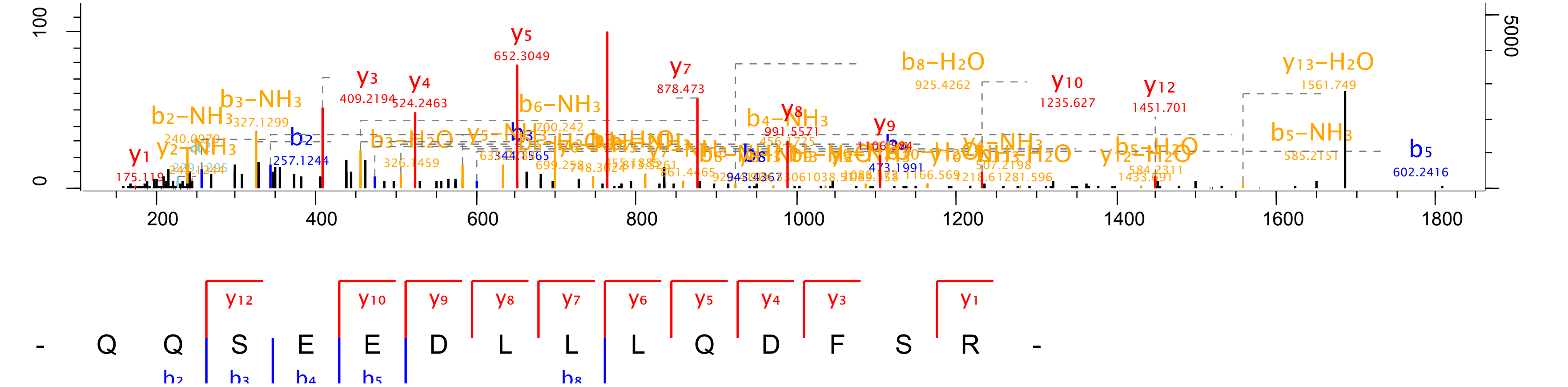

Raw file	Scan	Method	Score	m/z	Gene names
20141014_fract4_dyn_5ul_E4_01_583	33651	TOF; CID	51.73	659.36	CDC14B

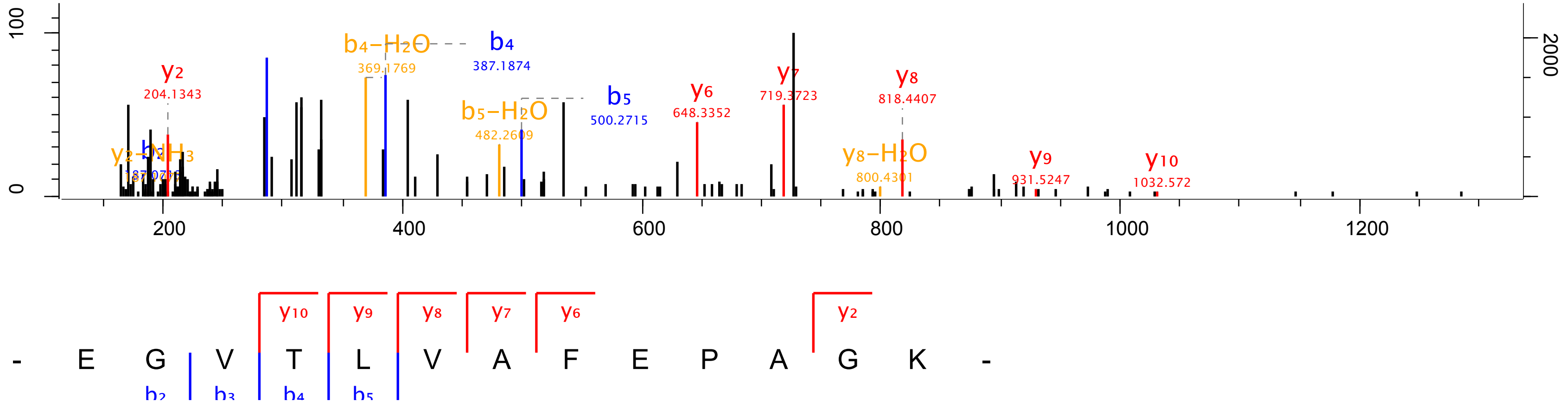

Raw file	Scan	Method	Score	m/z	Gene names
20141014_fract4_dyn_5ul_E4_01_583	34503	TOF; CID	77.66	568.8	MT-ND2

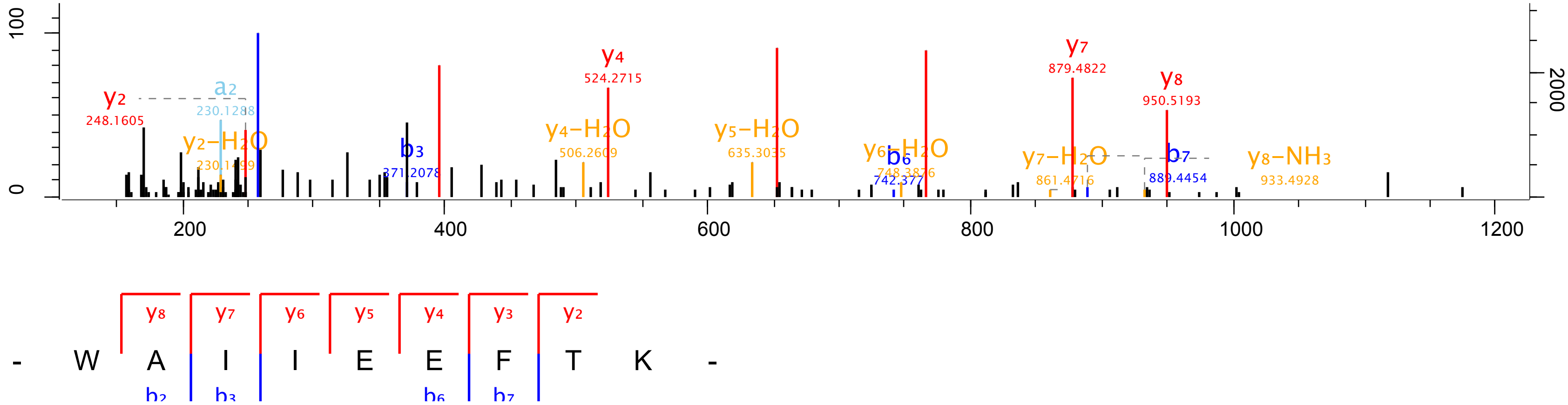

Raw file	Scan	Method	Score	m/z	Gene names
20141014_fract4_dyn_5ul_E4_01_583	38279	TOF; CID	59.25	953.5	C4orf46

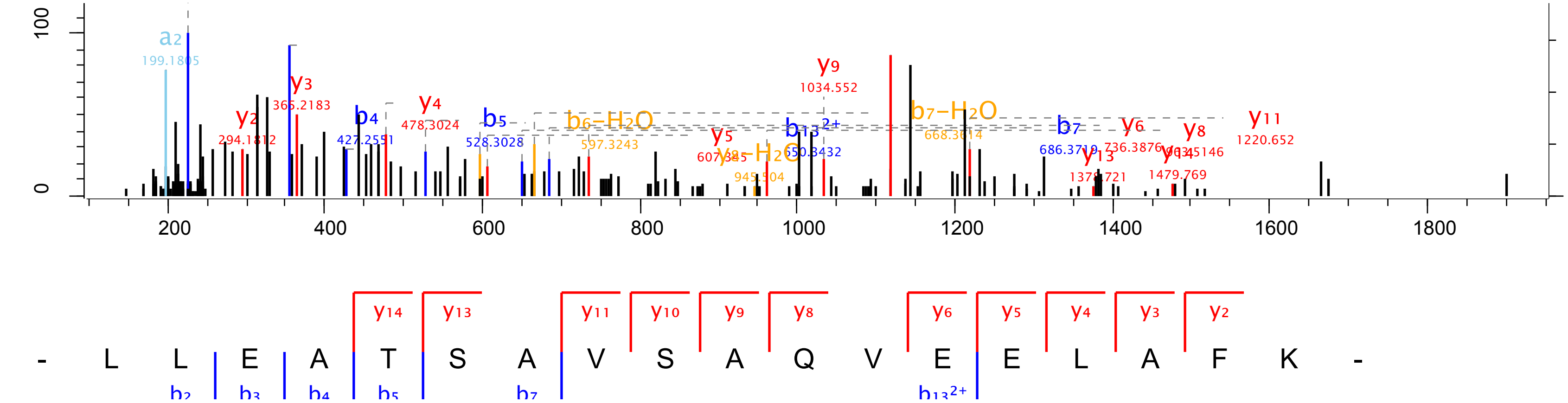

Raw file	Scan	Method	Score	m/z	Gene names
20141014_fract4_dyn_5ul_E4_01_583	38846	TOF; CID	73.93	754.9	BARX1

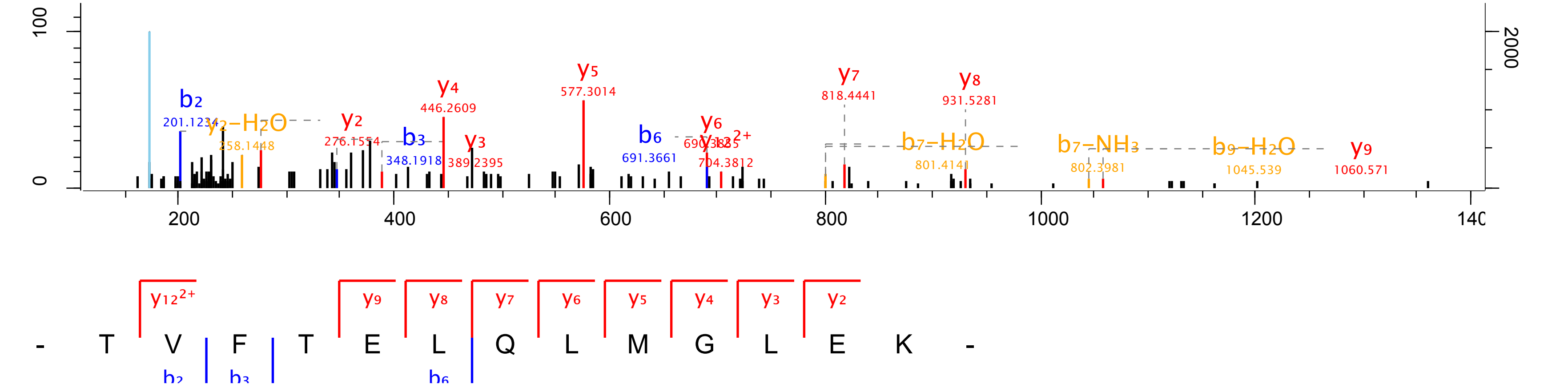

Raw file	Scan	Method	Score	m/z	Gene names
20141014_fract5_dyn_5ul_E5_01_584	7476	TOF; CID	139.28	429.24	VAMP5

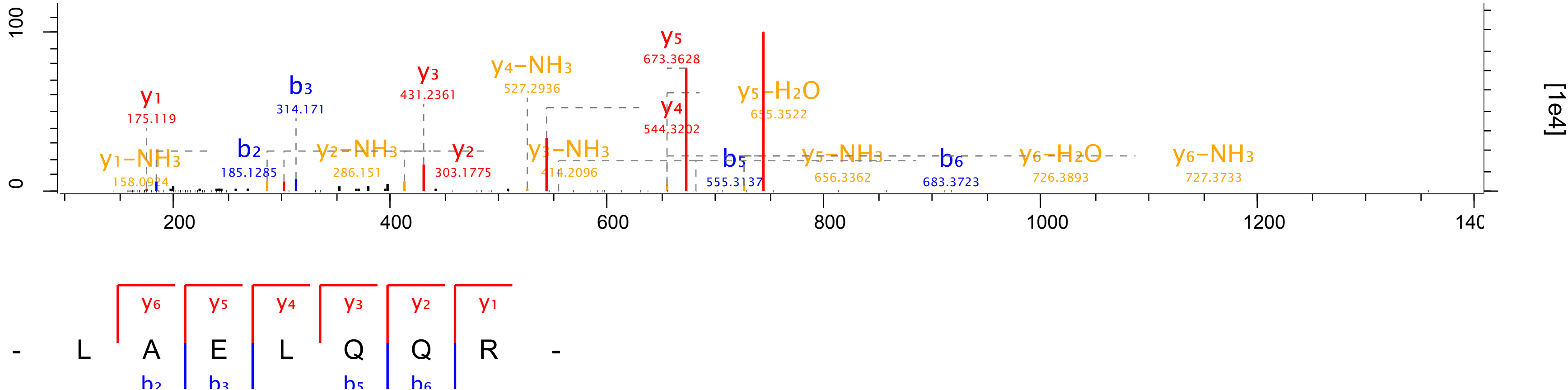

Raw file	Scan	Method	Score	m/z	Gene names
20141014_fract5_dyn_5ul_E5_01_584	9936	TOF; CID	134.66	432.73	TSPAN3

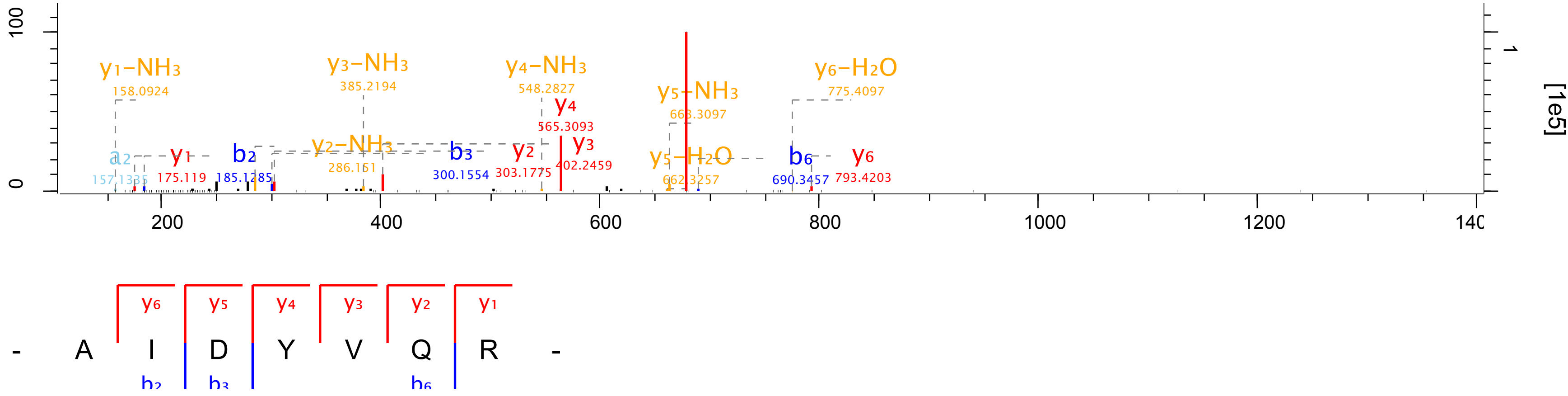

Raw file	Scan	Method	Score	m/z	Gene names
20141014_fract5_dyn_5ul_E5_01_584	12423	TOF; CID	135.1	640.8	C17orf89

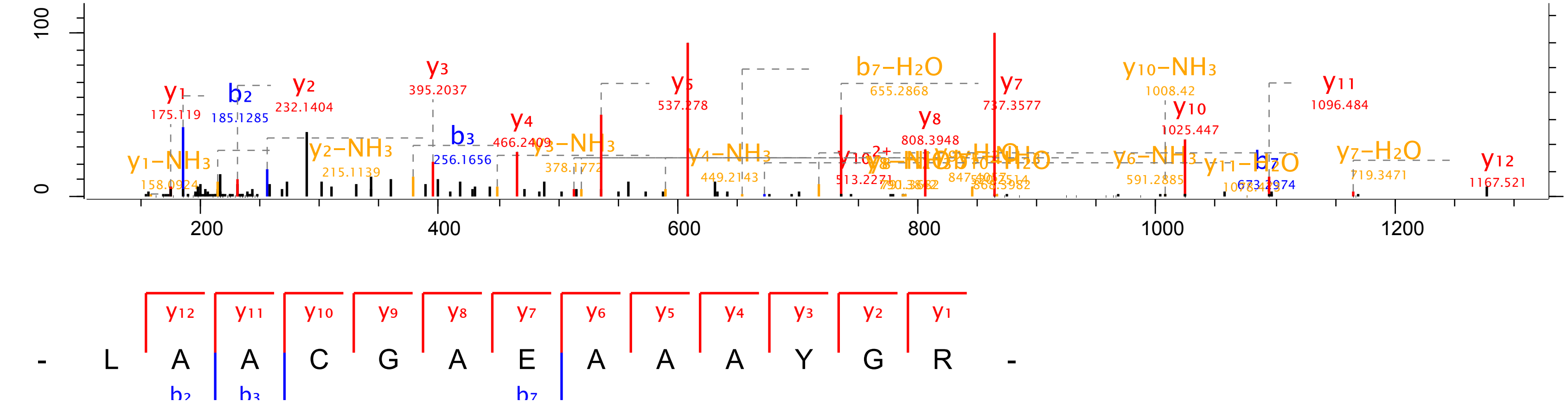

Raw file	Scan	Method	Score	m/z	Gene names
20141014_fract5_dyn_5ul_E5_01_584	12753	TOF; CID	66.99	654.82	MORC4

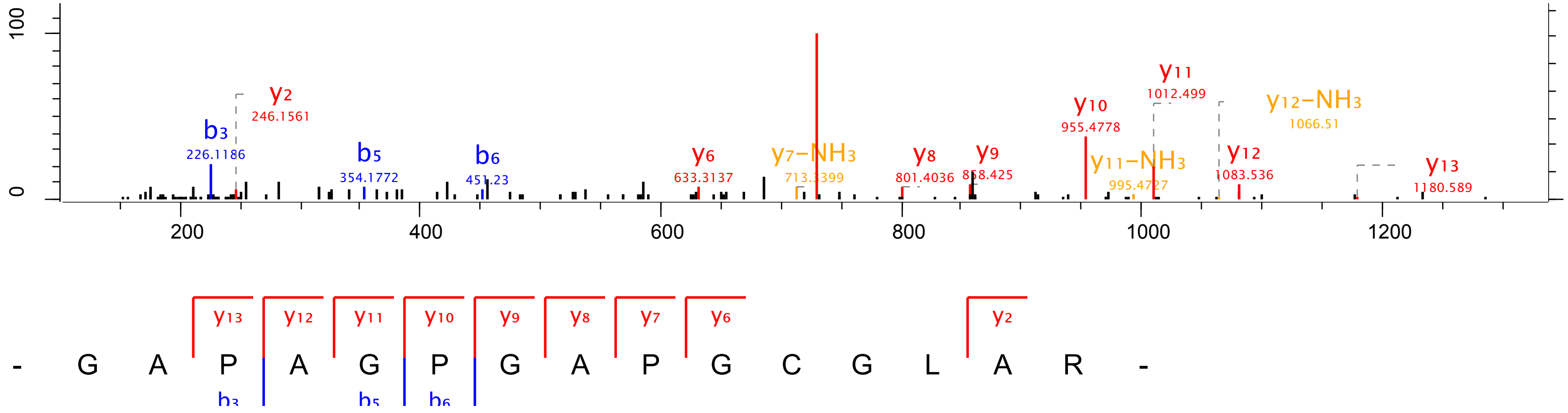

Raw file	Scan	Method	Score	m/z	Gene names
20141014_fract5_dyn_5ul_E5_01_584	13734	TOF; CID	48.61	511.28	IFI27L2

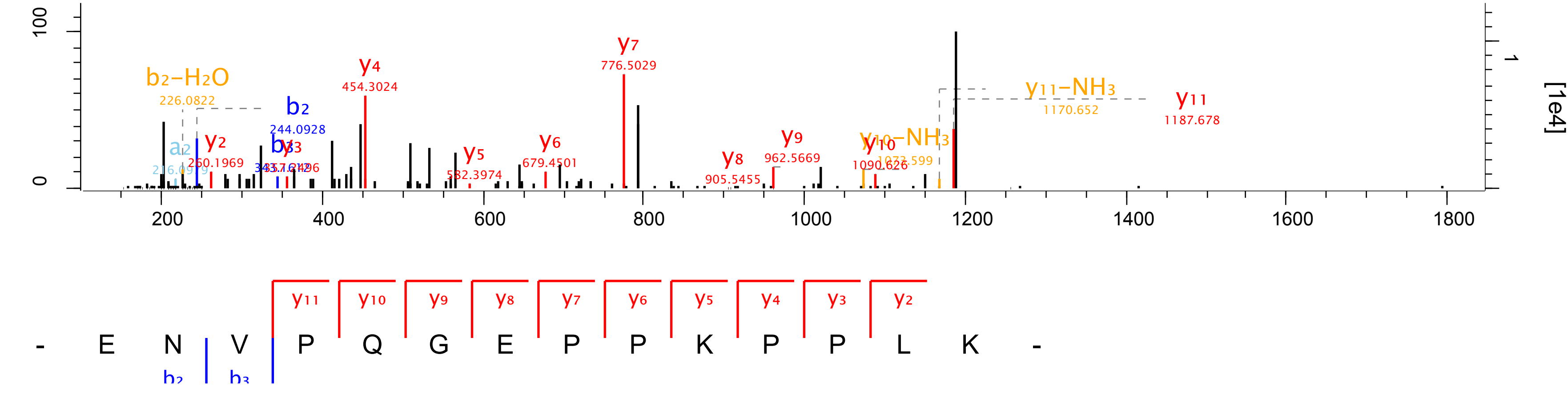

Raw file	Scan	Method	Score	m/z	Gene names
20141014_fract5_dyn_5ul_E5_01_584	15192	TOF; CID	63.29	698.84	ADIRF

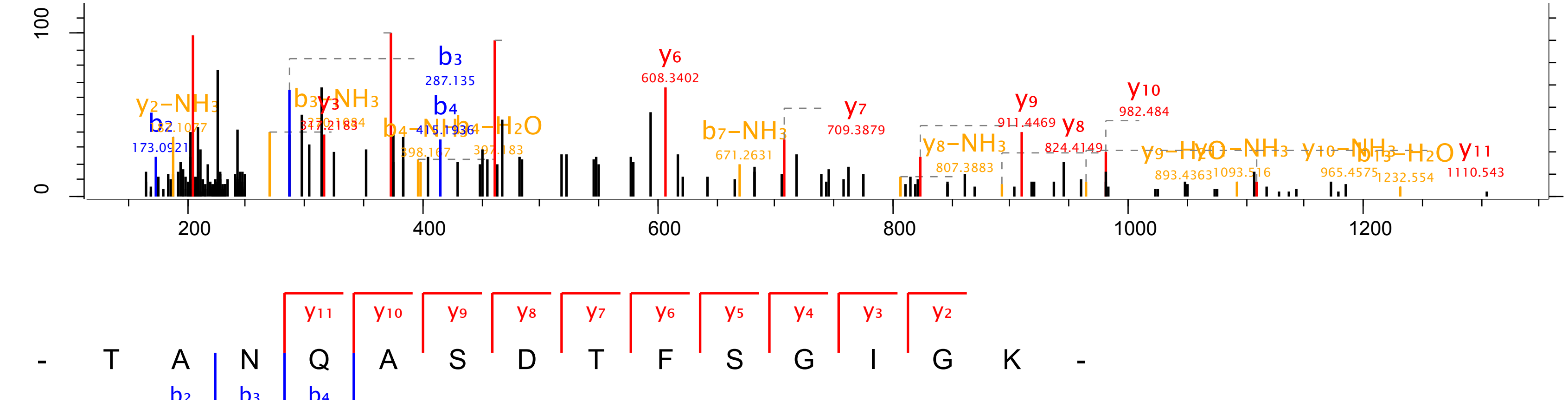

Raw file	Scan	Method	Score	m/z	Gene names
20141014_fract5_dyn_5ul_E5_01_584	21272	TOF; CID	143	451.25	BTBD16

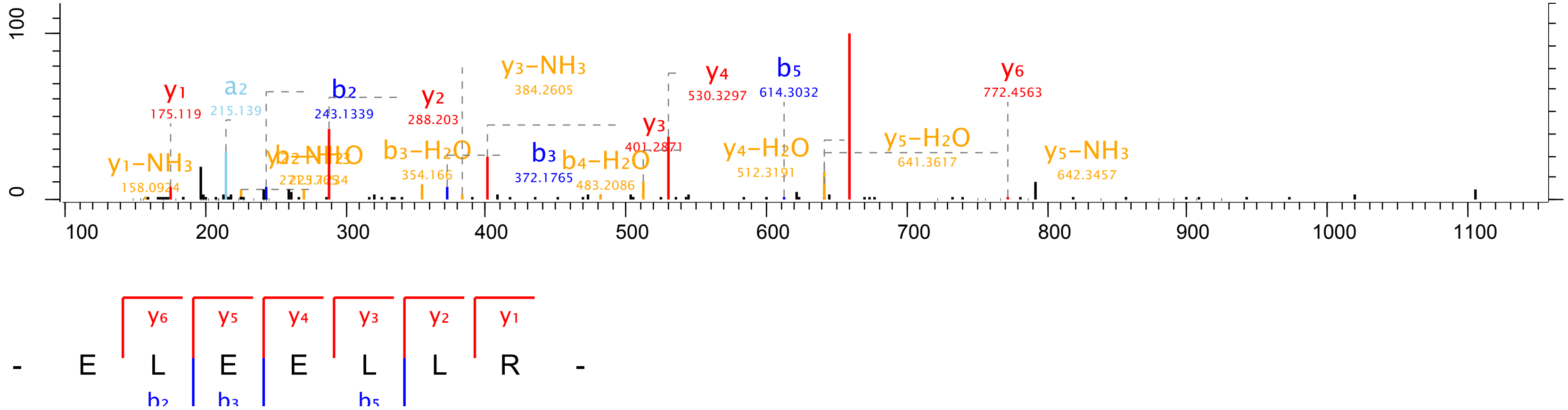

Raw file	Scan	Method	Score	m/z	Gene names
20141014_fract5_dyn_5ul_E5_01_584	23325	TOF; CID	72.34	648.29	PFDN4

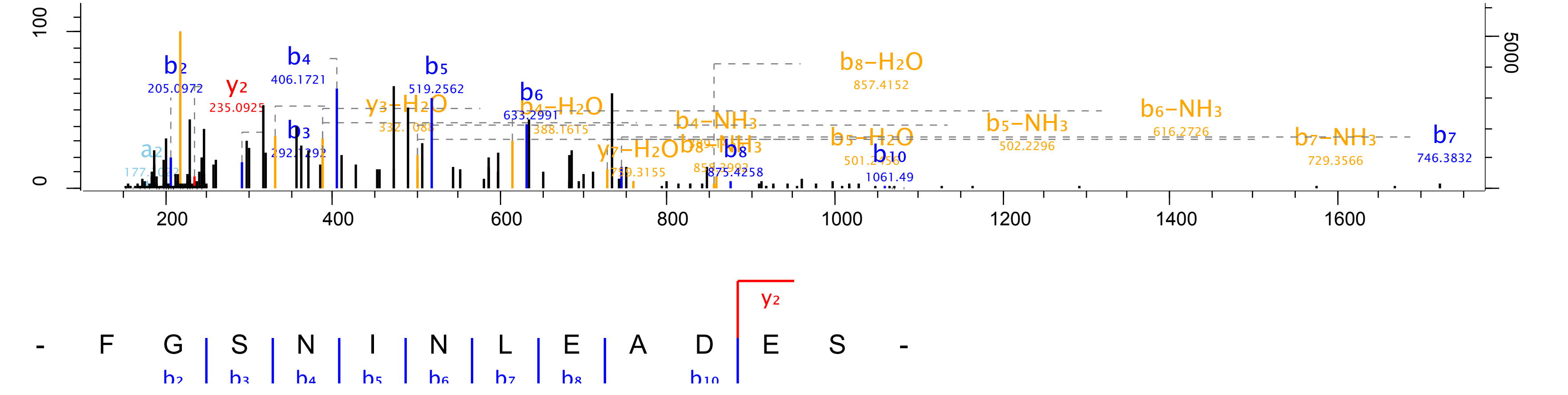

Raw file	Scan	Method	Score	m/z	Gene names
20141014_fract5_dyn_5ul_E5_01_584	23615	TOF; CID	97.27	793.89	PMAIP1

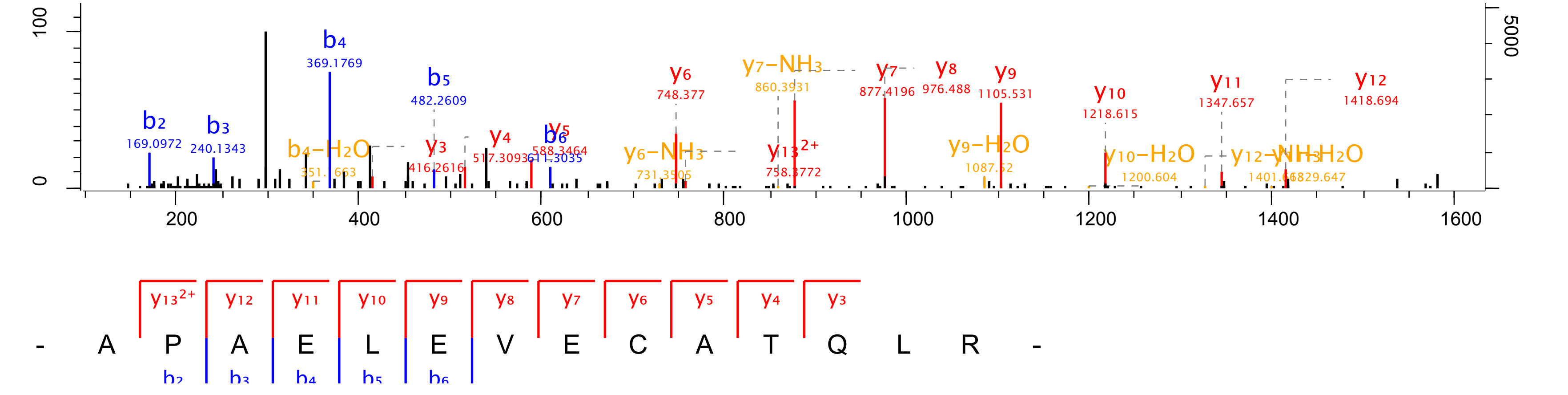

Raw file	Scan	Method	Score	m/z	Gene names
20141014_fract5_dyn_5ul_E5_01_584	27722	TOF; CID	148.28	412.22	SMDT1

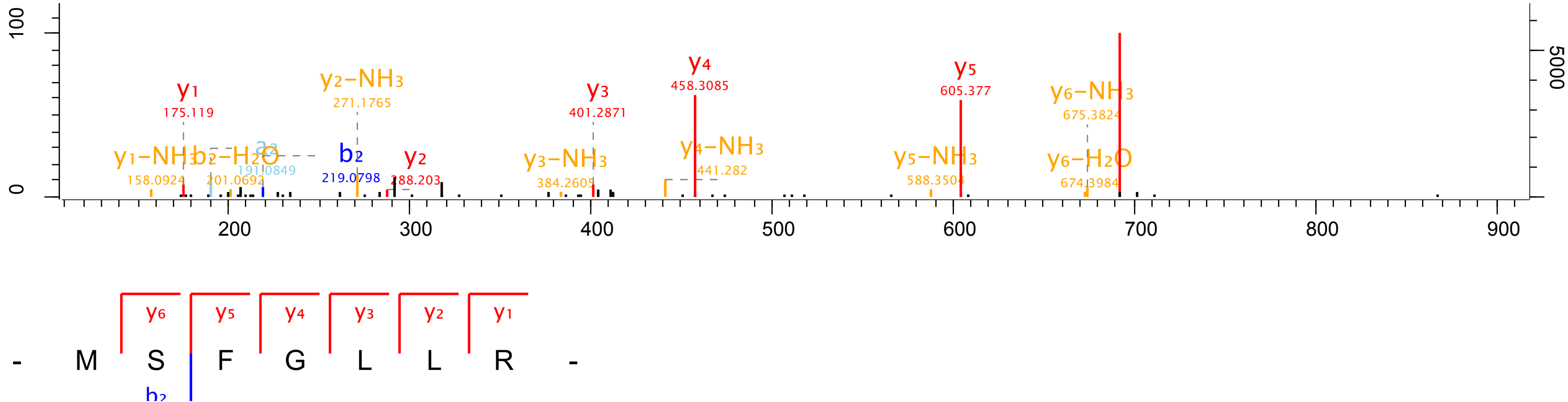

Raw file	Scan	Method	Score	m/z	Gene names
20141014_fract5_dyn_5ul_E5_01_584	28067	TOF; CID	143.28	504.72	TMEM258

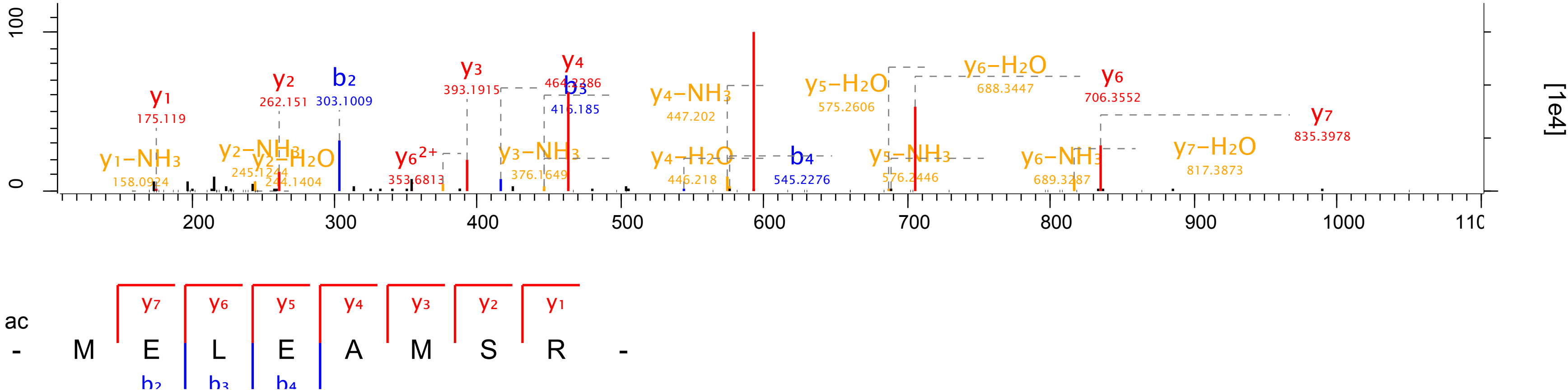

Raw file	Scan	Method	Score	m/z	Gene names
20141014_fract5_dyn_5ul_E5_01_584	30004	TOF; CID	106.04	581.32	ZDHHC12

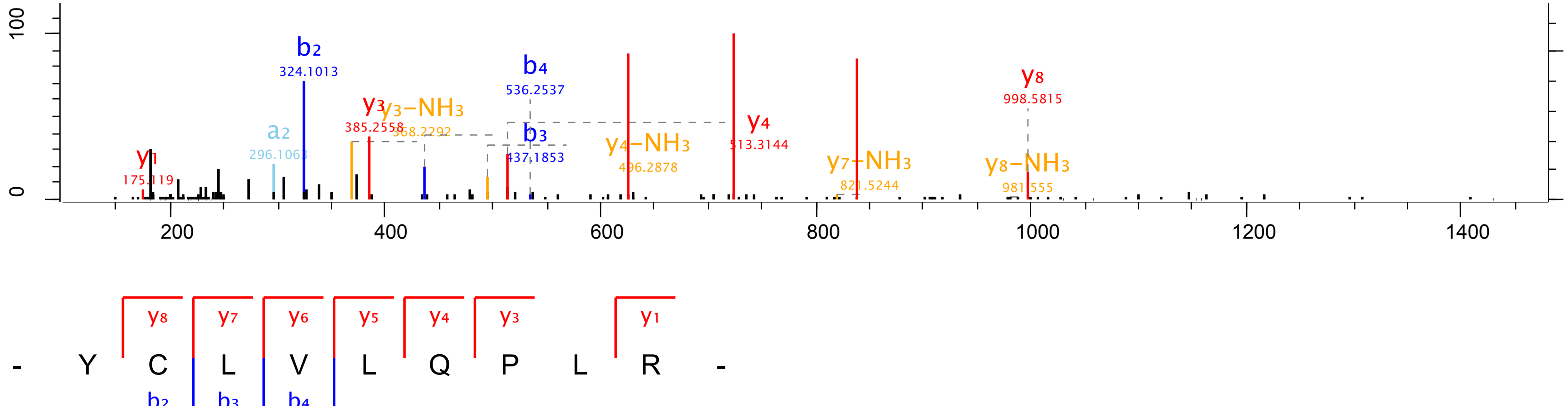

Raw file	Scan	Method	Score	m/z	Gene names
20141014_fract5_dyn_5ul_E5_01_584	31391	TOF; CID	61.94	725.35	PRR3

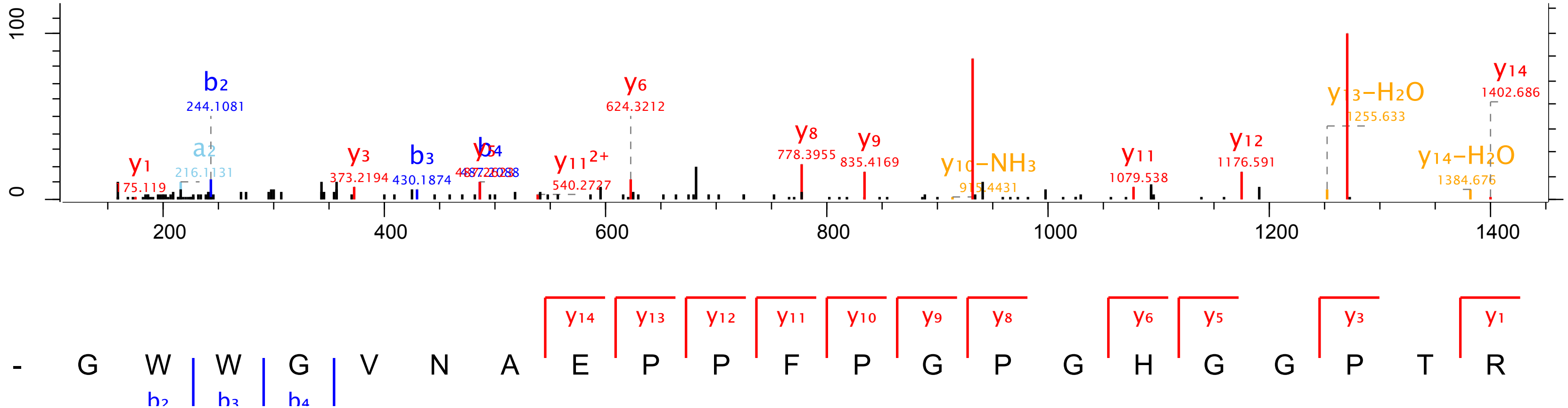

Raw file	Scan	Method	Score	m/z	Gene names
20141014_fract5_dyn_5ul_E5_01_584	32361	TOF; CID	72.33	522.8	ABCG8

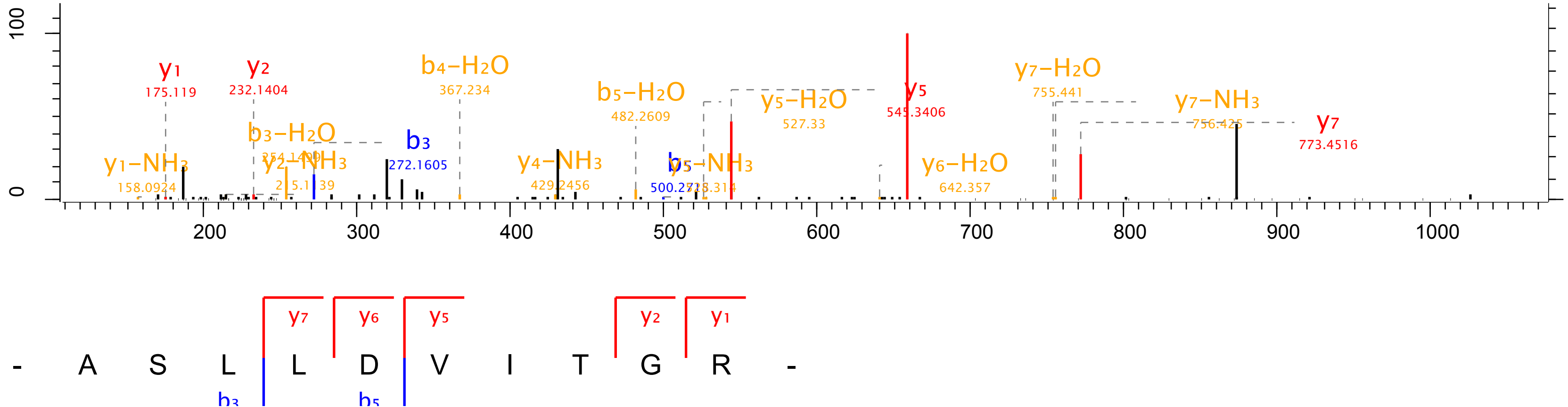

Raw file	Scan	Method	Score	m/z	Gene names
20141014_fract5_dyn_5ul_E5_01_584	33982	TOF; CID	90.05	577.35	SPIN4

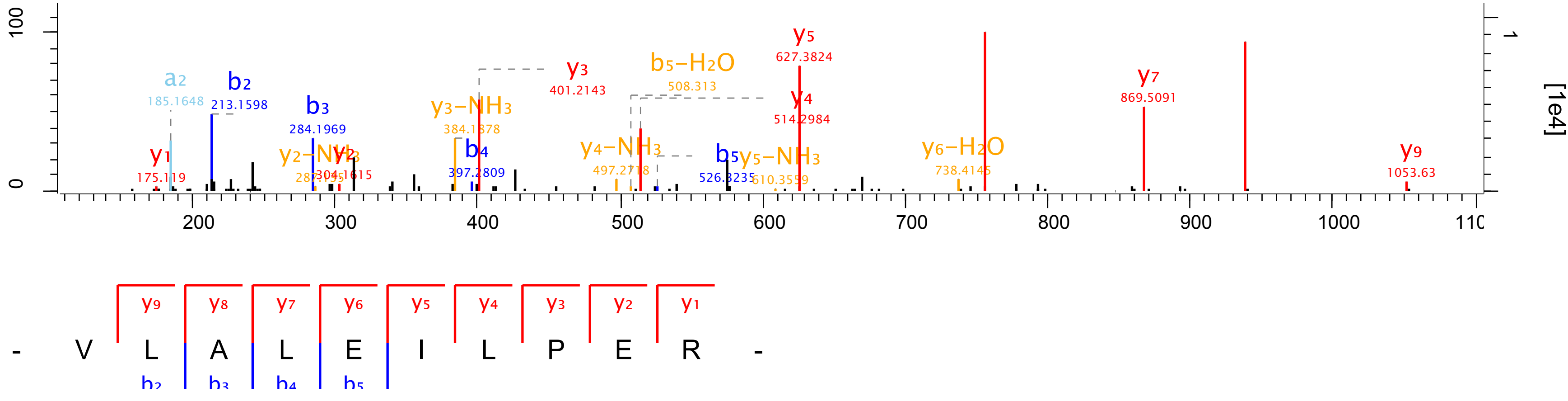

Raw file	Scan	Method	Score	m/z	Gene names
20141014_fract5_dyn_5ul_E5_01_584	34491	TOF; CID	59.73	650.32	RNF141

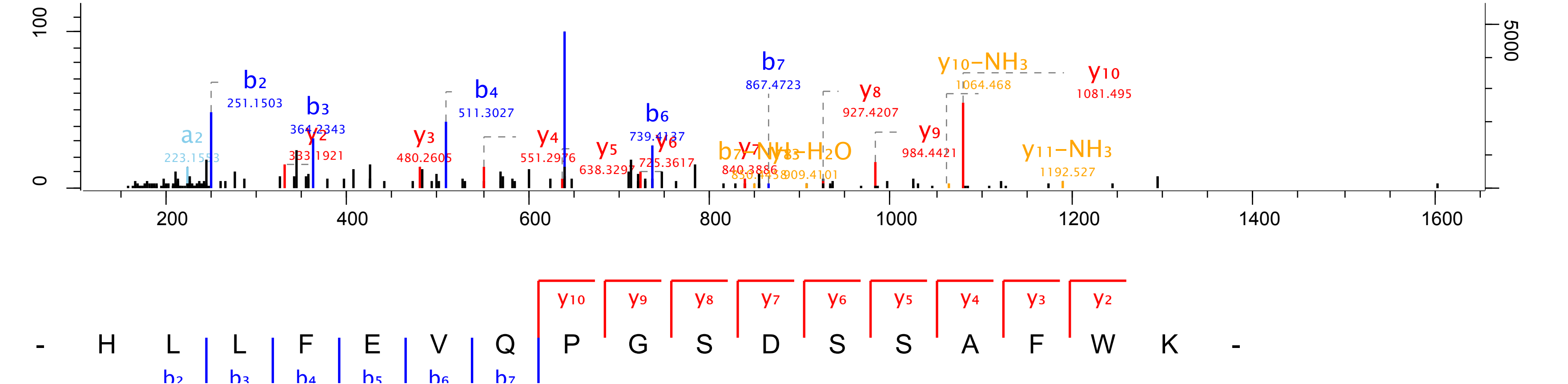

Raw file	Scan	Method	Score	m/z	Gene names
20141014_fract5_dyn_5ul_E5_01_584	34718	TOF; CID	35.54	860.41	ADPRM

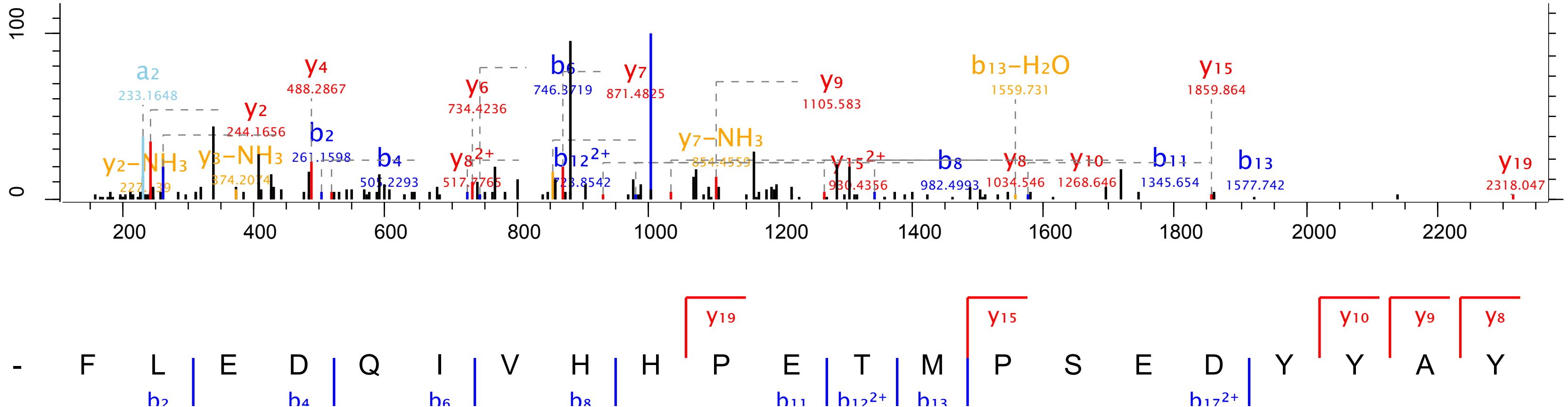

Raw file	Scan	Method	Score	m/z	Gene names
20141014_fract5_dyn_5ul_E5_01_584	36099	TOF; CID	80.75	500.31	IFI44

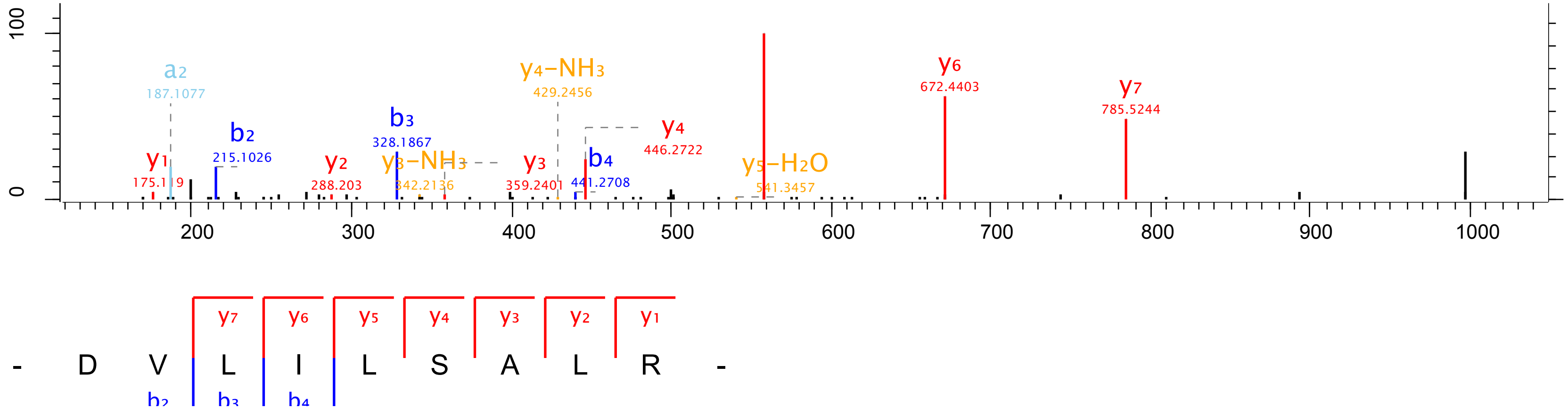

Raw file	Scan	Method	Score	m/z	Gene names
20141014_fract5_dyn_5ul_E5_01_584	36548	TOF; CID	90.41	750.9	TMEM128

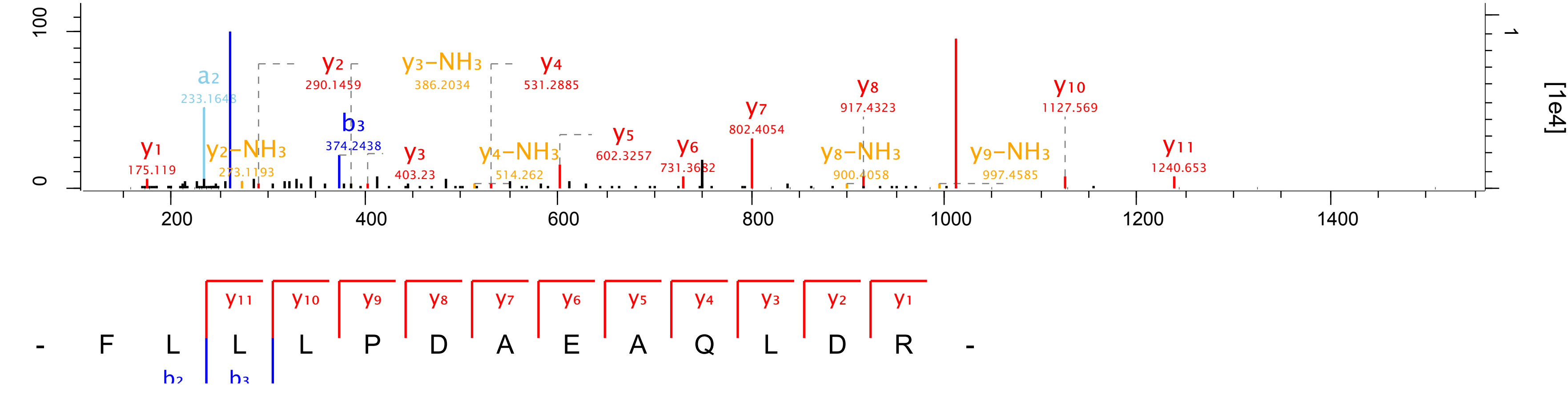

Raw file	Scan	Method	Score	m/z	Gene names
20141014_fract6_dyn_5ul_E6_01_585	4782	TOF; CID	52.39	449.22	ZNF526

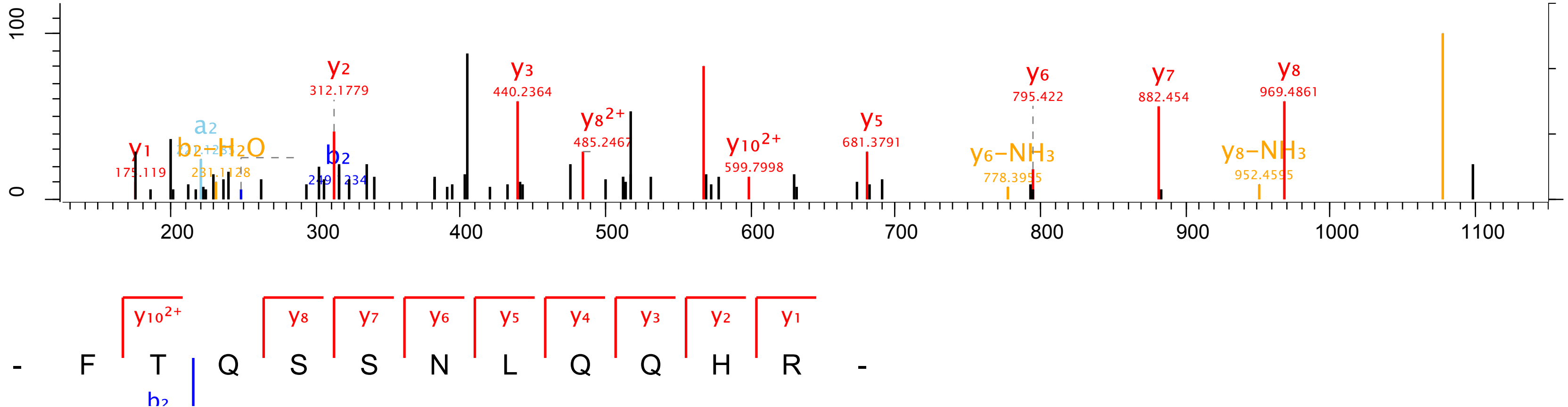

Raw file	Scan	Method	Score	m/z	Gene names
20141014_fract6_dyn_5ul_E6_01_585	5304	TOF; CID	71.88	497.59	ZNF689

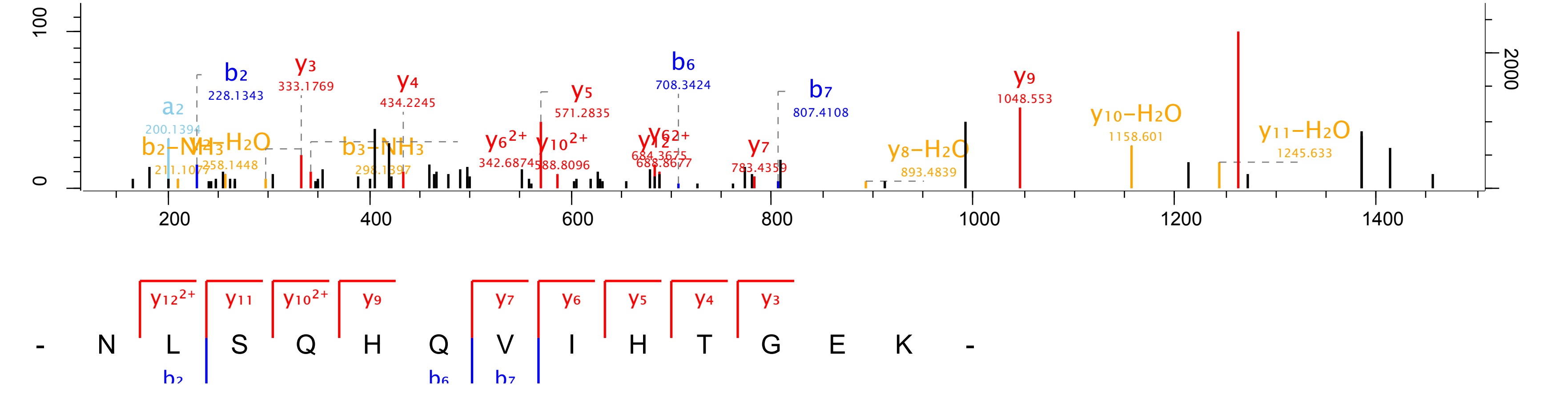

Raw file	Scan	Method	Score	m/z	Gene names
20141014_fract6_dyn_5ul_E6_01_585	5558	TOF; CID	84.17	464.21	ZSCAN29

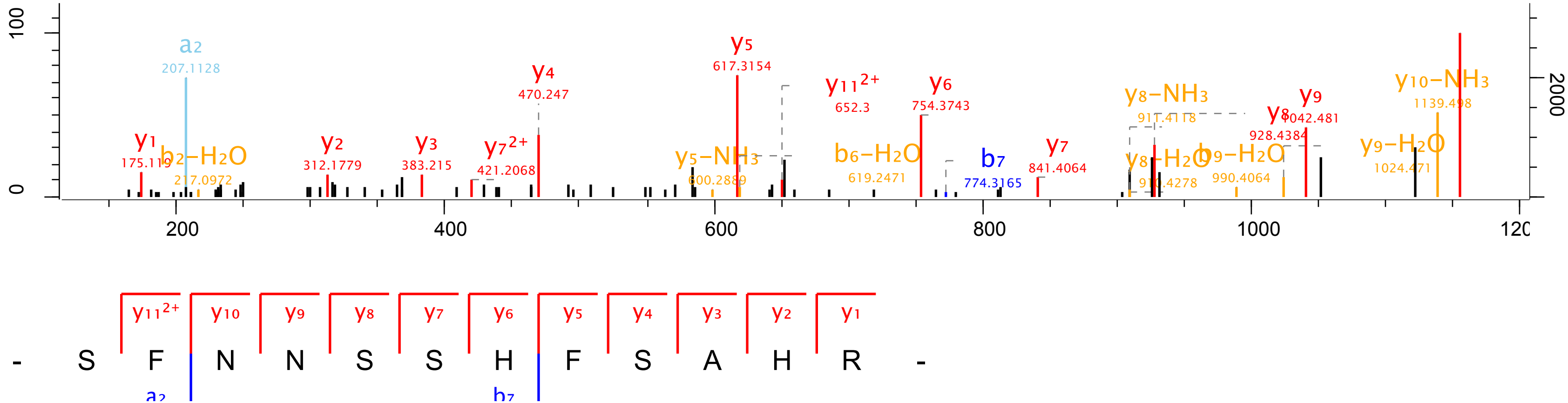

Raw file	Scan	Method	Score	m/z	Gene names
20141014_fract6_dyn_5ul_E6_01_585	9632	TOF; CID	111.01	476.27	ZNF584

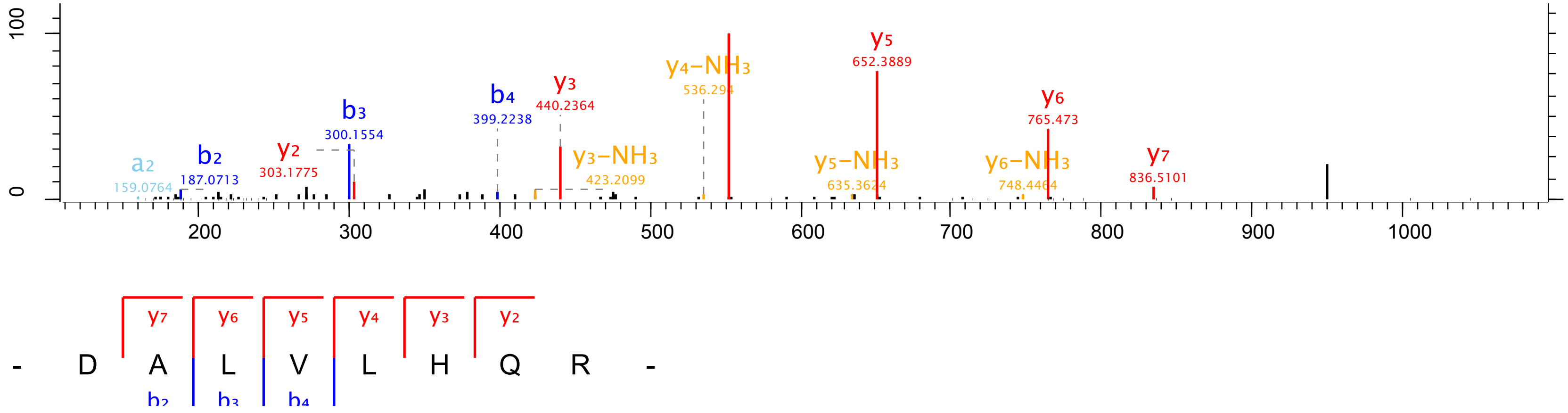

Raw file	Scan	Method	Score	m/z	Gene names
20141014_fract6_dyn_5ul_E6_01_585	9932	TOF; CID	113.99	617.32	HDGFRP2

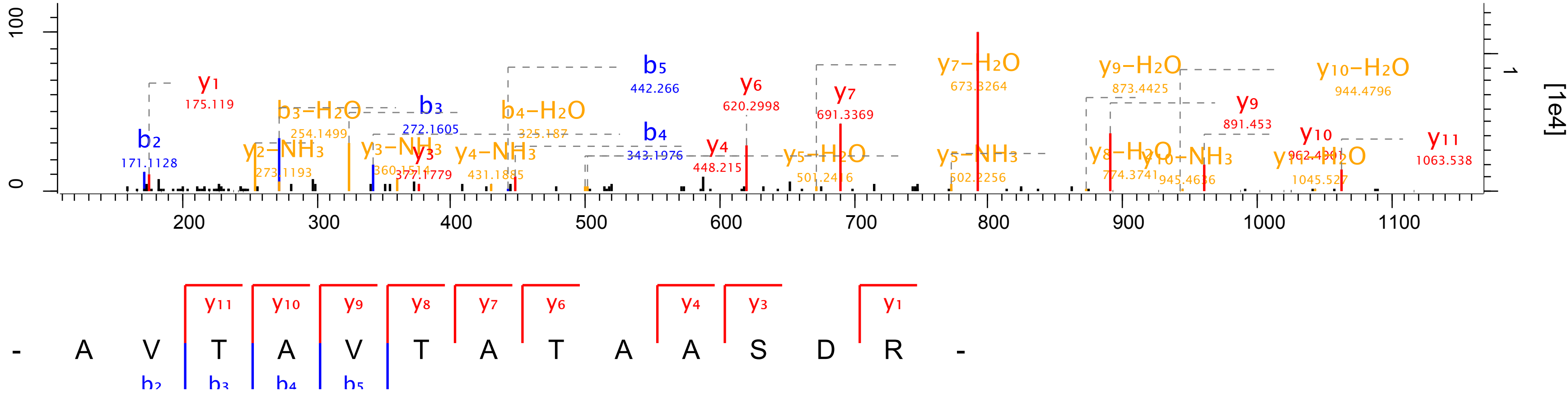

Raw file	Scan	Method	Score	m/z	Gene names
20141014_fract6_dyn_5ul_E6_01_585	11959	TOF; CID	62.86	592.66	C20orf24

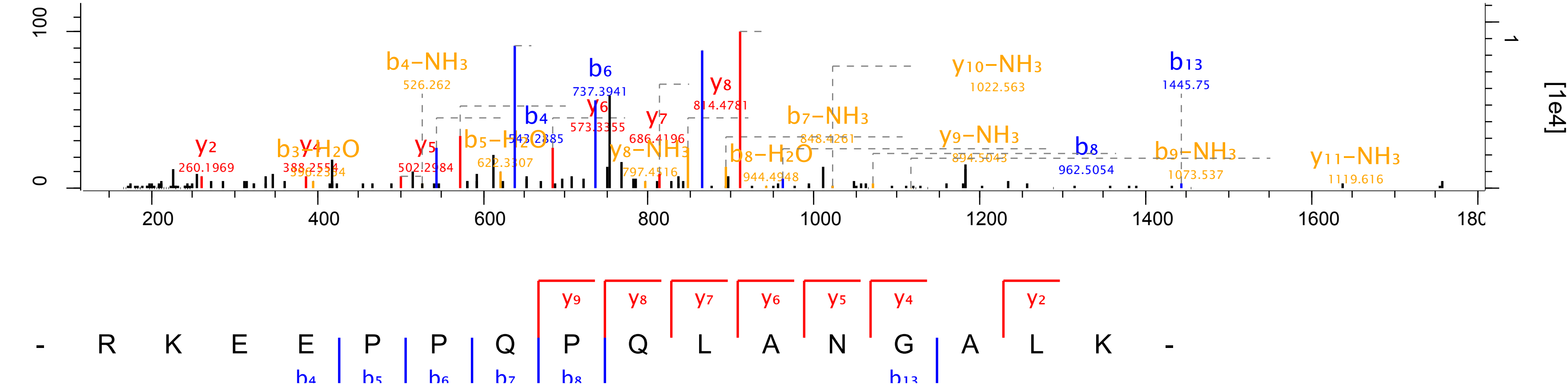

Raw file	Scan	Method	Score	m/z	Gene names
20141014_fract6_dyn_5ul_E6_01_585	12811	TOF; CID	96.74	607.83	SLC25A14

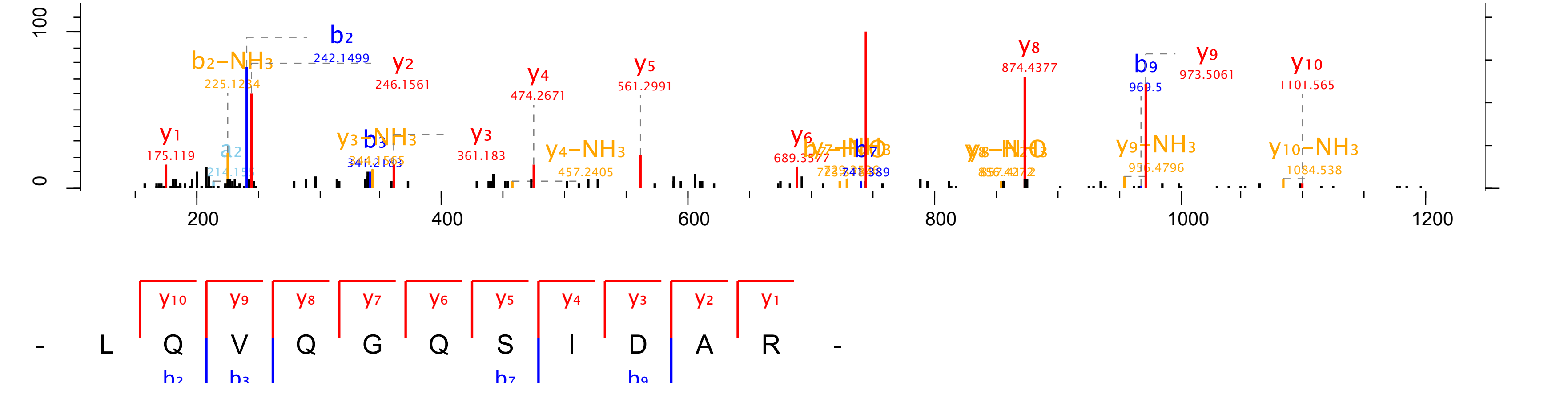

Raw file	Scan	Method	Score	m/z	Gene names
20141014_fract6_dyn_5ul_E6_01_585	13708	TOF; CID	123.79	638.29	CKS1B

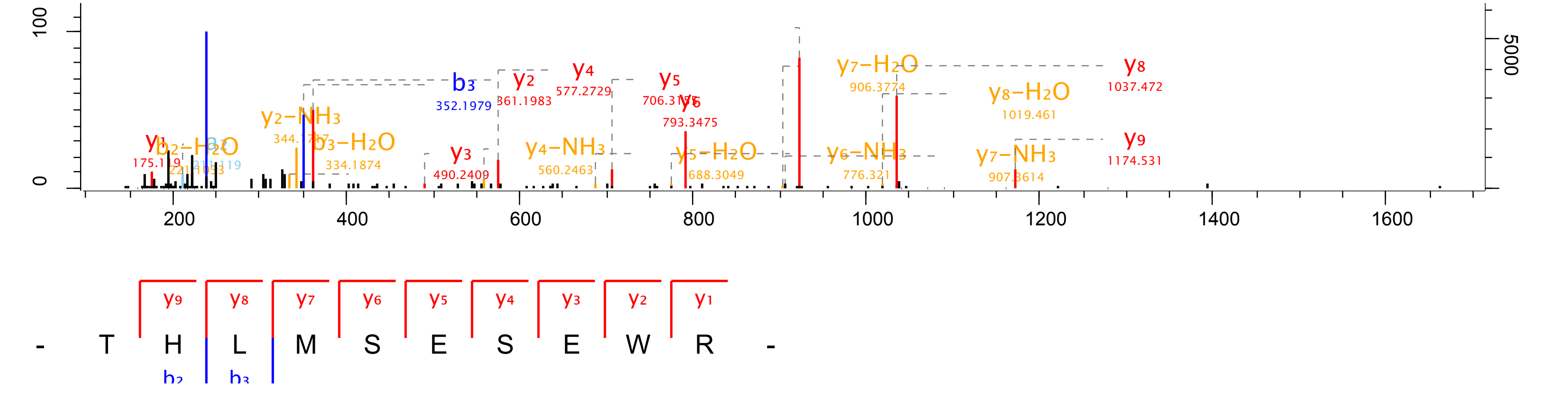

Raw file	Scan	Method	Score	m/z	Gene names
20141014_fract6_dyn_5ul_E6_01_585	18036	TOF; CID	77.22	905.46	TMEM44

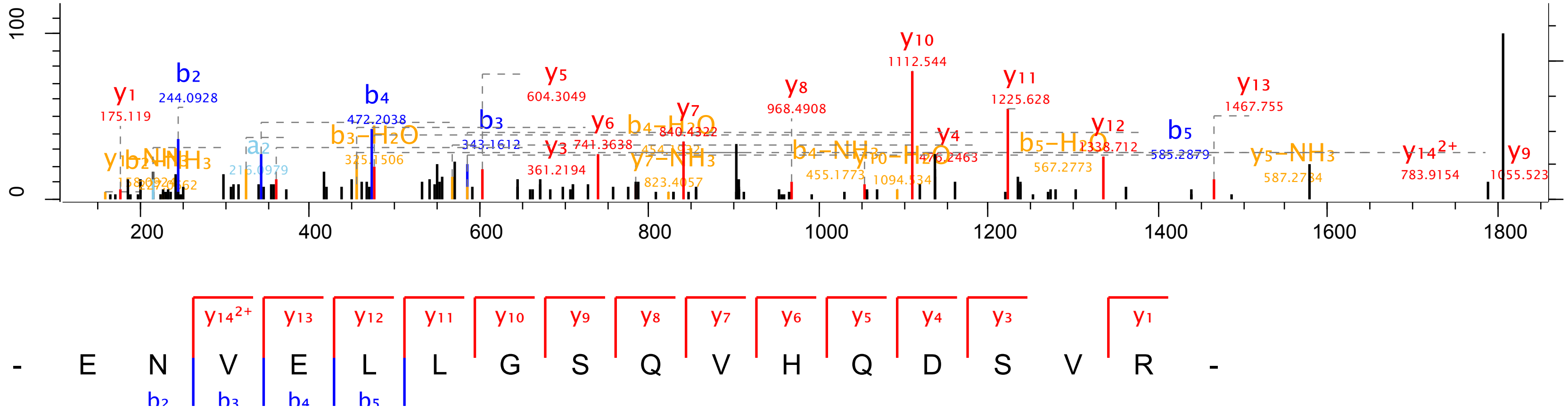

Raw file	Scan	Method	Score	m/z	Gene names
20141014_fract6_dyn_5ul_E6_01_585	26348	TOF; CID	61.96	802.06	CD68

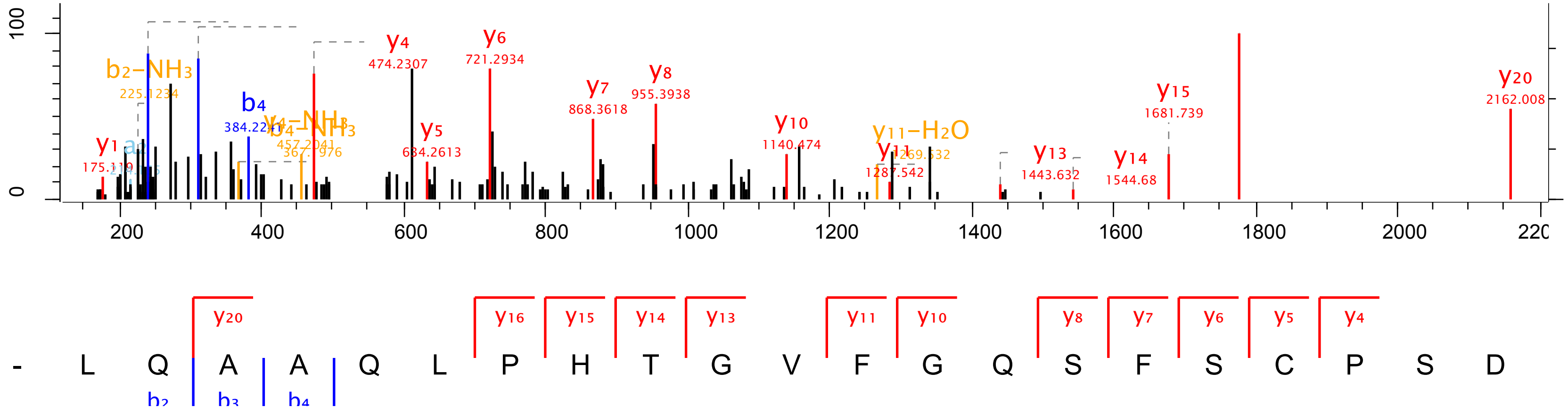

Raw file	Scan	Method	Score	m/z	Gene names
20141014_fract6_dyn_5ul_E6_01_585	33574	TOF; CID	91.24	874.79	ALDH8A1

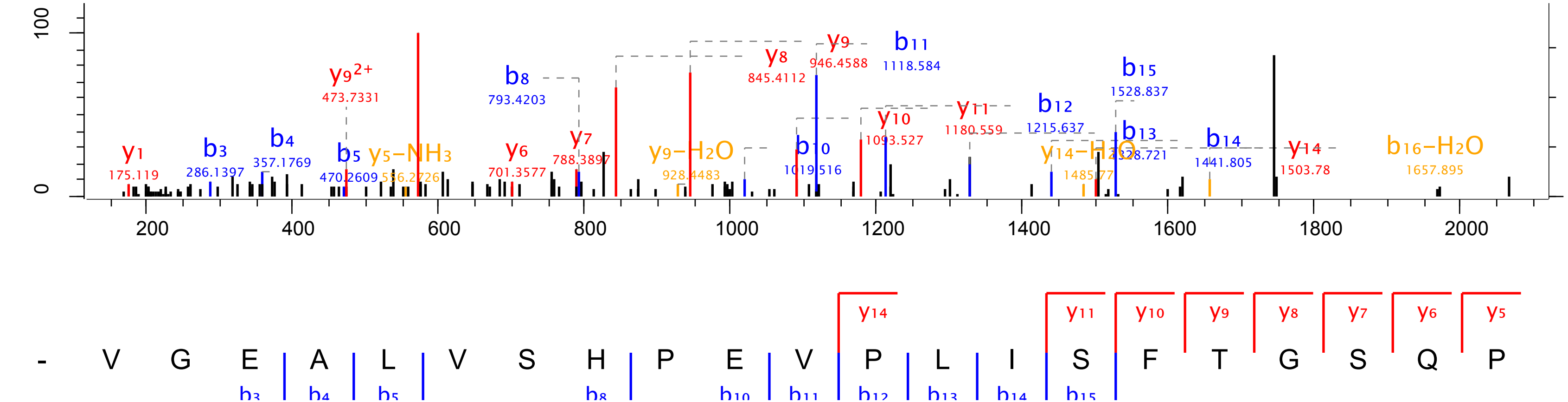

Raw file	Scan	Method	Score	m/z	Gene names
20141014_fract6_dyn_5ul_E6_01_585	33729	TOF; CID	88.6	739.38	PPAPDC1B

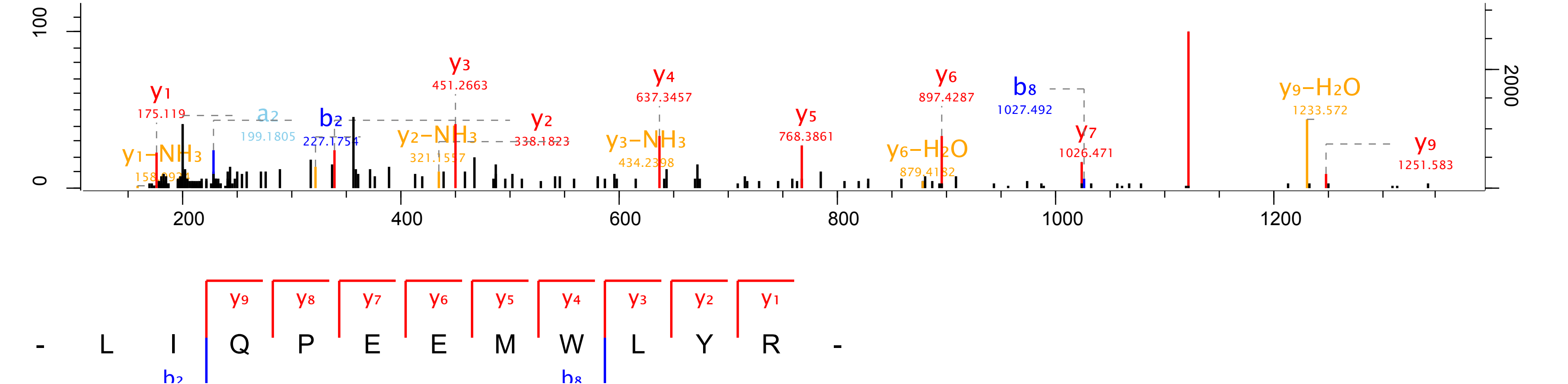

Raw file	Scan	Method	Score	m/z	Gene names
20141014_fract6_dyn_5ul_E6_01_585	34378	TOF; CID	111.01	996.5	PTTG1IP

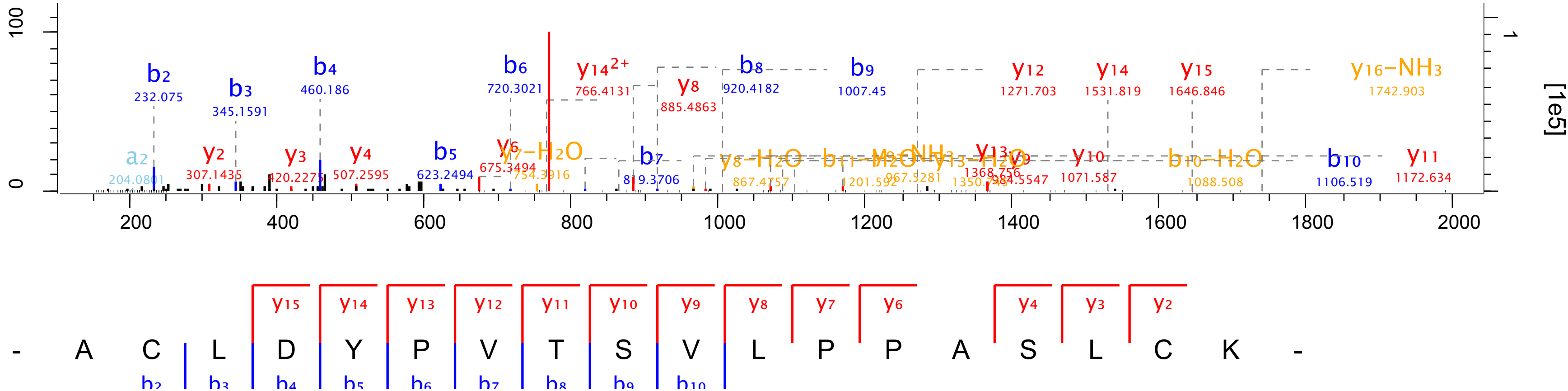

Raw file	Scan	Method	Score	m/z	Gene names
20141014_fract7_dyn_5ul_E7_01_586	4502	TOF; CID	74.48	481.23	ZSCAN21

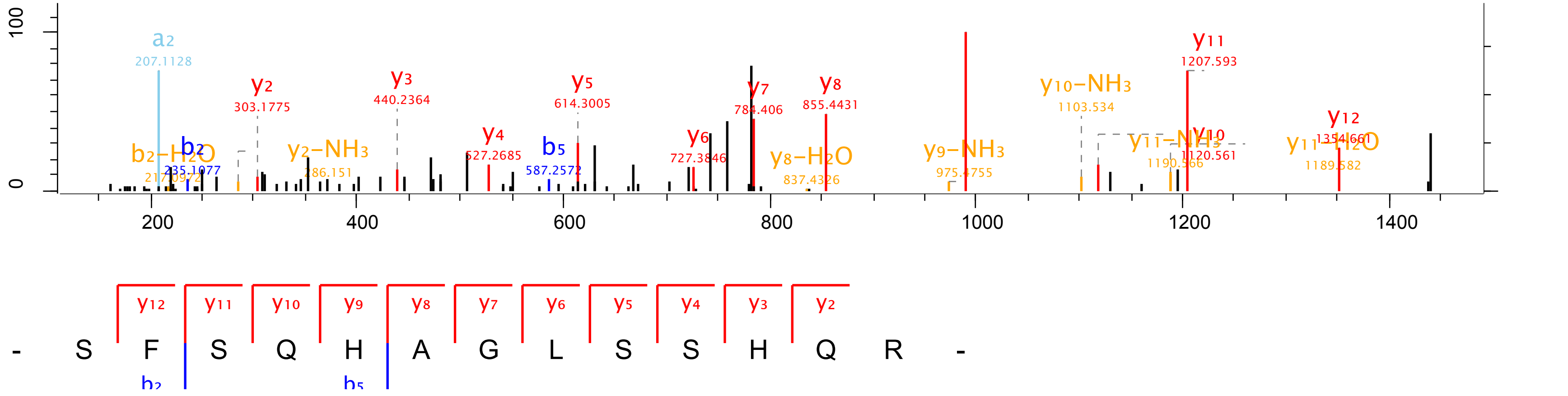

Raw file	Scan	Method	Score	m/z	Gene names
20141014_fract7_dyn_5ul_E7_01_586	4604	TOF; CID	55.72	506.59	STOX2;GDAP1

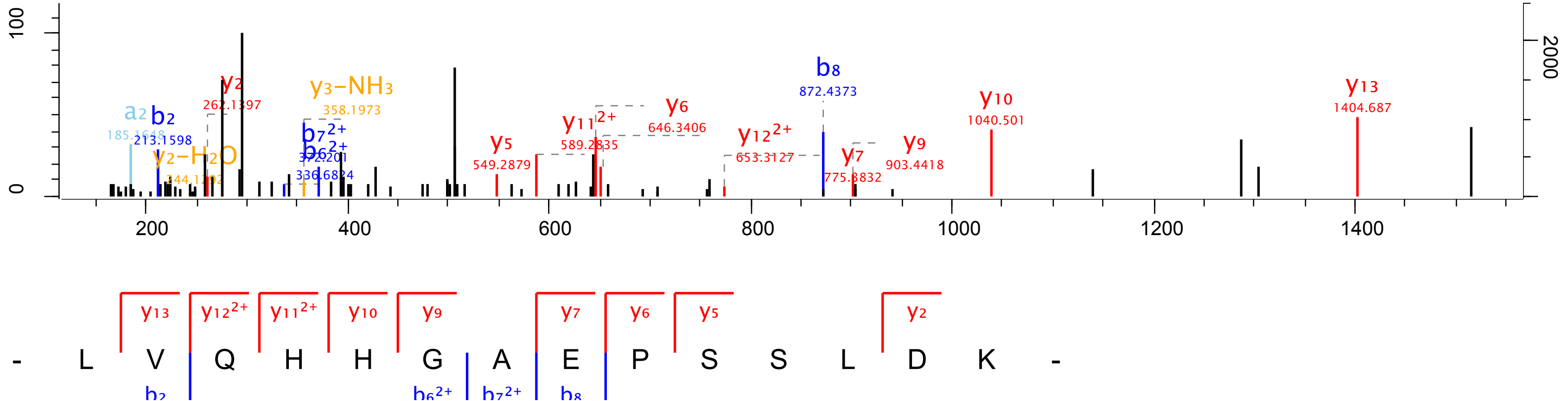

Raw file	Scan	Method	Score	m/z	Gene names
20141014_fract7_dyn_5ul_E7_01_586	7247	TOF; CID	121.86	629.33	CXCR4

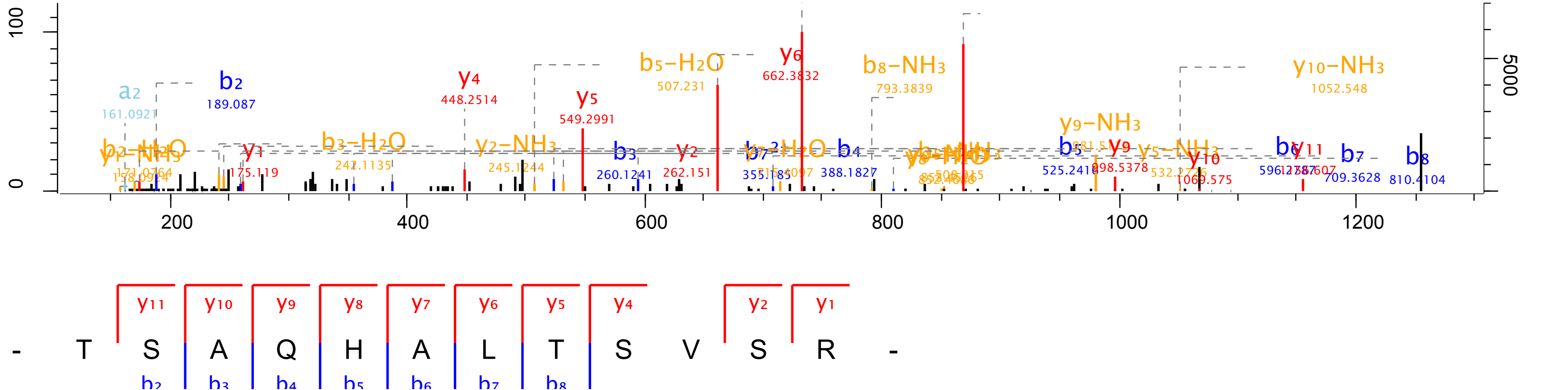

Raw file	Scan	Method	Score	m/z	Gene names
20141014_fract7_dyn_5ul_E7_01_586	10964	TOF; CID	78.94	538.28	ZNF670

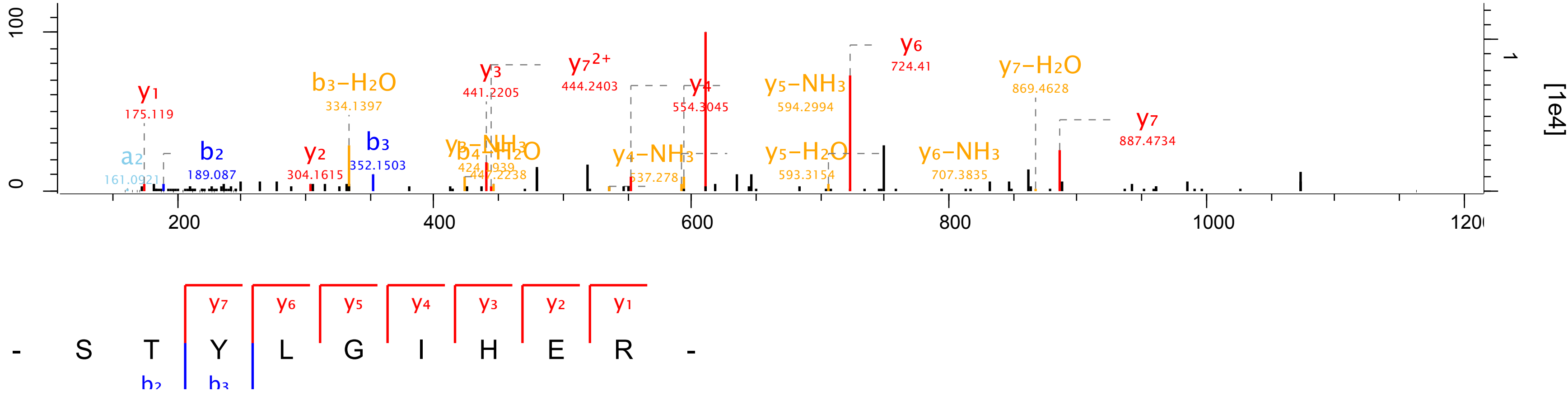

Raw file	Scan	Method	Score	m/z	Gene names
20141014_fract7_dyn_5ul_E7_01_586	12817	TOF; CID	77.6	619.29	USP53

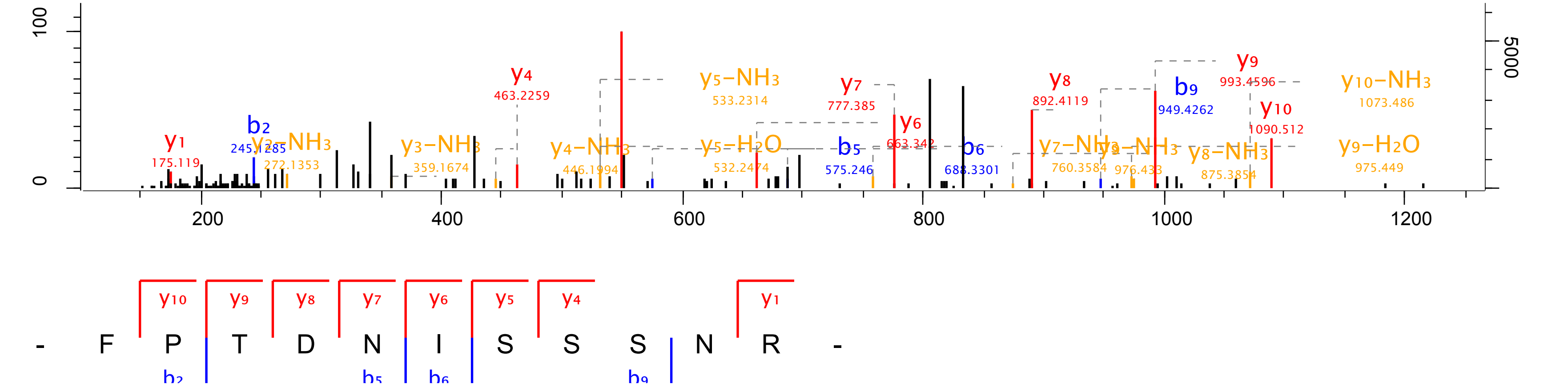

Raw file	Scan	Method	Score	m/z	Gene names
20141014_fract7_dyn_5ul_E7_01_586	17754	TOF; CID	49.63	453.23	UBXN2A

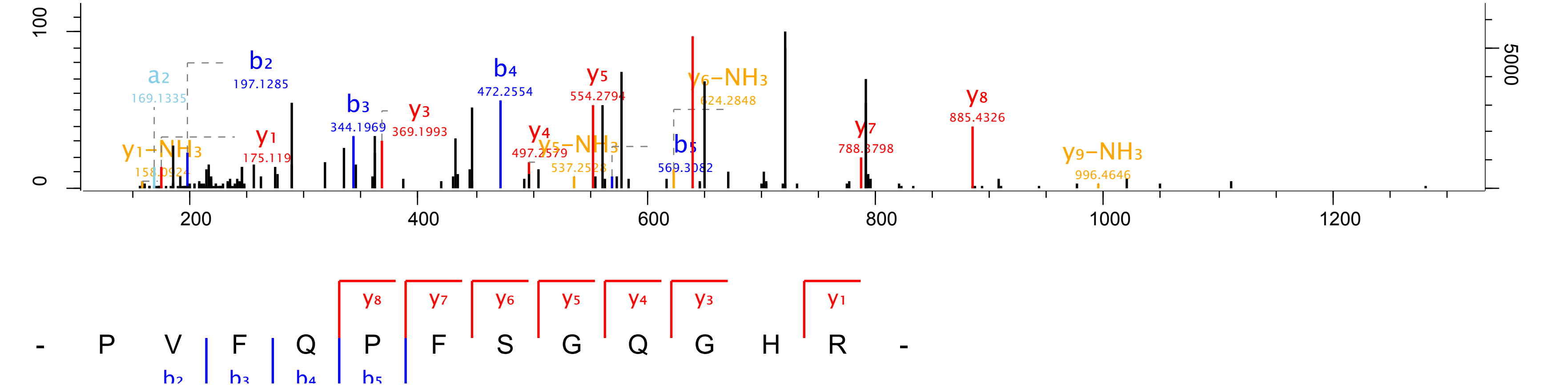

Raw file	Scan	Method	Score	m/z	Gene names
20141014_fract7_dyn_5ul_E7_01_586	18292	TOF; CID	103.13	777.89	C10orf35

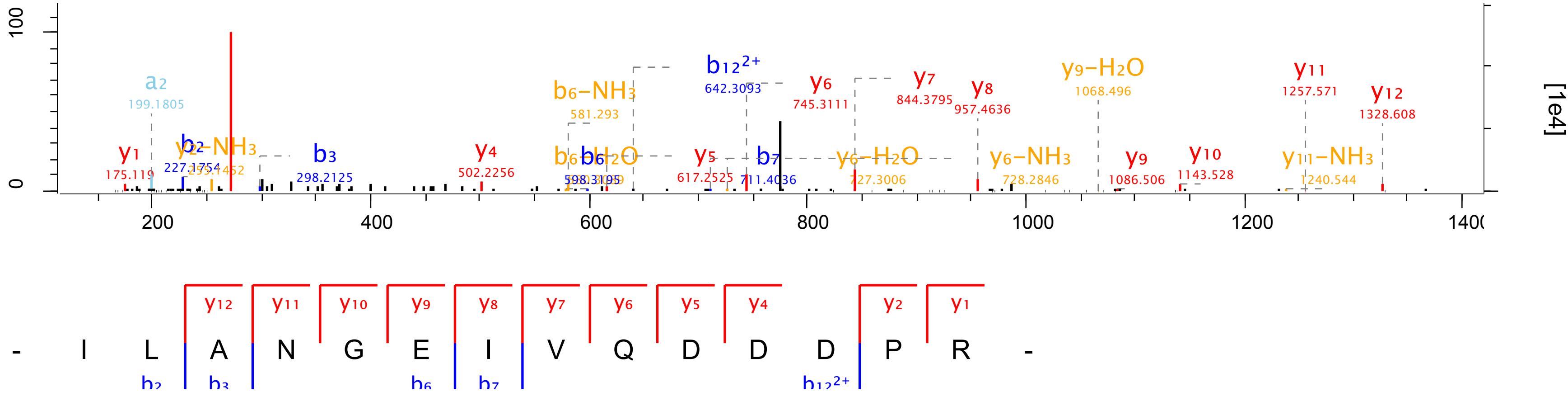

Raw file	Scan	Method	Score	m/z	Gene names
20141014_fract7_dyn_5ul_E7_01_586	20843	TOF; CID	67.93	611.8	TAF13

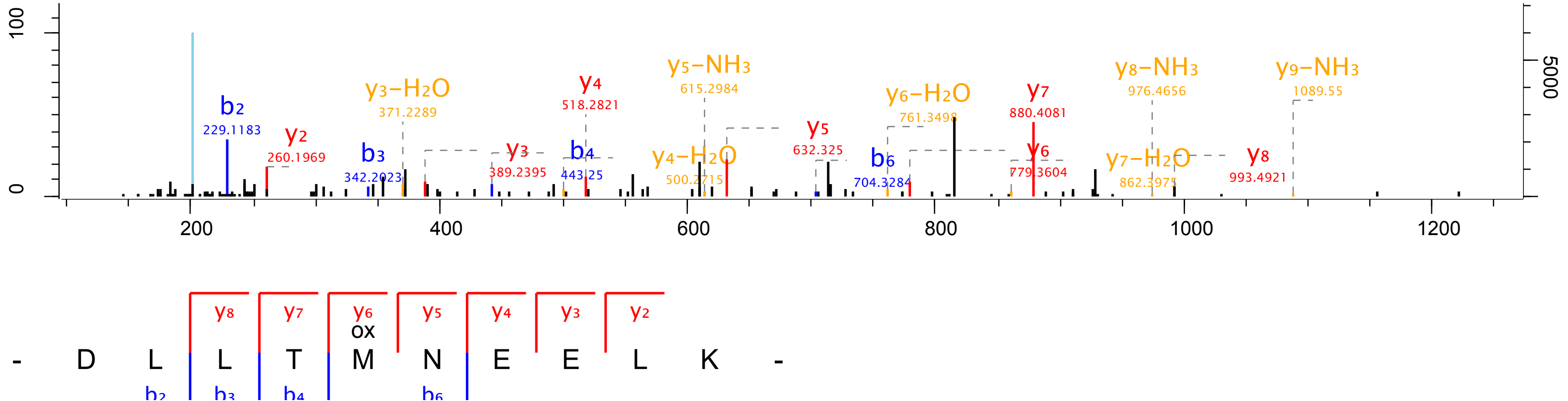

Raw file	Scan	Method	Score	m/z	Gene names
20141014_fract7_dyn_5ul_E7_01_586	20969	TOF; CID	80.74	926.46	LCORL

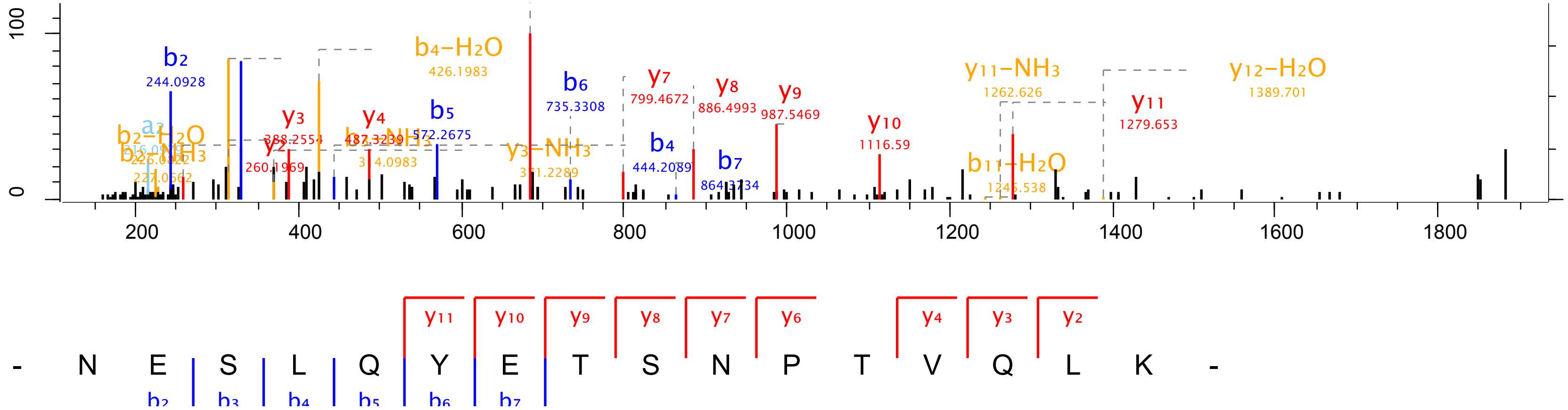

Raw file	Scan	Method	Score	m/z	Gene names
20141014_fract7_dyn_5ul_E7_01_586	24972	TOF; CID	85.36	926.46	HNRNPH1

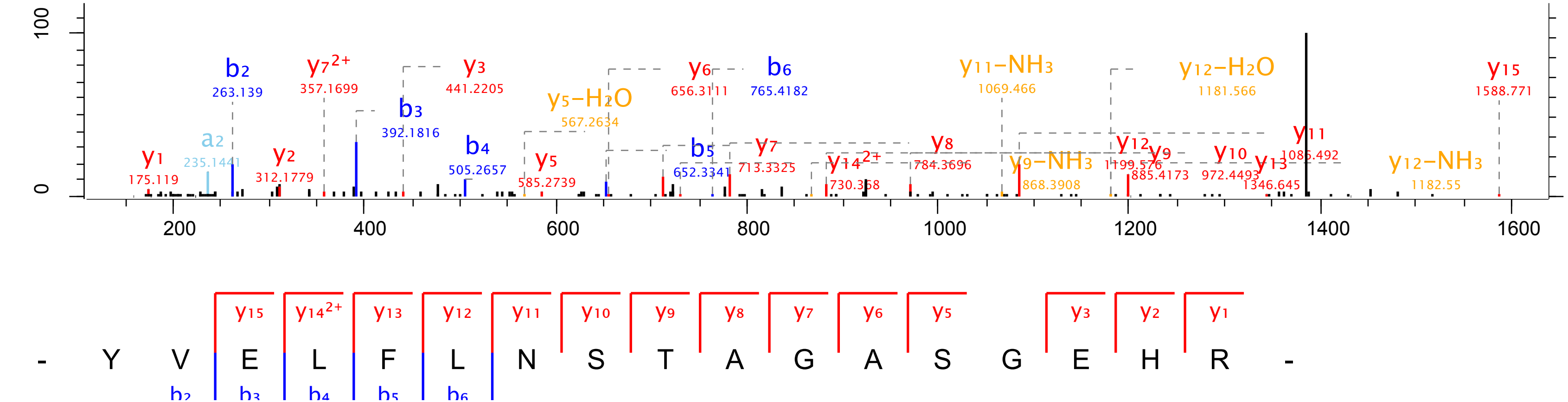

Raw file	Scan	Method	Score	m/z	Gene names
20141014_fract7_dyn_5ul_E7_01_586	25098	TOF; CID	92.86	692.86	PLEKHO2

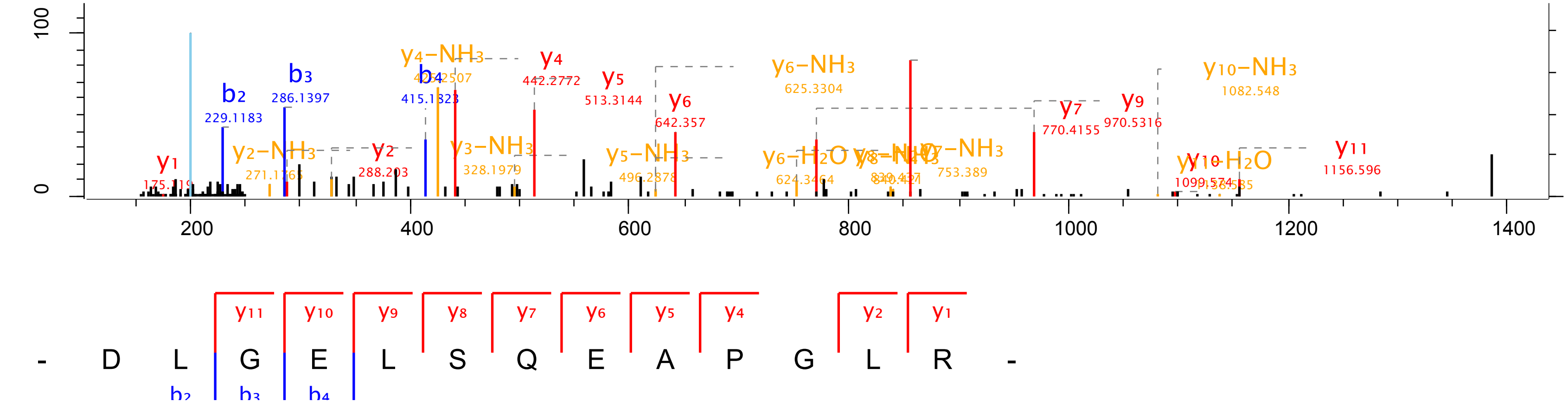

Raw file	Scan	Method	Score	m/z	Gene names
20141014_fract7_dyn_5ul_E7_01_586	29994	TOF; CID	63.29	778.4	TBC1D16

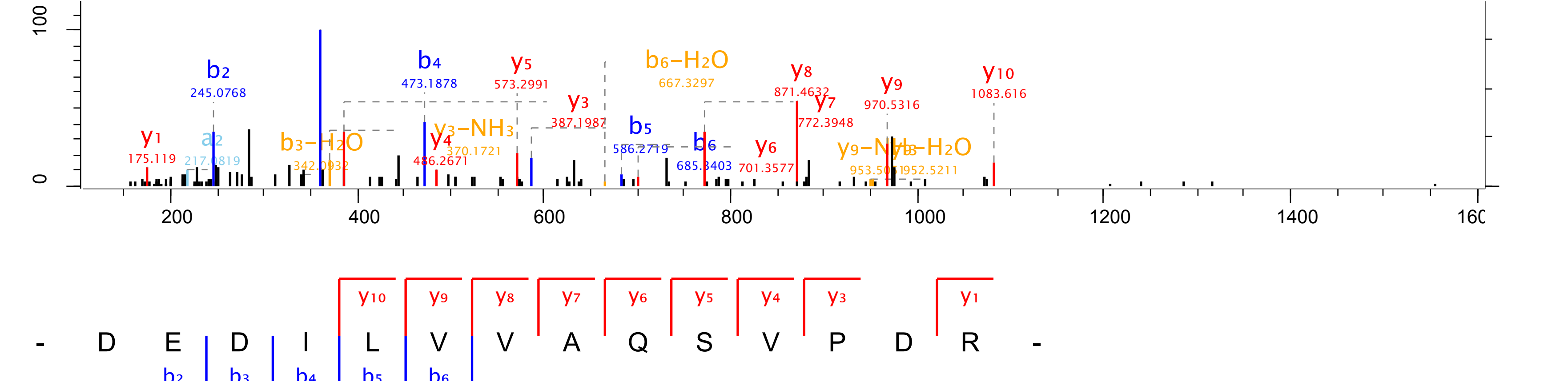

Raw file	Scan	Method	Score	m/z	Gene names
20141014_fract7_dyn_5ul_E7_01_586	31114	TOF; CID	39.34	560.32	GRAMD3

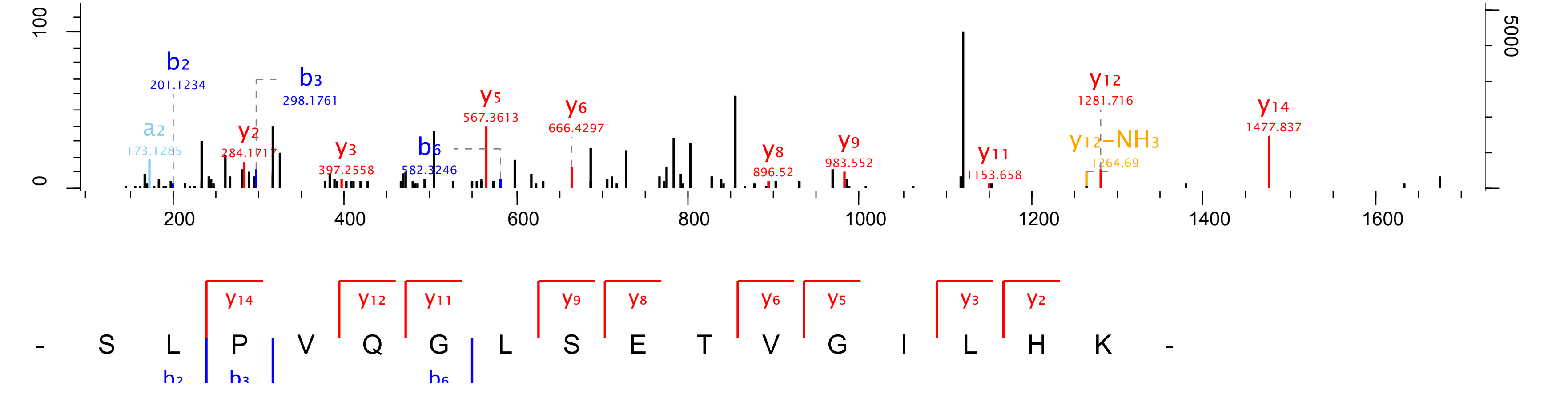

Raw file	Scan	Method	Score	m/z	Gene names
20141014_fract7_dyn_5ul_E7_01_586	34957	TOF; CID	63.16	839.42	GSTM4

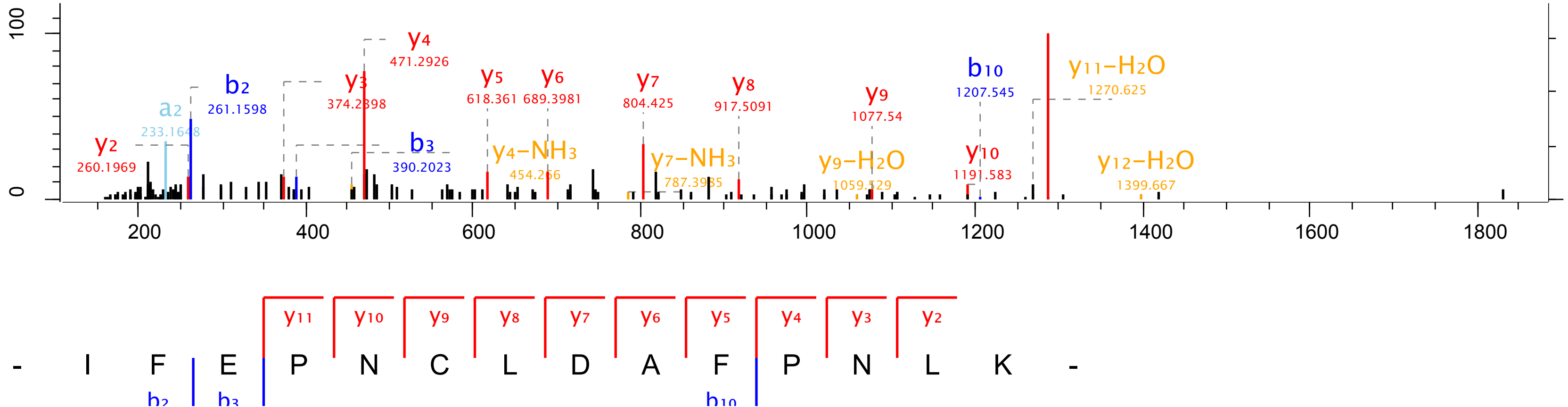

Raw file	Scan	Method	Score	m/z	Gene names
20141014_fract7_dyn_5ul_E7_01_586	37190	TOF; CID	93.23	892.49	PPP1R3F

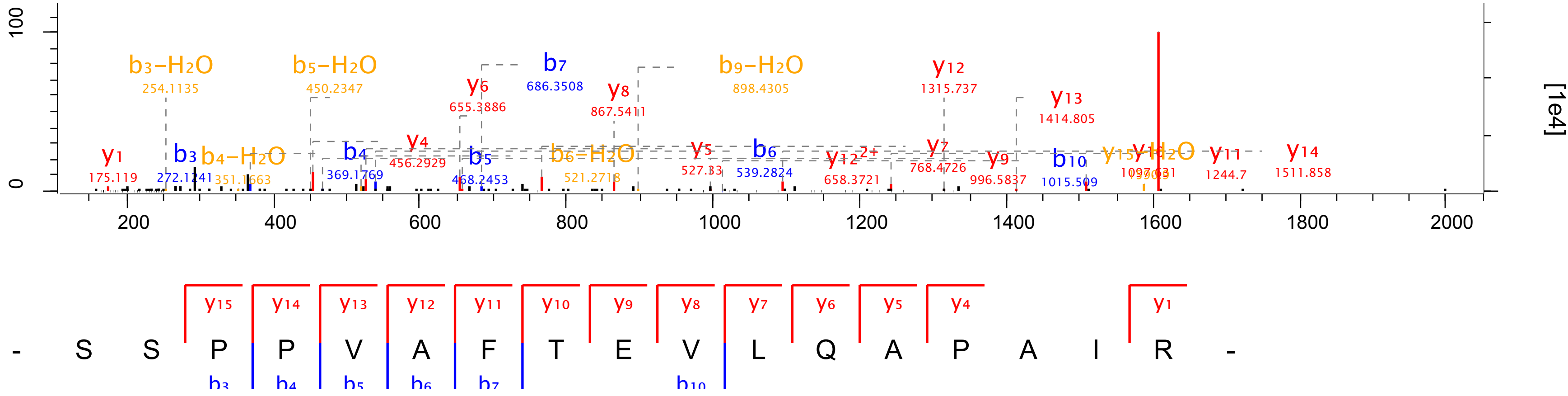

Raw file	Scan	Method	Score	m/z	Gene names
20141014_fract8_dyn_5ul_E8_01_587	9717	TOF; CID	72.2	547.28	ZNF675

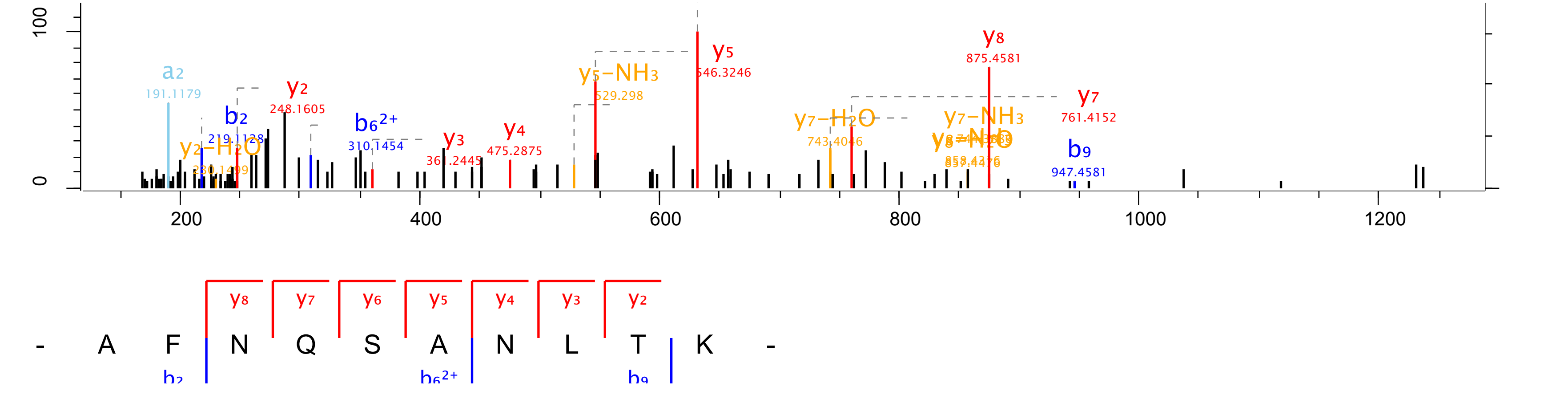

Raw file	Scan	Method	Score	m/z	Gene names
20141014_fract8_dyn_5ul_E8_01_587	9944	TOF; CID	49.04	546.75	LPHN1

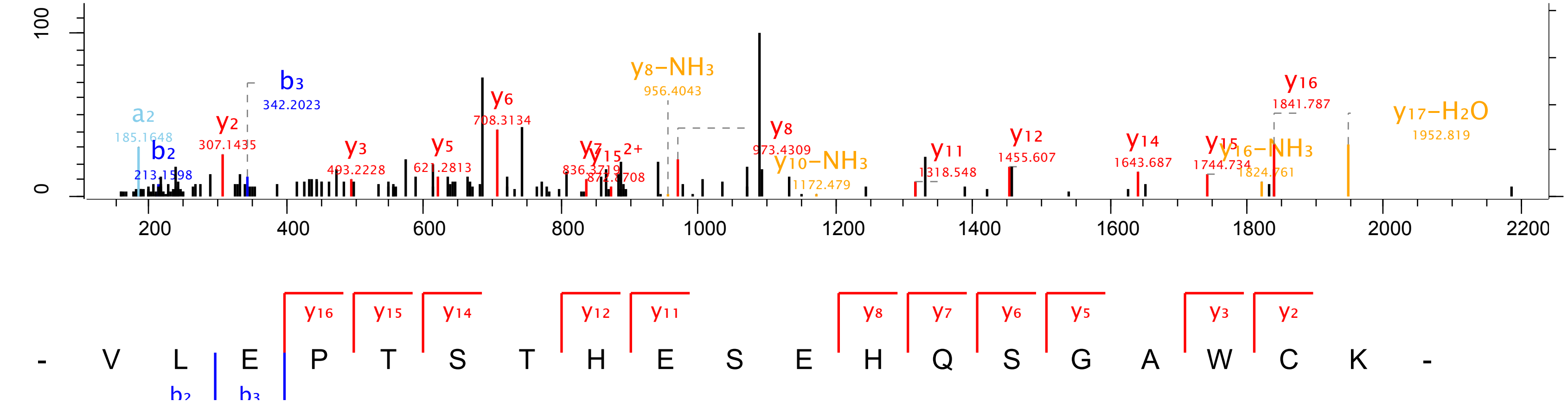

Raw file	Scan	Method	Score	m/z	Gene names
20141014_fract8_dyn_5ul_E8_01_587	13387	TOF; CID	59.2	578.79	TMEM254

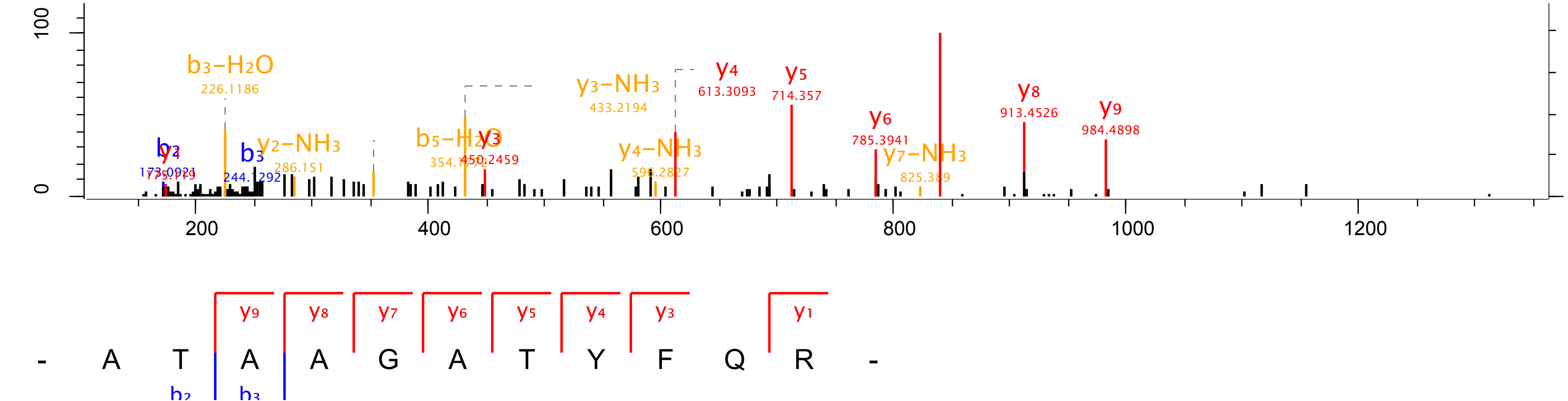

Raw file	Scan	Method	Score	m/z	Gene names
20141014_fract8_dyn_5ul_E8_01_587	13435	TOF; CID	68.52	656.35	UCP2

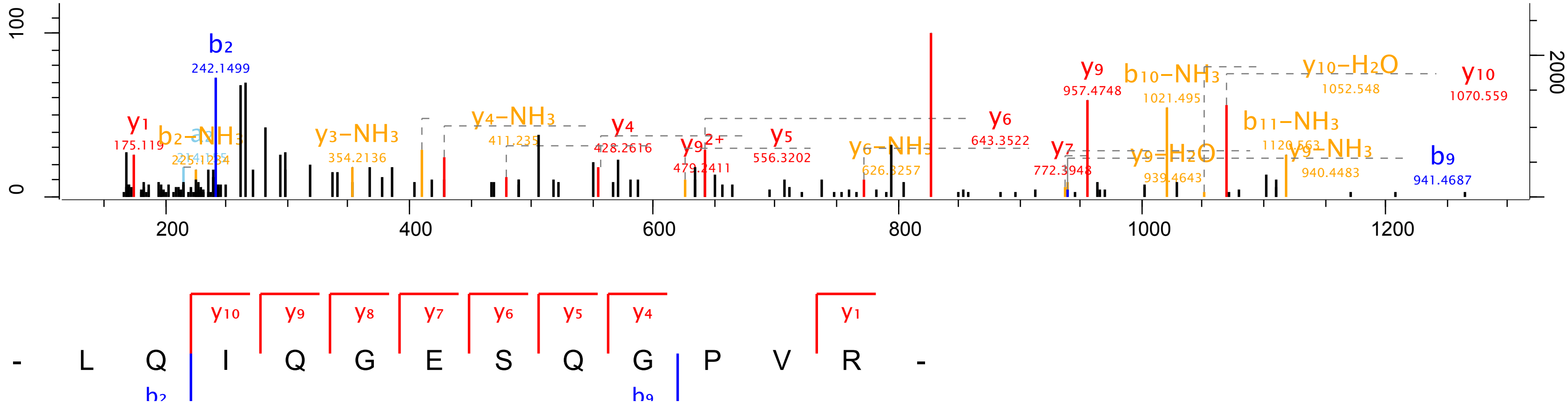

Raw file	Scan	Method	Score	m/z	Gene names
20141014_fract8_dyn_5ul_E8_01_587	16168	TOF; CID	63.61	624.78	C11orf83

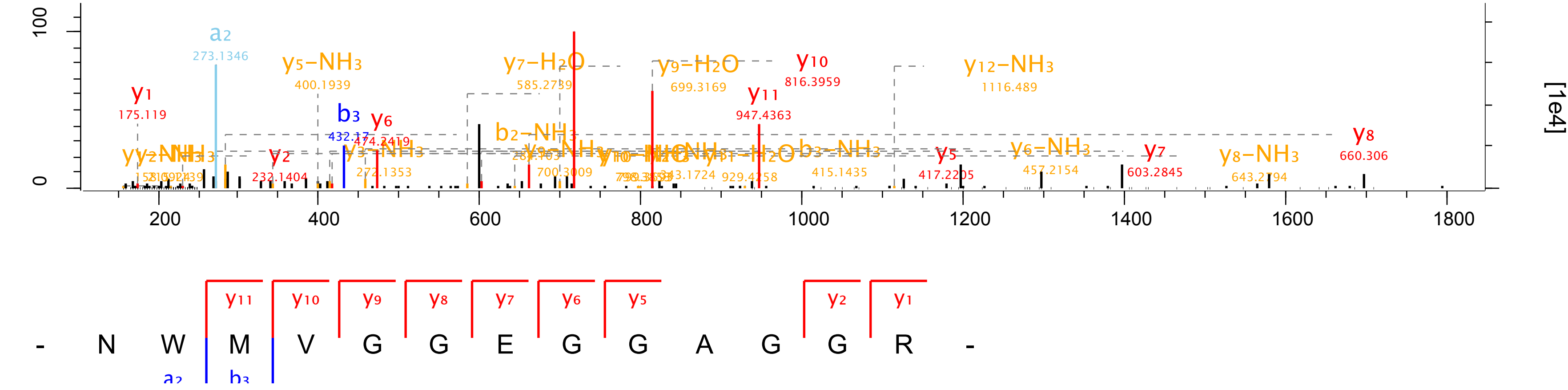

Raw file	Scan	Method	Score	m/z	Gene names
20141014_fract8_dyn_5ul_E8_01_587	17376	TOF; CID	110.41	539.26	NPDC1

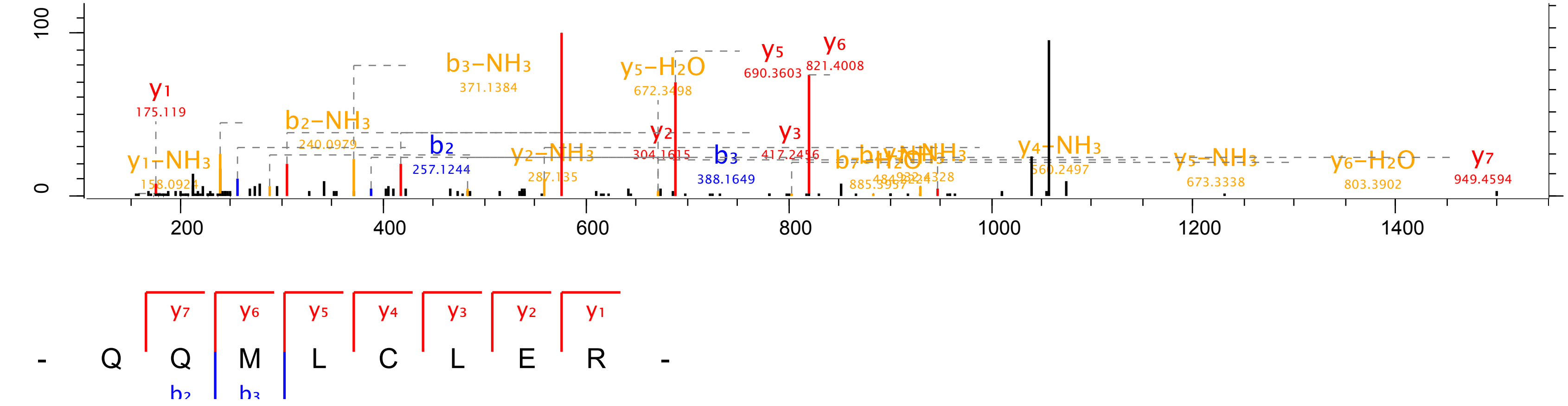

Raw file	Scan	Method	Score	m/z	Gene names
20141014_fract8_dyn_5ul_E8_01_587	17469	TOF; CID	94.82	566.29	C6orf57

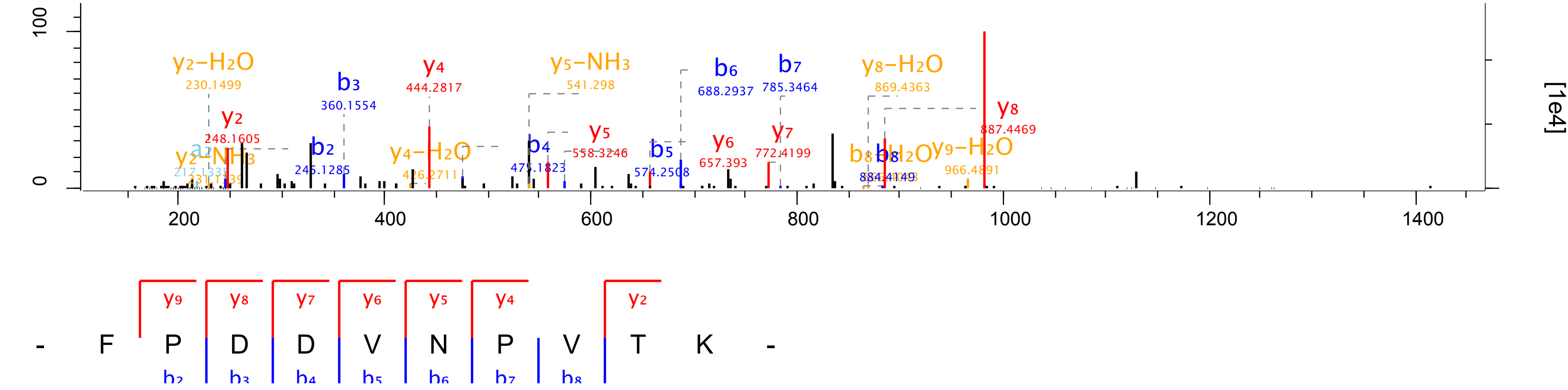

Raw file	Scan	Method	Score	m/z	Gene names
20141014_fract8_dyn_5ul_E8_01_587	23332	TOF; CID	61.11	670.36	CSTA

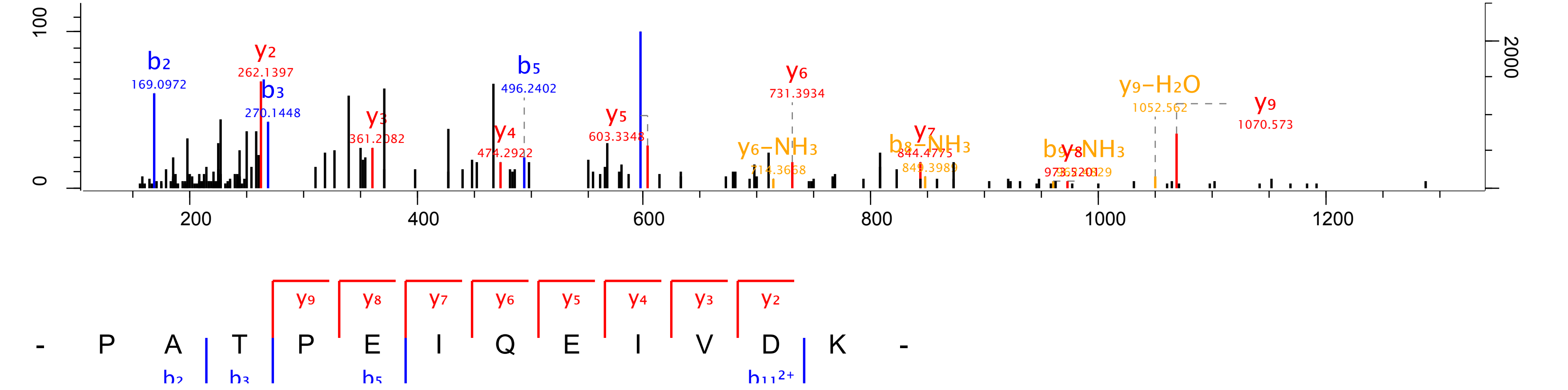

Raw file	Scan	Method	Score	m/z	Gene names
20141014_fract8_dyn_5ul_E8_01_587	24831	TOF; CID	60.33	676.35	ZNF202

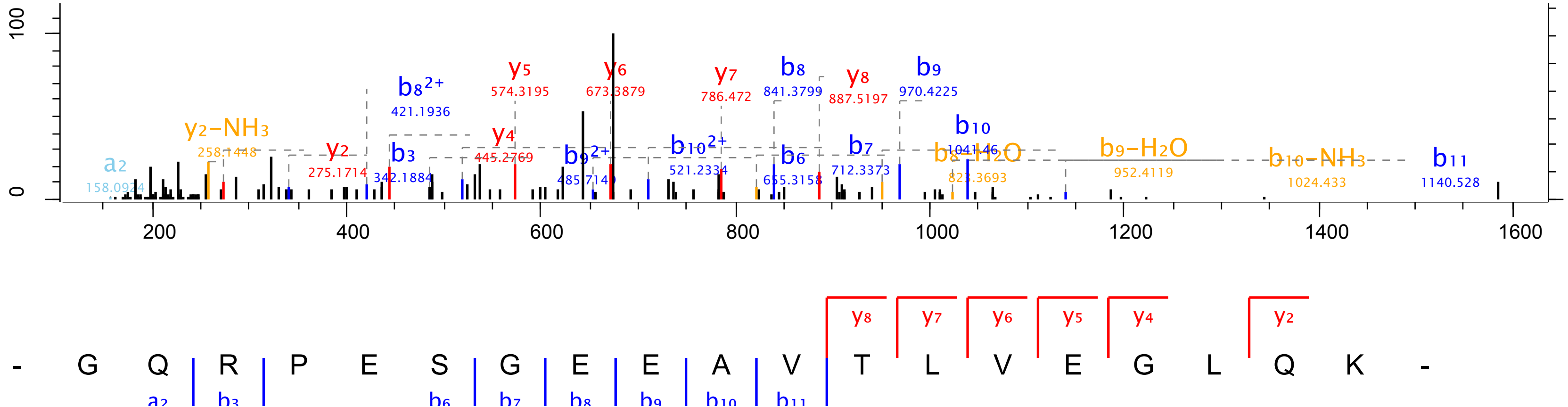

Raw file	Scan	Method	Score	m/z	Gene names
20141014_fract8_dyn_5ul_E8_01_587	25255	TOF; CID	67.38	582.29	TMLHE

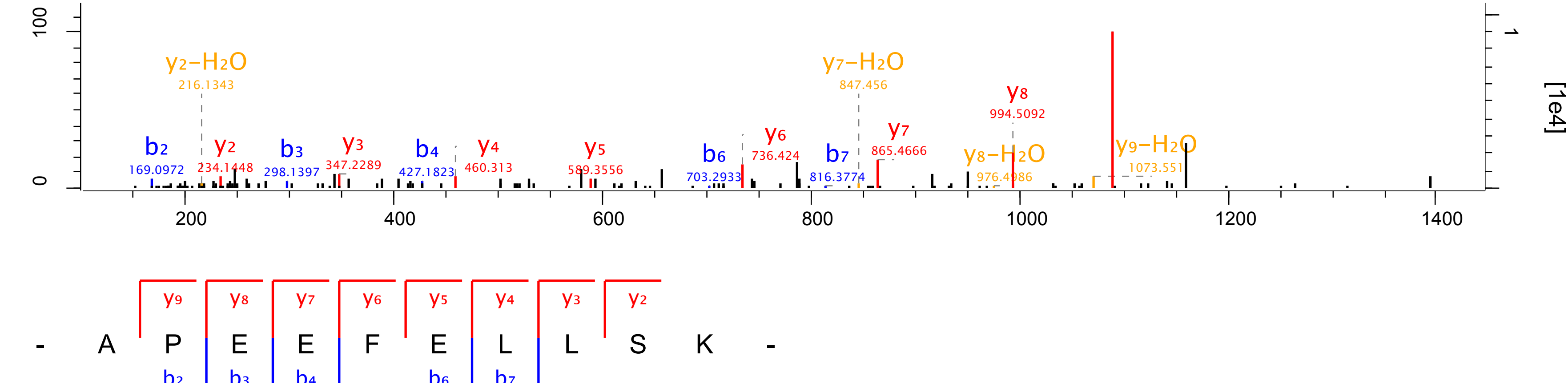

Raw file	Scan	Method	Score	m/z	Gene names
20141014_fract8_dyn_5ul_E8_01_587	25841	TOF; CID	102.07	502.27	PGM5

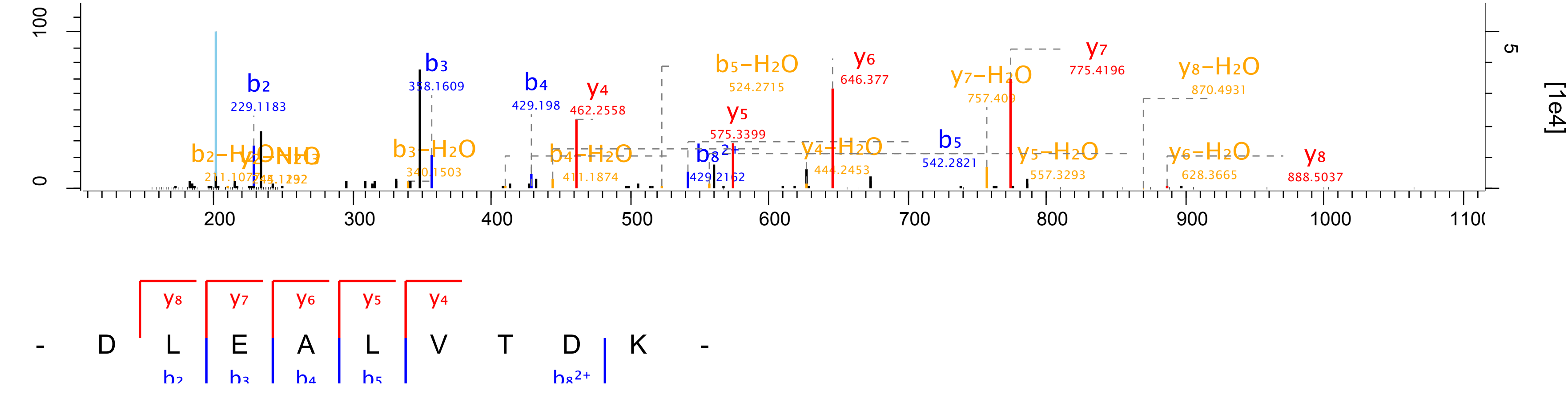

Raw file	Scan	Method	Score	m/z	Gene names
20141014_fract8_dyn_5ul_E8_01_587	26052	TOF; CID	114.28	546.32	BHLHB9

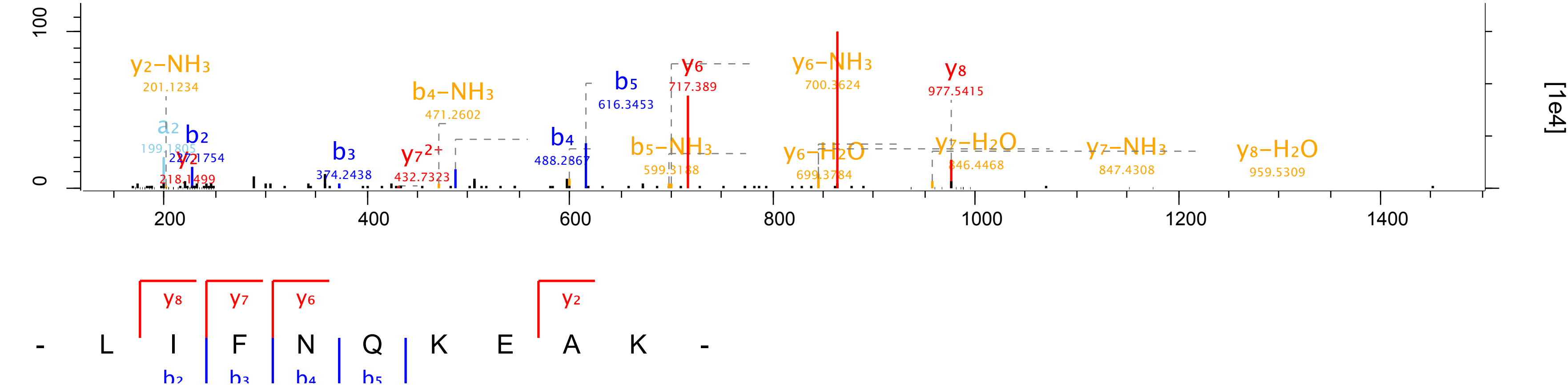

Raw file	Scan	Method	Score	m/z	Gene names
20141014_fract8_dyn_5ul_E8_01_587	29898	TOF; CID	113.42	1173.05	PKIG

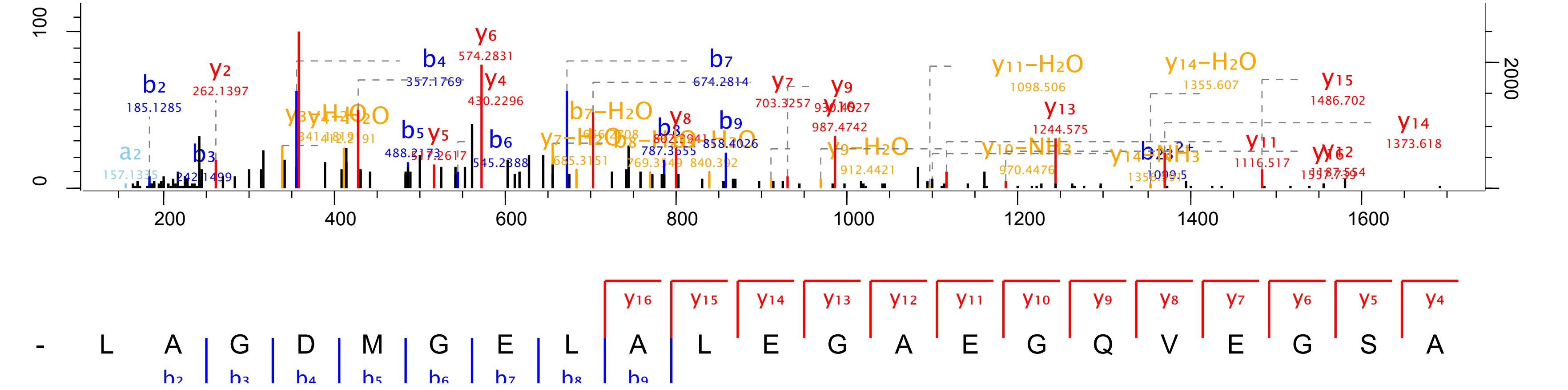

Raw file	Scan	Method	Score	m/z	Gene names
20141014_fract8_dyn_5ul_E8_01_587	31118	TOF; CID	49.54	869.42	MFSD8

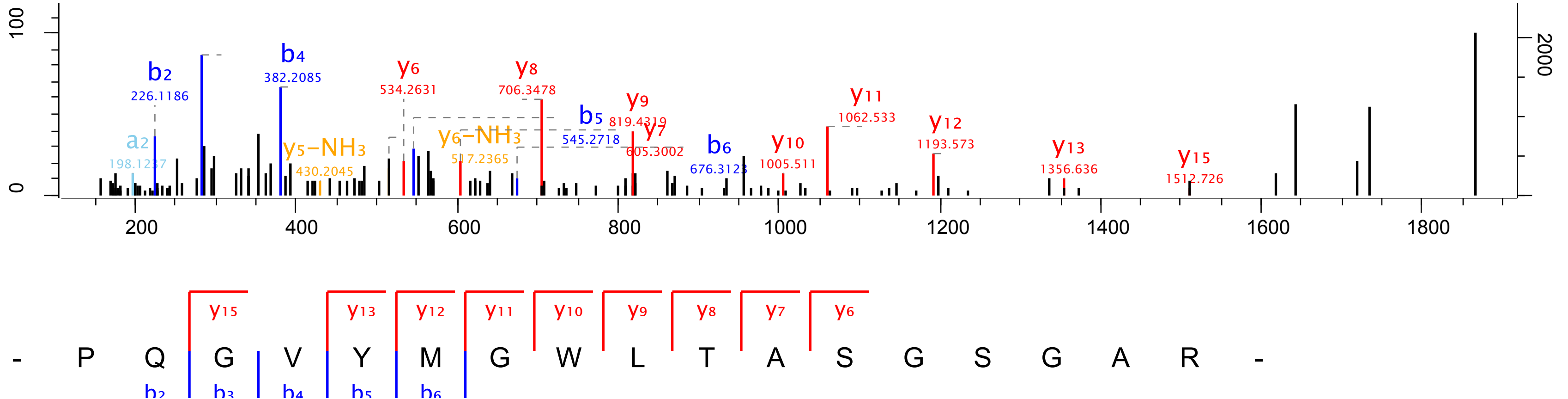

Raw file	Scan	Method	Score	m/z	Gene names
20141014_fract8_dyn_5ul_E8_01_587	36842	TOF; CID	80.24	497.58	KCTD7

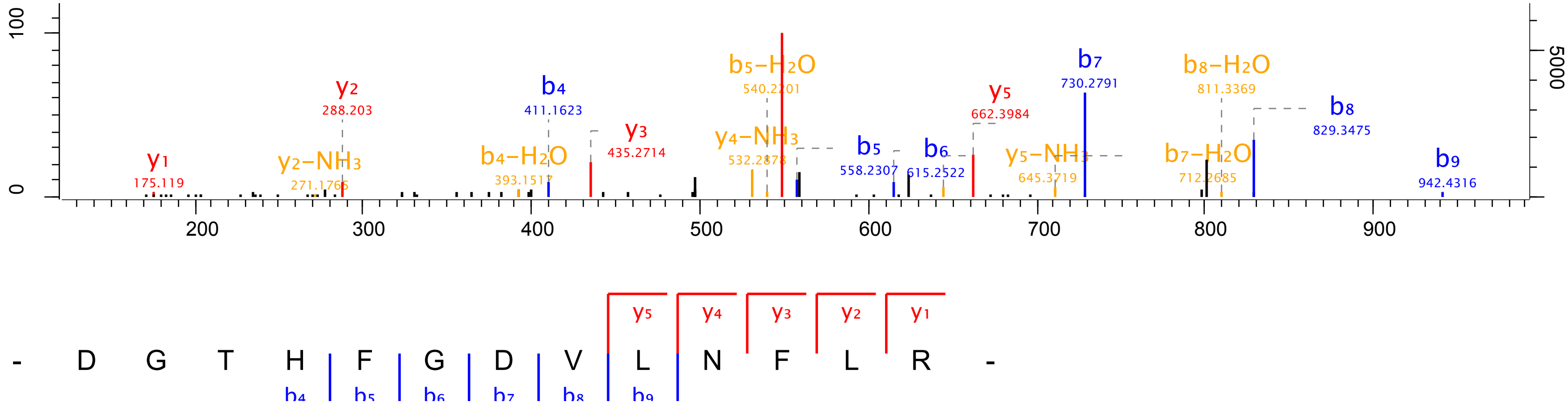

Raw file	Scan	Method	Score	m/z	Gene names
20141014_fract8_dyn_5ul_E8_01_587	38691	TOF; CID	54.28	729.39	IDNK

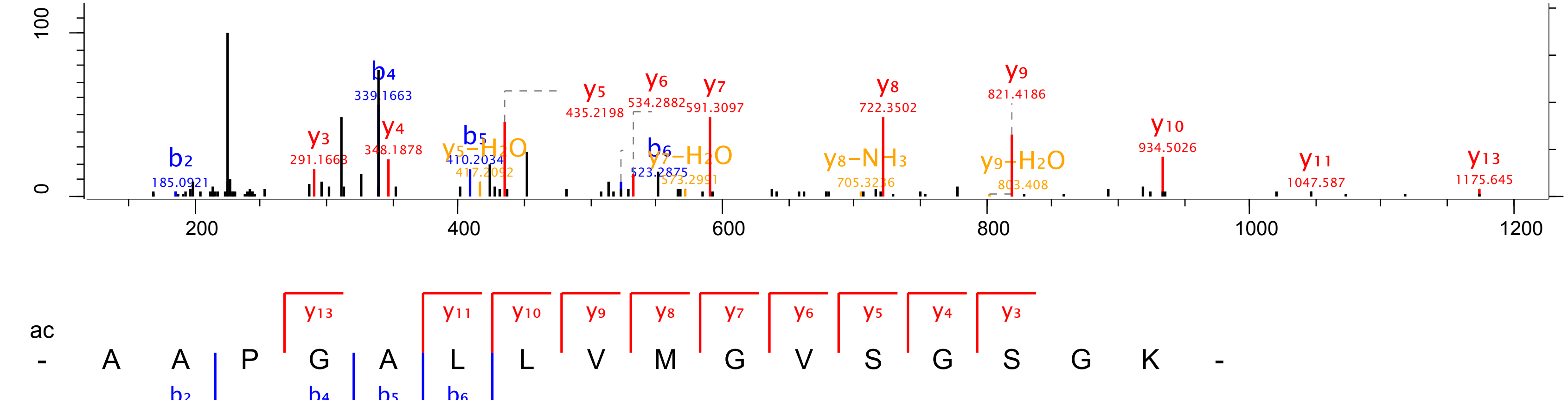

Raw file	Scan	Method	Score	m/z	Gene names
20141014_fract9_dyn_5ul_F1_01_588	4674	TOF; CID	91.55	521.78	SLC16A7

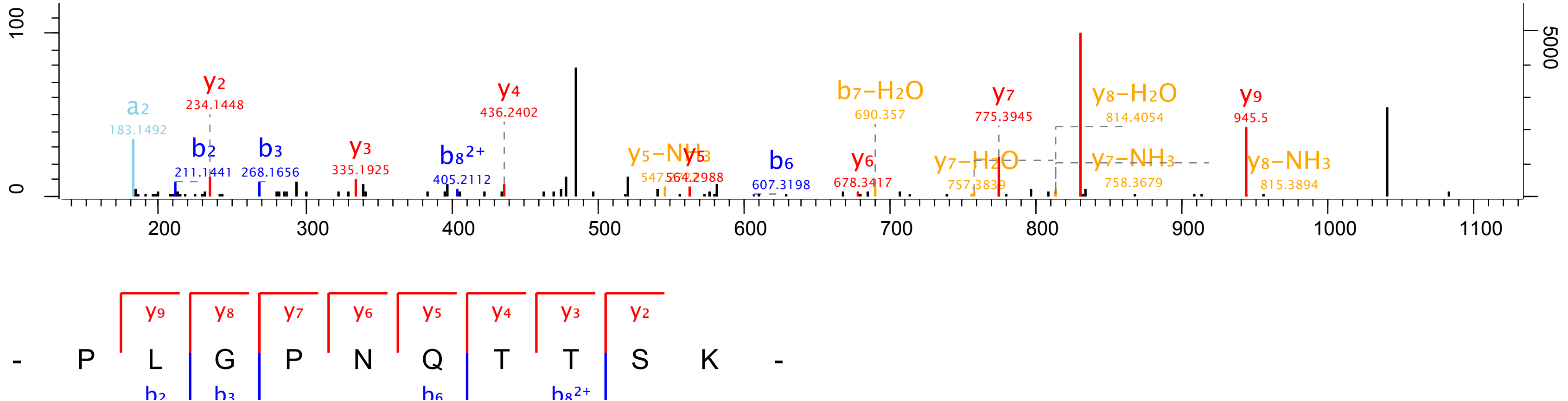

Raw file	Scan	Method	Score	m/z	Gene names
20141014_fract9_dyn_5ul_F1_01_588	9754	TOF; CID	37.46	690.32	PPAPDC2

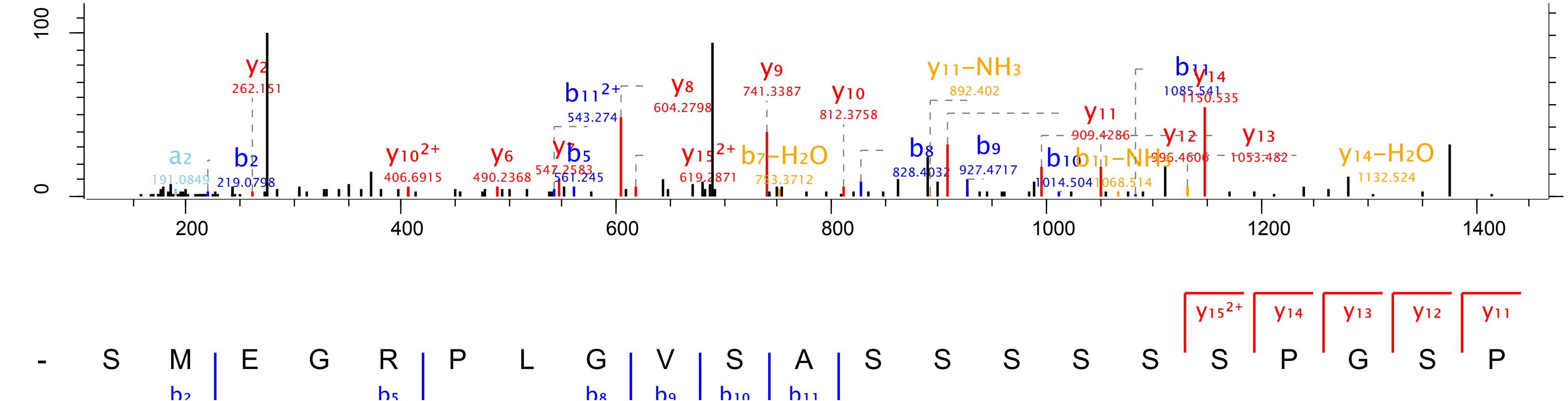

Raw file	Scan	Method	Score	m/z	Gene names
20141014_fract9_dyn_5ul_F1_01_588	11317	TOF; CID	133.81	438.18	CITED2

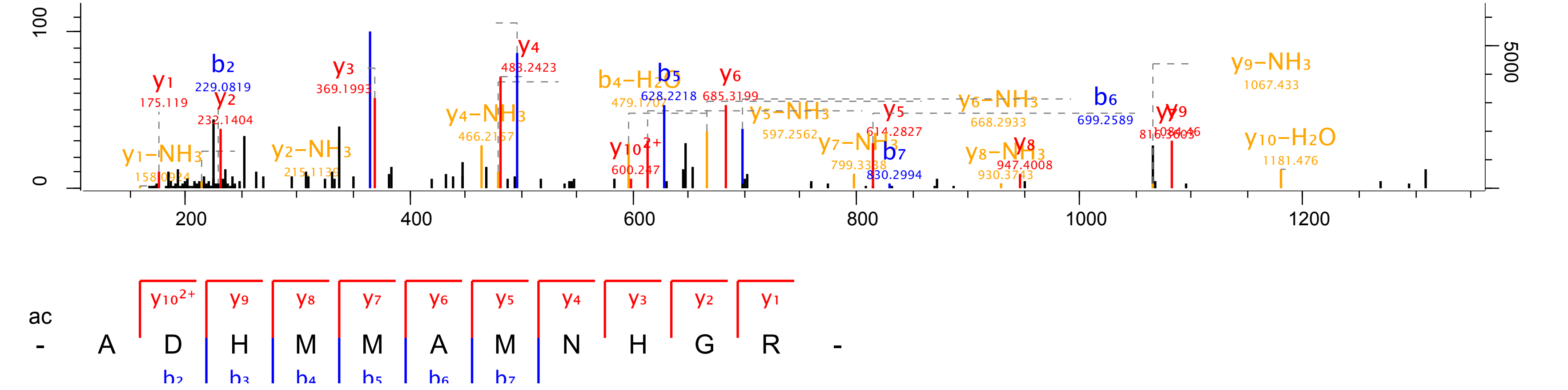

Raw file	Scan	Method	Score	m/z	Gene names
20141014_fract9_dyn_5ul_F1_01_588	12656	TOF; CID	90.14	600.83	SPATA24

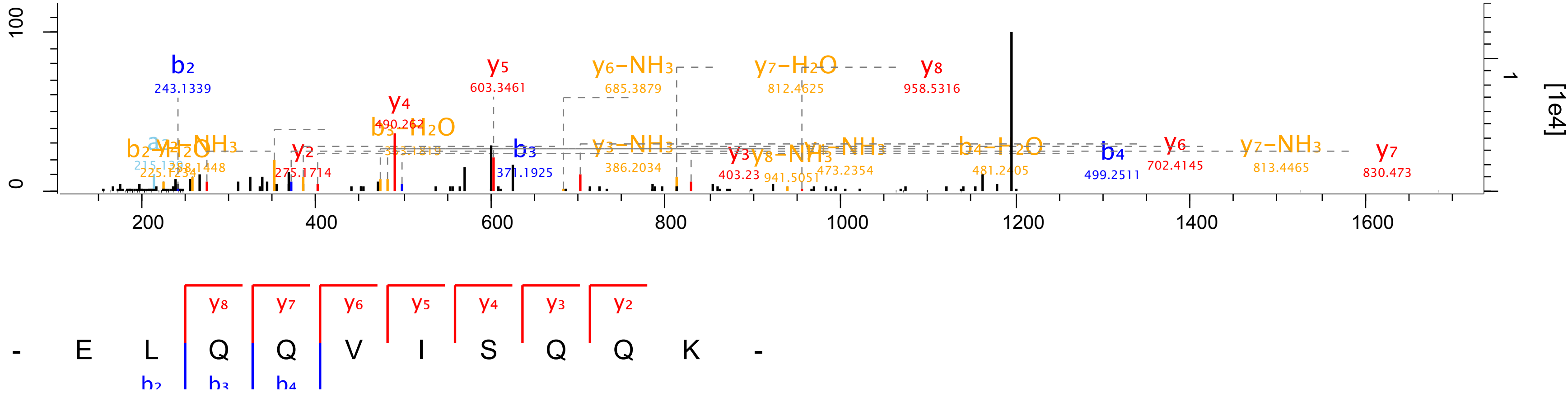

Raw file	Scan	Method	Score	m/z	Gene names
20141014_fract9_dyn_5ul_F1_01_588	13990	TOF; CID	59.73	759.36	IGFBP7

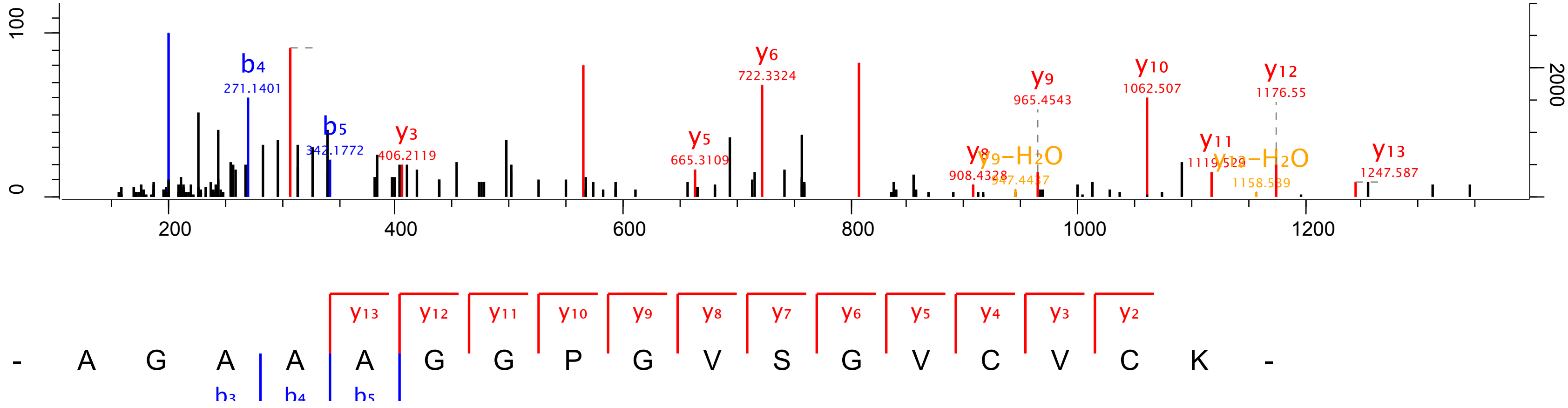

Raw file	Scan	Method	Score	m/z	Gene names
20141014_fract9_dyn_5ul_F1_01_588	15090	TOF; CID	83.62	492.24	TNFSF9

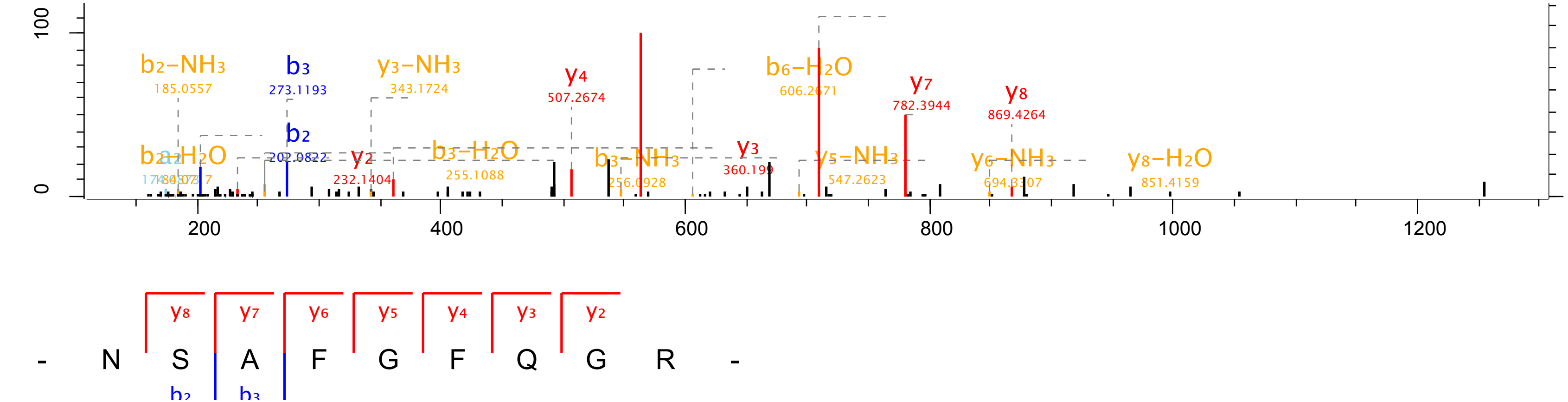

Raw file	Scan	Method	Score	m/z	Gene names
20141014_fract9_dyn_5ul_F1_01_588	15195	TOF; CID	102.46	454.25	ATP5E;ATP5EP2

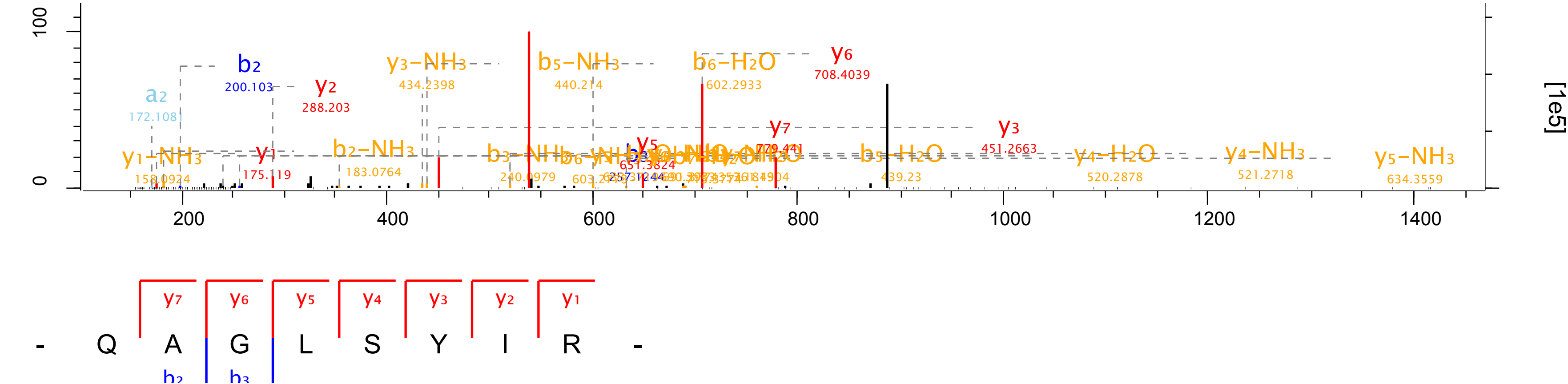

Raw file	Scan	Method	Score	m/z	Gene names
20141014_fract9_dyn_5ul_F1_01_588	16089	TOF; CID	143.42	824.4	SS18L1

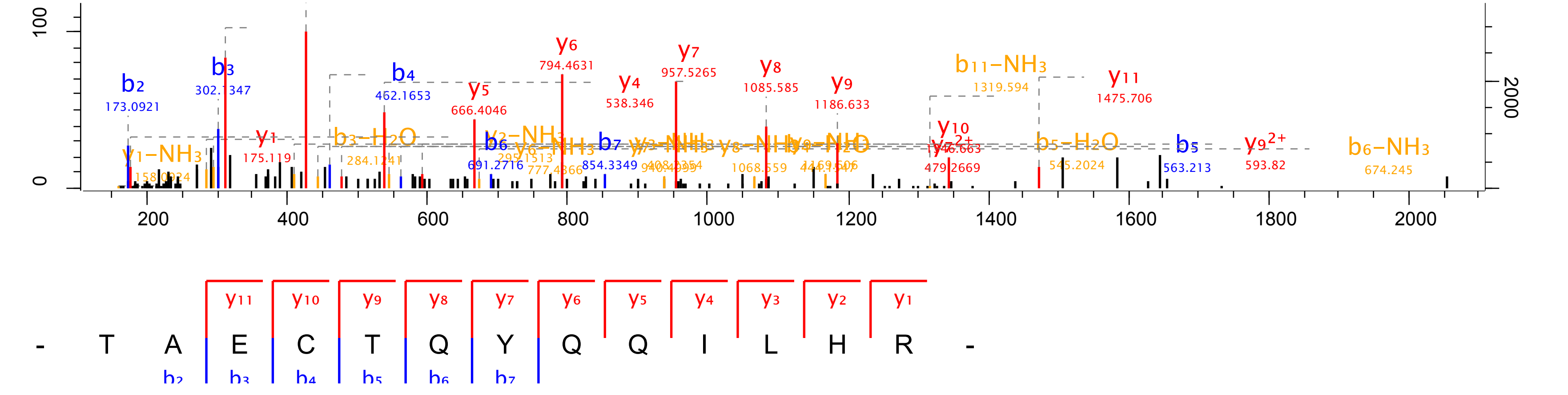

Raw file	Scan	Method	Score	m/z	Gene names
20141014_fract9_dyn_5ul_F1_01_588	18962	TOF; CID	110.44	850.9	ZNF239

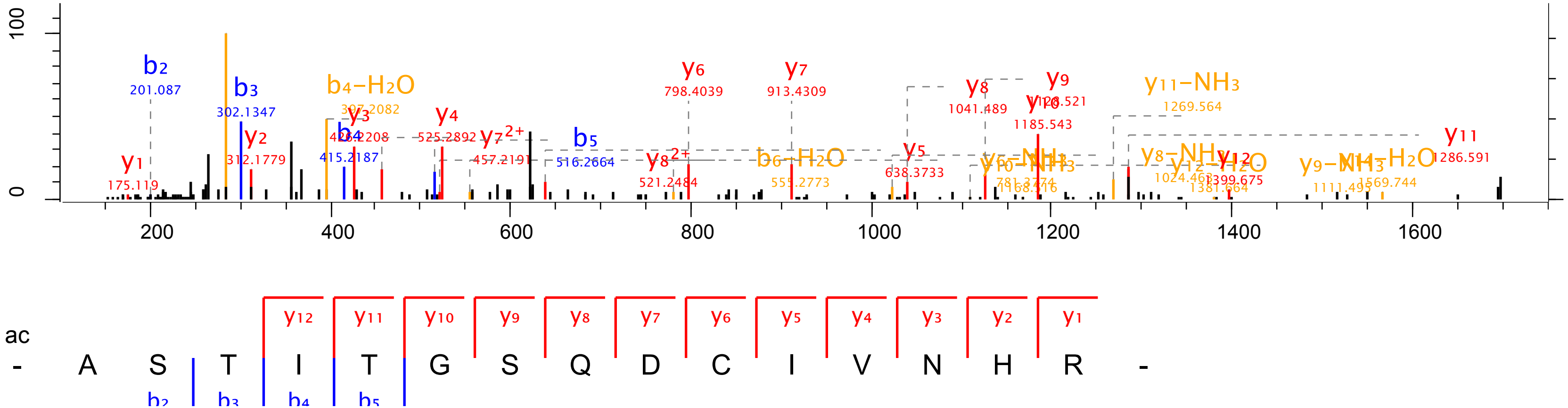

Raw file	Scan	Method	Score	m/z	Gene names
20141014_fract9_dyn_5ul_F1_01_588	23263	TOF; CID	41.36	957.78	SLC20A2

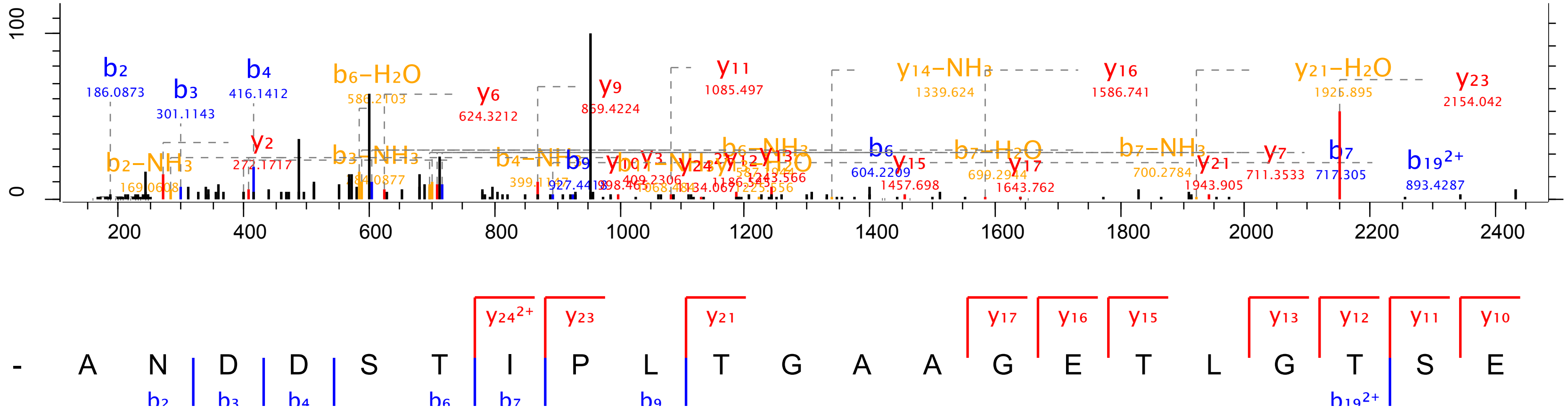

Raw file	Scan	Method	Score	m/z	Gene names
20141014_fract9_dyn_5ul_F1_01_588	23673	TOF; CID	140.14	661.32	SLC6A9

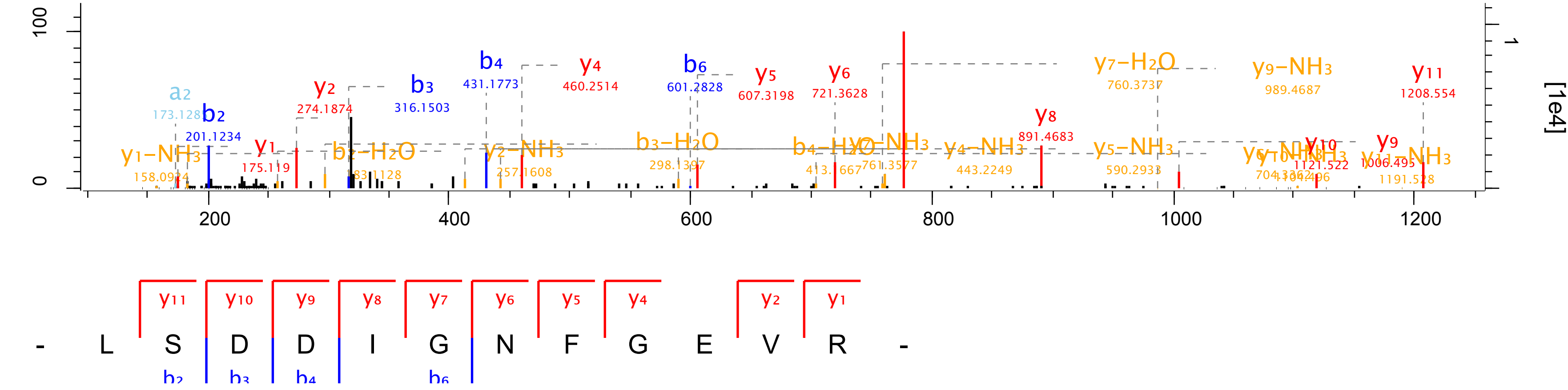

Raw file	Scan	Method	Score	m/z	Gene names
20141014_fract9_dyn_5ul_F1_01_588	25104	TOF; CID	93.32	632.34	CKLF

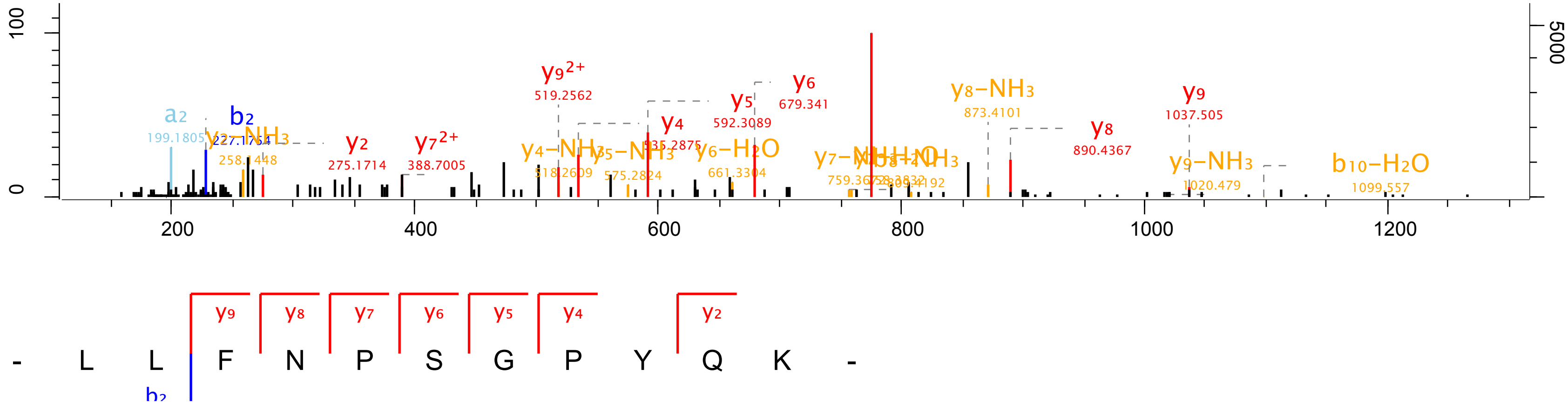

Raw file	Scan	Method	Score	m/z	Gene names
20141014_fract9_dyn_5ul_F1_01_588	29640	TOF; CID	57.53	442.61	FAM150B

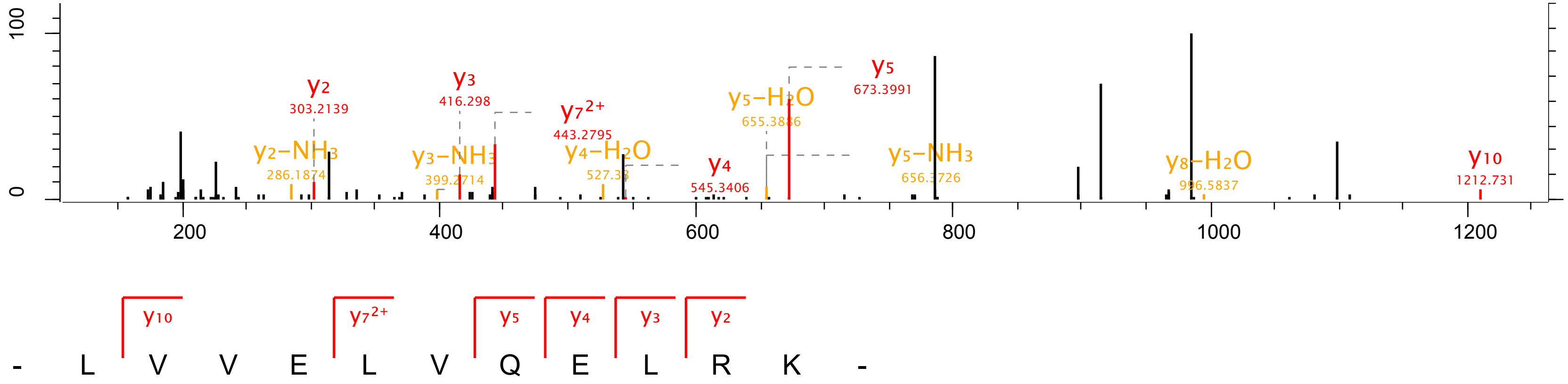

Raw file	Scan	Method	Score	m/z	Gene names
20141014_fract9_dyn_5ul_F1_01_588	29908	TOF; CID	64.45	865.99	KRTCAP2

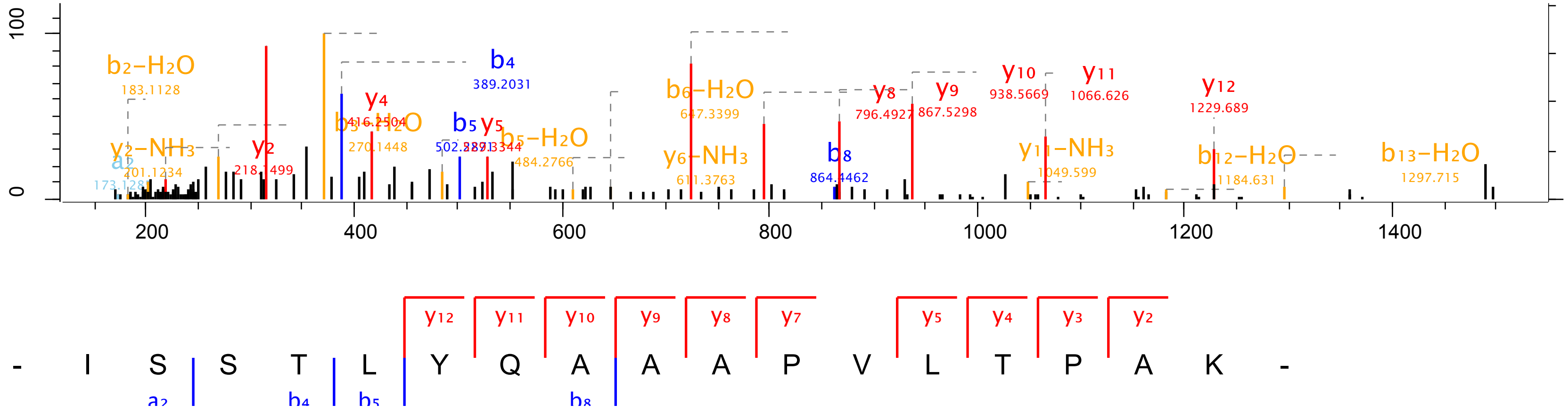

Raw file	Scan	Method	Score	m/z	Gene names
20141014_fract9_dyn_5ul_F1_01_588	30140	TOF; CID	111.95	529.28	SLC9A8

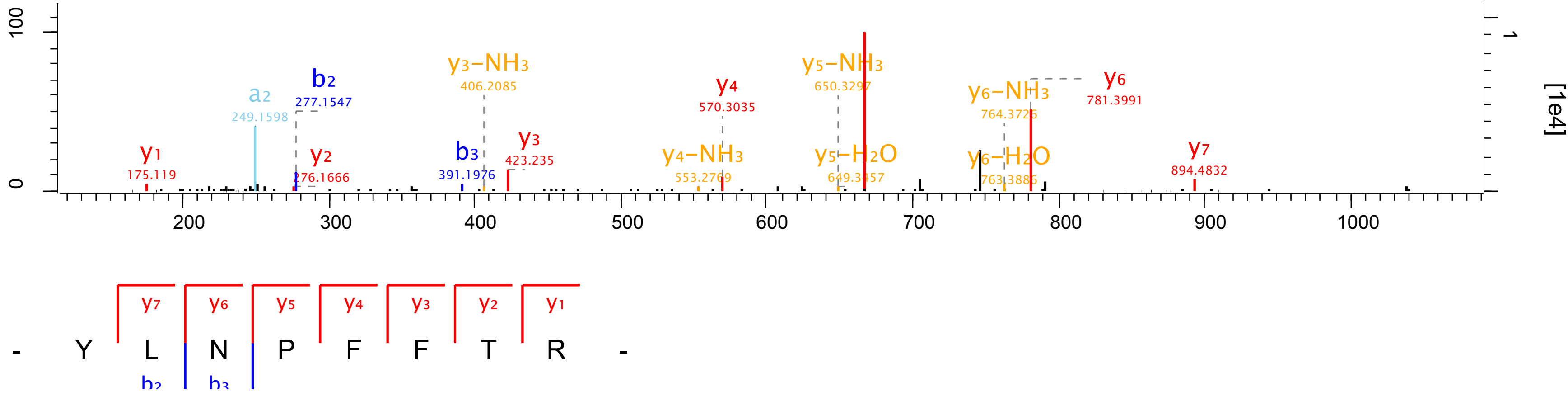

Raw file	Scan	Method	Score	m/z	Gene names
20141014_fract9_dyn_5ul_F1_01_588	30511	TOF; CID	108.43	624.86	RITA1

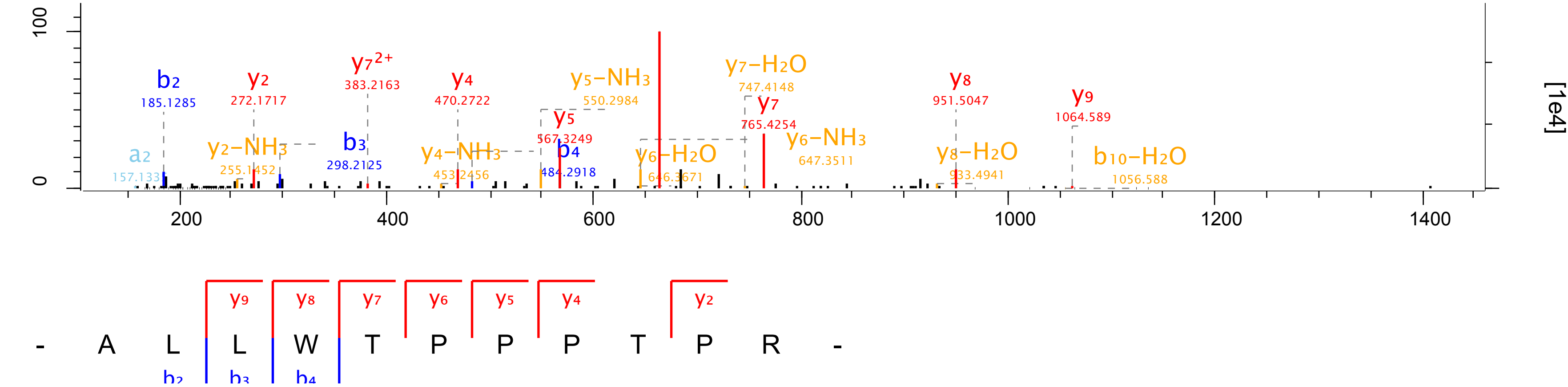

Raw file	Scan	Method	Score	m/z	Gene names
20141014_fract9_dyn_5ul_F1_01_588	31463	TOF; CID	86.8	765.36	CNIH4

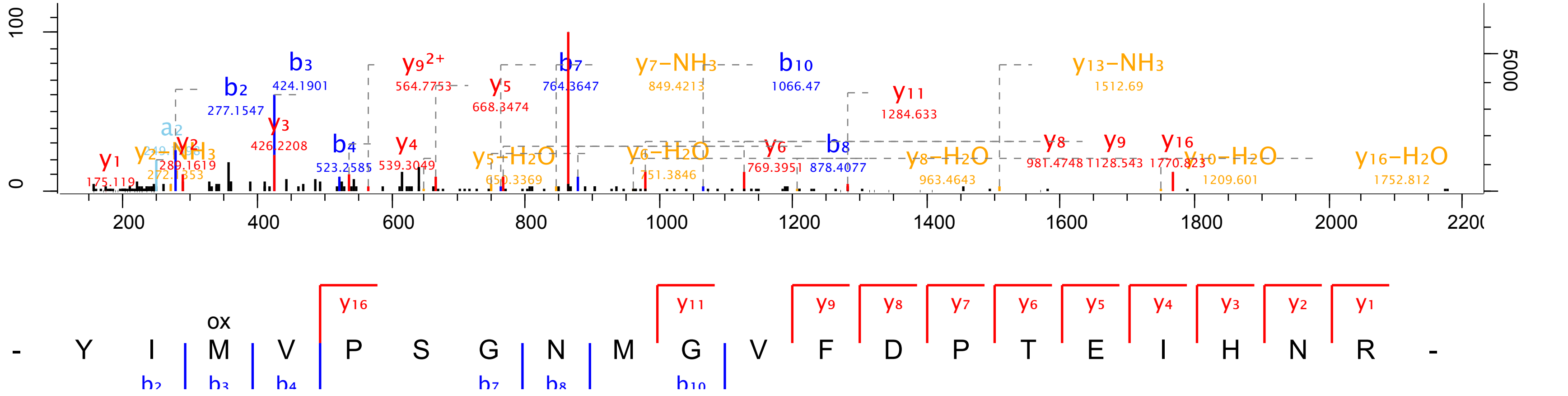

Raw file	Scan	Method	Score	m/z	Gene names
20141014_fract9_dyn_5ul_F1_01_588	31846	TOF; CID	50.9	764.84	ATF4

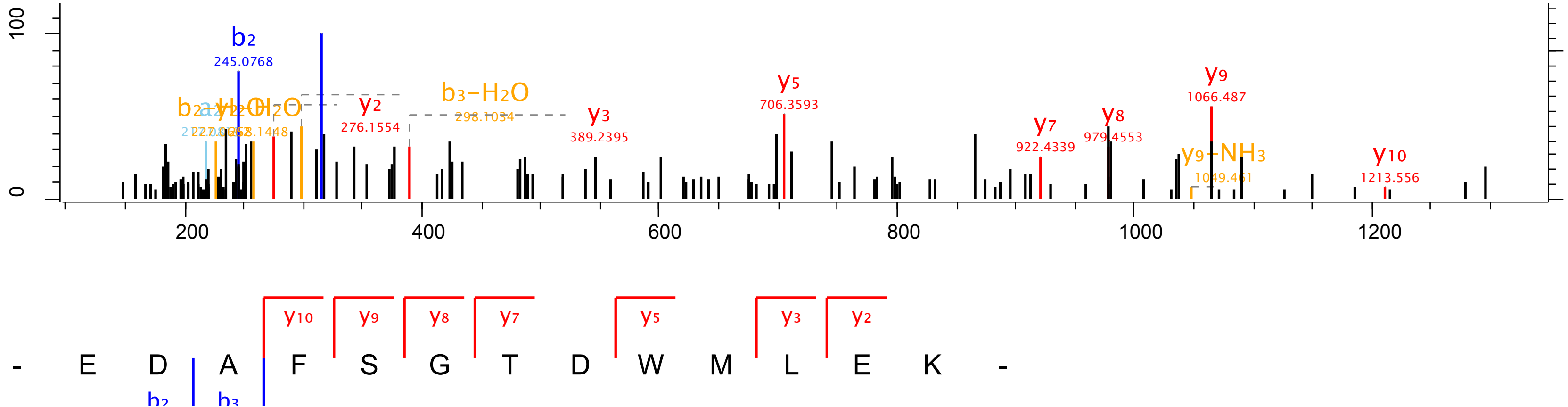

Raw file	Scan	Method	Score	m/z	Gene names
20141014_fract9_dyn_5ul_F1_01_588	32555	TOF; CID	53.45	757.67	EFNA5

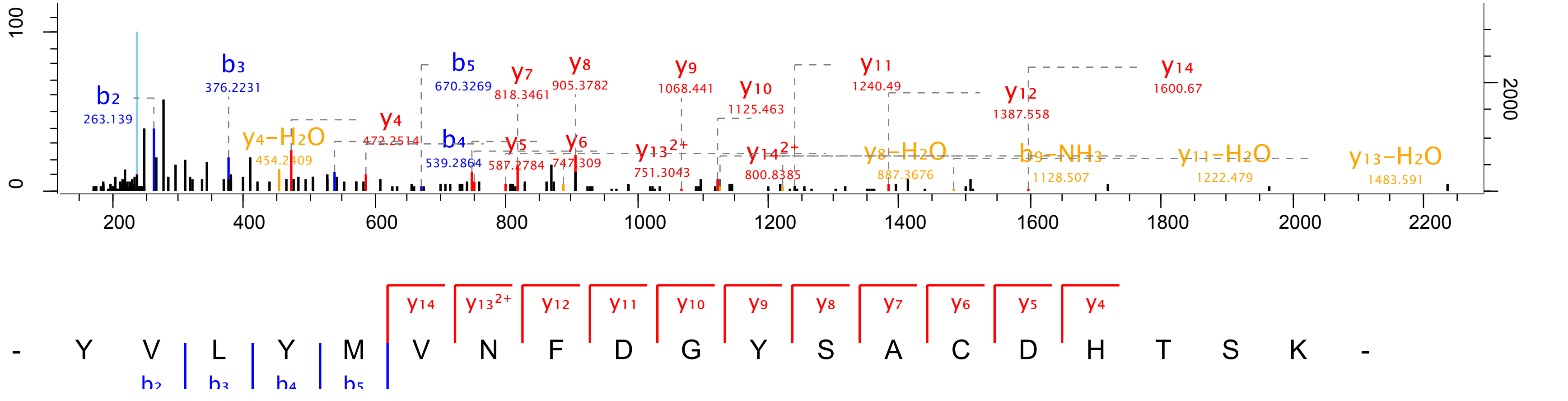

Raw file	Scan	Method	Score	m/z	Gene names
20141014_fract9_dyn_5ul_F1_01_588	36625	TOF; CID	70.26	847.47	MTERF2

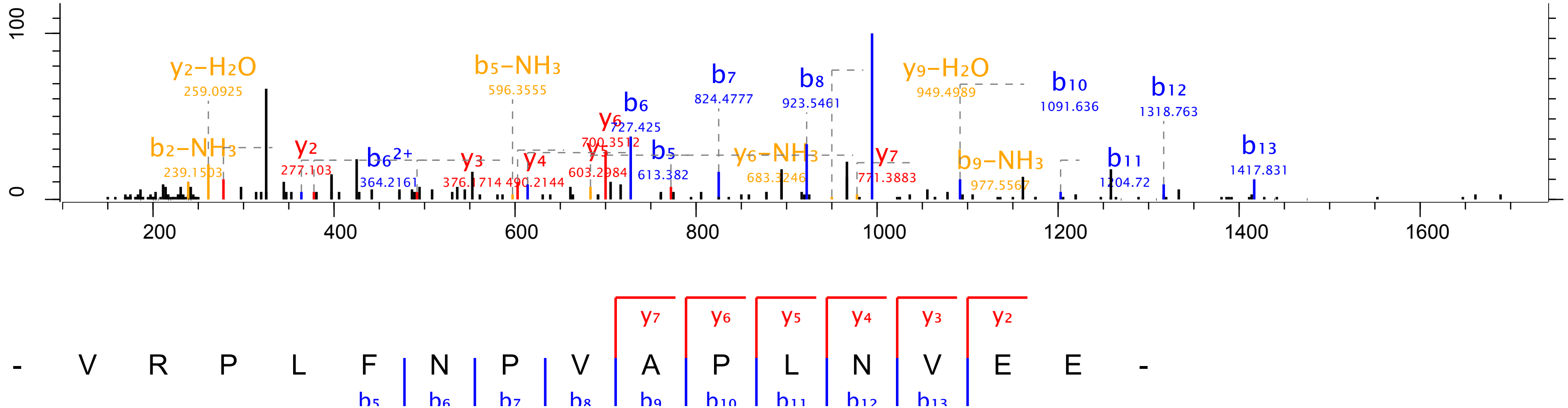

Raw file	Scan	Method	Score	m/z	Gene names
20141014_fract9_dyn_5ul_F1_01_588	37674	TOF; CID	57.17	673.35	PLAGL2

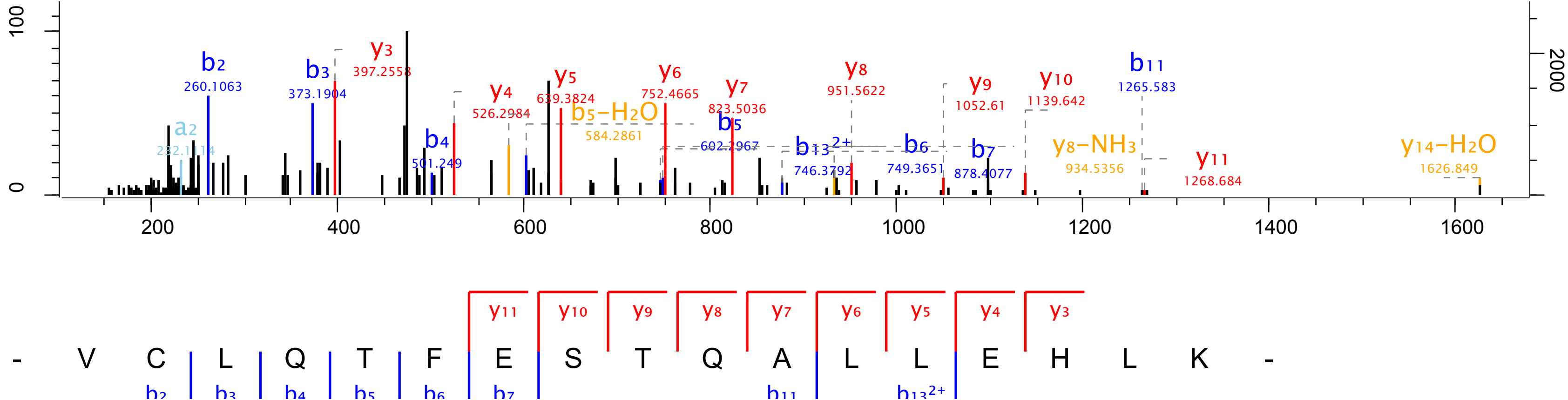

Raw file	Scan	Method	Score	m/z	Gene names
20141014_fract9_dyn_5ul_F1_01_588	38231	TOF; CID	84.94	710.36	KDELR1

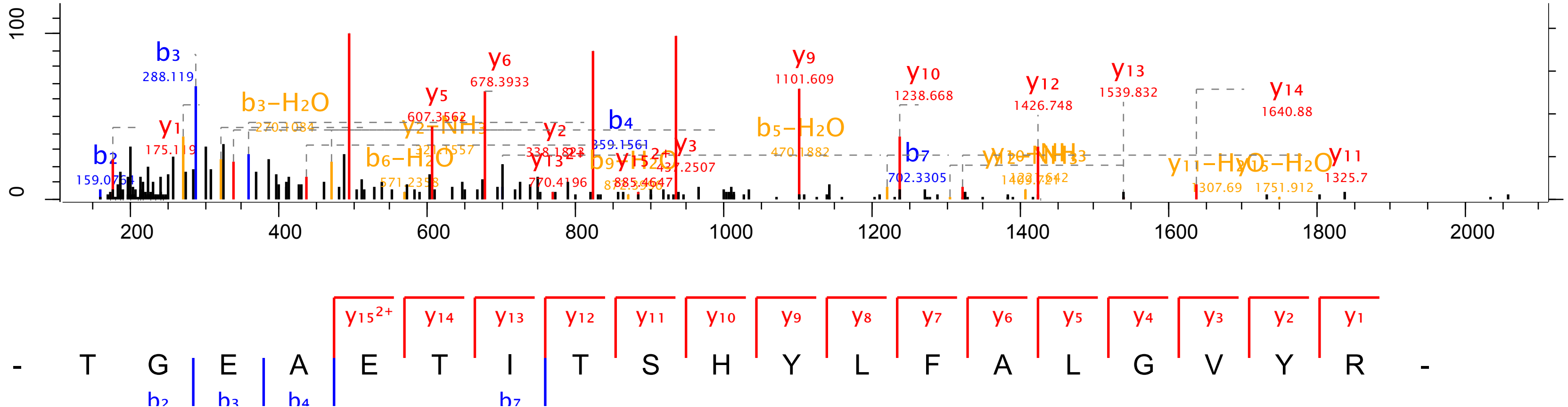

Raw file	Scan	Method	Score	m/z	Gene names
20141014_fract9_dyn_5ul_F1_01_588	38402	TOF; CID	34.04	1150.89	ZAN

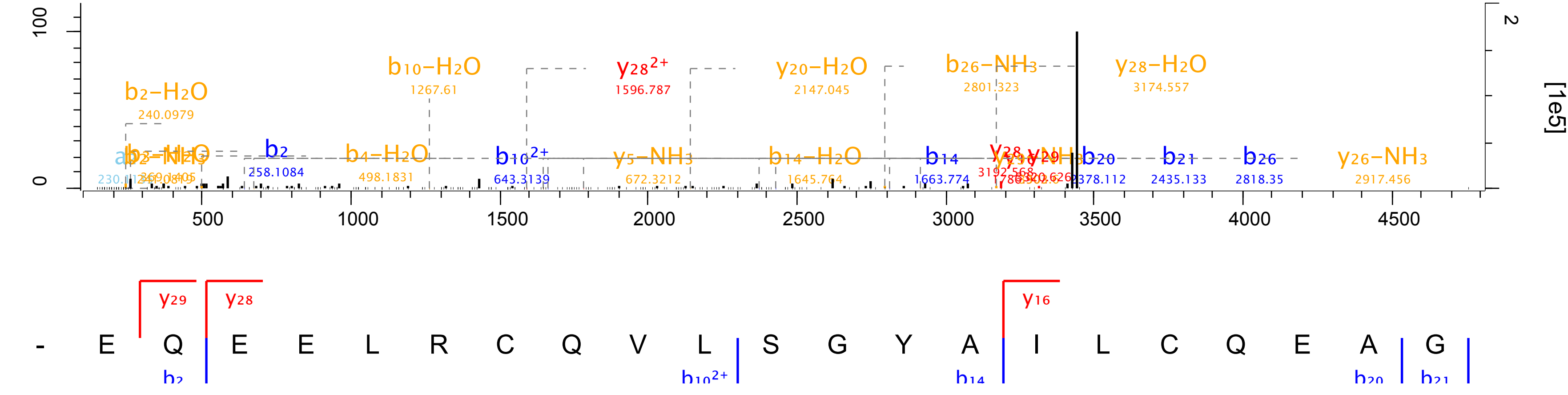

Raw file	Scan	Method	Score	m/z	Gene names
20141014_fract9_dyn_5ul_F1_01_588	38564	TOF; CID	31.29	969.7	CDC42SE1

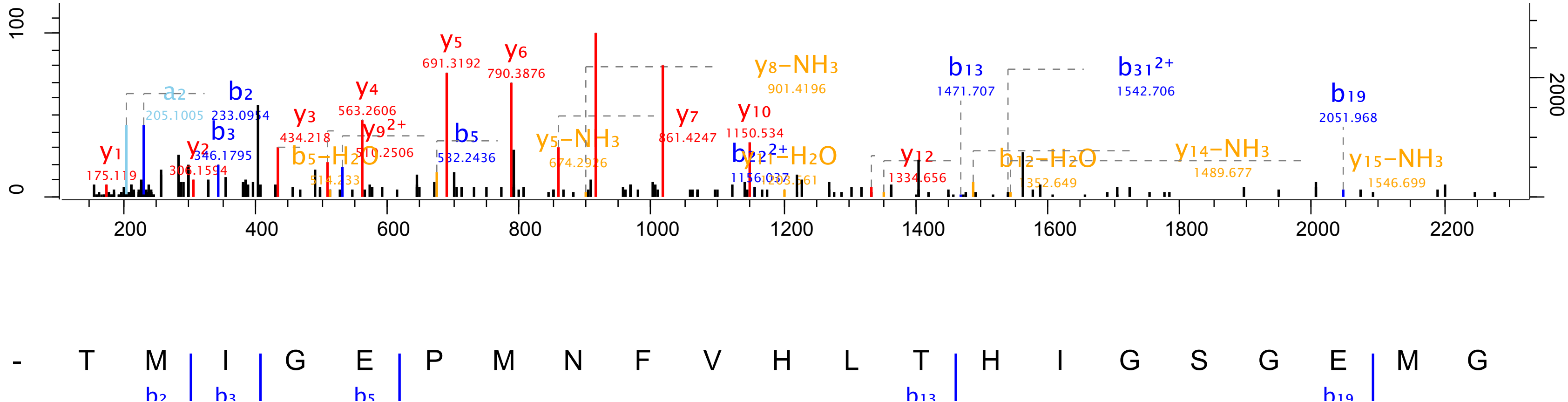

Raw file	Scan	Method	Score	m/z	Gene names
20141014_fract9_dyn_5ul_F1_01_588	39519	TOF; CID	75.02	836.94	ZNF273

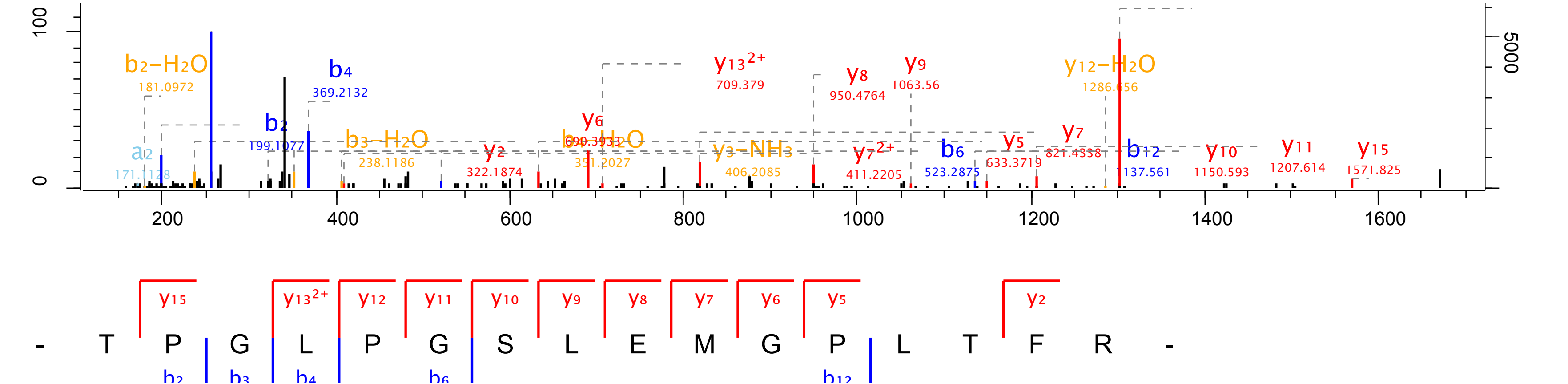

Raw file	Scan	Method	Score	m/z	Gene names
20141014_fract10_dyn_5ul_F2_01_589	4168	TOF; CID	64.1	465.23	CRAT

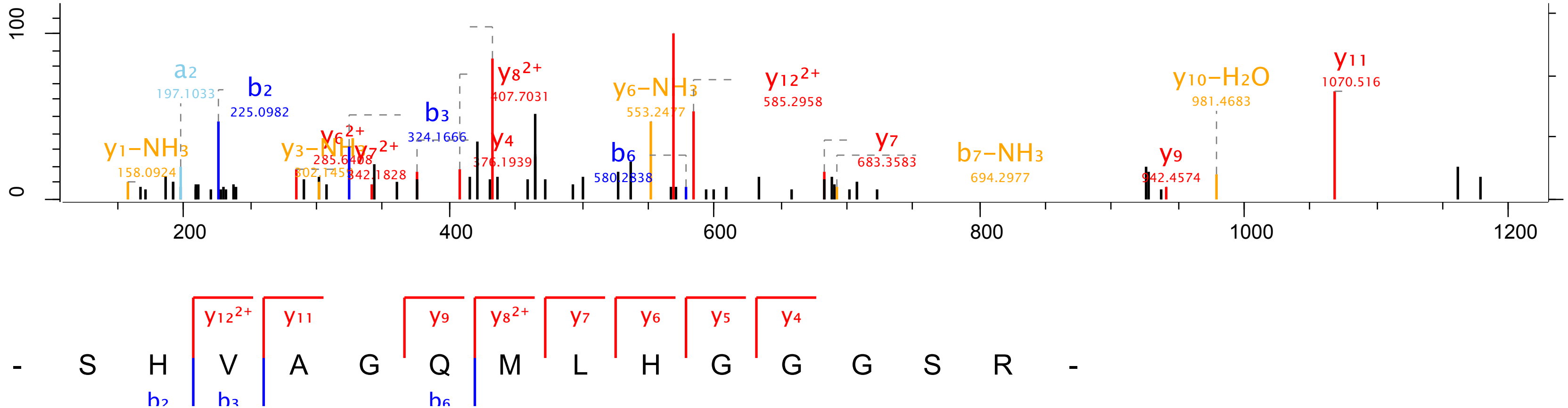

Raw file	Scan	Method	Score	m/z	Gene names
20141014_fract10_dyn_5ul_F2_01_589	11667	TOF; CID	91.09	529.31	RPGRIP1

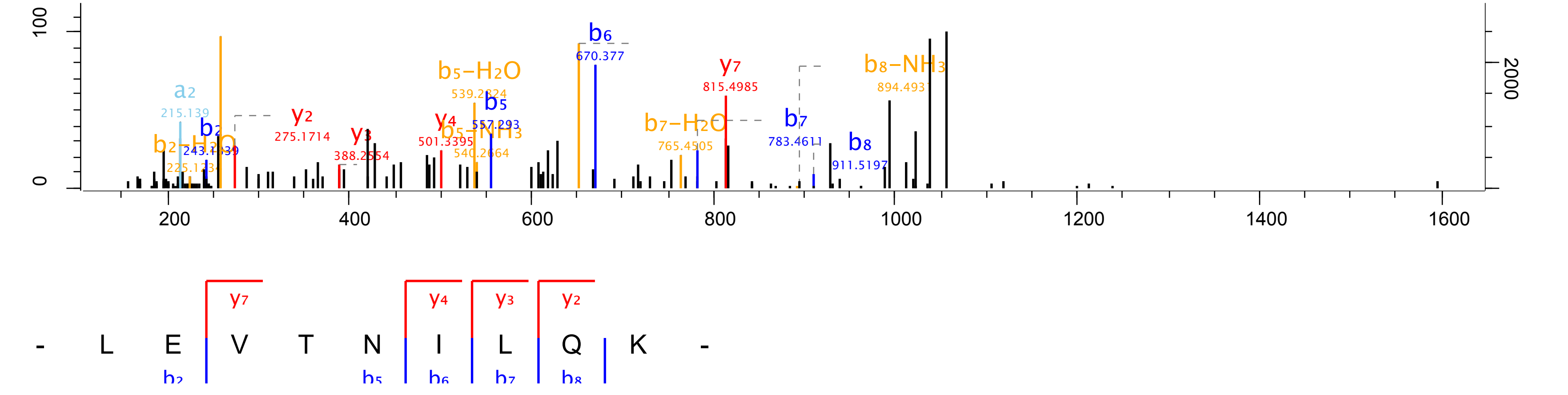

Raw file	Scan	Method	Score	m/z	Gene names
20141014_fract10_dyn_5ul_F2_01_589	16187	TOF; CID	86.46	432.9	NDUFC1

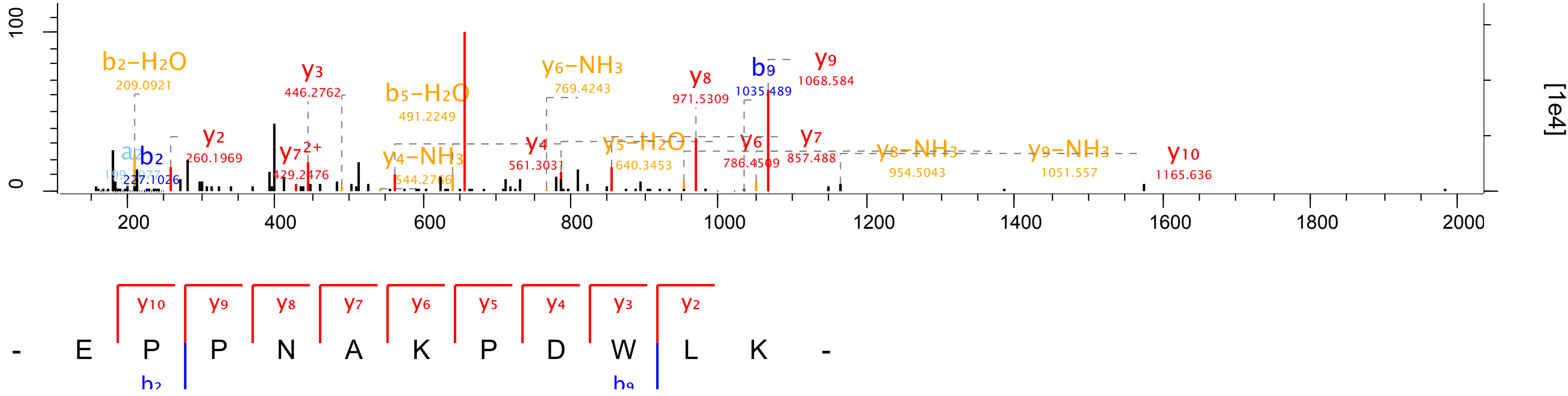

Raw file	Scan	Method	Score	m/z	Gene names
20141014_fract10_dyn_5ul_F2_01_589	19145	TOF; CID	75.82	421.23	GNG11

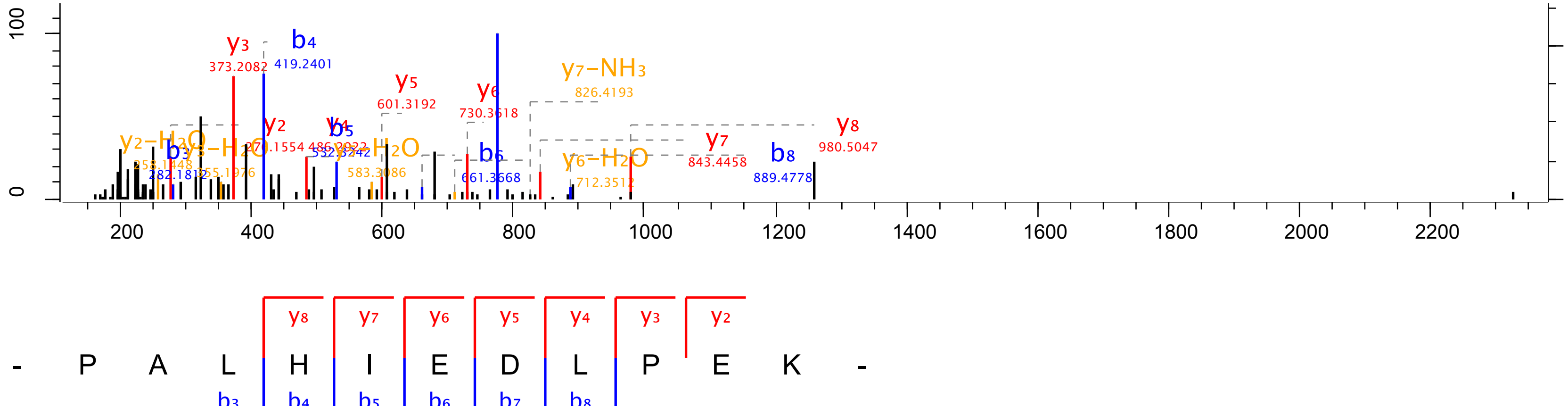

Raw file	Scan	Method	Score	m/z	Gene names
20141014_fract10_dyn_5ul_F2_01_589	22968	TOF; CID	91.7	688.83	SMIM4

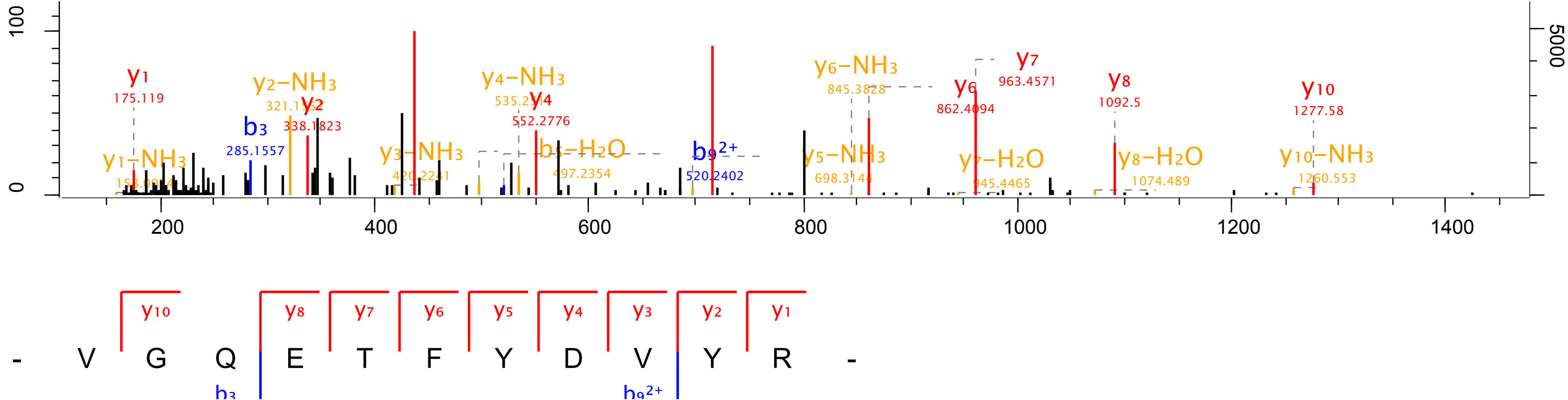

Raw file	Scan	Method	Score	m/z	Gene names
20141014_fract10_dyn_5ul_F2_01_589	23140	TOF; CID	71.08	617.3	TMEM104

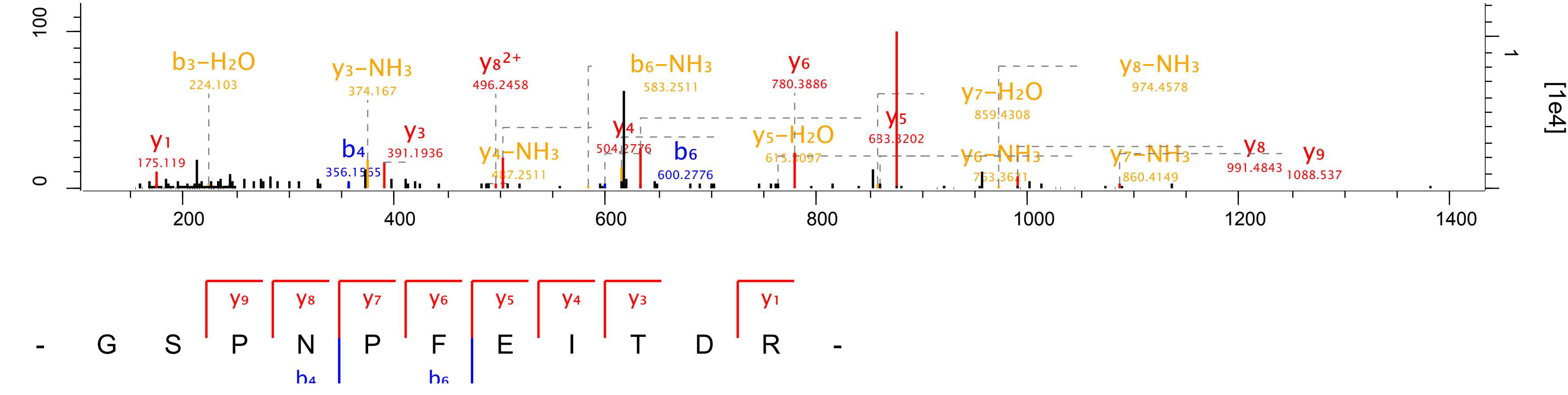

Raw file	Scan	Method	Score	m/z	Gene names
20141014_fract10_dyn_5ul_F2_01_589	26607	TOF; CID	74.92	558.32	STC2

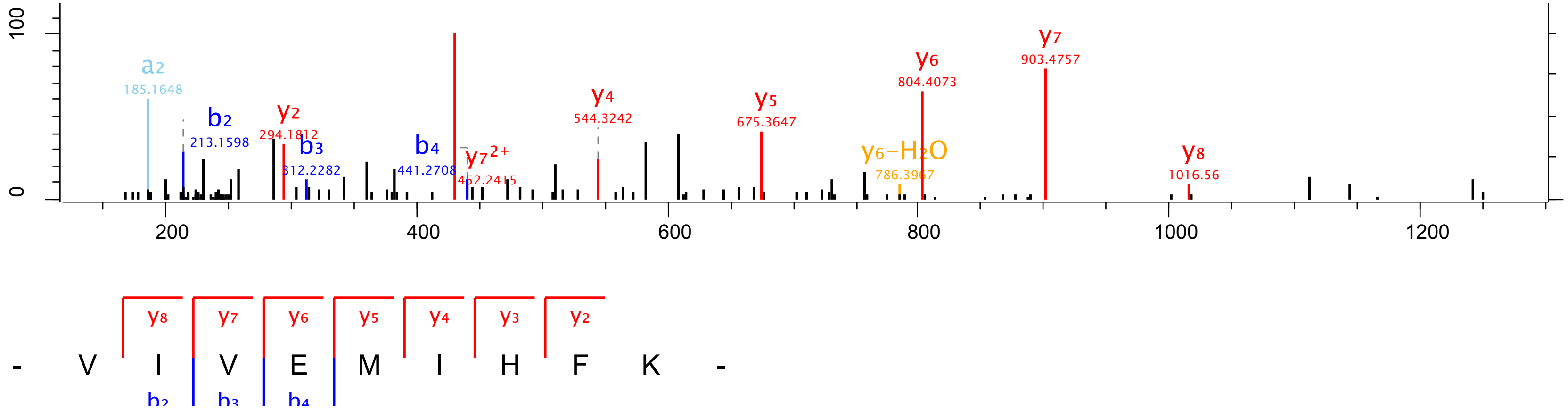

Raw file	Scan	Method	Score	m/z	Gene names
20141014_fract10_dyn_5ul_F2_01_589	27475	TOF; CID	80.51	757.92	C9orf91

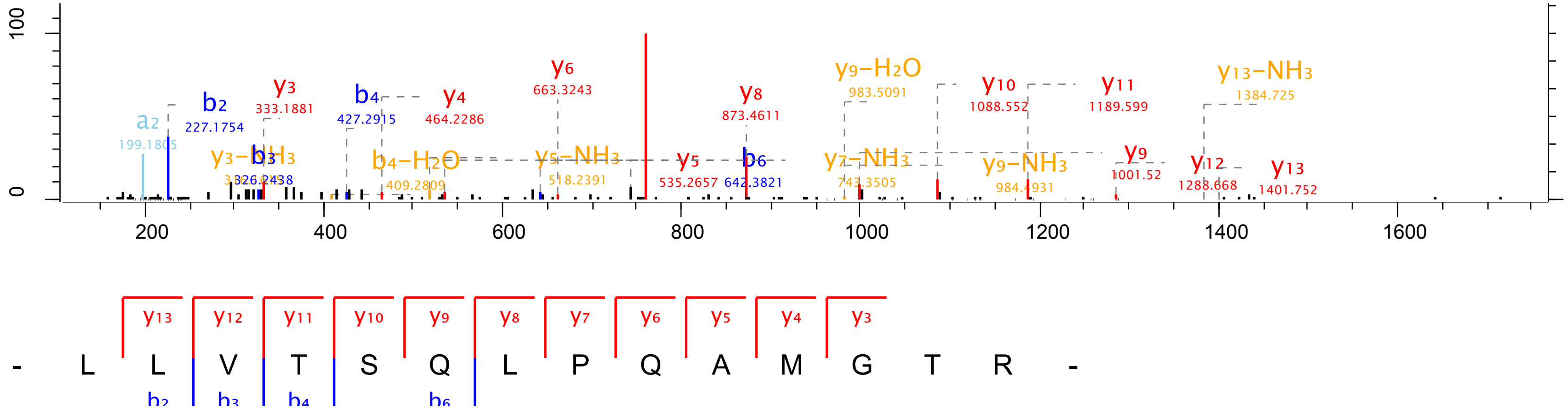

Raw file	Scan	Method	Score	m/z	Gene names
20141014_fract10_dyn_5ul_F2_01_589	27642	TOF; CID	118.22	894.45	DYNC1I1

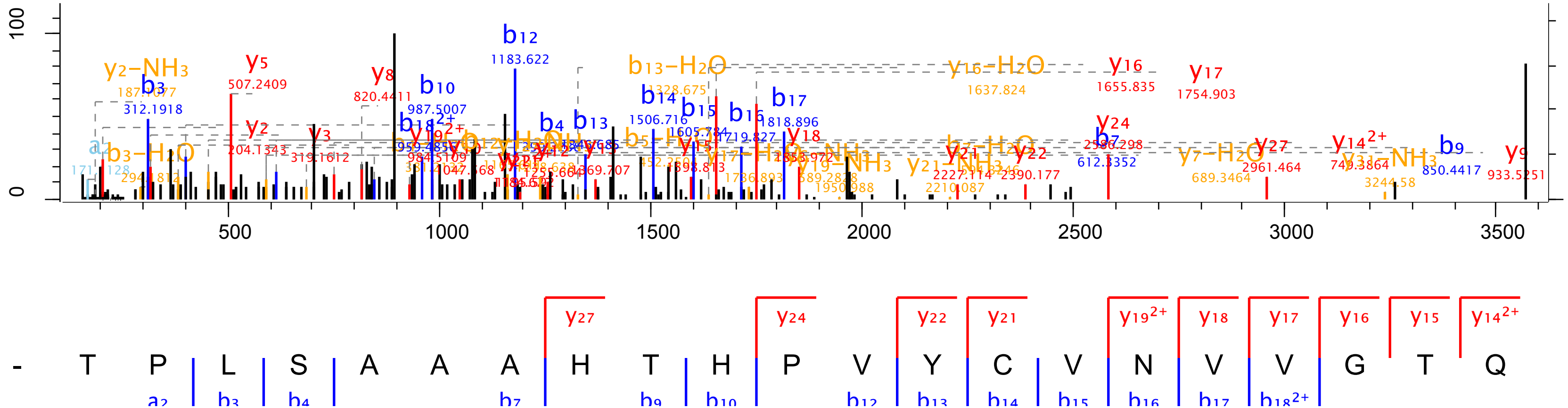

Raw file	Scan	Method	Score	m/z	Gene names
20141014_fract10_dyn_5ul_F2_01_589	32218	TOF; CID	170.77	928.44	MAPK1IP1L

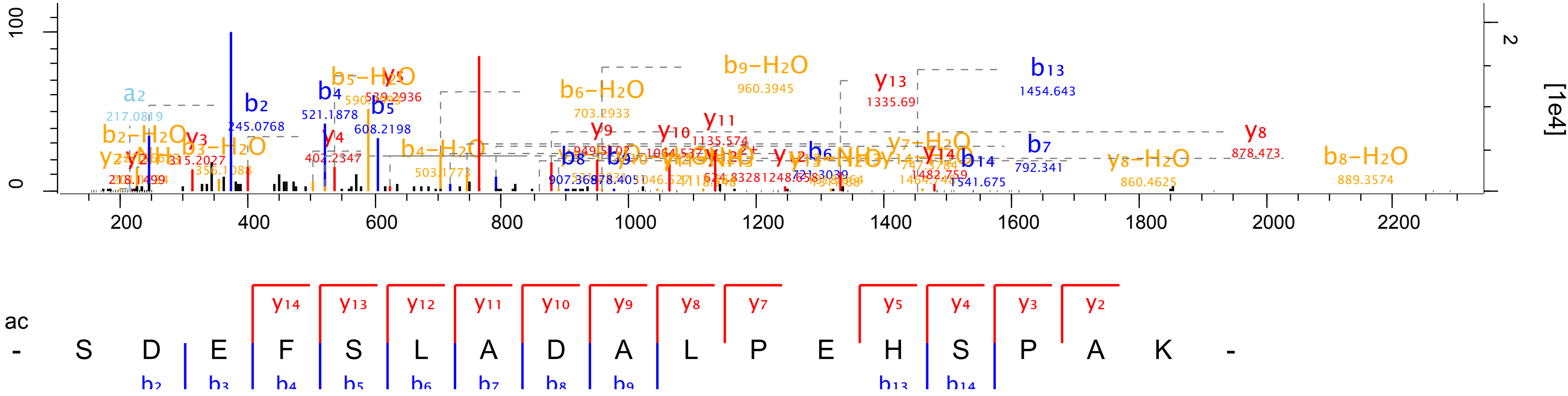

Raw file	Scan	Method	Score	m/z	Gene names
20141014_fract10_dyn_5ul_F2_01_589	33291	TOF; CID	140.48	629.84	GTF2A1;HIST1H4F

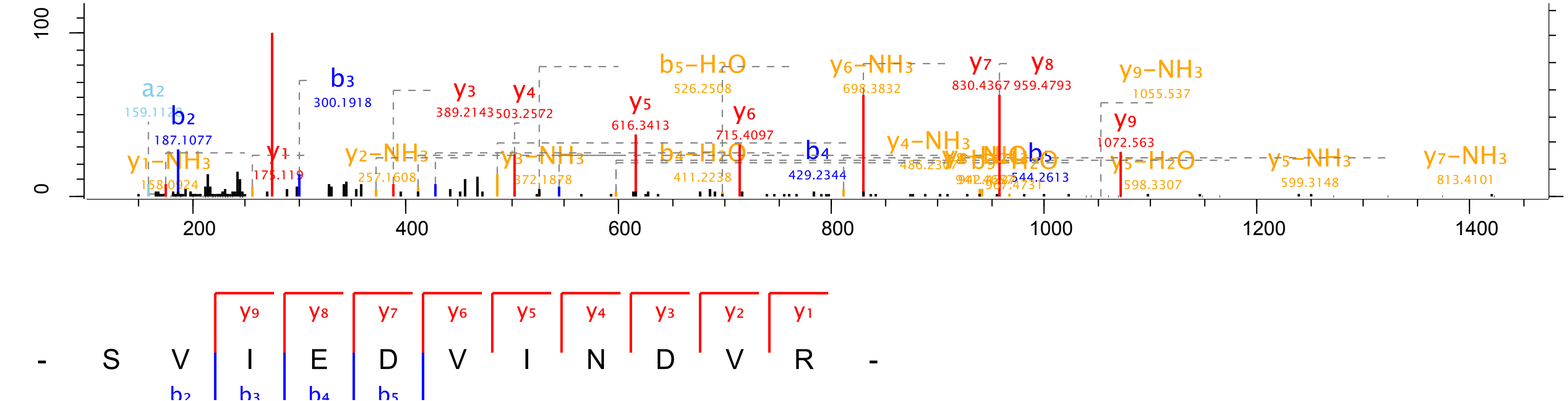

Raw file	Scan	Method	Score	m/z	Gene names
20141014_fract10_dyn_5ul_F2_01_589	35403	TOF; CID	68.24	642.67	SLC25A26

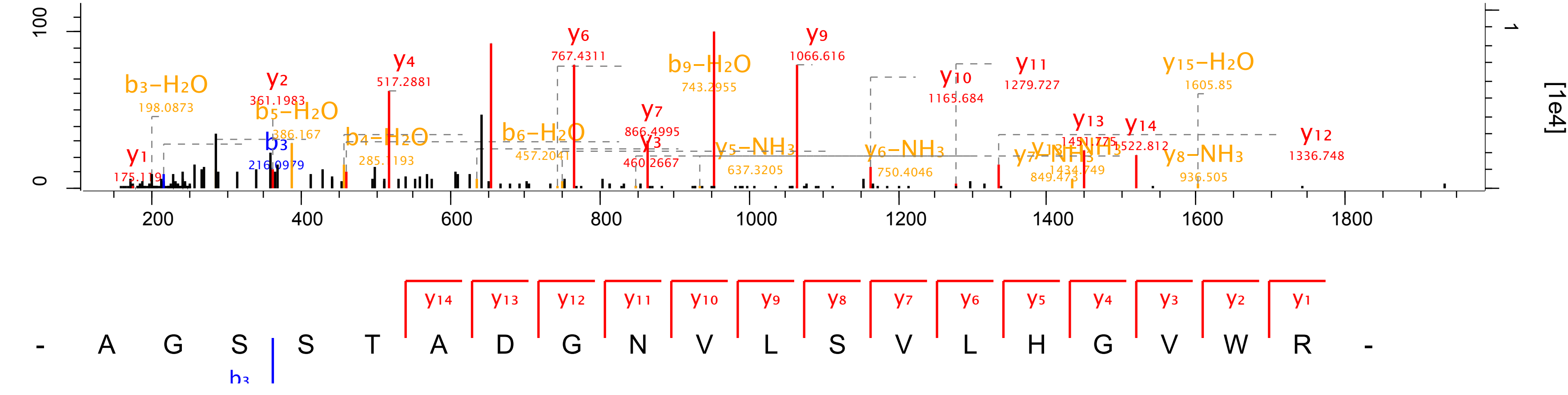

Raw file	Scan	Method	Score	m/z	Gene names
20141014_fract10_dyn_5ul_F2_01_589	37241	TOF; CID	118.37	1351.63	SMAGP

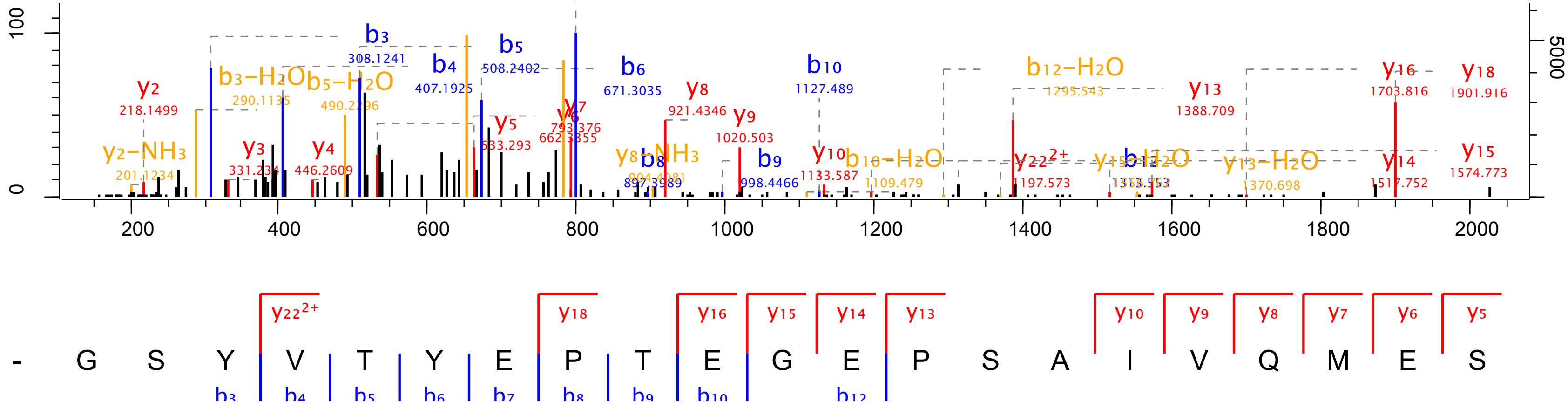

Raw file	Scan	Method	Score	m/z	Gene names
20141014_fract10_dyn_5ul_F2_01_589	40719	TOF; CID	71.67	1098.52	CNIH1

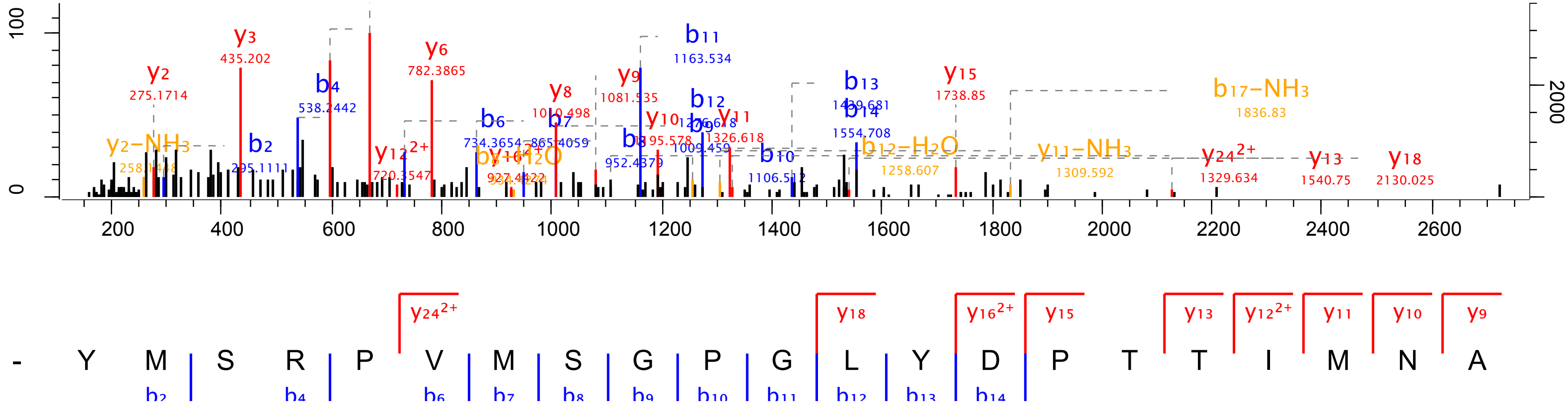

Raw file	Scan	Method	Score	m/z	Gene names
20141014_fract10_dyn_5ul_F2_01_589	41167	TOF; CID	36.55	1214.62	DAZAP2

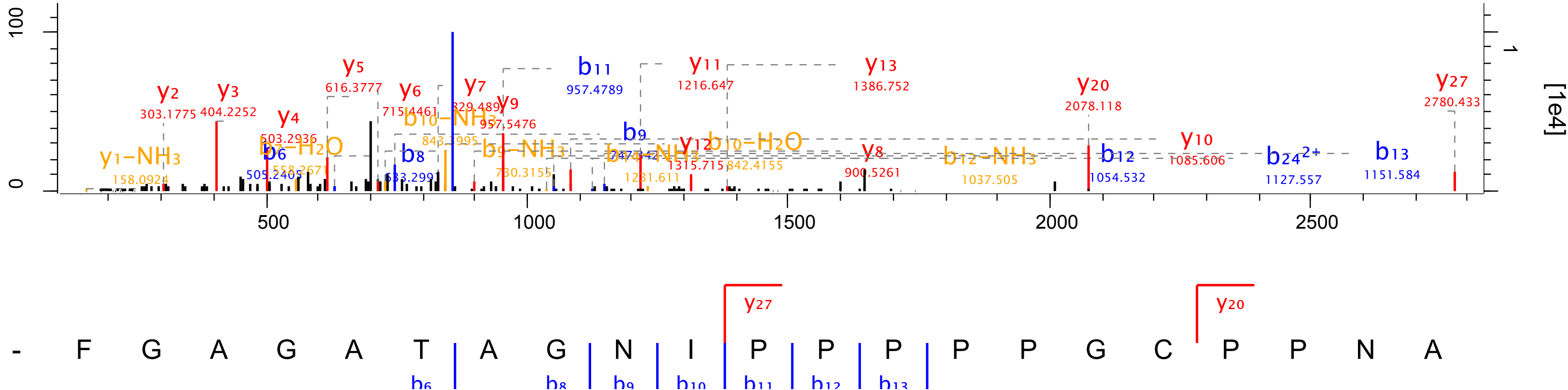

Raw file	Scan	Method	Score	m/z	Gene names
20141014_fract11_dyn_5ul_F3_01_590	6157	TOF; CID	101.56	446.23	NINJ1

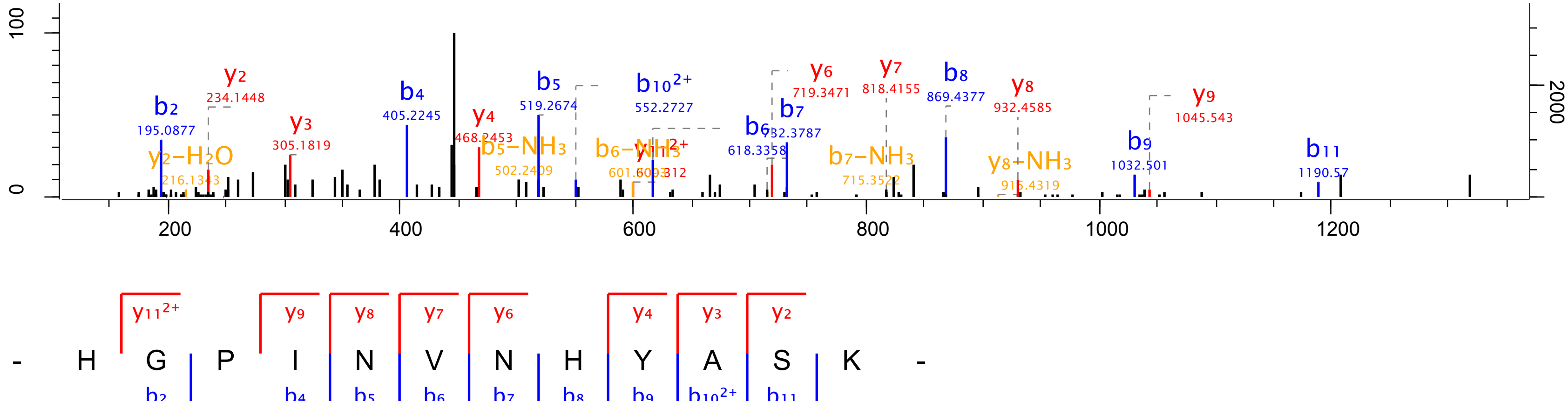

Raw file	Scan	Method	Score	m/z	Gene names
20141014_fract11_dyn_5ul_F3_01_590	10827	TOF; CID	43.04	847.43	C11orf49

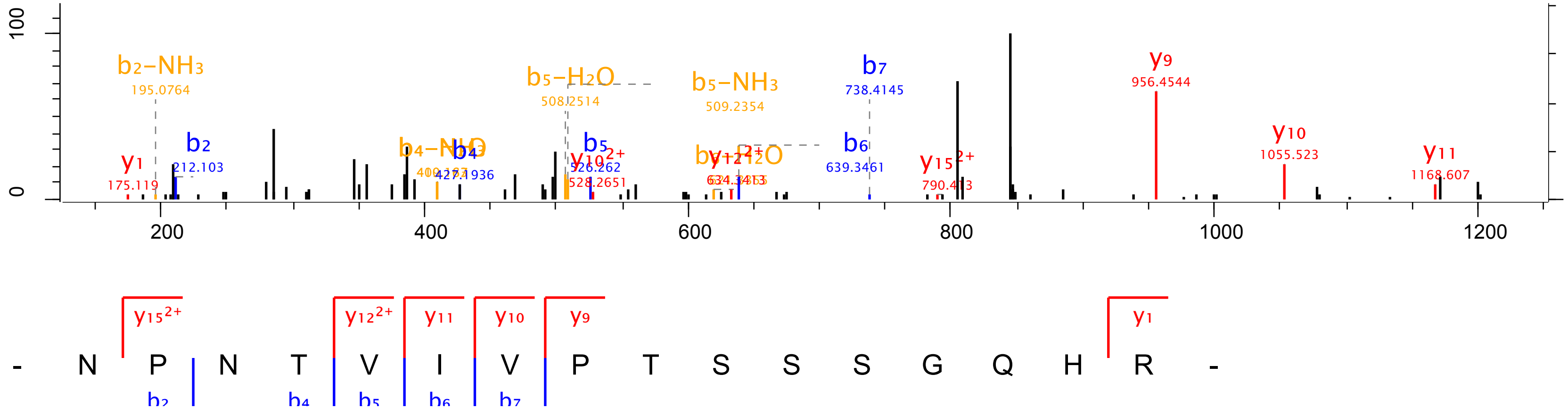

Raw file	Scan	Method	Score	m/z	Gene names
20141014_fract11_dyn_5ul_F3_01_590	11461	TOF; CID	54.34	638.8	PNMA3

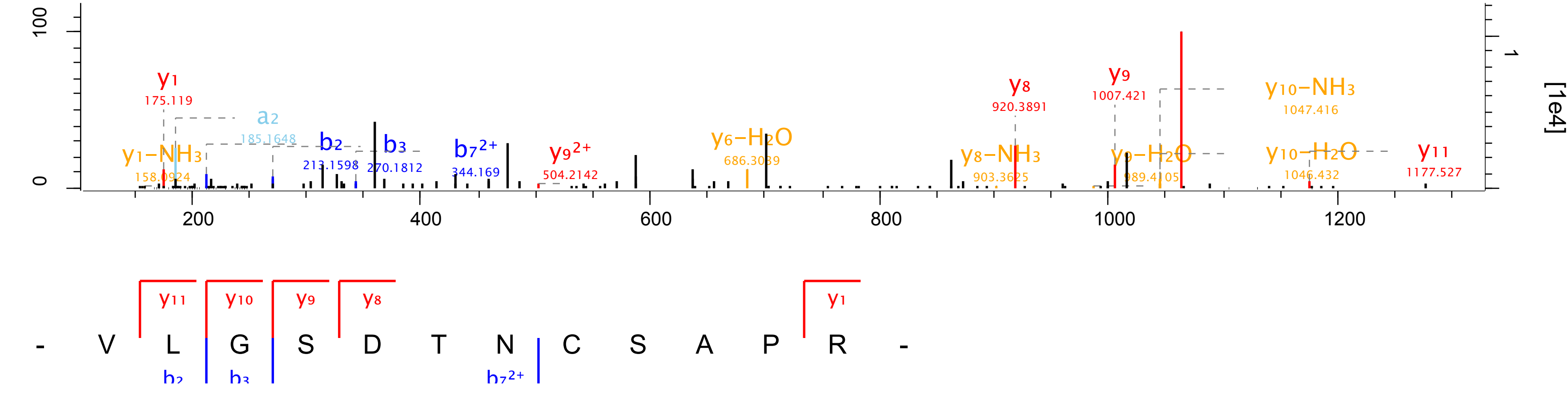

Raw file	Scan	Method	Score	m/z	Gene names
20141014_fract11_dyn_5ul_F3_01_590	12591	TOF; CID	58.89	463.02	ADAMTS15

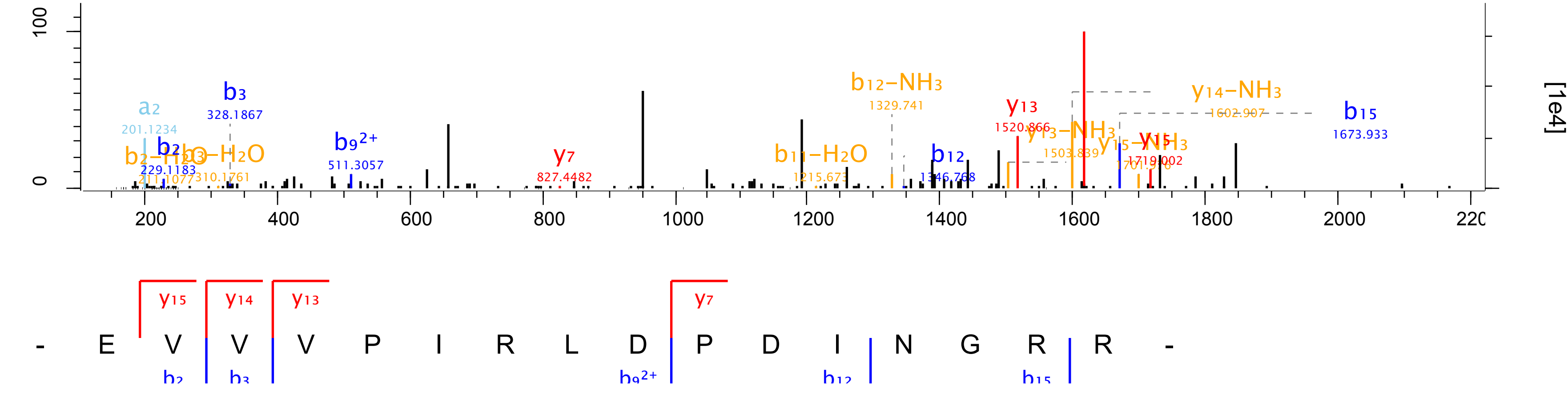

Raw file	Scan	Method	Score	m/z	Gene names
20141014_fract11_dyn_5ul_F3_01_590	14078	TOF; CID	152.34	497.26	AKR1D1

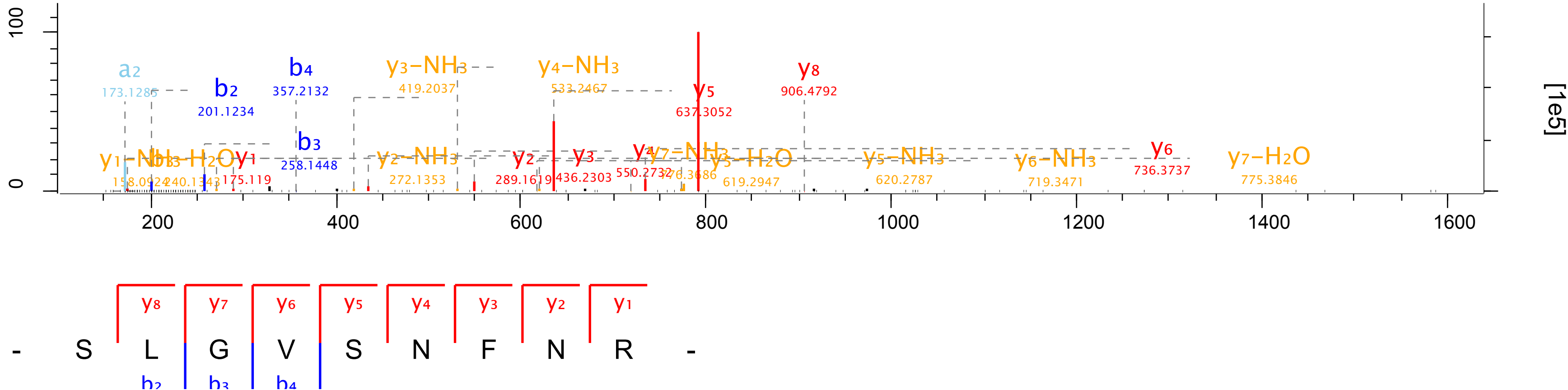

Raw file	Scan	Method	Score	m/z	Gene names
20141014_fract11_dyn_5ul_F3_01_590	15252	TOF; CID	101.43	490.26	ZBED8

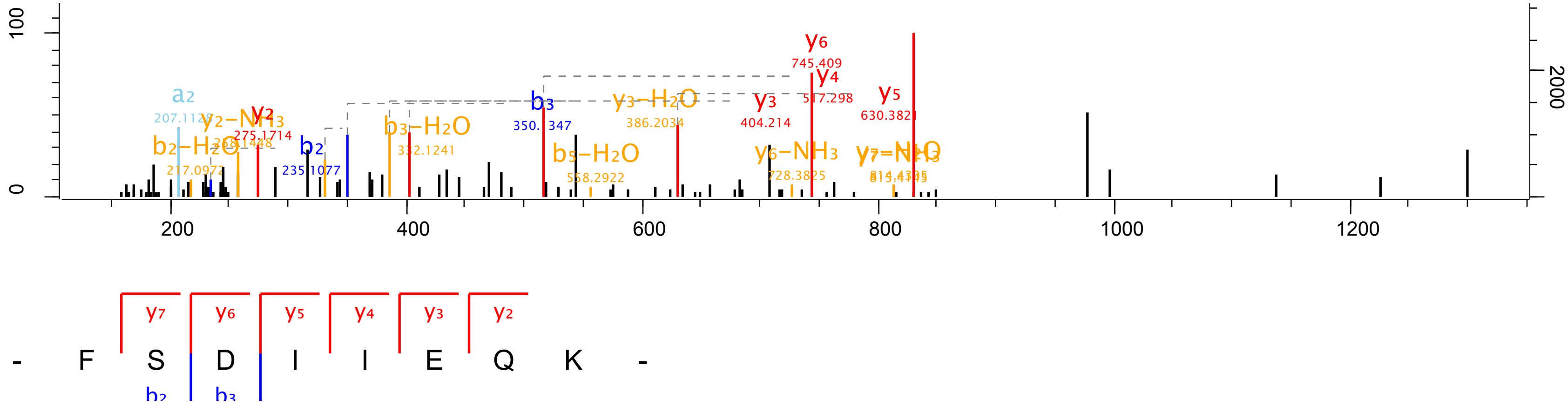

Raw file	Scan	Method	Score	m/z	Gene names
20141014_fract11_dyn_5ul_F3_01_590	15492	TOF; CID	122.33	617.82	SUMO2;SUMO3;SUMO4

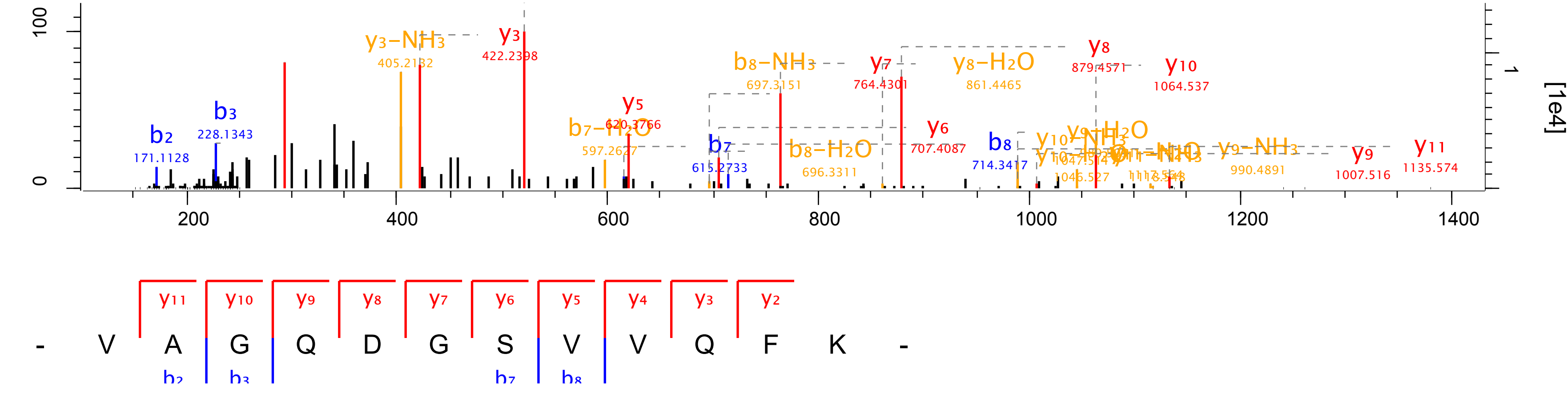

Raw file	Scan	Method	Score	m/z	Gene names
20141014_fract11_dyn_5ul_F3_01_590	17495	TOF; CID	72.29	470.25	CCDC88B

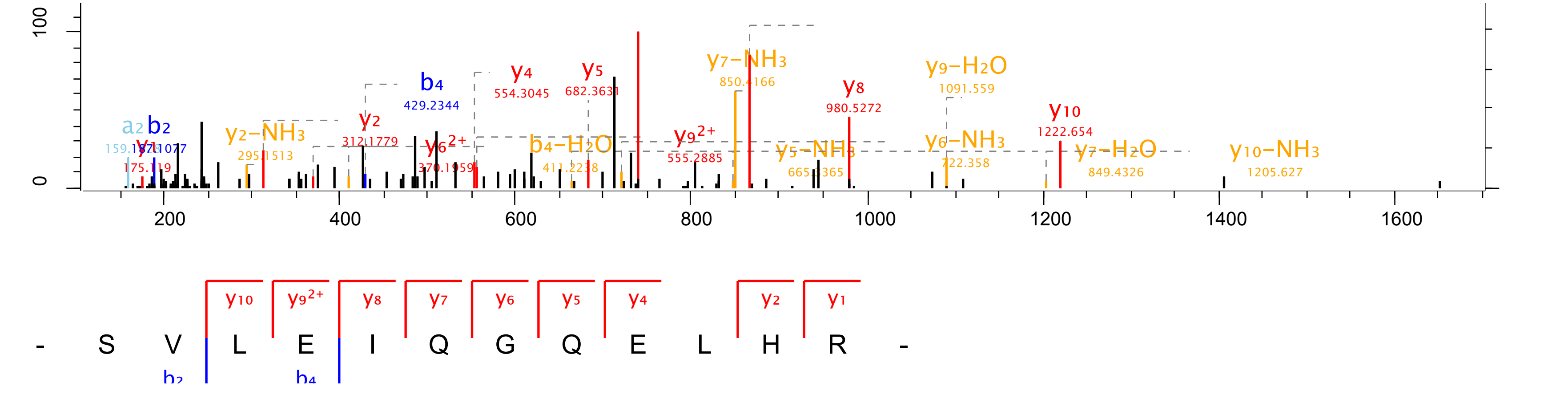

Raw file	Scan	Method	Score	m/z	Gene names
20141014_fract11_dyn_5ul_F3_01_590	17959	TOF; CID	145.82	888.45	NDUFV3

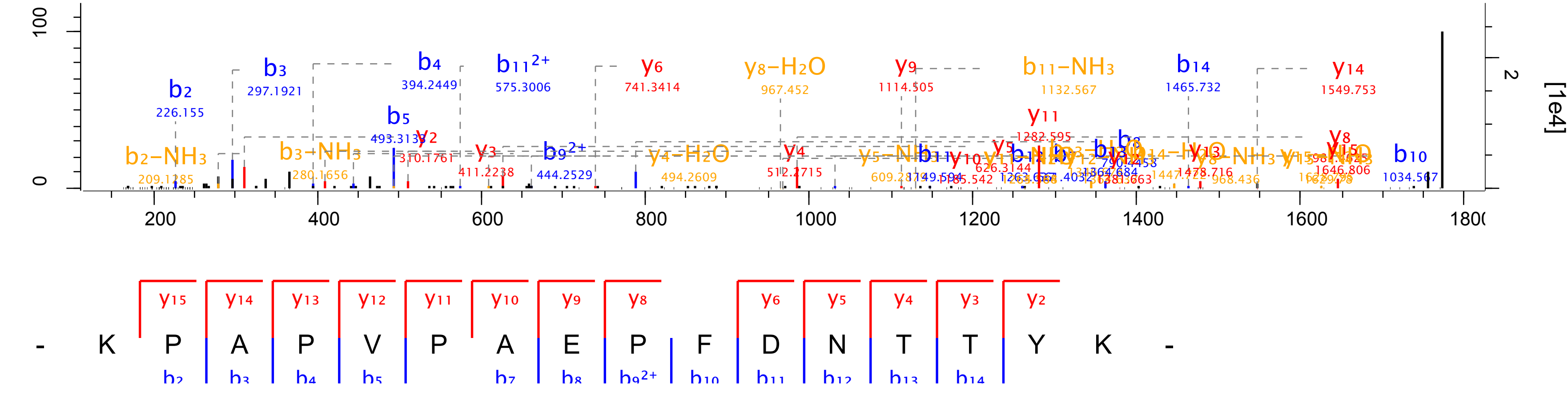

Raw file	Scan	Method	Score	m/z	Gene names
20141014_fract11_dyn_5ul_F3_01_590	19980	TOF; CID	108.47	627.81	SMIM19

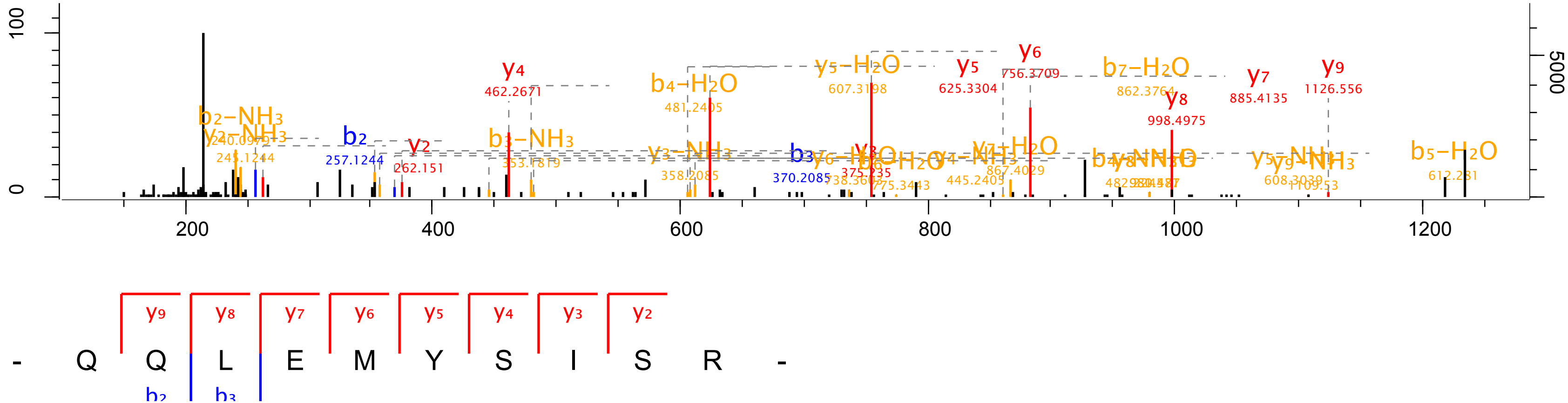

Raw file	Scan	Method	Score	m/z	Gene names
20141014_fract11_dyn_5ul_F3_01_590	21302	TOF; CID	49.92	751	POLR2K

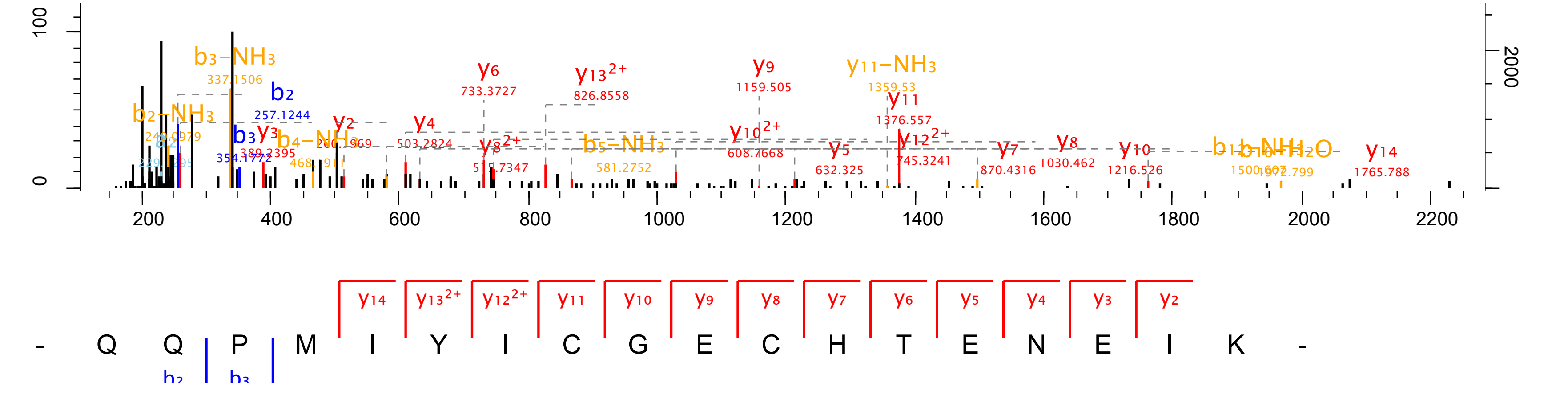

Raw file	Scan	Method	Score	m/z	Gene names
20141014_fract11_dyn_5ul_F3_01_590	24147	TOF; CID	78.51	858.37	MT-ND3

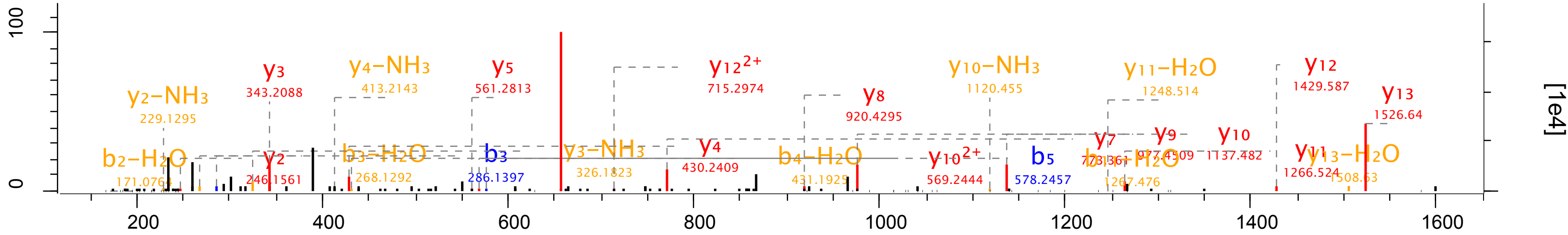

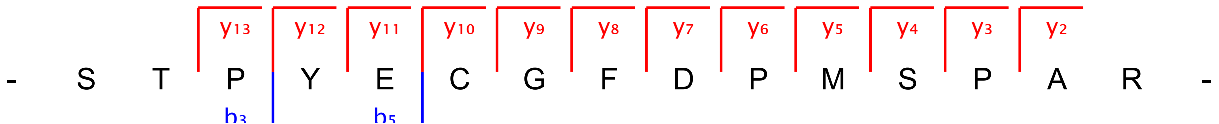

Raw file	Scan	Method	Score	m/z	Gene names
20141014_fract11_dyn_5ul_F3_01_590	24934	TOF; CID	107.32	497.28	MT-ND1

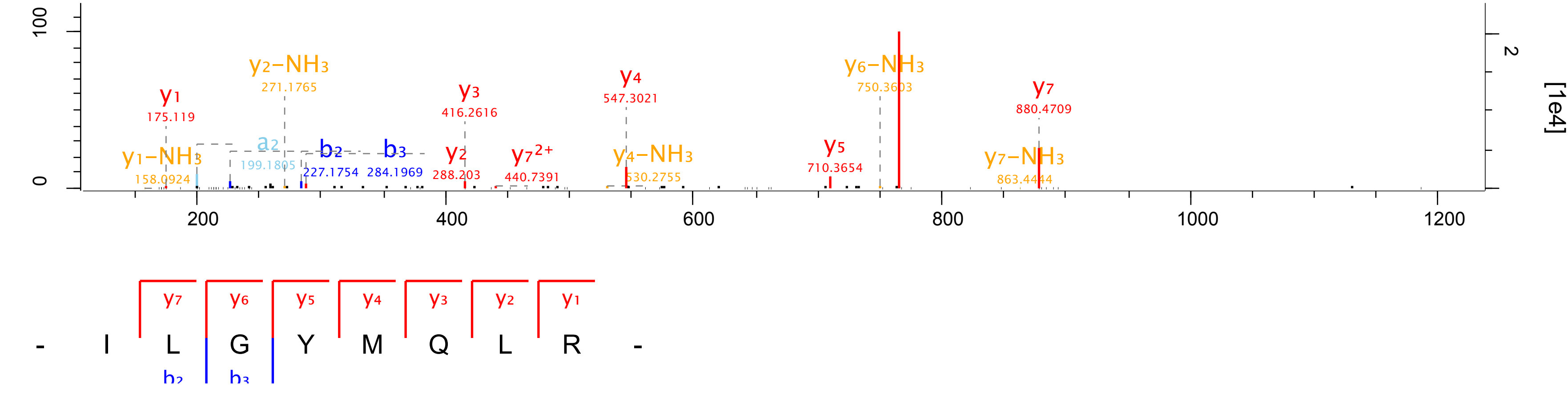

Raw file	Scan	Method	Score	m/z	Gene names
20141014_fract11_dyn_5ul_F3_01_590	25502	TOF; CID	98.1	496.3	BMP6

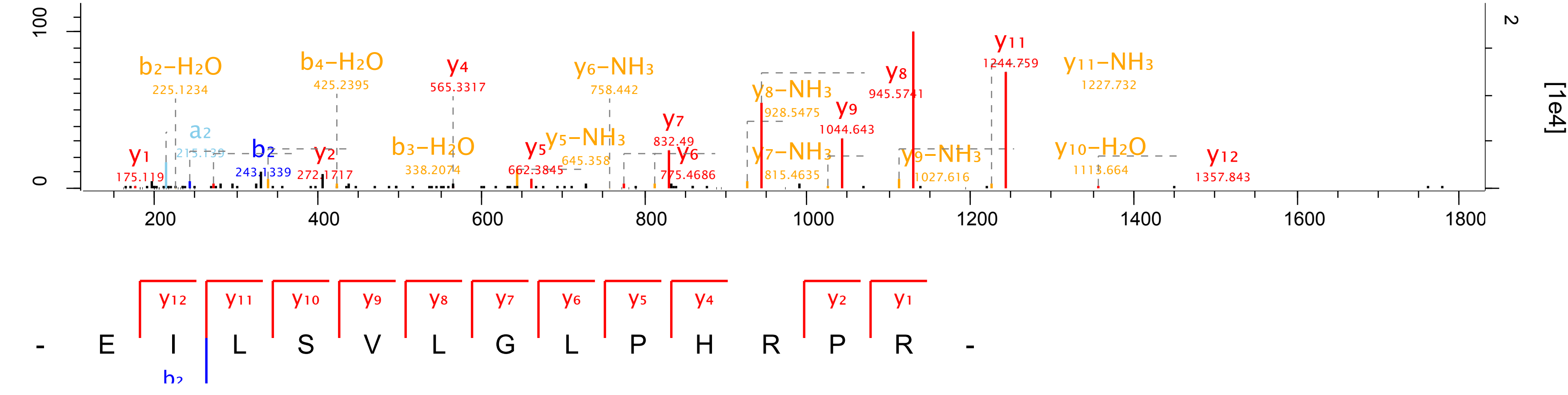

Raw file	Scan	Method	Score	m/z	Gene names
20141014_fract11_dyn_5ul_F3_01_590	27185	TOF; CID	157.86	966.93	CCDC23

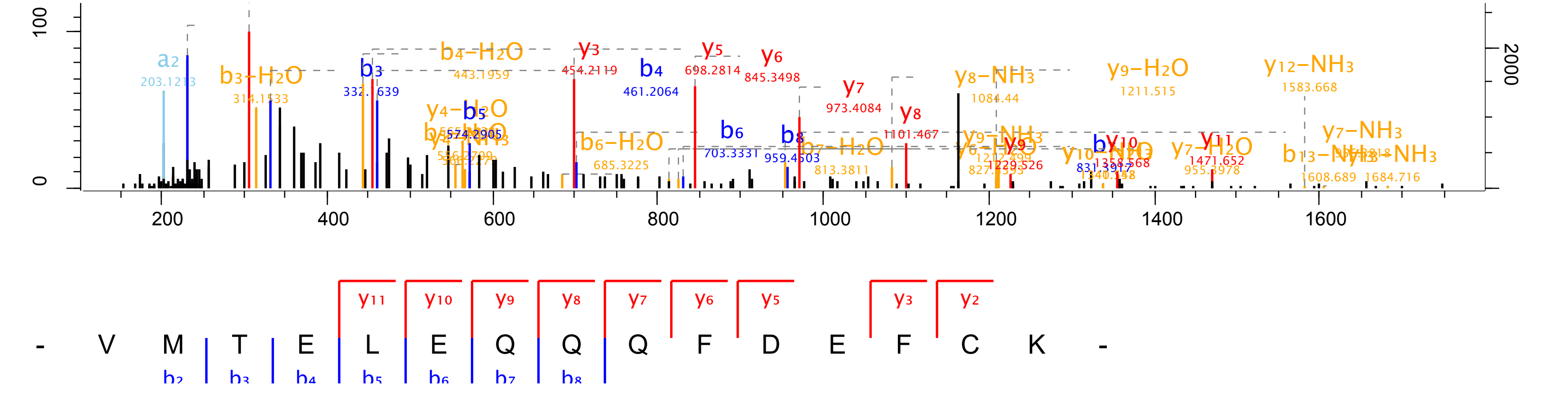

Raw file	Scan	Method	Score	m/z	Gene names
20141014_fract11_dyn_5ul_F3_01_590	28502	TOF; CID	120.46	452.77	MYLK

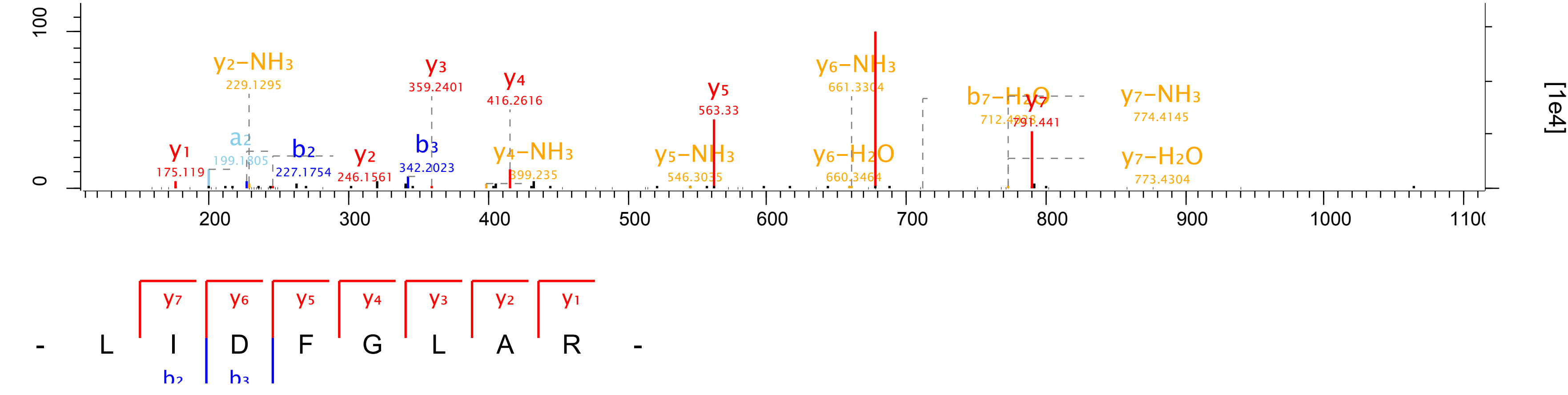

Raw file	Scan	Method	Score	m/z	Gene names
20141014_fract11_dyn_5ul_F3_01_590	30360	TOF; CID	66.92	501.83	PSCA

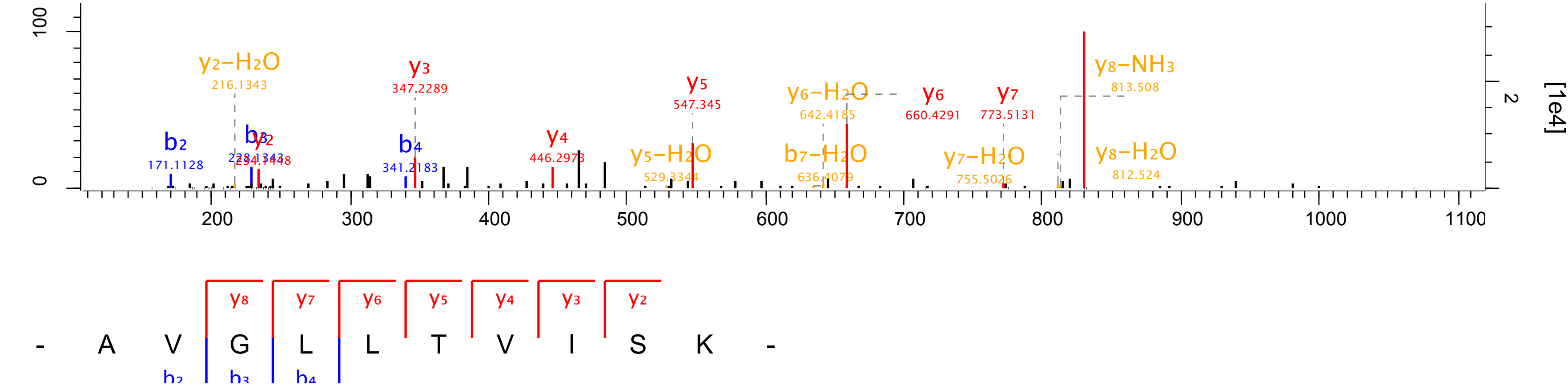

Raw file	Scan	Method	Score	m/z	Gene names
20141014_fract11_dyn_5ul_F3_01_590	30862	TOF; CID	68.54	493.61	TOR1AIP2

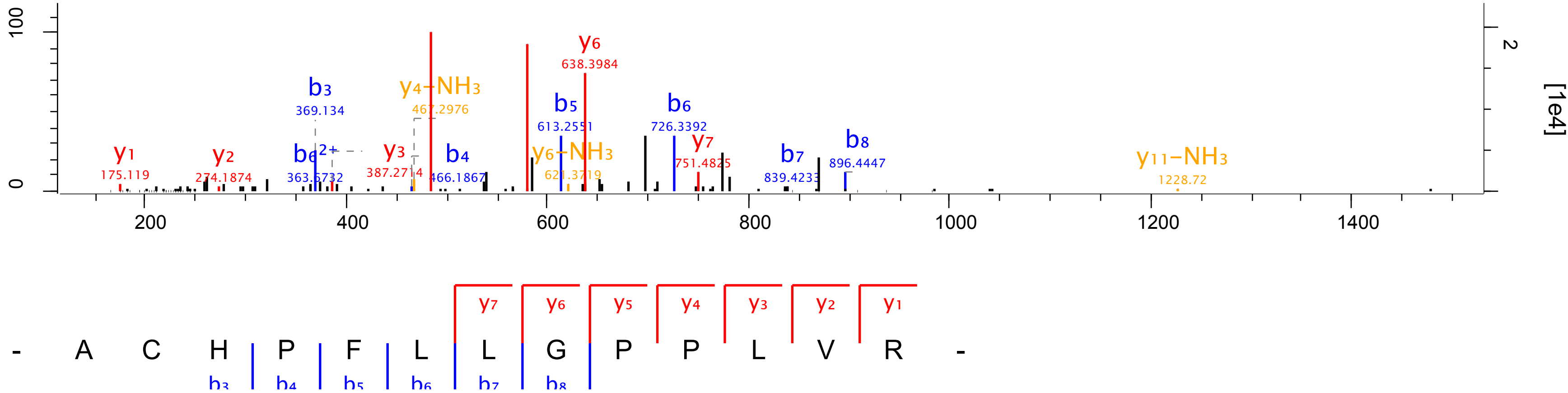

Raw file	Scan	Method	Score	m/z	Gene names
20141014_fract11_dyn_5ul_F3_01_590	31303	TOF; CID	107.09	738.41	TMEM56;TMEM56-RWDD3

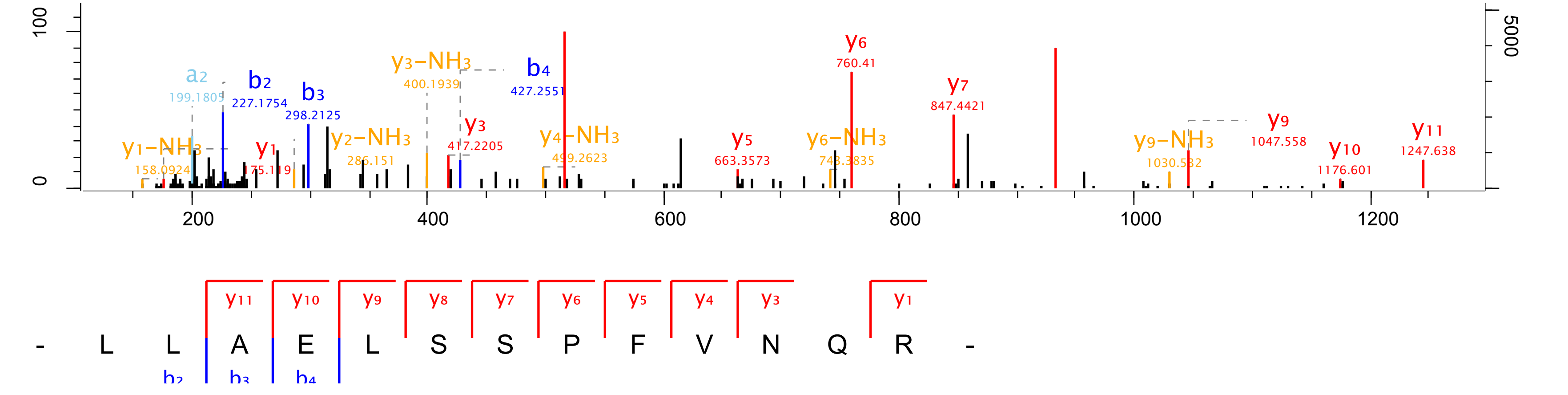

Raw file	Scan	Method	Score	m/z	Gene names
20141014_fract11_dyn_5ul_F3_01_590	35180	TOF; CID	120.46	560.32	KDELR3

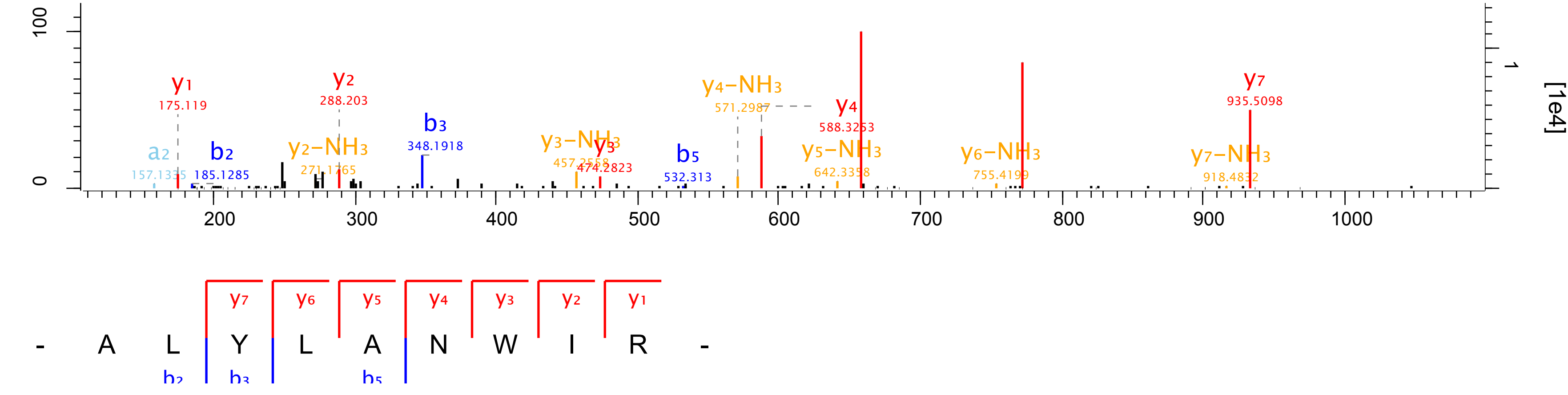

Raw file	Scan	Method	Score	m/z	Gene names
20141014_fract11_dyn_5ul_F3_01_590	36573	TOF; CID	78.44	665.35	DUSP19

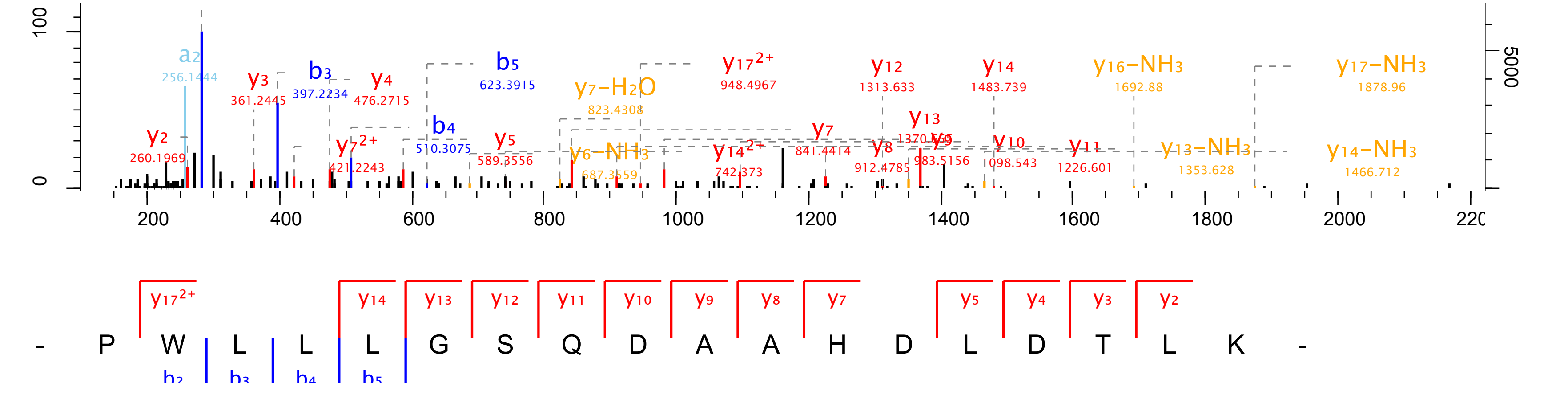

Raw file	Scan	Method	Score	m/z	Gene names
20141014_fract12_dyn_5ul_F4_01_591	5855	TOF; CID	76.06	499.21	ZNF580

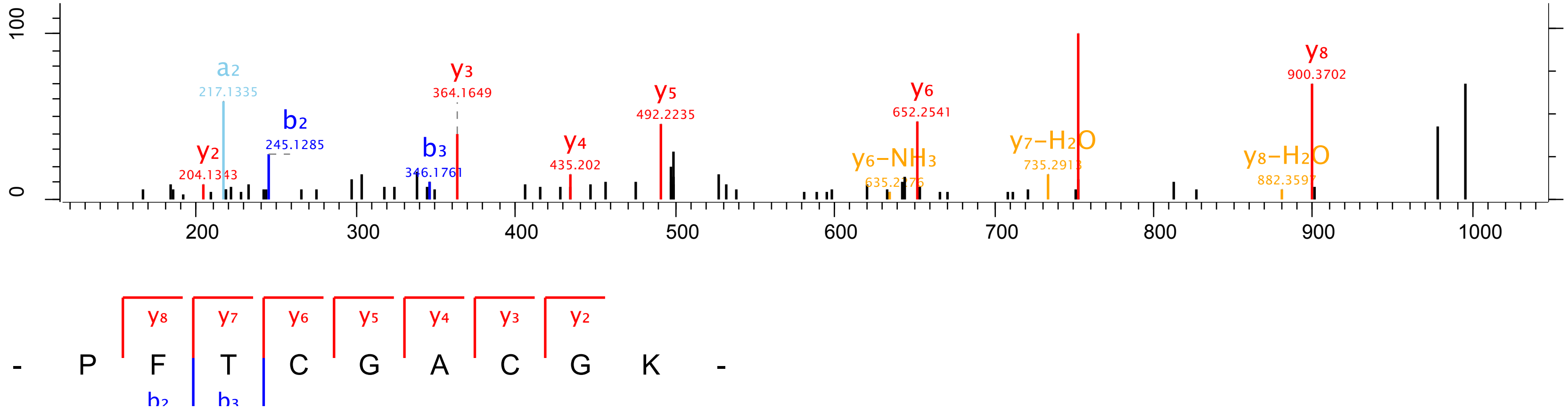

Raw file	Scan	Method	Score	m/z	Gene names
20141014_fract12_dyn_5ul_F4_01_591	7555	TOF; CID	95.36	464.78	TMEM185B;TMEM185A

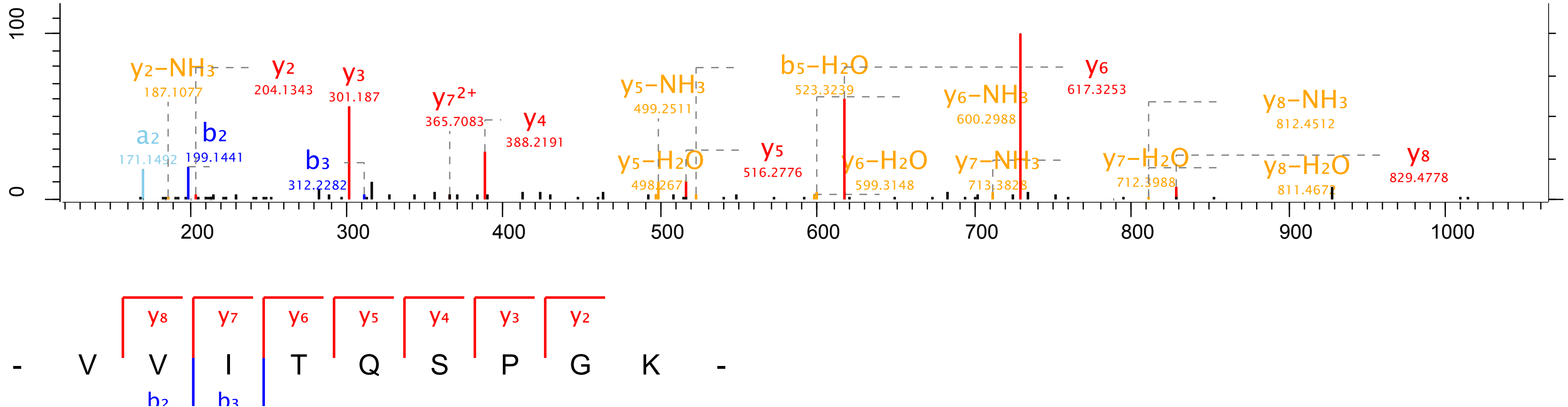

Raw file	Scan	Method	Score	m/z	Gene names
20141014_fract12_dyn_5ul_F4_01_591	7804	TOF; CID	50.79	385.55	TNRC6C

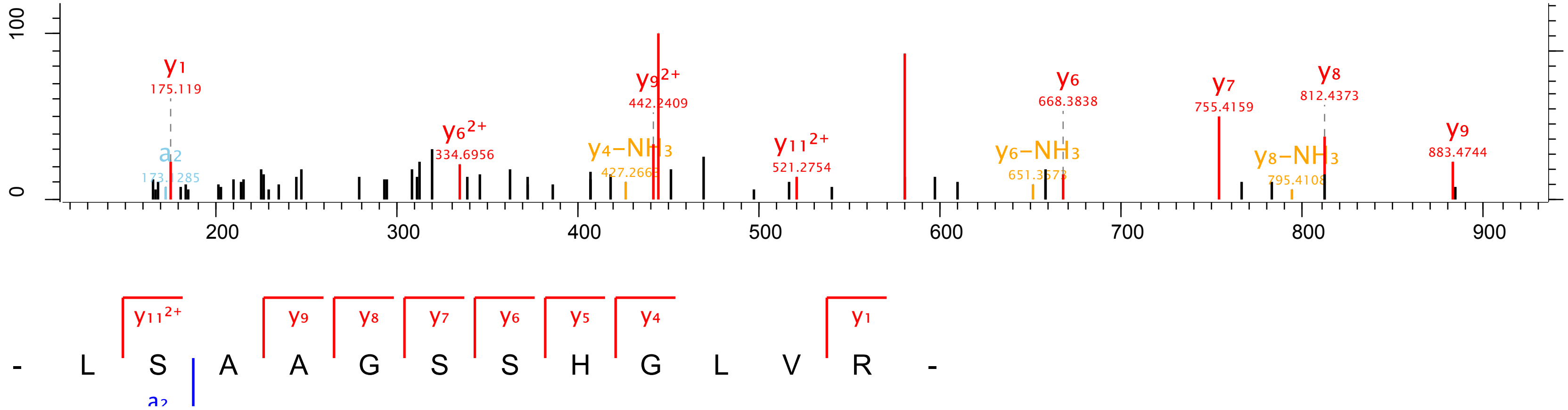

Raw file	Scan	Method	Score	m/z	Gene names
20141014_fract12_dyn_5ul_F4_01_591	12205	TOF; CID	75.91	570.63	HPS1

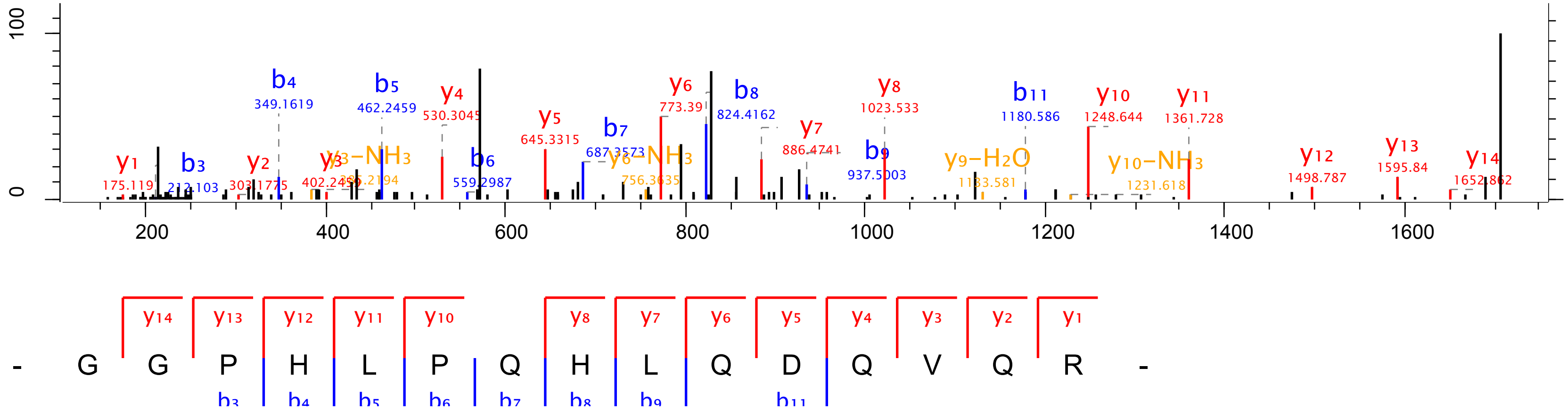

Raw file	Scan	Method	Score	m/z	Gene names
20141014_fract12_dyn_5ul_F4_01_591	12895	TOF; CID	151.07	586.8	SULT1E1

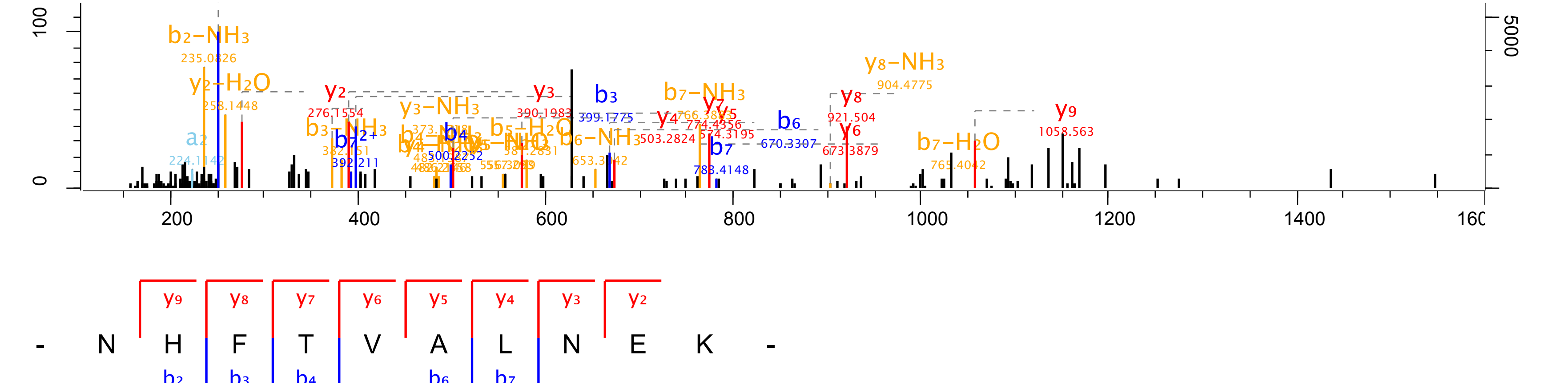

Raw file	Scan	Method	Score	m/z	Gene names
20141014_fract12_dyn_5ul_F4_01_591	14368	TOF; CID	69.03	434.25	RELL1

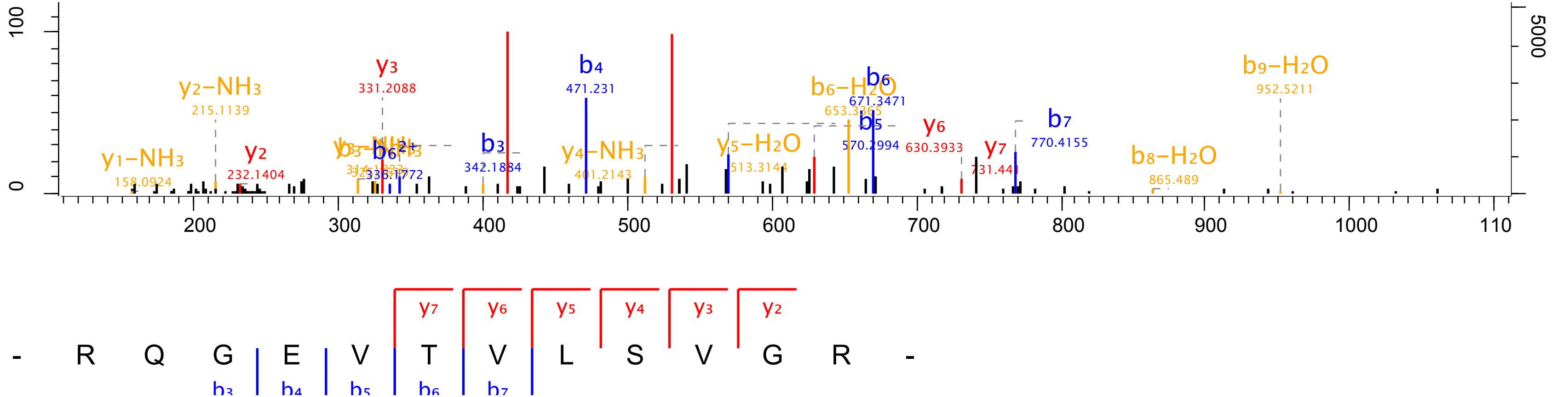

Raw file	Scan	Method	Score	m/z	Gene names
20141014_fract12_dyn_5ul_F4_01_591	14370	TOF; CID	74.61	578.82	MAP3K11

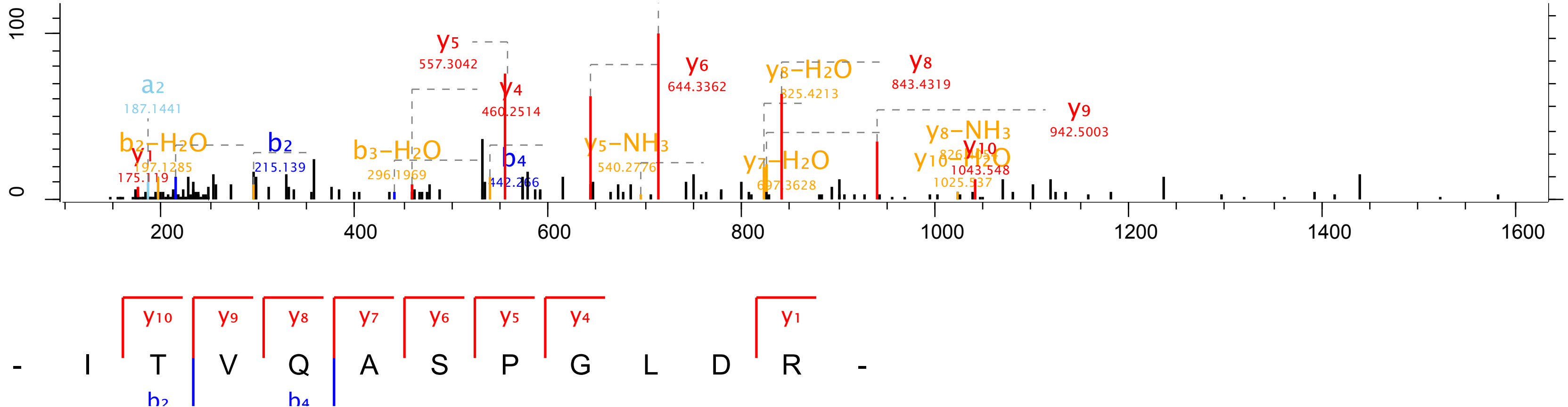

Raw file	Scan	Method	Score	m/z	Gene names
20141014_fract12_dyn_5ul_F4_01_591	15999	TOF; CID	93.6	479.27	SLIT1

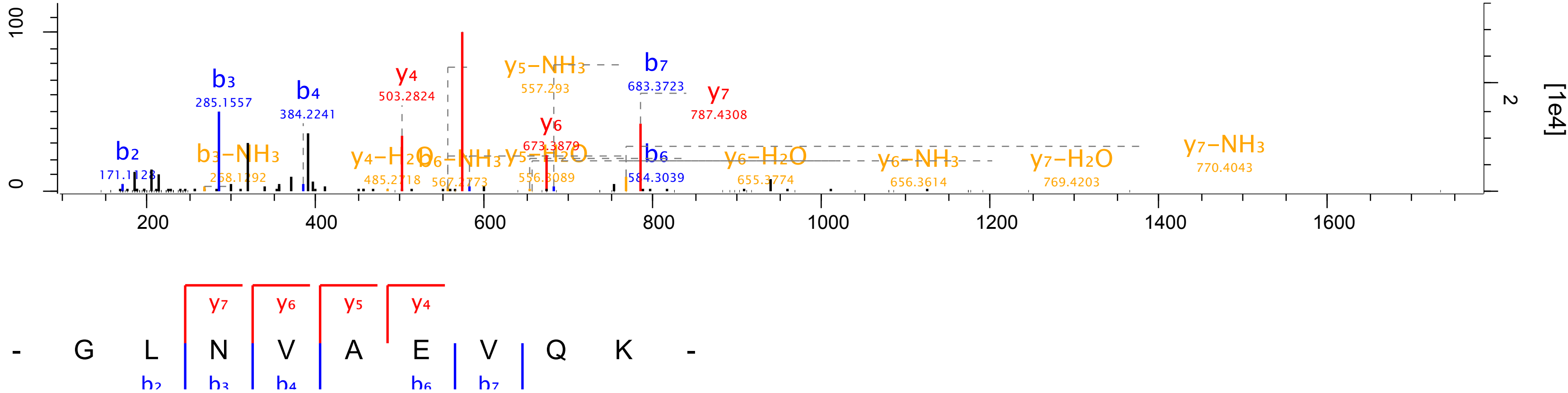

Raw file	Scan	Method	Score	m/z	Gene names
20141014_fract12_dyn_5ul_F4_01_591	23595	TOF; CID	89.23	802.89	C12orf49

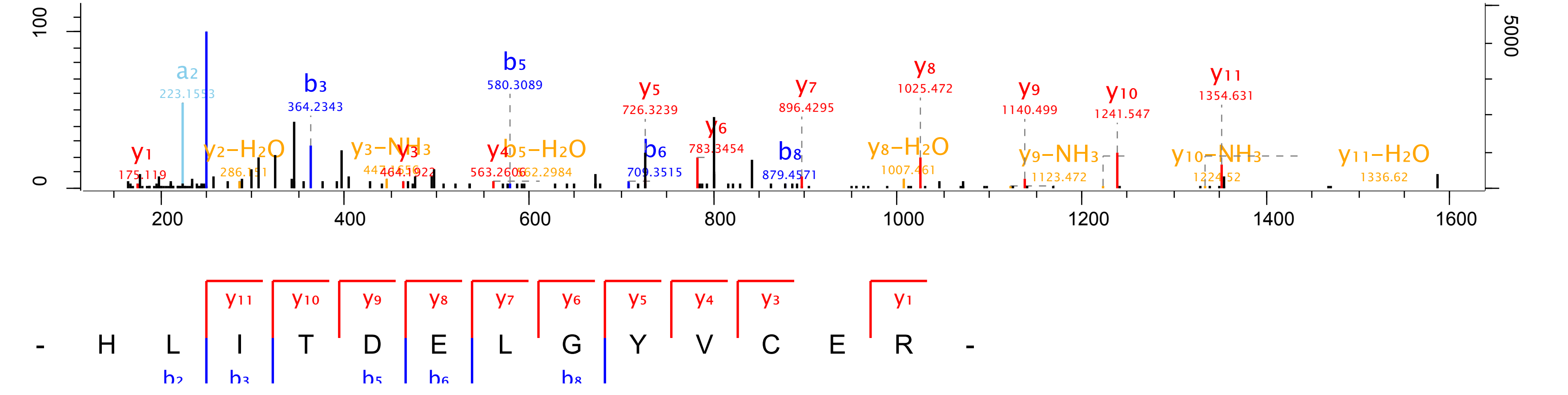

Raw file	Scan	Method	Score	m/z	Gene names
20141014_fract12_dyn_5ul_F4_01_591	25261	TOF; CID	43.59	486.95	TIGD1

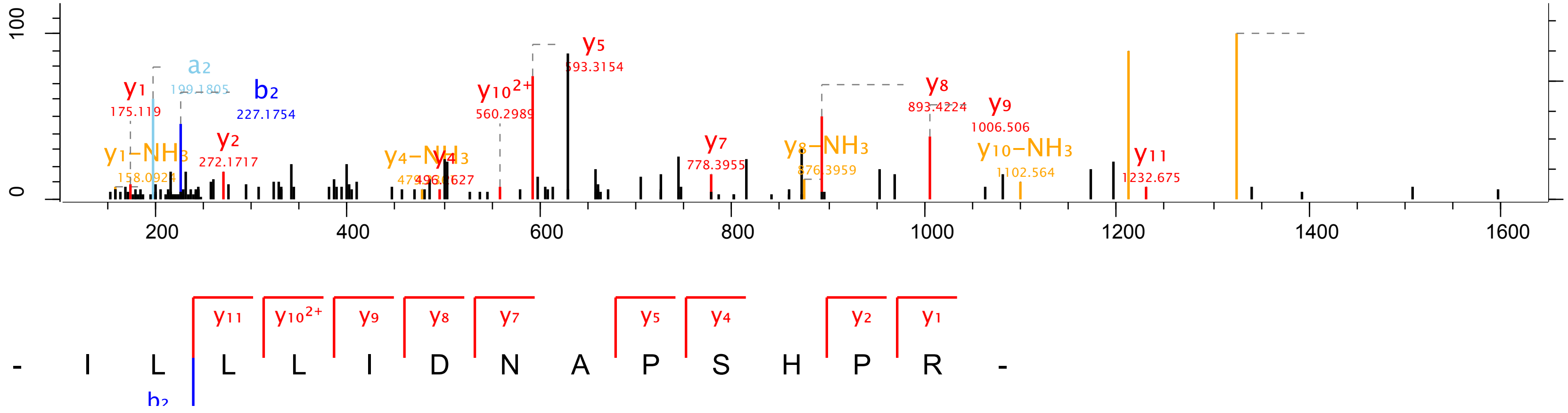

Raw file	Scan	Method	Score	m/z	Gene names
20141014_fract12_dyn_5ul_F4_01_591	25732	TOF; CID	59.23	655.86	USP40

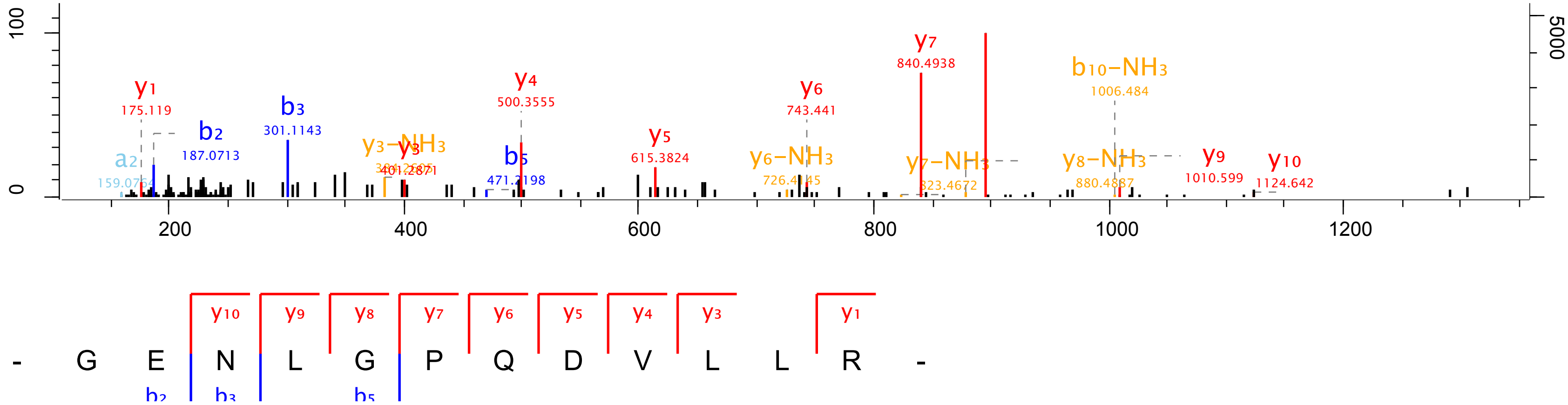

Raw file	Scan	Method	Score	m/z	Gene names
20141014_fract12_dyn_5ul_F4_01_591	28389	TOF; CID	121.56	710.84	SLC39A13

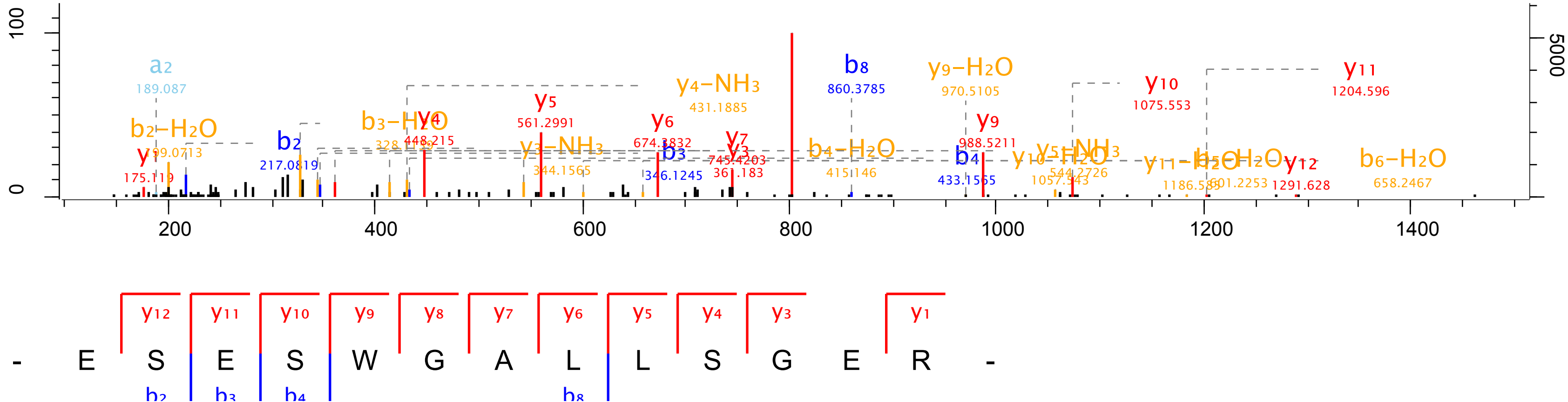

Raw file	Scan	Method	Score	m/z	Gene names
20141014_fract12_dyn_5ul_F4_01_591	28819	TOF; CID	112.36	622.37	MT-ATP6

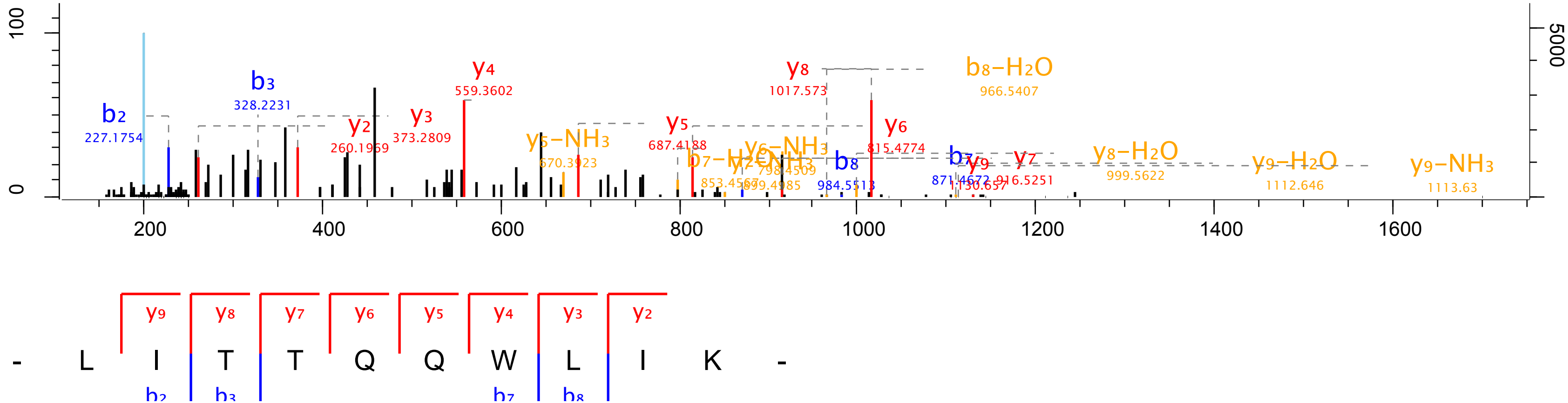

Raw file	Scan	Method	Score	m/z	Gene names
20141014_fract12_dyn_5ul_F4_01_591	30571	TOF; CID	69.88	555.31	ELF3

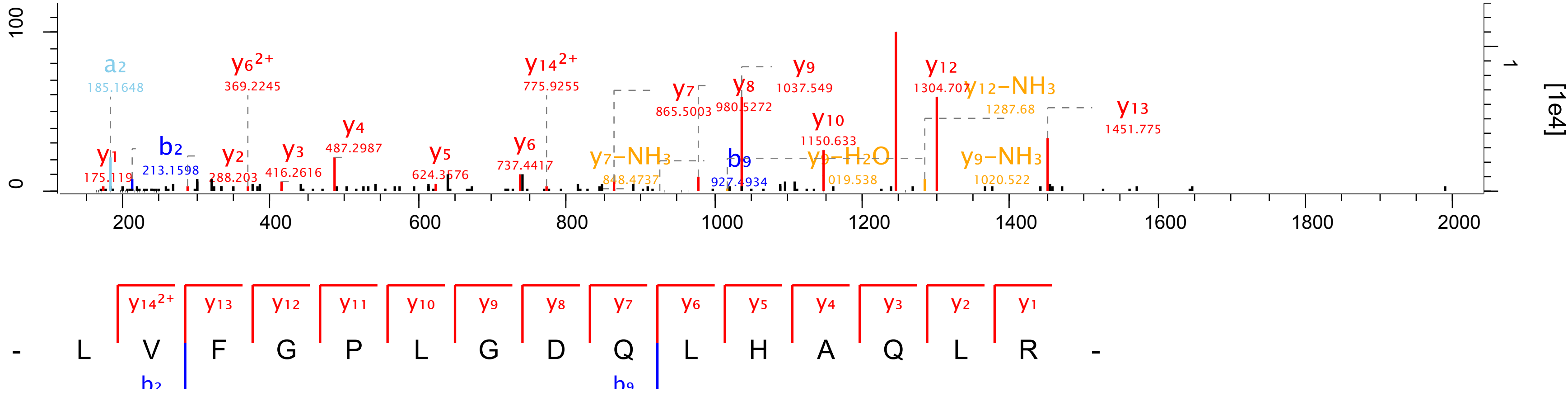

Raw file	Scan	Method	Score	m/z	Gene names
20141014_fract12_dyn_5ul_F4_01_591	34630	TOF; CID	77.22	574.99	ZMYND19

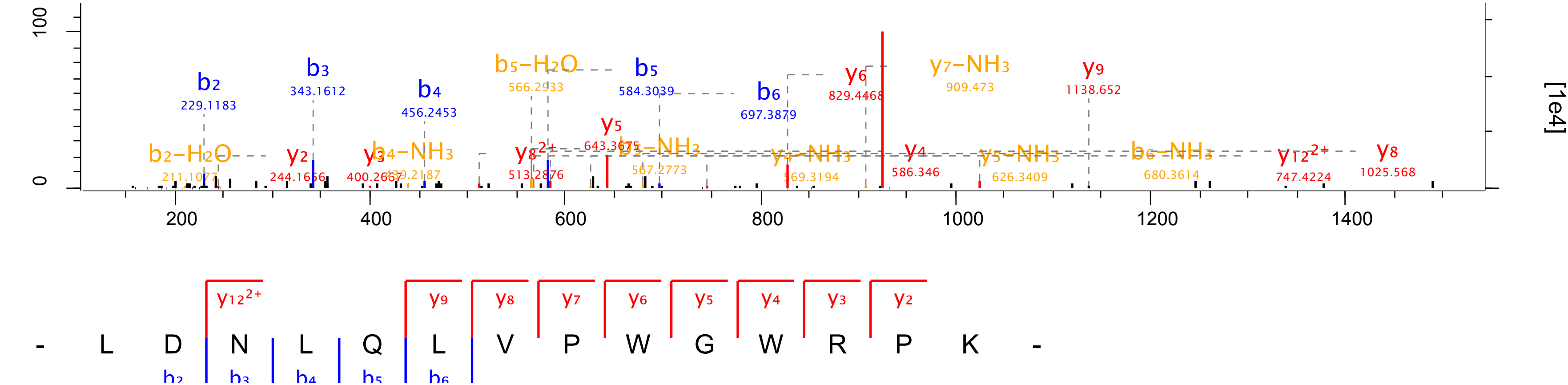

Raw file	Scan	Method	Score	m/z	Gene names
20141014_fract12_dyn_5ul_F4_01_591	34915	TOF; CID	140.2	666.09	SLC39A8

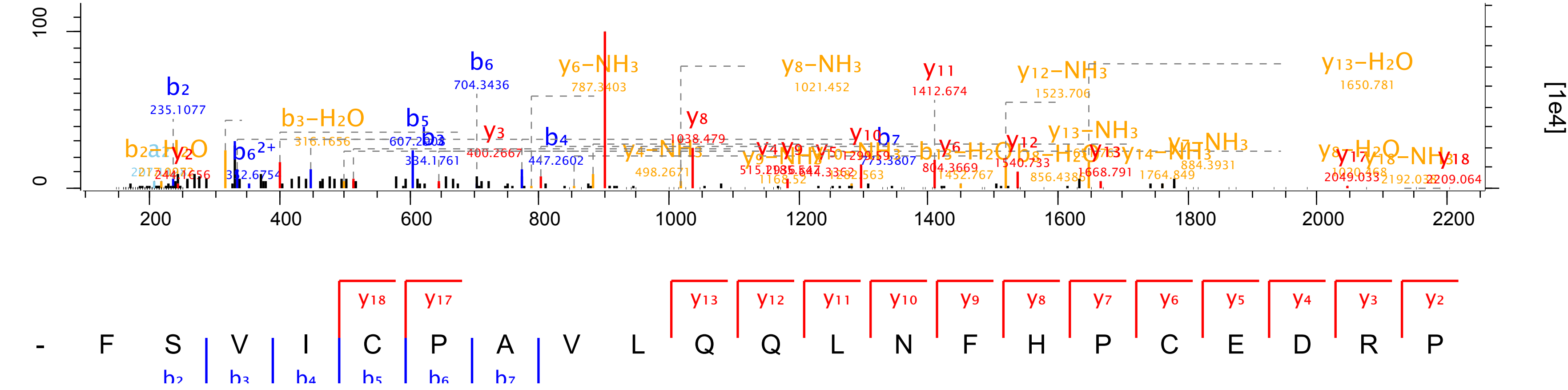

Raw file	Scan	Method	Score	m/z	Gene names
20141014_fract12_dyn_5ul_F4_01_591	35587	TOF; CID	88.28	881.47	C5orf28

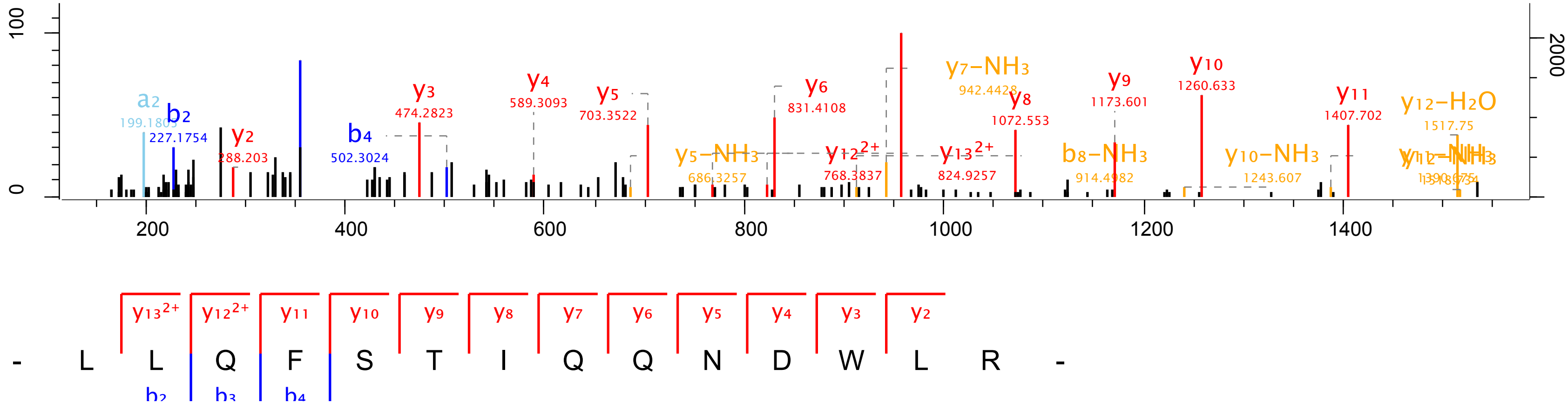

Raw file	Scan	Method	Score	m/z	Gene names
20141014_fract12_dyn_5ul_F4_01_591	37009	TOF; CID	41.26	546.79	S100A12

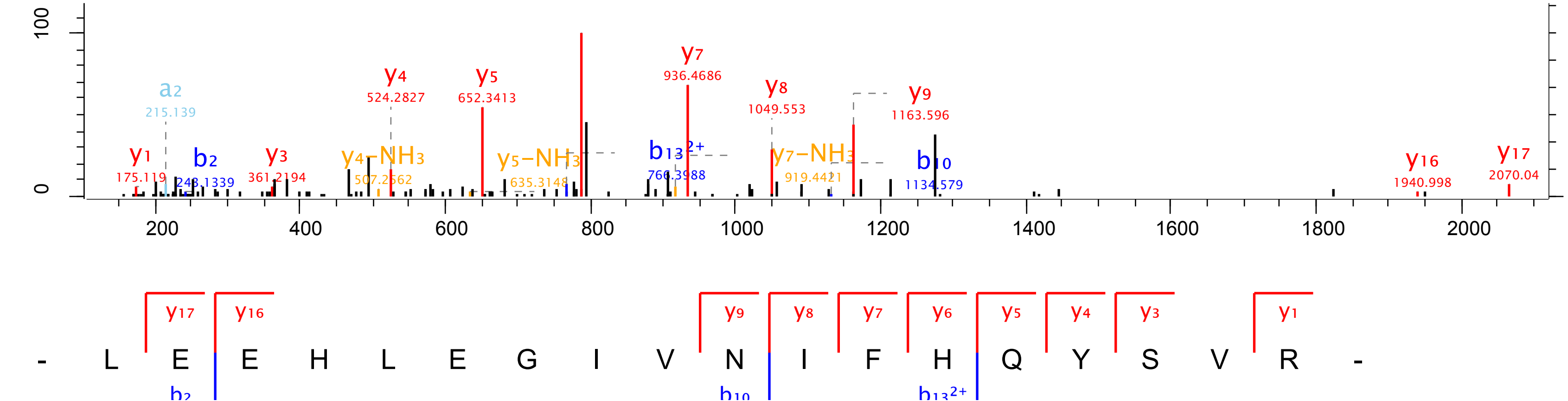

Raw file	Scan	Method	Score	m/z	Gene names
20141014_fract12_dyn_5ul_F4_01_591	38365	TOF; CID	128.22	821.44	ATP6V0D2

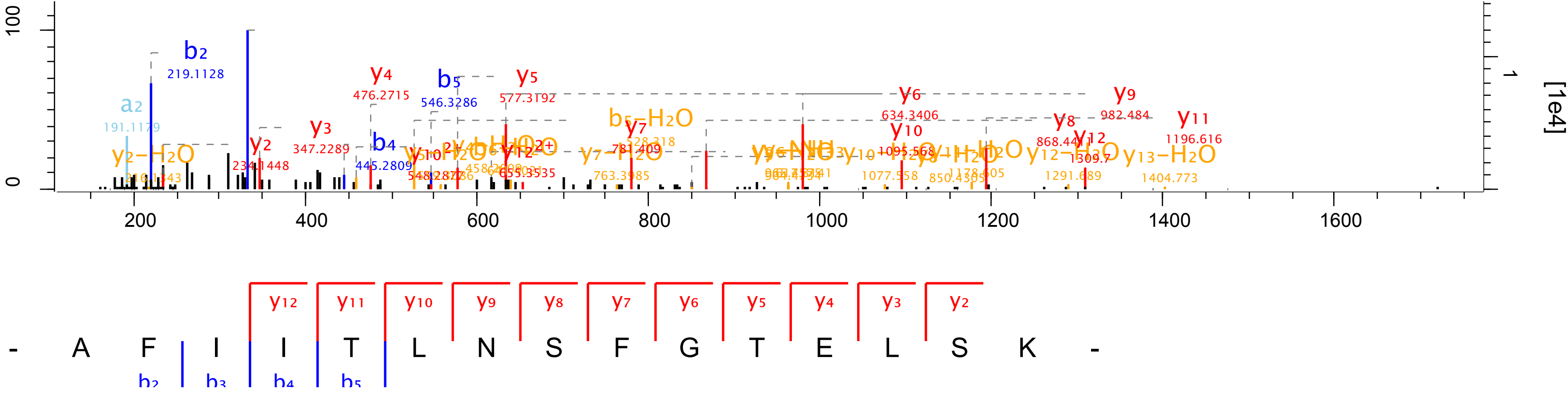

Raw file	Scan	Method	Score	m/z	Gene names
20141014_fract12_dyn_5ul_F4_01_591	38881	TOF; CID	53.3	712.44	SLC36A1

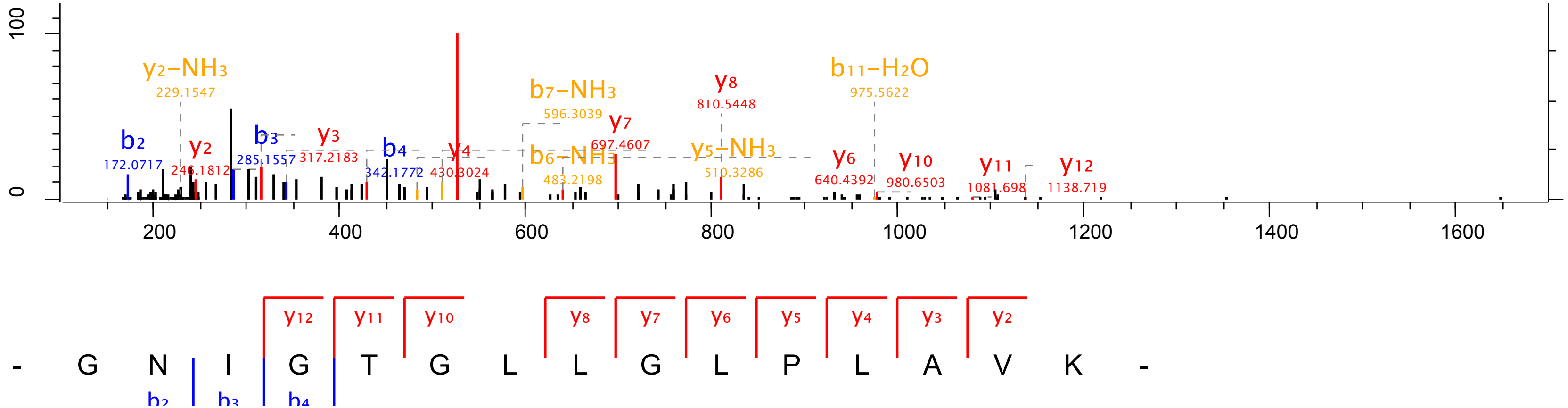

Raw file	Scan	Method	Score	m/z	Gene names
20141014_fract13_dyn_5ul_F5_01_592	13502	TOF; CID	103.13	485.91	SLC41A1

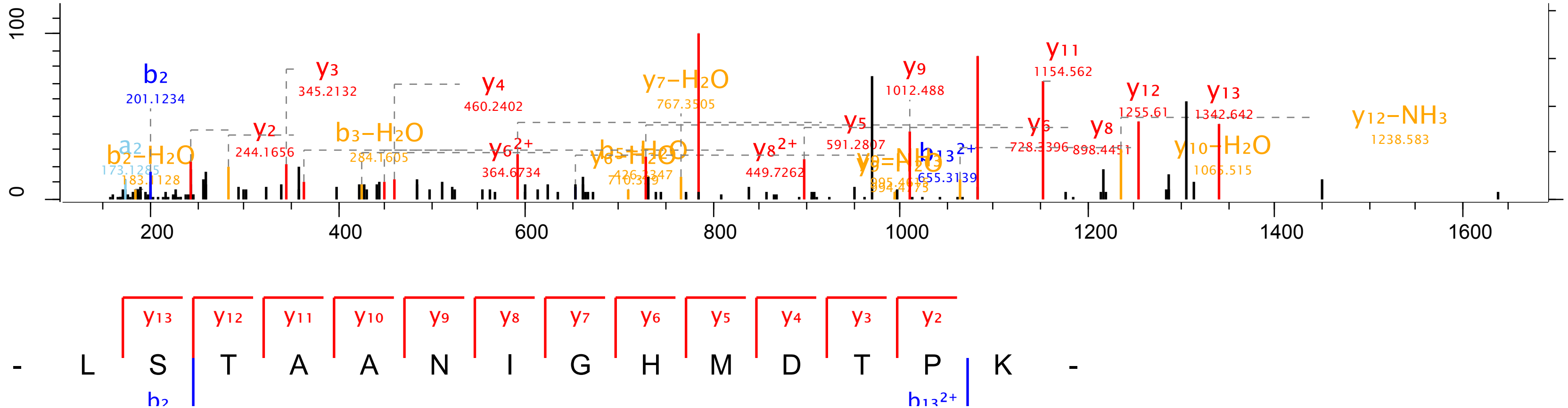

Raw file	Scan	Method	Score	m/z	Gene names
20141014_fract13_dyn_5ul_F5_01_592	18052	TOF; CID	106.14	744.38	DNAAF3

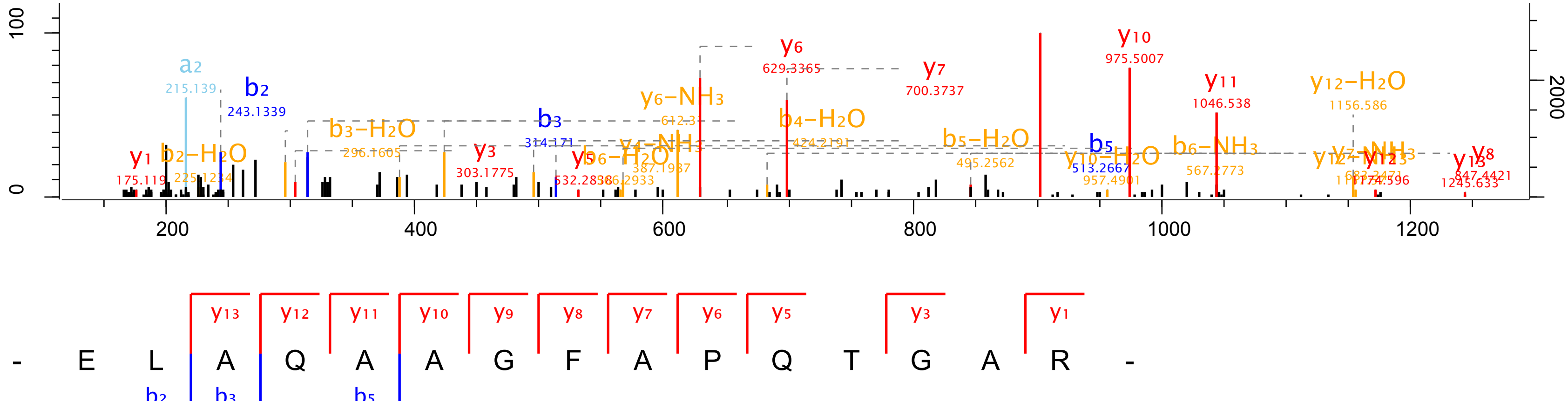

Raw file	Scan	Method	Score	m/z	Gene names
20141014_fract13_dyn_5ul_F5_01_592	19254	TOF; CID	71.69	877.46	KCTD2

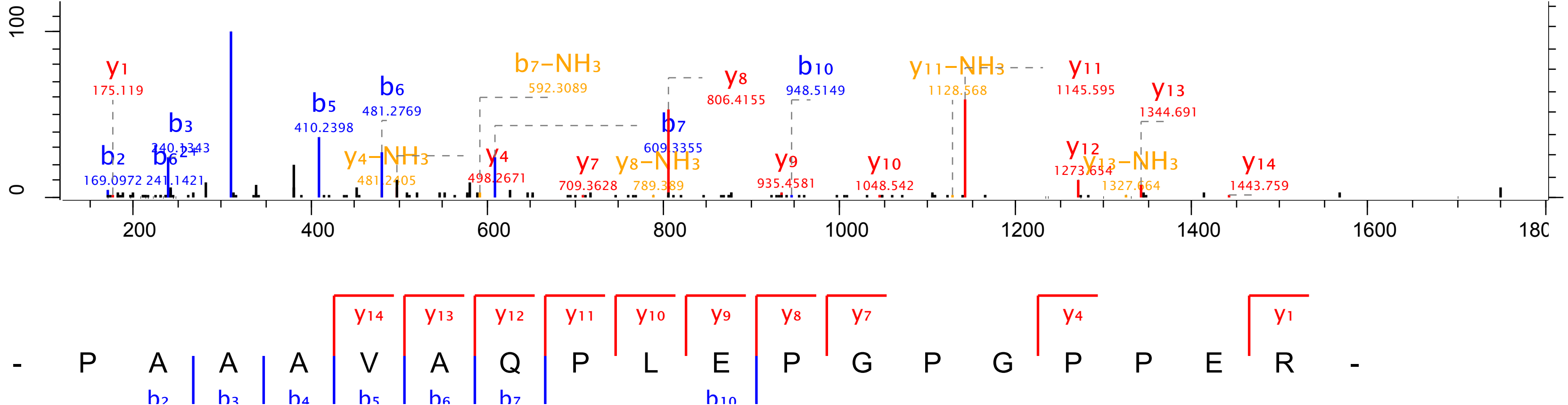

Raw file	Scan	Method	Score	m/z	Gene names
20141014_fract13_dyn_5ul_F5_01_592	19954	TOF; CID	61.17	672.32	IL17RA

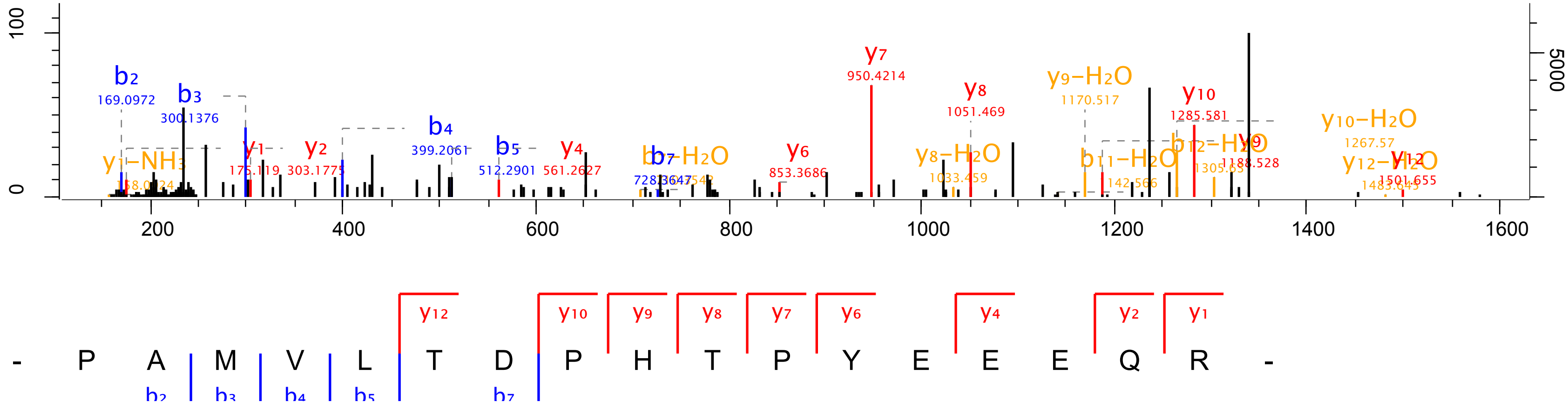

Raw file	Scan	Method	Score	m/z	Gene names
20141014_fract13_dyn_5ul_F5_01_592	21431	TOF; CID	60.97	765.82	SLC35F6

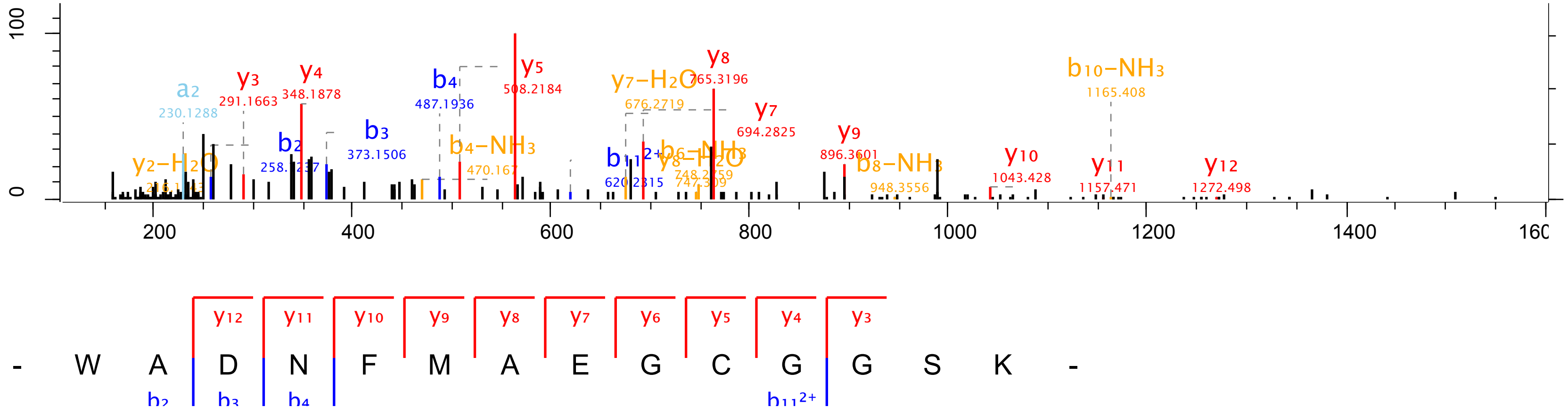

Raw file	Scan	Method	Score	m/z	Gene names
20141014_fract13_dyn_5ul_F5_01_592	22212	TOF; CID	62.29	521.28	AP1AR

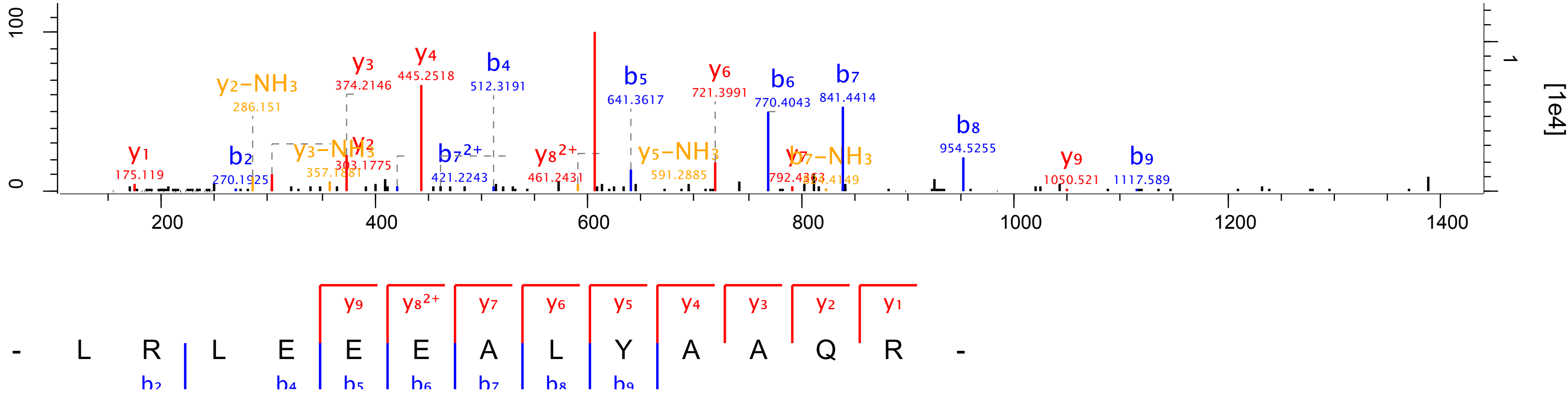

Raw file	Scan	Method	Score	m/z	Gene names
20141014_fract13_dyn_5ul_F5_01_592	22541	TOF; CID	58.02	972.42	LYPD3

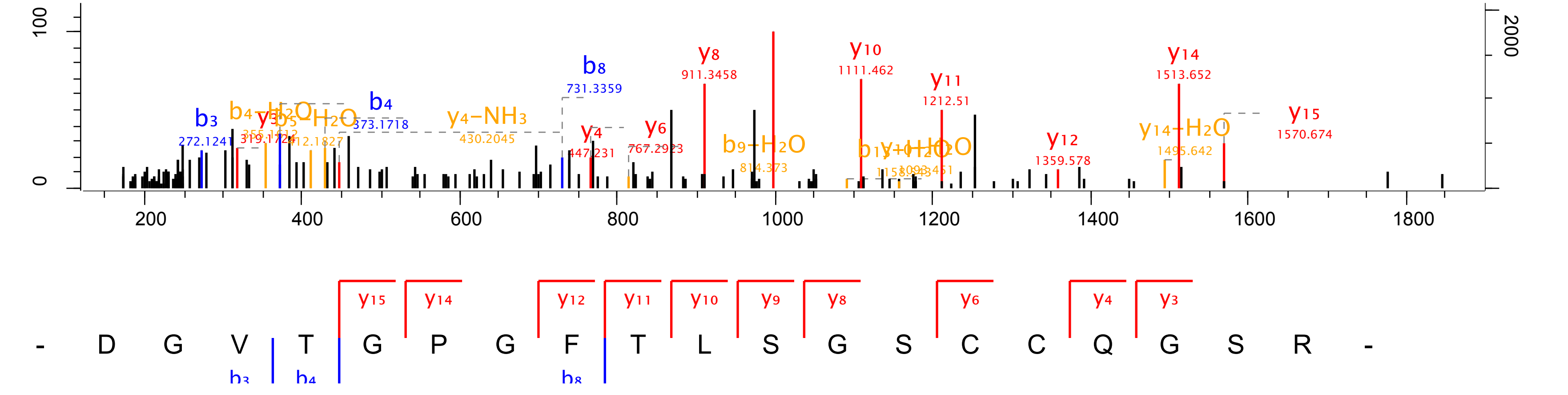

Raw file	Scan	Method	Score	m/z	Gene names
20141014_fract13_dyn_5ul_F5_01_592	23383	TOF; CID	33.96	773.85	TM2D2

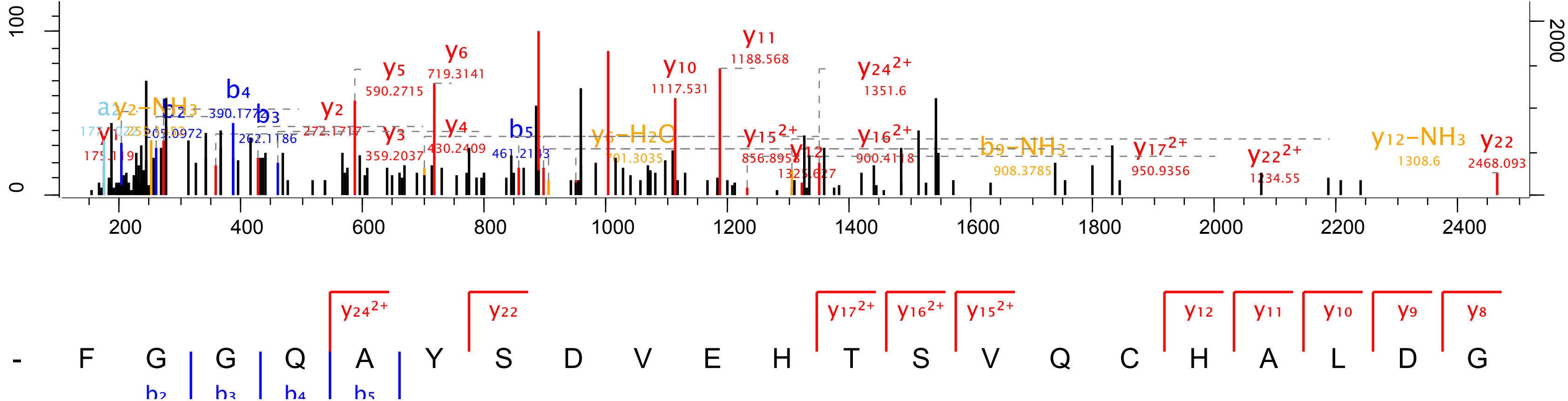

Raw file	Scan	Method	Score	m/z	Gene names
20141014_fract13_dyn_5ul_F5_01_592	24347	TOF; CID	42.09	773.88	METTL10

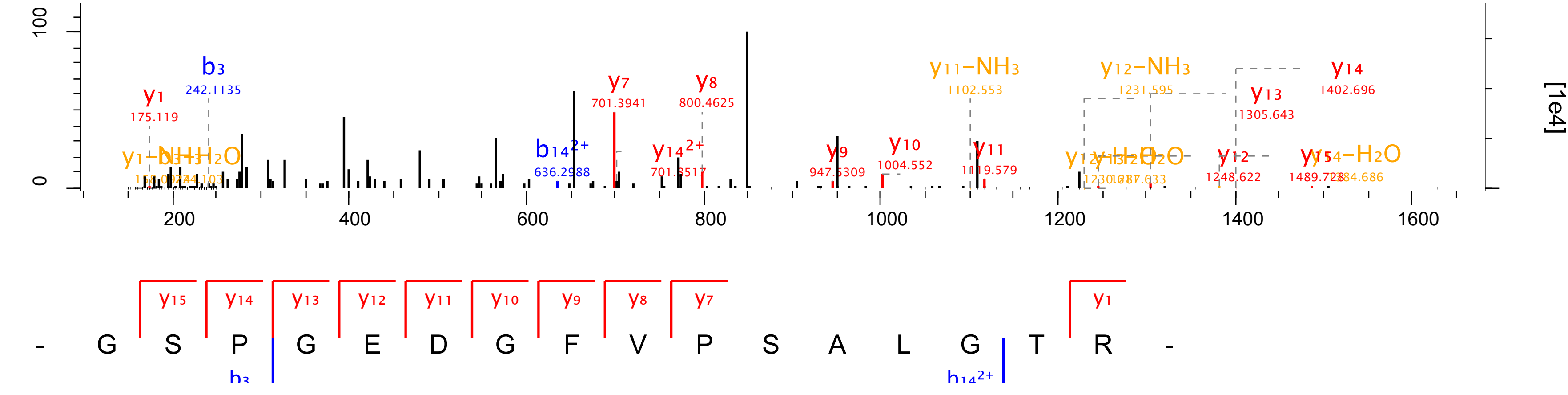

Raw file	Scan	Method	Score	m/z	Gene names
20141014_fract13_dyn_5ul_F5_01_592	24524	TOF; CID	48.22	728.87	ZNF655

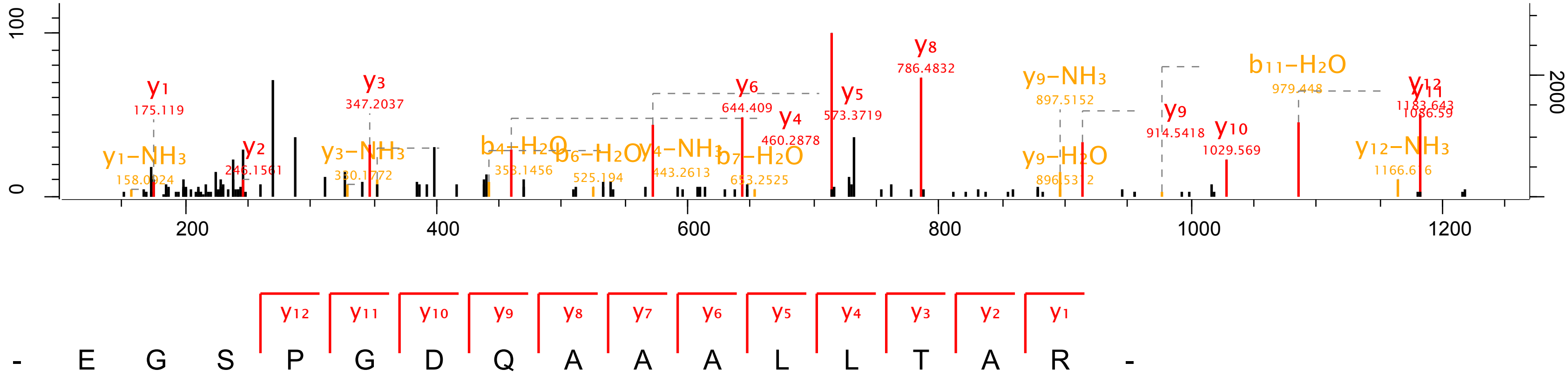

Raw file	Scan	Method	Score	m/z	Gene names
20141014_fract13_dyn_5ul_F5_01_592	24998	TOF; CID	81.3	477.82	BBS10

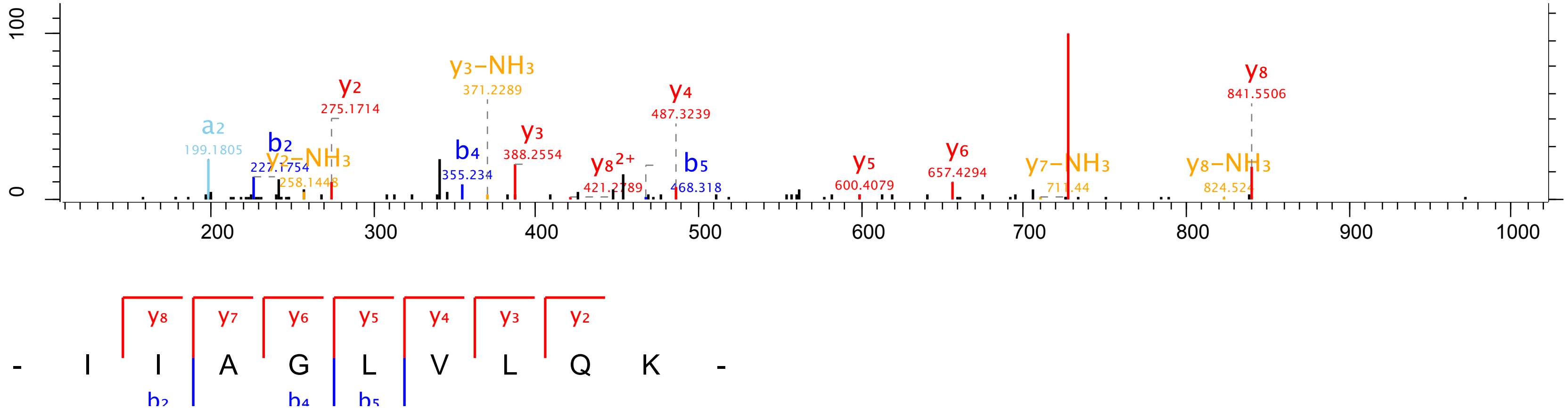

Raw file	Scan	Method	Score	m/z	Gene names
20141014_fract13_dyn_5ul_F5_01_592	27038	TOF; CID	62.09	580.31	GADD45A

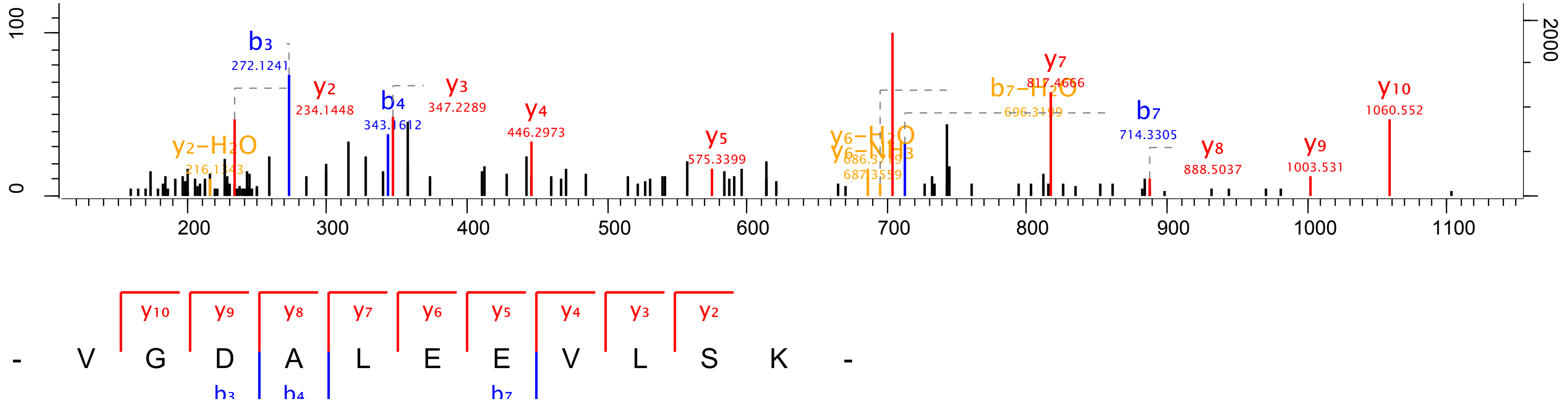

Raw file	Scan	Method	Score	m/z	Gene names
20141014_fract13_dyn_5ul_F5_01_592	27860	TOF; CID	103.08	543.8	CPA4

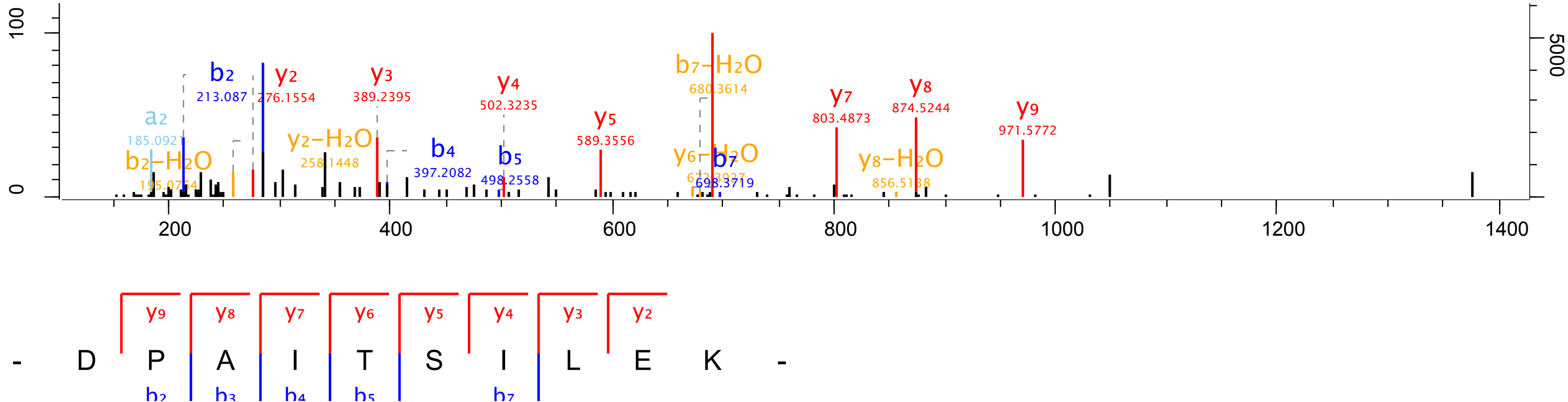

Raw file	Scan	Method	Score	m/z	Gene names
20141014_fract13_dyn_5ul_F5_01_592	29425	TOF; CID	78.67	800.41	ATMIN

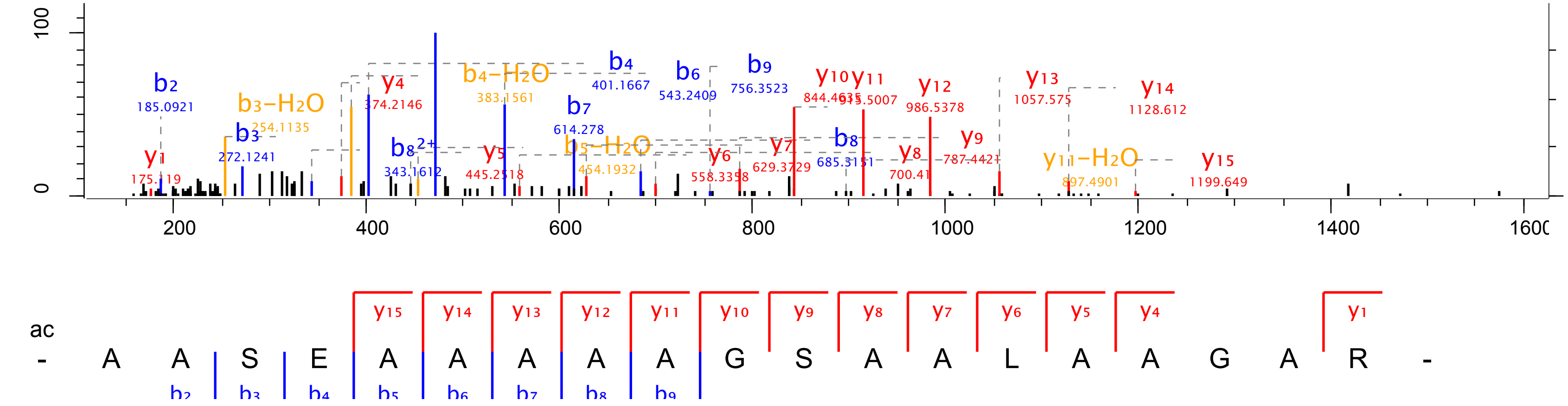

Raw file	Scan	Method	Score	m/z	Gene names
20141014_fract13_dyn_5ul_F5_01_592	30641	TOF; CID	72.32	588.8	PKD2

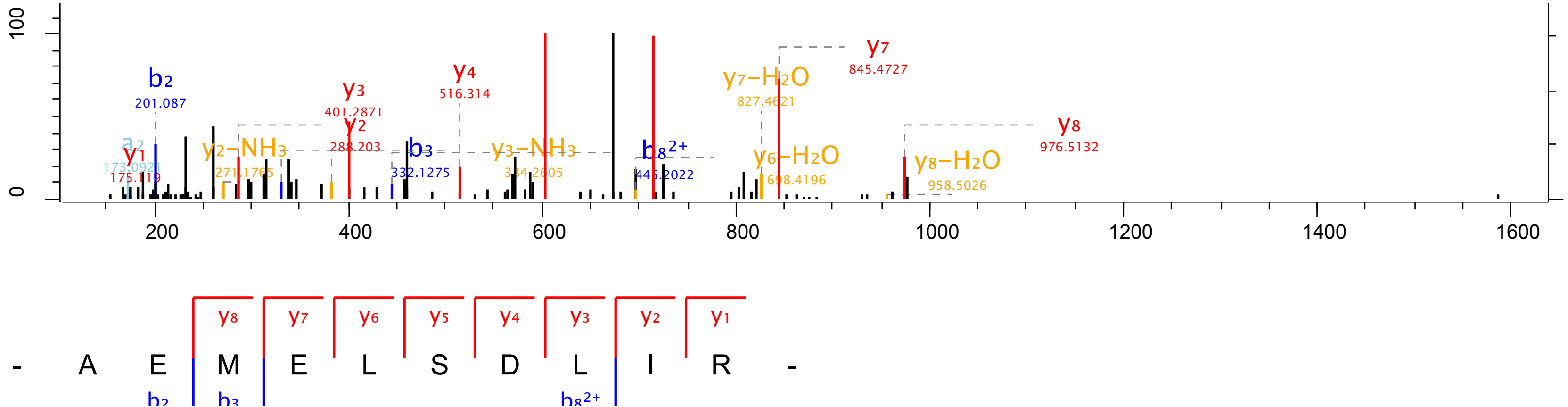

Raw file	Scan	Method	Score	m/z	Gene names
20141014_fract13_dyn_5ul_F5_01_592	31178	TOF; CID	118.51	836.38	WRB

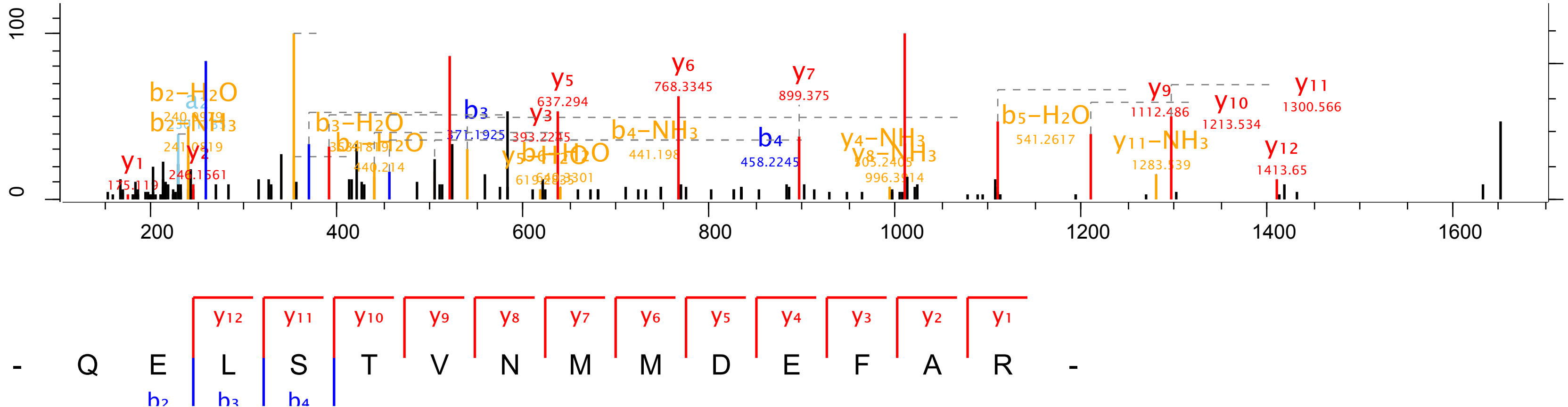

Raw file	Scan	Method	Score	m/z	Gene names
20141014_fract13_dyn_5ul_F5_01_592	32116	TOF; CID	104.05	600.84	ATG101

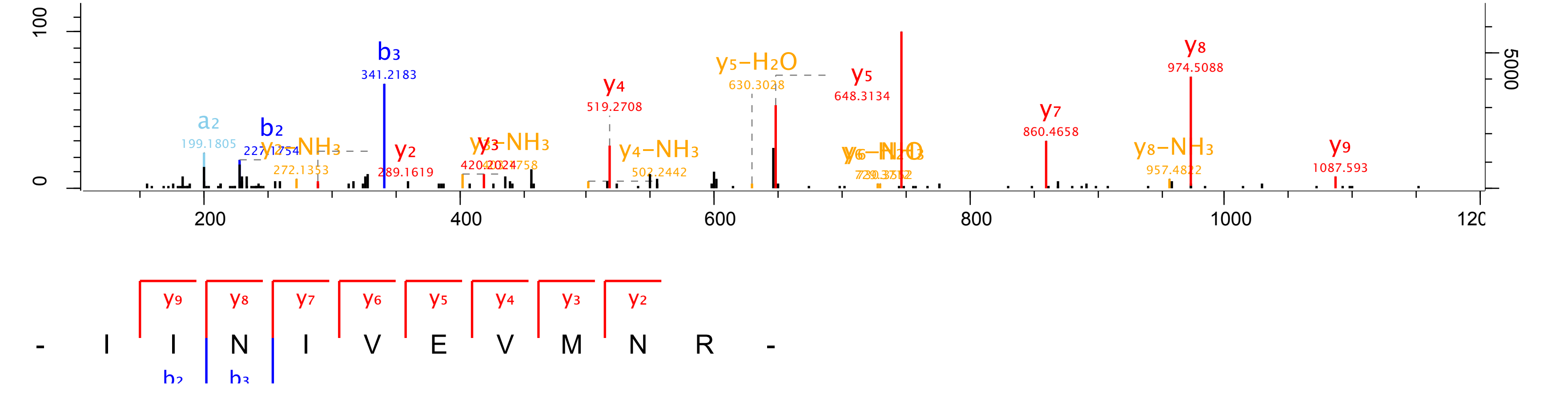

Raw file	Scan	Method	Score	m/z	Gene names
20141014_fract13_dyn_5ul_F5_01_592	32472	TOF; CID	60.2	707.41	MRI

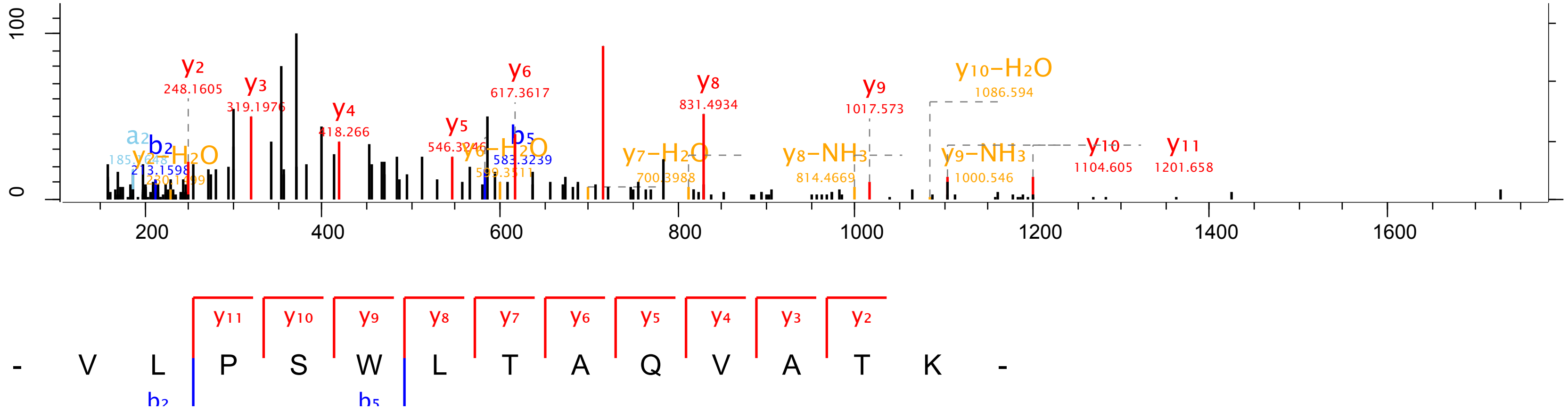

Raw file	Scan	Method	Score	m/z	Gene names
20141014_fract13_dyn_5ul_F5_01_592	32774	TOF; CID	55.72	745.38	PPARGC1B

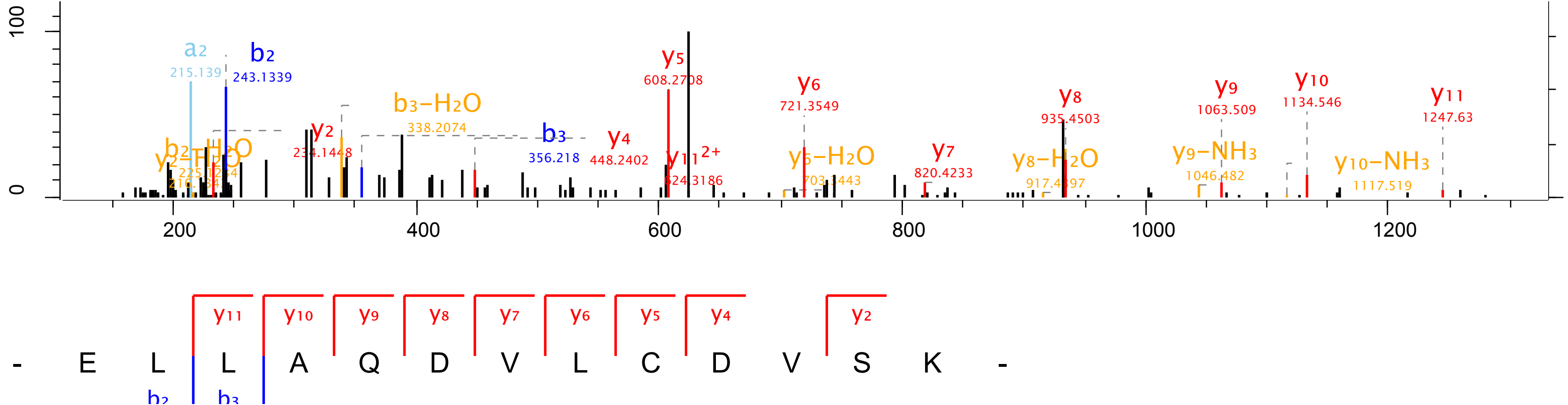

Raw file	Scan	Method	Score	m/z	Gene names
20141014_fract13_dyn_5ul_F5_01_592	33810	TOF; CID	63.19	499.94	METTL4

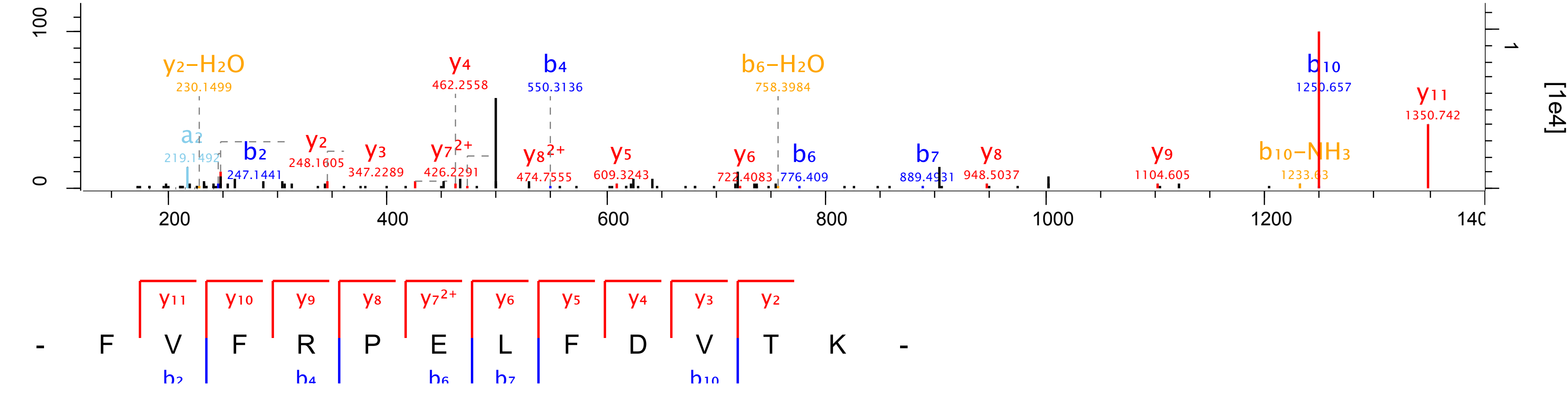

Raw file	Scan	Method	Score	m/z	Gene names
20141014_fract13_dyn_5ul_F5_01_592	34518	TOF; CID	45.44	576	DHRS3

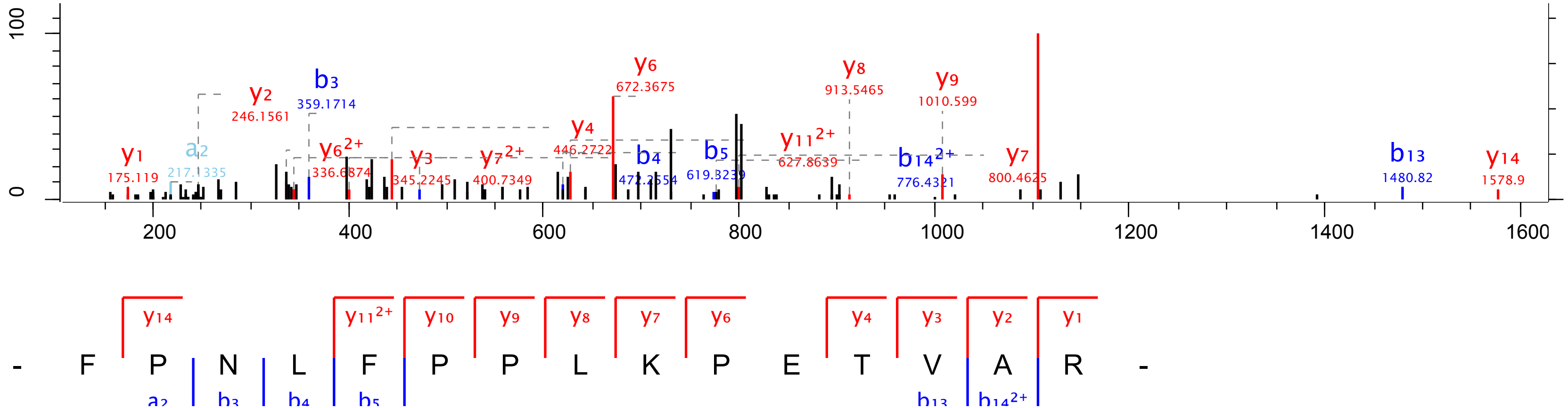

Raw file	Scan	Method	Score	m/z	Gene names
20141014_fract13_dyn_5ul_F5_01_592	34790	TOF; CID	79.34	571.32	G2E3

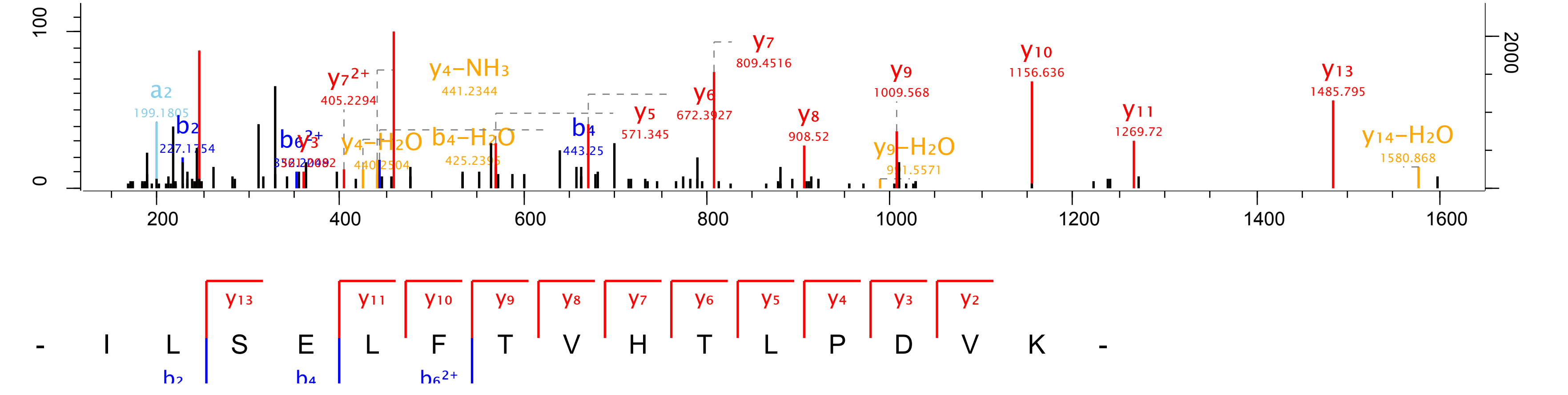

Raw file	Scan	Method	Score	m/z	Gene names
20141014_fract13_dyn_5ul_F5_01_592	35837	TOF; CID	81.3	493.31	SLC29A3

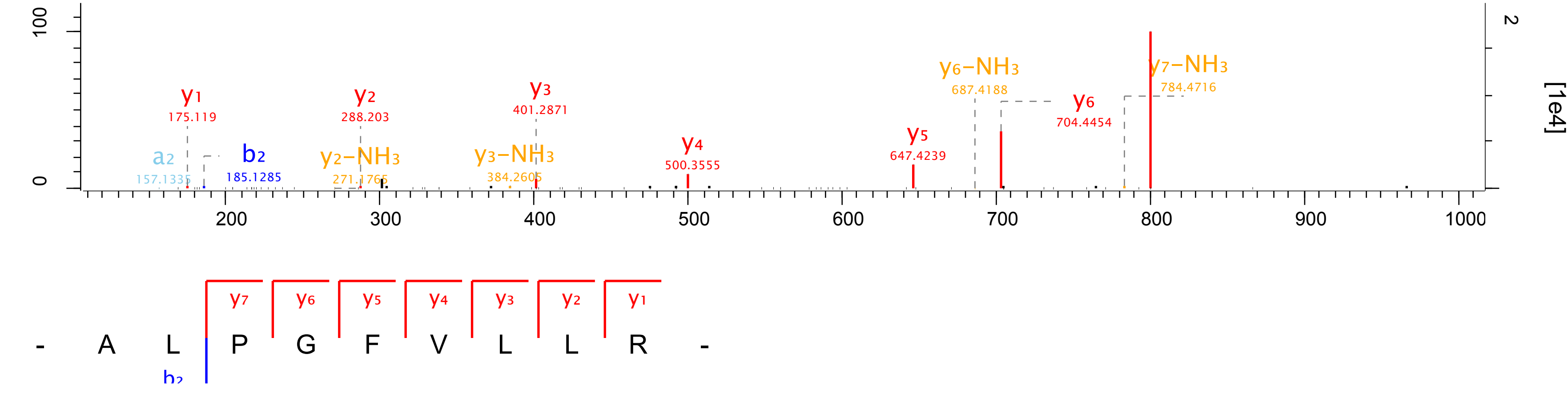

Raw file	Scan	Method	Score	m/z	Gene names
20141014_fract13_dyn_5ul_F5_01_592	37719	TOF; CID	60.55	748.44	SIM2;SIM1

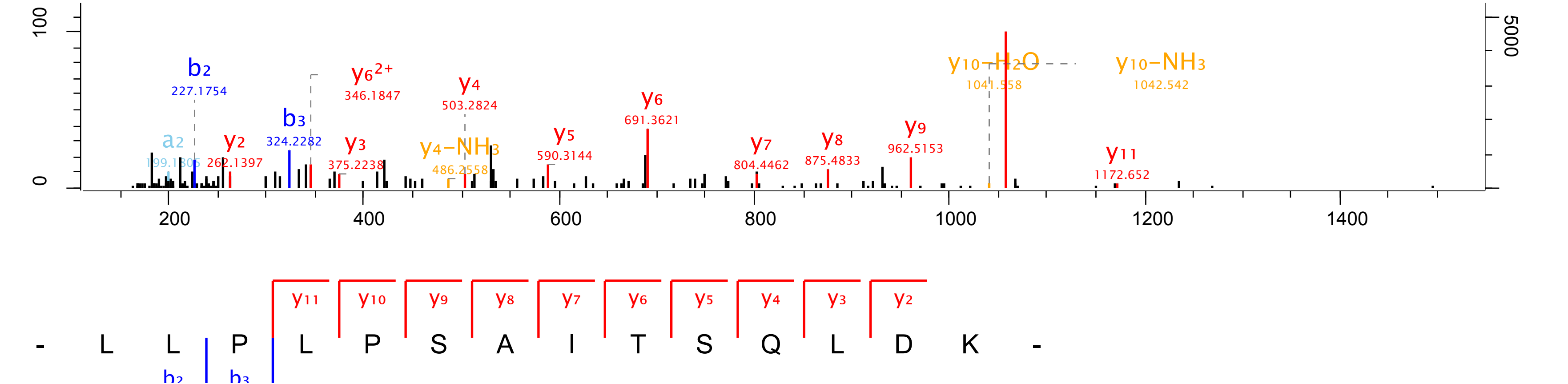

Raw file	Scan	Method	Score	m/z	Gene names
20141014_fract14_dyn_5ul_F6_01_593	10326	TOF; CID	65.18	412.9	ARRDC1-AS1

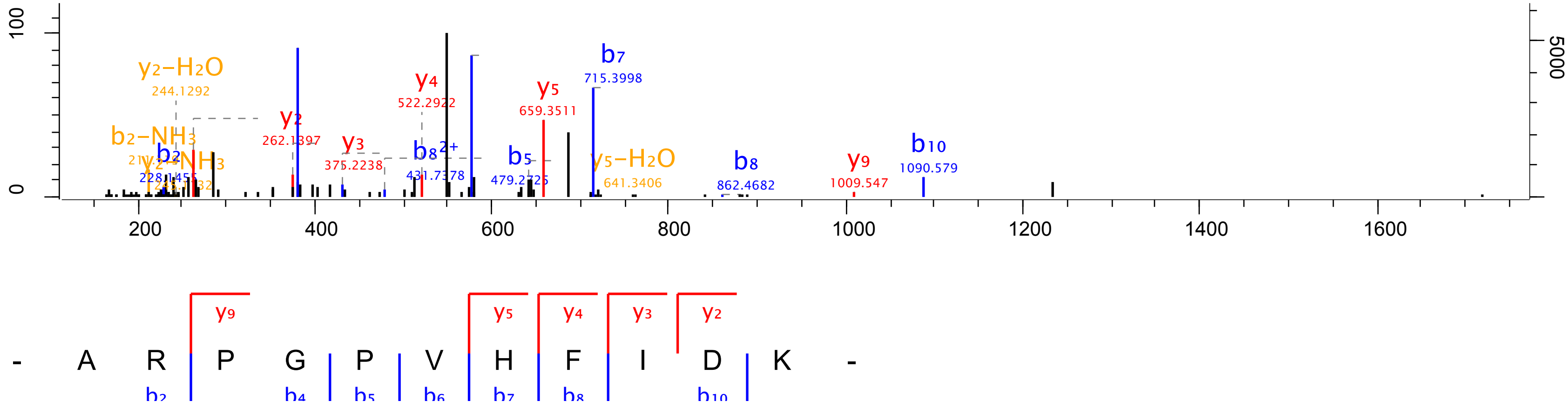

Raw file	Scan	Method	Score	m/z	Gene names
20141014_fract14_dyn_5ul_F6_01_593	11009	TOF; CID	68.52	430.91	DPY19L3

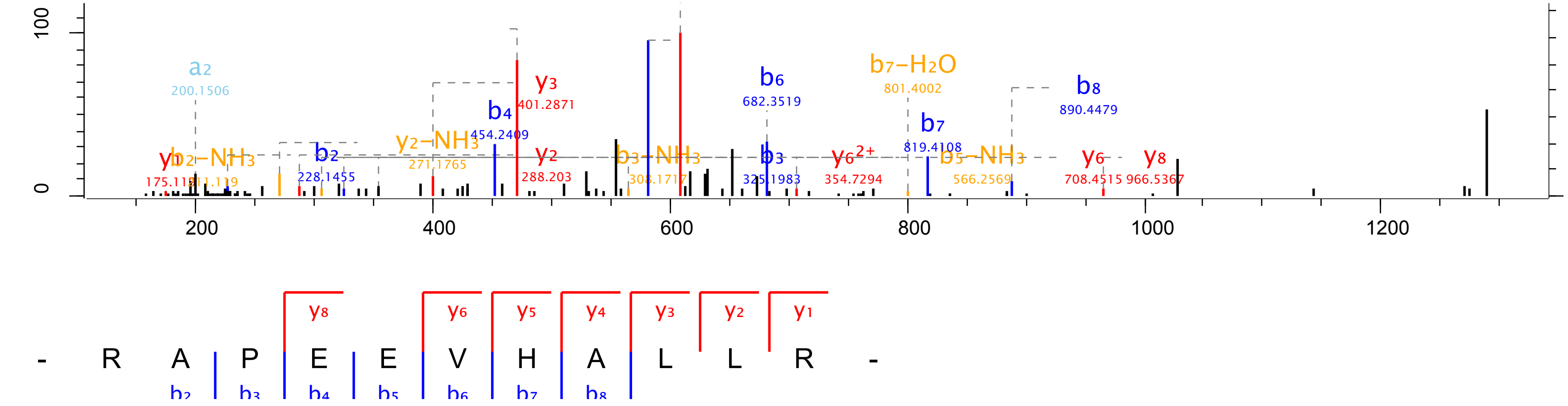

Raw file	Scan	Method	Score	m/z	Gene names
20141014_fract14_dyn_5ul_F6_01_593	11297	TOF; CID	90.38	690.33	MED11

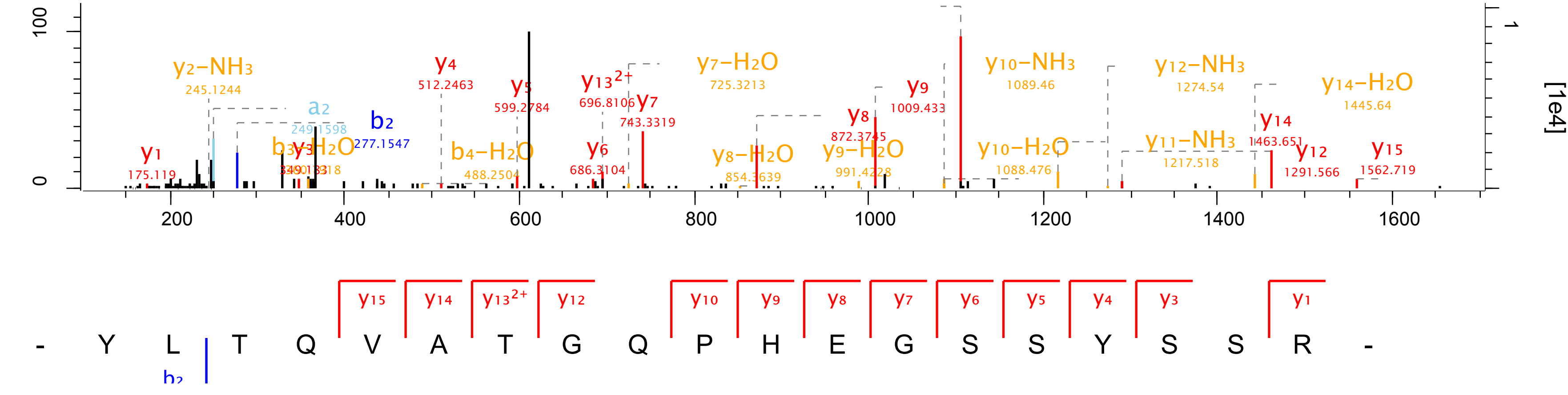

Raw file	Scan	Method	Score	m/z	Gene names
20141014_fract14_dyn_5ul_F6_01_593	12997	TOF; CID	118.74	420.73	ARMC7

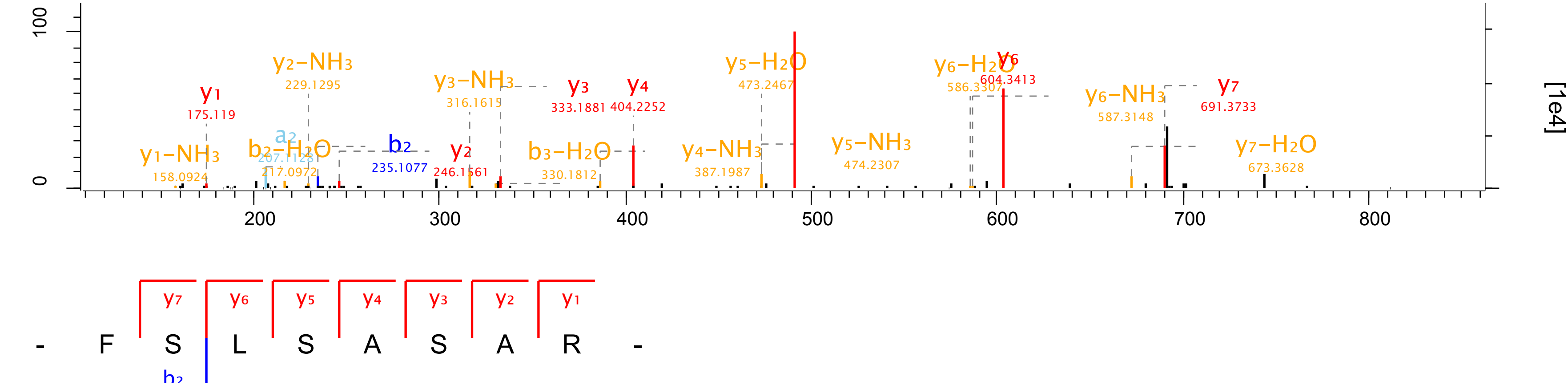

Raw file	Scan	Method	Score	m/z	Gene names
20141014_fract14_dyn_5ul_F6_01_593	13885	TOF; CID	88.16	569.79	SGMS2

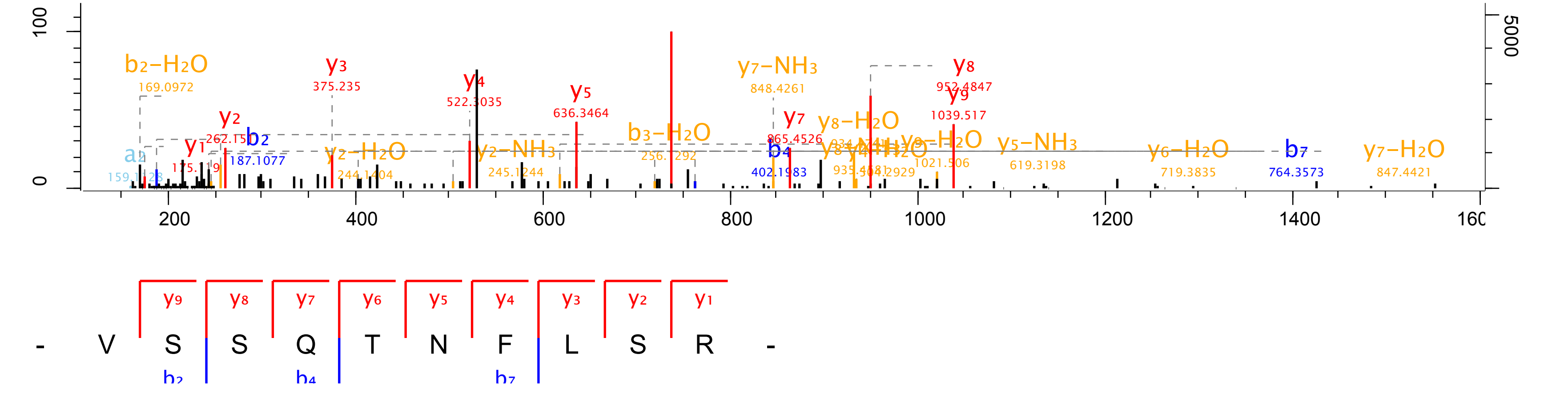

Raw file	Scan	Method	Score	m/z	Gene names
20141014_fract14_dyn_5ul_F6_01_593	18083	TOF; CID	116.37	587.78	MAX

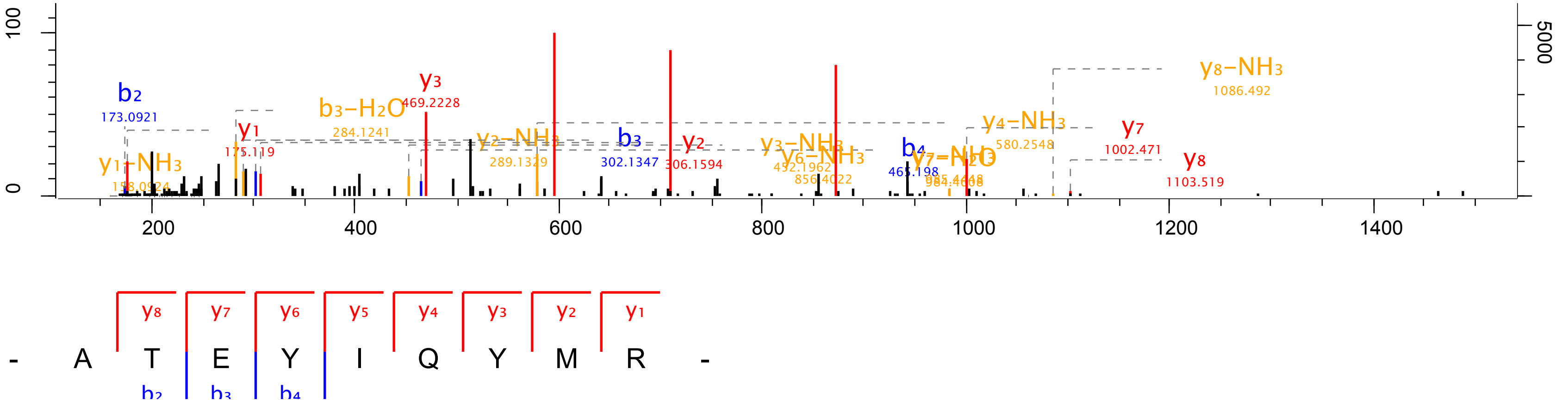

Raw file	Scan	Method	Score	m/z	Gene names
20141014_fract14_dyn_5ul_F6_01_593	20499	TOF; CID	79.15	540.81	ADAMTS14

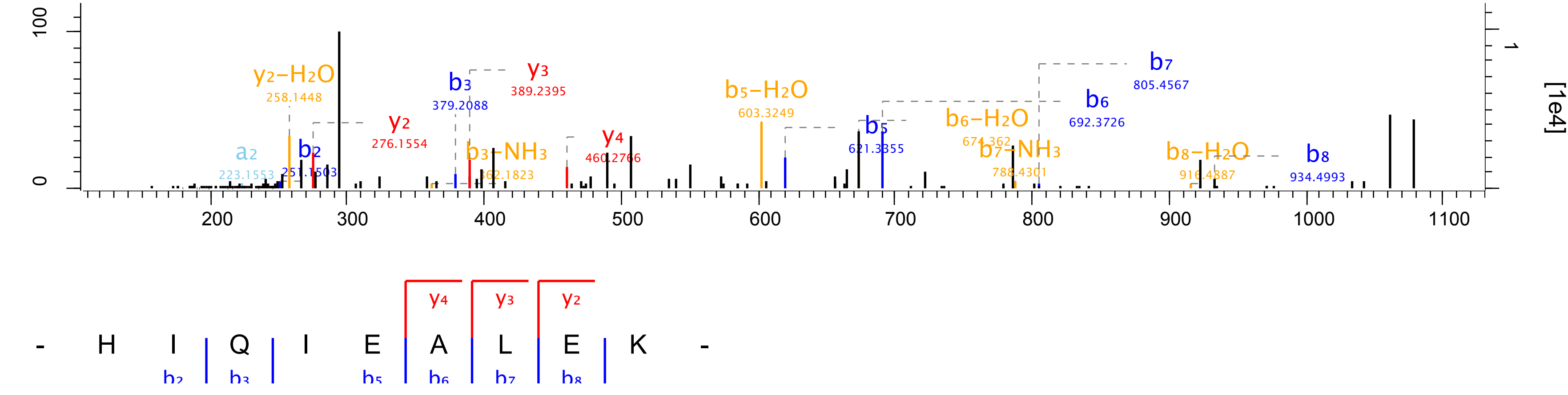

Raw file	Scan	Method	Score	m/z	Gene names
20141014_fract14_dyn_5ul_F6_01_593	22717	TOF; CID	64.12	743.34	OTX1

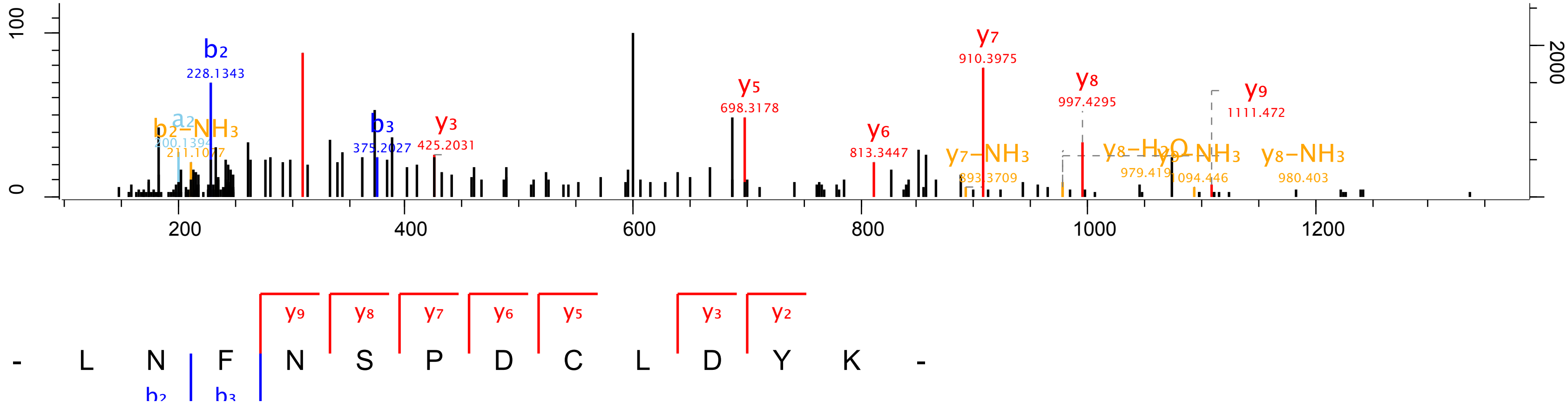

Raw file	Scan	Method	Score	m/z	Gene names
20141014_fract14_dyn_5ul_F6_01_593	24847	TOF; CID	118.96	928.97	HIATL1

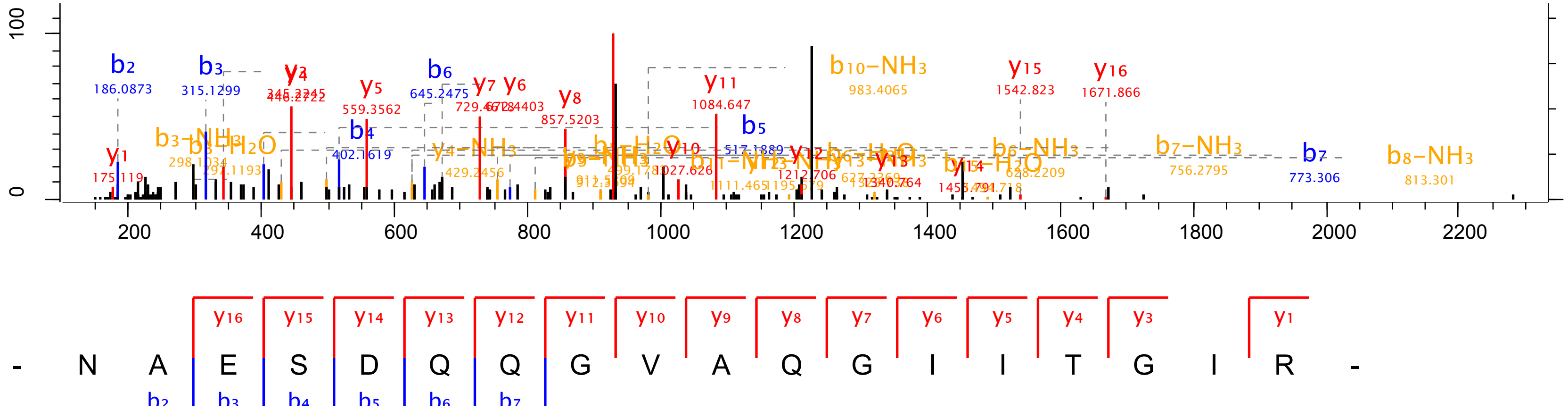

Raw file	Scan	Method	Score	m/z	Gene names
20141014_fract14_dyn_5ul_F6_01_593	30294	TOF; CID	69.85	813.9	PAG1

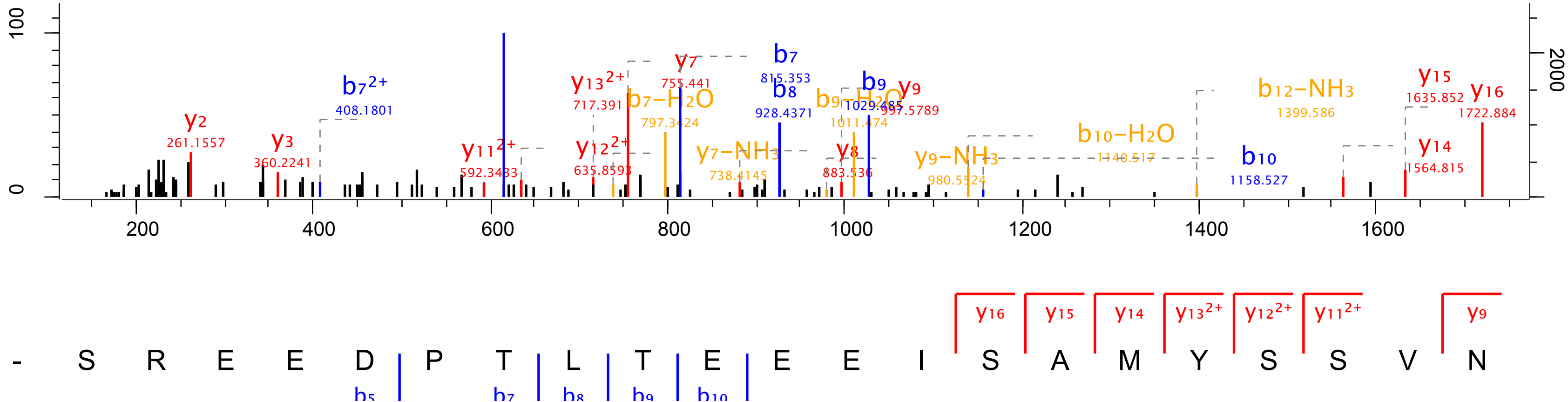

Raw file	Scan	Method	Score	m/z	Gene names
20141014_fract14_dyn_5ul_F6_01_593	35876	TOF; CID	78.81	770.42	NPL

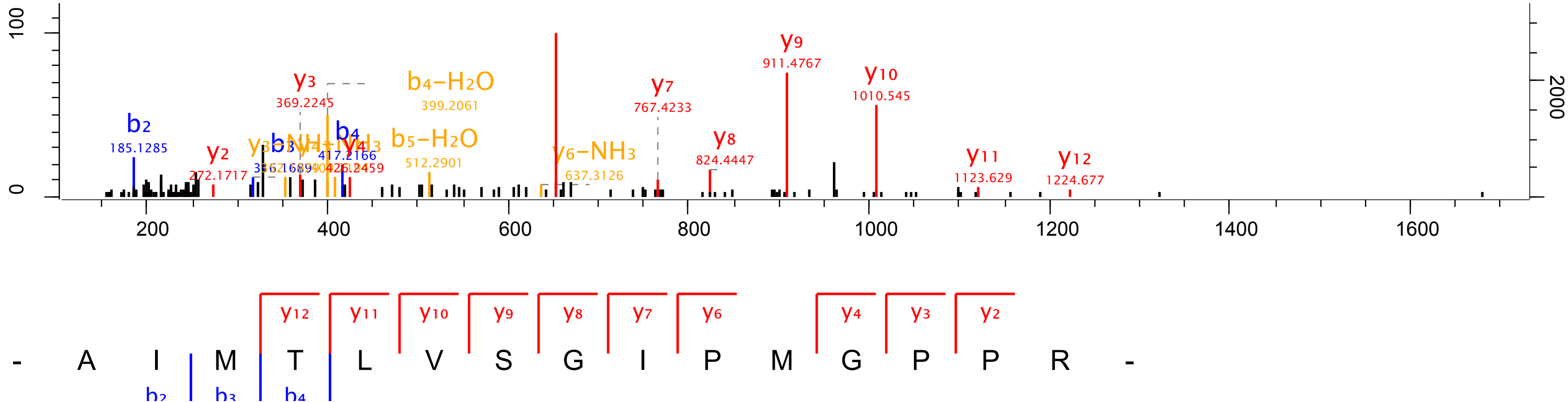

Raw file	Scan	Method	Score	m/z	Gene names
20141014_fract14_dyn_5ul_F6_01_593	35994	TOF; CID	100.02	837.36	PAIP2

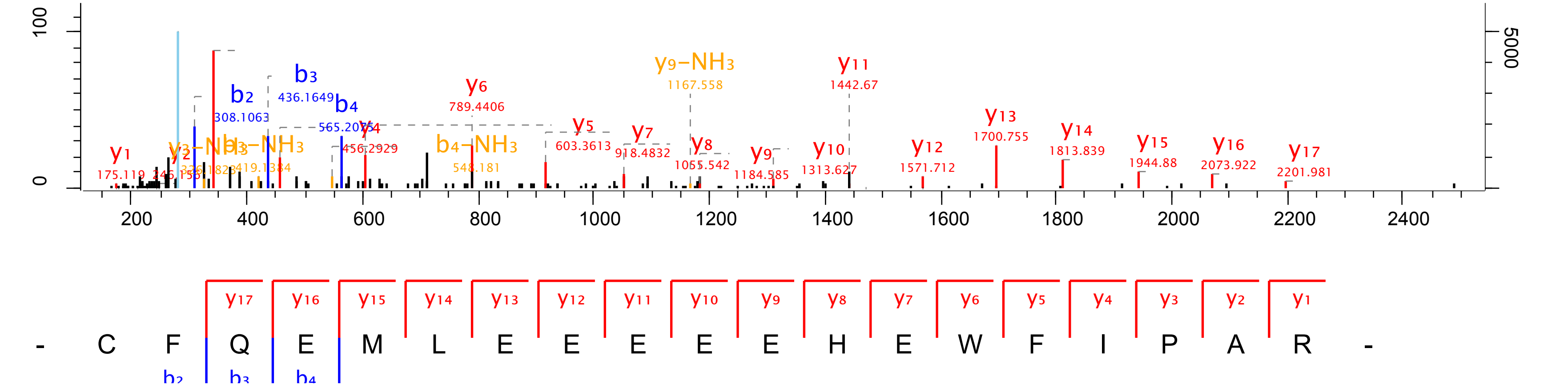

Raw file	Scan	Method	Score	m/z	Gene names
20141014_fract14_dyn_5ul_F6_01_593	36542	TOF; CID	98.16	588.36	KREMEN2

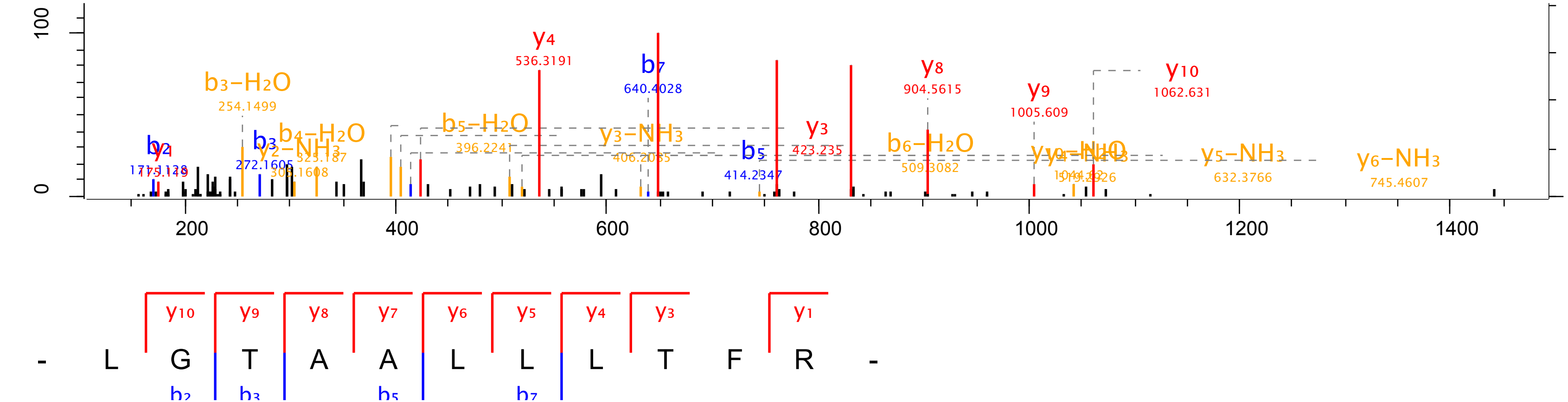

Raw file	Scan	Method	Score	m/z	Gene names
20141014_fract14_dyn_5ul_F6_01_593	39070	TOF; CID	96.03	517.8	TMEM167A

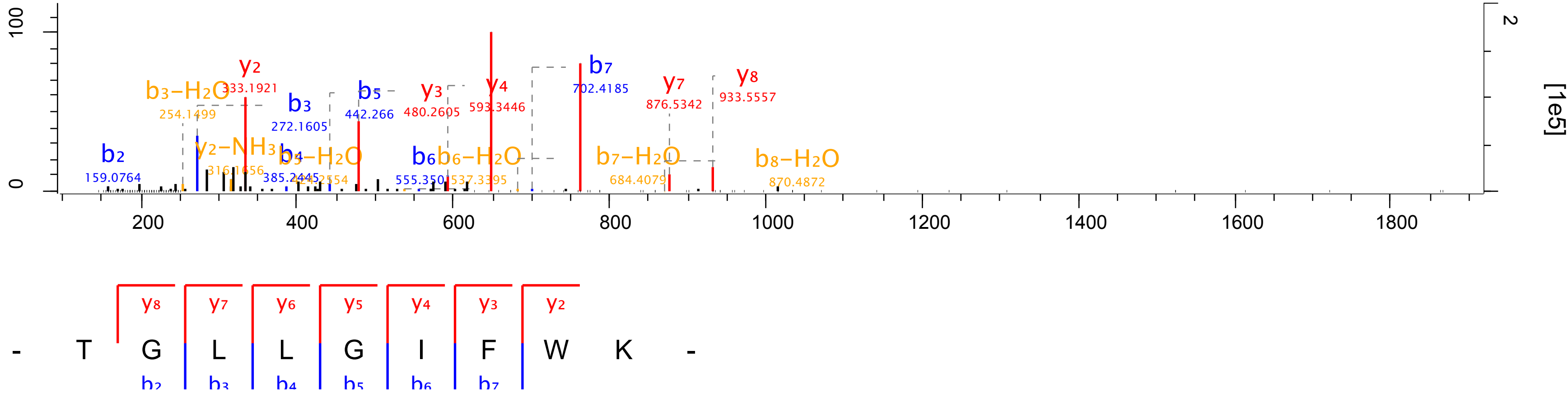

Raw file	Scan	Method	Score	m/z	Gene names
20141014_fract14_dyn_5ul_F6_01_593	39365	TOF; CID	74.17	579.31	FAM184B

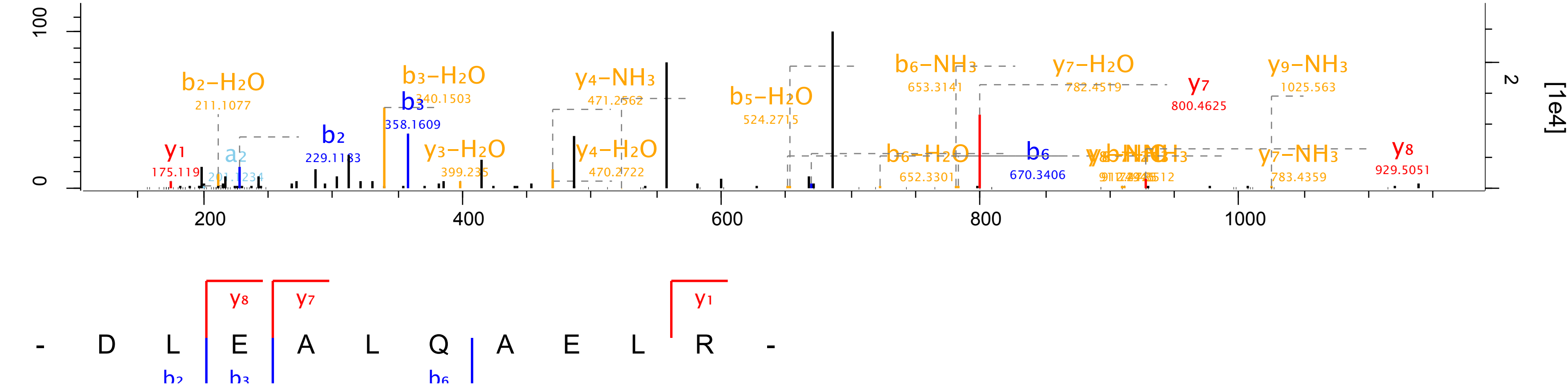

Raw file	Scan	Method	Score	m/z	Gene names
20141014_fract15_dyn_5ul_F7_01_594	13920	TOF; CID	69.37	521.3	CCDC17

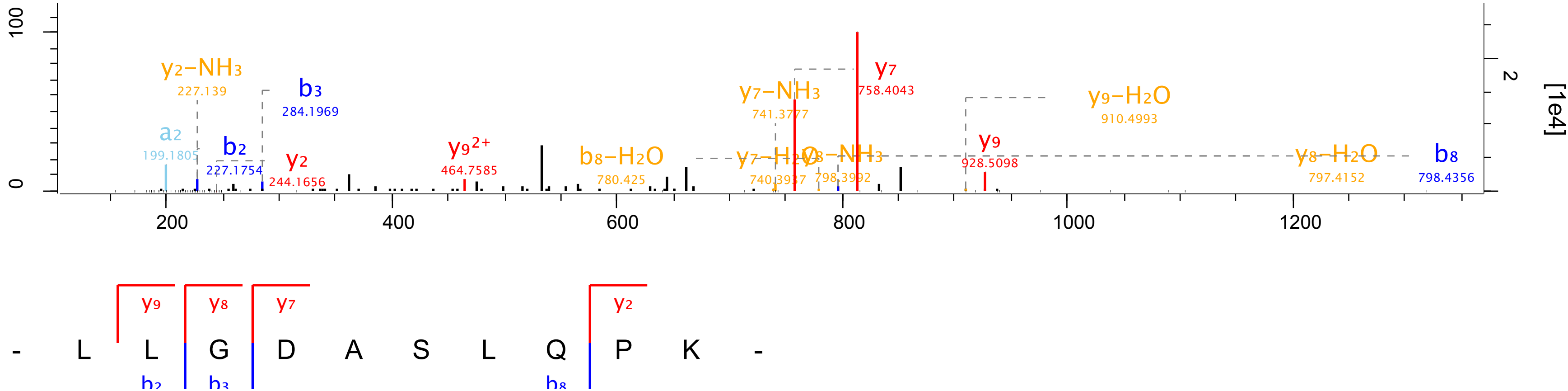

Raw file	Scan	Method	Score	m/z	Gene names
20141014_fract15_dyn_5ul_F7_01_594	20708	TOF; CID	99.01	524.28	COX7C

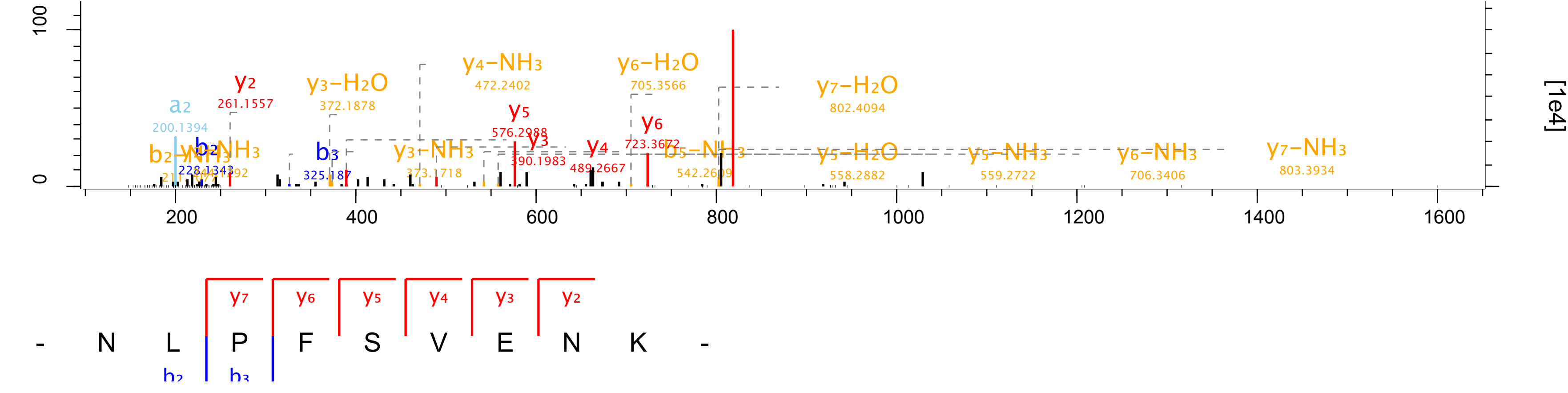

Raw file	Scan	Method	Score	m/z	Gene names
20141014_fract15_dyn_5ul_F7_01_594	24123	TOF; CID	69.03	691.34	TMEM164;RP13-360B22.2

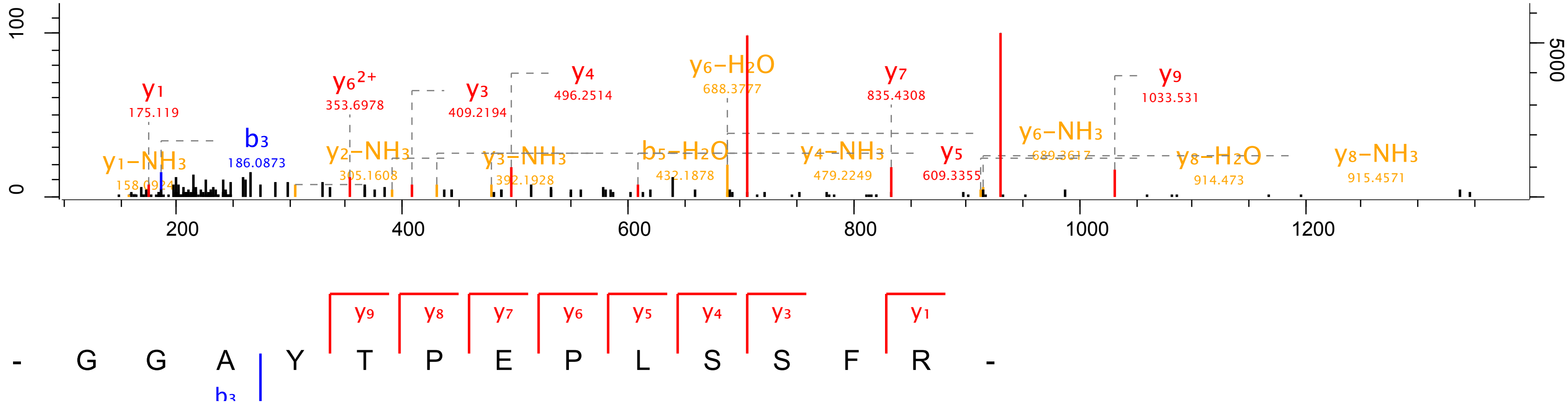

Raw file	Scan	Method	Score	m/z	Gene names
20141014_fract15_dyn_5ul_F7_01_594	30946	TOF; CID	63.73	916.95	TNIP3

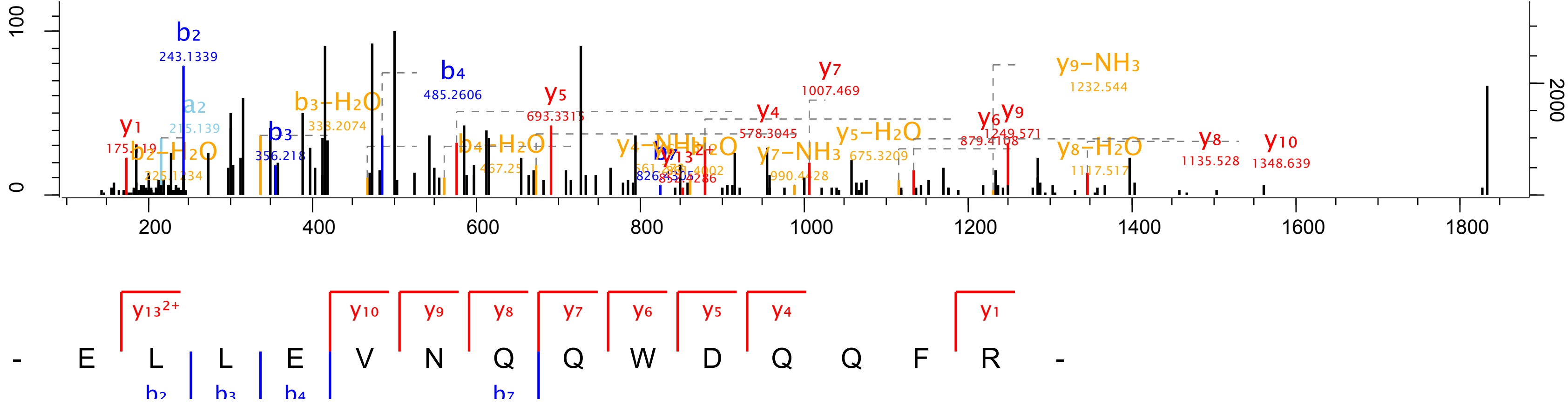

Raw file	Scan	Method	Score	m/z	Gene names
20141014_fract15_dyn_5ul_F7_01_594	32768	TOF; CID	54.86	920.93	DEAF1

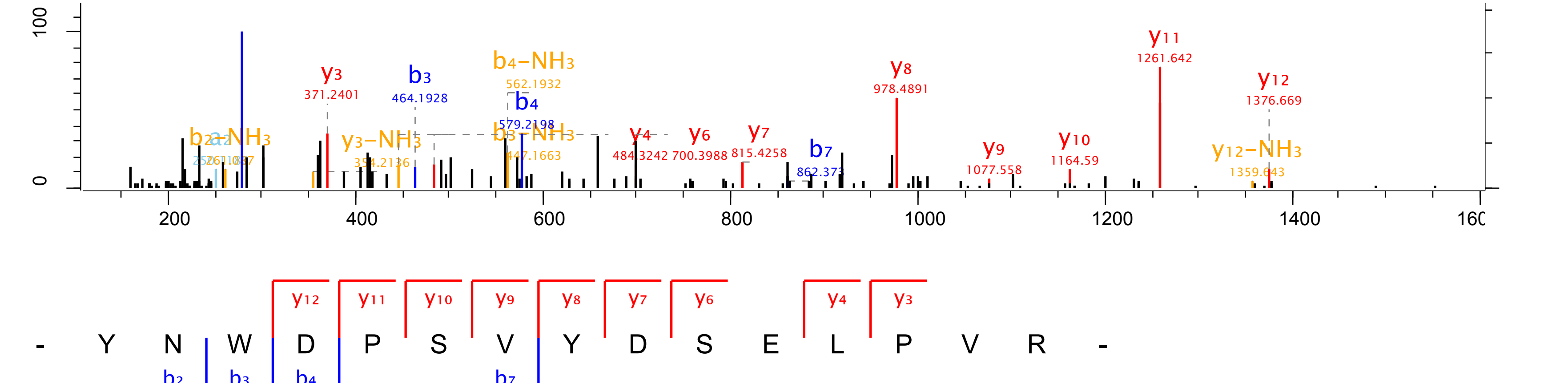

Raw file	Scan	Method	Score	m/z	Gene names
20141014_fract15_dyn_5ul_F7_01_594	34288	TOF; CID	88.39	935.51	VAMP1

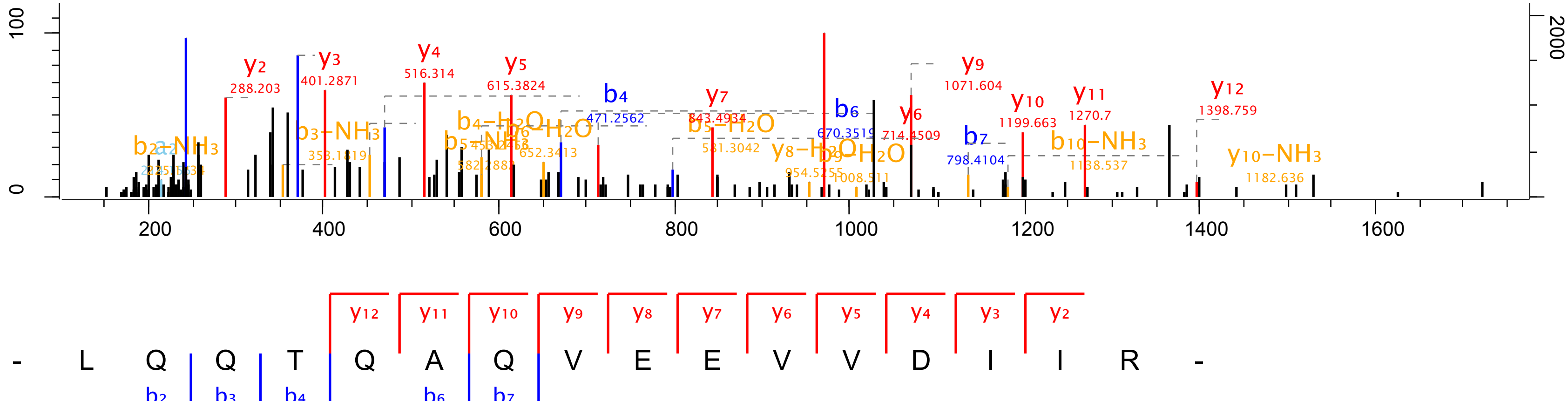

Raw file	Scan	Method	Score	m/z	Gene names
20141014_fract15_dyn_5ul_F7_01_594	34743	TOF; CID	119.62	806.9	NIPA1

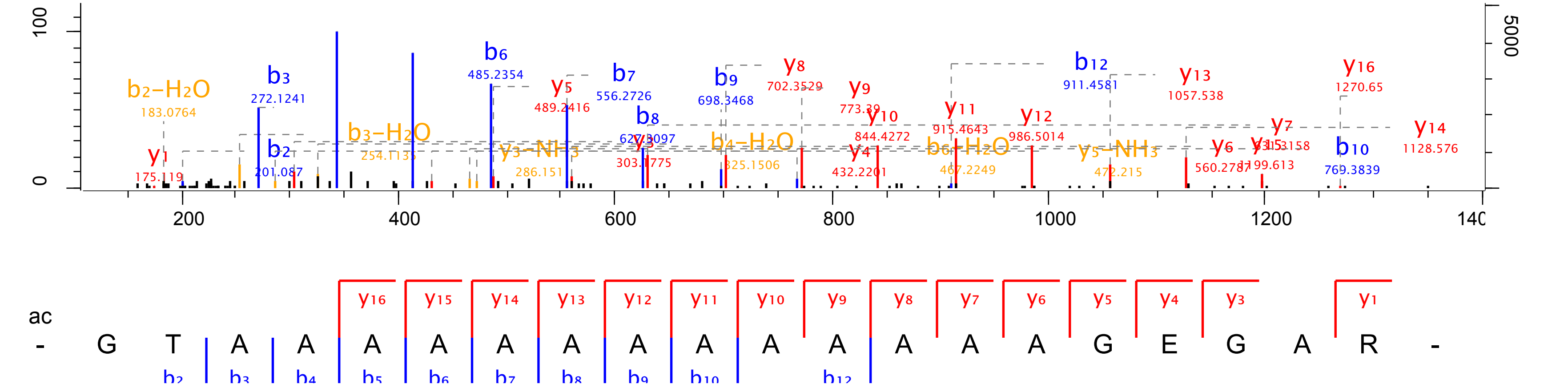

Raw file	Scan	Method	Score	m/z	Gene names
20141014_fract15_dyn_5ul_F7_01_594	36642	TOF; CID	90.86	741.41	VAV3

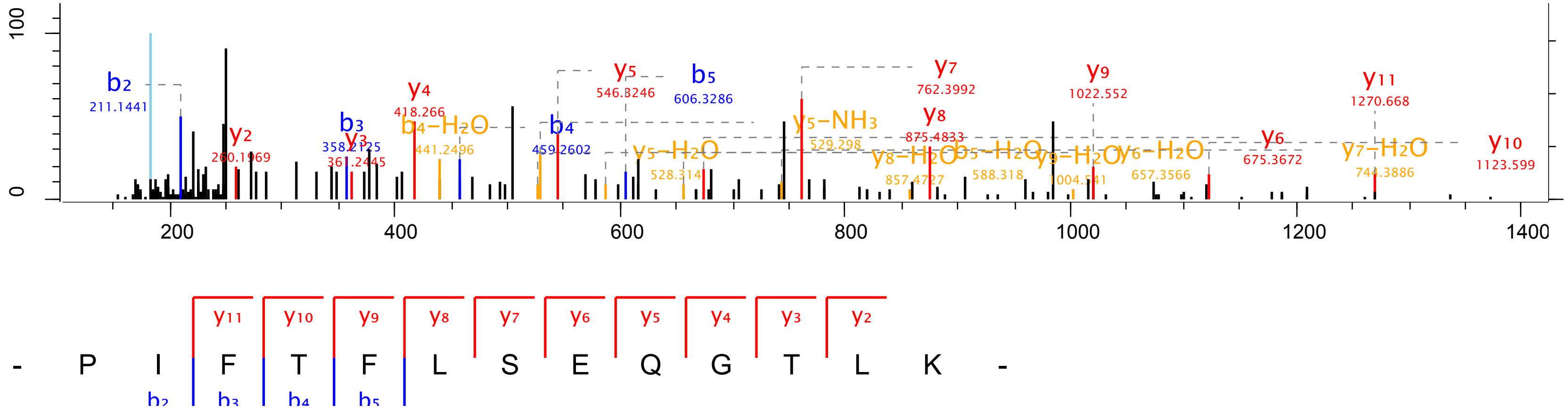

Raw file	Scan	Method	Score	m/z	Gene names
20141014_fract15_dyn_5ul_F7_01_594	39052	TOF; CID	91.66	630.99	CSAD

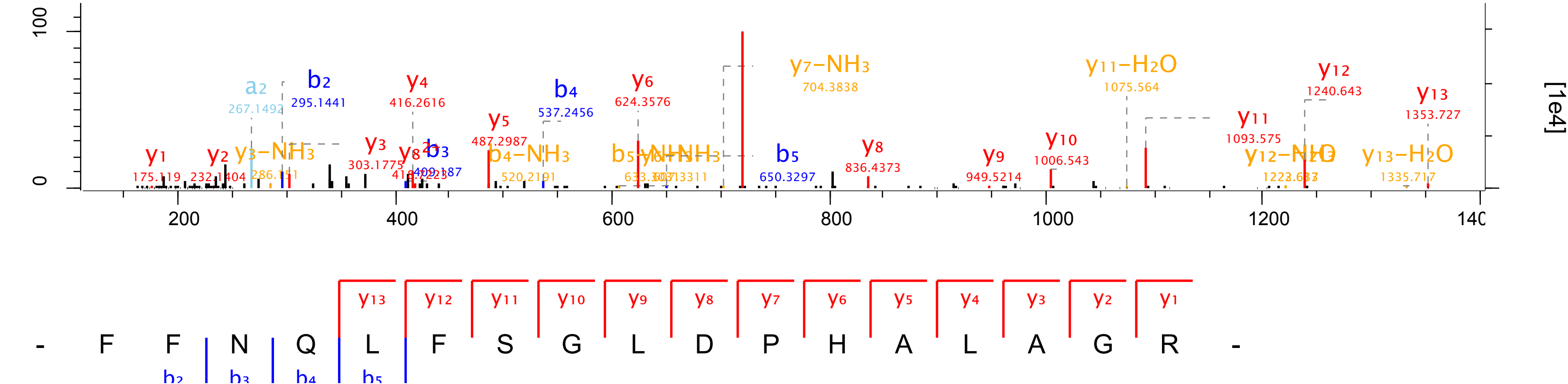

Raw file	Scan	Method	Score	m/z	Gene names
20141014_fract15_dyn_5ul_F7_01_594	41392	TOF; CID	86.8	1029.03	SLC50A1

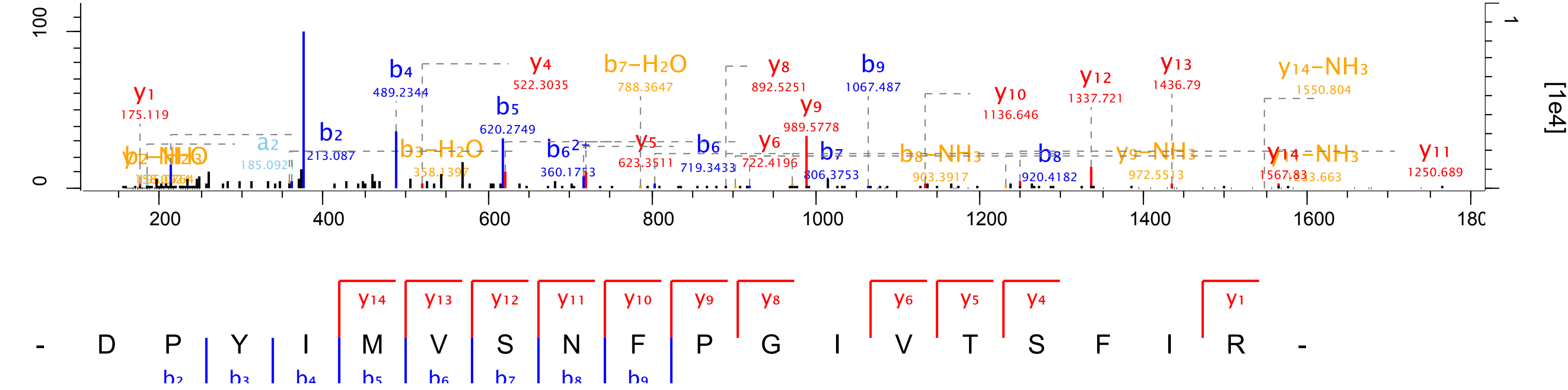

Raw file	Scan	Method	Score	m/z	Gene names
20141014_fract16_dyn_5ul_F8_01_595	8984	TOF; CID	94.4	637.81	FUNDC1

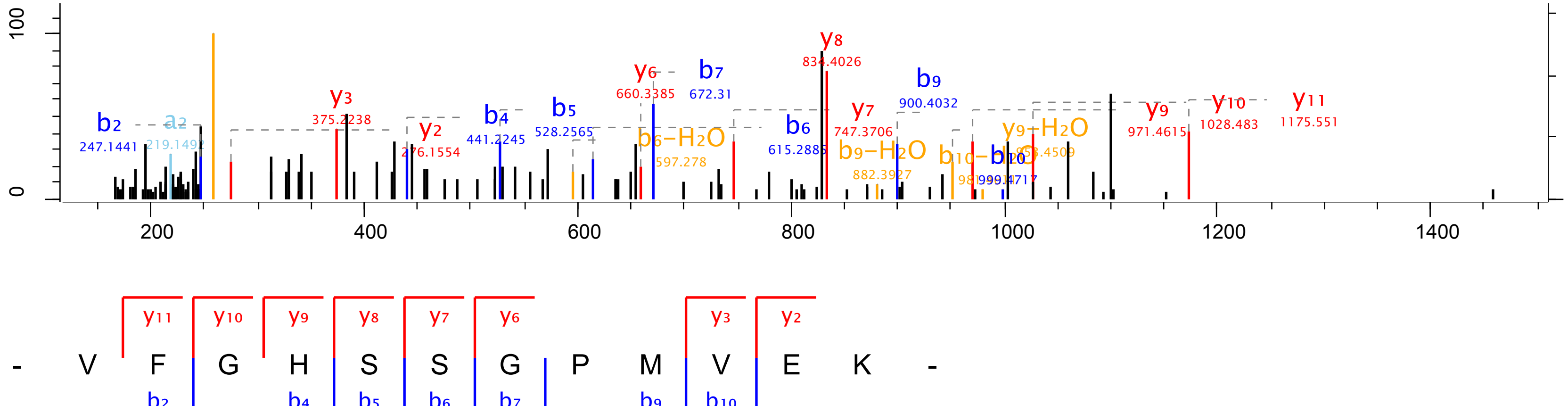

Raw file	Scan	Method	Score	m/z	Gene names
20141014_fract16_dyn_5ul_F8_01_595	13765	TOF; CID	88.5	522.8	TRAF5

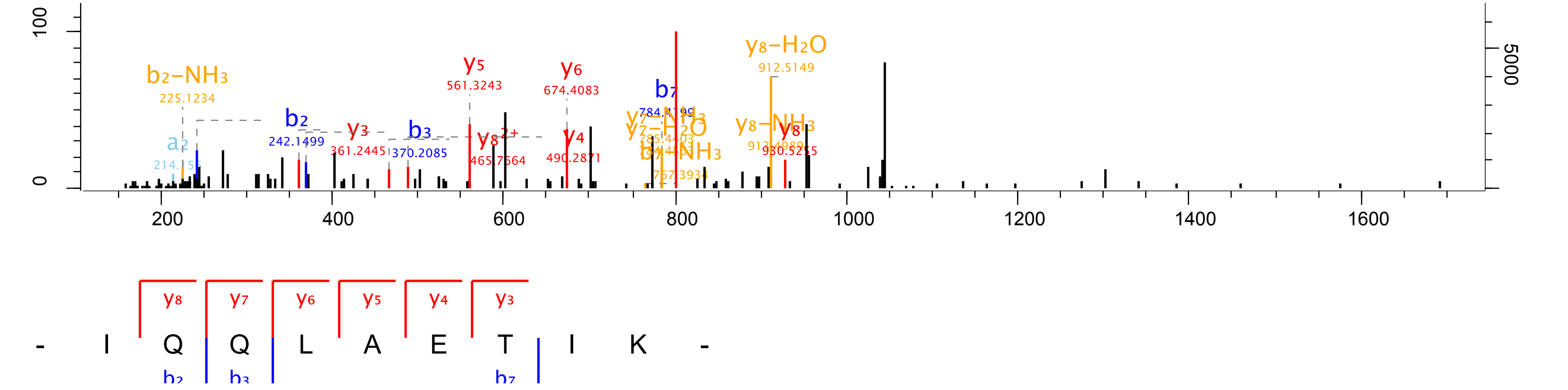

Raw file	Scan	Method	Score	m/z	Gene names
20141014_fract16_dyn_5ul_F8_01_595	15101	TOF; CID	65.28	631.78	CRLS1

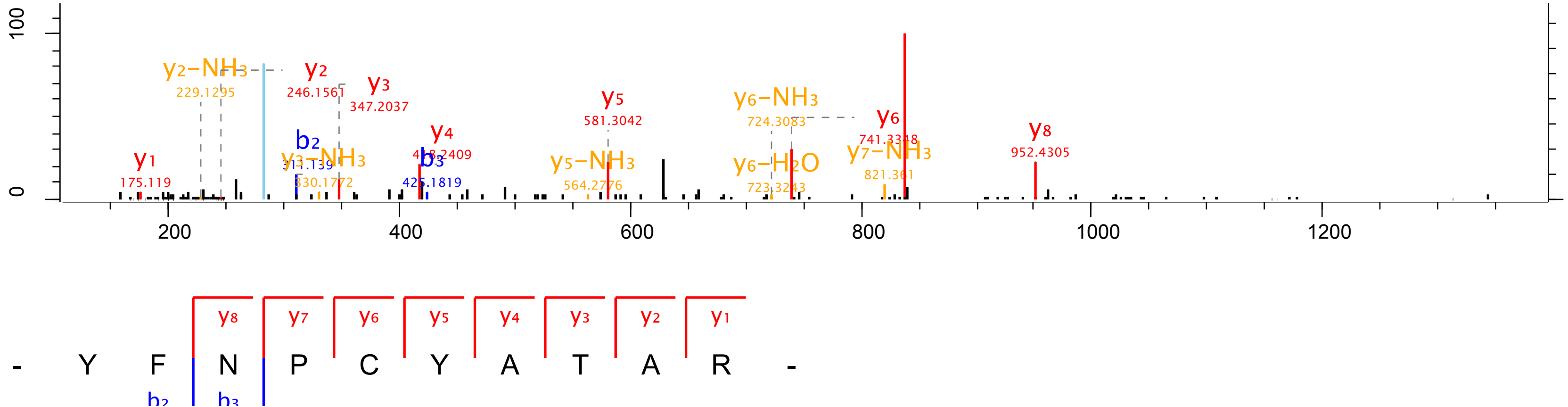

Raw file	Scan	Method	Score	m/z	Gene names
20141014_fract16_dyn_5ul_F8_01_595	15536	TOF; CID	61.41	504.27	ZNF385A;SOX6

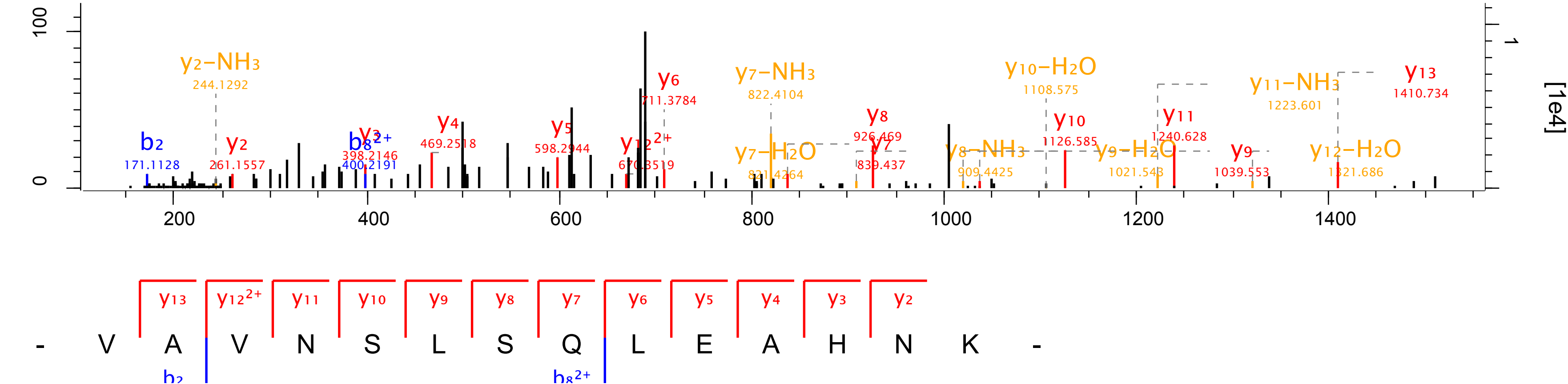

Raw file	Scan	Method	Score	m/z	Gene names
20141014_fract16_dyn_5ul_F8_01_595	15923	TOF; CID	89.43	602.33	DAZAP2

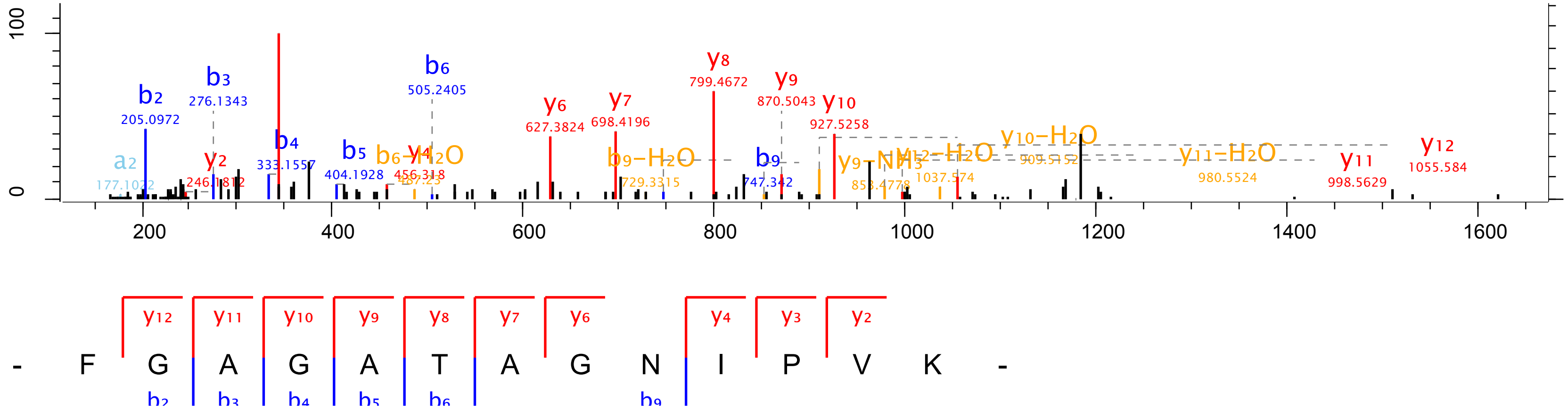

Raw file	Scan	Method	Score	m/z	Gene names
20141014_fract16_dyn_5ul_F8_01_595	18528	TOF; CID	104.26	705.36	TMEM69

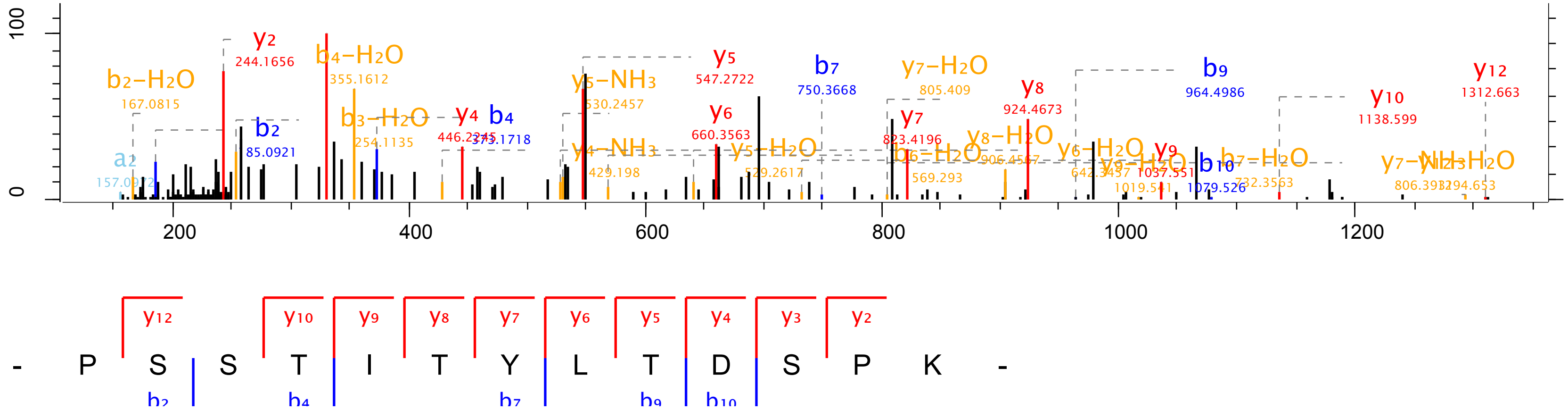

Raw file	Scan	Method	Score	m/z	Gene names
20141014_fract16_dyn_5ul_F8_01_595	19533	TOF; CID	68.95	1007.49	TMEM243

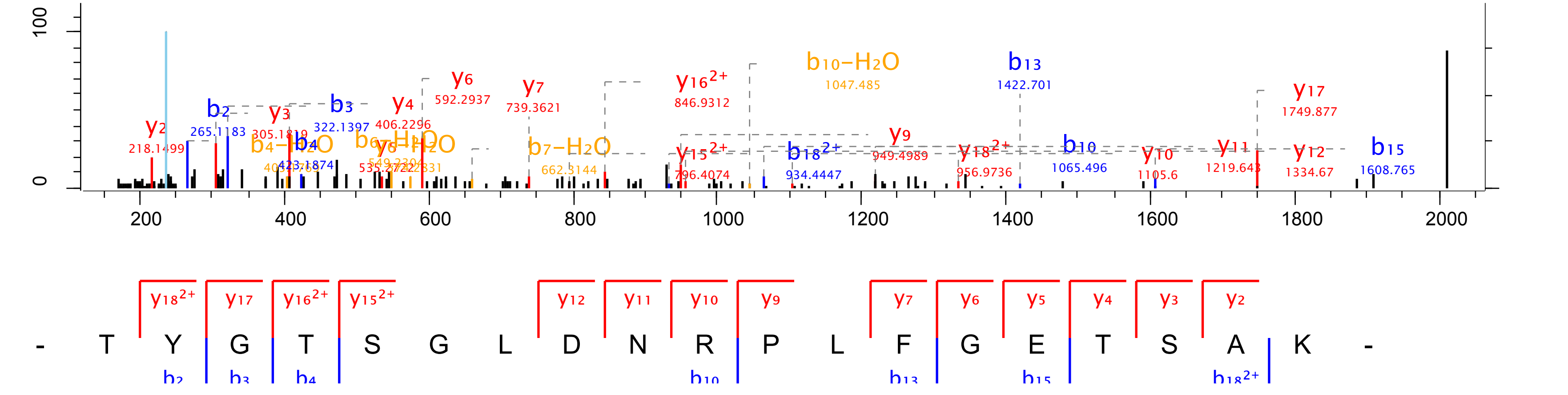

Raw file	Scan	Method	Score	m/z	Gene names
20141014_fract16_dyn_5ul_F8_01_595	20390	TOF; CID	78.51	709.89	DIRC2

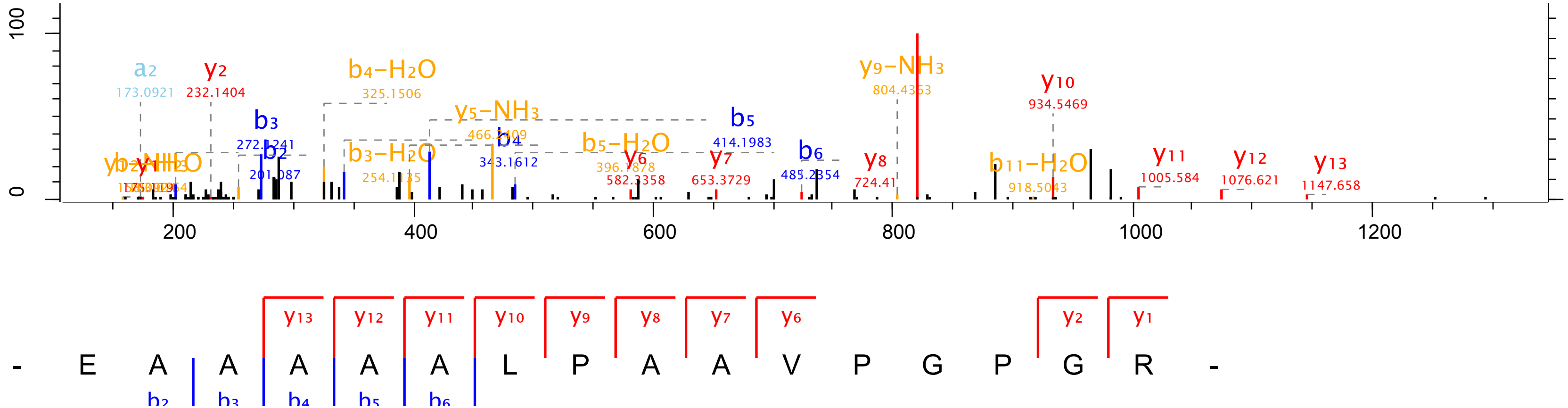

Raw file	Scan	Method	Score	m/z	Gene names
20141014_fract16_dyn_5ul_F8_01_595	25303	TOF; CID	72.21	730.38	DMBT1

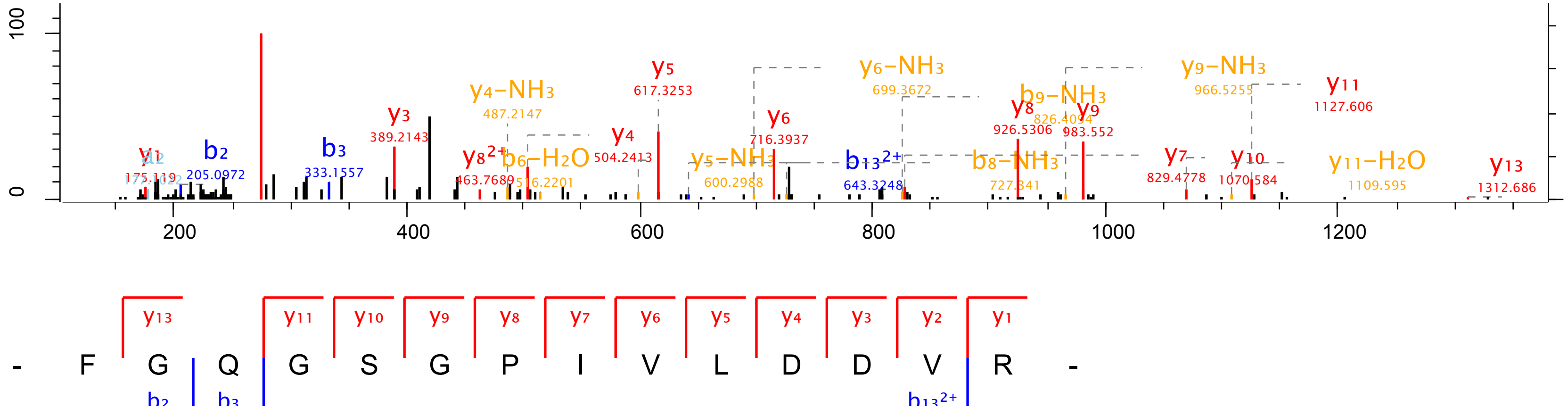

Raw file	Scan	Method	Score	m/z	Gene names
20141014_fract16_dyn_5ul_F8_01_595	28148	TOF; CID	81.97	1010.09	FAM199X

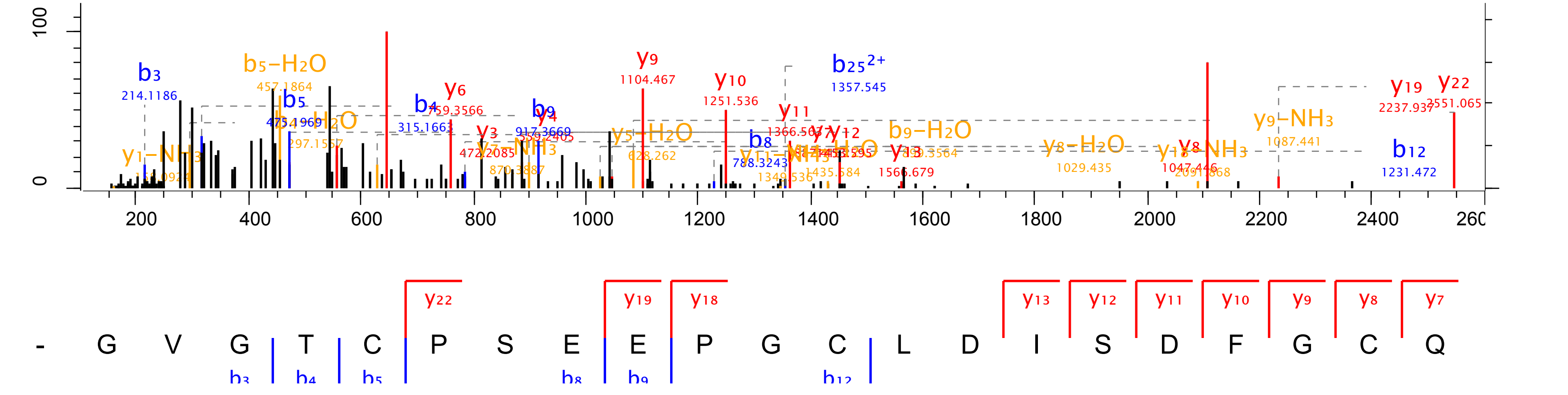

Raw file	Scan	Method	Score	m/z	Gene names
20141014_fract16_dyn_5ul_F8_01_595	32575	TOF; CID	91.4	955.46	CEP350

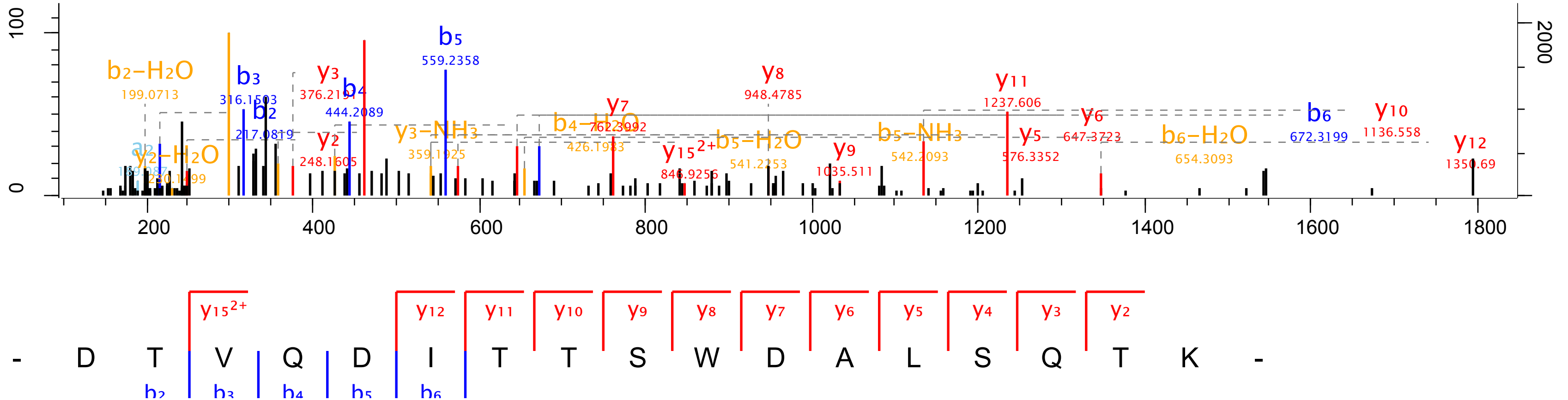

Raw file	Scan	Method	Score	m/z	Gene names
20141014_fract16_dyn_5ul_F8_01_595	34265	TOF; CID	59.37	860.4	SWI5

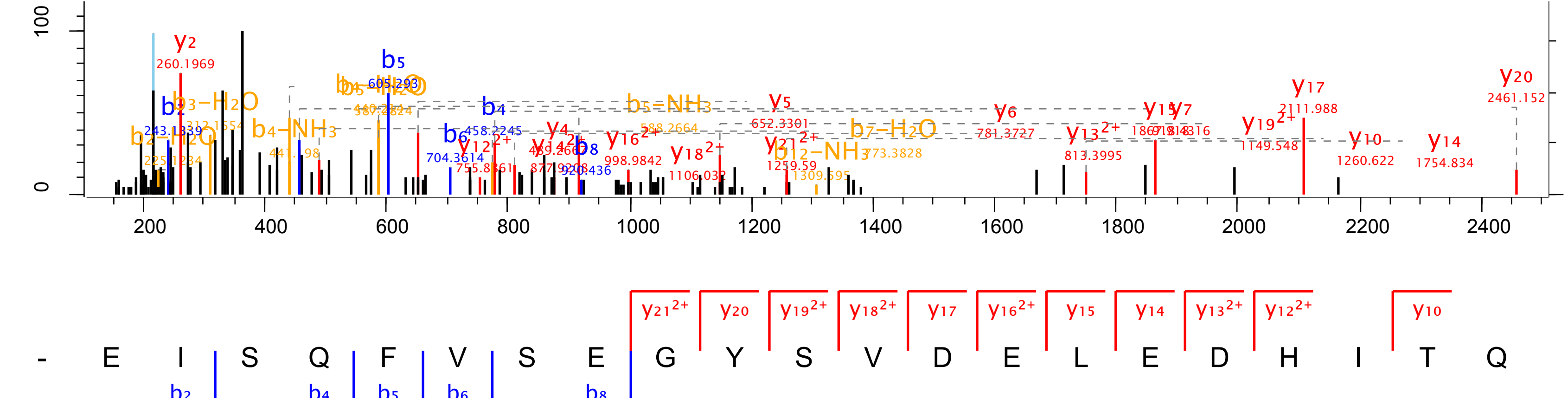

Raw file	Scan	Method	Score	m/z	Gene names
20141014_fract16_dyn_5ul_F8_01_595	37267	TOF; CID	66.83	898.01	R3HDM4

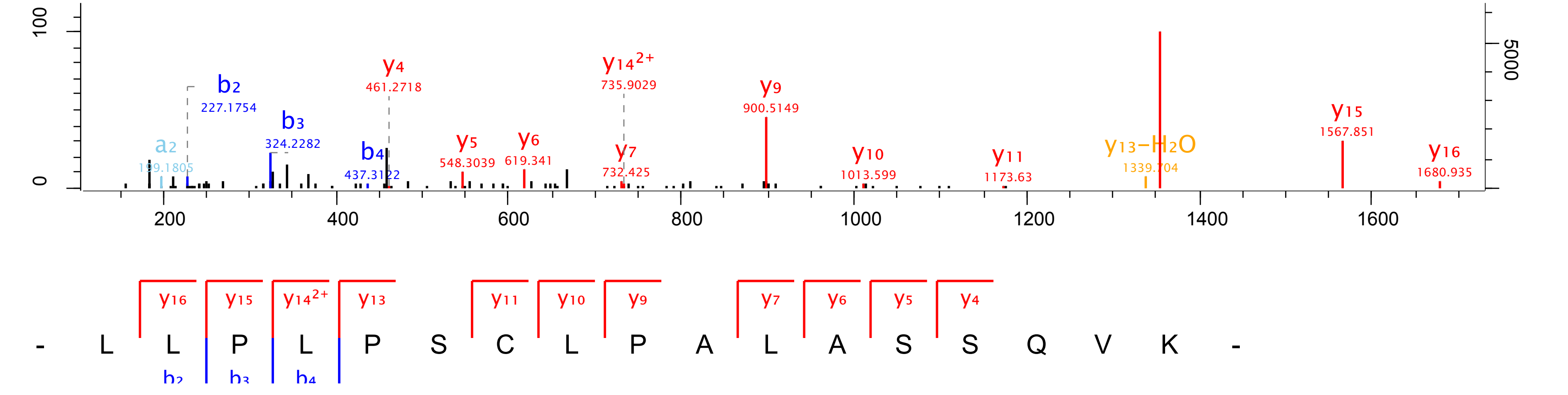

Raw file	Scan	Method	Score	m/z	Gene names
20141014_fract17_dyn_5ul_G1_01_596	11851	TOF; CID	110.08	689.87	FBXO33

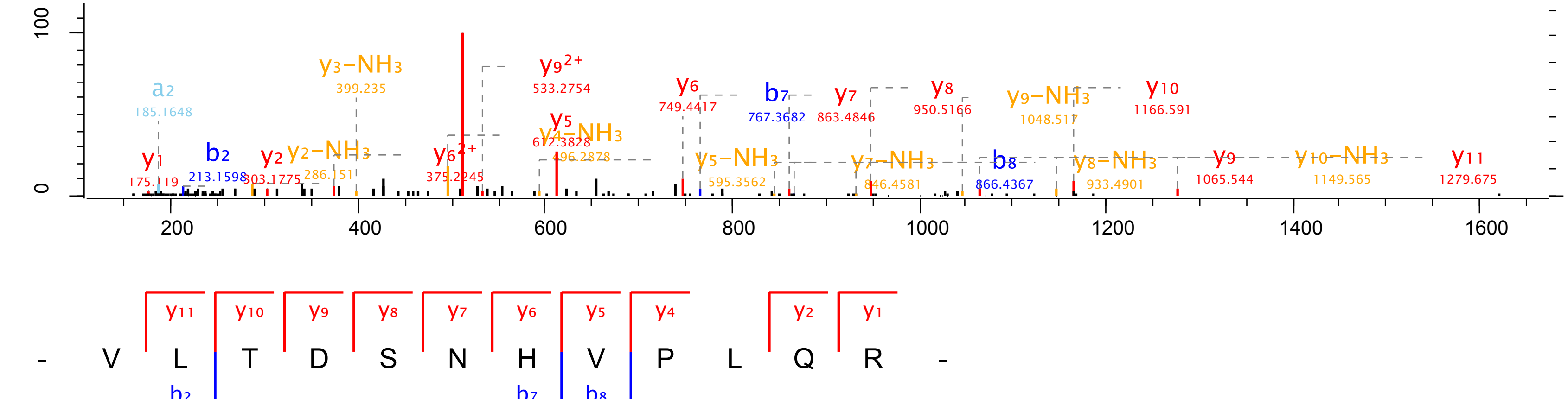

Raw file	Scan	Method	Score	m/z	Gene names
20141014_fract17_dyn_5ul_G1_01_596	19333	TOF; CID	91.96	553.79	C19orf43

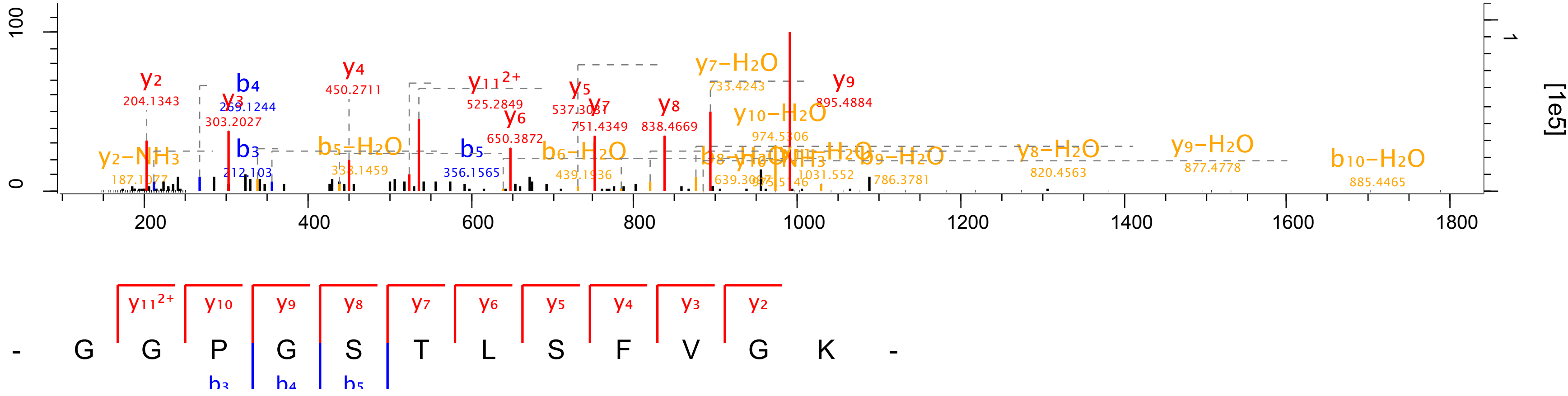

Raw file	Scan	Method	Score	m/z	Gene names
20141014_fract17_dyn_5ul_G1_01_596	20256	TOF; CID	52.17	563.64	ELF4

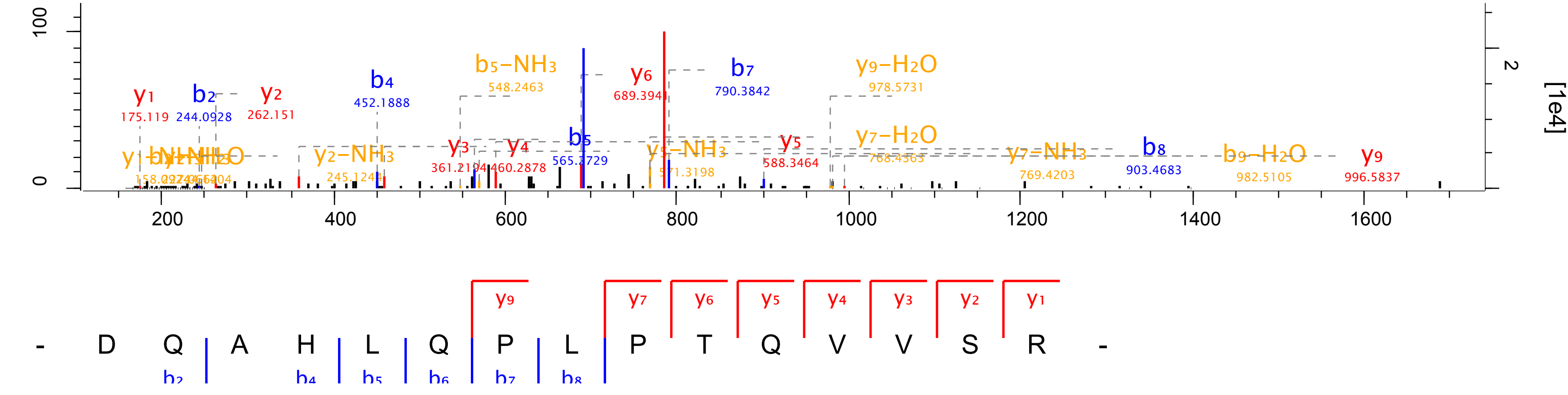

Raw file	Scan	Method	Score	m/z	Gene names
20141014_fract17_dyn_5ul_G1_01_596	23450	TOF; CID	79.28	622.34	PEX2

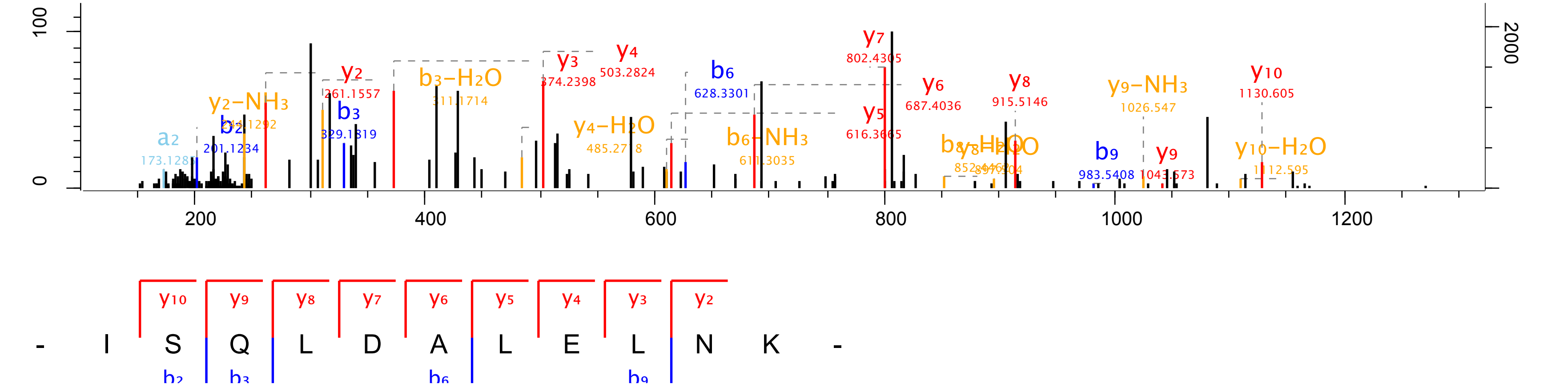

Raw file	Scan	Method	Score	m/z	Gene names
20141014_fract17_dyn_5ul_G1_01_596	23528	TOF; CID	62.03	813.07	LHFPL2

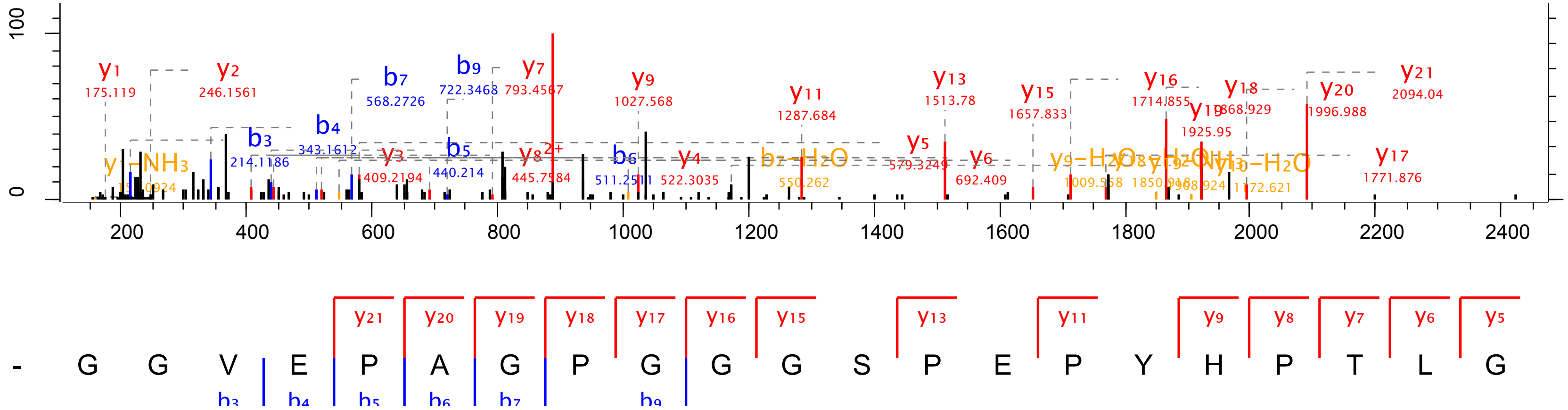

Raw file	Scan	Method	Score	m/z	Gene names
20141014_fract17_dyn_5ul_G1_01_596	23833	TOF; CID	98.16	587.81	SYNGR3

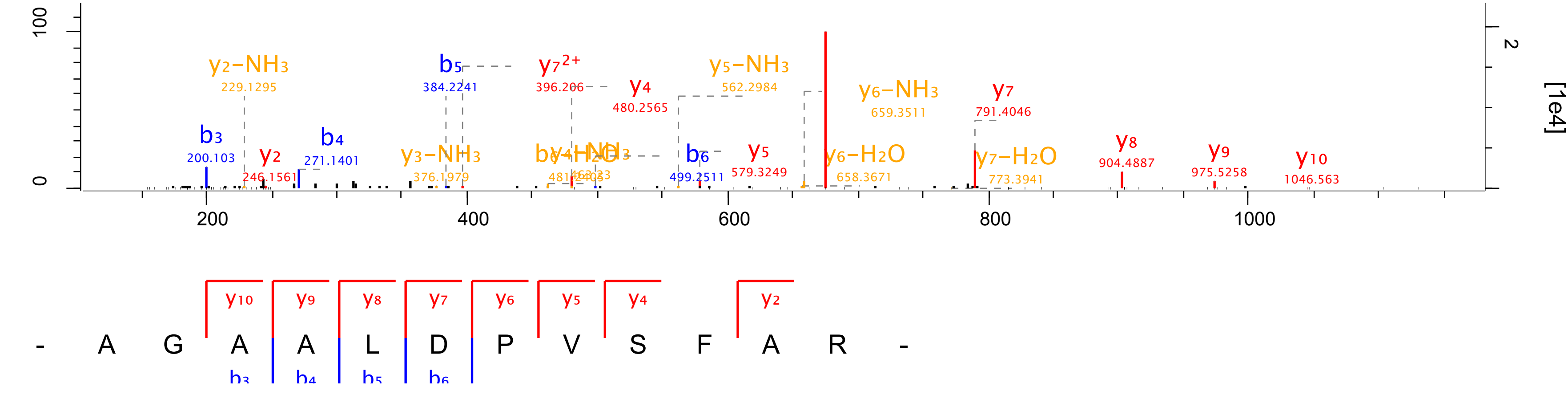

Raw file	Scan	Method	Score	m/z	Gene names
20141014_fract17_dyn_5ul_G1_01_596	27158	TOF; CID	64	930.47	HIAT1

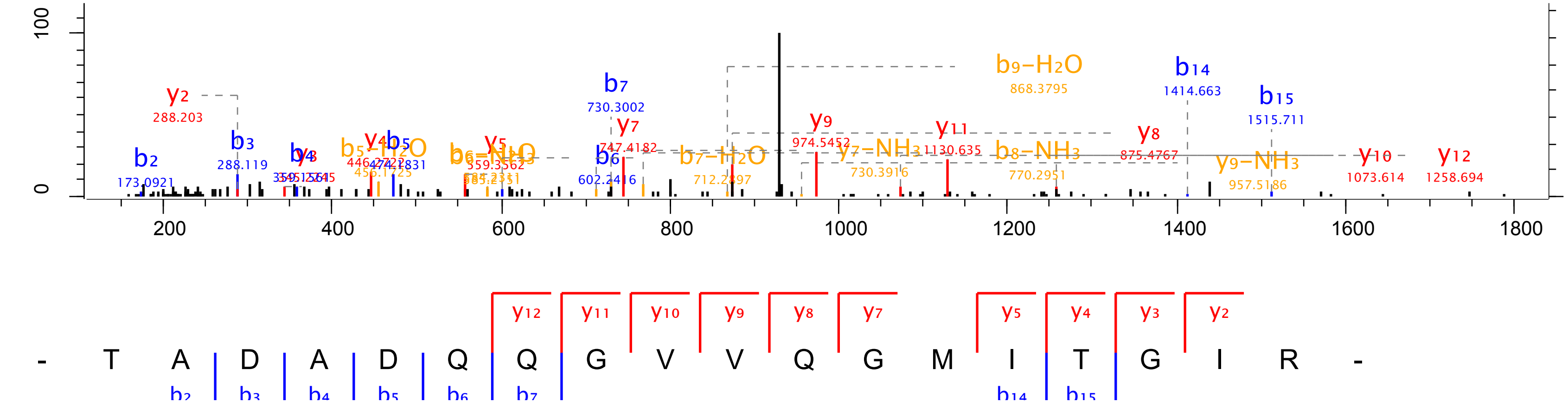

Raw file	Scan	Method	Score	m/z	Gene names
20141014_fract17_dyn_5ul_G1_01_596	27778	TOF; CID	58.89	862.4	PSENEN

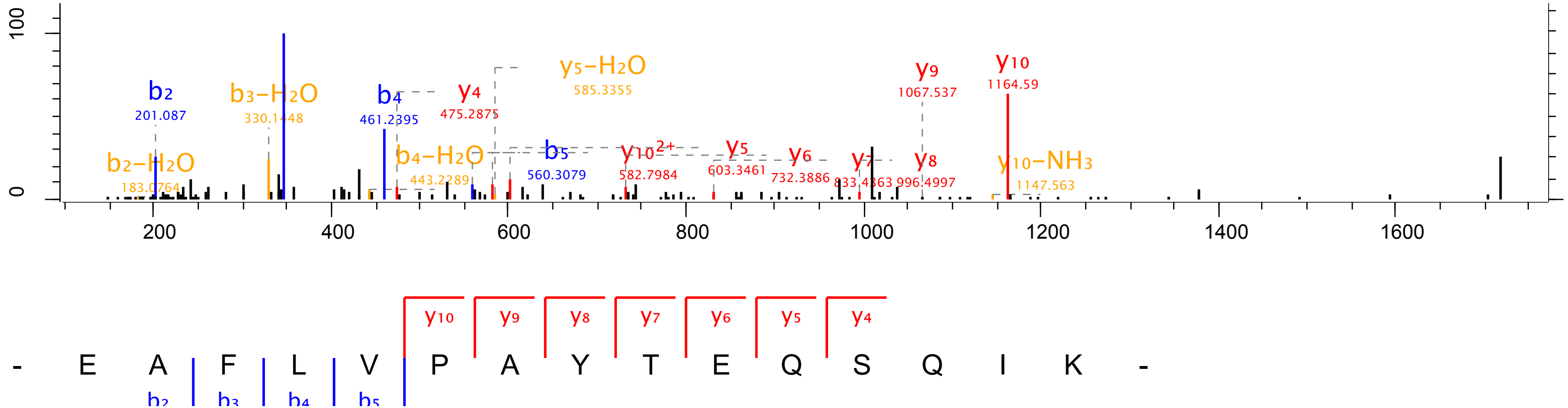

Raw file	Scan	Method	Score	m/z	Gene names
20141014_fract17_dyn_5ul_G1_01_596	29706	TOF; CID	67.88	848.91	WDR65

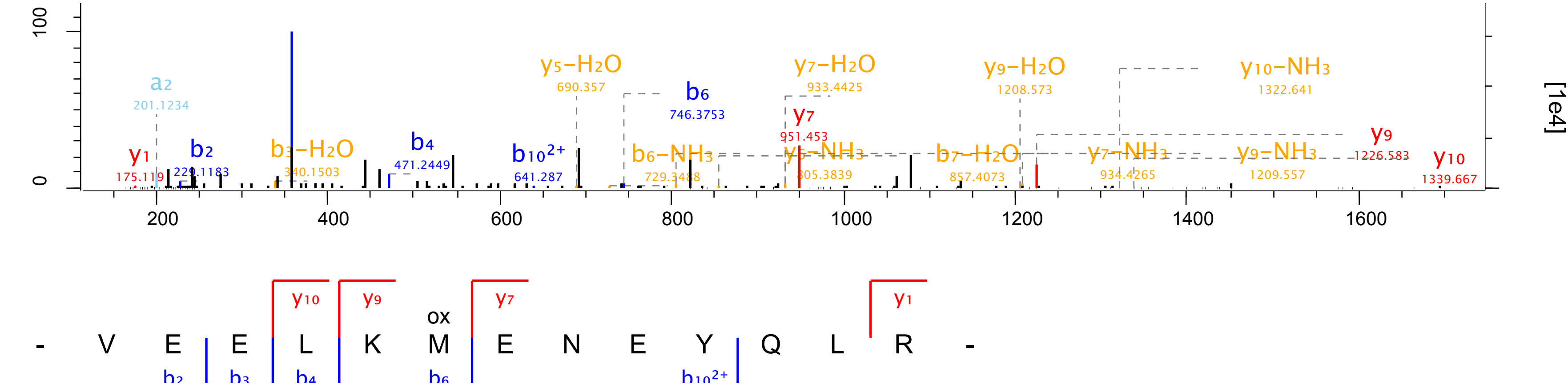

Raw file	Scan	Method	Score	m/z	Gene names
20141014_fract17_dyn_5ul_G1_01_596	31561	TOF; CID	53.24	712.84	DUSP18

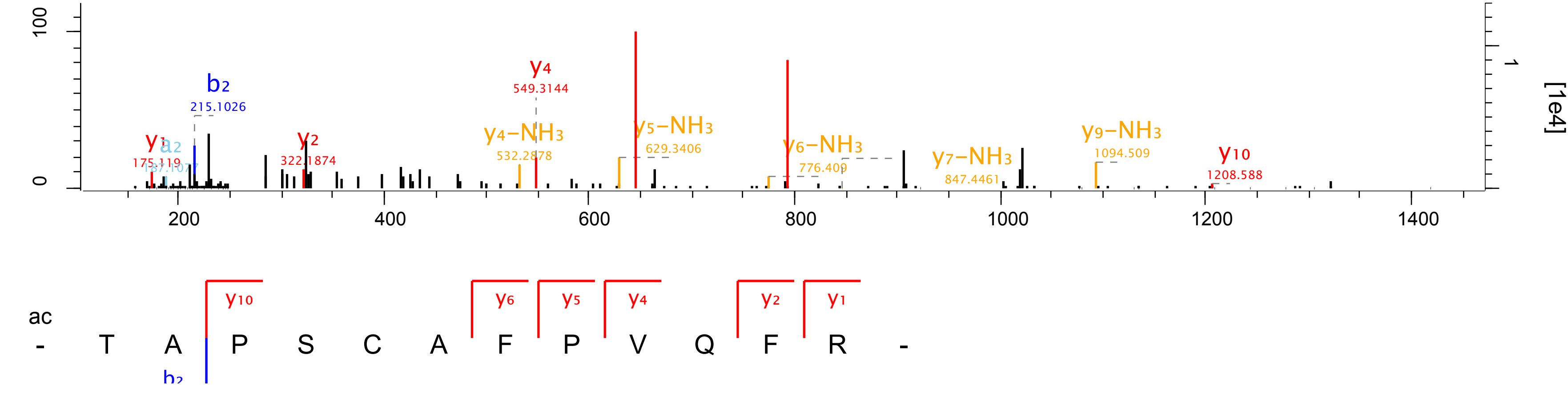

Raw file	Scan	Method	Score	m/z	Gene names
20141014_fract17_dyn_5ul_G1_01_596	34721	TOF; CID	149.73	1007.98	TMEM106C

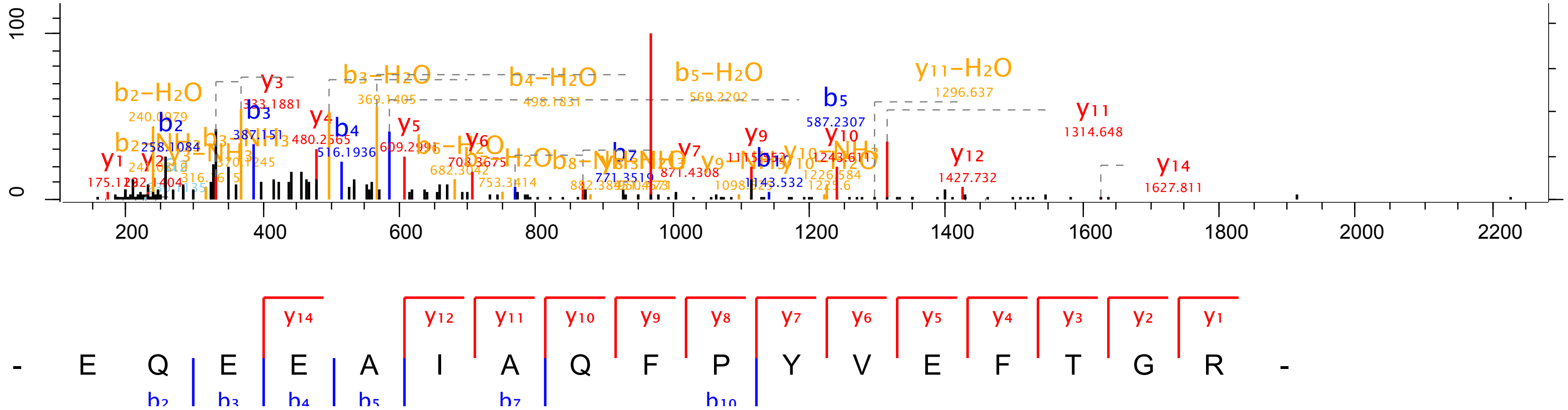

Raw file	Scan	Method	Score	m/z	Gene names
20141014_fract17_dyn_5ul_G1_01_596	36308	TOF; CID	38.72	743.38	ZNF512B

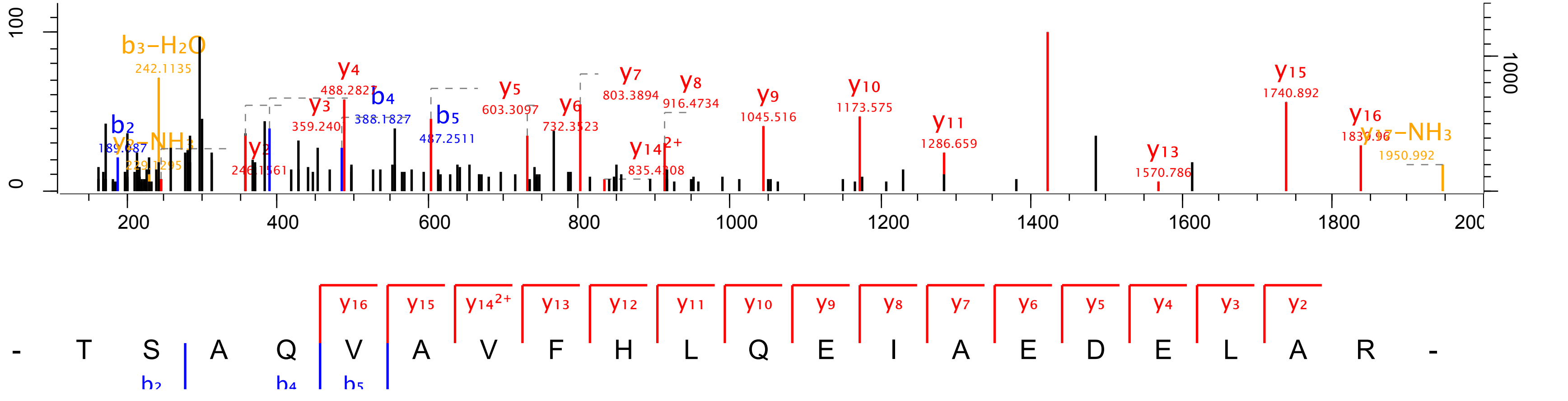

Raw file	Scan	Method	Score	m/z	Gene names
20141014_fract17_dyn_5ul_G1_01_596	38062	TOF; CID	52.46	1240.52	RNF11

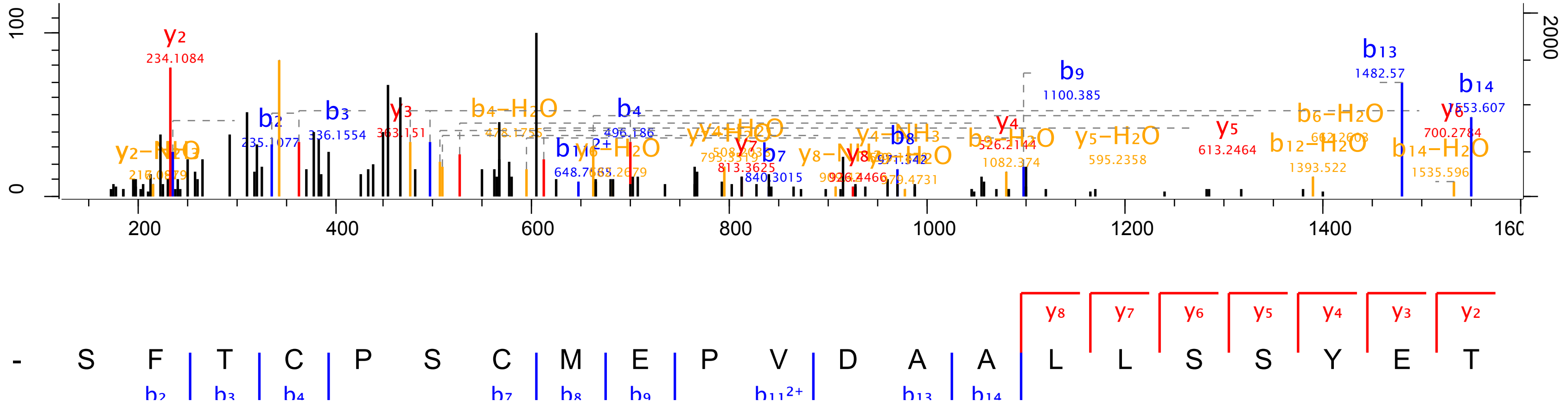

Raw file	Scan	Method	Score	m/z	Gene names
20141014_fract18_dyn_5ul_G2_01_597	8120	TOF; CID	56.2	457.24	ZFP62

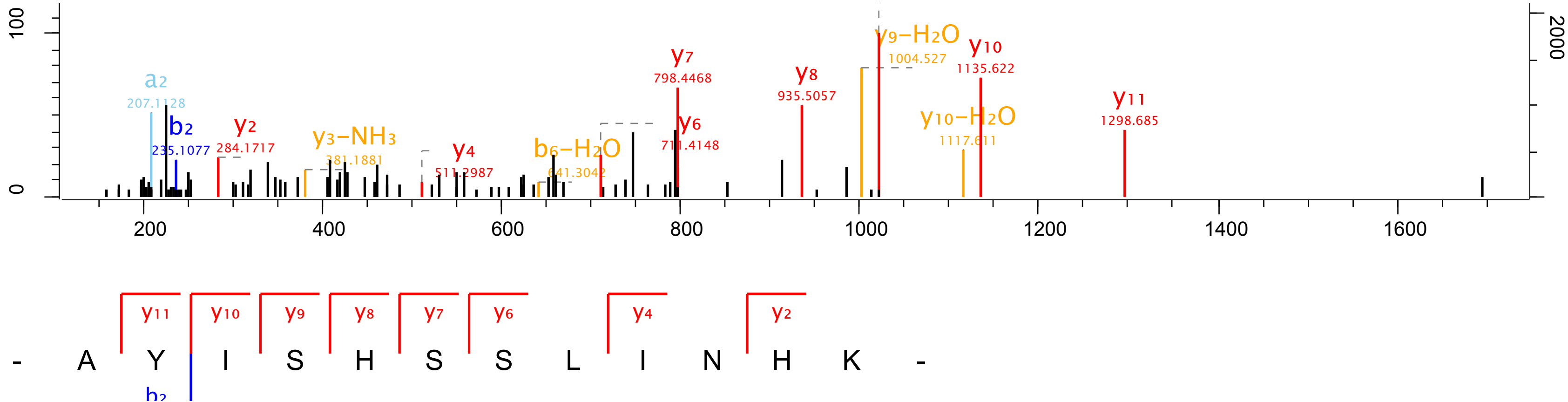

Raw file	Scan	Method	Score	m/z	Gene names
20141014_fract18_dyn_5ul_G2_01_597	12482	TOF; CID	80.23	487.78	CENPW

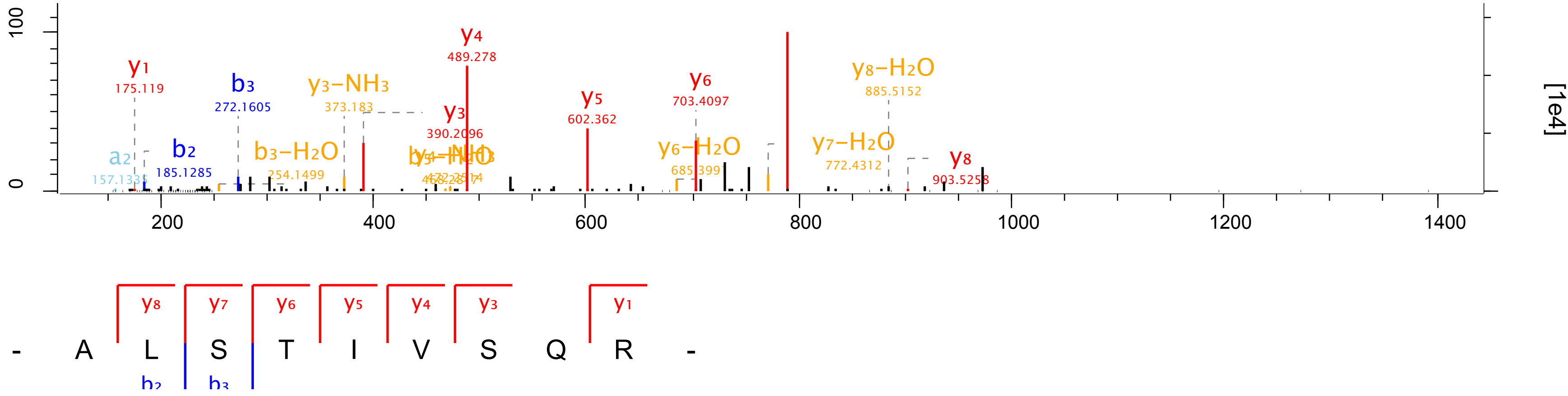

Raw file	Scan	Method	Score	m/z	Gene names
20141014_fract18_dyn_5ul_G2_01_597	16907	TOF; CID	106.04	494.79	GPNMB

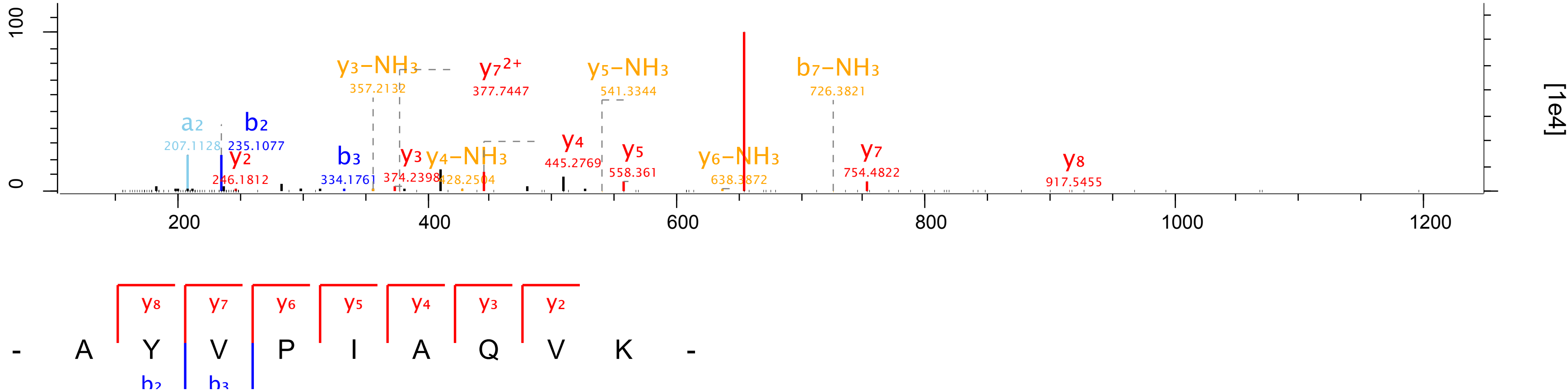

Raw file	Scan	Method	Score	m/z	Gene names
20141014_fract18_dyn_5ul_G2_01_597	17983	TOF; CID	120.92	476.24	MAPK15

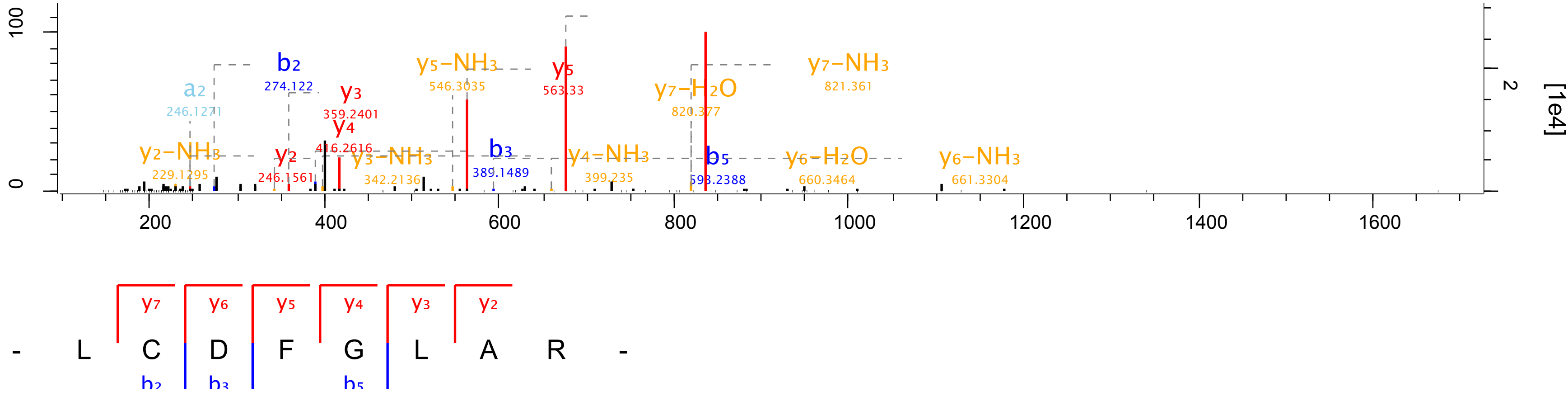

Raw file	Scan	Method	Score	m/z	Gene names
20141014_fract18_dyn_5ul_G2_01_597	23835	TOF; CID	83.95	542.78	HCLS1

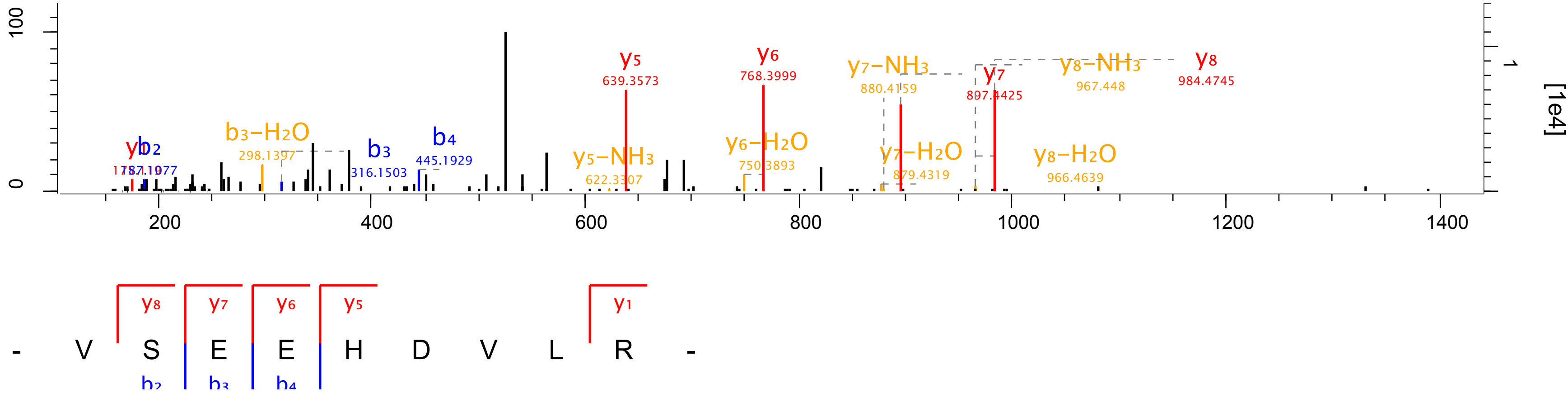

Raw file	Scan	Method	Score	m/z
20141014_fract18_dyn_5ul_G2_01_597	26720	TOF; CID	50.36	809.4

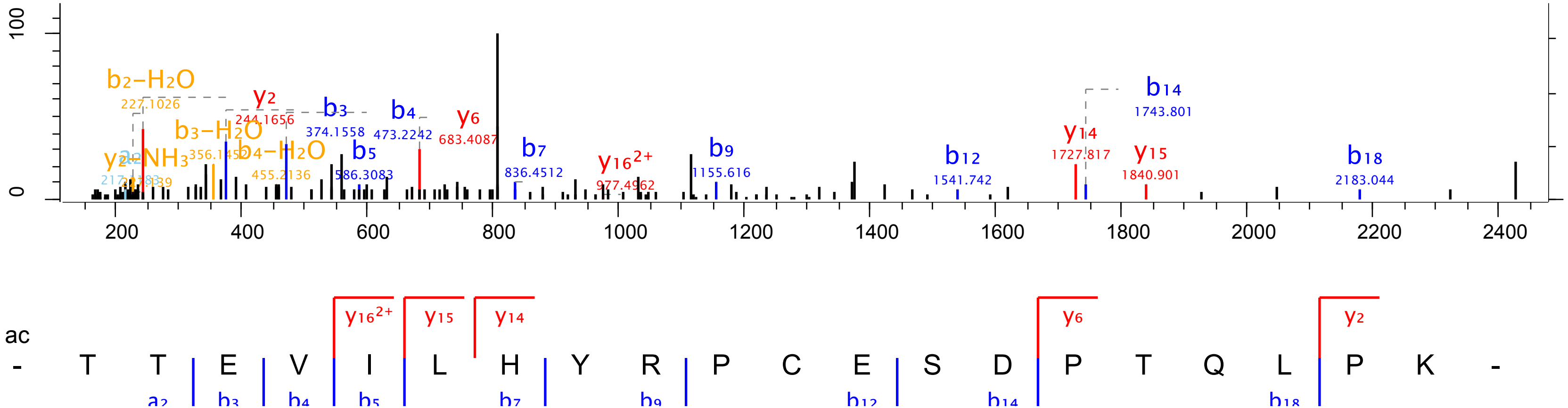

Raw file	Scan	Method	Score	m/z	Gene names
20141014_fract18_dyn_5ul_G2_01_597	27725	TOF; CID	57.94	1108.99	TMEM51

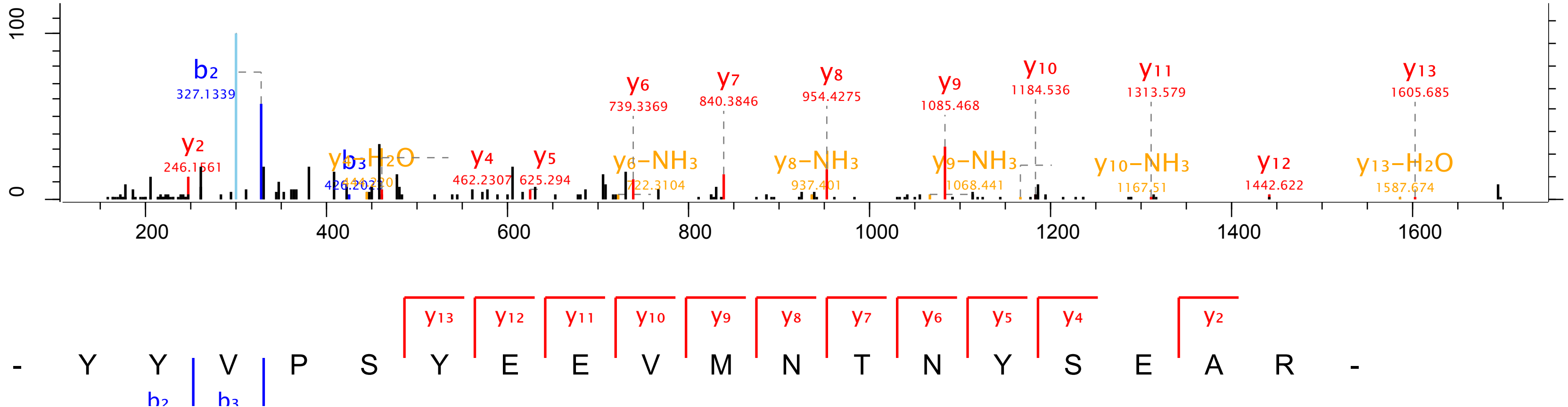

Raw file	Scan	Method	Score	m/z	Gene names
20141014_fract18_dyn_5ul_G2_01_597	28110	TOF; CID	59.8	702.37	VPS37C

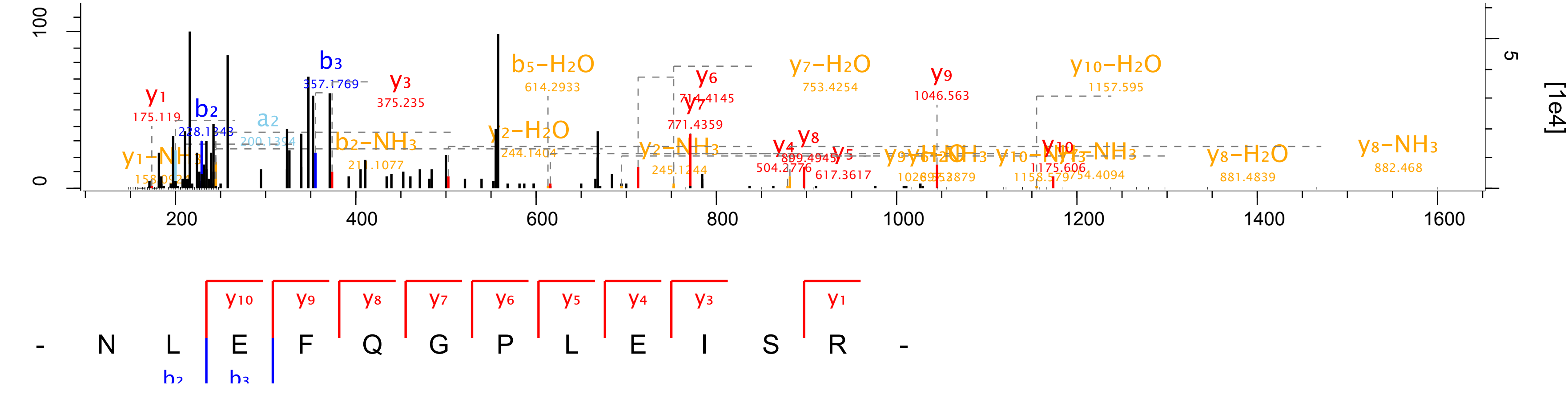

Raw file	Scan	Method	Score	m/z	Gene names
20141014_fract18_dyn_5ul_G2_01_597	28746	TOF; CID	62.1	777.33	TSEN15

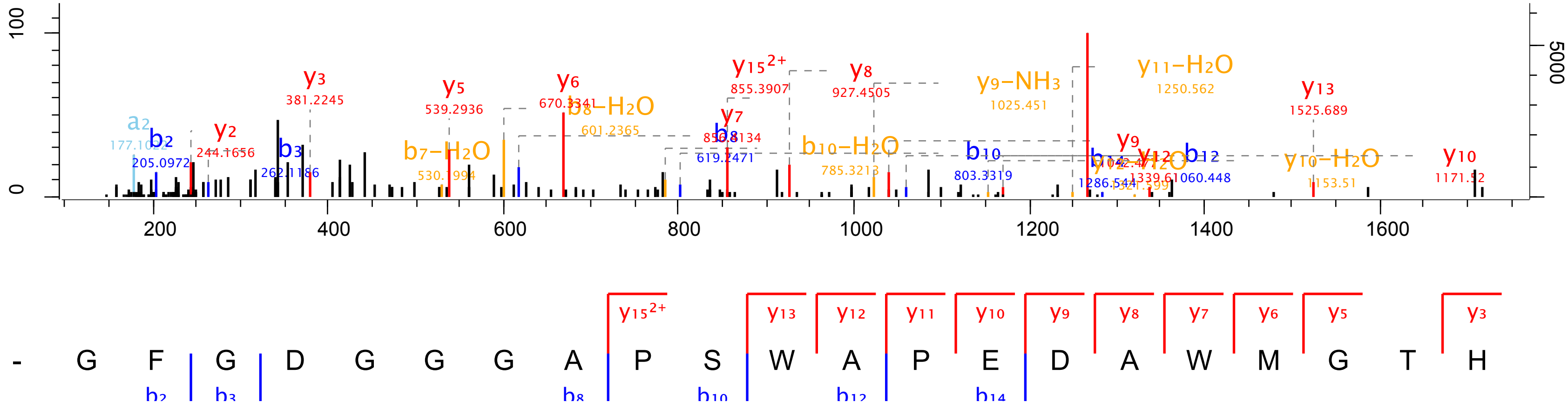

Raw file	Scan	Method	Score	m/z	Gene names
20141014_fract18_dyn_5ul_G2_01_597	29037	TOF; CID	68.24	990.51	UBE2W

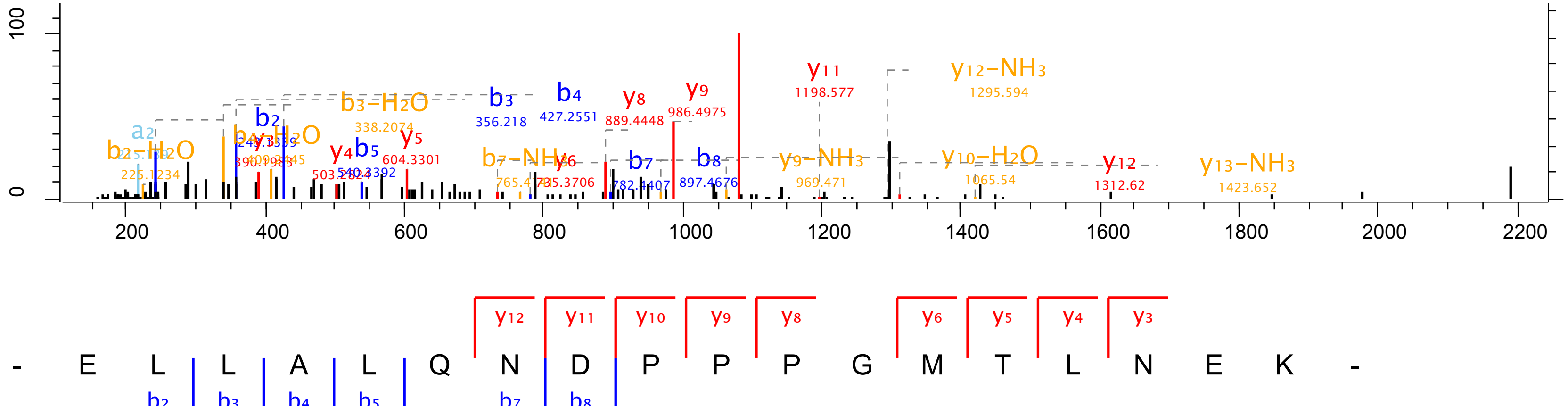

Raw file	Scan	Method	Score	m/z	Gene names
20141014_fract18_dyn_5ul_G2_01_597	29283	TOF; CID	204.53	917.11	EIF4EBP1

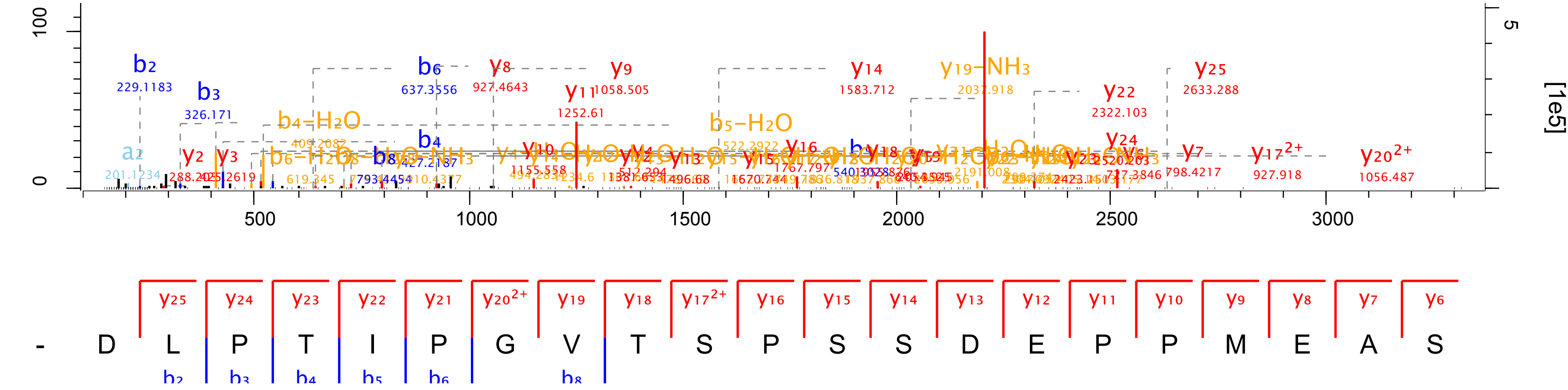

Raw file	Scan	Method	Score	m/z	Gene names
20141014_fract18_dyn_5ul_G2_01_597	29448	TOF; CID	82.45	899.98	BEGAIN

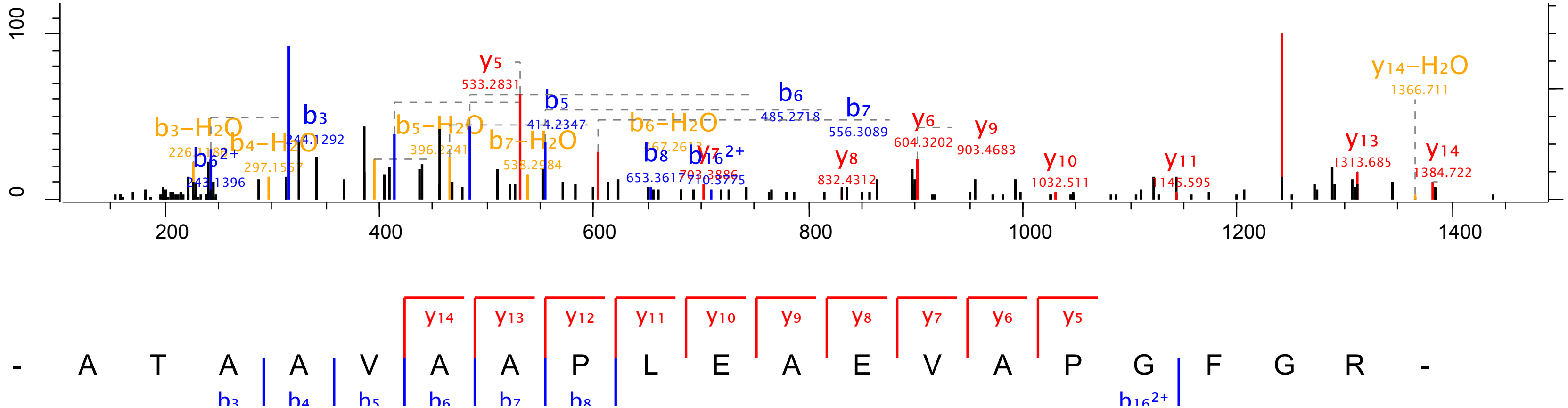

Raw file	Scan	Method	Score	m/z	Gene names
20141014_fract18_dyn_5ul_G2_01_597	30228	TOF; CID	108.72	728.87	MALSU1

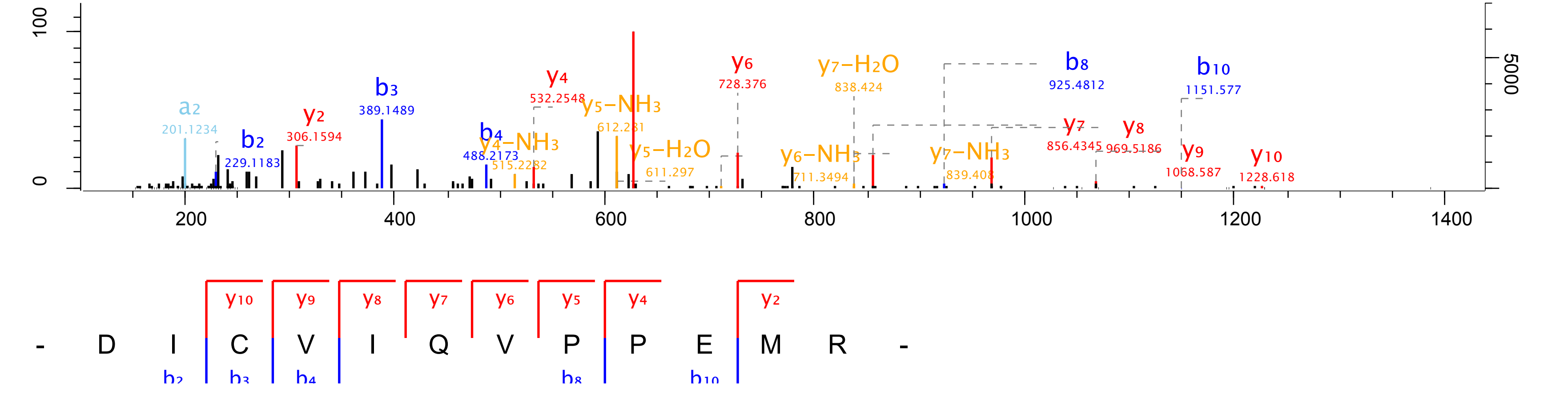

Raw file	Scan	Method	Score	m/z	Gene names
20141014_fract18_dyn_5ul_G2_01_597	31765	TOF; CID	47.04	805.93	PIP

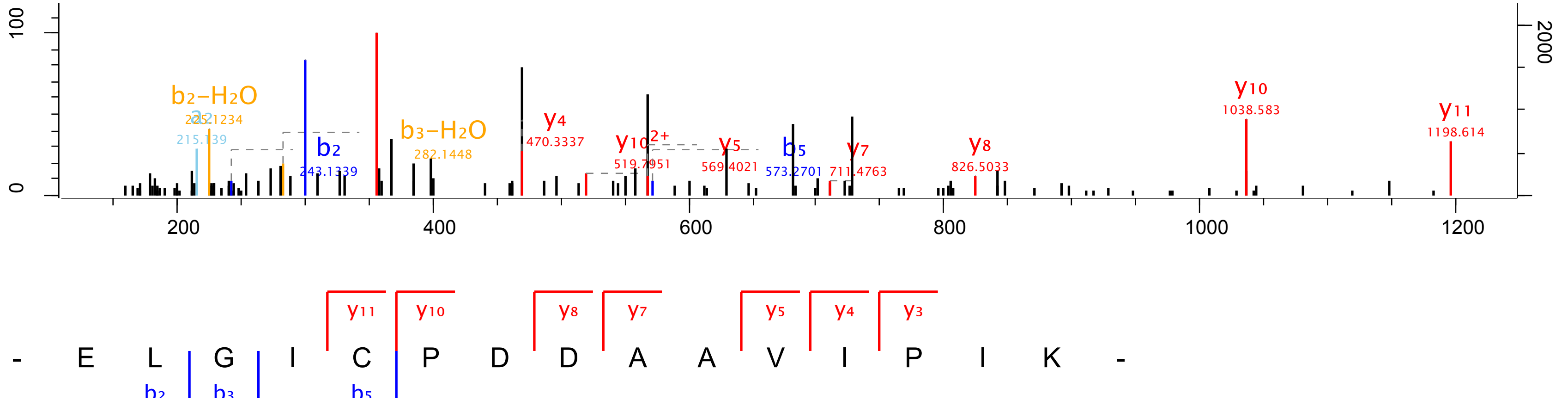

Raw file	Scan	Method	Score	m/z	Gene names
20141014_fract18_dyn_5ul_G2_01_597	32329	TOF; CID	96.1	968	EFHC1

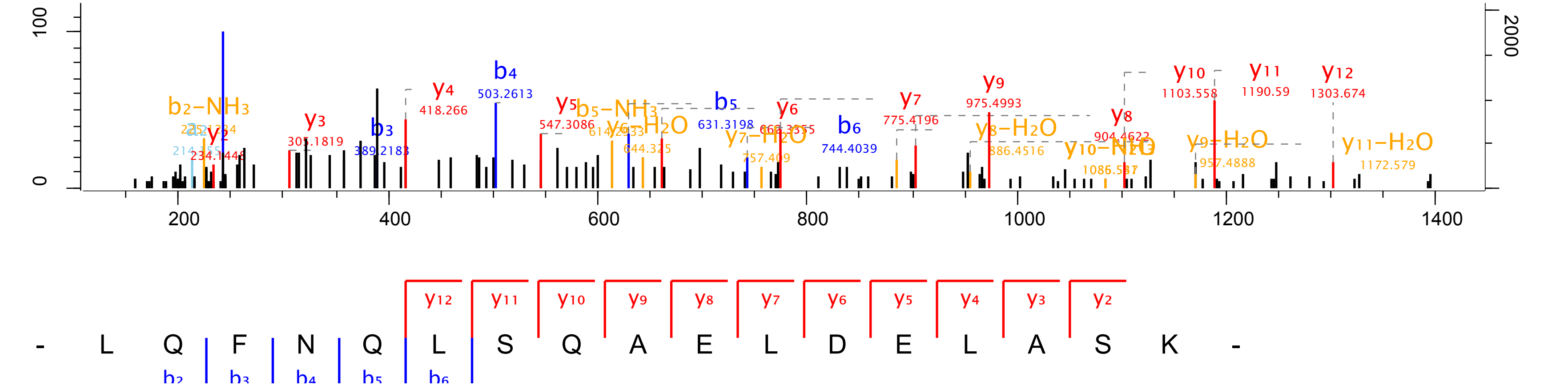

Raw file	Scan	Method	Score	m/z	Gene names
20141014_fract18_dyn_5ul_G2_01_597	33912	TOF; CID	78.44	948.42	TMEM222

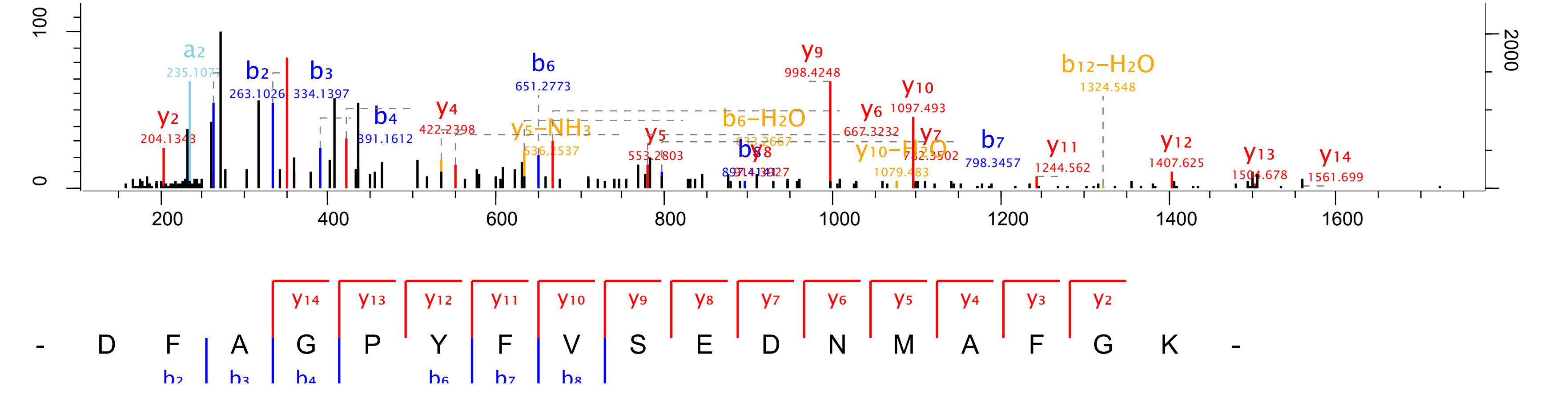

Raw file	Scan	Method	Score	m/z	Gene names
20141014_fract18_dyn_5ul_G2_01_597	34439	TOF; CID	60.55	808.43	NAA60

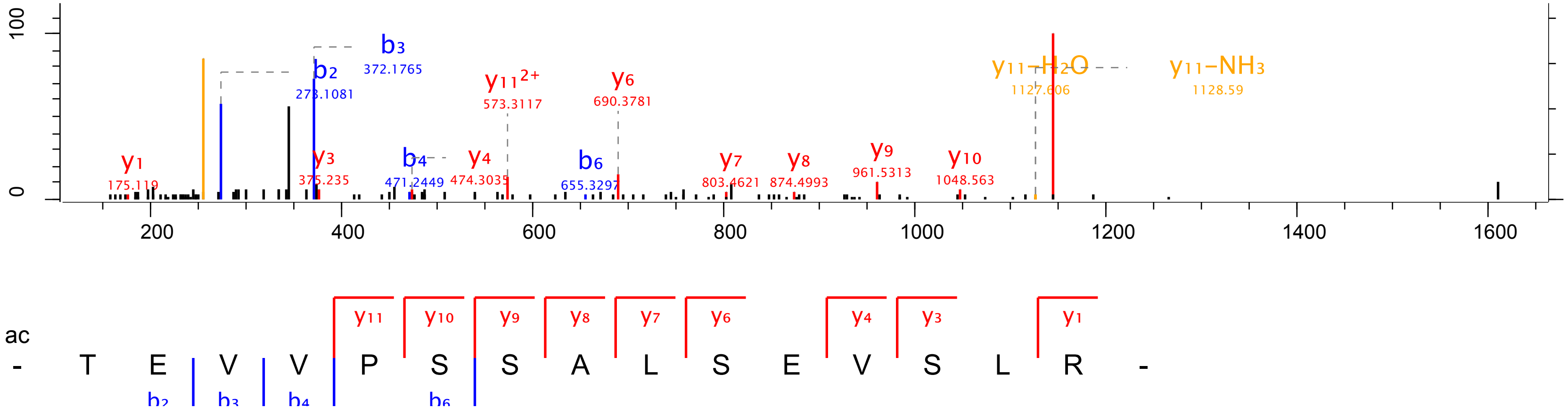

Raw file	Scan	Method	Score	m/z	Gene names
20141014_fract19_dyn_5ul_G3_01_598	10366	TOF; CID	55.04	530.25	MGAT5B

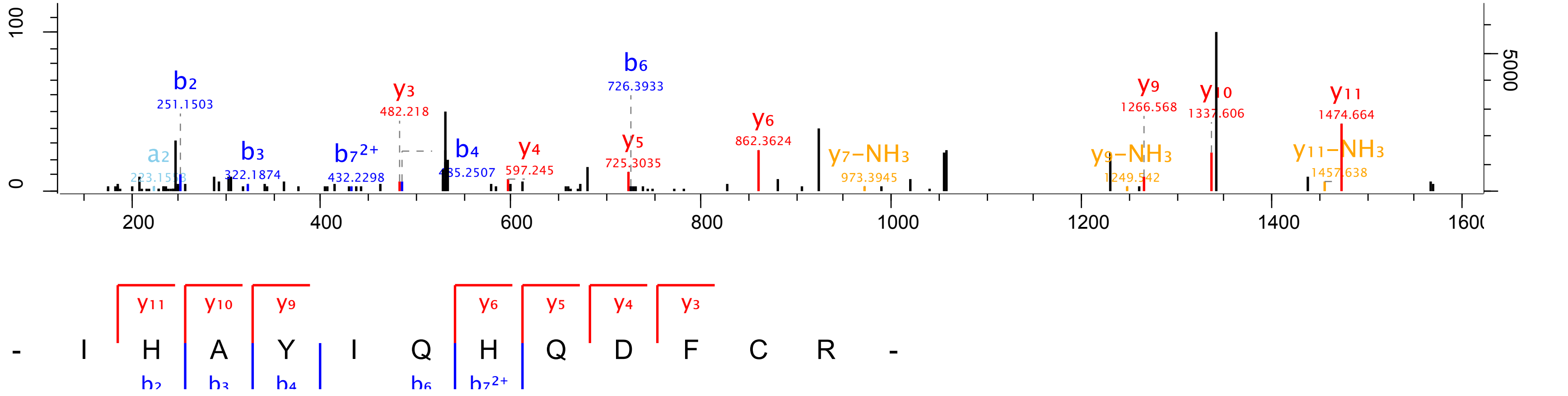

Raw file	Scan	Method	Score	m/z	Gene names
20141014_fract19_dyn_5ul_G3_01_598	14294	TOF; CID	44.81	514.26	TMEM53

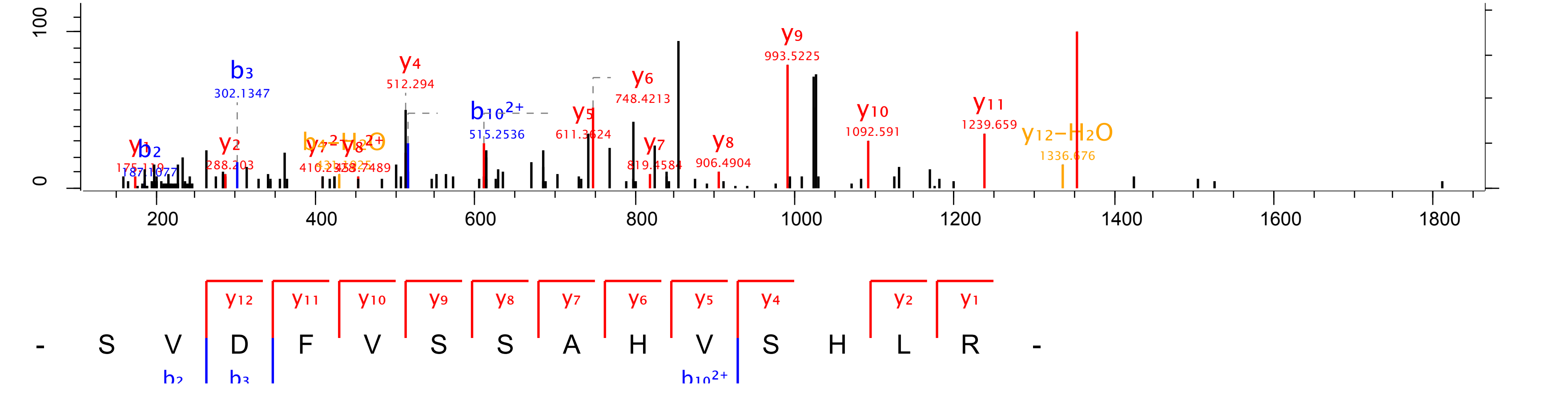

Raw file	Scan	Method	Score	m/z	Gene names
20141014_fract19_dyn_5ul_G3_01_598	18406	TOF; CID	34.4	731.36	ZDHHC24

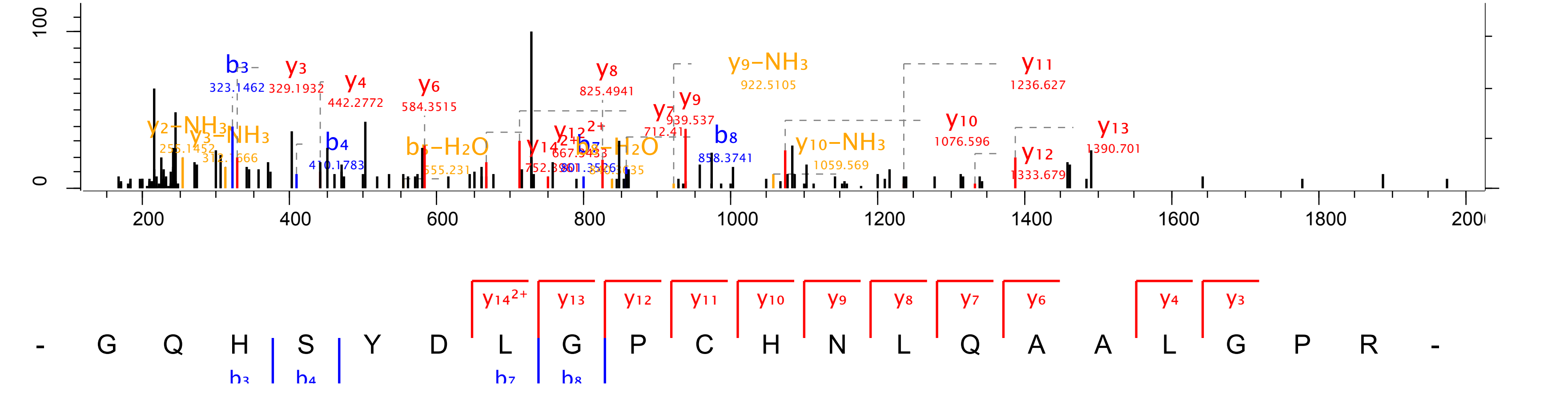

Raw file	Scan	Method	Score	m/z	Gene names
20141014_fract19_dyn_5ul_G3_01_598	22302	TOF; CID	70.78	885.42	DNASE1L1

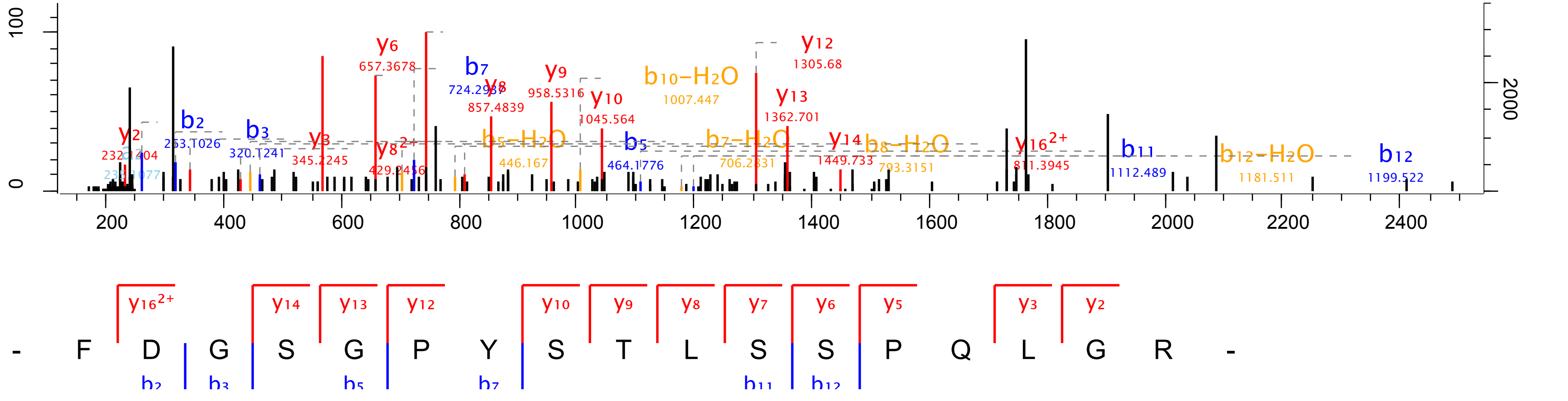

Raw file	Scan	Method	Score	m/z	Gene names
20141014_fract19_dyn_5ul_G3_01_598	22540	TOF; CID	99.57	538.29	S100P

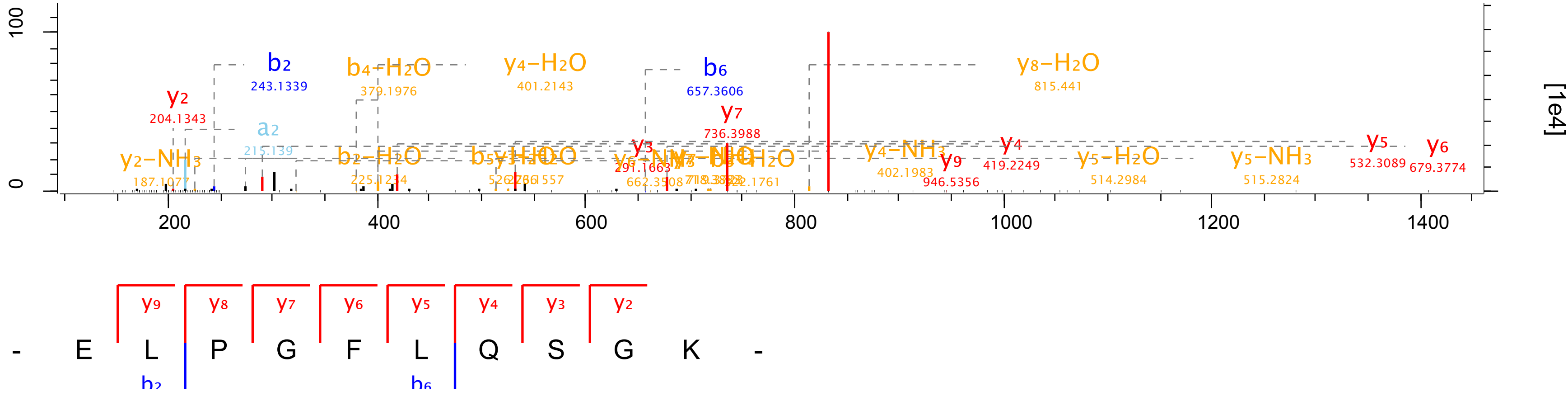

Raw file	Scan	Method	Score	m/z	Gene names
20141014_fract19_dyn_5ul_G3_01_598	27711	TOF; CID	50.96	1143.56	CDON

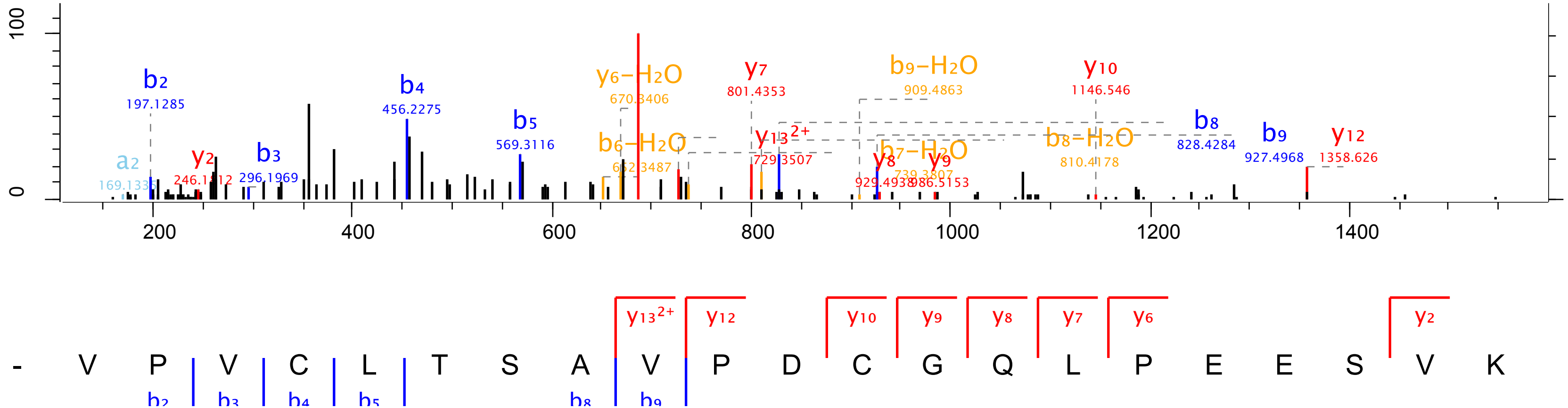

Raw file	Scan	Method	Score	m/z	Gene names
20141014_fract19_dyn_5ul_G3_01_598	32776	TOF; CID	69.75	899.47	RNF130

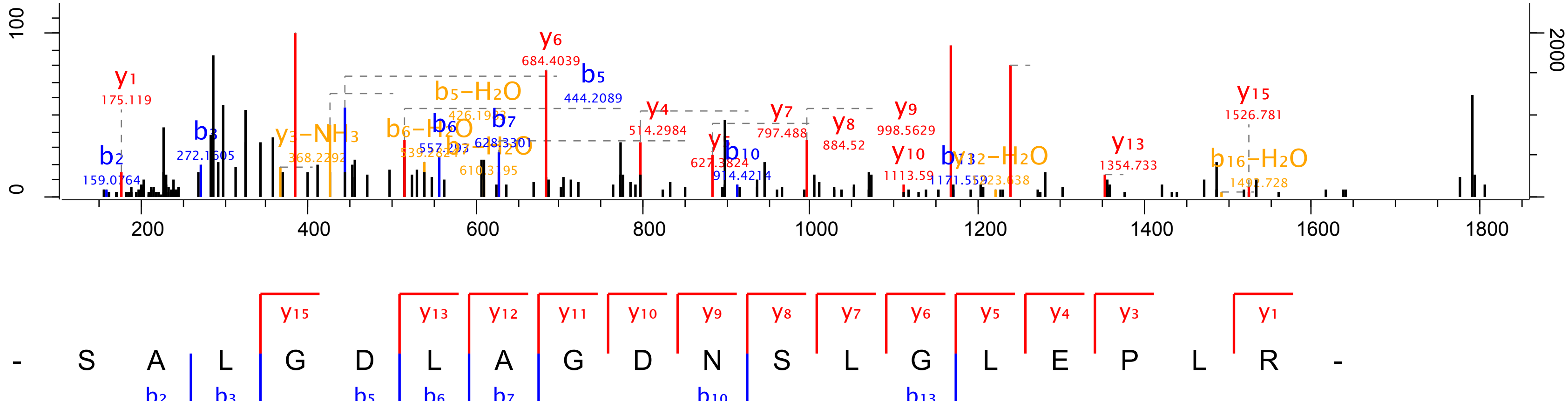

Raw file	Scan	Method	Score	m/z	Gene names
20141014_fract19_dyn_5ul_G3_01_598	38338	TOF; CID	74.54	894.45	CCDC70

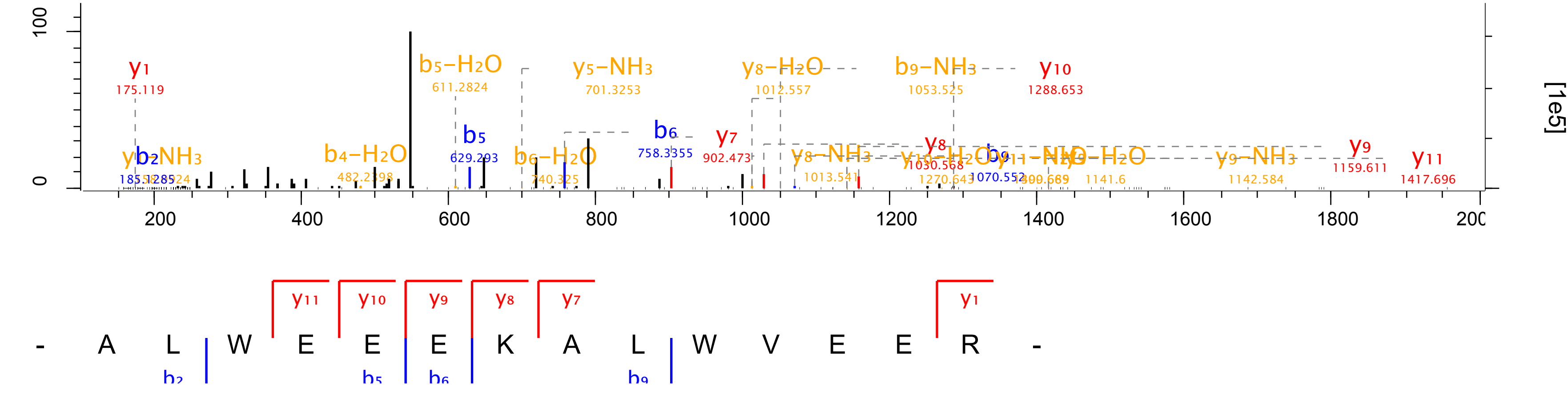

Raw file	Scan	Method	Score	m/z	Gene names
20141014_fract20_dyn_5ul_G4_01_599	15417	TOF; CID	63.62	384.57	ARHGAP29

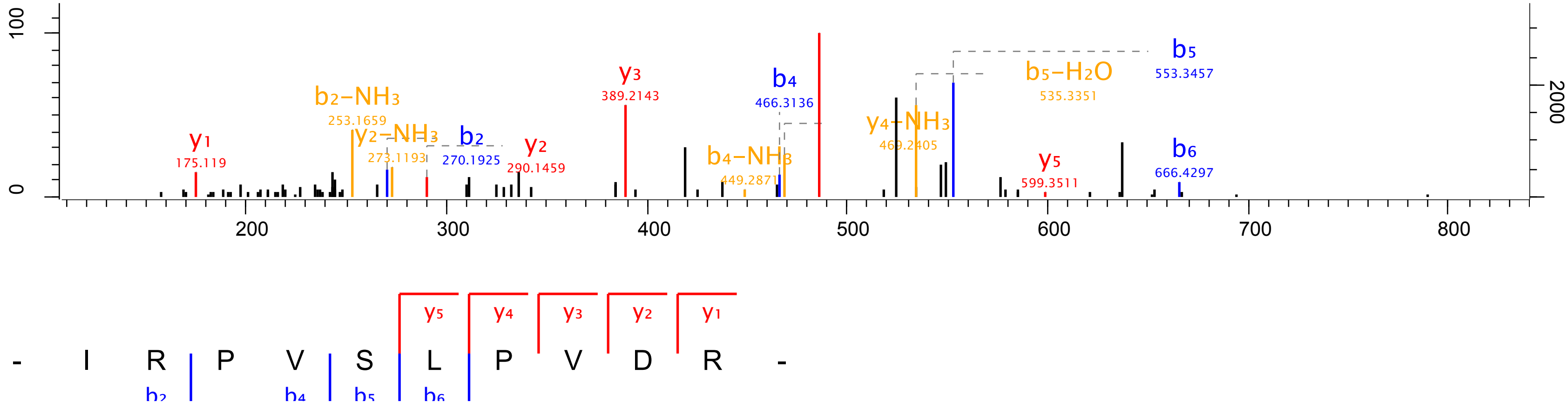

Raw file	Scan	Method	Score	m/z	Gene names
20141014_fract20_dyn_5ul_G4_01_599	16215	TOF; CID	44.69	610.33	SLC22A20

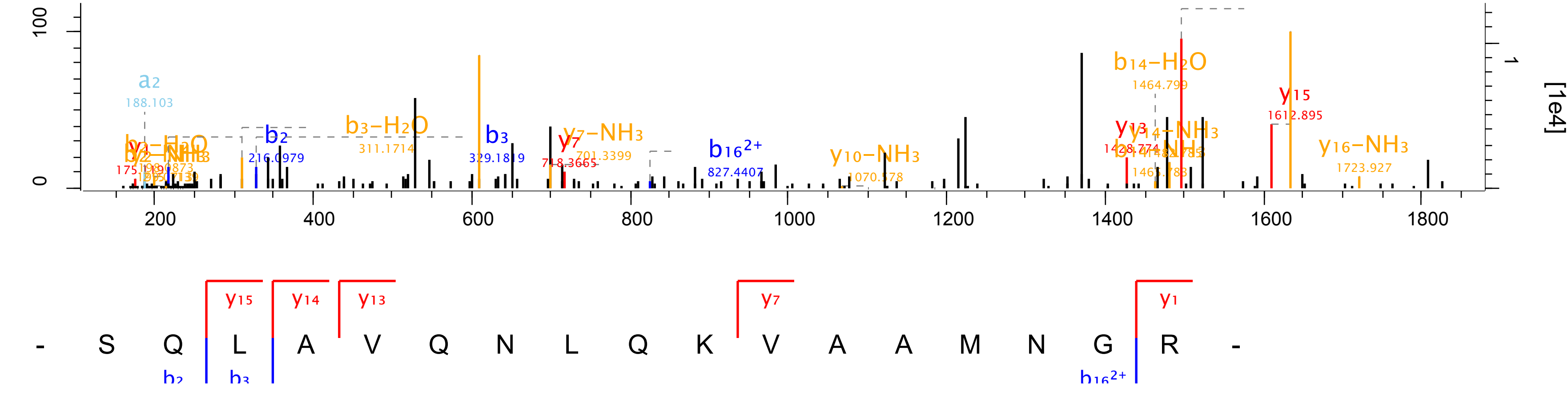

Raw file	Scan	Method	Score	m/z	Gene names
20141014_fract20_dyn_5ul_G4_01_599	24655	TOF; CID	52.07	682.35	ENTPD6

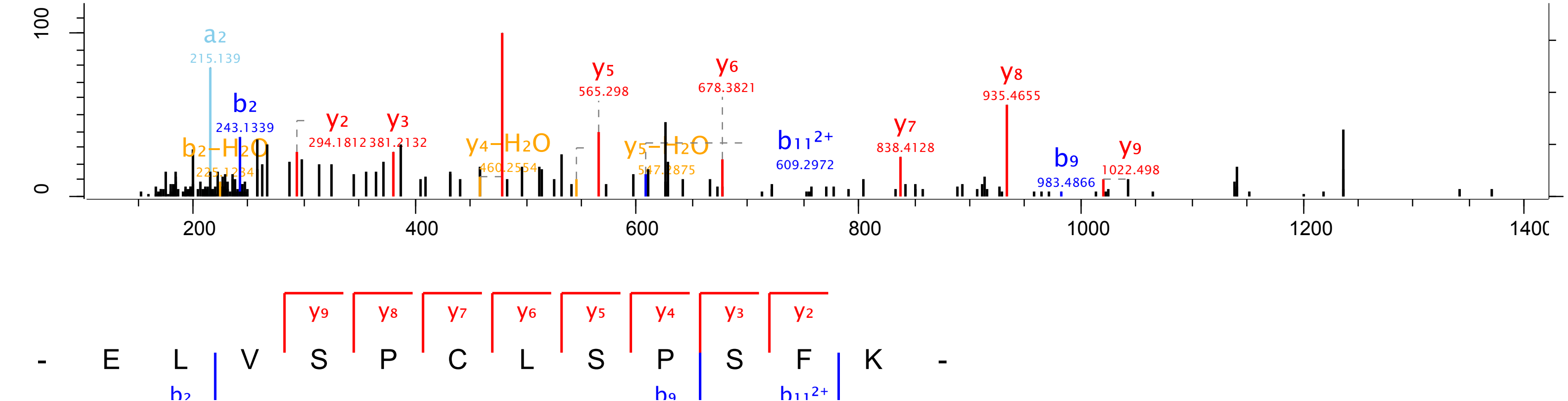

Raw file	Scan	Method	Score	m/z	Gene names
20141014_fract20_dyn_5ul_G4_01_599	28236	TOF; CID	98.94	731.88	SNAPC5

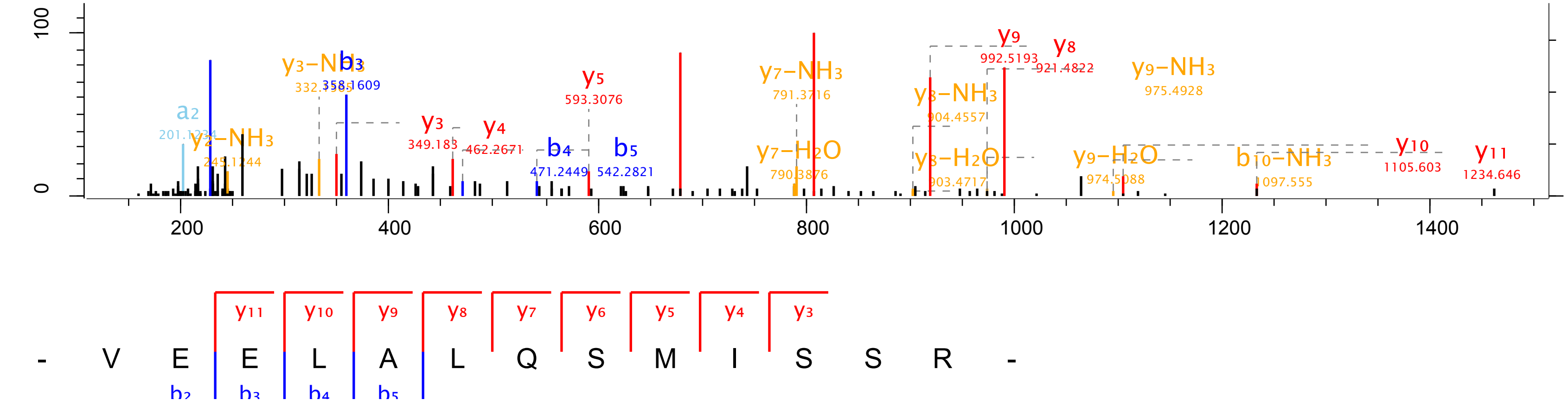

Raw file	Scan	Method	Score	m/z	Gene names
20141014_fract20_dyn_5ul_G4_01_599	28849	TOF; CID	80.54	980.47	FAM168A

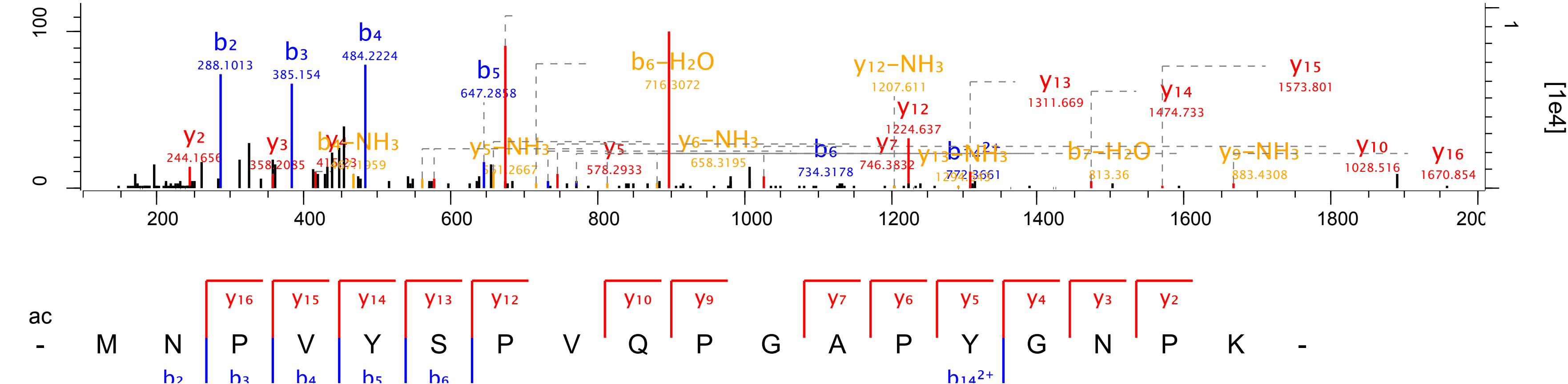

Raw file	Scan	Method	Score	m/z	Gene names
20141014_fract20_dyn_5ul_G4_01_599	29642	TOF; CID	93.18	609.33	GOLGA8A;GOLGA8B

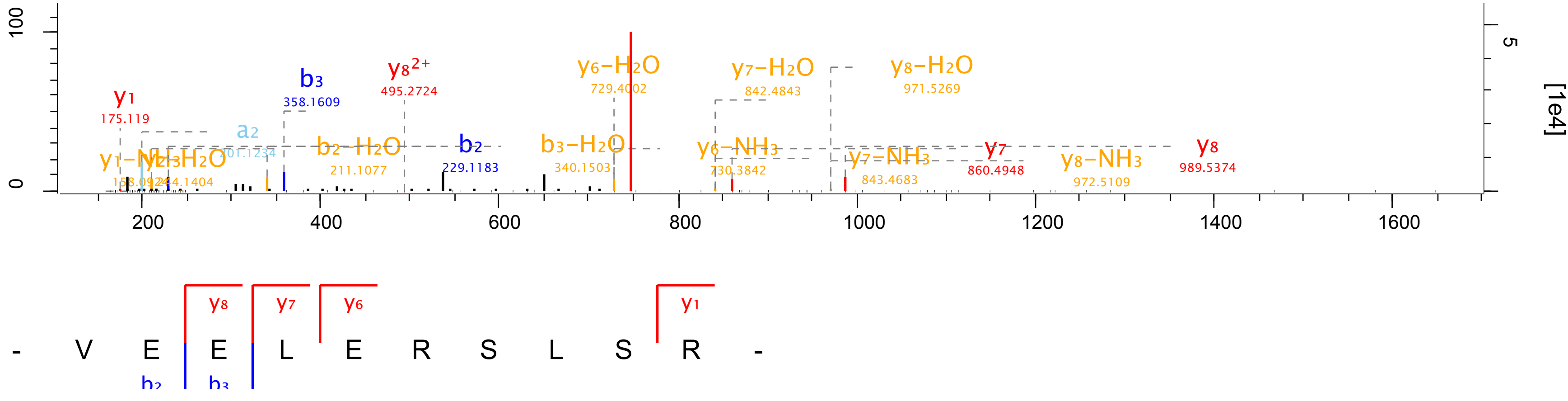

Raw file	Scan	Method	Score	m/z	Gene names
20141014_fract20_dyn_5ul_G4_01_599	31462	TOF; CID	100.43	840.44	COMMD7

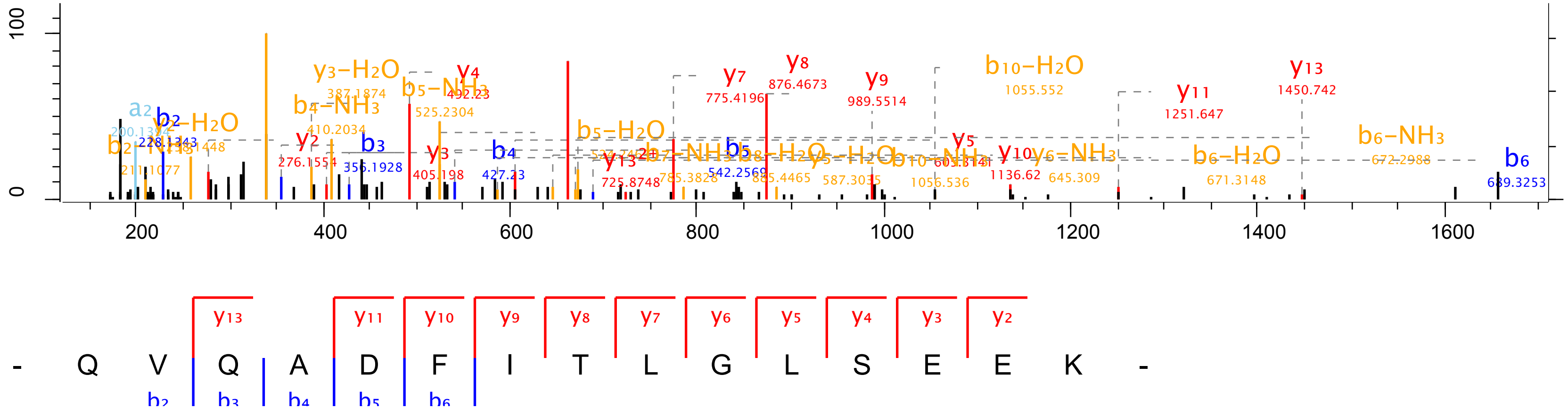

Raw file	Scan	Method	Score	m/z	Gene names
20141014_fract20_dyn_5ul_G4_01_599	33602	TOF; CID	36.08	881.42	SAYSD1

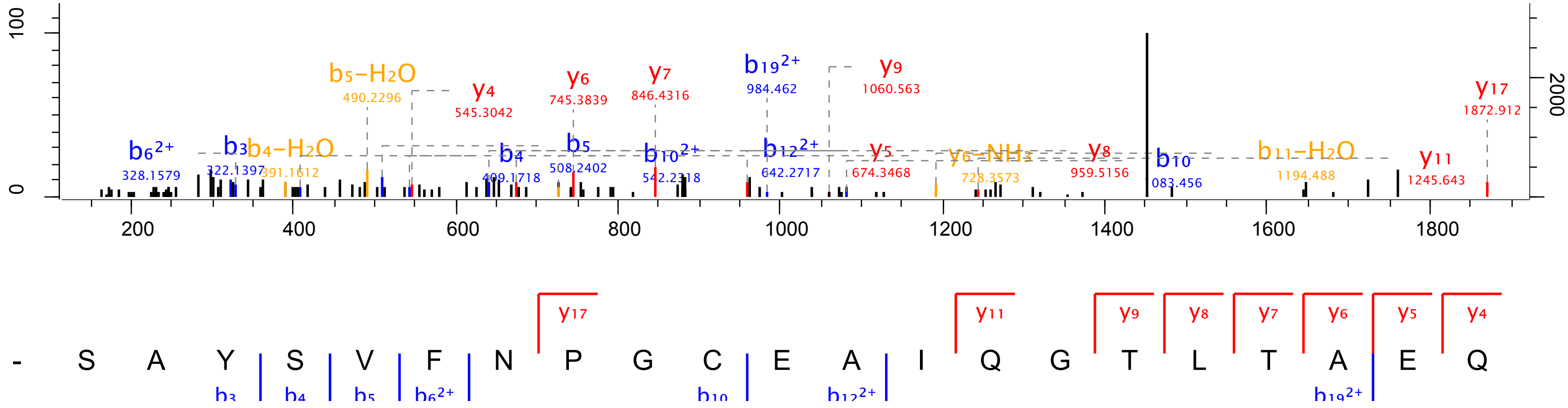

Raw file	Scan	Method	Score	m/z	Gene names
20141014_fract21_dyn_5ul_G5_01_600	6457	TOF; CID	138.37	433.76	SLC39A1

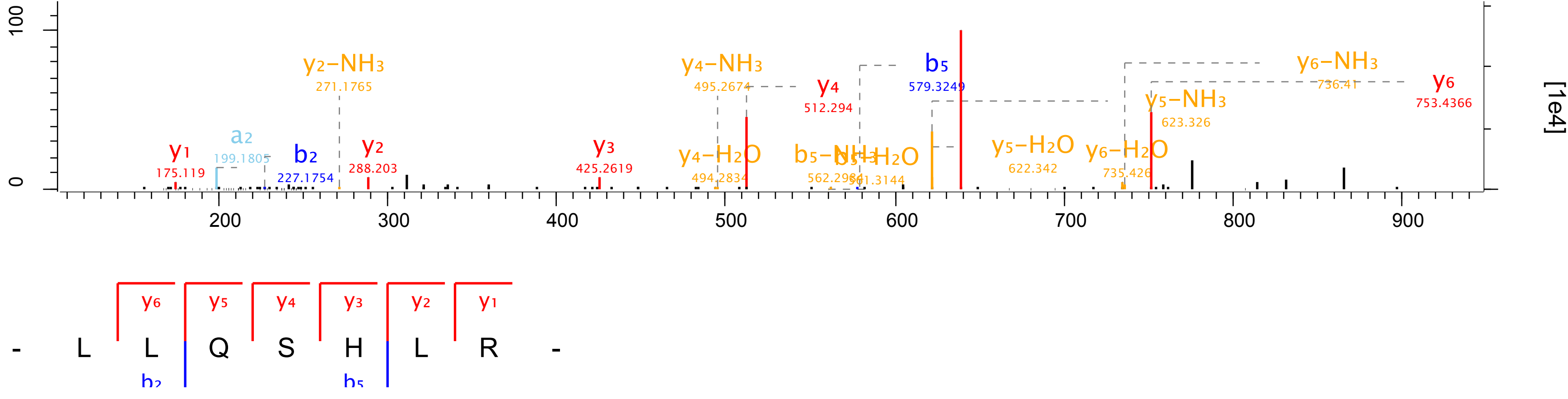

Raw file	Scan	Method	Score	m/z	Gene names
20141014_fract21_dyn_5ul_G5_01_600	13464	TOF; CID	91.66	578.84	KIAA1107

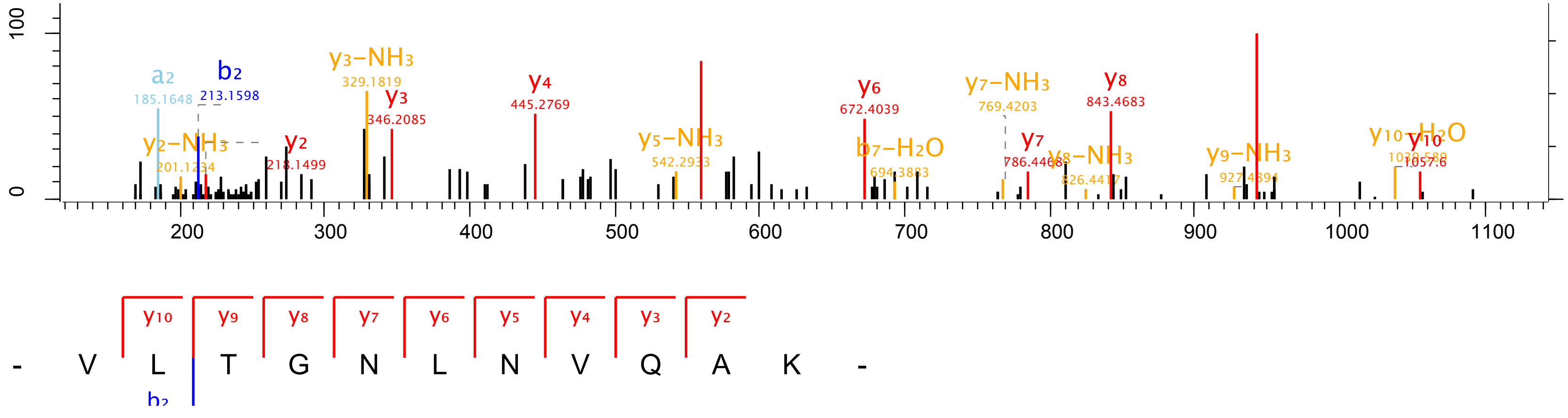

Raw file	Scan	Method	Score	m/z	Gene names
20141014_fract21_dyn_5ul_G5_01_600	15422	TOF; CID	50.9	606.83	AGBL5

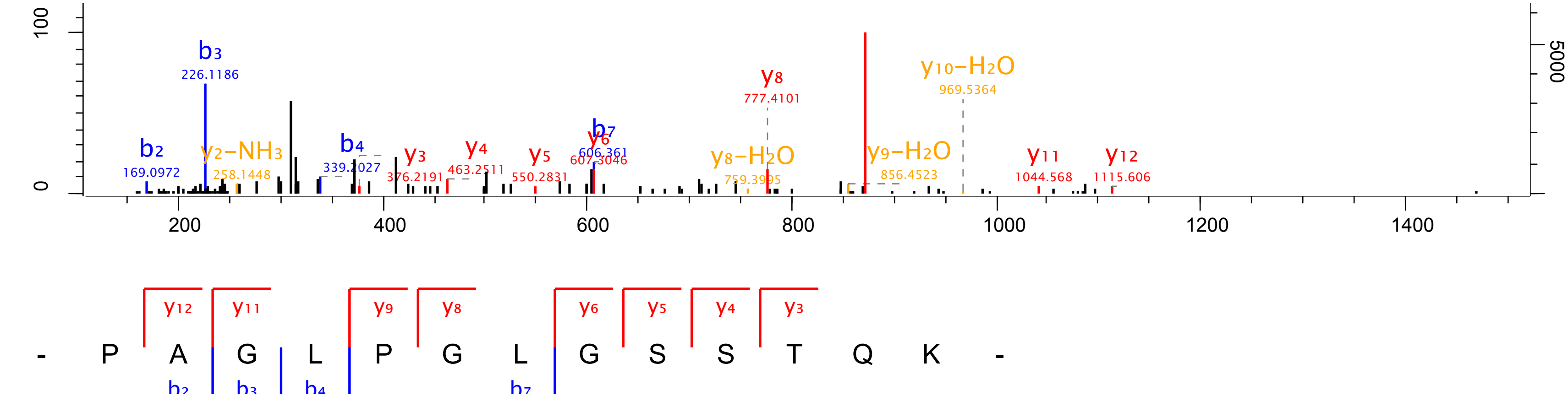

Raw file	Scan	Method	Score	m/z	Gene names
20141014_fract21_dyn_5ul_G5_01_600	15509	TOF; CID	90.15	525.79	ABLIM2

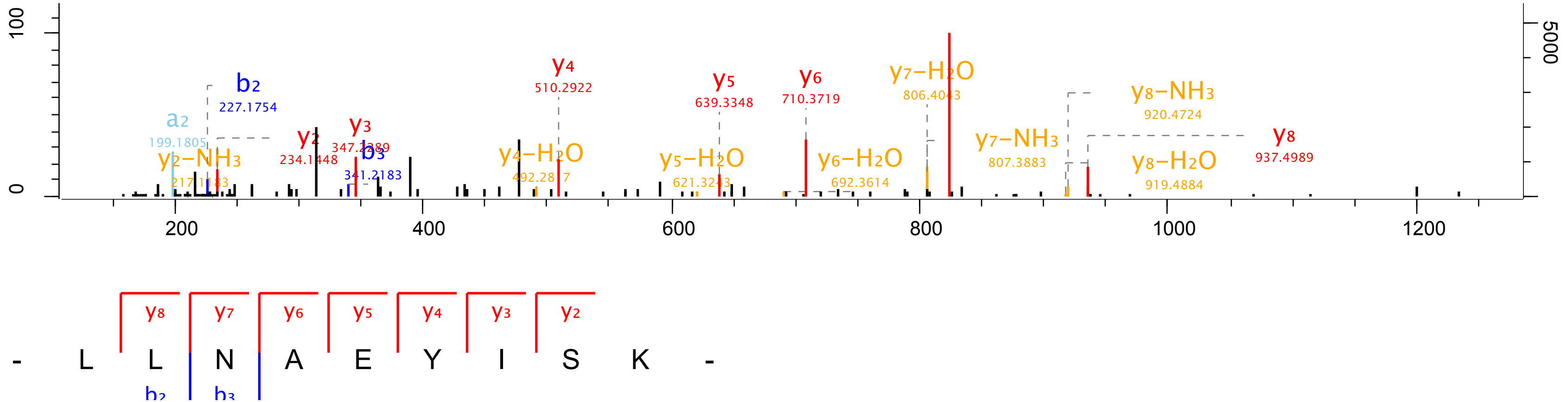

Raw file	Scan	Method	Score	m/z	Gene names
20141014_fract21_dyn_5ul_G5_01_600	16497	TOF; CID	76.22	377.23	NIPA2

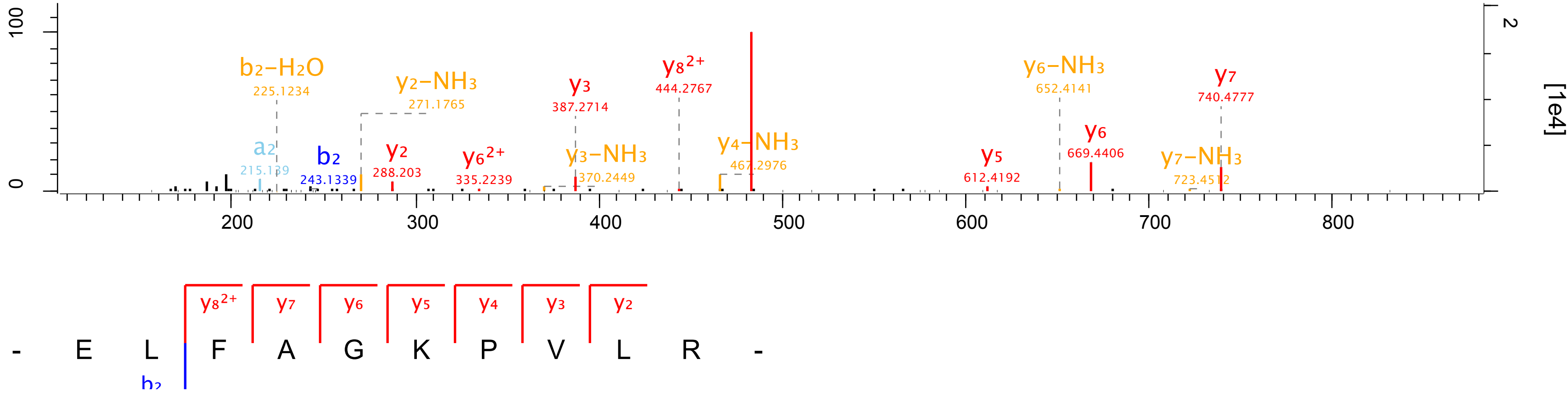

Raw file	Scan	Method	Score	m/z	Gene names
20141014_fract21_dyn_5ul_G5_01_600	17422	TOF; CID	121.9	664.33	ZNF706

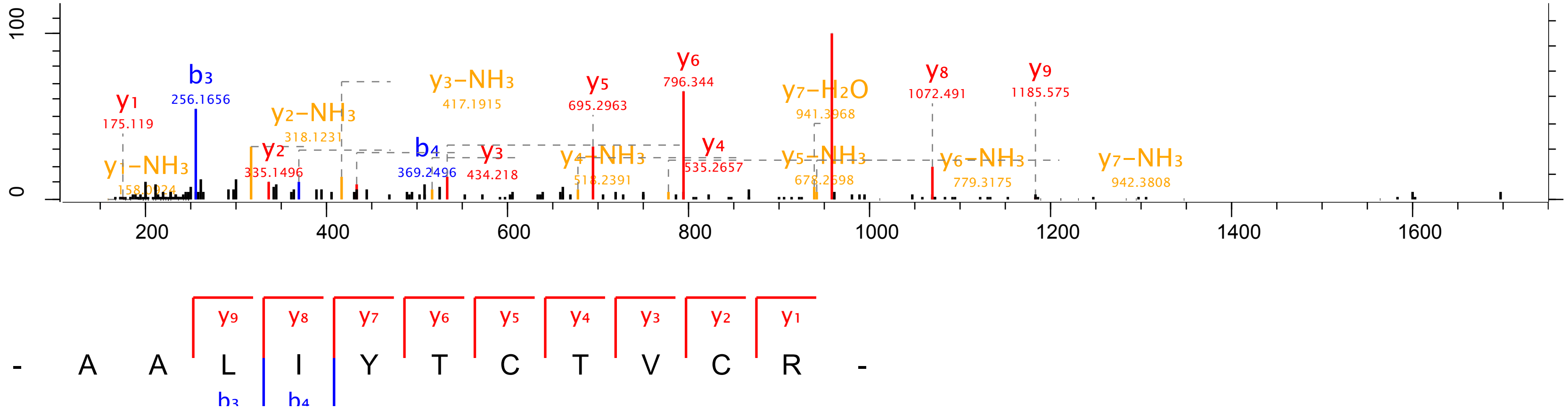

Raw file	Scan	Method	Score	m/z	Gene names
20141014_fract21_dyn_5ul_G5_01_600	19806	TOF; CID	50.18	461.74	ZBTB45

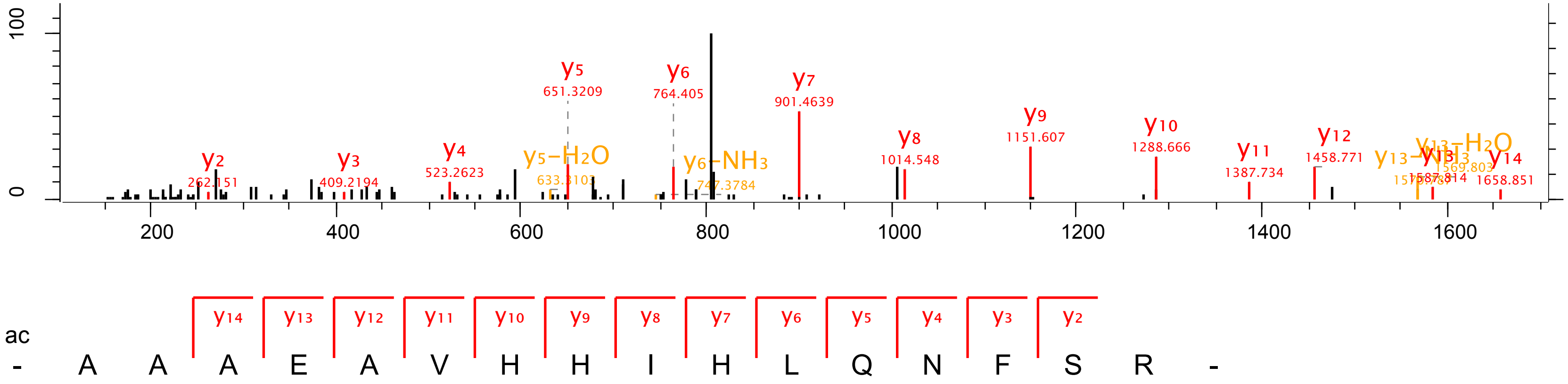

Raw file	Scan	Method	Score	m/z	Gene names
20141014_fract21_dyn_5ul_G5_01_600	22193	TOF; CID	78.4	636.86	TMEM219

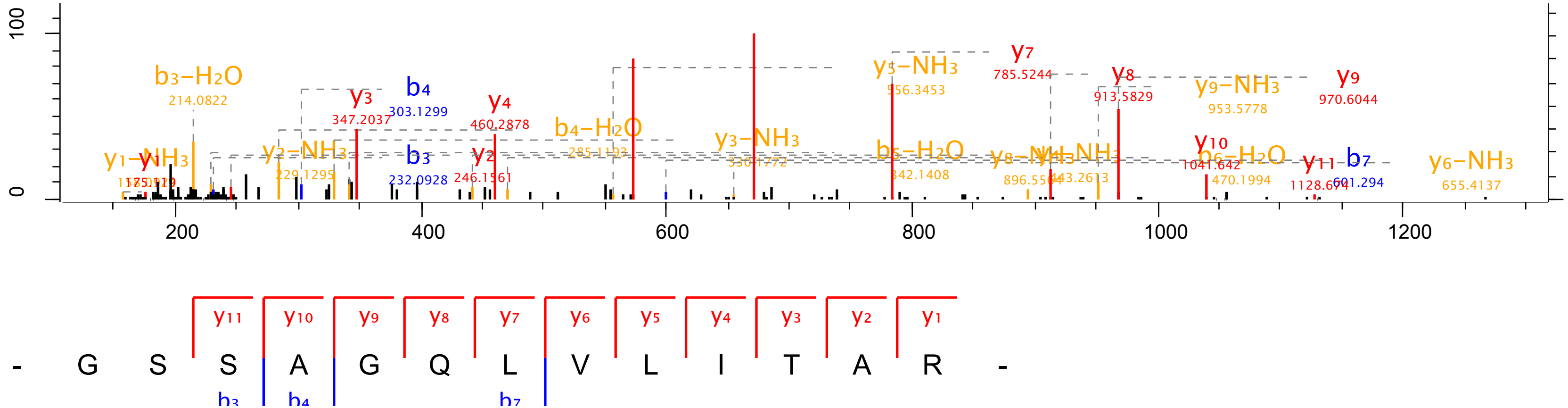

Raw file	Scan	Method	Score	m/z	Gene names
20141014_fract21_dyn_5ul_G5_01_600	22235	TOF; CID	36.92	998.51	LMO4

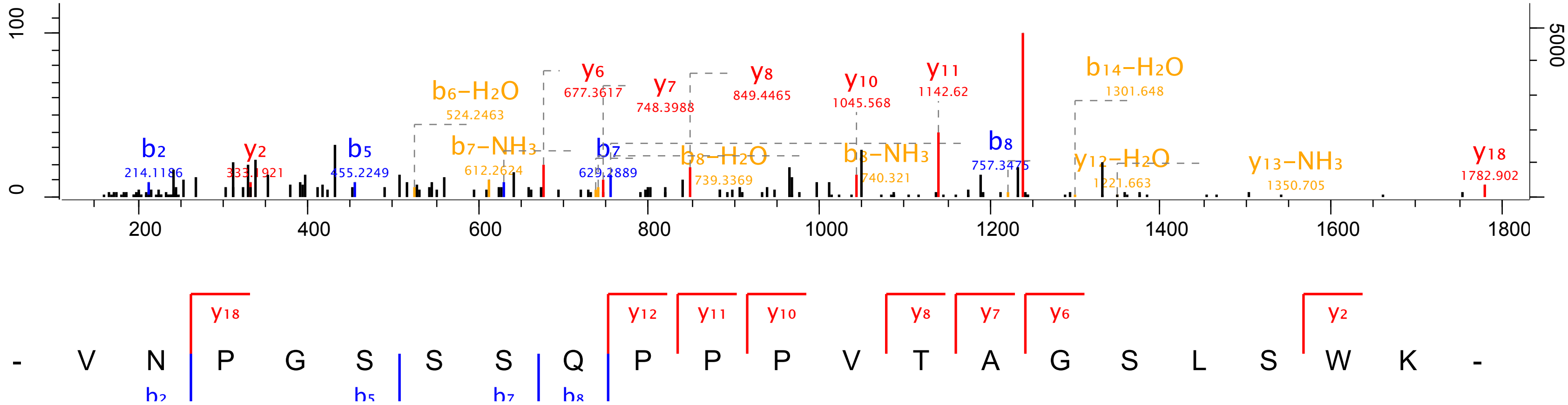

Raw file	Scan	Method	Score	m/z	Gene names
20141014_fract21_dyn_5ul_G5_01_600	28402	TOF; CID	63.68	585.64	TWSG1

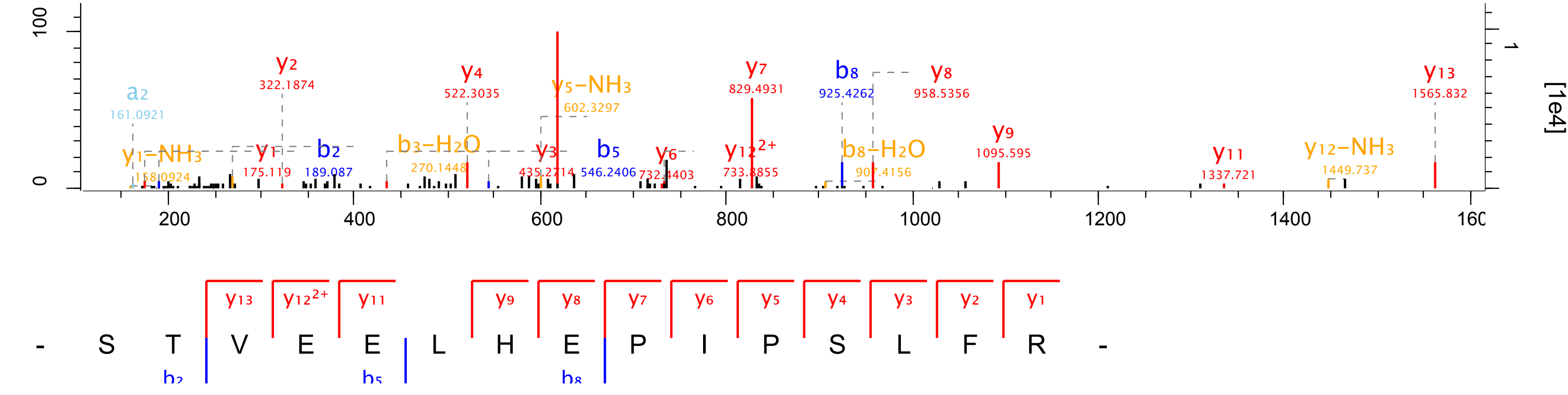

Raw file	Scan	Method	Score	m/z	Gene names
20141014_fract22_dyn_5ul_G6_01_601	8661	TOF; CID	63.06	457.74	ZNF780A

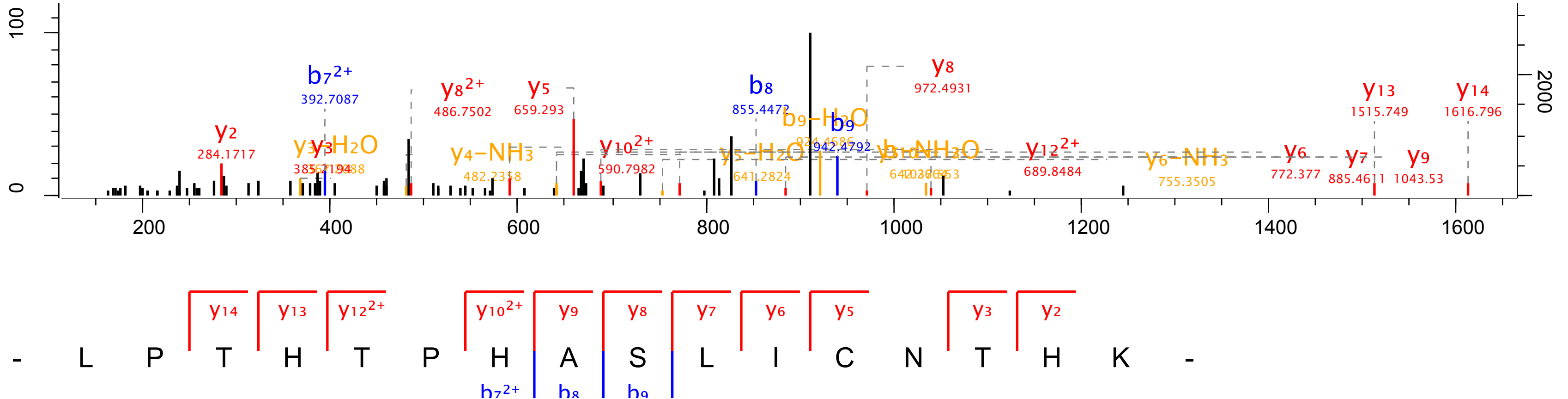

Raw file	Scan	Method	Score	m/z	Gene names
20141014_fract22_dyn_5ul_G6_01_601	14883	TOF; CID	42.32	515.28	SLC23A2

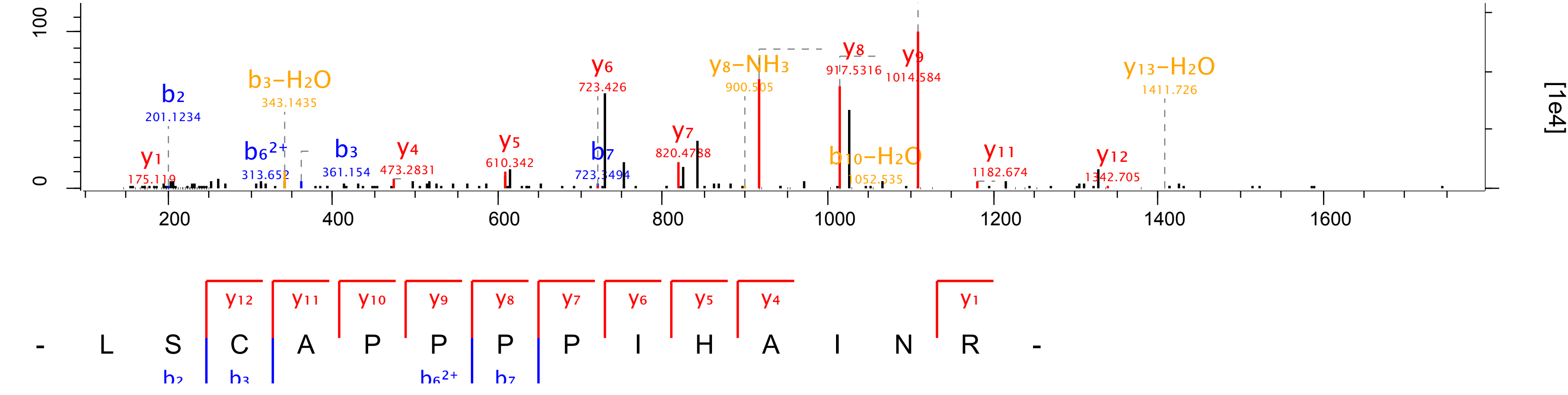

Raw file	Scan	Method	Score	m/z	Gene names
20141014_fract22_dyn_5ul_G6_01_601	16056	TOF; CID	114.72	721.39	AKAP7

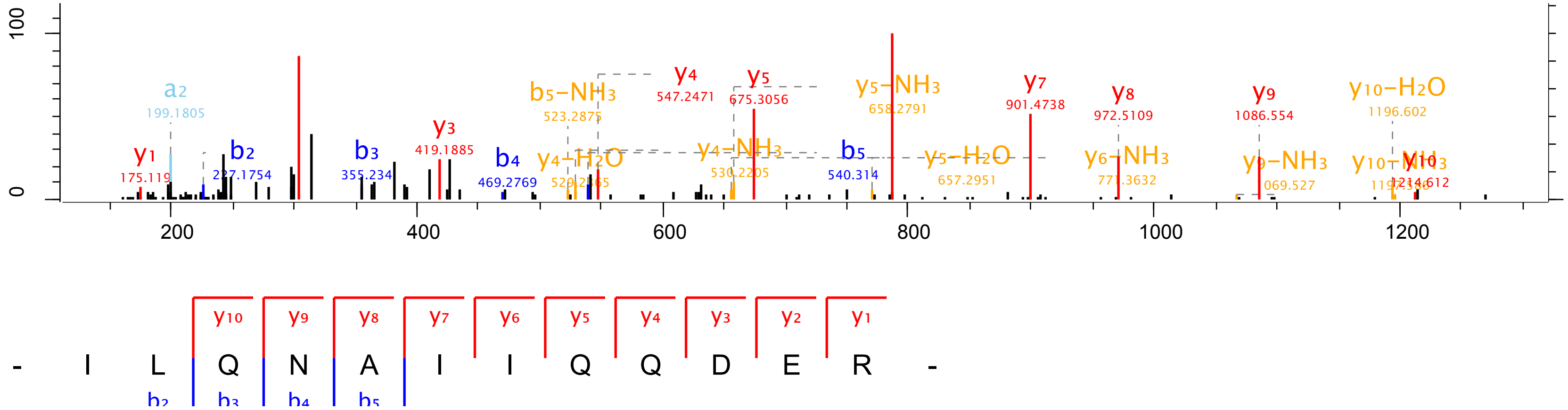

Raw file	Scan	Method	Score	m/z	Gene names
20141014_fract22_dyn_5ul_G6_01_601	19737	TOF; CID	105.03	826.89	FBXW2

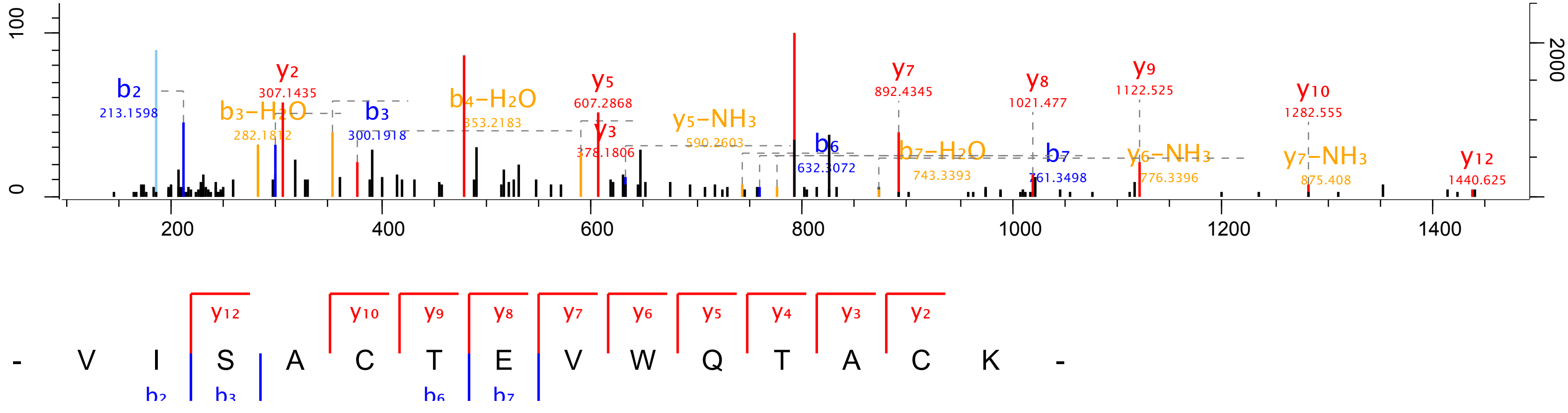

Raw file	Scan	Method	Score	m/z	Gene names
20141014_fract22_dyn_5ul_G6_01_601	22553	TOF; CID	105.9	734.65	ELOF1

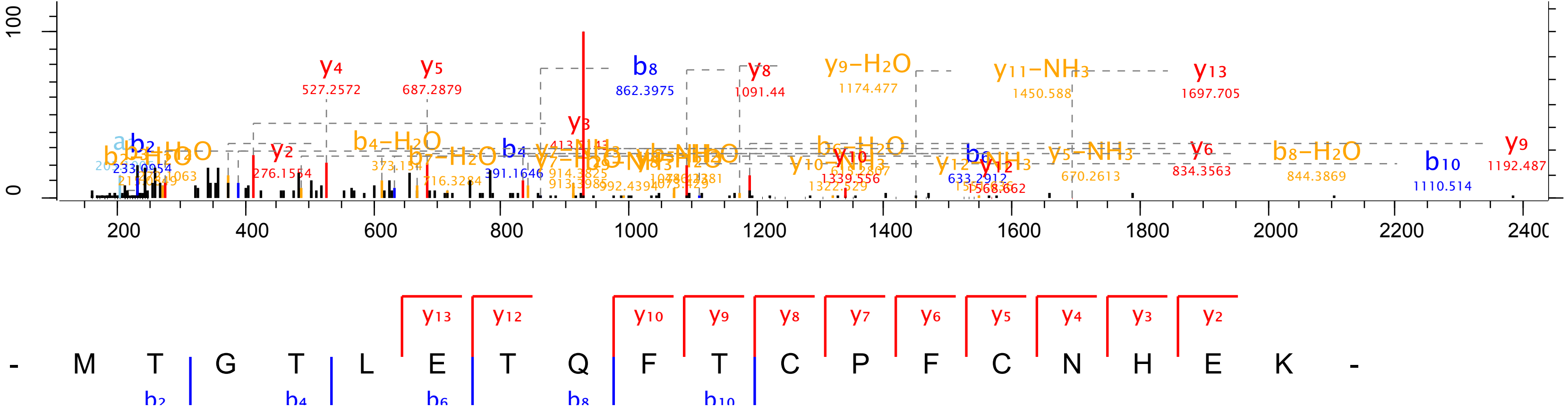

Raw file	Scan	Method	Score	m/z	Gene names
20141014_fract22_dyn_5ul_G6_01_601	24438	TOF; CID	103.55	519.82	OPN3

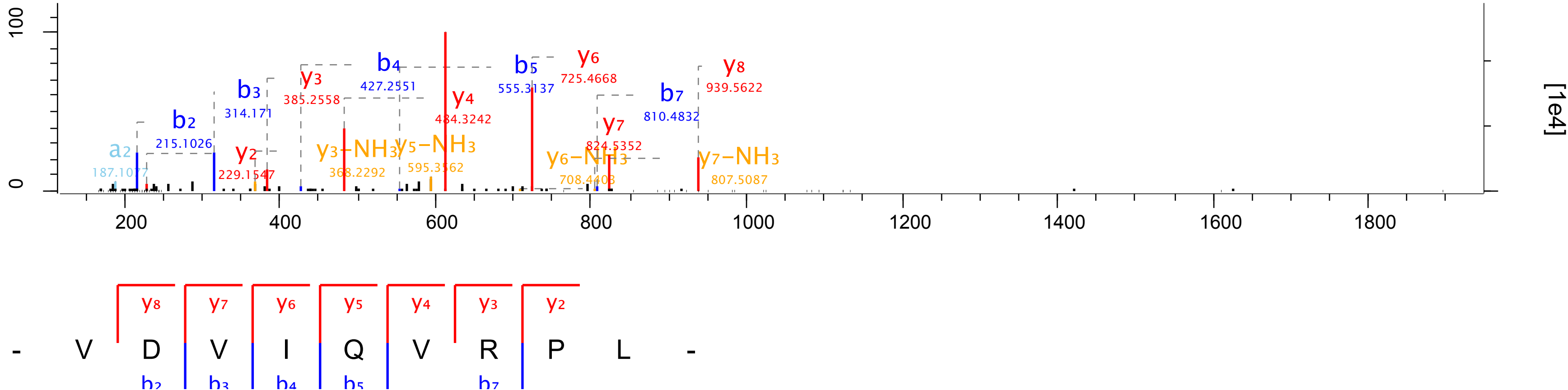

Raw file	Scan	Method	Score	m/z	Gene names
20141014_fract22_dyn_5ul_G6_01_601	32899	TOF; CID	68.26	813.89	TSPAN1

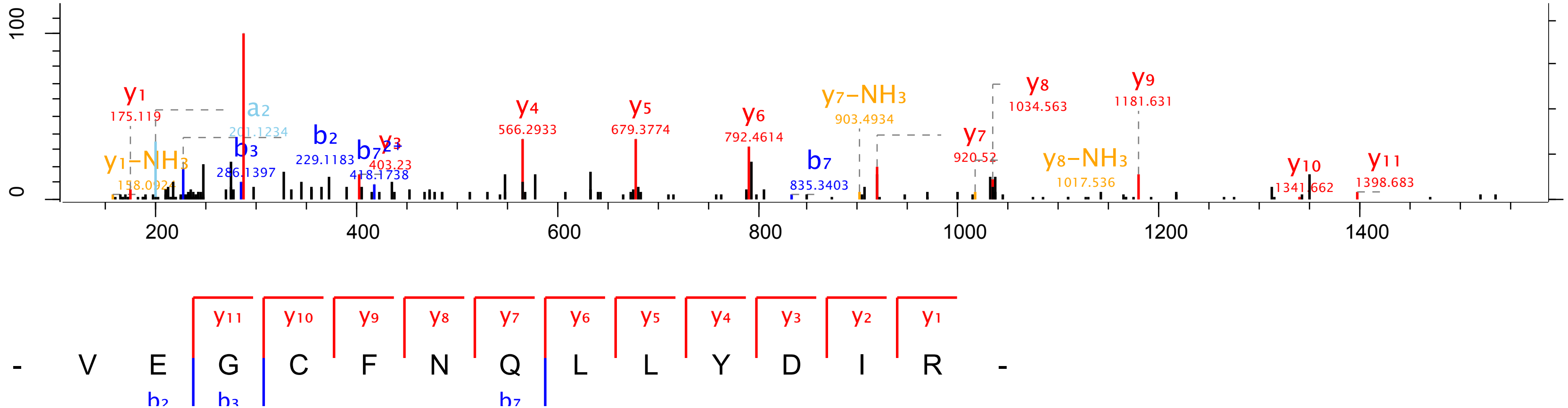

Raw file	Scan	Method	Score	m/z	Gene names
20141014_fract22_dyn_5ul_G6_01_601	33398	TOF; CID	72.58	928.51	PET117

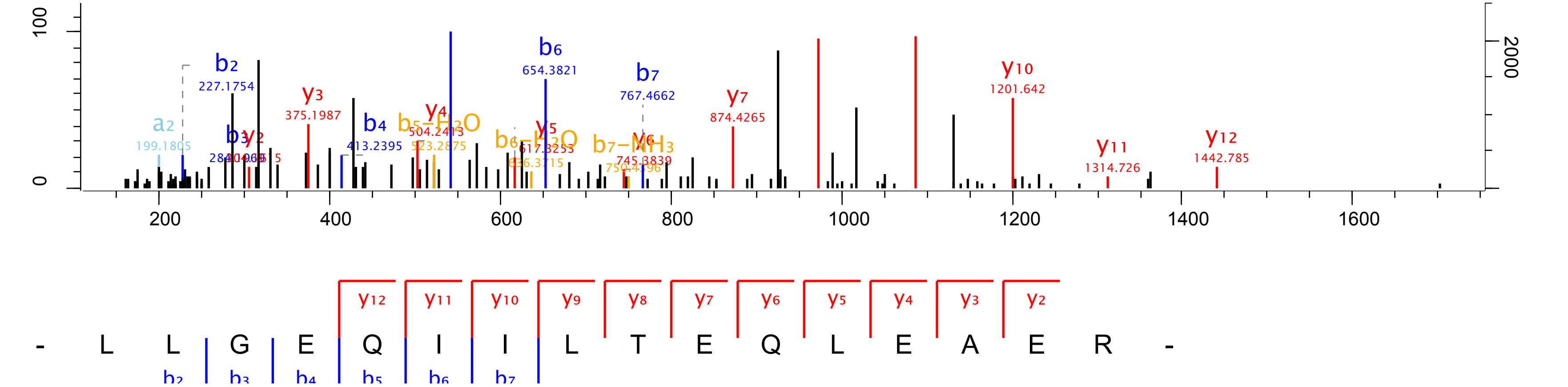

Raw file	Scan	Method	Score	m/z	Gene names
20141014_fract23_dyn_5ul_G7_01_602	8390	TOF; CID	90.61	527.61	ZNF34

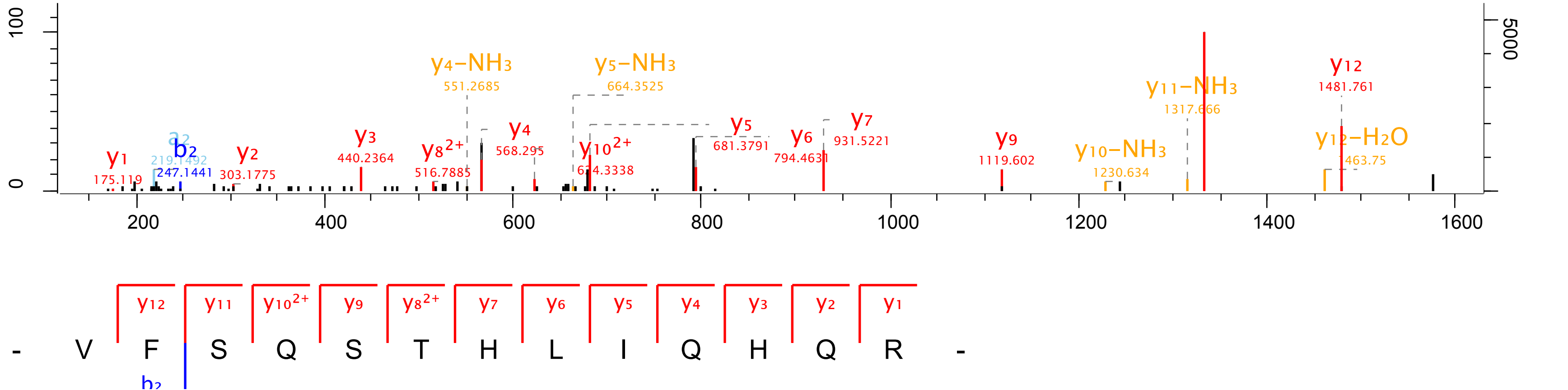

Raw file	Scan	Method	Score	m/z	Gene names
20141014_fract23_dyn_5ul_G7_01_602	10940	TOF; CID	103.74	467.77	ENTPD4

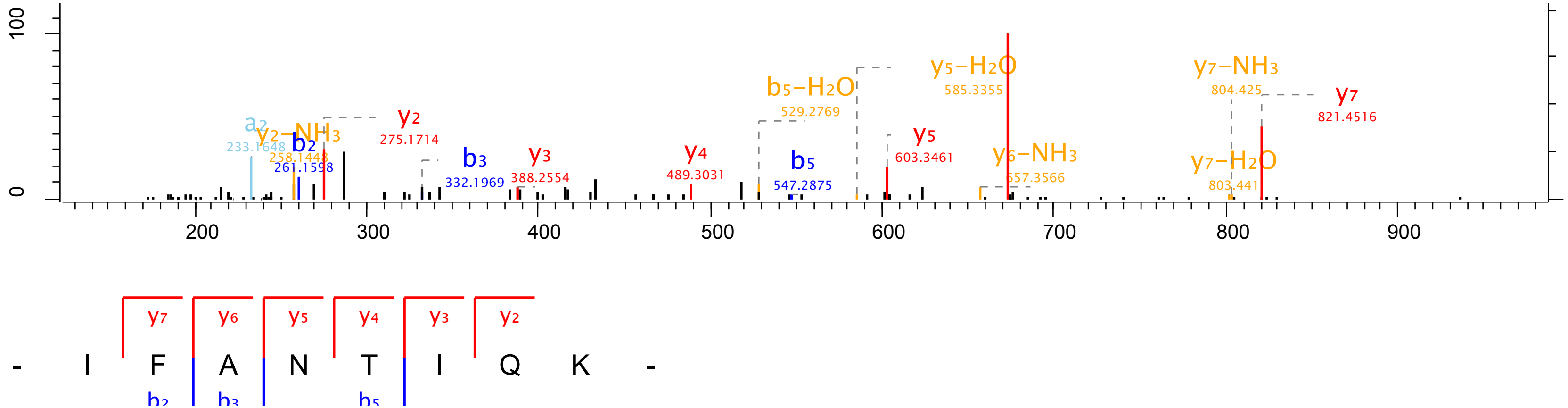

Raw file	Scan	Method	Score	m/z	Gene names
20141014_fract23_dyn_5ul_G7_01_602	11199	TOF; CID	87.18	582.33	MIDN

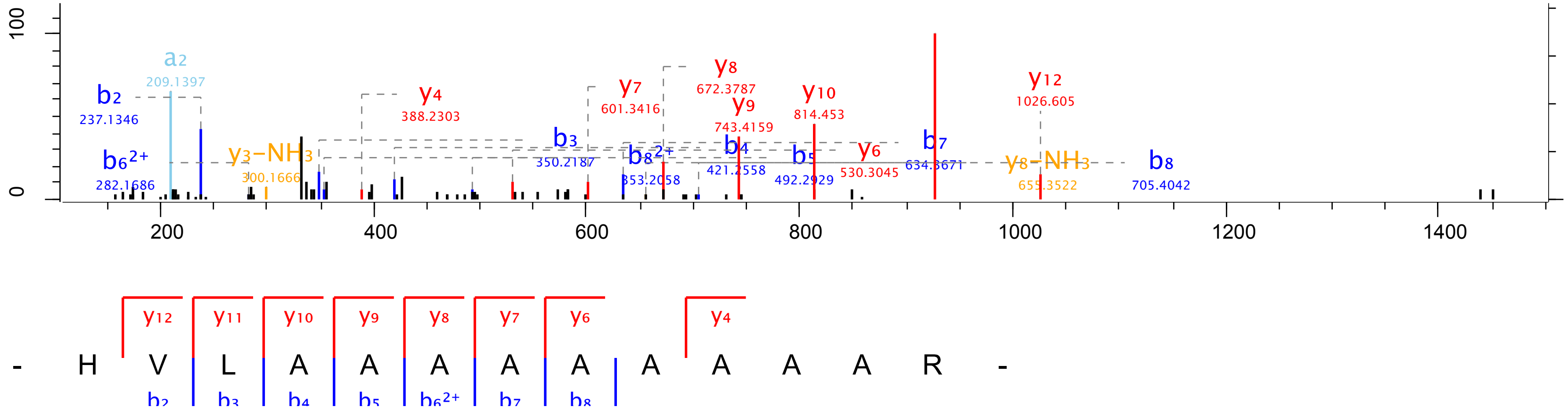

Raw file	Scan	Method	Score	m/z	Gene names
20141014_fract23_dyn_5ul_G7_01_602	12421	TOF; CID	55.66	587.65	RBL2

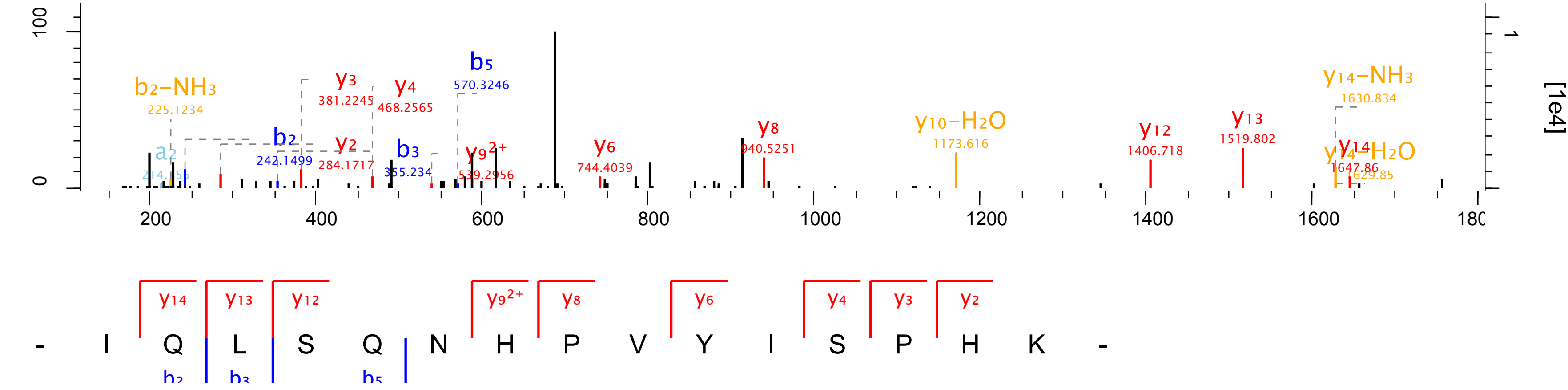

Raw file	Scan	Method	Score	m/z	Gene names
20141014_fract23_dyn_5ul_G7_01_602	13733	TOF; CID	94.12	625.86	FKRP

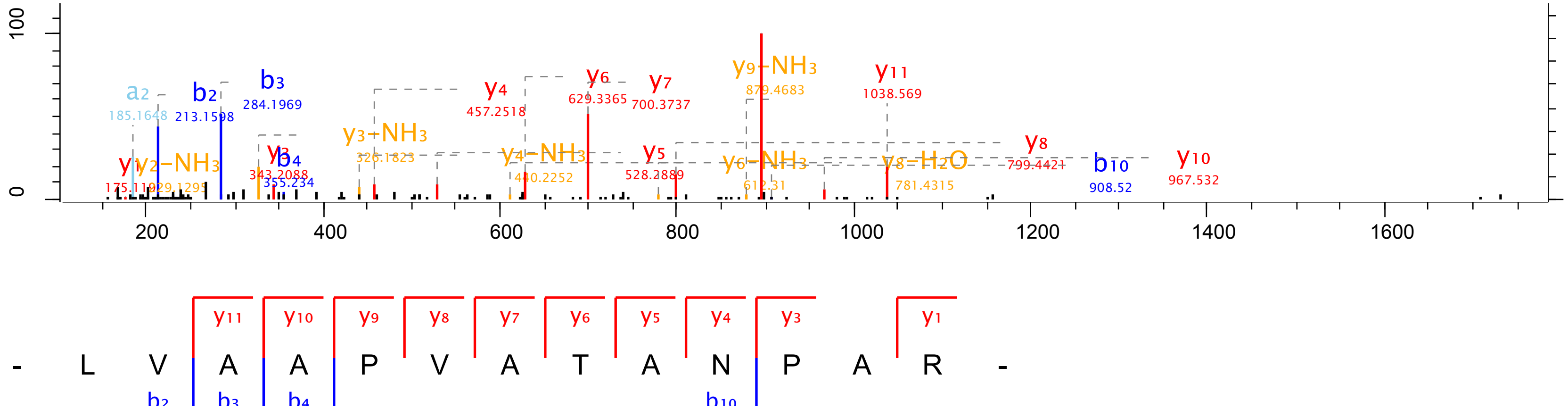

Raw file	Scan	Method	Score	m/z	Gene names
20141014_fract23_dyn_5ul_G7_01_602	16291	TOF; CID	117.1	469.76	APH1A

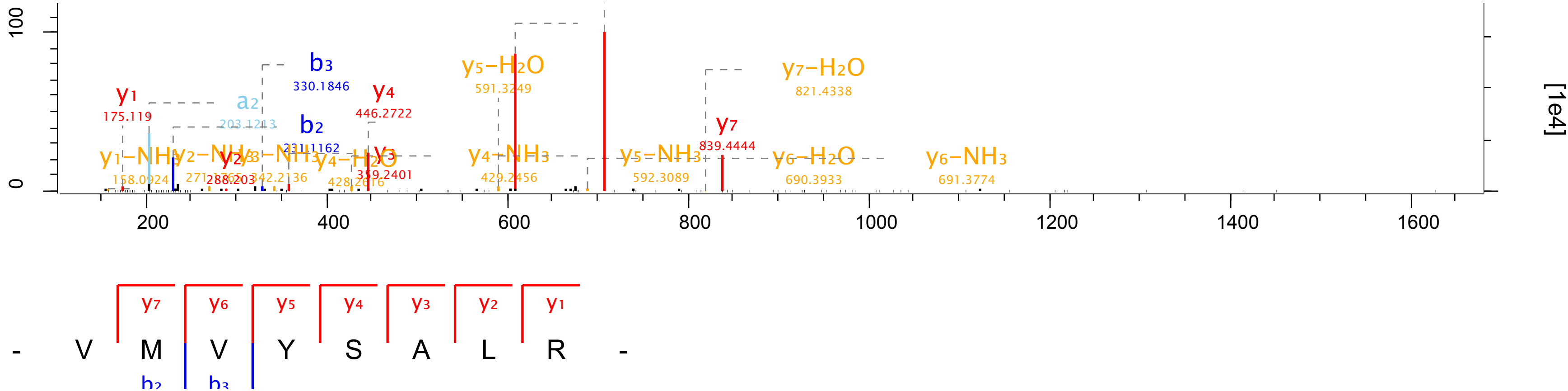

Raw file	Scan	Method	Score	m/z	Gene names
20141014_fract23_dyn_5ul_G7_01_602	17175	TOF; CID	75.5	632.35	TTBK2

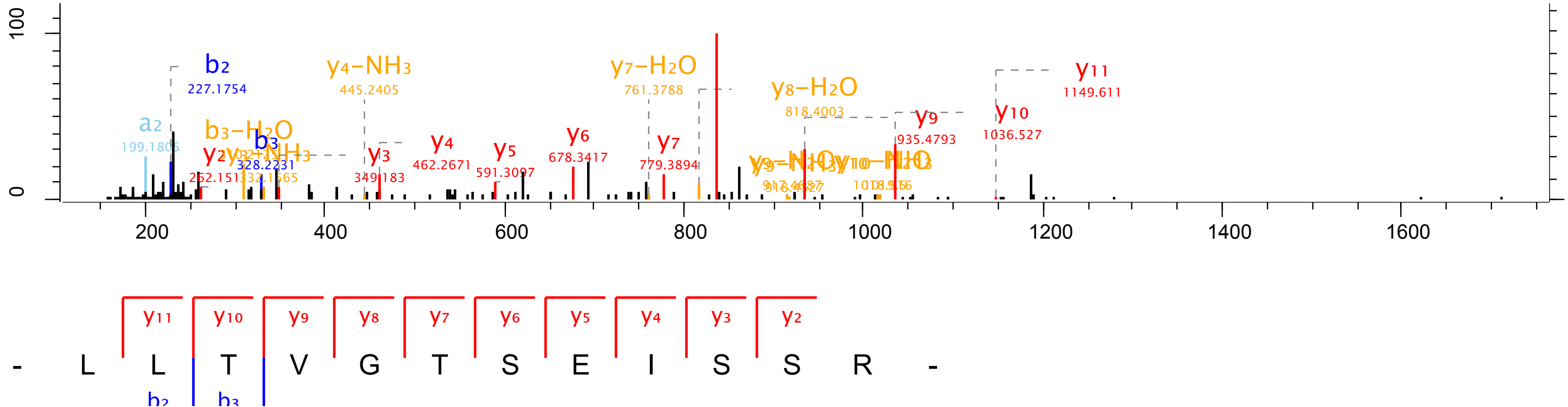

Raw file	Scan	Method	Score	m/z	Gene names
20141014_fract23_dyn_5ul_G7_01_602	17490	TOF; CID	99.97	602.3	GALNT11

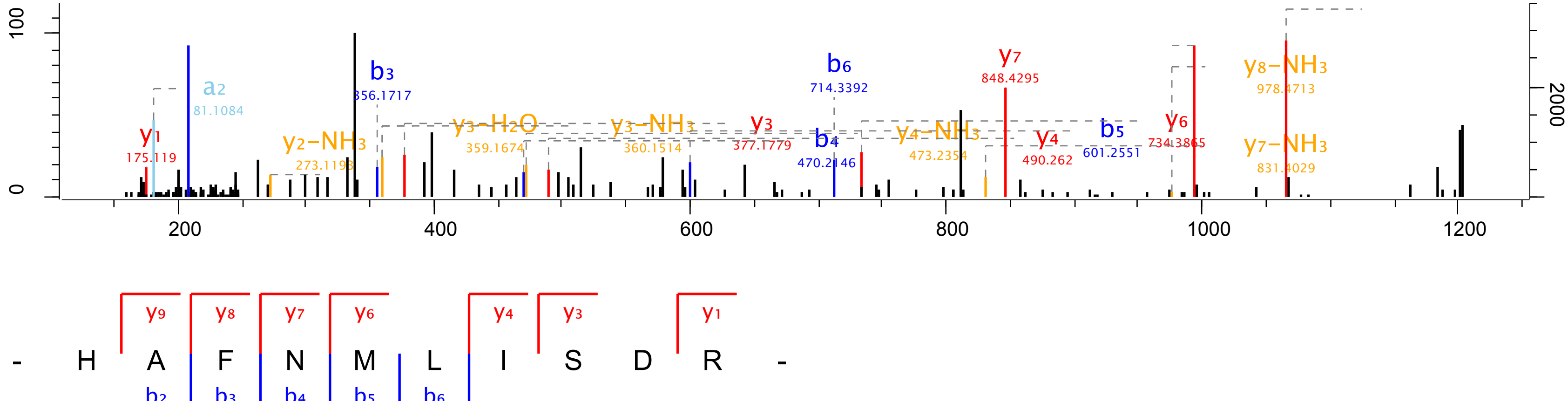

Raw file	Scan	Method	Score	m/z	Gene names
20141014_fract23_dyn_5ul_G7_01_602	22769	TOF; CID	149.59	849.38	PLP2

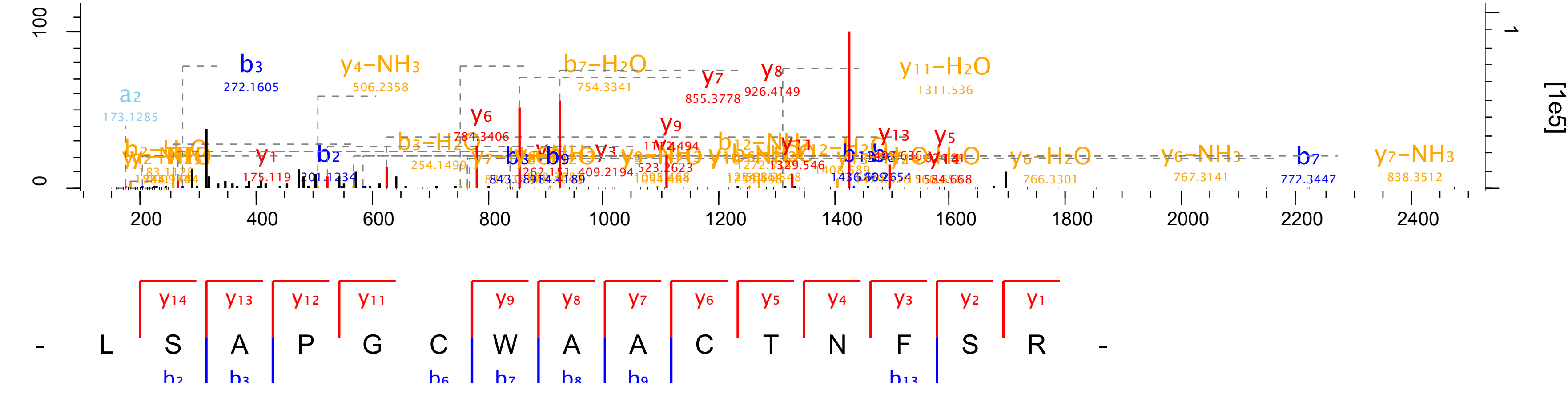

Raw file	Scan	Method	Score	m/z	Gene names
20141014_fract23_dyn_5ul_G7_01_602	23436	TOF; CID	81.7	1127.07	MYADM

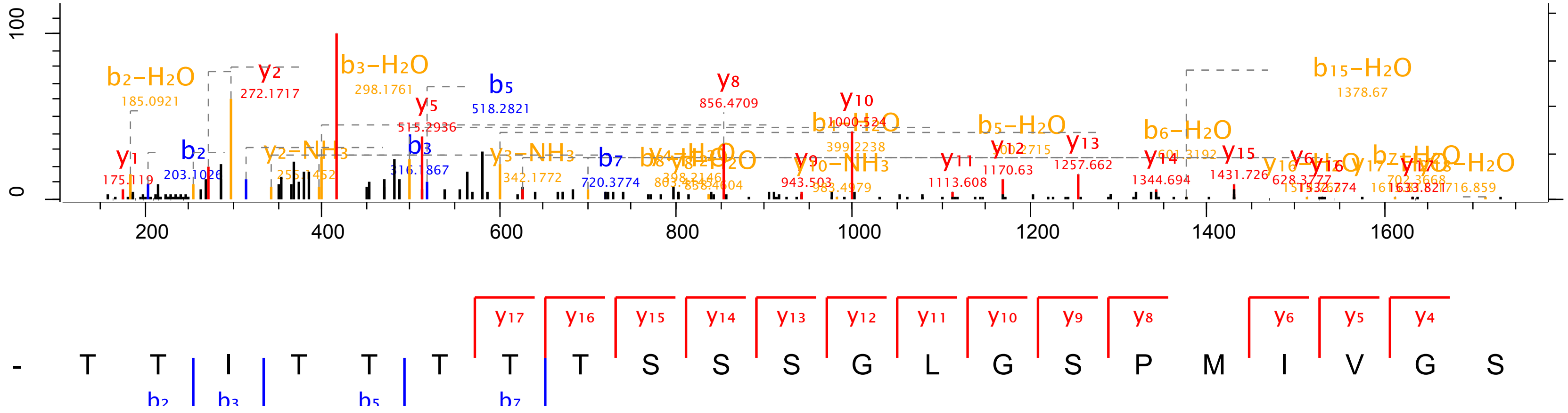

Raw file	Scan	Method	Score	m/z	Gene names
20141014_fract23_dyn_5ul_G7_01_602	25922	TOF; CID	74.44	836.41	TMEM39A

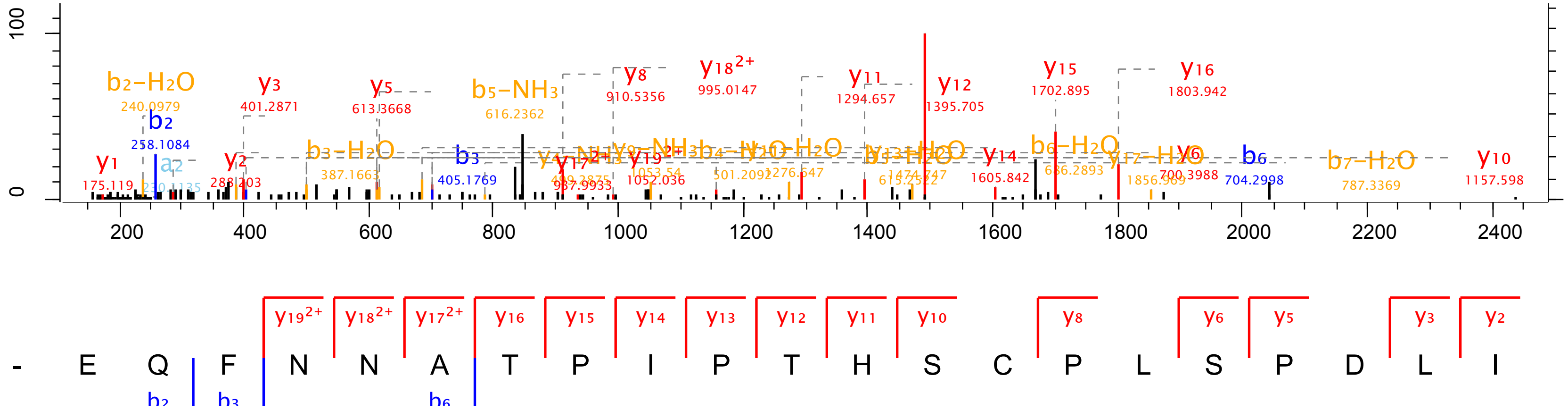

Raw file	Scan	Method	Score	m/z	Gene names
20141014_fract23_dyn_5ul_G7_01_602	30065	TOF; CID	93.65	714.39	MPV17

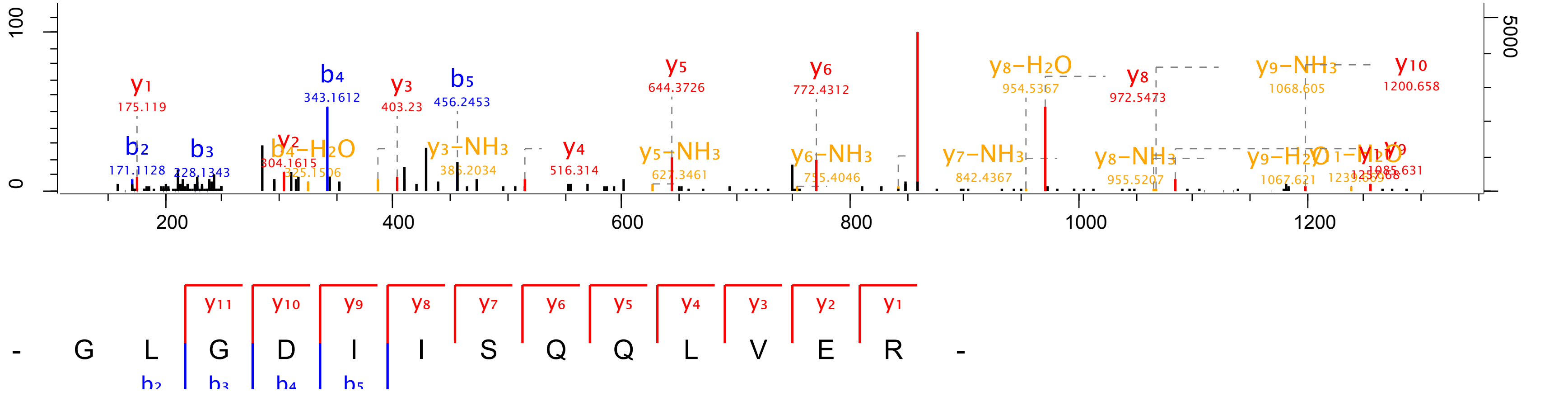

Raw file	Scan	Method	Score	m/z	Gene names
20141014_fract23_dyn_5ul_G7_01_602	30090	TOF; CID	56.13	908.73	TSPAN4

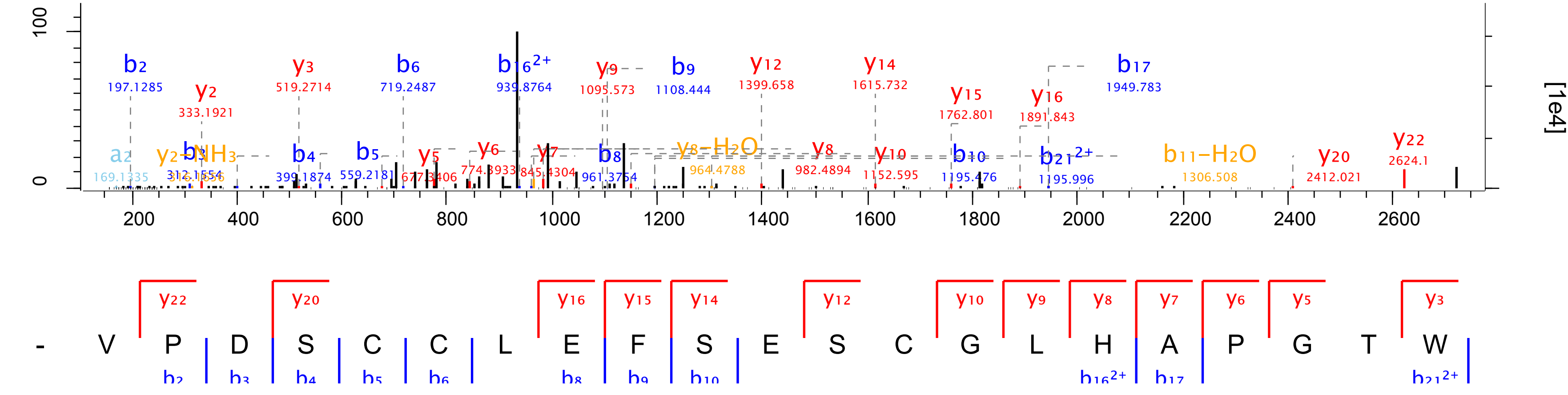

Raw file	Scan	Method	Score	m/z	Gene names
20141014_fract23_dyn_5ul_G7_01_602	35313	TOF; CID	65.84	698.87	PRKD2

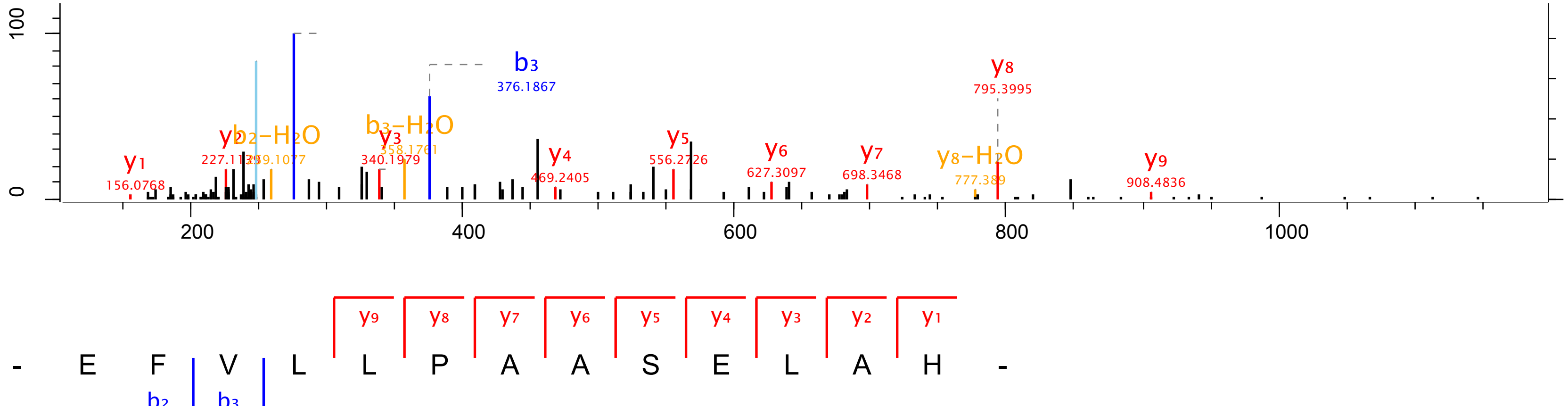

Raw file	Scan	Method	Score	m/z	Gene names
20141014_fract23_dyn_5ul_G7_01_602	37784	TOF; CID	51.71	1199.88	MYEOV2

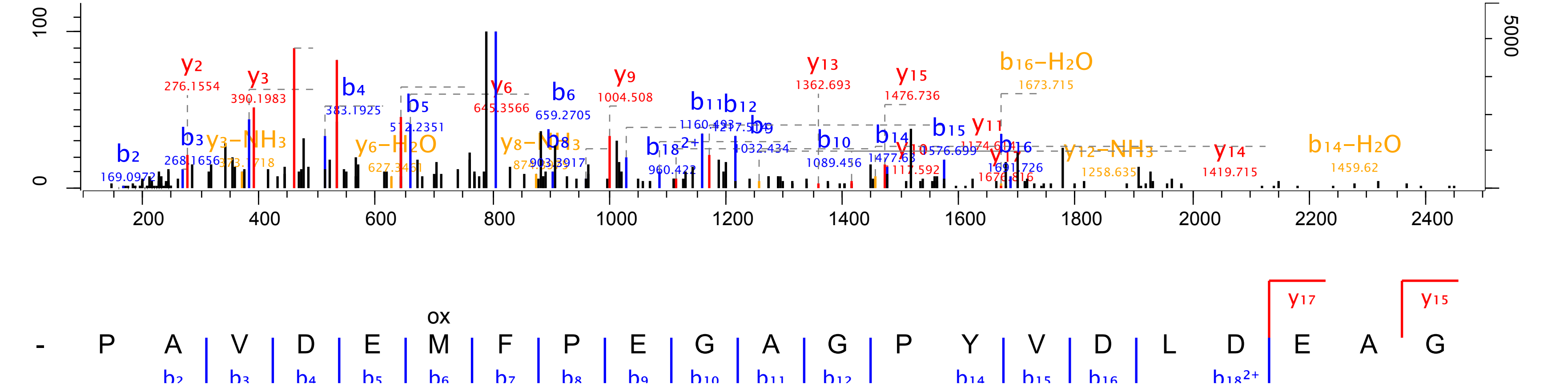

Raw file	Scan	Method	Score	m/z	Gene names
20141014_fract24_dyn_5ul_G8_01_603	12552	TOF; CID	95.5	528.81	TTC7B

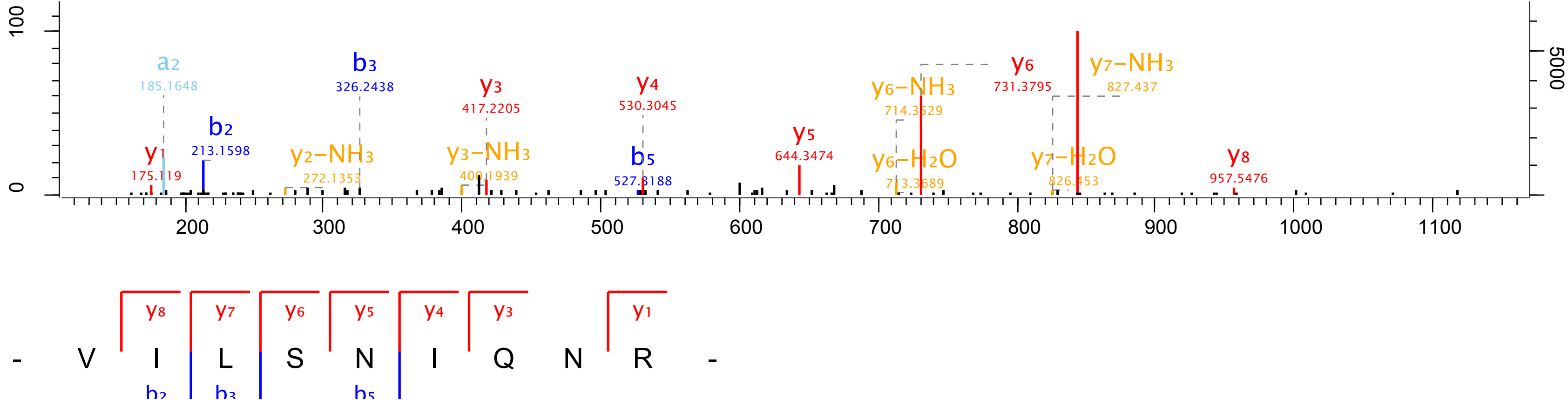

Raw file	Scan	Method	Score	m/z	Gene names
20141014_fract24_dyn_5ul_G8_01_603	16867	TOF; CID	69.35	483.56	PRNP

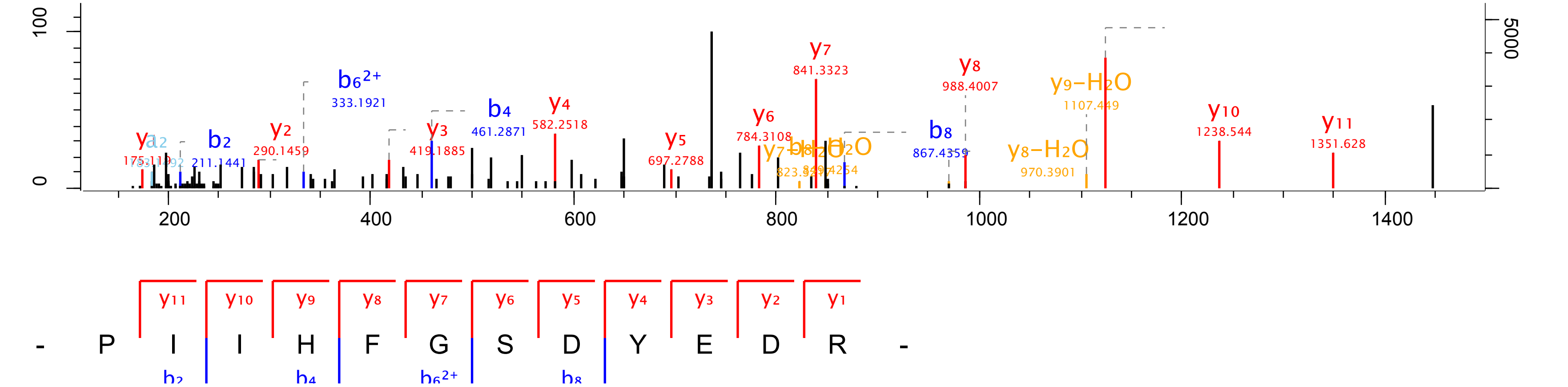

Raw file	Scan	Method	Score	m/z	Gene names
20141014_fract24_dyn_5ul_G8_01_603	18665	TOF; CID	69.72	638.32	PIGB

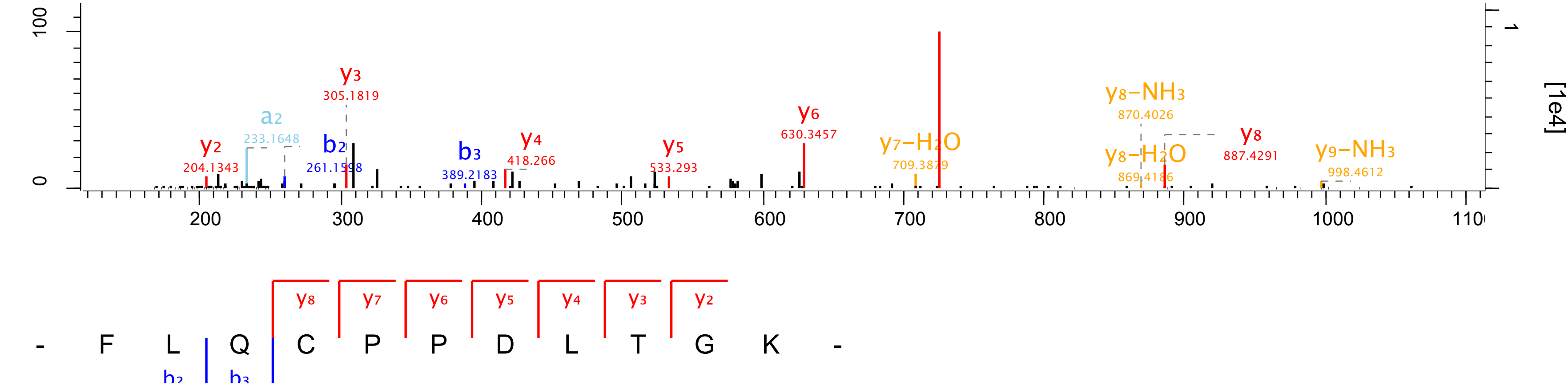

Raw file	Scan	Method	Score	m/z	Gene names
20141014_fract24_dyn_5ul_G8_01_603	20741	TOF; CID	68.04	800.44	ZCCHC10

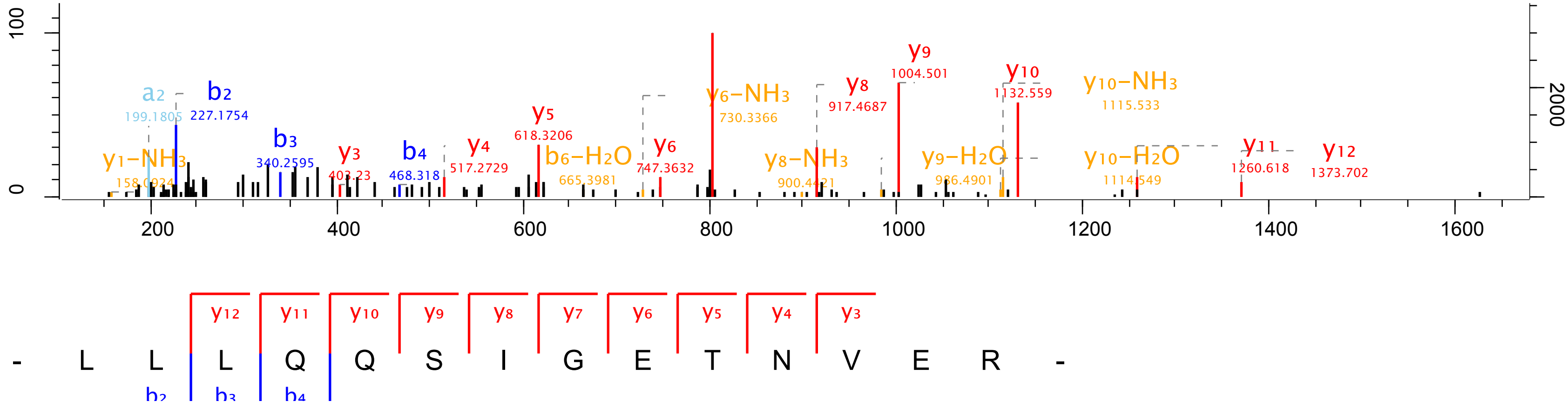

Raw file	Scan	Method	Score	m/z	Gene names
20141014_fract24_dyn_5ul_G8_01_603	21101	TOF; CID	81.63	507.96	EPM2A

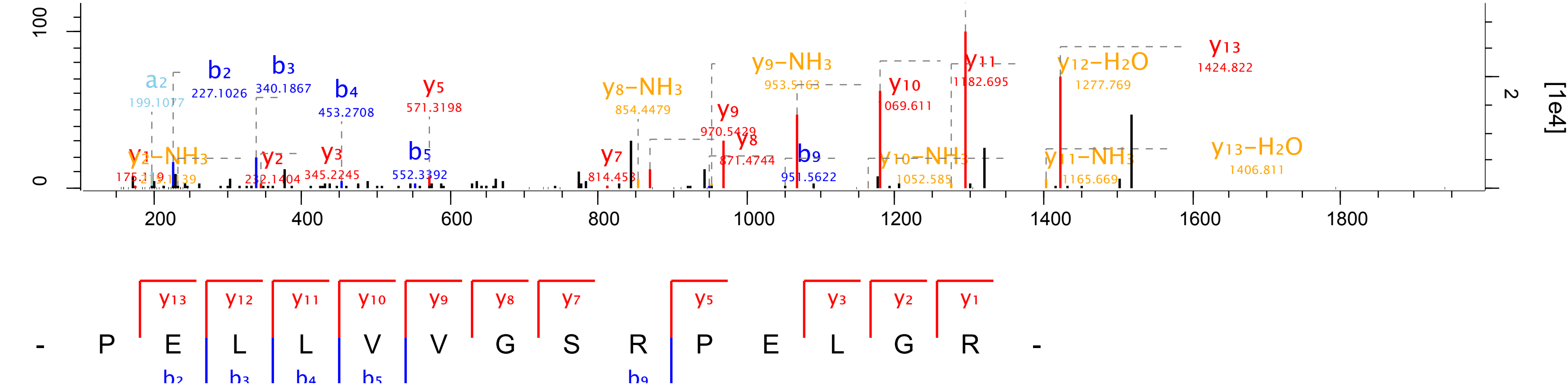

Raw file	Scan	Method	Score	m/z	Gene names
20141014_fract24_dyn_5ul_G8_01_603	27723	TOF; CID	84.51	891.45	TAF11

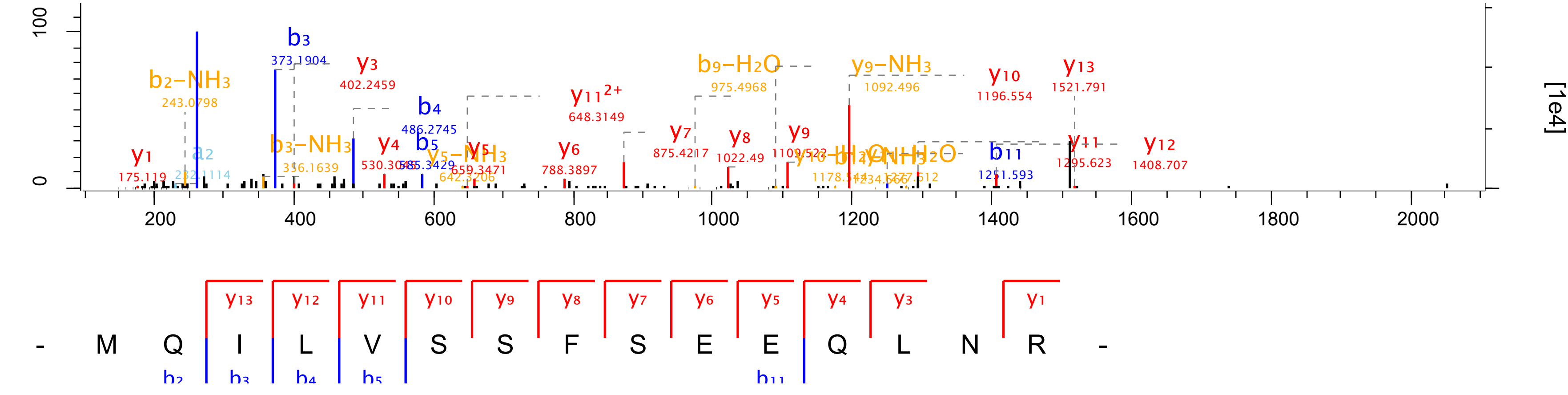

Raw file	Scan	Method	Score	m/z	Gene names
20141014_fract24_dyn_5ul_G8_01_603	32521	TOF; CID	91.55	670.33	LRRTM4

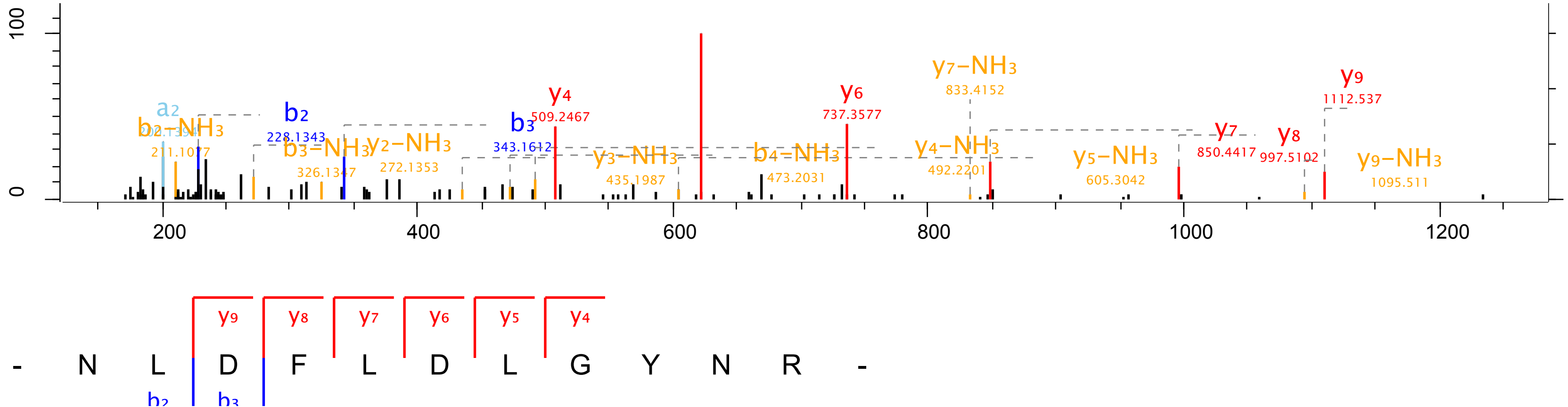

Raw file	Scan	Method	Score	m/z	Gene names
20141014_fract24_dyn_5ul_G8_01_603	32753	TOF; CID	58.69	803.41	CEP68

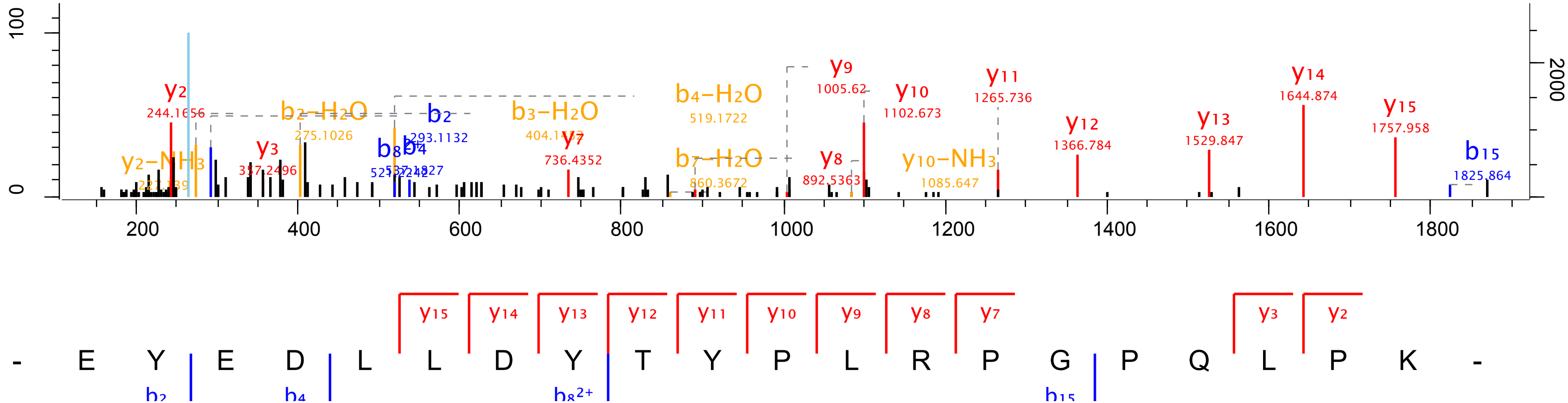

Raw file	Scan	Method	Score	m/z	Gene names
20141014_fract24_dyn_5ul_G8_01_603	33196	TOF; CID	52.07	712.36	SMIM15

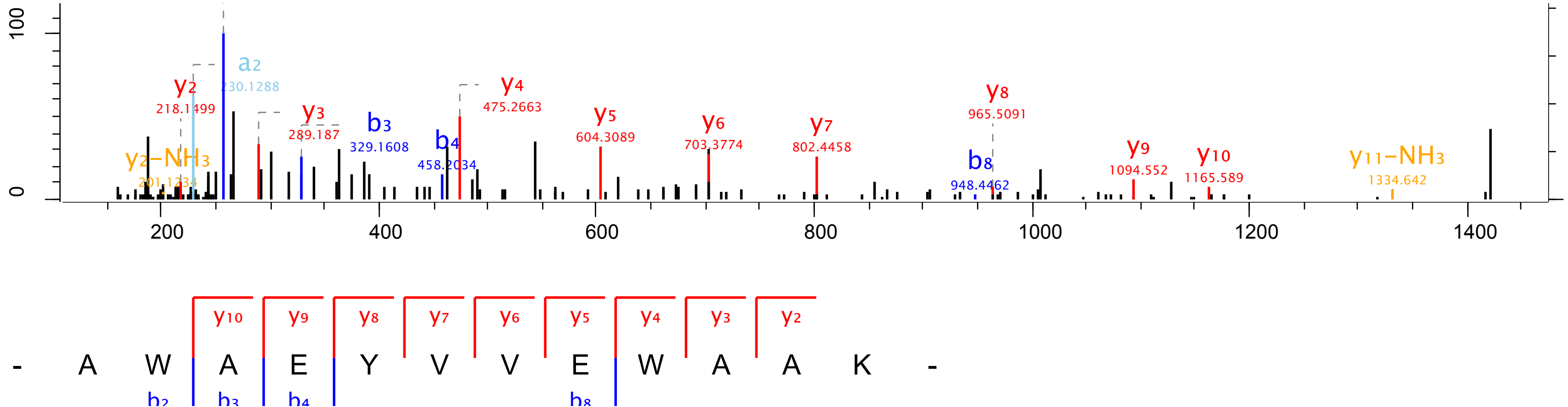

Raw file	Scan	Method	Score	m/z	Gene names
20141014_fract24_dyn_5ul_G8_01_603	34885	TOF; CID	103.83	814.95	RCE1

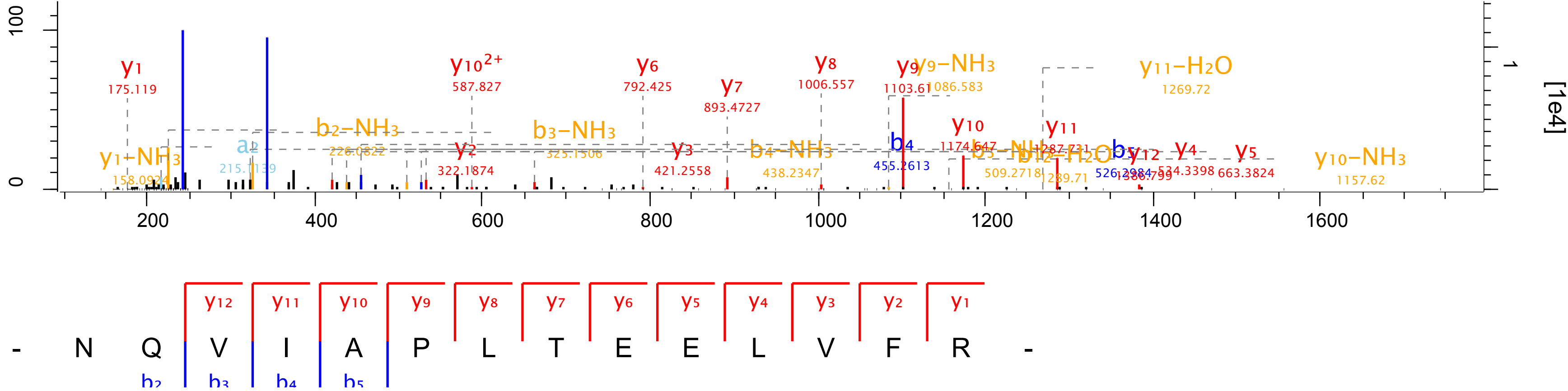

Supplement: Supplemental Data [file supp_M114.047407_mcp.M114.047407-12.pdf]
