# Supplementary material for: The Impact II, a Very High-Resolution Quadrupole Time-of-Flight Instrument (QTOF) for Deep Shotgun Proteomics
Source: Mol Cell Proteomics. 2015 May 19;14(7):2014–29. doi: 10.1074/mcp.M114.047407 (PMC4587313; doi:10.1074/mcp.M114.047407)

Raw file	Scan	Method	Score	m/z	Gene names
20140918_fract1_dyn_5ul_D1_01_367	5371	TOF; CID	131.06	330.19	HMGN4

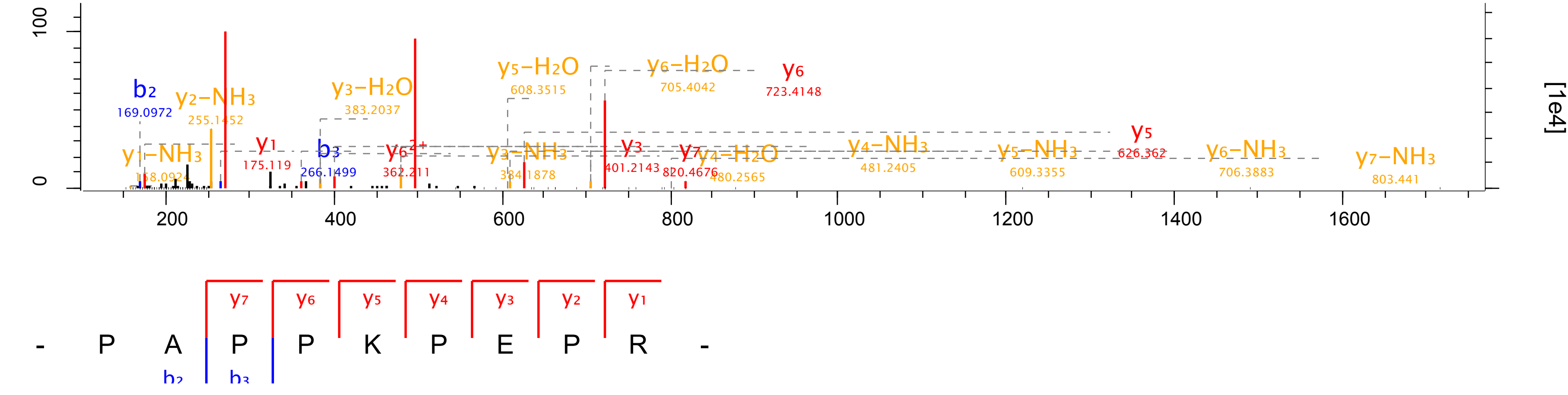

Raw file	Scan	Method	Score	m/z	Gene names
20140918_fract1_dyn_5ul_D1_01_367	14149	TOF; CID	91.31	538.29	PTMS

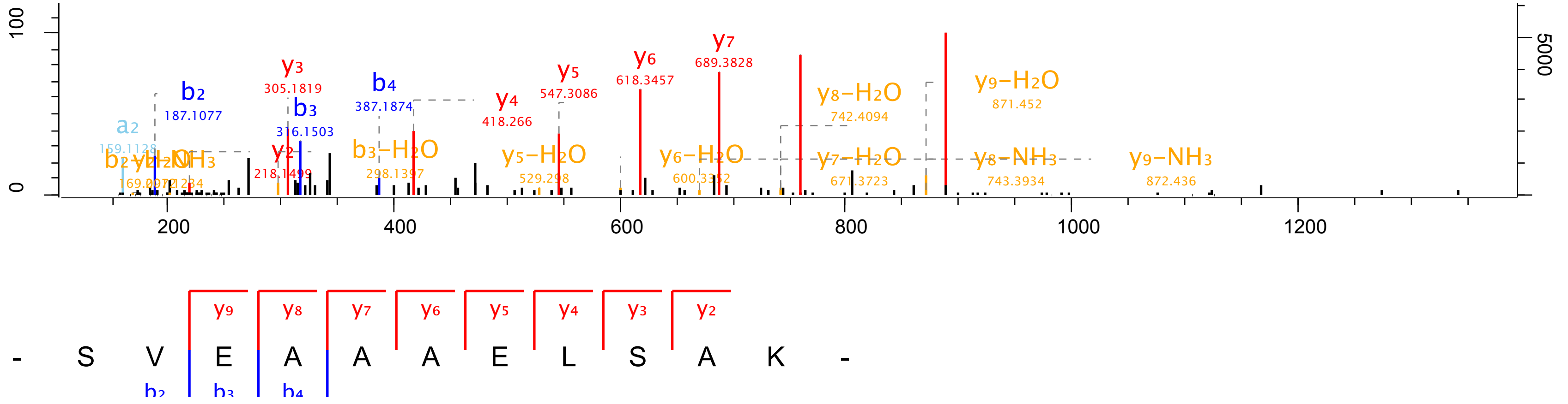

Raw file	Scan	Method	Score	m/z	Gene names
20140918_fract1_dyn_5ul_D1_01_367	19764	TOF; CID	58.71	576.26	GCA

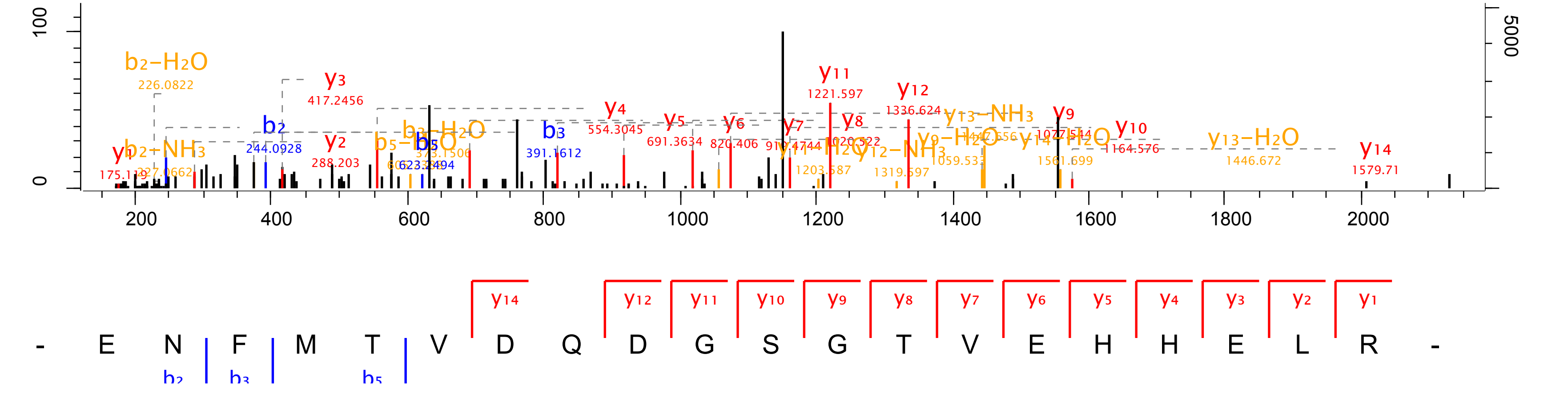

Raw file	Scan	Method	Score	m/z	Gene names
20140918_fract1_dyn_5ul_D1_01_367	25911	TOF; CID	106.4	597.31	FBXL17

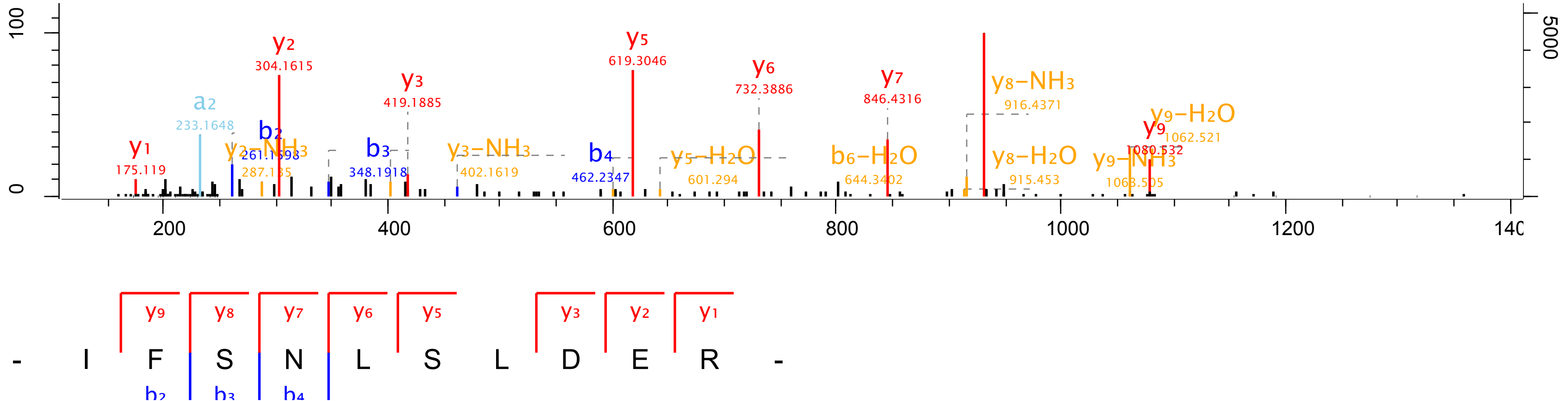

Raw file	Scan	Method	Score	m/z	Gene names
20140918_fract1_dyn_5ul_D1_01_367	27688	TOF; CID	115.78	640.34	METTL23

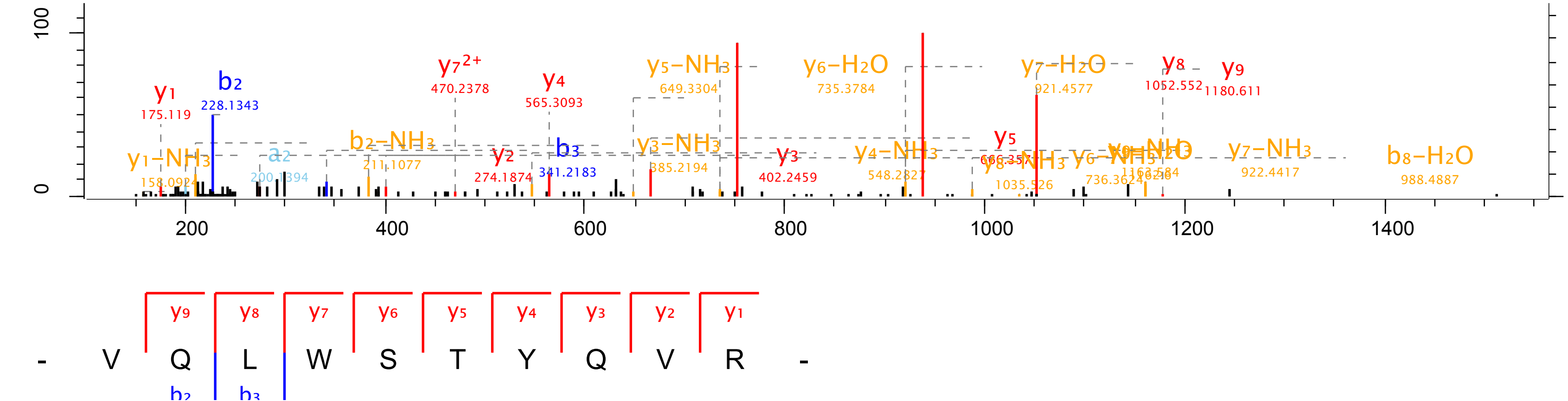

Raw file	Scan	Method	Score	m/z	Gene names
20140918_fract1_dyn_5ul_D1_01_367	29299	TOF; CID	95.27	469.25	CHAC1

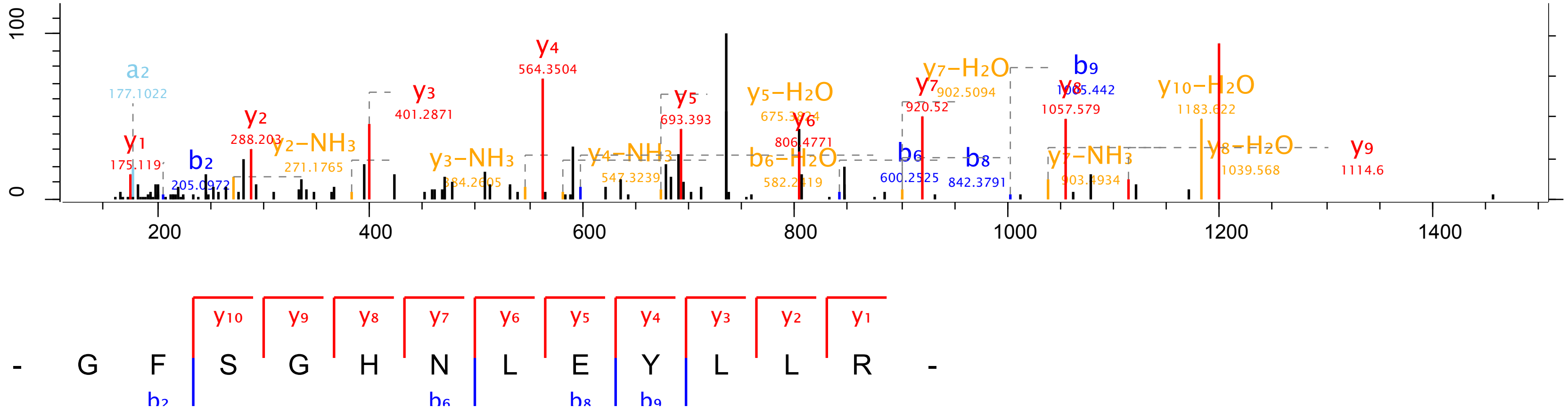

Raw file	Scan	Method	Score	m/z	Gene names
20140918_fract1_dyn_5ul_D1_01_367	33339	TOF; CID	47.56	772.74	PLLP

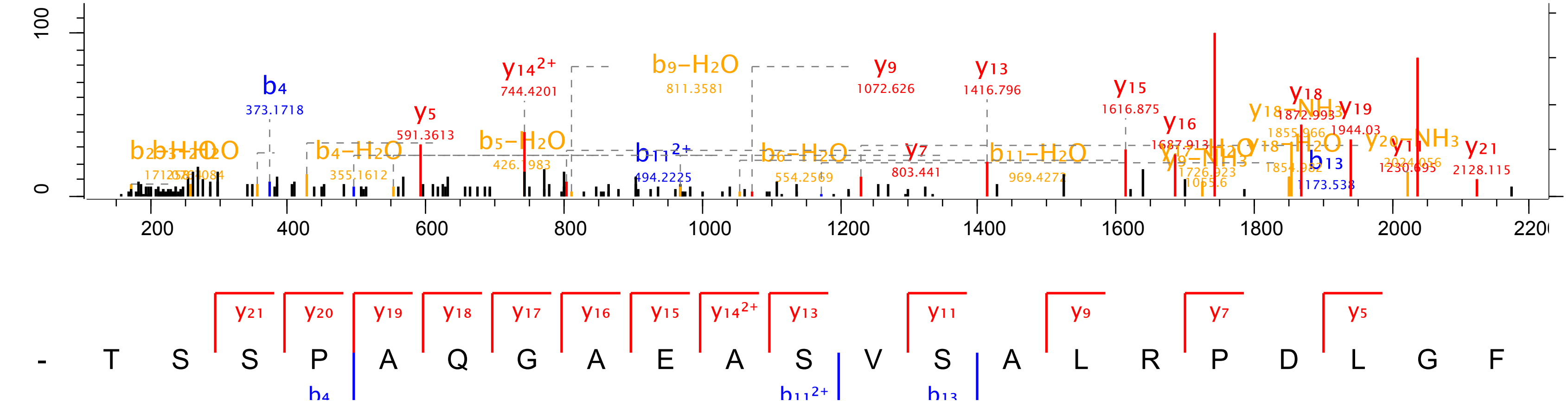

Raw file	Scan	Method	Score	m/z	Gene names
20140918_fract2_dyn_5ul_D2_01_369	8659	TOF; CID	60.17	478.76	EPM2A

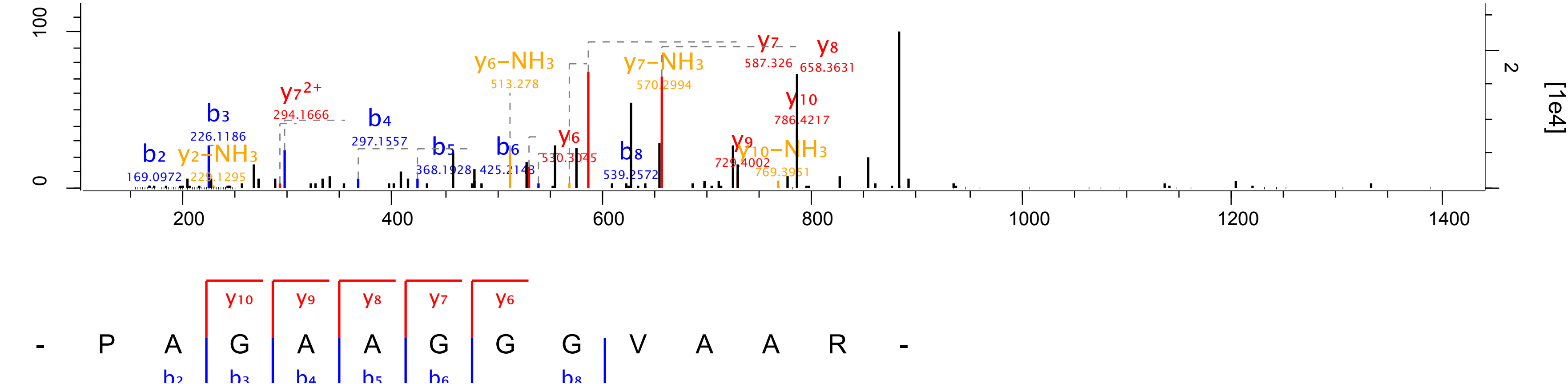

Raw file	Scan	Method	Score	m/z	Gene names
20140918_fract2_dyn_5ul_D2_01_369	13430	TOF; CID	130.56	720.31	LEPROTL1

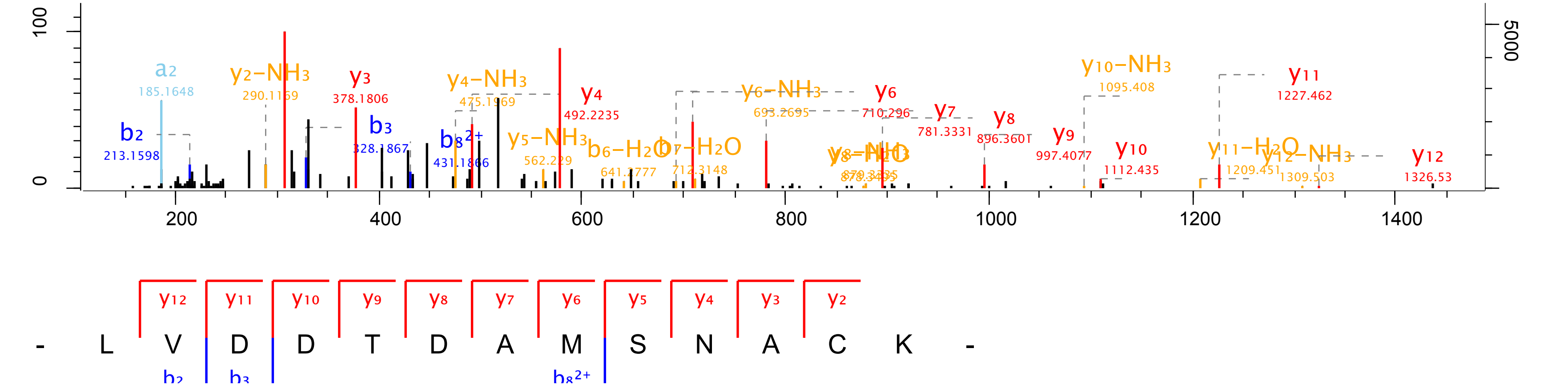

Raw file	Scan	Method	Score	m/z	Gene names
20140918_fract2_dyn_5ul_D2_01_369	18483	TOF; CID	101.65	509.26	PPP2R2A

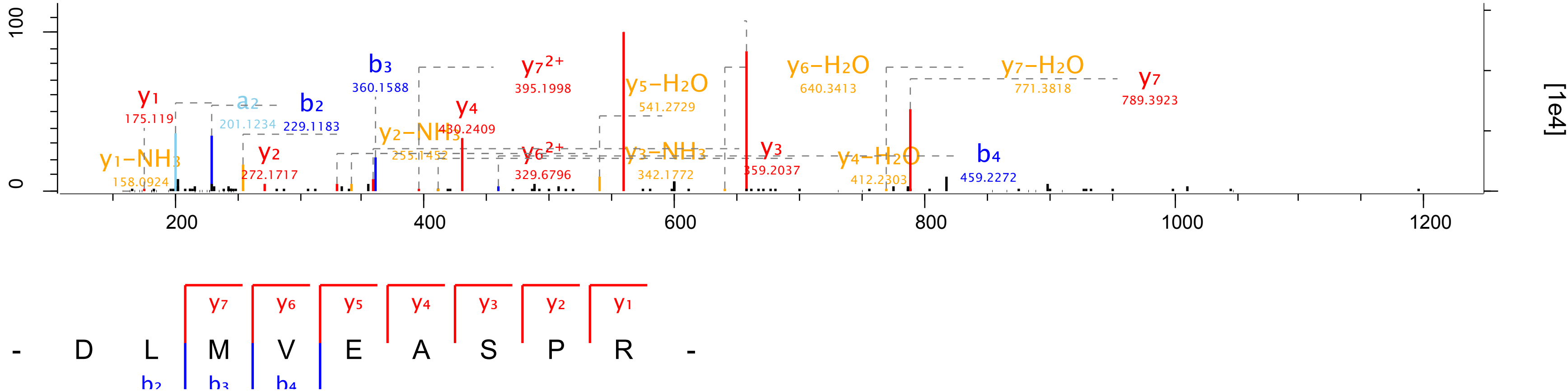

Raw file	Scan	Method	Score	m/z	Gene names
20140918_fract2_dyn_5ul_D2_01_369	41437	TOF; CID	147.64	1019.55	LSM5

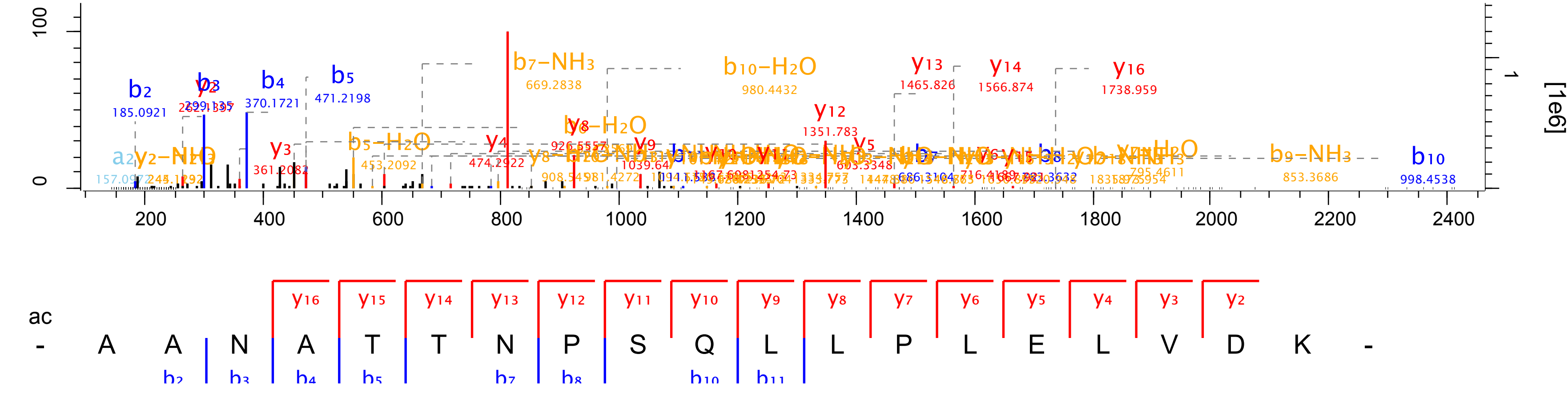

Raw file	Scan	Method	Score	m/z	Gene names
20140918_fract3_dyn_5ul_D3_01_370	12643	TOF; CID	39.39	627.31	CYBRD1

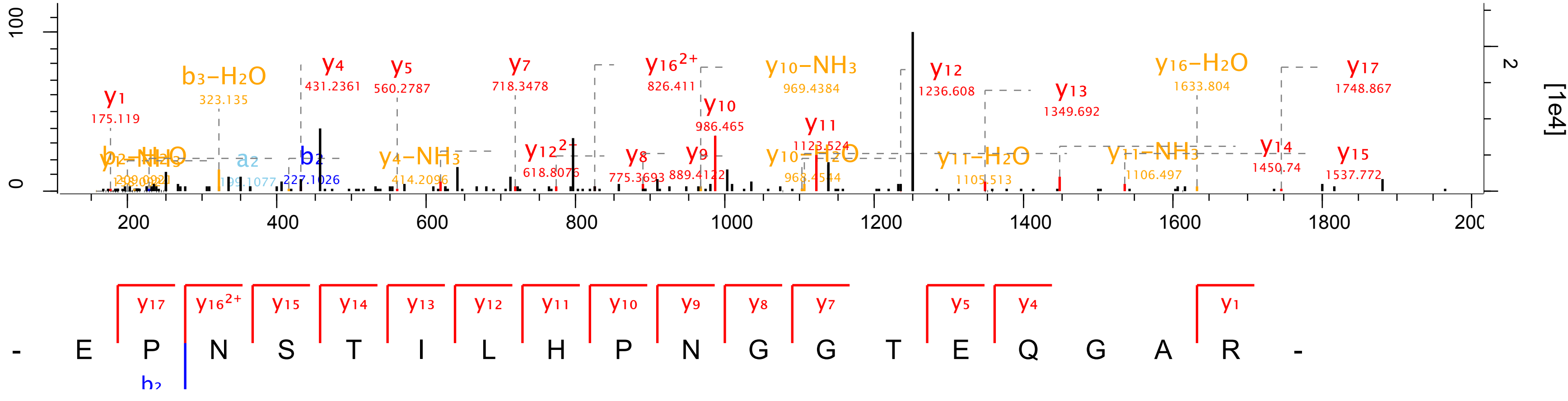

Raw file	Scan	Method	Score	m/z	Gene names
20140918_fract3_dyn_5ul_D3_01_370	18788	TOF; CID	136.81	858.93	APOC3

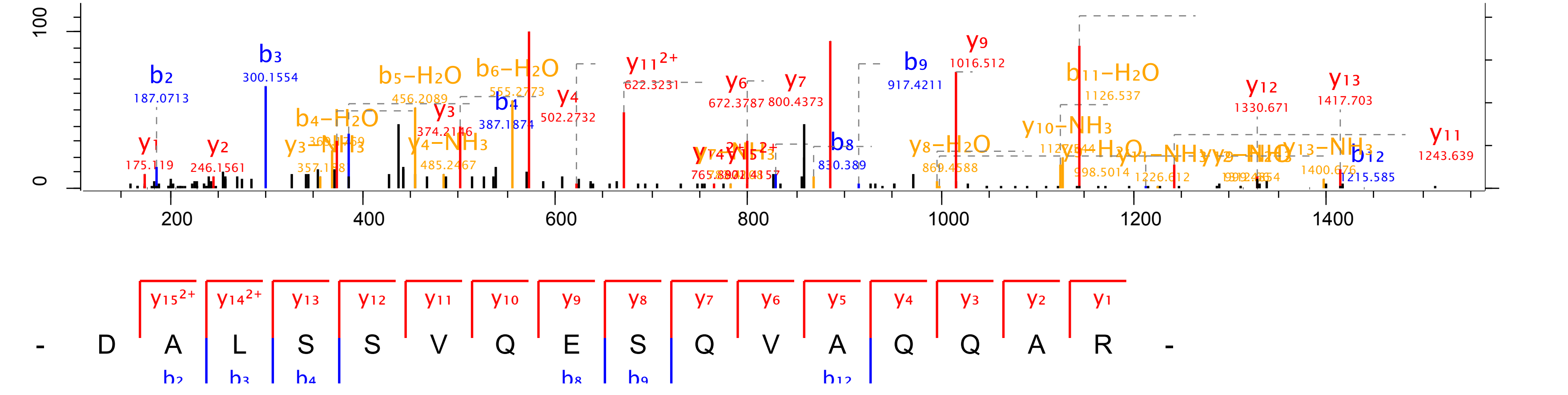

Raw file	Scan	Method	Score	m/z	Gene names
20140918_fract3_dyn_5ul_D3_01_370	27345	TOF; CID	89.3	586.84	KATNAL2

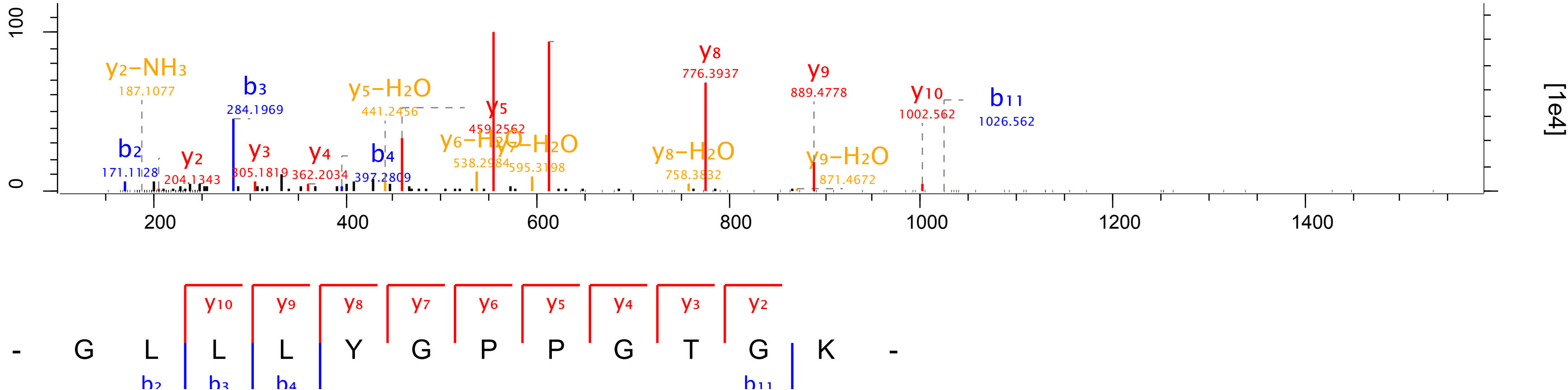

Raw file	Scan	Method	Score	m/z	Gene names
20140918_fract3_dyn_5ul_D3_01_370	35999	TOF; CID	113.71	568.8	MT-ND2

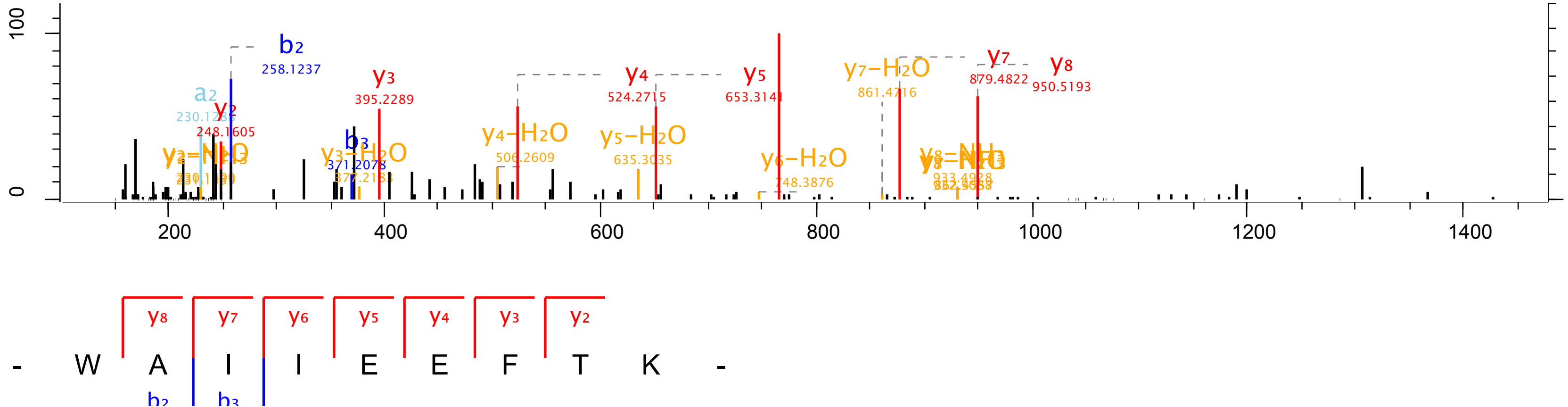

Raw file	Scan	Method	Score	m/z	Gene names
20140918_fract4_dyn_5ul_D4_01_371	7729	TOF; CID	109.48	476.77	ENTPD7

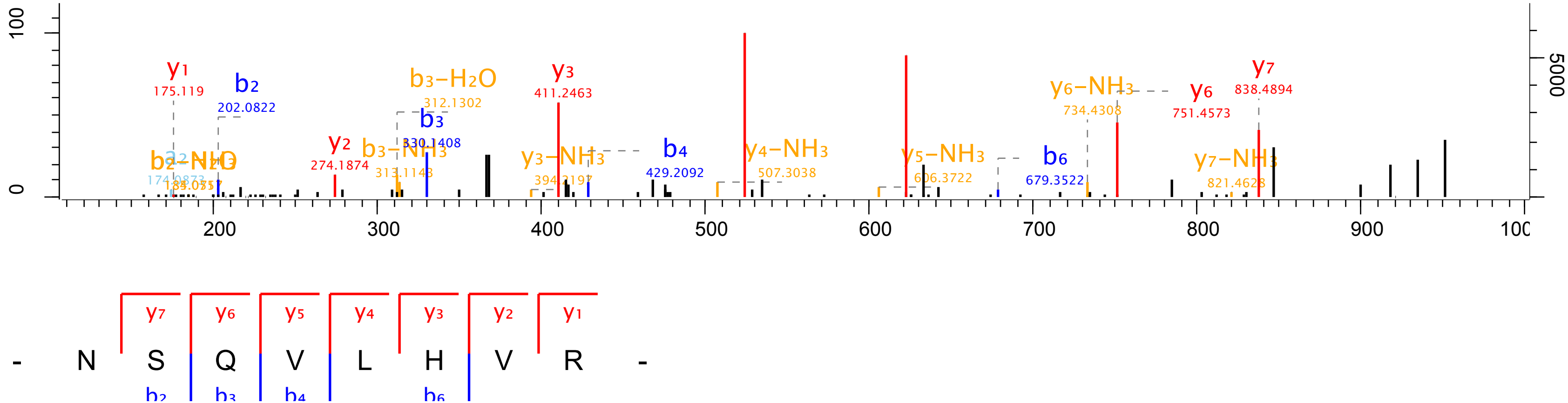

Raw file	Scan	Method	Score	m/z	Gene names
20140918_fract4_dyn_5ul_D4_01_371	8826	TOF; CID	90.79	464.54	DPM3

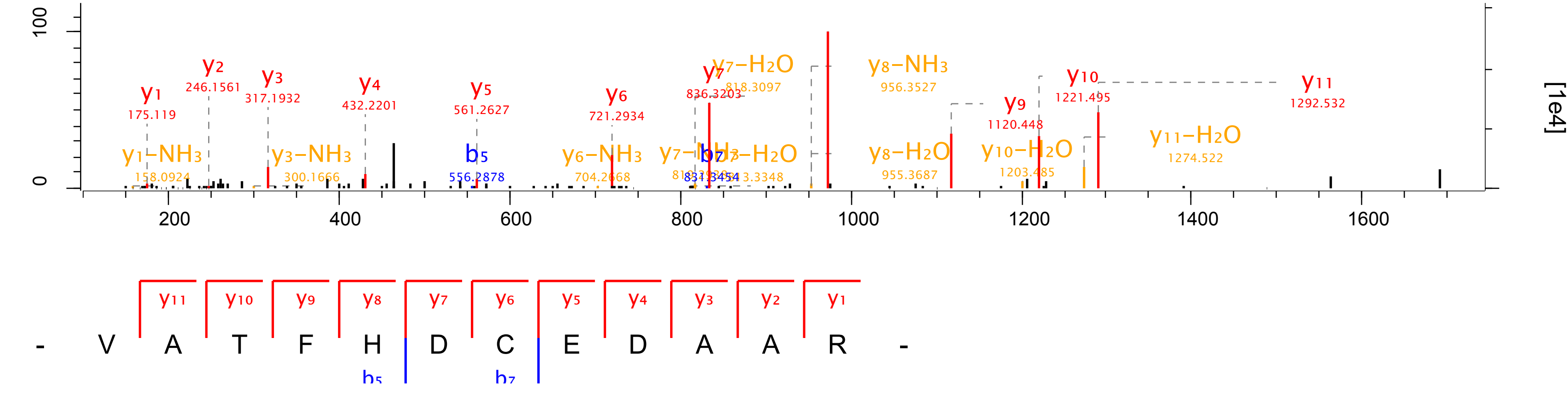

Raw file	Scan	Method	Score	m/z	Gene names
20140918_fract4_dyn_5ul_D4_01_371	13366	TOF; CID	151.98	459.56	CYB561D2

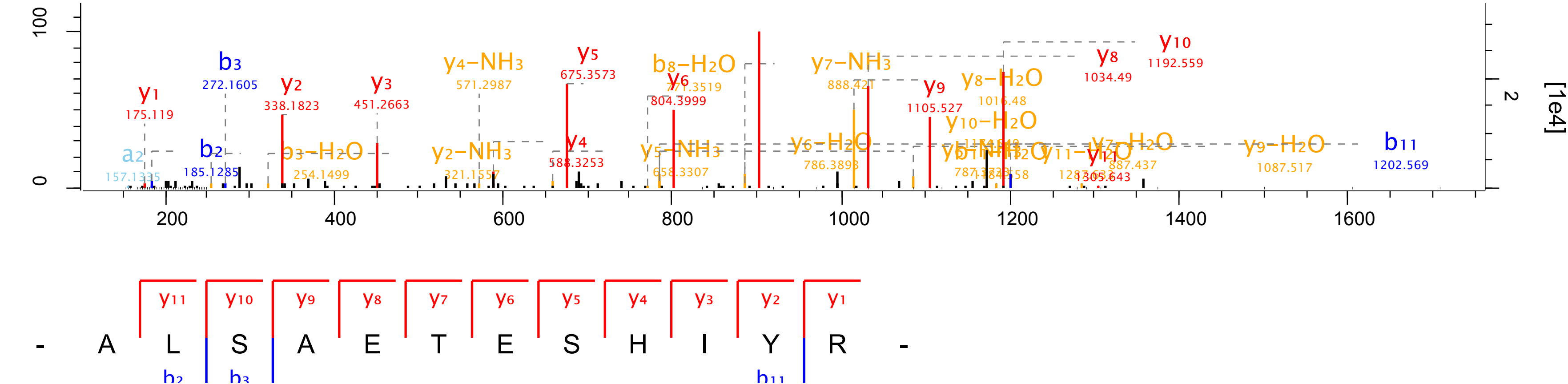

Raw file	Scan	Method	Score	m/z	Gene names
20140918_fract4_dyn_5ul_D4_01_371	22016	TOF; CID	119.87	532.62	SOCS3

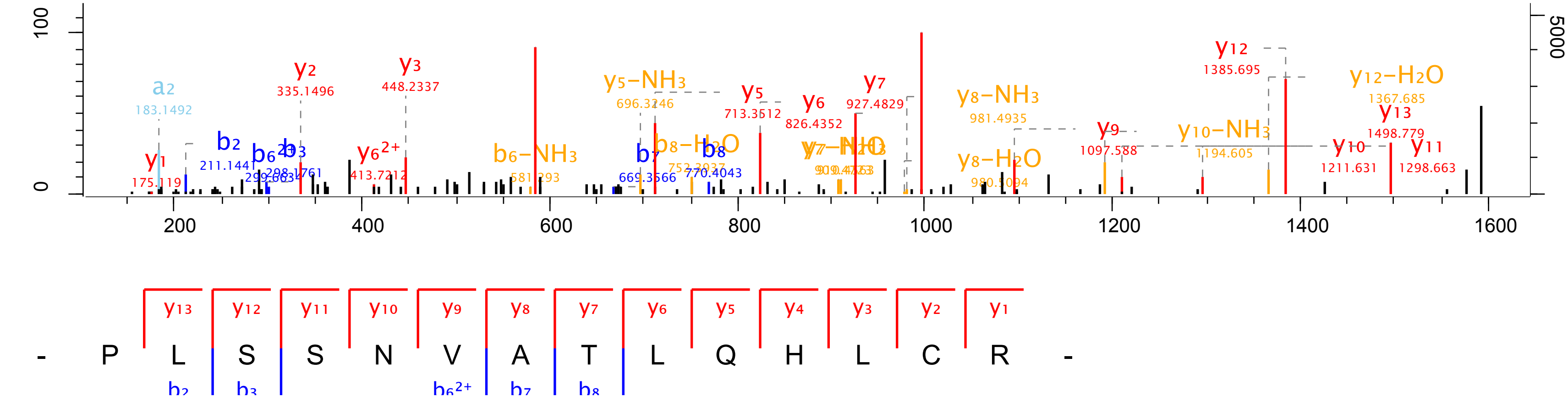

Raw file	Scan	Method	Score	m/z	Gene names
20140918_fract4_dyn_5ul_D4_01_371	32344	TOF; CID	66.83	668.65	PERP

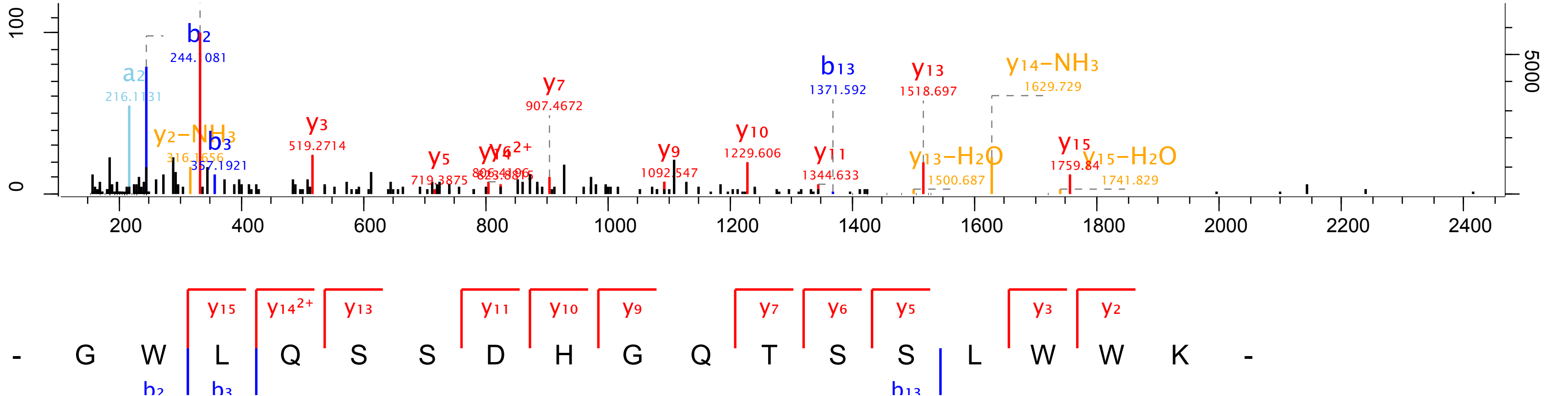

Raw file	Scan	Method	Score	m/z	Gene names
20140918_fract4_dyn_5ul_D4_01_371	38454	TOF; CID	141.58	797.94	CLDN12

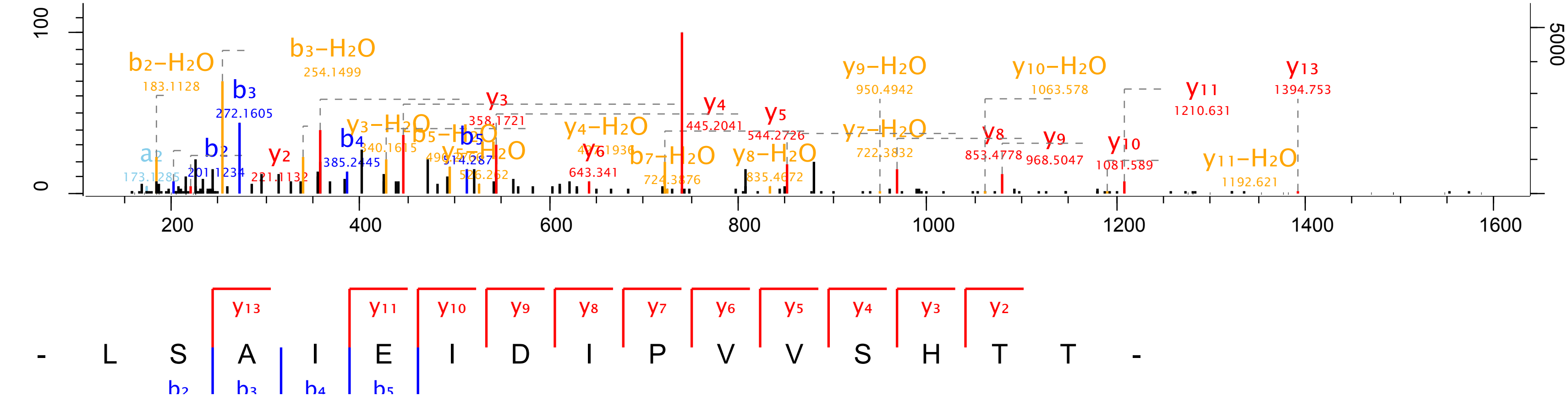

Raw file	Scan	Method	Score	m/z	Gene names
20140918_fract5_dyn_5ul_D5_01_372	14907	TOF; CID	94.02	510.62	IFI27L2

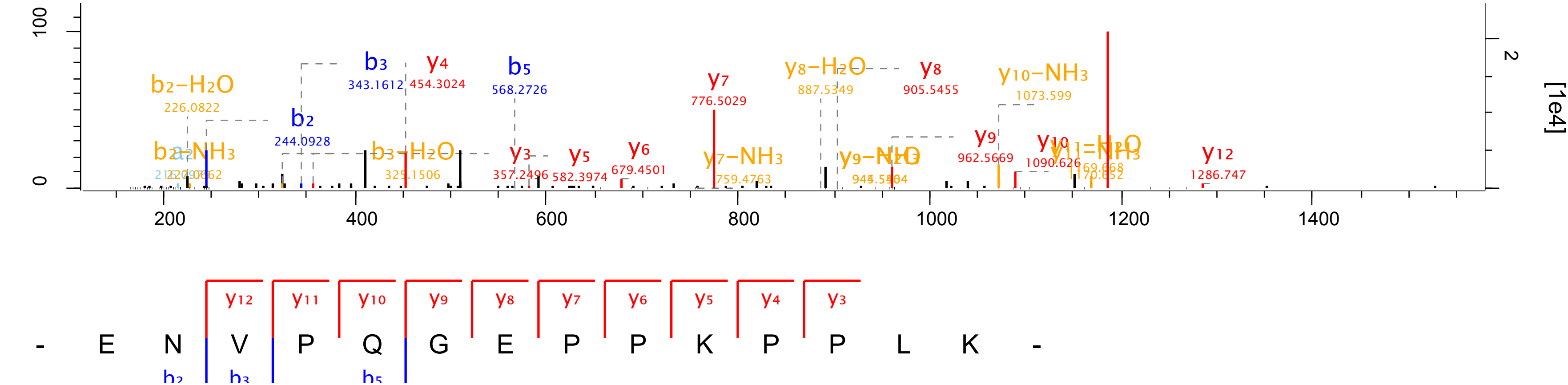

Raw file	Scan	Method	Score	m/z	Gene names
20140918_fract5_dyn_5ul_D5_01_372	30562	TOF; CID	100.02	735.03	UBALD1

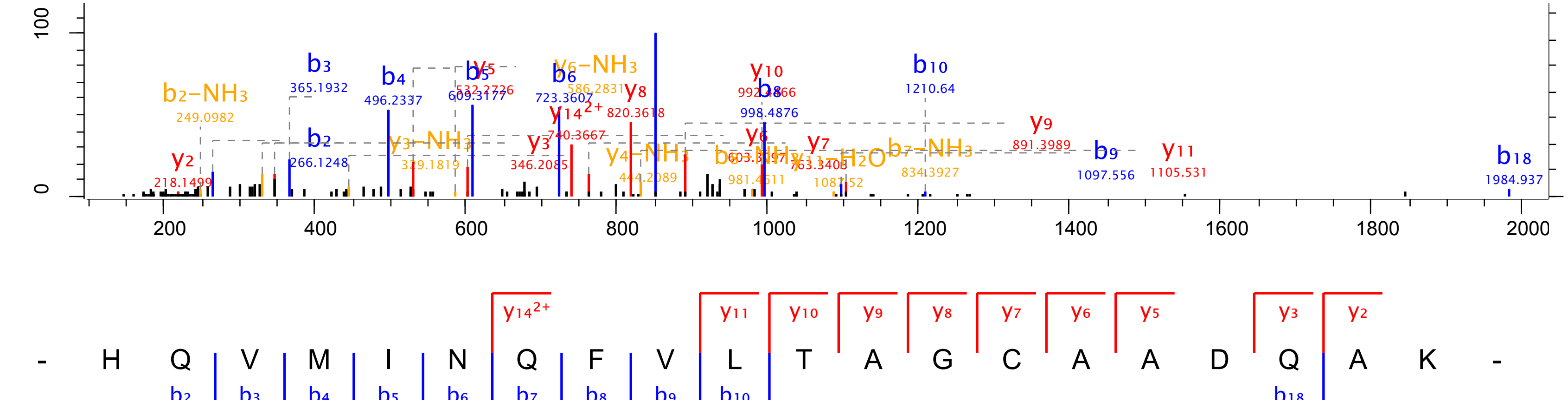

Raw file	Scan	Method	Score	m/z	Gene names
20140918_fract5_dyn_5ul_D5_01_372	33628	TOF; CID	64.1	773.43	PIGL

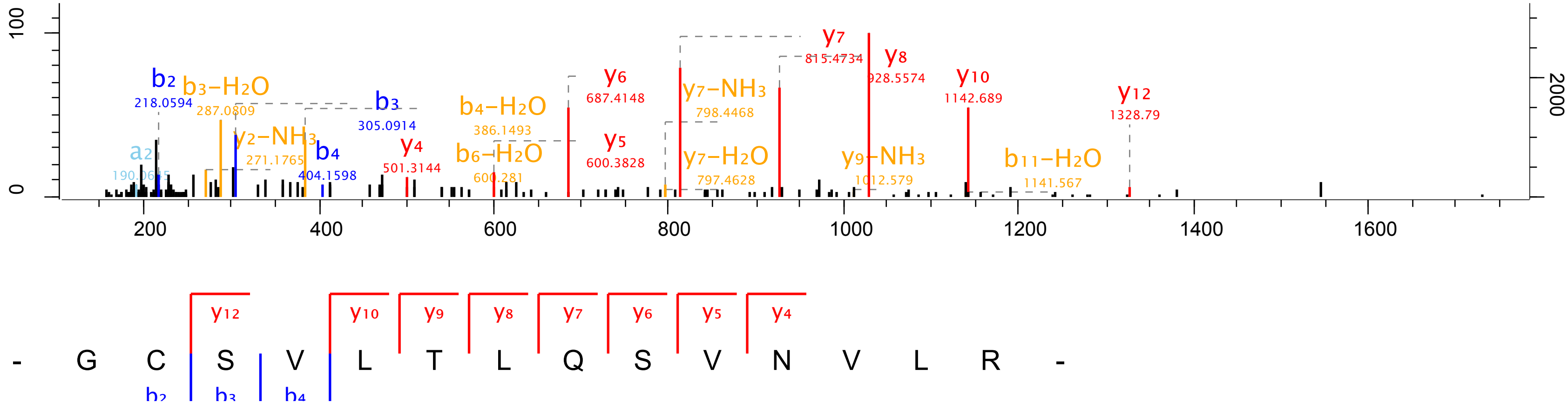

Raw file	Scan	Method	Score	m/z	Gene names
20140918_fract5_dyn_5ul_D5_01_372	34434	TOF; CID	59.23	659.36	CDC14B

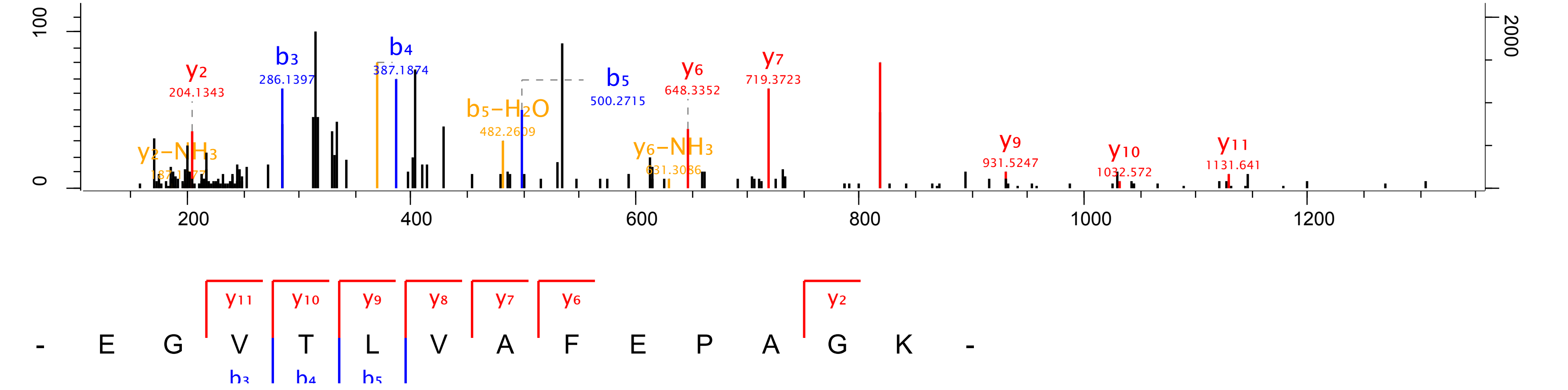

Raw file	Scan	Method	Score	m/z	Gene names
20140918_fract5_dyn_5ul_D5_01_372	37423	TOF; CID	54.09	557.29	IFT46

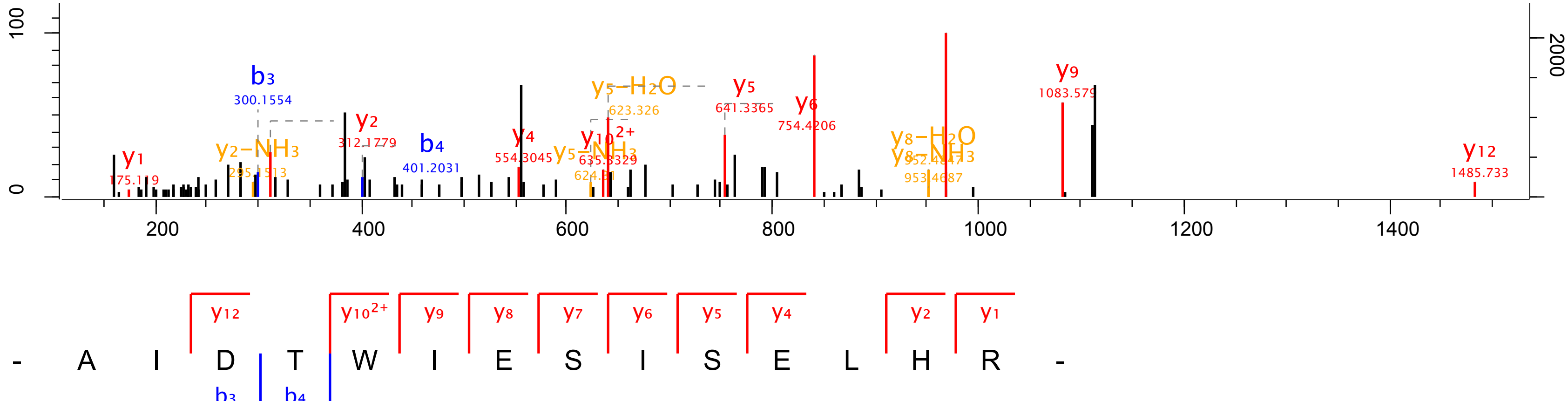

Raw file	Scan	Method	Score	m/z	Gene names
20140918_fract6_dyn_5ul_D6_01_373	7562	TOF; CID	91.07	566.3	ZKSCAN4

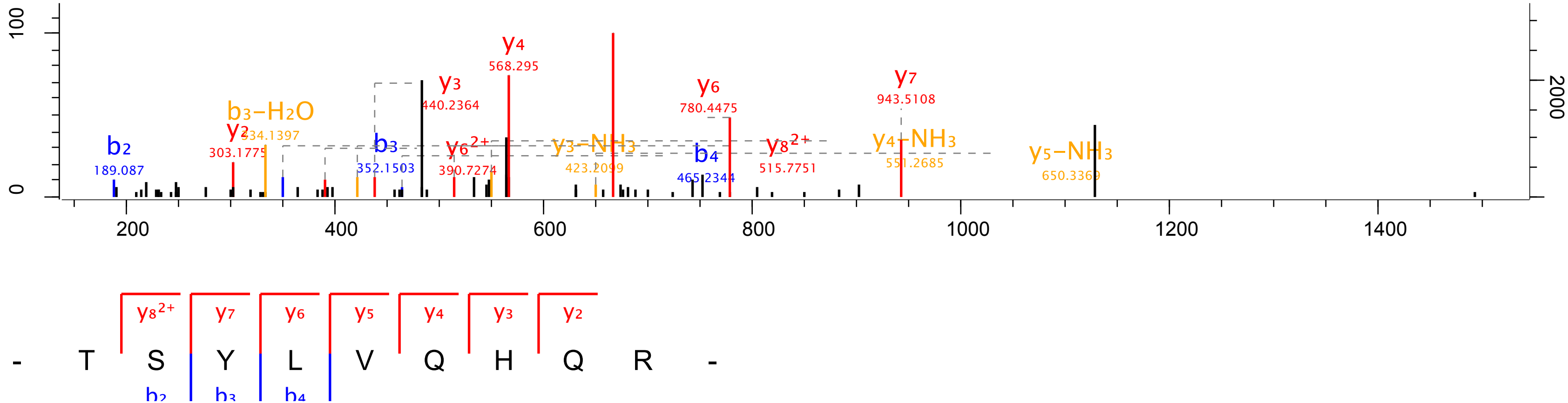

Raw file	Scan	Method	Score	m/z	Gene names
20140918_fract6_dyn_5ul_D6_01_373	13691	TOF; CID	133.42	607.83	SLC25A14

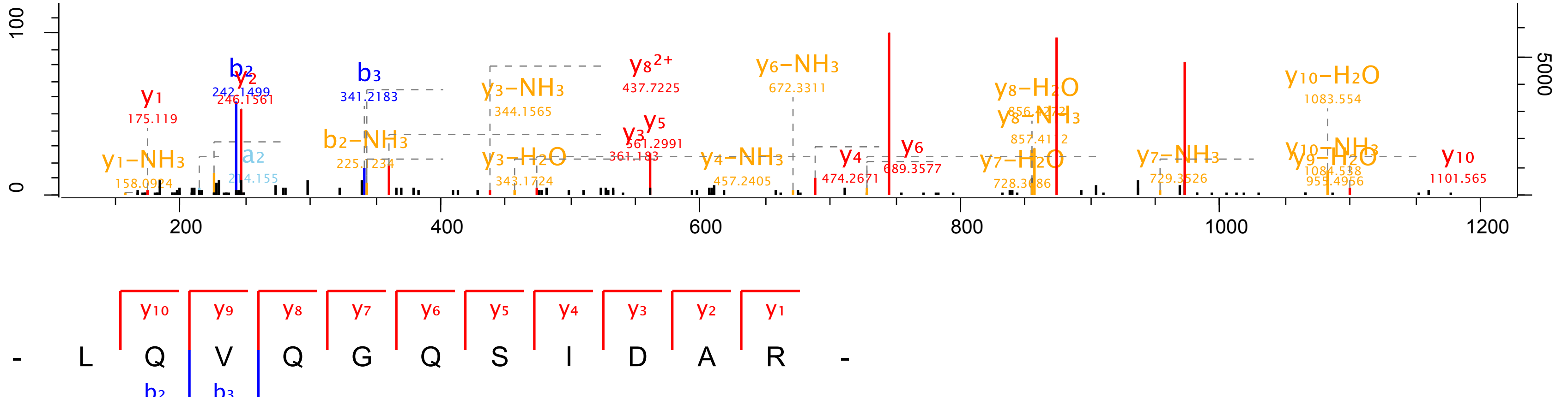

Raw file	Scan	Method	Score	m/z	Gene names
20140918_fract6_dyn_5ul_D6_01_373	21525	TOF; CID	54.87	1137.52	MDM2

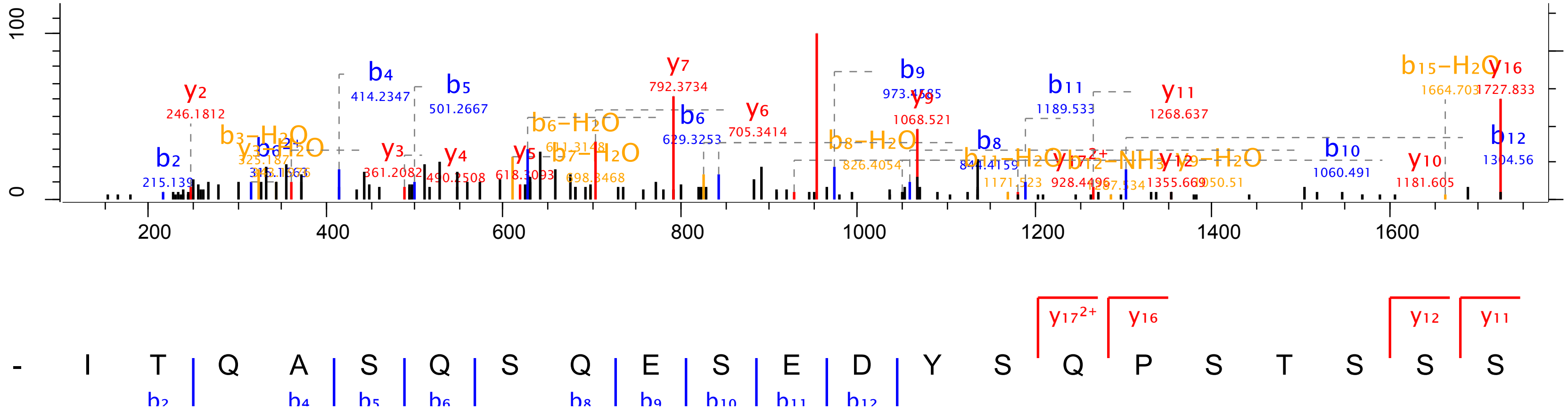

Raw file	Scan	Method	Score	m/z	Gene names
20140918_fract6_dyn_5ul_D6_01_373	22016	TOF; CID	102.52	548.28	SOCS2

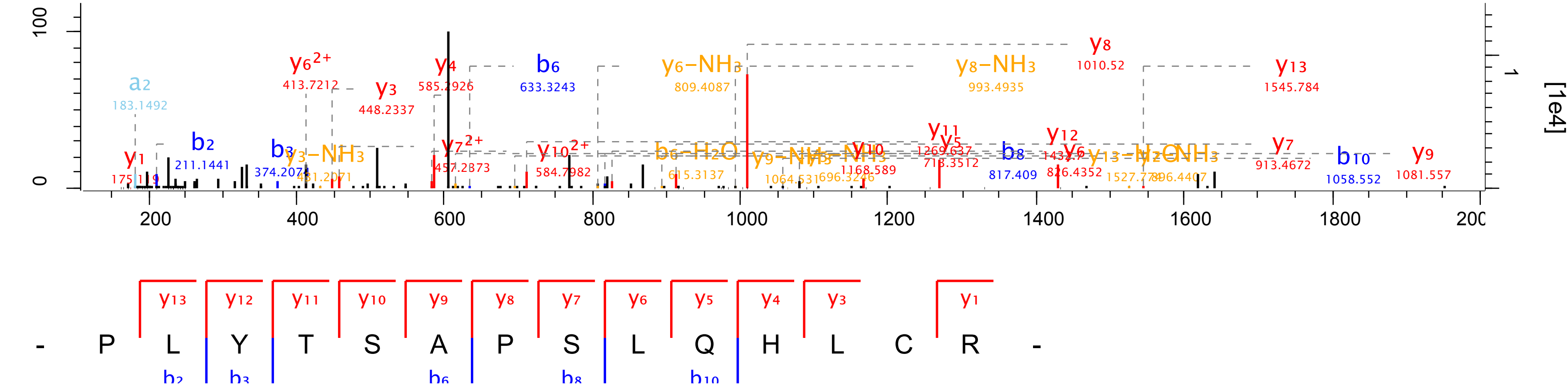

Raw file	Scan	Method	Score	m/z	Gene names
20140918_fract6_dyn_5ul_D6_01_373	22980	TOF; CID	93.16	499.31	ZBTB26

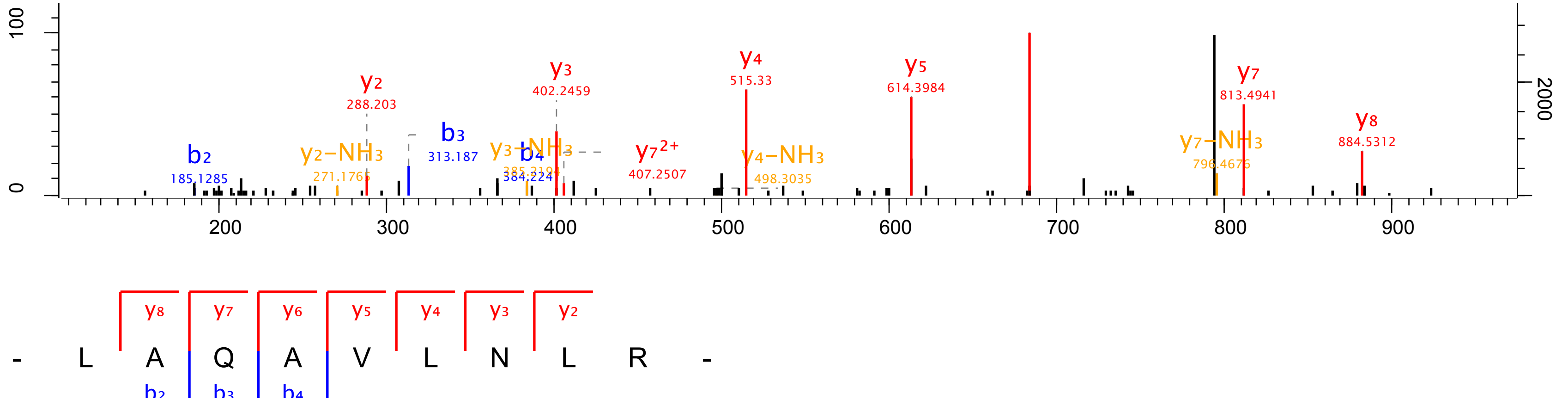

Raw file	Scan	Method	Score	m/z	Gene names
20140918_fract7_dyn_5ul_D7_01_393	19337	TOF; CID	73.93	799.01	PGAP3

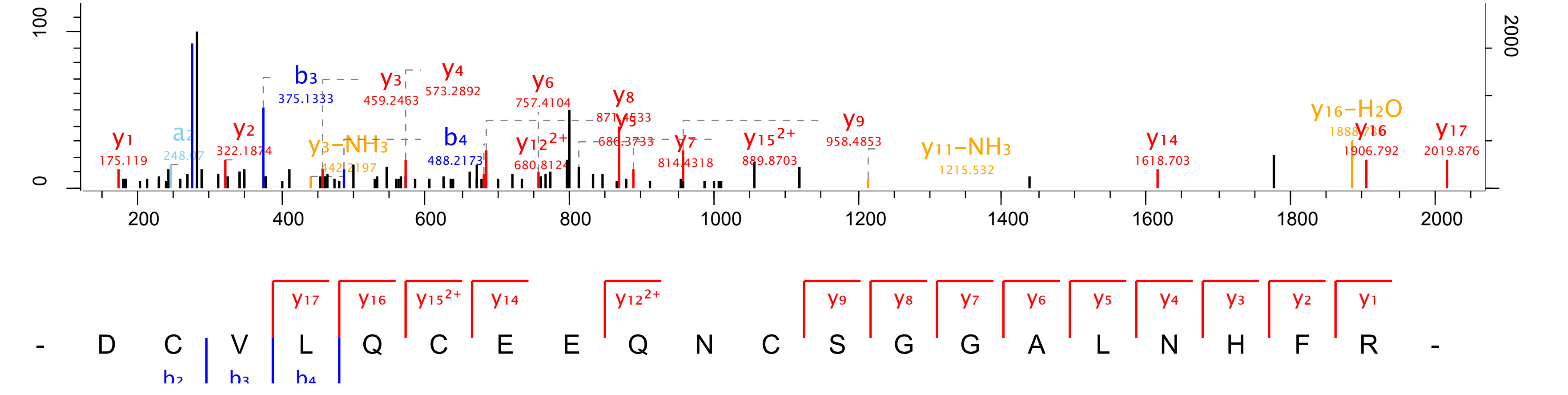

Raw file	Scan	Method	Score	m/z	Gene names
20140918_fract7_dyn_5ul_D7_01_393	26476	TOF; CID	100.45	946.97	PAGE1

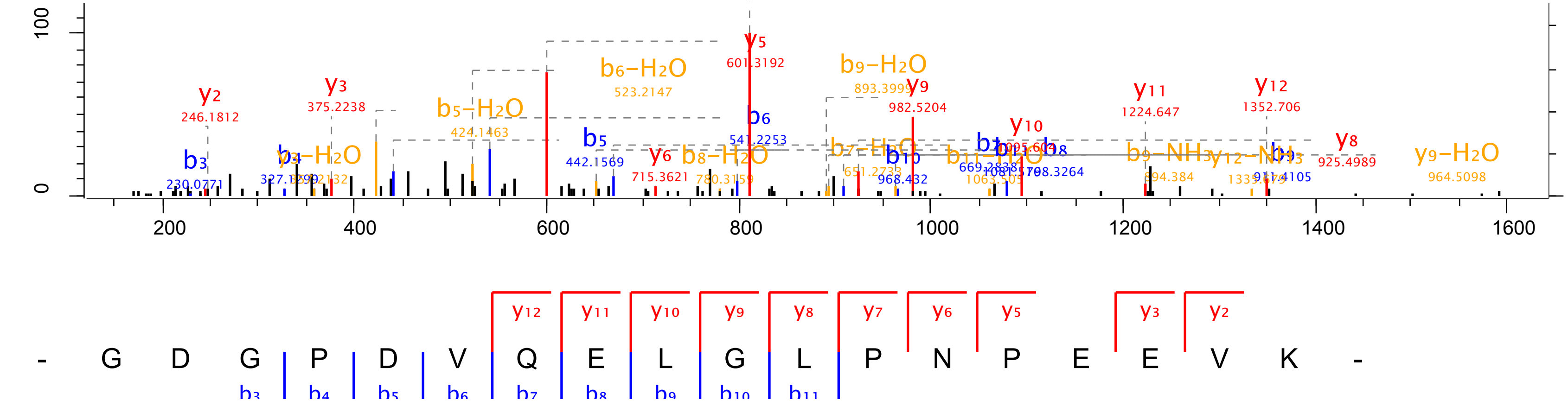

Raw file	Scan	Method	Score	m/z	Gene names
20140918_fract8_dyn_5ul_D8_01_394	17254	TOF; CID	90.15	517.27	CGRRF1

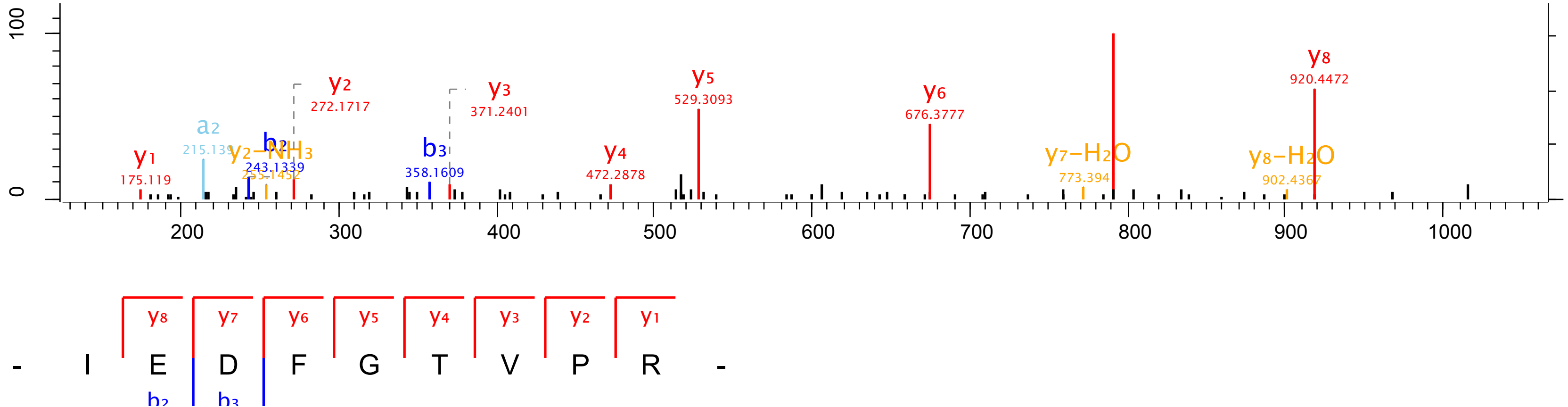

Raw file	Scan	Method	Score	m/z	Gene names
20140918_fract8_dyn_5ul_D8_01_394	23593	TOF; CID	76.28	595.28	RPS3

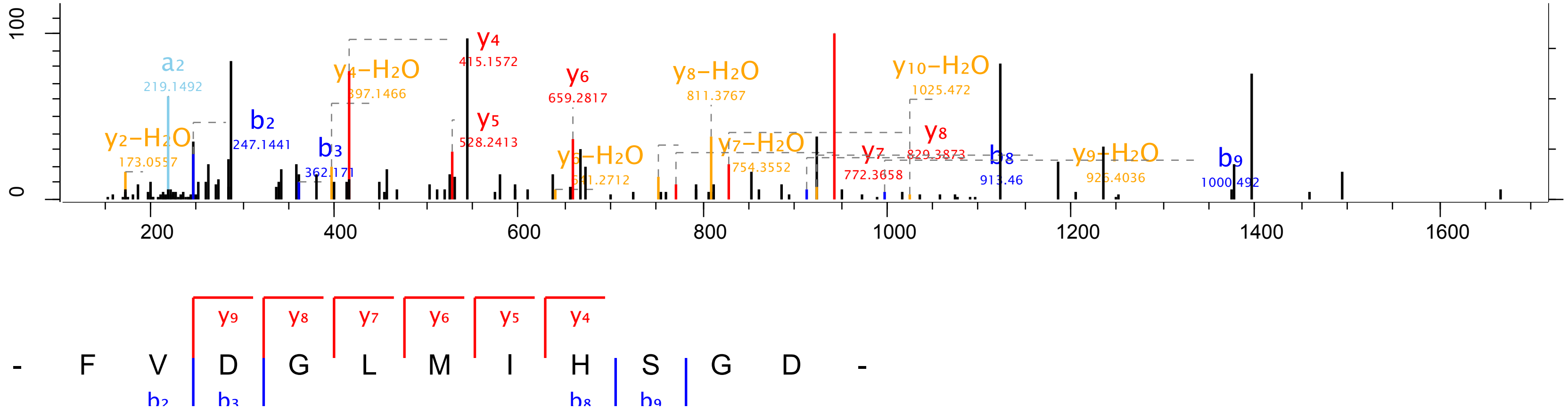

Raw file	Scan	Method	Score	m/z	Gene names
20140918_fract9_dyn_5ul_E1_01_377	14012	TOF; CID	110.38	600.83	SPATA24

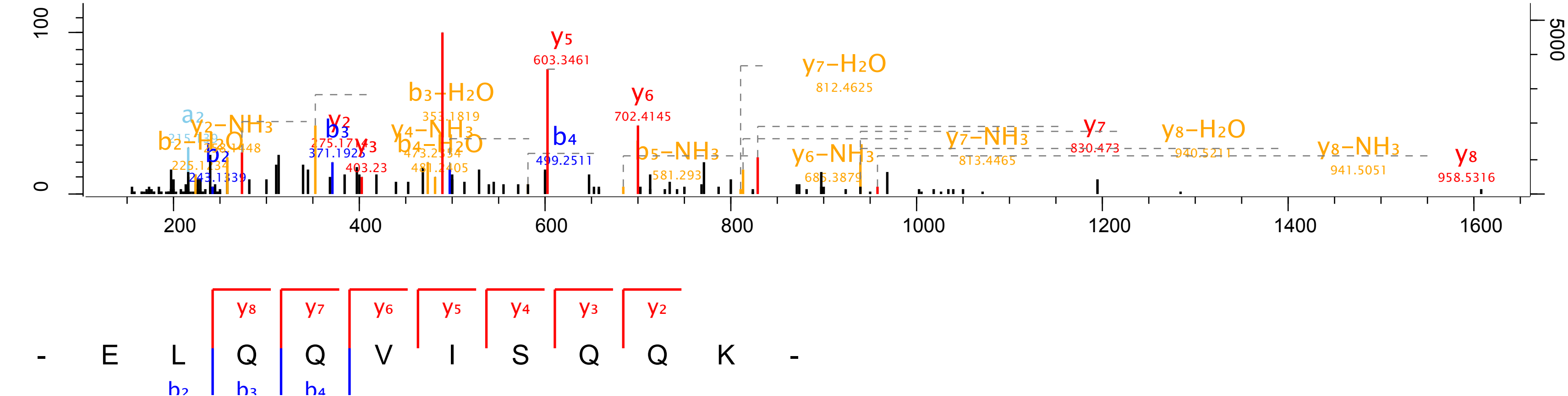

Raw file	Scan	Method	Score	m/z	Gene names
20140918_fract9_dyn_5ul_E1_01_377	15473	TOF; CID	41.91	599.55	COL16A1

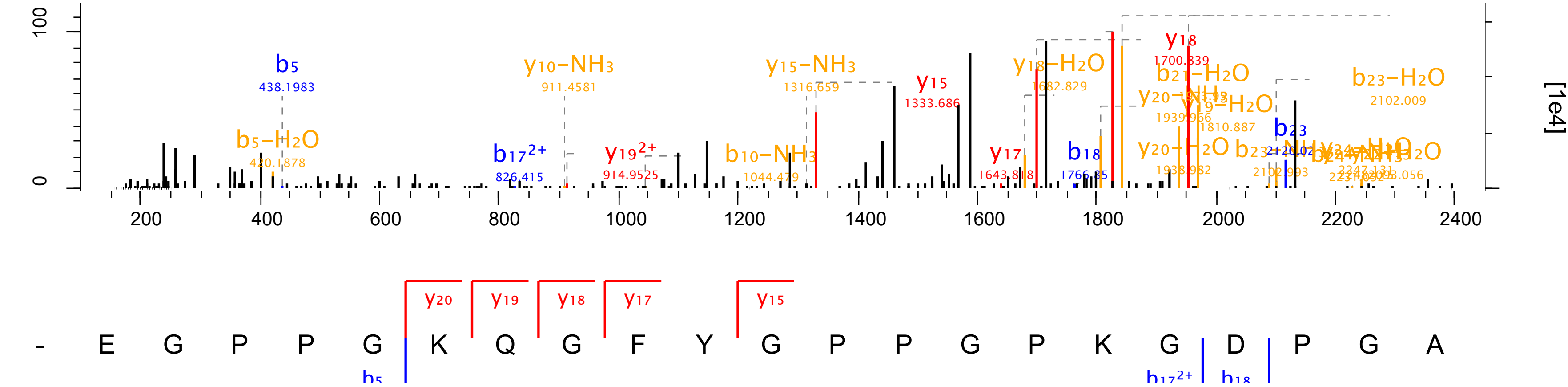

Raw file	Scan	Method	Score	m/z	Gene names
20140918_fract9_dyn_5ul_E1_01_377	21888	TOF; CID	72.29	698.36	TRAIP

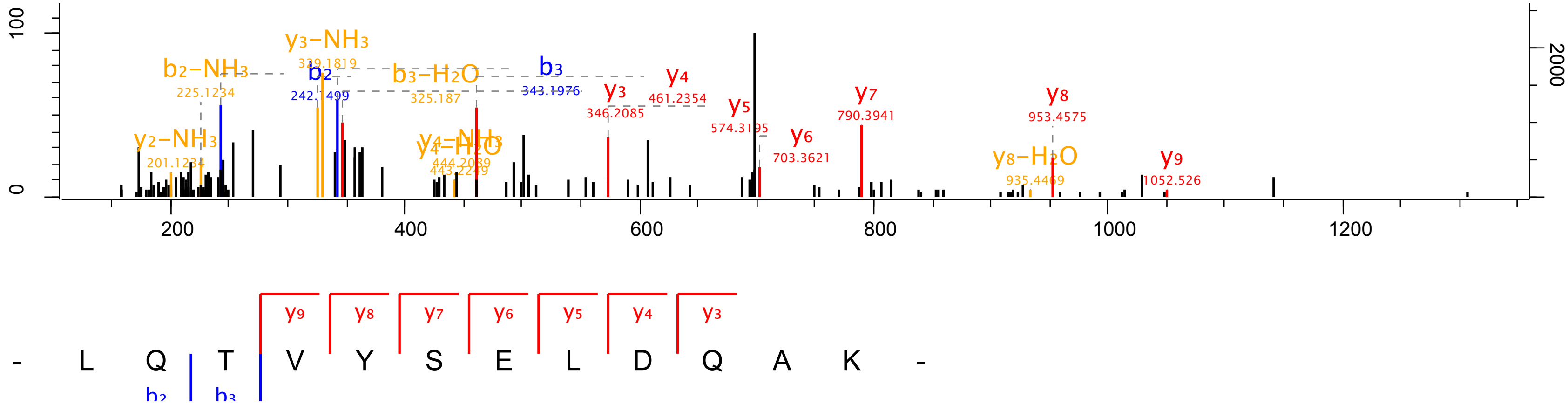

Raw file	Scan	Method	Score	m/z	Gene names
20140918_fract9_dyn_5ul_E1_01_377	29824	TOF; CID	59.23	827.4	SYCP1

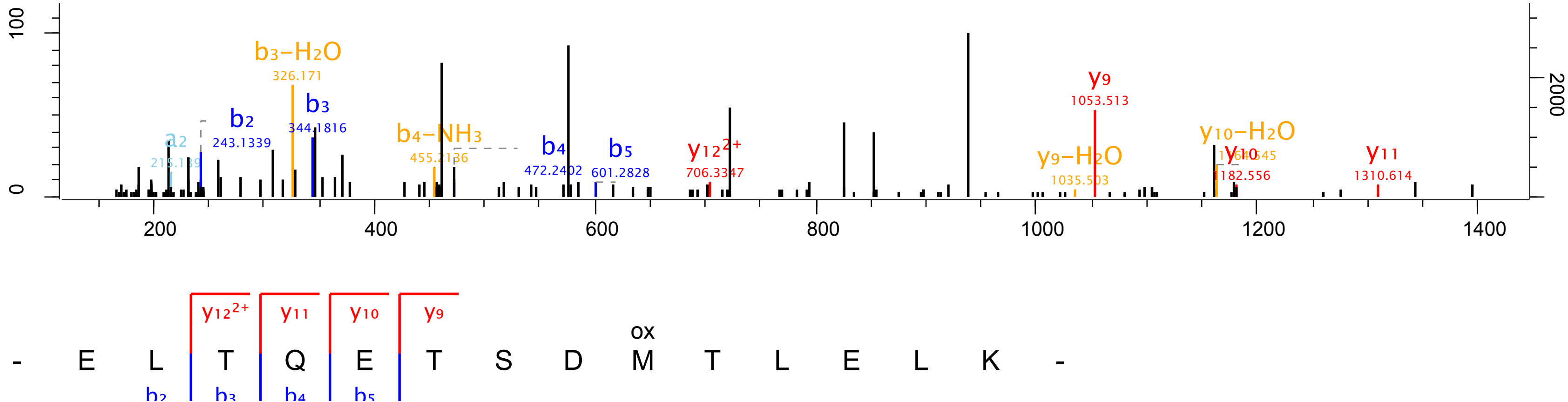

Raw file	Scan	Method	Score	m/z	Gene names
20140918_fract9_dyn_5ul_E1_01_377	29965	TOF; CID	121.44	765.37	CNIH4

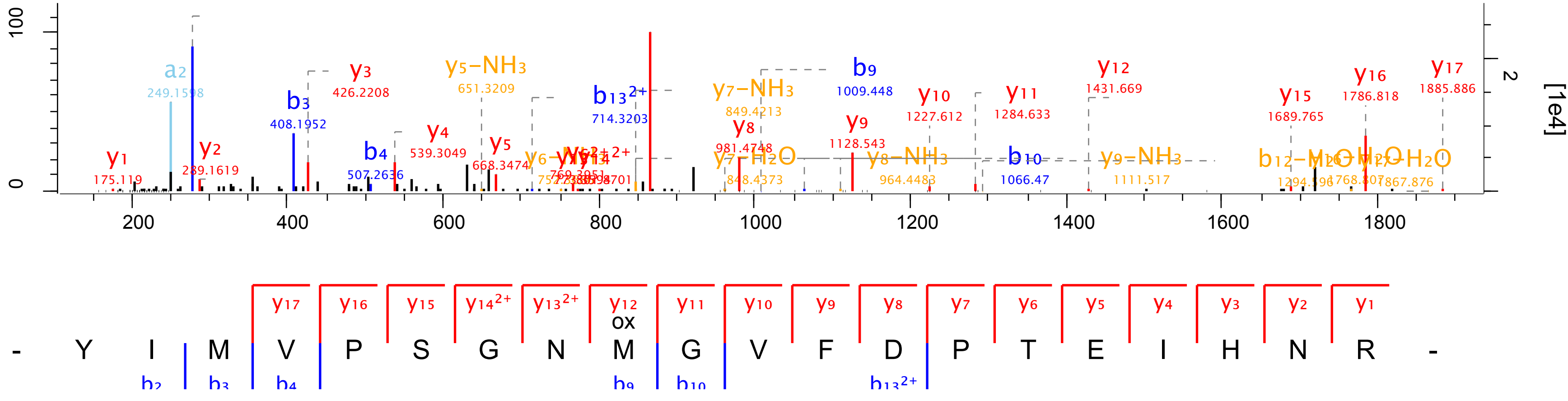

Raw file	Scan	Method	Score	m/z	Gene names
20140918_fract9_dyn_5ul_E1_01_377	38027	TOF; CID	61.13	673.02	PLAGL2

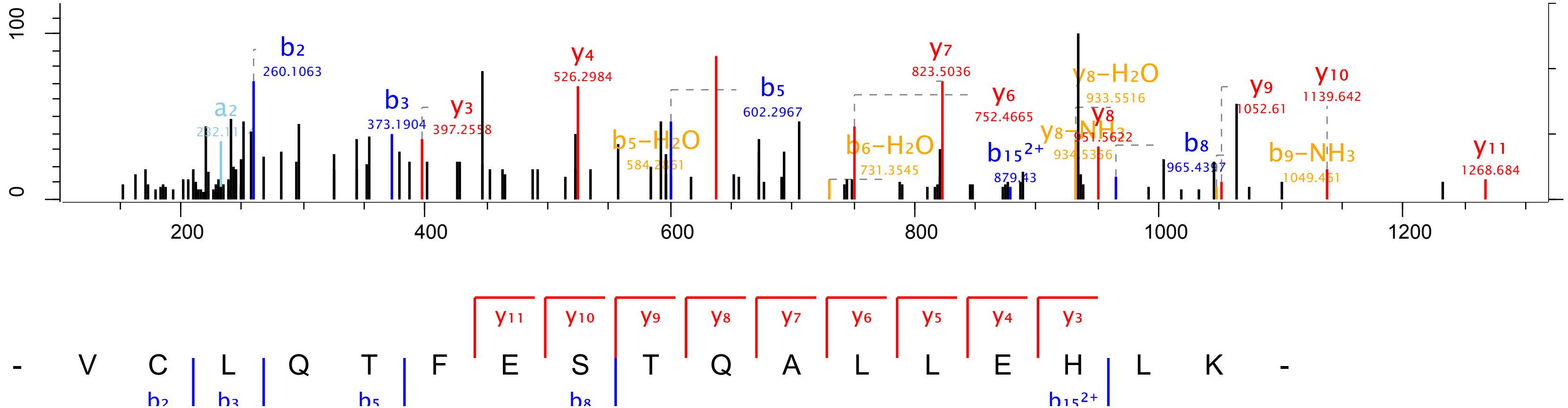

Raw file	Scan	Method	Score	m/z	Gene names
20140918_fract10_dyn_5ul_E2_01_378	6158	TOF; CID	75.32	465.23	CRAT

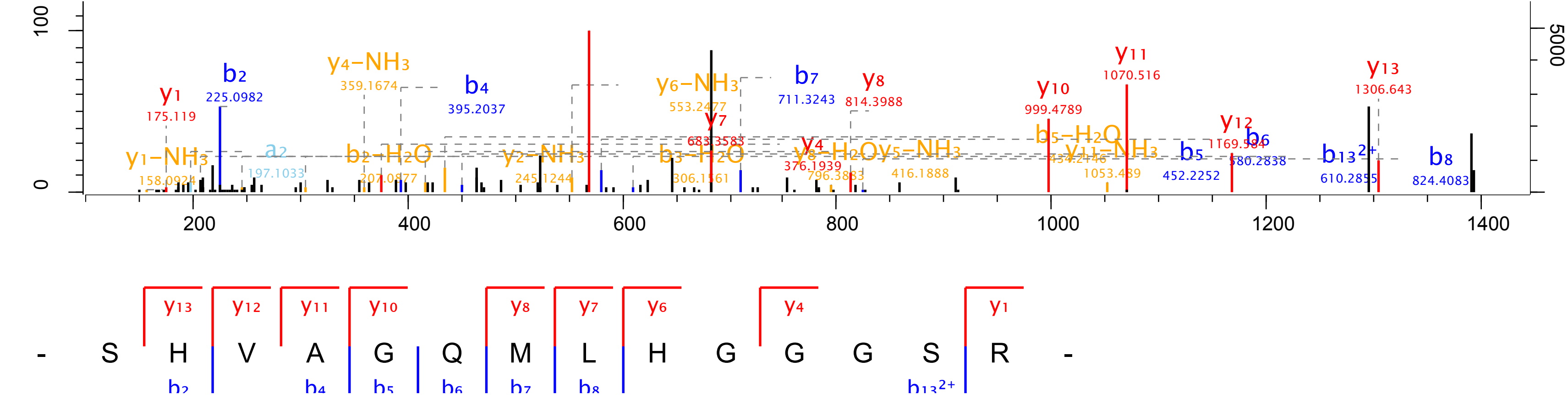

Raw file	Scan	Method	Score	m/z	Gene names
20140918_fract10_dyn_5ul_E2_01_378	10459	TOF; CID	125.74	545.32	PRICKLE2

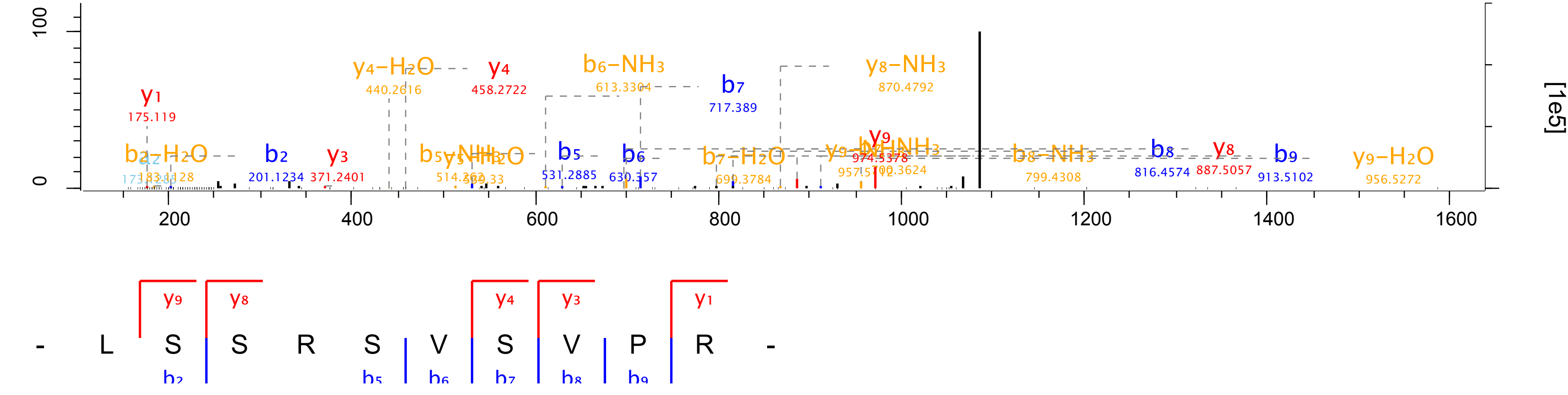

Raw file	Scan	Method	Score	m/z	Gene names
20140918_fract10_dyn_5ul_E2_01_378	26649	TOF; CID	43.33	979.49	OSBPL8

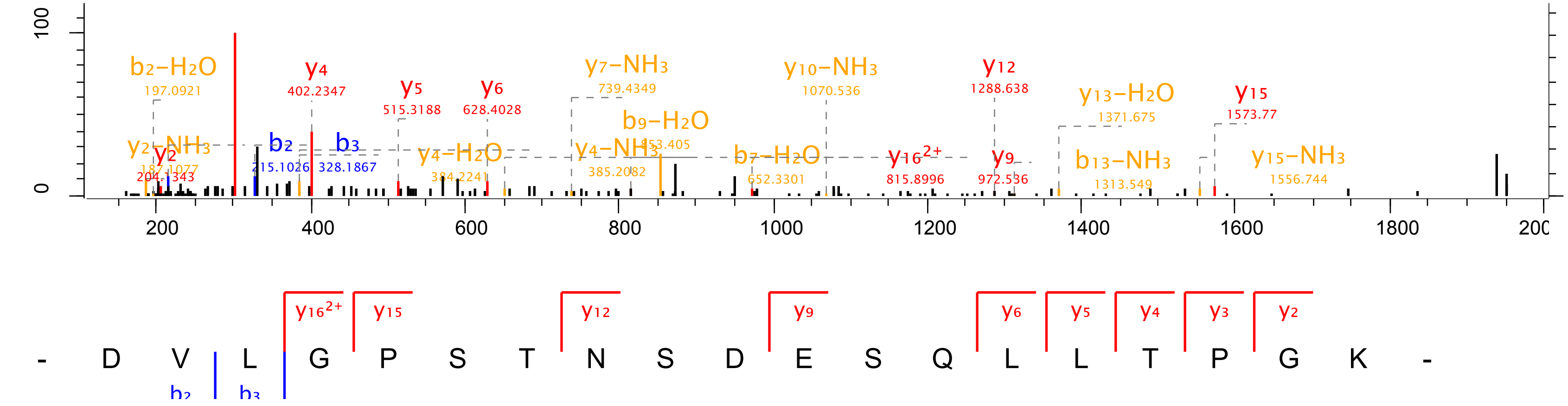

Raw file	Scan	Method	Score	m/z	Gene names
20140918_fract10_dyn_5ul_E2_01_378	30767	TOF; CID	120.9	865.99	KRTCAP2

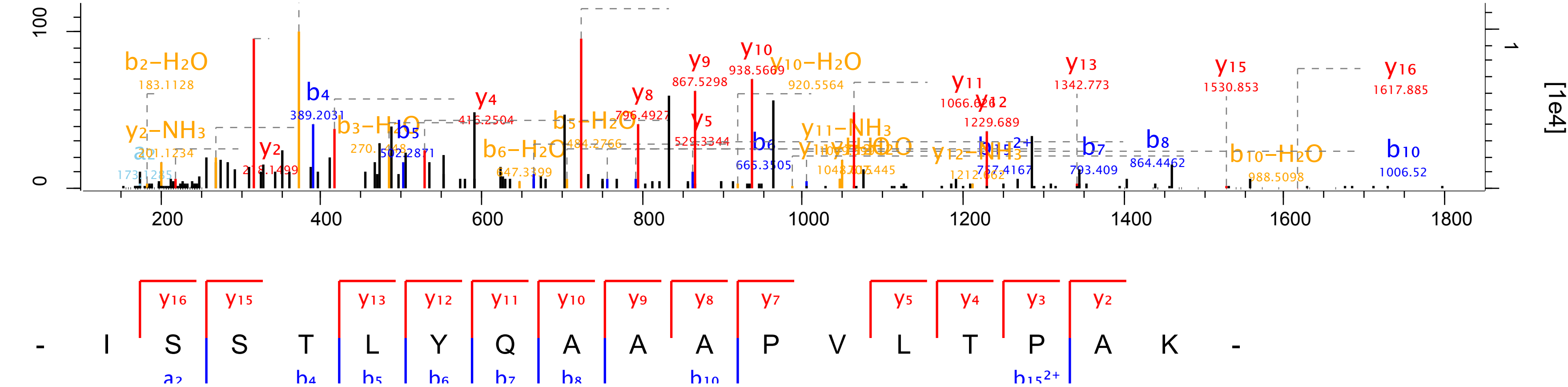

Raw file	Scan	Method	Score	m/z	Gene names
20140918_fract10_dyn_5ul_E2_01_378	34653	TOF; CID	137.89	814.92	CCDC167

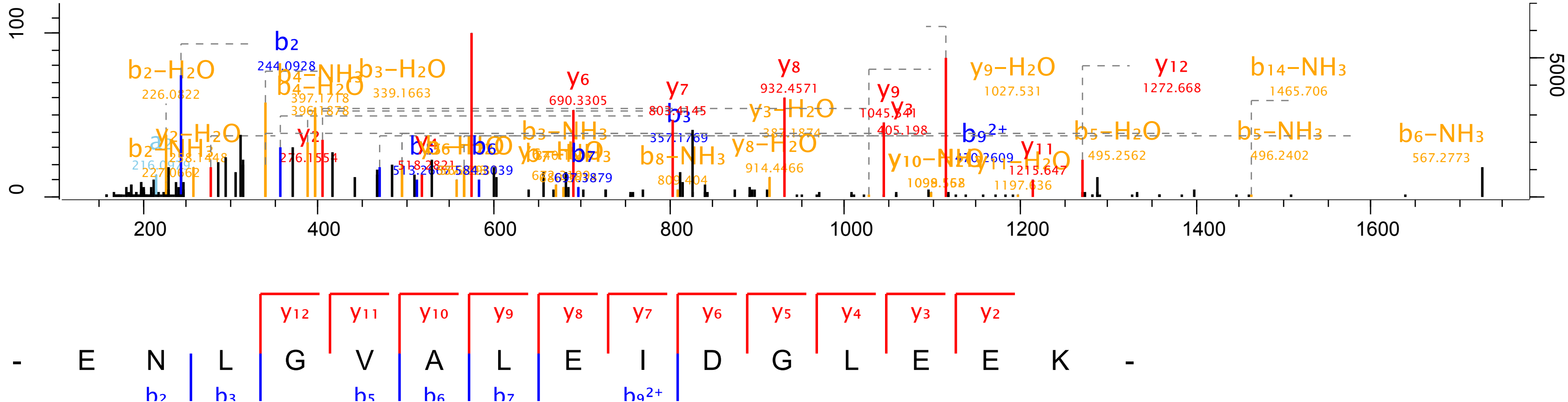

Raw file	Scan	Method	Score	m/z	Gene names
20140918_fract11_dyn_5ul_E3_01_379	9894	TOF; CID	74.53	368.21	SPATA24

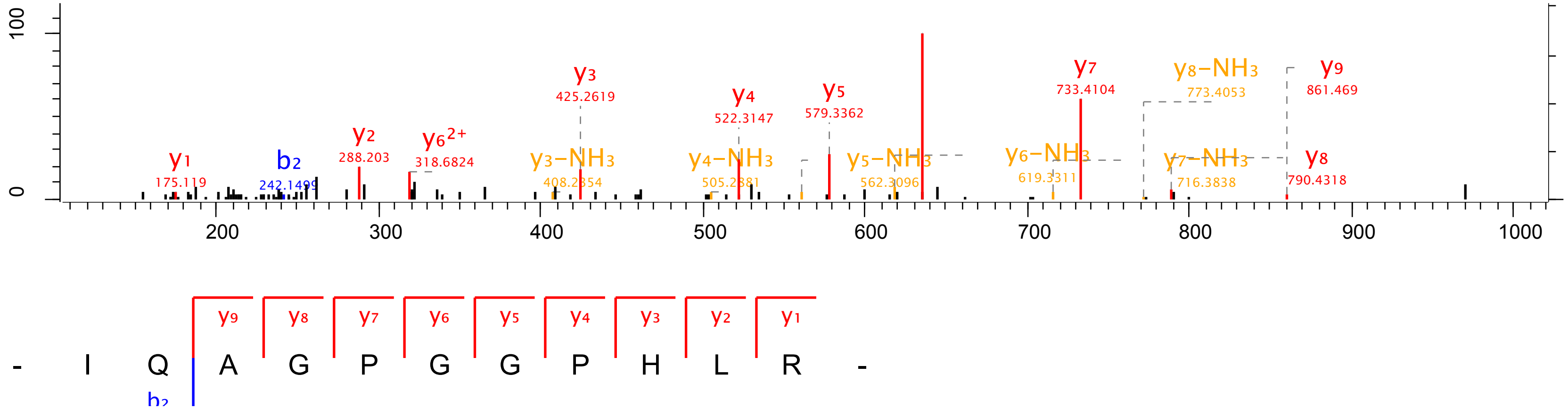

Raw file	Scan	Method	Score	m/z	Gene names
20140918_fract11_dyn_5ul_E3_01_379	20243	TOF; CID	61.41	513.28	MYEOV2

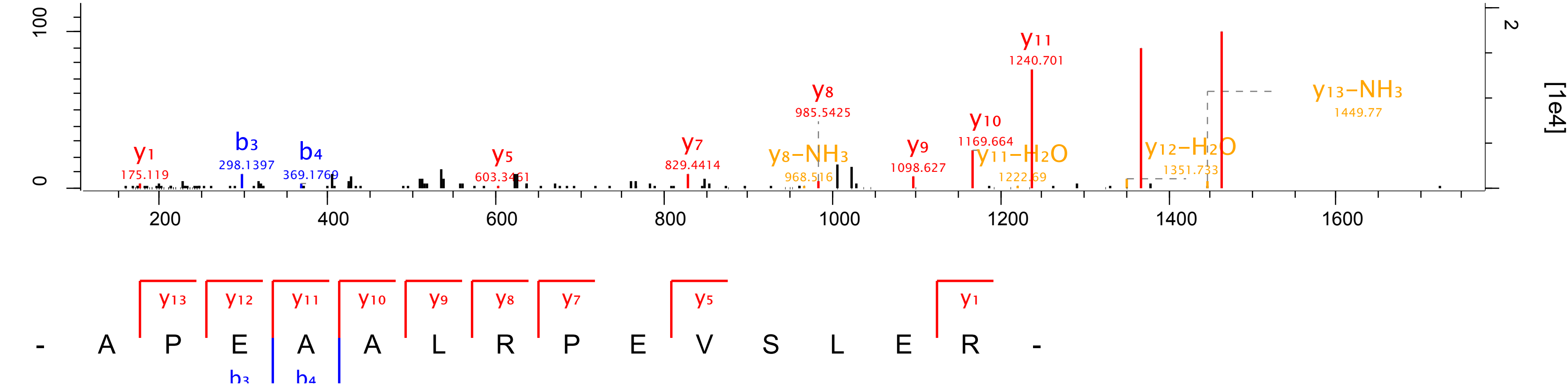

Raw file	Scan	Method	Score	m/z	Gene names
20140918_fract11_dyn_5ul_E3_01_379	24909	TOF; CID	83.82	857.86	MT-ND3

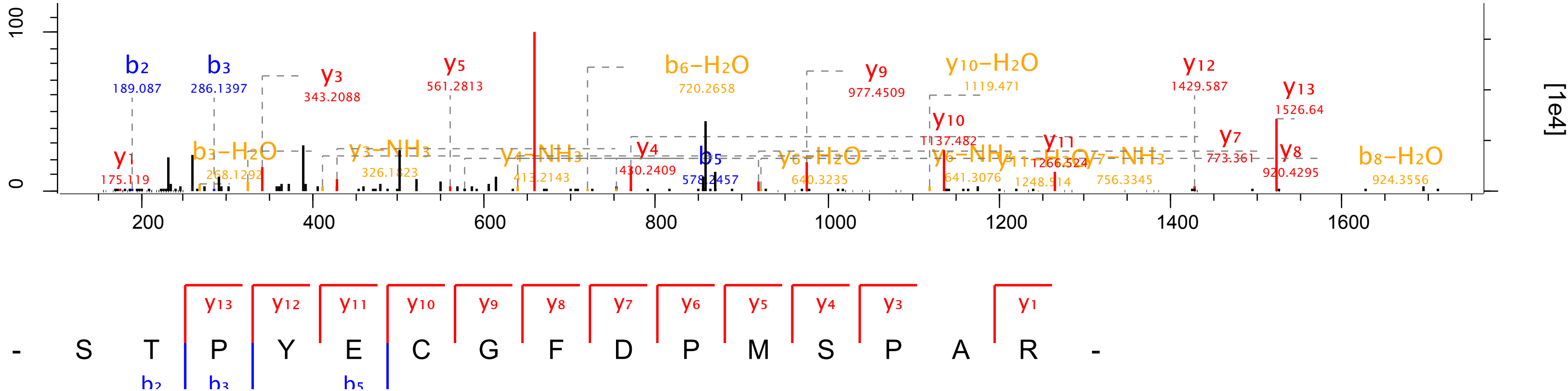

Raw file	Scan	Method	Score	m/z	Gene names
20140918_fract11_dyn_5ul_E3_01_379	32811	TOF; CID	69.72	493.28	TOR1AIP2

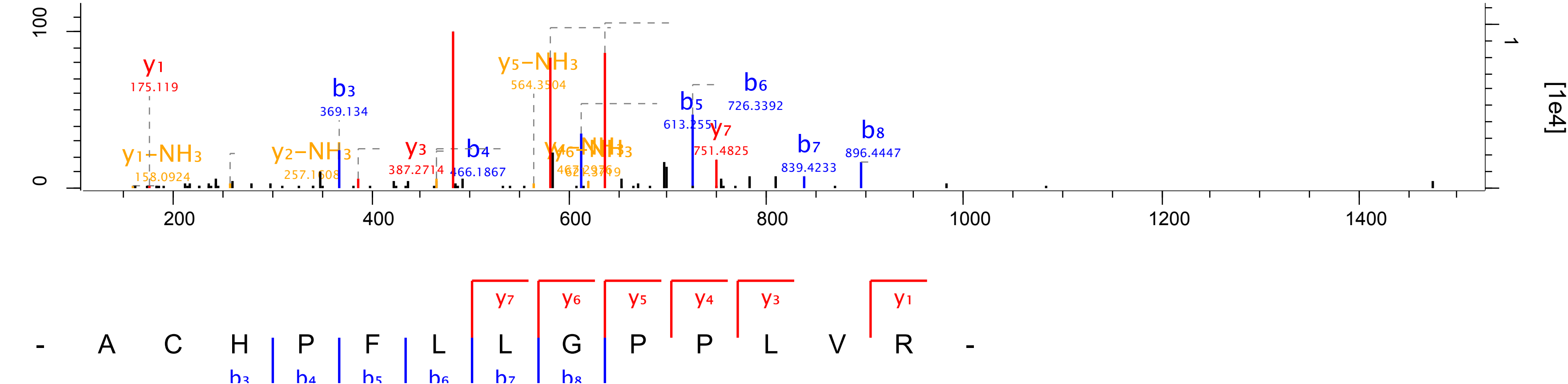

Raw file	Scan	Method	Score	m/z	Gene names
20140918_fract11_dyn_5ul_E3_01_379	36638	TOF; CID	139.88	560.32	KDELR3

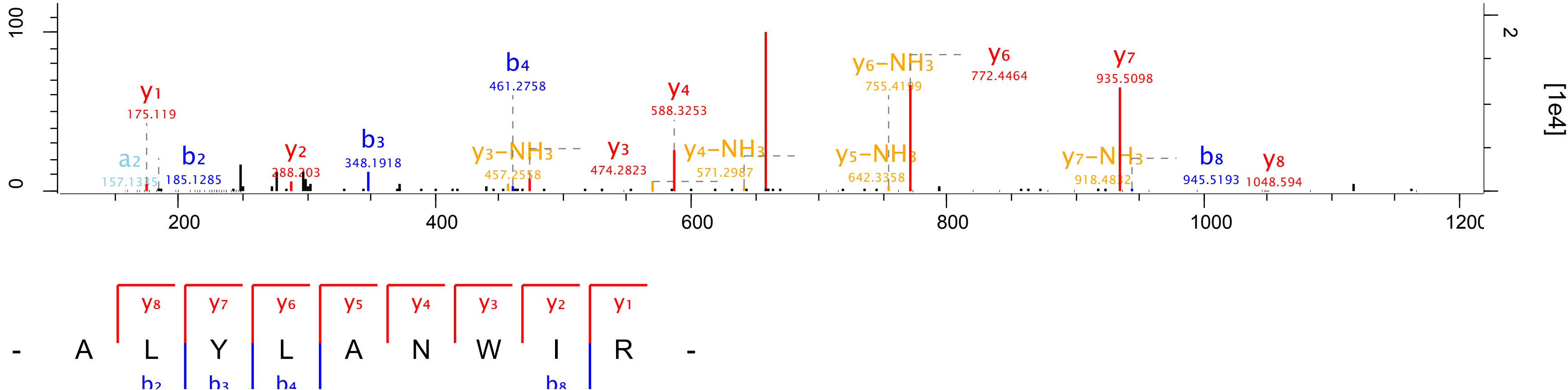

Raw file	Scan	Method	Score	m/z	Gene names
20140918_fract12_dyn_5ul_E4_01_380	6672	TOF; CID	87.64	499.22	ZNF580

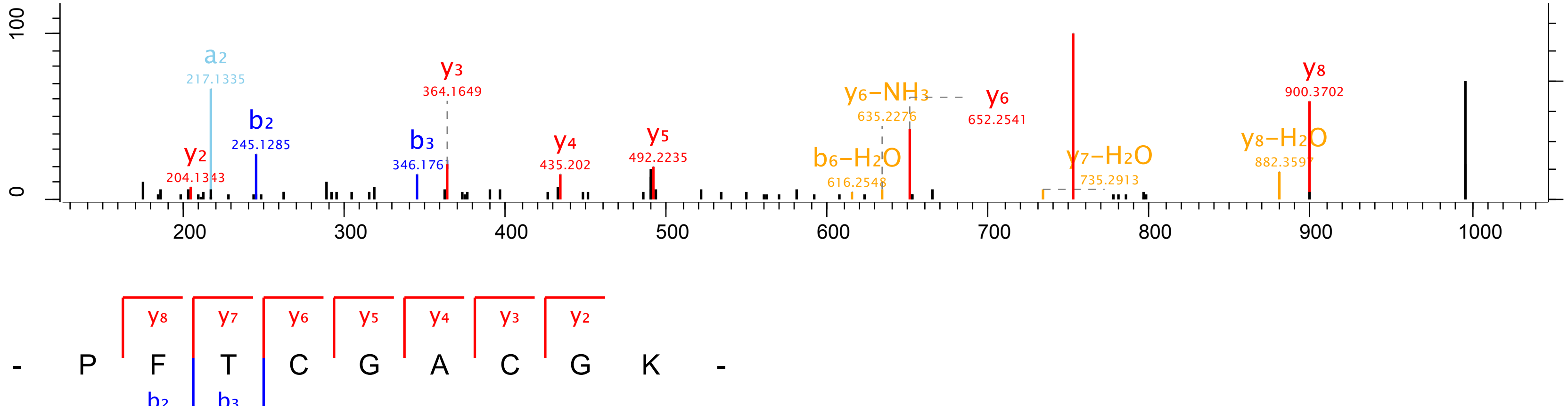

Raw file	Scan	Method	Score	m/z	Gene names
20140918_fract12_dyn_5ul_E4_01_380	11045	TOF; CID	64.22	705.87	SAMD10

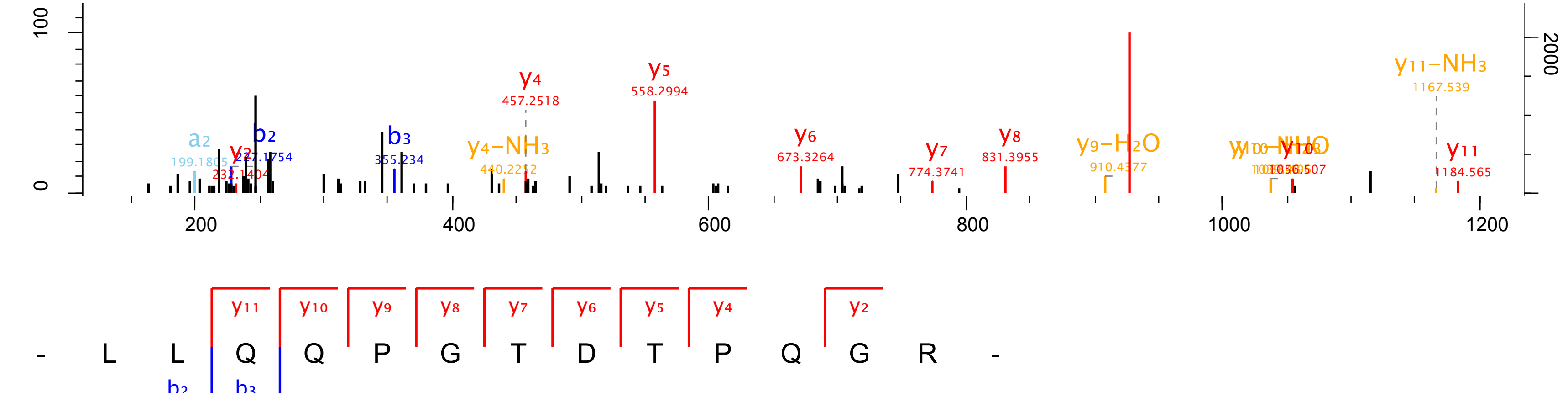

Raw file	Scan	Method	Score	m/z	Gene names
20140918_fract12_dyn_5ul_E4_01_380	21996	TOF; CID	89.35	524.25	BTN2A2

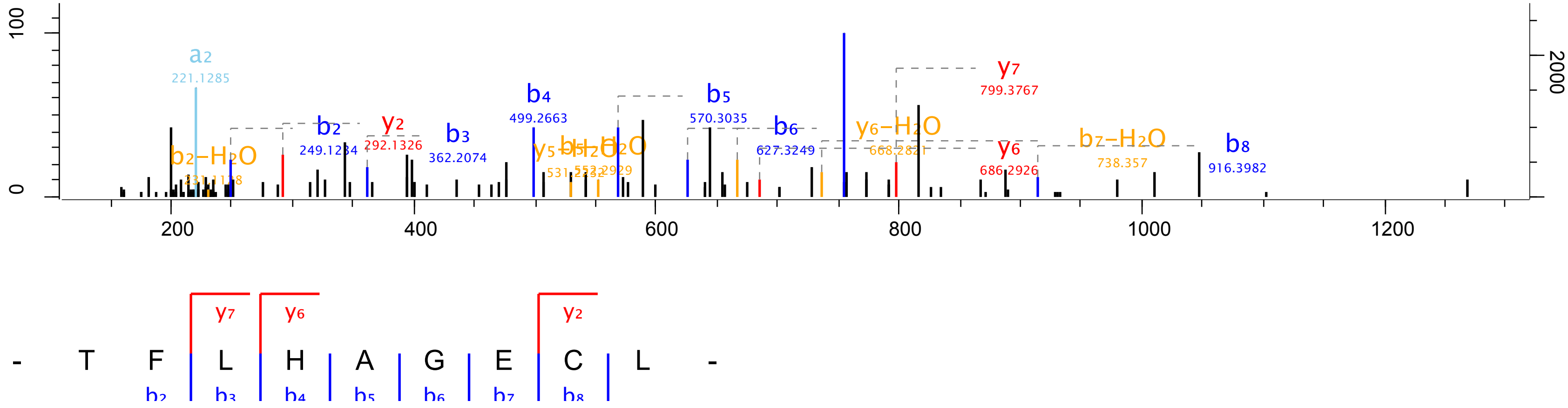

Raw file	Scan	Method	Score	m/z	Gene names
20140918_fract12_dyn_5ul_E4_01_380	26404	TOF; CID	90.79	486.95	TIGD1

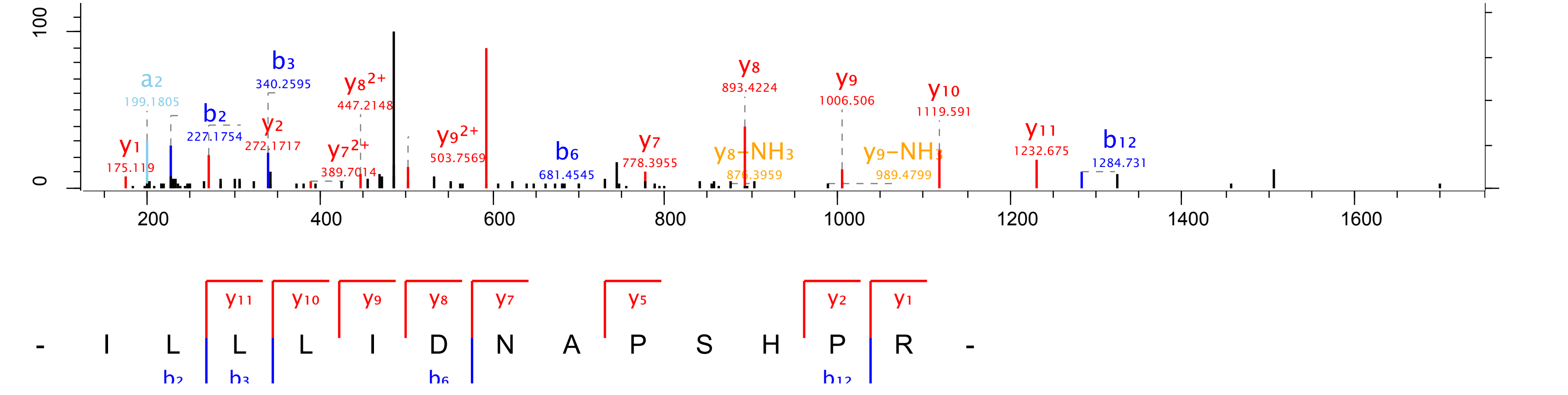

Raw file	Scan	Method	Score	m/z	Gene names
20140918_fract13_dyn_5ul_E5_01_381	22797	TOF; CID	63.49	972.43	LYPD3

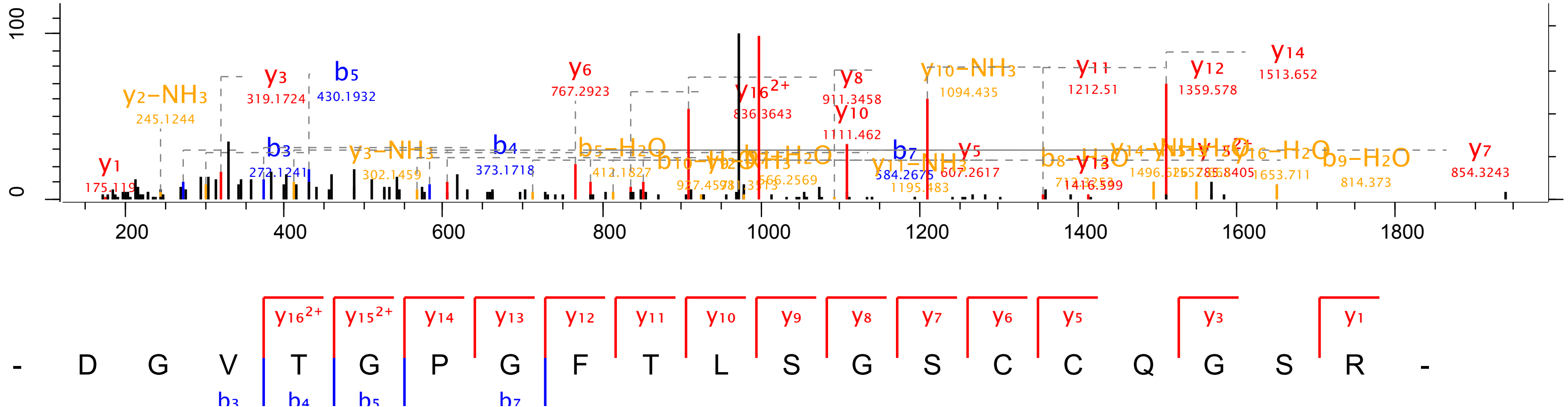

Raw file	Scan	Method	Score	m/z	Gene names
20140918_fract13_dyn_5ul_E5_01_381	27792	TOF; CID	73.08	580.31	GADD45A

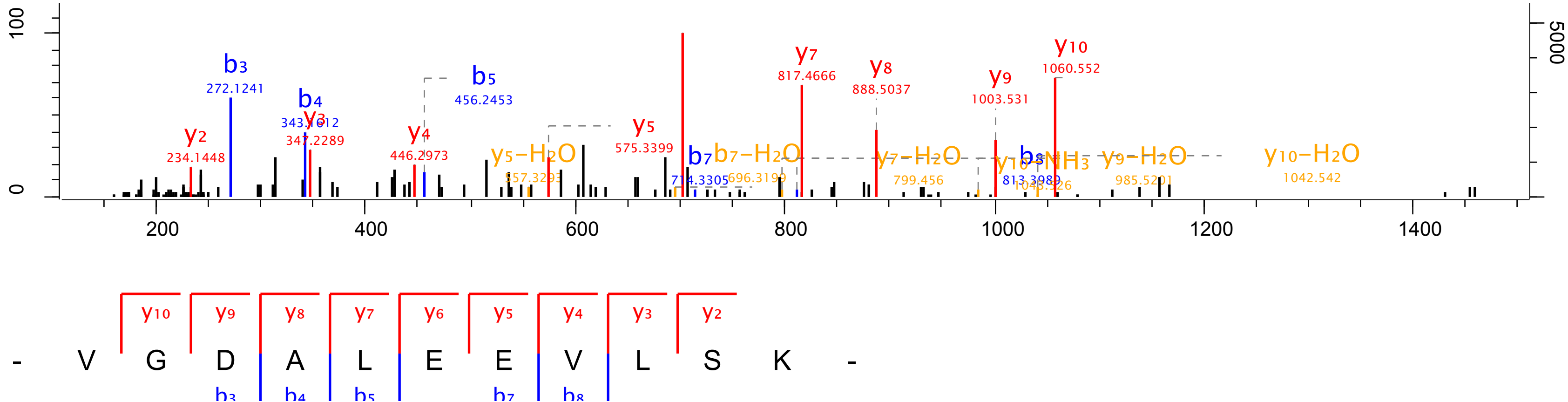

Raw file	Scan	Method	Score	m/z	Gene names
20140918_fract13_dyn_5ul_E5_01_381	33194	TOF; CID	74.99	745.38	PPARGC1B

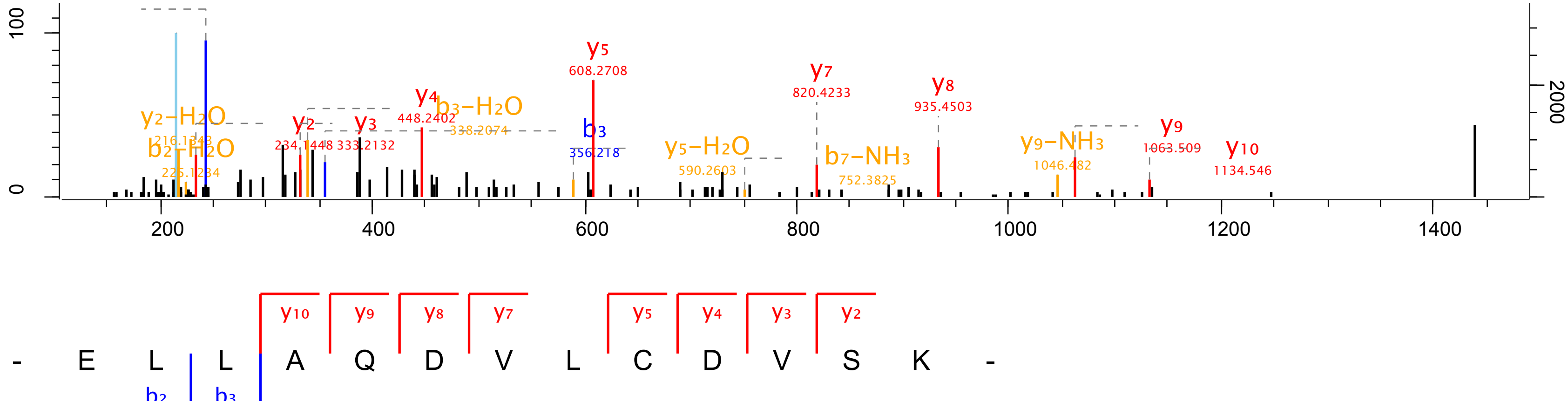

Raw file	Scan	Method	Score	m/z	Gene names
20140918_fract14_dyn_5ul_E6_01_382	13466	TOF; CID	103.69	430.92	DPY19L3

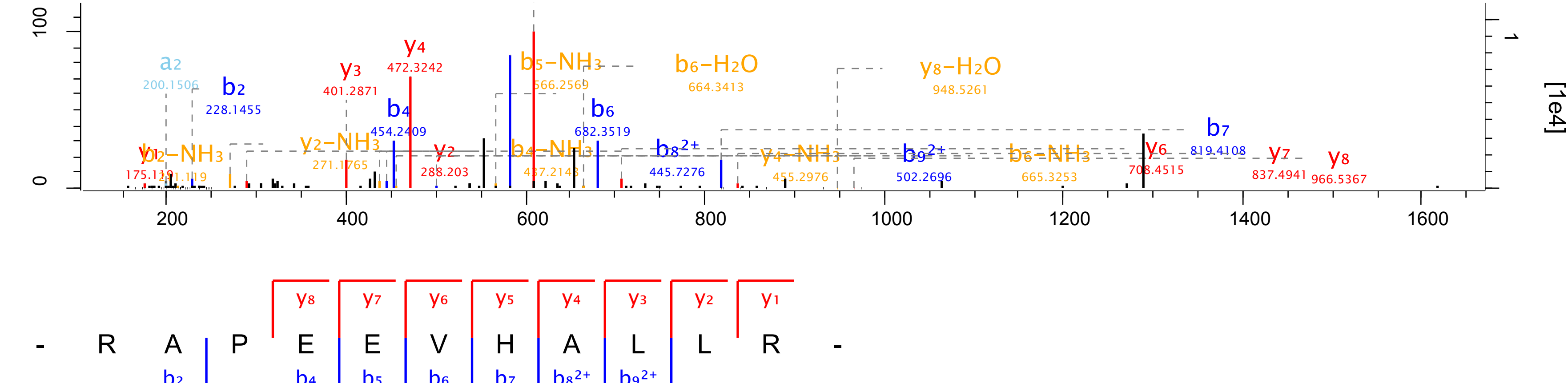

Raw file	Scan	Method	Score	m/z	Gene names
20140918_fract14_dyn_5ul_E6_01_382	20987	TOF; CID	69.72	588.31	ZDHHC14

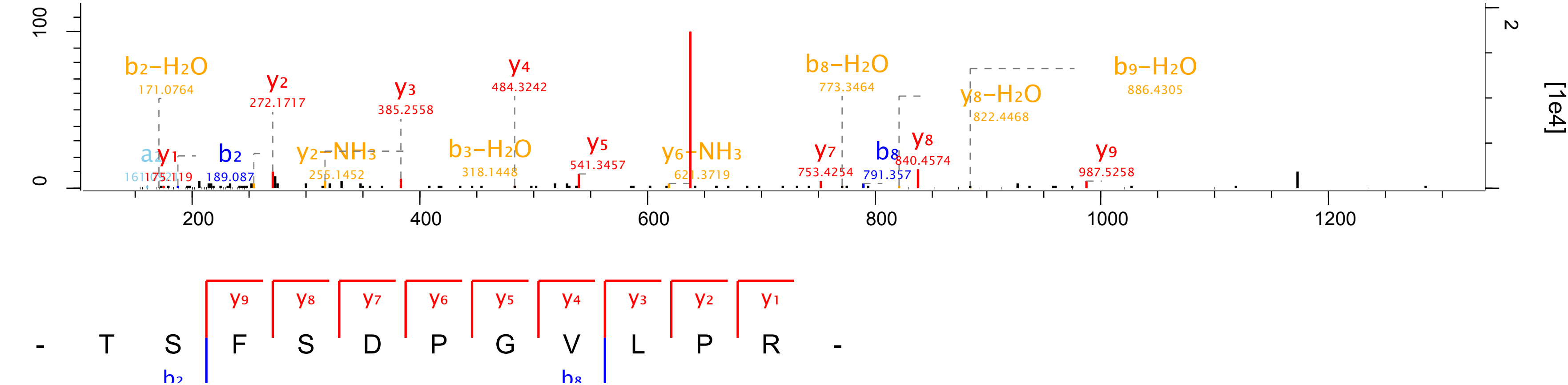

Raw file	Scan	Method	Score	m/z	Gene names
20140918_fract14_dyn_5ul_E6_01_382	25573	TOF; CID	163.45	929.48	HIATL1

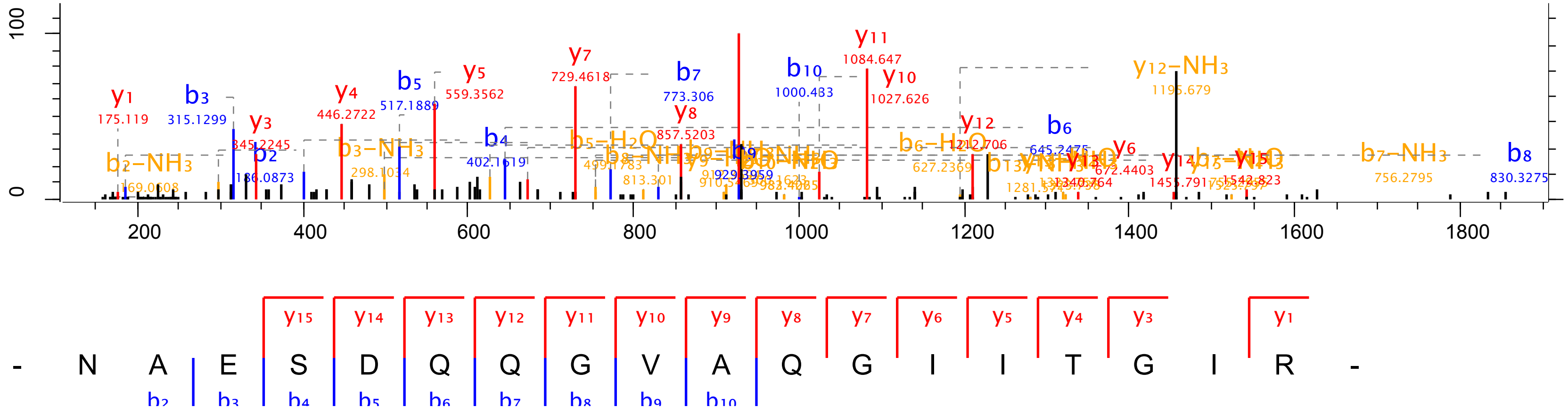

Raw file	Scan	Method	Score	m/z	Gene names
20140918_fract14_dyn_5ul_E6_01_382	34095	TOF; CID	37.88	730.02	PTPRH

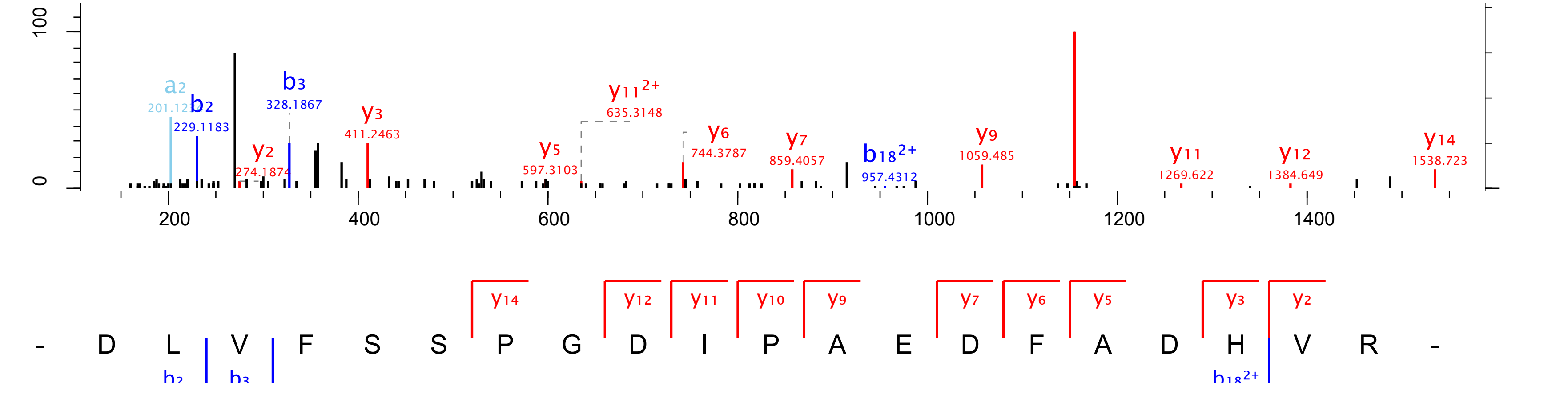

Raw file	Scan	Method	Score	m/z	Gene names
20140918_fract14_dyn_5ul_E6_01_382	38899	TOF; CID	73.07	907.8	HIGD2A

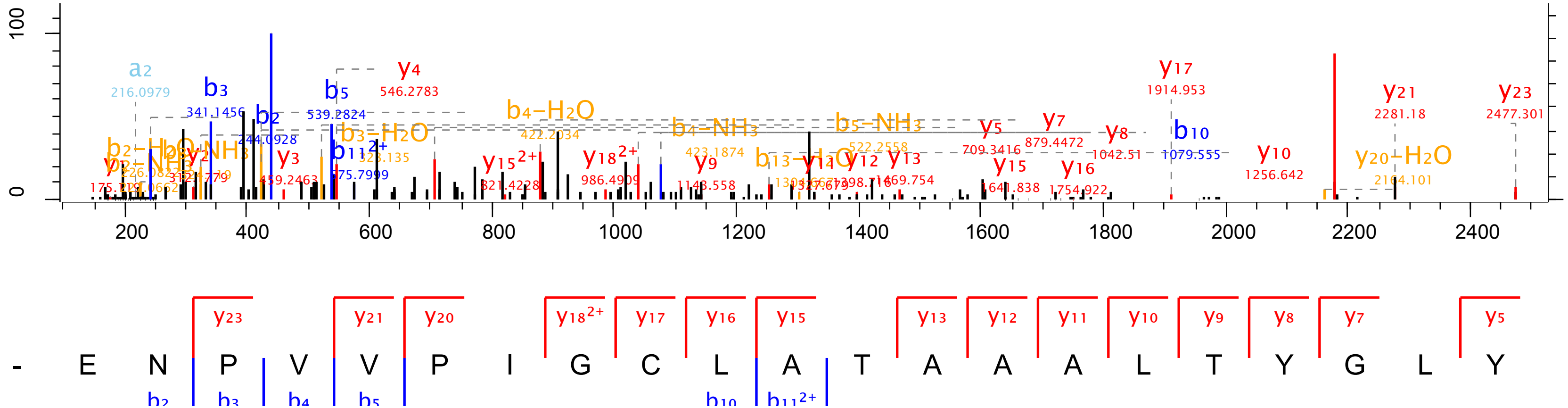

Raw file	Scan	Method	Score	m/z	Gene names
20140918_fract15_dyn_5ul_E7_01_383	10912	TOF; CID	109.72	613.83	TMC7

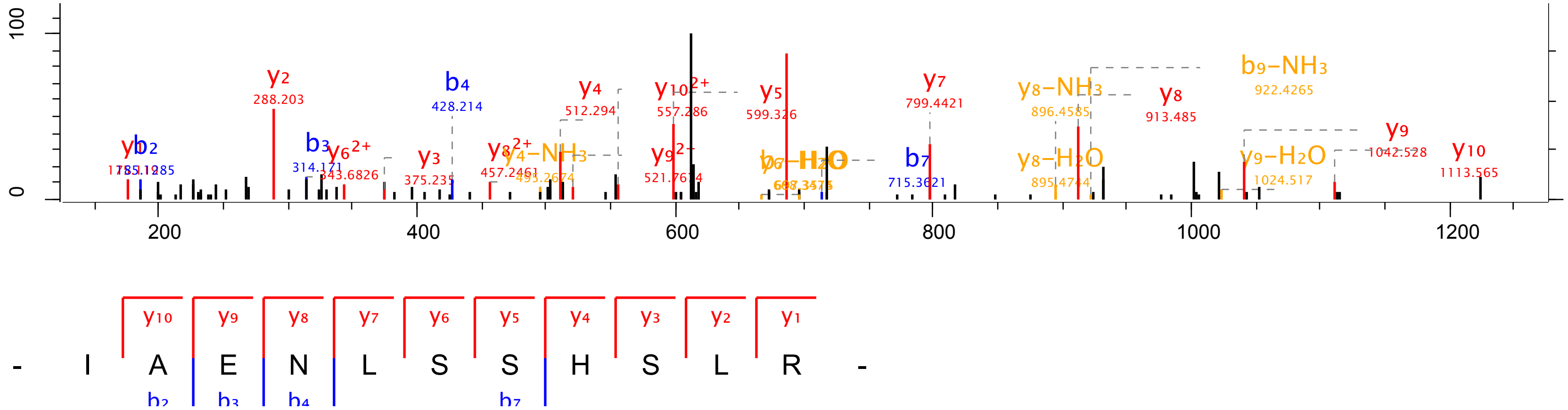

Raw file	Scan	Method	Score	m/z	Gene names
20140918_fract15_dyn_5ul_E7_01_383	35442	TOF; CID	179.74	935.51	VAMP1

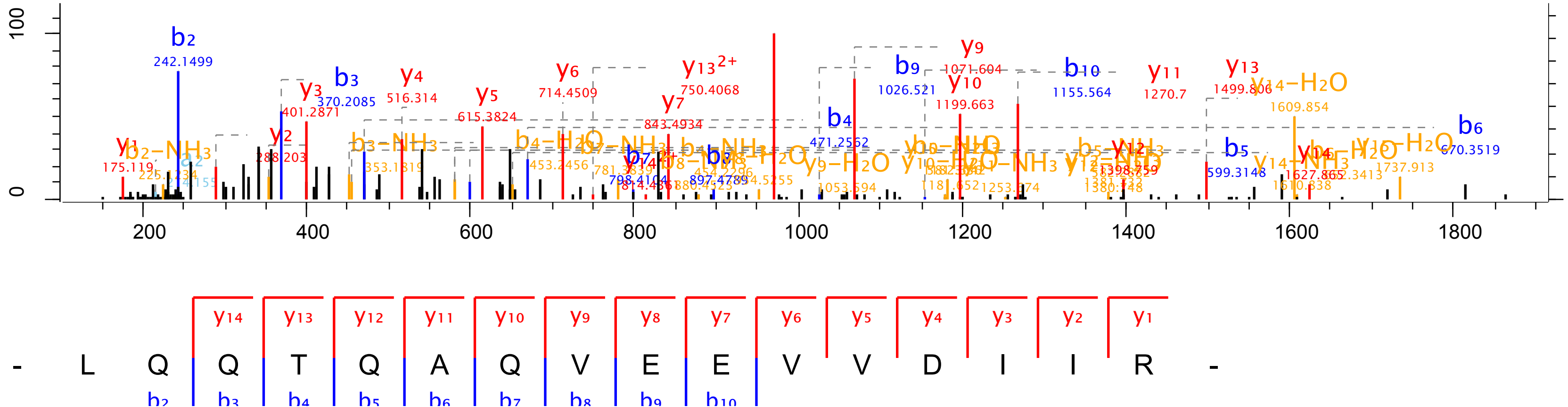

Raw file	Scan	Method	Score	m/z	Gene names
20140918_fract15_dyn_5ul_E7_01_383	36900	TOF; CID	125.74	615.86	ORAOV1

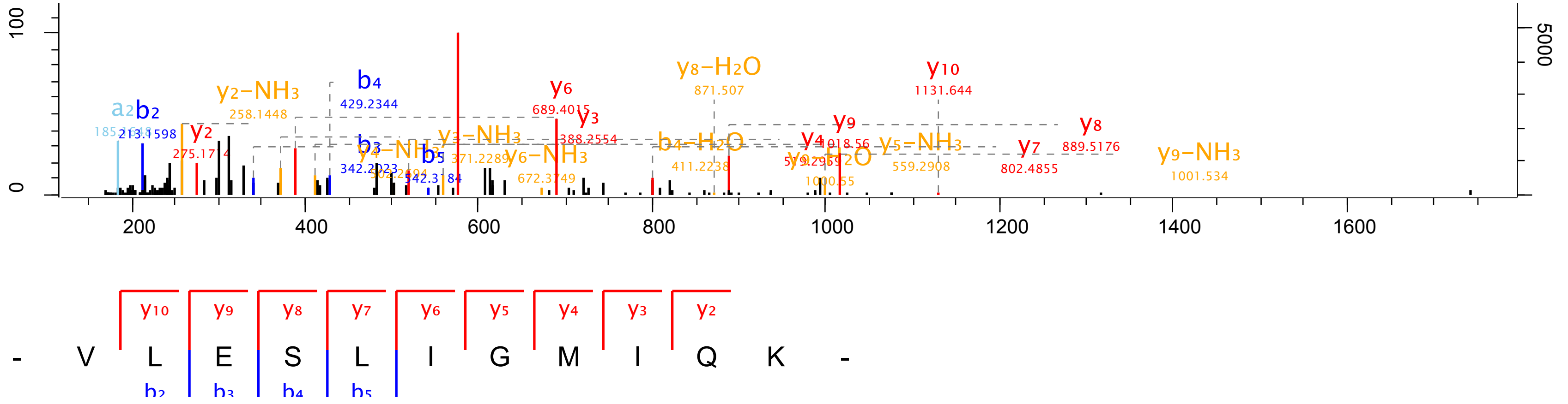

Raw file	Scan	Method	Score	m/z	Gene names
20140918_fract16_dyn_5ul_E8_01_384	10407	TOF; CID	108.72	637.81	FUNDC1

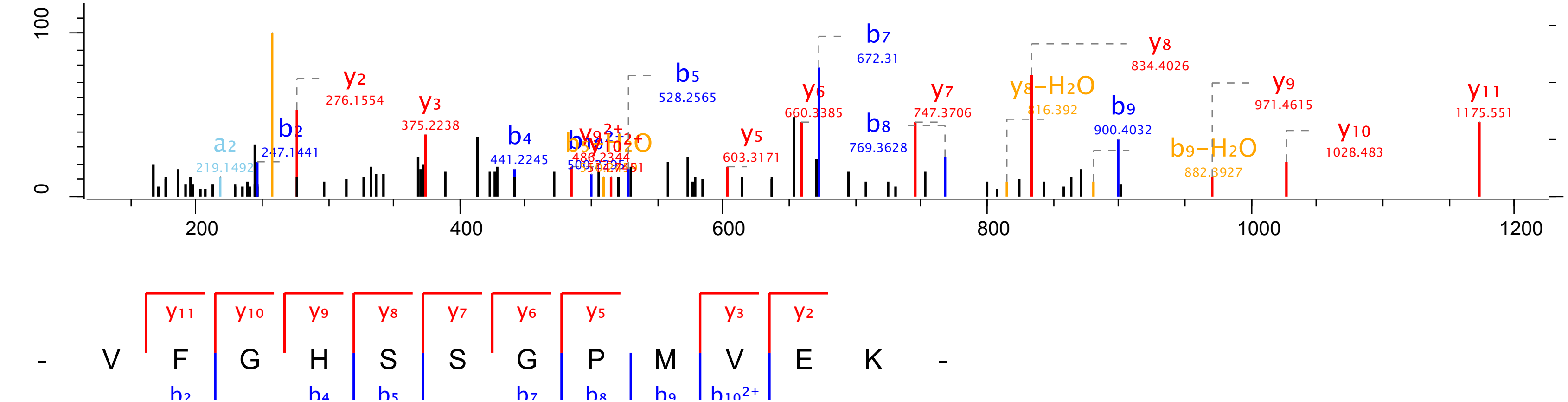

Raw file	Scan	Method	Score	m/z	Gene names
20140918_fract16_dyn_5ul_E8_01_384	15801	TOF; CID	130.41	631.79	CRLS1

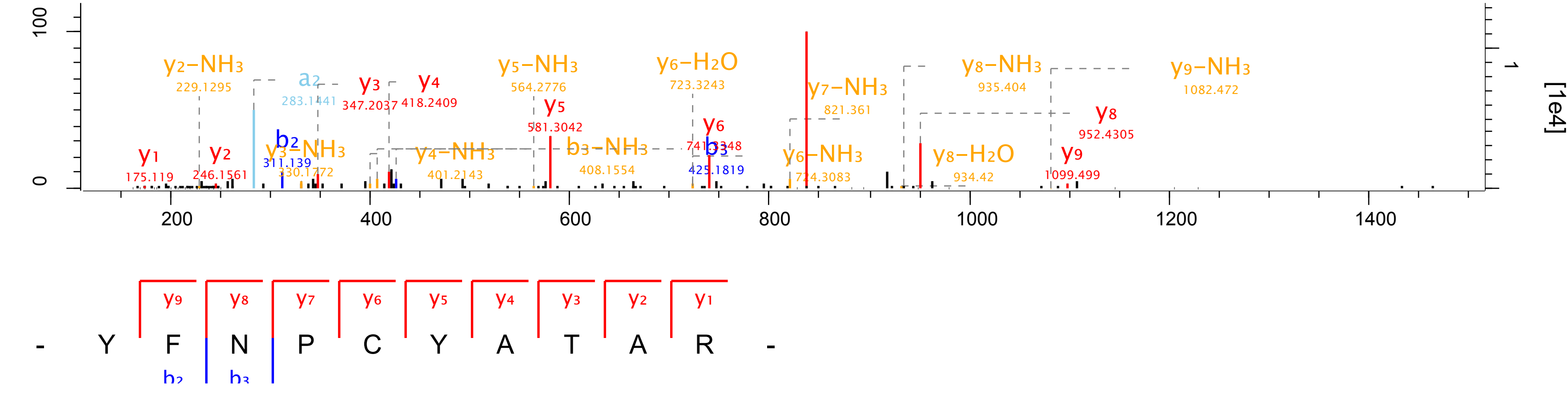

Raw file	Scan	Method	Score	m/z	Gene names
20140918_fract16_dyn_5ul_E8_01_384	19188	TOF; CID	147.26	705.36	TMEM69

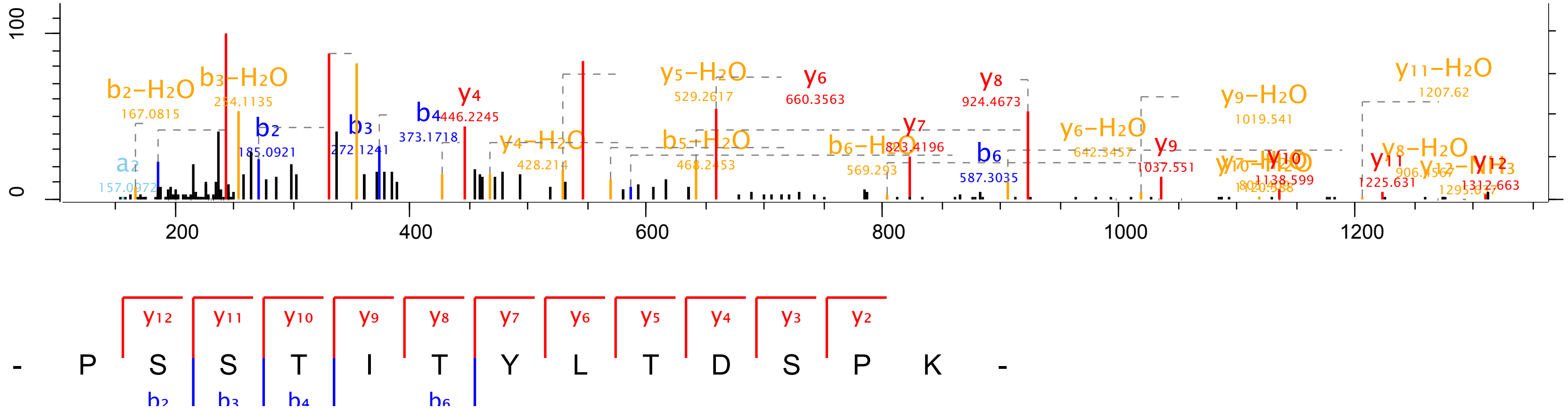

Raw file	Scan	Method	Score	m/z	Gene names
20140918_fract16_dyn_5ul_E8_01_384	21868	TOF; CID	99.82	517.78	TMEM18

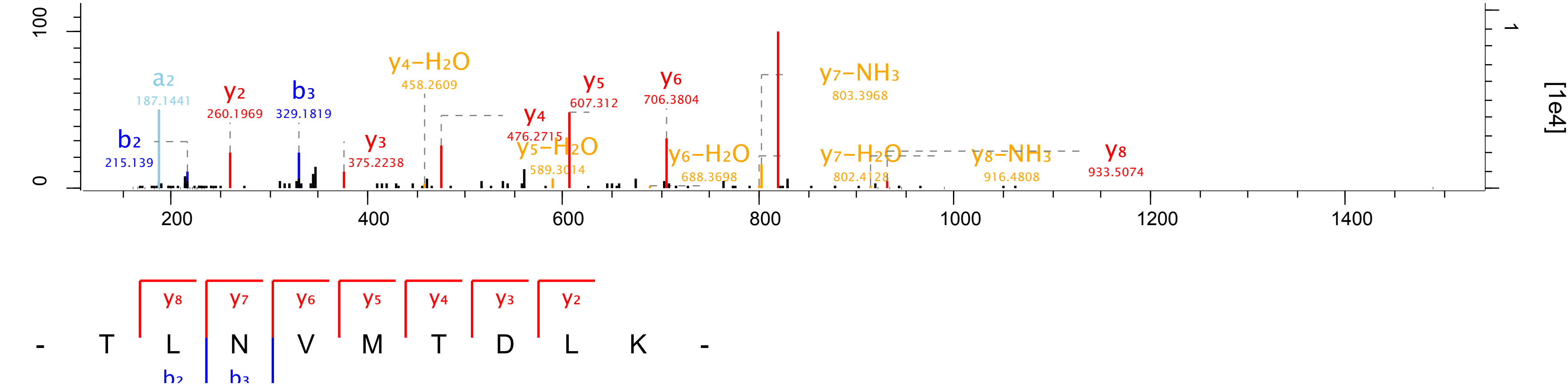

Raw file	Scan	Method	Score	m/z	Gene names
20140918_fract16_dyn_5ul_E8_01_384	25775	TOF; CID	74.96	648.83	GSKIP

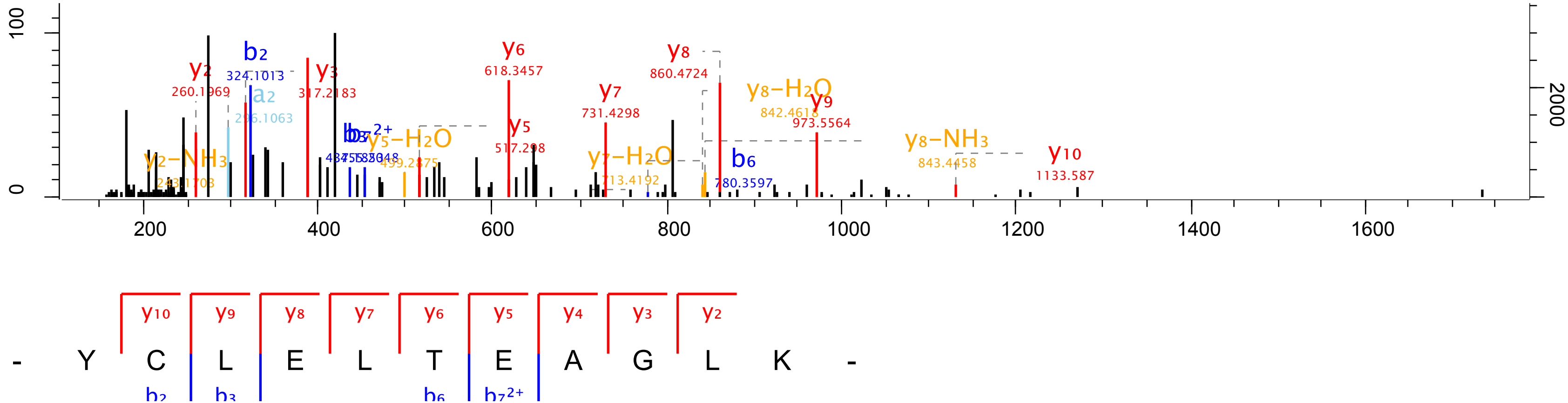

Raw file	Scan	Method	Score	m/z	Gene names
20140918_fract17_dyn_5ul_F1_01_385	20475	TOF; CID	68.58	563.64	ELF4

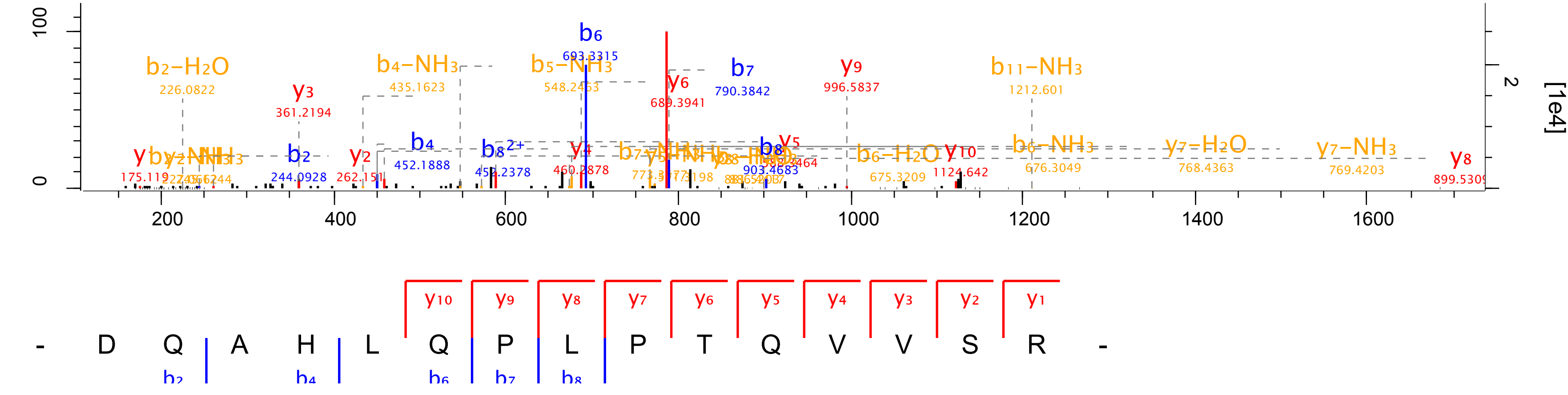

Raw file	Scan	Method	Score	m/z	Gene names
20140918_fract17_dyn_5ul_F1_01_385	21637	TOF; CID	76.33	637.84	FICD

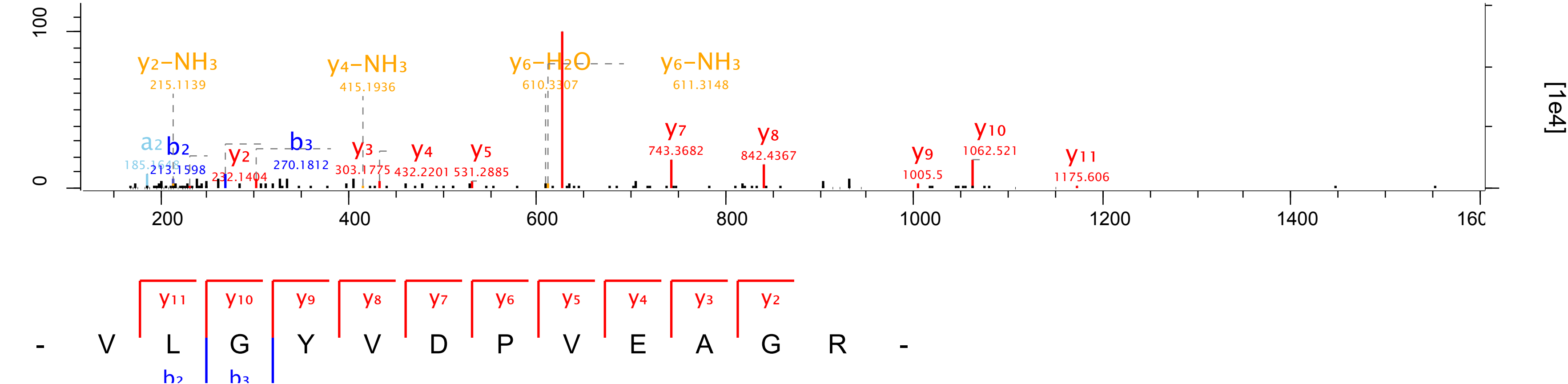

Raw file	Scan	Method	Score	m/z	Gene names
20140918_fract17_dyn_5ul_F1_01_385	27872	TOF; CID	65.37	862.46	PSENEN

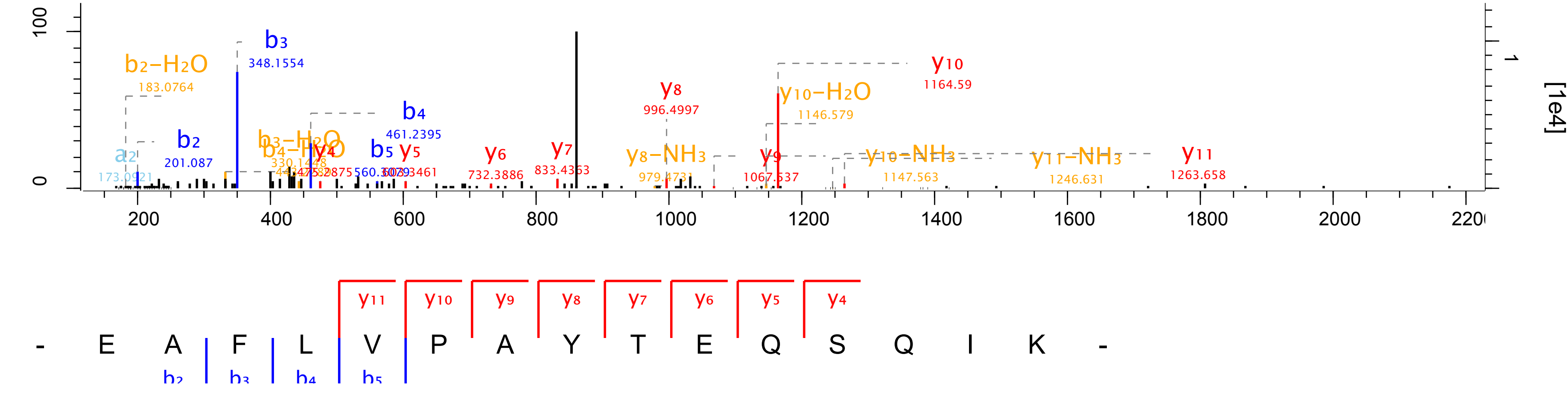

Raw file	Scan	Method	Score	m/z	Gene names
20140918_fract17_dyn_5ul_F1_01_385	28637	TOF; CID	76.3	1074.52	GGACT

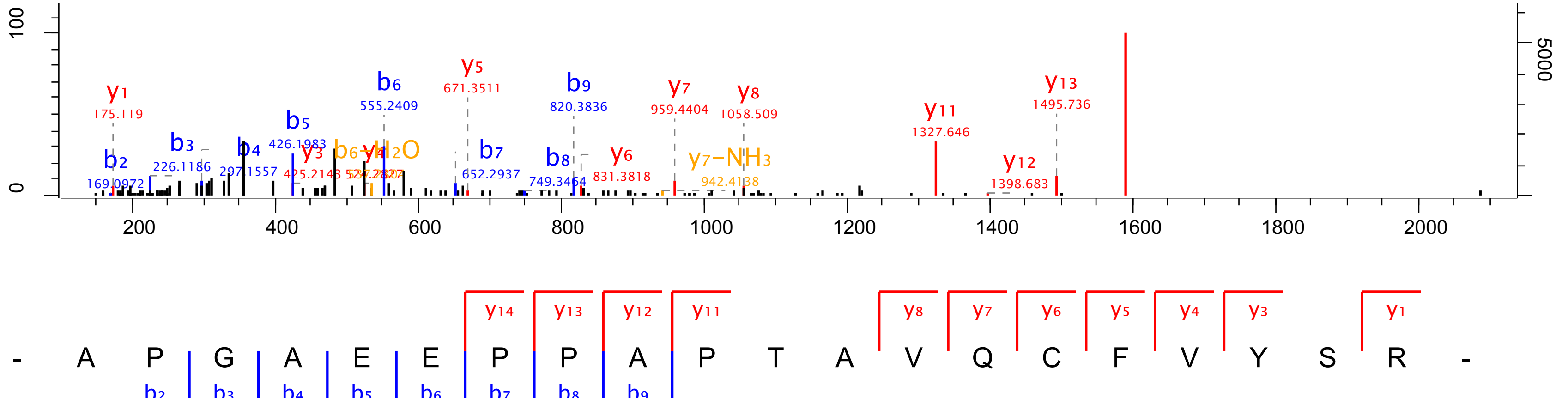

Raw file	Scan	Method	Score	m/z
20140918_fract17_dyn_5ul_F1_01_385	32221	TOF; CID	44.93	963.44

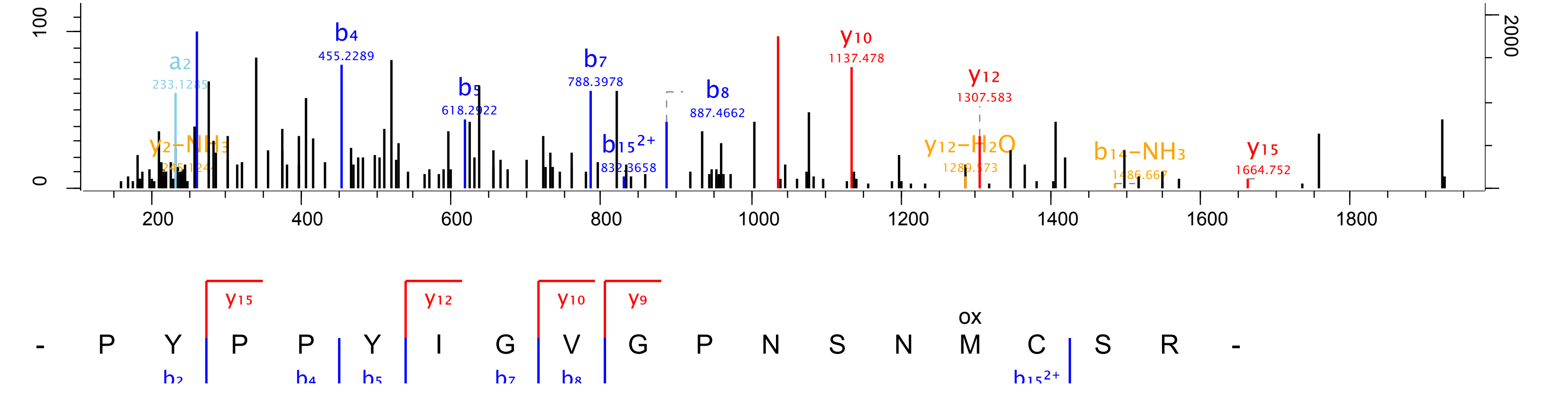

Raw file	Scan	Method	Score	m/z	Gene names
20140918_fract18_dyn_5ul_F2_01_386	19576	TOF; CID	72.85	436.91	LAMB2

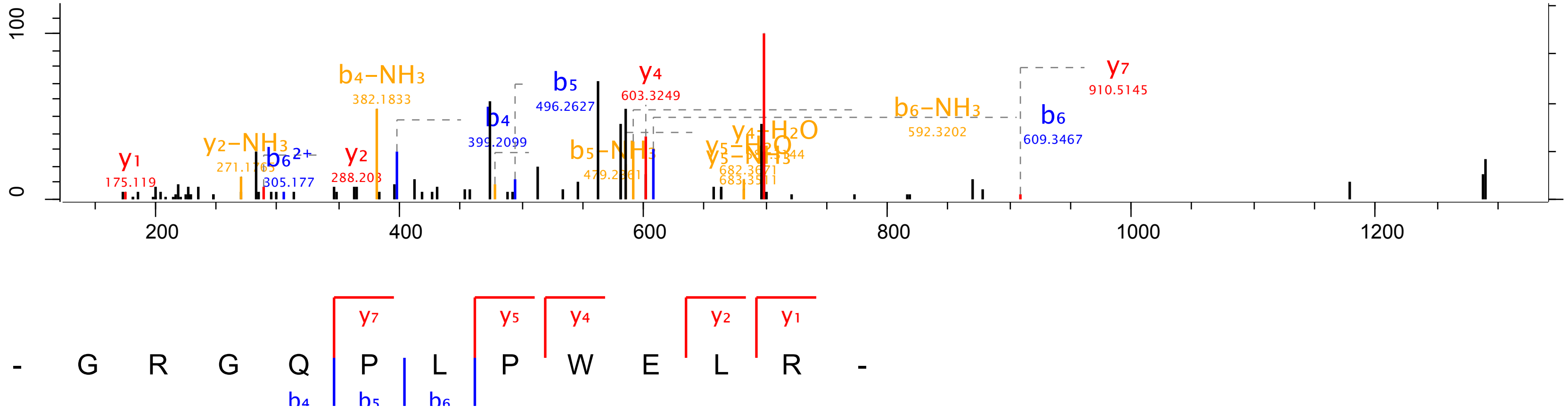

Raw file	Scan	Method	Score	m/z	Gene names
20140918_fract18_dyn_5ul_F2_01_386	27291	TOF; CID	55.55	830.4	C11orf74

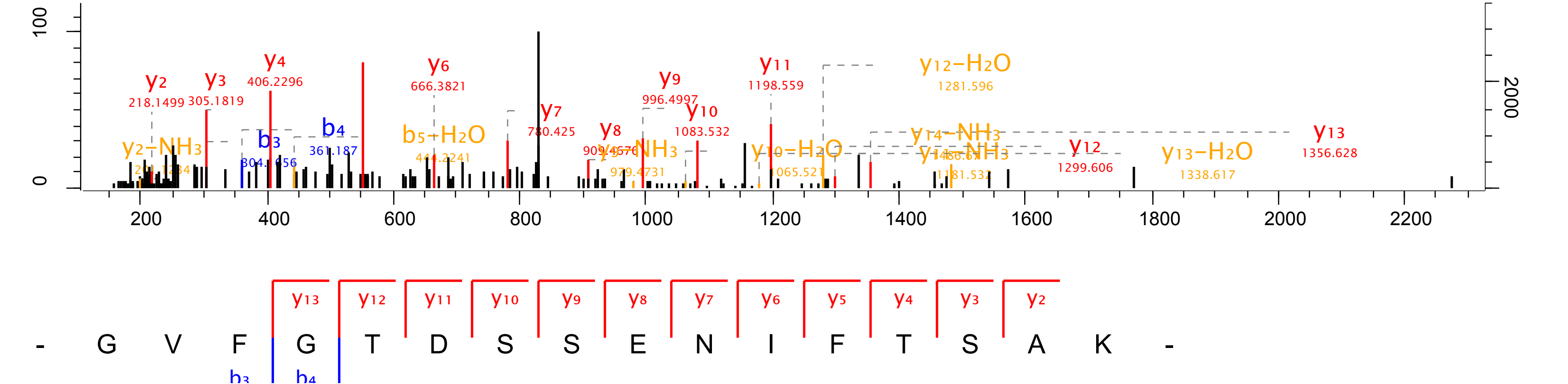

Raw file	Scan	Method	Score	m/z	Gene names
20140918_fract18_dyn_5ul_F2_01_386	29698	TOF; CID	103.42	1101.17	CEBPG

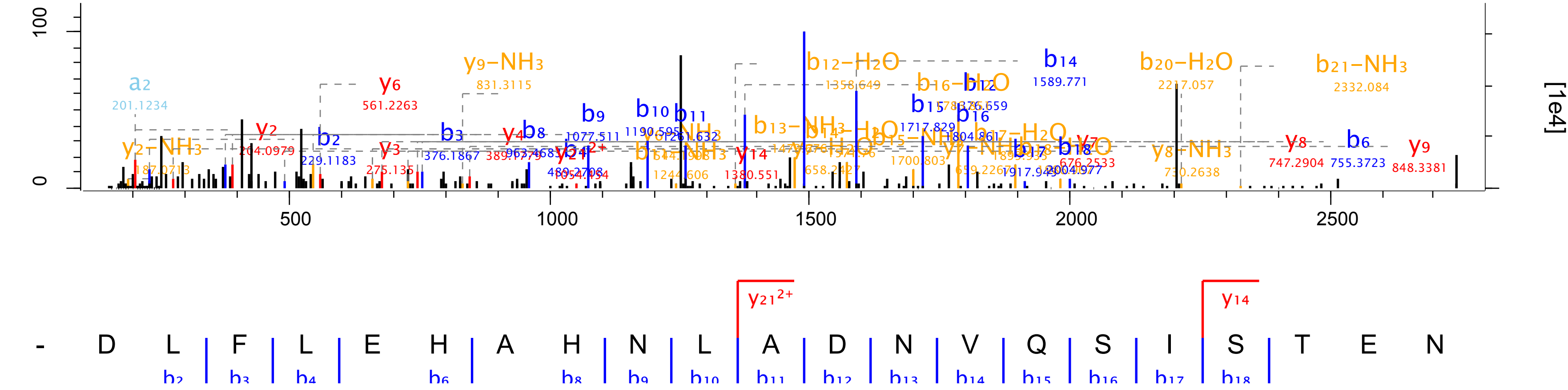

Raw file	Scan	Method	Score	m/z	Gene names
20140918_fract18_dyn_5ul_F2_01_386	30818	TOF; CID	70.94	633.81	BACE1

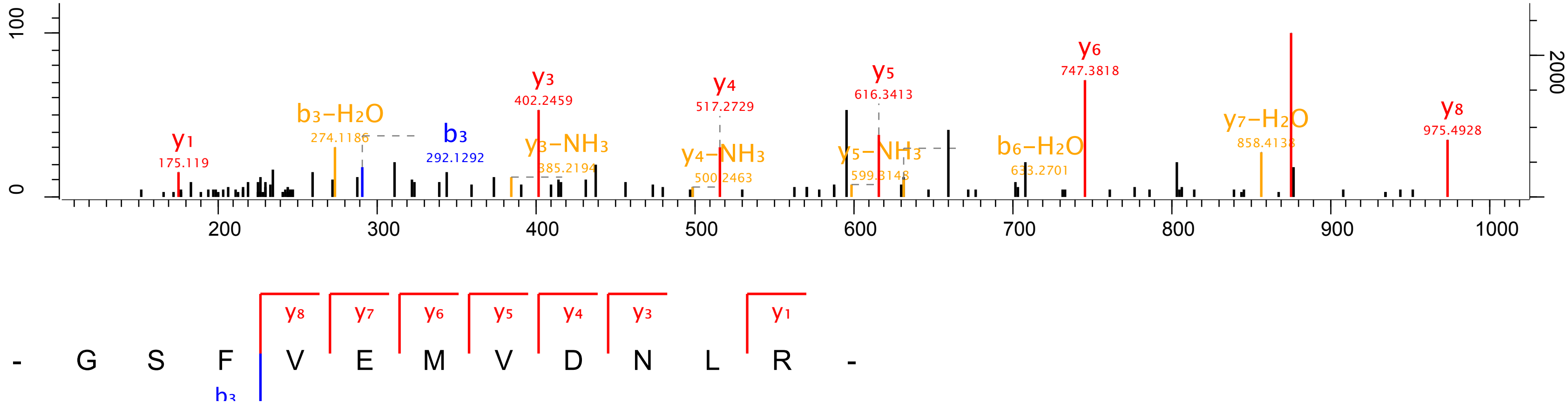

Raw file	Scan	Method	Score	m/z	Gene names
20140918_fract18_dyn_5ul_F2_01_386	35229	TOF; CID	77.78	742.89	AURKAIP1

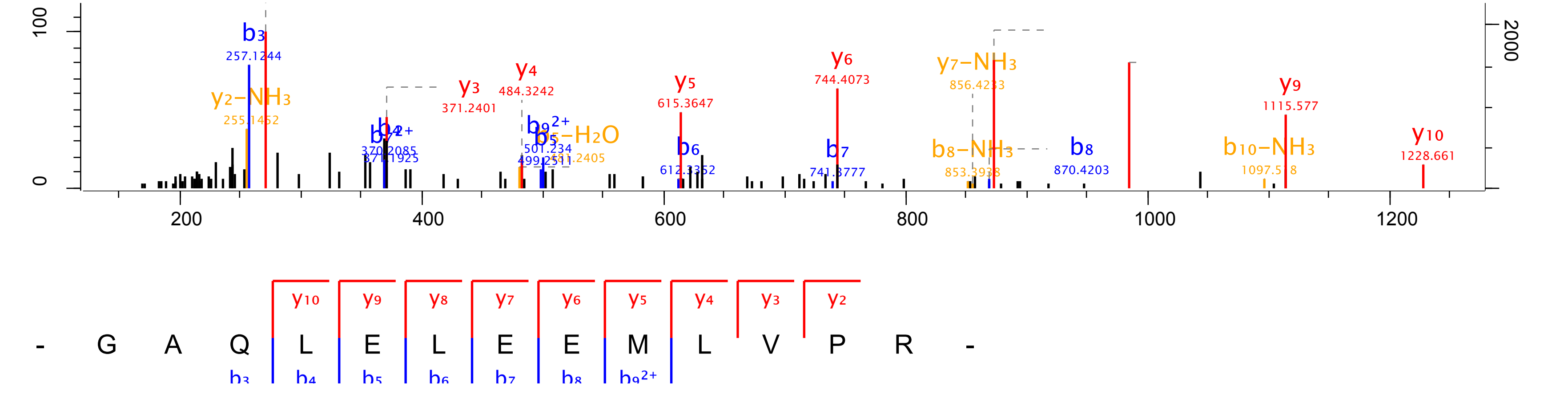

Raw file	Scan	Method	Score	m/z	Gene names
20140918_fract19_dyn_5ul_F3_01_387	18389	TOF; CID	147.52	476.24	MAPK15

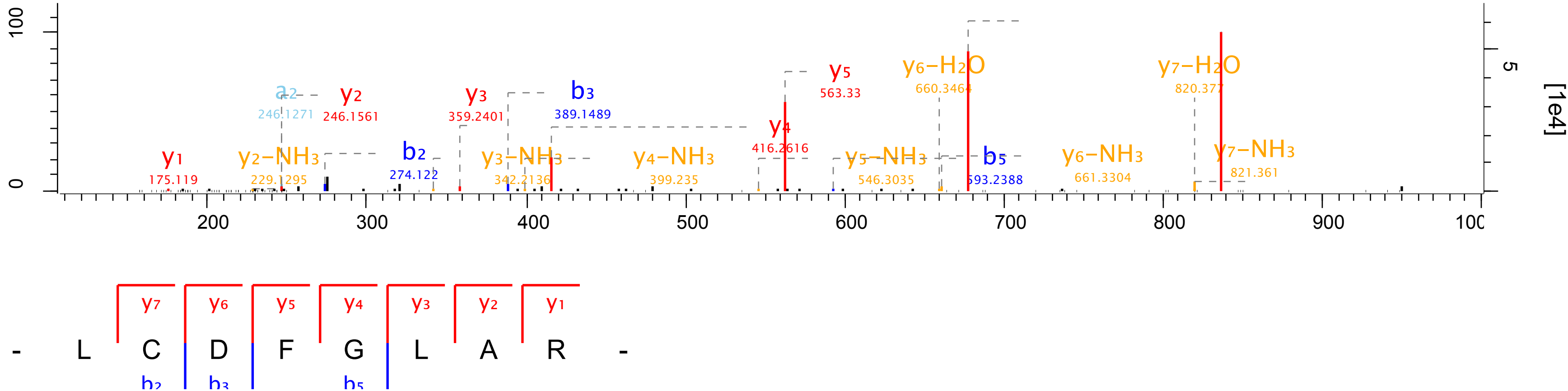

Raw file	Scan	Method	Score	m/z	Gene names
20140918_fract19_dyn_5ul_F3_01_387	19285	TOF; CID	95.41	731.36	ZDHHC24

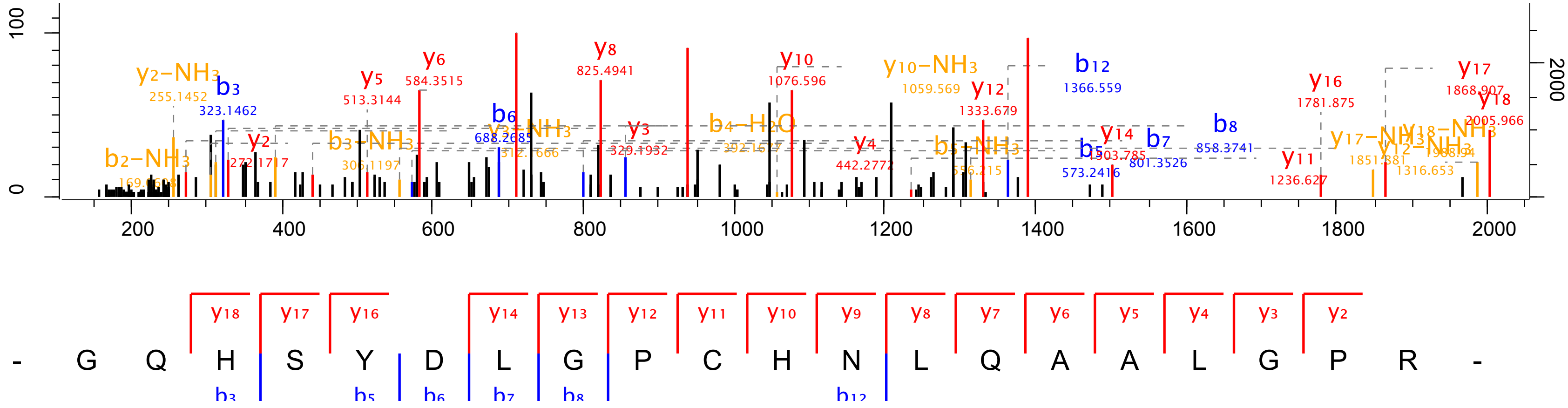

Raw file	Scan	Method	Score	m/z	Gene names
20140918_fract19_dyn_5ul_F3_01_387	24337	TOF; CID	79.09	771.36	FBXO17

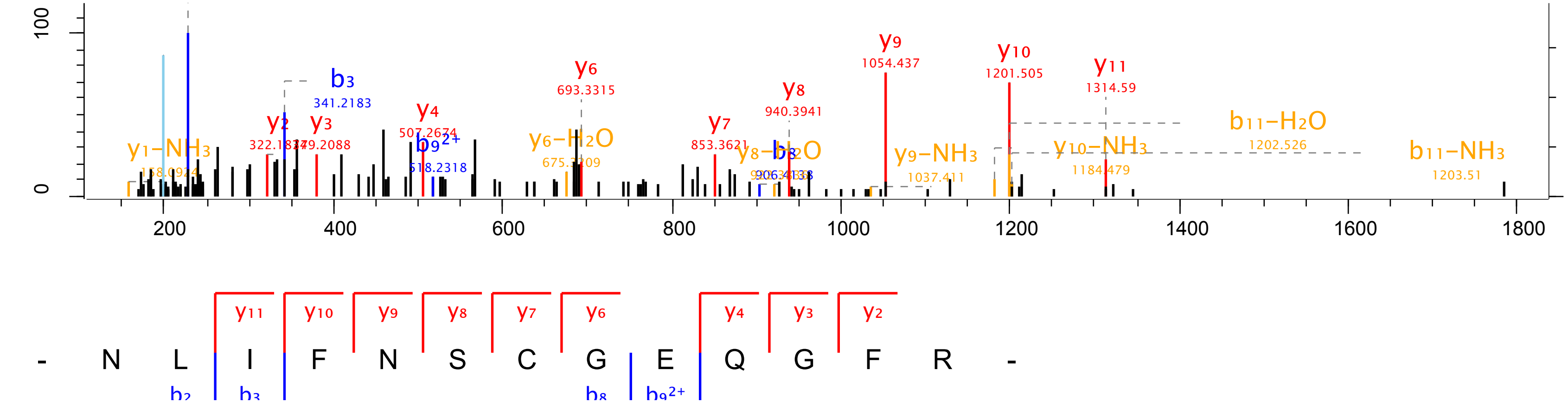

Raw file	Scan	Method	Score	m/z	Gene names
20140918_fract20_dyn_5ul_F4_01_388	16067	TOF; CID	93.18	505.78	NAIF1

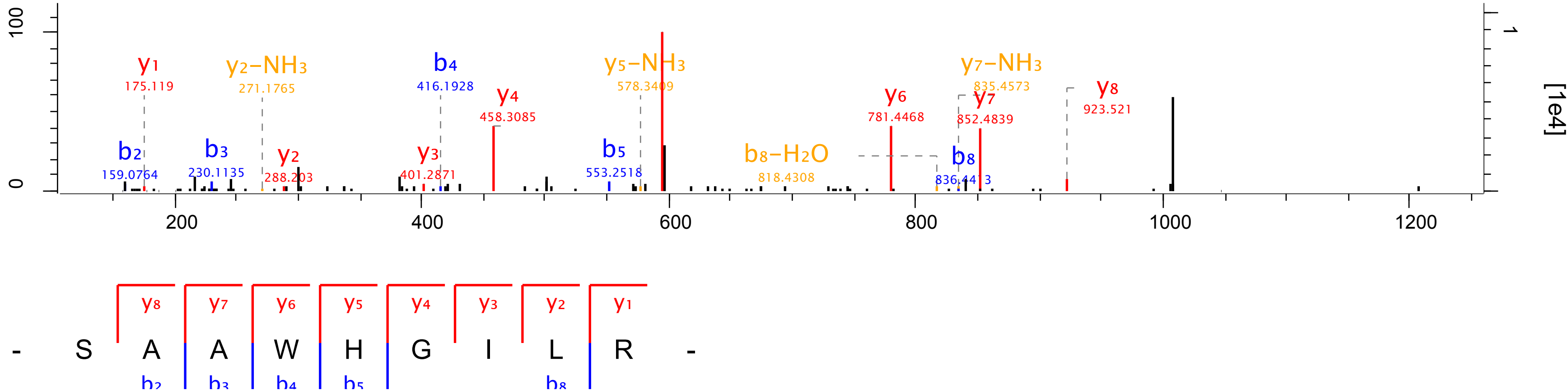

Raw file	Scan	Method	Score	m/z	Gene names
20140918_fract20_dyn_5ul_F4_01_388	17487	TOF; CID	30.94	624.83	ZNF831

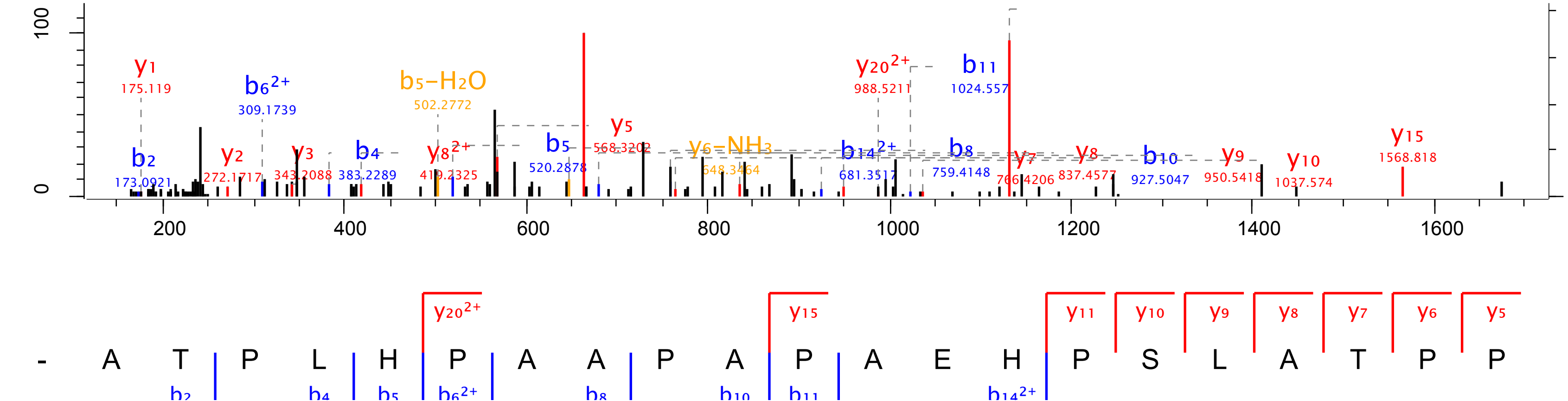

Raw file	Scan	Method	Score	m/z	Gene names
20140918_fract21_dyn_5ul_F5_01_389	24025	TOF; CID	52.48	741.03	ZBTB8A

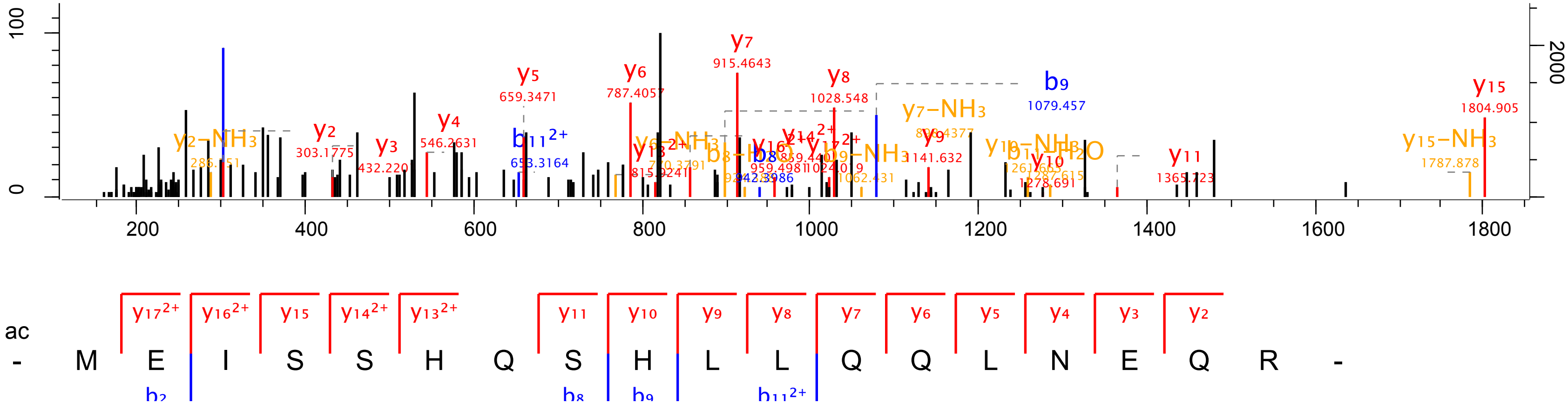

Raw file	Scan	Method	Score	m/z	Gene names
20140918_fract21_dyn_5ul_F5_01_389	24342	TOF; CID	109.11	486.29	LGI1

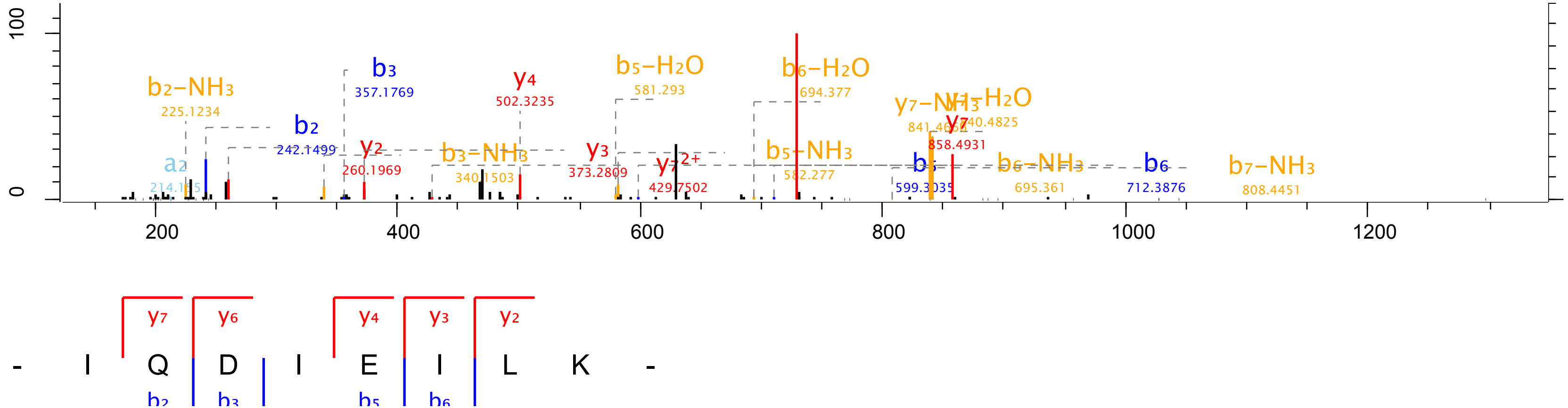

Raw file	Scan	Method	Score	m/z	Gene names
20140918_fract21_dyn_5ul_F5_01_389	27728	TOF; CID	117.93	731.88	SNAPC5

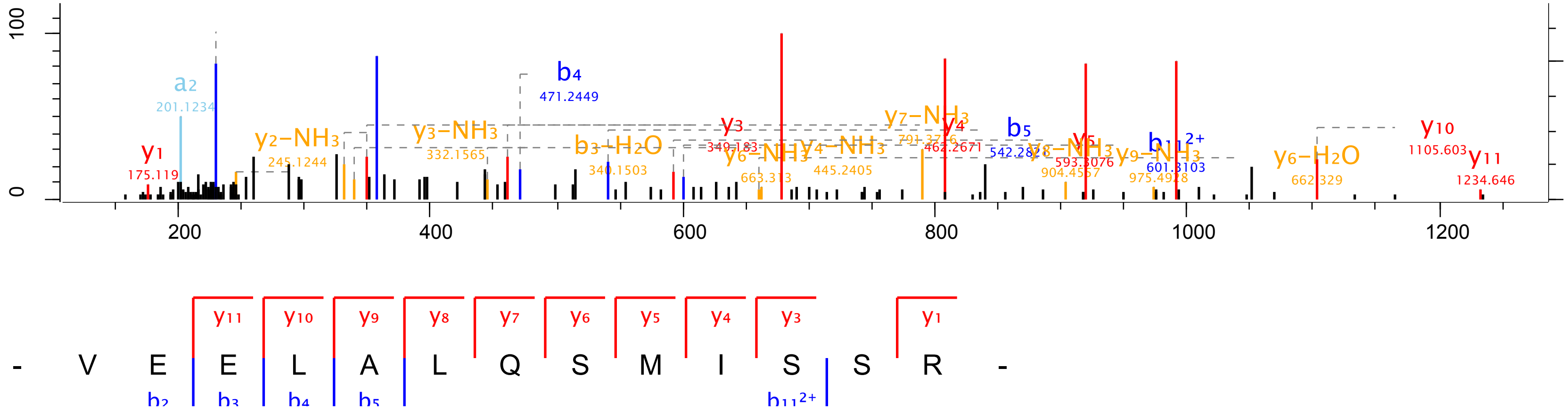

Raw file	Scan	Method	Score	m/z	Gene names
20140918_fract22_dyn_5ul_F6_01_390	12713	TOF; CID	54.95	604.31	HORMAD1

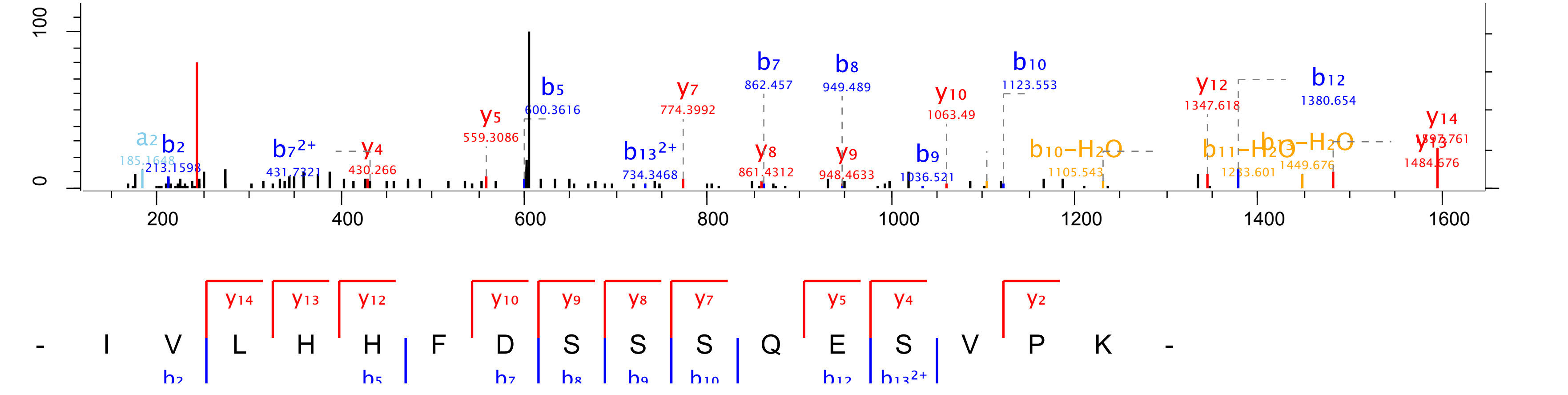

Raw file	Scan	Method	Score	m/z	Gene names
20140918_fract22_dyn_5ul_F6_01_390	32616	TOF; CID	102.52	813.9	TSPAN1

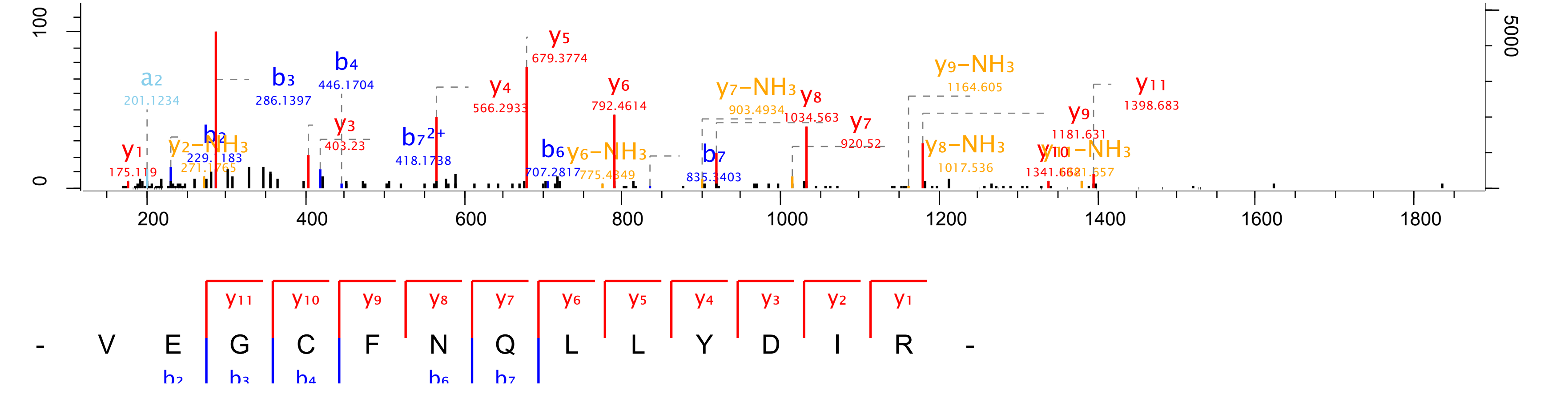

Raw file	Scan	Method	Score	m/z	Gene names
20140918_fract23_dyn_5ul_F7_01_391	14436	TOF; CID	96.74	625.86	FKRP

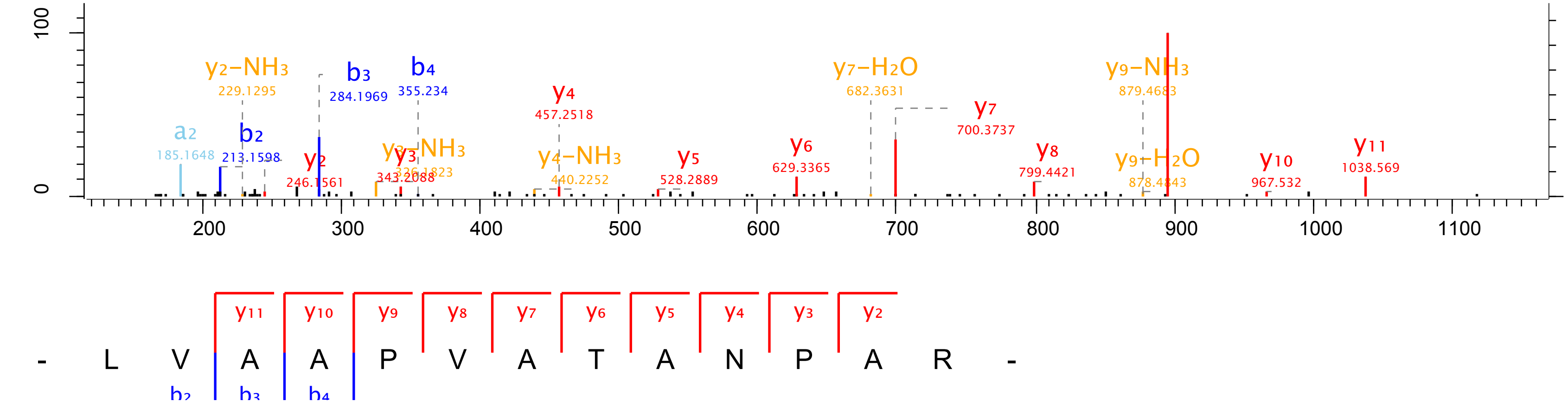

Raw file	Scan	Method	Score	m/z	Gene names
20140918_fract23_dyn_5ul_F7_01_391	16629	TOF; CID	84.51	569.32	ZNF219

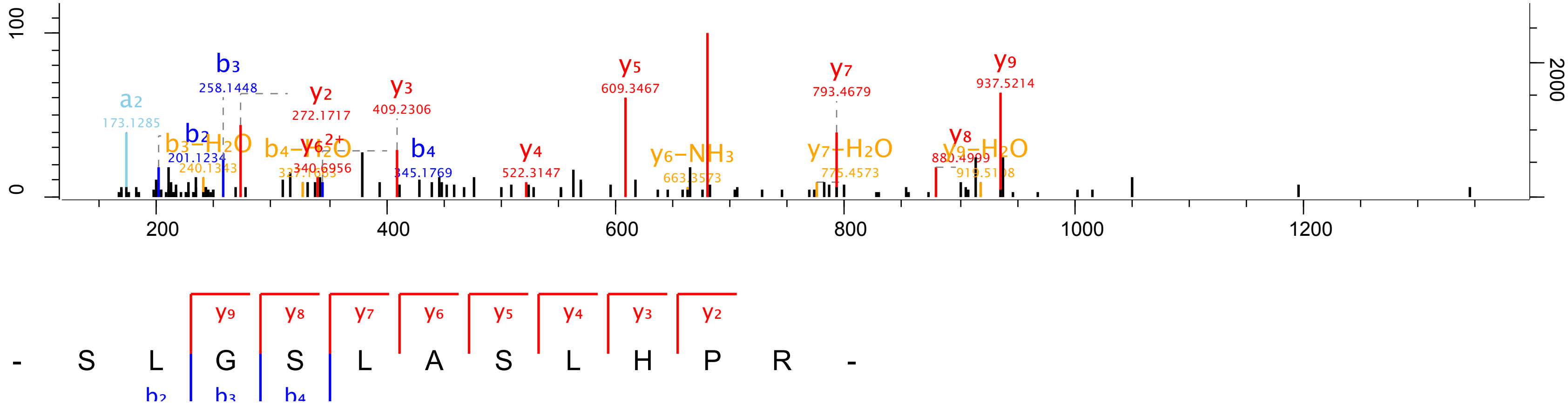

Raw file	Scan	Method	Score	m/z	Gene names
20140918_fract23_dyn_5ul_F7_01_391	18077	TOF; CID	76.07	734.85	STYXL1

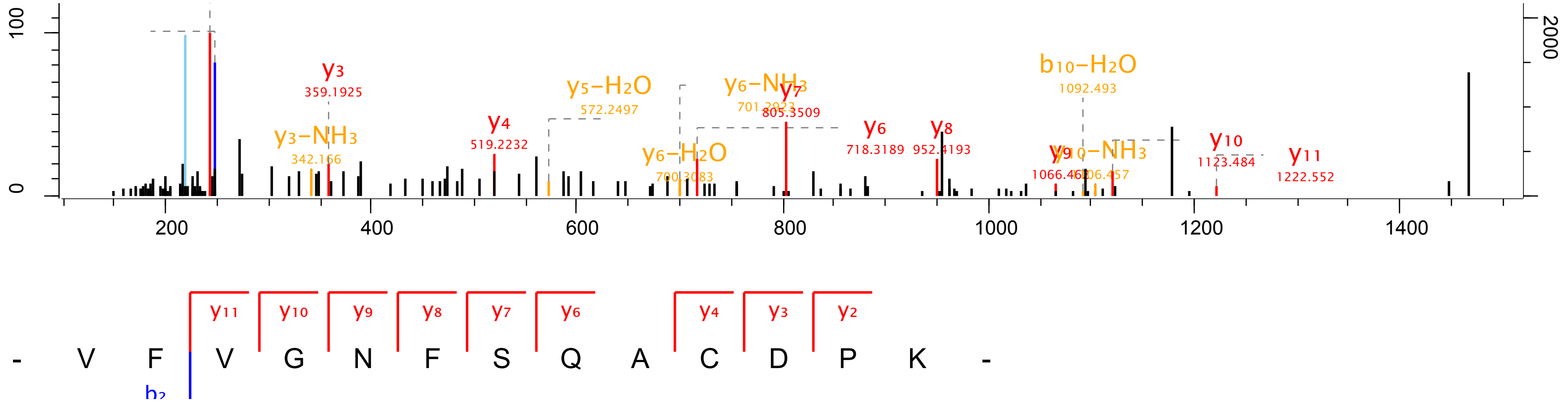

Raw file	Scan	Method	Score	m/z	Gene names
20140918_fract23_dyn_5ul_F7_01_391	21107	TOF; CID	80.37	800.44	ZCCHC10

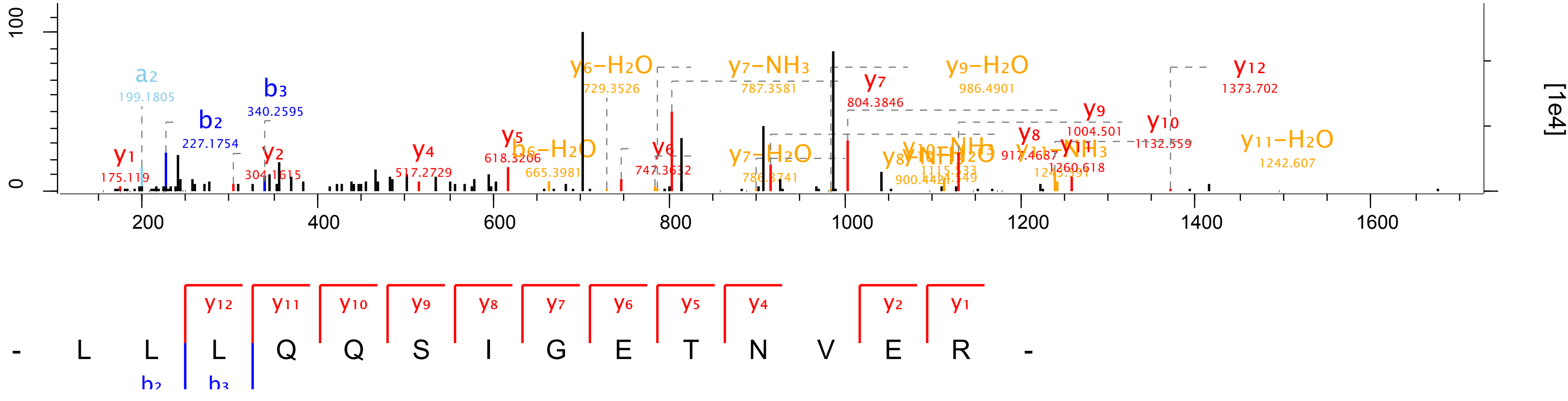

Raw file	Scan	Method	Score	m/z	Gene names
20140918_fract23_dyn_5ul_F7_01_391	30129	TOF; CID	69.7	908.73	TSPAN4

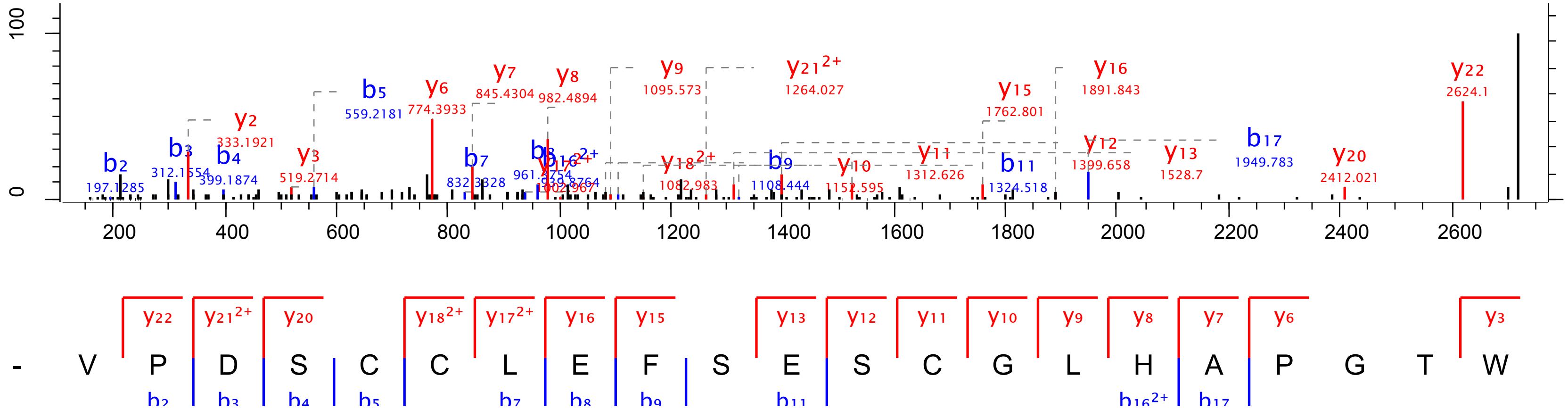

Raw file	Scan	Method	Score	m/z	Gene names
20140918_fract24_dyn_5ul_F8_01_392	20157	TOF; CID	42.12	762.34	PEG10

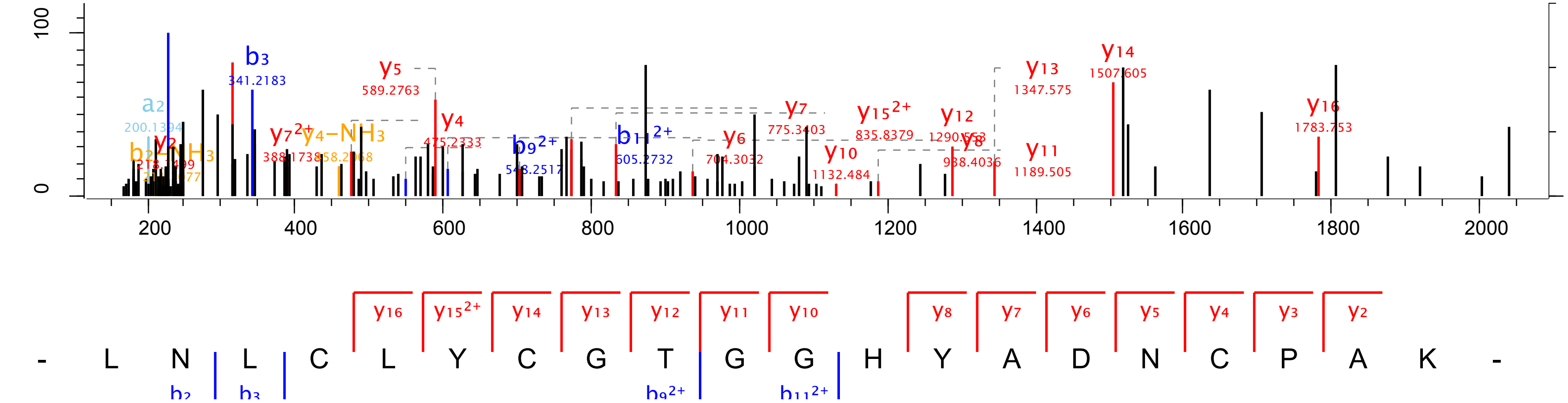

Raw file	Scan	Method	Score	m/z	Gene names
20140918_fract24_dyn_5ul_F8_01_392	29116	TOF; CID	139.58	512.29	FAM118A

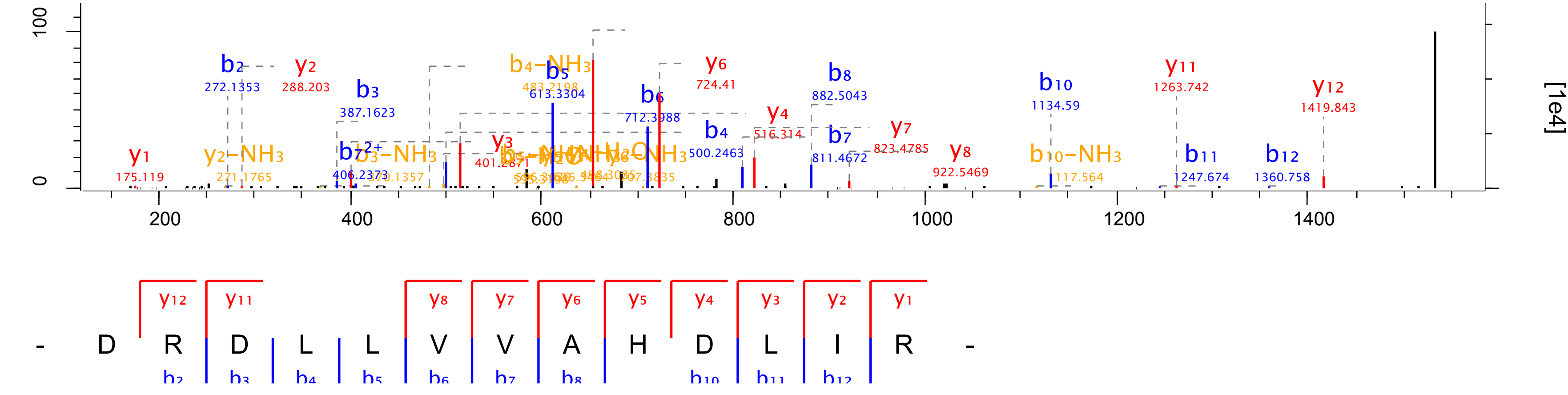

Raw file	Scan	Method	Score	m/z	Gene names
20140918_fract24_dyn_5ul_F8_01_392	34044	TOF; CID	83.23	711.86	SMIM15

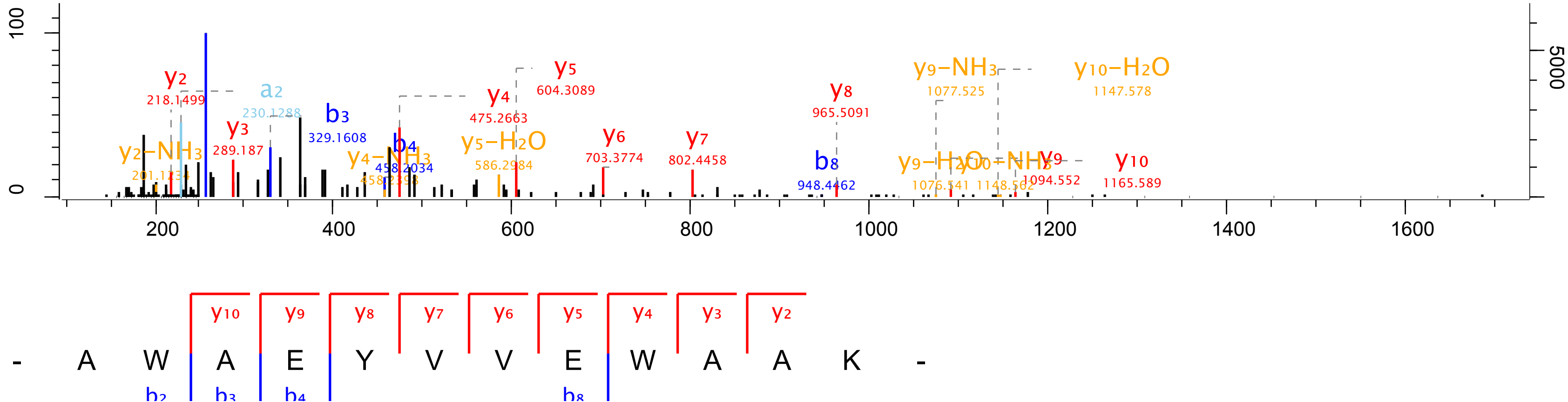

Raw file	Scan	Method	Score	m/z	Gene names
20140925_fract1_dyn_5ul_B1_01_436	3761	TOF; CID	88.5	538.76	TM4SF1

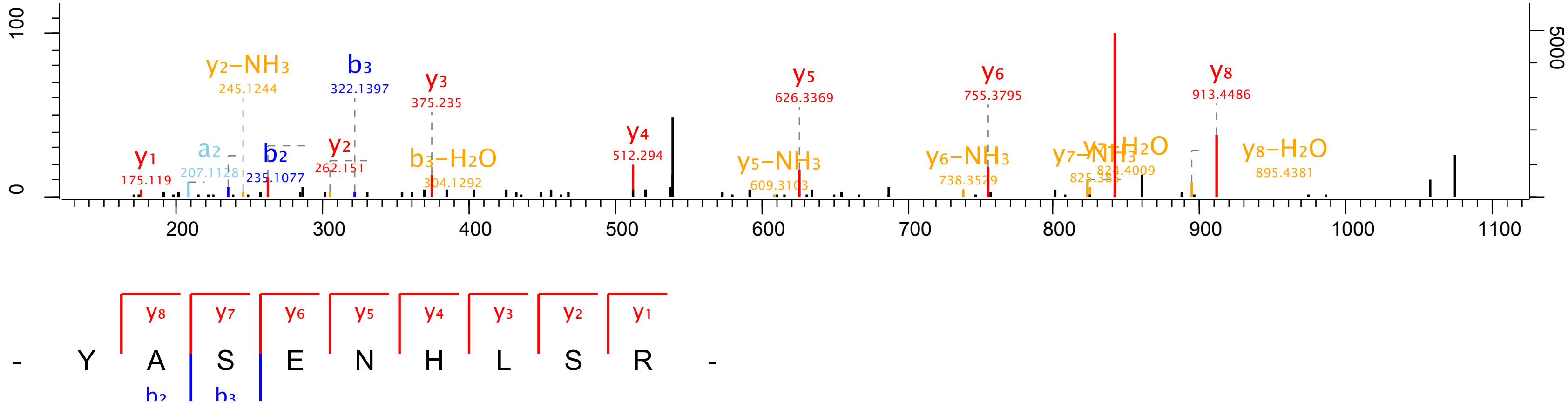

Raw file	Scan	Method	Score	m/z	Gene names
20140925_fract1_dyn_5ul_B1_01_436	17592	TOF; CID	81.8	726.36	SCNN1B

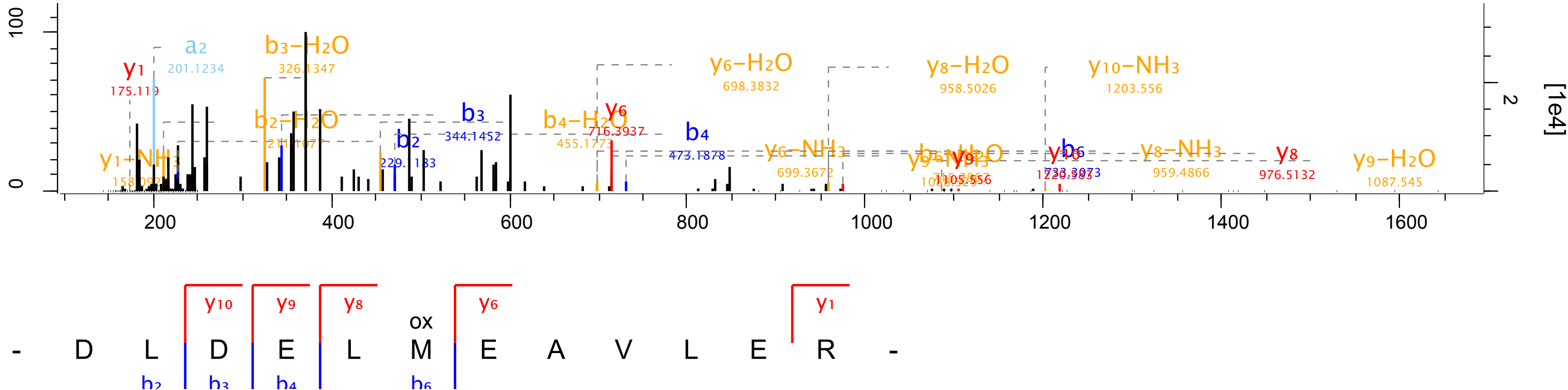

Raw file	Scan	Method	Score	m/z	Gene names
20140925_fract1_dyn_5ul_B1_01_436	30678	TOF; CID	61.28	999.02	GLYCTK

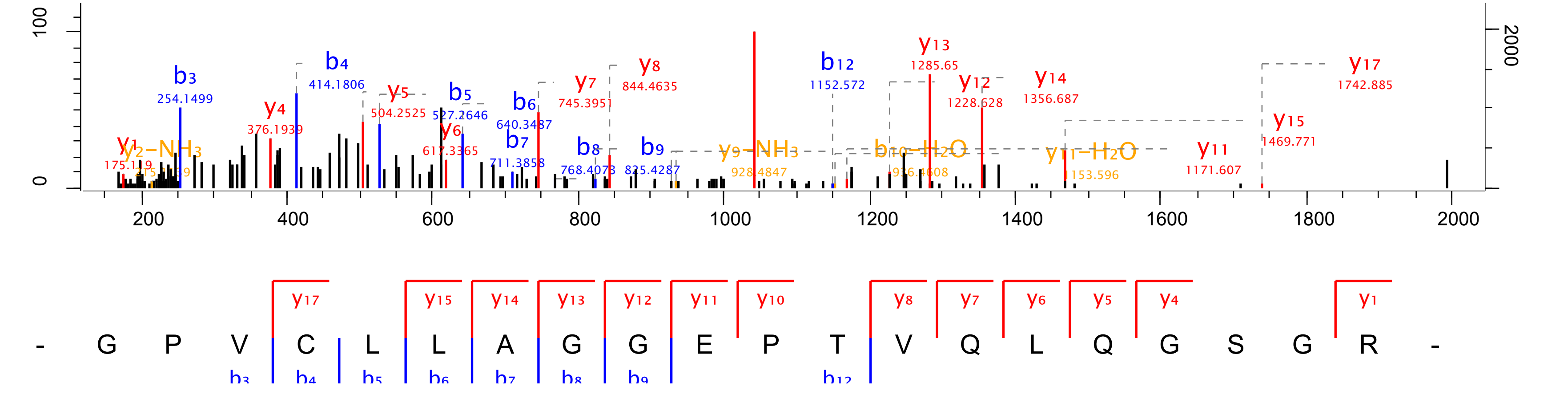

Raw file	Scan	Method	Score	m/z	Gene names
20140925_fract1_dyn_5ul_B1_01_436	37990	TOF; CID	101.3	613.31	TMEM234

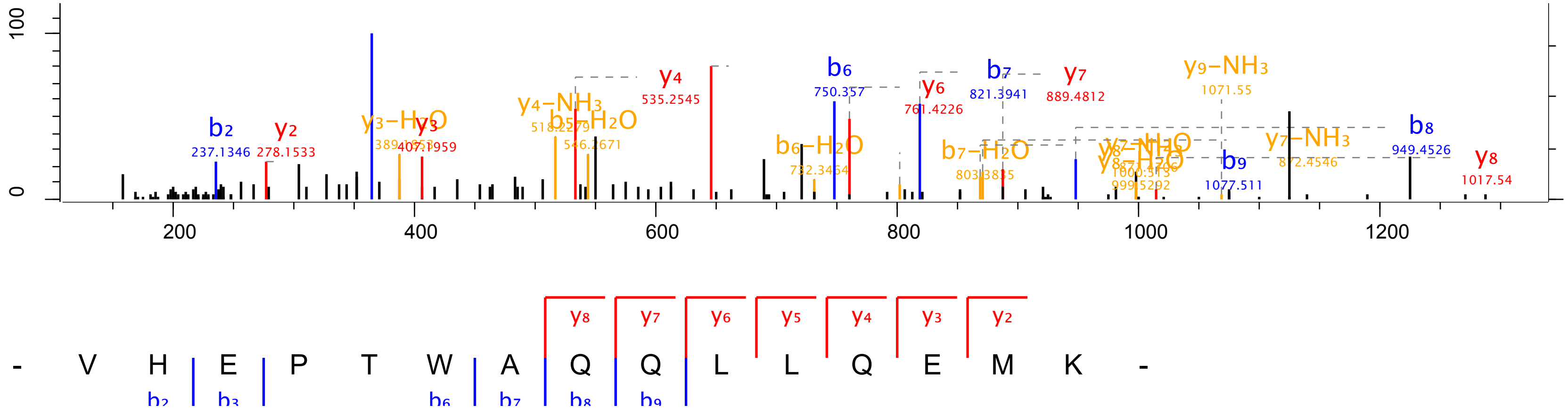

Raw file	Scan	Method	Score	m/z	Gene names
20140925_fract2_dyn_5ul_B2_01_437	11066	TOF; CID	79.64	660.32	NDFIP2

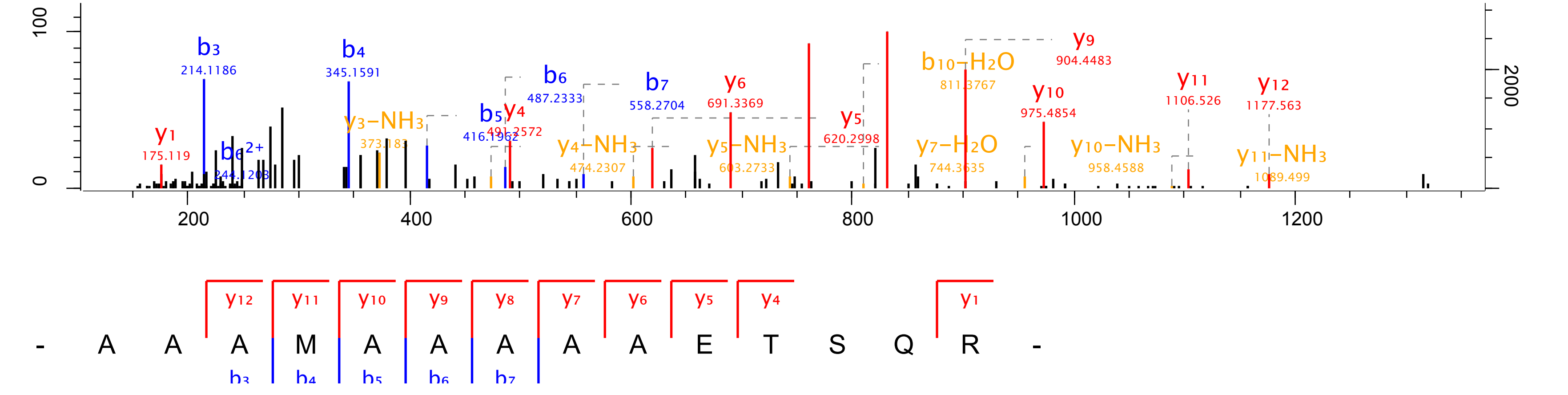

Raw file	Scan	Method	Score	m/z
20140925_fract2_dyn_5ul_B2_01_437	15478	TOF; CID	72.2	609.8

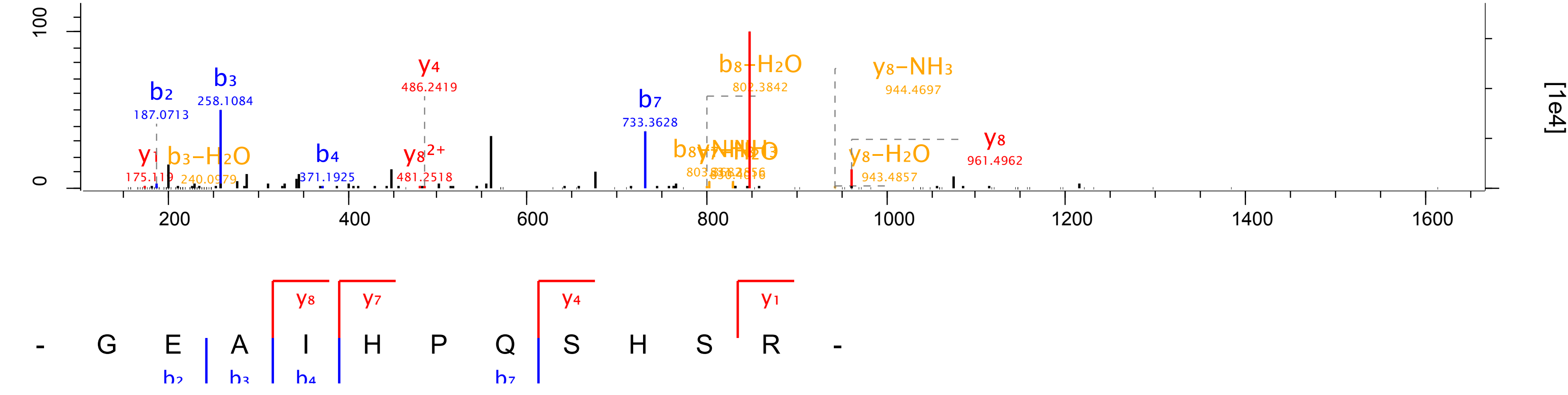

Raw file	Scan	Method	Score	m/z	Gene names
20140925_fract2_dyn_5ul_B2_01_437	25530	TOF; CID	101.32	756.86	FAM73A

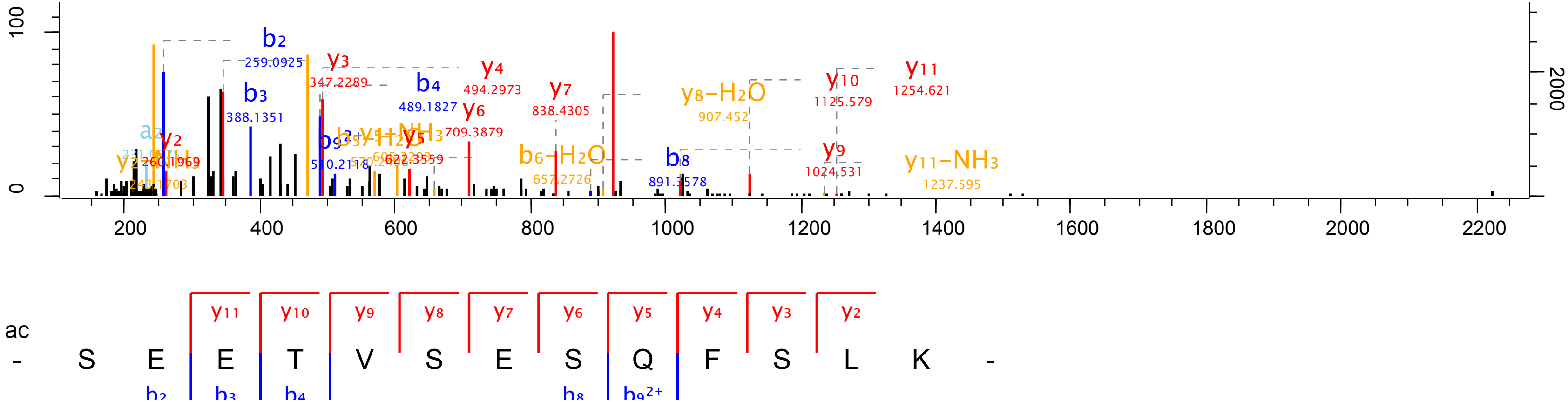

Raw file	Scan	Method	Score	m/z	Gene names
20140925_fract2_dyn_5ul_B2_01_437	37413	TOF; CID	73.92	972.02	SLC10A7

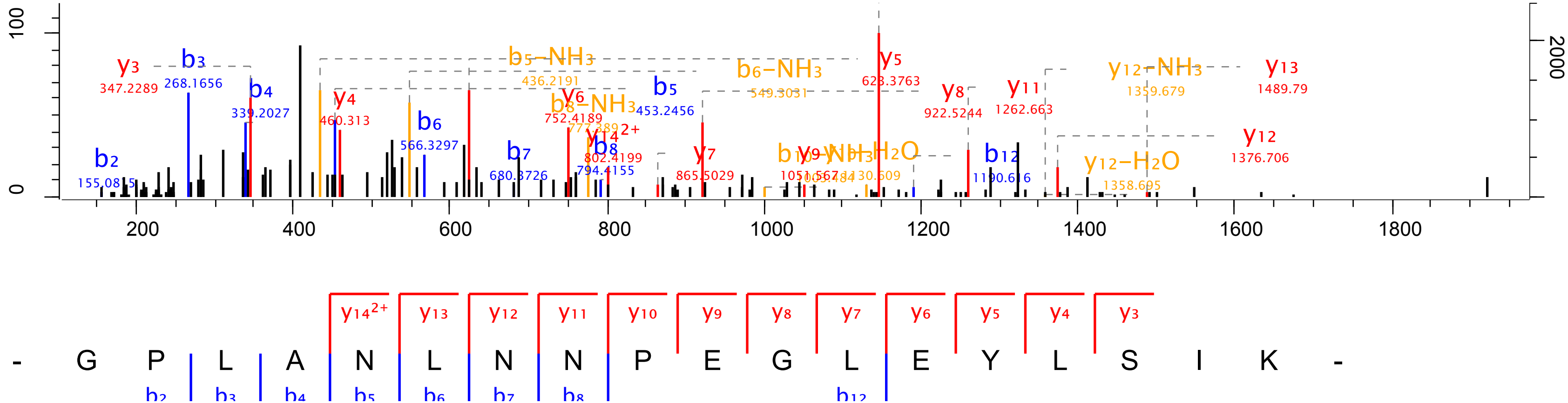

Raw file	Scan	Method	Score	m/z	Gene names
20140925_fract3_dyn_5ul_B3_01_438	14078	TOF; CID	122.33	630.83	TFDP2

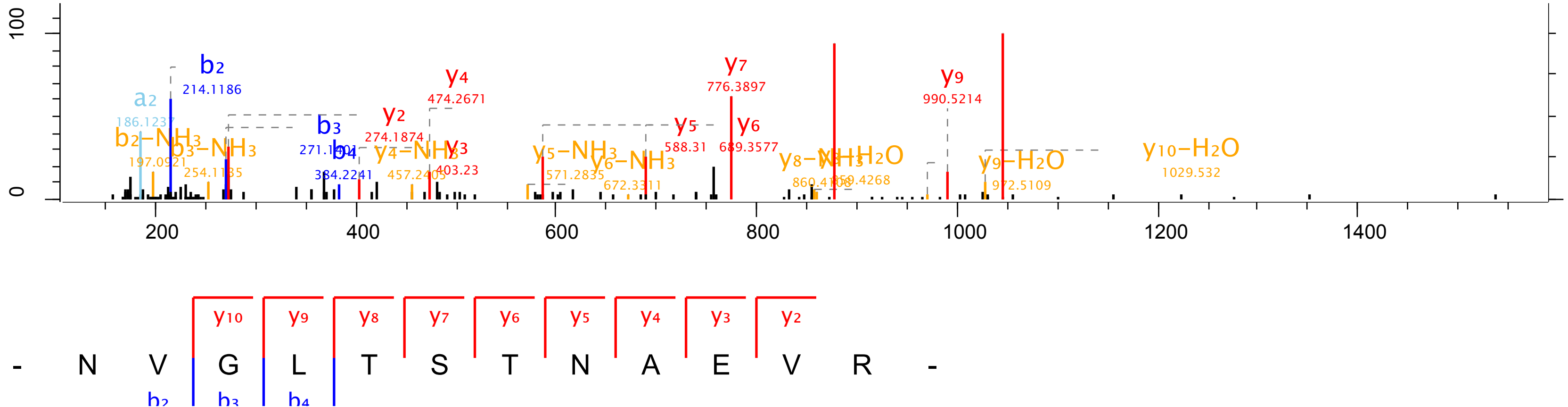

Raw file	Scan	Method	Score	m/z	Gene names
20140925_fract3_dyn_5ul_B3_01_438	17637	TOF; CID	33.32	778.38	FXYD5

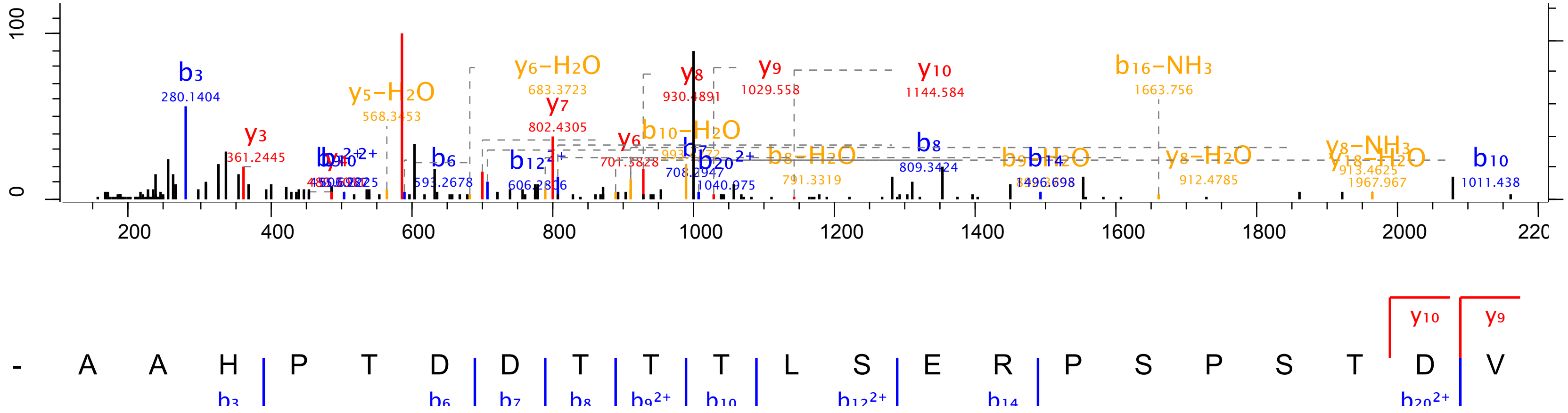

Raw file	Scan	Method	Score	m/z	Gene names
20140925_fract3_dyn_5ul_B3_01_438	32375	TOF; CID	92.26	996.91	CLDN7

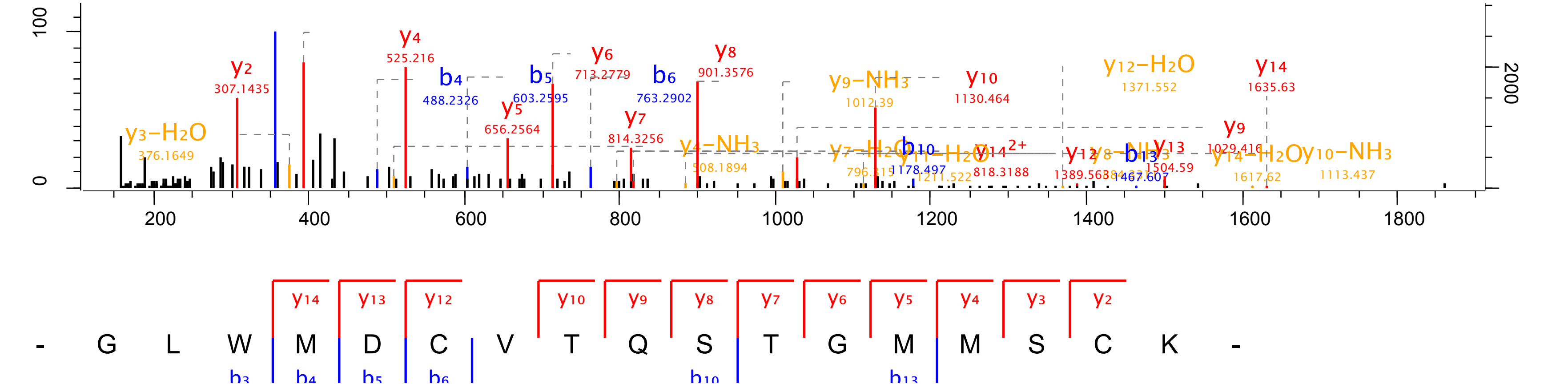

Raw file	Scan	Method	Score	m/z	Gene names
20140925_fract4_dyn_5ul_B4_01_439	3804	TOF; CID	116.37	438.2	SMOC2

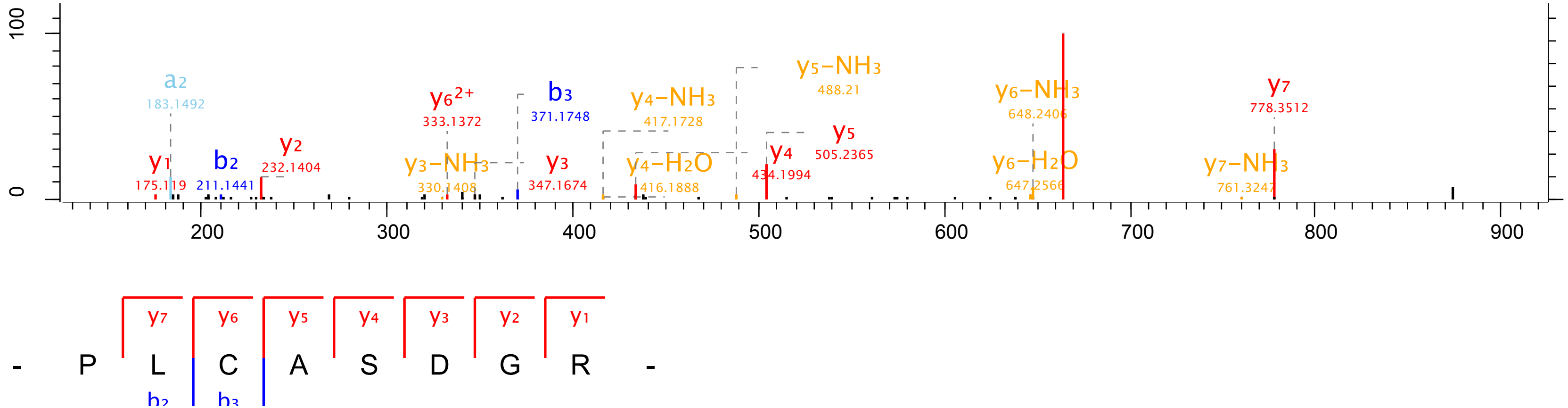

Raw file	Scan	Method	Score	m/z	Gene names
20140925_fract4_dyn_5ul_B4_01_439	12557	TOF; CID	144.1	577.32	PLEKHG4;CYB561A3

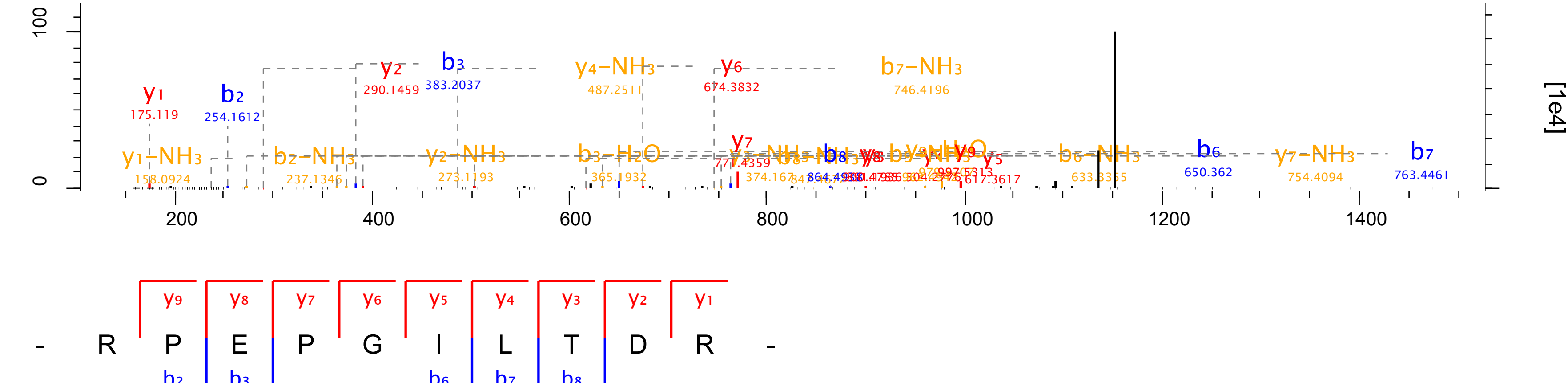

Raw file	Scan	Method	Score	m/z	Gene names
20140925_fract4_dyn_5ul_B4_01_439	15240	TOF; CID	80.32	634.8	MXD1

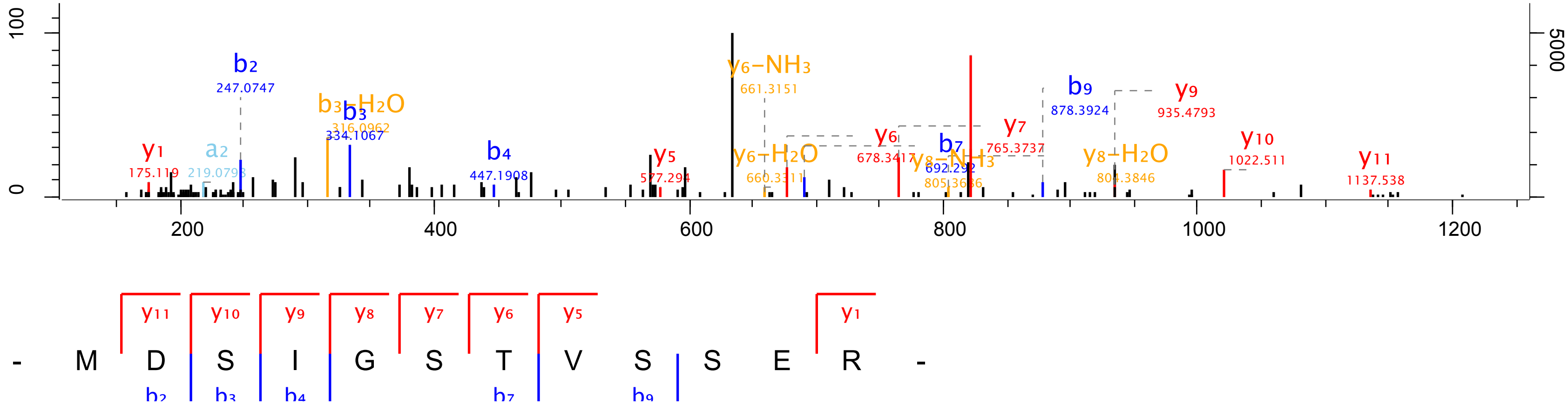

Raw file	Scan	Method	Score	m/z	Gene names
20140925_fract4_dyn_5ul_B4_01_439	17334	TOF; CID	85.81	658.84	ZMYM6NB

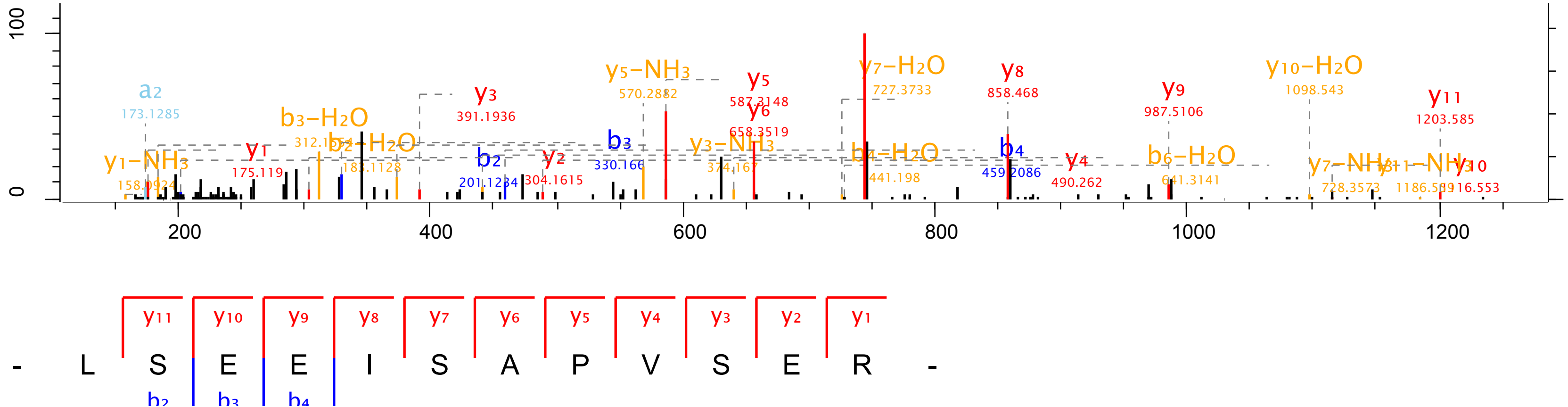

Raw file	Scan	Method	Score	m/z	Gene names
20140925_fract4_dyn_5ul_B4_01_439	32642	TOF; CID	244.37	854.42	SSR3

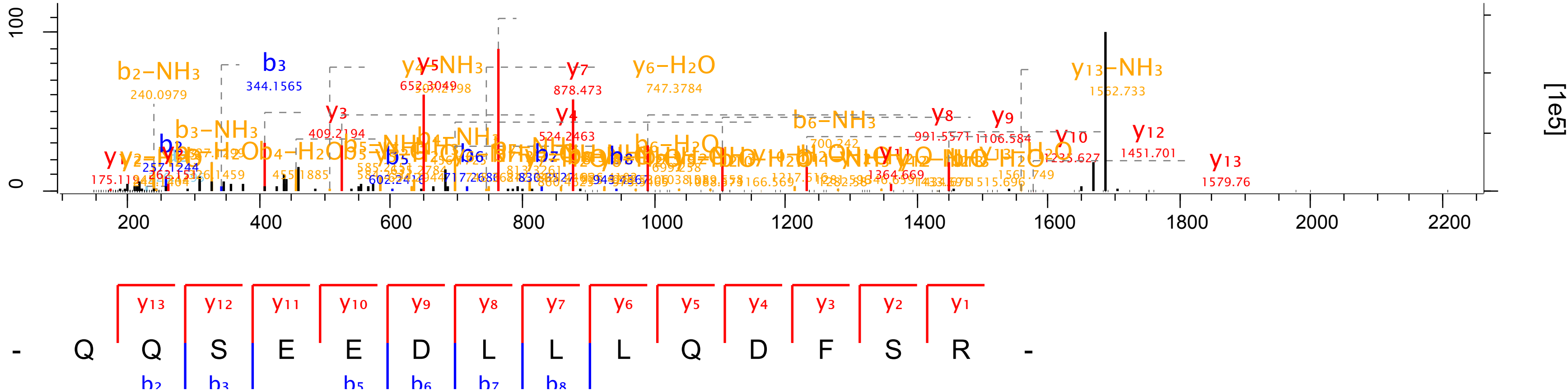

Raw file	Scan	Method	Score	m/z	Gene names
20140925_fract4_dyn_5ul_B4_01_439	36201	TOF; CID	65.72	917.44	CNBP

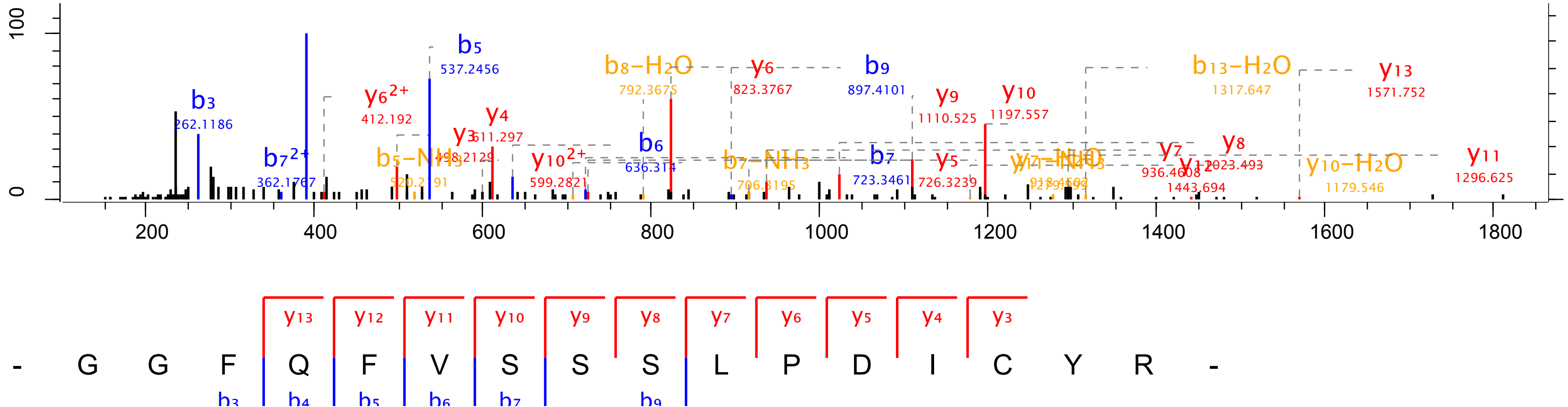

Raw file	Scan	Method	Score	m/z	Gene names
20140925_fract5_dyn_5ul_B5_01_440	10987	TOF; CID	94.11	408.21	TMEM187

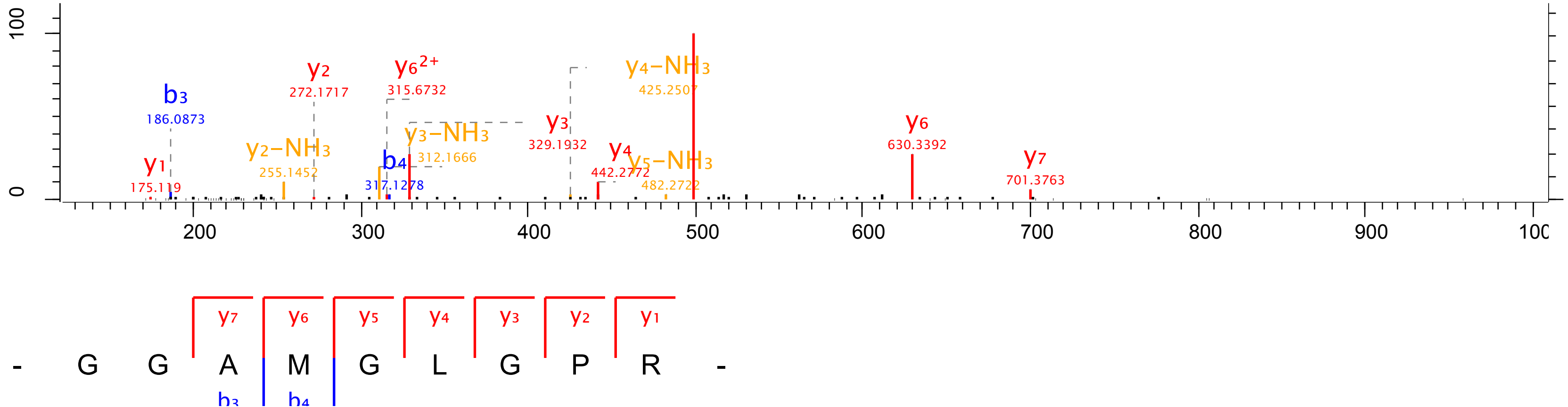

Raw file	Scan	Method	Score	m/z	Gene names
20140925_fract5_dyn_5ul_B5_01_440	14538	TOF; CID	142.43	425.86	CKS1B

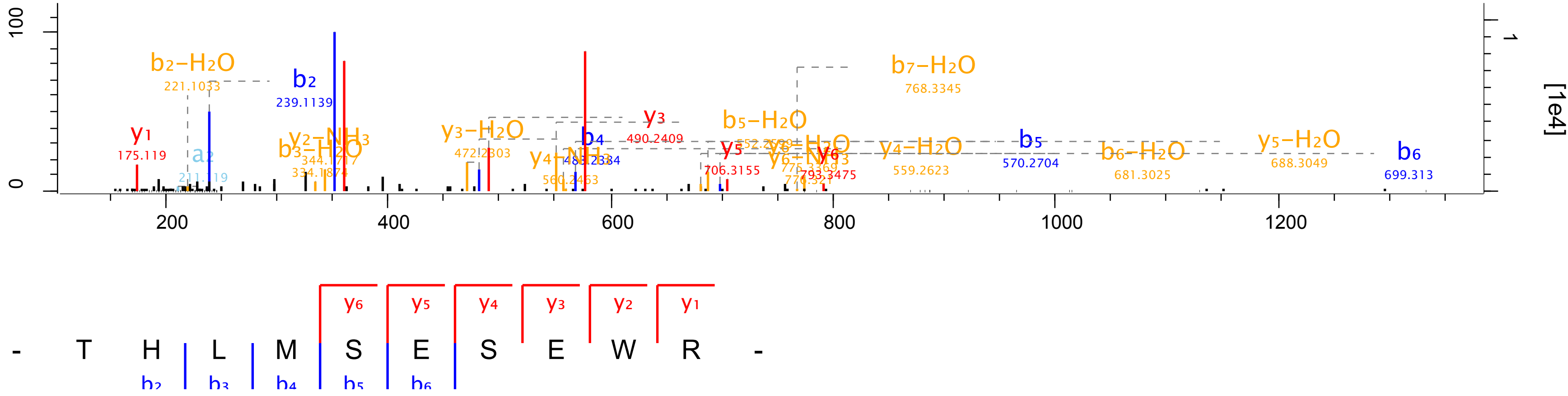

Raw file	Scan	Method	Score	m/z	Gene names
20140925_fract5_dyn_5ul_B5_01_440	29617	TOF; CID	58.89	687.86	FAM89B

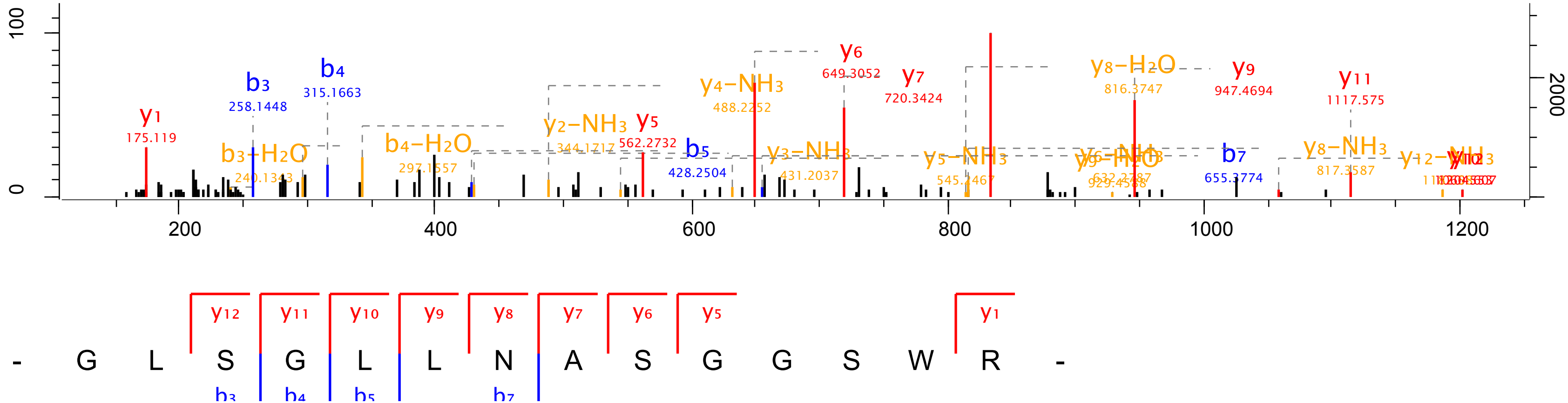

Raw file	Scan	Method	Score	m/z	Gene names
20140925_fract5_dyn_5ul_B5_01_440	34064	TOF; CID	117.7	576.85	SPIN4

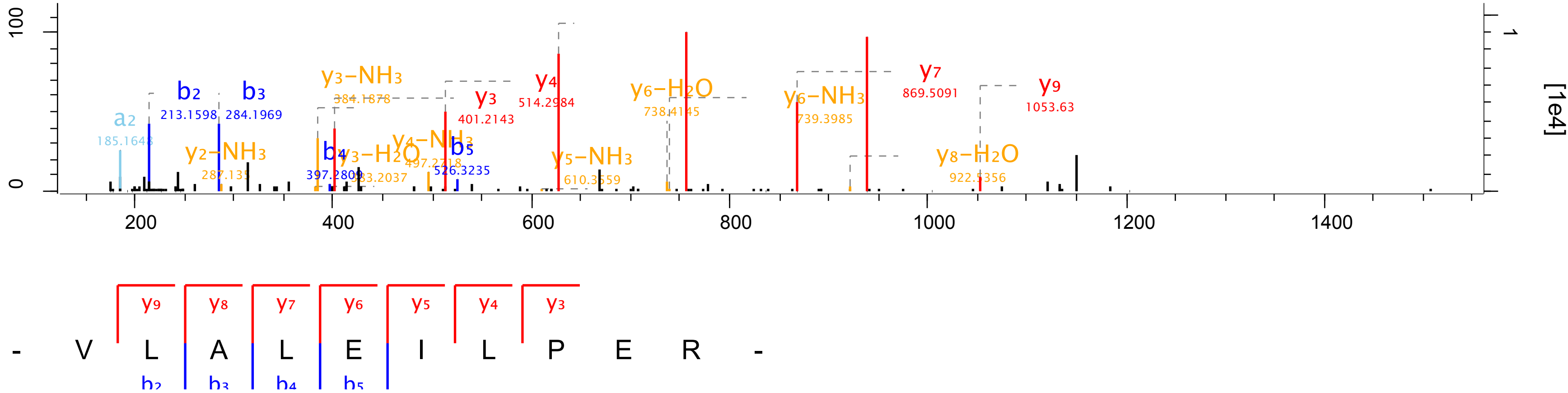

Raw file	Scan	Method	Score	m/z	Gene names
20140925_fract6_dyn_5ul_B6_01_441	10157	TOF; CID	118.76	617.32	HDGFRP2

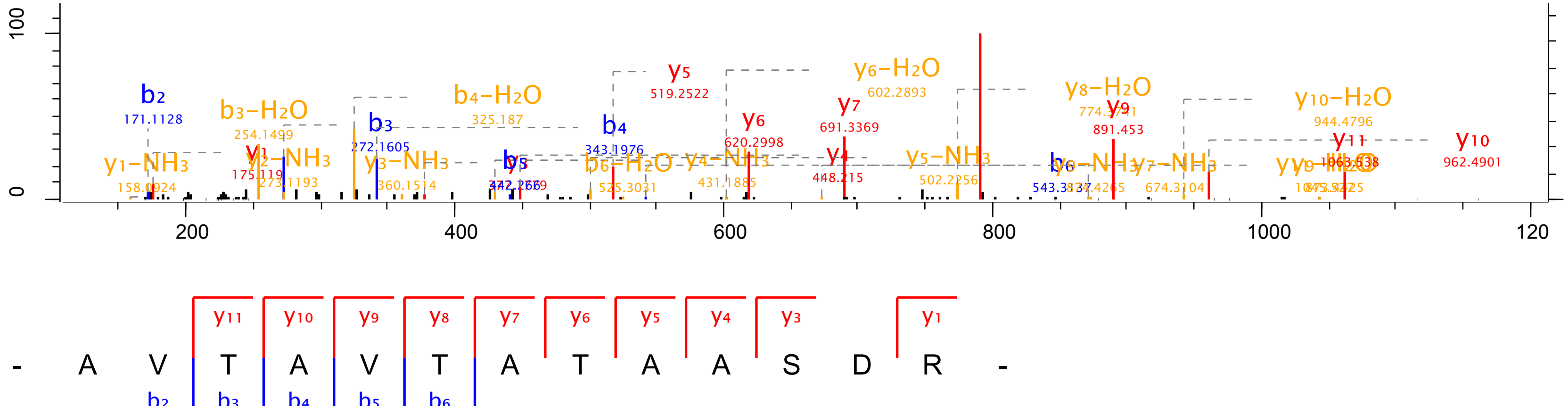

Raw file	Scan	Method	Score	m/z	Gene names
20140925_fract6_dyn_5ul_B6_01_441	22628	TOF; CID	86.8	461.23	ZKSCAN5

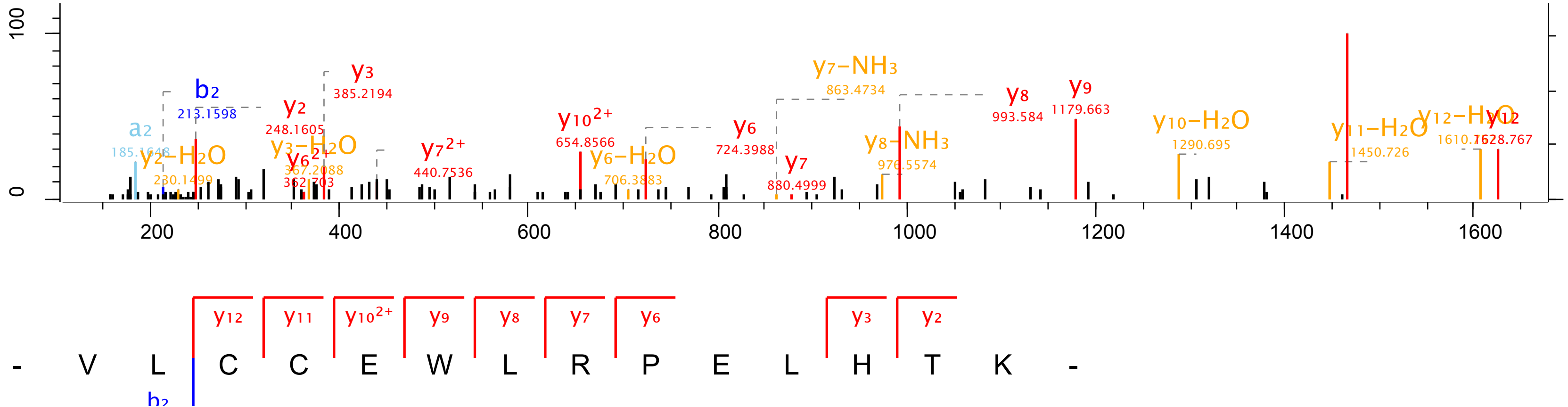

Raw file	Scan	Method	Score	m/z	Gene names
20140925_fract6_dyn_5ul_B6_01_441	26604	TOF; CID	73.83	556.29	FAM210B

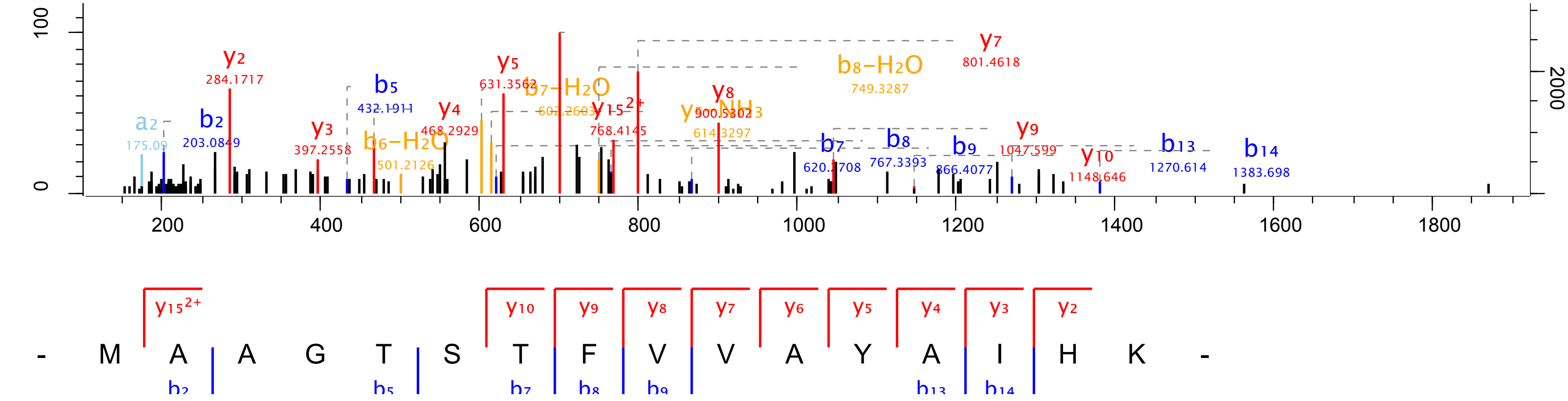

Raw file	Scan	Method	Score	m/z	Gene names
20140925_fract6_dyn_5ul_B6_01_441	29925	TOF; CID	67.23	725.03	UBALD2

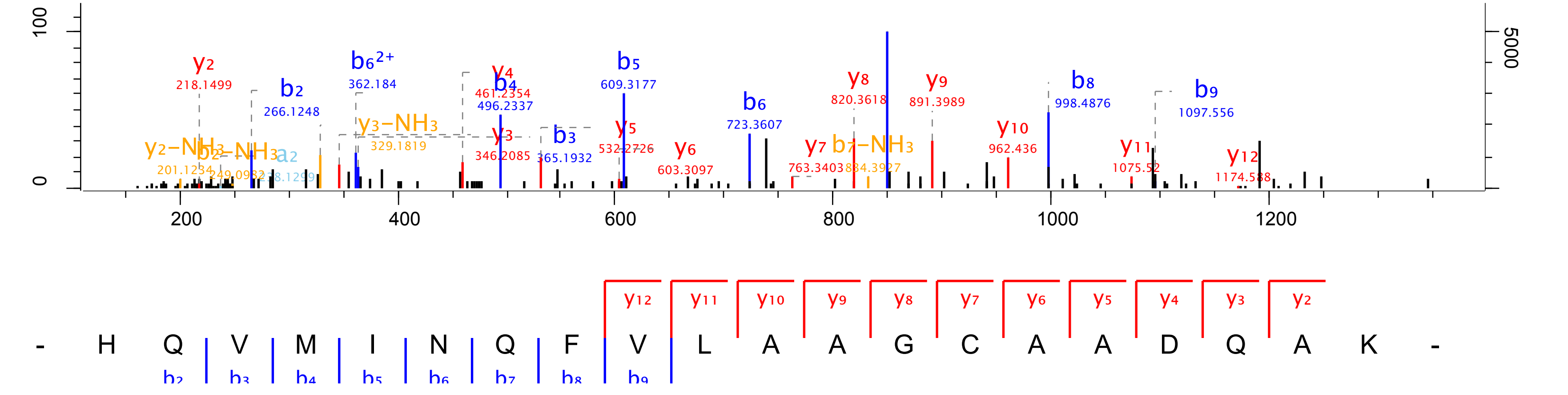

Raw file	Scan	Method	Score	m/z	Gene names
20140925_fract6_dyn_5ul_B6_01_441	34441	TOF; CID	60.54	866.47	TTC32

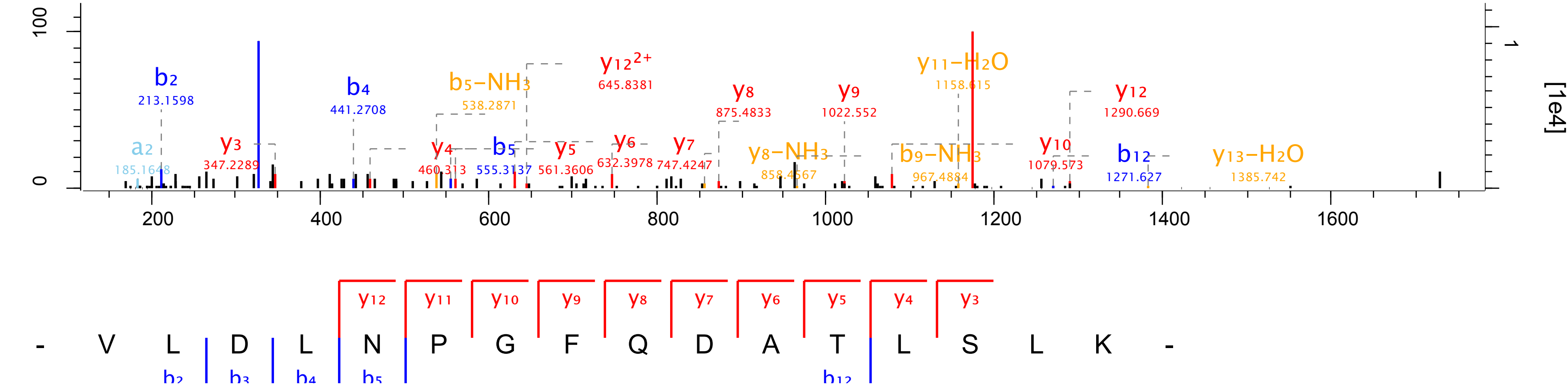

Raw file	Scan	Method	Score	m/z	Gene names
20140925_fract7_dyn_5ul_B7_01_442	11706	TOF; CID	66.15	690.34	DMTF1

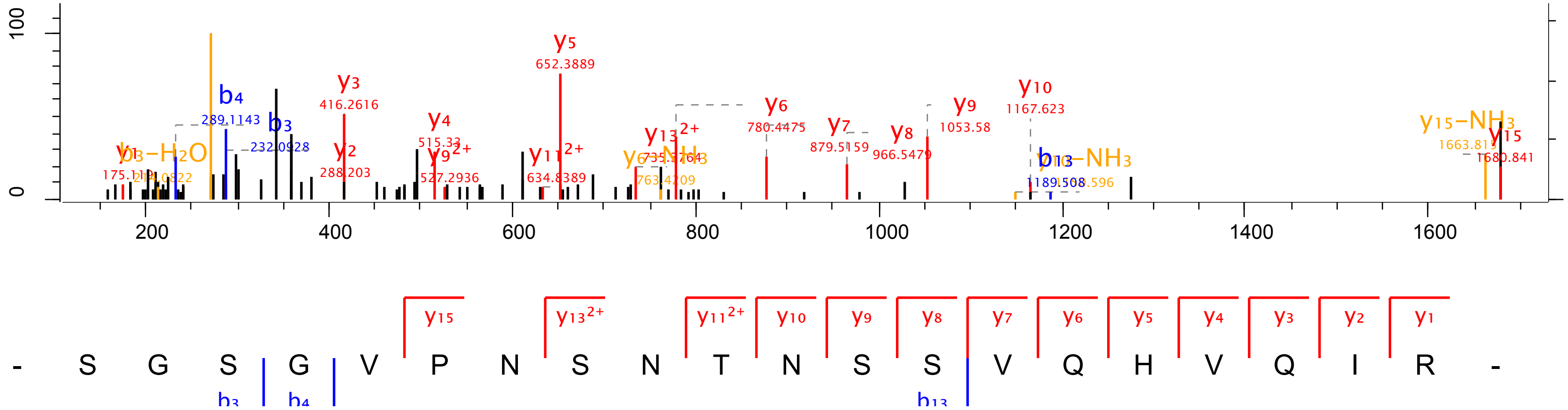

Raw file	Scan	Method	Score	m/z	Gene names
20140925_fract7_dyn_5ul_B7_01_442	17433	TOF; CID	49.93	452.9	UBXN2A

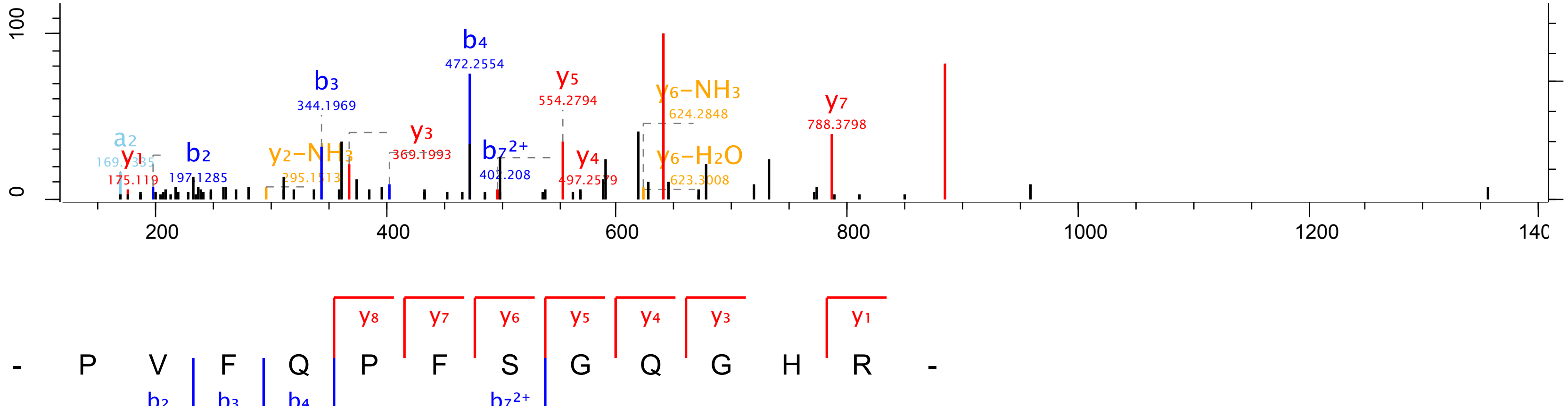

Raw file	Scan	Method	Score	m/z	Gene names
20140925_fract7_dyn_5ul_B7_01_442	19373	TOF; CID	42.71	678.33	ODF2L

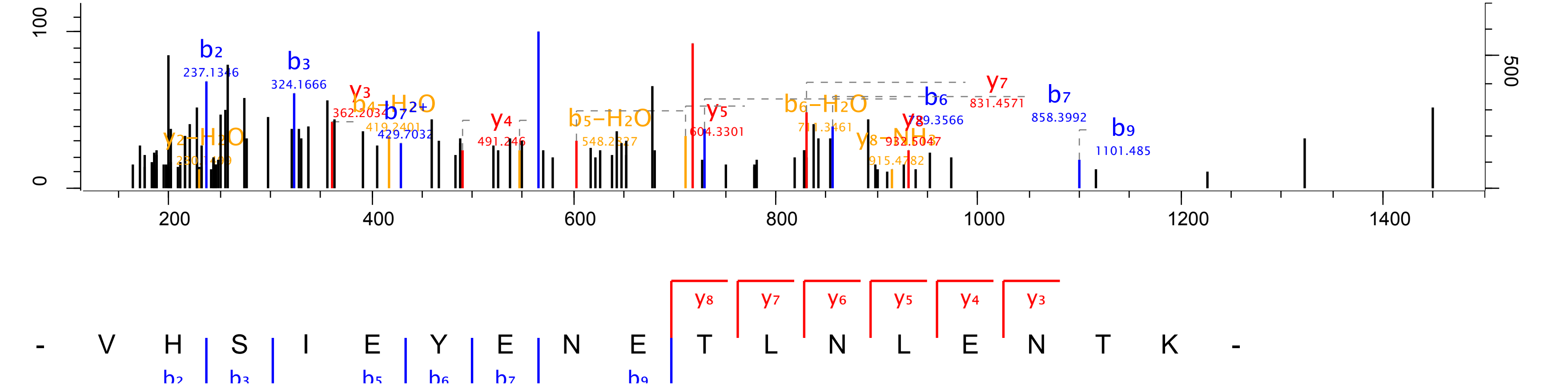

Raw file	Scan	Method	Score	m/z	Gene names
20140925_fract7_dyn_5ul_B7_01_442	23880	TOF; CID	105.53	617.97	HNRNPH1

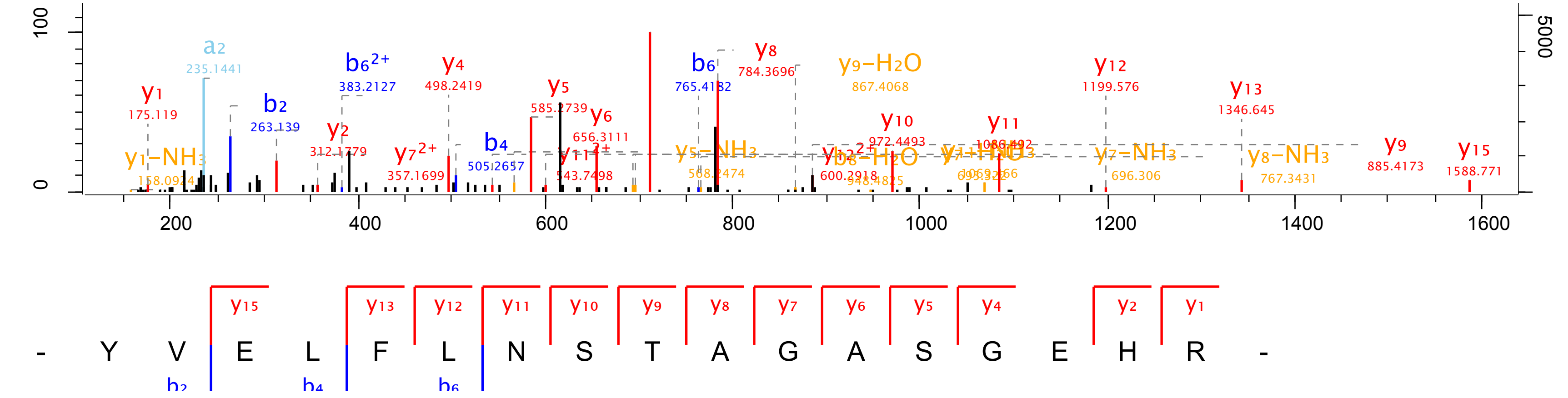

Raw file	Scan	Method	Score	m/z	Gene names
20140925_fract7_dyn_5ul_B7_01_442	24744	TOF; CID	105.98	603.31	TAF13

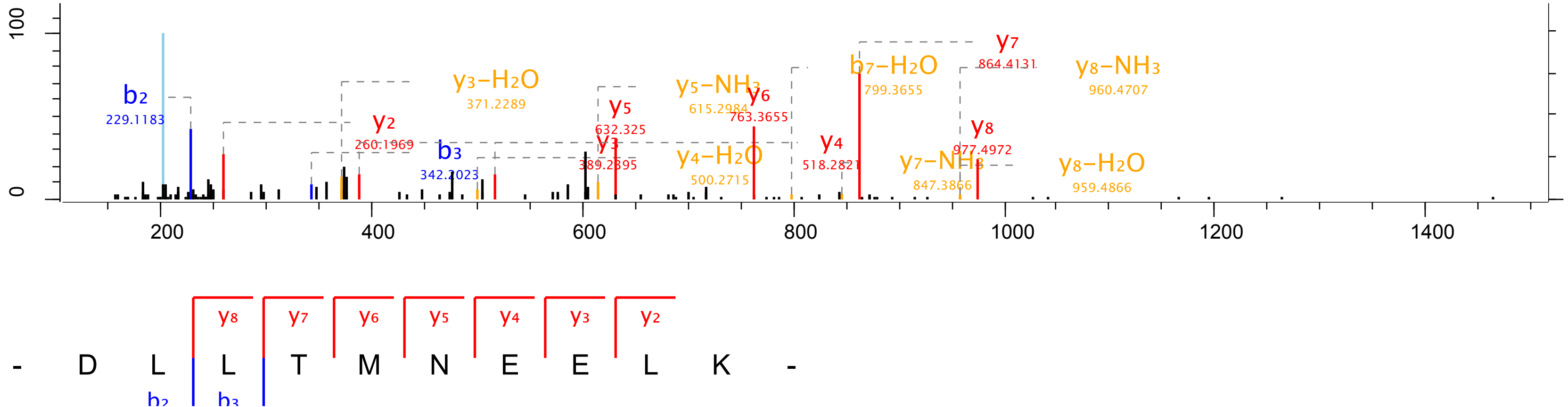

Raw file	Scan	Method	Score	m/z	Gene names
20140925_fract8_dyn_5ul_B8_01_443	5102	TOF; CID	79.47	313.85	ZNF212

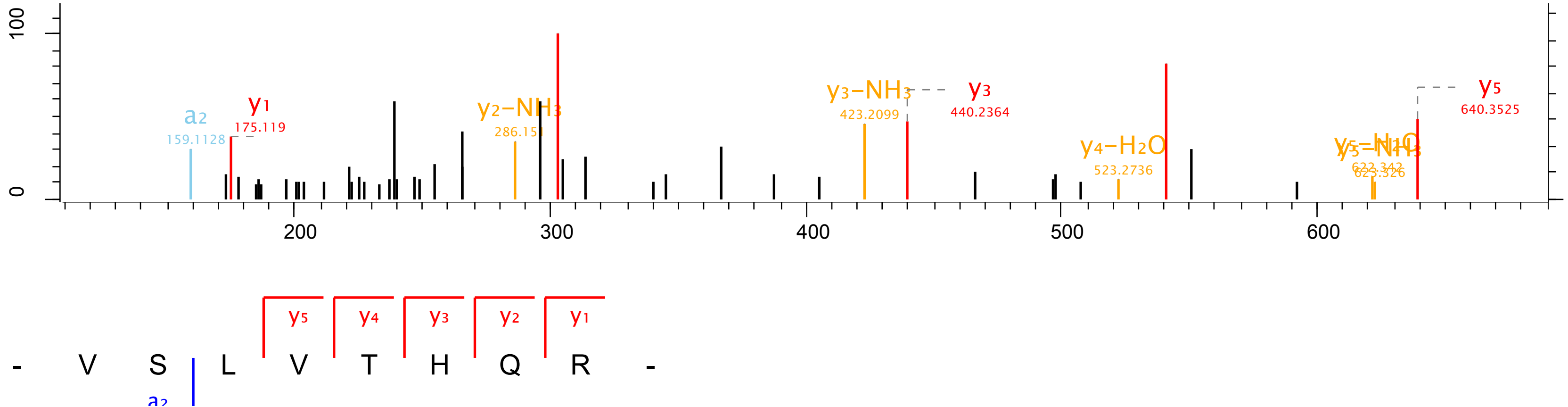

Raw file	Scan	Method	Score	m/z
20140925_fract8_dyn_5ul_B8_01_443	9549	TOF; CID	103.26	574.32

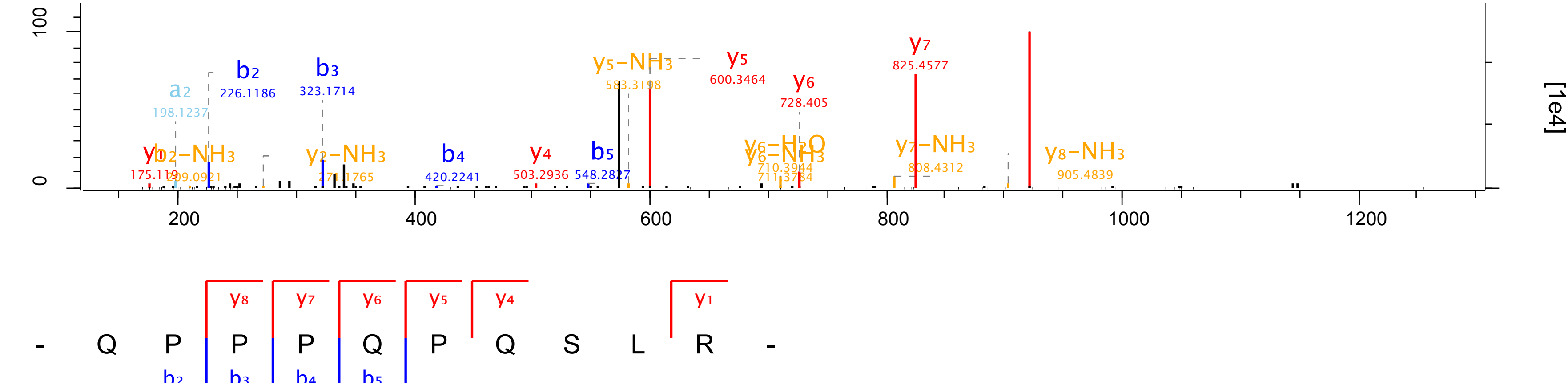

Raw file	Scan	Method	Score	m/z	Gene names
20140925_fract8_dyn_5ul_B8_01_443	12927	TOF; CID	79.12	339.16	AQP3

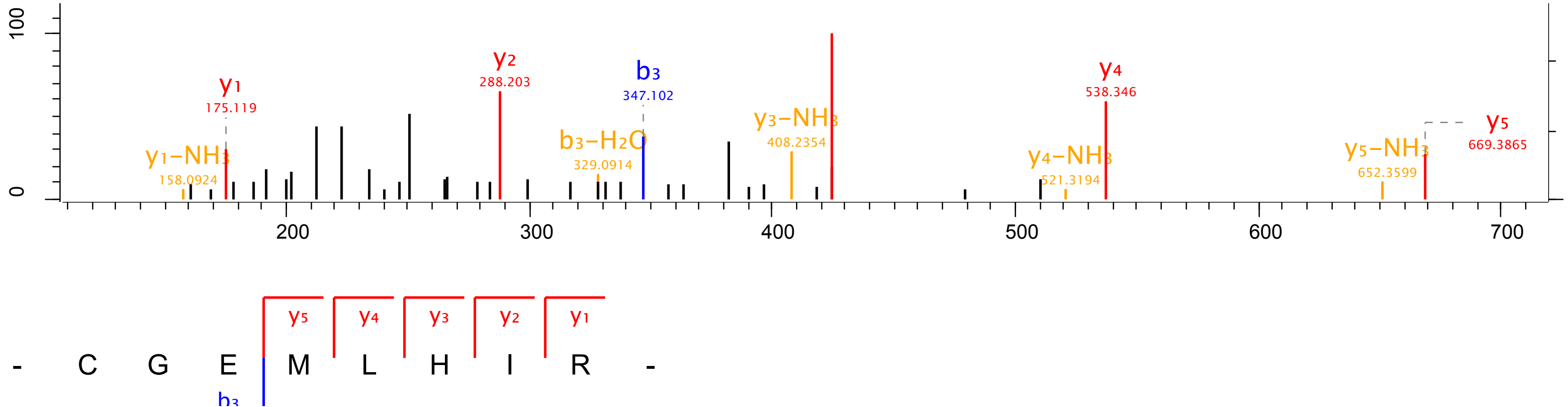

Raw file	Scan	Method	Score	m/z	Gene names
20140925_fract8_dyn_5ul_B8_01_443	13166	TOF; CID	97.73	578.79	TMEM254

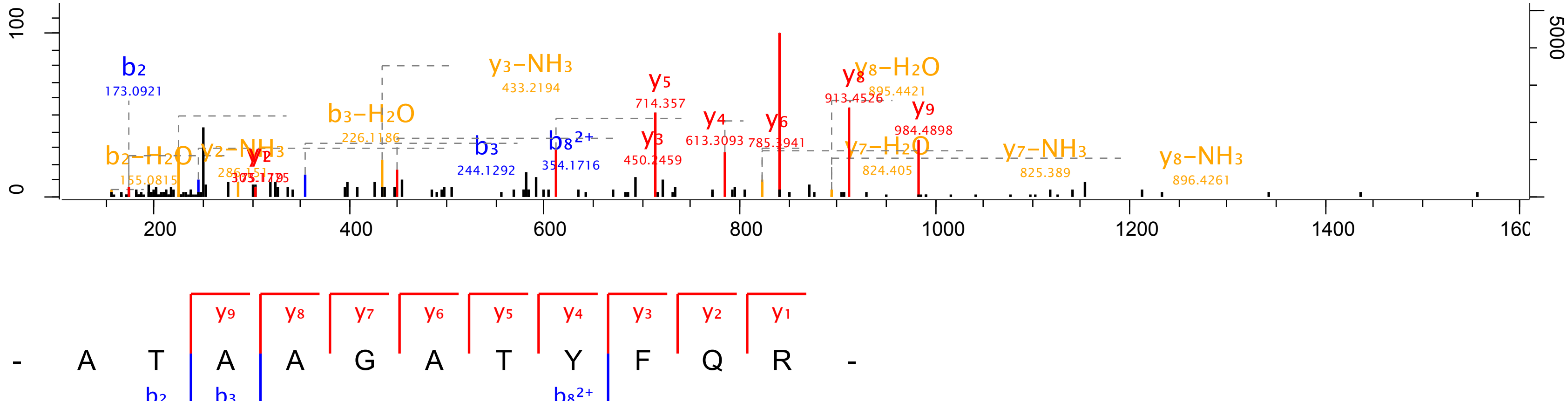

Raw file	Scan	Method	Score	m/z	Gene names
20140925_fract8_dyn_5ul_B8_01_443	14497	TOF; CID	107.82	874.92	TMEM181

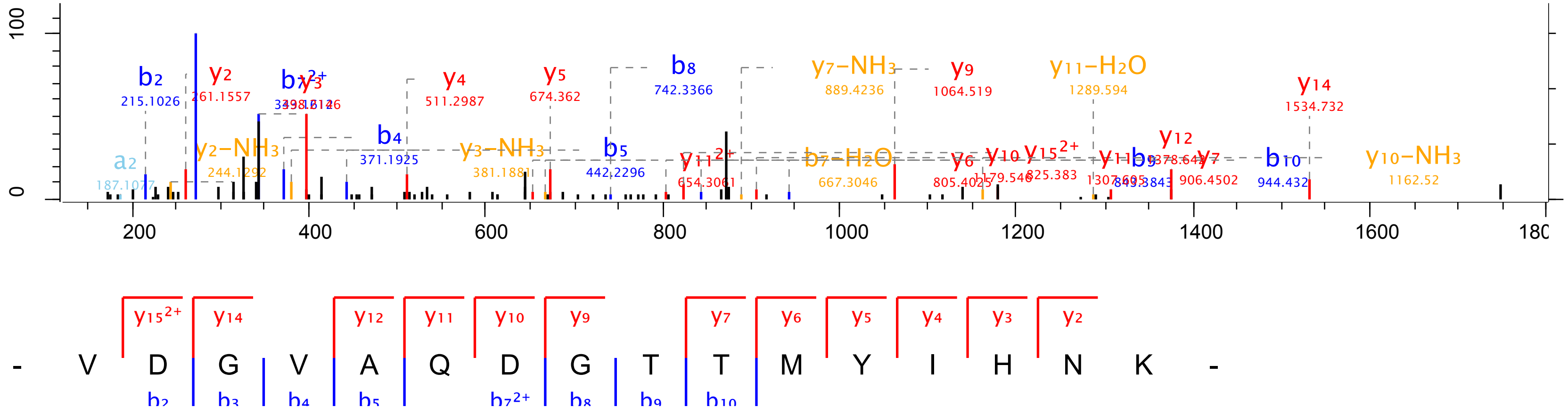

Raw file	Scan	Method	Score	m/z	Gene names
20140925_fract8_dyn_5ul_B8_01_443	23826	TOF; CID	45.42	798.38	ATXN7L2

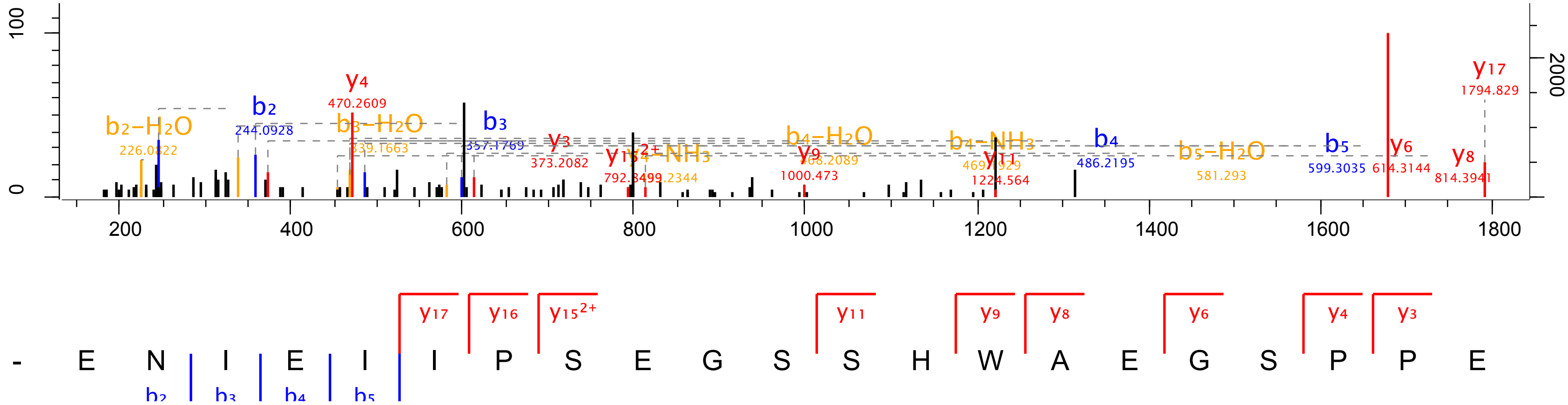

Raw file	Scan	Method	Score	m/z	Gene names
20140925_fract8_dyn_5ul_B8_01_443	24478	TOF; CID	75.82	658.85	PLAC1

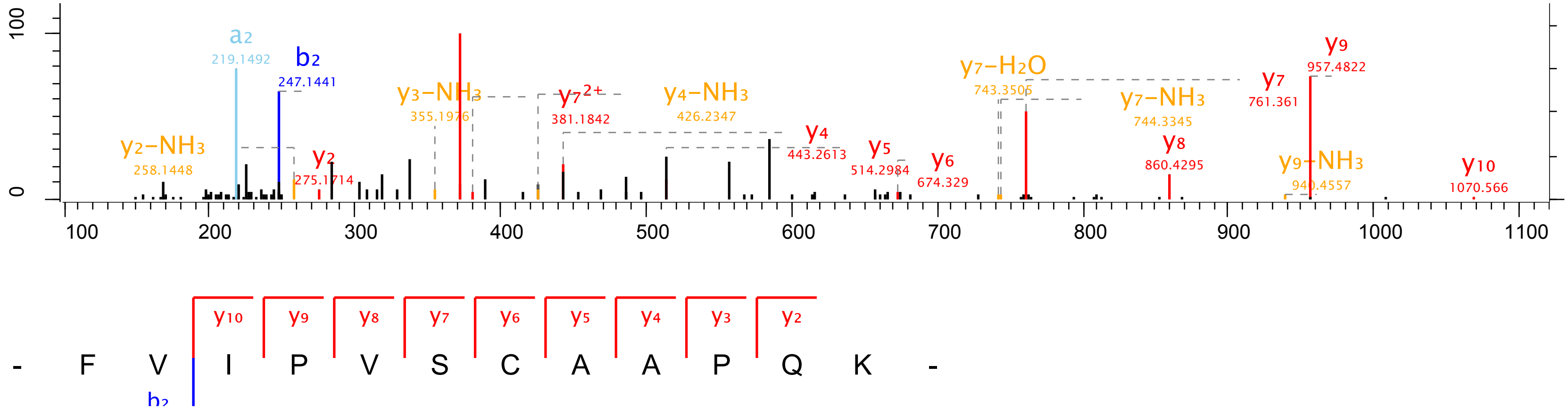

Raw file	Scan	Method	Score	m/z	Gene names
20140925_fract8_dyn_5ul_B8_01_443	30412	TOF; CID	64.27	869.43	MFSD8

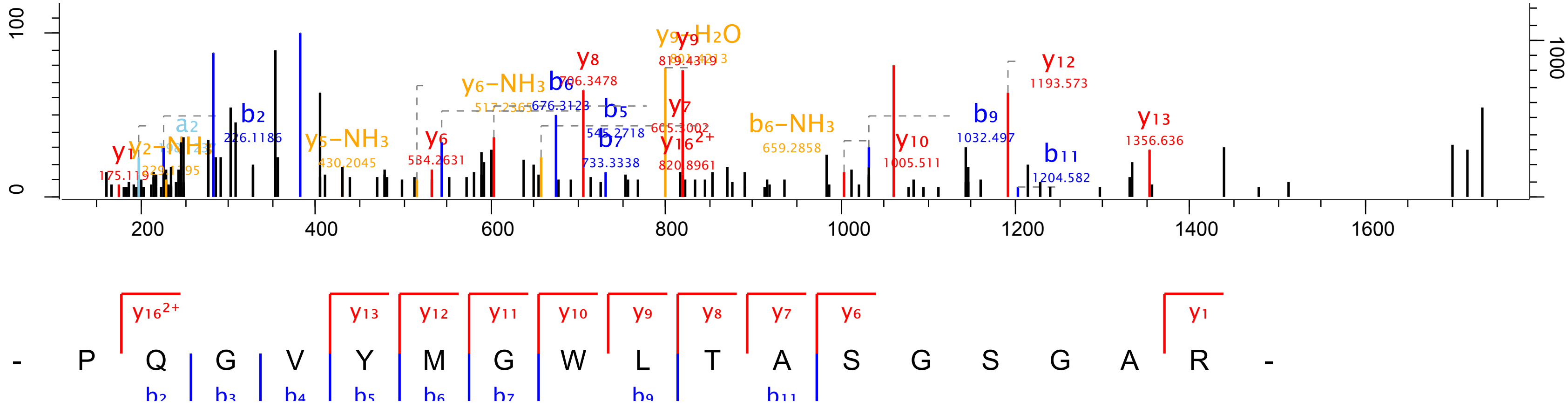

Raw file	Scan	Method	Score	m/z	Gene names
20140925_fract9_dyn_5ul_C1_01_444	3856	TOF; CID	59.23	453.58	SPOCK1

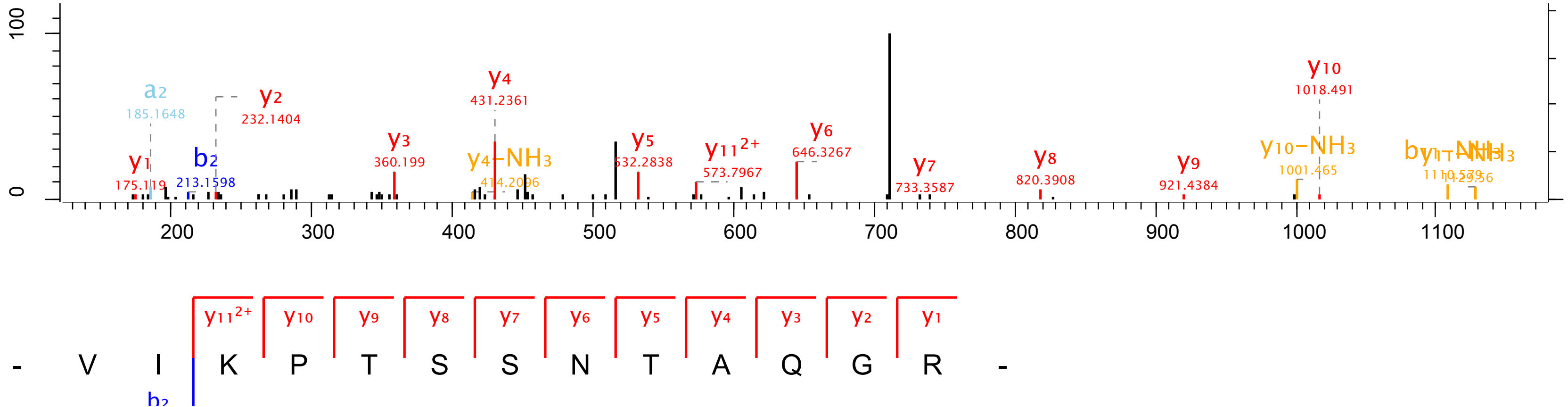

Raw file	Scan	Method	Score	m/z	Gene names
20140925_fract9_dyn_5ul_C1_01_444	25470	TOF; CID	65.37	749.36	MT-ND6

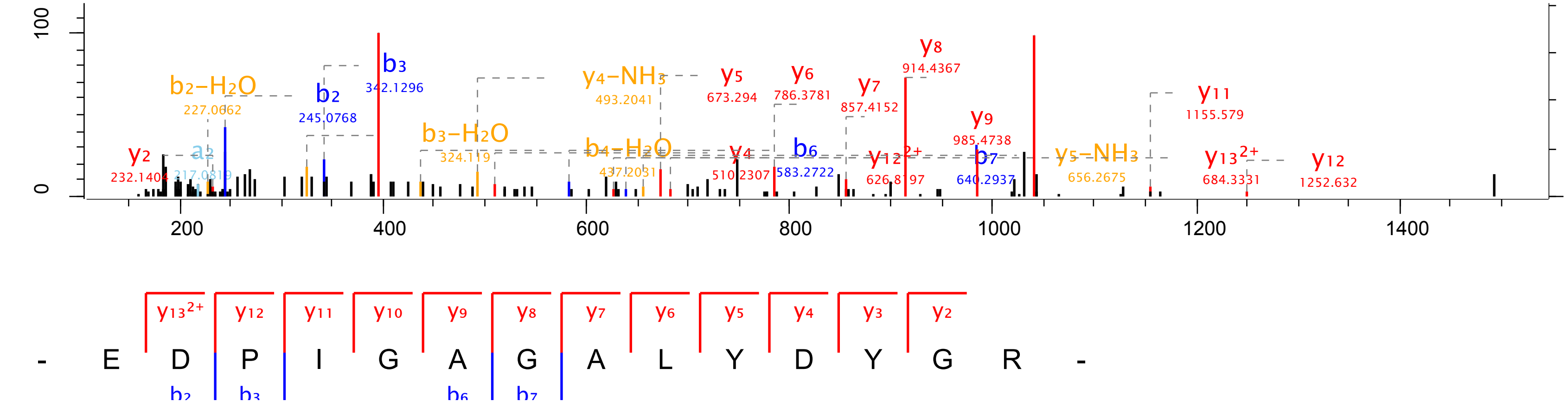

Raw file	Scan	Method	Score	m/z	Gene names
20140925_fract9_dyn_5ul_C1_01_444	29165	TOF; CID	87.36	1207.54	CMTM4

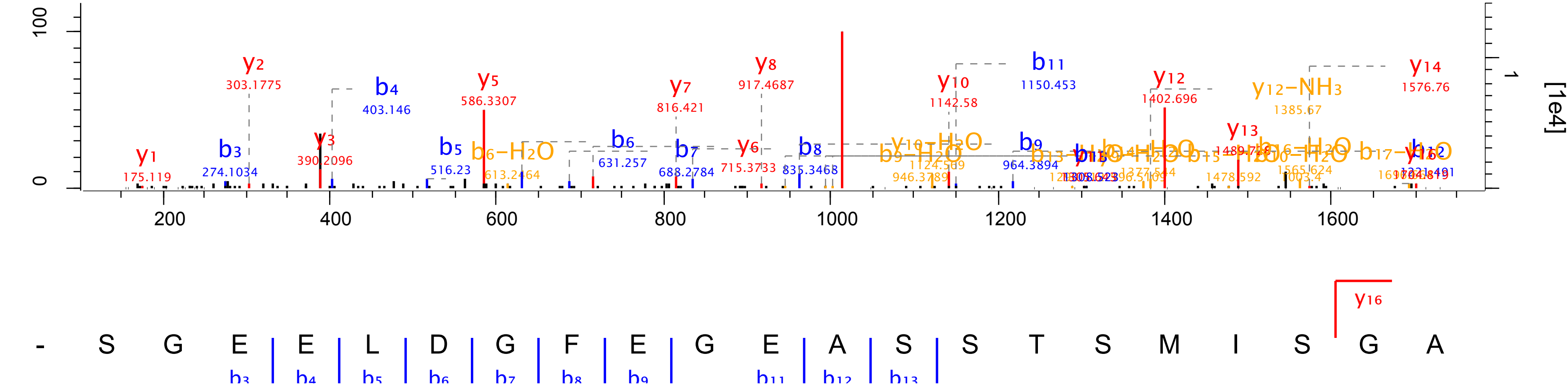

Raw file	Scan	Method	Score	m/z	Gene names
20140925_fract9_dyn_5ul_C1_01_444	37645	TOF; CID	104.88	1065.55	KDELR1

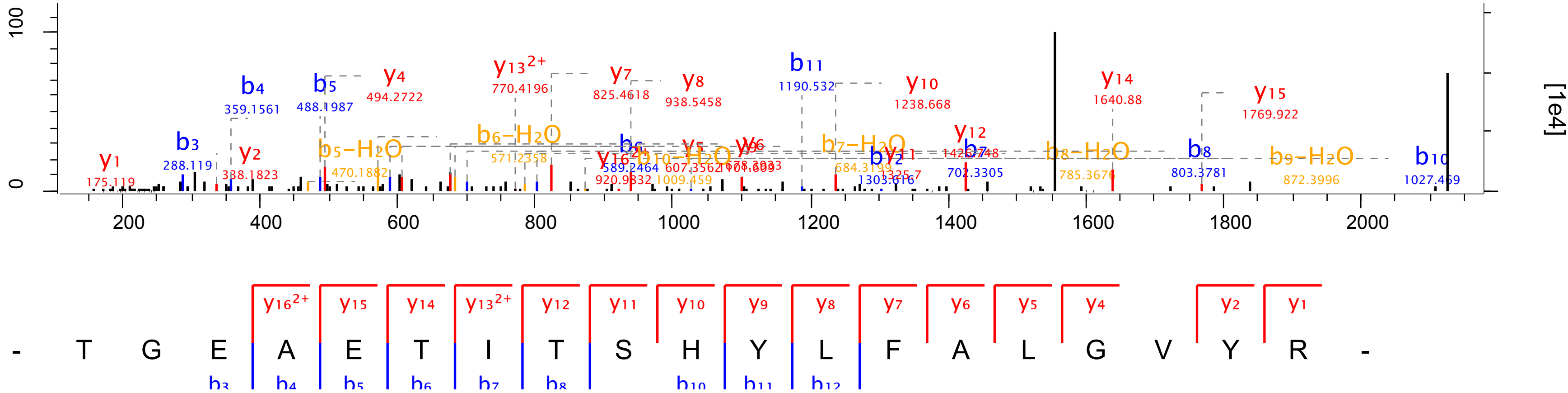

Raw file	Scan	Method	Score	m/z	Gene names
20140925_fract9_dyn_5ul_C1_01_444	37770	TOF; CID	79.84	1017.54	ZFAND2B

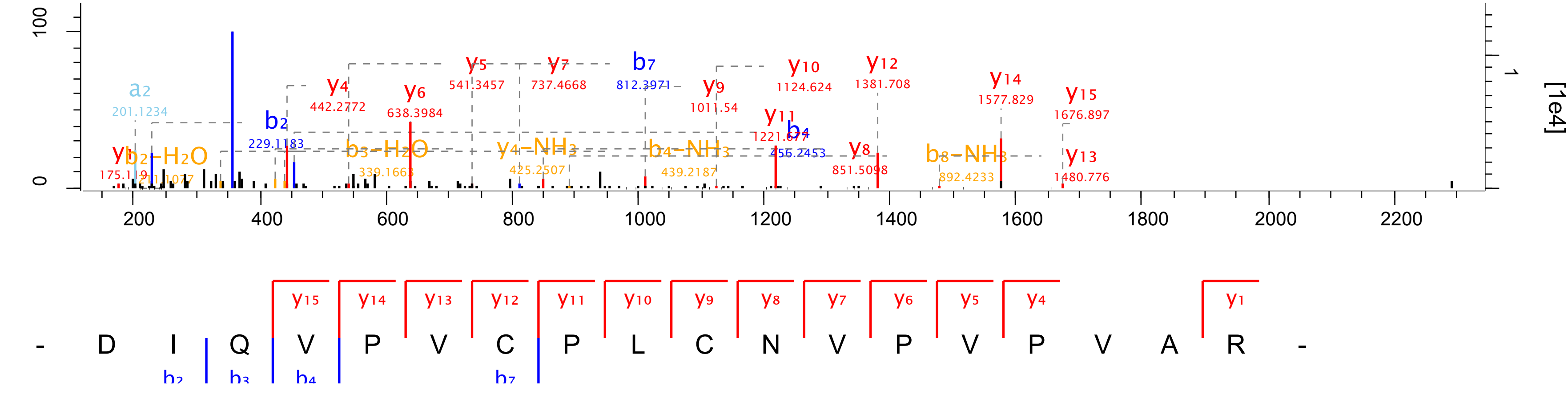

Raw file	Scan	Method	Score	m/z	Gene names
20140925_fract9_dyn_5ul_C1_01_444	38245	TOF; CID	49.83	969.45	CDC42SE1

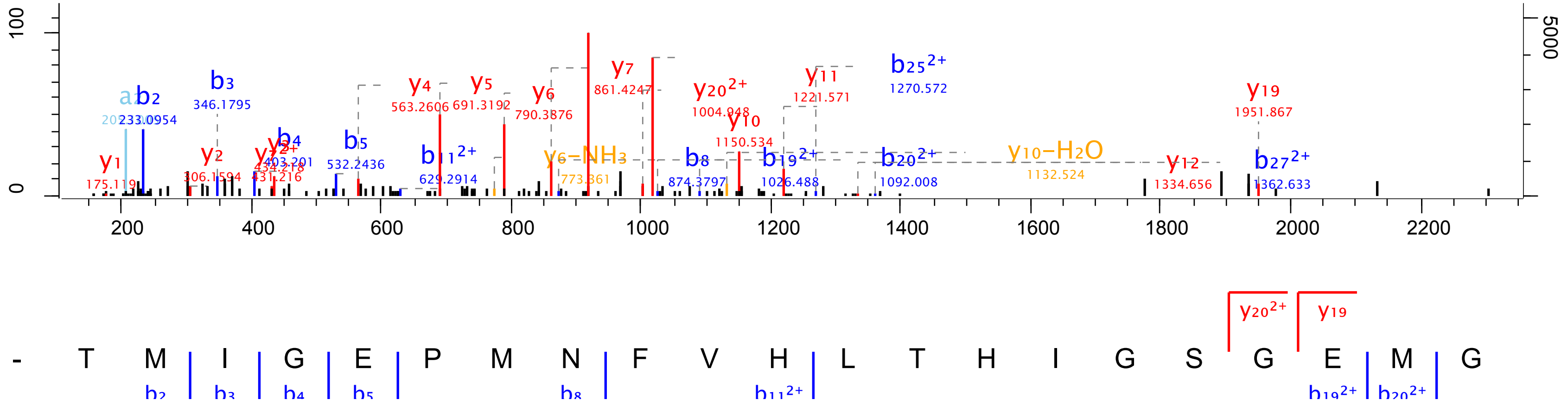

Raw file	Scan	Method	Score	m/z	Gene names
20140925_fract10_dyn_5ul_C2_01_445	6793	TOF; CID	196.27	446.23	NINJ1

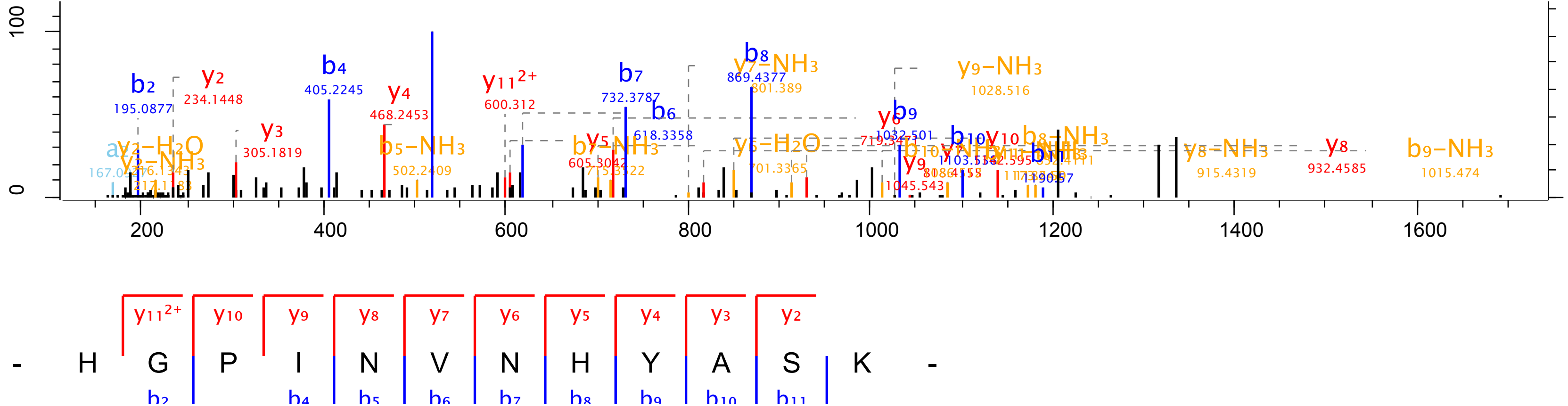

Raw file	Scan	Method	Score	m/z	Gene names
20140925_fract10_dyn_5ul_C2_01_445	12153	TOF; CID	93.6	529.32	RPGRIP1

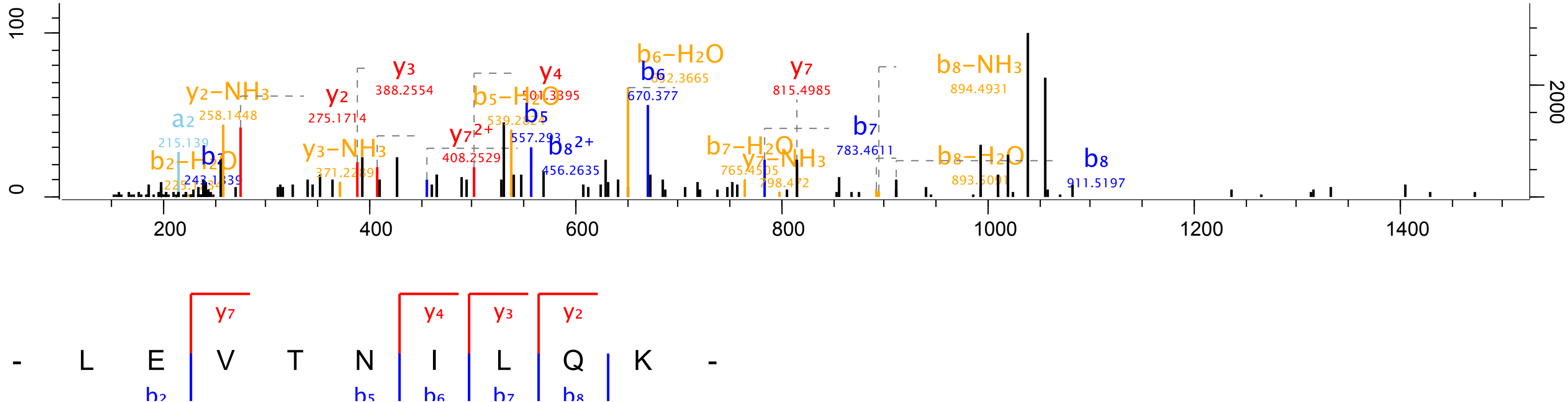

Raw file	Scan	Method	Score	m/z	Gene names
20140925_fract10_dyn_5ul_C2_01_445	33269	TOF; CID	147.62	629.83	GTF2A1;HIST1H4F

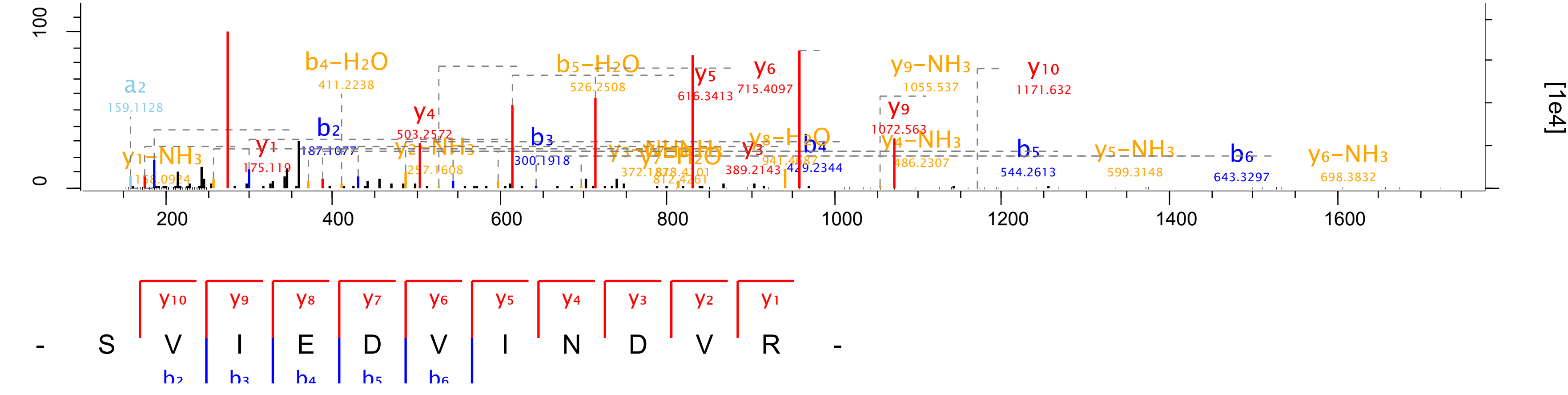

Raw file	Scan	Method	Score	m/z	Gene names
20140925_fract10_dyn_5ul_C2_01_445	40949	TOF; CID	106.28	1099.53	CNIH1

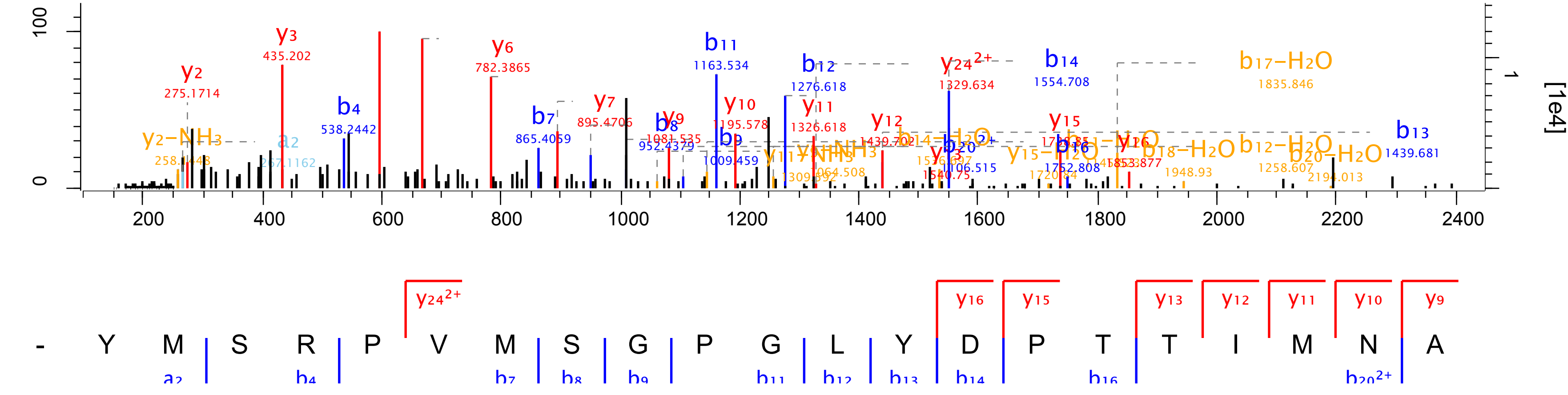

Raw file	Scan	Method	Score	m/z	Gene names
20140925_fract11_dyn_5ul_C3_01_446	16053	TOF; CID	168.85	617.83	SUMO2;SUMO3;SUMO4

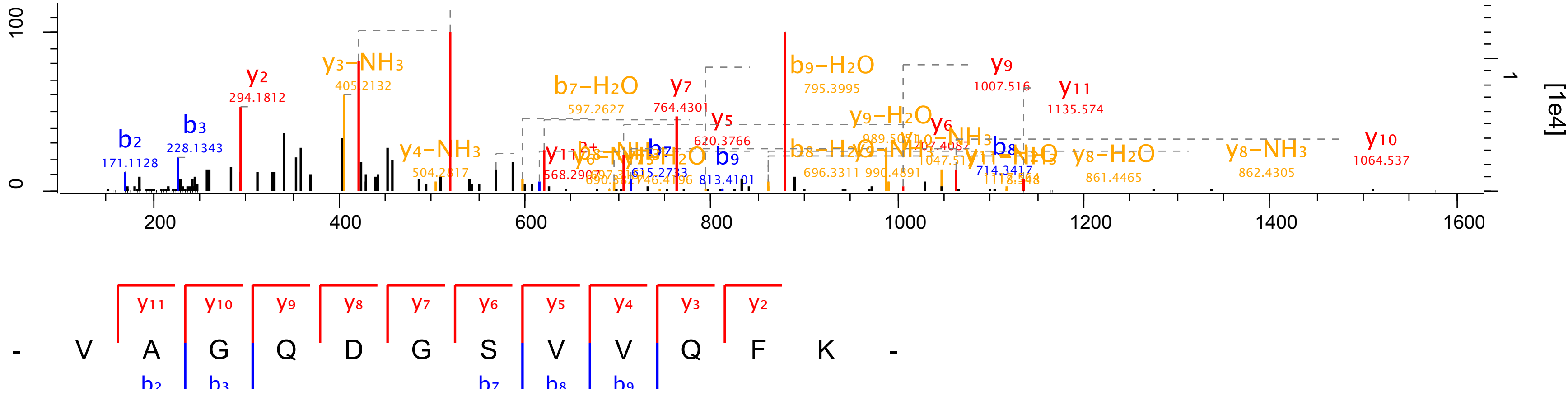

Raw file	Scan	Method	Score	m/z	Gene names
20140925_fract11_dyn_5ul_C3_01_446	28160	TOF; CID	90.71	597.99	DGCR2

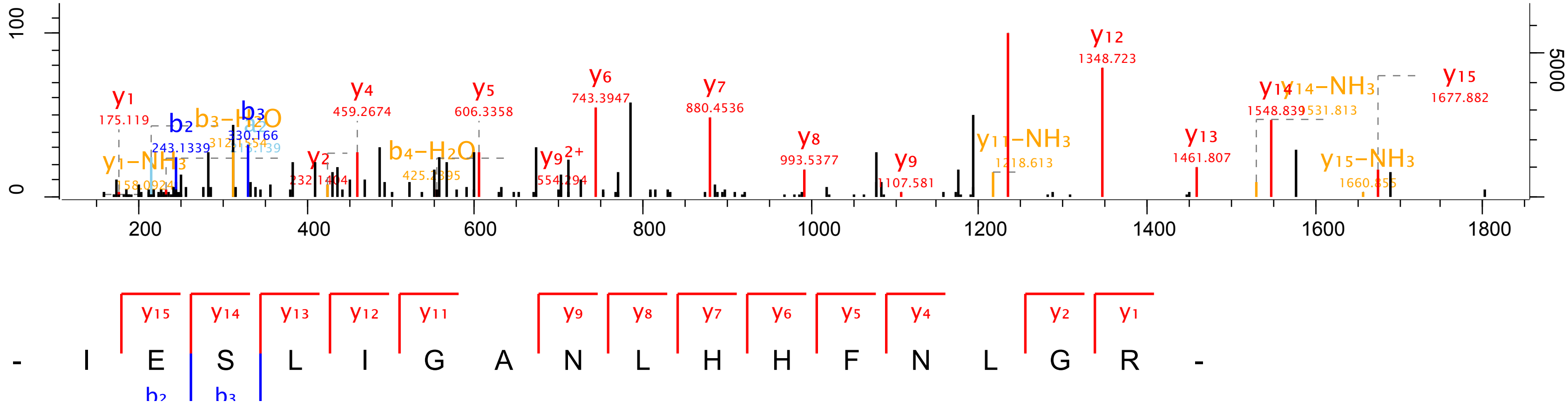

Raw file	Scan	Method	Score	m/z	Gene names
20140925_fract11_dyn_5ul_C3_01_446	29914	TOF; CID	114.97	623.38	MT-ATP6

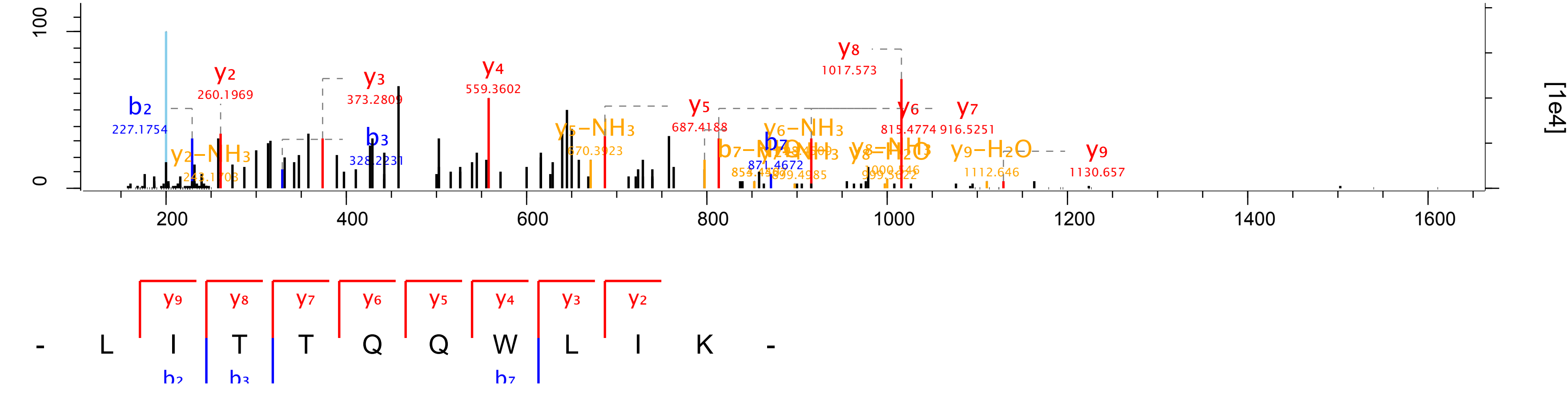

Raw file	Scan	Method	Score	m/z	Gene names
20140925_fract12_dyn_5ul_C4_01_447	10892	TOF; CID	67.38	543.8	SLC26A11

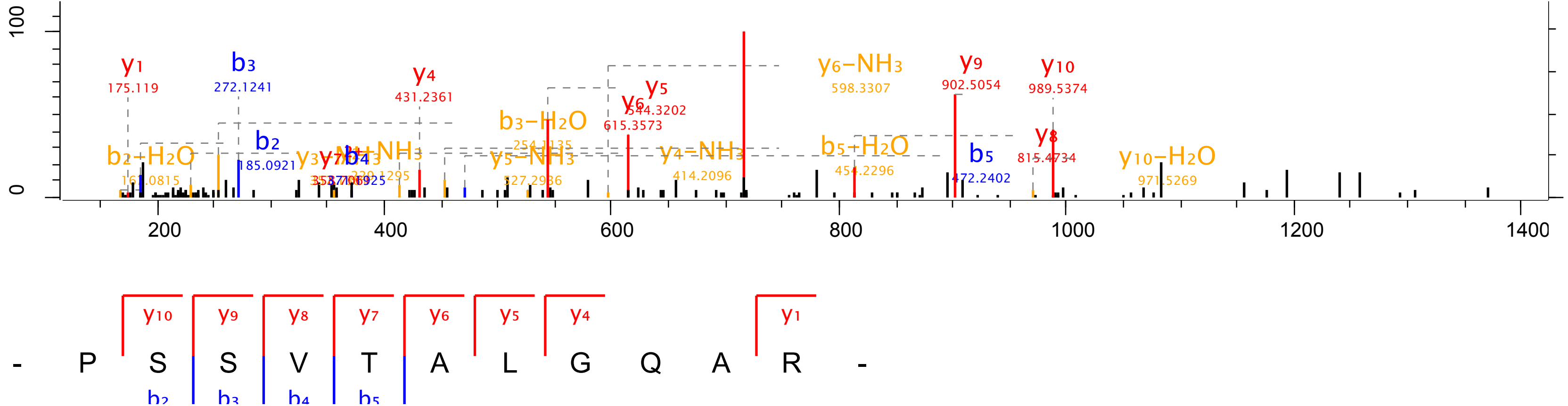

Raw file	Scan	Method	Score	m/z	Gene names
20140925_fract13_dyn_5ul_C5_01_448	8066	TOF; CID	107.65	463.92	ARNTL2

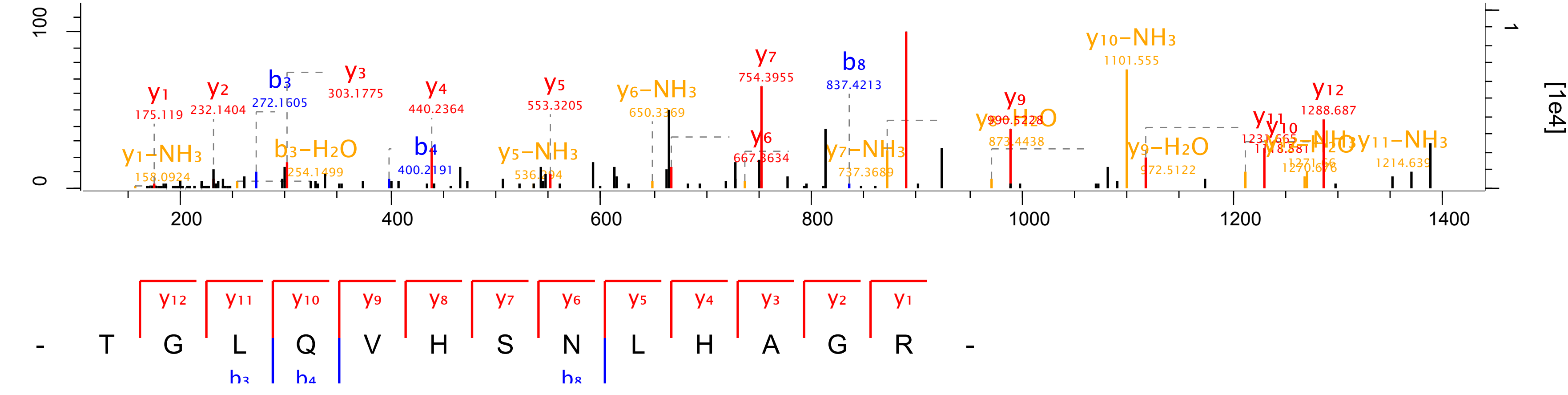

Raw file	Scan	Method	Score	m/z	Gene names
20140925_fract13_dyn_5ul_C5_01_448	22877	TOF; CID	73.39	767.89	PREP

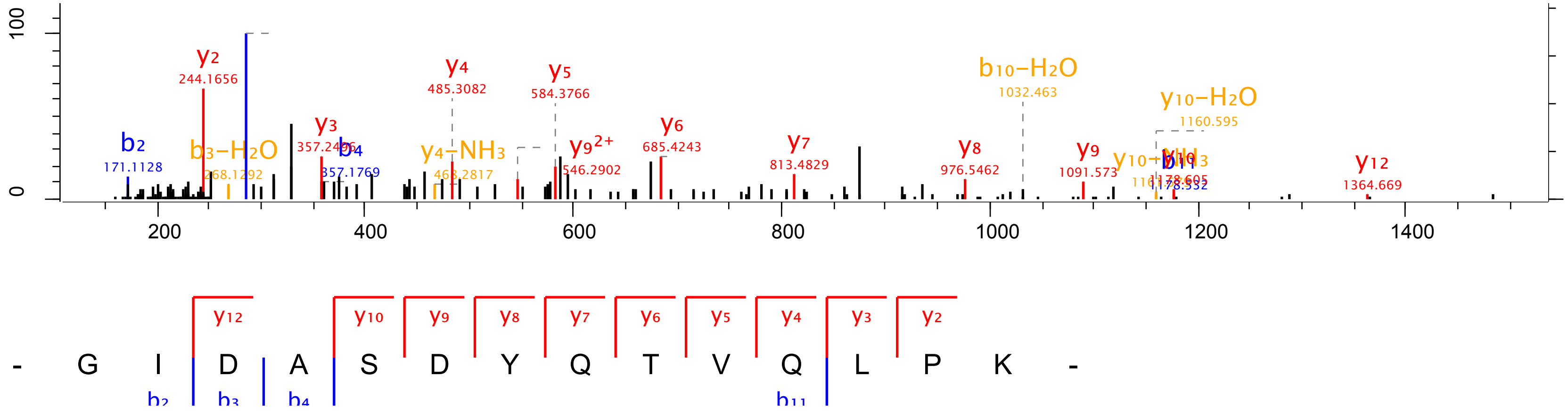

Raw file	Scan	Method	Score	m/z	Gene names
20140925_fract13_dyn_5ul_C5_01_448	23106	TOF; CID	56.37	773.85	TM2D2

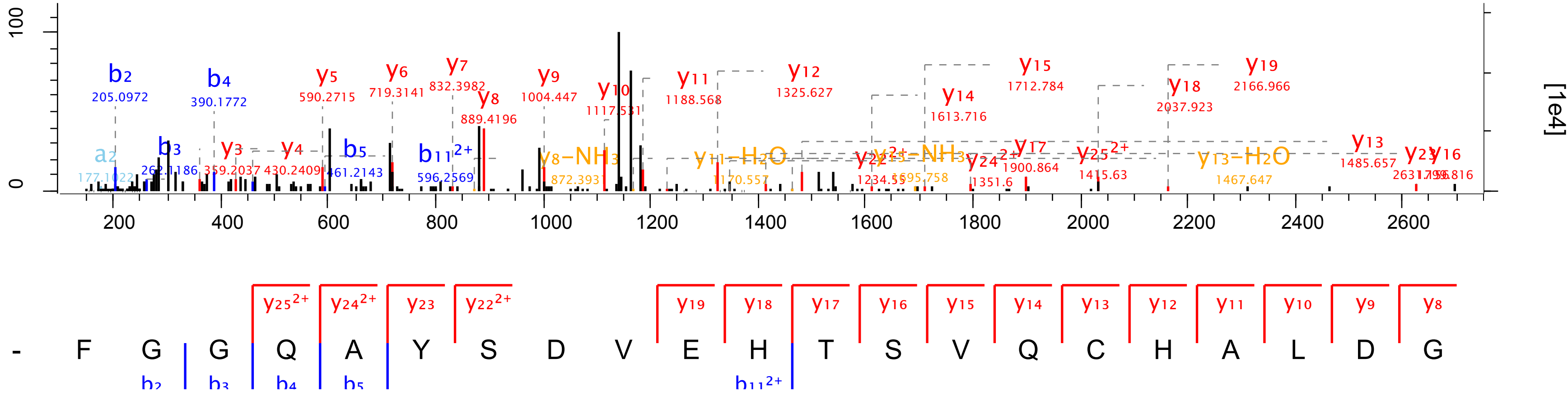

Raw file	Scan	Method	Score	m/z	Gene names
20140925_fract13_dyn_5ul_C5_01_448	36368	TOF; CID	68.75	879.92	TPM4

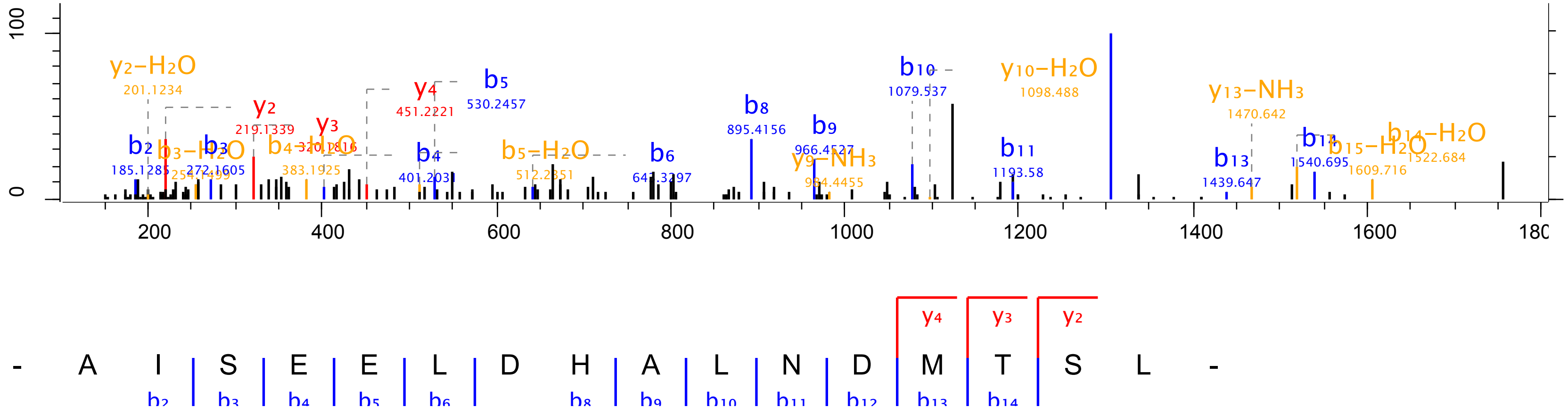

Raw file	Scan	Method	Score	m/z	Gene names
20140925_fract14_dyn_5ul_C6_01_449	8085	TOF; CID	68	521.27	GDF11

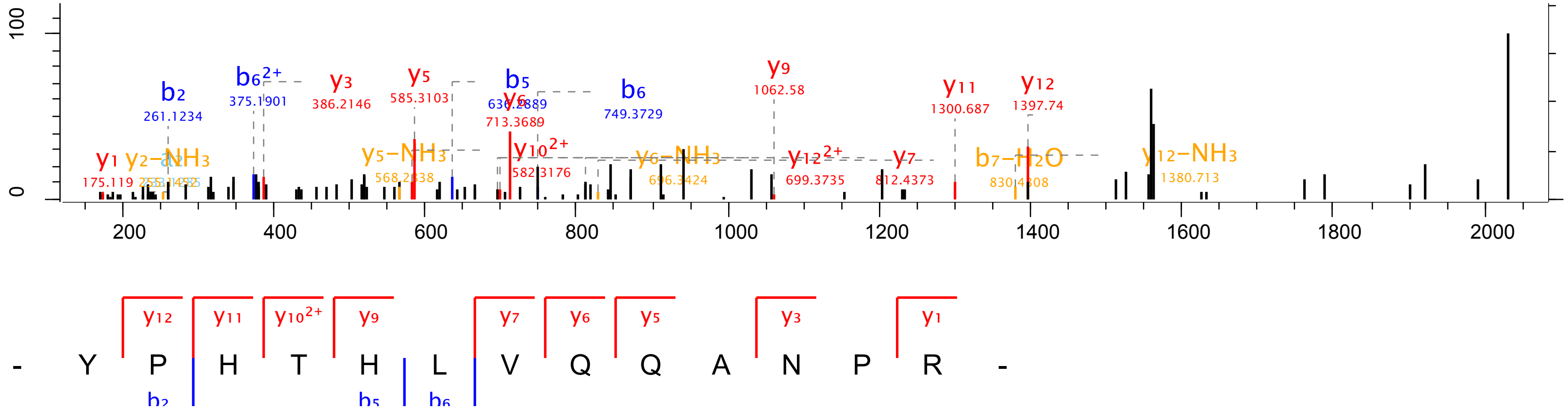

Raw file	Scan	Method	Score	m/z	Gene names
20140925_fract14_dyn_5ul_C6_01_449	38602	TOF; CID	128.74	620.02	GIPC2

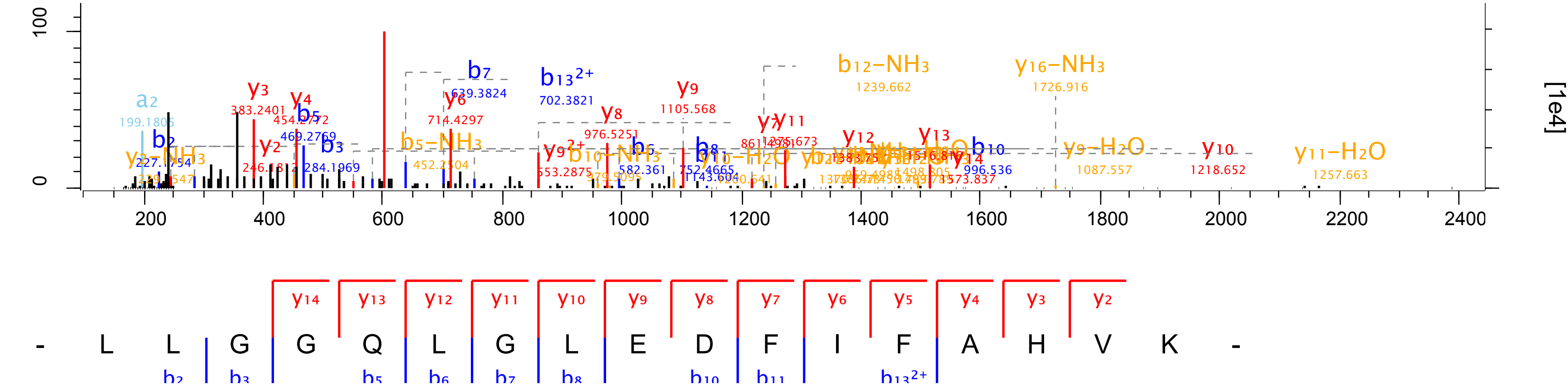

Raw file	Scan	Method	Score	m/z	Gene names
20140925_fract15_dyn_5ul_C7_01_450	27155	TOF; CID	96.14	629.85	C10orf90

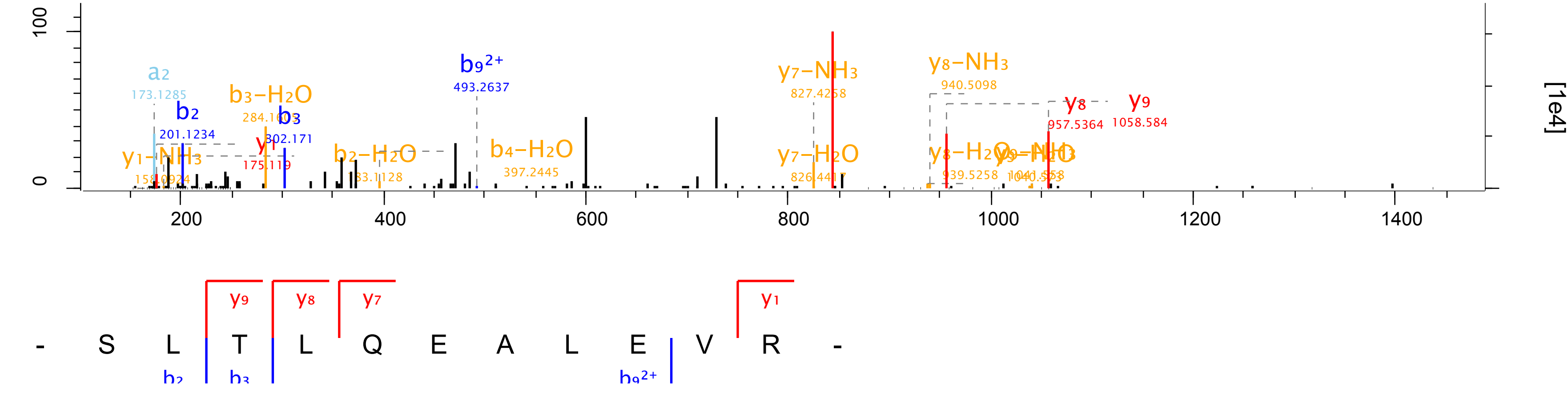

Raw file	Scan	Method	Score	m/z	Gene names
20140925_fract16_dyn_5ul_C8_01_451	18592	TOF; CID	81.57	1043.03	ZFP36

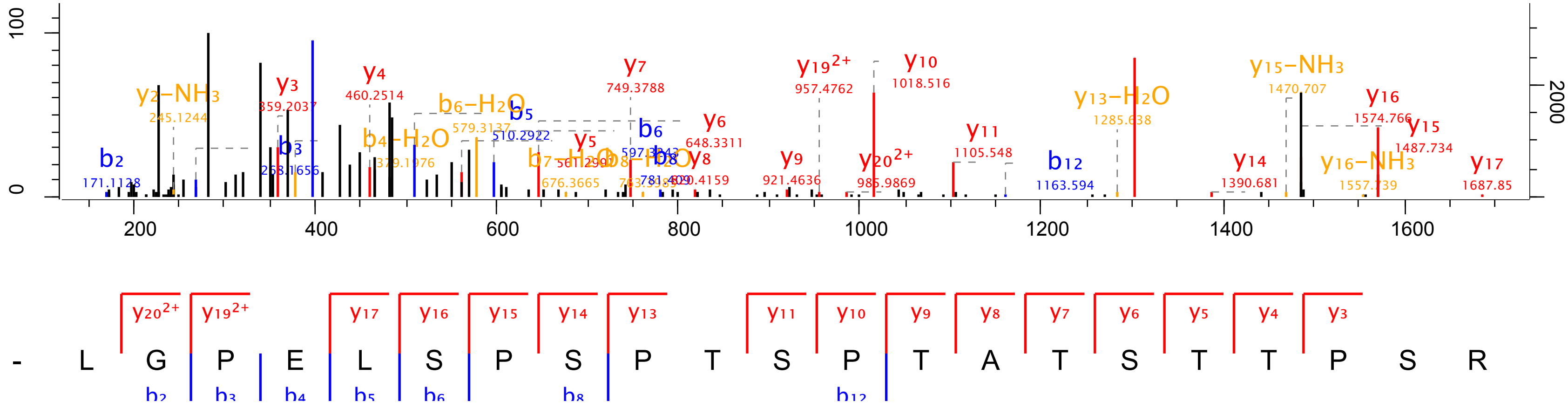

Raw file	Scan	Method	Score	m/z	Gene names
20140925_fract16_dyn_5ul_C8_01_451	19513	TOF; CID	93.37	672.33	TMEM243

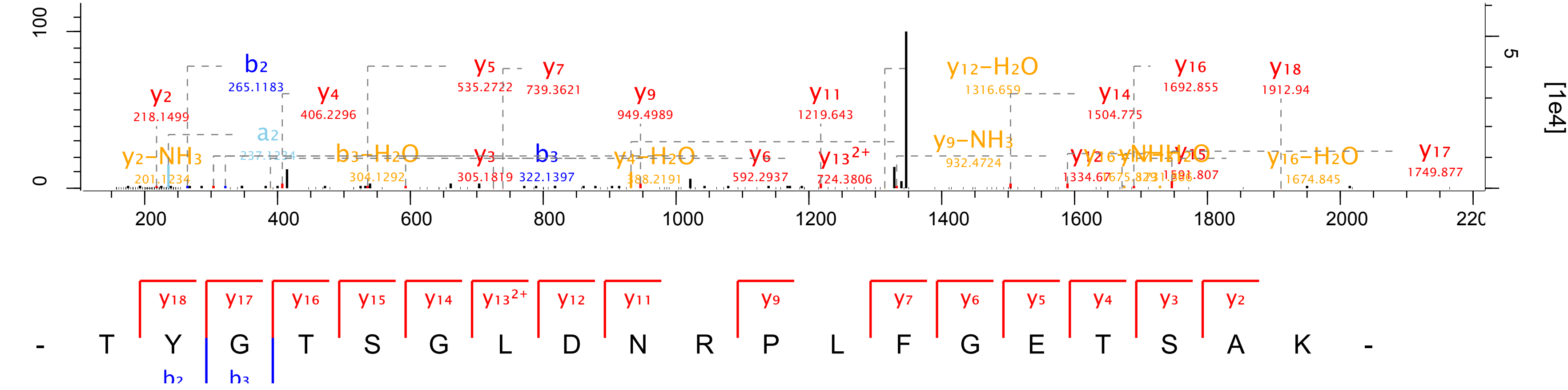

Raw file	Scan	Method	Score	m/z	Gene names
20140925_fract16_dyn_5ul_C8_01_451	19533	TOF; CID	137.05	524.28	COX7C

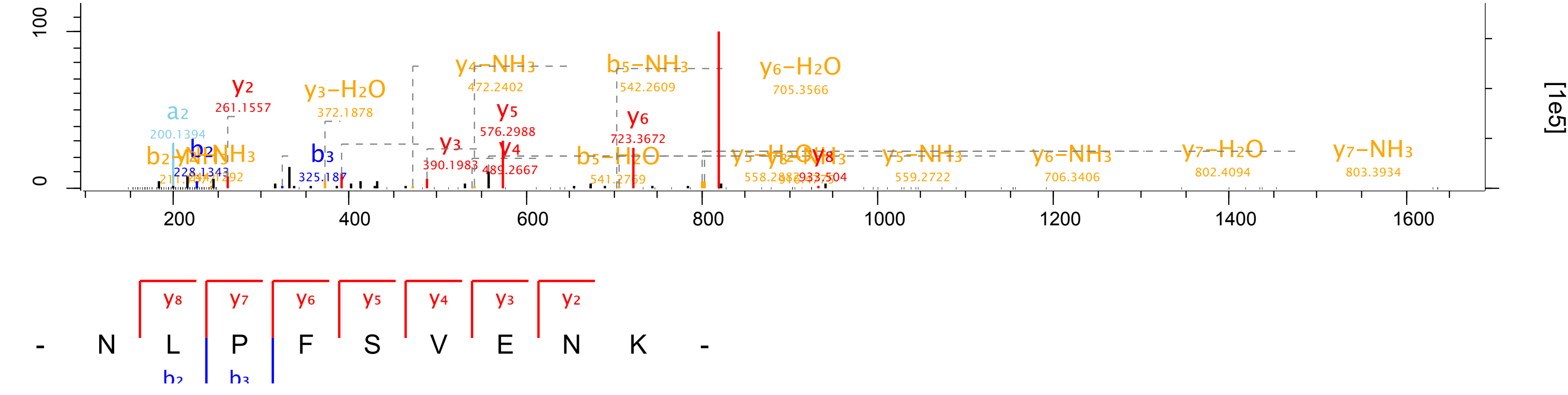

Raw file	Scan	Method	Score	m/z	Gene names
20140925_fract17_dyn_5ul_H1_01_452	23260	TOF; CID	99.45	813.07	LHFPL2

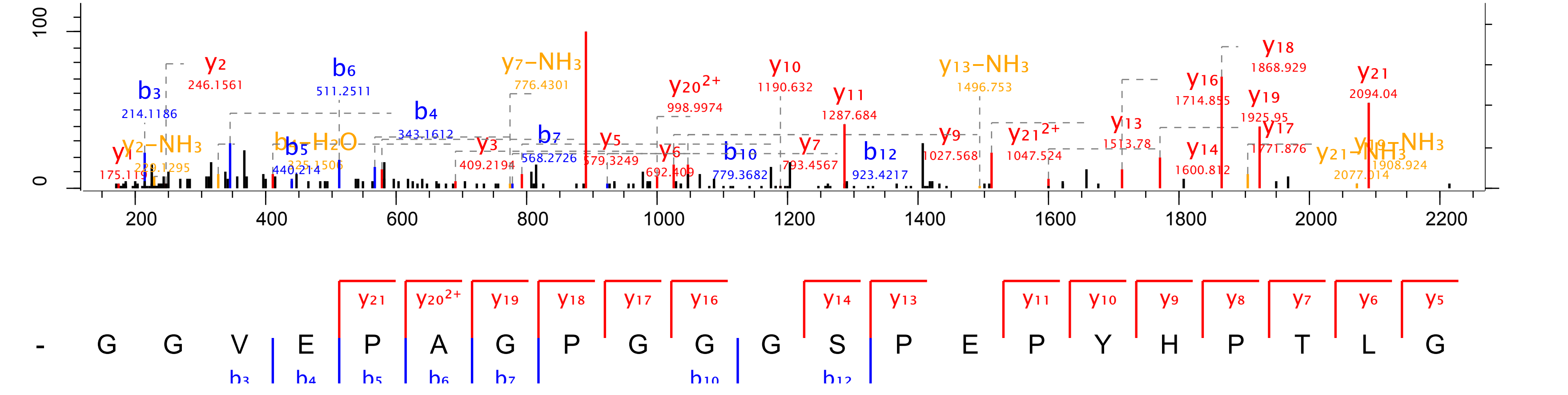

Raw file	Scan	Method	Score	m/z	Gene names
20140925_fract18_dyn_5ul_H2_01_453	5019	TOF; CID	72.29	436.25	ZNF707

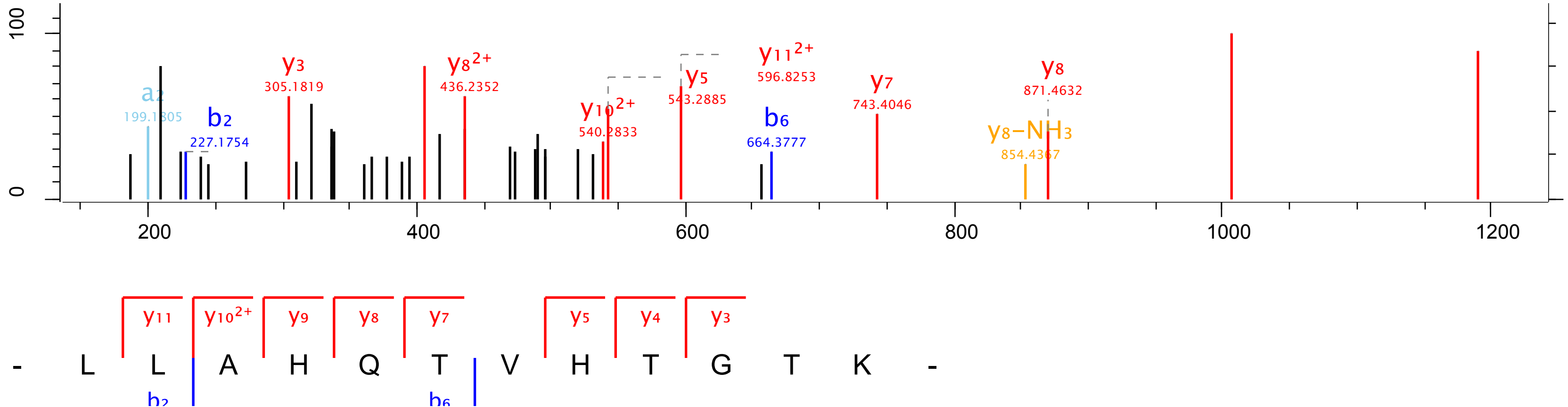

Raw file	Scan	Method	Score	m/z	Gene names
20140925_fract18_dyn_5ul_H2_01_453	8990	TOF; CID	123.67	457.24	ZFP62

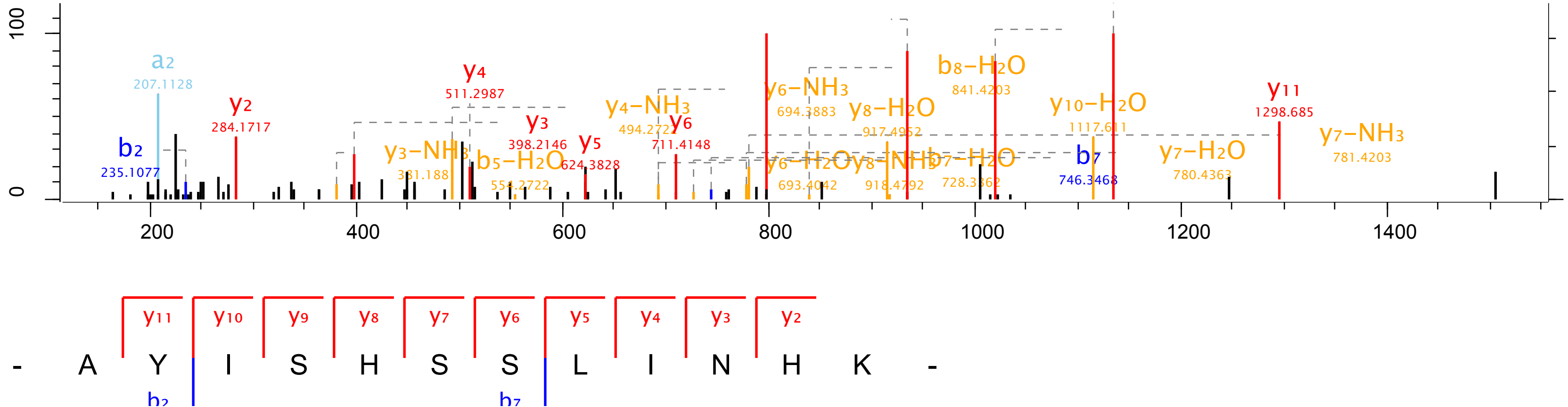

Raw file	Scan	Method	Score	m/z	Gene names
20140925_fract18_dyn_5ul_H2_01_453	11106	TOF; CID	63.32	362.2	RTN4R

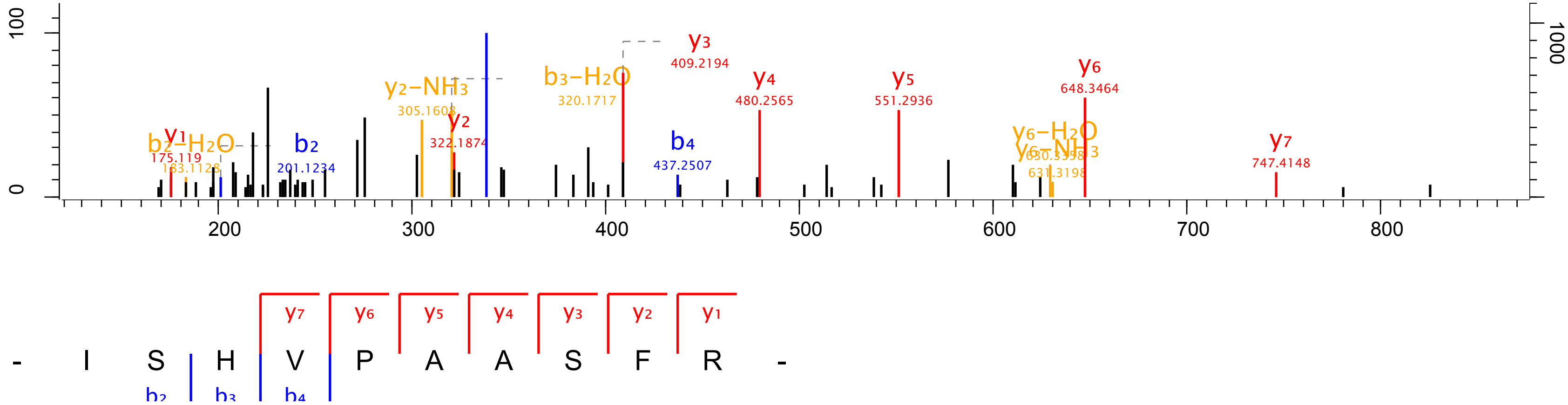

Raw file	Scan	Method	Score	m/z	Gene names
20140925_fract18_dyn_5ul_H2_01_453	15973	TOF; CID	81.3	365.55	ATAT1

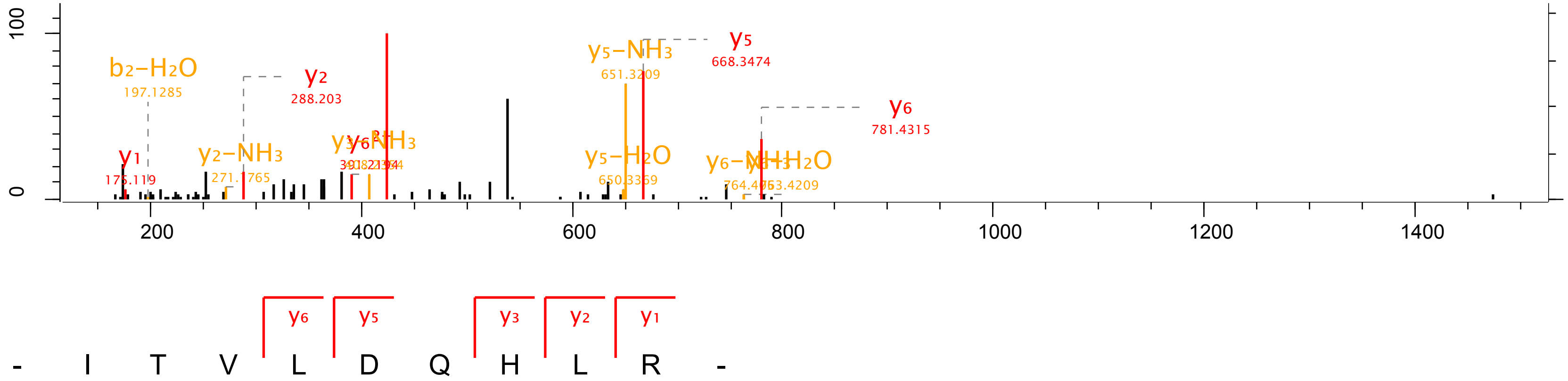

Raw file	Scan	Method	Score	m/z	Gene names
20140925_fract18_dyn_5ul_H2_01_453	26144	TOF; CID	106.28	941.45	FAM168B

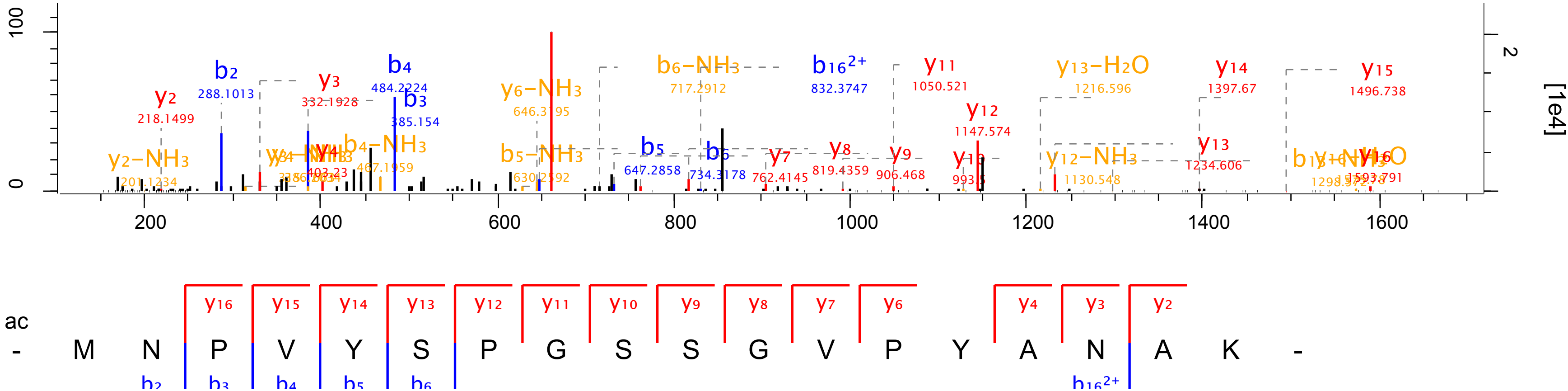

Raw file	Scan	Method	Score	m/z
20140925_fract18_dyn_5ul_H2_01_453	26801	TOF; CID	55.44	809.4

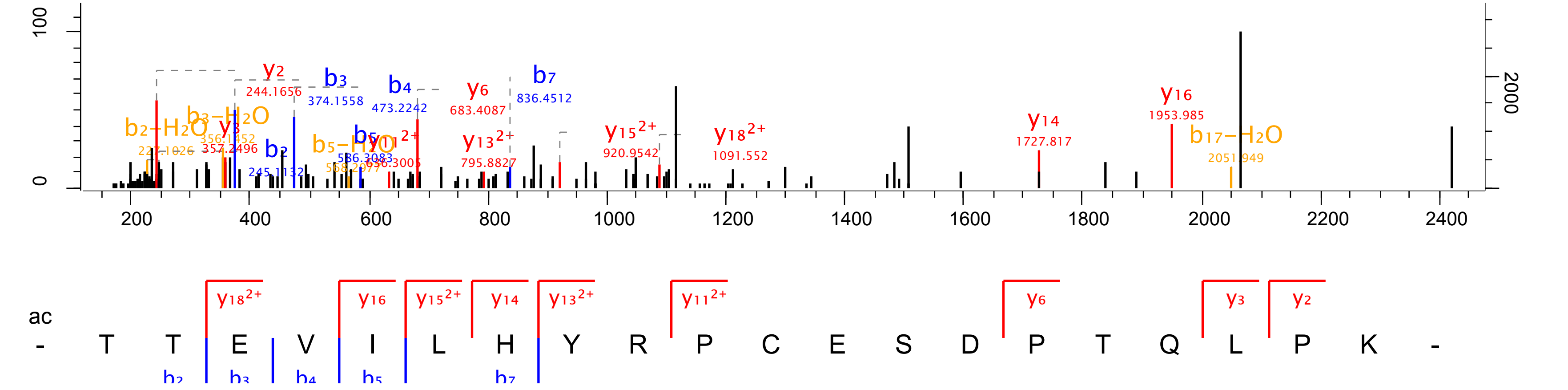

Raw file	Scan	Method	Score	m/z	Gene names
20140925_fract18_dyn_5ul_H2_01_453	33602	TOF; CID	77.75	808.43	NAA60

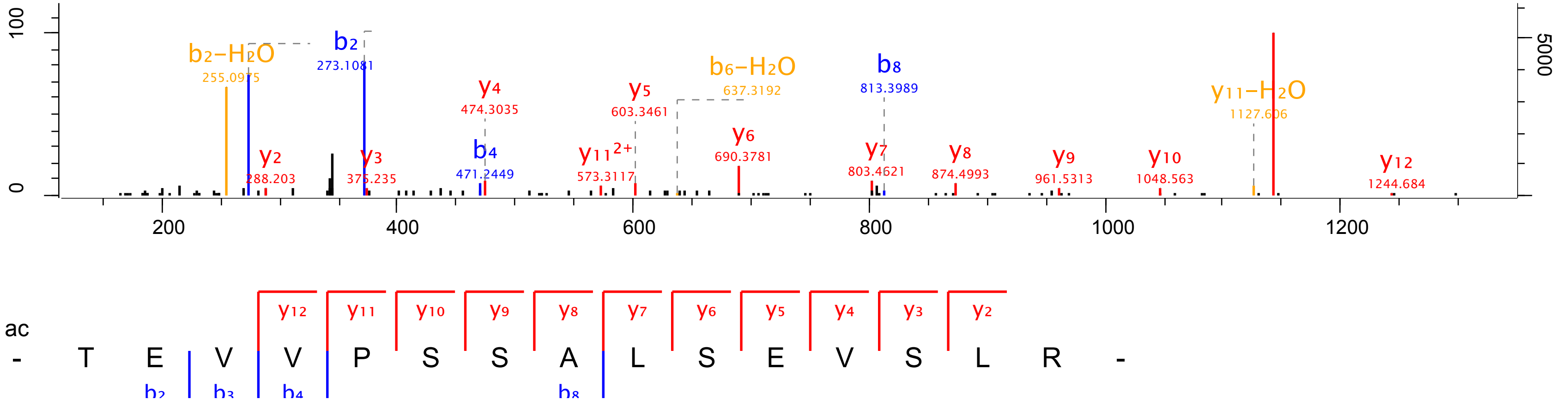

Raw file	Scan	Method	Score	m/z	Gene names
20140925_fract19_dyn_5ul_H3_01_454	6187	TOF; CID	79.12	348.87	ZNF12

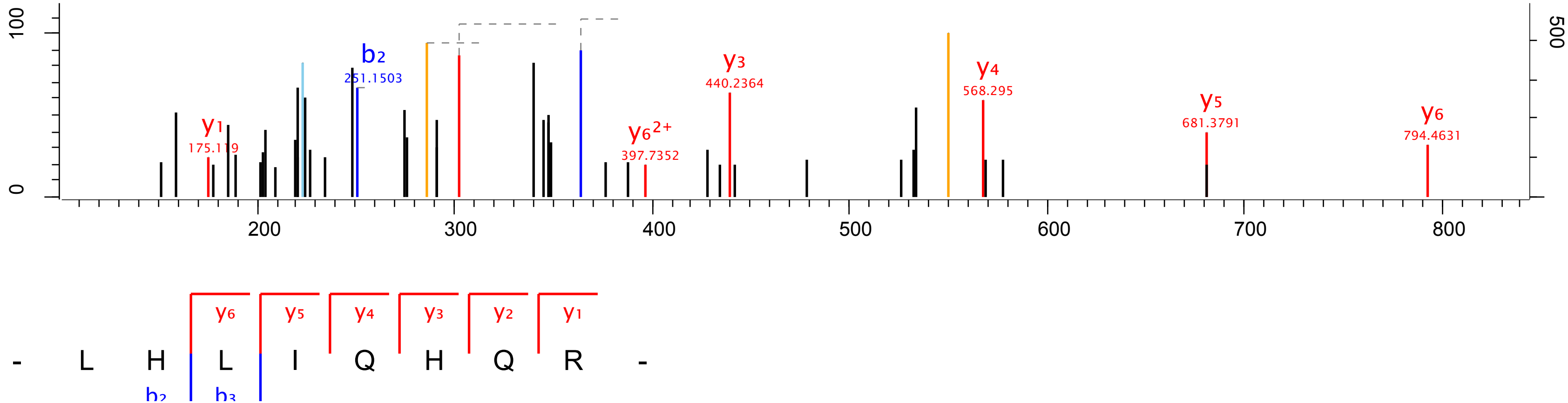

Raw file	Scan	Method	Score	m/z	Gene names
20140925_fract19_dyn_5ul_H3_01_454	14659	TOF; CID	49.19	514.27	TMEM53

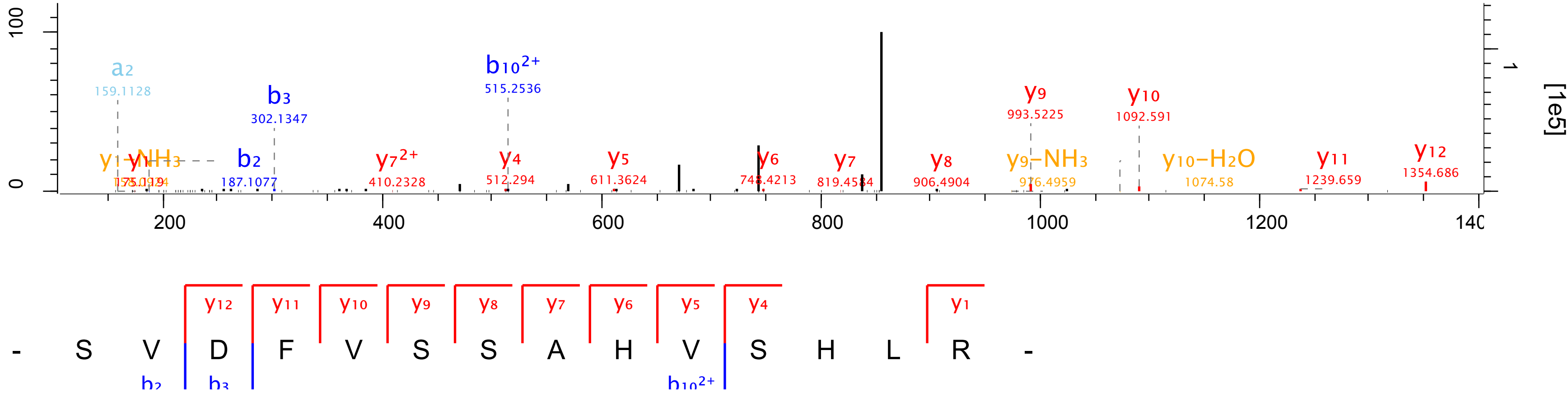

Raw file	Scan	Method	Score	m/z	Gene names
20140925_fract19_dyn_5ul_H3_01_454	15075	TOF; CID	90.15	499.8	DNAJB14

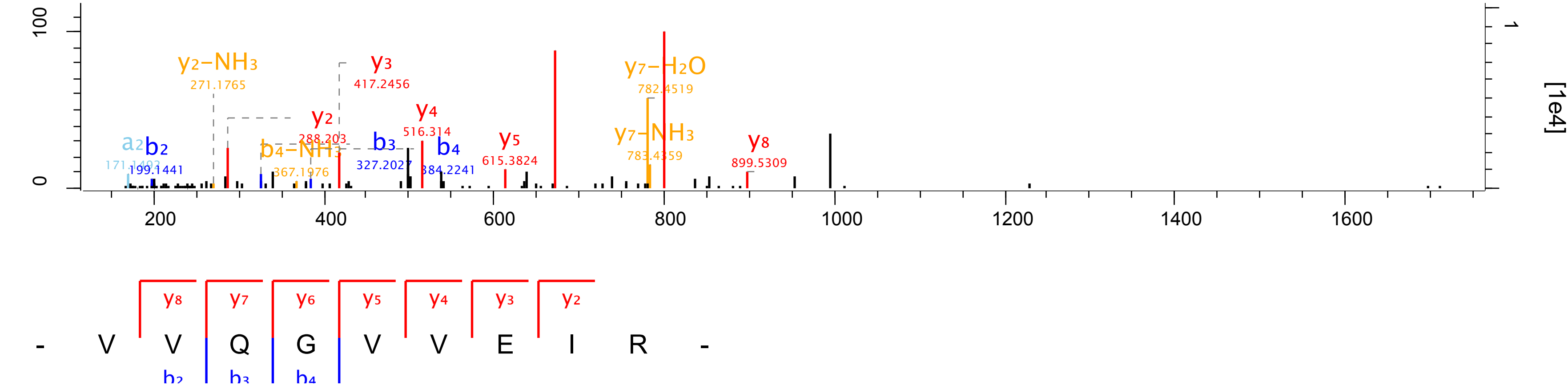

Raw file	Scan	Method	Score	m/z	Gene names
20140925_fract19_dyn_5ul_H3_01_454	18392	TOF; CID	130.04	510.78	MAP2K5

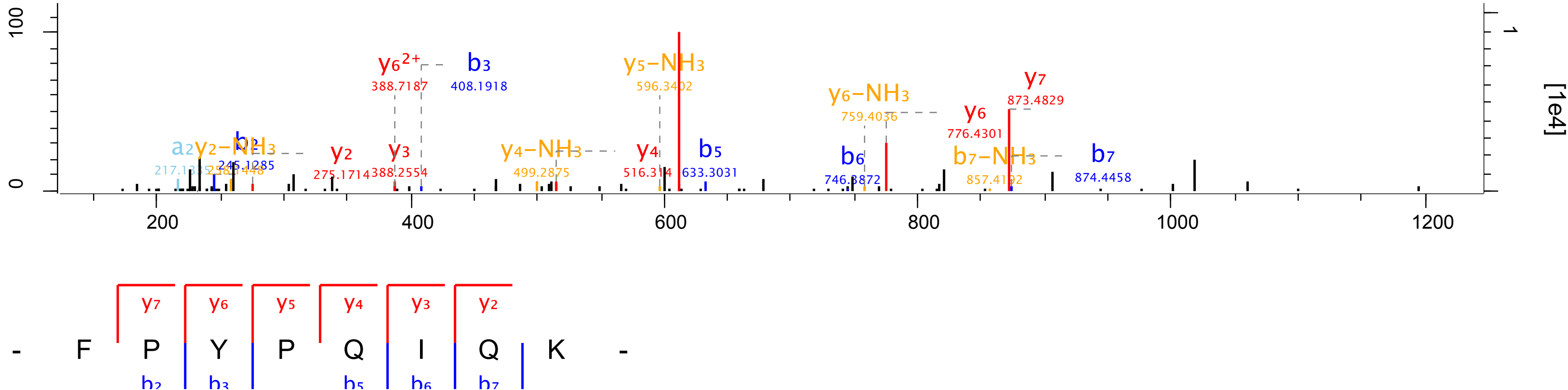

Raw file	Scan	Method	Score	m/z	Gene names
20140925_fract19_dyn_5ul_H3_01_454	26432	TOF; CID	42.64	712.7	SDC3

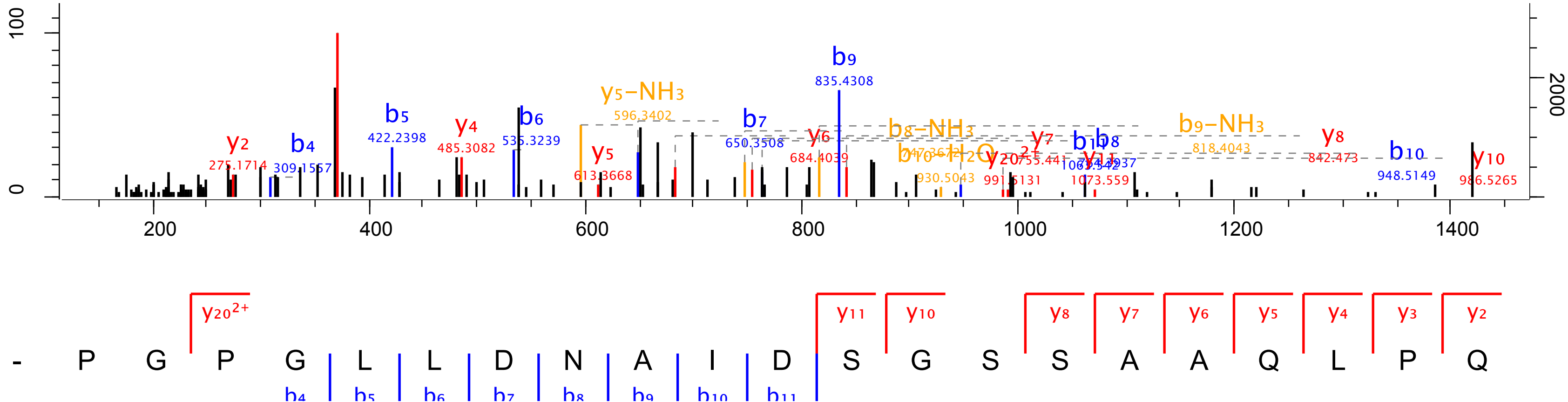

Raw file	Scan	Method	Score	m/z	Gene names
20140925_fract20_dyn_5ul_H4_01_455	16399	TOF; CID	46.89	610.33	SLC22A20

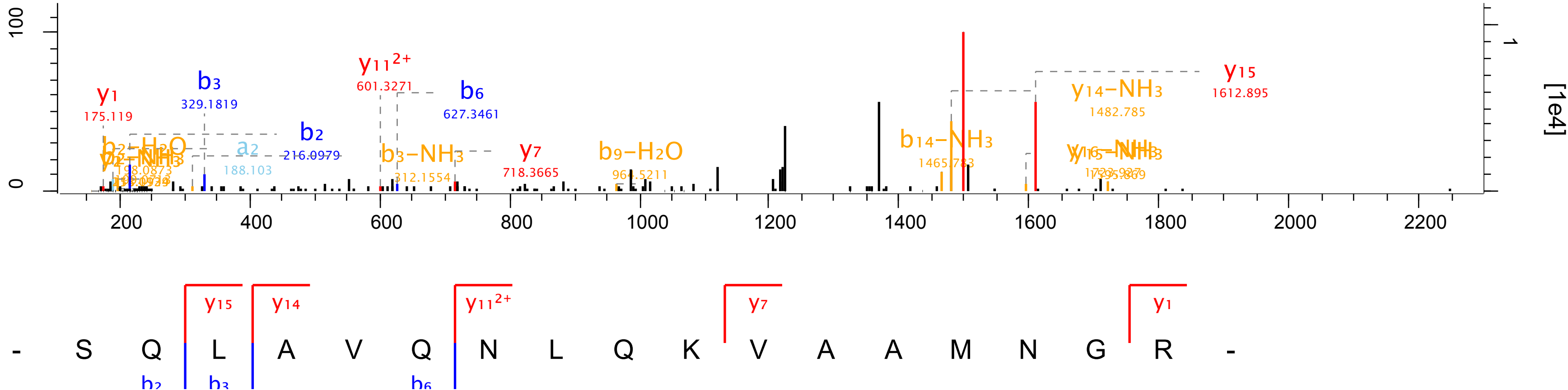

Raw file	Scan	Method	Score	m/z	Gene names
20140925_fract20_dyn_5ul_H4_01_455	23305	TOF; CID	48.9	867.95	FAM102A

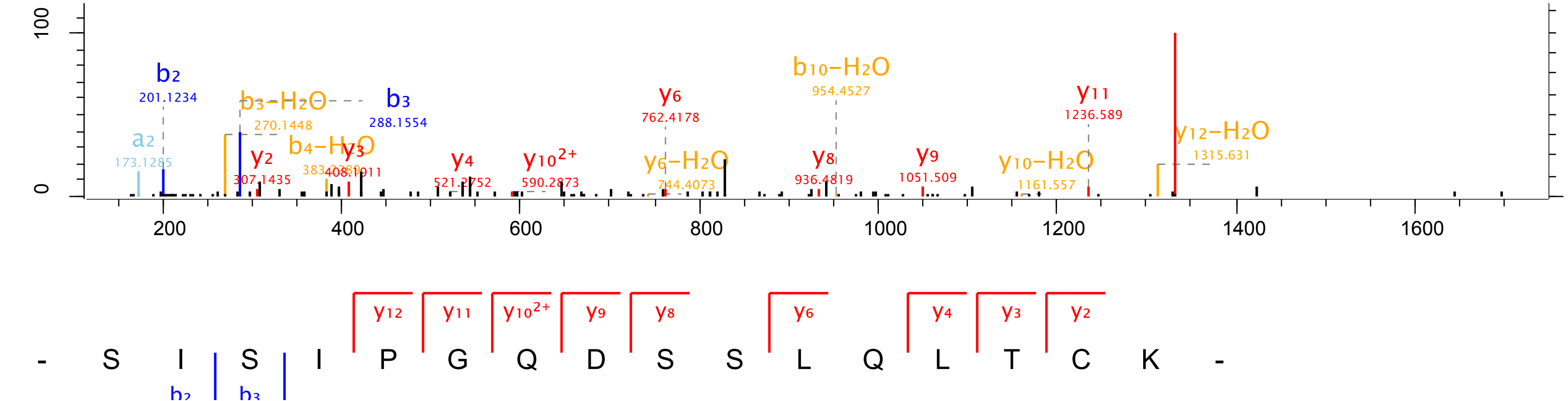

Raw file	Scan	Method	Score	m/z	Gene names
20140925_fract20_dyn_5ul_H4_01_455	31205	TOF; CID	68	673.36	CD82

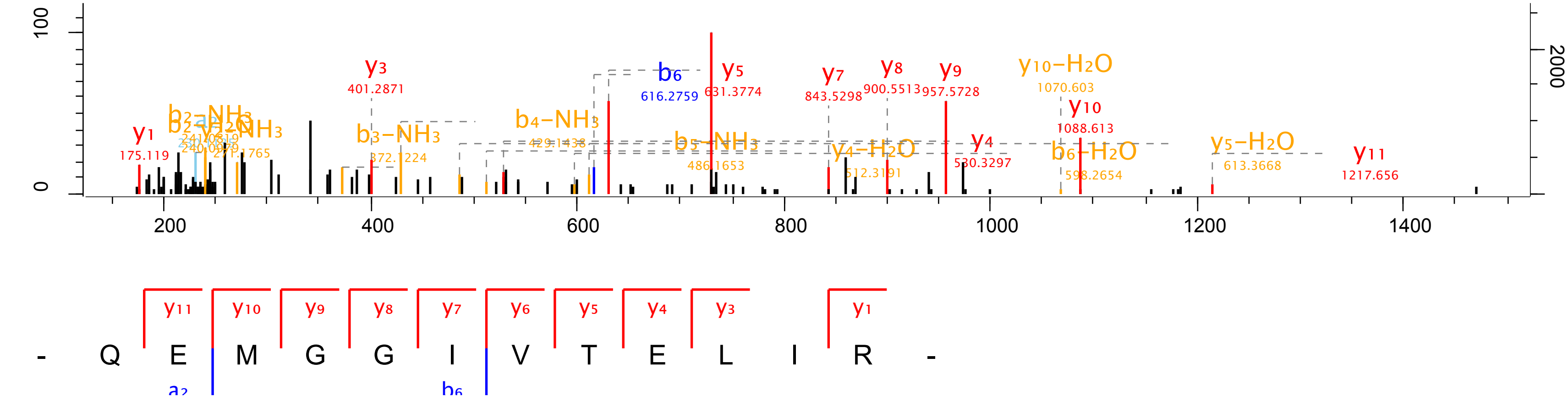

Raw file	Scan	Method	Score	m/z	Gene names
20140925_fract21_dyn_5ul_H5_01_456	16339	TOF; CID	115.71	565.34	NIPA2

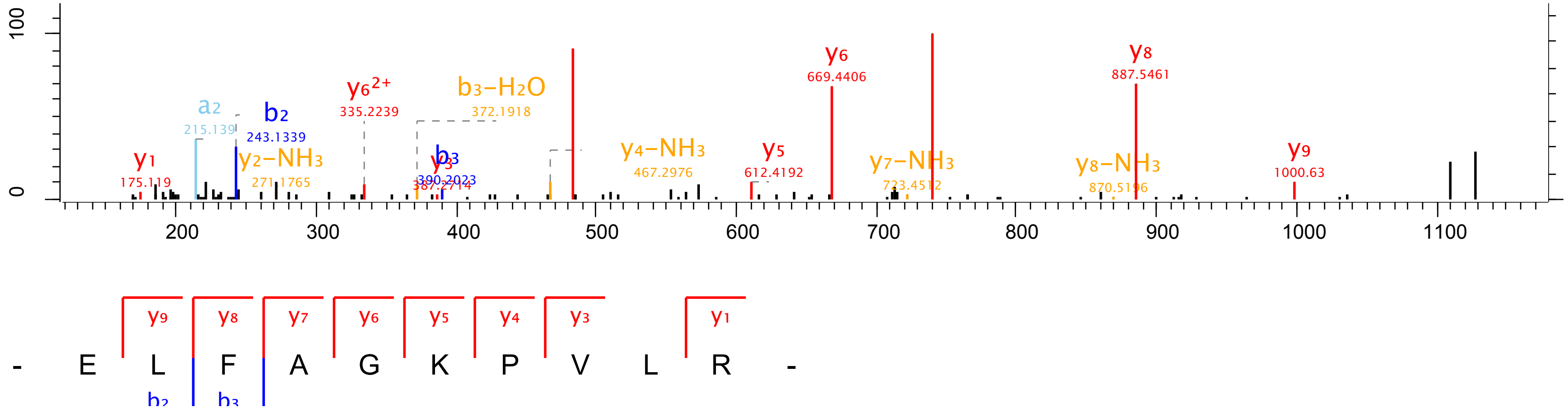

Raw file	Scan	Method	Score	m/z	Gene names
20140925_fract21_dyn_5ul_H5_01_456	24059	TOF; CID	91.62	501.6	HEMK1

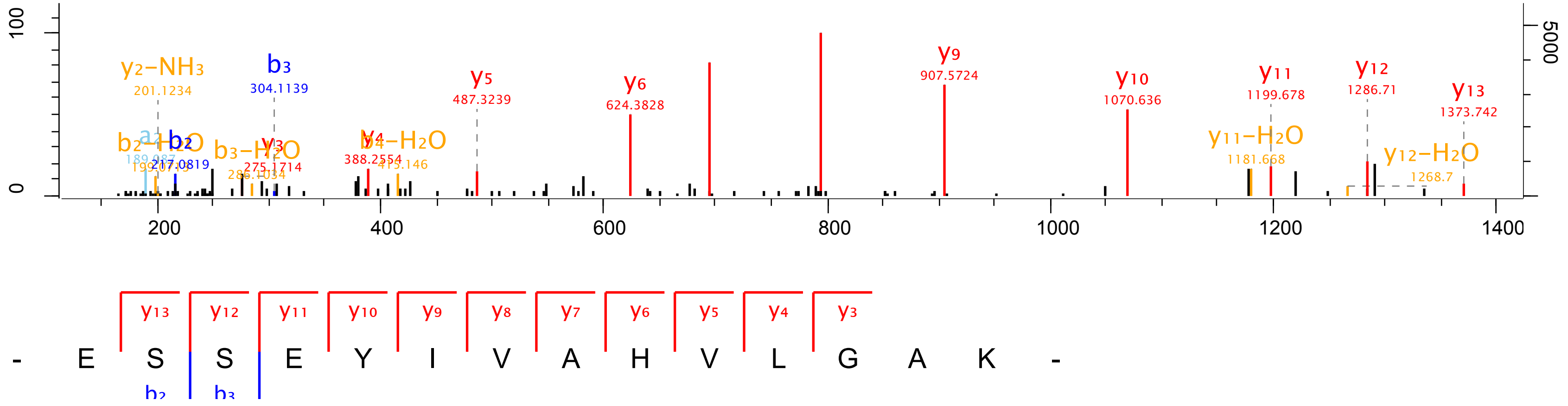

Raw file	Scan	Method	Score	m/z	Gene names
20140925_fract22_dyn_5ul_H6_01_457	21078	TOF; CID	120.55	509.28	NLK

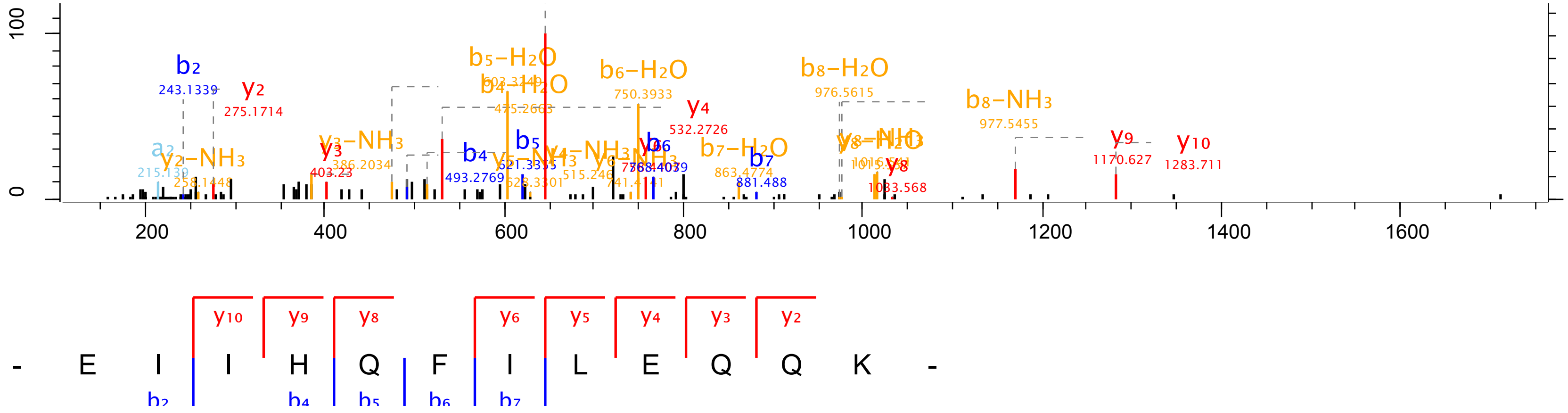

Raw file	Scan	Method	Score	m/z	Gene names
20140925_fract23_dyn_5ul_H7_01_458	10320	TOF; CID	76.23	450.27	SOX4

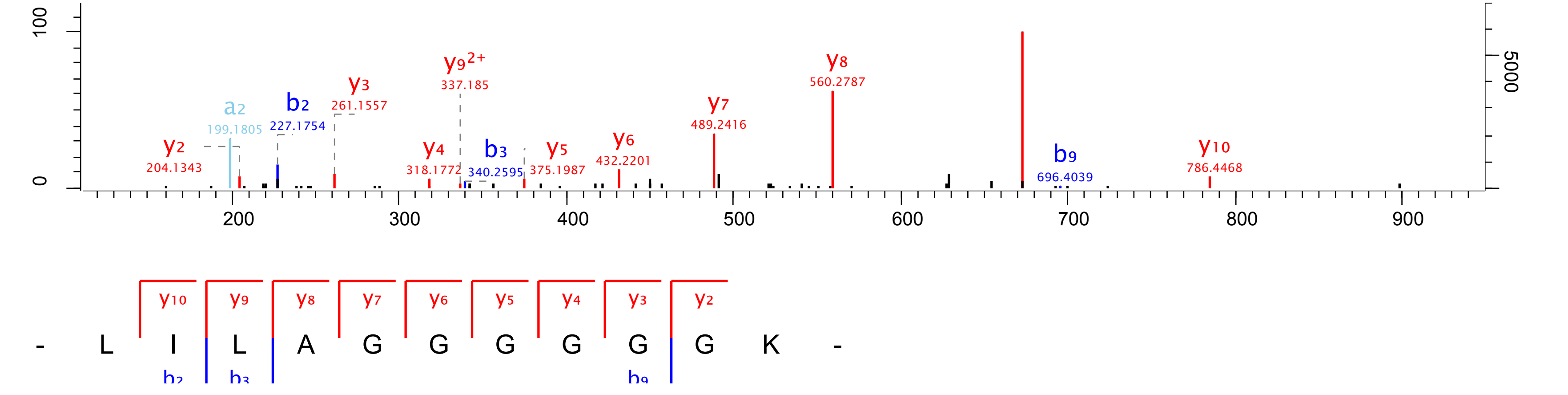

Raw file	Scan	Method	Score	m/z	Gene names
20140925_fract23_dyn_5ul_H7_01_458	11453	TOF; CID	134.48	554.76	ZNF341

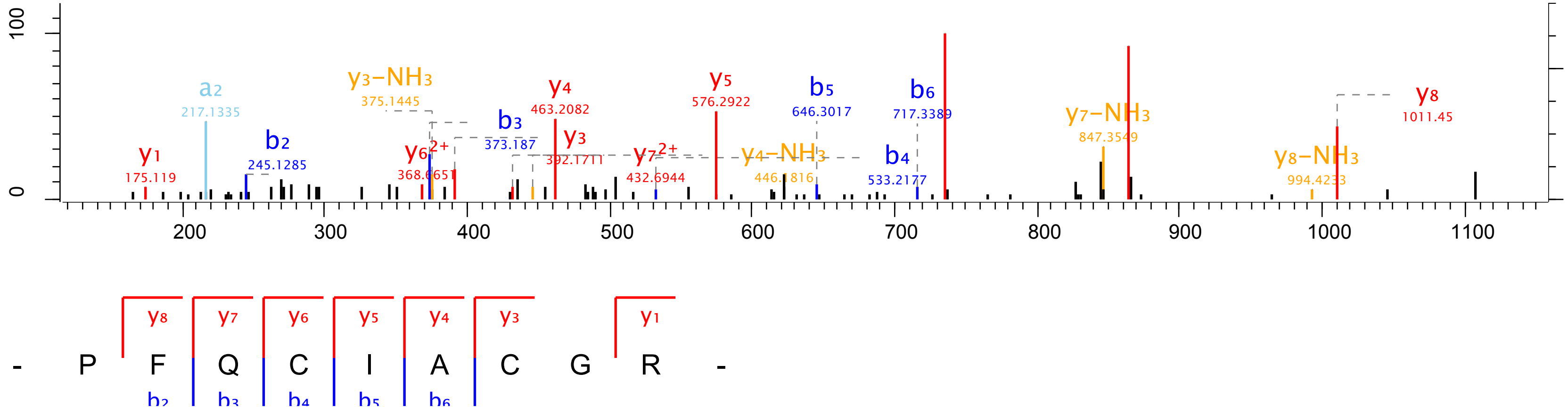

Raw file	Scan	Method	Score	m/z	Gene names
20140925_fract23_dyn_5ul_H7_01_458	28773	TOF; CID	43.7	1072.54	METTL12

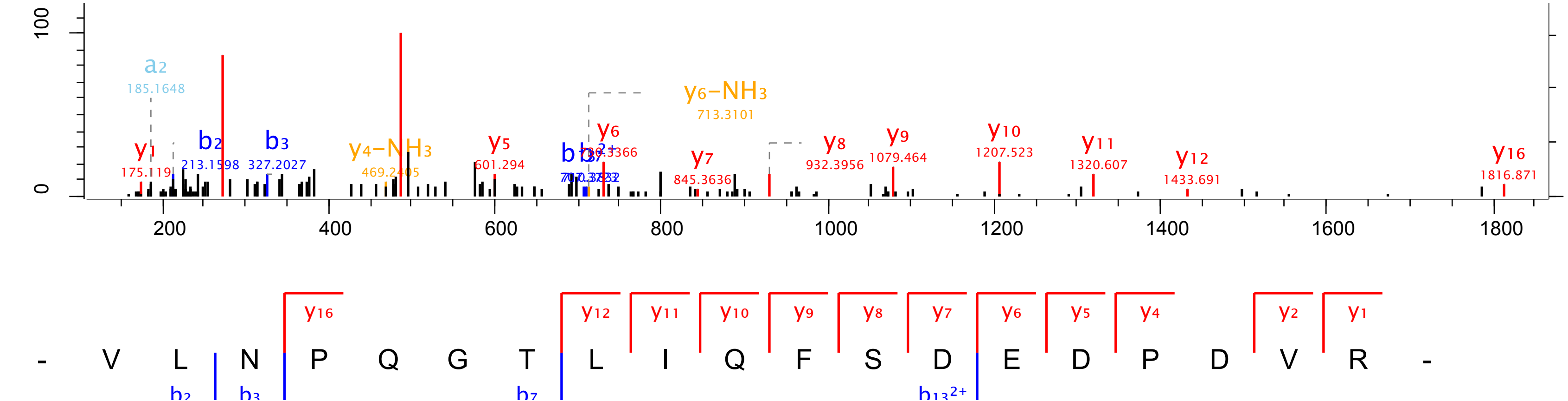

Raw file	Scan	Method	Score	m/z	Gene names
20140925_fract23_dyn_5ul_H7_01_458	33082	TOF; CID	107.75	961.13	SFT2D1

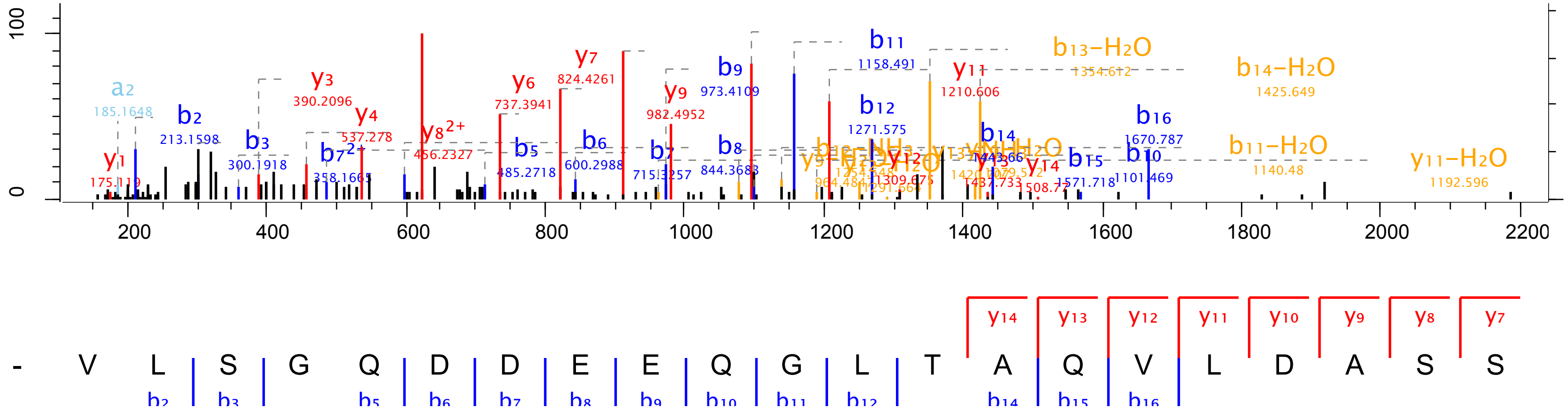

Raw file	Scan	Method	Score	m/z	Gene names
20140925_fract24_dyn_5ul_H8_01_459	9206	TOF; CID	74.79	570.63	RCBTB2

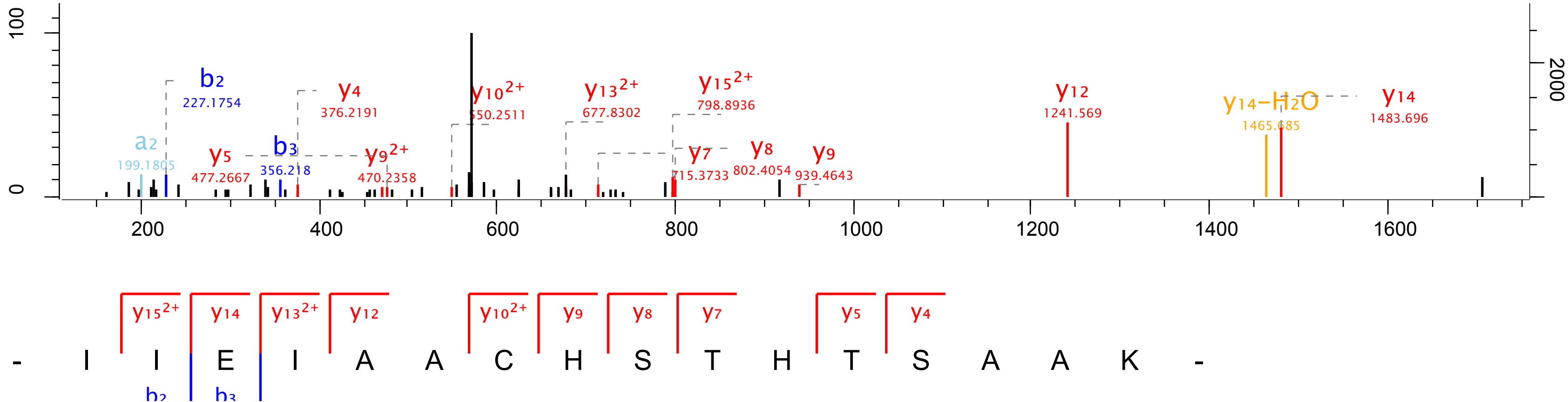

Raw file	Scan	Method	Score	m/z	Gene names
20140925_fract24_dyn_5ul_H8_01_459	19885	TOF; CID	53.45	552.98	FAM189B

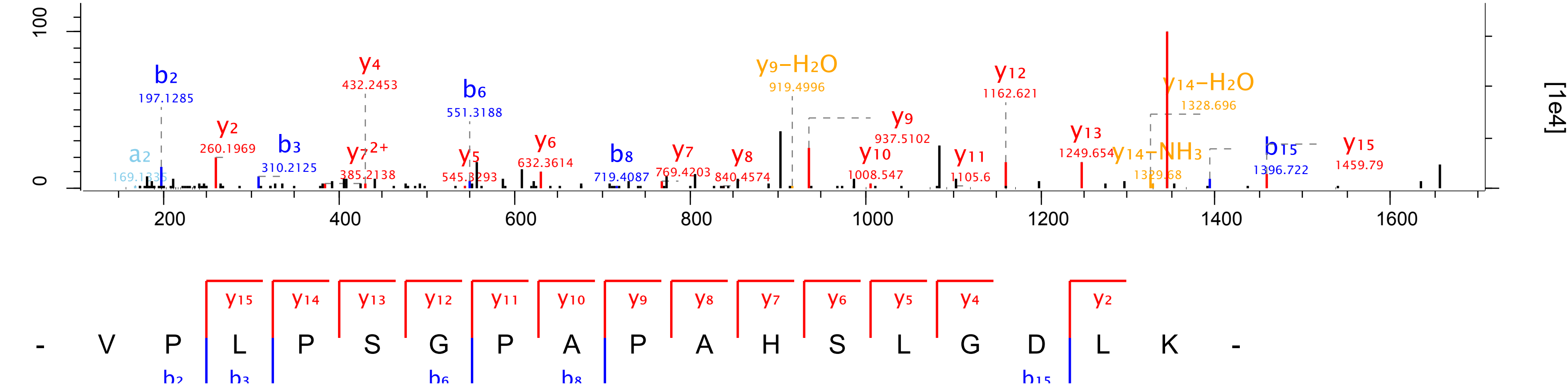

Raw file	Scan	Method	Score	m/z	Gene names
20141014_fract1_dyn_5ul_E1_01_580	27070	TOF; CID	80.24	699.87	DEPTOR

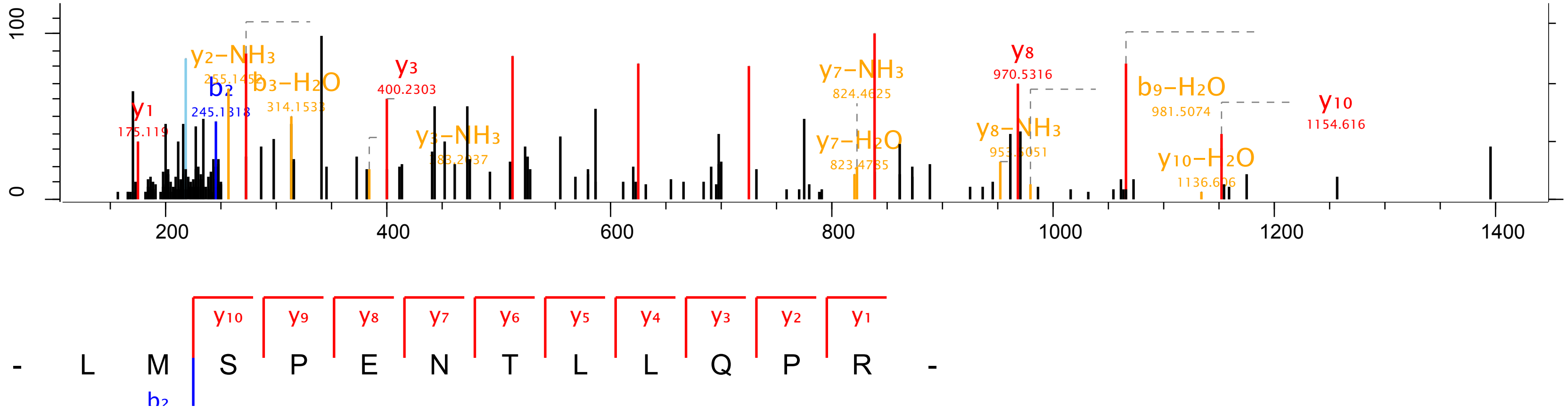

Raw file	Scan	Method	Score	m/z	Gene names
20141014_fract2_dyn_5ul_E2_01_581	3355	TOF; CID	81.1	390.53	BBIP1

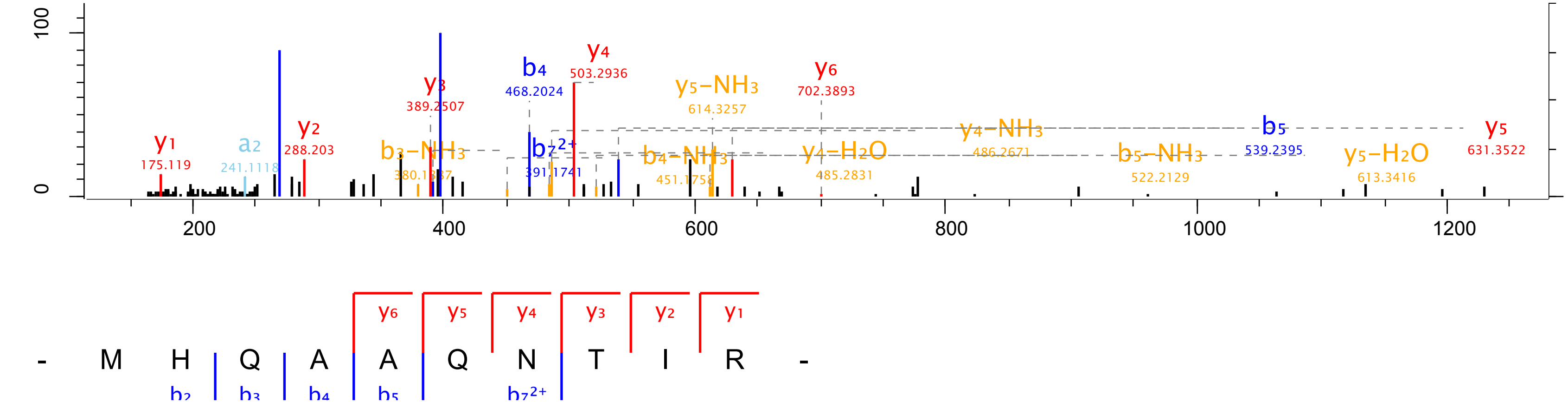

Raw file	Scan	Method	Score	m/z	Gene names
20141014_fract2_dyn_5ul_E2_01_581	8678	TOF; CID	52.25	482.24	ZNF165

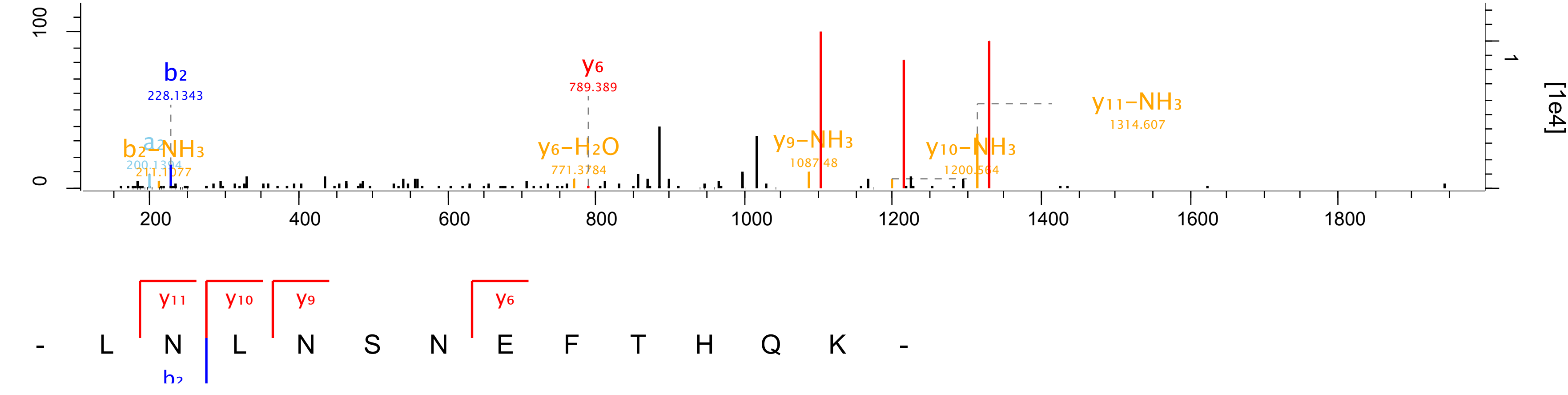

Raw file	Scan	Method	Score	m/z	Gene names
20141014_fract2_dyn_5ul_E2_01_581	11931	TOF; CID	87.18	502.25	C18orf32

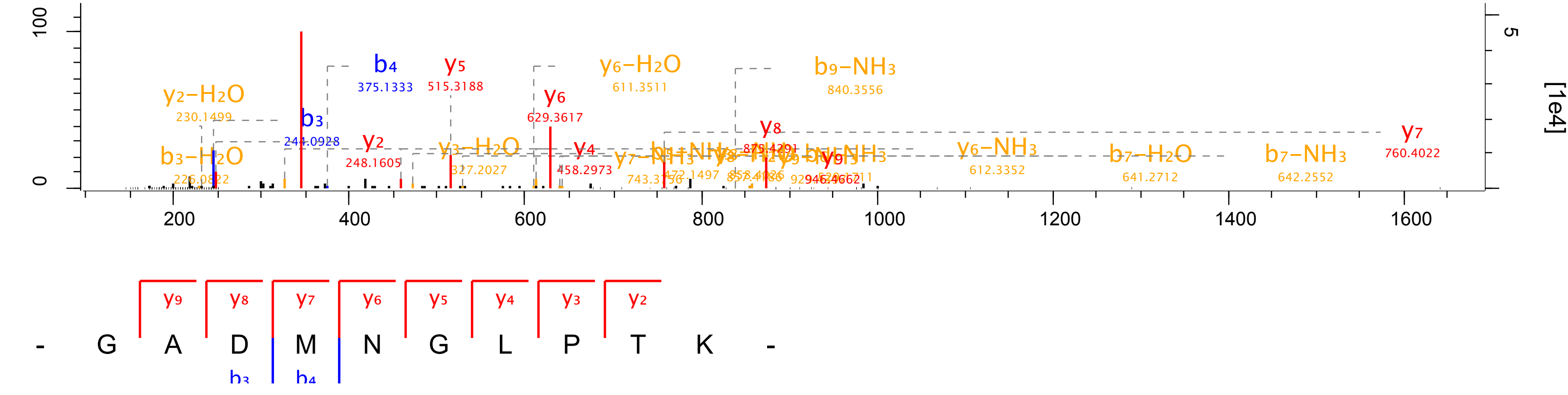

Raw file	Scan	Method	Score	m/z	Gene names
20141014_fract2_dyn_5ul_E2_01_581	33792	TOF; CID	136.83	582.95	TMEM138

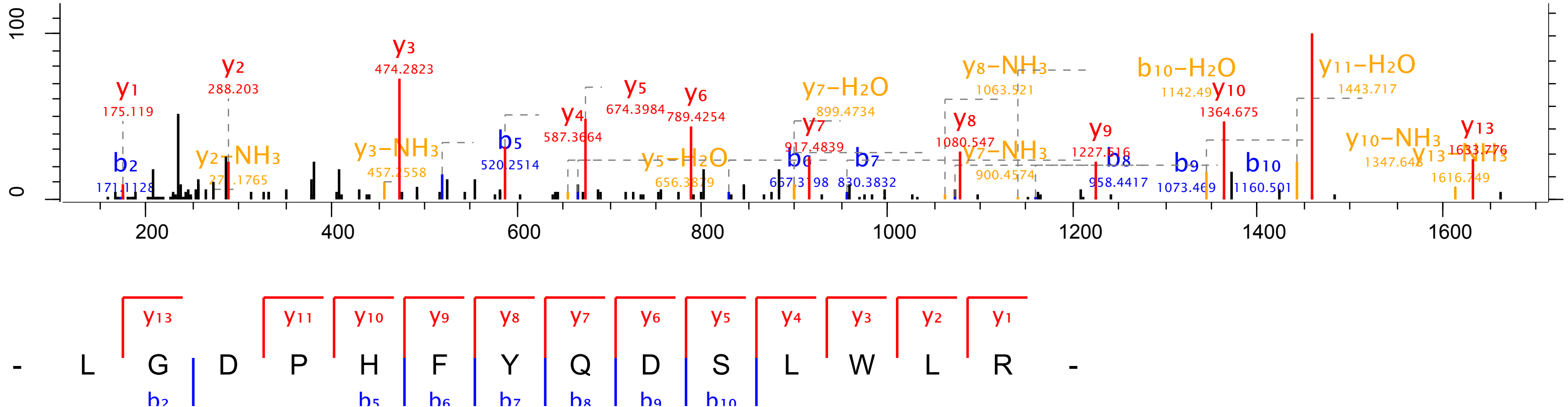

Raw file	Scan	Method	Score	m/z	Gene names
20141014_fract2_dyn_5ul_E2_01_581	35148	TOF; CID	90.15	530.29	PHLDA1

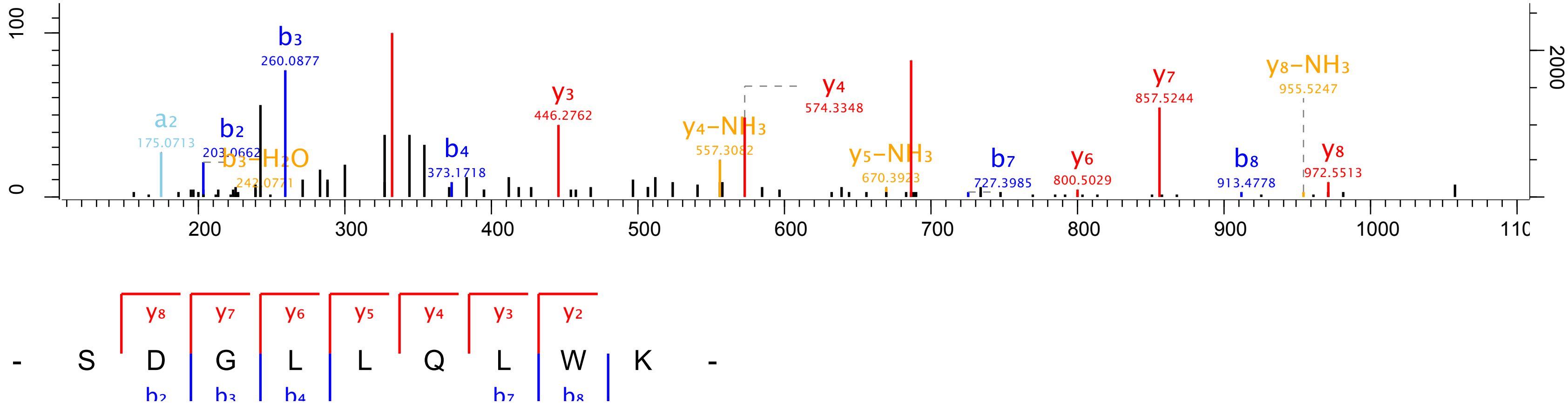

Raw file	Scan	Method	Score	m/z	Gene names
20141014_fract2_dyn_5ul_E2_01_581	37360	TOF; CID	82.65	516.97	STK17B

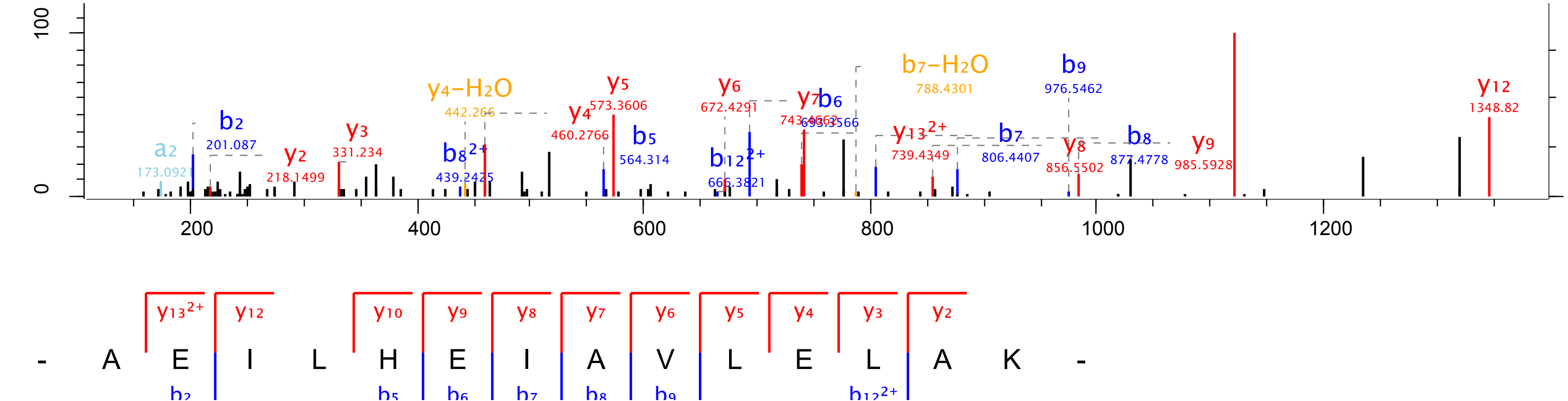

Raw file	Scan	Method	Score	m/z	Gene names
20141014_fract2_dyn_5ul_E2_01_581	38373	TOF; CID	142.43	628.32	SLC2A6

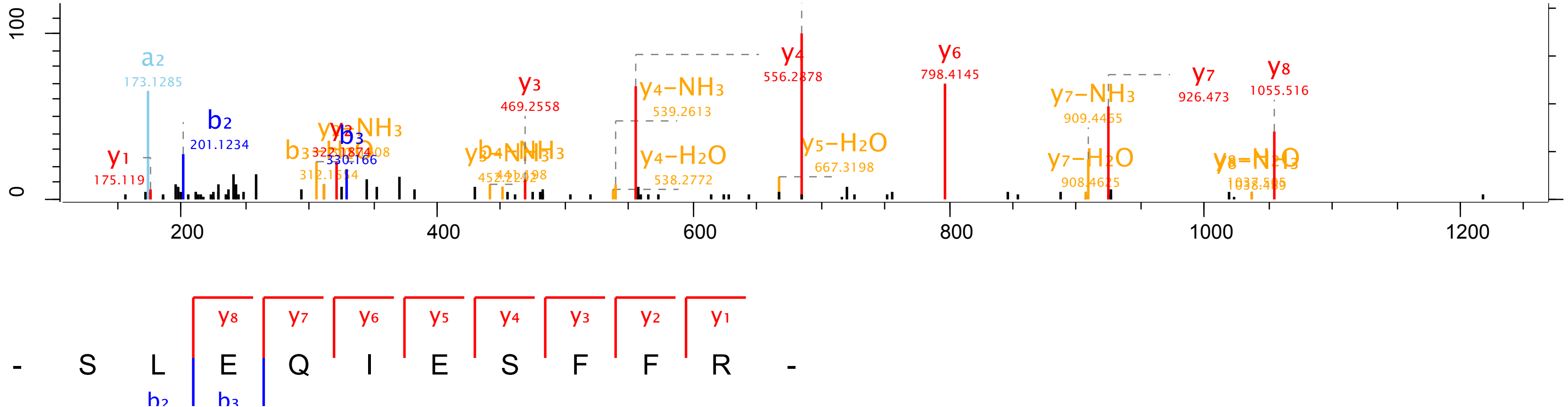

Raw file	Scan	Method	Score	m/z	Gene names
20141014_fract2_dyn_5ul_E2_01_581	41524	TOF; CID	43.73	1040.48	FUOM

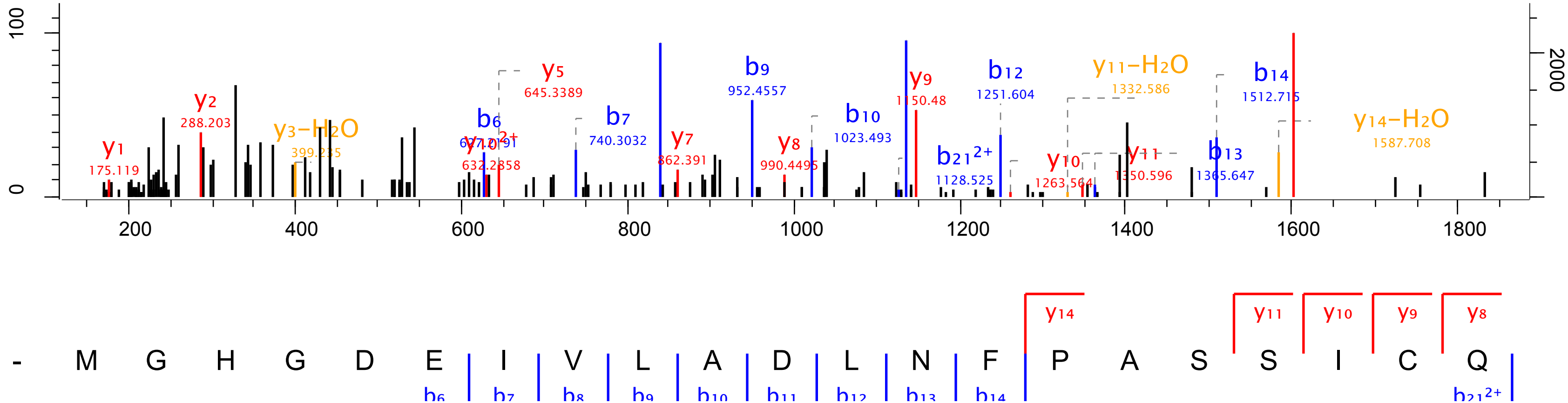

Raw file	Scan	Method	Score	m/z	Gene names
20141014_fract3_dyn_5ul_E3_01_582	7727	TOF; CID	104.01	601.79	C1orf53

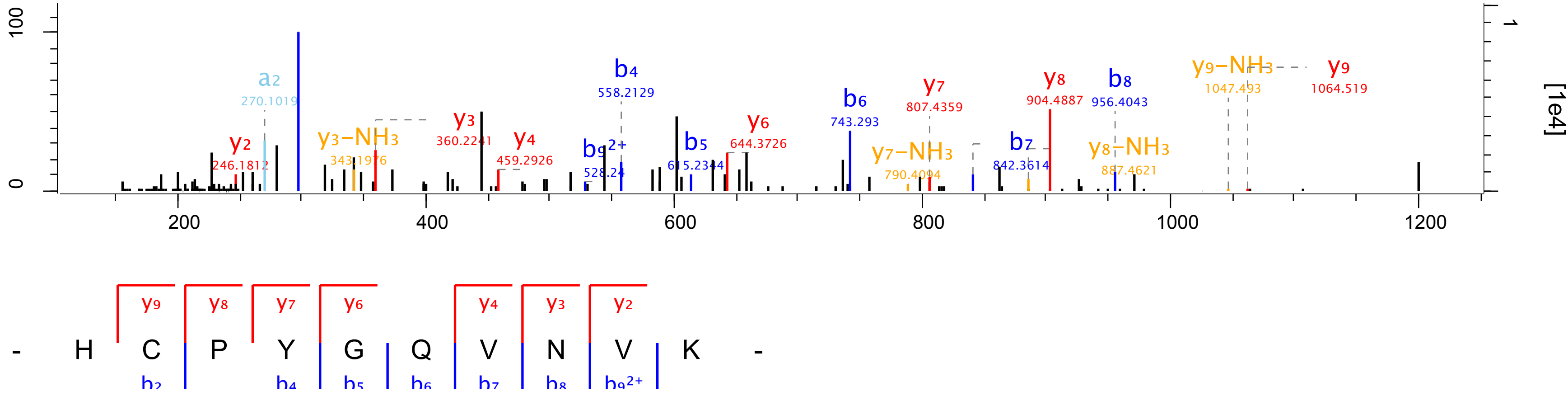

Raw file	Scan	Method	Score	m/z	Gene names
20141014_fract3_dyn_5ul_E3_01_582	21322	TOF; CID	79.49	764.63	SMIM14

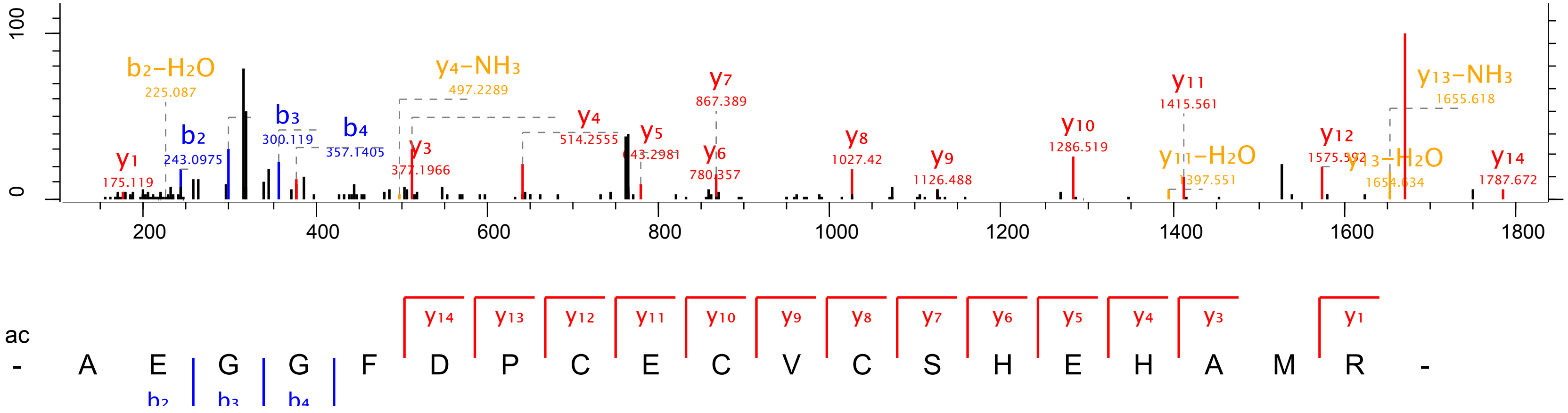

Raw file	Scan	Method	Score	m/z	Gene names
20141014_fract3_dyn_5ul_E3_01_582	28058	TOF; CID	58.32	487.59	TMEM231

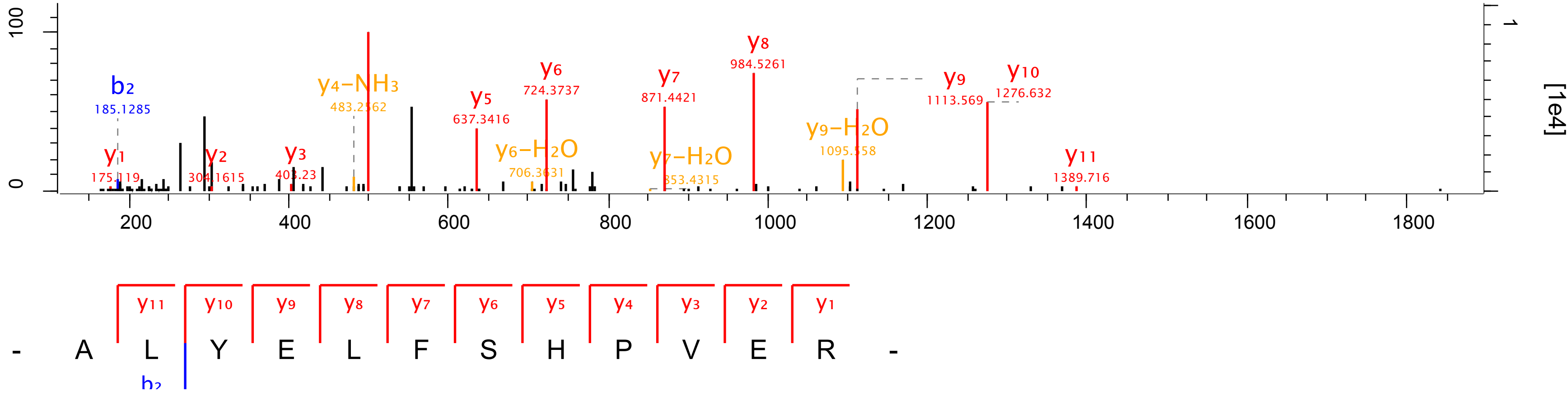

Raw file	Scan	Method	Score	m/z	Gene names
20141014_fract3_dyn_5ul_E3_01_582	38024	TOF; CID	65.04	777.4	TPRA1

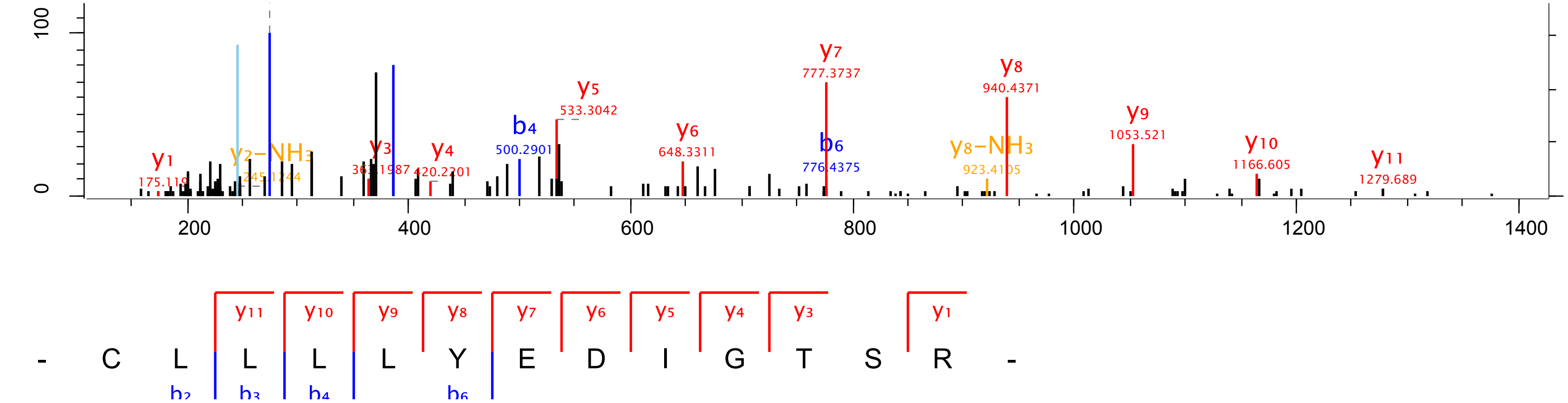

Raw file	Scan	Method	Score	m/z	Gene names
20141014_fract4_dyn_5ul_E4_01_583	19196	TOF; CID	91.28	650.82	SLC36A4

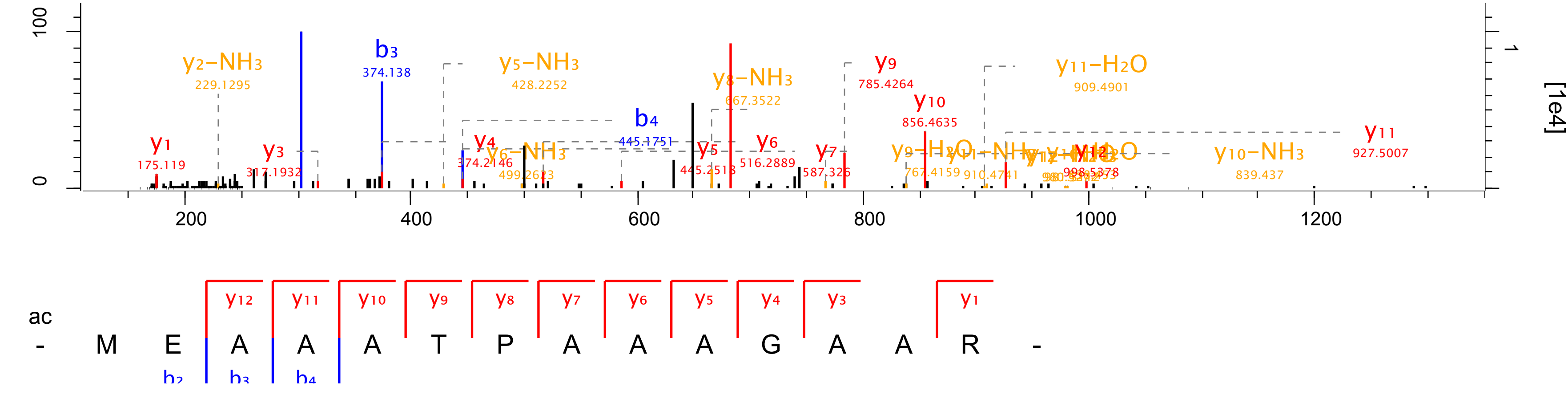

Raw file	Scan	Method	Score	m/z	Gene names
20141014_fract4_dyn_5ul_E4_01_583	29058	TOF; CID	86.18	572.33	BOK

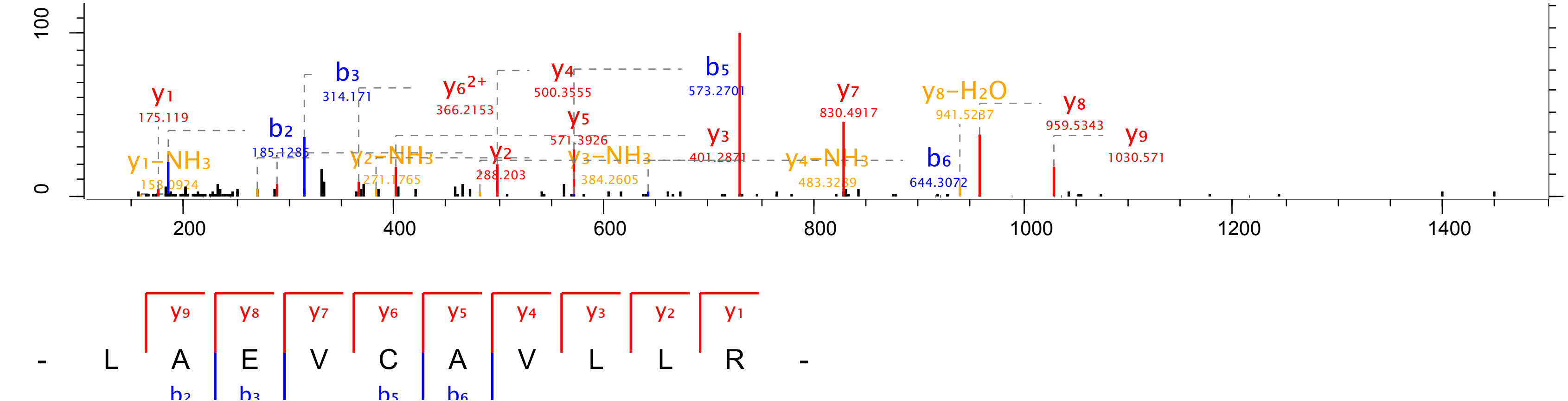

Raw file	Scan	Method	Score	m/z	Gene names
20141014_fract4_dyn_5ul_E4_01_583	38279	TOF; CID	59.25	953.5	C4orf46

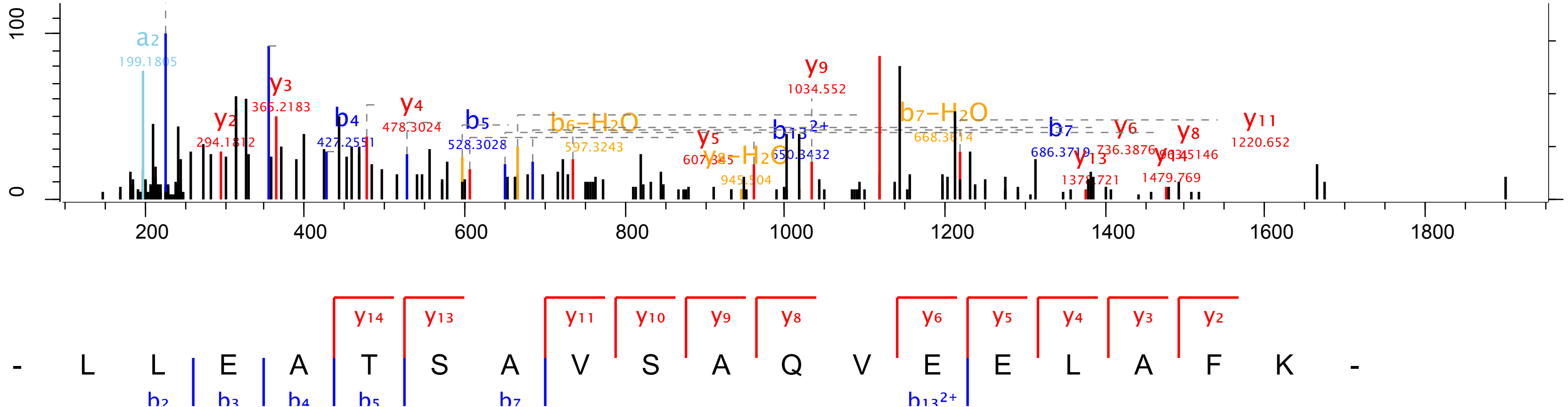

Raw file	Scan	Method	Score	m/z	Gene names
20141014_fract5_dyn_5ul_E5_01_584	12423	TOF; CID	135.1	640.8	C17orf89

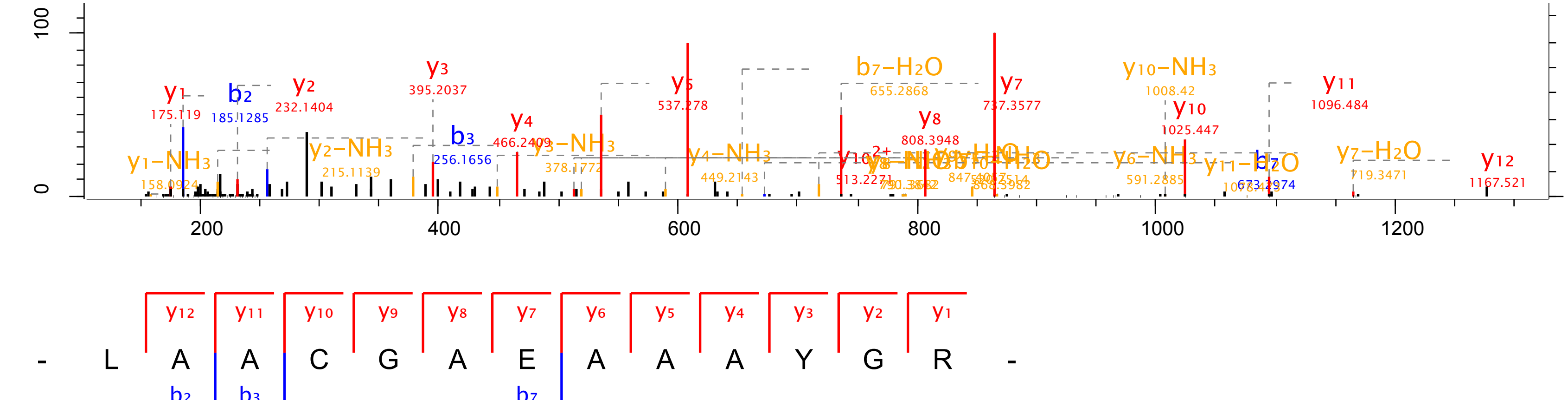

Raw file	Scan	Method	Score	m/z	Gene names
20141014_fract5_dyn_5ul_E5_01_584	23325	TOF; CID	72.34	648.29	PFDN4

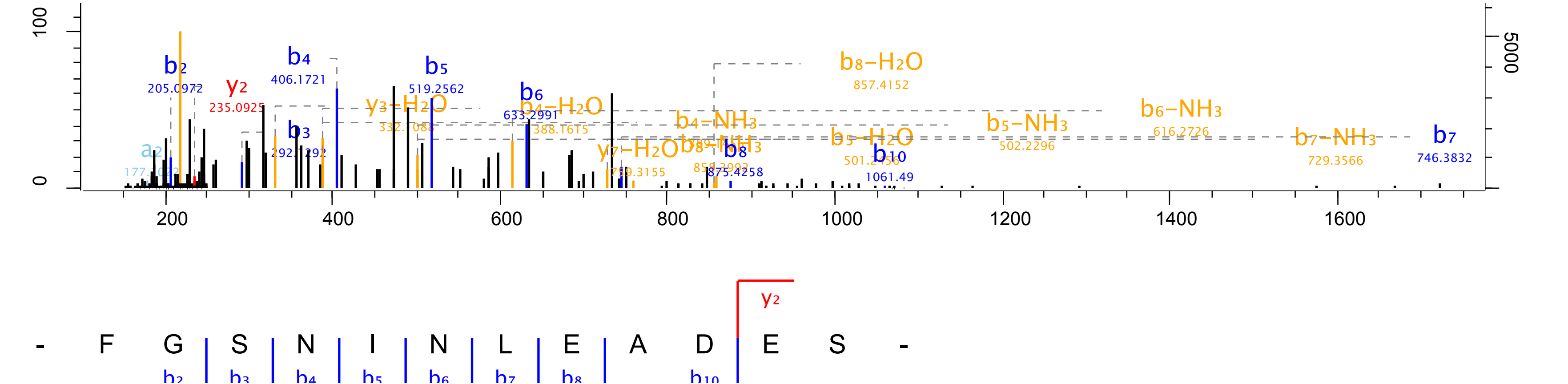

Raw file	Scan	Method	Score	m/z	Gene names
20141014_fract5_dyn_5ul_E5_01_584	28067	TOF; CID	143.28	504.72	TMEM258

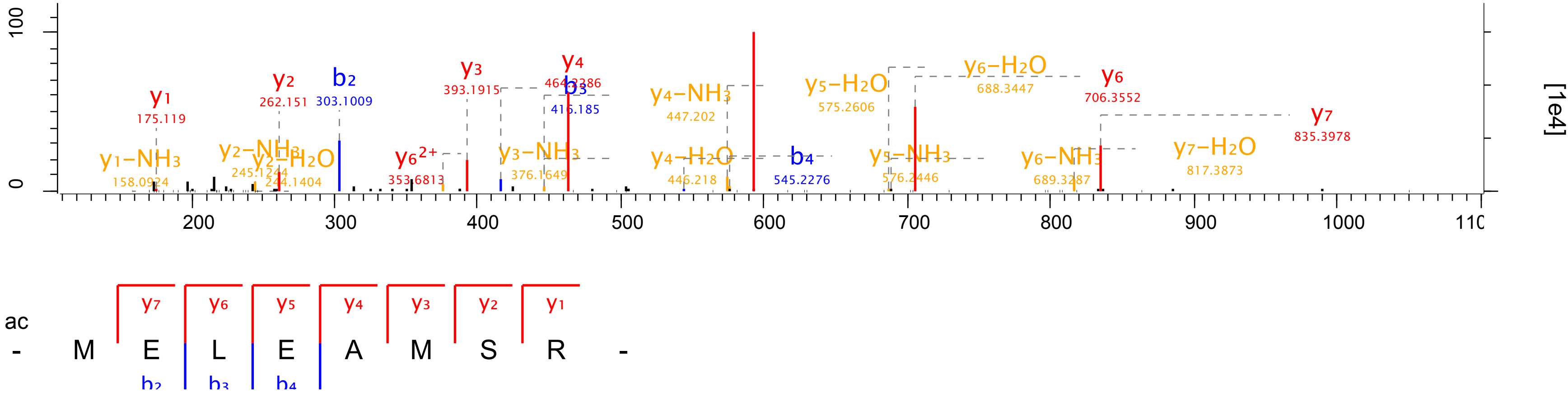

Raw file	Scan	Method	Score	m/z	Gene names
20141014_fract5_dyn_5ul_E5_01_584	31391	TOF; CID	61.94	725.35	PRR3

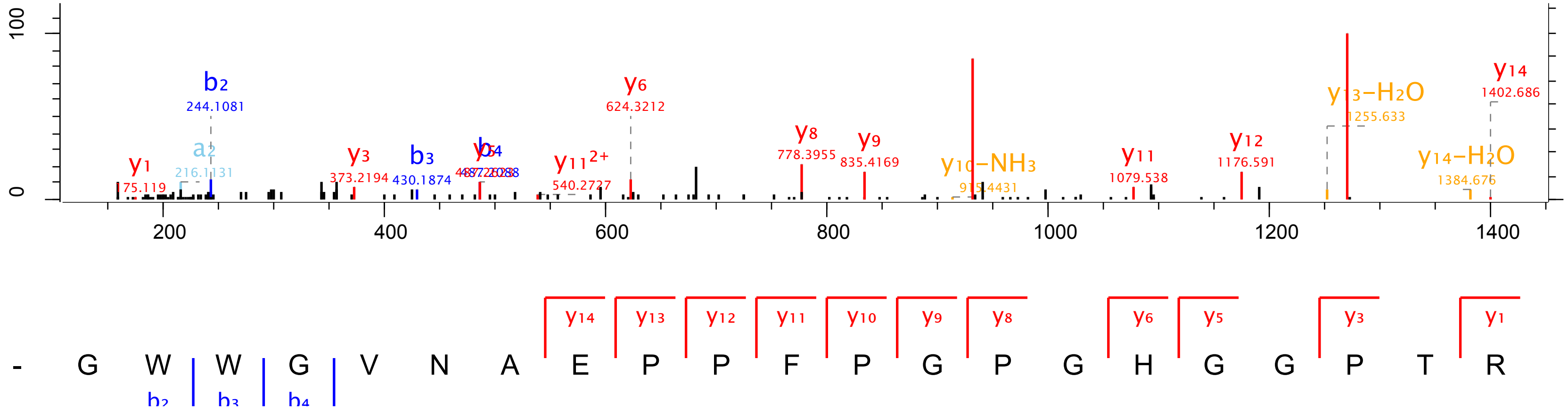

Raw file	Scan	Method	Score	m/z	Gene names
20141014_fract5_dyn_5ul_E5_01_584	34718	TOF; CID	35.54	860.41	ADPRM

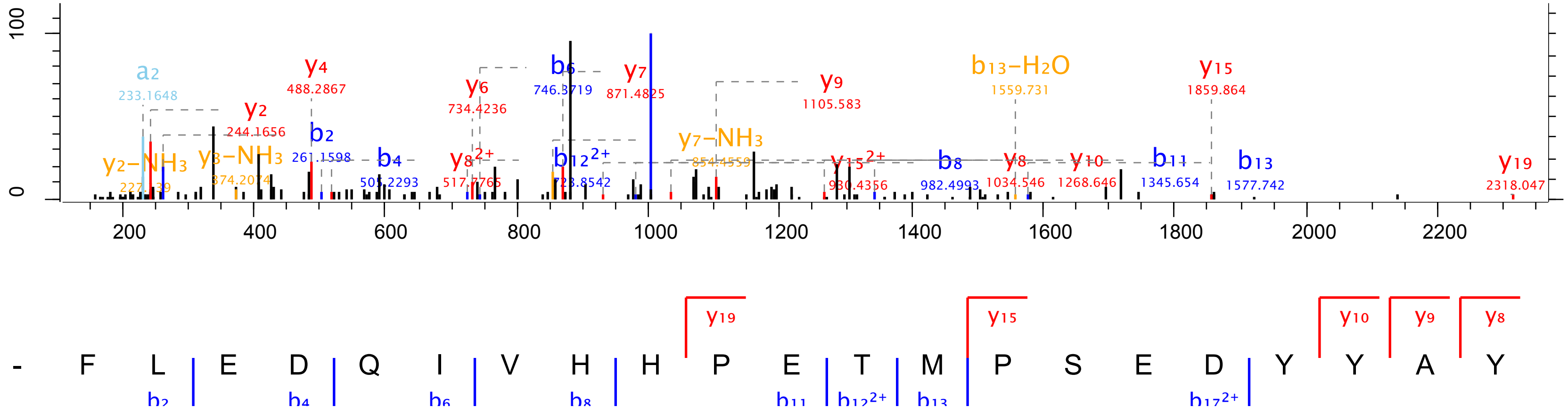

Raw file	Scan	Method	Score	m/z	Gene names
20141014_fract7_dyn_5ul_E7_01_586	4604	TOF; CID	55.72	506.59	STOX2;GDAP1

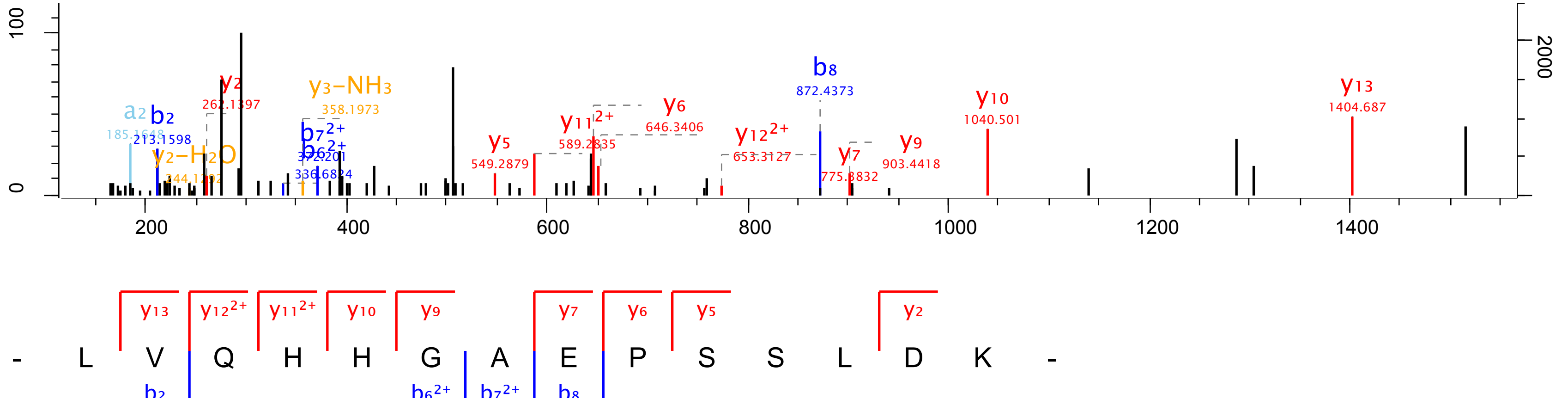

Raw file	Scan	Method	Score	m/z	Gene names
20141014_fract7_dyn_5ul_E7_01_586	7247	TOF; CID	121.86	629.33	CXCR4

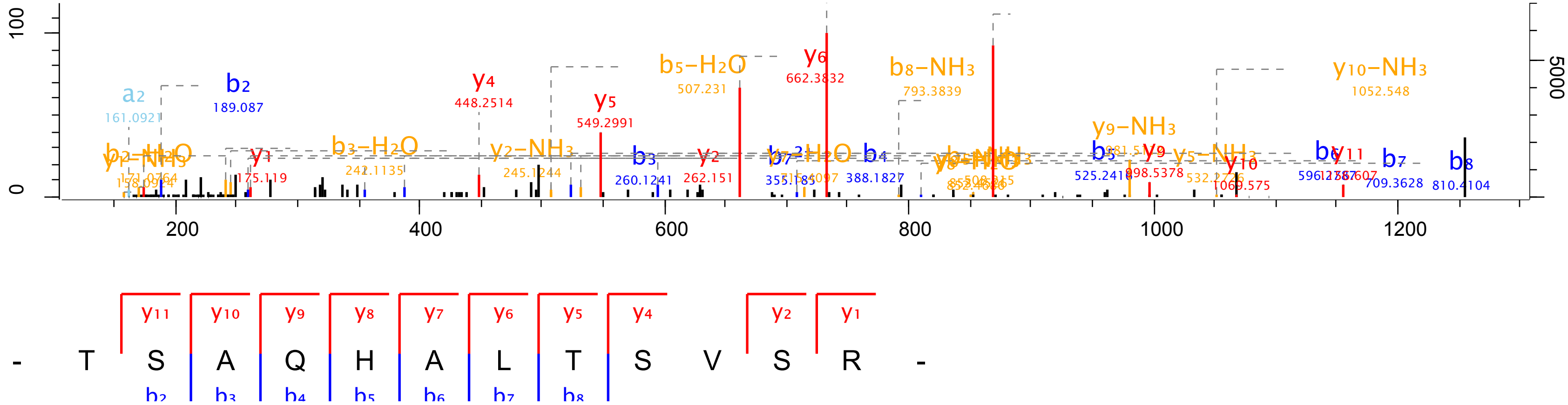

Raw file	Scan	Method	Score	m/z	Gene names
20141014_fract7_dyn_5ul_E7_01_586	12817	TOF; CID	77.6	619.29	USP53

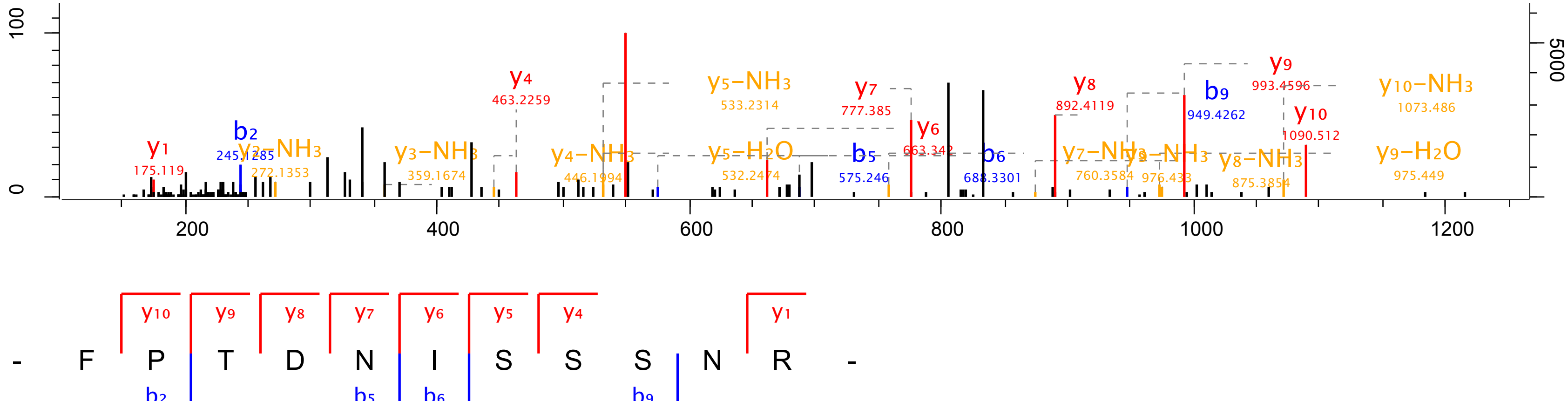

Raw file	Scan	Method	Score	m/z	Gene names
20141014_fract7_dyn_5ul_E7_01_586	29994	TOF; CID	63.29	778.4	TBC1D16

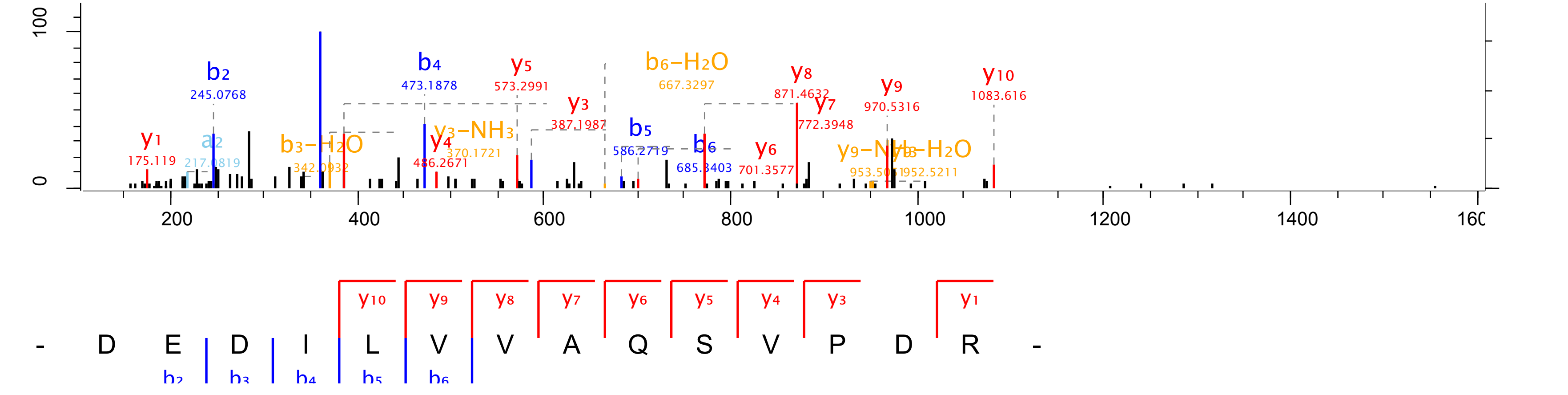

Raw file	Scan	Method	Score	m/z	Gene names
20141014_fract7_dyn_5ul_E7_01_586	37190	TOF; CID	93.23	892.49	PPP1R3F

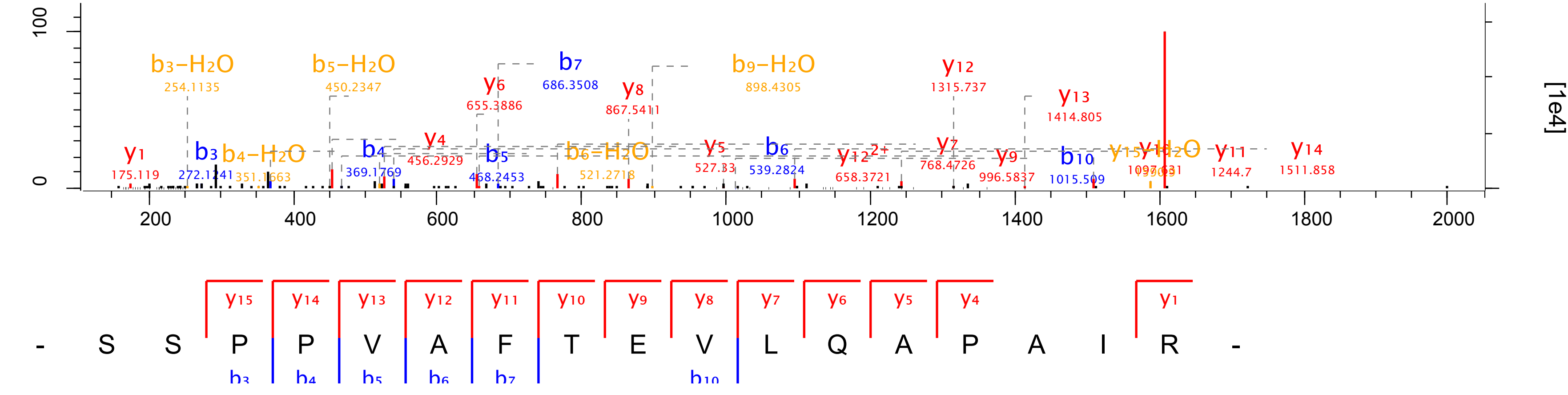

Raw file	Scan	Method	Score	m/z	Gene names
20141014_fract8_dyn_5ul_E8_01_587	13435	TOF; CID	68.52	656.35	UCP2

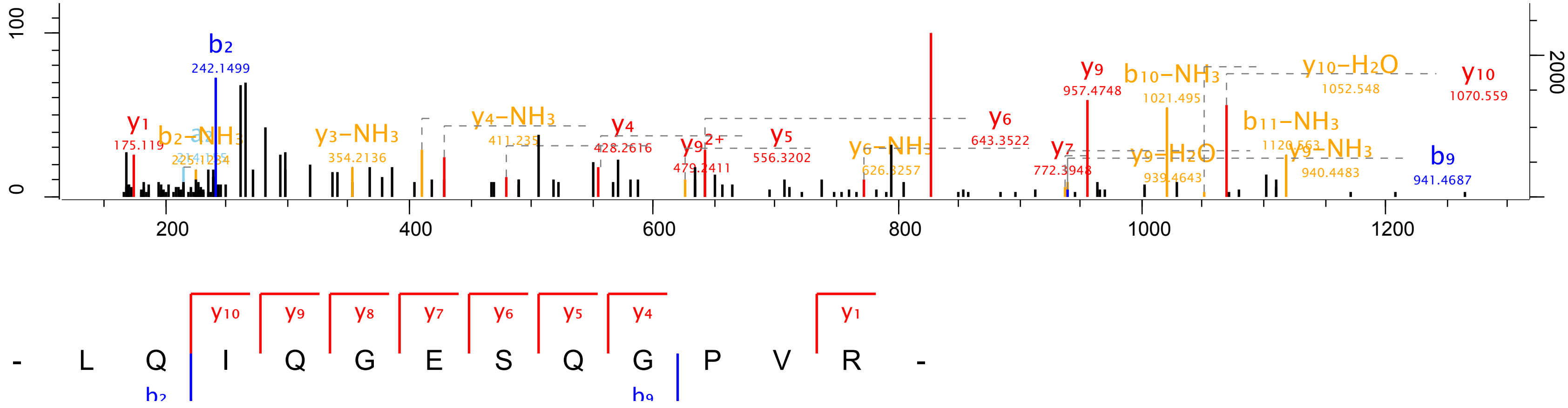

Raw file	Scan	Method	Score	m/z	Gene names
20141014_fract8_dyn_5ul_E8_01_587	17469	TOF; CID	94.82	566.29	C6orf57

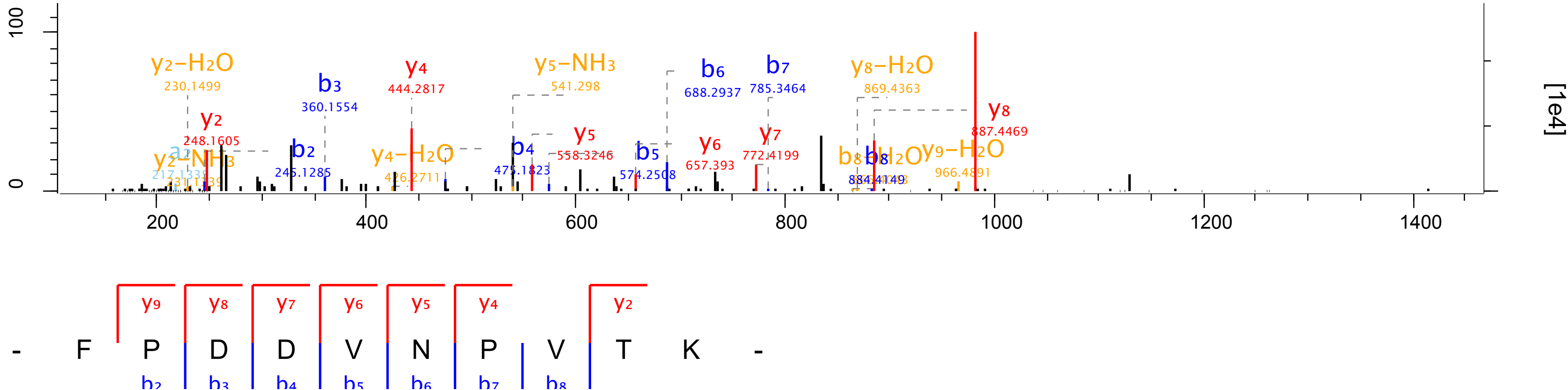

Raw file	Scan	Method	Score	m/z	Gene names
20141014_fract8_dyn_5ul_E8_01_587	23332	TOF; CID	61.11	670.36	CSTA

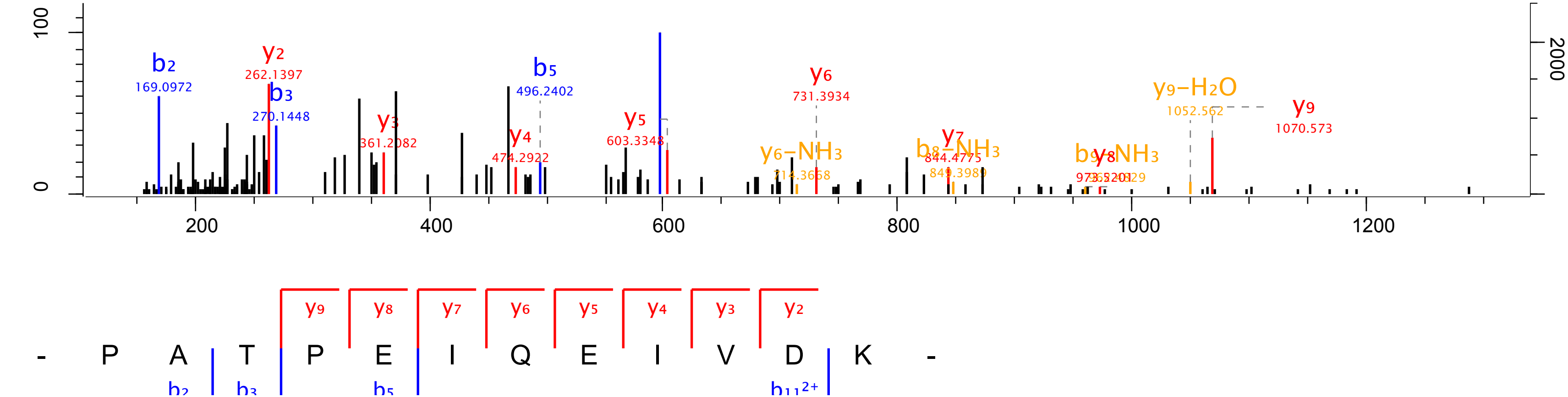

Raw file	Scan	Method	Score	m/z	Gene names
20141014_fract8_dyn_5ul_E8_01_587	25841	TOF; CID	102.07	502.27	PGM5

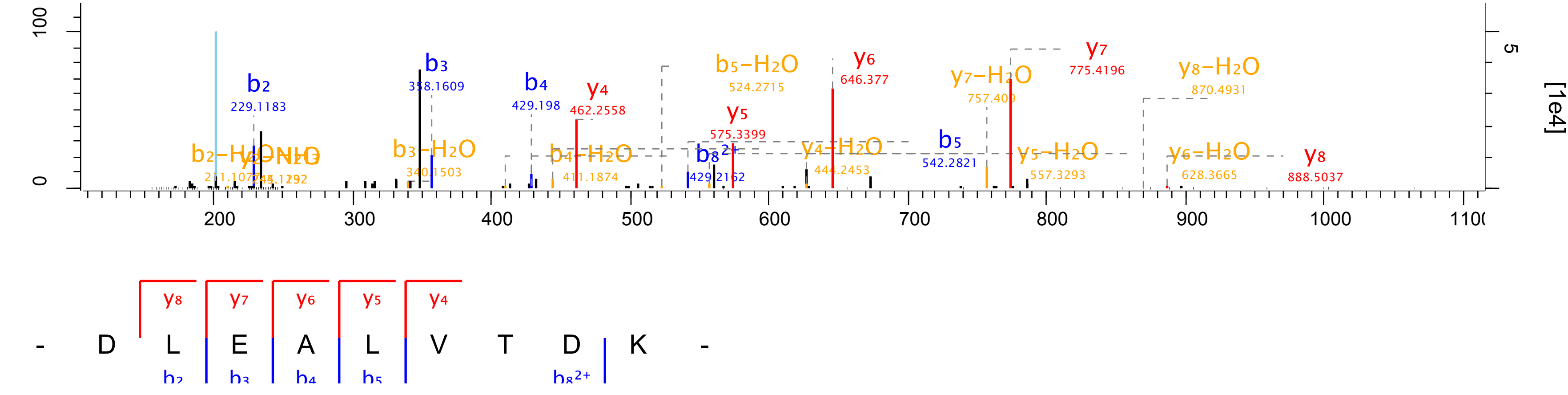

Raw file	Scan	Method	Score	m/z	Gene names
20141014_fract8_dyn_5ul_E8_01_587	29898	TOF; CID	113.42	1173.05	PKIG

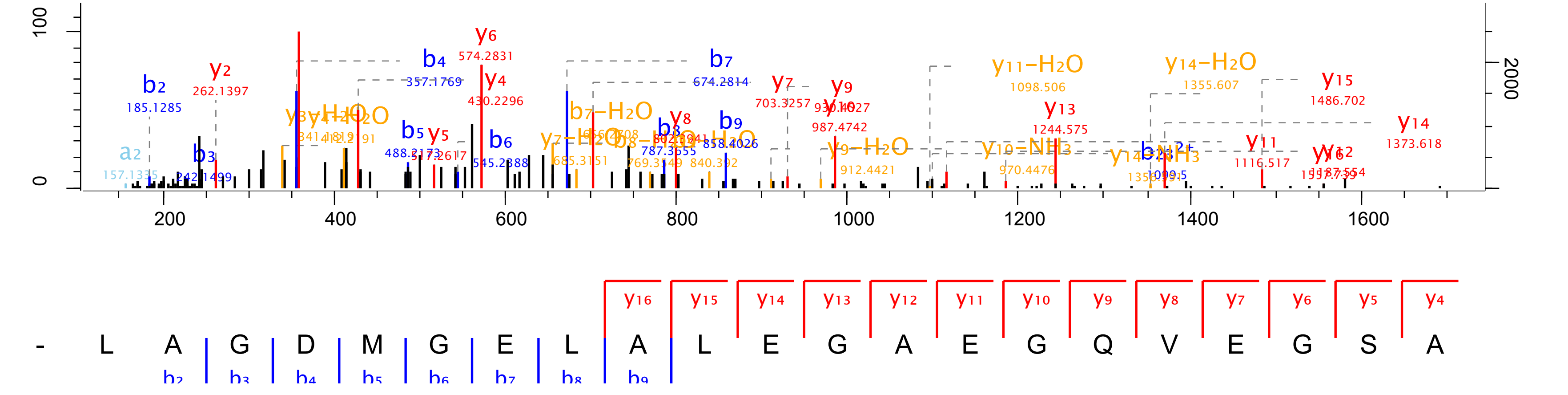

Raw file	Scan	Method	Score	m/z	Gene names
20141014_fract8_dyn_5ul_E8_01_587	36842	TOF; CID	80.24	497.58	KCTD7

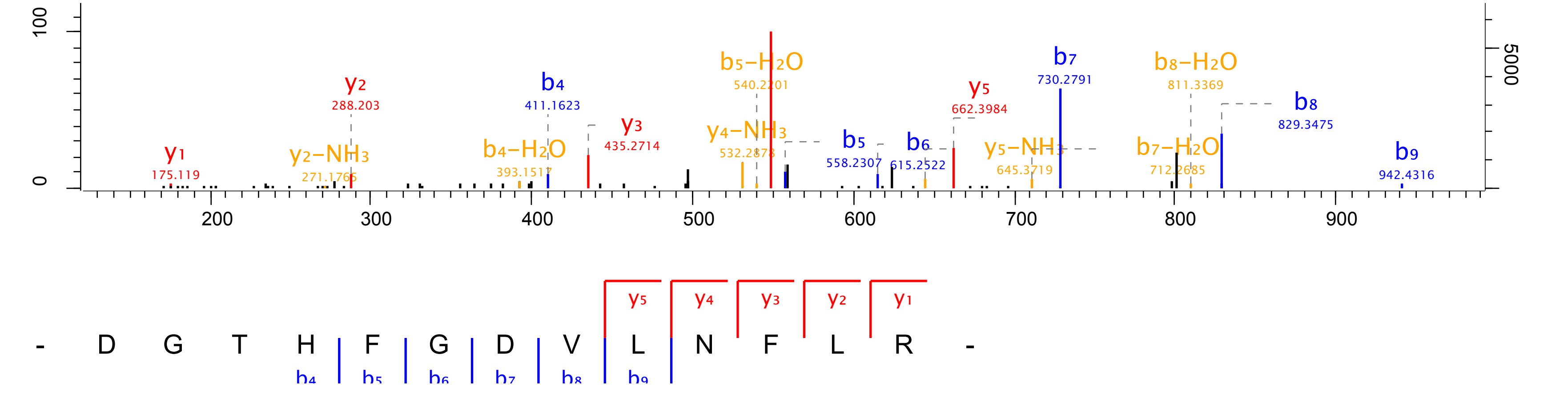

Raw file	Scan	Method	Score	m/z	Gene names
20141014_fract8_dyn_5ul_E8_01_587	38691	TOF; CID	54.28	729.39	IDNK

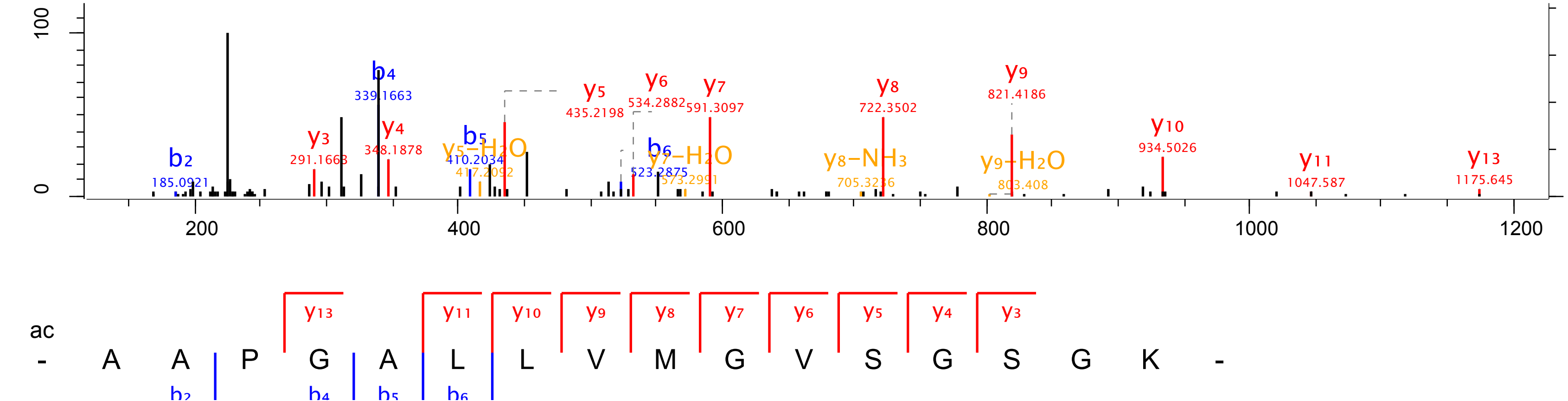

Raw file	Scan	Method	Score	m/z	Gene names
20141014_fract9_dyn_5ul_F1_01_588	25104	TOF; CID	93.32	632.34	CKLF

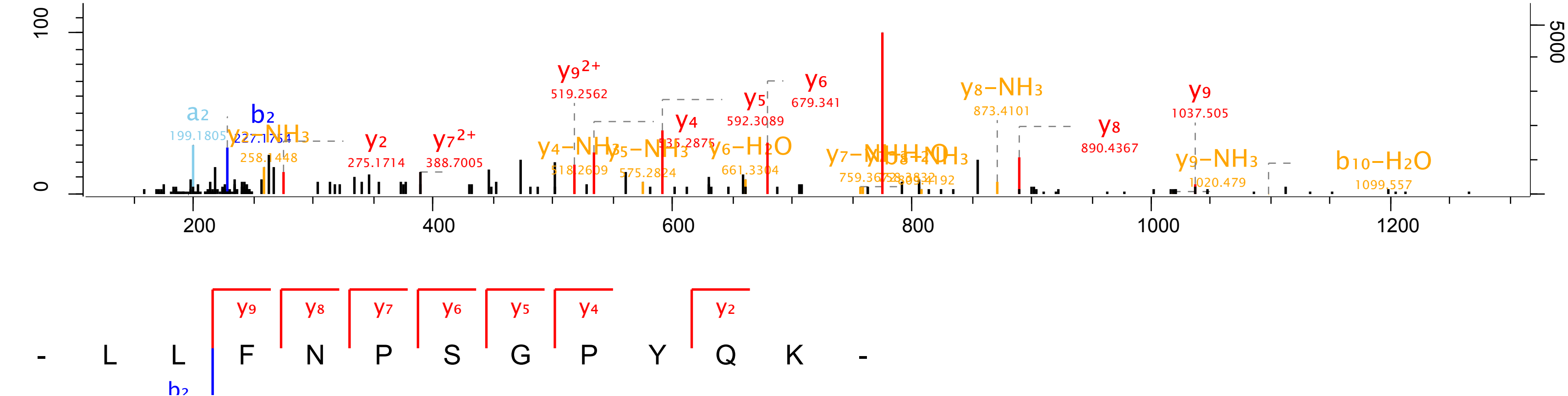

Raw file	Scan	Method	Score	m/z	Gene names
20141014_fract9_dyn_5ul_F1_01_588	30140	TOF; CID	111.95	529.28	SLC9A8

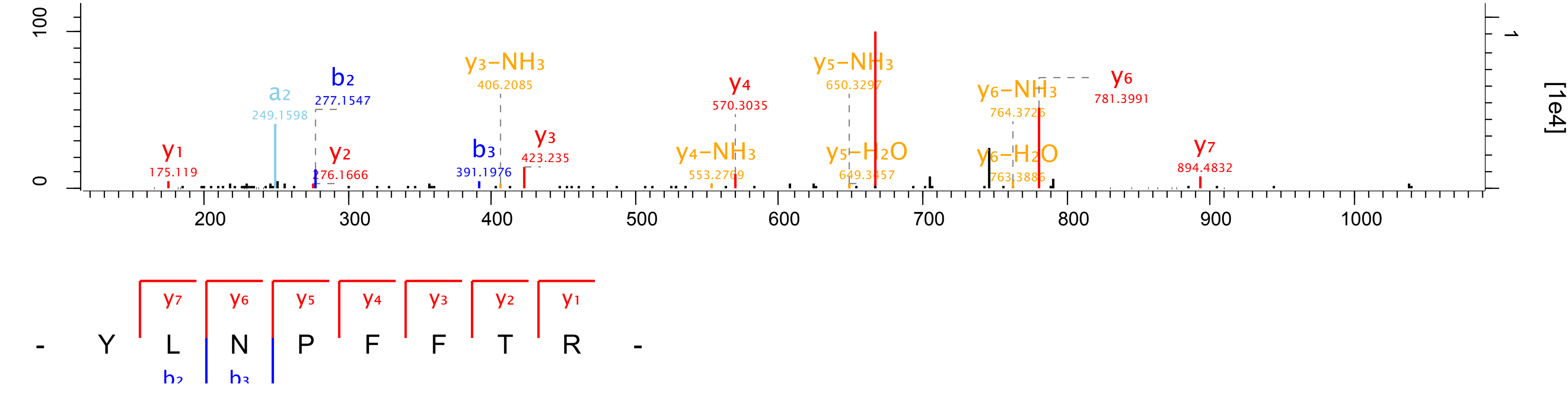

Raw file	Scan	Method	Score	m/z	Gene names
20141014_fract9_dyn_5ul_F1_01_588	38402	TOF; CID	34.04	1150.89	ZAN

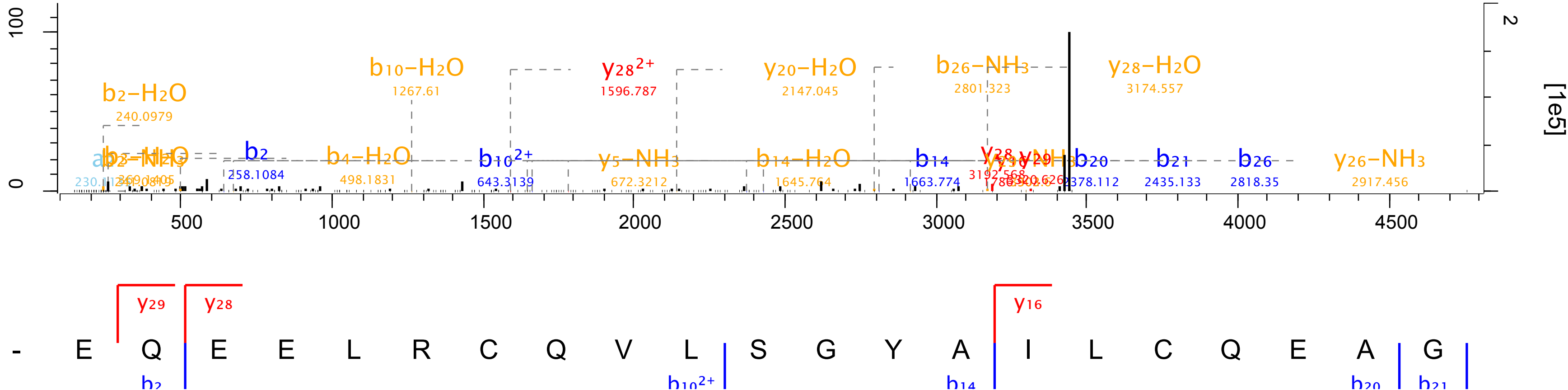

Raw file	Scan	Method	Score	m/z	Gene names
20141014_fract10_dyn_5ul_F2_01_589	16187	TOF; CID	86.46	432.9	NDUFC1

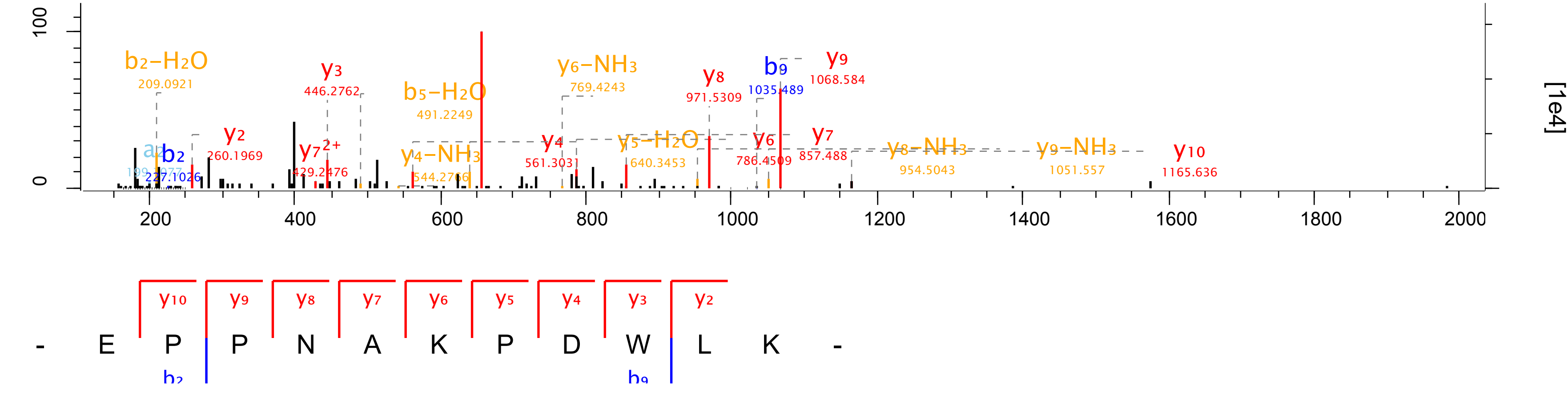

Raw file	Scan	Method	Score	m/z	Gene names
20141014_fract10_dyn_5ul_F2_01_589	23140	TOF; CID	71.08	617.3	TMEM104

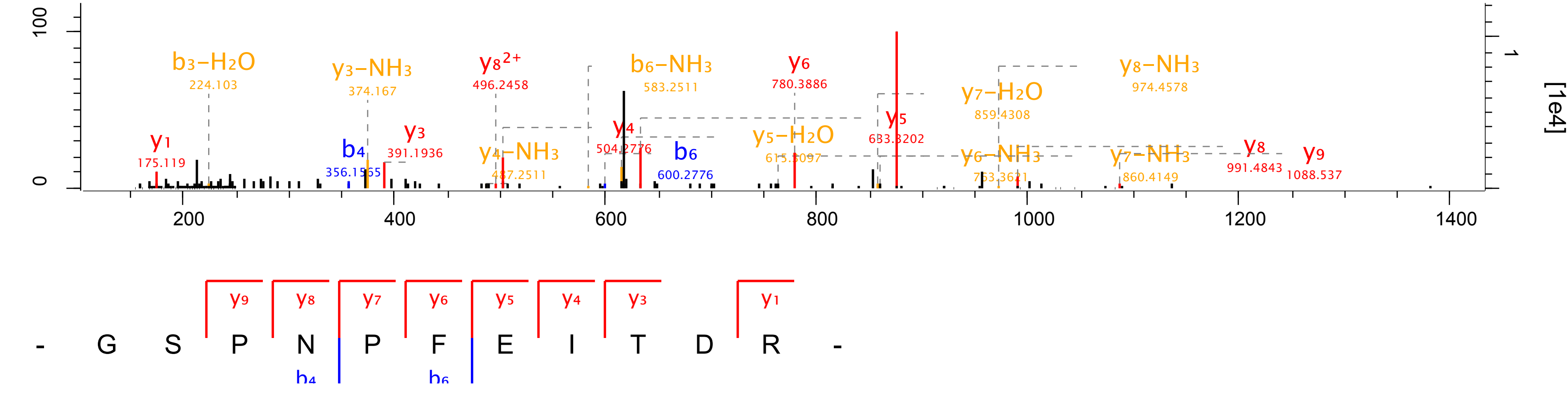

Raw file	Scan	Method	Score	m/z	Gene names
20141014_fract10_dyn_5ul_F2_01_589	37241	TOF; CID	118.37	1351.63	SMAGP

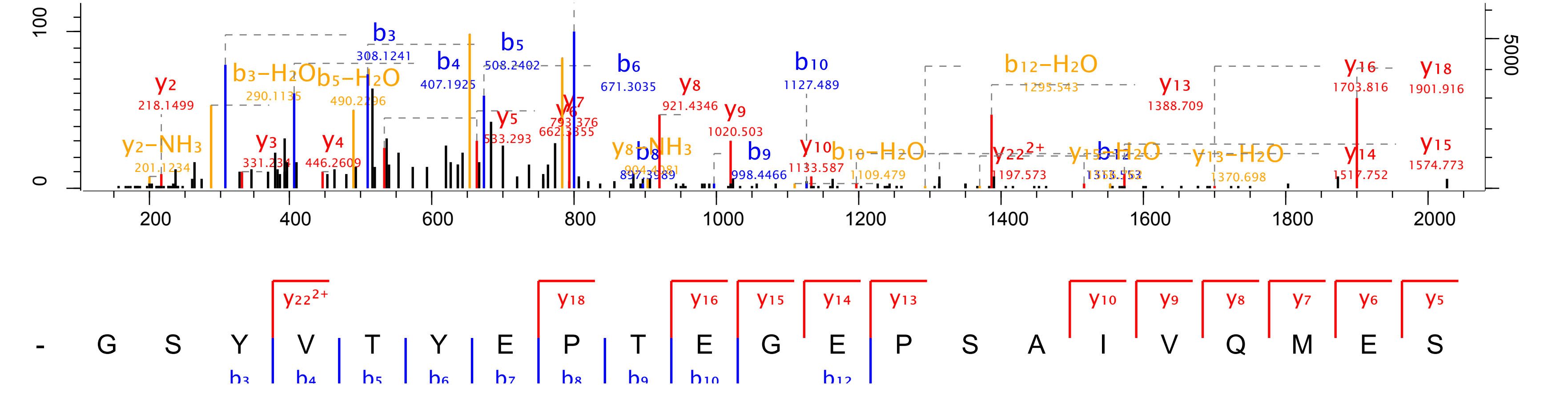

Raw file	Scan	Method	Score	m/z	Gene names
20141014_fract10_dyn_5ul_F2_01_589	41167	TOF; CID	36.55	1214.62	DAZAP2

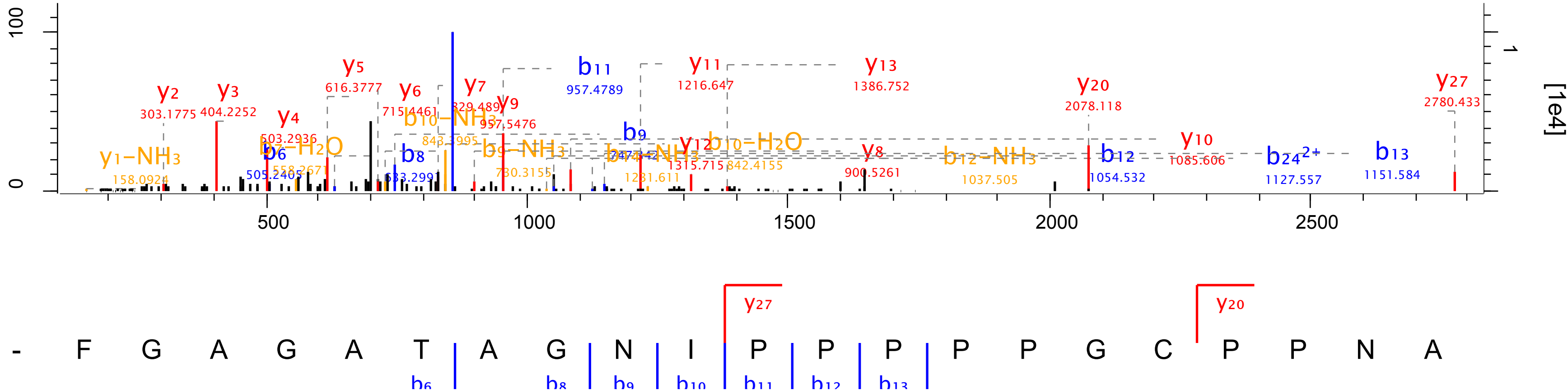

Raw file	Scan	Method	Score	m/z	Gene names
20141014_fract11_dyn_5ul_F3_01_590	12591	TOF; CID	58.89	463.02	ADAMTS15

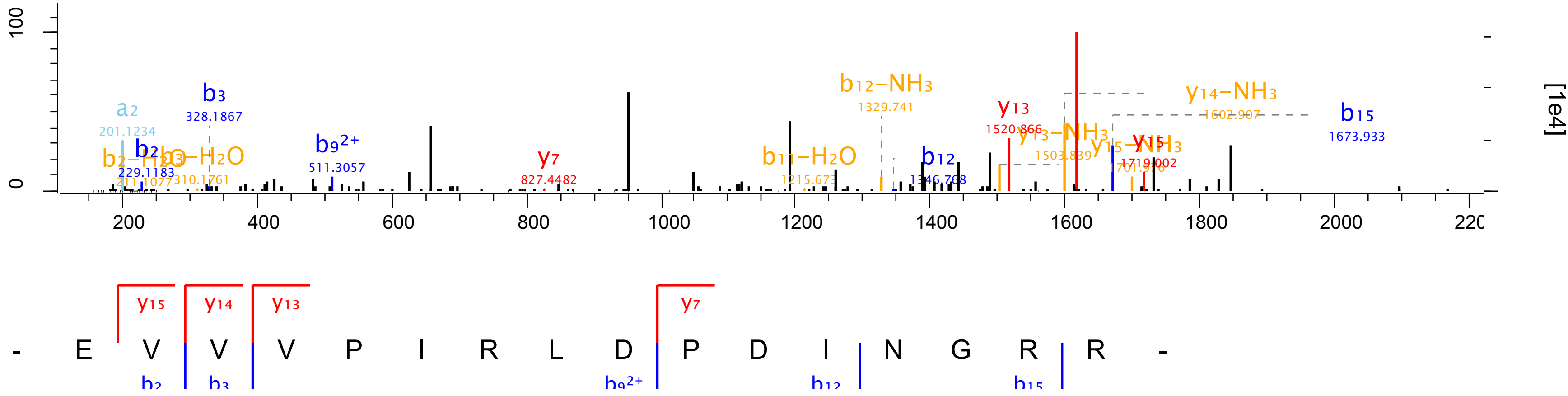

Raw file	Scan	Method	Score	m/z	Gene names
20141014_fract11_dyn_5ul_F3_01_590	14078	TOF; CID	152.34	497.26	AKR1D1

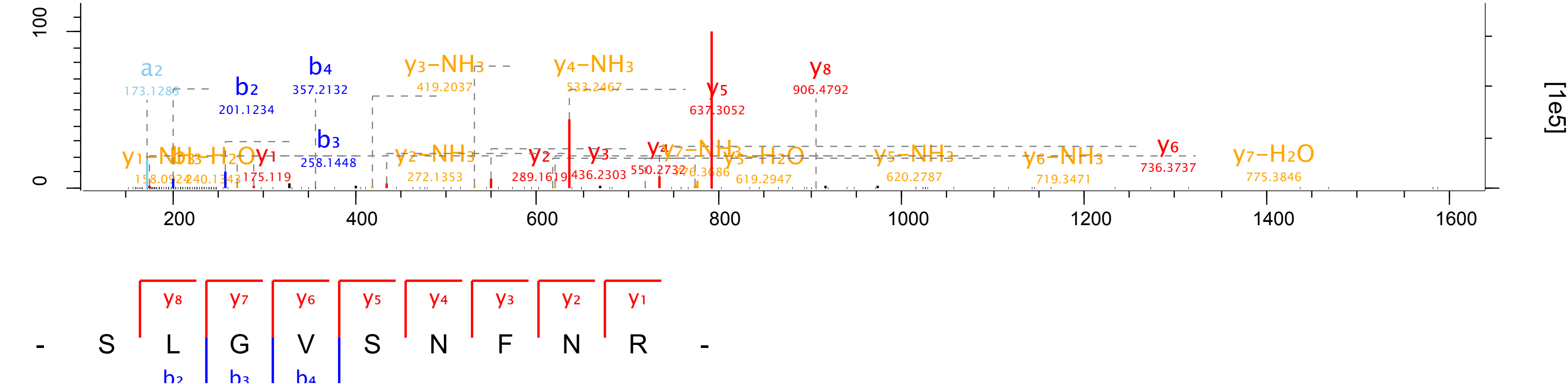

Raw file	Scan	Method	Score	m/z	Gene names
20141014_fract12_dyn_5ul_F4_01_591	12895	TOF; CID	151.07	586.8	SULT1E1

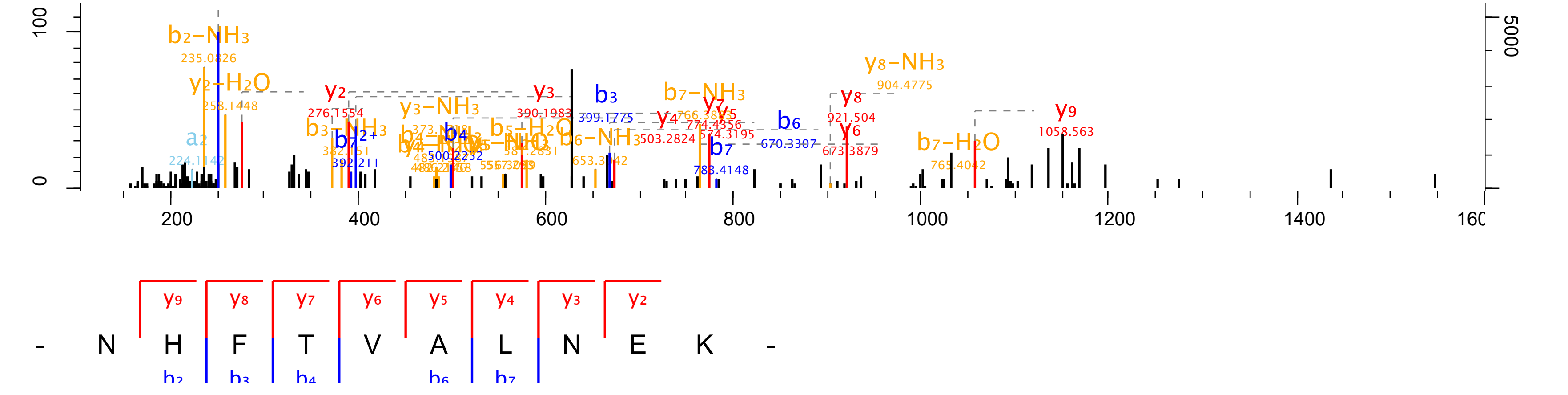

Raw file	Scan	Method	Score	m/z	Gene names
20141014_fract12_dyn_5ul_F4_01_591	15999	TOF; CID	93.6	479.27	SLIT1

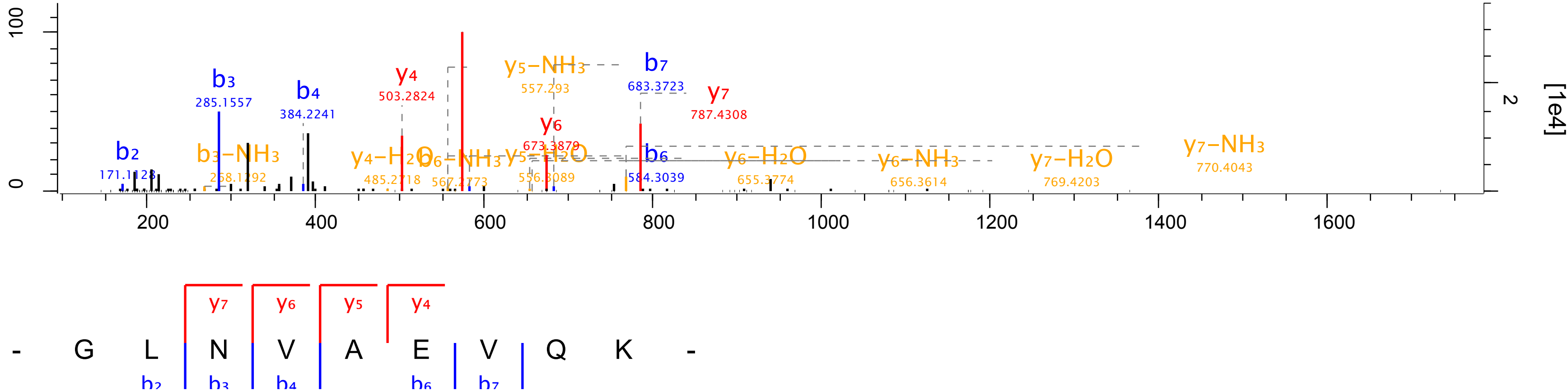

Raw file	Scan	Method	Score	m/z	Gene names
20141014_fract12_dyn_5ul_F4_01_591	28389	TOF; CID	121.56	710.84	SLC39A13

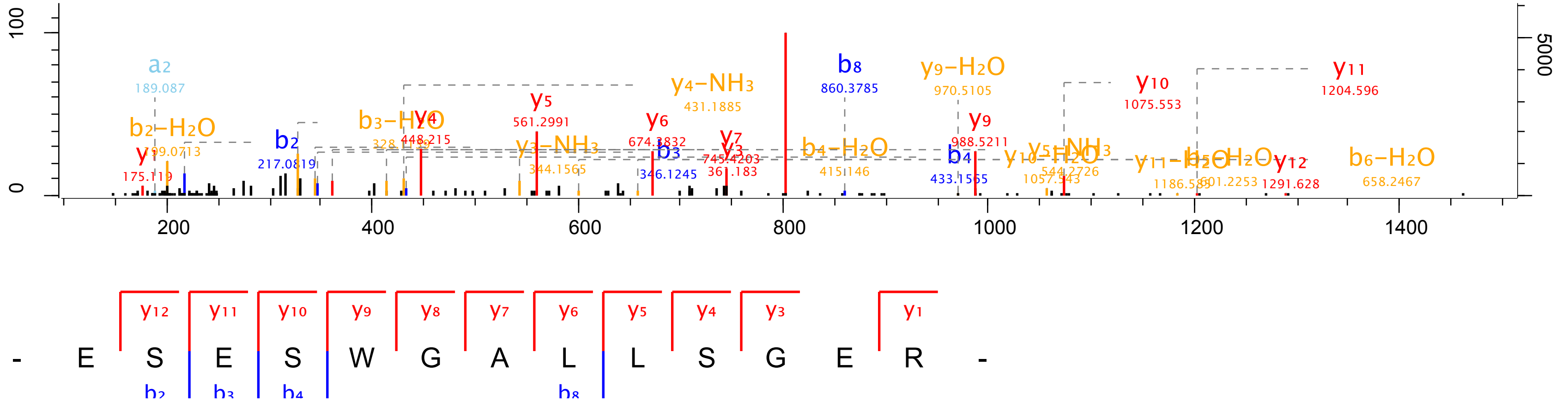

Raw file	Scan	Method	Score	m/z	Gene names
20141014_fract12_dyn_5ul_F4_01_591	34915	TOF; CID	140.2	666.09	SLC39A8

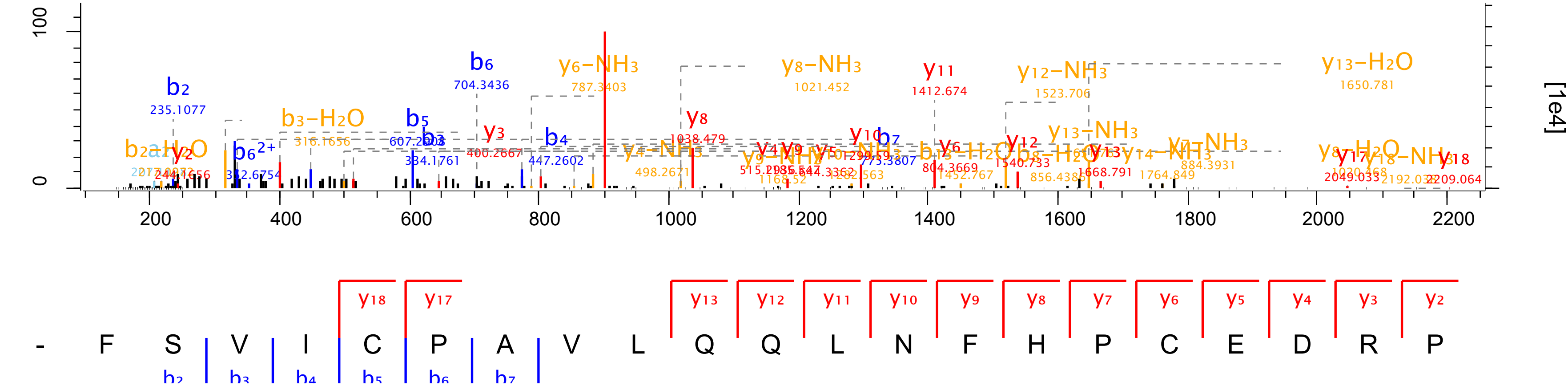

Raw file	Scan	Method	Score	m/z	Gene names
20141014_fract12_dyn_5ul_F4_01_591	37009	TOF; CID	41.26	546.79	S100A12

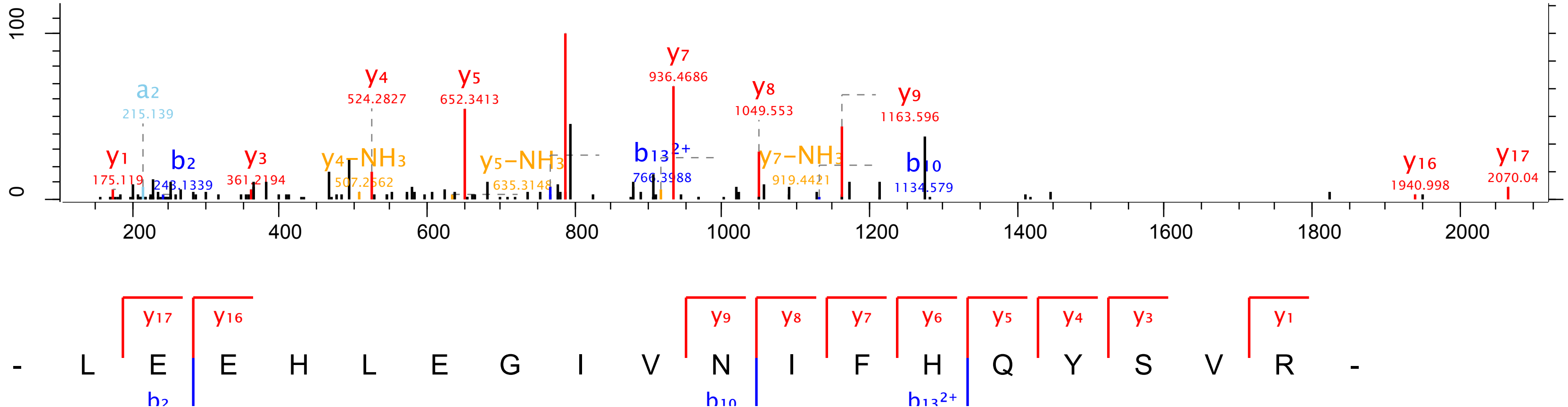

Raw file	Scan	Method	Score	m/z	Gene names
20141014_fract12_dyn_5ul_F4_01_591	38365	TOF; CID	128.22	821.44	ATP6V0D2

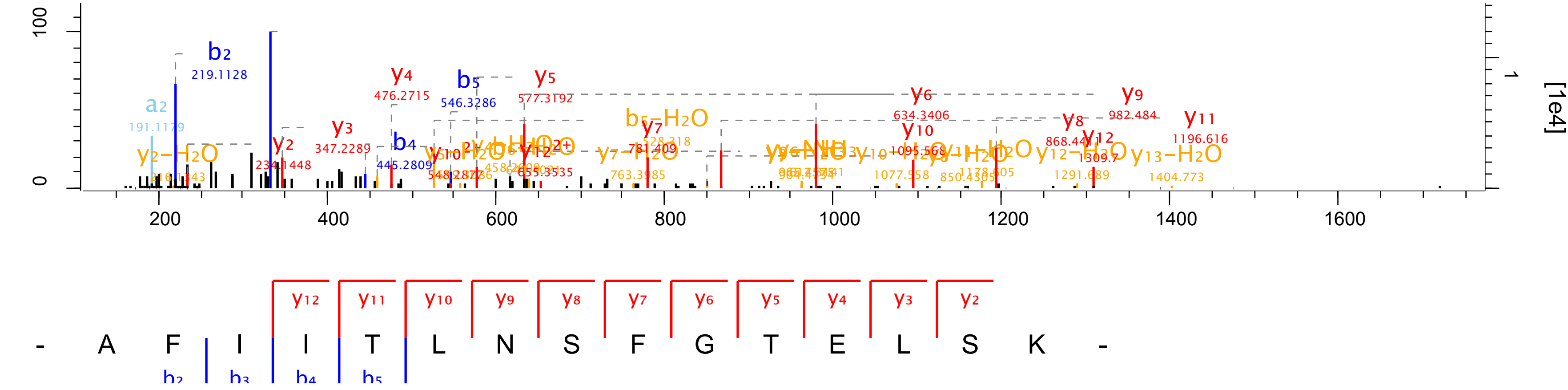

Raw file	Scan	Method	Score	m/z	Gene names
20141014_fract12_dyn_5ul_F4_01_591	38881	TOF; CID	53.3	712.44	SLC36A1

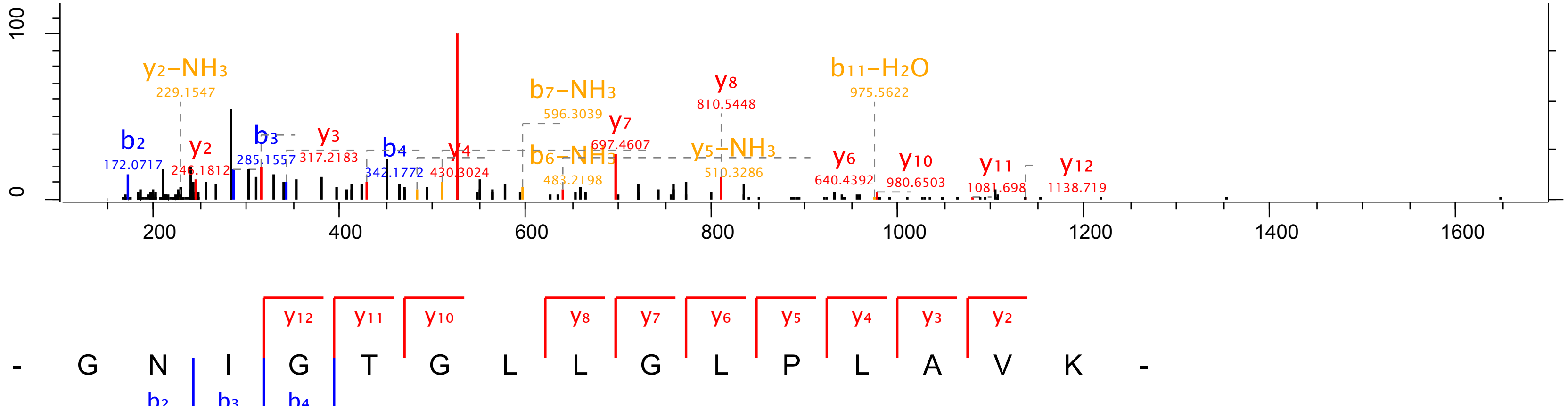

Raw file	Scan	Method	Score	m/z	Gene names
20141014_fract13_dyn_5ul_F5_01_592	29425	TOF; CID	78.67	800.41	ATMIN

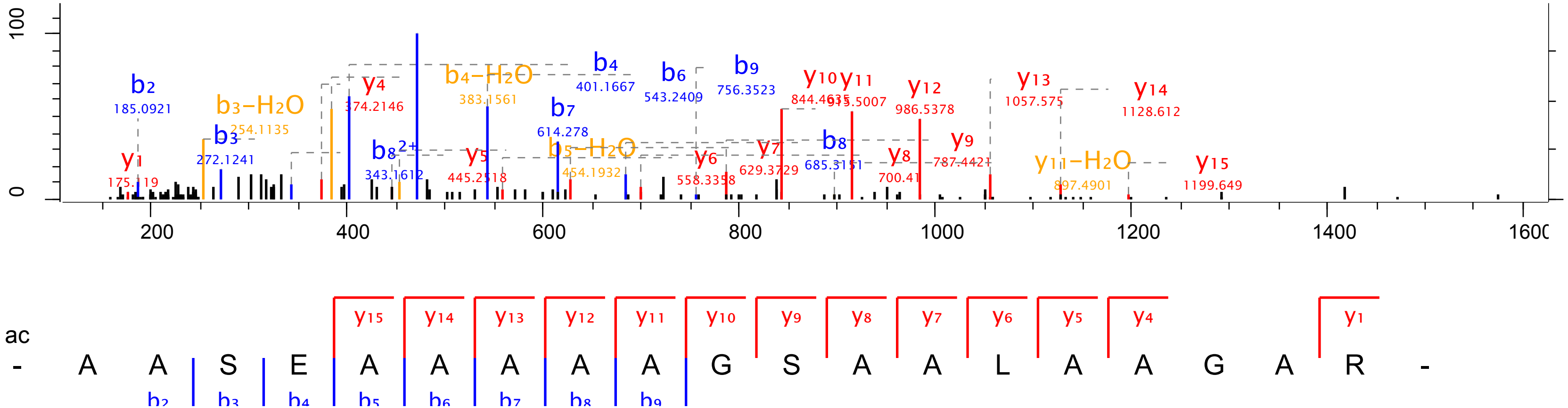

Raw file	Scan	Method	Score	m/z	Gene names
20141014_fract13_dyn_5ul_F5_01_592	37719	TOF; CID	60.55	748.44	SIM2;SIM1

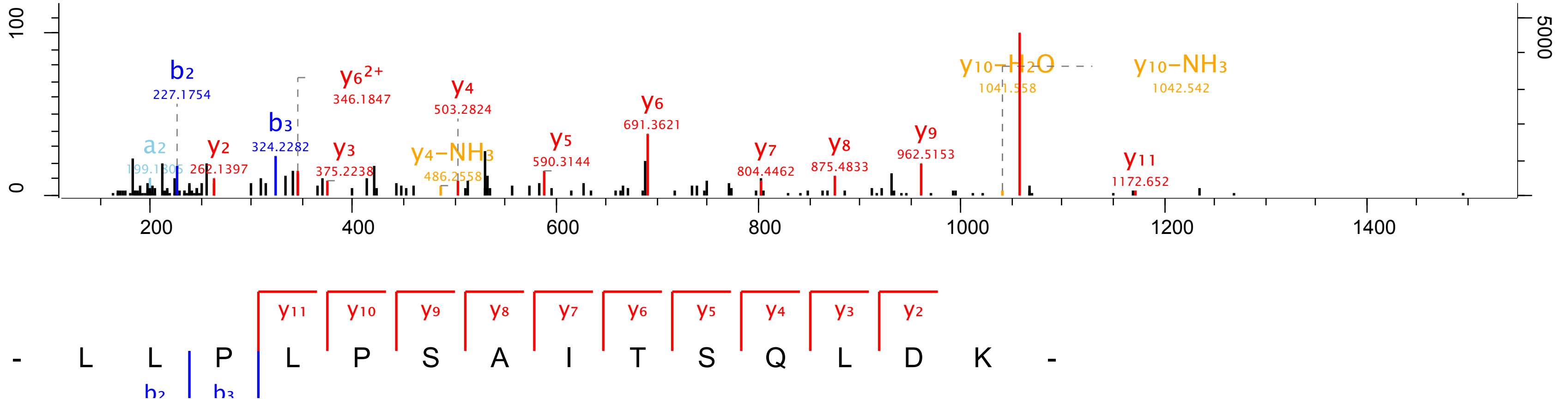

Raw file	Scan	Method	Score	m/z	Gene names
20141014_fract14_dyn_5ul_F6_01_593	10326	TOF; CID	65.18	412.9	ARRDC1-AS1

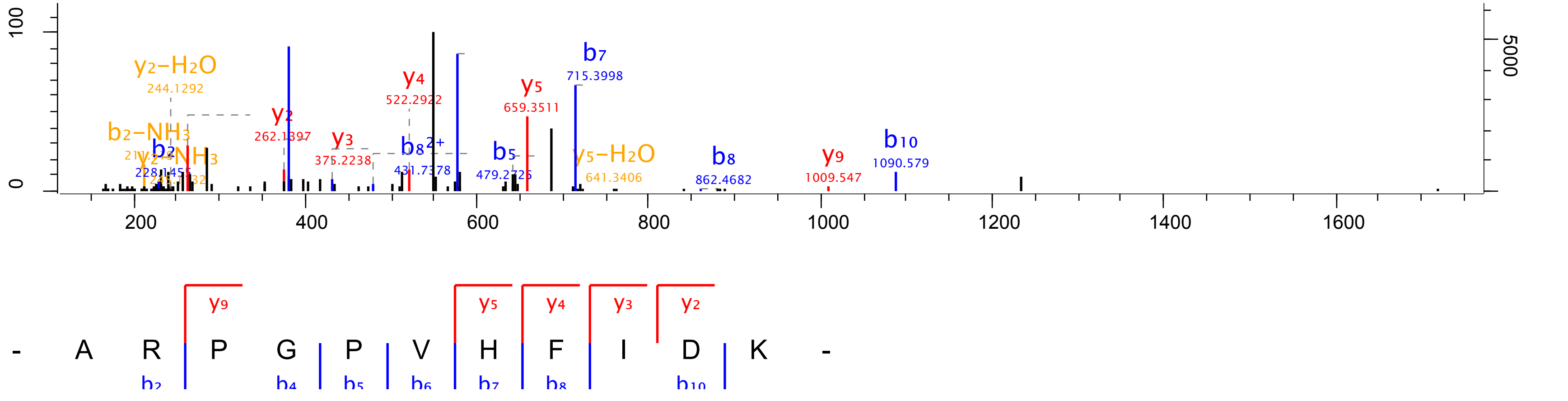

Raw file	Scan	Method	Score	m/z	Gene names
20141014_fract14_dyn_5ul_F6_01_593	35876	TOF; CID	78.81	770.42	NPL

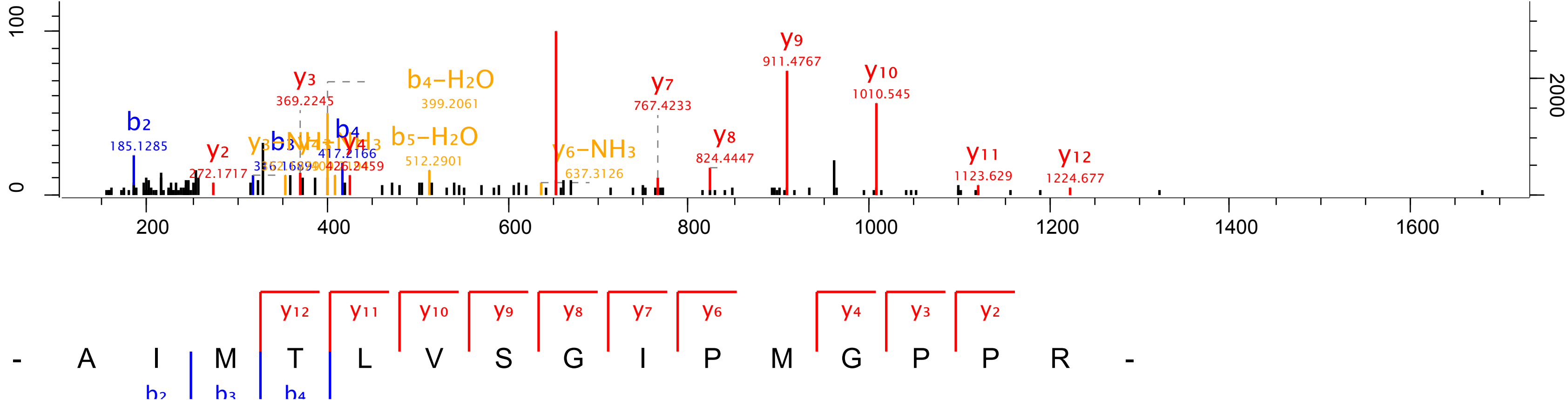

Raw file	Scan	Method	Score	m/z	Gene names
20141014_fract15_dyn_5ul_F7_01_594	41392	TOF; CID	86.8	1029.03	SLC50A1

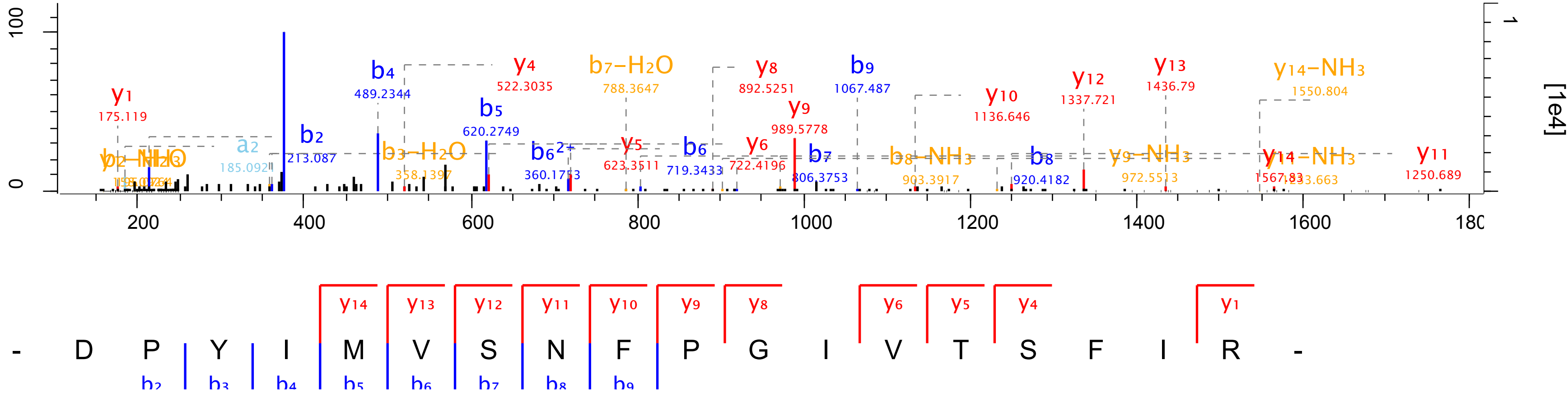

Raw file	Scan	Method	Score	m/z	Gene names
20141014_fract16_dyn_5ul_F8_01_595	15923	TOF; CID	89.43	602.33	DAZAP2

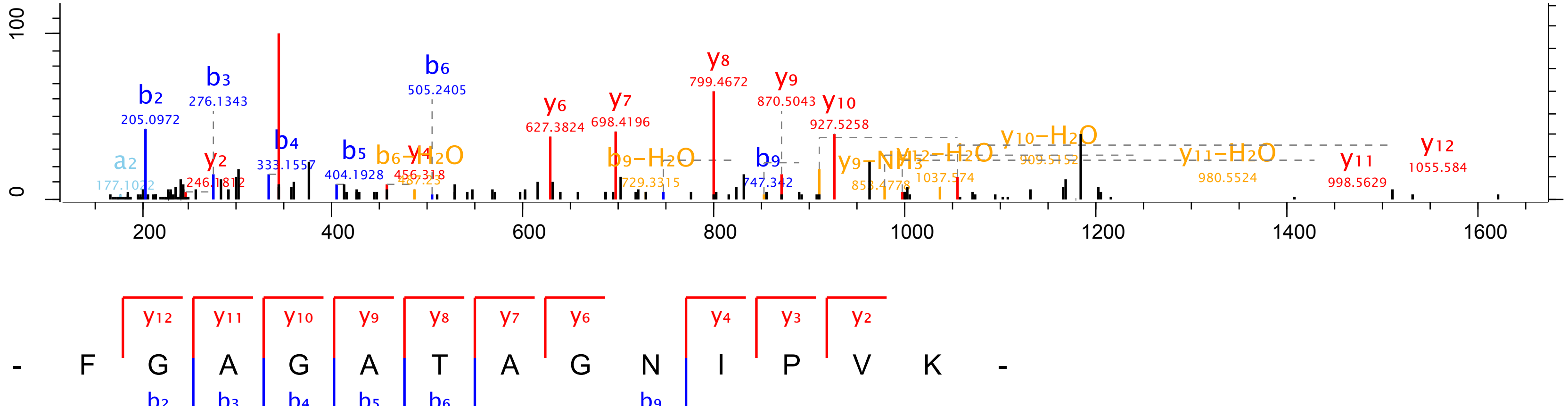

Raw file	Scan	Method	Score	m/z	Gene names
20141014_fract16_dyn_5ul_F8_01_595	28148	TOF; CID	81.97	1010.09	FAM199X

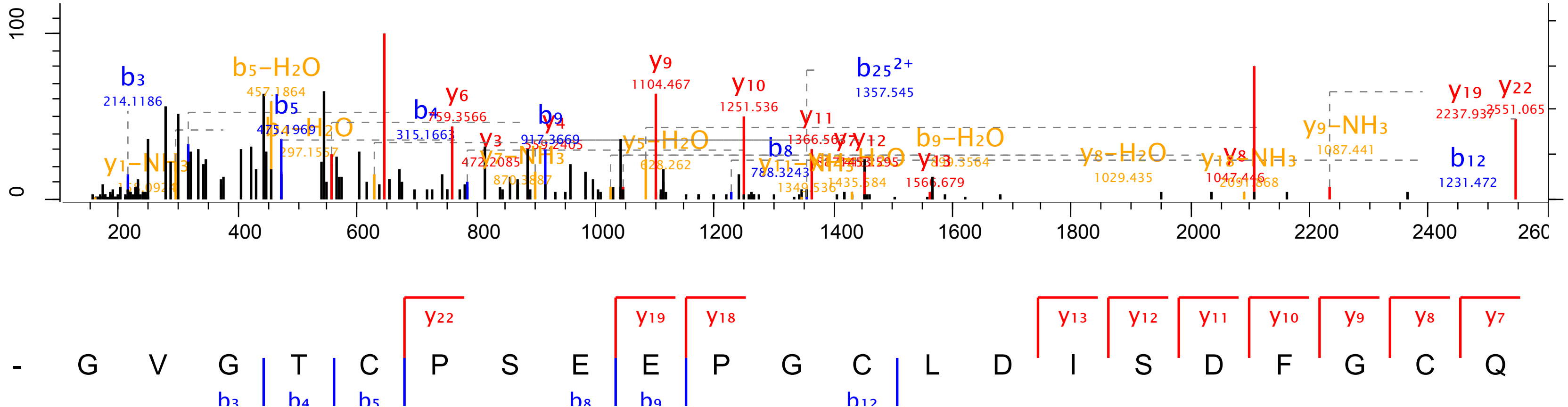

Raw file	Scan	Method	Score	m/z	Gene names
20141014_fract17_dyn_5ul_G1_01_596	19333	TOF; CID	91.96	553.79	C19orf43

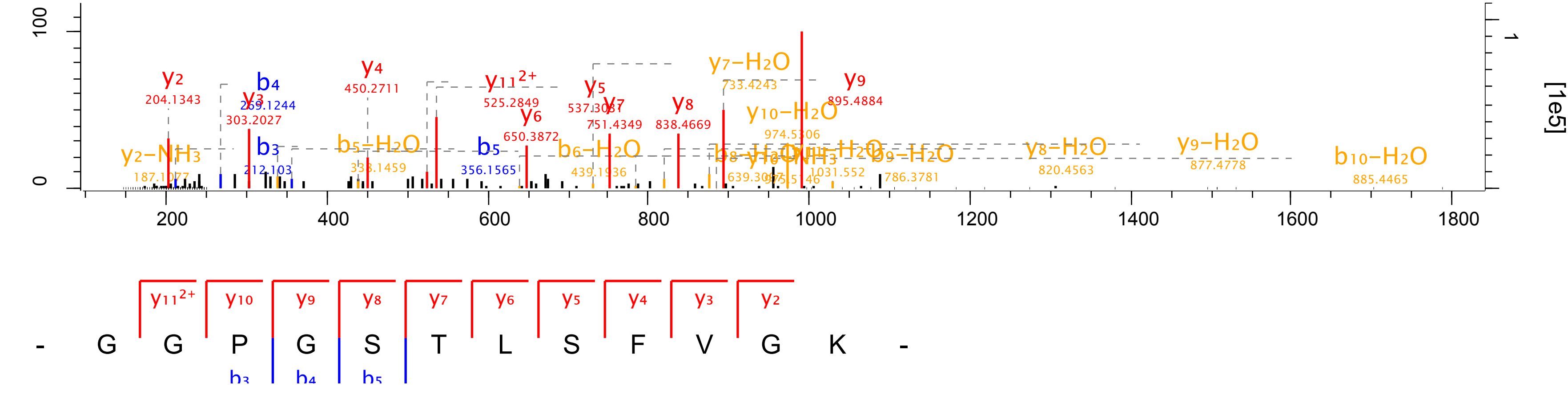

Raw file	Scan	Method	Score	m/z	Gene names
20141014_fract17_dyn_5ul_G1_01_596	23833	TOF; CID	98.16	587.81	SYNGR3

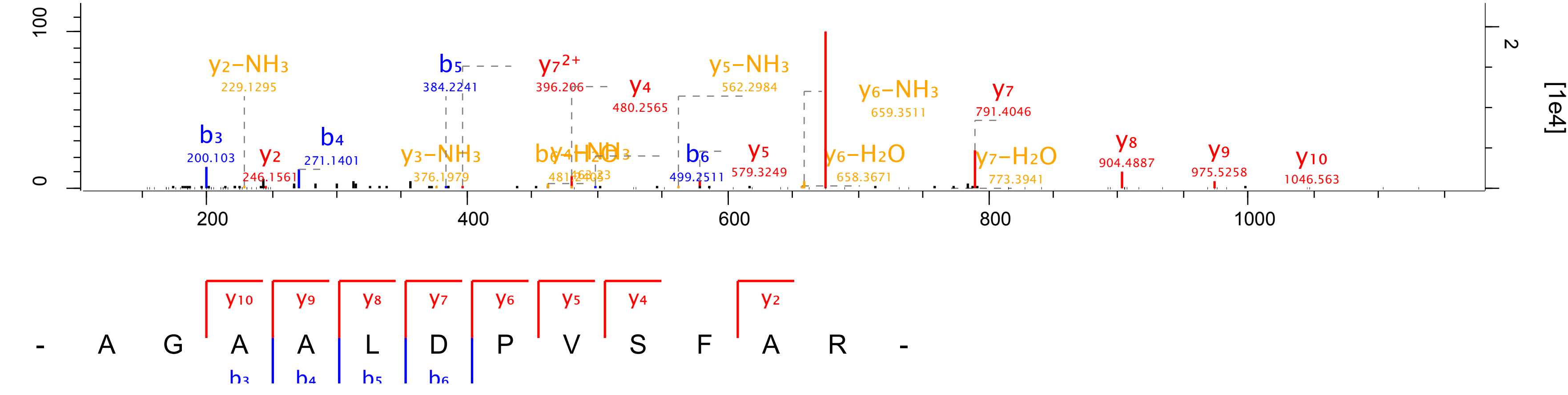

Raw file	Scan	Method	Score	m/z	Gene names
20141014_fract17_dyn_5ul_G1_01_596	27158	TOF; CID	64	930.47	HIAT1

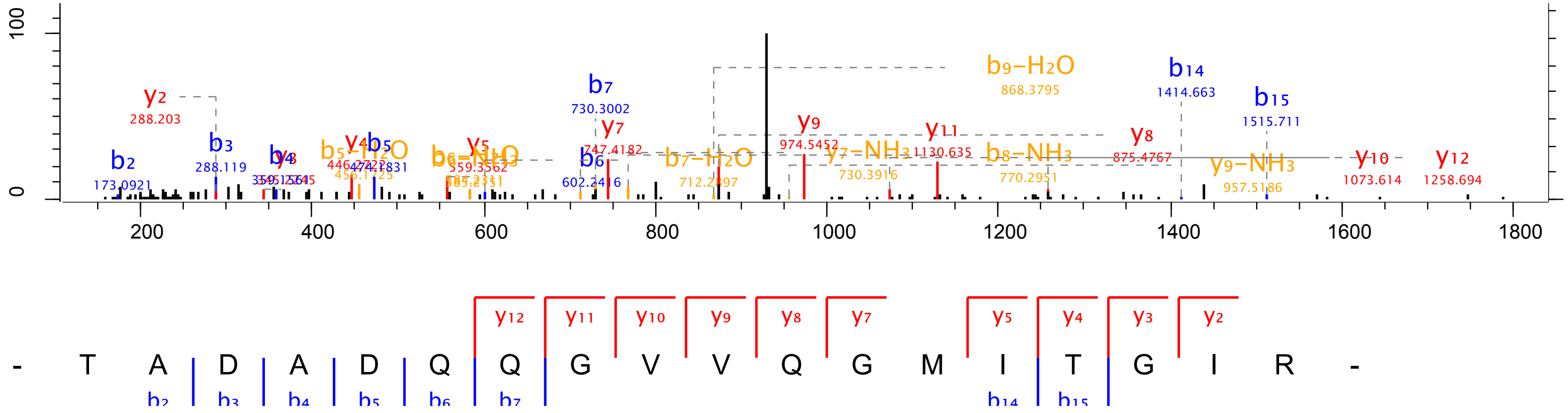

Raw file	Scan	Method	Score	m/z	Gene names
20141014_fract18_dyn_5ul_G2_01_597	29283	TOF; CID	204.53	917.11	EIF4EBP1

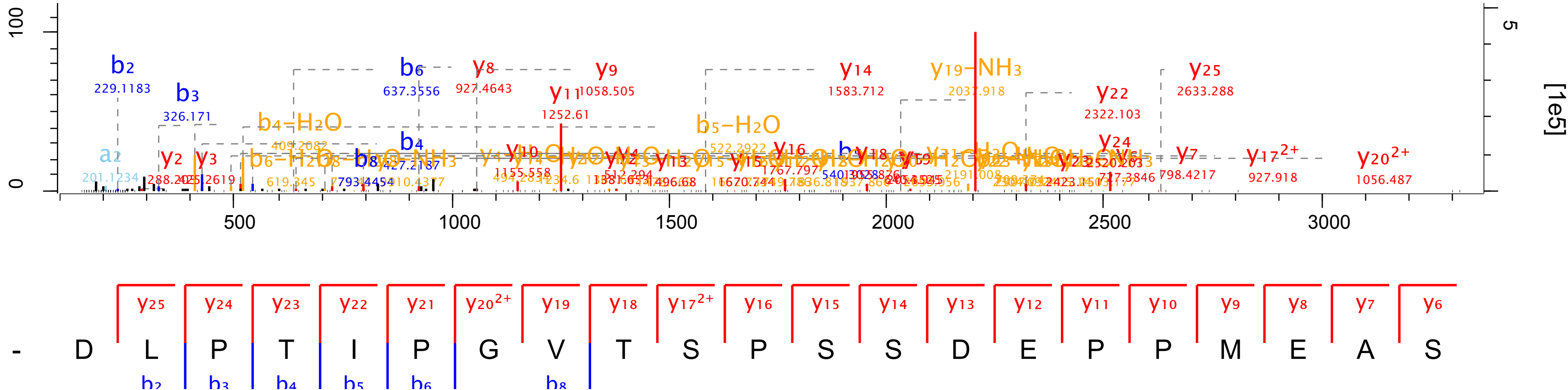

Raw file	Scan	Method	Score	m/z	Gene names
20141014_fract18_dyn_5ul_G2_01_597	32329	TOF; CID	96.1	968	EFHC1

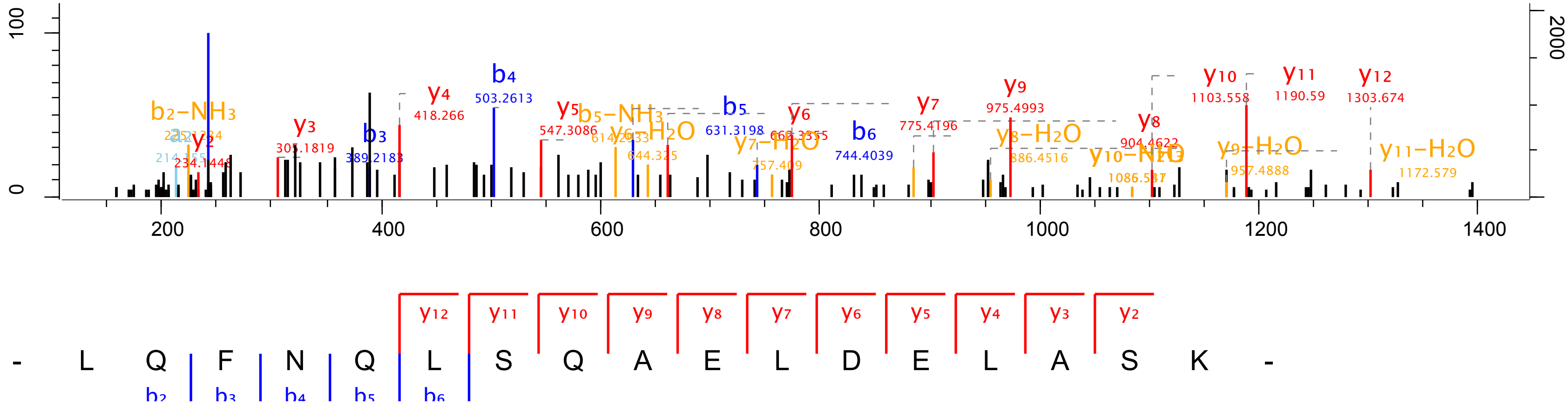

Raw file	Scan	Method	Score	m/z	Gene names
20141014_fract19_dyn_5ul_G3_01_598	10366	TOF; CID	55.04	530.25	MGAT5B

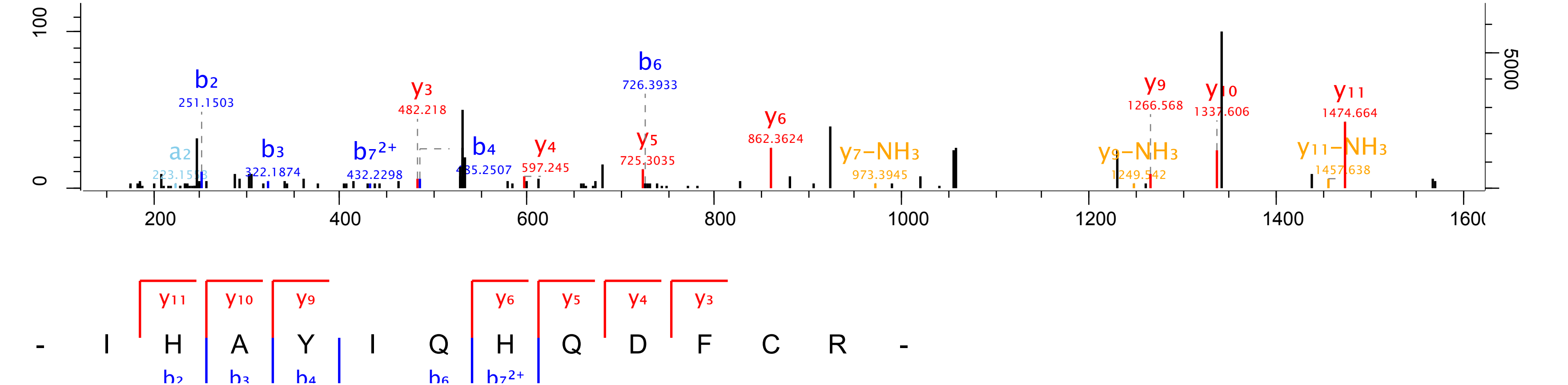

Raw file	Scan	Method	Score	m/z	Gene names
20141014_fract19_dyn_5ul_G3_01_598	38338	TOF; CID	74.54	894.45	CCDC70

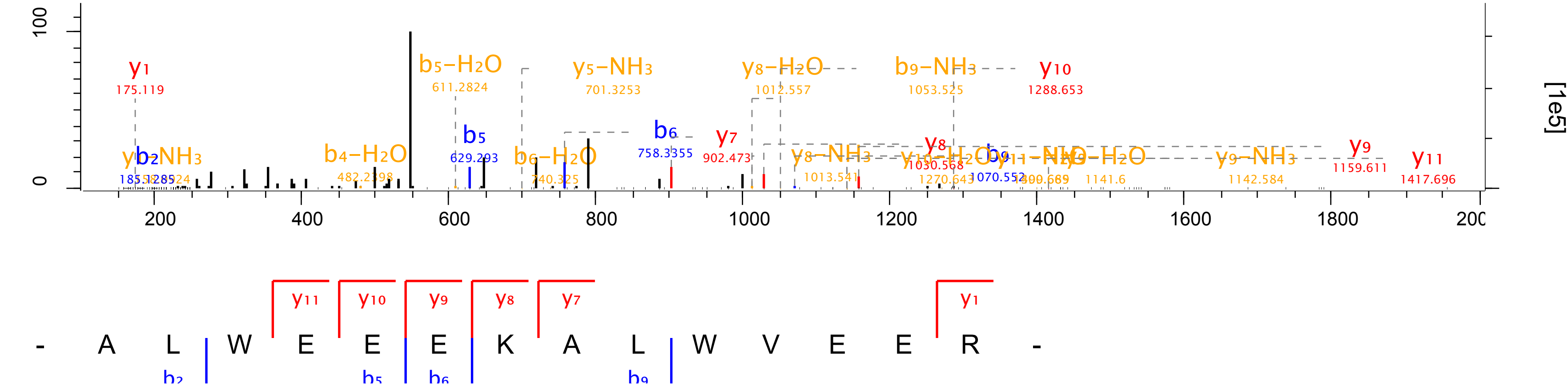

Raw file	Scan	Method	Score	m/z	Gene names
20141014_fract19_dyn_5ul_G3_01_598	38518	TOF; CID	84.75	565.02	SMIM11

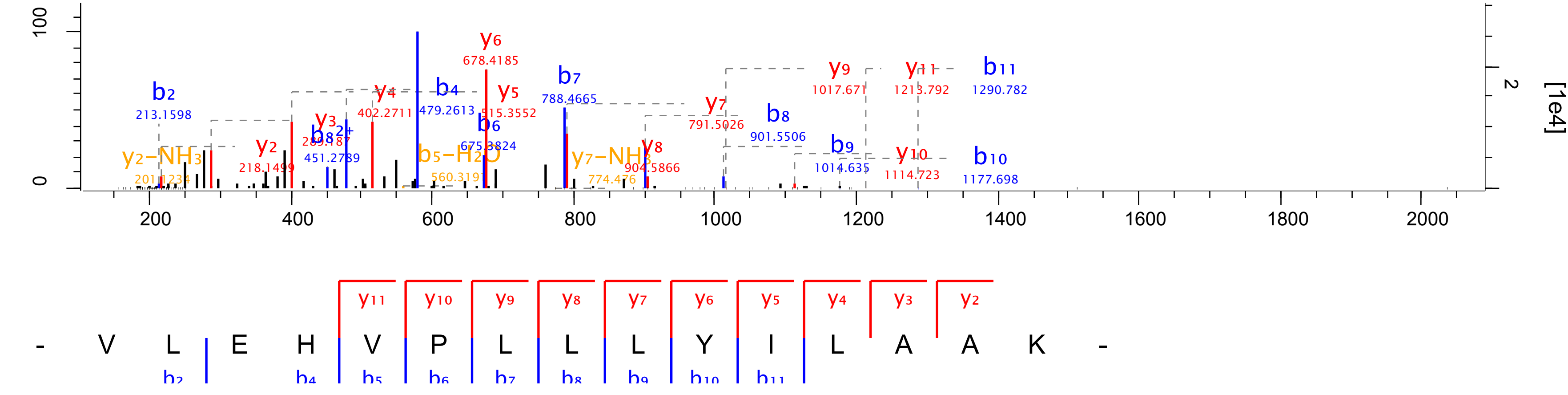

Raw file	Scan	Method	Score	m/z	Gene names
20141014_fract21_dyn_5ul_G5_01_600	28402	TOF; CID	63.68	585.64	TWSG1

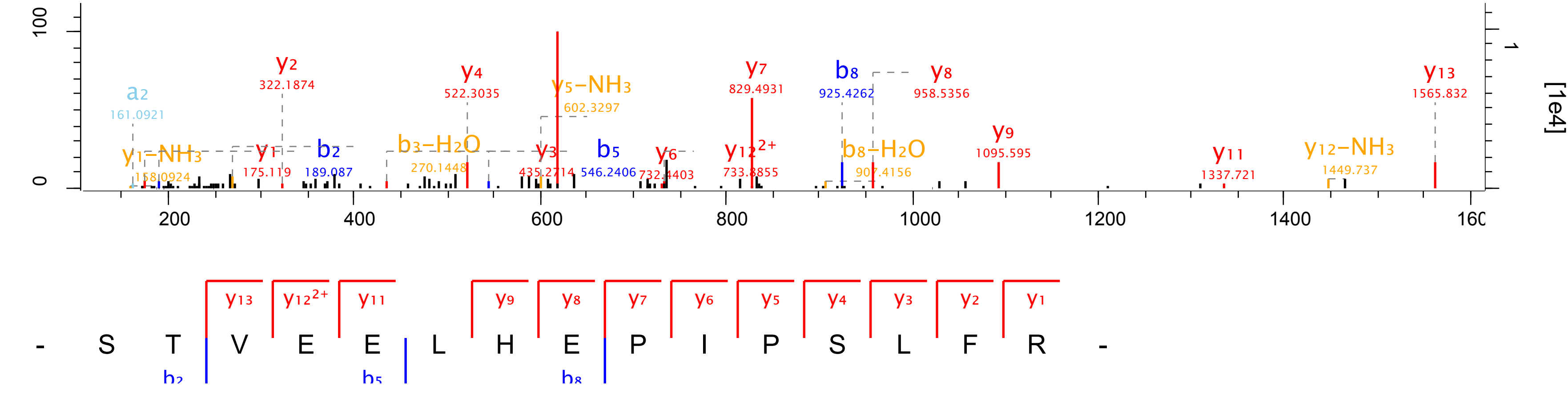

Raw file	Scan	Method	Score	m/z	Gene names
20141014_fract22_dyn_5ul_G6_01_601	22553	TOF; CID	105.9	734.65	ELOF1

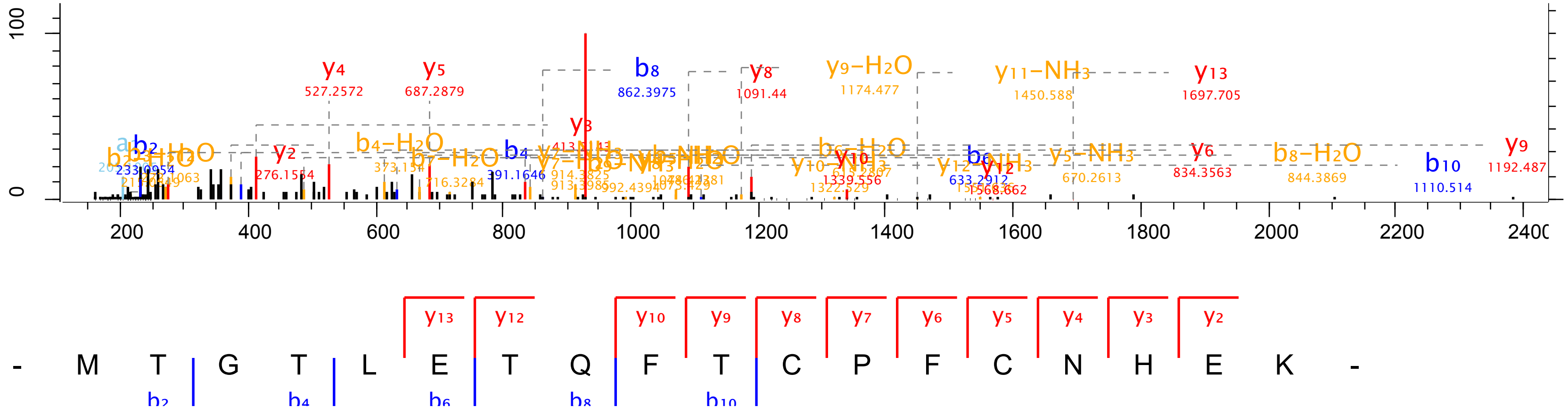

Raw file	Scan	Method	Score	m/z	Gene names
20141014_fract23_dyn_5ul_G7_01_602	16291	TOF; CID	117.1	469.76	APH1A

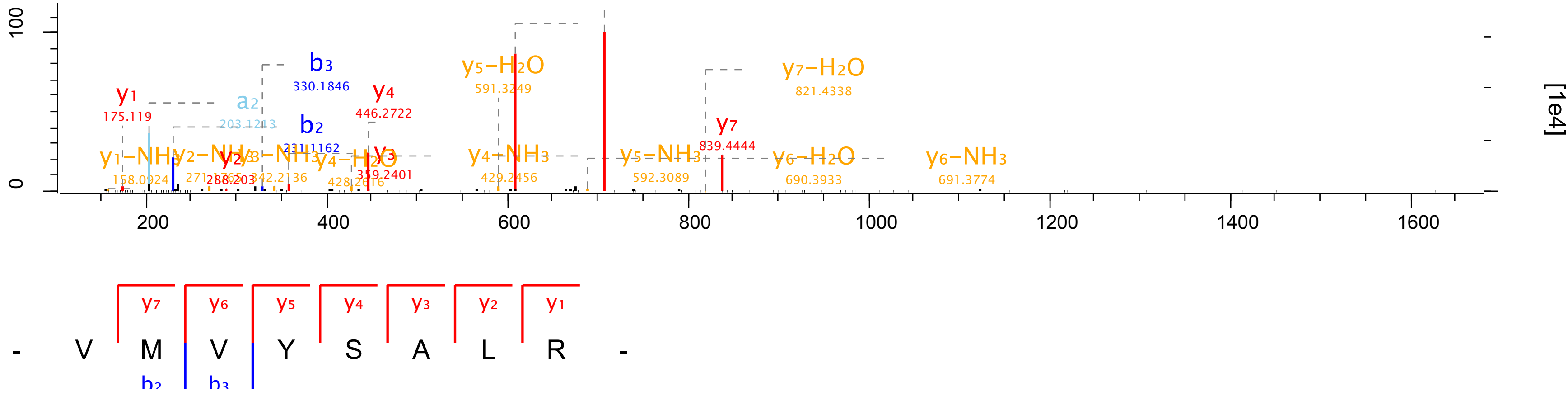

Raw file	Scan	Method	Score	m/z	Gene names
20141014_fract23_dyn_5ul_G7_01_602	17175	TOF; CID	75.5	632.35	TTBK2

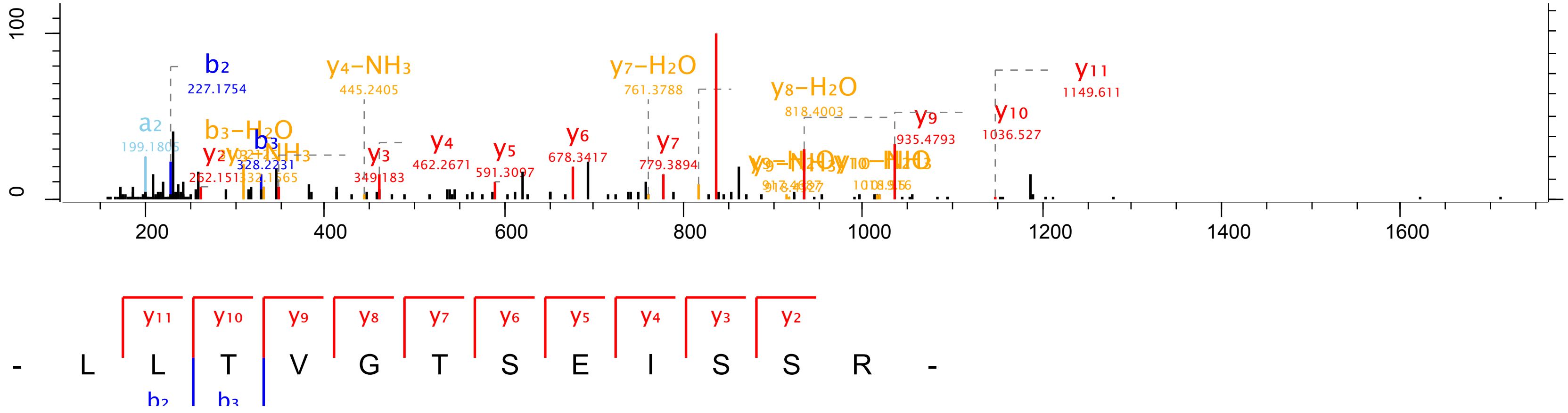

Raw file	Scan	Method	Score	m/z	Gene names
20141014_fract23_dyn_5ul_G7_01_602	35313	TOF; CID	65.84	698.87	PRKD2

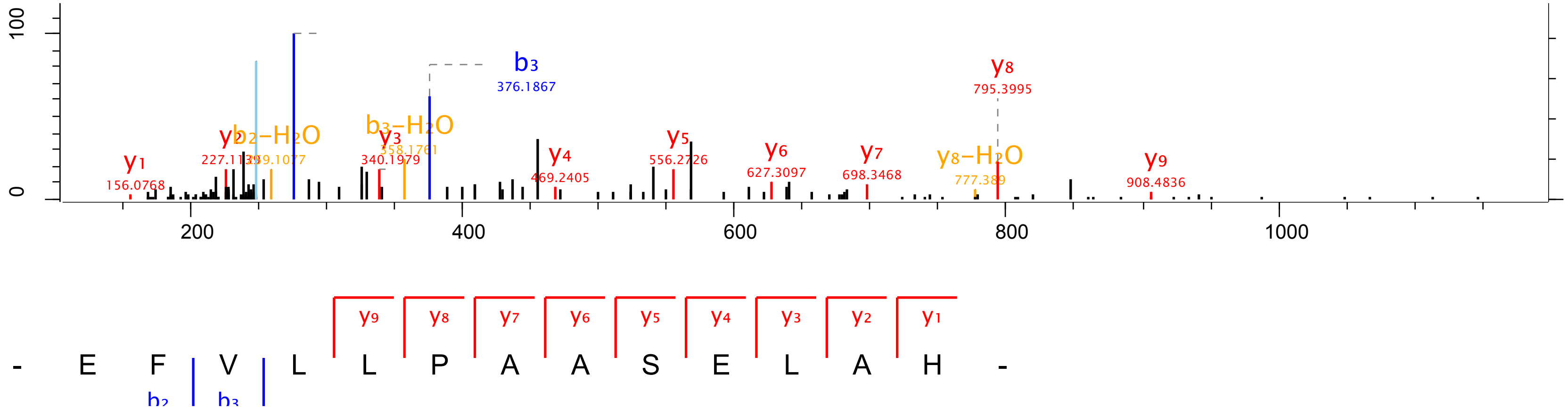

Raw file	Scan	Method	Score	m/z	Gene names
20141014_fract23_dyn_5ul_G7_01_602	37784	TOF; CID	51.71	1199.88	MYEOV2

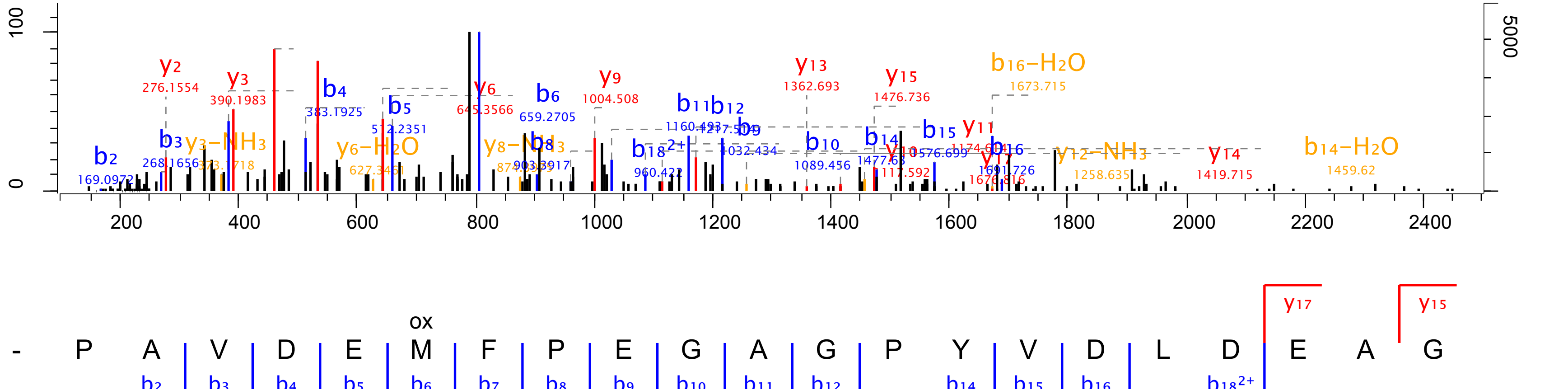

Raw file	Scan	Method	Score	m/z	Gene names
20141014_fract24_dyn_5ul_G8_01_603	12552	TOF; CID	95.5	528.81	TTC7B

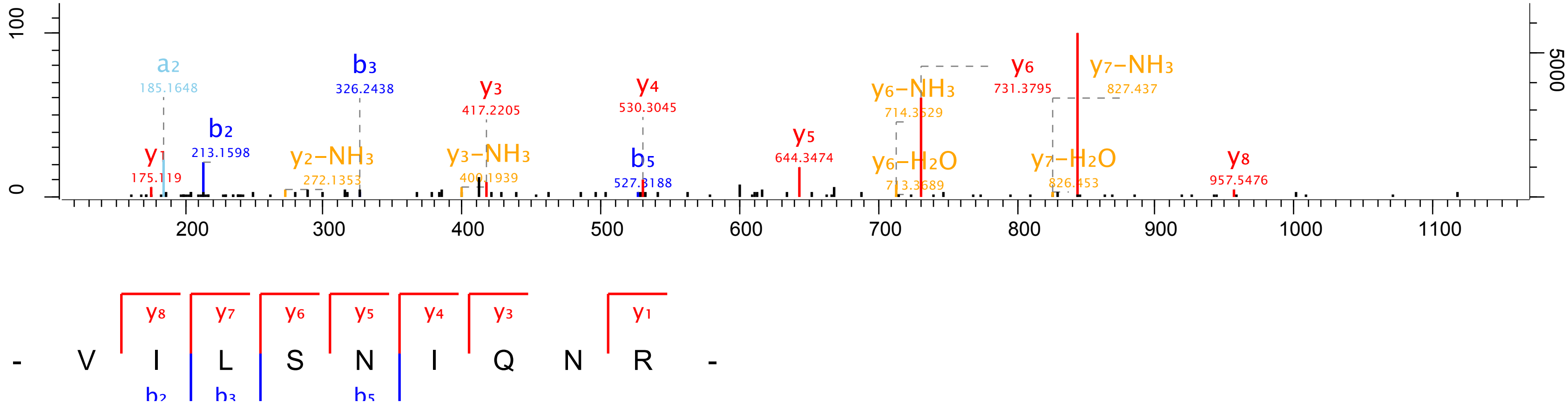

Supplement: Supplemental Data [file supp_M114.047407_mcp.M114.047407-13.pdf]
