# Supplementary material for: The Impact II, a Very High-Resolution Quadrupole Time-of-Flight Instrument (QTOF) for Deep Shotgun Proteomics
Source: Mol Cell Proteomics. 2015 May 19;14(7):2014–29. doi: 10.1074/mcp.M114.047407 (PMC4587313; doi:10.1074/mcp.M114.047407)

Raw file	Scan	Method	Score	m/z	Gene names
20150402_CerP14_Frac01_top_opt_B1_01_1810	11901	TOF; CID	90.79	487.9	Tgfa

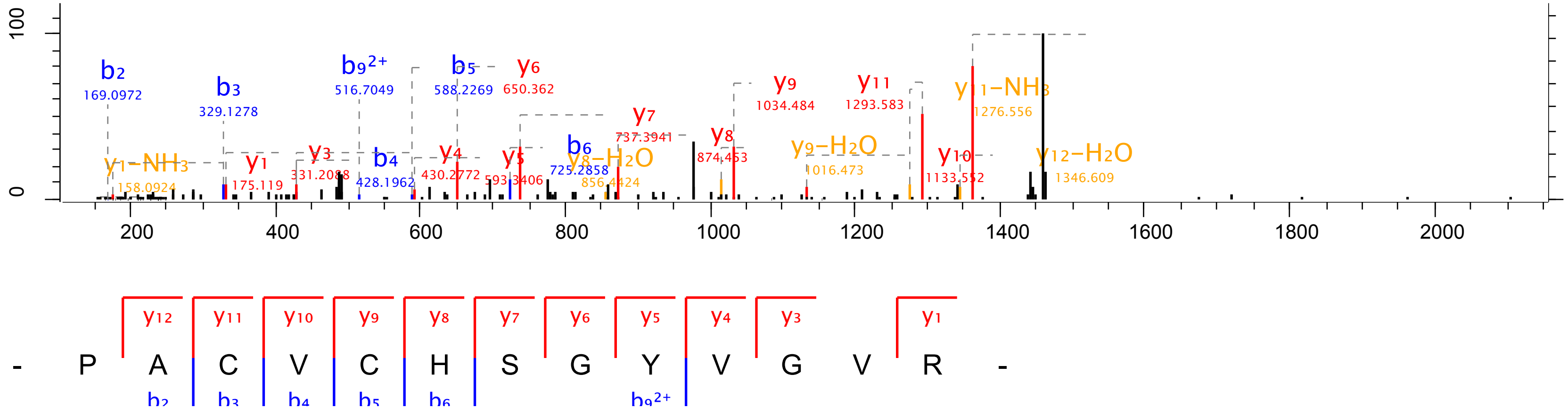

Raw file	Scan	Method	Score	m/z	Gene names
20150402_CerP14_Frac01_top_opt_B1_01_1810	13660	TOF; CID	51.29	394.89	Tmem158

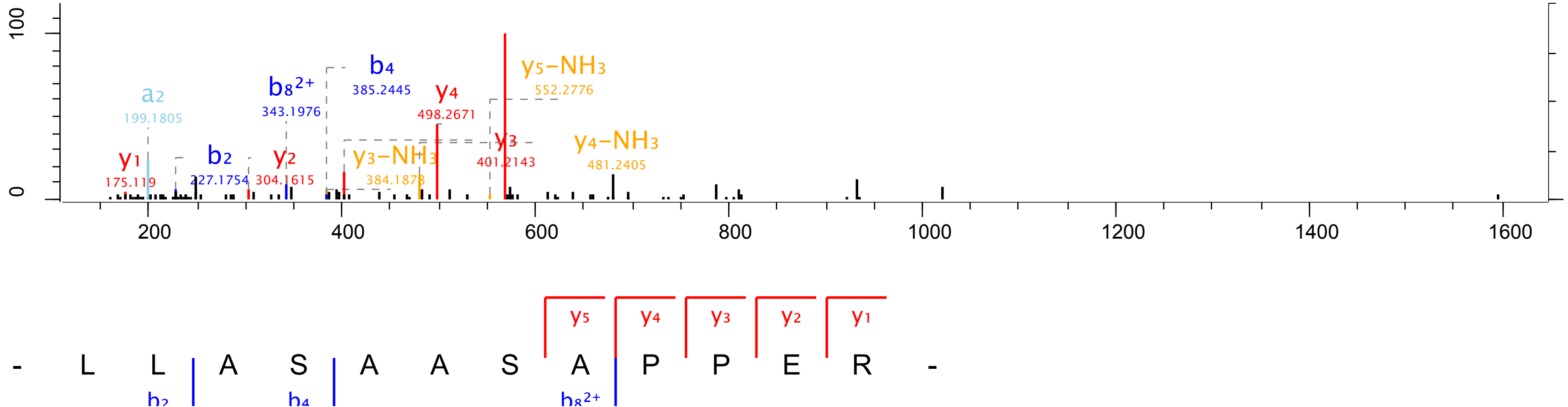

Raw file	Scan	Method	Score	m/z	Gene names
20150402_CerP14_Frac01_top_opt_B1_01_1810	14840	TOF; CID	75.82	660.34	Emid1

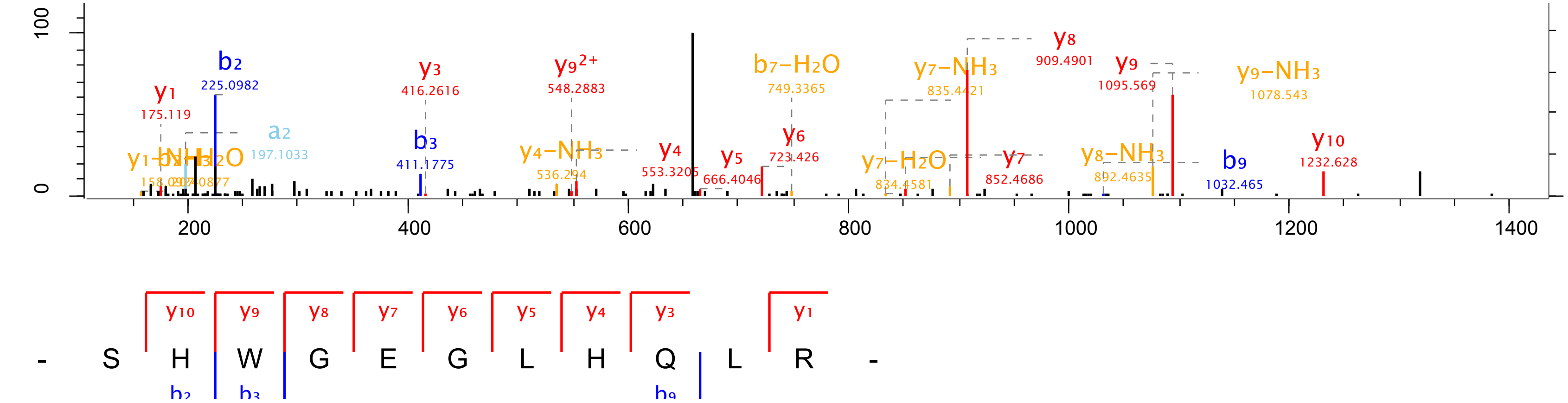

Raw file	Scan	Method	Score	m/z	Gene names
20150402_CerP14_Frac01_top_opt_B1_01_1810	15916	TOF; CID	83.79	658.35	Trnau1ap

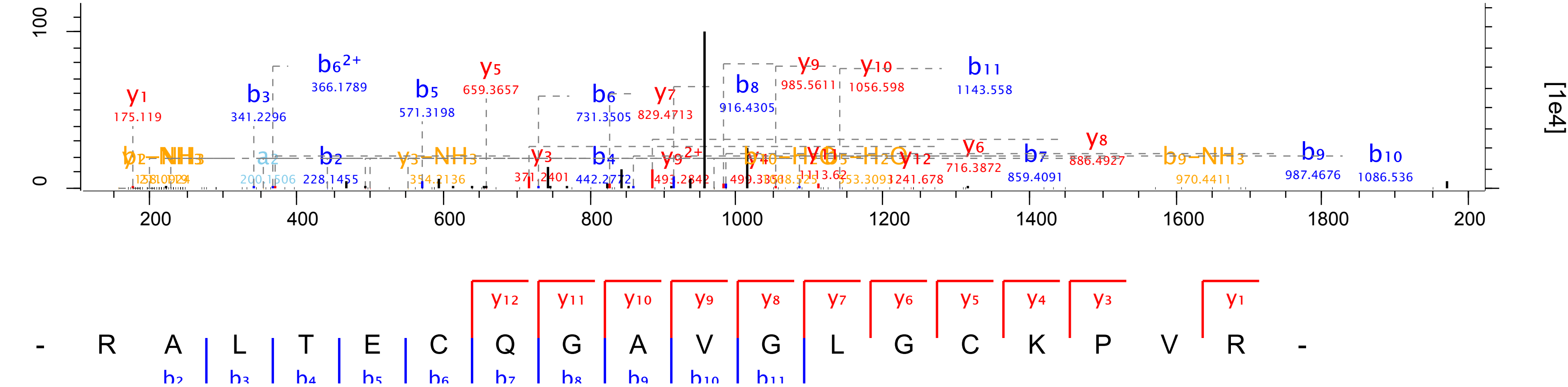

Raw file	Scan	Method	Score	m/z	Gene names
20150402_CerP14_Frac01_top_opt_B1_01_1810	17521	TOF; CID	71.5	337.53	Ccdc30

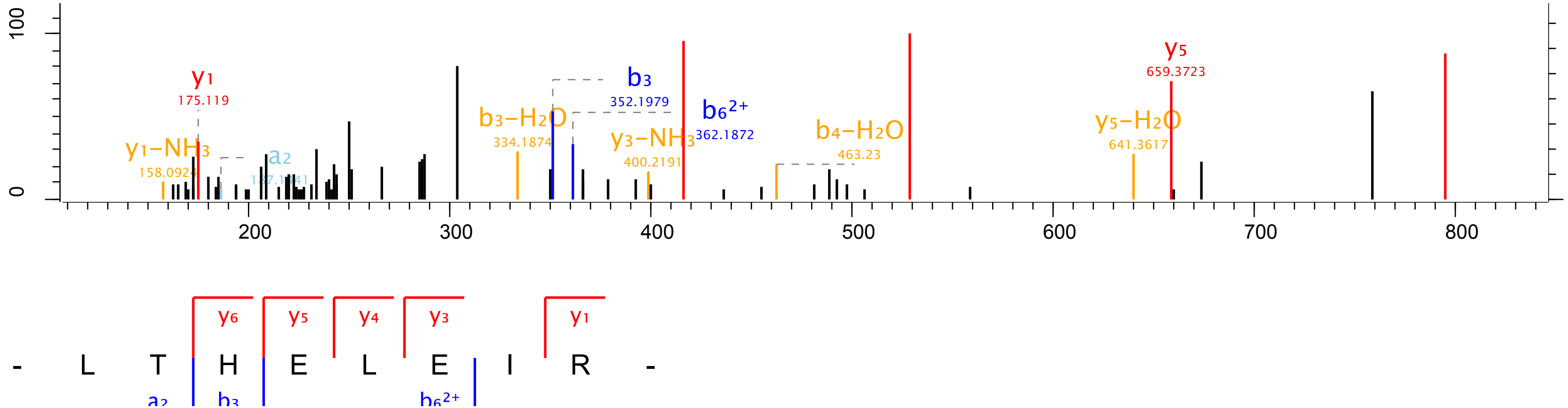

Raw file	Scan	Method	Score	m/z	Gene names
20150402_CerP14_Frac01_top_opt_B1_01_1810	18227	TOF; CID	70.06	396.22	Hrh1

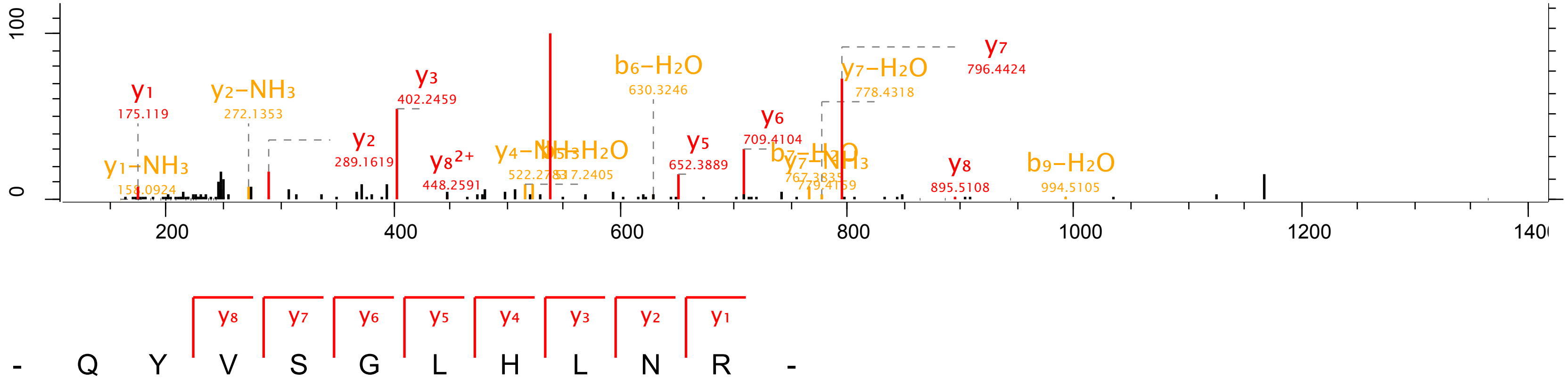

Raw file	Scan	Method	Score	m/z	Gene names
20150402_CerP14_Frac01_top_opt_B1_01_1810	21111	TOF; CID	48.53	491.27	Tlcd1

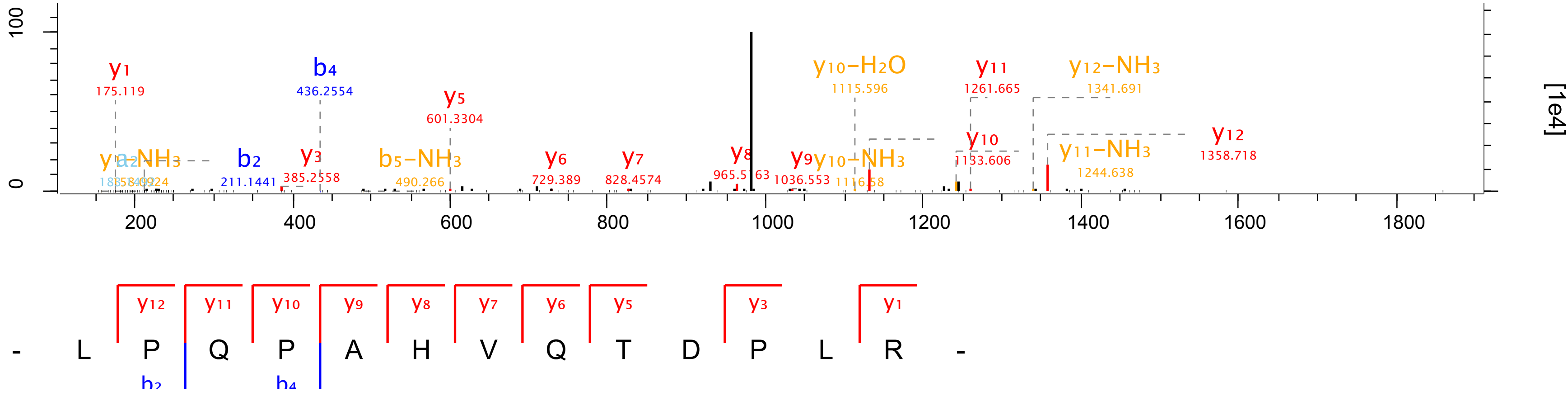

Raw file	Scan	Method	Score	m/z	Gene names
20150402_CerP14_Frac01_top_opt_B1_01_1810	22274	TOF; CID	57.61	883.43	Klf16

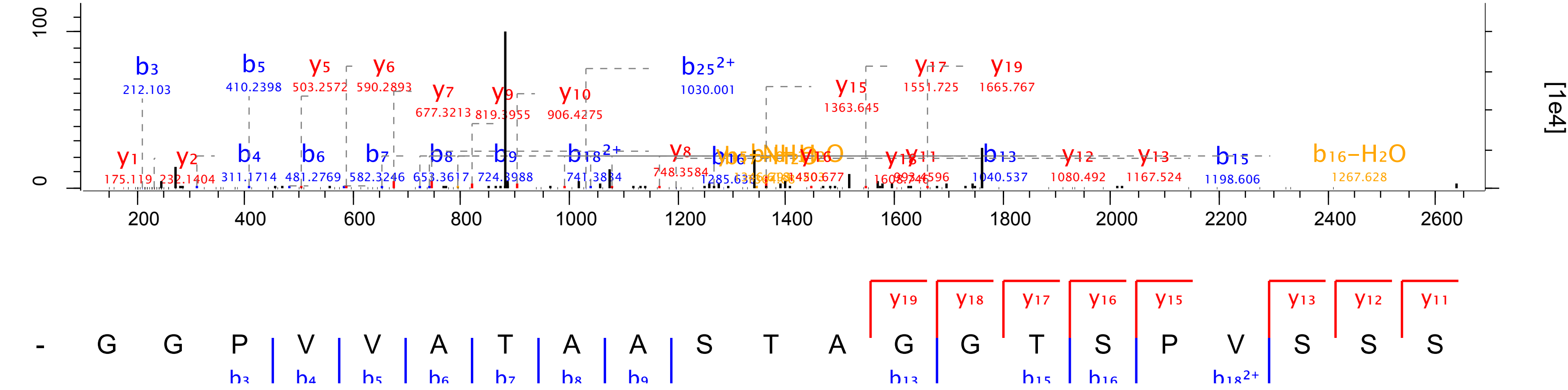

Raw file	Scan	Method	Score	m/z	Gene names
20150402_CerP14_Frac01_top_opt_B1_01_1810	22889	TOF; CID	63.69	648.3	Krtap15-1;Krtap15

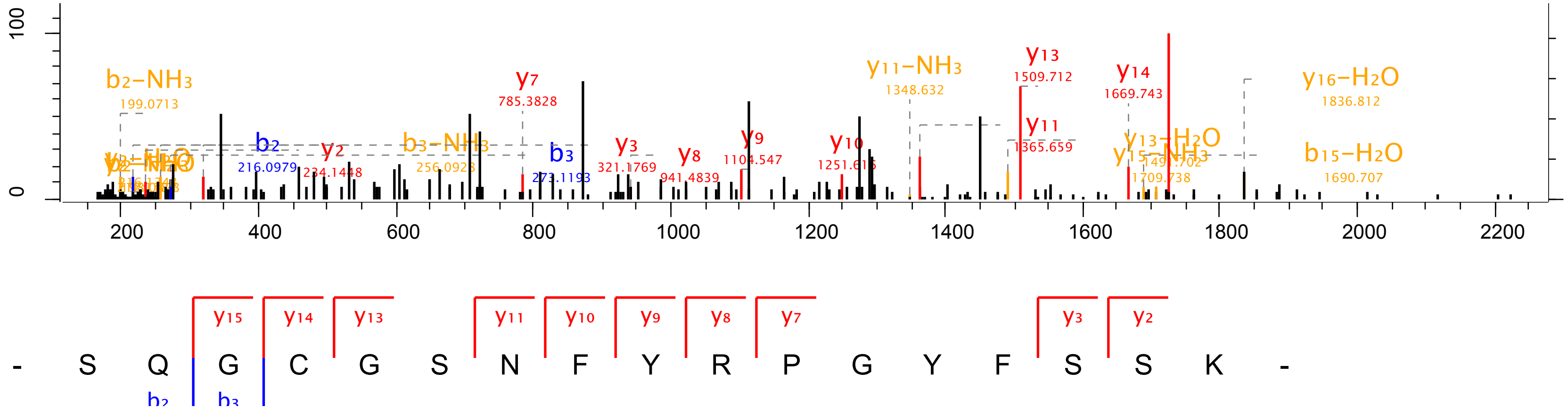

Raw file	Scan	Method	Score	m/z
20150402_CerP14_Frac01_top_opt_B1_01_1810	25390	TOF; CID	63.19	666.32

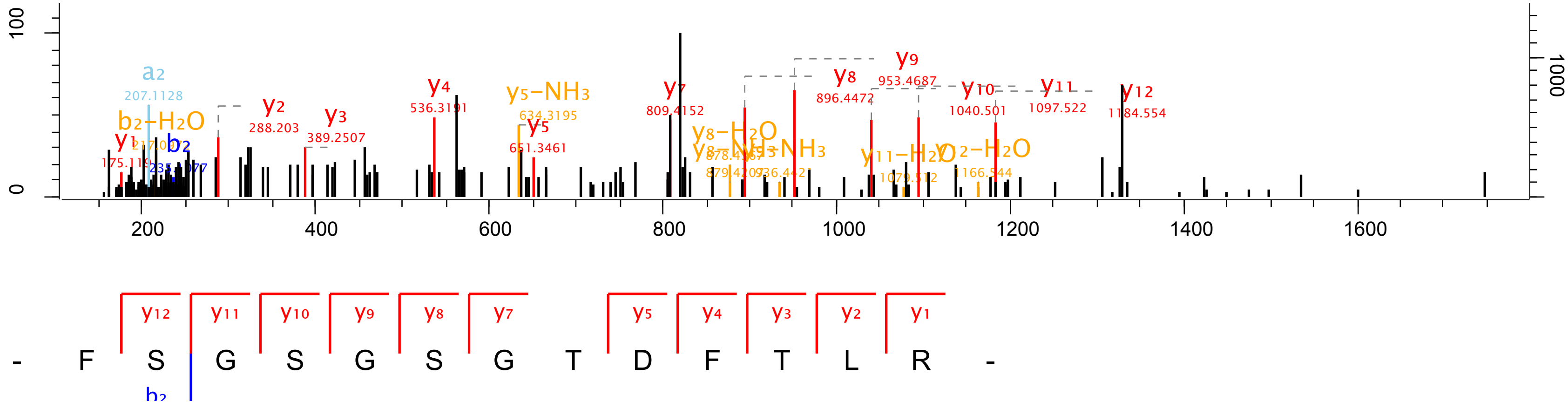

Raw file	Scan	Method	Score	m/z	Gene names
20150402_CerP14_Frac01_top_opt_B1_01_1810	26678	TOF; CID	99.14	481.26	Car9;Ca9

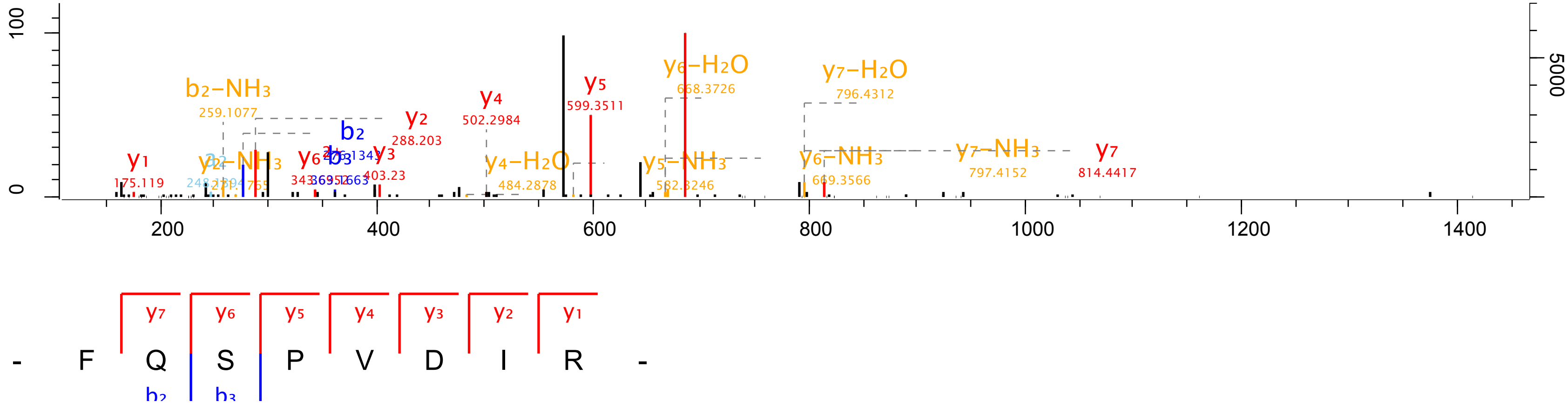

Raw file	Scan	Method	Score	m/z	Gene names
20150402_CerP14_Frac01_top_opt_B1_01_1810	26999	TOF; CID	88.5	548.27	Fktn

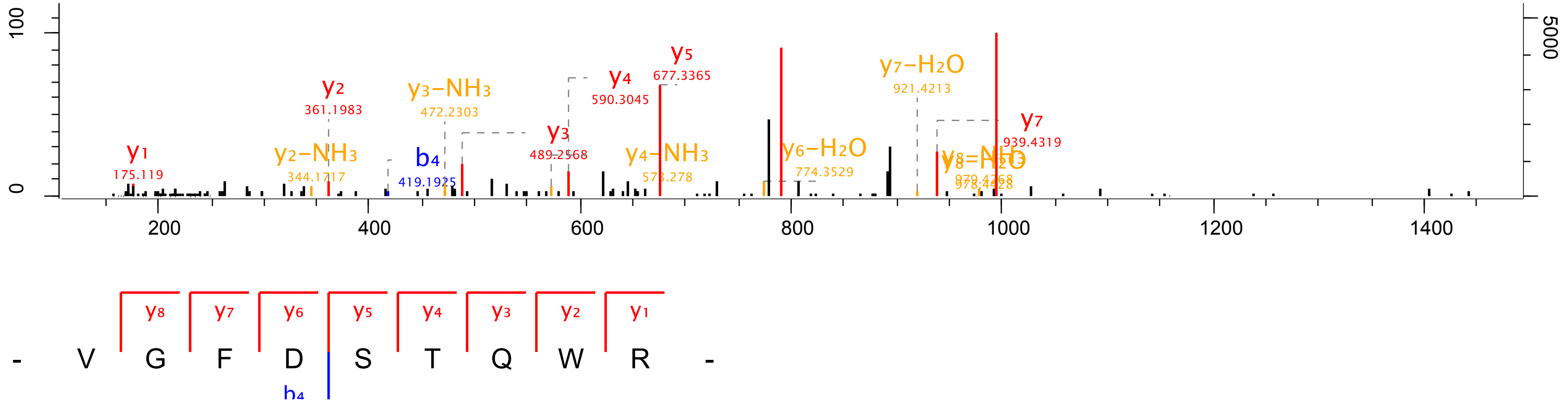

Raw file	Scan	Method	Score	m/z	Gene names
20150402_CerP14_Frac01_top_opt_B1_01_1810	28279	TOF; CID	66.56	542.32	Thsd1

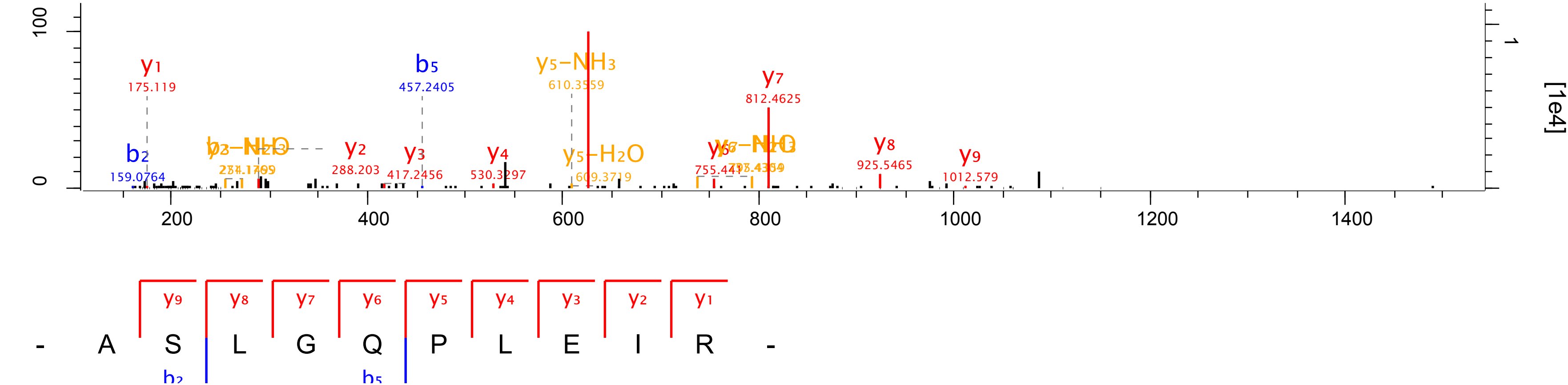

Raw file	Scan	Method	Score	m/z	Gene names
20150402_CerP14_Frac01_top_opt_B1_01_1810	28909	TOF; CID	82.42	533.8	Pacrg

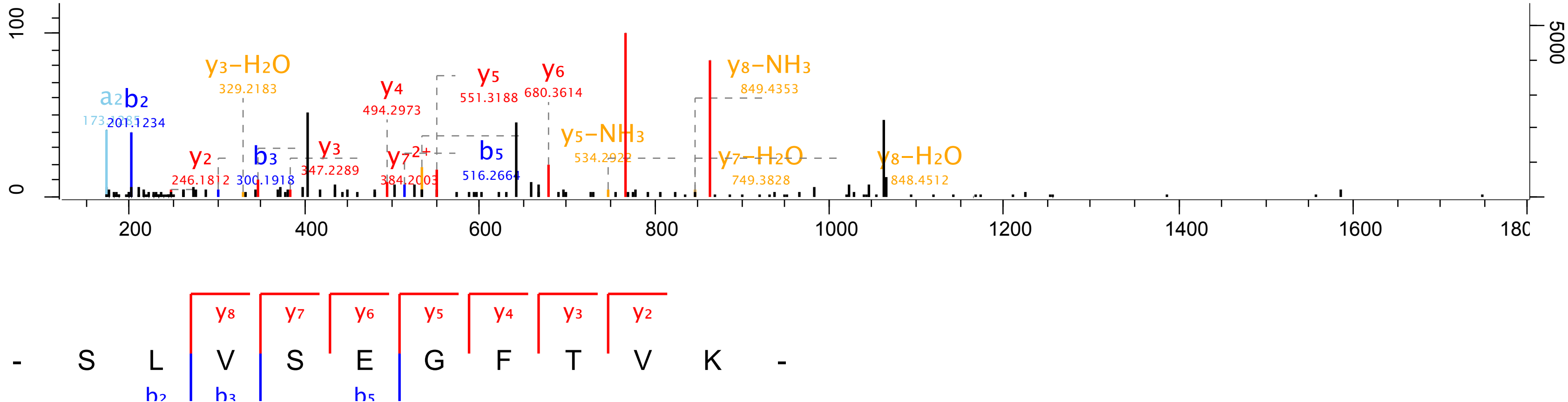

Raw file	Scan	Method	Score	m/z	Gene names
20150402_CerP14_Frac01_top_opt_B1_01_1810	29018	TOF; CID	96.96	638.32	Rnf167

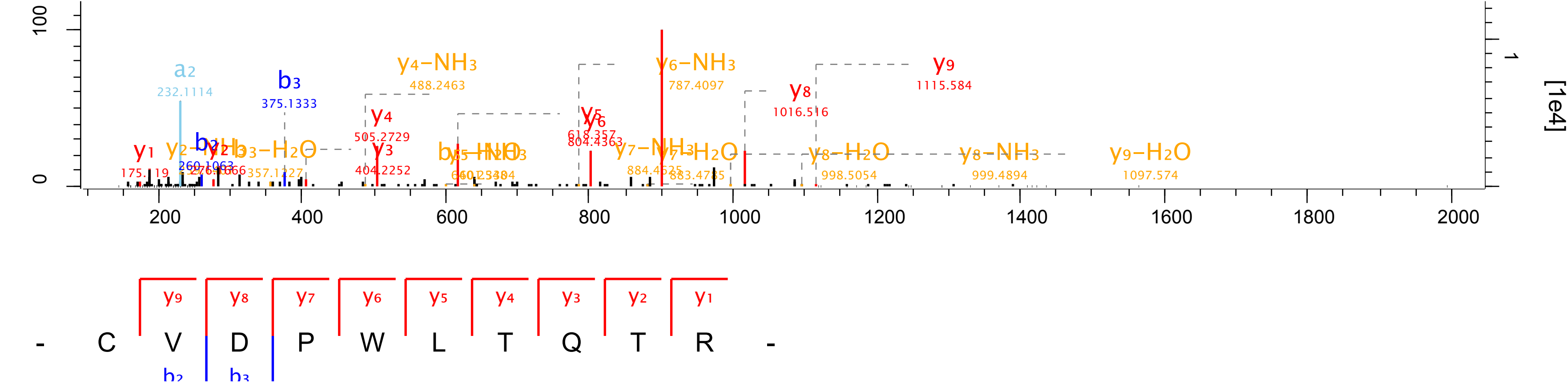

Raw file	Scan	Method	Score	m/z	Gene names
20150402_CerP14_Frac01_top_opt_B1_01_1810	33982	TOF; CID	113.4	565.97	Oxld1

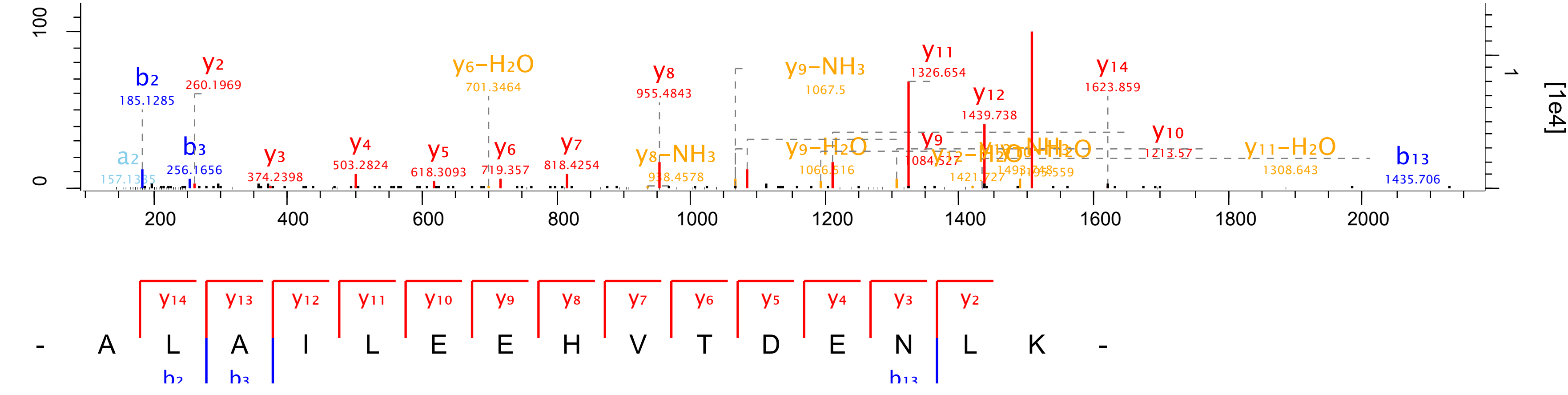

Raw file	Scan	Method	Score	m/z	Gene names
20150402_CerP14_Frac01_top_opt_B1_01_1810	34386	TOF; CID	52.93	796.42	Pcdh12

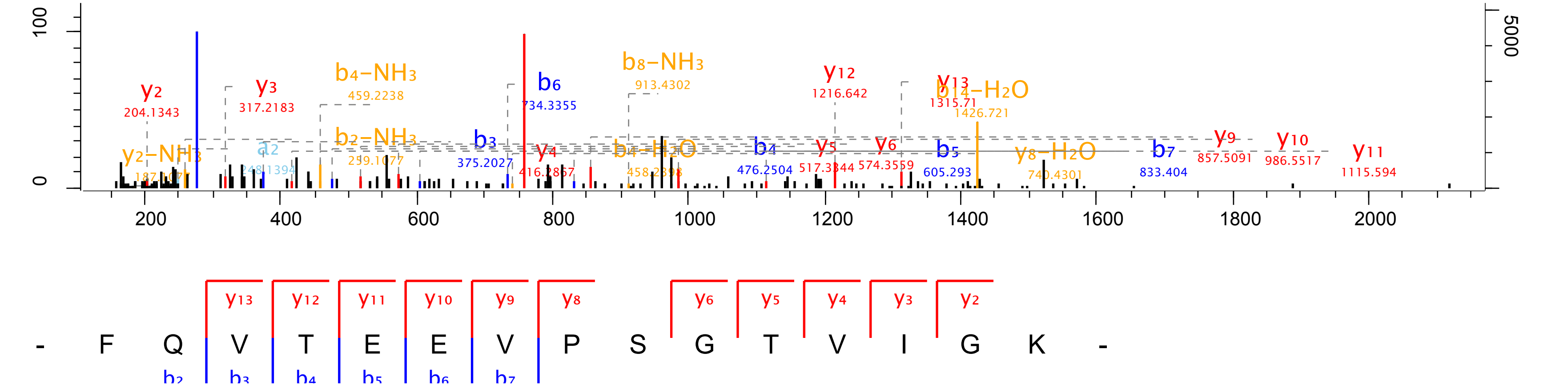

Raw file	Scan	Method	Score	m/z	Gene names
20150402_CerP14_Frac01_top_opt_B1_01_1810	34885	TOF; CID	53.68	944.45	Chst1

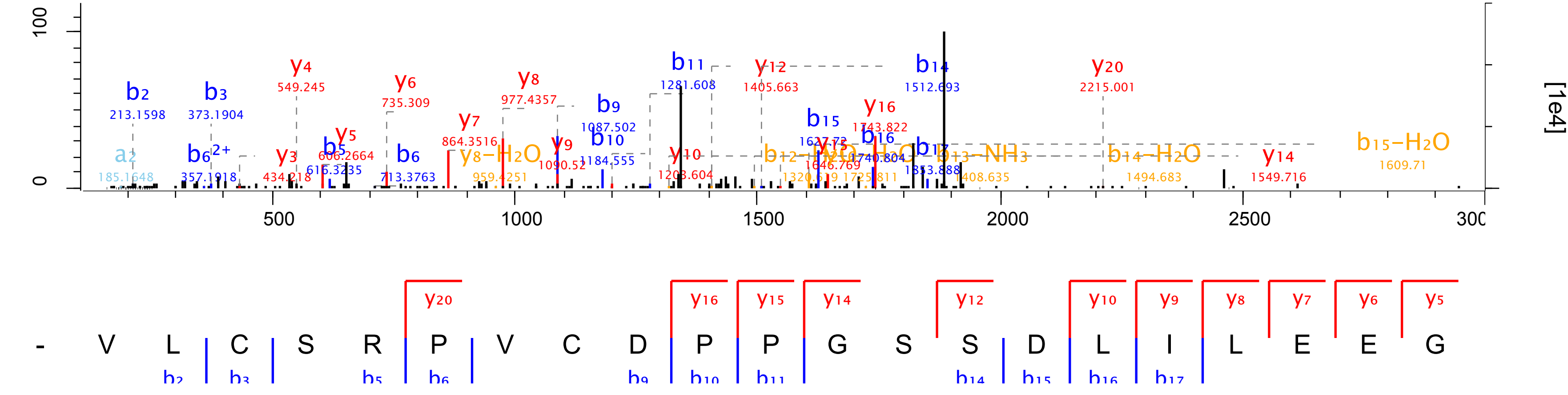

Raw file	Scan	Method	Score	m/z	Gene names
20150402_CerP14_Frac01_top_opt_B1_01_1810	36267	TOF; CID	68.97	741.89	Fcgr2;Fcgr2b

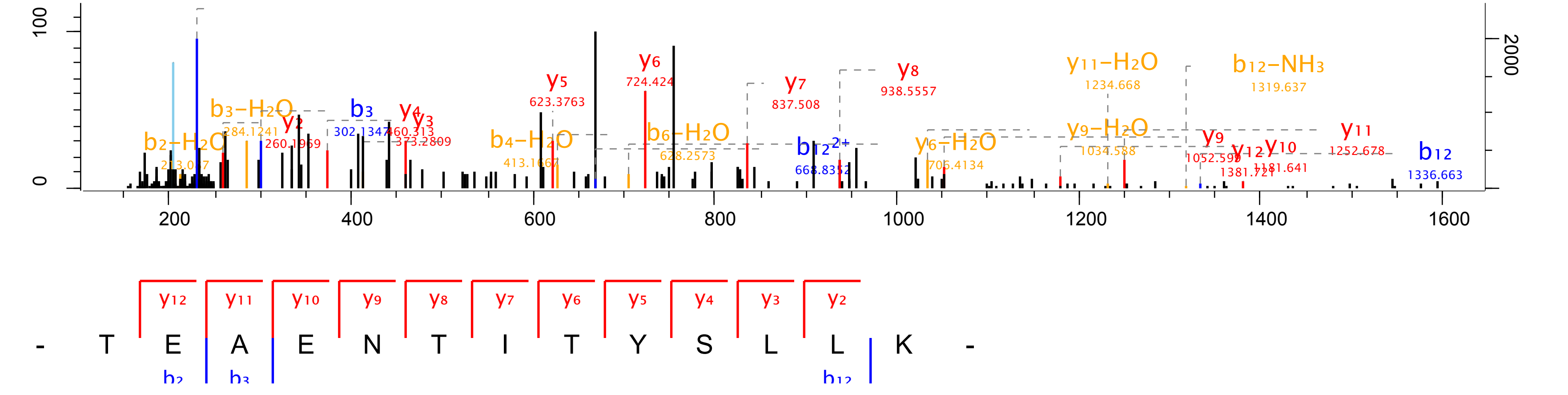

Raw file	Scan	Method	Score	m/z	Gene names
20150402_CerP14_Frac01_top_opt_B1_01_1810	36630	TOF; CID	71.38	346.91	Cnga2

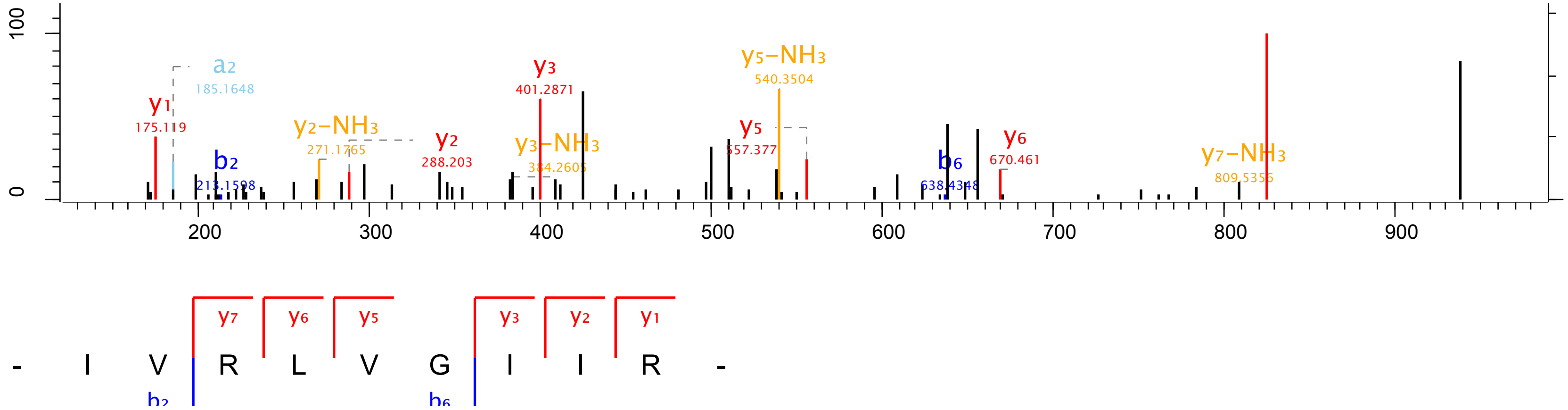

Raw file	Scan	Method	Score	m/z	Gene names
20150402_CerP14_Frac01_top_opt_B1_01_1810	38272	TOF; CID	72.29	657.87	Arhgap36

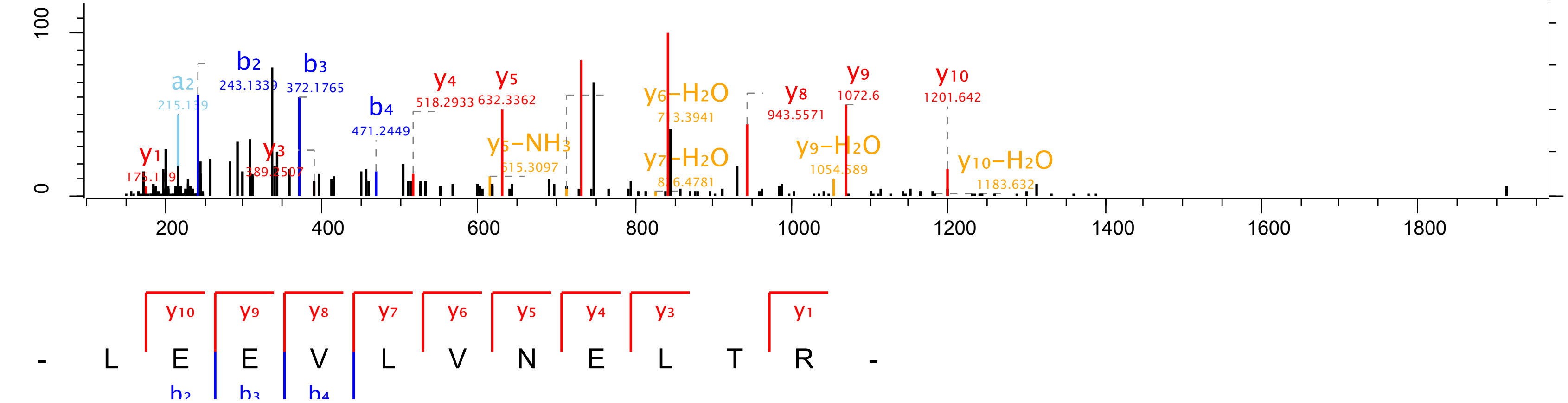

Raw file	Scan	Method	Score	m/z	Gene names
20150402_CerP14_Frac01_top_opt_B1_01_1810	39711	TOF; CID	58.32	658.85	Fam81a

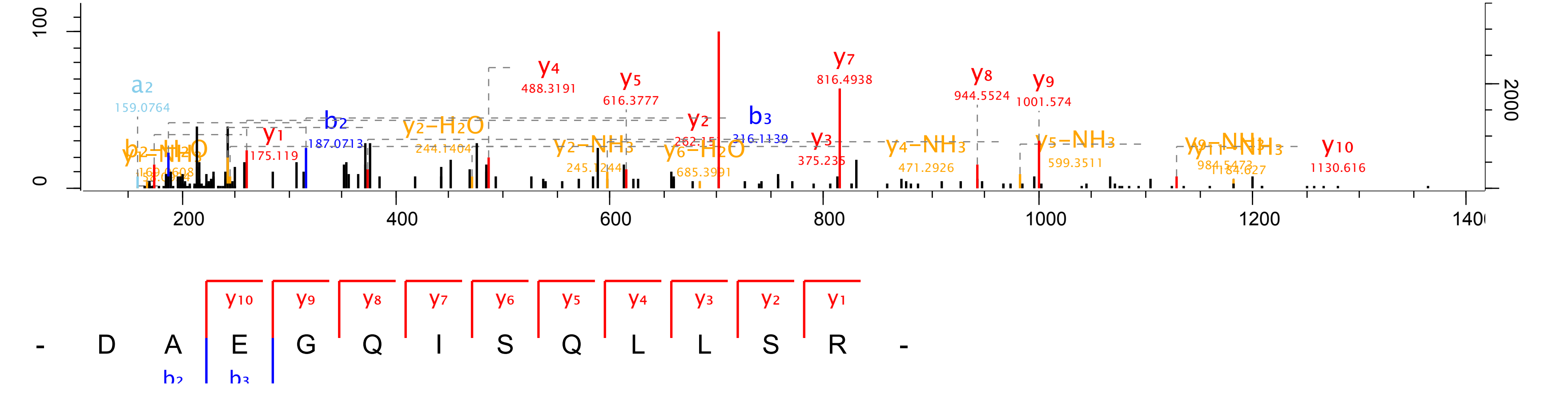

Raw file	Scan	Method	Score	m/z	Gene names
20150402_CerP14_Frac01_top_opt_B1_01_1810	43055	TOF; CID	118.63	1242.12	Tmem42

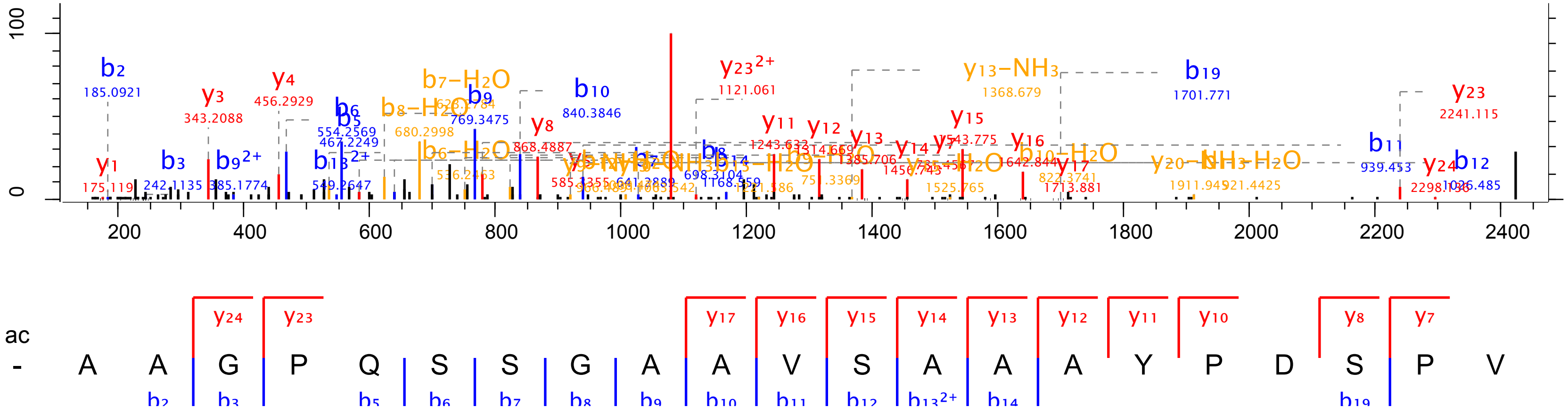

Raw file	Scan	Method	Score	m/z	Gene names
20150402_CerP14_Frac01_top_opt_B1_01_1810	43879	TOF; CID	211.92	882.78	Mapk1ip1l

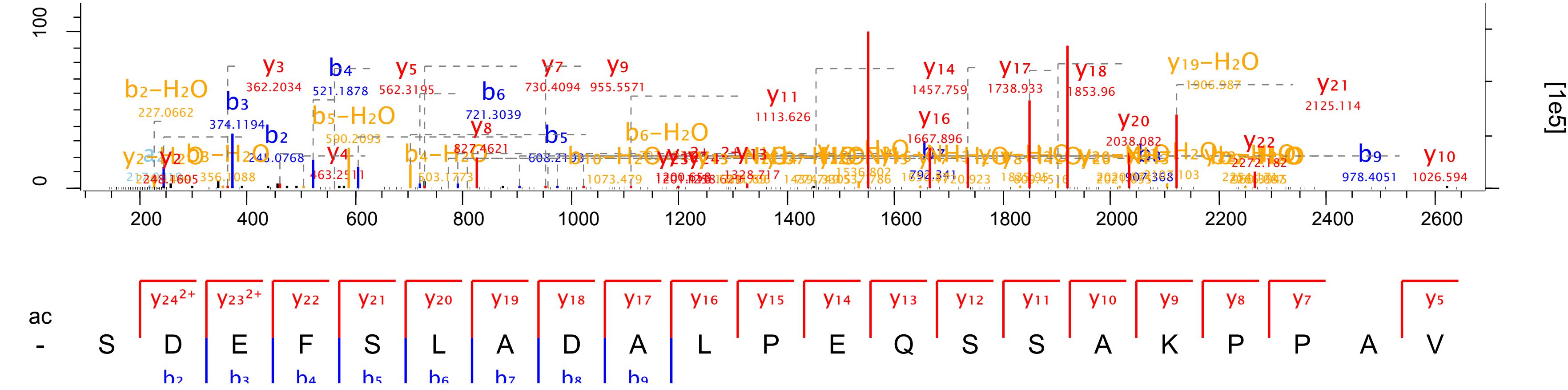

Raw file	Scan	Method	Score	m/z	Gene names
20150402_CerP14_Frac01_top_opt_B1_01_1810	45481	TOF; CID	93.18	967.75	Tmem208

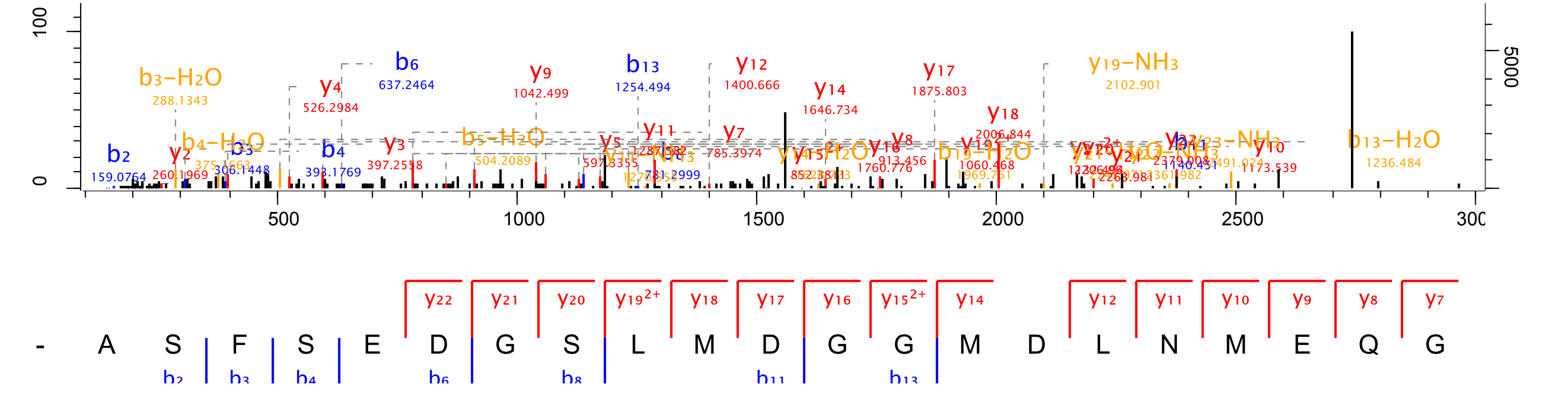

Raw file	Scan	Method	Score	m/z	Gene names
20150402_CerP14_Frac01_top_opt_B1_01_1810	46026	TOF; CID	52.18	682.36	Pole2

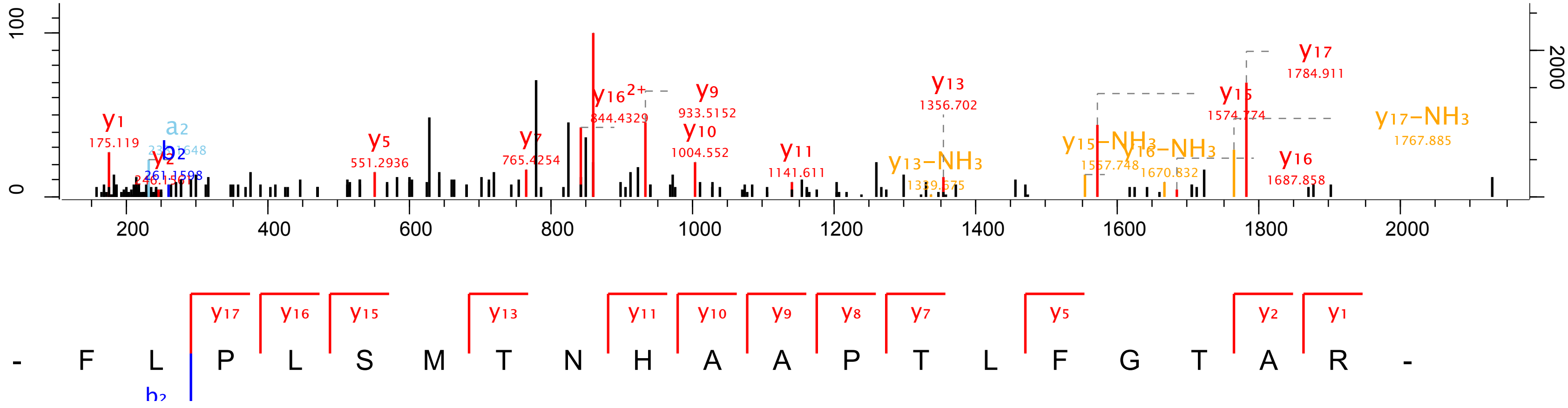

Raw file	Scan	Method	Score	m/z	Gene names
20150402_CerP14_Frac01_top_opt_B1_01_1810	52928	TOF; CID	64.5	1317.6	Fam174a

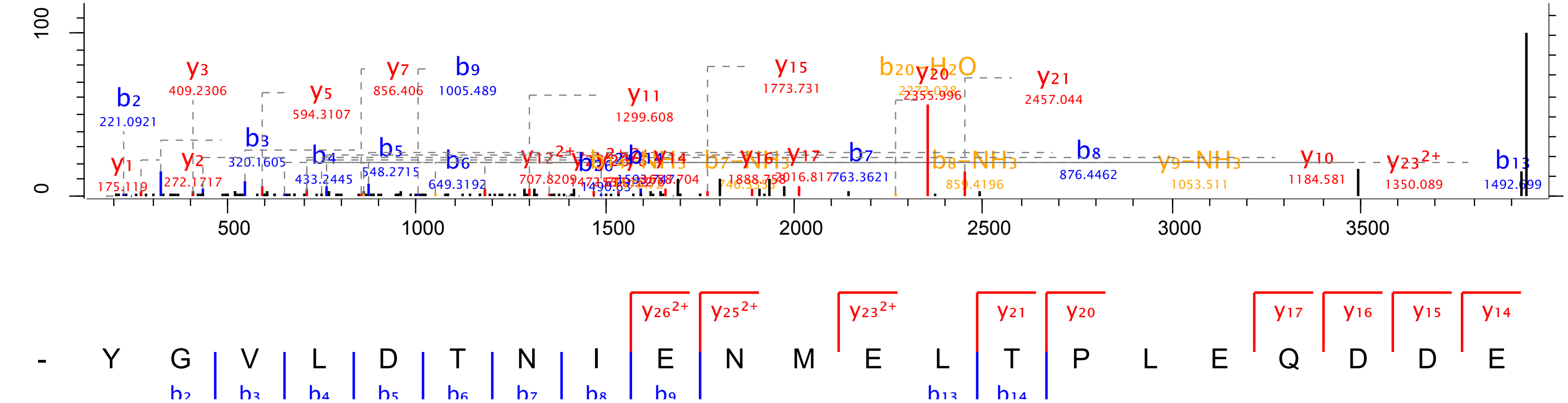

Raw file	Scan	Method	Score	m/z
20150402_CerP14_Frac01_top_opt_B1_01_1810	54428	TOF; CID	80.71	551.85

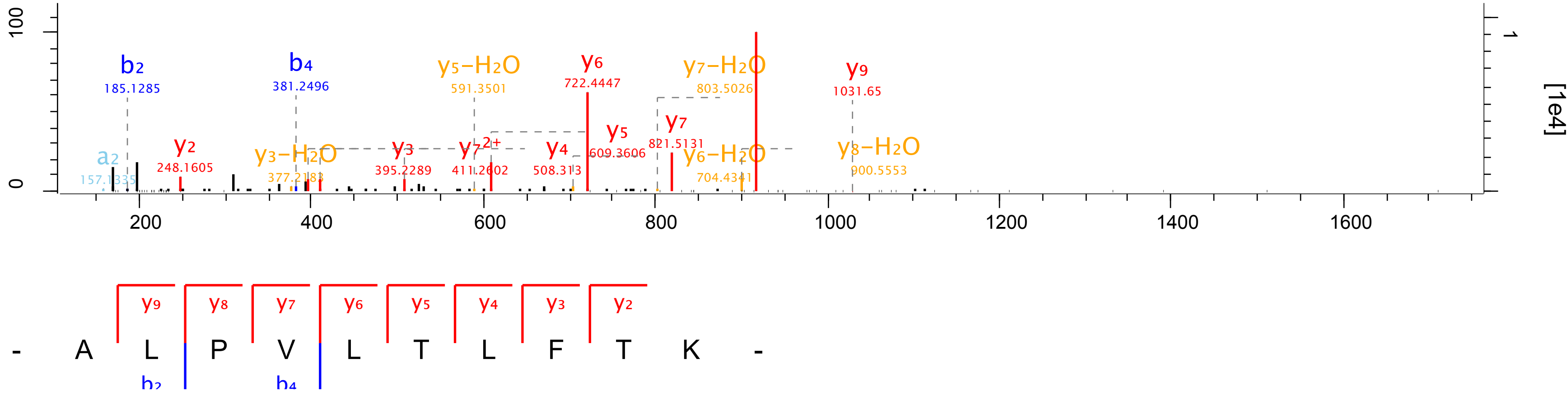

Raw file	Scan	Method	Score	m/z	Gene names
20150402_CerP14_Frac02_top_opt_B2_01_1811	15721	TOF; CID	139.31	447.76	Myf6

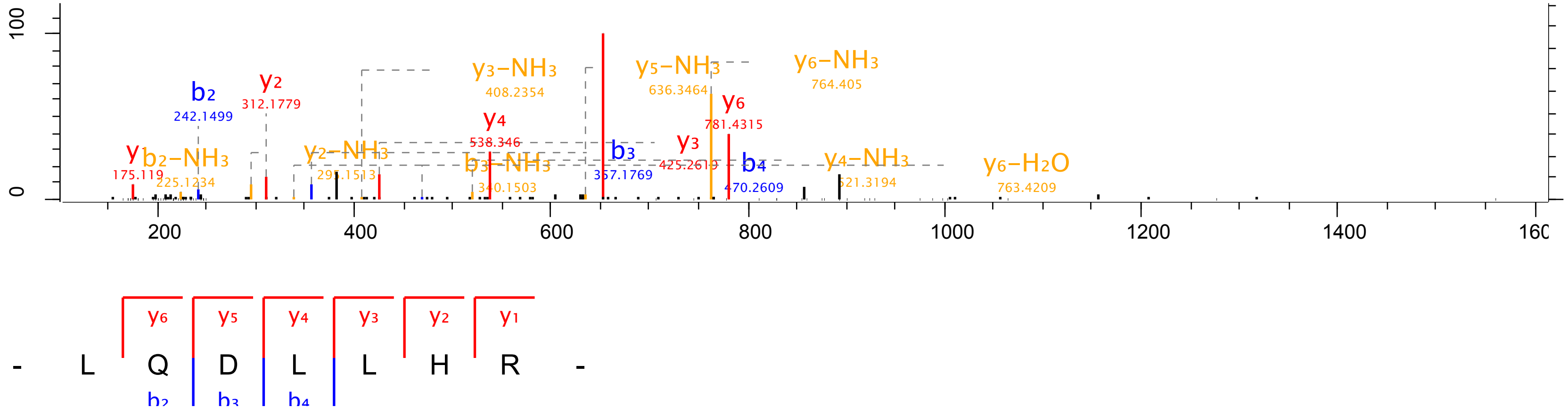

Raw file	Scan	Method	Score	m/z	Gene names
20150402_CerP14_Frac02_top_opt_B2_01_1811	16257	TOF; CID	75.55	647.3	Clec14a

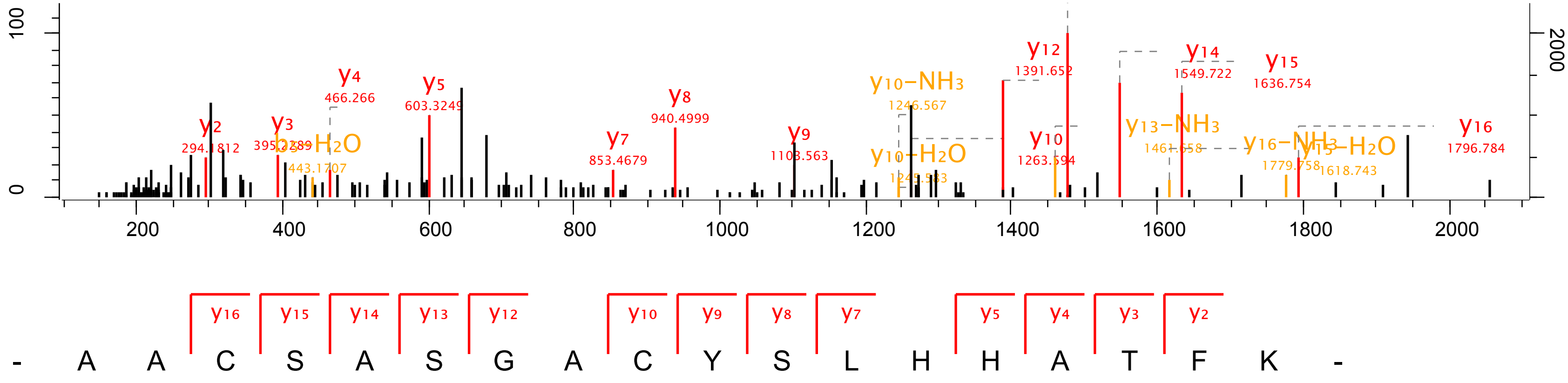

Raw file	Scan	Method	Score	m/z	Gene names
20150402_CerP14_Frac02_top_opt_B2_01_1811	16792	TOF; CID	104.24	565.32	Lpar4

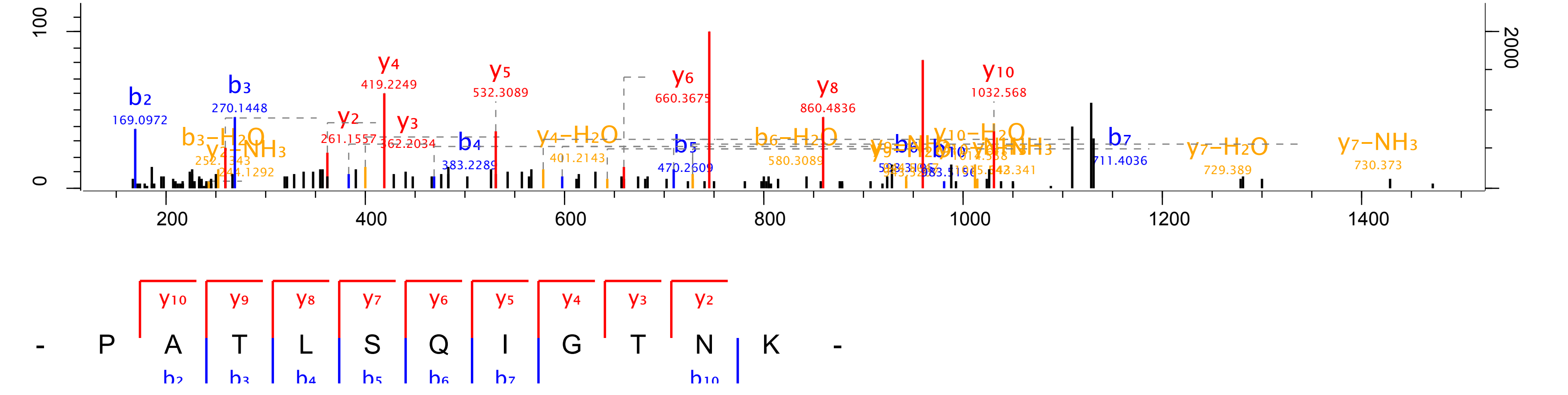

Raw file	Scan	Method	Score	m/z	Gene names
20150402_CerP14_Frac02_top_opt_B2_01_1811	18281	TOF; CID	91.08	591.98	Nmu

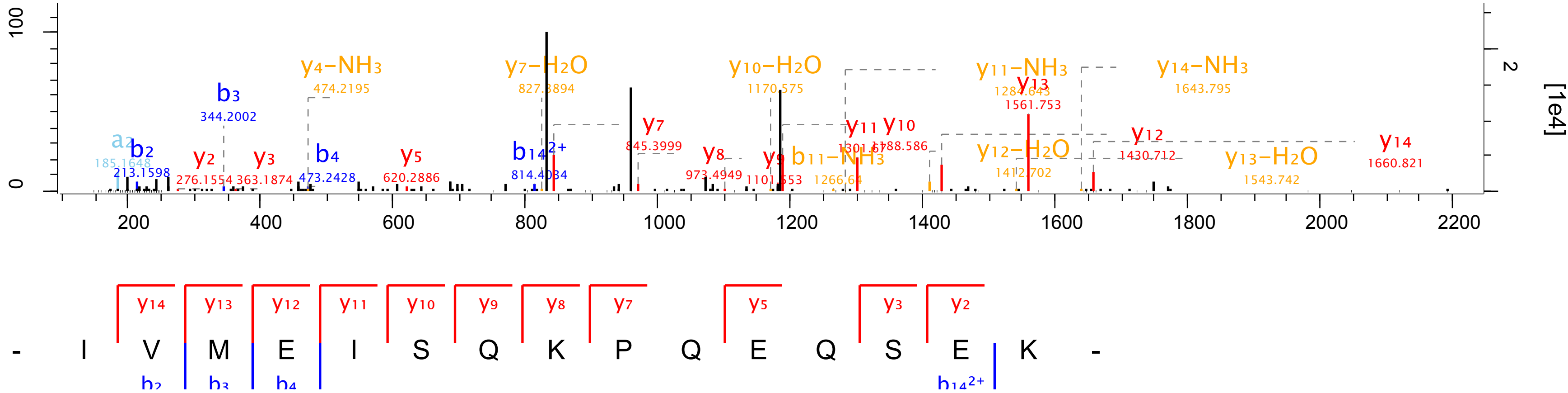

Raw file	Scan	Method	Score	m/z	Gene names
20150402_CerP14_Frac02_top_opt_B2_01_1811	24996	TOF; CID	74.17	520.77	Nabp2;Nabp1

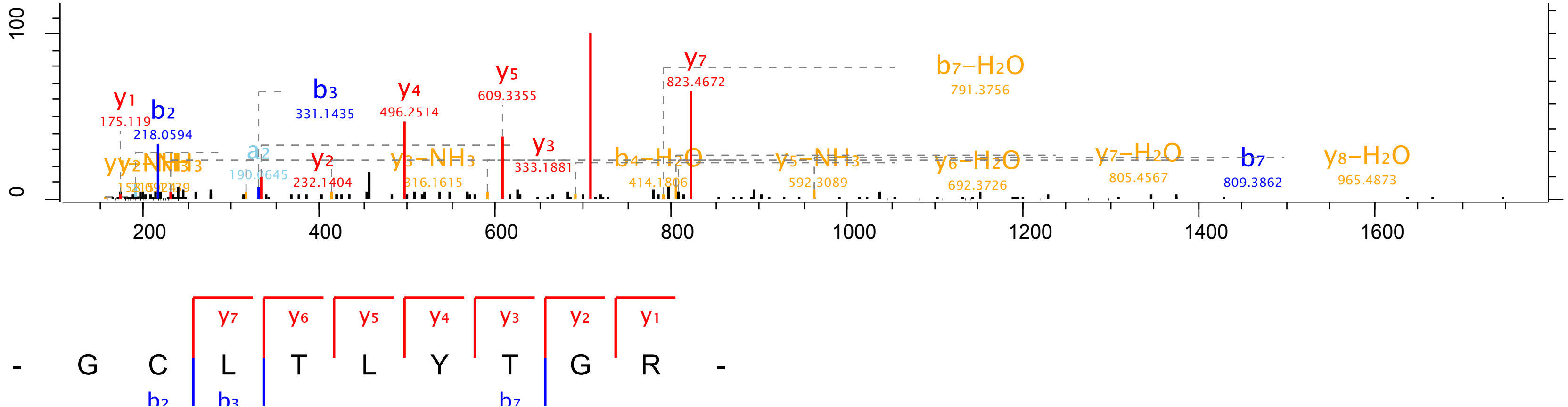

Raw file	Scan	Method	Score	m/z	Gene names
20150402_CerP14_Frac02_top_opt_B2_01_1811	25203	TOF; CID	51	814.42	Acvrl1

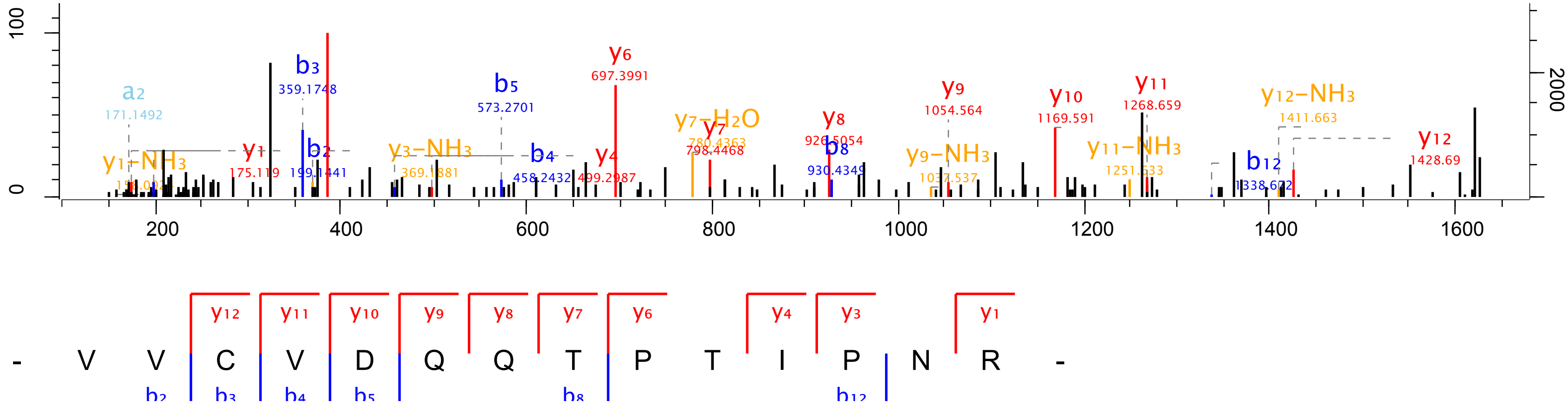

Raw file	Scan	Method	Score	m/z	Gene names
20150402_CerP14_Frac02_top_opt_B2_01_1811	25459	TOF; CID	166.45	705.33	Vkorc1

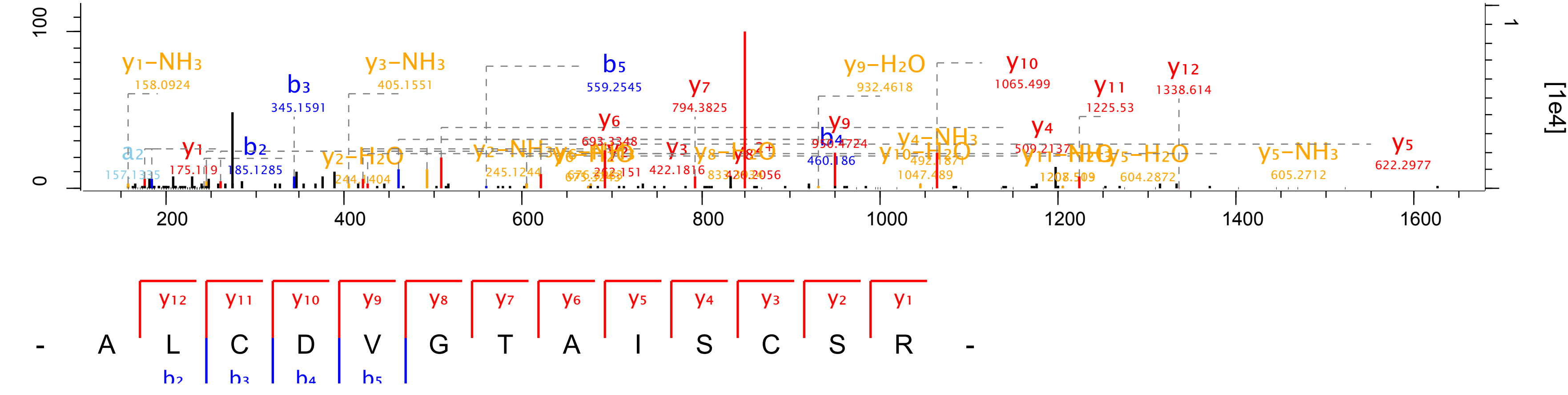

Raw file	Scan	Method	Score	m/z	Gene names
20150402_CerP14_Frac02_top_opt_B2_01_1811	27123	TOF; CID	60.97	700.38	4933426M11Rik

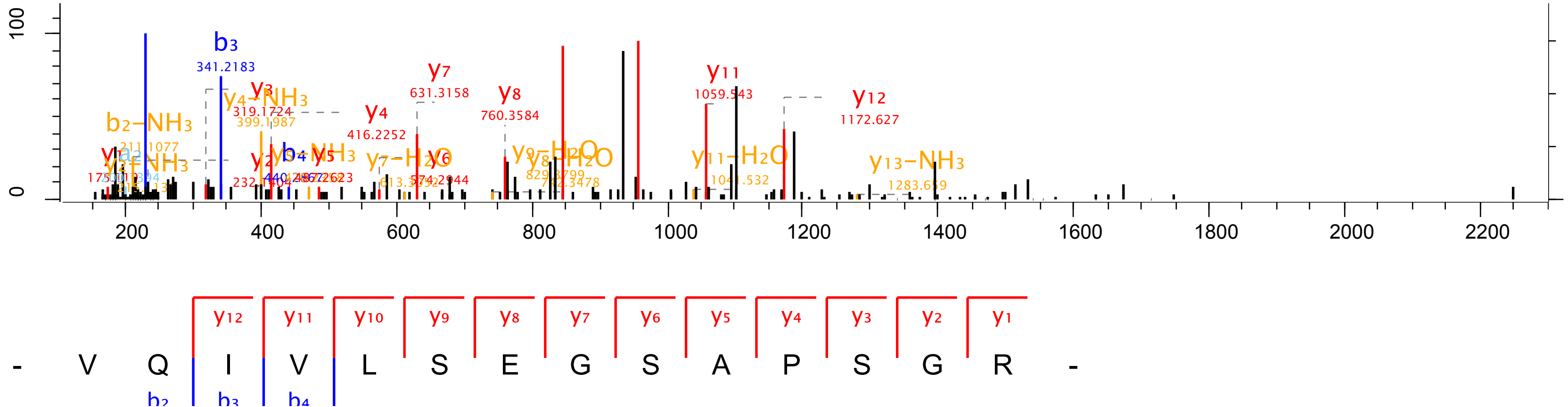

Raw file	Scan	Method	Score	m/z	Gene names
20150402_CerP14_Frac02_top_opt_B2_01_1811	27907	TOF; CID	65.18	628.86	Cep57

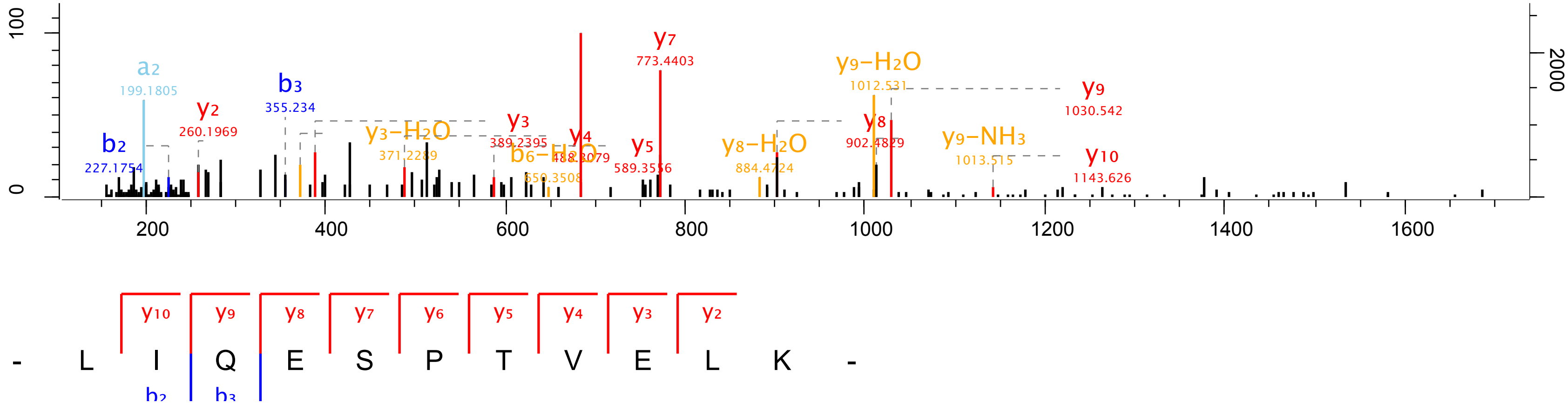

Raw file	Scan	Method	Score	m/z	Gene names
20150402_CerP14_Frac02_top_opt_B2_01_1811	33267	TOF; CID	77.06	544.31	March3

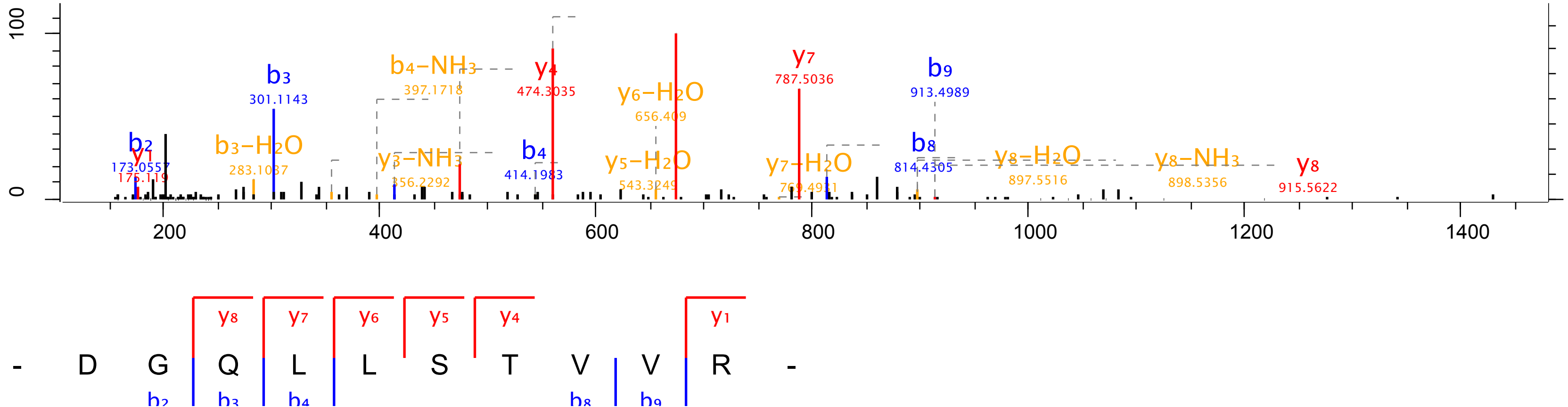

Raw file	Scan	Method	Score	m/z	Gene names
20150402_CerP14_Frac02_top_opt_B2_01_1811	33776	TOF; CID	54.9	810.42	Pramel7

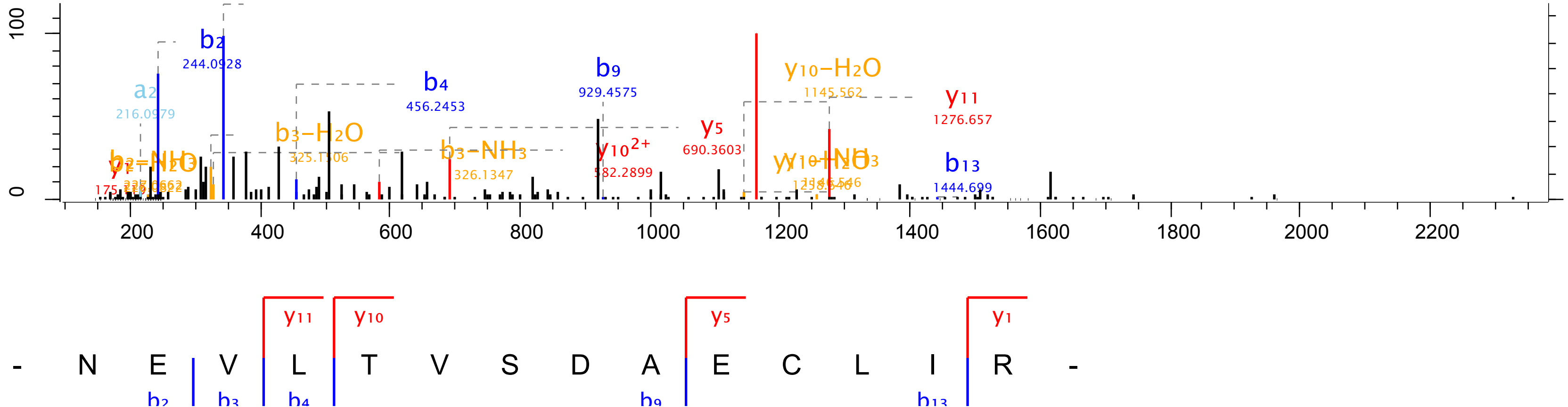

Raw file	Scan	Method	Score	m/z	Gene names
20150402_CerP14_Frac02_top_opt_B2_01_1811	36409	TOF; CID	45.22	839.42	Bcl11b

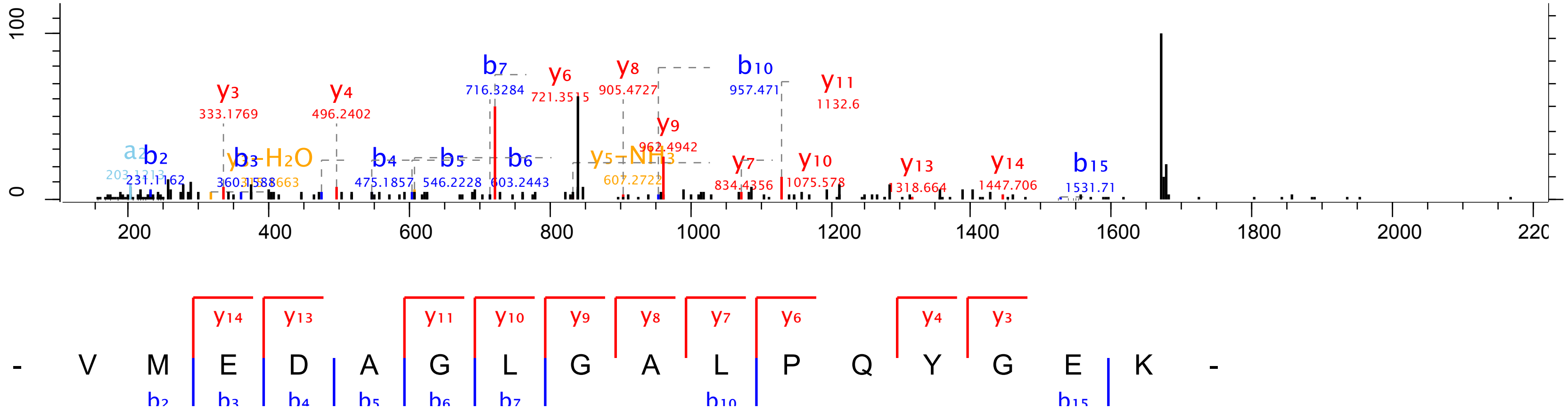

Raw file	Scan	Method	Score	m/z	Gene names
20150402_CerP14_Frac02_top_opt_B2_01_1811	40713	TOF; CID	48.28	858.77	H2-Ab1

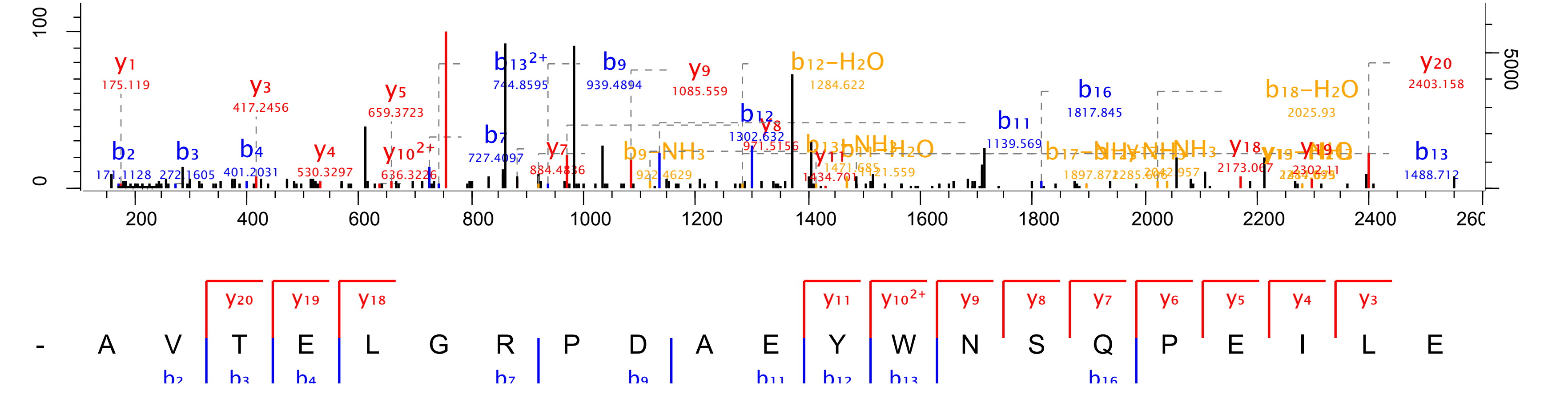

Raw file	Scan	Method	Score	m/z	Gene names
20150402_CerP14_Frac02_top_opt_B2_01_1811	46153	TOF; CID	41.48	883.14	Rreb1

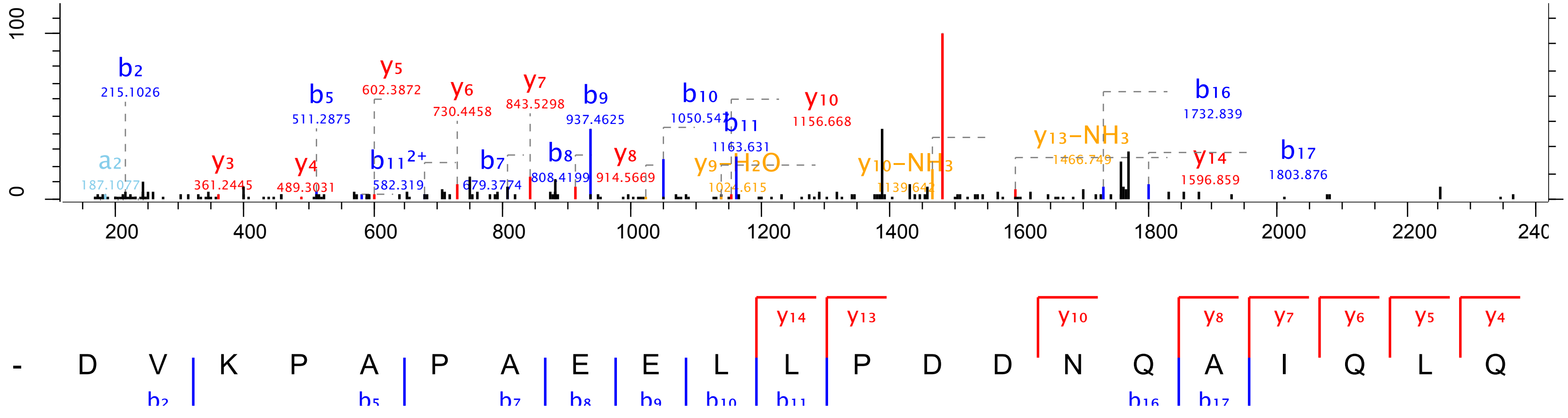

Raw file	Scan	Method	Score	m/z	Gene names
20150402_CerP14_Frac02_top_opt_B2_01_1811	54871	TOF; CID	55.1	726.97	Peg12

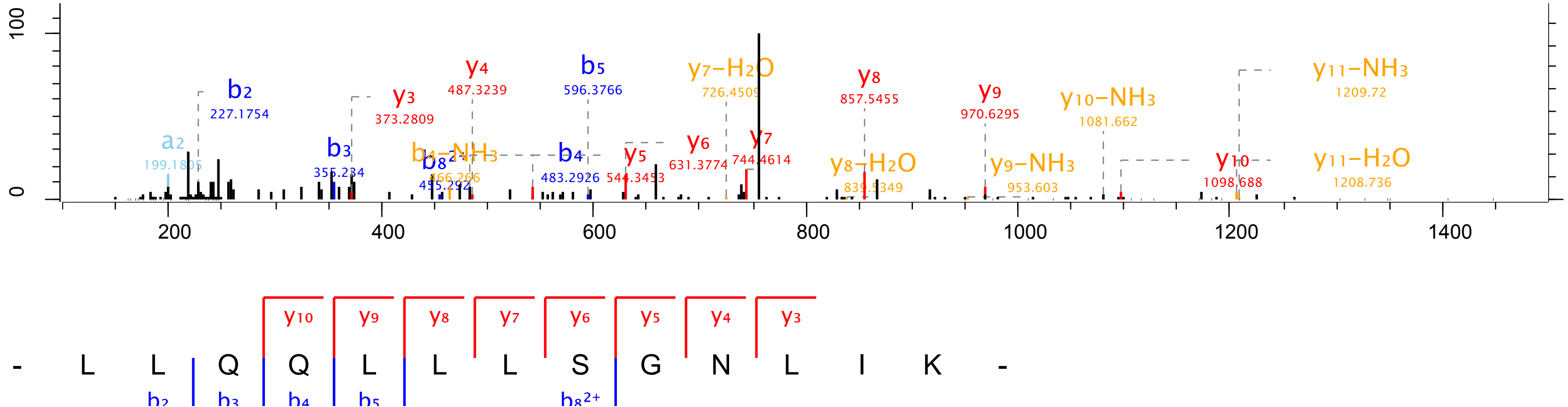

Raw file	Scan	Method	Score	m/z	Gene names
20150402_CerP14_Frac02_top_opt_B2_01_1811	57067	TOF; CID	136.02	605.87	Gm1604A

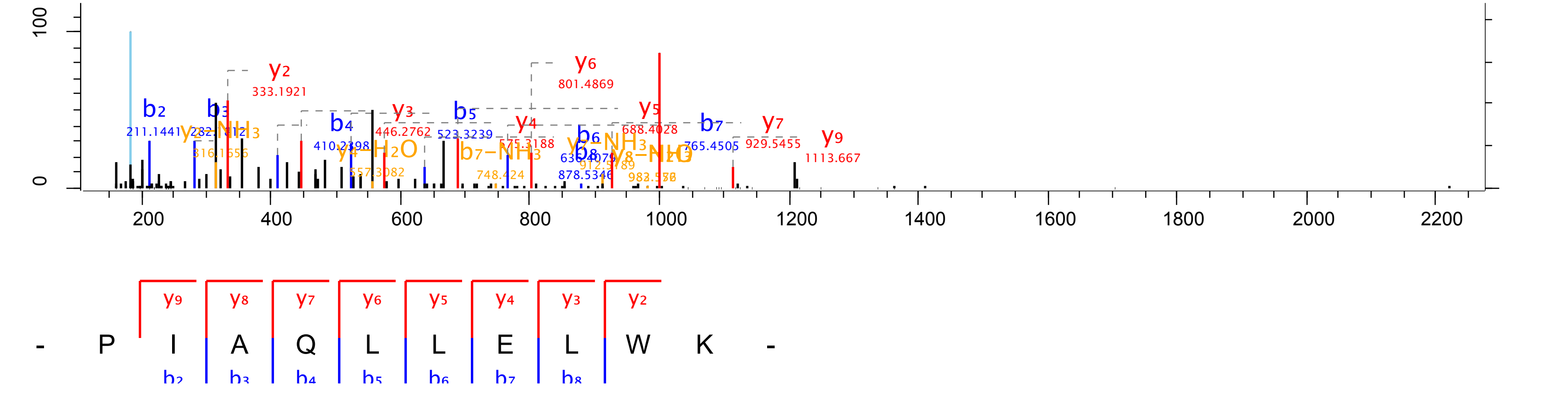

Raw file	Scan	Method	Score	m/z	Gene names
20150402_CerP14_Frac03_top_opt_B3_01_1812	8405	TOF; CID	73.28	767.36	Spn

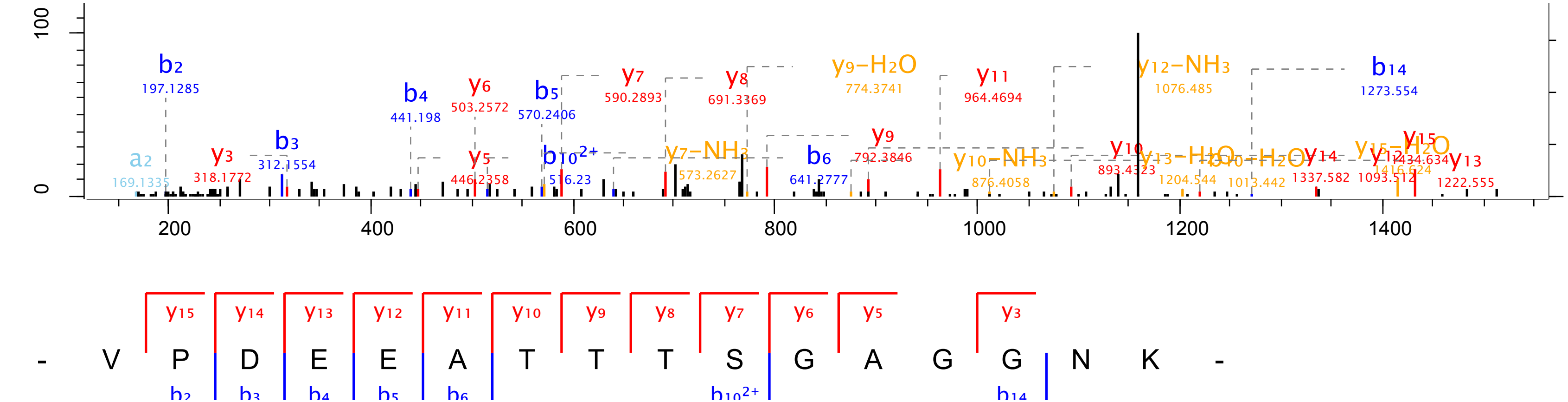

Raw file	Scan	Method	Score	m/z	Gene names
20150402_CerP14_Frac03_top_opt_B3_01_1812	13479	TOF; CID	73.17	903.88	Vsig8

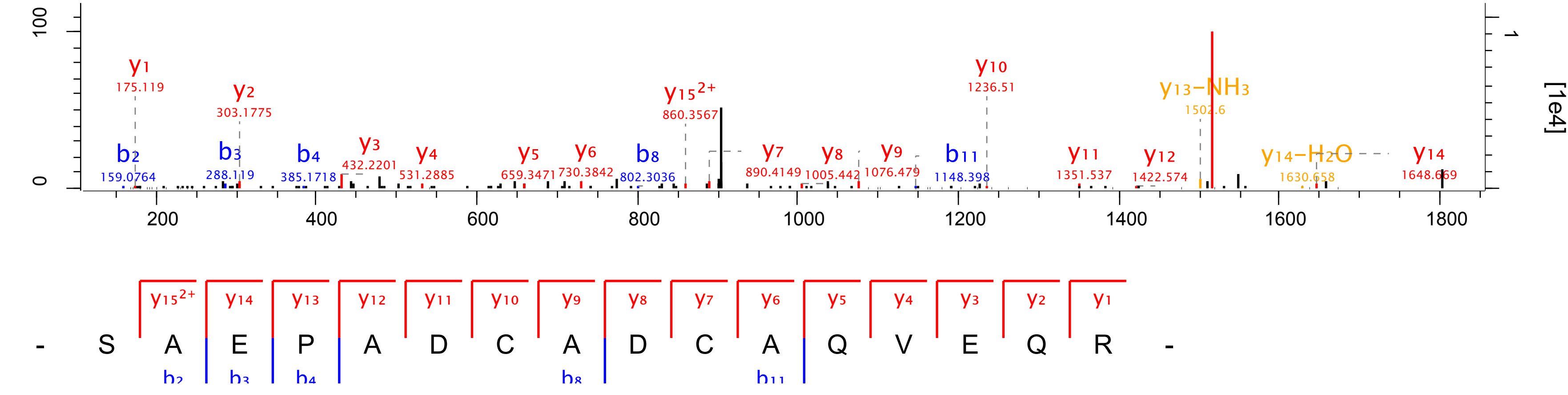

Raw file	Scan	Method	Score	m/z	Gene names
20150402_CerP14_Frac03_top_opt_B3_01_1812	14294	TOF; CID	57.97	462.25	Chst8

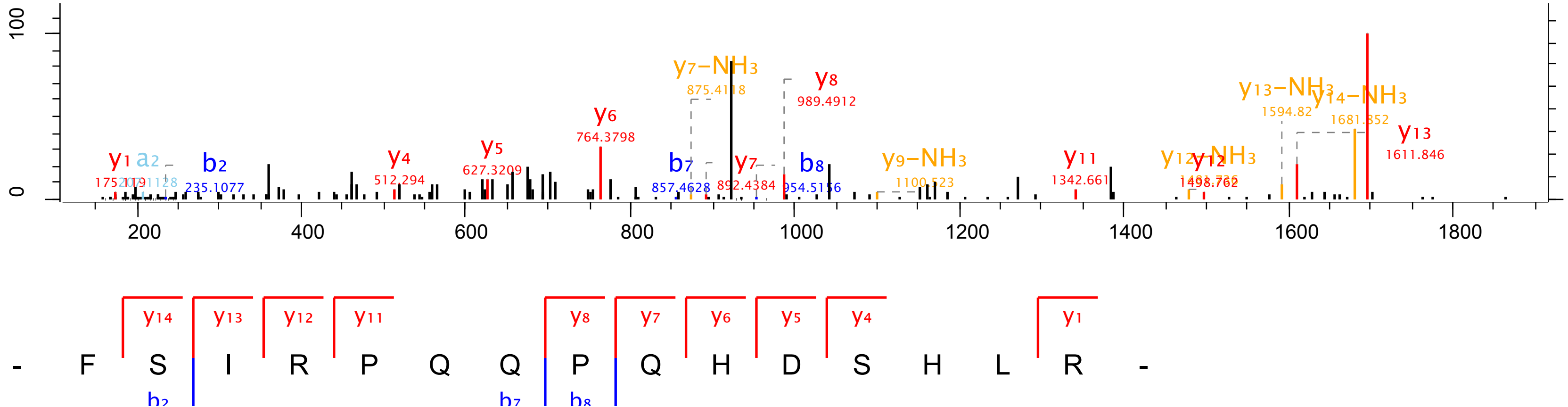

Raw file	Scan	Method	Score	m/z	Gene names
20150402_CerP14_Frac03_top_opt_B3_01_1812	20558	TOF; CID	80.69	644.36	4632428N05Rik

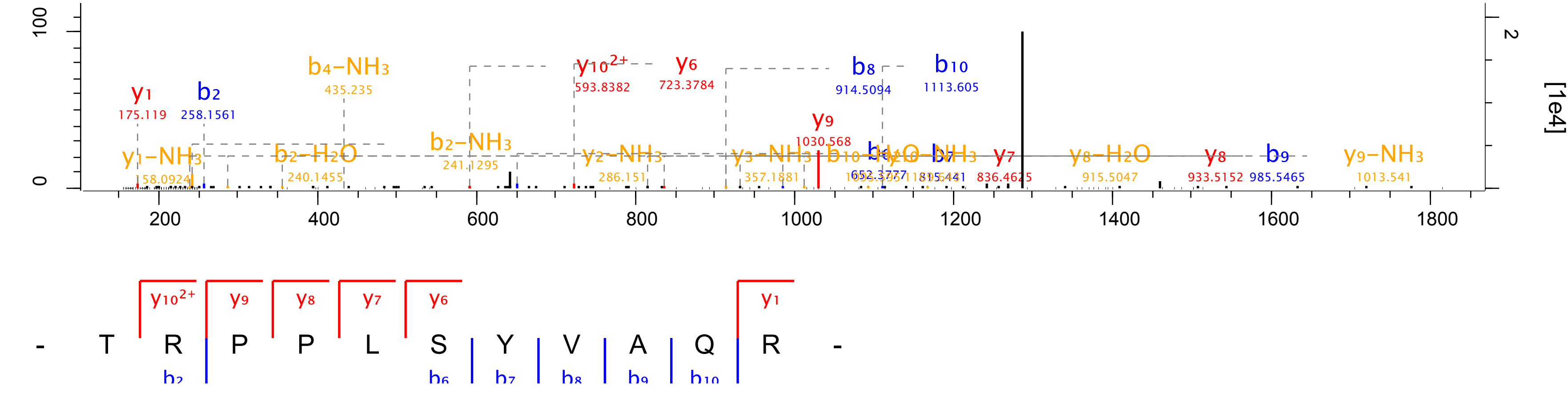

Raw file	Scan	Method	Score	m/z	Gene names
20150402_CerP14_Frac03_top_opt_B3_01_1812	21348	TOF; CID	45.08	622.32	Rnf122

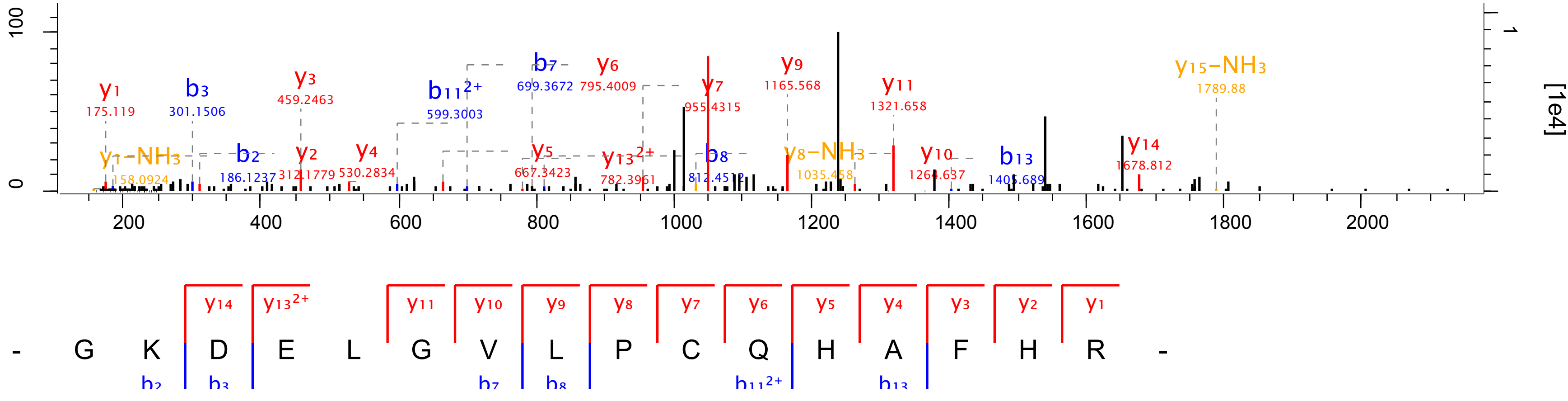

Raw file	Scan	Method	Score	m/z	Gene names
20150402_CerP14_Frac03_top_opt_B3_01_1812	29442	TOF; CID	64.57	852.45	Tbc1d31

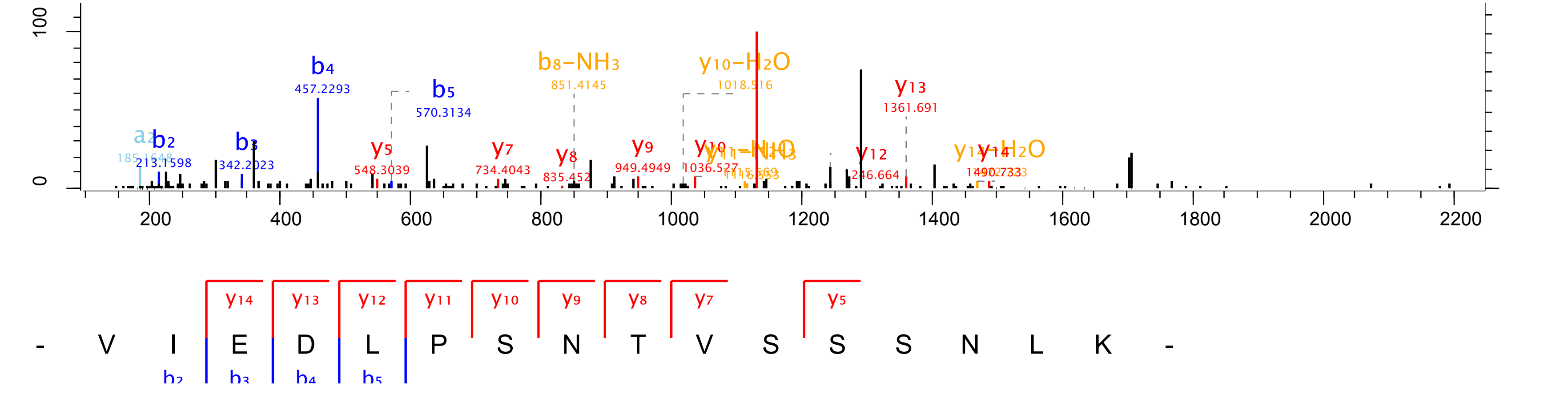

Raw file	Scan	Method	Score	m/z	Gene names
20150402_CerP14_Frac03_top_opt_B3_01_1812	32616	TOF; CID	110.39	697.83	Fam159b

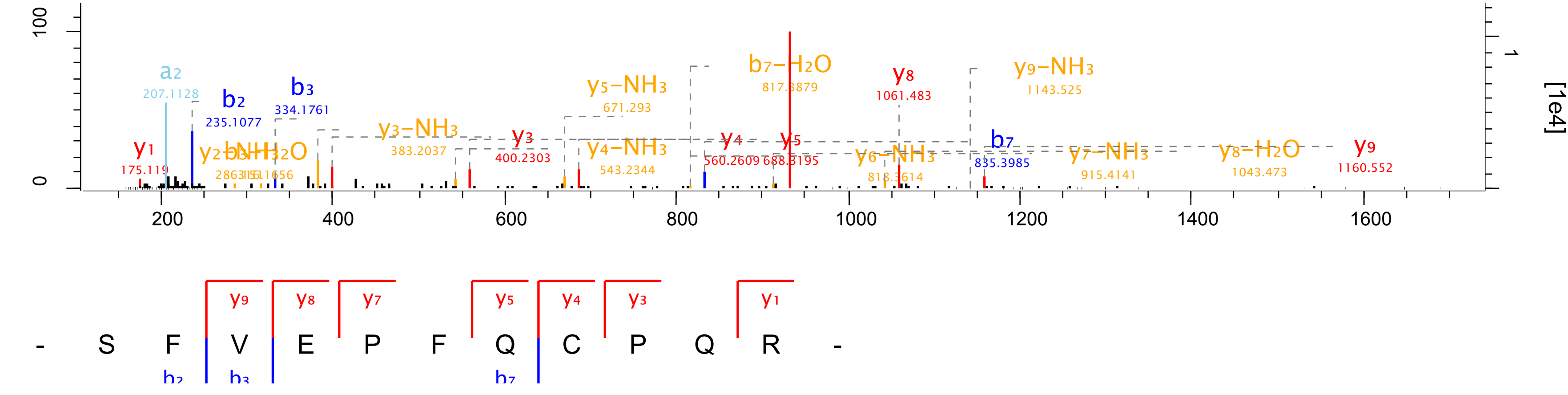

Raw file	Scan	Method	Score	m/z	Gene names
20150402_CerP14_Frac03_top_opt_B3_01_1812	33707	TOF; CID	65.23	578.99	Gal

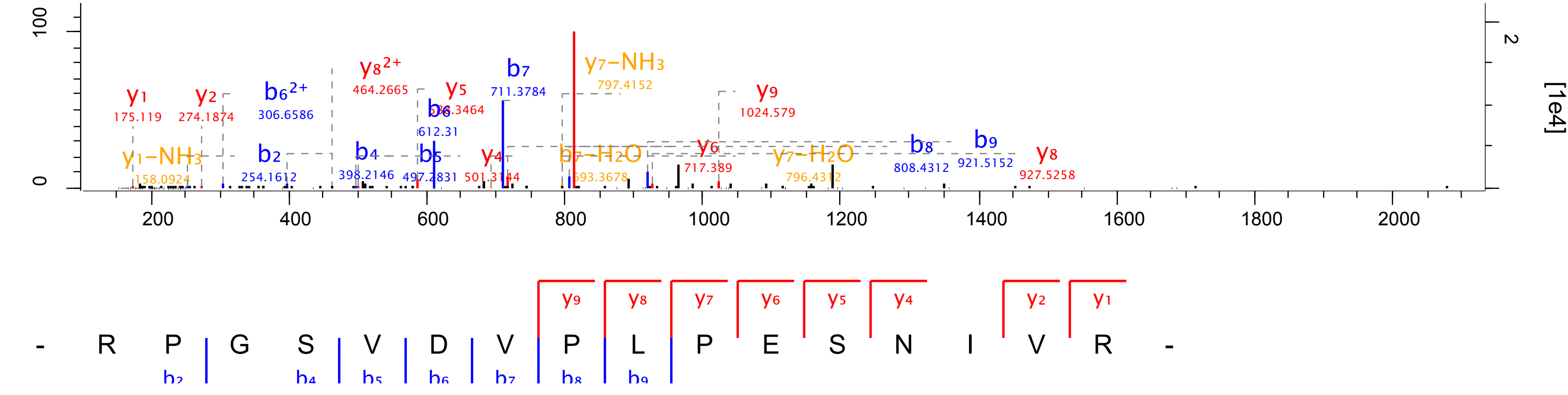

Raw file	Scan	Method	Score	m/z	Gene names
20150402_CerP14_Frac03_top_opt_B3_01_1812	34961	TOF; CID	55.72	580.28	Cdh16

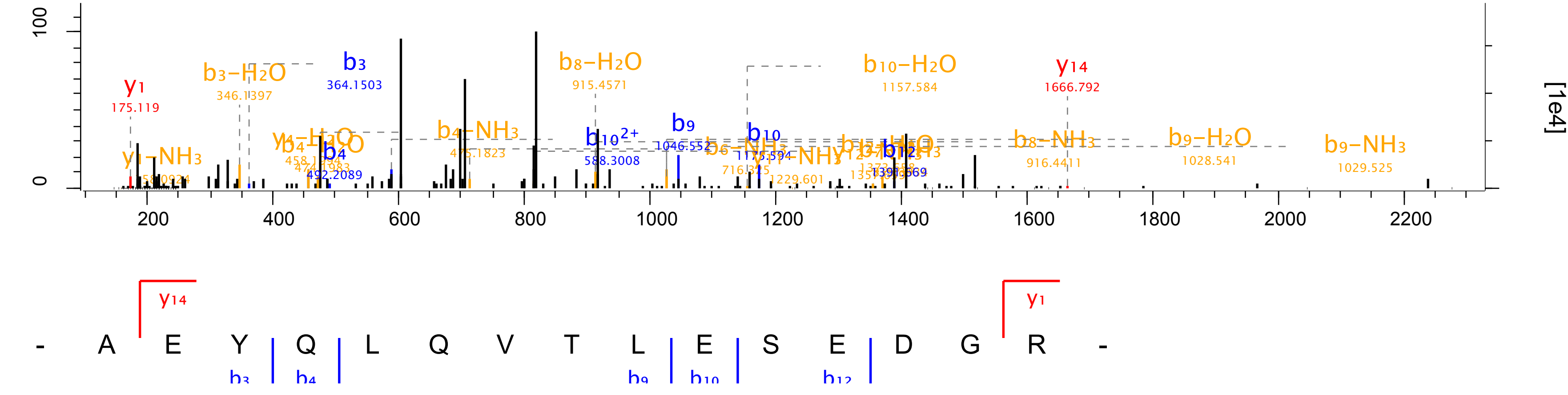

Raw file	Scan	Method	Score	m/z	Gene names
20150402_CerP14_Frac03_top_opt_B3_01_1812	37406	TOF; CID	38.99	1067.53	Apcs

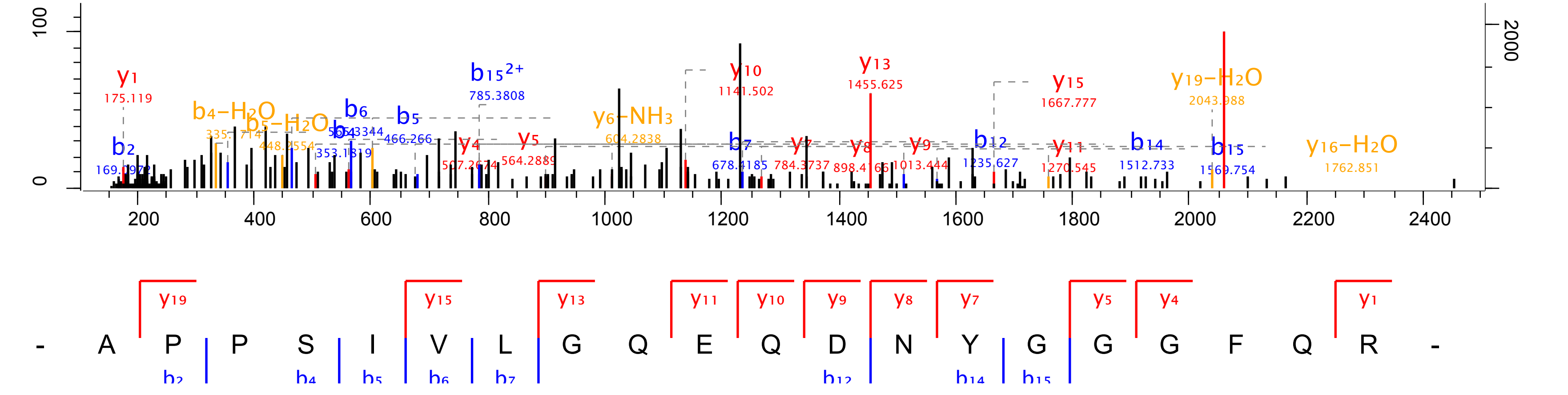

Raw file	Scan	Method	Score	m/z	Gene names
20150402_CerP14_Frac03_top_opt_B3_01_1812	40418	TOF; CID	90.05	588.32	Try10

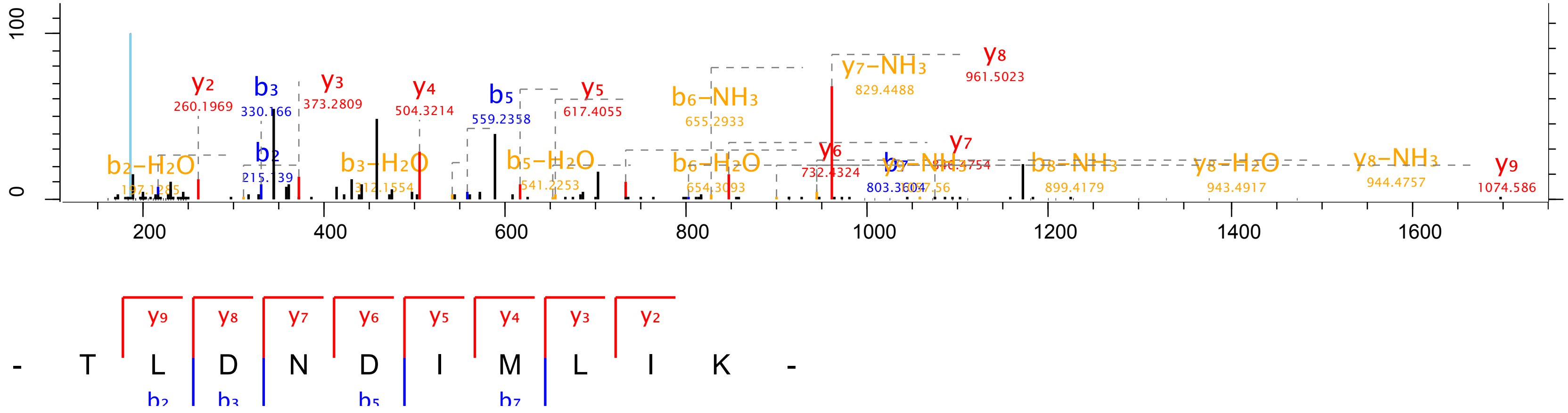

Raw file	Scan	Method	Score	m/z	Gene names
20150402_CerP14_Frac03_top_opt_B3_01_1812	42602	TOF; CID	51.01	700.87	Zswim6

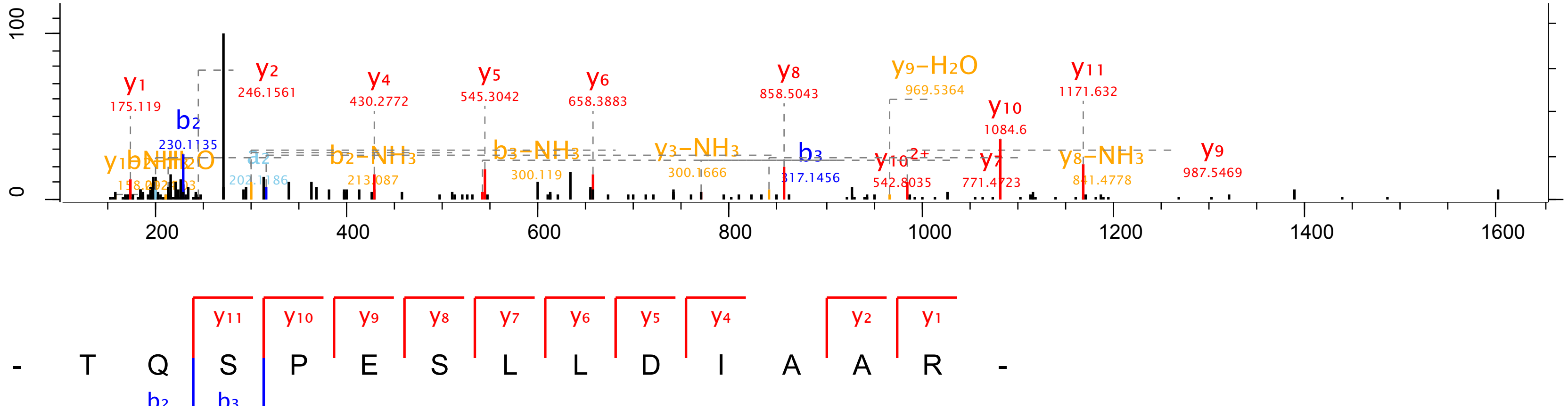

Raw file	Scan	Method	Score	m/z	Gene names
20150402_CerP14_Frac03_top_opt_B3_01_1812	43800	TOF; CID	50.96	1118.49	Nrsn2

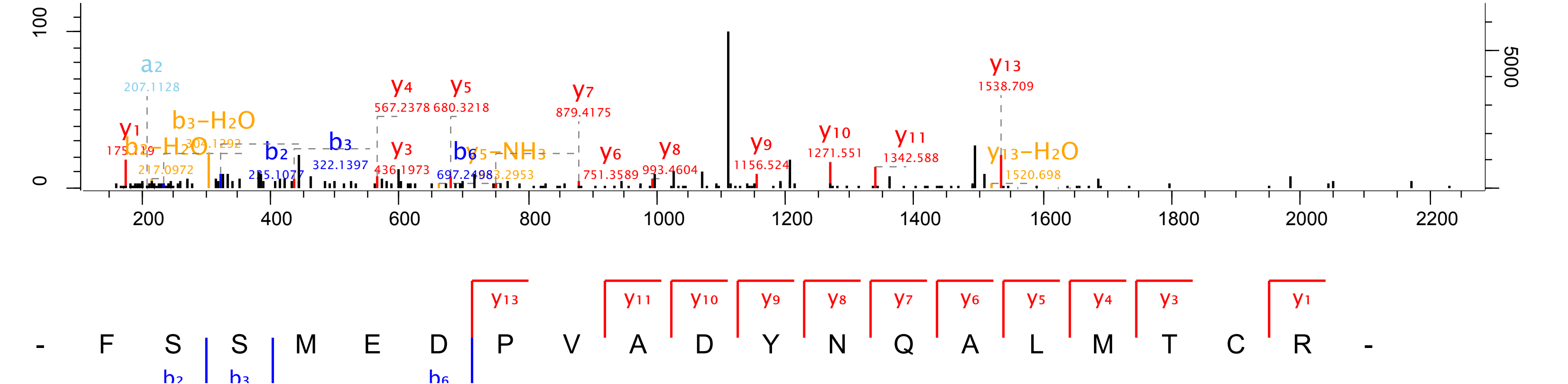

Raw file	Scan	Method	Score	m/z	Gene names
20150402_CerP14_Frac03_top_opt_B3_01_1812	47388	TOF; CID	80.69	593.81	Katnbl1

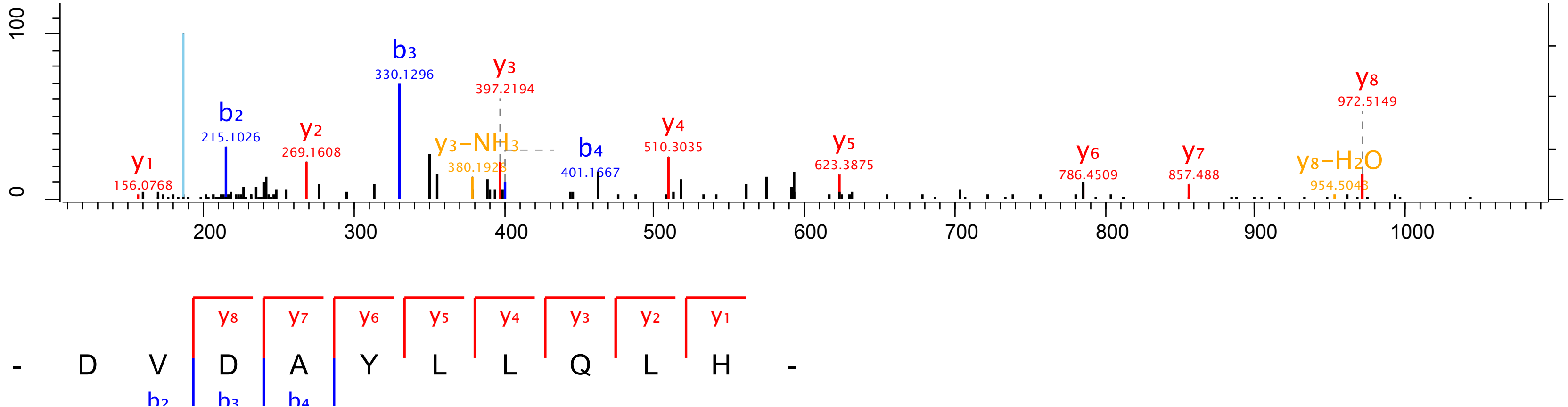

Raw file	Scan	Method	Score	m/z	Gene names
20150402_CerP14_Frac03_top_opt_B3_01_1812	48609	TOF; CID	114.64	840.43	Tmem38b

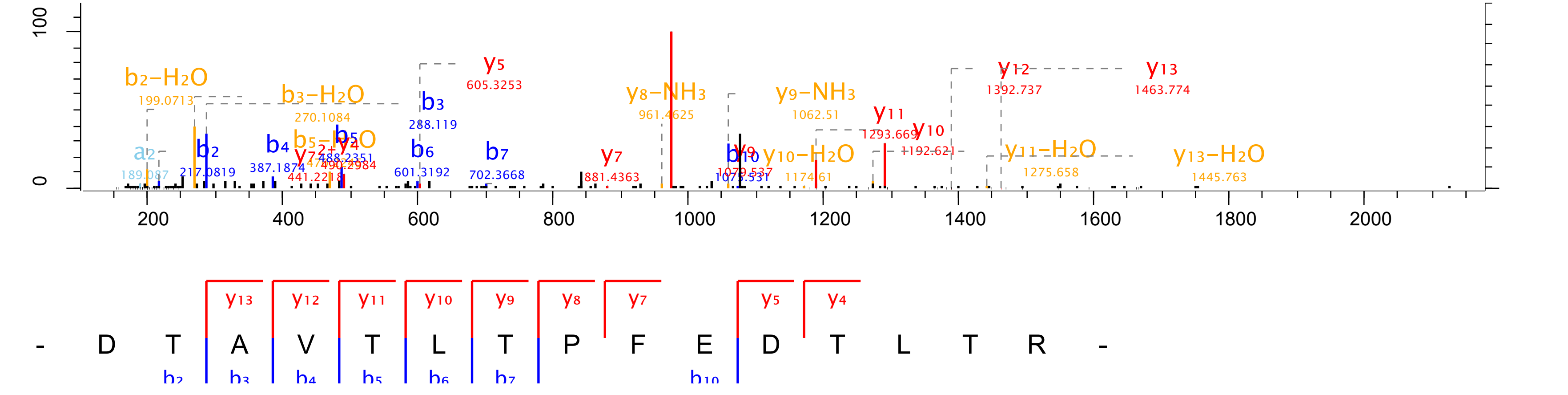

Raw file	Scan	Method	Score	m/z	Gene names
20150402_CerP14_Frac03_top_opt_B3_01_1812	50506	TOF; CID	125.97	665.88	Aqp11

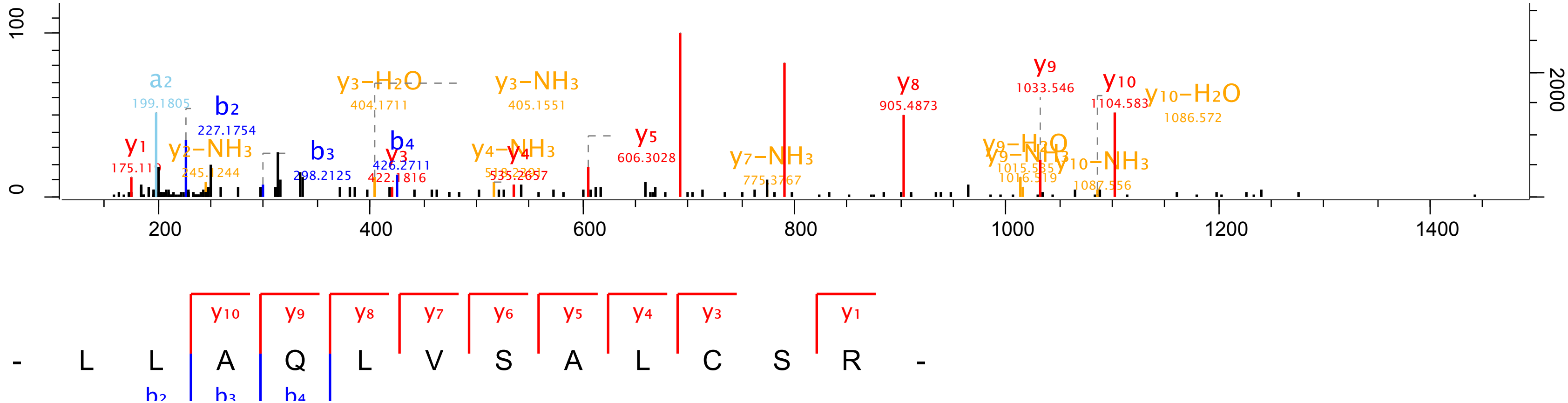

Raw file	Scan	Method	Score	m/z	Gene names
20150402_CerP14_Frac03_top_opt_B3_01_1812	53524	TOF; CID	81.45	554.34	Cln3

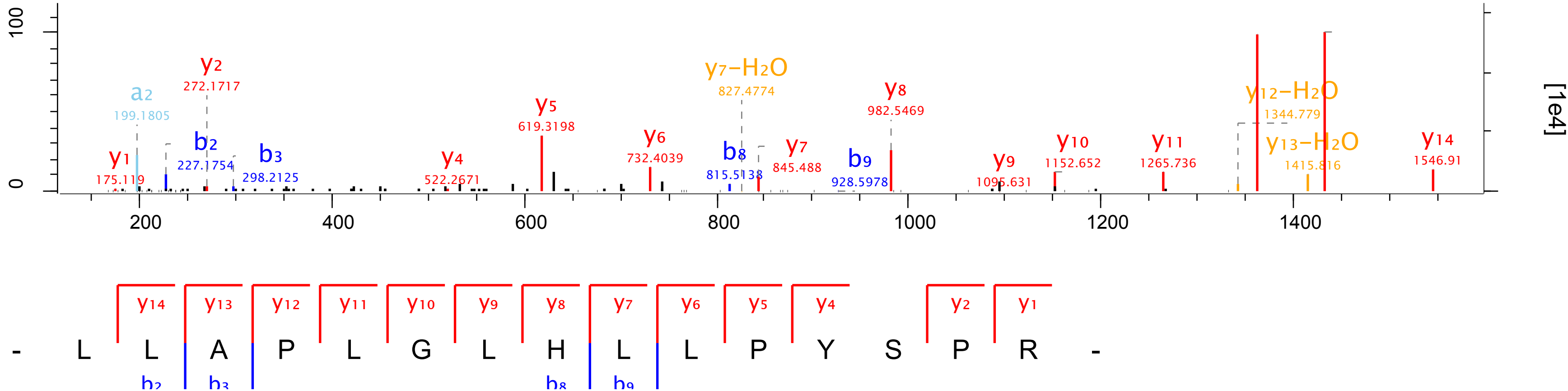

Raw file	Scan	Method	Score	m/z	Gene names
20150402_CerP14_Frac03_top_opt_B3_01_1812	55194	TOF; CID	75.39	612.35	Acrbp

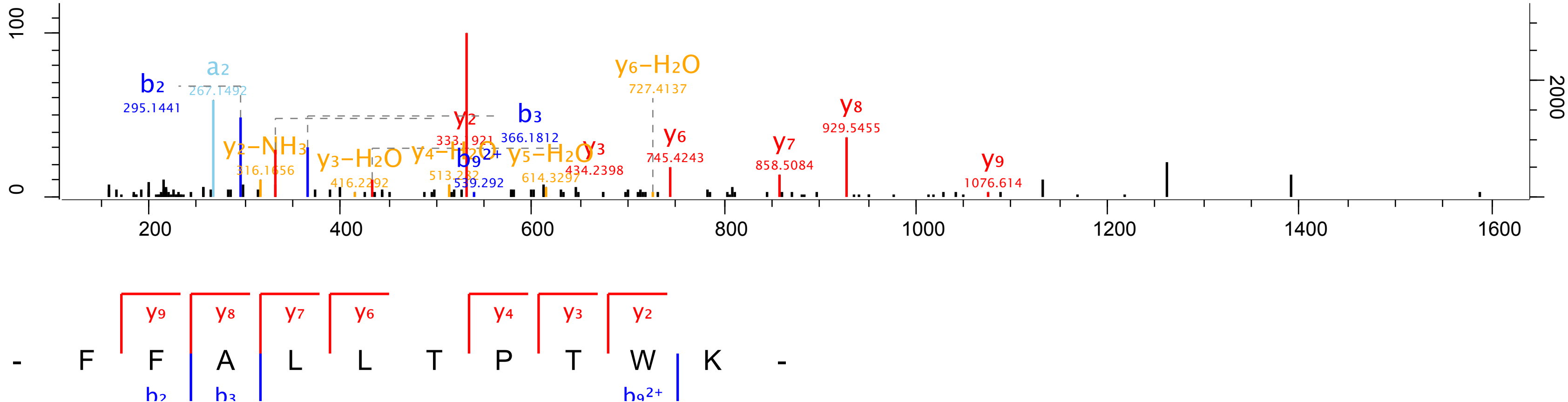

Raw file	Scan	Method	Score	m/z	Gene names
20150402_CerP14_Frac03_top_opt_B3_01_1812	55654	TOF; CID	57.05	1101.56	Poli

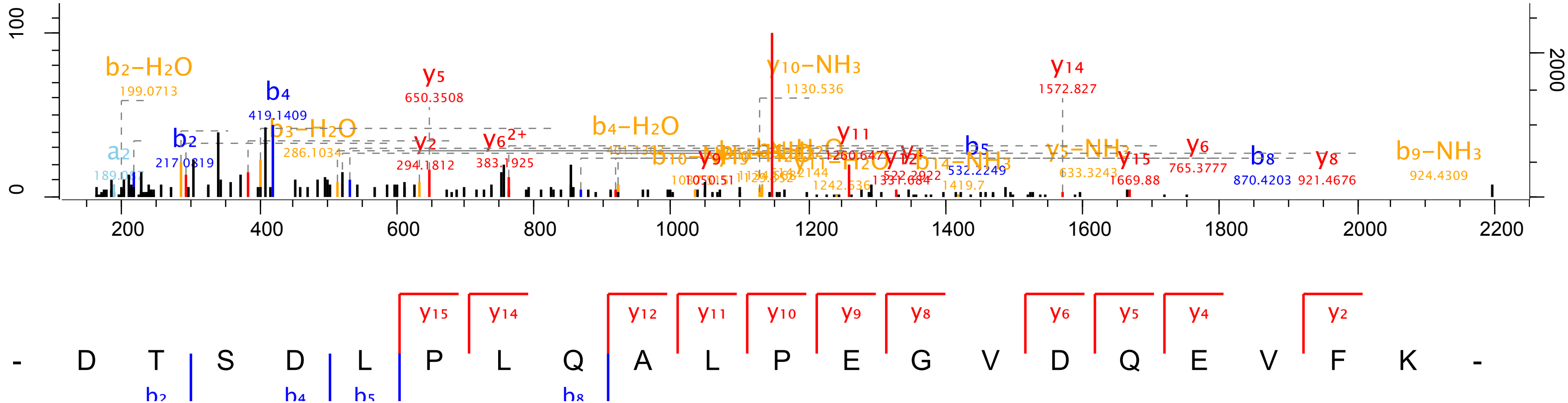

Raw file	Scan	Method	Score	m/z	Gene names
20150402_CerP14_Frac03_top_opt_B3_01_1812	57428	TOF; CID	34.26	886.46	Mmrn1

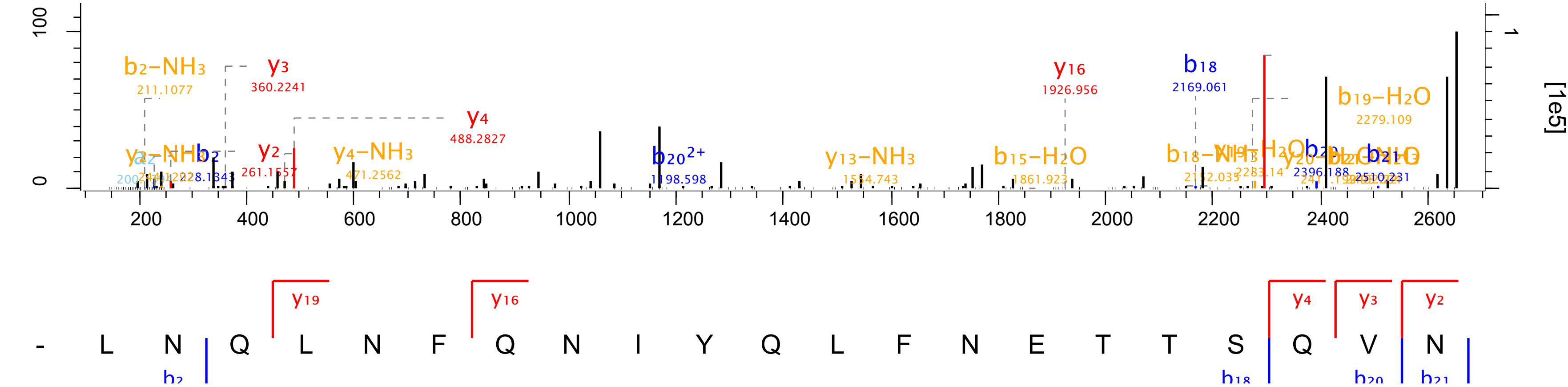

Raw file	Scan	Method	Score	m/z	Gene names
20150402_CerP14_Frac03_top_opt_B3_01_1812	58684	TOF; CID	74.61	660.94	Chst7

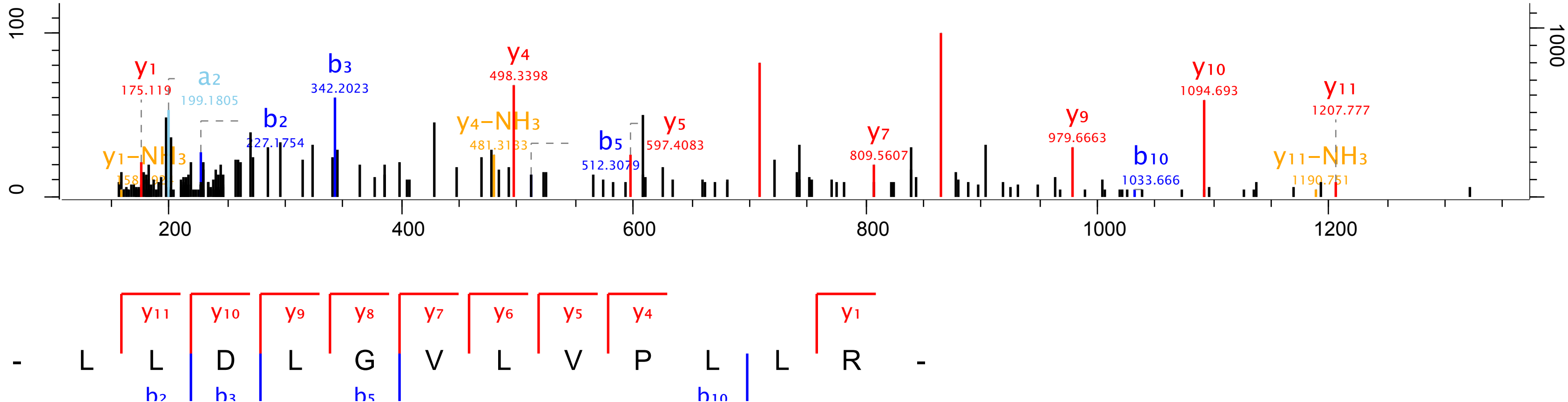

Raw file	Scan	Method	Score	m/z	Gene names
20150402_CerP14_Frac04_top_opt_B4_01_1813	4075	TOF; CID	80.92	370.52	Mfsd1

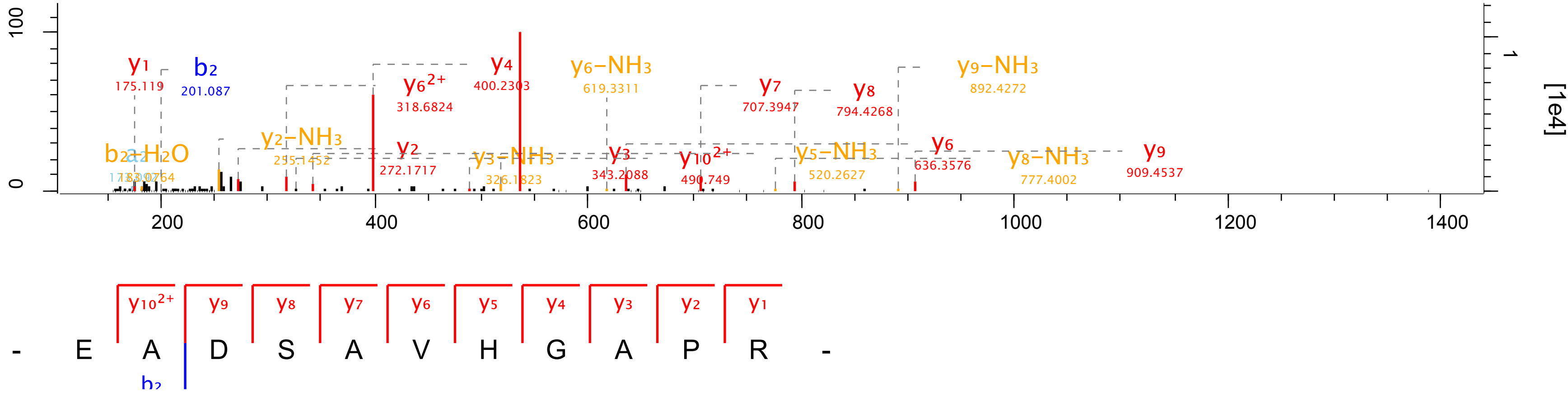

Raw file	Scan	Method	Score	m/z	Gene names
20150402_CerP14_Frac04_top_opt_B4_01_1813	5454	TOF; CID	91.87	358.19	Ccdc167

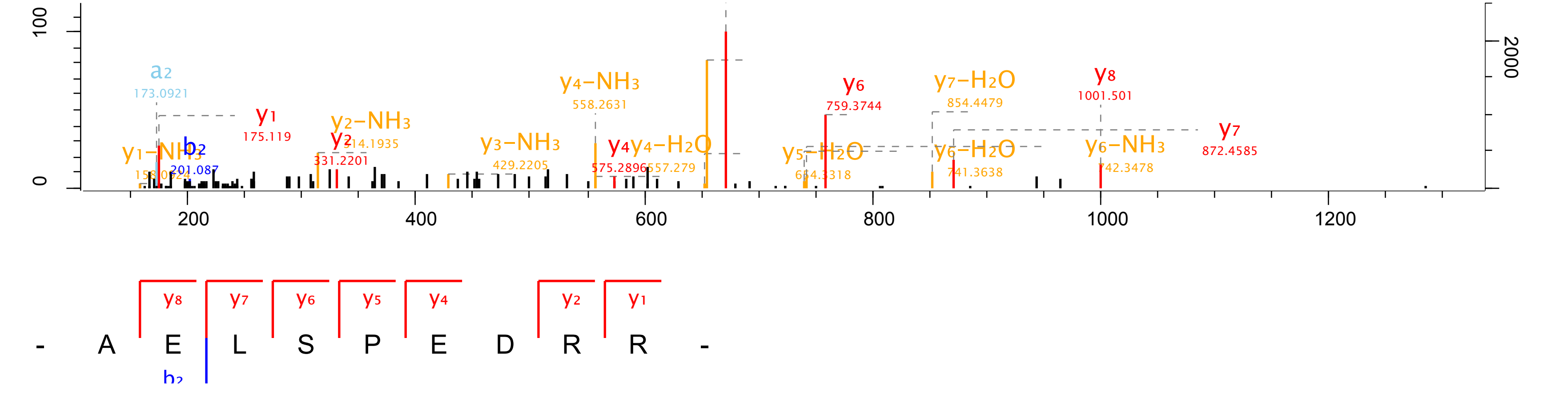

Raw file	Scan	Method	Score	m/z	Gene names
20150402_CerP14_Frac04_top_opt_B4_01_1813	10771	TOF; CID	81.77	532.23	Wdr83os

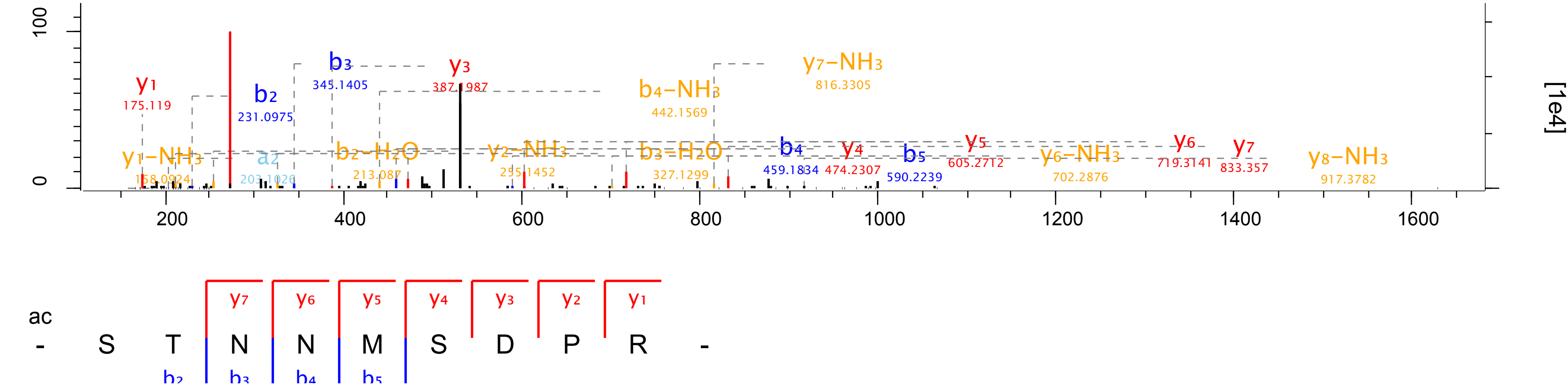

Raw file	Scan	Method	Score	m/z	Gene names
20150402_CerP14_Frac04_top_opt_B4_01_1813	15453	TOF; CID	73.06	721.31	Usp54

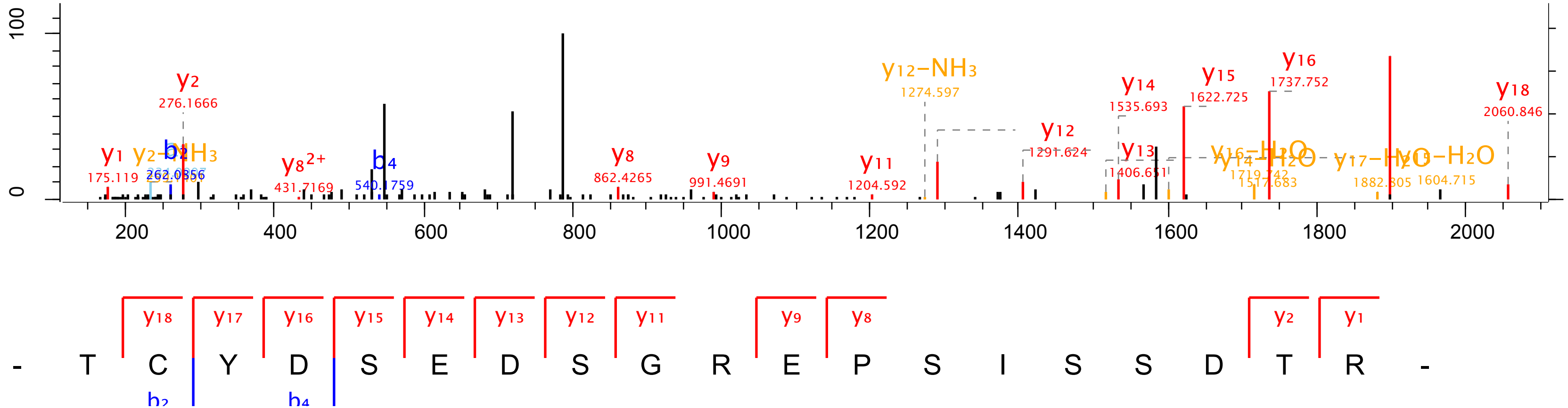

Raw file	Scan	Method	Score	m/z	Gene names
20150402_CerP14_Frac04_top_opt_B4_01_1813	19456	TOF; CID	69.79	407.24	Tmem108

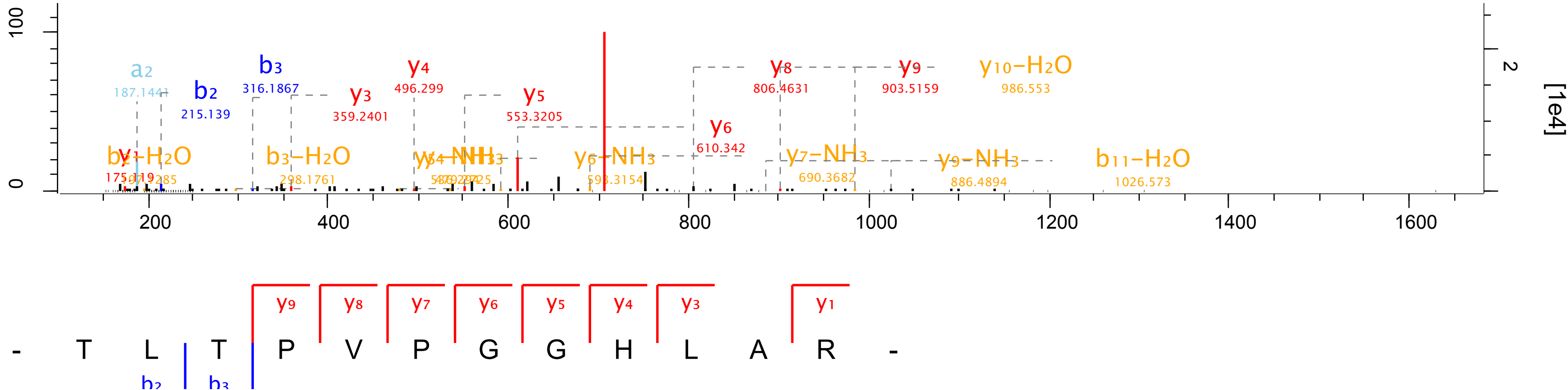

Raw file	Scan	Method	Score	m/z	Gene names
20150402_CerP14_Frac04_top_opt_B4_01_1813	20528	TOF; CID	115.78	385.9	Tmem179

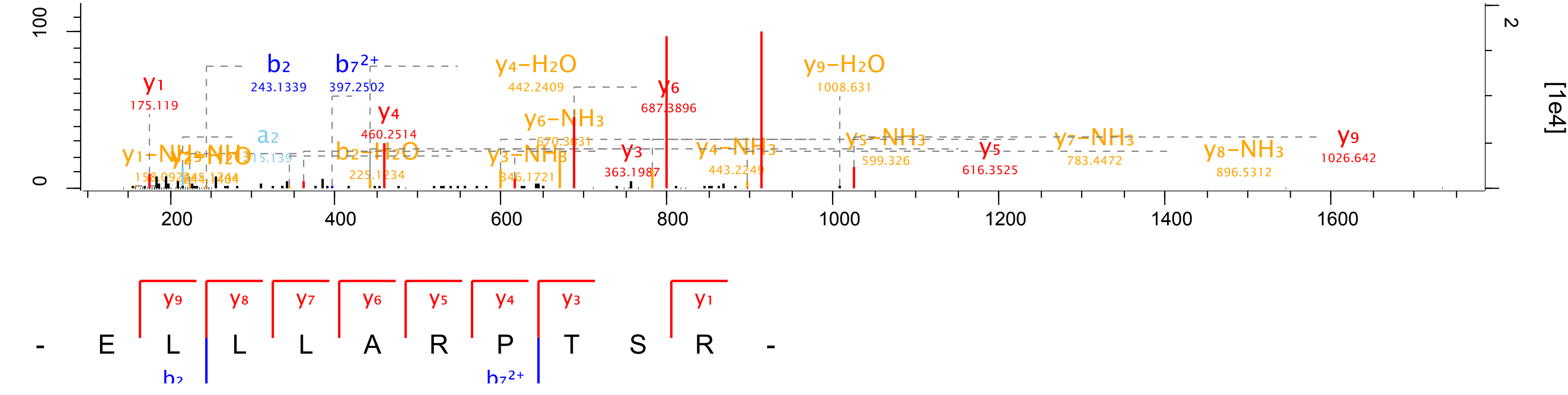

Raw file	Scan	Method	Score	m/z	Gene names
20150402_CerP14_Frac04_top_opt_B4_01_1813	26628	TOF; CID	54.81	571.3	Maml2

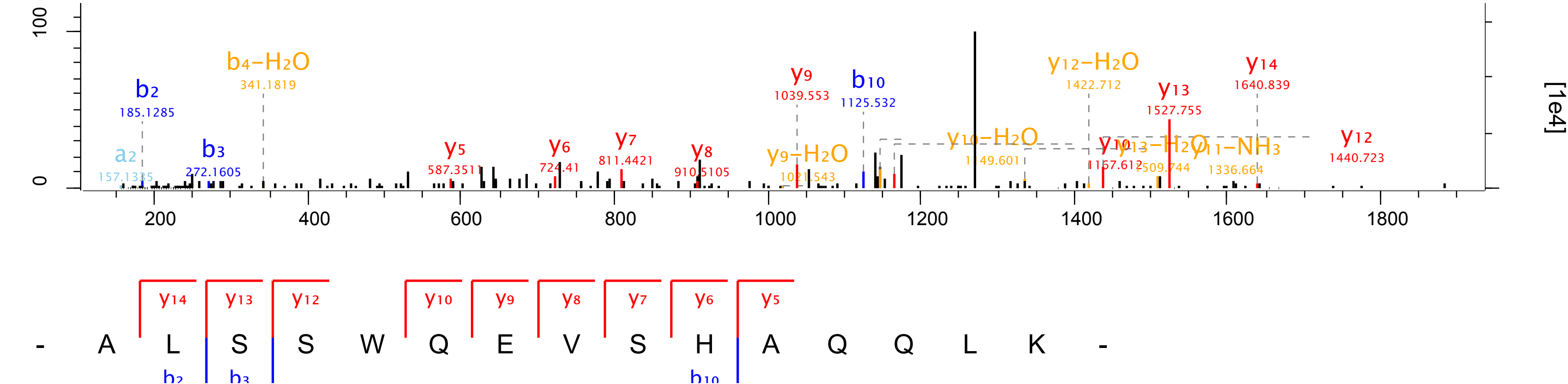

Raw file	Scan	Method	Score	m/z	Gene names
20150402_CerP14_Frac04_top_opt_B4_01_1813	29468	TOF; CID	44.44	872.45	Htr2a

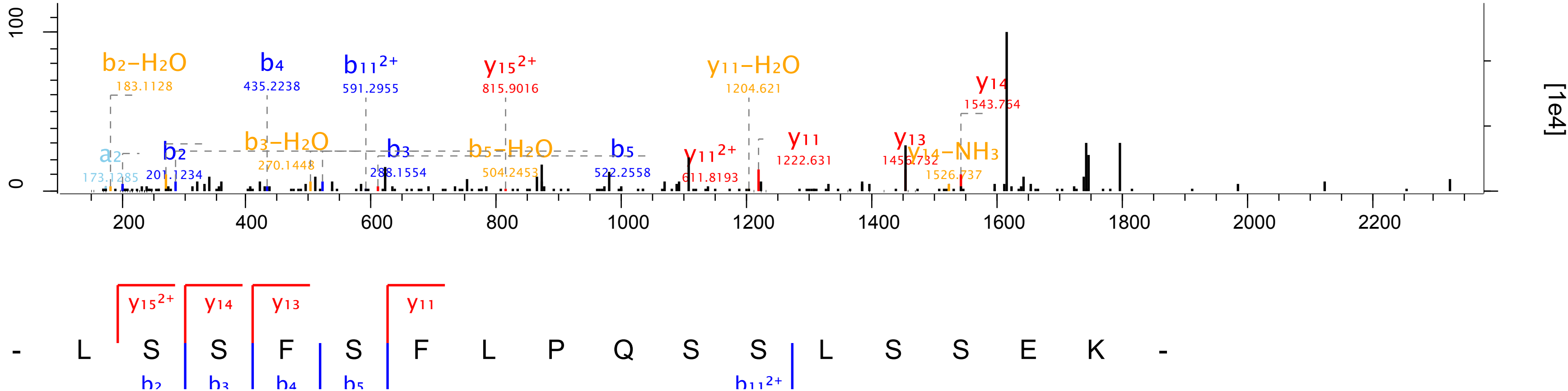

Raw file	Scan	Method	Score	m/z	Gene names
20150402_CerP14_Frac04_top_opt_B4_01_1813	31937	TOF; CID	162.79	792.43	Id4

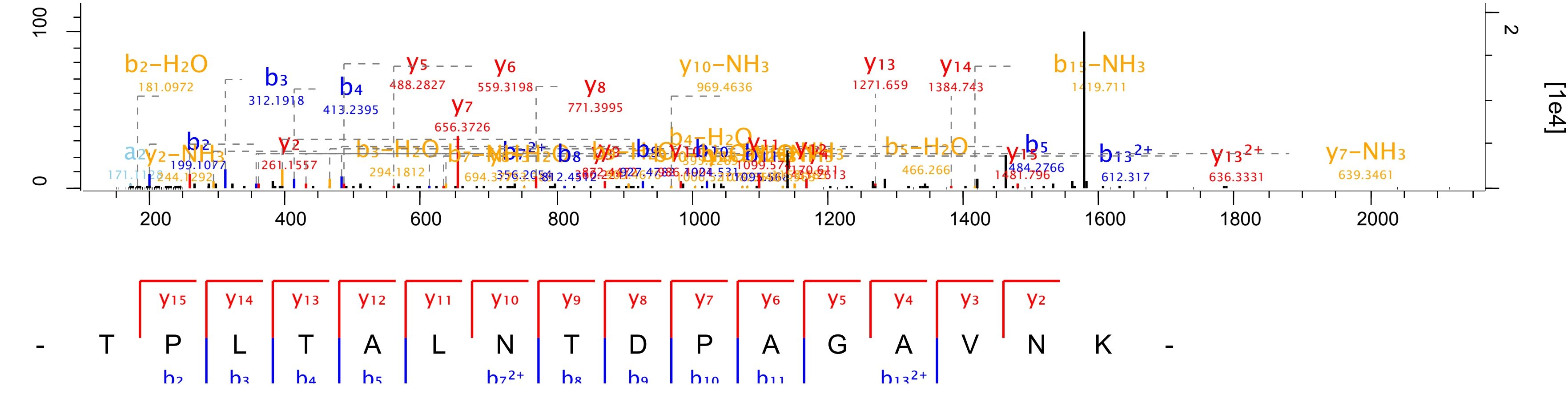

Raw file	Scan	Method	Score	m/z	Gene names
20150402_CerP14_Frac04_top_opt_B4_01_1813	32944	TOF; CID	66.27	434.24	Rarres2

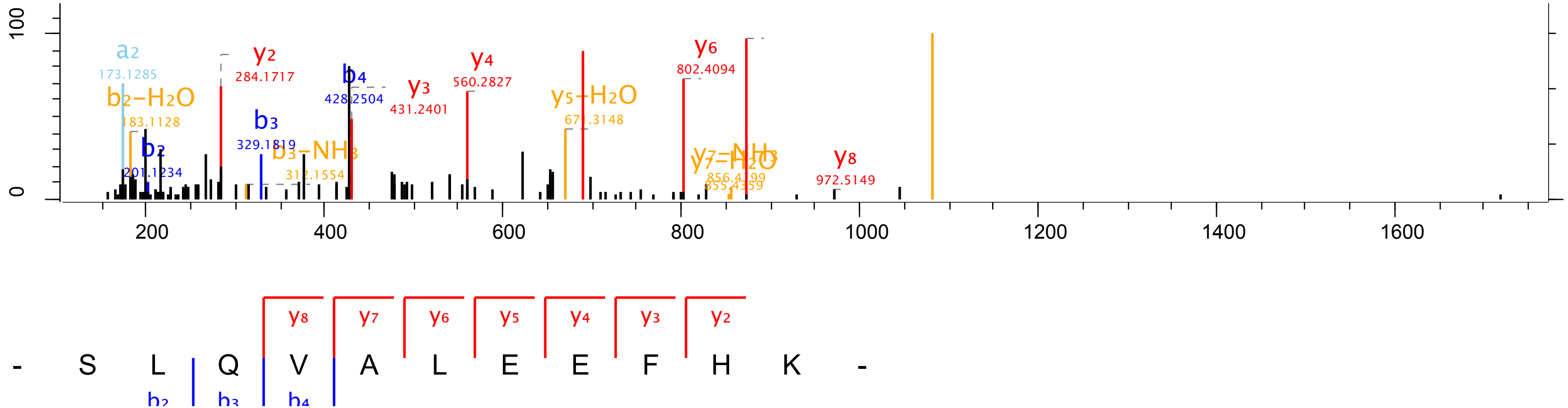

Raw file	Scan	Method	Score	m/z	Gene names
20150402_CerP14_Frac04_top_opt_B4_01_1813	34945	TOF; CID	81.92	752.88	Adipoq

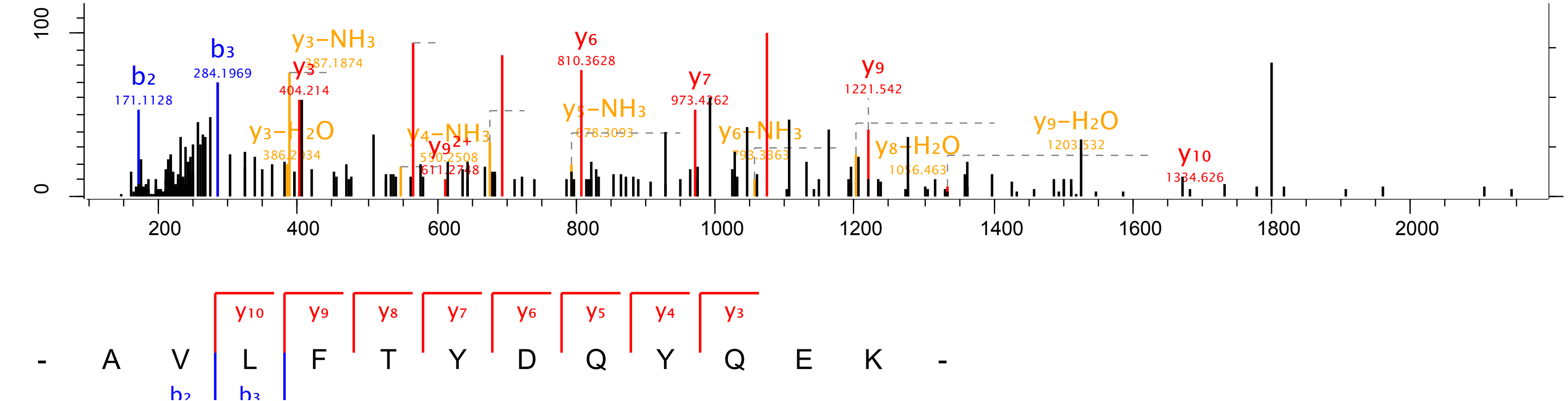

Raw file	Scan	Method	Score	m/z	Gene names
20150402_CerP14_Frac04_top_opt_B4_01_1813	35561	TOF; CID	94.36	707.85	Cd63

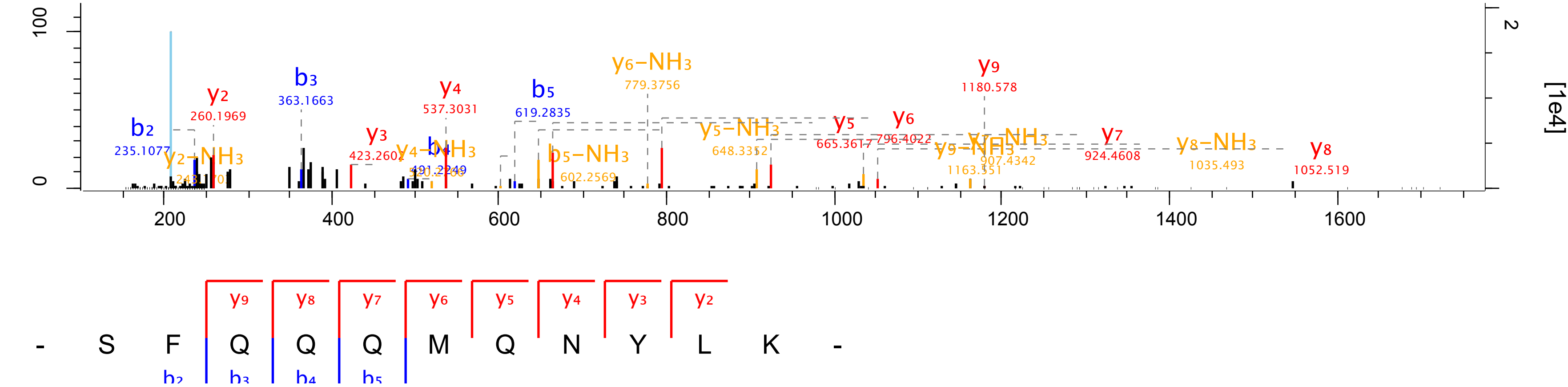

Raw file	Scan	Method	Score	m/z	Gene names
20150402_CerP14_Frac04_top_opt_B4_01_1813	38797	TOF; CID	45.89	756.07	Arhgef37

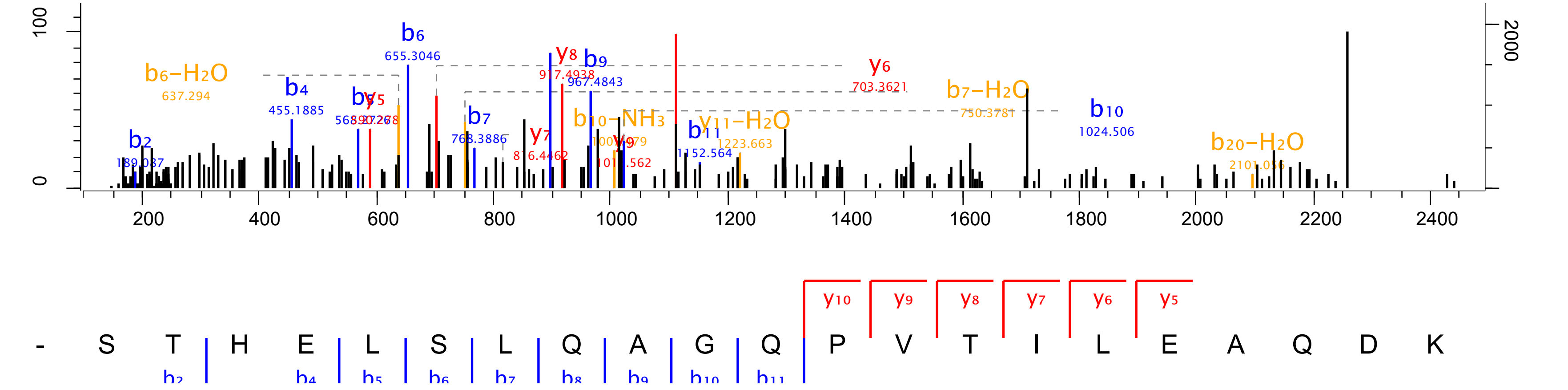

Raw file	Scan	Method	Score	m/z	Gene names
20150402_CerP14_Frac04_top_opt_B4_01_1813	40125	TOF; CID	130.56	930.47	Hiat1

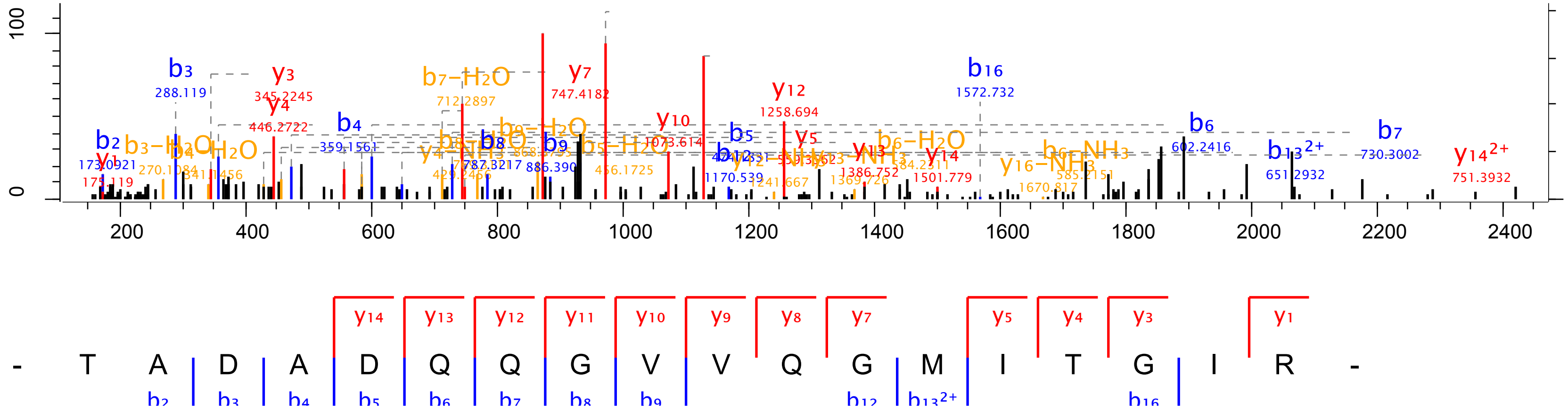

Raw file	Scan	Method	Score	m/z	Gene names
20150402_CerP14_Frac04_top_opt_B4_01_1813	40562	TOF; CID	48.05	709.37	Cep78

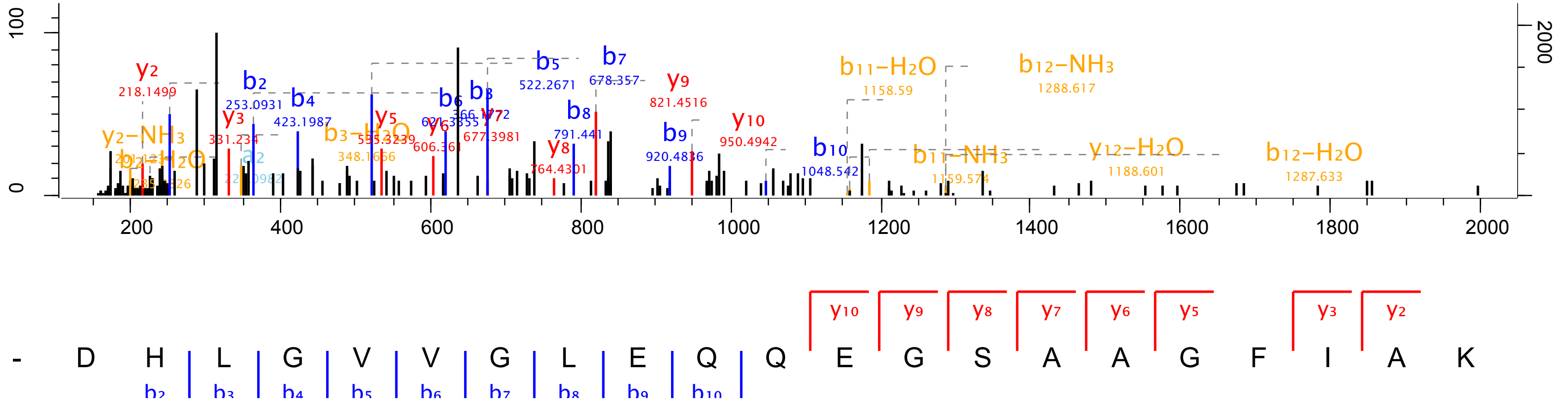

Raw file	Scan	Method	Score	m/z	Gene names
20150402_CerP14_Frac04_top_opt_B4_01_1813	41705	TOF; CID	42.88	1072.23	Rnf152

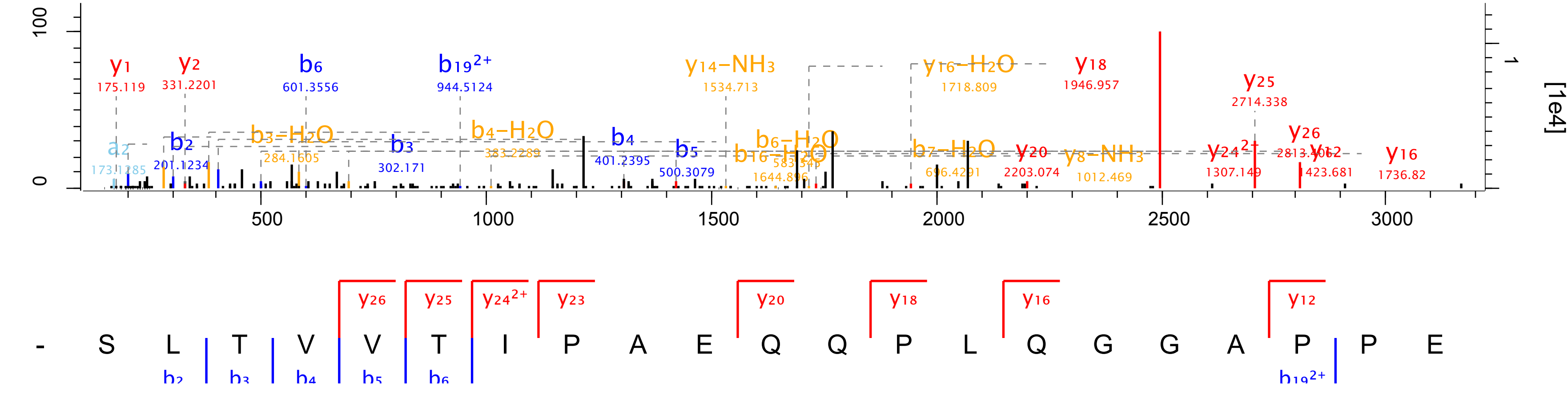

Raw file	Scan	Method	Score	m/z	Gene names
20150402_CerP14_Frac04_top_opt_B4_01_1813	47917	TOF; CID	98.1	771.36	Clec2d;Clec2j

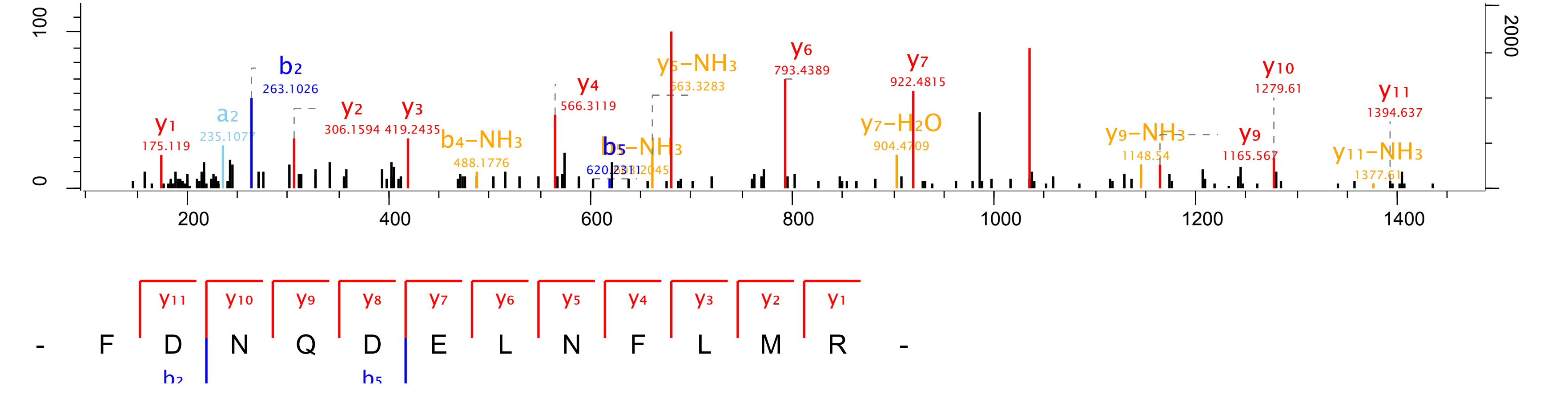

Raw file	Scan	Method	Score	m/z	Gene names
20150402_CerP14_Frac04_top_opt_B4_01_1813	51730	TOF; CID	38.74	831.41	Zfp41

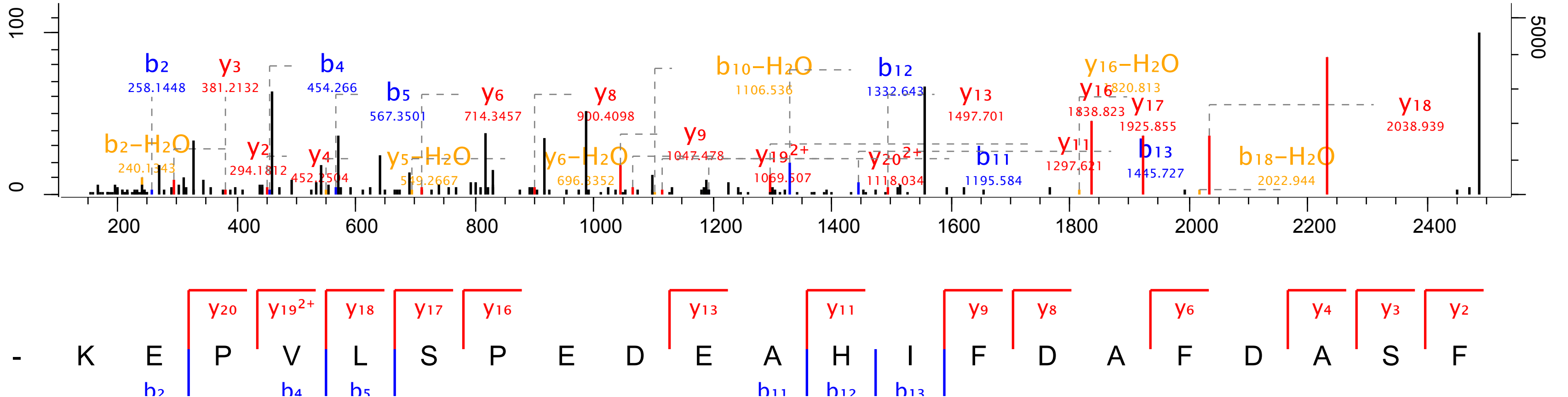

Raw file	Scan	Method	Score	m/z	Gene names
20150402_CerP14_Frac04_top_opt_B4_01_1813	53709	TOF; CID	42.06	1102.52	Zfp395

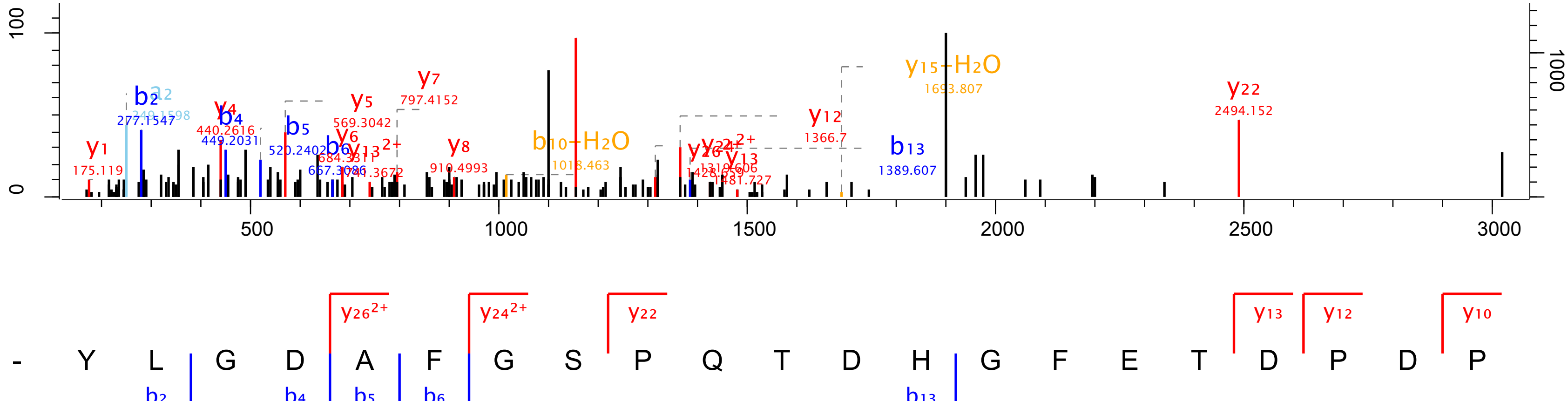

Raw file	Scan	Method	Score	m/z	Gene names
20150402_CerP14_Frac04_top_opt_B4_01_1813	58128	TOF; CID	58.35	864.42	AI413582

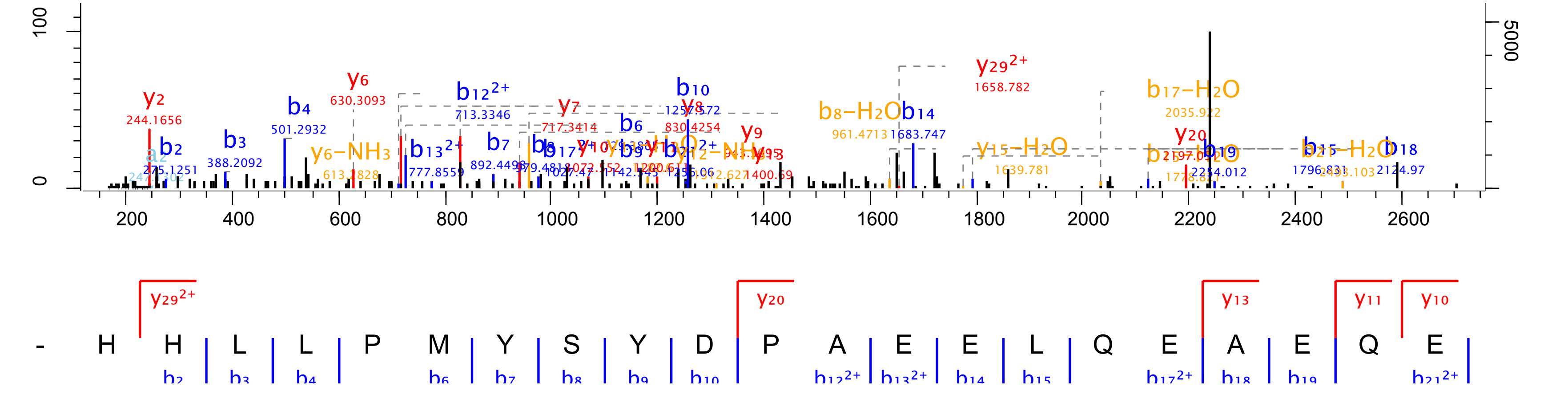

Raw file	Scan	Method	Score	m/z	Gene names
20150402_CerP14_Frac04_top_opt_B4_01_1813	58562	TOF; CID	65.42	665.69	Fndc9

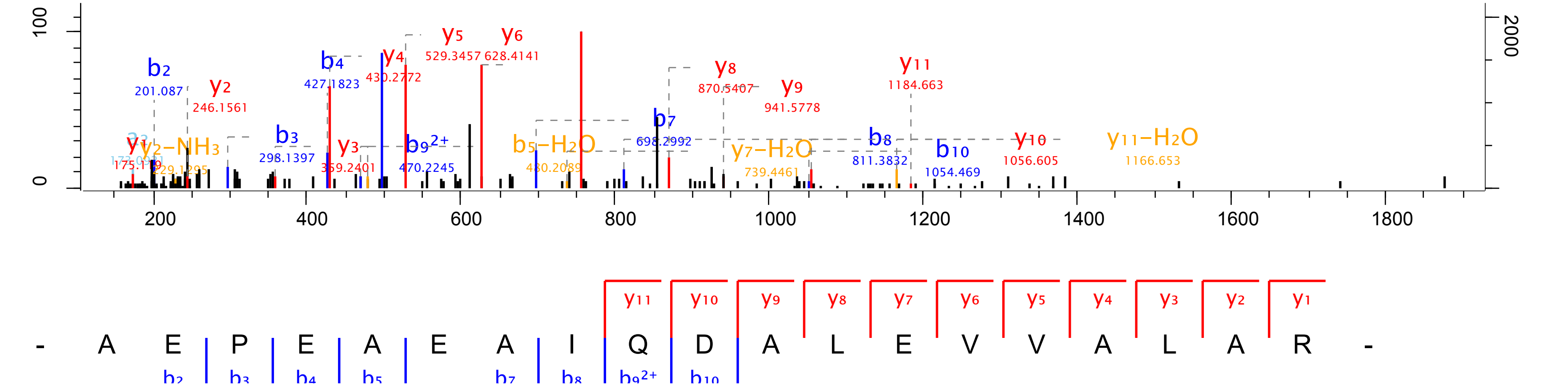

Raw file	Scan	Method	Score	m/z	Gene names
20150402_CerP14_Frac05_top_opt_B5_01_1814	7961	TOF; CID	66.06	520.26	Fnip2

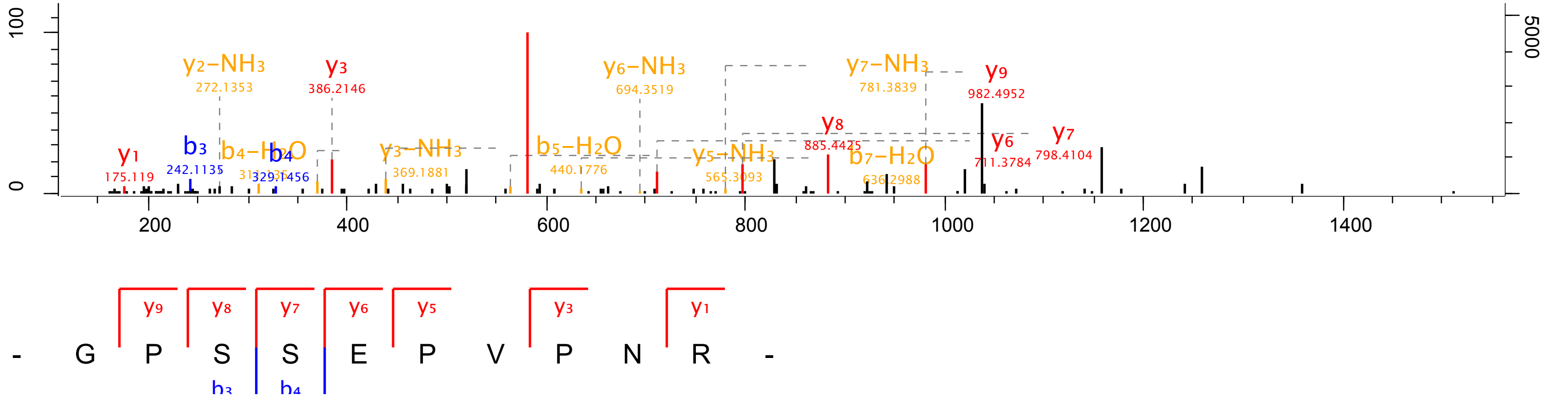

Raw file	Scan	Method	Score	m/z	Gene names
20150402_CerP14_Frac05_top_opt_B5_01_1814	10047	TOF; CID	111.79	733.82	Smim1

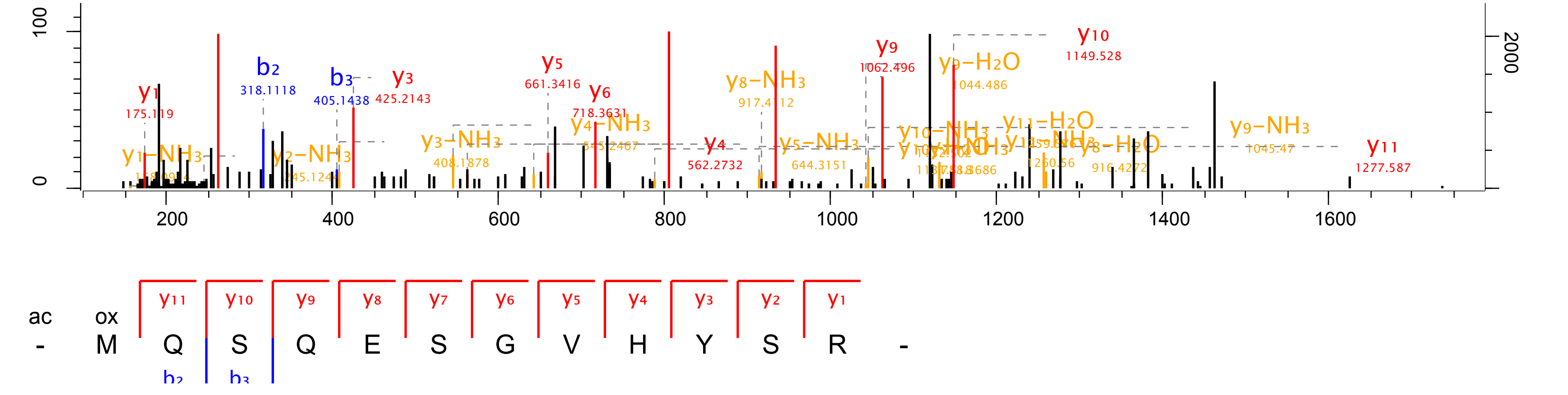

Raw file	Scan	Method	Score	m/z	Gene names
20150402_CerP14_Frac05_top_opt_B5_01_1814	11275	TOF; CID	75.59	714.82	Jtb

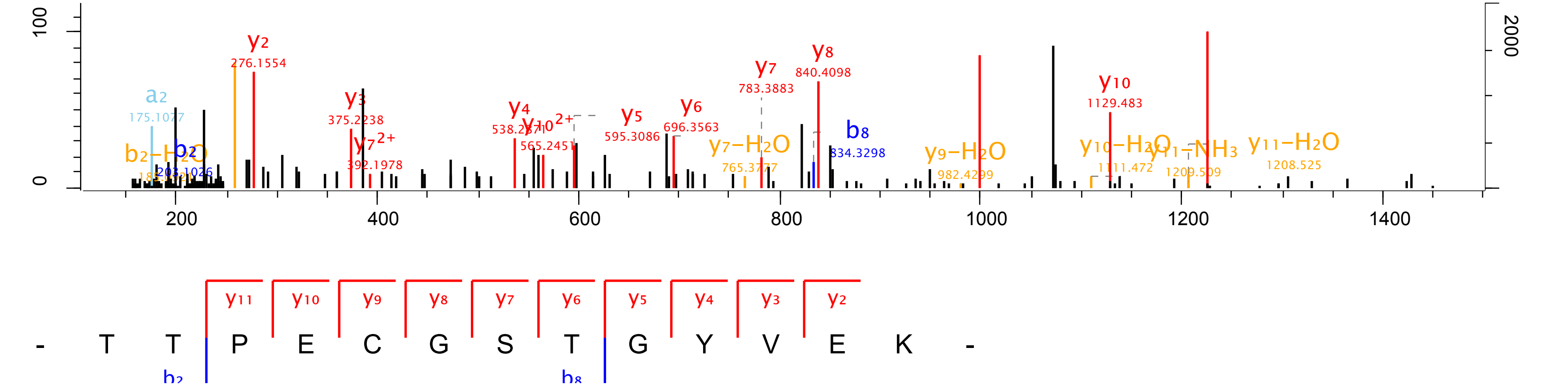

Raw file	Scan	Method	Score	m/z	Gene names
20150402_CerP14_Frac05_top_opt_B5_01_1814	21340	TOF; CID	103.31	928.44	Alkbh2

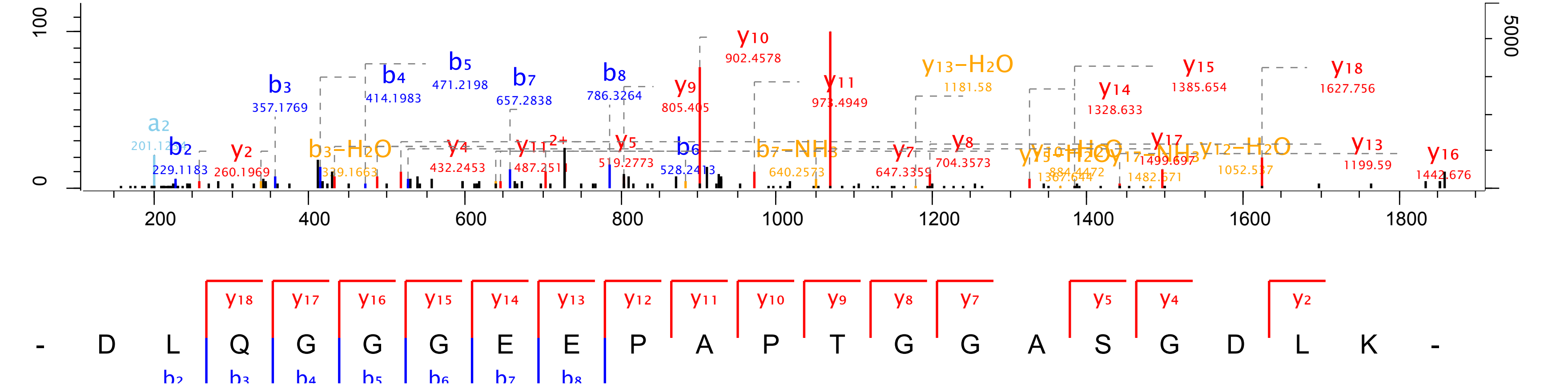

Raw file	Scan	Method	Score	m/z	Gene names
20150402_CerP14_Frac05_top_opt_B5_01_1814	26469	TOF; CID	59.2	405.58	Ccdc126

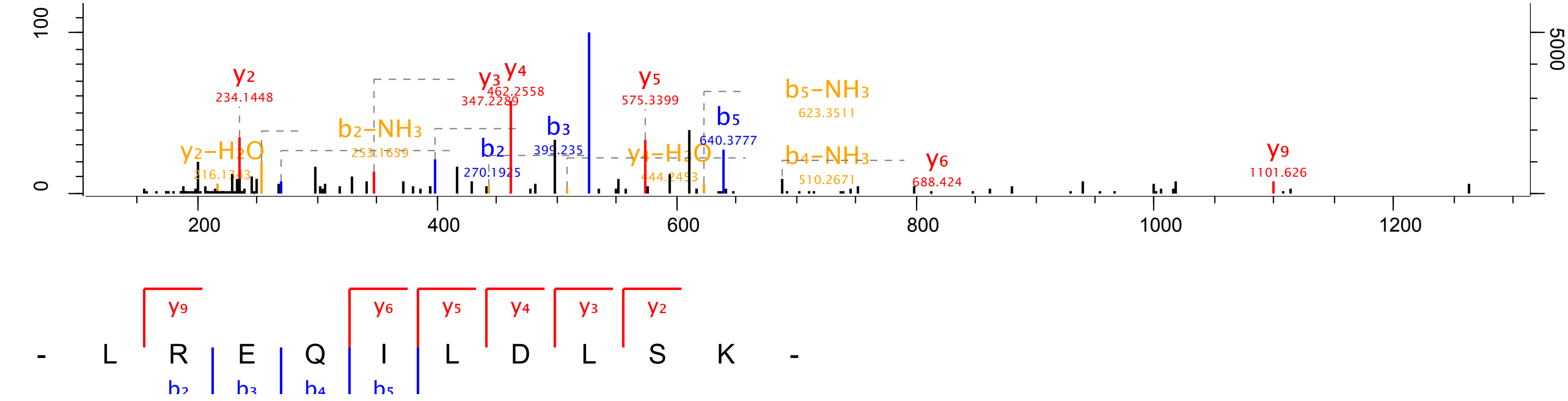

Raw file	Scan	Method	Score	m/z	Gene names
20150402_CerP14_Frac05_top_opt_B5_01_1814	28079	TOF; CID	122.18	476.24	Mapk15

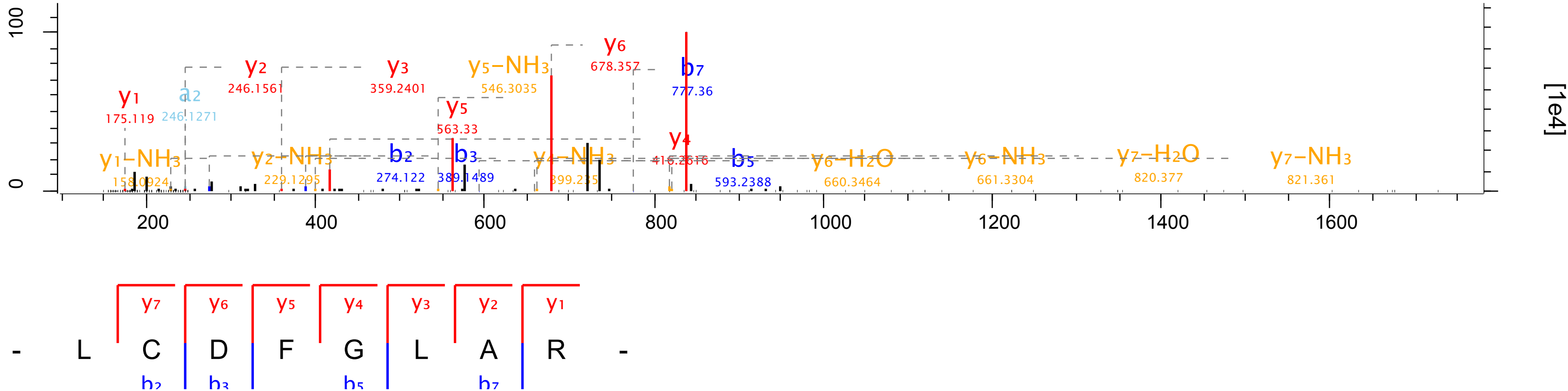

Raw file	Scan	Method	Score	m/z	Gene names
20150402_CerP14_Frac05_top_opt_B5_01_1814	28937	TOF; CID	84.48	642.85	Prg2

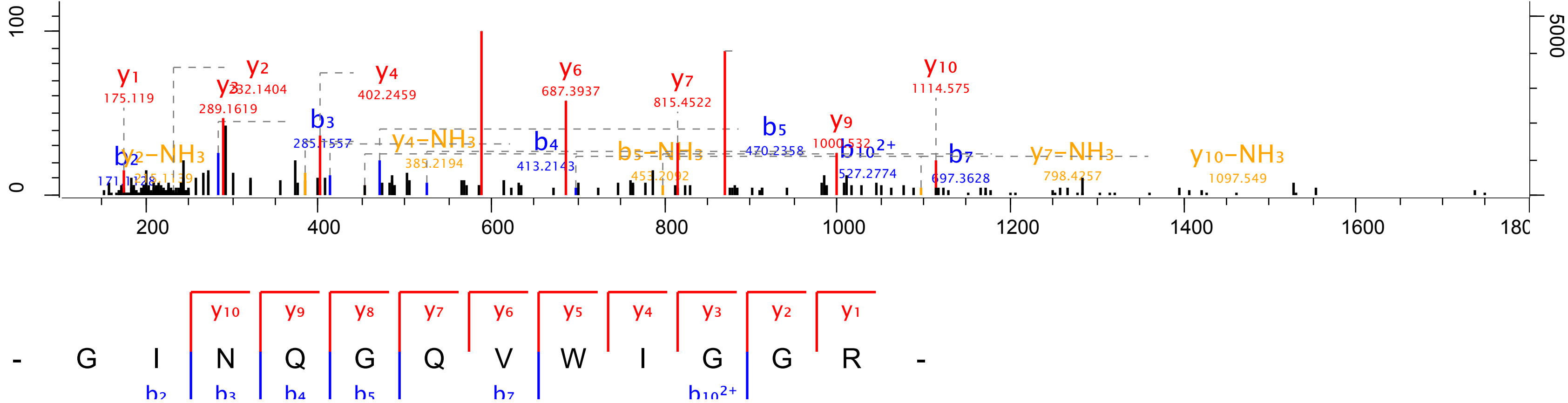

Raw file	Scan	Method	Score	m/z	Gene names
20150402_CerP14_Frac05_top_opt_B5_01_1814	29637	TOF; CID	71.81	687.85	Foxf1;Foxl2;Foxf2

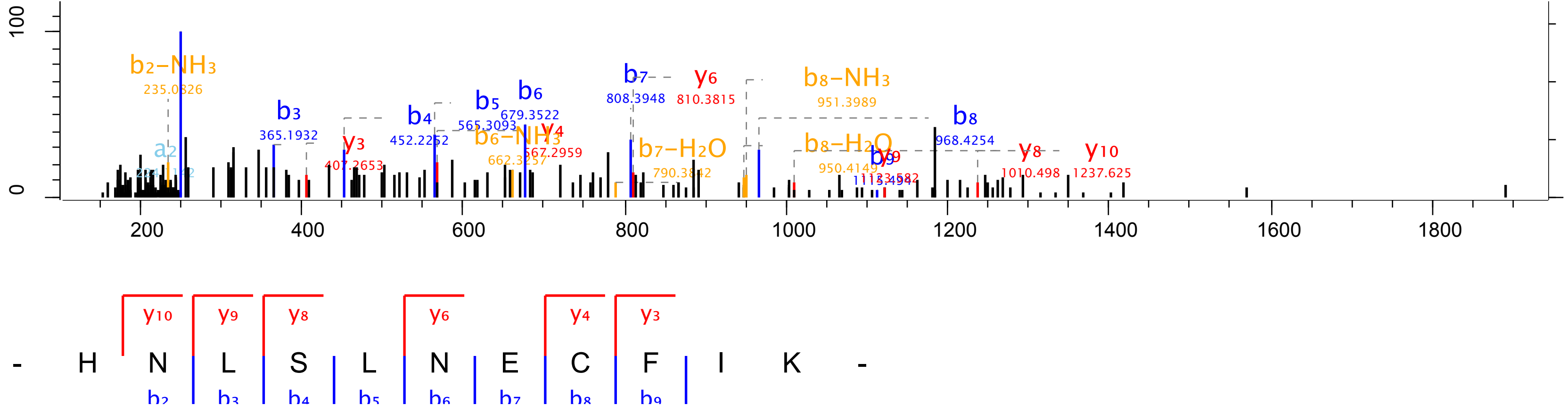

Raw file	Scan	Method	Score	m/z	Gene names
20150402_CerP14_Frac05_top_opt_B5_01_1814	36527	TOF; CID	56.12	606.3	Socs6

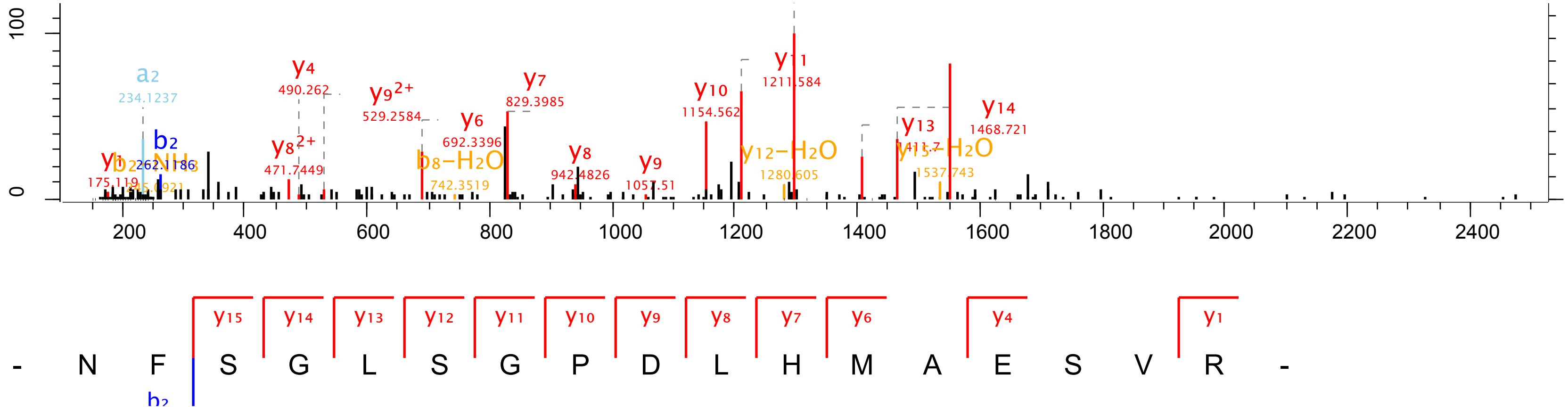

Raw file	Scan	Method	Score	m/z
20150402_CerP14_Frac05_top_opt_B5_01_1814	40631	TOF; CID	84.14	957.48

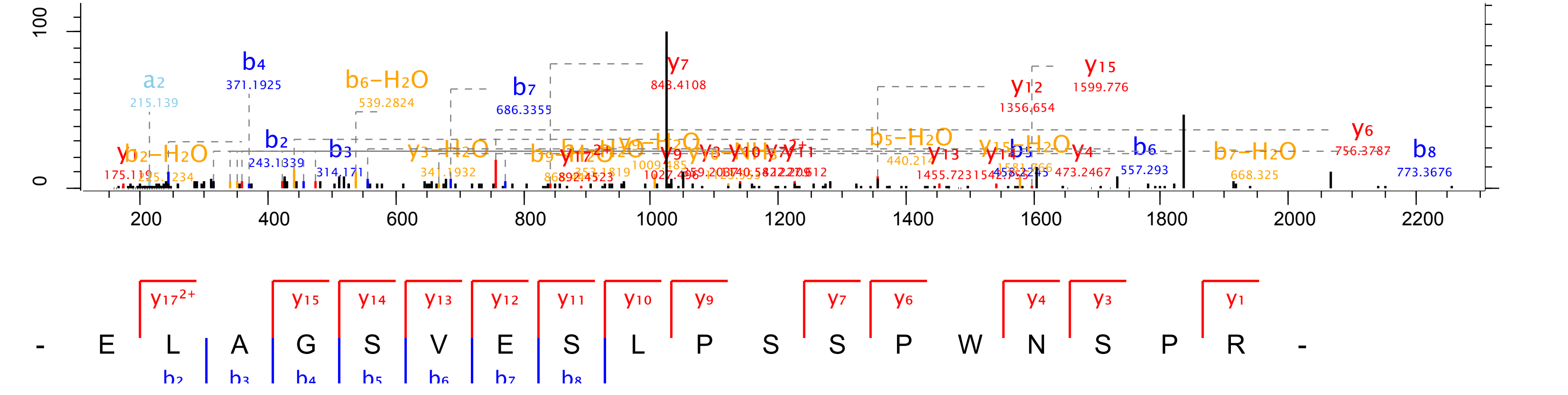

Raw file	Scan	Method	Score	m/z	Gene names
20150402_CerP14_Frac05_top_opt_B5_01_1814	41940	TOF; CID	69.92	714.84	Vamp5

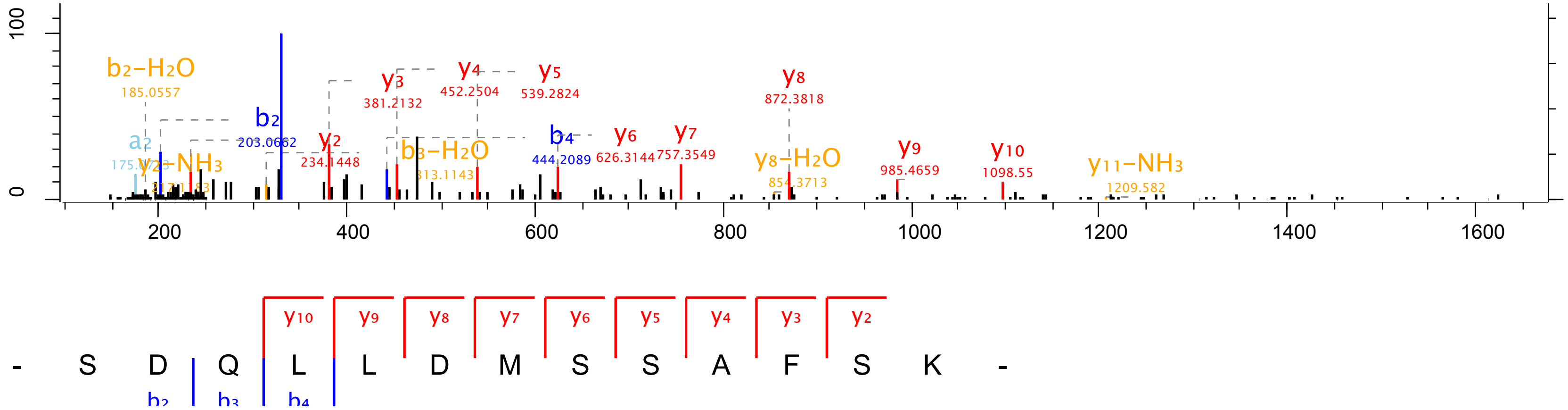

Raw file	Scan	Method	Score	m/z	Gene names
20150402_CerP14_Frac05_top_opt_B5_01_1814	45879	TOF; CID	36.32	828.39	Rassf6

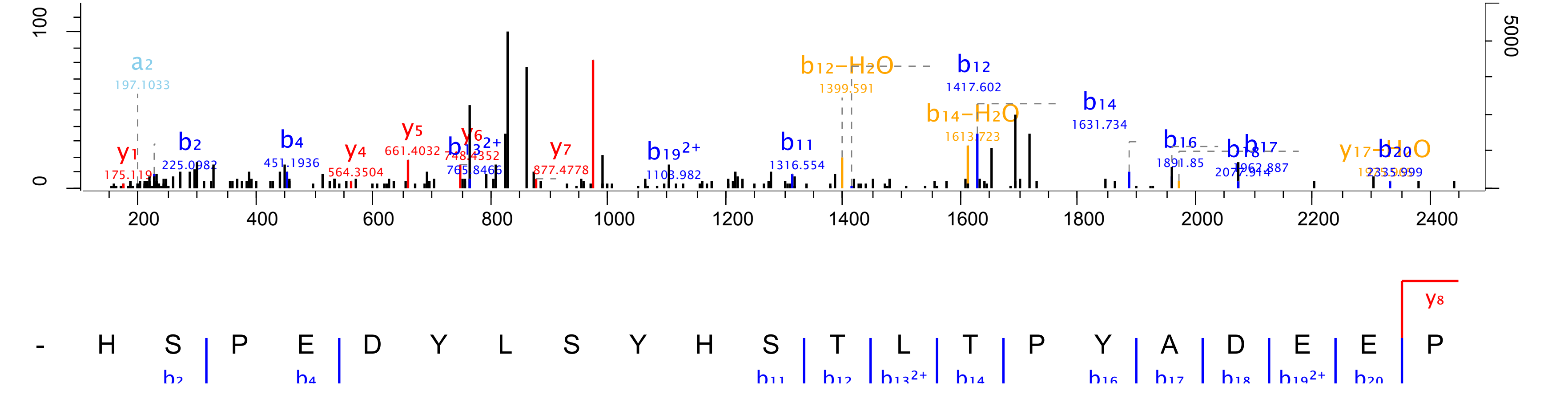

Raw file	Scan	Method	Score	m/z	Gene names
20150402_CerP14_Frac05_top_opt_B5_01_1814	46378	TOF; CID	59.54	724.05	Sytl4

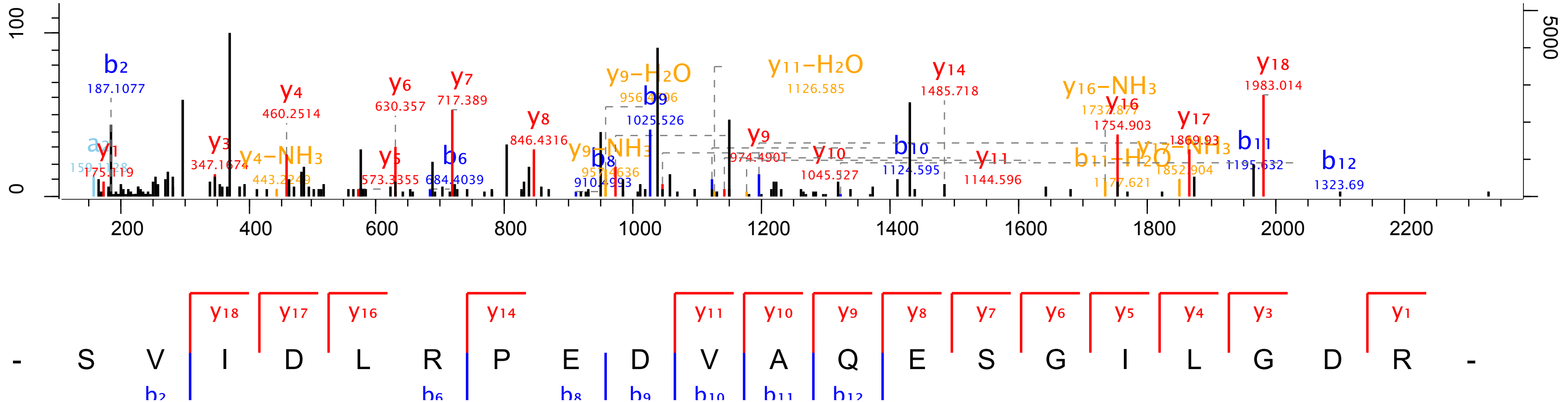

Raw file	Scan	Method	Score	m/z	Gene names
20150402_CerP14_Frac05_top_opt_B5_01_1814	47096	TOF; CID	48.97	825.35	Parm1

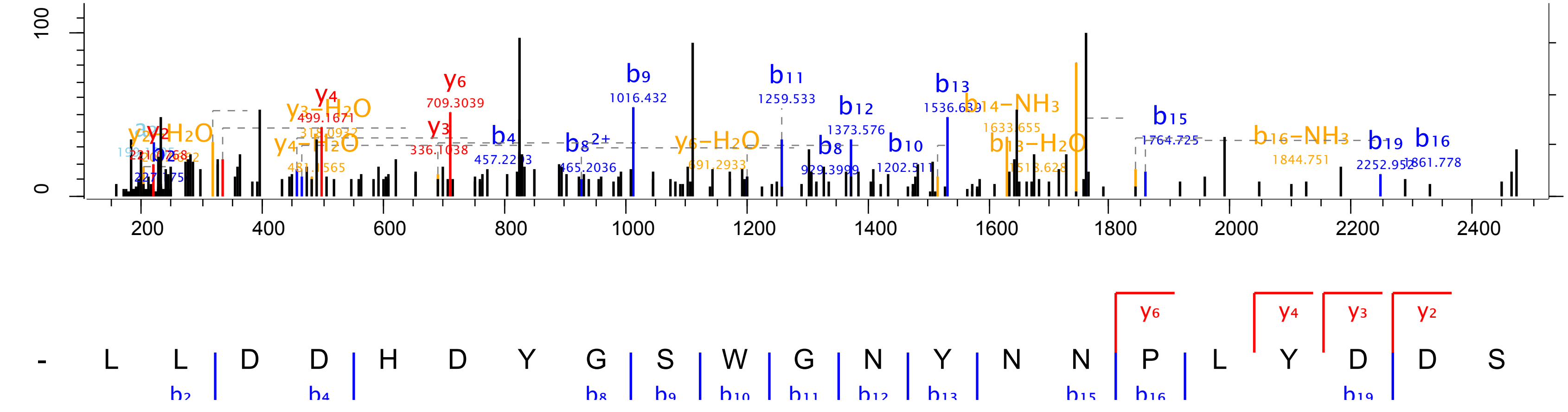

Raw file	Scan	Method	Score	m/z	Gene names
20150402_CerP14_Frac05_top_opt_B5_01_1814	56998	TOF; CID	54.77	878.96	Mcph1

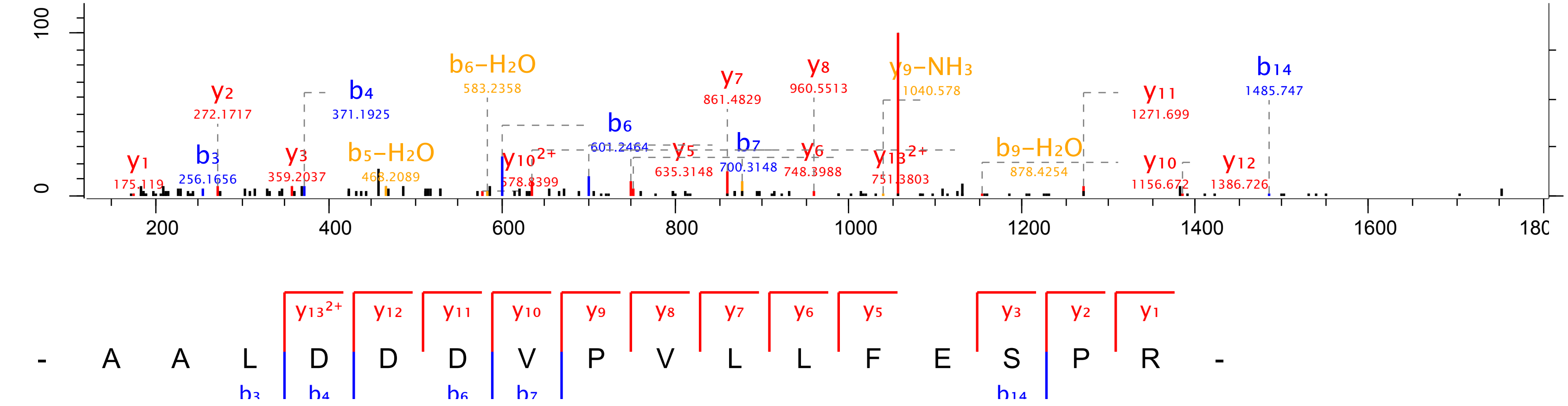

Raw file	Scan	Method	Score	m/z	Gene names
20150402_CerP14_Frac05_top_opt_B5_01_1814	57395	TOF; CID	58.89	585.68	Stk33

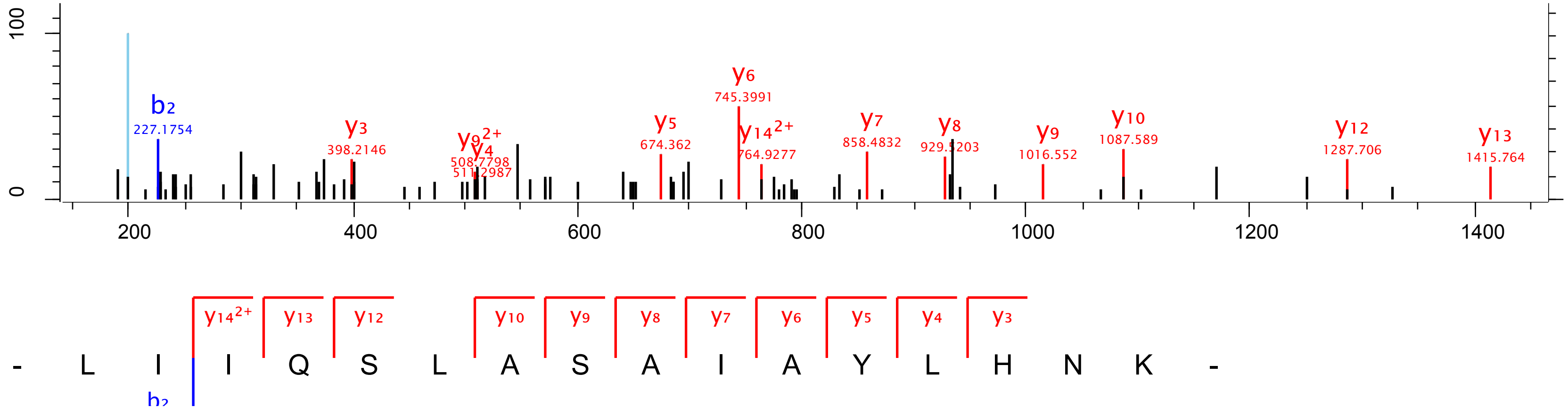

Raw file	Scan	Method	Score	m/z	Gene names
20150402_CerP14_Frac05_top_opt_B5_01_1814	58789	TOF; CID	111.11	796.12	Slc35d1

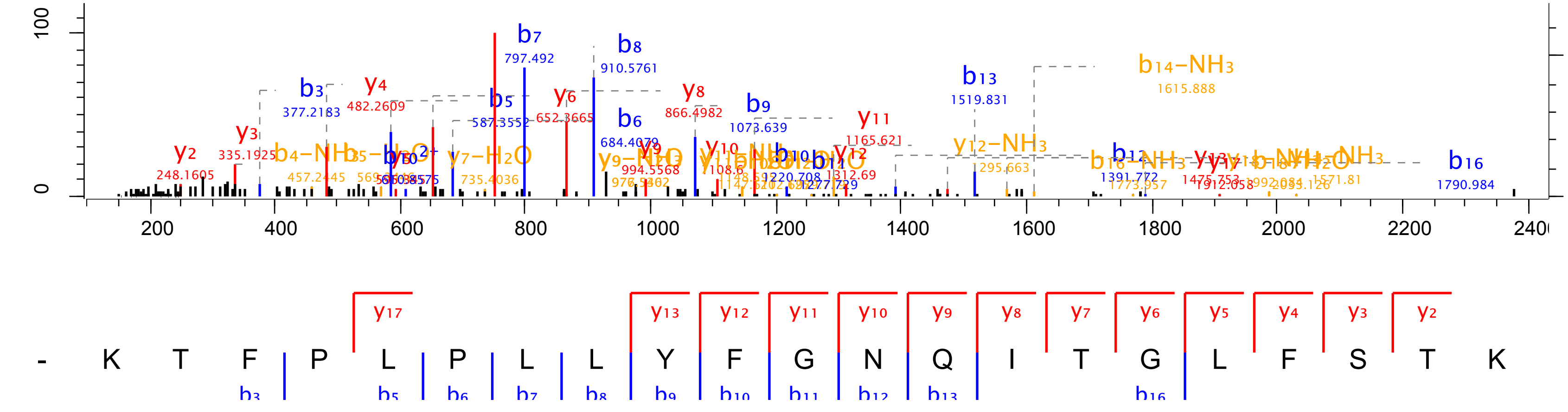

Raw file	Scan	Method	Score	m/z	Gene names
20150402_CerP14_Frac05_top_opt_B5_01_1814	59113	TOF; CID	130.47	769.49	P4ha3

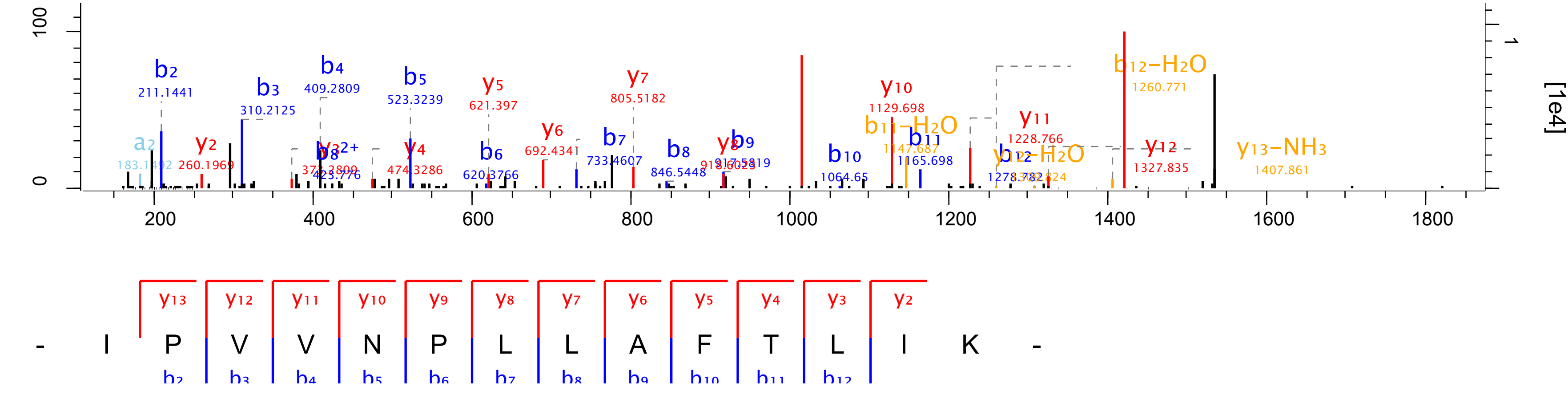

Raw file	Scan	Method	Score	m/z	Gene names
20150402_CerP14_Frac05_top_opt_B5_01_1814	59141	TOF; CID	139.86	919.98	Acer3

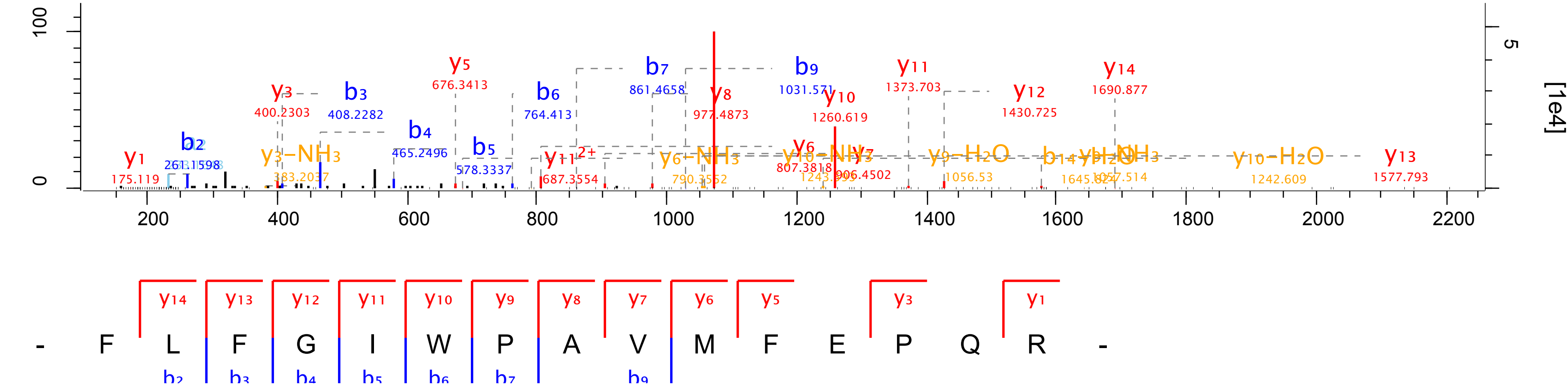

Raw file	Scan	Method	Score	m/z	Gene names
20150402_CerP14_Frac06_top_opt_B6_01_1815	5945	TOF; CID	155.86	731.26	Mt1

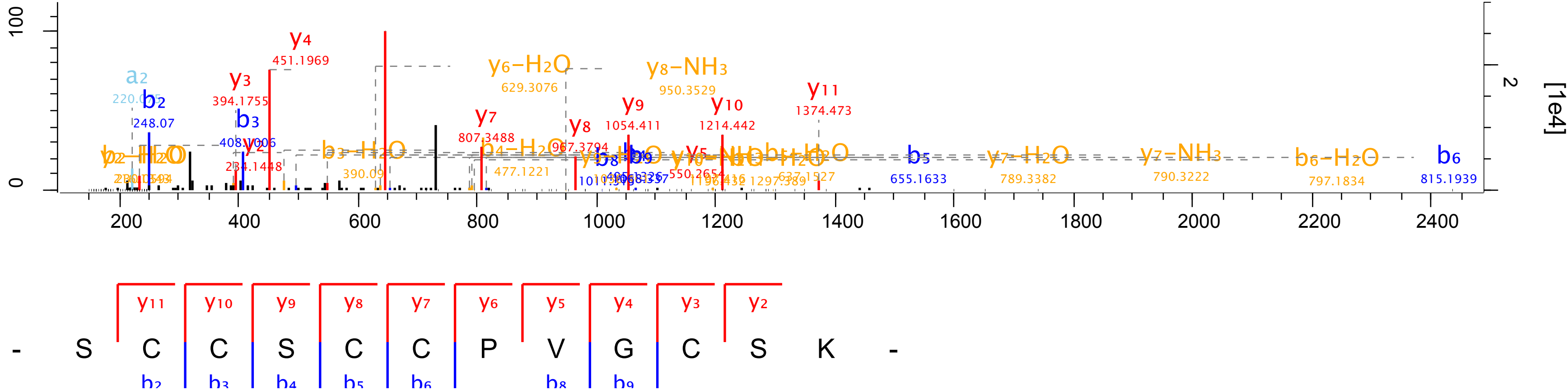

Raw file	Scan	Method	Score	m/z	Gene names
20150402_CerP14_Frac06_top_opt_B6_01_1815	9597	TOF; CID	68	692.8	Tspan31

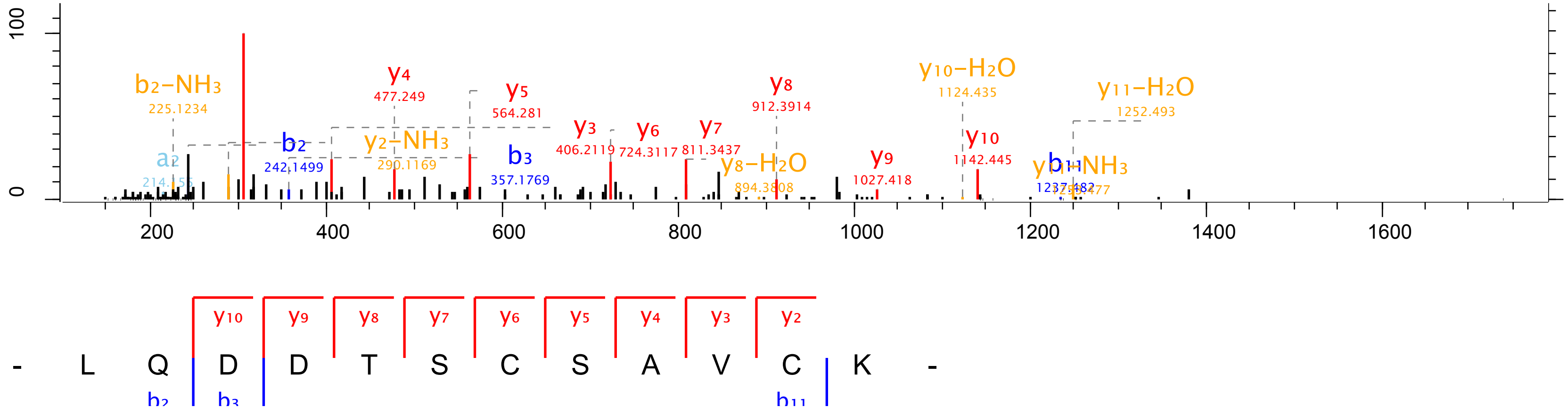

Raw file	Scan	Method	Score	m/z	Gene names
20150402_CerP14_Frac06_top_opt_B6_01_1815	25444	TOF; CID	72.93	540.29	Tm7sf3

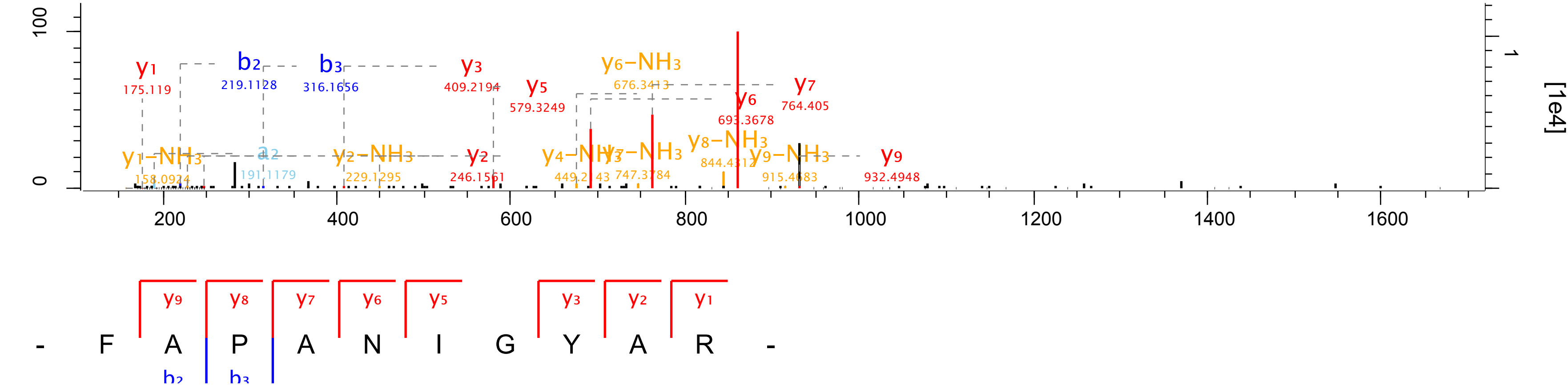

Raw file	Scan	Method	Score	m/z	Gene names
20150402_CerP14_Frac06_top_opt_B6_01_1815	26216	TOF; CID	166.07	436.58	Rpl39

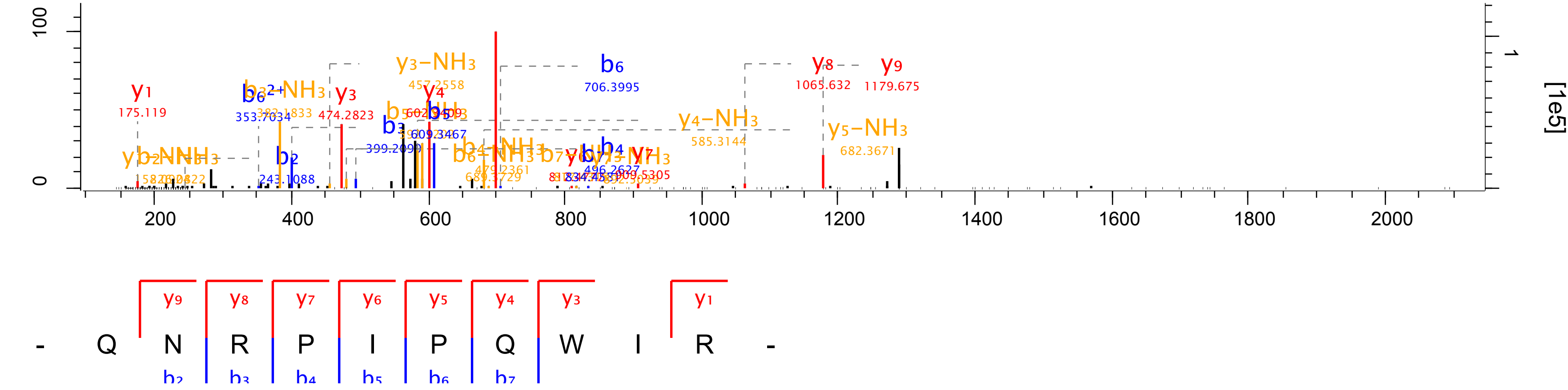

Raw file	Scan	Method	Score	m/z	Gene names
20150402_CerP14_Frac06_top_opt_B6_01_1815	26988	TOF; CID	60.55	532.3	Lrrc25

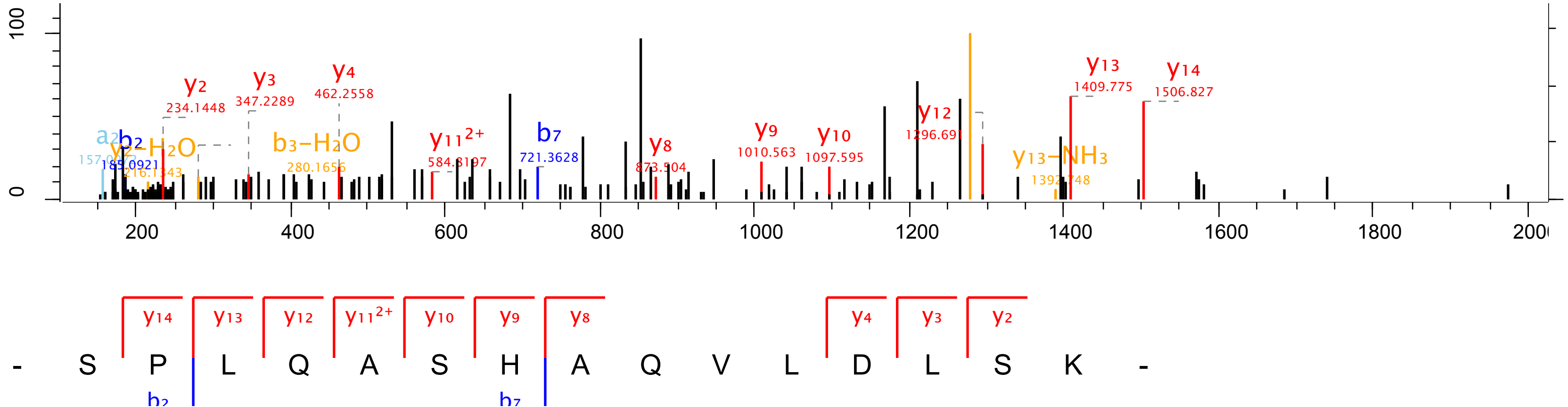

Raw file	Scan	Method	Score	m/z	Gene names
20150402_CerP14_Frac06_top_opt_B6_01_1815	27231	TOF; CID	65.95	845.89	Fblim1

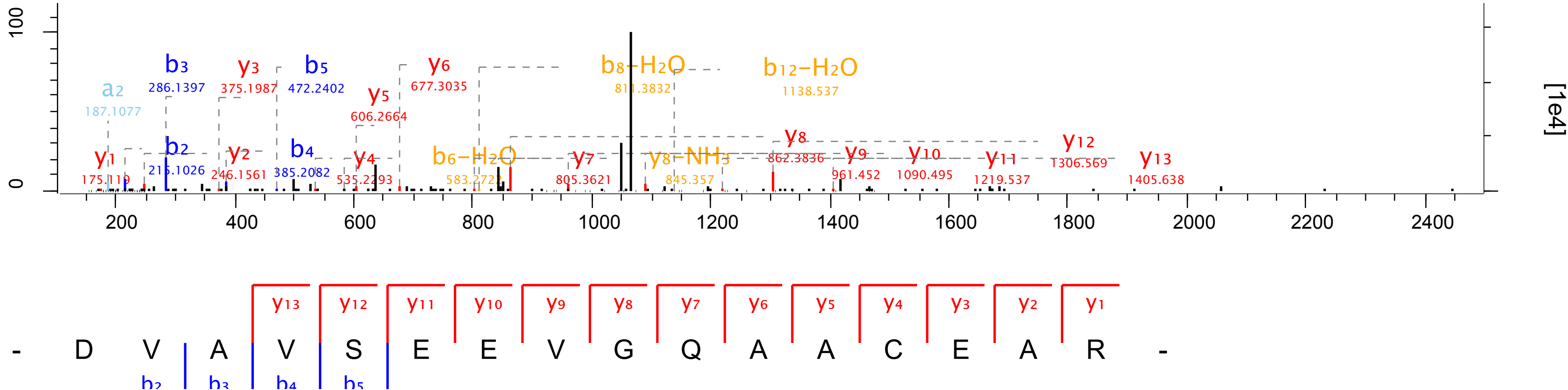

Raw file	Scan	Method	Score	m/z	Gene names
20150402_CerP14_Frac06_top_opt_B6_01_1815	31521	TOF; CID	68.52	692.87	Efcab7

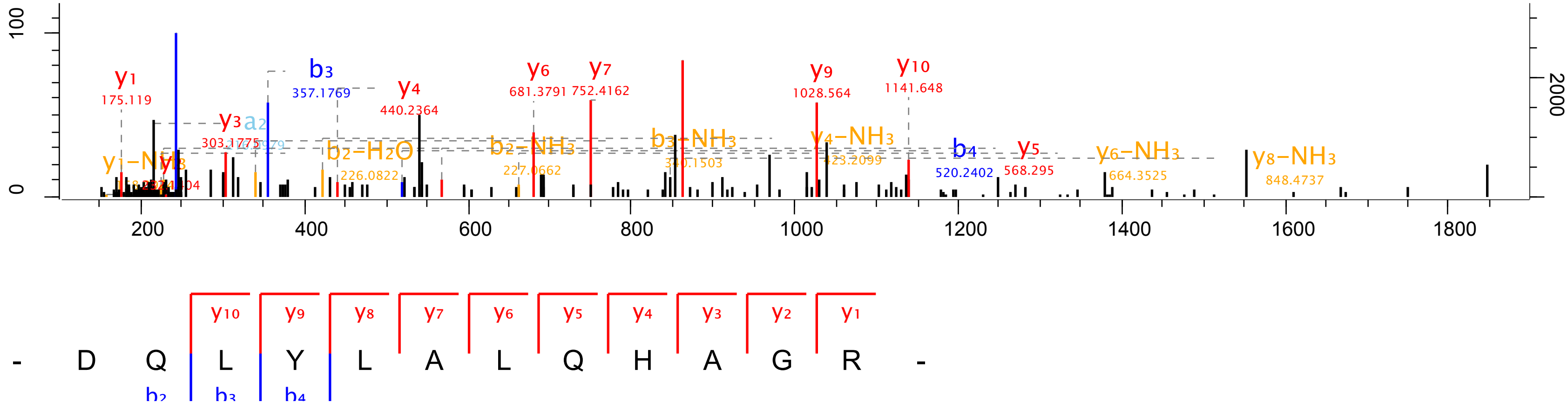

Raw file	Scan	Method	Score	m/z	Gene names
20150402_CerP14_Frac06_top_opt_B6_01_1815	32180	TOF; CID	93.48	703.39	Slc35f2

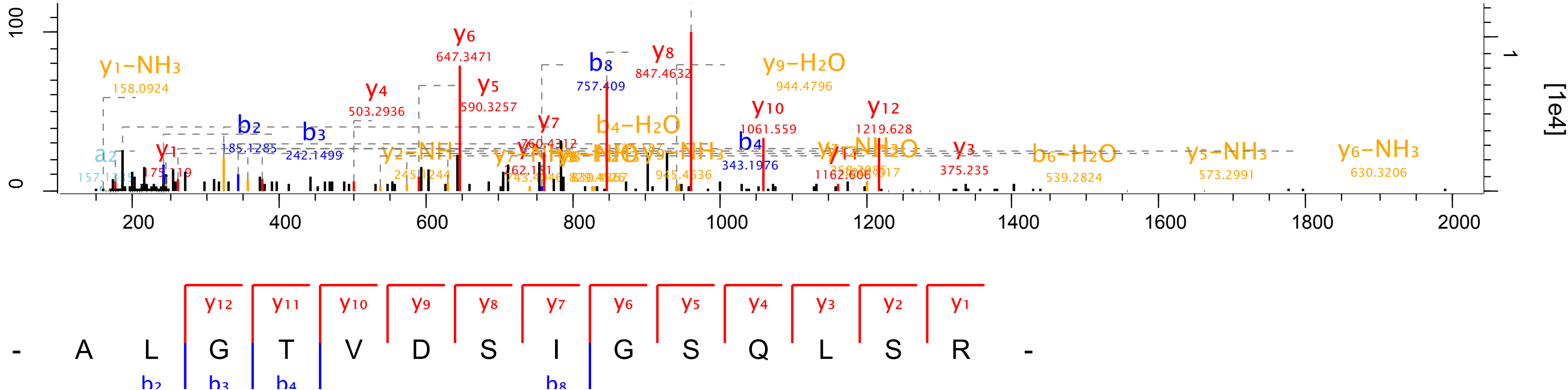

Raw file	Scan	Method	Score	m/z	Gene names
20150402_CerP14_Frac06_top_opt_B6_01_1815	35048	TOF; CID	64.55	879.75	Usp2

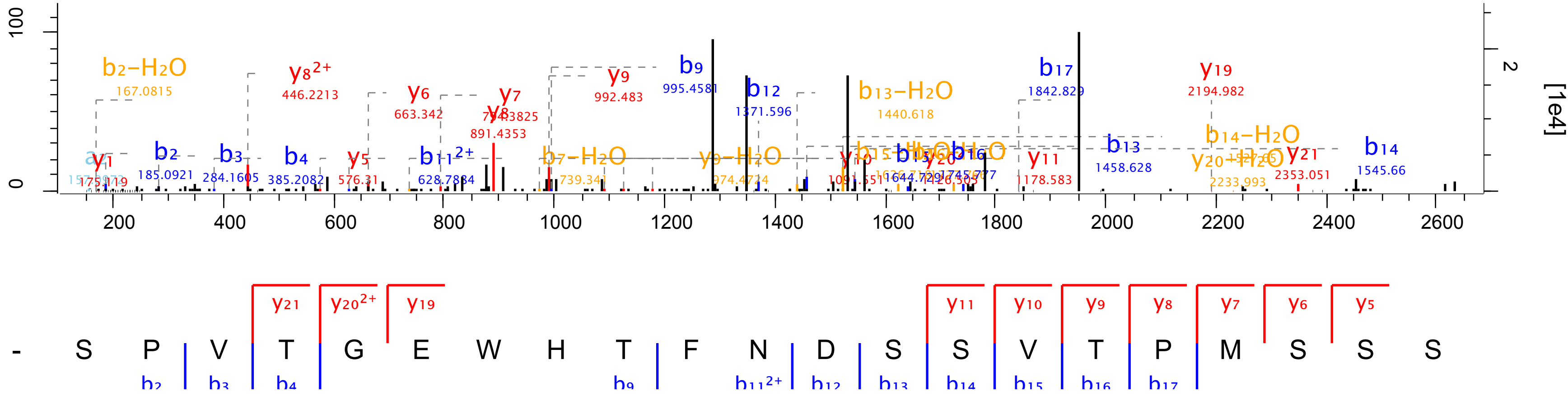

Raw file	Scan	Method	Score	m/z	Gene names
20150402_CerP14_Frac06_top_opt_B6_01_1815	37289	TOF; CID	92.26	866.95	Tmem242

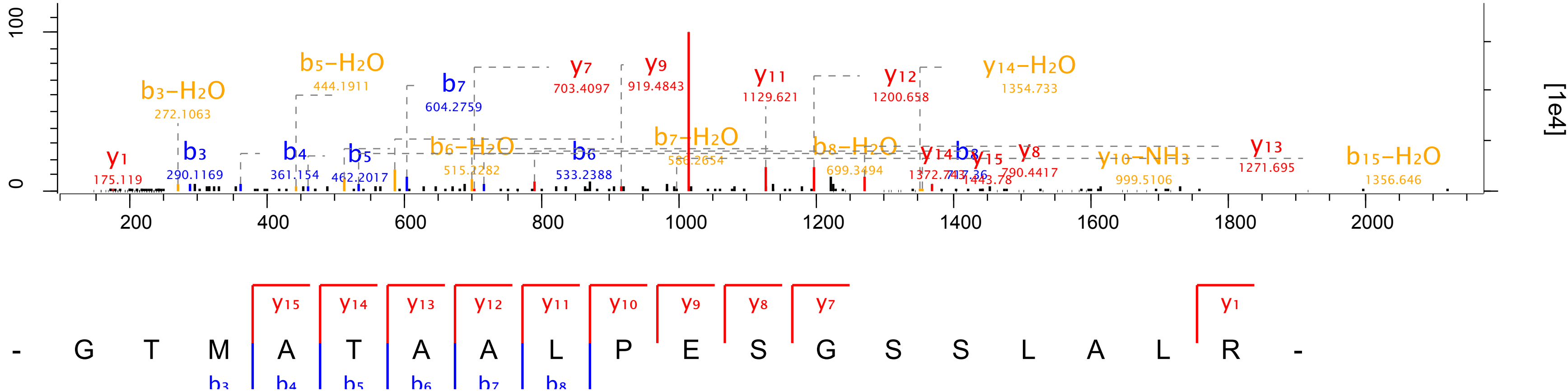

Raw file	Scan	Method	Score	m/z	Gene names
20150402_CerP14_Frac06_top_opt_B6_01_1815	41024	TOF; CID	78.15	523.3	Sypl2

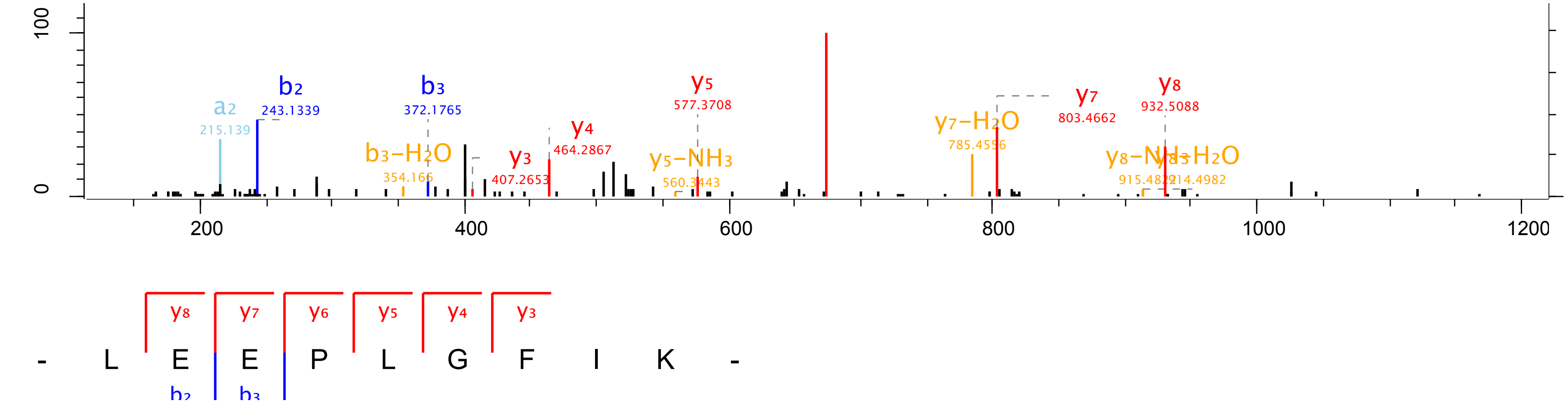

Raw file	Scan	Method	Score	m/z	Gene names
20150402_CerP14_Frac06_top_opt_B6_01_1815	41996	TOF; CID	70.78	878.46	Lcp2

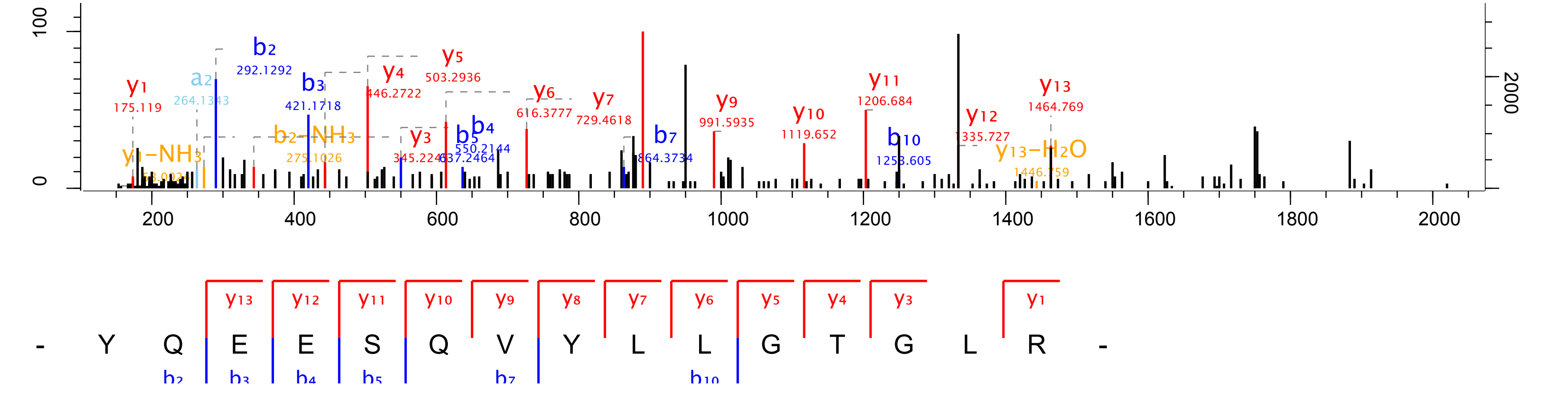

Raw file	Scan	Method	Score	m/z	Gene names
20150402_CerP14_Frac06_top_opt_B6_01_1815	42732	TOF; CID	78.32	657.35	Dleu7

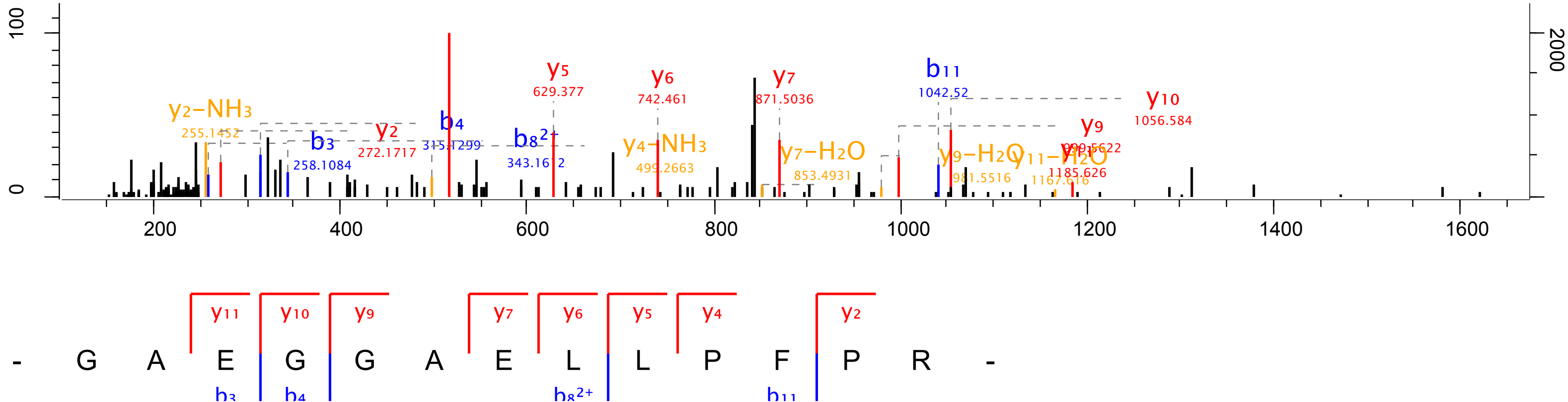

Raw file	Scan	Method	Score	m/z	Gene names
20150402_CerP14_Frac06_top_opt_B6_01_1815	44110	TOF; CID	41.37	1108.53	Cebpg

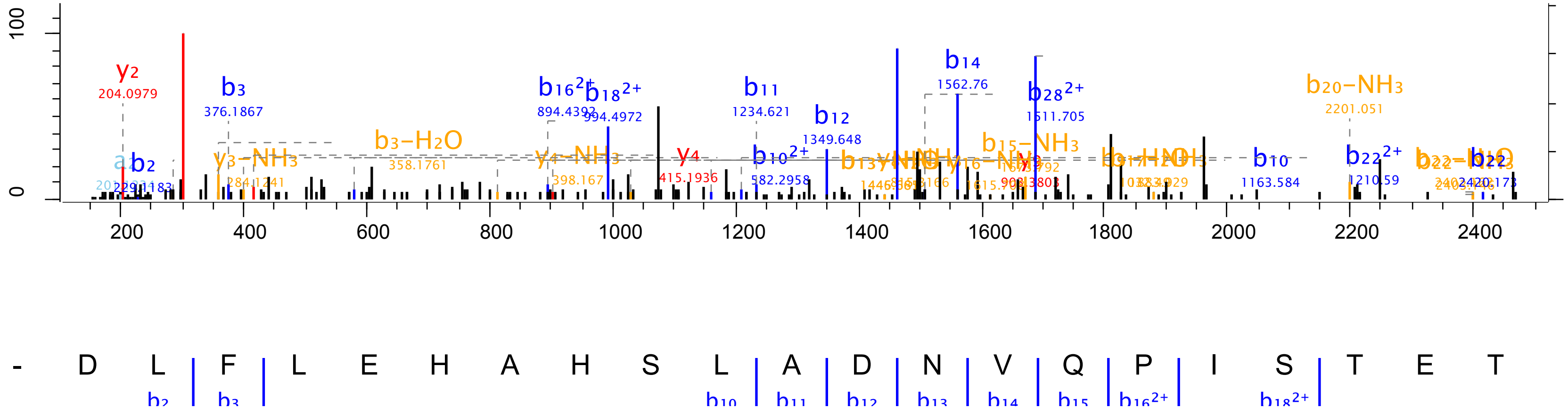

Raw file	Scan	Method	Score	m/z	Gene names
20150402_CerP14_Frac06_top_opt_B6_01_1815	44445	TOF; CID	37.37	923.4	Tspan4

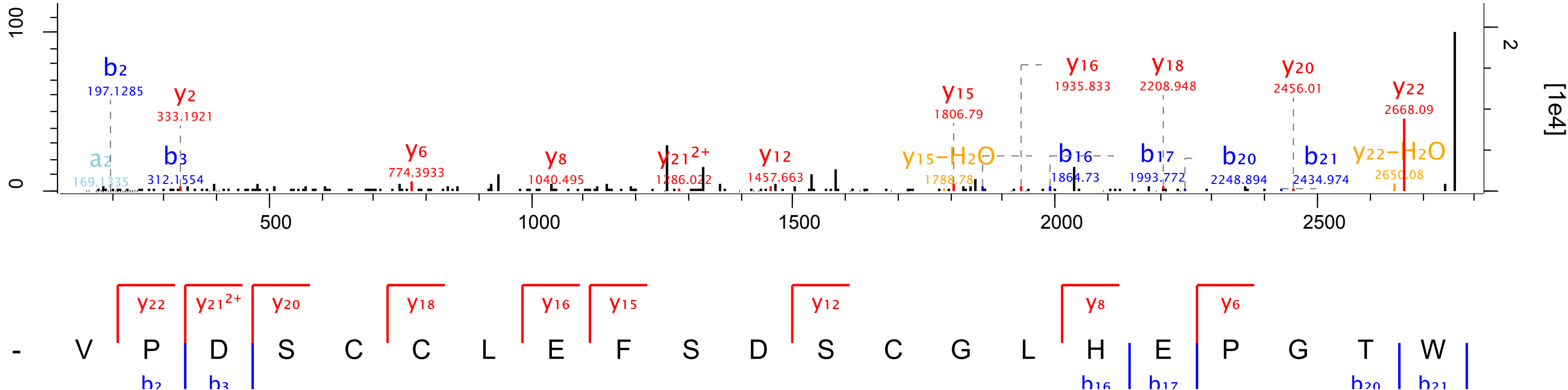

Raw file	Scan	Method	Score	m/z	Gene names
20150402_CerP14_Frac06_top_opt_B6_01_1815	56281	TOF; CID	45.99	785.42	Rsph4a

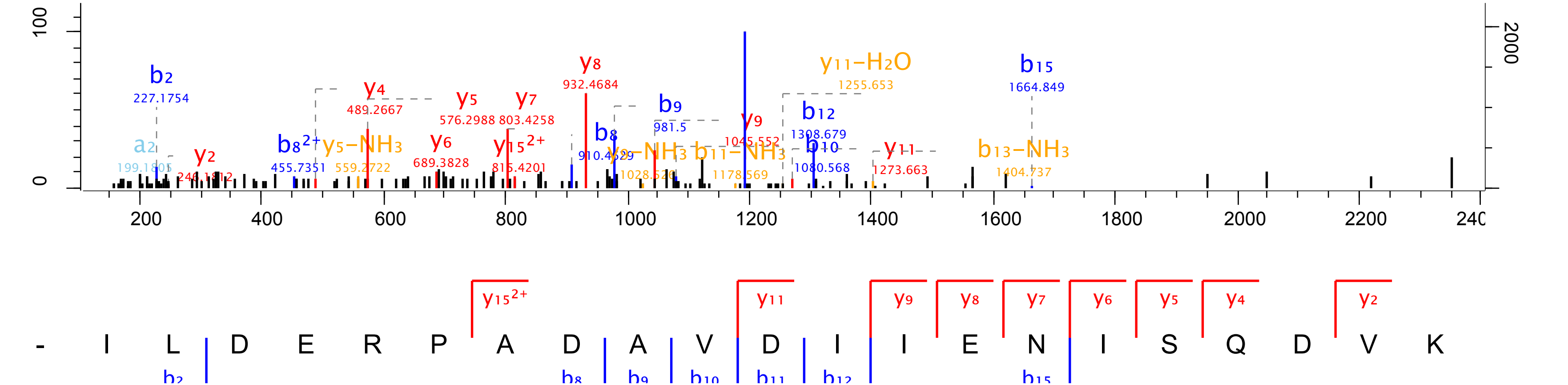

Raw file	Scan	Method	Score	m/z	Gene names
20150402_CerP14_Frac06_top_opt_B6_01_1815	58796	TOF; CID	60.69	657.32	Slc48a1

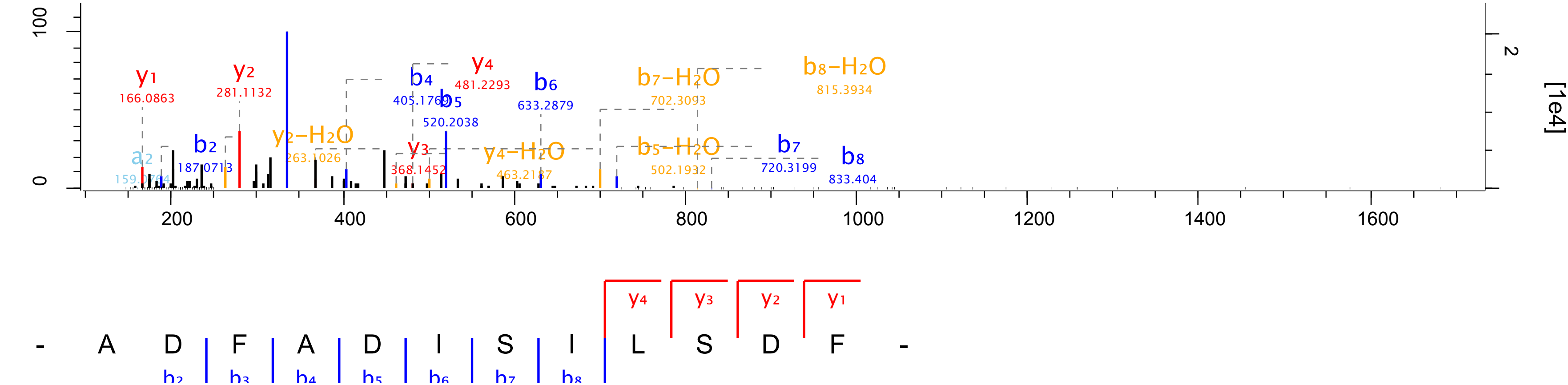

Raw file	Scan	Method	Score	m/z	Gene names
20150402_CerP14_Frac07_top_opt_B7_01_1816	10519	TOF; CID	97.72	716.81	Leprot

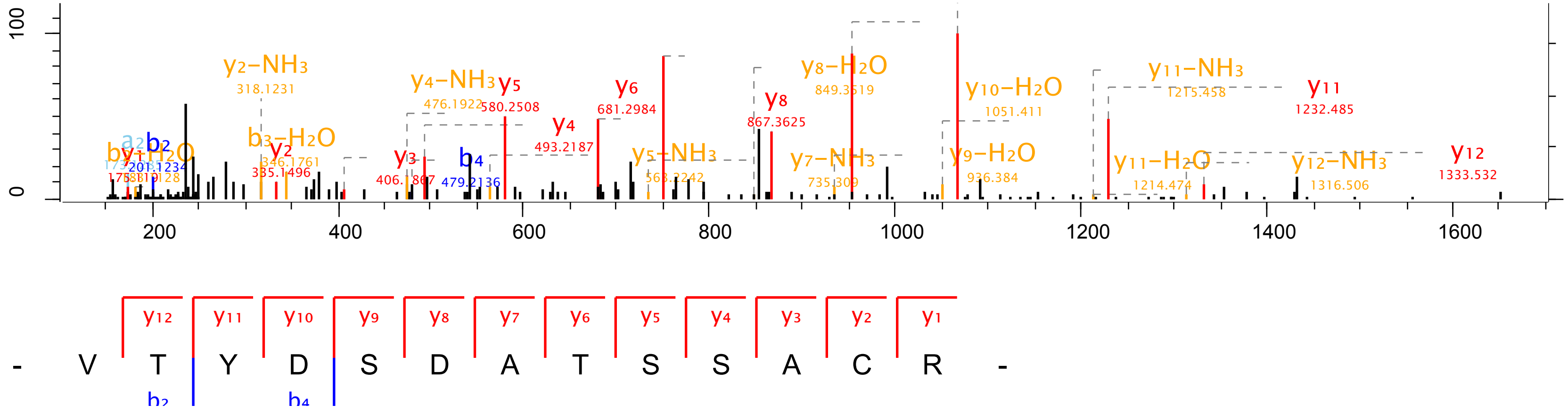

Raw file	Scan	Method	Score	m/z	Gene names
20150402_CerP14_Frac07_top_opt_B7_01_1816	11800	TOF; CID	96.07	627.3	Dbndd2

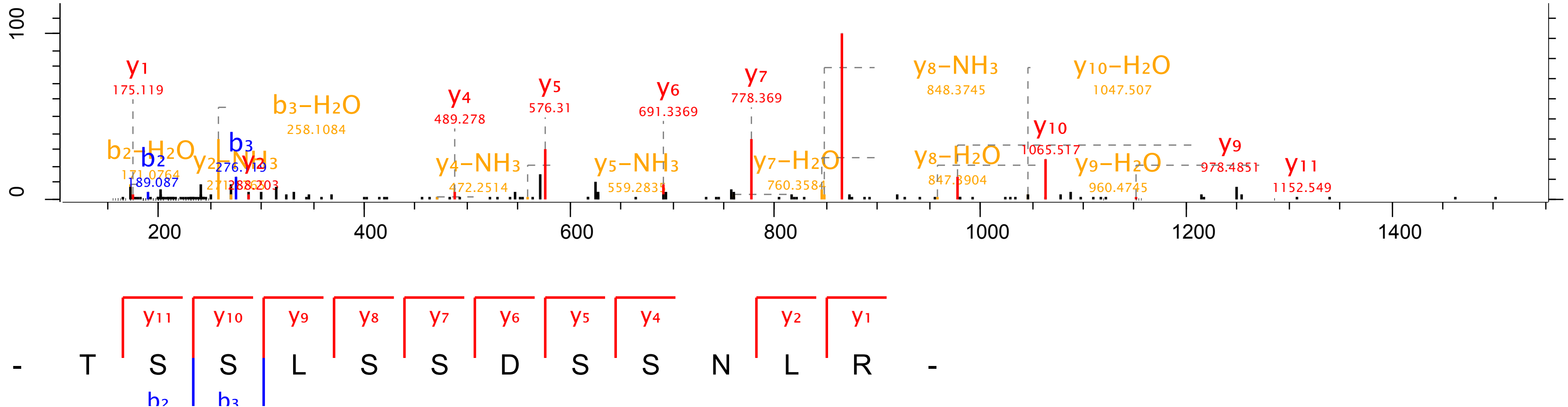

Raw file Sca Metl Scb m/: Gene names

20150402_CerP14_Frac 16; TOF 12; 56; Olfr1341;Olfr148;Olfr630;Olfr314;Olfr13;Olfr1415;Olfr1414;Olfr844;Olfr447;Olfr328;Olfr1416;Olfr845;Olfr169;Olfr711;Olfr224;

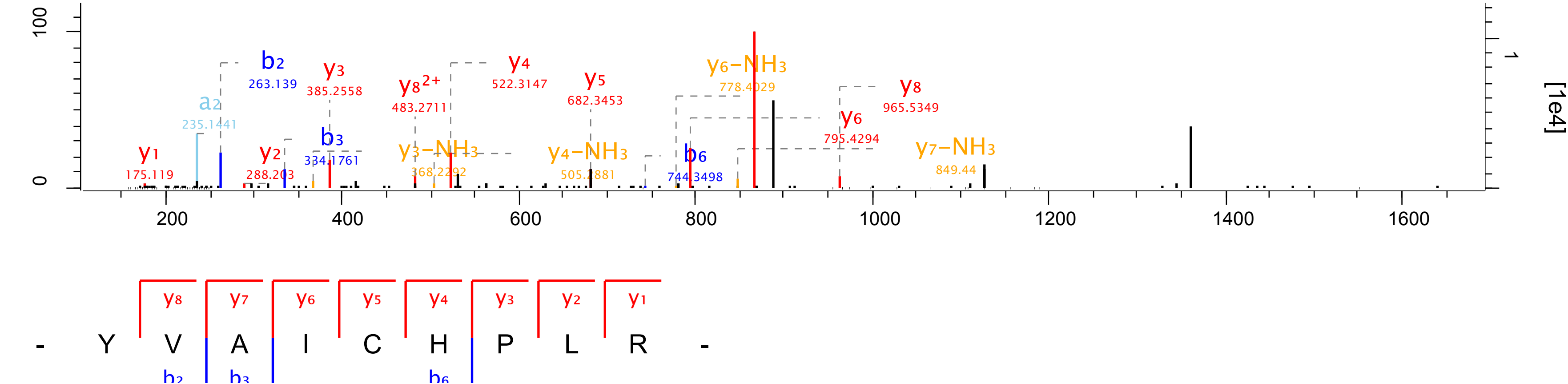

Raw file	Scan	Method	Score	m/z	Gene names
20150402_CerP14_Frac07_top_opt_B7_01_1816	17858	TOF; CID	74.46	501.77	Tex9

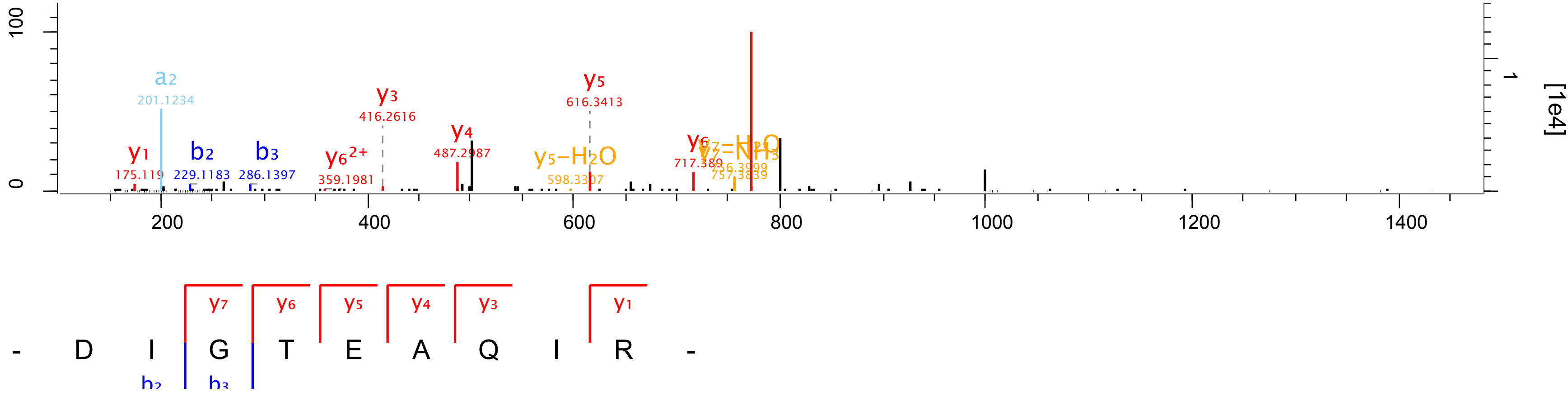

Raw file	Scan	Method	Score	m/z	Gene names
20150402_CerP14_Frac07_top_opt_B7_01_1816	20469	TOF; CID	109.79	661.32	Smoc1

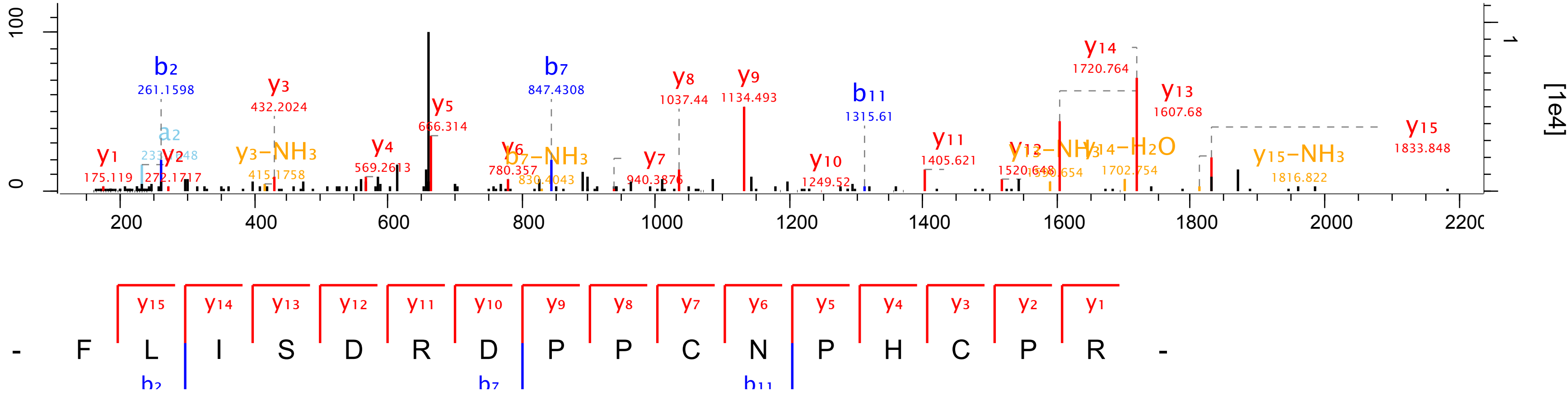

Raw file	Scan	Method	Score	m/z	Gene names
20150402_CerP14_Frac07_top_opt_B7_01_1816	20948	TOF; CID	59.12	790.37	Mfsd8

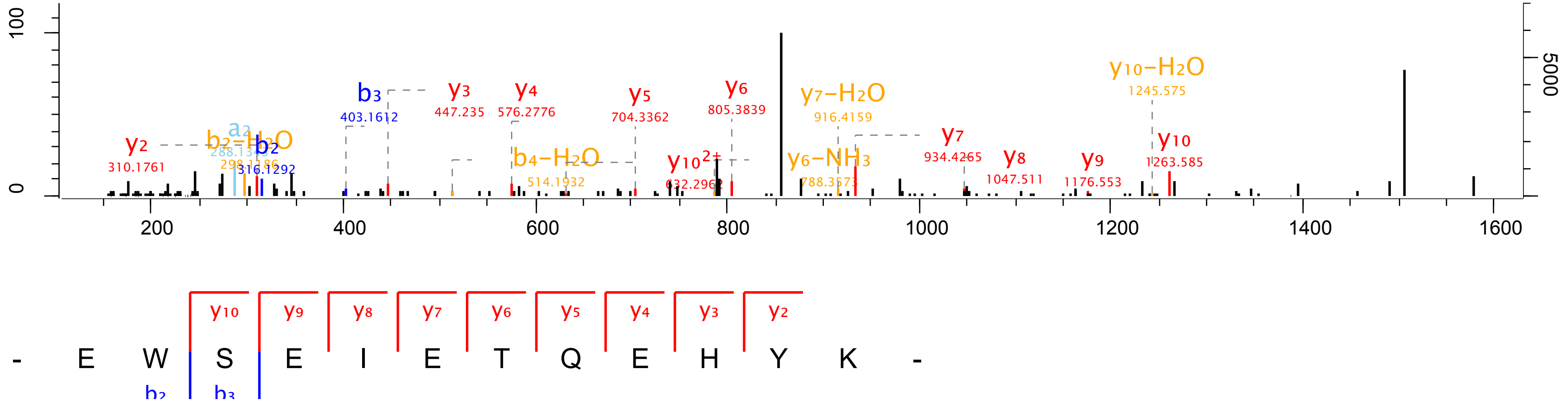

Raw file	Scan	Method	Score	m/z	Gene names
20150402_CerP14_Frac07_top_opt_B7_01_1816	27889	TOF; CID	91.31	614.35	Lrrc61

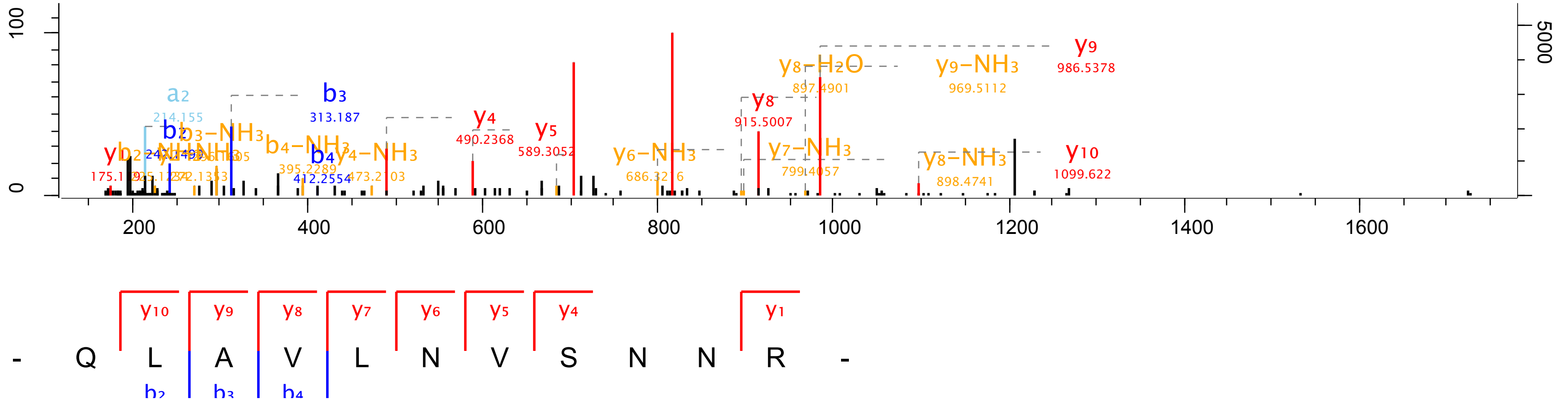

Raw file	Scan	Method	Score	m/z	Gene names
20150402_CerP14_Frac07_top_opt_B7_01_1816	36244	TOF; CID	49.3	903.96	2610020H08Rik

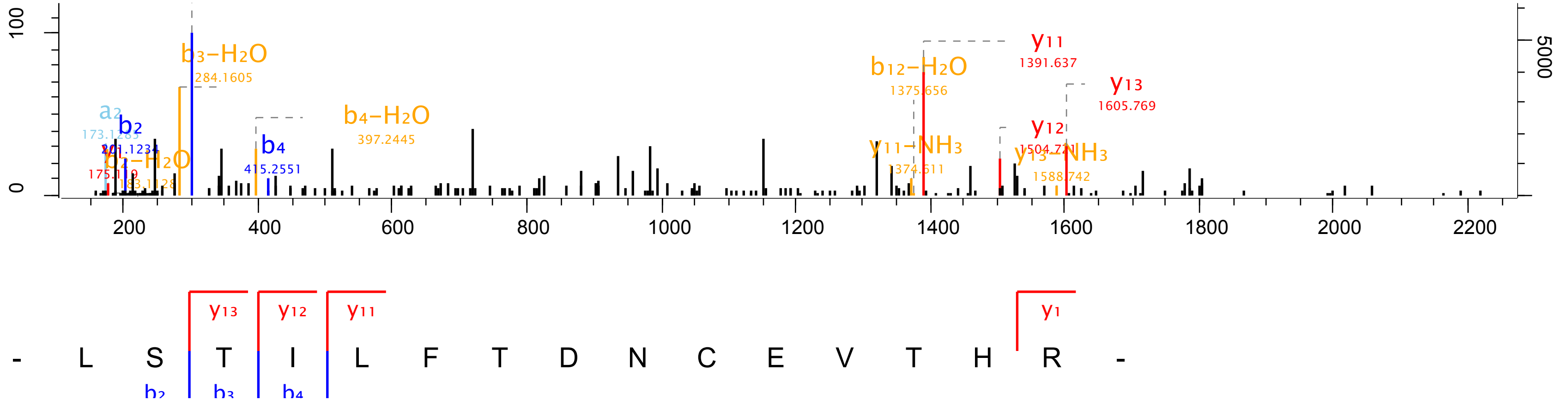

Raw file	Scan	Method	Score	m/z	Gene names
20150402_CerP14_Frac07_top_opt_B7_01_1816	36974	TOF; CID	60.16	658.4	Slmo2

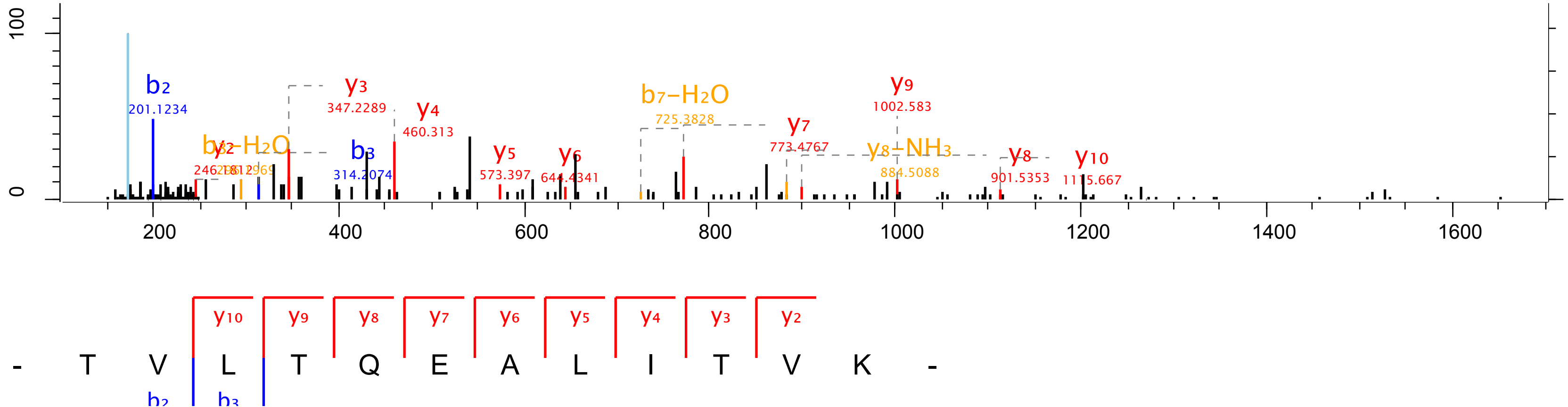

Raw file	Scan	Method	Score	m/z	Gene names
20150402_CerP14_Frac07_top_opt_B7_01_1816	37730	TOF; CID	47.93	819.41	Ercc6l

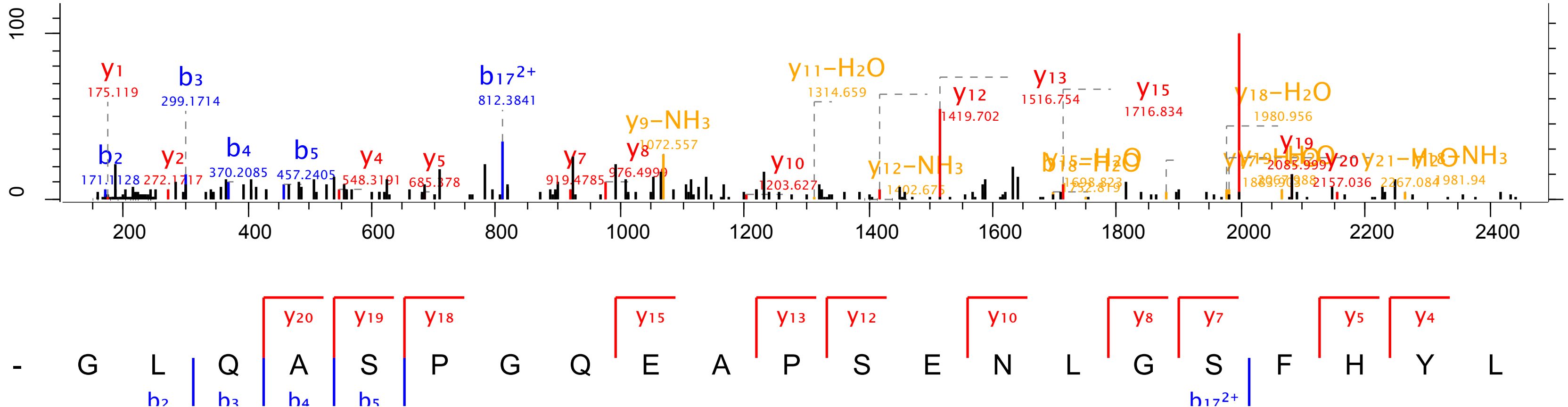

Raw file	Scan	Method	Score	m/z	Gene names
20150402_CerP14_Frac07_top_opt_B7_01_1816	38470	TOF; CID	85.81	619.34	Tmem69

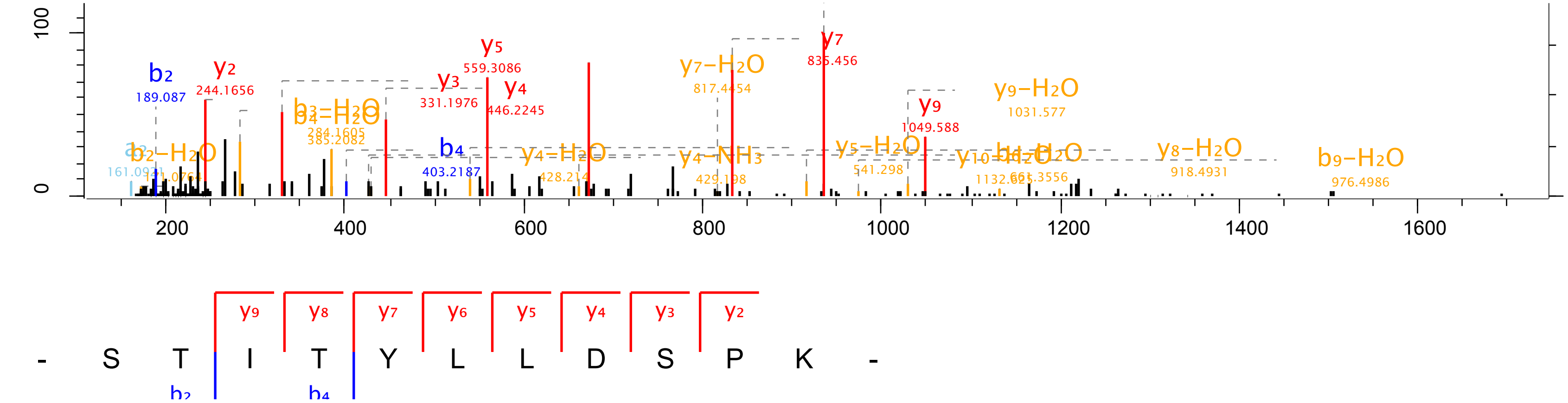

Raw file	Scan	Method	Score	m/z	Gene names
20150402_CerP14_Frac07_top_opt_B7_01_1816	41636	TOF; CID	92.47	599.31	Ficd

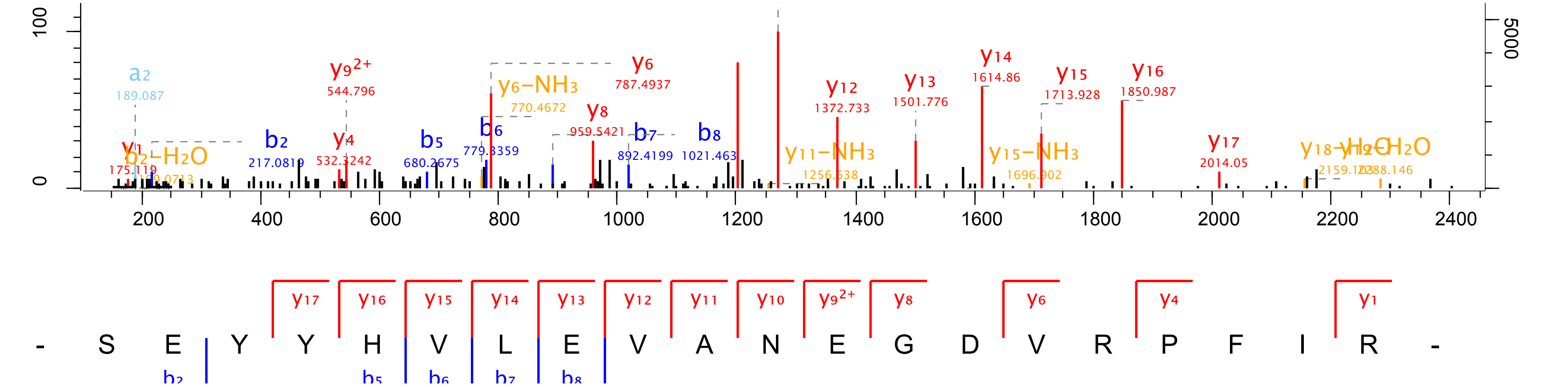

Raw file	Scan	Method	Score	m/z	Gene names
20150402_CerP14_Frac07_top_opt_B7_01_1816	43519	TOF; CID	70.68	638.35	Rnf19a

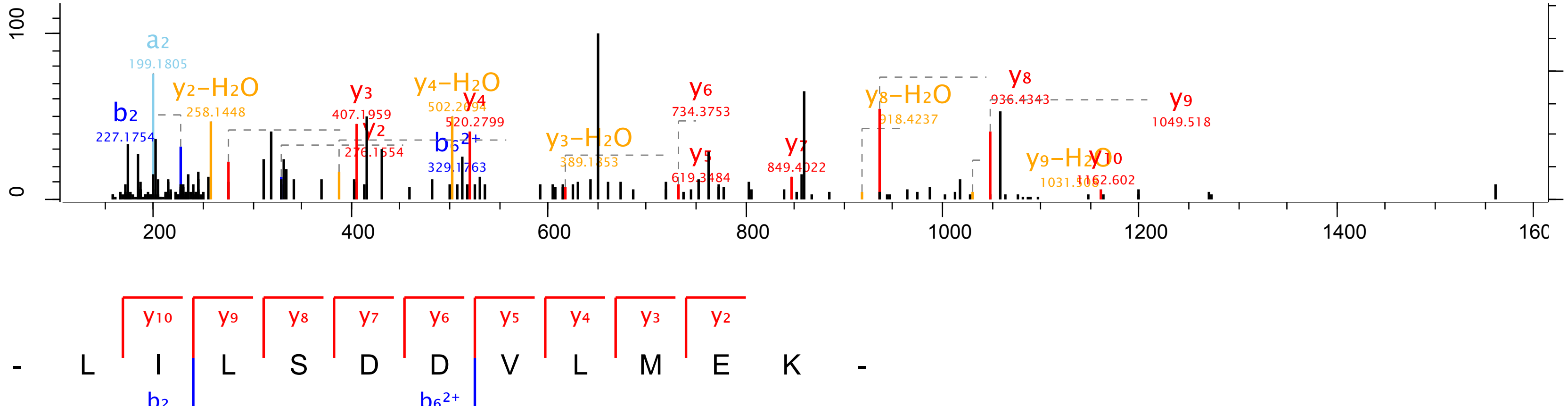

Raw file	Scan	Method	Score	m/z	Gene names
20150402_CerP14_Frac07_top_opt_B7_01_1816	44305	TOF; CID	42.47	888.44	Slc31a2

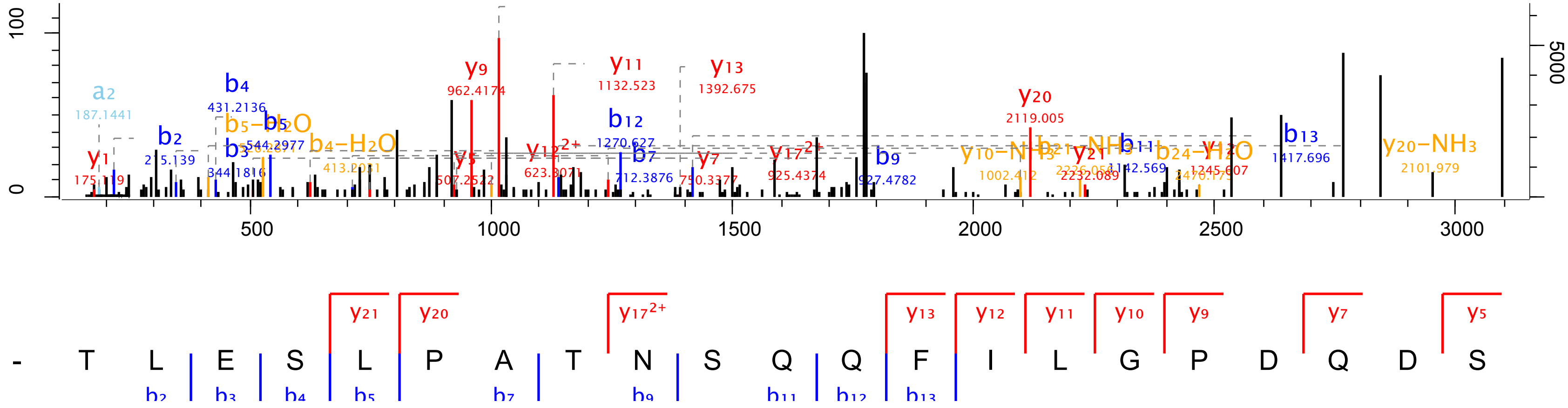

Raw file	Scan	Method	Score	m/z	Gene names
20150402_CerP14_Frac07_top_opt_B7_01_1816	48067	TOF; CID	80.01	644.34	Trim45

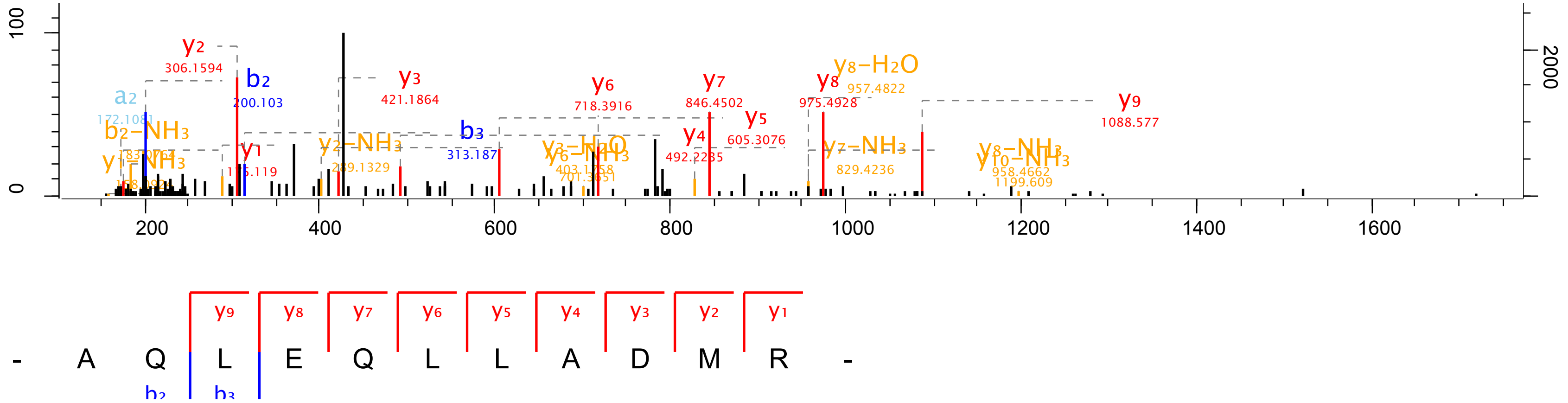

Raw file	Scan	Method	Score	m/z	Gene names
20150402_CerP14_Frac07_top_opt_B7_01_1816	51203	TOF; CID	63.66	700.86	Robo4

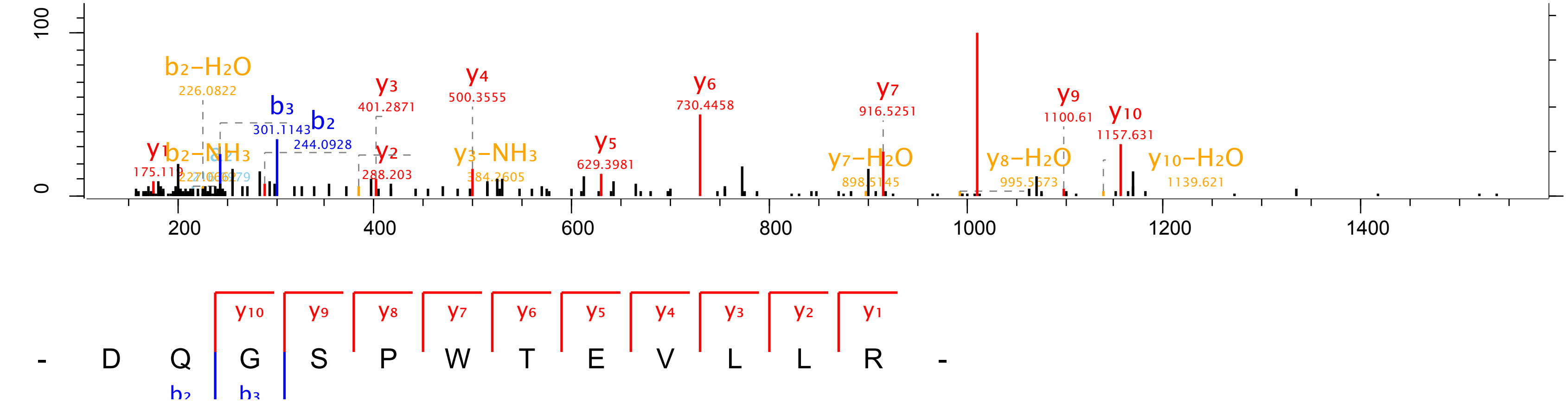

Raw file	Scan	Method	Score	m/z	Gene names
20150402_CerP14_Frac07_top_opt_B7_01_1816	55840	TOF; CID	75.53	1018.01	Lrrc55

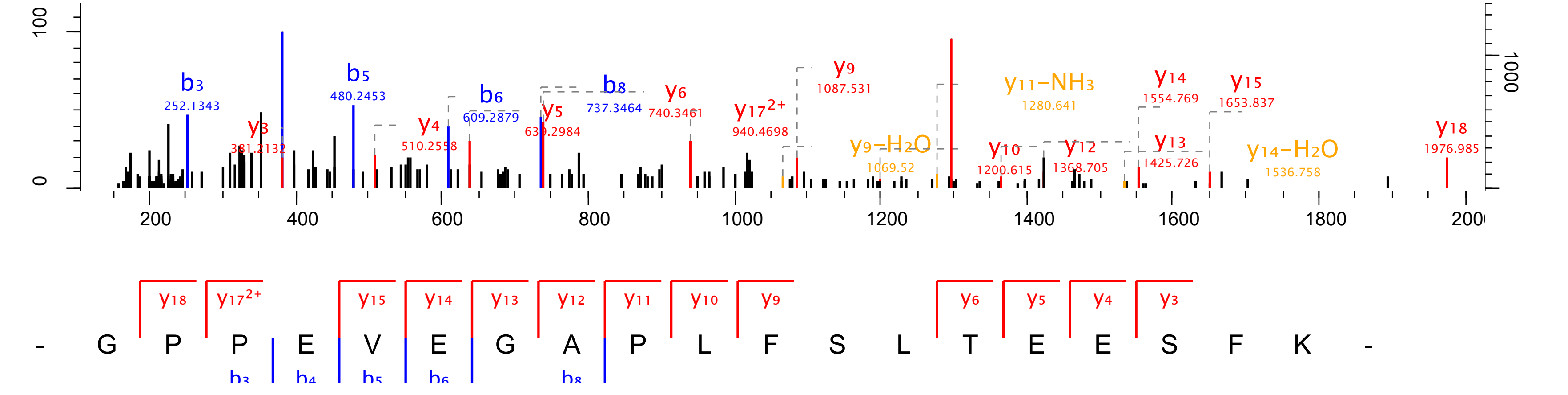

Raw file	Scan	Method	Score	m/z	Gene names
20150402_CerP14_Frac07_top_opt_B7_01_1816	59212	TOF; CID	85.29	645.07	Pet117

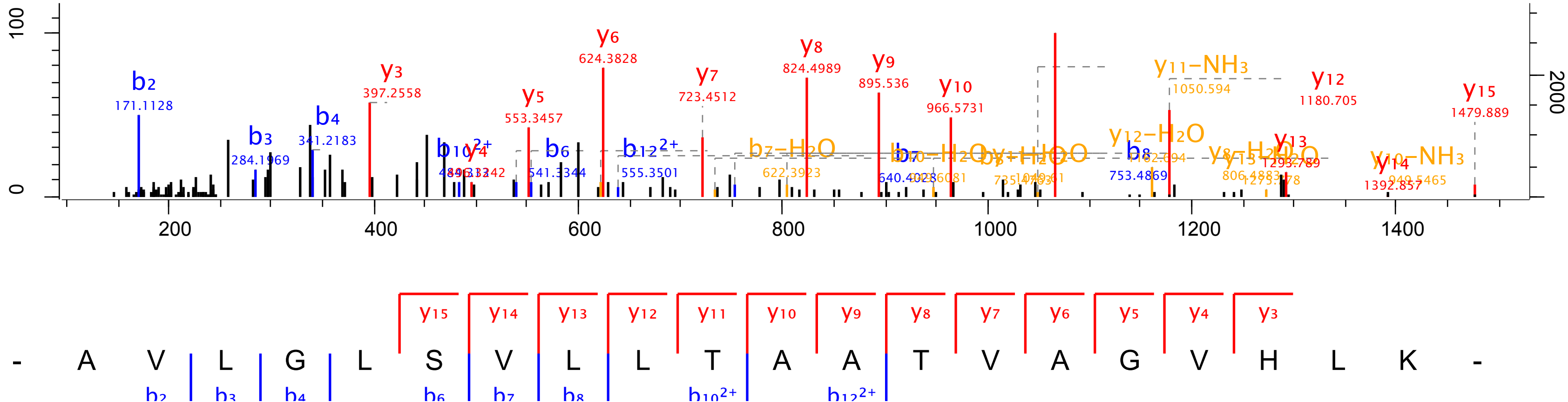

Raw file	Scan	Method	Score	m/z	Gene names
20150402_CerP14_Frac08_top_opt_B8_01_1817	4450	TOF; CID	76.3	860.44	Erdr1;Gm21887

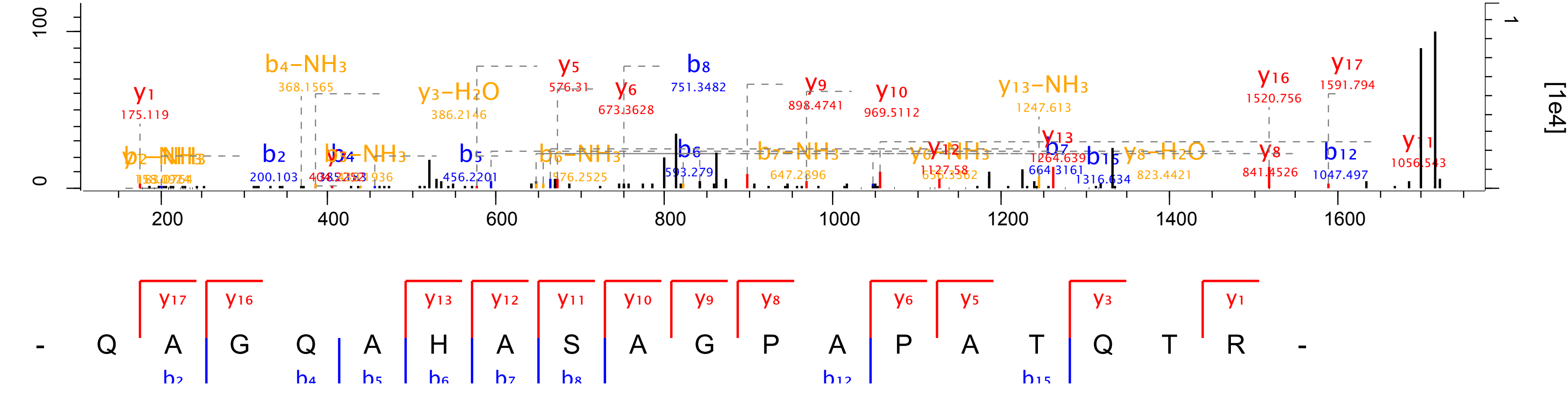

Raw file	Scan	Method	Score	m/z	Gene names
20150402_CerP14_Frac08_top_opt_B8_01_1817	9530	TOF; CID	132.32	541.28	Cnih2

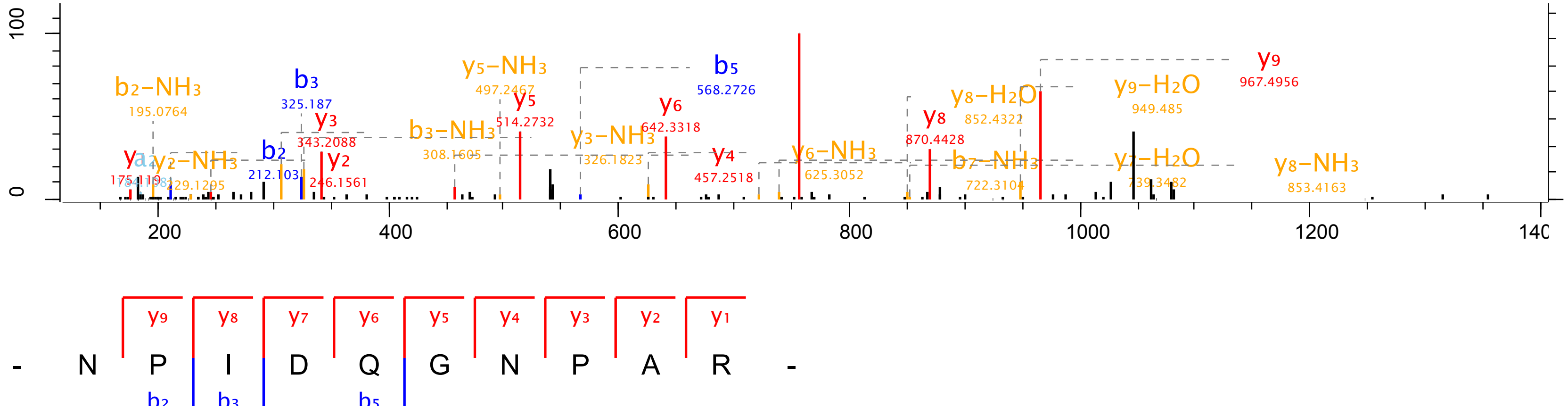

Raw file	Scan	Method	Score	m/z	Gene names
20150402_CerP14_Frac08_top_opt_B8_01_1817	18894	TOF; CID	58.63	604.65	Tusc1

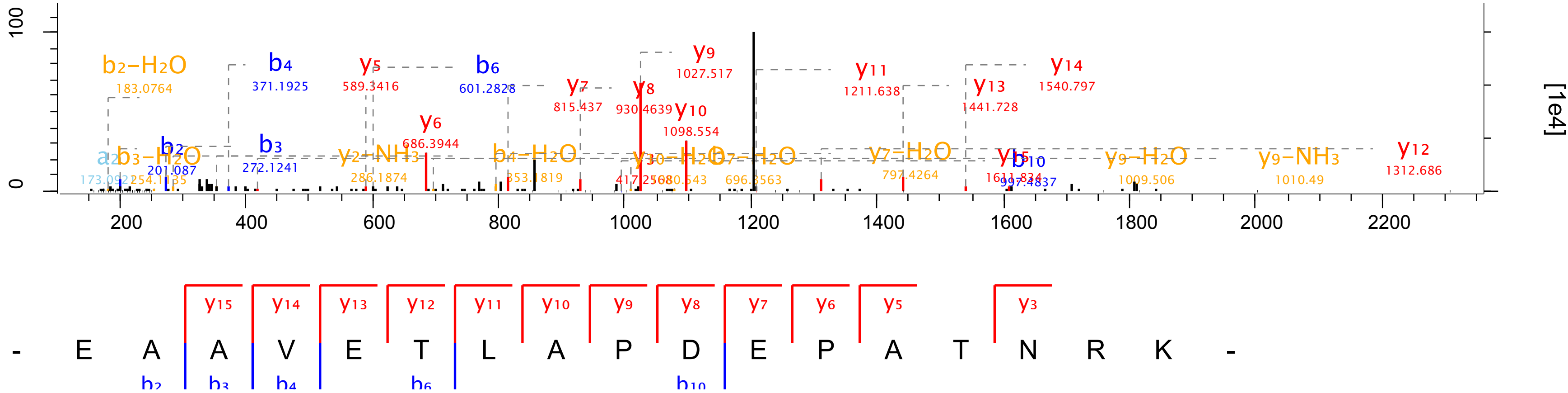

Raw file	Scan	Method	Score	m/z	Gene names
20150402_CerP14_Frac08_top_opt_B8_01_1817	25222	TOF; CID	80.96	688.9	Ipmk

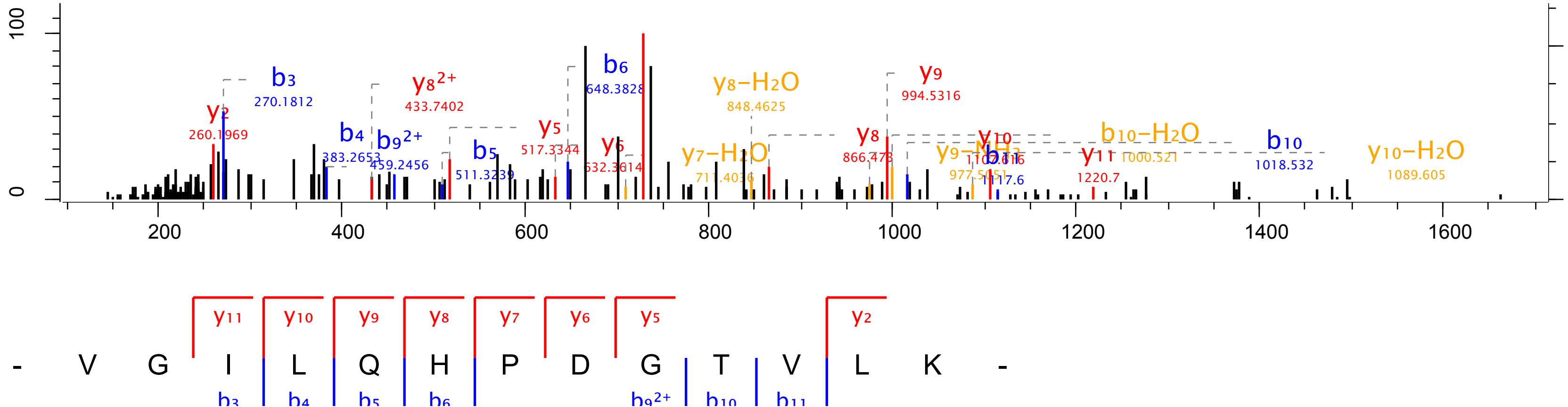

Raw file	Scan	Method	Score	m/z	Gene names
20150402_CerP14_Frac08_top_opt_B8_01_1817	26487	TOF; CID	69.48	912.4	Tspan5

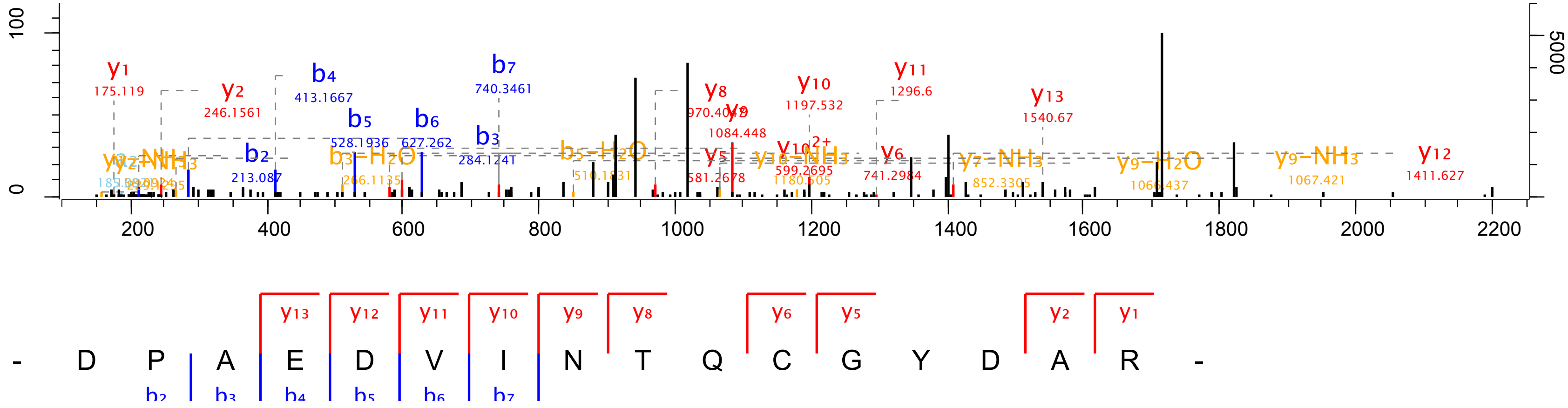

Raw file	Scan	Method	Score	m/z	Gene names
20150402_CerP14_Frac08_top_opt_B8_01_1817	27838	TOF; CID	72.89	525.29	Nr1h3;Nr1h2

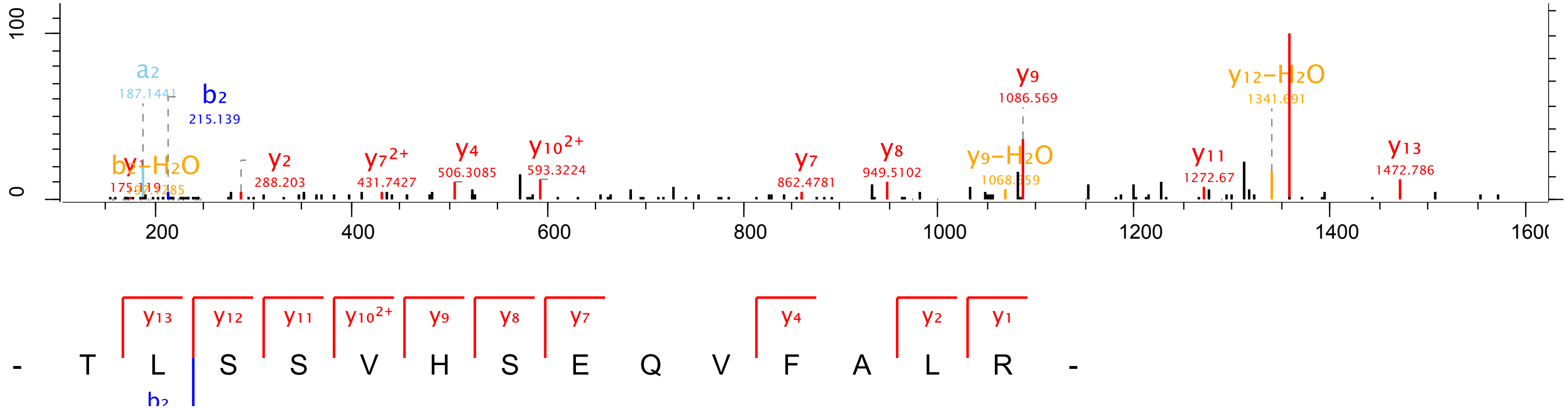

Raw file	Scan	Method	Score	m/z	Gene names
20150402_CerP14_Frac08_top_opt_B8_01_1817	31732	TOF; CID	58.04	722.88	Nkain1

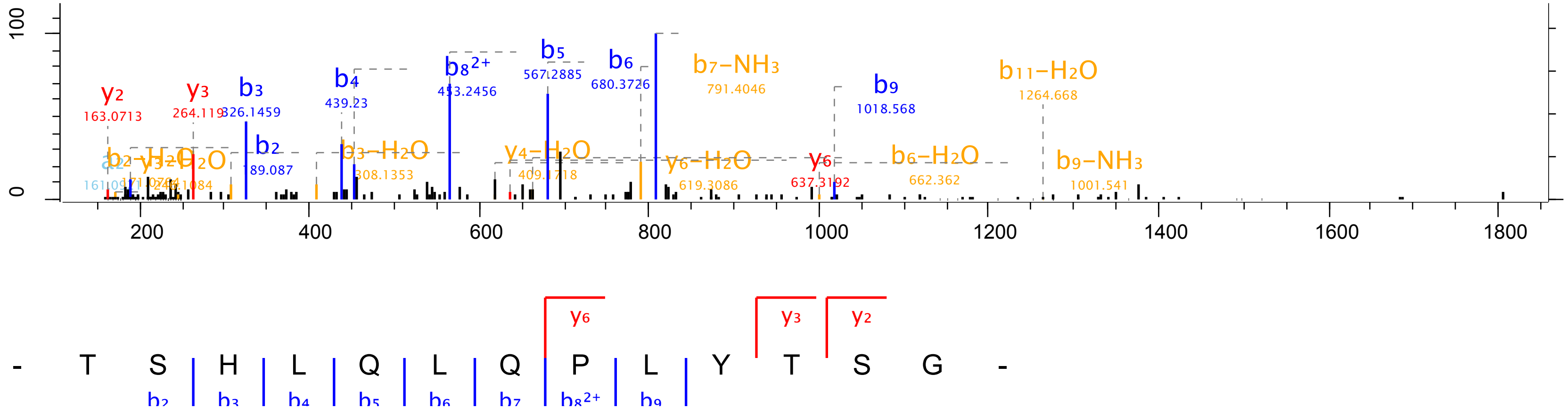

Raw file	Scan	Method	Score	m/z	Gene names
20150402_CerP14_Frac08_top_opt_B8_01_1817	35068	TOF; CID	38.99	780.91	Hyal2

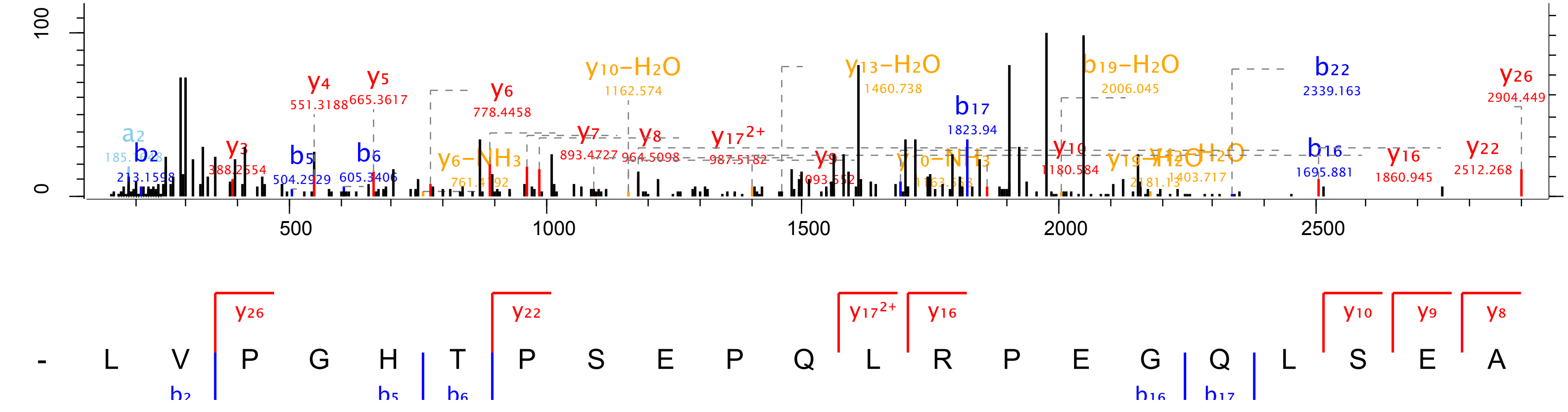

Raw file	Scan	Method	Score	m/z	Gene names
20150402_CerP14_Frac08_top_opt_B8_01_1817	40056	TOF; CID	90.15	537.79	Rnf138

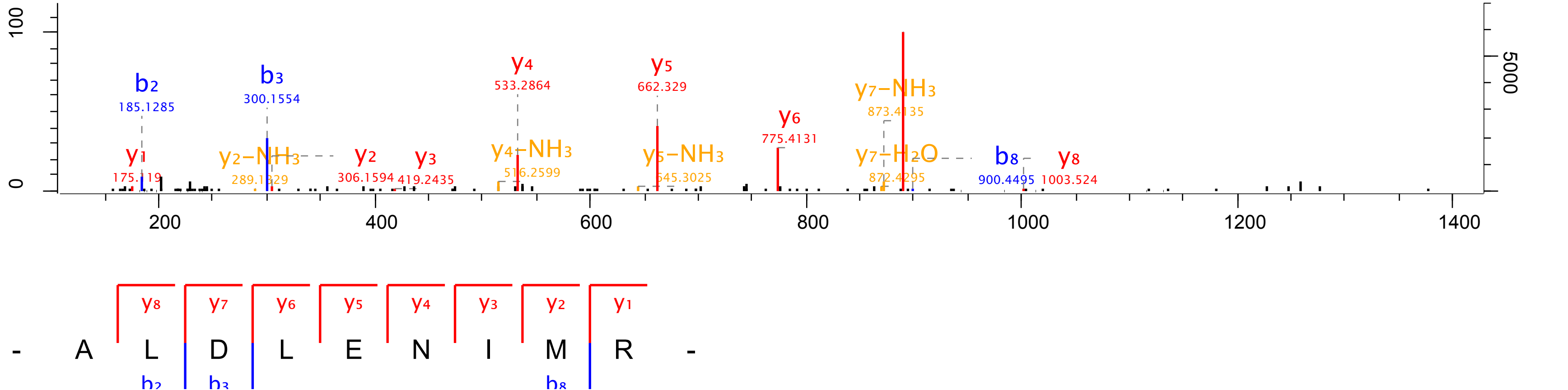

Raw file	Scan	Method	Score	m/z	Gene names
20150402_CerP14_Frac08_top_opt_B8_01_1817	47080	TOF; CID	48.91	929.48	Cdk5rap1

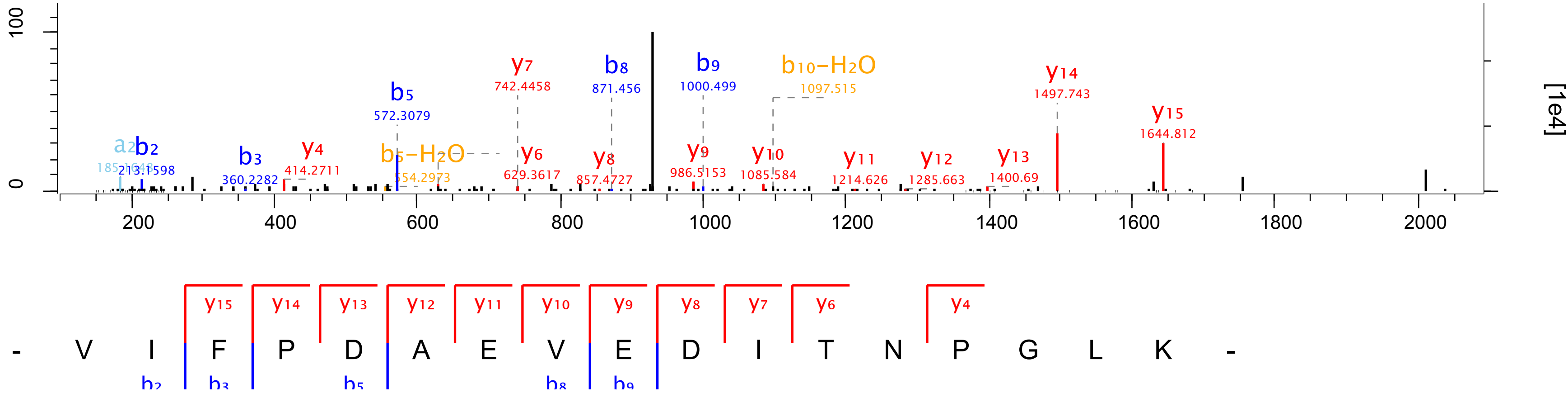

Raw file	Scan	Method	Score	m/z	Gene names
20150402_CerP14_Frac08_top_opt_B8_01_1817	47586	TOF; CID	43.95	953	Parp6

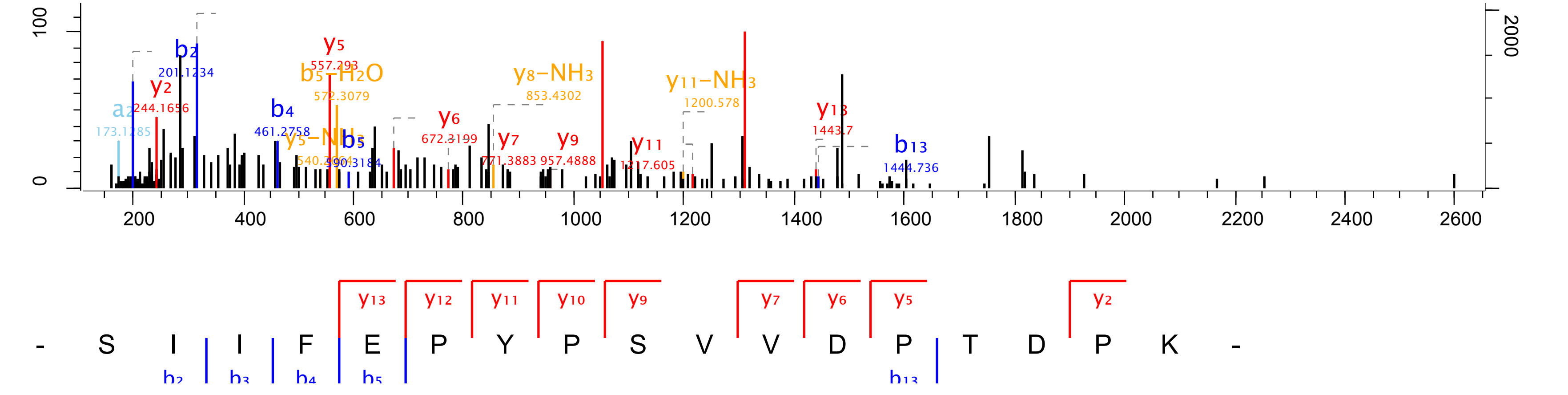

Raw file	Scan	Method	Score	m/z	Gene names
20150402_CerP14_Frac08_top_opt_B8_01_1817	51143	TOF; CID	94.77	667.86	Pcgf5

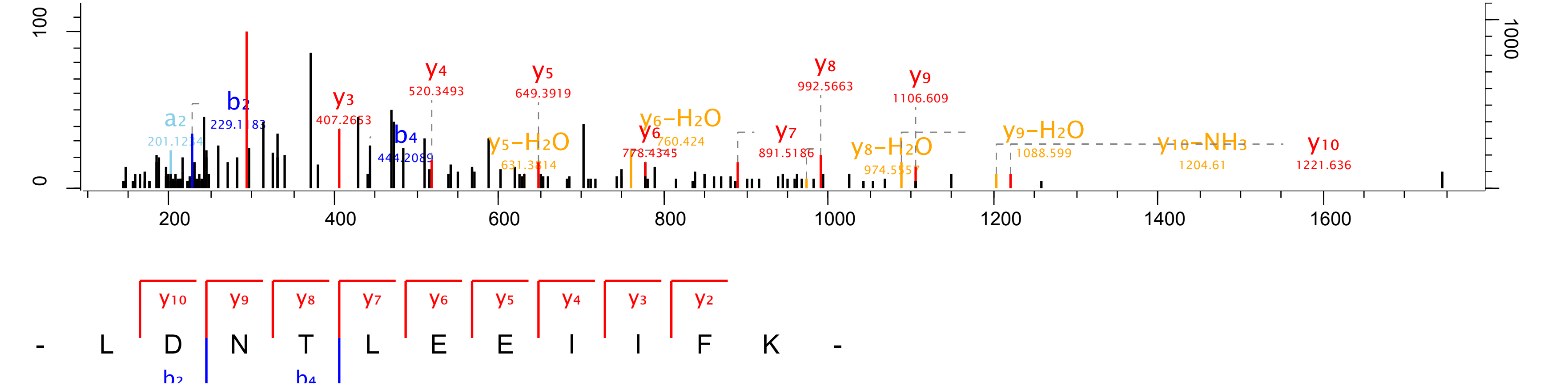

Raw file	Scan	Method	Score	m/z	Gene names
20150402_CerP14_Frac08_top_opt_B8_01_1817	54686	TOF; CID	68.41	866.96	Azin2

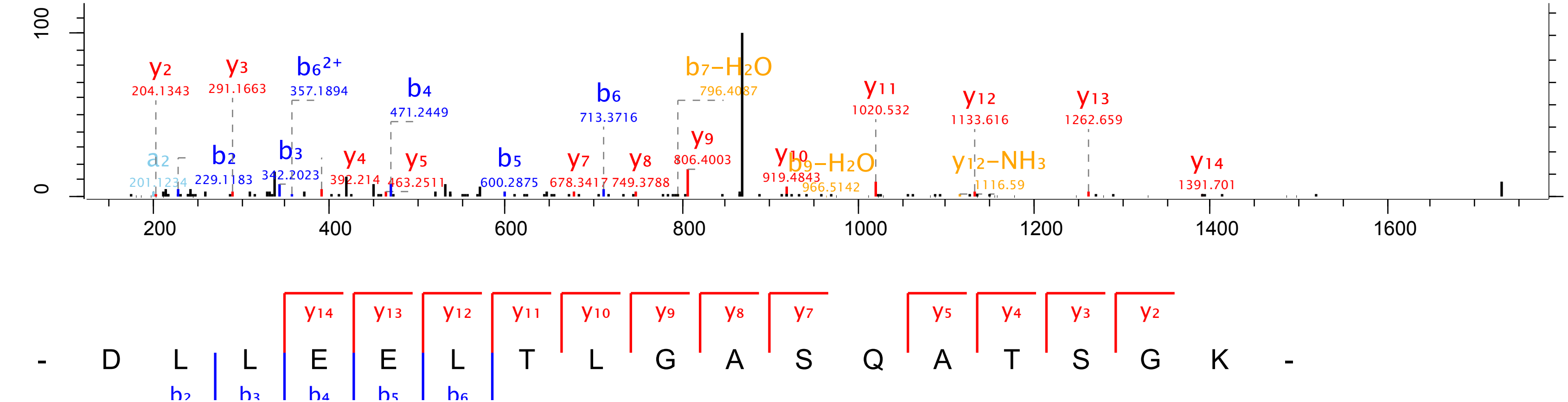

Raw file	Scan	Method	Score	m/z	Gene names
20150402_CerP14_Frac09_top_opt_B9_01_1818	8514	TOF; CID	78.26	475.71	Gm7367;Ubald2

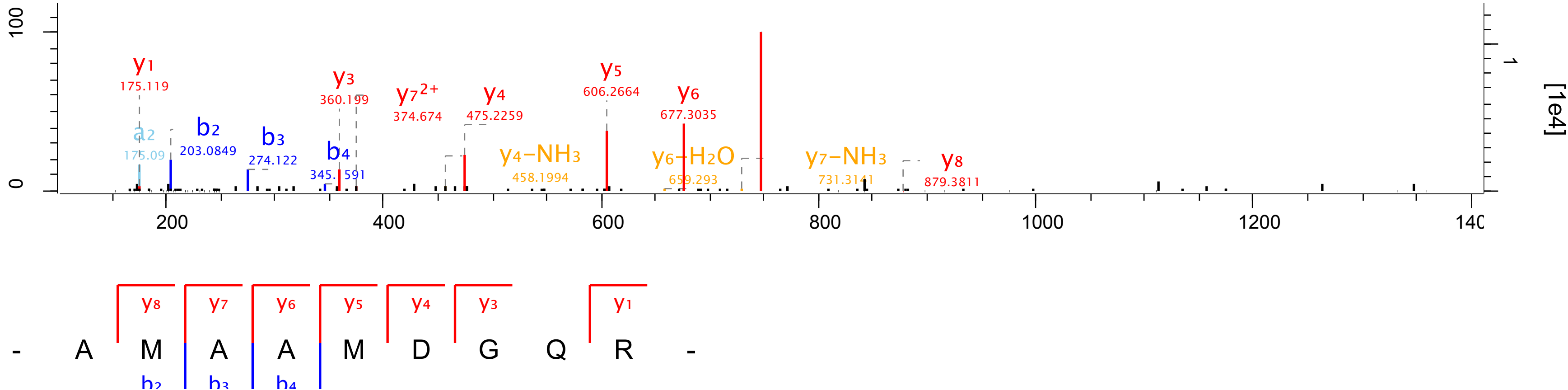

Raw file	Scan	Method	Score	m/z	Gene names
20150402_CerP14_Frac09_top_opt_B9_01_1818	9939	TOF; CID	83.18	572.31	Stc1

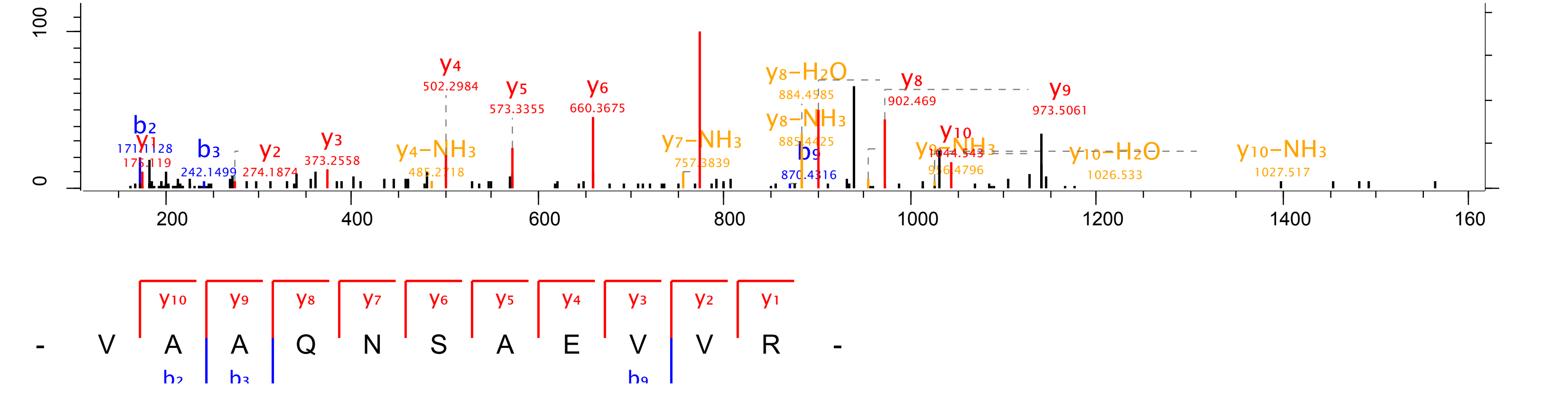

Raw file	Scan	Method	Score	m/z	Gene names
20150402_CerP14_Frac09_top_opt_B9_01_1818	9971	TOF; CID	66.99	612.83	Ank2

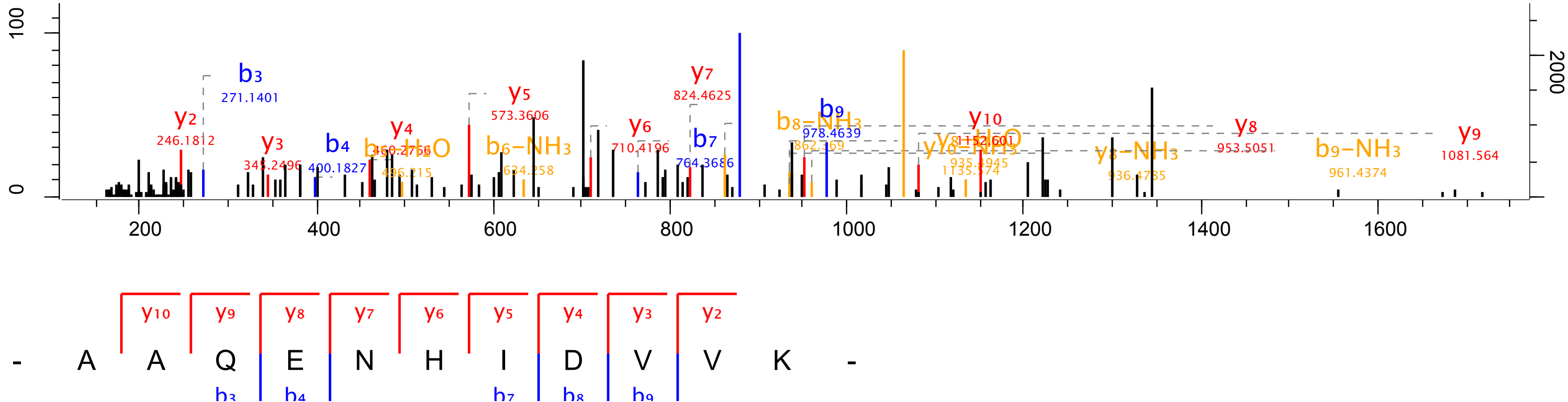

Raw file	Scan	Method	Score	m/z	Gene names
20150402_CerP14_Frac09_top_opt_B9_01_1818	18229	TOF; CID	196.8	896.37	Tspan7

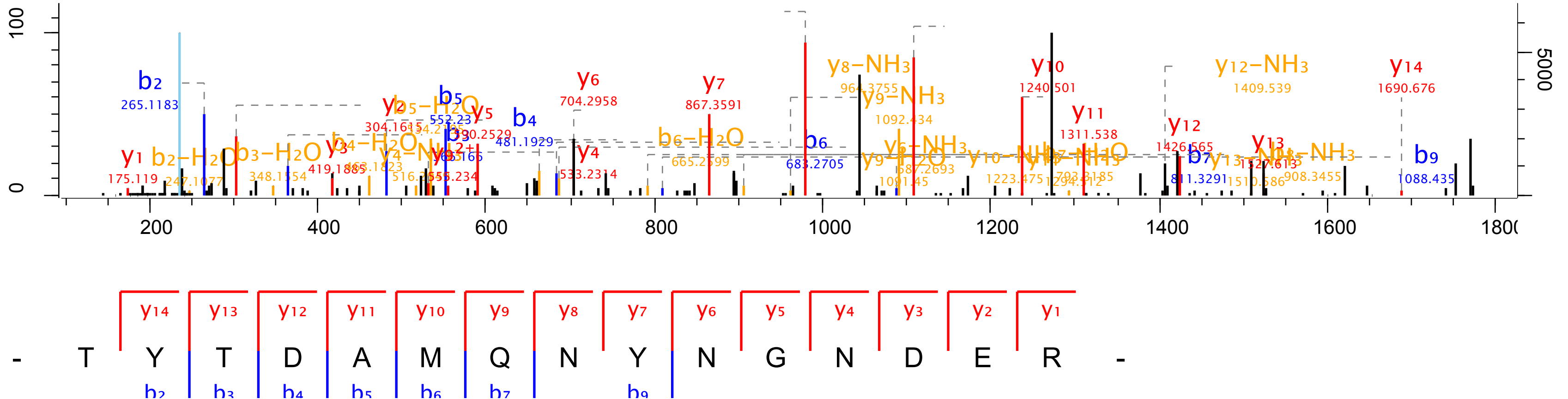

Raw file	Scan	Method	Score	m/z	Gene names
20150402_CerP14_Frac09_top_opt_B9_01_1818	21728	TOF; CID	67.1	542.27	Eaf1

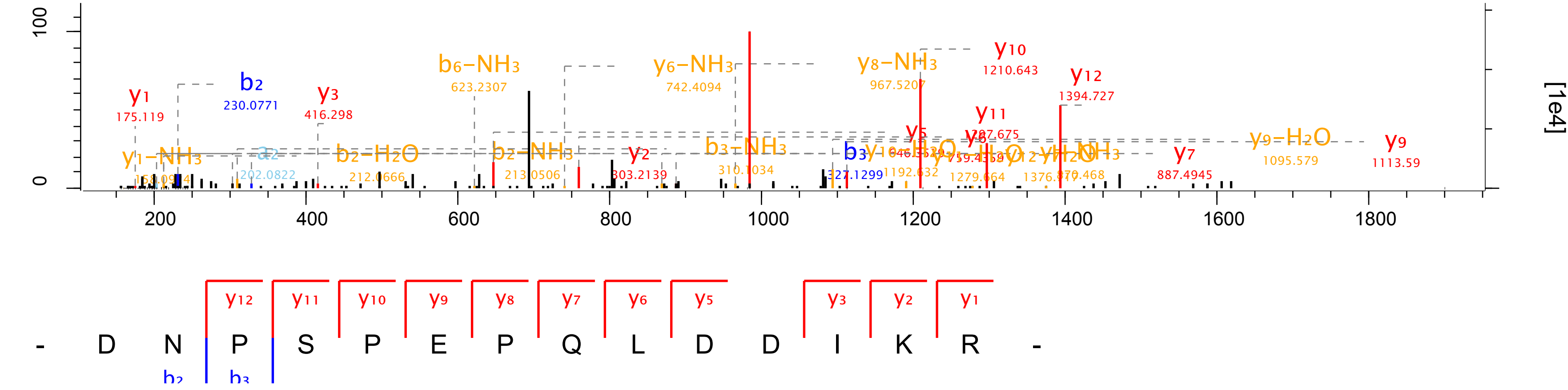

Raw file	Scan	Method	Score	m/z	Gene names
20150402_CerP14_Frac09_top_opt_B9_01_1818	23236	TOF; CID	93.35	652.63	Anapc11

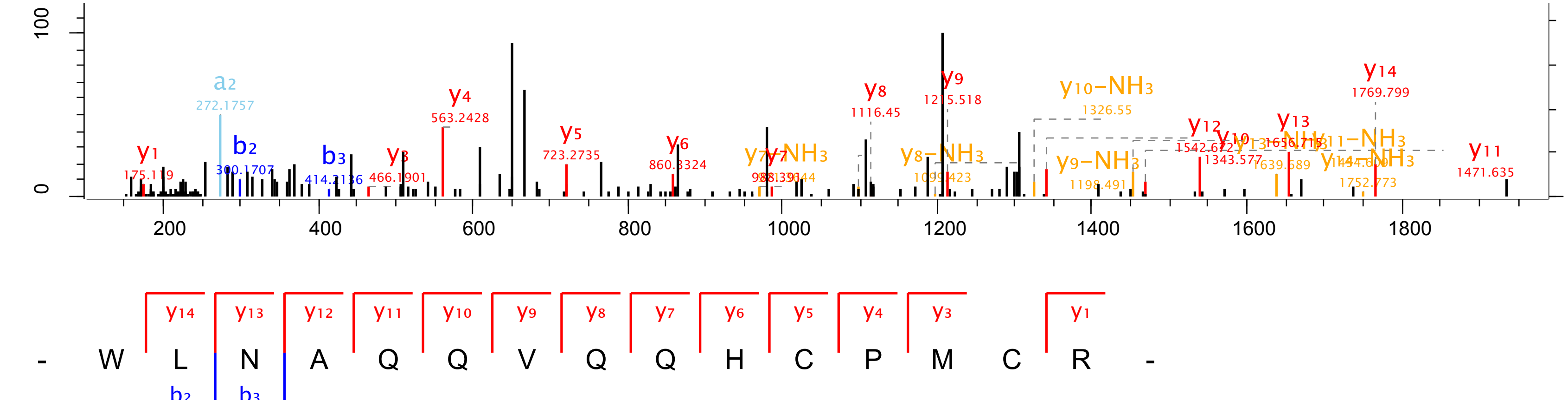

Raw file	Scan	Method	Score	m/z	Gene names
20150402_CerP14_Frac09_top_opt_B9_01_1818	25089	TOF; CID	82.5	517.62	Fam117a

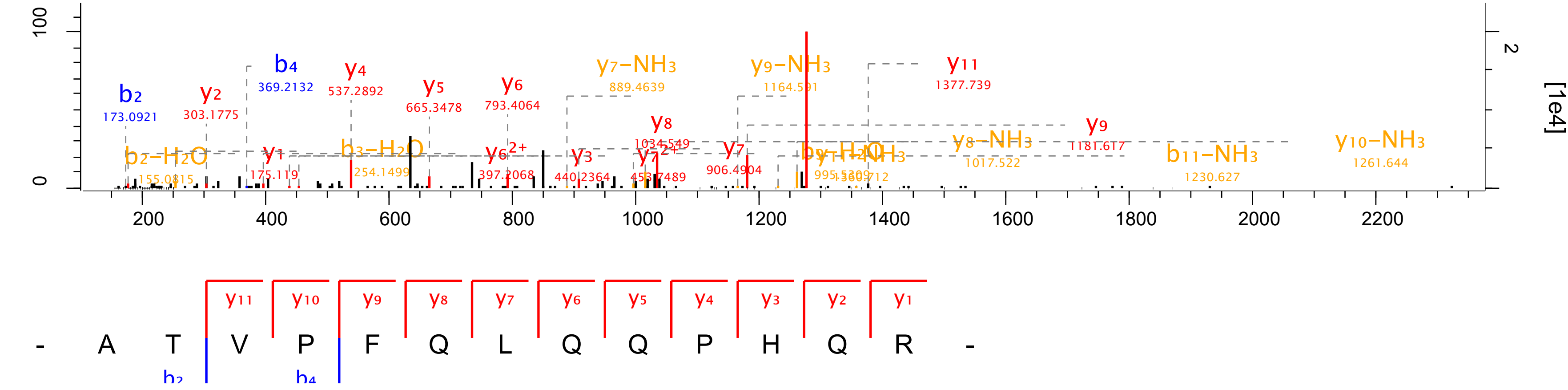

Raw file	Scan	Method	Score	m/z	Gene names
20150402_CerP14_Frac09_top_opt_B9_01_1818	28802	TOF; CID	55.26	437.25	March11

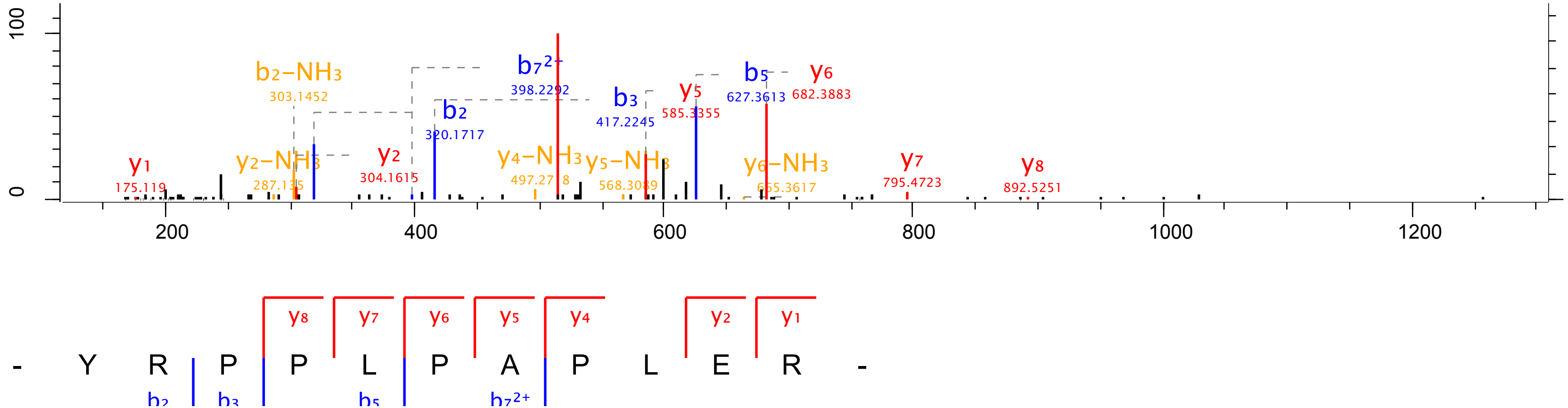

Raw file	Scan	Method	Score	m/z	Gene names
20150402_CerP14_Frac09_top_opt_B9_01_1818	33571	TOF; CID	34.17	788.4	Ptpn14

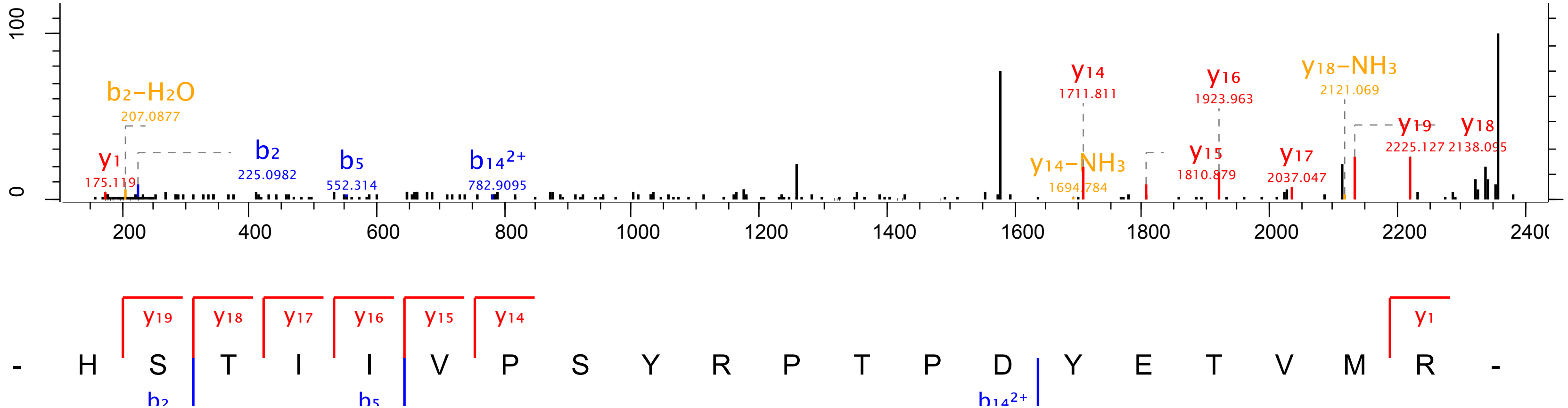

Raw file	Scan	Method	Score	m/z	Gene names
20150402_CerP14_Frac09_top_opt_B9_01_1818	34121	TOF; CID	106.57	800.07	Psmc3ip

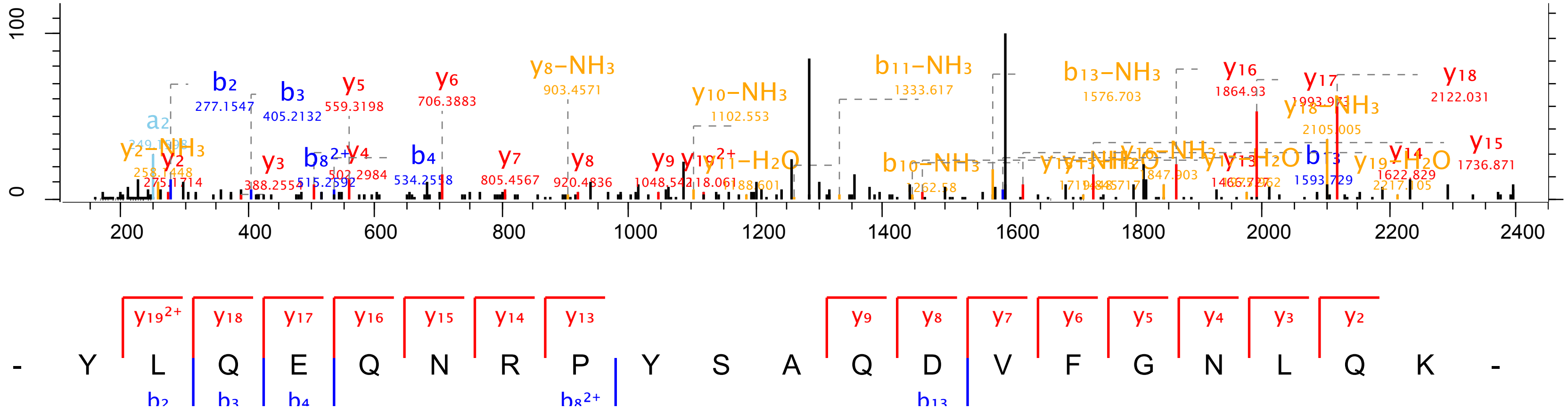

Raw file	Scan	Method	Score	m/z	Gene names
20150402_CerP14_Frac09_top_opt_B9_01_1818	36905	TOF; CID	202.13	881.41	Ly6e

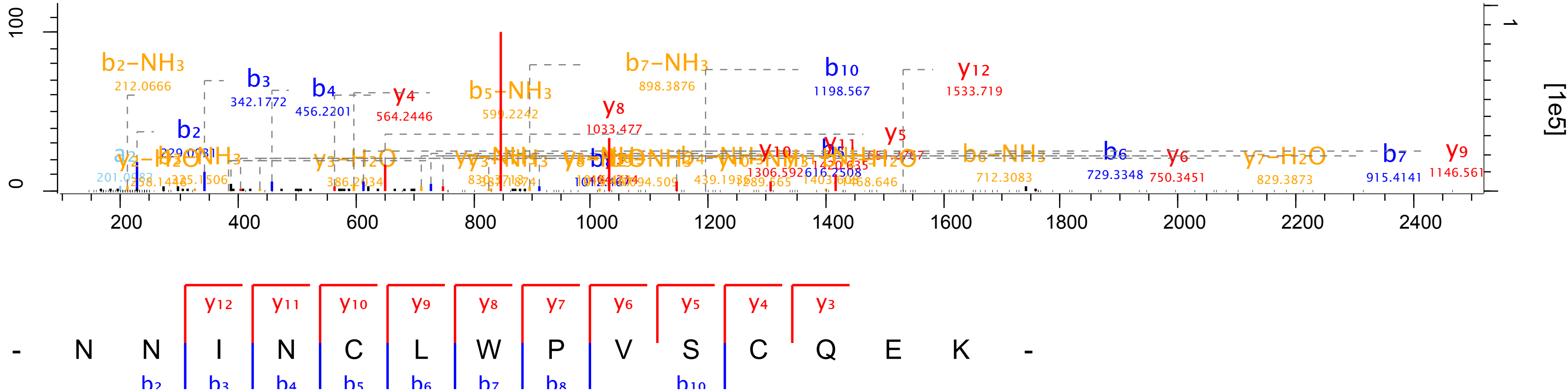

Raw file	Scan	Method	Score	m/z	Gene names
20150402_CerP14_Frac09_top_opt_B9_01_1818	45322	TOF; CID	76.07	677.39	Enkd1

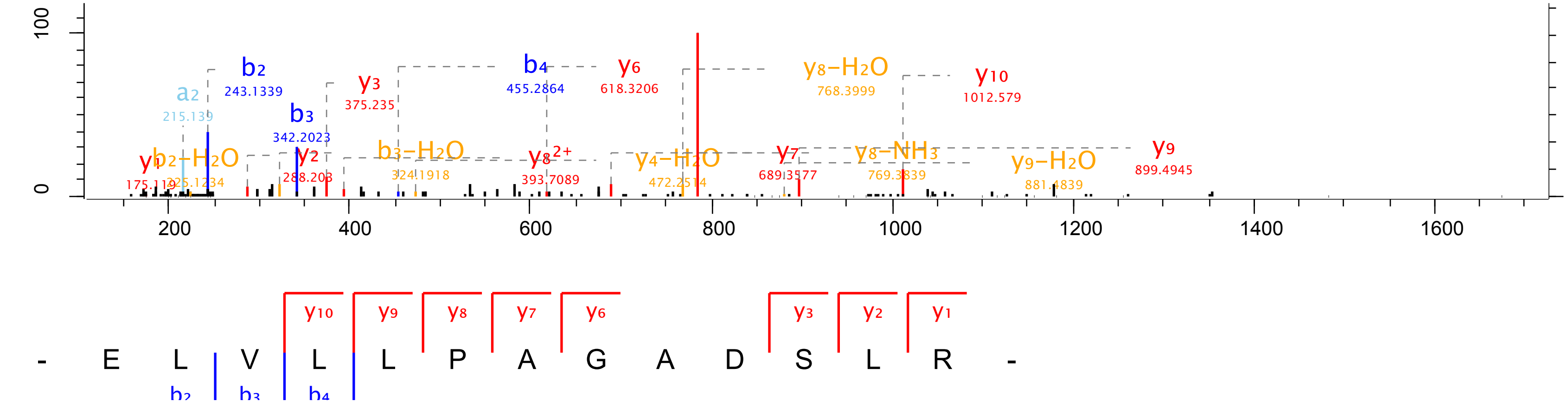

Raw file	Scan	Method	Score	m/z	Gene names
20150402_CerP14_Frac09_top_opt_B9_01_1818	47424	TOF; CID	64.54	882.43	Mitd1

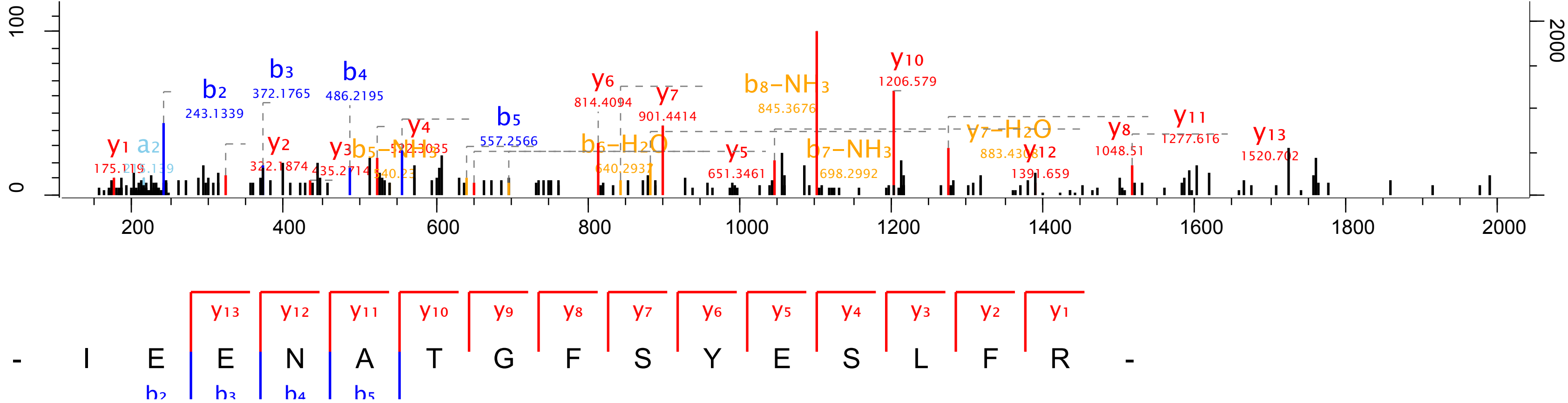

Raw file	Scan	Method	Score	m/z	Gene names
20150402_CerP14_Frac09_top_opt_B9_01_1818	54122	TOF; CID	64.52	894.44	B3galt1

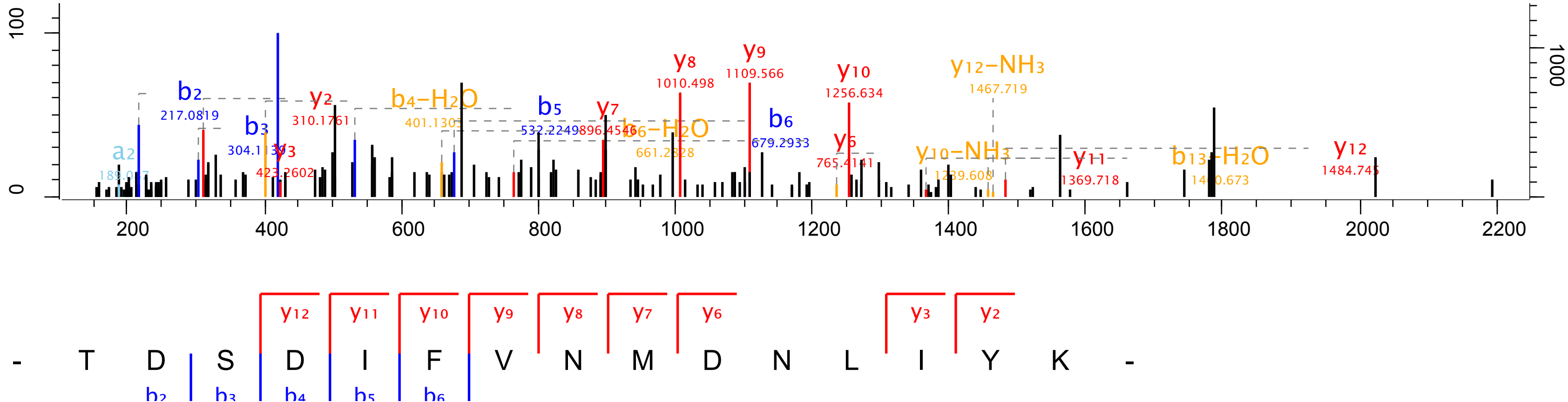

Raw file	Scan	Method	Score	m/z	Gene names
20150402_CerP14_Frac09_top_opt_B9_01_1818	56138	TOF; CID	59.91	1441.7	Lysmd4

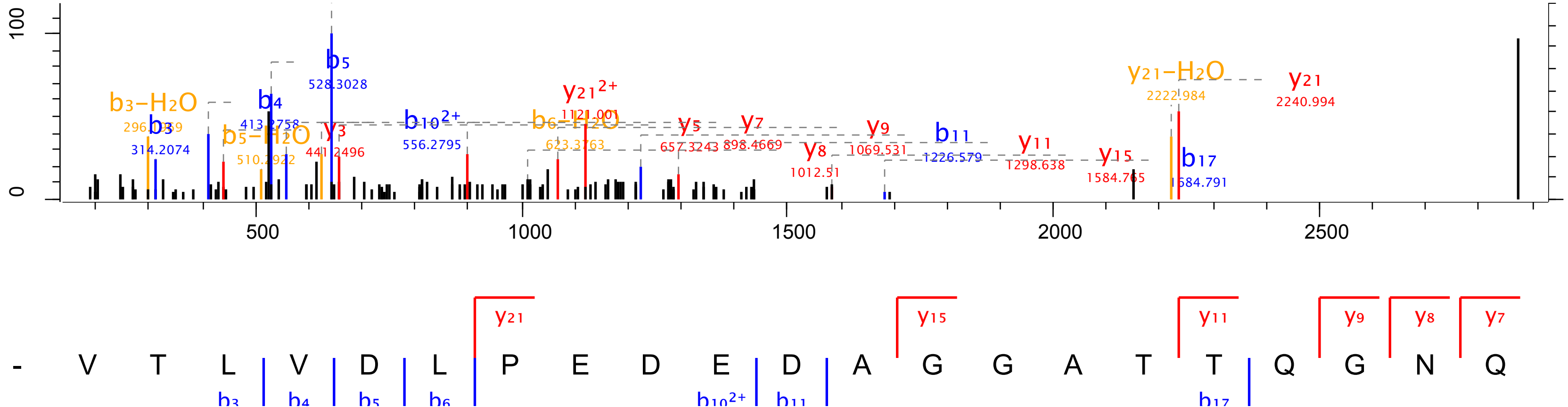

Raw file	Scan	Method	Score	m/z	Gene names
20150402_CerP14_Frac09_top_opt_B9_01_1818	57539	TOF; CID	38.36	1024.81	Dos

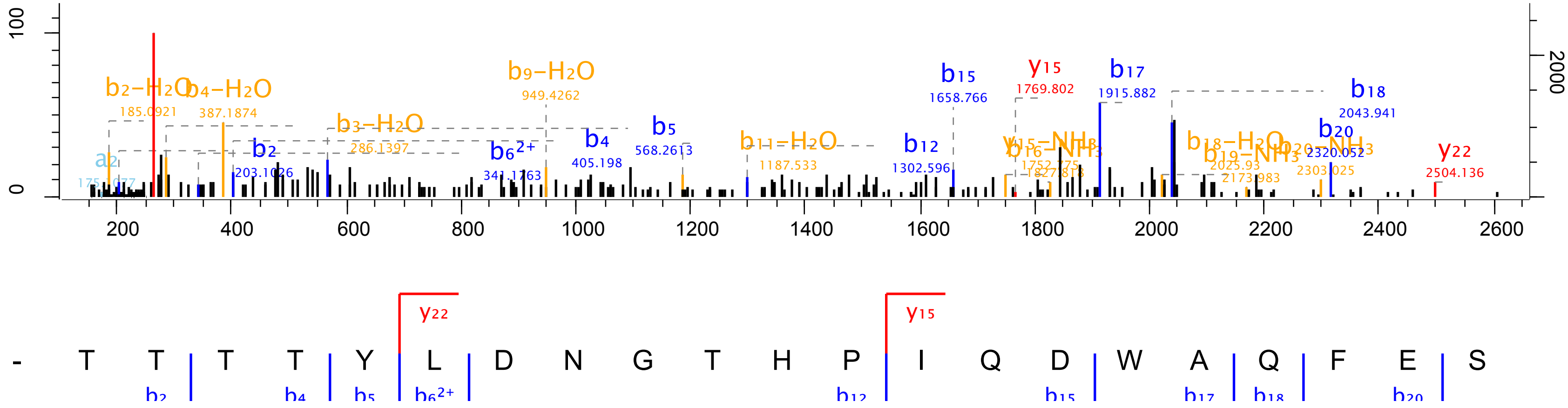

Raw file	Scan	Method	Score	m/z	Gene names
20150402_CerP14_Frac09_top_opt_B9_01_1818	58690	TOF; CID	85.61	1179.55	C1d

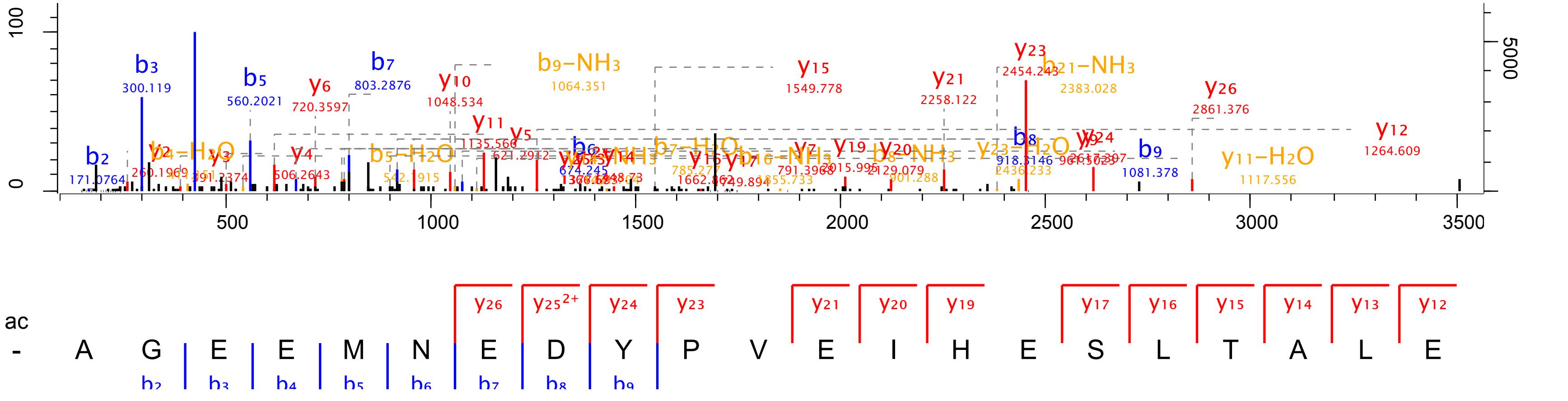

Raw file	Scan	Method	Score	m/z	Gene names
20150402_CerP14_Frac10_top_opt_B10_01_1819	7388	TOF; CID	88.82	438.72	Slc39a13

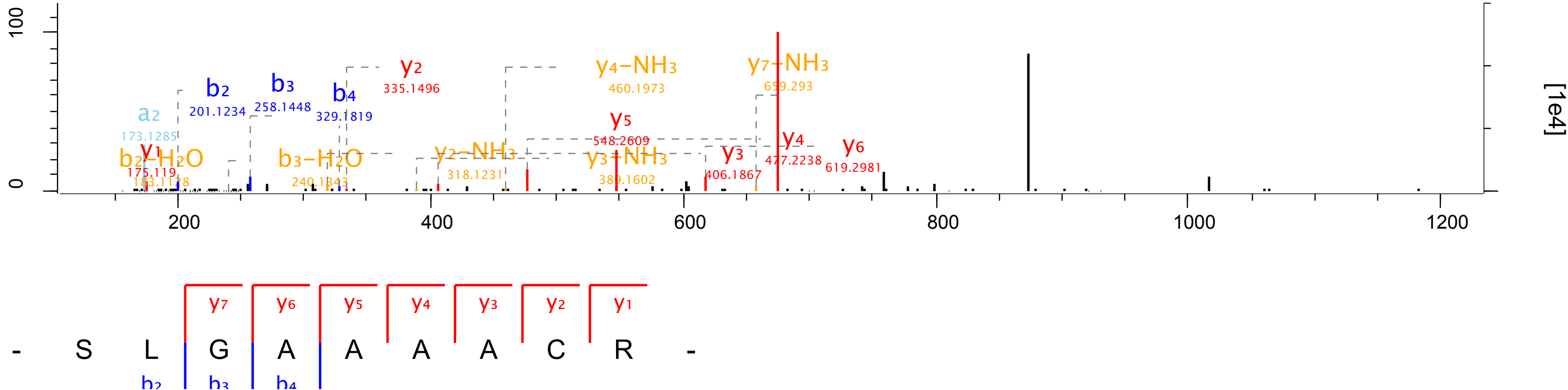

Raw file	Scan	Method	Score	m/z	Gene names
20150402_CerP14_Frac10_top_opt_B10_01_1819	11482	TOF; CID	71.38	810.89	Cplx3

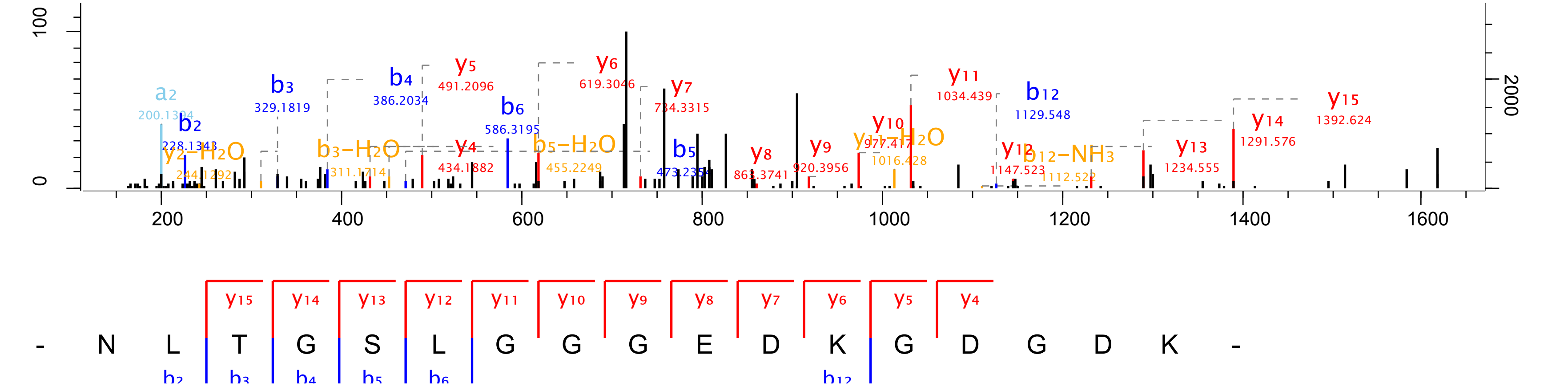

Raw file	Scan	Method	Score	m/z	Gene names
20150402_CerP14_Frac10_top_opt_B10_01_1819	12662	TOF; CID	69.09	839.42	Crls1

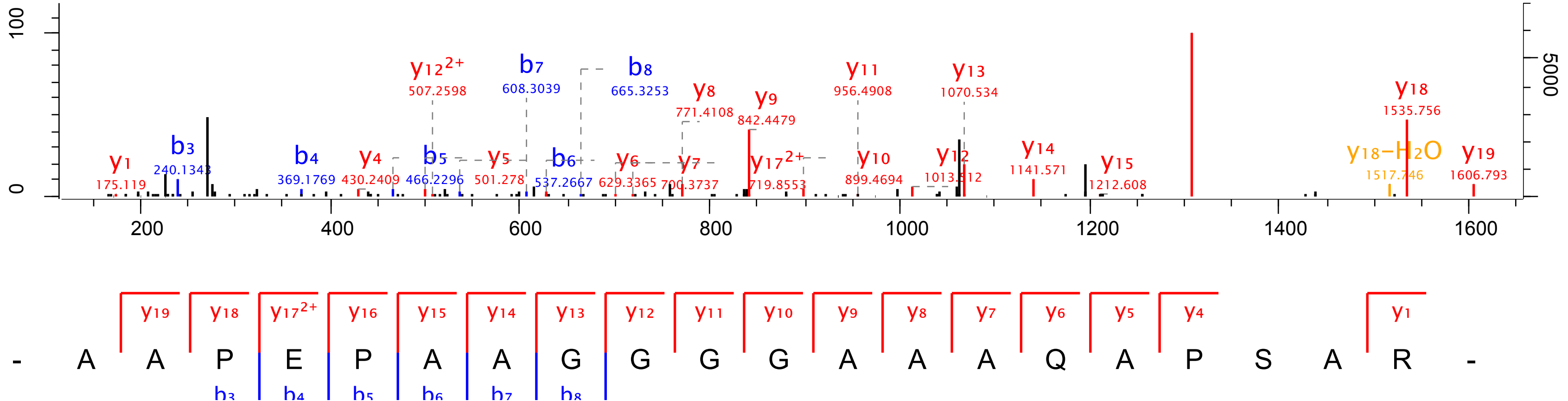

Raw file	Scan	Method	Score	m/z	Gene names
20150402_CerP14_Frac10_top_opt_B10_01_1819	16298	TOF; CID	57.43	492.91	Tmem119

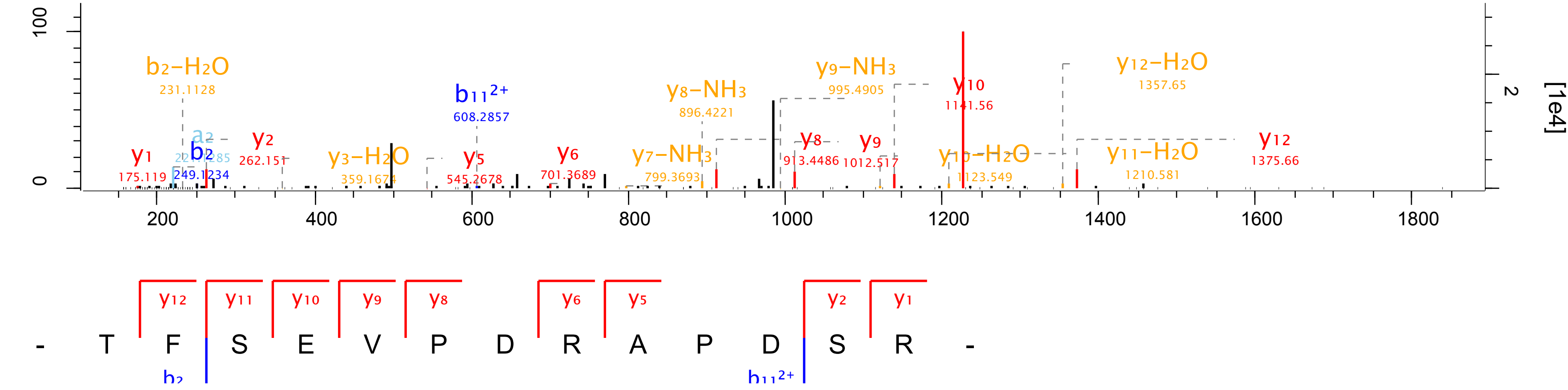

Raw file	Scan	Method	Score	m/z	Gene names
20150402_CerP14_Frac10_top_opt_B10_01_1819	21178	TOF; CID	70.41	617.96	Lyrm1

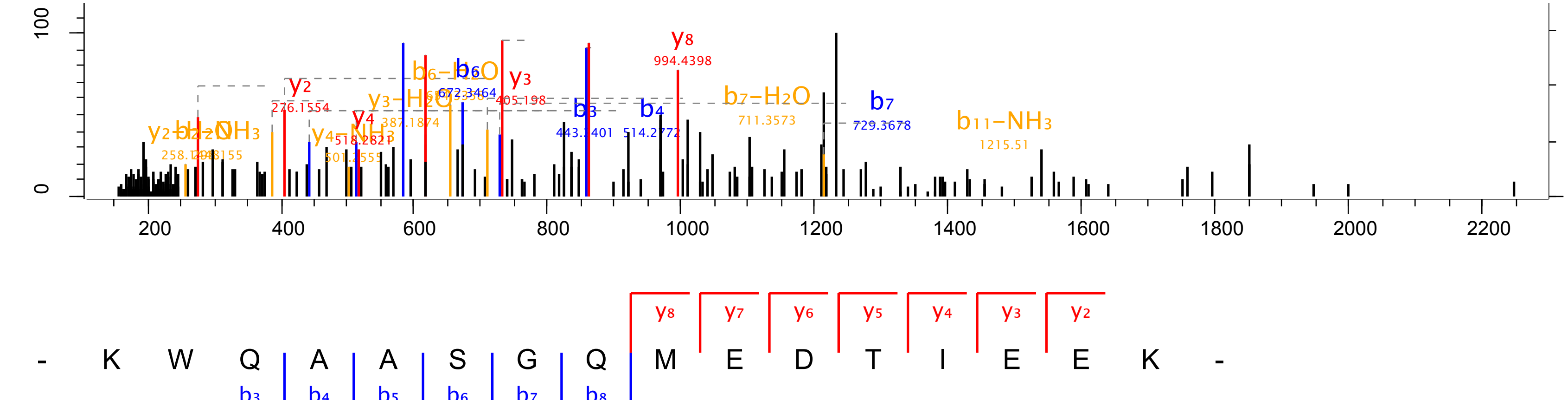

Raw file	Scan	Method	Score	m/z	Gene names
20150402_CerP14_Frac10_top_opt_B10_01_1819	21983	TOF; CID	96.67	515.3	Npy

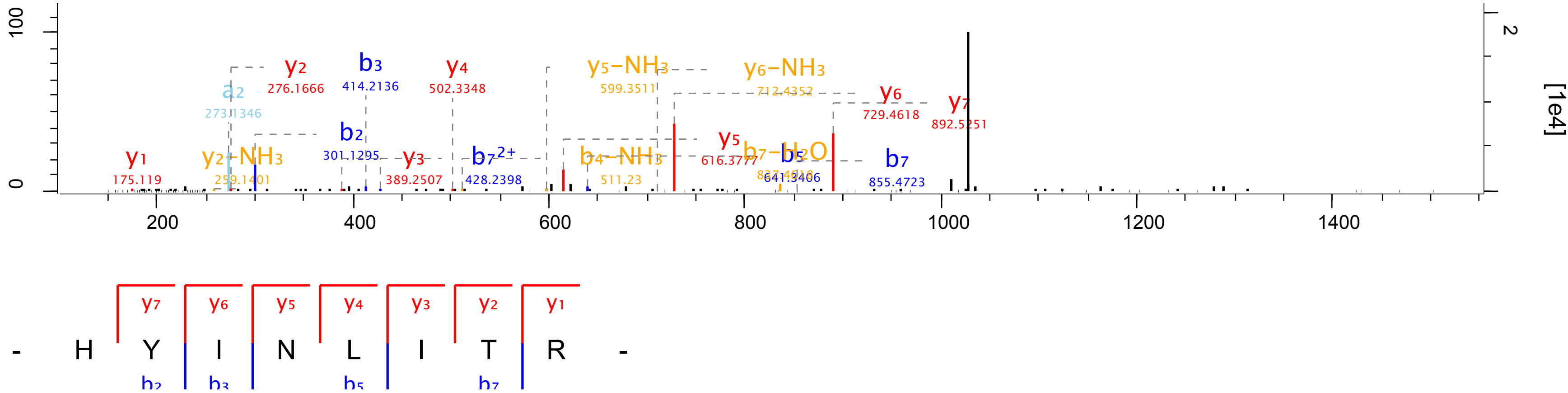

Raw file	Scan	Method	Score	m/z	Gene names
20150402_CerP14_Frac10_top_opt_B10_01_1819	23321	TOF; CID	44.74	736.32	Tmem179b

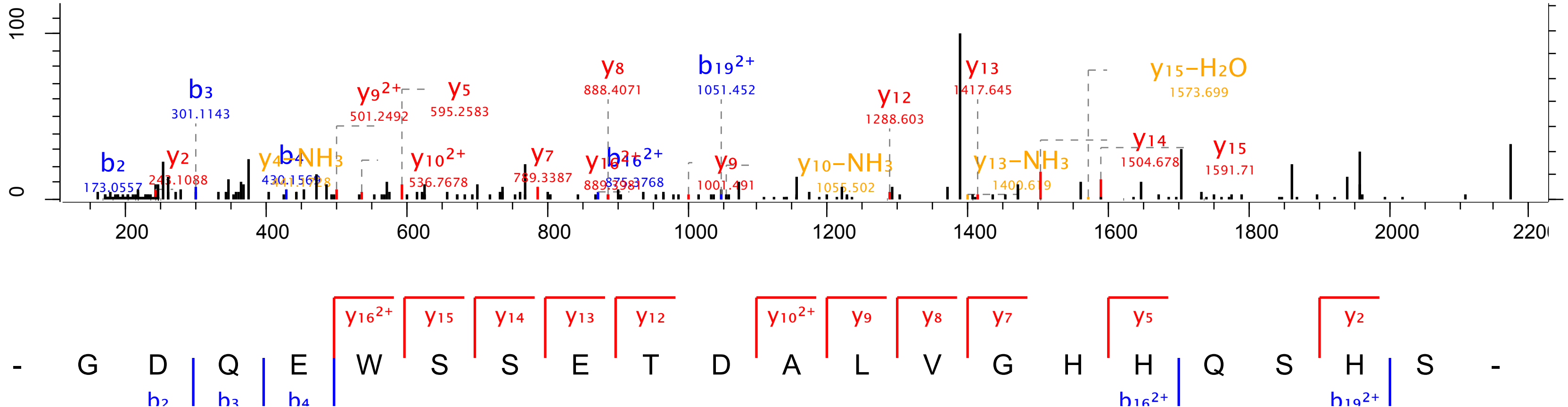

Raw file	Scan	Method	Score	m/z
20150402_CerP14_Frac10_top_opt_B10_01_1819	24243	TOF; CID	147.64	567.27

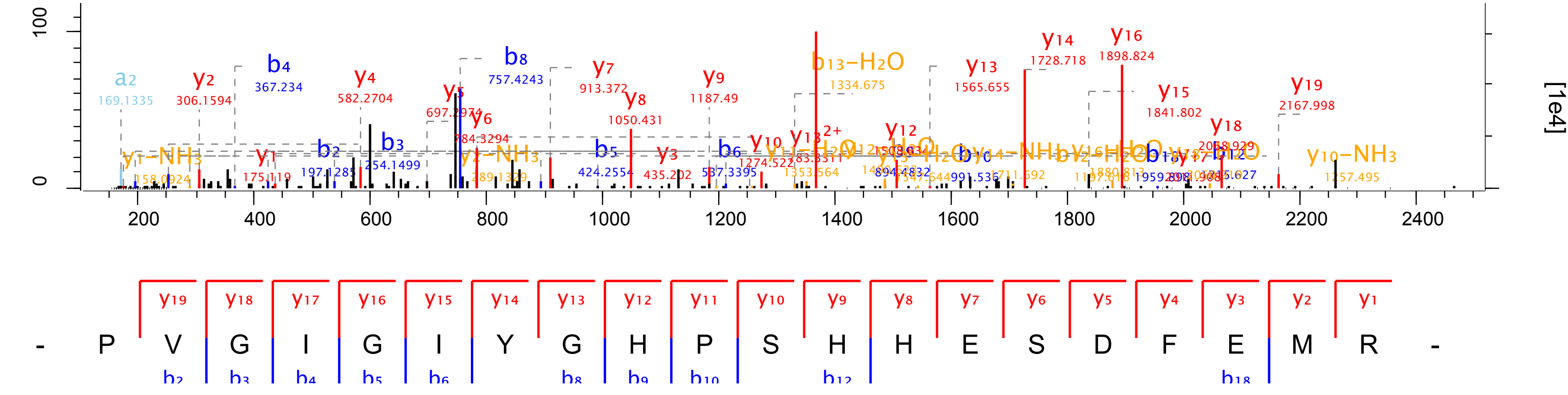

Raw file	Scan	Method	Score	m/z	Gene names
20150402_CerP14_Frac10_top_opt_B10_01_1819	24288	TOF; CID	56.94	557.78	Fxyd7

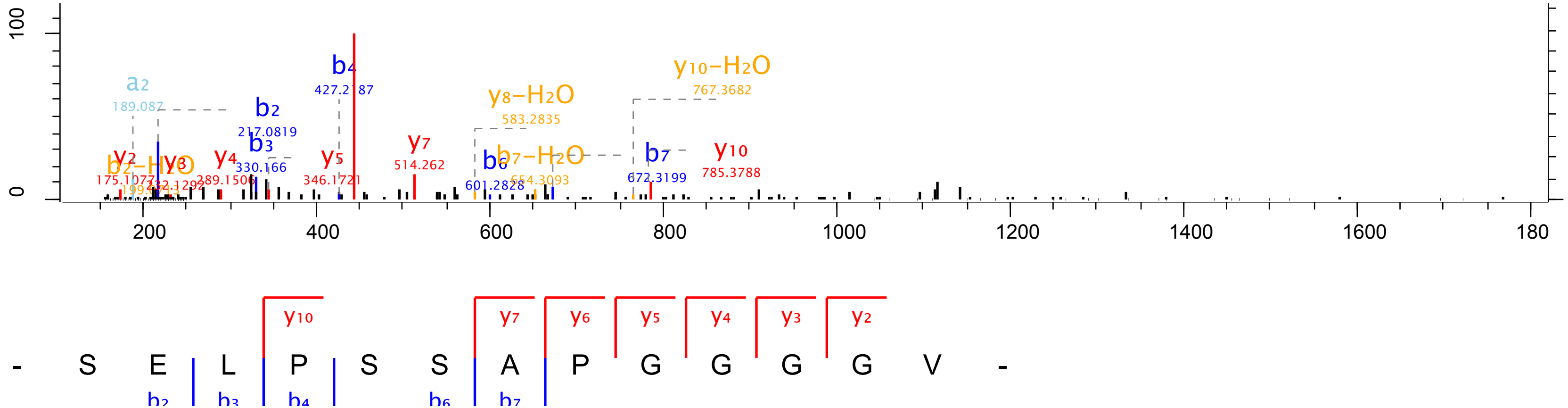

Raw file	Scan	Method	Score	m/z
20150402_CerP14_Frac10_top_opt_B10_01_1819	25434	TOF; CID	96.74	465.6

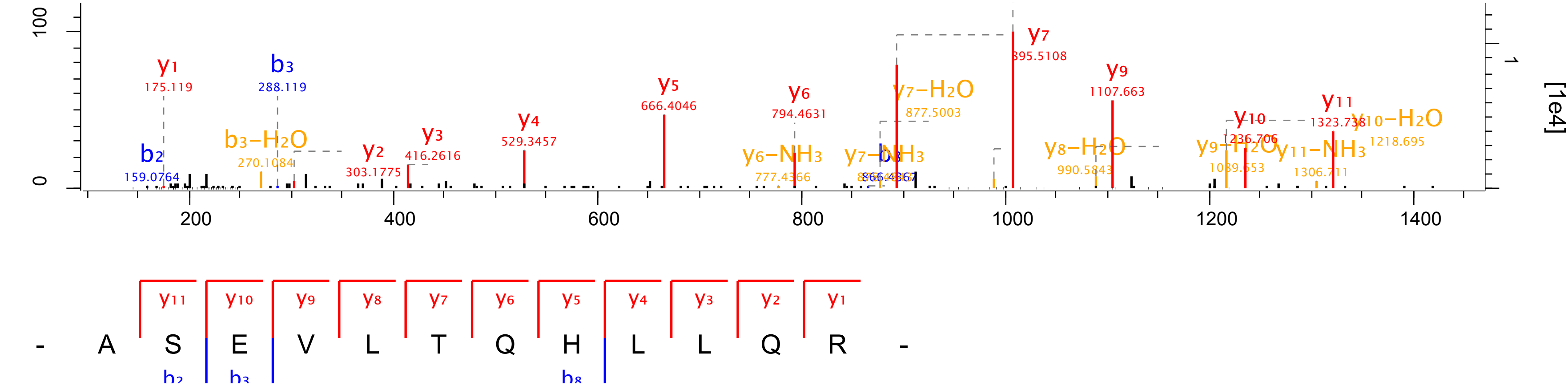

Raw file	Scan	Method	Score	m/z	Gene names
20150402_CerP14_Frac10_top_opt_B10_01_1819	25573	TOF; CID	79.2	544.8	Spata18

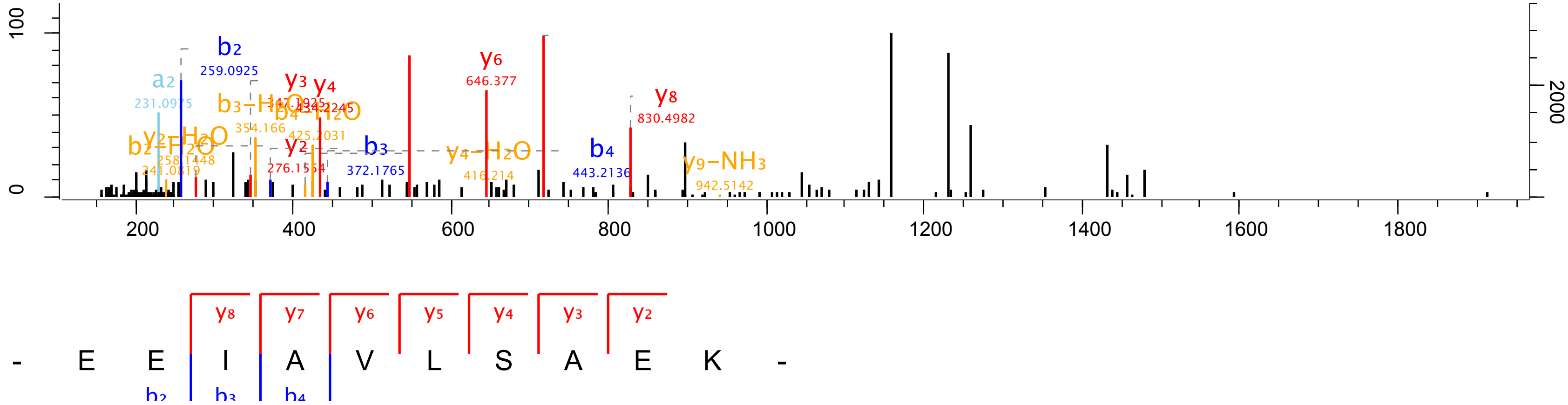

Raw file	Scan	Method	Score	m/z	Gene names
20150402_CerP14_Frac10_top_opt_B10_01_1819	26413	TOF; CID	67.9	365.55	Nmb

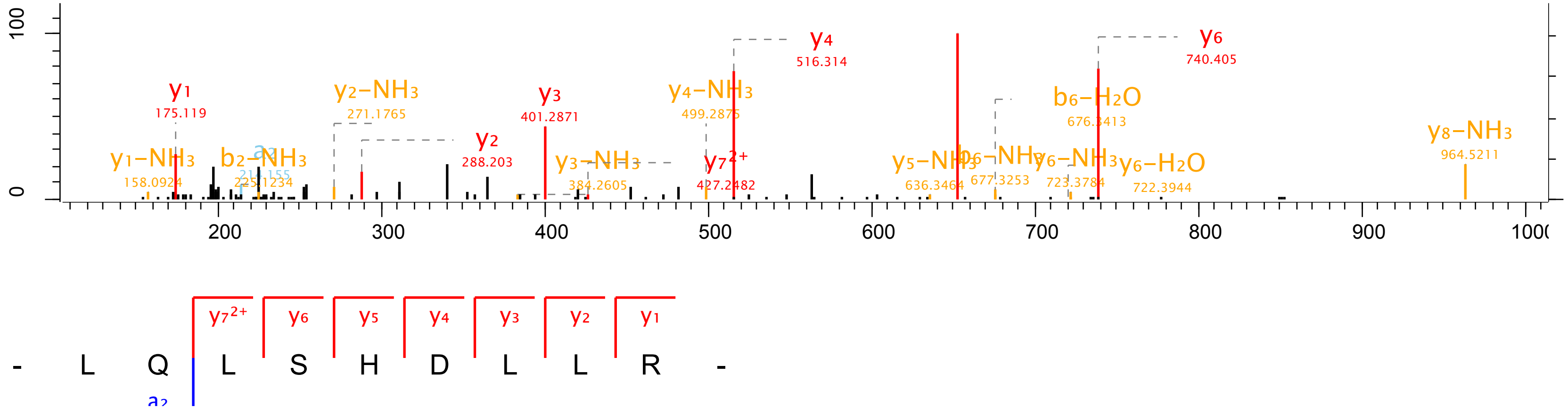

Raw file	Scan	Method	Score	m/z	Gene names
20150402_CerP14_Frac10_top_opt_B10_01_1819	31012	TOF; CID	82.18	714.39	Tmem219

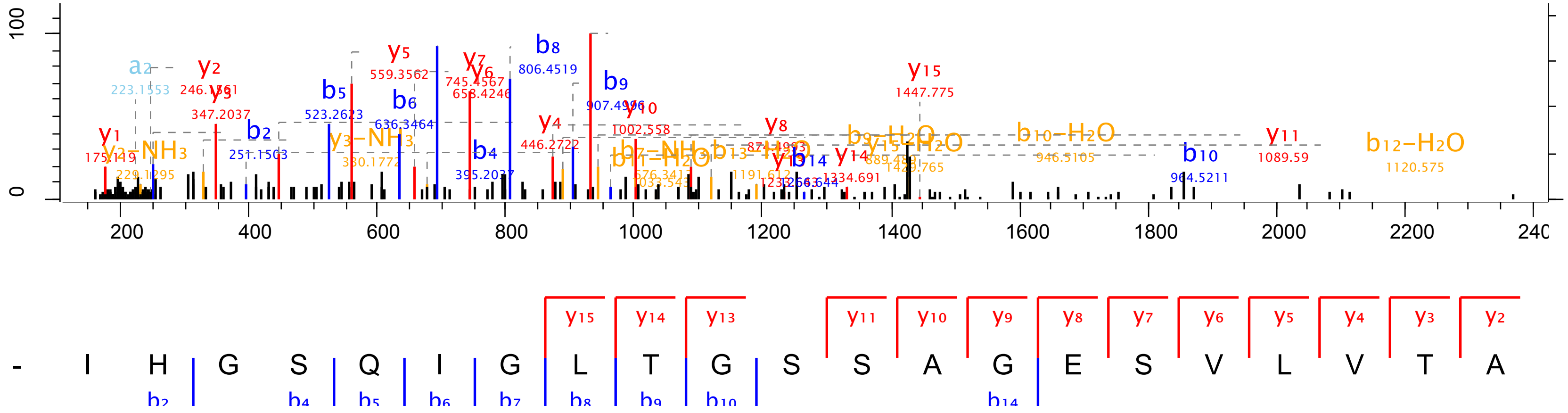

Raw file	Scan	Method	Score	m/z	Gene names
20150402_CerP14_Frac10_top_opt_B10_01_1819	34350	TOF; CID	69.93	615.98	Ube2c

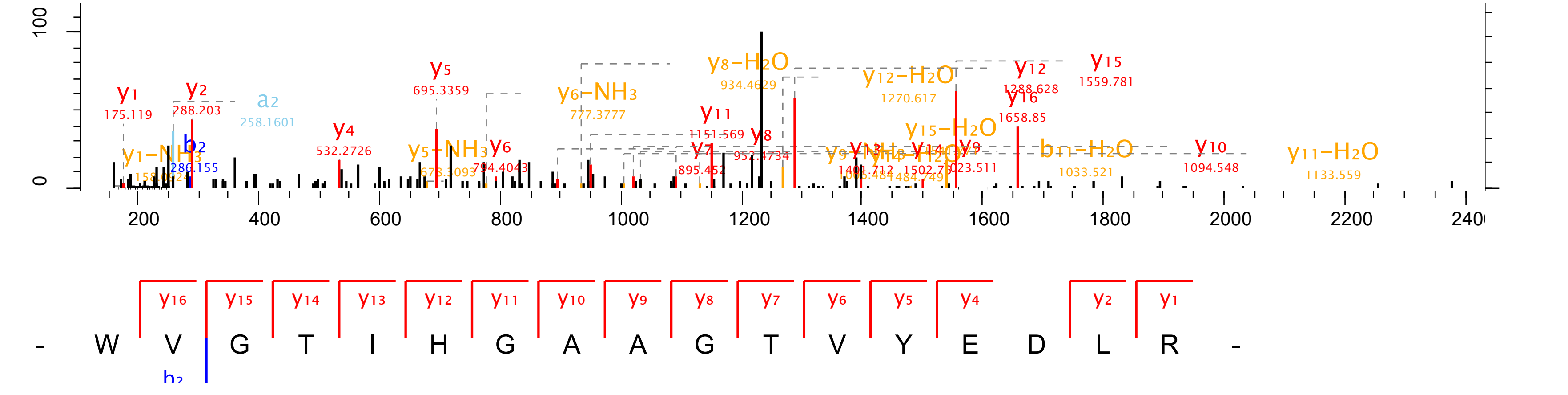

Raw file	Scan	Method	Score	m/z	Gene names
20150402_CerP14_Frac10_top_opt_B10_01_1819	36103	TOF; CID	86.75	800.74	Mbd5

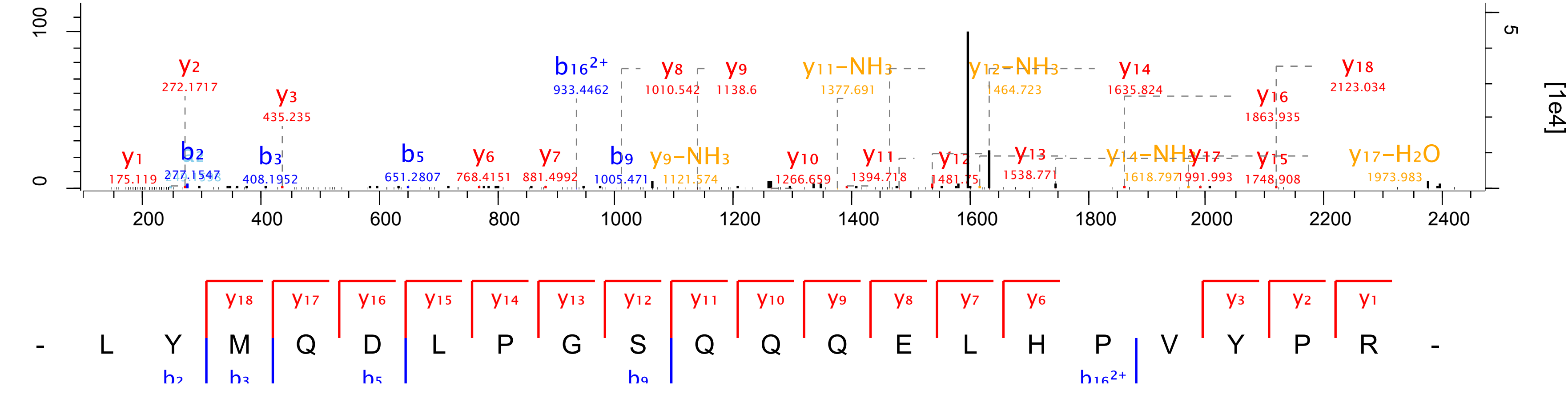

Raw file	Scan	Method	Score	m/z	Gene names
20150402_CerP14_Frac10_top_opt_B10_01_1819	36418	TOF; CID	76.85	575.32	Btg1

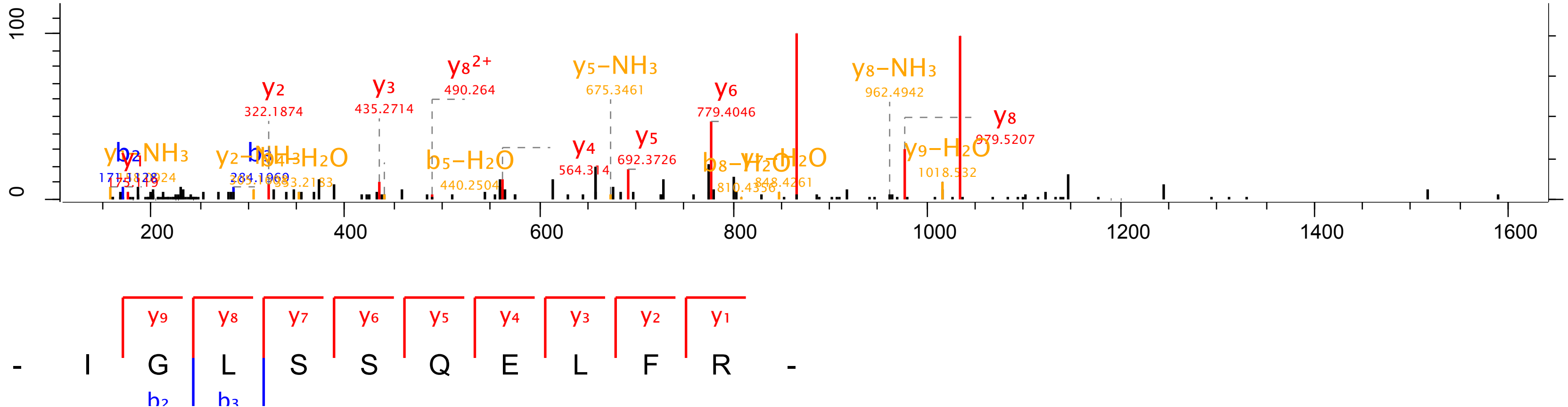

Raw file	Scan	Method	Score	m/z	Gene names
20150402_CerP14_Frac10_top_opt_B10_01_1819	37127	TOF; CID	46.43	1050.55	Cipc

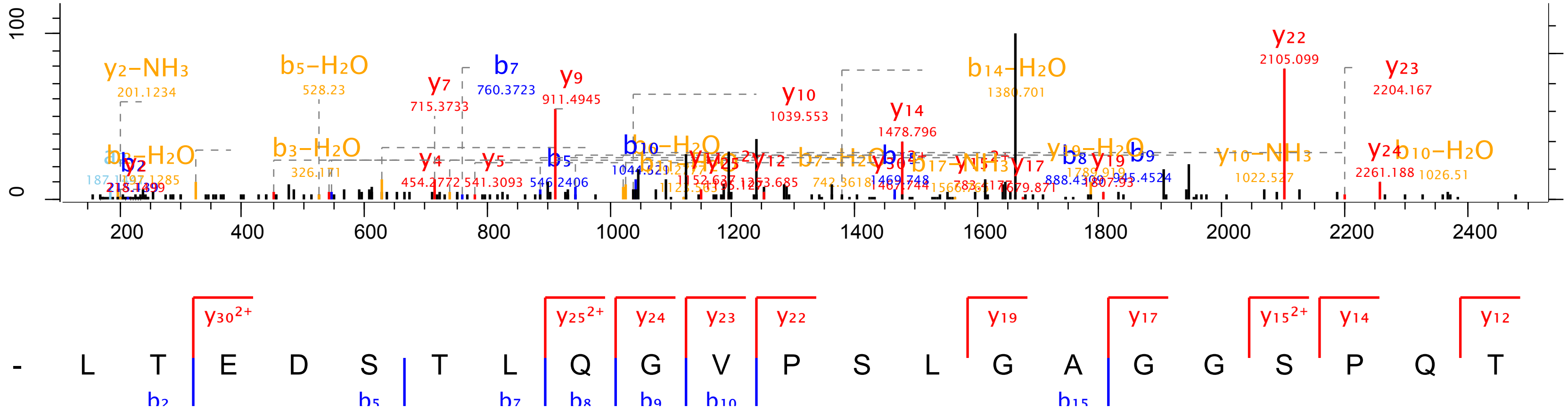

Raw file	Scan	Method	Score	m/z	Gene names
20150402_CerP14_Frac10_top_opt_B10_01_1819	45842	TOF; CID	45.62	642.34	Enpp3

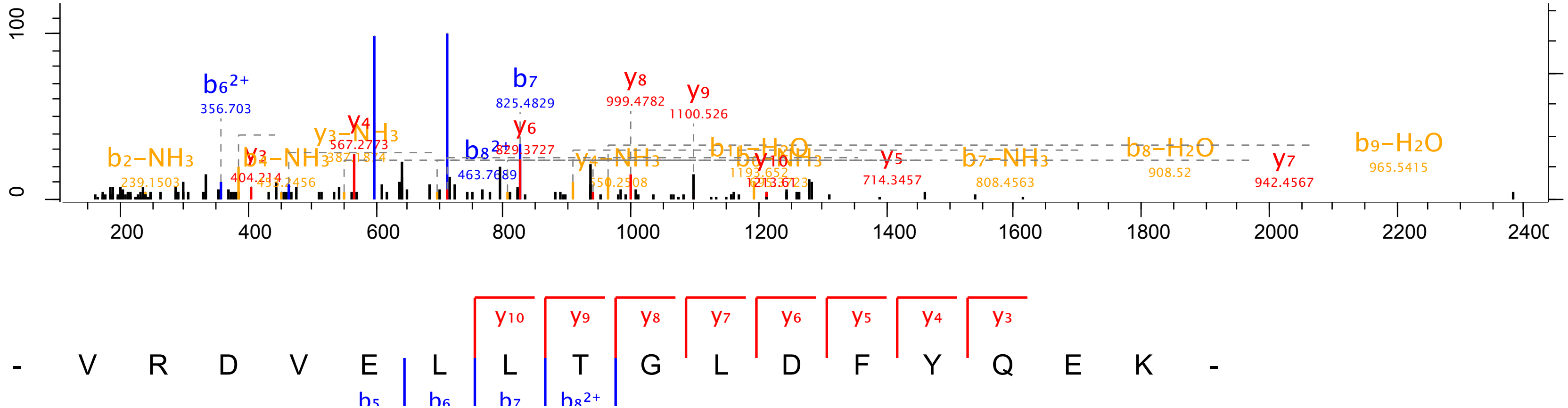

Raw file	Scan	Method	Score	m/z	Gene names
20150402_CerP14_Frac10_top_opt_B10_01_1819	45921	TOF; CID	62	644	Mal2

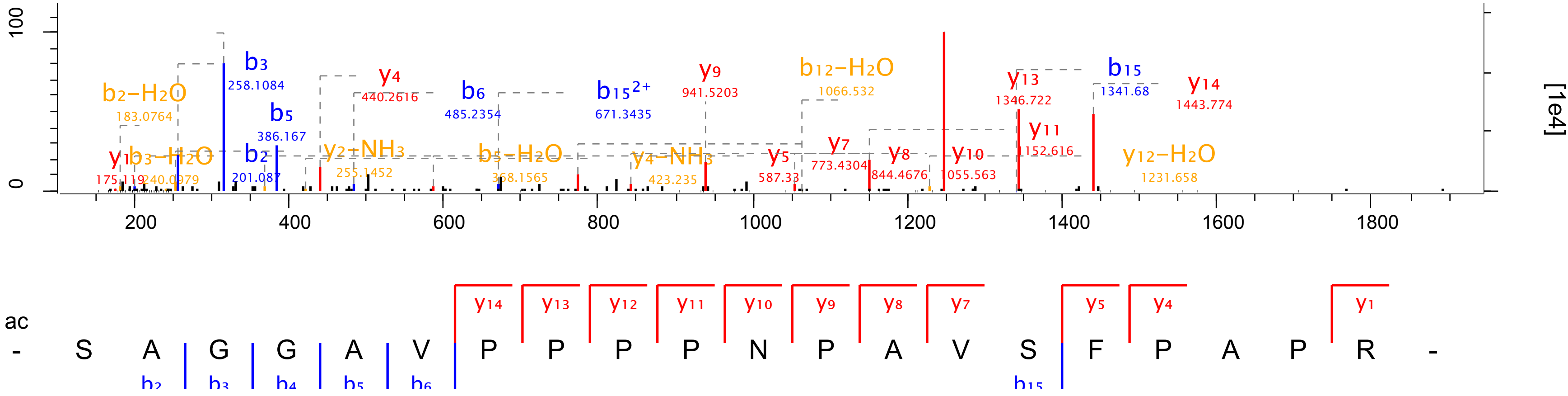

Raw file	Scan	Method	Score	m/z	Gene names
20150402_CerP14_Frac10_top_opt_B10_01_1819	49297	TOF; CID	94.69	758.9	Tac3

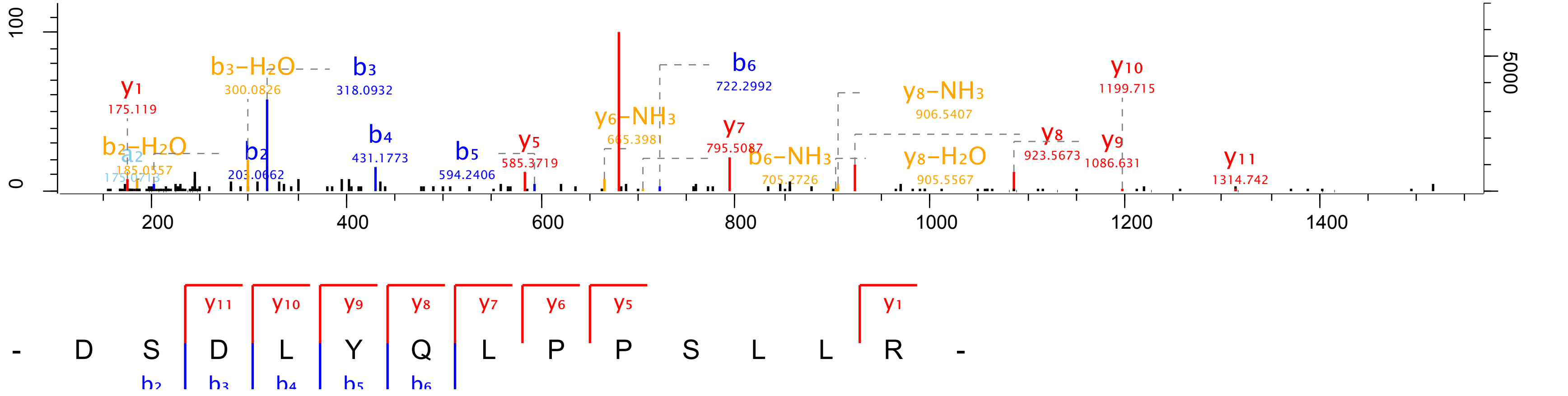

Raw file	Scan	Method	Score	m/z	Gene names
20150402_CerP14_Frac10_top_opt_B10_01_1819	50354	TOF; CID	98.94	495.94	Pcdh20

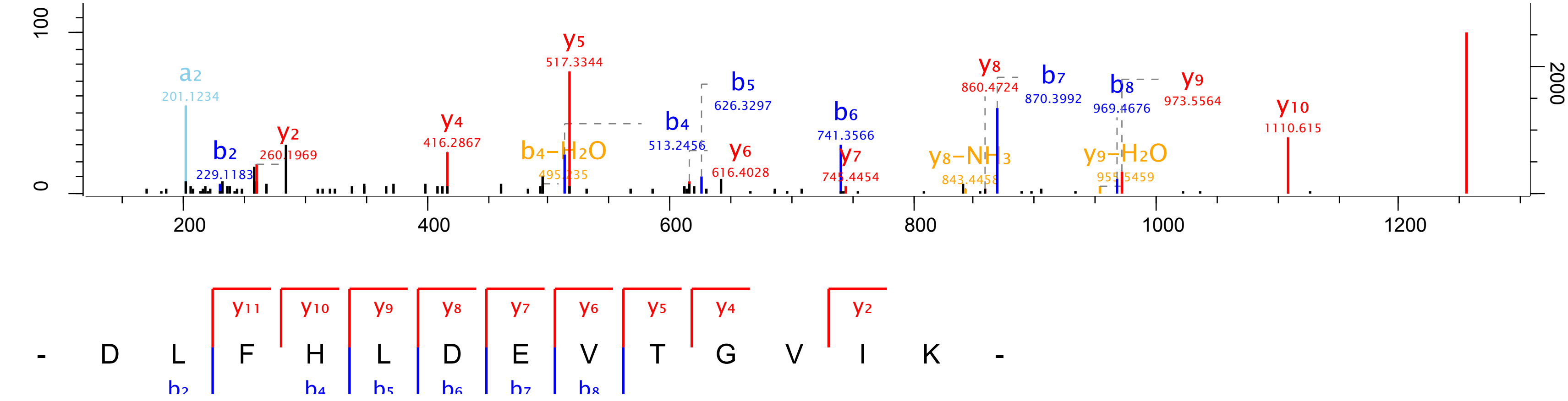

Raw file	Scan	Method	Score	m/z	Gene names
20150402_CerP14_Frac10_top_opt_B10_01_1819	56819	TOF; CID	34.15	705.72	Rnf217

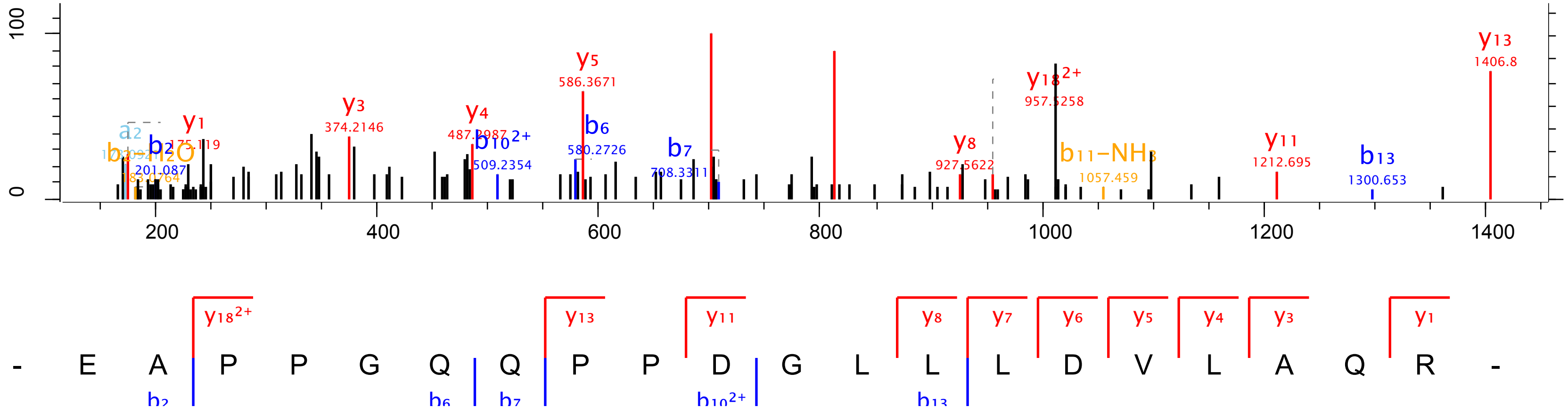

Raw file	Scan	Method	Score	m/z	Gene names
20150402_CerP14_Frac10_top_opt_B10_01_1819	56865	TOF; CID	102.21	984.5	Fads3

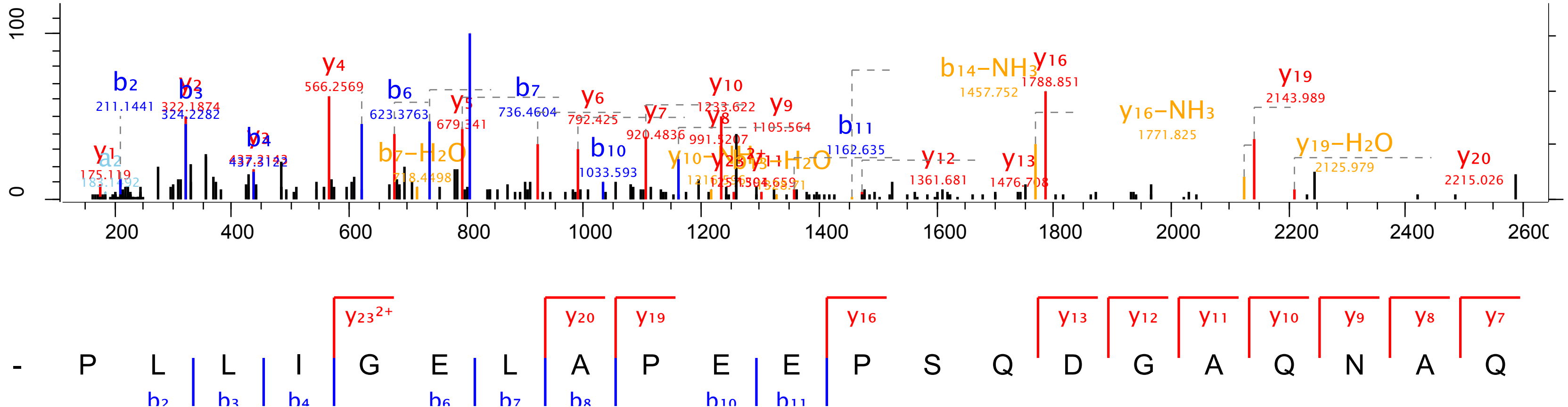

Raw file	Scan	Method	Score	m/z	Gene names
20150402_CerP14_Frac11_top_opt_B11_01_1820	3400	TOF; CID	76.06	498.27	Ankrd6

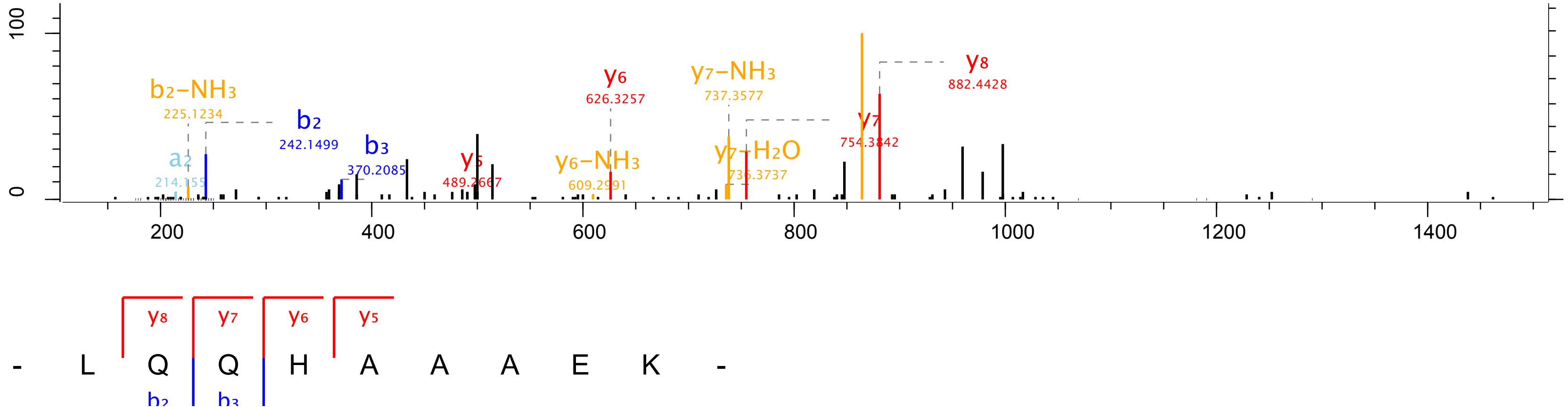

Raw file	Scan	Method	Score	m/z	Gene names
20150402_CerP14_Frac11_top_opt_B11_01_1820	7201	TOF; CID	68.54	553.78	Snrnp25

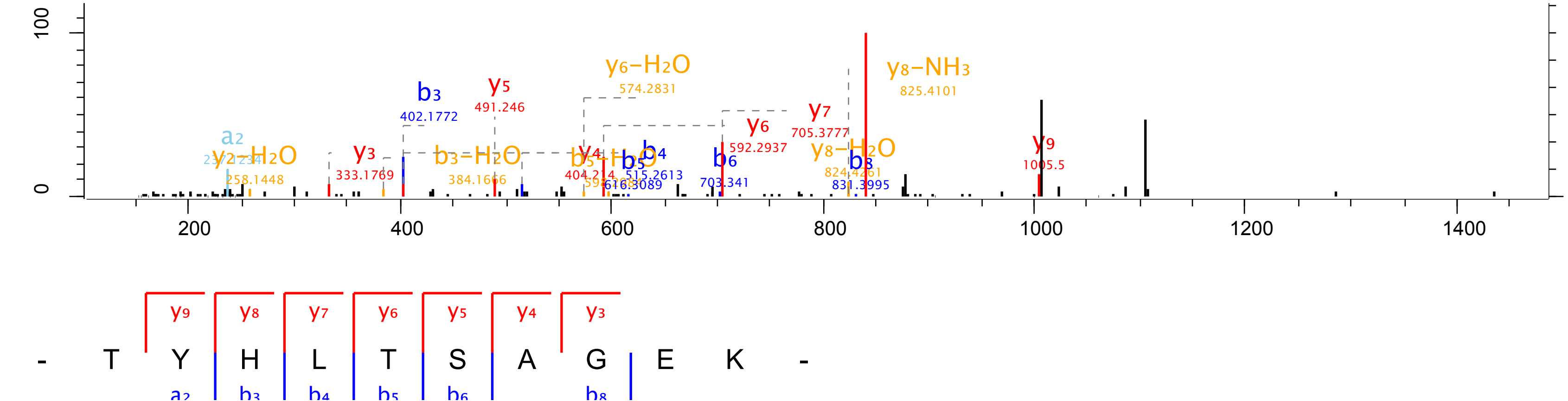

Raw file	Scan	Method	Score	m/z	Gene names
20150402_CerP14_Frac11_top_opt_B11_01_1820	11127	TOF; CID	67.55	614.33	Hps1

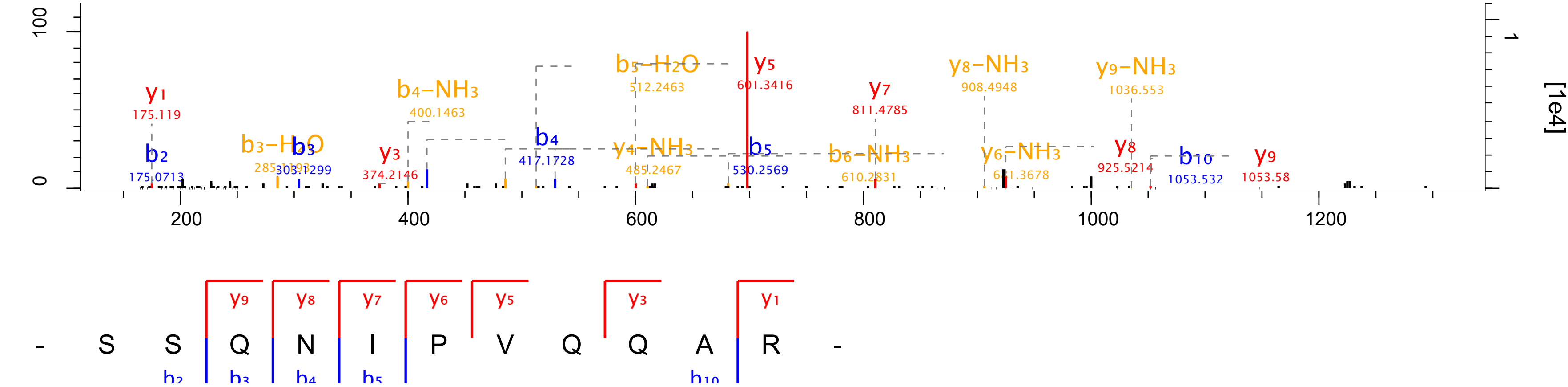

Raw file	Scan	Method	Score	m/z	Gene names
20150402_CerP14_Frac11_top_opt_B11_01_1820	14207	TOF; CID	49.59	758.37	Fzd9

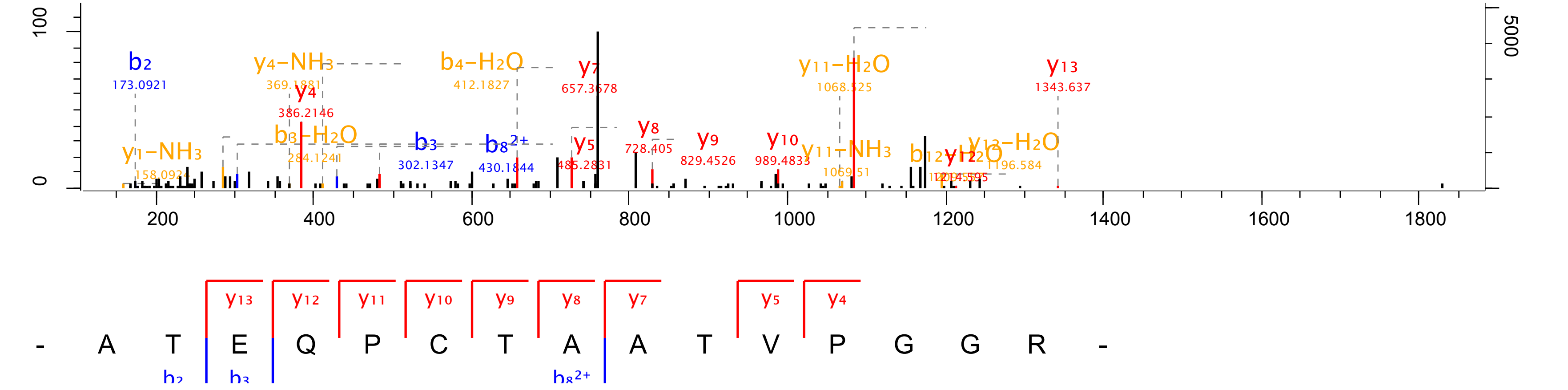

Raw file	Scan	Method	Score	m/z	Gene names
20150402_CerP14_Frac11_top_opt_B11_01_1820	16924	TOF; CID	68.04	789.32	Dlk1

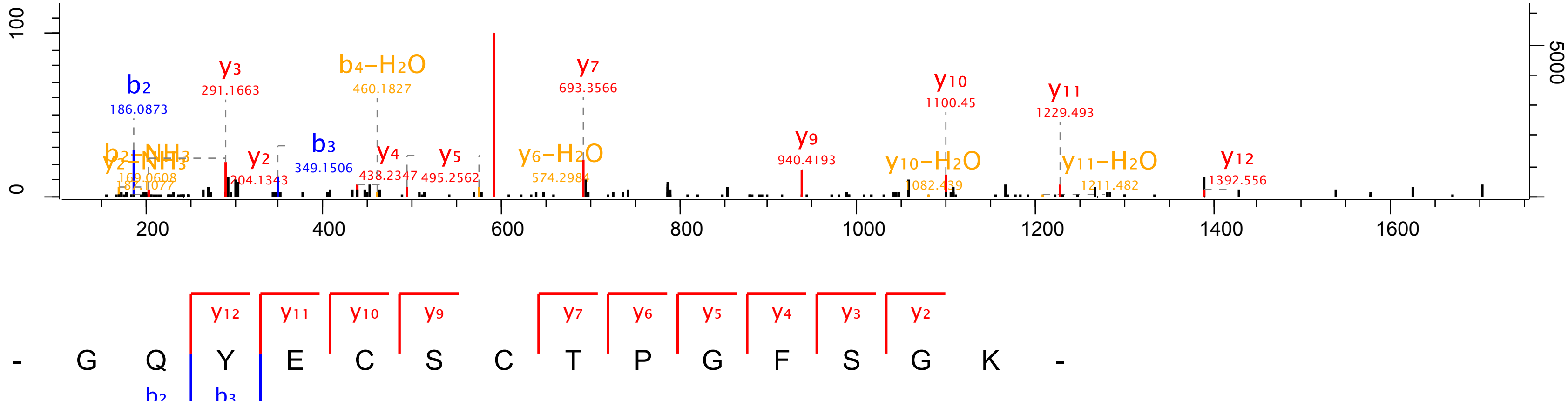

Raw file	Scan	Method	Score	m/z	Gene names
20150402_CerP14_Frac11_top_opt_B11_01_1820	22011	TOF; CID	56.79	532.24	Tmbim1

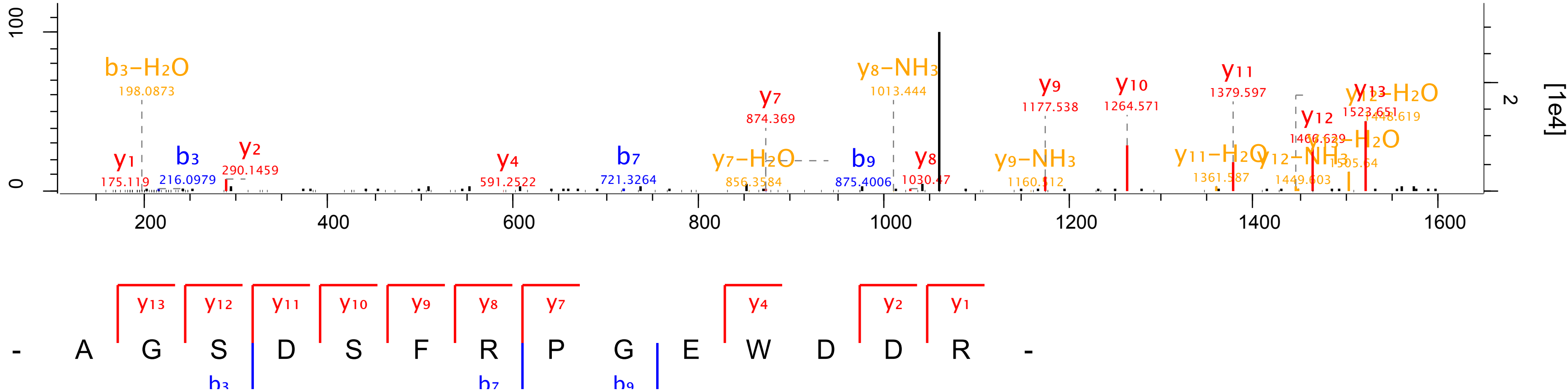

Raw file	Scan	Method	Score	m/z	Gene names
20150402_CerP14_Frac11_top_opt_B11_01_1820	24938	TOF; CID	55.75	825.88	Zbtb5

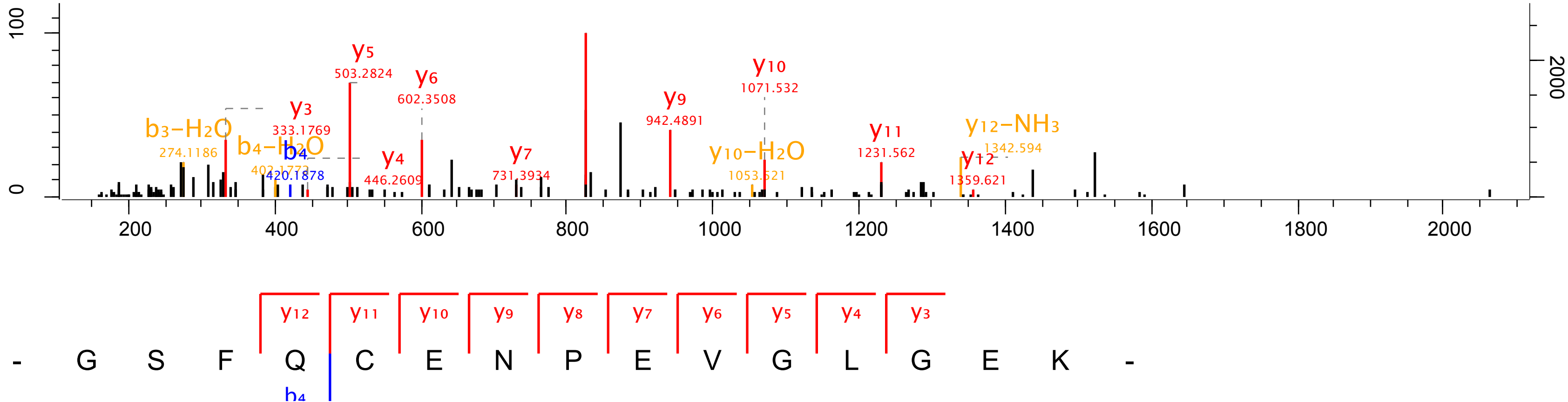

Raw file	Scan	Method	Score	m/z	Gene names
20150402_CerP14_Frac11_top_opt_B11_01_1820	26310	TOF; CID	69.86	626.83	Pigw

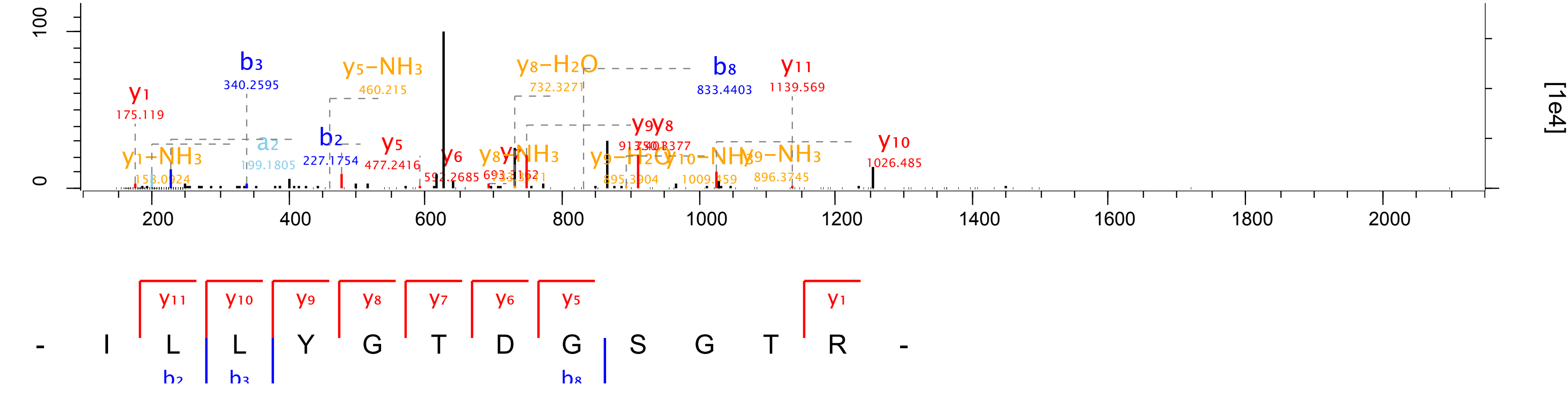

Raw file	Scan	Method	Score	m/z	Gene names
20150402_CerP14_Frac11_top_opt_B11_01_1820	26374	TOF; CID	133.86	832.9	A430005L14Rik

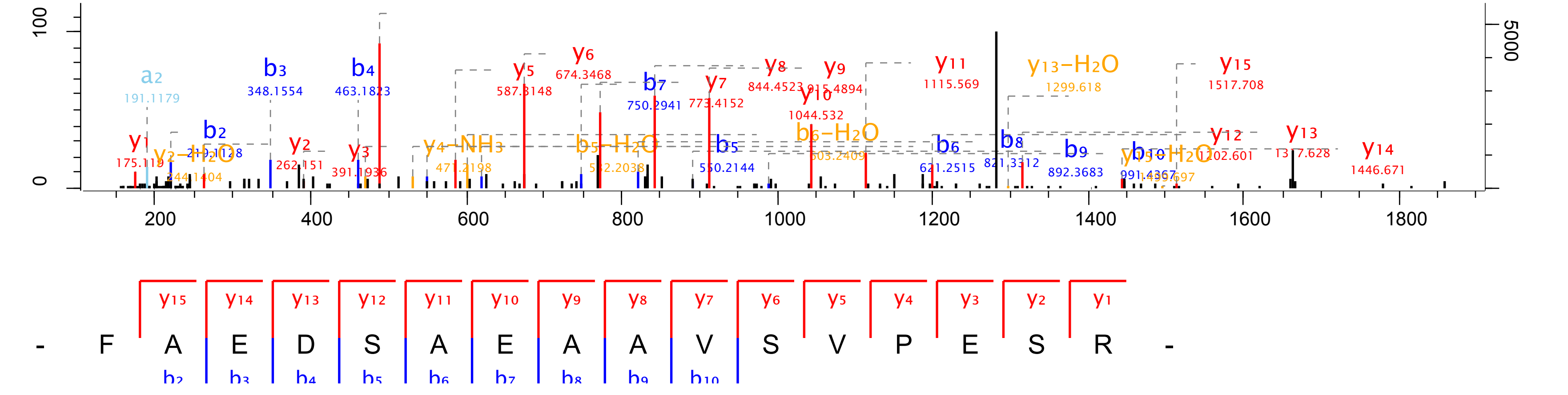

Raw file	Scan	Method	Score	m/z	Gene names
20150402_CerP14_Frac11_top_opt_B11_01_1820	28353	TOF; CID	58.25	480.77	Zfp688

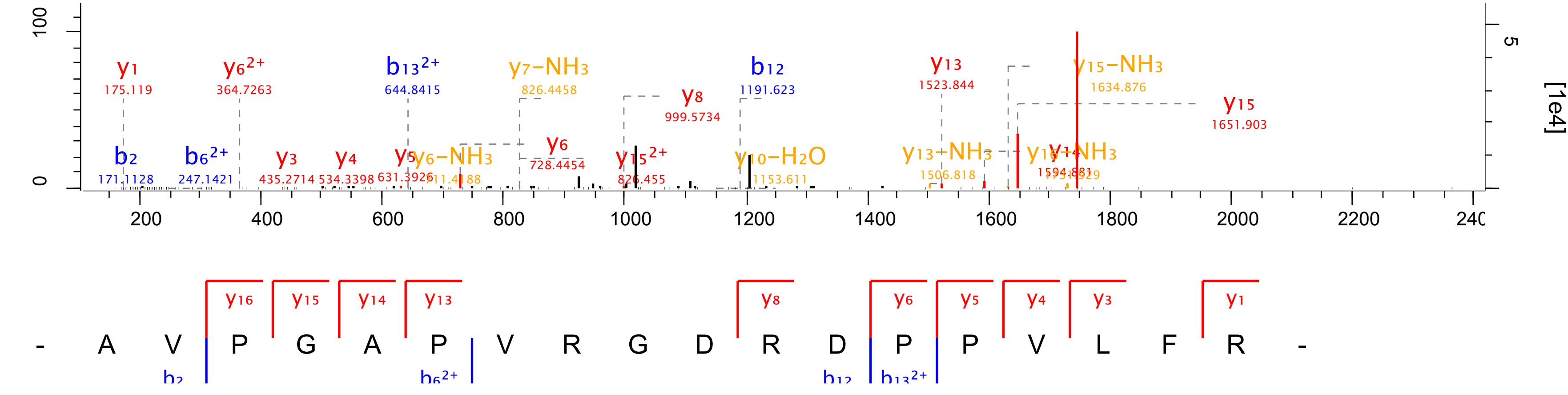

Raw file	Scan	Method	Score	m/z	Gene names
20150402_CerP14_Frac11_top_opt_B11_01_1820	28861	TOF; CID	100.23	561.31	Tmem251

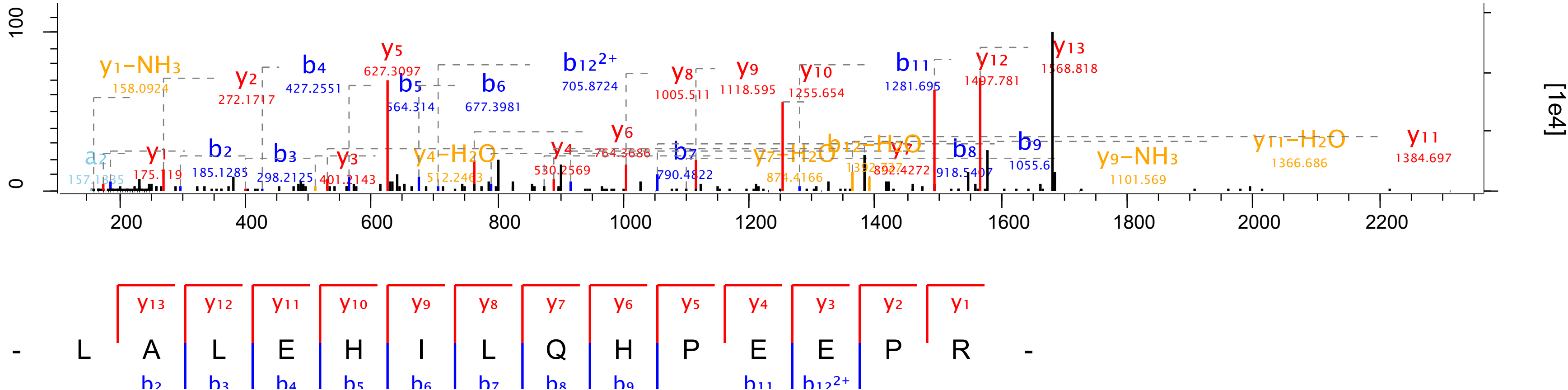

Raw file	Scan	Method	Score	m/z	Gene names
20150402_CerP14_Frac11_top_opt_B11_01_1820	28924	TOF; CID	53.17	565.28	Igf1

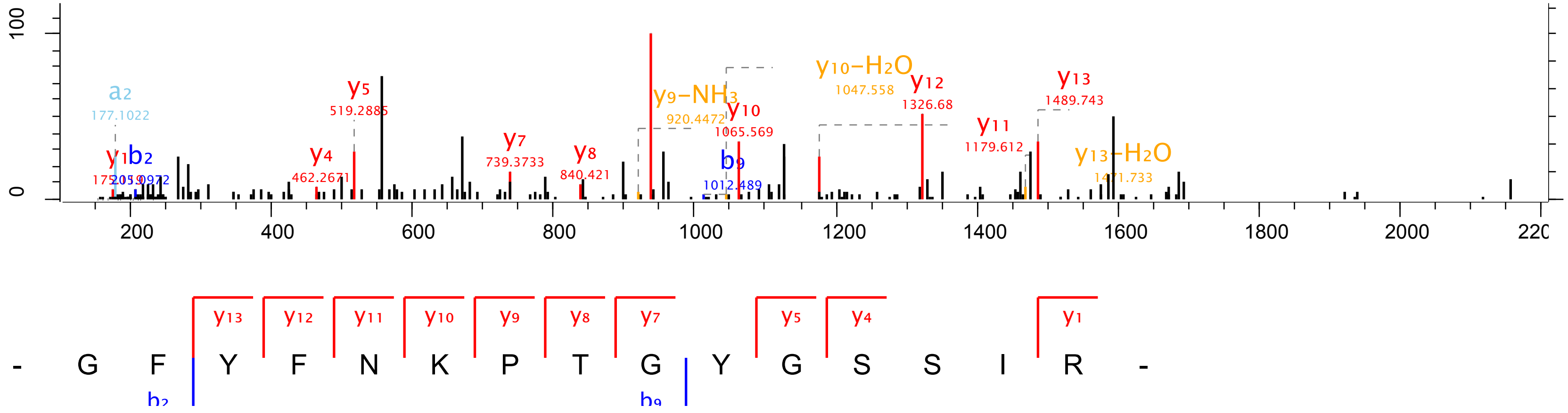

Raw file	Scan	Method	Score	m/z	Gene names
20150402_CerP14_Frac11_top_opt_B11_01_1820	31579	TOF; CID	65.22	737.89	Pou4f2;Pou4f3;Pou4f1

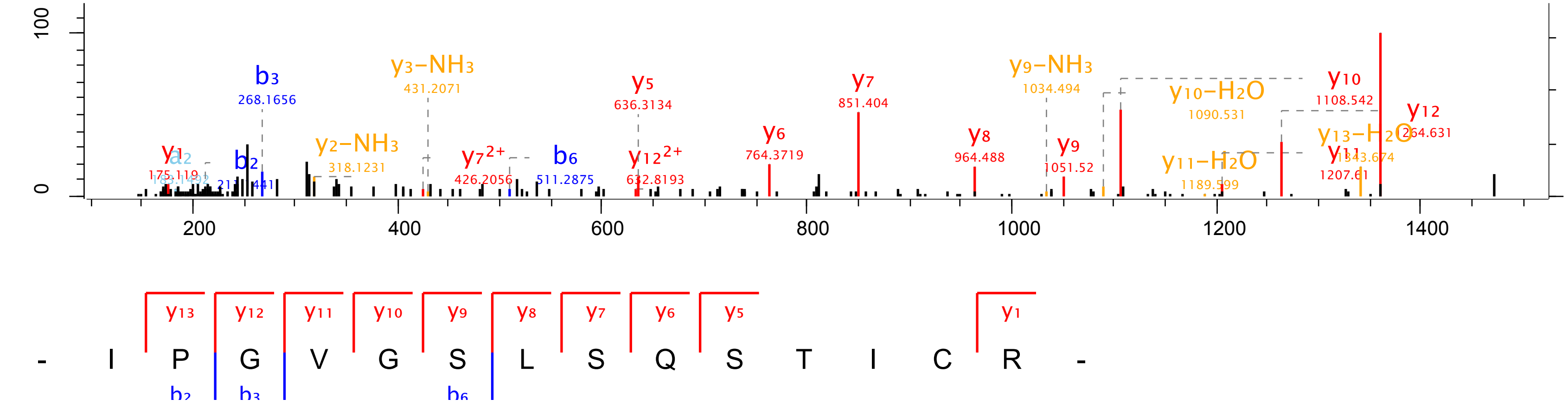

Raw file	Scan	Method	Score	m/z	Gene names
20150402_CerP14_Frac11_top_opt_B11_01_1820	36973	TOF; CID	52.17	632.33	Cpn2

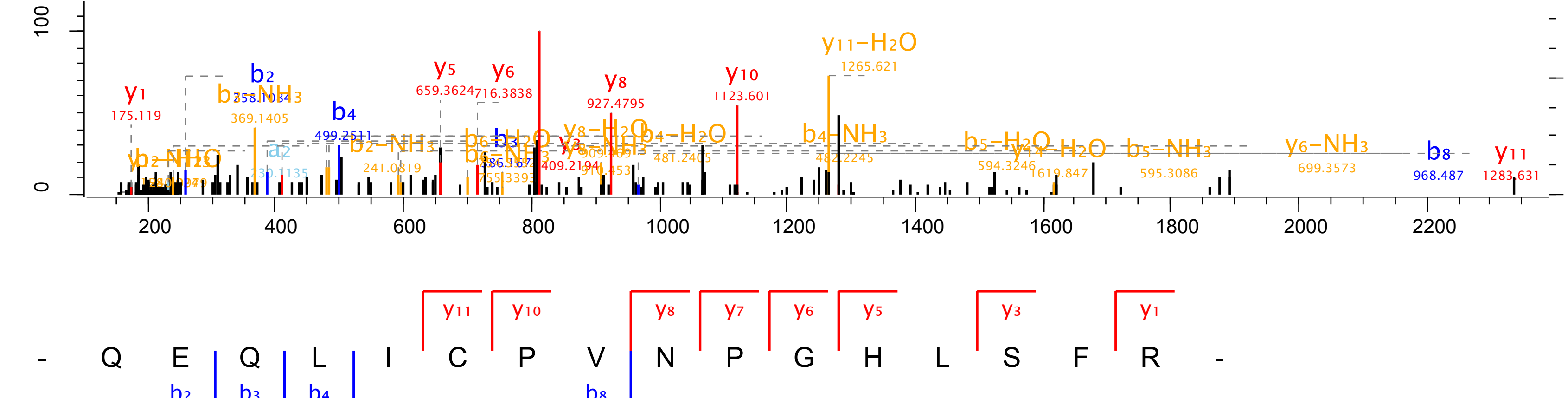

Raw file	Scan	Method	Score	m/z	Gene names
20150402_CerP14_Frac11_top_opt_B11_01_1820	41223	TOF; CID	69.07	807.43	9430015G10Rik

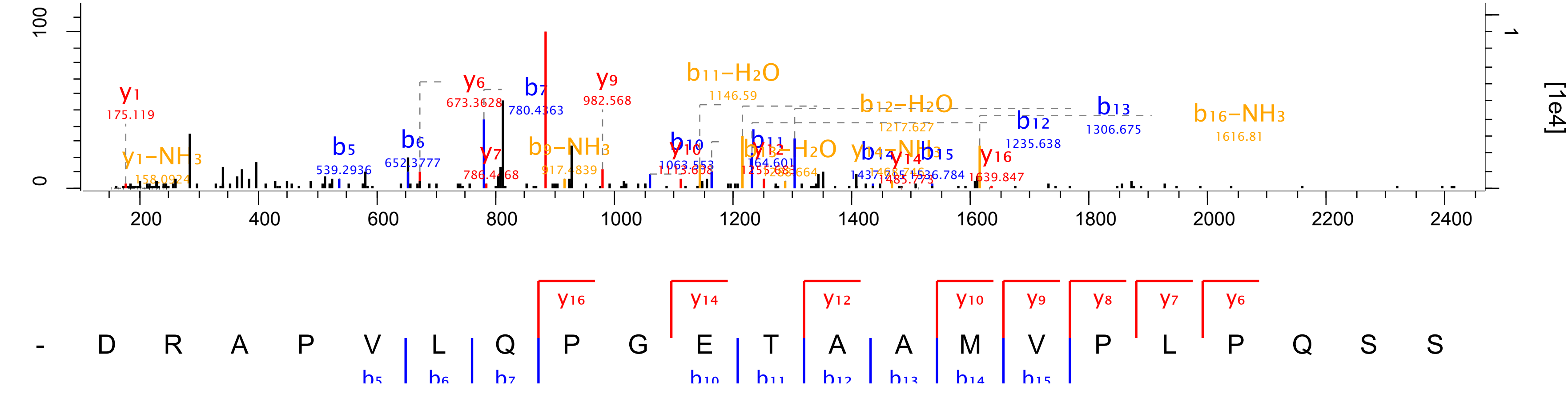

Raw file	Scan	Method	Score	m/z	Gene names
20150402_CerP14_Frac11_top_opt_B11_01_1820	41287	TOF; CID	67.38	647.86	Snn

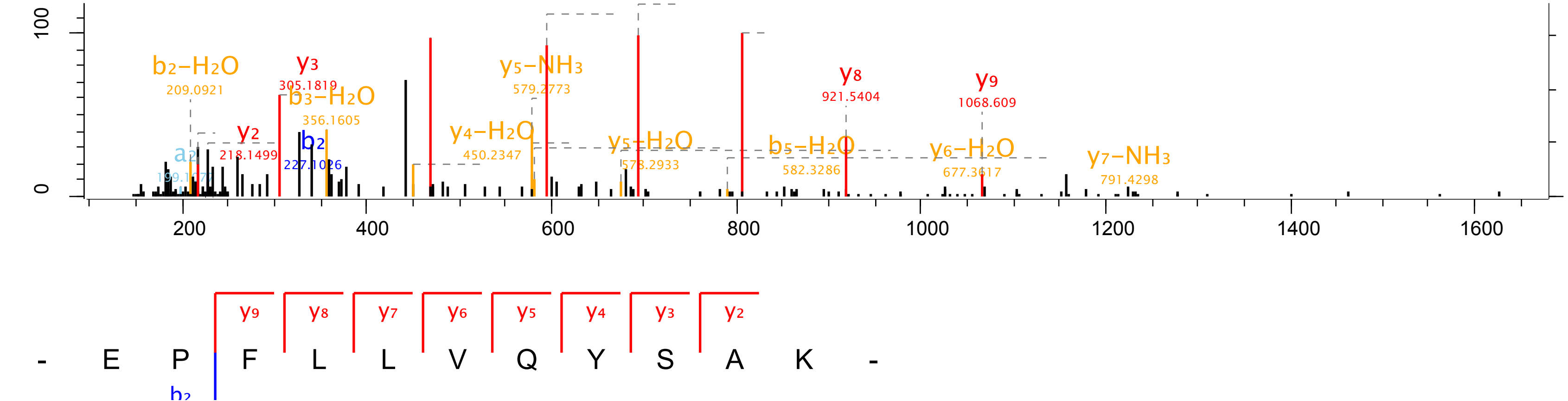

Raw file	Scan	Method	Score	m/z	Gene names
20150402_CerP14_Frac11_top_opt_B11_01_1820	50038	TOF; CID	95.5	472.28	Fgf11

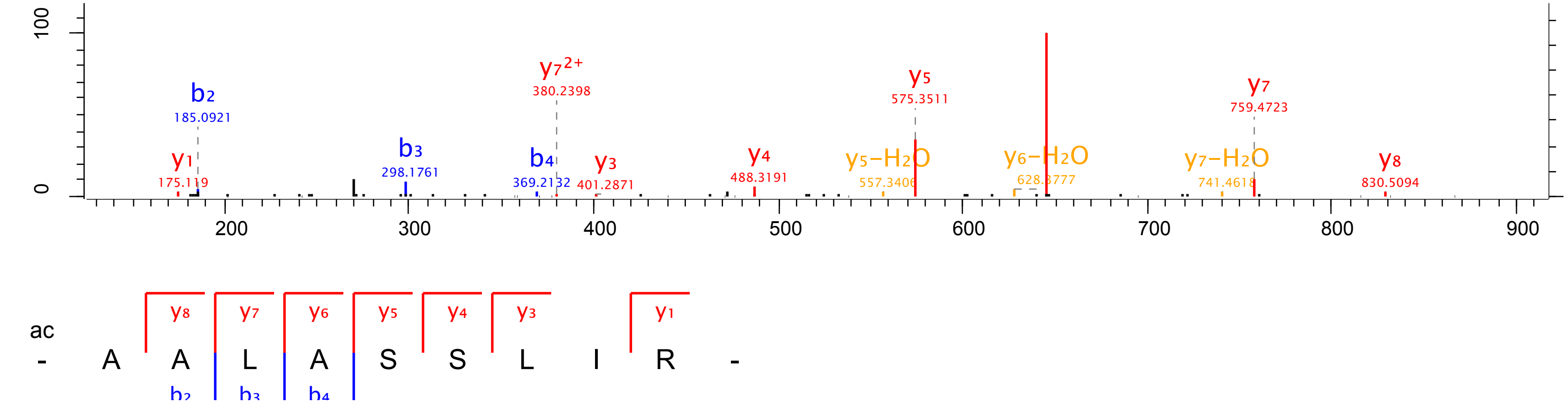

Raw file	Scan	Method	Score	m/z	Gene names
20150402_CerP14_Frac11_top_opt_B11_01_1820	50503	TOF; CID	52.17	1035.04	Rhox2a;Rhox2h;Rhox2c;Rhox2f;Rhox2b

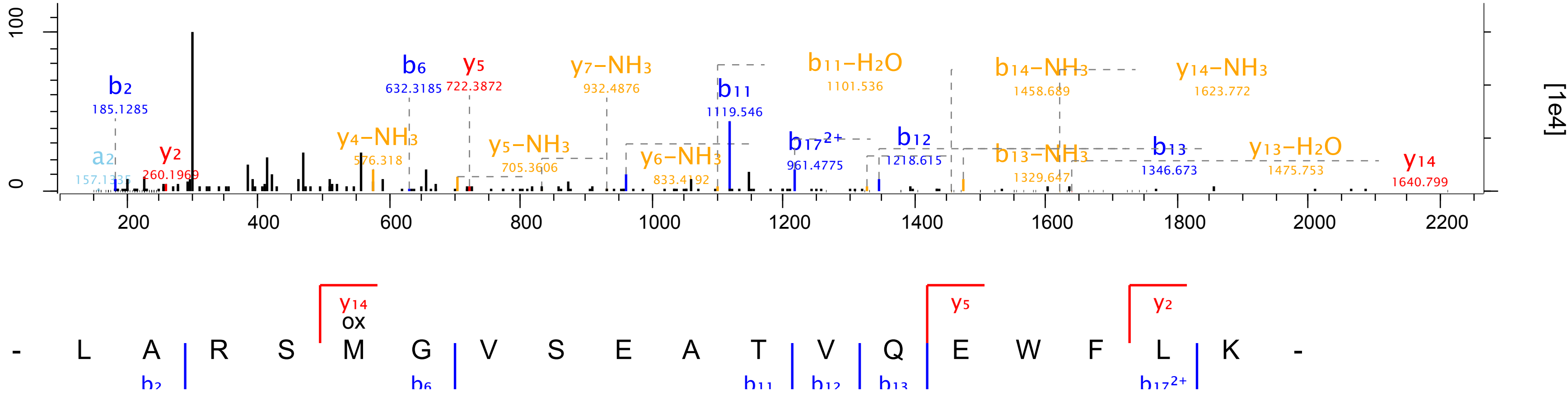

Raw file	Scan	Method	Score	m/z	Gene names
20150402_CerP14_Frac11_top_opt_B11_01_1820	51894	TOF; CID	204.96	1065.51	Agtrap

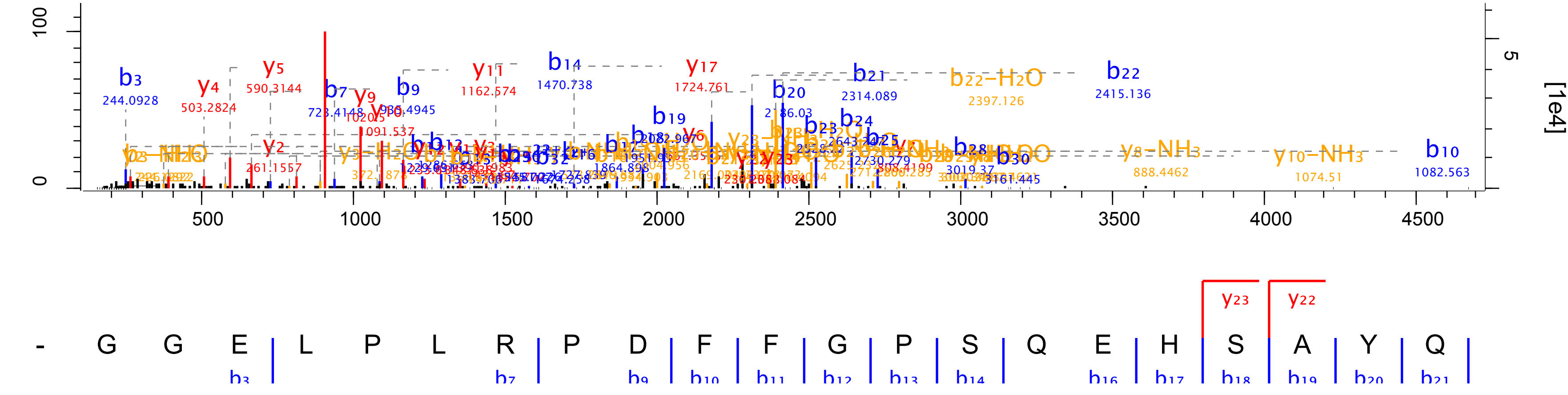

Raw file	Scan	Method	Score	m/z	Gene names
20150402_CerP14_Frac11_top_opt_B11_01_1820	54841	TOF; CID	53.55	1132.02	Caly

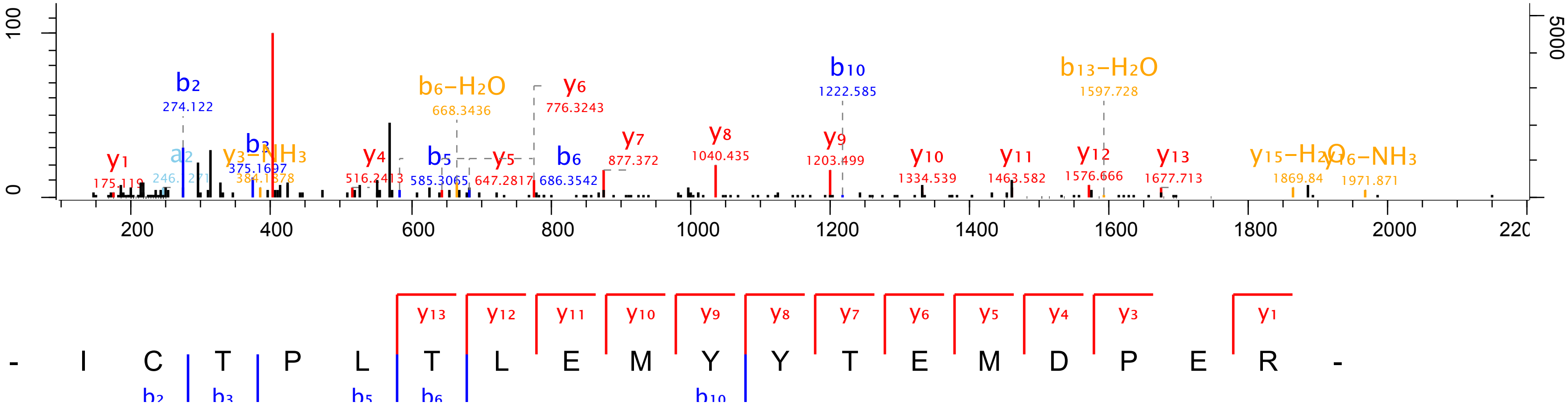

Raw file	Scan	Method	Score	m/z	Gene names
20150402_CerP14_Frac11_top_opt_B11_01_1820	57778	TOF; CID	113.76	754.85	Sec61g

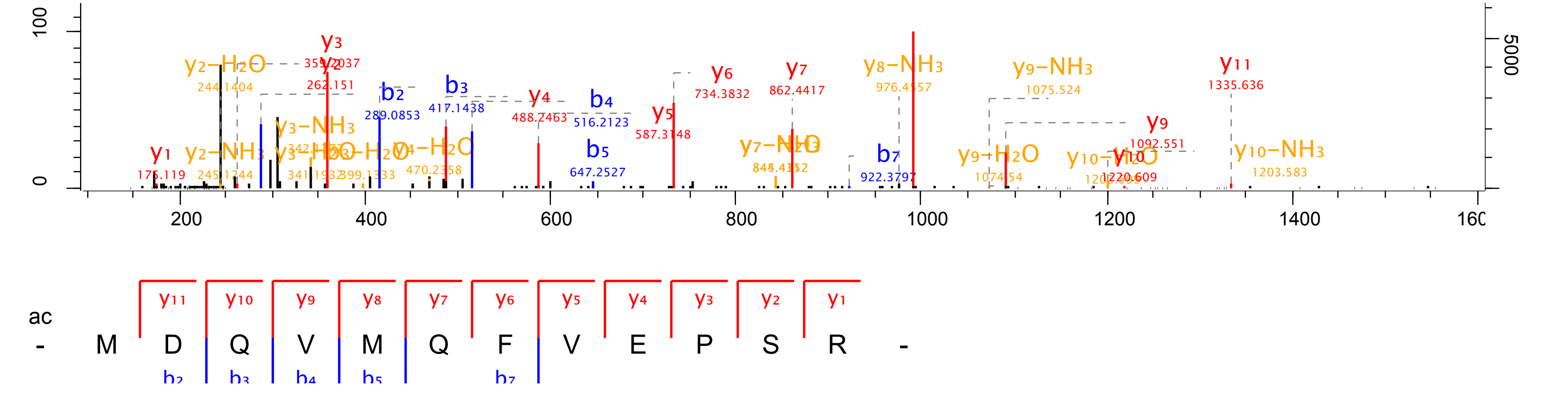

Raw file	Scan	Method	Score	m/z	Gene names
20150402_CerP14_Frac11_top_opt_B11_01_1820	58746	TOF; CID	47.64	903.47	Klrb1

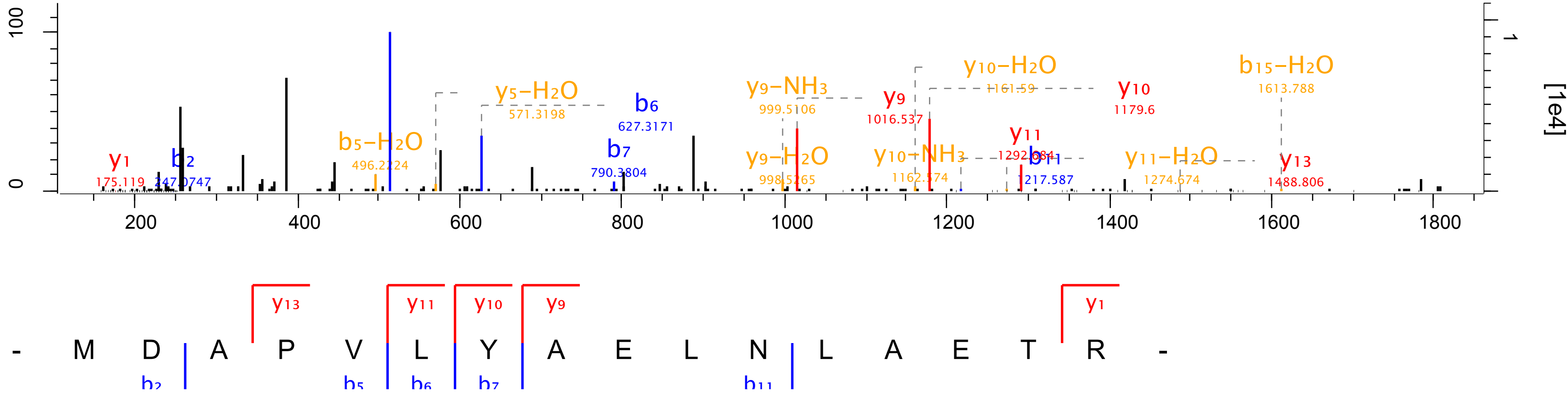

Raw file	Scan	Method	Score	m/z	Gene names
20150402_CerP14_Frac12_top_opt_B12_01_1821	11255	TOF; CID	135.09	589.63	Tsen15

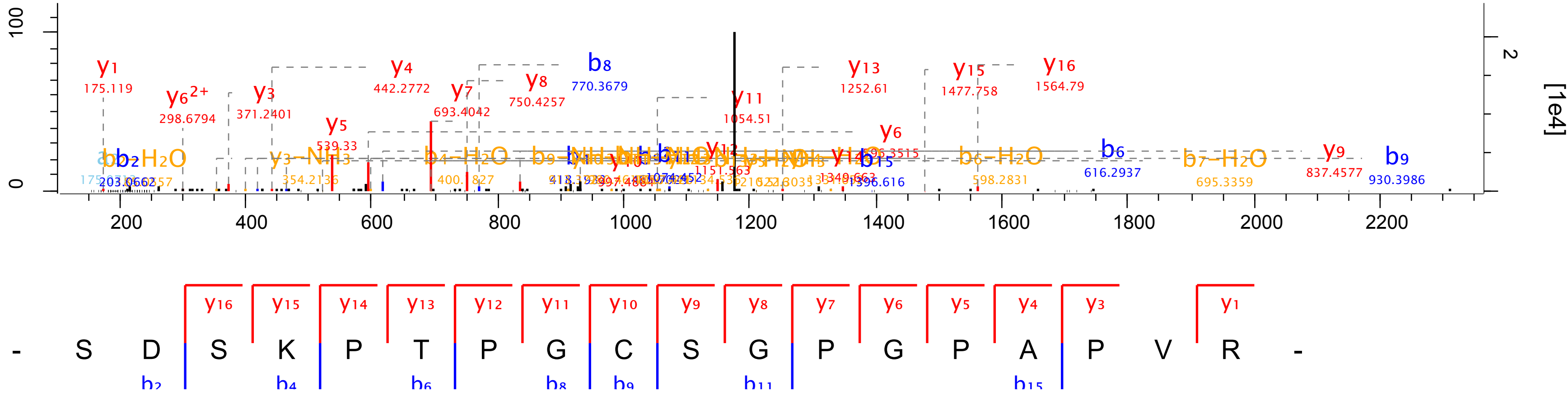

Raw file	Scan	Method	Score	m/z	Gene names
20150402_CerP14_Frac12_top_opt_B12_01_1821	13713	TOF; CID	57.71	863.93	Fndc5

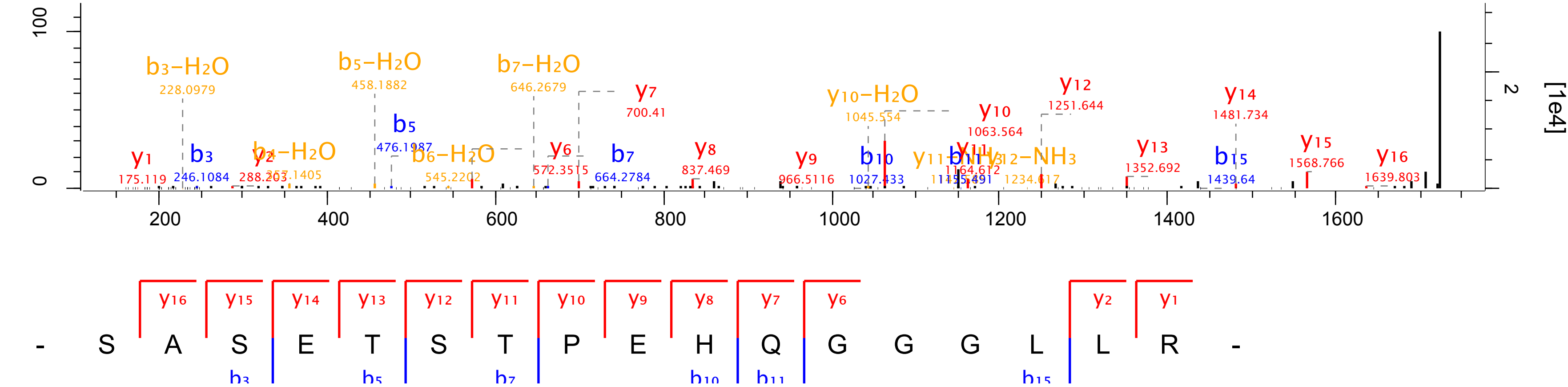

Raw file	Scan	Method	Score	m/z	Gene names
20150402_CerP14_Frac12_top_opt_B12_01_1821	15643	TOF; CID	62.09	656.84	Wnt7a

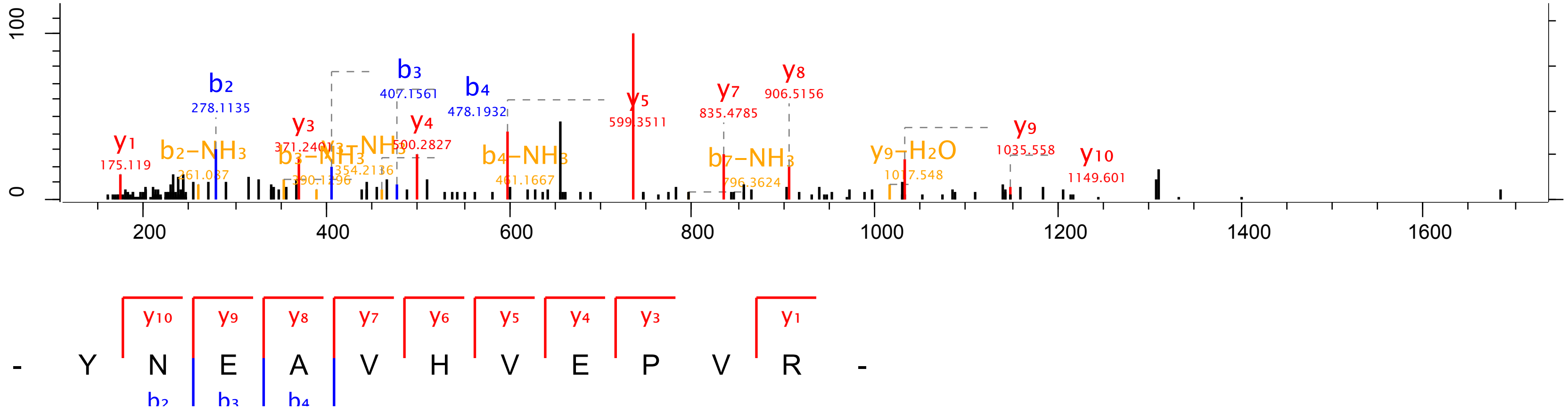

Raw file	Scan	Method	Score	m/z	Gene names
20150402_CerP14_Frac12_top_opt_B12_01_1821	16083	TOF; CID	61.82	599.96	E2f5

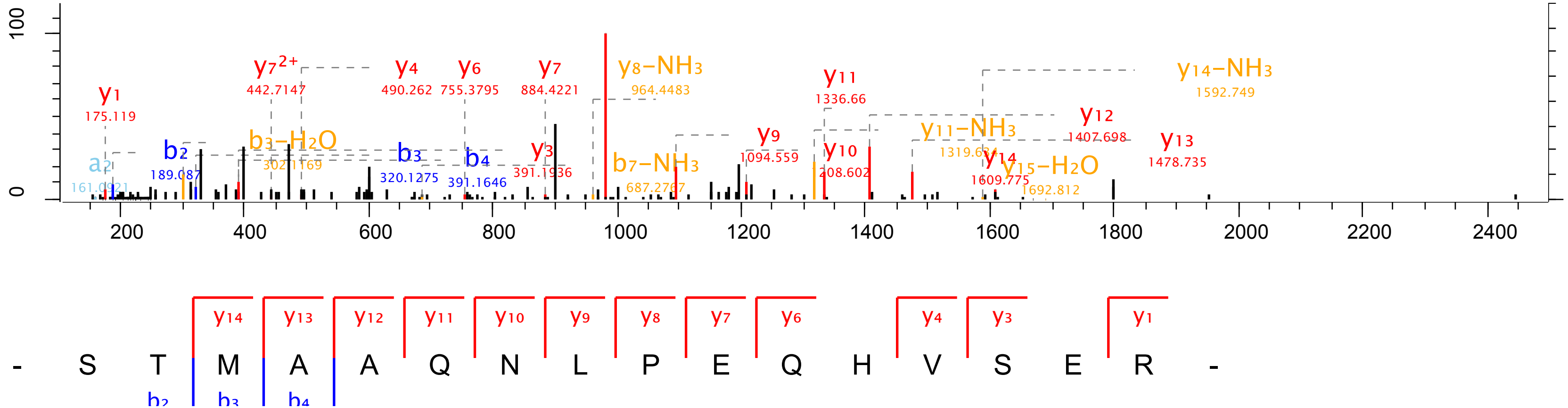

Raw file	Scan	Method	Score	m/z	Gene names
20150402_CerP14_Frac12_top_opt_B12_01_1821	16500	TOF; CID	85.54	720.32	Leprotl1

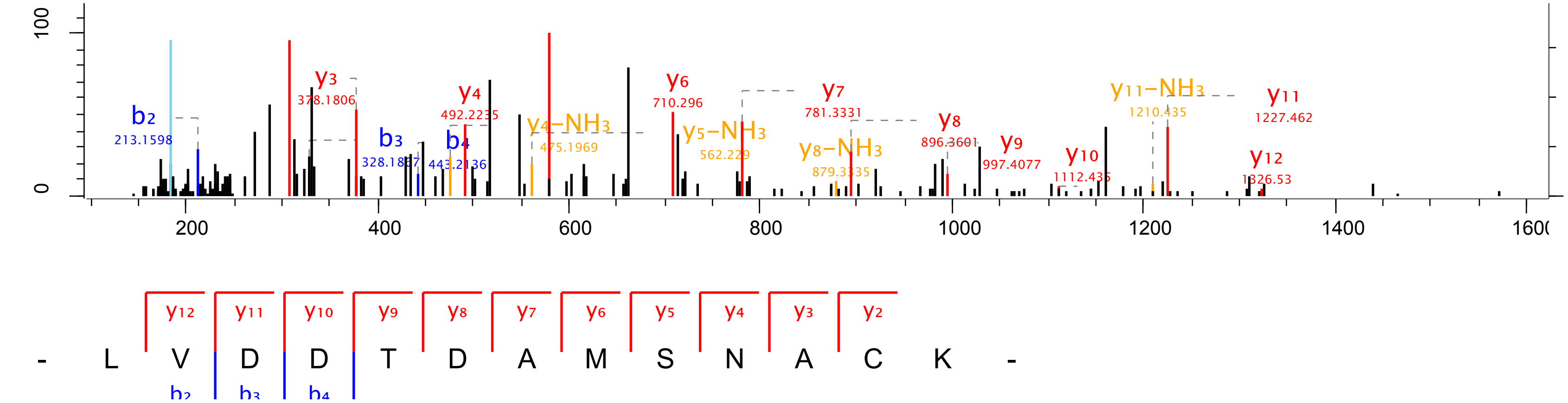

Raw file	Scan	Method	Score	m/z	Gene names
20150402_CerP14_Frac12_top_opt_B12_01_1821	19728	TOF; CID	67.52	666.32	Aph1b

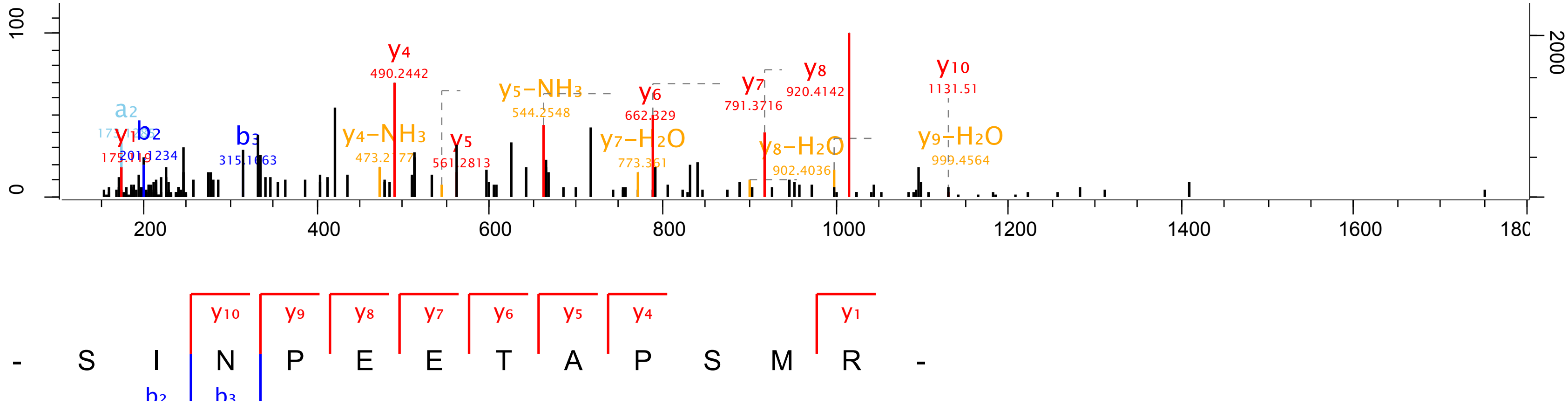

Raw file	Scan	Method	Score	m/z	Gene names
20150402_CerP14_Frac12_top_opt_B12_01_1821	31880	TOF; CID	69.72	454.56	Glp1r

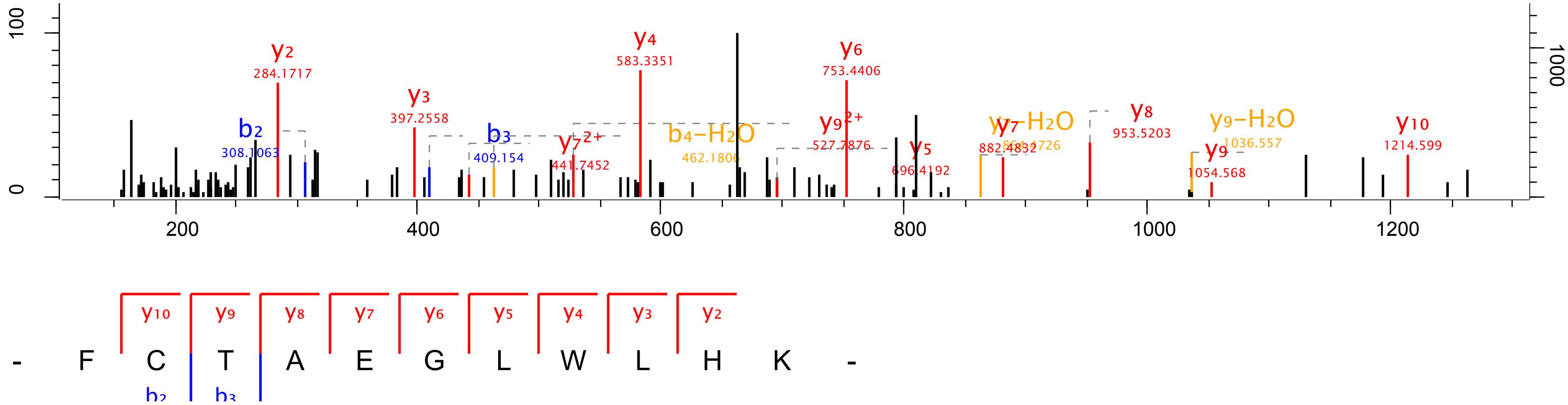

Raw file	Scan	Method	Score	m/z	Gene names
20150402_CerP14_Frac12_top_opt_B12_01_1821	31890	TOF; CID	56.42	710.31	Slc16a14

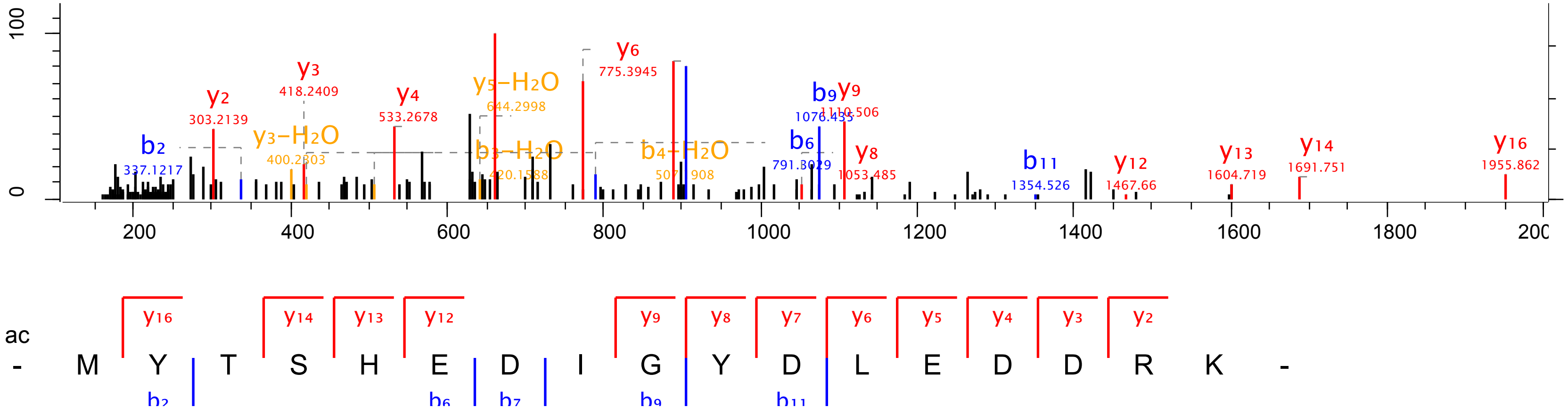

Raw file	Scan	Method	Score	m/z
20150402_CerP14_Frac12_top_opt_B12_01_1821	40020	TOF; CID	75.77	819.41

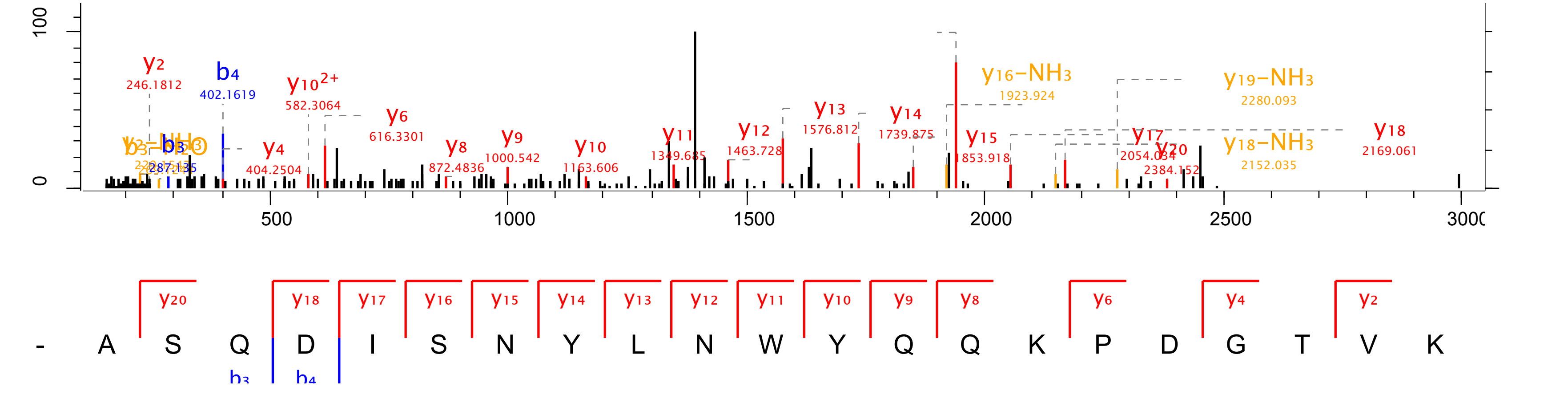

Raw file	Scan	Method	Score	m/z	Gene names
20150402_CerP14_Frac12_top_opt_B12_01_1821	40154	TOF; CID	58.17	658.87	Dscc1;DSCC1

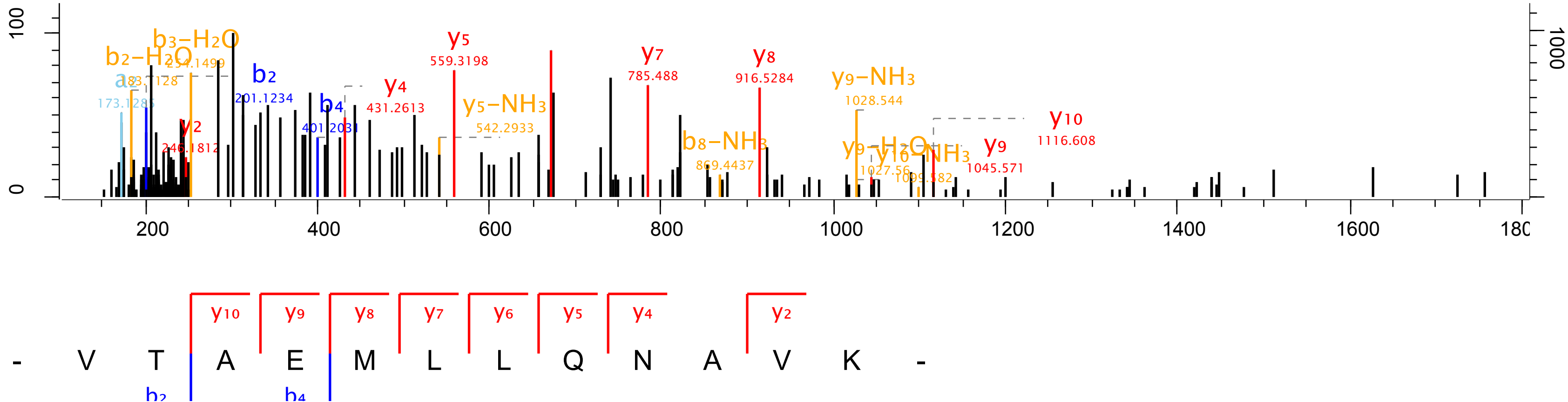

Raw file	Scan	Method	Score	m/z	Gene names
20150402_CerP14_Frac12_top_opt_B12_01_1821	40308	TOF; CID	129.85	629.82	Slc35b4

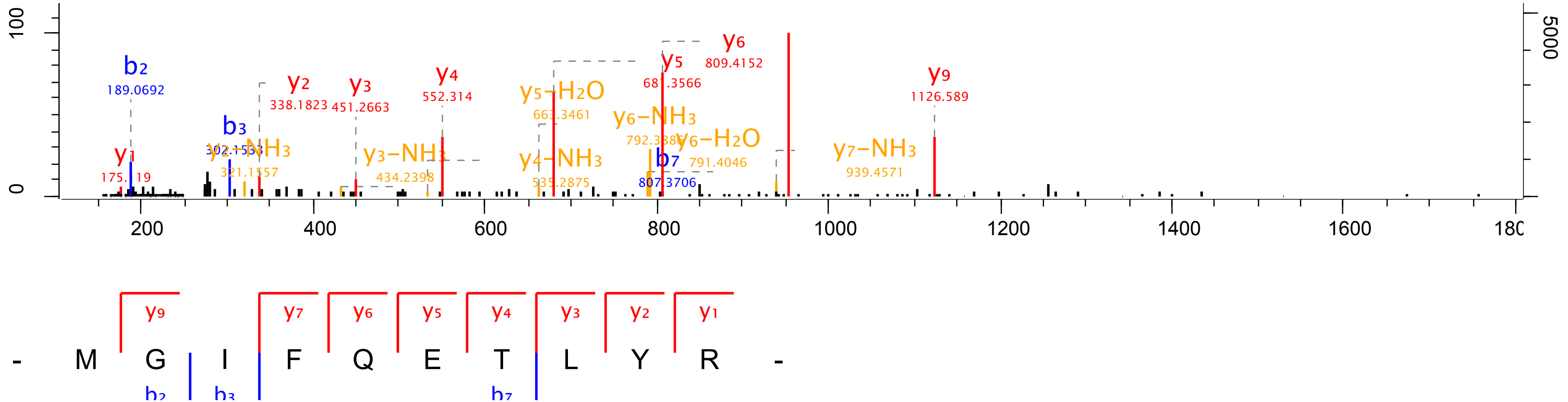

Raw file	Scan	Method	Score	m/z	Gene names
20150402_CerP14_Frac12_top_opt_B12_01_1821	44449	TOF; CID	57.37	762.05	Calcr

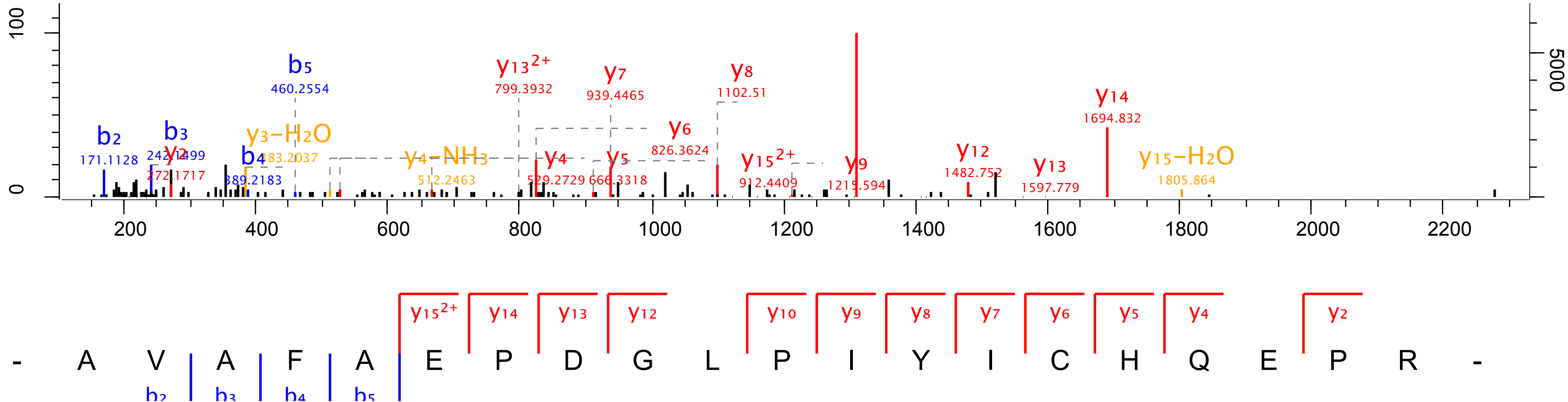

Raw file	Scan	Method	Score	m/z	Gene names
20150402_CerP14_Frac12_top_opt_B12_01_1821	50693	TOF; CID	58.25	1052.01	mt-Nd4l;Mtnd4l

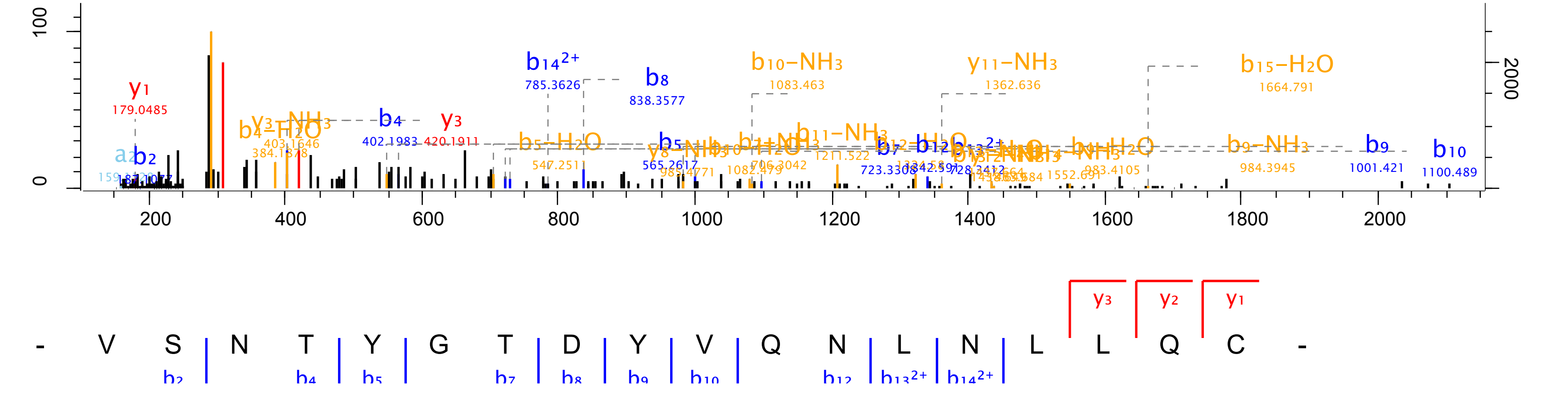

Raw file	Scan	Method	Score	m/z	Gene names
20150402_CerP14_Frac12_top_opt_B12_01_1821	52720	TOF; CID	58.71	645	Dapk2

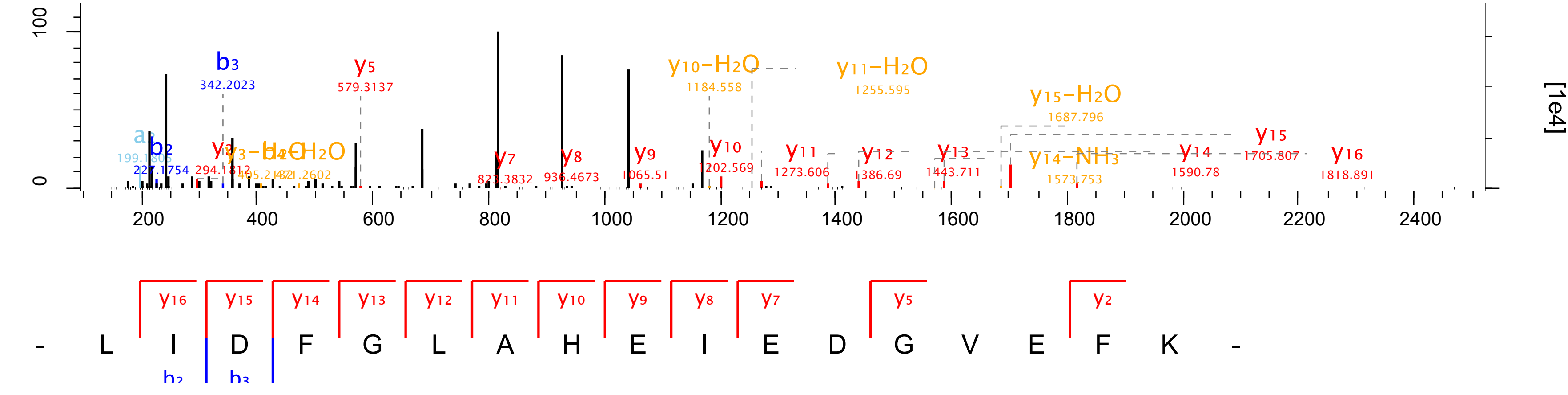

Raw file	Scan	Method	Score	m/z	Gene names
20150402_CerP14_Frac12_top_opt_B12_01_1821	54672	TOF; CID	45.92	1050.52	Ear10;Ear1;Ear2

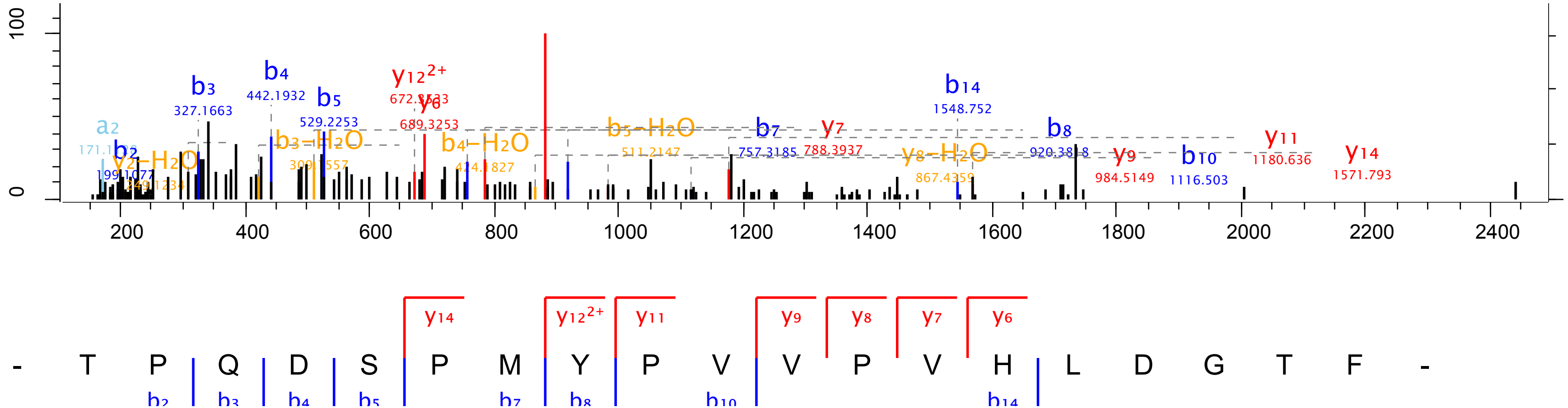

Raw file	Scan	Method	Score	m/z	Gene names
20150402_CerP14_Frac13_top_opt_C1_01_1822	5543	TOF; CID	85.29	494.26	Rhbdl3

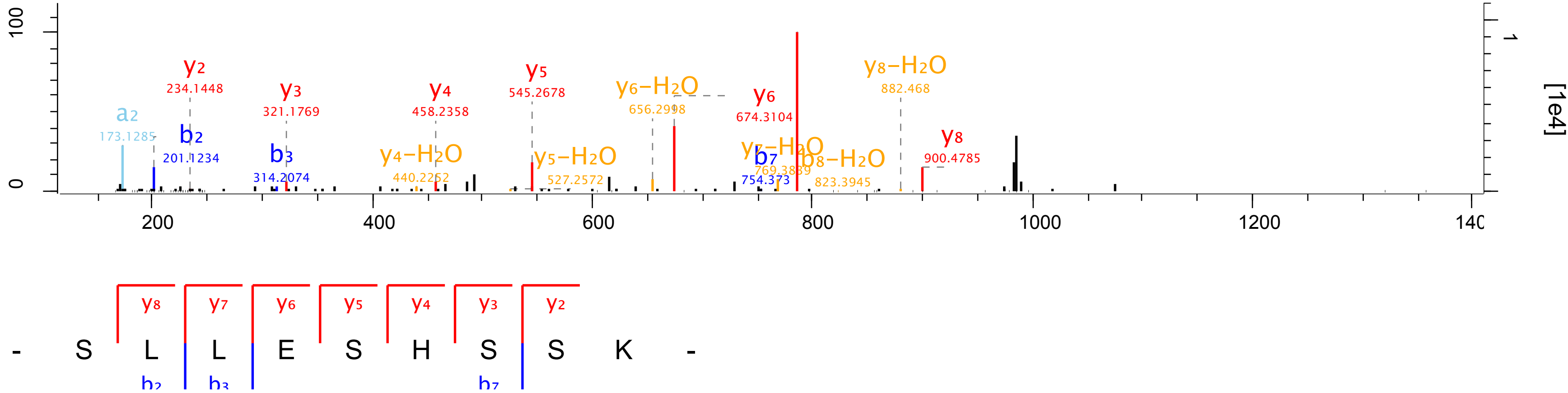

Raw file	Scan	Method	Score	m/z	Gene names
20150402_CerP14_Frac13_top_opt_C1_01_1822	6949	TOF; CID	155.64	797.91	mt-Co3

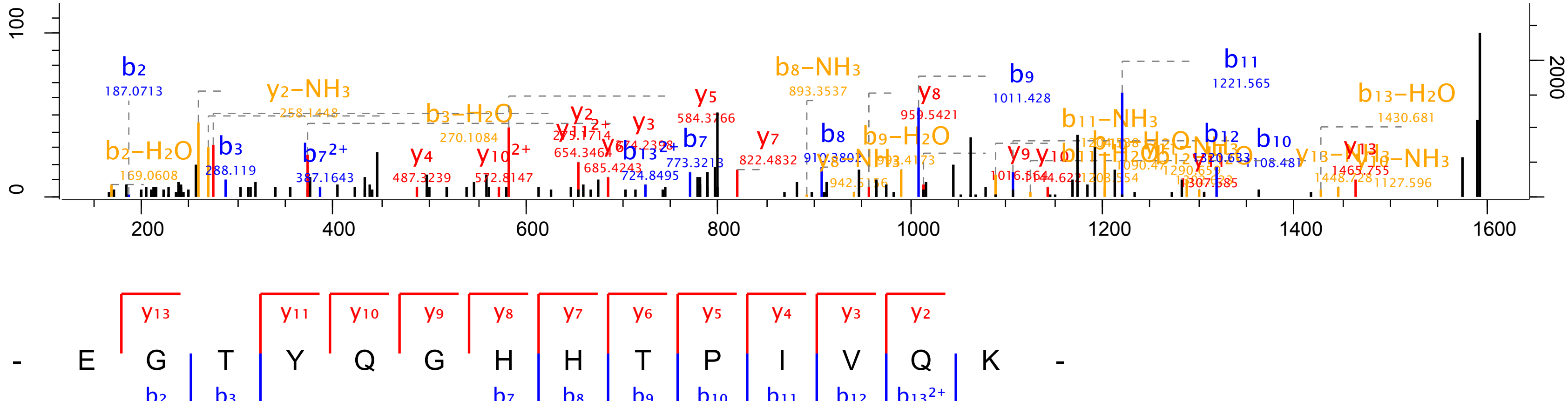

Raw file	Scan	Method	Score	m/z	Gene names
20150402_CerP14_Frac13_top_opt_C1_01_1822	9395	TOF; CID	97.24	525.62	Fam189a1

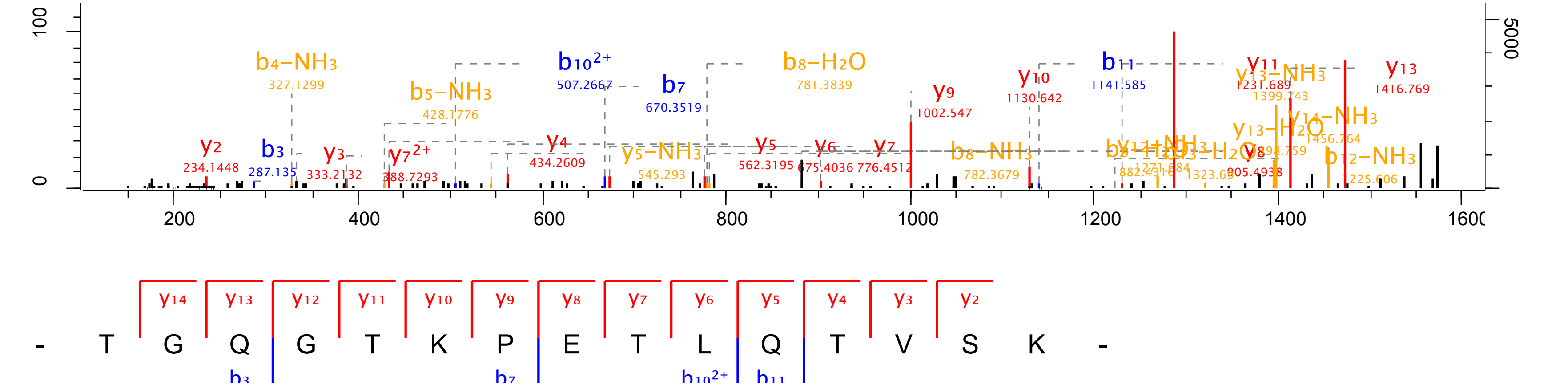

Raw file	Scan	Method	Score	m/z	Gene names
20150402_CerP14_Frac13_top_opt_C1_01_1822	9517	TOF; CID	79.2	521.79	Resp18

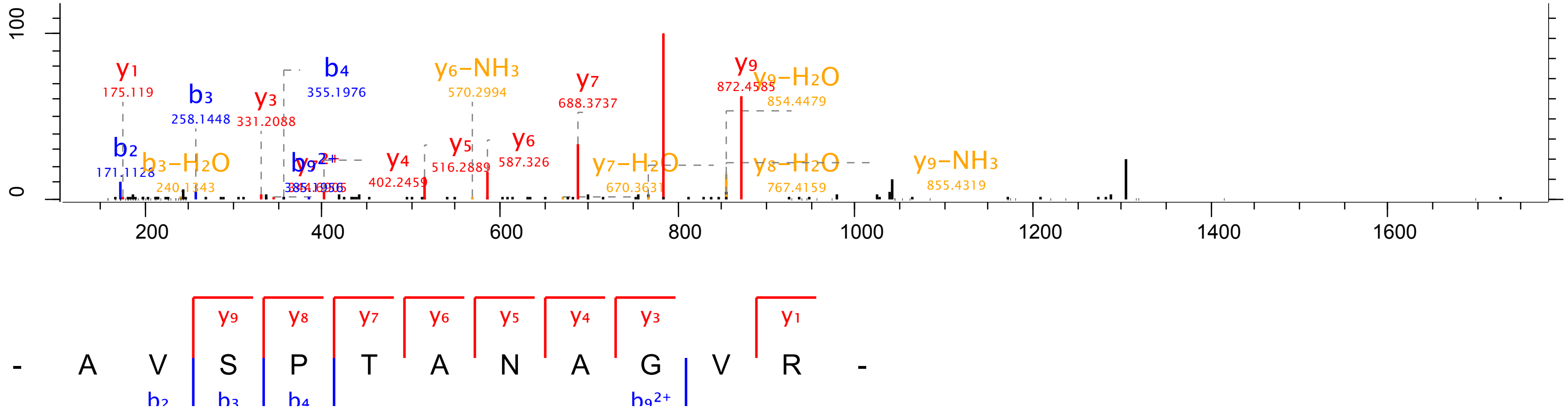

Raw file	Scan	Method	Score	m/z	Gene names
20150402_CerP14_Frac13_top_opt_C1_01_1822	13033	TOF; CID	99.34	594.81	Rnf4

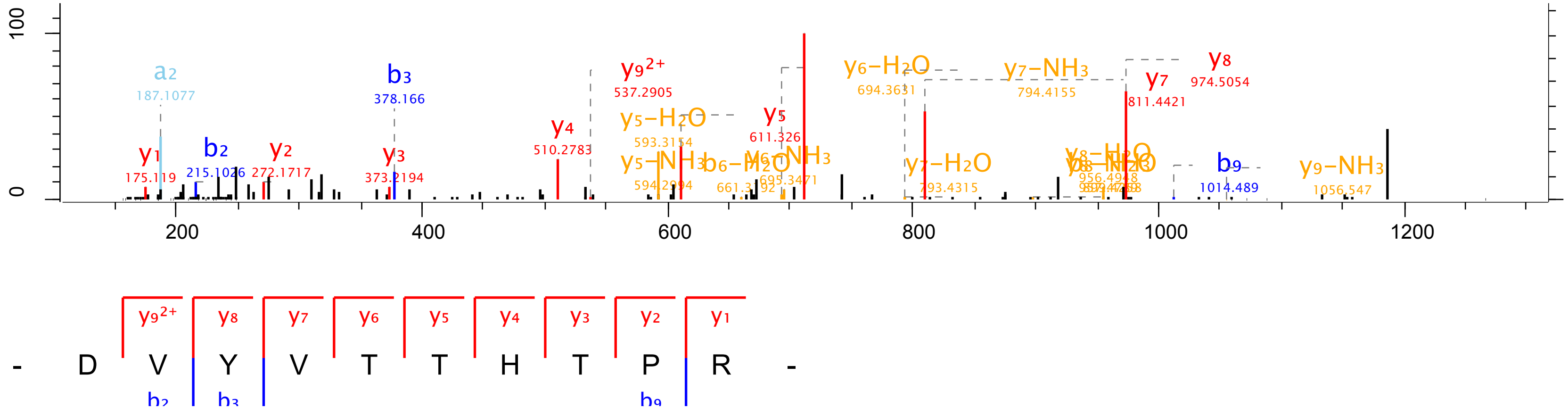

Raw file	Scan	Method	Score	m/z	Gene names
20150402_CerP14_Frac13_top_opt_C1_01_1822	15889	TOF; CID	99.75	443.59	Cd82

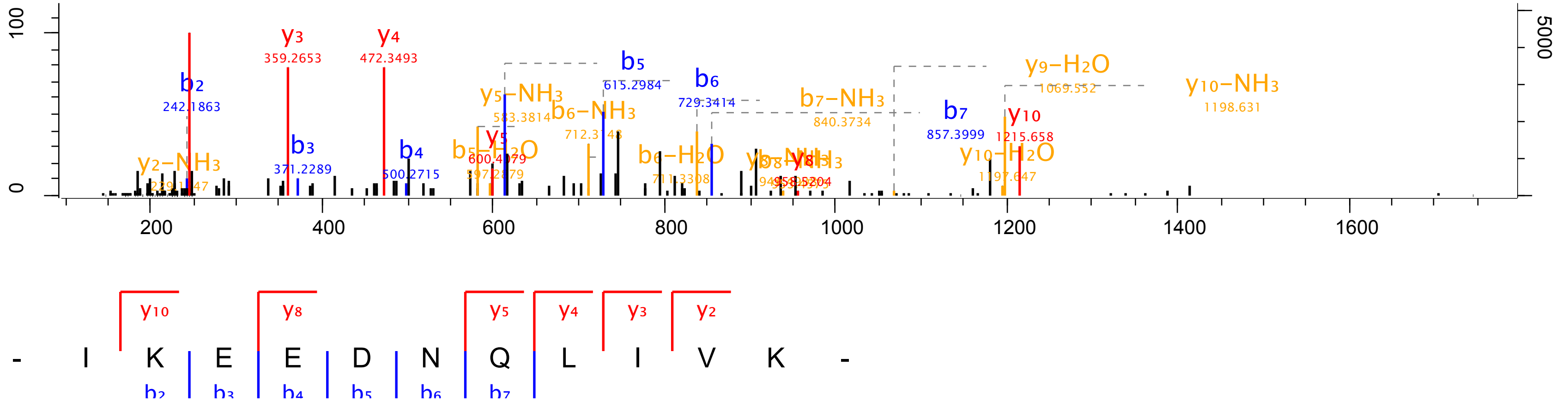

Raw file	Scan	Method	Score	m/z	Gene names
20150402_CerP14_Frac13_top_opt_C1_01_1822	19457	TOF; CID	69.49	1039.81	S1pr5

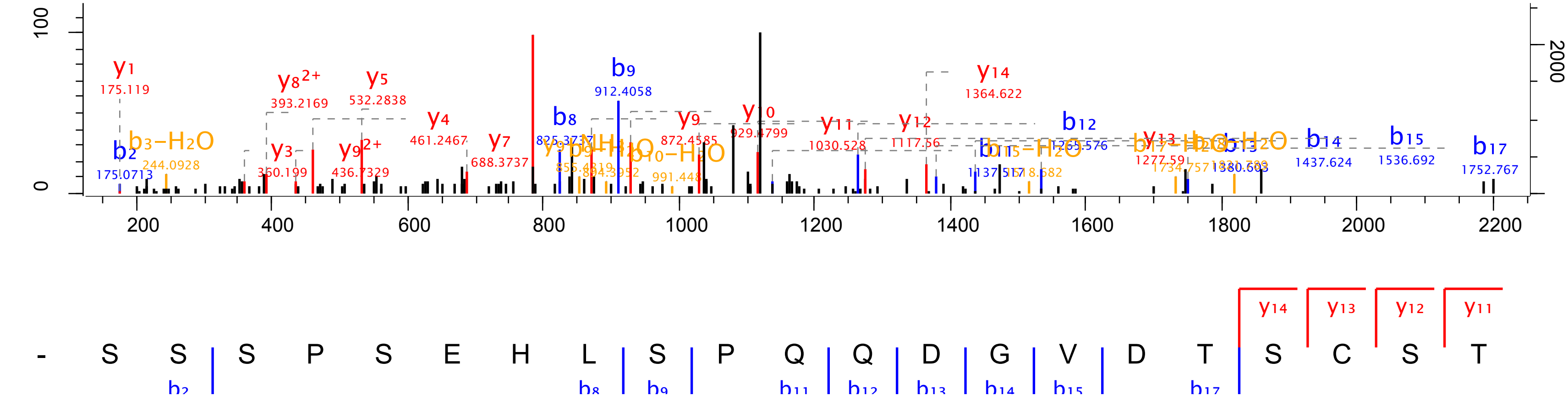

Raw file	Scan	Method	Score	m/z	Gene names
20150402_CerP14_Frac13_top_opt_C1_01_1822	21327	TOF; CID	96.67	541.62	Snurf

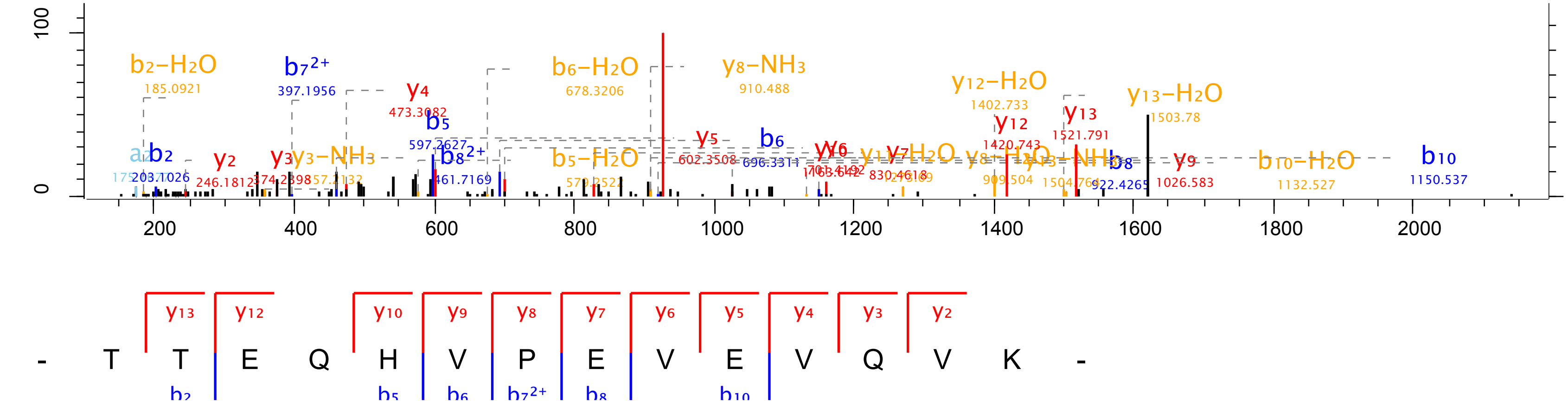

Raw file	Scan	Method	Score	m/z	Gene names
20150402_CerP14_Frac13_top_opt_C1_01_1822	26917	TOF; CID	73.23	377.23	Dnal4

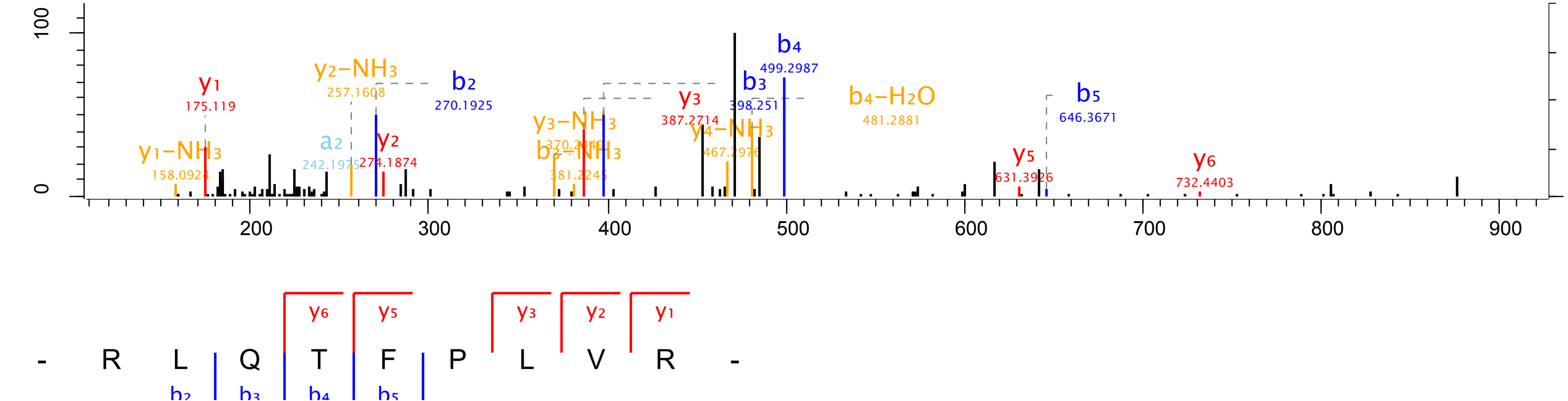

Raw file	Scan	Method	Score	m/z	Gene names
20150402_CerP14_Frac13_top_opt_C1_01_1822	31574	TOF; CID	59.2	647.82	Eva1a

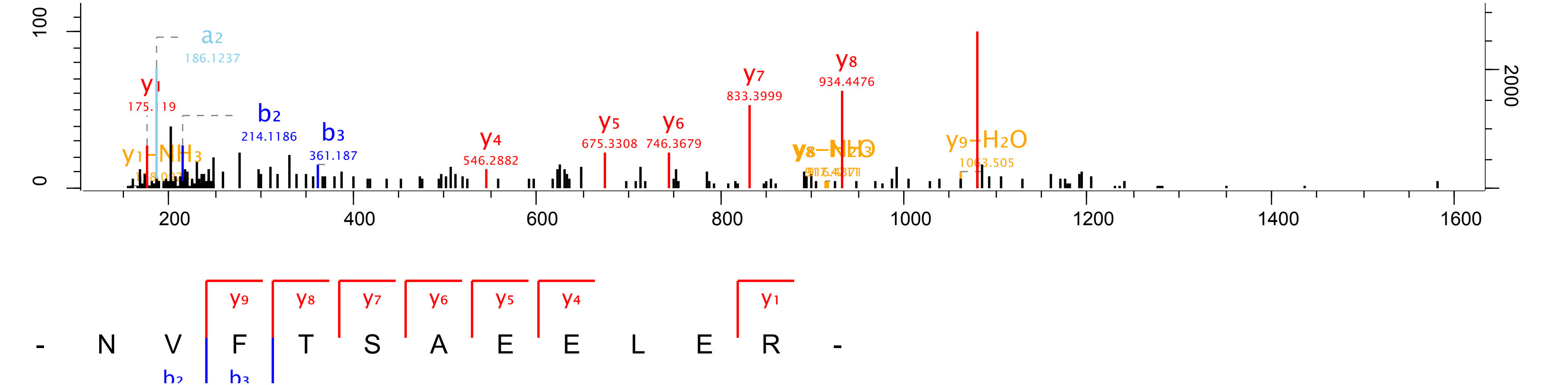

Raw file	Scan	Method	Score	m/z	Gene names
20150402_CerP14_Frac13_top_opt_C1_01_1822	32781	TOF; CID	69.35	556.27	Tacr1

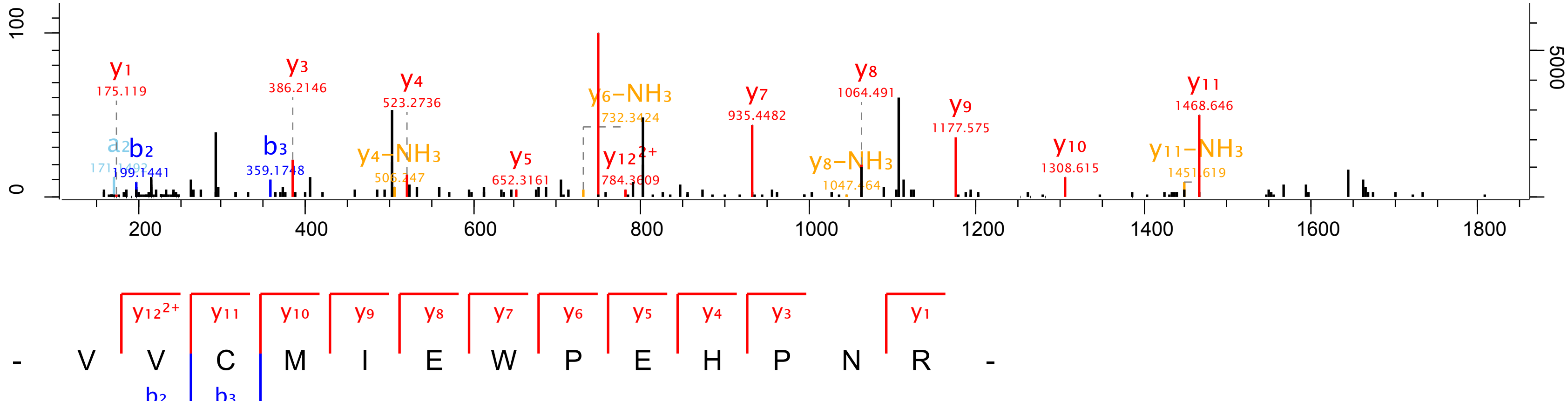

Raw file	Scan	Method	Score	m/z	Gene names
20150402_CerP14_Frac13_top_opt_C1_01_1822	33812	TOF; CID	42.53	670.04	Gpank1

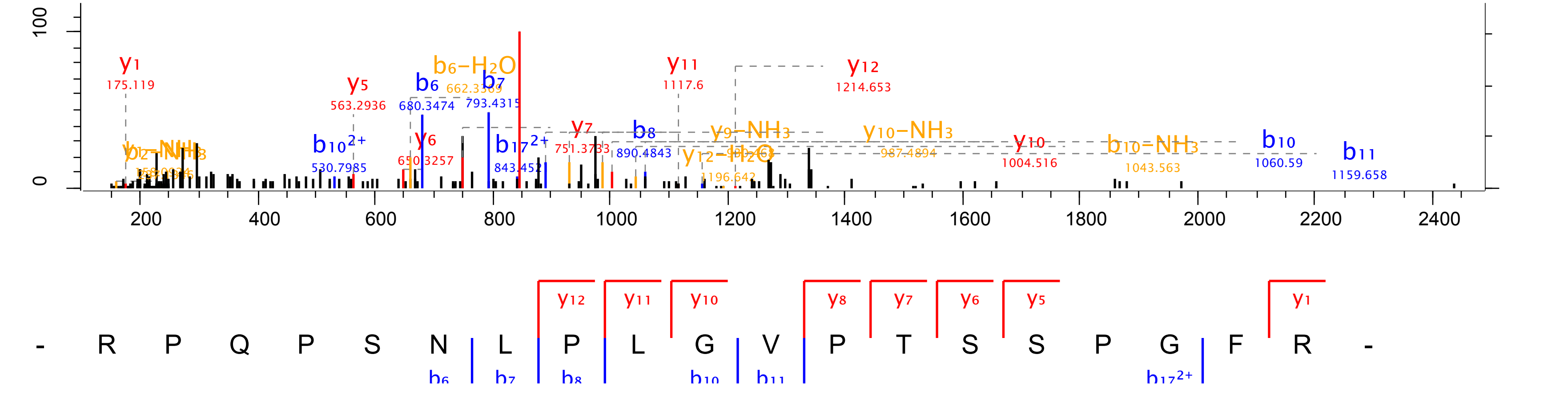

Raw file	Scan	Method	Score	m/z	Gene names
20150402_CerP14_Frac13_top_opt_C1_01_1822	34248	TOF; CID	66.27	525.79	Olfr1458

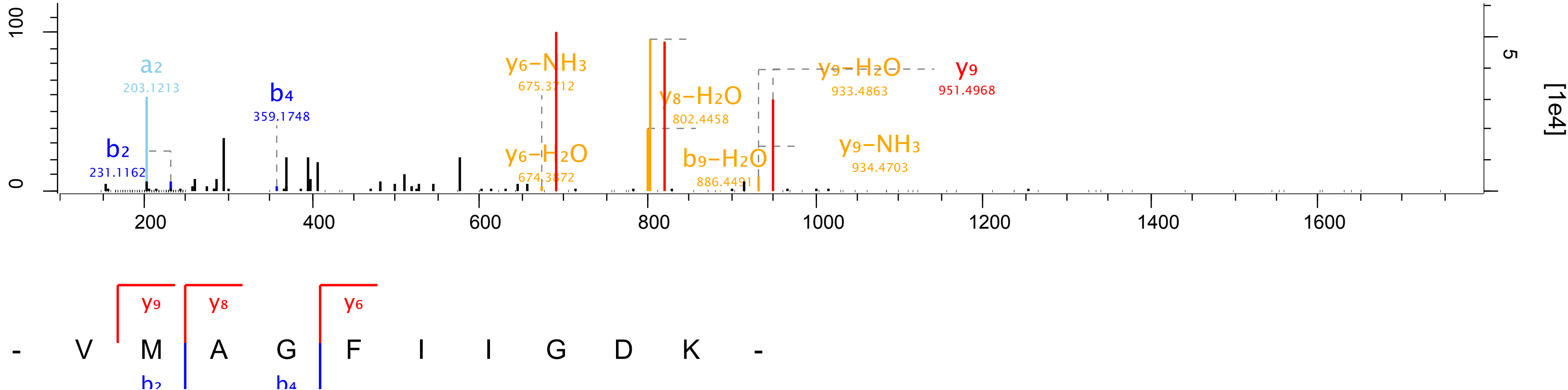

Raw file	Scan	Method	Score	m/z	Gene names
20150402_CerP14_Frac13_top_opt_C1_01_1822	38211	TOF; CID	60.09	1079.48	Cmtm3

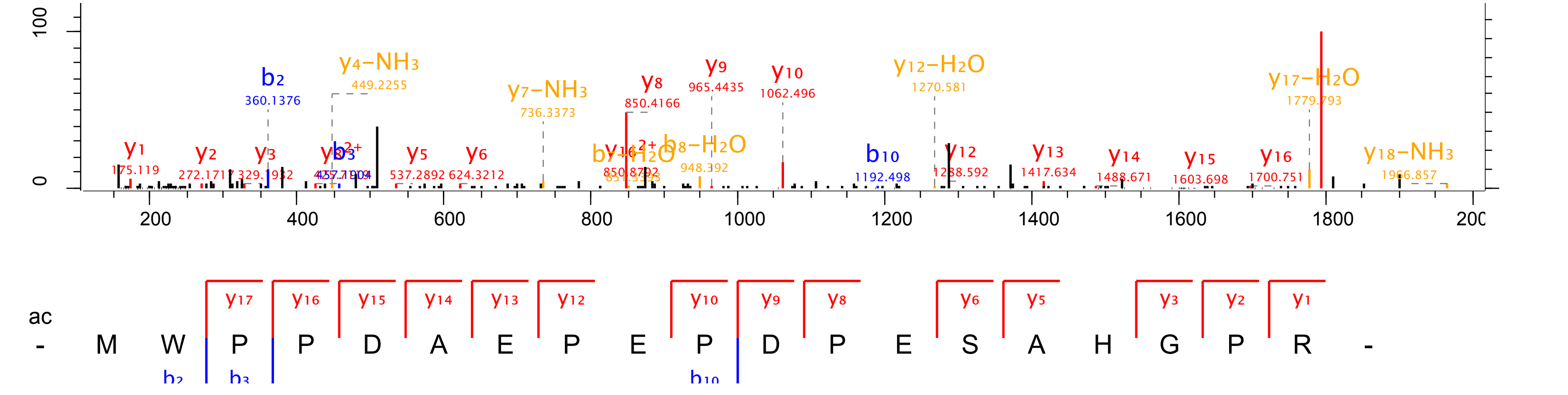

Raw file	Scan	Method	Score	m/z	Gene names
20150402_CerP14_Frac13_top_opt_C1_01_1822	40448	TOF; CID	74.14	971.49	Hemk1

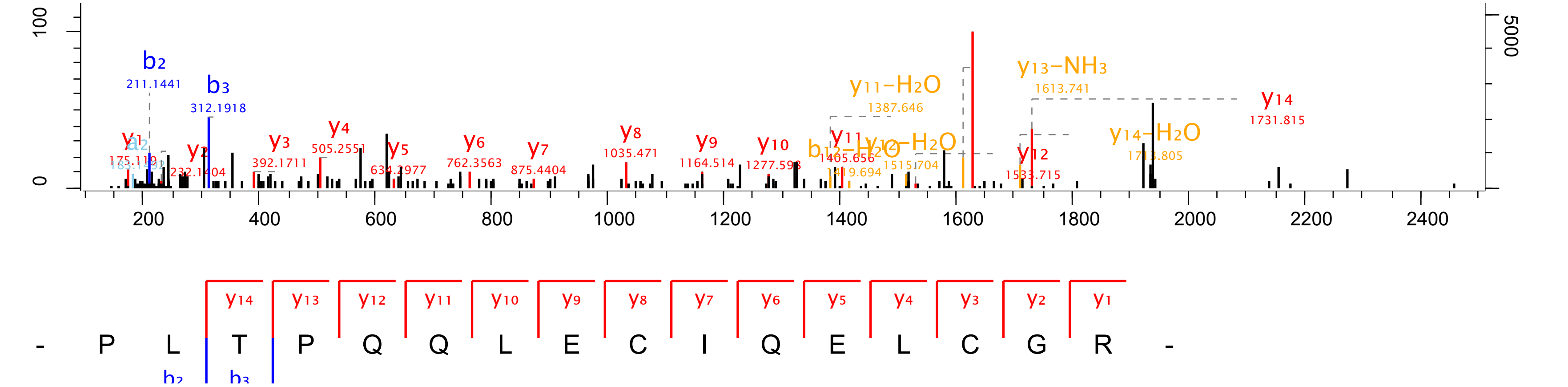

Raw file	Scan	Method	Score	m/z	Gene names
20150402_CerP14_Frac13_top_opt_C1_01_1822	41058	TOF; CID	29.71	926.45	Abl2

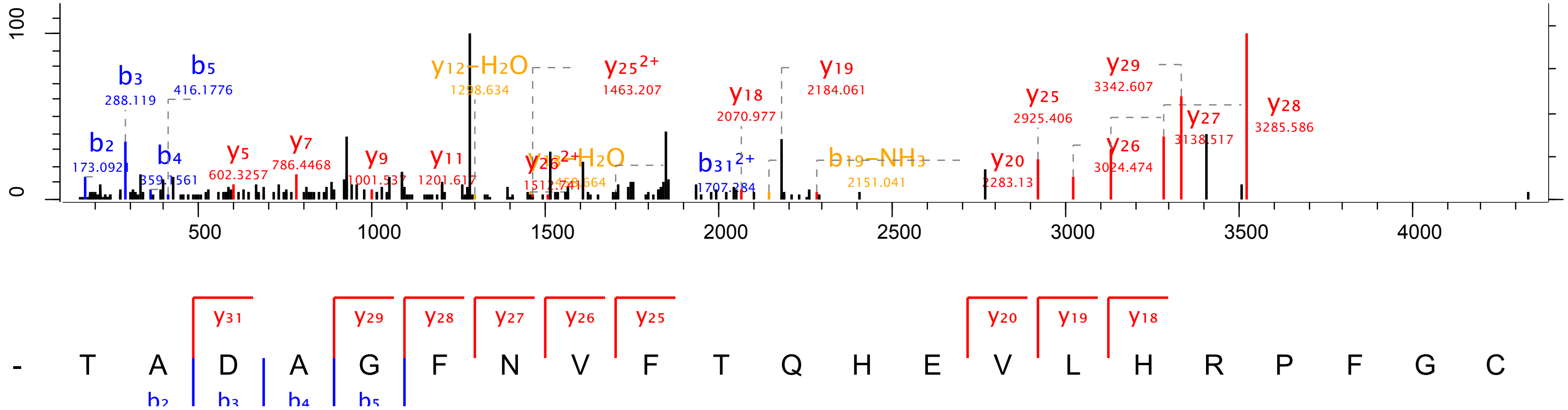

Raw file	Scan	Method	Score	m/z	Gene names
20150402_CerP14_Frac13_top_opt_C1_01_1822	43189	TOF; CID	53.45	652.36	Cdc42ep5

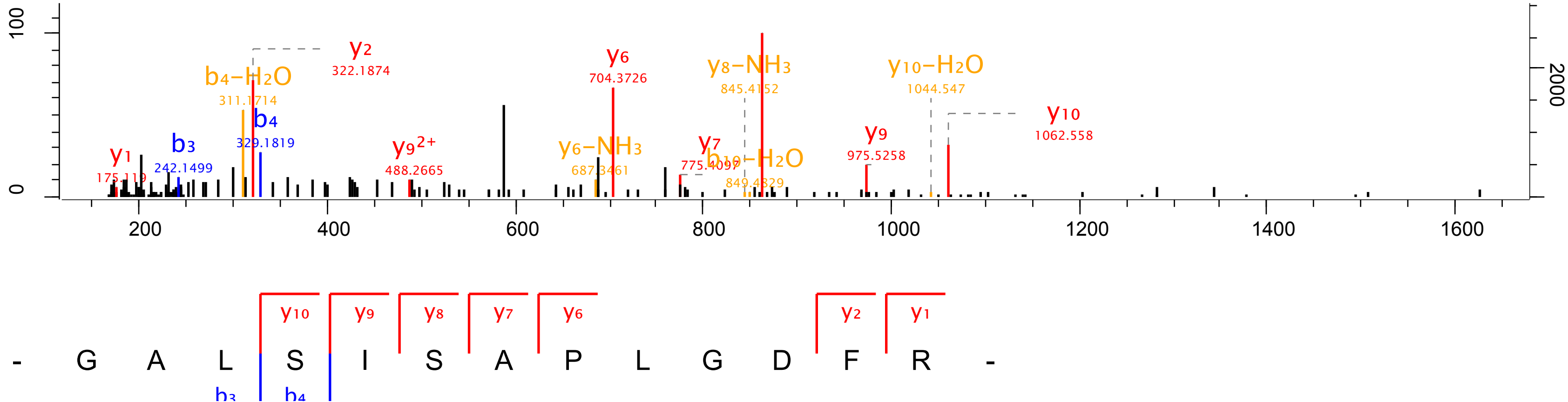

Raw file	Scan	Method	Score	m/z	Gene names
20150402_CerP14_Frac13_top_opt_C1_01_1822	47244	TOF; CID	107.09	749.92	Lrg1

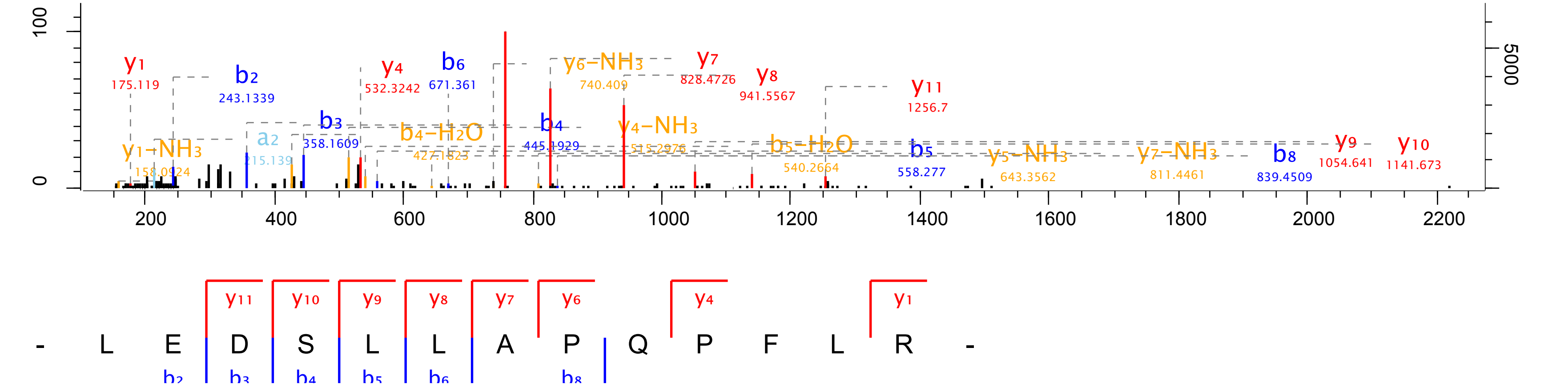

Raw file	Scan	Method	Score	m/z	Gene names
20150402_CerP14_Frac13_top_opt_C1_01_1822	50922	TOF; CID	64.3	869.99	Lsm5

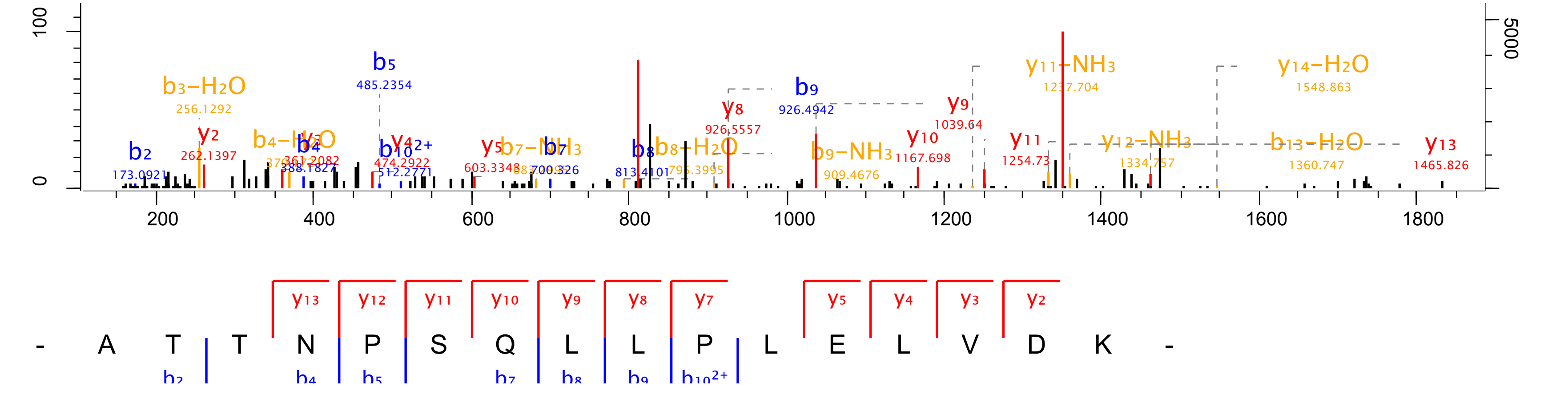

Raw file	Scan	Method	Score	m/z	Gene names
20150402_CerP14_Frac13_top_opt_C1_01_1822	51028	TOF; CID	64.12	650.88	Fos

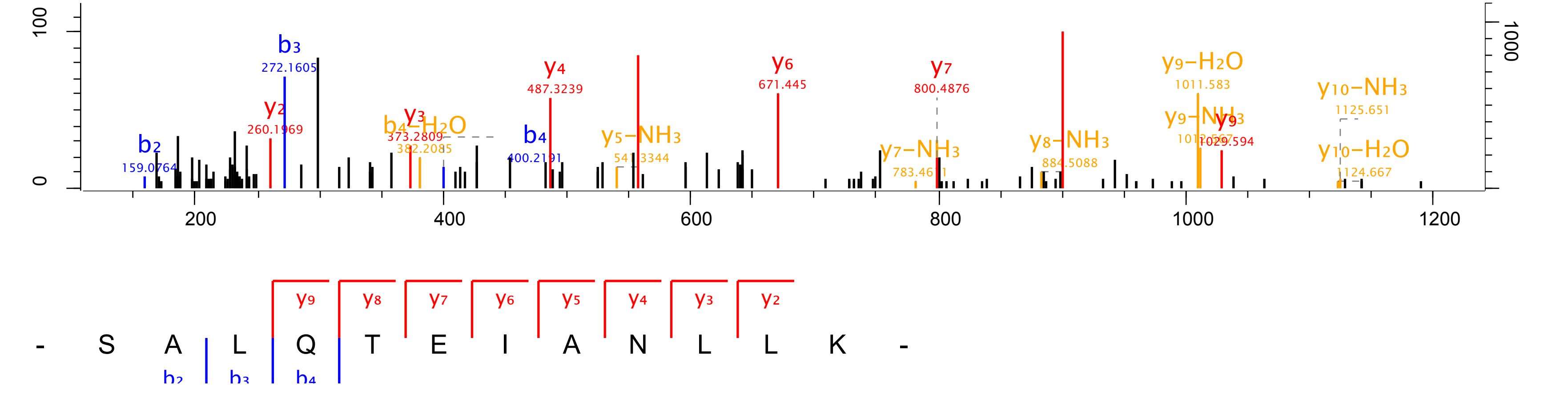

Raw file	Scan	Method	Score	m/z	Gene names
20150402_CerP14_Frac13_top_opt_C1_01_1822	53313	TOF; CID	82.59	903.49	C5

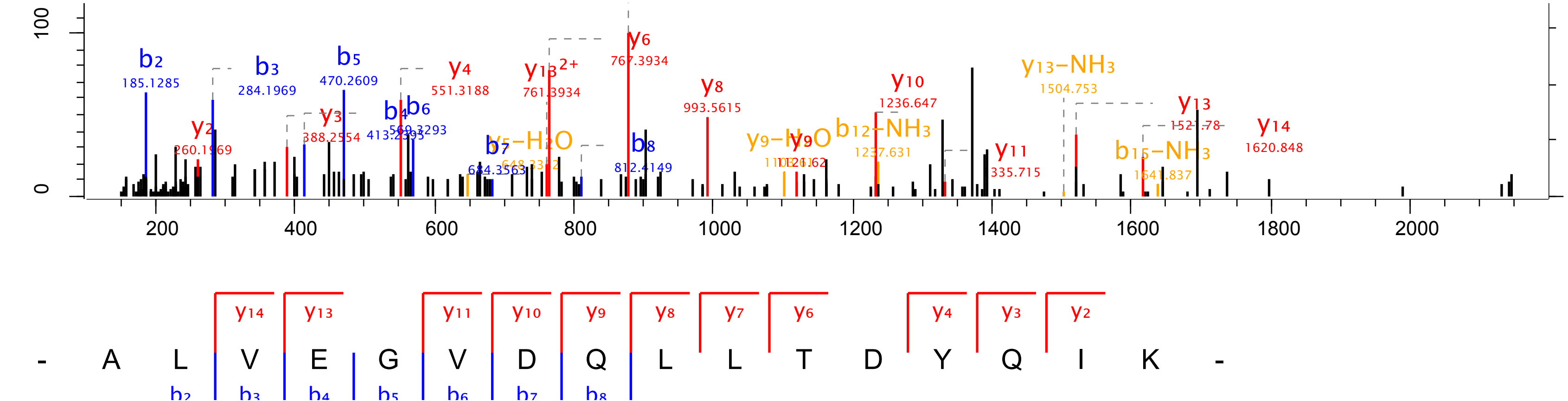

Raw file	Scan	Method	Score	m/z	Gene names
20150402_CerP14_Frac13_top_opt_C1_01_1822	54847	TOF; CID	81.88	925.77	Hyls1

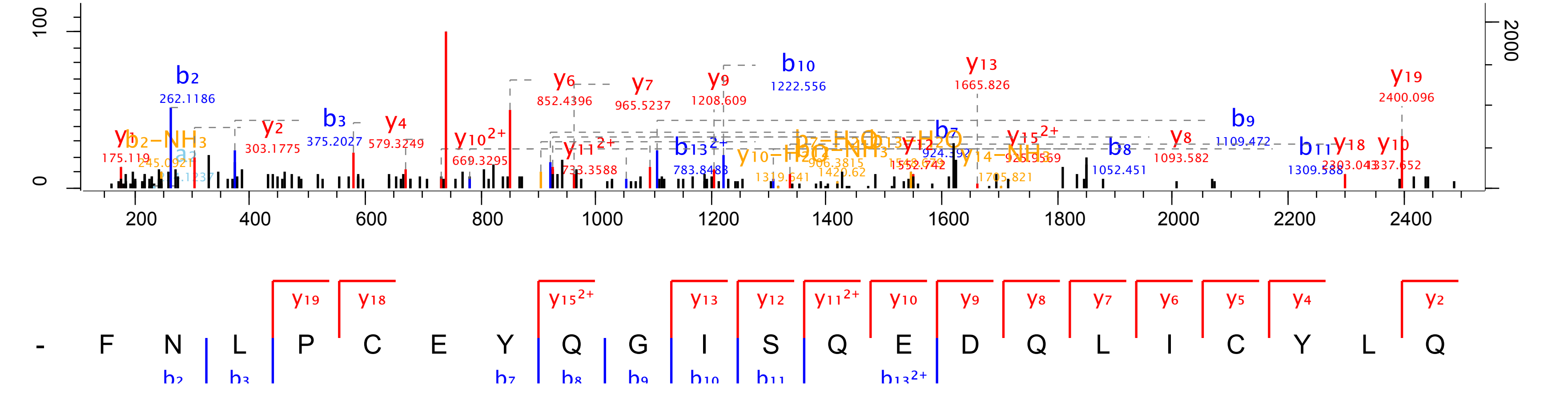

Raw file	Scan	Method	Score	m/z	Gene names
20150402_CerP14_Frac13_top_opt_C1_01_1822	56066	TOF; CID	84.75	869.45	Ube2j2

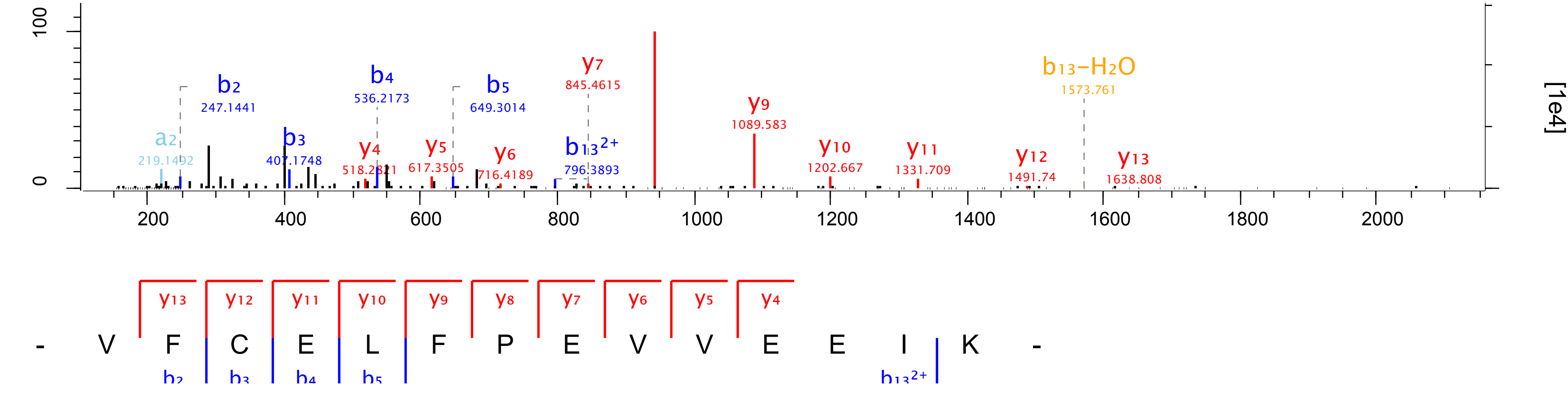

Raw file	Scan	Method	Score	m/z	Gene names
20150402_CerP14_Frac13_top_opt_C1_01_1822	56838	TOF; CID	90.94	680.04	Lsm5

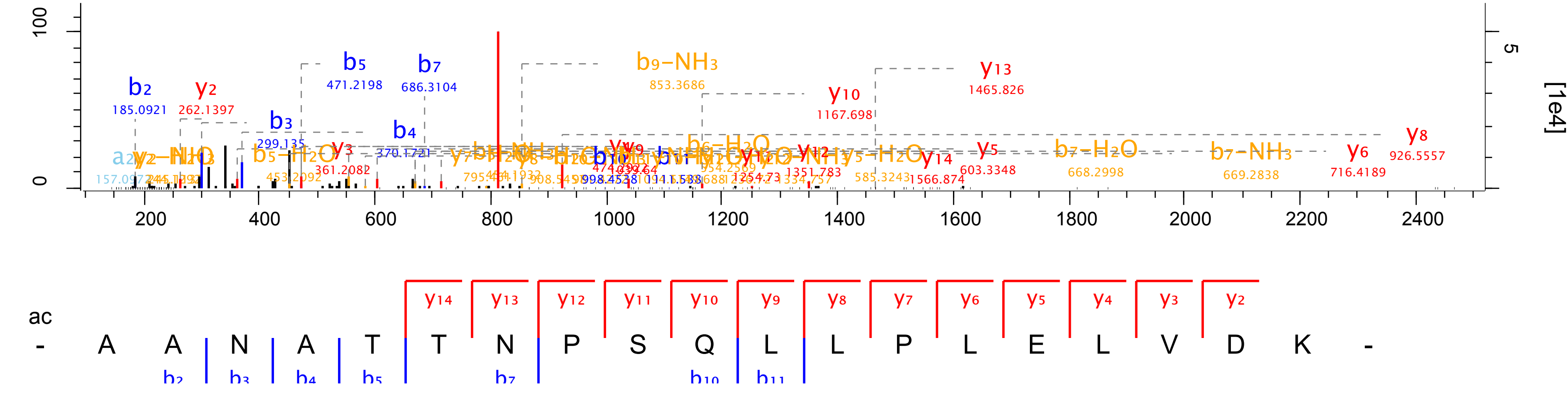

Raw file	Scan	Method	Score	m/z	Gene names
20150402_CerP14_Frac14_top_opt_C2_01_1823	10490	TOF; CID	100.93	655.84	Fyb

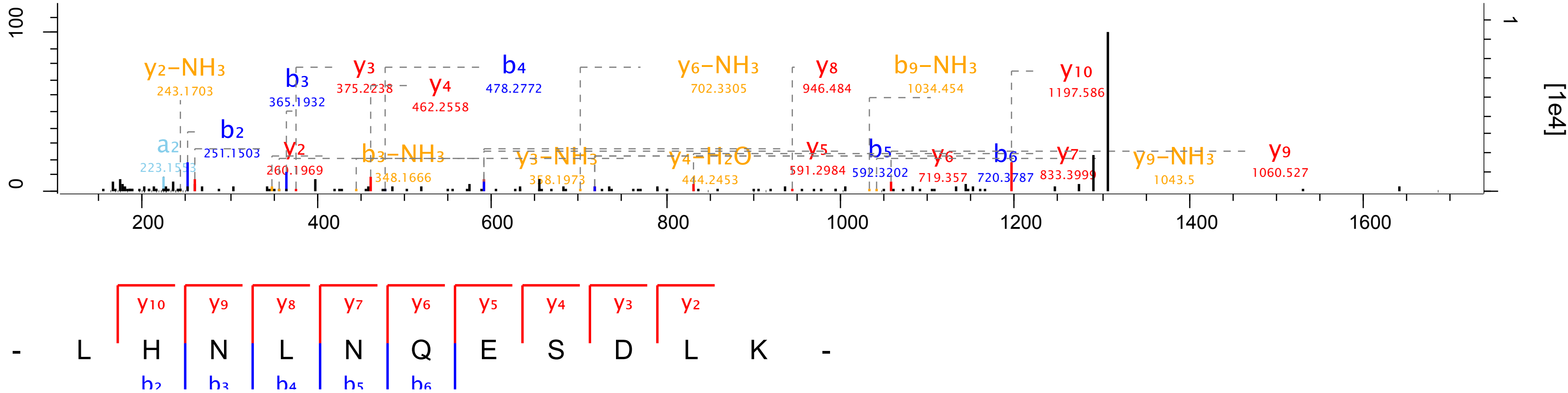

Raw file	Scan	Method	Score	m/z	Gene names
20150402_CerP14_Frac14_top_opt_C2_01_1823	14275	TOF; CID	95.85	535.94	Znhit1

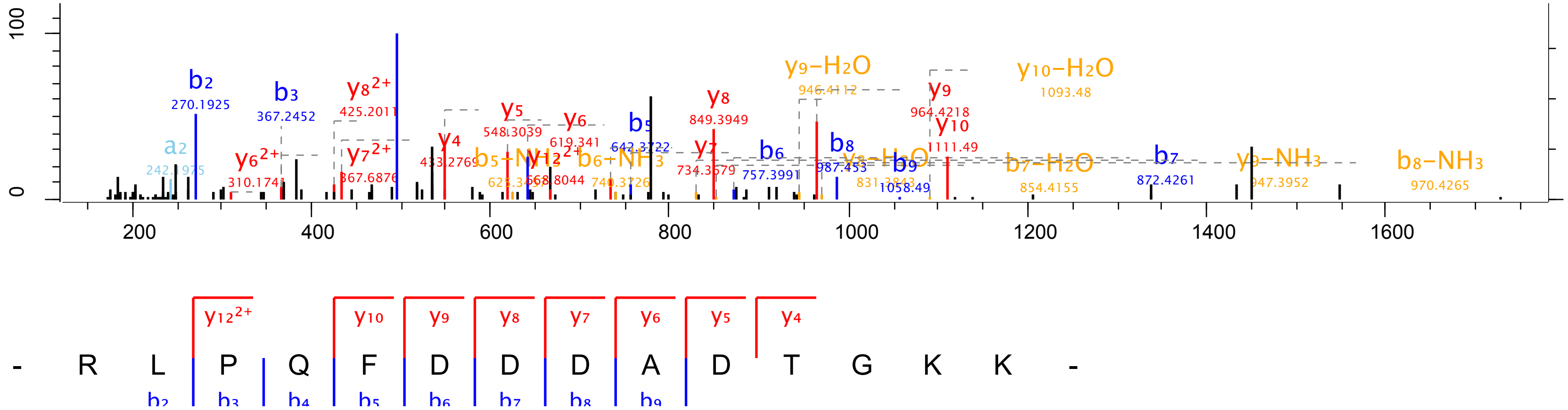

Raw file	Scan	Method	Score	m/z	Gene names
20150402_CerP14_Frac14_top_opt_C2_01_1823	20889	TOF; CID	79.49	906.45	Tfpt

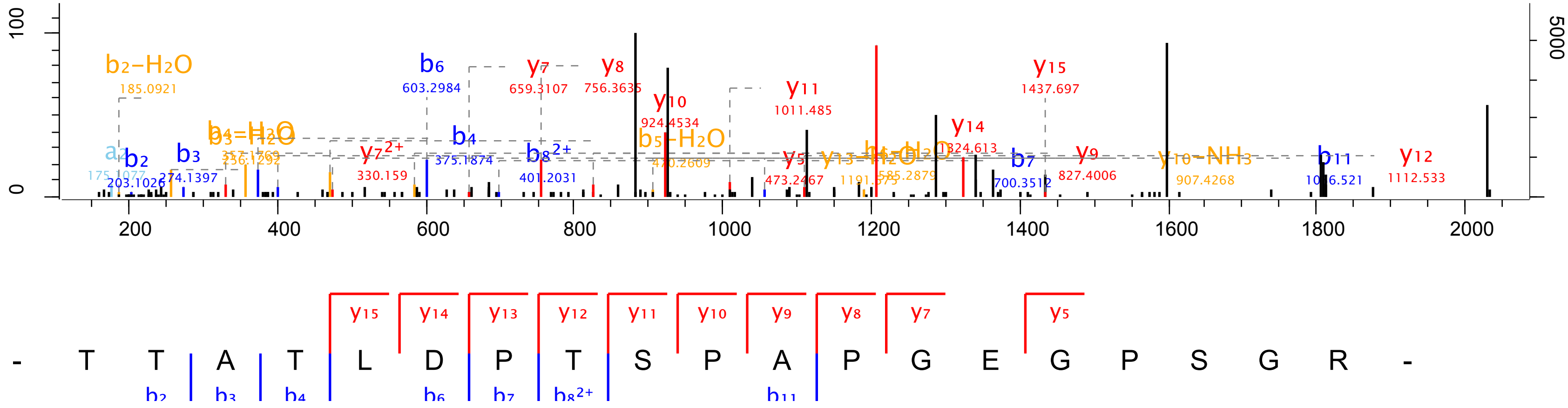

Raw file	Scan	Method	Score	m/z	Gene names
20150402_CerP14_Frac14_top_opt_C2_01_1823	24574	TOF; CID	106.42	473.77	Cd320

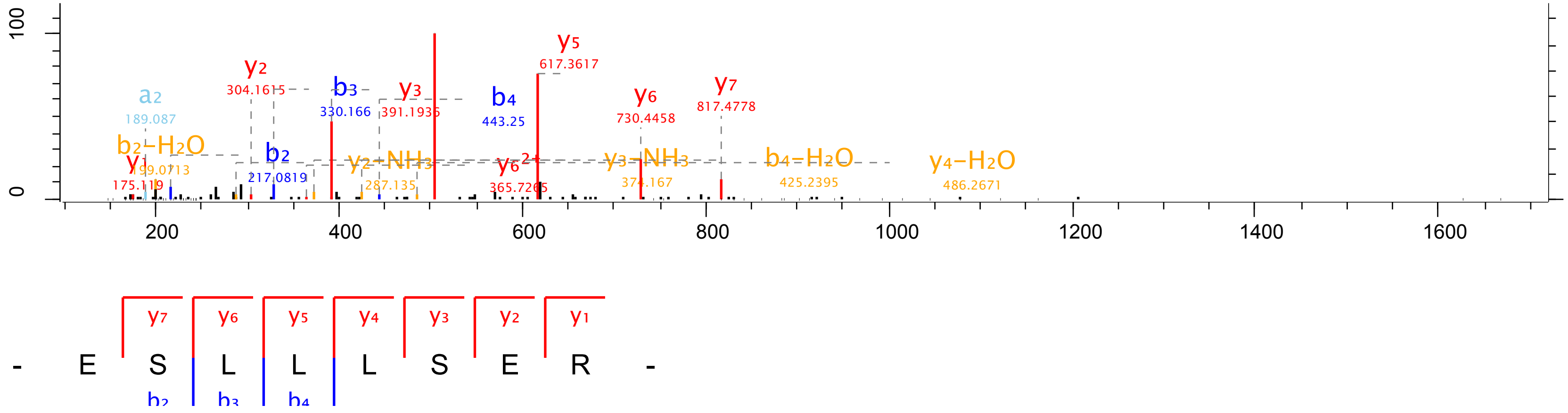

Raw file	Scan	Method	Score	m/z
20150402_CerP14_Frac14_top_opt_C2_01_1823	24718	TOF; CID	63.73	811.37

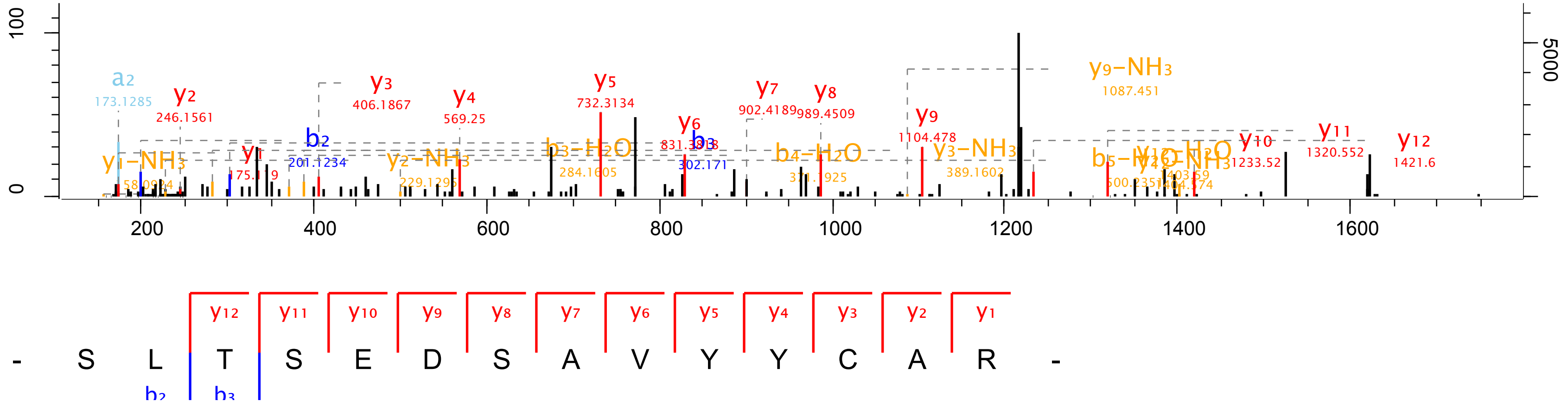

Raw file	Scan	Method	Score	m/z	Gene names
20150402_CerP14_Frac14_top_opt_C2_01_1823	24764	TOF; CID	91.31	644.85	Pgap2

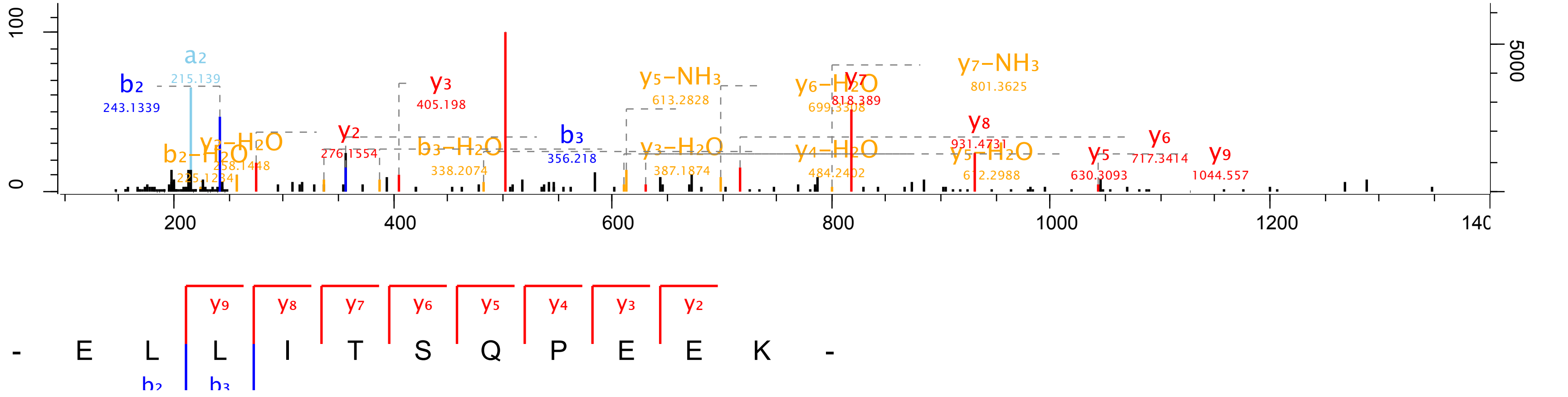

Raw file	Scan	Method	Score	m/z	Gene names
20150402_CerP14_Frac14_top_opt_C2_01_1823	27268	TOF; CID	43.34	459.25	Mynn

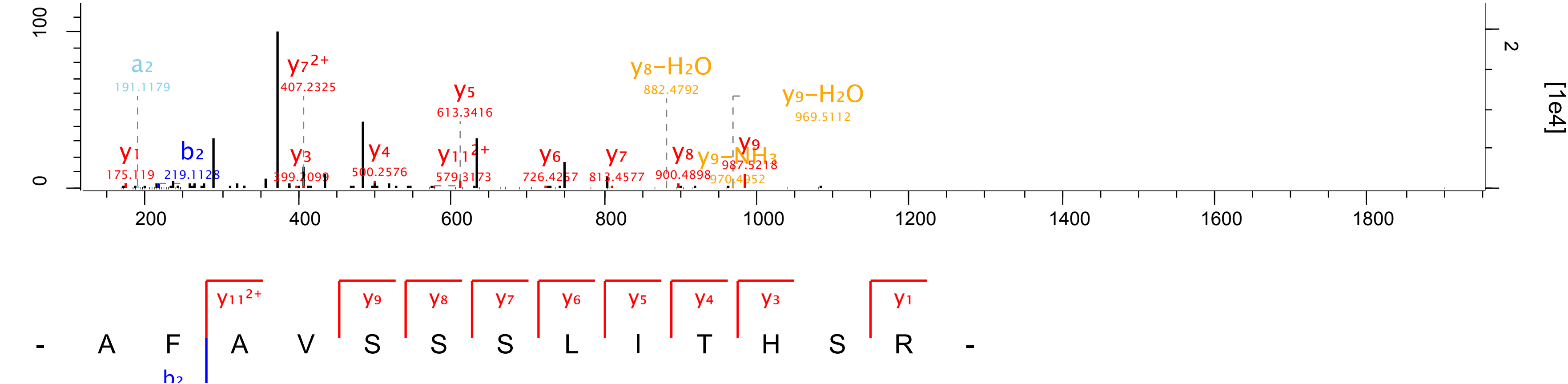

Raw file	Scan	Method	Score	m/z	Gene names
20150402_CerP14_Frac14_top_opt_C2_01_1823	30980	TOF; CID	130.77	410.24	Gm10647

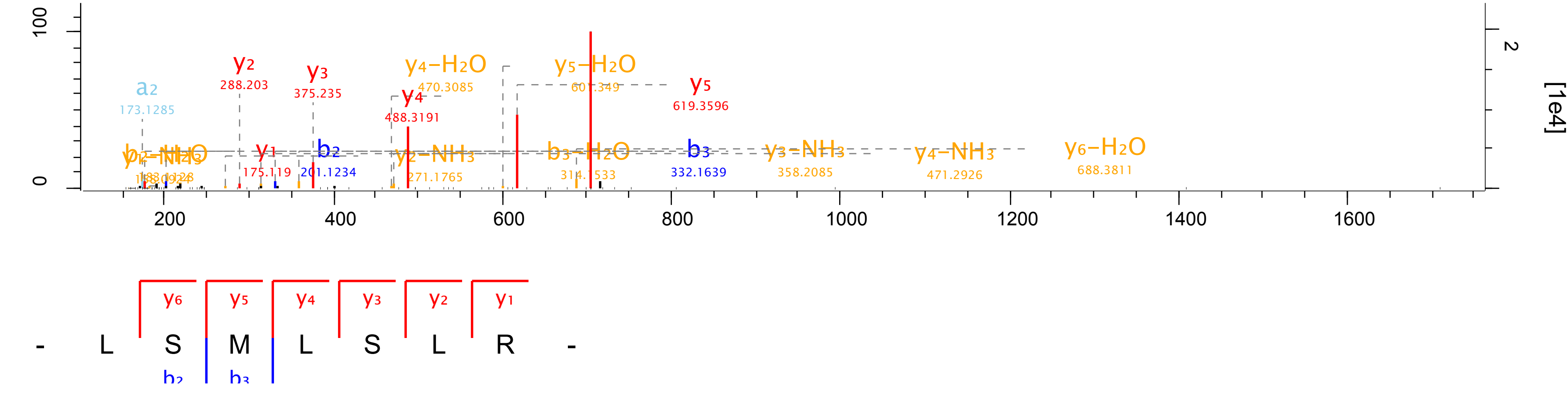

Raw file	Scan	Method	Score	m/z	Gene names
20150402_CerP14_Frac14_top_opt_C2_01_1823	33166	TOF; CID	79.09	494.26	Serinc3

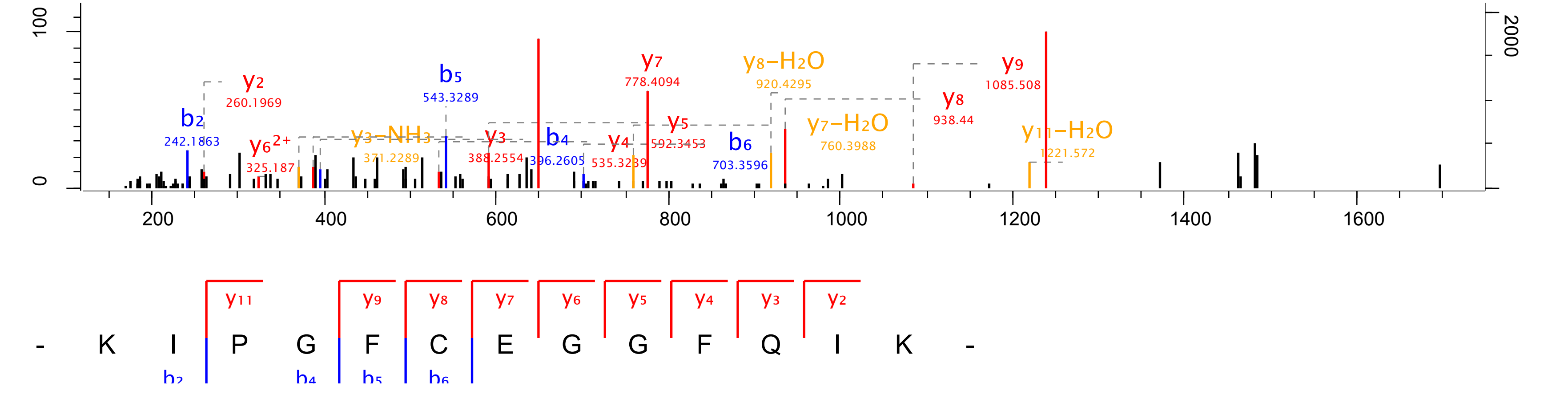

Raw file	Scan	Method	Score	m/z	Gene names
20150402_CerP14_Frac14_top_opt_C2_01_1823	35799	TOF; CID	44.55	888.95	Oprm1

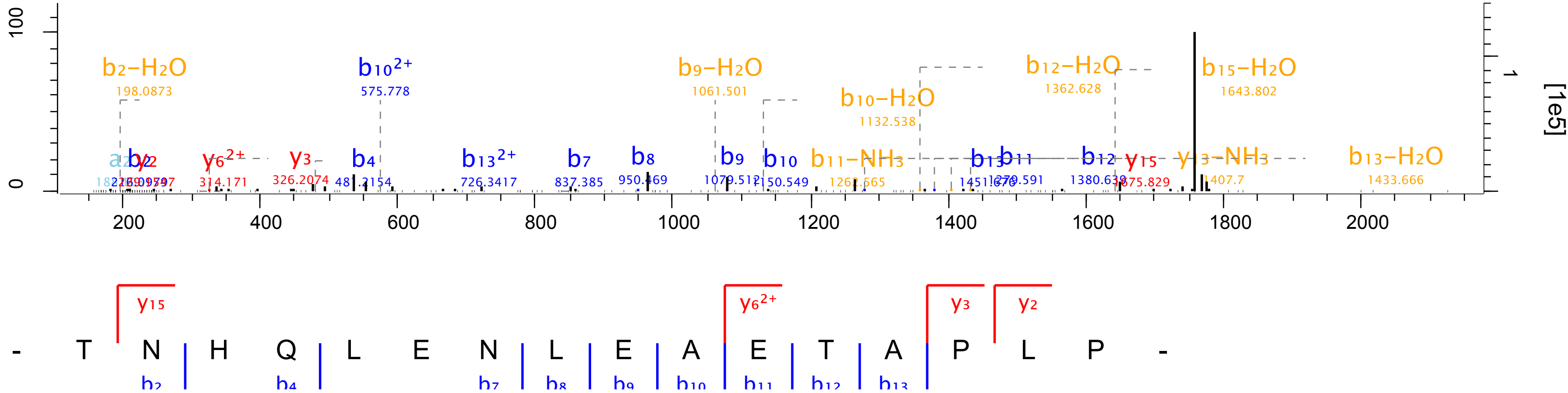

Raw file	Scan	Method	Score	m/z	Gene names
20150402_CerP14_Frac14_top_opt_C2_01_1823	36293	TOF; CID	90.83	587.84	Bok

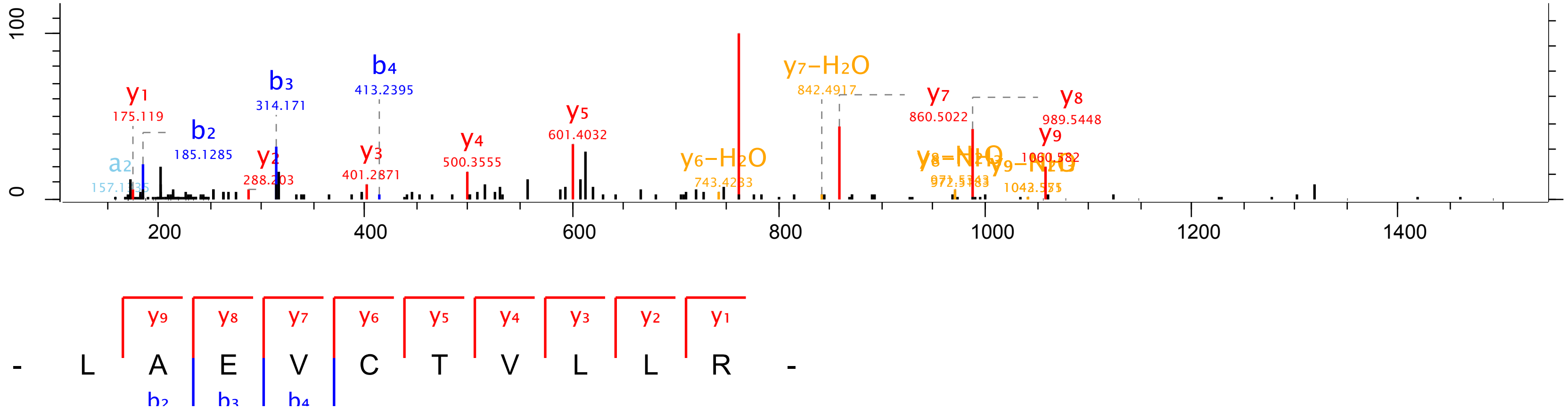

Raw file	Scan	Method	Score	m/z	Gene names
20150402_CerP14_Frac14_top_opt_C2_01_1823	37535	TOF; CID	79.15	426.57	Mlx

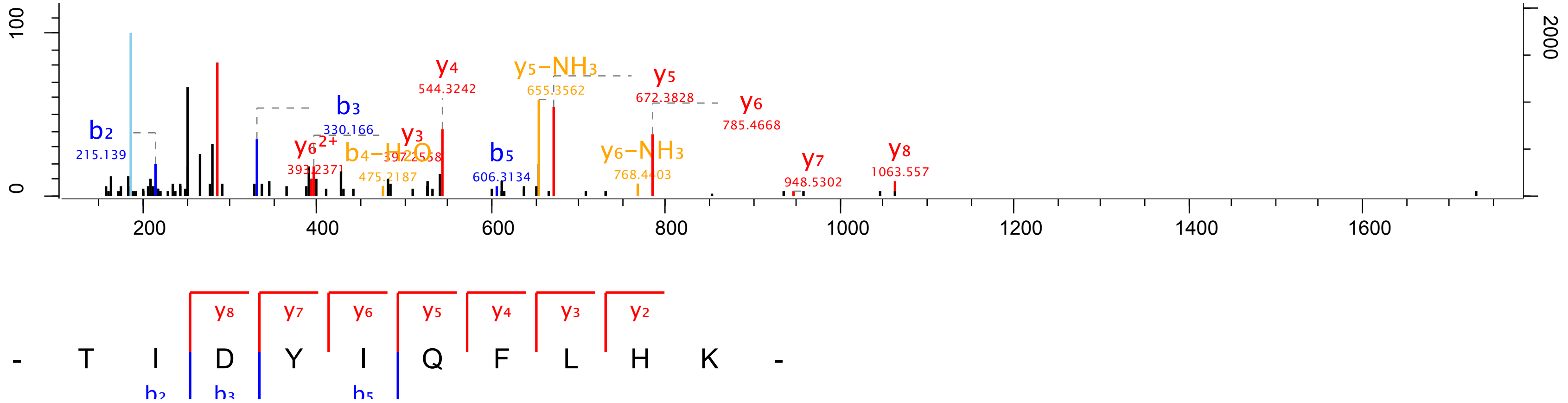

Raw file	Scan	Method	Score	m/z	Gene names
20150402_CerP14_Frac14_top_opt_C2_01_1823	43247	TOF; CID	55.53	569.95	Ssr3

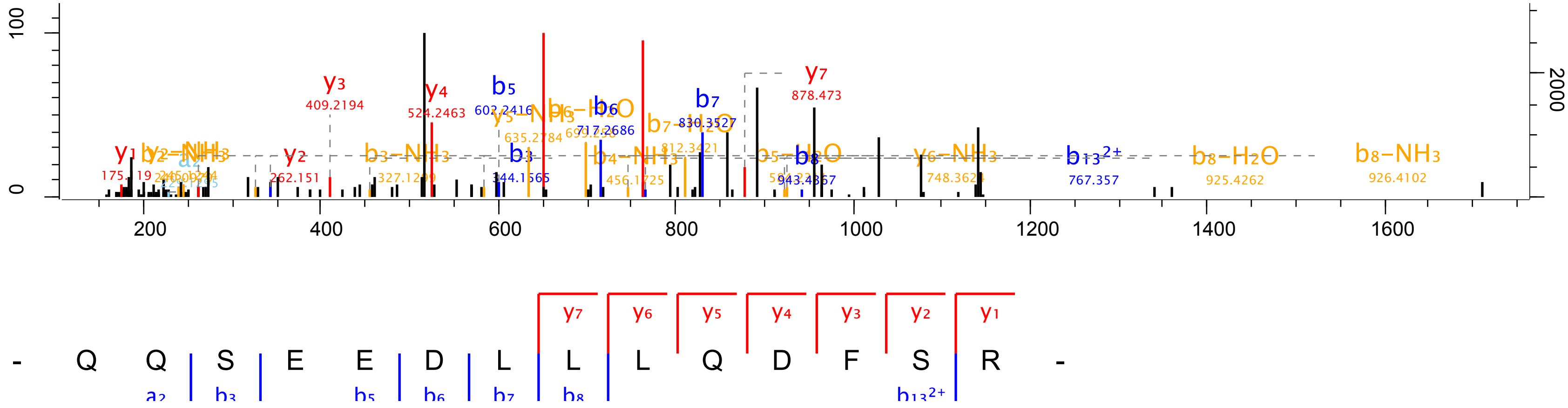

Raw file	Scan	Method	Score	m/z	Gene names
20150402_CerP14_Frac14_top_opt_C2_01_1823	49050	TOF; CID	112.08	790.93	Cldn12

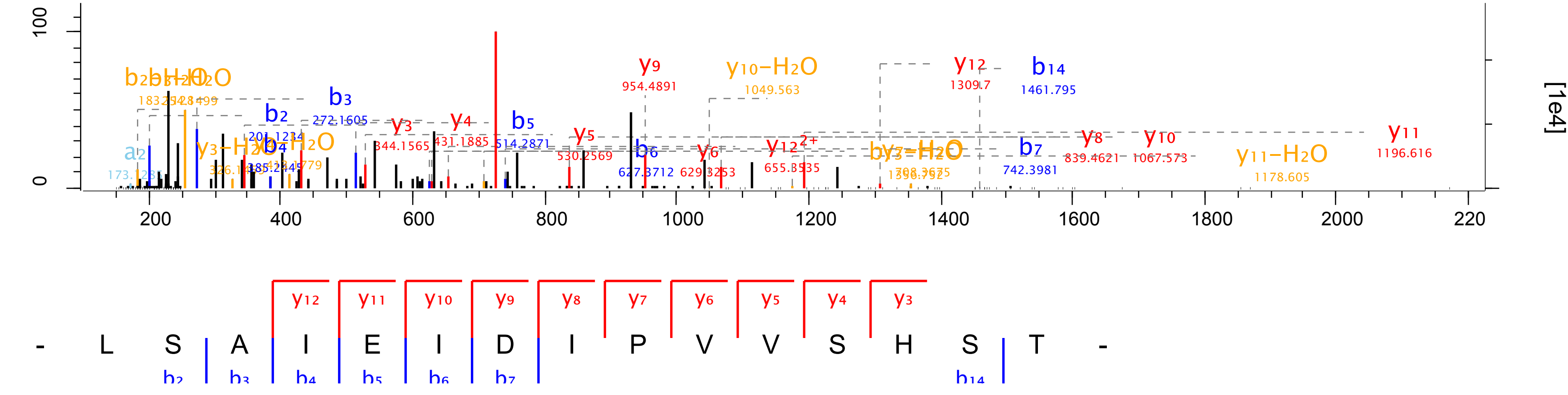

Raw file	Scan	Method	Score	m/z	Gene names
20150402_CerP14_Frac14_top_opt_C2_01_1823	49382	TOF; CID	77.22	888.46	Crybb3

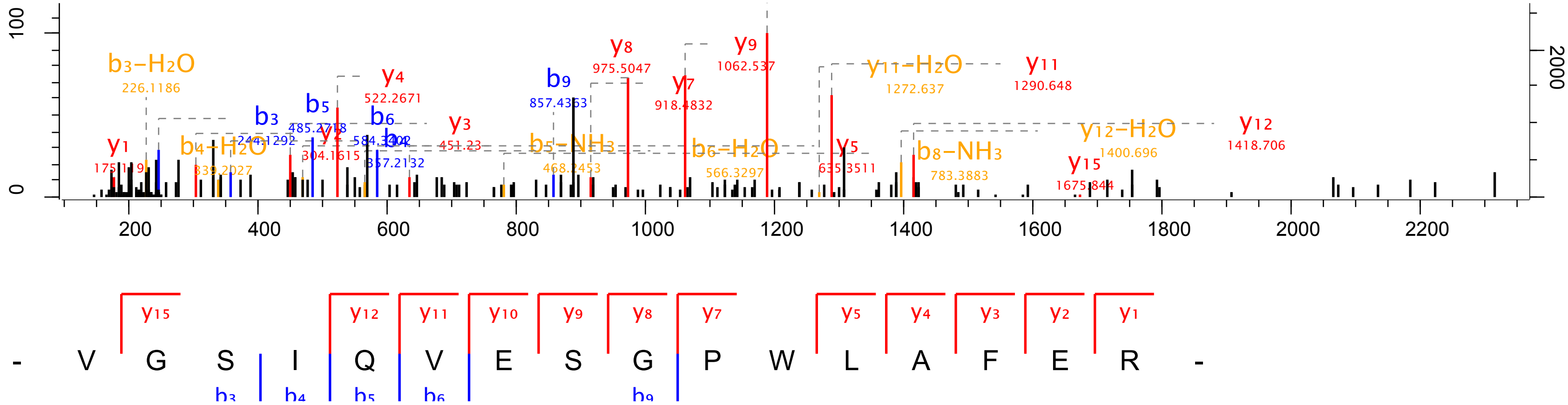

Raw file	Scan	Method	Score	m/z	Gene names
20150402_CerP14_Frac14_top_opt_C2_01_1823	53989	TOF; CID	93.23	650.32	Lpl

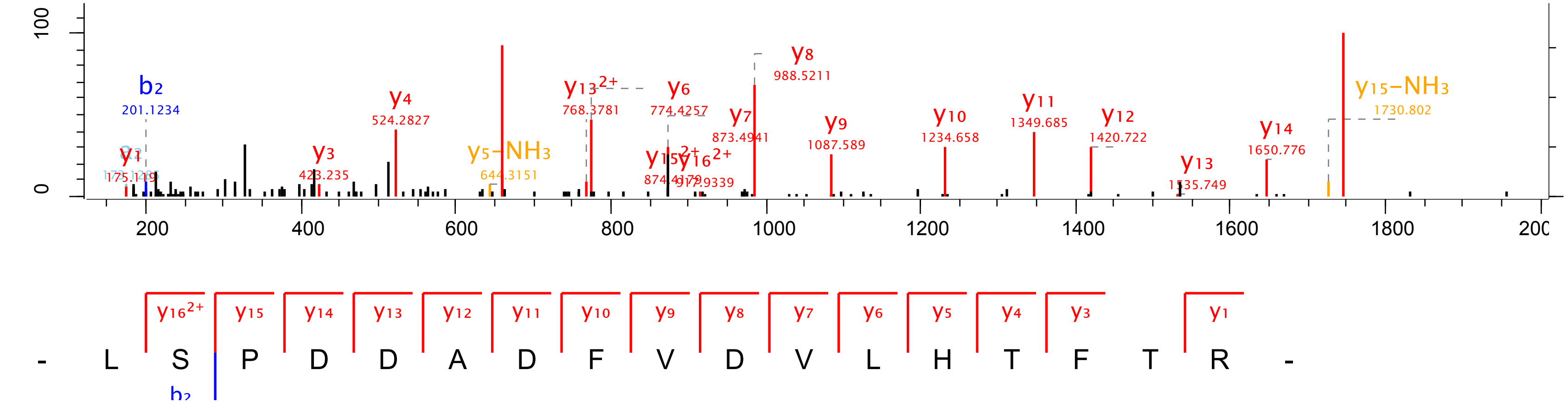

Raw file	Scan	Method	Score	m/z	Gene names
20150402_CerP14_Frac15_top_opt_C3_01_1824	3667	TOF; CID	80.1	644.37	Adra2b

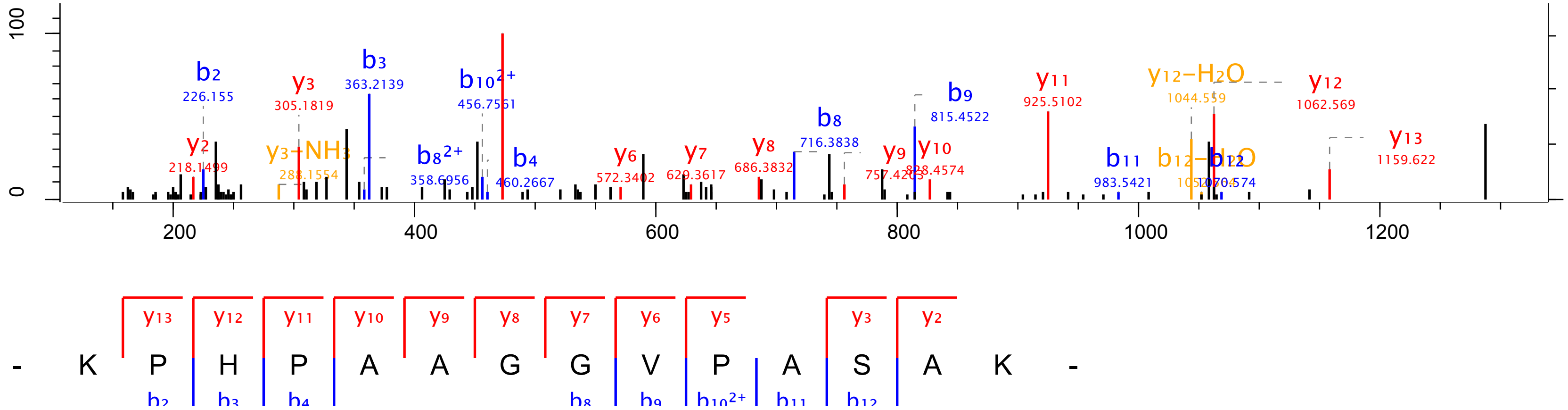

Raw file	Scan	Method	Score	m/z	Gene names
20150402_CerP14_Frac15_top_opt_C3_01_1824	8765	TOF; CID	48.38	616.31	Asnsd1

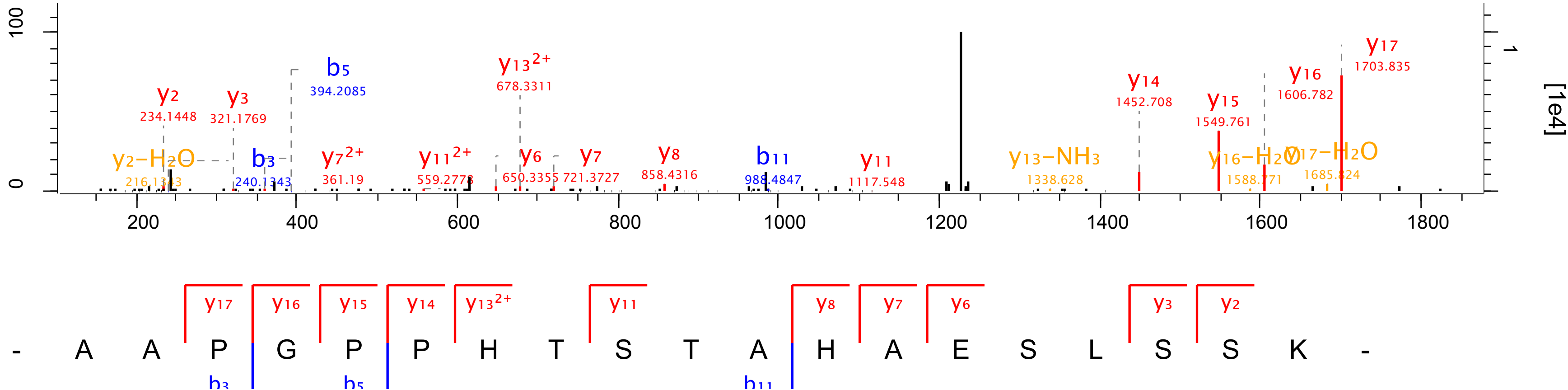

Raw file	Scan	Method	Score	m/z
20150402_CerP14_Frac15_top_opt_C3_01_1824	9956	TOF; CID	73.08	642.34

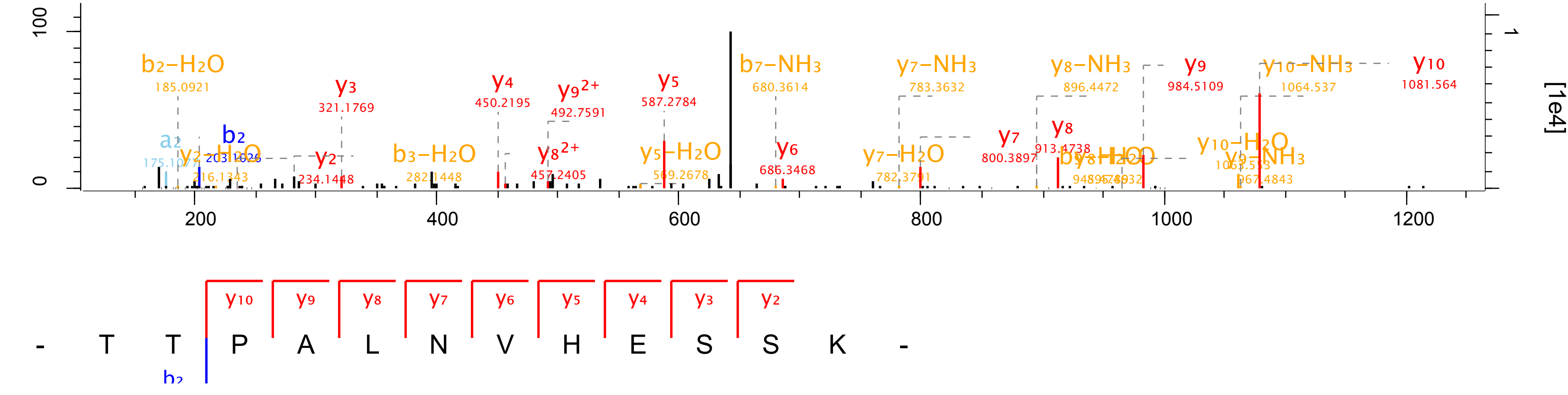

Raw file	Scan	Method	Score	m/z	Gene names
20150402_CerP14_Frac15_top_opt_C3_01_1824	13265	TOF; CID	86.29	465.23	Cnih3

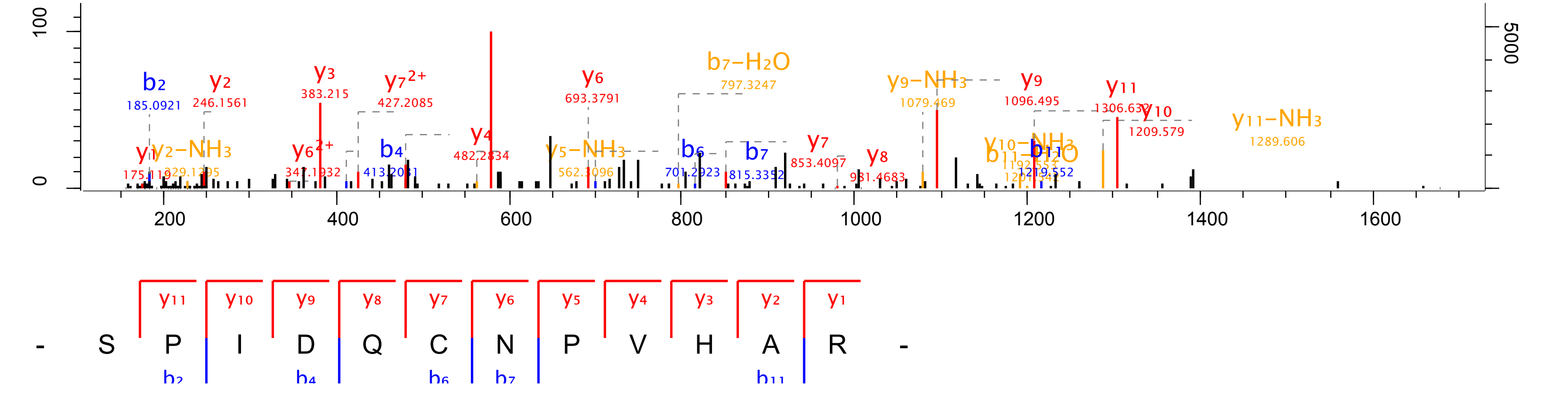

Raw file	Scan	Method	Score	m/z
20150402_CerP14_Frac15_top_opt_C3_01_1824	18657	TOF; CID	75.55	888.43

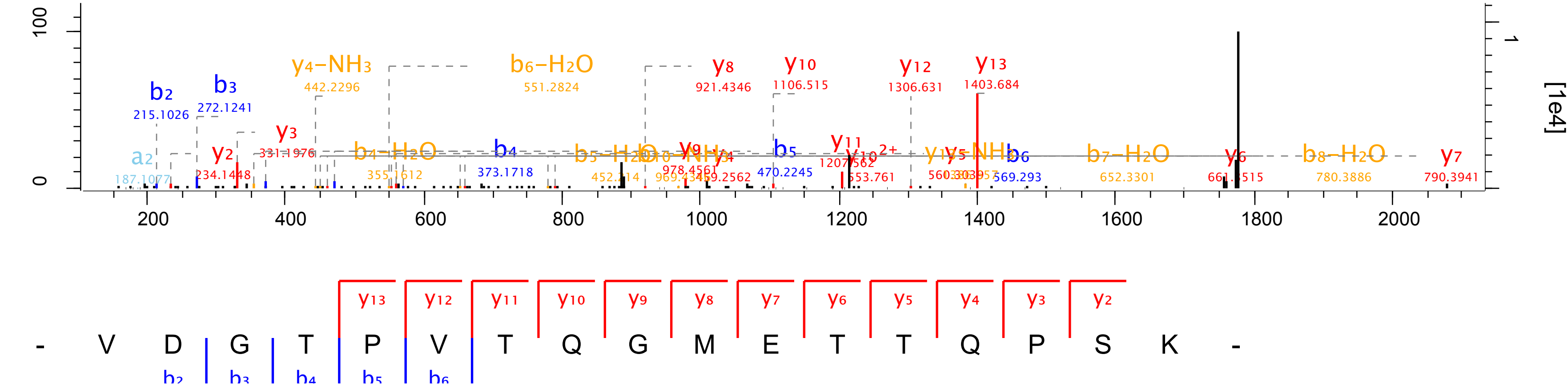

Raw file	Scan	Method	Score	m/z	Gene names
20150402_CerP14_Frac15_top_opt_C3_01_1824	24477	TOF; CID	37.18	444.25	Midn

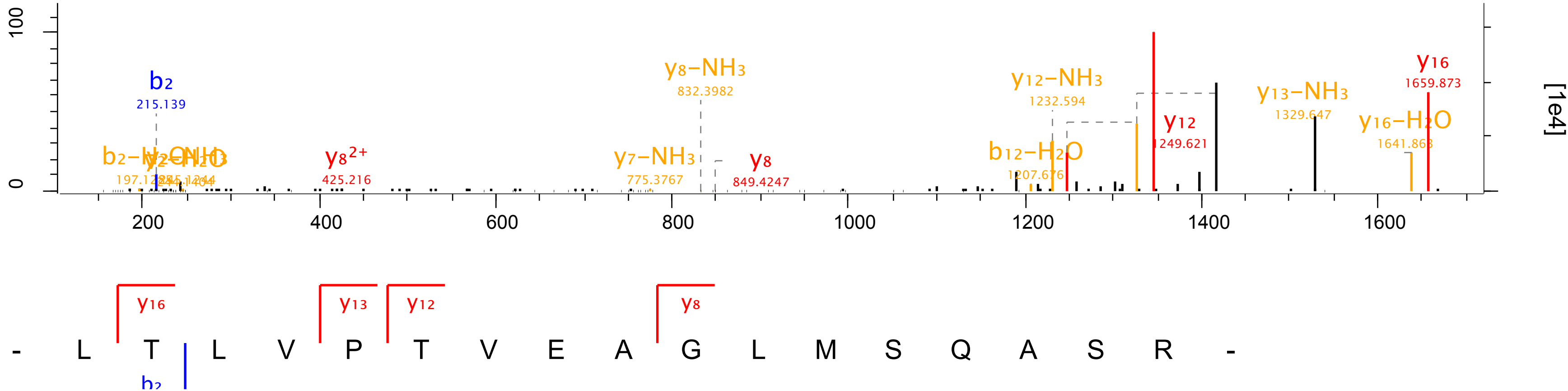

Raw file	Scan	Method	Score	m/z	Gene names
20150402_CerP14_Frac15_top_opt_C3_01_1824	30261	TOF; CID	84.51	554.84	Fbrs

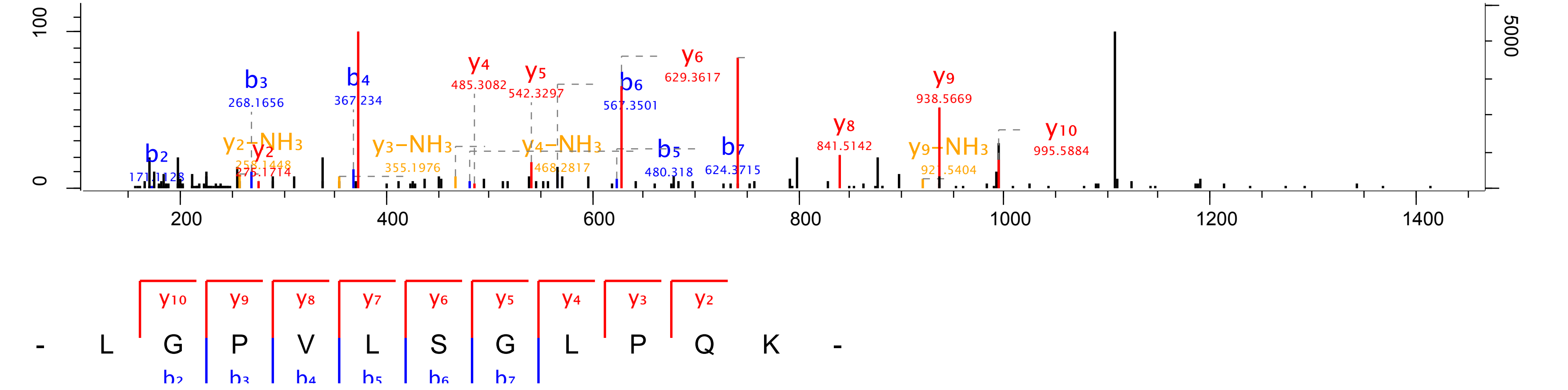

Raw file	Scan	Method	Score	m/z	Gene names
20150402_CerP14_Frac15_top_opt_C3_01_1824	32029	TOF; CID	62.78	880.14	Snapc4

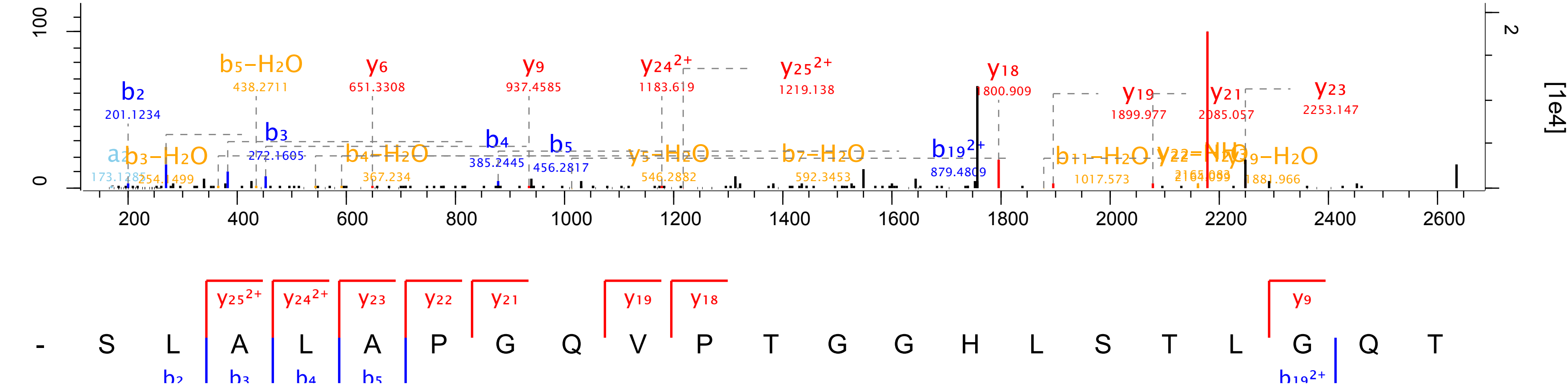

Raw file	Scan	Method	Score	m/z	Gene names
20150402_CerP14_Frac15_top_opt_C3_01_1824	34980	TOF; CID	34.72	847.76	Fam163a

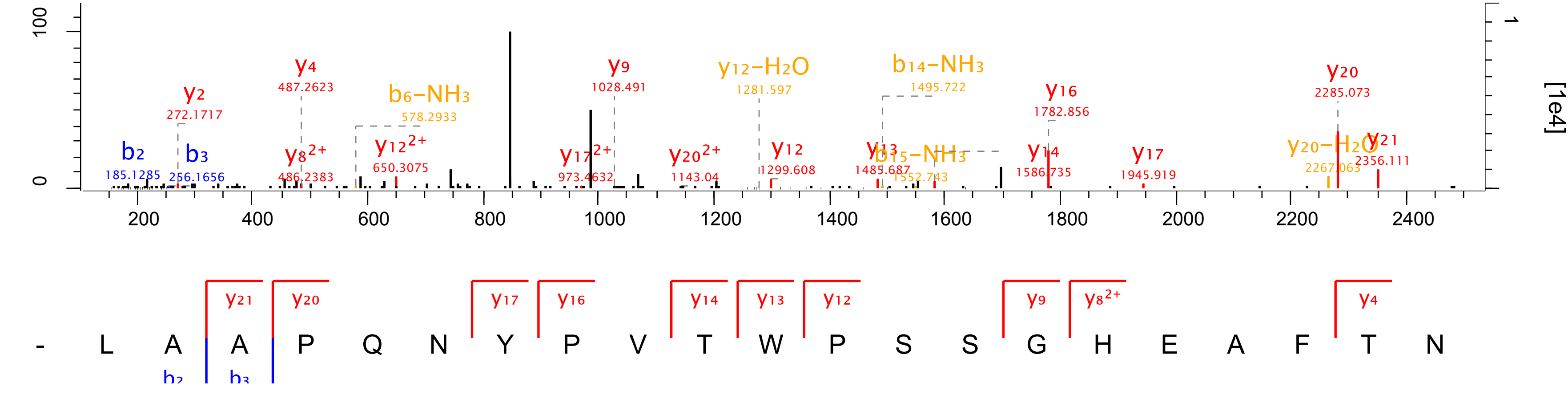

Raw file	Scan	Method	Score	m/z	Gene names
20150402_CerP14_Frac15_top_opt_C3_01_1824	38550	TOF; CID	117.25	692.89	A330050F15Rik

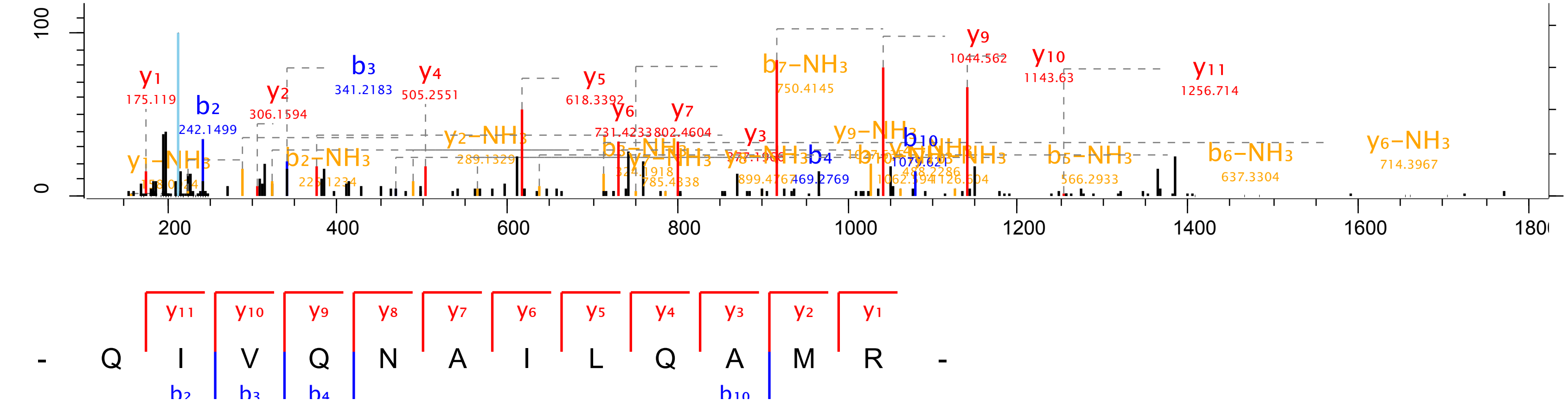

Raw file	Scan	Method	Score	m/z	Gene names
20150402_CerP14_Frac15_top_opt_C3_01_1824	44679	TOF; CID	50.78	882.46	Gpr22

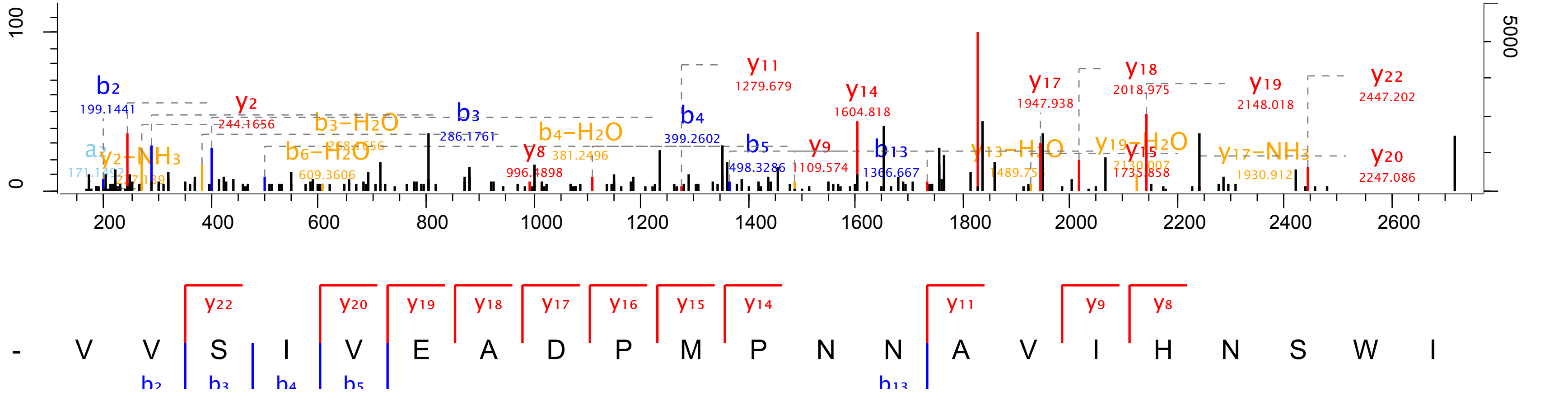

Raw file	Scan	Method	Score	m/z	Gene names
20150402_CerP14_Frac15_top_opt_C3_01_1824	54134	TOF; CID	44.68	843.12	Mbtps1

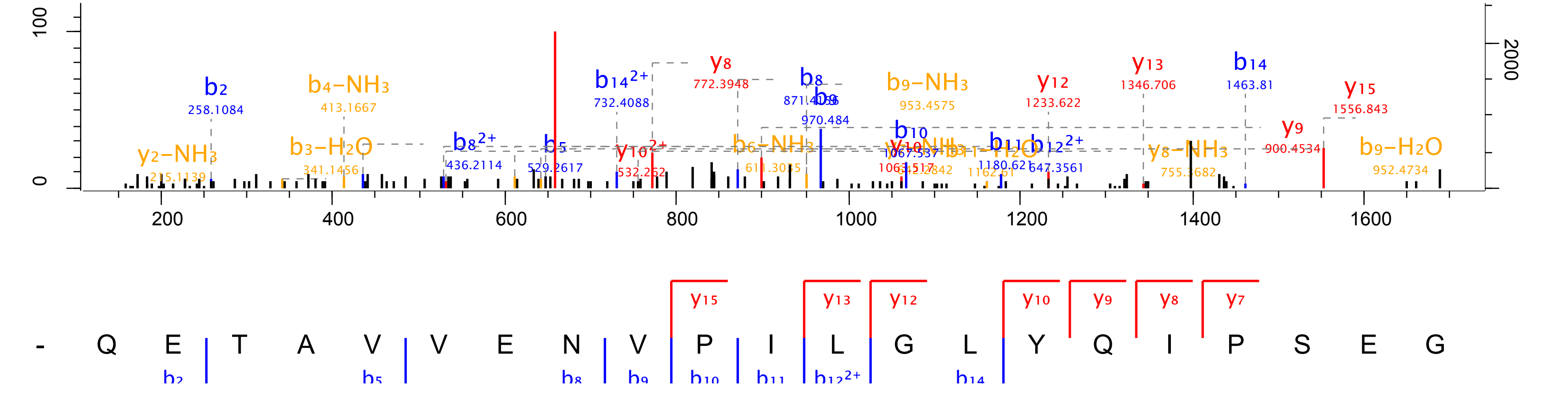

Raw file	Scan	Method	Score	m/z	Gene names
20150402_CerP14_Frac15_top_opt_C3_01_1824	56240	TOF; CID	61.42	801.92	Mkx

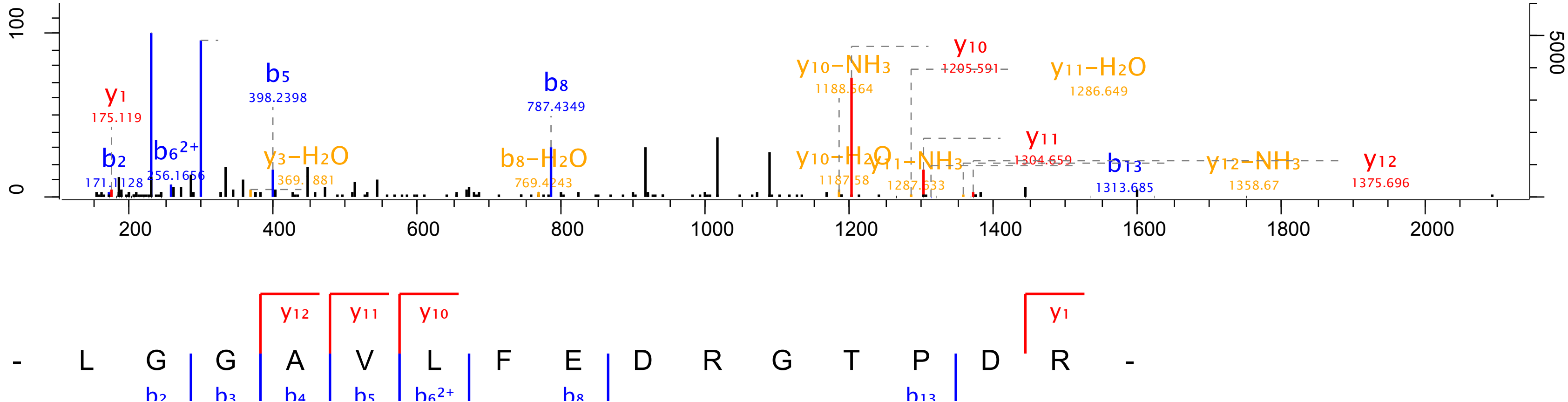

Raw file	Scan	Method	Score	m/z	Gene names
20150402_CerP14_Frac16_top_opt_C4_01_1825	11761	TOF; CID	120.57	677.85	Pex2

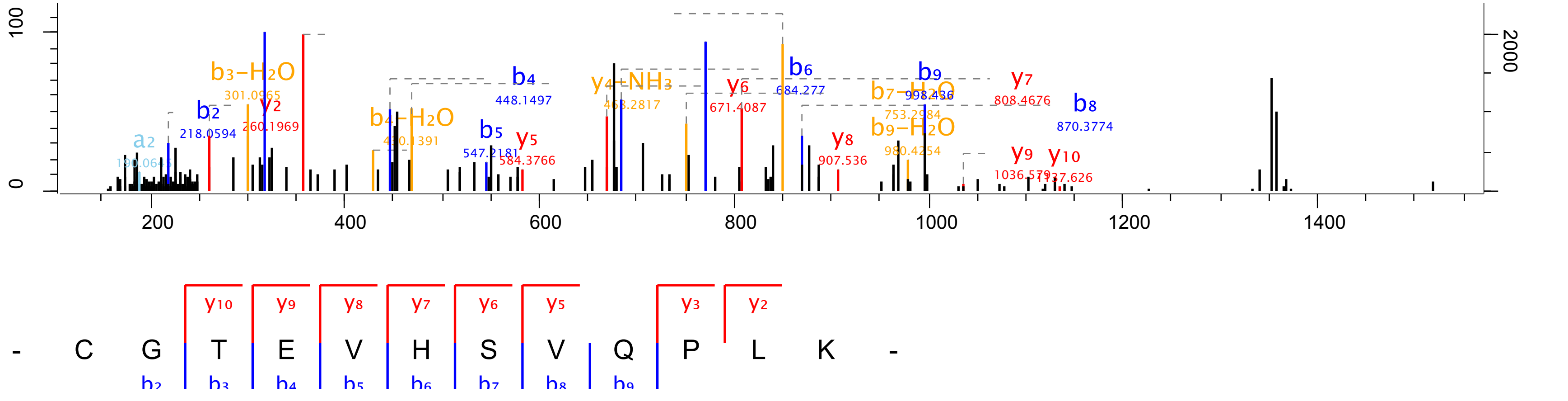

Raw file	Scan	Method	Score	m/z	Gene names
20150402_CerP14_Frac16_top_opt_C4_01_1825	12418	TOF; CID	44.54	510.28	Slc5a9

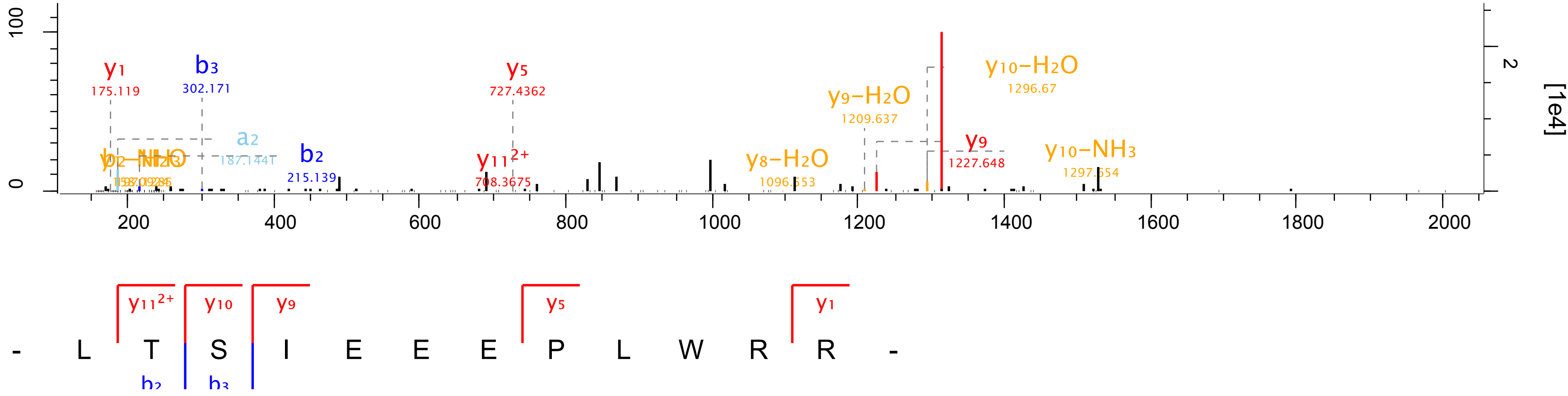

Raw file	Scan	Method	Score	m/z	Gene names
20150402_CerP14_Frac16_top_opt_C4_01_1825	14278	TOF; CID	83.4	749.37	Rin3

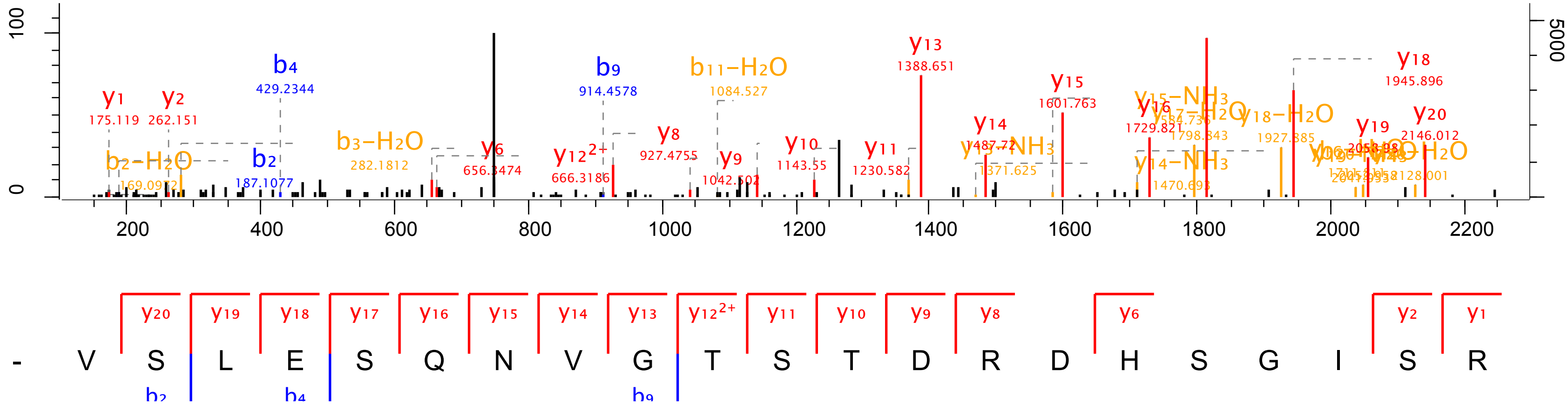

Raw file	Scan	Method	Score	m/z	Gene names
20150402_CerP14_Frac16_top_opt_C4_01_1825	15004	TOF; CID	76.07	620.33	Peli3

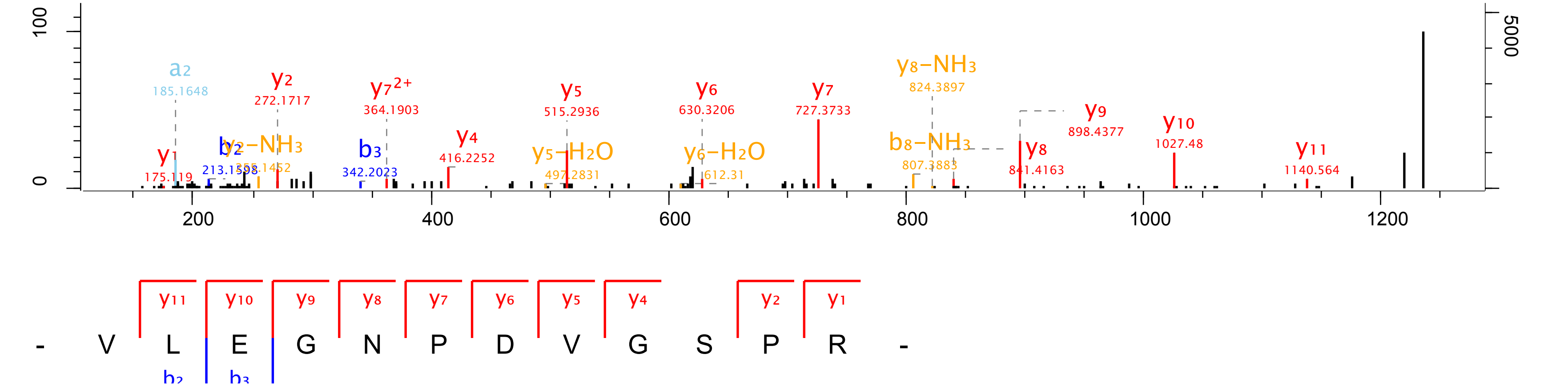

Raw file	Scan	Method	Score	m/z	Gene names
20150402_CerP14_Frac16_top_opt_C4_01_1825	17225	TOF; CID	143.89	515.81	Kcne1l

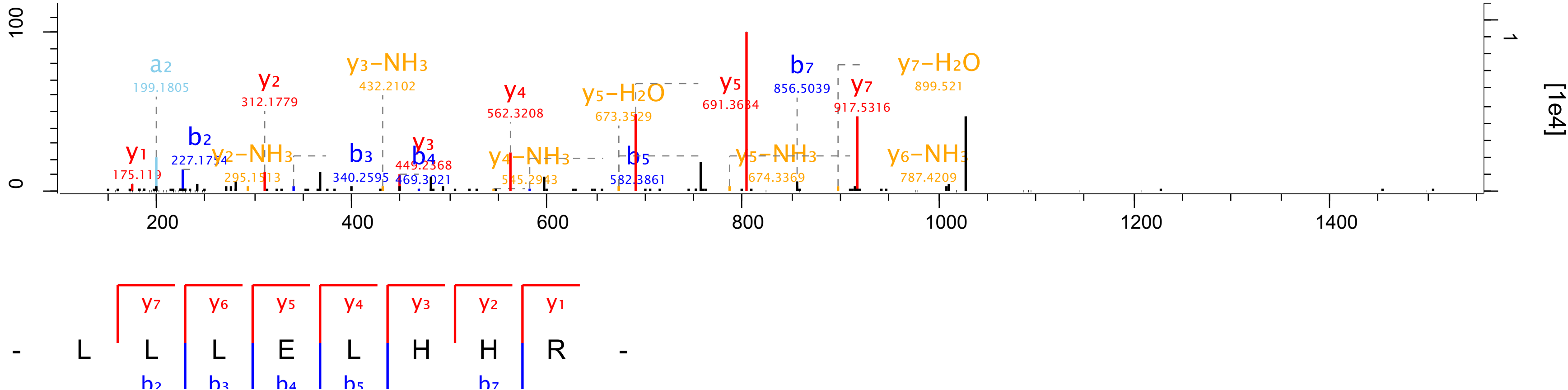

Raw file	Scan	Method	Score	m/z	Gene names
20150402_CerP14_Frac16_top_opt_C4_01_1825	17397	TOF; CID	82.42	638.3	Cks1b;Cks1brt

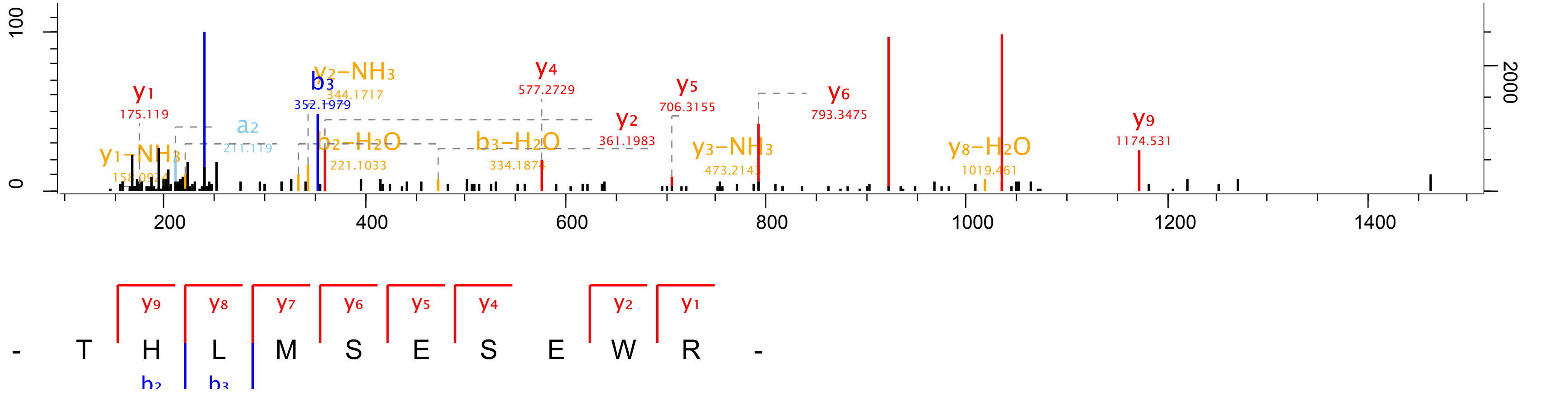

Raw file	Scan	Method	Score	m/z	Gene names
20150402_CerP14_Frac16_top_opt_C4_01_1825	18862	TOF; CID	81.02	613.8	Cebpzos

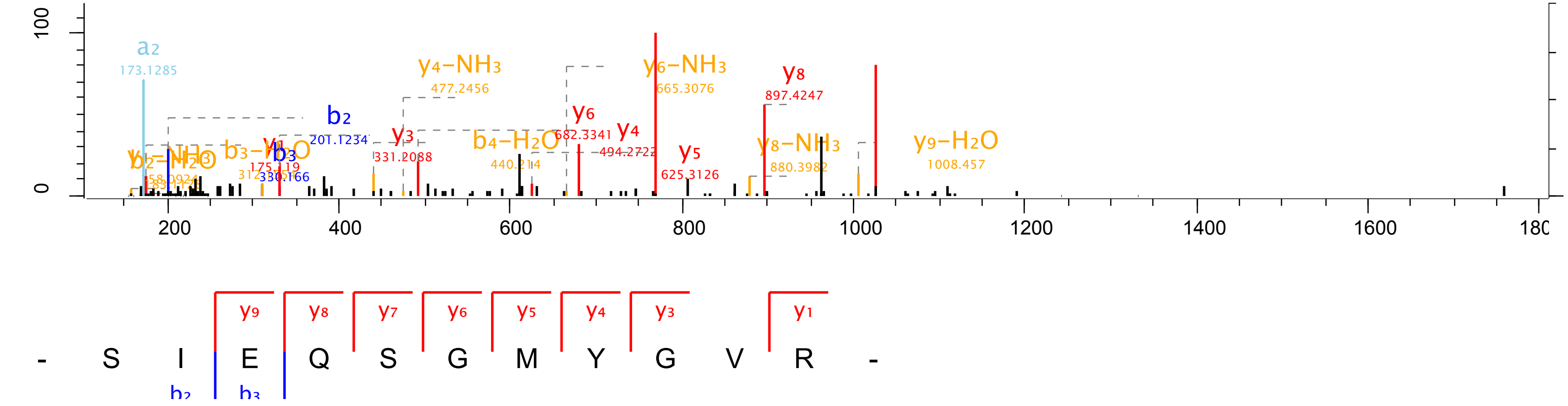

Raw file	Scan	Method	Score	m/z	Gene names
20150402_CerP14_Frac16_top_opt_C4_01_1825	20577	TOF; CID	51.57	657.33	Fbln2

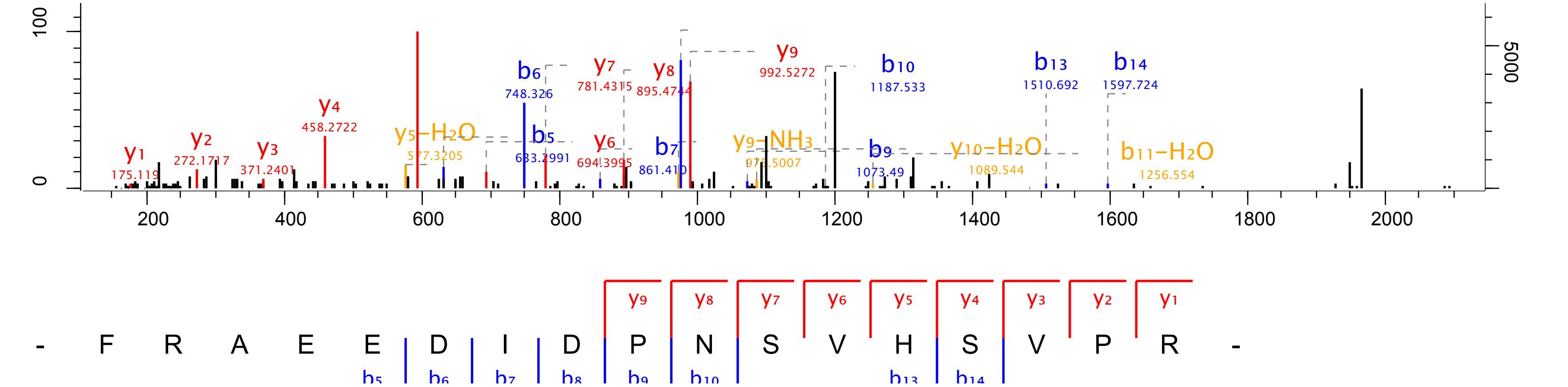

Raw file	Scan	Method	Score	m/z	Gene names
20150402_CerP14_Frac16_top_opt_C4_01_1825	22133	TOF; CID	76.15	935.42	Ramp2

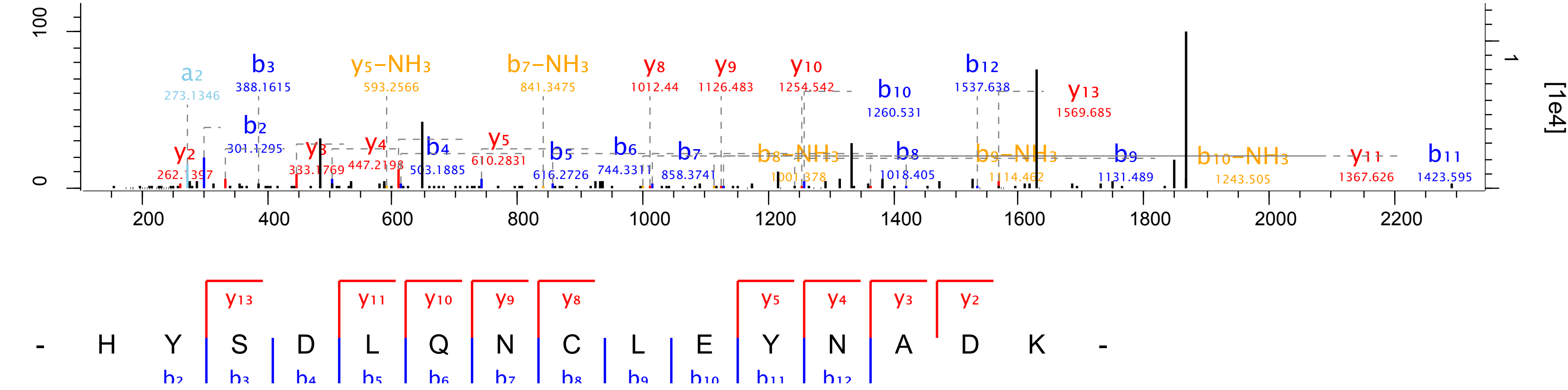

Raw file	Scan	Method	Score	m/z	Gene names
20150402_CerP14_Frac16_top_opt_C4_01_1825	26460	TOF; CID	113.14	891.4	Dph3

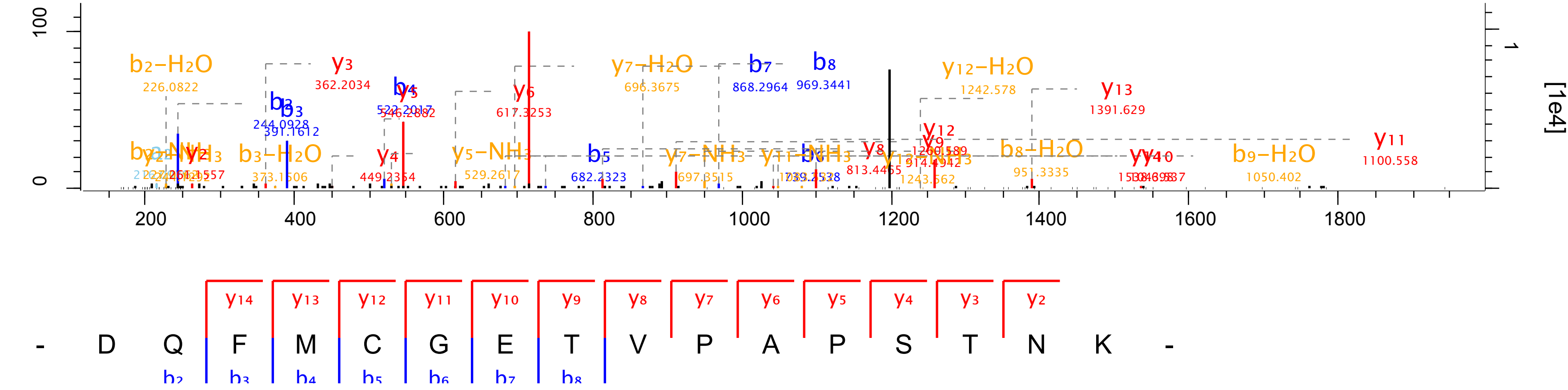

Raw file	Scan	Method	Score	m/z	Gene names
20150402_CerP14_Frac16_top_opt_C4_01_1825	30984	TOF; CID	59.71	505.91	Rrp15

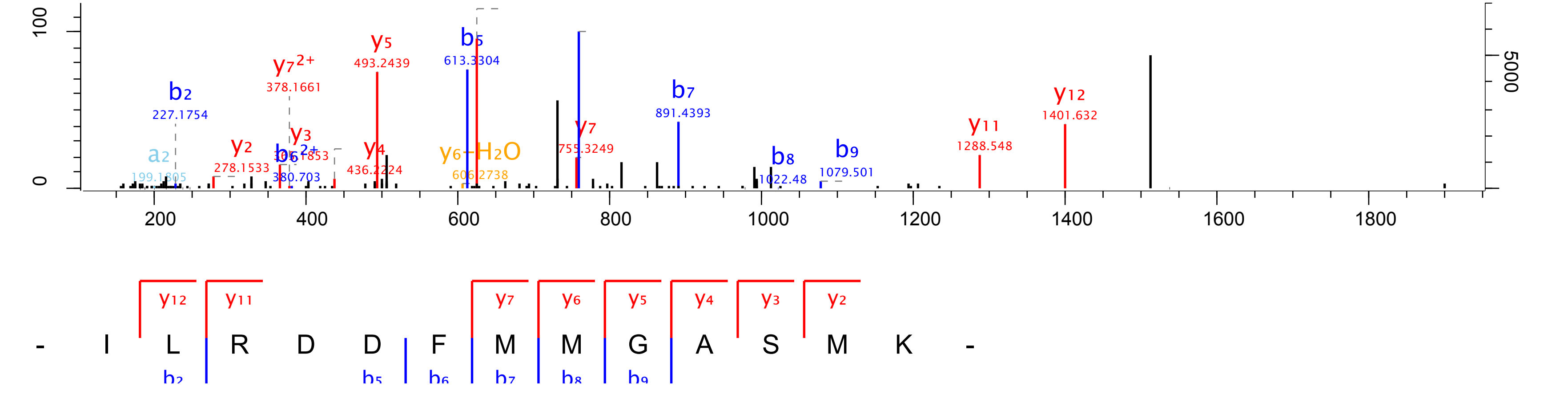

Raw file	Scan	Method	Score	m/z	Gene names
20150402_CerP14_Frac16_top_opt_C4_01_1825	33947	TOF; CID	53.45	729.35	Rsl24d1;Gm20509

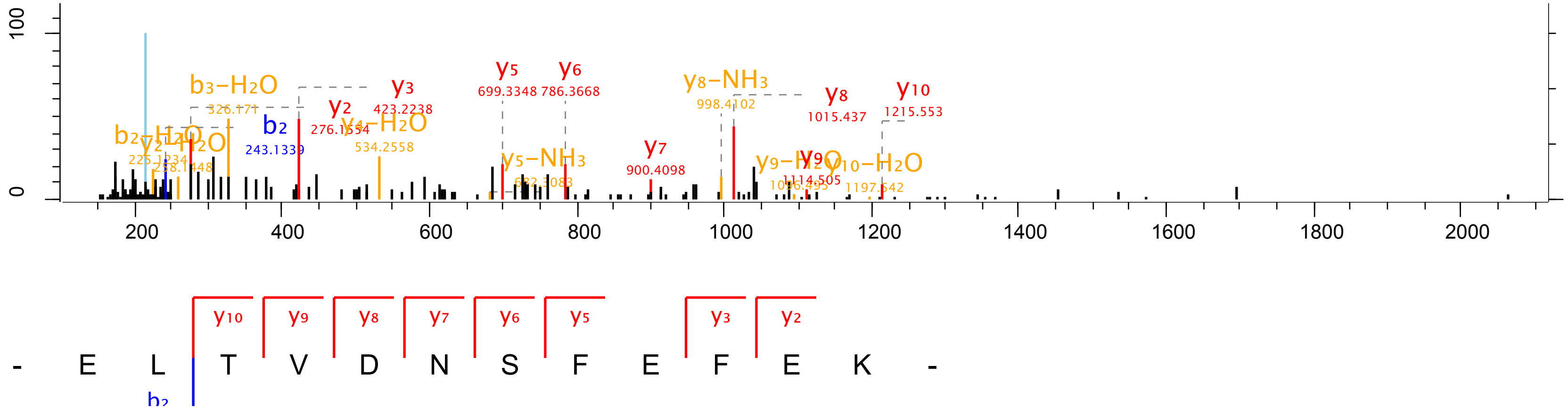

Raw file	Scan	Method	Score	m/z	Gene names
20150402_CerP14_Frac16_top_opt_C4_01_1825	35938	TOF; CID	70.94	592.83	Spag6

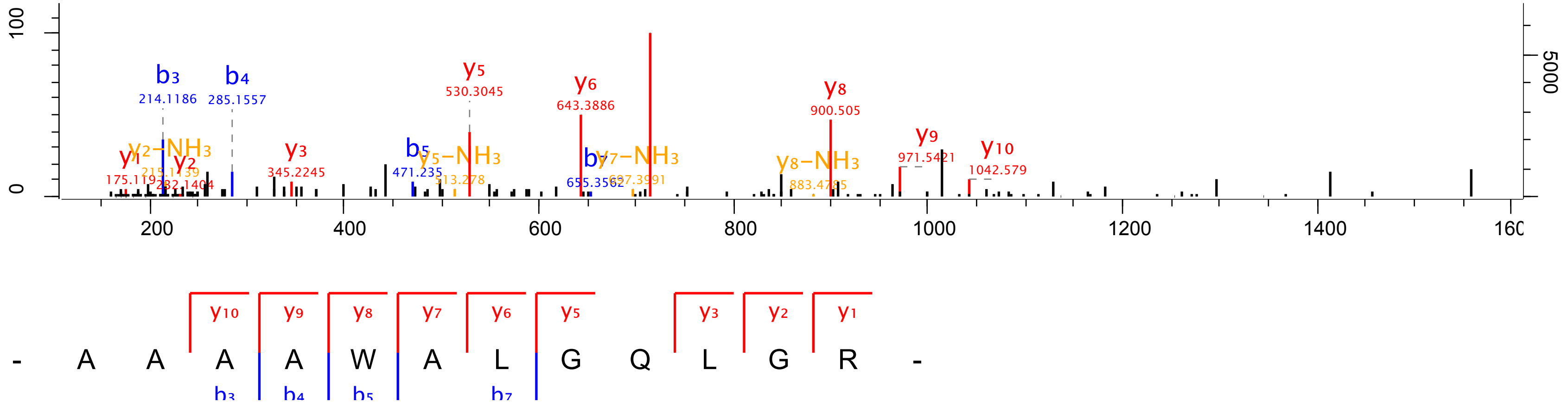

Raw file	Scan	Method	Score	m/z	Gene names
20150402_CerP14_Frac16_top_opt_C4_01_1825	37204	TOF; CID	107.69	504.73	Tmem258

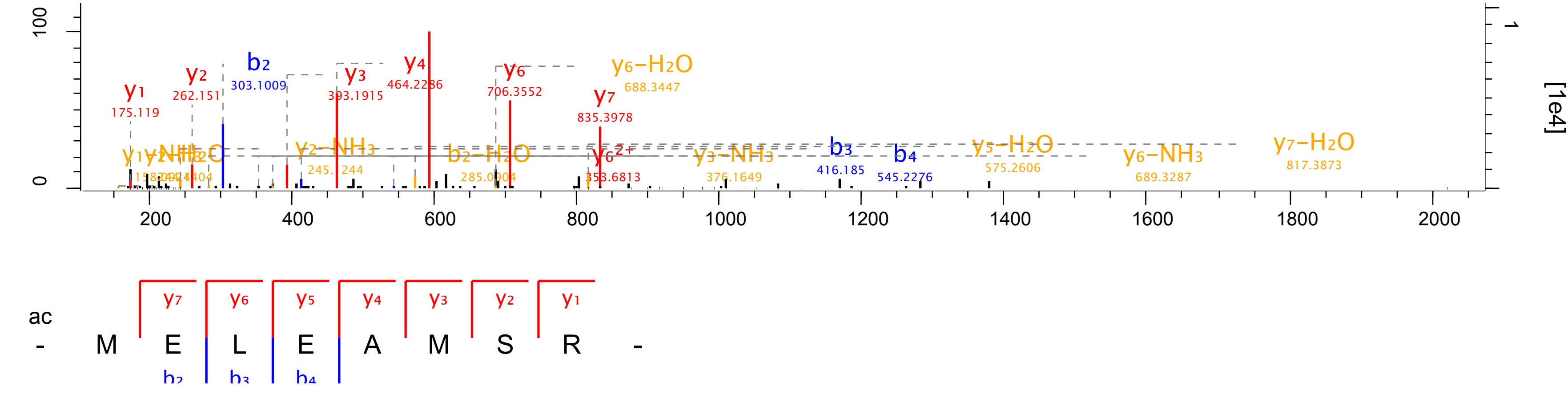

Raw file	Scan	Method	Score	m/z	Gene names
20150402_CerP14_Frac16_top_opt_C4_01_1825	37874	TOF; CID	104.47	705.7	Tmem38a

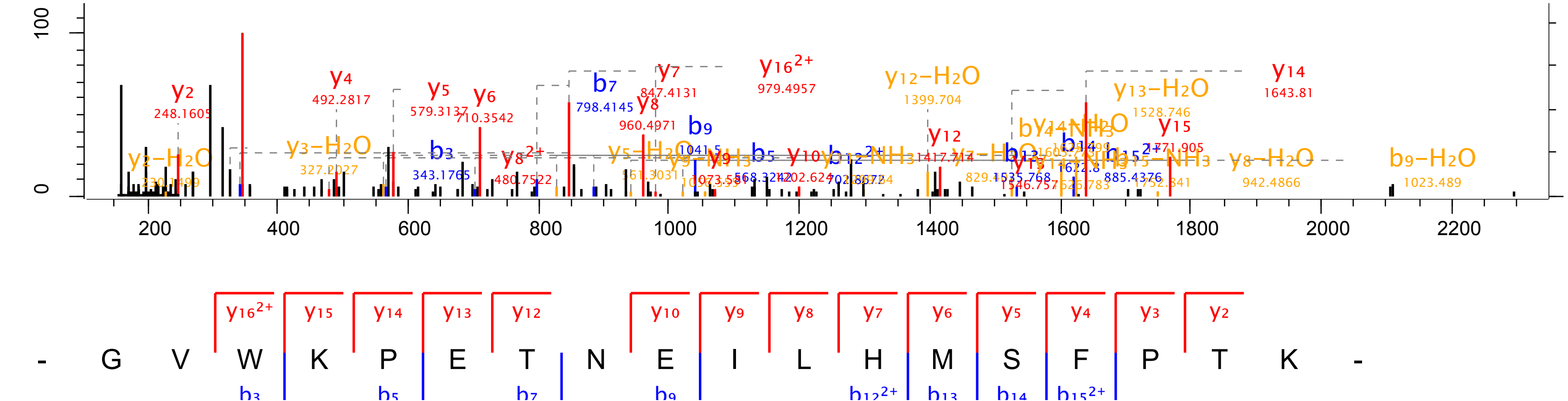

Raw file	Scan	Method	Score	m/z
20150402_CerP14_Frac16_top_opt_C4_01_1825	48486	TOF; CID	81.51	928.02

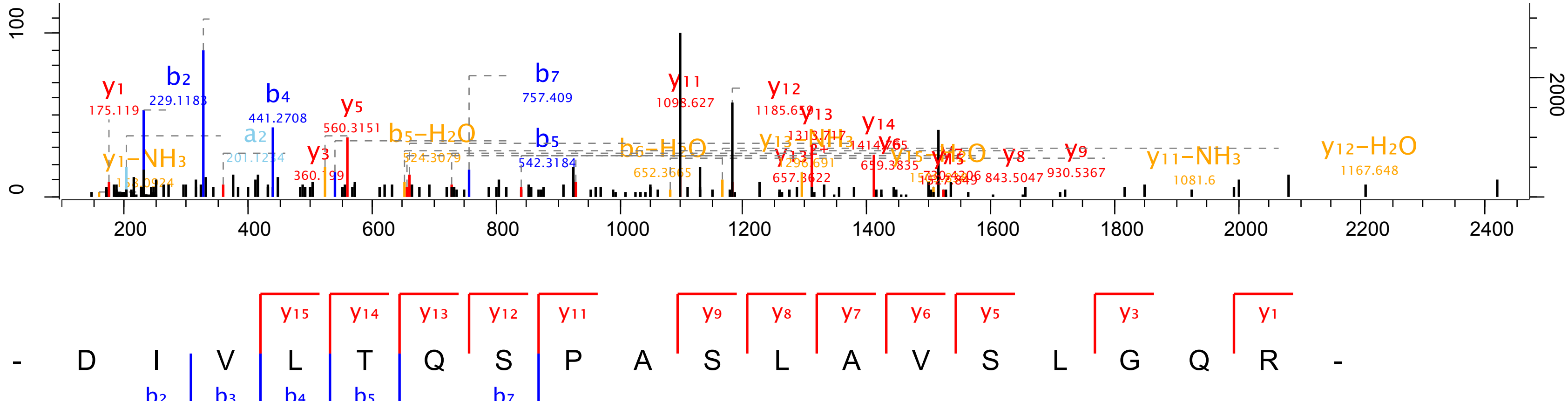

Raw file	Scan	Method	Score	m/z	Gene names
20150402_CerP14_Frac16_top_opt_C4_01_1825	54250	TOF; CID	74.44	785.43	Rbm48

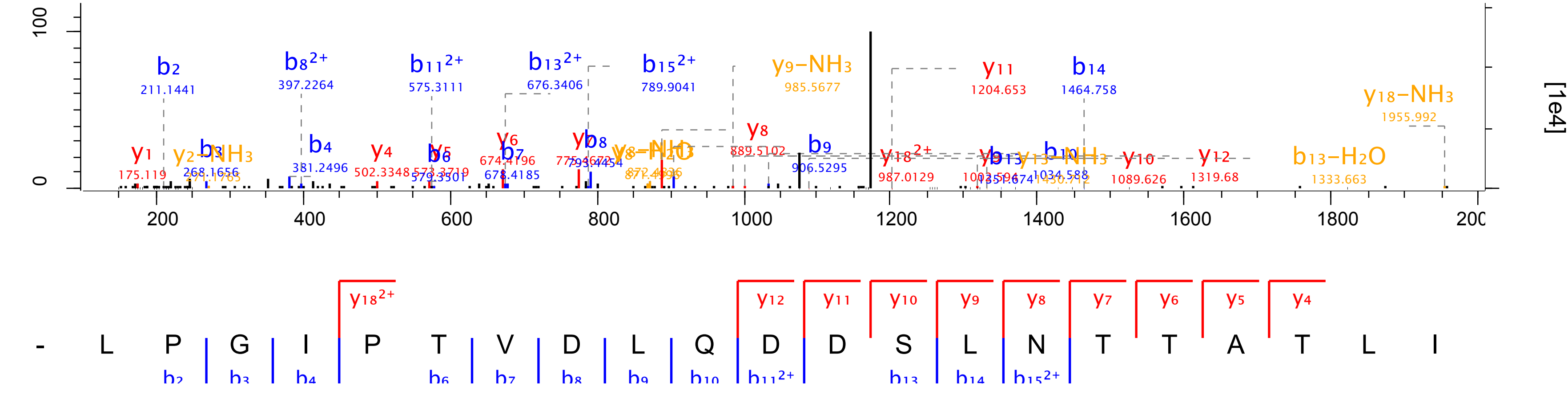

Raw file	Scan	Method	Score	m/z	Gene names
20150402_CerP14_Frac17_top_opt_C5_01_1826	13816	TOF; CID	97.97	515.28	Trhr

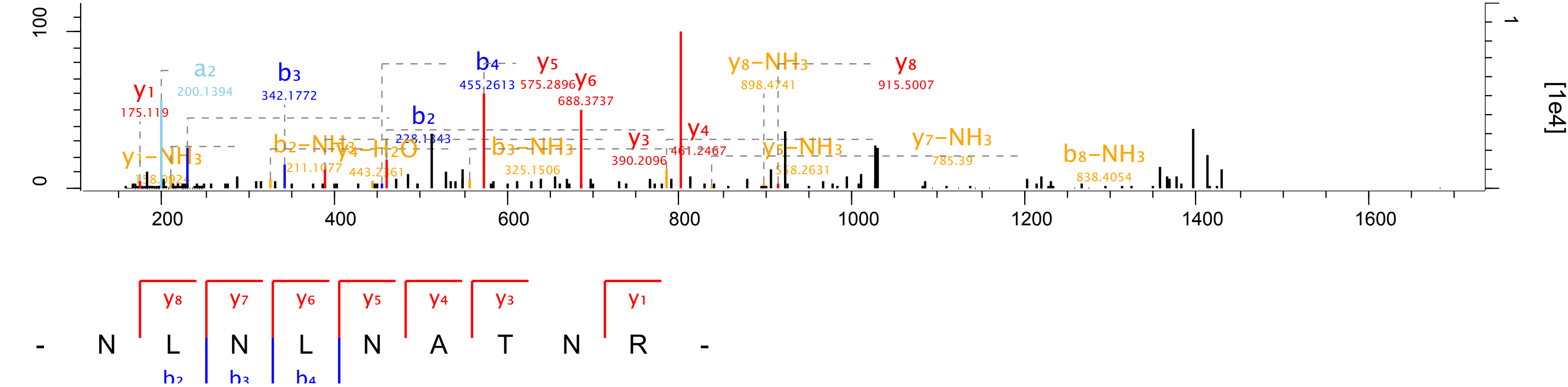

Raw file	Scan	Method	Score	m/z	Gene names
20150402_CerP14_Frac17_top_opt_C5_01_1826	14724	TOF; CID	86.14	493.78	Habp2

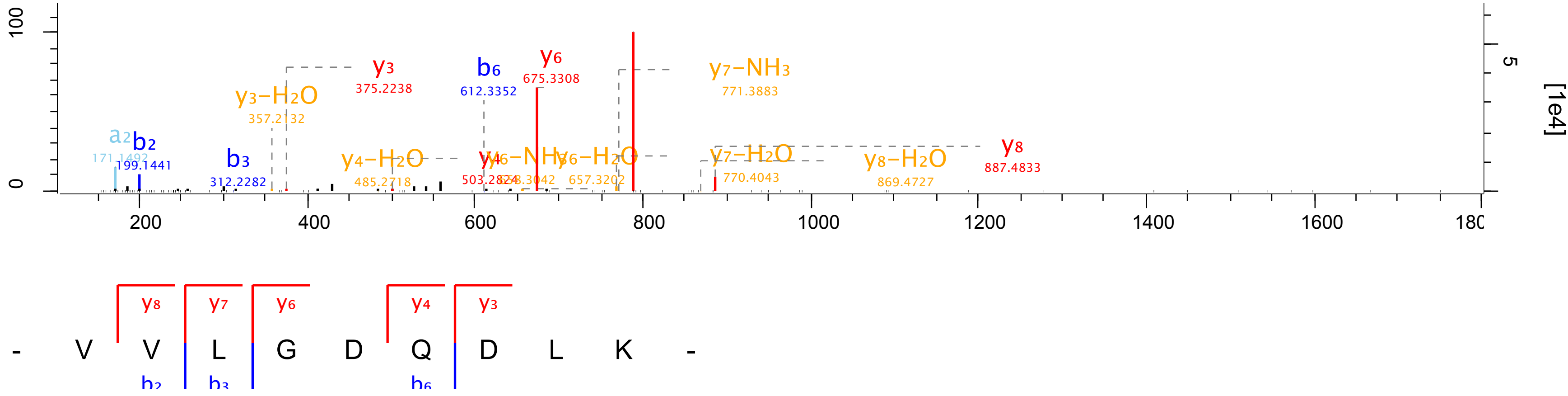

Raw file	Scan	Method	Score	m/z	Gene names
20150402_CerP14_Frac17_top_opt_C5_01_1826	20093	TOF; CID	81.57	629.82	Gemin7

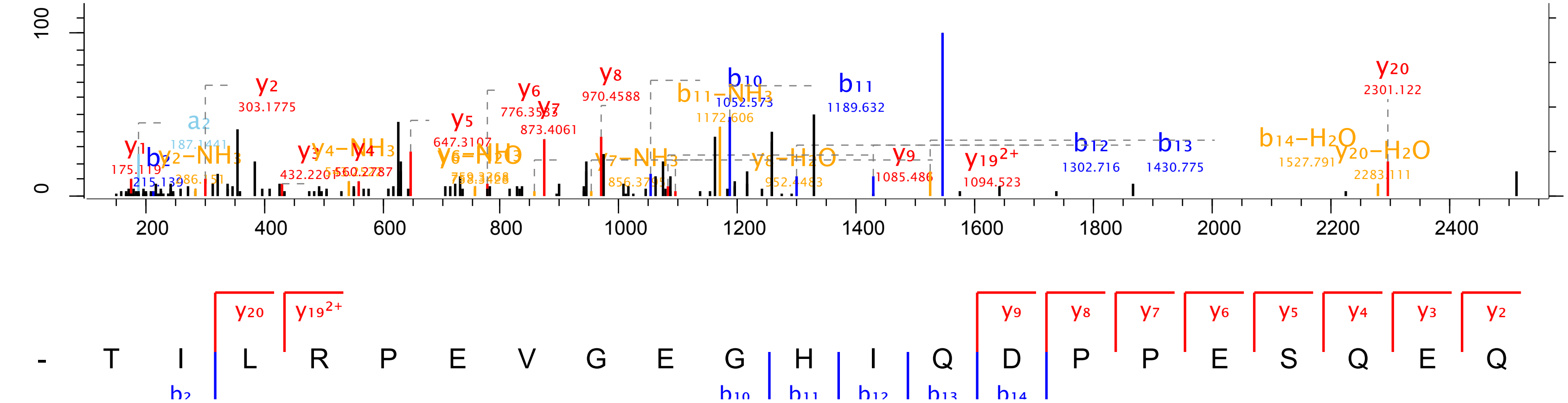

Raw file	Scan	Method	Score	m/z	Gene names
20150402_CerP14_Frac17_top_opt_C5_01_1826	22280	TOF; CID	54.86	858.9	Elovl6

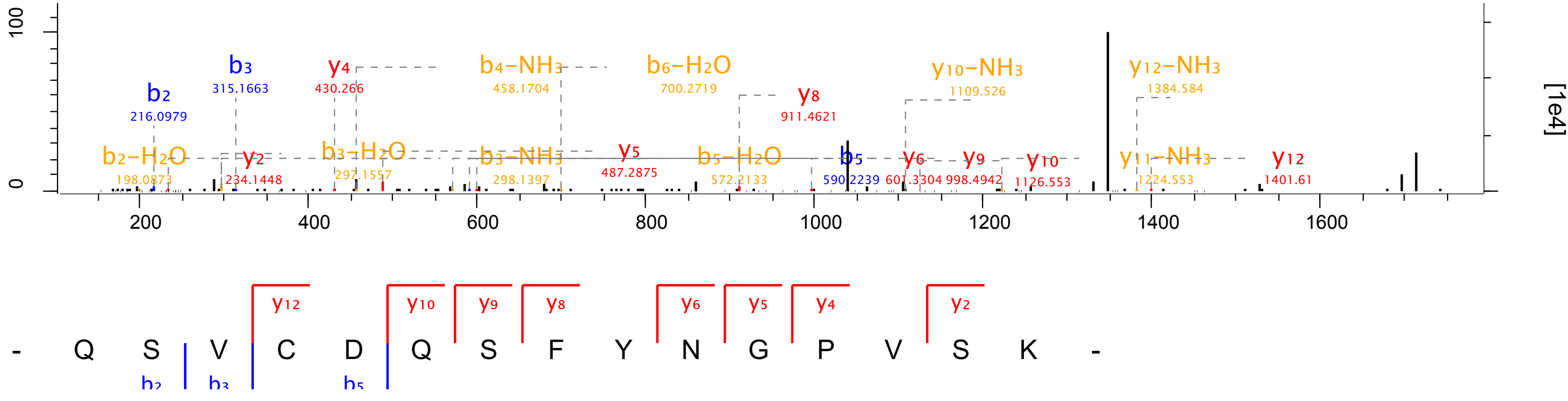

Raw file	Scan	Method	Score	m/z	Gene names
20150402_CerP14_Frac17_top_opt_C5_01_1826	24102	TOF; CID	142.54	836.36	mt-Nd3;Mtnd3

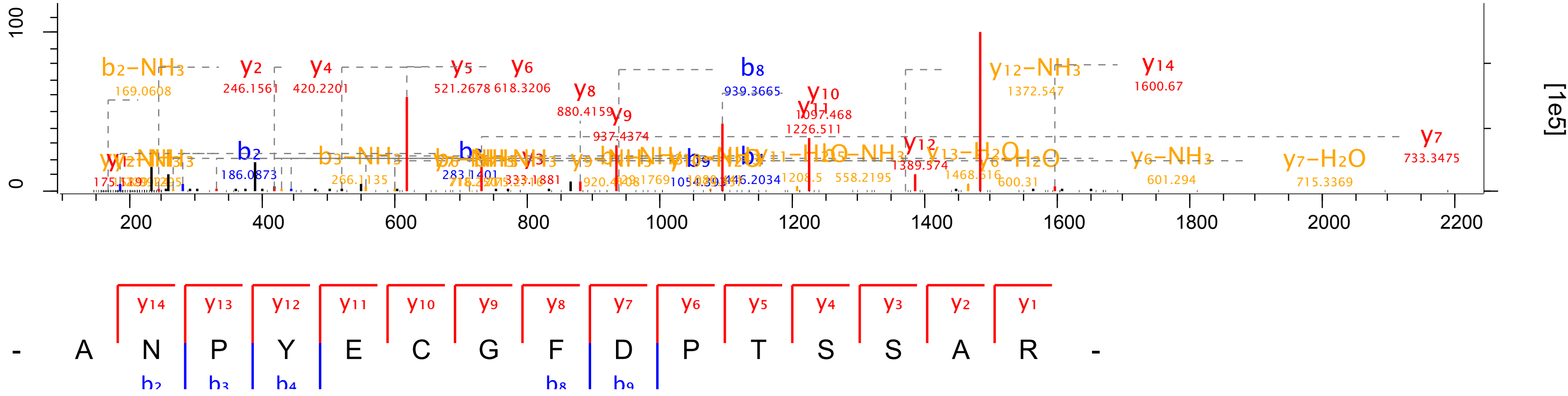

Raw file	Scan	Method	Score	m/z	Gene names
20150402_CerP14_Frac17_top_opt_C5_01_1826	27436	TOF; CID	63.49	542.31	Fam43a

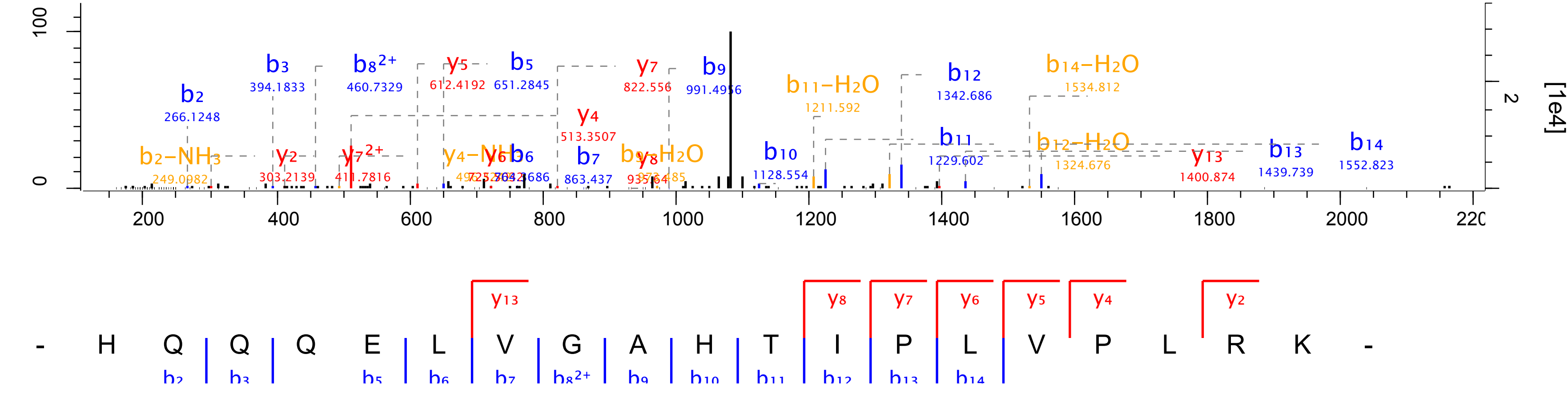

Raw file	Scan	Method	Score	m/z	Gene names
20150402_CerP14_Frac17_top_opt_C5_01_1826	35599	TOF; CID	77.03	881.5	Krtcap2

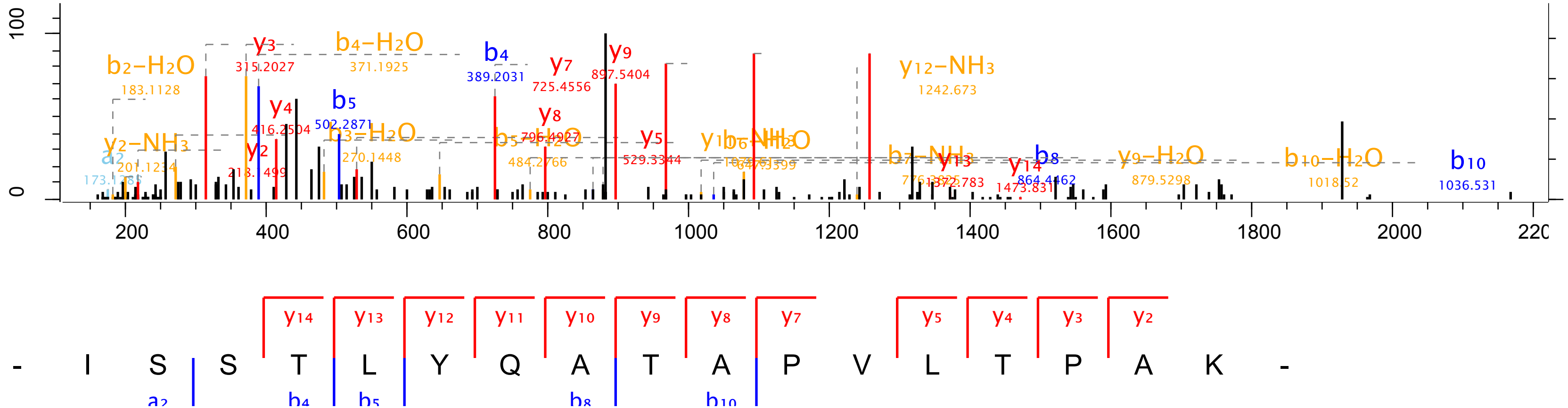

Raw file	Scan	Method	Score	m/z	Gene names
20150402_CerP14_Frac17_top_opt_C5_01_1826	38298	TOF; CID	83.76	844.47	Cxxc4

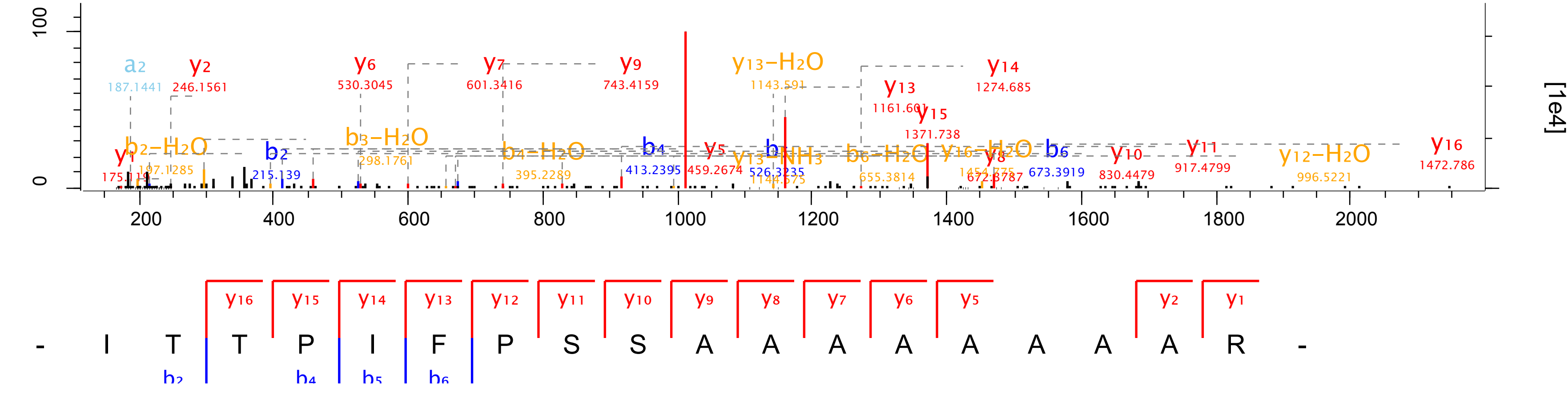

Raw file	Scan	Method	Score	m/z	Gene names
20150402_CerP14_Frac17_top_opt_C5_01_1826	46482	TOF; CID	74.66	971.05	Evi2a

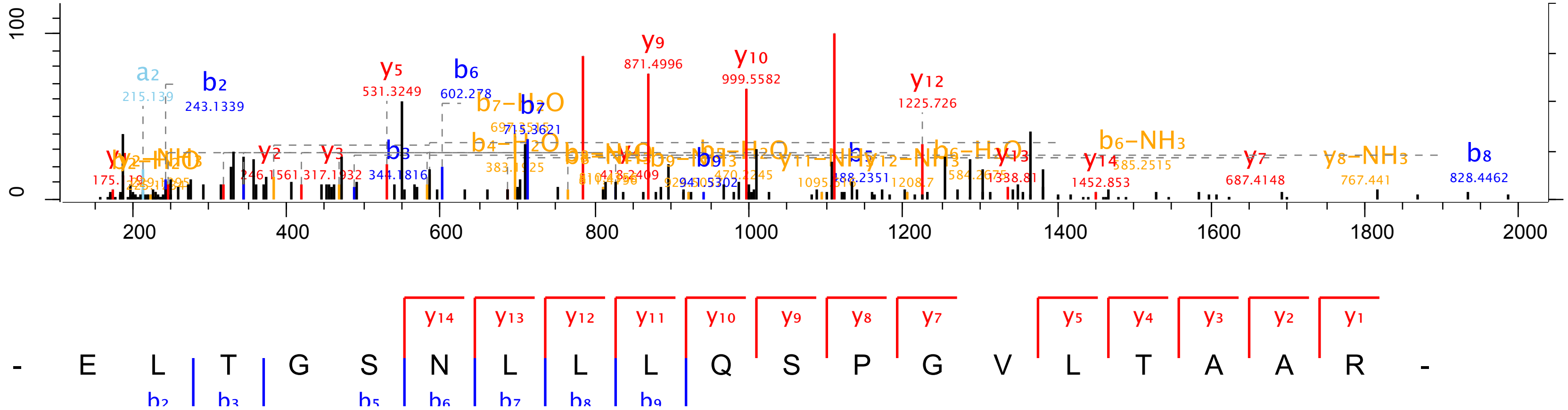

Raw file	Scan	Method	Score	m/z	Gene names
20150402_CerP14_Frac17_top_opt_C5_01_1826	48325	TOF; CID	59.23	766.4	Fam86;Fam86a

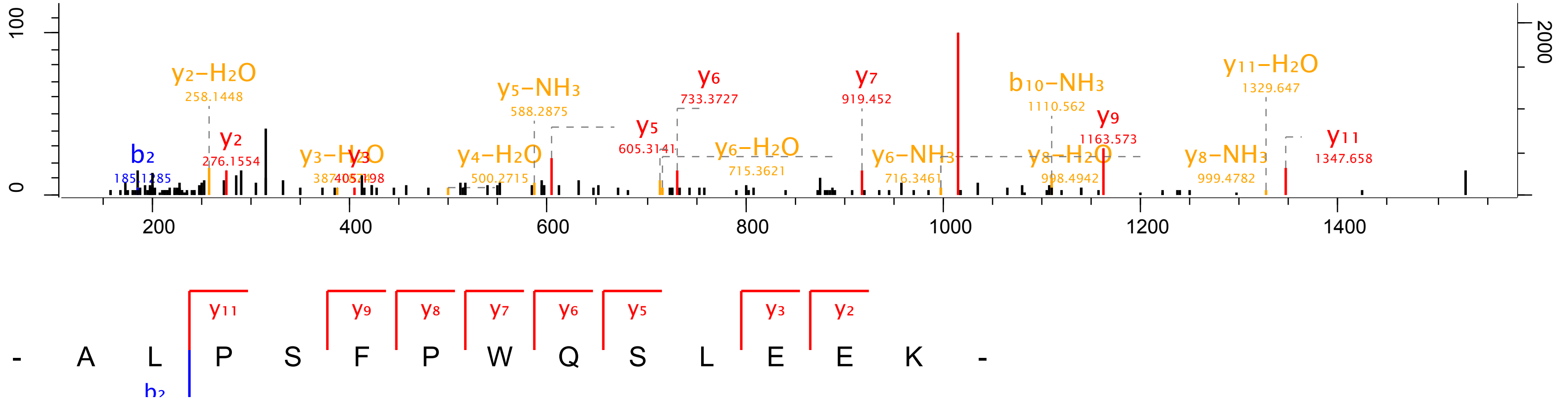

Raw file	Scan	Method	Score	m/z	Gene names
20150402_CerP14_Frac17_top_opt_C5_01_1826	48986	TOF; CID	33.71	836.4	Nmrk1

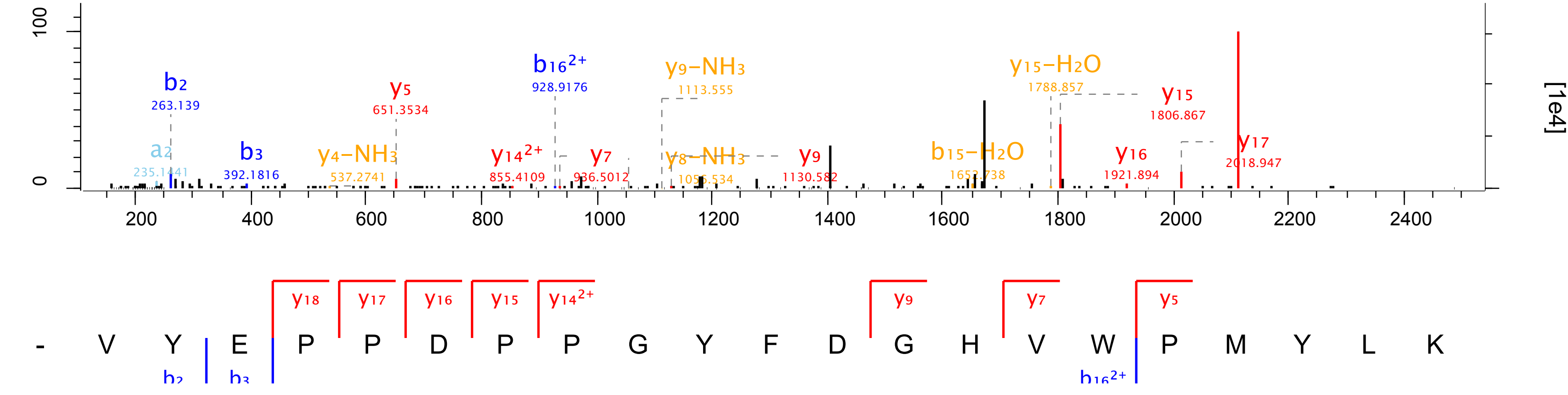

Raw file	Scan	Method	Score	m/z	Gene names
20150402_CerP14_Frac17_top_opt_C5_01_1826	49188	TOF; CID	81.71	497.59	Kctd7

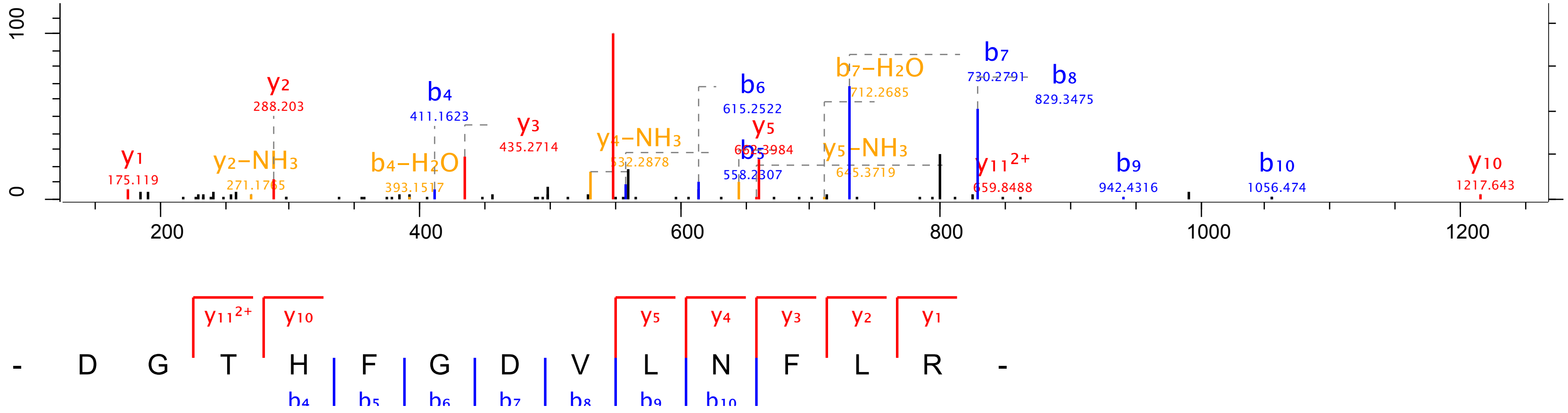

Raw file	Scan	Method	Score	m/z	Gene names
20150402_CerP14_Frac17_top_opt_C5_01_1826	50112	TOF; CID	32.8	707.38	Synpo2l

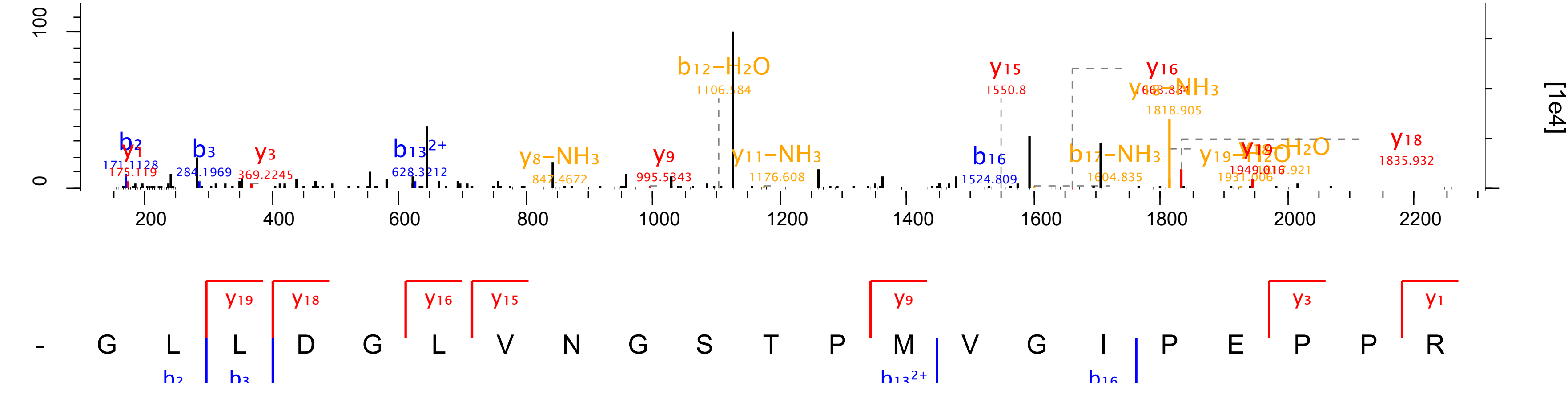

Raw file	Scan	Method	Score	m/z	Gene names
20150402_CerP14_Frac17_top_opt_C5_01_1826	54460	TOF; CID	105.96	1230.62	Armc7

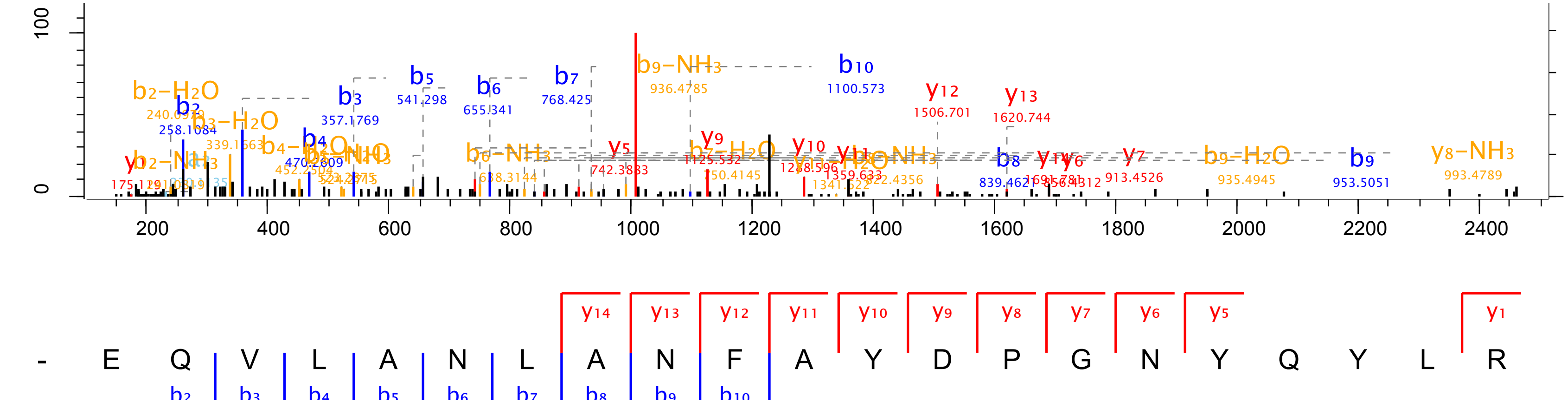

Raw file	Scan	Method	Score	m/z	Gene names
20150402_CerP14_Frac18_top_opt_C6_01_1827	6360	TOF; CID	95.36	535.77	Desi2

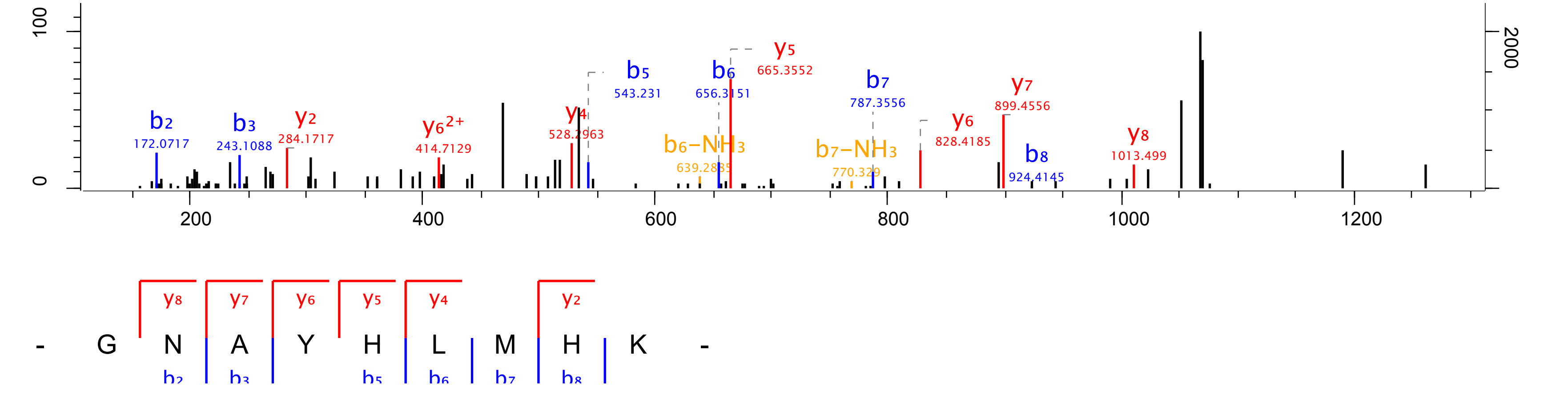

Raw file	Scan	Method	Score	m/z	Gene names
20150402_CerP14_Frac18_top_opt_C6_01_1827	18176	TOF; CID	88.02	644.85	Ropn1l

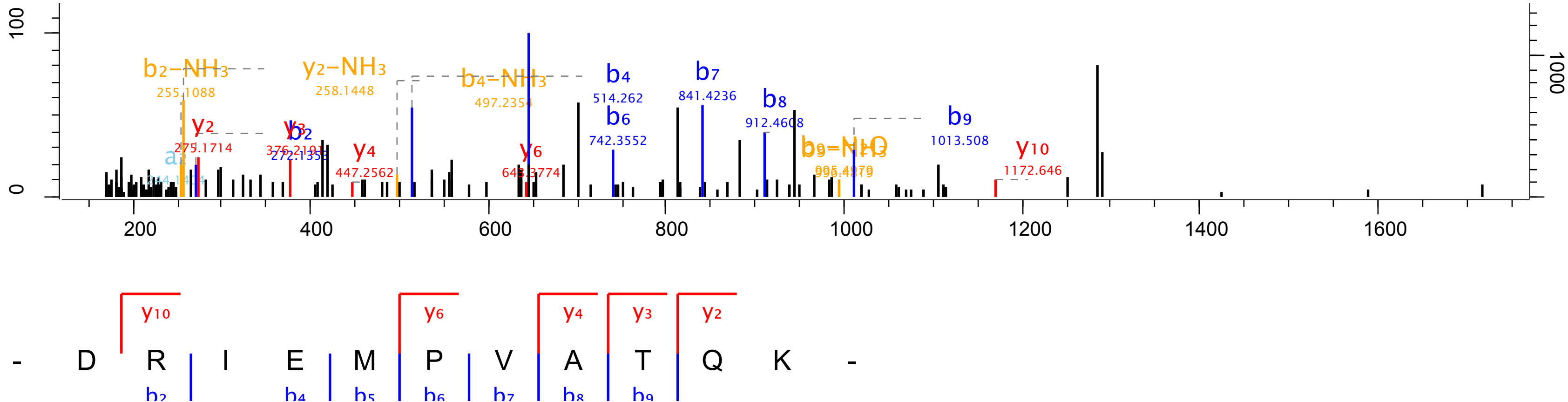

Raw file	Scan	Method	Score	m/z	Gene names
20150402_CerP14_Frac18_top_opt_C6_01_1827	27598	TOF; CID	53.45	705.36	Cldn5

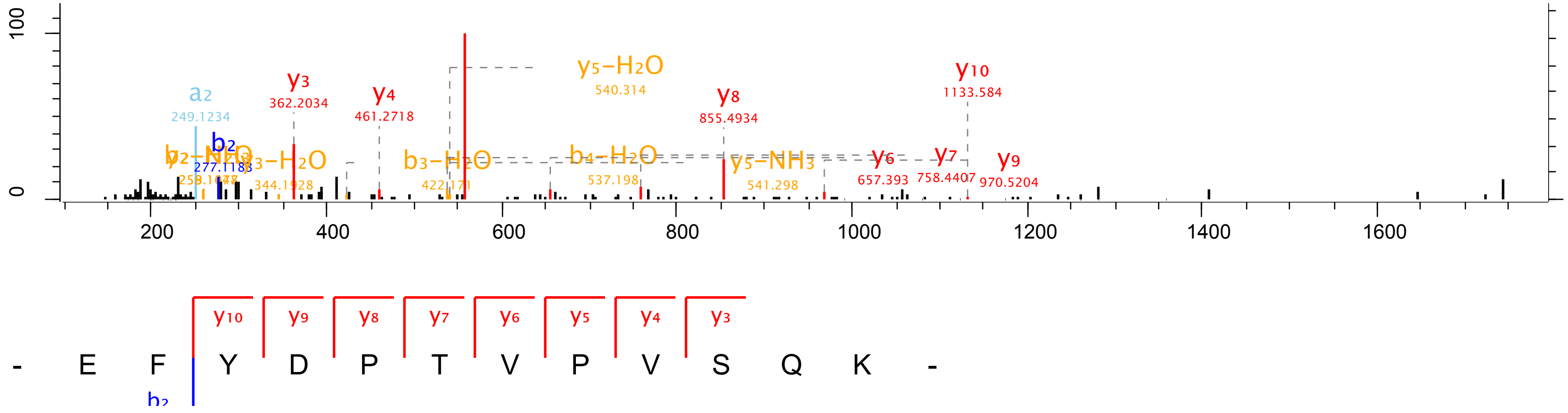

Raw file	Scan	Method	Score	m/z	Gene names
20150402_CerP14_Frac18_top_opt_C6_01_1827	29115	TOF; CID	79.49	622.34	Dhdds

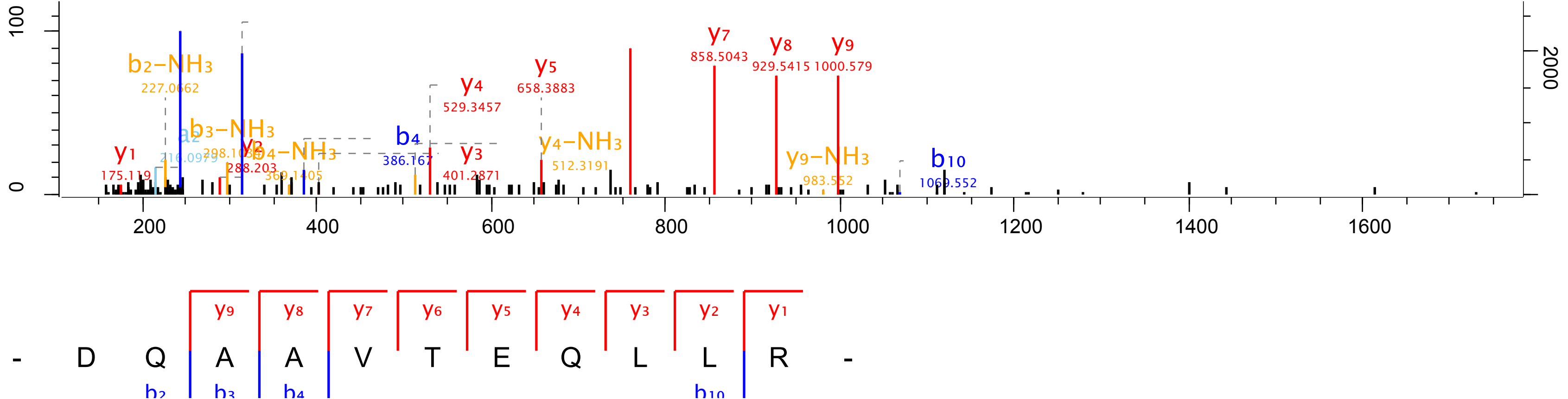

Raw file	Scan	Method	Score	m/z	Gene names
20150402_CerP14_Frac18_top_opt_C6_01_1827	29998	TOF; CID	72.34	643.35	4932411E22Rik

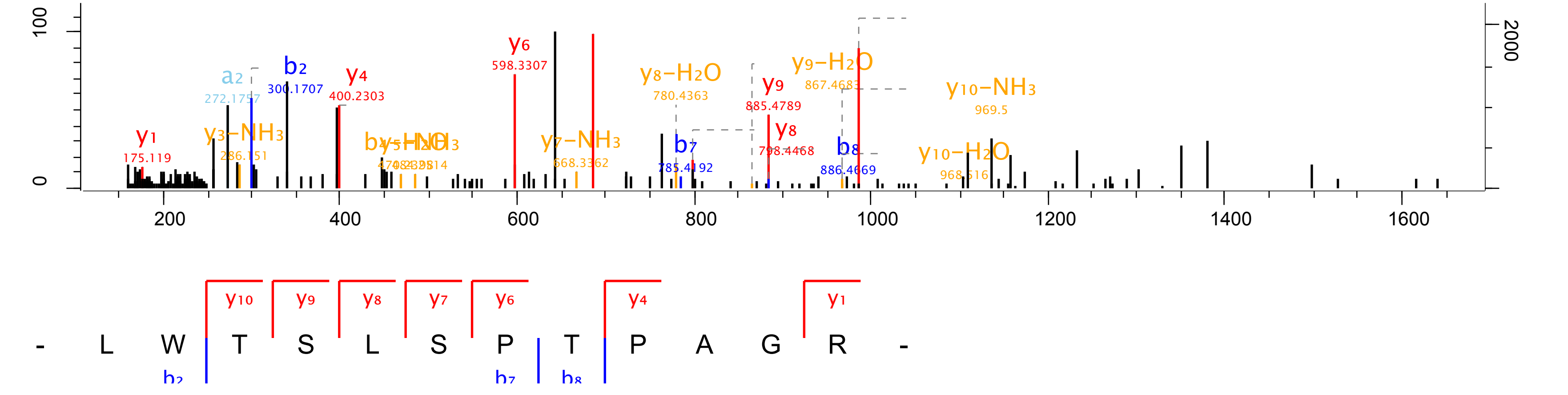

Raw file	Scan	Method	Score	m/z	Gene names
20150402_CerP14_Frac18_top_opt_C6_01_1827	32343	TOF; CID	50.78	897.46	Gpr137

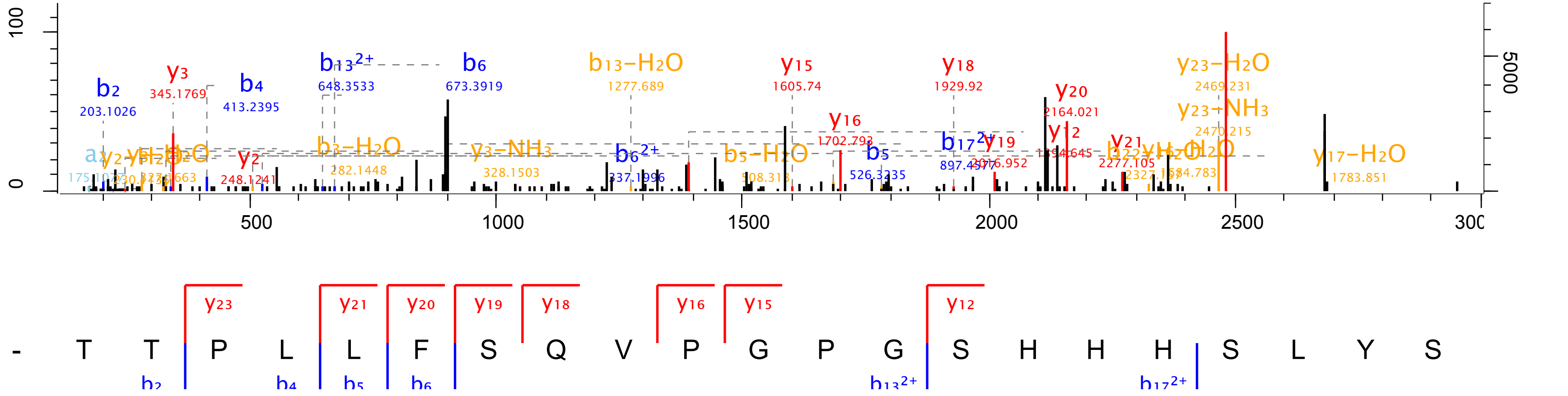

Raw file	Scan	Method	Score	m/z	Gene names
20150402_CerP14_Frac18_top_opt_C6_01_1827	40002	TOF; CID	80.69	538.31	Sgms1

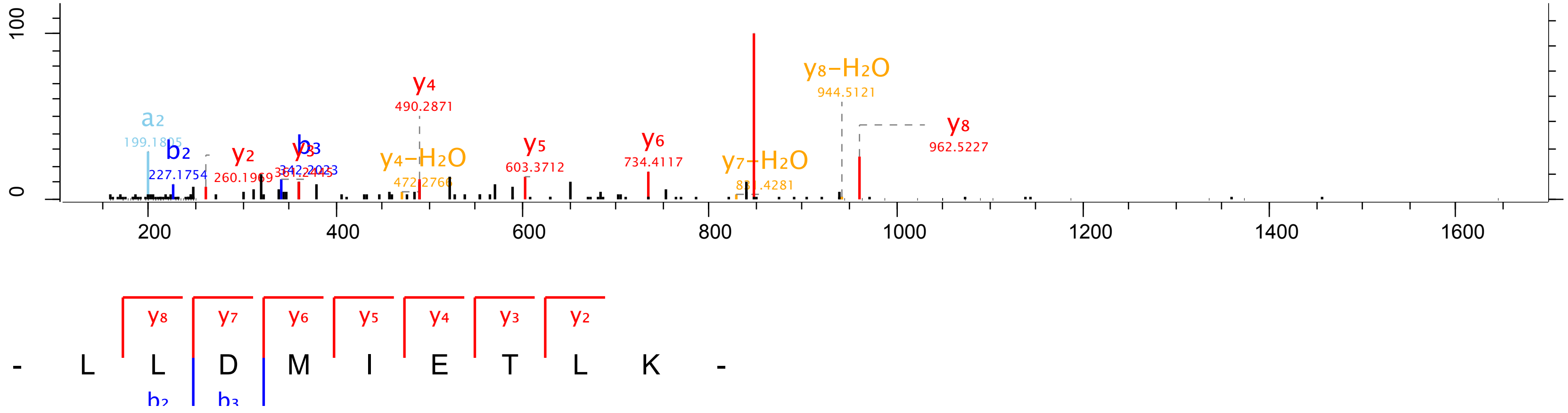

Raw file	Scan	Method	Score	m/z	Gene names
20150402_CerP14_Frac18_top_opt_C6_01_1827	40660	TOF; CID	72.93	527.81	Rnf26

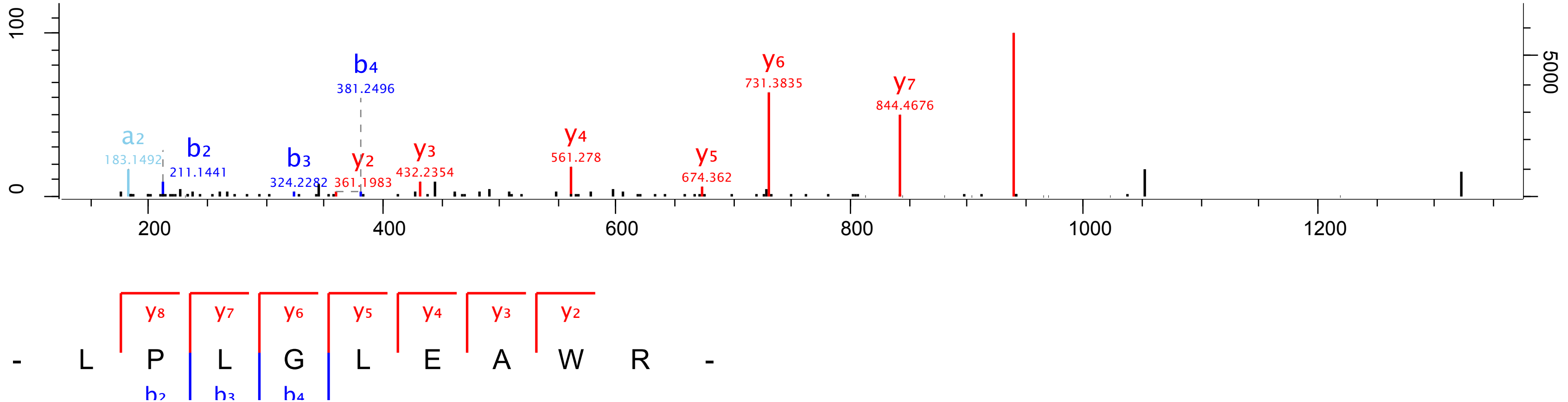

Raw file	Scan	Method	Score	m/z	Gene names
20150402_CerP14_Frac18_top_opt_C6_01_1827	44693	TOF; CID	66.47	857.46	Klf3

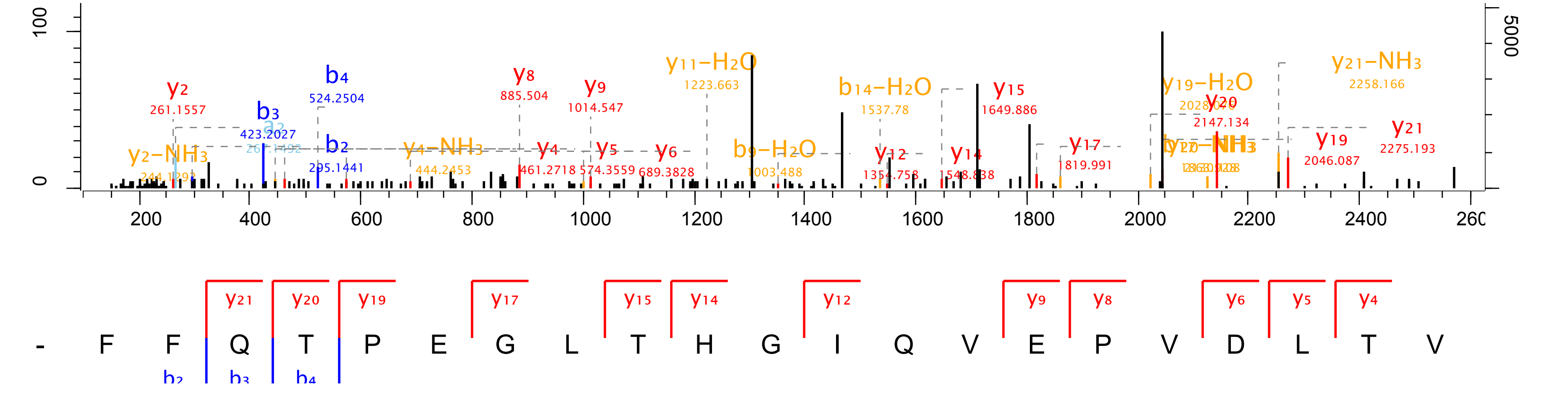

Raw file	Scan	Method	Score	m/z	Gene names
20150402_CerP14_Frac18_top_opt_C6_01_1827	51302	TOF; CID	90.41	744.92	Gins2

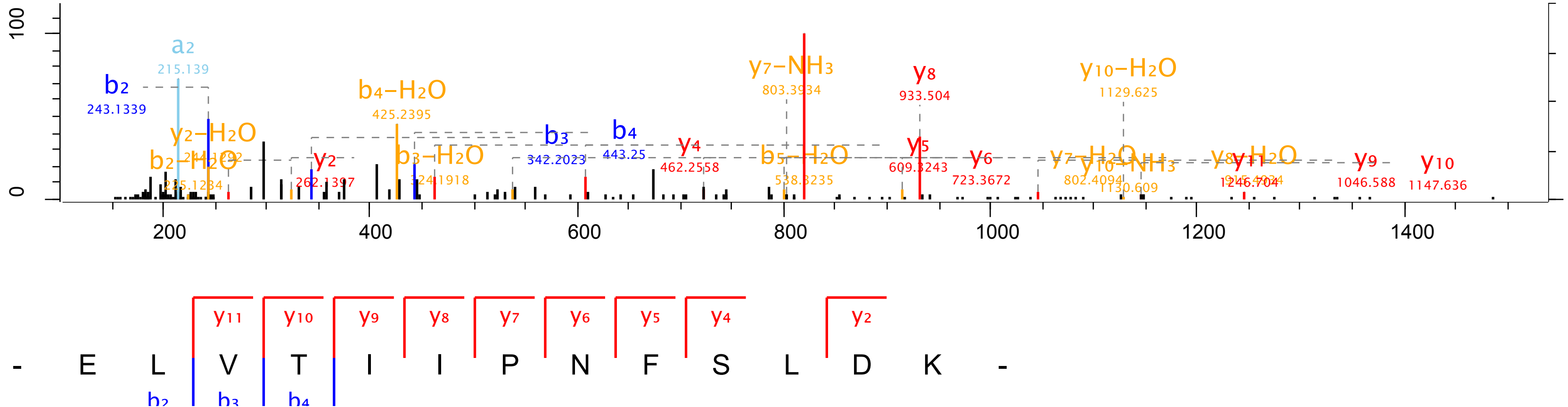

Raw file	Scan	Method	Score	m/z	Gene names
20150402_CerP14_Frac18_top_opt_C6_01_1827	52768	TOF; CID	59.54	886.81	Gpr153

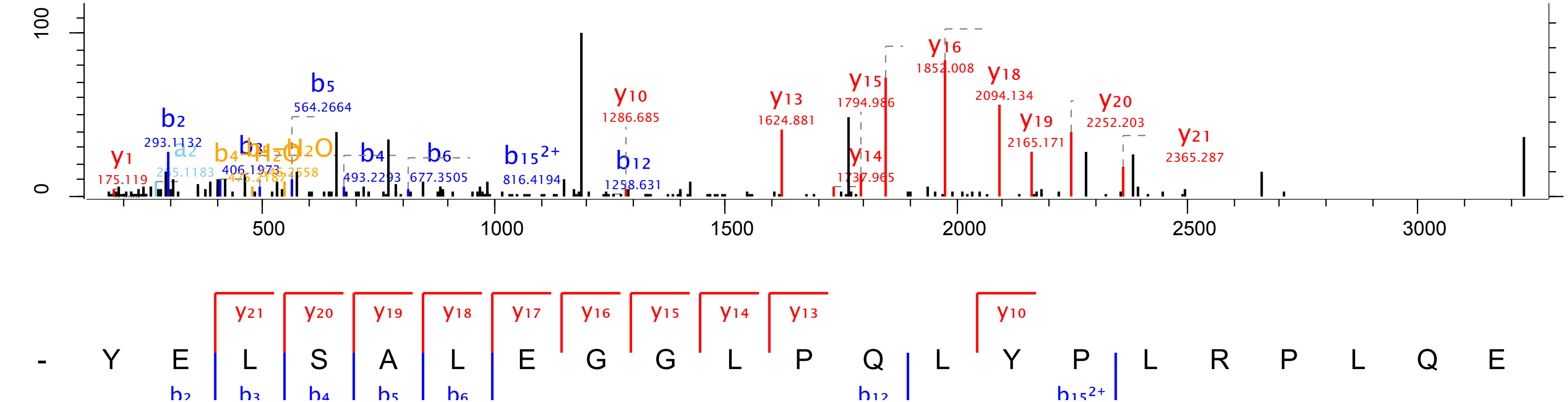

Raw file	Scan	Method	Score	m/z	Gene names
20150402_CerP14_Frac18_top_opt_C6_01_1827	53538	TOF; CID	120.06	665.9	Taf13

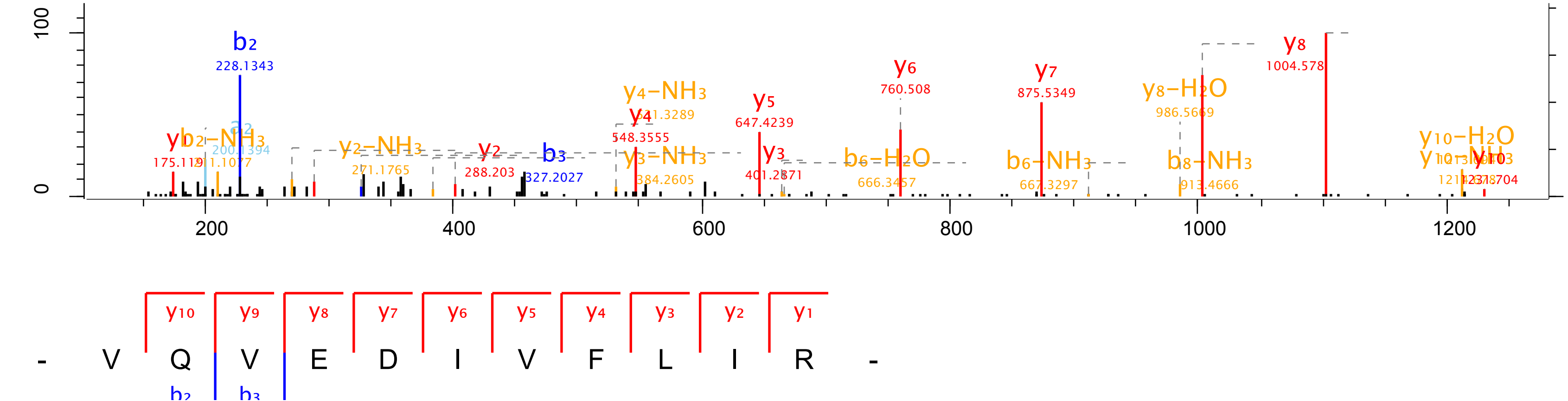

Raw file	Scan	Method	Score	m/z	Gene names
20150402_CerP14_Frac18_top_opt_C6_01_1827	56614	TOF; CID	83.69	871.44	Tomm7

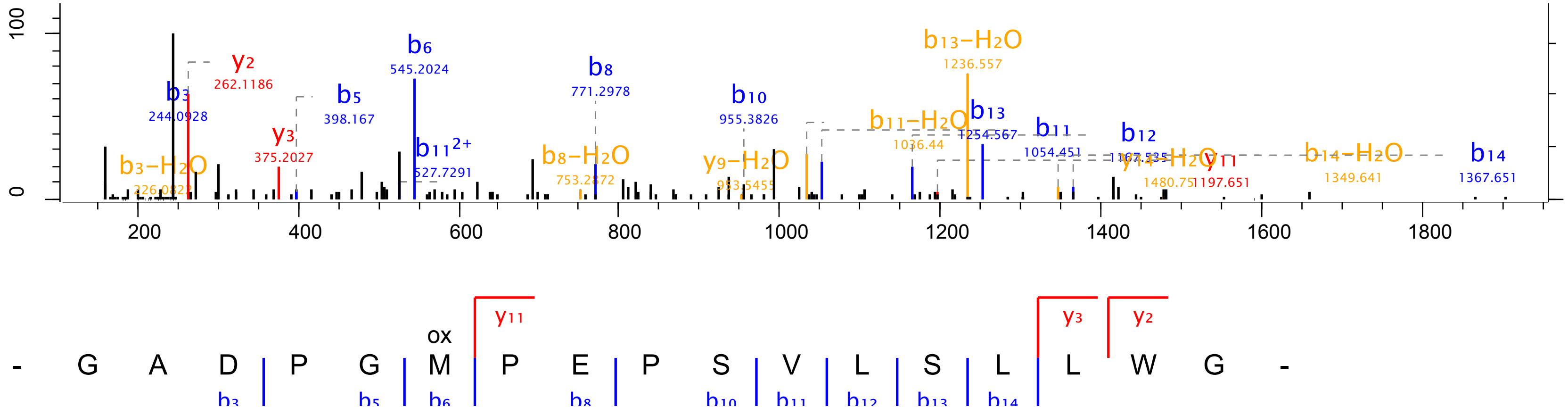

Raw file	Scan	Method	Score	m/z	Gene names
20150402_CerP14_Frac19_top_opt_C7_01_1828	3816	TOF; CID	110.53	577.79	Fut10

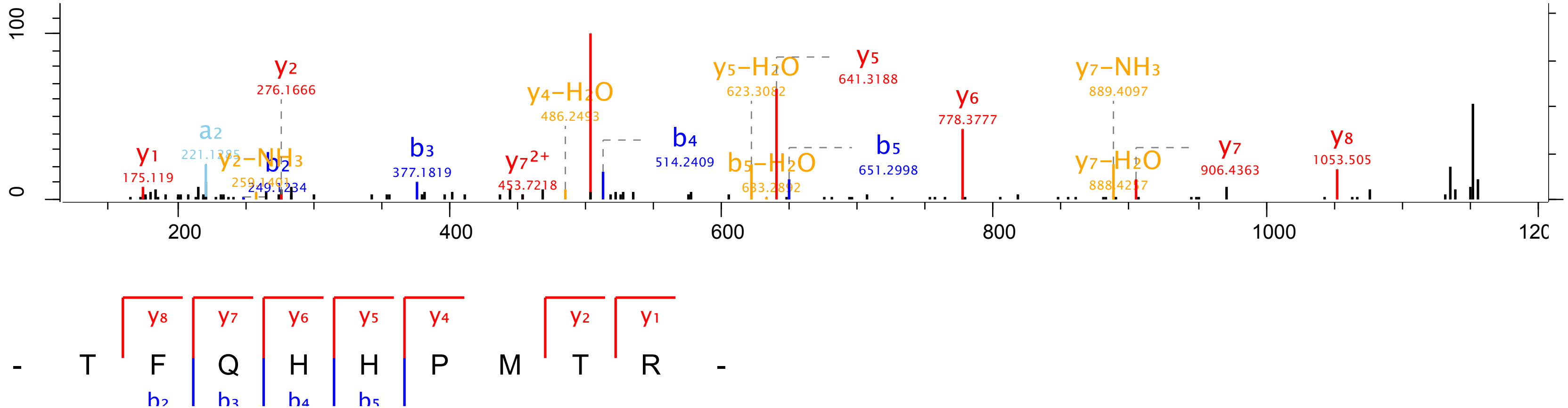

Raw file	Scan	Method	Score	m/z	Gene names
20150402_CerP14_Frac19_top_opt_C7_01_1828	10843	TOF; CID	50.09	466.24	Ifnl3

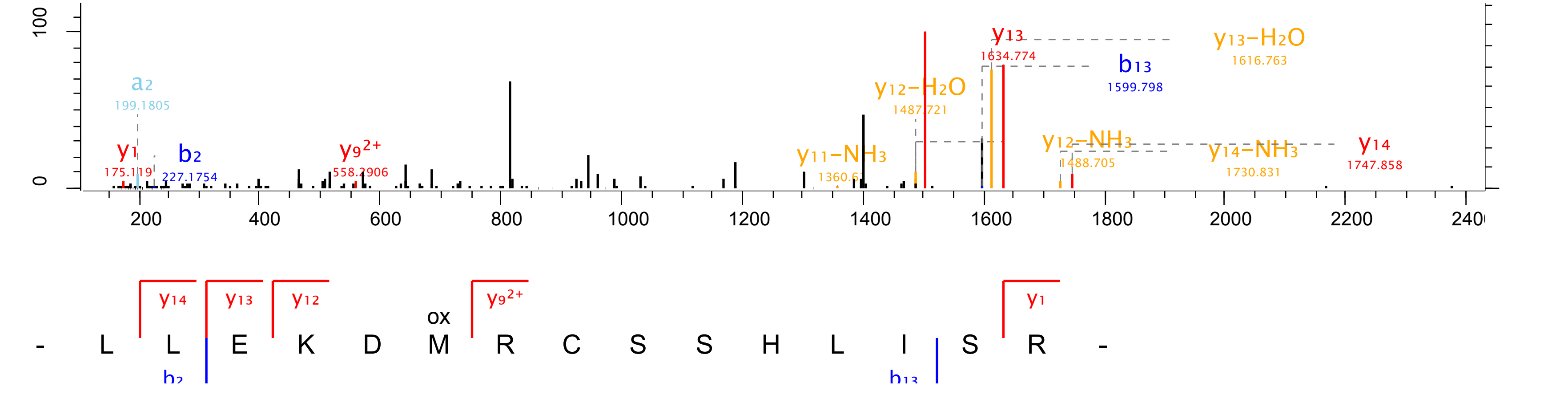

Raw file	Scan	Method	Score	m/z	Gene names
20150402_CerP14_Frac19_top_opt_C7_01_1828	14207	TOF; CID	95.48	438.19	Cited2

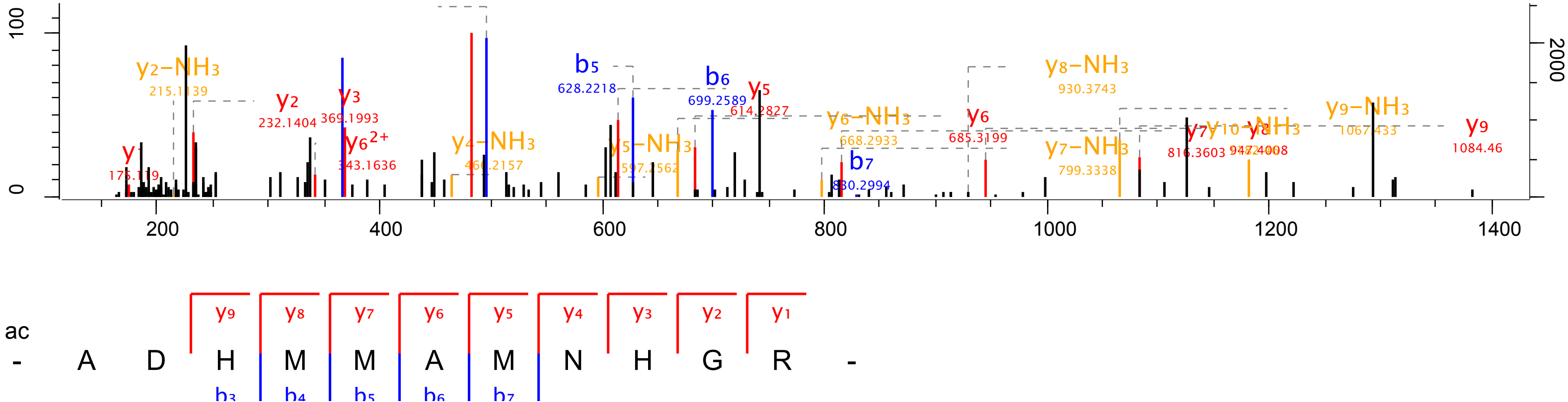

Raw file	Scan	Method	Score	m/z	Gene names
20150402_CerP14_Frac19_top_opt_C7_01_1828	14975	TOF; CID	73.84	691.36	Htr4

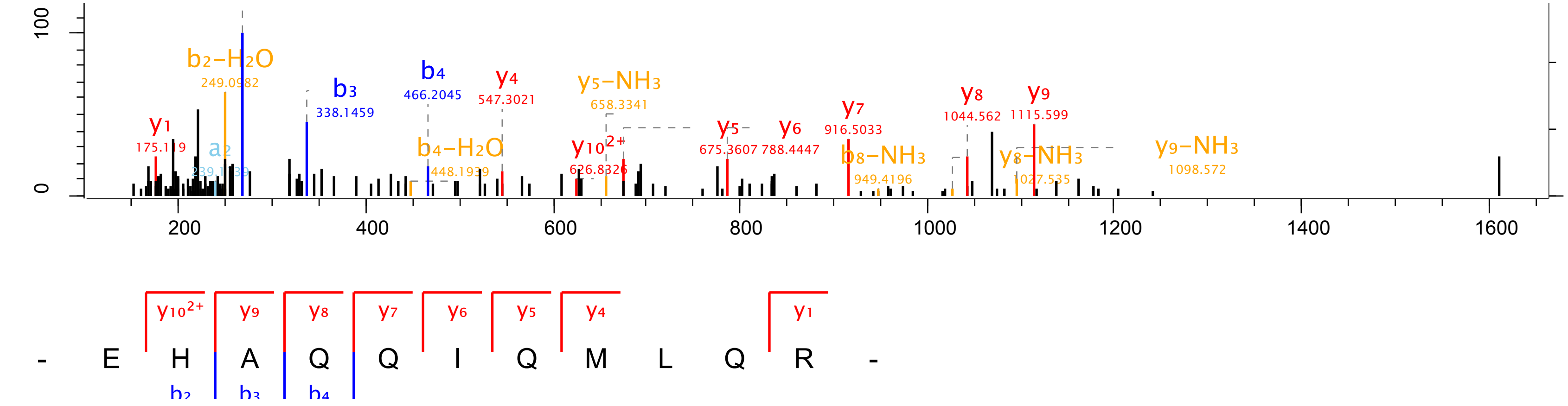

Raw file	Scan	Method	Score	m/z	Gene names
20150402_CerP14_Frac19_top_opt_C7_01_1828	16678	TOF; CID	59.2	668.81	Cryba2

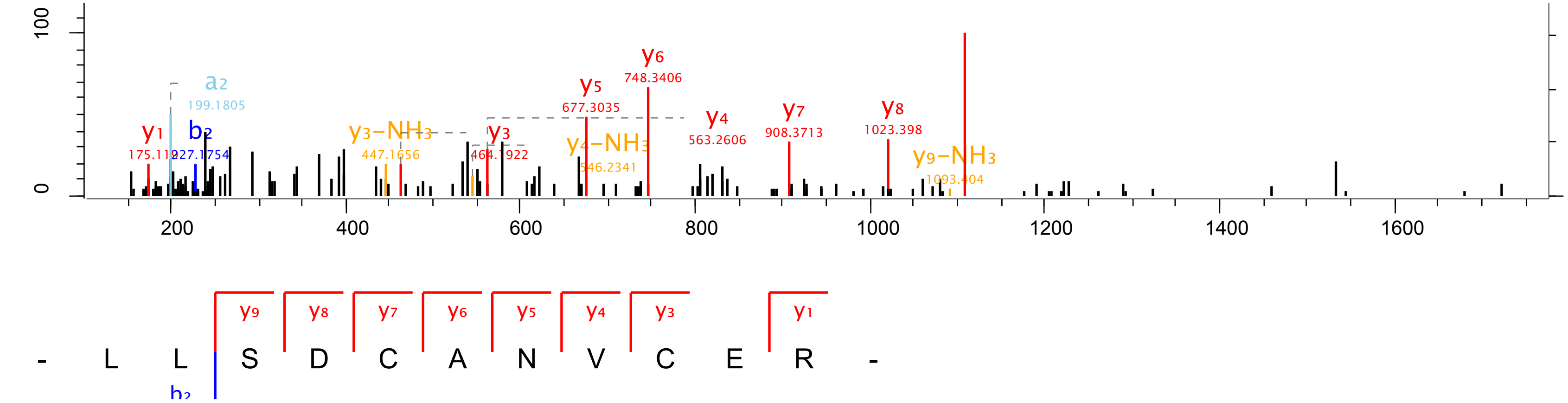

Raw file	Scan	Method	Score	m/z	Gene names
20150402_CerP14_Frac19_top_opt_C7_01_1828	16782	TOF; CID	98.04	514.79	Elk4

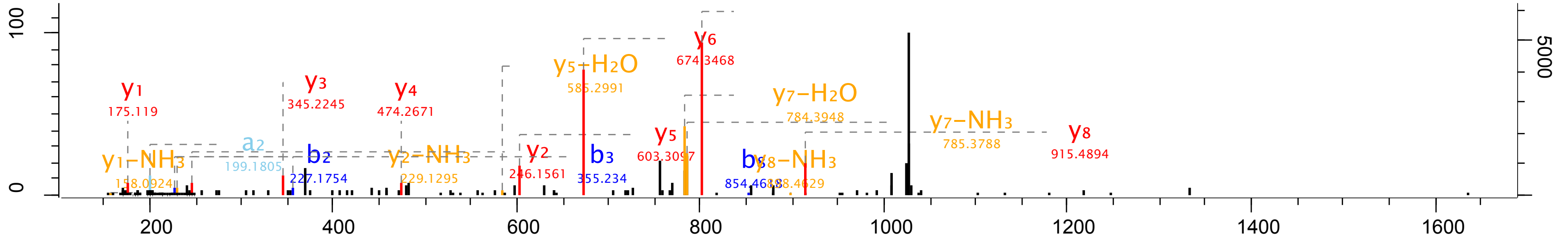

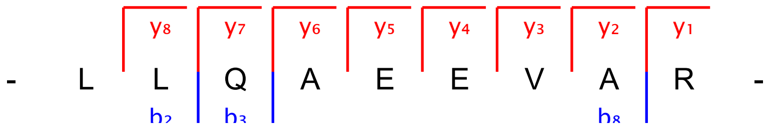

Raw file	Scan	Method	Score	m/z	Gene names
20150402_CerP14_Frac19_top_opt_C7_01_1828	17111	TOF; CID	110.44	717.85	Rsph1

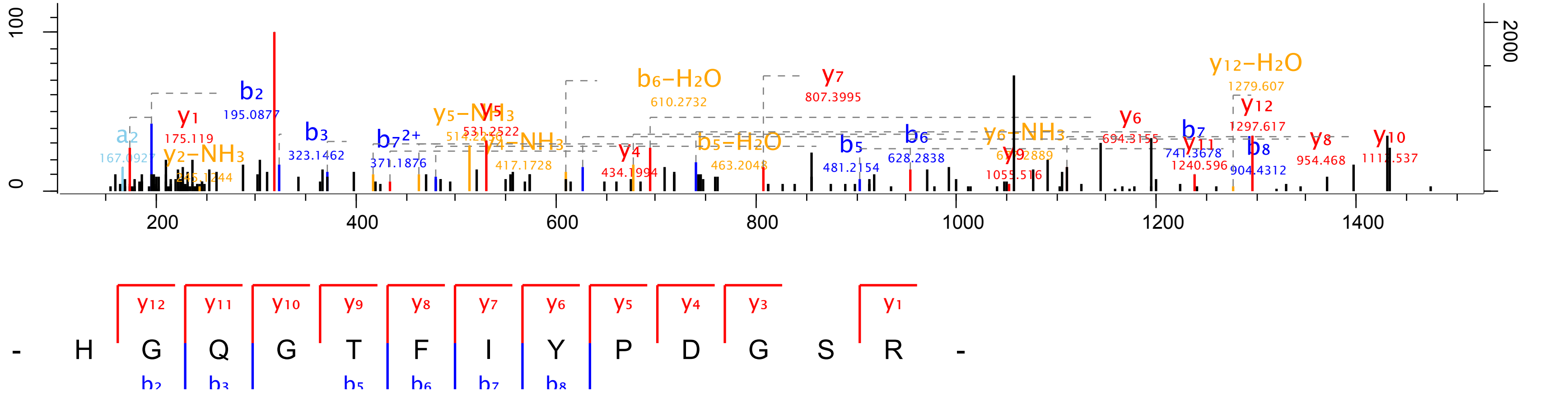

Raw file	Scan	Method	Score	m/z
20150402_CerP14_Frac19_top_opt_C7_01_1828	22068	TOF; CID	53.31	807.4

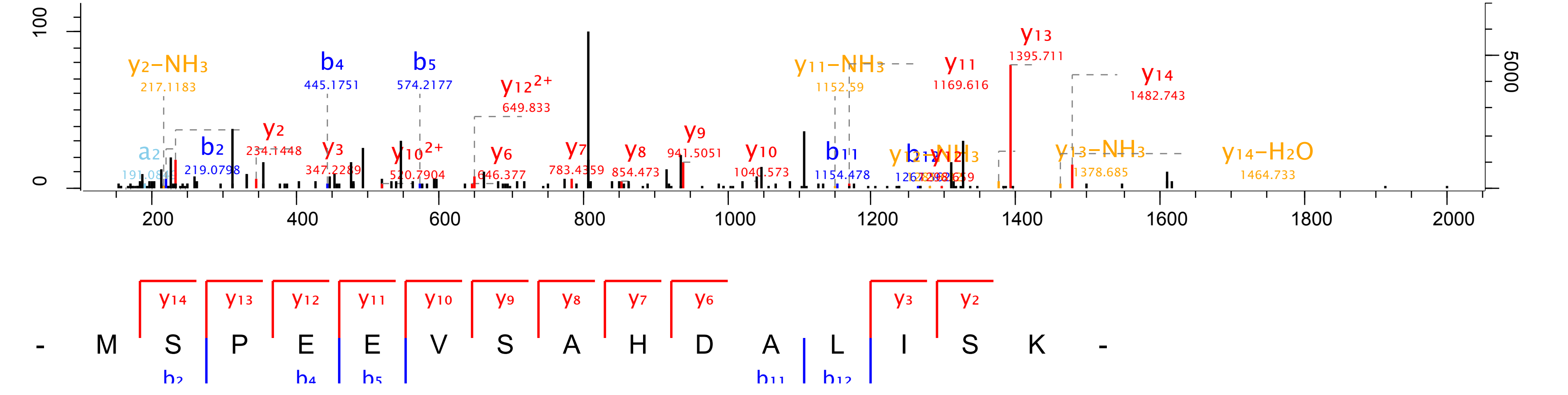

Raw file	Scan	Method	Score	m/z	Gene names
20150402_CerP14_Frac19_top_opt_C7_01_1828	23633	TOF; CID	47.08	749.39	Homez

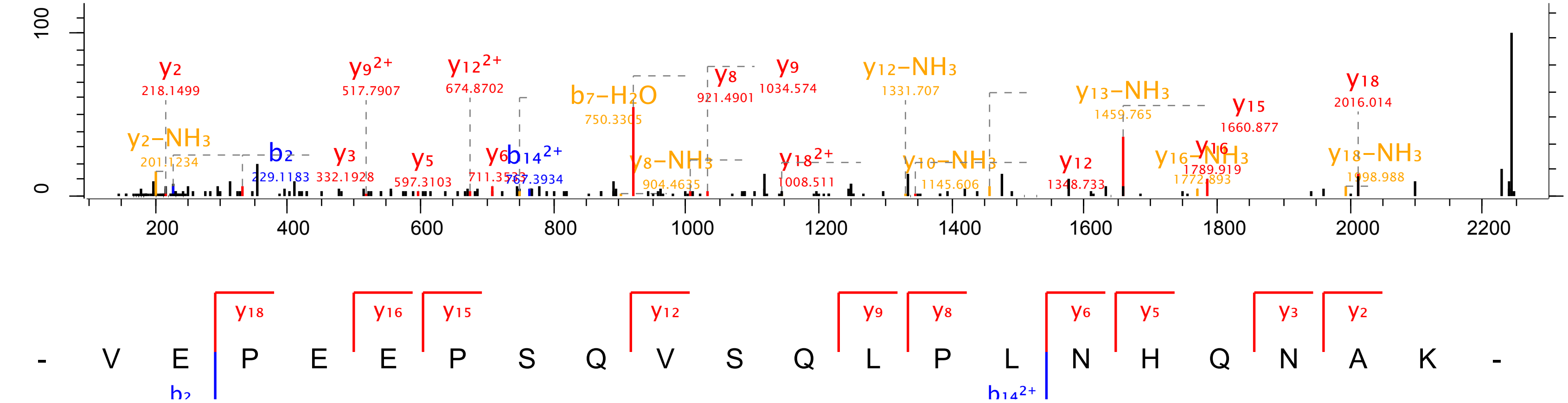

Raw file	Scan	Method	Score	m/z	Gene names
20150402_CerP14_Frac19_top_opt_C7_01_1828	23813	TOF; CID	48.91	838.44	Zmat1

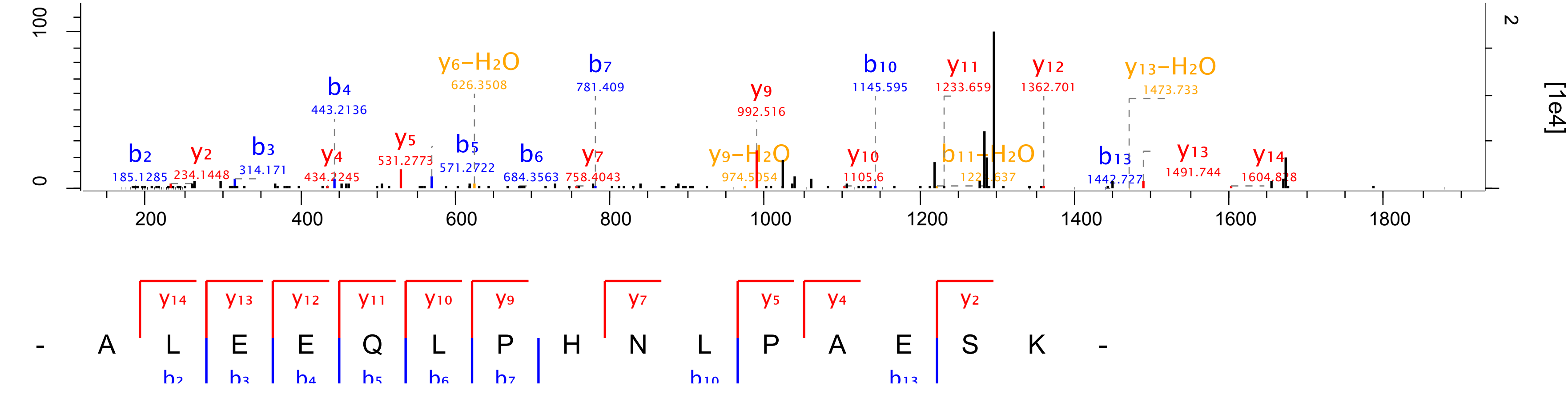

Raw file	Scan	Method	Score	m/z	Gene names
20150402_CerP14_Frac19_top_opt_C7_01_1828	29145	TOF; CID	124.12	723.87	Cyb561d2

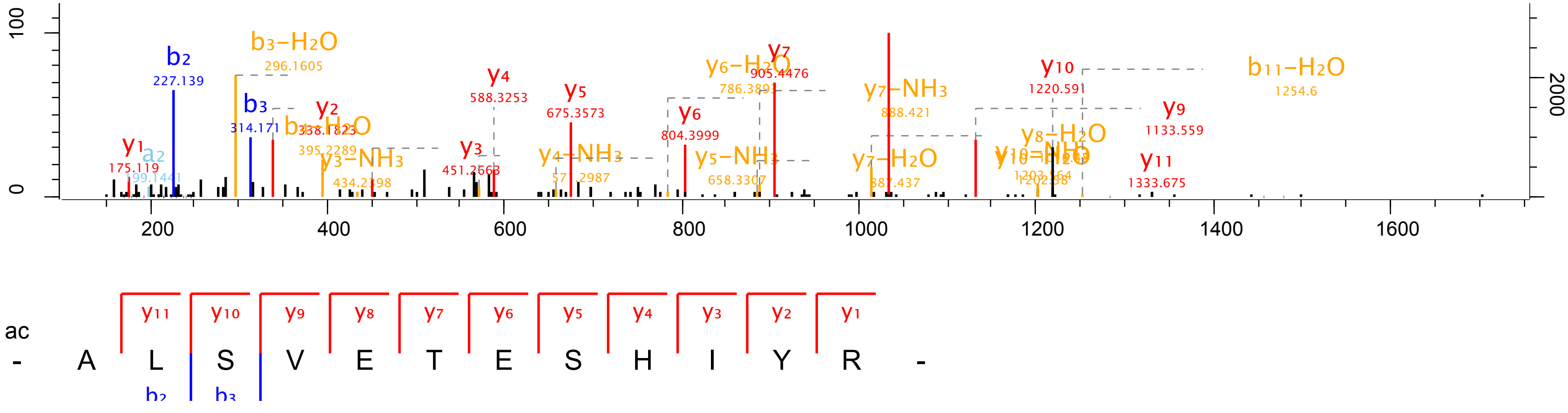

Raw file	Scan	Method	Score	m/z	Gene names
20150402_CerP14_Frac19_top_opt_C7_01_1828	29370	TOF; CID	104.06	442.78	Chrac1

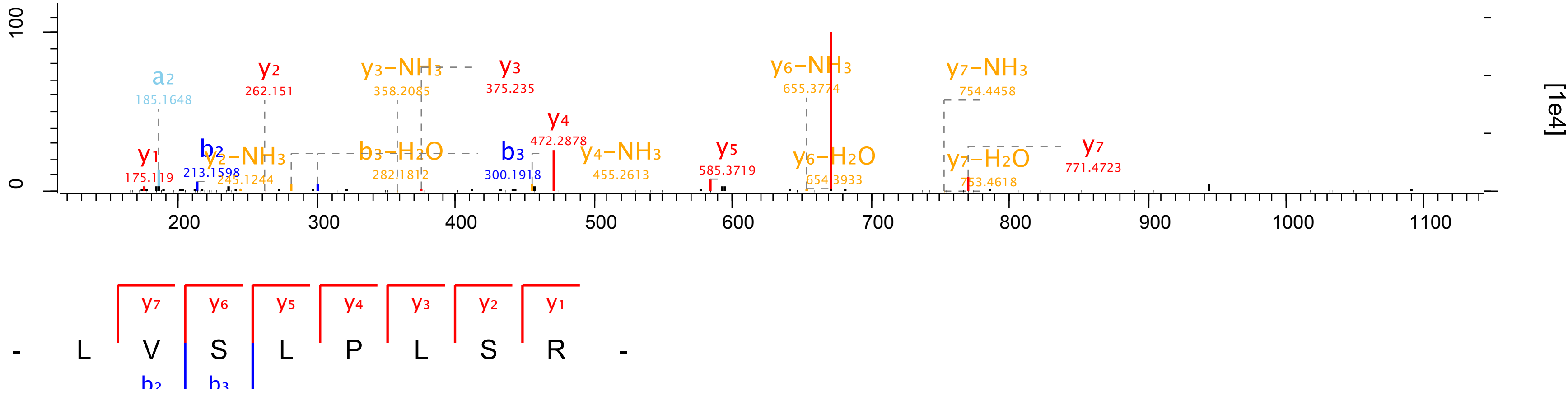

Raw file	Scan	Method	Score	m/z	Gene names
20150402_CerP14_Frac19_top_opt_C7_01_1828	31552	TOF; CID	68.54	570.86	Kcnj8

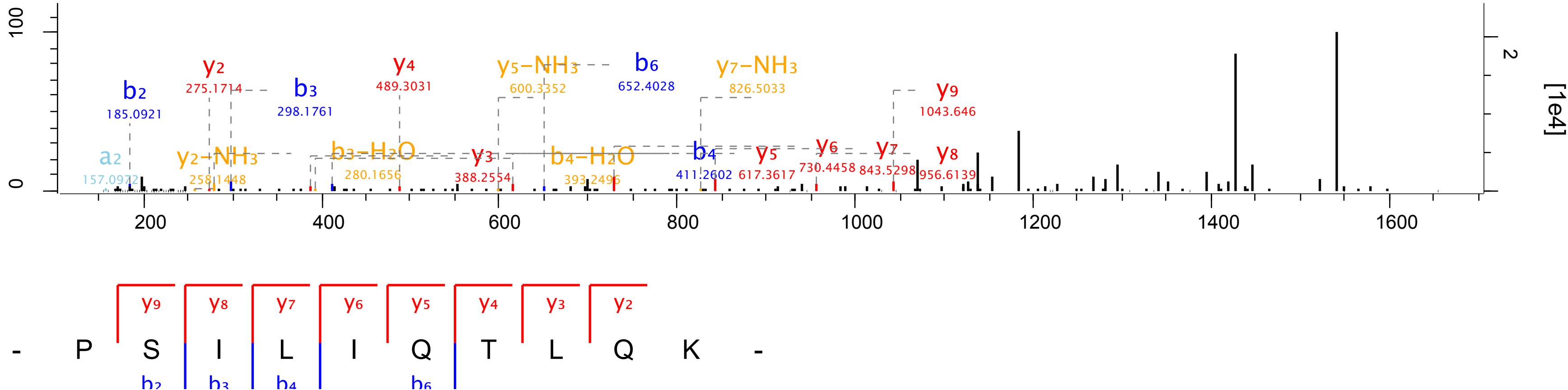

Raw file	Scan	Method	Score	m/z	Gene names
20150402_CerP14_Frac19_top_opt_C7_01_1828	31869	TOF; CID	98.1	713.34	Pcgf1

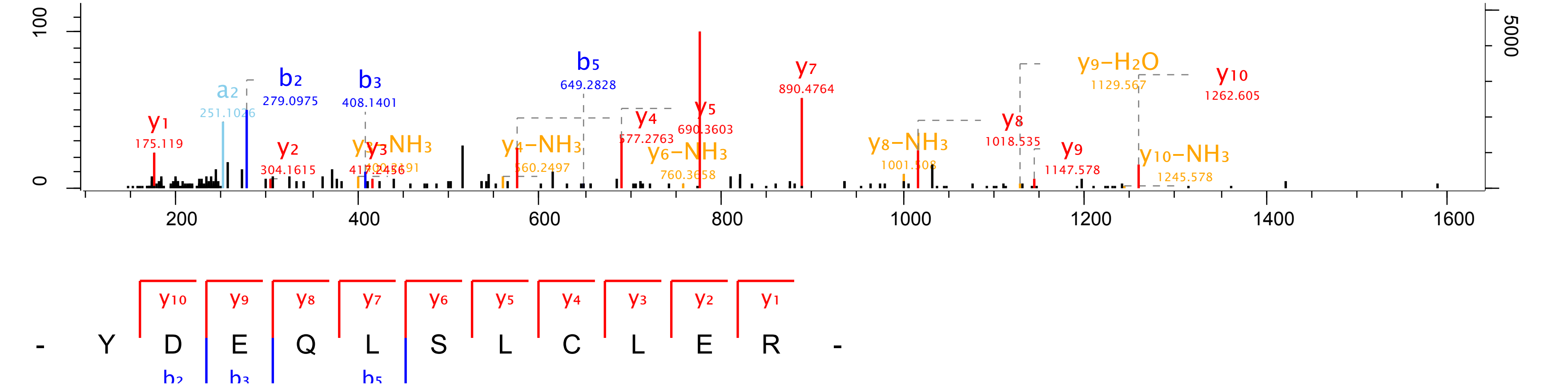

Raw file	Scan	Method	Score	m/z	Gene names
20150402_CerP14_Frac19_top_opt_C7_01_1828	34190	TOF; CID	111.45	557.89	S100a8

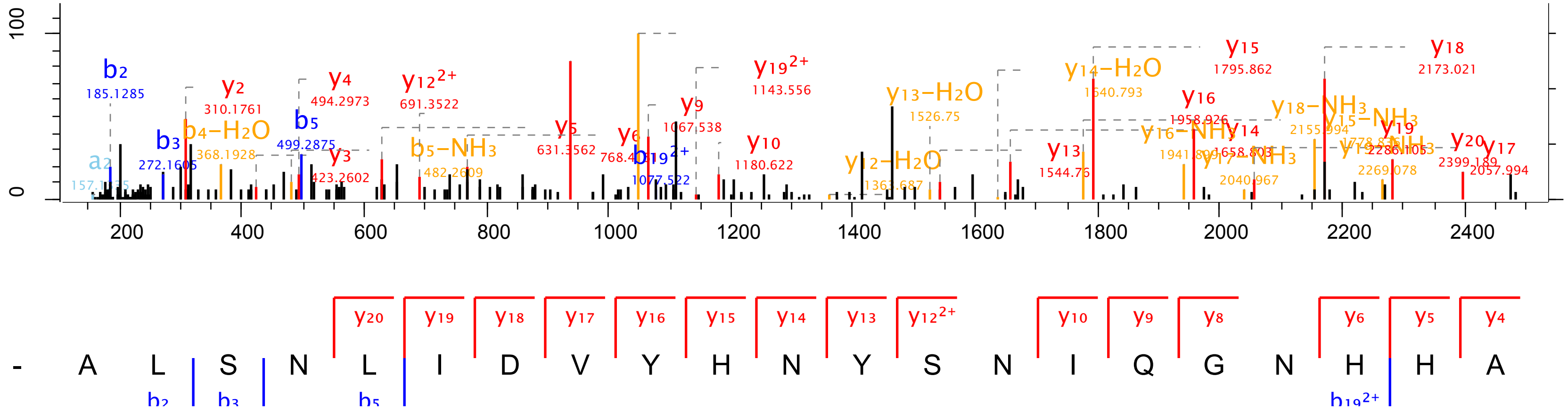

Raw file	Scan	Method	Score	m/z	Gene names
20150402_CerP14_Frac19_top_opt_C7_01_1828	36276	TOF; CID	83.95	509.31	Stra13

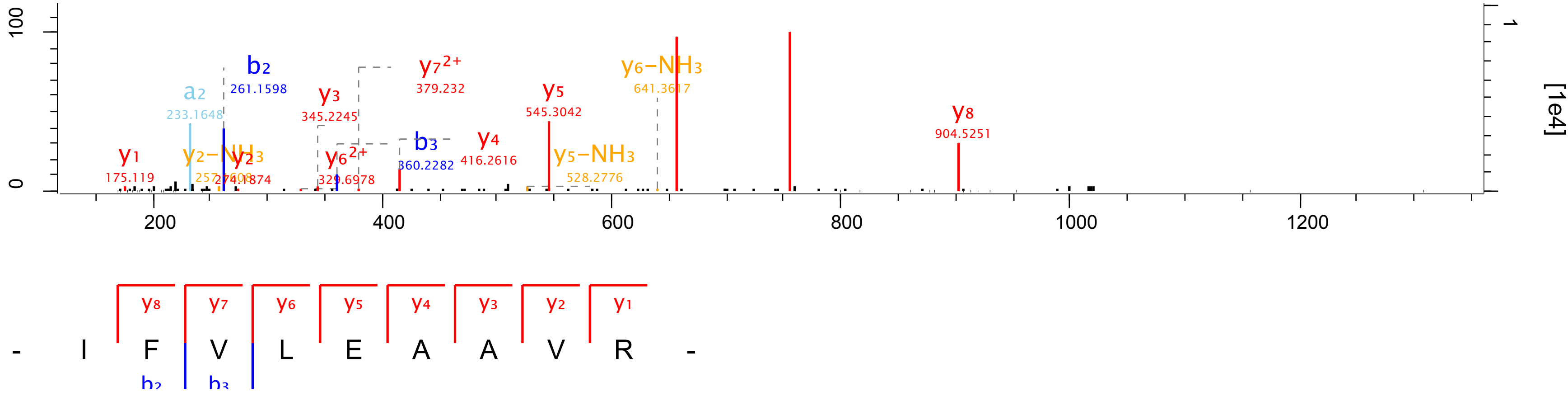

Raw file	Scan	Method	Score	m/z	Gene names
20150402_CerP14_Frac19_top_opt_C7_01_1828	39965	TOF; CID	125.45	708.05	Crhr1

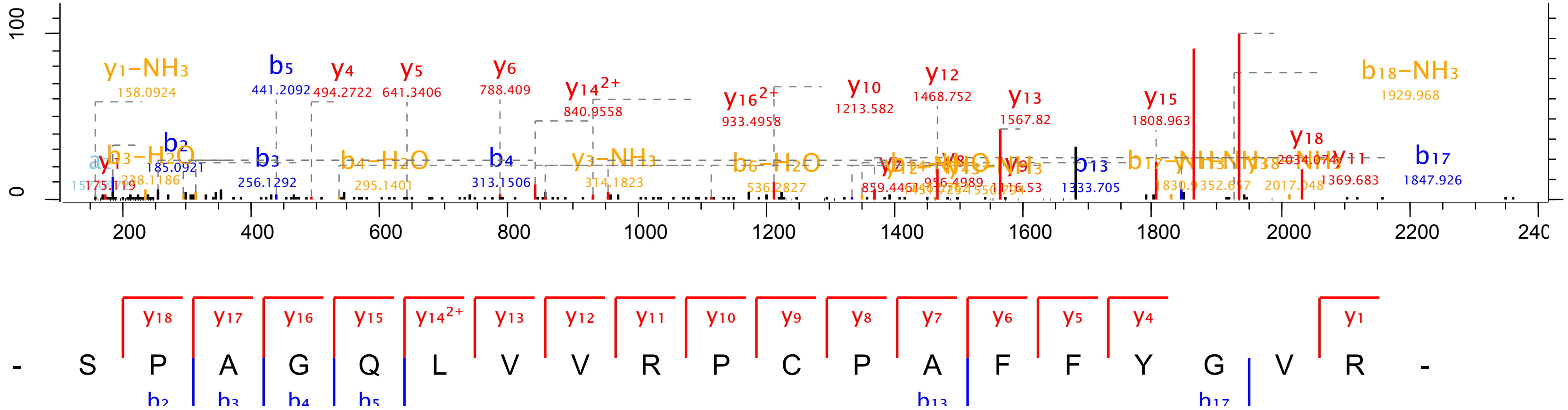

Raw file	Scan	Method	Score	m/z	Gene names
20150402_CerP14_Frac19_top_opt_C7_01_1828	43199	TOF; CID	54	1028.55	Sprn

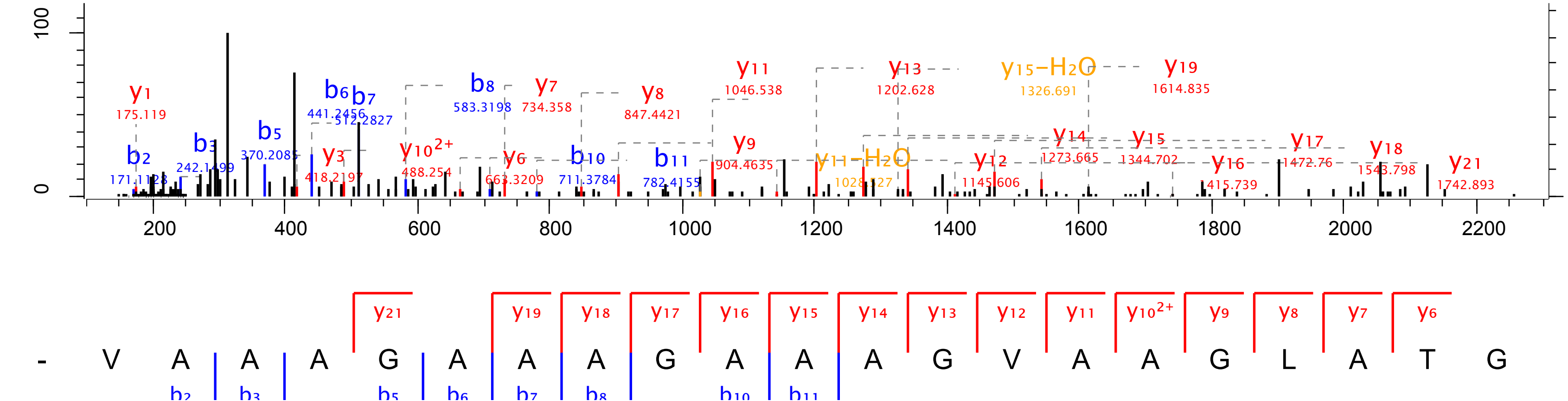

Raw file	Scan	Method	Score	m/z	Gene names
20150402_CerP14_Frac19_top_opt_C7_01_1828	43463	TOF; CID	70.94	614.33	Endov

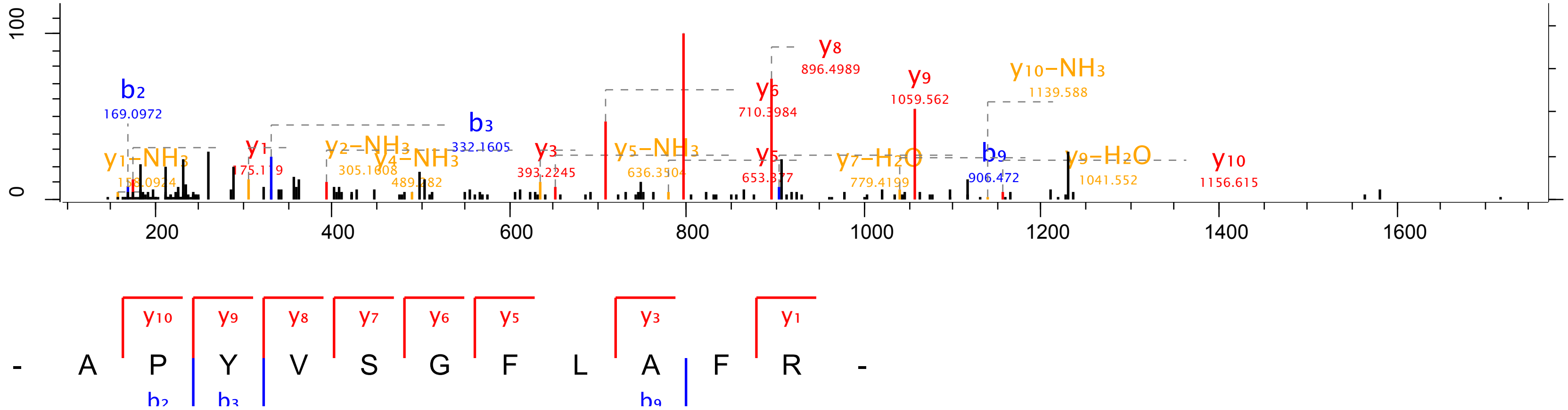

Raw file	Scan	Method	Score	m/z	Gene names
20150402_CerP14_Frac19_top_opt_C7_01_1828	45948	TOF; CID	55.45	842.43	Dusp7

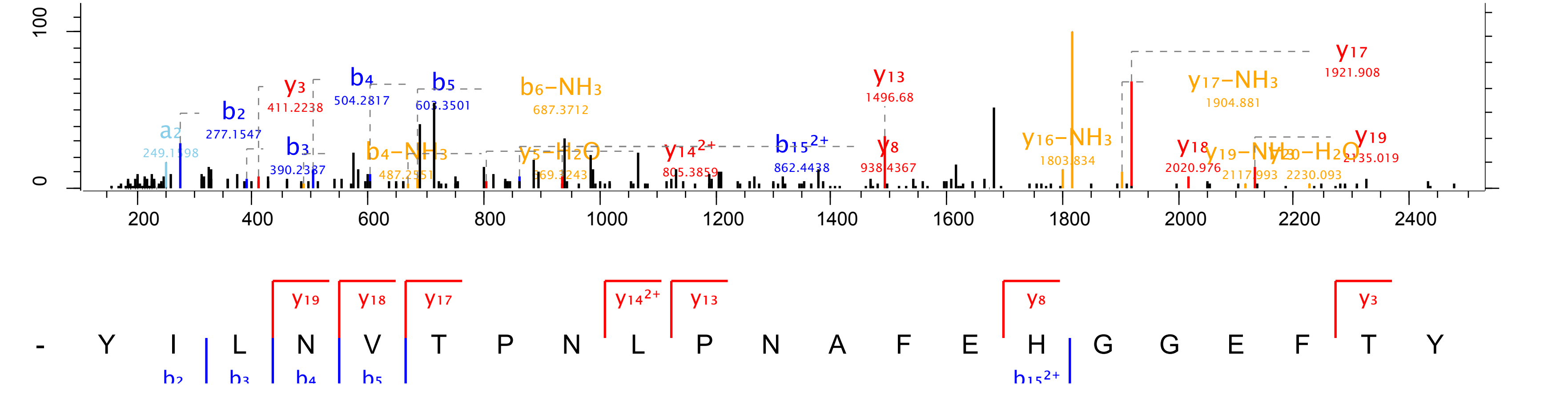

Raw file	Scan	Method	Score	m/z	Gene names
20150402_CerP14_Frac20_top_opt_C8_01_1829	3068	TOF; CID	100.02	543.8	Nppc

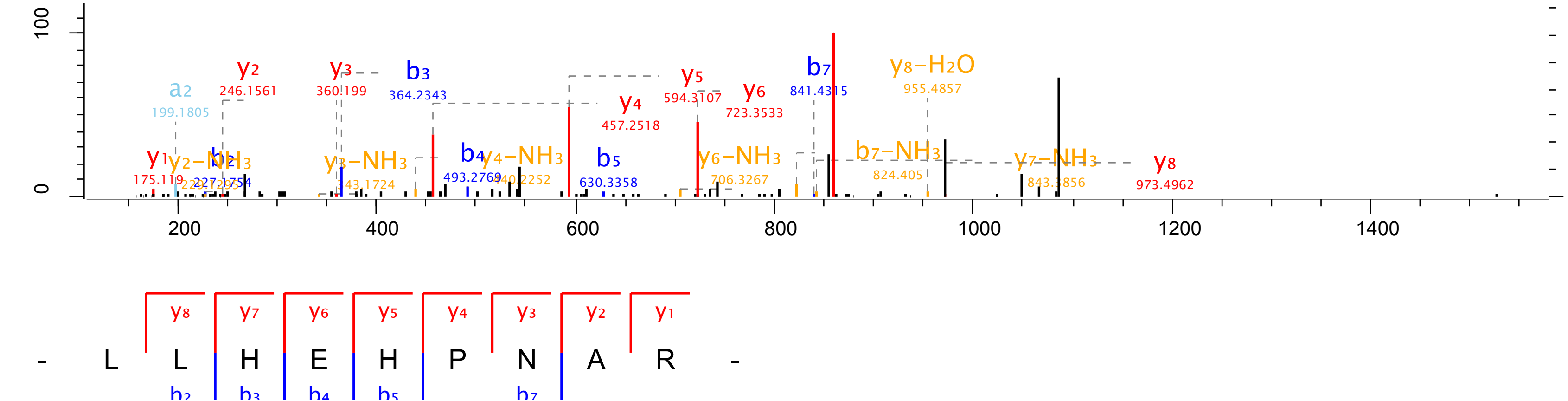

Raw file	Scan	Method	Score	m/z	Gene names
20150402_CerP14_Frac20_top_opt_C8_01_1829	9470	TOF; CID	85.68	338.87	Samd8

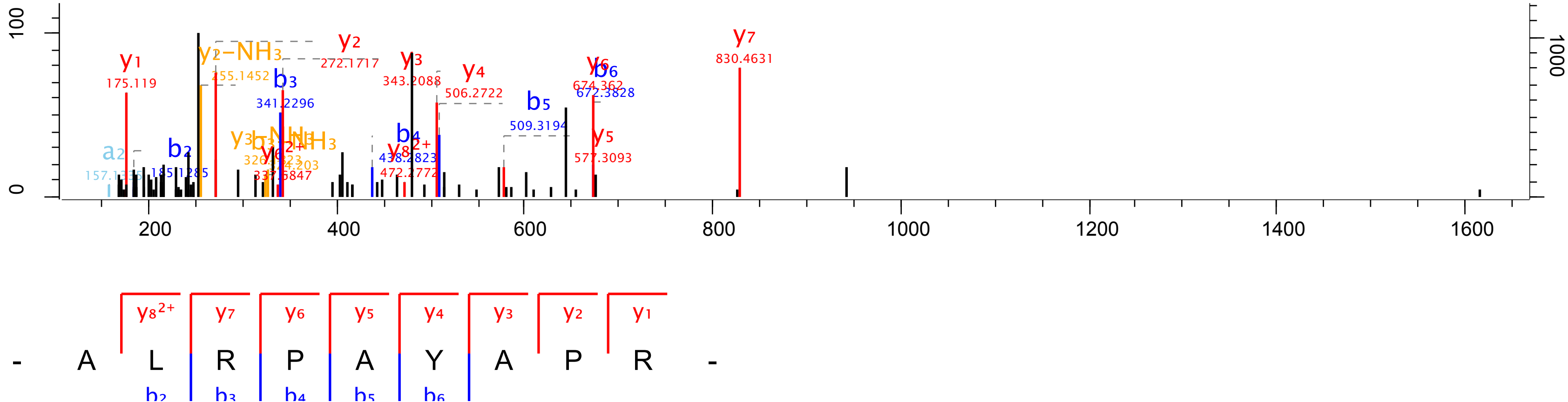

Raw file	Scan	Method	Score	m/z	Gene names
20150402_CerP14_Frac20_top_opt_C8_01_1829	11549	TOF; CID	86.67	472.27	Gtf2a2

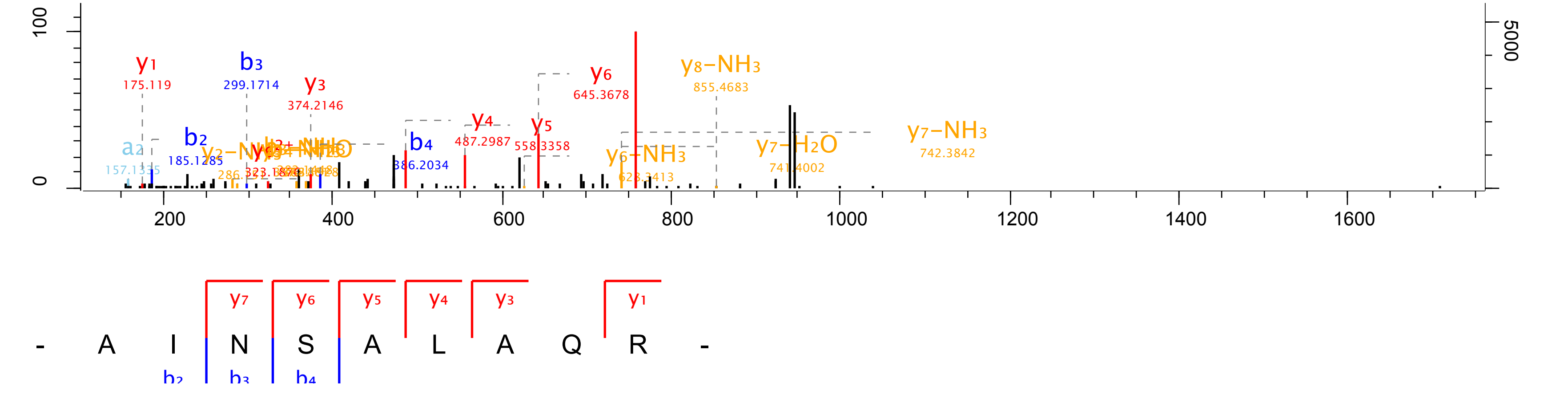

Raw file	Scan	Method	Score	m/z	Gene names
20150402_CerP14_Frac20_top_opt_C8_01_1829	12275	TOF; CID	84.74	511.79	Creb3

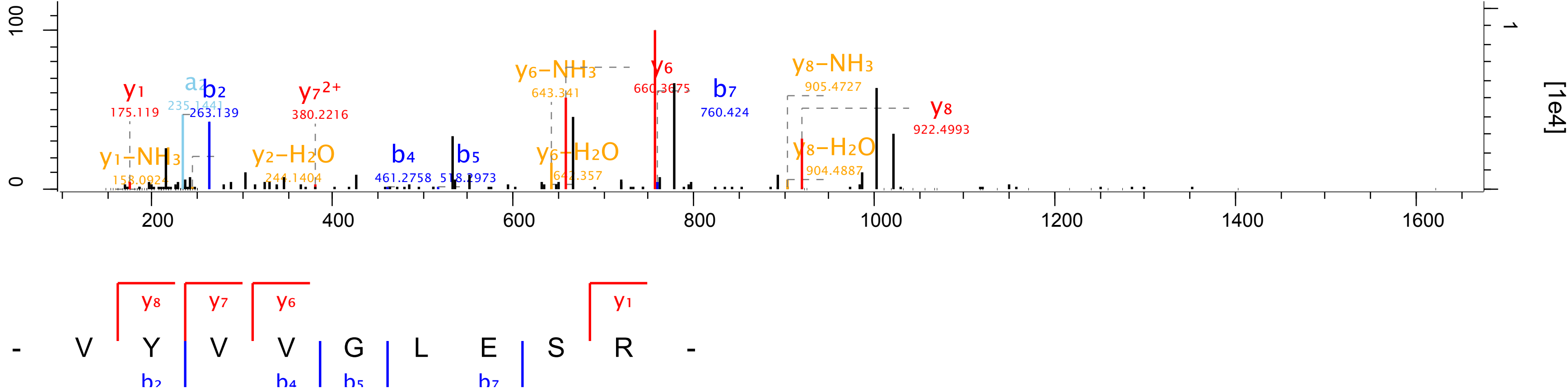

Raw file	Scan	Method	Score	m/z	Gene names
20150402_CerP14_Frac20_top_opt_C8_01_1829	16249	TOF; CID	90.41	643.33	Id2

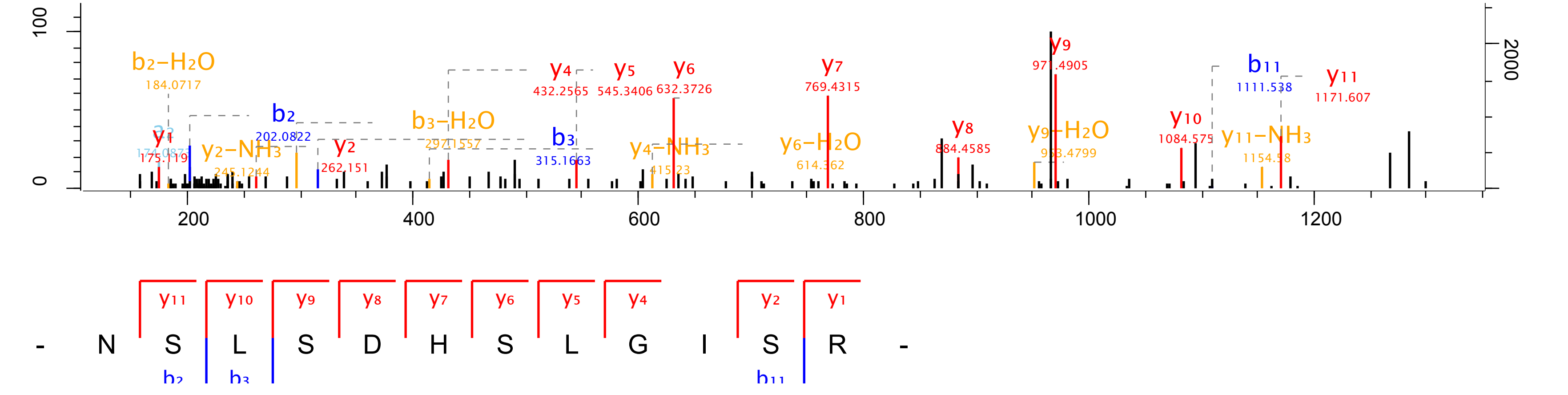

Raw file	Scan	Method	Score	m/z	Gene names
20150402_CerP14_Frac20_top_opt_C8_01_1829	21786	TOF; CID	67.38	543.82	St14

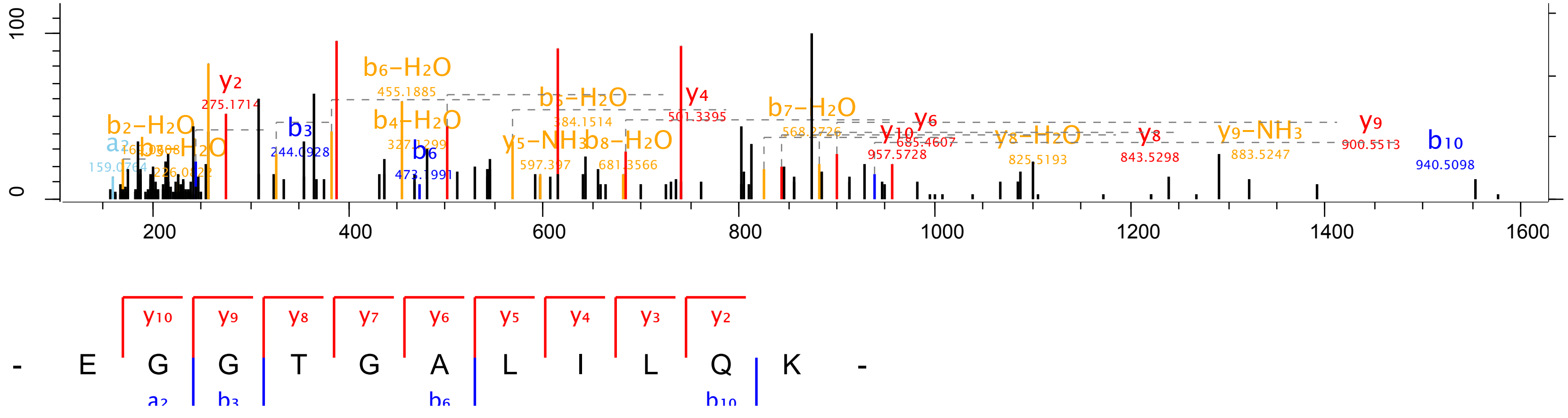

Raw file	Scan	Method	Score	m/z	Gene names
20150402_CerP14_Frac20_top_opt_C8_01_1829	23447	TOF; CID	58.32	563.31	Dusp8

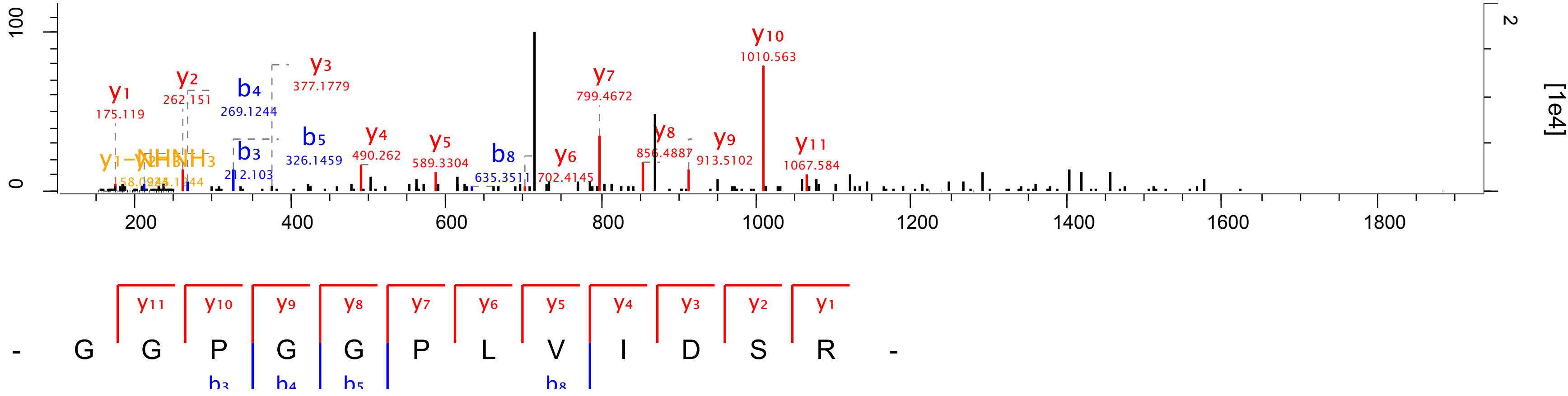

Raw file	Scan	Method	Score	m/z	Gene names
20150402_CerP14_Frac20_top_opt_C8_01_1829	26448	TOF; CID	93.78	690.71	Poc1a

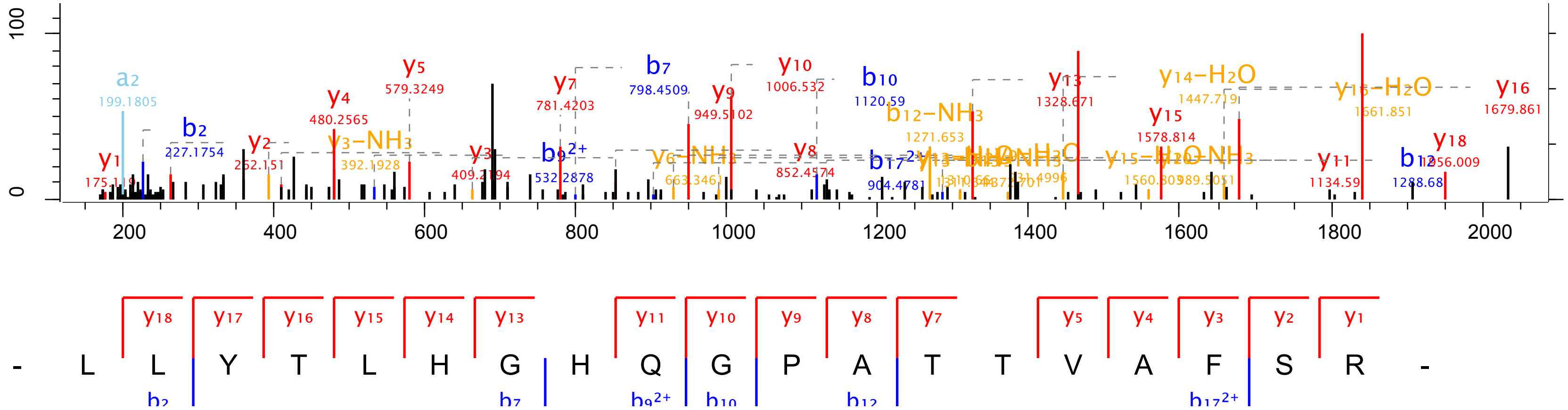

Raw file	Scan	Method	Score	m/z	Gene names
20150402_CerP14_Frac20_top_opt_C8_01_1829	32232	TOF; CID	89.62	522.61	Ptrh1

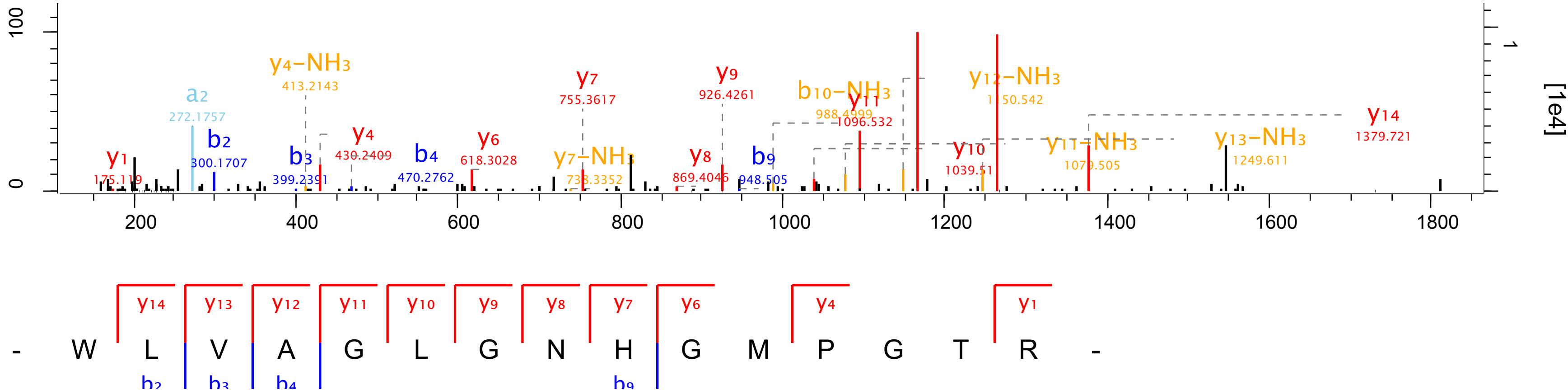

Raw file	Scan	Method	Score	m/z	Gene names
20150402_CerP14_Frac20_top_opt_C8_01_1829	33739	TOF; CID	121.21	772.07	Slc19a2

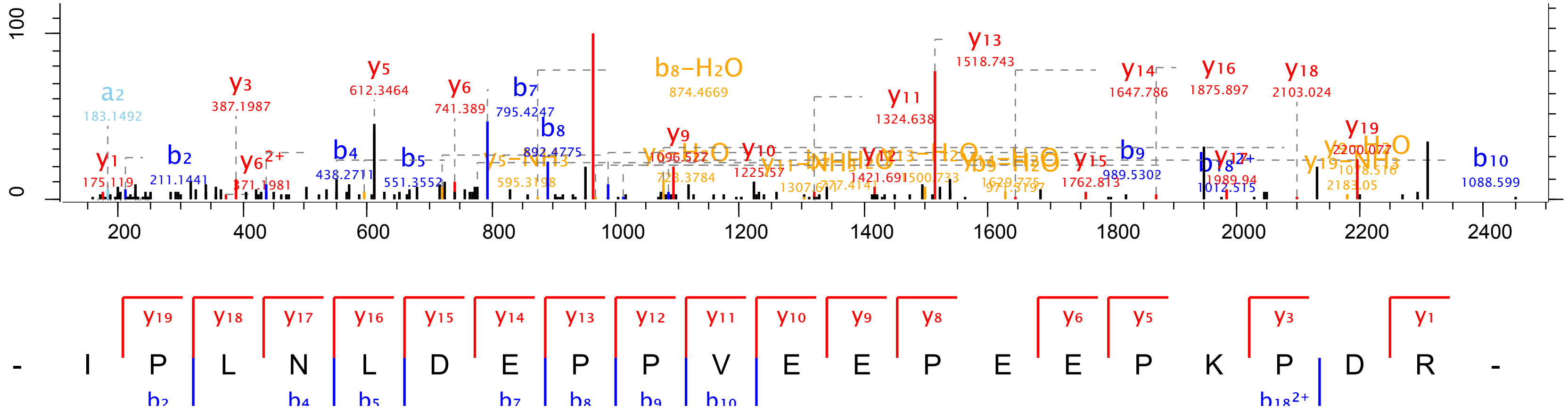

Raw file	Scan	Method	Score	m/z	Gene names
20150402_CerP14_Frac20_top_opt_C8_01_1829	34495	TOF; CID	101.53	519.28	Alg6

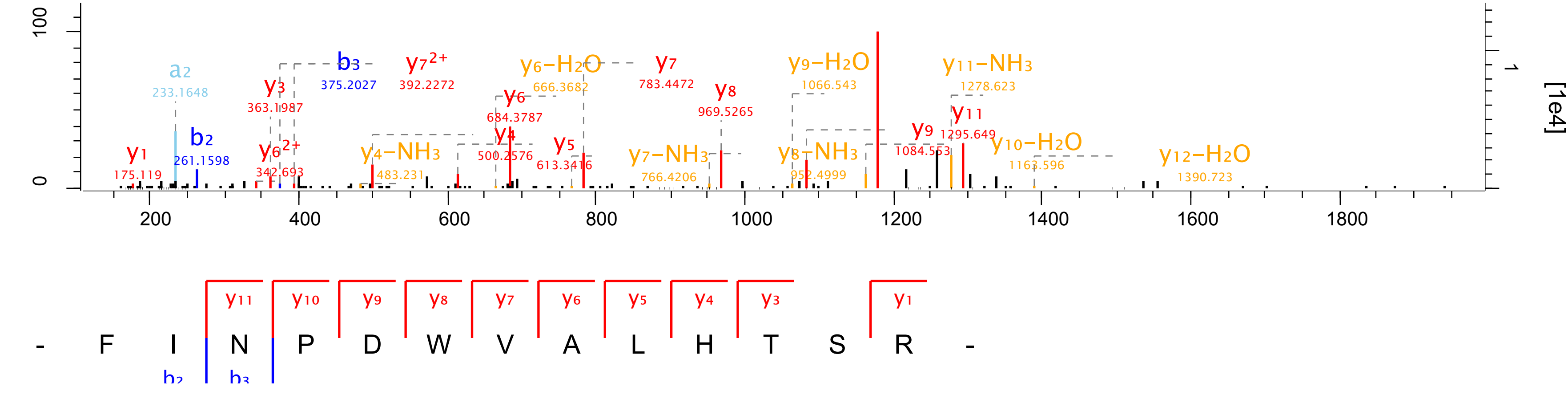

Raw file	Scan	Method	Score	m/z	Gene names
20150402_CerP14_Frac20_top_opt_C8_01_1829	37298	TOF; CID	46.46	1173.58	Kitlg

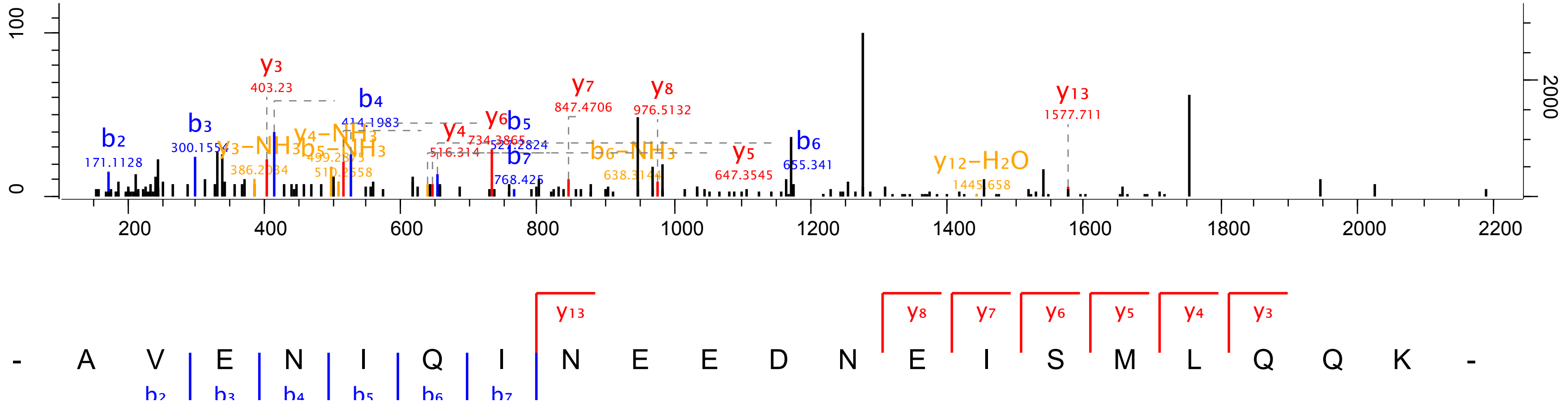

Raw file	Scan	Method	Score	m/z	Gene names
20150402_CerP14_Frac20_top_opt_C8_01_1829	37829	TOF; CID	43.68	492.95	Tor1aip2

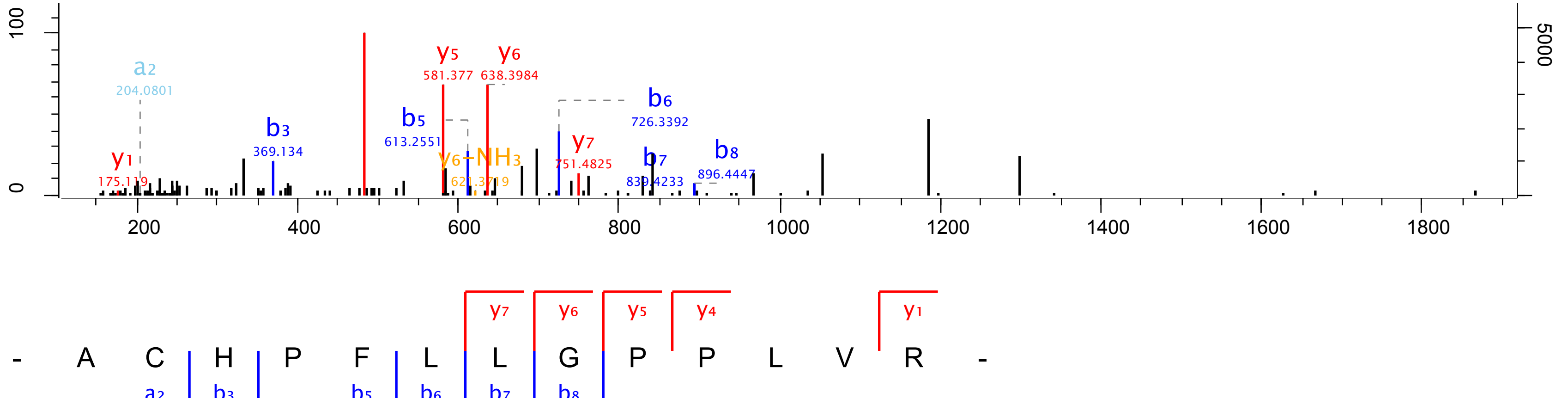

Raw file	Scan	Method	Score	m/z	Gene names
20150402_CerP14_Frac20_top_opt_C8_01_1829	40529	TOF; CID	102.52	690.42	Ak6

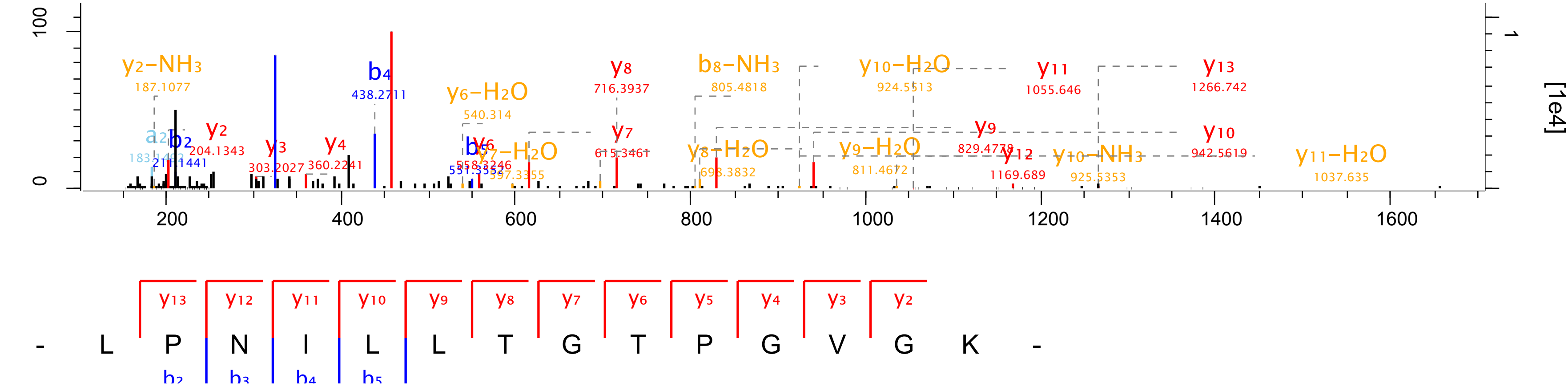

Raw file	Scan	Method	Score	m/z	Gene names
20150402_CerP14_Frac20_top_opt_C8_01_1829	40532	TOF; CID	146.54	465.3	Ttc23l

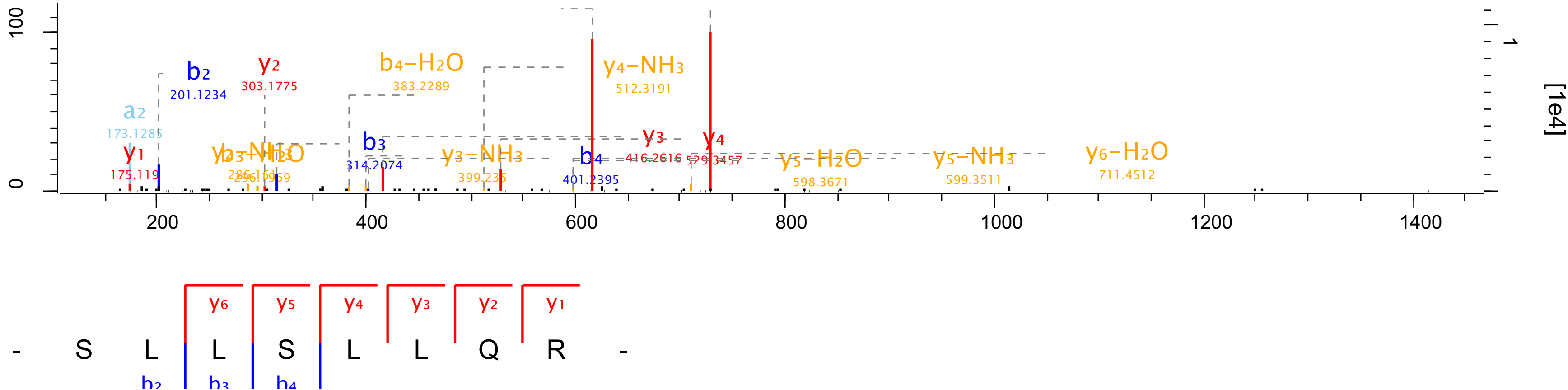

Raw file	Scan	Method	Score	m/z	Gene names
20150402_CerP14_Frac20_top_opt_C8_01_1829	41861	TOF; CID	97.43	506.34	Cox8a

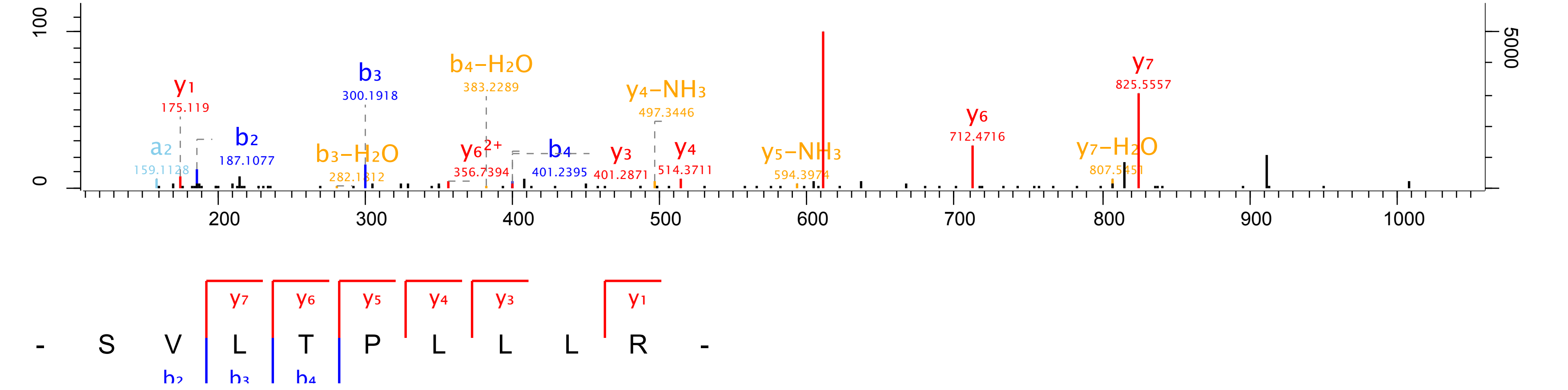

Raw file	Scan	Method	Score	m/z	Gene names
20150402_CerP14_Frac20_top_opt_C8_01_1829	44139	TOF; CID	50.3	569.32	Herpud1

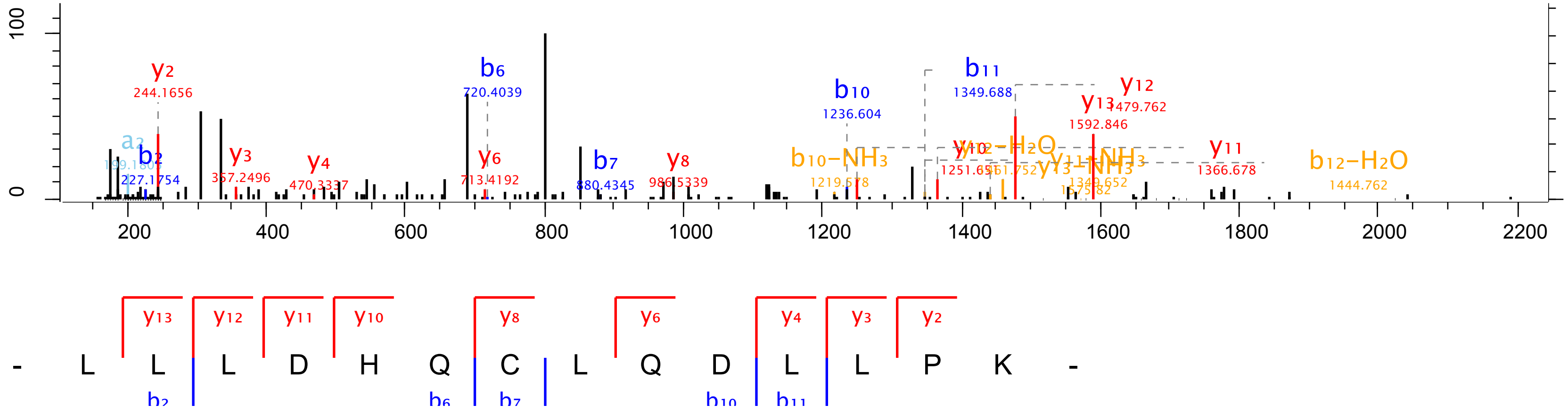

Raw file	Scan	Method	Score	m/z	Gene names
20150402_CerP14_Frac20_top_opt_C8_01_1829	56033	TOF; CID	64.21	1043.07	Mfap4

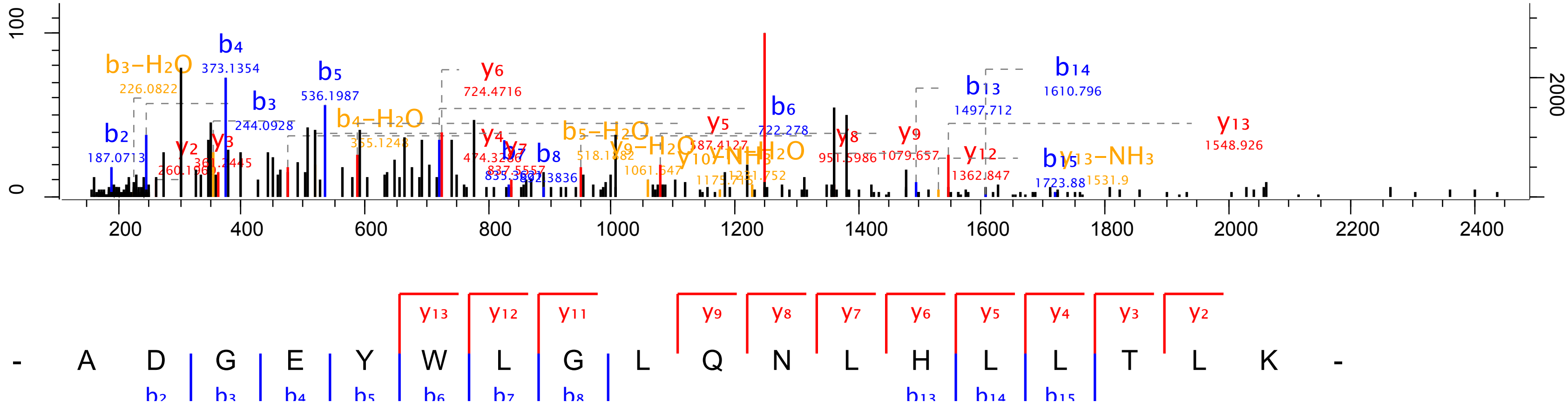

Raw file	Scan	Method	Score	m/z	Gene names
20150402_CerP14_Frac21_top_opt_C9_01_1830	3158	TOF; CID	116.88	353.55	Zscan25

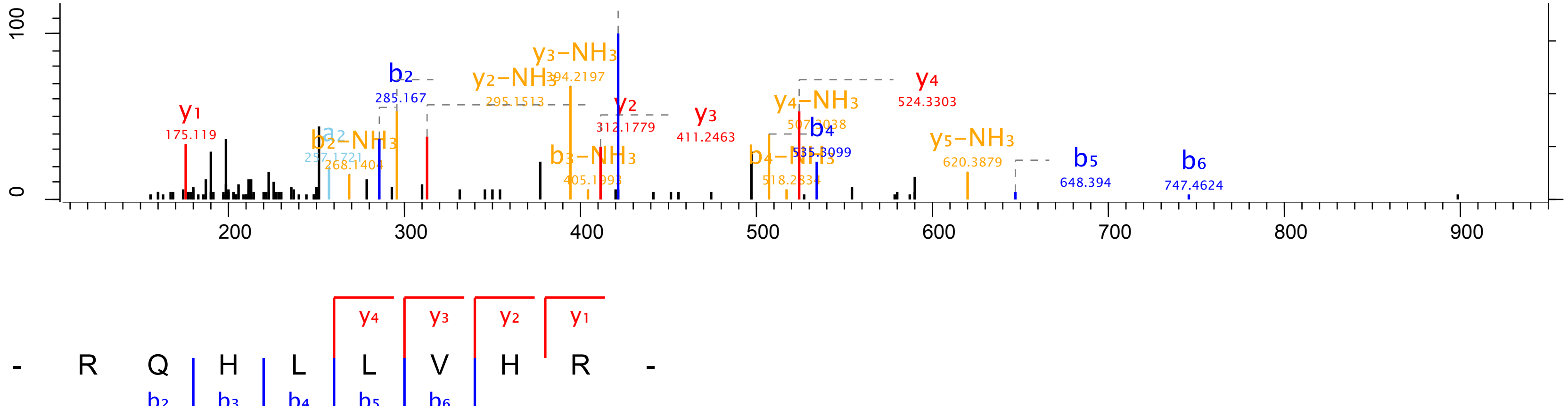

Raw file	Scan	Method	Score	m/z	Gene names
20150402_CerP14_Frac21_top_opt_C9_01_1830	10271	TOF; CID	113.71	511.76	1110059E24Rik

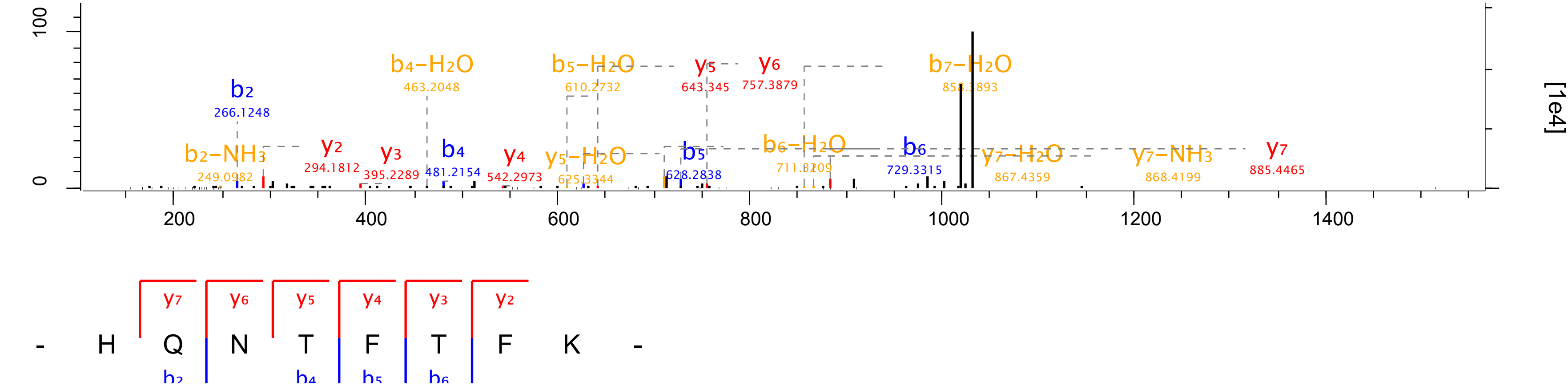

Raw file	Scan	Method	Score	m/z	Gene names
20150402_CerP14_Frac21_top_opt_C9_01_1830	15360	TOF; CID	63.62	687.32	Tet2

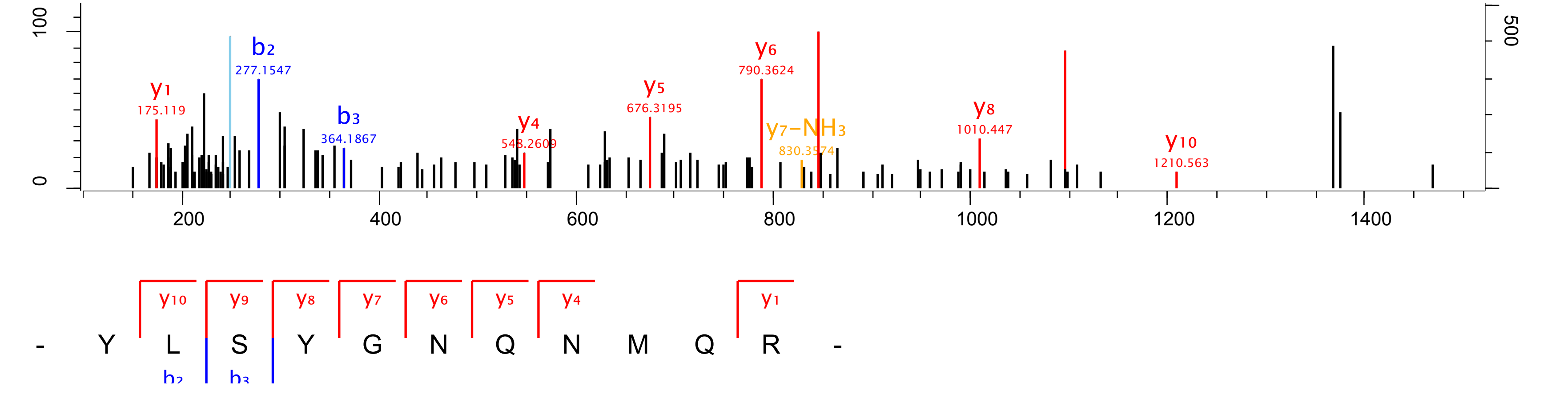

Raw file	Scan	Method	Score	m/z	Gene names
20150402_CerP14_Frac21_top_opt_C9_01_1830	15765	TOF; CID	89.91	675.36	Stap2

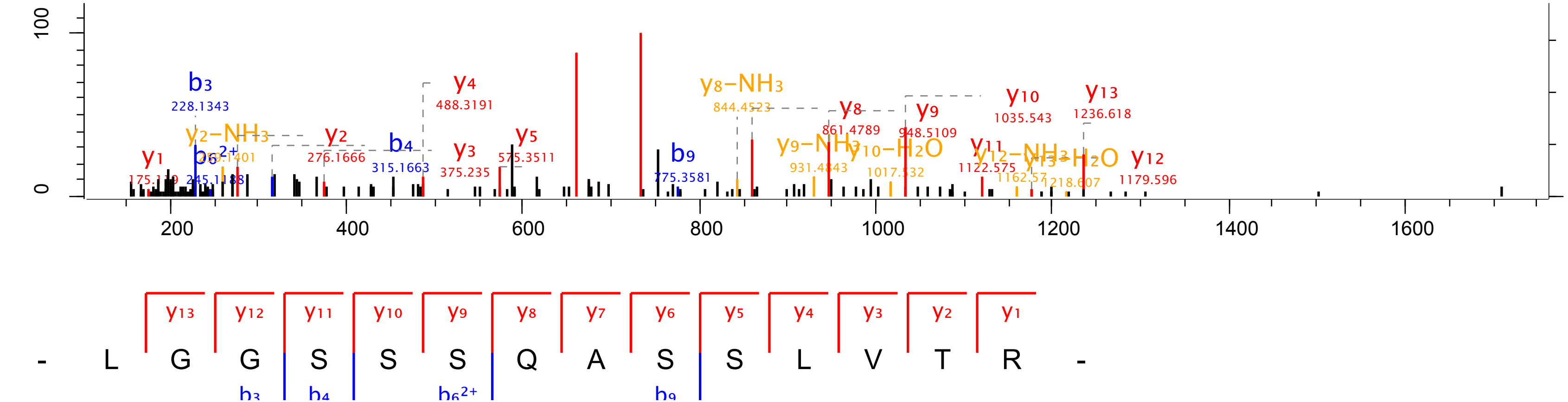

Raw file	Scan	Method	Score	m/z	Gene names
20150402_CerP14_Frac21_top_opt_C9_01_1830	16398	TOF; CID	56.85	794.89	Ngfr

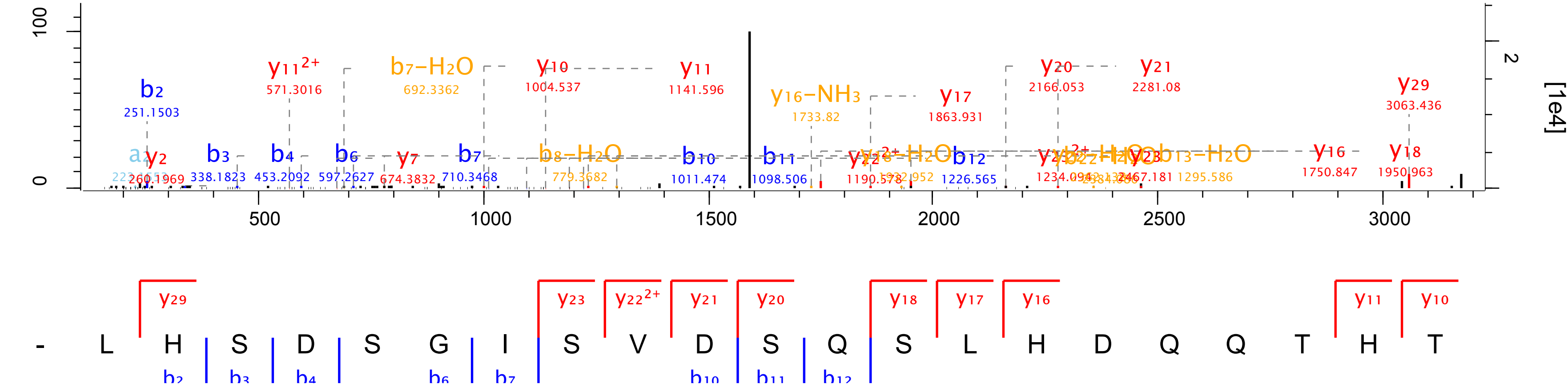

Raw file	Scan	Method	Score	m/z	Gene names
20150402_CerP14_Frac21_top_opt_C9_01_1830	17279	TOF; CID	89.03	543.81	Ly86

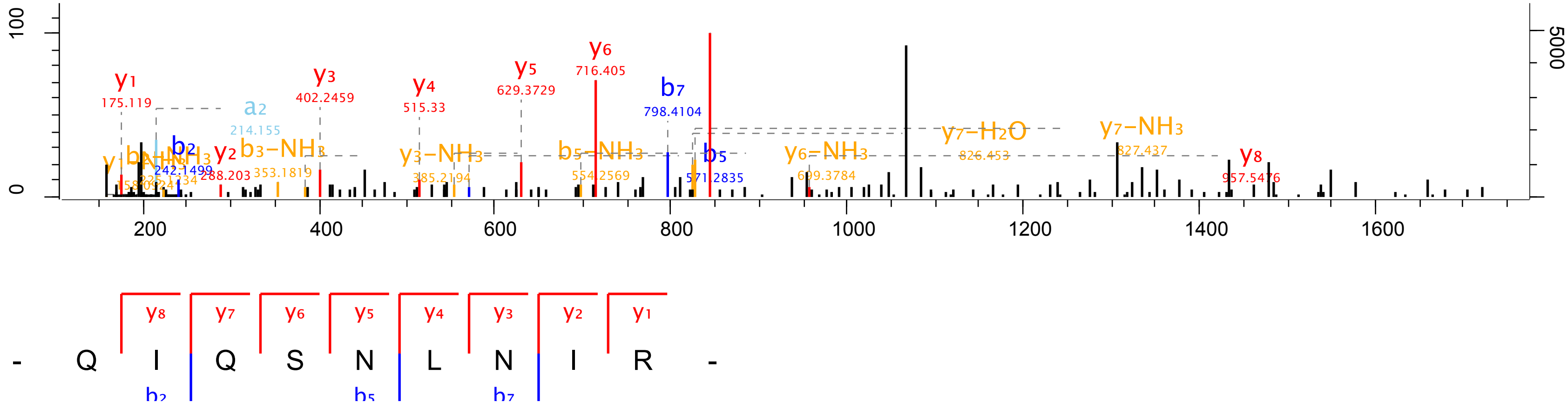

Raw file	Scan	Method	Score	m/z	Gene names
20150402_CerP14_Frac21_top_opt_C9_01_1830	17842	TOF; CID	92.89	897.76	Mplkip;Gm7102

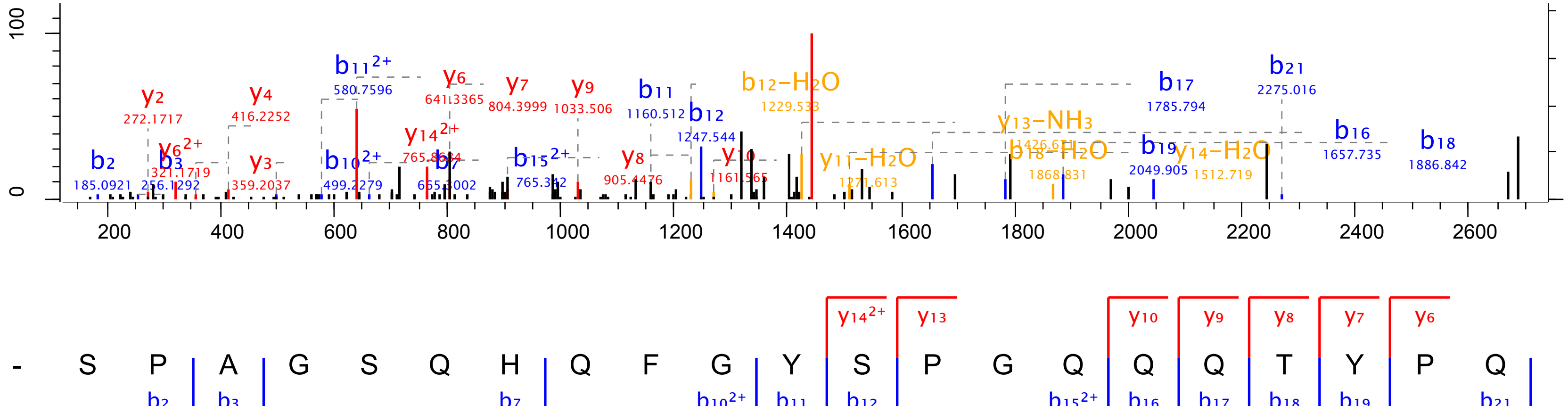

Raw file	Scan	Method	Score	m/z	Gene names
20150402_CerP14_Frac21_top_opt_C9_01_1830	20196	TOF; CID	57.81	986.01	Sox4

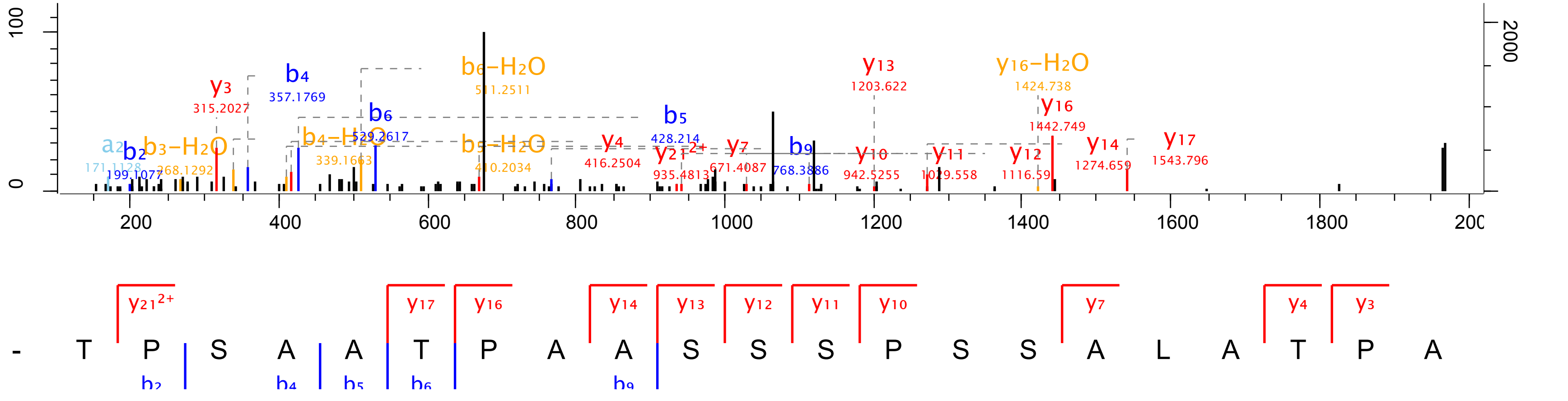

Raw file	Scan	Method	Score	m/z	Gene names
20150402_CerP14_Frac21_top_opt_C9_01_1830	26422	TOF; CID	41.62	751.37	Prr15

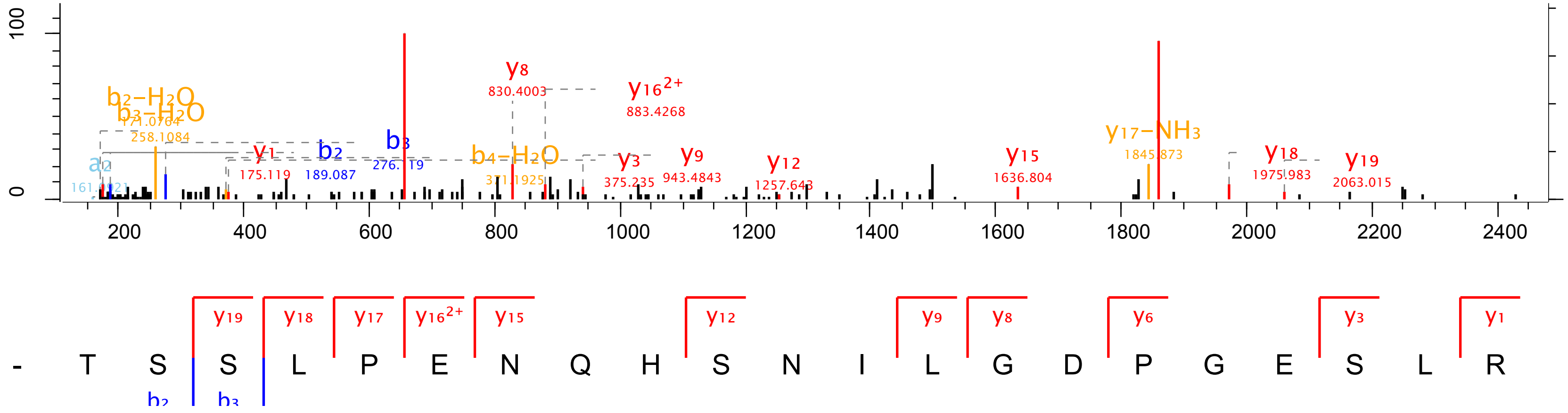

Raw file	Scan	Method	Score	m/z	Gene names
20150402_CerP14_Frac21_top_opt_C9_01_1830	27343	TOF; CID	92.39	787.7	Ppp1r15b

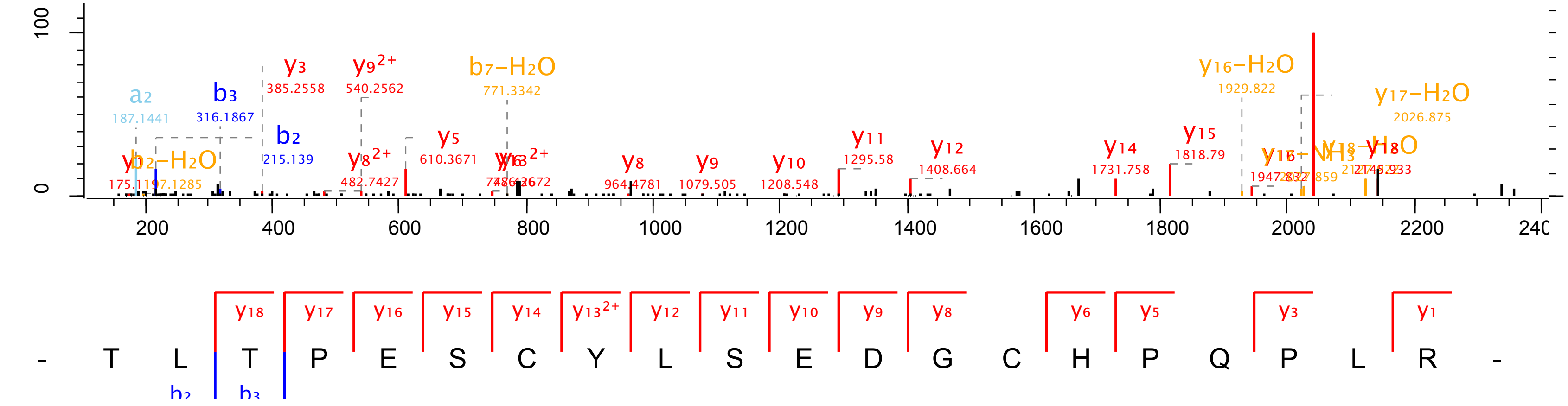

Raw file	Scan	Method	Score	m/z	Gene names
20150402_CerP14_Frac21_top_opt_C9_01_1830	27671	TOF; CID	68.66	495.78	Tonsl

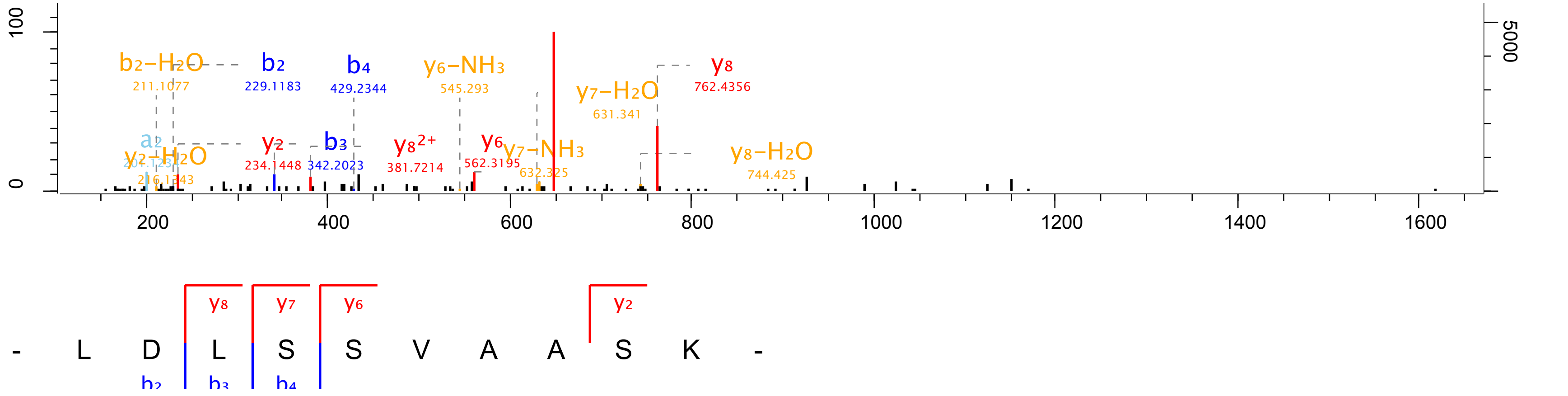

Raw file	Scan	Method	Score	m/z	Gene names
20150402_CerP14_Frac21_top_opt_C9_01_1830	31409	TOF; CID	61.26	795.88	Vip

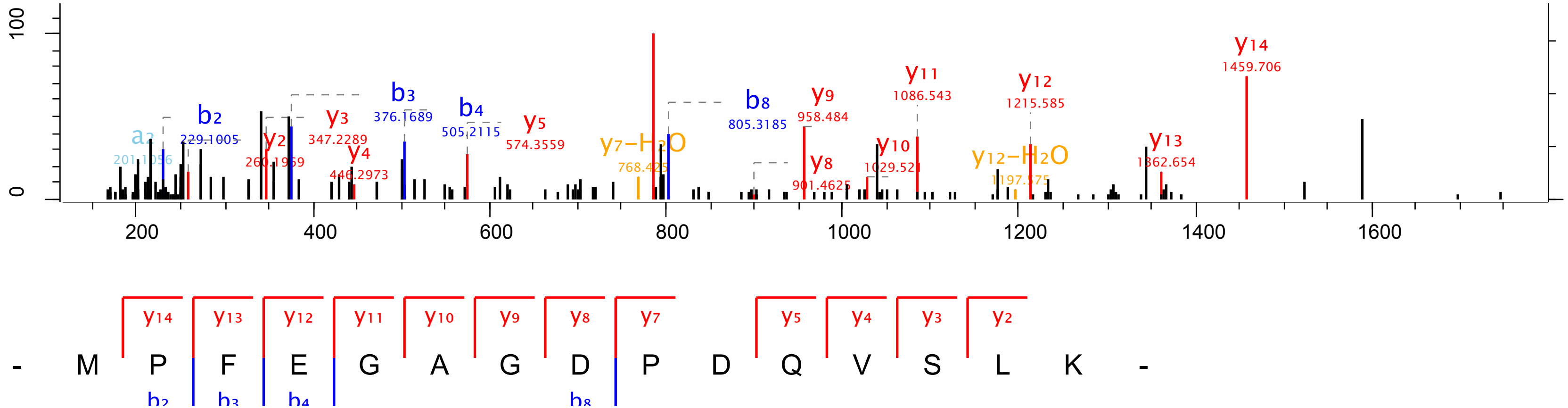

Raw file	Scan	Method	Score	m/z	Gene names
20150402_CerP14_Frac21_top_opt_C9_01_1830	35492	TOF; CID	71.6	635.64	Gtf3c6

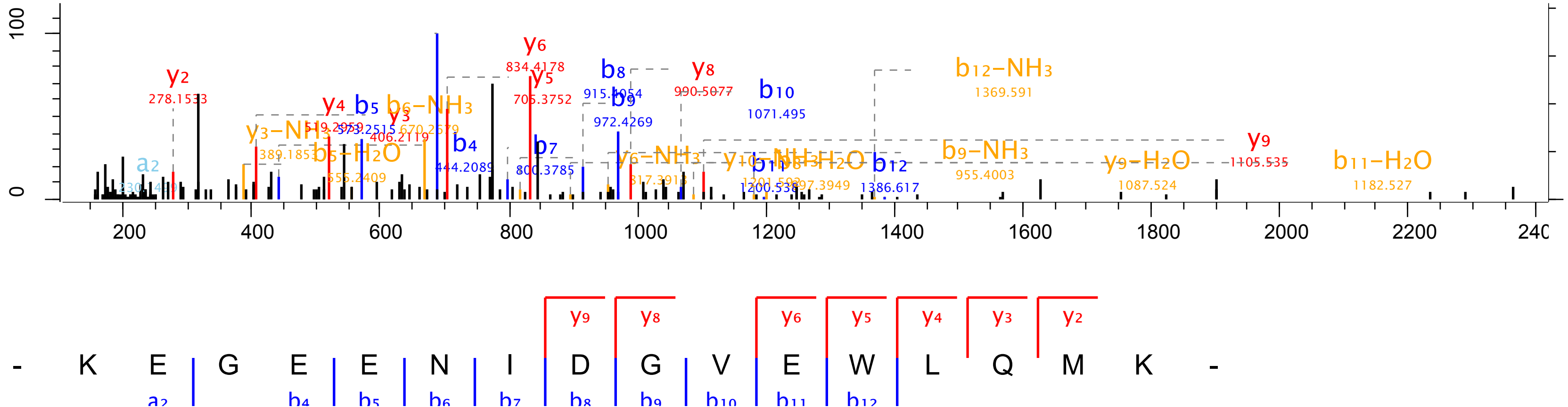

Raw file	Scan	Method	Score	m/z	Gene names
20150402_CerP14_Frac21_top_opt_C9_01_1830	35644	TOF; CID	67.33	673.87	Dusp16

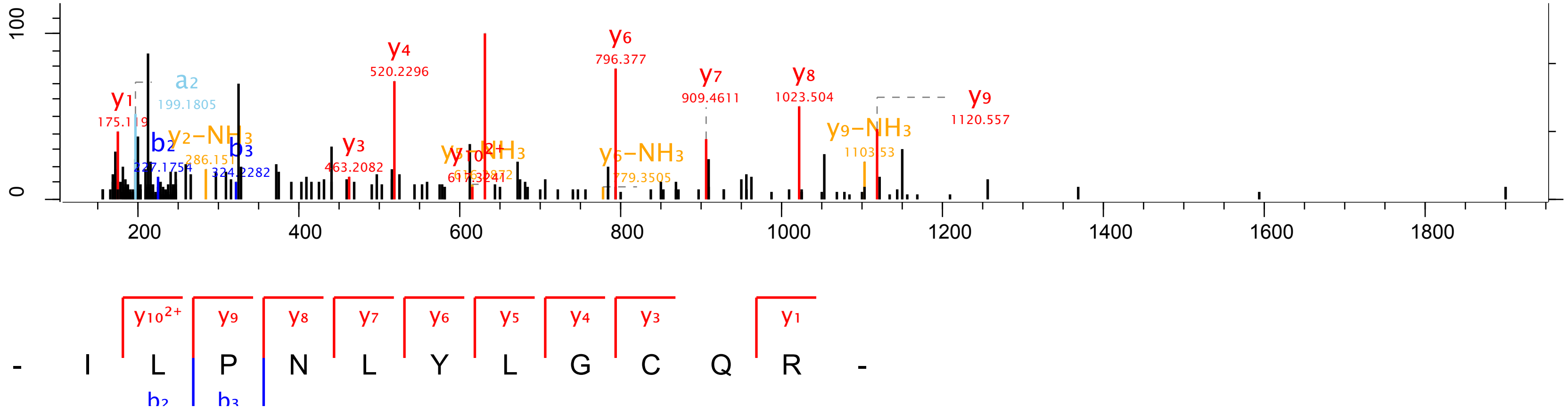

Raw file	Scan	Method	Score	m/z	Gene names
20150402_CerP14_Frac21_top_opt_C9_01_1830	35712	TOF; CID	86.67	518.8	Rhag

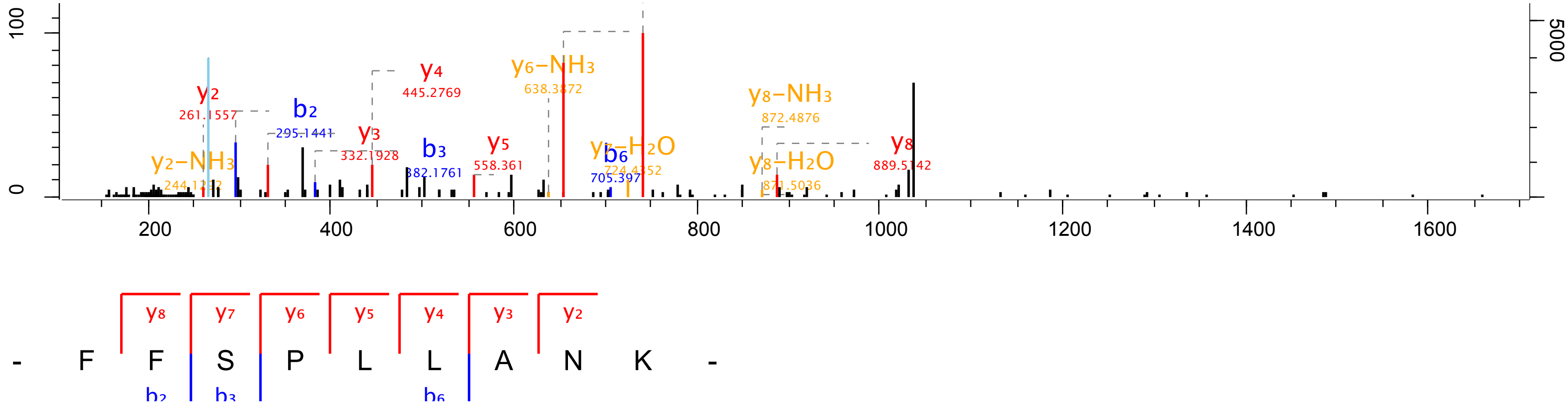

Raw file	Scan	Method	Score	m/z	Gene names
20150402_CerP14_Frac21_top_opt_C9_01_1830	35808	TOF; CID	55.05	1217.6	Sox11

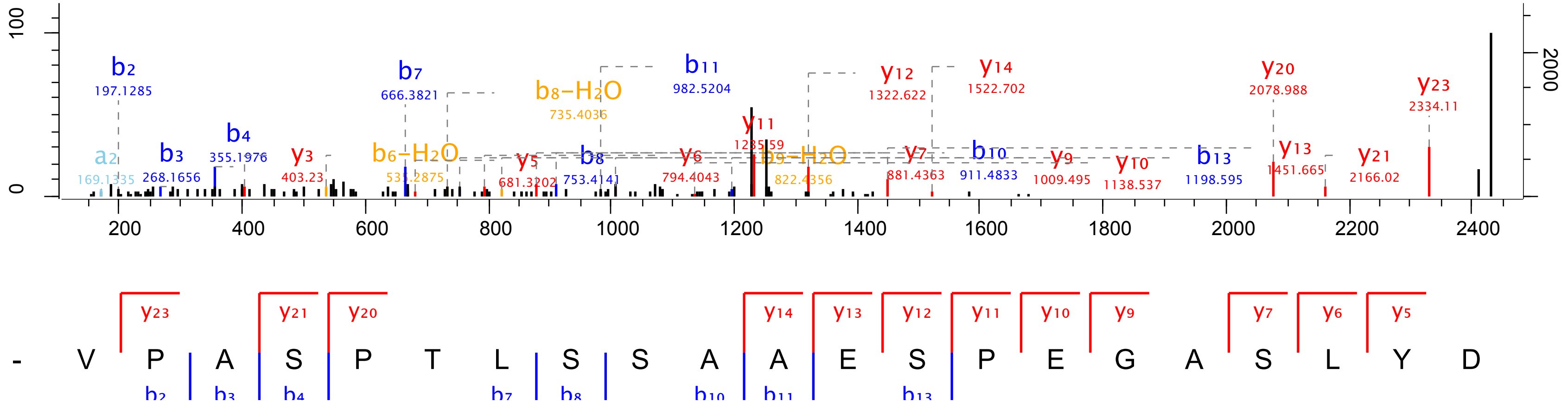

Raw file	Scan	Method	Score	m/z	Gene names
20150402_CerP14_Frac21_top_opt_C9_01_1830	40212	TOF; CID	93.62	478.58	Snapc3

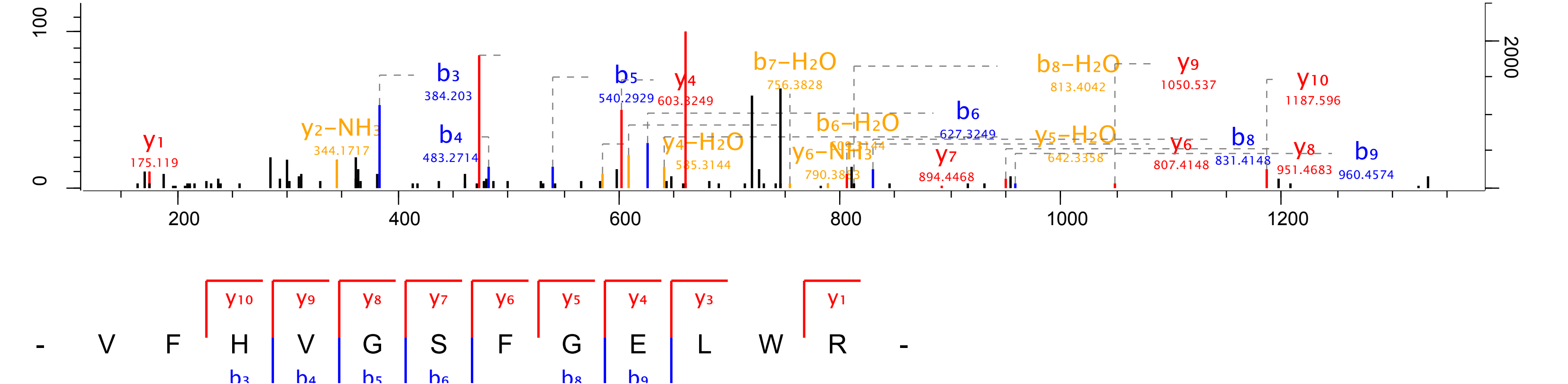

Raw file	Scan	Method	Score	m/z	Gene names
20150402_CerP14_Frac21_top_opt_C9_01_1830	40516	TOF; CID	79.09	495.97	Dnajb13

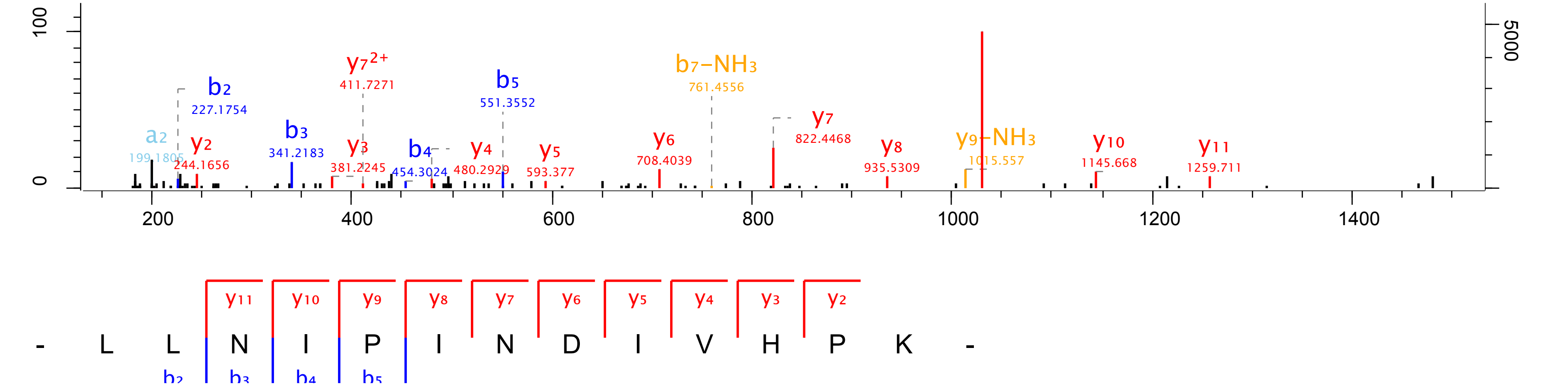

Raw file	Scan	Method	Score	m/z	Gene names
20150402_CerP14_Frac21_top_opt_C9_01_1830	42677	TOF; CID	72.88	543.84	Slco1a6

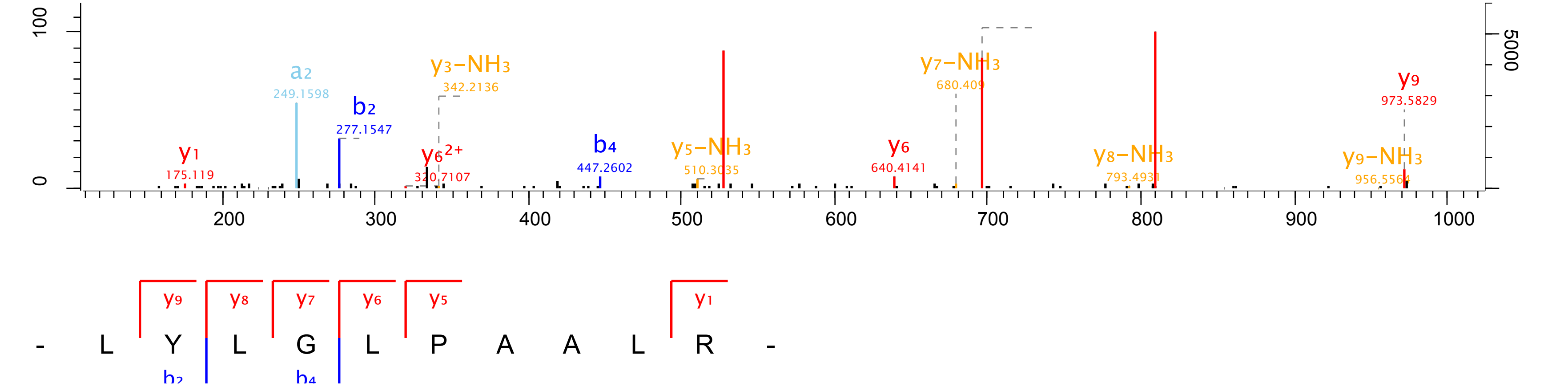

Raw file	Scan	Method	Score	m/z	Gene names
20150402_CerP14_Frac21_top_opt_C9_01_1830	42984	TOF; CID	97.43	560.33	Tifa

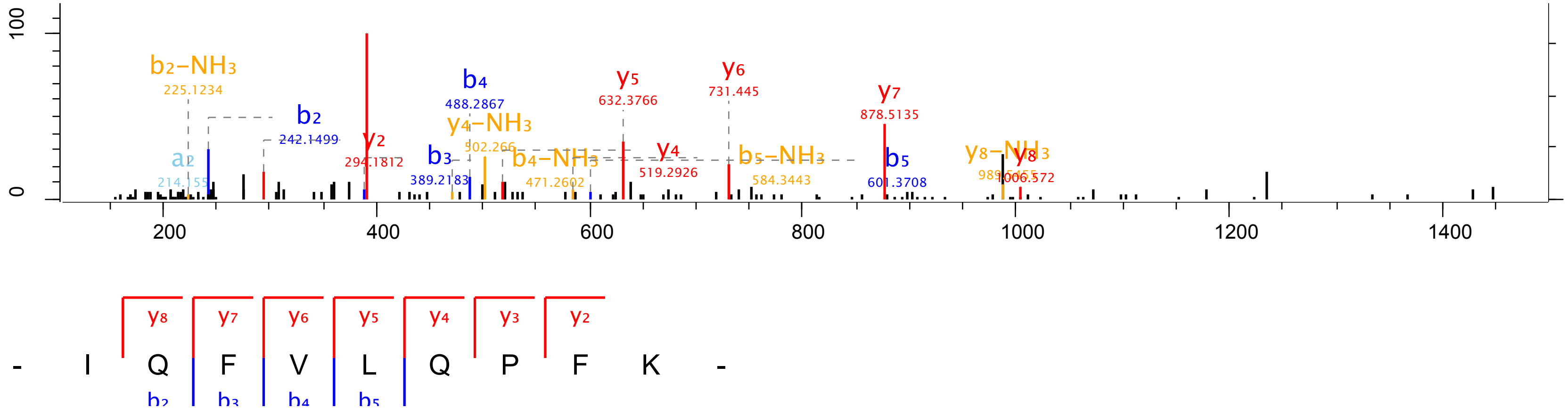

Raw file	Scan	Method	Score	m/z	Gene names
20150402_CerP14_Frac21_top_opt_C9_01_1830	44830	TOF; CID	159.26	754.71	Cnih4

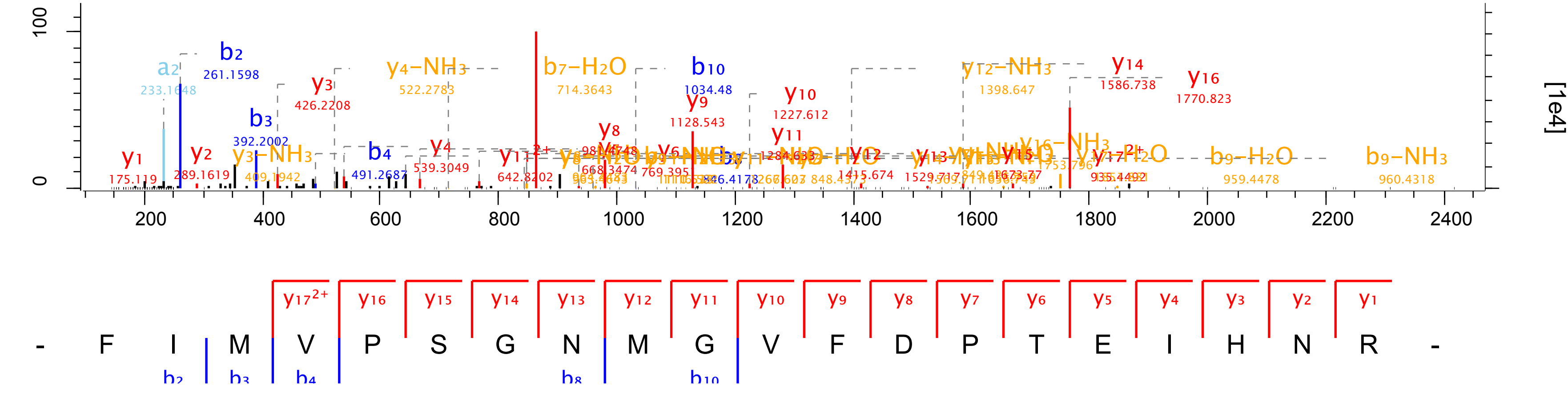

Raw file	Scan	Method	Score	m/z	Gene names
20150402_CerP14_Frac21_top_opt_C9_01_1830	46830	TOF; CID	36.67	736.9	Vgll4

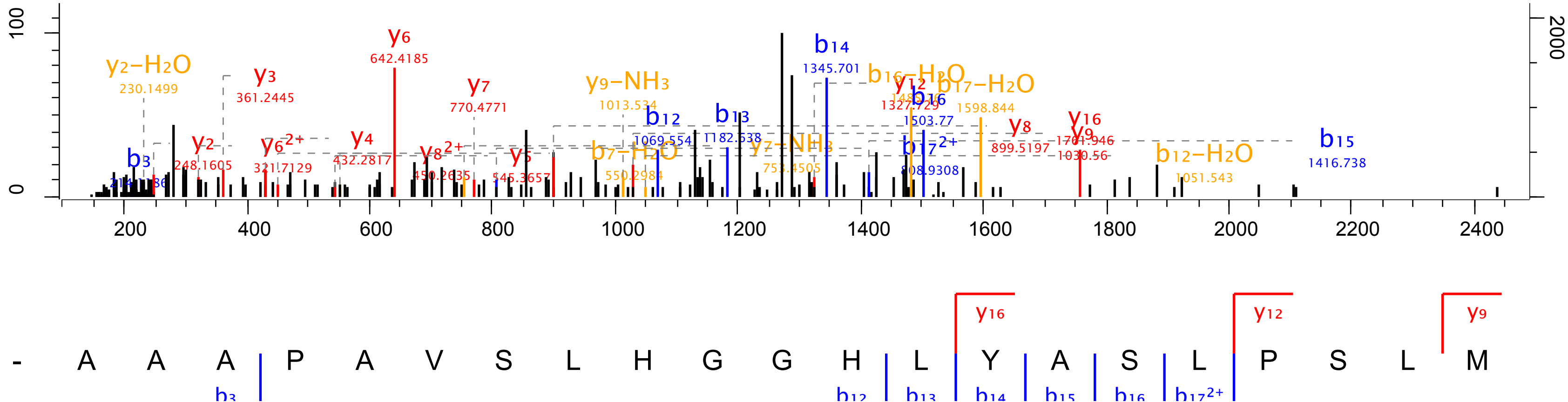

Raw file	Scan	Method	Score	m/z	Gene names
20150402_CerP14_Frac21_top_opt_C9_01_1830	48132	TOF; CID	63.29	572.3	Taf1c

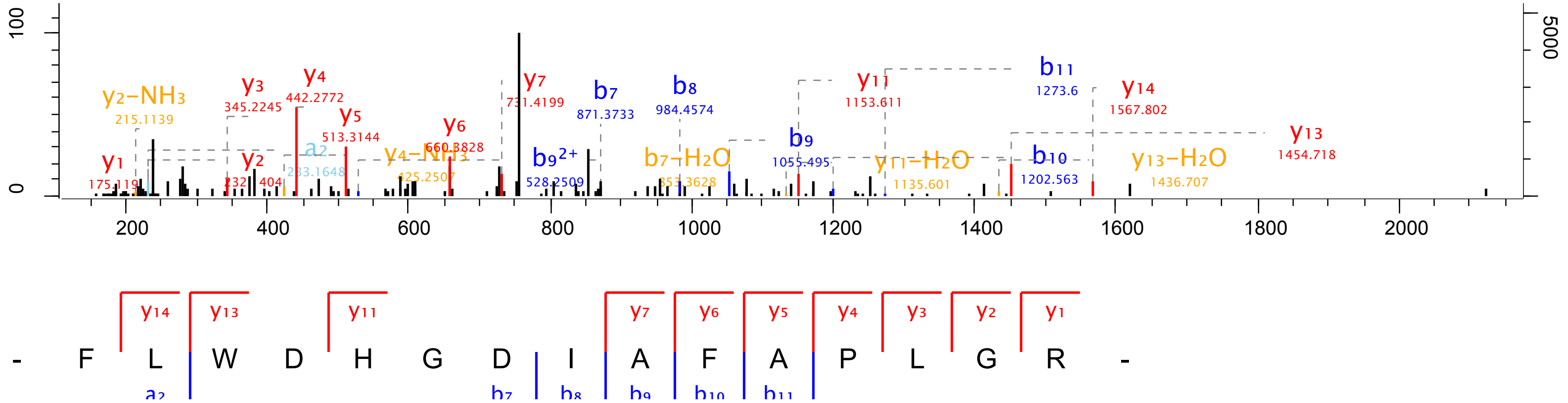

Raw file	Scan	Method	Score	m/z	Gene names
20150402_CerP14_Frac21_top_opt_C9_01_1830	48286	TOF; CID	80.41	829.13	Ppp1r1c

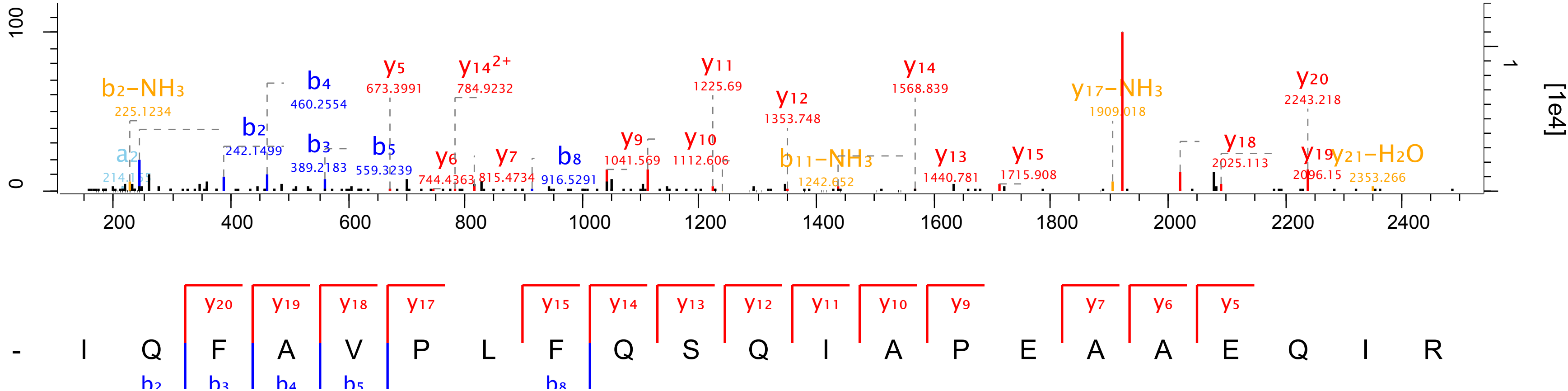

Raw file	Scan	Method	Score	m/z	Gene names
20150402_CerP14_Frac21_top_opt_C9_01_1830	48574	TOF; CID	65.29	694.42	S100g

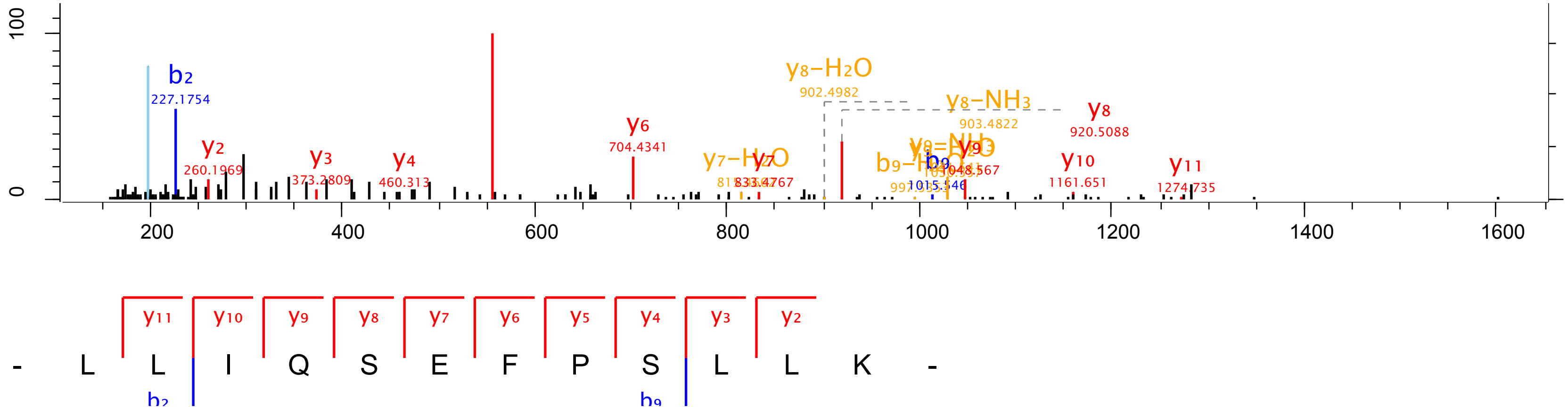

Raw file	Scan	Method	Score	m/z	Gene names
20150402_CerP14_Frac22_top_opt_C10_01_1831	5734	TOF; CID	44.51	514.26	Zfp324

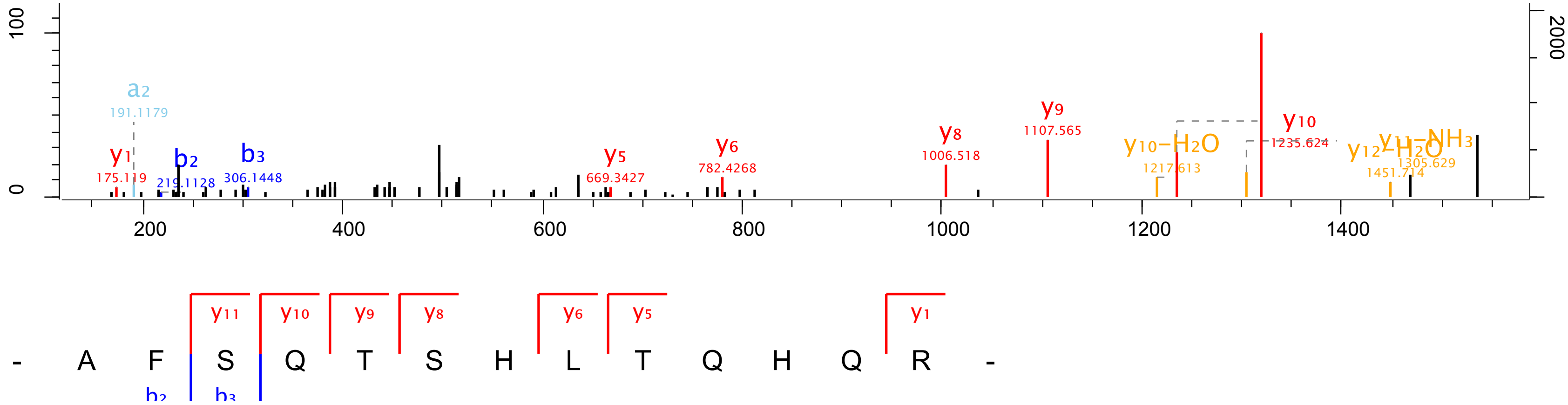

Raw file	Scan	Method	Score	m/z	Gene names
20150402_CerP14_Frac22_top_opt_C10_01_1831	21666	TOF; CID	48.38	667.3	Chrna7

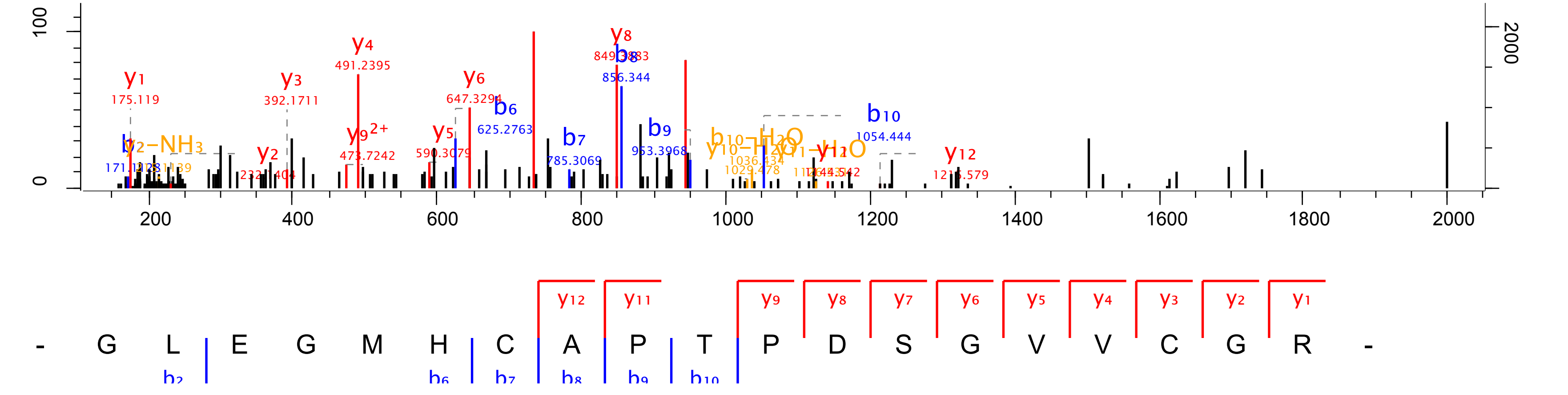

Raw file	Scan	Method	Score	m/z	Gene names
20150402_CerP14_Frac22_top_opt_C10_01_1831	22335	TOF; CID	113.07	710.89	Dirc2

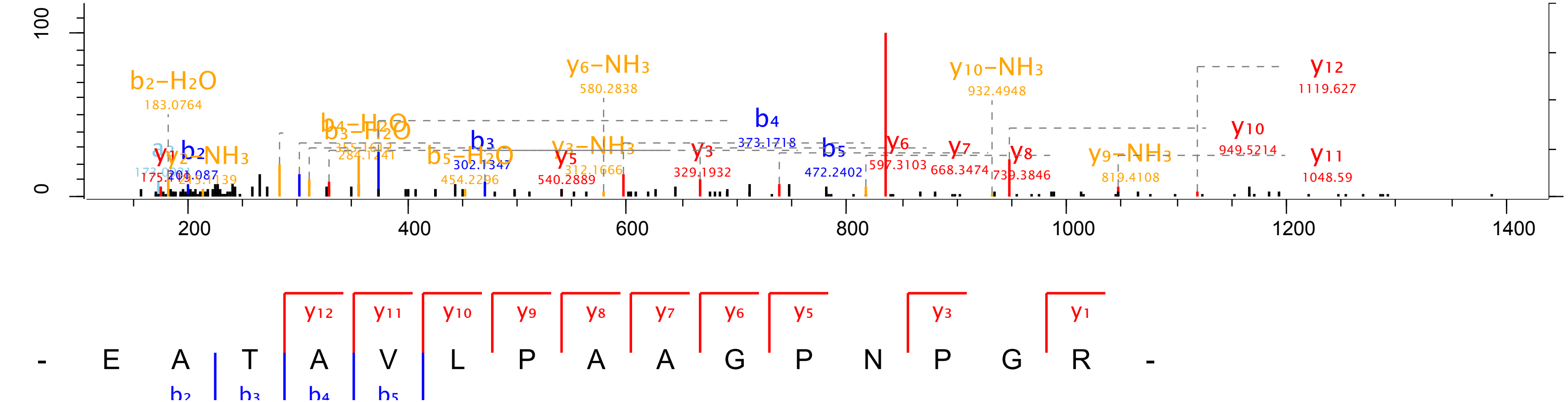

Raw file	Scan	Method	Score	m/z	Gene names
20150402_CerP14_Frac22_top_opt_C10_01_1831	25431	TOF; CID	91.78	651.35	Itgb3bp

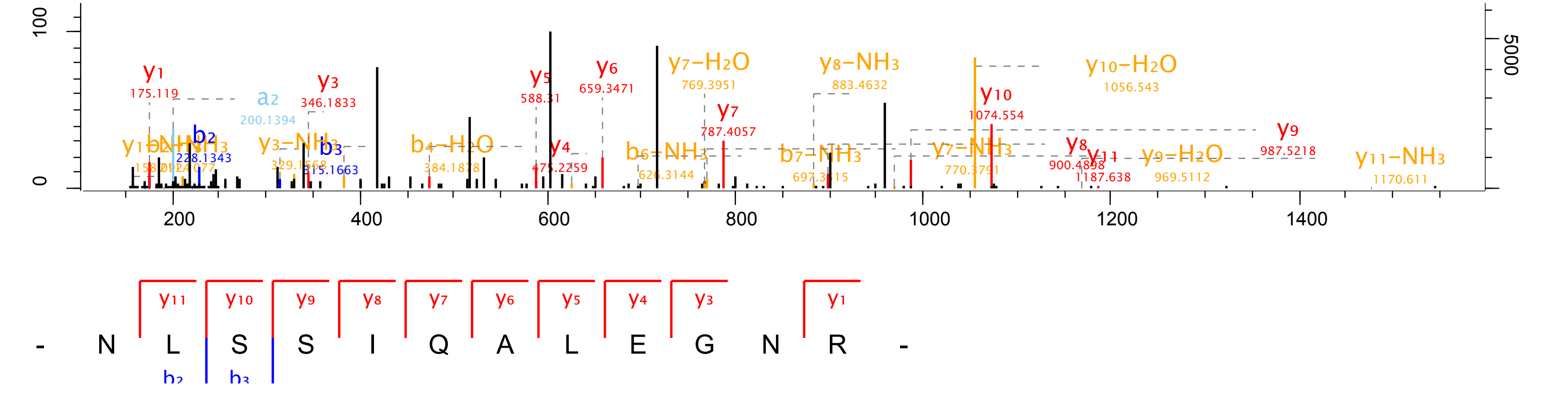

Raw file	Scan	Method	Score	m/z	Gene names
20150402_CerP14_Frac22_top_opt_C10_01_1831	30550	TOF; CID	88.18	553.81	Fkbp7

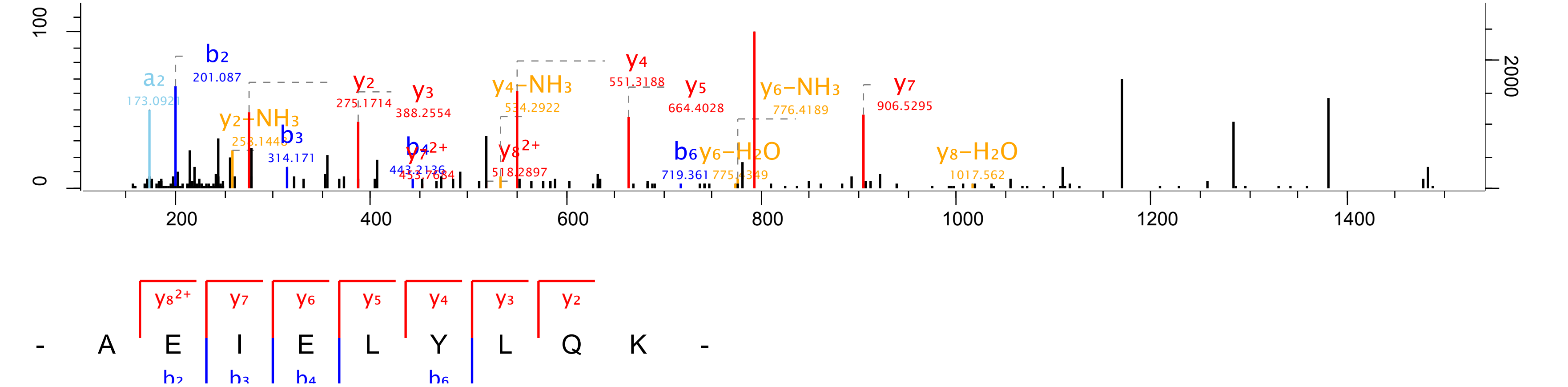

Raw file	Scan	Method	Score	m/z	Gene names
20150402_CerP14_Frac22_top_opt_C10_01_1831	37927	TOF; CID	112.48	966.46	Tmem128

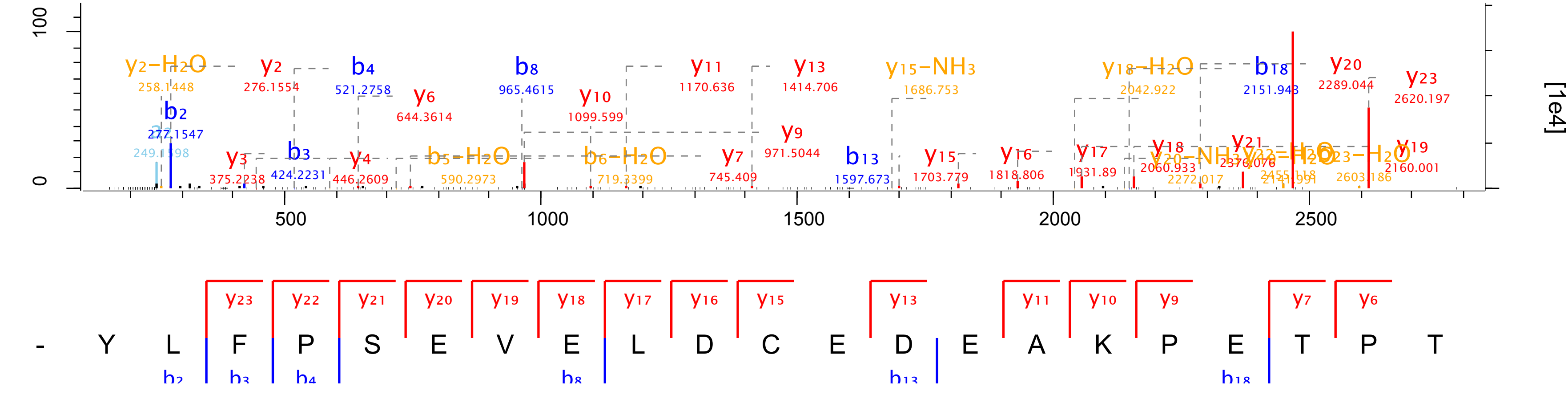

Raw file	Scan	Method	Score	m/z	Gene names
20150402_CerP14_Frac22_top_opt_C10_01_1831	40442	TOF; CID	45.22	914.46	Efna3

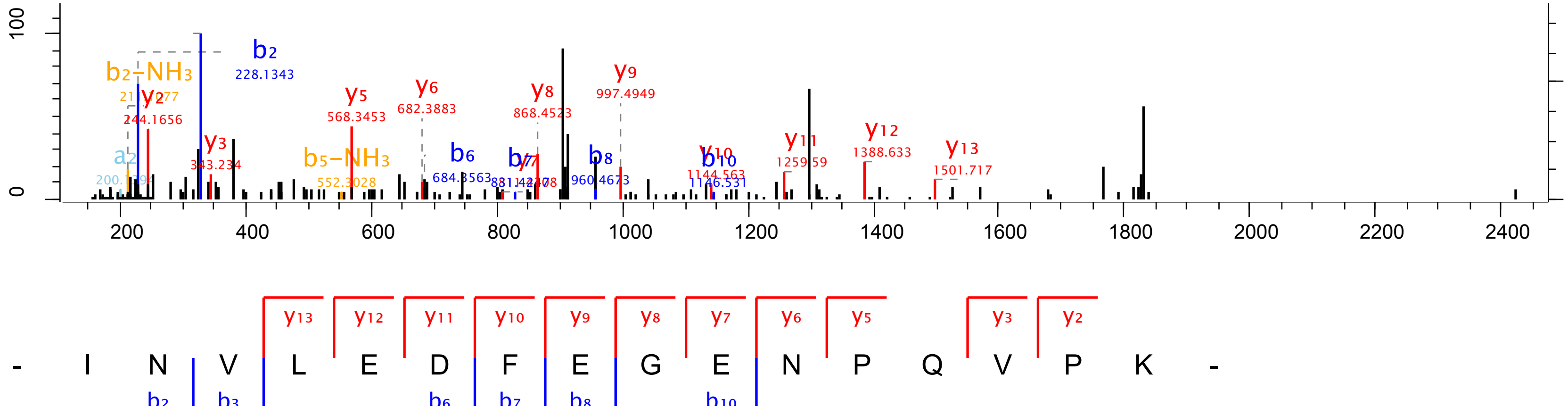

Raw file	Scan	Method	Score	m/z	Gene names
20150402_CerP14_Frac22_top_opt_C10_01_1831	45171	TOF; CID	107.9	489.79	Bcl2l11

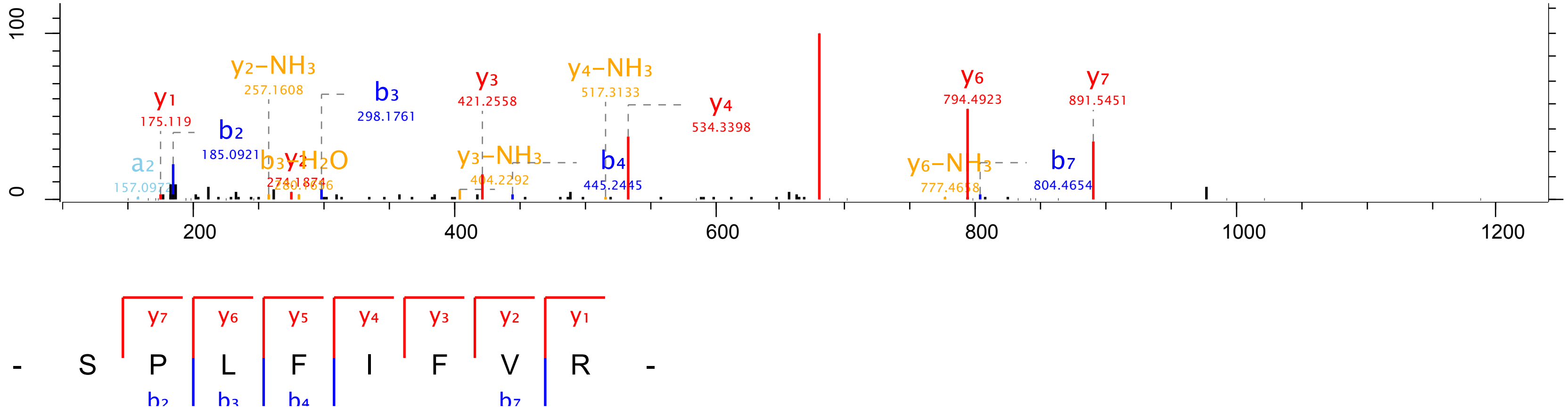

Raw file	Scan	Method	Score	m/z	Gene names
20150402_CerP14_Frac22_top_opt_C10_01_1831	48713	TOF; CID	59.67	810.94	Anapc16

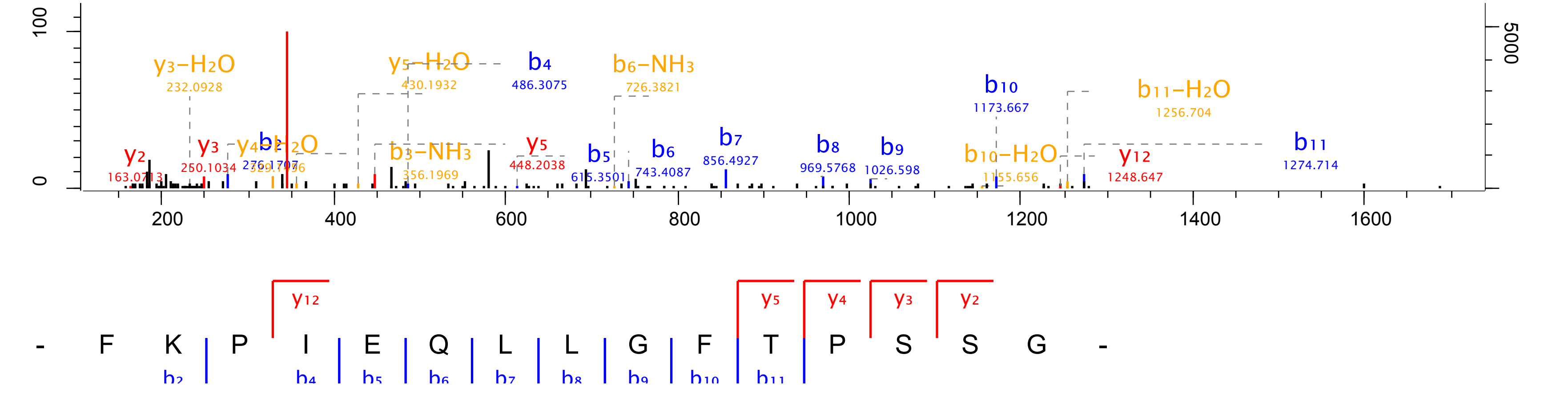

Raw file	Scan	Method	Score	m/z	Gene names
20150402_CerP14_Frac22_top_opt_C10_01_1831	55904	TOF; CID	78.67	656.7	S100a4

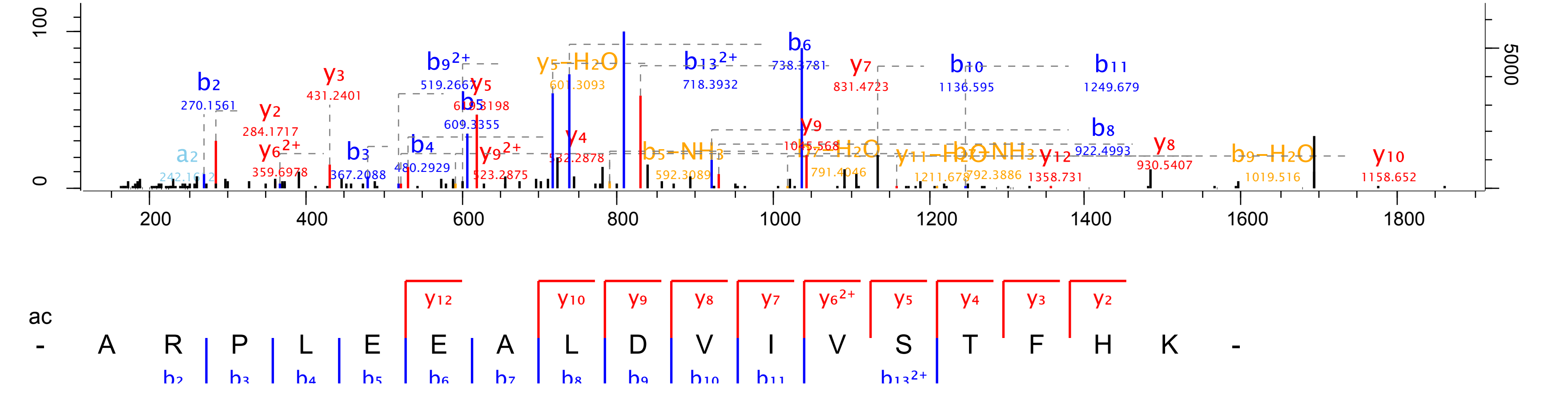

Raw file	Scan	Method	Score	m/z	Gene names
20150402_CerP14_Frac23_top_opt_C11_01_1832	4378	TOF; CID	125.72	475.91	Zfp959;Zfp119b

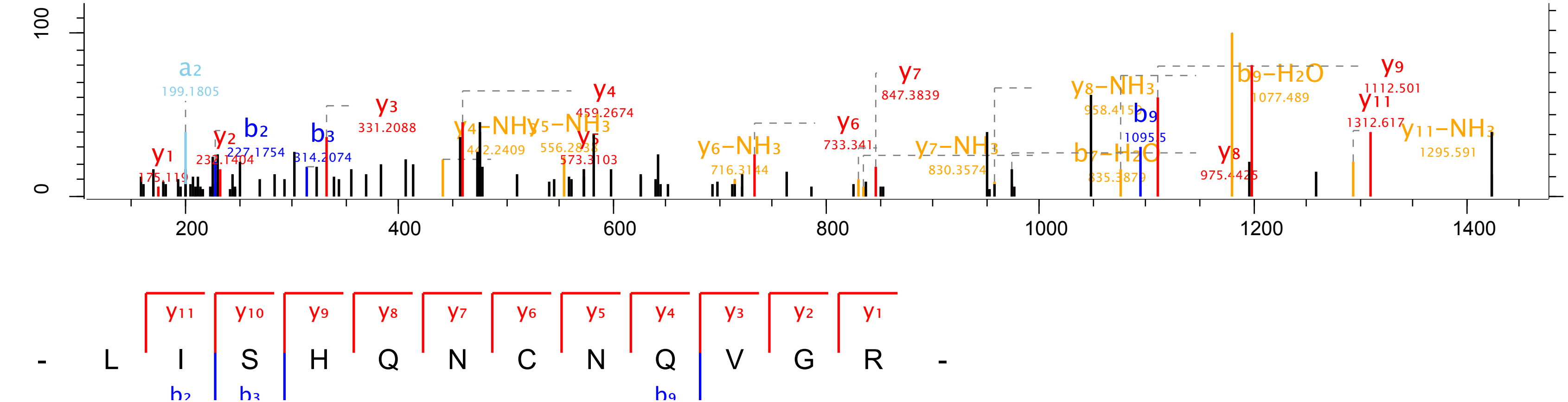

Raw file	Scan	Method	Score	m/z	Gene names
20150402_CerP14_Frac23_top_opt_C11_01_1832	5564	TOF; CID	54.26	397.55	Zfp772

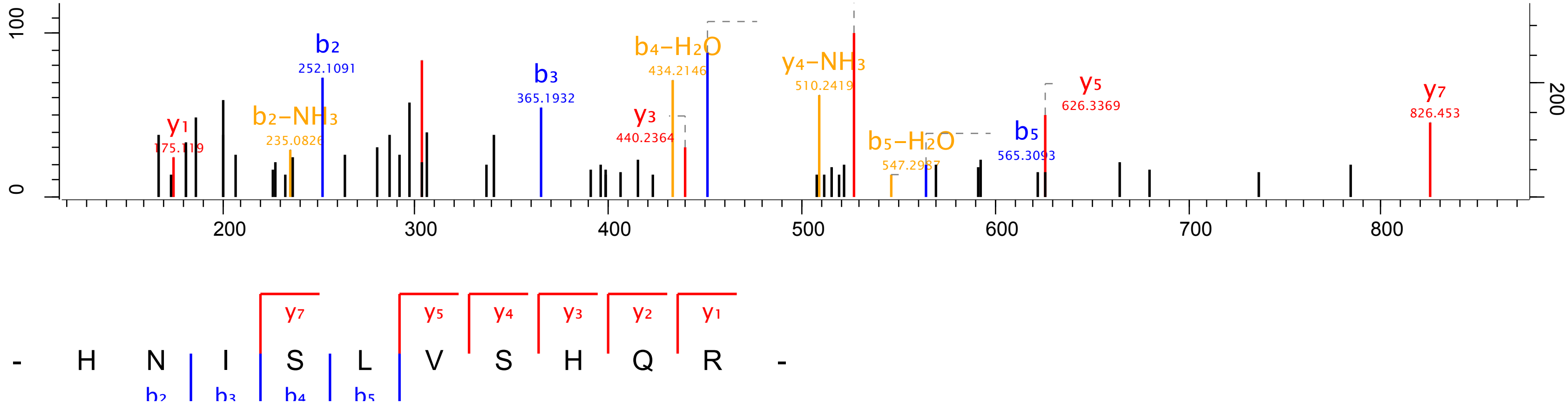

Raw file	Scan	Method	Score	m/z	Gene names
20150402_CerP14_Frac23_top_opt_C11_01_1832	6471	TOF; CID	63.82	366.54	F5

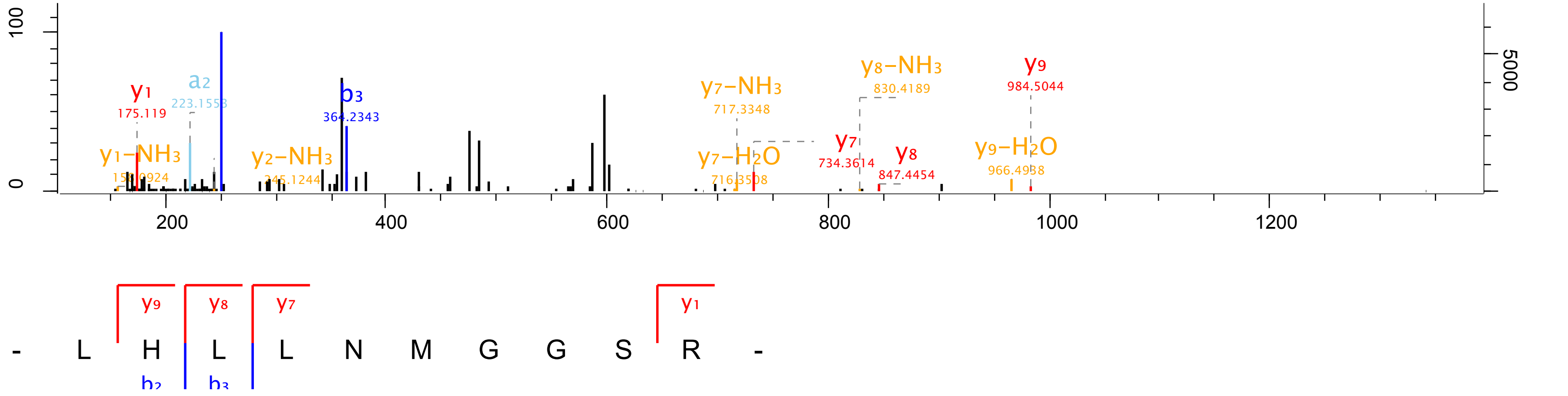

Raw file	Scan	Method	Score	m/z	Gene names
20150402_CerP14_Frac23_top_opt_C11_01_1832	8601	TOF; CID	100.11	707.39	Zbtb48

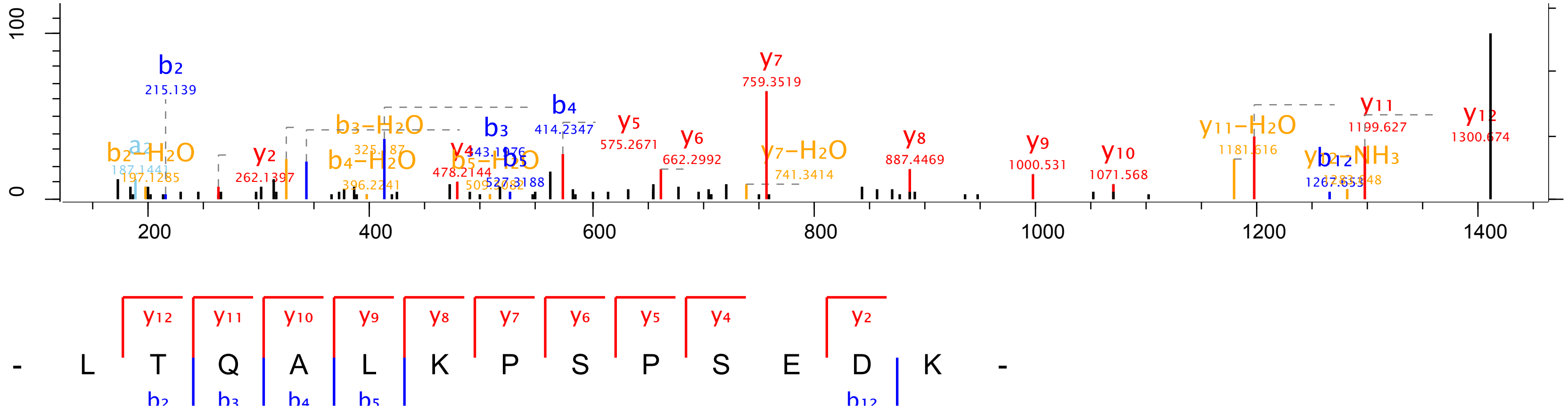

Raw file	Scan	Method	Score	m/z	Gene names
20150402_CerP14_Frac23_top_opt_C11_01_1832	8881	TOF; CID	39	503.74	Megf6

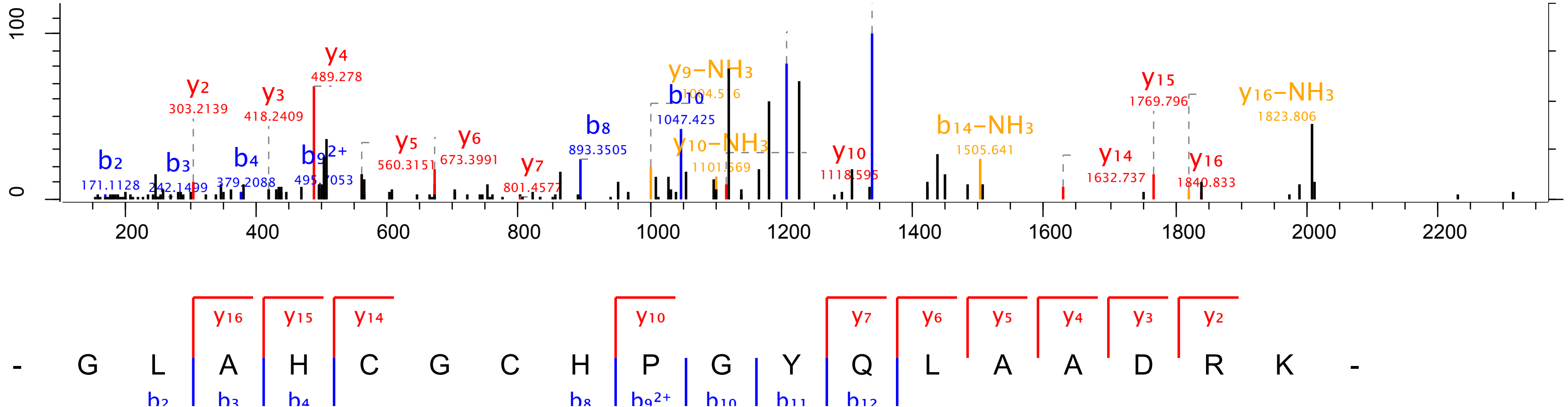

Raw file	Scan	Method	Score	m/z	Gene names
20150402_CerP14_Frac23_top_opt_C11_01_1832	11097	TOF; CID	142.64	436.77	Il19

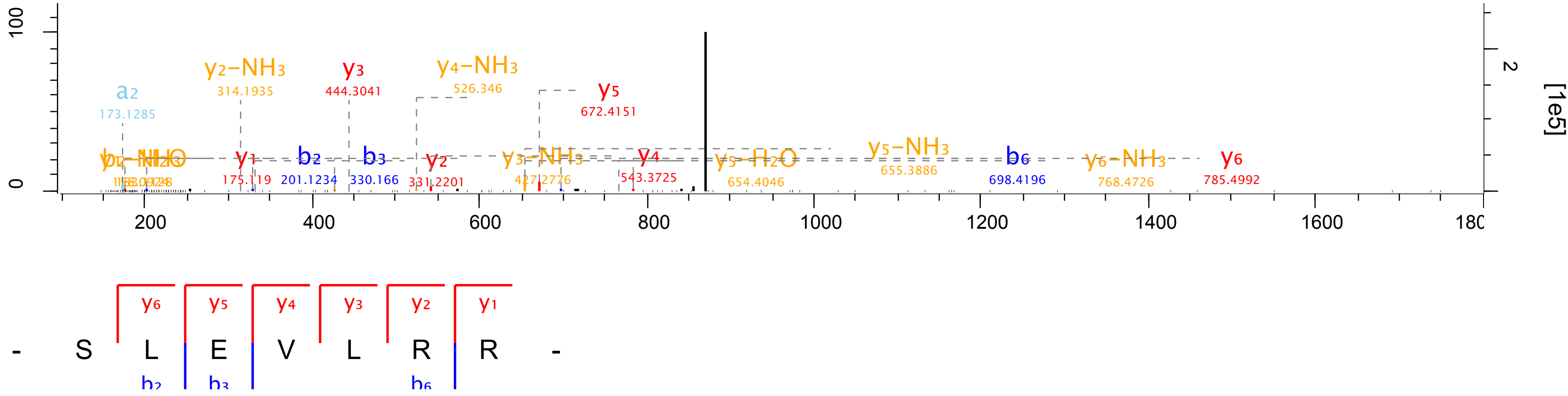

Raw file	Scan	Method	Score	m/z	Gene names
20150402_CerP14_Frac23_top_opt_C11_01_1832	13654	TOF; CID	56.94	688.87	Mypn

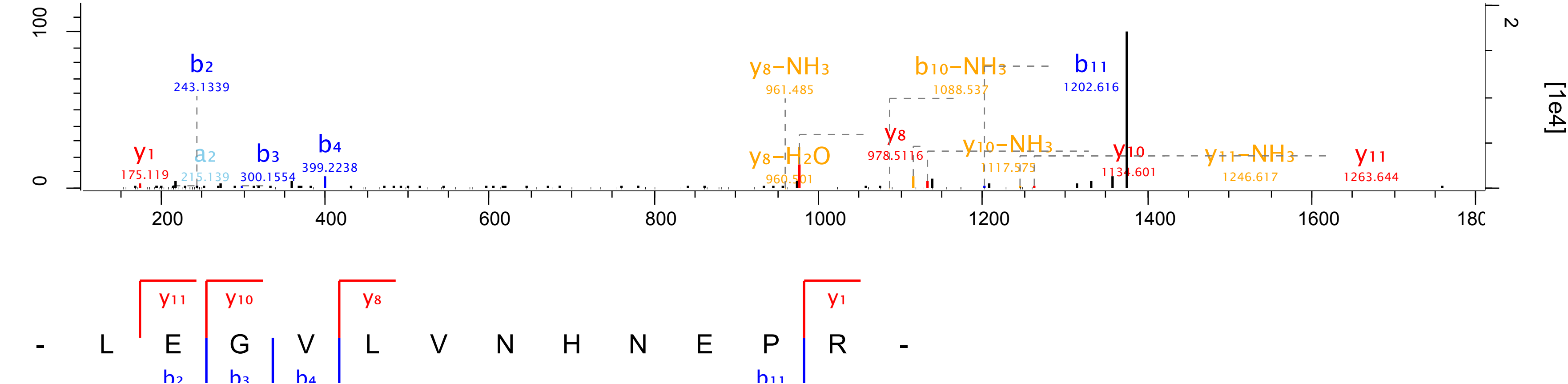

Raw file	Scan	Method	Score	m/z	Gene names
20150402_CerP14_Frac23_top_opt_C11_01_1832	21348	TOF; CID	72.34	440.23	Terf1

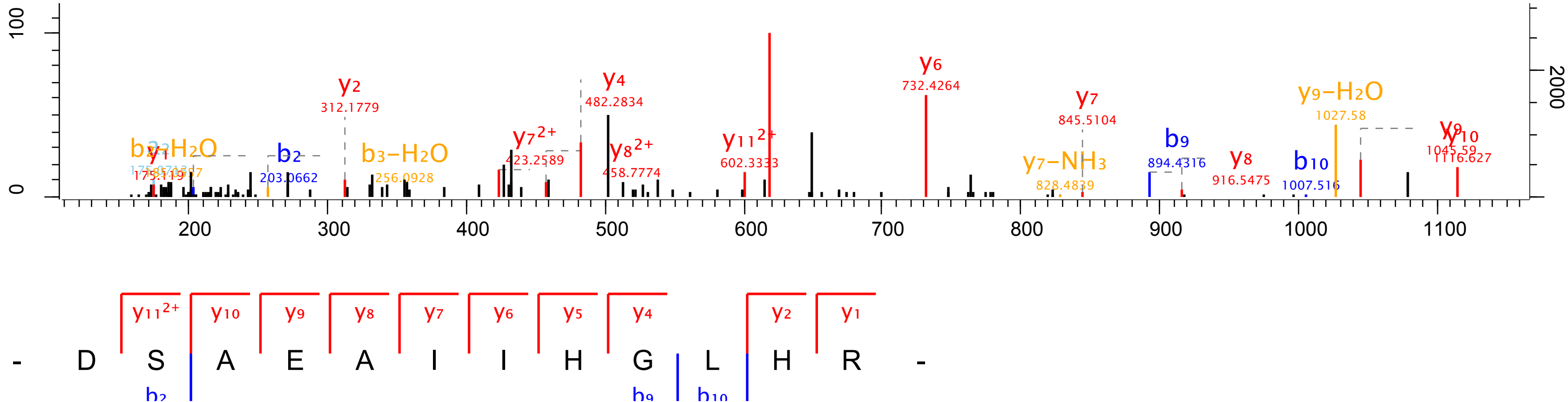

Raw file	Scan	Method	Score	m/z	Gene names
20150402_CerP14_Frac23_top_opt_C11_01_1832	25143	TOF; CID	103.01	852.44	Ptpn18

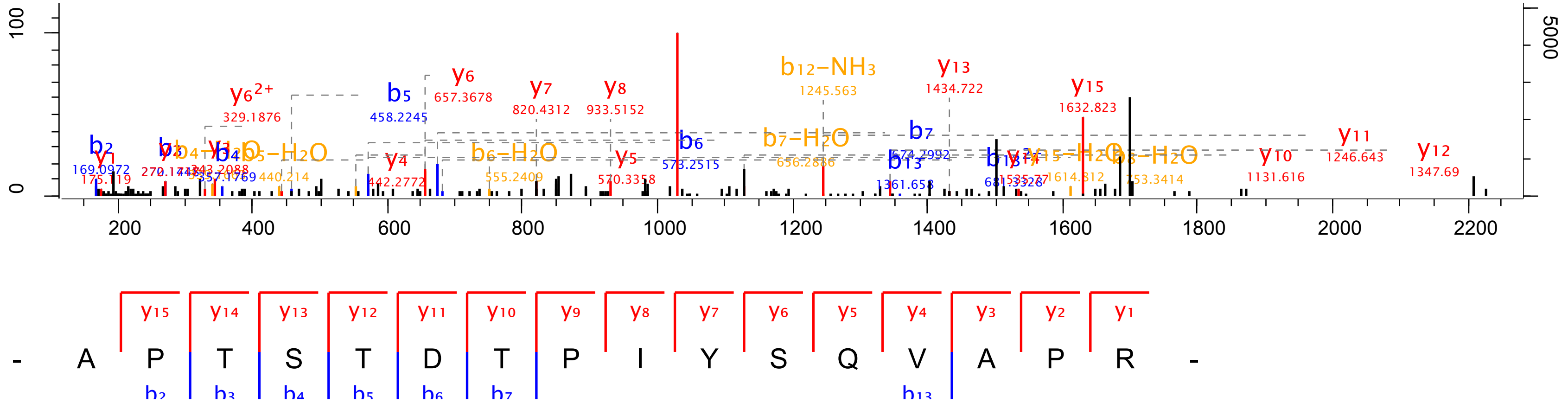

Raw file	Scan	Method	Score	m/z	Gene names
20150402_CerP14_Frac23_top_opt_C11_01_1832	27787	TOF; CID	35.54	836.39	Las2

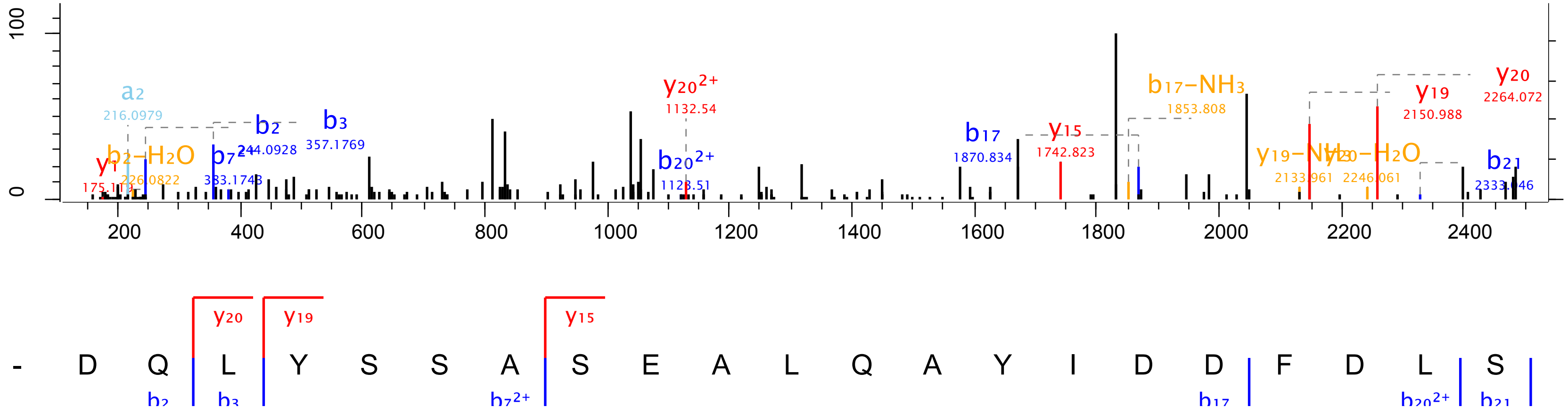

Raw file	Scan	Method	Score	m/z
20150402_CerP14_Frac23_top_opt_C11_01_1832	30882	TOF; CID	77.18	535.6

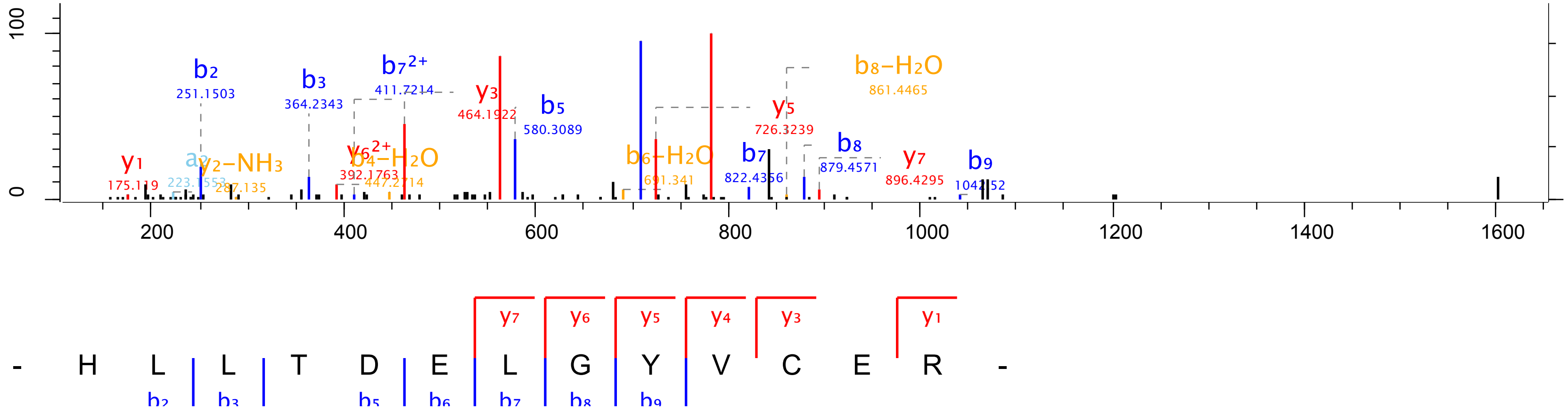

Raw file	Scan	Method	Score	m/z	Gene names
20150402_CerP14_Frac23_top_opt_C11_01_1832	30929	TOF; CID	56.68	692.01	Cd99

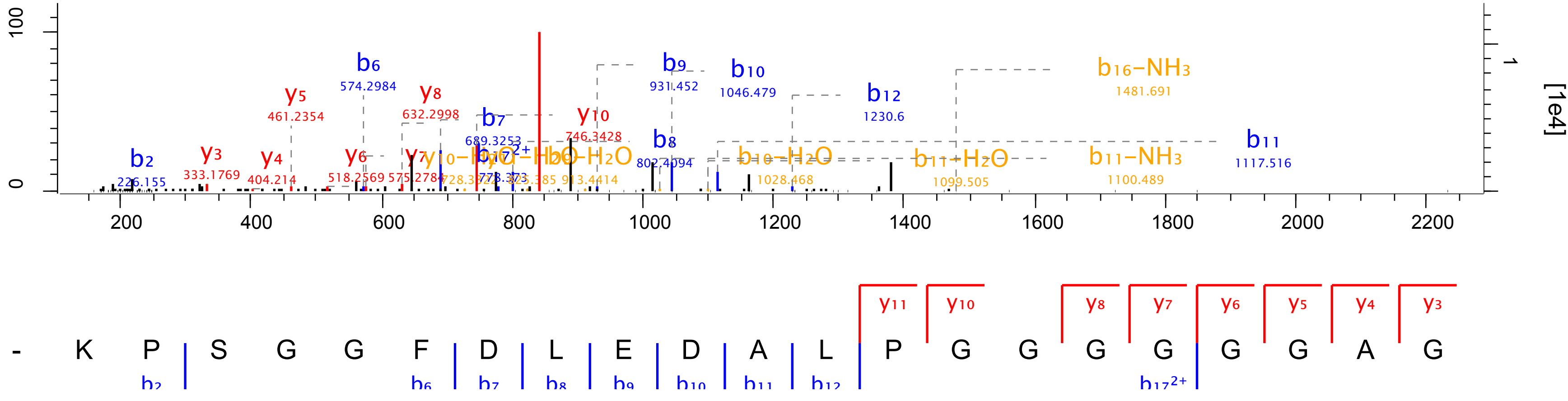

Raw file	Scan	Method	Score	m/z	Gene names
20150402_CerP14_Frac23_top_opt_C11_01_1832	32575	TOF; CID	68.54	603.33	Pnmt

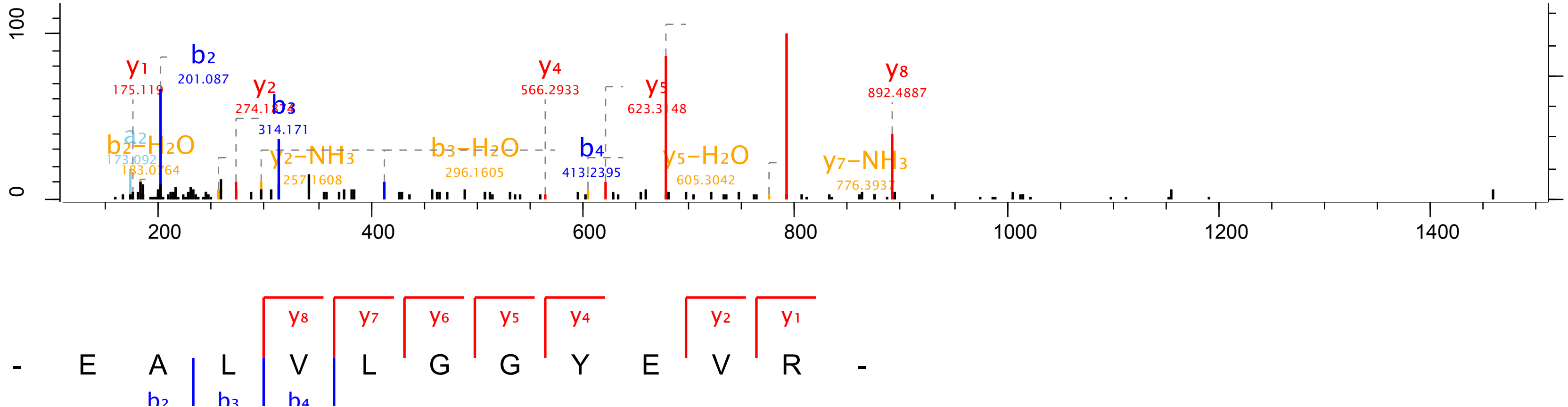

Raw file	Scan	Method	Score	m/z	Gene names
20150402_CerP14_Frac23_top_opt_C11_01_1832	32885	TOF; CID	41.8	863.41	Rdm1

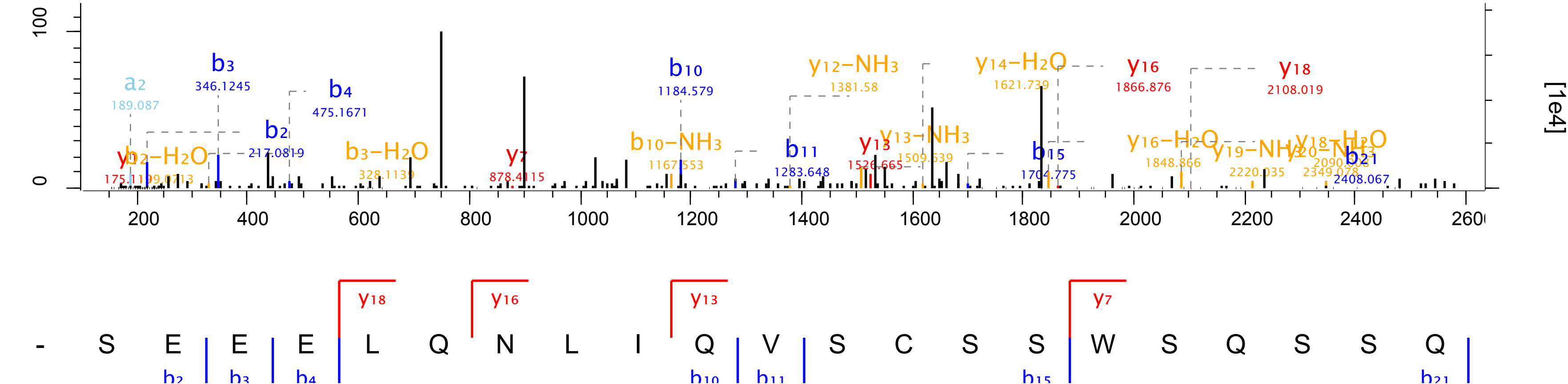

Raw file	Scan	Method	Score	m/z	Gene names
20150402_CerP14_Frac23_top_opt_C11_01_1832	36401	TOF; CID	69.03	715.87	Emilin1

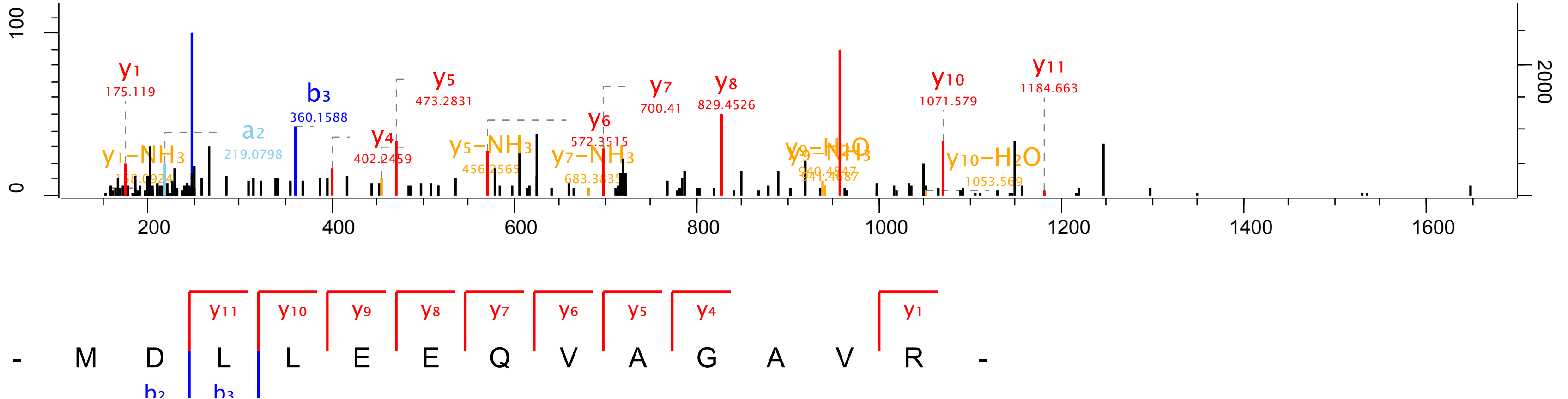

Raw file	Scan	Method	Score	m/z	Gene names
20150402_CerP14_Frac23_top_opt_C11_01_1832	36829	TOF; CID	72.34	675.34	BC052040

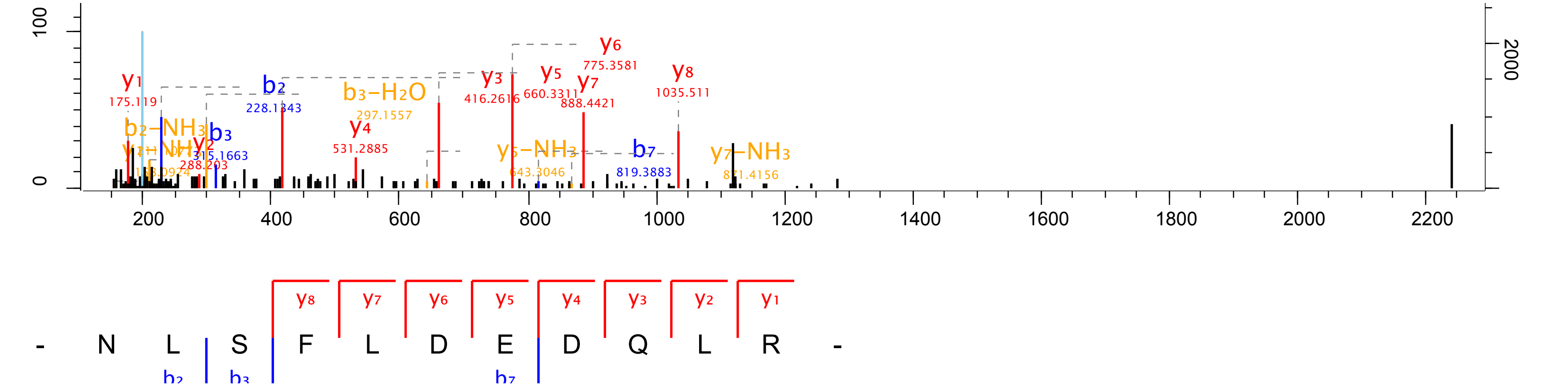

Raw file	Scan	Method	Score	m/z	Gene names
20150402_CerP14_Frac23_top_opt_C11_01_1832	37414	TOF; CID	129.37	872.41	Ltbr

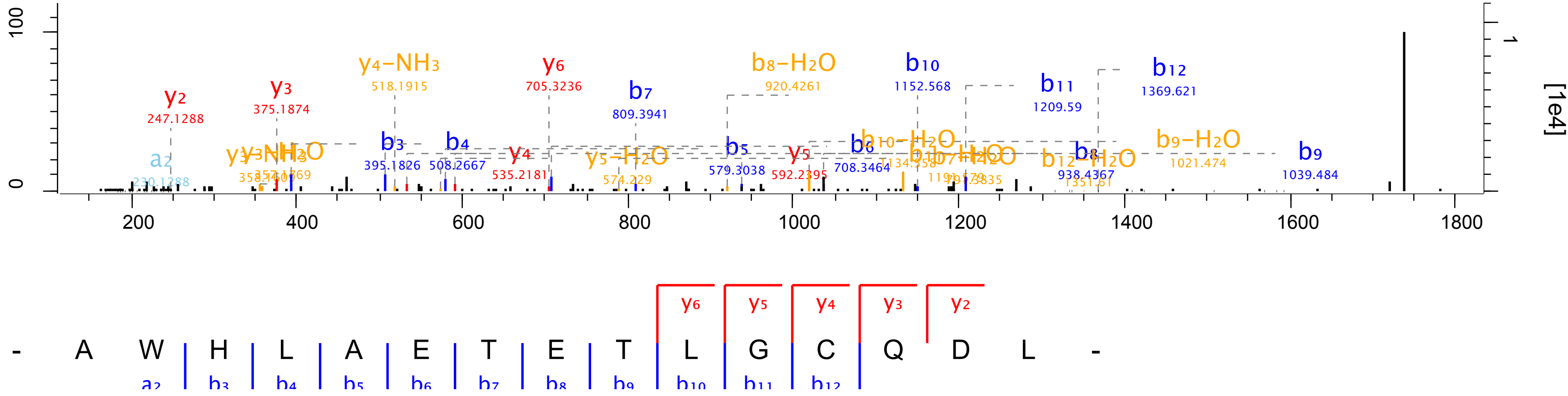

Raw file	Scan	Method	Score	m/z	Gene names
20150402_CerP14_Frac23_top_opt_C11_01_1832	44770	TOF; CID	127.36	627.7	Mppe1

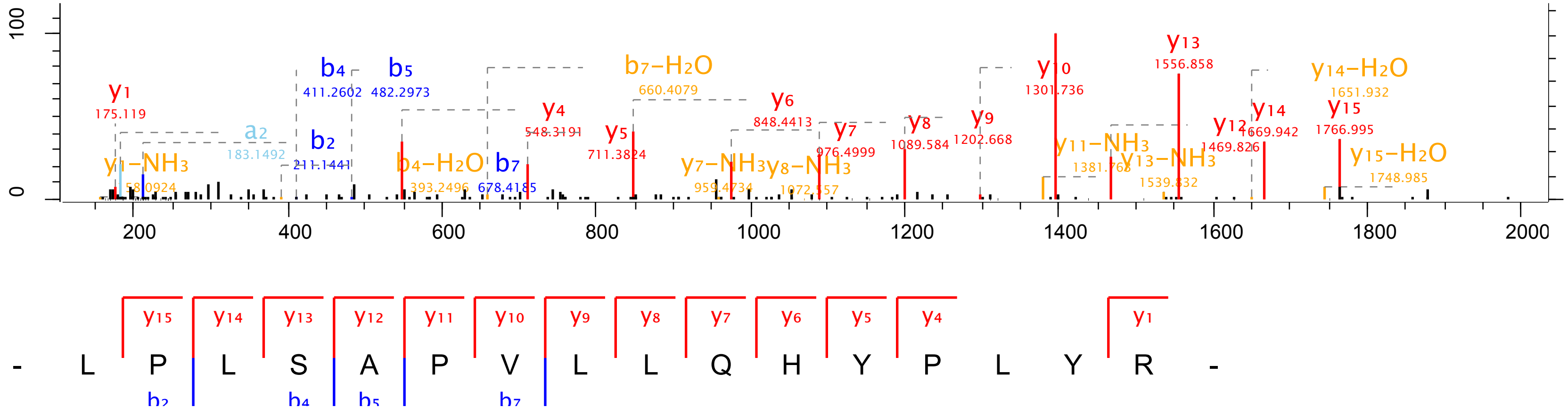

Raw file	Scan	Method	Score	m/z	Gene names
20150402_CerP14_Frac23_top_opt_C11_01_1832	45482	TOF; CID	38.26	685.72	B3gntl1

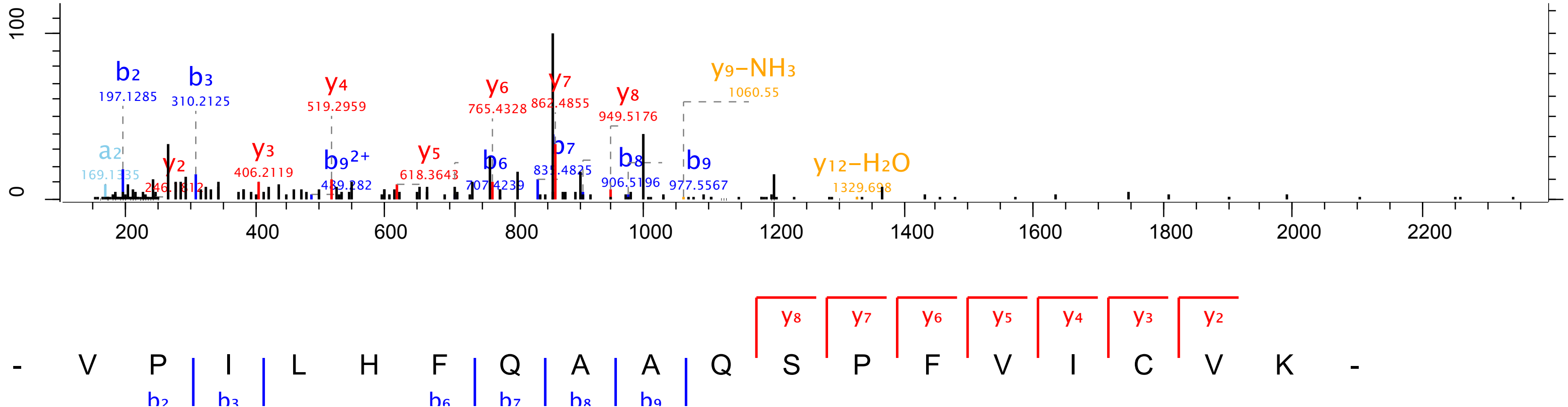

Raw file	Scan	Method	Score	m/z	Gene names
20150402_CerP14_Frac23_top_opt_C11_01_1832	49040	TOF; CID	62.86	871.48	Shh

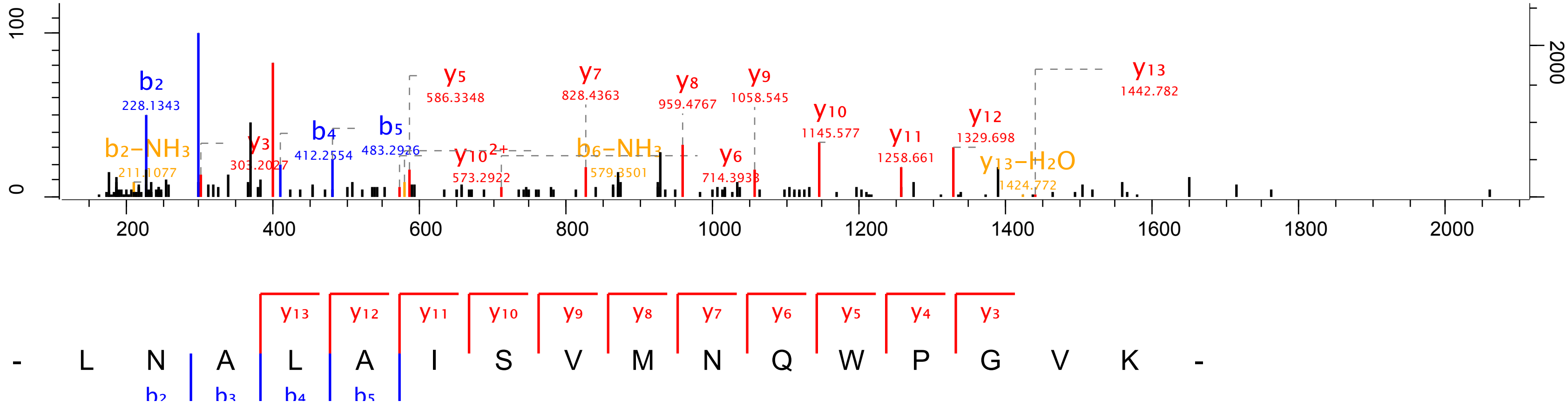

Raw file	Scan	Method	Score	m/z	Gene names
20150402_CerP14_Frac23_top_opt_C11_01_1832	49049	TOF; CID	61.28	749.42	Lrrc32

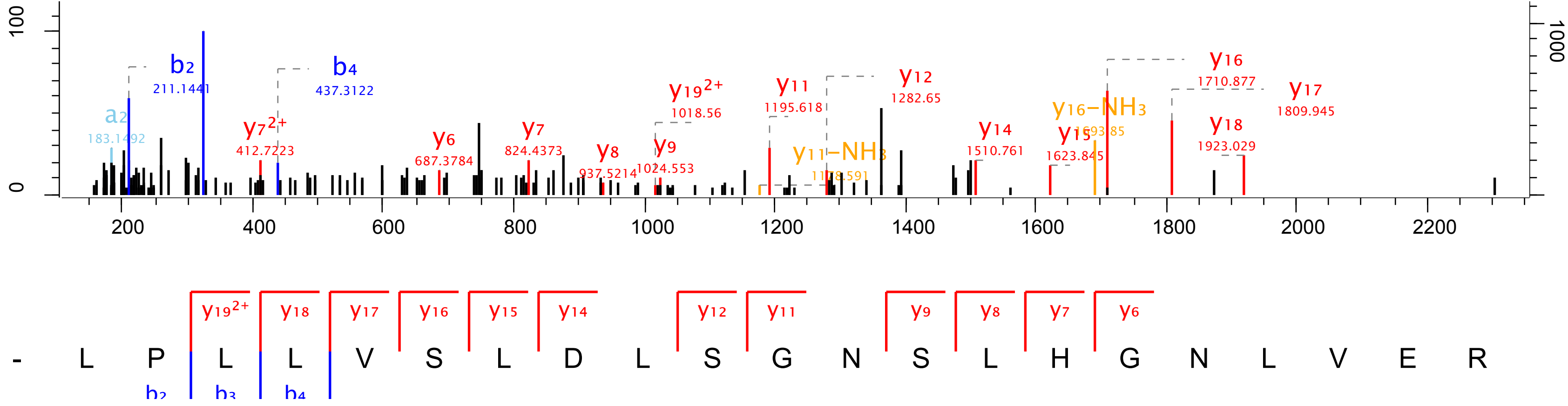

Raw file	Scan	Method	Score	m/z	Gene names
20150402_CerP14_Frac23_top_opt_C11_01_1832	49685	TOF; CID	65.04	787.92	Fjx1

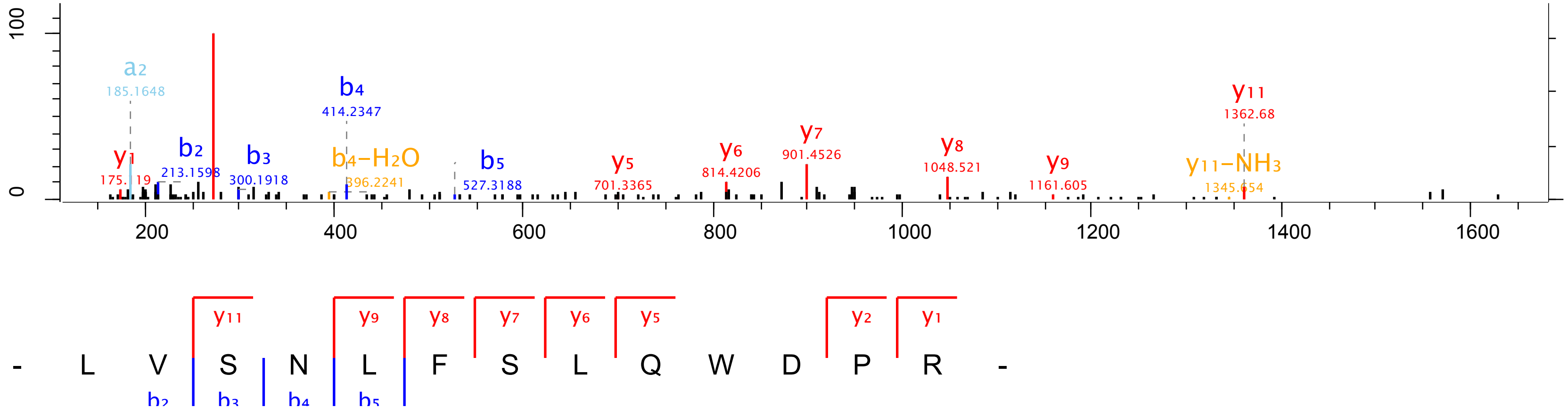

Raw file	Scan	Method	Score	m/z	Gene names
20150402_CerP14_Frac23_top_opt_C11_01_1832	50627	TOF; CID	86.46	651.88	Nfkbie

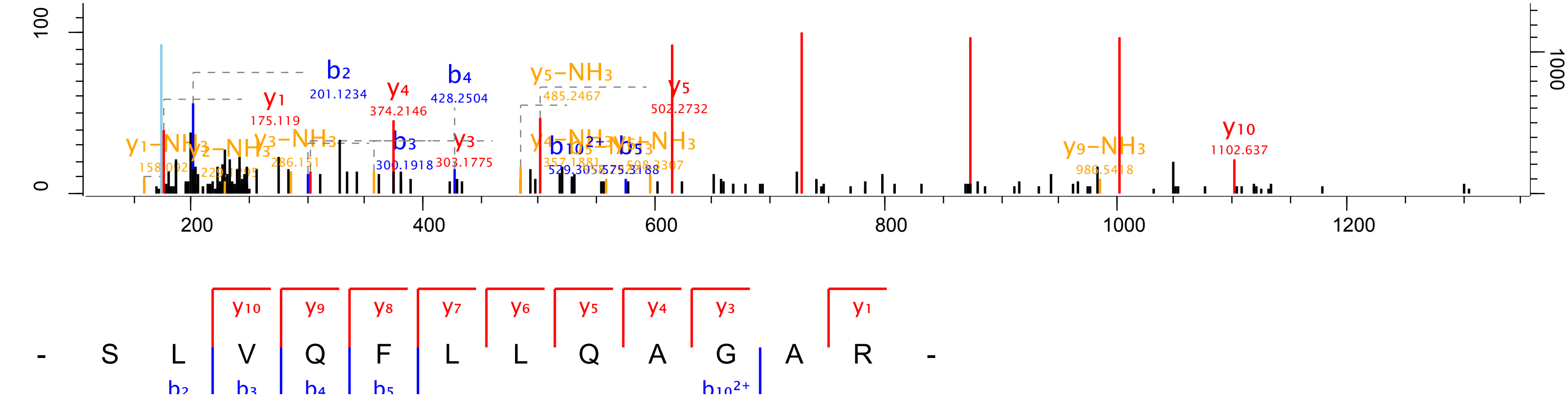

Raw file	Scan	Method	Score	m/z	Gene names
20150402_CerP14_Frac23_top_opt_C11_01_1832	52528	TOF; CID	198.95	620.02	Gipc2

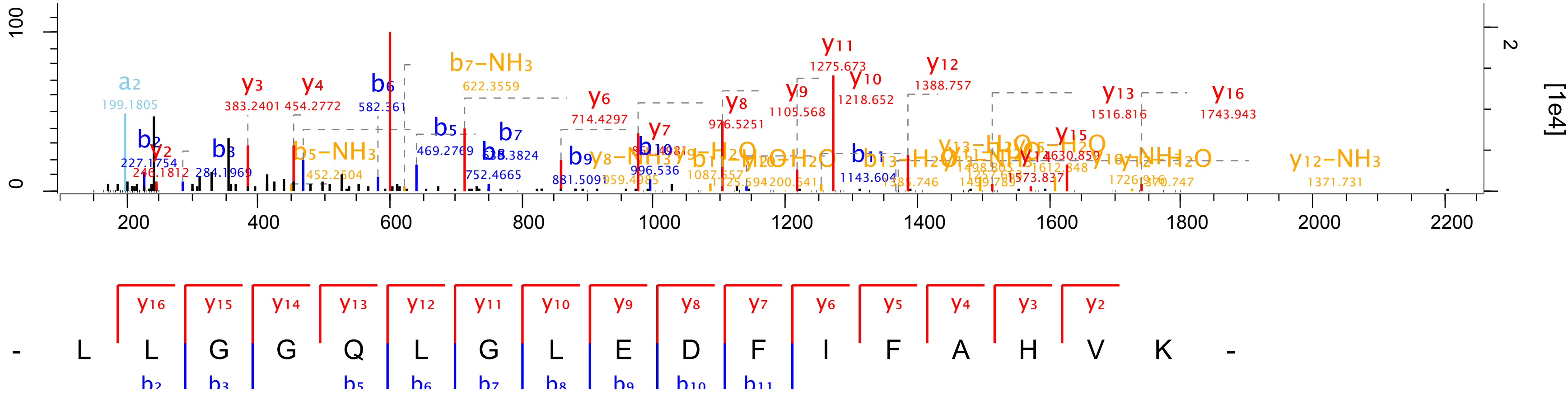

Raw file	Scan	Method	Score	m/z	Gene names
20150402_CerP14_Frac23_top_opt_C11_01_1832	55129	TOF; CID	138.22	589.84	Sec22c

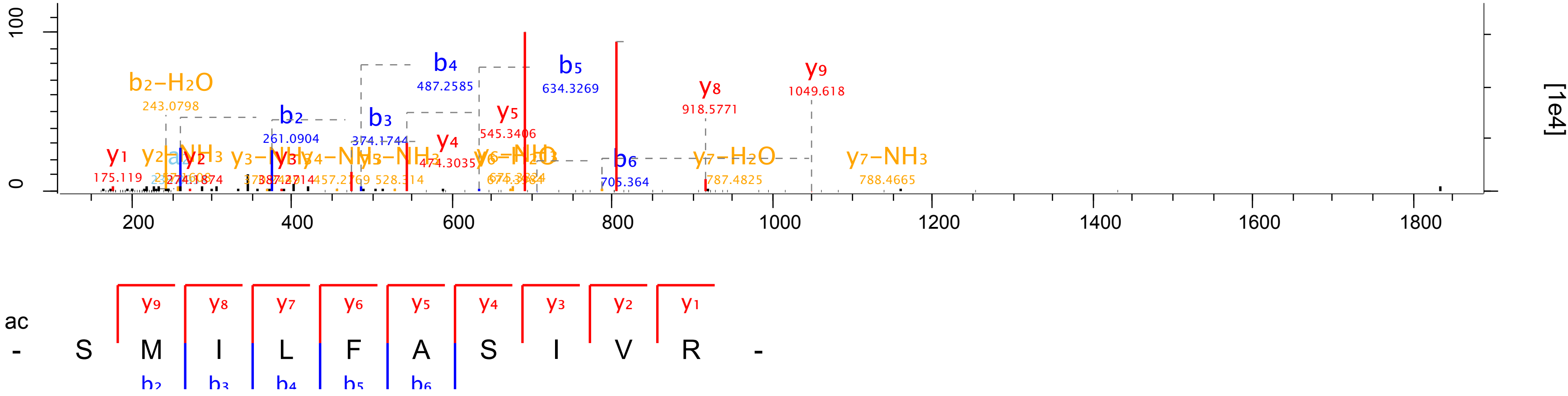

Raw file	Scan	Method	Score	m/z	Gene names
20150402_CerP14_Frac24_top_opt_C12_01_1833	6644	TOF; CID	131.12	434.54	Sf1

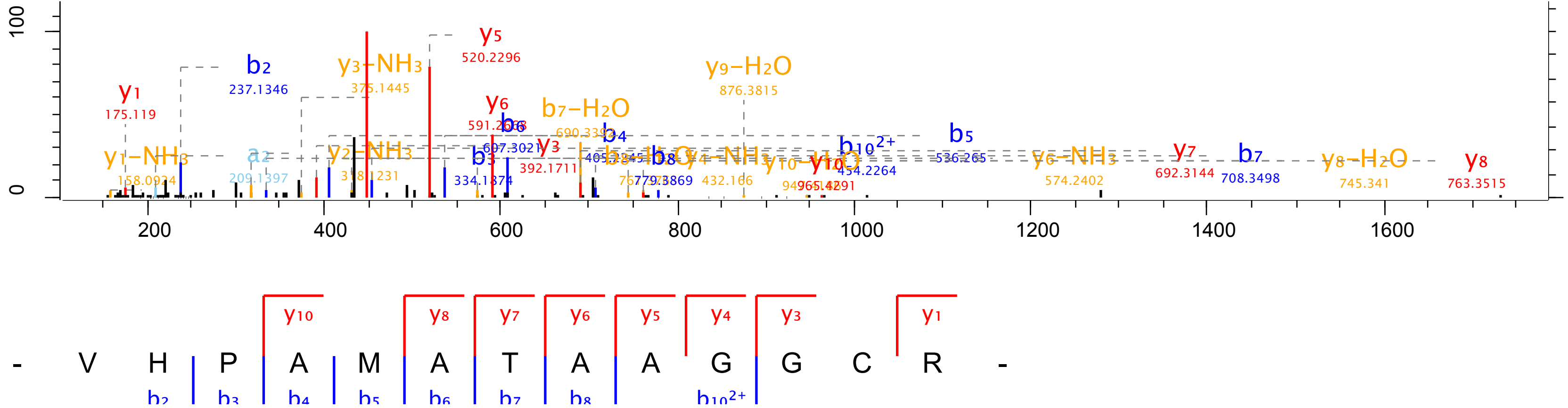

Raw file	Scan	Method	Score	m/z	Gene names
20150402_CerP14_Frac24_top_opt_C12_01_1833	6870	TOF; CID	81.26	581.81	Rhbdl1

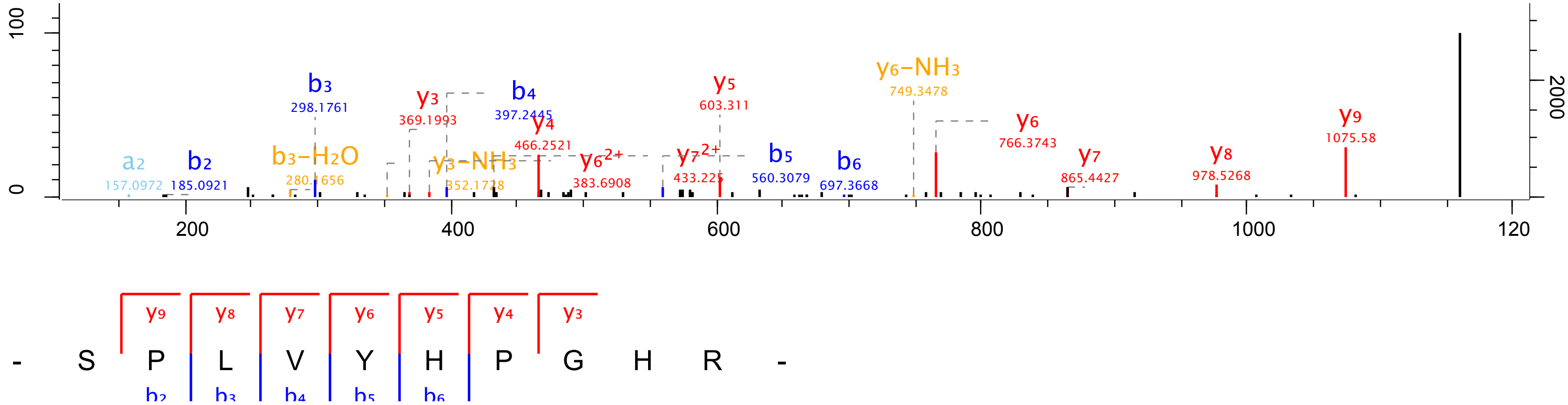

Raw file	Scan	Method	Score	m/z	Gene names
20150402_CerP14_Frac24_top_opt_C12_01_1833	14482	TOF; CID	158.79	587.33	Tktl1

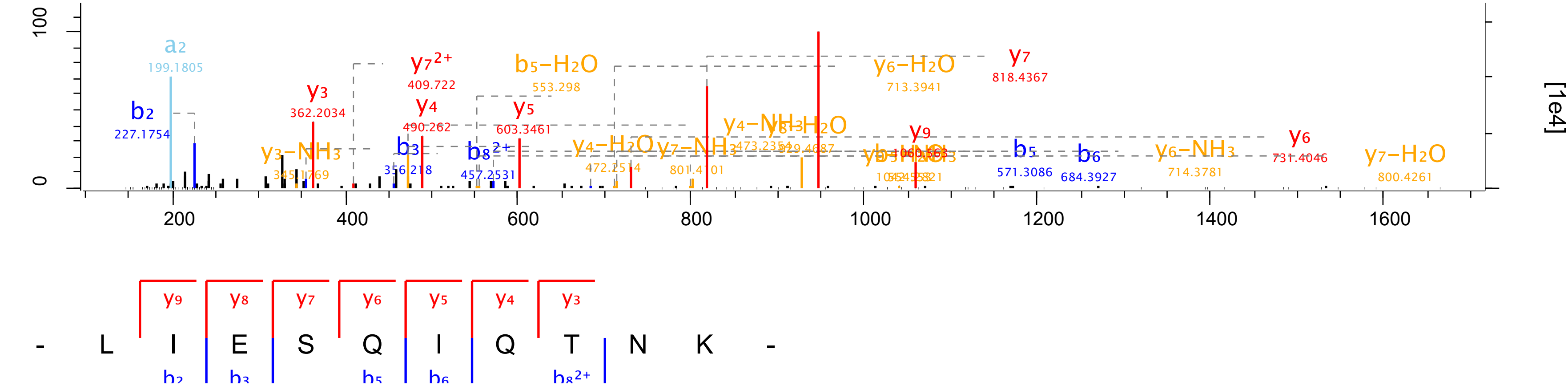

Raw file	Scan	Method	Score	m/z	Gene names
20150402_CerP14_Frac24_top_opt_C12_01_1833	21862	TOF; CID	145.25	536.81	Ikzf5

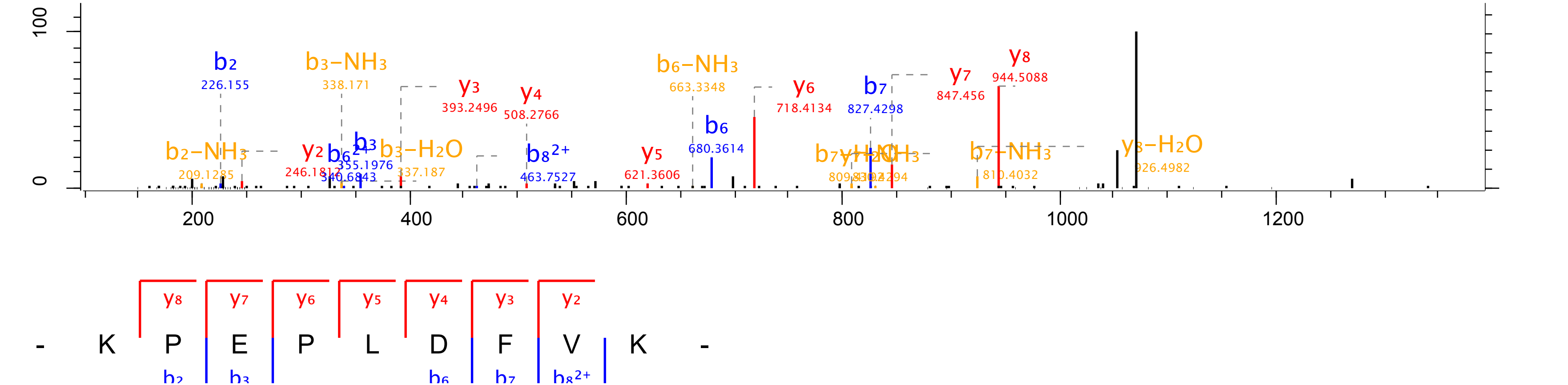

Raw file	Scan	Method	Score	m/z	Gene names
20150402_CerP14_Frac24_top_opt_C12_01_1833	23322	TOF; CID	97.69	652.32	Igkv1-115

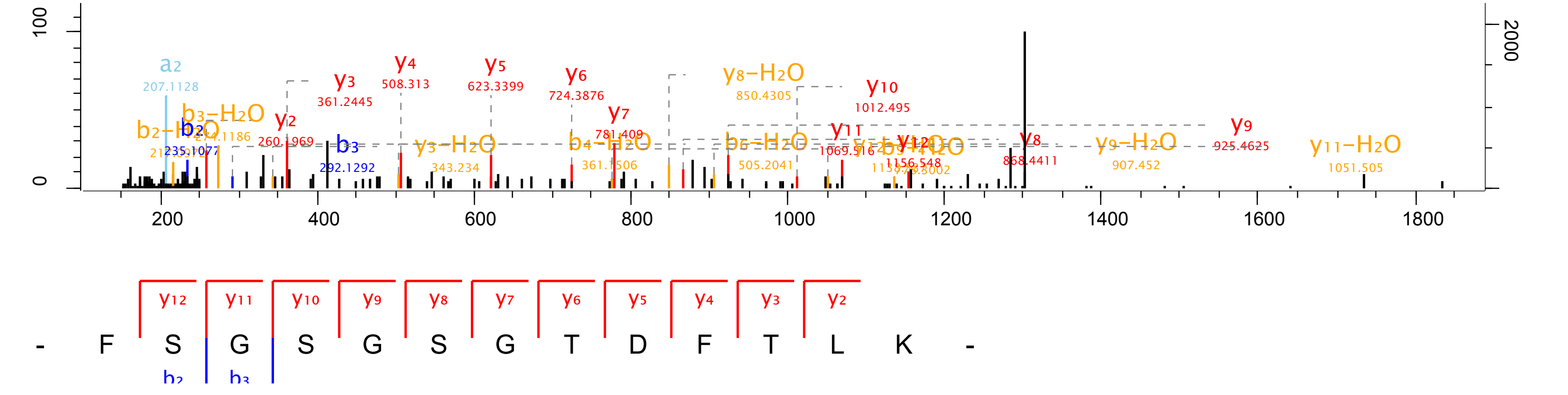

Raw file	Scan	Method	Score	m/z	Gene names
20150402_CerP14_Frac24_top_opt_C12_01_1833	33900	TOF; CID	69.08	528.28	Ccdc80

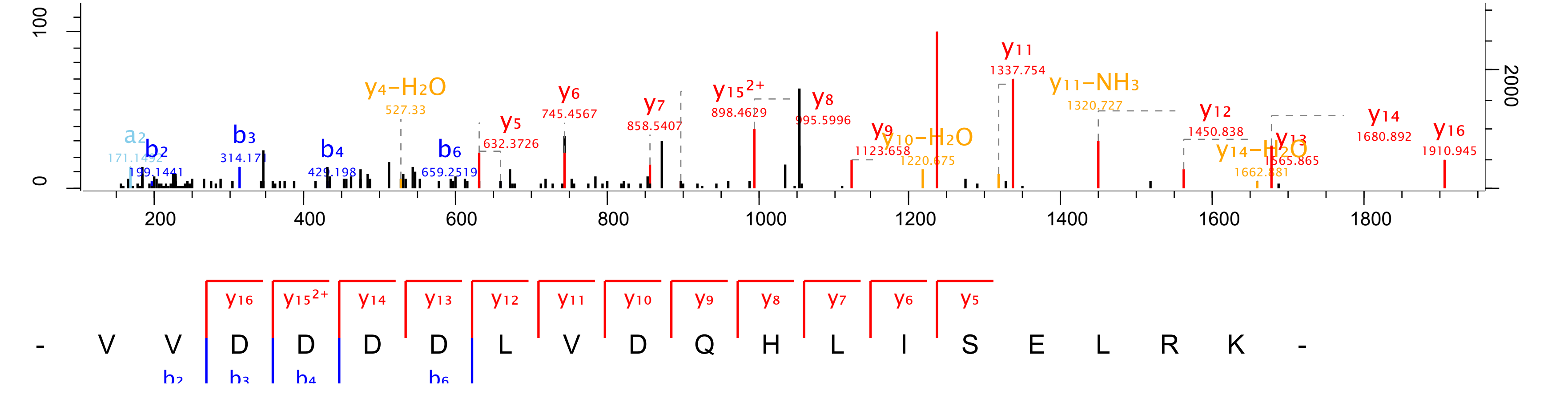

Raw file	Scan	Method	Score	m/z	Gene names
20150402_CerP14_Frac24_top_opt_C12_01_1833	34399	TOF; CID	87.81	579.81	Tmem125

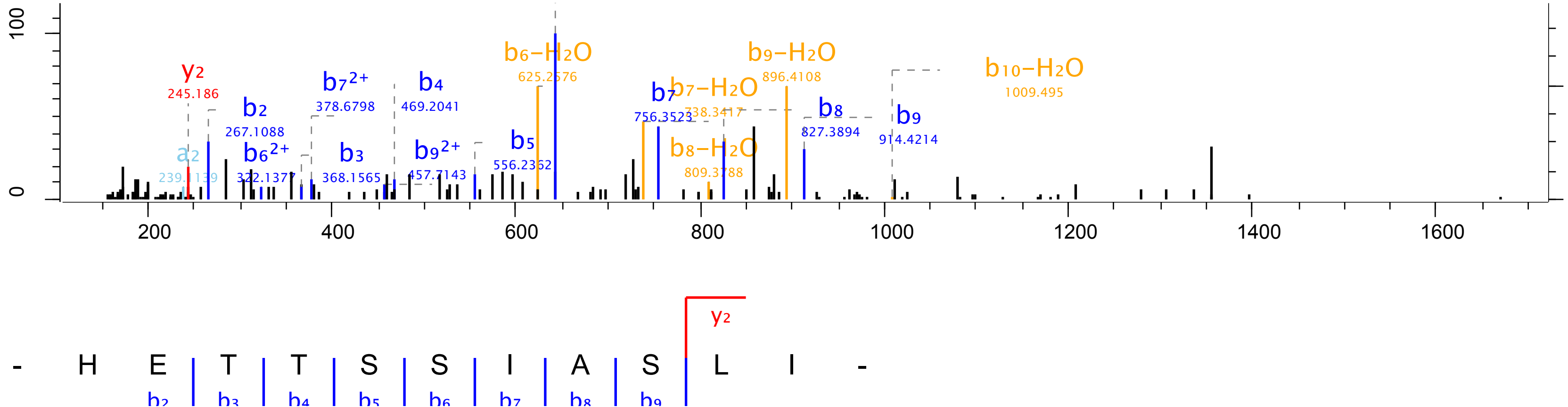

Raw file	Scan	Method	Score	m/z	Gene names
20150402_CerP14_Frac24_top_opt_C12_01_1833	36716	TOF; CID	79.49	501.96	Sigirr

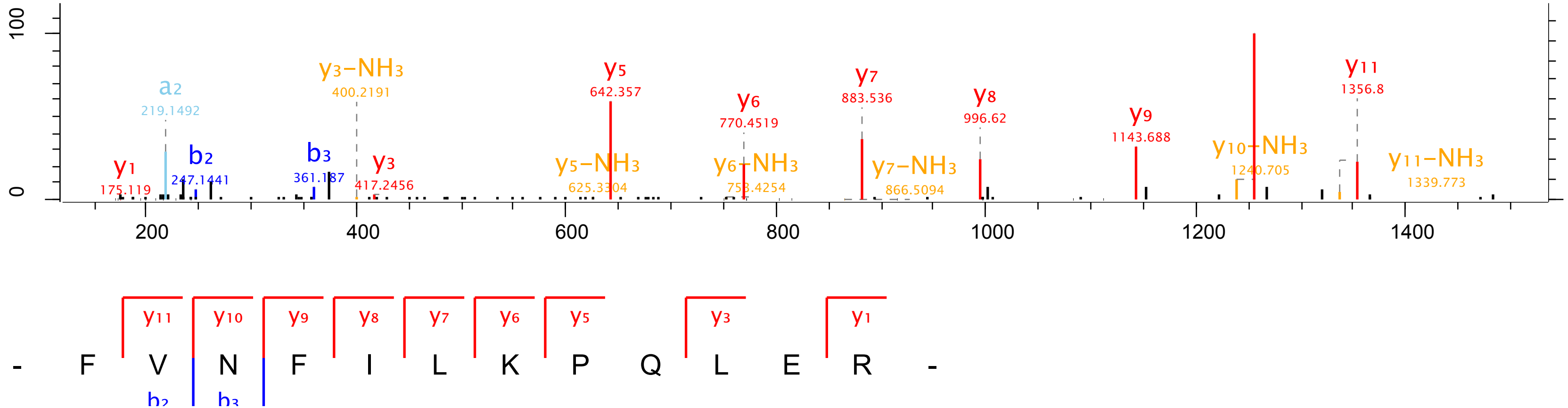

Raw file	Scan	Method	Score	m/z	Gene names
20150402_CerP14_Frac24_top_opt_C12_01_1833	48906	TOF; CID	65.37	844.46	Gpr146

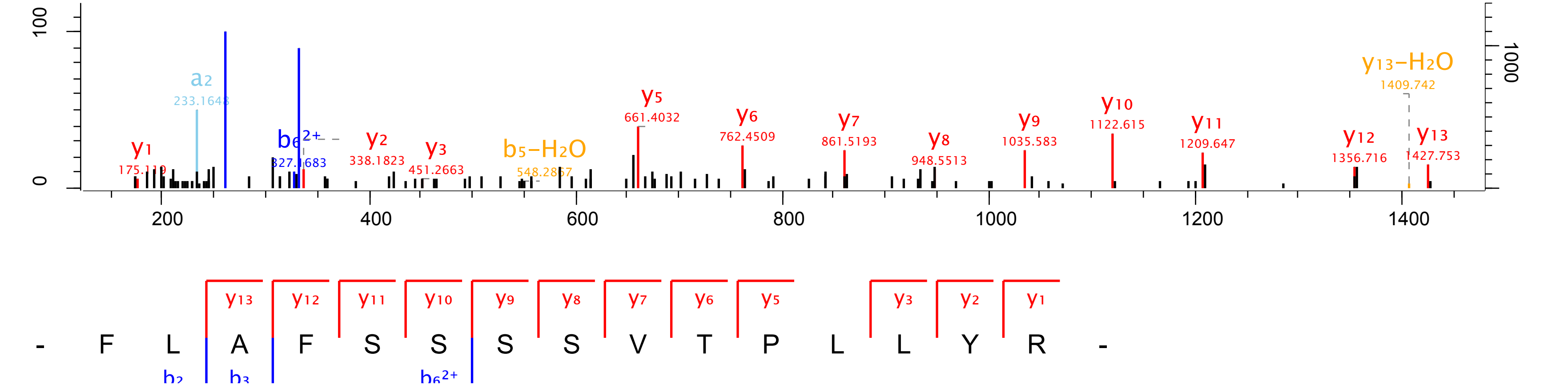

Raw file	Scan	Method	Score	m/z	Gene names
20150402_CerP14_Frac24_top_opt_C12_01_1833	50779	TOF; CID	36.92	804.43	Arid5b

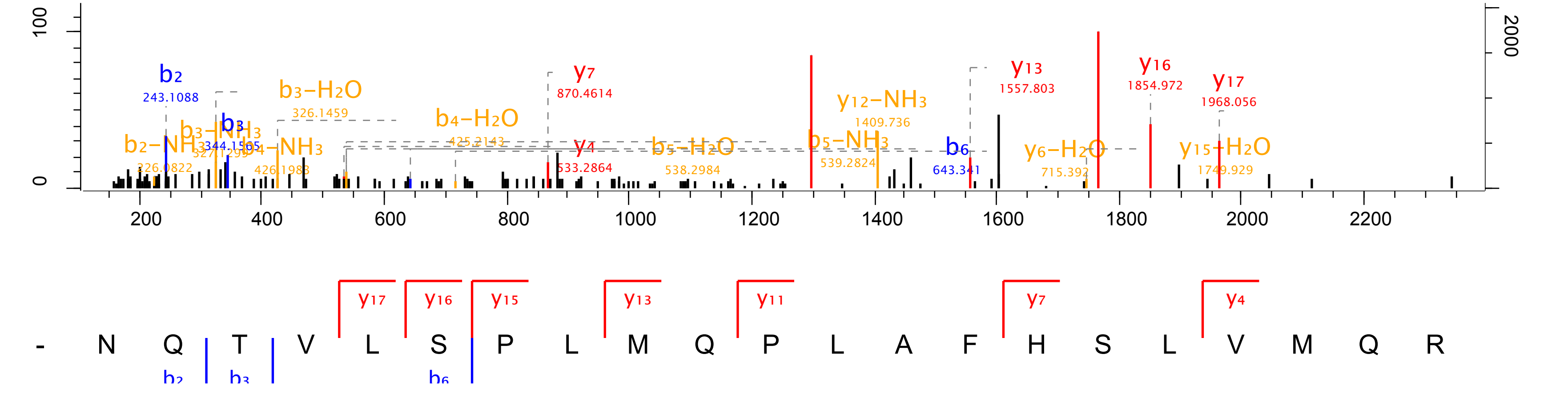

Raw file	Scan	Method	Score	m/z	Gene names
20150402_CerP14_Frac24_top_opt_C12_01_1833	50983	TOF; CID	44.34	817.46	Lgr5

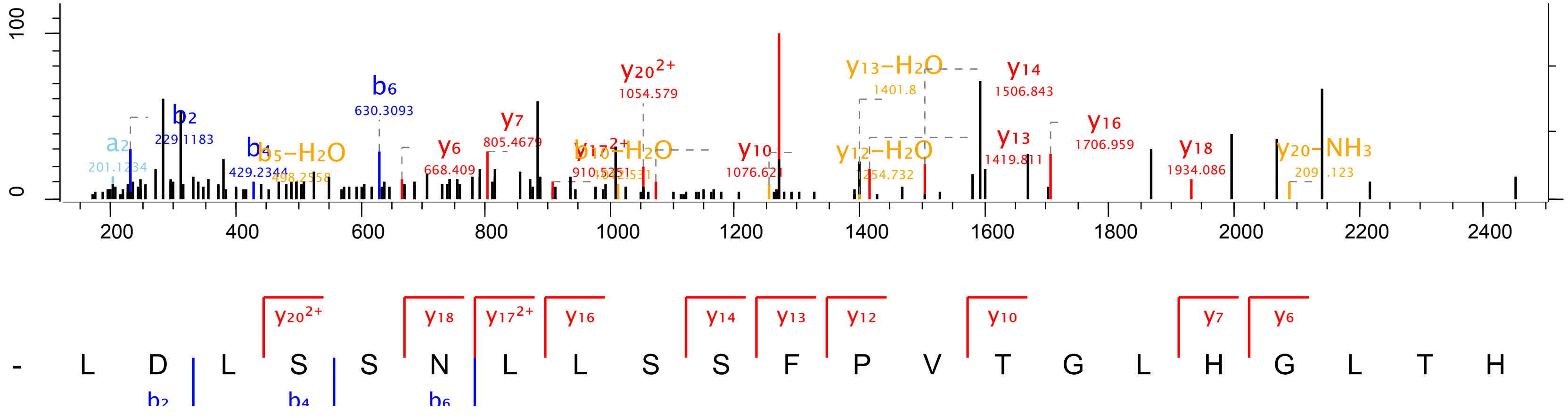

Supplement: Supplemental Data [file supp_M114.047407_mcp.M114.047407-14.pdf]
